# Transcriptome Profiling of a Toxic Dinoflagellate Reveals a Gene-Rich Protist and a Potential Impact on Gene Expression Due to Bacterial Presence

**DOI:** 10.1371/journal.pone.0009688

**Published:** 2010-03-12

**Authors:** Ahmed Moustafa, Andrew N. Evans, David M. Kulis, Jeremiah D. Hackett, Deana L. Erdner, Donald M. Anderson, Debashish Bhattacharya

**Affiliations:** 1 Ecology, Evolution and Natural Resources, Institute of Marine and Coastal Sciences, Rutgers, The State University of New Jersey, New Brunswick, New Jersey, United States of America; 2 Marine Science Institute, University of Texas at Austin, Port Aransas, Texas, United States of America; 3 Woods Hole Oceanographic Institution, Woods Hole, Massachusetts, United States of America; 4 Ecology and Evolutionary Biology Department, University of Arizona, Tucson, Arizona, United States of America; Cairo University, Egypt

## Abstract

**Background:**

Dinoflagellates are unicellular, often photosynthetic protists that play a major role in the dynamics of the Earth's oceans and climate. Sequencing of dinoflagellate nuclear DNA is thwarted by their massive genome sizes that are often several times that in humans. However, modern transcriptomic methods offer promising approaches to tackle this challenging system. Here, we used massively parallel signature sequencing (MPSS) to understand global transcriptional regulation patterns in Alexandrium tamarense cultures that were grown under four different conditions.

**Methodology/Principal Findings:**

We generated more than 40,000 unique short expression signatures gathered from the four conditions. Of these, about 11,000 signatures did not display detectable differential expression patterns. At a p-value < 1E-10, 1,124 signatures were differentially expressed in the three treatments, xenic, nitrogen-limited, and phosphorus-limited, compared to the nutrient-replete control, with the presence of bacteria explaining the largest set of these differentially expressed signatures.

**Conclusions/Significance:**

Among microbial eukaryotes, dinoflagellates contain the largest number of genes in their nuclear genomes. These genes occur in complex families, many of which have evolved *via* recent gene duplication events. Our expression data suggest that about 73% of the *Alexandrium* transcriptome shows no significant change in gene expression under the experimental conditions used here and may comprise a “core” component for this species. We report a fundamental shift in expression patterns in response to the presence of bacteria, highlighting the impact of biotic interaction on gene expression in dinoflagellates.

## Introduction

Dinoflagellates (Phylum Alveolata, Supergroup Chromalveolata) are unicellular protists that are among the most abundant phytoplankton in marine and freshwater ecosystems. Dinoflagellates display a range of lifestyles that together make these organisms of central ecological and economic importance. On the one hand, as oxygenic photosynthesizers, about 50% of the known species play a vital role in oxygen evolution and ocean primary production. On the other hand, some dinoflagellate species form massive toxic or non-toxic harmful algal blooms (commonly known as “red tides”) in the oceans, leading to negative impacts on human health, fisheries, and many other coastal resources.

Dinoflagellates can exhibit different trophic states, of which some are obligatory and others reflect rapid and transient responses to cellular or environmental conditions. Many dinoflagellates are able to exist autotrophically *via* photosynthesis in some stages of their lifecycle. However, there are also strict cases of heterotrophy due to the absence of plastids, as in *Protoperidinium* that feeds on other dinoflagellates [1] and *Paulsenella* that parasitizes diatoms [2]. In addition, alternation between autotrophy and heterotrophy; i.e., mixotrophy, exists in many dinoflagellates and is supported by the presence of food vacuoles and plastids in these taxa (e.g., *Alexandrium ostenfeldii* [3], [4]).

In dinoflagellates, sexuality and subsequent encystment play a key role in bloom dynamics [5]. Encystment allows dinoflagellates to survive unfavorable environmental conditions in the form of resistant cysts, which remain dormant for a mandatory period of several months and then germinate when conditions become favorable. The exponential proliferation of germinated cells results in blooms, which terminate through induction of encystment. Cysts can also be geographically dispersed, giving rise to blooms in regions with no previous history of that species [6], [7], [8], [9].

Although dinoflagellates follow a typical eukaryotic G1-S-G2-M cell cycle [10], they have genetic and cytological properties that distinguish them starkly from other eukaryotes. One of the most remarkable characteristics of dinoflagellates is the large amount of nuclear DNA. On average, algal and plant nuclei contain 0.5 pg/cell, however, in dinoflagellates, DNA content varies from 2.0 pg/cell as in *Amphidinium carterae* [11] to up to 200.0 pg/cell in *Lingulodinium polyedrum* (formerly *Gonyaulax polyedra*) [12], corresponding to ca. 200,000 Mb. Such a massive amount of DNA has made dinoflagellates a challenging system for complete genome sequencing approaches. However, modern transcriptomic methods provide promising strategies to gene discovery in dinoflagellates and an opportunity to address key questions about their ecology and life cycles.

Bacterial assemblages were shown to be associated with and attached to dinoflagellates [13] where their availability markedly affects different aspects of dinoflagellate life cycles such as the quantity of toxin that is produced [14], [15], level of motility [16], growth rate [15], [17], and bloom formation and termination [18]. To investigate the influence of the biotic interaction between dinoflagellates and associated bacterial communities, we prepared RNA from a xenic (X) strain of *Alexandrium tamarense* (hereafter, *Alexandrium*) and compared its expression profile to that of the nutrient-replete control condition (F) and nutrient-stressed cells under nitrogen (N) and phosphorus (P) limitation. A previous study [19] validated the utilization of “massively parallel signature sequencing” (MPSS) [20] to analyze transcriptional regulation in a closely related dinoflagellate (*Alexandrium fundyense*) and provided evidence for the complexity of the transcriptome, the presence of gene families, and the extent of transcriptional regulation. Here, we report the results of a comprehensive profiling of *Alexandrium* transcriptome using MPSS. Our results provide novel insights into the extent of gene richness, the dynamics of gene family evolution, the magnitude of transcriptional regulation, and the impact of the presence of bacteria on global gene expression patterns in dinoflagellates.

## Results and Discussion

Using MPSS, each sample resulted in a library of ∼3,000,000 short signature sequences, containing an average of 290,941 unique sequences (hereafter, simply signatures) with 1,073,382 signatures from all treatments. After screening for deterministic (i.e., absence of nucleotide ambiguities) and significantly expressed signatures (i.e., ≥4 signatures per million [TPM] in at least one library), we found between 38,000 – 39,000 usable signatures per culture treatment (Table 1). We identified 40,029 unique signatures when the data from all treatments were combined. In agreement with earlier findings [21], our data show that the most abundant transcripts among the examined conditions belong to families that encode chlorophyll *a*-*b* binding protein, histone family protein, S-adenosylmethionine synthetase, and S-adenosylhomocysteine hydrolase. Of a total of 40,029, only 18, 2, and 12 signatures were found exclusively in the nutrient-replete (control), N-depleted, and P-depleted cultures, respectively. In contrast, 487 signatures were found exclusively in the xenic culture, suggesting the presence of bacteria had the most significant impact on the transcriptome of *Alexandrium* under the conditions used here; i.e., exclusive transcription of 1.3% of the total number of transcribed genes. Our data also showed the expected transcriptional responses to nutrient limitation, in particular the up-regulation of genes involved in the pathways of cell-death and gamete formation, which will be discussed in detail elsewhere. Here, we focus on genome-wide aspects of dinoflagellate gene expression with a specific focus on the impact of associated bacteria on gene expression.

**Table 1:** Summary of *Alexandrium tamarense* MPSS signatures that were significant and reliable.

Condition	Common	Specific	Unique	>10	>100	>1000
**Nutrient-replete (Control)**	38633	18	38651	17041	895	35
**Nitrogen-limited**	38948	2	38950	14128	1180	45
**Phosphorus-limited**	38780	12	38792	14580	1068	38
**Xenic (bacterized)**	38078	487	38565	15213	878	39
**Average**	38610	130	38740	15241	1005	39
**Total**	39426	603	40029	23412	1843	61

Definitions of the column headers are as following: Common; signatures that are expressed under at least one other treatment, Specific; signatures that are exclusively expressed under the corresponding treatment, Unique; the total number of unique signatures expressed under the corresponding treatment, and >10, >100, and >1000; signatures with expression values at least 10, 100, and 1000 TPM, respectively.

### Gene Content and Gene Families

Previous MPSS analyses using well-annotated genomes have shown a strong correlation between the number of transcribed signatures and the total number of nuclear genes. In addition, these studies have demonstrated that as more libraries and conditions are examined, the number of unique signatures more closely represents the total number of predicted gene models in a genome. For example, in *Arabidopsis*, the number of annotated genes is 27,165 [22] and the number of unique MPSS signatures associated with protein coding regions gathered from 17 libraries is at least 29,569 [23]. Based on this correlation, we postulate that there are about 40,000 transcribed genes in *Alexandrium*, making it the most complex protist transcriptome yet described. It should be remembered, however, that although this number is relatively large compared to other free-living protists (e.g., 27,000 genes in the ciliate *Tetrahymena thermophila* [24] and 12,000 genes in the diatom *Phaeodactylum tricornutum* [25]), it does not account for the massive amount of nuclear DNA (ca. 150 Gb, estimated using pulse-field gel electrophoresis) in haploid *Alexandrium* cells. Clearly, gene number and genome size are uncoupled in these taxa. It is worth pointing out that in a recent genome size versus gene content regression study, dinoflagellates were predicted to contain 40,086 genes in the smallest genome and 92,013 genes in the largest [26].

**Figure 1:**
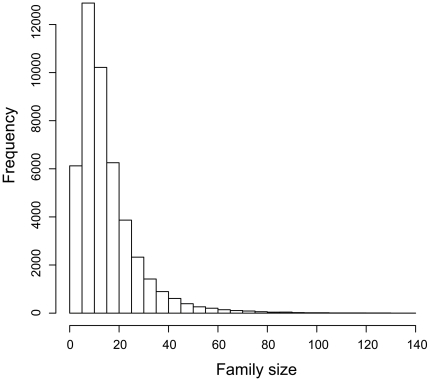
Distribution of gene family size with a maximum of five pairwise mismatches. Histogram of the extrapolated sizes of gene families and the frequency of each class of family size.

This unusually high number of transcribed genes in *Alexandrium* is unlikely to represent unique functional categories; rather many may comprise large gene families that arose by extensive gene duplication events. To address this hypothesis in a conservative fashion, we first identified 4,341 expressed sequence tags (ESTs) from this strain that match perfectly and uniquely the identified set of reliable and significant MPSS signatures. Then we used KEGG Orthology (KO) [27] to functionally cluster these ESTs into families, resulting in the assignment of 1,020 KO entries to 2,169 ESTs (Table 2). The largest gene family comprises 31 members that encode peptidylprolyl isomerase (EC 5.2.1.8; cyclophilin). Subsequently, we counted the number of pairwise mismatches between signatures that correspond to ESTs clustered into the same families and ESTs belonging to different families. By comparing the numbers of pairwise mismatches between signatures from the two groups, we found that five mismatches can distinguish significantly between the two categories with *p*-value < 1E-10. Thus, using five mismatches as the maximum number of pairwise mismatches between signatures to obtain a rough estimate of the genome-wide distribution of gene families, we found 56 families with more than 100 members and the largest family contains 139 members (Figure 1). The largest family with members of known function contains 81 members and encodes pyruvate kinase (EC 2.7.1.40). The second largest family of known function encodes ribosomal protein L27a and contains 74 members. However, using KO-predicted families, we found cases where signatures within the same families shared low to zero identity. These cases are interpreted as duplicated genes with a relatively ancient common ancestor and the accumulation of mutations in the 3′ UTR has erased the phylogenetic signal in the signature sequences.

**Table 2:** Gene families identified using KEGG orthology that have sizes >10 members.

Gene	Definition	Class	Size
**E5.2.1.8**	peptidylprolyl isomerase [EC:5.2.1.8]	Genetic Information Processing; Folding, Sorting and Degradation	31
**ANK**	ankyrin	Cellular Processes and Signaling; Cytoskeleton	29
**E2.5.1.18, gst**	glutathione S-transferase [EC:2.5.1.18]	Metabolism; Metabolism of Other Amino Acids; Glutathione metabolism	23
**fabD**	[acyl-carrier-protein] S-malonyltransferase [EC:2.3.1.39]	Metabolism; Lipid Metabolism; Fatty acid biosynthesis	19
**CALM**	calmodulin	Environmental Information Processing; Signal Transduction; Calcium	17
**fabG**	3-oxoacyl-[acyl-carrier protein] reductase [EC:1.1.1.100]	Metabolism; Lipid Metabolism; Fatty acid biosynthesis	16
**ATPF0C, atpE**	F-type H+-transporting ATPase subunit c [EC:3.6.3.14]	Metabolism; Energy Metabolism; Oxidative phosphorylation	15
**eEF-1A, ef1A**	elongation factor EF-1 alpha subunit [EC:3.6.5.3]	Genetic Information Processing; Translation	15
**dnaJ**	molecular chaperone DnaJ	Genetic Information Processing; Chaperones and	15
**E4.2.1.17, paaG**	enoyl-CoA hydratase [EC:4.2.1.17]	Metabolism; Carbohydrate Metabolism; Propanoate metabolism	14
**E1.14.11.16**	aspartate beta-hydroxylase [EC:1.14.11.16]	Unclassified; Metabolism; Other enzymes	13
**rluD**	ribosomal large subunit pseudouridine synthase D [EC:5.4.99.12]	Genetic Information Processing; Translation; Other translation	13
**HSPA1_8**	heat shock 70 kDa protein 1/8	Environmental Information Processing; Membrane Transport; Pores ion	12
**YWHA**	tyrosine 3-monooxygenase	Cellular Processes; Cell Growth and Death; Cell cycle	12
**RAB**	Rab family, other	Cellular Processes and Signaling; GTP-binding	12
**E1.1.1.37B, mdh**	malate dehydrogenase [EC:1.1.1.37]	Metabolism; Carbohydrate Metabolism; Citrate cycle (TCA cycle)	11
**E1.1.1.95, serA**	D-3-phosphoglycerate dehydrogenase [EC:1.1.1.95]	Metabolism; Amino Acid Metabolism; Glycine, serine and threonine	11
**GAPDH, gapA**	glyceraldehyde 3-phosphate dehydrogenase [EC:1.2.1.12]	Metabolism; Carbohydrate Metabolism; Glycolysis/Gluconeogenesis	11
**E3.1.3.16**	protein phosphatase [EC:3.1.3.16]	Unclassified; Metabolism; Other enzymes	11
**RP-L40e, RPL40**	large subunit ribosomal protein L40e	Genetic Information Processing; Translation; Ribosome	11

**Figure 2:**
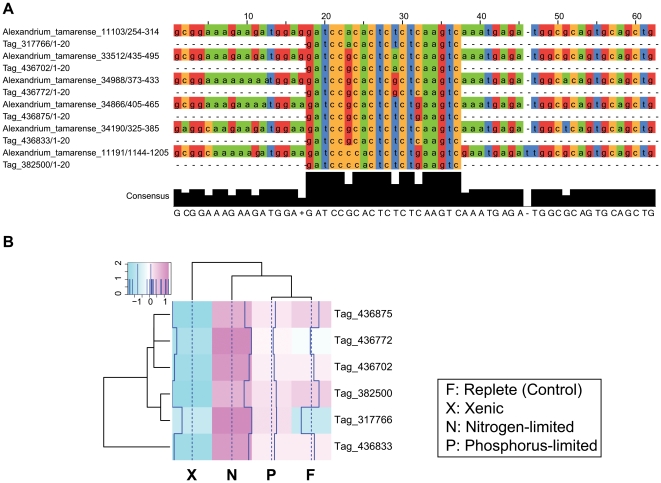
Co-regulation of elongation factor 1-α gene family members. (A) Multiple sequence alignment of six signatures and their matching ESTs. The six signatures contain one or two pairwise mismatches. The mismatches among the signatures co-segregate along with mismatches in the ESTs. (B) Heatmap of the expression of the six signatures.

Examining the *Alexandrium* expression data drew our attention to several examples of different genes that belong to the same family and exhibit similar transcriptional profiles. For example, three S-adenosylmethionine synthetase (SAMS) genes were down-regulated in the bacterized culture. Similarly, three serine hydroxymethyltransferase (SHMT) genes were also down-regulated under this treatment. Genes encoding light-harvesting chlorophyll binding proteins followed the same pattern. In addition, four members of the ubiquitin family were up-regulated under nutrient limitation. To examine this association between gene family members and gene expression, we identified six signatures with a single mismatch between each pair with each of the six signatures having perfect matches to ESTs that encode the alpha subunit of the eukaryote translation elongation factor (EF-1α). The multiple sequence alignment (Figure 2A) of the signatures and their matching ESTs shows co-segregation of the mismatches among the signatures along with mismatches among the ESTs, suggesting these mismatches are not due to sequencing errors. Next, we found that the expression values of these six signatures are strongly correlated (Figure 2B) with a general pattern of up-regulation under nitrogen limitation and down-regulation in the presence of bacteria; i.e., when both are compared to the nutrient-replete culture. Therefore, the expression profiles among members of this (and perhaps many other) gene family are strongly correlated. This suggests that gene family expansion in *Alexandrium* may be a general mechanism used to enhance transcript abundance. Searching for similar patterns of co-regulation among family members, we found several families of different sizes (2, 4, and 8 family members; Table **3**) that follow the same trend. In summary, our data indicate that dinoflagellate genomes contain large gene families with evidence for expression correlation among studied family members.

**Table 3:** Gene families with significant within-family co-regulated expression patterns.

Gene	Definition	*r* ^2^	*p*-value	Size
**atpE**	F-type H+-transporting ATPase subunit c [EC:3.6.3.14]	0.952	4.82E-02	8
**CETN2**	centrin-2	0.995	4.62E-03	2
**metK**	S-adenosylmethionine synthetase [EC:2.5.1.6]	0.994	5.73E-03	2
**petF**	ferredoxin	0.953	4.70E-02	2
**psaC**	photosystem I subunit VII	0.910	8.95E-02	2
**psaE**	photosystem I subunit IV	0.974	2.57E-02	2
**rbcL**	ribulose-bisphosphate carboxylase large chain [EC:4.1.1.39]	0.998	2.16E-03	4
**SMD2**	small nuclear ribonucleoprotein D2	0.927	7.33E-02	3
**tktB**	transketolase [EC:2.2.1.1]	0.992	8.12E-03	2
**YWHA**	tyrosine 3-monooxygenase	0.949	5.13E-02	2

### Bacterial Presence and Gene Expression

**Figure 3:**
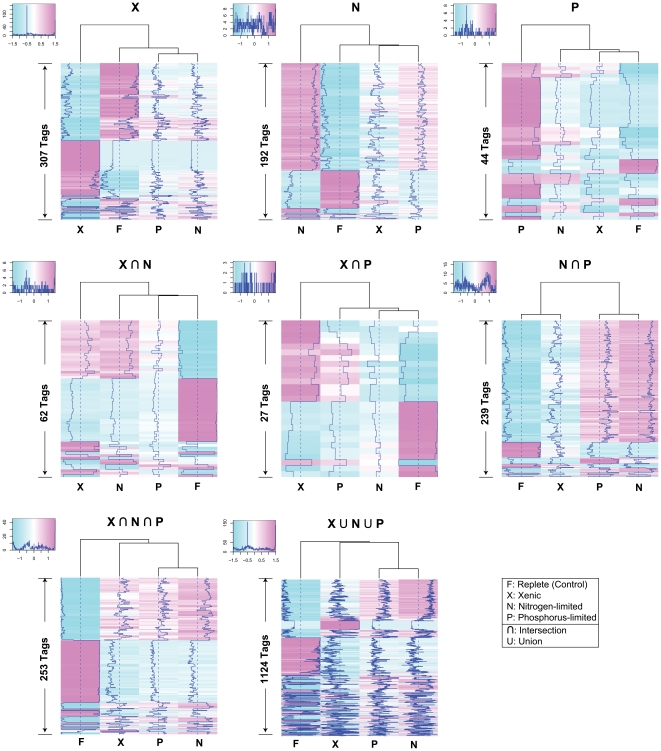
Differentially expressed signatures in response to three different culture treatments when compared to the control. Heatmap of the differentially expressed signatures under the three treatments (N, P, and X) compared to the control (F). The intersection between the treatments indicates signatures that showed significant differential expression patterns in two conditions out of the three or in the three conditions compared to the control.

Although complex and multi-species bacterial assemblages have been shown to be associated with dinoflagellates both in extra- and intra-cellular environments [28], [29], taxa appear to be limited to the Cytophaga-Flavobacterium-Bacteroides (CFB) group and the α- and γ- classes of Proteobacteria. In this study, we did not attempt to identify the prokaryotes present in the bacterized *Alexandrium* culture. Previous studies have shown however that members of the genera *Roseobacter* (α-Proteobacteria) and *Alteromonas* (γ-Proteobacteria) are the dominant bacterial groups associated with *Alexandrium* sp. [30]. Here, we focused on the effect of the presence of bacteria in the culture on gene expression in the dinoflagellate. To identify transcriptionally regulated genes, we used the nutrient-replete culture as the control condition and identified signatures that were significantly up or down regulated in the other conditions using Fisher's test. At *p*-value < 1E-10, we found 1,124 signatures that were differentially expressed among the three (xenic, N-limited, P-limited) treatments compared to the control (Figure 3). By relaxing the *p*-value to a relatively more permissive threshold of 0.05 to detect even slight changes in expression among treatments, we identified ca. 11,000 differentially expressed signatures, indicating that about 29,000 signatures are consistently expressed with non-significant differences under the culture conditions used here. In dramatic contrast to a recent study which showed that about 6% of the expressed genes in rice are uniformly expressed, housekeeping genes [31], our results suggest that about 73% of the *Alexandrium* transcriptome comprises a “core” component and 27% comprises the regulated component, under differing cellular or environmental conditions. Of the 1,124 signatures, 307 (27%) were differentially expressed in the xenic culture, of which 119 and 188 were up- and down-regulated, respectively. Of these differentially regulated transcripts, two sets of genes stand out because they are collectively involved in the regulation of two important cellular processes, the methionine-homocysteine cycle and photosynthesis.

#### Methionine-Homocysteine Cycle

The majority of the signatures that showed a significant expression change in the xenic culture were down-regulated. Of these, three signatures match perfectly (i.e., 20/20 matching nucleotides) three different ESTs encoding S-adenosylmethionine synthetase (SAMS; EC 2.5.1.6). Although the log2 fold-change ratios were not dramatic for these signatures, 0.7 (4038/2534), 2.0 (579/142), and 1.5 (826/286), expression differences were statistically significant with p-values < 1E-10, respectively. SAMS catalyzes the synthesis of S-adenosylmethionine (SAM) from methionine and ATP [32], [33] and is vital for prokaryotic and eukaryotic cellular growth and proliferation. SAM is the primary methyl group (CH3) donor and a precursor for the biosynthesis of polyamines [34]. In saxitoxin-producing microorganisms, e.g., Alexandrium and the cyanobacterium Anabaena circinalis, SAM is thought to act as an alkylating agent in the biosynthesis of saxitoxin [35], [36]. Given such a critical role for SAM, the observed significant decrease in the transcriptional level of three different SAMS-encoding genes in Alexandrium in the presence of the bacterial community may potentially be of biological significance. A similar interaction between Amoeba proteus and its proteobacterial Legionella-like symbionts was shown to repress the transcription of amoebal host SAMS genes [37], [38]. It was proposed that plasmids from the bacterial symbionts [39] transfer defective copies of SAMS to the nuclear genome of the amoeba host, thereby repressing transcription of native SAMS. This establishes complete dependence of the amoeba on symbiont supply of bacterial SAMS, with removal of the latter resulting in host death [37]. Although such an irreversible repression of host SAMS activity in the presence of bacteria has not been previously reported in dinoflagellates, a possible, albeit speculative, explanation for our result is that bacterial effectors employ a mechanism that “transiently” down-regulates the transcription of Alexandrium SAMS. With regard to SAMS, among the significantly down-regulated genes is S-adenosylhomocysteine hydrolase (SAHH) with a fold-change of 1.13 (636/291) and p-value of 1.92E-28. SAHH is a key player in the methionine cycle by catalyzing the reversible hydrolysis of S-adenosylhomocysteine (SAH) to homocysteine (HCY) and adenosine [40]. This takes place after the transfer of the methyl group from SAM to an acceptor and the conversion of SAM to SAH in SAM-dependent methylation reactions [41]. Although preliminary, these results begin to demonstrate the significant impact of bacterial presence on Alexandrium via the regulation of key enzymes that share metabolic connections.

#### Photosynthesis

The second set of genes that were significantly affected by the presence of bacteria in the Alexandrium culture is those involved in photosynthesis. These genes are categorized into two groups (Table 4). The first is primarily associated with light absorption and carbon fixation and were down-regulated, whereas the second group was up-regulated. Members of the latter group play a role in photoprotection and response to light stress. Among the down-regulated genes are three signatures that match three different ESTs that encode light-harvesting chlorophyll binding proteins. In addition, a transcript encoding a transketolase (EC 2.2.1.1) was down regulated by a fold-change ratio of 2.5 (173/31) and a p-value of 3.24E-23. Transketolase plays an important role in cellular metabolism through the catalysis of two opposing reactions in the pentose phosphate pathway, the primary source for nicotinamide adenine dinucleotide phosphate (NADPH) and five-carbon sugars, the precursor for nucleotides and carbohydrates in the cell [42]. In photosynthetic organisms, transketolase performs a similar enzymatic function in the Calvin Cycle (CC), the core of carbon fixation in plants, algae, and photosynthetic bacteria [43]. A minor reduction (less than 40%) of the transcription of transketolase in plants has a dramatic effect on the regeneration of ribulose-1,5-bisphosphate (RuBP), which fixes the carbon from carbon dioxide into six-carbon intermediates in the CC, a reaction that is catalyzed by ribulose bisphosphate carboxylase (RuBisCO). This decrease in RuBP regeneration causes a significant inhibition of photosynthesis and, subsequently, leads to a fourfold decrease in the growth rate in the plant cells [44]. Such a significant decrease in the growth rate was not observed in the Alexandrium xenic culture (see Materials and Methods) when compared to the control, suggesting that Alexandrium cells do not depend solely on photosynthesis for energy production. Interestingly, RuBisCO was down regulated by a fold-change ratio of 3.37 and p-value of 2.65E-230, providing strong evidence of a decrease in carbon fixation because of the presence of bacteria. Another photosynthesis-related gene that was down regulated in the presence of bacteria is ascorbate peroxidase (APX). APX scavenges oxidative radicals (e.g., hydrogen peroxide, H2O2) by reducing hydrogen peroxide to water and oxidizes ascorbate (vitamin C) to dehydroascorbate [45]. The expression of APX is linearly correlated with photosynthetic electron flow in Arabidopsis [46]. Although the role of antioxidants is traditionally expected to be in response to oxidative stress, reactive oxygen species (ROS; e.g., hydrogen peroxide) and ROS-scavenging molecules (e.g., APX) are also involved in transcriptional regulation [47], [48]. Therefore, the down-regulation of APX in the bacterized culture could be a response to the decrease of photosynthetic activity or, conversely, is a mechanism to reduce photosynthetic activity.

**Table 4:** Photosynthesis-related genes that are significantly differentially expressed in the presence of bacteria.

Signature ID	Definition	Fold Ratio	*p*-value
**845164**	Ribulose bisphosphate carboxylase (RuBisCO)	−3.37	2.65E-230
**90074**	Chloroplast light harvesting complex protein	−0.55	1.88E-53
**761359**	Chloroplast light harvesting complex protein	−0.54	5.73E-29
**948885**	Transketolase	−2.48	3.24E-23
**294382**	Chloroplast ascorbate peroxidase	−1.74	2.45E-12
**569915**	Peridinin-chl a protein precursor	4.1	1.11E-21
**570798**	Peridinin-chl a protein precursor	3.75	2.69E-21
**1018239**	Chloroplast photosystem II 12 kDa extrinsic protein	1.68	4.76E-21
**846848**	Photosystem I iron-sulfur center (PsaC)	2.04	3.74E-17
**571472**	Peridinin-chl a protein precursor	1.92	7.72E-16
**873883**	Ribonucleotide reductase (Ferritin)	0.96	1.88E-13
**626619**	Peroxiredoxin	1.46	1.02E-11
**17398**	Chloroplast cytochrome b559 subunit beta	1.88	1.34E-11

In contrast, the presence of bacteria led to a significant up-regulation of eight signatures that are involved in photoprotection. Of these, three signatures match perfectly a family of ESTs that encode peridinin chlorophyll protein (PCP). PCP is a dinoflagellate-specific light-harvesting complex that is water-soluble and uses carotenoid (four peridinins to one chlorophyll *a*) as the absorption pigment in the blue-green region of the spectrum [49], [50]. In plants and algae, chlorophylls *a* and *b* are light-harvesting pigments and carotenoids are primarily involved in the protection from high or excess light. In dinoflagellates, carotenoids are the major light absorption pigments [51]. However, given the apparent general inhibition of photosynthesis through the down-regulation of RuBisCO, light harvesting proteins, and transketolase, the up-regulation of genes encoding PCP proteins is likely to provide photoprotection to the plastid, in response to a decrease in the efficiency of photosynthesis. Additionally, one of the genes up-regulated in response to the presence of bacteria is peroxiredoxin (EC 1.11.1.15), a major antioxidant enzyme in the cell. Peroxiredoxins reduce and detoxify ROS in redox reactions in which they act as the electron acceptor [52].

Based on this pattern of differential expression in the presence of bacteria, we hypothesize that interactions between *Alexandrium* and associated bacterial communities affect the trophic state of *Alexandrium* by reducing photosynthetic activity. In contrast, there is an enhanced expression of photoprotection and oxidative stress response genes. However, our data do not clarify the mechanism that *Alexandrium* uses to acquire nutrients from bacteria, if indeed that is what is happening in these cultures. Therefore, this relationship could be phagotrophic, similar to the induction of phagotrophy in the dinoflagellate *Heterocapsa triquetra via* nutrient depletion [53], or a mutualistic relationship that also provides benefits to the bacteria; e.g., protection from predators. In a recent study, Fagerberg and co-authors described a stimulation of growth in *Alexandrium minutum* by high molecular weight dissolved organic matter, highlighting the potential use of organic nitrogen from these large molecules and the ability of *Alexandrium* to switch from autotrophy to osmotrophy [54].

In summary, our work provides insights into genome-wide responses of *Alexandrium* to differing environmental conditions. Our data show that dinoflagellates contain the largest number of nuclear genes known among unicellular eukaryotes, which occur in complex gene families, many of which have evolved *via* recent duplication events. The expression data suggest that about 73% of the *Alexandrium* transcriptome is uniformly transcribed independent of the environmental conditions used in our study. Finally, the presence of bacteria in culture has a significant impact on gene expression in *Alexndrium*, regulating key metabolic processes such as photosynthesis. Although preliminary, these data form a set of hypotheses that can be tested by using a larger variety of culture manipulations followed by validation of RNA and protein expression levels. Of highest urgency is to validate the significance of biotic interactions on gene expression in marine microbial communities. Other key results are that a majority of genes are uniformly expressed in dinoflagellates and that these taxa can transiently switch from heterotrophy to phototrophy in response to the environment.

## Materials and Methods

### Cultures

Alexandrium tamarense strain CCMP1598 was used for all treatments except for the xenic culture, for which the bacterized clone, CCMP1493, was used. Strain CCMP1493 was first isolated from a germinated cyst from Daya Bay, east of Hong Kong (latitude +22°17′60.00″ and longitude +114°17′60.00″) and identified by Enrique Balech in 1991. It was deposited in the Center for Culture of Marine Phytoplankton by Donald M. Anderson in 1992. The growth rates were f/2 – 0.4 divisions per day, f/40 N, and f/40 P – 0.095 divisions per day, and 0.37 divisions per day in the xenic treatment. In each of these treatments, the in vivo fluorescence was monitored daily, and the culture was harvested when the division rates were consistent for several days.

### Expressed Sequence Tag and 454 Transcript Sequencing

Total RNA was extracted from cultures of CCMP 1598 grown under replete (f/2), nitrogen-limited (f/40 N), and phosphorus-limited (f/40 P) conditions as described above, using the Nucleospin RNA II purification kit (Clontech Laboratories, Mountain View, CA, USA) according to the manufacturer's protocol. A start and a normalized directionally cloned (3′ *Not*I-5′*Eco*R1) cDNA library was constructed from the pooled RNA as previously described [55]. The complete set of existing EST clones derived from a previous study of *Alexandrium tamarense* CCMP1598 [21] was then used in a DNA hybridization protocol with the normalized library [56] to generate a subtracted cDNA library for single-pass 3′ EST sequencing. We generated a total of 11,171 ESTs using Sanger sequencing of the subtracted library which were processed as previously described [57]. The clustering, which relied on the 3′ UTR regions to facilitate accuracy, was done using UIcluster v3.0.5 [58]. This procedure resulted in a total non-redundant “unigene” set of 6,723 unique clusters. These data were combined with the existing unigenes described by Hackett et al. [21] and clustered using CAP3 [59] with a 95% cutoff identity between overlapping reads to avoid over-assembly that could mask biologically significant differences among closely related gene families. This second round of clustering resulted in a Sanger-based database of 12,329 unigenes from *Alexandrium*. We also generated EST data from *Alexandrium* using ‘454’ pyrosequencing. For this procedure, equimolar amounts of total RNA from each condition were pooled and cDNA was synthesized from 1 µg of total RNA using the Clontech Super SMART PCR cDNA synthesis kit following the manufacturer's instructions with the following modifications. Second-strand cDNA synthesis was done with a single round of primer extension using a 5′ trans-spliced leader primer conjugated to Clontech's primer IIA sequence to select for full-length dinoflagellate transcripts. All dinoflagellate transcripts contain an identical 5′ trans-spliced leader sequence on mature mRNAs [60]. The product of this single round of primer extension was purified using the Qiagen PCR purification kit to remove the spliced-leader primers and the cDNA was amplified by PCR using the Clontech primer IIA according to the Clontech cDNA synthesis protocol. A single microtitre plate of 454 Titanium sequencing was done at the Arizona Genome Institute (Tucson, AZ, USA) using 5 µg of amplified cDNA. These data were assembled using gsAssembler (Roche NimbleGen, Inc., Madison, WI, USA) into contigs representing *Alexandrium* cDNAs. The 12,329 unigenes generated using Sanger sequencing were co-assembled with the 454-derived contigs under Seqman (DNASTAR, Madison, WI, USA) using the default settings into a total of 35,431 dinoflagellate unigenes. The combined Sanger and 454 EST data were annotated using a best-hit approach against Pfam (version 23.0) [61] with blastx.

### Massively Parallel Signature Sequencing (MPSS)

The same sources of mRNA used to construct the cDNA libraries were also used to generate the MPSS libraries to ensure comparability between the EST sequences and MPSS data. Additionally, mRNA was extracted from cultures of CCMP1493 grown under replete (f/2) for the xenic condition. The cDNAs were captured according to Illumina's protocols as described in Erdner and Anderson [19] and Brenner et al. [20]. Briefly, the cDNA was digested with *Dpn*II and then amplified using PCR. Each cDNA was tagged by a 32-base synthetic oligonucleotide. The tagged cDNAs were then hybridized to their complementary 32-base tags that were covalently attached to microbeads. Each bead has only a single type of tag, but it is present in excess and generally, about 100,000 copies of a cDNA can be bound to a single bead. The result was a library of microbeads, in which each bead contained about 100,000 identical copies of a cDNA fragment that was derived from a particular mRNA. Libraries of approximately 2 million microbeads were loaded into flow cells for sequencing, which was performed simultaneously on each microbead in a cell. The result was a 21 bp signature sequence for every bead (hence every mRNA species) in the sample. The sequences from one or more flow cells (each containing a portion of the sample) were combined to form a set of 350,000 signatures. All of the signature sequences in a data set were then identified and compared to all other signature sequences, and identical sequences were grouped and counted.

### Expression Data Analysis

Using blastn, MPSS signatures were matched to the assembled unigenes. To increase the sensitivity of the blastn search, the option of filtering low complexity regions was disabled, the word size was set to two nucleotides, and the expected *e*-value was relaxed to 1E+3. The search results were validated such that no more than three mismatches between a signature and a matching EST were allowed and a perfect match within the four nucleotides of the *Dpn*II site (GATC) was necessary. For each signature, the matching ESTs were ordered by the identity scores rather than original *e*-value-based by blast. A matching EST with the maximum identity score and minimum *e*-value was designated as the most likely signature-matching EST. The annotations of the matching ESTs were directly transferred to the signatures. The unigene set generated by this study, including all previous EST data available from *Alexandrium* can be accessed from the public project web site: http://dbdata.rutgers.edu/alexbase. This web site also provides the MPSS expression data and matching ESTs. The combined Sanger/454 sequencing derived unigene set and the MPSS tag expression data over the different culture conditions are also available as supplementary files Figure S1 and S2, respectively.

### Differential Expression Analyses

Signature frequencies were transformed to transcript per million (TPM) normalized values, where a signature-normalized value equals the signature frequency divided by the sum of the frequencies of all signatures in a library. Signatures with ambiguous nucleotides (i.e., other than A, C, T, and G) or repeats of sizes (string of the same nucleotide) >7 nucleotides were excluded. Additionally, signatures with frequencies less than 4 TPM under all conditions were also discarded. Pairwise Fisher's exact tests [62] were performed to determine the statistical significance of the differential expression patterns between the different treatments. Considering two libraries 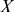 and 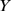 of 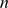 signatures with frequencies for signature 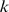 is 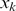 and 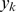 respectively, then the 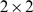 matrix (i.e., the contingency table) for the Fisher's test was prepared as following,
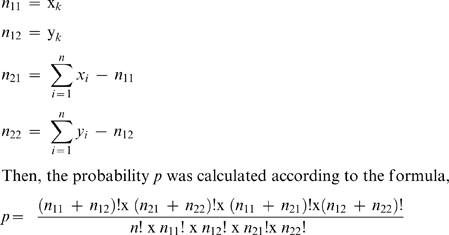



Finally, the probability was adjusted using the BH method [63] to control the false discovery rate. To determine the differentially expressed signatures, we used *p*-value < 1E-10 as a consistent threshold between all pairwise comparisons.

## Supporting Information

Figure S1 The set of unigenes derived from the dinoflagellate Alexandrium tamarense CCMP1598 using Sanger and 454 sequencing of cDNA.
(19.86 MB PDF)

Figure S2 The unique set of Alexandrium MPSS signatures derived from this work and their expression levels under the different culture conditions that were studied.
(71.88 MB PDF)
