# Supplementary material for: Transcriptome Profiling of a Toxic Dinoflagellate Reveals a Gene-Rich Protist and a Potential Impact on Gene Expression Due to Bacterial Presence
Source: PLoS One. 2010 Mar 12;5(3):e9688. doi: 10.1371/journal.pone.0009688 (PMC2837391; doi:10.1371/journal.pone.0009688)
Supplement: Figure S1 — The set of unigenes derived from the dinoflagellate Alexandrium tamarense CCMP1598 using Sanger and 454 sequencing of cDNA. (19.86 MB PDF) [file pone.0009688.s001.pdf]

>at00001
GTCTCTTCGACGCAAGCCGGCCGTGGTCGACCCGGTCTCCGTCGGCAGCACGAGGTCAGG
CAGCACCACGCCAGCGGCAGAAGAGGCCGCAGGACTGGCGGCAGCGCCAACGACCCCATC
GGCGACCAGGGAGAAGTCGGACTCAAAAATGTTCTCGATATCCGTCTTCACGTCGTCATC
GTCGGCGGCCATGTCATGCAGTATAACGCCAGAAGCCGGCTGCACCGTCTCAGTGGTGAG
GAACACGCCAGCGGACTCACACAAGACATCAGAACGAGGTCCCTCAACTGGCACATCAGT
GGAGCACACACCCATCCTGGAGGACAAGGTCACCCTCAAATCATTCTTCAGCAGCCGCAC
CGACGCCACAGTAATATGTCTCATTACATTGTAAGTGTCATCCAGTTGCTGCAACTTCCG
ACGCATGCCAGTGGAGATGAGCTGCTGCTTGCATGCCA
>at00002
TTTTTTTTTTTTGTTTTTCCACTCCGCTGACTCCTCCTCCGCCAGTCTTCAAGGGCCGAC
TCCAGTGCTCTTGTTCACTGCAAGTTGAAGCCTCATGAAGAGTACTTCCTCATGCTTTCT
TCTGCCAATCGAACGATGCAGCCTGAACGGAGGACTCGCCCGTGTACCCAAGTTTCTCCT
TCCAGCCAGGGTAGATCTGGTCCGCATCCTTCTGGAAGGTCAGGAAGGCACCCTTGAACT
CCTCATGGGTCTTGGCATACTCAATGACGCCGTCCGACAGGCTTATGTCGCCATATGTTC
CAGCTTTCCAGAGCTTCCACGCCTCTTCACCCTGACGGCACGAGGTTGCACCTTGCTTGG
GGCCATCCTTGTGACCAAAGGCACCGCCGCCAGCAGTCAGGATGACGTTGGAATGGCCCA
AGTTCTCAAAGAACGCCGGCAGTCTGAGAGCGTTCATGCCACCTGAAATGATGGGGGTGG
TCTGGACCATCCCCTCCCACTGCTGATGGTAGTAGGGTCCATCGGCCTCGTCCTCCTGCA
ACATGAAGGAAATGTTTTTGTCGGAGGCATCACCCTCCATTTTTCCGAAGCTCATTGTTC
CAACATGGATTCCGCTGGCAACAATGACGCGCGACAGCTTGGTGTGCACAAAAGCGGTGT
AGCCGCGCTGAGTCTGCGGGCTGGTCACAGCTCCGTGACCTGCGCGGTGGTAGTGCAAAA
ACTGCTTCGGAAAATTCTCC
>at00003
GCCTCAGTCCAGACCTGGATGTACGTCCTTCGCGGAGCTTGTGCCAGGCCAAGACCACTT
CGTCGGGGTGGTGGTGGCACAAGACCGCGGCTGGCCCGCAGTAGACCTCGCTGCGGGCGC
CGCTGAGGCGCCGCCAAGCGTGGAGACCTTCCTCCAGACCGCGGCCGCCGGCCCCGAAGC
TCGGGGTGCGGAGGGACCTGCCGACCCAACACTTTGCCGGAATGAGCCCGAAGAAGCGCA
GCTTGAGCTCGGCAGCACTGCTGAAGCCTGCAGTCCGCGGCTCCTCGGTCAACGCTCGCG
GAAGGTCTCCTCGACTCTGCCATCTTCCGCCGGGCCTGCGGTGGCAACCCGCGAGCCGAT
>at00004
CCATAGCTAGCATCAAGCGGCTAATGGGTCGGATACTGCCACAACTcCAGATTCCCATTG
GAGATGAAGTGTCTGAAGCCATGACTGGAGGCTGAATGGAAGCCTCCTGCGCGAGGCTCC
TGCTCACATCTGGTGCGACGAGCATGCTGAACGAAGTGGTGGCTTTGCCGAGCGAACTCA
GCGGAGTCTCTGGCCGTTGATCATTGAGGCTACGAGTTGACAGCTCTCTCTTTCTTGCGC
AGATGTTGCGTCGAGGCCAGGCCAACTGCTCCACCATCTGGACT
>at00005
CTTGCCAACATCAGCCAGTGCTTTGTGATCGGGCGGGCGGTGGTGTCAAGCTTGGTTGGG
GCTCGGGGGCTGGAGCTGTGCCCGACAGCGCTACTGTGTTTGATGCCCACAGTTGAGAAG
CTTATCGGCTTGGCGACAAGCATGACGAACGCGCAGTGCCTGCGCGAAGGCTCAGTATTT
ATGTGGACAGGGGATGCTAGCAAGCGAGTGTTGGAGTCCACCTGGGAGGTCCTGCGCGAC
ATCAAGCGTGTTCAGTGATTTCATGCTGAAGCTCAAGGAGGGACGCCGTTCTGCTGTGTG
CACATAGACATCCACAGAGTGCAC
>at00006
TTTTTTTTTTTTTTTTTTGGNTCGTCAGGCCTAGCACCAATTAAAAAGTTGTTGTCAACA
GGATCCCCCGATGGGACCAACACCATGCCCAAGTCCATGTGCAAGCCAGCGGAGACGGCC
TAGGCCCATGCCCCCGCCGTCAGAGGATGGGTAGGTTGCTGTGTAGACCTTCGACATGAT
GGTCCAGAAACCTGGTGCACTTCGTCCAAGTTGCACCTTCAAACCACAGTCTCTGCCAAT
GAACTCTGAGCAGGGGTTCGCAAGAGTGTTCATGCCAAGCATGCCGCCCTGCACAAGCAC
AGCCTCTGCTGAAACCAGCCCTTTTCATTGTGAAAGCGGCACCGCAGCGTGCCAGCTTCT
GTTGCTTGATGCTCCCCGCGAAGTACTCCCTCTGATGCTCTCAATGGCTTTCCAGGTGCA
CTCTTGCTCACAAGCTGCTCCTCCCCGCCACTTTTGGGCACAAAGAACAGCTCGTACGCC
ATGTCTTGCGAGCAGACGCGCACTTCCCAAGCGATCGCCGCTGCTGAGGACACCTGCAGA
AGCTCTACTGTAGCAGACGAGCGCGGCCCGACACTGGCAACCTGGCAGCGCCCTTGGAGC
TGCCGGCCGGGAGCTCCCGGCGGCGCCAGCATGCCGCCCATGTCCTCTGGCAAGACCTCC

GGAGGGACGTGGTCGAACAGCAGCGCATGATCTGCCTCTCCCAATATGATGAACTTCTTC
CTGGTCTTCTCANGGATGAAGATGGAA
>at00007
CAGCTGCTGACCTCGAGCGAGATGCGGAGCTTGGGCGCGGACGTCACCAGAGACAGAGCG
CTCTCCCACTGGACGCTCTTCTTGCACGCAAGCACAGCAGCGTTGCAGCTACTCCCGCGA
GGTGTAGCAGACGGCTCGCGGCTAGGCGGGGCAATCGACAGTGGCTTCCGCCCGGCGTAG
AGCTTGCTGGCCCCTGCTCGCCAGTCTTTCGAGGACTTTGAGGCAACCAGAGTACCCTGA
ATCGCCATAGCGCCGCCTGGACACTCCGTGGCTGTACTGGCCCTCTTCCATGCTGTCAAA
GCTGGAACCAACACCTGCTATCGCTTTGCACGTGTCGGAGCATGCCCCTGGGCAGGCAGC
AAGCCAGGCAAGCGGCAGGGCCACCATGGCTCC
>at00008
TTGTTTTTTTTCTTTTTTTTTTGTTTTGCCTCTGGTCTGCTGTGTAGTCCGGTCTTGGCT
GGAAAAAGCCGTGGCCCCACCAGCACGAACCTACAGAACCCCCTCCACCCACTTACCCGA
CTCCATGAGCGCCTGCTTCTTCGCGATGATGTCCCCAAAGTAGAGGCCAAGGCCTTCGAA
CGAGACCTCGCCTGGCAAGAGGACCCCGGTGGGCTTGTCCTCCTTGGATCGCCAGTAAGG
GACCAAGTCGTTCGGCACGCCCGGTCCCCTGCCGAAGTCCTTGAGGAAGGGCGATGTGCG
GTTCCCCATGGACTCCAGCGCAGCGATTGCCGCGTTGCGCACGGCTGGCATGGTGTCGTT
GAGCAAGTCCTCGATCTCCGGCTGGCACTCGGCACCCGCCTCGCCCATGTTTGCGAGGGA
CTCGCACACTGCCGCTCGCACGGCCGCATCCTCGTCCGTGACCATCGTCGACACGACGCT
GGCGTAGTTCTGGGCATTCTCTCCCAGCGCTGATAGCACCTTCACTGCCGCAGCCCTCAC
GTCGGCGACAGGATCGTTGGTGAGCTTGAAGACCTCATGGGCATGCTCCTCCGCAGCCTC
TCCCATCGCGCCGACCGCCTGCACTGCATACATGCGAACCGTGAATGCCGAGTCCTCGAA
TGCTTCGACAAGACGGGCCAGGGCCTCAGTGTCTTCGATCTGCCCAAGTGCGAAGCAGGC
CATTGCCCGCACAGGAAACGCATCGTCCTCCAGGCAGCCCATCAGTGCGCTGACTTCCCC
CTTTGCCTTCGACTTCAGAGTAGCCAACGCTTCAGCTGCGCAGAGCCGGACCTCCCAACT
GGCATCGTTCAGAGCCTCGGAAATCTTCGCAATGCGTGCCTCGTCGCCAATGTGAGCCAG
TGCAGTGATAGCTGCAGCCTTCGGCCGCCGCATCTGTGGCGGCGGGCGCCTGCTGCCGCC
GCCGATGACGAGTGCCAGGCCAGACTGATCTTCGCCGTCGTCGGTAAGGAGCGAGACCAG
GTCCT
>at00009
TGATGGCTTCTCCTACCTCCACAGCCAGTCACAAAGCTCACCACGACATCAAGCCCAACA
ACATTGTTTTCATGAAGAAATGGGATGGCAAAGATGCTGACCAAACACTCAAGATCATCG
ATTTTGGCCTGGCTAATCAATACAGCAGCGCCACCGCAACGAGCACCGAGTTTTGTGGCA
CGCTGCAGTATTTGGCTCCAGAAGTCCTGATGAACG
>at00010
TTTTTTTTTTTTTTTTTTTTTCTGCGAACAGCCAGACATCATCATGGTTGTGCTCCAAG
AGAACAGGGCTATCAATCACGCACAATCCATGGGACAAAGATGCGTAACAAATAAAGCAC
ACAACAAACCTCCCCGTTGAGGCATAACCGCCCTGTGCAAACGAGAAGCTCTGAAGCATG
TGAAACAGCAGCTCTACCGTGGGGAGACCTCACCTGGGGGAAAAGGCCGATTCGACAAGC
CAGAGCCATGGACAGGCATCCAGCGCCACGACGGCGCGCGCGTTGAGAGCGGGAGGAGAA
TTGTTGGGCAAGCCCTGATAAGTCCTTCAACCCTCGGACGCAGCCATACCAGGCAAGGGC
ATCTTCCACACTCTGAGCTTGAGCAAGATTTTCTTGATGTCCCTCTTCGAGGAAGACTGT
AACTCAAATTAGCTGTCTCGTACAGACCCCAAGATCAAGCCCCAGAACAACCACGTCCAA
GTCCTTTTCTTGCCTCTCCGCCACCTTGACTTTAAGCTGCAGATTGTCGTCCGACCCAGC
CTGCTTGGCCTTGTCCGACGCGATGCGCGAGACACCGTCTGACTTTATGCGCGAGACACC
ATCCGACTTCAAGCGCGCAACGGAGGCGTCCGACTTGGTTTTCAGGATTGCCGTCTCGCC
CGACATCCTACTTGCCAAGTTTCCGATCTGAGCCTCGATGACCGCGATTCGCT
>at00011
CACCAGCAGTCTCAGACGTGTCCAATTTGGTGGTCTCCTGCAGTGCGACCTTTCCCAGCT
GCAGTGCAGACTGAGGGGGAGGGCAGCTCGCCGACAATGGGCACTGCTTGCGGCCAGGCC
TGTATGGATGCAATGACAGGCCAGGCGGGGAGCCCAAACATACGCCGCGACCCGTTGGAT
CTGCCCTCTTGCGAGGAACCGAATCGCGCAGACCGTGTACAATGTAATGGGTGCGGCCAG
GCCCGTAA
>at00012
AACGCGCAGATCTGAGCCATCTCCGCCAGCGTCCTGGGCGCCGCAGCAGGCAGCACCTGC

AGCAGCTGCAGGGCCATGGCCGCGCCGAGCTTGTTGCCAGGTCGCATTCGAGTAACTTTT
GCCTCCACGTAGCGCATCCTCTTCACCCTCAGCCTTCCCTTCTCGAACTGCTCCCTTTCC
CACTCCTCCATCTCGACAGGGTCCATGGTGCGCCACTGTTTCTTGGGCTCTGGATCTGGG
GGCGGAGGCTTCTCGCGGAGGCGCCCATCCTCCCTGGCAACGTCATCCCTGATAATGATC
TTGATCTTGTGGTCAACAGGCAGGCCATCCACCAGGGATTGGGAAGCTGTCTGCGTG
>at00013
TTGTTTTTTTTTCTTTTTGATGGTATAATCTGCTCCGTCGCTCAGCGCACTGCCGCCCCT
GCAACCATGGGTCTGTGCCGGGCACCCAGGGCCTGATTGCTATTCTGATCTCACTGGCTC
AGCCACACGAGCCTGAATGGCATCAGGCCAGGGACGAGGAGTCCACGGAAGTGGGAAGGT
GGGCTGCCACCTCCTCTGTCAGTGCCTTCATAGAGTAGCCCCCATGCCAATCGTAGAAGC
AAGGGTAGTGCTTCTGCTTGATCTCGAAGCCCAGGGACTTGTACAGCTCGATGGCCGGCA
AGTTGCATGCGCGCACATCCAGGGACATGAACTTCCGGCCCGCCCACGCCAGCTGGACTG
CGGCCCCTGCAACCAGCGCCTTGGCCAAGCCGCGGCCTTGGCAGCGGGGGTCCACGGAGA
GGTCGTCGGCGTAATCGTCGTGGAGGGCCATGAAGCCGACGAGCTCATCCGAGCCGGCGC
AGCGGCACTCCAGCACCTTGTGACCATGCCTGGAGCCGCAGGCAGGGTGGTAGGGGTACG
CACGCAGCCGGAGCTGCATGTGCCTCTCTTGGAAGACCCCTGGAACCAAACGGAAACCGC
CAGGTGCCTCCGCCTGAAGCTGCTGAAATCGCAGGCGGCTCTCATTGGTCACCTGGGCCA
GTGCGTCTTCCAGCGACAGGCTGGTGGCGTAAACGGCCACGAATGTGAGGCGCTGCTCAA
GGCTGCGGAGGACGAGCTCCTCTTTGGCACCCGAGGCCAGGGCCAGGGCGCGGGCAGCTT
CGACAAGGGCCTGGGCATGGGCAGCTTTGAAGGAGTGGACCGTGGCAAGAGCCCTGGCCT
CCCTGAAGTCCTCGCACCAGTCTGCAAGCCTCCGCCTGGCCACTGGCTTGCCAAGAGAGG
CCTCTGCAAGCGCGGCTGCGAGCTCTGCAAAAGCTGCCCTCATCTCCGCAGGCACGCCTA
ACGCCTCCGCCTCCTCCGCAGCCGCGGCCGCGGCAGCTGCCTCCGCAGTCGCGCTTGCCC
CACTCATCGTGCCGCGCGTCCAGGTGAGGGAAAGGCGCCACCGCGGGCAAAGGTAGACTT
GAGCCAAAATGGCTACGG
>at00014
TTTTTTTTTTTTTTTTTGCAGCCATGAGTCTCCCAAGGTATGATGCAGTGCAGTGCAGA
GTGCAGCTGCAGGCACTGATCTCTCTAGCAACTTTGGCACAGAGTCGACCCCCTCTGCTT
TGCTGTGCCGCGGGCGCATCTTGAGCAGGCGAATCCAGCACTCAGACGATGACGGGCAGC
TGGCCAGAGCCTTTGCTGGCCAGGCGCCGGCTTGGATTGGGATTTCCCAATATCGTGGGA
GCCGTGACATTCACCTTCCCGCAGGATAACGGAAAGTCCGCGCCGTACCCGACGATGGAA
GTGAATACCGTCAAAACACTGGCGATCTCCATTGAGCAAGTGGCTTGTAGGTTGGTTGGG
GCGCAGGTGGTCGCGAGCATGCTTGCATACATAGCCGTGAAATACAAGGCCCCGAAAATA
TTTGTGATGCTGACCGTGCAGTAGATTTTGCTCGTCTCGTTGTTCATCTGTACTTTATGG
CAGTCCTGCATCGCATCATGGAACGAGGTCATTGCGCGGATGAAGTACATAGGCGTATGC
AAACTGGCGACAAAGCACATGGCTATCTCAGCAGAGTTGACCGCCGCCAATGTGTGGTTG
GCTTGCGCGTCATACAGCTTCCGGTGCAGGTCNCGAACAGTCTTGTTGTAATCTCTGACC
GTACCCTGGAGGAACTTGTACTGGTCCTCGTGCCGAATTCT
>at00015
TTTTTTTTTTTTTTTTTGACGACAAGACTAACCGAAGAGTAAAGAATGCCAAGAACGGA
TCATCAGACAAGTAATTAATGACGCTGGCATGGGCTTTCCATGCTTACATGAGTATACAC
AATCAAAAGAGTCCAAGGGAAAAGACGCTCCTTCGAAGCGTTTGGACGTGCGACCCTCTC
AAACCCCTGCCAGGGATCAGTGAGCCTGCTCAATCAGGGGGTGTCCACACCCTCAGCACG
GGGCTTTGCAAAGTGTTCCAAGATAGGCCACCATGGGTCATTGCGCTTGACGCATACAAC
GTTTGACAAACCAGCCCACTCCCACACATCCTCCCAACAGTACCTTACGCTGATCAAGAC
CCCAATCCCACCAGCCCAGTAACAGTCGAAACCGAGAAAATCGAGTTGGTCGATCACCGA
CTTCAGGCTTATCCTCGCCCACTCGGTGCCCTCGAGGTCCCGATGCACCTCAAACTCCAA
GTAGCGAACCCTCCGCAAGGTAGCGTTAGCTCCCCGGAGCACGGCGGGATCATGGCCTTC
AGCGTCAATGTGGAGGATGTCCACGCGAGGCAAGCGGAGCTCGGCCACAACGTGGTCCAC
AGTGGACACCTTCACGAAATCCTCGGGCTTCTGTGGGTCGGCGATGAAGCCCATACCGAA
GCCATGCGCTTCCGTACCGACCGCTGCGTTGGGAAATGCAGCACTTTCGCTGGGATGGTC
CGAAAGTGCAGCGCGGACAACATGGAACGCTCCGTGCGGCCCAGAAGTGCCATAGCCCAG
CTGCTGTGAAGTCCGCTGCAGCAGATCAACGTTGGCCTGCATAGGCTCGACGCATATGCC
GACGGGGGGGCTCTGACTGAGACCCTGTGGCTTCTCTTCGGTCGGTATGTCTCCGACCTT
ACCAAATTTATTGTTCAAGTTGGGAGGACAGTCAAACCATCCATCTTCTCCAACCTGTCC

AGCCACAGCTGCCACCCACGCAGAATAGTTCCAAAAGCTGCCAGAGGGATCCCACCTTTC
CATCCACGCAAGAGCATCCCCCCTTTGTTGCAGCCAACATCTATGATGATCTTGTCCGGC
GATGGATCTGCCCAAACCATGACGTCAACCCAAGGCGTACTGGGACAGCGATATCGTAAA
GGTTGAGTGGATTGAACGGCAGAACTTGCCAAGGACACTGCCTACCCACGGTTGTTCGCA
GCAAACCCTGCCAGTCCTGTGTGTGACATTTCGTCACCAAATGAATCAAGCTCGCAACGG
CAACAATCGCTGCAGCATGGCGCAATGCCGACATCTTAGCCTTCTTGATCCAACTGGCTT
GAGCCAAAATGGAACTAACGG
>at00016
TTTTTTTTTTTTTTTTTGCATGTGCAAACATGGAACCATCCAGGACGTCAAGCATCAAG
ATGACTTCATCAATCATTGCTGATGTGCAGTGGACCGGCGATTTGTACACCAAGAGCCCA
CAAAATTGGCGCCGTGCCACGTCAGTACAGCACTCAAAGGCCTGTCACTTGCGCCTGTGT
TGCTGCGCTACAGCACTCACCATAAAAGAGTAACTCTATACACAAATGAGTAGAGAGACG
GCATCCACAAGCCTATACATGTTTGTCGGCATCCGCTCGACTGTCTGGGAGGGTGTGGCA
ACACTTATAGCTCAGCATGCCACCCCTCCGCTTTGTTGATGTCGTCATTTTCCACAAGCT
CTCCAACAGTAAGCTCTGCCCTGGCCATGGACACATTCGAACCCTGGATGAAGGCTACAC
CTCGCGTAAGATGACCCGCATTCGCGCGGAGCCTGGGACGTCGGTGCAAGGAGAGTGCCG
GTGTTGCCTTGGCCATGTGCACCAAGAGCGGGCCACCTGGCTCCATCGACCGTGCGGGCG
AGGCCCTCGTGCCGAAT
>at00017
TTTTTTTTTTTTTTTTTAACTTACTGAATGTCATGAGACCATTCGTGAAAGGAAAGGCT
TTGCTACCCGCAGTCGCAGTCCCGCATCTCACAGCCAAGTTCAAGTCACCTCTGAGCATG
TGCACCCAAGCGTGTCTGCAGGGCGGGCGTCGGTACCCCACCTAGTGTGTGCTCGTAGTC
TTGAAATCCTATATGCCATGAGGCGCCACGGTCTGTCTGAGTGACAACGGGATTGTTTGC
CGGCATGCGCTCCCACACACCTGCATCTCGGACCTCCTTAAGACTGGCCTGGTCCAAATG
TCGTATCAGCTCGTCGGTCCGGACTGATCTCTCTGCCGGCTTCGGGTCTGGCGTGCCGCC
AAGGGTGTGCTCGTACTCGCCAAAGGGGACGTGCCATGCGGCACCCTTGTCCGACTGCGT
GACAATGGGATTGTTCCTGGGCATGTGCTCCCACAAGCCCGCGCCCCGGATCTGCTCGCG
CGACGCTGTGTCCATCTGGCGGAAGAGGTCTTCCGTGCGATGCGGTCTGTCCACTGGCTT
CAGGTCAGGCACTCCACCCAAGGTGTGCTCGTGGTCGGCAAAGGGAATGTGCCATGCGGC
GCCATTGTCTGTCTGCGTGACGACAGGGTTCTTTGCAGGCATGCGAGCCCACAGGCCCTC
TTTCTGAAGCTCCTTGACCGAGATGGCATCGAGGGTGGTCATCAGAGAAGGATCCTGGCG
GCACCGCTGATCGCTGGTGCTCGTGTGAGCAGGATAACCTCCTGGCCCATCGTGAAAGCT
GATGTGCCCACAAGGCAGCGAATGAGTCGCGCCGGTGCTGCCTAATGTCCGTTGGGACCC
ATACGCTGGTGCAGCTGCCTGCACCAGTGGCCGGGTCGCGCCACCATGCTTAGCCAAGCG
CTCTGCACGCACCTCCTCTGCGGTGCCTGCGGCGTGAGTGCTCCGATCAGCACGCACTCC
GGAGCTCTGTGGCCGCACAGCCTTCGGCTGTGGCTTCGACCCTGAGGCAGGACTCGCCAG
CTGCCGGATCCGGGCAGCACGCAGATCTGCTGAAGTCAAGGCGCTGGAACTTCTGGGGTC
CGACAAGTTGCACCCGAAGGAGGGACCGGCCGAAAGCTCAGAGGTGGCGCAGTCTGCGTC
CGACGGCACGCAAGTCGTGAGGCCGGCAGGCGAGGCGGCGTCATCGGCAGTAGCGGCGCC
CCTGCGGCTCGGGGCTGAAGCCGTCTGCGGCCTGGGTGCCACACCGGCAGCACTTGGGCC
GCTGCGTCGGGGTATGCTCTTCGAAGCTGGGCTTGTAACTGCACTCTTGGTTGCACTCTT
CGCGGCTCTCTTGGCCACGGGGGTGACTTCACTAGACGCTCCGCCCCTCCTCATCTGTTG
GAGGCGGGCAGCGCGCAGCTCGGCAGAGCTCAGCACTCCACAGCTGCCGAGGTCCAAAAG
GTTGCCCGTGTCCCAAGTGGCGGCCGACGTTGTCGCACTCTCTGTGTTGTCGGACGGCAC
GCGAGTGGAGCAGCCTGACAGCGAGGCGTTATCGTCAGCAGCGGAGCCAGTGTATGGGTG
GGCAGCCTTTGGTGCCTTCATCGGAGAATCCACGCCAGCAGTATGGCCAGGCCTGCTCAT
GGTGGAAAAGCGCAAAAGGGTTGCACGAGCAAGAAGGACAATGCTCCGCAGGGCTGGCAC
AGCTCCTGGACGCCTCCAGCAGCCAACAACTACAGTGGGACCAATAGGGACCGGTGGGGA
CACAGGGGCCTTGAGCCAAAATGGCTACGG
>at00018
CCGCCAGTAGAGTCTTGAAAGAGGAACGACTCGCCGTCATCAGTGAATATTGGGTAGAAG
TTCCAGACCAGATGGTCAGTTGCATAGTCCCAGAGGATCTCGCCAGTGCTTGCGTTCAGG
CCCGCCGCTTTGCAAGCTCCTCCCCCAACAGGTTCATGCGTCTTTACCACAACGACTCCT
TTGTTAGCAGCCACTGCGCCGATATCGCCCCCAAGTGTACTACAGTTGCAAGTGACGTGG
CACTTAGCCGCTTGCGTGGTCTTCCAAGAGCACATTGGTGCTGACTTCCACACCCTGATG

CCTGACTCCATGCTGACAGCC
>at00019
CAAGCGCCGCTCGGGGCCAGGCGCAGCTGTCGTTGGCGGGAGGTTGTACGTCTGTGGCGG
CGAGGACATGCCACAGCGAGAATGCCACAGCTCTGTGGAGTGGCTAGACCTGTCGGTTCC
TCCGGACAAGGCTGGCCTGCAAGGAGCCTGGCGCTTGTTGCCCATCACAATGTCAGCCAT
GCGGCGCCATTGCGTTGCTGCAGCAATCCCCGGCAAGACGGGAAGGAGGAGCTCTTCATC
TGCGGTGGCTTCCACACCCCCGACCGGCATCGACTATGGGGTGCTCGACAGCGCAGACGT
CATCGAAGTCCATGGGCAGCACGGCAGCAGGCAGCGGTCTGCACCGCCCATGCCGAGTCC
GCGGGCCGCACGCTGGAGCCAGCCTTGGTGGCAGGGTTTACGTGTGCGGAGGCTTTGGCG
GAAGCCACTTCATGGAGCGAACATGCTCGCCACGGTAGACTGCTACGATCCAATGCTCGG
AGTCTGGTCCTCTTGCGCGCCTATGCCACAACCACGTGCGCAGTTTGTCAGGCCGTAGTC
GTGGCGGGCTGCCTCTACATACTGGGCGGCAAATCCCCGCCCATTGACGCCAAGAGCGCC
AGCGACCCGGTCTGCTATTCGCGCAGCGTCTTCTGCTTGAACCCTGCCACCGAGGAGTGT
GGGGCATGTGCCTTGGGGAACTGGTGCACGGGCGAGAGACGTTCAGCGCCGTGNCCGTGT
GGAATGGAGCCGTGCGGACGGGAGANCTGGCGGAGTGAGANGCCTTTGANAGNGCTGGCA
AAGGCCATTGCGAAGACC
>at00020
TTGTTTTTTTTTCTTTTTTTTTGTCACTGGGCGATTAGAAGGGACATCGCTGCACATCTC
TAGCCAAGACAGAGAACATGCATGCGGAGGCGCATCAACCTCCTACCATCTCCAGACGCA
GACTCTGATCGGGTGCAGAGGTGCAGTGCGTCCGGCAGTGGCTTTGAAATGTTTGCAAAG
CCGCAGGCGACTTGCTCCGGGGAATGCACACGACACAAGATACCACTGAGGCATCACCCG
CAGAGGACCTCGCTCGCGCAACATTGGCGAGGAGGACTTTGGGCAAGCATGTCCGTGCGG
CACGTCAGGCGACGGTACTGCCGTTGCAGATGAGCCACGGCTTCTCGGGGCAGTCCAGGC
CGGCCTTGCAACACAGGATCTTGCAAGACGTGCTGCAGTCCCCTTGCGGCTTGTACATGT
CGCAGGAGGCGTCGGCAGCGGGGCATCCGTCTGAGCCGCCCAGACCGCAGTAGTCCTGGC
CCTTGATGCAGAAGCAGGTGTACGCGGCGCCGATCACCCAGTTGCCCAAGGGCTCCCCGA
TGCCGCAGCTGAGGCACACGACCTTTGTGTACACCGAGTCTGAAGAGGTCGGCTTGGAAG
GCACGCCGCTCCCAAGAGAGGAGAACCAAGCGCCATGCTCCGGGGACGCGGCCATCCTCT
TCTGCTGCGGCGCTGCCAGCTCCTCATCCAGCATCCCCAAGTTCTCCATAAGCTGCTCCT
GCGCCGGGGCCGCCAGCACGCCGTTGGTGCGAGTGAACTTGGCCGCCGCCATCTCGCAGG
GTGGTCTCTCCGGGGGAGGGGCCGGCAAAGGAGGTTCCGAAGTGACGCCGGGGGTCTGCT
TGAGCCAAAATGGCTACGG
>at00021
CCTGCCAGAAGGACTCGAGCCGTTGGGAAATAGTGGCCTGCCATTCCTCTTCCAACTCAG
GCCATTTCAACCTGGGAACGGCCAGCGCTGCCGCCCAAGCAGTGGCTCGGCCCGCAGCCT
GCCGCGCGTTCCGCGCCCACAGTGGACGGTGGAGGGGGATTGCCGGAGGTGGAGGCAGCT
TGAGCCCATTTGAAACCGTGGCACGGGAACAAAAAAAAAANAAAAN
>at00022
TTTTTTTTTTTTTTTTTTCACCGCGGCAACAAGGCAGGACCGCCCCACGGCGCCAAGGGC
CTCTCAGACCAGGGAGCGCCAGGTCAGGACCAGCGCCGTGATGTTGTCGTCGGAGTTGGC
CGCGTAGGTCTTGCGCAGAAGGTGCTCCGCAGCCGAAACCGGGTCCGCTTGCGCCGCGCA
CGTGGCCACCGCGTCCTCGTTGCTGACGACGTCCCAGACGCCGTCGCTGCCGAGGACCAG
GAAGCGGTCCGTCGTGGGCTCCAGGTCCACGACACGGACCTCAGGCACCGCCGTGATGAG
GCCACCTGGCGCCACGCCCGCGCACTCGTAGCGCTCCGCCTCCTTCAGCAGCAGGTCCCC
GAAGGCGCGCGAGACGGCGAGGCCCCAGCGCGCGATCACCTGGCCGCCGAACTTGGCAGG
GCCGGGGATGAAGGCGCGCCAGACTCCCTCAAAGTCCACGATGCCACCCCTTTGCTCGAT
TTCGCGCTGCTCGTCGGGCCGGCTGGGCTTGTGG
>at00023
AATGTGGAGGCTCGAGATTGTGCCATTGTTGGCGATGTTATGCAGAAGAAGCTTGACAGT
TCTAGCGACTTCATTGATGCTTTGGACCTCGAGAGTCTTGAAAGTTTGCAGGAGATCGTG
TTGGCTAATCCTAAGCACCTGCTGACCGACACTACGATTCGCAAGTACTCTGAGCTTCTT
CCCGAGTTTANGCTGGAGATTCGGGTTCGTCAGAAGCGGCTTTCTGACACACGTGCGGTC
TTGCTGAGTAAGCTGAAGATGGCCTGCGCTTGGAGAATGGTACGATGTTCAAGAACAAGG
TCACAGACGCATTGGAAGTTGAAGCGCAAGCAGGCGATGCAAGATGCATTAGCAGAACAA
GC

>at00024
AGGACGACCTCGAGAAGGTCGCGGGCTTCCTCCGCGCCCACGGCGGCAAGCTGCTGCAAG
GTGAGCATGCGGAGTCCTACCTCCTCCTTGACTGCCTTGAGAAGGAGATGAACGGTGAGC
ACAGCGCGATGATCCGGTCTTCTCGGCAGTACCAGCTGCTCTNCCAGCTTCGCGAGTTCT
CGCGCGCGGCTGGACGACCGGCCCGCGATGCAGTCAAcCCTGTCTTCCAGCGTCTTTTGG
ATCACGAGNCTACAAAGGAGTCCTTCGACGAGACAGTCAGCGACTTTGTCAAGCGAGTTG
AGAAGCGCGCTGTAGAGAAGAAGAAGGAGATGGACGCCGAGGAGGAGGAGGCAAGCGGAC
CGGGACCAGGGGGCTTGGACCCCATGGAGGTCTTCCGGTCGCTGCCGGGGGACATGCAGG
CAGCCTTCGAGGCCAAGGACCTGCAGAGGTTGCAGGCGGCCGTGGAGGCCTTGCCGGAGG
AGGAGGCCAAGTACCACCTGAAGCGCTGCGAGGAGAGCGGATTATGGGTGCCGAACC
>at00025
TTTTTTTTTTTTTTTTTTGACCTTCATCCAACTGAAGAAGGGCTTGTAAGCTGTACATGA
CCCCAGTCTCCATTTCCTACTACCGCACCAAAACTGTGAGCACGGGAGAGGAGGCGGCAA
GCAGCCACCTCAGTAAGTGCCAGAAGCGCTCCAGCCTGCAAACAGTTAGCTTCTCACTTC
TTGCCGCCTTTGGACCCCTTGTCCTGCTTGGGCTTCTCTGCCTTCTCGTCCTCCAGCTTG
TTCACAAAGGGCAGGAACTCTTGGCCAAGCAAGAACGGCACTAAGACACGGGGGACACGG
ACCCCATCTGCCTCTTGATAGTTCTCAACGATGCTGCAGAGGGCACGCTCCGTGGCGCAG
AGTGTGCTGTTCAGCATGTGGCAGAAGGGGTCCTCCGGCCTGAATCCGCATTTGGTTCGC
ATCGCTCGTGCCTGGTAGTCCAAGCAGTTGGAGCAGGAGACCAACTCTCGGTACTTGCCC
TTCCCCTCGTTGTCACCGGGGAACCAGCCCTCCAGATCGTACTTCTTAGCTGCGGCATTG
TTGAGCTCCCCTGAGACAATGGCAACAGTTCTGTATGGGATGCCAAGAGATTGGTAGAAC
TCCTCAGCCATGTTAATCATCTTCTCGTGCTCCTCCCAAGAATTCTCTGGCTCAGTCAGC
ACGAACTGCTCAATCTTCTCGAACTGGTGCACGCGGAAGATTCCACGGATGTCACGGCCT
GAGCTGCCCGCCTCACGCCTGAAGCAGGAGGACAGGCCAACATACCGGAGAGGATACTTT
GCCCTGTCAAGATTCTGTCCCTTGTGGAACGCCGAGATCGGCTGCTCAGATGCTGCGATC
AGGTACTTGTCAAGCTGGGGCTCGTCCTTGTCCTCAATGACCTTGTACAGCACATCATCG
TAATCTGCGAGCTCGGCTGTCTTGGCCATGAGGTCCTTCTTCATCATGAAGGGCGGCTGG
AGTGGCGTGTAGCCACGCATGCTAAGGAAAGCCAGGCCATAGTTGATGAGTGCCTGATTC
AGCAGCACACCTGGCCCAGTGAGGAAGTAGCCGCGGCTGCCAAAGACTTCGTGGCCGGCG
GAGAAGTTGACAGCGCCGATCATTTCAAAAAGCTCAAAGTGAGGCCGGAACCCATTTGTC
TGATACTTTATGCCATCAAACGAACGTGGCACTCCCCACATGGAGACCACCTTGTTGTCT
TTGTCCTCATCTTGTGACACAGGTACACTGTCATGCACAAGGTTTCCGATGCGGCCCAGC
TTCAGGTCGCGCTTCTCAAGCAGCTCGTCCGAGAGCTTGTCAAGCTCGACACTTTCCCCC
TCCAGCCTGGTCTTCTCTGCAACCAAGTCCTCGCACTTGTCCTGGCCTTTGGAGGTCTTC
TTGCGCTCGGTGATGGCCGCTTGCACCTTTCCTATCTCTTTCCGCTTCTGGTCGGTGTTG
TACTTGGCGACGACCCACTCCTTGTCCAGTTCGATCACCTCGTCAACAAGTTTTCCATCA
CGAAAGCGTCGGCGCTCTGATTCACGCACCATGTCAGGGTCACCGCCCTTGTCCTTGCGA
AGCAACTCAATGCCAATAGTCATGGCTCTAAAACTTGACCGGGCAGGCGTGCAAGCCTGC
CAAGCTCGAAGTGCAGGCCGCAGAATTTGGAATGGTGGGGTTGGCTGAAATCCCAAAAGC
GGGCCTTGAACTGCAGAGACCTTGAGCCAAAATGGCTACGG
>at00026
ATTGGGCTGCGATATCCCTGCCCAGATAGTCCTGCGCCTTCCGTTGCCGCCGCAGATCGC
CGAGGCTTTGTTTGTAGTGGTGCACACTGTCAGCGTATGGCTGGGGGATTTCAATTGGCT
TCTCTGGGGTGGTCCTTACACGAGGACTCACGACGTGTACGCCTTCGAGGCCGATACCGG
CACACTTCTGTGGACTTGGGAGGGCCCCACGAACCGCCGCATGTGCAATCGGGGCGACGA
GGACGGAGCTGTTGCGAGGGCCTATAGAGGATATCGACGGGTATGCTGTTCAACGCCCTG
GGGTCAGCCGCGAATCGGCGCAGACGGATCCATCTACATCCCCAACGAGAACGGCGACTT
CTTCGCGATTAGGGATGCGGACCGGAAACGGCCACATCGACAATGACAAGGAGGTGTCCG
TCTATCACACCGGGGCAACATTCCCACACTGCGGAACAGCTCATGCTCCTGGCATGATTG
TCGCTGTGAACTTCGACGGGTCTTGTTGGAAGTCCG
>at00027
CGCTGCTTCAGGTCGCCTTGCTTGCCATGCGTGTCCGTGAAAGTGAACCGCCAGTTTGGA
TTCACCTTGTCATTCCACAAGCCAATGTTTGGGTCGAAGGCAAAGAAGAAACCACCACAA
CGGAGCCCAGGAAGGGGCGGGGGGAGGTAAGGACGCCGCAGGCGAGCCGGGCGTATGCCC
TGCAGAATCAACAGGTGGTGCATCACTGGCTGCAGCCTTGTCAGACGACTGAACTGTTGT

AGGCGTCAAGGGTGTCTCTGCAGATTCCGCCGAGGGTGGCGGCGGTGCAGTGGTGGGAGT
TGCAGAGGCGGCAAAGCTGCGTGCCTGGCCCATGACACATTGCCTTTGCCAAACCAACGC
TGCACCGCGGAACCAT
>at00028
AAGAGGGATCGGATGTGGCCATTGCCAACTCGATCAGCCCCAAGGTCTATGATGAGCTGG
TGAAGGCGGGAATGGTGCACTGCGGAACACTGACGTCGCCTCTGATCTTTGCAGTAATGA
GtTCCTTGATCGTCAACCTCTACAACAAGAGCCAGTACTACGCGTACGAGTGCGTCAGCA
TTAACGAGAATCTTGTNGCGATCGTGTACGCAATGATCTATTGGATTGCGGCGCTCGGCC
TTTTTGTGATGGACtATTGTCTTCTTCCGCGGTGCGCAGGTGTTGGATTTTTTTCTCGAG
GTGCATGTGTTCGTACCTTCGCGAGGACGCATGGCTTCTAGTGTTCACGTTGGCCTTCGT
CGACTTGGTCTACACTTCCTGCTACTTCATCAAGCACGATGGTATGGCAGTCTTGACACA
GATTGGCAATTGCTCATGAGATTGTGACTCCAGTCTTTGCACATAACTCGTGGACCTTTG
TCACAGCCGTCCCCAAGGG
>at00029
AGTTCTCTTGGAGACCTTCAGTTTTCAACGGTCCGTGAATGATCAAGCGCGTTGCTGCCA
ACACCACGCGCTTTTGGAAAACTGTTCCGAGCCGCCCCCCTGCGCCCATCATGTCCGCCA
GCCGAGGAAGCGCCAGCCCGCATCTGGCCGAAGCTCAACGGGCCTCTGGCCCCGGACCTT
GGGGTCCCCCTTCTTGCCCAGGAAGCCCGCTCCCTGCTTCAGGGCCTCCAGGATGAAGTC
GTCCCCCCCGTGCCAGGGAGCCTCGACGCGGTTGCGGTGTTGTGACCGCTGACCCTCGTC
ACTGTCCCGTGCTTCGCAAAGTGCATGTAAGCCCCTTGCGCGGACGCTGTCGTACTCACG
CTTCTGGTCCGATGTTGGCCTCGGGAATTCCTCCTCCAT
>at00030
TTGATGGTCACCTTGGGCAGCACGCCGAAGGTTCAGGTTGGCCCAACTTGTCCTTGAACA
GCAGGCTCCAGATGGGCGGAGTGCCCTTGAAGAAGCGGCGCGCCTGCGCCGCTCGGTTCA
GGTTCATAAGGCAGAGGGCAGACTCCTTGAGCTTGCTCCTTCTCGAAAGGTGCGGCTGCA
CGATGGACAGCCACGAAGAAGGTGTTCAC
>at00031
TTTTTTTTTTTTTTTTTACTAAACCTCCAGGCTCCGCCTCTACCGCTACGCGTCAAGGC
GTCCATGTGTACATTGAGCCCCTCCAAAAAAACCAGTCATTCCTGGAGAGCGGAGCCCTC
TCAGAGGCTCTTCCACAATGCCTTCACCCGGTCAGCGTACCCCTTGGGCAGCAGGTCCTC
GTTGTCCAGGTCGTCCGGCTGGGGAATGGGGAAGAGGTTCTCCTGGTCGCTCCAAGCGTT
CAGCCGGGCCTTCACGAACATGGTGGACAGCGGGTCCGCCTCATAGGTCTTCATGTGGTA
CTTGGTCCACTTCAGCAGGCCCGGCTGCGCATTCTGCCCCACAGTGAAAGGACGGCCGTT
CACCTGCACGCGACCCTTGTTGACCTTCTCAGGGAAGACGCCGTCTGCGGGGTGCAGGAA
GATGACAGTGCCGTCCTTCTTGAGGCGGTAGATCTTGTAGTTCTCGATCTTCATCTTCCG
CAGCTGCTTGCCCAGAGAGATGCATGCCTCCTTCCTCCTGAAGTAGCAAAGCCTCGTGCC
>at00032
GCGACCAAACTGACCACTCACAGCGTCCAGCTCTCCGGAAAATGGGTCATGTTGAATGGA
GTCCCTGGGAATGCATGTGCTGTCCTTAAGATAGCTCAGGACCTTCCCAGTCGCCCCCACC
ATGCCTCGTAGAAGGCTGCATCACTAGGAGAGGTCGCCTGCTCGAGCGGCCACCCCGGGT
CTACTCTGAGCCAGTATCGTCGTCGTACACGCGCTGATACAACAGCTTATACTCAGGATA
TATCTGCTTTTGCTGTATACCACAA
>at00033
CCGTAGCCATTTTGGCTCAAGAAGGTAGAATGCGCGTTCCAGCGCAAGATGTCTGCAGTC
CCACAGACCATGCCCACCTCGCTTGCCGCAAGCCATGGTATTCCAACTAGAAGATGCTGA
GCAGGATGCAGATGTCAGAGGACGGATCTCAATTGCTACCTACATTATCCTTTTCGTCCT
CAAGGTGTCGTTGGCCGCCTCCGTTGACACATCGGAGTTCCAGAAGCGGATTGCCCAGAG
ATGGGCCATACTCTTCGGGGTAATGTGCCAGTTTGTGGTATCTCCCTTCTGTGGTGCTCT
GAGCGTGTGGCTCCTTGGCATTCCTTTCATACCTGGCGTGTTGCTTTTGGTAATTACTAG
CTGCCCTGGTGGGTGCTTCTCCAACTGGCTGTGCCAGCTTGTCAATGGGGACTTGGCGAT
GAGCGTTGCGATGACTGGGTGCAGCACCCTTGTGGGCGTGGCCGTGCTCCCAACCAACGT
CTATGCCTACACTTGGCTCGTCTACTCCAAGCAGGTGCTTTCTTCGCATCAGTGGTGTGG
AATTGTCTCCTCGGTGGCAACAGTTCTCGTAGCCCTATGTATTGGTCTTGCGATAAGCAA
CTACCTTGGCAGTGAGAGATGGCGCGTGCGCTTCGGCCACTTTGGTACGGTCTGTGGGCT
GTTTGTCTTCGCGTTCTCTGCGTTCGAGTCCACTAACCCGGAGACAACGACGATGCCCAT

CTGGAAGAGGCCCTGGACTTTCTACGCGGCAGTGGCCCTCCCGGTGATCCTGACGAGCCT
CCTCATGATGGTGGTGACCTCTGTTCCCCACTGCAGGCTACAACCTCCAGAACGTGTTGC
TGTGGTCATCGAGGCCGTGTACCAAAACACAGCCCTAGCTTCGGCCACAGCAGTGCAGCT
CTTCACTGGCCGGCACTTGGGGGATGCCATGGGCCTCGTGGTGCTCTACCAGCTGTGCCA
GGGGGCGGTGCTCCTGCTCTTTGGCATCTGTGCTCACTACGCTCACTGGACCCTTGCCTC
GCCCGAGGAGACCTCGCT
>at00034
CCTGTACAGCTTGCGTACAACCTTGGGATAGTGTTCAATCCCGGACTCGTCAACATCCTC
CTTGACCTTCATCTTGCCTTGCACATAGAGATCCAGCAACTCCGCCATGCACTGCGCAAA
CTCAGAAACATGGTCGAAACATATGAAGCCCTGCACCTTGATGCGCCGCATGAGGATCAT
TTGGTATTGCTGCACGCTCACGGGATCGCTGTCGCTGGCGTTGTACCTGGAGATGTGGCC
GCAATACGCAATGCGACCGAATCTGTTCATCTTCGCAAGGACAGTATCGAGAGTCTTGCC
ACCAACATTGTCGAAATAGGCATCAACCCCCAATGGGCAGCAGCGTGCGAGCCCCTGTGC
CAGACTTTCGGTCTTGTAGTTGATAGCAGCATCCAAGTGCAAATCTTCCaAAAAGCCGCT
TGATCTTCCTGTCGCTGCCCGCGAGGCCCACGACCGTCATGCCTCGGGCCTTCGCGAGCT
GGGCAGCCACGGAGCCCACCGCCCCGGCGGCGCTGGACACCACCATCGTCTGGCCGGGCC
TCGGCTCGCAGATGTTGACGCCGACCCAGGCGGCGAGGCCAACTGCGGCGCTGAACAGCG
AGAGGTTGAGGCTCAGCGGGATGCCGGGGATGATGGGGTCCAGGGCCGCTTCAGGCACGC
AGCAATACTCTTGAACACCGCTGGGAGTAAGCACCCAAGTGCCCGGCTGGATTCTCCCAG
TGGTCGACTTCAAAACCTTGCCGATGGCGCTGGCCCGCATGCAAGTGCCAAGGCCCACGC
AAGCCATGTATTGCGGCCGATCCGAGGCCCAGATACGATGTGACGGATCAAGCGAGATAA
GGATGGTCTTCACGAGTGCCTCACCCTGGTGCGGCACTGGGATCACATCGGTGACCACCT
CGAAGTTGGAGTCTTGCAAGTCGCCTTGTGGGCGGCTCACCAATATCACGCGCTTGCCGC
CACCGCGGATTCCAACATTGTCAGCAATTGCCTTGTCACCCGCGTCCTGCTCGCCGCT
>at00035
CTAGCAAGGGGATCTAGTGATGCGTGCGCGGCCACTGGGTTGCCCTGCAGCGGAAGGGCC
GGGAATGGATGTATGCAGCTGGCCCTGCCGTCCAGTTCGGTGCGCTGGACGCCCGCGTGG
CGCAGCTGGAAGTCGCTGTCCAGCGCGTGGGCGACGAGGTTGAGGCGCGAGCTCGCCGCG
CAATGGAGCGCGGCCTGCACCGGTCGGAACATTGCCTGGCTTGCCCGAGACAGATGAGCG
CGCTTGCAGGGACAGCTTTGGCTAAGGAAAAAAAAAAAAAAAAAANAAAAAAAAAAAAAN
AAAAAAAAAAAAAN
>at00036
GCCCGATAGTGCACTGCAGAGCAGATGGCCACCGGTGGGCTGATATATCTGCAGGCTGGA
ACCATACCGCACAAGCACCGAAGATGCTTTTGCAAACCACACCCTTTGATCCGTGAACTT
CTCCCAGCATTGTGTGGCGAAACGCTGTTCGTGCGGCGGAGGGCTGACCAGTGTCATCGT
GCAACACTTCTTGTAGTCAAAGTCGTCGTCCCAGCAAACTTGGTTTCCATTTGGAGGTGT
GCAACAAAACTTGTAATCGAAACCGCCTTGCCAGCAAACTGGGTTCCCATCACTGGCTTG
TAGCCCAGACGCCTCAGGAAAGAGCTTCTGACAGCCTGGTATGTGCTGGCAAGCAAAGTC
CGACTCTGTGATGTCCTCGTCACTTTGGCCGCCTGTCAAGTGAACAGCAAGGAAGGGCAA
GAAAACTTGGCAACCAGCCAGCATCTCTTGTACAGCGCGCTCAGAAGTCTACGATGTCAG
TCTCT
>at00037
CGCCTTTTTCACTGCGTTTCACAAAAACTCCTTCGTGCACTTTCTCTCTAGCCGCAGTGC
CTCCGACTGCTGGCCTTTGGCCATGCTTCTAGCAGCGGACATGGCCTCAGTCCTCGGGTC
CGAGGCCGGCCTTGGAGGAGCCGCTGCCAGACTCTCCGATGCGGATGACCTGGCGGAAGG
CCACGCGCCAGACGTTGAAGAGAGCCCAGGAGATGGCGCCCAGGGGGAGTGCCAGCACGG
CCAGGATGCGAAGGTCAAAGCCCTCCTCCTCGTCCTCCAGCGCCAGCACGGCCACCGGCT
GCGCGGCGACGGCGGCCGCCGCCAAGGCGGCTGCCCCAGTGGTGCCGAGCGCCGGCTGCT
GCAAGCGTGGGGTGGCTGCCGGCGAGGGCACGAAGGCTGGCACCAAGCGGGAGGCCAGGC
AAGCAGCGGCAAGCGCCAGGAGGAGCGCTGCCAGGGGTGAGCGAGCGCGTGCCATTGTCT
GCAAGGCTGGGCAGCGGTGGGGGGCTGGGGTCAAAAATCACCTCTGTGCCGACACTCTCC
TCGTGCC
>at00038
TTGCCGCAGGTCGCACTGACACGCCTTGTCACTGGTGTTCAGCTTCTTGCCTGGTGCTTG
CTATATAACAGTGGCGACAAAAGTTCTTGCTGCCCTGCAGACGCATACACTCAGCAGAGG

TGTGTCTCTGGCTGCTTCTCTACCCTGGCTGCTGTATGTCACGTCCGCCATCTTTGTGGG
CGTGTACCGCGGAGACGAGCTGTCACTCAGAGGCGACATACTGTATGCGCCCCTCTTTCA
GTGCGCGGTCCGCATGCTGAAGGGTTTGCTGACGGTGCTTCTAATATATCTGAAGAACAC
ACCTTTGAGGTGTAACACGGGGGGTGCTGCTCATCCAGCTGTCCCTGCTTCTGTGCCTCC
GCAGAATGCACGCATGCTCGGTGCAGGCTGTCACAGAGACTCGCATATGCCTGATCGCTG
CGACCAGCTGGGTCGGCATTTGCAGCCTTGCCATGGAGATCTTTGAAGGAA
>at00039
AGCAGTCCACTAGCGGCAACGAGTACAGGCTTAGGCCATGTATAGCCGCTGCTGCCGCCG
CAGCTGAGGCGGCACAGCGCGGGTGCTCATAGGGGCGCCGGGCTCCTTTCCTGGTTGCCC
TAGAGCCTGCGGGGGCTGTTCCCTCCTGAGGAGGGCCTCCTGCTGGTTTGAAGCCATCTT
GGCTGGGTTTGGCTGTATGGCCAAAGCAGGGTGCAACGCTCGGTCCTGGTGTCGGCTTCG
ACAGGAAGCACACTTGTTAGACACCTTTTTTCGTGACCTTTGCACTCCAGCGGACTTGAA
CTTGAACTTGAACTTCCCATTCCA
>at00040
AGAAGTGTTCGGCATACTCAGCCCAGAGGCAAACGTTGCCTCTGTGGTCGCTCTGAAGGC
TGCAGAGCTGCTGGTAGCTCCTTGNNNNNNGNGAAAGACTTGCGCAGGTCAGGCTTGCCA
CAGTCGTGACAAGGGTTCGTGGACACGGCCGACGAGTCTTTGGGAATTGCGTGCGAACGC
TTTGNACGTGaAGGGaCaACCTGCTGACCGTCACTACAAGCTGTCGCCAAACATTTCTGA
GTCGCGCAGGCTGCGTCAGCAAGCTCGCGAGTGCACAGTTCTCAAAGCCTTTAGATGGAG
GTCTTGGGTGTAAGGGTAGATAATGTTTGTTACTCAAAGCCATTGCTTGAGGTTGTCATC
TTGTTACATCCGCAGTTCTCGGGACAAGGCGGCATTTGCATCACACACAAGGAGGAGGCT
CACAGGGCCGTCCGGGGCCTCTCCACCGATACCCTGGGATCTTTGAGGAGACCCCTGTGG
TGAGTGCGCAG
>at00041
TCAAGTGGCGGGTGGCACACCTAATCAGAGGACGCCCAGCCCTAGGACGCGCCCTCCAGG
TAGGGTGGCTTGCCATGGCACTAGAGCTTAGAGAAGGGGAAGGCATGACCAGGTGCCAGG
TGCCGACCCATGCAGAGCATGCCCGGATGGCGGCCATGGCCAATTCTGAGGTCTTGGGGG
CCATGCTGGAGGGGCTGGGGTCGAGGCTGAGCGGAGGAAGCGCTGGACAGCAAGCGGGGC
CTCGGAGCGGAGGAGGGGGCGCTGGAGGCGGAGGCATCGTGGGCCGCCGGGCGGCTGACG
GCCGGGGCTGGGGCGTAGGAAGTCCGGTGAGGGGAGTGGCGCGTGCGGCGGAGCTTGGGA
AGTTGCAAGCTGCAAGTCTGCATAAGGAAAAAAAAAAAAAAAAAAAANAAAAAAAAAAAAA
ANAAAAAAAAAAAAAANAAAAAAAAAAANAAAAAAAAAANAAAAAAAAANAAAAAAANAAAA
ANAAAANAAANAANAA
>at00042
TCCAGGAGTCAGAATGTTCCCAACGGTCTTGCGGTGGTTGCGCTGCCGCTCCATAGCAGC
ATCCCAAGCTGACTCGTTTACCAGTCGACTGTTCGGCTTTGTGATTGCACTCCCATCAGG
CGCGGCGACTCGGCATGTATGCTCCATCAACCAGGGTGCCTGGTCTTCAATGACAGTGGC
CTGTGCAGAGAGTCGATCTTTGCGTCGATCCAGGTTGAGCACATAGACTGGCAGTTCCTT
CAGCTCTGGGCATGATGACAATGATTCGGCGGTGCCGTTGTGAAGCCTCAGAGCTACCAC
TTTCCACAGCGGGGACGACAGGCAGTACAGTATCAGGAAGCGACTGCACAGCAACATCTG
CGCATTGCTGAGCCTCTGAAATCTTGAGCCAAAATGGCTACGG
>at00043
GNNNNNNNNNNNNNNNNNNNNNNNNNNNNCCACACCAATCGCCATGGCCTTCTCCTCCCCC
CCCCCCGTCACAGCTCGTCGTTTCTCGGCTGCGCGCTGCGTTCGGGGTCGTGGAGCTTCC
TGCCACTGCGCCGTCTCGTCTCGGGCTCGTCCCCCACGTACTCCACGGCCTTGACGTACT
TGTGCGTCCGGCACTTCTCAGGGTCCTCCTCCTTGCACTTCCTGAGCGCGCCATCCAGGG
AGGAGAAGGCGGCCTCGCATCTATCCGCATCGTGGAACCGCAAGATGACATGCCAATCAA
AGCCATCAATGACTTCATCAGGCCTGTACTCCGCCTTGAGGGCCTCAATGGCCTCCCTGT
TCCACTCAGGATCTGAGCCCTCCCAATGGAGTCTCGGCAAGATAGTGCAAGGGATCTCTG
GCACTACCCAGCCCTCCTCCGGTGGTGGCGCGTCTGGCCCGTCGCAGGGCTCGCTCGTGG
CGGCCATGCGGACGTGCGCAAAGTTGACCACCCTGCTGGCCGCGTCCCCGTCGTCCCAGG
TCAGGAAGAAGTTGCCGTCGGTGTCGGGGCCGGAGTACACCCTGGCGGGGTAGTAGCACT
CCTCCATGTCCACCCGGCACTTCGTGATGCCCATGCCACCTCCCAGGTCCACCT
>at00044
TTGGCCGCCGCGGTATCGATTTCCAAGGGCAGTGCGTGCTCAGGGTTGCGGAGCCCATTC

AGCTCCCCGAGGCATGGACGATCTCGGTTTGGACATTGGCGCCCATCGACACTGGCAAGC
CCTACAGGAATTTACTTGATGACACCGGTGACAACCGCATTGTTGTGGCGATTACGGGAA
GGAAGATCGGTGACTACAACAAGGGTACCTTTGTCAACTACGACCCTGGCACGCTCCCCG
CCGGCTGGCATCACATTGCGGCCGTTGGCGCTGAAGGTTCTGTCAGCTACTACGTTGACG
GGGCGCTTGTGGGTCGCCACGCAGGCCAGACACAGGGGCCGCATTAGTACTGTGGGGAAT
CGTGGTGATGGGCAGTGCCCCGAATCCTGGGGGGTGATGTCAGACCTCCGCATATTTGGG
TCTGCAGCGAGTGCAGAGCAGGTCGAAGCATTGGTCGGAGCTCGTGATTACAAATCCTTG
ATTGGCATGTGCTCCC
>at00045
TTTTTTTTTTTTTTTTTTGGAATGCCATCGTCCTGGATTCTCGGGGCCAAAACGCATTTG
GCGGCGTTCCACTCAACGTCCTCGAAAGGAGGAAGTCATGGGGAAAACTCGATCATGCAG
GCACGGGTCGGCGAGCCAGCCCGCAACCTACCCATCGGACCCAGTCGGCGTCATGTAGAA
TATCCTCGCGTCGCTCGACCGGGAGGCGCCGCGGCGCAGCAGCAGGTTCAAGACGTCCAG
ATCGCCAGCGCGCTGCGCGTAGAAGAGCGGCGGATAGCCGTCTGCCGAGTTCTGGTTGAC
CTCGGCCTTGCGGTCGAGCAGCAGACTGCACATGTCGGCGCTGTCCCCGACCGCAGCAAC
GTGCAGCGCTGTCCATCCGTTGCCGCCTCCGGCGTTCGGGTCCGCTCGGGCGGCCAGCAA
GGCTCGCGCGGCCTTGAGGGAGCCCACTTGCACCGCCTTCATCAGCAAAGTCCTGCTCGC
ACAGTCCCTCTCCTGCAGCACCTCTGGCCGAGCCAGAA
>at00046
GCCATTTGTGCTACAAGGACGTGCAGTCCACGACTTTTTGATTAGCAGAGCTTGATCTTC
GCTCATGGCTGCNTCCTCGTCTGAGGACACCATCGATACCACGCTCCAGTACAAGGATGG
CATTCCACCGGACCAgGGTcGTTCCAGATATTCGTCCAGACCTTAACGTTTGGCTTGATC
ACCGTAGACGTGAAGGCATCCAACACCATCGACTGCGTCAGGAAGAAAATCCACGACAAA
TTAGACACGATGTTCACCGGCTATGATGTGGAACTCCCTATACAGTATGAGCAGCACATG
GTCTACGCAGGCAAGCAGCTGGAGGATTGCCACACAGTCGGCTACTACAGCATCCAGAAG
GAGTCAACAGTGCACATGACCTCAGGCTTGGCTGGAGAAGCAGGCAAGAGAGGTCGTTCA
AGCAAGGATGATGAGGAT
>at00047
TTGTTTTTTTTCTTTTTTTTTTCTCTGTCGGTGCTGCGCAAGGGCGTCTCCGGCATTGCA
GTCTTGTCAAAAGAGGCGCCACGCGAAACAAGAAAGCGTCGCGCATTTCAAGGCCAACAC
GGCCAAACATTATTGCCACGAGTCGACACTGTTGACGACTCTAGTTAAACTTATATTTCG
AGCACCGCGGCGGCGGCCTGTACACACGCGTCCGCGCTGCTCTGTTCACGCCAGGAGCAG
TGGCCCGAGGCACATGATCCCCTCACTTCCATGGGGTGCGGAACATCTTGCCGCAGTAGG
CCAGGCCAGACTCCTCCTCAATGCGCAGCAACTGGTTGTACTTGGCCAGGCGCTCGCTCC
TGCAGGGGGCCCCGGTCTTCAGATGGCCGGTGCCCAGACCGACCGTCAAGTCCGCGATGA
AGGTGTCCTCGGTCTCGCCAGAGCGGTGCGAGACGAAAACGCCCCAGCCGTTCGTCACGC
AGAGCTTCCACGCTGCGATGGACTCAGTGACGGAGCCGATCTGATTGACCTTCAAGAGCA
TCGCGTTGGTGGCCTTCTTGTCCAGGCCCATCTGCACCCGCTTCACGTTGGTGCAGTAGA
GGTCGTCACCCACAATCTCCACGCGCTCGCCCACCGCCTCCGTGAGCTGGGCATGGCCCT
CAAAGTCTTCCTCTGCGAAGGGGTCCTCGAGGAGGGCGATGGGGTACTTCTCGCAGAAGC
CCTTGTAGGTCTCAACCATCTGCTCGCGGGTACGCTTGTTCTCAGGCTTTGGGCTCGAGT
TCTTGAAATCCAGGTCGTAGACGCTCTCGTCGCCCTTCCAGAACTCCGAAGATGCCGGGT
CGGAGCCGATGAGGATCTTGCCTGTATGCCCCGCGGCCTCGATCGCCTCCATCAGTAGCT
TCAGGCCCTCCTCAGGGTCCGACACCGCCGGGGCAAAGCCGCCCTCGTCGCCTACAGCCG
TGGAGTCGAGGCCGTACTTCTTTTTGATGATGCCCTTCAGTGTGTGGTACGTCTCGGAGC
CGAGTTGCATGGCATCCGCAAAGCTGGACGCCCCCACGGGGATGAGGAAAAACTCCTGGA
AGGCCAGGAAGTTGCCAGCATGCACGCCACCGTTGATGACATTGAAGCAAGGCACTGGCA
TGATCATCTTGGGCCCGCCCGCCAGCTGGTTGATGTACTCGTAGAGGGGCAGGGACTTAT
GCGCTGCCCCCGCGCGGCAGCAGGCCATCGAGACCCCCAGGATGGCGTTTGCGCCGAGCT
TAGACTTGTTGTCCGTGCCGTCTAGCGCAAGCATGGCGTTGTCAACGCCCTCCTGGTCAG
TGGGGTCCTTGCCAGCAAGTGCTGGCCCAATGACGTCCTTAACGTTCGCAACCGCCTTCG
TCACGCCCTTGCCCAGGAAGGCCTTGCCACCATCCCGCAGCTCGCAAGCCTCATTCTCCC
CCGTGCTGGCACCACTGGGCACCTGCGCCCTGAAGAGACCCTTCTCCGTCGTCACCTCCA
CCTCCACTGTGGGGTTGCCACGGCTGTCAAAAACCTGACGCGAATGGATCTTCTCGATCG
GCATTGTGCTTCAAAGTCTTGGGCTTGCTGTTGCACCGCTCTCAACGCTCTCTTATGGAG

CCAAATTAGTTTGAGCTAAACTGTCTTGAGCCCCTACTGGCTTGAGCCAAAATGGCTACG
G
>at00048
GACGTCATGTCTTGCTTCCAGTCAATCCGTTGTCAGTTNGGTGCAGGATNNCCGAGNTCT
CGTTTGATATTGTACCGCCGCNGCCATGGCTTGGANGCGCGCACTCGCCCAGCTNGCCTT
CTTGNNGTTNTCGCCNGTGGCCCGCGCNTTGCGTGCCAGCTCTGGCTGCCTCGGCCTCAG
CGCCGGCGCCTTCCCNTTGAACGTGGACCAGCTCCTCGAGNAGAGCAANTACANCAAGGC
CAAGtTTNTTTGCCAAGTTGAANGCAATGGGGATCAACTCTTCGATCCGCATGACCGCGC
CCACCGGGCTTCGCNTGCCGAACACATGCCTCGCCCAGCAGGGGAAGcCCCCgGGCAGCA
CGTCGTCTTgTGtGGNGATTGCATGGCNCGCATGTCCTATGGAGCCTACTTGCGCAACGA
AGGGCAT
>at00049
GTTGAAGCCACAAATGACGCCCCCACGTGCGTTGACCTTCCCTCCGCTGAACCATGCCTT
GCTCCCCATGACGTAACCACCTGGGCAACAGCCTGTGGAGGAGCGCTCAGCCACCTCGAC
CTCCTCATCCCACCGCTTCTGGTCCCAGTCCGCCTGCTTCACGCACTTGCCGAACTCCGA
GCCGTCCGCGAGGGAGTCAAGTGTCAGCGTTGACGTCTGAGGCGGCTCCGGCGTGGTGGT
CGGGCTGCGTCGGCGCCGCCACCACTTGTCGATGATGCCGTTGTCGATCTCGCCCAGGGT
TGTAGGTCTTGCGCTGTGCCGCTCATACCCGGCAGCAAGAAGATATACACCGACCAGAGC
CAGCGCAGCACTGGCAATGGATACTCCAAGGCGCCTGAATTGAAAGCGCCTTGGCACCTC
CTCCTCCGGAGGGACCAAGCGAGCGGACACCTCGTCGTCGGAACACTCCATCCCACAATC
GCAAGCGGCTGTGGATAGGACACCTGCTGACGGCTGGCGGAACTTGAGCCAAAATGGCTA
CGG
>at00050
AGCCTGCAGTGGCCACGTCGAGCCAGAGCAGGTGTGTGGCGTAGGCCTCGGTCACCCCTG
GGGAGCTCTGGACCACAAAGCTCAGGACGGAGAGCAAGGCCACAAGCTCCAGCAGCCTTT
GGTATGACCCAGCGCCCGGTGTCCGGAGTCTCCCGTCGAGCAGGTCCAGAAGCCACGCTC
GCGTGAACTTTGCAGGACGCCTCGAGAATCTGCGTCTGACCTCAGATCTTGAGTGAGTGA
GGGAGTGATTGGACACGCTGTCGCTGACATTCCTCCAAAGGGCTTTGGGCTTGCTTGTCT
TGCGTCCGAGAAGTGGCGTGCGGAGGTCGGCCATGACTGCCCCTGCGTAAAATGCAGGCA
AGCAGTGCTTGAATCAAGATGGCTGCCATGAGATCAGAACCCAAACCATCGCCATGGCGT
TGGCATAAGCAAACACGAGGC
>at00051
ATCCTGGTCTTCGACATGGGCGGCGGCACCTTCGACGTCTCCATCCTGGCCATCCAGGAC
ACGGTCTTCGAGGTCCGCTCCACGGCCGGCGACCCGCACCTGGGCGGCGAGGACTTCGAC
AACCGGATGCTGAGCCACTGCATCGGGGAGTTCCGCAGGAAGCACAAGTCGGACCCCACG
CGCAACCAGAGGGCCCTCAGGCGCCTGCGGACGCAGTGCGAGCGCGCGAAGCGCCAGCTC
TCCACCCAGACTTCCGTGGCCATAGAGGTCGACTCGCTCCACGAGGGGATCGACTTCAGC
CTGCGATGTCCCGGGCGAAGTTCGAGGAGCTCAACATGGACTACTTCAAGAAGGCCATGG
AGCCCGTGGCGCAGTGCCTCGAGGACGCGAAGATGGACAAGTCCAAGATCAAGGAAATTG
TCATGGTCGGAGGCTCCACTCGAATTCCAAAGGTGCAGCAGATGATCCAGCAGTA
>at00052
CCGTTTTGGGCCCGCGGTGAAGGCCTTGGCACCCCTCGCCGTCCCAGCCTGCCACCCGCC
CAGCACAATGTTCGCGGCCCGGTGCTGCATGGCGCTTCTTGCGTCGGTGCCGCTCGCAGC
TGGCCATGGCTTCATCATGGAGCCACCTGCACGGAACATGATGAGCTCCTCGAAGAATGG
CAACTGCCCACATTGTGGGAATGGTGGTAGCAAGGGTTCTCCAGGTTCAGTCTGTGGCGA
TGGGAACCAGTGGCCTGCCGGCAGCAACTACGTGGCAAGCGTGCAGCCGCCGCAGAGAAC
ATACACAGCA
>at00053
CCGTAGCCATTTTGGCTCAAGCTCATGCCAGCAGCAAAGGCCTACCCGCCCCCTTCCCTC
CAAGTCGGTGATCTCCGCTACCCACTGGGGGTGCTCGCCGCCGATGGTGCGCCGCCGCTG
CAGGAGGTGTTGAGGAGGCCACGCGGGTGAGCGCAGCAACGGCTTGGAGAGGCGGGATAC
GATGAGCAGCTGGCAACAGGCAATATCAGCGGATGTGCGAAAGGGCAAGAAGCGGGTGCC
GGCCTGTCCTGTCGTCATGAACCGGAACCAGGAGAAGAACATCAAGACGGGCCGTGAGGA
CCCCGAGGTGACTGCTGGCAGTGCGAGGGGTGCGGCCACAAGATGCCGGAGTCCTACTAC
CGGTGCGAC

>at00054
TGTTTAAGAACATCCACCGCCCGTCTATTCGCTCTCCTCCTCAGACGAAGTCACCTCTCC
GTGGCGTACCTCTGACTCCTCATACTGCTGCCTCTTCTCAAGAAAGATGAACCAGGTGGA
CAGGGCCAACGGCACGATGCTCAGGCCAATCAACCAAAACTCACCAGTGGTTGTCAAAAT
CACCATCCCGGATGCTCCGGACAAAGACAACGGACAGGATCTGCAGATAAATCGTCTGGA
AGACACGGCCCAGCAGATTCTTCCATGCAACGCCATTGGCGATCAGTGCTGGCTGTGTCT
GTAAGGCATGAGGAATCGCTCCAATGCCCCCGACACTGCAGATTCGCAAGGCGATACCGT
GCAAGGTCATCTCCGCATA
>at00055
TTGTTTTTTTTTCTTTTTTTTTTGGTTCGACACATCGCTGACTGCCTCGCGTCCCAGCTA
CCGGCATTTCGTTGTCCAAGCAGGCGCTCAGCACAACCCTGCAGAGTCAGATGCGGGATC
ACAATTCACCCAACTTCCTGTACTTCTGGAAGCTGTCAAACAGGGCCGAGGCAACCTTGT
CAGAGGCCAGATCCTATCCGCTCACTGCAATTGTGTTGAACGATACGCGAGGAAGAGCAC
AAGGGGGTGCCAAAGATGGACACGACCACGTTCACTATGGCAAGGATCAGGAAGACGTTG
TTGCCCTTCTGCTGTCCGCCAGACTCGAGCAACGTCTCGCCGGCGGGAGTTGTGTTCTTG
GACTTCATGTACTTCTTGCAGAACATCCAGGCCATCACGTAGCTAAACACCAAGAGGAGC
ATGAACGGGACCGCAGTAGAATCAGAGTTGTGCGGTCCGAGTTCCCTCCAGCTCCA
>at00056
CCATTTCCAGCTGATCAAAAGACAAGTGCACTTTGGTCCCTCTAGTTTCCTTGACGTGCA
CCTCTGTCACCCATTGGTTTCAGGATCTAGCCATTTTTAGACTCTGATTGCATCCACTGG
ATTTGGGCTTGGCATCAGCTACGGCAACCTGGTCATTTTTGCTGGTACGTTCAGCGTTTC
TCCGATCAGGTGCGCTTATCTCCTGCAGGTCTGGACCTCGTCGTCTTCTCCACGCTCGGC
CGCATTGCAGCAGGCCCCCtTTTTCCAGCGCCACTGCCGTTACTTGCTTGACTGCGTTAC
TCGCATTCACTGGTTGaAGCGGATTGCNGTATTGATTTCGATCTCAGGTGCAACTGCGGA
TTCTGTGTGTCAAGTTGTTGCGTTTGTCTTCAAGTTCGGCCGCTGACTTGGGCTTGCTCT
TTGTTGGTGTGTGGAGGGCTTCGACGACGGATCGCCTACTGGACGCTCTGCTGTCACCCT
TAACGCCTACGTTGGTGGACGAGTCCACGTTGCTTGACAGCGGCACTACTGCAACCCAGC
TGGATTCGACGGCGACTTTGCAAGAAGCGACTCTCTGGAGGAGGTCTTCAA
>at00057
CCGTAGCCATTTTGGCTCAAGTGCACAGGCTCAGGCAGTGCTCTCCTACTTGATGGTTGG
AGGTTGGGGTAGGTGGCGGAAGCTGCTACTGCTTCATTGTTCTGTGATGTCTTGCCGTCC
AATTGTGATTGCATTCATCCTTCCCGAAGCTTGGCTCTAGTGTGAGGGCGAAGCACCAAG
ACTGGCTCCCAAGGCCAGCAGAGCCATGGACACAGTTTTTTTAATTCCTCATCGTGCTG
GTGGGTGCGGAGCCCAGCATATTGAA
>at00058
TTTTTTTTTTTTTTTTTTGAAGCGCCTGTCACTTTACAGTGCACAAACACACGAATGCGG
CCCCCCTCTCGTAATCCTACGTAAGCCGAGTCACATCACTTCTTCTTGCCACCTCCAGCC
GCATCCTCCTTACGCTCGCAAGAAATGACCTTCCCGAGCATGACAACGCCATTCCCATCC
ATGAAAGCAACACGAGAGAGACCCTCGCAGTTCTTGAACGTGTCGCACACAAGCGGCTGC
TGGGGCTGGAAGCTGCACTGTGCCATCTCATTCGATTTGAGCGAGTGCGGATCTTCCATC
TTCTTCCCGCCTGTTTCTTTACCCATCTTCCACTTCAGCTTGGAGATGCGGCACGCGGAG
CGACCACACCTGACGAAGCCAATCGGTGAGTAGCCAACCTTGATCTCATTGGGGATATCC
AGGACCTGGATCTGGGCGTCGAACTCGCGTGTCTGACCCAGAGTGGTATCCTTCTTGTAA
ACCATGACGTCACCAGAGCGCGGCATGTTGTTCTTGTCCAGTCCCTTAATGTTCAAACCA
ACATTGTCACCGGGATTCGCANAATCCACGCGCTGGTGGTGCATCTCAACCGTGAACACC
TTGCCAGTGCAAGGGTTGGNAGCTGTGTGTGTGGG
>at00059
CTGCAGTCAGCGCCACCTCTTGCCCAGGTGGTTCTAGTTCTGCAGCCATGGTGCAGGAGG
GTGAGAGCCTCGGCAATGCCATGCGACAAGAGCCAGTGGTGCCAGGAACGCACAGCAAGG
CGACTGGTGCAGGCAGTTCAATCATTGGCCTGCTAGAAGTTGTTGAAAGCGACTTTGCCA
AGAACTTGGCCGCGGAGGAGGTGCAGGAGGACGATGCAGAGGTCGAGTACCAGAAGACGA
CGCAGGTCAACAAGGTGACCACAAAACTCAAGGATCAG
>at00060
GCAAGAAATTCGACAGGCACGCAACTCCCCAATTACCGTAACTTCCAATGTGGATCTGAT
TACGTTTGGACACTCATGACAACAAACGATGTGGTTGCCAGAATATTTGGGCTATGCATC

AGCATGTACTCGCTCATCAGTATTGGTTGGCTGGTATACTGGTACGCACTAAAGCGTTCC
ACCGCGCCGCCCAAGTTGATCAGGGTGTATCGAGTGGGCCTGCTGTGCTGCTCTTGGTCT
TCCATGTCAGTGGGCATGCACGTGCTTAACAAAGTCCTTATGAGCAACCTTGCGGTACCA
GCACTCGTTTCCACGGCACAGATGCTGTTGGCAGTCTTGATCACGGGCCCGTCTTCTGCA
GTGACATCTTCAGGCTAGACCGCAAGGTGTTGGGTAGCTGGATGGTCGTGCCGATGTTCT
TGCGGCGATGCTGTCG
>at00061
TTTTTTTTTTTTTTTTTCGTGCTCGCGTCCGTAGAGCCCGCCGTGCACCTTTCAGCCGT
CGCTACCATGAGCTGGCTGTCACCGCGTCTCCGAATGCGCGGGTCTTGGAGCATCTTCCT
CGCAGTCCACCGACCGCGAGTACTGGGATGCCATGTCCAGGGTAGCCTTCCCTGCTGCCA
GCCTGCGCTCTGCCTGCCGTTTGGGCCCAGAATCCAGGAAGTTCGGCACCACGGGGCGGT
GGAAGCAGCTTTCGAACCACATCATGTCCGAGTAGATGTGCTCATCCCGGAGCATCACCT
CACTCTGCCCGTACTTGCCGTCCCCAATCGTGGGGTGCTGCAGGTGCGTCAAATGTGCAC
GGATCTGATGGTGCCGGCCTGTGAGAATCTTGATGACTAAGAGGGTATCAAGGTTGTCTG
GATCTTGCTGTGGCCACGCATGCACCTGGCTCTTCAAGAAAGTCCGTGCAGGTGCACCGG
TCGAACAGACGTAGCTTTTCATGTTCACCTTGTCGATGCTCAGTCGCCCGCAAACATCTT
CCTGCCGCGGGCTGAGGGGATTGACGCACACAACAACATACTCGCGGCACATGGCGTGTG
TG
>at00062
TTTTTTTTTTTTTTTTTCACTGGCTCAAAACCTCGCGTTCGGAAAATACATCCTGTNGG
AGCAGTCATGAATCGCTCACACCGGTGAGCGCTCTTGCTATCTGGCTCACTATGTCTGGC
CGTTGAACACGAACATAGTGTCGCAACTCGCAATAGCCATCAGTCCCGGTGCCAGTGAGA
CGCCACCGCTGCTGAACGCAGCTTCCGCATCGTAAGTCGAGGTCACCTTGACACCGGGGC
CATCGCTGGCGCGTGTGAGGTGGTAGATGAATCCGTCAAGCATGCCGACGTAGTAGTTGC
CGTTGCTGTCCAGCGTTGGGTTGCCGACCGGATTCGGGAGGCAAAGCCCTGCTCCCGAGA
GTATCCGCTCGTACTCTCCCCGGAACAGGCCCCCTCCCCAGGCTGGGGGCGCCCACGTCC
ATAGCACCTTGCCCGTCTCGGCGTCCATGCCCCAGACCTCTGGCGTATGGCTGGGAAATC
CCCAGAGGTACTGCGCGTTGTTGCCGAGCATGTGCGTGACCTCCTCGTGCC
>at00063
CTTCTTTCCCACCAGGTGATATTTGAATATTTGCCCGCCAGGCTGCGCGGGTCTACGCCT
CCCTTCTGCGCTTCAGCAAGTGATGGGCTCCACACCCAAGAGCTCACATCCCCGATGCCG
CATGCTCCGTCTGTGACGAATTGGCTCCAGAGGCAAGCCACATGCTTCTCCCTCATTGTC
ACTGGACATCCCACCCAGGCCCCTCACCTGCACTCATGTAGGCATGGCGCAAACGCTCCT
TCTGGATC
>at00064
TTTTTTTTTTTTTTTTTCAGGCCATCGAGTCCATGCCACGAACTCCATGCATTGGACCG
CTTGCCAACCGCAACCAGCCCCATCAATGTCACCATCTAGTGAATCGCGTCGACAGCAGA
CTGTACATGCCCAAAACTAAGCACACCCGCGGCGCACGCCTGTATACCGCACACGCGTGC
ATGCACGAGAGCATCGTACACAATGCCTGGAACTCAGATCAAGTCGGCAATGTCATTGGG
CATCTCTTCGATCTGGGTATGATAGTACCTCTCAAGGTCCTTCATGGTTCGGACGTCGTT
GTTCGTGACAAAGTTGATTGCCACACCCTTTCGGCCGAACCGACCACTCCGCCCAATGCG
ATGCAAGTAGTTCTCCATGTTTGATGGAAGGTCGTAGTTGATCACAAGGGACACTTGCTG
CACGTCGATGCCGCGAGCAAGCAAGTCTGTGGAGATGAGAACACGCGAAGAGCCAGAGCG
GAATTCGCGCATGACCAAGTCACGCTCCTTCTGATCTAGCTCCGCGTGCATGGTTGAGAC
GGTGAAGTCACGCTTCGTCAGCTGGTCCTGAATGAAGTCCACCTTACGGCGCGTATTGCA
GTAGATGATGCACTGTGTGATCGTCAGTGTCTCGTACAAGTCGACAAGGGTGTCAACCTT
CCATTCCTCCTTCTCGATGGCCACGTAGAACTGGCGGATACCCTCCAGAGTCAGCTCGTC
CTTCTTCACAAGGATCCTGACCGCGTCACGCATGAACTTGCTGGTCATCTCCAGAATCTC
CGGGGCCATGGTGGCAGAGAAAAGACACACCTGGACTTGAGCTGGCAAGCACTTGAAGAT
GTCGTAGATCTGGTCCTTGAACCCACGCGACATCAGCTCGTCTGCCTCGTCCAAGACAAA
TACTTGAAGATCATCCACCCTAAGGTTGCGCTTGCTCACCATGTCGAAGACACGGCCGGG
CGTGCCCACTACAAGGTGCTGGCCATCCCGGAGCTTATCGATGTCGTCCCGCACGGACGT
GCCGCCAATGCACACATGTGCTCGGATGTTGCAATAGTCGCCCAGCGCCAAGGCGACCTT
GTGGATCTGGTTGGCAAGCTCCCGGGTCGGTGCGAGAATGAGCGCCTGAGTTGCCCTCAA
CGAGAAGTCGATGCGCTGCAGGGCGCCGATTGTGAACGTGGCTGTCTTCCCGGTTCCGGA

CTGCGCTTGGCCGATTGTGTCGCGGCCATCAAGAATGGGCTTGATGCCCCGCTGCTGGAT
AGCGCTTGGCTTCTCGAAGCCGTAGGAGTAGATGCCGCGCAACAAGTTCTCGTCAAGGTC
GTAATCGTCAAAGGACTCGAAGACAGGGTACTCCTCTGCACCAGTGACCGTGATACCCTC
GGATGACATGATGAACGTCTGGAGACCAACGCTGCACACAGAAGTATTGCAAGCTCAGGG
CCACCAAGGCTTGAGCCAAAATGGCTACGGA
>at00065
ATTCGGTATTACATTTAAATTCGGAAATTCATAGGAATTCAATATATCATAATCAACATT
AGAAGGAGAATCATGGCCCATACCCCATTGAAACACATTAGTAGCTTGAAACAAAAAATG
GCATACTAACTTTATACATTGAAGGAAAATGATTAAATCTATACAAAGATGGCTGCCATG
TATGATAATAATATTTATTCTTCAATAAATTATATTTACGACGCAAAGCAAATCTACGAC
GTTTATTAAT
>at00066
AGGGAGTTCGGCCTGTCAACGTGCCAGGCCACGCGGTCAGCGGCCACGGAGGCGCCGGCA
TGGATCTTACAGCACACGTCCCCAAAGCTGAAACCGCCCTCGACCAGGCGCCTCACCAAA
GAGCCGCTCGGGGCCTCGGCCGCCAAAAGATGGAGCGACTCCTGGTTGACCCGCCTCGCG
GCCTCGCAGAAGGCGCGAAGGCGCACCGGAGCCGCTGGCCTGGAGAAGTCTGAGCCGGAC
CGAGGGAACATGCATTTGAGCCCCTCGTGTGTGGGCAGGCCATGGAAGTAGTCCTCGTAG
CACGTGTGCCCAGGACGTTTGGCATGGTCGACACGTTGGAACCCAAGGCCTGGGAGCCTC
TCCTCAGATGCGCAGAAGATCTCCTCCTGGAGGAGGTACTCGCGAAGCTCCTCGCTGATA
CTCCCGAGGTCTTGAAGCCCAACGCACTCGAACCCAACAATAGCTGACCAGGATTGCATG
GCATTGGAGCACGTGCTGCTCG
>at00067
TTGCACCACGGACCTGTGCAACAACGCGACCTCCCCTCGCGAACGAGCCGGATGTGGAGT
CGCCAAGCGAGTGCTATGCTGATGGCCCCGGTGGGGCTCTCATCAAGTACAGCATGACTG
CCGTTCCCGATGGAGCCTGCGCCAGCTACACATTTATGGGCAAGGTGGCCCACGTCCCCG
TCTCCAAGANNNCGTGNGANACCATGAAGaCCATGCCGGCTANGTACGNAGACCTCACGT
GTTGCACCACGGACCTGTGCAATGGGGACTGACCTGGGATGCACGCTTGCATGCATGGAT
TTTGTGAAGCGTTTAGATACTCACCCGCCGGTCAAAGCGCACGGGGTCTTGGACAGGACC
ATGTAAAGATTTTGCCCACGCATCAACCTTCACGAGAAGGATTTGCGTGTGCGTG
>at00068
CCGTAGCCATTTTGGCTCAAGCTGCTCGCTTTGCAACTTGTCTTCTAGTATTGGTGACCT
CACTCCCTTGCCGTCAGGTGCAGAGCAATGGCCGGGCGTTTGATGTTGACAGTGCTGTGC
TCGCTTGTGCTCCTGCATTCCGCCAGCAGTCACGCCTTGGAGGAGAGCAATGAGACGGCT
CATGAACAAATGCAGGTTGTACAGCGCAGATTGCAAGCTGGTGTGTCCTGTGATCGAAAC
GCTGCGGAGAAGTGCCGGTGGAAGCTCTACATGCCGTTCAAGTACCCATACCAGAAGAAC
TACGCGATC
>at00069
TTTTTTTTTTTTTGTTTTCCACGGGCCTGTTCCACAGCTCCGCCCTTTTTGTAAATATTT
CAAGCCGGTCCGCAATCCAGCAAAACACCCTGTAAGTAACTGTCTGCAAGCACCTCATCT
GGGGAGTGTGCTGGCTTCCTGCATCGACGCTGGGATGATCAGCTCAGAGAATCTCGCGTC
AAGCTGGAAAGAGCTGGACAACAGCAACCGCAGCTGCTGTTGCCCTCCGCTCCCGAGCTG
CCTTGCAGACTTGCTCGCTCCACTATGAAGTCTTTGACCATTGCGTACACCGCACAGGAT
GCCATCTAGCATCAAGCGCGGCGCCAAGTCTCAATGCTCAGAGAAGACGTTCTTGTTGGT
AATTTGTAACGCATAACACAGCATGTTGACCCTAGCCCACTCGACTTTTCGGTCAACGGA
ACGCATGCTGCAGGCGTCTCTGGCTCTCGGGAGCGGCTGGAACATGCCGGCGTCTTGAAT
CGCAAGGCCAGCTGTATCTTCGTCCTCAAGCGGCTGGGATCGTAGGACTGCTCCATCACT
GGAGGGTACTCGGGTGCCGTCTGCAACCGTGCACCAATGCTTCGGCGTGCTGTCCGTGTC
TGCCTCTGGCTTCCTGGTCTGTTGGAGGCACGCCGTGCCTTTGCTCGGCGAGCACCAGCT
GCAAGACGCGCTGGTCTTGAGCATGCCTTTGGTGATGTTGGCGGCACTGCCTCTGTGAAG
GACTTGGCAGCCATGGCAGGAGGCAAGGCCGAAGCTGGCGCAGCGGGTTGCTCTCGCACC
ACTGCGTGACGGGGAAGGTGCGTGGGCAATGTGACGGCCGGCAAAGATCCATCCTGTGGA
CATGGTGACATCAAGCAAGGAGGCAATGTGCGGACATGCGTCTCCATGACAGGGGGATCG
GCTGGCAAGGGTTGTGCTGCCGCGCTCGGTGCAAGATGCGGCACGGTGCCCATCTCCACC
ATGGCCCTGCACTGAACGACTTGCTGACGTGTGGCAGAACGGGCAGTTTTGCCTGATGGC
CTGCACAAGCCACGCGTAGCACAGGCACACAAGAGCAAGGCGCAGCTCACAGAGCTCCAG

GGTAGTGCCACTGAACCAGTGCCATGAGTTCGACATGGTAAGCTGCTGGCCTGCTTGTTG
ACACGCGTGACTGCAAAGTTGGAGTTGG
>at00070
TGCAGCCGCGAGGTCACGGCGGCAGACCTGGAGGCTGACAAGGCGGGTAGTCTCAGCGAG
GCTGCGGTAGACCGCTTGCTTGGTGGTCCAGCAACTGGCTTCAACCTCACAGACGAGGAC
CGGGAGCTTTTTGACAAGGCGCTTCGCGGCGAGATGGTGAACGCAGAGGCTCCATCAATC
CTTGGGATCCAGGTCCACAGGGTCCCTTGGTGGCTTCAGATACTCTACATGGTGGGTGTA
GTTGCAGCTGTGAGCTGGGTGGTATTACTTGTCGTCCAGCGCTTGACAGCCAGGGGAGAA
GGGAGAAGGCCAAGGCAAAGGACGAGAAAAAGGNGGGTAAG
>at00071
CCAATGGTGTAGATGTCGTACAAGTAAGAGGAGCCCTCACCCCTTCCTCGCTGCACCTTG
GAGTGTGGATCCAGTGCCTGGAAAATCTCGAATGCACACCAGACCCTCGTATAGCACACC
CTGCCGCTGTCCAAGACAGAGAGTACGCCCACTGACAACTCCATGGCCTTCCTGAACGAG
CTCTCCTCGGGGTCATCCCCGAGGTCAGCGTCGACCCTCCACTGGTTGTTCGCATACGCA
CAGACCCAATAGCCGGCCTCTGGGGGCAGCTGGCGGTCCGCCGCATGCTGCCTGACGCAG
CGCACGAATGAGTACACTGGCCCCCCCCACAATGGCTCACGAACCAGGCTGGCCGCTGTG
GGGCGTGGCGATCAGCTCGACGAAGCTGCACTGCCGGCTGGCCGTGGCCGGCCTCACGAG
GTAGTCCACCGCGTCGTACAGATTCACAGTGTGGGGCTCC
>at00072
AACTGCGTGGCAAAGTACTTTGACACCCGGAACCGAATGCTCGGAGAGGACTACAGCACC
AAGCTTGCTGCACACCTAGCACATCGGATGTATCAGcCCGCGCCGCATCTATCACGAGAT
CAAcGCGCTACGAGAAGGAACGGACGCCAATAAGTCGACATACTGGGTGTTATTTGAGCT
CACGTGGAGAGATTTCTATAGGTTCTTCACGCTGAAACATGGGAACCGGGTCTTCCATTC
TGGCGGTACGGTCGGTGCACGGTCCAGTTGGTCACAGGATCCTGAGGCATTAAAGCGCTG
GAAAGAGGGCCGAACTGGCTGGCCACTGGTGGATGCCAACATGCGCGAACTAGCAGCCAC
GGGCTTCATGTCGAATCGCGGGCGCCAGAACGTGGCTTCCTTCTTGGCGCTGGACCCTGG
CCTGGACTGGCGCGCTGGAGCGGACCACTTCGAGTCGCTCTTGGTGGACTATGACGTGTC
CTCCAACTGGGGCAACTGGGTGGCTGCTGCAGGGTTGACCGGTGGCCGCGTGAACAAGTT
CAACATCACGAAGCAGGGCTTCGACTA
>at00073
CCGCATGCTCAACGTCCTCCTCGGGCGCGTATTTCTGGGCAGGGCTGCCGTGGCAGCGGC
CGTGCCGACGCAGCCGCATGGGCGGCAGAGCACCGCAGGGTTCGAGTGGATGGACCTCGG
CCCCAAGTCGAACACGTCTGTCTCCTCCATCCTCATGCCGCCATCCTCGACCGGTGGCAA
GCTGGCCATCGAGTTTTTCCACCGCATGGACCACCTTGGCGAAGGGACGTCCCCGGACAC
CCTCATGAGAGGCTACCAGTACATGGGTTTGAGCAACAGGGCCGGTGAGCGGATGCAGCG
TAGATCGCCGATAGGCTACTACGGCCTGATTGCGCCCGACGCCAAGTTGTAGACCGGCGC
AGTCTGTGCCCAGTAGCCGGCGCGCAGGGGTCATTGATTTCGATGGCCACAAAGCTTGAT
GACAGCATTAGAATCATGGACTTGCTCCAATT
>at00074
GCAGAGTCCGTAGCCATTTTGGCTCAAGCTCAAGGGCGCCTTTGCAGGGCTGGGGTGGGG
TCCTGAGGCTGCCGAGTCCCGGTCGCATGCGATGATCGAGGTAATCTGCAATGACCGGAT
TGGCCGCAAGATCCGTGTGAAGTGCAACGCAGACGACACAATTGGCGACCTGAAGAAGCT
CATCGCGGCCCAGTGCGGTACCCGGTGGGAGAAGATCCGCATCCAGAAGTGGTACAACAT
TTACAAGGACCACATCACCCTGGAAGACTATGAGATCAAAGACGGAATGGGTCTGGAGCT
GTACTACAACTGATCAGCCGACGCATGCCTCGGAGCAGCCTGCGCTTGGTGGCTGCCCGG
AGCCCTCTCGCAGCTGTACAGACGTGGCGGGCGCGGAAAGAG
>at00075
TTGTTTTTTTCTTTTTTTTCGCGGCATGCCGGCTGCCCTGACAGCAAGCCGTCACTGGT
CACTCAGGACGCCCCTGCGAGCAACACACGCAACGCAGCAAGCTCAGCAAGCCCAGCAGT
ACCCCTGAGCGCCTGCGCGCCAGCCAGCACCTGAGCTTCGCAGAGCTGGCGGCGGGGTCC
ACGGGCACCACGGCGGCCTGGGGCTCCTGACTCGTGCAGAGGGCACATTCCAAGGCTGTG
CTGACACCTTGCAACACCTCGCCAAAGTGCATGAGGTTGGCGTCCGGTCGACGTGTGCAA
AAGGCTTTCCACTCCAAGCACATCTTGTGCACAACAGCCACAAAGAGTTCGCCGAAGACC
TCGAACTCACTCTCCGCGCTGGGCAGCTGCCGCATGAAGTTTTTCAGCGCCAAGTTGCAC
GCAGGCCGCGCATTGGGCACCGGGGCAACGATGGACTGGGCATTGAGCTGCAGCTGCACG

ATGAGAAGCCCACAAACATTGATACAAGCCGCAGAGAAGAGCATCTCGCTGTGACCCTGC
CGGGCGTCCTCGGCCCAGCGCAGCGCCTCCTCGCGGTGCCTGTCCGCAAGGAAGCAGAGG
CATTTCAGGGCCAAGAAGCCGCCACCGCGGAAGTCTGTCCGCGGGTCGTCCGCCTGGAAG
CCAAGCTCCCGCCACCGCCCGGAACTGGTCTCGTACTCGCCCCCGAGCATTGTGTCCCAA
TAGCTCCGCAAGGCCGCCTCATGCATGGCGCTCTCCTTGTCAAAGGGTATTGCAGCCAAG
TCAAGTAGCCGCTGGTGTGCTTCGACCTCATCAGCAGTGAAGGCAACCGCGTCCTCCAGA
CATCGAGCCCAGCTGGGGCCGGCGGCCCGCGGCTGCAGACTCGAGCGGCCGGCTCGAGGC
AGCAGAGGCTCCTCCTCATGAGGAGCATTTAACTTGACTGCCTGCACCATCACGGGCCAG
CAAGGATGGAGGCGGCCGGATGCTCAATTTCGGCTGGCTGGTGACATGAGTACTGGTCGA
GCCACTTGAGCCAAAATGGCTACG
>at00076
GTATTATTCTGGGCGCAGTTCAGGAGGCGTAGGGTGTGCAGTTGGCATCGCCCGTGCGGC
ACTGCATCAAGTCCATCCGCCACACCTCCATCTCCACAATCACGAACACCTCAGCATGCC
CACACGCATCTGGATTGTCCGGAGATGGCAAAGCGTCGCACATGATTGTGGGATCGCCCG
AGCTGATGACGGCCTCAATCGCACTCCTCACCTCTGGCTCCAGCTGCTGACTCATTGAGT
CTGTAGCAACNTCGGAAACNCTCCTGGCCTNAGGCGCCAGCTGCTGAGCGGCCTTGCTGA
TG
>at00077
AGGATGTTGAGGTCAACGTTGATACGAACGCCAAGAAGGCCTATGAAGCTGACTACTGGA
AGGGCCTGTTGGATGCACAGGGTAAGGTTGATGGCACCTATTACTGAATGTAGCTTCTGC
CTTGTCCACCTCGGATGAGTCTCGCACTGAACTTCTCAGTGGCGTCCGGAGCAAAAAAAA
GAAAAAAAACAA
>at00078
AACGCGTGTTCGCGTCCAGCGCGTCCCGATTGTCGAGCTGCAAGTTGATGATGCCGTCGT
ATCGTTCGCGGACAAGGTCCAGGGCGCCAAACGGGAGGTCATGGTCCACATAGGGCCTCA
CGTTTTCGCCCAAGACATAGAACTCCGCGCGTGCGATCCTGTCAAAGACCTGCTGTGCGT
GCGCCTCCACCGTCATCATCCACTGGTGGGCGTCACTCGGGCGCCAGCCTTCTGCAGCGG
CCGCGTCCTCATCTGCGGCTGCGCCGACCTCGGCCTCCATCCTTGCCACATTCCTTCTGG
ATGTCTGGTCCAGGCCGGATCTCACCTG
>at00079
CCNTTTTGGCCCAAGCAGCGGAGCAGTCTTGCGCAAAGGCCGGCGGGTGTATCTTCGGAA
CCATGGACATGGACCTGCTGCACCCGGACCCCAcGGGAGGAGAAGGCCAAGCACAAGCTC
AAGCGCATGATCCAGTcCGCCCAACAGCTTCTTCATGGACGTTAAGTGCCCAGGCTGCCT
GCAGATCACCACGGTGTACAGCCACGCGCAGACGGTGGTGCTCTGCGGCAACTGCAACGT
GATGCTCTGCCAGCCTACGGGAGGCCTCGCGAGGCTCACA
>at00080
TCTTTTCGTGCAGATCCTCGCAAGTCTCTTGCATTCCCAGACCAAGGACCTCCTGAACTG
CCTCCTTCAATGGCCACCTACTTACATCAAACTGAAAGATGGAAGACTCACTTTGGATGT
GCTCCAGCCATGGCGGAATCCGTTCAGTTAAACCGTGAGAAGCCCTCAAGGCAGCTACTG
CTTGCGTCTGCTTCCGTGCCTGCAGGCAATAGGACTCAGCTATGTGCGGAGGAACATCAT
GCGAGTACGAACAAGACTTTCCCCAGCGGCACCGGCGCCCCCACACGGCGTGTGAGCAGA
GTGGTCGACCAGTGGCATCGACTAGGGCTCCCTGGATTCCCGGCTCGGCAGCCTCGGGAG
ATACGTTCTCAGTCATCCTGATGCCCAATCCGCAGCCTGCTCTACCGGCCTCAGCTAAAA
GGCGCCTGTCACTTCCGTCAAGCTATACTCGTCAGCCTTGCTGATGGCATATCGGTTGTA
GGACTCTACGT
>at00081
GTAGGAGGGGCCTTGGGTGCTGCACTCGGACTTGTCCCTGCCGTCTTTACCTTTGGCTGT
CGATCCCAATCAGTGCGGCCATGGGTGGCGGCGCAGGTCTTGTCGTCGGCACCACAGTTG
GCGGGACGGTTGGTGCCGTGGCGGCGGTGCGGCCGGCTACGGCCTCTACTCCAGGAAGGA
GGACATCGGCATGTGTGCCGCTTACGTCAAGGCAAAGGCCGCAGAGGCATCGGAGCTCGT
CAGGGGCAAGTCCACAGCCGCGTACGACTGTGCCGTTCAGCAGCTGACTTCACCAAGGCG
CAGGCATACGCCTCTGCCGAGTACGTGGCGGAGAAGGCCTCTG
>at00082
TTTTTTTTTTTTTTTTTGCTCTTGAGGGCCTCAACATCGTCCCTACGGGTACAGCAACA
TGCTGTCGAGCTTGGGCCTGTTCTTGCAGAGGAGCTTCTTTCCAAAACGCTGCTCGACTT

GGGCAGCCTCAGCCGCGAACCGCCGCAGCGCAGCCTGGTAGACGCGCTGGTCTACCTCCG
TCAGCCGCCGCACCAGTCGGACATCCTCCGCGGCGAGGCTCTCGGTGCCGTTCTCAGTGG
GCTTCCCGGAGCGGTGGTCGAAGTAGCTTCCCCGGAACGAGGCCCAGGCCTTGGGATCTT
CGCAGTCACACCACCGAGGGGTAAGCCTTTTTGATACTTCACGTGCAGGAGGCACATGGA
CTCTTGATAGAAGTCCGTCAGGCCGACGAAGGCTACCCCCGTTTGCGAGTCGGCGACGCG
CTGCATCGCCACCTGCTCGCGCACCGCAAAGTGCCCAGACCTGTCCGCCAGCACCTCCGT
ATCCAGAGCGGGGGTGGAACGGGCGCACCGGGACGCCTGGCAGGAGAGGGCGCGTGCTTG
CAAGTTGATGGGAATGTAGCAGTGGAAGCTGTTGTTGGCGGTGCTCGGCTCGTCTCCCAC
GCGGACCCAGTGCTGGATCCATTCCATGAAGGACCCTGGGAGGTTATGCGGCTTGCACCA
GCTGTCCAAATTCTTGTGGCAGTGGTTCCACTGCGAAAGGACATGCGCCACTGGCTCCCG
CATGAAGGTGACCACATCCCATGACGGCGGAGCACGAGTCCGGGTCGGGAAGCAACACTC
TTGCTCCCTGTAGCTGCGGCGGGGAGCCTGCCGGCGGATGTCTCTACTGACGGACGCCCC
ACCTGTCTTTGCAATGTGATGGAAGTAAATTCCGCCAACGGCCCCTTGGCTGCCTGCGTT
GGAATCACGCTGGAATGCAAGAGCAATGGCGCGTGGAGAGTAACATACCTCCTGGACCGC
AAGACCATGCTGCCGGGGGAAAGAGTACGGATGATATTTATCCACTGGTCGAAACAAGCT
CTCCTTGAGCCAAAATGGCTACGG
>at00083
TTCTTTCACATCATCGTTCGGATCAGCCAGGCACCTGTCTTGGCCTTGGAAGCCACGAGG
CTCTGGATTCTAGCAATGGTTCATTTTTCAAGCTGCACGGAATGGCATGCCACTGTCAGG
CAGCGCCTTTAGGTATGCTTTCAAGCTTCGACCGATGCGAATGAGTGTTTTATCTAAACG
TGGCCCTCTGCTTCAGTCTCCTTGAGCTTGCATCCAGCTCTCCTTTGGCGACTGACGGCC
TCTGTGGGGGTACCCTCAACCACGCGAGCTCTTGATTCGGTACCGATGAG
>at00084
TTTTTTTTTTTTTTTTTACAAAAGATCAGACTCACACTCGCCATGACCAGAATCTCTGG
CGCAAAGTGACCATAACTGAAAACTGGACACCTTGCCATCCCATAATGGCAGTGTCAATC
AAAGTCAACAAATCAATCTACACACTGAATTCGTCAGTCCGGGCCGGACTGGACACTTTG
AAATCTTCCTGGAACCAGATGCGTCAAGACCTTCTATTCATAAAGACAGCATCAGCCTGC
TTTGCATGCCTATATCGCATCGGGGTTACCCAACTGACAGGGCCAGCATTGGGCACCTTG
GAGTAGCTAACGCTGAGGTTGTCAACATGCAGGCACAAAACTGGATAATGCGAGCCCACG
CCCTTGGCAGAGGCTCCGCCTACCAACGACTGGTAACTTGCAATCACTGTACACAAGGGC
ACGCTGCAGCACCCGACGTATTCGCGGCCACACCGCTCCTCCATGAGGGACAACTAGAGG
CGGGGCTCCTGGTCATTCGAGCGAAGCCCTCCACACGCAAAGGTGGCAACGTGTACAAAA
CCGGNCATCTTTGCAGCCCCTGCGCTTGCCGTGGTACTTGCAGGCCTTGGCGCAGAGGTA
TGGGTGGCCGATGGGACCCACCGAGGGGGCCTCAAGTGAGATGGGGCCCGCCCACAAGCT
GGCGGAGGCGGCGGCGTCCTGGAGTGTCTCGCAAGCGGCACGTGGCC
>at00085
GGGAGAACGCCCCCCCTAGGTGGCGCTGCTCTGCGGGGCGCACTCCACGTGCCCCAAGAA
GGCTGCGCAAGATGCGCTCACGAGAGCCACGCCACCGGCGAGGATCAGGCAGAGGCAGTC
AAAACCTCCTCGGGTCAGAATCAGCACGCCGATGGAAGGCCCGATGGCACTGCCCGCCGT
CATGGCCAACTCCGTGGCGGTCATGGCCTCGCCGTAGCCCCGGCCGTGCGCGACCGCCAG
ATCCGACATCAGGGAGATGGAGGGCGCATCGGCGATGGATATGGCCATGGAGCTGGTGGC
GACAGCGACGACGATGGGCAGCACAAAACCTGCAGGATAGAACCTCGCGCAGGCGGAGCA
GCAAGCCAGGCTTGCCCCCAGCAAGAGCGGACTCAGGATCATGATGGCCCTTCTCTTCCG
CGGCTCGCCCGCCATCTTGCCTCCCCAAGGGGGAAACGACCATCTGCAGCAGGCTGGCTG
GCACGGTGATGAGGCCGATCTCCTGCGTGCTCGCCCCTTTCGCGTGCATGTCCAACGAGC
CTGCGCAGAGCAAGGCCGCCTGCGCACCGGAAATGATGAGCAGGGTGATGCTGATGACCC
AAGACCGGACGCCAACCACTGCGTAGACCCCGAAGAGGCGGACCAAGATGCCGGGGCTGG
CGTCGCTCGGCGGCTCCTCCAGCAAGGGCTCTTTGGGAGTGAAGGGGACCTCAGGCAGTC
TCCGAAGCACAATG
>at00086
CCGTAGCCATTTTGGCTCAAGGGCTGTGTAGGATCATCCAGTCACGCTGCCAACATCCTG
GCTGCCATGGCAATATCCTTGCATGTCACGCTCTTGAGTGGGCGGGCAGAGCTTGTACAG
CTCTTGCCCGGGGAGACGCTGGGAGCTCTGCAGCGAAAGGCAGAGGGAGCCCTTGGGACA
GCTGTGCATGGACTCGTCACTGCAGATGGCCGAGTTCTGGATACGCTCTCGGCCCCCATC
AGCGAGTGCGGCGTCGAGGACGGCAACACCTTGACGGCACTGGCGAGGCCACCGGGCCCG

CAGATCTTTCCTGGCAGGTATGCCTTCGGCGCCATCCGTTGTGACGGCTCCGTCGTGGCT
TGGGGCAGCCCGGAGCACGGCGGAGACTGTTCCGCGGTCAGGGAGCAGCTGCGCAGCGGG
GTGTGCCGCCTCTTCTGCAGGCGCGGCTCCGCGTTCGCGGCGCTGATGCAGGACAGCTCC
GTGGTCACCTGGGGCTCCCCGCGCTTTGGTGGTGACAGCTCGGCAATCAAGGCACAGCTG
ACAGAGGTGGAGGACATTTTCTGCTCCGACGGCGCATTTGCTGCTTTGAAGCGAGGTGGT
GCAGTGGTGACCTGGGGCCACAGCGACTTTGGAGGCGACAGCACGAGTGTGCAGCCACAA
CTTGCAGCTGGAGTTTCTCACATCCATGCAGGCAGGACTGCCTT
>at00087
TTAAAAGCGCGGTAAGTCTGGCCATCATGGAGGCCAGACTGCGTACGAGAACACGCGATT
TGACGAGAGGACAATTCGAAATGATTGTCCTTTACGTTCTCCCACCTGTGCAGCGGCAGC
AGGAGGAGCAGCTGTGTACGAGTTTTGCTCTGGAGAGAGGGGGTTAACCTCGCCGTGCGC
CGCCGGCAGC
>at00088
GAACAGAGCCTCTCTCACGCAGCGAAGAACCTGGCCACAACTGGCCAGCCGAAGCTTCCA
CAAAGGCCGGCGTGCTTCGAGACGTCCCCGAGCGTGTCCTTGAGCTTCCGCATCGCAGCC
GCGGGCGCGAAGTGGTCCCCCACCGCAACCTGCTCAAGCTCCAGCTTGCGCAAGGTT
>at00089
TGACTGGGCCCGAGCAGCCCTCCTCCTGGGCGGCGCTCCGCCCGCGCGCCGCGGCGGCCA
TGCCAACCCACCAAGAGGTGATCGCGGAGAACCCTCTGGAGGCCCCTGCGCCCTTCTGGG
ACAATGTTTACTACGCGTTCAATGGGTTTGCAGCCTTCGTCATCATGGCCTCCGTCCTGG
CACCTTTGTGTGCTGTTGCCTCTGCCTCATCGAGAAGGTAGAGAACATGGAGAACCGGAG
GACAGGACAGCCC
>at00090
TTTTTTTTTTTTTTTTTTGACCCCCAGAGAGAATAAACAAGCTGCCCTCATGTTGTGGTT
CCACCTCATGGTGATTACACGCCGAGACTTCTGAGAGCCTGGGAGCAATCGCATGCCCTC
TGAACAATGACGTGCTGCGCGCGAGAATGGTGAAATGCGGCTCTACATCCGGAGGCTTCA
TTGAAACAACCTCAGCGCACAAAAGTTTGCCCTTCTTCTATGACGTGCTACGAAGCCAAG
AGCCCAACTTGACCGGCAAATGCATCTTCGCGTGTTCAGAGAACTAGCATATCAACAATC
ACCCTGTGGACAAAGCACGGGTTGCACTGCTGGGACAGGGCCAGACAGCGCTCATGCAGG
GCCAAAGGTCTCTTCGAAGGCTACCCGCTCCACGCGATAGACCTCCGTGAATGCGGCCTC
CTTCTCGTCAGCCCAACGTGCCACGAGGTAGTCACCCGGCTCGAGAAGCATTGGCGTGCC
CCAGTTGGCGAGGAAGTGGCCGCCAGGGGGAATGTCCCCAGCCTCAACTTCCAGAGCCAG
CACCGGTACACCCTCGTGCC
>at00091
CCGTAGCCATTTTGGCTCAAGCCATTCTGGTTCAAGCCCTGTTGATTCACGCCGCCTCGG
CACAATCACTCCTTGTCGCAGTAAGTTTCACCTCCCGTCCTTCGGAGGGCACAGCTGCCT
ATGGATAACTCTTGCCCATGGTATGAAGTGAGCAAGCAGTGCTGGACATGGAGCTACATT
GCCACAGCGGTTTTTCCGTACATCATGCGAGGCTGCATCGACGCAGCCACAAGCAAGCTC
ACAGAGGTGGCGGGGGGGGCCTTTGCAGGATGCTTGAAGGTATTGGGCGGCGGGGCCGGC
TTGAAATATACTTTCGCTCGTGTCGTACAGCATGGAAGTACATTCGGCTGGTCCCAGATT
CTGGAGCAGGCGTCCAGCAAGGAGGCGCTTATGCCTGCATTTGCTCGTCTGATCTTCTGG
CACTTGAGTCAACCTGCAGGTTACTTCTACATTCTCTTTTGGTGTATTTCGGCACTGGAA
AACCACGAGATGATGTTGGCAGTGATTGTGCTGGTACGTGAGGCATTGTATGTCATGGTG
ACCATGCTGTTGGCGATACGTTACCCCTCGTTCCTCTTCCTCGTACCTGGGGATGTGATT
CAATGGATGTGGTTTGTGGCTTCGCCAGAGAAGATCGTATTCCTAACCTTGTTTCAAATT
ATGCATAACGACACCTCACGCCGCGGCAATTGGATAAAGGGCCTCATGCTGACGATTCTC
ACAGTGGTATTCGATTGTGCAGCCGTTTGCGGCCTCGTGATGGGCATCTACACGGGAGCG
CCGGCGGCGCTGGTGATCGGTTACGCGGTGGTAGCTGCAAGCACGCCCGTGAGCATGTTT
CTAGCTTTCAGAGGTTTGTCCGGCAACGACCTGGATGATGGTCTGTAGTCTCCATTGGTA
ACTTACTCCTGAG
>at00092
TTTGTTTTTTTTTCTTTTTTTTTTTGTCTAGCTGGAGCGAGCAGCAGGCCAATCTGGTGA
CAGCGACAGCAAGGAAGTCCGAAGCAAGAGCATTCTACTGAAAGGCGGCCATGCTCCTCA
GGCCCTGAAGCAAGACGGCGGTGCCTGCAATCTACGCCTGTCGCCGGCCCTCAGCCTCCG
AGCTCGCTGCCTGGCGCCCCTGTCGGCGCCCCGGCCCCCTGGCAAGCTCCATGGCTGCTG

CGCGCTCCTCTTGCGCCGCCAAGCCTGCCAGCCCCAAGCCATCGCAGAGTGGGCATGGGC
CCTCCAGTGGGT
>at00093
GCCGCTATGGTGGCTCGAGTGCCTTCCGACCAGAGTTCCGGGGTTGATCTTTGCCACTTC
CCTCCTGCGGTGCCTGGCGCCCCGCCTCCGGACCACTCCTTGGTCGCCATGGGTTGCGCT
GCTTCACGAGCACGCGCGATGGCCATGACGCCGACGCAGAAACGTGGCCGACACGGTGTG
GAGGCTGCACCCAGCACCCTCGTCCGTGGTGTTGCTGCGCGACCGCTGCAGCGGTCTGAG
GTCTCACTGCCGCGGCCACGGGCCGGGGACTTTGCATGTTGTGAGGCTTGGTGCGAGGTC
AGTTCCTCGCCTCGATCTCCCCGGCGCGCATGGCCCCCGCGGCCACCCTTACGCATGGTG
GCAGCTG
>at00094
CCGTAGCCATTTTGGCTCAAGGCCAGCGCTCAGGCGGCCAGGGGTCCCCGAGTAGGCCCG
CCATGGCAGAGGACGTGGCGGAGGAGGGCGGGGCGGAGGCCCAGCTGCCAAAGAATTATG
CCATGGGCGTCGACTTAGGCACAACCTACTCCTGCGTGGGAGTCTTCGAGGACGGCGAGG
TGCAGATCATTGCCAACGACCAGGGCAACCGCACCACGCCCTCTTACGTTGCCTGGACCG
ACGACGAGCGCCTGCTGGGGGACGCTGCCAAGAACCAGGTGGCCAGCAACCCCACAAACA
CGGTCTTCGACGCCAAGAGGCTGATCGGCAGGAAGTTCAAAGACCCCATTGTCCAGGCAG
ACATCAAGCTGTGGCCCTTCAAGGTGGTCTCCGACGGATCCAAGGACGACAAGCCGCTGA
TTGAGGTCCTGTACCAGGGCAAGGAGAAGAGGTTCCACCCGGAGGAGGTCTCCTCCATGG
TCCTGACCAAGATGAAGCAGACCGCCGAGTCCTTCCTTGGCCAGCGCGTGCGCGACGCCG
TGGTCACCGTGCCCGCCTACTTCAACGACTCGCAGAGGCAGGCCACGAAGGACGCTGGCG
AGATCGCTGGTTTGAACGTCCTCCGTATCGTGAATGAGCCCACCGCGGCTGCCATCGCCT
ACGGCCTGGACAAGAGCGGCACTGAGCGTACCGAGGATGTGAACATCCTGGTCTTCGACA
TGGGGGGTGGGACTTTCGACGTGTCGATCCTGACCATTCAGGACACAGTCTTCGAGGTGC
GCGCGACCGCCGGCGACCCGCACCTCGGCGGCGAGGACTTCGACAATCGCATGCTGAGCC
ACTGTATCGCCGAGTTCCGACGGAAGCACCGGTCCGACCCCACCCGGAACCAGCGCGCCC
TCCGCCCGGCTGCGGACGCAGTGCGAGCGCG
>at00095
GTAGGGCCGGAAGGAGCCAATGGCCGGGAAGGTCTGGCTCACGTCCCAGCCGAGGCGCCA
CTTGCACCATGTGAGGATGATCAGGTAGTCAGCTCCCACAGCGCACACGAGGACAAAGAC
CGAGACATCTATGAGCAGCAAGCTCTCAAAGGCCTTGACGACGTCCAGCCGGATCTTCTT
CCGCAGGTCCGGCTGCAGCACCTTGGCGACCATCGAGCCAAAGCAGTACAACGCCCAGGC
CTCATACATATCTGCCAAAGCAAAGTTCGACTCAAAGCTCTCCATCAGTAAGTTCTTCTT
CTCCGTATCGGTCCTGTTGAAGCAGTTCAGCGGATCGGCAAAGTGGCCACGCATGTTGGA
CCACATGATGCAAACATTCTTCGCGGCAAGGATGGAATAGACCATGGGCAGTAGCAGCAC
CTGTATGGCCACGTCCCTTGGCAACGTCACCATGGCAGAGCCAGCCACGAGC
>at00096
TGTTACATCGTTGCGGCCATTGAAGAGCGCCCCCATGGCATGTGCAAATAATGTCGCTCA
ACTGCGGTACCTCGATCGAGGCCTGGAGAAGTACGCTCTGGCAGGACATGAAAACGACAG
TGCTGGTGCTACACTTGCTGTGGCCTCAGGATCAAGTCTTGCTGTCCGCGAGTACGAGGA
CAAGCTCAAGGCAATTGAGCTCCAACGCCGAGGCAGTGAGAAAGCTGAGATTGCTGCCAG
ATTGGGCAGGTCGGAGCGGTGGGTCCAGAAGTGGTGGAAGCAAGAGCCTCAGTTGCTGAA
TCGGCCACCTGGTGCACAGAATGCTGTCTTCCAGAAAACTCCGTTGGCCGGATTCGGATG
>at00097
ACCCGGCCCACCCCGCGAACGCGATCGCCGAAGGCGGCTTGGAAGTCTTCGAAGGGCACC
GTTCGGCCCGCTGCGCGCTCTTCCTGAACGCGGTGCTGGTTGATACACAGGCAGCAGATC
CAGATGTAGGTACGCCTCTTGCCCAGACCAGCAGGATCCGTCTCACAGTATTGAGCGAGG
GCTCCTACGATGTCGCCCACACTGTAGCCCCAAGTGTAGCTGAGCATGGCAGAGGCCAAA
CCAGCATGGTCCCGTCCTCTGATGGCGTCAACGTATGAGGCGCCTTGCTTACCACTACGT
GGGCTGATCTGGCCTTCTCCCTTTGGACGGATGACCAATGGCTCCACTTCATAGACCTTG
GCCTCGGGTCCAAGACCCACTGATTGGACAGACTGCAGGAAGTCGTTCTCAAGATGGTGG
ACACTGACCCCTCTGAAGGGGTTGCCCACGGATGGATCGTCTTCGCGTGCCATCTGCCAG
CAGCTGACAGGC
>at00098
TTTTTTTTTTTTTTTTTCCAGCAAGGGCCTTTCAGTCTTGCAGCCATATGCCATGGAAC

GCCCATATGGCACGCGCCCAGGCAAGCTGATCCTTGCGACAGGATGGTTTTCCAAGTTCT
GGGCATCTAAGATCCGGAACTCGCTTCCACCATCAGCTCCTTCCGTGAAGGTGAAGCCAA
CTAGCCAGCCATCGTCCTCTGAGGTACTGATGGGCCTCGGCACGAAGGACCACTCACCAC
AAAAGGTACCTGGGGGCAATTGGTGTTTGACCACAGCCCCATTTCTCAAGTCTACGCGCG
CAGCGCCGTTGAAGTGCGGCGATCCACGCTGCCGCTGGAATTCTGCTGCGACTGTTGATG
CAAAGGCAATGCTCTGCGGCTTCCCCAGGAAAAGCGGATGCATGGAAGGGAACTCCAAGG
CATCTGCGCAATTCGGGCGCTCAGAAGCACGGGGGGTCTTTCAGGTCGAGCGTCAATTC
>at00099
TTTTTTTTTTTTTTTTTGCTTCCGTAATTTCAAAAAGTCGCTGCACTCTGCGGAAGGGT
GGCTCACATGAGAAGCCATCTTCTGTCACAACATGCATGCTGTACATCAATCACCAAGCT
GCCTGAAGGCTGCTGGCTCTCACTTGGGGTTGTAGGGGAATGGGGAGTTTGCAGAGTAGT
AGTTGCCGTATTTGCTCTCCCTCAGAAAGCCGCTTTTCTTCCACTCCGGGATCTTTGCAA
GAAGCGCTTCGTTGGGCTTAGGGGGGTAGTTCTTGATTGGGTAGGTCCCGTTGTCCATGT
CAGGGATGGATGAGATCGGGGAGTACTGGTCCATGCCAAACTGCTTGTAGCGGCCAGCGC
CCGTGGTGGGAATGCTGAACTTCTTGTCCGGTGGGGATGAGAACTGCCGGTCAGCCCAGA
TCTTGAACTTTGCCTTGTTCCACGCCTCTTGCTCCGCCTGGGACTGGCCTGTGCGAATGT
CATATTCTGCTGGTGCAGCGACCTCCGCAGCCTTCGCTGTGTTTGGCAAGCCAACACCTG
CCAGTCCAACCATGAGGCCCAAGGCCAAGCCAAGCACAATGCCCTGCGTGGTTCGGCCTT
CAGTTTGCTGCGGTTCCACTGTATCGGACGTGATGATCGGGAGGCTCACCTCACGCAGCT
CCGCTCCGGCACTGGGGATGTGGCGCGCACCTGGTGCCAGTTGCAAGAAGAGCGGGCCGC
TGACAGACAAGGCCACCGCAAACGCAGCAGCTGCCAGAAGCCCCGAGCCTTTTGGTGTCA
TTGCT
>at00100
TTTTTTTTTTTTTTTTTAACTCTTTCTCCGGCACACGGAGGTGCCAAACTCGGTGCAGG
CTATGCGGCCGCGGCGGCCGCAGGCGATGCTGCGTAGAGGTGCACGGAGCCGTACGCGAA
GCTGGTGATGCTCCGCACAGGCAGGCCCTCTTCGGCCATGGACTTGGCAAAGTCCAGGGG
GCTGGGGGAATTTTGAGAATGGACTTCTCCAGGTACTCATACTCCTCGCCGCCAGAGCCC
AGGGTCGCTATCCTGCCGATGGCGGGCACGACGTGCGTGATGAAGGTGCGCGCTATGCGG
GAGAGCAGGGTCTTGCCGTCCGGCAGCGCAAACTCCAGGATGCAGACGCGGCTCGACAAC
CTGCTCCGCAGGACCAGGCGCATCTCGCGTAGCGCCCGGGCGCGGTCCGGCACGTTCCGG
ATCCCGAAGGACATGGATATCTTGTCGATGGATCCGGGAGGCCACCCCNNTGCGGTCGAT
GACGCCAGGGTGCCCTCTGCATCGATGCCACGCACTGTGGTAAGGTTCTGGGCATCCCCC
TTCTACGAGCCGCACGACGCCCTCGAGGCCCTGCATCTCCACCTTGGCAACGCCGCGGCG
CAGCATCTCAGAGCTGGGGGTCCAGGCCCAAGACTGAGTCAGCTGGTGCTGCGGCCCCCT
CGACTGAAGACTGGCGCAAACGCTTGCCCGCGAGGATGCTGACATCAGCCGTGCCTGTGG
CAAGATCCAGGACCC
>at00101
GCTGCCATTCCAGTTCAAGGGAACATGTCACTTCCCACTTCGAAGTGTAAGCAACCCGCC
TCGGAGGGCTCCGAACGGAGGCTCTTCCTTCATCCTGTTCACGTCGAGCAGGGAGACCTC
GTGCCTGTAACAGGTGATGTGCTTCAAGAGGAGTTTGTGATCTCTGAATGTGATGTCACT
CAAAGGGTCTCGGCCCTCAAATCAGTGAACTTCCATGAATTGCCTAGCGTTGACGTTGCA
TCTGGCAATCTCCTGCCATGCTGCCTCATTCTAGCGAGAGCACCTGCTTGATGCCGGTGC
TT
>at00102
TTTTTTTTTTTTTTTTTGGCTGCGCAGGCCGCTGGGCACACAGCCGCAACAGCAGCAGC
AGCAGCTGCTGCCGCGCTTCCACTTCGCCCGCCTACAGAGACACAGGACGGCCCGCAGAG
CCCCCCCCCACTCCAGCTCGCGCCCGCCGCGCGACCCCTCCCCCTCACGCGGCCCCCTGA
GCGAGCTGCTCGTAGTCGGCGGAAGGGCCCGTGAGGACCTCAACGCCATCCTCTGTGATG
AGGATCGTGTGCTCGAACTGGGCCGACCGCGCGTTGGTGAGCGTCTGAATGGTCCAGTCG
TCGTCCCACTGCTCGTAGGAGTCGTCGCCGTGCTCCACGAGGATGGGCTCCACCGTGAAG
GTCATGCCGGGCAGCATTGGACCCTGATCAAGGTCGTTGACACAGGGAATCACCTCCGGC
GCGCCATGAAAGTAGTCCCCAATGCCGTGGCCGACGAAGAGGGGGCTGGCATAGCAGCCC
GCAGCATCGGCGACGCCCTGGATGCGCTTGCCCACCTCCCTGAAGTCCACGCCGGGACCG
CAGGCCGCGATGCCGGCGGCCGTGGCCCGGGAGCCCGCTCGGCAGAGCTCCAGGCCCTCG
GGGTCGGGGGTGCCTGCAAGGAACATGGAACTGGTGTCCCCGTGAAAGCCTTCCANGAAG

ACCGTAACGTNCATATTCAGGATGT
>at00103
AAGGGCGTTGGGCCTGCATTATTCGACAGGCACTTTTGGTTTTCAGGAAGGGCGGCATTC
CATGGCTACTTCGGCAGATCACAGCTGTTGGACAGACCGAGAATCCCCATGTCAGTTGTC
CTATCCTGGTGATCTCAGCCTTGACCATCACAGCCTTCTGGAACACCACCAGCAGCTTGA
GTACCTGGGCTGAAGCCGATCAGACTGTCGCCGAACTGGTGGCGGCCCAGAAGCTCATCA
ATCTTGTGATGCATGAGCCGAAGTGGAAATGTGTGAATGCAATACTTTCCAGGGCTAGGG
TCATCCGGAATTCGACCCGGGTACTTTGGTCCGG
>at00104
TTTTTTTTTTTTTTTTTGCACTCAGACGAGCCAATGCATGCGAGATTGCTTGACCCTCA
ACTGTGAGTCTCCGGCCAAGAGTTTGTCAAATATCGAAAAACTGCCCACCGCAAGCGCTA
GAGAAATTAAGCCGATGGTAAGGCACCTACCTTCCGGAGGCCCTCTGTATGCGATGGGCA
CGGGTCAGGGCGTCCTGCTGCCTCAGCCTGCAAGCTGAGGTCGTAGAACGCGCCATTGCG
CCATTCGAACTCCTTCACGGCCGTCGGGAAGTGCGCGACACTCCGTGCGTATGCGGCCAG
CCTGTCGCAGAGCACGCCGTGGTCCCACTTCACGTCAGGGTACGCAGCCGTGAGGGCCGT
GGCAAGCTCGCTGATGAGGCTGTCCACTTCCTGCCGATGGCCGGCCTCGACCTCTCCAAC
CTTGCACCCTTCATGAAGTGCCCCGGCAAGCATGTAGGCCGCAATCCAGAGGTTCTTCTC
CCAGTAGGCCTGAGTGAAGGCCGGCTCGGCAAGGACGCGACATGCGAGGTTGGCCTTGGT
CAAGCGGCTCTCCACCTCTCCAGCCCACTTGCCGGTGGCGTTCACTGCGCTCAAGCCATC
GGGATCGGTGTCGGTCTTCCCGTCGAGGGGCGCTCGCCTTCT
>at00105
TTTTTTTTTTTTTTTTTTTTTTTGCATGCACGACTCTGACAGAGTCTCTCCCCAGGAG
CACTGCAACAGGGCTCTTCTCGTTCACTTTGCAGAAACTGTACAACATGAATGGAGACCT
TAACAGGCAGCTCGAAAACTAGCCTCTCACCCTTCCGAAAACGGACTCACATCGGTCCAC
AGGACTCAAATACTACCTCAGAAATCGACGTCCAATGCAAAGCCTTTGACGTCCTCCTGT
CCTCCTTCCTTGTTACTCACGGAAGACATGACCCCTGCCTTCTGGTACTCGCCAACACGC
TTCTCAAAGAAGTTTGTCTTCCCCTGCAACGAGATCAGCTCCATCCAATCGAACGGATTG
CAAGCTCCAAAGAGCTTGGAATGACCAAGAGCAGTTAACAGCCGGTCAGCCACAAACTCA
ATATACTTTGTCATCAGTTCGCTGTTCATGCCGATGAGATCGCATGACAACGCCTCACAG
ATGAAGCTGCGCTCCACTTCCACGGCGGTCCTCACAATGCTATGGACAACATCCTCTGGG
AGCTTGTTCTCCAGCATTCCGTAGATGAGGCATGCAAACTCCGCATGCAGACCCTCATCG
CGAGAGATCAGCTCGTTGGAGAAAGTGAGGCCAGGCATGAGACCACGCTTCTTCAGCCAG
TAAATGGCACAGAAGGAACCACTGAAGAGGATGCCCTCGACACACGCGAAGGCGACGACA
CGCTCAGCGAAACTGTTTTCCTTGTTCATCCATTGCACAGCCCAATCCGCCTTCTGCTGG
ACAGGTGGCATGGTGTGGATTGCATCAAACACGGCATTCTTCTCGGCCGGGTCCCGAATG
>at00106
TTTTTTTTTTTTTTTTTGGTAAACGCTATGCAGTACCCACTGTCGAAGCCCCCTCACCG
CACCAGTGTCATGATCTCCTCTGCCAGCTCCGGCGTGCAAGCGCTGAATTGGCTCCGGCC
CATGAGGTCCAGCTCTGCGCTTCCTCCATTGAAGAGGTCGCGCGTCACGCCACCAGCCTC
AGAGACGATGAGCTTGCCAGCTGCGAAGTCCCATATCTTGGGGCCCTTGCCGCGTTCCCA
GTCGCCGTGCTCGAAGGCTGCACTCAGCCGGCCCGCTGCCACATAGGCCATGTTGAGGCC
AGTGGAGCCAGCCATGCGGACACTGCGGACGCCCGCGCGCATGACCTTCGCCAGTGCGCC
GGTGACCAGCGCTATGGACGCCTCGTCGCGGCCGTACCCTATCTCCATGCTGACGACGGC
GTGGGCAAGCTTCTGGGCCGAGGACCCTTCAGGCGGAGCGCGCCCGGCAGGTTCGGCCTC
TCCAGATAGGCCCCGCCGCCCTTCGTGGCCCAGAAGAGCTCATCCGCGATGGGGTCGTAG
ACCACTCCCACGCAGGGCTCTCCGCCCTCGAGTGTCATCGATCCATCTGCAACAGTGACA
ATGTTGTAGGCGGAGATGTCCCGCTTGGAGCCACCTAGGCCGCGCACCTTGACCTGCGAT
GAACTTGCTCTCTGCACCTGAAATGCCGGTGACCCACTTGGTGCACTTTCCAGAGGACAT
GCAGAACTTGGAATTGTGGAAGGGACATTTGAGCTCCGGCCCGTCCGGGCCAATCTCAAT
CTGCCCGTCCACCATGGACTTGTTCATGTGCGGGCACACTCCGTCAACAGGCGAAGACCT
TGCCACCGATCCTTGGCGAGGACAATGGGGCCATCAGGGCCTTCCACGGTCGCCCGCTCA
CCCTCATTCATGAAAACCGCCGCACGGGGCAACGCAACCCCTCGGAGTGGCAATGTCCGG
CTGCAGCCGAAGAAACGCTTGGCGGTCGGGGCCAGCATTGCTCTGAGTTCCGGGCGCAAC
TCAAGCAATACCTGCTGGAGGCCCCCCTGTTCTGGATCACACACGCGCAGCTTGAGCCCG
AGTAGCTTGAGCCAAAATGGCTACGG

>at00107
TTTTTTTTTTTTTTTTGTAGGCCTTCAAAGTACGCATCTTGTGGAAGTCAGATGGCGCCT
AGGCACAATTTTGCGCCGACAGTCTTGGCAAAGTGGATCACGAATTAGGGGCAACAGTTG
CTAAAGGCAAACGACTGCCGGGCACATATGCAGGCTCTCGCAAACAGCAGGAAAAACGCA
CGCCACATCCTACTCATGCTGGACAGTGATGCGGGGTCGTGCCCTTCCCATCTATCCAGA
AGTGCGGCTGGGGGATGCATTCGTACTTTCCACCGTTGCGAGATATTATTTGGTGGTCCG
CAGCGCACCAAGACGCCTTTATGCAACTGCTGCCTTGACTTGATTCTCTTGGACGTTGCT
CCAGTCCTCCATGCTCCAGACGTCGTCGCCCTGCGCCGCCTCCTCCTCCAGGTCTGCCAG
CTTGTCGGGCGCGACCTCTGCTTCAGGCTCGTCGCAGGTGTCGAGGTTGATTTCCTGCTT
GCCCCCCTCCTTGCCCAGCATCGAGACGACGTGCGCCTGGACCATCGCGAGCGACGACCG
CGACTTCATGATCATCTGCCGCTCGCGCGCGAGAGCCTCCTCCGTGCGGACCAGACTGTC
GGCCCGGCCCTCCAGCTCCTCGTGCACCATCTTCAGCTCGGCGGCCTGTGCTTGACGCAG
CTCCTCCTCCTCTTTCTGCTGCCACTCCACCAGGTTCTTCTGCTTCTGCAGCTCTTCCTT
CTGCGCCTCGAGCTCGGCCTGCCTGG
>at00108
CCATGCGGCGCAAAAGAGGATTGCTTTTCCTCTGGTAATGAAAATCCATATCCCATCCAT
GGCCTTGCATAGGTCCTTGAATGGCGGACAGAGCTGCGGAAGGGAGAGCTGCTAAAGCTC
GCAGGCGAAGGCGCACGAGTGGTACGGACGCTAGGAAAGTCATGATGGAGGAGGGGGTCC
TGGCGCAGGATTTCCTAGATACAGCCTCCAAGCTGCCAGGGGACTCTTCAGATGAGGACT
TTGAGGTTGAGAAGGATGAGGAGACGAAGACAAAGGAGAAGGAC
>at00109
TTTTTTTTTTTTTTTTTAGTCCGCCGCCGAGCCTGCTTTCTCATCAGCTGCAGACTCGG
CCACAGAGCCCGCTTTCTCGTCAGCGGCCGACTCGGCCACAGCGGAGCCCGCTTTCTCGT
CAGCCGCTGACTCAGCCACAGAGCGTGCGTTCTCGTCAGCCGCCGACTCGGCCACAGCGG
AGCCAGCTTTCTTGTCAGCCGCCGACTCAGCCACAGAGCCTGCGTTCTCGTCAGCCGCCG
ACTCGGCCACAGCGGAGCCAGCTTTCTCATCAGCCGCCGACTCGGCCACAGAGCCGCCGC
TGCCCAGCCCAGCGTCCAGTGACGCGGCAAGCTCCCAGAGGGAGGCCCGTGCATCTTGGG
CGCTCCCGCGCCAGCGGAAGTCGGCCGCGGAGCGGCCGTCAAAGTCCCCCACAGCGCGGC
GGTAGATGGCCGCCTCTGCCGCCTCGTGGACGCATAGGTGCCATGTCCTCTGCCGGTCCG
CTTGTGCTCCGTCTGCCGGCTGCCGGAGGCGGGTCTCGAATGCCATCCCAGCTATCGCCA
GCTCTGGGTGCACGCCCTCTCTGAGAAAGCTTGCCCACAAGAGACTGGACACCCCTGACT
GCTGATGCAGCG
>at00110
CTTCATGAAAAAGTTGGTGCAGATCCTTGCGTAAGTGGAGGTGTTCATGAGGTTTTTGAG
TACAAGGCTGGCACCTTGGTGATGCTTTGTGAGGGCCAGCTTATGTCGGACATCAGTGCT
TACATTTGTCCTGCCGCTCATCAGGAAGTAGAACTCCATACCCTGGCCGCAAGTCACCTC
GCCCGACTTGTAGACGTCATCCCAGTCCTTGCAAACACACGCAAGGGTGCTCTCTGTAGT
ATTGTCCACGTGCGAGACACGCAATGCCTGGTTCTCAGCGAGGAGGAGGGCGCAGAGCGT
TGCAATGGTTGCTCGCGCCTGTGCCATTCTTGCTGCTCTGCAAGCACTCGGTGGTGGTAG
AGACGAAGGGTGCGGTGTGATTGGCGGAACTGCTTGAGCTTGAGCCAAAATGGCTACGG
>at00111
TTTTTTTTTTTTTTTTTGCTGCTGCCTGTAATAAAAGGCTGCCTCCTCCTCCTCCGGAC
GGCACCTCCTCCGGCCCTCACCCACTCGAAGGCTCCTGGGCGACAGGATCGACCTCAAGA
CGGCGGTTGCCCGCCGGCTGCATGTTGGGGCCCTCATGACTGAAGTCAGGCATGCAGGTC
CCCGCACGGTCGTCCAGCTTCCGAAAGCAGTGGGCTGCGTAGGCTGCCACCTCCCACACC
CTACCAGGCTCCCGAGCATGGTGCTCATAGTACCCCGACCACCTGCAATTCCTCTTCGGC
TCGAAGCCCGAGGCCGAAGACCAGATGAGCAAGGGGCCATCTCGGCCAAAGTCTCCGTTG
GAGAGGCTGTGCCACCGTGCCATGCGCTCTTCTGGGTACGCAAGGGCTGCCTGCACATAG
CGAACTCCGTCATGTATGGCATCCCAGCCGAAGTGCGTGTGGCCAAAGACATGCAGCGCC
GGCCGCAGGGCATTGATGCGTTCTCTGAGGAAGTTGGAGCCAGACGCCTTGGCTAGCGAT
GGGAGCATGAGGAACCGCTTCTCAAGAAGCAGCTCCAGCCTCGGAAGGAAGTGCGAGAAA
GTCACCAAGGGCTCTGATGAGTCGAAGTCCTCTAAGCGCGTGAGTACACCCATGCTATCA
TTGAGCTGGTCAACTGTGCGAGCGGCCGAGTCGTCATACATGGAGCTGCCCCCCGGAAAC
CTGCACCCCTTGTAGTCGGTCATCACATTCTCCGCCTTCGGAACCTTCCATCCCTGTATG
TCAGGCTCCGTGTCGAAGCTTGGGTGGTGCCATGAGAGGAGAGGGCAGACCACGACCGCA

TGAGAACCCGTGTGGCCGCCAAGACGGCGCGGCTCTGTCACAACTCCAAGCTTGCGGCAA
AGCTGCATGATCCGATGCAGCTTCGCCAGGCTGTCTGCTACGCCGTCCGCCTCATCGTCG
TCGGTAAGCCAAAGGTCGTGATTCCCGGGGGTGAAGAATACCGTCCCGAATCTCTTCGTC
AAGGCCCGCAGAGTCGCTTCCAGAACCTCCAGATCCTCAGAAACGTCGCCTGCTACGATG
AGCGCGTCTCGCTTATAGTCGCTGGCTGAGAGACCCTCAACCCAGGCAACGTTCTCAGGT
ATGTCAGTGTGGATGTCGGAGACCGCCCAAAGCCTCACCATCGCCCTGCTCAGATGGGCC
CCTGTCCAAACTAAACGCCCTTGAGCCGAGATCGCATGAGCCTTGAGCCAAAATGGCTAC
GG
>at00112
GACGACGAGGAAGGTGAGGATGGTTGATTACTGCGCTATCGGTGAAGGGCGCTCTTGTTT
GGTACCGCGGTCGAGAGAGCAAGTGCAATGCCTATACTGGCATGCTGCTTGCCTGTGAGG
CTTCAACACACATGGCTGTGGCTGGCTGCACTGAAAAATGCACATCGTCACTGCCCGCGT
TCGCGCAGCTGTCCGCGCCTCGTGGAAGATGTTATCAATCAGGTGGTAGATGATGCTCTG
GATGAGGTTTTTCCCTGCAACGCCGAAGTCTGTCACGACGTTGAAGAGGTCCAGCGCGCC
TCAACGTCTTTCTGCTGTGGCTGCTTCTGCTGCCAAGCGGAGGTAA
>at00113
GGAAGTCCAGCTGCAAGTAGGGATCATCAAAGTCTACGCTGACAGCTTCCTCTTGGAGCT
TGCCCAGCATCTTCATGCGGCTCTGCGCCTGCTTGGCCATCTTCTTGTGACCATGACCGA
ATCTGGCGATGAACTGCTTTAAGTGGGCGACCTTGCGCTGCTGCTGCTGTGCAATGGCCT
CCTGGTTTGAGCGCTGCTCAGAGCGTACTCGCAGGAAGGTGTCATAGTTGCCTCCGTAGT
ACCAAAGTCTCTTTCGGTGGAGGAAGATCGTGTGCGTGGTGCACTCATTCAGGAAGGACC
GGTCGTGCGAGACAATGACCACAGTCTTCTCCCAGGTCTTCAAGAACTCTTCAAGCCATG
TCACCGCATGCACGTCGAGGTGGTTTGTTGGCTCATCCAACAAGAGAAGGTCTGGCGACA
CGAAGAGTGACTGGGCCAAGGCAATGCGCATGCGCCACCCGCCACTGAACTCCTTTGTAG
GCCGCTTCTGGTCCCCTGCGCTGAAGCCCAAGCCCGCTAGGATTGTCCCAGCGCGTGCCT
CCGCGTCTTCACTGCTCATGTCGTCTAGGCGCTCGTAAACCTCCATGAGGCCCACGCCAT
GGACCTTGGGCTCCTCGCCGGACTCGCCAACCTCGGCCGTCGCGAGTTCCTTCTCGATCT
TCAGAAGCCACTCGCGCTCGAGGTCCGCCTTGAGTACGCTCTCCAGCACCGGCGTGTCAT
CGCCCTTGATCTCCTGCTCGACGTGCATGATCAGTATGTGCTTGGGGACGGGCACGCGCC
GTCGCGCTCGAAGATCGCGCGCAGCAGCGTGCTCTTGCCCACGCCGTTGCGCCCGATGAG
GCCGTACTTGTGGCCATGCGCGAGCCTGAGCGAGGCGTTGTACAGGAGCTCCTGGCGGCC
GGCGAAGACCACGATCTCGTTGCACTCGACGTCCATGGAGACTCCAGTCTTGCTGGGGCC
TCCGGGCCC
>at00114
GAAGCATTTCTGCGGGGAGCAAGACGCTTAGTTCCTAACCTTTGTTGCGACTTTCCCTCC
ACGATCTTCTTCAGCTCCAACATGGCGTAGCAAAGGCAGTAAGACGACTTCAGCTGTAGC
GTCGTGAGCGGGTAAGCAACAGCATCCGCCTCGGTGAACACCTGGAGGTCTTCATCAGGT
ATTGCTTGCGGTCTCGATGTCTCGCCGACAACCTCATGGCCGTAGAGCTCGTAGAATCCG
GGGGCTAGAGTACACGACGTCCATGGTGGTGTGGCAGAGCACTAGTCTGAATGCAGGTTT
TGNGTCCATGATCCGCTTAGCGATGGCCCACAAGATGGTGGAAAGCCCGTGCGCAAAGG
>at00115
TTTTTTTTTTTTTTTTTCGCGACGGCTGTGCGCAAATGGCTGCAAAGTCGCAGGTACAA
GAACGGACAAGGGGAATGGGCAATTCAGAGTACGTCACGTCCTCACTCTTGGCGACGTGC
AAGGATGTTGTGGATGCTCTCGATCAATTGGGAGACCGGAACGCATGCGCTCCAGCCAAA
CCCACAGCAATGCCAACAACAATGCCTCCCAAAACCTCCGGCTTCTGGTGACCGAGATTC
GTTTCCAAGAGGCTCGGCTGGTTGATCGCGTCCCGTGATAACCAGGCCTTCTTCTTGGGA
CTGAGATCTTCCAGCGTGACACGAGCTTGGTGCCCTTCTGGCAAGTTGGCTGCCAGTTGG
TTCAAGACTGCCGCGTGATGCCCGGCTCTAAGCCGCACATGGCAGGCGTCGTACATAACT
ATCGCGGCCAACACGGCCGCCATGGCGAACTCGTCACTTCGCCAACCTGAGTTGACGCCC
ACGCTCGTCGCCAGCCCGACAACGCAGGCAGTGTGGCTTGACGGCATGCCACCAGACCCC
CAAGCTCGTGAGAGGAGGAAGACCCATGCGTAGCTAGGTGCGTCAGTGGCTTTGCCAGCT
GCGCGAAGAGGATCCCGAGGACCGCAGAGGCC
>at00116
CGTACAGGTCTGTAGTCATTGCCAAGGCTGTCAGTTGCGCGCGCAGGGACGAAACATCAC
CACCATGAGGCCCAACACATTCAGGTATGACATTGCACGATCAAGTTCTCCTCCCTGCTT

GCGCAAGAGCAAGGACAAGTCCTCACTGCATCTCCTGCATAACCCTCCCAGGCCATACTC
CCGCGCAGCTTTAGTGCTGCCCAGAGCTCGTGTGCTGGCTATTCCTGTTGCCTCCAAAAA
TGGTCTCTGCTGACCTGCGCCCATATGCAAGTGAGGTTGTGCAGGACTGGCTGATACAGC
CATCTCACTACAACCTCTGTCAGCTAAGAAAGTAAAGATCGCAGCACTCCCATCGGCGAT
GACTACGTAAGCGAGGAGCGCCACTCTGCATCCGCAACCATCTTGTCAAGCTCTGACCCT
TTTGCGCCCGATGCTCTGCAGATCTTGACGATGCATCCGTGCTTGATGTTGGCTTTGATC
GCTTCTGGGTATTCGTTCAGGGTTCTTGACATGCCCAACAGCCGCTCACCCAGT
>at00117
CCGTTAGTCCATTTTGGCTCAAGCGTTTTCCGGGCCGAGCCTTTCCCGTCAATTTTACGT
TGGCACTTCCGGCCCCATGGACGCCATCTCTGCTGACTGGGGTGCTGATGGGCAAGAAGT
ATTCACCTTGGCAAACCGGAGAGCGATGCTCATCATGTGTCTCGCACCATGGCCCTTCTA
TTCTTGGGGCTGCCTTTGCTCCGCCATGGGCGTCCCTGTCCTTCACCTGGACGTGCATAA
TCTTTGCAGCGGCATCGACGATGGCTCTCACGTTGAGCCGAATCGGA
>at00118
TCGAGCGTGCACGTTCACGCTACCTGGAGCAGCAGGCGTTCGGGCGGTCTTGGGCTCGCG
GAAGCTTCGAGGACTACGTGGGCAGGGAGTTGCCTCAGCTGCAAGGCTGCATGCGAGAGG
CGCACGCCAATGACGAGAAGCAGTTGCGCTGCGCAGGGAAGCACAACATCCTTGGCCTCT
CGCTGTATGATGCTGGCATCAAAGTCTGGCTGAAGCAGTTCCACCCTGCGGATCTCCTCG
TCACCTACCTGGATCATATGGCGGAGGCGCCGCAGGAGGTGCTGAATGCCATCCACGCGC
ATTTGGGGCTTCAGAATTACACG
>at00119
CCGTAGCCATTTTGGCTCAAGTTTGGCTCAAGCTGAGAGGGCTTTTGTCTACTCCTCATT
CTTATCTCGGCAGTGACCTGGACCGGTGCTAGAAGAGAATGCCGGATATTTGGCTGGAGA
AGTACAGGCCCATCAAGTTCGAAGACGTAGTTGGCAACGTTGGGGCTGTTGGTATCCTCA
AGGCACATGCGGCCGACGGTACGTTTCCGCATCTGCTTCTCAGTGGCCCACCAGGGATGT
GGGAAGACCACTGTGGTTCACTGCCTGGCAAGGGAGCATCTGGGCGAGCACTATGAGCAG
GGTTGCATAGAGCTCAACGCTTCCGATGATCGCGGCATCGACGTCGTGCGGGAGAAGATC
AAGGGCTTCGCACAGCAGAAAGTGACGCTGCCCGAGGGCAAGCTCAAGGTGATCATCCTT
GATGAGGCCGACAGACAAAAGACGGGCGCCGCGCAGCAGGCGATGAGGAGGACGATGGAG
ATCTTCAGCCAGACCACTCGCTTCGCTCTGGCGTGCAACATCTCCTCGAAGATAATAGAG
CCGATTCAGTCCCGTTGCGCCATCCTGCGTTTCAGCAGAATAGCGGATGAGGATCTCGTC
CAGCGCTTGAAGTACGTGCTCGAGAAGGAGGGGGCTCCGTACGACCAGTCTGGGTTAGAG
GCCTTGATATTCTCCGCGGAGGGGGACATGCGAAACGTCCTCAACGGTGCTCAGTCGACA
TTCAATGCCTTCGGGACGATCACGAAAGAGACCGTCTTCAGGATGAACGATCAGCCCCAG
CCAGAAAAGCTCCATGGCTGTCTGCGAGCTGCTTTGCGATCAGACTGGAAGGCAGCCTTC
CAACCTGTTCATGAGATCTTCAAGCAGGGATACAGCTCCACAGACATCATATCCACATTC
GCGCGGGTGGCAAAGTCATCAGACGCTCCAGAGCACATCAAGCTGGAGTGGCTGAAAGAG
ATCGGCCTGACTCACATGCGTGTGACAGCTGGGACTCAGGGACTCTTGCAGCTCGATGCC
ATGGTCTCAAAGCTCGTGCTCGCTGCTGGAAAATGATGATCTAGGGATGGGGTTATGCAC
ATTCTCAACGACATGCAACTAGTGCATGCTGATGGCATCCGACCAATGAGTGTTGCTCAT
CTCATTGGCTTCTGACACGAGGGGGAGTTTCGCACACCAGTGTTTGGACCCGGAAGCAAT
TTATCATCG
>at00120
TTTTTTTTTTTTTTTTTCCAACGCAAGTCCAAGGGAGAGTCGGCCGTCGGAGCGCCGCC
CGAACCAGTCCTGCCCCGGAGCGCCTCGGGCGAGGGGGCGCAGCTCTGCCGCGGGCCGCC
GCGGGCCCGCCGCCGTGCCGGCCCGGGGCTCCGGCGCTCAGGAGTGCCCCGAGGGCGCGG
CGTCGGTCAGGCCCTCCGGCACGGCGCCCGGCAGCGCCTCCTCGAACAGGGCGGGGACCA
GGCCCCGTTTGTTGCCAAGGGCCTTGCGCAAGGTGCGCGCGCGGGGACTCAAGTGGTAGT
GGTCGTCGTCCACGCCCTTCATCAGCATGAAGTCGTGGAAGGAGCCCTCCAGCTGCTCCC
GCTGGTAGTGCGCGATGCCGTTCAGCGCCCCCTGGAGGTCCACGGCGGCCATGCCCAGCG
CGAAGGTCCAGGGGGAGAAGAGCGCGGCCTCGTCCCCGCCGGCGCCCAGCGGCGCGGGCG
CCGCCGCGCCCCAGCCGCGCGCCGGTGCGTCCCCCGCGCCGGCCCGCGCGAGGCGGCACC
GGACCCTCTCGGTGCACTGCAGCCGCTCGGTGCCCATCGCGAAGGCCTGGTGCGCCAGGT
ACTCGAGGAACTGCACGGAGAGCCGCTCGAGCTTGCACTCCC
>at00121

TTTTTTTTTCTTTTTTTTTTGCCTCTGGCCCATGTAATCGTGCTCAGAGCCAACTGCACA
AGGAAAACTGCCGCTGCGCCCATATGAGCACACAAGCAATGACAGACACTTCGGTAAAGG
CCCAGACGAATGGACACACAAGCGCCACTGAATGATGATTGGTTACTTTGGGGGAGGTGT
CTATGCACACAGCCCGCAAGAGGACCTGAAGATTGCCTTTCAAACATGGACCCCATGTGA
GCAGTAAAAATGAACGCCTGTCAGAGCTCACCCAATTAGAGACGGCAACGCGGCCGTGAG
GCCTCGATTTCTGCAATGAGGCGCATGATCTCCTCAGGCGAGTTGTGGTCCTCAAGGCAC
TCCCAAATCTCCTCGATGCTTGCGTCTGCGTAAATCTCCTTGAGAGAAGGGAGCACCATG
CAATCAACCCGTTGAGGCCTCGCGCCCTGCCAGCTGCAAGTCTCTGTAGCGGTGCCCTTG
ACAGCATCCGCGTCTTGATCAGGGCCTTTGCTCGGCTCTCCTCCTTTGGAGGCATGCCAA
GACTTGGCATCCCGTGCAAAAGAGGCGACCTCATGGCGTCCGTCCCCTGGTGCATCTGCG
TCGGTGGAGTAGGTGTGTACTGCAATGTGAAGTCCCTTTGCTGCGCTCAGGAAGCTCGCG
CGCAAGCTCTCATCTGCGTCAATGCGGTAACGGCGCATTGGCATGTGAGGCGCCTCGCGC
AGCGCGCAGCGCTCCACCTCAGATGCCATGAGGGCGTCTGTTGGAGTGCTCTGCATGGCT
CTTGAGTGATGTATGCTGGCAGTGGGTGCCCCTCCTGGGTGCCGCGAGGCCTGGGGCCGA
AGCAGCTTGAGCCAAATGGCTACGG
>at00122
TTTTTTTTTTNTTTTTTTTTTTTTTTTTTGTTTGATGCAGTATGCTTAAGATTAGTGCT
ATGGGCATGGTACAATCGGGAGAACAAGAAGCAGGCTTCACTCAAAGATCTTCAGAAGGG
CAAACGCCATCGATCCCTGAAGCTCTGTAAACTTGAGGTACCGGACGCCGTCACACACTT
CATGGCGAAAGCTTTCCTCGCGCAAGTCCCCGCTAGGGACAACATCGCCAGATGAAGCAG
GCCCTGCACACGCAAGAGAATCGCTTGACGAATCCAGATTCGGACAACAATGATACACTC
TCCTCGCCCAATCTAACTCCAGCTGTAAACATCTCCAGCCACTGTCAACGCCAGACCGGC
CTTCTGCCGCTGCCACCAAGCTGGCCACTGCCACCGGCGGCGCTTGCGTCGGCTTCAAAT
ACTTGCAGAGCTGAAGCTGCGCAGAGGAGCAGCGGTCGCCACTCCACGAGAACTCTACGA
GGGAGATTCTTCGGTGCCTCCCCATGTCAAGTACAAACTCCTCGGGAGGTGACACCGGGG
ACAGTGCCAGCACGTGGTAGGCAGCCGCGTGCCTGCATGACCTTGGGGTGACAAGGATTG
TAGGCTCCAGCCCATGAGTCTTCCCTTTCAGGTCATGACGTGCCCAATTCCAGGTGCTGA
GAAAGTCCTCAGCTAGGACCAGCTTTCTGCTTGCTTCACGCGGCTCACCTTTGCAGGCCC
GCCATTC
>at00123
CCCAGATCCAAACGAAGGCGTCCTCGAGCGCGGCTCTGCAGCCTACGGACGCAGCGCCCG
GCGCCAGGGGCTCGAAGCGGGGGAGCGGCGCCGTCCGCCTGAAGGCCAAGCCGGCCAGCG
CCAAGGCTGGCGGCCAGATGAAGCGCAAGAAGTCCCCCGTGCGCAGGGTCAGGGAGACCA
GCACCAGGGGCCACCAGGAGGCAGATGCGGAGGCCCTGCGCAACGCGGAGGCCCTGCGCG
ACGCGGAGGCCCTGCGCGACGCGGAGGCCCTGCGCGAGAGCCTGATCAAGAATGTAGTGG
TCGAGTTCGCTGGCACGAAATGCAGCGACGGGAGAAGAGGTGTATTCTGGCATCGCGGCG
GTTGGCAGGTTCAAGTCAGGGTGGACGGCAAGGACCTCTGTGGTGGCCGGTTCC
>at00124
CGTAGCCATTTTGGCTCAAGCCATTTGGGTTTCAGCAGTTTTTGAGAGGGGCGCCGCCTT
TTTGAAGCCTCGGTTCCACCATGCCGTCTACGGCCCCCAGCTTTGCCGGCTTATCCGGCA
TGGCTCTGGCATCTGCCACCCTTTGCTCTCCGAGTGCTGCCTTCGTCACTCCATCCGCGG
TGCACACTAGAGCTGTTGCTGCAAGTACGGCCTCGGCGGCAGAGCGCCTTCAGCAGCCAG
AGTTTGGCGCAACAGCCGGCTTGGCCACTGCTGCTTGCGCTCTGGCAGTAGCAGGTGGCA
CTGCAAAGTTTCTTCGCAGGCGCTCTGTAACCTCAACCAAGGTTCTCCAGAAGCCTATTC
CCGTCATCAATTGGCAGGTGGACGC
>at00125
GAGCCCACGGCCGACGAGCCCACCAACTCCTGGGCTGAGCCAAATGCGGGCGGACGTCGA
GAGAAGAGTGACGAGGATCTCTACGGGCTCCGACCGACTCTCCAACTTCCCCTCGGCCCT
CTCAATGCGGCCATGACGCACTCCGCAGGGGCCGCAGCGGGGCGCTGTGCGGAGACCGGT
GCCTGTCTGGCTCCGGCGAGTCCTCTGGCGCTCTGCTGCCGCTGGGGCTGCACGTCCACC
TTGGTGCCCCTCAAAGTCTGCAACACTGCACGCCCACCAAGGGGAACACTGCCTGCTAAG
GCCGCCGCGCTTTCAGTTTGCATTGCCGTGGCTGCTATTGCTGTTTGCTTGCACTGGTGC
CTCAAAGGCATCTCGATGTTCTTTCTCACCCTTCTTTTCAT
>at00126
CCGTAGCCATTTTGGTTCAAGAGTCTGGTGCTACTCCTGACATCAGGATTGGAGGCCTAT

CATGGCTTGGCGCATTGGCGTTGGTGTTCTGGCAGTGGTTGAACTAGCCTGTGCAGCCGG
GGAGGTGCTGAATGTGACGCTGAGAGATGATGCCCGGAACCGTGACGTGACAGCCACTGT
CTGTATGAGAGGTCAATCCCAGCAGGACTTGTACGTGTTTGCGCATGGTGGTGGTCTCCA
TGCCGCAGACTACGAGTACCTGTGCGACGGCAAGTTCCCAGGAGTTTTTGCACGCCTCGT
GAGCCCTACCAGCGACGGCCCTATGGATCTCGTCCCCACGGCAATGGATGTGCTCTTCCT
TGCGCAGGCACTGCCGAAGCAAAGCAGCGACAACGCATCATCTCCACTGCATGGGCGCCT
CACGAATGCCGTCGTGCTTGCTGGTCATAGCATGGGAGGTGCCGCGGTCTTGCTTGCTGG
CGCAGAGGCTCCTGAGGCTGTCAAG
>at00127
GTTCATTTTTGTTGGTCTCTCTCGTGGGCCTCCCTGCATCCGCACAGTTGCTCCATCAAG
CGCTCGGGTGCTTCACTTCAGCTTCTTCTTGGGGCGCAGGAAGTTCGTGTGGCCGCACTT
CTTCTTGCGGCAGTTCACGGCACGAGCCGGCAGCCGCGCGTAGCACTTCCGGCAGATCAT
CTTCTCACAGTTGTACTTACGCGCAAGGACGGCCAACGAAGGCTCAATGACACCTCCACG
CAGACGCAGGACAAGATGCAGCGTGGACTCCTTCTGGATGTTGTAGTCGCTGAGGGTGTC
GCCCATCCTCAAGCTGCTTCCCAGCGAAGATCAATCGCTGCTGATCTGGCGGGATGCCTT
CCTTATCTTGGATCTTGGCCTTCACATTGTCGATCGTGTCCGAAGCCTCCACGTCCAGAG
TGATGGTCTTGCCTGTGAGGGTCTTCACAAAGATCTGCATGGCGCAGGGTGGTGGGGCCA
AGGCAACAACTTGCTCTGGCCAGCCCCGCTGGATCTTGAGCCAAAATGGCTACGGACTCT
GCGTTGATA
>at00128
GTCGGTTCAGTTGAGTCCCAGATGTGCACTTATTTATGGAGCAATATCATGAGGTTGAAC
CCAATTTGTGGTCAGCCGATGGAATTGTTGCTATGCAGCAGTTCGAGTTATGGCGAATAC
ACCGCAGTCTGAATCTACTATACCAAGGTGAGCCAGAACGCTTTGAACTATTCATCGCAA
GATGTCCGCGGCTTATCTCCGGATTTTCGCTGGATGCCATTCGTTACGGTGTTCCGCCGT
TGCCTCGGCTCCACCTGCCAGTTATTGAGCGGTGTCCTCGTGGGAGGATCCTGGGTGGAA
CTGTTATGTGTACCGGGTCAAGTGGCGTTGGAAGGCTGAGGTGTTGGATGCAC
>at00129
TTGTTTTTTTCTTTTTTTTTGCTTTTTGTAATTTCTTGCAAAGAAGTGCATCCTCCCTA
GCGGTGCCAACCTAATCAACAACCTGCAGCAAGCACTAATCCCAGGGCAGTTGGTCACAC
GTCCTCCATTGGTGGCTCCTCGGCCTTGCGGCGCCTTCGCATCTCACCACCTTCAGTCTC
GATCCGCTCTTTGTGCGACGCGGTCCCGAGGACCTTCATCTCGACCTTGTCGCTGATCTC
GTTGATTTTCGAATCGACCGAGAGCACCATGGACCGGAGCATGCTGTTGCCCGTGAACCT
GAAGTTCAGGAAGTCGATGAAGAACATGATTTCAATGATCCCACCGAGCCAGGACTGGAT
GTCCGAGTAGTGCGGCATTTGGATCTTCTTGTAGATCCAGTTCAGCGCATAGAAGACCCG
GTAGCCACCCAAGGAGATCACGTACAAGCTGGTGGCCCAGTCACGGCCATGACGTTCTCG
GTAGCAGAAGATGTACTGCGGTACCATGGCAAAGCCCTCGATGAACTCTGAGAAGGTCCA
CAGGACCTCAAGCATCGAGTACTCGTTTGTGAACAAGAGCGCAGCGGTGGCACACGGCAG
GATGATCACGGACAAGTTGCATGTGTCGTGCCTCCGCTCGTAGGTCGCATCAAGCTGCCA
GAAAAGGAGCAGCACAATGATGGAGGTGCCGATGTACGTCAGCTTGAAGAACACGAGGTA
GGCAGTCTGTGAGTGATCGAACAGGTCGAGGTATCTGGTCGTGAACACGAGGAAGTACAT
GAGCTGCGTACTCCTCGAGATACCCGCAACTGACTTGTTCTTGGCGATTGTGGCGAGCAA
CACGATGATGCCAGTTAAGTGCAGGTAGTCTCCAACGTAGCGGAAAATATTCCAGGAATA
GGCGGTGAAGTTGGCAAAATTTGACGTGCCATGGAGCTTGTCCATGGAGTCAATGGCGAT
CTTTTCCGGTGAACCTGCAGCCATGGCTGTGAGACTTAAAAGTGCGGCTGCGCAGAATGA
TACCCCCTTCCCTCCTAAACCGTGAACCAACGCTTGAGCCAAAATGGCTACGG
>at00130
GCATTGGCGATTCCGACATTTTCTTCCGAGAACGTCGCACTGCTTCCCTCATCCACGCCG
ACCAGGTGCATTGGCGGATTCCGACATTTTCTTCCGAGGACGTCGCACTAGCTTCCTTCA
TCCACACCGACCGGAGCACTTGGCCGGTCGGGGACATCCAGGAAAGTGAAAGTGATCGGC
AAAACTATTTTGTGAGGTTGCCTGTGGAGATCAGAGACATCTCAAGAAGTGAATTCGCTC
GAAGCCGCTTTACCATCGTGGCTGTTGGATCAGAGCAGTGTGCACTTACGCCTCTGACTC
TGGTGTGATTTTTCAAGCACGAAGCTCGGGCACTTTGATGTCAGCATGGCTGGCACTGTC
ATATGTCAAGCGCACATGATCGACTGACAAATGGGCGGTATATGCGACCTGTTTATGGAT
CAGCGATTGGGTCGCTGACGGAGGTGTGAGCAAACAAAAAAAAAGAAAAAAAA
>at00131

ACGCGTTCAGGGTGGACGGGTTCACAAGGCCCGGCCAGCACTGCCTCACACATAATGTGC
CAGAGAGGACAAAACATACGATGTCCTGGCTGAGGCTCAAAACCTGATCTGCGCCGGAGT
GGCCGCGCTGGATTATGATGATCAGAGCGACCAGGACTACCATTGTCAACACCACGCACA
TTTTGCGGAGTGCTCTGACCCGCATGTCAGCTAGTGTTTGCTGTACTTCCTCGTCAAACT
CGTGCGACCGAGTAGCCGAGCCAGTGAGGCAGCGTCGGCCTAAGGCTTGGAGCTCCGCCA
AGGAATGGCGGATCTTGGCAATCAGCCACCACCTCTGTTTAACGAAGGTGCGAAAGGCCA
GTGCGAGGTGAGCTGTAATCAGGATGAAGGCCAACGCGAAGCTCACGCGAGAATAACCCG
ACGACTGCGGCTGCATAATGGGTTCTGCAAAGGCAACTCGCTACGTGTGCTGCGCCGC
>at00132
GTTGGATGGTGGGGGTGACATATACGCAAGTGGCCGCGGGCAGTCGCCACACAGTGCTGC
TCCGGAGTGATGGCACAGCAGTGGCCTTGCGGCTCTAACGGCTCGGGCAAGTGCAGCATA
CCACCCCTGGACTGCGGGGTTACATATACGCAAGTGGCCGCGGGCGCTTATCACACGGTG
CTGCTCACTAGCGACGGTGAAGGCTGTGGCTTGCGGTTCAATTGGTCTGGGCAGTGCCAG
ATTCCATCCCGCCAGACATGGGGCAGAGCTTCTTA
>at00133
GCATTGTGCACGCAGGTATGCATCTGTACAACTATTCGCGCTCACAGGCAAAAACCCATG
CACACAGATGCATTGGCCCAGGTGCGCTTACCCAATCCAGGCCTTTCAGCCTCAGCCCTG
CTCATCAACGGCGACCGCCGCCGCAACACGCGCCGCGTGCTCAGCGGAGACAGCGGCCCA
GAGGCGGTCGCGCTCGGCCGCGGAGAGGTCCTCAAGCGGCATGAAGAAGGTGTCTCGCAC
CATGGCAAACCACTCCTCACGCGAGGTGGCCGTACACAAGATCTCGTACCCCTGCAGCTC
AGGGTGCGACCGGCGCAGAACGCAGCCAAGAGTGAGAGCACACCTCGCTCAG
>at00134
GGATCGGAGTGACTGCTGCGATGGTATTGCCTTCCTCTGCCTTGCTCAGCTCCATGGGCC
ACTCTTTGGCCCTGTTGCCGGTTGAGCGGCGCCCATCTGCGCCCAACGGGGTCATCCTGC
TGGGAGGTATCCAGCTCAGACGTGGCCTGATGTTAGGCACCGCTTCGGATTGTGACCTCG
GCCACGCCTCCCTTCGCTTCTGGTGAAGCCGCTTGCGTGGAGATGGGGGGCGTTGGTTAT
ACCTGAAGCGAATTCCAACTGTGACATTCAGATGTCAAGAGC
>at00135
TTTTTTTTTTTTTTTTTGCCAGCGGTCTACTTACCTGTGCACCTGTACACTGGACTCCT
CTAAGCACATGCTTGGAGCCTCTTGCTCTGATACCTTTCGGGCACATGGAGGGAAAAGCG
CACCAACACTGAGGGCAGGCTTTGCCTCAATCTGAATCTGCCACAATCAAGCCTGGCACC
AGGGGGGTCGGGCCCCAACAATCTTCCCATTTAGGTACCAGACATGACCGCAGACAATAA
CACACCGGCCAAGGTCGGGCAGTAAATGCCAGCAAAATGCAGTGTGGCAAAAGTGGGAGC
AGCTTGCTCGCTCCTGCTTTCTGCAAGAAACACTGCCACGGATTAAAAGTTGTAGGGAGG
CTGAGGGTTGCATCCTTCCCAGATGCATGCTCCGGTCGTTTTGTAAGCACAAGCATAGCT
ATGTTTGAGCATTCTCATGCAGAGGGGGTTAGGCTGATCCATGCCCAACCCATGAGACAA
GAGCAATCTCCTCATCCGTGTGGACTTCATCACTGCACCAATTTGTACAGCACCTGGTTT
GGCAGACGGGTCAGCCACTTGTCGTTCATCAGCGGGTCTTGCTTCTCGACCTGCCTCTGC
CTCAGAAAGCGGTGACCGAATTCTCAAAGATCGGCTTCGACACGTAGCCAAAGCACATCG
TGCGCGCCATGCCAGGGGGCACCGATGCGCGGCCATGCATGACACGCCAGTTGTTCATGG
CCACGATGGAGTGCTCCGGCCAGTCAAAGTTGCAGATGAACTGCGGCTGCTCCACAGGTT
GGTCCAGAGCCTGTAGGCT
>at00136
AGGCATCTCGGTCTCCACCCGCCTCTCCACTAGCGGATGTATGGGACTGCACAATTGGAG
TTTGTACCGGCGGCGATTGCTCATCAGCTTCATTTGCCTCCCTGGCCTTGGGATGATGGC
AAGAACGCTTCACACATCGGTACACAGTACTTTCGTGCGTGGTCCTCGCGTTTGTCCCCA
GCAGACGGTCCACGTACGACGCGCAGGCAGTCAGCCGGAGGGTCTGCCAAGTCGAGCCGT
CCCCGACGAGCCCGCTAGGAAGGCCCACCGCGTCCATTTCAGGGGAGTCTGCGTGGAGGT
GGCTGATGGGGAGCTGCTGCGGTCCGCCTGCTGCGGAGGGGCTCTCGCCACNCAACGGCA
ACTCGAAGCTCATCAACTGCCGCGGTCTGGGCACATGCGGCACCTGCGCCGTCCGCATCT
CTGGGCCCGTGACGCCTGCCGAGTGGACGTCCATGGAGAGGTTTCGGCTCCAGCTCCCGC
CACACCGGCCACCGCAGAACCAGGAGTTGCGCTTGGCTTGCCAGTGCAGAGTTCGCGGTG
ACCTGCAACTGCAGAAGCGTGGGGGTTTCTGGGGGGAAGACGTGGACTTGTCTGAGGAGC
CAGCTGCCGCTTTCAGCACATACCTGGGAGAGCTCGAGTACATCATGGACAGAGGCGGCC
CATCGGACGAGTGGTCAAAGACACCTCGCCAGTCTGATGGTGATTGACGCCTGTGACAGT

CTCTGCGAAGCTTTCTTTGAGTGGTGCTAGTGACGA
>at00137
TGTGATTGTTCAAGATGGCCGGGCTTGCCTTGCTTTGCCCTTCTTATGCAGATGAGATCT
GACACGATGCTCGTACCTGCTGGTCAGCTTTCCGCCCGGTTTCCTTGCAAGAAGGACGGG
ATGCGACGGATATACGACCTGCAGCCAGGCCTGCTGGTGCAGGGCGGAGCTGATGACGTC
CGGGAGTTCTATGATGCAGTGGCGAAGGGATGCGATGAGAGCGTCGTCATTGTCAACT
>at00138
CCGTAGCCATTTTGGCTCAAGAGCTCTCCGCGCTTGCCCAACAAGTGTGTCGCACCATTT
CCCGCAAAGTTGCTTTCCAGCCAGCTCAGGCACGATGTCTGGAATTGGGATTGAGGGGGC
ATCTGCAGGCCACTTGCCGGCAGATGCCACCCTCTCAGCTTCCTGTGCCGCGGCGGGTAA
ACTTCCACCACCAGACGTTGCTGTCTGCGGAAGCCCACAGTCTTGCGGCGCTCACACGCC
CTTGTCCCGGGCGAGTACTGCTCCCTCAACTCCTTTGCTCTCGTTCAGCCTCGACGGAGA
TGTCCATGGAAGTCGCTTCGTCCGTTGCATCTCCGGAACCTCAACGTTACTTTCGGAGCC
GCTCTTTGAGG
>at00139
TTTTTTTTTTTTTGTTTGTCCATATACTTACACGACCCCTGGTAGGTAGCATTCTCCCC
TCTGTCCAAAAGAATACCTCAACAGCCACCCTCGCGGCTGCCAACAGGTAAGCGGAGATG
CGCGTAGCTTGCATACAAGCGCGAAAATCTCCAGCAGAGAACGCTTGTGTAGCAAAGGTT
GCCAGTGCCTGAACGGCGCTCTGGCATCAGCATGGCCATCGCTGTCTTGCCAGAAACAGA
AACGTGAATTAGCCTTGCATATCTGCTGCCAAGAGGTCAACCTAGCCCTTGCAGCCGGAC
AAAAAACAGCAAAAATTAGCATCAAACAAGCCTCAGCTCCTCATGCACTGCCGCGCGCGA
GAACATGTGCCAGATGTGGCCTCAGACGTGGCCTCCCCCTCCCTTGGTCCTGAGGGCAAC
TTACGTGGGAAAAGGCCAAAGCCCCGGCTGAGGAGCATGCCAAGGATCCGTGGCAGCAGC
AACCTCTTTCCTGAGACGACCATGAGCAGGGCGTCCCAGACCATCATCGGTGGGCTCTTG
TCCTGGACGAGGCCCTTGTTCACAAAGGGCGAGATCACCACGCCCTTCTGCACGTTGGGT
ATCTTGGAGAGGTTTCCAATGAATTCGAGCCCACTCTGGGGCTTCTCGCGGCTGTATGAG
CCCGGGGCGCCGCCCCATACGAGGAGGGGGTTGGGGTAGCCCAGGGCCGCCCACATGTTC
ACAGCCACTACGCCGTANTTGAGCTCACGGATGACGGCGTCCAGCACGTCGTCCCGTGTG
TCGGGGTGGACAGGGTGCAGG
>at00140
CATTTCAGCTTATGGGACATCCTCGACGTCATTGTCAGGGCAGATGACCCTCCTCGGTGC
CACGGAGGTGTCCCTAGATGACCTGATATGTTTCTCCAAGCTGCGGTCCTCCTTACAATG
GTCGCCAACAAGCTGGATGAGCAGGATGCTGCCATCCAAGCAGCTGCGC
>at00141
TTTTTTTTTTTTTTTCTTTTTTTTTTTTTTCTTTTTTTTTGTAAGGGTGGATACCATCA
CTGCTGTTCAAACAACTGTGCACAGTCTGTGTGGCATCACCTGAGAGGCAAGGCAATAAC
AAAAGCAGTTTCGACTGGCAAAAGCGTCCGCTCTTACGGAGGTGGCTGAATTCAGCATGC
ATCGCAGTCAAGCCAGTCAGCCCGAGGCTGCTGTAGCACTCGAGCTCTCCCGAAGCCTGA
GGCACTCTGACTTTACCGCGCGGGGGAAGTGGTACTCGAATGAAAAGTTGCCGATGCCGA
GGACGATGCGCATGCCGACACCACTGTCGTGCCACCCAGCAAATACCTCGCCACCCATGG
ACAGCCCTGCATCGGCCTTGCCGGCCGCGCCGACCATTCGGTAGCCCTGGGTGTCGCACC
AACCAAGACACATCTGGCCACTGACGGCCAATCCTGCAGATGCGATGACGCGCAACGACA
TCGGTTCCACACTGGGGGCAGCATTCGACCCCTCTGTCTTCGCCGACGTGGATGCCCCAC
TCAATACATGGTTGAGGTCGTCAACGTTCACGCCCAGAGACTGCGCAATCGAGCCGATTC
TGGAACCTGCCTCGGTGGTGGAGTCACTGAGGAGGCTCTGAGCTCGTTCGAGCGCAAGCC
CGGCTCCCGACCAGATGGCCTTTGCCTGTTGAGACCCGCAAGTGCCAAGCCTGTCCGCGC
ACTTGGTTCTGTCCAGACCAAGCATTTGCATCGCAGATGTGATGGCAGAGGCTGCCCCGG
AGCCGGCCTCCGCAGCGTCGTTGCCGATCTTCAGCCTGATGTTTGCATGCAGCTCTTCCA
GCGAGTGGCCGGTGCTCTTTGCATCTGCAAAGGCCTTGCAACCCCAATCAAACTTCAAGC
CGTCGCGGACGTCGCTGAATCCTGCCTCAGCCTTGAAGTGTCCAACCTGCCCTCCAAACT
TCATTTGTGGCCGGAGCTCCACGTCGCCAGCCCGAAAGGTCCAAGGTCCCAAGTGGAGCT
CCCATGGGGTAGCTGTCTCCGCCCCGTCCTGTCTAGCCATTGGATGGCTGATGCTCTCAT
TGCTCACTGAGTTGCTTGCCATGACGATGGCAGAGGTACCAGAGATAAGGGACGCACTTG
GAAGAGTGAGCTTGAGCCAAAATGGCTACGG
>at00142

GCCAGCCCTTCCAAGCTTCTGGCATGACTGTGGCACCATGGTGAGGCGTGAGCCTCTTCC
CGAAGAAGTCCTTCATGAAGTGGTCCGAAGCGCAATCATCGCTGTGGCACCAGTTCCGGT
GGCCGTAGAAGGACGGGATGCAGTAATGCTCGTCCATCCGACGTTGCCACATGCGGACCA
CACCTCGCTCCGCAGCGGTGCTGCCGACCATCGGCGGCTTTGGGAACTGCTCTTCGATGT
ACTCCATCATGGCAATTGTCTCGGCCAGCACAGTGCCATCATTCAGGACAAACCAAGGCA
CNCACGGTGAGGGTCATCTTCAGGCACTCCTCGGAACGGTTCTCCAGCGCCGGCAGATCG
ACGTAGTGCTCAATGGAGGAGA
>at00143
CCTCTTATGACTCATGGACCAGTACTCCACGACCTGGCCCACCACCAGGCACCTGGCGGC
CTCCTTCCCCCCAATGCCGGCGGCCTCTGGAGCCAAGCGGAGCTTCCCTGCCTGCTCCTC
AACTGCGAGCCACGCACCGCGCTTCACGTCGATCTGCACGGCCCCATCTTCCTTGCGCAC
TTCGACGATGCGGCACGGAATCCAATTGCCGTTGGTGGCCGAAAAATCTCAACGGCCTGA
CCGCAGGCAAAATGGCTGCCTCCGACAGCTGTTGCACCCGGCAAGTCCGCGTGAACATAC
CTACATCGTCTTCCGCGATTGCACTTCCCTACTGCAAAATCTTACATGGATGGCTCGATG
GCGGCGCATGCAGGAAACGGCATTTCTCGCCGTCACGA
>at00144
GCCTGAAGCGAAATCAGCCAAGGTACCATGAGGTGATCAAGCACCGACGTGGAGTTCATC
ACTGGAATGGCCATAGGCTCACCCTGCACGATTCGCTTTGTAACTGGCCCAAGATCGTCT
CTCTCTGGTCCCAGGCCCGAGTAGATACGATCTAACCAGCCCAACTGGATTCTGACCATT
TCCTTTCGCTTCTTGATGGCTCCGGCTTTGCCATCCTCTGACATGAAGTACCAGTCGTTG
ATCTGCGCTGGCTCCACTGTCATCTCAGGTATCATTGG
>at00145
TTTTTTTTTTTTTTTGCTCGAACTGTGTACTGTCGACAGTCCAAGACTAGACACGGCTCC
CAAGAACACAACTTTATTCTGTTACAACAGCAAGCTGTGCATGCGCTCCCTGTTTGTCTT
CATACATCTTCCTTCAAGCACTACAGCTTCGACTCACGGAAGGGACAGACGGAGAGAAAT
CCGAACTTTGGGGAGGCCAGTCACAAATAAGCAATGGGCTGTTCACCAAACATCCTTGCG
GCCCAAGGCGCACTGCCAATCTGCATCAGAGCTACCTGATCAGTCCACCTTGCATCACAG
CCTAGACAGGGCCTCGCCATCCAAGAGGTCGAAGAAACTCAGCGATCATTGTCGGTGAGT
CGCTGAGGCTGCGTGCGCAATCACCGAGGCAGGAGGGCCAGTGCCGGGCAAACCGCCGAG
CTCCGCTGATTGCTTGCTAGCTCGACACTTGCCCTTGATGCTGACGAGTCAGGCTAGGCT
GCTCTGGTGCCACT
>at00146
TTGTTTTTTTTTCTTTTTTTTTTGCTTTTGTCAGTTGGCTTGGTCACGCTGCATGAATC
CAAGTCCACACTCGTCAAAGGATTCGCCCGAGATGGTCACGCGGCCAGCCTCCTCCAGAC
TTTGCAGCTTCTTGCGAAACAGCTTGAAGTGCTCGTGCGCCCTATGCTCGCGGAAGTACG
CAACCTT
>at00147
TTTTTTTTTTTTTTTTTTGAGTCGGGGCAAGGTCTGACCTCTCATGCACTGGTGCCATGC
TTGCTCCGAAGCACTGAGCTTCTCACAGCGATATACACCCACATGCTCAGGTGTGAACCT
TGCTTCACCTCTCCTGTTCTGCCGCGGGTGCATGGCCAGCATCTGGCTCCTCGCCACCTT
CCTCCACTGGAAGGTTGCGGCCTGAATGCTCTGAATCGCCAGCTGGTGCTGGCAGGACAA
CAGAGGCAAGGATCGCGGCTAGCAAGATGACAATGATGGTGGCGCAGGAAGCGAGCATCC
CAATCTCAACGTAGCCGGACAAGATCAACTTGACGCCAATGAGCACGAGGACAATGCCAA
CGCCGTATCCCAGAAATCGGAACATGCTGGACAAGATGCTCACAACAGTGTACAAGCTCC
TCAGAACAAACATGGCAAATGCACTTGAGGACAAGTTGATGAAGAAGCTGATGCTCTCGG
TCCAGCCTGTGATTGAGGATATCTTTGCAGTAACACTATCGACGGCAAACACGACATCTA
TGATCCCAAGCGTGATCACCACTAAGAAAAGCGGGGTCACCTTCAGTCTGGTCTTTCCGG
AAACACTTGTCGGCATGGGTCCTCGATCAGGAAACTCACTGACGTGCCCGCATTCAACGA
CGGAGTGAGGAATGCCTCTGTCTGTCCCCATCTCAGACATCTGCATCTCTAGCGCATCCA
TGGCGTTGCCAAGCACTGCTGGACGGGGCTGCCTAACCTCTATCTCAGGCACACGGACAA
AAAGTCTGCCTCGTCAGCATAGCAGTCATGCACAGGCAAGACTCGCGCAACGCATTGGA
CCAGCGGATTTTTTGCGGGGTCATCCTCGTCCTCGTCGTCCGAGCTCCGCAAGGTCTTGA
TGCCTGAGTAGATGAGAGCAAGGCCAAAAAAACAGCGCGCAAGAAAGCCCATACTCAACA
TCTCAGTTCCCACCCAAAAGAACACGAGACGGAGCAAGACAGCTGCACT
>at00148

CCGTAGCCATTTTGGCTCAAGTGTCATCTGGGCTCATGCCCCGTTGGCGTCGGGAGCTGC
TGTGGAACGTCCGCCGTGCAGGCTTCGGGCGTGGGCTCGTTTCGGCCAGGCTCCGCTGGA
GGAAGGGCACGCTGCGCAATGAGCCTTTGATTCGAAGAGGAGGGCGTAGCAGCGCTCATT
TTCCATTGATGTGAAGAGGACGGGGGGCGTAGCATCGCTCATTTGCCATGGATTTGAAGA
GGAGTGGAGCGTGCAGGGATTGCGATGTTG
>at00149
CAACCACTGGATCGCCTTGAGACTTGAGGAGCATGTGAAGCTTGTAGAGCTCTTCCACAC
TGCAGCATGCGTCCTGCGCGCCGTTGGCGACGAATATAGGTGGCCCGTGACTCAGGTCAA
AAGCGTCGGGATCCGCCGAGAATTGTGCGAGAGCGCCGCCGACTCCGGGGTAGAGGAGCA
GCAGGAAATCAGGCTTTGGGCTTG
>at00150
CCGTAGCCATTTTGGCTCAAGCCACCGCTGCCCAAGCGAAGTGCCAGACGTCGCACTTGC
ACCCGAAGAGGGGGCCAGGCTAGGCAGCGCTGTTCATAAGGAGGCCCGCTGACTGCGATG
CCCGCCAGCAGCGGCCCGAGCACAAAGGCGTCGGTGCACCCGCTCTTCCTGCGCGACTAC
TGCGTCTTTATCCTCATCACCCTCGCTCTGACCGTTGGCATCTTACGTGGACTCTTTGGC
CCCAACTTCCTGGAAGACCCTTACTAGCACGCTGCCGATGCATGACATTTACTCGAAAAC
GTGGGTGTTTGGCGGTCTGCACTTGCAATCTTCCCGCTGGAGGGATCATGAGGCCTGCGC
ACGCCGGGCTGTCCATCACACTGAAGAGCTCCACTACATGCCAAACTTTCGGGAGGTATA
TTGGATGAGCAGGCCGTACAGTGGGTAGTCCATGGGGTTTGTTAGCGATGCCGTCTGGAA
GGCAGGCAGACTATCTGTGCAGTTGTAGTCGTGAGGGATGCTGCAAGGGTTTGCAGACTG
TGGGAGAGCGCGGCAGCGTGTACCGGAACAAAAAAAAAAGAAAAAAAAAAACAA
>at00151
GTTTTTTTTTCTTTTTTTTGCATCTCAGAAACATCAATAACAGTCACTCTCAACCGGGAC
GCTGGAATACACCTCATTGGTGGTTGTTCGGCAGGCCAGGCCCTCTGGCCTTCCTCGACT
ACGGCAGCCCATTCAGATGTCAAAAGCCGTGCGGTCATCTCCAAGTTGGAGTGGCGCCCT
GGCTCACGGATCAGATGAGGATCCGGCCTCTGACTGAGTCCGTGGTATTCGTGGTGGAAA
AACCACCGGTGGGCAGCGTAGAGGACACTGGCGGCTCCTTGAGAGCCACTTCGTGATGCA
CTCAGAGTGAAACGTGTGGTTGCATGGCAACTGTAATACGTCTTCATCAGGTTTGACGTC
ACTAAGGCAGATGTCGCAGGTGGGGTCGAAATCGTTCGTGCCGTCATCTGATTGGCCCAA
CTTC
>at00152
TTTTTTTTTTTTTTTTTTTTTTTCTCTCTATTGTTCACCCCTTCTGCCCTCCTCTCGCGC
GAAGGGGGGCAAAGTTGAAATAATTATGCAAGGCATGCGCCTAGAAATGAGGATTCGGAG
CGCTGCTGGCCAGCGAGCGACATGCGAGCATGCACCGGCCAAGAGCGCTCACATCAAGGA
CTGCCTGGAGCACAGCCCCCGGAGCATGTGTAGTCCGGGATCCACCTCAACCTGTTCCTC
CATGAGGCCAGGATCGAGGAGTGATGGCTGCCACTGTTGGTGGACACGACCGGATCCTCC
TGGCGGGGCCTCCGGCTTACAGCTGCCAGACACGAAGGCGCCGTCGGCGCTGCAGTGCGT
AGCGGAAAACCGCTCCAGGTCCTCAGGAGCGTCGACAAAGATGTAACGCCTGGGAAGCCG
GGGACAAGCGACGGAGCTGCTCCAGAACATGAAGTCCAGGTAGTAGCGCTTGCGCGACTC
CAAGTGCCGCCCGCCGTCCTCTTTCTTGTCTGCCGGGTCGAGCTCGTACATCGCCTCGTG
NNTGGGNTCGTCCTCGAAGAAGCGCGCCATGGAGACTGCCGTAACCGATCTTGAAGTTCC
AGCGCAGCAAGTCGATGACCTGCGAGACGCCCCAGAAGCACATGGTGTATGTGTCCTGCG
TGTTGATGCTCGAGCAGACCCCTTCGATGGCTTTGGTGTACTCTGCCAGCCCCCATCTCC
TTCCGTTCAAAGTTGTGGCCGCTCTCCTTGAGGCCGCGAAGTTCACCCTGGAGCGGTGGA
GGCGGCGACCCAGCGGGAGTG
>at00153
TTTTTTTTTCTTTTTTTTTTTTTCCCCTTCGGACACTCCCATCACGAAAGCTGCTGACTGA
GCTTGTTTGGCCGACACTAAACACCTATCGCAAACAACCAAATGCTTACCTATGTCGGTG
AAGCCACTGCGCAAAGACTTGGGGCACTGTATTCCCCTCGCGCCGCCTCTTCAAAAAAGG
TGACCAAAACAGCACTACAACTATCTGCAAGCAGACATGACACCACTGCTGCTCACGGTG
TTTGCACCGCATTCTGGGTCGATCATGACCGAGCTGTCGTCCAGTGTCAGGCCTGTCGGG
CCGTTTACGACGCTGGCTGGCATTAACATCCGAGCAAAGCCTGAGGAGCAAGGGAGACGC
TCACAGCTTCCGGGCCACAAACACCTCCGCTGGCAAAAACCGCGCCCTACCCTTGTCGTT
TGCACTGCTGCCTAAGTCCTCCGACACGTGAGCACCACCAGTTCCGTGATCCTCTTCACC
CTTCTCGACAAGTTTCTGGGGCTTCTACGGTTCGCAACTCCACCCGCTCACAGTGCTCCA

TGCAGAAGCCCTGCCTCTCGCACGCCCCCGTTATGAAGGACCGCGAGTGCGCGAAGCGCT
CGCTCTCGCGCTCCACCCAGCCTTGGTCGCACTCGCCCTCCTCCATGCTTTCAGTCGTGA
ACGTCAGCGTGCCGCCCTGGCGCAGTGCGGTGGCGCAAGCTTGCAGCACCCTCTCCACAC
TCCAGACGTAGATGAAGACGTCTCCCGCGATGATGTGGTCCACTGAGCACGGCGGCTGCT
TCGAGAGGCCGACGGCAGCATCTGCCGCCAGCAACTCGTCGTAGAGCTCCTTCTCGCGAG
CCACCTCCAACATGCCCTCGGAGAGGTCGACGCCGATGAGCCGGGAGGCGAAGGGCCGCA
CCTTTGCGCCGCAGAGGCCGGTCCCGCAACCCAAGTCGACGACGACGCCAAGGTCACCCG
GCCCTTCAGCCTTAGCAAACTGCCCGATCATGCTTGCGATTATGGCTGGTCCACGATAAT
CCAACTTTCCCACCAGATGATCTTCGAAGGCGGCGCATAGTCGTCGTATAACTCCTCGAC
GGCTTGCCGATGCAGTAACGCCCGGGTGTCCATGGGTGTGGCAATGTCTGCACAACAAGA
ACGGGCTGCAACAACGTTCCGTGGACTTGAACCAACGCTCATGCTTGAGCCAAAATGGCT
ACGG
>at00154
TTGTTTTTTTCTTTTTTTTTTGTCGTCCAGCAGTGCCCCGCCACCGTCATGCATCCGCA
GACGACGTCACGGCCGAGAGGCCTGGGCTCGCTCATCGGGCATCTCGCCGTCGAGCGGGG
CGTTGCCTTGGGCAGAGGGGTCCATGGACGGGTAGTAGGGGCGGTAGATGTCGGCCGAGC
CCGCGGCCCCGGCAGAGGGCATGCCCGCTCCGGCCATCGATCCGGCAGCCGCCGCAGACG
CCTCCGACTGCTTCGTGCTCTGCGGGCGCCCCCACATCAAGTTCACGCGCTTGCCCTTGA
TGTCCAGGATGCGGTGGAGCTGGGATGCCGCCTGCTCCGCGACCTCGCGCTTGGCGAAGG
TCACGAAGGCGCAGCACTGTCGCTGCACCATGCGGATGCTAGAGACCTCGCCGAAGACGT
AGAACTTGTCCCGGACGTCCTTCTCGCGCACGCTGGGCTCCAGACCACCGAGGTAGAGCG
TGGTGATGGACTTGTCCTCGGGAGGGAGCAGCGTCATCTCCTCGTCGGCGCGGCGCAGCA
GCTTGTTCGCAACGGGGTCGTTCTCTCCGTGGAATCTGTCCCGCAGCTTCTGGTTGGCGA
GCTCGCCACCCTCGGGCAGCTCGTGCCTGAAAGGACAGTCCTGGCCTCGGTTGCACGCAC
CCTTCACGTAGAAGGTGCAGATCCTGGCCTCGTTTCTCTTGTAGTACGGGGTCATCCTGG
CCAGCCGCTGCAGCATGGGGTGTGCAGAGACCTTCCCATATGGCAGGTCATTGCCGTCGG
CTGCCTTCTGCGCCTCCTGGAGCTGGTAATCGCGGCCGACGCGCGAGGCAGGAAGCGCGA
TCTTGTTCTGGCCCTGCTCCTCAAGGAACTTGTCCCGCACTTGCACAGGCAAGCCAAACT
CGAGATCGAAGAGACAAGTCTGGCAGACGTTCTTTATCCGAGCGCAACTCTGGCATATTA
TGGTAGCCTTGTAGCGGCCCCGGGTTCCTGCCTTCCAGCGGAATATGGTGAATGGCCGCA
CACAGATCTTGCATTCAACGCCGTAGTCTTCCTTCTGCATGCGAACATAAGGATTGTCGC
CAAGGCATGTCTCACAGAGAATTGGGAATGCGCTCTTCTCCCATCCTTCTTTGTTGACTG
TTGCAGCAATGCGATATCCTTTCTCAGACATCGCAGCCTGTGTCTTACATACCCTCTTGC
TGCATCTTGAGCCAAAAATGGCTACGG
>at00155
TTGGGCTTTTCGTTTTGTAAAGCATCCCCCCGTCTCCCAAAGAGAGGCGTGAACTTCTTT
GCACTTCTTTGCCTCGACCCAACAAAGCTCAGCAGCCATGGCGATCAGCAACAAGAAGCT
GCAGAAGATCATGACCTTGCCAATCAACCAGATCTTCAGATATCTGCAGAACAGGAGCCG
TGTGCAGGTCTGGTTGCACGAGCAGGCGGACTTGCGGATTGAGGGACGGATCTTGGGGTT
CGATGAGTACATGAACCTCGTGGTTGATGACGCAGAGGAGGTCCTCGTGAAGAAGAAGAC
CAGGCGCGCCATCGGCCGCATCTTGCTCAAGGGTGACAACATCTGCCTCATGATGAACAC
TGGAGCGTGAATTGCTGGCA
>at00156
TTTTTTTTTTTTTTTTTTCGTCTAGCTCTCCACCTCTCAAATGAGAGAGAGCAGGCCCGC
AAGTCCGTGGTTCAGTCATTGCAAGTGGAGGCGCGTACTGACCGCGCGCCTCTGCAGCCC
GGCCGGCAGCGATGTTCACGGCCTTGGAAGTGGCTACGCCAGCCGACCTCGCGGGGCAGA
GCTGCGGCTCAGCCCTCCTCGTCGCCCTCGTCGAAGTCGTCCCCCTGCGAGCCCGCGTCG
TCCTCCTGAGGGTTGGGCTCACCCTGCTCCGAGAGGTCTGCCGGCTCGTCCATGACGTCC
TCGGGCACCAAGGGCAGGAAGGGCGAGGTACCGCGCAGCGGCAGCCCGCTCTCCTTGTCT
GGCGGCACGAGGCTGCCGCACTTGACGCCGTATCCCACGTAGAGGTTGGCGAACCTGGAG
CCCTGCGCGACGGCCACAGCGCCCGGCCAAATCAGCGAGCGCACGGCCAGGGCGCAGTAG
CTCTTGGCATCGTCGCCGGCGGAGTACTTGCCCTGGTCCCCGTGGGCCTTGATGGACACG
CCGGGGAGGTCTCGGCGCCTTCCGGCTTCAGCTCCTGGAGGTCCTCCTCGATGCCCTCCA
GCATCTCGTGCCCGTTCTCGTTGTCCTGCTGCTTCTGCAAGTCNCTCACCACCTCCTCGC
TGATGAGCCCCTCACCAGCAAGGGCCTCCAACTTCTCCAGGTCTGGCCACGCACTCT

>at00157
TTTTTTTTTTTTTTTCATGACACCGCAACACACACGAATCGATGACGCGCGTCGTAACAC
ATGGAAAGACCAAAAATGGCACGCTGGCTGACTCATGCAGCCGTCAGCCGACAAGAGATG
AACCCATCCAGGTCCTGGTCGGAGAGGAATAGCACGCAACTACGAGCGCCTGGGCTTCTT
GTGAGCCACACACTGCTCCACAGCTGTTCGCGTGCCCCTCAAAGTGCTGGAGCGTGCTCT
TGGCTCCCCCATCCTCTTCGCGTGGAACAGTGTCCAGTGGTGACAAGGCAGGATCGACAC
CAGTCTCCAGAATTATCCTCGTGCC
>at00158
CGCGCAGACGTGGAGGGTCCAGGCAGTAAGTCGCGCCGAGCCCCTTGGCTCTCGCAAGTC
GTTCCGGAGCACTTGACGCCATGCGCTTGCTGCTGAGCTCGCGCGCGAGCCCTGAGGCGG
CCATGGATGAAGTGCTGCCGCTGTTCGAAGCTGTCAAGAGCAACAGGCCAGCGGCTGCAG
CCATGCTGCTGGAGGCGCATGCCTCGGTCAACGCCACTCGAAGGGGAGCAGGGGAGCACA
GCGCTGCACATGGCGTGCCGCGGAGGTCATCTGCCAGCTGTGGAGTTGATGCTGGCAGTG
GAGGGCAGAAGTCAACGCTGTGAACGAGAA
>at00159
TTTTTTTTTTTTTTTTTGAAACTTCGCGATGTCCACCACCAACACACGTCCCAACGTCC
CAAGAATTCCAATGTCAAAACAATTAGAGAAGTTGGATCAGCGATAGGGGCCTGAGCACA
CGCTTAAGAGCCAGCCCACCTAGATGCTGGGTGCCATCATTTCGGAAAAGTGGCTTCGAC
ATCCACGGGATGCGGTGCATCAACCCCCAAGCTGGGATCCGGTGAGCATGCACACCAATG
GCGTGGATTCTCTGCCACTTCCTGGGAACTATGAATTTCACGTGCAAGCAGACATGTGCT
GCCAGTGAGGCTGCGCTGACAAACACGTTAAATGCCATGACCTGTCCTGCAGCTTTGACC
CTTCAAAGCGAAGGAGCCCTCGAGGGCCCTTTTCCCGCTTCAGAGTCAGTGGACTGCTCC
CGCGGCCTTGGATTCCGAACATGGGTGTGACCCCACGGGCTCTTGTATGCCTGCAGCTTT
GCCTTGACAGTTCGCAGAGCCTCTTCGTGCTGAGCCGACCTTGGCATCACACCACGCTGT
TGCCGGGCACCTCGTGCC
>at00160
AGGCTTGCGTCATGCGGCTGAAGCTCATGAAGTTGATCTCGGAGGCGGAAGTGAGAGGAG
AGGTGGAGGCGCTGGCAGCAGACAAGGGCACCTTCTTCGGCTAGAGTGGACTGATGCTTG
CAGCTTTGGCCAAAAGCTGCACCCGCTTGAGGAGGAATGAAGTCTGCCAGTCTTATCACT
AAGGTTCAGTGATCATAATGGACCTCAGCGAAGCAGCGGCATCATTGTCTGGTAAGAGAG
GTCCAGGTGCCGGAGCCGGACCAGAGCACCAGCCGTGACTCAAGTGAATTGACGCTTCTC
GCTTGGGCCAGTGGCTGCAGCTGCTTGAAGGAAAAGG
>at00161
GAGAGCAGCATTGTCGTGGGAGGAACTGAGCCAGTTCAGCAGAAGTTGCCTCTTCTGCCA
GTTTGGGTGAGCGCTGCGGCTTGCGGCGGGCATTGGCCTCTACCTCAGCCTCTTCCATGG
ACGTGAAGACCAAGCTGGGCTGGTTTCCGCTTTTCCCCGTCACGCGGCGTTTCTTACAGA
CTGCCGGCATCTTGAAGTGGCCGGGCATGGCAGCTCGACGCCCGCGCAATGATGCTGGTC
GCACAAGAAGGGGCTGATAGCGCAGCAGCCGGCATTAGT
>at00162
NTTTTTTTTTTTNTTTTTTTTTTNTTTTTTTTTTTTTTATTTAATTACGGTACGCTCCA
CTTTCCGTGCGGATTTCACCTAAACAGTCTCTCTTGGACTGAACTTGCTCTGAAATCGAG
TGCGCCTGTAAACTGGATCCAGGTATCGTTCAAACGAAGCGCCATGGGATGAAGGAGAGT
CAAAGGGTGCCTTATCGGCTTAGAAGGAAAAAATCAGCCAGGCCCTCAAACCGATCTCAG
ACCATTCCTGCAGCGATGCCTCACATCACAGGCCGATACAAGGCACTGGCAGAGAAGAGC
GCAATCAGGATGTAGTGCCACACCTCATGCAGGCGCATGAAGATCCAGTAGCTCCGCTCG
ACAATGCAAGTGCCGTCTGCTTCGCGCTTGATTCCACATGGATAGACCTTGCGGAAGTAG
TTCCCCAGTGCGCAAGAAGTCCACGCCACCGCGAAGCCGCCCATCACAGCCAGAAAGTCT
GCGATGCGAGGGCGCTGCTTGATTGCGAAGAGGCAGGTGGCGGGGGCAACCAGGCTGACG
CAGACCGCCCGGTCGATCAGCCGCGTGAGGTTGTTCCACCGGTCCGTGCCCAGGACCTGG
GGCAGAGCGCCCTTCTCCTGGATCTGTTTCGCGACCCAGTCCTCGGACTTCNCAACGGNC
AGGGCCGTCTGATCGTAGCGCCTGCCCTTGCCGTCCTCAAGCACCCCGGNCCACACGCAG
TACGCGTCGCACAGTGGGCTCGAGACTGAGACGCAGAG
>at00163
CCGTAGCCATTTTGGCTCAAGCTGTTTTGGCCCCGTTTTGAGTCCGAACCTCTCTGCCAC
TTCTGAGGTGTGGTTCTGTTCTCCGTTTTTTAACGATGCCGGCTAACTCGGAGAAGGAGC

ATTTGTCAATTGTCATCTGCGGCCATGTCGACAGTGGGAAGAGCACGACCACCGGACGCT
TGATTTTTGAGCTTGGTGGGCTCCCTGAGCGTGAGCTGGACAAGCTCAAGCAGGAGGCTG
AGCGTCTGGGCAAGGGCTCTTTTGCCTTTGCCTTCTACATGGACAGGCAAAAGGAAGAGC
GTGAGAGAGGCGTGACCATCGCATGCACCACCAAAGAGTTCTACACTGATAAGTGGCATT
ACACCATCATTGACGCGCCAGGACACCGTGACTTCATCAAGAATATGATCACAGGCGCGT
CACAGGCAGACGTTGCCCTCATCATGGTGCCCGCAGATGGAAACTTCACGACTGCTATTG
CAAAGGGCAACCACAAGGCAGGTGAGATCCAGGGTCAGACCCGGCAGCATTCACGGCTCA
TCAATCTGCTCGGGGTGAAGCAGATTTGCATCGGTGTGAACAAGATGGATTGCGACACAG
CCGCCTATAAGCAGTCCCGACTATGATGAAATTGCCAATGAGATGAAGAGCATGCTTGTA
AAAGTTGGTTGGAAGAAGGAGTTCATCGACCAAGAACACACCTGTGGTGCCGATTTCCGG
ACTGGATGGGCGATAATCTGTTGAAAAAATCCGACAACATGGGATGGTGGAAGGGCCAGG
AGATCGAGTACGGAAAGGAGACCATCCATGTCGACACCGTGTTCGATGTGCTCGACAAGC
TGTGCCGCGTGCCAGAGCGGCCTGTCAGTGCGCCCATGCGCATGCCAATCTCAGGCATCT
ACAAGATCAAGGGTGTCGGCGACGTCCTGGCTGGCCGTGTGGAGCAGGGCGTGGTGAAGC
CTGGCGAAGAGGTCGTATTCCTGCCAACACACACAGCATCCAATCCATGCACCGGCAAGG
TGTTCACAGTCGAGATGCACCACCAGCGCGTGGACTTTGCAAATCCTGGCGACTAACGTT
GGCTTGAACATCAAGGGACTCGACAAGAACAACATGCCCCGCTCCGGGGACGTCATGGTC
TACAAGAAGGACACCACACTTGGTCAGACGACGTGAATTCGACGCACAGATCCAGGTCCT
GGATATCCCGAACGAGATCAAGGTCGGCTATTCACCAATTGGCTTTGTCAGGTGTGGTCG
CTCAGCATGCCGCATTTCCAAGCTGAAATGGAAGATGGGCAAGGAGACCGGCGGAAAGAA
AATGGAAGATCCGCACTCTCTCAAGTCAAATGAGATGGCGCAGTGCAGCTTCCAGCCTCA
GCAGCCGCTCGTGTGCGACACATTCAAAAATTGCGAGGGTCTTTCTCGTGTTGCCTTCAT
GGACGGGAATGGTGTTGTCATGCTTGGCAAAGTTATTTCTTGCGAGAGGAAGGAGGATGC
GGCTGGAGGCGGCAAAAAGAAGTGATGAGATGGCTTGGCTCACTTAGAGATGGCTTGGCT
CACTTCTGTCGACTGAAGGATAAATGGCAGTTTCATGCACTGGAGCATCCTGTATAGTAA
TGGGTGCTGAAAGTCGTTCTGTGTGTTTCCTGTCTCCTACCTTTGATACTCAGGCATGCC
AGGTCCGAGGAAAAAAAAAAAAAGAAAAAAAAAACAA
>at00164
TCATCGTTGCAAGCGGAGCAGCACTCCCAGTTGAAGGTCACGCTGTCAGGCGCCACGCTG
CGGATGTCGTGCAGCAGCTCTGCCCATGGAGACGGGGCTACTGGCGCTTGCCAGTCTCCG
GCGAGGTACTGGCGGAACTCGAACACGTCTGAGCAGCGCATGAAGTACGTGCTCACCCTC
TCGCCTGGAACCGTTCTCGGCTCGATCTGCTTGAAGTTCTCGTCGTATGATCGCCCCCTCA
GAGATGGTGACGAGGACTGCGACAACTTTGTCCTGCTCCTGCCCCTTATTGACAAACAGC
TTCGTGGGAGCTCCAGTGCTCTTCCGTCCTGTGCTTTCGGGCTTGGCGGCTTTGGACTTG
CCGAAGCCAAACTTCAAGCACAAGCAAGGCATCGTCGTGAGGTGCGCAATGAAGTGCACG
CAAGACAAAGTCAGGGAACCACCAGCAC
>at00165
GTTTCATCAGGCACATCACAATCACAGCAGGACAAACGACTTGCAGCGGCCCAGAGcCCT
CCGAGCATGACCGCCTAATAACATCTTCTGCCGGGACGAGGTCAGCCAGCATGCGGCTTC
ACTCACACATCATGAGGGTCAACGCTTTAGAGCTCTCGATGCAGCATACCGCACGTGCGA
GTCCTTGGACTGCGCGTGCAGCTCACTGAGGGCAGCATGTACCGAGACCCGGAGGGCCTC
AAGCGCCTGCTGATGCGCCACCTCGCCGCTCTTGATGTCCCTGCCCAGACTGCCCCAGAG
CCTGGCCAGCTCCTGCACGCGAGCAGCTGAAGGGCCGCCCTTGTCCCTGAGGGCCGTCTT
CGCTGCCAAGCGGACGCGCACGCACTCGTGGAAGCGCCCCAGTACTTCGATTCTCCGGAC
CATGCAGGCTTGCACGGGCCGCTCGAGCAGTTCCAGCCATGCGATGAG
>at00166
TTTTTTTTTTTTTTTTTGAATCTTGTTATCCTTGCCGCGAGACAATGCTAAGCTCAACA
ACCACATGTGCTGCGATACAACCTAAAAAACTATGTCTACATTGGCTTTCGCCAACCCTC
AGCGGCTACCAATGCTCCAGCTAGACATGGCCGCCTGCATCTGGGGAGTGCTCCCGCATT
GACACTCCACAACATGGCCGCTGACATGCTAGCTCAGCCTACACGGAGCATTGTCAGTTG
ACAATGGAAAAGACTGGAGATGTAAAACCTGCCTACTCTACATGGAACTGGGCTCATGCA
TGACACAGTGCAATCCCGCTGCCTGCACTGATGGCAAAGCCCCACTTCTGGTCTTCACCG
CCTCAGGCTGTTGATCCAAGATCTTCCGCTGTTTCAAGCGCCTTGCAACAGCCAGAGGTT
CCGGTGAGAGCGTATCCCTTGACGTCCGCCGCGCGCGCTTCCGCTTCTCTGAGGAAGGCG
CAGCACCAGATGGTGGCGT

>at00167
TTTTTTTTTTTTTTTTTTGCCACAGCACACTGCAAGGGGGTTAACACTCCACTTCGGGTG
GGGTCTGGCACCACCACTTGATCCACCGCCAACCCCAATCTGCAATGGGAAAAAGCAGAG
GAGAGTTTCCTGCATCAACTGCGCCACGCACCCATGCCGGGAAAGGCTCACTGACACGCA
ACGAACCAGCCAGTTCTTCTGCAAAGGTAATGCAGTTCCTCTTGATCGGATGGTATGATC
TTGCAGGCCAGTTGTCCATGGCAGCAGCGATGACATCTCGAATCTCAGCCTCAGAAAGAG
GCGTCTCACCCAGGCATAGGGACTCCCGGTAGATGTGCACC
>at00168
CGTTTGCTTGAGTTTGTTGCTGCCCCTGCCGTTCCCATGTGGGCTCTCCTTGCCTTTGCC
GTTACTCCGCTACTCGCCGGTGGACAGTCAATAAATGCTGTATGCCTGCTAAGGCACTGT
GCCGAGCAAGTCGCAAGAGCCATGCTGCAGCCGAGGTTCCTGCAGATGACTCTATGTGAG
AATGGATGCAGCCAAGTGTAGGACGAAGACGTGACGCCCGAGAAGGTGCACTACCAGAAC
TGCACGACCAAGTGCGCGCTGACCTACGAGACGGGAAGCGGGAGACGCCTTCCTTGCGTG
TGCTATGGAC
>at00169
TTTTTTTTTTTTTTTTCTGAAAGACATTCATGAGAAAACACTCCAGCTGTTCAACTGTCG
CCACGCGCGCAAGTTGAAGACACTCCTGCCCGCGGCCGCGACGTCAGCGCTTCAGAGCGC
GGCGATGGCGGCGAAAGAGGTGATCAGGAAGGTGTCGACGTCAGGGAGAAGCTCCTGCGA
GGAGCCTGCCTGCTCCGACCTCCGGCTCTGCCCTCCTCTGGAGGCAGCTAGCCCATGAAC
TTCCGCGTCTTGATCAGCTCAATCCTCTGGATCGTCTCCTCAGGCATGTCCAAGTGATCT
GTTACGTTCAGGTTCTTGGCAACCCACTCCTGGTCCTTTGCGCTTGTGTAGACGTAAGAC
TCCCACTCCTCTGGCTTGAAGGAGAGTTCGCTATCGATCAGGACCTTGCCAAGATTTTTG
GCTTCCTGGCAGCTGCCAGCATCCAGGACATCCCACTTGTTGTTGTCATCCACCCGTTTG
ACCATGTACTTGTAGTCAGCAAAAGGGTTGGGCTGCACTTTCCTGATGTTGTCCATGAGG
AAGAGTCTTCTGCGCAGAGTCGGCTCCATGGCCCAGGACTTGGCCTGCAGCTTCCGGATG
ATGGGGTTGTCGCTGGGCTCCGGCGGCAGGTCGGTGTCCGGAAACTCGTTCTTCTCGGCG
TTTCCTGGCTTCAAGTAGGGGAGAGTAAACTGGTCAGCGCGTGCGGAGGGGGCCACTCCG
GC
>at00170
TTGTTTTTTTTTCTTTTTTTTTTCTCGGTAACGGGTCATGGCTATGCACAGGTCCGCCTA
CGCATCTTCCAACTCGTGTCTCTCAAAGTGCATGTAGAGTGGACAGAGCGAGTTGGCCTC
GCCATTGATTCTTGCATGTCCTTGAATTTGCATGCGCTGCATCACGGTGAGAGAGCAGAT
CTGTACAATCAATCCGGAGTTGCCTGGCAAGCGCACGCCAAGAGAAGGATATGCTCAGCG
GGATCAGAGCATGTAGAATACAGAGTCTGCAGCATGGTTGCCAGTGATGTCGGGGCAGCA
TCGATGAGCACTGCCATCAGAAGGCCCAGAGCGAACCCCAGAGAGCCACCAATGAGCGTT
GACACTTGGCTGCTGGCCGCGGGAACGACTGGAACCAACAGAGCAGACCCAGCGATGGAT
CCAACCACGGCGGTAAAGAAGACCAAGCACCATAGCTGCGGCAGGCCGCTGACACAGCAC
TCGCCAAGCCGCGCAAAGTCCTCAAACTCTGACATGGCTCCGGACTTCGCTGTTGACTTG
TCGACGATGCTTGCAGTGAGCCTTCTTCTCCTGCAGGCTGAGGGATCAAATGCCCCAGCG
CCGCATGCCCACTCGCTGTACTTGCGCTTTCCGCGGCATGTGCTGCCTTTCAGCCTCAAG
CTCAGCGTCTGCACGTCCTGTAGAGCATGAGGAACATCCAGGCCCCTTCGCTTCTCAAAG
GGCTCAGCAAGCTGGAGCAGCGCCGGGGCTTTCGCTGCGCCGTCCATGCATGCCATGTCC
CCCCGTGCTCAGCGCTGCTCGACTTGAGCCAAAATGGCTACGG
>at00171
TGGGTTAGAGTATCGAGCTAGCAGGTCCATGCGGCAAGGGCCGCCGCTGTTGGTAAGTTG
CGTGAGGCTGGCGCGCACCCAACGCCCTGTCAAGGCACCAGCGCCGTGCCAAGCAGCATC
AGGGCCTGGGACCACCTGGGTGCGCCTGAGATATTTTGTCCTGGATCAGCTGCCGCGCTG
ACGCGGTCTGCTGGAAGTCACCGCTGATTCGCACGGTGCTGTAGCCCATGTCCTTGGTGG
ACTGGTCAATCTTGACACTAGCACCGCTCTGGAGCTCGATCTCCTTCAGGACGATCCCAG
CTTTACCCAACAGCCACCCGACCCACTTCTGCTCAACCTGCATCTCAGTCTCCGGGTTTT
GCCCCGCCAAGGCCTCTGTGGCTGCGGCCGCCACCTTGCCAAGGGCTGCAAGGCCATCTG
CGGGGACCGCTGGTGCCGCGGAGGACGTTGTTGGCGTCACTAAAGAGAGAGAGGCCTGGA
TGCGCTGCTGAGCCAGCTGTATGGACATGGCGCTGCCAGAGACTTTCACAGTACTGAAGC
CCATGTCCTTGCAGGACTGGTCTATGCTGATCTTTGCCCCTGTCTCAGCCTCTATCTCCT
TCACAGTCTTCCCTCCGCTGCCCAGGAGCCAACCTACCCACTTTTGCTCAATCTTCATCT

CTATCGCAGAGACGGCAGAGCTCAGCTCCAGCAGCGGCAGTATGTTCCTCAGCAGGTTGG
TGGCCACGCTGTTGCCCTGCTGGCCCTGGGCACGGCTGAGCGATGCCGCAATCTGCTGCG
CTATCCCCGTGAGGGCAGCGGCACCAGCTGCTTGGCTGCCAGTGCTGCCGATGCCACCCA
GCTCGTTGCCCCCATTCGAGATGCGTACCAGACCCTCCCCGGAGGGGTTTACTCGCCTCA
GCTTCTCGCCCATCTGCTCGAAGGCCGCTGCCGTCTGCTCGGGAGTTCCGTTGATGGTCG
CAATGCTATAACCTGAAGCCTTGGTTTCTTGGTTAATCGA
>at00172
TTTTTTTTTTTTTTTTTTGATCTTTACCGCGGTTTGGAACAGGTCATGTTTCACCCAACA
CGGAACTTTGCTATGCAGAATGCCGACCTCCAGTCATGGCATTGCATTCAGAAGTCTGTA
TGCCGACCTCCAGTCATGGCATTGCATTCAAAAGTCTGTACATACACTGTCCGCACCTAC
TGTAAGTGTTCAGCTTGAACGCTGATGGCCAGCAACTGGACCTATTCATCCTCTGTAGTC
TGAACGGCAGGATGTGCGTCCCCCGACCGCCCCAATCTCTGGCCCAGGAACTCCACCTTG
CCACTTGCCAGATCATAGATCCCTCCATGAACCTCCACCTCGCCCAACTCTACTTTGGAA
CGGAGAAGTTTGCTGTACTCGAGGAGTTTGTCAATGGTGTTAAAGACGTTCACCTTCACG
GCGTGTGCCGCCACGGNGTCCTCCTCCGCCCCCGCCCCGAGCTCCGAGGCCGCCTGCAGG
GCCACTGGCGCGAGGCCGTGGAGCATGGAGTCCAGGAGTGAGGCCTTCCCGCCCCCCCCG
GCCGGGAGGGTTTCCCGCCGGCCNGCGAGCATGGGCTTTGTGGCGCCGGCGATGGCGCCG
CACCTGGTGTGTCCA
>at00173
CCGTAGCCATTTTGGCTCAAGCCATTTTGGTTCAAGACTGCCAGGATCATAGGTCAGCAT
TGCTTCTCCTTGCTACTGAATGCCGGTGGATGGCGACGGGCACAAGGCGGAAGAAGCTAT
CCAAGAGGAAGTAGACTACAAGCCGGTCTCCGACAAGGAGATCCTAGAAGATGAGAGTTT
GCTGAAGGTCTGTGGAAGCAGCTATGATGCCGCCGACTCCCTCTCATTGCTCAATGAGGC
CATCAAATCAGGTAAACTGAGCCTTGCTGATCGCAACTTGGCAGACGAGTCGTACATGAG
GGTCATCAAGTCGATTGGAATCATTGCGAAGATCAAGAACCTCTTGCCGAAGCGCACATC
AGTCCTGCCGATCACGGAGGTTGACATCGCTGGGAATACCCTCCTCTCGCTTACGCCACA
CCCCGTTACAGGTGCTCCAGAGTTGTCGGCAAAGTCGTTGCAGCCGCTGCAGCTCACTGT
GCAGTTTGTTCGCGTCACAACGGACGCAAAAGTTGTTCGGCTTGATGGCTGCAATCTGCA
GGGAACAAGCCACGACAAAAATGACAAGGTGGAGCAAGAGGTGATCCGTCTTGTGAAGAA
GTTTGGAGCGGGCAAGGATGCACGTTATGCGCGCGAGGTCTCTCTGGTCGGGAACAAGTT
CGAGTCTGAGTTTGCAAAGAAGATCATCGAGGCAGCATTCTGGGAGCGGGCCCGCGATCC
AGACAAAGAGAATCCTCGGAAGCTGCTGCTGGACCTTCGCAGGAACCGGATACGATCCCC
TCAGCAGCTTATAGACGAGCTTCGTGCGGGCAGGAATGCGGGAGGATCCATCAGTGTGGC
GACTACTGAGGACCCCGCAGAGGAGCGCGAGAAGGCACTCATTGTAGTCGACTTGACTGA
TCAGGTCAATAGGTCAGTCACGCCGGTCAGGGGTGTCCGTGAGCCAGTGACGAGACCTTC
CCGGCCTTCTCCTCCCCGGCAACGCTCGGAGTCCCCGGCTCCGCGGAAGCAGCCTTCTCC
GCGGAAGCAGTCTCCTCGCAGGCAGAGGCAATCTCCACGCAGGCAGTCCCCTCGCAGGCA
GTCTCCGCGCGGGCAGTCCCCTCGCAAGCAGTCCCCGTGCAGGAAGTCCCCCCCTGCAGC
ACGGCAGCAGTCACCTCGAAAGCGAAAGCGGGAACCGTCAAACATGGACTCCAGATCACC
AGAGAGGCAGCCATCTCCGCGTGGCCGTGGTCGCTCAGCGTCCAGGTGCGCACGGTCGAG
GAGTGGAGGCCAGCGTAGTGTCAGCCCAGCGCAAGGTGGACAGCGTGGGGGGCATGGCGA
CCGACATGGGGGACGTTCGCAAAGCCAGGTGTCATACATGTACGAAGACTCCCGGAGCCC
ATCACCCGTTGCTCACCGCCGCCCGCGGCGCCGTCGCCGCCGCCACCATCACCGCCACCA
CCGGCACCGACGCCATGCCAAGAGCCGAAGAGCGCCTTCCAAGTCCTACTCGTACTCTGT
AAGCCCCGAACCCCGCCGCCGTGGCCACGGCCGACACAAGAGACACCGGCGCCATTAGAG
CATGTAGATCATGGCTTGGGACCAATCGTTTGCGGTTTGGACTCGCTGATGAAGGGAAAG
TAAAAACAAAAAAAAAAGAAAAAAAAAACAA
>at00174
TTTTTTTTTTTTTTTTTTCGTCTGCGAGGCAGCCCTCAAAACACGATGCGCCGGCAGCCT
CCCCGGCCGAAGCTCGCTCCCGGGGCCCTCTCACTCCTCCTCCGGCGGCGGGTAGACGTA
GGGGTTGTGCGCGTAGAGCTTGCCCTGGTTGATGTAGTTCCAGATGTGGTCCCACATGCG
ACTCCAGTAGTAGCCGCCGACAATGCCACCGCAGACCATGAATCCGTAGAAGCGCGGGCT
GCTCTTGAGGAAGACCTCGTAAATCTTTGTGGAGTTGAAGGCGCGGAAGAACCAGTCCAG
CGAGTCCTTGCCCTGGCGAGCCTCGTGCC
>at00175

CCGTAGCCATTTTGGCTCAAGCCATTGTCGGTCAAGTCGCTTGCACGCCTTTCAGCCAGC
GCCCGGGATCGCGAAGATGGCAACGACTCAGTCGCGCGCTCGCCGCACGGCAGGGCGGCT
GCTCCCAGGAGCTGTGGCCCTTGTCATGATGTTGGCCGCGGCAGGGCTCGCCTTCTCCGC
GCCAGCCTTGCCGCGGGCGGCCCCTCCAACGCTGCTCACTGGACTGACAGGGCAGAGCTT
CAGTGGCCCACAGTTCACCCCGTCCACCGAGGCCCAAGACCTTTCGAGGCACGAGGCCAT
GCGCGCGCTGCAGGGGGCAGAGCGCGAGGCAGCCCCGCCAGAGGCAGCTGGAGGATGCTG
TCCGGGCTTGGCTCGATTGTCGGTCTCGCGCTCGGTGCTCGCCGCCGATTCCGCAAGGCT
GGGGGCCTCCGCGCCGAGGACGCTGAGGCAGCGGTTGTCGTTGCCGAGGAGGAGAGCGGC
CTTAAGAAGGCCGCCACGCTGTTCTGCAACCTCTTTGCCGTGTGGCTCGCCATTGCCGCC
GTCGCGGCTGTGCAGGTGCCCGCCACGTTCACGTGGGTCAAGGCCTCCTACTTCACCGGG
CTCCTGGGCTTGCTGATGTTCAGCGTCGGCATCACCACGACCATCGACGACTTCAAGGCA
TGCTTCACACGGCCCGGGGCCATCGCGATCAACTTCATTTCCTGCTACGGCATCATGCCC
GTGCTTGCATACGTCCTGGCCAAGATGATCGGGGCCGAGGGCGCAATCCTCGCCGGCCTG
GTGCTCGTGGGCTCCATCAACGGAGGCCAGGCCTCGAACCTCTGCACGCTGATCGCCGGC
GGCGACGTGGCCTTGAGCGTCCTGATGACGACCTCCACGACGCTGGGCTGCATCTTCATG
ACTCCGACCATCTGCAAGCTGGTCCTCGGCACCGTGGTGCCCGTGGACGCCATGGGCATC
GTGGTCTCGACCTTCCAGGTGGTGCTCGCTCCCATCTTCCTGGGCGTGCTCGCGAACACG
CTCGCGCCAGGGGTCTGCAAGTCCGTGGCCCCCTTCACGCCGGTCATCGGCGTCATCGCC
ACGTGCTCCTCGTGGGCGCCTCCGTGGCCAAGTGCGCAGGCCCCATCCTCGGAGCCGGCC
TCCCGCTGCAGCTGGCGTGCATGGCGCTCCACCTCGTGGGCGGCATCCTGGGCTTCTTCG
CCACGAAGCTCACAGGCTACGACGAGCGCACCTGCCGCACGGTGGCCATCGAGACCGCGA
TGAAGAGCAGCGCTTTCGGCTTCCTGCTGGCCTCCCTGCACTTCGGCGCGTTCAACGTGC
GCGTGCCGTCTGCCGTGAGCGTGGTCTGGATGGCAATCACAGGCAGCCTGCTCGCGGTCT
ACTGGAAGGGCAAGCCCACGCCAGCGTGAGGCAGGGAGGGGTCAGGAGGCGCGTGACCGG
TGCAGAGGCATTGGCCCTGCACCCGGCACGCCTTTTTCCGACGGCATTCGTTTCCGGAAT
TTGATCAGCCGAAGTTCCTTGGAGCGCTCGACGGCTCATGAAGGAGCGCTGTGCAGCGCC
AGACCGATGGCTCCCGCCGACTGGTCCGAAAAAAGCGTCGTCTTACAAAAAAAAAAAAGAA
AAAAAAAACAA
>at00176
CCGTAGCCATTTTGGCTCAAGCCATTTTTGATCGAGGTACTTTTGATTCAGAACTCCTTA
AGTCTTCTTCTTGGCAGCGTTGCAGAGCCATGGAGACACTCAGGACGCCGCTCACCGAGA
TGCTCGGCATCAAGTACCCCATCATGCTTGCGGGCATGGGGAGCGTCTCCGGCCACAGCC
TCGTCGCGGCTGTCAGCAACGCCGGGGGCATTGGCTCCCTCGGCGGCGCGACCTTTGAGC
TCGAGGGCCTGAGGAAGGAGATCAGGGAGGTGAAGAAGCTCCTGAAGCCAGGAGCCCCAT
TCGGCGTCGACCTGCTCATCCCCAAGGTC
>at00177
AGGATGCCGAAGGGCTCGGCGCTTGTGGCCTTACGTACGGTCGGCAAAGAACATGAAGTG
GGTCTCACCGTCGGAGCAGGAGACTATCTTGAAGAAGATGCCCGTGTTTGTGCCTGGTGA
CAAGGTGCACGTCTGGAGCATCAGCGGCGGCTGCTGGGT
>at00178
TCTGCAGTTTCACCCTTGCAAAATGGAGCATCCAACGCACGTTGCACCTGTCCCATGTAA
TTCGCGCAGACTTGGCCTCCACGCGAAACCAATTATCATTGCATTTACGCTGCCAGTCAT
AGGCCTCAGAGCTTCTTCCACGCCGATGCCCATTAACGTTCTTACCAGGAGCCGGGTCGC
GAGTAAGGTATGCTGTGAACTGGTCATCATGACGCCTTGCGATCTTCTCGAACATCTTGC
CTCGTCGTGAGGGGGTCCAATCATCAAATGGAACCCCGCGGTATGCAGCGCTGGTGCTGA
CTTGGCAAAATCTGCTGGAAGAACGGTATACATGCCAAGATCTGAGCTGAACTCTGGAGA
CACAGAGCAGAGGTAAAGGTGAAACCATGCCTGCAGAACATGCTGCCCTCCTCAATGCGC
TCGGTGGTAGCCAGAGCGCACAGAGAAGAACTCGCTCCA
>at00179
CGGTGCACGCCGTTTCGCAGGCCGCTTGCTCGGCTTCTTGGGCTTTCGCGCCTCCCTTTC
TTCCTTCACCCTCGCCAGCATCTCTGCCCTGAGCACCTCGAAGTGCTGCCGCAGTTCATC
TGACTCACCGTCCGCTGCCGCTTCGAACGCGTCAAACACCTGTCGATGGAAGTCCAGAGC
CGTGGCCAGCACCTGTGTGGTGGGTGTCACTATCCGTACGCTGTTACCACCTCTGTCCAC
AACAAAGCTGAGGCGAAGCTCCACCTGCCCGTCGGCTGCACGGCTCCGGGCCATCTCTGC
CAGCGCTTCTTCTGCAGCTCTTGCAAAGGTCATGCCGCACCTCATCAACTCCTGCAGGCG

GCGCTTAGCCTGCACAAGGCTAATGTGCGCCTCGAGGACAGTCTCCAGAGTATGGATTGC
C
>at00180
CACCTTCCTGGACATTAATcCGTCTTGTATGGCCGCCTGTCGTTGGTGCCNGGTGCCTGC
CTCGCGGaAAAAAgGGGTaCAAAGCCAGGGACAGCGAGACAACGCCGGCGCCACCGGGAG
CTGCTCAGAGCTCCAAGCGACACAGGGCCTGGTCCAACAGGAACTTGGCTGCTTCGTGTG
TCCTGAGGAGACCAAAGGCTGCATGCCGGTGCCGACACACGCGCGATGTCATGACTGGCA
AGGACTTGCCGCATCTTGAGGCACTCATGCTTCAGACAAGCAGG
>at00181
ACGCCATCGCCGTCCTCGAGGCCTTCTACAAGGACAGCGGCATGGTGCCCAAGGAGTCCT
GGGAGCTCCTGCAGCGCGGCGTGACGCTGCCCGCGGAGCCGAGCACCTGGGACGCCTCGT
ACACCGGGACCACGGATCCCAAGGCCCAGCCTGACGGCATCATCACGGTGCTGAAGCGGG
TCTCCGCGGACTTCGCGTCGATGGAGGCGCAGACGCTTGCGCAGGAGGAGTCGGACCA
>at00182
TTTTTTTTTTTTTTTTTGCACGCATAGTCCTTCACATTACGTATTGCTTCTCCGACTTC
AATGCCTTCTGTGAGACGACATCGTCACCAGAAACTGTACACCAGGTGCTCAATGTCGTC
AGGACTTGCGATGTTGTCAACTACTGAGGCTTGCATTCATCCTGTCCAGGACGAGGTCGC
GGATGTGAATGTTGTCCTCGGTCTCGCAATCGGGGTGCAGGCCGCTCGACGTGCCATTCA
CCTCCAAGATGTACTCCTGGCCAGTGCCCTCCTCCACGATGGCGTCAACCGTCAGGATGT
CGAGCCCCCCCAACAGCTCGGCCGCAGCCTCTGCCCAGCCTCGCCAACGCTCGGTGAGCT
CAACCTCCTCCATAATTGAAGTCCCCGTGTTTGTCTTCCACTCACCCGAGATGCCGGTCC
GCCGAAAAGCACGATAGTGCTCGCCGATCTTCTGAATGCGCAAGTCGACCTCCCCACGGA
TGAAGGGCTCCACCATGCAATGCTCCTGCGGCATCATGTGGAGCACGGATCGGAAGTCGC
TCATCTGGTGGTGGTCGCTTATGAGCATCTTGCCGCAGCCCGCGTGCGCCGAGCCGACCT
TCACCACGGCGGGGAAGGTGTAGCCGTACATGAGTGAGCGGTGCGAGGAGGCAAAGTGCT
GGGGCACCACGGGGAACCTCTCCTTGCCCAGCCGCCGCTGAAGTCTGTGAAGCTGCCCCT
GCACCAGTGGCCGCTCGCAGCAGAGGAGGATGGACTGCAGAGAGTTCACAGATGGGGCGT
CCGCAAAGAGCAGGCCGTTCAGGAGACCTCGGCCGTCAAAGTCGGGCGTCTTGACCTCGT
TCCTGACCAAGAGGAAGTCTGGCTGCACGGTCTTTGGGTACCTCTCCTCCGGCTGCCTGG
AGGCACTCGTGGCAGGCTTCCGGACGTCGACACAGAGGCCTGCAGAGCTGTAGGTGTCTG
CAAAGACCTGAAGGTCGTCCCATCCTGCCTGGACGACGCGCACCGGGCGTCCATCGCGCA
GCGTCTGACCCTTGAACAGCTCAGGCCAGTCATACTTGTCTGCCTGCACCACTAGGAGGG
TGGCCGGCTTCTGTGCGCTGGCCGGCCTCGTCTGCTCGGGCTCTTCAGCCGTGAAGAACC
TGCGGAGCATTGCAGGCAAAAGGGGGTGGCGCCAGGCTAGAGTTGATCCAACAGCTGCTT
GAGCCAAAATGGCTACGG
>at00183
TTGTTTTTTTTCTTTTTTTTTTGGGGACCAAGCATGTGCTTGAACGCAGTCGAAAATCAG
ACACATCCACGTGCCAACCAGCAAGAGCCAGCCGAATGTGGTCAGGTGAAGAAACTTAGG
CGAGGCAATCCGAAGAATGTGGGGGATTCAAATGCAGATCAACATGCTCAAAGAGTCTCC
ACGATGAGCCCCTGTCCGATGGTCTTCCCACCCTCGCGCATGGCAAAGCGCATGCCTTTC
TCGATGGGCGTGCCAGCGATGAGCTCGCAGTTGATGGTGACATCGTCGCCAGGCATCGCC
ATCTGCACCTCGTTACCATCAGAGCTTGCCATGGTCTCGATCTTCCCTGTCACGTCACAA
GTGCGGAAGTAGAAGACTGGCATATATCCTGGCATGACCGGATTGGAACGGCCACCCTCG
TCCTTCTTGGACAGGTAGATGTTGGCCTTGAACTTGGTGTGTGTTGAGGTTGCCCCCGGT
GCGCAGATGACCTGCCCACGCACAATGGCGCTCCTTTCGATACCTCGGCAGAGGAGACCC
ACTGTGTAGCCTGCCGGCCCCTCGGGGAGGTCAGTGTTGAACATGCGGATGCCAGTGACC
ACAGACTTCTGGGGCTTCTTGCCGCGGCCAAGGATCTCGATCTCGTCGCCCTTCTTGACG
ACACCCTGCTCCACGCGGCCCGTGGCGACAGTACCACGGCCAGGAATGGAGAAGACGTCC
TCAACAGCAAGCAGGAAGGGCTTGTCAGTCTCGCGCTCAGGAAGATCAACGTACTCGTCA
ACGGTCTCCATGAGCTCCAAAATCTTGTCACACCACTTGTCATCACCCTTCTTAATGCCA
GGGTTTGCCTTCATCGCCTCGAGGGCCTGCAATGCCGAGCCCTGGATGAAAGGAGTGTCA
TCGCCGGGGAAACCGTACTGGCTGAGCATCTCACGAGTCTCAAGCTCAACAAGTTCCAGC
AACTCCTCATCATCCATGACATCCACCTTGTTCATGAAGCATACAAGCTTGGGCACGCCA
ACCTGCTTTGACAAGAGAATGTGCTCGCGCGTTTGGGCCATGGGGCCGTCTGGAGATGAG
ATAACAAGAACACCACCGTCCATCTGGCAAGCACCAGTAATCATGTTCTTGACATAATCG

GCGTGGCCGGGGCAGTCCACATGGCAGTAGTGACGAGTCTCGGTCTCGTACTCAATGTGG
GAGGCGTTGATAGTGATGCCGCGGGCACGCTCCTCTGGTGCATTATCAATCTCCTCATAG
GTCTTTTGCTTTGTATCCTGGGTAGTGCTGAACTGCGCACAGACGAGCGAGATGGCAGCT
GAGAGCGTGGTCTTCCCGTGGTCAACATGCCCGACTGTGCCAAGATTGACGTGGTCCTTA
GCATGTCTCACAATGGCAGCGGCACGTGTGGACCGAGACCTTCTTCCGGCACTGCCGACG
GCCATGACACCAGCTGCAACCAAGGCAAAGGTGCCAACTGGCAGTAGCCCTACTGCTGCA
CGCTGGTCTGCAAAACTTGACGCACTGCCCTTGAAGAGCACTGTTGACAGCTCTCCAGTC
TCAACATTCAAACGTCCCGAAGGACTGCCAGGCAGCGAAAACGCAAATGAGGGGATTCTC
AGCAATACCACAAGGCCAGCAAAGCCAAGAAAGCAGGCTGCCAAGTGGCCGCTTGCTTTG
CGTGCCATGCCTGAACAGCGAGGGCGGGTGGTCCGCTGGATGCAAAACCCAAATTGCCTG
ATCTTGAGCCAAAATGGCTACGG
>at00184
GACCTTGTACGGGCGCGTCGTGTGCTTGAAGCGCTCCGACCCCATCCACGCCGCCATGCC
CTGCACGCGCGTCGCGAAGTCGGCGTCCTCCGAGGCGCTCGTCCCGCGCATCTCCACGCC
GCCGGCGACCGAGGCATGGCTGGCGAGGATGTGGCGTTGGACCGTCAAGACGCGGTGGGC
CATGCTGGAGAAGAGAGGGGCCTGGGCCTCCGGACACGCGCTTCGCACCCTGAGCAGAAA
CAGCTTGAGCCAAAATGGCTACGG
>at00185
TTTTTTTTTTTTTTTTTAACTGGCATGTGGTTGGCCACCACTGTCCACTCAGCCGCATG
CCAGGAAGGAGAAGACGTTCAGACCTCCAAATGGAGATAAACTTGAAGAGGGCTGGGCCA
AACTCGACAGCGCACAGCGCACACAAGGACTTTGCGTGGCGTTCTTGATCAGTTCCTACA
AATCACATAAAGGACATGCCAGATGACAAACTGCCAAGTGGCGTGGGCTTCCCTCATCTG
AGGCGGCCGGAACTGCGGATGCTCTGGTGCCCGCCACGAGGTGCATGATGGCTGGCGCGT
TGCCCCTGCTGGGCATCGTAGGTGCTCGCGCGCCCATGCTGGCCGTGGTGGCCACTCATG
CGGCCAGTGCTGTGGTGAGCCTGATGAAACGAGTGGGCGACGGCGCCGCTCTGTANCAGC
AGCTCTATAACCTCCCGCTTGT
>at00186
TAGTCCTATTTTTGGTCTCAAGACATTTGTCTCCAGCTTCAGCGGTCCTCCTTTCAAGGC
CACGCCCCTCCNNAGTTTTTCCagGTAcGCCATTTTGNNGCtNTCAAGcAtCNAgTTNTT
GTCTTCCACGCTCTTCAGCCGGTTCGCTCCCCTTATTCTAAGGTCCACAGCGCCACGCGG
GCGCtACTTAGGGACTTGgACTTCCAGCGTACTAGTGGAAGGCAATGAACGGAGAAAGGG
CAAAAGGAAGGACCATACGACGGACCCAAGGATCGGACGGATGGCACGGCTCCTGGGCAC
TTCGTCAATGCCGTAGCTTCCCCGTTTGCTCGTTGGTCGGGCCGGTGGGCGCGTAGCCCG
TTGACGAAGTTTACAACGTTTCCATCGGTTGCCGGAAGGGATCAAGCGAAGAGACCGTCA
AAGTCTGCCGATCTGACGAAGTAACAAGGGACCAATGGTGAGTAGGCAGGGAAGGGCGCG
TACGACCCATGCATCAACGGATCCGCCGGCCCGCGCCCGAAAGGTGGACTACGGTCGTAG
GGGCCTATGGGCAGCCCCTGACGACGGCAGTCTCGAGGGCCGTGAACTGGTTCAAGATTG
CCTGTTGCAGAAGAGGATTCAACCCAAAAAAAAAGAAAAAAAAATTGGGGGGCCCCCCCT
GCC
>at00187
CCGTAGCCATTTTGGCTCAAGGCCGTTTCAGGTCAAGAGTAGCGTTTCTGATACCTTACC
AGAAAAGGTCAGCATCGACGTTGTCGGCCCCACTTAGATGTGGAATCTGCTGAACCTGCT
TGCCGTGCTAGTCCATGCCCAGGGAGCAGAACACAGGACGCTGCCGCTGAGCGATGCTTC
GGTACTTATTCATCGCTTCACACTGGCCAAACCAGTCACCACTGACAAGAGGCGTGAATT
GGATCTGCAAGATGAAGATGCTGCCATGCCCACCGGAGCCGAGGACCCCAATCCGATGGA
GGCTTGGGTAAAGGAGCAAGCTGTACGAAAGAAAAAAAAAGAAAAAAAAAACAA
>at00188
TTTTTTTTTTTTTTTTTCCACTCTCACTGGCTGCCACAGCTGTGGGTTCTTTGATGTTT
GCTGAACCACCCTCCTCCTAGCGCATGCATGAAAGCTGACGGCAAACCCCTGCGAATATC
GGCTGTACAAAACATGCATCAGCGGAAGGCCTCCACCAAAGGGACTGGACCGGTGTCAGC
ACTACCTGCTCCCAATAGCTGTACAGAAGCTTGCTCAAGATATTATGATAACGCCCGTTG
CAGCTCTCTGCGCACGGGCGAAGGAACAAAAGGCTGTACCGCAGCACATCCCGCTCAGCT
GCCTTCACTTAGGCGGCTCCTCAAGCGTGTGGTCATTATGTTCATGTAACCGTGCCCTCT
TAGTGGCCTCATTTTCCTCCTCGTCCTATCGTTGTGCGCTCAGCGCCCTAGAGGCTCAAC
GCTGGTGTGTGCAGCGCAACTCATCTGCCCTTGCCAGACAGCAGGTGACAGCAGTGGCAT

AGTGGCGACGACGTCACGTTCAGAGTGCTCATGAACTCTTGCAGCAGGCTCAGCCTCTTG
CGCTCACAGTGCACAAAGCCCCCGCATCCAGTCCTCTGCATAACCACGCCAGCGGTCCGG
CGACATGCCCTTCACCGCGAGCCAATAGTCGGAATCTTGCCAGCCGTGCCCGCCATCTGG
AAAGGCGTACATCGTGACCTCGTGCC
>at00189
ATGGTCAGCAGCATCGTCGTCCCGCCCATCGCGACACCGAGACCACCGGCCAGCCCTTCT
TGAGAGCTAGCATCCGTGCCGAAAGACAGGATCATGATGATCTCTGGCAAGCCCCCAGAA
GTGGCGTAATGAGAGTGTCCTGGTACTTCTCAAGGCTTGGGATGAGGTTCCCGAGCTTCA
CGGCGTCATTCATCTTGGTCCCTGCGCCATAGAAGTTGTAGAGCACGATGCCCCCGAGCA
AGCACAGCTGCTGGAAGCTGGCCTGCTTCGCCGCGCAAGACTCGGGGAAGATCATTGTCG
AAGTGGAGAGCGCCCGTCCTTTGGGCTAGGACCCTCGCGCTGCCTGGAGCTCAAACAG
>at00190
TTGTTTTTTTTCTTTTTTTTTTCTTTTCACACAATCACTGTGAGTCATGTCATTGCTTC
CGAAGGTCAAGCGAAGCGAGAACTCGTTCATCAACTACACTGCAGACAGGCCACGCCACG
AGCAGTAAGAATTCTGCCTACCCCCAGATGGTCAGATACTGCAGACAGGGCTCGTCAACC
TCGGAAGGTTCACATCACGCCGCTTCACTCTCGTCTGGAAATCGCGGGCCCATCGGATCC
TCGTAGTCATCGTCCCAACCGGCATCAAGCCCTGAGTCTGGCACGTCTTCTGCCAGGCCT
GGCTTGGAGTTGAAGTGCCCTACTGCCCTGGGGCCATCCAGCTGCGGCACATACGGGGCT
GCCGTACGGCATTGGCGCAGGCTGTCCCAGTCTAGCGCTGGCTGTCGGAACCAAGCGTGC
CCGTGCAAGCTCTCGGCCCCTCCCTTCAGGAGCGGCAGGCGCTGACTTGGCACTGGCCGG
CAGAGACGCCCGCACCAGGTCCACCCAGGGGCGCTCGTGGCC
>at00191
TTTTTTTTTTTTTTNTTTTTTTTTTTTNTTTTTTTTTNTTTTTTNNNTNTNTNTNNGCN
CACGTTGAAATGACGCGGGCAGAGCGATACGGCCAAAAACTTTGTGCTGCACGCACGACA
GGGAGCGCTCCACACTACCCATGGGGAGAAGGAAGAGGCCACTCAGCCACGCACCGTGGG
CACGGCCCAGTGACTCGGACGCGTGGGGACTCTGCAAACTGAGAGGCCCATGCCTGCCTC
ACACGAGGGTCTTGTTGATCAGATTGTTCATGAAGTCGTCCGGCCTCTCGCCCCGATCCT
CGCGCTGCAAGGGCTCCCCACAGGACTCGAACCAGGTGACGGCATGGTTCCGCTCGCCGT
TGAATGTCTTCCGCCCATGAAGGACGCGGTAGTTGTCCAAGAGCACTACATCGCCCTCTT
TCATCATGACGGAGATGATGTTCTTCTCGCAGACCTCGTTCACATGGCGCACATCCTCGG
GACTGATGCGGCCAAGGTCGCCGTGGTAGACATCAGTCATGCCGACCTCCGGCACCGTAC
AAGGCCGGCGGTCACGGAGGTAGCGGCTGTGGTTGTGCATGTTGCAGAACCAGACGGGCT
GCCCTGTCACAGGGTGCCTGTTGATCGGGGACTGGGCGTGCTCAATGGCCTGCAGGCGCC
AAGGGTTGGAGGGGTCGGCGCCGTAGACCATCTCCAAGTCCATGTCGAACTTGGGCGCCA
CCATCTTCAAGATCATGTCCTGCAGCGAGTCCTTGACGCTGCCTTTGACANACTCAGGAA
GCATGTCGACAAATTCANGTCCAGGTTGCTGACCGAGATCCGGACTTTGCGCTCGTACAA
GCGCCGC
>at00192
CTCGTCCGCGTAGGGAAAAAGTCCATGTTGAGTCTTTTTCGTTTGTTCGTCCTGCGGGCG
AGTGCGGGCGAGGAGGATGTCGACGACTACGAGAGCGGCAGTGACAGCTGGGTCCCTTTT
GGGCGCTGGGGACCTCGCGTCGGCATCCCCTTGCTGAGTTGTGCGTAGCGTAGCCGTGCT
TCATGTTACGCACTTTTTTGTACCCTGGGGCGTGTTCTTGCAAACGCAATGTTAACACCC
CAAGGTGAAGCTTGCGGGGAAACAGGTAGAGAGGAAAGACTTTCACCCTCCAGCCACACG
GAGCTGGGTGCTCCTTGCAGATTCCATGTGTGGCCTCCAGCCACACGGATCTGGGTGCTC
TTTGTGGGAACTGACATACTTCTCTGGAATAAAAAAAAAAGAAAAAAAAACAA
>at00193
GGCACGAGGCTTGCAACTCTGGGCTGGATTGCTACTGACCTTGGGGTCCGCTTTCCGGGC
GAGGTCTTTCAAAACGTTTCCACCGTTGCGGCACACGACAAGATGGTGGAGGCCGGCATC
ATGGGCCCCTTCCTGGCGACGATCGCCATGTATGAAATCTATGGTGGGTGGCTGTGCTTG
GAAGGCTTTGAGGGCAACGTTAACCGTGAAGCAGGCGACTTCTTCCTGGGCAAGAACTTC
CTGCCGAAGGATGAGGCTCAGGCCAAGAGCATGAAGCTGAAGGAGTTGGAGAACGGCCGC
CTCGCAATGCTTGCTTTCAGCGGCATTGCCACACAAGCTGTTCTGACGGGCAAGACCTGG
CCCTTTTTTTTGAAGAATGTTTCATTCATGAAGGCACGTGGTTGGCAAGATGAGCAGCGG
CGGTCACCAGTCACAATGTGCTGACATATACTGGAGCAACTCGAAAAAAAAAAAAAAAAA
A

>at00194
CAATGAATACTCCGCCAACGTGATGTCTTCACAGGCCAAGCAATAGTTAAGGAGAGAGCC
ACTGTTTGTGCCCAATCACAAACGGCCACTCCTCGAATGGAGTCTGGCGTCACGCAGACC
AAAGGACAGACGCCGGCCATCACTCGTGCTTGCAATCACGCGCTACGCATCAATACCGTC
AAGTGCAAAGCCAGCGTTGCTGTGAGGGCGGCGTCTTTTTTGTCACTGGACNGTATTGTG
CNTCAGNATGGCGTCNCNCATGTACNGCCGTGCANATGGANCAGTCGTGACAGCCGAGCT
TNCGCTCGCGCACAGTGCCCCAGNGTAGTNGCATNGGACCGTGCGGTGCCCTCACCTCCC
CATGAGCTCCTTGACTTTGGCTAACAGNGCCTTGCCTTCAGGGCCCTTGTTGAAGGCCAC
AAAGCCTGTGATTCCCAAGGCCTTACGNGCAGCGGCNGTAGCCTTTGCCCAGGGGTTGCT
CTTNCCCCGNGCTGAGAGCTTCCTGGATACAAACTTGCCCTTCTTGTTCCTGATGATATC
CGAAGCCTTCAGGCCACCAACCGTCTTCTCCCTTGCACCTTGTAGACCATGGACTTGGA
>at00195
TTGTTTTTTTTCTTTTTTTTGGTCGGCAAGGGGCCCGCAACTTCTGTCAATCCTGACAG
GGGGGCCAGATCATCAGCTGTGATCCCGGCCATAAGCAAAGCAGAGGGGAAAAAGGAGGT
CCATTACCAGCCGGATTGCACCGGTGCAATCGTGGTAAAGGCTAAACCTTTACTTCCTCG
AATCATGAGCGCACTCAAGAGCCTGCCTGTCTGTCTTTTTTATCCGCACCTCTTCGACAG
GATTCACGTGCTGACTGGATGGGGACTGCGGAATCTGTTACCACCATTGCTGCCCCCTCC
CAACCGA
>at00196
TTTTTTTTTTTTTTTCTGCATCACACGAGCAGGAAGCCACATCTCGCAGATCCTGACAT
GGAACCTCGCGTGCTGCATGCCATCACCGAAGACTAGCAGACCTGTGGGAGAAGCATTCC
TCTGAAAAGACCTCACTGAGTAGGCAAGAGTCTGGCATCACTGTGAGCTGCAGCCGTCAT
CGCAACTACCTCTACCGGCATTGTGAGGCGGCTAGCAAGATGCCAACTAAAAACTTCCTG
CTAGATGCAGGGATCTGAACAGCCCTGATGGGCACAGCTGCGCCGAAGAATAACCGGCCT
CAGCCGGCTGCACCTACATCCTTCGGCTGTCTGCGCACCTCAGCCTCCGCTTCCAGCTGC
AGCATCTCCGAAACGATGCGCGTGCAGGCGGCTTCGCCGACAGGGCCAAAGTTGATCTCG
CCGTGGCCGAGGTCCGGGAACCAGAGGACCCTGAGCGAGTCCAGGCGGCGCCGCTGCTTG
TAGGAGGTAAGGTACCGCCTCACGGAGTGGCTCGGCACGATGGAGTCGAGGCCGCAGAGG
ACAACCAGTGTGGGCCCCGGCAGCTCCTCCGGCCAGAGCTGGTTCTGGAACCAGAAGAAG
TTCCGCGAGAGGCTGTGGGCGATGTAGAGCTCCTTGGCCAGGAAGTAGTTGATGAGCAGT
TGCGTGGGATCGGTGGGCTGCCTGTACATGAAGTTGTAGCACACATCAGGCTTGATGAGA
AGGAAGCAGACAGGGTCGATGAACGTCATCATGCTGACAAATCTGGCGCTTCCCTTGCAT
>at00197
TCACCGACGCGACTCTGAATACTCGCAGTACCTGCAACACCAGCGGTACTAGACACGCGC
GTCATGCACAAGTGCATCAATGTCATAACTTCCTACATCCTGTACTCCGGCGCGTTGCAA
ACCAGATGCGAAGCCCACCCCAAAGGCTCACCTGCAACTGCCGCCGGGCATGCAGAGCCA
TGACACCCAGCCAATGATTCGAATGGCTGCCCCCCTCGACTCTCTGATTGCAAGGGATGT
AAACATACTGAATGCCGCCCGTGCAAACAGTGAGTCAGCAAGCTCGACGGCTGGGCATCA
CCCTGTTCTAGGAAGTCCATGCCTGCTCCTGCGCATGGGGCCTCAGGAGGATGACCAAAC
AAGTAATGCTTCACATAAGG
>at00198
GCTCTTCATCATGCCTATGGCCTGCAGCCGGTCCAAGTCCCAGATCCGCAGGATCCCGTC
CTCGCCGCCGCTGAGGGCGCGCTGAGACTGCCGATCGATGTCGGATGGCACGACAGGCAG
CGCCCCTTGTCCAGCGAGAGGCACTCCGAACATTCGCCAGACTTGAGGTTCCACAGGCGC
AAAGTCCCGTCGTAGCAGAAGGAGACCGTGCGATGGGGGAGCGCCGCCGAAGCGCTCATG
TCCAGATCGGGCACGCGCACCAGAGTGACCAGCGCCGGCGTTTCGCAGCGCGCGCCCACA
TCCCACAAGCTCTGCGCGTCTTCCATCAAGTCCGCGCCGAACGCCAGCCGCTGCTCTCTG
CTGGGGATGCCCAGCTCCTCTTCCACTCTTCGCTTCAATGACTCCACCGATGCAGCGGCT
GGTAAGTAGAGCTCGGTGACGATGCCGGAAGCTGGAGTGGTAA
>at00199
TTTTTTTTTCTTTTTTTTTGATTGGTTGGCTGATTTTGATTGTTGACCGAGGTGGAAATC
CCACTGTTCAGAAAAAATCGGGACCACTGCTCAGACTCGATGGCTGCCTGAAACAGCTCAG
CATCTTCAGCCACACATTGAACAATGGAGGTGTTCGCAGTTTGGCGCACACCATAGTGCA
CTTCCCAGTCTTCACTGCTGGCTGCGCTGGTGAGCTCTGGAGAAATGAGGCGGCACACAA
AGGCCGTGCACTTCGTGTCGGGTCGGTCCCCATGGACCTGTGTCGAACTCTCGGCAATGT

AGCCATATGTGACTACTCTTACCTGCTGCGGCTGGCCCCAGCTGACGATGACGTGGGCAG
GCTGGAGGAAGACGGGCACCAACTCCAGCACTCCGGTAAAGTGGAGCCCAGTGCTGATTG
CAGTGC
>at00200
TTTTTTTTTTTTTTTTTGGCGAGACTGCCAATCGCAAAGTCACGGCTGCAAACCTGCAA
CGACGCTTTATGCGGCCATCCATTGGCTGTCTCAGCACGTTTCCACAAGATTATCCTCTG
CAAGGTGTGTGGCGTAGAAGAGGGAGAGGTGAAAAATCATCAGCACTGGCCATGATGATG
GCGAGAAGCGATATGCCGGGTCGCAAGTACACACTTGGAGTCGGCAACACAAGCGAAGGT
TGGCTCGGCCTTGACAGTTGCAGACTCCAGCAGCCGGCGAATGCTGTGAAGGATCCAGCC
AGGGCTGGTGCACCCTCGGCAAGACGTCTGTAATGGTCCCCCTCCCTCTCCTCTGCGCTC
ACTCGTAGGATGCAACCTTCTTGCTCAGGATGTCCTGCAGAGGCCCAAGCATCTTGCTGA
AGGACACACGGCTCATCGAGAGGACCTTGGCTGTCTCGCTGCTCACGATGACACTGGCGG
CGCGGGGCTGGTTCTTGAGAAGCGCGAGCTCACCGAAGTAGTCGCCGGGCCTTGTAGTCC
ATGTACCTTGACGTCGTTCTTCGTGGCATAGAGAGAGCCGTCCTCCACGATGTAGAACTT
GTCGCCCGGCTCGTCCTGCTTGACAATGACATCGCCCTGGATGCGCGTCTCCGACTTGAG
GGCGTCGGCGATCTGCGAACGCTCGTAGTCGCCGAGCGACGAGATAAGGGCAACGCTCTT
GAGGAAGGCGTCGTACTTGTTCCGCCGCTTCATGGCAGCATCCTTCACAATGTGGTTGAA
GGTTTCACGGTCGAGCTGCCAGCACACACATTCATCCTTTGCGATGACCGTGGCCGCGCG
TGGGCAGTTGTAGAGCAGGGCAAGCTCTCCGAACACGTCGCCGGGCACGCATGTCTTGAC
CACCTTTTCCTCTCCATTGATCGTCTTGCTGCAGTCCAGCGAGCCCTTCTCGATGACAAA
GAGGTAGTCTCCGTTCTCTCCCTCCACAATGACCTTCTTCCCATTCTCAAATGTCACCTC
CTTCATGGCCATGAGGATGGTCTCCATGTCCTGCGACTCCAGCCCCGAGAACATGAAGCT
CTTCTGCAGCGTCGCCTTCAGCCGCTCCTTCTGCTCGTCCGTCTTGCTGTGCGTCGGCGC
CGTGAACTGCTGCTTCTTGTTCCACTGGCCGTACGCCTCCGCACTGACGGACTGCCGGGC
CCGTCCCATCTGCTGCTCGGGCTTCTTGAAGCTCTCGGGGATCTCGTCGCAGTCCTCGTC
CTCATCGTCCTCCTCATCCTCGTCCGCGTCCGCGGGGGCATTCTTGTCCTCCTTGATGAC
AGTGCCAGCCTCTTCGAGGCAGCCTGTGAGCTGCTTGAGCTCCTGCTTCAGCATGGCGTT
CGTCTGCGCGATGGACTTTGTGCTGGTCATGGCGACGCCGCTGCGCTTCCGGAGCCAGTT
GATCATGAATTCTGG
>at00201
CGAAGCAAGGTATCGCGGAAGGTGCCGAGAAGAAGGCAACGGCTGAGGGTGATCTCCAGA
CCACATCAGCAGAGCTCAAAGAGGATACGGTTGCTCTTGCAGACCTCCATCACAACTGCA
TGACAAAGGCACAGACGGTAGAGGCAGAGACGAAGAGCCGGGGGGAGGAGCTCAAGGCTC
TTGCGGCCGCAAAGCAGGCCATCACGGACAGCACTGCCGGCGCAGCAAAGCTCTCGTACG
GCTTCAATCAGGTGGCGCTCCTCCAAGTGTCCCGCGCTGGACTTGCTTCCGGCGCGGACC
TTGCGAACTTCGAAGCCGTGCGGTTCATCCGCGACCTTGCGCACAAGCACCACTCGTCTG
CACTTGCACAGCTTGCGGCTCGCGTGGCTTCAGTCAC
>at00202
GATTCAAGCGTTATTGGTGTTCCAGGCACAGCCCCCCGTGAGAATGAATCATGGCAGAGA
GGTCCTGGGAGGTCATCCAAAGTCTGACAGTGGACGGGATCCCTGTGCAAATTGGTCAAG
AGCGCGCTTTCGGCGTCCTTACCCGAGCACCTCTGCCTGGGTGCGTGCGTGCGAGAGCAC
CTTTTTCAGGATGGCCGACTGTTCTT
>at00203
TTGTTTTTTTTTTTCTTTTTTTTTTTGTTTCTTATCGCAAAGAGAGGCACAGTCAGATGC
GCACTAAAACGTGTCCACTTAAAACCTCGTCTCATGAGAAGCGCACGCGAACCCGCATGA
GACGTTCCCAGTCAGCCTCCGGATGGTTCATACGTATGTCCGTGCTCACCTCATGCGGCT
CGCCATCGATGTGCACATACCGCGCGCCGCCTCGAACATCGCTGTCGCTCTCCGGCTGTG
CCCGCAAACGCTCTTGGGCTGGTCCCTCCTTCAGAGCTGAGCCTGGCGTGGGCAAGCCAC
GCATACCGTACTCCCAAGGCCTCAGTGCATCCACAGCCTGGGTACCTCCAGAGAGTATGT
CGGAGACCGGGATCTCAAACACTGCCGTGGGCCGCTCCTGCCCAGCTTTCTGACTTCCCT
CCTCCTCTTCGGTTTGCCTGCACTGGCAGTGGATCACGTCTCCCACCACCGTCCAGGTCC
CGCTCCGAGACTTCGTGTTCGCAAAGTGCACGGCGGTGATGACGCACTCCGTCTGCCACC
TGTGCTGTGGGAGGAACTTGAAGGTGTAGAGCACCTGCTGCTCGAAGTCGGTGCCCTCCG
CAAGCAGGGAGCTGTGGCGGTAGCTGACGCAGCCTTGCCGGTGCAAGGTGGCTCCGAGGC
AGATCCTGGGCTCCAGATTCAGGGACCTCCGAAGGAAGGCTCGCCAGTGCCTGCAGCAGA

GGCAGCCGCGAGGCTCGCAAACAAAGGAAAGAACCTGCGCGAGGGTGCGCTCGTCAAGCT
CCGCCATCGCCCGATGCGGTGCACGGCGGCTTGAGATGCCACGCCTCGAGCCAAGAATGG
CAGCAAACCTTGAGCCAAAATGGCTACGG
>at00204
CTCGTAGGCAGCGTTGCTGGTGGAACCCTCAGCGATGCATACATAGCAAGGTGCTCGCCT
GATCGTCCTCTTGTCGGGAGCCGCTTGGAGGTGGCGATGTGCTTCATCCTTGGGCTCGTG
GTTTCCCTGGGCGTCTTGGTTGCCATTCCAGCCCAACATACAGGCACACCAGCTACCGCA
TGCGCACTGTTTG
>at00205
CAAGCAAGCTTGTCTGAAGCATCCAAGCTGGAGAAGCTGAGATCGCTGCAGGTGATGGGA
TCTTGTTGCTCCGGCTGCTTTCAGGGCTTGGGCCAGCCGGAAAAGGCTCCATTGCAGGCA
GCTGCGGAGCCAGAGCCATTGCCAGAGGGCTTCGAGAGCTGGAGTGAGGCCGATAAGATG
ATGTACGAGCAAAGTGTGCGTGGTGGGCGCTGAGTGCTGCACTTGGCCCAGTTTGGAGTT
CTGGGAGTGGCCTTCAAGCAGTGAATGATATATGAGCCCACGTGAACGTGAGCTTGCCAG
CGGCACATTTCGCTCTGTGACACGATCTCGACCGTGACTGAAGTGGGGCCTGCCAGAAGG
GGCTGACGCAGTTTTGCTGATG
>at00206
TTTTTTTTTTTTTTTCTTGAAGGCGCATTTCATTGTGCACACGCACTGATCAACTGCCCA
AGGAAGGCCAACACGGTCCAGCGCCGCCGAGGAAAACGCGCTGGCCATAGTTGCCGCACG
AGGGCATTGCAGCGCAGAGCATGGGATTGCCATCGGCTCCGCATGCACTGACTGCGAGCA
GGTGTGGCCATACAGTCAGATCGGTACCCATTTCCGAAATCGTGTTTGACATCAGGAAGA
CAGATATCGCAGCTGGGGACTTGCAGCATGCACGGCCAGGGCGGGCAACTCAATTAGTAC
ATTGTTGATCCCCGCTGTCCAAACATCCGGTAGCGCGACTCAGGAGCCAGACCATACTTG
TGCTCATTCTCCTGCAGCACTCTGGCTCTTGTGGAAGCACTGAGCGTCTTCAGCTTTGTG
AAGGCCATGTCAAAGTGCACTGCCGTCTTCAGGCCGTTCGCGCCCTCCGCCCAGAGCTTG
ACGGCAGAGCACGCGTCCAGGACGCTGAAGAAGCCGCGAATGATGCCCAGGCCTTCAGGC
CAGAAGGGCTCCATGAGGCAGTCGCCGACAAGCACCGCTAACAACGAATCGTCCTCGACG
CCCATAGAATCAACACCGGGAGGTGGCACCACCTTGACCCCGTCGTGGGAGCGTTTCATC
TTGGAGAAGTCGAAGAGCCTTGGACCCTCGTCGGCATAGCCCGGGAAGCGCCCGCCGGCG
TCCTCGAGGATG
>at00207
TTTTTTTTTTTTTTTTTTGGATGCCGGACACCACATGGTCAGAAGTATGTCACTGGCGGT
GAGAAGTGGTCGTCCCGGGAGTCGTCCCCTCTCTCATCACTTCCCTCAAATGGCCGCTCA
CTGTACTCATGCTCAGCCGGCTGCGGGTTGGGCTCCACGTGCCTGACGTGCTCTCTAGGC
CGAGGAGTGGCATGCCTCCTCTTTGGCTCAGAGGCTTGATCAGAACCGAAGCTTCCCGCC
AGGAGCTGTTCTCGGTATTCTGCCCGGAGCTCTCGTCCTCGTTGCCTCTGGCCCAATGCG
TCACACGCATCCATGGCAGCGCGGTATGCCGCCATGTCCGGCAACAGACCATTGTGCACC
ATCTCGTCAAACGCCTGCAGTGTGTCGCGGAACGCCTCGCTCTCCTCGGTGGCGGNGAAG
CATGCGAGCCGCGCTCCTCGTGC
>at00208
TTTTTTTTTTTTTTTTTGAACCCACACTGGCGATGATCAAGTCACAAACTCATGTCCCA
GCACTGTAAACAGTTCTGCCGCAGTCTGTGAGCTGCTCACAGCACATGGCTCAGTACACA
ATTCGAACAACGCCTGATCGCCGTGGGTCCAACGACTGGGCTGCACAGGAGGTCAAAGTA
ACGATGTCTGGCCTCTTGCATCGCGTCCACTACCTGAACGGTACGCTAATCTGCACGGCA
AGCCGACGGAGTCTCAGGACGTCAAGGAGGCTCGGCCTCAGCCTGGCGCATCACTTCCAT
CACCTTTTCGGAGCAGCTCATCTTGCCCTCCACCTGCAGGCAGACTTCGCTGTCGGTGCC
GAACCACGTGGCGAAGATCGTGTGCGCCCTGTATGTCAGACCTCCTCGGTGCAGCCGAGC
TTGCTTCGCAGGTAAGCGCAATGTTCGGTGTCGCGGCCAAAGCAAGTGGCCGTGATCCTG
TGTGCCTCAGAGTAGCTCGTCTGTGCCAGCTGCATCTTGGCAATGT
>at00209
TTGTTTTTTTCTTTTTTTTTTCTTCTGCTCCATCACATGGAACTCCACAAGACAATCACG
AGGTCAGTTGTAAATCCGTGGGGCCGCCATCGAGACCAGGTGGGTAGCTCCCCCCTACTG
GGGACTCCGGAAGACCCGCACCAAACCAGCTCCAGGCCGATGCGTGAGCTGCGAGGACCG
ATGGGCTCAGGAGCCCTCTTGGATGACGAACGCCTTGGTAGGCAGCTCTGCTCTGCCTCC
GACGAGTTGCTCAAGTGCCACGATCGTGCCTTCTTGGTCAGGTCGGACTGCGGATACCAT

CAAGAGCGAGGGACGGGAACACTCAGCAGCCAAAGTGTCAGTGCATAGCGCTTGGCGTAT
GCTGGCAGCACCTCATGCATCACCACGCGTGATGGAAAGAGAACGGCCGTCCCACCGCAC
GGACGAATGTCGATGGTGCTCTTAGCTGTGGCCCCGCTCAGATCAGATGTCTCGGTGTCT
ATGTGACACCTAAGCTGGCCACCATCCCTGGAGTCCTGAAAGCCAGTTGGGTTCACGTAG
GCTATTGCAGTCAGCGTGCGGTAGTTGACCCAGCGCTGCCCGCCTGTGGCACTGCTGCTA
ATGAGTTCGTTGTCTCGGTGCGCAGCGTAGTGTGCACCCTGTCCGTCATAAATTGCGGCC
ATGCCTTGCTGTGGACCTAAGAGCTGTTCACCATCTGGCCACTGCAGATCCGAGGCCGCA
GCCTCCAAGCGTCGCAACAGTGTCAAACACCTTGGTGGACNGACTGGTGTGCCCCCTCTG
AGGTCAAAGAAGGTGACCTTGTCCTGAACGTAGAAAACCTTTAAAAAANCC
>at00210
ACAAGACCCTCAGGGAGTACCACCAGAACGCCGACACTCTCCGGCGGAGGGGGTTCTTGC
GAGGCCTGAACCACTTTGCGATCGAAGCTGATGCGACTTTGCCCGCCCTTCTGGACGCGC
TCGCCGAATTGGAGTCGGACAGCCTGCGCGAACTCAGCTTCGACATGATAGTGGACGATG
CCTCCCACTTCTCCCAAGATATCATCAGAAGTTTCGAGCTGCTCTTTCCTAGCAGGTTCC
TCAAGCCCAGTGGTCGATATGTAATAGAAGATCTTGACGCAAAGCATGGTCCGGACAACC
AGCACGTGGTGCGGAGGTATTTTCAGAACTTGTCCTCTGCC
>at00211
CCGTAGCCATTTTGGCTCAAGGCCTTTTGGCCAGGAATGGCTGGGCGCCATTTGGCGCAG
TCCTCCCTGTGCACCGAGGACCGTCTGGTTGGCGGCCACACTGCCGTCTGGGGCTCCTAC
TATGACAGGGAGCAGAAGAGCTGGGGCTATGCCTGTTGTCGGCGCACGGAGCAGAACGCG
CCCTGCCCACTCAAGGAGGCCGAACCCGCGGCCCCCGTCTCTGCTGAGGACGCTCTGAGC
GACGCAGCCTCAGCCGCGCATTCGGAGTGGAACGCCACCAAGCTGCTGGAGGAGGCGCCG
ATGGAGGAGTTGGGAGCCGCGGGACGCGCACCTCTCGGACGAGGAGTACCTCACGCACTT
CGTTCGGCACTGGTT
>at00212
CGCCATCTACGCCTTCAGGGGCTCCAGCCGCGGCCTGGAGGCCGTGGCCACCCTCCTGAT
CTACTTCGGGGCGCAGTCCGGCATGAACATCTATATGAAGAGCGTGATGTCCAAGGTGGT
GGTGGACGCAGAGGAGGACCTCAAGGGCGTGCCCATCGGCTTCCTGCTGACCTCTCTCCA
GCAGCTCGTGGCCTTCGTCATCTTCTGCTGCTTCCTCCTGGCCAGCCGACTGCTGAACAG
CGGGTATAAGCCGAAGAAGCTGTCCTCCAAGAAGGAGTACTTTGCCGTGCTGTGCTTCAG
TGCTACGTTTGCCTTGACATTGGGCTGAATAATTTTGGGCTCTCCCTGGTGGCAATCTCC
ATCAACTTGCTCATCCGCT
>at00213
TTTTTTTTTTTTTTTTTGCACCGACTGCAAATATATCCGCCTTCCGAACCATTGTAATG
CATCAACAGCATACGCTGCCCTTGCTTGGGTGAACAATCATGCTTTGACATCGCAAAGCT
TTCCAATCTCCTATACGAGATAAAGATGGTGGCAGTGAAATCCATCAATCTCACATACCA
CCGGCTTGCAGTGGCACCACAATCTGTACAGTGTGTTAGCTAATTCAGGCCACGCACTTT
GTGGCTTGGGTCACCGGATAGTGCGTGCCGGCCTTGTTCACGCAGCACTTGCTGCCTTTT
GCAGCGCACACATCACCGCAGCACGAGCTTCTCTTGCTGCAGGCAAAGCTATTGCCATAT
CGATTCTTGCAGCANNGTGCACCCGCTCCGACGCAGATGTCTCCACAGCACGAAGAGTCA
GGGCTGCACTGGGATTTTT
>at00214
TTTTTTTTTTTTTTCCTTGCTATCATTCTAGTTAAGGGCCTGGGCGCACACCTTCATCTG
GTCAGGCCGAGAGCAAGCCCCCCCAACGCCGCGCAGAGCGCTCAGCGGAGAACGGCCCTG
GCCTCAGCCTCGGCTCCGGGCCGCGCTTGCCCCGGGAGTGCAAGATCCGGACCTTGTGCT
CGACCGCCTCGTCGCACCAGAAGCGCTCGCCGTCAGGGAAGAAGCGCACAGAGCTCGAGC
CAGGAGGAACGAAGATAGTGCAGCTCAGCCTCAGCCAGCCCGCATTTGGGCTGCTGCGCC
CGTTGATCACCAGAAAGGGGAACCGACCATCGTCACGCTGGTGCAGATGCGGGCGAAGGA
TGGCACCTTTGTATGGTCAAGCCAATCGCGAGCCCAACCACCGTTGTGCGAAGTACTCTG
CAGAGAACAGCCGTGGCCTCACCTTCGGCTCTGGCTCTCGCTCCCCTGCGGAGTACAGGC
CAGGATGGAACACGTCCAAGTAAACAGAGACTCGGAAGTCGTCGCCCGTGTACACGACCT
TGTGCTCGACGGTCTCGTCAAACCAGAACCTCGTGCC
>at00215
TCATATCCAGGAACCCCACATCCAGCTTGGACATCTTCGTCCACTGCTCGTGGCCGAATC
TCGCGTCGCACATCTTCTCGGCCTCTCCGAGGGTCGTGGAAGGTGTGATCTCAAAGTTGT

CACAGTGGCCAAATGTGAAGCAGTAGTGGGCGTCCACGGACTGGATGGCCGTGGAGATGC
GGTTCTGGGCCTGGTGCATGAGCCTGCCGTACCCTCTCAAGTCGCCCTGTTGACAATGAA
GGGCGGCGTCAATGTACTGGGGGGATTCGCACCAGGTGTTGTAGTCTGCGTGCCCATCCG
CGGTGGCAATCGGATTCTCGCCCTGGGGCTTCCGTCCGAGATGGCAGTTGAACGGCGGCC
GGCATGCCGCCGGGAATGCTGTGCAGTTGACCTTGCCGGGGCGGCTGCTGGAGCAGGGTG
T
>at00216
GTCAGGATCAAGTAGCCGTCAAAGGTTTTTAAGTTTTGTTTCGCAGCCCTCAAAGCTCGG
CCATGCATCCTGGCGAGCATCCAGCTAATCAGAGCATCGCAGAGTTCTATGTTGGCAAGG
TGGTCTTTGTGACAGGATGTACTGGGTTTGTGGGTAAAGTGTTGCTGGAGAAGTTGCTTT
GGGAGCTTTGCACTCCGGCCTTCTCGAAGTCCCCCAGCACATATGCAGGCCCAGTCATCA
AGCTGCTGATTCGGTCATCACGGGCGGCATCGGCACAGGAGCGGCTGCAA
>at00217
CCCGTAGCCATTTTGGCTCAAGGAGCGTCTCTGCCATGGGGTAGCCCCCTCGCCCCAAGC
CCACAGGTGAGTTGCTTGAATGCAGGCAGTTGAGATTGGCAACGTGAATGCCGAGCCGGA
ACAGTCCCAGAATGGGCTGCTCCACATGAACACCAGCGCTCGACTGGAAGTTGCCAAGGA
AGGTGCGTCCCTGATTCCAGTCATGTGGTGCGCGACAGGTTGCTGCTGCTTTTGGATACC
GATGCTTTTCTTCTTCGCAGCTGCGAATGTCCTGCAGAGTTGCGAACAGGACCTGTCTAT
ATTCCTCAAGGTATATGGGTTGGTAAGTATATTGCTGGCCCCATGCATGCAGACGATGAT
CATAGCTGCGGCTTGGGCTGGAAGCGAGACCTGCTTTAAGTTTGCTGGGCGCATCCATCT
CCTCACCTCTGCCTTGAGCTTAGGCTTGATGGCTGCTGGGTGGGTCTTCTGGCACGAGAC
CACGGACACCGCTTGCTATGATCCAGATGGAGCCCACGACCTCGGGGACATCAATCCAAG
GACGCTGCTCTTTGTGTGCGTGATCATTGGCACTGCCGCATCTACGTGTGTTGCGTGCGG
CGTGCTCTGCAGCATCTGTGGACTTGTCGGCCGCACTGCTGCTTATCAGGACTAGTGATC
GCGAGGGCACGGGAGTCGGGCCAAACCGGGCCAGCTCAGATTTACCTGGCACAGACGCCC
TGAGCAATTGCACCTCCCAACAGGAACGTCTCCCACCAGCGGAGACGGGATCAGGCACGC
ATGCATCGAGATTACATTTGATTGGCCATGCCTAGATGCCTTATTTTGCGCTACAGCTTT
GTGCTGATGATCTCGCCCAGCACCAGTCTTGGCCTCGGTGCTTGTACACTGCTTCCAACG
CATGTGCGTACACATGCGAAAGAGCTCTTTCCGTTTCAAGCTGGTTGCACATTGATGGCC
GTTGCAGTTGATGGCAGTCAGTGGTGACAGAGGAGGCTGTTGCC
>at00218
CCGTAGCCATTTTGGCTCAAGGCCCAGGAAGCAACGCGGCCCGTGGCGATATTCTCCCAC
AGGAACATGTGGAGTGCCGATACTTGTTTGATGGACTTGACTCGAGCCTCATATGACATC
CGCCATCTCAACCTACCATCAAGACTGCACAGTATCTGCCGCGATCTGTCGTCTCCGAAT
GGAACGTGTCGGGGACCCGTGGCCGCAGAGAGGCAGCATTGGCGCCATGGAGGTCGTTTA
CTGCATTGCGTTCCTGTGCAACACTTC
>at00219
TTTTTTTTTTTTTTTTTGAACACCAGTCTGGGACCTTCCAGAATCATCACTTGCAACTC
GCTGTCCAAGCTCACTGTTGAAGCTCGATGTACCTGACCATCCGAAAGTCAGGACGGTCC
TTGCCCCCCTTTTACAAACAAACACCAGGCACTGCTTCCAAAAAGGAAGACTCTGCCAGT
CTTAGGGCTGAGTCTAGACCGCATCGAGAGGCAGCCTGATGAGACTGACATCTCAAAGCT
CGATGCCGCTTGGGCAGCCAACCGACAGGATGAGCAACGGGCTCCTCAGAGCAACTAAGG
TGCGGTCTCAAACGTGAATTGAACGTGTATCTACATAGGCATGCATGGGCAGCACAAAGA
CGTTGCTGAAATCAAGTGTCAAAACGTCCTTAGACAGGAACGCCACGACGTTCTGCCTTC
GGAAAGCGGCTACCCTCTTAGTCCTGCTAAGCACCTATGAGGCCACTGCACGATGAAGGG
CAACCGGATATTGGCAAAGTGCCGCACCGGAGCGCACACCTCATCATTAAAGACGGTGCC
TGCCTCAATCATTAAAGACTGTGCCTCCACACGC
>at00220
TTTTTTTTTTTTTTTTTCCTGTGCGTGGCATGCAACTGTACAATCAAGGCTCACGCCGA
TATCAGCAGTCAAGTGCCCCACTCCATTTCCACTCAGAAGATGACGCCTGGGAAGGCAGC
GCAGAGCAGGCTGAGCAAGACGAGCTAGCTGGAGCCGGGAAGAGATGCGGCGCTGACGCG
CAGATCCACAAGAGGCGAGCTGAGCGTACCCACCTTGCTCAACGCTCAGTTCAGTCCCTG
GGTTGGTATCATCACTGGAGCAGAGCGCCTGCTCTGGGCACGAAGTCGTAGTAGACGATG
CACTCGCCGCCAAAGACGCCGCACTGCTCCAGCGTCTTGTCACGGGAGACAATCTCCTCG
GGGTGGAAGCGATTCACGCAGACGGACAAGTCACTGATGGAGCCACCGTGTCGGTCGATG

ATGCGGTCGACGACATAGGATATCTTTGTGGAGGGAGTGCACTCGAGCTTGAACTCCAGG
AGACTGCACACAGGCGCCGCCAGCTTCACCGTCGCCACGACCTTTCGTTCGTCCTCTCGC
GACACGGGCTCCGGGTGCTTGCCCTCGTAAGGGGACTTCTCAGGAACGGCACTGGCATTG
GCCTTCTTGCCCATGACAGCTCACGGCCGCCCCCGCGTGCCTGCAGGTCGCTCGGACGCC
ACCCCCGAAAACTTGGGCTGATCTCG
>at00221
CCGTAGCCATTTTGGCTCAAGCCCTCCTGGCTGAAGGGCAGGGATGTGGATCGGGTCTAG
GGGGCCAACCGAGCATGTGGAGGAGAAAGAAGGGCTCGCCTGTTTCCTCCGGGCCACCCT
TGGAGCCGGTTGAGGAGGGCGCAGCGGAGCAGGCCGTGGAGCCCGCATTGGAGCCTCAGG
ATCCGGACCCCAGCGTCCCACTCCCCAATGCTCCCGACAGTCCCCAGCATGATCTGCCGG
AGCCCACGGCGGTGGCCCAAAGCATCAACCCCGCAAGCCCCCAGAGCGCCGAAGCCTTCG
ACTCGCTGTCCTTCGGCGTGGACCTGATGGCCGCGCGGGCACGGCGCAAGAGGCTGGCAC
GGGGGGAACCGGAGCCGCCGAGACGAATCGCGGACCGGAGGGCCGACATGGCGGACACCT
CAGGGCGGCAACTTCAGGACTATTTGACGGCCGCCTTGGCTGAGGAGCCGAAGGATGAAT
CGAAGAGAGGTCTGATGGAGATTTTCACCGAGTCGGTCCGCTCTCTGGATTTCAAAAATC
GCTCCCACAGATGTTTACACTGCCTGCGCGGCTGCCCTACGAGAACAGCTGAGTGGACGA
GAAGAAGAGCTGCAGATGTCGATGAGTCAGTGCACCGGTGGAAGACATCAAGGTTGGGTC
GTAGAGTCCAGGACGAGTTGGTGCGTATGGCCGTGGAGGCGGGGGACTTGCCACCTTCAG
CACTGCCCCGTCGCTGCTGCCGTGGCCATGTCTCTGATGAGGACAAAGAGAAGCTGCGGT
TCTCCCCGCCATACGTGGTGGAGGCTTTTCGCAGCGTACGTCCACGGCCGAAGAAGGCCT
TCATGGAGAAGGTCCTGGACGGAGAGATTTCACTTGATGAAACTCTTTGCGTGCTGATAG
CTTTCACGCTGCTCGTCACGCTCGTGGTCCTCTTCCTGTACCTGGGGCTGGCGTCTGCCG
TCTTCTCTGCCAAGGACGTTGCTGCGGATGCCCTCCAAGGGACGATCGTGGACGCCAGCA
CTGGACTCACAACGGTCGGGGCTGCGGCAACTGTAGCACTGAAGCCGCTGTGGAACTACC
CGTCCTTGTCCCTTGATGAGCTGCGGAGGGTGCAGGACGTTCTGTTCGTGCACAACGGGG
CCACCCACGTGCTGCACGTGGCCACAGTCTCGCGGACGTCGCCTGGCACCGTCCTGCTGA
AGGCAGTGGACAGCTCGACGGTACGCGTGGAGCCCAGCGGCCGGAGCTTCTGGCAGCGCG
TCGGGCACCCGGAGGTCCTTCTCGAGGCCATGGAGTCGTGGCGGACGGCGCCCGGGAACG
CTGAGGTCGACTGGCTGACGTCTGGGGTGCTGGCAACGCAGGTGACGGTCCCGATGTGAT
GCAGTCCCACGTGCGTCGCCGGCAGGTGACAGTCCCGATGTGATGCTGCCCACGGCGTTG
CCGACGAGGCAAACGCAGCGCCTGTTAGGTGGACAGTGCTGTTGACCTCCACCTCCCGCA
CTCCCTACCCGTTTGTCTCGCGCGGCGTCACGATCCTGACTTGGCTTCAAGCTGATCTTA
CCTTCCCCTTGTAGGATTCCTGATGTCCTTGCACTTGCAGCATTGATGGCCAAAAAAAAA
GAAAAAAAAACAA
>at00222
TTTTTTTTTTTTTTTTGGAGACCCGACACGCGAGCCACTGCCTTCACCCGATGCCTGAGC
CAGAAGCAATGATTTGTTTGGGGGATTTTTCTTTCAAGTATGCAGCGCGCGCCGCTTGCT
TCTCCGATAAGCTCAATGCGCCCTCCACATTCCCACAGCTGTGAACCTGCTGCTCAAAAG
CGGTGCATGGCGTGCCCCGCAGCAACGCCGTACCAGCAGGCAGGTCTGTCGGACTTGAGG
GCGTCCAGCTTGGAAACGGAGCTGTGTGCGGAGGCAAATTCCATCCAGCAACTCTTAGAT
GTTGATCGGGGAGAACGCAGCACCAGTGGTTGGTTGACATGTCTCGCCCGGCTTGCCCCC
GGGTGGTGTCATGCACACTCCATTAATGCAATAATGGTCCATCCCCTCTTCCTTGGAGTT
GAAGAAATCGCCGAGGTGAGCACCGCAATTAGAGCAACCAACTTCACCCTCCGCACCGAG
GCCCAAGTCAGTAACAATACCACGCTCCTGCACGCCATACCCAGGACCGCGGTGACAGAC
TGCTGGCTGACCATCTGGGCCATCGAGTGGCGGTGAGTGAAGGCTGGGCCAGGGCCATTG
CTCAGCCGGGAGCTGGTCAAACTGCTTGGACGGCAGCCAGAGGGGAGCGCCGCAACAAGC
ACATCGGAACACACCCTGCGAGTCCTTGAAATATGGCGCTTTCTCCCCGGGCATGCAGCG
TCGCTGCTGCAACTCGGCCAGCGCAGTGCGCGTGATGCACACAGGCTC
>at00223
TTTTTTTTTTTTTTTTTTCCAACACTCAGGTATGTGTAGCCATGGATCTTTGCACCCGGA
CTCAGTGAAAGCCCACCTCGTCAAGTCAGAAAAAGCTGTGATCCCTCGTCACTATCTGAC
AGGAAACGTAAAGATGCCTGGCTTCAACATTTCTGAGTCCAGGAAGTCGTAGTCGTCAAA
GTCTACTTGAGGGGAGCCACGCCACCTTTCTGCCTCTGCACCAAAGTCATGCGCTCGCGC
TGCTGGCGCGCCCGCAACCTTTGGGTTGTAGGCACCAAGCCCGGGCGTGTGGTTCAAGGC
CCCTTTCTTTTTGGGGAAGCGGGGCACCATGGCAAGGAAGCCCTCCGAGTCCGGTGGATA

ATGTTCTGGCCGCCGCTGGTCAACCACGTCATAGGTCCCGGGCGCCGGGCTACCCGCGTC
TGCACCTGCGTCCTTCCCAGCGTTGGAACAACCCCAGCGCAACAAAGCTCGGCCAGGCCG
ATCTTGCACCTCACTGCCTCCGCGCGCAAAGGCCTGAAACCGGTCCGCGCGGGATCCAAA
GCTGGTGTCCACGTCAATCTTGGGCACTGCAACAGCTGCTGCCAGTGTGCCCCTGCGGCT
GTCATAGCTTCCGGGGCCTGGCCAGCCGGGGGAGGCTGAGGCCCAGCGACTGCCCCGCCC
>at00224
CTTGCCGTATAGCGAGGTCATTTGGCAGGCTGCTGACTCCACTCTCGCAGCCCCTGGCAA
ATTCAGCGCCCTGCCGCATGGAATTGGGCCCCGTGACGGGTTGCTGATCCCGCCCTCGCA
GCCTCCTGGAAGTTCAGCCTCTTGCCATGGAGCGTGGTCCAGCGGCGGGTTGCTGATCCA
TCTCTTGCAGCCACCTGGGAGCTCATCCTCTGGCGTTGGCTGTGGAATGGACNCTTTGAC
AAGGCGTGATGATATTGGCTATCGTTCGTACTCGTACTATCCGTCTCTGCCTG
>at00225
CCGTAGCCATTTTGGCTCAAGCCGTTCCGGCTCTTGTGCAGCGTGTGCTGGCATTGGGAG
GCCTCCGGGCCGTGCATGGCACTTGACGTCATGCAGGCCACCAGCAGCTGCAAAACATCG
TCCATGGGAGCGGTTGCACACTGGTGTTGCCCGGCATGCTTCTGGTGGCCTCAGCGTTTT
TGCTCGCGTACCGGCATGCCATGGAGGACCGCTACGACCTGCCACTCAAGGGCTTCCTGG
CCAACATCTTGCTGCAGATGATCGCC
>at00226
CTCATCTTCTGCCAGGTGTCATTGGCCTCTCCNGCACCCTGGAATGGTATGGCGGGAAAA
ACAGTGCTTCATGCTCACTGCATATTCACAATGAANACAGAAATCATGAAAGAGCATCTC
ATCATAcCGCCACAGCATGTGCCATGTGCAGCTGTGACtATTTGATTGAATGTAGGTGAG
CACGCTTTGTACTAGTTATTGCCCAAAATCAGCTTAAGCATCAGCCCGCCAGGATAGCCT
CTCACTCCGCGAGCTCAGCCTGTCTGGCAGCTTTGTCCTCCTCTCCCTCCGCAATCAGCA
CTCGCAGGTCGCTCCGCTGCG
>at00227
TTTTTTTTTTTTTTTCTCCCGACATGCCCTAGTCCAGGAGGTCAACTATGTCCACGCTG
TCCTTCTGCATGCCCGTATCCCGCATCATCTCTAGATGTCTCAGAAGTGGACTCTTGAAG
AGATCCTTCACTTTGTTCTGTGCCAAGAAGTCCTCCACTAACCGAGGCAGATCGTCGTTG
CTGACAAACTCAATCTGCGAGGCCCTGTCATCGGAGACCTGGACACTCAATTGAAAGACC
TGAGATTGAGTTGCCGGAGGAGGCACCGACTGCTCTGGTGGAAGCAAGGTTGGCGCCCTG
CCCTGTGTGTCTCGTTGTCCAGCTGGGCCGCCGGAGTCGTAATCCTCAAAGTTTGCATCA
TCCGCGGAATCGGGCGCTGGCCTTGCCACCGGTGTCGGACCCCCAAGCCGCTGACCCTTG
ACAGCAACGGCTTCCGCTCCGCACTCGCCGCTCCCCCCGAAAGTGGCAGCAGCACTCGGC
TTCGCTGGGCTTGCCGGTCGCACGGGCTCCTCAGGCCCAGGTTCCAAGCGCAGTTGCAAG
CTGTGATAGCCTTCCTGCGCCACGGAGTCGCCGGTGCAGTCGCGGCTCTTGGTCGCCGCG
GTGCGCTCGAGGCTGCTCAGCGCGTGCGAGAGGCGCATGAGGGACCCCTCGTCATCGCTG
TCCATGCTCTGGGAGCGCTTCCGCTCCAGTGCTTGACTGGTCTCCTGCAGTNGCACGCGG
CTGGCGCCATGTCGTATT
>at00228
CAATGCTCATCGATTCGCGGTCTTCCACATCCTCCTCCTCGTGCTTCTTGACGTGCCCAT
ACCACTGTGCCTTGACTGCCTCCGCGACTGTGAAGGCCTCCTTCTGCAGTTTGAACTCGT
CCTCCGCAGACTTTTATGGTTCTTGCAGCTTGTCCACTTTTTTTCCGCACCAGACTCTGC
CTGTCGACGCACATCTTGTGGTAAGCAACCGCCTTCGTGTACTTCTTGTGCGCCTCCTCC
CTCGTGGGTTCCCTGTTTCTGAAGGCGCGGGCCTTCTTCGTGACAATGTCTATGATGTCC
TTGTCCCCGAAGTTTGCTGACTAGCTTGACGAGCTGCTCGAGACCG
>at00229
TGAAGAACCTGCTCACTGAAGATGACCCTGTATACAGCACCTCGGACAAGCTTTACGCCG
TGTCTTCTCGCATGTTTCCGTTCTTGCGTATTCTGGCCGTGGGGCTTGCCGGCCTCTTCG
CTTTGTATTTCAAGCTGAAGTCGGTGGCGTTTCTGTACAAATTGCCCTTGAACCGATGGC
ATCGCAAGTACTGGCTCACATTCATTGGCTTCGTGTACCAGGTGTCAGGTCTGATTCCCG
TGTCCTCGCTGAAGAAGGAGACGATTCTGTTCTTCATCTTCACCGGCGAGGACGCGGAGA
TGCACGTCAGCGAGTACCGCGCCATGCAGAAGTTCAACGCGAGGCTCTACAAGGCGATCT
TCAGGACTTGCGGCACTTTGCGCGGACTTGTTGTCGGGGTGACGATCTCGCACATCGACT
TGCAGAAGATCGTCATCACTGAGCGTGCACGGGGGAGTCATGCTAAGGTAGCTTGACATG
CAAAGGTGCGGCTGCCACGAAGGCTCCAGCCCAG

>at00230
CGTGCACCCAGTCCTCTCCCTCCTCCTCCCGGTTCCTCCAGACAAAGATGCCGGGGGTGG
GCTGCCCGTCCCAGCCCGAGAGCTCAGCTTCGTTTGCCGGGTCTTGCGCGCGCCACCAGG
CGGCCTTCGCCTCGCCTACGGTCCGAGTTCGGAGGTCGCCCCTGCTAGCGGCCTGGATGT
TGGGCCACAGGAAGCGGACGAGCTGCTGCTCGCTTGGCGCCCGCTGGCCCAGCCAGTACA
TCCATGACCCCAAGTCCGGGCCTTCTACCGAGATGACTGCATCGGCCCAGAAGAGCCTGA
GCGCGGCGTAGGCTGGGGCCACCCCCAGGCACTTGGCCATCT
>at00231
CTTTTTTTTTNTTTTTTTTTTTTTGTTCGCGGCCTGGCGACTCTTCGGGCACCGCAGCA
CCTCTTTAGAGGCGCTCACCGGGTCCCTTCAGAGTCGGCCTATCATTTGCTAACCTCCGT
CGTCACCGCCGCTACCGCCACAACCACTGGTTCCGGCATCTTGGCCACCACCAGCATCGT
CTCCGCTGGCCAGTTCGCTCCCCACAGGGCCGTATGCAGTCCGACTGCTGCCGCCGCCGT
CCCTGCGGACGAAGGCGGCGAGCGCCAGAGCCACGGCCACCAGCAGTGCCACCTGCCACA
GGGGAAACTGCTGGAGGTGCCCCTGCCCGTAGGTG
>at00232
TCGGCGAGAGGCCGCACTCCACCAAGTCTTGGTCACGGGCAGCGGGCATTTCGAAACTTT
TGACAGCGATACTGGCGCATAGGACGACATGGATGCCAAAGGCTACGCTTTAGATGACTT
GTGGGCCACCAGTTACAGTCTGTGGGTGATCCTGGACGACGAGCAGCTTCGAGGTGACAG
CATAGTGCATGTGAAGCACACCACGGAAGCTGCGGTCAATTGGCTGCTGACGAACCCACA
GGCTGGCACTGGCACCAAGCAGGGCATGCAAGTGCAGTCGGCACTGAAGAGCTTACGGAC
CCTGTGCTAGCAGCTCTGGGCGCGACGACTTGTGCACTGCGTCGGCATCA
>at00233
TTTTTTTTTTTTTTTTTTCCAGAAACTTAACAATTCAGAGGTACAGCAATTCCACCATTC
TGCTCATAGCACAATATGTCAAAAGCAGGGCCCTCACTCAGGAGGCACAACCTCTCACTA
CTCCTGAGCCCACCGCCTAGAGCTCCGTCTTCGCCTTGGCGTTGTGCGCCTTCACGGACT
TCATCATGCCGAGGCCCGAATTCTTGATCGCCTCAATAGCGTCGTCCTTCTTCTTCGACT
CCTCTTGGTACTGCTTGTTCAAGCCCTCGACGAACGTCTTGAAGTCAGCCTCAAGCTTCT
CCATCTCATCCGTCTTCTCCTTTACTTGAGCATCGAGATCCGCAGCGGACATTGCCATGA
GCTTCTCGAGCTGTGCCTTCTTCTCTGCGTCACAGAGGTCCATGTGGGCTGGCCCACACG
ATGGGCCCAAGCTCTTTTCTGCAAACTCCTTGAGCGCCTGGTAGGAGCGCCCTCCTTTGT
AGTCTTCCAGAGCGTTTGCCTCGTGCC
>at00234
TTTTTTTTTTTTGTTTTGTCAGCGGCAACTTTCACCATGCAAACAAGTGCAAACTGTAC
AAGAACACCTCCAGCAGCTCCACAGCAAGAACCGGCACGAGCATTATGTTGGAGGTCAAT
CGCTCACCTGTCTGATCATGCCGGCGTCAAGCAAGCAGCAATGCGCCAGAGAATGCAATT
TCAGGCGACTCGTGTGCTCCACTGTACTCCATGACAGTAGGCGAAAAACACTGCCCCCAA
GTTTCCGACTGAAGATCTCATCGTATATAAGACCGTCTGGTCTCTGCCCTCCCAGAAACT
GGATACCTAGGGGTCTTCTGCCCCTGGCCTCCAAGTGCGGCTGTCTCATTGTCCAAGTGG
AGTAAATGAGGGGAGCAGCAGTGGCACAGTCTGCCCAGGAGCGGCAACACATAGCAACCA
CTGACATGGCAAGCAGCAGTTTGGTGGAGAGAAACTTCCTCAAGCAACGAATTGCTGCTT
CTTGCCCTTTGGGCCCTTGACCTGCACATTGTGGGTGGGGGCTGCAAGTGCCCTCGTTGG
CGGCGGGAAGGAGATGCGAGTTCTACCTGACAAGAGCACCGGACGATATTCATCGGGAAC
AGACTCGTGCATGAGTGGGCTGTTCCTGTAGCGCTTCAGATGCGCCGGAAGGCCCTGGAG
GGGCACACTCCAGCTCACGCGGCAGATCTT
>at00235
TTGTTTTTTTTTCTTTTTTTTTTTCTGACAAAACAAGGGAGGCAATTAAAAACCGGTCGCT
TCACATGAACGTCTCGCACGTCTGCAGCGCTCACCCTCTGATCAAGTCCAGAGTCCAGCA
GTTGAACGCCCCTCCAAACTCGAAAGCAGAAGAAAAGTCCATCTCAATGCAGTCCGCTCC
GCATTCCTCTCGCAGGAACTTGACTGTTTCCTTCTCTCCCCTTTCTAGGATGATCTTCTT
AGGGCCTATTGGAAGGAAGTTCAGGTTCAATCCCGAGGAGCAGAAGTCCTCCGGAGAGAC
CAGGACGCTTCGCCAGGGCGCCTCCAACAGGTTCCAGTCCCTCTCCTTGAACCAATCAAA
GACCTCCTTACTGGGCCTGTGAGCCGGATCCGGAGAATAGAGGATGGTGCCCTCCTCAAT
GGCGGAGAACTTCGCATCGAGGTGGTAGTAGGACGTTCCGGTCTCGAAGGTCATCTTGTG
AAGGTCGATGCTCTCCTTCCGCAGGATTCTGTTCAATCAGTTGAATCCTTTCGCGTTGCT
GTACCCCCCGGTCTGCATG

>at00236
GTCTCCGGATGACAAGACCAAAGTCAAGGACCGtCtAtTGGtTCTACGCGAGCTCCCAGG
ACGCCATCAAGAAGAAGCTcCCCgGGGCATCATGAAGGCCATCCAGGCCAACGACAGGTC
AGAGGTGGACGAAGCAGAAGTCCACAAGGAGATGANGCGGTGAGCCCGCAGCCTGAGGGC
CGCGCCCCGATTCTGAGCCACCACTGGCTGAGGCGGGCGGCCACGCTCGGCACAGGAGGC
CGCGGGACCGGACGTAA
>at00237
GCACCTTGGAAGCAAGGCATCTTTATCGGCTGTGGTCCCATGGAGCCGCCGTGCAGCAAG
CTCTGCGACACGTCATCTGGCTCTCCATGGAAAACTGGCGGCTTGCCCTTCGGCTCCGCA
GCTCGAAATCATGCAGCAGCAGGCCTCTTGATCCTCGCTGGCGCCACAAGACCCCGGCAG
GGCCTCCCGAGATTCGTGCCACTGCTCTTCGCAGTGAAGCTAGCAGCATGCGAGAGGACC
GTCCATACAGGGCCGTGGAAGGAGTACGATCT
>at00238
CCATTACTTCTTACACACCCCCCGAGCTAAGTAGGAGGTTGGCTGGCTTAGAGCCCATGC
CGAGGCATTTGCCATCGCTAGCCAACGCCATCAGCAACTTGAAGGCCGAGCACCGCAAGC
AGGCCGAGGCAATTGCGAAGCAGCAGCAACTGATCGAGGAGCTCAGGAGCACAAGTTGCG
CACTGGCCGAGCGCCACGCGGAGCTTGAGGCCATCAGACCTCTGCAGGTTGCAGGGGTGG
CTCCGCCGGATGCTGGGCAGGCCGGCCAGGCTGGCTCAGAGAGGTCCGAGATCCGAGGAC
GAGTCGACGTTTTCGTCATCACAGCCTCTGCGGATGCGACTGCACAGATG
>at00239
TTTTTTTTTTTTTTTTTGTATTCGCTGTAAAATTGATGTCTGAAAATTCCAGCCCAAGT
ATCTCGATGGATCAAACCCCAACCCTGGGCTCTCGCCCACCACTCACTACGCCAGGCCGG
CGACAGCCGCTGTGGCTGCGGCTACGCCGCGTAGGGCAGAGACGAGTGGTGCGCAACAAT
CCGCAGCTTGCGGTCCCTGCCCTTGAAGAAGGCCCAAGTCTTGTCCACCTTGGTGACAGC
GCCGGTCTTGTCGGTTATGAGGACGTTGCCCACCGTGACGGCCATGTTGCCTTCCCTGAA
GATGGCGCTGTTGACAATCTCCACCTTGCGCCAACCCTTGAGCGCAAAGCCCGAGTCCTG
CGGGAAGGCCGCATCGCCGCCCACAAAGTAGGCCAAGGCACCTTCGCGCGTGGTGCGGAA
GGTCTGGCTCCCGCCAGCGAGTGTGGGCTTGAAGAGCACTGGCCCATAGTTGTATCCATA
CGCTGAGTCGATCACCTGCTCCGCCAAAGCCTTGGCCGCTGCGAAGCCCTGAGCCTCATA
GGTCATGGAGATGCTCACCAGGGCATCCCCCCAACCCTTCTGAGCAGCCAGCACCTCCTC
CTCCGTGATCTTGGAACGCAGCTTGACGTTATCACCTTTCAAGAAGGTCCAGCTCTTGTC
CACCACTGTGAAAATCAAAAACACCACGGCCAAGCACGCAATCCTGGAGGAGCGCATGCT
GCACGTCGAGGAGACTCCAAGCTTTCGCGCTTACCTCGTGAAAGTGTGGCCTTGAGCCTC
>at00240
GCAATAGCCTACCTCGAGCTTGCCAAACTAACGCGTGTTTCTGACGCCAATGTCCAATGC
AGTTGAGTCGGACGCGGTTGAAGACAGTCTTTTGTCCGATGTTTTGTGTGGTGGGGCGCG
ACTTCCTTGCAACGAGCCTCCTGGTGTCGCCTCTTCGCATGAACGGCAGAGGCAGAGGTG
CAAAGGCTGTGCTTGGCGTTTCACCCTGGTCATGTTCTCACTTCTTCCTGTGCTATGGCT
TGCCTGGCTTTCCGTTTGCGGATTTCCCCAGGCCAATGGAGATACGATCGTTGGATGCGG
TGAGCCTTGCTTCAAAGCGGGCTGAGGTGATGCGCAACTTTGCAAACAGTGCAGTCTACC
CATCGATTGGTGCCTTCAACTTTTCGATCGGATCTAGGTTGAAGGAGTTGAGGTTTG
>at00241
CATTATCAAGGCTAGCTCTTAATTGGTACTTGGCACTTGCACCTGGCGCCATGGCCAGGC
GCTGCCGGTGGTCGTGCTGTAGGTTAGCTCCCTTCTTCTACTGGTGCCTCGTCACGTTCT
GCTTCATTGACGGTTTGCTCGTCAATAGACCTTACCAGACCGACTTTCCAACTTGGGCTC
TTGTGATGCTGGCTACAGGGCAGGTTGGCAAATTCTGGTGTTATGTCGCTGTGACGACCC
CTGTCTATCTCTGGTGTCGAGGGATTCCCAAGATATCTTGGTTATCCTGTTTGAGGCTGC
TTCTCTTGATTGCGTCATTGGTGTTGCCGCTGGCACCCGTCTTCGCAGTGGCAGACCTAG
CAACGCAGCTGCCAGAGCGGCTGGCTGCAGGCTTTC
>at00242
TTTTTTTCTTTTTCTTTTTTTTTTTCTAACCTCTGACATGTTTTGCTGCTCTGAGGTATA
CATCACTCTCACTGGCAGCACACCGCTCTTAAGAAAATCACACGCACACGGCGGCGCACT
GCTGTCGACAGGGGACTGCCGAGCACTGCGCGGCTCCGACCCTCTTGGCACTGAGGCTAC
ACCATGCTGGTGTCAGCCACCTGCGCCTGTTCGCAGCCCTCTCAGTTGAAGAAGTAGCTG
GAGAAGAGGACCCTCGTGCC

>at00243
TTTTTTTTTTTTTTTTTTCGCCTTGAGAGTTGCACAATGCCGGCTCTGTGGGCAAGAGAA
CTTGCATGTTGCAGGCATTATGGGACGCCCAATCTTTGTGCTGGGCATGCCCTGCATGAT
GAGAAGAACAAGGAGCTCTTGATACTGTGTGCAGGTGACATCAGCATGGGACACTCAGCT
CTTCACCCTTACGGCTGTCTAAGCAGATCCCCACGCTCCCTGGTCCTGTTGCACTCGTGC
CCCAGGTCATGTGGAGGGACACTGCCAATGTTCTCTGCAAGCGGGTCGGTGATCTCACAT
GGCTTCCAGTTGAAGCGGTCGTGCACCGGATCGTCAAAGTTTGGGGCATACGACGGCTGG
GCCCAAAGGTGCATGGCCTGGGCGTCTTCGAGCCGCTTCCGGGCCTCGAAGCAGGCACCT
GCAGCCTCCAGCTCCGCGATGAGGCACTCGGAGGGGACCTCGCACGTGATGGTTCGCCCA
GGGCCGTAGTGCGGTAGGAGCGCCGCCAGGAGAAAGCTCGCGATGGCGAGGGGGGCCACC
AGGATGGCCGCCGGAGAGTCCTGTAGGGCCAACGCGAAAATCACCACGAGCTGGCCGACC
ATCATGCCTATGCAAGCGCCCCGGAAGGCGCTGACTGCGAGCGCACCGCCACTGTCGAAG
GAGGTCTTCCAGTAGAACTTCAGGAGGTACTTGGACGTCACGATGTTGAGCAGAAAGTAC
AGAAGCCCGAAGAGTGATGCAAGTGGCGCAATCATCGAGTAGCTCATGCAGACAACGCAG
ATGAACAGGTCGCTGGC
>at00244
TTTTTTTTTTTTTTTTTTACATGCCTCAAGTCTGAGAAGCCACAGCTGTCTGTCCCACGC
CGCTCGCAGCACTTTGGGGTGGGAGGGGGAGGCCCCTGGACCCCCCGAAAGGGAGCTACC
CCGTCCCGGCTAGAGCCTCCGCTGGACGGGCCGCAGCGCCAGAAGACGACGCCTCTGCGG
CCAGTCTCGCGTACTCCTCCTCGAGAGAGGGCGCCGAGGCCTTGGAGCCGCCGAGGCCGC
CAGCAAGGGCCTCCGCAGCCATCTCCTCCTCCATCCTCGCGTACTCCTCGTTGAGCTCGT
CGTCCGCGGTACCATCCACCATCGATGCGCTGCCCAGGGCCTCGCCGATGGCCTTCATCT
CGTCCGCTAGGTCCTCCATGTCGTCTCTCAGCTTGTCAACCTGCCCGACGTTGATCCGAT
CCGCCTGCTGCTTCAGCTGCGCCTGGCCCGCTTTCATGGCTTCGACGGCAGTGAGGGTGA
TGTCGGCCTGCTCCTGCTGAAACTTCAGGGTCTCCACGTTGAACTGTGTCCCCATCAGCT
GGTCCCTCTGCTGCTCGTACATCTTCTTGCGCTTCATGGCCTGCATCGCCCTCTGCTTGG
CTGTGGGGTTGCTGCCGCCCTTGGCCACGTGCATCCTGATCTCTTCGTCTGCCTTGGAGA
TCATCTTATCAAGATTCTGGATCTGGCCTTCTATCTTCGTGGCAGCATCGTCGAGGCTTG
GCGCTTCGGCGGGCTTCTTCTCCTCCTGGCTCCTGCCAAACAACCGATGCATTGCACGAG
CTATCTAAACTCGCGGATGGCCAAGGGCTCCGCGCAGACCCTTGG
>at00245
TGCAGGCCGCGAGGCTTGTGCAGATCCCGACCCATGACGAGGCTAAGGAGATAACAATGT
CGTTCCTGCTCACGAGTTGGCACGTTCCGTGTCTCGGCAGCGACTGTTGCCCCTGGCACG
CTCGCGTCAACGCGGGGATCGAGACGCTGAGCAAGCTCACGCGCTGTCCTTGTACCCCGG
AGCACGCGGGGGAGACGTCGTGGCAGTGCAGGCGCTGCGGCATCTTGAACCAGGAGTACA
GGCGGAAGAAGAGCTCGGGCGGCTGCAAACTCTGCGTAGACTTCTTGAACATCCAGCCAG
AACTTGTCCAGACAAACGTCCAGATGGCACTCTGAAGGCCCGGCTTGGCGATAATCCACA
TGCGGTCTCTTAGGGTCAGCGCACGCTTTTCCATTGTACATCGGCTCGAAGTGTAATGTT
GGGATTCCAGACAATGATATGGAATTG
>at00246
CCGTAGCCATTTTTGGCTCAAGCTTTTTTATCAAGCCCCAACCCCCTTAAGTATGTCGCA
GGAGGTCGCACGTTACACGATCATGCTGAAGAGCCATGGCACTGCTGTTGCATGTGGGAT
GATGCTGAGGCAGTGCAGCATTCCAGCCTTGGCTGCTGGAGCCACCTACACACAAGTTGC
TGCA
>at00247
CCGTAGCCATTTTGGCTCAAGCATGGTCCCAGCGAGCGCCCGAGCTGGGAGGAGGGTTCA
GAGAGGACCATCATGGCGTCGAAGCCCAAGTACGCCCAGCTGCACCAGCATCCGCCTCCA
CTGAGGGCGCCGAAGATCCCCATGAAGGAGCTAGCGCTCTCGCTGTTGCTTTTGTCTGTG
GGCAGCACGTGCCTCCTTTGCGGTGCCAGCGTCTTCTGGCGCACATCATTGTCGGATTCC
CTGCCATTCACGATGATGGGGGCGCTGTGCTTCATCCCCGGCGCGTACCATAGCTTCATC
TTCTTGATGATCTTCAGAAAGGTCCCTGGCTTCTCGTATGACATGATCACCAGCTTCAGT
AACTGACAACGGCAATGGCTTGCTTGTCCACAGTGACTGTACGAGTAGTCTTGCAGCCAC
A
>at00248
GCCCCACGAGGGATGGACACCTGAAGATGCTGTTCCTCATCAAGTGCTCGTACTTGTCCC

AAGCCCTCTCGCGTTGGCCATCATTCAGGGCAGCAGTGGCACGGAACTTGGCAAACTGGC
TGTGGAGGCCAGCCCAGTCCAGCCCAGTCCGAAGCAGGCAATTCCTGCCAACTGCTGAGG
ATCTTGATGGAGTCGAAGCCCTCAATCTCCTTCCCCCGCTTCCTCTTCCCCTCCACATCC
GAAAACCCACTGGGTCGTGCAGTATTGCCAGCCCTCTGAGTGGCATGAAGTGCCTGAACT
TTGTAGAAGACAAGGAAGGGTGGGCTCCGCATGCTGGATGGCTACGCCGAGGTTGTTGGA
ATGAAAGCCATTGTGTGGTCTAACCCTCTGTGAGCTTGCAGTGCTTTACCGATGTCCACG
AGCCTTCCACAGAGGGAAGGATCGATAACCAGGGCACCGAGCATCCTCTCGATCTTCTCA
GCC
>at00249
TTTTTTTTTTTTTTTTGTCAATACATGATCTTTTGGAAAGCACGTTGCAGGTGCTTGCCA
ACACAGTGACAGAAATCGTTCAAGGTCAATCCCTTGAAATCTCAAGGCGGGTCGTGCAAG
CCACCCTCAAACTCTTGCCAGCAACGGGCAGCAATATCAAGTGCAGATGCAGTTGGGCGG
CGTCTCACAGGGGAAGAGTCTTCTCTGTGCGTGCCTTTCAGAAATATCTCTTGCTCCATG
CCAGAAGCAGCCTGACAATGTGTGACATCGTCAGCAGGACGGCTTGGGCGTGATGCTGCT
CTGACTGTAAACGGAGTTACTACATGCGTGCATTCACCGCTACTTGATGAAGCGTCACCG
CCAGCACCGCGGCTGCTGAGCTTGCGACAACTTGCGGTGCTCCTGCTGCTTCCACTCTGG
GTTGCCCTGCCTCCTGTGGGATCAGAGCCACTCGACAGAGAATTCCAAGCCTCGTGCC
>at00250
GGGAACCTGCCAAAGGGCGTACCCCCCCTGCACAAGAGGTGGAACAACGCGATGGACTTC
AGCGAGTTCTCACTGGCTACTCTCGCCGAGTATCACTGAGGGGTGTTGTAGGCGTGCTTG
TGACACGATGACCCGAAGTGATGAGCTATATTCATTGTTTGTTATAATGTGAAGATTTAG
TATATGTATTACCTTAGCATCAGTTTATTCATTGTTATGTTACATGTATGGTGTATGGGT
TTGTGTTCTGTCTGTGCATTTCCCCTTGATTCTATGTCGGCTAC
>at00251
TTTTTTTTTTTTTTTTTCAAGGCTCATACCATGGCCATTGAAATCCTACATTCGGCTAT
ACTGGACCAGACCCAGTAATGGCCCACAGGCTGGTCACTGCGTATCGCTCGCCCTGCATG
GGAGGTAGCCAGCCCAAGCCAACCAAGCCAATTGCATTTCGAATTGCCGTCGTCGCCGCA
GTCGTCCGCCTATGCCCCAGCCCATCCCCAAGCGGGCGCCCAAGCGCATGCACGCATGCA
TCCCCCTGGCCTCTCAAGGACACGCCGCCCCTCGCTGCACGTTCAAGACCGCCTCGGGGT
GCCGGCCGCCCCCGGCATCTGGCCAGAGATGGCCCACAGCTCCTCGTTCTCCCGGAGGAT
TGCCTCCAACTGCCCCTGCCCCCTGTCCAGGGCATCCCTGTGGGCTGCCTCCACGGATGC
CAGTTGCGCCTCGTGCTCCAGCTTCACTCTCCTGAGGCGAGACTCCACCTCCTCTTGCTC
CTCGTGCC
>at00252
TTTTTTTTTTTTTTTTTGCCCCCCCCCCCCCCCCAACCCGCCGCACCCTGGAGCCGCGG
AACGCGCCCAGGCCGCCGCCCCCCAACAAATGGCCGGGGCACACGAGGGGATCTTAGGCC
AAAGCTCCGCGCACCCCTTGGCCACCGGCCCCTGGATGGCCGAGCCCTTCAACCCCCCCT
TGTCGTTGACAAAGACGCCGGCATTGTCCTCGAAGTAAACGAAAATGCCGTCCTTGCGGG
GCCAGCACTTCTTCGGGGGGATGACCACTCCCTTCAAGACCTTCTTCCGCAACTCAGGGT
TGCCCTTCTTGCAAGTACAAAGGACCATGTCGCCAGGCCCGCACTTGGGGATCTTGTTCA
GG
>at00253
TTTTTTTTTTTTTTTTCTGTACACACGTTGGATCCTCTGCTCCCGTTGCTCCACCAACGT
ATGAGGCCGACACAAGCACGAGAAGGGAAAACGCAATCCTCCATCAACGGGGCAGATGTT
CAAAGACCAAGCCTTGTCTGCAGAACGTCTCAGACCTAATGCTGATTCATTTGGTCACTC
ATGATCTGGCAAGAATCTTTGCCAGCGGCTTACCTACGGTGATGAGAAGTCCAACCATAT
CTCCAGCCCCACTCCTCAGAAAGGTGCACACCTTCGACATAGAAGAAGTAGAGGGAGTGC
ACCTGGGGAGCCTCATCACCAGTGAGCTTCACAAGAATTGCTTCAGGGTCGTCGGTCATG
TCGATAACGCCCTTGTAACGCTTCTCCTTGTGGAAAAACACCATGGCCTCTCTATCACGC
GCGCTCGTGAAGACGCCTTCGATGGACGCATACTGGAGGTCGATGCTCTTCCAGTCACCT
GCGGTGTCCCGCTTGCACTTGGCCAGAAGCTGGTCACGTGGTTTCTCCTCAAGCCCGTCC
TTCTTCCCAGATCCGGTGGGCAAGCTCTGGACCACCCACGTCTCCGTGCCAGCGGAGGGG
AGCGCGGCCTCGAAGCCCGCGCAGTCGTGGCCGTTGAGCTGGCGCAGCTTCGCCTGGACG
ACTCCGTCCACGGCATCCTTCAGCTGCCCCCGCTCCGGCGACTCCGCGTAGAGCCTGAAC
TCCGAGATGCGGAGCTCTCCCCCCTGCTTGATCACGTGCGCGAACTGCGCCCAGCGGGCC

TTCTCCACGCGGGCCTCGGGGGCGCTCCGCCGGAAGACCTCGTAGAGCGCCAAGCC
>at00254
GGCGCTCAAGGACCAGTTCACGAAGAACGACGGCGCCCTGCCCCTGCCGGTCATGAAGAA
CTCGAAGACCGTCGACCNCCGCGACAAGGCGTCCACCCAAGGTCCTGCAGCTGGAGACCG
CCATGGGCGCGGCCATCGAGTGCTTCCCCGGGGCCACCGCGCTCGTCATCCCGCGCTCGC
GCTTCGCGCCCGTGAAGACCACCAACGACCTCTTCGCCCTCCGCTCCGACGCCTACGTCC
TGACGCCGGACTGCCGCATCATCCTCGCCGAGTCCCGCGGCGGCGTGCCCCCCAACATCA
AGCTCGACGGCATGTACAAGTTCACCGACGCCATGGAGAAGCTCATCCCCGAGGGCCCGC
CCTCGCTCATCGACTGCAAGAAGCTCGTCATCGAGGGTGACGTGGTGTTTGCCAAGGATG
TCGTGATCAAGGGCGAGGTGACCATCAAGAACGCAGGCGGGGGGTCAAAGGTGGTGGCTG
CTGGCACGTACGAGAACACGACGGTCGAGCTGTGAGTGCTAACCCTGGAGTCCGGACGGG
GCACAAGAGCCTGCATTCAAGGCAGTGTGGTGCTGATGGGCCCACACCTTGCTCCGCTCG
CTGACGACTCAGCTCAACTTGTTTAGCGCGACCACATCTGAAAGGCCGTAGCTGAGAATA
GCTGAGGCTTGCAAACCAGAGATCTGCACTTGCGTGGTCACAGAAACATGGAGCTCTTGG
ATTGGAGTCCAACAGACAAAAAAAAAAGAAAAAAAAAACAA
>at00255
TTTTTTTTTTTTTTTTTAATTTCCGCAGGGAGGCCACGGCCGAGACTCCAGGGAAGGCG
CCAAGCCCCTCAGCTCTTGAAGACATACATGGGGCCCCAGCAAGGGCTGGCAGCCAGCAG
CCCTGGAGCCAAGGAGGGGCTGCCGCGGAAGACGTTGTGGGTCTTGAAGGTGGAGACTTC
GCTGTCCTGGATACGGCCGTCCCCATTCGCGTCCCTGATGGCATAAAGGTTGCCGGTGTT
GCCGCTCGCGGCGTAGACCGTGCCGTCGGCCCCGATGGTCGGAACCGCCCAGGGCTGCGG
CCGGCAGCTAACGTCGTGGCGGGAGTTGACGACGTAGCGCTCTAACCGCGCCGGCAGGCG
CTCCTCCTCGCCGGCGGAGGCCCAGAGGCCCCAGGGCTCCTCCTCCCAGTGCCAGAGCTC
GGCGCCGGTCTCGGCGTCGAGCGCGAGGAGGTGGTTGGACGTGCTGGGGACCTGAAAGAG
CCGCCTCAGCCCGCCCCACCGCAGCATCAGGCGGGGGATCAGAAGCCTGAGCCACCGCGG
CAGCCACTGCTCGCCCAGGGCTATCTGAGGCTCCCGCGTGGGCACGCCCGCACCAACGAA
GACGGCCATGCGGGAGCCTGGTCCATCAATGAAGCCCACGGCTGGGTACTGCTGGCCGCT
GGTCNCTGGCAACAACCGTCTTGCNCACAGGACCTCGCCATCGTCGACCCTGTANNGCAT
GATCAAGCCGTTCACGAAGAGGCAAGCGAACAAATCACACGGNGGGGATGGTCCTGTTGG
GCCAGCT
>at00256
CCGTAGCCATTTTGGCTCAAGACGCAGCAGCCAAATGCTGGGCTGTGCCTGCGCCATGGA
GGGCTGGACGCTGATGCGCATGCAGCCCTTGGGGTGTGTCCCCTTGCAGTCTCACAGGAT
ACTTCCATTTGAGCCAGTCGCAGTAGGCTCTGCTGGTCCAAATGAGCGGTTGCACGTGGT
CGGGGCACCAACTGCGCGGCCAGCAGAGCAGAGGTGTGGAAACCAGCGCCTCATGGCTCC
CCTAGCTTTGGTCGGAGTGGGACTCTCCACAACTGTTCATTGGTCAATGATCCGGCGCAG
ACGGCGATCTGGCACCAAAGTACAATTACATGCGCTCAGCGGTTCCAAGTCCCATGATAC
TGACGTCGTTGTCATTGGAAGTGGTCTTGGGGGTTTGAGCTGCGCTGGCGTGCTGGCGGC
TGCTGGCCGTCGCG
>at00257
TGGTCCAGTGGCACCGACGAGGGCGCGAGGCCTTCATTCCAACGCCGCCCTCCATTTCGC
GCCCATGGCGCTACAGCGGCGTCTGCGCCCGCAGGTGGGGCGCGACACTTGGCGATCCCA
GGTCTTGCAGGCACTCGTGGTCGCGGGGGTGCTTGGCGCCACGAGCTGGCAGGGCCGGTG
GGTGTGTTCAGGGACTTACCCTTTTGCA
>at00258
GCAGCGCTCCTCCCTCTGCGACTCCTCCAGCGCCCAAGCCACAGGCTCCTCGCGCAAGAC
CACCTCATCGGCAGCGATCTGCCTTGTAGCTACGAGGGCACGCCCCTTGTCTCGCATACG
CGCAATCCTGAATGGTGCATCCTCAGGAATACATGCAGTCAAAATCCCAGGTGCCTCTTC
CCGCGTATCCCTCTGACGAGCAGCTGCGCGGTGCCCAGGCCATGCAGTGTGACATGCATT
GGAGAAGCAGCTGCTCCCAGTTGTGCACCTACTGTGCCGCCAGCATTGCCAGAGTGCCCA
TGGCATCTCTGGCGTGCTGGTGCGGAAGTGCATGAGATGTTTCACAGATCAACTGGGATG
GGACATGGGAAAAGACCGATCATACAAGGAAGCACGGTGCCGAAACTCAC
>at00259
CGCAGCCTGTGCGAGGCGATGAGCCGCTGGAACCTCGCAATGACACCAGGAGCTTGCTGC
TCCGTGCACGAACGCGCAAAGGTGGCCAGGGACCTCTGCCAAGTGGTGCTCAGCAACTCC

TGCGGGTCGGAGTCCTTCCGGTGCCTCAATGGCGCGCAGTGCCCGCGCTGCGGCGTACTT
GGCGTAGAGCTCGGAAAGAGGTGCGACATGTGTGAGGAGAGCGTCGGCATGCCAGGACAC
CCATGACTTGGAAGGCGCGAGGACGATGTGGGGAGGCAGTGTAGCTTGCGAGAGCACTCC
GCTTGGACGGCTACTGAGCCAAAGGGCCCGGCAA
>at00260
TGTTTTTTTCCTTTTTTTTGAGTGCGAGCAGGCGAGCGTCTCCCGCTCCTGGGCACTCCC
TTGCACGCAAGTGCACTTGTGCGCTCCTGCGCGAAAAAAGGGGGCTTCTCAGCGTCTCGG
CTTGGTGCCATCTACGTGCTTCAGGTCCGCGGCGGCCACAGGCCGAAGGGCTCCAGAACG
AGGGTCCTCCTCCCAGAACTCGTGGGCTTCGTCGCCGTCTTCGTCCCGGTAGAGGCCGGC
GCGCTCTATAGGAGGAGCTCTGCCAGGAGCAGCTGTCCTGCAGGACT
>at00261
ACTACCAGTCTNCACAGCAGGCAAACAGGCCCTCCGTCCTTCGCACCGCCGCCCTGCCCT
CCTTCACTTGCTGAGCATCAGCAAGCTCGCGTCCTCCCTCATAAGCTGCGCCGCAAGCTT
TCGCTCTTCCACCTGGGCCGCGGAGCCAGCGTCGGAGGAGGcCCACAgGGTCTCCTCgGG
AGATgGTCAGAAGTTCTGACGTGGCAAGNTCGCTCAGCTCTGTCAGCGGATGACTGCTCA
TTATCCACCAATCAAGCTTCTGCAGACTCGCAAGG
>at00262
CCGTAGCCATTTTGGCTCAAGCCATTTGGCTTCGTCACTGGGCCGGCTGGCGTCGGGCCA
TGAACCCTGGGGCGGTGGCGCCATGCAGCCGATTTACAAGACGGTCGCCGATGCGGGTGC
AGACCGCAGAAGCAGGCTGATGCGGACCGCCTGAGTGAGACCGCGCCTGCGGCCGAGGGA
GTTGAGGCCCCAAGGAAGAAGAAGCGAAAGGGAGGCAGAGCGCGTAGAAAAAAAAAAAGAA
AAAAAAA
>at00263
ATGGGGCGTCATTTTGGGATCGCTCTCCCGTCAGATTGGGATCGTGCCGAAATCGCCTTG
GAGACCACGTCATTCCCAGCATGCAGCCCCCTCCAGTGGTCAGAGATTTCCAGCAGCGCC
CTGTCGCGCTGATCATCCTCCAGGGCACCGTCCTCTTCACAGAGTCCAATCAAGNTC
>at00264
AAGGCAGCACCGGAGAACCAGGTGCTGGGCATGCGGGTAGCGTGATTCTAAAAAGGGCCG
TTCTCTATTCAAATCTGATTGAGCTCGTTGTGCAAACGATGGTTGGAGGAATGTTTATGC
TGGCAAAGGTTAACCCGAATGAAGAGGCCGCCCCTCCTCTTCCTCATTCGCAGGTGCTCA
CTCTGATGTGCATCAAGTATGCGTTTGAGCTGCTCTCGGACTTTGTCATCATCGCCCTGG
GGCTCGGCATGGACGAGATCCTCGACCCCGGCATGGAAGGTACGTGGATGACGTTGGAAA
CGAGCGTACTTGCATCTGCCTGTTTCCTGGCAATAATTTGGGCACTCGAAACCCATGTCT
TGACCTTTCAGTTTCTGT
>at00265
CCGTAGCCATTTTGGCTCAAGACTTCACTTGGCATTGCAGGCCGTAGTGAAGCAATGATG
TTGGAGCGGGCAGCCCTGGCATTCCTGGCAGTCCATGCCGTGGTGGACGCGAGCCTCATG
GTCCACAAGAAGTGCCAGGGGCACACGTGCAACTCACCGGAGTTCCCCCTGCTGGATTGG
GACCCAGTGACGCAAACATGTCATTGCCGAGCACATCCATGCCACCATGATCAGAATGCA
GACGGGAATCGCGTCATTCACTCTTGCGGCGCCGACCAGCCATTCCTCAGCTTCTCCTAC
ACAGCCGACAAGAACTTGGTTTGCGAGTGCCTCATGAATGCTGCCGCTGGGAGCGTCTAC
ATT
>at00266
TGCAAAGTTTGAGTGCGCTGGTTAGCGACAGCAGATCTGGACTTGTTTCGGTCCACGCCT
GCTTGCCGAGCGCAAGAATCTGCTTGCTGCGGCCGTGGACGACACCAAAGCCGTACTTTC
CCCTGCAGCATGCCGCTAACGCTGTGAGGAAGACAGACTGCAGAATCGCATCAGAAGGCA
CGCCgGCGATTCGGTCTGCAtTTCTCGCACCTGCAGAAGAGCGTCCCACAACGTGAACCT
CCGCAGCGCTTCATAGCGACTGTAATGATGCTTGAGTCCGCCTGGCCACCGTCGATAGCT
TCTTGCAAATGACGAAGTAGCTCTCCGGGANACTCAGAGTCTGGAATGCGCACACGATAC
TGCG
>at00267
TTTTTTTTTTTTTTTTTTGGCAGACATCGCTCAGCAATGACTTGCGAGGTTTCGACGTGC
GTCCAGGTCTCTGCGTGGCACTCAGGCCTCTCGGCTCAGAAGTTGATCTCATCGCGCTCA
GCTCTCCGATTCAAGTCGGCACTCAGGACCTCCTGAATACGAGCAAAGTAGTTGTCCTCC
ATGTGCTCCCCGACAGCTTGGGTGCCAGTGCTCAGCACTCGCCGAAGTTCCGCTACCTCG

TTGTCGTCCCATGAGCTCCAGATGTCGCCGTCCGAGCGCACCAGGGCCGAACTCCTCCCG
TCTCCGTTGCCTTTGCTCAGGAGGGCCTCGACCTCCAAGGAGGCTGCCTCCGCCGTCTCC
TTTGCTGCCGCTTCCTGGAGGCCTTGTGTCCTCCCTCTGACAATGAGCTCCTTGTTTGTT
GGGTCCAGCTCAACGCCAATTCCAGCATGGCGGAAACCAAAAAGCGTAGTGGGCGGCACA
GTCGGGATGATATCTGACTTACTGGAAGGTCCGCCAGGCTCGCGGCACCCATCGATCATA
GACTGCACAGAACTTTGCACTTCNCACTCGGGGTGCACCANAGGTGCATGCAACCACATC
TGCACCCTTCAGCGGTGAAGCCTCGTGCC
>at00268
GGGGTCTGGAGGTTCCCATGCACCCTCTCCCCATGACGGCGCGGCCGGCATGCAGCAGTC
CAGCCGCGCAGTGTCAAGCAAGTTTACGGGAGGCAGGTATTTAATGGCACAGGACATTGA
GCTCCGTGGACAAACATGTAAGAGGTCAAGGCGCGGCACTTGTGGGTCTTGGTTATGCGT
GGAGCCTTGCACGAGCAAAGACAGGCCGCTCCCAATTTAGCACTCTCTGGCTCTGTGCTT
GGATGGTCCTTGCATTTGGCATAATGCCCGGAATCAAAGCTTCCAGAGGTGACGAAGCGA
GAGATGCTTGATAGCCTTAGCAGGCCTGGCTGATCCTGG
>at00269
TGGAGTTGGCCATCGGCCCCAGGTCTATTCCCACAGTACGGCGCGGGGCTCGTGGCAGTC
TCGGGCAACGCCACAGGCGAGATGCCGGCTCAGATAGGAGGCCTCGCCCCGAACATCACG
GTGGAGGACTTTGTTATGCCAGAGTTTGGTCCACAGGCCGTCACTTGGGATGGCGATGTG
GACTTGCGCCATGGCTTGGTTGCAGGAAAGCTGTTGGTCACCCGTGCCGTGGATGAGACA
AGCATCTCGCACTACAACGTGTACTGGAGCAATTCTTCTGGTGTGCGCGGGCCAAAGGCG
GGCAGTATACCCTCTATCGGATTCAGGATGCCGAAGTGCGTTGGTCCAACGTGCT
>at00270
GGGCTCCCGCCACCAGCTCTTGCCGACGTACTTCCTCTTGATGAACTCCTTGACCCGCTC
GGTCTGGCTGCTGTCAGGCTCTGGGAACTCGTTGGCAGTCCATCGCGCCAGCCAGGCGGC
GGCCGCGCGCGCGTTGCCGCCGGCCTCCAGCGCCTCGAGCTCCTTGACGGACCAGTCGGA
GAGGCTGATGCCCTTGATCTTGTGCCCGAAGCTCCGGTGGATGCCGCTACACGTCTGGCA
GACGAACGTCTGGAACGTCAGGCATATGTATGGTCGGGGACCCCCGC
>at00271
TTTTTTTTTTTTTTTTTCGAATTTTCAGAGACGTCTCCGCGGCATGGCGCAGTGTGCAT
CGGCCCCCTCTCTATCGCAGGCTCGCGAAAGAGCGGTCCACCCACGCAAAGGCTTCGTCG
AGGCTACTTGCACCCCGGAGGGCGGAGAGACCTGGCTGGTAGACGCGCGCCATGTCCGCC
TTGAGACGGTCGCGGACCTCGTTCCAGTGCTCCGGAGTCCGCTGGCCCATGATCCGCTCG
CCCTCTTCTTGGGACATGCCGGCCAAGGGCTTCTTGAGGCCAAACACGAACTCGTACGAA
CCCGGCCAGAACGTCAAGAGCTCCAAACTTTCTACCAAGGCACTGTAGACAAAGGCGGGC
GTGTCCTTAGCCATGAAGTCAGCGAGCACAATGATGGCCCCCTCATTCAGGAAAGGTCTC
ACTATCTCAAGCTGCTTGAGGGTGCGCTCCCAACCTTTGGCAGAGTCGGTGCACCAGAAG
TCCAGCCCCTGATTCCTCCACCCCGAACGCAGCTTCTTCGGGCTGTCCCTCGTGCC
>at00272
TTTGTTTTTTTTTTCTTTTTTTTTCTTTGTAACGCAACGCTTGGAAGTGCACGCCCAGTG
GCGTGCAAATGTGGTGACCTGCTGGGGCTGTGCCGGCAGGTCATGCCTTGGGTTTCTTCT
CCAACATCTCCACAAGGTTCTTGAGAGGCTCCTTGGAGTCGGGAACACCCTTGAATGTGC
CCTCTTTCCAGACCTTCTTTGCACCGCCAATCGAGCCGACTGTAATGTCCTTCTTCTGTG
TAGGCTTTGGTCCCGCAAGGAGGTCCTCCGGCTTCAGGGGGATGCCTGAGGGCGAGTAGG
TCTCAGAACCAAGCGTCCGGAGGCTGCCCCCGGTGTCGCGCTTGGTGCTGTACTTGAGGC
TGGGCATGGCCGGGCGCTGCCAGGGGACGTTGGCCTGCACCAGCCTGGGCACATACGCGT
GGGCACCCATGTAAAAGACCACACCCTCATCCTCCTCAACGACGTACTGGGGGATGGTCT
CGACCATTGCTCCTAAAATAAGGGCTGCCGGGCAGACCTGAGGGTAGCCTGGAGTATCCT
GAGCCTGGGAGGCTTGAGCCAAAATGGCTACGG
>at00273
TTGTTTTTTTTTCTTTTTGGACGAGACCAATCATCATGGCTTACGCTCTCTGGAAGCAGC
ATCCCCTGCGGCGAAGCAGGCATGCACATAGAGCTGCTCCCCGCGCTTCCCAGTATCACC
AACCTTCACAAACGGATGCGAACGGCATGCGGACCAGGACGAGTGGCGTTGCAGCTGCCA
CTCCAGTCTCAGTTCTGGCCCATTGAAGGTGATTGAAAGCAGCACACCACCGGGCCGTAA
GACTCTTCGCGCCTCCCCTATAGCCTCGGCCAGGAGCGCCGCGTTGGACTCAAGGGCATC
CAAAGTGCTCTTGTCGATAATGACATCAAAACTTCCCGTGCCAAAGTCCAGCCGAGAGGC

ATTCATGAGTTCCCACCGCATGTGCGGCAAGGAGGCGCCAAGACGATTCCGCATTGTGGC
CAGCACGTGCTCACTGCTGTCTATGTTCACGATGTCCTGGAAGCCCTCCCTGTAGAGCCG
CTCAGGCATCTGAGCCTCGTGCC
>at00274
CCGTCGGCGCATCGTATCCCAGACAGTCCAGCTCACGTGGTCCTCGCCGCCGGTCCTGTG
GGCAATGAGCTCGGCCGCGCTGTCACAGCCTCACATGGTCACGGCGTAGCGCCTCATCGG
GAAGTTGCTGAACTTGGTCTTTGTCTTCTGTGTCTTCGCGTATTTCTGCTCAGGCTTCAA
TCTCCGCCTGATAGCACGGGTCTTCTTGGGGCGCAAGTCCAAGGGCACATACTTCTTACC
CTTCCACTGCTTCCGCGCTTCCGCCTTCTGCTTCTGGTTATAGACCGTAAGAATCCTGGC
AATGGACTTCCGCACAATCTTAATCTTTGCCAGCTTGGATGCTGCGCCGCCAGCTACCTT
GGCAACACGGAGCTGTGCGAGCTCGGCCTTCATCTCATCAAGTTCCTTCAACAGTTCCTT
GGAAGTCTTGGTGCGGAGCTCATATGCCTTCACGCGGACCATCTTGGCTTGATCGCAGAG
AAGCTAAGAAGAAACTTCCAGCTGCCCTAAACCTGGGCCCAAACGCCTTGAGCCAAAATG
GCTACGGACTCTGATACCACTGCTT
>at00275
TTTTTTTTTTTTTTTTTGAATTCCGTGAGCCTGGCGTGCCAACATCGGAGGTATGAGAC
GGAAATGCTGAGCGTCCAAAAGCACCTTTCACTACACAGACTATACAGTGCACACGCACA
TAAGACCGCCACAAATCCTGTTGTCAATCCACAAAAACCAAACCACTTCGCATCACTTCT
TCTTGCCACCTCCAGCTGCGTCCTCCTTCCGTTCGCAAGAGATAACCTTCCCCAGCATGA
CAACACCGTTCCCGTCCATGAACGCAACGCGAGAGAGACCCTCGCAATTCTTGAAGGTGT
CGCAAACAAGCGGCTGCTGAGGCTGGAAGCTACTGTGCCATCTCATTTGACTTGAGAGAG
TGCGGATCCTCCATCTTTTTTCCGCCGGTCTCCTTGCCCATCTTCCACTTGAGCTTGGAA
ATACGGCATGCAGAGCGACCACACCTCACGAAACCAATTGGTGAATAACCAACCTTGATC
TCATTCGGGATATCCAGGACCTGGATCTGGGCGTCGAACTCGCGCGTCTGACCCAGAGTG
GTGTCCTTCTTGTAAACCATGACGTCACCAGAGCGTGGCATGTTGTTCTTGTCCAGTCCC
TTGATGTTCANGCCAACATTGTCACCAGGATTTGCAAAGTCCACCCGCTGGTGGTGCATC
TCAACTGTGAACACCTTTGCAGTGCATGGATTGGAGGCTGTGTGCGTTGG
>at00276
AACACTGATGCCGACCAGTTGCAGAAGATATTCAAGGTCCTCGGCACGCCGAGCAACGAC
ACCTGGCCGACAATCACAGAGCTTCCAGACTGGAAGCCAGACTTCCCGACGTATGAGCTG
CAGCCGTGGGCCCACATCGTGCCCAACCTTGATCCGACTGGCGTTGACTTGCTGTCGAAG
ATGCTCCAGTATCACCCGGACAAGCGAGTGGCTGGAAGCTTGCAATGGAGCACGAGTATT
CCGGGGTCTGAGCGAGGCGATCAAGACATGAAGTGATGGCCGTCTCCCGTGAAGGGTTGT
CCGGTACCTAGGTTTTGGAGTACGCTCATGAAGCCAGCAGTAGTGCTTTAC
>at00277
TTTTTTTTTTTTTTTTTAGGCTTCTCCCGATCTGCTGCCGAGATTCACTCAAAGCTCAA
AGTACGAGAGATGCTCTCTTTGCACATTGCTGTACATAGTTGTGCAAGACCTGTCCATGC
ATCTCGCCGGCTCTTATCCTGGGAATGGTGGTTACCGTGTCCGTCGGCGGACGAAAGAAA
CAGAGAGAGAGAGAGAGAGAGAGAGATGCAGATACTTGTCGTGTTTGGCAGCTCTTGAATTC
GTCGCTACCCAGACGTCCCATCTGGAAGCACACTCGCTGATGAGTGGTGGCTCGAGCTGC
AAGACATGCGCCGCCTGGATACGAAGCTGCCAACAGAGAACGTGCTGAGGCGCCTGCTCC
GAGACTCCCAAGGCACCTCAATCACCCGGTCCAGCTCGCGGAAGACGCTCGCTGCCATCT
GGCGTGCCTCCTCCTCCCCGTCGGATACGTCTGTCAGCTCACTGCTGCAGGCTGCCTGGT
TGAGGCTCTCCACAAACTTCGTGGCATAGTTGAAGCCATGCAGTGTTGGAAAGGTGCTCA
TGATGTTGGCCACTCGCTGCGCATCAAGTGAGAGCAAGCGCGACTCGGTCAAGGCACGCA
GTGTGCCCTGGTGCGCCACCTTGTCCAGAGCAACCTTCAGCCACCCACTGCTTGGCCGTC
ACGTTCTGCTGAACCTCTGCACCCTGACGTAGACGCCTCGTGCCGAATTC
>at00278
GTCTCTTCTTCCGCCATGGCGCCCTCGTCCTCGGGTGTTATGCCCAGGATCTTGCTGATT
TGACTGAAGACCCCACCTGAAGAGCGCCGCGCGAGCATCATGCCGCAATAGTCAATCTTC
TCCACCGCATACTGCGCGTGCGAGGGCATGACGCTGAGGGCAGCTCCCACGCAGTCTGCG
TGCGTCACCAAGAGGAAGTTCCGCTTCGTGGTGATGCTCCTCTGCAAGTGAGTCCGGAAG
CGGTTCGCGGAACCNCGTGCCGAGCCGTCCGCGGATCTTCGGGCCACTTGGGCCCAGGGC
CCCCC
>at00279

CGCCTTGGGTCAAATGTTGTTGTAGCGATAACGGCGGCTACCGAGGATGCGATCGTGGCA
CTTCAGTTATGGAGCCACTGGTACTGCCACGGCGCACAACAGTCACCACCACGGGCACAA
ACACAAGGAGTGCGACCGCCACAGAGCAAGAGATGCTTGGACGAACCTGTGCAACCCCTTG
CGACACATCTGCGCCAAGACCGCCTCGAGCAGAAGGCGTCACGAGCCGATGCCATCAAG
>at00280
TTTTTTTTTTTTTTTTTTTTTTTGTTCACAGCATTCGCGGCTGACTTTCAGGCGACGC
TGCCGATGGCAGCTTGCCCATGCCAACTTTGGAACACCTCCTGGGGCTGTGTAGTTGTTC
CCTTTCTGCCCTACTCCTCGACCATGAAAGGCTTGTCCAGCTTGTCGGTGTAGTGCAAGG
CGGAGACTGGGGGCTTGTCGAGGTAGTACTCTGGGAAGTCGGGGTCGTGTGAGTCGCGCA
TCCAGACCCAGTATGGGTGCCAAGGCAGGTTGTACTGCGCGATCCAGGTGCCACCACGCT
CCGTACGACGGCGGTCAAGGTAGCGGGTGACGGGGTTGGTGTTCTCAATACGCTCGCGCT
CCTTGGTTGCCTGCATCCACTCGATGGTGTAGCGCTTGGGCTTGATGCCAATACTGCCGG
AGTTCTGGAAGATGACCATGGTCACCAGGGCCCAGAGCACGAGGTACGGCGTCACCACCC
ACATGGGGAACTTCTGGAGCCAGGCCCAGTAGGGAGGATAGTCCTGAGCAAAGTCGCCAT
AGTACACCGTGTGGAAGACACCTGCGGGCAGGAGAGACCAGTGCTCCCAGTCGTAGCCGT
GGTCCCAGATGGGCTGGTGCAGGGGGTACTTCTTCGGGGAGCCGTCGGGTTCAAACTCCG
GCGGTGGCGGCGGCGGCAAGTGCAAGAACGGCGGGGAGCCGCCAAGCCGGCTGGCCGAGA
TGGCGATGCTGCGCCTGCTGCCCGCCTGTGCGAGCGCGTGCTGGCGTGCCCTCAGCAGCA
ACATGGGCCCTGCGCCACAGGGAGGGGAATGCGAAGAGAGCTCGGGGC
>at00281
TGGGGGCAGCTGAACCAGCAGCCATAGTTCCACCACAAGCCCGTACACACATTGATCCTT
CGTCGTTTTTGTTGGATCTGATGCCACAACAGACACCTTGCGTCACTTCTGCATCGGTTA
TGATGTACCGTGACGACGCACGGCGTCGCATGCGTTCTGGATTCACGGATGGAGCTGTAC
TGCCATCTTCACGTTGGACGCGGATGTGCTCATCAAGAAAAAACGCCTTGGTCATGCTCC
AGCAGACTTACACCAGCCCGACTGCGAACGCCTGCACTTGCAAACCGAGGCATCAGACGT
CGGTTCCTTCGTCGAGTGCAAAAGCAAGACTCGGAAAAATGCCAAAGCTCCTGCAAACAT
GTTAAGCATGCTGGAG
>at00282
TTGTTTTTTTTTTCTTTTTTTTTTGGCGTGCAACTGTATTCACTTGCCAATGAACGATCC
AGTTGTCGTCCTGAAGTTGTCTCTGGTCACTGCTAAATCTTATCCCATGGGATAGTGATT
GGAATCAAAGCGTCAGCACGAATTCGTGGATTCTTGAGCGTCTTGAATTTCGCCCACAAT
TGTTGCAAAGCACTCTTCGTACAGTGCGGATCGTTGGGTAGATCTCGCCATGCGTCTTTG
AGAAAGGCCTGCTGTGTTTCACCATGAGCAGCCTGGCTTGCCATCTTGCCTTGGCAGTAT
TGCATCCGTGAAATCGACTCCCTGTAAAACGAGAAAGGTATCTCCATACTGCTATCCGTG
ATTCTCGCCAATCCCCAAAACTTTTCAATCCATTGCGACACCAAGCAAGACTCATAATGG
ATCAAAGCCAGAGGTGGCATCGAATGTGATGTTGCGTGGTGTGAGAGATTGTACTCAAGA
GTTCTTGCGTCGCTGCCGTCCGCATGCCTCCAAAAGTGAACACCCCACTCTGTCATGTTC
GCAGACACCCTTGCGGCCGCCTTTCCATTCGCGTATCCTGCAAACTGGTCCCACTTCGGA
TTCATCTTCCCAGTATGTCGGAAGCACCTCTCGCCATCCGCTGAATCATACACGGCTTCA
AGATTCTGCACGCGCACTTGATCGACATCCTTGGGCACACGCGCGAGGACTTCACCAACT
TTAGCCTGAGAAGGGGAGTAGAGGAGCTCATCATCGTCAATGTGGAAAAGCCAAACAAGA
CCCATTGCGTTTGCCAACGGCAAAGCGCGATCCATCACCAGTTCCTGTCTGTGGCGCAAC
GTAACGAAGTTGTTTGCCGGTTGAGGCTCGGTGAACTCATGCCATACCGTGATTCGAGAC
CTCACGGCCGGAGATAACGAGCCCCATGTACTGTTCCAATCGGGGGTGTTCTCCACGTCC
AGAAAAATGTGCGCCACGCCGACGTAGTCCAAGTGATACCTGGCCCAATCTGCAAAGTCT
GCCGGCTGCCTGGTCAAAGTAACGATCCCCACTCGCGGCAACCGGTCCAGAGCGCTCCGG
ACCTGCAGTGTGGCGCTCTCGTCGCCCGACGGCAAGTCGGTCGCTCCGCCGGGGCAGGCA
TGACTGTAGCCCTTGGACTGGCGGCATGCGGTCTGAGGGCAAGCACCTTGCCCAGCGAAT
GCATTGCCAGTGATGATGGCAACGATGACGTGCGCAAGGCTTCCGCTCGCCATGTTCGAA
GTGAGAGTCACGTAGATCTTAGCGTGAGGCCTTGAGCCAAAATGGCTACGG
>at00283
TTTTTTTTTTTTTTTTTGAAGCACAAAGAAAAAACAATGGCAAGTCCACAGGGATCAGT
ACCTGCGTTTCAAAGGTAAACCAAGATGGCGACGTCTCAGCGCCATTGACCGGGCACCTC
ATCTGCCTCGTCCTCATACTCCACCTGCACGTCGGCGACCTGCTCATCGCCATACGCCTC
TGAGGCATCTCCAAACTCCTCAGTCTGGTTGAAATCGGGATCTTGTGCTGCCGAACCAAG

TTCGCGGTCGTACTGGTCGAGGAAAGCTTTGAGGAAGGGTGCCTCATGCCTGTTTGGATC
TCGAATACCTGCTCCCTTGGCGTCGCAGTATCTCCACCAGGAGGCTTTGTCATCGTCGCT
CGTCCGCTGAATCCTCTTGATGCGCTCCACGAAGCCAGCTTTCTTGGGGTCGTCGGAACC
CCAGCTGCCCCCGCGCTGCGCGTAGGGCCCTCCTCGACCCCAGCTGCCTTTGCCTCCCCA
GGCATCGAATCCTCCCATTGGGTCATATGGCCCAAATGGCCCGAAAGCTCCCGGTGTAGG
CCAGCACATTGGCATTCCCCAGTCATAACCTCCGTAGCCAGATCCGGGGCCATAATGAGA
GCTTGAGGCAGCCATCCACTCGTCATAGCTAGATTTGAAGAGTTCGATGTAGCGGCTCCC
AACCTTCTCATTCTGCTTCTCTTCCATGGCGCTTGAAGCAGACTCTTCATCCGGGAAAAT
GACGAATGCCTCCCCCTGAGGGCCTGCCATCGCTTCCGATACCAAAGACCACACTGGAAG
CGTCCACACCGTAACCGCCAAGGAACTTCACAACGTCACTTGGCCCGGAGGTGAAGGGCA
GCCCCCGTAGCCTCAGAGTTGGCCCACCGGCACGCGACCCACTGCTGCAGCCTCCAAAGG
ATGCCCTGGCCGTCTTGCCTTTCGCCTGGCTCCACTCGTCCATGCTCGCCCTGAACAGCT
CCACGTAGCGGCTGCCCAAGTTCTGGTGCTGCTTCTCCGAAAAAGCAAGCTCTGCATCAC
CGCTACTTCGGAATCTGACGTAGGCCTCACCTGAAGGCCTACCCTCAGGGCTCATGGTGA
AGAGCACTTGTGACTCCTCGACGTCGTATTCCGCGAGGAAGTCTACGACCTCAGCGGCCT
TGCAAGAGAAGGGCAACCCTCGCAGCCGCAGAATGGTGCCCGTCTCGGCCCTGCCAGGGT
GGACACTAGCGCCCGCCGATGAGGCCTCTGGCATCTCACCCATGTCATACGCATTGAGGA
AACGTCTCAAGAAGTCGATGTTGTGGCGGCTGGGGTCGCGAGTTCCAGTGTTTTGCGAGT
CGCAGAAGCTCCACCACTTTTGCTTCTCCTCATCACCAGAGCGCTGGATTGCCTTCACCC
GCGAGACCAGAGCCTCTTTGTCAGGATCCTCAGAGTTGTCCCAGCCACGAGCCATGCCGC
CGTGCGACGGGTACATTGGCCCGTAGCATCCTCCCTTTGGCCAGGCACCGAATGGGCCAA
AGGCTGGTCCGCCATAGCCACCGGCACCCCAGGGATCACCCCTTGCTGGTCCATAACCGC
CGAATCGAGGTCCCATCATTCCGCCGCCGCCCTTGCCATTCCACATGCCCGACCGCAGTA
CCACCCCTCCCAAAACCCTTGAGCCAAAATGGCTACGG
>at00284
TTTTTTTTTTTTTTTTTCCCCCGACAATGGACCTCCATACTGCGCCACGTCTGTGCGGA
AGTGGATGAAGAACTCGAGCAGAAGACCAACTGCCAGCATCGAGAAGCCAAGGGTGAGCG
AGACCGTGGCATAGCTCATGCCAACGACCTTTTCCACCAGCACTGGCGCAATCAACTCTC
CCTCGTCTGAGGATGTCTGTGGCGCCTTTCGCAAGGGCCTCGCATCGACATAATGGGGCA
GTTGCTTATCCGTGAAGATCTTGTCTACCACACCAACAAGAAGCGTAGCAAGGATGCCGC
CGATCAGCAAGCCCCACGCACTTCTCACTCCCCCGCCCAAGCAAGGAGCCAGCATCGTCG
GCCCGCTTCCAGCAATCGCCATGCCCATGCCCAAAAGTGTTAGACCAAACGTCACTCGCC
CCAGCCCGTAGCTCTTCTTGGAACGCCCCCGAGTTGCCGCGAACGTTTCTGGGGCTATCA
TGGACATAAGCCCCTGGACCATCATGGAGCTGCCAACTGCTCCTATGAACATCTTGAGCA
CGTACAAAGTTCTTGAAAACGAACTGGTCGGCCAGAACTTTGGGATAGATGACGAAGGCA
CTTCACTGTGACTGAAGCCTTGGCATGCTGCCAAGGCAAGCCAGATTGCAATCCATGCCA
TGTCGCTGAGTGCAATGTACTTCTTGTGCTGCGATGCAGGTGCCTGGTCAGCCGCGCACA
GTGTGTCCGTCTCGTCACTGTCTGGACCCATTACACTGGCAAACTCTGGGTCCTCCTTTG
TGATAAGACCATTCACAGCCATCTTCTCGGAAATGCAAAATACAGCTTGGGGCTTTGAGG
CAGA
>at00285
TTTTTTTTTTTTTTTTTGGGATCGGCAAGTACCAATGCAACGGACAGCTGCTTACTTAC
ATGCCTTGCGAACATCATGGAGTGCACAACGATGGACCGCACTAGGGCTACAAGCACTAC
GTAAAAGTGAGCTTCTGTAAAGCTTTTGCTCAAACAGACCGCGCAGAACGCGCGCAACGA
GCCTGATGAGTGGAAAAACCTCATCTGATGAGGTGTGCAAAAAATTATGGCATCTGCCTC
TTGCATGGTGGGTGGGGTCTACACGAGCACCAGATTTGTCACGGTAGGCTTGGTGATTGC
ACTGTGGTTTGCTGATGCGGCGTCTGCCACTGCGGCGTGGGTCTTGCAGCGGCCGTCTGG
CTCGTACAATTCGTAAGCCTCCTCCAGCAACCAATATGACTCAACGGGGCCTTCCGGAAA
GTAGCTTCGGATGCGAGCATCTGCGATGCGTGCCCGCTCGTCCCATAGCGCCACAGCGGT
CAATGGGTCATCCAAGCGCGGGTCTGCGGGGTCAATCTCCAGGTGTTCAGCCCAGATCTG
ACGACGCAAGGAGCGCGCAAACCCATCGCCGTGCAGACCAACCGCCAAATCCAAGTCGTT
GGTGAACCCGGAACGCTCGATGCCAGCACTACCGACGATGGCGTAGTCATCGTCCGCAAG
GTAGTTCTTGGAGTGGAGGTAGATGACATCCCTCACGTCCTTTGGACGAGTCACGCCAGA
CCGCTTCAGCGTGAAAATGTGGACCTTTCTGCGCCAACGTTGCATTTTCTGTGAGCAGCC
GCAGTGACTGGTACTGCGTTCGTCGGCGCAGGTCTTCGCCGACTTTCACATCTAAGCCCA

GAAACTTCACGTCGAAGACGCTTGCATCATCTGTCACGATGATGACAGCCTCCAGCCGAG
GCAAGGCCTCGGCTACTGCAGCCATGGCCTCGGGAAAGAAGACAAACTGGTCCTCGATGT
AGAGGTACCGCTGGGCGCTGCCTACCATCTTGTAGAACGCCGCC
>at00286
TTTTTTTTTTTTTTTTTGCTTTTGCGATGTATTGCACAGATGCTGAAGACTCCAGTCGG
TTTCAAAAGTCAGTTTCTGAGTGTCTGCATGGCCCTCTGTGGATCGCACCTGTACAGCGA
CTGGGCCCACTGATGCAAACGACTCACCTCAATCACACATACACACTCTTAGCCCTTCAT
GTGTCATTGGCTTCGAGCGGAAGCGTGCGGCTCGACTCGAACTGCCGTCTCACATACTCA
GAACCTCCTGCAAGAAACTCGACTATGTTGGCACGTGCGCTCGGCGGAAACCTCTGCAGG
GTACCGTTCTCCACCAAGCGAACCATCGCCCTGACTTTGTCTGCGTAGTTTACGGTGACA
GCCTCTGAGCTGGGTGTGGGAGCGCCCTGCCGCAGGACGAAGAAGCCGAGGACCGTGCGC
TGCCCGCCATCGGGGGAGCTGCACCGCAAGGACGTCATGCCATGGTAGGCCGCGGCGTTG
TCGAAGGCAACTGCCGTGCCGGGCCTGATGGGAACGGTCTTCGAGACTGGGAACTTCGAG
CCGCAGCCTCCGCGCATGACAACGGTGATCTCGAGATCGCCACCTGTCAACGCCTCATCA
ACAGCTGGGTAGT
>at00287
TTCGGCTCAAGCTTAATGACGATCTAGGNCACAGCCCCTNGGCCCGGGCACTCCTCTGGG
CAANTTGCCCTCAGCGCTCAACCTNTCCATNGCCTTCATGGATCTGNCGTCGNTCCTGCT
GCTGCTCTGCCTCACGGCGGNGCAGGCTCTGCCGCCGCTGGACGGCGGCTGCCAAGAGGC
CGGCAAAGGCTCTTCCTGCCCCAGAGGAGGCAGATCAGAAGCCGGAGAGGTGTGGAGTTG
CTGCAGTGGCAGGA
>at00288
CCGTAGCCATTTTGGCTCAAGCTATTTTGACAAGATCTTTCTTTTGCTGAGCTACAAGTT
TATTGCAGATCTAGCCATGTGGAGCTCCCTGCTCGCGATGGCCCTCGCTTTTTCTGTGGC
GGTCGGAGAATCTCCGTTGCCGGCCCTGCCGCATGATGCCGCTGTCATTGATGATGAGTG
CCTCGTGACCCCTGGTGGCGGAGGTGGTGTGCCTTGAATGCCCTCCAGCTCCGCTCCACT
GAGCACCGTGGCTTGTCAGACGGAGAGTACGGACGCAGACGACAGGGACGCAGACGGCGC
TGGGACAGCCGACGCAGAGAACCTGGCAACCTCAACTGCCCTGCTGTGCCAGGGAACTGA
CAAATCAGGGCTCGCGGCGCCGTTCCCTCAAAGGC
>at00289
TTTTTTTTTTTTTTTTTTGGGCTGGCAGAAGCCAGTCATACCAAATCCATGCGCTCACGT
CTCGAGCAGGTAGAGCAGCCCTTGGCACCTAGCAGGGGCTCACCCAGTCCTTGCCATAGG
CCTTGCCAGAGTCCTTTCTGTAGCCCCTGCCATAGTTCTTGCCGCAGCCCTCAGGTGGAG
CAGCCCTTGGCACCTAGTAGGGGCCCGACTTGCCCTTGCCGTAGGACTTGCCGTAGTCCT
TGCCATAGCCCTTGCCGGCACCCTTGTTCCGGTTATCACACTTGAAGGCGACTTCGATGG
GGTTCTCGAGGCCCTCCGCGATGTTGCCGTTGAGGTTCTCCACGACCCACTTGGCCTCGG
CGACGTTGGCAAACTCCACGAGACCTGCTTGAGTGTCCTTGCCCTTGCTCGGCATCATCT
TGCACCACGTGACGGTGCCGTAGGCCTCAAAGATGCTCTTCAGCTTCTCGTCCTCGAGAC
CCGCGGGAAGGCCTGCGATGAAGATGTTGGTGTTCTCGCCGGCCATTGCTGCACCTACTC
ACGGAGCGGCACCCAAAACGGAGCTTGAAGAAGCTTCCCTCGTGCC
>at00290
AAAGTAGACACCGTTCCACACGGAGTTCCTGCAGATGGTGACCTGCAGGCCCACAAAGAG
CGCCAGGGGGCCCTCCTGCCGCAGGATCGAGGTCAGGGCGTGGAAGGTGTTCTTATACCG
CGACAGGAATTCCGAAGACTGCAACCTGACCTTGACGACCTGGAAGGGGGTCACGGTCAC
GGCCTCAGGGACGGAGCAGAATAGGGCGCTCAAGAACTCCACTCCCATGCCGGGACCGCC
CATCTTGCCTTCAAGATGCTTCTTGGCAAAGTCATAGGAGCTGTACATGGCCGAGTTCTT
GGGGACCATGCTGGACATCTCCGGAAGCATCCCCCGGTAGAACCGCCTGATGCCGCCTTC
CCTGTACACATCACGGAAGGCCACCAGCAACGAAGGGTTGGTCCCATGCTGCAGCTGAAA
CCGCGTCTTCAGCATGTCAACGGCTGCACGACCGTGCATTCCGTCATCCCG
>at00291
TTTTTTTTTTTTTTGTTTCGCAAGGTTCCTTGAGGGTCATGAGGCGCATTGGGCTCTCTG
AGTCGTTTGACAGCTACGCCGTGTAAGTCAGCCTGCACAAGGCGGCGAAAGTAAAGGCGG
CAAAAAAAGAGCTCGGGCCTGCCCCTTCCAACAGATTAGACCCCCACCTCCCCTATTTGC
CGTGGTGTCTCCGCGCCATGTCCAATTCATCCCATCTGTAGGGCATTGAAGCGGCTCCTG
CCATGCTGGGCTGTGCAATTCCCTGATACCAGGGATTTGGTGCAGCAGGACGATGCTAAA

GATTACAGGCGCATCATTGCAATCCAGAAGAGCACTGTCGACCTATGCGAGCCCTCCTCC
ACGCCTCACTGTCGATGGCTCGAGGCAATCAACTCGTTATGTCGGTCATTGGCTGCCGTC
TGCATGCAGCACCAACATCGACAGACATCCGCGGTGGCGTCGGACAGTCGAAGCCAACAC
TGAGGCCTCCTCTTGAAAGGCTGGATTCAATGCAACCACTTGCTGCTCCACCTCAGCCGA
AGGAGGCAGAATCTACCGAAGGGCGCGAATCTACCGAAGGGCGCCGCTCTGTCTTCTCGC
TCTTCTTTGAGTCAGCTTTCAGCTCATTCTCCCAAACCTTGAGCATAACTGCCTTGCACC
TGGTGCGGGTCAAAATCAACATCCATGTGCAGCAGCAACACCCGAGCCCCGACAAGCTGA
TAACAATCACCTCCGAAGATTGGCGACTTCCACTCTTCGCTGCCCGAACCTACGTCACGA
ATGGCAACTCCTCTAGCGAATGGGCCCTCTCCATGAGCGTTGCACGCAACTGCGCCCAGC
CTTTGCGCCGTGCCGGGTATGACGGTGCCGCCCTGTATGGCGACTCCAGGCCTCGTGCCG
>at00292
GGCTTTCTTTATCTTTCCTCAGATCACNCCGCCAGCGCNCACATGGcCGcCCCCACTGCA
GCCACAANNCNCANGCGCTTGNCCNGNAGCGAGGCCGCAATGCTNCGGNCAGACAgGTCG
GACNCAGANTNNCAGCCCAAGCGCGTCTCGGCCACGTCCACGAGCACGCTGGCNGCCTTG
GGGACGCTCAGCTGGAAGGCAGNCNTCACNTTGGTCAcGCaTGGCCANGGTGGCGTTCAG
ANACTTCGCAANNTTCGGNGCCACGTCCGGCGCGAAGCCCGCAATCATGGGGAGTGCTTG
GGGGAGCATTCCCTTGAGCTGCTCCAGCTGGCCGGGAGCCTGCTCGGTCATGAAGCTCGA
CGTCGCGGTNAGGTTGGCTCCCATGGGCTGGAACTTGGCGCAGAACGTGGCCTCGTCACT
GaATaGaAGGaCTTTTaGATCCCGGGCAANANGACAAGGTTGTGAACGGTNGAGGACACC
TGNTCCTTCGTAAGGAACCGGTCATTTGAAGCATAGGCTCTGACATGTTCCTCAGCTTGC
CAGGTGGCAGGAGACGCATGAACTGTCGAGAGTCCACGACCACGTCTGCAGCCATCTGTG
CAAGCGCTTGTGACTGCTTCACgGAGATAATAcCTCCATCGCCGTGAGCACCTCAGCCCT
GTTAGTGGCGTTCGTTGCCATGACTGACAGATTTGTAGCATCCTTGTACAAGTGCTCAGA
ATAGGCAAGCAGACCTGTTGACATGTTGCGTAGCGCTTCAAACATGATGTCTTCAAGGGT
CTTGTTCCGGAAGAGGCCCAAATACTCCATGCCGATTGCCATCATGTCCTCCATGGACAT
GTTGGGCATGCTCATGGGCATTGTGCCCATTGTGCTTGGCAACTG
>at00293
TTTTTTTTTTTTTTTTTGCTCTTTCTGACACGCTTCATATGCAGAATCGCGTCAAGCCA
AAAGTCAAACGAGGACAATTTTACCTCCTCGTTTCCGTGCACACAGCTTGGAACCTGGGT
CAATGCCACACCAGGTCTGCTGTGAACTGCTGCAATCTAAGCTAGCTGCGCTATGTTGGC
CTGGATGTACGACCATGTCCGTAGAGACGAGCAAAATTCTCTACTCTAGATCGATCATAG
ATGTCGGCACGTTCAATGCCGCCTCGTGCC
>at00294
TGTTTTTTTTTCTTTTTGTTTTGAGAACTCCATGCTGCACCCCACAGCTAAGGGTTGCG
GAGCCCAGCCTCAATTCTGGAAGAGCGCGTATCAGACCTGTGCGCATCACTTTTCCGCGT
GGCGGATCCTGGAGTAGTAAAGTGGTCCATGCTGAGCCATCTGGTTGGGGAGGATCATAG
TGCCGTGGGCCGTCGCCATTGCTCCTTGAATGGGCTGATCTTTTGCAGCTTGAACTGCCT
CAAACTCACCGTTCATGCGCCGTAGGAACCTCTTCACCGCCTCGTCGTTCTCCCGCTCCT
GCAGTGACGTTGTGCAATACACGATCGTTCCGCCTGGTCGCAGCAGCGCACCGGCACAAC
GCAGAAGTATCTCTATCAGCTCTGTCTTCCTCTTGAGCTTTGATGCATCGCGCTCCGCCT
CCATCCTGTTGTTCTGTCCTCCAAATGCCTGCGCGCGAGCGTCGGTGCACGGAGGGTCGA
CCAAGATCTTGTCGAAGGGCCCCAACCGCTGCAGCGCCAGGGGCACCTTCTCCGAGACCT
CCCCCTTGGTCACGGTCACGGGGCCACCCTTTGCCAGCAGCTCTGACGGCAGGAACGATG
AGAGCATACTTTCCAGCAGCGTACTTCGCACCTTGTTCGGCTCGTTGCAGACCAGCCGCC
CCCGGGCCTGGCCCCCGGGCCGGTCCAGCTGCCCCGCCTCGACGCCCACCTGCTCCAGCA
GGGACTGCTCGGGCGGGCCGCTATTCTGGGGTGCGAAGAGCATGGAGGCTATCACAAGAG
CCTTGGCGCCAGGCCCTGCACAAAGATCCAGAACCACCTCCCCTGGCTGCAGATCCAAGG
CATGTGCAGCAAATGCAGAGGCACCGGCCGCGGCTGCGCCCCCTCTCCTGGTCATGAAGG
TGGCCGCCGAGCGGAAGTCCGCGGCCTTGCGGTCCTCGGGGGGCTCGTCCGGGCAGTCGT
AGATCATAGGAAAAGGTATCTCCTCGAACTCTGTGAGGTCTTGCGCCGCACGGATACGGT
CCTGCACCTCCTGCGTTGCGAAGGGGTTGACCAAGGCAAGGTGCTCAATGGGAGCTCGCA
AGCCAGCCTCAACGTACATGTCGAAGCGCTGGGGCTCATAAGACTGGCCAGGCGCGGCTG
CTTGCGCATGTCTGTCCTTCATCTTGAAGCAGGTCAAGCCGAAGTCTTCGGCTGCATCCT
CCATGCATAGCTCCGCTATATTAAAGAGGGTTGGGTCGCCAGGGTTGACCGCCAACTTCA
TGGCCCGGGCTCAGGAAGATATGCTTAACAAGTGTCTTGAGCTTGAGACCTTGA

>at00295
GACTCTGAGTTCATGTGCAAGTTGGCCGGGTCCGTCAACGCCTCGGCCTTCTCGAAGAAC
TTGCTGGAGAGCTCTGTGTTGCCCTCTTTCAAGTTCTTCATGCCGAGGGTGTTGTAGCCT
ACGGCCAGCCGCTCGAACAGCGTGGAAAGAACCGTGTCCTGCATCTGCTGCACGGTCTCC
TGCTCTGCTCTCAGCACCGCCTCCAGGCACCTCACCGCCATGTCGTGGTCCCCGGCCGCC
TGCAGCTGCACAAACTCCCTCTCCGCGTCCACGAAGGGTCTCAGGGGGTCGTTGGGCTCC
ACGGACTCCATGACCTCGATCTTCTCCATGG
>at00296
TTGTTTTTTTTTCTTTTTTTTTTGTCAGTTCACTGGAGTACGGAGTTGTTGTCACTCAGC
CATGCTTCTCCAAGGAGGGCAACTGGGGTCTCCCGGTGCAGGTCTGTACTCCAGCTGGAG
CAGCGCTCGCTGCTTGATCTGCTTTAGGAGACTTTATTCTCGAAAAACCTTCCGAAAGGT
CTTGACCTCCATGTGCCCTCTGCCGAGCGCACCAGCCCATGTAGTGCTCAGGTCTCTGTC
TCCAAGCCTCGGGTCTCTGGGATCTCTCTGCCTCTGGACCTATTGTCCTGCGGTCTCCAC
CATGGCAACATCAGTCCCACGGGATGGTGGTGGTCGAGAGGTAAGCCCTGCAGCTGCAGA
AGACTCCTCAAGACACCCAGGGATATCCCGGAGGCTCCTCGCATCCGCGATGCCATCAGA
CTCCTTCGCCTGCGCGTGCTTCAGCCAGGCATAGCCAGCAACGCCAATGCTGGAGACTGC
AAAGCCAGCCATTTGCGCAGAGGATAACTGCTCGTTGTCTTTGATCACGAAGAAGAGGAC
GATGATCACATTGTGTACTGCGGACAAAATCGAGACCGTCAATGGCGAAGTCAGCTGAAC
AAGCTGGAGCTCAAAGATCAGAATACCAGTTACACCGAATCCAAATGAGATGAATACGGA
CATGGGCGGTGCACTCCAATCGCTCTCGAAAAAGGGCAGGAAGCACATGGCAACGATGCC
TGTGACAGGCTGCGCTACGACCGCAATCTGCATCTTTGAAGGGGTCAGAGAGCCGGAGGA
GCCATTTTCATGCGAACCTGAGTGTCCATGGCCAAGAAGCAACTTCATGAGCGAGTACTT
CAATGCATAGGCAATGCTCGATGCCAGCTGCAAGCAGATCCCAGTTGCTGAACCACGCCA
GCTCCCCTTCCCGCAAAGTGCAGTGACGACTCCACCACTCGCCACCAGCGCTAGCACAGG
AACCAGAGCAAGATTACCGCGGCAACGGCAGCCAAAGAGGCACGATTGTGACCGCTTGTC
CACGCCGAGCAGTGCAGCGACAAAGAACGTGACGAGAACGTTGAAACCATGGAGTGCCGT
CTTCAGACCACCGCCGATCGAGTACAGAGCCGAGTTGGTCAAGTTCCGCTCGAGGGCTGA
TAGCACCGATATCAGCAGCACGACGCGCCACTTCGCGCGCAATGGCTGAAGACCCTCAGA
CAAAGCGCAGGGTGCAACAATGAGGGTGCCGACTATCCCGTTGCCATAGACGCTGAGCAT
TGACTTGTGTGTTGCATGTTTCACCGCAGCCACCACCCATATAGCACAGGCAAACCAGAG
GATCATCACCAGCCACCACTGTCCCCATGCCCAGCGCAAGCCACTCTGCAAATGTGTTCG
GCAGGTCACCATCGTCTCAGGCATGAAGCAGCCTCAGGGGGCGCGGAAAAATGCGATGGC
ACTCGGGTTCTAGGCTTGAGCCAAAATGGCTACGG
>at00297
TGCCGTTTGTTGGCGACGGCCACGCACGGCAAGGAGATGGAGAATGTTGTGGCACTGCAC
TCGAGACCTCACTCACTGGACAGTTCAGACTAAGCGTCATCAAGCCTGTTCCATTGCCGT
CCGCCGTCTGTAGCGGATGTCAGTTGCTCCCCGTGCGAGCTCCTTTAAAGCGTCCGCGTG
CCGAGACAGCCGACGCCATTATCACAATGGCAGTTGACGACCATGGATGGCTCGGCTCTG
CTGCAAATGCCGCATTGCAAGACATGCTCGAGTGGCTGTCTGAACTCAAGCCCTCCTTGG
CTCGTGCAGACGCCTATTGCTTCTTGTCGGTGGCCGCTGACGTGCGAGTGACGCAGCTGG
TTAACAGTCCGACAAAGGGAGCGCATGTGGTGCTGGAG
>at00298
CTGACCTCTTTCCACGTGACCACCAGCACAGCACGTGGGGCAATCTGCCCACGCGCCTCT
GCGAAGAGCCACCTGGCAAAGTGGGCATGGCTCCGGCAGTACTGACACTTCTGTTTAAAC
CGAAGTTTGGCTGACTCGTGGCCAGGCTCGAACCCACAGAAGACAGTCACCGCCTCAAAG
CTGACAGCCTCACTAAGCATGCATCTGGCTTCCTGACGTCCACTTCCGTTGAGCGAACCC
TCCACTGCCTGCGGCCGAGAAACATACTCAAGCATGTGAGATGGTGTTTGCTCTGCTTGG
GTAAGTTCCGTAACCGTGGAATGCAAGTCTCCTGCAGAAGCCATGTCGACTTGCTGCATC
GTGAGCGGTCGCTGGTGTACCTGCCACTGCAGCTCAGCTGGCACGCAAGA
>at00299
ACAGTCGCCACATGTTCCAGCTGCTTGTCTATTCTAGGGCCCTTGGCCCATACCGAATTC
TCCCGCTATGGCTGTTGCGGCAGCTGCCGCAGCAGCAATGCCACCACCAGGAACTGCGCC
TTCCTCGATGGGCTTCAGTGGTGGAGGCCAGGTTGCAGCACCACTGTGGCCTGAGCCTAA
CACCCAGGGACCAGAGGTTTGGGGCCTTGCACAGCCTCAGTGGCCAGCTCAGCCAGCGCC
>at00300

TTGTTTTTTTTTCTTTTTTTTTTGTCTCGTACCAAACACTCATACGGTGAACATTGCCCG
CAAGGTTGAGTGCTTCTGTACAGCCAAGGGTGCATCAGTGGCGTGAAAGCACCTATCAAA
CGCATCAGGTAGATCGCTTCTGAAACCAGAGCACACCCATGGAGCCCCTCACGTCACATA
ACTCAATCCGGAATTCTGTGCCTGGATCAGGACGCAGCGTCCACAAATGAGTGAACCCAT
CCTTCAGCCTTCTGAGTGGCACCAGCTTCTGGGAGCAATGTGCCACCTGCGACAGGAATC
TCGGCAGCGGCGTCGTACCCCGCTCTCAAGCCAGCCTCGAATGCATAGCTGAGGGAGAGG
CCGACGGCATACACCACCACACTTGCAGATCATGTCACCTATTCTTTTCCCCTGCGTACG
ACACGAGCTGGCCAGTGAGCCCATTCACCTCCACCAGGGTCCCCAGTCGAACTCCATTGG
TGGCCGCATGCTCAAGTGCCATTCGGTCAGGGGAAGGGGCTAGATGCCTTGGGATGGAAA
AACCCAATGCTCGAGCCTTGAGCCAAAATGGCTACGG
>at00301
CGTAGCCATTTTGGCTCAAGGCTAGCCTTAGTGCCTAGGTTTTCACGAGCCTGCGTGTGG
GAGCAGGCATCATTGCGATGCGCAATCTATGCAGTGTGACAAATGCACTCTCCTGCCTCA
TTCTGACCATTGCAGTTGTGGTGGGATTGGCTTGGACTTACTGGCCAAAGTACAATTATG
CATACCTCATCTCCAATAACAAGGTGCCCATGTC
>at00302
TTGTTTTTTTTTCTTTTTTTTTTCTTTGTTAGTCCTGCCCGCCAGTCTGTCTCCCGCCTG
TAATGTGGGACACCATCAGGCCCGGTTCTAAGCGGCCATCCATCTTCAGGACCATAGCAC
CCCTCTCGGGATCCACGATCACTTCCTCCTCGACGCCATTCACATCAACCTTGACTGTTC
CCATCTGGTCAAAGACAGTCACCCTGTCAAACTCTGGGATAGTAATGCCCATCATGTCGA
TATCCTTGACCGTCGACGACATCGCTGCAAGAAGCAGCGGGCTCGAGGCGCGCCAGTTGT
GCACCCAGGATCAGACGACGCCAGCCTCCTCTCCGCCGAGGACCATGCGCAGCGGAGCGC
GCGCGCGGG
>at00303
TTTTTTTTTTTTTTTTTTGACAATCGAGTCAAGCACCAGAATCCCGCAAGCACGCGCCAC
ATCCGCTCGCGCCCAGCAGCTGCGATGCAAGCGCCGTCGCGCATCCCTCAGCCCGTCTTC
TTCTTCTCCAAGCGGTCCATGGCCTTCGCTAGCTTCCTCATCGACCTTGCCGAGCGTTCT
TTCGGTGGACGTTCACGCATGAAGTGCCGCAATGCCCAGAAACCTGCGCCGAAGACACCT
CCAATCACGAGCACGGTGTACGCAACCTTGGCGGGGGTCGACATGCTGCGGCCGATGATG
CCCACGGCGCCGGGCATGTCTTCGGGCCGCATCTCCTTCGCGAGCCTGGCCTGCTCCTCG
TAAATCACGGACTCCAGCAGCTTGTACTGCTGCGAAGTTGGACGTGGCGGGGATGATGAC
TGCCTGAAGAGGGCCTCCTCGACATCTCGCAGCAACCGACCGCCACCCACGACCTTGGCG
ACCGAAACATTGTCAATGCTGGAATAGCAAGACATGACCATGGAGAAGAGCACCTGGCTA
GTCGCCTGCTCCTCAGACATCTCCGGTAACCCAACTGCTCCAGCTGCAGCTGCTCCTTCT
CTTTGAGCCATGAGACCGCCGACGGCCGTTTCCACCTCATCAGGATTGTCGGCCATTCGG
GCCCGAGTGGCTGTCAGGCACGCTCTCATTCGCTTTCGCCGGGAGCTCTTGTCCAATCTC
CTGTCCACGTCGGACGTCGCAGCCGTACC
>at00304
CCTCGGAACTGTACGCCAACGTCTGCAGGACTTGGCCTGTCACCATGCCGGGGAGACATC
CTTCGCTGCTGGTCATCAAGGCCAGACTGCATGCCTCCAATGCAGCGAAGGCCACGAGGA
AGTTCCAACGCTGCCCTGGGCCTGCAGAGTCAGCTCCACGTCCGATTGGGAGTGCACAGA
GCACCCCAACAGCCGCAATGCCAGACTTGATGAGTCCCAGCAAGCGCGCGCTGGCTACCT
TCTGCAGAGCAAAGTCATATGTTCCACTCA
>at00305
TTTTTTTTTTTTTTTTTTCACCCGAAGCTGATGATCTAAGCTCGCGCCCGCACTCCCGTG
CCAGTTCGTACAGGCAGATGGCAGCTGCGTGCGCAACGTTCAAAGATTCACCCTGCTGCT
GAGGAACGCAGATCCTAGCGTCCGCCAGCTCAATGGCCCTCTCAGACACGCCCAGGTCCT
CGTGCCCCAGGACGAGGGCCCAGTCCCTGCTGCCGCGCGGCTCGTGCGGCGCCACCCGCG
TGGAGAAGCGCTCCTCCGCCGCGTAAATCCGGATGCCGAGGGAGCGCATCTCCAGCAGGG
CGTCCTCCAGCCGGTCGGCCTGGTGGAAGTCCATGAAGTAGAGCCAGCCCATGGACACGC
GGCAGGCGCGGCCGCTCAAGTTGCCCCACGCCCCCCTCGAAGCCACCACCGAGGTCACTC
CCAGGTGGAAGGCCGTCCGCAGTATCTGCCCCACGTTCTGCGCGGAGCCGATGCCGTCCA
GCACCACGATGGGGGGCCGGAGCCGCGGGATCGGCACCGTCACGGGGAAGGCGACCACGT
ANTTGCAGGTGGCCGTCTGGTCGGTGCCCTCCTGGGAGAACTCCGCTGCGATGAGGGCGG
CCTCGTGCC

>at00306
AAATGGGGAACAGATGCGAGCTGGAATTTAACTCATTGGCAGCCCCCTTGGATCCCGTTG
CCATCACGCGCTTCGATAAGTTTCTCCGTACTTCAGAACACGTCCCGAATCCAGAGTGCT
GGGATGGCATGTCCTGTATGCGTGGTATCTGGTTCACATCTAACGCCAACAAGGGCTCTG
TGCTACGTTGCCTGTTGGAGCCTGGTAGGCACGTCGTCTTTGCAGACGACAGTCTTCGCC
ATGTCAATGCAGTTACTGCTTCACTTGATGGTCATGCAGCATCGGTGGACACACTGCACT
TCACTGCGGCCACTGCTGCTGCAAAGCGAAAGCTAGACCCCACCAGTTGTGACCATGCAA
TGGCCGGCCATGTCGCAGCATTGTTTGCAGAGGGCGACCCAGCCATCATCGGTCTTGTGC
AGAGACGGCAGCTTTTTCTGTCTAGATTCATCGCCCAGCAATTGGTTATTGAAGAGGGGT
TGAAAGGTAAAATGGACTCCGACCTGCAGCAGCTTGCTTTGGCTTTGAGGAGTGATGATC
CCTCCAATCCTCAATCGTAGAGGGGGGGACCAATTGCAGCAATCTGGACATGGCACCTTG
CTGTCTGTGAGATGAGAGGATTTGCAGCAACCCAAAAAAAAAAGAAAAAAAAAACAA
>at00307
GGCCAAGCAAGATCACGCTCCGATGCGGTGAGGGAGTGTTGGCGCCATGGGGCGAAAGAA
CGCATTGTCTGAGGCTCAGAAGGAGGCTGCCACATTGGAGAGAGGTGGCGAATGGAATTG
GAACCGGCTTGCTCGTCTACGGATTTTCAATTGCGGCCCTCGGTGTGATAGGTGCTGGTC
TCCACGGCTTTGAGAAGAAGGCAATGCACTCCCTCTACTCAGGCTTGGGATGCCTGTTTT
TCATGCTGATTTGTTTGCCCTCCTCAAGTTCAGCAACCACAGGACGATCGCTGTGTGTGC
CGGCGCTGCCTCTGTTGCGGCTGAGTTCCTCTTCCTCATAATCTTTGCCATCCAGGCTGT
TAGGTCGCGAAGGGATCCAGCCAAGCAAGATCGTTTCTACCTGTTTGTCGCCATGGAATG
CGCCACCGTCATTGGGCTGTTACTGACATTCAAGCCCTTGCTGCCAATGAATCGTAGGAG
CCGAAAGGTGAGGACACAGTGACCACGATGCGTGAGGTTGGCCGGGCATATCCTTTCTCG
GCTTCGTAGGGGTTGCTGAGCCGCAAGTGAGCTTGTGCGTAGAGATTAGTGCCGCTACAG
TACTGCGAAGTATCGCTTGCCTGTATGATACCGCAAAAAGAAAAGGAATGCTCTGTGCCA
TCGA
>at00308
TTTTTTTTTTTTTTGATCAGACGCATGCTCAAGCGTGCGGCTCCCCCGAAGCGAGAGGG
ACAAGGCCAAGGGTAGCTAGCTGCAGCTTACAGCAGGCTGCAAGCACCAGAGCCCAGCAA
CGACAAGGGCGCGCTCACCCGCCGCAGCCTCCGCAGCCGCCGCAGCCTCCGCAGCCTCCG
CAGCCGCCGCAGCCGCCGCAGCCGCCGCAGCCACCGCAGCCACCTCCGGCGGCGGCATCA
TCCCTTCGCCGCTCCTCGCTGGGGTTGGCCATCATCAGGTAGCCAGCAGACAGCACTCCA
GCACCTCCCGCCACGCCGGCCAGGGCCACCAGGCCCTCGCCGCTGCTGCCCTCCGCGGAG
CTGCCGGCGCGGCCCTGGCCGATGGGGACGCCCATGACCTGGATGACGGTCTCCGTCGCT
TCGCCGCCCGCGGCCACCAGGAGGGCGTGCTTCCTCTGGTCGGTCGCCCAGGACGGGTAC
AGCGAGAAGCTGGCGGGGGGCGGCTGCAGCTTCTGACCCGGCTGCAGGGTGCCGATGGGG
GCGCCGATGGCCGCCTTGTCCTGGTTCGCAGCCTTGAAGCGGGGCTGGAGGAGCACGAAG
AGGGTAGCAAGGCCCAGGCCAACGGGGCAGGTTGGCACCAGCGCCCCACTTCTGCAATCC
AGTCCGCCCGAG
>at00309
GCGTGGTTGAGACCTGGCCTCCTTGCGTTGCCACCCACCCGCGAGCGCAATGATCCTCAG
CCACGGCTCATGCGGTCACCAAAGTCTGGCCATGCTCGTGCATCAGCATGGTAGTGGCAA
CTTGCCCCCACTTGCTTCTACCCTGCAGAGCCGGAAGTGCATCTTCAGCTCCAAGGGCTT
CTGGCCAGTGTCGGTTGAGTCGATCGGATGCCGGAGTTCCCTGACTATGTGCCTTCATCC
AAAA
>at00310
TTTTTTTTTTTTTTTTCTGAGCAAGCATCAGAATGAGCGACACTTGCGTAATTGATGCTG
AACACGGACACGATGACCAGCATTGAATCAGCAAGCCTCACACTTNGGCCTTACCGGCCG
GCATCGTGGAACCAGGCTTCAATGCAGTTCCTTTTGAAGATCTACTACTGGATCCCGGTC
GCGCGCTTTGCCGGTTGTAGATGTCGGACAGAATCCTCATGAATGCCTCCTCTACATTTG
TGGAGTCCAAAGCTGAGGTCTCGATACAACTTGAAAGACTGGAGCGCTCCACAAAGGCCT
GAGCCTCCTGCTGTGAGACTTCCCGCTGCTCGTGCAAGTCGCTCTTGTTGCCCACAACTA
GCACGACGATGTCCTGGTCAGTGCGTTCCCGGAGCTCCTTCAACCAGCGCTCAAGGCTGT
CGAAGGAGGTGCGCCTGGAGATGTCGTANACGAGGAGCGCGCCCACAGCGCCCCTGTAGT
AGGCGCCCGCGATGGCGTGGTAGCGCTCCTGGCCCGCGGTGTCNCAGACCTGCGCCTCGT
GCC

>at00311
TTTTTTTTTCTTTTTTTTAAAGGCAAACAGAGGTTATGATCTCGGACATGGCGTGACATG
GTCAAAAGACTCAAGCGGCCGCTAAGTGCCAGCGCAAAAAAGAAGGGCACCTTGAAATCT
GCCACTCCAGAACAGATGACACTAGACTAGAACCACTGTCGATAAATTCGCAGGCATAAT
CGACTGCAAATCTGCGAGCTTCCAAGGATGGCGGACGGTGATGTAGTCCTGCTTGAAGAT
TCCAAAGCTCTCGTCATCGCCTTTGCCGCCGGAGTGCTGGTAGCCGTCAAGAGCCCAATA
TGCCCAGCCAAGGTTTGGGTGCTTCTTCAACAGCTGAATGGTGAGATTCCAGGTTCGGTC
TGCTTTGTTGGTGCCAAACTCGCCAAGCCAGAATGGAGCCGTGAAGGCCATCCCCTCTGT
GGTGACATATGTCTCGTACTGCCGGACGGATTTCTCGAAGGCAGAGAACGTTCTTGTAGC
CAGAAAGCCGTCCCAGCTGTAGAGGTGGCCAGAGTACACGAGGCGGTTGGCCACGGACAA
TCGGACCGGGTGCTTCTGAGCGTGGCACTCTGTGCACAGCGCGTTCGCGTCTGCGTGCGG
CGCTGCGCCGGCCAAGAGGGAGTTTGCAAAGTCGAGGCCCTCCACGATGATCAGCAGGTC
GGAGTTCACCCCCAGGACAGCGTTGCCGCACTTCTCCGCTGCCATCGCCCAGTCGGTCTC
TGCGTTCCCGTCGCCCCAAGTAGGCTTCACCTTGCCCGCCTGCCGCAGCTCGTTGCGGAG
GTCGGCGCCGACGACGCGCTGGTTAGTCTTGTAGCGCTGGGCCAGATCCACCCACATCTG
TTGCCACTTGCTCTCCGGGTACGTGGGGGTGTGCCAAAGTCCGTTCCCATCGTCCAAGCT
GCAGCACCAGCCCGCCTCACTGATGTGGTTGTTGAGGATGACCATCACACCGCGTGAAGT
GAGCGCCTCCACAACTGCATCGAAGGCATGCATTGCCGGGCGGCCGCCGAGGGACGGATT
TGCGGCGACAGCAGTCGGGTGCACAGTAAGGTTCTTGTAATAGAGGTCATTGGAGAAGAC
CAGGCGCACACAATTGAAAACCCATGGCTGCCACACTTGCAGCAATGTCACCAAGAGGG
>at00312
GACCGAAGAGTACGCTGTCACGGAGATCCTTTGGACTATGTCCATCTGGCTGGAGAGCGT
GGCCATCTTGCCGCAGCTGGTGCTGCTACAGCAGATCCGCGAGGTGGAGAACCTCACCTC
AGACTTTGTGGGTGCAATGGGCGCGTACCGCGCCTTCTACATTCTGAACTGGATCTATCG
CTACTTTGCAGATGACTACGTGAACTGGGTCGGCTGGATCGGCGGGCTGCTCCAGACGGT
GCTTTATGCTGACTTCTTCTATTACTTCGCGAAGAGCAAGTGGTATGGCAACAAGCTCAT
CCTCCCAGTTGCCTCCTGAGGCGAGCCACAAACGCGGGAGTTCTTGTCACAGGGGCTGCG
CTGAAGCGCGCAGAGGCTCTAGAAAAA
>at00313
TTTTTTTTTTTTTTTTTACGGCTCATGGCATGGCATGCCCATACCACCACCACTTCCCT
CCCTAGAGTGGCTCGAAGGGTCCCAGAGGATGACCAACTCACAAGTACCCCATGGACTTG
GAGAGGATCCAGCCTCGCTCGATGCCCTGTGGCAGTGAGGGCAAGGGGCTAGCATCAGGT
GGCGCAGGAGCCGCCTTCAGTGGCGGCTCTGGCTTCTCCACACCTCCAGACTGCACCGGA
ATAGAGTTCAAGACGCTCGCATGTTCAGTGATCCGCATGCACGTCCCACAGGAATACCTA
GACCTCGCAGCCTCTGCTGGGTCGTCTAGCAACACTTCACGGCGGAAGATGGCCTCCAGT
GCGCCGCTTCCAGAGGAGCACGCCTCCCGCGCGTCGCTGTGGAATGACTCCGGCAGTTTG
ATCTTCTCAAGCCAAGCGTCTGGAAAGTCACCTAATGTGCGGTATTTGGCTTCGAACCAT
GCTAGACCGCAGACTGGGAAATGGAGGATGCATGGATCACTGCACTCTCGATAGGCCTCC
ACATCCATTCGCCTCATGTCAGCCAAGGCCGTGCAAGAGCGCTTACCTGTGGGAAGTTGC
CAGAGGTGCTGGCTCCGTGGCTTGGCCCCCAGTCCAGCCCTGCATACGCTCTTACCGTTG
TCATAGAAAAGCAGATACTGACCTCTCCGAGAGCGGTCCATCCAAAAGCGCAAGCCCTGG
CGAGCCTCGGCACTCAGAGGCACCACGAAGTGGTGGCGCCTGAAGAGCGTCGTGGTGCCG
AAATAGTCAAGCGTTTCCGCAACCTCCGGCACACCCTCATGGTTGAGATATGTCATCTGG
TCCACACCATCTGCTGCAAGAGCCTGGAAGTGTGGCTCCACCGACGTTTCACGGGTGAAG
AAGAGCTCGTCAGAGTCTAAGCAGAC
>at00314
CCGTAGCCATTTTGGCTCAAGCCATTGTGGTGTGGCTCAAGTAACATGGGCTTGGACGGA
ATAGTAGGACCAAGGGAGGGCGGCAGTAGAGGCAGTGATGTTGCGAGCCTTGGGCGGGTG
TGTGTGCAGGCTGCTGTCATGGCTTGGATGGTGTTGCGCATTCTTTGCTTTTCTCATCGC
GCTTGGCGCTTTCATACAGGCCGGCACTTGCCTCCTTGTGCGCTGCGAGTGGCAGCCTTG
CTTAGCAAGCGGCGGCATGGTCTCAAACACAACAAGCGGTCTGAAGGTCATATTGCCCAG
TGTGGGTCAGACTGGCACGACCTCCGTACACTCCGCGCTGGCCACCA
>at00315
TTTTTTTTTTTTTTTTTGAACCGATCTTTCAAGTTGCCTCGAATCGACTCGGAGCTGAT
CTCCAGCCCAAATTCTGTCGCGTTCTGCAGCTCCGCTGCCTGCGCGCAGTCCTCGGGCGC

CTCACGCCGCGAGCGCGCCTCTCCACACGGCGCCCGCGCAGAAGTCCGCGCGGCCGTGGA
CGAGCTCCCAGGGCAGCACCTCCCAGGCGCCCGTGGCGGGGTCAAAGCGCTGGACGGCGC
GCTCGGCCGGGCTGCCGGGGAGCACGGAGCTGTAGCCGCCCAGGAGGAGGAGCCGGCCGG
CCACGACGGCCGCGGCGGCGCAGCTCCGCGCGAGCGGCATGGGTGGGGAGGCCGACCAGG
CCTCGGTCGCTGCGTCGTAGCAGGCCGTGGAGAGGGCAACCCCGTCCCCTGCATGACCTC
CGCACGCGAAGACTCGCCCACGGAGGCAGGCCACGACGTGGCTCATCCGCGGCCCCGGCA
TGGGCGGCAGGCCCCGGAGCCTCGGGCCCTGCTCCCGGAGCTCCAGGACCTCGGCGGAGG
CGAGTGCTCCGGCGACCTCGGCGTCCCAGCTGTGCGGGGTGTCCCTCCCGCCGCAGAAGA
AGAGGTCCTGGCGGCCTCCTCCACCTATTGCTGCCGCGGCTGAGCCGCCACGGCGCTTGT
TCATCGAGGCAAGTTGCTCCCACTTGCGGCTTGCGAGAACAAACGCCACGCTGACATGCC
AAGCTCCCAATAGCTGCCGCAGACATAGAAGCATCCATCCAGCATGGCTCCTGCACCGCC
AGCTGAGGACA
>at00316
TTTTTTTTTTTTTTTGTTCTCATTGCCATTGCAGAGCTTTGGAGTCCATGGCGATCCCGA
TGCGCAGTCGGCACGCACGCACATCTTGAAAATATTACAGCATCTCTTTGCAGATCAACG
GAAGCAATGGACAGCATCGATGCAAGACATCAAAAGATGCGTGGAAAAAGCTGGGTCAGT
GCCCCTCGGCCACTGCCCTGCACTCACCTTTGCCCCGTAAACTGCTGTAGGTGTCTCAGA
CCACGATGTCAATCTGCTGGTGTGGTGGTCGCGTGCATTGGGCGTTGCAGGTCACAATCT
TGGCGAGGTTCTGGTCGCGCAACAGCTCAATGCCTGCGCGCCCGCACGTGTCCGCCATCT
GGGCAGCGCACTTGGCTGCCGCCCTGAAACCTTGGGGTCCGTTCAGGGAAGCGGGGGCAC
ACTTATAGGCCACTGCTGGCAGATCTGTGAAGAACACAGCACGGCAAGTGCGGTCAATCA
AACACTTCGAGGCATGCAGCCCACAACTGCCTGCGATGCAGAGTGCCTTTGCCAGGGGAT
TCTTCCGCTTGTAGTAGCAATTGCTGATGCACGTGCTCAACTCCTTGTCGAAGGCCCTCT
TGGCTTGGTCAGTGACCATCTTCCAGACGTCAGACGACTCTTGCATGATGCCGTCGATCC
AGCAACGCAGCTCGCTCTGGCAAGAAGGGTCCCCCATGCAGTCCCAGAGTGTCTGGCCGC
ACCCCTGGACACCGTCTATCAGGCACGTCGCCATGGAGCGGATGTCCTTT
>at00317
GTGCCGAGGCCGCCAGTGAGGCCAACAACGCGACCGGGGCCAAGGAAGAGCTGGCTGCGG
CCGACGAAGGCCGCAGACTACAGACAGGTGACAGTGAGATGTGGATGATCGGAGGTGTAT
TCATGAAGTATTACATCACCATCTTTGACTTCGACCACGCCAGAATGGGCTTCGCAAAGC
CTAAGCAGCGGGTTGTGCCGCCGGCCTTTGGGCAGGACCAGCTCTGGGAGATGGGTCCCC
TGCCTTGGGACCTCAGAGGTTGGAGCCGGAGGACGGCGGTGTTGCGACTGCTTGTGCTTC
CAGCTCTTGGCATCGTGGCCGCCTGTTGTGTGGCTGTGCTGTTTCTACTCGTTGGGTGGA
GAACGTTGTCTGCGCGAGGAGGGC
>at00318
TTTTTTTTTTTTTTTTTTGGTGTTTGAATCGCATGATGACGTGCCCGTGGACCGATGCAT
GATCTTGCTCACAACAATGCACTGTGGCATGAAGGAGGCGCGACAGGCAACAGTTAAAGC
AAGCAGTCACATGCCGACAACCTTCCCTGACAACGCGTTGATGTGCATCTGAGCCTCGGC
ATTCCCATTCGCAGCTGCCG
>at00319
CCATTGAGCGCACAGTTTGCCTCCCTGCCAGCAAGAGCAAGAGGGGGCGCCATGGCAACC
ACAAACGGCGGCACCTCCAGGCCCAGGACGCCTTTGCCGGAGATCGACAGCGCCGGCTTC
CTGAAGTTCCTGCCAGTCTTCTGCGCTTCCTGGCGGTGCCTCGCGCGATCGGC
>at00320
TTGTTTTTTTTTTTCTTTTTTTTTTTTTGGGGTCAGAAGGCCGCAGCAGCGCCGCTTCC
GAGGCGGTCGGCTTCGCGCGTCAGCAAGGGAAAAGAAAAGCTGCAGGCGGTTCGCCCCCT
CAACTGCGGAGGGACGCCCATCCCAAGGAAGTCTCTAAGAGGGGGGGAGGAGCGATCGAT
CGCAGTGGCCGCAGCAGCCTCACTTCGCCATCATCATCCTTTTGAACTCGTCGTAGTTGA
TCAGCCCGTCCCCGTCCCCGTCGGCCTCTCGGATCATCTCGTCGACCTCGCTGTCTGAGA
GCTTCTCCCCCAAATTCGTCATGGAGTCCCTGAGCTCGAAGCCACTGATGAAGCCGTCGC
CGTCGCGATCAAACACCTTGAAGGCCTCGATGAGTTCCTCCTCCGTGTCGGTGTCCTTCA
TTTTTCTGGCCATGAGCGAAAGGAAGTCGGGAAAGTCGATGGGGCCATGCTCATCTGCAT
TGACCTCGCTGATCATGTTCCGAAGGTCCTGCTCCGTCGGGTTCTGGCCCAGTGCGTTGA
GCATCGTCCCTAGCTCTTTCAGGGAGAGCCGGCCGTCCTTGTCATCGTCGAACATGTTGA
AGGTCTCTTTGAACTCATCGATCTGTTCCTCGATAAGCTGATCGGCCATGGGGGCGAGGG

GCACTGAAGGCGCGGCCCCCGCGGAGCTGTGAGAGGGCGAGCTTGAGCCAAAATGGCTAC
GG
>at00321
CACCATCCAGCAGTGCAGCTTGACCCCGCCATGGCGCCACACGTGGGCTTGGCGGCCGCC
GTGGTGCTGGCGGCGGCGTGCAGCGTGGGAGACGCAGGGACGGCGAGCAAGGCTACAAAG
CCTGACTGGGTTGTGCAAGGCGTGCAGCTGGACCCTGCCTGCGGCACTTGCTTCCCAGCC
AATCGCAAGGCATGCAGCAAGGAAGTGTGGGACAATTGCTGTGCCCAGGAGGTCATTTGC
CACGACTACCACTGCCGAGGCGAAATCCTGGGCGAGCTTCAGCCAGGCACTCGAGTCCGC
GTCCGGGCTGACTGCACGTCTGACGAT
>at00322
TTTTTTTTTTTTTTTTTTGCACGATCTCGTGACTGAAGTGCAGGGCCGGCTGCGGCACTT
GCGAACCAGTCGCCAAGCTGCGCTGGCCCGACATCCACGGCAGGCACCCTGGCACGGCTC
CGTGCAATCCCACAGAAGCCACTGACTGGACAGCCCAGCCTCAGACAAAGCGCACGGCGG
GCCGTTGTGACACGCGTCGCCCGCCCGTCGGGGAGCTGCCTCCGCTGCCACTCAGCAGAG
GTGCGGCCGAAGGCGCTCGCGGACAAGCAGACGGACGCTCCTGCCGTGGACGAAGCCCAA
GGAGGGCCGTTAAAGAACTTCGCGTCGCCAGCCCGTCGGAGGGCCGCCTCCACGGAGGCG
CCCGCGGACGAGAAGAGCGACACTCCTGCTACACTCTGCAATCACAGAGCAAAAGAATTG
CACCGCGGAGAACTGCAAACGAGACCCTCGGAAATGTCATGGCACGAGCTCGTTGGAGCC
AGCATGTCATCGGGGGCACGTCCTCCGGCAAGCGGCGTCACCCTGTCGGCGGGCGTCTCG
TCTGGCTCCTCGGACGGCAGCGTCGGTATGACCCGGCGGCCCTGGTGGAAGCTCAAGGAA
GCGATGACGCAGATACCGCAGCCCAGCAGCTTCACGTACGTGGGCGCCGTGCTGCCCACT
GCGCTCTCGATGGCATAGAGGACCGAGATCATGCTCGAGTTCGCAACGCTGGTCGCCACC
GGGGATGCAAGCATGGACGAGGGCATGAGGGATACCTCCGTAAGGGACAGGAGGAGGGCT
GTCAGCAGCAGCTCCGGGAGGTTCGAGAAGCCTCACCCGCGTGCTGCACGCCCGTGGCGA
GCCAGGTCACGAGCGCCA
>at00323
CCGTAGCCATTTTGGCTCAAGGTGAGGCCTGCAAGTGAAGATCCCATCAGATGCAGGCAG
CACTCTGTGATCCTTGAATTCATGAATTCACCTGCCGTCGGGCGCAGATGGATTCGCATC
CATGCCGGAGGCAATGGTGTGGTGCGAATGCGCCTGTCCAGTTCGTTCAAGCGCACGGTG
GCTGTGATTGTTGTGATGTCCGCCTTGTCGCACGCCTTGCGCAAGCTACGCTCCGGCCGT
GTGCTTGGTGATTCAGCATTGGTGCCGAGCCAGCAGTTGCCAGGCTGGAGATCATGCTCA
GACGTGCTGCGCATG
>at00324
TCAGAAGGCTGATGCAAGTCAGTCACACACCTCAACAGCTTGACAGCTTTACAACCTCGG
CCAACTGCAGCCGCCCATTGCTCGCCGCGCATGGGACGGTCTTGCAAGTTCTTGGAGATG
CCTCGCAAGCTCTGGAAAATGCAGACCCAAGACGGCGTGCATCCTGGGCGGGGATTCGGC
TCGCAGTGCTGGCCGAGTGAATCATCCGCTGTGTGCCACCGAGGTCTGTTTCACACCGTG
TCCTAAGGCTCTCAGTGAAGGCACGGCATTGGAGCAGAGACCAGATCGGCTGCACAGTGA
CGCAGCCTGGCCATCTGCGGCAATGCAGCTCGAGGTTCGTTTAATCAGGTTTTTCACTCA
TGGCTTGATGGGCCCCCTGGGCACACTTGCAAGTCATGCAGCGCTGGCATTTCGATACCG
ACCCTCCTCCTCATCCAAACTTGATCGGGAACTTCGGCCGCGCGTACACGTTGCATACTC
CGGGCGGGCACCTCAAGCACCTCCCACGCATCATGCCAAAGTAGTCCTCGACCGGTTCTG
GAAGGTTCGCCAAGACCTCCGCGAGCAGCGCCTCTTGCCGAGCCTTCTCGGCCTTGGCCG
CCTCTTGCTCCTTGGCTCTCCTCCTCGCGGCCGCCTCCTCATCCATGGCCTTCTTCCT
>at00325
TTTTTTTTTTTTTTTTTTGACGCTCCGTCAGCATAAGTGAGGGCAAACCATTGAGCTTAG
AGCCCCGGCTCTTTCCACTGCGGAGCAGTTGAAAAGTGCCAATGAACTACCAAATGTCGA
GATGGCGTGTAATGCTATCAAGCCTGCAACCTGCACACTATTGGAGGTTTCCGGCTGGTC
TTGTGCACAAATTACGCACATGCTCACTCACCCACAGTACATGCCCTCACACGGTTGCTG
TCCGAGTGCTGTGCAAATCAGCATGGCTGGGTCTGCATCCTGGTTTTGACTTTCCCTGCT
CCGCTCCCATGGACACAAATATACTTTGTAATCTTTGCTGT
>at00326
GAAGATGGGTAACCCTTGGGATGCTGTGTTGCTTGATGACGACGTGGATGAGGTTGTGGA
GTACACAGCAGATGTGGACTACTGCGAGTATGAGGTCCGCAAGGAAGGCTGGACCAAGGG
CCTCAAGCGGAACATCGGTGCGTGCATAGCTCGCGGCGCAGTCCTTGCTCATTGCGATGA

GGGCTGCCTCTACGCTCCGGACTACCTCACCAAGATGCATGCGGAGATCGCAAGACTGGC
AGACGGAGGTGGCACTTCCAGTCCCGCTGCAATTGCTCTGAGCAGGTGGTACACCATGGC
TGTCAAGGATCAGGACTTCCGCCTTGTTGACTTGCGGCAGAGGGAGCCCATGTGGGACTT
GTTCAACAGGAACCCGGCCACTGAGCAGGCGAAGGAGCAGTTCAACTTTGGCTTCGGGCA
CATGTACACGCGCGCGGCAT
>at00327
GTGGTGAATGACGAGCGGGGCTTCGCGTGGCCCTTGATCGGGGGGGGGCTTTCCGCAGC
AGCCGCGGCATGGCATGCAGGAGCAGGAGAACTCCCAGAAGCATTGCAGACAGCGTTCTG
GCCGCCATTGCTAGTATGCTTCTTGCTGGCCCGCTGCTACAAGATGCTCAAGCTGGGCGG
GGGAAGCCGGGTAGTTGATCAGTGTGGTGCACCGTGAGAGTTATTGCTGT
>at00328
GCAGCTCAAGGAGAGACATCCCAAAGCTGCAAGCTTCTTGGGAACTTGCAGACAAGATCG
TCACTGATGACAAGTTTGCCTTGAAGATTGCTTCGAGACTAGTCGAGAACATTCCCGTGA
AGATGATGAACGAGGCTGGTCTGATGGTTGTCGCCACCAAGGAGTTCCAGAAGGGTAACT
TCATCGTCATACGTGTTGACATCGAGCACGTGGACGTCCACAGGATGGTGATGCTCAAGG
GTGGAGAAGAGAAGGCCAAGGCGGTGGACCTCCTGATCACGCTGGCGGGCCTGCTGCCGC
CCCGCTTCGGGAAGGACCTGGAGTACCTGCTCCGAGCCCGCATCGCCGCGGGCCTGCTGC
AGAAGATGCCGGAGCAGATCACGCGGCAGATGCAGGACCTGGGGGGCGTGGAGGTTGACG
TGGAGGCGAGGCGCAAGGACGCGGAGGCGGAGTTCCTCTTCGCCGCGCTGCGGCAGCTGG
AGGAGGTGTACGGCAGAGGGGAGAACGAGGGGCGCGAGAAAGTTGCCTTGTACGTGTGAA
CATGTGCAGGAGCGCTGACTTTTGAGTGCCAGAGCAGAAGCCTCACGCATAGTTACCAGG
ttCCCCtAGtTGCCAGTGGCAGTGCTGGCGCCtTTTTTCCTTCGGtTTCGCGAGTGTACA
GAGGGACTTTCAGGCGGCGGCCAGATGAAAAAAAAAAAAAAAA
>at00329
TTTTTTTTTTTTTTGGTTCGGGGAGCATACTCATGACCTCTGGGATTCACTCTCATCCGA
CCTCCATGCACACCTGAAAAGATGCATACCTGTACGTTAGAAATCGCCTTAGGAGGTGGC
GCATGCACTCAATGCATTCAGAAACCACGAAAAAAGTGTGCAAAACAGGAACCGCGAGTG
CCGCGCTGAATCAAGGGCCCACCGCCGTCAGAATTTATCTGCAGAGCGAGAGCAACGCCG
AGCATCATCAGAACTGGGGGCCACTGGGAACGTCGGACTCCTCATCGGACTCCGAGCTGC
TGACTGACGCCGACGACGTAAGTGCGGACTCCTCACTAATGCTTGTTGAGAAAGAAGACA
ACAGAAATCGCAGCCCCAAGTCGTTCTCAATCCAAGCCATGTCAGCCAGGCTTCTGCAGC
TCAAACCCACAGACACACTGAATGTCAAGAAGGTGACATTGCTAAAGACAACCTGAAAGG
CTGCGTAGGCATCAACCAAAAGAGTGGGAAGCGCCTCCAACGAAATCTCCGTGTGCTTCA
GGTATAGGTATTCGTCTGAATACATGCCCAGCTTGAAGGACTCTCTTGCGGCAAAGTAAA
GTTTCTGTGGACCGCCTCGGTGAAATTCCTTCCACGAGCTGAAGAGAGCAAATATCGTCT
TGACCGCGGCACAATAGACGTGACCGCCAGCAATCAGCGCGTACATGTTCCAGGCATCGT
TCACCATGTCAATGTACATCAGCAAGACGTCAAGAAAGATGAGTTTCGACACAGACCGTC
CGGTGGCCATGCCAGGCCGCA
>at00330
TTTTTTTTTTTTTTTCCTGGCTACCTGTACATGAATTCATGAGGCCAGTGAAAACTCTTG
TGAGAAAAATGTGCATGTTGGATAGGTAGGCAGTCGAAGACTGGACAAGGCGCTGAAAAC
CCAGGGATTCCTGAAAATCAGGATTGCTGAGCAGTTCCCTACCGGCATTGAGCTGCCTTG
AGCTGCCTTGGACTACCTTGAACCAGCTCAGAGCAGAAGCTCACACACCACGAGGATTGA
GAAAGCTGGAGTCTCCGGCCTTCTCCGGCTTGGGTGTGATCACAAGCGGCATGACGCTGT
CCCAATCGCCGGTGATGACGTCACAGCAGGGCTTGAAGATGCCATTTGTGGTGCGCTCGC
GGTGCCAGCACGTGCCGCACCACCCATCAATACAGCCATACTTCAACTCCCTCCCCGCTG
CTGCTTCAGCAGCAGCAGAGAGCCGACAATTACGGCGCACTGTCATGGCAAAGCCCTGTG
GCATCAACACTATCTTGACAGGGCCCTTCCTGGTGTTGCGCATGAGGGTCAGGGTGATCT
CATCCCTAGTGTCTTTCGAAACAATGTCAATGACATCTTGAATCGTCCCGTCCTCAACAC
TTTCACCATTGACCGCCAGGACCTGGTCACCTTCAAGTATCCACATGCCACTGCCTGGCT
CATCATCAACGCAGACTTTCCTGTTGAGCTTGTCGGCGTTCCCGTCCTCTGAAATCTCGC
TGATGCCTATGCCTTTCTTCTGAGGGAAATTCTCCATTTTCACACCCAGCGGCTTGGGAA
CGACTACGTCAAAGATGGCACGGTCGCCACCAACCCGTTTGACCTTCCTGTACCACTCCG
TCCTGGTTGTCACGCGCATGATCGTACATGCTCGCTGCCAGCGCTGTTGCATCCGTGCAG
TCCTTGCTGCTCCGACAGCCAAGGTGGCCGCGAATGCTCCTGCAAGGGGCACGGCCAGGA

GTGCCAAAGGGTACATGGGCGTGCTTGTTGCTGGAGGCTTCAGGACGTCGCTAATGGCAT
GCGTGGCAACGTGGCCACGCAAGCTTCGTGCGGCTGGAGGAGTACCAACACTGAGGAAGG
CAGCAGAAGCATGGAGGACACCGAAGATCATCACGGCAACAGCAATGGCCGCGGCCAGCC
ACGAAACTGTCCGAGCTGCCATGGTCGATGGGTCCTCAGAGGAGAGTATACGCTGCAAGG
CACGGTGTCTGCATGCTTTGCTCCTGAGCAAGCTTGAGCTAAAAGCTTGAGCCAAAATGG
CTACGG
>at00331
TAGTCCTATTTTTGGTCTCCAGCTATTTTGGtCTCACGTGCCAAAGCCAAGCTTGTCTCC
TGTTTGACCCTGCTTCATGTTGCGGAACATGAATTTCCGCTCCGTCATCCTGCTGCtTCC
TCATCGCTCCAAACGCAGCCAAGAGGGCgGaAGCTGATGCGGaAGAGGGTGTCCGCTGAG
CTGGATTTGGACCTGGATGCGGCGAAGATTTCAGGCCCCCAGACGGAGGACATGCTCCGC
CTTCCTAAATGCTATTGTTCGGGCTGTCAAGGACAAGT
>at00332
GGCAGGAATACTAGGCGCTCTGGCGCCGCAGCGGCTGCTGCCAGCAGAGCTCCTCCCCAG
CGCGGCCCGAAGGAGAGGCCGCCTTGAGGAGCCGGCAACTGGACAGGAAGAAACTCAGCG
ACTGGCCGAGAGCAGGTCCCAAGTACAGCTTCGCCAAGAGAGCTGTCGCCAAGCCTGGCC
GNGCTGCGCTGCTCGTAGGATTCCCATGGATGCGGTGCAGATGTCAATGCCAGGGGCTCT
GGA
>at00333
TTTTTTTTTTTTTTTTTGCACACTGCGAAGATCAAGGTTGGTGAGTGGCCAACACCTCG
CGTCTGTACAGAGCTGTACATATGAAACCGGAAACCGATCCTGTAGGAACTGGCTTGCTC
CTTGCCATTGCGTTTGATAAGGTCACTGTCCAGCCGCCTCGCCTTCCCTGTGCACGTCTC
CTCGATCCTCCGGGAGTGCCGAAGCCACCACACGGCACGAGGTCACCATACAGATCCAGC
TGGCATCGCCCCTTAGTCCTCCTCAATGACCCTCGCTTTCTAACAGTACTCGCTGTCCTC
GTCCTCCGCCTCCTCCGACTCGTCCACTTCCGAGTCCTCCGACTCGTCCACCTCCGAGTC
ATCTCCCTCAGCCACCTTCTGGACCTCCTTTGTGCTCTTCTCTGTCGTGTCCCTGAGGCC
GACCCACAGGTTCCGCGCAGCCACCGTGACGACCTCGCAAGGATCCCCGCAGAGTGCCTC
GAGGACCGGAGGCAGGTCGTCAAAGCTGCAGTGCCTCTCCCCGAGGGCCTGAACCACGAT
GAGCCGCGCCGACGGCATGGAGGAGGCAGCGAGCACTCCGAGGACTTCGTCGGAGAGGTC
CCGCGCCACCAACTGGGGCAGCTCCGGTGGCTCCAAGGCGTTGGCCTCGCAGAGCGCCTC
GTGCCGATT
>at00334
TTGTTTTTTTTTCTTTTTTTTTTACGCTGCATGCCAAGACTGGCTACTCCACTTGCAGGT
CGGCATACCGGAAATGATCATTCTGAGTGGTCTTAAACTCCATATCTGCACTGTATTGGC
ACTCAGACACGTGCCAGGATCGGGTCCTGGCAACAAAAAAACTGGGTGAAGCAGAAAATT
GCGGTATTATAAGAGGAGCCTAGAGCAGTGAGGACCGCGATGACGTCAGGAGCGGCCCAT
CAACTTGCAAGGCTGTTTCAGCTACCCCACGAACACATAGCCATCGCCTCACTCCGCTCC
CTCCTCCTCCTCACCATCACCTAACTCTACAATACGCATCATCTTTTCATGGACAACAGC
TGCTCACGTCGACAACCGCAGCATCCCATTCTTCAAAACATCGGTGATGCATTGGCGGCC
CACCTCCTCTGCGGCCGGGCATGGCCCGTCTTCCACTCCATGGGCTTGCTCGGCACAGCT
GCGGGTGGGAGTGCCGGCAGAAGGCAGACAGTTTCCTTCGCCTTCATGACACGGAAGACT
CCGACTGACCTTGGCCTGGAGGAGTGTCCTTGAGGTAGTGGGGCTGTAGGTGCCCAGCAG
ACTTTGGCGTTGCTGCTCCACACCGCGGAGCCTGCCTGCATGGCGGAGCCTGCCTGCATG
GCGGCGGTGTGAGGCTGGGCTTCGCCACGCGAGGGAGCTGGACTCGGAACCCCGCTGCGA
GCCAGGGCAGGCTGCGACGGCGCGAGGTCAAGCATCATGGCCGGCATGGAGGCAGCCTGA
GCAGCGGAAGGCGCTTCGTCAAACGCCCGGCGGCAGTCCAGCGCATTGCATGGAGCCCGG
CACAGGATGCCTGATGGCGGCTCCGTCTGTGGCATGGACGTCTCACCTGCTGGGGCTCTC
TCTCCCATGTGATGGAGCTCGACGCCA
>at00335
AGCAAGTGGTAAACGACCTCGCAAGGCTGGGTTCCCAGTTGGCCGCACTGGGAGAGAAGC
GCGTGAGCGCATCTGCTGGCATTGCTTCGTACGACGCTCAGCGCAAGGAGGTCGAAAGGT
TGTTAGAAATTGCCACTCAACAATTCACGGCACAGAGGTACAAGAACCAGCAGGAGCTTA
CCAGGCGCAAGAATGACCTGGCTGTCTTTGACTTCATTCTGAACCTGACAGCATGTCAGG
ACGAGGGTGAGGCATTCCTGCAGCGTGGAAAGGGCGGCTCGTCAAGGCCGCAAGTGAAAG
TTTGTAGTACAGATGATGGTGTCAGGTTGAACTTCAATAACCCACAGCTGCAGGCCCAGG

TTGAGCGCATGATGACA
>at00336
CCGTAGCCATTTTGGCTCAAGTGATGATTTGATTAGTTGACTCTGCCGCATTTGGATCAC
GTGGGAGGCATGCAGCTCAGCCAATGCCGTGGCCGCTATTGGCCTTCTGCTGGGACAGCC
GCTCGGAGCTCACCCGCGAGCTCTGCTGCCATGGGCCGAGGTCGGCCGAAGTCTACCGCC
GGTGCTTCGACTTAGTCTACACACCTGAGGAGTGTTGCCCGCCGGATCATCTTGGTGGGG
CTTTGCGCGGCGCTCGCCGGCCAGCGTGCCGGAGCCGCTCCGGGCACCACAGCCTAGGCA
GAGGTTGTCACACCCGTCCTTCAGCCGGGCACAGAGGACCTTTGAGCGGAGGCATGCCGA
TTGCACCAAGGTGATCCCGTACGTGTGGACCAGCGGCAACGGCAACCAGGTCACCTTGAA
GCTGCGCGTCTTCAAGCGCTCTCTGATGGTCGCCACAG
>at00337
AGTTGTGCATGCATTGTATCAACTTGCATATATACAACACTGCTCTCTCACCGAGAAGTA
ACTGCTGGTCACTGTACAATCTTATCCCATGGAATAGCGATCGGAATCAGTGAGTCGGCG
CTGATTCGTGGATTCTGGAGCGTCTTGAACGTTGCCCAGAGCCGTTGCAGCGCACGTTTC
GTGCAGTTCGGATCGTTGGTAGAGCTTGCCACGTGTCAGCGAAGGGAGATATGCTGCATT
TCAACGAGAGATACCAGGCTTGCCATCTTGCCTTGACACTCTTGCATCCTTGCAATCGA
>at00338
CCGTAGCCATTTTGGCTCAAGACTCGTGGCTCCGACTGCTGAAACCAGCTTGCAACCCCA
ATCCGCAGAGTCCACCAGCGCTGCAATGGATCTCATGAAGAACGTTGACTTAGCCTCTGC
CGGGAGGTGCGTCCTCTACGGTCTGGGGGTCATTGGGATGCTGGGGATGTACGGAGTCAT
TCAGGAGCGGATCATGTCGGAGCCATACGAGGGGAATGAATTCTTCAGAACCTCAGTGTT
CTTAGTCTTCTGCAACAGGATGGTGGCGATCGTGTATGCCCTGTGCATGGTCAAATACAA
TGGCGAGTCGTTTGCGAACAAGGCCCCCTTGTGGAAGTACTTGGCAGTATCCATGTCGAA
CGTGGCTGCCACGTGGTGCCAGTACGAGGCACTCAAGTATGTCTCATTCCCTGTCCAGAT
GCTTGGGAAGAGTTTCAAGATGATGCCAGTCATGATCG
>at00339
CCATCTCGGACAGGCGACCAAGAGGGTATGATCTGCGTTCCCATAACATGCCAGTCCCCA
ACTGATTTCTTGAAAGTCTTGTCCTCGCCTGACATCGGCAGAATCTGGCCAACCCTTGCT
TGGAGCGACGAAAACAGGAGGCCACTCAGCCTACTTCGCACTGGTACAACTCTCAATACC
CACATGATGTTAGAGACATCCTGTGCCTGCATGGACCCAACAACCTGCTGAGCTCGTGTG
TATTGCTGGGGCACCCAATCCAGTAAGTAAGGCTCGTGCCTGCGAAGACTTCCAATTGCC
CACAGAGAATTAGCCACTTCTCGCGCACTCATTGCGCCTGTCATGCCGGGAATCACTTCA
GCAAGAAGCGGCAACAAGCGTCTGACATCGGGCAGCTTCGTGTACAGCACCGCAACCGCC
CAGAAGATGTTTGTCACCGTTCGTGCTTCCACGGCATCACCTTTGGCAACCAGTCCTTCT
GTATACGAGATGATGGTGTAGTACTCGTGACTCTTGTGCGCCTCTTCAGGAATCTCGCGA
ATGTAACGTGCAAGCTGACAGAGCACAGCACTGGCATGAAAGTGATTGAACTCTATCCCA
TTCTCGAGCCCTTCATCCAGTTGATGCAGTACATCCTCAGCAGACACAGCATCCATGATC
GACTGCGTCAGTGCCTTCTGCCGCCCCTCTTCTCGTTGAGCCGATGACGAGAGCCATCTT
ACAGGCACATTCGCAGATGCCTCTCGATGGAAACAAACTGAGGCGTGCACAAGGCTTGCA
GGGTGACCAAATCTAAAGGAGTCGCTCTGGCAAATAATTGTCCTGAAGATCCTGCTACTC
AAGCGGTTGCGCAGAGGCAACATTGAAATCATGGTTTGGCACAACCTACATAAGATGGCT
AGGCCAAGCCGAGATTGACCGCCTTCTGGCGATTTGGCTCAAGCTAAGATGGCTACGGAG
TCTTGAGCCAAAATGGCTACGG
>at00340
GCTGGTCCCCCCTCGAACCACGCGAGGGCGGAGGTCGCGACCTCGAAGAAGCCACCGGAG
CAGCCGGTCTCGCTGCCGCCCCAGCAGCTGTGGCGGTCCAGCGCGCCAAGGTCCATGCCC
ACCGCCTGCAGCTCGCGGCGCCGGCGCTGCAGGATCTCGCAGACCCACTTGGTGTTGTAC
GCGTAGGTCTCTGGGGAGACGCACGGAGGCGATAGGCATCCAGGCAGTGAAGAATGTGGA
AGCCTTCAGTCAAGGACAAATACGCCTCGTACTCGCCATTGC
>at00341
TTTTTTTTTTTTTTTTTTCNCCACACACAGACTCCCATGTGTTATTTTCTTCCGGTGCAT
CTCAAAGCTCCATGCAAGGCCACCCCACAGAATCCTGCGATTATGGTCCACTCTACACCT
AATCGATGATGTCGCTGTTGCTGTAATTCTGCCCGTGGAATACCGGCCCCGTCTGTCTGG
GCGTGGACATGGTACTCCCTGGCATAAGCAGAGTCTGTGGAGCCGCATCGACACAGTGAG
AGCTTGATGATGGCGGAGATATACTCAGCGATTCGCGTGGCCTCTCGAATGCTTTCCTGG

AGGCGGCCTGCACTTGGTACCAGGCAGTGTGCACCAACTTGAGAACCTCTGGAGGTAGGG
CCTCTGACAGCCTTCCAAGGGCCTGCTGCACGTGCTCCTTGCCGGTGCCTCCTGCCACGG
CGCAGGCCGCCCCCGCCGCCAGCACGGCCGCGAGCCGCGCGCGCAGCGCAAGCCCCAGGG
CCCAGACCCCGGCCTCCCGGGCAGCATGGAGAACGACGGTGGC
>at00342
GCGGCCTCTGCAGCATTGTGGCCGAACACCTGCCAGCCGATTGTCAAGCCCAAGCCCATG
AGCATGCACCTCACCAGCGCTGCCATCAACTGAGCAGCACCATTGAGCACGGAGCCGTAC
TTGACCTCGTAGGCCCCATAAATCAGCTCACATCCCGGGAGGTAAAGCAGCAGAGCGCAG
CCCCAGAGCACTGGCACGTGACACAAAGGTACCTGTATGACATGCTTCCAGACAAGAGGT
GTGACGATCCCGACGACCAGAGAGCATAGTACAAGCTCTAAATTGGCCAGGCCGCTGCGT
CTGCAGAAGTGCATTGTGCCCAAGGATAC
>at00343
TGGAGGCCCTGCCGCTCTGGCGCGTCTGCTTTGTCGTCGTTGGGGCGTACATTCTCCTCT
TCTTGCTCGCTGCGGGTGTCGGCCTTCTGCTGCACGCGATGACGCGCGGTGCATACCGGT
CTGGTCAGTCAGGGCGGTTGGCGGAGGAGGCAAGCAGCAGCGATTGAGAGGGAGACGTGC
AGGTGAGAGCGACAGCGCTCTCTTTCATCTGCGGTGATGCTGGCCTTCAGGAGGCGAGAC
AGCCACCTTGCGGAGAGTGAGGTCACATTGGGTCTGCGCTTCGTCTACAGAAGTGCTGTT
GGACTAGTCTGATGGTCGGGTGGGCAGTGAAG
>at00344
TTGGCCCACTCCTCCGCTGTGAAGAACGTCTCGAGGGCCACGTCCTTCAGCGCCAAGGAC
TCCCTGCTGCCAAAGTAGCGGCTGAGCACGGACTCCGCCGTCTGGTACGCGTGATAGGAC
TGCGGCAGGTCGTTGTCCGGGTCGGACTCGTCGACGATGGTGTCCAGGAAGCGCATCATG
TCGTCCACGGACATCACCGCCTTGTCCAGCTTCCGGTAGTCGGCGAGCTTCGCGCGCACG
CCCTCTACAGTGTGCCGGCTCTGGATGTCAAAGTACGTGCGCTCCACGCGGCTCTGAAAG
GCTGAGCTCTCGTAGTTGCGGAAGCCCG
>at00345
TTTTTTTTTTTTTTTTTCACCTGGTCGGCGCCTCACCAAGGCTTCCGATGTCTGTGTCT
GGAAAAACTTGCTCAAGGGACCAACAATCTGCCATGAGCAACTAAACATCGTTCAAAAGG
CTGATGGATCTAATCACAGATGGCTGCGGTATGCAGTCAAAAGGAGCGCGACAAGAACTG
ACAGTGGGATGACAGCACTTATGGCTGCGCTTGCATCAAAACAGAGAGGATGGCGTTACC
AATCCTACAGCTTGCTGGGGTGCAATTCAAACCACCAACCCCGTCCAATTGCAGTCGACA
CAGCTCTTTTGCCAGCCAGAGGAGAAACAATACGATCGTTAATGCAGGATCTGAGATCTG
TCAGGTCCTGGGTGCGAGCCCTGCTTTGCTGGGCATCGATATTCTTGATGGCCTCTTCGA
GCTGCCTTTTATTCTCAAAGATGGTCTGCTCAAGCTGCCTCTTGTTTTCGTGGATAATCT
GCTCGAGCTTTTCTTGTCCTGACTGCGTGCCTTAATTTGTTTGACATCCTTTGCGCTGAT
GGCTTTCTCAAGCATCAGCATGGCTGCCTTGAGTTGCTTCGCGTCATTGATCGGGGCCCT
GCTTTGCCTGGCAACCCTCTTACAGATGGTGCTTCCGAGCATGCACATAGTGCCAATAAT
CATGGTCGCCCCACCCAAATCTCGCTGCACCTTCATGCGCTGAAGCTCATGCCGCACGCT
CTGGATCGTGATTTGCCGCTTTGCGAGCTCGCCGCGTATCACGCGGTTCCAGGCATGGAA
GACCCTGCTCAGGCAGGATCCAAATGCTCCGTGGCGATTGTTCGGCGTGTGCACTGCCGC
AACCCACAAGAGAAAAGCTTCGCGCTGGCAGCGCTCGTGGCGGAACTGCTCGGCCAGCTG
CTCAAACCCGCGCTTCGTCGCCTCCATAATAGCCTGCCTGCCTTGTCTCAGCCAACGCAT
CTGCTCTGATCGCTTCCCATCCTCCATGAGTTGTACGAACCTCTCGCATTCCTCCTCAAA
CCTCTCGTACTCTTCCTCCGGCTGCCAAATCGCATACTCGAGGCATGCTGTGTTTGGGAG
TGCCTTGCCGCCTGGCAAGACCTTTCCAGAGTCAGCGGGCAACTGCTCACATAGCTGTGA
TGCCTGCATTGCCATGGACCTGTCCCTATGCCTGCACATGTTCCACCAACATGCCGGGTT
CAATTCCCCATCTGTGGCGACATAGGCAACAGGACCTGAACTGCCAGCATATGCTTGAGC
CAAAATGGCTACGG
>at00346
TTGTTTTTTTTCTTTTTTTTTGGGTCTGGACGCAAGCCAACCTGGACACAAGTTCAGACG
GGCCGGCTACTTCTAGTCAAGTGTGCAGGCCGCTCATGAGCTGCCCAATGGACTGCTGCT
GCTCCCGCGCGGCGTTGGCCTGCTGCTTCTTGAGGATCCGGAAGCCGTTCTGCAAGGCTT
TGAGTTTGGTGCTGTTGTCCTGCATGGTTTCGTTGACGCCCTGCAGGCGAGAGCGTGCCT
TCGCTATCAGCGACCGGACCTCTGCCTCGACGTCCTTCCGGCCCTCCGCGTCCTGCCTCT
CGCCATGCGCGGCCTCCGCGGCCTTCCGGGGGCTGTTCGCCTCCTCCGCCTCGCCCTCCT

CGTCGGCCGCCCCCTCCTTCGGCATGGCGCCCCGCAGGGCAAGCGGGGCGCCGTCCGCAC
CCGCTTGAGCCAAAATGGCTACGG
>at00347
TTTTTTTTTTTTTTTTTTTCTTACACCCACTGCAGAGTAGGTGCATCTGACACAGTTCCC
GCGACAGACAGGAGCAGTTTGCGGTACCTGGCCTGCAAGGACGCATGCCGCGCCTCAGGA
TCCAAATGCTTCTCACAGCTTGCGTCCTGCGCCGCGGCAGCCGTGGCACGCTTGTCGCCC
ACCTCCGTCAGGCTGCCGAGGGCTTGCGCCGCCGCACGCCTCACTCCGGAGCAGGGGTCC
TCCAGGCGTGCCGCGGCCATGCCGATCGCACGCTCGTCCCCTCTGGCGGCGACCTTCGCC
AGCGCCTGCAGCGCAGCGCGCCTCCCGTCGGAGCCGGCATCTTCGAGGCAGGAGGCGAGC
GCAGCCATGGCAGGCTCGCTGCCCTTGCCGGCCATCTCAACCACGGCCCTGACTGCGGCT
TGACGCACGGACGAGTTGCCATCCCGCAGGCAGGCACTCGCGGCGTAGATGGCATGCGAG
TCGCCGTGCTTGGCCATGGCGACCAATGCATGCAGCGCTGCGAGGCGTACGCAGAACTCT
CGGTCTTCAAGACAAGTACTTGCGAGAGCAATGGCATGGACACACCCCTTGTCGGCCACA
TCTATCAGTGCCTTCAGCATTGCGCACCTCACATCGACACTGCTGTCCACTACCAATCCC
TTTAAAGCACCAACTGCGCGGGCATCCTCCTTTCCAGCTACTTTTGCAACAGCCGAAGCA
GCGACGCATCTTACACCGGAGTGCAAGTCCTTCAGCCCCATGTGGGCCACATCAAGTGCA
GC
>at00348
GTGCCTGGCCTGGCTGCTCGAGGTCCAGAAGGACCTCCAGGGTCAGAAGCCCACGAAGGA
GCTCCTCACGCAGTACGCCAAGAAGACCCTGGAGTCCGGCAAGGTCATCCCCGGCTACGG
TCACGCAGTGCTCCGCAAGCCCGACCCGCGCTACATGCTCGAGAGGGAGTTCGCGCTCAA
GAACACGCCCGAGGACCCGCTCTTCCAGCTCGTGGACGCCTGCGACCACGCCATCCCGCC
GGTGCTGGAGGCGACGGGCAAGGTGAAGAACCCCTACCGAACGTGGACTGCCACAGTGGC
CAGCTCATGTACTTCTACAACCTCCGGGAGCAGAACTACTACACCGTGGTCTTCGCGGTC
TCCCGCTCGCTGGGCTGCATGGCGCAGTACGTCTGG
>at00349
CCTGGAGCATCAACTGTGAGCCCCCCCAGCTGTCCCTGCAGCTTGTAAATCTTGAGAAGC
ATGGGAGCACATGCCTCACCTACCAAGTTCACTGCATGGTACCTCGACTTGTAGCTCACT
GTCCACTTATCCAGCCAGAGGCTTTGCCCATGGCCATCCACTAGCTATTGGCGGCCTCCA
GCATCTTTGCACAATACAAATCCAGGAAGCACCGTCTTACACCACTCAACTTGCTGATTG
GATACATCATGCAGGTAGCCCCATCCTCGTTGCCAGTGCAAGAGAGACCACCCCGCTTCA
TGATACACTGCAGT
>at00350
TGGACACCATGGTCCTCTGTCGCGGCACGGCAGCCTGCGCCGTCCTGCACCGAGGGGGAG
GAAGGGCCGCGAGCCTTGGAGCTGAACCTACCACTGGCGCCGGGCGCGTACTGCGACGTG
GTCCAGTCTGACGCCAACGGATGCCCCAAGATCTTGGTGGGGGAGGGCGGCGCCACACGC
CTGCAGGTGCCGCCTCTTGGTGCAGTGGCTCTTCACGTCGGTGCGCGCAGGGGCACTTCC
TCCTTCAGGACTGCCAGCTCCTTCCTCCGCGCGGGCTGACCTGAGCTCGCGGGGCCCTAT
TTCCGTGCGCGCCACTGGGTGGTGGCCTTCCGGCCGAGGTGAGGGAGTGCCGGACTCGGC
CTCGACGCAAGGAGGGAGCCTGCAGTGACGG
>at00351
TAGCAGGTGGCTCCACGAGTGTCTGAAAGGCGCCCCCGCATGGCAGCAGCGGCCTCTCCT
CTAGCGACGCATCCCGCAGCTCCCAAAGCTCTCGCTCTCCAAACCACTTCACGGTGTGCA
GCGGACCTGTTAGGCACCAGCGACGGCCAGTGGCCTCAGTGGCGGCCTCAAGCAGGCCAC
GCGGCGAGCCGAAGGCCTTGTACGAACTGCGCAAGTCCCGTTGGCCCGGCAGCTCCACGA
AGTGCAGGGATGCAAGGTTGAGAAGTCCAGCCAGCAACCGAACAAGCCCTTCTGGCTCGG
GCGCGCCAGGGTTGGGGTACTGACGGCGGGACAGTCGGTGGACGTGGTGCACGACAGAGA
GCAGCAACATCACGTCGCAAAGCCGGTGGGTGGAATCTGCCAGGGAGCAGACGCGCGCGT
AGTCCCAGACCTCGGGGGCGACCCTGCAATTCCGAAGGCCAAGCGGCTTGCCCACTGGAG
GTGCGTCTGCACTGCCTCGGTGAGGAGTATCTCAGGTGCCCCGCCCTCGAGCCCAGCCTT
GCCATTCCCTACGCCTATCGAGCCCTCGACTCCAACGACCTGGGCATTGGGATGCCGCAA
TGCCGCCTGCAGTGAGAAGAAGCCATGGTTGGAGCCAACGTCCACCAGGCGGAATGGTGC
CCCGCGAGGGCCGGCTCCGTAAGACTGGTCCAAGCGAAGTCGGATGGCTTCCCAGCGGCC
AATGAAGTGTTCCCATGGCCGCTCCACGGACCCACCGGCGTAGGTGAACTCTGACTTCCA
TTTTCCTAAGTCCTCCCGCAAGCAGGCGCGGCGAAGCTGAGCTCTCTGGAGCCTTCTGCT

CGCGCGTCCACGAGTGCAGCATGCCCAGTCTCTGCCTCGCAGCGCCCAGCATCTGCCTTG
CAGCGCAGCGGTGGCAATGCTCGCTGCTGACAGTGCAACCCACTGACGCTGCAGCAGGAA
ACACAGAGCCAAGACAATAGAGGCAGGGCGTAGAATGTGTTGCATGGTGCAGAGTCTTGA
GGCCGGATGTTCT
>at00352
CCGTTTGGGCTCAAGGCCTTCACGTGGCCTTGCTTGAGATTCANCCCGTTGCCTTGTAGA
TTTTCGTGTCATGTCGGGGAGTCTGTTGATGGCTTGCGCACCACGCAACTGGATCTTGCT
GTCCNTTGCCTTGCTGCTNCCATTCCTGTGTCNAGCCACACCCATGACCGATCTGCCGGC
GATTGGGGTGAGTGAGAGTCGCGCTAGCCCCAGCTTGCGCGGGAATGCTGTTCCTCTCTG
GTGGCTGTCTTGGTTTCCAGATGCCTTTGCATACTACTCCTACTGGGTGCCGAAGATAAA
TGCTATGGGTGACAGAGTNCGTGCTGGCANCAACAAGAGCGAGGCAGACCCGCTTTGGGA
TCAGGCGGTGGACTTGGTGCGCAATGCCACTCAGGCCACAGCGCCAGGCAGGCGTTTGCG
TCGTGTGC
>at00353
TTTTTTTTTTTTTTTTGTCCGTCCAAATGGGTCCACGCCTGGTCGAAACACGCCTCTGGC
GAGTACCTAGAATGACATCCAACATCTGAGGCCAATCTGCACTGTACACTTCCTGAGTTG
CCACAAGGCAGATCGAGGAGGCGCATGGCACTCGGCGTCATTCGCGCTCTGTGCTCCTGC
CTCCTGAATTTGGGGCCTCTTCTGGGCACCCTGTTCAAAAAGAAGCTGACAGATGGTTAG
GGCTCCGAGGCAGGCTTGGGGTGGCGGATGATGATGCTCACTGGCAAATACTCACTGCCT
CAAGCATTGGCCCCCAGGCTCTGGGCCACCATCCCCGGGGCAGCGAACAAAGTTGGGCTG
GCTTGCTTCACCCAGGCGCGTGGCTCCATGCTTGAAGAAGCCAGTACTGACCACAAGCCA
CCCGGCCGGTTGGCGCCCTCAGAGAGGCTTGCGGCCGACCATGAGCCACGCGGGCGTGAA
GATGCCCTGGGCACCTCCGCGCGCACAGCCAACCCCGCCAGCCTGGAGCATGACCTGTGT
CTTCACCGTGCCCTCAGGCACGGCCCCCAGCCACTCGAGCAAGCGCAGAATCAAGGGCAT
CAGCTTGAACATGACGGGGTTGAATTGGAACCTCGGCCACTTCAGCGGGTTCCACGACGG
TGTCAGCGGCACGTACCACGCCTCCCCACCACTCAGGTGCCCGTCCAGTGCGCAATCGCG
CACCTCCAGGAGCTCGAAGCCAGCATCGCAGAGCGCCTTTGTGCAGACCGAGGTGTGTAC
GAGGTCGGGCAGCCCATCACCGATCTCGATGTCCCTCTTGATCTGCTGGTGGTCGGTCAT
GCTTGCATCGTACTTGTCTGTCAGGCACCACTCATAACAGGCAAACACAGCTCCAGGCTT
GAGGACGCGAAGGATCTCACTGTAGACCTTTGCACGGTCGGGCGCGTGGCACGTGGACTC
AATGGCGTAAACACCGTCAAAGGAGGCGGCAGCAAAGGGCAGCTGCATAAAGTCGGCCTG
GACGAGCTCGACCTGGTCGCGGAGGCCCTCGCGGGCGGAAAGGGTGTTGCCGCGGTCGAC
TTGAAACTTGTTGATGGTCACAGCCTTGATGTCTGCACCGGTGAAGCGAGCAATGTTTCG
AGCTGGTCCACCAATGCCGCAGCCGCAGTCCAAGACCTTGGCGCCTTGGCCGACCCCCAA
ACGACCGGCAAGGTAATACTCGTGCCGCAGGATGGACTGGCGGAAGGACTCGCCGGCCCG
CCGGTCTGCAAAGTGGAAGCTGGTGCCCCAGCCCCACTCGTAGAACTCTGTCGCCAGATC
ATAGTAGGAGTCGACTAAAGTGGCGTACTCTGAGTTGCGGTCGTCGGCCGATTTGTTGGT
GCCAAAGAGCTCGTTGTAGCCGTCAATCTTCCCATGCTTCTTCGAGCGTGACATTGAAGA
CAAAAAGGCTGAGGTGTGCGCCAGCCCT
>at00354
TTTTTTTTTTTTTTTTTTGTCAAATCGTCCCTACCACCAACACCATCCTGCACCTGTC
TACGCCAACAGGCAGGTCAGCCGCCGCAAGCGGAGGCACATGGACTCTTGTCCACGTCCA
TACCTGCGCATCTACTTGATCAGAGGGTTGGCAAAGAGCAGCACCAGGGCAATGACGAGT
CCGTAGATCGTCAGCGACTCCATGAACGCCAGTGACAGCAGCAGCACGCCGCGGAGATCA
TCGGCCACCTCTGGCTGCCGGCTGATGCCGTCGATGCAGCGGCCCGAGGCGATGCCCTGG
CCAACGCCCGAGCCGATGGCCGCGAGGCCGATGGCGAAGCCGGCGCCAACGGCCGACAAG
GCTGGGATCCAGACGCTGCCCTCGTCGGCATGTGCTGNCTGTGGGTGCGGCGACAGCCGC
GGCACTCAATGCCGCAGTGGGAAGCCTGGCCGCAGACACCTCCATGCTTGAGGCAGGCAA
CTTATGAGCGGCGGGAATGAAGGCGCAGCCGAGGGAGACCAATGCCATGCCGGCAAGAGC
CACGGCGCCAGTGAGGATCATGGAACGAGGTGCGGCCATCTTTGCCGCAGTTGCCTGAGC
ACAGGAGGGGCAGAAGGAGCAGGAGGAAGGACTGATCCA
>at00355
TTTTTTTTTTTTTTTTCTGAGACTCTGCGCCGCATTCGTCATCAGAGGCCAAGCTATGCA
GGAAAAGATAACAGCATAGCCATCCCTTGAGTTCGCATCAGCCTAGAGCAGAGCATTTGT
ACTGGATCATGGCGTAGTGGTTGAAGACCTCCAGCACCCGCCAGTAGAAAGGTGAGATGC

ACGTTGGGTCCTGGTCCCCTGTGTGCTTGCAGTGCATCCAAGGCTTGCTCATGCCGGAGA
AGTGAAGAATCCGAGCTTGCGTGACGCAGGTGTCCGGCCACGGCATGACGTTGAAGACGT
GATACAGGTGGTGCTGTATGTTCCACCTCCAATCCAGAAGTTCGATGCCAAGAGTGCCGT
TGAATAAGATGTTGACCGCCGCCTGGTCGCCCTCGAAGCCTCTGGCGATTCGCACCAGCT
CACGGATGGGCTTGGAGAAGCCCTGCGACCTCCACTGTTCGAGGTCCAACACCATGACAC
CCGTATTGAAGACGGAGTTCTCGGGGTTGCGTGGCGCGAAGCTCTGGTTCTGCATCCCCG
CCTTGACGCCCGACTGCTTCAGGTAGTACTGCAGGGGGCAGCAGGGCTTGACGAACTCCT
CAGAAAAGGGCACCATAGGATAGCTACACCACTCTAATGCGGCCGCGACGGGCTGCCGCA
TGCGCATCTGGTAGAGAGGGCTGACATCTGACTTCACAATGGTGT
>at00356
TTGTTTTTTTTTTCTTTTTTTTTGTTCTTTGTTGGGACTTCTGAGCATCCACCCATGCTG
AGTCCACCTCCTGAGAAGCACTATGATATCTGACCGTGGCAAGCGCGCAAGGGCCCACTG
CCTCCATCGGCCAGCATGCGACTGCCACATTTGGAAAGAGTGCCGGAGTGGGGCACATAC
GAACAAGCGGCAGGAACACAGGAGAACGAGCAAATACCACACAAAGTTGTTGGTCTGTGC
TCGCGAGTGCAACCTTCCCTTAAGAGACTCCGCGCCTACGGCCGCGCGAGCCACACCGGA
GGTGTCACGCCAGGCCCGTAGATGGAGCCGATCCGCTTCGCCTGGAGTTGGCCCCGCGTC
ACGCCGCCCACGGCGCTCTTGGAGAATGAGGGGAAGGTGGCGGGCACAACCTTGTTCAAG
CCAGGCAGAGGCACGCTTGCACGCACGTAGTTGCCAAGCTCGCCACTGGCCGAACCCCCA
TATGAGTCGACACTGCCAGCGCTTGCGTCAGCGGCCTCCATGAGGGCTGGGATGCCGGCC
TTGACCAGCTTGTCCTGTGCGCTCATCCTCGCGGCTTTGCTATTCTCCAAGAAGGGCTTG
GGGCTGCGCGGCTCGTCGCTTGAAACTTGAGCCAAAATGGCTACGG
>at00357
CTTCGTTGAGGAGGCCTTCCTCCGACGACTGCGCGCTCCTGCACAATGCGGGTACAGATT
GGTACGAGAGCACCTCAGAGGCAAGTAGGCACTGCGACCAACTCCGAACGCCTCTAACGG
GCTTGCCGCTGGAGATCACGCTGCGATCCGCCGAAGATCCGTGGGTGGTGGCGGGCCGCA
TCACCGGCTGCACACATAACTTCGGCGCCACAGCCGGTGACCACACCGTCTCCGCTGTGG
TATGGCTCCTCGTTGGTTCCGCCTTTCTGCTCTCCGGTGTGTACTCCTTCTTCGTGGCCG
CAAGGTACACGTGGGAGCAGTTCGGAAGCAAGCGCGTCTATGCGGAGCAGGTAGGAGATC
CTGAACCAGTCGGGAACCCGTCGTGATCCTGTCCTATGGCTCCTCTTCGGCAGCGTCATC
ATACA
>at00358
TTTTTTTTTTTTTTTGCTCGCACACTGCACCTTCACCCGCCTCCCCTCGCAGTCGCACCC
CCCCCAACCGGGTGCTCTGCGCGTCAGGGCCCAACCCGCCACGCAAGGAAGAACACACCA
GCTGCCCGTGCCGCTCAGGGTAAAGCCCCGGTGCCGTTGCCGTCGTGGTCGCTGTCCGAG
TCGCCCTCGCTCTCCCAGCCCCCGCCCGCGGGGGGGAGGGCCAACAGCGGGGGCTCCTCC
TCGCCAAGCGCCGGGGGCGCGGGGGCGGGCGCGGCGGCTCGGCGCGCATCGCGGAGGGCG
AGGCCGCGGCGGGTGGTGTCCAGGTGCTCCTCCAAGAGGTCGCAAAGAGCGGCCACGGAG
AAGTCCCAGGGCGCCTGTATGAAGCCCGCCCCTCGTGCC
>at00359
CCGTAGCCATTTTGGCTCAAGGGGGTTTGCGAGCCCGGGCCATTGTGGCAACGATCCGCA
TCCTGTAGCCAGGTGTGCGCCCGCCATGCCTTTGGTCGACAAGCTCTTCGACGGCCCCAT
CGACATCGTGGGGGACATCCACGGCGAGATCGGCGGCCTCTGCCAGCTGCTCTTCCGGCT
GGGCTACGACGGGCTGGGGAGGCATGCAGAGGGCCGCCGCCTTGTCTTCGTAGGCGACCT
GGTGGACCGGGGGCCCGACTCGCCGGCGGTGGTGCGGCTGGTCCGGAGCCTCGTGCAGCG
CGGCGCCGCCCAGATGGTCCTCGGGAACCACGAGATCAACCTCATTCGACGGGCCAAGAA
GCACGGAAACCATTGGTTTTGGGGCGAGACTGAGCAGCTCTGCAACAAGTCGCCCGAGGC
CAAGCCAGGCATCGCGTTGCCAGAGACTGCCTCCTTCCAGGTTCTTGAAGATCCTGATCG
GCAGGAGGAGTATATCAGCTTCTTCCGTAGCCTCCCCCTCGTCCTCGAGCGGCCTGGCCT
CACGGTGCTCCACGCCATGCACC
>at00360
GAACGGCGACATGGAGGAGGAGGAGCTCCGAGCCACGATGCGTGAGGAGTGGGGCTTCGC
CGTGGAAGGTGGCATCGCACCGGTCATCCTCTCAGAGCTTGGCTGCCCCGCCGACAACGA
GCACGAGATGGCTTGGTTGGAGCGCGTGACGCGTTACCTGCGTGAGACGGACGCGGACTT
CTTCTACTGGCCCCTGAACGTGGGGCCGAAGCCCGGGCCGAGAGGATCGCCTCCATCAGG
GCCCCGCGGCGGCGATGAGCACTACGGTTTCCTCACGCCCGACTGGTCGCCCAAGTGGGA

CGACCCCAGGCTAGCGCTGCTGAGGCCACTGTGCCCTGGCCCAAGTGAGCGGGACAAAGA
GGCCTGAGGGCAGGCTGCGGACCTGATGTTCCT
>at00361
GGCATCGTGTGCGCGTCCAATGCCGGCGGCCTCCGGGGCTGCGAATCCTGGGGCCTGTCG
GATTACTGGATTTGCGTGCGGATGCGGACTTGTCTGCGATGCGGGCTGCTCGCCGCGGTa
CTGGCCCTGAGCCAGGCTCAGCTTGCTGAGGCGCGTAATGTGCTGGTGTGGCTCGGGGAC
AAGTCGGCTTGCATCGTCGTTCAGCAACCTCAAGCCCTGGGTTTTGAGGCTGCCGCTGCT
CAGGGTCACGGTCCTGAAAAAAGCATATTGCTGGGCATGGGAGCTTCACTTTCTAATGGT
CATGATATGCGTCATGACGACGAGCTGGAACGTA
>at00362
CCGCCTTCGAGAGATTCTGGACATACCTGTCGATGTTGCGTCTGCGGATGGCTGTGAATT
TGTCCAGCTTGCGCAGCTGTGCAAGACCGAAGGCGGCACACATCTCGCAGGCCTTCATGT
TGTAGCCCACGGCGCCGTACAGGAACTTGAAGTCGTAGTCGATGCCGTCCACGCTGTGCC
CGAAGCGCTCCGACATGTCCTCTGAATTGTTGCCGATGCGGCCCCAATCCCGATACATCA
AGGCCTTTTCCCTGAGTGCTGGGTCGTTGAACATGACCACCCCACCAAGTCCGCCTGCTG
TGATGATGTGGGAGGCGTAGA
>at00363
CCGTAGCCATTTTGGCTCAAGAAGTGGTTACGACCTAGCACCTGGAACGCAGCCCATCCT
CGCTTTGTTGTTTCTCAACAAAATGATCCGTTCTGCTCTCCGCACGCCAGCTGCCAATGC
CAGTCGCGCGCGTTGCTTCAGCAGCATTTTCACACCAGCGGCCGCCCTCGACGGGCGGGA
GTTTGACTTCATTGTTGTCGGTGCCGGCAGTGCCGGCTGCAGGCTCGCTCACCGCCTTTC
GGAGCAGGGGTCGGCCTCCGTGTTGCTCGTCGAGGCCGGCGTCGAGAGCCAGCCGCGCAT
CCCAGGCACGGACTTGCAGGCAAAGCGCACGCCAATAGCCCATCTCCAGCTCCACCGGCA
GCCGGAGACCGACTGGAACTTCCGCACTGCGCCCCAGAGGC
>at00364
NNNNNNNNNNNNNNNNNNNNNGCGGGGCGTGTACTGGTTTGCGCTGTGAATTCGAAGCTT
GCTGCAAACACTGCAAGCAGCCCTGTACAATTTGCTTCCGCATGTAAAATGGCAAGACTA
TACTAAAGGAGGGCAGAAAGGATCACCACTCACAGGAACATACGAAACACAATACACTGC
GCCGCATAGCGAGTGGTACTTGAAATCTGGCTTCACAGTTGAAAGTAGCTGGCGTTGCAT
ATCTAAGACCAGACACGCTGATTCAATGCAACATTAAAATATAAATGTAAATGCTCATTC
CGCCGCTGTGGGCGTGAGCAGCTGACCTCGAGATCAAGTGAAGTAGGTCCGGCCGATCCT
ATCTCCACCTGGTGCGACGTATCGTCTATGTACACACCGCCATGGATACGCCCATCCGTG
CTCTTCGACAGCTCCGCGATCGGGACCTTGAGGGCAAACTGCTGGAACTGCTCCTCGAAG
AGCTGCTGCTGGATGAAGAGACGCAGGCAGTGCTAACGTTGGACGTCTCGCCGGCGCCTG
CAGGTTTGCACTCCCTGGCCGCCCCAGAGA
>at00365
TTTTTTTTTTTTTCTTTAAATTTACAAGACATGTACCCACTAATCTTACTGGAACAAAT
AATAAAACCCACACTGGTACTGAGAGATAGAGATTCATAAACACCTGTACATCTTCAATT
GCTAAATCTTTTGCGAGGTCTTGACCTGCTAATGATAATATCCCGACTTGTCTTCCTAGA
CTCATTGGAGATCCACAAAATTGTTTTAATATTTCTACAAGCCTCATACCTTTGGCTAAT
CTATTTTTGGTTTCGCTTCCTAAATCCGCAGCAAATTGTGAAAATGATTGTAATTCANCA
NATTGAGCAAGCTCTAGCTTATTTGAACCCTGCACTAATTTCATCCCATCCCACTGAGCT
GCTGAACCCACACGTGTTACAGATAATCCANCATCT
>at00366
TTTTTTTTTTTTTTTTTCCCTCACCACTGTGCCAACGAATGAAGCTCTGGTGTCTGAGA
CTCAAGACATGAAAACTGCCAACACGGCAGGCGCAGCAGCACGCTAAGCCTGATGCGTGC
GCGATGGATCAGCTGCTTGGGTACAAAACTGTGCACCAGCCCCACCAACATAGGACTGTC
ATGCGAGCTCATCAATAGAAACCATCCGGCATCCACATCCACCTCTCGTAGGCAGAGCAG
CTCCACTGCTCCACCAACACCTCCTGCTAGACCCAGTCAGCCCTCACCCCTCAGGCTGCA
GTCTTCTGGCGCTTGGCCCAGGCACTGCCGTAGTCGATCAGGCCAGCCTTTGCCAGGCTC
TTGGCCTCCACAGCTGAGACCTCCAGGTAGCCCTGCGAGCTCACCGGGTTCAGGCCCTCG
GAGGCCTCCATGGAGGAGTCGGCCTCAAAGTACACACTGTGGGGGCACTCAGTCAACGAG
AGGAAGTTCATGTGCCCCTTGTAGAGCCCGAGCTTCACCGTGCCGTCCGCTGGCTCCGCA
AACTGCCAAATGGCGGTGAGCGCGGCGCGCGTGGAGGGGTCCAAGTACCGCCCGTCGTAG
ATCTGGTCAGAGACGTGAGAGGACAGATGCTCGAAGAGCTTTGTCGCCCGGCGGTCGAGC

ACAGCCTGGTAGACGCAGGTGATCCTCGTGCC
>at00367
TTTTTTTTTTTTTTTTTTCCCCTCCAACAGCCATGCACTACCTAAAATAGAATTCTACCT
ATACCTAGGTATTCTTTCAGATAACTGATAAGCTTTATCGCATAACATCGAAGTTAAATC
ATCTATTCCACGTATTGTTCGAAACCACCACCAGAACATTTAAGTTTCACAAGTGAAAGC
GTACTCCTTAGGTTAAGAACCTTAATTTGTTAAAAATAGATCCAATCTACAATAAGCCAT
AATTTATCACTTGAATTAATGGGGTATCTAATCCCTGTCACACCTCAAGGAATAGTGAAT
AGAATATCTACCTTTGAAAGCTACCAATATCAAATAAATTTAATACCAATTATATCTACA
TTTCACTCTTTCCATTAACATTTCTACACCACATCAAATCTAGAATCACTCTCCTTAATC
AAATCACACTGATAATAAATTCTCAACATTCACATTCTAAATCACTCTTTACACCTAATC
AACCTATAGAACGTTCACACAACTTGCTTTACCGCTGCTGCTGGCACAAGATTAACCGGT
ATTCATCCAATTGAATGAAAGCTTTACAACACTTATTAAAACACCCT
>at00368
TTACGTATCCTCTCGATGGCAGCAGAGCGCGATCCGGAGGCACTGCTCCGATGCTGCCGC
CCCAGCCGCGGGCGTGCCAACGCGCGTGGACTTGCGCAATACTTGATCAACAGTCTATTT
TGGAAACGATCTGATGTCTTGTTGCCGCAAGCCCGGCCATCCGCCCAAGGG
>at00369
TTTTTTTTTTTTTTTTTTACGACTTTCACGACTACGGTGTTAAGCTCAGGTGGCGCGCAG
TTCACGGAATGTCAGCTGCCGAGATGTGACAGGAATCTGGGCTTGCCTGCTGAACTCTGT
GCCCGTCCAGAAGCGACCGCCGATGCCGGAGGAGCCAAAGGACCACTGCGGCCTCCGTGA
AAACTGGCAGTGCAAGGCGTTGTAGTCAGGCTGAGCCTGAGGGTGTTCCCGCCTGCTGTT
CCTCACGATGGGCGACTTGGCAGCGCTCTGCTCTGCTGAGACCTCCGTGCCTGCTGTCGC
AAGTGCCTTGGGGGTCCTTTTGGGGGGCTCCTCGTAGGGGTATGTCTGCGAGAATGGCGA
GGCATTGGTTGAGTGCCGTATGTGAGGCATGGCAATCTGGGCGAAGGATGGGTCGTCGTG
GAACCGTCTCTCTTGGCCCCAGCCGTTGAGCACCGTCGTGCACGCGCTGTCCTTCGACGA
GGCCACGTAGCTCGGGGCGGGGTTCAAGTTCGACGGGTGGCCGGTGATCTCTCGGCAGCG
CCCAAGGCTTCTGTCAGCTCCGGCTGTCTGCCTCGGACTGCCAAAAGTGAACACCGGGTT
GGTCCTCCTGACGGAGGTGATCTGCCTCCCGCACGACTCCAAGTGCAGCCTCTCGTGGGC
CTCGCCGCGCGACTTCTGCAGCGCGAGCAGCTTGTCAGGAGGGAGTCTCGGCTTCCGCGC
GTTCTCTGCGCCCGCTGGAGCCGCTCCGCCCGCTGGAGCGGCTGGTGAAGCCGCTCCCCC
TGGCTGGGCCGAGTCCGCCCCGCCAGCCCCACCGGTGTTCGAAGGTGCCGACGCGTCCTC
TCCGGGAGCAGACATGGCTGGCCCAGTGCCCCCGGCCCCCCCAGCTTGGGGTTGAGGGAG
GGCTCATGGGCCCATGCCCGCTCCGGACAGGGGCCTTGAGCCAAAATGGCTACGG
>at00370
GCTGTGCCCCGCTTCCAGCGTCCCCATGCACTCGCAACTGCTCAAGTCCCAGACACGCAG
CTCGGTATCTCCGGCGCCGCTGACAGCAATGGCCTCGGGCACCCGCACCGCAATCACAGG
CTGACTGCAACTCACGGCACCAAAAGACCTCTCATCGGGAAGGACACGGCCTTCTTGCAC
CAAGCGCACTGCCCTCCCGGCACAGCCCAAGAGGCCGGACACATGCCTTCGAAGCTCACC
CACTGTATCGCCCGCCCATGCATCTAGGCAAAGGGGGCCCCGCTGAGCAGCCTCACTTCC
GGGTACGCCGCATCGGCCATGCGGATGCAAGGCGTGCAAGGGCGAGGGCGCCTGATAACT
GGAGTCT
>at00371
NNNNNNNNNNNNNNNNNNNNNCCCGGCTTGCTCCACACTGAGCGGAACTGTCAAAAGCTT
GGCGCAATCTTCCAGACTGCTGGCCGAAGGAACAGTACCGGCAACATTGTCAGCAGCAAC
TGCTGCACGATGGCGCAACTCCTGCAACGTGGCTTCTGCCTGTTGACGTGAATGAAGATG
CAATCCCTGGGCATTTGCCTCGAGAAGGAGCTCCAAGATATCCAGGTTGTCAGGCTGGGT
TCGCAGAGCAAGGTCCAAGGGTGCTTCTTGCGGTAGGCGCGTTGCGCGTTTGTGAACCGC
CGCCGGCACCGCTGCAGGACTTGGATCCGCGTTTGAGACCAGCAAGGCACGGACGACTGA
TGCATGCCCACCAAGCGTGGCCCAATGCAAGGCCGTCCGGTGCTGCTTATCACGCACATC
CAGCTCAGCACGAGCCTCTGCTGCAAGCCGCAAAGAGTCTGCACTAGCTGATCTTGCTGC
GTAGTGAAGAAGGCCCTGTCGGGAGTTGTCGCGCACTCGGGGCGGGGTTCCAAACTCGAG
AAGCGCCTGCAAGGCTGCGACTGCACCGGCAGCGGCAGCTGCATGCGCGGGCACAGCCCC
TGCCTCGGTTGTGGCTAAGATGCGCGCACCCCCATCAACCAGAGCCCGAAGCGCTTCCAC
ATTGCCGCGCGCAGCAGCCCAGTGTAAAGGTGAGGTTCCTCTTTCACGGGGCCCGAGAGC
GCCGTTGGCACAAGCACGGGCGTCCAGCAAACCACGCACCAAGGCAACCCTGTCTCTTCG

AATGGGCGCCTCCAGAGCTGTTGACAGAACAACTTCACGTCTTCACCCNGAGCTGGTGCC
CTGACACTGGGGCCAGCTCCATGCTCGGAGTCTGCAGCTG
>at00372
TTGTTTTTTTTCTTTTTTTTTCATGCACAAGCGTACGCGTAGACGAGGACAGCTCCAGTT
CCACTCGTCGGCAGCTCGGCCCCCAGGTCAGACCACGCTCCAGAGGGACTGCAGCGTGGT
GCCCTCCTTGAGGCGCTCCACGGCCACTCCCAGCTCGGGGCTGAAGACCGGCTCTCTGTC
GATCTCCTTCTCGCCGTCGGGCTGGTACAGCAGGCACGGCTCGATGTCGTCGCCCTCGAC
GATCTCCACGTCGTCCTCGGTGCGAGGGACGAAGTTGTCCTTCGGATCCCCCTGCCTCTC
CTCGATGGTGTACTCCACGCCGAAGATGGGCGTGGAGCTGAAGATCTTCCAGAGGTTCAG
GATCTGCGTGTAGACCTCCTCCAGATGGTCCACGGGGTCGGCGCGGAAGCCCAGGATGTA
GCCGCCGCTGGACACCGTGGTCTCGATGACCAGGGCCTGGCCGAACTTCGAGTTGCGGAC
GTTGATGCTCTTCATCTGGAGGTAGGGGATGGAGACGTTGAAGTTCTCCGCCAGCACCGC
GAACCACACCGTCCGCACGTTCGTGATGATGAAGGTGCCTAAATTGCCCTGGTCCGACGA
CAGGTTCCAGATGCCCCCGATCTTCTCGTAGACCTGCTCGTGGGGCAGCATGACGAGCTC
CTTGTCGCGGATGATGGCGCCGCGGAGCTTCAGCTCTCGGTAGAGCTTGGTGGTCTCGTA
GCTCTTGAAGACAGCCTGCACGGTGGTGAAGAGCCGTGGGCTGTTCTTGACCAGGGAAGT
GAAGATGAATTCGAAGCGGGTGGAGTTGAAGCGCGTCATGACGAAGAGGGCCTGGCAGTT
GCCCCTCAGCTTCGAGTTCACCTGCCGGATGTTCACGGAGGAGATGCAGGTGAAGCCAAT
GCTCAGGTTCGTGCGCGCGGACTTGGCGGACGTCCAGATGAGGCGCAGGTTCGTGACGAT
GAGGATGCCCCGCTCGCCGTTGTTCCCCTTGGTGTCCTCCACCGAGTTGATGGAGTCTAT
CTGGAACTCTCCTCTGCGGAGGCGGAGGGCAGACTGAGCCTGGTCGAACTTGATCTCGCG
GTCCTGCCACACCTCCTCAAACTGCCCATCCGACATGAGGCTCTCCCCCTCTCGCCCGAA
GGCGAAGGGCCGCCCAGTGCCGAGCCCACCCCACGCCCGCCAGCCTAGTGCCGGACTGCT
GCCATGGCCTTGAGCCAAAATGGCTACGG
>at00373
TTTTTTTTTTTTTTTTTTTCGCTCCATCAATTCTTGAGTTGCCTCAGAGCATCCGACTT
TGGGGAAAGACAAAGCCGCCACCAAACCTCGGTGGCAGCGGCACGCGTCGCCTCCCCAGC
CTCAGCCCATCGCCATTGAAGGCGCGGTGCAAAACCCGCGACTGCATGCGCAGGGCCTGG
GAGGGAAACATGCCCTCCGGAATGGGATACTGGTTAGCCGCGAAACGATATGCACGCTCG
TAGGGCGGCGGCTCGGGATACTCATTGTCGCTGCGGAGCTGCCGTATGTATGCGTCGCGC
AACAGTTCAACCCAAGCGTCGTCGTCCTGGTCCAGAGGCAGACCCTCTGGCATGACCTCA
TCACTGGGCACGGAGCCGCCACTTCTCTCCACGGCGCCCTCTTCCGGAGAAGGGCACGAA
TTGCCAGGGCCGGACGCGGGCGCTGAGTCCTTCATCTCCTGGTCCTCGTCGGGGTCCACC
TCCAGATTCTGTGGCCTCACCGCCTTCGCCGCTGCAGTGGAGGCCAAGAAGACTTGCCAC
GAGCCCAGCTCCTCGTGCC
>at00374
TTTTTTTTTTTTTTTTTCCACAATGGTCAAATAGATGAGGTGCGGCTTTAAATCCCTCG
CAGGTTCGCTGTCTGAGCATGCCTGCGCTGAATGTGGCGCAGGCCATTAGTATCCGAGGT
ATCTGTCACCTCAGCATATCGCCTACAAACACAAAAAGTGTGAAAGGCTAAGAAATACCT
GTTGCCTGTGCACAGAGTGCACGGCCCTGTTCCGCACCGATCATGCACATAAGTGCTAGT
GGACATGCAAAGCGAGCAGATTGGAGTGCGCACATGATCTTGGACCTTAAATCAAAGTAC
TGAGGTCCCTGGCGCAGATCCTCCGTCTGTACAACTCTGCCCTTTCCGTGGCGCAAGCGA
CTCATCTCCGAGGTGTCTGTCACCTTGGGACATCGCCTGCGGTTGCTATGAAAACTTGAC
TGCCTCTGCAGGTAAGCAGGACCCTGACATGAGTCAATCTGAACAGAGCTGCACACTGTC
ACCTGCCCTACGCCTCTTGGACACAAGTGGGTGCGCTGACTGTGCGCCCCAGTCCTGTAG
GTTCTTGGGCCCTTGTTGTNACAGAGCTGCTCTGCTGGGAGTCACCTCGTGCC
>at00375
TTTTTTTTTTTTTTATTGTCCCGACTTGGTGATGGTAATGGCACTGCTTTTGGAGTGTG
CACTCCTCGCAACACCATTGTAAGTCAAAAGCGCTGGTCGACACACTGCCGGCCCAAGCG
GCAGCAGCGGCGACAACAGCAGCTGGACACCTGCAGTGCTCGAAGGTTTGACTCGCGTGT
CAACCTTTCGGAAAAGCTCACCGCAGGAAGGTGACCTGCCAACTGCAATCACCCAACTGG
GCCTCGGCCCTCTGGGACTTTGGGCCAGGCCAGGCTTCACGCAACACGTTGGTTGGGCCC
CACTGACTTGCAAACAGCTGCTGCACATCTGCACGGTTGCTGCCGTCAGCGAGGTTGAAC
TGGTCGCCATCCGTCCAATTGCTCCCTAGCTGGTGTCCGAAGGGATCATGCCAGTAATCG
TAAACCTGGGAGCCATTGATGTGGCGCCCTATGCCGCGTGCATGGTGGTAGTGGANGCCN

NTNTCTCTNTGNTTCTTCAAGTGCGTGTTGCCCACAAANAGGTCGTCNATGTTCGCCACC
TCGAANGCNGCGTGGAAGAACTGCTTGCCCTTTCCATGGCCTATTGGGCTGATTACCGTC
GTTGCGGATCAAGAAGATGCTGT
>at00376
CCGTAGCCATTTTGGCTCAAGCCAATGTGGTTCAAGCCTGAAAAGGAGCTTATGAGAGAG
GGAGGATGAAGGTATCCTTCGCCCATCCACTCTCGTGCCTGCGCAGAATCGTACACCCCG
CGGGCAGTCTGCGGTGTGCCTCAAGTTCCGCCGGAGGGCCCCGCTCGTTGGCAAGTGCAG
CTCTGCAGAAGGAGTCGTCTTCTCGAGGAACGCTGCATAGCAGACCAGTTGGCGCGCGGA
ACTTATCGCTGGACGAGGCGCTGTACTTGGCTAAGGAGGCGGGATATTGGACGACACGTG
AAGTGCAGCTTCAGATTGTCCGCTCTCCGCCGTTCTCAAGCAAGGAGCGGCCATTCTTGC
TCGAGAACTTCTTCGAGCTAGCTGACCAAGATGTTGACATTGATAGCAGTCCCATGGAGG
ACGGAGGCGAGGAGGACGAGCACGCGGGCGACGAACAGACTCCGCCCTCGAACCTGGCAA
GTCCTGCCATCCACGGCGCCATCCAGAAGTACTTCAAGTCAGCGCAGCCAGCCAAGTTCT
GGCTCGGCGAGGAGGAGTGGCTCTCGCGCGCCGAGGGCCGGGGCACCCGCAAGCGCGCCA
GCGCCCACGTGGTCATCTGCCGGGGCACGGGCGTCTTCAAGGTCAACGGGGAGTCCGACA
TGTTCGCCCGGTGGCCACACTACTACCACCGCTTCGACGTCTGCCAGCCCTTCAAGCTGA
CGGGCACCGCCGGGCTCTACGACGTCTTCGTGGAGGTCCGTGGTGGAGGCCTTGGCGGAC
AGGCGGGGGCCACGCGGCTGGCCGTGGCGAGGGCGCTGCTGGCGGCCAACCCTGGCTGCC
ACGAGGCTCTGCAGAGAGGCTTCTGCCTGCTGGAGGACACGAGGCAGAAGATGTCGAAGA
TGCCGGGGAGAGCCAGCAGGCGGGCATCCTTCGCTTGGACGAAGCGGTGAGGCGTCCACG
CTGTTGCAGGCAAGTGAGGCGAGCACCGCAGTGCTGGATTCGAGAGTCGCCCTGGGAAAT
TCAAAAAAAAAAGAAAAAAAAACAA
>at00377
TTGTTTTTTTTTCTTTTTTTTTGTCATCGTCCATGCCTGAGGTCTTATCAGGGTGATAGA
CCAGCGACAGCCTTTTGTACGCTGCCTTGATCTCAGCCTGCGTAGCCATTTCCATCACTC
CAAGAGTGCCATAGTAGTCGACGAACTTGTCGTACTTGAAGGACTTCGGCGCAGTGAAGT
CGCTATCTTGAGGGAGCCATGTGCAGTCAATGCGCATCAGCTCTTGGCTTTTCTGCGTCA
TGACGGTCTTG
>at00378
TTGTTTTTTTTTCTTTTTTTTTTTTTTTTTCTTTTTTTTTTGCGCCACCTCACAAAAAA
CTTGGCAAGTACCAGTACCTCTCCGTGCCGCCTCATCGGCTTGCAACCGCGCTATCGGCT
GGCACAGATCAGACACCAACAGCAGATCTCATCCCTGCTTGCCGCGCCTCTGTGGAAAAC
ATGGTGAAGCACATCACGAATGCATCCCTGGCTTCAAGGTCAGGCAGTCTGAAGGTGATG
CACTCCTGTGTATTCAGCGCCAAGGTCACGGATAGGTCATCCAAAGGGGCTTGGAAACCT
ACGCCGACCTCACTGTTGCGCCCATCTGTCCCCACAAAGATCTCGTCAATGGCAGCCAAC
GGAATGTTTCGGCCCTGCTTCTCAGAGGCCTTTGCCTTGATTTTGAGCGTGTCCAGCGTG
CGGCTCAAGGTTGCAAGTACACTTCGGACGTGCCCAGAGCTGAGGACAACATGGAGCTTC
TTGCCCTTCACCATGGCCTTCACGAACTCCTGGATCCTCTTCTTGGCCTCGGCGCGCTGC
TCCTTTGTCATCTTCTCATAAGCGAGGGAGCCCTGTGACGAGACGCTGGAGCTGCCGTTG
GAGTGGCTCGTCTCGGATCGCTCCTCCCGCAGCATGGCCAGAGCCTCCTCCCGCACCGCG
TCCCTCCAGCAGGCCTCCTCTCTGTCGGCATCCCTCCACGCGGCGGTCAGGGGCCGCGAG
GACTCGGCTGCGGCAGAGCTGGAGCTGCTGTGGGAGGCCGACGGGGCCACCACTGCCTTG
CGCTTGACGACCTTCGCGGCGCCGTCCTTTATCGGCGCGCTGCAAGGACATACGCCGGGC
CCCTGCCCCATAGCGTCATGCTGGCGGGGCCTTGGGAGCGCGCGGCGCCGGCCTTGAGCC
AAAATGGCTACGG
>at00379
CCAAGCGTTCCTTCTAGCAGGTACAGAGCTGTGGAAATCATTGCTTGACTTGATGGTTCC
TCTTCAGAGTGGCCCGTGGAGTGCTGCCGTAGTGAGGGACAGACCAGCATCCGGACGAAG
AAGCCGAGCCAGGCAGACGGCGATTAGATGGGCACAGTGCCAAGGCTTGGCCTTGCGAAA
CGCTCTGAGGTGTGATTCGTCATCGAACGACGTTGAGTCTGGATCTGGTATGATTGCGTT
CATCGGTCAGAGTGTTCAGAAGATGGCAAAGCCATGGTTGGAGATGCAGCGGCAGTTTAT
GGAACAGGTTACAGCGATACTTGAGAGGTTTGCGCAGATGGTGGAGAAGATGGTTGTCCG
GATGCAGGAGCTTG
>at00380
GTAGTTCCAAGACCTCGAGGAAGAGGCCAAGTTTTTGCCATTGGCTACTGGTCATCAAGT

GTCTGCTTGACCCTTTCCAGGCAGCTAACTGCAGAGCTCAAGTAGCCTCCACCAAACAAG
TTGTAGTGGTTCAGCTGGTGGTAGGCCTCATACAACGGGTAGCGCTCGTCGAATCCTGGA
TCCTCCGGAATCAGCTCCCGGTAGCCACTCCAGAAGTCTCTACTGAGGGACGCTGCCCAC
GACATTCCCCATTCTGCCTCATGGTGACCGTAATAAACAGCAGGGTCGAAGATAGTAGGC
CTGCCGTCCGCCGAACCCATGTTGCCGCTCCAAAGGTCTCCATGCAGCACCGATGGGTAA
ACCTTGATGCCTTTGAAGAGATCCCCCAGTCTGGGAGCTATGGATGCGAACAAAGAGCTT
GCATAGCTGTCGCCGGCCAGCTGCACTTGGTAGCGCAGTCGCCGCTCACGGTAGAACTCA
ACCCAGTCGTCACACCAGGCGTTGCGCTGCGGCGTCTCCCCGATCGTGTTGTCGACCTCG
AAGCCGAAGCGTGAACTGCCTTGGTGTAAGTGCATCGATGCCATTGCGCGCCCAAACGCT
TTGTCGTCACTGCGGCTTCCGAGTCTGAGGTACTCCAAGCAATGAACGAGCCCCGGCCTC
CAGAGCGAGGGCCCACATGGGTACACCTTGGGTATGCGCATGAGTCCCGCCTCGTGCGTA
GCATCATACATGGCTTTTAAGCCAAGAGCTTCGCCCTTAAACATCTTGTCCACGCTGCGG
GATGACGTCTTCACGAACAGCTCGGTACCAGAGCTGAGCGTCACTTTGTAGAAAGATGAC
CAATCGGAGCTGCCGAGAGGCTTGGACTCTTGGACTTGGCCTAGCCCTGCCTCTGCTGCC
CAGTCAGCTACGTCATCCATTGGCGATGCTGCACGGGGTACTCTAGAACATGCACCACGC
TGCCACCGATGAGGCTGGCACGAATGAGGTGCGACGAATGTGACGCCTGTAAAAGAAGCA
ACCCAGCAACATGTCAAAGCCGAAGCAAGCACGTGTGTTGAGAGCTTCCGACAGCGAGAC
ATGGACCCAGGCGCTGGAGCGTCAACAAATCGGCTAAGATGTAACTGGTTTGGGGACATA
GCGAAACAATCCAAACCAGCGTGCAAGCTGGGAAAGCTTGAGCCAAAATGGCTACGG
>at00381
TGCTGGTGGATAGAAAGGAAATTGCAACACCTTCGTTCTATTTCCGCCAAGCTCCCTGCG
GTATCTGAGCTGGGAGATTTTGGGCCTTACGTTTGTGGCCTACGACATCGTCACGATTCC
GCTGCAGATCTTTGACCTTCCCCGGACCACATTCCTCTTCCTTCTCTCATGGCTGGTCCG
TTGCTTCTGGACTTGCGACATCCTCATTTCCCTTCCTTACAGGTTATGTGTCTCCAAAGG
GCCTCGTTGAGTTGCGGGCTGCAGGGTGGCAAGTAA
>at00382
CCGCTGCAGCTTAGTCATGGGAGCCCTGACAATGGGAGCCCGACCGCGGACCGGCTGGCT
GGGAATGGTCGCGGTAGCGGCGGCCGTTGCTGCTTCTGCGTCATTGGCGCTGCAGATGCT
GCGGCTGCGCGCACGGCGGAAGCGGCGAGGGCCGCCAGTGGAGAAGCCACCGGAAGGCCA
GCGTCAGCCGCTACCCAGCATACAGGGCCGCGCGCGGGAATAGGAACAACAGAGCGGTGG
CGCGCAACGTGGAGGGGT
>at00383
GAAAAGATGTTGCTGACGACAGCATCCACGTCGAGCTCGGTAAATAAGTCGCTGGTGACC
ACCACCGCTGGGGGTGCACCTGCAGCTTGCGGCACGTGATCCAGAAAAAGACATGGCATC
GCAAACTCGCCAACGATGGACTCGGAGAGGAGCGTCAGGGCATCGCAGAACACTTTGTCC
AAACAGCTCCACCTTGGGCGGCCGATCCTCCAAGAACGTGTCTCGCAACCTAACCGTAAC
ACTTTGGTGGCAAGCCACATGAGGGTCCGGCGAGGGAGAAGTCCTCAAACGCAAAGCGGA
TGAGCCGCCACGTACCCGATTGACAAGCCAATCTGCCTGTGCTCGCACTCCAGGCGGCAG
CTGCGCGAGCCACTGTTGGCACCGAGTCGCAGGTTCAGCTGCTGCCCATGAAGAGAAGAG
GGCACGGGCTGACAAACGAAACTGGCTTGGACCACACTGGC
>at00384
CCGTAGCCATTTTGGCTCAAGGGATATATCCGACACTCCACACCGCTGGGCCTGCCAGCT
CGCCTCGCCAAGGCCATCCCTCCAGCAAAGATGGTTAAGAGCACGAAGTTCAAGGAGGAC
CCCGTCCCTTGACCGCAAAGATCTACCAGTACCAGATCGTGCGGCCGTGCTGCTCCCACG
CCCAAGAACCCCACTCCGAAGATCTTTCAGCATGCGGCTCTTCGCGAAGAACGCTGGTGC
TGGCCAAAGTCGAGGTTCTGGTACTTCATGAAGAAGCTCAACAAGGCCAAGAAGAGCGGC
GGCGAGCTGCTCGGCGGTGAACGAGCTCAAGGACCGCGGCCCCACGAAGGTCAAGAACTA
CGGCATCTGGCTGACGCTACCAGAGCCGCACGAACACGCACAACATGTACAAGGAGTACC
GCGATGTCAACATCAATGGAGCCGTTGGGCGGATGTATTCTGAGATGGCGGGGCGCCACC
GTGCACAGCCGGGCTCGATCCAGATCATCAACACCGCGCAGCTCCCAGCTTCCAAGTGCC
GGCGCGAGCATGTCAAGGAGATGCATAACTCCAAGTTGAAGTTCCCCATCGTCACGAAGA
TGCCAATGGCGGTCAAGAAGTTGCGCAGCACCTTTACAGCCAGCCGGCCCATGACCTTTG
TCCAGTAAGTGGCCCATGACTTTTGCGCAGTGAGCATGGCAAGACGACGTAAACAACGAA
AAAAAAAAGAAAAAAAAAACAA
>at00385

TTTTTTTTTTTTTTTTTTGGCGCAATCAGCAGCAATGCAGCAGGACCCGCCCGCGCATGT
ACATGCGCGCGCGCGCGCGCGCTCGCGCGCGCGAGAGAGCGAGGCCCACTTCCTACCGCG
GGCCTCAGGCCCCTCTCTCGAGGAGCGCCTCGTCCCCGCTGCTGCCGGTGTCCTCCGCGG
AGTCCGCGGCGGCGGCTGCCAGCAGCTGGACCTTGGCGGCCTCGGGGTCGTAGACCTGCT
CCAGCAGCACGCCCAGGCCGACGGAGGTCCCATCGGTCAGGGCCCACGCCTCCCGGGGGG
CGTCCGAAATATCTGCCCAGGTCATCTCCGAGAGCCGGAGCCAAGGGCCGATGGTCTCGC
CTGTCACCACCAGCTCGTCGCCGGCGTAGAAGACGTCCAGGACCTTGCCCTGCGTTGCGG
GCCTGTCTCGAACAGCTATGCGCGGATTGTGGACCACGCGCCACACGGCTAGACCGCGCT
CCTCTGCGCGGCGGAGGGCCATGGATTTCGAGGGCGCATTGAGCCTCGGGTCGTCCAGCG
GCAGCAGCAGGGGCGGCCGGAAGGTCTCGGGCGGCATGGGCTCGATGGTCCAGGCGGCCA
TGGTGAAGGCCCCGGCCCCGTGGATGTCCCA
>at00386
TGTCAATAGATCTGGAAGCCATGCTGAGTTGGCCACTCCGCAACCAATTCAAATGCGTGC
ATAGGCAACAACTTGGGGAGCATGGTATTGCCCTGGTGGCTGCTGCATGCATGGAGAAAT
CAGGGCTCCTGCAGCAACGGCCGTTGTCGACACGACCAGCAAGACAGTCATATGATCTCC
GTTGATATGCTCGTTTGCGCACGGGGTCCCTGCACAATGGAGATGGGTGCGGTCAGCAAT
GTACTGTGTGTCGCAGATCTTGCAACCGGACCCTATCAGGAGCTTGGACTTCATCGGAAC
ATCCGCTTTGTTCCTGTCCCAGAAGTCAGTTGCGCCGGAGACCTGCGTCATGTCCGCTGC
TGGCATGAAGCTGTGTTCGTCGCCAATGTGCAAAGGNGCGCTGTGTCTGTGGTGCAGCAG
CGCC
>at00387
CTTTGGGCTCCGGCAGATCTTCCGAATTGTGGACAGGCCGTGAAGAGTCACTCGGCCCGG
CTTCAATCGCTTTGCTGGAAGTATGGGCTCTGTCTCTCGATTGCGGAGTATTAAAGAGCT
CGCCAAGGAGCCCGTGAGATTGCTGCTTACTGGCCTCGACGCCGTCGGGAAGACCCTCAT
CCTCCGCCAGATAAAGAGGCCTGAAGACAAGATTGTGACGACCGTCTCAACGATCGGCTT
CAGTTTGGAGACCATGCGGTACGACGAGATCAGCTTCACTACTTGGGGCGTTGGTGGCCC
GGGGACAGGGAGTCGGTCCACTACCCCGGCGCATCTGGCAGGGCGTCCACGGAGTCATCT
TCGT
>at00388
TTGTTTTTTTTTCTTTTTTTTTGCTGTCATGTGGGGGCAGCAGAATCTTGCAAGTTCTCC
GCTGCAAGACTGTGATCATAGAGCAGCCTCTGCAACCTCAGGCAGGCCGCTGTCCAAGTC
ATCCATGAAGTCCTGACGGAACCATCGCCATGTGAGTGACACTCGGCATGCCGCCACCGG
CTGCACCTCGTGCATGAGCTCATAGCGTGCATTGCCCTGGAGGCACACGGCGAGGCCCGG
CGTCTCCTGAACACGAAAGCCTCGCTGCATGCGCAGGGCCACGCCTTGTGCTCTGGCAAC
TACGGTGCTGTCTCTCTCAGAGAGGCGGAGGCCATCGCT
>at00389
TTTTTTTTTTTTTTTTTTGATGCGCAGATCGCGCCACTTGCGGAACTTGTCCTGAGCAAG
TCCCTCGGAAACAAAAAACCCTCGAGTGTACAATGTGGCATGACTCCTTTGCGCCGTCGG
TGGATCTCACTTGCCGTTCTGCTTCTGGATGTAAGCAGCAATGTGCATGAAGAAGGAGAT
CGGCAAGAACAGCGACTCCACAATGTAGGACACGTTGAAATTCTCCCCCGCATCCACGCG
GGCCCACTGCGGATCGATGAAGAGGAACTGGATGTTCCCGTAGAGCCCAATAGCGAAGAA
CAGCATGGAGAGGAAGGCAAGCTCGTTGGAGCTGAAGGTCTTGAGGTTGCCCGTGGCATT
GCTGGTCATCTTGCCGTTCTTGAACATGGGGGTCCCTGGGTAGTCGGGCAGGAAGCCCTG
GAGCTTGTCCTCATGCCAGTACATGGGGCCCTTGAGGTACTCCGCCGTGTAGTCCTCGTG
CC
>at00390
TCCGCAGCCGCCTCCCTGCAAGCCCGCCCGGCCACTTCCGTCCCTCCGCGGACCTTGCCA
TGGGCAGGCGGCGCTGGCCCCTGCTCGTCTTGGCGGCTGCGGTCTGGGCTTGCAGTGCAT
GGGGAGGAGTCGTTGCTTGGACAGGTTTTTGCAAACACTGGCAGCTGGCCGCATGCGTCC
AGAACAGGGGCGACAGGAGCGGCTACGCGTTCGTTGCGTGGACGCCGGAGGCCTGGACTG
CATGCATCACTTGTGCAGGACAATCCCCATATCCAGGTTGAGGATGACGACCTCGAGATC
TTCAGACCGCCTCAGGAGCGCCGCCGGGGCGCCGGAGCCATAGCTCTTGTCCGAGCCGTT
GCGTTCGCCTCACCTTGTACGGCTTGGGCATGTTCTTCAGCCCTGCCATTCTCGTGGCGC
TGCCC
>at00391

CCGTAGCCATTTTGGCTCAAGCTGGCATATGGTTCAGCACACCTTGTCACGCTGATTTTT
CTTCCCCATGATGGAGCCTCCATGCGACGGAGCGCAGTGGAAGCCAGACCAGAACAGCGA
CGACTCGAGCACATGCATTGACGAGGAGACTGCCAGCACCCGAAGCGACACCGTCATGCC
TTCTCTCGTCCGGTGACCGCAAAGTGGTCCCACAGGCGCCCAAGCTGTCCTCGCGGACAG
TGCGCACGGACTTGGATTGGTGGCTCGAGTGGGAGTCCTTCAACAGGTACCATGAGTATG
AGACCTATCCAACGAGGCCTCATGCCGCCAGCAGAAAGACTGTTCGGTCCAGCGCGGACA
CCGAAGCCAAGTGGACCGCAGGCCGGATTCCAGAGAATGCCAGCTTCCAGACTCCCGTCA
AGGCAGCACAGCAAGCCGATGCTGGGAAGGACGCACTGAAGAAGAACAGATTTCTCATCA
GTTTGTAGTGGTGATATGTGATGAGGCCGTGCAGAAGCGCGCGCGCAACAAGAGAGAGAC
GTAGTGCTTCTGATTTGTAGCGCGCTAAGCTTGTGTGGACCTCGCGAGTAATGATCACTC
GCTGAGAGAGTTTGTTCTTGCCGTTAGACTGTTGGTTCCTCTGGCGCTTACGTGCACGCC
GTGGAACAGACTGGGAGTTACAGTAATCTCAGAGCGGCATACCAAAAAAAAAGAAAAAAA
AACAA
>at00392
GACGAGGAAGAACGCGATACCGCACATCATGACCGCATGCCAGAGCGAATGGCGCCAGCG
CCACTGCTCCGGCGTGCTGGAGGACTGCGAGTACGCGAGCAGTCCAGCAACTACGAAGAG
GTTCAGCACCGTGAGCAGGGGGACGCAGAGGTATGCCCGGTAGGTGAGGTAGATCGTGAA
GGACACCGCAACCCACCGGTCAATGACGTTCCAGACGGTGCCAATGTAGGCAAAGTCCGC
CAGCAGCGAGCAGCACGTCACCACCAGGAGCCAAGTGCCCGTCAGTGTGTCCTCGTTGGC
CAGCGCTAGGGGAGCCGGCGCGGCCAGCAACAGGCAAGAGGTGCCCACGAGCCAGTGCTG
GTGCTCGTGGGGCTTGGCATTGGAAGAGAGGAGGCCGCTGAGGAACCTTCTGGGCCACGC
TGGCTCACCATCTGCAGCCATGCGCCCGGAGAGCTCGCGCAAAAGGGTGGCTTGTGGGGC
CTCCTCCCCTCTCCCTGGCCTGAGCCGTATGCAGCTCTTGAGCCAAAATGGCTACGG
>at00393
GTTCCTTGCACCAGGCACACGAGCTCCGCGGGTAAGTGCAGACGCACCAACGAGCGCGCG
AGCATGACCGCGCCATCGATGTATTCCTGCTTGTCGCCCCCTGCGTAGGTGACGATGGCG
CGGTGGCGCCCTGCGCTCGGCCACGGCCATCTGTACATGCGTGCGGCATCGAGGCGCTGG
CCTAGAGCGTTCGACCATGACCCTGGGCAGCCCGCTGCGGCGGCTTCGGGGGAGCTTGCT
CCGGGGACGTCACGGCGCAGCGCAAACGCTTCTCGCAGAGCCAGCAGCAACAACAACAGT
CTCCACCACACAAGAGCCGGCATCAGCTCGCCGGCCGCGAGGTCCCTGCTTGCA
>at00394
GCCCAAGGGGCCTCCGAGGCTTGTTCACTTCTCACCTTCTCAGCCATGCCGCTGGAGCAG
CCGAAGAAGCCCGTTGGCGGTGCCTATGGCGCCTTCCTTGCCAAGCACCGCCCAGAGTTT
GCTGAAGCAACGAAGGGCCAGAAGGCATCAGCCATCAGCAAGATGGCCGGCGAGAAGTGG
AAGCTGCTCAGTGAGGCAGACAAAAAGCCATACGAGAAGGAGTATGCCGTCGCGAAGGCA
AAGTATGAGAAGGACATGAAGGCCTTCCTCGAGGGCGGCGGGGAGAAGAAGCCGGGCCAG
CGGGCCTTGGCCACGCAGAAGCGCA
>at00395
GGTTCTTGCCTCCCTTCCCGAGAATGAAGGCCGCATCGTCATCGGTGATCTTCATGGTGT
CAACGCCGTAGTCGTCTGGCTTCTTGTTCCTTGGCGCTGCATCTTCGAAACTCCGGCTTC
GGCTGCGATCTCGGCTGCGAGCACGGTCTCGGCTGCGGCTCGGTTCACGGCTGTCGCGGC
CACCGCCATGGCCCGGCTCAGGCCCGTAGTCACGTCCTCCCATGGTCTGGCACTTGTTGG
GTGCACCTGGAGAGCACTCGGCCTCGTGCCAATGGC
>at00396
GGAGCGTGTCCAGCCCACGCTCATGCTGCATCATGGAGCCGTGTACTGTGATGAGGCCGG
CTGGGTAGTTCAGGTCCTCTTGGAGCTGCGAAAGCGTCTGAGCCATGTCGCTGTTGACGA
TGACGGTCGCCCCGGCGCGCAGCAGCCCGCGGCAGATGCCGCGCCCGATGACGCCGGCGC
CGCCGACTACCCAGGCCACTGAACCCTCCAAGGAGGGCACGCCTGAGTGAGATGCTGCAG
CGGCTGCCAACGGCCTCGCCGTCCTCATGGCGAGCTCCGGCAAGCCGCCTGGCAGGCA
>at00397
GGAGACAGCAAGTCCCATGTGCTGTTCCAGCCTGGGGTGTTCTCCAAGCGCAGGAAGATG
TGCTCCACCCCAACGTGGTCCAAGTGGTACCTGGCCCAGTCTTCGAAGTCTGCTGGCTCC
CGGGTCATCGTCACAATCCCCACGCGTGCCGAACGGCCCAGCTCCCTGCGCACCTGCAAA
GCAGCGCTCTCGTCTCGTCGTGGTGCCCAGCTGATGCAAAGCCAGCGATGAGAGCGGCAG
AGATGCGCAGC

>at00398
TTTTTTTTTCTTTTTTTTACGTTGCGAGGACCCTTCTATCGAAGTCAACTGCTCAAATTC
ACTACCACATTGGCATCGGGGGCCCGACGCTGACAGCGCTTCAGGCGAAAGCAACGACCC
CACGCAGAACCCAGCCGATACGACGAGGGGTCAACAGGAAAGCCAGCCTCAGCCCTTCCC
GGCCCCTAACGCCTCCAGCTCCTCCACAGATCGGTCCATCTCTTGCAGGATGAGGTTCTC
TCCCTGCCCCAGCTCCTCCCGCGCAGTTGTCCTCAGCTCCCTCAGCGCCTTCACGGCCTC
CGTCACTTGATCTTTGCCAAGCTCGGTCCTGACGCGCATTTCAAGCTGACGGCAACGGCT
GCGGCGCTCGTCCCTCCGGCGCTGCTCGAACGCCGTCGCTGTGCAGGCAGAGCAGGCGCA
GGAGAACTCCAGCGCGGACCGAAGGCCCTTGCGCCGCGCGTCCGTGGTCTGCCAGAGGTC
GATGTACGACGTGACCACCTCCTCGCCCTTCGCGACGGCTCTCAGCGCTTGCACCTCCTG
CCTTCCGGAGGCCGCGGAGAAGG
>at00399
GTGCTGCGTTTCGGCCCTCGACGCACTTTGCGAAGAAGATGAAGTGGCTCACGTCATCCG
GACGCTTGTGGAGCTTCGAATGCAGCATGTTGAGGTGAGCAGCGACTTCCGCTGGATTGA
CCGTTGACGGGGAGATCCGTGGTCAGACTTGTTGCTCTTGAGTTCTTCAAGATAGTCAAC
AAGCCCT
>at00400
CCGTAGCCATTTTTGGCTCAAGTCTTGGCCCAGGTGCTTGTGCCCACGTCCTCTCGTTGC
CCAGCGCAGGTTTTGCAATTGTGTGCACAGAATGGCGGTCAGCGGTCACCAGCTCCTGGG
CCGTCTGCTCAACGTGCGCGCGTCGTGCCGTGGTCATGGCCTGACATCATCAATGGTGGG
CACCCTCAGGCCTCGCACCACCCGTGGCATGGCAGCAGCGGCAGGGCTGAAGCAAACGCT
GGTGCATGACCAGCATGTGGCGGACGGTGGCAAGATGGTGGACTTTGCAGGCTATTCGAT
GCCAGTCCAATATACCAAGAAAGATTCTCCTCACTCACTGAGCATCATCGAATCAACAAA
GTGGACCCGGGAAAGCGCTTCGCTCTTTGATGTGAGCCACATGTGCAGCATCCGCTGGAC
CGGCAAGGATGCCATCGACTTCGTGGAGCGAGTCACCACCGCCGACGTGCACGGGCTGGG
AGCCATGAAGGGAACTCTGAGTGTCATTCTGAATGAGGCTGGCGGTGTAATCGATGACAC
GATTGTCTCGAAGTGCATCTCCAAGGAACACGGTGAGCACGTGTACCAGGTGATCAATGC
AGGTTGCGCACCGAAGGACCTCAAGCACTTTGAAGCGGAGCTGGGGAAGTTCGGCGGCGA
CGTCAAGATGGAGGTGCTTTGGGATGACCGTGGCCTCTTTGCGCTTCAGGGGCCCAAGGC
AGTCGAGGTGATTCAGCGCCTCAGCCCCTCTGTGGACCTCTCGGCCGTTTCCTTCGGCCA
GCTGGTGTGGCTGCCGATCGAAG
>at00401
TTTTTTTTTTTTTTTTTACGATCAGTTCGGCCCTGTCGCAACCATCTTCATGATGGGCA
CCCAACGACTCTCACGAGGAGTCACATCAATGACATTAGGCAGCTACTGGCCGCTCACCC
CCACGGTGATGCTGCCTAATGTCGACCCAAATCGACATGCCAGCATGTACAGAAAGCTCA
TGTTGCAGCAAAGATCACGCGACTTGCTCAAGGCTCCTGGGTACACTAAAGCCTCTTTCC
GCGTGGCCACTCACCTCACAGGCAGAGGAAGTGATCGTCGTCAATGGTTGTGAAGATCTC
GCCGGCGTCAATGAGCTTCTGCAGGGCCGCGATCACGTCGTCTGCTGGCGTGGAGCTGAT
GTGGCTGCAGACTGCAGCAACAGACACACCTTCAGGCTTGCCCTCCCCTTCTTTCTGGAG
GAATCTAAGCACAGCCTTCTTGAGCCCCGCACCCGAAAGGCGCTCTTTGGGCGCATCTGC
AGCGGCTGTCACCGCCACGGGCGCCAAGCTCTCCAGCTTTTGCGGCGTGACCTCAGCCTC
CATTATCGCCTTTTTCTTCGGCGCTGGGCTCACGGGGTCGGCCTGGCCTTTCTGGAGCTT
CAAGGCAGCATAGGCGGCCTCTATCATGTGGTAGCTGACTTCACCTGCCGACTGCACCAG
GCTCATCCCTGCCACCGCANAGTGGACTTCGGGTGCCGTTCGCACAGACCCGAAGAACTC
ACGTAGCGCC
>at00402
TTTTTTTTTTTTTTTTTGCTGGGGCGGGTTTAAAGAGCGATGTTGTGAAGTAGCCTCAA
TATGTTTCAAGAGTGCCGCCCAGTGAGCATCTTGACGCAAGACATCACCAGAGCCAAAGA
TAATCAAACCACGTCGTGCGCGGGTCACCGCAACATTCAGCCGCCGCCAATCCTCGAGGA
AGCCCAGGCGCCTGCTGCGAACAAGGCTCAGCAGTGCCACCTCCCTCTCAGCACCCTGGA
ATCCGTCGACCGTCTGGACGCAAAGACCTCTGTCAGAAAAGTTGCGCTGCAGAAGGCGCC
GGAGGTGCTCGGCCTGTGCCCTGTAAGGACTAAGGATGGCCACATCAGAAGCGGCAATAT
CTTGTGCTTGGAGCAAGCCCCGCAGCACATGCACGAGCATCCTGGCTTCTGCAATGTTGA
TCAATGACTTTCATGAGGGCCAGGCGCTTCCCTGCTCTTGGCCCCGCTTGTGTCTATAAA
GGCCATGGGGCCAGATGAGGGCCATTCAAAGCCTCGTGGTGGCTTGTCTAGGACAAAGCT

GCCAGAAACCAGCTTGCCTCCATAGAACCGATTGGAAGCAAATGCAGCGATGTGGGGGTG
CATGCGGTACTGCTCTTCCAGCATTCTTGGCTGGATGCCACAAAGAATGAATCGAACGAA
CAAGGACACCCCAAGGCCCGCATCGGCTGCGGCGGAACTCAATACCGTCGGTGGTAGCTG
ATTCT
>at00403
TTTTTTTTTTTTTTTTGTCTGCCCGCGCGCTCTGGCAGGCCCGCGCCGAAGCGTCTCAAC
TCAAGGTGCCTGCCGCCGATCACGTGCTGAAGAGGTACGTCTCCTCGCCGTTCGCCACCT
CCACGCGAGCCCAGGGGAGCAGCAGCTGGAACTGGCGCAGGGCCCAAATGCAAGAGATGC
AGGGAGCAGTGGACACAAAGAGGCAGACTCTGCCTTCCGCCGANNNNNNNNNNNNNNNNN
NNNNNNNNTGCCACGCCAGGTCCATAATCGCGCTCAGCAGCTGGAACTCGCTGCACAGGC
TCCTGTCCACCAGCCCGCTGATCGGTGAGGCCAGGGGCCTCAAAGACGTCGGCGGTGAGG
CAACTCCGCGGTCGCGGCGCGTGGACTCGTCTCCCGGCCGCTCCCTCCGCCCGTTCTCCC
TCGTGAGCGAGCCGGCGAGCGGCGAGCCGCGGCCGGGCAGCCTCAGCTCGTACTCCGCAT
AGCTGAACACCCTCCGGTGCACCAGCGCCGCCCCTGACAGCGCGCTCACGGCCTCCCTGC
CCCGCCGGTCCTCGCTGCCCGCGCCTGCGCTGAGGCAGATCTGCTGCGCGGCGCGCGCGC
AGAAGTCCGCGGGCGGCTCCGCCAGGCCCAGGCGCCTGAAAACGAAGCGCGTGCCCCAAG
CCCCAAAGTTATCCACGCGGATGTCGTGGACAAACTGTTGGAAGGCGGCTGCGGAGCTGG
GGTCCAAGGG
>at00404
TTTTCTTTTTTTTTTTTTCCANTAGAGAAATCACAGCGAAGAATGGAGATTCTCAATGTT
TGAAAGCAACCTGCCGTACGGCCTACACTCTAGCTGGCAATGCCAAATAAAATGCAAGGA
TAAGCGTGTCACCTCTTGCTCTTGTTGGCGATGGCTTTCAAGAGGCGCTTTCGCCAAGCC
GGGTTCTCCGCCTTCAACCAGCCAACCTTGTGGACTGGTCTCTTCCAGAGGGTCTCCTCC
GCAGCCTTCCGAACCATCTCGTTGTCGTCTCTCTTCAAGCGAACTAAAGCTGCCCTGTGC
AGGTACTGCTCTGGGCCAAGCTTTCCGAGTGCCTCACAAGCAACCAATCGCACCCGCCAG
TCCTCATGCTCCAGCTGGTCGCCTATCACCCGAGAGTAGACATGAGCAGGCGTACCAAAG
CGACCAAGTGCCTCAATCAACAGCACTTTCTCGAAGGGATCCGTGTCCTCCTTCTTCAAT
TCATGGGCAAGCCTTGCAATGAAGGGGTCCGTGTTCCACCGGTCTAACTTCCCCAGCTTC
TCCAGTGACAAGGCTGCGTCCCATCGCACCTTGTAGTCCTCGTCATACAGCGCGTCCAGA
AGCGCCTCCATCAGAGCAGGGCTTGCCTTCGTGCATGTCCTCCAGATGCTGTATACGGCG
GCCCTTCGAGTCTCAACTTCGTCGCTTGACAACGCCTCAACGTGGGGCTTCATGTTGGCG
TCCACCTCCCAGACCCG
>at00405
CCGTAGCCATTTTGGCTCAAGATTGCTCAGTGCACAACTTCCCACGACCACTCGCCTCCC
CAGCTTGCGCAATGGCCGCAGCTATGTGGACAAGCAAGCTGGGAAACAAGTACCCAAAGG
ACGAGATTCAGCGTCAGGATGCTGCCACCCAGCGTGAGCTCAAGCGTCTGCGCGGATTGA
AAGCTTGTGGCAACCACCTTTGTGCTGATTGCGGCGCTCAGGACAGCTCCTGGGCCTCCG
TCTCACATGGCGTCTTCATCTGTGTGACTTGCTCCGACGTGCATCGGTCTGTTGGAACCC
ATGTGAGCAAGGTGAAGGGCTGCACAGGCACCTATCTCTGGGGACCTGACGAGATCGAGC
TGATGCAGACCAGGGGAAACAGCTGGGCAGATGCAATGTACGGTGCCGAGAAGGCGGCCC
CCAGCACAAGCAAAGAAGAGAAGCAGCGGTTTGTGCTCCGGAAGTACCAGGAGCGTGCTA
GCAAGGCGACTGCGCACGCAACTCCGATTGTACGGAGCTCCAAAGCCTCGGCACCCACGC
GGACCAGCGAGGCCACCTTGCCGCCGCGGCCTGAACCCCAGCCACAGGCATTGTCTTTGG
TTGTGCAGCAGCCGCAGGCCGTGCAGCCCCAGGCCAGGCAATCGGCGCCATTGGCAAAGA
GGGCGGAGATCGCGGATTCAGTTTTCGAGGACCTCTTTGGCGAGTCGTTGCGACAGGAGC
AGCCGCTTGCACAGGCTCCCTTGCACTTCACGAAATCTGACGACTTGGAGGCATTCCTGG
AGTCTGCATTTAGCGATGTGTGCTTCACGCTGATGCCACCGCACGCACAGCCGCGGGTGC
AAGAGGCCGTTGGCCCGGTGCTCGGGGGCATTGTTGGATAGTGAGGTGGGAAGCTGTGCA
GGTGCAGGAGGATTGCTTCGCCCATCCTTGAAGCGCGCTGTGTACAGCTTAGCTCGCTGC
GCGGCAAGACAGGCTCAGTGGCGGGTTAGGGACGGTGAGATGCTTTGGGCAGTGTGGGGA
TTGC
>at00406
TTTTTTTTTTTTTTTTTTGCAATCGAGTAGTAGTTTTGTAAGGCAATGTTACACCTCAAG
GATCTCGATGCCTGTGCAATTCAAATAGGGGCACACCACGAGGCACTCAACACATCACAG
GAGCACTGGCTGGGGCATACCAAGCTCCCCCTTGCGGCCACGGCGTCATTGAGCCTGTTT

ACAGCCTCCGCAAACTCCGGCGAGGAAGCATTGGCAGAGGCATTGAAGTTCCAGAAGGAG
GCAGCGGCAACATAGCCACGAGGCCAGATCATCCCACCAACGCTCCTTGCAAAAAACTCG
TCAGCTGAAGGTGGGAAGAGCATGTGGCCCACTGGCGGTGGACCATGATAGGCACCGCAC
TGATTCACGTTGCAGTAGCGGTCGCTCCACATTGACACCTCGCCACCAAGCAGGAGATGG
TGCTGCGAGTATGGCACCCCAGAGCCGATGTCATAGTGGCACCTTGACAACCCCTGCGAA
CCTGCTGGTGCAGGATCCGTGAAGTAGAAGTGTGCAGAGGCCGACTCAACCGCGCGTCGT
CCAGTTTTCGTTATCGAAGAAGCATTGTGGACAGACCATGCATTAATGCCTCGTGCC
>at00407
CAGTTGTGGGATGTCTCCCGTTCAGATACGTTCGCAGAGCCGAGTTGCCGCTCGGATCCT
GCATCTGGTGCTCCGAGCACCACTGACTCGCCAGTGCTCTGCTGCCTTGACGAGGTGGTG
TGCACATGCCGTCAAGTGCCGCGGACAGGAGGCTTTACATGCCGAGCGCAAGAGAGGCCT
GTTGGAAGTGCAGCGGCTGGAGGCTGCACATGGTGCCACCTTGCGGGCTGAGGAACGTGC
AATCACTCATGTTCGAGCAGAGGCGAAACTGCAAGAGGATCTGCGCAAGATTGAGTCTGA
GCGTCGCCAGCGCATTGAGGTACACGAGGGGTGCTGAG
>at00408
GCGAGCCGCCCCCACAGCCGCGGCGCGGTAACCACAGCATAGTGTGCGGCATCTGCACGC
AGACGCGGCCTTGCGTGCGACTGTCGCCGTGTGGTCACCTGCTCTGCATAGAGTGCTCTG
AGCGCAGCACCACCGAATGTCCCTTCTGCCGGCAGTGCATCCACGAGCACCAGGACGTGT
TCGAACCTTGACAGAGTTCTTTGGCGCAGCCCTGCTGTTGGAGGTGGGCTGAGCAGCGCT
CTGCATGCTCTTCTGATGATAACTCCGCATGGTAGATGCGTTGACATGTGCACCATGCGC
GTGCACAGCACGCACAGCTTGTCCAGAAGACTGCGGCCTGTCTCTCAAGTTGTCGGCTGT
GTGTGCCGGATGACGTCAGCAT
>at00409
CCTGCCTGCCGCAGCCTCCACAGAGGCCGCCCTCGCGGCAGCCCTGCTTCGCGAGAGGGC
CCTGGGCCAGACGTCGCACTTTGCGCCCTACGTCGCGTCGCTGCCGCGGGGCAGCGACCT
CGCGCCAGGCCACCCGCTCTTCTGGCCGGACGGCTTGCGGGCGGAGGACCTGCTCGCTGG
TGCTCGGCAGATGCCGGGAGGACATCGCAGCGCTCGTCGCATCTGGGGTGGCTGGCAATG
AGGAAGAGGCACGATGGGCTTTGGCTGTCGTCGACTCGCGTGCGTTCACTTTCCAGCCGG
ACACACCAAACGAGCAGTTGGCGCTCGTGCCTCTTCTCGACATGCTTAACACATTCTCCC
TGCTGCAGGATGACGAGGCCCTCTGGCAATGCACCTTCACTCGGAAGGCACTGCAGC
>at00410
TTTTTTTTTTTTTTTTCTCGGGAGAGAAAACATGTCATCAGCAACTGGGCAATCAACGGG
TGTGCTAGTCACTCTGTCCATAGACGTGGCTTAGCCCCGTGTCCAAACTGAACCGCTGCT
CCACTCGATCCGTCCACCTGACTCGGGGACCCAACGCCCCAGCTGCTTGAAAAGCCTAGC
AGCACCGTCAACGCAAACGGTCTCAGCTCTTACATGCTCTACCTGCTTCAGAGCGGGATG
CTGGATTGCGAGCGGGCCCTCTGGCGCGGCTTGCATGATTTCAATAGCTCCTCCAGCTGC
GTTTCGGAAGACACCCAAGAGATCGGATAACTGCATCTGGGTATAGTGCTGACAGAAGTG
CACCTCCAGATCTGCGCGAGTTGTTCGGAGAGTGCTGCCACGATCTTCCAGCTCAACTTC
GGAAGTCGACGTGCCATCCAAATTGCTCTTGCATAAGAGCACTCCAGGGTTCTTGACAAT
GCCGCGGAGCAACGACAAACCATTGCTGTGCTCCGATGCGTCCTCGTCAATCACTGCCCC
GAATGCACCAGGCACCATGTCTGGTTCAACAGCTAGGACCCAGTGCTGGGGACGGAGGTC
TGCATTGCCAGTCCATGAACCCGACCGCGACAGCGTTCTCCCAACGGGGTCCTGGTGGAC
AAGCAAGAATGCCCCTTAGGACCCCCCTTGCAATCAAGCCATGTCCCGCCGAAGCACAAC
TCACTACACCTGTCCCTCGTGCCGAAT
>at00411
TTGTTTTTTTCTTTTTTTTGTCTAGTCATGGCCTCTCCCTTGTAGCTTCAGAGGGCCCAC
TGGAGAAGTCAGCGCTAAGCCTCCATGGAGCCCTCTTCACTTTCCTCCAGCATGGCCTCG
TACCGAGCGCGGTAGCGCTCCTGCTCTTCTGCCAATTCTTCGTTCTCTCTGATGAGCCGC
TTCATCTCCTTGCTCCTTGAAGCGATCTCCGCACCCAGCCAGCGCAGCGCTTGGAACTCA
CCCTGCACGTCCTCCACGGAGAACAACACCGCATCGTGGCCGCCCTCTTGTGCCAGTTCT
AGGTCCTCTATTCTGGCAGCCTCCAGCTGCGCAAGCAGCTCCTGGGCATCGTCCACGTCC
AGCCACTCCACCTGCCCCAGTTCAGCCACGTCCGCCAACCGAGCCTGCCCGCCGGAGTCC
CAGACCACGATGGCCTCTCCGCTGGCAAGCTCGCTGAGAG
>at00412
GGCAGCTGTAAACCCTTGCATCTCCTCCTTCGACGCCACGGCTCTCATGCCTGTCTTGGC

ACTGTATTCTTTCACTTGCAGTGGGTTGGTGGTGCCGGAGACATGCGGATAGCGGCAGTC
CCTGGGATAGGCATGATGCATCCACTGAGCAAAGAGCCGGCCGTGGAGCGGCACTTCACC
CTGATGGTATGCGGCCACTTCGCTCAGACGGCGCAAGAGTGCATCGGAGGGCGTCCTGTT
AGCGACTGGATCCTCAGACGGCATTCCCGACAGCGCGCGCGCAATGCTGTCCACGCTGGC
GGTGGGAGCTCCAAGCTGAAGCTCCAAGTGGTCCATTAGCTCCTCGCACTCGTCAAGACA
GCACACCGAGTA
>at00413
TTTTTTTTTTTTTTTTTACCCAGACATGCCTTGTTTTGGGAAAAAACACTCACCGTCCG
GCTCAGGCCGGTCCTTGCCTCAGGGCTCGGTCCCGTGAGTGTGCACAGAGGTGGGCCTAT
GTGACTTTCCGAATTGGCTCGCGTTGCCCAATTCAATGCACGTGCCCGCGCTTGCAGGAA
CGTCGGCTGGTGTGGGGCACAGCTGCCCGTTTGTTCCACAGGGGTACGGGAAATTTCCCC
CGCCACAGTTGCCACTTCCGGGGCAGGGTTTAGTGCAGGGTGCTGTGATCTCCATCACAT
TGTGGTGGCACTTGCAAAGAGCTGTCGACTTGCAGGAGTCTGAGACTGGAGTGTGATCGG
GCACGCCCGTGTCGAACATGCTCATCAGTCGGCCGATCTTCTGCTTCCCGACCGGGCGGC
CACCGACACACGTCAGAGTCAAGACTTCTTCTCTCGAGAGGTTCTCGTAGAAGGCAAAGG
TCCCGTCAGACTGCCTCCCAAACTTGGGCTCCAGCTTCTTTGGCTTGGCCCAAGTCACGA
GGCAATCGCGGAAGGAGCGGCTGTTGAAGACTGCGCTAAGGATTTCCGGGTCCGACCGGT
TGAAATCGCCCTTTGCCGCTGCCCCCAAACAGATTCCCATGCTGCTGAAGTCCCAAACAG
GAAATCCAAAGTCTATGCGGATGTCGTTTGCGTTCGTGGCTTGCTCCGTGGACT
>at00414
TTTTTTTTTTTTTTTTTCCCCACGATATTGGTGATCCCAAAGAGCAGGCCTGAGGGGCT
GCCCATGGTTGCAAAGGAGGTGATGCAAGTCTCTCCAGAGGCAAAGTTACAACCGGAGGA
CTCGTAGTGCTCCGTGCTACTCACGTCGATACAGTCCATGGACGTCGCACGGCAGTAGCT
CGTGCCTGTGAGGAGGTCCTGCGCGGCGGCGCTGCAGCAGAAGTCGTCGAGGCCCCGCTT
CGTAAATGAGCAGGACTTCCCAGTGTCCTCAAAATTCCCAGTGGTGGTGGCCCTGTAGCA
AGTGCCGTCGTTCAATATGTAGGAGCCAAGGGAGGGGAACTGCGCGGCATTTGCCATGTT
GGTCTCGGGCTGGGTCGCCCTGGCAAATGCGTGCAGGGACGCGTCCTCCAGGCCATGGAA
CACCTTGCCGGGGCTGCCGTAGAGACCACTATCGTCCCCGGAGCCCGCATACACGGAGAA
GGTGAAGACAATGAGCATCAGGAAGATTACCGTGGTGTGGACGTATGAGGCAAAGAATGT
GGCCTTCAGCCCCCCGTGCATGGTGTAGATCCAGCAGGAGAGCAGTGGGATGAGGAAGGC
GGCCCAAATCTTCGACATTCCCGTGAGGTCCGCGATCGTCGCAGAGCCACCCAACAGAAG
CATTGCGGTGACGATCATGTTGGCCATCAGGGCANNGCATATCATGATGATGTGCGTCAC
CGTGCCGAAGCGCGCTTGAC
>at00415
AACGCAGAGTCCGTAGCCATTTTGGCTCAAGTGAGTCATGTCGGTTCAAGCCAAGCAGCC
CACGGCAATCAATACCATACTTGACAGGCCTGGTTCCCGCGTTCCGAACACTACCACTTG
ATCTCTGGCGTTGCCATGGCTCTCTTTCGGCGCGCTGCTCTGGTAGTGGCTGTCATGTCC
ACACAGCTTCTTCAGGCGGAGTCAAAGGAGGAGAATTGTCAAGCAGAGACGTCCCCAAAG
ATGGAGGTTGATGCCAACCTGCTGCTGCAGAAGCACAGTCAAATGAAGAAGGGCGTCATT
GGCACCAGACCGCACTATTCATTGGAAGTCCCAAAGGTCGAACTGATCGCTGGACGCTTG
GAGGGCAAAGGTCACGTCATGCAGTCCAAGGCCGGGCTGAACTATTCCTCATCCACGACG
TTCTGTTACGAGAGAGACAGCTCGCAGCTTGGTAATTGTTGGGCCTCCAAGCCCGACCCC
GCCACCACGCCCGCGTGCACCGCCGACTCTGGAACGACGCCGTGCCTCTGCAGCAGCACT
GCCTGCGACGTGGATGCTCTGGATCAGGATCCGCCGACCACAGGGAGGTGCGAGCAGAAG
GAGATCTACGTGCCGGGGGGGAGGAGCACACCTCGAGCAGTGGCGCTCATGACGTTACAG
GCTCTGGAGAGGAGGTCTACTGCAAGGCCATGGTCAAGAACGATCAGTGGGCGCAGCTGC
ACCTCGTCAAGCTGCCCTCGGACCCTCCCGCGCAGAACGGAGCCAGCTACTGGCAGTGCT
GGCCGCCCTACACCTGGCACCAAGGCA
>at00416
TTTTTTTTTTTTTTTTTGCGCCAACTGGCATGGAACACAGATGCTTCATCAGCATCACG
TCGAAGGCCTCGGGGGCGGAAGGCCGCCGGGCGTCCGATTCTTCCTCCCCGCGCTCACTT
CCAGACGCTCCTGACCTCGGAAGAGCTGTCGGCGTTGATCACGCACTGCGAGTGCATGCG
GATGCCCTTCCAGCCTGGCTGCATCCACTCCCACTTCTTCCAGTCGTCCCAGTCGATCTT
GGAAGCGTCCTGCAGGTTTGGCAACTGCCGGGGCTGCATCCTCGGGGGCAAGTCCTTCGT
GGGCACGTTGTAAAACTCCTGGTGGAGCTTGTCTTCGACGATGCGCCTCACCGCGGCAAA

GTCCTCAAAACCCTCAGACATTGCTGCGCTTCCCGGCTGGTGGGGCGTCGCTTTGCCAGG
GGTTCCTCGTGCC
>at00417
CAACGGCGCTGGGATTGAACATGTCAGGATTGAGCTTCTGTCTCATGCTCCACCATGGCT
CCCCGTGGTCGACCAAAGCGGCAACGGCTTCATACTTGTTCTGCTTCATGTACCGGACAA
ACGGCCACGTCTCCGCGCCCATCCCATGGGGATAGCCACCCTCTCTGCGGAAAATCTCCA
AGAAAGCATTTGGGTCATAGCAGATAACTTCGGGCCCCGCAGTGTGCATTCGAACCAGAG
GTCCGTATTTCTCAAAGTATGTCTTGAACAGCTCTGGATGCTTGTTGGCACCATCATGGC
TGAGAAAATCCGGGAGGCTGCCAATCAAAGGCCATCTCCGTGGTCCCGGCATCAAGTCCC
AAGATTTGTGCGGTGAGCCCT
>at00418
GCACGAGGCAAGCCGCAACGTGCAGAGTTTCAAGGAAGCCCACAACCCCCACACTGGCAA
TGGTGAGGTACTGCCAGGAGCCCAAGAATCCCACCAAGTCATGCAAGGCCATGGGTGTCG
ACCTCCGCGTGCACTACAAGAACACCTACGAGACCGCCAACGCAATCAAGGGCATGAAGC
TCAGGGACGCGCAGAACTACCTCGAGGACGTCTGCGAGAAGAGGCGGTGCGTGCCCTTCA
GGAAGTACACCGGCGGCATCGGCCGTCACGGCCCAGGCCAAGGCCTTCAAGACCACACAG
GGCCGTTGGCCCATCAAGTCAGCCAAGATCCTCATCGACCTCCTGAGCAACGCCGAAAGC
AACGCCGAGTTCAAGAACCTCGACACGGACAACCTCTTCATCGAGCACATCCACGTCAAC
TGCGCACAGCAAGGACGCCGCCGCACATACCGCGCCCACGGCCGCATCAACGCATACATG
TCCTCGCCCTGCCACGTGCAGATGATCCTGTCAGAGAAGGAGGAGCCGGTGGAGAAGCCC
AAGGAGGAGGCCAAGCCAAAGAGGTTCACCAAGAGGCAACTGGCGATCCGCCGCCTCAAG
GTTGGTGGAGCACCAGAATGTCTGAGGCATCCGAGGTCTGTGGCATTTGAGGCAGGGCTT
GATATGGCGGAGAGACGGTAAGCGGAGCGCCGAGCATAGAAAGCTATGTTGTCAAAACAA
AAAAAAAAAAAAGAAAAAAAAAAAAACAAA
>at00419
TTTTTTTTTTTTTTTTTAATCGCAACAACTAGCAGCCCGCCACTTCTCTCGTGGACATG
CTCCTACCAAGCCCCCCTCATGCCGCCCCGGCTCCATCCGCGCTCATCCCATGGACCTCT
ACGCGGCCGTGGGCCCTTTGGAAAGGTCTCCAACCAGCGCCGCCACAATGCCGCCCGTGA
CATCTGTGCCTCGTGCC
>at00420
CACCTTTGACTTTTCTGAGGCGGAGGGTCTTGACAAGGAGTGGACGGCTCGTGCTTGGTA
TGTGGTGAACAATGCAGAGGTCTTCATGACCGAGGGCAACGTGCTGGTGCCGCACGTCGA
CATGGGCGTGGAAGCTCGGGAGCAGGAGCTCGGCAAGAACGGCGTGGTGGACGTCCCCAT
GACCAGGCCTGACCACCCCATGGTCAAGTACGCGTCAGCGTTCACTCGCAACTTCGACCT
CATCGCAGAAAGGAAGAGCGTGGTCTACCACCTCAGAGAAGTTGCAAAAGCTTCTGTCCT
TGCCAAGTTCCTTGTGGAAGCCGAGATCCACGTCGACGATACATGGTTCAGCCTGGTGGA
CTGGGCCACGGTGAAGGAGGCGGCGGCGGACTGCTGCCTGGA
>at00421
GCGCTGTGGTACTGGTCGTTGATTGCCATTGTGTTCATGGTGGTCCTGAACATGGTCTTG
GGGGTCCTAGTGGACGCAGCAATCTCCGCGGGCAGCGACACCCAGATCACTCTGTCCCAG
CAGCTCTACCTCACGCTCTCGTCCCCGCTGCAGTGCTTCGTCCCAAGGCGGGGCGGGGGC
GAGGGGGCTCCCCAGCGCCACCTCGAACCGGTGCCCGAGCTGACGGCGGCCGAGCTGAGG
CATGGCAAGTCCCACTGGAGTCGACAGGTGAGCCTTGACTTGTCTCTGAACCCGCGCCTT
CCCTCCGAGGGACCGCAAGCGGGGGCCGCTGATCCATAGAGCCAATGGCGCGCTGCTGCT
GCTGCAGCTGCTGCTGCTACTGCGTGAGCCAGACAGTGGCCGTG
>at00422
TTGTTTTTTTTTCTTTTTTTTTTCATGCATTCAGCATTCCTGCATGAAGAGACCGCGGTC
CGCAGGGCATGCAACTGGTCATCAAGACTACCCCTCCTCACAACTAAGGCTAAAATGGAG
CCTGAAGGCGCCCTTGCCACTGCTACTGCCCCGGTACTGTACAGGCAATGAGGCTCTGCA
ACAAACAAGGGGATCGCCGTGCATGGCCGCTGCCCCGCGCACACAGAAACTGCTTGCTCA
GGAGGCATTGACCAACGGGTGCAATTCCGGTCGCGCTGGAAACGGGACCGACAAGTCCAC
ACGCCCTGTGCAGTAGCCTTTCTTGTACTCCAAAACCGTGTAATCCGGGTGCTGCTGGAG
GAACAACGGCACGCACTCGGCACTGAGCTCGTAGCCCTTCATTCCCTGCCTCATGTCCGG
GCATGCGAAGAAGTGGCTTCTGCTAAGGTACTTCCCGGACATGTCGCACCCGACGTACTC
GAAGTCGTACTCCGTGCCCGCTTCTGGGCAGGGGACGACGGCAAAGTTCACGACCTCCTC

TGCGGGAGA
>at00423
CTGGCTCGAAGCGTGTGCCCGTGCATCCGTAGATGGGCCAATGGTGACGTGCCTCGTGAG
TGAATGTGCTGCTGACAACCTCAGCACTGATGGAGTCAAGGGGAGCGGCACCTTTGCGCT
GCCAAGCCCAAAGCGAGCAAAGAGGCTGTACTCCGAGGAGGAGGTGGAAGCTCGCGTGCA
GG
>at00424
TTTTTTTTTTTTTTTTTCCGGCAGGAAAGAACGCTTCCCATGCAAGTGCCTACACAAGG
GCAAGGGGTCAGCACACAGCAGAGACAGCACCGCGCACTCATTGCATGCCCTCTTTTACC
TCTCCTGGTGATGCCCAAAGGGCATGCGGACTGTGCTGCACACAAGCGCCCACGAGCTCA
GCAGCAGGGAGACCATGACGCTCTGCCTCCCGGGCGCTCCGCTTGGGGTGTACGTGTTGC
ACGTCGAGCCGCAGGTGGCCTGGCTCTGGGCTGCGCAGGTGCTGTTCGCGTAGGACATGC
GGTTGAGGACCTCGGGCTGGTCGAGGCCCATGATGGACAGGAGCTCGAACTCCAGCGTGA
TCGGATGGCCAGAGCAGCGGCCATCGCCCCAGTAGCATTCAGGCCATCGTGCTTGGCACG
CGTCGGCGCTTCTGGCCTCGCCGCAGGCAGCGTGCCGCCGGAAGAGCAATCGAAGCGGGC
AATCCTCGCCCGTCACCGAGATCAGCGCGTCCACGGTGCGGAGCGTGCAGCGCCCGCCGG
ATCCATCCATCCTCGTGCC
>at00425
TTTTTTTTTTTTTTTTTGCCGTGTCATGGTACAAGCATATGCAGTGTTTGGTAGTGGTC
TAAGAACCCTGCACAATGGACTCACGACCCCTGAACCCATGGCTGGGCATCAGAGAAAGA
TGGCACCTAAGAGGCTACGCCAGAGTGCAACAGTCCGACACTTCATGCAGTCATGCACAG
GCATCTCTGGAGTGTCCTGTGCCTGTGCTACAACGTTTGCAGAGGCAAATCGCACTGGCA
ACATCACAAACGCTGCAGTAGCAGCAGCACCACCACCACCGCCAAAGGATGAACCAGAAA
CCTTTGCCGATCGGAAAGGAGCACCGAACATGCCCTTGGCAGAGTCTGCATCTTTAGGAA
GCTTGATATATGCCGAGGTCTCTTCCAAAGGGCCTCTCGCGTAATGCACTGCCACTGCCG
GCTCATCACTGCCGACACACCGATTGAATGCGCCCTCGTGGTCAGCGAACGTGAAGAAGT
GGCAATGCACGTCTGCATCACACCTCTCCCGGCACTCCTGCAGTGGATCGTCACGGAAGG
GCCCTGGCCAGGTGCAAGTGCTGCGGGACGACTTCGCGGGGAGTATCGGACCGCCAAGGC
TGCTCGGCTCCGACATGACAGTGGCGTCACATCTCACATTCATGGCCGGCCGCAGGTGGC
CCCTCCCTACCACGTGACACTCGGAGTGCGGCAGGAAACGGTATCCATCCAGCTGCTCCA
GCGCTGCGAGCGCAAGGCGACCAGGAGCCATGGTTGGTGCTCGAGGCAAGCTCACGCGCA
GGGGCCTATCCTTCGATAGGCGTTCTGGACCCTTGAGCCAAAATGGCTACGG
>at00426
TTTTTTTTTTTTTTTTTTTGGATACGCAGACTGGGCCGTTGCTCGAGGCAGAGTGAAG
CGTAAACCGCTACACGCACAGCTCGTGCACCCCCGCAAGGCTCACGCGAGCGGTCCCCCA
CGCCCTCTGCGCTGCGGTCGTATGCCAAGATCCTCAGCCATCTGCGCCAGCACCGGCCAC
AGCAGCGTCCAGCGCTCGTCCGTGTCCGGCGGCCCCAGCTCCGGCTCGAGCGGCGGCGAC
GACTCGACCAATGCGGGGTGTGGAGCATCGAGAATAAGGCTTGAACGTGGAGCGTCTCCT
TCGTACAGGAGCTCGTGCTCGAAGCTCTCGTCGAACCACCAGCACCGGCCCTTGTCTTCG
GCGCCGTAGGCAAGCTCCGCAGCCCCCGGGAAACGGAGCCGTGAAAGCGAGCCCTCGGCT
GCCAGCAGGGGGCAAAGCAGCCGCAGCCGGCCCTGCTGCGGGCTGTGGTGCCGCGCGATG
CGCGTCCGCGGGGGGTGGACCTCAGTAATGCCCACGCGCGACAACGGCACGCCGCTCTTG
CGCAACCGCACCATGAGATTACAGGCCCGCGGGAAGACGTCNCANAAGCAGACCTCCTCC
TCGTGCTTCTTGGGGTCNCAGACCGTGTACTCCGCCCACTTGCCCCAGGCGCTGCTCGGC
GGGTCCGTGGCGGCGAACTGTAGTGGC
>at00427
CCGTAGCCATTTTGGCTCAAGCTTCGCTCAAGCCCCTCAGCTCGTGTCCGCCACCCTTAT
TTGTAAAGCTCCGGCAGCCATGGGGTCCAACTCCATGCCCGTGCGCATTGCGCACGGCTG
GGCCATCGCATGGCTGGACTTCTACCAGACGCCAGCCATCGTGACCGAGGGTGTCGTCTC
AGAGGTGAAGGGATTCTTCAGGGAGATGGTCCGCCCGAAGGAGAACTACCCCCACAACCA
CGTGAACCCCCAGACCGCCGAGACCAACCGTCTTGGCCGCGCCAAGCCGTACTCTGCTGG
AGACGACGAGTGGGCGTTCAAGGGCTGCAGCTGAGCGCCGAGGGGCTTGCCCTTGTGGCT
GGATTGTGAGAGCCCTGTGCGTGTTGATTGTCCAGTATGGTTGAGCGAATAAAAAAAAAA
>at00428
AGCCGCAGGGACGCCAGGGCGCCCAGGGCCAGGTCCCAGCGCCCAGCTTTCTCGCAAGAG

CTGAGGGACGCATTGTAGGCGATCAAGTCCAGTGACACACAGGCCGATCGCATTCTGTGT
AAGACACCGAAAGCTGATTGCCACTGCTCTCCCTTGGCATACGCAATGATTGTGGTCTTG
CAAATGACCTCATCAGGAACCAACCGCCAGGGAGTGGACCTGCGGCTCCGAATGCAGGCT
CCGATCCTCCAGGAGCAACGCCGCCTCCGTTCCCGCAAGGCAGCAGCCGCGAAGCGCCAT
GGCTGCAAGTCCATTGCGCCGAGCCATGTGGACGGGAGTGGTGGCAGACCGGGCACTCTT
GGC
>at00429
TTGTTTTTTTTTCTTTTTTTTTTGGACCTCGCGTCACCTTCCCCGCTGCCATTCTCTGTC
CACGCGCCGGTGGCCATGTCCCCTCTGCAGCTCCCTTGCTCACTTGCGCTTCTTCTCAAG
CTTCCTCTTGTAGAAGGTCAGCTCCTCCCCTTCCAGCACGTAGCCGTCGACGCGGCCGCT
CTGGCCCGGCCTCGAGGAGATGCAGGCGAGGAGCCGGCCCCCCTGGAACTGGTCCTCGAT
GGCCGGGTCCAGCTTCTGCTTGGCCTCGCGGTTCTTGCGCTTGGCGAGGACGTGCCTGCA
AGCCTTCAGCGGGTCGTCCTTGTCGGCCTCCTTCTTGCCTGCGTCCTTCTTGCCGGCGTC
CTTCTTGCCGGCGTCCTTCTTGCCGGCGTCCTTCTTGCCGCCGTCCTTCTTCTCGTCGGC
CTCCTTCTCGCCGGCCTCCTCCTTCTTGGGCCCCTTGCTCTTGCGGCCAAGATCCACGCC
GTACCTCCGCAGGTACCACTGCTTGAAAGGCTGGCAGTCCACCTGGATGATGGCGTTCTT
GCAGAGCGTCTTCGTGCGCACCAGCTCGTTGCTCGAGGCGTTGTAGACCACGTCGATGAT
GCGCGTCTTCCGGGAGCAGCACTCGGAGCCCCAGGCGATGTTGCCTGCGTCGAGGCGCAG
CGCGCGGTACTTCATGTTGCCGCCGCGGCCGCGGACGGGCCGCACG
>at00430
CCTGTTCAACCAACTTTCACCGTTGGTCACCGGTGGTCAAGCCGCGCGTCCGGGCCAGCT
TGTGCTGGCCGCGTGCGGCCCTAAGATGTTGGGGCCATGCACCAACCCCAAAAGAGCGTG
AGGTTTCGAGCAGCCAACCCTGCTGTCCAGGCTGTGCGCTCGTCTGTGCTCTTGACAAAA
TAAATTCGAATGCAAATGTTGCCAGTCGCTTGCGGGTGAGCGGCTGACGCCATCATAGGA
TTCTCACTGGCCTCGATCCCCACGGCACCGTGTGCACCACACGGCCAAACAAGCAGCGTC
TCCAATCACTGGCCTCGATCCCCACGGCACCGTGTGCACCACGCGGCCAAACAAGCAGCA
GCTCCAATCAGGTCTTGTGCCTATTCCACCAGGTTGGTGTTATGCCCTTGTCAAAGTTCC
TCCTCTGCAGGCTCGTCCTCTTCTTCGGAAAGTTCCAGCTCAGTCCGCCCCAGGTGAGCC
CCGTCCTGGACCAATGCTGCACCTAGGGTCTTGTGGCGCCGGGCCAGCCTCAGGCTCCGC
TGACGGACTTGAGCCAGCAGGGGTCCGCTGCACTGGCAGAGGATCGCCTCGTTGCAAGGC
CACCATTTGTAGCCCTGCGGGCACTTTGCGCAATCGTCGTCCGTGACACCCCTGTTCAGG
CCTGGTGTTGCCACACAGGTCACAACACCCGGCGCTGGCACTGGTGCAGCCTGGGGGTCT
GGCTCTGGCTCGGGCTCAGGCTCCGGCATGACAGGCTGAGTCTCT
>at00431
TTTTTTTTTTTTTTTTTTCGGGCAGACACAATACGCACAATAAGGTTCTGTACACCATCC
TCCATGCACACAGTCCTTACGTGGCATCATCCACTCTGGACTGCGCTCAGCACTACCCAG
CTGGGACCAACGCCTCATTTGAAAGGTCGTTTGCCCAAGGTGCANAAGCCGCGCCTGCCG
CCTTGACCCCGCAGGGTGAACGTCGCTTGCCTGTGCCAAGTCTCGGAGGCGGTCTTGCTG
GCTCTGGC
>at00432
TTTTTTTTTTTTTTTTTTCGAAGGATCGCGCATCAACGTCCTCAAAGTCCTGCCACGGCC
CAGTCTTCCTGGAACTTTTGGAGGATGGCACGCTCAGAGTTCTCTTTGATAATGACCTTC
CAGCCATAGGAGCACCGCCTCACACGCAGGCGTGCACGGCCTTCCACTGGCTCCTCTGCA
ATGAACTCCAGGCCGCCCTGTTCTAGATCTTTGAACTCCCTTCCGACGGGTGTGCCCAGC
GGCCTGAACTTGAAGACGACCCCGGTGGGCGTGTTGCGGAGCTGCACTGGCGTCTTCAGG
CCCCATCGCAGTGTGTCCTTCATCGAGCCTCCCTCATCGGCGGTTTTGTACCACTCGGCA
GCCCAACTCTGAGCAAAGATGGCCGCGCGCGCTGCGCTGTTGACCTCTCTGCGCCAGAGC
TCTGGGCCGATGTAGGGCAGCATCAGCTCCGGCCAAGCTGTGGCTGGGGCCTCCCCGGAG
CAGGGCTCATCCACCACACAAAGGTTTGTGTCCACGGACAGTGCCT
>at00433
CCGTAGCCATTTTGGCTCAAGCTCGGAAAGGAGGAGGTCCAACTTGGCAGGCACAGAGTT
CCAGGGCGTGATGATGGCGCGGGCAGCTCGGACGATATTGGCCCTTGTCTGACCCGGCTT
GACCGGCCTGAAGAGGGACTGAGCGGCAGCCATCGCCGTAAGAAGGGTTTTGGTATCTGA
AAGCTGTGACCGATGCCACCGAAATGCTTGGGCATCGTCTGCACCATGATGCCACGGCTT
CGCGCTCCGTCTCGTCGCAAAGGTACAGCAGGCGGCGGGCAAAGGCAAGCAGCATTCCAT

CGCCTGGCTGCAAACGTGCGCACTGGGGAATTGCTGCATGTACCGACGGCGTGTGTCATA
GAGCTGGCATCTTGGACGGAAGCCAAGCTACAATAGAAAAAAAAAAGAAAAAAAAACAA
>at00434
ACGGAGGCATGTTTGCGCAGAATCCTGATTCTTGCACATGCAAAGAGACAGAAAGGAGAC
ACTACAGAGAGAGAGAGAGAGAGGCTATCGCTATGAGGTCAAAGTTGATCGTGTTGAGCC
ACCTCGCTCAGCGCTTGGCTTGGGAGCAGGTCCCAATGCTTCTGCCACTGCTTGTGCAAG
ATCTGAAAAGCTGGATGATCTCTTAGCCACAACCAGCTGCTCAGCC
>at00435
AGGCATCGAACATGCGGCAAGTCGCGGCGCTGCCGCGGCATGTAGCCGCACCTTCACGGA
AGGCCTCAGCGGCGTCGCAGCAAATGCGGACGATGGCCCCGCGGGTCTCTGCACAGGTGC
TGGAAGAAGGAGGCCAGGCGGTGGCGAGGCCCCAGAATACGCGAGCGCCGGAAGCTCTGC
CGACTCCGACTCTGGATACGGCCGTGCCGAAGAGGACCCTCAGCAAGGACACATCCACGA
GGATAACTCAGCAGGCGGCACTTGCCGGCAGCTTCGTCCGGACGTCGTTCAGCCACCCAG
AGCTGGGCACGGGGGCTTTCGCCCAGATGGTTGAGGTCCCAGAGCCCCCCAACTGCAACG
GCAGCAGAGACCCCGTCAGCGTTTCCAACCACAGT
>at00436
CAGCAATGCGGCACGCTTACTGGCTGAAGGCACCTGCGGCGACGTCATCTGAACCATATG
TACGCGGCGACGCCTTGCCCTTGGGCATCAGGCCAACTGCAGCGACACTTGGAAAGCAGC
AACTCCTGTTAGCCCGTTGACTGCAGTGGCATGGCAGCTGAAGCACCCTCTACAGAGGTG
CCCTTTCCCTCTGAGACTGGTGCTCCTGAAGAGGAGCCGGCCAGGGCCCCCGAAGATCCA
GCAACTGTGAAACGCTTCGGGCAGGCGCAAACTCGTCCGGCGGCCTTTGCTGCTCTGAGC
TCCACACACGCTAACGAATGCCATAGGACAAGTATGAGGTCCCAGTCTT
>at00437
CGTAGCCATTTTGGCTCAAGCCATTTGCGCTCGGTGCACCTGGCCTGCCCAGCCCTCCGG
AAGCTGGCAGCCGCCCCCGGCGCCGCCAGCAGTTGATGTCCTCGCTGCTCGTCAAGGGCT
TCCATGGGGTAGCGGTGGCGACAACGGTGCCTTTCGGCCTGATTGCCATCTTCGCCCCGA
ACCCTGAGAAGGTCAAGTTTGACAGCAGAAGCGTGTCTCTGCCGCTGCCGACGCCGCTTC
TCCACATCTTCGGCACCTTCTGCCATCTCACCATGGTGTGCCACGAGTGCATCTGCGGGT
ACTTCGTCTTCGCATCGCTGCGCGACCATGGCATCATCCCTGCCAAGCCTCCGAGCTTGG
CGGAGAAGGCCGTCGGGCAGGAGGACCTGTTTTTCTCGAGCCTCCATGTTGCAAGCCTCT
TTGTCCTCGTCGTGTTCTGGAGCGCCATGGTGGTTGACCCGTTGCTTTTCATCGACCCCG
AGATGATGAAGTTCCCCCATGACGCCCCCCGCTCCGTACTCTACAAGACCCTCTTCCGGA
TGCTCTGGTCTCCAGCGAGCGGCGAGGAGGCAGCCTTCTTCCACTTCATGAGATACATCC
ACACCTGGAGCACGCTGCTCGTCACCGTGGAGGCGGCGATGGTGGAGCACCCCCTGGCCG
ACTGGGCTTCTGAGGCCTCGACTCTGGTGTCCTATTGCAGCAAGTACTTGGCTTGGAATC
TGTTTACATGGTGGCTGCTGCGACATCCGCCCTACCCTTGGCAACTGCAGTTCTGGAAGA
GGGGCATGGCGCAAGGGCTCCTGGCGCATGGCCTTTGCTTCGTTTGGGCGTTTGCCCTCC
TGACATACAGTCGGGCATGGAGAGGTAGTGGCGGCACTTTTCTCGGGCGAGTTCTGCTGT
CACTGCCCCTCGTGATTTTCGTCTGGGCCTCGCATGGTGGCTTTCCCATGAAGTCAGTCT
TAGCCCGGTATCATGGCGCGGAGTTGAAGCCTTGAGTGCGAATCTCTCCGAACATGTACG
CATGCATGCGTGCATGCGCCAGCTGAAAGCGCTGGCATCATTCTGAGCATGCTCAGAGCT
GTCTAGCGATGACTTTTGGGCCCTT
>at00438
TTTTTTTTTTTTTTTTTTCGCTAGGTACGTGTCGGCAAATGAATGTCAGGAGAACGGCGA
GGCACGCCCCGGGGACGTGCTAGCGCCACCCTCAGAGCACCTCTAAGAGGAGACATGCAC
GATGGGGAAGCTGACCGGGATCGAACCCTCGCCGGTCTTCGTGTCCTGGCAGCGCCCGGG
CACCTGCTCAGGTCCTCACAGGTGGCGGCATGGGCGGCGAGGTCGACGAGACAGGCGGCT
GCGGCGGCCAAGGTGGGCTGGTGCCGTTGCCCGGGGGGAACGTGACGTTCGGCNGGCCGG
NGGGCCGGGGAGGACGGGCTGCGGCGGGCCGCTGGTGCTGGGCGGGAGGGTGCCCGGGGC
TCCGGTGGTGCTGCTG
>at00439
TTTTTTTTTTTTTTTTTTCCCAGGGTCAGTGCACGGGTGATGCCTTCAAATTCCGACGCT
GTCCTTTCTTCTCCTAGGGCACAGCCAGCTGATCTGTACTCCGTGCGCTGAATTCGGCTG
AGTACGATGAGCCCAGGACCTCAGGCGACAGCGAGCTCTTAGACCAGAGTTTGCTCTGGA
ATGTCAGAGCGGCATCGTCATGATACAGTGGCGATAAGCCAGTGGAGCTCGCTGCTGCAG

TACAGCGGAATTCCGACAACCGCTCCCGCTCGTGGCCAAGGTCGCCCCACAGGACCACGG
AGCCATCGCCCCTGACGGCGGCGAAGACCCTCTCCGCCGAGTGCAACTGCCTCACTCCGC
TTGCCAGCTGGTGCCAGACCTCAAGGCTGTTCCCTCCATAGTCACCGTGGCCCCAAGTCA
CCACCGAGCCATTGGCCTTCAGCGCCGCAAACGCCCAGCGCGTGGAGGAGACGTGCGCTA
CGCCCTCGAAGAAGGTGCTCCTGAACTCGTGCGCAGTTGCCTCCCAAGTCGGAGTCTCCC
CATGCGACCACTGCACCGCCATAGGTGAGTGCGGCGAAGGCACGGTCCGTGGAGCAGATA
TGCCGCACGCCCTTTGCCAGAAGGCCCTCCACAGCCTCGCTGTTGCCGCCTGCACTCTGG
CTGCCCCATGTCACCACGAAGCCCGTGTCTTTGACAGCGGCAAAAGCCTCAGAGGTGGAG
TAGAGGCACTTCACGCCATTCGCGAGCGCCTTGCGAACAGCTGTGCTGTCCCCACCTCTG
TACCAGCTGCCCCACGTGACAACGGAGCCGTCGGCCTTGAGTGCCGCAAAGGCCGAGCCT
>at00440
TTTTTTTTTTTTTTTTTCGCGATCTTTCGTGGGCATTGTGTGCTACGTCAGTCCTGCAA
TGCTTTGTCCGCGATCGGTTGCAATCAGCTAGGCGGCTCCTCTAGACACTCAGCCCTTGC
ACTGGTCTCTACTCCCCGCGGTGCAAGGCCCACAACGATCCACGCCCTGGTAGGACCCTG
TCAGCGGCCTTCCGCAATAGGCCAGGTAGGCCTCCACACTGCAACAGCAGGGGGTGACTT
GAAAGCTGGCTGAGAACCAAGCCACGGTTCTGAGCGTTCACGCTCAACGGGAGGCCCCCA
GCAGCCTCCCCAGCAGCCTCCGAGAGGTCCCCCACGACCAGCGCCCGGTGCACTCGCCAG
AGGAAGAGCCGCGCGCAGTCTGTGGGCCGCAAAGGCTCCAGGGGGAAGGCCACCACCTTG
TGCGCGCCCAAAGCTTGGAATGTGGCTTCCCGGGCTCCAAACACCAACACGACGCGCTCG
GTTCGGAGCAGAATCTCGGTCATGACCTTCCTCATCTCCTCGACGGTCGACGGATCTTCC
AACAGCAAGCTCAGGCCATCGATGATCACCAGCACCTTGAGGCCACCAGCCTCCAACTTC
GAAAGTCCTTGCACGACCCTTGTGCGGAGGAAGCGTGCAT
>at00441
TTTTTTTTTTTTTTTTTCACTGTCACTGTCACTCTCGAGGTATCAGACTTCGATTCCAC
CTTCAGCTACATCCCAACTTGTGNTGGGCCGCCTTCCATAACACAAATCTAATCCCAGGA
ATCCGGGGCAGGAGCACGTAGGCACCTGAAAGCAAGTCGCGACCACGGTAGCTACCGTGC
CCAGTTGCAAGCAAAGCGAAAGGCAACTGCATCAAACAGACCAGAAAGGCAAGAGAGGCC
ACAGAAAAGAGGCCCTCTGCGAGGGGGGGACAGGGCGCTGGCAAGAGGTGGAAGCGGTGC
TCAGCCGAGCGCTGCCCTGATCATGTCAGCGCTGCCGCCATTGTGCGCGCCTCGTGCC
>at00442
GCCCCAGGCCGCCGAGGGCACGGCGCGATGGCCGGCGCGGCAGCGGCCAATGGCGAGGCT
GCGGCGCCGGCTGCGAAGGCGGGCTTTCTGAGCTTCCCGGAGGAATTCACCTTTGGTGCG
GCCTCGTCGGCATATCAGATTGAGGGCGCTTGGAATGAAGGCGGCCGCGCCCCCTCGATC
TGGGACGACCTGACCCCACTTTTCCCCGGGTGTTGGGTCTGCAGCATGGTGACGTTGCAT
GCGACCATTACCATCGGTTTCGAGATGATGTTCGAATCATGGCGAAATTGGGCTTGAAGG
CCTACCGATTTTCCATATGCTGGCCTCGCTTGATACCCGAGGGTGAGGGCAAGGTGAACC
CTGAGGGCGTGAACTTCTATTCGGACCTTATAGATGCACTGGTGGAAAATGGTATTGCGC
CGTGGGTGACACTCCTCCACTGGGATCTCCCATCGAAGCTCAACGGTGA
>at00443
TTTTTTTTTTTTTTTTTCCATTCAAAACTAGAACTGTGCTCCTCACGATGGATCGCCAC
CCGATTGGGATGTCCTCACTTCTGCTTTTGGCCCCCACACTTCTGCTTTTAGCTCCCTGA
CTTCTGCTCTTACCAGGGCTGACTGCTGATGCTGCCCCACGGTGGATGGCGGTCCCATTG
GGATTCCCTCACTTCTGCTTGTAGCTCCCTCACTTCTGCTATGAAAAGAACTGACCGCTG
CGGCTGCTCGACGATGAATTGCGATCAAGATAGCCTCACATCGACTCGTAGATCCCTCAC
CTCTGCTTTCAGCTCCCCCACTTTTCTTCTTATCAGGGCTGACTGCTGATTTTGCGCTTG
GAGGAGAGCCGAAGAAATCTCCTGTGTGGAGCTATTGCCACCATACAGAGTAATGTGAGA
ATGGGGTATGACGTGCAGTTGAGCTGAGCTGCATGGCACCACCACTGCATCTGGTCTCGG
CTTGATGGGGCAATTTTCAGTTCGACTTTTTCCTTTTCCTTTTCCAGCAGCTGGACTGGC
TGTCTGGTCGCGTACGCATGCGCGACGCGCCCACAGCACAGGAAAGTACCGTGGTCCTGC
TTGGAGCCGATACTCACGTAACATAGATACGCCATAGAAGACCGAGAGCCATCCATCTTG
CAGAGATATAGACCAAATGCCGCCTGTGGCTAATGCATTGTGCTGCATGCCGATGCTGTT
CCGAGACAAATGGCTTCCAGCGCAGTCTGGCTTCTCTGACATGAGGAGTGATGCATCTGG
GGGTCCTGCGTCCGAGTCCTGAAGCCAANCCTTTAAAGGATTGAATGCGCGGCGTCTCGG
AATGGGTGGAAAGTCTAGTGACAACTTCCGAGACAAAGCATCTGCACGATACCTCCATGA
CCATGCTCCCTTGCCCTTTCCGCTTCCCTTTCCCGCGTTCTTCTCTTTCCTGCTCAACAC

TGGCAAATTGTGACGCTGAACATCTGAGTTTGCAGTTTCTAGCACGACTCCTAAACTCAT
CATCTGGCTTCCACTGCCCCTCTATGCGCACTGACNCCTTCCAGTTGAGTGCGTTGGAAA
CGTGGAATGGCCAACGTTCCCAGGCAGCCTTCCCTTTTGGAGTTAAATGCCGAAGTTCAA
ATGTACTTCCGCCGCTGCACCGCAAATGCAGACACCTCCTCAAGACCATCACACCATCCA
CGGAACATTCCTGTATCCAAGGAATAGCTTGCACTGACTGG
>at00444
GGTCTCATCGCGTGACCACGCAGTGGAACCCCGCACTGCATCCACAGCTGCAGATCCTTG
CTTTTCAGCTGGTTACAGCATGGCCTTTGGCTCCACGTTGGCGTTTGCATGGAGTTTGTC
CGTTCTTTGCTTCTTCGTCGGGTGCCGAGGCGCATCCTTCTTGGATGAGCTTGCAGATCT
GGATGACAACTTGGCACTGCTCCAGCAGAGTATGAAGCCGCGCAGGCGCCAACCCCGTGA
TGATGCGGTCCCGTTCCCATATACTGGGTATATGCCATCTGATTATTACCTTTCCGTGGA
AATTGATTGCGCAACCCACCCTGCCCTGTGTTCACCACCTTTCAACTGCAATCAGATCAA
CCAGACTGTGCTCGCCCAGTGGTTCCAGGAGGGTTGGGCCAAAGGTGGTCGACCAAATTG
GTCGACATGGTGCATAGATTATCGGTACATGTACGAGTCATATATCACCAAGTGTGCGGC
CGGTGATCTTGATGGGGCTGCGCTTGAGAAATACCAGTTGGCAATTGCTGGCCATTATGG
TCCAGACATAGACGAAATGGAAGCCAGTGTCTGCTTTATGGATGGGCACTGCGCAAACAC
AGCTGTCACAAACAGCACCACGCTTGCGGAGGCCATCCAAATGTGTGATGACCGATTCGG
CCGCGACGCATGGACAAGCTTTGGTAAAAAGGACTCCCCTCCTGAAGACAAAATTGGCTT
CACTTGGGAAGAGCTCCCAAACATGCACGATGGCTTCACAGACCCAAAGCAGAGCAGGTC
GATCGCGCTGGCAGCATGCGCTATGGGCAACTATCATTGTGACATTCGATATTGCCAGCA
TTATTGCAAGAACGAGCACTACGTGCGTAAGTATGCCTACCTCGAGAAGAAGTTTGTGCA
GACCAAGTGATCAATCTTTTGTGACCTTGGTGTGTCACCGCCTCTGCCTGACAAAGACAC
GTCAAGACAACCAAAAAAAAGAAAAAAAAAACAA
>at00445
TCCCATGGTGCGACTGGCAGTTCGAGGCAGTCGACCAGCTGGAGCTGGTGGCGCGCATCC
GCGCAGCCAAATTGAAGATTCTGCGGGAGGGCTGGGGGAAGCTGAGGCGCATGGAGCGAA
AGTGGGCAGTGCTTGCGGACACACCGGCGGAGTTAGACGGCTTCATGAAGGCTTGCGGCG
CCGGCTCTGCCAGGCCCACGGCTCCTTCAGACCACCAGCTGCTCGCGATGATCCTCGGAG
GCCGGCAGGGCCTCAGCGAGGAGGAGGCGGAGGGAGGGAGGGACTTTGAGGGAGTTCCGG
GCGCACGTGTGGCCCATGTTGTGACGCCTGCCAGGCGGTTCGGCTTACAGAGGCTGCATA
GCCTCGGCATGCTGCGCTCCTCCGTTCCAAG
>at00446
CACCAGCGCCGCGAGCGAGTTCGTGTCGTTCGAGAAGGACGGCTCCCCGTACTTCTTCAG
GTCCTTGTAGAGCTGGCCCTCGTGGAGAAATTCGCGGGCGTGCCACTGGAGGCAGAATTT
GAAGTCAACCTTGTCGATGTTTTGCCGGCGCATCTCCTCCTTCACGGCGTCGCTGAAGAT
GCGCCAACGCAAGCTCTTGCGTATCTTCTTGGGCTGGATGATGCGCTGCTCGACCTGCTG
GCTGTGCATGCGATTGCTCTGCGGATCCCAGAAGGCCTTGCAGACAATGTTCTTCTTGCT
GAAGCCGGCATGCAGATGCCGCGCAATGAGGCCCAGCGCTCGCTGTCGGTAGTCCTGCAA
GTCCGTGATGAGGTCGCGGAACTCCTTGGTGTCCACCAGAGGCTGGGACTTGTCCGTCCA
CTCGCCCTTCGCCAGGGCCTGCGGCAGCTTGTGGGAGATGACGCCGGTGAGCATCAAGTA
GTAGAGGCTGCCCGGCAGTTGCTCGCCCATGATTGCGCTGAAGTCGTGCGGCACTTGCGG
AGCGGGGGCTCCGGGCATCATGGGCCCCAGAGGCCGCACCTCGTTCTCTGGCATGTGATA
CACCGGCGAGCTCTGCACCAGCACCTTGCAAATGCAGTACCCGTCGATGTGGTCGTTCTT
CATCTCCTCGGTGGGGAACGTCTGCATCCAGTTGATCAGTGGGGCCTGCTTGATGATGTG
TACTGCAGCGTCAAAATTGAATCGAGCGTTGCCTGGTGCAGGCTGGAAATGTCCGAGGTT
AAGGAAGGGGTACGTAAGGCAATACTCTGTGCCAGCCAGCAAACAAGCATCTATGAACTG
ATCCTTGCTGATCTGCCACTTGGTCAAGACAACATCAAGATCAACCCAGTCAACCCCCTG
GTGGAAGAAATCAATGTGGATGACGACCCGCGGGATGCTGTAAAGCAAGAGACCGGGAGG
GCCAAACACTGTCTGTACTATCCCCAGCTCGCAGAAGTGTGCGAGCTGGGCGCCTGCAAA
ATACGGTGCTTGCAACACTTCGCAGCCCTTCTGCTTCAAGTGGTGAAAGATGAAGTATAC
AAAATCACCGTTGATGCGGCTGGTGGAAACGGCAAAACACCTCTGCGCCTCAACCTTCTG
GCCCAGCATCAAGTGAGTCCAGGCAGCCTCCATTTGCTGGTCCATGCGACTGATAAACAT
CGAGTGCTGCGGGCTGGGCGCCATGCCCTGGAACACAAAGATGGGAGAAATATTGTGCCT
TCTAAACAAGTCCAGCTCCTTGTCGACAAAGCCAAAGATCCCGGGGGG
>at00447

TTACCACAGGGGTCGATGGAGACCCTCAGTATTTCTGATCCGGAGCCTGCTGATGGAGTT
GGAAAACGTGCGGTCAAAGGGCAGCTCTATGACAAGAAGTCCCACATGCATGTCGGCTGC
TGGGCGTATGAGCTTCCAATGGACGGCATCAGCCTGGGTGCGCTGCAGCGCGACTTGGCG
GGGTCGGTGATGTGGTCCGGCGACTTCTGGAGGGACTACGCCTTCTTCGTCGCCAACAAT
CACCCCCTCGCCGGCATCCTCCTCTCGCACCCTCAGCACCCCTATTCAAAGCTGGAGCGC
ATTGGCGTTAACGTGTTTTCCGTGTCGTTGACCCTCTTTGCGGACGTCATCGAGTGTCAC
C
>at00448
TCTGGTCGAGGCACCTCCAGCATAGGAATTCGCGCTTGGCAGAGCACATCCGCCAGTGGA
ACTGGATGGAAGCTCCAAAGACGGCCAGCGATGTAATGTAGTAGACGATCACACCCAGCG
CGTAGAGCGCTCGCGGCCAAGGCATGTCCAGGACCGTGTCCTGGGATGACAGCCACACAG
CAAATCGGTAGAGGGTGTACGCGTATGCGAGAATGTGCGTCCAGATGTTGCCCAGCTCAT
TGTGGCAAGAGCAGAAGTGCCTCAGGCACGGTCGTTCCAAGCGGTAGTGCTGGCAGATGA
ACGGGCGGCGCTGGTACTCTGGCACCTCCTCGGCGGCGCCCAGGAGCCGAAGCTTCCGTT
GCTTCGCCATGGCAGGGCCGCCGCGGGGGCAGTGCAGGGCGAGCCCAAGGCCTTGAGCCA
AAATGGCTACGG
>at00449
ACTCCGACGGCAGAAATGAGGTGGGTATAGACGAGGTTGTGATAGGCTTCCTCGAGATGA
AAGGTGCTGCCAAGGGAGCAGACGTTGTTATTCTCAGAAACAACGTCACGAAGTTGACTC
AGAAGTTCTCCGCCTTCATGGCCGAGACAGCAAGGACCCTTGAAAAGCTCCGAGAACAGG
TGGAACTGTCCCGAGAAAGTGTGGAACTGTCCCGAAGGGACTTATTCATAGTTGGGGGTG
GCCTTGATACTTTTTCATTGACCAAGCAGTTTGAGGCGTCCTCTGCCGCAGTGGAATGCC
CACCTCCCAGTTCATGGGATGATGACGGGAGTCTTAGAGAAATTTGAGGCACGATGCTTG
ATAGACTTGGAGGGCATGGCGCCTGTGTGCCATCCGCGATTGTGTCATCCGCGATT
>at00450
GACGTGACCAGGGTTCCCGCCGACGAAGGTCTCCATGGCCGAGCGCNTGAAGGGCAAGAA
GACCATCGTCGTCGGCCTGCCGGGCGCCTTCACGCCCGTCTGATCCAACCAGCAGGTGCC
TGGGTACCTGGCCAAGCAGGCCGAGCTGAAGGCGAAGGGCGTCGACGAGGTCCTGGTCTA
CTGCGTCAACGACGGCGCCGTCATGGAGGGCTGGGCCCGCGAGCAGAAGGTCGCCGGCTC
CATGGTCTCCTTCCTCGCGGACACGCGCGGAGAGCTCACGAAGGCCTTGGACCTGGTCCT
GGACGCGGAGCCGGCCATGGCAGTGCTGGGCAACGCCAGGTGCAAGCGCTTCTCCATCCT
CGTCGAGGACGGCGTGATCAAGGCCCTCAACGTGGCCGGGGACGGCGTGCCCGATGAGGA
GACCTTCGCGGAGAAGGTCCTCACGCAGTGCTAAGGTGGATGGGAGTCTGCTGCCGCGGC
GAGGGCAGCGGAGACGGCTTGCCGGGCGCCGGAGGCGGAGCTCCGGTCGTGAAGAGGCGC
CGGCTTGGTGAGCGTGTGGATCCCCTCTGCCCGCAGCATGGGCGGGAGGGGCAGCGCGTG
AGAAGCCGGCGGCTCGTGGCGGCGATCGCGCAGCACCGCCTGACGCACCTGCTCCGAGCG
ATGAGGCGGCATGGGCTCGCTCTCTCGCTCCCGCAATTTTTGCGAGGCCCGTTGATTGC
CGCAGGGGCGGACAGAAGAACGTTCCATTACCGTCATCTTCGAAAAGTAATGCTACAAAA
AAAAAGAAAAAAAAAACAA
>at00451
TTGCTGTGACAAGACACCTTCTTCGAGATATTTCCTCCACTGTCGTGCACTCAACGTCAA
CACTCATTTGTTCTGCGGCTCGTTCACGTATGCATATCGTGCTCTCGTTGTTCCGCTTCT
TCTGACGGTATACATCCGCCGCCACCCTCAGAAGAGAGTTTGACTGTCGAACAAATCTAC
CACGACTTGAATATATCCGCTCTCAACGTTCTGGACAGCCTTGCCATTATTTGTTACTGC
AACAGCTGTAGCGATGATGCGTTCGACAGGCATAGCTAATTCTTGACTTGATCCTCCATA
TGAAAAAGCTGGCGCAAGTGGGAACATGCACACATTGTGATCGTCATACAAAGTATATGA
TACCAGTGATGTATAAATTTCTGCTGAAGACGTTGTTGTTCATGTATT
>at00452
CGCGCCTCTTGTGGTTCGTGCAACGCTGGCATGGTGCAGCGGATATGGGCAACAATTGAG
ACAATGTGTGCCAGAGGAGGTTTAAGCTTAACTGCACGATCCCGTGAAGTGACCATGGTG
AGCCAGGACATTGAGATTGTGAACCTGGAGCGGCCTCTTCTGCTCGAACGACTTGAAGTT
CAGTGATGTCTCTGGTGGTCC
>at00453
CAAGAATGGCAGGGCCTCCAAAGCTTCCTCGAACGCCCTCCCCAGAAGGTGGAACAACTG
AAAGCGCCGGATCAACTTCATGAACTGCAGCGCCGGCAGCAGCAGAATGAAGACATTAGC

CTCATGTGGGAAGTAGGTATTTGGCAGCGCGAGGCCGATCGCAAGGCGGGCGACGCCGGC
CAAGATGGCAGCGAGGTCGAGAAGGTTGAACACGGACCTGAAGAACGCGCACGAGCTCGG
GGAGATGGCAAAGCGGACCGCAAGTTCCCCGATAAACACGAGGTTGAAGGCGATGTCCAG
CGCCGAGGCGCCCATGGGTCCGAGCATGTCGCTACGCCGCGCCTGGAAGNGGCTGGCGAC
GGTGCAGAGGGTGATGGTCAAGGACATCGCCG
>at00454
TTTTTTTTTTTTTTTTTCAAGTCCCCACGCGATGTCCGCAACCCTCTTCAGGGAAGCAG
GCACTGAGAGAGACAGCTGGCTCCCAACCCTGCGCAAAAGACGCATGCAAATGCGTCTG
TAGTGCTGCCGCCGGCGAAGCCACTGCGTGCTCAGTTCTCGCCGCTACTGCTCCTGGTGC
CAAGGCGGCAGGCGCCGGAAACACAAGAACGCGTGCCCACGCACAGAGAC
>at00455
CCGTAGCCATTTTGGCTCAAGCTTTAAGGCAAGGGCCTCGAGCCGCTTTTGCTTGGCGTG
TGGGCACTCGTTGTGATGGGGAAGCTGTGCGGTGTTTGTTGCACCGTACTCTTGCATATT
TTGGCGTTTGCATGCTGGACAGGCAAGGCAGTGGCAGATGCGGATGAAGCTGTTTGTGCC
GCAGGCGCTTCCTGCATGCCAGGGGGTGGAAGGCGCCAGAGAGTCCTTACATTTGGCCAA
TGGCACGTGGGCCGATTGGGTCGCATGCCACTTCCCCATACCAGGGACCACGCAACCTCT
CAACGTCACTCGCTTGGAAATGGAATCAGCCGGGAGGCCGGTTCAGTGGCGGAGCCAGCA
ATGGAGCCATGCTGATTGACGACAAAAA
>at00456
TTTTTTTTTTTTTTTTTGCAAGCACCCAAGGAAGCCCATGGGTTAAGATGTAAGAGCGC
AGTCTTCTCATCCAAGCCATCCCATCACTGAACGGCCAGGTCCTTCGCCATCTCAGGCGC
GGAGGACGCAGACGCCGCCCGCTTCCTTGATCTTGGACTCTGCCTCCTTTGTGAAGTATC
TTGCCTTGACGATCACAGGCTGCTGCGGAATGTGGCCCTTGCCCATGACCTTCATGTAGC
CCTTCTTCACCACGTCAATCACAGGAGCCTTGTCAGTCTTGTCCTTGTACTCCAAGCGGA
CACTCTCTGGTACGAGAGACCAAAGCCTCTCCACGTTGATGGTGGGGATGACCCTGCTGT
GCTTGATGAGGTGGAAGTTCCGCATACCGACCTTTCCGAAGTAGCCT
>at00457
TTTTTTTTTTTTTTTTTGCATGGCAAGACCTCGCCATACGTTGTTTGGGCCCGCAAATC
AGCTCGGGTCGTGCTCGCCCTGAATTCTTCTCGACCCTGATGCGCCTCGCCTGCTAAAAG
AAGCCCTCGACCTTCCACGGCTGATGATTGCGCAATCAGGAGGGCAGCTGAGCTGCCCGC
GTTTCGGTGTTTGCCCAGCTGTTCACTCAGTCGCGGTCTCAAAGACAAGACTCTCGGGTG
CACGACCTAGTGGCCCGTGTGGCCATGACCGGGCGCGCGCTGGCGCCCGCAGACAATGCG
CATGCGCGTGCACGCATTCACGCATGCACGCAGTCCAGACAGTGCCACCTGCCTTGCCAG
CGCCCGGTGTTCAGGAGCTGGGTGGGGCAGGGAGGGAGGGTGAGACAAGGGGCAGCGGAG
GCCCTGACGCCTAGCGGGAGGTCAGAGGCGCCAGGAGGGCCCTCTCCAGCTTNGCGGCGG
NCGGAGGCTTCGCGCAGCGCCTGTCCACGGACGGCAGGCCCATGAGCTCGCTCACCGGCA
ACCGGGC
>at00458
TTTTTTTTTTTTTTTTTGCAAGGTCAGCATCAGCTCTTGCACAAGGCCAGCTTCGCTGC
CAGCGGCTACAGTGCATGCAACGTCCCCCCTCCCTCCCCTGTGCCCTCTGCCTCCGCGCG
GGTGCCGGCGCCCTCGCTCACCTCGGCAGCTGGCGCCGCGTCCTCTCCCGCCGAACCGAA
GCTGCTGGGGAAGCCGTCGGAGTCGCTGCTGCCGCCGTCCTGCGACGGCTCTGCGCAGCG
CCCGAAGATCTGCGGCTGGCTCGTCGGCGCGGTGCCGTTGCCCATGGGCCGCTTGCTGGG
ACGGCCCTTGCTCCACTTGACCTCGAGGGTGATGGCATAGCGCTCGGAAGTGACTTCACC
TCCGCCCTCCTGCGATGGGAAGTAGGCACTGCACTGAAGGCAGATCCTCTTCGCATCGGT
GCCGTTGCCCGGCCACGTGTCTATCTCGAACTCAAGCTCTGCGTCGTATGGGACATGCAG
GTACCCACTGGAGATGCCGTCCAGGCACGACCGGAGCCGGGCTTGAAACGGTTCTGGATC
CGGCAGGAGCTCCGTGAGCCTGGGCATCGCAGAGACGCTGCCGGTCAAGTCCAAGACGGC
CGCGAACTCCTTGGTGATGTTGACAATCTGCATGGTCCCCGAATTGACCATCACGGCCCA
GTTCTTCTTGCTGACGTCCTCCACTTC
>at00459
GCCAATGCACGCACACACAAACACAGAGAACTTGTGCCGCCACCTTCCCCACCCAGCCTT
CTGGGCTTGTAGTTTCCGCCAGAGAAAGGCTGGCCGCCACTATGATCATAGTAGTCAGTA
CACAGAGAGACGCACCCCGGTGAGGCGCAGCTCCGCAACATGCTGAAATGTTTGACTGCG
CAAAGAATGAGGCTCCTGCCAGCAAGTACGCCCAATAGTAAGGTCCCCAACACCATGCAG

GGAGGCTGTCAAGCTGAAGAGGAAGCAGGCCACAGAAGTAGATCGTAGGGCAAACGACCC
AAGCATCGCTATCCTCGTGAGGAAGCAGAACTTGCCTGAA
>at00460
TTTTTTTTTTTTTTTTTTCGATCTGACGTTTCTGCGCACGCGCACGCGCACGCACATTGA
CTCTTCCAGCGCCGAAGAACGCAGAAAAAAACACATCCCATGCCCCTCACTGCATATTGT
ATGCGCGCTGAGGCATAGCGCCGGCACTTCGCCAGCTGCTCCGCGCCGGATTTCCCGGCG
CCGCCGAGCGCCGAGACTGCGTTGGCTGCGGGTAGAAGCTGCGGCTCGGGACCGGCTCGT
ACTGGCTCGGGTACGCAGAGCCCTGCGGCGCATACATGCCGTACCTGTCCCCAGGATCCT
GGTAGCCCATGCTGGCGGGAGGTGCGAAGCCGTTTCCAGCGCCAGCCGACCTCGAGTAGC
CGCCTCTCTGGCTCGCGTACGCGGACTGCTCGTAGCCGTCCTCGTACGTGTAGCCTGCCT
TGCCCACGCTCTGCCGGGCTCCGGCGCCCCCTCCGGCCGCGAGCTTGCCGGCGTTCCAGC
AGCAGGCAAAGTACAGGAACACAACGGCCACAAGAGGAATCACGCAGACGGCCGCAANAG
CGCTCGGCCCTCGAGATCCCACAGCGCGGACACTACTTGCCACGTTCGATGCGTGCGTGA
AAACCGCTCTCAGGGCCGATGGAGCAGCCTCAGCCTCGTAGGGTTTTGCCAGGTCGTGCA
AGAGTATGCGGAGCCTGC
>at00461
GCACTGGGACGGGCTCAGCGCGCGACGGGGCTTTTAGTCTCCTCCTCGCTGGAGGAGGCT
GCACTGCTGTCCTCCTCGCTGTCCCCGAGCGAGGAGGCTGCATCCTCGCTGTCCCAAGTG
CACACGCGCCCATTGCAGCTGTGGTAGTCCGTGCTCTCATCATCGCTGCACTGTATGCTG
TCGTCGTCGCTCGCGTCAAGCAGCAGCCTCCAGGGCTTCCTGCTGCTCATCGGCTTGCTT
TCAGACTTTGGAGGCACGGCAATGGCGATCCCGGTCGGCGAAGCGCACTTCGACAAGTGC
TGCGGCAGCAATGCGTGGAAGAGGTTGTCAACGGTG
>at00462
AAAAGAAGGGGCTGTAAAGATGGCCGCCTATGCGTGCGGTGCCACGCTTGCGTGTGGAGG
CACTGTCTTCAATGATGAAGTGTGCGCTCCAGTGCGGCACTTTGCCAATATCCGGTTGAC
CTTCATCATGCAGTGGCCTCATAGGTGCTTAGCAGGACTAAAAGGGTAGTCGCTTTCCGA
AGGCAGAACGTCGTGGCGTTCCTGTCTAAGGACATTTTAACATTTGATTCCAGAAGCGTC
GTTGTGCTGCCCATGCAT
>at00463
CCACCACGCCTTGCAAGTGCCTCGTTCCTACTTGCACAGATTGAGCCAACATGCGTGAGC
ATGCTACCTGCACCTGCGACTGCAGAGGGAACGTCCGCATTGCGTCAATTGCCAGCTCCT
GACCACCAAGATCTACCAAAAGCTCCTGCAAGCTGGAGTCCGAGTCGACTTCACCGGCCG
ACATGTAGCATCCAAATAGCTGCAATGTGCTGTTGCCGGGTGAACTCTTCATCGACGATA
TGATGAACCTGATCCGATCTGGCCGGTCACCTCCTGTTGTGCCCGGTCCGCGCCCATCAA
GTCGATGTGTTTGGACCAGCGAGACGATGTCGGCGGCCTCTGCCAGGCTcCCGTCCACGG
CTGCAGAAGCGGTGGAGGCTCCAGGACACCATGCTGCAAGGAGCAGCGCAAGCACGGCGT
TCTTCATGCTTGGCCAGATGGCTGCGGCATCGTGTAGTTCGGTGTCTGGCTGGGATGAGA
GTTCTGCCTGCAGTGG
>at00464
TTTTTTGTNCgNTCNNNTgTTNTTTTGAGGCACGCAGACCTCCGATGAGGCCAAAGGCTC
CTCTGGCTTCACCCCAACTTGCTCAGCGCAGGAAACTTGCTCTCAAACTCCTCCCACGTG
TATGTCTTGCCAGAGCATTGCCCTTGGATAGGGGCATAGCCCTTGCGCGCGGCGTAGCAG
ATATCGGCGGGCTCCATATCGAAGTCGACAAAGTCCTTCCCCTGCCTTCCCTGCTCCGAG
TAGTCGACTTCCAGACCCCAAGGGGAAGAGGTCGCTAAGGCTGAGACTTACATCCCTACG
GAAAGTCCGGTCCGTGTAGATAAGCCCGTGCTCCTTCGTGTCCCACGCTTCTGCATTGAA
TCGCACCTCCAATGACCCTGTCAAGTACTCGTCGTCGCCTTCCTCATCCTCGGACCCCAC
AGGTTACCGAAAGCCGGCCTCATCCACCCAGACCGTCCAAGGTCGCAACGCGCTTGGTCC
AGTACCCGGTNTTCGCCGTCCGTGCTAGCCTT
>at00465
TGTATGTCATCCGTCGGTTGGTCTTCAACGACAGCAGGGCCTGCTGCTCGTGCAGACAGC
ACGCGGCCTGGCGGCAATTGGCGAGATAGGCGTCGTCCTCCATGCCGATATCCTTCATTG
AGTTTAGGACTGAACGAGCTTCGGGGTGTTCAAAACAATCATGATGCCCGTGTTCACTTC
GGCATCCGGATAGTCTGTTGTCACCATAACATGCGTTCGCACCTCTCTGGGATCGGGACC
CTTTGTTTGACTCTCTAGTACCTTGTCAAATGCGTGGACGCCTTGGTGGACCATGTTTGA
AATCTCCATGTAGCGCTCGATCATGTTGCCAAAGAAGTTTGTGATCACGACGTCAGAATC

CAGCCAGACAATGACCTGGTTGTCCTTGTCATCCATTTCGCACAATAGCAACAATACTTT
GTACCAATACGGACCCCAATGGCGACCGTTGCTATCTCGCACTGCTTGGTACATACGGTA
GCGGTAACCCATGGCATCTGCATAGGGACCGTGGCTTTCGCGAGCCACCCGTCTGAAAAC
GTGATCAGCATCATTGGCCACGGTCTCATCGATCGCCGAAATGATCAAAATGCCATTGCG
TTTCAACCCAGGCCAGACATGAGGCCATGGGGTACTTTCCGGCATCCCACTTTGAGGAGC
ACTGGCGTAGTGCCTGACAACCCACCTAGGCTCCAGCAA
>at00466
TATGGCTTCTGCAACGCGCGCCCAGAGTCTAGTTGGGTCCTCTTGCTCCGCATGTGCCTG
GAAGACTTCCGCCAGTTCGTGAGGCTGGACAGTCATCAACCATGAGGCTGCATGCTGTGG
CAATGCAGCCATGCCCAACATCACAATGGCTCCATCCAGGCGGACGTCCTTCGGTACGAC
CTGGACGAGGTCCGC
>at00467
TTGTTTTTTTTTCTTTTTTTTTTAGAATCCCTCATCATCACTGCAGATGTGCGCATGTG
AGTGCACGCAGACGGACCAACATCCAGTCTCCAGGCCTCACTCGCGCGGCGAATGGCGAG
TGCCCCCTGAGCAACTTTGGTTGGCCCCGTCAAGGCAGCTAATAGCCCCCGGTCTCAGCC
GGACTTCCTGCGCTTCAGCGCACCCAGCTGCTGGGCCTTGTGGGCGCGCTCGCGTACGGA
TGTCAGCTTCACCTTGAGCTCCTGCTCGAACTGACTCTCGTCAAGCTTGGAGTTCGCGAG
GTCCTTGAGCTGCGCCTCATATGCCGTGACGAGGAGGTCATACAGCTCAGGGACTTCCTT
CTTCATGAAGTTTGCGTAGCATGGGCAGGTGTCCTTGGGTCGCAAGTCGATCAAGGCACC
TTGCGAGCTCCGCATGAGGCCCCGGAACTGCGAGAGCTCGTAGACCTTCTTGCGGCCCTG
TTTGTCCTCCTCGGGCTCCCACTCCCACTTCTCGATGGGGACCTTTCGCAGCGACCGCTT
TGCCAGCTCGGCCTGATAGTACTCCTCGTTGGTTGGTGGCCAGTCCGGCAGTGCATACCA
CCAGCGGCAGAGAAGCTCTGCCACAACCTGCTCCTTGACGGACCGCTGGCGCTTCTGCAC
GGCCCCTCCTCCATCCTCCGGCATCCCGTCCTCGGCATCTTTATCCAGGATCTTCATCTT
TGCCTTCTTGCCAACAGACGAGTTGGACCCTTTCCTGCGAGGGGGCTTGGGCTTATCGTC
CGAGTCGGAGCCTGAGCTGTCTCCGGAGTCGGAGCTGCTGGAACTACTCGAGCTTCCAGC
AGCTTTCTTCCTCGGTGTCGGCTTTCCACCACCGGGCTTTCCGCCTGCCTTGGGGGAGCC
TCTCGGCGGTCCCTGTGCCTTGCCAGCGGGAGAACTGCCAGCTTTGGCCTTGGGGCTCGG
CTTCGTCTTGCTAATGTCCGCCAAGGATGCCAATGGAGTGTCGTCGTCAAGCTTCAATGG
CTTTGCCAAAGCGGACAGCGGGGCATCGTCATCCATTGATCCCTCAGCAACTGTATCCTC
GTCCCCAAGTTAGCCAGCTCTGGAGCCAAGAAGGCTTGAGCCAAAATGGCTACGGA
>at00468
TTTTTTTTTTTTTTGTTCTTCAAACATGGCATGACGGTGCCACCGTCAGGAGTGCTGCC
CCAGGACTGGGGGCGTGCAGCACAAGCTGCTTGCAAGTGGGCAGAACGCCCAAGCAGTGG
AGAGAATCATGCACGGGGGCCAATTCTTTGATACGGCATTCTGGGCCTCATGCCAGGCCG
TGCCTGCCAGCAACGCCGGGCCGGGCAGGCCCAGGCCGGGCCAAGAGAGAGGAGGCGTCT
TGGCAGGTAGGAGGCCAGCAGACNCTCACCGACCATGGATCTTAGGCAGATGGTCTGCTC
AGCAATGTCTGGTTAGCGTTGCCCTCGTAGTACTTGGTGTCGTACCCCGCTTTCCAGCGG
CGGAGGCGCACATCGGCCTGCTCCTTGTGGCCAGCAAAGTTCTCGTAGCCGCAGAGCCTA
GAGCAGTAGTCTCCGACCTCCACGGACGCCTCCTTGGAGATGTGCTGGTACGTGTGGGTC
TTTATAAACTTGCCAACCCACAGGCCGCCAGTGTAGCGGCCGGCAGTCTGGGTGGGCAGC
GTGTGGTTTGTGCCGATCACCTTGTCGCCGTAGGCAACGTTGGTCTCCACGCCCAGGAAG
AGACCGCCATAATTCCGCCAGGTTCC
>at00469
CCGTAGCCATTTTGGCTCAAGCCGTGGCCATTTCAGCTGAAGGGCTTTCTTAGTTGCTGT
AGAACTGCGGTTGCCCGCAACCATGCCTTCAAAGGTGAGCACCTTGGCTGTGCTTGGCGC
TGGCACCTATGGTGCCACAACTGTCCTGAGAGCATTCGTGGCACCAGGGCAAGCAGCCCC
ACAACAGGACCAGAGGGAGCAGGTGCGGCACTTGCGCAGGCAGCCGCAGCAGGCTCTTCA
GCAGGAGCAGCCAGAACAGGCGGCAGAGGTCCGTACTCCCAGTGGCGCATGGGCCTTGGG
GGCAGCGGCCATTGCGGTGGGAGTGGGAGCAGCAGGAAGGCATGCACCGCGTCGCGGGCA
TGTCGGGCGCAAGGCCACTGCGACACTGGAGAACCCACCCGCGTTTGC
>at00470
TTGCCGTTTCGAGGCCCGAACAAGCAAAACAATGCTTCCAGCTGTTGTACCACACCTGCC
ACTCTAGGTGGCTGTCCCTGCCATGGGTTGGACCTCGCCTGACAAACGGTATCTCAAAGC
TAGCGTTCGTAAGCTGCATGCCCTGCGCCGCTGCCTCTGGGCGTGCTGGCTGCGGACGCG

GCGGAGCCAACAGGTT
>at00471
TTTATTTTTTTTTTTCTTGCATGCTCGACTTTGTGAGCGACACCGCTGCCAACATCACGC
AGTTGGTCAGGAGTGGATCGTGGGTCTGTACAATGAAGCTTCGACGCTTCGAACTCTGAG
CACCGAGAGAGAGAGGATTGCCAATCTGCAGACGGGCATCTGCAGGGAGCCCGCGTTTCT
TGATAAACAGGAGGCGTCCTTAAAAAACAGTTGTTGTTCGCAGGAGCACAAGCTCCAAGT
CAGCACTTGTGCACAACGTAAGCAAGAGATAGCGCCCATCCACTGCATGTGAAGACGCGG
CTGTAGCATCCCAGGTCCATTCCGAGACGTACACCGCGACCGCACCCAGTACCTTCCACA
AGTACCAGGTACAATGAGCCACCCAAGCTCGGCTCAAATAGTTCGCACGTGTTGGTCACA
AGACCTACCAGTAGGTCTGCAATTTCAGAAATTAAAAAATAGTTGAGTGGGGCCCATGCT
GCATGAAGACAAGACTGCTTCCCGCTGCGTAGGTGTAGGCGGCACGCAAGCGTGTGCCCA
GATTCGACAATCAAGCGTCAGCTGGGATCCACGCCGCATGGAAGCCATATGGCACTCTGC
TGGGTAGCTGTAGGCGGCACACGGGCGCGTCGCCAAAGTGCTGCGCATCGATCACCAAGA
CCTCAGAGCGAGCCTGGGTCTCGTTATGCGCGTGCACCAGAAGGTAACCATCATCCTCCA
NNACAGCCTGGGCTCCGACACGAGGA
>at00472
TGACACAGGGCAGTGGCCTTTGTAGGTAACAACACCACCAAAGTTGAAACCATTCTTTGC
CGCAAAGGCCATGTACCAGATGAGTGAGATCGCTCGCTCACCAAGTCCGTCTCCTCTGGA
TGGTATGAATAGCCGGGGCGCGTGATCGCCATTTAACTTGCATGCTGGACATTGCAGCGG
TTGGTAGAGACCGCCACTGCAGATACGGCCAGTAGAAGACCCACCATTGTGATCACGGAC
TGCCTTTGTGCTTCGAAACAGGGACGGATGAGTATGACAATTTGGAGGAGCCTCAAGCCG
CAAGCAGCGAGCTGGCATTGGAAGCGACACCACCTGTACGAGCAGAAGCTGGCACGCCCT
CTGCAGCAGCACCATCCCGGAATGCAGAAACCACTGACATGATGGAATTCAGTACCGCT
>at00473
TCTGGCCACAAAAAGAGAAGGGCATTGGCACTCTGTGCATGGAGTATGACGCCGGCAACA
GATAATGGATCAAATAAGACAATGCTGAAGCAGAGGACAAACTCATACTGGGCACAATTG
ACATCATGATCGGCGTGCTGATGACTCAGGGACCGAGACGCATGATTGACATATGTGAGG
ACAGCTACGACACGATTGTCGCCGTCGTCGAAAAACNTGGCAAGGAGCGAGGTCAAATAC
CTCGGCGACAACCCAGAAGACACATGGATGTACCCTACGATGCGGAAGCAGCTCATCCTC
ACGCGTGCCGAGCCAAAATATTACAAGCTTGGCAGACAGCAGCAAAGAGCCAGCGCGAGC
AGAATGGAGCCATTGAGCTCTGCCT
>at00474
TCGTCCGGTGGCTGGCCTGTGAAGTTGACGCACGCCCCCTCGCCAAGATCCTGAAACATG
TCCTCGTCGATGAATTCCGATTCCACGCGTGCGAAGGAGTCCGCAGCCTGGCAGTACTCG
TTCTGGGTTGGGAGGTGTGCCTCTGGAGCCTTCAAAGCGAGAGTCTCTGCCCTCGGTGGG
CGCATAAGCACGAGAACAAGGCCCACAGTCGCGGCGGCAAGAAGCAGCCGCCACCAATGT
CTGCGGATCGACTTGCAGCTATGAGGAAGATCGTCAGCAGCTATCAGCTGTCGAGACTCT
GAGCAGAGCGCGTCTCTCTCGTCGCCTGAAGGCTCAGGAGACTCTGACAAACACCGGGAA
AGCATCCGCAATGTATCTGG
>at00475
TTGTTTTTTTTTCTTTTTTTTTTGTCCACGCCAGACGCTCCCACAAGAATGCCGCAGAAC
TGTTCAACCACATGCATCTAATTAGCAAAAGGCGCGGCTGCTCCATAGTGAACAGTTGTC
CGGCCGCACCAACTGCATGTGCCTGTTGCTGAATAGGTAGCACCAACAGCTCATAATGCC
AGCGAGCCAGCTACTGCATGGGGAGTTTGCCCCGATGTTTCTGCCAGCGCGTTGTCAGGG
CAGCAAGGTTGCATGAAAGCTCCGCGAAGCCAGTCGTGATATGGCCCCAAATCGGTACGG
CACCAGTGAGGACCTCATGCGTCTGACTGCTGGCAGATCGCTGAGCTTTGTCCTTGATAG
CAGATGCCAGCGCGTGCCTACCGCACATCGCTGCCCGAGCCCGGATATGATCGGCACAGG
GGTGTCTGACTATTCCGTCTCTGCCGCGTCTTGAGCCAAAATGGCTACGG
>at00476
GGCCTGGCTCAAGCCTTCGTTTTCGCATCATTGCGATTGCTACAGTGCACGCGCTGAGCT
TGTAGAGCTGGCACGTTCATGCACAGTCCCGAAGATTGGACGAGCCTGGCAACTTGATTA
CCAACCCCGTGTACATGGAGCCCGCGTCCAGCACCGCCAAAGGCGAGGGCCAACGCCATG
GAGGTGTGTCTGCGTCCAGCACCGCCACAGGTGAGGGCCAAAGCCATGGGGGTTGAGAAT
GAGGCTAACACTGAACAGCATTGCCTGACTGAAGATTGCAACACAGGCAAGCAAGACTTC
CACGACCCACCAACGGAGGAGAGCGACTCTTCAGAGGACTCCGAGAAGATATCCGAAGAC

AGCACC
>at00477
TTTTTTTTTTTTTTTTTTCATTCTTGTTCAGCCTTTTCATTGCCAGGCTGAGGCCGGCAA
GCTGCATTGAGCTGCTCGAGCACATCAGCTGTCCTCTGTACACTGTTGCTGCTCAGAGCT
GCCCATCGCTGACACGAATAGCTGCAGCACGACACCACCTGGCAAGCACGTAGAGGCGAG
GGAGACTGGTGTCAGAGGCGCGCGAGATAATGTCCACCGTCGACGGCCAACATCGACAGC
AAGATGGCATCAAGACCGCTTCACTGCGCTAAGAGACCGGCACCAGTGGAAGTGTTTCCA
CTTCACCTTTCTGAGCTGCCTCCAGCCACCATGCTCCCAAAAAGCGAGTCACAGACCTCT
TGCCGCCCTCGCCGGGGAAGAGGAGCTTCACGTGGCCTTCCTCCCCAAGATCCTCAGGGA
TCTCCACCACTTCACAAACATCGCCATGTCGCTGAAAGTCCCAGTCTGGCTGGTTGACAA
TTCGCAACCGGCTGCCCACACGAAGATCAAGCGATAGGTTGCTGCCAGGCTGGCGGCGGC
CATCTGATCTATAAGGCAGTCCCGCAAGGACTCGACCTTCCATTCGTTCGTGGGTAGATC
TCTCGGGCGCCACAGGGAGGTCCAGTTCTGCGGCGAGAAGGGCCATGACTTCGTCAATGG
GTGCGAAGATCCGGAGGGAGGAGAGCTTATCGTATTGCGTCTGCTGAAGTGCCACTATAA
CAGTGCCCAAGGCATCGCAGCGGTGAGCACGTGCAGCACGCTGAGCTGGAGAGACGGCCA
TGCGATCGGCGTTCATTCCAACCATGGAGGTTCCGAGCGCAAGACAAAGATCGGCCTTTT
CCTCCCACTCCAGCATCCATTCGATCAGATCCTGCCTCAAGCTGCCGTCCATTGGAACGA
CGGGATTCGAGGGGTCCCACCAGGCGCCGTGCACCTCGTTCAGGTCTTGCTGTGGGAAGC
CTGCCTTCTGGGGCAGACCGTCATGGTTCTGCTGCACCCAATGCTTCAAGAATCCTTCCC
TGTGCAGCGCCACGAGCGTTCGGTGCGCTAGTGTGGGCTGCGCCAGCCAGGGTGAGATGT
GCTTTCCAGTGGCAGAGCGGCTCACAGAGTCCTGGACCTTTGTCGCGTAGTCTGAGATGC
CCGAGGCTGTGCTGATGCCCGCACCGGTGTAGGCCACGCAGGCCTCGCTGCGGCGCACGA
GGCTTGCAAGCAGCTTGATCTTTTCCCGCAAGACGTCGGGCTCATCAAGGAACTCATGCG
CCTGCATGCTCTTATATCCTGGCCTTGCCTCCTCTGCGCAGGCGCGCGCGATGCGAGGCA
TAGGCCAGGAAGGGTTGCCAAGCATCCCATGTGCCGCAATCGTCTCGGCTGACATGGGCG
TG
>at00478
CCGTAGCCATTTTGGCTCAAGCTTCTATTCCATTGGTCTTTGTTAAGCACTCGTGCTTTA
TGTTTGGGCTTCCTCATCCTGTACATACCTGTCCATCTCGGCTTGGGCTGGTCGCAATGC
TTTACGCATCGAAGCTGCTTCTCTTTGTGGCCACTGCCTTTGCTATCTGTGGTGCGAGCC
CAGCAAGTCCCCAGCAGAGCACTTGTGCTGCAGAGGGTGGCAGTTGTGGCACCGGCGACA
ACCCATCATTCATCCAGAAGCCGCTTGCCAAGGATGTAAGCCTCACTGTTTCCCAGGGCC
GTGCCCTGAAATGCAAACGTAAAGACGTGTGTAATAAAGTGGCCCTCACAAAGACTGATC
CTGAAGCAAGGTGCAACGACAACACAAAGGCGTTCTTTACTATGCACCCGCAAAAGACCC
AGCGTCAAAGACATGGTTTATCTACCAACGAGGTGGTGACTGGTGCTATGATCAGAACAG
CTGCAGTGGACGTTCTCCAGAGTTGATGTCTTCAGCTGCTTGGCCAGAAATCGAGAGTTT
TCCCAAAGGTTCTTTCCTCAACTACGATGGCTCGTTCGAGGCGCACCGG
>at00479
CGCTCCCCGGAGGCCCAAGGTCCCGCGCTGTGCCGCGCCCAAGGCGCCGATGCGAGTGCT
AGCAATCGGCGCGGTGGCGGCTCAGTCCTCGGCCGCGTCCGCCTTGACCTCCGCGGTGAG
GGTGTTCACTGCGTCGTCCGCCTCCGCGTGCACCATGGCCACGCCGGCGTCGTCGTCGCC
ATCACCATGCAACTCCATGAGCTTCGTAATGTCAAACTTGGGCTTCTTCAGGACCTTTAC
CTTGCGGACCAGCACATCCTTCAGTGGGAAAATACCCTGGGTCTGCTTCTCAATCTCCTT
CCCGATGGACTCCGGGATGAGCTTCTTCACCAGCTCACGCAGCTGAACCTTCCCGGCCTC
GGCGATCATGACGTCCACCATCTTCTTGCGGATGCGCCGGATCTGCGCACTCAGCGCGTA
GCAGTTGGACTTGACCTGATCCGGCCGCCGCTTGGTGAAGGCGATGACAAACATGCGCAC
GACGTAGCCGTCGGATGTCTTCACGTCGGTGTGCGCCTCGATGAGGGTCTGCCACTTGCG
GATCAGGGAGCACTCCTTGTCGCGCGTGAGAGACATGCCGTGGAAGTCCGTGAGGCAGGA
CCTGCCCTGGACCTCCTCGATGCAGAGCTTGATCTTTTTGTAGGCCTGGTCCTCGTCGTT
GTTGAGGTCGGCCAAATTCACCTCCAGGACGCGGNCCTTCAGCTCCTCCGTGGCGATCTT
TGTGCCNTGCGTCTTCGTGACGAGCGTCTTGCCGCAGTTCCGAATGCTGAAGAGGCTGGG
CGCCTTGATGTCGTACCA
>at00480
GATATTGACCGACTGAGAGGAGTGTGCGCCGCGGCAGTGTCACGAGTCCGGGTACCTTCG
CTCCAGGACCTTATCGATGACAGCACTCTGCAGCGCGTTGGCTTTCTCCTGGTTCCAACC

TTCGGGCACCTTGGGCAGTGACTGGTCCCGCGGCCTGACCCAGATATCGCCCCAGGCCTG
CTTCACCTCGAAGGTGTGCATGGCCGGCGTGTCGGGAGGCTGCGCCAGCGAGGCGTCCCA
GAGGCAGGCCCCGGTCACGGCGTCGAAGAGGTACGCGTGCGCTGGGCAGGGAGACGACTG
GGCCACTCCCGACGTCCTCGATGTCGCCTCCCAGGAGGTCGATGCCGGCATGG
>at00481
TTTTTTTTTTTTTTTTTCCAGGCTCACAGCGCATTCCGCTGCCTGCATCCGCTGAGCAC
CACAGCTTGTGCCGCCAACAACCTCCGAAAAGCAGCGCGGAGGCTCCAACATCTGTTTCT
GTACCAAGGCTGGAGCGCGCCGTACTGAGGGCAGGGATCAAAGTCCGGAGGAGCGCCCAC
GGCATCAAGGCTGCGATGTCGCGGCCGGCGTTGTTGGCACTGCGGCATGGGCGGAGGCCA
TGGCTGCATCAGCGTCCTCCTCTGCCTGCAGACGTCGGGCCAGGACCTCATCCTCCGCAA
GCTGCGCGGCGGTGCCCGTGACCGCTTCGTCTTCCTCTTGGTCTGCCCGAAGGGGCTCAG
CTGGACCCTCCTCCGTCCGCACTGCGCCAGCTGCCCCTGTGGTCTCCTGGCGGCCAGCTG
CCCTGGCACTTGCCACGGCCGCGGCGCTGGCAACCGCCAGGCGCAGTGCACGGACAGGCA
CAAGGGTGCTGGCGACAGTAACGCCCGCCACGGTGGAGGCCAGAGCCGGGACGGCGCTTC
TGGCGGTCCAGCGCTGCTGGGCCTCCGACTGTGGTGGTCGCTCCAGCTGTGGCTCCTGCT
CCTCTTGACGCGGCGCTGACTGCTCCAGCTGGTCTTGCGCTGGCTGCGGTGGGGAAGGCT
CCTCCTGCGCAG
>at00482
TTTTTTTTTTTTTTTTTCAAACACAGCAGAGGTGCCAATTGAAATCCTGATGCGACGAG
ACCGAGCTCCACATCAAGATATTAGAGAATCTGACAGGGTCAGACACAAATCCAAGCCAT
GCAAGTTGTCCTCGAACAGGACGTGAGAAGTCATCTTAGCAGACAGATGAAACAAATCCA
CCACAAAATGTCACACGCGGGAACGGAGAGGGGCTCAACTAAAAGGTTGGTTGGCTGCCT
CAGACTGCCTGTTCCTCTGCGGCTCTTTCAAAGGGCACCGTGCAAAAGAAAACTGTATGC
TTCCTCTGCGGCCCAGCCCAACACCAGGGCTGAGTAGGCCGCCGGCAAGACAAGATGGAC
ACTGCACAATTTAAACATGTGTGACTCCAGCTGCCTCTATCTCACTTCTTCTTGCGCAAA
GACCACCACCCGTGCCTCCCCATCAACTTCTGCAGTCGGCCACTGCGCTTCTCCTTCATG
GTCGGCAGCTCCAAGTGGCGAGCCTCATCACGATCATCCTCGGCGACACCTGGGGGGGAG
TGCCTGCCCTGCTCTGGCAGGACACTCATAGGTCCTCTGGTGTCGCCCTGGCCCCCATCG
CGTCTTTGGTGCTCCTGCTGGAGCTGAGCCACGAGCATGGCTTGAGAAGACATGTCGCGG
ATCAATTCTGCCTCTTGGAGGATCTCCT
>at00483
ACTTATCCTTCTTGACAAAGTCGATAGCAATGTCAATCCAATGCGTCTTCAGGATGGGCT
TGTACGTTTCCTGCTTCTCCTCCGTGTACTGGAAGTAACCAACAGCATTGCTGACCGTGA
CCCATATGGAGGTAGAGGCCACCTTTGCGGGCGTGAGAATCAGCGCTGACGAGGTCCTGG
GGTCAAACATCAGCTGGTCGAGCGGAGCCTCTTGCGTCTTGACATGGCGCAAGGCCTCAC
CATTCTTCAAGGAGAGCACAGAGAGGATGGCCAAGAGGATGGCCAACCACCGCCACATCG
GAACGGCTAACCCTTTCGCCACGAAGTTCAATATCAGACCATAGCTGAGGGTGACTCAAG
AACGGTGCTTGGGATTGGCACTGCTCGTACCTGTGAGT
>at00484
NNNNNNNNNNNNNNNNNNNNNGGTAATTCTCTTTTCTGTCTGTAATCTTTTTCTCTTTTG
GATAATTATCGTATGGGTCTCGTGTTTGGGATCACAGATTATTTCCTCTTGTTTCTTATT
GATATTGAATTCTTAATCTTTGGGTTAATAGCGTCCAGCGGCAGTTTCTTTTCAATGGTC
TCTTATTCTTAGCGCTGGACAATAGCTCATGAGTAAACGCCAAGCCAATTCGGATCACCC
TTAGAATCGATTCATGAGCTGTAACACTCTTCATTTAATGGCATCATCGATTCTTTCATG
CAACATCTCTTGGCGTCCAGCAGCAGTTTCTTTTCAATGGTCTCTTATTCTTAGCGCTGG
ACAATAGCTCATGCCTGGCAACAAACCCAAATCACGATCACCAAACCAGTCTTGACCATC
AGCTGTTTTTCCTTCATTCCTTTCAAGATTCTGTTCCTGATTGATGGTCTAGGTTTGGAT
AAGATTACAGAAAGACATGGAAGATGAACCACACGCATGATCAAGACCTTTCCATTCTAT
CAAGCAGTAATCCATGCGTGTTTGGTTTGAAAATCATTCATTTCTTGTCATATGCTAATA
ATAATATCGTTTGAAAGAAATTTCTTAAGGATTCTAATGATATTACAAAGAGTTAAGAGC
TGTATCAAATCTTAGACCAGGCGTTAACAGTCTACTGGCTCTCTCTTCTTTCTCTCCATC
ACTATCAATTCTTAACGCCTTTACGGTCTGTCAATCTTTTCTCTTGAAGAAATCTTAAGA
ATCTTGTGGATGGTCATTTGATTCCCAATTCTTTATCTCTTGCATGTAATTCCTCTTTTT
CTTAGGTATTTTCATGTAATTATCTGTCTTTTTCATCATAATAATCTTCATGTCAGTAAT
TCCTGTATGGTTAGAAGGTGGATCATTTTCTTTCAGATCTTCAGTTTGTGATCCACCAGA

TAGTTATTCAAAAAATGGAAAATTACTTCATGGTTTAAGGTTCTTGGTGATTGAATTCTT
GTTTCTTATTTTCTTTTCTCTCTTTCATTAGTAACCTTAAGAACTTCAGAAATTCATTTA
TTTTATAATTTACAACTTTTCTATTGGCATTTTCTCTGTGATTTGATTCCTGTCATTGGG
ATCTTCATGTGAATCGGTTCATGATAGCGTTCAAGCTCCAGAATCATTTGATGGTGACCG
CGCGTGATTGAGGATTGGTCACAGGAACACGCGCTTAATGCCGGTCTTTTCCTGGGAGCA
TTTCTTGGACCGGCTTTTTCTTCCTCTGGTTCATGGTTCTCATCTCTTTCTTCTCTTTTT
CCTTTAATTTCTATCTTTTTGGAATGGTGTTATCCCGAAGCCGTTTCATGCTATCATTTC
ACTCTTCTTTTTGGGATCTTTCAATCGGTTAAGGGATTCTTGGTTTATGATATGCTGAAA
ACCTTTCGGTGAGTCATTACACCATTTCATTCACCAGCTTTTATAAACACGCGTGGTGTC
TTCATGGGACTCACCTTCATATTTTTCATCTTTTTCTCACAGTAATTCTTGAAGATTTCT
AAAATAATTGGTTGAATTTGTTAGGCTCTTTCCCTTATTCTCTCTAGTGTATTGGGAATG
AATAAAAGTAAAGCCATATTATAATCTCTTATCACTTATTATTAGTAAAATTTAATAGAG
ATAGAGTATTGGAACAGGATTAAGGAAAGACATTCTAGAGATCACTTCTTTATTCAAGTA
ATTTCTCTTTCAGATGGTTAAGATCTTCAAACAAATAATATAATTTCTCTAAGTTTATAA
CTATCCTAATATATTACAGATGATTATAGAAGAGAAGAAAGGAAAATAATAGCTGTGTAC
TAATAACTTATCGTACCTGTCTAGAATCCTTTTCAATCTTGATTCATACTCTTATTAACA
GGAAGAATATAATATCACACTAATAATCTCTTACATTCTGTGAGCTACAATATTTGATAT
CATCTTAGGATAATACAAGCATATTACAGGTAGATATTGGGGAGACCTTCAAACATTCTG
TCCATCTTCTTTTTCAAATCGTAAAATAATTCTGAAGGATTCTAATAATTACAGTTTCTA
AGGATTCATATAATTTCCAATAATTTTCTTCTCTTTCACATATGATTCTCGTGATTGGAA
ATATTATTACTGGCATATGAGAGAAAATCAATATGTTTCCAAAGNNNNNNNNNNNNNNNN
NNNNNNN
>at00485
TTTTTTTTTTTTTTTTCTAAGGAAAGAGCAGCAGTAGTCACCCTCAGCCTTGCCACGGTC
ACTTCGCCGCGGCTCAGGCCGCGGAGCTCGCGAGCACCACTCGGTCGATGAACTCCTCGT
AGGCCACGACGCCCGTGGTGCCCCGGTCTGCCCAGGCCAGGAGCTCCTCCCGCGCGGGAG
GCGCGAGCGCCGGGCAGAGGGCCTCCAAGAGGCCGCCGAGGTCGTCCTTGCGGATGCCGC
CCTTGCCCTCGCCGTCCCAGGCCCGGAAGCTCCGCTTCAGCTCCTGCGCCAGGCGGTCGG
GGCCAGATGCCAAGCTCACGGCCACGACGCTAAAGACCGCCCGCTCGGTGGTGGGCGGCA
GCATGAGCTCGACGTTCTCGGCGACCTTGAGGCTGAACACTTTCATCGTCGAGGCAGTAT
CTCCCCAGCTCGGGGCAGAGTTTTCCGTTTGCCATCCAGCCTCTGGGAGAGACTCGAGGA
AGCCACCACTTCTGGCGCTACCACCCTTGTACTCAGCCTCCACTATCACGTAAATCCGAC
ATGCGCCACTGGCAGTCACCCGCAACGTGGTTCCGGTTGGCATGGAATCGTGTGGTCCTT
GAAAAAGTATGCCACCTGTCATGAAGGTGGGCACCCACGCCCACCGATTCTGCTTGTCGC
TCCAGGCCACCGCTGTCTCGACCATTGGGGCCCGGCAATATTCCAGGCCCAAGGTTGTTT
CCATGCTGGCATCAAAGGCCTCCAGGTCGACTTTTCACGACAGCATGANGACGGCACCCT
CCTCAGCTATTGTTGGCANGCAAAGCACCTCTCGAGCAGTGACCCGCTTCGCAAAAACAG
CCAGCTTGGGTT
>at00486
TTTGTTTTTTTTTCTTTTTTTTTTGGATCCAAAGAGATGAGTGGTCACACGAGAACGTCT
CCGCAAACTCAGGAATCTCCCAGCGCGAGCCTCAGGCCTCTTCACCCGGTGCGTAGTACA
CCTTTGCTGGCAAGGAGCCCAAGAAGCGGTCGCGCGCAAACTCGGCGTTGCGCCCGGTCA
CAATGTCGCCGACTGCACGGCCAACGAAAGTGCCGAAGTAGAACATGAACGGTGCAGCAA
GCAGTCTGGCCTGGTAATTGTAGCGCCGGAAGGACCAATAGGACAGGCCGGCCATGGTGA
AGCCGCCGGCCCAAAGGAAGTTCTGGGACACCACGTGTTTGTGCACGGACTCCTGGCGCC
GCCTTACAATCTGGGCGACAGGGCTGTTGTCCTCCGCATCAAGCTTCTTCGTGAGCACTG
CGCGCATGTCACCCTTGTCCAACTTGCTGTAATGAATGTGGAACATAGGAGCCGTCAAGA
GCAGCCCAGTCCACTCGAACATCCCAAGCCTGCGCCTTCCCACGCTCATCTTGTTCTCGA
GGATTGTGATGTCGGAGGGAACGACTAAGTTGGCTGCCATGTCCCAAAAATGCCAGGACT
GGTGCACGGCGATCAGAAAGGAATACCGACTGCCAATGCAACTTTGCAACACACTTGAGC
CAAAATGGCTACGG
>at00487
TTTTTTTTTTTTTTTTTTACCATCCACGCTCCCGATCATTTCAGGCCAACGGCCGCACGC
ATGTGGCTCACATCCTCAGCACGCAACCAGGCGTTGGCCACAAACTTCTCGGCACCATCC
ACCGGCAGGGAGGCGTGCATCGTCCGTGGGTCCGGCTGGCCGTCCGGGCGCACGTTCGGC

CACACGAGCGCGGCGCCGAGGCGCGGTCGCACGCGCACGCCCAGGTGCTCGAAGTGGGTC
TCGCCTCCATCACCTTCATCGAGCTCGTTGAGGTAGATGAGCACAGTACAATGCCTCTGG
CCACCAACGAGCATGGAGTTCTCCGCGAGTGGGTCAGGCTGATCCACTGCGAACCTCACG
TCTACGTCTTGGATGTAGTCGTAGTGCGCTTTGTGCTGCTCGTCGCTGACGTAGTGGACG
CAATGCAGCGACTCGCAGTGGCCCGCTGGCAGGCCCACGAGCTTCGCGGCCCGGCCACGG
ACCTCCGAAACGAGTGGGTGCCCTCCGGCCTCGCTTTTGTTGAGGACGGCCACGGAGACC
TTGCGGACCGCGTTCTCGGTGCGCTGCCAGCCCTGGGAGCTGGCGAGGTCCTGCAACTGC
CTACATTCCGCGGGCGTCGCANACGAGCGCAGCAGGATGATTGGTGGCTCGTAGCAGTGG
ACCTGGGCGTCCTGCCTCCGCGCCAGCGCATAGAGCCGCTCGAAGAGGAGGTCCGTCACG
GCGGGGGGGCAGCTCACGAACCTCGGGAAGTAGCCTTGCTCCGCCCATTCCGGTCTGAGT
CGGTGCACGGACCACGTCAGCCTCAGCCACTCCACGGACGTGAAGTCGCCCGGCTGGAGC
GC
>at00488
CGATGAGCATCATCGCTGCATGCTGGCCCACGTCCAACGCCGGCCCGTAAGGCCCGTCAA
CCTGGATGTCGAACGTGGCGCCGGTCTCCTTCGCCGTCGTAGCCAGTGAGTAGAGTCTGT
CGGTGAAGGTGCCTGGGCCCATGCTCTTGATGCACATCCGTGCAACTCCATCATGCGGCG
CACTGGCGAGCGAGAAAGGATGCCCATTCCCACAGAGATATGGCAGGGACCCTCAGCCAA
GCGAACTGCGCAGGCTGAGAATGGCTCCAGTCCATGCGGAGTTCCGTAATACCCCCGTCT
GCCTGGTACACTTCGATTCCCTGCTCGAGCAGCCGAGCTGGTGGTGCG
>at00489
GGGCGGCGTAGCAGGAGGTGCCGTGCGATTGTCCCATGGCACGGACGGGTCTACTGTCTT
GGCACAGGCCGCGAGCTCTTCGGGGGCGGGCGGCCGGCATCGGTGCCTGCGCAGCGGCCG
CACACCCTTGAGGAGCGCGCCGACGGAGTGTACTTGCGCTTGCCGCTGGGGCAGCCGGCG
TCTGGCGACGGGAGTTGCAAAGGTCCGTCCTACCCATCCGACGAGTTTGCTCTCGAGGCT
GGGTCGACCGCGAGTGAGCCGCATCGCGGCCCAAACCGCGATCCACGCTGGAAGCTCTCC
TTGCAGCTGGAGCAGCACCGAAGTCAAAGCCGAAGGTAGCACCGGCAGCACATGAACTTG
CCAAGGCCACTGGGGATGATACTGCTGCAGG
>at00490
TTTTTTTTTTTTTTTTTTGCTTGCCCCACATAAGCCATTGCTCCCGCAGAAGTTGACATG
CTCCCAGAGGGACGCTCGCGCGCTTGAAAAAGAGCGGCGGCACCGCCGCCGGACTGGGTC
CGTGGCGTGTCAGCCGATCGGCTGCCAGCCTCGACACCGCTGCCCTGCCGGCCTCGGCAC
TTCAGTCTTCCTTCGACTGGTGGATGTACTTCACGTGAAGGTTGAGTGCGTGCTGGGTGT
CGAAGCTCTGGTTGCAAGTCTGGCACTTGAAGGCCTTGTCCTGGCCGTTCACCTTGGGAC
CCTCCGGCGACGCGCGCACAGGAGCCTGGCGCAGAGGCGCGCAGGGCTTGCAGACCTTGA
GCGTCTCAGGTGCCGGGGGGGCCATGGCTGCCGAGATGCAGCGGCACGCTTGTGCGAGGG
CTCGTGTGCAGGGGAGCTCCAGCCTCGTGCC
>at00491
GCACGTGCTGCTTGTGACTGGCTCCTTCCCAGCATTGGAACCTTTGTAGTTGGAGAAGCT
AGTCCCGTTTATTAGCGTGCACTGCGGTGCCACCAAGCGCCTGGGACCAAGTGCCTCTTT
GAGTACATCCAGTGCAAGAGAATGAGGCCAACGCGGTTGCCAAGCCGAATTCGCCTTGCA
TGAGGGCTTGGACCTAGTCAAATGGGGGGTGGCCGGACAGCTGCTGATGATGAAGGGCTG
CCCCTGCGCTAGATGGCGCATCATCTTTTGTATGCTGTTGACCTTGCTCGGGTGGTAACG
GGGGGTGTCGCAGACGCCTGAGAGGGCTGCGTCGTGCGTCGTGGCTGCAGTGCGGTAAGC
TGCGCCAAGTGACGCGTGCAGCGCACCCC
>at00492
TCCGTTTGCCTTCTTTTTCTTCTTCTTCGAGTTCTCGTCGTCCTCCTCATCCTCGTCCTC
CTCTCTTCTCTTCCTTTGGGGCTGCCTTTGGCCTCCCCTTCTTCTTCGCCTCCTCCTCCT
TCCTCCTCCTCGTGCCGTCAGCCCCTTTCGCCGACTTTGACCGTTGAGTAGTCTCGGAGT
CTGATCGCCTTTTGGATTCTACTTTAGCCTCCATGTGAGCG
>at00493
TTTTTTTTTTTTTTTTTTGCACTGCAAGGCCTTTACGTGGTCCCGCTGGCATGTCCCGCT
GGCAGGTCCCCCCCGACAGGCCCAGCCGGCTCAGAAGCCGTTGCGGCCCCAGACGACGAG
TGCGATGGACATGCTGCCCAGAGCCGTGAGGAAGACAAAGCCCAGGTCAGGGTTGCTGAG
GGGGTCCATGGCCATGCCCAGCGAGCTGCCAACCGGCTCCGCGGCGCCCGCGGCCATGGG
TGCCAAGCCAGCAGCAACTGCGGCGGCCGCTGCCTTCTGCTGGCGGCGGCGCGACGCCCA

GCCGAGCACCATGCCCAGGCCGCAGCCCAATGCGGCCAAGTCCAACGGCCCCGCGCTTGC
GCCCTCTGCCGGCAGCTCACCACTTTCCGCGCCAAGGACAGGGGTGGCCACCAAACGATC
GCGGGAGCTGCTCGCCACCGCACTGCCCTTTGGAGCAGGCTGCACGAAGGCAACGGTGGG
AGCCAAGAGGCAGGCAACCGCAAGGACTGTGGCGCGCATGGCCATCTGCTGGGCTGCTCG
GGCTTGGGAGCGCGTGGAGGCAGGAGAGCAGCTTTTTGAAACCTCGTGCC
>at00494
CCGTATTTGCTTGAGCAGGACTTCCTACGGCAACATTCGCCCACCTGCCGGCAGCACAAG
CGCCGGGGGCATGTGATGTCGGGaACATGGGCAGTGAGGAGCTGCTTTGGAGAGCCGTGT
CCGAGGTGGTGAAGCCCATCGCCCACTTGGAGCGCGAGCTGGAGACAGTGAAGCTCGAGA
AGAAGATCCGCGACTACTTCAAGAAGGCCGCCAAGGGGATCGAATTCTCCGGGAAGTCCT
GGGAGGCCATGGTCCAGGGA
>at00495
TTTTTTTTCTTTTTTTTGCATATCCTGGATTTGGTGTAGCATGCCTCTCATGCACGAGTC
TGCAGAGAAAGGCGGACTACTCCTCCACAACTCGCATGCGACAGGCCACTGTACCGGCGT
GCAAGGCGAGTTGGGCGGAGCCCAAAGGTCTTCTGTGTACACGGGCCGACCTCCTTCTCT
GCCTTGCTCACAGCAACTTCTCCAGGACGGCCTGCAAGTTGCCCTCGTTGGCTGCGAGCA
ACTGCGCGTTCAGCTCACGGTTGGTGAAGCCCATGGCCTCGAGCGACTGCAGGTGTTCCT
CCTCGCGCGAGGCTTCTGCGGGCGGCGCACTGGGCACTGGCTCGGATACATCCATCACCT
CCGCCTCCTGCGCTGGCTCCGACGCTTCCGGAGTACCCTCGCCTGCGACTGCAGCCTCGG
CGTGGCGGACAAAGTTGGCGATCTCCGGCAACTCGTCTTCGGCAATGCCATGCTGTGCTG
CGAAGCGAACTGCAACCTGCTCAGGTGCCTCGCCGCGGCTCCATGAGATGGTAAGGCGGC
GGCCATCGGCCACCTCCACAGGGAAGGTGAGGCTGGGGCCCTGAGCCTTCGGCTGCTCAG
ACTGCAGGTGTTCCTCCTCGCGCGCGGATTCTGTGGGCGGCGCACTCGACGCTGGCTCGG
ATACATCCATCACCTCCGCCTCCTGCGCTGGCTGCGACGCTTCCGGAGTGCCTCGCCTGC
CACTCTCGTGCCGAATTCT
>at00496
GCATGCGTCGAAGCCAGTGCAGCTGACAAGCTGCTGCTCCAATGACTCCAGCATCTCAAC
GTACCCTTTCGTCTGGCTTGCTGGTGCAAATGGATGCATGTTACTGATCTCCGGCCAGCT
GCAGGGTGCCAGAGACGAGACCGAATTCAACTTCATCGTGCAGGAGCCCAGTGAGATCAT
GGACTTGTCCAAAGCAAGATCCTTCAACTGGAGTGTATGCATGTAGCGCATCATCTCTGT
CTCCGAGTAGATGCTGTTGAAG
>at00497
TGTGGGCATCAGAAGAGTACACGGTATATCTCCCACAAACAGAAGCTGCGCGCAGAATTG
TTACCTGCCACGGGGACTTCACGTGGGGCAACGTCTTGCGCACGGGAGCTGGTGAGCTCA
AAGTGATCGACTTTGATAACGCCTTGGCCTGCTGTGCCGCCATGGATGTGTGTTGGACCT
TTGCCACATGGTGCACTCAGTCAAGGGAGGAGCAGAGACGTTTCATTCAAGGCTACCTTG
CTGAAGCTGGCTTCCCCTCTGATGCCGAAAGTGTTGACGCGTTCGACACCGACGTGCAGA
TTTTTCTCTGACGCTTGCCGGTGCTGTTGCCTCCAACGTCATTCCACGAAAGGAGGCCC
TTGCAAAAGGTTCACCTGCTGTTCGCCGGCTCTGTGCGGAGGTGGCTTCCAAGTATCGAG
CTGATGCTGCGTTCCGAACGGTGTGGAGGCGACTCCAGCCTCTCCTGG
>at00498
TTGTTTTTTTTTCTTTTTTTTTGTCGTGCGGCACTCTCATGAGCTGGGACAGTGTCTTCG
CACGTCCACAACAACATACTGGGCGAGATCAGTAAGCAGACACAACTCTACGCGCTAGAT
ATCTACCAGTGACGCTCCGCTAGGGGGGCACTGCAATACTCTCAAAGCTGTCCCAGGGAG
CCAGTGGTCCTGATTTAAGCTGCAAACGTAAATGACACGATTGATGGGTGGCATGCGCTA
CATTGAGTGTTCGCCTTCGCCCTCCATGACCCATTCAAACCTTCAGCGCCTTGCCGCAAA
GGACTGTGTAAAGGCCGGCAGCTCAGCCTCCAGAAAGATGCACATCTCGAGTAGCCTAGC
CGATAGCACTTGATTCTGCTGCCCCGCGTCCAGCAGTGCGAAGCCCGCGTCGAAAGCTTC
GGCCGTCAGCTCCGGCAGCTCACCCGCGAACTCGCCAATGAAGATGCCGCCGCTGATGGG
AAGGAGCCGCAGATGCGGCCGGCCCGAGGTCGCGAACTCAGCCAGGACGGCGCGGTACGC
CTGCGCCAACTCGGCGAGTGCCTCCCCGCGCGAGAGTGGACGCCCACGGAAGTCGGGCCC
GATGGAGTGGATGCACAGCTTGCCCTCGTGCC
>at00499
TTTTTTTTTTTTTTTTTTGATGTTCATTCCCTTCCCAATCCGGTTCCCGTGCCCCCCATC
CACCCCTCACTGGTCCAGGATGCCCTCCTGGGTCTTCTCCAGCAGCCGCGTCCAGAGCCC

GCTGTCCAAGTTTCGGCCGATGAAGACGATCTCGGACCTCCGCTCTGCACCCGAAGGCCA
GGCGCCGCGCGGCTCGAACATGCACATGTCGTGCACGCCCTGCAGCACGGCCCGCTTCAC
GGCGCCGCTGGGCTCCTTGACGCAGACGACGCCCTTGTAGCGGTAGAGGTCCACTGCGTG
CTCGAAACGTAGCGAGTTCATCACCTGCATGAAGCGGTCCAAGACCAGCGGCTTGCCCTC
GAGCGCCACCGCGAAGGTCGCCACGCCAGTGTCATGCCGGCTCTGCTGCGGTCGAAAGGG
TGCGCAGAGCTGAAGCGAGCCTCGCGACTGGGTCCGGGCCCTCTTGACGGGCGGCTCCTG
GGGCTCTGGTTCACCCAGGTCTCGGAGGACCCTGTCCAGGCTGAAGCTCTCAGAGTGCAG
AAGCTGATCCAGAGGGACCTGATCTGGTCTGGTTGCAAGGCTGCAGCGGTAGATGGGGCA
AACCTGATTGATACAACGAATCTCCTTCTCGACCTCCTGCAGAGTCGCCTCACTCACAGA
ATCCACCTTATTCAAGATCAGTCTGTCGGCGAAGCCAACCTGTTGCGCCGACTCGTTCAC
AGCTCCCCGAGAGCGCTGTCTTCTAAGCTGCTCTATGAAATGATGGGCATCAACCACGGT
CAGGACGCCATCCATGCGTGTTCGTTCGCGAAGCTCGTCCTCTGCGTAGAACGTCTTGCA
AACCGGTCCAGGATCTGCAAGCCCCGTTGTCTCAATGAGGATCCCATCAAGCGGAGGCTT
CTCTTGACCTGCGCCTTGCGCACCACCTGCAGCCAGGTTGTTGGCCTCCTCCATGTCCAC
ACGATTCAGAATTTGCTTGATTGCATTGATTAGGTCATTCCTCACAGTACAGCAAAGGCA
TCCATTGTCGAGGACAGTGATAGCCTCTTCTGTTCGCGTGGAAAGCTCCTTGTCGTTCAA
TAGCTGATTGTCGATACCAATCTGTCCGACCTCGTTTTCGATTACGGCGTACCTCTTGCC
ATGCTCCGCCCGCAAAATGTAGTTGAGGAGGGTTGTCTTTCCAGCTCCGAGAAATCCAGT
AAGCACAACCACAGGTACTTTACGAGCATCCCCAGCCTGGCGTTCTGCTACTAGTGCAGC
CGCCAACTCGTTCCGTGCTCGCGCCAACGCATCCTTTGCTCGGGGATCTATGTCGCCACC
AGCTCCACAGCAGTCTCTAAATCCTGAAGTGCCCGAGCAGGTTGTGCCAGTACCAAGAAG
CCAATACCTCGCTCGGTCAATATCTCGGAGAATCTGACAACGGCCTCAGTATTGCCTCCA
AGAGCCTTGAATGCACGACCAAACGATTTAGGGTCTGTGAAGAATGTAGTTGTGTCAATG
GGCAGGGTGTGCACGCTGTGGAGCTCTGCAAAGGCTTTCATCTCAAGCATCACAATCTTC
ATCCCCTCAATAGGCTGCCCCGTCGCCATGAGTCGCTGTGCCTCAGCACGCCGCGCCACC
CAACCGTCATTGGCACCTGCTACCTCCATCGCTCTGGGCGTAGACTTGAAGCTTTGAACC
AAACTTGAGCCAAAATGGCTACGG
>at00500
TGTAACACGAAGCCCCGGAAGGTGCCTGACCGGTTTTGAACGGAAAAAAAAACAATCCCA
CCGCCATAACCATCTAGTGGGGCTTGAGTTGCCTGTTCTGTCTGTGGGCGACCACTTGTA
GCGCCTTGATGTTATCTTGCAATTTCCTGATGATGTCGTGCAACTCAGCCAGTGAACAAT
CCTTGAGCTCTGGTCGCTTGGCTTCCAATTCCAGCTCCGCAGCACAGAGGTCTCCGGCGT
CTCCCAGCGACTCCGCCTGCTTCTTTATCCTCTCCCTTGAGCCTCTTCTGCTTTT
>at00501
GCTACGAGATTGTGCAGCTGCATAAGCAACCATGGGAGGGGAGTCAGCTCCAGCCACCAG
CCATGGGTCAAGGGCATTCAACCTCTGCTTCACGATTCTAACATGCGCAGCCAAAGCATT
GTACTTGGAACGCATGCTACAAATGCGGTGGGCCACGTAGCCAACCCAGCAATGTATCAG
TAATGGCAGCGAATAACTTCCAGCACAGTATCACGCGGCGAGCGCGAGACGTCCAGCGCC
GAGCTCAAGTATCAAGAGGACAGGTGAAGGGGCATCACGTTGGCTTGAGCCAAAATGGCT
TGAGCCAAAATGGCTACGG
>at00502
TTTTTTTTTTTTTTTTTCCATGAACTCAACCTTGGGAGCGATACTCCTATTGTCTCTCC
TGTCAGTCCATCAGATCGCCCCTTCTCGGCAAGGCAAGGAGCGATCGCTCCTGCCACTCA
CTACATTTTCTGCATGTCCAGCAACCCGAAAAAAAAGCTTCGCTAGCCGGGCCGGCAACG
AGCTGCTGTCCTCAATCCTTCATACAAAGGCGCGCCTCAAGAACTCGTTGAAGCGCTGGG
CATATCGCCCAGGAGGGCAGCATGACACACCACGGCCATCGTAGCGCATTGCTTTGAAGT
GGTGCTCCAGCTTCTTCGCTGAATCGTAAGGGGTGAGGATGTCGATGATGCCAAAGAAGT
AAAGAGACTTCCTGTCGGAAGAGAGCATCCCACCCATGTCGCGCTGGTGCACCGGGACTT
CGGGGCCTTCGGATGGATTCATAGTTCCGCCATGATTCTGATGACTCATGCCTGCACTGA
TGGGCGAACCGAGGTGGTCCGAGCCAGTGACTGCTCCCTTGCTGCCGGGCAAAGCTCCGG
AATCTGAGCAGGCAAGCCTGTCGGATGCCCTTTCACTGGCAGTTTCGCTGACATTGTCAT
CCTGAGCCGCACCGGCCGCCGCGGGGACAGCGCCAGTGCCCATGTCGTAAATGCCCAGCA
ACAGCGAGTAGTCTATGATGTTGTTGTCACGGAGGAAGCTACTGTCACTTTCTATTTGCT
GCATCAGCTTGGCTCTCCGCTCCTCGCCAATTCTAATGGTCTCGTTTGCCTGTGTGAAGT
CACATCCTTCAGTGCACGCTCGAATCAACTGTC

>at00503
TTTTTTTTTTTTTATTTTGGTCTTGTCTTCTGTTGGCAGTTCTATAAACAACGGCTTCAA
TGAAAAAAAAAGATGAGTGTGGAGCGTGCTTATGACACATGCAGTGGAAACGCGTAGTGTG
CCCACGCATCAGTCGTGAAGATGTGCAAGCATGGGCCAGCCGAGAAACAGTGCAGTGAGA
AGACAGCATGCTGCCCAAGATGCTCTGCAGGCTGTGGGGTCGAGTTGCCCAGACAGTCAA
GGCCTAAGCTGGCGATAAGGCAACCGCGATCCAACCGCAGTTCGGCATCTCAAACTTGCG
GGCAACACCTCCGAGCGGCCATGTGGTGTCAGGTCCCCACGCGTCCCTGACTTGCGTTCT
CGGAAGTCCATCTGCCCAGGCTTCGCGATTCATGAGATTGTTGATCGTGCCCCAGTGGCT
CACAGTAGCTACCATGGATTCTGGTCTCTCGGCAAGCCAACATCTGAAGGCGACAAGCCG
TGATGCATCAGCAACATGAGCTCTCCAGCCGTCGCCCCGTGCCCAGCTTGCGTCCCATGA
TTCACGCCACTCGGCTGTTGCATCATCCGACAGCGCGGCCTCGATGACACCCCAACACGG
AGATGACCGCAGTTCTTTGGAGCTGCGGAGCTCAGCTACTGGCCTGCCGCTGTTCTCAAA
GACATCGTTGCTGTACCGATCACGAAGCTCCGG
>at00504
GTCAGCGGCAACTGTAACCATGCAATCGATGCAACTGTACAAGAACACCTCCAGCAGCTC
CACTGCAAGAACCGGCATGAGCATTACGCGGGTGGTCAATCGCTCACCTGTCTGATCATG
CCGGCGTCAAGCAAGCAGCAATGCGCCAGAGAATTCAATTTCAGGTGGCTCGTGCGCTCC
ACTGTACTCCATGACAATAGGCAAAAAGAACCGCCCCTGAGTTTCCGACTGAAGTTCTAT
CGTATATAAGACCGTCTGGTCTCTGCCCTCCTTGAAGTTGGATACCTAGGGGTCTTCTGC
CCCCGGCCTGAAAGTATGGCTGTCGCATTGTCCAAGTAAACCGAATAAGGGGAGCAGCTG
TGGCAAAGTATGCCCATCAGCGGCAATGCATGGCGGCCACTGGACATGGCAGCAGCAGCT
TGGTGGAGAGAAACTTCCTCAAGCAAGAAATTGCTGCTTCTTGCCCTTTGGGCCCTTGGC
CTGCACATTGTGGGTGGGGGCTGCAAGTGCCCTCGTTGGCGGCGGGAAGGAGATGCGAGT
TCTGCCTGAAAAGAGCACCGGACGATATTCATCGGGAACGGACTCGTGCATGAGCGGGCT
GTTCCTGTAGCGCTTCAGATGCGCCTGAAGGCCCTGGAGGGGCACACTCCAGCTCACGCG
GCAGATCTTTGCACTTGGGAAGATCACTTCTTGTAGCCGTCAAAGACGCTCCAGAATCGC
GACACTACAGAGGGATTCACCAAGTTGATAAACGCATAACCAAAGCCAGCTCTGGTCTTG
AAGTCCATGGGCAAGTAGACGAAATCATACTCACCCGCAAAGCCCTCGGCATCGAGCATC
CGCAAGAGCATCTGCCTTCTGT
>at00505
TTTTTTTTTTCTTTTTTTTTTTTTTTTTTTTTTTTTTGAATGAAAATGGTGTCTGCAA
GTACGCGGTCCAGGACTCTTGCTGCTCTAAGCGAGGCTTGCTCAAGTGCGTTGGCCAAAA
AGATGCAGAAGTCAGACTCTCCCGACCTAGGCAGTCAGCTTCTCATAGACCTCCCGCACC
TTCAGGGCGATGATCGCCTGGAGCAAGCCGGTGCCATTCTGCGAGCCTGGGCTTGACTTC
AGCTTCACCATGGCCTCGGTGAGTTCACCGGGCTTGGATGTCAGACCCTCAATGCTGTCC
TCGATCTCCTTCACCTGAGCCTCCATGTTCTCCATGTCGATCTCACCGTCCATCTCCACA
ACTGTCACCGGATTGCCGAACCACTCGTCGTCGTACATCTTCAGAGCCGCCCCGGGCTTG
CTGCCTGTGAGCGCGAGGTCCTTCTTCAGCGAGATCGCCGGGAACTTGCCCCCCTTCTTC
TGGATCAGCTTCACCTTCAAGTAAGGCAGACCCTGGTCTGATGGCTCGTACCGGAGAACG
ACGTCGGCATCCGCTTTCTGGGGATCGACATACTTGGCAAAGTCCGGCAGGCGCTTCTCG
ATGTCTTCGCGCACCTGATCCTCTGTCCATCCGCGCTCCGCAACATCCCTCTGCACCTTC
CACGCAAATTTCACGTCATTGACAATGTCAATGTAGATGCCCAAGTCCAGCTGCGATCGC
GCCTTCTCGTCATAGATGGGGTGAAGGCCTAAACACCATAACCTTGTTAGGCTCAATGAG
CTCCGGCGGGTCCTTGTTGCCCGTGTCATGGTTGTAGATGGGCTTGTACACCGCCTTGCC
CTCCTTGAGCGCCTCGATCTGAGTACCCATCAGGGCAAAGTCGTTCTCCCGCTGCGTCCA
GGGCAGTGAGACCAGTCTCCTTTCGGCCCGCACGGTCGTTCGTGTGGTAGTCGTCGAGGC
AGATCACCGTCATCATGTCTCCAATGGCAGTGTGTCCTGGGGTGACCTCTGTGCCGAGAG
CCCCCAAGATCCGCTTGAGGAAGGTGGACTTGCCGCACCCGGAGTCTGCTGCCACGCCGA
TGATCACGGGCATGTTGTCAAGCTGCTTCGTCGCGGGCAGGATGGGCACCATGTCGTCCG
TCCAGGCGTGAGTGGGGTAGAGCTGGATGCCGTCCACCTCGCGCAGCTTGGCCTCTGCCT
CTGGGGAGGGCCATGTAAGGCCGGTGGGCACACTGTTCGCGCTCGCCCGCAGAGCGACGC
CCTGGATGCGGGACGAGGCGTGCACACTGGACGTGCGTGGTGCCACGAAGGCCACTTGGG
ACAACAAGGCCAATCCCGCAAGGGCTGCGCACAAGGGCAGGAGGATCGAGCGGGATTTTG
CCATGGCCCCTATGCTGGATGACCAAAACCAACCCTGGAGCCAAAGGCTTGAGCCAAAAT
GGCTACGG

>at00506
CAATTTCTGGATGCCACTGACCGATTACTCCAGGACCGGCACGACGCTCTGGGTTGAGTC
CGCGCCCGGTGCCGGCGACTTCCGGCCGATGGAGGTGGAGTATGGCGCCATCGTGGCCTT
CCATGGCACGCTCTGCCGCCACAGCGTCCCGCCCAACGCATCCGCCTGCACACGCGTCTC
GGTGGACTTCCGCGTGGGCGTGGGCGCCTTTTACGACAGCTCCTGGGCCCTCGAAGGTAT
TGGTCATGCGCATGGTAGGAGGCTTTGCACTGTGTAGACCTCGCGTCGTGAGGGCGGTGA
TGGTCGGGACCAAAGAAAAAAAAAAANAAAA
>at00507
TTTTTTTTTTTTTTTTTTCCTTCACGCTGTTCGCATCATCTCCTCAGGTCAAGTCCACGT
CCGCTGAATATTCCCCCGCGGTCAAAGCAGTGGGGCATCGTCAAACACGACGCAGGGAGA
CCCAACGAAGCTGCTGCCAGAAGAGGGCAATGGACACGGTGAGCATCCTCAATGGCCAGT
TCTTGACCAGCGGTCGCAGGCAGCTCTGTAGGCGTTGCCTTGATTGTGAAGCCGCAGAAC
TGACAGACGTGTGTGTCGCGGTCAGACTTGTTGCTGCATTGTCTGTAAACCTTCCACGTC
GAAGAAGCCTCTGTCAAGGACATGAAGGGTCCGGGGAAATACCTCTCTCGCTGACCTGCT
GTCTGTCGCTGTCGCAGATTAAGCAGGCAGTAGATGAAGCCACCACAAACAGCTGCGCAC
ACGTTCAGCATGATCATAAACTGGTCTGTGCCTCGTGCC
>at00508
TGTACCGCCATCCTTTCACCTAGAGGCACGTGATTGCTACGTCGAGCACATGGCTGAAGG
GGAGGAGGTCCAGAAGCAGAAGCTCAAGGACTTGCTGGAGAGTGTGGCCATGCTCCGCAC
GCCAGAAGAGGTCGATGCGCAGCTTCGAAATGGATCTGTGATCCGGCTAGATGCGGTTCG
GGAGTCCCTACGGCTGGGGAGATGGGCACCACCAAGGCCACTCGTGGAGAGCATGGCGGC
AAGGCTTAAGAGGCGCCCAACGAGTGAGGGCATGGACCGCATCAAGAAGaATGaCCaAGC
CGCAGAAGCTGAGCTGCTGTTCGCTTCCAGCGGTGGTCTAATGAAGCAGCTAGACGCTGG
CTTAGATGCCATTTCTCTGCGCCTTGAGAGTGTTGGATGGGCCGCTTGTACGCTTGGNGT
ACCCACCGGGCTCATCAAGCGGGCCTTGGAGGAAGCACAGAAGGTTGAGGGCAAGATGGA
AGCCGGCACGACCGTTGTCAAGACCATGTCATTG
>at00509
GGGCTCTCCTCAGCCTTGGGTGCGCTCTTGCGGGCTTTCAGACTGACAGCAACGGGTTCC
TCCTCGTGGATCAGCAAATGTGTTGCAGCATCCCTGGCTCTTCAGAGTCACCCTCAATCT
TTGCGGGTGGTGACGTTGCCTCTGTGGTGGGGCATGAACGGCCAAAGGCAGGTGTCTTTG
CGGTCATGGCGGGCATGGTGCTCTGGATCAACATCCGCGCCACACTC
>at00510
TAGAAGCCTCGTCTCGGGTAGGCGAGCGTAGGTTTTTTCGGTTTAACTTTAAGGTTTTTC
GTTTTGGTTTTTCCGTTTCTTAGAAGAGGTCGCCGGCGATGACAAAGTCTAAGGGTACGC
GGGACCAACTGGAGAGCTCCACGATGCGCGCGTCCGCAAGCTCAGGCTTGGCCAAGTTGG
CTTCTTGGAGAGCCTGCAGATGGAGTGGCACTA
>at00511
TTGTTTTTTTTTCTTTTTTTCTTTGCATGAGCATCTCAACGGTTGCATGCAGCCATTTTA
GACCAGCACGGCAGCAGCCGGCGTGTTGTGACTTCACCAGCTCCATGGATCGGCGATGAC
CAGCTGGATGCCCTCACGAGTGGAGCTCAGTGAAACTCTCAACCCTATCAAATCGCTCTG
TCCAGCCCTGATGCTCACCCTGAAGCAAGCGACGCACCTCTTGTAGGAGGCCTTTGACGC
CGTCGCCGAAGCGTGCTGACATCGCACGGACAAAGCGGCAAGTTCCCTCGAAGGCCGCAT
CGCATTGCTGGACAGACTGGAAGAGCTTGTCCACGCGCGGCAAGGAGATGTGCGGATCCA
GGTCGCACTTGTTGGCAATCACGCCGCAGGGCTTCAAAGCCATCTCCCGCGAGAACGCCG
CAACCTCCCTCTGGAGGCACAGCAAGTCCTCGTATGGCTCTGCCGAGCGCGCCACGTCTA
TGACGTAGAGCAAGACGCGCGTGCGTTCGAGGTGCCTGAGGAACTCATGGCCGAGGCCCT
CGCCTCTGTGCGCGTTCTCGACGAGGCCCGGCACATCGGCCATGGTGAAGCCCGACCCGT
CGAACGAACTCGACATGGCCTACGTAGGGAGCCACGGT
>at00512
CGATGAGAGCTTCAAAGATCCTCGGGGTCGTCGTCGCAAGGCGTGTGGAGGGCGCATGTT
CACCGTGCGCGGGCTGAGCGGCGATGCTCTGCTCGGCCCCTGCGAGGTGGAGCCGGGAAC
TTCGGTCAGCGCCGTGAAGGCCCGGCTGCTGAAGGCCTTGGCGCCAGCGGAGCCGACCTA
cCGTGGAACTGCTGCACGGANCAGCTGTNCTCGCGGACAATgGTCCAGCTTGAGGACGTG
GTCGAAGGCgGGAGCGTGGAGCTTGTAGCGGCTGTCAACGCATACCTCCTGCCGACGGAG
GACCGCGTACGCATCCCTGGAGGAGCTCTCTGCAGCACGCCCTCTGGGGGCGCACTTGGC

GGCCTTCCTTTCAGAGGAGCCCGACGCCGCCAGGGCCGACCGCGCCGTGATGTTGGCGGC
CGTGAGGCGCTTCGGCGACAACCTGCGTTATGCTCACGGCGCGCTGCTGGACGACGTTGA
GCTGGTCTCCGCCGCGGTCGGGGACTTCTGGCCGGCCGTGCGGCATGCCAGCGGCAGGAC
TACCGCGCGGACCGCCAGTTCATGCACCCGC
>at00513
TTTTTTTTTTTTTTTTTGATGTCCGAATACATGCCTGCAAGCAGGCCTGCCAGCCCTCC
TTACGCCCCACGTCGTCCTCACTACGCCGAGCTGCCGCTCGATGCGGTGAGGCCCCAAGG
CCGTGGGACGCCAGGCGAGCTAGCGCAAGGTGGCGTTGAGGATGAGGCACGGGGAGCCAG
AGAGCGAAGCCTCGCTGAGGAAGAGCAGCTCGTCAGGGGACACCGGCTGCAGCACCGCCG
CCCCCTCCGGGCACTCGCCCTGCCCCCTCTCCGTGGCCACGCCCGTGAGCTCCACCCTGC
GCGCCTCCAGGGGCGCCAGCCAGGCGTTGAGCGCCGCGGCGGGCGAGGGGAAGTCCGCGC
GGAGCCAGGCGGCGACCTTGTGCGACGCCAGCAAGGCATAGACGTCCCGCGTCACCGCGA
AGAACTGAGGCGGCAGCCACAGCGAGGGCTCGCCTGGTCCGTGCAGCGGGTCCGTCAGGC
GTACCCAGCCGCCAAGGATCGTGCGGATGGGCGGGTCCAGCTGCATGAAGGCGCCTACAA
GACGCTCCACATGCACGAACGTGTCCTGCTCCCTCGTGCCGAATTCTT
>at00514
GACATCTTGAAAGGAGTGATTTGAGCATTTGCCGTTCCAGCAGCTTTCAATCATTGGTAT
TGTGTCCGTTGGGCTTGGTCAGGGGCTGACGTTGGCAGGCTGGTCAGCGAGGCTCGTTGT
GTCCTCGGTATGACCTCACAAGAATTGGAGAGCTTGCCTGTCCGGGATCTCAAGGCACGT
ACAGGGAT
>at00515
CCCCATGGGCTCAGATTTCCCACAATTGGCGTGGAGCTCCATCTCATGCCCATAGTCCAC
GGCTCCGAGTTTCCGGTGTCGAGGACGACACACCCCTTCCCATGGGACAGGGTCACGGGC
TTGGCCTTGGAGGAGCGGCGACCGACTCCCAGGGCTCCGACGGGGGCNCCGTCGGCTGAA
CCTGGTGCCCCAGGCCCTCTCATGCTTCNGACGCCTTGCTGAATGGCAGGACGGGAGAAT
CTCTGTCGATGAGAGCCAGTCCACGTCAAGCCGTTGTACTTGAAGAGCACCGCCTGCAAA
GTCTTTGACATCAATCTCCATGAGGTCCGATAGGAAGGCAAAAACGTTGAGCGGTACTAC
TGCTTGTGC
>at00516
NNNNNNNNNNNNNNNNNNNNNCACAAGGCATAGCTGGATTTCCTCGCGCGCGCTGTGCT
AACCCTACACAGTCTGTGGGCAGCCAACCTGTCTTGGCAACATCAGAGCTCAGTCAGGCC
TTCTCTGGCTGGTGCGGTCTCCTCGTCCGACGACAGCAGGTCCATGACCGGCACGCCCTC
CGCCGTCCTCTTGGTCTGGTCCTGGCACGCGGCAAAGACGTCCATGGGGATGTCATCGTT
GTAAAAGGAGTAGCTGGGGCCCTCTACAGCAGCCGCGGCCTTCTTGGCCTTCTTCTCGCT
CTGCGCGGGCGCGCCCGCAGCATCCGCCTCCTCCTCCTCCTCGCCCTCCCCAAGGGGGTC
CGGGCCCAACGTCCGGTCCTGCAGCTTCCTCACCGCCTTCCTCGCCTCCGGCCCTGGCCT
CGCCAGCAGCGGCCCCGTGGCCTCCAGCAGCCGCGAGGCCATGGCCCACTCCTGCATGCG
CTCAGCGACCTCGCCAGCCTGCTCCCCTTCGGCGCGAGGAGGCGGGTCTGCGAACAGCAG
CGCCAGCAGCGTCTCCCACAGCCGCGCGTGCTGCGGGGAGCGGGGTTGGCCGTCCTTCGC
GCCCGCCGCCAAGTGCCGC
>at00517
CTTCTTCCACGATTTGGGGCGTCGGTGGAATACCACTTCATGCTTGCCTCCATCAGCATC
TGGCTGGCCATCCGCCGTGTTATTGTTCACACCGTTGGCAAAAGAGACATTCTCAAACAC
GTGCTGCTGTGCTTCCTCCGATGCAATGCTCGTCGGCTTAAAACCGCACATGCGCCGTCC
CTGGCACGGCCTCTTGACACCCTTATTGGCCTTGGCACTGTCCGTATGCAATCCTTGCAA
TCAATGTGGCGGGTCCTAGCATTTAAAAAGGCTATCGGATAGTCGGGTCAAATCCGGTCA
GGTCGATGACTCGGGTTGGGTCGGGCCGGGGCCAGGCCGTCCGTCTGTGCGTACGACCGT
CGGACCGTCCGTCCATCCCCCTGCCCGCCGGCCCGCCGGCCCGCCCGCCCGTCCGTCCGC
CCGTC
>at00518
TGGTCATGATTGACACCGTGCAGCTGGCAAAGCTTTCGCCCCACTTATCCTTTCACTGGA
GGTCACATTTGACATGGATTGAAGGCACCTTGACCGGACACAGCAGCCGCTGCTGAAGTA
GATTGGATCATGGATGCTGGCCACTGCCCTGAGTACTCCACTGGAGGTTGAGGCGATACG
GCCTCACTCCTGTGAGATTTAGAGCCTTTGCCGCCATGCCATGGAGTCGATTTGTATTTT
GTTGGCCATGACCATGATATGCT

>at00519
TATCTGAAATCAGCTGGTATGTCCAGTTTTTCTCTTGCCTAGGCTTGCAAGGCCTTACGT
CCCTCTGGCACAAAAGTGGCAACGTGGACGCTATCGAGGTCAATGTTTACAAGTATGTTG
GCAGATGCCCCCTCGAGCAAGACAGTTCCAGCTCGCTTGCCATCGCATGCGATCCAAGAT
CTGCGGTGGATTGGCAGCACGATTCCAGAGAACTCAGTCCCTTGCTCGAGACGTTTGAGC
GCAGCGACAACAGCAGCCTCCAGGTCGGCCGGTGCTGGCTGCACGCTGGTGAGCGCCAAT
GACTGCACC
>at00520
CCGTAGCCATTTTGGCTCAAGCGCCTAGTAGCTGAAGGATTCCCCTTGGTTGCACTGCAT
GTCCTCACGTTCAACCATGTTGCCTAATGCTGCGCATCGTGGCATTGCTTGGGCTTTCCT
CTTCAGGGACACCTTCATGCGGGTACCTCTTGCACTGACGCCATCGAAGAGGTCTTCCTG
TTCCCAAACCTCGCCGAGCTCAAACACGGCGCGCAGGCAAAGGAACTGAACACGGACACT
TGCTCCTGCCTCAACTGGAGCACGCTCTACCATGAGAGCGTGGTCAAGTGCGGCGACGGT
GAGGAGCTCTCTGGCCCGGGCATTGCTCCGCGGGGAGCTGACGGCCTCTGCGTACACAAG
TCCCCAG
>at00521
TTTTTTTTTTTTTTTTTGCTGGAGGGTGTACGTAGTGACCTAGGTCAATCATTGGATCA
ACCTGATATCCCACTGTACAGCAGCCGGGCATGAGAAGTGTCCTGACGCACCTGGTGCCT
TCATCACTTTGACGATTTCGAACCTGTGGTGTGGCCCTGTCCAGGTCGAGCTCCGCTGGG
GAAGCTCCAGCAGCTGGGAGCATATACAAAAGTTGGGCGCCAAAAAATTGGGCGTCAAAA
GCAGTCAGGAGACGTGCCGCCCTCTCCACTTGGATGCTGTGGTGCAGCCTGTCTGCTAAG
ATTGAGCACAGCCGGCAACGTTCCGGCAGCTGGGCCGCAGCAGAGTGCGGCTGTGCTCCC
ACGCTAGCAGACCTTCAAACAGGGCGACTGAGCGGGCCTTGGCCGCTGCCGTTGCTCCCT
CGCATCTCCAGCTCCAGCAAAGCGCCTATCACGCCGCCCGCCGCAAGCAGCCGGAGGTCG
ACGTGGCAGCGCATCGGGCACTCCGCCTTCTGGGCGAGCACTCGTCCACGCACTGGGCGT
GGTAGTGGTGGCCGCAAGGCAGGTAACGGACCTTGTCACCAGGGTCGAAGTCCCCAGGCA
GATCGTGCACTGTGGTCTCGTCGGC
>at00522
TTTTTTTTTTTTTTTTTGATGCGTGAAGCCATGTACGAGCCCAAAGCTCAGCCACAGCT
GTCGCGGTGTTCATCACTGTGTTGCCAATAGGCTTCTGCTCTGTGAACGTACGATGGCCA
CGGGTCGTTCTGATCATGCCTAAGGTGTGCATGTGATGATCTCTGTACAAGCACTCAAGG
GGCTCAAGGCTGATCAGGGAGTTCATCGCTGTGTTGCCAATAGGCCTCTGCTCTGTGAAC
GACGATGTCCGCAGGATGCTCTCTGTACAAGCACTCAAAGGCCTCAGGGCTGATCAAAGC
GTTTGAGAGGGTTGCTGTGGTTGGGGCGGCGAGGAGCGGGCGGTGGGCCAGCAGCGATGT
TTGCTGAAGAGCAACAAAAAGCAGATGGTGTCCGCGCCTGGCCGTGCAAGGAGCTGGCAG
CCTGCTGCTCACCGATCCTTGCTCTTCGCAATTCTATGTAAGTTCTTCGTACCTTGCACA
ACATGCCTGATGTCATATCGAAGAGGTGCCCTGCCCAGTCCAGGTTGTCATCGTCTCCGA
GTAGCCCTTGTTCATCAGATCATAAGATCATTGCTCAGGGAGTTCGCGTGGCCAGAGTCA
GCATTTGAAAAGCTGGGGAACGTGTCTCAGCTAACACACAGGTATGGACTGGTCAACGGC
AGCCACGTTGACGGCAGCCACATCCACAGCAGCTGATTGGGAATGTTCACCAGCGACTTC
GGCTGCTTGAGCTTTGCCCCCTGGTTCCACCTCACCTGTGCTGCATGTAGCCGCTACAGG
TGTAGCACTTTCAGTGGTGGGCTCCGCTACGCTGCCAGCTGATGACACTGGGGGGGTGGT
CAGGCTCAGGTATGCCACTATGAACTCTTCAACCCTCTTTGGCCAGCTGACCCTGGCACA
AGGCGAAACAGGGGCAACAGAGACAGCACGCTCAGCCGTACTCTGATCAGCAAATTCGAC
GAAGGCAAACCCCTTGTGATCATCGTTGCGTTTAAGGTTTGCCACAGTTCCATAATGCCG
AACGAGGTGCTCTGTCAGCTTACTGCTCTTCAGGGATTTTGGTACTGTGACAAATACCGT
GCGGAAGTCACCTGGCACAGGGAAATCCCTTGTAGCACCCTTGGTGGGTGAAGCACTAGC
CAGATGACACTCCAGCCGCTTCCGTGACACCTGCGCTATCAATTCCATATCCTCTGTGTC
>at00523
TTTTTTTTTTTTTTTTTAACACACGAACACGCAAATATAGATATGTCTTCATCTTCAGT
GCACTTTGCCACTCTCACCGGCCCTCACAGCTTGCTCTTGGGCAGTGCCTGCGCCGCCGT
GTCAGCCCTGCGCAAATGCGTGTAAACCAGCTCCTTGAGCTCACTGCGAAGCGGATAGAT
GTCGGGTGAAAAGATCTGAGTGAGGAAGACAACCGACACCCTCCCCCTGGGATCCGCCCA
GAAGATGGTTGACGCCATGCCTCCCCACTCGATGCTGCCGACACCAGGCCCAAGCGATCG
GCCCCGGCCCTCGGGGTCCACAGCAACAACCACGCCCAGGCCATGGCCATAGCCTGGGTC

CTCCTTGCCACGCCCGCCCACCAGCCTCATAGAAGGATGAGTCAAGCACCCCGGCGGCCG
CAAGTCCGTGGCCATGAGCTCTGCCGTGCCTGGCTGGAGGATGCGTCGCCCAGCGCCAGG
ACAAGAGCCACCCTTGGCGGAGGCACTCCGGTGAACAACACCCAGTCATCGAGCGTGGAC
AGGAGGCCGCCGCCCGGCTCAAGTACAGCGGGACCGGCAACAGTGTAGTCGTTGGGCCCC
TCCGAGAAGCGGCCGTACATGAAGTGCCCCTTCAGCATGCGAGGCGTCTCACTGAAGTCG
CTCTTGAAGAAGTGCGAGCCATACTTGGCGATGGCAGGGTAGTTGGCCGAGNTAAGGTGA
ACGAGCCTGGCTTTCATCTC
>at00524
GGCGGCGTCAGGCTCAGGCCACCCGCCGTCCGGCAGCCTTCCCGGCGCTCGATTCCCGCG
CCTCTGGACCGCTGCCGGCGAGGCTTCCCAGCTACGAGCCATGGCCTCCAGCTTGCAGAT
GGAGAGGGCAACAGAGGTCATTGTCCGCATGATTTTTCTCGCCTTGACTTTGGCGT
>at00525
TTTTTTTTTTTTTTTTTGGGACAAGGGGCATTTCAAAATGTTCAACCTTCTTGTCCCAA
AAGTGGCAAGGATGGGGGGACTGGCCCACCAACCCTCCTGGGGAGCCCAAAGTGCTGGCG
TACAGGACCACGTGGCGAGGATCCTCTGACGGGCTGTGGGCTGCTGCAACGAAAGCTGAG
CCGCTCCACGGTTGTGCTCCTTGCTCTCGGCGTGCTGCCGTTGGCACTGCTGCCGGAGCG
TCCGCCGAGCACCGAGTTCGGGCGCGGCCGCGGAAAATCCTTCTGCTGCGCGAGGTGCTG
GTCCTTCTGCTGTGCAAGGTGCTGCTCTCGGAGTGAGCGCTTTGCCATGCTAACACCCTG
ACTGCGCGCTGCAACAACGGGCTGTGGTGACCCCGCATCAATTGGCCTCCGCCGTTGGCT
GCTGCGCATGGACGCGGGAAACGGGGCGGCCCCTCCCGGCCCTGCAGGCCCTGGACCGGC
GTCTCCGCCTCGTGCC
>at00526
CCGTAGCCATTTTGGCTCAAGCCTCGCGCGCCCGGCCCCGCTCCAAGATGGCCCCGCGCC
TTCCCGCTGCTGCCGCCTCGCCGCCCGTCTGCAGCCATGAGCGTCACCGGGTACATGTAC
AACATCAACGCCACGAAGAAGCTCGGCGAGCTCGAAATCAAGGAAGGCCTTGAGGGCAAC
CGGTCCTGGCATCACCAGTTCCGCAACTCGGCCTACGTTTACATCGGTGGCCTGCATCAA
GGCCTCACGGAGGGCGACGTTGCCATTATGTTTGCTCAGTTTGGTGAGATCGTGGATGTC
AACCTGGTCCGGGACAAGTCCACGGGCAAGTCCAAGGGCTTCGCCTTCCTGTGCTATGAG
GACCAGCGGAGCACAATCCTGGCCGTGGACAACATGAACGGCTACCAGCTCCTGAGCCGG
ACGCTGCGGGTCGACCACGTGGACAAGTACAAGGCGCCGAAGGTCCTGGACGAGGAGGAG
AAGGACGAGAACGGGGACCCCAAGCTGCTCGAGTACAAGGCCACGGGTGCGGAGGGCGTC
GGGTCGGGCGCCTACAACGTGCTGCCCTCCCAGCGCAAGATCGCGGAGGTGCAGGAGCAG
CGGCGCGCCAAGCCAGCGGGGCAGGCGCCCCCAGAGGACGAGGACGAGGCCTGGGCAAAG
GCCTTCGAGGACGGCCTCAGAGCCCAGGCGGCCAGCGGCGGCAAGAGTAAGAAG
>at00527
CCGTTAGCCATTTTTGGCTCAAGCCAGTTTGGTCCAAGAGCATTGTGGCCCCAGCACCCT
CTCACAACGGTGGGCCATGGTGGTGGCAGGAGGTCAGCAATCGAGCGGCAATGGCAGGAA
GAGACAGTGCAAGAATGCAATGCGGCGCCAGGCCAGACATGCATTGAGCCTCATCTCTGG
TGCGTACTTCGTGCTGGTGGCCCTCTGGTCTCCAATGCAGCTTCTCTTCTCATCCCCCTC
ATACATGAAGCTATCAAGGCGTACTCCGATTTTGACATCGTCTTTGATGACCAGTGCGGC
GGCCCCGTTTTGCGGACCAACTCCAGAATCGCCCTTGCCAGCAAGCCTTTGCAGCCAACA
GCGCTCGTCTTTGCCCATGGCTGGCACTGCCGTTGCAGCCCCCGTTGGTTGGATGGATCC
CCGGCGGTGGACTGGAGCTCCCCCATCTCCCGCAGAGCGGATGTCACCA
>at00528
TTTTTTTTTTTTTTTTTTCCGGTGTGTACGCTCTGTCCTCACCAGCGTGAGCTCCAACAC
TGCCCGTGTAGGACAGTATGCCCTTCAGGACAAGCAAGCACAGTGTACACAGCTGACGGA
CCCCGAAACTAACATGCCTGCCGGAATGAAAGCGCGATGCAGGATTCTCCCCGATGCTCG
CAAGGTCCGCAGCCGCGTGGTGTTCAGAGCCCCACTTGCAAGTGGAAGATGTCAGGGGTC
CAGAACATGTTGCCGTGGCATCGAGGCTGCATCGTCAATCTCCATTGTTGCGCGAGTATC
TCACTCTGTCCGCCCTGGGGCTTTATAAAAGGATGGACAGCGTCAAAACTCAAACCTATG
AAAAAGAGACCTGCGGCGAGCCACTGCTGAGGAACTGTTTGATGCGCAAGTGGTCGTTGT
TGTGGCTGCTAGTGCTTTCCT
>at00529
TTTTTTTTTTTTTTTTTTGGAGCGCGGCCATTTCCTGCTGCCACAACAGTTCGCGTGGCG
TAGCATCAGCATGCACAAGCTGCGTTACATTCCTTTCAGGCTGCTGTCACAGCATCTTGC

GCAGGGCACGCTCATAGTACCACGGTATGTGCTGGGCAGACTTCTTTGCGGCCAGCTCCG
CCATTCGCTCGGGCAGCTCACTTTGGAGTACCCGATCGATCAGGGCATTTCGCTTGACTG
ACTTCTTCTTGGCCTCCAGGACGAGGGCGGCACCCACAGTCCGGATGAGCAGGCTTTGCA
TCCGGAAGTGCTGCGTCATGCCGAAGGGGTTCTCCGCCCGGCCCTTCTCGCATGGCTCGC
TGCCCCGCGGGGAGTTCGGCGGTGGATCCACTGCCTGGATGAGTCCCATGCTGACGAGCT
TCCTGGTGGCCCACAAGAGCCGGAGGAGGTCGAAGTGCGTGGCACCGCCCCACTGCGAGC
AGGTGCTCGCTGCGAGGTCGGGCAGCGAAAAGGTGTTCTCGAAGCAGGTGCTGACCTTGA
GGGCCGCTGACTGGATGGGGTCCAGGCTCTCCACCCGACAGACGGTGGCGCCGACCATGG
CCGTGTGGTTCCACAACGCGATGTCCACCGAG
>at00530
TTTTTTTTTTTTTTTTTCGGCTTGATCCTCCCGCTGCGCACGGGCAATGATGCAAATGC
AACGACTCATAGCTACGATCGTGGAGCTGTTGAGCTCAGGCCGATCACTGGAAGAGCGGC
TCGATCGCCGGCATCGACGCCTCCCGGCCACGGGCTGCTCTGACCGAAGGTGAACGCCGC
CCACAGCACGAAGACGGCCAGCCCGGAGGCGGTCGCGGGGAGGAGCAGGACAGCCAGCAG
CGCGGCCAATGCGAAGGACACCTCCCCGAGGTTGCCCTGGAGATAGCCCGCCGCGGCGGC
CGCGGAGCCGCTCACCTGCGCCGCCACGTAGCCGGGCTGCACGAGCTCGCCGTAGACCCA
GCCTGGGACCACGTAGAGGAGCAGCACCGCCGCTGCGAGCTTCGCCGCCTCTCGAAAAAA
TGGCCCTCGAGGAAGGATTCGGTCCCTCGACCGCGGCCCAGCCGATGATGAGCGCAAAGG
TCCCTGTCGCAAACGGATGATCCGAGACGAACGTCGACAACCAGGCAGCTACAGCAGGGA
AGCCTTGAGACGGCGCCTCCGGGGTGCCAGCGAGACTGCCCTCCAAAGAGGAAGGAAGTG
CACGCAACGGGGCGACGGCACGGCAGCCACCCTGAGGCTGCCCCACCTGAGGCACACAAG
CGCGAAGCCGTCCTGGGGGTGCAGGAAGGATCAGCCCAGTTGCCGGCAACGGCCGCAGAG
AGGCAAACGTCTCCAAAGCTGATCTAAGGACGATGGCTACAACAAGAAACAACCTGACGA
TGCAGTGGCCCGCCGAGTTACGGCAGAACTGAGCTTGCCCTCGTTGCAGAAAGGTACCAG
CCTCCTGCCTGCGGAGATGGTACAGGCACTGAGGAGCGAAGGATGTACAACTGCCAACTG
CAAAGGCGTTCAAGTCAGGCAGTGCACCGACGCAGAGCCCGCCAGATGCAGTGTCCATGA
TGACTTGCAACCTACTGGAGAGCCTCTTGGCCGACTTCAGCCAAACTTGAGCCAAAATGG
CTACGG
>at00531
TTTTTTTTTTTTTTGCTGTCGGAAACTGCGCACCTGTGGCTGGGCTCTGACAGCTAACT
ATTGTTCATGTGTTCTGAGTGGCACACTGGATGACAATCCGATATGCCTGAATCAAGGTA
TAGATCAAAATGCAAGCTTATGGAAAGTTCTGGCTCAACCTCATTGCATGGTGTCTAATT
GTGCAGGGACTGGGAAGCATGCAGCTCAGTGCTTGAACAGATATGCACCGCGCAAGGAAA
CAGTATCGATTTAATGCACACCGTGCTCCCATGAGGCCATGTGCAAAAATCCAGGTCACA
AACTATCTCTAGCCGTTGCATTGGTGTAGCCCTGCCTCTCCTGCCTCATGAGATAAAAGT
GAGTCCTCCGGCCTTCCAGTCAACACACCCAAGGAAAACATGGTGTGAAGCAAGGAGGGT
TGCAGTCATTCCGCTGTCTAGAGTATTCATGAAACCAAAGGGAGGTAAGTGAGACAAGCA
GTGCACACTGGAGACCGTCCGAGCATCCTTCACTCTTCCCGGGTGGGGTGGGGCGGAAGA
GGAGGAGGCAGGAAGCCCTGGTCCTTTGCAAAGGGCATGGCTTCAGGCTTCTGAGAGCGC
GATCCAGCTTGGGCAAGGGAGGTCGCAGCTGCAGCGGCTCTCCTGAAAGTTGAAGTCCAA
GGAACAAGCGCTGACAAGGGACACAGCCACAGTCTACGTCCAGAAAAAAGGACAGCATAC
AGAAGCAAATGCACGGCAGCGAGTGACCAGCACGGTATCCCAGACGTGACCCACACTGCA
GGAACAGCACAGGCTGACCAGGAGCAATCCCACCCCAGGGCTGCCGATCGAAGCGCCTCT
GGCGGATCGGAGCATCAAAAGCCGATGCTGTGACCATCGCTGATGCATTGATCCTGTGCA
GCTAGCAAGTTGCTCTCTCACAAGGCAGTACTCGGGCCCTCCTACCATGGCAGCGCCCAC
ATAAAATCTGAAGTAGTTTCTGTCTCATGGGGACTCGTCGCCGCTGCTTGGTGGAACGAG
CCCCATTGGATCTGGAATCTGGGGTGTCTGAGGCATCAGTGGGTAGCCTCCATCAGCAGG
TGTTGTCACGTTGGACCTCGTTGCTGAGACTGCTCGGCGCAGCTGAGCACGGACTCTGAT
GTCATCATAGAATCGATTCAAGCCCGTCAACTCCTCACTGTGGCGCTCGTCAGCCAACCT
TTGCTGTACGCCTTGCACCGACGACGCCTGCGAAGTGCGCTCTGCAATCGAGTCTTCCAC
CCACTTCTGGTCCGCGCTGATCGGCTTCACACCACGCGCCTGAAGCTTCTTGC
>at00532
GCCGAGTACCTCAAGCTGCGCATGAGCGCGATTGCTCCAAACCTGACGTACATGGTTGGC
GAGCTCGTGGGCGCCCGGCTCATCTCACAGGCCGGATCCCTGATGAGCCTCGCAAAGCAC
CCTTCGAGCACCGTCCAGATCCTGGGTGCGGAGAAGGCGCTCTTCCGCGCTCTGAAGACG

AAGAAGAGCACGCCCAAGTACGGCCTGATCTACCACGCGTCCCTGGTGGGCCAGTCCGCG
CCGAACCTCAAGGGGAAGATCTCGCGCGTCCTCGCCGCGAAGCTGTCCCTCTGCTGCCGC
ATGGACGCCCTGGGCGACCAGACTGAGCCAACCCTAGGGAAGGAGTTCAAGGAGTACGTA
GAAAAGCGGCTTGCAACCTTGGAGGAGACTGGCATGAGGTCCCTCACCAAGGGCATCAGC
AGACCCGCCACAGGCAAGCAGGAGAAGAGGACGGCCACCACCAAGGCGGGTGTTGCTCAG
TACAACCAAGCGGAGGATGCCGTGGAGGAGCCCCCCCCAAGAAGAAGAAGGCGAGGGAGG
CAGAGGCAGAGGCCGAGGGCGCCGAGGAGCCGGCGCCGCGAGAGAAGAAGAAGAAGCGCA
AGGCATCGGAGGATGCGGAGGCGGGCGAGGCAGAGCCAGAGGCCGAGGCGGAGGAGGGCA
AGAAGAAAGAAAAAAAAAGAAAAAAAAAAACAA
>at00533
TTTTTTTTTTTTTTTTTTGAGCATGACTTAGGCAAGTCTCTGCCCCCAACCTCACTCACC
GAGTGGTGCACATGGGGCAACAAGCGCCGGCTGCGCAGTGCACCGTGTCTCCGACACGGC
AGCTCGGCTTGAAGCAGCGCACGGCGTTGCTGCCGGGACCGCGGCAGGTCGAAGGCGCGC
TATCGCACTGCTCGACGGCGAAGCCGGTGGCGTTGTACTCCACGACCGCGTGGCGGTCCG
CTGCCACGACGTGGTCTGGTGCCCAACAGTAGCCGCAGCAAGTGCCGTCCACCTTGAGCT
CGAAGGGTGTCGGACAAGAGTACGAGCCGCACTCGATCTTGGAGCATGCGGGCGTGAGGG
CCGTGTTGCCGAAACTGTCCTCTGCGCGGTACTCCATGTCAAGACTCACCTCGTCCGGCG
TGTCCCGGGAGCGCATGCGCGCGCCAAATGCGGCGGCCACAAG
>at00534
GTAGAAGAGCACCGCGACGCTCCTCACGTCGTGGTGTCTGCGCCAGGCAGCGGATGTACT
TGCCAGAAATCGTCCTGGGGTGCTGGGTGTACCCCCGAAAGCCGTCCAGCAGGATGTCCG
TCTCGTAGGCAGGCAGGGAGGCCACGCACGCCTCCAGGTATCGCGCAAGCCTCTCGACGC
TGCGCGCCTCGTGCGGCAAGCCAGAGCCCGGCCTTCAGTGAGCCGNTCCACTTCCCGACG
CGTG
>at00535
TGCTTTGCTGGGGGACTCCTTGCCGAGGACGTCGAAGTGCGTGGCCATGAGGGAGCTGAA
CTCCTTCAAGAATGACGTGACTTCCGGATCCTCCTGATACTTTGCCTTTGCCTTCTCAGG
GTCCGTTTGCATGAGCTGGATAGCCTCCATGACCCGAGGGTTGGAGAGGCCCTTCATGAG
GTCGGGCCTGCACTGCAGGGCCTGCATGAGCTGGGGCGTCATCCACGACTGGTCGCTCTG
CAGCTTCCTGACCCCCTGCTGCAGCGCCTGCTGCACCTCTGGCAGCTTGAGGCTCTCTCT
CCTAGCAGTCTCCGCATCACCAGCTCCAGCGATGTATGGGATGCTCTCCTGCTTCGCGCT
ACCTTTCTCCGCCGATGCCTGTCCATTGCTGCTGCGCCTCGTGCCCG
>at00536
TTTTTTTTTTTTTTTTTCAACAAGCTCGACTCTTCAATGAAGTATCAGACTCAACCAAA
GATAAGCACGGAAGCGATTGGTGAAGTAGAGCACGCGAAGTAAAGAGAGGAGGAGCACCG
CCACCGGGACGCAGTAGGAGCACGGCTTCTTACGCAACATGCGACAGAACGTCGGCAGCA
ATTGCATGATGCCATACATGTTCAGCGCAAATGCCAGCGTGAGCGAACGACAGGCCTTCT
TTAACTCATCATCGCCGGTCTCCAGGAAAGCACGCAAGACGTGGAATATGGCGAGGAGCT
TCGCGAATGCAAGTGGCAGCTTGACCAGAACAGAGGCCGCCAGCCTGCTGCGCTGCTCAA
TGTCACCGGCATTTGTCCGGAAGCAGACGCCAATGAACTGCAAGTTCCCGGCGTCACATG
CCTCAGCCATCAATCCGTGCATCGCCTCCTGCTCCGATGATGGGTGTGGCAAGCCGTTGA
CCTTGGACATCCAGTGTCCGACAAGGTAGAAATGGATGCAAGCGCTTCCGATCAACAGCA
CCACGAGCGATAACCATAAGAAGGTAAAATCATGGAAAGCCTCGCGATTCGCCAAGTATA
CCGTGCCTGCCGCATAGGAGGAGCTGACCACTGCGACATAGTTCGGGGCANAGCTCAAGA
ACTTCCACCACCATCTCGAGACACATGTCTTCACGCCGCAG
>at00537
CACGAAGCGCGTGCCCCAATGCTTCGTTGCCAGCTGCCTGAGATGCTTGTCCATGATCTC
GCACCGCCACGTGCTTTCGCGCCAGAAGTGCACAACGATCTTGTCGCTCTTCTTCGCGAC
ATCGAAGAATTCCTTCTCCTCGATCATCTCGTACTCGCCATGCCCAGCGGCCATCTTCTG
TTTCCTGTCTGCATGCGTTGCCTTCATTTGCTCAAGTCGCTTTCTTCTGATATCCTCCAG
ATCATCCTGTTTAAGCCGATCCAGGCGCGATATCTCCTCATCGATCTCAGCCTCCTTTTC
TTTTGCTGCGGCCACGACCACGTCAGCAATGACCTCGAGGTTCTTCTCATACAAGCTCAT
GGCGCAACAGCTTTCGGGGCTCAAACTCTTTGGGTGAGACTACTTGCTGTGATGGGAATT
CGGGACTCTTGAGCCAAAATGGCTACGG
>at00538

CACTTGAAACCGATGATCGAAAACATGCTCCAGAGTTTAGTCCCCGGCTCGCATACTGGA
CAGTGTCAGTTGGCAGACTCGGTATCCGTGGATGAGGTGCAGGCAGTTTTCAACCCTGAA
GTTTGGAGACGCTATTGCCTGCGCAAGCACGAGATCGCTCGCGGAATCAAGTCGCACTGG
GACTGTCCGTGGGTGCGAGATGAAATCCAGGAGTTGAGAACGATCCACGACCACATGCGG
AGTGCGAGGTGTGATGTCGCAGCGAACGAAGTCATGCTACTTCATGGCACTGACCCAAAC
AGCGCTCACGATATCACAAAGCAGGGCTTTGACCACCGCCTCTCGGGGAGGGAGTTGTAC
GGGCCAGGAGTGTATTTCACAGCCGACTTC
>at00539
TTTTTTTTTTTTTTTTTACCCGTTGGCACTCTTGCGTCCAAGGGTGACTCAAGACCGGT
CTTGACAGGAAAAGGGGCCACAGACGTGCTGATGTCCATGTGGCTCATGTACACGGTATG
CTGGGGGGGATTGACCCCTGTTCATGTTCCTACTAGCTCAGGAGACACTGCCCTATGTGC
AAGGCCCTATTGAGTCATTACTGCTCATGAAACTTCACCTGGGCTGGAGGCCAGACAAAA
AATTCAAGAAAACACCGGGCATCACAGCAACCGCTTCAGATAGGTATACTGGATCTTGCA
CCATTGGCGTTCATGCAATCTGAGTGCAATAGTCGCCGTCACCAACGTCACCGCTTCCAC
CAGCCTCGCATCCCGGCACCAACGTGCTGCTGGATGAACGTTGGAAACACCGCAAACCCT
CAGCACACTGGGCATCGTTGTCGCAATCGCCTTGGCACTTCTGGCAGGGGCTCTCCTCTG
TGCAACCACTGTCGCCACTGTACTGAAGGGCGAAGTGAGCGCAATAGTCGGTACTTCCAC
TGTCACCACTGCCGCCAGCCTCACATCCGGGAACCAATGAGCTGGACGAAGAGCGCTGGA
AGCAGCGCAAATTCCCGGCGCACTGAGCATCATGATCGCAGTCGCCCTCGCACTCTCGGC
ATGGGCGTTCTTCAGTGCAACCGCTGCTACCCAAGTTTTGAAGGGTAAGCTGAGTGCAAT
AGTCGCCT
>at00540
TCTGGAGGCGGACCTCGTAGCGCTCGTGACCTTTCGCGCCAGGTTACCTGGCAGCAATCG
CCAGGTGACCTGGCAGCAGTCATGCCGTCGCAAGGTGCAGCGCCTGTCACCAGCCTAGGA
GAGAGAGGCTACAAGGATGGAAGTGCTGGGAGGGCAAGTTGGGTTCATGGTTGGATCCGA
AGTCCGACCTCGCCGCGCTCATCACCGACCCAgGGGGATGTGGCGACAATGTCTTGATTG
TTTGAGCANATTCTAGTACGNAAAGCATCGGCCTGACTGCTTTTTANCGTGCTTTGAAGC
TTTGAGGTATCAAGCAGGTCTGTGGAGACAGCTGATACGCCCGTTaNNTTTTTAGtTCCC
GCTTTGAGTCGCTACCttTTTTTTCGGTATCTAACCTCGCACTGCTGGTCCtNCCCGTCC
CANTGACAGGCTACTTGCCTGGAGATATaCCAAAGAATGACGCCTTACCA
>at00541
CCGTAGCCATTTTGGCTCAAGCAAGCTTGGCGGTGCTCAAGCTCACTGGATGAAGTGGGC
AGCTTGGTCTCACATACATGGTGCGCGCTCGCTCACTTCCGCCACTGCGCCAAGCCGTCC
TAACGGCGCGGCGATTTGAGACAAGGCTTCCCCAGGAGTCGTCTGTTGAAGCGCTGCACT
TGTCTTCAACTTGGCAGGTCTCCGAAGCATACAGGCAGAGGGAGGCAGAGTCCCGCTTGC
ATATGCCTGTTGAGCAGGACGATTTCCAGAGGAGGGTGGAATCTCGTCAGCTTTCAGTGA
GAACAGGACG
>at00542
GCGGACGACCTCTGGTCTCTGGGCTTCGTGCTCCTCGAGCTCTGCGCCGGCCACTTGCCC
TGGAACTTCTACAAGAGCCAGGCGCAGGCCGGCCTGGGCCTGCAGCGAAGGCCCAGGCCA
AGCAGCGCGTCCTCGAGGAGAAGCTGAAGGTCGTGTCCATGGCGCGGCGGGCCGACCGCA
AGCCCTCGGGCTTCCTGCGGAGCGTTCCGGCGCCCATCGTCGAGTTCCTGCGGGCGGTCG
ACAAGCTCAGCTACGCGGACGTTCCCGACTACGACTTCTTGGTGTCCCGGCTCCGGAGCA
TCGGCACGGAGGAGGCGCGGAGGCAGGCCGCCAAGCGCGAGCTCCTCCCCTCCGACACGA
TCTGTGGCTTCAGCCTCCGCGACGTCATGCGGCTCCCGGGAGGCCCGACGCCGCGCAGGC
CGCCAGAAGTCGTCCGCGAAGAGAGGCGAAGGCTGCCGACGGGAGGAGGCCACCACGGCG
GACACGCGCGACTCGAA
>at00543
TTTTTTTTTTTTTTTTTACATCTGCGACCATCCATCAAGGGGGCATCCCAATGCTGAAG
TACAGCGCTCGTGCAGCTCTGAAGAGGATGCCTGCAGCTCACCGCCTCGGGCCTTGCTCA
CTCCAGCACGAGCTTGATGATGGGCGAGATGGTGAAGCGCACCGGCTCGCTGCGTCTGGT
GGCGCTGCCGGAGGTCTTGGCGCCCGGCCGGGGAGAGTCGGGCACCGCCTCCACGGACGT
CCACTGATCCGGCCTTGGAGGAGTTGGGCTGCGGCTCAACCAAGGCAACAACGGATTGTC
CGGAAGTATGGCTGAGACTGGAGGTAAGGAGTCCAGCGCATCGTCTGGTTCAGGACTTGG
CGACTTCAGGTTGATGGGTCCGTCGGACTCCTCCGCAGATTGGCTGCTTGACACCTCGAC

CAAATCCGGTGGAGGCGAGGGTGGGGCCTCTGGTGGAGGACTGGCACTCCGATCTGACTC
GCGAGGAGGGCTGGCACTCCGATCTGACTCGCGAGGAGGGCTGGCACTCCGATCTGACTC
GCGAGGAGGGCTGGCACTCCGCTCTGATTCAGGAGGAGGGCTGCGACTCCGCTCTGATTC
AGGAGGAGGACTGCGACTCCGCTCTGATGCCTGAGGAGGACTGCGACCCTCGTGCCGAAT
TCT
>at00544
TTTTTTTTTTTTTTGTTTGTGCGATATCACTCACTGCTCATGATGAAGCTTTGCAATGCA
TTCGCGAGAGAAACCATGCATCACGAAGCCCATCTGCTTGTGAGATTGACATGCCTGAAA
GCCTGCGAGCTCCACTCTTCCGGCTCATAACAGATACAACAGGCAGATGAGATCCACTCC
TCCATTCCAGATGCTCGGTGAAACATGCCAAGCCGCCATGGAGAAGTGAAGGTCTGACCA
ACATCATCCAGAGCAAGGATGCTATTACCATAACTCTCATGTCAGGCTGGAGAGGACGTC
TTGGAATCTCAGGTAAATGCACACCTGGACTCCAAAGTAGTTCACACCCTGGCTGCACTC
TCGACGTTTGATAAGTCGACGTCCTTTTCTGAAGAGCTCCACTCTTCGAACATGGTACAC
AAGAAGAGCTGCTCTCTTCGAACGACTGTACACAAGACGAGCAACACACATCCACACATC
ACAGTGACCATCATCGCAGGCTGGCAAAGCCTTCTTCGACAAAGGGTGGCGAGGCCTTCT
CGGAAATCTCAGCTAAATACAGACCCGGATTCCCAGAGGTTCACACCATGCCTTGGCTCT
CAACGTTTCAAACGTCGATGCCCTCTCTGAAGAGCTCCACTCTTTGGAATATGGTACGCA
AGAAGAGCTCCGCTCTTCGAGCCAAA
>at00545
CGCAGTAGACGCGGTGGGAGGCACCGTGACGAACTGCACCGACTTCCTGCCCGCGGCAAG
TGGTCCTGCACCTGCAGTGGAGTGGCTCCGCGTGCGGCTGTCGGCTGAGCCAGAGAACCA
CTTGCGCTTGGCGCTGACGTTCCGCTACGTGCAGGCTCGGGTCCGCCGATTCTTTGGTAT
TCCGCTGGGTGACAGGCGCATCACCCTGACGCTACCAGTGCCAGGGCCGCTGCTGACCCG
TGTGATTTCGTTCTTCACCAGAAGGCCCCCGCCACGACCATATTTCGACATCCTCTACAT
AGATGACCAGCTTCGGGTGCATCGCACCGGCGAGGGGAATCTCTTTGTGCAGCGACGTGC
AGGAGCCGAGCCTGTTGCCTGAGAAGGGCCATTCGTGCTGGTTAGACCGTTTGCAGCGCT
GCAGAGATTGGCACGAGTGGACATAG
>at00546
TTTTTTTTTTTTTTTTTTAATCCGCCCCACCCTGATGCAATGAACACGTGGGGGCAGTGT
GAAATATCAGAGCCTCGAAGCGAGAACCTGAACTGTCAGAGCCTCGAAGCGAGAACCTGG
TCTCGTAGACGTCGTAGACTTGTCAAATTCCGCTAAGCTAAGTGAGGTCGCTCAGTCCAT
GGGGTTGAAGATGCATCGGGTCAGGGTACCATCCTCGCGCTGGGCGACCCCCACGACCAT
GTTCACGGTCATGGCCTCCTCCCCCCCGGGCCCCGTCAGCACCGCCACCAGCAGGCCGTT
GACCTGCGCCCGGATGTCGTTGGGCACCCCTGACACGTCGAACCGCGGGCATGTGAAAGC
CATCGCATTGTGCCCAGGGACATAGTCGGTGAGGGGGGTGTTGCCAACTTCTATGATCAT
CCGCCGTTTCGACGTGTCGACCACGTAAGTCACCCTCCAGAAGTATCCGCTCAGGGGCGT
GTCAGTGCCTCGTGCC
>at00547
TTTTTTTTTTTTTTTTGTATTCCCCCCTACGACACATGCCTTCAGCAAAGTGCGGGTGCC
AAAGTGGCTGCCAGAGTACATGTCTGCGGCATGTCAGCCATGAAAGCAGATCTGTGACTA
CACCAACATCGCCACGCCAGCGGGCTCACAAATTGAAGAGCTCGTCAAAGCTGCAGACTT
CCACATTGGACACATTCAAGGCCGTGGCAGCATCTGGCATGGATGTTGACGTTGCAGGAG
CGCCAGGGCTGTGCGTGTGTTGCCCTACCTCCCGCACTCTGGGCTCACTGCTCCGGCGTC
CGACGGTTTCACTTGCAGAGAAGGTAGCCTTGGCGCCGGCCTCTCGAGCCTTGACCTCAT
CGTGCGGTGTGCTCGATGCGGAGGTAGAGGGCTCTGAAGCTGTGTGCCTTGTCCTCAGGC
GTCTTGCAGAGGTCCTTTGGCCCCCCGCTAGCTGCGCAGCCTTGCAAGCTATCGCGGGCC
CAATCACGAGCCACCTCCAGTACTGCAGCAGCTTCCGCATCGGTGTCTGCCCGTTGCAGA
TCCCGGTGTAATGCAGGATCCTCATTGTCAACTCGTCAGTTTCGGGCTTTCCTCCTTGGA
GTTCGAGCCTGCCATGTCTGCTTGACCCGTGCTTTGGGAGTCATCCCTGGTCACCAGGCA
ATTCTTGCTGCTGCCACAGGAGCTGCGGCCAGCGTCAGGCATGGCATCCCATGTGTGTCC
GGCGCGATGCGGACTGCAAATTTGGACAGTGCTGCTAGGGTTGCGTCTAGCAGCCACCTG
ATTCTGGGCTGCACAGTTCTTTGGCTCCAATAGACCATCATCTTCCGCGTCGTCTTGTGG
AT
>at00548
CCTGAAGCTCCACAAGTCCAAGCTCCAGGGCCGGAAGCTCAACGTCGAGCGCACCGCCCG

GGGCGGCGGCAACAGCGACGCGCGCAAGGACGAGCTGGATAACCTGCGCTCCTCGAGGGA
CAGCGCGCGCATCGAAGACATGAGGAAGATTTCTTCAAGGCGGTGAAGGCGGTGAAGAA
GGCGGCGAGGGAGGAGGCCAGCGAGGCCCCGGCCGGCGAGGACGAGGCCGAGGCCGGCGG
GGACGGAGGCAAGCCGCCCCCGCGCAAGGGCCTCCTGCGCTCGGATGCGGACGACGGCGT
GCTGGAGCTCCTGGGGACGGTGCCGGAGAAGGTCGCACTTCAGAAGCTGCTGGAGTTCGC
TTCTCAGCCAATGATCAAGCCAATACACAATCGCCCCGCCTTCCTCATGACCACCTTGCG
CAAGCTCCTGCAGGGCGCAAAGACAGATCCTGAAGAGAAGGACCAAAAGGTGTGATGTGT
GTACGCACAGTGGGCTTCTGCTTGTCTCTCCTTGCGTAA
>at00549
TTGTTTTTTTTTTCTTTTTTTTTTGTTACGATACTGGCGCCCACTCCGAGCCGCTCCCGT
CCAGGGCCTCCCCATCCCCACGCCGCCGGCGCTTGACTCAGTCCTCCTCGCCGGTGGCCG
CCTGGACGGCCACGGTGTCCAGCTCGCGCTGGCCCTCCTTGGTGATGCAGCGGCCGCCAT
TCTCGTGCTGCTCGACGAGGCCCATCTCCTCCAGCTGCTGCAGGACGTAGCGGCAGATCT
TCCCGGAGCCCCTCGCGAACTTCTTCGTGCAGGTGCCGCGGCGGTGCTGGGAGCCGTAGA
TCTTGCGGAAGCCACCGACGCCAGTGCCGCCCTTGATGTAGAGCTTCCTGACCATGGAGG
CCGTCCGCACGTAGAGCCAGTCGGGGTTGTAGGGCGCGAGCTCCTTGGACTTGCCGGTCT
TCACGACGTCGGCCCACTTGGGGATCTCGAACCTGCCCTGACGCTTCAGATGTTGCGAGA
AGGCCTTGATGAATGTGGCGTCGTCGATGTCCTTGACTGTGGTGATGGTCTGGTTCTCCT
TGAACATGTCGTCGTAGAGAGAGCAGGCCATTCCGAAGAGAGCCGCGAGGAACTGCCGCA
GGTGCGAGGAGGGGGGGCTGGATTGGCAGGCACCGAGCCCTGACGGCTTGAGCCAAAATG
GCTACGG
>at00550
TTTTTTTTTTTTTTTTTTCCAGCAACTATCTCTTACCCGCCATTTGGTCTGGGACTAAAG
TCTATACTGCCGCGGCTCACAGTCATGGCCAGGATCTCATCCTCCGTTAGCGCCTTGTAC
ATCGCAGACTTTCGGCCCGCGACGCCGTTGACGCGCAGGATGGCGTCACCGTCCCTGATG
GCGCGGTCGGGGTGGGCCATGTTCCAGTCAGCTATGAGGCCCTCCGGCTTCACCGTCTGT
ATTCTCAACTGGGGCCTCCTTGGAGTGGCCGCAGATGTCTATGCCCAGAGTCCATTCGGA
GGTTGTACGCACCAGGAGGACCTTGAAGGTCGGCAAGGTGACGTTCTTATTCTTCCGGAC
GACCTGGTGCGCCTTCTCCTCCTTGAAGCTCAGAGGCCGGCCCACCGGCGGGAAGGCGTG
CCTCNNGCCTCGCTGCTGACATTCTTCTCGTAGCAGCACTTCGCAAAGTCCATCGGCGTC
CCTCCCCCCAGCGCATGCGTGCGGGGTAAGCCGACCAAATCTCCGCCCCCATACGGGGGG
GGAGTGGGCAG
>at00551
TTGTTTTTTTTCTTTTTTTTTCTTGTCCTGTCCTCGCTGTTGCCCTCTTTCGTGCCCTC
TTTCCTATCATCGTGTTTGTCTTGATCGGTGGCATCACCTTTGATCCCCAACAACGATCT
CCCTGACTTCTCTTTCAACACATCTGTCCGAGACTTCACCTTCAATATGTCAGGTGTGTG
CGACTCCTCCTTGGCACGCAAGGTGTAGAGCACGCACGCAGTGCACGAGGCTGCAAGGCT
TGCGAGCCCGAGGATGCCGTGCCTATTCACGGCCTCCTCCGAAGGCGGCCACGCGAAGAT
GGTCGACCTGCCGATCTGGGCCCCCAGAGCGGTGCCGATGGCCGCCATTGTCGTCTGCGG
AGCGGCTCTGGGGGTGGGGACGCGGGCGGCACCTTGAGCCAAAATGGCTACGG
>at00552
CGTAGCCATTTGGCTCAAGGTGTCGGCCCCTCCCCAAGCCCCTCCTGCTCGAAGACTGC
CTCCGCTTAGTGACCATGGGCGTGTCCGCCGAGCTCCTCTGGGAGTGCGTGAAGGACTCC
TCCTCCTTCATCCGCAAGAGCCGAAACGCCCCCGTCATGACCGCAGAGCCCAACAACCTC
TGCGGCCTCAACAGCTTCAAGTACAGCGGTCTCGCAGGCAGGAGGCCGCTCGACGTGAGC
GTGCAGACGACGGGCAAGAAGCAGACGATCATGCTCATGAAGAAGCCGAAGAGCCGCAAG
GCCCTGCGGAGACCGAAGATGTTCGTCGGCACCGGCATCAAGAAGGAGACCAAGAAGGGC
GTTGAGGCCATCGCTAAGGAGACCGACGGCGTCTTCTACCGGAAGGACTTGAAGGACCTG
GCCGTGCGGAAGTACCTGAAGATCAAGAGGTCCTTCAGGAAGAACAAGGGCGTCCCTGCG
CTCAGGAAGGCCAAGGCCGCCTCGAGCTGAGCCGGCTAGGCGCTGCCGCATGGAAGGAGA
CGTGTGCTGTAGGGAGCCGCGGGCTGAACAAAAAAAAAGAAAAAAAAAAACAA
>at00553
TTTTTTTTTTTTTTTTTTCATAAGCCACGCCCCATGACAACCTCCACTGGGGTGGGTCAC
GGAGAGGGGGAGAGCCAAGAGTCGATGGACTAAGATGCAACAAAGTAGTTGGGCCCGCAT
TACGACGAGGAGCTCCATGTGCCAATGCGGCAGAGGTGCTCGTCGACTCACAGAACGCAA

TTACTGTACATCCCGCACAAATAAATTGACACTCCGAATTAAATGCCACGCGGGAGTGGC
TGGCGCACCTGTACAACCTAACATTGTCCAAGCAATTGCCTTCCCAATGCCGGTGGATCC
AAGAGCTGCCAGTGTTCTAAGGTGTACATGGCTTGCACTGGAGTGACCTCAGTGTGGCGG
TCGAGCATGCGATACCGGCGAACCATCGAAGCAGGCACCGCCTTGGTGTCGCCGAGCTGC
GACCAGCCTTGCCCCTGCATGCGGTGTGCACAGCAGCCTCGGTCATGGCTNNNNNNNNNN
NNNNNNNNNNNNACGCACGAAGGGCATGTGGTCCGGGTTGCGGATCCGGCGGTAGCGCCG
CGTGCACCACTGNNCGTGTTGTCCGTGAGGCCCTGCACTGACGCGGGCGAGACCCGAAGG
CACGCACTGGAGAG
>at00554
TTGTTTTTTTTTTCTTTTTTTTTGGTCAGATAGTGACTTGTTGTCCGCTTCTAAATCAAC
TATTTAATGAGCATGAGGCAAACCCTCAGGCTAGACAGTCAAAAAGTTCAGCGCACTAAA
AGTATGGCTCGTGGCAGCAGCAAGCTTCACTGGCTCACTTCAGCTCCGTCGATGCAGCAA
AGTCCATGCCGCTTTGGATCTCCTCTTTCAAGACTGCGACCGCTTCGGCCAACCGTCCCT
GCTCATACTGGTTCAGCTCGCCGATGCCGTGGACCTTCTCGACGCCCTTCGTGCCGAAGG
TCACCTTGCTCGTGAAGTAGGGCACCTCCGGGACCACGTCCGACAGCACGTACGCGCACT
CCGTGGAGGGCGTTCCGTTCAGGCCCGCCAGGATCGCCTTCCCCAGCCTGGCCCCGGCAA
AGGCCATGGAGAGCGTCGCGCTGCCCTTGCCGTTCTTCGCGTTCACCACATCGGTCCCAG
CGTCCTGAACGTGCTTGTCCAGAGCAGGTATGTCGGACTCCGGGATCTTGGCCGCGTGCT
TGTCCTGGGAGAAGATTGGCGTGATGGTCACACCGGCGTGGCCTCCGATCACCGGCACGT
CGACGTCGTCAGGATCAGCGCCTGTGATCTCGCTGACGAACTTGTTCGCCCTGACGACGT
CCAGCGGTGGTGATGCCCACGATCTTCATGGGATCCAGGCCCTTCTTCCGATACAGCTCT
GCCATGGCGGGGACCACGGAGTTGACTGGGTTCACGATGAGGGCAACCACCGCCTCTGGG
CAGTGGTTCGCGCAGGCCTCGACCACCTCCTTGGCAATGCCCGCGTTGACCTTGAAGAGG
TCATCGCGCGTCTGCCCCGGCTTCCTGGGCATGCCGGCGGGCACGAGCACGAGGCTGCAG
CCGCTGAGGCAGTCGCCCAGCTTGTCGGTGGCCGTCTCCTCAAGGCTCTTCATGTAGCCC
GTGACCTTCACCTTCTTCGCGATGTGGTTGAGGTCGGCGGCGACTCCGGCCGGCGGCACC
ATCGCGATGCTGAGATCGTAGACGGAGAGCTCACCGACGTTGTCGTCGAGGGCCAAGAGC
AGAGACAAGGGCTGGCCAATGCCACCTGAGCCGCCGCACACGCAGACCTTGAAGTTGCCC
TTCTTCGCTGAACTCGCCATGAAGGCAGCTGCGACGAGCACCTCGATCAAAATCGCTTCT
TGAGCCAAAATGGCTACGG
>at00555
TTGTTTTTTTTCTTTTTTTTTTGGACCGTGGCAAACCATCGACATGCACCGCTCAAAGCA
CACCAATCGCTGGTTGCTTTTGCAAGTCATTAGTGGAGGCGCGCGCGGATTGCCTTCTGT
GCGCCCTCAGAACATTGCATAGCCCACGGTGGCTCACCCCCGCATGCCGGCCTTCCGTGC
CTGCTCGCCCTCTTGCATCATCGCATCCTGCTCCGATATGTGGTGGAGGACAGCGCGCTT
GCTGGTCTCCTGCAGCTCCCCCGAGATCATTATCTCGTCGAGGATGTAGTACGCCTTGTG
GAAGTTGAAGATCAGGTCCAGCTCGCACACGTTACCGAAGTAGCGGTCCAGACACTCGAC
AAAGTGATGCACCGACTCCAAGGCCAGCAACTCGTTGTCAGTGTTTTCGATACATGCAAT
GAAGAACAAGCTTGCGTAGCGCTTGTAGCAGAGCTTATAGTCACGCCACTCCAGGAAGTT
GCAGACCTTTGGTGAGCGGCTGAGGATCAAGCTGCTCACCTCCTTGACTGTCCGAAACTG
GTCACGCTCCAAGCATGGTACAAACCACTGCTTCAGCCGTGTCTTCCCCTGCCGGCTGAT
GAGCAGCACGAACTGGATCATCAGACGCACAAGATGCCCTCTGCTTGGAGATAAGACTCA
ATCGGAAGGCGGGCTCTGTACTGGGGCAAGACCCCTGAACCGAGAAG
>at00556
TTTTTTTTTTTTTTTTCTCTTTGGACTTGCAAGCGCCGGAGCCAGTCCTCCTCCCCACTT
ACTGCCCCATGCGCAGTCACCCCATGAACAGACCCTCAGCGCTGTTCGAGCTGCCCAGGC
ACAGCCGATCAGAGGGCCGTGTTCGGTGCGAAGAGGTGGCGAAGCTGCGGCATGTGCGCC
ATCTTCACGGCCTCATCCCACAGCTCCACGTCCACCTCGCCCCCAGGCAAGGGCACAACC
CCCGCCGCAGCCAGCACGCCTTCGCCCGCCTTCGTGCCCACGACCTCGACGAAGCCGCCA
GTCTGCGGCAGCTCGCACTCCAGAGCCAGCGCCAGGGAGCCGCCGTCCGTCGTGCACNAG
GACGCGCCGCTGCGGGTCGTCCGGGGACGGGCGAGAGGGCGGCGCCCACGCGCACGGCGG
CGCCCTGTGGCGTCGGCTGGGAA
>at00557
TTTTTTTTTTTTTTTTTTGCTTTCGAAGAGCATGAACAAGTGCAATCAAGGTGCACGATG
GACACAAGAACCAAAATTGTCCAGGCGCAAGGCCCAACGCGCACTTGCCCAGCTCCATTC

CCGCCTCACTCAAGGCCAACCCTGCGCTTGCGGTCACGCCTGCAGAGCCAGCGCCCACTG
AGCGGTGTCCAGACGGAGGACCAAGGACAGCAGCGCCTGGCCGCACCGCCACCCCGCGCC
TCCCTCGCCCCTCCCCTCCGCGGCAGAGTGCCCGTGGTCGACCCCGGAGCCGGTCCGCTC
TTGGAGGGAGGCGGCACGGGCTCGGCGCGCGCTGCCCTGGCCGCTCCGTCGCAAGAGGGC
CTCGGCGGCCGTCGTGCCACAACGGCCCTTGCCAGCTGAGAGCGCACGGCGGCTCAGGCA
ATGACCATCAGTGGGAAGACCTCCAAGCACCGCTCCTTGAACTTGGTCCAGGCGGGCGAG
TCCTTCCCGTGCTCCTCTGCGAGGTACGCGAGCATCTTGTTGCAGTCCTTCAGCGCGCAG
GCGCCCTGCGCGGACCAGCCCGCCAGGAAGTCNCCTCGTGCC
>at00558
CCGTAGCCATTTTGGCTCAAGCCCACAGCGATTTGGGCTCAAGATCTTTGGCGTCGTGCA
GTTTGCCCTGGCCTCTTTCCAGTACTCAGCTTGCTAGATGATCTCCTCGGCGGCTCTCAT
CCTGTGGCTGGCCGTTCCAGCTGCAGGAAGTGGTTCCGACATGACCAGCACAGTGATGGG
CATGGCCACCAAAATGATGTCTGGCAGTCATGAGCGGTGCCTTGGCCCGACTTGCTGCAA
ACAGTCCACTTGCTACAGGGGCCCAGGCGTGGGTTGCAACAGTGCCCGCGGGAAGACCAA
GTGCATCGGTGCAGGTCTTTTCCCACCCACCAAAGGCATGTGCAGTTGCGAGATTGGCAT
CTGCGGGGACGATGGGAAGTGTCCTGATGCTCCAGGGAGGTCGTCGCAATCGAGCTAT
>at00559
GGTTCGACGACGTCGAGGCCAAGTTCCACAGGGACGAGCTTTAGATCTAAACCAGCAGAG
CCTTTTGCCATTGCACGTATTTCGCGCTCTGGCGATTCCTCCGACGACACGTTAGTGCCA
GCCAATGCCGCGCAGGACAGCACAGCAGCTCACAGAACAGTGCACTTTCTGGCATGCTTG
CCATAGGCCTTGGTCACCCCCAGCTTGGGTGTCCCGTGCGCATAGAGACTGAAGGTCTGC
AGTTTTTCTTGTACAGGCCTCAAGCATCTTGTGCAGACGAGGCGCGGCGTTGCCTATAGC
AG
>at00560
AGTTCCCCACGCTGGTGCCCACGCTGATGAGGACGATGCTGAGGCCGCCGATGAGCTGCT
GCTGCTGCGCCTGGATCTTGCCCATCTTCACGACCTCCGCGTCCGCCGCCACCGTGTAGT
CGTCGATCCTCCTCGTCAGCTCCACCTTCGCCGCCTTGAGCAGCTTCAGCAGGTCCTCGG
GGGGGTCGTCGAAGTGGTTGATGTCTTGCAGCTGCCGGGCCACGGCGACGTTCCTCCGGA
TCACCTTGTTGGAGAAGATCTGCTCAAGGATCAGGCGTCCCCGCTCCGTCTGCATCCGGC
CGTAGCCCATGTAGAGCGTGAGCCACGTGTCCGTGAGTTCCGTCCAGGTGGTCTTCAGCA
CCGGCTGGT
>at00561
CCGTAGCCATTTTGGCTCAAGCCATTTCGGCTCCAGCGTGAGTTGTTCTTGCCAGTTGCA
GCGTGCATCCGGAACAAAGTTCTGCGCAAGGCAGCGAGTGTTCCGCCAGGCCCTCATCCT
CGTGGCTCTCGCCGTGGCTCCCGTTGCGGCCTTCGTGCCACCGGGAGCGCAGCCGGTGAG
CGCACCCCGTGGCCTTGGTGCACGCCTTCCGGTCGTGGCAGGTGAGGCCTCTGACGCCTA
CGCATCTCAGACTGATGCAACAATGCCGTGGCCTGGTTGCCTGCTTGCAGGTCTTGGCCT
CGGCTACGCTGCTGCGGCCGCTGGCGCCTTCACGCGCCAGTCGCGCACAGCCTGTGCTGC
GGAGGCAAAGCCCAAGGTTGCCGCGGCCAGGACACCTGTGGCCTATCCAATCTTCACCTT
CCGCTGGCTTGCCGTGCACGCAATCACGATCCCAACGGTCTTCTTCCTTGGCGCAATCAG
CTCGATGCAGTTCATCCAGCGTTGAGCAGGTGGGCTCGCGTGAAGCAACGCGTGCTGCGG
GATGGCAGGTTTTTGATTCGGGATCGGTCGAAGAGAGGTTTCCTCGTGCACCATCAGGAT
TTGCTACGACATGCAGCTGCCATTGAGTGTTAGAGCATTGCAACCAAAAAAAGAAAAAAA
AAACAAA
>at00562
GTCCTGGCCCCTTGGTCGTTTTGCTAGGGATCCTCTGCTTCGTGCTGCAGTACTATCTCA
GTGGCTTACTCTTAGGCCCCTTGGGCGTTCTGGGCCCCAATCTGGCTTGGTGGCTATCCA
GTGGAGCAGGGCTTCTGCTGTGGGTTGCAGCCGTGGCACATTGGTACTTCTTTGATGGCA
CCCAAACAGGGTTCTTGGTATCGACTCTCACTGCAATCGGAGGTCCAGCCATCGAGATAG
CACTTCTGAACGCCCCTGGCTGGGACTTGTATGCATATGCATCAGCGGACATCTTCGGCT
TCCCGTCTTGGGCCATTGCGGTTTACTTCTGTGGTGGTCCAGCAGTTGGAAACTTGGCAC
GTGCCGCACTTCAGTTTCTGGATGGTGCTGTGAACAAAAAAAAAAAAAAAAAANAAAAAAA
AAANAAAAAAAAAAAANAAAAAAAAAAN
>at00563
TTGTTTTTTTTTCTTTTTTTGTTGCTGATCAGCTGTTGGCCACACAGATGTGGAAGCAAA

ATGCCAGTGAAGTCCAAGTGCCCTGCCCATGCAAGACACAGCATCCACCGACGGAAAACT
CTTCTGTCGCACCTGTACATGTGCATCCTGTACATGTAGCATTTACACCGACCACAGTGT
GGCTGTCATGAGTTGTCAACTGGCGACGAGACGACCAAGCAAGTTGGGGCCCGTGGCAAA
GACAGCATCGGGGACAATGGCGGCAACAACGGCCGACCGTCTATCTGGCCTCATCACTTC
AGGGCAACAAGACTGGAGGTGGTCCCTTCACAGCCTGCTGTTGCGATGTATGCTGACATC
CACTTGCGCGCCCATCTTTCTCAGAGCATCTGCCAAG
>at00564
TTTTTTTTTTTTTTTTTTCAACGCTCACACGTCGTGAACCGCCTGGACTGGGGCATCCTC
CTCTCCAGAGCTCCGGTAGAAAATAACAGAGTACCGCTCTCCAACGTAACCGGATACCCA
ATGCGGGAACCTCCCATCGAAGCACGCCAAGCGACCGCGCGTCTCGATGGCGCGCACCAC
AAACGCACTCTCCTCCACGCAGAGCTTGCCCCCGCCCTCGTCGAAGTCGCCCAGCGATAG
CGCGTACTGGACCGAAAGGTCGTTCTTGTCCACGTGGGGGCTCCCGCGGAAGTTCTTGGA
GACGGCTATCGCCGTGAAGTGAAAGCCGGCTGCCTTGGCGGAGGCGGCCCGGAGCAGCGC
CTCGGACAGCTCCCAAATTCTTCGGCGCACAGCCCGGGGGTCTCCTTCGCGCCATCGGGG
CTGCACCTTCAGCTCGTAGGGCCGCTTCAGCACAGCGTAGCCCGAAGCGTGAACGGCCGG
TCTTACGTTCTCCTTCCAGTCCATCATCCGCAAAGCCTCCAGCAACTGCTCCGTGGCATG
CTCCGGCAGTGGAACGCCCGCGTCATGGACAACCTTGCGAGGCTGCGAGTCGGCCCTTGC
CGCGTATCCTGCGCAGAGGGCATCCANAAGCTCCACTCTGCGAGCTGCTTTGAACCCTCG
AAC
>at00565
TTGTTTTTTTTTTTCTTTTTTTTTTTCGAGCACGCCCTCAACGCGTTCAGTTCCCGAGGA
CGCTAGCGCCGGATCCTAGCGTGACAGCTGTGGGTAGGATGGTCACGAGATCCTCGGAAC
TCCCCGGGTGAACATCGCTCAAGCTACATGATCGTCGCTGTCATCATCCTGGAGGCCGTC
TCTGGCCTACCAAATGTGCCTAGTGCCGCTTCTTCGCACGTGCACCATTGGTGGCTGCCC
ATCAGTTGAAGTCGACAGTCCTGGACAAACTATCCCTATCTGCTGCTCTCCAGAGTTCCG
GGGAGTCTGGCACACCACAGAAAACTGCGCGTGCAGGCCTGCCGCATCCTGAGGTACCAG
GGTTATCATCTGTAACAGATGAAGGACGCAAAAAACCATTTCTTCGCATGGCACTTGCTT
TGAGCAACACTCATTGCGGCTTTCTGAAACGTGGTCTGTCACCGATCAACGAGCCGCTCA
ATCACCATCTGCAACGTATCGTCCCTCTTGCACACCTCGGCTAGCTCCTGCGCATTGCCG
GAGACTCCGTTGACTATAATAACCCGGTCATCCTTTAACACCTCCTTACCTGGGTTGTTC
TTGTTCCACTCGCCTATGAGGCCGTCATTGACCTTGTCAATCACGAGGGCAACGCCATCG
GAAAGATCAACATCAACGCCCAAACGAGGACCACCTTGAGCTTTACTCACCATGACTTGG
AACTCGACCTTCCCGGTGCCGGGCTGCTGGGCTGTGGCAGCTGGGGCTGCAGTACCCTTG
ACCATCTCCTCATCAGTTGGCAGGTTCTTTGGGCGCATGGCACTGGCATCAGCTCCATTG
TCTTTGGCCTCTTGCCCGGCACAGCACTTGGCCCCCATGTCGGCCACCGAGTCGCAGAAC
TTGAAGTGAAAGCGGGAGTTGCCCAGATCCGGGAGTGCCGGGATGCAGGCTTGCACTGGC
AGTGTCCCGACACTTGGTTCGAGCCAACCGCCTTGAGCCAAAATGGCTACGG
>at00566
TTTTTTTTTTTTTTTTTTCAGCCTTCAAATCCATCTCTCTAAGCGCGCACCGCCAATGCG
CTGTACATTAGGAGGCACCCATACGGCGCCAAGCCCACGTGCATCAGACCATGCCCCCCA
TCCGTCAAGCAAGTGGTCGGCCACGAAGAAGAATGTGAAGAGCCCAGCGCAGGCGATGCA
AGCGCACAGTCGCCAGAAGTCACCGCGTGCAGCCATGGGGGCCAAGTACATAATGATCCC
AATGCCCACGCGTTGGACGCGATGGTGTCTCAACTCTGTGGCACTCGGGGCCCAGAGTCG
CACGACTGCATACACGACGAAAGGCAGGCACGACGCAGATCGACGTACACCGCCGACTGG
GACAGGGCCCATGCAGAAGTGATGCAGCCAATGAAGATGAAAGACTGGTCCAAGCATCGC
CAAGGTCGAAGATCCATGCCTGGCCGGTAGATGCTCAGGGCGCAGGCGGAGTGGTAAGAC
ACAGAGGCGACCAGGTGGGCGGCAAAGCCGGCCAGCAGAACCCATCCGCAAGTAAAGCAA
CTCCGCGCTCCAGAGCTCTGCCTCTCGTGCAGGACGGCAAACGATGCCAAGGTCGGCAAG
AACATGGTCACCGCTGAAAATATCTCACGCACCACTAGCCCGGTGCCCCCTTCACCAGGG
CCCTTGAGCAGAGGCGCCTTCAGTGCCTCCGCCTCTGATGCATTGCAAAGAGAGGGTCCG
TCTCCATCCTGGGCGTTGAACTCCTCGATGACACGT
>at00567
CTGTTCCTCCTGCTTTCTCTTGAGCTCTGCCTCCTCGGCAGCTTTCCGCTTGGCCTCCTC
CTCAGCGGCGTTCTGCTTTTTGAGCGCAGCCTCCTCTGCTGCATGCTGCTCCACCTCCTT
CACCTCCTCCTTTGTCGCTGCCTGGGGCTCAGCCTGCTCTGCCTTGGCGCCAGGTGCCTC

TGCAGGGTCCTTCGCCGAAGCCGCAAGCCCAGCTAGAAAGGCACGCACGGCCTCTTTGCC
TCGCTCGGTTGCCAGCTGCAGT
>at00568
TTGTTTTTTTTTCTTTTTTTTTTGTTGAGTGTTGACGGTGGAGCAGATCTACTGCATGGT
GTTCAGGACCTGCTCGTAGAAGATGTCGCTAGTAATTGGTGCCGCTGTGGGCACAGGTGT
AGCGGCTGGATCTGCGTGAGGAAGGGCATGAGGAAATGCAGGTATTGGAGGCACTGGGGC
AACGGATGCTCCTGAATGTGCAGCTGCGGCGACTGGTAGGATCTGCGGATGAAGCACTCG
CCGATGTCCGAC
>at00569
TTGTTTTTTTTTCTTTTTTTTTTCCGTCTACTTCGCAAATGACGGTGCGGGAAGTTCGGC
CGCCAGTGTCTCAATTGAGCTGAAATCCCAGTCGTTGTACTCTTTCAGCGTGCCGATCAC
TTTATACTTCGATGTGAAGTGCTTGTACCATCCAGCAAGGGACTTCACCTGCAGCTCCGA
AAAGTCGTCAAGCTTGAAGTCCAAGCGGTTTGCATCCTGCGCTTTGAGAGACACGGTTGC
CATCGAAAAGGTCGTGTCCCTCCCGGCCCAGAGCTTGCCGTAGCCTTCTCCGATTTTGAA
GTTGTCACTGGAAGATACGTCGATGACTTTGCCACATACGCCCAAGCACATTGGCAACCG
TACCCCGTTCCACTGCGCCAGAGATTCCAATGTGAAGACACCACCTTCCGGAAGTTGCTC
CGAGTGCTCAGGCATCCATTTTACGGGCTCTTGCTCCTCGGGGCTGCTTGCCGCAGCTTG
CGTTGAGCTTTGCCCAACCTGCGCAGGTGTATCCGCATTGCCACCCTCNGGTGCACGAAA
TACACACTCCAGTACCACCAGCCCTCGCTTTCGCTGCTTGCTTCTTTGTGAAACGTGAAT
TGGGCTTCTCAACTTTACAGGTGCTGCAGACGGGCATGGTTTCTAAACCTCCGCTGTTAC
ACGACGCAAAAAGAGCCAAATGGCTTGAGCCAAAAATGGCTACGG
>at00570
TCCCATGCGCAACTTCGTCCGCTGCTACCGAATATCATGGTTCCTGACGAACGGATGGTA
CTCGCCTGGGCCTCAGGACCAGGCTCCGCGAACTGAACAAGTGGCCGCCGCCGGAGAAGT
ACAAGAAGGACGACTGGCTGGCATGGACGCAGGCAGACGCCGAGGTGAACCGCGTGATCC
TGGACTACTGCCAGAGCATGCGCCAGCAGCTGAAGGACACCATGGACTCTGCCACGGCAG
AGAACTTCCGGAAGTCCAAAGACCCCACGGACCAACTCTTGTGGCACGTACGCGGCGAGG
AGTCCAAGACGTTCAAAGAATGCATAGCCCTGGCTAAGCAGGTGAGAACGTAGGCATGGC
GGGTCCAGCTCCGGGGCATCAGGCTGGACAGTGTAGCCAGAGACTCTCAGCCAGGAAGGT
CAATGACAACCGACAAC
>at00571
TTTTTTTTTTTTTTTTGTCAACACCGAGGGTCAAGAACATTCTGACCAAGATCTATCTGT
ACACGTTCTCTTGCAAAAGAGTGCTCACTACACAAACTGTCATGGACATACCATCTGATG
CCATGCATGCCAAAGTGTAGTGAAGCAATGGGAGAGGCAAGGCTGCACTCTGCTTTCCAC
GCGACATTCACAGCGCGTGGCCAAGACCTAGCGCAAAAGGGAGCATGGCTCTACCTGGCA
TGCAACATCGCCTTCATTAAAGGCCAGCTTCCAATGATGTAAAGCAGTGCGTCAACTGTC
CAAAGCCCCGAACTCAGCGCTGAAGCAAGAAAGGATGTCCAAAGTTTGCCGTATACAACG
ACAACAGCTCCCAAGAGAAAGACCAGATCAGCCCATCCTACCAAGTCAATGGTCCTGCCG
CATACCCGGTACATCAGTGGATCCTTCTCTTCCGATGGCGTGAAAGCACAAAAATGCCAG
TGAGCAACAAGCCCCAGGCACGCGTCTAGCACGAAGAGAAGCGCAGCCGTCCAGTCGCAA
GCCATAACAAATAAGACAGTGTCAATCACTCTGGCGTTTAAGGCACCTACTAACTCGTGG
CCTACCACGTTTGGATCAAACAGCGACTCTAGAAGGTAAGCAATGCTAGGCACAAGGAAG
GAAGACGAATTGACCAGAACTCCCAGTCGACACCATGAAAACGGGCTGCGATGGAAGTGG
ACGGTTGAGGTGCTTCTGGATTCACCGCTGAGCACGCACTTGCGCGAGTCTGTCGCGCCA
GCAAGGCCCCGCCANATGAAATCAACGAGGGAGTCCAGCACAAAGAACACAGCACCCGAG
AAATAGAGCCANGAACATTGCACCGCAATTCCCACTTGGCAGATGGGAACTGCTGCGTTG
TACAAGAGCATTGCCATCAAGAACACCACAGAGCTTGCCACACCACTTCCCAAGAATGAG
ATCCACCAACGGCTGCATCCCTTCACTNGTNTGAAGTCCTGTGTGGTCTCTTGGTCATCG
NGGCTGAAGTCCGTGGCGGCCTCTNCATCAGAANTGCCAGANTGGGANCTTTCTGGGCGG
TTGTCACNAGCCATTNTTCTGGGCANCAAGNGCACAGAATCAAACGCTCANGCAAAAGAT
GACGCTGATG
>at00572
TTGTTTTTTTTTCTTTTTTTTTGTTTTCTTCTTCTGTCACTGATGATCCAAGGTCACGCA
GCTTAAAACAGTCCGCCTCTCTGATGGCACTGTACATCTCGATCATGTTTGAAGTGGCCA
CACGCCCTGCAAAGCGGTCCTGTCACGAGGTGGAGTGATCGACATCCTCTTGCAAGACAA

TCCAGTCATCATCATAGAGTTGTTCGAGGTCTGTCGTGACAACGCATTCTTCTTCGGCAC
TGTCGGCAGGGCGTGCTGCACATGGTGTCTCAATAGGCGCCTTCATTGCCACGGTCGCTG
AAGGCAGCTGTCCAGGCAAACGCACAGATGCGAGCATGCTCCTCAAACGCTCCGATTCTG
GGGAGGTGGCTGCCATCAAGGCTTGTTCACGGGCTGCAGCGGCAACAACATGGAGGCGGC
CCACCTCGTCATGGACTGAGACCTGCCTGCGCCGAGCAGAACAGAGCGCTTGCAAGCGTT
CCCGCTGTTCAGCTGCAGCAGTAGCCGCCGCAGCACATGCCACAGCAGCGTCGTTCACTC
CAGCCCAGCCTGTTGGTGCCATGCTCAATCAAATGCCGAACAAAGCGATATGGACTGGAG
CCTTGAGCCACAGTGGCTTGAGCCAAAATGGCTACGG
>at00573
GCCTCGTCGGCGGCAGCGCCTGCCAACGTCACGATGTAGGTGCCGCCACTCCCGGTGTCG
TCGAGCTGGAAGATGAACGCCACCGCCAGGCTGTTCTGGAGGAAGTCCATCGGCGTCTCG
GACGTCATGAGGATGAGGGGCGTGGACAGGGCGACGACGCTCCGCAGGATCTCGTTGATG
ACGAAGGCCATGGCGCAGCGCGCCGCCAGTGTGGTCCTGCTGATGCTCATTGCCTTCGGC
TCCTTGCCTGCGACGTCCTGCGCCAGGGCGATCTGTGTCCAGTTCTTCAGCAGGTATGCC
CACAGGGACCCGTGGAAAGCGCGCCCCATAGAGCTGTCAGCGCCCCTGCCGAACATGGAC
GCCATCTGCACCGCCACGATCGACCCGAACCAGTATCCATACGACACGGGGTCGTTCTCG
ATGTCCAGCTTCATCAGAGAC
>at00574
NNNNNNNNNNNNNNNNNNNNNGAGAATACGAACCAATGCTCGGAGATGAGCGTTTTTTT
TTTTTTTTTCTTTGTTCCGCCTTGGTCAAGAGACTTGCTGCTCTCTGTCGAATTAGATTG
CGATCCATGATAAATGCTGACTCAACACATTCTACCACTCTGATCACTTTGCCATCATCA
TCTTCACGAACTCCTCGTAGTTGATTTGCCCGTCTCCATCCACGTCGGCCTCTCGGATCA
TCTCGTCAACCTCCTCGTCTGTAAGTTTCTCACCCAGATTGGTCATGACATGCCGCAGTT
CGGCAGCGCTGATGAAGCCATTGCCATCACGGTCAAACACTTTGAAGGCCTCAATGAGCT
CCTCCTCCGTGTCCGTGTCCTTCATCTTCCGGGCCATGAGGGAAAGGAACTCGGGGAAGT
CAATGGTCCCGTTGCCATCAGCATCAACCTCGTTGATCATGTCTTGCAACTCCGCCTCAG
TGGGGTTCTGGCCGAGGGATCGCATCACTGTTCCCAACTCCTTCGTCGTGATGGTGCCAT
CTCCGTCCTTGTCAAATAAGCTGAAGGCCTCCTTGAATTCGGCAATCTGTTCCTCCGTGA
GCTGGTCAGCCATACTTGGTGGGCACAAGGGAAATGGCGAAAATCAGAGGCAGCTGGAAG
CGCGATTGGCACCCTCGTGCGAATTC
>at00575
GGCGTTCTGGTCCACTTCTGAATCTGCCCTGACACTCTCAACTCGCCCGCTGTACAGAGT
GGCGTGCTATGCCCAAATCAAAGAGGCAATGCCCGAGGAGACGCGCTCCGCCCCGTCATC
GCAAAAAAACATGCAAGCTCGCATTGCCGGGAAGGTGATGCTGATGTCAGACAATGCGCT
TCGTGGTAAGAGCACCTCGGGCAGAACGCCCATGAATCCACGTGGCAAGTGGAGACCGCA
AGACGCTGCTGACCGCTCAGGCCGACTTGGCTCTGAGGGGAGAGTCGGTGTACAGAGCCC
AGTCACCCCAGGCAGAGCCAGTGAGCCCGTCCTGGAAGAACATCCCGATGATCGCCATCA
TGGCCAGGCGGCCGTTTGCCAGCTCTGCGTTCAAGCGCTTTGTCCTCAGCTCGGTGTCGT
CGGTCGCGAGCAGCGGGGGCTTGAAGCCATAGTCCCCAGGGGCCGGGTTGCTCCGGCCCA
AGTCGTTGCTGCACTCGCAGAAAAATGCGTAGGCCAGAATCTGATACAAACCAGCAGTTG
GGATCTTGGAGACCGCCGCGAGGCCATTGGGAATGTCTTCAAACTTCAAATCCATGGACG
TAGACAAGTAGCCAGGAAACTTTCCTGTGATCTCGGGCGTGATGTATCCCATCGTTGCGA
GCATGCTGATGCGGCCGTGCTTT
>at00576
TTTTTTTTTTTTTTTTTTAAGCATGCCACTCCCGCAACTCACAACTGCAGAGCAGTCAAC
AGACTTCCTGTACTACATGGACCTTGGCCTAGCCGGGCCTCAGGACTTCCAGCCGTAGTT
TCTCGGGATGGTCTTGGGGAGCGAAGTCGGAAAGCGACCGGATTTGTCGTGCTGCATGGC
AAAGTTCACAGCAGCTGCGTTCCGCCCATGCACTTCAACGGAGCAAAGTGAAGCTCCACA
CTCGCCGACTTCGCAAGTCCACACCTCCCCGTGTCCGGTCTGCGAGTGCACGGCCAAGAA
GTCGATGCGAGTGCACTGGGGCGGCTCCACCGACTCGGTCTGGAACCAGAGCAGCCACTG
GTCAACCAAGCTCATGGCCTCCTGCTCCGCGGAGTGCCGAGCGTCCGCGTCTCCCTTGAA
CAGCATCCGTGTCACGGCCGTGTCGGGCACCGCCGTCGCGGAGGCGACGGGGAAGCCT
>at00577
TTTTTTTTTTTTTTTTTTACAAGCAGACTGGGGACTGCACGCCGGGAAGTGCCTGTAAGG
ATCGGACGCGGCCAGAAACCTCAACGAGGGCGTGGATTGCCACGTCCGCAGGGACACCAG

CATCTGGAAAGCTCAGCTTCATGCAACCATCCATGAAGACCGTGAGCCCACCACTCGGCG
TCACCAGCAAGCCCAGAACGGAGCCTTCTGCAAAGTCCAGTGATCTGCCACTCACGCCTT
CACCAAGGTCTGACACCTGCTGGCAAGCCTTCTTTCGGCTCGCTGGCGGCGCTGGAAGGC
TTGGGGGCTCAACGCTGGCATTCACACAGAGGAAGCCACTTGTATCAACAGACCAGCAGT
GCGGCACCTCCCGAGCTCTCCTCACTGAATGCCGGATCTCAGACGGCACGGAACCTGTGA
CACCCAAGACAAGGCCTGCTGAGGATGGCGGTGGCTCGGCCCGGGAGGGACGCTCGACTA
TCAGCTCCGGGGCTAAGACTGAAATAGGGCAAGCCTCTGTGAAGAGGTTGCGAATACGCA
CTTCGAAGTAGCGGCCCGCACCAGCTGCAGGAGGCCGCAAGGGCAAGTCCGAGATCACGA
CTGCCTGTGGCCTGCTCATGTCTTGCCTCACCAGCATAGTACCTCCATTGAGAAGTGCAA
AGTTGTGTCCTGAGCGTGGGTCCTCCCCGTCCACTCGGCAGAACTTGCTCCACGTCGGCA
AGCGGCGTCGGGTGATTGGCATGCGGCCATCACGTGGTGCTGAAACCGGCGTGGCAACGC
TCGGTTGCTGTGCCTCAGTCTGATGTCTTGTGTCGACACATGGTTGCGCTGATGCAGCTG
TAGTATCATCATGATGTGAACGTCCAATTGACANTTGTTCTGCTTCAGGAGCATGGGCAC
TTTCGTGCTCTTGTGGGCATCAGTG
>at00578
TTTTTTTTTTTTTTTTTCCTCTTGCTGCAGCACCGCCCTTGGGGACTCCTCGCTGCGCC
CGCGAGACCCCCGAAGGCGACACTCAGCAGGTCTTGCGCCATTTCTCGCCCATCGGCACG
GCAACCTGATCATGCTGTCTTGCACACATGCTGCAGCAATCGCCTGTGTGATAGTTGTGT
TCATTGAGGCAGAGCTTGTGGAATTCGTCATGCTCGAAATGTTTCACTGCGTCGTCGCAC
GACGTCCCCCCACCAACCCATCCTGTCAGATGCTTACCCATCATAGGTCTCCGGTCGGTG
GCCAGCCACCTCTCGCCTTGTTGGAGGCAGCAACACTTGTAGTCGAGCCCTTCTGGGCAC
GGGTCCGCAGTCTCGGCCAGGCTACTGGCCATCTCCCCTTCCTCGGTCTCGCCGCGCCAC
GTCAAGCCGGGCGTGGCTTGGAGGAACAGGAGGGCGAGCACGACTGTCTTTGCGGCTGGC
GTCGTCATCTTGACTCCTCTTTGTAGAGGCAGATCTTGAAAGTTGCGTGGGGCTTCACAT
CCGGCAGCCGCTCCTTCCCCACGGGCTCGGTCCTCTCCCGCTCGCACGTCGTGAGGCCCC
TGTCGGAAGGGCGCCGCTTGGCGGCAGTCCACTTGTCGCCGTACTGGAGGCAGCAGCACT
TGTACCTCGTCTCTTTATAGAAGCAGGGCGTC
>at00579
CACGGCCTTCTGAACATTACTGCGCCACTCCTCGAACCAGCAACACCCCCACTCTTTCGG
TCCGTCATAGATGTTGTGGCACCAGCATTTCCCCGTGTCCATATTTTTCACATGGCTGCC
AAATCGTTTGTCGCCTTTCGGCAAAAAGACAGTTGCTGCACTCAAGTGCTTGGAGTGTTG
CGCCGCGTCATGCTCGGCGTCCATAGCATGAGCGTAGATTCTCTTGCTGTGGAAGTGCCT
TTTGCCGTCATCGTCGACAACAGCAACTAAAGTATCCTTCCAGTCGGGACGCCTTGCATG
AGCGTACACATGGTCGACAGCTCCTCGTACTTCCCTTTCTGCTTGTGTGGCGTTCCTGAC
AATCATGTCCCACTCATTCTTGTACATGCCGGGGAACGACACTAGTGTGCGCACCTCCTT
TCCTTCCACAGGGTTTATCCAACCATTGAATGATATGTCTTGTCGACTGCCATCACACGC
ATGCAATGACTCATCCACTTCTGGCCTGCAGCACGCCGACGCAGAATGACCCATTGCTGC
AAAAGGAGAGCACAATGGCAATTGACCTACAACCTTGAGCCAAAATGGCTACGG
>at00580
TATGATTCTCAGCAGACACTGTCTTCAATGCCTCCCGGAGTTCCTCTAGCTGATGTTCCG
TGGCTTCTGTGGCTGCTTGATGCGTTCTCCTCTCTTGTTGCAACTGCAACTCCAGGCGAG
CCATGGCTTCTGTGGCTCCTTGATGCGCGCCCCTCAGTTGCTGCGACTGCGACTCACGAC
AAGCGTGTGCTTTGCTTTGCTGTTCAAGTGCTTGTTGAGCATCCGCACGCACTTTCTGCT
CTTGTTGCAGCTTCAACTGAAGAGATGTGTATGCTCTCATCTGCTGTTCCAGTCTCC
>at00581
CCGTAGCCATTTTGGCTCAAGGCTGCGGCTTTGTGGCTGTTCCACCGGCAGTTGCGCCAT
TTCATTGTTTGGCATAACTGCAACGGGGGCTGCCCTTCCACCTATCTTCTTGTGTCTGCT
GTGCATCTGGATGGCAGCCCAATTTGGACCCCAGAGGCGTGCCGTGAAGCACCGCACCAC
AGGTCTGACGGTAATCGTGTGCCAAGAGACTTCCGCAAGCGAGAAGTTACAGCCGGTCTT
GCACAGACTGGGGTATCGCGACATCATCTACTTCTCTTCGGCGCAAGTCTTAGCGAGGTG
GCTGATCAACCGGCATCATGGCGGCGGCATCATGCTGGTCACGGGCTGGCGTGAGGCAAA
GCCTTGTGCTGCTGCTATCCATGCCGTGCGCAGCGGACACTTGGAGCGCCTTCGCCCGGA
CGCGAAACTGCTTCGCTTGACACTGCGT
>at00582
TCAAGGGCAAACTGTAGGGCTGCAAGTATGAGCATCAAAGTCACGACGGCCACTTCTCGC

ATCTGCGGGGTCAGGAGCCTCAGAACTGAGGGCATCTTGGCAGCTGTTGCCACCACAATG
GAGGTACCCATGCATGTGGCCGCAGTTAGGAAGTTCAATCTCCAAGTGCTCTCAGCTCTG
ACACCAGTCAGCTCTGGGGAGGCCAAGGATGCCATGACGATGAGAGATGCAACCGTGGAA
GCCAGGCTACCCCGGAATACATTGCGTGCAAGCATGTCGGCATGCAATTCGACAAAGTGG
CACTCCGTGTCAGCGTTGAGAAACCTCATCGACAACCAGCGCCGTGGATGAACTGCATAG
CTCGACATCGTGTAGTTGGAGTGACTTTCTTCTACGCGCAAACTAGATGAATAGTAAGTG
CGGGGTCCAAGACCCTGGTCCAAGACCTTGCGCATCTCGACGGTCCACAACCCTGGTCCA
AGACCCTTGCGCATCTCGACTCAAATAGC
>at00583
TTGTTTTTTTTTCTTTTTTTTTTTTCTCACAAGCACGGCCCATCCGGTTCCAAAACACCT
CTAATGGGGGCAGCACCTGAATGGAAATGCGTGCTTTCTAACGAATGCAATCAACGTAAG
GCATGACTTACGTTCAAGAGCCGGGAGGCATCACTTGTTCTCCTTCAGGAAGTTTTCAAA
AGCCTCTTTGTAATCTGGATGCCAACGTGATAGTGGAGGCCGGTCTTGGGTGATGTCCTG
CATCGGCCATGTCATTCGCAAGTTGTCAGTAGCGCGGTCGACGTCGTTGTCGGGACAGAT
TATATAGAAGTGGTCCTTTGCAAGTTCTTGAAACATGTACTCTACAACTTGTTTGGGCAT
CCAGGCCCCACCAGCTGGTTTATCCTCATGAAAAAAGACGTCATTTGGGTCAAAAGCCTG
CCCAGCTGCCTTCGCAGCATCGCGCTTAGACTTCAGCATTATGGACGTGTTGGTCCAACC
AGGGACAAGCAACGCAGCGGACAGCTTTGAATCGGCTGCACCGCGCAATTCGTGCTGGAG
CCCTTCCGTGTACGCTTTGAGGGCAGCCTTGCTCACGTTGTAGGCCAAGTTTCCTGGTGG
CATAGTGATGCCTTGCTTGGAGCCGGTGTTCACAATCAGCCCAGGTGCACCACTCTTCTG
CATCATCGGTACAAATGCCTGGCACCCATGGATTGGCCCCCAGGTGTTGACATCAATTGT
CTTGTGCCAGGTTTCCAGGGGGGCCAGAGCACCACCACCTGCCCCAACTCCTGCATTGTT
CATCAGGAAATTCACTGCACCATTGACGGCAAACGCCTTTTCCTTGAGAGCTTCTAGCGC
CTTCGGGTCTGCGACATCAGTTGGAACTGGAACCACAAGATCAGGTGATGCTGCAACTGA
CTGGACCACCTTTGATGCAGCATTCAGCTCCTCTTCATCAACATCCGCCATGAAAACTTT
CATGCCCAGCGTGGCACAGTGCTCTGCAACAGCCTTGCCTATTCCAGACGATGCGCCGGT
GATCACCCCCACTTTGCCTGGTGCAAAAATATCTTTCAGGCGAGCTGCTGCCATTGCACG
GTAGTGCTCAAAGCCGGGGGAGACAAGGCCAGGATATTTGTTCAAGGTCCTTGACCCATA
AGTGCCACTTGAGCCAAAATGGCTACGG
>at00584
TTTTTTTTTTTTTTTCTTGCTCTTTCGGGATGCCAGCGGACGCTGCCGTCAAAGCTGCCG
CCTGCGGTCTGTACATTAAAGTTCACACTCCGCTCATCTCCACATGGGAGTGTCGGGAGG
CGGAATGTGGAACACACTCAACTGCTGGAACTGCTTGTAACAGATAAGAAGCATTCTATC
AAGGCTTGACTGGCCCTGCATCAGGTGGAAAATGTGTGGGTACGTCCAAGATGTAGCAAA
CCGGTATCTACATCAAGCCACTGTACAACCCAGAACGTTCAAGAAACTGGCACTGTCCAC
TGCCAAGGCCACCTTACTGTTCAATCCCTAATAGCAGATGCCGCACAGCAGAGCGCATGG
AACACAATGGTGGCGCTTCCCTTCTCTTTTGGGTGTCTATGATGGGGTGTAGTAGACAAT
CCACTGTGGGTATGCACCACCCTGGCCAACAACACGCATAGTGGTGTCGTCGCGCTTCAC
CTCATATTTGGAAACAAGGCACACAAACACCTCGTGCC
>at00585
TTTTTTTTTTTTTTTTTCATTCTTCTCCTGCCTATAGCGCTGTTTGCAAGCCTGCAACC
TGAACAAAAACTCACCGTCATGCAGTGCCCTCTTCAGCTGAATGGTCCGCGGTTTGCCAC
CTCGGACCCAGATTACCTGTCCAGCTGCCTTTCCAGGTTGTATGAGGCTCTGCACGCGAT
CCTGGATGTCTTGCTCGGACATGTAATGCAGCTGCTGTTTCAAGGCTTGGACGACTTGGG
ACACGCCGACCGACTGGAAGCCACGGATCACGCCGACCGCCTGCTCTGCAGTGCAGAGGC
TCAAGCCCCGGTCTGTGCACATTTGCTGCGCGAGCTGAAACTTCAGGCCGAAGACGAATG
CCGTGCTGGCGTCAATGCGGACTTGCTGCGGAGAGCGGCGTGCGCTGCTGGCTGGGCAGC
ATACAGCGTCAGGCAAGTTGGAGTCATGCGGTAGACACCATCCTTGCTTGGCATTGACTT
TGCGGAGATCAGGGCGGATCACACTCTGGCAGAGCACCTTCTCTCGGTACTCACGGATCA
GCTGACAAGCACAGAAGAAATTTCCCACGTTGCGGATGGCAGCGAAGAAGTTGCCCATGA
GCATCCTGGCTTGGTACTGGCTGTAGCCCTGGAATTCAATGAACGTGCGTCTCAGCGGGC
TCTTGGTCTGCCACCAAGCGTTGCGTGTGATGGTTGCAGAGAGGTCGGGCCAAGAAGTAT
GCACCACCCTCNNGTGCG
>at00586
CTTCAGAGCCTCTGGATCCCAATGACTTGTCCTGCGCAAAGGCAAAGGTACTCCTTCCGC

ACCTGCCGTCCTGCAAACTGCGCCTTCAGCCACTGCGTGGCCCCGCCATGGGCACTGCCC
AAAGGAAGAGGCATCACACCAGAAGTTGGGGCATCCAAGCGCGAAACAAAGGTGACATCA
CTCGAGTAGCCATTTGCCTTCAGAAGGGCCGAAAAGTCGTCGCGCAGCTTTTCACTTGTC
ATTCCGGGTGGCTTGAATAGTGCTATCACACCGGGAGCTTG
>at00587
TTGTTTTTTTTCTTTTTTTTTTGGTCAAGTCAGAGCGGACTCTTGAACATCTACAATGG
TGGCTTGCCTCATCTAGTCCCTCTGAGCAACACTTCACGCTCATCGTCTGAGAGCCTGTC
GCCGGGATACTCAGCCAGATCAATGGTGTTCTCCGCAACCTCTGCAAAAGATGCAGCACC
CATCTCGATACACCACCTGTCCACAAGTGCAAAATGCCGCTCCAATCCGCGAGCCCAGAG
CCAATCACGCAAGCCCTCAACGCTCTCTGAGGCTGACGCCGCACTCCCTCCCTCCAAGTC
CTCCTCTGCCGATGCTCGCAAGCAAGTCTGACCGCTGCGAGACCTCCAAC
>at00588
GGCACGAGGCGCGTACCTCGAGCCCTACGGCTACGCGGCGGCGGACTTCCGGACCTGGCG
CGTGGCGCCAGGCGCCGCGGCCCCCGCGGGCCGGCGGCCGGCCCCGCGCTTCGGCCTGGG
CGTCGCCGGCCACGAGGAGATCAACGGCCACACGTGGTACACGCTCGCGTGCGCGCTCGT
GGCCGCGGGCGCCCGCATGGACTGGAGCGTGCAGCGCAGGCTCGTGCAGCTCCGCGAGAA
CCTCCACGACGCCGTGCAGGGCGAGCTCGGGGACGCCTACGCGAAGCACTTTGCGAACGC
GCCCTTCGCCCACAAGGGCGGCTTCCCCGGGACGACTGCGCGGCTCCACAGCTGGTGCGG
GGCCCTGGCCTCCTGCATAAACACTGGGCATACGCCGCCATCTCTTGTGGCCCTGGCGCT
TCATTTCCTTGAAGCCCCTGAGATAGATAGGTTTGGAGGTGAGGGCATAGACCTCAGTGA
TATCGAGCTTTCTTTTGACATGGACGATGACGAGGACTGAACCTTCGGATTCTCTGAGCG
GGGCCTCAGGTGTAGGCTAGTGCATGTCAGCTGTGTAGAGAGGCCAACCACCTGAAACTG
AAGCTCGGGTCGTGGTCACGAGCCCAAAAAAAAAAAAAAAAAA
>at00589
TTGTTTTTTTTCTTTTTTTTTTGGCGCACCGAGGTCACCCACTTCCCTCCAATGTTGACCA
CTCCTTGCTTCCTAACCTTGCCACACACCCTTGCCTCCTGTGGACGAGGCCTAAGCATGG
GCGGCCCGCTACGACGCCCCACTTCAGTCCCTCTGGTTGCAAAGCAGCTTGAGGACCTTG
GGCGTCCTGAAGAGCCTGAAGCCCTTGGCGGTCGGGAGGAGCGGATACACGTCCTCGAAG
AAGTAGTAGACGTGGCCCACCGCGATGCCCATGAGGTGGTCCTGTATGTTCCCACCCATG
AGCAGCGAGATCCAGGCCAGTACCCACGGAAGGTAGGGAGCCCTGACGGCGAAGAAGAGC
ACCTGCATGCGAGCGGTGGAGTTCCGCCTCCCCCAAACGTAGGTCATGACGTCAATAATG
GCTCCATTGATGAAGTACGAGTTCCCGCAGAAGTAGGTCAGGAAGAGCAGCATGGCGGCC
CCGCAGATGAGCATCCAGAGGAAGTCGGCGGACCTGCTGTGGAACGCCACGTCCTCCAGC
GAGGAGCAGTAGAAGACCAAGACATAGGTGTTCCAAAAGAACGGGAGGCCGAACGTCCCA
AAGAAGAGGAAGCATGTCACGAGCCTCCACAGCTGCAGCTCATAGATGATGAGCTGCCAG
TTGAGGTACAGCGAGAAGGGTGACAGCACATCCAGGGAGCAGAGCAGCATCAATGTCACG
GAGGTGAGGATGAGGCATCGCGTCACAGGAGGCTGGTGCCCGAGAATGGAGTTCATGGTC
CGGGTCTTGTCACACTGGCTTCACGCATGGAGCAATTCCCTCTACGCCGCCAGGGGCCAA
GGAGGGGGTGGCTGTGACCAACTTGAGCTTGCAACGCTTGAGCCAAAATGGCTACGG
>at00590
GGGCCATTGCTATTTCTACGACCAGATATCGATGACGTGAGGATTACAGTGTTGTGGAGA
CATGTCGAGGGAGGAGGCCAAGGAGCTGCTGGAGAAACGAGAGATTGACACCGACTTTTA
CACATCTCTGCGAGTGTGTGGGAGAATGTGCAACTGATCTTGCAGACTTTGCGAACAGTG
ACGTTGTCGTGGAATATTTAATTATACTGTCCTCTGACGAGGTTTGCCGGGATCTCGCAG
CACGGACCTTCGCTCGTTCTCCTAAAACACCCCGAAGGGTTTCATCAGCGACTACCCTGT
GACAACAACAAGAGCAATTTTGAAGGAGGATAATGAGGAACATAACCTTGATCATGATGT
CTTCAAGTTGGAA
>at00591
TTTTTTTTTTTTTTTTTTGGGCGGCGCAGAGCTTGACTTGAAGTGTGGGAGGTGAGAGTA
TGAGCAGCCTCTCCTCCCGCCTCATCGCCTCCCCTCTACAGATCATAATCGTGCGGCCCC
TACCCCCAGCGCAAGAGCATCTTGCAGGGTACCCATCTCCTTGACGATGGCCGCAGCCGC
GCGCACCATCGGCAGGGCAAGAGCAGCGCCACTGCCCTCACCCAGCCGCAGCCCCATGCG
GATCGCAGGAGTGGCGCCCAGTGACTGCTCCAGAATTTCGCCGCTGCGTCCAGCGCTCGG
CTCCTCAGCGAGGCCATGGGCGAAGAGCATGGCCCGGCGGCACGGCGGATCCATGCGGCA
CGCGCAGAGGGCAGCGACGGCAGATATGGAGCCATCGACGATGGCGACGATGCCCAGGCG

CGCTGCCTCCATATATGCACCGGCCATGGCGGCAACCTCCAGCCCACCCAGGCAGCGGAG
CGCCTCGCGAGCCTGCACCCGTAGTTGGTCCTGGCTCGCTGAGCCCCCACGCGTCGGAGC
CACGGCGTGCCTGTGCACGGCGAGCGCTTGCTCCACGGTGCGGGTTTTGTGCGCGAGTCC
GTCGTCGTCGAGTCCAGTGCCACGCCCACAGCATTCCGCCGCGTCAGCCCCGGTGAGTGC
GGCGAGCAGCGCCGCTGCAGCAGTAGTGTTGCCGGATGCAACCTCGCCGATGCACACAAC
ACGTGCGGCGCGCTCAGCTTCTGGCTCGT
>at00592
CCCACAAGAGGATGTTTTTTCTTTTTTTTGATACCGCCAACAATTAAGGTCCACACGCAG
GGGTTCTCCAAGGCAGCAGCACCAGCAACGGCAACCACTCCGCGACTCAGTACGGCTGGT
ATTTGTGCTTGCCCTTGCCTTTGTCCCCACCTCCGTAGGAGTCCTTTGAAGCGAGTCGAG
GGCCGTTCCACCTTGAGCTCTCCCTCGCCTGGCCGCAGCTCGTACCCTGATGCCATCACC
TGTATGCATGCGTCTGCTTGAGCAGGCGTGGTGTAATGAACGAAGGCGCAGACCTGATCA
CTCCTGGACTTGTTCCGAAGGATGCTCACTTCCTCCACCTTGCCATACGTTTTGCACAGG
TCTTCGATCATCTGGGGAGTGCTGTCTCCGGGAAGGTTGCCCACCCAGAGCTTTGCACCC
ACATCTCTGCCACTGATTGGATCTCTGGCTCCACCTGTGTCACCATTAGGGGCCCAGTGC
CGCCCATTGGGTTGTGTCCTTCCGTTCTCGTCGCACCGGATGCCCCCACCGTTTTGGGTG
AGGCCTCCTGAACCCTAACCTCTGCTC
>at00593
TAGTCCTATTTTTGGTCTTCTAAGGNCtTCGAGTTCCAAGCTACAGATTCAATCCTCCCT
CCAAGATGGCAGCCAAGGGAAAGAAGGCCAAGGGCAAGAAGAAGTCCGAGTCCGTGAAGA
CGGCTGCGGACNCCAAAGCCACGGNGCCCGTGTCGGCTGAGGAGCAGAAGCGCCGGTCGG
ACCAGCGCGCGCTTCAGGAGCTGGTGCTgGCGGCTgGAGGAAGGAGGGCGCCACCGGACC
AGGAGATCAAGAAGCAGAAGTTCAAGCTCAAGACCGACCTGATGTACACCATGGCGGAGC
GGAGGGAGTACTGGAAGCAGAAGAAGAAGGAGAACTTCGAGGTGAAGAAGGCACTCGGTG
GAGACGGGAAGAAGGAGGAGGA
>at00594
CGGCAAAGTCCGCGTCCGCGAGCAGATCCTCAGACGCAAAGCGCAGCGCATTGCGGTCCT
TGCGTGCAGCGGAGAGGACAATCACACGATCAGAATGGAGCTTTGGGGCCGCGTACTCCA
GTGCCTCCAACACGAAGCCCCGGTCCGCGCGCAGGGCCTTGTGCGCCTGCTTCAGCGCCT
CAGCGTCGCGCTCCACAGCTGCGAGCACCCCATCAGGATCCTGGCGGAATTCTCGCTCTA
CGGCATCTAGGTCGCCCTCCTGCGCCCTGATCAGCGCTGTCCTCCTCCTGAACAGCTCGT
CCCCGAGCATCTTCCCCCAGGGCCCAAGCTGGGGACGCCAGGGCCTGCGGACCGGCCCGT
GGGCCCC
>at00595
CCGTAGCCATTTTGGCTCAAGCCAATTTGGTTCAAGGTACGTGCATTACTTGTTTGACAG
CGTTCCTCCGAAGCCCGCCTGCGATGGGACCCTCTCGTTCTCCCCTTGGATGGCTTGCTC
TTCTATACATCGTGTCGGTATTCTCCGAGGAAGTTGGCGCAGGCGGCAACCTGGGAGCAT
CACCGGCAGCCGAAGCGCTCGCAGCCGATGACGCATGCTCGGAAGCAAGTGGCGAGCCGG
CCTGCGCACTGAGTGCGCTGCAAAAGCGTGGGGTGGCAGAGGCAGGGCAGTCGCTGCCAG
CAT
>at00596
TTTTTTTTTTTTTTTTTTGGACGTGGTGGGAATCTACAAGTCGCAACAGCATCACCAAGG
TCTCCTTTGAGAGCATCGCGGCAGGAGTCTACACAAGCCCACCAACGTCCTAGCAGAAAT
TACAGCAAGCTGGCCAAGGCTGGGAAGGAAGCCTGCGGCCATCCCGCGCTACTGTCGTGG
CTCGTTGCACTCACCTCCCTACGCGAATTGCTGGCATGGAGCTGGCGCGACTGTGGCTCC
CTTCGGAGGATGAAACTTTGTATCGCGTGCGTGGATCCAAGCCGTATGCTTGCTCTTCAG
GTCGCAACACTGCTTGGCGCGTCTTCGCTGCCAGTGACTTCAGCTGCCGATATGCAAGTT
CAAAGTGGTTTGCCGCCTCCTTGTCGTCACCGGTCTCAATCCAAACCTTTGCAGCAGAAG
CCAGATCGAGCGCCGCGAAAAAACCTCGCACTATACCCAAGCCCTCGGGCCAGAAAGGTT
CAATCAATGCATCGCCGCAGAGGCCCACGAGCATCCTTGCAGGCCTGCCCCCCGTGCTGG
CCGGGGCCTCGACCACACGAAATCCGTTCGCAGCCCGTCTGGTCTTGGAGAAGTCGAAGA
GAGACGGATTGCCAACCGGCGAGTCGAGCGTCTCCGCTGGAAGGGGGCCCTGCGTCGGCT
TCCAGGGGAACGCGCACACTGCACGCGCCACTCGCCAGAGCGCGGCCTCGTCCAGCGATG
CCAGNNTNNTGCNGAANTCTNCGCAACCTCTGCACCGAGCACGCCCGCCTGCTGGAGGCT
CTTTCGAGTGGGGGTCATGACGAAGTAGTGCGTCTGCGAGGCGATGTAGCAGACGATGTT

CTCCAGCGCCACGCCCGTCTCCTGCTCGCAGTCTTTGAAGAGCTGCTCGTAAAAACTGCC
GTGCTAGGGAGAAGGACCGCATTCGCTTCTCAGCGACGGACTGCCGATTGGTAAAGTTTT
GCGACGAGTCCAAGGGCCGCTTCCTTGCGAAGGCCGCTGTTCTGGACCAGGTCTACACAG
TGGGCCTGAGCCACAGAGCCGCGTGGCCCATCTGCACCAACAAGGATGGACGCACCTCGC
AGCTGGCCGGGGGC
>at00597
AGCCGGTGGAGCGTTCTCGCTTCAGCTAGGTCGCCCTCTCACAGCCATGGGTTGTTCATC
AAGCAAAAGCGAGGACGCAGGTGTAGATGAAGAGCACTTGCAGTTCTTGTCAAGGGTGCG
CCTCTTCAAGCGCCTCCCCAAGCCAGACCATACTGCTCTTGCGAAAGCAATGGTGCAGAA
GAAGTTTGAAAGCGGAAGGTCATTATCAAGCAGGGTGATGAGGGCAACGAGTTCTTCGTG
ATTAGTGGTGGCGAGTGCA
>at00598
CCGTAGCCATTTTGGCTCAAGCCATTTGGCTCTAGGCTCACTGCAGCACTCCCTGGCATT
TCACCCACCTCCGCTCATACCCCAAGCTCAGTCAAGATGAAGCGTGGGCGTGGCGGCAAG
CAGGCCAAGCTGAAGATGTCCGTGGGCTGCGCCGTGGCCACTCTGATGAACTGCGCAGAC
AACAGCGGTGCGAAGAACCTCTACATCATGGCCGTGAAGGGCGTGGGCGGCCGGCTGAAC
AAGATCCCCAAGTGCGGGCCCGGCGACATGGTCCTTTGCACCTGCAAGAAGGGCAAGCCC
GAGCTGCGCAAGAAGGTCCTGAAGGGCAAGGGCGTGGTCATTCGCCAGAAGAAGTGCTGG
CGCCGCAAGGACGGGATCTTCGTGTACTTCGAGGACAACGCCGGCGTCATCGTCAACGAC
AAGGGCGAGATGAAGGGGCTCAGCCATCCAGGGACCTGTCGCCAAGGAGTGCGCCGAGCT
CTGGCCGAAGATCGCCTCCTGCGCGCCCGCCATCTGCTGAGGGTGGCAGCGTGGCTGGGA
GTCTGGGGATGTGGTGTCGGAGCAGTGCTGCGAGGGAGGGCAACGACCAAAAAAAAAGAA
AAAAAACAA
>at00599
CTGTTGTGAGTCCATTGTTTCCGCCATTCGGGCCTTGTCTGTGGAGTTAATCACACCTTG
AGCACTGCTGTGGCGGTACTCCATGCCGTCCCAATCCAAGCAGCCATCGCAGCCGCCAGT
ACCATCTTTATACTTTAGGCAATCGTGGAAGCCAAGACGGAGGACTGTAGCAGGGGTAAA
CGAGCTGTACCAGCTTGCCCTGAACCACGACCCATGGCCGAAATAGGAATTCGCCGCCTC
CCACACGAGAGTGTGCGGCTCGTTGAACATTCGCTGGATCTTGGCCCGAACGATCAGCAT
CTCCTCGGCCGTCCAGTTCGCTCCAGGTGTGCCGGGAATGTAGGTGG
>at00600
TTGTCCTTGCACCCATACATGCATGCACATATTTCCGCAGAGGTGGGTTGGAGCTTGTTT
CTCGCTGCTTGCCAACCGATGTTTTCTCCCCGAGCCGTTTAGCTTGCGGAAGGGAACTTA
GGCAGCATGGCAGAATCACAAGCGCCACACCTCATGCACATTGTTGGTAAAGCCCACAGT
ATTGCAAGCTTATCCCGGCTTTGAGGCTGGATCAGCATTTTGCTCGGTGCACAGACATCG
AAAGCCAGATAACCACCTCCTCCCGGAC
>at00601
TCCGTAGCCATTTTGGCTCAAGCTCCTCTCGCCTGGGCGGGCGGCCGCGAGGGCGGCGGC
GGCTCCGGCGCCGAGGCATGCCCTGGTAGGCTCCCCCCCTTGCCCTCTGTGCGCAGCTGG
CTGGCAGGAGCTTTCCGTCCGCCGCTGCCTGCACAGATGGAGACAGAAGTGCAGGCAATC
AAGGTGGAGTTCAGCGATGTTCTTGTCATCACACTGGCCGCTGTGCTCTTGGCCCGGGTC
TTCCGCCGAACCTACAGCTTCCGATCACTGCTATGGTGGAAGGAGGACTGAGGCGTGAGG
CCGCGCTGCTGTCTGGCGAGAGGCACTCGGTGAAAGGGGCGGCAGACTGTTA
>at00602
TTTTTTTTTTTTTTTTTTAAACGAGTAGGTGTCTGCCGATGTGCAACAACATACCTGCAA
GTGGCGTGCTGGACTGCCCACGTCAGTCATGCTCGACCTTCTGCACATCTGTCTTGAAGG
CCTTCCGTTCCTGAGCAACAATGGGCGGCCAGAGGCAGTAACCGCGTGGGCGCATCGTGT
AATCGGTTTCCCCGGGCCTGGGCGGGCGAAGGCCCTCGCGCAGGGACTCATCATTCAGAT
GCGTGTCGATCTGCCAGTACATGGGCACGCAGACCTCCAGCGCTGCCTTTGCGCCGGCTT
CGTTGATGAGGTAGGCAGTGGTCTCTCGGAAGCCCTTGTAGAGCCGCCTGACTCCCTCAG
CCACTTGGTACTCGTGCTGCCTGTGCAGCAAGTCCTGCCCACCTAGGTAGATCAGCTCCC
AGTCATCGGGGACGTGGGACAGGCGACTGGAGAGGAGGCCCTCGCAGAAGCCAGGCAGGA
ACTGGCAGTCGTCCTCCACCACCATGTAGACATCCGACGGGCTGGCACCGTCCTCATGCT
GCCGAGACAGGATGTCCCTCCAGATCATCATGTGAGAGAGGGCGCAACCCAGGCCTCCGT
CTGTAAGGTCGGTGCCGAAAAGCTTCTGCTCATCCGGCAGGTAGTAGCGAGGCATCGCCT

CCTTGGCGACGAGGCCCATCTCTGCGAGCTGCGGCACGTCGAGCGTGCGGCCGTCCACCG
CGCTCCAGCGTTGCACGGGCAGCCGCAAATGGGTAAACTGCTGCTGCATGAAGCTCCAAC
GCTCGGGCCGGCGGTCAAGGTTGATGCAATAGACATGGCGCAATGACTGCCATGGCAATG
CAGCCCTCCCCACGTGCAAGGCCCTTGTGCATGGCTCCATGACCGCAAGCTGGCGGCGTC
TGTGCATCCGGCGCCTCTGCAGCGGCAGCTGCACGGCCGCAAAGCAACCCGCCAACAGCA
AACTACCGCTGCTGCCTCTGCCCCCCACGGAGGAGAACCGACTCTGCCGACACGGCCTCA
CCCTCGGCACTGACGGCCCTGCACGGGACAGTGGCGAGGACGCCACCAGGGGCACAGAAA
GCATTGAGGCCCCAGGAGCAAAATTGACGGCGCAAGCCTTGAGACCAAAAATAGGACTAC
GG
>at00603
AGTGTGTGAAGTAGATCCTGTCCTGCTGCTTGTTGGAGATATCGCCCCAGCTGAACTGAA
CAATGTAGGACGAAACAAGGATGAAGGTCCTGCCGAGGTCTTTGGTGAGGTAGAGCATGT
GGTTGCAAGGGCCCTGATCGTCCTGCTCGTCCTCAGCGTCAGCATCCCTTCTTCCTGCTG
GACTTTGCCTCACACGCGTCCGTCCAGGTGGAAAGTAGTGCCCA
>at00604
CCATCTCGGCTCAAGGTGGTTCGAGACCGGTCTGAAATTGTCCCAGTTCCTTTGGTCGTC
TCCCCGCTGCCAGCATTGCCCTCCGCAACTTCCTGCATTGGGCAGCTTATCCCATGGAGA
ACGCCACGGGTGCGGAGACGCTTGGGAATGTCGCCTGGGCGCGGGTCCGGAAGCTCGCTC
GCATCGGACACCGGATCCGGATCCTTCCGGAGTGCCCCCGACGACCATCGGACGAGGCCA
CTGAGGTCTGCGTCGCCCTGAGCCTGGACGGGTCGCCCATGGG
>at00605
ACTTAAATGCAAATTCAAACTGTAATGTTTTGCTGGGACCGAAGGTCTGGACTTAGCTTC
GCTGAAGAGCTTTAAAAATACAATCTCTTTCCTTATAGCACGCCTGGATGACCGCAGGCT
TGGCAAAGTGTCTTCATTGATGAGCAAGTATACAAGGCTTGCAGTTATGATTGAGGTCAG
AACGAGCATGTTCTGCCGCGGTTGCTGCTGAGGGCAGGCCGCGTGCCCACGCACAGCCGG
CGCCCCCTACCCCGTCCAGCCTGGCAGTCCACACCATGTCACAGCTTCCGGTGTGGTTAA
CTT
>at00606
CGCCCGCAGCCTCGCCGCGGCCTTGCTGCCGGCCGGGCATGGCAGACCGGAGGAGGCAGG
GCACACTCGCCTCAGGAGGTATCGCCAGCTGGCAGCGAAGCCGGCAGTGCCCAGGCTGGG
CTGAGCTAGCCGCCCCGCGCCCCATCCGACAGCAATCAAAACCAGTGGGGTCACGCGGCG
AAAGCTTGCTCCAGGCGAACGGTGCCAGCCGCCCATAGCAGAGATGTCCGGTGCCATGGG
CCCCCCGCGCGCTCTCATTGGCTCGTGGGCCTGCATGTCTCGCGTCTCCGTCTGT
>at00607
ACGTACGACATGACCGGTGATGGAACGATCACCACGCATGAGTCGAAAGGGGTGATGCTT
TACTTCGGGCAGAACTTCGATGAGGCAGGGATGCAAGACTTGATCGATGATTTCAACGAG
GACACGGATGGGATAATTGACTTCCCTGAGTTCCTGTCCATCATGGCCAGCGACGTTGGG
AGCGCCGATTCTGGACAGGGGGAGCCAGACTGCCCATGATAATATCACCATGGACTGGGG
GGGGCAATACTGCCCATGAGATTATCTCCGGGAAAGCCTTTACTGGCGTATTGGATCAAG
AGGAGGAGCAAATGATCCTCCTTCACGTTGTGAAGTGCCCGAAAAACTTGTGATGGATGC
TGAACGTGTGCCCCCCGNTGACTTCAACAGAGGCCAGCCAAGG
>at00608
TTTTTTTTTTTTTTTTTGAGACAAAAGAAGATCGAGAATCTCTCTGTAGTTCCATCGAT
CACAGAGTCCAGCTGTGCTTCAATCACCTCTCCTTGCAGCCGCGAGACACAAAGTCTCGA
AGGCCGCCCGAGTTCTTGCGGAGCAGCCCCAGGATGCGGCCATCCTTCAGGCCCTTCTCG
GCGCAGCGTTGGCAAGCCGTCAGGCCGTCCGGCACTCCCGCGCAGGCCTGGCCGGCCTCG
AGTCGCTTCCTCAGGATCCCATCGCCCTCTCCCCCCGTGAGCCAGCCAGCAAGTCCTTCG
ATCGCCTCAGCGGACTTGCTGCCAAGCTTGCCGTTGCCATGAGCGAGGATGTCCACCGCG
AGCAGGCAGACGGCCTTCATCGCAAAGTTTGGGCAGAGCTTCGCCAGACGAGCGAGCGCG
GGCCCAGCCACCTCGTCGTCGGGCGAGGCGGCATCCGTCCTCGCATATATCAGTGCATTG
CTCCGAGCAGCCTCGTCCTGCGCAGAGTTCCCCGGGCCGGAGGACGGTGTANCGAGATCC
CGATTCCGAGCGGCAGAAGCAAAAGCGGCGGTCTTGTCTCCGGCTGCAACTGCAATCTCC
GTGATCTTGCACCGCTTCCCGCCAGCTCCAAGAACCTGCAATGCAGCACCCGTCGAGTCA
GGCGCTTGCAGAGCAG
>at00609

CCGTAGCCATTTTGGCTCAAGTGGCCCAAGCTTGGCGGAACCTCCCGCCGGTCAATTCAC
GCCTCCTCCACCTACCACCTGCCCTCGCGCCCATGGCGGACGGGGGCTCCGAGGCTGCCC
CAGAGGGTGGCGCGCAGGAGAAGGACCCCGAGAAGGGCGGTCCGAAAGAGGAGGAGCGGG
AGACATGGCTCCGAAGCTGCTGCTACGGCTTCCTGGACCAGCTCGTGATCGTCGCCCGCT
TCTTCGTCTCTTGCTTCAGGGCATTGCGCGCTGCTGCCGGTTGCGCTTCTACC
>at00610
TTTCTTTTTTTTTTTTTTTCTTTGGGCACACGTTGAAATGACGCGGGCAGAGCGATCCG
GCCAAAAACTTTGTGCTGCACGCACGACGGGGAGCGCTCCGCACTACCCATGGGAGAAGG
AAGAGGCCACTCAGCCACGCACCGTGGGCACGGCCCAGTGGTTCGAACGACTGGGGACTC
TGCACACTGAGAGGCCCATGCCTGCCTCACACGAGGGTCTTGTTGATCAGATTGTTCATG
AAGTCGTCCGGCCTCTCGCCCCGATCCTCGCGCTGCAAGGGCTCCCCACAGGACTCGAAC
CAGGTGACGGCATGGTTCCGCTCGCCGTTGAATGTCTTCCGCCCATGAAGGACGCGGTAG
TTGTCCAAGAGCACTACATCGCCCTCTTTCATCATGACGGAGATGATGTTCTTCTCGCAG
ACCTCGTTCACATGGCGCACATCCTCGGGACTGATGCGGCCAAGGTCGCCGTGGTAGACA
TCAGTCATGCCGACCTCCGGCACCGTACAAGGCCGGCGGTCACGGAGGTAGCGGCTGTGG
TTGTGCATGTTGCAGAACCAGACGGGCTGCCCTGTCACAGGGTGCCTGTTGATCGGGGAC
TGGGCGTGCTCAATGGCCTGCAGGCGCCAAGGGTTGGAGGGGTCGGCGCCGTANACCATC
TCCAAGTCCATGTAGAACTTGGGCGCCACCATCTTCAAGATCATGTCCTGCAGCGAGTCC
TTGACGCTGCCTTTG
>at00611
ATGATGCTTGGCTGTGCCTACATGCACGGCAGGGGCAGGTCAACCTTCCGCTGTCGTGGT
GTCTGGAGCCACGTATGGTGCCCAGCTCGGCGCACCTTGCGCAAAACGCTCATGGAAGAC
CCTAGCATAAGCCTCCCACTCTGTGGGGCCATAGAGGGAGGCGGCATCGCCTTTGACACC
TTTCCTCAGGAACTGGAAGCCCTTCCTTCCAAGAGACAGATACCGGCTGGAACTGGTCCG
CATTCGCCTTCATGTGCCCGAAGTCGAAGGTGGGAAGCAACTCGTCGAAGTCGGTGTCGG
ATAAGGCTAGGTTCAAGTGTGCAGCCACTCGCTCTACGCAAGGCCTCAGGTTTGCCAGCA
TGTCCTCATAAGATACAAAGAGGACGCGCGAGTCCCCAGCGGCCTGATCCCATGAGGCCA
AGTGGTCCGTCCAGCGGCCAAACTCTATCTTGCCTGCGACCCAACGCTGATGGAATTCCG
CAAAGGTGCCCTCGAAGACAAATCGGTCGCCGGCCTTGGTTCGCTGGTTGCTCAAGTGGT
GGAAGAAGGAGCAGCAAGCGTCAGGCCCATCCCGGCACACGTAGATGTACTTCGTCGCTC
GTTCTTTGGCATCATCTCCCAGCGCGGGTGCGTGTTGAACATGCGGCGCCCGATCGCTCG
GTGTCCGTCCTCGACGTGCGAGGCCAATTGCCCAGGCCCGCGCCAGGTGGGGATCGATCT
CGAAGAACGGCGTGCGCTCGCTGACATGCCCGCCGGGCGGGGGCATCCCTCGCGAGCGCA
GGACGAGGGAGCCGACAATGTGCTGCATCCATGTGGTGCCTGACTTCGGATAGCTGGAGA
TGAACACATCCTCCGCGAAGTCCTCGGGCGGCGGAGGCAGAGACCGGCACCATTCCAGGG
TCTGCGCCGAGGAGACGTCCAGCAATGGGAAGGGCCGGTCCTCGTCCGCAGCTGGACCCT
CCATCACGCTGGCGCGAGACTAGGCCAAAAGACAACGTGCCGCTCCTTGAGCCAAAATGG
CTACGG
>at00612
TGCGTGCCCACTTGACTTTTGCCCTTCTGTTTGTCTAGTCCCTATTACGCTGCGGTGTGC
CAGAGTGTTTGCCCGTCCCTTCACATATGTGGCATACTGTGGGGCCACCAGAGCTGGCGT
CAAACGGATACTGCTTAAGGGAGCAACAGGCGGTGCCTGCCTGGCATCGCCTGTCCAGGC
AGGCCACGCGGCTCCTGTCCAGAAAGAAGGCGAGGGCACACCTCCGCTGGAGTCTCCTCC
TGGCTTTGTCATTGCCAACCAGTGTCGTTGACAGTGCAAGGGGGCTGTGGTCGCACATCA
TCTCATATTTGTTGATGGCGCCATATCCATGACCAACTGTCCAGGCTCGCAGCAGTCGTG
GCCAACTGGATTTGTGAATGACATGATCTCGCTGCTTGGGACTCACGCGTCTAGACATGC
>at00613
CGCTGGTTGCCCCCGCTCCCGCAACCCCTGTCCTCGTTGGCAGCTGCGCTTTGCAGGACT
TGCCTGCGACAGGCAGTACGGCGACGGCCCCGACTTGGGAGCGATGGCTGCCTTGCTGGG
GCTGATGTCCCCGAGGCGCTCGCCGAGCAGTGGCTTCCGCTCGCCCCGCGCGGAGGGCCG
CGCCACGCTCAGCAACTTTGGCTGATGCGATGGTTCAGCTACCTCAAGAAGCTCTGGTCC
ATGGTGTGGTCCAAGAACGGAGGATAGTTCTGGC
>at00614
TAGTCCTATTTTTGGTCTTCAAGCCACTTGGCTCAAGCTGTCTCGGCTTCATCGTGGCAC
ACCTAGAGTCCCCGCTTGGGAAGGAGGCTGCCAGGCATGGCGGACCAgTCACTTTGTCCA

GTGACCATGGCTACGTGATGATCTCAGCAGGTCTCATCGCGATGGAGGCCCTCGGCATGG
GCGCTGGCGTCATGGGACTGCGCAAGAAGTACTTTGAGTCGGAGAGTTCAAGAAGTCCCT
CCGAG
>at00615
TTTTTTTTTTTTTTTTTGCTTGCAAGCCAACCTGACGCCGAGCCCATGGCCAATACGGG
AGGCCGTCTCACACCAATCCCGTCACCCCTGCCTCCAGCATGGCGCCGACAACATCTGTC
TGGCCTTGATCTCCCTGCCACTTCATCAAGTTCAGAGAGCCTCTTCCAGCGCCTTTCCGC
CTCTTGGACGGGGGGCCCGAACTGGTGGGATCGAGCCATGTGTTGTAGAGTCCCTCCGCG
TTCTGCCAGATGAAGGGCCAGCCGCTGGGTGGTCGGCCGGTGCTGAGGGTCAGCGCCGCG
GGCACGCAGGAGAAGGGGTGGTCGTCGCCAAATGGCGCCACGAGCTTCTGGAAGAGCCGG
GTAATGACTTCGGGCGTGAGCACCACCTCGGAATTCTCGATGGACTGCCAGCACCTGCCC
ATGCTGTTCTCCATCCATCGGCCCACAAAGGCCTCTACACGCTGCTGGTCGGAGATCCTC
CACTCAGCGCAGGCCTCGGTGTAGGAGGCCTCGTGGGAAGCTTTCAGCGCCTTGTAAACC
TTGTTGCCGATGGGCTCATCGCCAAAGACTTCCTGCGCGGAGTCCCACATGGCCTTTTCG
GTCAGGATGCCATCCATCAGTTCCTCATACTTTAGCTGTTGCCATCTGCTCCATCTCCGG
ATAGTTGGCGCGAGGAGGGCAATGACTTACCCTGACGATTTCCCAAATCGCGGCGCAGAT
GGTTGCAGACAGGTCCAACTCGAAGAACCACGGCCTGTCGCCGCACCGCCGCGGAGTAGC
CCTGCATGGCGCCCTCGATGTACTGCGTCGCGGCTTCCGGCCAGGGCATGCTCAGCAGGT
CAGGGGACGTGCCCGCCTTGTAGAAGTACTTCACAATGCGCTTTGTCATCTCATTCAGGG
TCCACTCCGAGTCCTCCTGAGTGAGCTGCTTTGCAGCATTCCACCCTCGTGCC
>at00616
CAAAGAGATGGCAATTCCGTCACGGCAAACCCGGGGTCAAGAGCTGTGAAGGGCAGCCGA
GGTACGGCCTAGGGCCCGTGTTCTGTAGGTGCCCTCTGCAGGTCGCCCCTCCTGCTTCCA
TGCATCGCACCACACAAGGGGAAGGGCACCGGCTGAGAATCCGATACCTCTCACGACTGG
TGACTTCAGGCCCCGTCTCTTTCGTGAAGAGCAGGGAGGCTACTAGTGGCAAGTTCAATT
GGTAT
>at00617
TTTTTTTTTTTTTTCTTACTTTACCATCCTTGAGCAAGTGACGGCGGGGCCCTGCTATG
CCTCTTGCGTGAGCGTGGCCCGAAATCCGCCTTCCAACTCTCCGCGATACAAGTTCCGGC
CGATAAACACGAGCCTGTTCTTCCTGGGCTCGCCTTCCGGCCACTCGCCCTGAGGCTGGC
CGCCAAAGATCATGTGGATGCCCTGGAACACAAAGGGGCTCGAGCAGCCCTTCACGGCCA
GCACCCCCTTCGTCCTGAAGATGTCTGTGCCCCTCTCCTTCAACAGGCCCGCTATCCACG
CGTTCAGCCTCTCCAGGTCGCACTCGCCCCCGCACTCGATGCCGACGGAGCTCACCGTGC
TGTCGTGCTGGTGCTCCTGATCGTCGCGCAAGAACTCCGCGTCGGCCTCGAGCACCCTGT
CCAGCGAGAAGCCGCGAATGCCCAAGATCTCGCCAAGGTCCACGACGGACTGCTGCGTGT
GGACGATCGGAGCGACACGGTTGATCTTCCGAATCTCGGCCTCGACCTCCGCCAAGGCCT
CGGCCGAGACGAGATCGACCTTGTTCAGGAGGATCCGGTCTGCAAAGGCCACCTGCTCCA
CAGCCTCGTTCTCCACTCCTTCTGGCTTCACCTCGCGCAAGTGCTGCAGCACGTGCTTGG
CATCCACCACCGTGATGATGCCGTCCAGCACCATCCGGGCCTGTATGTAGTCGTCGGCAA
AGAACGTCTGCGCCACGGGCGCAGGGTCCGCCAGGCCGGTGGTCTCGATGAGGACGCCGT
CGAGCCGTTGGCCTCCAGTGCTGTGCGTTGGA
>at00618
TTGTTTTTTTTTCTTTTTTTTTTGTCACAGAAACCGCCCCTCAGAGTCAGCACAGATGG
CGATTTTGCAGATACTGTCCCAAGGGTTGCGCCCGCATGCACAGGCTCACAGGTACCCGT
CTGGATAGCGCCACCGGCTCATGAGCTCCGAATATCTGCAGCGATTCTGGGGGAGCCCGG
CCTTCTTAAGGCACCTGTTCTCGGCCATGCCCAGCCAGAGCATCAGCTCCCGCCTGCTAG
ACACGGAGGGGCGGCCATAGTCTCGCAGCGCCTCCTTGAAGCAGTTGGCATAGGGCCCTT
CTCGGCACAGGTCCTGGAACTTGCCGTAGAAGGTGTCTAGAGCAGCCTGCTCGTCCTTCG
CGGGGCTCTCCGGCGCGTACGCCGCGGTCGTCCAGAGCAGCAGCCACTGCGCGCTGCAGA
TGTCCTTAGTCTCGTTGGTCTCAGAGAGGCTTGCCATCAGGGGCGCACGGCCCCTTCCCA
AGCCGCGGCGCCGAGGC
>at00619
TTTTTTTTTTTTTTTTTTGATGCGATGCCAAGTGATTCCACGGCCGAGTGTTCTGCTCCC
CGCACTCCCCTTTGCACCCACTCACCCAAGCATGAGCTGCTTTCCCCAGCCAGTGGATGT
ACCCATCCAAGCGTCTTCAAGGTTTGGCAAGCCCCCGCTGCCAAGGCGCCTCTCCCAGGC

TCGCTTCCCCAACATTCACTGGTCAGGCAGCTCTCCAACACAGCGGTGGAGAGCGGGTCT
GCTTTTGGACAGCTTTCCCGCCTAGCGGTGCGCGACGTCGTCAGAGTGTGGCACGTCGGA
TCCGATGTTCCACGTCTGTGCCTGACGTACTGCCGCAGGCTTGACCGAGTAGGCCAGGAT
GGCTCCCTCCTTGATGTGCCATGCCAAGAAGTTGTCGACCGGCATGTCCAACGGCCGCAT
TCGCAAAAGCTTCCTCGCGGCCTCCGGCCACACCAAGTAGCCAACAGTCGTCCAAACGTA
GTGCGGCTCCATCAGGACCTTGCCCACGCGCTTGGGGGTGCCCCGGTCCTTGCTGCTCAA
GAAGATCATGCCCGTGCCCTCCGGGGCCTCTTTGACAGCCTGTGCGAGCGATGCAGTGAA
AGAGGGCAAGGCAGCGGCATCGTCCTCAAGAACCAGCAACGGGTGCTGCCGCCTGGCGGC
CAGCTTCCACGCCGCCACGTGCGAGGCGCAGCAGCCCCGCTCTCCGGGAGACATCTTGAT
GGTGACCGGCTTGTACCAGAAGAGAGCGTGGACAGCCTGGGCGTGCTCACTTCTGCGCGA
CGT
>at00620
TTTTTTTTTTTTTTTGTCTTCGACCCCTTGATTGATCTTCAGAAATCGCATGCCTCTCG
TCAGAAAACTGCAGCGAGCTTAATTACACATCTGTCGCCGAGCTCGCTGCCACAGCTTGC
ACTTACCTTGAACGGTGTGCATGCTCTATTGCATATGCAATGGCTGCCTTCATGACAAAT
TTAGTGCACGGGAGCGCTCAGCGGCCATCACTGGGGAGGCTGCGCCTGCCCATTATGAGC
ACCTCCATGGCTCACTGGCATAGCAAACATCTGGATGCCTGCCTGCTGACCCGCCGCCCA
TCCCTCGAAGCATGCCGGTGGCTGTCCCGGAGTGTTGTAGCACTGCATGTCGTGAACCGA
GGGTGGCTGCATGGGAAACATCGGTGGTTGCATGGGTGCGGCGAACCAGCCGGGTCTGGG
AGCCTCTCCTGGCACTAGTGCTCCTGGCATAGTGGCCGGGCCGGTGAAGGCTCCACCGCA
TGCAAGCGACCACGGTGGCAATGGCGGCATGGAGCCCTGAGGTGGAGCCGGCGCCCCCAT
CATGCCAAGCATTGGGTGCATCGCGTGCGATGGGCTCGGCATGCCAAAGCCCTGGTGCTC
CATTCCGTGGGGTGACCCGCAGGGGCTGCCCATGACGCTGCCTGCAAGGCTGCCGGGGAT
GCTGGCGGCCAGAGAANCATGCAGCATCTGCCCCATTGGCGTGAAGGATGCCTGACTCTG
GGGTGTGCCATCA
>at00621
TTGTTGCGAGACTGAAGCGCGTCACTATTGCACCACTATGTTTAGGTGCACCACAAGTAT
AGAAATACCCGTCCATGTGGGCCCCGGCGCAATATATCCAGCCGGTGACAAGTGGCTCCA
ACACCTCCCATGTCCCGGTGGACACAGTAAGGCACTCGGCACTCCTCAGGGCGTCAGAGC
CCGCGCCGTTTTGATCAAAGCCACCGAAGACGTAGAGCTGATAGCCCGTGAAGATGGCGT
CCGTGCATACCTTGGAGCTTCTGGAAACCTCCCTGAGCCTCAGGAGTTCCTCCCTGCCGC
CGCAGAACTTCTGCACCCGACTCAGAAGCCCGCGCTGGAGGAGGTTCGGAAGCCTGGCGC
GGTAACCGGCCCAGGGGCCCGCGCCGCCGCGCGGCTTCGAACTTGCCCTTGAAGAACTTT
GCGGCAGCCCCTGCCGGAGTCTTTTTCTCGCTGCCCAGGCAGTGCAGGAGCCGGTCAAAA
CTCCATTGCGCAGCCTCCGGTGCTGGCGCCGGAGCTGGTTGAAACTCTCGCGGTTAACCG
CGGCAGGCTGCCCTTCCATCATCAGGCACTGTACCGGTCGGAGGACAACCTTCGAGGCCT
CCGCTCAATGCGAGCGAGGCAGCTTGCAGAGTGGGGAGGGGTTGGCCAGAATGCGGGAGC
CACATGGCTTGAGCCAAAATGGCTACGG
>at00622
CGGCTGGGGAGGAGGAGGACGACGACGATGACGAGGTCATCGACGTGCCCGAGGACTGGC
AGCCGCAGAGCAAGCAGGGCGGAGGTGAAGATGCTGGCGTGGCTGGCACACTGAAAGGTG
TGCAGTGGGGTTCTGTAGACCAGACCTTGTCGTCCTTGCAGGCCAAGTGATCGCCCCTAG
GCGATTATGGGCGGCCTCATCGGCTGACGGCGATGGCCACGTAGCTACGATGTAGGATGT
GCCGCATTACATTTTATGACGTCACACGATATTGGGCCTGGGGTGCAGAATTCCGTGATG
TCAGTGACTGCTGTTCGCTGCGGCGCTCTTCACGCACGCCGTTCACCGATTTACGGGGGA
CCCCCCGGTCACCGAGTTTT
>at00623
GCCCTTTAGGAGGAGCCAACGTGGGCTCTCAATGAGAATGCCATGGGAGAAGATGACAAC
CAGTGCGGGGGCAGCGACGAGGGCGAGCAGCAACCTGAATGCCATGGCAGGGTAGACGGA
TCCCAGAACCACAGAGACGATTTGCGAAAGCAAGACTCCCAAGGTGCCGAGGACCGGCGC
CCAGCAGCAGAGCTGGCCCCGGCAGTCGGCAGGAGAGATCTCGGAGATGTACACGTTCAC
AGCCACCGAGGACATGCCAACCCCGAGCCCTGTGACGGAGCGGCCGACAACCAGCACCAA
GTCGCTTGATGCCATGCCCATCATTATGGCACCTGCCACATATAGAGCGGCGACCGAGAG
CAAGGTGATCTTGCGGCCGACCATGTCTGCTGTGGAGCTGCCGAGTGAGCCAACAGTAGC
ACCCACGAGCAGCGAGCTGATGACGCTGTCAATGTGCGTGAGCC

>at00624
TTTTTTTTTTTTTTCTTTCGATCTGTAGGTAGTACTCTGCCTCATCTCAATGTCGAATTG
AGGCATGCATGGACATCAAATGGGACGTTGGGAAAATTGTAGGCGTGCATGTGCGTTGCA
GCATGTTCTTCCTGCGCTGGCCCAGTCAGATGTGATTGGAGCACCTTATGAACTTCCAGC
AGTCCTTGAGGCATAGCGCTCGACGGTCCAGTCACGGGCACGCTCGTTCAGCTCGTTGTT
CACAAGCCCGCCAACTCCGTATCCCCAGAAAAAGCGAAACATACGAACACCGGTTGCTTC
TGGGTATCTGCTCATCACAGCACGCATGCGCTCCCCCAGTGTCGCCTTGGGCGTGTTGTA
GAAGTTCAAACCGAGCAGGTGAATGGGCGTGATGATGGTCTGCATACCGCAGGGCGTGGC
GAGCTGAGCTACCGTGTCGGCGCTCTCTTGCTCCATCCCCGAGGCCGACTCGATTATGTC
TGCCACCATCGCCGGCAGGATGAATCCTGCGCCGATAATCAGAGCATCACGGAACAAGAA
GGTACCATACGTCGCCAAGGGGACCGCGCGCTTCACCTCCTTCACAGATTCTGCGGCCTT
TGAGAACATCTTTGCAAAGGCGGCGTCTTTGAGGATTGAAACTGTCGTGAAAGCGCCTGT
CACGCCGATCAATTTCATGGAGTTCTGCGTTGCCGGATCCAGATTGTAGCGCTCCGACGT
AACATCAACAAGGTTGTCCGCACCGTAAATGGTGGTGCACACGCCCATTATCATCCAGAA
AGGAGCCGATCTGACCATGGCAAGCGGTCGCGTCAGCATGTCACGAGCCACCATCTTCAA
GGCGTGACCCAACGGCATGCCGGCAGCAGCTTGGGTGACGCACTTGTCGACAGCCATGCT
AACAGGCGCCACAGACATTGCCGAAAGCACGGAGCAGCCGGCAAACACAGCTGCATCAGC
CTGCCACGGTATCTTTGCCAAAGCCTCTGCGGGACGACCTGGTGCCGGACCACTGTCACC
GCCCATGTTCAGCAAATCGCTGCGACAGAAGGAAGCAATCAATCCGCGTGCTTGGGCCTT
GGGCAAAAATGTCTTGAGCCAAAATGGCTACGG
>at00625
TTTTTTTTTTTTTTTTTCCATCAAGACGAGTGAAAGATGCGCGTGCACGGCAAAGTGCT
CGCTCCAAAATGGCACTGGAGATCAGAGTCAAGCGAGTCAGAAAGCATACATCAGCTTGA
CTGTGCATGGAACGCCAAGAATGACCGGATCATATTGACACTTTCTGAACAGTCTGCGCG
CCGGAAGCCACACTCTGTCGAGAGTTCAAACGCAAAAACGGCCAGCAGATCAAGCATGCT
GCCAGCAGATCAATCCTTCCGCGCACGCATCAGACAACAGTCTCCGACACTCTGGAAAGC
AACCCATTCGATGACGTCGGCGGATAGACAGGACTGCACACACCCTGAGATGGGTGGAAA
TTCTCCGGATTGCATGTGCACGCCGGTGTAGTGTCTTTGGAACAGCAGACCCTGAGCCGA
TCATCCTTCCCCAAGCTAATCTGGTTGGGGACTTGCACCAACGTCGCTTCGTGCTCAAGT
TTGGCTTGCCTGATCTCTTCCATAGCCAGTTGGTACTCAGAACTGCTCGAATCCAACGGG
TACATCTCACCTGGATCCTCGTGCC
>at00626
TTTTTTTTTTTTTTTTTGGAGCGGGAACACGCGAGGCGCATCTGTCGCTCAAGCCGGAC
CTCCCCGGTGCCCGCAAGTGCGCCGCGAGGGGCCCCAGCCAGCGCGCGGCAGAGCCGCCG
CAGGTGTGCGGCACTGCCTCAAGCACCTGCGGGCGCGCCCTGGCGCACCGACGCGTCTAG
GCCGGCGGGGGATGCCGTGATTGTCCACGCGGCGCAGGCACGCGCGGAAAGGGGCCGCGG
GCGGAGCCGGGCCGGGAGCGGGCCCGCCCCGCTCGTCTTGACGAGGCCGGGCGAGCCGCC
GAGGGCTCGGGGCCCCTCGACGGAGCCGGGGCGGGCGCCGCGGGGGCTCGCCCCCCGCGG
CGGACGCCGGGGGCCAAGAAGCCGGCCGAGCTCGCCGAGCGCCAGGCGCGGTCATGCAGC
GGGCCGCTTGGCGACGAGTGCAAGGTGCCTGGCCGGCGCCGAAGCTCAGTCCAGTATCTT
GTCCGCAAGTGGCATGTCGATCTTCAAGTCTTCCTCGTCGATCTCGATGTCCAGCTCTCC
ATCATACTGCACCATCATGCTGAGCACGTCGTGAACAACCCTGACCTGCGCGGCGAGCTC
GCCGATCTCGGGGTCGTCGAAGAGCTTCAGCGTCTTCGTGCGGCACTTCTTCTTCGTCTT
CGGCCGCTGCAGCACGACCCGCCGGAGGGCGTCGCGCTCGAAGA
>at00627
TTTTTTTTTTTTTTTTTTGTCGATAACGCACATTTGACCAGGCATGGATCTTGCAAACC
TACCGGTACCCATGCTTGTATGAAACTGAAGCCTAATCAGCCGCTTGCATCTTGCATGCA
AGGACCTCTGGAAAAAAAAAAACGACCGATGCACAGATAATTCTTAGCCTTGCTCTCAAC
GTCGCGAGCTCCGGAGGACTCAGTCGTACAAGCTCTCCCACTGAAGCAAACGGCTCTTCA
GTGGATCGCCCTTGGTGATGCTGACGAGACCGCTGGAAATCTGGAAGCGCTCGGCGGCCC
GCTCCGCCACGGAGGCCGGTAGTGGGGCGTCCGAAGTCCGGACGAAGCCCAGCCGCCGCA
TCTCGTGCTCGTACACCTCCATGCCAGCGCGGTCATGCTCCGGGACGACTGCGGCCACCA
CGGCTCCGACCTCCGCCTGCGCGGCCGCCGTCCTCGCCAGCAGAAGGCCGCCGAGCTCCG
TGTACACCGCGTCTGCCCACAGCACCACCTGGGCCTTCTCCGCCGCCGGAGGCCGCTTGC
TCCTCAGGCCTGAATGGTCTCTCCAGTCCAAGCGCAGGACCTGCACACCAAATCTGCCAG

CATTCTGGGAGGCCGTCTCCAGCACTTCGGACACAAAGTCGGTGGCGGCCACGCGCTTGG
CGCCCACGTGCGCGGCGACGATGGAGGGCACGGCCGCGCACCCGCACCCAAGCTCGAGGA
CGCGTCTGCCACGGACTGGCAAAAGTCCTTGCCAAAGGCACTCCCCCAAAAGCAGTCCGC
AGCCT
>at00628
ATGGTGTTGGCTGCACCTTTCGAGATCCCAAATCTGACTCGAGACTTGCTTGCAGACAAG
TGAAATGATGGACATGAACTTCGGCATCCCCATTGATGTCCTTGAAAATGTTGGAAGTGG
GATGCATCGCTGCACCTTTTAATCCGGTGTCACTTGATGGTGCTGTTCTGGGCGAAAATA
CATGATCGCCATCAGTTGTCGCAAACATGCGAATATCCCATATGCCAGTTGACAATACAG
TCAATCAGACTTGTTCACTCAATCCCTCGTATGCCAATCGTAAATGACAGGGATGTGCAA
AGACTTGGATTGGCCGGCACTTCCGATCTCCATCCAATGATGGGAACATCATCATCATCA
AGCATTGTATCGTTCATGATTGCACATAGAAAGTTGCC
>at00629
TGCGCCAGTACAACCCTCCGGCTCTGGCCCAGCTGGCGCGGGAGCAGAGTCTGGAGCTAG
GCCTCCTCAACAAGATGAGCTACCCCTTGACCGAGTACAGGTGGAAGTACGTGTCCAGTT
TCTGGGGAGGCCACATGGACGAAATCAAGACCGTGCCACCGGAGCTGGGGAGCGTCACCT
TGGCTGACCTCAAGGATTACTTGAGGCCGCGCTGGGGGCACCGGGGCCGCGAGATCGACG
CGGAGATGTCCAAGGAGCTGCAGGCCATCGCGGACAGCAACGTGCCCACCACCTTCGACA
CTGCGAAGGACAAGCACCCGCCTCACGTGATTGTGCAGAACCGCACTGTCTGGATTGTGC
TTCCCAACATGAAGCCGACTCTCTTGTGCGCCTACAACTGCTGATTGCGGCCCACCGTGC
TCTGCACCTCAAGCTTGTACAAAGGGTTGGCTTTTCACTGCCGCCAACTGGAAAGACCGC
GTCATGTGTCAGCTCA
>at00630
TTTTTTTTTTTTTTTTCTCCCCCCACGCACGCGCAGAGTTCCCTGTCACAAGGGAATCAA
GAACAGTTACACTAACCCCATCATGTCCACTAATGCATGACGCCGGGAGGCATCGGCACT
ACCCCCGGGTCAAGGTTGCTGTCTGATAGCCCGCCACCGCCTTTCAGCCGCAGTCTGATG
TCCCCGATGGCCTCTTTCAAAAAGTCCATGTCTGACTGTAGCCGATCCTCTTTACCAAGT
GACTGCATCTTTTTCCACATCCGGATCGTCCGGATGGGCTGCTCGCCTTCCATGCCATCT
CATGACTACGTGCAAGACAAGGGCGATACCAAAGATGAATGCAGAAACCACCACTGTCCC
AACAAGTCCACTGACGGGCATTGGATCCAGCTCTCCGAGTCCCTCTGCCGGGCAGTTCTC
CATAGGCTGCGGAAACAGATTCGAATATTTGTTCTTTGCCTGTTGCAACACGTCGCCCGT
TGTACTGTGGACTTGCGCCCAACTCACCGCGTGCTGGACCTCTTTTCTAATTGGAATTGC
GACTGGCACCGTCAAAAGGATTTCACGGCTGCCAGCCTTGGTGAGCTTGCAATCACGCCT
AACGTCTAGTTCGCCACCTCGCTGAGGGCAATATGGCGTTGACCCAGGAGAGAGACCGTC
GCAGTCCATTCTCACTCGATCGCCAGAGTACATTAAGTTCAAACCGTTTTCCGACATGAT
GGCAACATCAGCCTGCCCNTNGTGCNATGTACTTAGGACACTGGTAATAGGGTGCTTCAA
CAAGGCTTGCCGTGGGATAGCGCTGGGAAATCATGCCCGCAATGGCCCGAGCGACACAAA
TCCTCTTCCTG
>at00631
GAGCCAAAGCTTGACCCACTTGGTTTTCTTCACGCAGGGTGGTCTACCGTTGCCGCAGGA
ATGGTCATAACGATCGTCGACGCTTCCTTGGTCCTTGCCATGTGCCATCGTTGGCTTGCC
ATGTGCCATCGTTGGCACTGCTTTGCGTCTCACCCCAAGTGCCCTCACAGGGACGATATG
CATTGTGCACCAAGCTGGCCACAAATGTCAAGTCAGACGGAGCAGACCCCCCAGCTGGCG
GTGGGAGATGGCAAGCAGCCCCACGCGCCAGGAGCCCACTGAAAACTTGCTCGGCCGCCG
CCTCTGACAATGTCCACTCGAGAGTGCCCGCGTCATCGTCAAGCTGCGCGATGCTTCGTT
TGACCCATTGCTCTGTAAAGGCTTCGGAGGCAGCCTGCACGGTCATCTGGTTGAAATCGC
CGCACTGCCACACACTGCG
>at00632
TTGTTTTTTTTTTTTTTTGGCGCTGCTCTGGTTAGAGGCCCGCGTACATGTTCACCACTC
TCCTGCACCTCATCTGTACCTATGCCATGCACGCCGATCCTGGTCAAAGAATGTGCACTC
CGCTGTAAATTCGTCGGTGCCCCCCAGAACTCCCTCTGCCCGGAACTCCTCGACGCATCT
CATCAGGTGGCGCTTGCCGTTGCCATTGAAGTGCAAGACCGCAGGTGCCCGGTCACTGCC
AATGCCCACGAGGCCAGGTGGTGAGAGCCCCTCCAAGGAGCGAGACGGTGCCTGGTATCC
TGGCACCGGAGGGCGAACGCCCTCGTCAGCACCCAGATCGGCCTCTGATCCCTGTCTGGC
AAAGTGAGCTCCCCTGCAGAGCGGCCGTTCCAGGTCGTGGAACTCATCATGGCCGTGGAG

CCCCAAGAAGAGCTGAGCGGAAGCGTCAACAAAGCCGAGCGTCTGATTCCTGAGGAGCAG
CAACTGCAGCAAAGCCTGGTCGTGGAACTCGCGCGTTTCTTGATAAAGCTTGAAGAATCG
CTGCAACACGCGCCGCAGGGATGATGCAGTGCTCATGAACGTCCCTGCACAGAGGAACGG
AAAGCGTTCCAAAGGCTTGGAGCTGCTGATGCCACCACCTCCGCTGCTGCTTCTGCCCTC
CCCTCGTGCC
>at00633
GGAGGGATCAGAGTTCCACGTGTCTGCGCGGAAGTTTTGAATGACATAGCCCTGGCCAGC
TGGCGACAGTTGGCAGAGCTGCCCGCGTGCACTGTCTTGTAGCTGGTTGCGATTGCAGAA
ATCCTCCCTCGCCACCTCGAGGACATCCGCAGGGACTTGCCTCCCGGACTCGTGCGCCAC
CGTCAGGATCAGGAAGACGTCGCAGTTTGGCAGGGTCTCCGACTGCGCGAAGGATGCCTG
CAAGCTCGCAGGGTCCGTCGGTGGCAGCGGCAGAACCGTCCAAAGCTCCGCATTGACCTG
CGGCAGTGCTGAACTCCAGACCGTCTGGCACTGGCCCGTGGACTGCTCGTACTCGTAGAG
AATACCAGTGGGCTGGTTCCAGATGTAGAGATAGCCACTCTCGCTGTGAAGGTAGAAGCC
CTTCATGGTTGCGCTATCGATCTGCCATCTGTCCTTGGCGTTGCGGAAGGCGCGGAACAT
CATCTTCAGCTCGGCACCGGTCGGGGGCGATGCCTCCACTGCCGGTGCTGGCACCTCGGC
TGCACCCTGGACTGCAGCCATGGCCGAGGCGGAGCTATGCCTTCGAGGCCAAGGGCTTTG
GCCTTGAGCCAAAA
>at00634
TTTTTTTTTTTTTTTTTTGCAAAAGGAAACACTTGTGCGCACACAGGCCGTTGGAGCAAG
GATGAACCGACATGCGATGGAAAACCCTACACGACTGACGACGCAAACCTGGTCTGCATC
CAGCGAACCCGCAGCGCAATCTCACCGACTGGATTCGATGCATCAACGCAGTCGCCCTGG
GCGACGAGAGTCACCTCGGCTACTCCGTGGTCGCGCGTCGCTCCGGCGGTGTCGTCGAGG
ATGAGGAAGCAGCGACCCGGAGTACCGAGCTGGTTCCGGACGTCGATCGCAGCCCTGGAG
CCACCACGGCAGAGCACGTCGCACGGCCACGGCAGCCGCAGGACGCCCTCGTCGTCCGCG
ATCCACTGCGAGCCCCGCGCGGCACCGCGCTGGGCGCGCCCCAGCCCCAGATCGTGGAGG
CACACCCTCGGTGCGGGCTGGTCCTCGCGGGGCAGCGGGCCCCAAGGGTGGGGCCAGCAC
CTTCAGCTCGAT
>at00635
TTTTTTTTTTTTTTTTTTGGGCTCGAGGAGCTTCATGAGGCGAGTACACCAACACGACGC
CAGCACAATGGGCTGCATCAATGCAAGCCCGGCTGCGGTCAAGAACGGAAAACGTAACAA
AGTGAGCCCACCAATGCGAGTTGCTACTCGCATGTCCAGGTTTCCCTGGACTTGTGGTGG
GACGTGGCATGGGACAGGATGAAACTAGTCCCGTGGAAATGTCAAACGTACTCTTGCAAT
TGTACGAGGTGGTATTCATGCTAACGCATCCTGCACTCAAGGCAAATCTGCTGGACGCAG
AGCTTTGCTCGGGCTTCATGTGAGGGCTGCATGGCGCCACTCAGCAGCCCTGGGCAAGCT
CCTGGATGTAGGTGATGTCGTCCCATGGGTGCCTGTAGAGGGGCTGCTTCAGCCTCAACT
GGTCAAGGTAGTGGGCAATGAAGCCCGTAGAACGGCCCAGGACAAAGAGTCCATTCAGCG
TGCCAAACGCCATCAAGTCATTGCACTCCTCCTGCGAGAAAGCGCCGCATGACCGGATCA
TATCAACAAAGGCGACCGCAATGCAGCCATCGACGTTGAGAATCAGGTTGGCGCGCTTGC
GTGTGGTCAACTGCTCAACAGCAAGGGCGAAATCCAGGACAGGTGTGGACTTGAAGTTCT
TCTTAGCATAGCCCTTAATGATTTCCACCCGCTGGTCCGGGTTTGCCAGAGACTTTATTC
TGTGCCCAATGNCCATGATCAGCTGGTTGGTCTTTTTCATCTTCTCANTGAAGTCCTTTG
CGCTGACGCCGCTGTCGTATGCGTCGGAGAACATCTTCGCAGCGTCGTCCAGAGC
>at00636
GTCATCATCCTCATCCACGGCACAACCGTTCTCGGTGATGATGATGCCCCCAGACGGCGA
ATACTCGTTCTGGATCCAAGTGACCAAGCGGCCCAGGCCCCATGGGACGATGTCCCAGCC
GATGTCGGTTTTCGGCCAAGACGGGTCGACTTGATTCTTCACCTCTTGGTCCTGAAAGAA
GCTCTTCGGGTTGCTCTGAGGATCAGCCGTGACATAATCCGAGGAGTAGTGGTTCAGAGC
GAAGAAGTCCGCAGAGCCTTTGACCAGCTTCTGCTCCTCCGGCGTGAAGCTCGGCAGGCG
GTCTCCGCAGCGCATCCGCATCGTCACCGGGTACTGGCCCTTGTAGAGGGGCTCCGCGAA
CCAGCCCAGCTACCAGTCCAGCGCCCGGCGCTGCGCGGCGA
>at00637
TGTTCTCCTTCTTGACGGGCGGGGCTTCGCCACCGCTCGCTGGCACTGCATCGCCGCCCT
CCCCGCCCGCGTCCTCGTCGCTCAAGCTGTCCACGTCGCCGTCGTCCGCCCCGCCTTTTG
GGCCGGAGGGCTGCAGGAGGAGGTCCATGTTCGCCAGCGTGGAGGAGCTCCGGCTGCCCC
GATCGCTCGAGATGCTGGCGTCGTCCGAGGCAGTGTTCGAGGGGGGAGCCGACTTCGGCC

TGCGTAGGAAGCTCCATCCGTCCTGGGTGTCGACAATGCTGAGGAAGGTGTTCTTCTCTG
TGTACTGGTACGTTTGGGACAAGTCCACTTGCTTCAGAGCGGCAGCCTTCTGGCCAGCCG
AGCCCGCTGTCTTGCCCCCGGCTCTGTCGTCGCCCATGGGCCGCAGATCTCAGTGGGAAG
GCAGGTGGCTCTTGGGGGTCAAGGAAAGGGACGGAGCCAAGCTGTGTGGCTTGAGCCAAA
ATGGCTACGG
>at00638
TACTGCGGGCCGACGGCACATTGGAGATCCGGAAGACGATGGTGTTGCAGGGCTGCTCTC
GGCAAATGGATTTTCCTGTGTCTTTGACTCAAGACCGGCGAGCAGCAACTTTGGCTCTCG
GTACACGCCAGCATTTACGCATTGGACAGGCACGACGGTGGACTACATGTATGTCCATGA
TGTCACGCCACCCAGTGTTGAGGTGGTGGGTGCCTTCGTTTGCACACCACTCTCTCAGAC
CACCTCCCACTGGTGGTGGACCTTCGATTCTTTGGAGATTCCCCGATTGATGACCATTGC
TGAATCGCGAGCA
>at00639
TTTTTTTTTTTTTTTTTTGCAAATGTCACTGCCCTCTCCCAAGACACGTCCGTAGAATAA
CCACTGAAGCCGAACCAGGCCAGGCTGCGAGTGAAGCCTGGTGTGGACAGCTATTTCGTC
AAGTGGGCAGTGCCATGCACCTGAAAGTATACCCATAAGCCCTCCACGGGAAGCGTGACG
AGGGGGGCCTGCATTCAGTGCACCCAGGTTGTTGCTCCCTCAGATGTTCTTGCCAGCCAC
AATTCAAGGCCGTGCATGGACAGATCGTCTTTGGCAGCTCCTTCTCGGGAGAAGGACATG
CCTGGATCCGAGCATACTCACCTATCCGGGGCGTCACTCCAGGCTGCCAACGCAACACTC
GCCCGTTGGAGTGACAAGCTTCATGGTCATGCCCATCCGAGAATTGAGCACGGGAGCCAT
GCATGGTAAAAGCATGTGGCCATTCACAAACGAGCCACCAGCTCACACTCACCAGGGCAG
AGGTGCCATTACTTGTCAGACCCCGCAGGTGGTACTGGATCATGAGTGACAACTCCTTGC
AACTGTGTCTTCACTGAGGATCCGCACCATGTCTGGCTCCCCTTTGGCTTGGGCATCGTT
CGCGGGGGTGTCGCCCTTGTGGTTCTGCACGCTGACGTCAGCCCCCGCCTCCACANGTAG
CTTCGCCCGCTGCGGCCGTACCCATGCCGCGGCGT
>at00640
TTGGGCTCCAGCTGAAGGCAGGCATGTGCCACGGATAAGTCAGGCCATGCAGACGCTGCA
CTAAGGAGCACATTGCCTCGCAGCGGCAAACTTGGCTCCCCCCCCCCCGCATGGCGGAGG
TCCTCGTCCCTGTGCAGGTGTGCGGCCGCAGAGAGTCGCTGCCGGTCTCCCGCCTCTCCG
CCCAGGACCTGGCGCATGCGGTCGAGCGCTGCTTCGGCGTGAGGCCACCCTTCCTCTTCG
CGGACCACCGAGGGCGCCCGCTCCCCGACGACCGTGCCATTGCCTTGTGCGTTCAGGCCG
GCGAGACGCTCGTGGTCAAGCTCACAGAGGGTGTCAT
>at00641
CCGTAGCCATTTTGGCTCAAGTCTTCCCAGTACCCCCCGTGGAATCAGCTGGAGGCAATG
GTGTTCACACTGGAGGCTCTTTATTTGGGGACGCCAAAGTCAGAAGAGGAGATTCAGGCT
GACATTGAGGCACCAGCAGGGAAGGCGGGCAAGGACAAGGGCCTCGCATTGCTCGACGCC
GCGGAGGCCTACCGCATCCGGGAGGACGCCCAGCAGGCTTTCACGACAGTGAAGGAGGCC
CTGGCCATTTTCCGCGAGCAGGGTGCGGATGAGCTCGAGGCGCTGGGCTTGTGCTCGTGG
CGAGGGTGCGCATGGGTGGGGCCTACGGCCTTGAGGCACTGCAAGCGGCGAAGAAGGCAT
TGAAGCTCTACAAGGAGCTCGGAGACACC
>at00642
GACGAAGCCGACAAGACCAAGGCCCTGGCGGACACCCAGCAGCTGGACGACGACACGACT
GCGCAGCTCCAGGCGGACGAGGTCTTCTTCGCGGACACCAAGGCCGCCTGCAAGAAGAAG
GCCCTGGAGTGGTCGGAGCGCTCGCGGCTCCGCACGGAGGAGCTCCAGGGCCTCCGCAAG
GCCGTCGAGATCCTCTCCAGCTCCGGCGCGGAGCAGACCTTCGTCAACGCCACGACCACC
TTCCTGCAGCTCGCCTCCAGCGGCCGCGGCCGGCGCAGCGGCGTACAAGAAGCTCCAGAG
CCTGGCGCACGAGCACCGGGACCTGGCCTTGGCGAAGCTCGCTGCGGAGCTGAAGTCAGG
CGGCCACTTCGACGAGGTCATCGCCTCCATCGACAAGATGATCGAGCTGCTGCGTGCGGA
GGAGCAGGCGGACATCGA
>at00643
TGCGAACAATGCAGCTGTGAGCCAGTACGTCTTCTCGTTGTTCATCTTCAAGTGCATGAT
CACGCCCTTCGTATTCGCCTCGACCTTCTTGTCGTTCTCCGGCACTCGTCGACGCCTGCG
CAAGTCTAGCCTCCACCACCGCCTGCACAGACCCCCAAGGATCTTACCCATCTCCTCTGT
GATCACCCGCAAGGAATATGTATCGAGCCGAACAGAGCGGAGATCATCAGCCTTAGAAAG
TCCAGCATCATCTTGTTCGCAATCGGTCATTCCGTTCTGTATCATAGACCAGAGCATGAA

CTTCACCTTGCGGTTTCGATCTCTACGTTTCCTGCTGAGAGACACTGCATCATAACGAGA
AGCCATCAGCT
>at00644
TTTTTTTTTTTTTTTTTTCATCTGCAGTCGGCCACGCTTGCGTATCCAGTCGTCAAAGTG
GGCCGTCTCGCGAAAACTTCCGTGTGGGCTTCAGATTAAACTTTCCACCCAAAGACGAGG
TGGCGGATCGGTATGGCCTCTGCAGCGCGAAAATGGCAATCTGCTCCACCGACCTCCGCA
GAGGCCGCCAAGGCCTCACGGATGGGGAATACTTGCCTCTCTGTACTTTGCTGCGCTGTC
AAGAGGTCATGGGTAGGCGAGGAAGAGCCACACTGAAAGAGAACGACCGAAAACGTTTCT
TGGAATGGAAATCGTTGGCCAGGCCAACGCTAGTGATTCAAATGGCCTTAGACCTAGCCA
AGACGGACTTTGTGGACTCAGCTTCTGTTGGCTTGCACCAAGAGTCCCTCACACAGCTGC
CAGTTTTTCCGGGACTTGGGTGTACTCGGCCACAATCTCGCGTAGCCGGTCACCGGGCAG
AGTCTCCAACTCCACCAACTCGTCGGCAATCTTGTCAAGGCATGCACGGTTCTGTGTGAG
CAGCGTGATGGCGTTGATGAAGCACTCATCGGAGATGTTGCGGATGGCGTCATCGATCTT
GCTACTGAGCTCCTCAGAGTAGTTCGGCCCCATGAAGCCGCCATCATCAATCGCAATGGA
ACCCACTTCTGACATTCCGAACTGTGTAACCATGGCACGCGCCATGCGTTCCACTTGCTG
CAAGTCACCACCCTGCACACTCGTCACTGAGAGCTG
>at00645
TTTTTTTTTTTTTTTTTTTCTCTCTCTCTCTCCTTCTGCCACGGCGCCCCGGTTCCTCCA
CTTAAAGGAAAACTGTCNAAAGAAGTGGGCACCACGCAGAGCCCCGTAACAACTCTAAAC
TATGGCTGCGCTGTACCCCCGCAGCCCCCACAGAGTGAACGGGGCCGCCCGCGCAGGCAC
AGGGCGCCCGCCTCTCCCGAGGGAGGAACGGCAGGTCGCCCGAGCCCTTGGGACCGATGG
CGCTGCGCACTCACAGGTACGCAGGCCATTGACACCTCCCGGAATTCAGGAGGAATGGTA
GGCTGGAGCGCCAGGCCGCCAGCTACCACCGAATTGCCGTGCTTGCAACTTTTACAAACC
ATGGATTGCGGTGCACCAGGCTCATGGCGTCCACATGCCTCACGGCATCGCCGAGCAAAC
ACTGCCCCCTCCAGGGCAATGCATGACGCCAGCCTGGGAGAGCGTCGTGGGCGTGCTCAC
ACAGCTTGCATACATGCATGCAACGCATTCCCTGTACAAATGCACCCTGCCAGCATCACT
ACAGAATCCTATCAGTTCAACCTTGCCTTTCTTGTCCTGGATCCCTGCCTTGACATTGTC
AATGGTGTCAGGGGCTTCTGCATGGGGCCTGACGAAGACCTGCACGCTTCCGCGCATGCG
GAGCACAGGATGCAGCGTGGACTTCTTCCGGTCGTTGTAGTCGGACAGAACAATGACGCC
GTGGCCATCTGGGAACATGCAGCGGTCGACCTGCGCCTTGATGTTGTCCACCTTTGATGC
CGGCGCGCCCCCCGCAGTTCAAGCATCCCGATATTGTTGTCAAAGCGGCCAATGTCCTTG
ACAATGGCAATGTGCTTCGTCTTCCTCACGTGCCCCAGAGTGATGATCTTGAAATTGCCC
GTG
>at00646
CCGTAGCCATTTTGGCTCAAGGTCCGCAGCCATTCCGGCTCAAGCTCAGGGATGCACATG
GTCAGTTCCGCTTATGTGGCAGGCTGCCTCATGAATGCTTGCCTCGGTTGACAAGTTGCT
TGCACTTCTCGGCGTTGCCCTTTGCGTTGTCTTCCTGGCCTCCAGATCCGGCTTTGGATA
CTTGACCAGGGTAACGTCGCCTCCACAAGGGCTGGCACCATCGCCTCCACAAGGGCTGGC
GCCAGCCTGGAGCCCGCTGGTTGAACGTGCACAGGTCGTTGGGCCATGGAAGGAGTGGCG
GCAGCCCACGGAGTTTGTCGTAGTGGTGGCGCAGCAGCGTAGCGGCAGCTCGAACCTCAC
ATTGGAGCTTTCTCGCCGACACCTTTGTCTTTACGACTGCAACGAGTTCTTTAGTAGTCG
TCCGCCACCGGACTGTGTCCGACCTTTGAAAGAAGCCGGTCAGGGCCGGAGGTCGCCTCT
GGCAAGCATGCGGACAGTACGAGAACATCTTTGCAGTGCCGGGATGCCAAAGAATTATCA
AAACCCCAACGTCCCTGATGACGTCAAGACGCAGTGCTTCAAGGGATGCGTCACCACAAT
CAAGCTCTTCGGTCCACACACGGTGCATGGAGAGAAGTTGTTCGAGCTTCTGCGTTACGG
CGGCACGCGTGTTGTCGTGCTTGAGCGCAACCCTGCCAAGCGCAAGTGCAGCTTGGACTG
GGCGAAGCAAACGGGGGATTGGGGCACCGACCCCTCCAAGCATACGGGGAAGAGGCCTCC
TTGCCCCGCACAGGCCCCAGCAGATTTCCTGAAGAAACACCAACAATGGTATGCGGAGGT
GCGTGGGTGGCTCTCCAATAACGGCATCCCACGATTTGAGGTCAATGCCGATGCTTACAT
GGGCGATTTGGCAAATGTGTTGATGGACATACAGCGTTTTGCCTCCTTGTAGAGCAGGCA
AGCTGCATCATGTGTCTCAAACTTGACCGGCATGTTACGAGAAAGATTGTGGCCGTA
>at00647
TTGTTTTTTTTTTCTTTTTTTTTTTCTTTGTTTGTCTGCCTTCCTCAGTGCCTTCTGCAG
GCATTGAATCATTGAGTTTGGCTCAGGCTAGACTGGACCTCGAAGCAATGTATTGGCCGC
ACTTGCGGGAATGCCCGGAAAGTGTTCCCAAATCTTGATTCTGCTCCAGGCCCGCAACAT

CCCCTAGGCTAGGCTGCCTGGCAGCGCTCGATCTTTTGGAGCATCTGCTCTGCAAGAGTC
AAAAGATGTGCGTCACCATCCTTCTTTGCCAACTCCACCATCTCAGATGCAGGTCCCAGT
GCCTCATCGTACCGGCGGAGAACGAGGTAAATGTCGGTAGAGTGAAGCAGTAATTGTGCC
CTGCCATCAGCCTCTTCGTTACCCAGAAGTTGAAGTGCCCTGTTTGCCGCCGTAAGTGCA
TCCTCGTATTTCCCTTTCGCGCTGTGCGCCTGCGTGAGCGCACCAAGGGCAGTAGCCTGG
AGTCCACGATCACCAAGGTCTCGCGCCAAGGCCAAAGCTGCTTCAGCCTCTTGCGCTGCC
TCCTCAGGCCGGCGTTGCCCTTGGGGTCCGGACAGCAGGCTGTGTACGAGCAACAATCGG
ACAGAGACCTCTGCAGTGGTCGTTTTGCCTGACGCTGAAAACTTGCGACTTGCATTTCGA
GCATACTGCTGTGCAATCTGCAGCTGACCTTTCTCAGAATAGGATCTTGCGACCATGGCA
AGGGCACAAGCCCACCAAAACGCCTCACCCTTTGCCTCGAAAAGCTCAGTGGCCTCCCGT
GCTGCTTGCAGGCTCTCGCGATGCGCTTCCAGCCCAAGGTGTGCGGCCGAGGAGGCCAGC
AGAGAGATTCCTTCAGCCACAGGATCGCTCGCCTCGCGGCAACGCTTGCACAGTGCACTG
GCCTCTCCTAGAACCTCAGCTGCACGGCCGGCCGCGACATGTTCCTCGGCCAGGCCTTGC
AGCGCCTCTGCCGCTGCACGAACATCGCCAGCGGCCGCGAAGGCATTCACCGGGGCTGCG
CCACTCCCACCGACCGCACCAGCATCATTTGTGCGACCGCGGGCGAGGCGGAGAGCCGAA
ATGGCACGCAACACACGTCTCTCGGCCTCTGCATCACGCGGCTCTGCAGCGAAGAACCGT
AGAGCATGACGTGCAAATCGCTCGGCCTGTGCTAGCTGGCCCACTGCTGCATACACTGAT
GTCAGTGCCTCTAGCATGATGGCTTCACCATTCCGGAGGCCGGCCTCCTGGAAGGCCTCG
CGAGCATCTCGAGCCGTCCGCAATGCCGCCTCATGCTGGCCAAGCTGCAACAGGGCGTCC
ACACAGCAGGACGCTGCTTCTGCCTCTTCAGGCGCGCGAGGATGCATCGCCAGGAAGAGG
TCCAATGCCTCATCTGCAGATTCCAGTGCTAGCTCTGGCTTGCCTGCCTGAAGGTGCAAC
TGTGCCAAGGTCTTTAGCGCTTGGGCTGTGGCTCTCAGGTCACCGATCTCTTTAAACAGA
GCTGAGGCTTCCGTTGCGGCACTGATTGCCTTGCTGGCGGTGGCGCCTCCCCCCGGAAGA
GAACTCTGGGCCGAGGAGAGGAGTTCCTTTGCCATGGCATCCTTGTCACTTGAATCTCTG
GACTTGGTGCCACTCATCTTGCGCAGTCGCTTGATACCTCCTACCACTGGCTTGAGCCAA
AATGGCTACGG
>at00648
AAACAGGCACCTGCCATCATGAAGCGGTCATCTCATTGCAGCAACGAAGATGTTGAGTCT
TCCAACACCGAGGTGGTCGGCTGGTCCTTGCAGCACCCGCAGCACCTGCAGCACCTGCAG
CATGTGCAGCACCCGCAGCACCCGCAGAGCATCCGCGCCAGCACCATGGCTCGGCCTACA
GCAACCGGCTCAGGGTGGCACTGCCCCTTGGCCAGCGCCTTTTTCCCCAGGTGAGAGCTG
ATGTGGCGTTCGGAATGGCGATTTTGCTCGAAACAAAAAAAAANAAAAAAANAAAAAAANAA
AAAANAAAAAAANAAAAAA
>at00649
TTGTTTTTTTCTTTTTTTTTGCCTAGGCAGAGACCTCCAACGAGCGCGCAGTTGCTCACT
TTTGGTCCCGCCGGCTGGTCCAAAAAATCGTGCGCTCACTGCGCTGGCTCTGCATGGGGG
CATGTAAGTAACCAACCAGCCAGTGAAGCGCCATCTAACGGAACGGCATGGTAGCATTAT
GGCAACATCATTCTGCCCATGTCGACCCCCACACGTGCTCGGAGGCACCCGGACGGAGCG
TAGGTCGCGCCCTCACGCGGATGTCCCGAGCCTGTTCTGCCAGAAGTGGGGCGCGTTCTG
TTCGGAGAGCTCCTTGATCTTCTCTTTTGCCGTGACGGTGCTTCCCTTTGGCTTGACCTT
CTCCCAGGCCTCTTCCAAGCCTTGCTCATAGTCGTCCCTGTTGATGAGACCGTTGAGGGG
ATCCTTGATGTCCTTGAAGTAGGGGTGCTCCCTCCATGCAGCAGTGATTGCTTGGGACTT
CTTGGAGCCAGGGATCAACTGGTAGAAGGCGTCGTCCGAGAAGGAGTACAGCACGAAGGA
GCCGAACACCACTGCCGGCACGACGAGCGTCGTCAGCGGCACGAGGATGGCGGACCACCT
CCGGGAGTCGCCATACTTGGCCTCCGCATCCTCTGAGAGCATAGTCAGCACCGGAAGTGC
TGCAGACGCAGCCACTGCCGGGTTGAACTGTGGCCCTGTGACGGGGGCTGGTGCTGCCTG
TGGTGACGGCACGAATGCAACCTCCAGCAGCGGGACGCAGACGAGCACGATGGCCACTCC
GGCCGCAAAACCCAGGAGGCTGGAGCTGCGGCGCCGGGTGCCGGCCATGTGTGCTGGGGA
GGCTGCCTCTTGGCGATGAGGGGGACAGCGAGGGCGGAGACCGGGCAAGCACTTGAGCCA
AAATGGCTACGG
>at00650
TTGTTTTTTTTTTTTTTTTTTGTTCTGGCACGGATGCCTATGGTCAAGGAGTCGGCAAGT
TCAGTCACGGGAGCGATGAAAATGACGTATCTGGTGAAAATGATTCCCGAGCCAAAGGAA
ACTAGCAACAAGATCAATCATGCCAATGCATGGCAAAACAAACGACTCGTGCGTCTTGCA
GCGACGTCTAAGTGGCCTTACCTTCACCCCTCCACGGTCCCGCCCAATTCACGTCTTCCG

ATGAGAAGGGGAGGCTCGCTGCCTTCTGGAGAATACAGCCTGTGCAGCATACATCACTTG
CCCTGCTGAGACCGCAAGAACTTTGTGCTGTGGCTGATTATGCCCTGGTTGCTCCGCGGG
TCACAAGGCGCGCCGGTGAAATGGACAGCATTTGTTGTCATGAATCGCCTTTTTTCGCAC
TTGAACATCCCATGGTCAAAGTCTACCGCTGAGGAGTTCTTGAAGGGCACCGGAGCATCA
TCTACTGCCAATCGATTCCGGTATGAGTTCGGGTTGTAAGGGGCCAATGGCTTTCCGTCC
ATGGATGCATAGCAATACGGTGCCTCCCTGAACTTGTGCTGGTGCTCGGAAACGGTATCA
AACCGAAGCCTAAGGCTTGCAGCTGATCTGCTTCTTGGCCCATGGGGTAGCCCCAAAATA
GTTCCCGCCACACTGACGTAACCTGTGGCGGAAGCAACCTTTGATCCCTTGAGGTAGTTT
GCTCCGGCTTCCTGGGTGGTGCGCTTCGTCAGCTCCGTCATGGCAGTGACAAGCAAGGCA
CTGGGGGCGTTGAAGCCGAGTGCTCAGAATGGCGTGCACAGCGACACCTTCAGCCAAATG
GCTTGAGCCAAAATGGCTACGG
>at00651
TTNTTTTTTTTTTCTTTTTTTTTTTTTTTCCCACAACACTGCGTGCCAAGCAAGTAACA
CCCGTCTGGAGCAGAATCTGAGGTGACAAAGTTTGTACATTTGCGAGCAGCTAGCAAGCC
TGGCGGGCCTGCATCAGAAGGAGATGTTGAAAGAGCTGCCGCAGCCGCACTTACTGTCCG
CGAGCTTGTTCTTTACGACCTGGAAGGACGAGCGTATCATCTCCTCGACGTAATCGACCT
CCGAGTCCTCCAAGAAAGACATCGATACTTCGTCCACCACAAGTCGGGCACCATCCTGCT
CGACCACGAGGCTTCCCGGAGCGCCGTCCGCTGCATCCACGAGGTCGAACGCGTAGGAGT
AGCCCGAGCAGCCGCCACTGTTCACCGTCAGGCGCAGGACGGACGGCAGCTGCTGCTCCT
TCAGGCTCTTGAGCTTCCGGACCGCCCCGTCGTGCAGCCGGATGGGCGGCTTCGGGGCGT
CCTCGGCGGACGAGGACATGGCGTCGAAGGCGAAGTGCCTGGCTCTGACCGTCTGAAGGG
GGGCCGCCACGGGGCGGCCGCTGGGGAGACCTCGCCGGAGGGCGTTGCTCAGCAGGCCCG
AGGCCCTCATGGCCTCTGTCACGGTGTGCAGGCGCCGCGGCTTTGTGGACGCACTTGGCC
CCAGATGGCTTGAGCCAAAATGGCTACGG
>at00652
TTTTTTTTTTTTTTTTTCCACGTCCTTGCATAGTTCTCAGGCGAATTCAGAGTCAAACA
GCATGTTGCTGCTTGAGCAGATGTTCTTGGCGTGTTGGCAAGGCACGTCGAACGTGCTCA
GAACTCGTCGCCGTACCCCTCCTCTTCACCCTCACCCTCGGCAGTTTCGATGCCGACTTC
CTCGTAGTCTTTCTCAAGCGCAGCAAGGTCCTCGCGGGCCTCAGAGAACTCGCCCTCCTC
CATCCCTTCGCCCACATACCAATGTACAAAAGCCCGCTTTGAGTACATCAGGTCGAACTT
GTGGTCAATGCGGGAGAAGACCTCGGCGATGGCGGTTGAATTGCTGATCATGCAGCACGC
CCGCATGACTTTGGCCAAGTCGCCTCCTGGAACCACTGTGGGTGGCTGGTAGTTGATGCC
GCACTTGAAACCGGTGGGGCACCAGTCCACAAACTGAATCGTGCGCTTCGTCTTGATGGT
AGCAACTGCCGCATTAACATCCTTGGGCACAACGTCACCGCGATACATCATGCAGCATGC
CATGTACTTGCCGTGGCGCGGGTCGCATTTCACATACATTGAAGCCTCGTGCC
>at00653
GCGGGGCTCAAGACGAGCCTCCTGTGCTCCGTTCAACATGCCAAGTCGAGTCCTTACCGC
GAGCGCTTCAGCGGAAAGCCGGCAGCAAGTTCTTGGCCCTCATGGCCTTCGAGCGGATGG
AGAAATGCAAGGGCAGCCAGTAGCCTGGCGAGGGGTAGGGCATGTTGATCTGAATGCCGC
CCAGCAGCACGAGGTGGCCGGGGCCGTAGTCTGTGTTGCTTATCTTCAACATCTCCTCCT
CCACAGCCTTGTACGCCTTCATGACGAGGTCAATCATTGGCTCTTCGGAGTCGGACACCT
CCGGGACGTACTGCCTCAGCTTCTCGCGCAGCCAGCTCTGCTGCATGTCGGCGGCGTCGG
CGCGCATGTTCGCGCCAGAGGTAAGCTGGCTGTAGGCGGCGACAACCGCCCCGCATGACG
TGGAGGCATCGCTCTGGCCAATACGCAGGAACTTGCCTGGCTCGCCGTCTGGAGTGAAGC
CGATGTGAGGGCCAAAAACGATGAGGACGTTTCCGTTGTCCGGCACATGGTGTGAGAAGG
CCACAAAACCGGTCTTCCCGACATAGGGAGCACCGCCAATGCCACCAAGGGGGAAGCTCC
TTCCATAGTACTTTGTCAGGAGCGTGGAAAGGTGCCCAGGGTCGCCGTTGATCTCATCAG
AGCAGATTGACTGGCCATAGATGGTGTTGCTCTTCTTGAGGGCGTACCGCTTCACTGTAG
AGAAGAGAGTAGACATCACATCAGGACCCCTCATGGCGCCCGGGAAGGC
>at00654
TTCCGTTTTTTTTTAGTTTTTTCCTATTTTGGCTCAAGGGCNAGATCTGACANTNAcANG
AGGCACNCACGGGAACNANGCCTGAGCAATGGCCGTCGGcAANGAAcCAAGCGTCTGACC
AAGGGTGGCGGCAAGAAGGGCAACaAGaAGaAGGCGGGCGANCCNTTCCTCAAGANGGAG
TGGTACGACATCAAGGCGCCCAGCCTCTTCAGCATTCGCGACTGCGGTAAGACCCTTGTC
ACGAAGACCCAGGGAACCAAGATCGCTACTGAGGAACTGAAAGGCCGCGTGTTGGAGGTG

AACCTGGCCGACCTGAACAACGACGAGGACCAGGCCTACAAGAAGATTAAGCTCTGCATC
GAGGAAGTCCAGGGCAGGTCTTGCCTCACAGATTTCCATGGCATGTCGCTCACCCGCGAC
AAGGATGTTCCTGATCCGGAAGTGGTAGACCCTGGTATTGGACGGCCGACACCGTAC
>at00655
TTTTTTTTTTTTTTTTTGCGTGTTCAACCACTTGGGACCACTGCGGGCGGCGCTGGAAC
TCTGGTGCACATGCCTTCCCACTCAGCAGAGATGTCCCTATCACCACACAGAAATGCACT
CCAGATCCCGGACAAGAACCACTGTAACAGCAGCCGATACAACTGCAGCGCCATGCCATG
GCAAGCGTACCTAAATCACGACGCGCTCTTCTTGATGCAAAAGCAAACGAAAGCGCCACA
GCAAGTTACTGTCTTGGCAATCCAGAGGGGTTGAACAACGCCGTGGGTCAAAGTAAGGAT
AAAACAATGGGTAATTTGTGTTCCAGAGGTTGTTATGGAGGTTCGCTGCCATACCGTGAA
CGCTGCCGCGAGGAAGTCGCGAAGTGCCTGTGCCAGAACGAGCTGCCGCCTCCTTGTATG
AAGCCGGCAAAGGGTTCCCAACTGGAAAGTCATCTGTGATGGGGTTCATGACTGCGGAAT
CCAGCGATTCAATGGCTAAACCTCCTGCAGTCGTCTGGACCACAGCCCCCGGCCAGACGC
CATGGGTGTACTGGTTCCCCCCGTCCAGCACACCCTCAGGGTCTATCACTTGTCCAAGCT
TCTGGAGATGCCAAGCAGAGCCACCCCCANGTGGAGCCAATGGCGGTGCAGGCCTAAAGG
TCACCATGGTCGCCTCACCGATCATTGTTGGCAGCTTTCCCACAGTTGTAAAG
>at00656
GAGCGCCCCAGCCACCATTCTCCTAAACTTATTTTGGTTCCATTTCGTTCGACTCATCCA
AATAAGCGTGCTCATGATGCGATGGAAGGCTTTGACTCCCGGAAACCTGGCTTTCCTCTG
CTTGGTCTCCTCCTTGAGAGCGTGCGGAGACATCGCGACTTGGCGTCTGTTCCTTGCTTG
CACTCGAGTGCGGCGACGACTCTGAGGCTTCCTCATCGAGTGGCTGATTGATTGCTCGCC
AATGCCACTTGCTAGGCTTTGGGCTTCTCTCTAGTCGCCCGTCCTCTTCCAAGCGGTACA
AAGCTGGGTTCACGCTGCTCTTGGTGGCATCTCTGCCAGCAACCTTCTTCGCGATGGCCA
AAGTGGACATCCAATCACCTCTTCGAGAACAGACAGTACA
>at00657
ATAATATTATAATGTGTTTTTAAATGCTTTCGTAAGCACTTCGCTCCGTTTCTTGGCACA
ATTGTGAAGAAAGCATTTGATTGGAGTTACCTATAAAAGTTCCCCTATCGACGTATTTCA
GCAGCTGAATGAATGAATGATGACAAGAAAATTTGATGAGGAGTGACGACAGTCGAGGGA
TATAATTCAAGCTCCATCTGATCCTATAGAAAAGGTTATCTGAAGTTTAGTGTTTTTAGT
TGCTTAAATGTGGATATATTTTTCTATCTATCCATTATTGTGATGTAACTGCGCCTAGTG
GCACGGCTGCTCATATTAGTATGTACGCTTGTTGCTGCTTCTCTATTCGTTTTTAATATA
TTAGCGGTGTGCAGGTGTCGCAGCTGAAATTCTACAATTTCTCGTTTAAATATAATGTGT
TTCGTTTACAAAAAAAAGAAAAAAAAA
>at00658
TTTTTTTTTTTTTTTTTGACTCATCAAGGCCACGCCACAACGGCTCATGCCGCCCTCCA
CACTGTACTGCTTCTATCCCCTTATGTAGCACCACACATGACGAGGACAAGTTTGAAAAA
GTCAGGCTTTAAATGTCGGTCAGCAGAGTCGCTCAACGGCACGTCTATCGCTGCCAAAGC
CACGCCAACAGTCGACCCCACTGTCAATTCAGCTTGTCACAAACCGCAAGATGTGGACCT
AAAAACGTGTGTCAGCTCAAAGAGCGTGCCGAGGGCATGGTCCCGAGCAGCAAAGGTTAC
ATCTGCGCTGAGTGCAAGCCGAAGCCACGGTCCTGCCGCAGACACACAACAGCTGACCAG
TGCTCTGCGGACCTTTCCCCTTTCCCTTGCCTCCCTTGCCAGGCCCCGCCACTGCTCTAC
CCTTTTTGCCCCCCTTGATGCCTTCAAATACTCCGTCTGATCTCCTGTCTCTCTGCTCCG
GAGCTCTGCTCCTGCCTTTTCCGACACCAGCCCCTTTTCCATGCGCCCGGGGGGGAGCAT
GCTGCGCAGCTACCGCAGCGAGCTCTTGTTCAGAANATGCAGTGGCCTCGATGGCAGCAC
GCAAGCCGTCCTTGTCCCGANGCTCGTTCCGCATCTTGGCTCTGCGCTTCTTGCCGTAGG
CAGGGGACATGT
>at00659
CCGTAGCCATTTTGGCTCAAGGCGTTTTGATGTTTGTCGTTGTTTTGCTTGGATGTGTGT
TTAGTGAAGCTCGCTCACCGCTCGCATGGCATAACCTCCTGAACGTCTGCCCCTGAGAAC
TGCCGTGGGCAGAAAAGCCACGTGCTTGGACAACCCCTTGGCTCAACGCAGCAGTGCTCC
GATGTTGCTTTGCTATTTCTAAAGGATCTTGTAGCTCCAGCTGCTCCGAAAAAAGTTATG
GCGCGGGCCGCAGCCGTCCTCGGTGGATCGCTGGCCGGTGCGCACGTGCTGCTTGGAGGT
GGTGGTGTAATCTTGATGGCTTTGCCCACGCCGTGGAGACGCCTGTTCCAAGCGCGAGGC
ACGCCTGCTGACGACGGCCTTCAATTTGAGTCGTTGCGTCTGGCAGACGGCTGTGCAGCA
TGGCTAATTGGCGGCGGTGCACCTGGCAGCCAGAGGACTGCTGTCTGCCTTTGCCATGGG

AGGTCGAGGAGCAAGGCCTGGCTCAGGCCGCTCATCAGTGCCTTGGCGCCTGAGCATGAC
GTGATGGCGTTTGACTTTCCGAGCCATGGCGAAAAACCNGTATGGTTTCACAACGGTTGG
GCTGCGAGAGGCAGACACCGTGGACCGTGCCTTGGAGTTGCTTGCCGAGCGCGGCTATGA
ACGAGTCTTGCTCTACGGTTGCAGCATGGGTGGGGCCGCGGCGATTTTCAGCCAGGGCCG
GCAGCCCAACCCTCGCGTTCGAGGGCTCGTCACGGACGGCACATTTGCAGCGCTCAGTGA
AGTGGTAGACAGCAGAGGAGGCTGGCTTCCCCGGTACTTGCGGCTGGCGGCGCTTGCCGT
GGCAGGCAAGCTGGTGGGCTACGATCCCTGGACTGTGCGGCCGATTGATCATGTGGCAGA
CATCAGAGTCCCCCTGCTGCTGCTGCACGGCGATAAGGACCACCTCGCGCCTCCGGATTC
CGCAGCCCGCATGGCAGGCCGAAGCTCTCAGGCCAGAGCAGCCACGTACCATGGCCGACA
TGACGAGCCAGGCAATCCTGCGGTGCAGGCAGCAGTACTTGCCTTTGCCAGGGAAGTCTT
GTGAGGGAAGGGCAACAAGTCTACACCTGCAGACACACGCATGCACAATAGACCACCGCG
CAGGCACTACAGTCATGGAGCTGAAGACTTGCAATTTGGTTCCGGGTGGC
>at00660
TTTTTTTTTTTTTTTTCTGTGCGCCCTCCAAATGAAACCAGGTCTCTCATGCGCTGCCTC
AAGCGTGCCAACCGCTGCTCTACGACTGCACCTTCTTCACGGGGCCGGTGCATGGTCAGA
TGCGTGACTCCGAAACTGTGTTTGAAGCACGCAGTCTGACACGGGGTGCAACTTGAAGCA
TTTTATGTACAGCTAGCCTGCTTGGTACAGAAAAGCATGTACAGATTCAATATCTGCACT
CGTGGCACTACCAAGAGAAACTGCCAAAAGCAAAATTCGGCATCCATCGGACCAGCATGC
AGCTAGGACACCTCCGTGTGTCCTATGTGCCACTGCAAAACTTACATTCCGCAAGATCGC
ATGTCGCCTGCGAATGTTAACTATCACACTGTTAATCGACTG
>at00661
CCGTAGCCATTTTGGCTCAAGCCATCTTGGCCCAGGGGCTTCCGACCTTTACAGCCCCAT
CGGCGTGGCGGCCCTGTCTCATGCGTTCCTCGCTCCGCTGCCTGGCCTCCTGGGCCTATC
AGGGCCATCGGCCCCAGGTTGACAAGGCTGCCTACGTGGCGCCCACGGCGGCACTCATCG
GCCGGGTGCGCTGCGAGGCCGAGAGTTCTGTGTGGTTCGGGGCCGTGCTGCGCGGCGACA
ACCAGGACATCGTCGTGGGCCGGCGGGCGAACGTCCAGGATAACGCCGTGGTCCACGTCG
ACGCCGAGGCGCCGTGCCACATCGGCGAGGGCGTCACCGTGGGCCACCTGGCGATGCTGC
ACGGGTGCACGGTGGGCGAGAACACGCTGGTGGGCATCGGCGCCGTCATCCTGAACCACA
CTGTCGTGGGCAAGAACTGCCTGATAGCTGCAGGCACCTTCATCGGTGAGAGGAAAACCA
TCCCAGACAACAGCGTCGTCATGGGCTCCCCTGGCAAAGTGGTGAAGCAGGTCTCTGAGG
AGATGGCGGCATCTTTCCGCCGCAACGCTGCCACCTACGTGGAGAAGATCTCCAAGTACT
CAGATCTGGAGTTGCTGGAATAGCACGAATGGTGTTTGCATGCTTCAATGAAGGCTGCAG
CGTCTTGCTCATGGAGAGGTGGCTTCCTGCCCAAAGTGCTCAGGGAATGTACATGCACAT
GTGAGCTCCTGTTGTAATAAAAAAAAAAAGAAAAAAAAAACAA
>at00662
GCGTCGACCCCTCGGATCCTCGGCGCTGCCTGGCGGTGCCGAGGGGCTTCTACGACGGAG
GCCTCCTCTCTCTGGTCTTGCGCTCCTTGCGCCGCGTGCCCGACAGACGGCGGATGCTCC
GTCGAGCAGCGGAGACTGTCCGGCCAGGGGGGCGCCTCGTGGTCGTAGAGAAGAACAAGC
TTGCGTCGCTCGTCAAGTACCAGGGCCCTTGGGACAAGCCCTGGCAAGATGCGGGCCTGC
AGCGGCGGCAGACCATCTCGGGGCCGCGCGCCGTGCTCGTCAACGTCTTTGAGCGTGTGG
AGCCGGAGGGCGATGGGCCGTGATGCGCGGAGCCATGGGATGGGGGGGAGGAGGCGCAAC
GCGTCCTGCAGCAGGGCACTGCCGTTGGCAGGCTTCTGCACACACGCAAAAGAAAAAAAA
AAAAAAAAAANAAAAAAAAAAAAAAANAAAAAAAAAAA
>at00663
TTTTTTTTTTTTTTTTTTCAAAGAAATCGTGCAGAGACACATGGAGGGGCGATGAAGGCC
TCACGGCGCTGCCGGCTCGAGCAGCTTGCGGCTACGTGACGACATGTGCGCCGCCTCCGC
GGCCGCCGTTCGGAGACACTTGTCGTCCTCCTCGTCCTCAGCCACGGCTGAGGTCACGGC
TCGCTCCCGGGAGAGGTAGGCACCCTTGTCCGACACCTGCCAGAGGAGCTGCCCAACCCA
GTCTGCGACGAGGACCCAGCCTCCTCGCTGACAAAGGGTCGGGGGCAGACCGGGCAACCA
GACAGGGTCCAGCTTCGCCCACAGGCCACCGTCGCAGACCTCCATAGCGTCAGTGGCAGC
AACCTGCATGCACTCTGGTAACAGGGAGACCTCCGCGCCCAGGAGGCCTGCGGCTTCCAG
CGGCCGCCACACGAGCTTGTCCAGCCGAGCTTCGTAGACAGTCCGGNGCTTGTTGGTAGC
GACGTCTGCCCAGTGCAGGTCCGCATCGACGTGATGAAGCAGCGAGCCTAGTTCCGTATA
GAAGACGTTGGGGACTTCGAGGCGGTCACACTCGCCT
>at00664

CCGTAGCCATTTTGGCTCAAGCCGCCATTTCGGCCCAAGCTCTTCGCGGTGCTCGCTCCG
TGCCGCACATGGGCAGCCGCTGGCGCGCCGGATACGCTCCGGCCTCAGGGCTGTTCCGTC
GGCCCTCGGTCGCCGTGGCCTCCTTTGCGGCAACCCAGGTGGCCGCTGTTGCCCTGGAGC
CGCTTGCCGGCGTCCAGTGGCACACTCCGCTGACCGTGGGCTATGGCCTGGCAGCTGGGG
GCTACTGCTTCGCCAGTCAGGCCCTCCACGCTGACCTGGCTCACCTCCGCAGTGGCGCCA
TCGCCGCACTGCCGGACGCCGCACAGCGGGCGCGTGCGGCGGCGCAGCTGGGTAGAGG
>at00665
CAAACTCAATGCCCTGCTCGCGGCTCAGTACCCGGTGGCTGTAATACAAGTCCACATAGT
CGAGCCCCAGCCGCTCGAGGGAGCCAAGCAGCGCTGCCTTGACGGTCTCGTAGTCGGACT
TATTTTTGCGGATCAGCGGCATAAACTTAGTGGCCACTGTGAACGAGTCCCGCGGCACGG
TGCGGAAAAAGGCGCCAAGCTGGGACTCATTGAACACGGTGTCCGGTGTGACTGGGCCGT
TGAACGGACCCGACTTGTACACCTCTGCCGTGTCGAAGTGGCGGCAGCCGGCCGCGTGCA
CGCCCTGGAAAAAGGCCAGTGCCTTCGCCGGCTCCAGCGGCGTCCCGTTGTCATACTGCC
CAGAGACCGACATGCACCCGAGCCCAAGGCCGCTGGCCTCCTCCAGCACGCCGCCAAGAA
CCACCATTGCTGAGTGTTGGCTGCGGTGAGAAGCCGCCTTGGAGTGGGCAGGATGTGGCC
TGGCAGCTTGGCTGCTCACCAAATGCTTGAGCCAAAATGGCTACGG
>at00666
TTTTTTTTTTTTTTTTTTCGCACATACGCACCCAGGCAAACAATGCTGCGTGCATAAATC
GACTTTCCCGCCCTCACATCTTCATGAGCGTGTACGTCTGTGTCTCAATCTGGCTGATCC
CGTCGATCTTCCCAAGCTCCGCAAGGAATTCCTCCTCCGGTTCAAATTTCTTGCCATCCC
AGTCGGCAAACTTGATGGCAGGGAGAGAGGTGATGACCTTGAAGTCCTTGCACGCGCCGC
ACACCACCCGCTGCACCTCGATGCAGCCGTCCACGATCTTGATCTTCTCCAGGATGAGCG
ACAGCGCCTTCTGCGCCAGCTCCAGGGACTTCTTGTCGCAGTCGTCCGACCACTTGCAGC
GCCACTCGCGCGCGATGACGCCAAAGGTCACACCCGGCGCGATCTCGCCCATGGTGGGCA
AGCACTTGCAAAGCAGGACACAGGGGGGATACTCAGCTTGAGATCACGCGACTCGTGGAA
CTGGGGCTCCACAATTAGCACCACTGAAATGATCAGCTTGAGCAAGATGCCTGCGG
>at00667
TTTTTTTTTCTTTTTTTTTTTTTTTTTCTTTTTTTTTTCCTTTTTCCAAAGCTACATCAGG
GCATGATCCATATTCCATGACGGAAAAAAGAGGCGAGGGCAGGAGAATGTGCATGTCTTT
GCGTCTATACATCACACAGGAGCCGGCGGCCACATCTGGCTCACTTGCACCTTTTGCAAG
TCGGCACTTCCCTGCTGCAACCATCAATTCTCATGGCCATTCTAGTCAGCCTGCCGTTTC
CACCTCTTAGATCGAATTCAGTAATCAACAGGTTCTTGTCGCCGCATCGAGCAGGAGATG
AAAAAGTTTGCCGCGACGACTTTTACGACCTCGTCCATGTGGGCATCGAGCACATTGAGG
TTCTTGCACATCTGAGCCAGGTCGGACTTGCCTTCTGGGAGATCGGACTGCAGGCCGACT
ATGAAGGGCTGCTCTTCTCCAAGCTTGCCACCCATGCTCAGGACCTTCAGGTAGAACATG
TTGTTGAACAGCGTCCCGTCCTTCCGGGCGTTCTTCTGCACGCAGAACAGCTCGTCGCCG
GGCCTGCCCTTCGGCATCCAGGACTCGCGCTGGCCCTGCGGGACCCAGTAGTCCTTGCCG
TCCTTGACGGCCAAGCAGAAGGCCTTCGCATGCTGCCGCATGGCGGTGTCTATGAGGGCT
GGCGGCACAGGGTCCACGAGGAACCTGCAGTTACGACCAACAATCTCCTTCATCGTGTAG
CCACAGAGAGTGCCGAAACCTGAGGAGCAGCCGACAAGCGGACAACCCTCTACCTTTGGA
TCGGCAATGGTAAACGAGAACTTCAGGCCCAGCAAGCTGTCTCCGGAGCTGCCGAAGATT
CGGTCCACCGTCTCCTGAAGCTGCTGCGCCTTCTCCGCCGTCCAGTTGTTGGCCGCGCCG
CTCTCCTGCTGCACGTCTTCGGGTAGGTCACTGGCAGTGGGGGACGAGCTGCAGCAGCCG
CCCATTGGCTTTGCCCCTACACGTTCGTCACTGGGACGACGACTCACAGAGAGGAGATGT
TCAAGCCCTCGACGGCTTGAGCCAAAATGGCTTGAGCCAAAATGGCTACGG
>at00668
TTTTTTTTTTTTTTTTTTGCACTCTTGCCGGCTATCGTCCGCTCCTTCTCTCAGAGCCCT
CAGATCATGACCGTGAGCACTCTGAGAAGCGCACCTCTAACACGCCTCAAACGTGGCACA
TCAGGCACAGCAGGCTGGAGGTCACCGCCCCGAGGCAAACGCAGCAGCGGCGCTGGCGGC
CTTCAAGTCCGTCGGCTGTACATAGTCGGACTTGGCAGGAGCCCCATCCACGTCTGTGCC
TGGTCCTTCAGCATCGAGTGGGGCTGGAGGCTGTTCGTCAACACTGTCAGCAGTCTCAGC
AGCCCCAACAGCCTCGTCAGCCACAGCATCCTGAGCCGCAGCGCCCGGTCGGGGGTCTAG
ACCGCCAGCCGCAGGATCGCCAGCGGGAACGGCATGGGTCGCCGGCGACTGAGGCAGCTC
GCCGCGGGCCGCGCCTCGTGCC
>at00669

TTGTTTTTTTTCTTTTTTTTTGGTCTTGCAGCTCTGTGGCCTGGGCACCAGAGGGGCCTT
CTCGGCCAAGCGTGAGCCATGCCGGCGCCTTCACCTGCCGCCGCCGGCACTGCCTCACTG
GTTCTCGTCATCCTCGTCGGGCGTGACGGTGAGGTCCAGGTCCGAGCCAGTGATGGTCCG
CCACATGCGCAGCACCTCGGAGGACGCGTCCTCGAAGTGGGAGGTGGGGTCGTAGGAGCT
GGTGACGAAGACCTGGTCCGCCGTGCCGTCCGCCAGCGGGACCTTGTACTCGCTGACCTG
CACGAACTTCTCCAGGATCGGCCCTAGGAGCTTCAAGGCAGGCTGGATCTCCTTCTCAGG
CTCACCTGTCTCCACGACGGTGCTGACAATGGCCACGTACTTGTCCTTGGCAGCGATCCT
GTGCGCCCACGAGACCATCATGATGTAGATGTCGTTCTTCCTCCCCAGCTGATTCTGCGG
CATGATGATCTGGCAGGATCTTGCCGGCTGCCCCTCCTTCGTCCTTGTGTCGGGGATGGG
GGCCCCCAAGATGCAGATGGCGCGGACGATGAGGGACGTGATCTGGACCTTTGCCGGCTC
TGCATAGGAGGGGTCGCAGATCACCATCTTGCACTTGGCGACTTCCTCACCCGATTTCAC
GCCGCAGACCTTGCCTGCGTCATCGTACACAAAGCCATCCACAGGCTTGTTCAGCATGTA
GGTCCCAGCGTTGATGGCTGCCAAGCGTGAGAAGCCTTCAGGCAGCCCGCCCAGGCCATA
GATCGGATAGATGAACGGCGATCCCCCGTACCGCAGCAGCGACTGCAGGTAGAGCTTGCA
CTTCTGGATGGTCGACCCGCAGGCCTTGCTCATGTAGTCGTCGTTGTGGTGCAGGGCCAC
GGCGTGGCCGATGAAGTCGATGGTGCTCTTCTGCAGGCCAAACTTCTCGTAGACCTGCGC
CATGGTGTGCCTCTTGGCGTCCAGGTCCTGGTGGGTCGACACGTCGCTGTCCTCCCAGTT
GCCGACGAACTGGACGAAGTTGATGAACCTCGGCTTCTCCAGGAGGCCCATGAGGGAGGA
CTTGAGGCCCTCCTGCGCCGTGGTGGGCACGCTGTGGATGGCCTTCGCGCTGCTGAAGAG
CCCTCCTTCCTGGGGCTGGTAGACGTAGCTCCTGTCGCAGGACATCCACTCCAGATACCG
CGAGACCTTCGTCTTCTGCAGGATCTTGACCAGGTCGCCCTCAGCCATCACGAACTTCGG
CACGAGGTCGACGTTCCAGTCGCGGTTGGCGCCGAACTCGCTCGGAGGCGCGGTGCCTGG
CCGGAACTTCTCCCAGAGGTTGGTGATGTTCAGGGAGGCGCACTCGCCGCCGTAGTAGTT
GTTCCTGTCGAGGTGCAGGACCTTCTTGCCGGCCACGGAGAGGAGCCCGCTGAGGATGCA
CTCCTTCAGGCCCGTGCCGCAGACCACAACATCGTACTCCTCGTCCATCGCGGCAGGCGT
GTGGGGTTAGCTCCCAGCCTGCATGGCAGCTCCGCGTGGCAACGTCCGTGCCGAGC
>at00670
TCCTGCGCTCCTTCGAGGCCGAGCGCCTGCGCGAGCGCGACTTCCGGGGGCTGCGGTGCT
TGCCCCGCGACCTCGACCGCCGGTCCAGGGCCCGGCGCCGGGACTTGCTCCGCGCGCCGC
GCGAGCGGGACCGCTTCTTGTCATCTTTGATGGCCTTCACTGCACCCATGGCATCTGCGA
ACCCAACTGATGAGGCTGCCACAGCGGTGGAACCAGACCCCTCGGAGTTTGTCAGCGCCA
GTGGTCCGCCCGGTGGCATGCCACCAGGAAGATTTGGGAAAGGATTGTTGTAGCTCTGCA
TCGGGACCGCTGATGCTGCTCCAGCACCCTTGCAAGTCCGGCCTGGAGCACAGATCAAAC
GACCAGTGCCATCATGGACAAGGCTGTCGAGGCTACGCATCTTCCCATGGAGGGCGCACA
CCATAGTCGCCTGTGTCGAAGGAACGCCTGCACCCGCACCCTTGCATTCAAAGCCAGGCA
TGCAAATTGTGCGCCCGTACGGGTCGATGCACAGGTACTTTGAGTTTCTAATCTTCCCAT
GTGCAGAGCACATGGCGTCCGCCCGGCCCTCCACGGTGCCCCCCGGCCCTGCGCTGGTCT
TGCACTCGTTGCCCGGCGCGCAGACGAGACGCCCGTCGCCGTTGTTGACCAGGCAGTCGA
TGCTCCTGAACTTGCCGTGAACTGCGCAGGCCACCTGGCTCTTGAAGGAGTTCGCTCCAG
GCCCACCGGTCCAGTCGCCCGTGGTTCCGTCAGTG
>at00671
AGGGCATTCTGTGCCAGCGACTGTGCCAAACTTCTCCAGGGCTCGCGGATTGGGATTGAA
GGCAGCCAACTCCTCAGCCAGCGCCACGACACTGTCTGCAGCGCTCCCAAGCTCCTCGGA
GATGCGGTAGAACGCACCATCACTTGCCAAGTTGTGAGGCGCCCGCGACTTCTTGTCAAG
CTCTCCACCGCGCACACCAAAGTCCACCGGCGTTGCGCCCTCTCGCCCCTGCACCTCCTT
GAAGTGTGCAATTGAGCTGATCAATGCTTGCTTAGCTTGCAACCTGCCCTCCTCAGGGTC
CTCCTCCGCTGAAGCCTCTGTGCGCACACCCGATGACCCCCGCCGTGCCGTCTTCCCCAC
ACTGTACACCAGAAGCCTTGGGCGGGAACTGCGAGCTCCTCCGGAAGAAGCGCGGCCAAA
CTTCCATGTCTGTCCTACACAGAAGCTCAAAGCATCTGCAGCTTGGCGGGCCAGACAGGC
AAGGGCTATCAGTGCTAAGGCCGACCTGCCGCAGAGCGCCACATGACGGCTGCTCCG
>at00672
TTTTTTTTTTTTTTTTTTCAAATTGCTATCGATTACAGGTCAATAGATCTGGCATCACCT
GAATGAGGCAGCTTGCCAGCGCAACTCTCACAAGTTGCTTCTTGCCGCTTTCCGTGCTGC
CATCTCTTCCATCCACGCAGCCTCGACCCTGGGAAACTCGTCTCTGTACCATCGCTGGGT
TACCTGGGGGAAAAGCTTCATGCGATCGTACGCGTAATCCAAGCCGCGGTCTCGTTGAAC

GATGCGAGTTCCATCGAACGGCTCCAAAGGGATGTCAAATCGAGGCAGCCGGAAATATGG
CAGGCTCACGCGGCGGCGCTGGACCTTCAGCGCGTTCACGCGGTGCATCGTGGCAATCAT
GCGGCCACTGCTGAGCTTTTGCAGGGTGCCGCCGATATTCACGACAATGGAGCCAGGAAT
CACCGGCACGTCGATCCACTTCTTCAAACCTGGGGAGTAAACTTCAAGCCCGGGCTGGTC
GGCAATCAGCAGGGTCACGAATGGCGCTCCGTCTACATGTGCACCGCCACCTCGACGGCC
CTGCTGATCACGCATGATCTGGACGCGCTCGTCGTCTGTGAACTCATCCCTTGCGTGCAT
GTGGTTCATGGAG
>at00673
TTTTTNTTTTTTTTTTTCCTTTTTGATCCTGCTTGGGTGGTCAACTGACATCAGATGGCA
GGCATCCTGTAGCCTCTGTCGGATCAAGCCCTGATGCCACTCGAATATGGCAATTGTGGT
CGAAAATCGACGGCGTGGCAGTGGCAGCGAGCCGTGATGACGCCAGGCGCTGCAACAAAT
TGGAGACCTCCTACGCCTGGGTGGAAGCAATGATGTCGTCGATCAACTGTTGCGGGAGTG
GCCCGCCCTTCGCAATGAACGGCGCGCAGTACGAATCCGAGTTCAGCCTACAAACGTCCC
ACCGACGACCTTCGGGAATCCACTTCTCATCGATGTATGGTGGTGAGGACGCTGTGAGTG
TCTCTGCG
>at00674
TCTCCGACTCAGAAGCAGTGGTATTCTAACGTCTAGTAGTTCCGTTAGTCCTATTTTTGG
TCTTCTAAGCTGGCCCAAGCTCCCCCTTGCGCCCCGCGGCTCCGCTTGAGCCCAACCCGG
CCCCATGGCGACCTCCAAGCCCCTCGGATCCGCATCCGGCCCTGGCGGTGACAACAGCGT
GGTGATCCTGCCCTCCTGCCTCTTCCACGTGGGCAACACCCAGCGCGAGCAGGACCTCTT
GAATCTGCCGCCCCCAGCTTGCTCCTCAAATCCTGGTGCCAGCAGCCTCTTCGCCCTCCG
CACGAAGACGGCCATGGGCGTGCAGACGAGCTTCGCCACGCGGAACCTTGCCCCTGTAGT
CAGTGCCCGGAGGGTCGAGCCGCTGCGGAGGTGAGCGGCCGGTAGCGGCGG
>at00675
TTTTTTTTTTTTTTTTTTGGCCAACTAAAAGTCGCTGTCGTTGTCTGTAGAACACTCGCC
CCCGCCCGCCGGCGCGGCCGCGCTCCCCGCGGCGCCGCCCGCCGCGGGGCCGAGCCGCGG
CGGCCCTACTCCGAGCCCGAGCTCTCGTCGTCCTCCGCGTCCGCCTCCCGGCCGCGGAGC
ACCGTGGAGGAGGAGACGCGGCAGAGCGGGCAGGGCATCTGCCGCCGGAGCATCTGCGCG
GAGCACTCGCTGCATAAGAGGTGGCGGCAGGAGAGCCACAGATCCCAGCGGTCGTTCTCC
GACTGGCAGATCCAGCATTTGTTGTCCTCATAATCCTCAAAAGATGACTCGATAAGGCGG
TCCCTCTCCATATCAAACGGACGCTCGAGGACGATTACACGAGCTCCGCCTATCCACAGA
ACTGAACGCCGCTTACCGACAAACTCACAATCCCTCTGGGAGACGCTGCCGCCATCAAAA
TACCCGCGGCCAGAACCCATCTGCGGCGCTGTTTCCACGCTTTGGAGAAGCCAGTCGTGC
ACCTGACGCCTCCAATCCATATTGTCGTGCAAGTCTGCTAGCCGTTGCCAACCCTCGTGC
CGAATTCT
>at00676
GTCATCCAGCAATTGCAAGACCTCATCGATGGTGTGCTCTCCCCGGCGTACCTTGGATGT
CGCATTCGGATCTTCCAGCAAGTCAAGCACCTCACTGATGTTGCGCTTCAGCTTCCGCTG
CGGGGCAGACAGCGGAGCTGGAGCTCGCGACTCCTCGCCAGCAGCTGGATCCGTGACCTC
AACTGTGCTGAGCAGTAGATCAGGAAGCTTCGCGACCAGTTCGGGTTGGTGGAGCAGCGC
CTCTGCGTAGAGCTCCTCTCCCATTCGCACCCACATGCCCAGGGAGCGCTGCATCAAGTC
CGCGTCGGACGATGCCATTGCTGCTTAGGAGTGGCCTGGGTCAAATCAGGCCTGACAAAA
TCTTGAGCCAAAATGGCTACGG
>at00677
CCGTAGCCATTTTGGCTCAAGGGAGCCGCTCCCTCGCTTCGCCAATACAGCCCCCAGTCG
GCGGCTGGCTGGACGTCTGTAAGACTCATCTTGAGGAGACACCAGCCCCGTTGCAACCAT
GGCGACGCCTGGACAGTTGCAGATGTACAACAAGCTGGAGGTGGGCGAGATGTTTGGCCT
CGACCACCGGGCGATGGATTCTCGAAGTGGCGAGATACACCGGATACATGTGTACCCCGA
TGAGAAGGTCCCCAACGCCTGCACCTACGTCATCTGGCTCGAGGACCACACGCTGGGCAA
CATCCTTCGAATGGAGCTGCTGCGGAACGAGTCTGTCCTCTTCGTCGGCTACAAGGTCCC
CACCCTCTCAACCACATGATCGAGCTGCGCATCCAGACACTGCCCAAGTCCTCCCCGGAG
CTGGCGCTGAGGCACGCGATCGCGAACCTCAAGACGGAGTGCAAGTCCATGCTCGACCAG
TTCGACGAGGGCGTGCGGGAGTTCAAGGAGGCGGTCGCCAAGACTCAGCCGCCCCCGGTG
CCGCCCGCCGGCGTCCTCACCGCCGGCCCGCCA
>at00678

TTTTTTTTTTTTTTTTTTCCCAACGCAAAATGACCTGGCCATCCGGTAAGACCTGCAAGT
CGGGATGATGCCGAGCGAATACCTGCGGATGGCTACGAGCCAAGCTCTTGAACGCATTCA
TACCATAGGAGGGAATTAAGCAGTTGGTGTCCTGCCTCGGACGTTCTGATGCCACGGCAT
CAGACTCACTCCTGCTGGAGACAGGGGGTGCGCTCGATGGGTGAATGACGGCCTCCAAGG
AGGGGTTCCAAAAGAAGGGTGCTGACAGACGTCCTCCACGGCCCACTGGAGGCCTCTGAA
CTCTATGTACTGTGGAGAGGAAGGAGCCGCCAAAAAGGAGCTGCACCACCTCTCCGAGGT
TACACACAATTGAGCCATGAATTGGCTCTGCGGCCACCCATTGACCGCTGCCGTTGAGCA
CCTCGAGGCCGCCGATGTCATCTTGCAATAGAAGCGTGAGAAATCCAGAGTCGCTGTGCG
CACCCACGCCCAAACTAGCCGTCTCTTCGACGCCCAGTGCCTCCTCTGTGGCTGGGTAGT
GGACCAGCTTTGCCTGTATGTGGGGCGTCTTGAAGAGGTCATCGAGAGCATGCGCCTCCA
AGCCCAACGAGGATGCAAGGGCCCGAGTCACACTCCAAGAAAGCTCCTCCATCTGCTGCA
ACCACTGGCTGACGATGGCGCGAAACTCCGGCGGTCCTGCTGGCCACTGGTTAGGCCCTC
GCAGGCGCGTGAAGAGCGGGGCGTCCGTCAGCGCTACTGGCACCGGCTCCTCACGGCCAA
ACTCGATCTGCTCGCGCTCGTCGCGGCGCCCAGCGGTGTTCTCAACGCCGAGCCGCATGT
ATCCACGGAACTGCGGCGAGTGGCAGTAGTCCATGCGGCTCTTCTCCTCAGAGGGCAGCG
CAAAGAAGGCGCGAGCGACACGGAGGACGTCGTCATGCAGCTGGACGGTCTGCGGCGGCA
GCCCGGCGAGCACGAAGAAGCCGACGTTGGAGCAGGCGCTTCGCATGTCAGCGACAAACT
GCGGGAAGTCGCTCTCCATAGTCCTGTAATCCAACACCGGAAGTGCCTGGAAATGTGGAG
GGGCACTTGGCGCCCCAGCTACGCCACGCCGACTCGAAAGCTGCGCGGCGTTGTGGGCCG
CCATGGGCAGTGCTCTGGCAGAGGCTAGCCGCACAATGCGCGGCGCCAGCATAAGAACGG
CAGCTTCACCACGAAGGACGAGCTCGCGGAAAAATGCAAGCCCTCAGCCACAATGGCTTG
AGCCAAAATGGCTACGG
>at00679
TTTTTTTTTTTTTTTTTTCACCTGCCACAATGCTTATGCACTTCTTCTGCTATAATTGCA
TTAATCTCTCTAGCGCGGGGCTGCTTTCGCGGTACCGAATGTCCCTCATCGTGCTCAAGG
AACAACACCTTTGTGTACATGTCTGCACCCTTGAGCTTGAGAGCGCTTCTCGTCTTGTCA
CCTTGCCCCACAACCAAAATCGTCGGCTGCGGTGAGCGCTCGCGGAACACATATCTCTCG
CCATTGGTGATGCCGCCGCTAAAAAATACGCTGAGCCTCCATGGCACAGGCCTGCCTTGC
TTTCGAAGCTGCTCAGTATAGAGAGCAGCAAGGTTTGCGCCTCCCGAAAAGGCAACGACC
ACATCGACGGGTGCCCGCGTCGACAACACCTCTTCGAGGCACTGGAACCCTTCGTCGAGC
TTGAGCAGGTGTTGAGGAGATGCTCCTTCTCCTGACTGCACATACCACTCCCTGAAAGGG
CAGGCCCCTTGAGATCTTGATCTCCAGCTCAGTGCGCTCCGAATCATGATACTCCGTTCC
ATCCGATATTGGATGCCACTCCAGTGGAGCCTCCGTCCACAGCCACTCCACTTCCGAGCC
CAGGGACGACACCAATCCAGACAGCATGAACTTCAAGATGTTGGAATTTGCCGCCGCTCC
GTGCAATGCCAAGATGCGTATCTTTCCCTCGTCCCCAATCGCCGTCAACATGCAATGGTG
CCACCAGCCCTGCCCCGGCAGCAAGTGGCGGGTCTTCTCTTTGCATTAATCCTTCCCGTC
TTTCAACCTTATGCTCACCCAGCCTTCGTTGGCCCAAGCCCGGCGCATAATCGCAGGT
>at00680
TTGTTTTTTTTCTTTTTTCCTAGCGCTAGGTTCTTGACTTCCTTAGCACCTCCCGTCAAA
GTGCACATCACGCCCCAGTGGCACACCACGAAGACCATGCTCACGGTCGCCCCAGTGGCA
CGCGAATCCCGCACGTGGTTTGCCAGTGTCCAATGGATCATGTTTTTCCTCCAGACCATT
TCCTTGTTGAAGACTCTTGTTGGGAGAATTCCGAGCCTTGCGGCCTCGCTTCCGCCCTTC
ATCAACACTGCGCCCCGACTCGCCGGGGTCCACCAGTCAGCGTTCGCCACGACTAAATTG
CGCACTCTGGAAGAGAGAGAAGATGTGCGACTGAGATTCGTCGGCG
>at00681
ACGGACAGCAGGGGTACACCTCGCCTGTGACGGAGTCTCGCCGACCGCAGTCCCAGCGCC
CGTACTCGGGCGTTCCATGCGCTGGCGCGGGCGGGAAGCTGCCGCAGATGCTGGAGTGTT
GGTTCACGCAGAAAGCGCAGATCCAATATGTGCGCCGGAGCCCGCCATTCTGCGCGAGCT
GCCTTTCGAGCAGGTCCTGGCCGCCCTCGAGGAGACACTGCGCCGCTTGGCCATAGTCCA
GGANGTCCAGNGCNTCNGCGACCACCGNGGCGACGAGGTGCACGAACANGCNGTCCCAGG
TGTGCGTAACGAGNGTCTCCGGCAAACNGNNGTTNCCATGCCGTNCCGNATCGACAAAGG
CTTGGCCGCCNCATCCGACGCGGCTCATCGGGATGACCGCCTGCCTGACCACGTCTCGCG
TGGTGTGGGCTGACGGCTNGaAGCCAGGCATGTGCTCGCgGACCCAGACTTTTTGTAGAA
CTCCAGCAGCCGACTGACGTCGATGCTTCTTGTGGCCAGGCGTTGTACAGTCTCATCCCA
AACAATTGCCTCCTCGCCTCCTGGCCGCAGACTGCTGCTCCTGCAAAGCAGCATCTCCTG

AGGGCCGCTCTGCTCTGCGAGATCCTGCTCCCTGAAAAAGCCAGCAGTCCAAAACATGCA
CGAAAAGTCGACGAGG
>at00682
TTTTTTTTTTTTTTTTCTGTTGCCACCCCCAAGGCGCACCGCTGTGGTCACAGATCGCGA
GTCACGCAAGGGCCTTCTACAGCGAGGCGCGGCCTGCTTGCCTGCCCCCCTGCGTCGGCC
TCCTGTGCGGCAAGACGTGAAGCAAAAATCCTGTGTCCATTCAGACCACAGCGAGCCACT
CAGACCAGACCAAGCCACTCAGACCACAGCAAAGCCACTCAGACCCCAGCGAGCCAGTCA
GACCACAGCGAGCCAGTCAGACCCCAGCGAGCCGCTCCTTCTCACGCTTCTCCAAGTTGG
AGAGGAGCATCGTGCTTCTGAAGGACAGGTTCTTTGTCTTCTCGATGACCTGCTGGTAAA
TGCTGGGCGGGGCCTTCGACATGACCACCCGGCTCTTCTCCGAGTCGATGCGGGCATCGA
GCTTAGCGTTCTGGATCAGCTTCACGATCCACAGCTCCGCCTCCTCTGCCTCCATGTTCA
GTTTGGAGGCGATCATGCGAATGTTGATGCACTGATGGATCCCTCGTGCC
>at00683
TTTTTTTTTTTTTTTTTGCGTGCAAAGTCTTTTACACGATCACTGTACATGGTCAACAT
CCAGGTGTGCTGGCACGGCCCACCACCCCTCCGCGAACGACGCTGGCAGTGTCTCACTCC
GTTGGTGTGTGCCACTGCGTGACATTCGTCGCGCAATTCCAGTAGTAGTGGCTGCCATCA
TCGGCAAGGAGCTGCACCCATCCTTCAGCTGGCAACTCCCACTGCGTGCGCCCAGTTGAC
ATCTCGTGATAATACCGCGTCTGGTCTGCAGTCAGCAGCTCTTCCCATCTGACCACTCCG
CTGGCAGAGTGGCCGCTTGCTTCACCAGCAGCGCCGCTCGCAGCAGCCCGAGCAGCGACG
GCAGGCGTCTCAGCCTCTGCAGCTGATGGCTGCACCTGCGAAGGCTGCAGGCTGCTGTGC
CCAGCAACACCTGCAGCAGCTGCCTCGCCATGCAGCTGCTTTGACACCGACGATGGTGCT
GCACGCTCCGGTGCATCGCCACCGAGCGCACCGGCACTGAGCGTCTCAGCTGCGGCTCTC
CCTGACTTGGCAGAACTCTCTGCCTCTGAGGTCTGTGGCACTGGCGCTATAGAGGGCTCC
GGGGGGATCTCTGGGACAGTGGGGGAACTCCTGCTCTCTGCCGACTGCTCCGGTGCTGAG
GGGATGGCGGCAAGTGCCTCGTCCGCCCGAAGTCTGGCTTCCGCTCCAGTGCTGGGAACG
TTGGCTGGTGACTCGGGAGCCGCATGGCGTGCTTCTGCCTCGTTTGC
>at00684
TTTTTTTTTTTTTTTTTCGCCTCCGTGTCTGGTTACTGTTCTTCTGCTGCCGGATGCTG
CCTTGGGAAGCGTGTGTCTGCTCATGTCGCTAGCACAGTGTCGAAGGCAAGAAACTGGTA
CTGATCATCTTAGAACGTGTTCTCCAGAGAATCCCCCCTGAGGAACCAGAAGTAATACAG
CGTGCCCAGCCATCCGTGGTACGGAGGCCCAAGGGAATGACGTTGTGATCCTTTAGAAAG
AACAGGCTCCATGGNGACACCCCAAAGAAAGGCAGTGGCTCCAACCAGCCATTGATGTGG
CCAGTGCACCAAGGCCGAAAGCAGCTGCAGATAACAGTGTGCATACGCTGGTGCTGAGCA
GGCCGAACGCCTTGCAACGCAGTGCATCAGTGAGCACTGTTTGCTCGTCCGAGCCCATGA
AGCAGAAGTTCAGGTTGCGAGGTAACCATGCTCTGCACAAGGATCTGCTGCAACACTCCC
CAGAAAGGGTAGAGCAGAAATATCACCCAGAGGTGCCAGTTGTCAACGAAGATAGGCCTT
CCCAAGGCTTGGCAAAGGATGGCGAGCGCCGGGCCAGCGATGGCCGCGAAGCAAGCCTCG
TGCNGAAAGAAGCTTTGCCGCAACTGCCTCATGCTGCAACTGAGGCCCCATGACTTGGAG
ACTTGCCGCCAGCCATGCCGCATGAGCTGGAACATGAACCACGCACACCACCAACCTGCG
CATGCTGCTATGAACATCCAAGGTCTTGGGAAAGCCTTCAAAAAATGAACAGAACTCCGG
TGGCTATGCATGCCACCGTTTCGATGAAGCGCTCCCACAATGAAGGTCCAGAGAACAGCT
TGACTGCCTCGCATGCTGGACTGCTACCCTTGCATTCTCGTACCCCATCC
>at00685
ATGGCGAACCAGCTGGAGGAGGACGCCAAGCGCTACGGCACTGTGACGGTCCGCACGGCC
CACGGCATGGCCTTCGCGCGGTATCTGCCGGACGAGGCGTGAGCGAGGGCGGCGCTCGGA
GAGCGTGGTGCGCCGCGCAGGCGCGGAGTGCCCGCGGATGCAGAGGGCAAGCGCGCCTGA
GGGGCAAAAAAAAAAAAAAAAA
>at00686
TTGTTTTTTTTCTTTTTTTTTGCAGCCGAGGCCGGCCCAGACCTTCATCCAGACGTCTGT
CTGCAGCCACCCTCAGCCTCCGTGCGTGGCCCGCCAGACTGAGCGGATGGTGTCCCGCAC
CTCCATGGGCTGTAAGTCAAGGAGAAGCTGGGCAGGACATTTCCCATCCGTATTGTCTTC
CTTCATCATGTCTTCCGCCTCGTCGCGACCCATCAATGCTTCGCACACTGGTATGTGCTG
GCCATTGATCGCGATGTGCAAGGCATTGTCGCCGGCTTTGTCTTTCACCGTGACGTCCGC
CTTTGCCTCGAGCAAGATCTTCACGGCCTGCTCTCTGCCGCTACTACATGCCCGCAGAAG
GGGCGTGCCTCCATGCTTGTCGCGAAGGTTCCTCTTGGCCCCGGCGTGGAGCAGGAGCTG

CAAGACCTCGGCATGGCCCTTGGAAGCCGCGAAGTGCAGCGAAGTTGCTCCTGAGGAAGT
GCTGGCGTTGGCGTCCACCTCCGCTTCTAGCAGCGCGCAGACCAGTGGAGCATCCCCCCT
GCTTGCGCAAGAATGGAGAGGAGCCCAGCCCTCCTCGTCCTCGACGTTGGGGTCCGCCTT
CTTCGACAGGAGGAACTCTACCACCTCGCGGTGCCCGGCTGCGCAGGCGCGGTGCAGGGC
GGTCCGCCCGTCCTCGTCGCGCTTGCGCATCCGCTTGGGCAGTTCCGCCTCATCGAGCGC
GCGGATGGCATCCAGGTCGCCGGCCTCCGCATGCTGCAGCAGAGGCTCCAGCGGCCGCTC
CATGGCCGTCCGCTCTCCCCTTCCTCCCCTTGACAGCACTTGAGCCAAAATGGCTACGG
>at00687
TTTTTTTTTTTTTTTTTAGCTCCTTCATTTGGTCATGGCATTCCAAGTCCTTCAGAGAA
AAAAAGCTTTGGAGCATCTCAGTTGGCGGCGTCAGCCACACTTCTTGTCGCTCATGAAAT
GGGAGGCATGGGATAGAGGGGAGGCTGCACGTAGGGCGTAGGCGGGAAGGGGTTGCTCCA
AGTTTTAACGCCATCGCAGATGACATTGCCAAAGCAAGGCTTCGCGATGCCTGCAAAGTA
GTCACCAGTGAGCAGGTCTTGGGAGATCAGGCCAATAATACCCATCATTGCCAGACGGCC
GTTATTCAGCTCCTTGAGTTGGTTGTCCTTCCACTCGGGCTCCTCATCGTACAGGACCGG
GTCCCAGCCCAAAATGTCCCATGGCTTGTCTGCCGGGGGCTTGCAGACGTAGGTGGTGAG
CCACTCCAGGCACATGATGGTGAAGATTAGCTGGAAGGCGCCGCCAATCGGCAGCTGTGT
TACATTCACGAGCGGGTCGTCCGAGACCCGCATGCGCGCGGCCCCGTCGCCGATGGGGTG
CCAGCCGCCCGCGACGTGGAACCAGCCAAGGACGGCAAGCATGCAGACACGGCCATGCTT
CAACTCAACGGCACGCCAATGAAGCAGCTGTTCGTCAGACTTGCCCTTAGCGAGTCCTGC
AGGATCCCAGAAGTCATTCGGCATGGGGCCANCCGGTGCCTCGAGCACCTTTGGNCCTGC
ACGCAGCTGTGTGGAGGANGGCTGCACGTCAAGGGCAGCTCGGGGAATCCACATTTTCGG
GGCGGTGGGTGCAATGAAGCCGTTCTGCCAGATCATGCCCATGATCGCCATCATGGGCAG
ACGGCCATTCGCAAGCTCCGAGT
>at00688
TTTTTTTTTTTTTTTTTGCCGCCTCTCGCCATTGCTGCACACCAGAGCGACGCCCTAGA
GCATAGCATCCAGCTGGAGCCCACCCCGCAGGCCAAGTATGGCAAATCCCATCATGTGAC
CGCCTGGGACTCTTTGACCTCGAACCTCTTCAGCTGCCGCGCGGGGCTCGCCTCCGCGGA
GATTTGGAGGAGGTAGCCCTCGGCCGTGTGCTCCTCGCTCTCGATCTTTGCCATGGGCGG
GGGGCCACGCAGCCGGGCGTACTGGGCGGACTCCGTGTAGGGGATCAGCAGCCGGAGCCT
CTCCGCCCCGAAGCTCGTGCGCTCCAGCAGCGTGGCCTCGATTGCGTCTGAGAGGGCAAA
AAGACCTATTCCACTGCGCGCTGAGATCTGGCAATGGCCAACAACGCCGGGCCACGGGAA
TTCCTCGTGCC
>at00689
GACCGCGATGGTGACCGTGGTGGTGACCGTGGTGATGGCTGTCGCGGTGGCGAACACTTG
AGGAGCGCCAGTCTTCAAGGGCTTCTTCTTGTCGACTGGAGCCACGCCAAGGACGGGTTG
GGTATGGATCCTCGTTGTGCCGCCCATGGGAGCGGCGCCAGGGTCGGTAGCTTGACCCAG
CTGAGTCGCTGATGCTCTGCGCAGGTTGCCCACACCTCTGCCACCGCCAGACGCGCGCGT
GCTCCGCGTCCCAAGCCGTGTCCGGGACCCAGGCAATGGCGTCATCCCCCAGCCATTGCA
AGGACAGGCGGCCGTGCGTGCCCCACTGCCAGCGTTGCCTGCTTTGATCCCAATGGATGG
TAAAGTTGCGCGTTCCACGAGCATCACTCCTGGTTATCCGCAGACCATGGATGACGTACT
GCTCGCGTGGGCAGTCGACATTCATCCAGGTTCCCTCCAACTCCTCGATAGGCCGCATGT
AGTTTCCACCACTGCCCACCTTTGCTGAGTCACCTTGGCTCGAGTCACTATGCCAGGCAG
AGGCCTCATGTGGGCAGGAGATGCTGCCGTGCCTTGGTGGAGCCACCAGCATTGCGCCTA
GTCTCTGGCTCTCGCTCTCTCTCTCTCTTCAAAAGTCCTTGAGCCAAAA
>at00690
TTTTTTTTTTTTTTTGTCTTTTGTCTCTGTTCAGTTCAAAAACAGGCTCATTCAGCCGG
GCGTGAATGAGAGACCTGGCTGGTTCCAATGCTGCGCACATGTTCACCGAGACTGTACAT
GTACACAGAGGCACACTCCACAAGTGGACCCAAATCAATACACGGCTGAAGATCCACCGG
CCAGCCCTGTACGAACTTACTGCGGTAGAGGTGTCGATTGAAGTCATGCAAAATGACTAG
GCCCGTAGAAGCATGTCATGCACATCAGCACAGCCTTTAGCTGGTGAAGGAATGTCATCT
TCTACCCAACCTTTAGTTGGTGCGGGCTGGAGGGGACATGCCCCTCGAGTACATCATGCG
CAGAGCCTCGTGCC
>at00691
GTCTTGCCCGCCACGAGTCAGCGCCAGCTGCAAAGGTTGTGGACGCCAGTGGGCAAGGCG
TGCAAGGGTCGTTGCCCACTTCAGCCAGTTGGCGTGGCAAACAGGTTCGCAACTCTGCCC

AGACGGAAGAACCTCCAAAGCCGAGCACTGTGGACCATGCTCGCCATTCTGGGGCTCAGG
AGCCTGACTCAAGTTGCACGGCTTTGGCATGCAAGCCACCGTGCTGAGGCTCCCGTCGCT
GCACTCGGCCGGGCCGCTTGGCCTGTAGCCCTCCAAGCAGCCCGGCCGGCACGCGGCGCC
GGAGGGAAGAGTCCGTGGGCAGTCTCCAGTCTCCATGGTCCGGGC
>at00692
TTTTTTTTTTTTTTTTTCAGAGAGCATCTCTCACAGAGAAACTGCCACGAGGGCAGAGT
CGGATCAGGCACAGAGCGCTACCTACGAGTACATCTGGGACCGAACAACTTGGCCGAAAC
CAGCCCTCCACCTCACAGCTCCTTGAGGCTCGCCCAGCCGCTGTACTCAGCCCCAGGCGG
GGGGCACCAAGGCCGCAATTGCAGTCTCTGTGCTGCCAGCGGAAGCCGTTTGTATGCACG
GTAGTCGCGGTCGTGTGTGTAGGAGAGGCACTGCTGGTCCGGCGGATCCTCTTCACGGTG
GTAAACGGGGTCCTTGCGGTGCCGCGGGTGCTCGAGGCGCCACCTCGCGTTGTGCGACCA
GACAATGAGGTTCCTGCGCCGGCCCGACGCGATGTCGTCGGCGCCGTGGCGCCGCGAGCC
CAGGTGCAGCACCGCGCGGCCGATCTCGTGGCGGTAGGTGTGCGTCAGCTTGCGGTGGGC
CGGAGTTCCGATCTGGCCACAGAACGACAGCGTGGCTCCCGTGAACTCGTCGCCCAAGCA
CACATTGAACGTGACATCCGAGTCATCCGTGTGCATATCAAGACCGAGGTCCTCGCTCGC
CTGGTAGCGAACAATGAAGGAGTGATGGGTGTCGAACTTGCTGCCTTCGTCAGGAAAAAG
CCGCCTGGCCAACGGGCATATGTACTGCTGCCTCGTGCCGAATTCT
>at00693
CCACTTGGGGCCTAGCGCACCTTTGGCGAGTATCCCTCGTTTGTCTGTGACTACAGACCT
CCACCCTTGCTGTGCAGTCTTCCCAGCGCAATGGTCCgAATCGGTCCTCTGCGCGATGAT
GCTACCCTTCTTGATGGCATACCGTGAGGAGTCAGATACACTCTCGCTCGACATGGTGAA
CGTGAACTCGATATGGAGGGATGCGATGGTTGAGGGAACCAGCGAGCAGACCAACCGCAC
ACGTGCAACCGTCTTTCAGTTGCCCGCGGTCTGGACACGGAAGTTCACCCAAACGAAGCT
CCTTGGGAAAGGAGGCTTCGGCGAGGTGTGGCTATGCCGATTGAATTGCAGATCC
>at00694
TTTTGGCTAAGTATTGCCTTCTCGTCCCAAAAAGCTTGTGGGTCAGCAATGTCTGAGGGC
TTCGTCGCCGCCTTGCTCCCGGAGAGCCTCCTCCATGTTGGAGGCTTCTTGGACATGATG
GACTTGGCGTTCTTGGAGCAGGCATGCTCGCTCCATCTTCGCATCTGTGGTCAGCTCTGG
GAGGGGGCAGCCTCGCGTGTTCTCTCACAAACTGCCCTTTGGAGTCCACACGCTCACAGT
GCGGTGGAAAAGCTCCAAGAGCAACGCACTTCATGGAAACAATTTGTACAGGAATTGAAG
TTGCTGAGGCGGACGATGTTCTCACCAGAGGCATGGAGACCATGGATCCGTCAAGAGTCC
TTCAGCAGCTTGAAAGTTGTTCCTCGCTTTCAAGATCCAACCGT
>at00695
TTTTTTTTTTTTTTTTTGAATCGGACTCGCGCACTTACCGCGGTCCGCGGTTGTCCCAA
CCCCGAGCTCGTCACCGCACCACGTTCTTGGATGTATCGGCATTGGGCCGCAGCGTCTGC
GCCAAGGCGATGAGCCGCTTCAAGGAGTGCAAGTAGGTCTGCACCTCTGGTAAGTCCAAG
GGAGCCAGTGCGGCATTCTGCCGCTGCATGTGCCCCTGCGCAGCCCGCGCTTTGCGGGTT
GGCGCCTCTGGGCCGCCAGTGAGGCCCATCACATCAGACCGCCAGCGCACGCGTCCTAGC
GTGATCTCTAGAAAGGCCTCCGCGGCTGCTATGTACTCCTGTGCCGCCGTCAAGGCCACC
AGCTTCTCCACCTGCTCCAAGATGACCTGCTCCAACTGGGGAAATTCATCGGGCTCAACA
TAGTGCTCGTCCGGCCGCTTGCACTTCCGGCACGCCACTCTCTCGCCGTTGTTCTTGTAG
CGACAGTGAGGACACTCCCAGTCATCGCGCTCGGTCAGGACCTTGGGCTTGCAGGAATAG
GATCCGCGGCGACGGCGTGCGTCCCAGCGGGGCAAGGTCCGCAAGAGTGGCCGCAAGCCC
CTCGTGCC
>at00696
CGCCGGAGCTTCTGTCGCAGATCTGCGTTTGTACTCAGTGCCAGATACAATGCAAGTGAG
TTCTCCAGCCTACCAGCCTATTTCTCAGACAGCTCCCGGTGCCCGGGCCAATCACTGTCA
GGGTAATAGTGGCTCTTTTTCATGAATTTAAGCAAGGTAACAGTAGGGCCATCAACTGGC
CACACATCAGGATTAATTGCGTTGTCGCCAAAGCCCGCGTAGTGGCAAGTGAAGAGCATA
TCACGGGCCACCATCCATTCACCAGCCTCATAATTCCTATAGGCCATGGCGAAGCGCTGG
AAGAATTCTGTCGAGAATGTCGAACGC
>at00697
GCGGCCAAGTACTTCGGCATCGCCGCGCTGGGGCCGCCCGCGTTCAGCAGCAACTCCGGA
CTGTTCAAGGACCTGCCGGACCACGTCGACTGCGTGGCTCACTCCGTCTCCGGCCTGCGC
TGGCTGCACTGGAACTGGCCCTCGTATCTGGGCGCCCTGCTGGTCTGCAACGGCTGGTTT

GAACTGTGCGTGCGGGTGGTGGAGCCGGCGGTTACTGGAAGTCGGGAATAAACAAGGATG
GGCCAGCAGCACCTCCCAGGACGGAAAGCCGCCTCTCCGCGACCCTCAAACCCCTGACCA
GTCTCTGCTCAGTCTGCGGCTGGCCCGAGAGCGTGCCGACGCTCGCGGGGCACAGCGCTG
AGAATTTTTGAATTTGGGAATTTTCCCGCGATGTTTGAAGTTTGTGTGCTTTTTGGA
>at00698
TTTTTTTTTTTTTTTTTACTCTGCGCAGATAAGCATGACTCTCAAGCTACACCCCTGGG
CCTCAGAACGCGGCGCCTTTATCTCCAAGGCGGCGAATCCGCGACATTATGACAGCATCT
GGGTTGCGCACATTCTCGTCAAGTATGAAAGAATTCTTCCCACCATCTGTTCCCATAACC
TCGCATGCGTCGCGCTTGCTCAGCTGGTACAGGGCATCTTGGGTCCGCTCATCAACCCGA
TTCTTGCGGATGAAATCCTTCAGGTCGTGCTGCCAGTCGCCCGTGCCGCCCTCGCAGCCG
CCAGCGTTTCCGCCGCCGCCTCCGCCGGCTGAGCGACCTTCCCGGCCCCGCGGCGGGGGG
GCGCCGCCGTAGTCCTCCCGCCGCCCGTGCGAGCGACCGCGTCGTCGGCCGCGGTCTTCA
CGGTGGTGGTCCCGTTCCCTCCGGCGGTGCCTGTGGCCGTGCCGGTGCCTCCGGCTGCGA
TGTCGCCTCCTGCTTCGGCTCCGGCTTTCAGAATAGTCCTCAGTGTAGGAGTAGTCGTCC
GAAGGGCTGCGATGGCGCCTCGTGCC
>at00699
TGGTTGtTTTTCtAGGtTCCCtACtTAAGATCTGGCCGGAAGGCTGGCTCCTCCACTTCT
GAAGCATCCAATCTCGCCTGCCGCGATGTGGGCATATGGCTTTCGCAGTCCATGCCAcCT
cGCACAGCAACAGCTGAAATTGAGCGGTCCCGACCATATGCCGCACCCATCTCAGCAACC
TCTACCTCTTTCAGGTTGGTATTGGTCATCTCTGCTCGGCTGCTGCGGCTTTCCGCAGCC
TGCAGCAGCGTCTCTATCTCCAGATCTGCTTGAAGACATAAAACG
>at00700
TTTTGAGCCTCGAGGCTGATCCCTCCTGCTAACGCATGCGTCTTCCCCTTGCGTTACAGG
GCCAGCGCTAAGAATGAGGCGTTGCCGGCGCTCGGCACACCGTTCCATGGCAGTCACACT
CGTGGGGGCAGCGTCAATTGCAGGTTCCTTCGCAGCCGCCTTCACTCTGGGGCGCAGGCC
TTGGCGCTTCGGAGGTGTGCTTCGTCCATTGTCTTGTTCTACCACTTCGCTGCTGGCAGC
ACAGCTGGACACTGAAACGGCCATTGACCAGAGTCAACTTTTGAGCACGAT
>at00701
TAGTCCTATTTTTGGTCTTCTAAGGTCCTATTAGTCCATTTTGGCTTAACAAGCGCATGT
TCATTAGTACCTGGCCCTTGGCATTGCAGAGGTTCCTCCCTCGCTGTCGGAGGGATGCCG
CTCGATGCGCAAAAGAAGTCGAAGCTGAGGTCCAAGTTTGCCCAGCTGAACAAGAACgGG
GACAAGTTCCTTGATCTGCGGGAGNTCACCTCGCTCTTGCGCCGGGGCAACCCGGACATG
ACCGACGGGGAGCTGCGCATGCTCTACGACGACATCGACAAG
>at00702
CCGTAGCCATTTTGGCTCAAGGCTGTTCCAGTTTGCCAGCTTTTTGAGCCATGGATCACA
CCGTTCTGGAACGCCTTGGACTCAGTTGGACTGACACTGTTTGCGAGGCAAATGAGAAGC
GCGTGTGTGAGTATGATCGACAGCTAGAAGAGGCCATCGATTATGCGGACAAGGGCTACA
AAGCTGACTGGGAAAACCGCGTGCCCGCTGGGGTGAGCGATCTAGGAACATGGATGGTGG
AGAACAGACGTGTCTATGAAGCAGGGTTTGTCACAGATGAACGCAACACGCAGCTTGCGG
TGTCCATCCCCATAGACGAGCCGTTGCGCAGCCTGCTTCTCAGGCCCGAGATGATGTTGT
GAATAGCCTGTTGCTCTCGTCCGGCGCGCACGGT
>at00703
TAGCGTCGGAGATGAGCTCTCGGTCTGTGCGGAGCTCCGGTACGGCGTAGCGCAGCGCCT
CGAAGTCCTGCTGCAGGGCCTCCAAGACCACGGCCCGCTCAGCCTTCAGCTCCCCGGAGG
CGAACTGCAGCGCCTTCCAGGTCCTCCCCACGGTGGCCAGGACCACCTCCCGGTCCGCCC
TCAGCGCCTCGCCCACGAGCTCCAGGGCCCAGCCCTCCTGGCCGCAGGCCGCGAGCACGA
CCTCGCGGTCTGCCTTCAGGTCCTCGGACGCGTGCTCCAGCGCCCAGCCATCCTGTCGCA
CGGCCTCGAGGACCAGG
>at00704
CGCACTTTGGACGTTGGTTGGTAGTTGTTTGCCTTGCCCTCCCTGTTCAGGACAATTCAT
CTTCGTTGGGCGTGGAACACTCCGCGCATGAGTCGCTGCAGCTCGAGCGGTCTCGGTCAC
GATGCCCTCATGGAAAGTTGAACNATGACTGCAAGATCTGCAANGGCTGTCCGCATNGAA
AGCCTTGGCGNAAGTGTGGCACNTGCNACGGCTGTCCGCACGGNANGGTGAGGTGCGTCT
GCNGNGTNTGNAACGGATGCCCNCATGGGAAGCTCAAGTCNGATTGCAANGTCTGCCGNG
GATGCCCCCATGGNAGGGTCAGGCGGATGTGTGGTGCTTGCACNGGATGCCCGCATGGCA

AGGTGAAGCACAACTGNGNTGTTTGCANGGGGTGTCCACANGGNANGANGANGCCTGATT
GCAGNNCATGCANTGGATGCCANCACgGAAGGCCaAAGAAAGtTTGCCGAGTGTGTAACG
GATGCCCTCACGGGaAGCGGAAGTATGACTGtCGAGTaGTGTCATGGCTGCCTTCACGGA
AAGAAGAAGTCCCAGTGCAAAGTCTGCAGTGGCTGCGTCCATGGAGTGCTTAGGTACAGG
TGCAACTTGTGCAGCCGGTGCCCGCATGGAGAAGTGAAGCACCAGTGCCTGGCGTGCAAT
GGTTCTAAACCGGGAAGCTGACGATCTGCTTGCTGCACACTGGGTGGCAAATGGCACGTG
CTGGTGTGATA
>at00705
CATGTGTTCAAGCTTGTCAGTCCTTCGGTCTATTCACGGACGTTGTGAACCAAAGATGGC
ATTGACGGTGTCTGATACAAAGGAGAGCACCATTCTCAATTGGCGCAACCCTCTAAAGAA
AAACACGGTGGTGTGGCACAATGACTCCTTCGCTTCAGGGAAATTCCGTGATGCGTACAT
TGGTTTCAAGACCATCCACTCCACCAAACCCTGGACAGAAAGGTCGTTGTCAAGCGACTG
CGTGACGTAACTTGTTGGGAAGAGCCCAATTGGGAAAATGACATGAAGCATGGAAGAAAG
CGAAGGAGTTCGTGGAAGC
>at00706
TTTTTTTTTTTTTTTGTTGTGCCTTTCGGCCAATATAATTCTTTCACAGAATCACCGGAA
AAAGGCAAAGTCGAGCGCTACAGCATGCAAATCCTCGAAAGCCAGGGCCTCCAGCCCAGA
CGTCCATGCACCACAAAGTCGGCAACCTGAGAATCGAACGTTTAGCGCGACATCAATCCA
CATGCCAACCAACAGCGGGCGCGGACGAGGGGAAAGAGGAACTGACGGACGACGGCCGCC
GACAGACCCTTGGAGCCAGCAGCTGATAAGCAACCAGACCCAAGGAGCCGCAGCCCAACA
GCCATGCAGCCCCCACTGCATGCATGCAGGTGAAGGTGTATGCAAGGCCTCCGTGCCATG
GTCAACATGTGGGCATGTAGACATGTGGCCAAAGTCTCTTTGGACTCTTGTTCAGGCAAG
CTTAGCCGGGCCGTAAAAGATGGCGCAAATGACATCCTCCCGGCCCTGGGAAAATCACGT
CTGCCTGCCAAACTCGTCCTTCGGTTTGATGCCTACGATGTACTGGAAGCCGTTGCCATT
CTTGTACAGCAGCGACCATGCCGCAAGGGTCTCGGGCAAGAGGCCATAGAAAAACATTGT
GATAGACGCGCCGAAAAGGAAGTCACTCTGGATGAAGAGGTAAAGCTTGCACCTCTCCCA
CCAGCTCAGGGCATCTGGACCCTTGGCGGAGCCTTGCACGTCCGGAGCCGCTGACTCGGA
GGCAGCGACCTC
>at00707
TTTTTTTTTTTTTTTTTAGCGTGGCTCCATGTTTTTTTTTTTTTTTTTTCTCACTCCC
ACAGCACTACAGGATCTGCGCAAAGAAGCGCACCGCTGTGGTTGTGGGATCGCTATCCTG
CTCTCTACGCTGCTCAGTAACGGTACGTGTCACCCTTGAAGGGGCCGTCTATCTTGACGC
CAATGTAGTCCGCCTGCTCCTTGGTAAGCTTCGTCAGTTCGGCACCCAATGCTGGCAGGT
GGAGGCTGGCCACCTTTTCATCCAACTCCTTGGGAAGAAGGTATACCTCGTTCTTGTAGC
CCTTGTTCTCCTTCCAATTCTTGAGCAAGTCAAGCTGTCCCAAGACCTGGTTGGTGAAGG
AGCAAGACATGACGAAGGAGGGATGGCCTGTGGCGCAGCCCAGGTTCAACAGACGGCCGG
AGGCAAGAACAATGATGCCGTGGCCATCTGGGAATATGAAGCGGTCGACCTGTGCTTTGA
TGTTTTCCACCTTGATGCCTGGCATCCCTTCCAGTTCAGCCATCTCAATCTCGTTGTCAA
AGTGGCCAATGTTTCCGACAATGGCATTGTTCTTCATCTTTTTCATATGCTCCAGAGTGA
TGATCTTGAAGTTGCCCGTGGTCGTTGTGAAAATGTCAACCTCCCCAACTACAGACTCCA
GAGTGACAACCTGGAAGCCCTCCATGCACGCCTGGAGCGCGCAGATTGGGTCAATCTCAG
TGATCAAGACACGGGCTCCGGCTCCGCGCATGGCAAAGGCACAGCCCTTTGCAACATCGC
CATAGCCGCAGATGAGGGCACGCTTGCCCCCGATCATGACATCTGTGGCACGCATGATGC
CATCCGGCAAGGAGTGACGGCATCCGTACACATTATCGAACTTGGACTTTGTCACGCAGT
CGTTCACATTGATGGCGGGAAAAAGCAATTCCCCCTTGGCAGCCATCTCCTTCAACCTGT
GCACACCAGTGGTGGTCTCCTCGCTAACACCATGGCAAGCCTTGGCCATCCGGGTGTACT
TGGTCTTGTCCGCTGGAATGGAATCCTTCAGCAACTGCAGGATGACTTTGAGTTCCGCAT
TATCGGTGCTGGCAGGGTCGGGCAGGGACCCATCCTTAGCATACTTCTCCTCAGACTCCT
TGCCCTTGTGGATCAGCAGGGTCGCATCGCCACCGTCATCCACAAGCAAGTCGCAGCCGT
CGGCACCCGGCACTGTCATCATCTGCTCAGTACACCACCAGTACTCGGTAAGGGTCTCGC
C
>at00708
TTTTTTTTTTTTTTTTTTCATGGACCATGGGTCATTGCCATTCTGCCAAGGGTCGGGCTT
CACAACAGCTATGATGGCAGCCCCATGCAAGGCCTGCATGGGCTCAGAAGGAGCTGGGGC
ACCAGAACTGCCACTCGGCTGATCCAGATGTTTCTCCAGTGCCTCTTTCTCTGTTGCTCG

ACGCACATGCTCCATGGACCTCGGGTCGCGACACGCCCTCGCCTGCTGCCCACAGAGGTG
GGGTGGGTCATCGGGCGCTGCTGAATCCACGTCTTGCGAAGCGCGCTCTGGTGCAGCACT
CTGTCCGACGCCTCCGGTTCCACGCCATCCGCTGCACCGCGGCCCTGCTCGTCAAGACCT
GTGTCGAGACCTGCAGTTCGTGTCGGCGTCCCAAGCGGGCGCAATCCGGCCCTTGACGTG
CAACTCGACGAAGCGCCTGAGGAACCTGGAACCTCGCAGTTCTTGCGGCAGGGTCGCGTA
TGCCGTCCGGAGCCGTGAACTTGCGCCAAGCGACCGCGACTCGCTGGTCGCTGCGCTGCC
CCTGGCGTATGCGGTCCACGAGCTCACGTTTTCTAGCATCTTCATCGCTGTCCTCTGCGC
ACTCGGAGTCGCTCCGCTGCTCCGCGTAAGACAAGGACGCCATGGCTGAAGAAACGGCCA
GAAGCGCCACAGACTCCTTCATGTGCCTGGCATAACTGACTGCAGCGGAAAAGTGTCGGG
AGCCTGCTGAGCTTCCGCTGCTCCTCGTGC
>at00709
TTTTTTTTTTTTTTTTTGCCTGGACGCCAGGGCCCGAGGAGCTCCGCGCGCGTGCGCGC
CGCGCCGCGCCGCGCCGCGCGGCGCCGCGCCGCGCGGGGGCGCCGGCCTTCAGGCCCGTG
CCCTCCGCGAGCAGCGGGGCCACGGACCCCCGGCCCGTGCTCACAATGAGCTCCCGCTTG
TCCAGATGCGTGATCGTCATTCGGACTCCGGTGAGAGCCTCCAGCAGGCCGATCTCACGC
GCCAGGAAGAGGTCTGCTCCGCGTCGTCGGAAGTCAGAATGCTCCTGCTGATGAATGATG
AAGACCAGGTCGCCGGGTGCGAGGCCAGGATGCTCGTCGCCCCTCCCGGCCACGACTATC
CTGTGGCCGTGCGGGGCGCCCTTCTCGATGTAGACCTCCACGACCTCGCGGACCCTCCTG
ACCCGTCGGTGCCGGCCCGCGCCGGCGCAGGCGGCGCAGCGGGCAGTTCCGCGCGGTAGG
CCGGCTGCTCGACGGGCGGAGCCGCCGCACTGCTCGCACTCCTGCAGGTCCGCACGCTCG
TCCAGGACGCTACGGCGGTCGGCG
>at00710
TTCAAGCTGTATCAAGATGGCCTCCGAGATGGAGATCCAGAGCGAGGCAGAACAAGGTTT
CGGCCAGACAGACGAGGGAGGGACGCTGATTCAAAGGAACTGCACAATCCTCCAAGTTTG
GCAAGGATGGTGACTTCAAGGCGGGCACGCGAGCACTGGCATTGAGGTCAGAGCGGGCGT
CACGTGCTCCTCCATGCCGCGGAAGCGGCGGCGCGCAGCTCGAGTGCTGGGGATTGCGGT
GGGCTCATCGGCGACTGCGATTCGAGAGGCG
>at00711
TTTTTTTTTTTTTTTTTCACGCAGAGAAGATCAGCAGGCACAATGCCACCTGCTTTCTC
CACTGGAGATGGCCAGGGTTTTCGTAAAAGTATTGCTCGCCCAGCGAATCGTCCCAACCC
GAGACAGTCCGGTGCCTGCTGCCATCGGGGCTTCTCTTGGGACATACTACGACGAGTTGC
TGCTATCGCATTCTATCTTGCATCTTCTCAGCGGCCCCTTGGGCAGCTCTTGGAGGCTCC
CAGGTAGTGTGTTACGAGCAGCCTGGAAGTGCACTTGGAGAAACTTGGGGCACCGTCTCA
AAACTGTGAGGATGCAGACGCCTGCCCAGCCCGATCACAAAAATGTGCTTCAGGGTTCAG
AGGAACCGGTCTGGCGCTCAGAAACCTTCGTGAGCCCGGCCGGCTCACAAACCCTTGTTG
CCGTAGACCTTTGTCACCGGCTGGCCCTTGGAGTCCTTCAAGTTCTGGAACTCTGGCTCC
AGACTTGACACACTGATCTCCATCGTCTGTGGAAGCAAGGCCAATGCCACCGGCAACATG
CCGGCAAGGCACACTGTGATGAGGCCGAGCTCGGCTGCGATGGCAACTGGCCGAACCTTC
AAGAACGGGGACAACGCCTTCATCGCAACGACCGGCAGCACCATGGGAGCGATCGGCAGG
AAGCAGGAACGTGAGAGCACGTCTGCAGGACACAATCTGCCCCGCTTCTGCGACTTGCAA
G
>at00712
AGTGGTTTACAGCCCCTTTGGCATGCAGTGACGAGTCTGCGAGGCTGCTCATGTTGGAGG
GTTCACAGAGCCCTCAGCTGTACCAGCAGTGTTGGTCGAAGCAAGTGGTTATACAGGATG
TCGGGCCTTGTGAATCTTTCATCGGTCCGAGGCTGGTGCTCAAACACGCAGAGTTTGATG
ATCATTGGCATCAACGTCATCACCGACACTACGGCTGTATCTTAGATCAACAATTTGACC
AGTGTTTTGCAAGATGCATCTGTTAAGAAAAAAAAAAAAAAAAAA
>at00713
TAGTTGGTTCAAGGTCCTCATGCAGGTTCCTCCTCGCTGTTTGCGGCCATTGACGGTGCA
ACGTAGAAATTCACATGACTGCACGATTGGCATACTCTAGCGCGGCAGCCTCAGCAATCA
AGGCCACAGTCGGTATTCGATTGCCCCCACACAGTCCGGAAAGAGTAAGACAATCGAGGA
ACTACACCTGATCCAGGGTAACCCTTCACCTTCCGCCAAGGGCTATTTGAAAGTGGGCAA
CGGGAAGCAATCCCAGACGACCGAGCCAAAGCTGCTGCCGGCAATCCCGATCAGCTATTT
GAAGGTGAACACATGGGAGCCTCCAGATGCTGGCTGCGCAGCAAGATTGCGGACATGAAG
TCGAGAAGAGCAGATGGCACCC

>at00714
CCGTAGCCATTTTGGCTCAAGCAGTGTCGGGCTCCAACGAGACTTGCTCTTGCGACCCGT
GCGTAAACCGCTCGTCATGGCAAGCACGAGCACCACCAAGGCGTCGAAGGCGACAGAGTT
AGTGAATGCCTTCAACATGTTCGTCGGACAGGACTGCCCGCCCGGGCCGAAGCTGCTTCC
CTGGTACTGCGTCATCAACCTTCAGAAAGGCGGGACTCTGCCCTACACGCTCGCGCTGAT
GTGGTACTACGGCGTCTGGGGCACCGCGCTCGAAGTGCCCTGCCTGTTCTATGCCGCTGC
TCATGGCAGCTACGGCTTGGCATGGCTGACGAAGCACAGGCTCTTTCCAGACCCGCAGTG
GGAGGCCAAGGTGACGGTCATGGGGTCCGTGAATTCCTTCCTGTTCGTGCTCGGGCCGTA
TTGGCTGGCGCCGTGGC
>at00715
GCGACCTACCCGCTTGCTCTGACCTTCAAGTTTGCCTCCAAGATCCTCCCGAAATTCAGT
ATTTCGGAGGAAAACTTCTTCGACTGTGACGGCTGCACGCCTGAGATCGCAGAGCGCCGC
AAGAAGGCACTGAAGGAGCTGAGCGAGAAGTGGAAGGCCAAGTACCCCAAGTGCCACGAA
TTCTCGGCGGAGCTGAAGAAGATGATTTCCGACCTTCGATTCACCTCCGGGCGCTGCTTC
CCAGCCTTCAACAGTGTCGTGCAGAGCTACCTGGATCCGTCGATGGCT
>at00716
TTGTTTTTTTTCTTTTTTTTGAGTGCTTCAGAATGCAAACCGAAGGGCAGTCAACATGCT
CCCATCCACCGACCGACTGACTCAGTTCATGTTGACCTAAAGTGGATAGCAACATTAACG
CCAACTTTCGATAGCTCGTTGAAAAAGTTCCCTATGCGGCTTCTTGCAGCCGATCGCCTC
CAGCATGACTTCGACCTCGTCGGGCCGCATGGTCCGCATCACTTCGGAGTCGTATCCGTT
CTCCTCGAGTGCCATGGCATACTCGCCGAGGCCCCAGTTAGCGAGCAGTGCCTTCATGTG
GCGGGCTTTCGAAGGCGTTCGGCTGTTAGAGCGCGATCTGCTGCAAGAGGACCTTTGCTG
TGAGACCCTGGGGTTCATGTTGACGCCCTCCACTTCCGTCCTCGTGCTCGCAG
>at00717
TTTTTTTTTTTTTTTTCTCTTCAAACCATCCACTGCCCTGGCGCTCTCGTTGCGAAAGGC
ACAGACCTCAAAAGAAAGCGTCGCCCGGAGCCGTGCGCCAAGCTCCGATTGGGTCAAAAG
CCCTGACTCCCAGTCAAAGTCGTGGGCGAGTAACTCACCTGACCGCTGTGCTGAAGATCC
GCTCCTCCTGCTCACACCACAAGCGCAGCACTGGGCGCCTCAACCTTAGGGTAGGAGGCG
CAGTCAGCAAGCCCGCACTTGTTCTTGTTCCGCACGATCCTGAAGTAGCCTTCCTTGAAG
TCGTTCTGGAGGTCGCCCTTGATGGCTCCGAGCTGCAAGGCCTCCTTCGGCGTCGTCGCC
ACCTTGTACTTCTCGTACCGCTCGTACGACTGTGTTCCAACCTTCTTCGGGTTCCGCACG
TCGAAGGCTATTGAGTCACTCCCCGAAGGCATTGCACCGCCACTGCCGCGATCCCTGCGG
GCGAGCCCTGATCCAGAGGATGGGCCATCACCGCTGGGCCCGGCCGCGCG
>at00718
TTGTTTTTTTTCTTTTTTTTTTTGACTGGGACCGCCTGCGCGTCGGAGGTTAGTTCTCGC
AAGCCGGCTCAAGATAGCTGCACAAACAACGATAGGCACACGCCTCTACAGAATCGGCAC
AGAACAACTATGCTCCAAACTGTAAATAAATTCTGCGCAGAACGCGCCAGCTGGCACCCT
TGGTGCTTTGTCCAGTGTGCTGACCAAGCTACAAAGTCCTATTGGGGCAGGTTGACTTCT
GCGCTCTCACAAATTGTGCCAAAGCCTTGGGCTCTTCATATGTACATGCATGGATCTTAC
AGGATGCTGCAGCAAGTCAGACACTCACCAACTTGCCGATGCAATCTTGGCCCCTTTGAC
CATCGACTGCAACTTTGCATAGACGATCTCTGTGGGCACGTTCATGGCGCCGGCGGCAAG
TCGAGAAGCCACAATCCGTGCAGTGCCATCACCTGCTCAGGGCCGACCAAGCGGCAGTCG
ATAATTCATGAGCCGCTGCAGCAATGAGCCTGCTGGGTGCTCGATGTGGCTCGTTGTGGT
GTCCAGCACGCCGCGGCATAATCACCTTGCCCTCGGGCAGCTTTGGCTCGTGGTGGTGTC
CAGCACGCCCGGCATAATCACCTTGCCCTCGGGCAGCTTCGTGGTCTCAAAGACCTCCCA
TTCGTGGCCGTGGCCAGGGTTGCAGGCTTCCACGCTCAAGTACTTTGGCTTGGCCGTCAG
CACCAAGTCAATGATGTCGGCAAGCGGCACGTCGTGGTGGTGTGGGCCGGGATAATTCCC
CCAGCAGACGTGCATGCGGATGCGGTCCGCTGGGATGTCTGCCAGCGCCTCGTTGAGGAT
GCGCACTGCCGCACCGGATGCGGCCAGGAACTCCTCCAGCGAGCTGTCCTTGAACTTAGT
GTGGCGGCCCATGCCCAGGTCTGGGCAATCCACCTGTAAGGTGAGCCCAGATGCGTAGAT
GGCATCGTACTCCCTCTTCATTGCCTTGCCAAGGGCCTCCAGGTACTTGGTGTGGTCCTT
GAAGTAGTCATCCTCAAAGAAGTTTGCGAGAGTGCCCGGCGAGGGCGAGGAGAGGAACAC
ATCCTCCAACGCCACCCCTTTTGCCTTGGCACCCTGCCACGACTCGTTCGATCTCCTCTT
GCAGGTCCGCAAGGCCCTCGTCCGTGTAGGCAATCTCGCCACTGCATGCTGGGTTTTGAA
CGTCGGTGTTCTTTGTCAGCGTAAGA

>at00719
TTTTTTTTTTTTGTTTTTGCGGCGTCATATCAGTCAAAAGGATGCGGATCTCTACGCCGC
ATCCCCGGCCGATCTTTCAGCCAATGCCTCATTCCTCTATCTGCCGACCTTCACCCTGCC
GGCCAGTCCGGAAGAAGGGAATCAACAATTGTGCACTCACTGGGGACAGTTCTGCACGCA
CTCGGGAGCGTCGCGGACCTCCCAGACTGTCGAGTTGTTGCTGATGACCCCGGAAGGTGT
CCAGCTTGGTGCTGGCACCCACGAATGCGCCAGTGCCGGTGATGTCCTTGAAGCTGAGCT
GGCCCTTCATCTCAGCCGGGCCACTCCAGGGATAGGCCATCAGAACGATCATCTGGTAGT
CCAGCTTCAGCTGCTTGGCGAAGGCGAAGAGCTGCTCGGGAGGGAGTTCGCTGAGCTTGC
GGTCGCCGCTGCCACAACTTACTTGAAGGAAGTGGAAGGGGTGACGGCCTTGCCCCCGTT
GAGGTCCTGGCAGGCCGAGCTGACATAGCACCACGTGCCGGGGTAGGGGACGGGCTCCGG
GCCCTTGGACTTCAACGCCTTGACGCACATGGTGTGGTCCTGGTGGGGGAAGAACGCCGA
GTTGGGCACACCAGGGAAGCTGTGGCAGAACTCGACGTCCGCGTGCTGGGCGAGCTCCGG
GTCGGTCAGCTCCAGGCCGTCGCCGCACTGCGCCCTGCCGCTCCGGTAGGTGGTGGCCCA
GTTCAGGCAGGAGCAGGCATCGGCGGCGCTTGCCTCCTGGGGGGGCCCTCTCAGCGGCCT
GCGCTTCATGCAAGGGAGCTTGCAGGTCCATGGCGTGCTGGAGGAGGTCCACCTGCAGCA
CCTCCGCGTCCTCGGGGCACGCCTCCTCGCTGGCCAGCGCAGCGCCCAAGAGGCCGACCA
GCAGGGAGCGCGCCATCGCGCAGGCCATGGCGCTGCGCGTTGCGGACGGGGGCTGAGCGT
CCCGCGGGGTCCTGGGGCGCGGAGGTAGAC
>at00720
ACGAAAAGGCTTGGACTCGGAAATAGCGAGTGCGAAGAAAGAGGTCGACACATTGACAAA
GCGAATTGGCTTCGTGACGACGTCCGAGGCCGATCTAGCCACGAAGGAGCTTAAACTACC
TCGATACGTGAAGCTGAAGGATTTCTACCGCAACGCGAATGGGAGCGACAGGGCGTTGTT
GAAGAAGTACTTTGATGCAAATGGGTGTCTTGACAGTTTTTTTGGGTCGAAGCAGAAGTG
GTAACTCACTCCGGACGAGAAGCACGTCTGACTTTGGTAGGAGCTAGAGCTGTCCACATC
TGTCAATCTTCCAGGAGTTAAAACGTGTGCGAATCAGTTGAGGCATCATTAGGAGCAAAC
CATCGCATGCTTGAGGCAGGGCAACGTGTTCCCGAAATC
>at00721
TCCTCGGCGCACAACACTGCCCACCCCTTCAAAGACTAGTATTGTCAGGCCTCGCGGCCA
CGTGACCGCCTGGCCCAGGACCTGGCCCTTAGCCAAGTCGCTCAGAAGCCGACAGTCGCA
AAGCGGCTGGCAAAGTCCTCGACCTCCGCCTTCAGCTTCAGGAGGCTGCCGGGGGGGGCT
CTCGGCGAGCACCTTCTTGAACTCCGTGAGCTTGGGGCCGCTCTGCTTCTGGACCTCAGC
GGCCACGTCGACCGCGTCCCCGATGAACTCGCCGATGCGCTTGAAGTCCTCCTCCATCAG
GCCGCGCGAGGTCATGGCGGGCGTGCCGACGCGGAGGCCCGTTGGGGTTCATGGCGCT
>at00722
TCGGAGAGTCCGAAAAGGCTTTCCTTCTTGATCTGTTGCTTCCGGTATTCAGTCGCCGGT
GGGACTGCAGCGAAGAAGCCGACCCTGGCATGCTGGCGGGACTGAGTGAAGAGCTCAAGT
CTAGCCATAAGGAAGTCAGAGGTCTCCTGGCGGCCCTCCACACAAACATGTTGGCACTGC
AGAGTGTTCCCACTCAAGCTCCAGCCACTGCATAGGCAGTTACACATGCTGTAACGGCAC
TGTGATGATCACCAGGGGGGCCTGCAGAGCGGCCACCCCAAGGTACACATGCCTGTCTCC
TCCCATGGCATGAATTTAAGGAAGTGGAGCACACCTTATTCATGGGCTTGGGCAGCAATG
GCACCCTTCTCAGACTTTACAGTTGACACCAAACATTCCAGTTGAGAGACGGTCAT
>at00723
TTTTTTTTTTTTTTTTCTCCAGGGTCACAGGCCAAATATCATGATCAGGGGGGACCCTTC
CCTACCCCGCAGCAGCTCGGCGGCAGCTCAAGACTTGACCTTCTCGCCGTAGCCCTCGCA
CTGCACGTCCAGCCACGCCCAGACATCATCCACTCGACGCTGAAGCTGGGAGAAGAGGCC
TCTGCACTGCCACTCCACCCCGAACCAGGCTGCACCAGCCGCGGCGCCAATGGCCGAGCC
TACAGCCAGCTGCGCTGACGAGTGCACGCCGAGATGGTGGCCGGGACACCACCACCAGAC
AGGTCCAAGCCACGAGTGCCAAGTACATCAGAGCCTTCAGTGCCCATGGGCACCGCACAC
GGGCCCAAACCCACAAGGACAGGTGCACGGCCAAGAACGTGCTGAAAGCGGCATGCTCGC
TGGGCATGCCAGGGCCCTCGCGGTAGCTGCCCGTTGGCCTGGGCTGCTGGATCAGCTCCT
TCAAGGCAGCGCACGCCACATCCACCAGCACAGCTCCCAGCAATGCCCACGCCCAAGCCA
GCCTCCGCGTCGTCACAAGGGCCCCCAGCAGAGCGATGCCTGCAAAGATGGGAGTCAAGG
TTGCCAAGGCCAAGACCTTGCTCAGGAGGTTGCCATCCTCATACAGCACATACGTGAGCC
CCAGCGGCTTCCGCACGGCCATTGCCTCGCAAGCAACCGTTTAATGTATCTGCATCATAC
CACCTTGAGCCAAAATGGCTACGG

>at00724
GTCCTTTGTCTTGAAAGACCACACATGTCCGCGAGTGCCACTCCAGAAGGGACTAAGGCA
GTATCGAATGCTATGGAAGTCAGCCGCGTGAATTTAGAGTGATTCTTATTCCCGAAAGGT
CCTTGTCCTGCACAATGTCGGTAGGCACGCACATCCAGATGGGACCTCGCAACCTGCTTC
AACGAGTACCGCCAAGCACACAGGCCC
>at00725
CTCGAGCCCGCCGCTGCTGATGATGTGCCCGGAGCCTGCAGCCTGTGTTGCGTGCGACTT
GGCGTCAGCCGCAGCGTCAGCCGCGTCAGCGATGGCAGGTGCTGCAGCTGCGCCAGGTCA
GTAGCGGCCAGCGATGACTGGCGCTGACCGATGACGACAGCAGCCACAGCGTCGCCGGTC
ACGCCTGTTTTCACTGTATCCGATGCCCAGCACAGAGGCAGCAGCTGAGAGGCGCTGTGC
GGACAACCTGCAGCGCGATGGTGCGATCTGTGTAGCAGCCACGGTAACGGCAATGCTGGC
GACGGGGCTGGCTCGTCGCTGGTCCTGCATCTTGCGCAGAACTGTTTTGCACCGGCTTCT
AATGACACCTTGAGGACTGTCCGCGTGGAAC
>at00726
TTTTTTTTTTTTTTTTTCACATGCTCTTCCAATCGAACTGGCGTAGCTCTTGCTGTACA
CTGATAGGGAAGCCAGGCTGGGCCCATAGTGGAACGATGCCGAGCCTGAAAATGATGTCA
TTGCAACTGATAGCGTTGGCTCTCAGCCTAGACGGGGAGTCCGACCAAGGGCACTCATGG
CAGAGGCAATGGGGAGTTGCCAAGCTGTGCATCCCTGCATTTAAACAAAGTTATACCTTT
GGTGTGAACTCAACCGAGCCTCCATTTAAAATGCACCTAAGTCTCCAACTCTGGTGCCGC
TGCGCCTCTAGGGCGTCTCTAAGTCCTTGAGCGGCATGTACACCTTCTTTCCAATATCGG
CAAACTTGCTGCTCATCTCAGCCATGCCCTCCTCGATGGCCTTCTCCGTATCAGCCCCGT
AACCCCTTTCTGCCGCATAGGCCCGCACATCGTCAGTGATCTTCATTGAGCAGAACTTGG
GGCCGCACATGGAGCAGAAGTGCGCGCTCTTGCCGCTGTCCGTGGGAATGGTCTCGTCAT
GCATCTCGCGCGCGGTGACAGGGTCCAGCGCGAGGTTAAACTGGTCATTCCATCGGAACT
CGAAGCGCGCCTTTGACAGGGCATCATCCCAAGCCTGCGCTCCCAGGGTGCTCCTTGGCA
AGGTCAGCCGCATGCGCTGCAATGCGGTAGGCGATGATGCCCGCCTTCACGTCATCGCGG
TTCGGCAGGCCGAGGTGCTCCTTTGGCGTCACGTAGCAAAGCATAGCCGTGCCCATGGCG
CCGATGTTGGCAGCGCCGATGGCACTGGTGATGTGGTCGTANCCGGGTGCGATGTCTGTG
GTGAG
>at00727
GTCGTTACAGACAACATTGGAGGGAGGATGGCAGCCATGGGCGGATGAAAGCGGACGAGT
ATGCGAGGTATGTGCGGACATCTGGATTGTATGAGAGTTCCTTCCATTGGGGGCACGATG
ATGGGATTGAGGTCTCAGAAAGGGCGGGCTGTGATGGGTCCTCTACAGTGATGACGTTGG
CATCGGGAACACCCCCAGTTGAGGAAAGGATTCTCCCTGCATGCCAAAGCATGCCTGAAA
TGTCCTCTGATCCGAGGGGGCGCAGCGATCGCGAAGAAGAGAACCCATCCAGACAGAGGC
GCTGGTGGCGTGCAAAGCCCCAGTCGCGCCCTTGCCCTGCTGTCAGCGAGTCCAC
>at00728
ATGTCTCCAGAACAACAGACGCGCCACATTCTTGGACCCGCAAGCTGGTCCATCACTACA
CCCAAGCGGCGGCTTGCCAAGTCATTCGCCAGTCTCCAAGCCTCCATCAGGAGAGAAAAG
TCTGAGGTGCTCAAGTCCTGGCGGCAGTCTCTGTCAAGCCCGCGGCCAAATCCAATCCGC
CTCCCAGAATAAGCAGTCTCCGATGCGAACACTGACAACAGCCAGTCGAAGCAACTCAGC
TTTGCCTCAGCTAGGCCACCTCTCCCCCAAGTCCGTGGATCCTGTGCATCCAAGCCACTC
TCCCTCAAGCCATCGATGGATTATCTGCTCGCGAATTACATCTGCCAGCTTCTCA
>at00729
AGCGCAGCTGCTCCATGCTGGGCGTCGGGAGGTCGGAGCGGAGGGCCACGACCTCACGGT
AGAGCCGCGCTGCCTCCGCATGTTCGCGCAAGGTTGCCAGCCGCCTTGCTCCAAGGCCCT
TAATGGCTGTATAGCCATCATCTGCGGCCGCCAGGATATTCTCGAGATCACCGTATTCCT
CGAGGAGCCGCATCGCTATCCCTCGTGCT
>at00730
GGTTCAAGCGGTCTCATTCCCAAGTCTCCCGTTGTGCATAGGTTTGTGCGGCCATGGCTG
ACAGCAGTCCGCCGCTCTGGGGTCCCGGATTGGTGAAGGTCGAGTTATGGGACGGTATCA
TCGAGGAGGTGTACGTCGACGAGAAGACAGGGCCGAAGGTGCTCAGGATGGACGGCAGCT
ACATCGACTTCCGCTGTAGCGTCAACCAGCCCGACATGACGGCCTGCAGCGAGCAGGAGT
ACGGCCCGGCGTGGACGGATCTCTCCTCCACGAAGCAGACCTGCATCATGCACATCGTCG
CGAAGGTGAACAGGAAGATGAAGGAGCGCTTCGGGCACGTGTGACCGGACTGCCGCCGCA

GCGCCAGGGGCTGTGAGCGCGCCAGTCTTCCAAGGCTCCTCCGGACGTCTTCTGCAGCGA
GCGCACCTGGGCTTCGCCCTGAAAAAGAAATACAAAAAAAAAGAAAAAAAAACAA
>at00731
TCATCAGACCTGACACCCTTTGTGTCGATCAAGATAATACTCTTCGGCAACGACTCCTCA
CTTTCATGGGAGCATTGCCAAAGACTGGTACGATAGTGCGCCGCATCATCTCGTGGTGCG
TCAGTCCAGTTTAGATCATCCCCATATCGATGTTGACTTTCGCGCGCGTCAAGATCGCAC
GCCCTCTGTTGCGGAGGCACCACCTCTACAAACGTCATGTTCCTGTCGATCTCCTCCTGA
CCAACTTTGCGAACGCCCTCGTATCCATGCTTACCACCAAGGAGCCTGTTGATCAGCTCA
GACTTTCCGGCGTTCTGGCGCCCGATGACCGCAACGAACGTGCGACTTCCTGGTCTTGGG
CCCTCCCACTTCCACTTGCTAGCAAGCTTGTACAAGGTACCGAGGTCGGAGTCCAGTTCC
TGCTGAAGAGCGTCTGCTCCAGCAGATCTTGCCAGAGACCGACGCTGACTGTGCACGCGA
CCAGCGTACAGATTGCCGAGCAGGCGGGAGGAGCTGCTGGCATGGGGCCATTGCAGGCGA
AAGAGACCAGACTGAACGCAGGACATGATCCAGCATTCACTCCTGATTGCGGCTTGAGCC
AAAATGGCTACGG
>at00732
TTTTTTTTTTTTTTTTTGACCACGCTGACTCTCCAACACAGCCAACATCAGTGTCACCA
TGCTCGGCATCAGCTCCTCAGTGCCACCTAGCTGTAAATTGCCACTTCAAGATTGCATCC
CACGGTGAGTGACTCTACACACCTATGAAACATAGCCACCCCTTCCTCCGGCGGCCATAT
CCTACCCGGTCCATCAGAAGACCACTCGCCCATCAGGATGCTGGAATGCTCCTGGTTGTG
GATGCGCGACAGAGAGCCTCTCGATGCGTGTTCAGGCAAGTGACACTGCAGAACCGCCCG
CCGCACGTGGGGCACGCGTACCTGCCAAGGACAAAGAACAGGAGTCGCACAAGGGGCGTG
GCGGCAACGGCGATGGGCCAACCTCGCAGGAGAAGAAGGTCACATCACGCTGGGGGTTGT
TTGCCTCCTCCGCCATGATCTCCTCCAGCGACCTCCGTCGCAACTTCTTGCGGTGCTCAC
CACGCCGGGCTGCGCGGCCGCCGCCCCTGGGAACACGCCTGCGCCGCTCGCCAAAGTCGC
TGCCACTGCCGCTCTCGCTGTCAGATTGCCCCCAAGGGTCAGCGGTGCTGCGGCCACGCC
CAGCACAGTCAGTCTCCAGCGAGAGCAGGCGATCCGAACCAAGCTTGTTGACCTCGGNCC
CCGGNCCCGCCCGCTTCGGCAGCTTCGCGGCGCTGCCCTGCTGCAGCANGCGGCGCGAGC
GGCGCACGGGCGAGGCCGTGCGTGCTGC
>at00733
GTCTATCAAAAGCCCAAGAATGCGCACTGCGCGGAAGACCGTCTGCTTGCACTCCGCGGC
CTCGTCCTCCCTCTTCTGCTGCTGCTTCACCTCCTTCTCCTCCTTCTCCTTCATCCTCGG
CCACGGCATCGCCTTCGGCGCCACCTGCTCTGCCTCGAGGCCGCCACTCGTCTTCCCCGC
TGGGTCCAATTGCTCCTTCTTCTCATCTTGCTGCTTCGCTTCTGCTCTGCCACCATCCTC
TGGCTCCTTTTT
>at00734
CCCTTTGCTTTGGCTGAGGAAATTCCACTGCGACACGATCCCCACACGGTACAGAACCAG
CAGGCTTGATGCATCCAGTGCCGACTTGAGATTCCACAGAACCACAGGATGGTTTGTGAC
AATGTGCACAGCCATGAGCATCAGGTATGTAAAAAAAAAAAAAAAAA
>at00735
CGACGATGTAACAGAGCGAGACCTCCTAGTTCGGTTCAGGTGCTGGTTGTGCCTGTTCCC
CGACGCCGGCGGATAACGGATCCGAGGGCAGCCTCCACCCAAGAGGAAAGCGTCAAAGGG
GTTGGGCAGCAGCATGTCGGGGTCGTACGACAACCATGGTCTCTTCGGGTTGGTCTTCCG
GATGGAAAGCCTGTCCCCTGTTGCTTCGTTGTTCTGAGGAGCAAGAGCAGCTCTGCGGCG
ATGGGCGATTAACTCCTCGCGGAGCTCATCCACAATGGGCAGGCCGCTGGGGCCGACTGC
CGCGACTGCGCCCCCCCTGCTCCAGGCCCTCCAGCAGCGCCGCTTTCCGGCCGCATTCGTC
GCACGTGCAGACAGCCCTCGGCAAGGGTTGCTCTTCTATGACCGACGACATGGTCGACTA
CGGAAAACCATCAACCCCTTTGGAGGGGTAGGACTCCACCCATGACAATCGGCCTTGAGC
CAAAATGGCTACGG
>at00736
TTTTTTTTTTTTTTTTTGCGTGTTCCCCCTCTGGCAAAATCAGACGCTCCACAGCTTCT
GACGGTGTACGTTAATTAGCTCCTGAAGCATGGTCCTCGCTAGTTCTAACCAAGTCGGCA
ATGAGGACTGCGGAGGGTAGAGGCATGCTCTCAGATGGTGTGGGACATGTGCCCAATGTA
TCCAGCAGCTGCCCCCCCTGTGGTGTGTGTTGTGACATGCCCCCTTGGCCCTGTCAACCAA
AAATCTGTACACAAATCCAAGACACAATCTGTACCAGAATGCCCACATTCGAGATGGCTA
AGTCAGTCCATACTGAAGCACACTGTCAGCATGGCCAGACAATTTGACCATCATGATCAT

TGCCTTGATCCTGATCCCCACACTGTATCTCATCATGAACGACAGCAAGGTGCTCAGCCT
CATTGGATTTGCACTTCCAGCTCTTCGTCGAGCTTCAGGACGTAGTCCAGTGCATCCTTG
GGCGAAGGCTGCGAGACGACAAGCTGGCACAACCAAGTTGCAAGACCATCCTCGCGGCCA
ACCACCGTCGTACCAGCATGCACGAGGGGATCTGACGAGATGACGCCATTGCGAGTCCGC
AAGGCTTCGAAGCACTCTGTGCTAAGGAGCTTGCCCGAGCGTCGTGCGTTGTANAAGGAG
TAGCCCACACAAGTGAGTGGCTGCCTTGGCACTGGAGGCAGTGCA
>at00737
GTTGCAGTTACCTTCTCAATGGCAACATGGTCCTACTTCTTGCAGCACAGCCAGCAATTC
AGCAACGACATCGTCAGATCAGACGGTGGCGCAGTGCAATATGGGAAGCTTTTTCCTTCG
CCGGTTGCATCCGGTCCACATGGAGCGATGGACTGCAAGTCTTCGGCAGCCGCCCTTCAG
CGTCAGGTGCTCGTCCGCCCTTGGGTNCCCAAGGGTGTTTTCC
>at00738
TTTTTTTTTTTTTTTCTTGCCCTGGACCCGAAGCCGCTCGCGCAGCGCTCCGCCCGCGGA
GGCCCCCCATCAGCCGAGAGGCCTCCTCACCAAGCGCCCAGGGTGGTCCACCGCTTTCCC
GGCTCCTCTCCGTAGGGCTCCATCACGACTTCCTGAGCCTGCACCCCCTCCACGGAGGCC
TTTATGATCTTCACCTCCCTGCCATACTGGTATGGACCCACCGGTTTCGCAAACTTCCGC
TTCTTTTTCTCCGGCGTGCGGCGGACGTCTGTGCTAGCAACACCAAGCTCCTTGCAGAGC
TTTGCACGCCCACGCTGGCCGAGGGCTCGGTCCAAAGTGCTCAGTGTGAAGGCCGAGGGC
TCAGCGCCGCTCGCCACCATCTCTCGGAAGACGAGCTCGGCGCCGCTGCGGCCCCGCTGC
TTCCTTGGGGACTTCTTGGCGTTTGCGTGGGCAAGAATCGCCCAAGACCAGGCCATGGCA
TCAGGTTGGAGCCCGATCTTTTGCATCGATGCTATGACCTCTTGCGCCTCTTCGGCGCCT
CGTGCC
>at00739
GGGGCGGAGCACTGCACGGTGGCGGACCCGGACGCGTCGCCCTGGGCGAGGACCCGCCCG
AGTCGACGGTGCTGAAGGCGAAGCCCAAAGGGCGGCTTCCCCCAGTCGCCCGTTCCCGTC
CTCGCCCCCGCCGGCCTCGCCCGGCCACGAGGGCGACTCCGTA
>at00740
TTTTTTTTTTTTTTTTTAGCCACCCAAGCTTCTTCCCGCCGTAGTCCTCCCCGTCATAC
TTCAGCGCCGCTTGCACGCCGGCCTCGTCTGCGAAGCTGAGGAAGGCGAAGCCACGAGAC
TTTCCGGTCACCTTATCAGTGAGGAGGTTCACGTCTACTACCTTCCCACACTCCTCAAAG
TCTTTTTGGAGGACCTTCGCGGTGCAAGACCAGGGAATGCCACCGACAAAAACAGTCAGG
AGCTCCGGCGCCAGCTGGGCCTTGTCTGATGGCTTCTTGTCAGTGTTGTCCTCTGCAGTG
TGTTTCTTCTTGGCCTTCTTCGAAGGCTTATCCTCGTCGCCGTTCTCCTCCTCAACCTCT
TCTCTTCTGCCTTTCCTTTTCTTGGCAGAGGGCTTCTCTTCGTCTACGTCGGCATCGGGG
GCGTCCACTTCGGGCACGTTTGAGGCCTCAGCTTGCCTCTGCTTTTTTCTGTTCTTTCCT
CCGGTCGAAGCTGCTGCAGTAGCAGTCTCAAGGTCCGCATCAGGATCGGCCTCGGCAGCC
TCTTCCGCCTGCTCTCTCCTCTTGCTCTTCTTTGCCTTGACTGAGGTGGCTTCAGCCTGG
GGCTCCGAAGCCTCCTCTGCCTGCTCTTTCCTCTTCACCTTCCCTCGTGCGAATTCT
>at00741
GTACACCAGCTTTGACAATGCCGCCAAGGCCTTTGCCAACTTGGGGTCACACAGCTGAGC
TGCGTCCTCGGCTCCGTATGACACGGTGACTTTTGCGCCGTATAGACGGAAGTCACCAAT
TGCTGCGGCAGGCCTCAGCCCCAGCGTTGCCAACAGTGCAAGCAAGCACCACATGACTGG
GGAGTTGCCCTGCTGCTAATTGTAAGGCGGTCTTTGGCCCCAAACGGTGCCCATGGCCTC
GCTGCTCTTCAGCCCATGAGCATGAACCAGAATGCTTGAGCCAAAATGGCTACGG
>at00742
TTTTTTTTTTTTTTTTTTGGCAATGAGCCGGCACCTGATGAAACTCTGTTCACCGGGTGC
CTGCTCCACACAATCATACCACAAGCCCAATCCCTAGATCTTATCTCCGCTTTCCTGTCT
TCCTCCCCTTCCCTCCTCCCTCCCCCCGCGGGCGTGGGAGGGGGTGAGCCAAGGCGAGGG
AAGGCTCATGGCTGAGCCAAGGACCGCGGTGCTTAGCCGTGACGGCTCCGTGCTGTGTGG
TTATAATCCAGATGCTGGGTCTGCGGGTCCCCACGGCCAAACGAAGAGCTCAAGATCTCC
TCGTCTGGTGCCTTGCAGTCCATACCCAGCTGAAGAACTCCATATACTTGCTGGGAGTTC
TTCTTATGGGGGAGCTGGATGAACGCGCCTTCCAGGGGGCCTTGCACGACGCGGCCGCAC
GCCAGCAGCTCCCAAGGCGGATCAAGCTCGGCTGAAGGAGGGTACCGCCGCCCACCAGGG
CGGCGGAAGAGCGTGCGGGCGCCGTTGCTTGGCCTGACCAGAAAGCGCTGCACGGTCACT
TTGTGCAAGTCCCCAAGGTCGCTCAGAGCACCAGAATGTCGGAGTGACAAGCGCGCCTCC

GCGGCGACTGCCACAGCAAACCCCGACCATGTTGGGTCCAGGCGCACGCGTACGGTGTCC
AAGAGGTAGCGGAGCGCCGTGAGCGCCTCGGCAGGGTCCAAGTGCACGATCGCCTGCTCG
ATCAAGACGGCAAAGTTCATCCAATATCTCCCGCGGTGCGGCACC
>at00743
ACTTGAAGTAGCGCACCATCGCAGCCGTCCACGCCACGAAGATGGCTGTGTAAATCACTG
TGTACCAGCCAGCAATCTGCATGGCACTGGCCTCGTAGGTCGGGAGGTAGCCGTGGGCAC
TCCTGACGGCGACGCTGCCCGTGAACGTTGCCTCGCTGAAGTTGCCACAGTTCGAGAGCA
TGAGG
>at00744
TTTTTTTTTTTTTTTTCTAGACGTGTGTAATGCTTCAAGTTGTCTCGGATTTCTGATTGA
TAACACAAGAGACAGAGAGATAACCACCAAACAGGATTCAGGTTTGGAAGCAGCTGTTAT
TATGTTCCTATTATCTCTTCAATCACGAGAATCATATGTGAAAGAGAAGAAAAAGAACAA
GTGAAAAGAGATTATGTCATGCGTCAAGGAATTGGGAACTTGTCAACGGCTTTTCAATCT
CTCAGTGACAGGTGAATGACTTGAAATCAAATACGAATGCCCCCAACTATCCCTATTAAT
CATTACCTCAATTCTAGAAACCAACAAAAAGAATCATGATCTTAATTATTATTCCATGCT
AATGTATTCAAGGCCAAACACCTGCTTGAAACACTCTGATTTCCTCAAAGTAAATGTTCT
CAATAACACACCACACAGTCAAGTGCAGTTGTGCTTTCAAGATAAATGCTCAGGCTGTGC
CAAATACTCACCCAGAGGGCGGACCAGCCATCCTCAGCAGAAATCCAACTACGAGCTTTT
TAACCGCAACAACTTTAATATACGCTATTGGAGCTGGAATTACCGCGGCTGCTGGCACCA
GACTTGCCCTCCAATTGATACTTATAGAAAGGTTTGATGTCCAATCATTCCACCTCGTGC
CG
>at00745
CGAGAAGATCGCCGCAAAGGAGAAGACTTGGGACACACAGGAGAGCGCTCGCTTGCTGGC
CTTGAGCAAGCAGGCCAAGGAGGAGGCAGCTGCTCACAGTGCCGCACTAGCTACGGGGAA
ATCGCATGCTTGGGAGGAGCTGCGCTCGGCGGCGGCGGAGAGATCCGAGAAGCAGAGGGC
CGAGGCCGAGGTGCAGCGCGGGAGGGAGGAGAAGATCCGCCTGCGGGAGCAGCAGCGGCG
GGCCAGGGCGGCGGAGGACGCCCGGCGCCTCCGGACAGACCCCTCGGCCAAGTCTAAGGC
CGTAGTTCGGGCCTTCGTGGAGCGCGCGCAACTGGACTCCAACACTGTCAGCGTCCTGAG
CACGGGTCTGTGGAGCCTCAGTTCAGCCACTTTGGCGCCAGCACTCACGACATCCTCTGA
CGACACTGCATGCGGAGCAGCGCACAGGCACCTGTCCTTG
>at00746
TGTTGACATGCCTTGAGAAGCGGACTAGATGTGGACGCATCCGTCGCCCATCCCGTGTCA
GCAATGGCGCACCTCTTGGTGTGGCAGCTGCTGAACGCCGCCATCGCCACGGCCGGTCTG
AGTACCTCACTCAGGCTGACGTCCTGCGATCCGGGTT
>at00747
TTTTTTTTTTTTTTTTTTCAGTAGCACAGACCGACAACATGCACGATCCACATCATGCAT
TCCTGGGTCCAAGACTTCGGCCCCGAAACAAACGGAACGACCCAAGCTTCAGGCCCTCAA
TGCATCACCATGAACTGGCATTCATCCCTCCCCCTGTGCTTGTAGGGTGCAACGACCATA
GGCATCCACCAGGAAAAAGCAAGAATGGCATGCACCAGAAAGGCCCTCGTCTAAGAATGT
CGACCCAAGATCCTGGCTCTCCGTGCCTCCCTCTTGGACTGTGCACTTCTCACAAACTGA
TCCACCACAAGAGCTACTTCCATGGGCAGTTGCTCCAACGTCAGGCGCGTGCCCCAGAGC
AGCTCGAGCTCGACGTCGCGCCGCCCGTCCTCCAAGTCGATGTACCCGCGGACGTGGCGC
ACGTCCTTGAACTGCACGGTCTTCTGCGCTTCGCTGCCCCGCACCTTCACTCCTGGGCCC
AGTGGCCACGACAGCTCTATCTCGGACGCGGTGATGCGGTAGTCCGACCACTTGCCCCCC
CCGAAGGCGGCTGCGACCCCGTGTAGAGGAGCCTGTGCTCATACTCATGAGCAGCCCGGG
CCTTCTCTTGCTTCGCCCTCATCTTCTCGTAACGGGACACCTTCTTCTTGGGCCCTGCCG
GTGGTGCCTTTGGCTTGGGCTGAGGCACGGAGTCCAGAATCGCCTGTGCTGCACGAATCA
CCTGAGTCATCATGTATCCAGAGCCCGTTGGGCACACGTCTGGGTGGTACACACGCACAA
GCTCTCGATATCGTGCTTTGACCTCACTGCGGGTTGCCTCGCTAGACAAACCCATCACTG
CCCAAGGGTCCGCCTGAAGACCGTCCACTGTTGTGGTGTGCCCTCGCAAGGATGTAAACT
TGATAAGCCTCCGTCTGGTGTGGCCAGGGAAACTTCCACCGGCTACGGAAAATAAGNNGG
GGACACCACTGCGACCTTGACGGCCAAGACAACAACAAGCACCGCGATGCAAATGTACAA
AATTAAGGTTCGGCATCCCCCAGGGTGATTGCGTACACCAGTGCCCACACACTTGCCAGC
TCTCCGTCCTGCAGCCATGGCAAGATTGTGTACAAGAATTGAGNGGAGAGCAGCAACTAG
GATTCAAAGTCAGGCGGAGCCAATATGG

>at00748
TTTTTTTTTTTTTTTTTTGCCGCCCCTGCTTAAGATGCTGTGATCAACAGCAATCCTGCA
GTCTGTCCGGGGCCAGCGTGCGTTCACTTCTTCCCCTTGAAGTCCTTGCTCTTCCCTCCC
GCGGCGCCGCCCTTTTGCGGCTTTCCCGCCGCGGGCTGTACCTTTGCCTTCGCCTTGCCA
GCTGCCTTGGCTGCAGGTGCTGACGCCTTCTTTGTGGCCACGCGCGCCTTGGCCTCCTTG
GCCGCCGCCTCCTTTGCCTGCTGGCGGATCTCCGGCTTCTGCGCCTTCTTCTTCTTCAAA
TCATCAAGCGACAAGCCGACAATGGCCTTCTGGAACTTCTGGACCTTCCGTGTGCGTTTC
TTCACCTGCTCGTTGGCGGTGCCCTTCTTGTTCATGCGGCGCCAAGCCTGTGTCCAGCGC
AGCTTCACCGGCTTGATCCTCTGATGGAAGAGACTCTCTGCTTTCTTGCTGATGAAGAAG
CTGACCTTGCCATCCTTGGCGATGAACCTCTGGCCGCTGCCAGCCTCGTGCC
>at00749
CCGTAGCCATTTTGGCTCAAGCATCACAGGTCCGTCACCCGACTAGTCCCTGACACATTT
GCCCGATACCGAGCCCTATATGCCTCATGCAAGATGATGCAGCGAGGCCGCAGCCGCACG
TGCCCTATGGCGTCTGCTGCCTCACCTACGGATGTGGGGTCGTGGGGATGCTGCTCCTCT
ATGGGCTCTGGCAGGAGCGGATCATGGCCTACCCCTACGACGGCGAGTACTTCGCGGGCT
CCACCGTGCTCGTGCTCTACAACCGCCTCTTCGGCGTGGCCTTCGCGCTGGTCATGACGT
ATCCTATGTCAGAAGAGATGACGTGTGCAGCTCCAATCTGGAAGTACGTCTTCATCTCCG
TGACTGCGGTCGCGGCCAGCATATGCCAGTACGAGGCCTTGAAGTACATATCGTTCACTG
TGCAGATGCTTGGCAA
>at00750
TTTTTTTTTTTTTTTTTTGCCTCCGAGTGGCCTCTGAAGAATTGCAGACCTGGCCATGTC
CAGAGAGACTCCATCTGGTTGGTCTCCATGCCATCAAAAGGCGCTGAGAATGTTCAGTTC
ATCCTCACTCGCCCTCAATTGGGACGAAGTCGTGCCGAGATTCGGCGAAGCGTCAGACGA
CGAGAGTGCGCCGAGCCTTGATCCGATCAGATGGAGCTGGCTCAGGATCTTCCTGTTGTC
GTGCAAGAGCGCCAAGACGTCCTGGGACCTTGCCGTGCCCGTCATCCGGAGGATCCCTTC
GAGGAACTCCTCGAAGGAGACCTGGCCATCTCCATCGTCCAGCAGTTTAAATAGAGACTC
CATCTCGTGGGGCTCGAACTCGATGGACGCGAAGAGCGCCTTGGCTTCAGGGTTTCTGGA
GAGGACTCTGATCTCCTGCCAGTCGATCATACCGTCGCCCGTGGTGTCGGCCATGCGGTA
AATCTCCAGGAGCTTTGCTGCGAAGATCTCCCTTTCTTGCGCTTTCCGCCTGAGCATCAT
GTCCTGGTCGTTGGCGGCCACCGCCAGTGTGTCCTTGAGGAAAAGCGCCGTGATGATCCG
AATGACAGCAAAGACCACGCCGCCGACGTACACCGCAAAGTAGAAGGCGTAGAAGACCGA
AACTTCTCCACCAGCGGCGTCACCCAAGTCGGCCAGCCGCCGCTGAACGTGATCTCAAAC
ATCGTCCAGAGAGCTTTGGACGCGCTGCCGTAGTTTCCTTCCACCCACATGCGGTCGTCG
CGCAGCCTGGTGTCGTCCCTC
>at00751
CCTTGTTCAGGAACAGCGCGGGGGGGCTGGTGTGCTAGGAGACTGAAGTGGCCAAGAGGC
AGAGGAAATTGGCTCAGAGAGATGCTCTCCTTCCCTCTGGGTGGAATCTGTGGCTCCTAG
TGCCGCTGCATTGTCTCTGGGCTGTCCCTGAGCGGTGACACTGTGTGGCCATTAGCATGT
GGTTGCAGGATCCTCATGAATTACGGACAAGTTTGAGGTACGTTGGATCACCTTGGGGAC
TGCGCAGTGGCAAGAGATGGAGGGCTGGCGCGCGGCAGGGTAGGCAGGGGCTCGGTGGAG
AGCCGGAGGCGGGGGCCGGGCAGGGCGCGTTGCGATGCGATGGAGCTATTGACAGACTGG
TGCAAAGGCGTTGGACTTACC
>at00752
TTTTTTTTTTTTTTTTTTGGATCAGCCCAGCCAGATGCCACTTCCGACGTCCCCAGATGC
TGTGTCCGACGGCCTCTCGCGCGGAGGCTGCCCCCCACGTCGGCGCACAGCGGCGGGAAC
ATGCAATCTGGTGCATTGTCTGTCGTTGGCAGACTAAGAAAGAGATACAGCCCGCTCAGG
AGGTGGACGACGCGAGAGGCACAAGGTTCGTCTCCATCTGCCTGAGCTTACCCTGCTTGT
CGAACTCCATCGTGTAGGAAGCTCCCCCTTCCAACGTGTAGGTGCGCGTGTCCCCTGCGC
CGCGGACCTCCCAGTTGCGGCTGCGCATGAAGGAGTCGATGGCGAGCGCGTGGTTTTGGA
TCCACCCGGTGGACAGGGCCTGCTGGATGGACGTGGACACCCGCAAGGCGTCTCCCGCCG
TCTGCGGCGACCGCGGTGGGAAGTTCCGGTCCACAATGAGCATCACCGTTGAGCCTGAGG
CTGTCAGCGGTGTCAGGGCATAGGCAGTCACGCCCAGCAATCCGGATGCAACTGCTGCGA
TCTGCTTGCCAGTCGCATGTTCCTTGGGCACCATCCCTTCCGTGAACTCCGGCGGCGCGG
CCTCCCCCCTTGACCAGCCCTCGCCCGAGCGCATGGCCTGGGCAGATCGCAGCTGCGAGG
CGCTCAACCCCGGAATGAGTCCTTTGGNCGCCCACGCCCACGTGAAGCTCCCATCCACGG

NCTCCACGCCCAAAGCTTCGGGCTTGCCTC
>at00753
ACGCCTGGGTGAAAACTCTGAGCGTGCCCATGAGGAACCTTGGCCGCCCCCAGGGTACAT
CCGTTCCTCGATGCTGCCAGAGTTTCACTCTAGCTCGGCGCGAGAGCGCTTCGTGACCAC
GCACCCCTTTTGCCGGATCCCCGTGACCTGGCGAGACCCCTCAGCAAGGGCAC
>at00754
CGCAGGCCGGACGCGTTGCGTCCGCCGCGGGAGCGACCTACATGCCCGGGCAGGCCCGGG
CGTTCCCCTCCGCTCCTCTCCCCTGGGCGTCCCACTCCTCCTCGGCGATGGGGATGCCCT
TGAGCCTGAACGCCTTCCAATCCTCCCCCGCAAGGTCTGCGAGCCGGTCGAACCGCGCCT
GGTGTCCTCGGATGTAGTCCTTCTCAAAGATGTTCAGGCATACCTCTTGCGTCGGAACCT
CAACATCTGCCCCGAAGGCCTCCCGAAGCGAAGTCCAGTTGCTCTTAAAGTCCTTTGAAT
AATCGATGGCGTATTTCAGCTTGGCCAAGATCTCTGCAATATCCTTGGGCTCGCCGTCAA
TGTTGTCCAGCGTAGTGATGAACATGACGGCGGTCAGGTCCTTGACGAAGTCCAGGGGCC
CCTCCACGCAGACCATGATGGGCACCGTGTAGAGGATGATGTTCCGGGCCACGGAGTTCA
CGGTGAAGTCCATGAAGCACCGAAGTCGCCACTCGAGCTCGAACTTCATGTCGAGGATCC
AAAACACCTTGTGCCAGAGGGAACCGACATGCTCCAGGCTCTCCTCGAGGCACTCGTCCG
CAAGGCGTCGTCCTTGAGCACGGCGTTGATCTTCCCCTCGTGCCGAATTCT
>at00755
GCGGCCGCCCATATGAGGTTTTTTTTTACCACGCGAGCGGCAGCCCTCAGAGCAGCTCGG
CCGCTGCAAAAACCAGGCGGTGGGCGCTCTTGCCTCCCCTCAGTAGTAGGTGCCGTCCAC
CTTGCCCTGCGCATCCAACAGGCCCTTCCAGTAGTCAGCTTCGTAAGCCTTCTTGGCGTT
CGTGTCGATGTTGACCTCGACATCCTCGCCGAAGAAGCTCGTGCGAGCGTCAAGCACGGC
GAGGTTCCTCGGCTCCGTGATGAAGCCGGTCAGCGGCCCGATGATCGGCGGCGAGGGGCA
CCAGTCGATGAGCTCGCCGGTCTTGGTCGAGAAGGAGGAGCCGTGCAGGGGGCAGACGAT
GATGTCGCCGATGACGTCGGCGCCCTCGCTCATGGGCGTGCCGAGGTGGGGGCAGAGGTT
GCCCACGCAGAAGAGGGCGCCGGCCTGGTCCTTGCCGATCAGCACGTCGTTGCCGCCCAA
AGTCTTGGCCACGAGCTGACCTGGCGCGATCTGGCTGGACTTGAGGCCCGTCTTCAGCCA
CTGGAAGTTGGGCAGGATCTTCTTCGTGGTGTAGCGCGCCGCCGTGGCCGCCGGCGCCG
>at00756
TTTTTTTTTTTTTTTTTCCTCCCCGACCACAAGAGCGACAATCTGTTGGCAGCGGGTCA
AGAGAAAGTTTCGCTGCTCATCGAAGCACGCTGCCCGCGCTTGCGCCCAAAAGTCCAAAA
TTCGATGAAAGCGATGGCAACCCAAATGCAGCAAACGAGACAGCTGTCAAGGGTATTCCA
ACATGTGCGGTCGTGCATGCCTGCGGAGCATGCCGCACAGCCTACAGTGTCCCCCACCCC
TCCCTACGCCAGCAGCGGCCTAAGACCACCTGAGGCCTTGCGCCACGGCCTTGCCCCCAA
GGCCAGGGCAGCGGCCACAGCGCCCAAAAGGGCCGAGCCGGCGGCCATGCCCATGGTCGA
GGCCGGGGCGGCGGAGGAGCGCG
>at00757
CATCACCCTTTTTGCCCGAGAGAGCAGCGCGGGAAAGCCACCGCATGTGGCCTGAGGTCT
GCCATCATGGCCTTGGCGGTAATGGGGGCTGCCGATGTTGCCCGGCTCCAGAAGGTGCTC
AGCTGCGCCTTCCAGCGCTACTTCACGATGAGGTGCACTTCTGGAAGCATGACCCTTGGC
CAGGTGGAAGAAGTGATGATGAGAGTTGCCGGCCTCAGCAAGTCCAACGCCCAGCT
>at00758
TCAGCGGCAGCAGCTCCGGGCTCCTGCACCTCTGGGACCTCGACTCCGGCGAGCGGCTGG
CAGAGCTGGCCGGCCACTCGGGCCTCGTGCGCTGCGTGGCGGCCGACTGGGCTGCCCAGA
AGGCCCTCAGCGGAGGAGATGACCGGTCGGTGCGGCTCTGGGACCTGAAGCTCGGAACTC
TGGTGCGGCAGCTGCCGGGGCACACGAGCCGCGTTACGTTTGTGGCGATGTGAGGCGCAG
GTCATGATGGGATAAGAGCCCGGTGACAGGGTGGTCTGGAAGGCGCTGGGTGCTGTCCAG
TTACCAAAAAAAAAAAGAAAAAAAAACAA
>at00759
CCGTAGCCATTTTGGCTCAAGCTATTTCAGCACAACTCCCTGTACATGCCCTACCATCAG
TTCGCGGCATGTCGCTGAGCCTCCGACGCCTGGGCAGCGCGCTCGGCGCGCGGGTGCTGA
CGTCTCTCCGGGACTGCGACCCGAAGGAAGTCCTACGGCAGGTGCACATGAACGGGGTGC
TGGTCTTTGAGGGCGTGTCCTGGACGCAGCCTGAGATGGTGCGGTGGTTCGCCCGGCTGG
CCGCGCCGCTGCCCCAGCGGGGGCGCGAGACGGCCGGGGGACTGCGAAGAGGTCTTCGTG
GTGTCTGACCACGGCGAGGCCTCGGCCTCGCCCTTCCTCT

>at00760
TTGTTTTTTTTTCTTTTTTTTTGCAGTCACCTCAACTCAAGCACACCATCTCACAGTCA
CGTCTATCCAAGCCCCCCTTCACCTGCTCAGCGTGCAAGTCCCGTTCAGGACCCTGCCCT
TAGCACAGTCGCCCGAGCTGTAGCCTCCTTTGTTTGCGTGGCATACCTGCTTGAAGCTGC
ACCCCTTCATGCCGAGCCACAGCAACAAGTCGTCAAAGTGCTTGGTGTCGAAATCCTCCA
CGCGGAGCAGCTTGGCCTGATCGGCGTGCTTCTTCTGGAATTCCGTCAACAGCTGCGTGA
CTGCCAAGATGTTCGCACGGGAAAGGAAGGTGGAAGGCCACCCGTGCTGCTCCATTGACC
GAACTTGCTCTTCGACGTTCCTTGTATTTACGATGAACCTGCCGCACGGGAGCAGCTTGA
GAATGAAGTCAAGCGACGTGAGCTCCCCCCAACGGATCTCTTTGAACCCCAAGGTGTGGA
CGCCGGGCGGTATCGCGCCGAGCGAAGCTTTGGCGACCGACTGCATAGCACACAACACGG
CCCTCTCGTCGATGGCAGGGTGCTGCCAAGCTCCACCTGGACTGCTTGGCTGCCACCCCT
GCCGTTCCAGTTTCTCATGATAGTCCGCGAGTTCTTTGACAAGGAGCTCGTTCTCCCCAG
TGAGGAACACACCGTCTGCCGCGTTCATCATATTGAGCAAAGTTGTGGAGCCTGTCCTGC
CACCTGCACCCATGATGACCAACCATCTGTCCTGGCACAAGCTGCAGTTGCTGGTTGCGT
TGTCGAGCGCCCTCTCGAGGATCCTGGCCTCCAATGTCTGCAAGGCCGTGAAAGGCTTCT
CCGGATGATGCAGATATCGGGAGGAAAACGACGTGAACGCAATTTGCAGTAACACCCGGG
GTGCCATCGCTGCGGCACTGACTTGTACATTAGCAAGCGCGCAGTGCGGTCTGCAGCCAA
AGTGGCTTGAGCCAAAATGGCTACGG
>at00761
TTGTTTTTTTTCTTTTTTTTTTATTTCGTTCCAGCTGCCACTGCTCAGCACGTTGGCCC
AACTAGCATGCAAGAGCGCGGGGATCCTGCCAAATGCACGCACCTAGCGCGGCCTCCCCA
ATTGCTCTGACAAATTACGGCGCAAAGGGAGGATGCACCTCAACTGCAAAGAGCGACGAC
CCCTGTCCAATACCAGTCAGAGTCTGACGTGCGGCGGGCTAACCCAATGGCATGGTGGAG
GCTTTTGGGATGAAGCGGTGGCCCGATGTGGGGATGTTGGTGCCCAAGTCACAGAGCTGC
CTTTCTGGTCGAGCCCCCACTTCTTCGACACGGCCTCCTTCATGACAAATCATGAGCAAC
CGCCACCTGTGGTAGCTCTGGTCTGCAGAACATTGGTGCTCACTCACATATTTGAGAGCG
ACCTTCTCTGTTATCTCGGCCATGGTGTCAGTTGGCGCCAGCTTCACCTCCACTTCGACG
ATTTGTCCCATGATCTCGCGCGATCCGTTGATGAAGGTGTACACCGAGCCCGTCTCGTCG
CTCTCCTTCGGAGGCATCTTCGGGCCGAGGGTATACAGAATGGCGTCCAACACCTTNCGA
GAGGGTGAAGGCTGGACTCCAGCCATTCCTCTCCATCATTTNGGATGGCAATCGTGCCAT
CAGGCAAGACGGCGGGGTGATCGATCGCCGCAGCGAGCTGCACACGGATCGGCGCGAAAG
GATAGTCCCCCGGGTACTTGACGGTGAGCACCAGCTCGCTTCCTTCGTGCGGAGTTCCAG
GCGGACCCTTCAGAGTCACCTGGCACTCAAAGGGGTTCTCTGGGTCACCTGCGCAGGAGA
CGAAGCCCGATGGCGGGTTCTGATTCAAGTCTTCCAACTCCTTTCTAATCCGTTTCTCGG
CCATCCCCTCAGAGTAATACACGCTCGAGAGCTGCAACTGGCGAGGCACAAAAAAGAAGT
CCCGTCAAGCTTGTCTGGAGCCAAAATGGCTTGAGCCAAAATGGCTACGG
>at00762
AGCGTCACCCATCTTTTGCGCTTCGACGACCCCTCCTGCTGTAGAGGAAGCGGATGCACG
AGAGGTGTTTACCAGCAGCCGCAACTGTCGAGTCCACGTGCTCTTCCGCCGGGTGCGGCA
TTGGTGAGCCCCGGCTGCGGCGCAACCCAGTACTCCAGCGGCAATGGGTGAGCTGCCGTA
GACAGTGTTTGACCTGATGAAGGTGGCCTCAGTTGCCACCCCGACTGCCCCTGGCAACGC
AGGCAACCTCAAGGCACCAAGGCCGTGCCTTGGCAACGGCACCCATGCATTGCCGCAGAT
GCGGCTGGCCACCACCATTGCGGCCAGCTGGGCCCCTTCTCCACCAATGGAGAAGGGGCC
AAGGAAGAACGGCACCTGCCTTAGTG
>at00763
GGCATTCTCAAGTACTTTGGCATGAAGCCGGATGGATTTGGCAGAAAGAACGAGTTTGCG
CAGTTGGCTTGCGCAATGGGAAATTGGCATTGCGACGTAATTTATTGTCGGCAATTCTAC
TGCAACAGCGAGTCTTGGGTCAAGAAGTATGGCTACAAGGCAGTGTTTGGCGGGATGCCT
GACCCAGAGGAGACTCTTTAGGACCACAAGGCTCTCTCGTGGCAACGACGGCGGCGTGCT
GGTGCTCGGGTGGCTGGCCACCATGTGTCAGCGACTACGACGTAGTCACTCGAGGTCTCC
TGTTCTGTTGCGACACCGCAGCACACTTCAGCTTGCTCGTGTGATCAGTGTGGCTGACGG
ACAAAGAACGCTTGGGCGGCGGCATTTCCGGAGGGCTGGGCTGTGGTT
>at00764
CCTTCTTCAGGGCAGAGCCGCGGTCGGGCACGGGAACCAGTCCATGTGTAGGTCCTAGCC
TACCCTGCTGGCGCGACGGAGGCAGAGCCTGGCGGACACCATAATATGGCAAGTTGCCCC

AGAATCGCGTGATGTCAGCGCAGGGCGGGCTTGACGACTGGGATCCGTTTGCAGACCCGG
CAGATGCACCTGCGGCTGGAGAGGGCCTTGACGCCTGGGATCCGCTTGCAGACCCGGCAG
ATGCACCTGCAGATGCTGTGAAGGCGTGGCGGCCCGGGCCGGGCCGCAGGCCGCCGCCTT
CCTTTCGGATTCCGAGAGCGGGAGCGGCGAGGCGGAGCCGGC
>at00765
TTGTTCGAGCCAAAGCTCATGGTGCTGCCAGCAGAGCAGCCGCCCGAGCCTCCTGGCTGG
CCCGAGCCCACTCCGGAACTCCCGGGGCCGAAGCTCGTCGCGCTCTGGCACTGAGCCGCC
CCGTTCATGCTCGAGCCACCAGGCCCAAAGCTGGTGCCGCTGTTCCCTGCCCCATTCGTG
CTCGGGCCACTGGACCGAAGCTAGTGCCGCCGTTTCCAGGGCCAAAGCTCGTT
>at00766
TTCGTAAGTACATTTTGGTTCGTCCAAGACCTCGTTTGGTTCATCCGGACCGAAGGCGAT
TTTTTGGTTTTTCGGGGTCCGGGTTCCTTTACCGAACGGTACCCGTTCCCGTCGACGACC
GACCTAGTTCGGTCCGGTCGGTAGTTTTAGGTTTTCGGACTACGTCCCGACCCCAACCCA
ACGACGACCTTAACCTTTACGTTGGTTCGTTCGTCCGACCTACCTTACCGTTAGGGTCGG
TCGTACCGAACCGAAGTACTACTAGGTAACCGGAAACCGACCGGACCGGAAGG
>at00767
TTTTTTTTTTTTTTGTTTGTTCAAATACTTACACGACCCCTGTAGGTAGCATTCTCCCCT
CTGTCCAAAAGAATACCTCAACAGCCACCCTCGCGGTTGCCAAAAGGTAAGCGGAAATGC
GCGTAGCGTGCATACAAGCGCGAAAATCTCCAGCAGAGAGCGCTTGTGCAGCACAGGTTG
CCAGTGCCTGAACGGCGCTCTAGCATCAGCATGGCCGTTACTGGCTGTCTTGCCAGAAAC
AGAGACGTGAATTAGCCTTGCATATCTGCTGCCAGAGGTCAACGTAGCCCTTGCAGCCGG
ACAAAAAACAACAAAAAATAACATCAAACAAGCCTCAGCTCCTCATGCACTGCCGCGCGC
GAAAACGTGTGCCAAATGTGGCCTCCCCTTCCCTCGGTCCTGAGGGCAACTTACGTGGGA
AAAGGCCAAAGCCCCGGCTGAGGAGCATGCCAAGGATCCGTGGAAGCAGCAACCTCTTTC
CTGAGACCACCATGAGCAGGGCGTCCCAGACCATCATCGGTGGGCTCTTGTCCTGGACGA
GGCCCTTGTTCACAAAGGGCGAGATCACCACGCCCTTCTGCACCTTGGGTATCTTGGAGA
GGTTTCCAATGAATTCGAGCCCACTCTGGGGCTTCTCGCGGCTGTATGAGCCCGGGGCGC
CGCCCCATACGAGGAGGGGGTTGGGGTAGCCCAGGGCCGCCCACATGTTCACAGCCACTA
CGCCGT
>at00768
GGCGACAACCGCACGCCAGCGTCAATAGCACGGTTGAACCACTTCTCTGCCGCAGACAAG
TCCGAAGCCTTGGCTGCTGCGTCAATGAGGGCAGTGAAAGTGAGCGCATTTGGCCGGACC
CCGCTTTCAGCCGCTCGACCAAACCAAAGTTCTGCACCCGTCAGGTTGCCACGTTTCGCT
GCTGCATCTATAAACGTGGTGAAGAGCACTACGCCCGGCTCATGGCCTGCATCAAGTAAA
GCTGTGAACCAATGCTCCGCTGCCAACATATTGCCCTTCTTCCCGGCCAGGTCGATCATC
ATGCTGTATGCC
>at00769
ACCTCGAGATTTTTTTTTTTTTTTTTTTTGATGCGTGGATTTGCATGGCTGCAAGCACG
ACGAGTTGCGATGGCCCCACCACTGTACAGAGTGAAGCATTCATCGACAATCGCAGGCAG
CGAACGAGGCGTTGTGCAGCAGCGTCACTCTGGCCTCTAAAAGCTTGCAAAAGAAACGAC
ATCCTGACGGCCCGATTGTTGAGCCAATCAATGAAAATACTGCTGTGATCTTGTACTGTA
GCAAAATCTTCGAGGACGATCTAGCCAGATCACCCTATGCTGTAGGTTCAGAGCGAGTCC
GGATCTGCTGTATCAGTTCATGGACAAACACTCGCACAAGAACTCAAGGTTCAGGATAAT
GATGATCCAGGCTACGGCCAAGATAGGGAGCAGGGTTGTGGCGACTCTGCTGTTCGTTCC
AGATTGTTTCGGCACTGCGCGTCTCTGTATGATGGACCAGGCACATCGGCCCAGCCGCCC
GTTGCAGGCAGAGCGTCTCCGTCCCTGCGCAGCAGTGCTCTAGCAGTAAGAGTGGGATGT
CGTGGTCGTCGTGCACGAGACGTAGTGCCAGCCCCTCTTGCAGCAGGAGATCCTAGTATG
GTGGCCGCAGAAATTGCCGACATGGTGCTCCTCGCAGAATTGGTTGTAGCCGCCAGGCGG
GGTCCATGGGTACCAACCAGGATGGTTGCCCCCTGGGTAGCCAGGCTGGAATGGGTGGTC
GGACGCGCCGTGCCAACCGCAGGAGGTCGAGTGCTCCGCCGAGCCGCACTCTTTATAGAA
CAAGCCTGACCGGCAGCAGCGGATGCGCGTGGTCTTCTGGCAAAAGAACCCGACGTGATG
CGTCTGGCAGAAAGACGAGCTACCCCACCAGCTGTTCTCAGCGGCCGCAGTGAGGGCCTC
CCAGCCGGCGACGGCAGGTCGCGGGTGCTCGGCCTCCGAGTCATGGGGCTCCTCATCGAA
GGGTCCGATGTCGCTGCCTCGTGCC
>at00770

CAACGGCCGACTAAGCCAGTCACCAGTTCCCCCGAGGAATGGGCAAGGAAGAGTTGCCAA
AGTGCGCAGCAATCGTCGCTCTCAGAGGCCAACAGGTAAACTCAAAAGACCTGCACGACA
TGTTGGAAAGCATCTTTACAAGAAATTGCGTAATTGCGCACGAGGTGAATTTTGGAACCA
AACAGGATGCCAGTTTGCTGCCAGGACGTCAGTTCGCCAAAGATGACGTGCCGCTGCTGG
GATGTTCATATCACAGCGACATGCTGGAATTAGGCCAGCTAGCATCGTCATGGCGCTCAC
TGCTCAAGCTATTCCACCGGGCACGTGCTGATCGACTTCAAGCCGTCAGAACCAACCGTA
GGCCACACGCACATGTACAAGACATGTGCGTGAATGCCTCCAGGGTGGACACAACTACGG
CCCAGTTCATTGCCAAGAAGAGCGCCCAGGTCTTTGTTTCGCGCGCTTTTGCCATGCACG
TGCACAACGGGTTAGCCTCCTCACCAAGGAATACCACCGTCATGAAGTCGGAGATAAGCT
CTGACCCTCGTGCC
>at00771
TAGTTCCTTATTTTGGTCTCAAGTNNtTTGCCTCgGCTCCTCNCATCCAATTGCTGNTGC
CTGGGATAGCACTACCAGTGTCATGTCAAAGCAAGNNTCAAACGTGGATAATACCATTAC
CATCTCTTGTATTCAGGCGCTGACNGGCGAGCACCTTGCTCNAGTCTCTTGTCGCACAGN
TGAAACCATTGCTGGCTTTCGTGCCTTGCTCTCCACGAGCCTGGGCTCGCATGCTCACCG
AAGAATCATCGCAGAAGGGCGCTTGCTTTGTGATACTCAGACATTGGTAGCAGCTGGAGT
TGTTGATGG
>at00772
GCATGTCTGTGCGGTCGGTGACACATGCAAGAGTATTTACGGCAAGACGTCGCTCTTCAG
GGTCGGATCAGGCAGCTCAAGATGGCAAGGAATTGCTAAACCAGTTTGCACCTTCTGACA
AGTTCCGATGCTACTGCGCATCTCCTCTGAGTGCCATCCTATTCCTTGTTCTTCCAATCA
CGCCATTCCTGACCTTCAGGTGGTGTGGGCCTGTCCTTACCTTCATAGTCAGCCCTGGCC
ACTTCGCCCCAATTGGTGGAAAGAACAGTACCACCAGAGGTCTGGAAGGACTTGTTCATG
GCACGACGTGTTTCATCATCTGCCCTTTCGTAGATCTCCTTGAAGAGCTTGTTCAAGGCC
TCATCCCCTCCTGGCTTTTCGTGCTTTAGCTCCGTGTCGATGTCCCTGTCGATCTGACTC
CAGTCACGCTTTTGCTTGCTAGATGTTGGGTAAGCTGGCTGGTCCGGACTGTACGCAATC
TCCTCAACCTGTTCAAGGTCCCTCCATTGGACACCCGCGTTCTTCTTTGCCAGAGAAATC
TCGACTTTCACTTTGGACACCTCACAGCGGCACTCCGCCGGCAGCACGTCATCGAAGAGG
GCCAGGTCGAGGTGGTACTCCTCGTCGGCGCTGCCAGGCAGGGGAAAGGCCAGGGAGAGC
TCCCGGTCCCCAAACTCCGCCTTACAGGAGTCCTGCGGCACCTCCTTGGCAAAAATGGTG
ACGAAGACGTGCGTGTTGCTTTGGTACCACTCTCTCTTCATCTGCTTCCGGCCTGAGATG
GAGAGCTCCTTTGCAGGGGCATCTGGCTGGCTTCTGCTTTCTTCGGCCTCCTTCTTCACG
CACGCCTCGCTAGGCCGATCTTCCACCTCTGGTACAGCTGGCTTGGGTGCAGACGTCCCC
AACGCAGCCTTGGATGCTGCGGCTGCGATGCCACCTAATGGTAGAACTGAACCCGAGAGC
TCAGCATCACACTTCCGCAGCCACATGGCTCGTGGCGCCTTGGAAGCTGGAGATGCCTGC
AAGGACTCTTCGAACGCTTTCTTGGCGTTGGCAATGTCACCAATGTAGAACAAGGCAATA
CCCCTCCAGTAGTGTGCCGTGTGACTTTTGGGGTCAAGCTTCAAGGCCTCATCAGCATCA
TCCAGTGCTTCCTTGAAATTTCCCAGCTTTAGGTATGCAGCTGCACGACATCTTCGAAAC
TCCGCGCAATCCTGCAGCGTCACTGCACATGTGTAGTGCTTCAGCGCCTCCTCATACTCT
TCATTGACATAGTGCACGTTAGCGCTGCCGAACAGAGATGCTGCATCCATGGGGCTTTCT
GCAACCCTTCCCGGCGTCTCAGATAGGAAGCCCAAACACTTGAGCCAAAATGGCTACGG
>at00773
CAAGTGCTTCCTCGTGTATCAGGCATAGAAGACCTATGATCTGGGCTTTAGCCCTCCTGT
CCATACCGGCAGTTTCACTCGCAGACCGCGAACGCGGAGCCACCGTGTCAGACCGTACGG
CAACCGTAGTCCAGCACTCTGGCAGGATCGTCGAGACGAAGCGCGGCGATGGCGATGCCG
GCACTGCGGGCGGCTCCAGCCCCGAGGCGCTGAGCTACACTTCCATGCATGCCCTGACGA
AGGAGCTCAGGTCGACTGCGGACGGCGTGAACAAGGAGATAGCAAGGGGGCTGACTGCGC
ATGCGAAGGCTGCCGTCAAAGCAGGCGAGGCCTACG
>at00774
CGCCACGCGTGACAGGGCGGAGGCCATGCCGCTCACCAGAGTCGAAGAACACCGCACGAC
CAACGCTTGGCCTCAGCAGGTGTTCACCACTGGCTTCGTCCACAACATCAGAAGGTCCTC
CTTCCGCCGGCCCCTCGAAGAAAAACTCGCCTCCATCAAAGTCAGTTCCTTGCTCACTGA
GCCAAATCACCGCTGAAATGTGCCGCTGGCGGAGATAGTCGCGACAGTCATCACTGTGCC
CCGGCAGCGATGACCCTGGCAGCCACGCTACAGTGACAGTGGTCTCAGGCCACAGCTCGC
CAAAGGCGTCAAAGTGCTCCTCCGCAGCATCCCGCGCGAGCTCTCGCGCCCGCAGCAGTG

CCGGCCAGGCCCAAGCAGGACAAGCCAGGAGTGTTGCTGAAGTGCACTTGGGGCGGTAGC
CAGGCACAGCGCAGATTCGGTGTACTAAGGCGAGCTCTTGACATAGTTCCACGGGCAGCA
AGTGATCCACAACATGGCGCCGCAAGGGTGCTGACTGCTTAGCTGCCACGGTCATCACGC
ACGCTCTCGCTGATGTCAGAGGCCGCTTGAGCCAAAATGGCTACGG
>at00775
ATTCCTGTTCACTGGCTATGTCGAAGTAGCTGTCGATGTTCAGATAAGATCTCTCCTCGT
TGTTGAACAGTGTGAGATTCTGCTCAACTCCGTCAGCGATGAGGCGCATGGTGGTGGACC
AATTGTAGGACGGTAGCATCAGGTAGTGGAAGAGAGAGAAGGCTACAAAGAAGATGATGA
ACTCAGCTGCTCGACATGCGGTGTCTGCTCTCTTCTGCGCCTTCTTCTTTTTCCCGGACT
CGATCAAATCATAGAGCGAGGGGATGTCTTCCATCTCGATCTTTGCATCCCCGAACTCTG
GATCCCCGTACTTCGCCACGGTGTCTTTCAGATCCTGGGCAAGCTTCTTCGCGTCGAACG
TGAAGTCGTGCCAGTCCTCGCCTTT
>at00776
TTTTTTTTTTTTTTTTTGGCAAGAGAATCTGGCACTCATGACTCGTTGATTCTACTGCT
CTTTGGCTTCTGTCGTGCGGAGTGCAAATGCACTCGCAGAATAAATCCAAATTACGGAGG
CCTGATGCACTACAATGCGGAAACGCTGAAGCCTCAGTTGCACAACAGGAACTCATTCAT
GGACTCCGAGCGCGGCGACTTGACCCCTGCACCTGGGCGAGCGGCGTGTCCTTGCGCGCC
CCGCCGACGTTCACGGAGCCAGATGTCTGCCGCGCGGACTCCGCCCTGCGCGTGCTGCTC
ACCACGGCCGAGCCGGAGATCTTGGGGCTCCCCGTGCGGGCCACGGAGACCGACAGCGGC
TCGGCCAACCACTTTCGTGGGCTTGGAGCCTTCCAGTCCGACTGCAGCACCTGTGGCGGC
TGCTGAACTGGCATTAGCATCTGCTCCTTGTGAGAGGAGCTCCGGGAATACATCAGCTCG
TCTGCTTCCCTCCTCTCGCTGCCCGCCTGCGAGCCCGCGCCCGCGGCGCTCGGCGCGGAG
GACGCCGCGCTCTGCCGCCGCACGTCGTCGACCAGCGACGCAAGCTGCTGGCGAAGTGCC
TGAGCCGTTTCCACCGGGGTAGTGTTCTGGAGTGGGATGTTGGAAGGCACGTGGACACCC
ATGGGCTGTCCGACTACNTGTGCATCCAGCCTCTCCATGCGCTCGGGAAGTCTGCTC
>at00777
ATTTTTTTTTTTTTTTTTGGGCGAGGCAGCGCGTCACTTGGCGAGCTCACTCCGGATCCA
CGGCTCAAGCCCCCCGCAGGCGATCAATTCTTGGGCCGCGGATCCGAGAGGCACCAAAGG
GAACGCGCCGGCCTGTACGCCGTCCGCGTCGGTCAGAGTGACCTTCCAGGCTGAAAGGTC
CAGGCGTGCCATGAGGCCGGTGCGCACGGTGGGCCTGTCGATGGCGGCCTCGGTGCCCGA
GCCGAAGTGGGCCCTGAGGAAGGCCACCAGGGCGGGGCTCTCGAAGAGCGGCAACCCGTT
GTTAAGGGCGTTTCGCGTGTACGTGGCGGAGTAGGAGCTGGCAAGCAGCGACGTGACGCC
CAGGTGCTTCAGGCACTGCGCCGCCTGCTCTCGGCTGGATCCCGTGCCAAAGTTGCTGCC
GGACGCCAGGATGGGGTTACCGCATGTCACGGCAAGGCTGCGGAACTGAGGATCGTAGTT
CTCCATGGGCACTCCCGCCATCTCCTCAGGGGAAATGTCCCCTCGTGCC
>at00778
AGCGGGTGGAAACGCTCAAATGCGCCCTCGAAGAATAGCTCCTCCAACTTGCCGTGGTGG
CACCTTGCCTGTGCTGCCACTGCTGCATTGCTGTTTGCAAGGGATAGCGGGAAGCGATCA
GGCAGGAGCTCGTCGAGCATTTTCATTATTTGAAAAACTGCCACCTGTCACAGACAGTAC
GCGCCCGCTTTTTAGATTCTAGCAGCACGAGTGTTGGGAAGATGTAACCATTGGCCGAAG
TCAATCTCCGCCAGTATGGGAACCAGTTGGCGCTCAAGTCATATGGCACCCCCTTTGCCA
CACAGCTTAAAAATAGCACACTGAGTCCAGGGCGAGTGAGCCACCCCAAAGATATAGGCG
CGATACTTGGAGGCGGAAGCAACATCGTGACCCGTCATGATGATTATGAACCTACTTCGT
TGCAGAGCTCAGAAGATACTAGCTGCTGGCGTGCTGCACAA
>at00779
GGTTCTCGGCCTCCTTGTGCTGTACGGCATCATCCTGCGACTGGAACAATTTGTACCTTT
GCCGCTTCTTTTCGTACTCCTCCTCAACGACGGCAAGGTCCTTGTCCAGCCTCGCCAGCC
TCTGCTGCTCCAGCTCCAGCTTCCTGGTCGTCGCCTCGGTGTCCACGTGCATGCGGGTGA
GGTGGTCCATCATGTAGAGCTTGTAGGAGCCACCGCCGATCTGGGCACCAGCGGAGAGGA
ACCTCTTGCTGGAGCTGAAGCTAATGGTATCCAGGGACGTGACCTCCCCGGACCTCTGCG
AGGGCTCGCGCGTGGACTGCTGCAGCGAATCATTCGGCGATGCGATCTTCTTCGACATCG
CAGCGCTGCCTTGGA
>at00780
AAAGACTTAGACAATAGGACTACAAACGGTAATTAGTTACCTAATTACTTAATCAGCTCT
TGCAGGACTTGGGCCGCATCCCTGCGGCCAGCCCGCACTTTGCGGTGATTGTGAATGGTT

GTTTGACAGGAAGCACTTGCTTGCAGGGTCTATGCTGGCTCCTGGCTTTCTGGACGCGCG
AAAAGCACGTGCAGTTCTCACCGCACAAGATACCAAGACGGACGGAGGCGGACTTCTCGG
TGCTCTTTGCTTGTGACCCTGGTCGTACCGCAGTTGGGGACATCCTCACACGAGAGGTG
CTAACTGTCCTCAGGACAAGGCCTGGGGTAAGCTTTCCATGAGTTGTACGAGGCCGTTCG
TGAGAAGATGTGGCGCTGCAGGCGTCA
>at00781
TTTTTTTTTTTTTTTTTAGCTCAGAGCTCATCTTCACGCGCGTCTCGGTGTGGCTGCCC
ATTGCCCTCTGAGGAGCCGTCCGCCATTGGGTGCTCACCATCGCACGGCTGGGAGGCGTT
GAGATGCTCCGCCAAGCAACCAAGCACGGAGCCATGTACACCCCGCAGGCGCACGGCCAG
TGCGCAGCCTGTGAAGCCAGCCTCATCAACGCGGGAGCCGAACGCACGGAGGGCCTTTGA
TCGGCAGCCTGCCCACTTCTCTGTCATCATCTCGGGCAGCTTGATCTGAACAGCGCAGCG
TCGAAACGGACCATCGCCATCGCACTTCTGGAGGTCCCAGGCCTCAGCCTGCATATCGAC
ACCGTTGCCGAGGCTGAAAGCCAACGTGGGCACGTGCGATAGCAGGAATGACGGAACGCT
GAACTGTTCGAGAATCAGACTCACGCGTAACCTCTGGGGCCCAGGCTCGGAGGGGGTCTC
ATCGCCAAGTGTCGGGCGAACGGCGTGCGGAAGTGTGCCATTGAACTGGGCCAACCGCCC
CGGCCGTGGAAACAGCCGCGCGGCCAAGTGCGGCTTCAGGAGGCGTTCACTCGACTCCAC
TGCGCAATCAGCGTCGTGCTGGCAGAACCGAGGATCGTTCAGCTTTGGCATGATGTCGCG
CGTTTGCCGAAGGCTGTGGGAGAAGACTCGAGTTCTCCACC
>at00782
TGCAGCTCCGGCATCGCTTGCAGCGTGTCGTTCACCATGGGGAGCACCTCATCCATCAGC
ATCGTGTCGACGTTGCCGCAGAAGTGCAGACCATTGGTGTCAGTGACATTTTTCACGGTC
CGCGCGACATATTCCTTGGTGTAGTTTGCAGCCTGCGTCAGGAGAGGGCCTGCCGCTTTC
GCAAGGTAGGACTGCTCGTCCGGCAAGGCGAGGAGTGCCTTCTCGAAGCTCCCCTGCAGC
TTCTCCACCAGCGAGGTGACGATCTGTCGCTGCGCCACGAAGAATTGGTTGATAAGCTGC
GGCAGCTCCTTCTCTGTGCTGTTGGCACTCGAACGCAACAGCTCGCTCACCTTCTTGTCC
GTGCGCGAGTAGTAGGAAACAACTGCCTGCTTCAGCTCAAGCACGCTCGT
>at00783
TTTTTTTTTTTTTTTTTGACAGCACCAAGCCGCGCGACGCAAGGGTCCGCGGAGTCAAA
AGCCATGCAAAGGACACGGACCAACCCCTCGTAAATGCTGTACAGCTATTTGATTATTTC
CATCTGCCTAGTGCAGGAAAAATGTTGCCAATGTTGGCAGAGGCTACACTTGGCACACAC
AAACGGCCAGGCCCAGAAAAACCAGAGCCGCCCTTAGCGAAGAACAGCCAGAGGCCGTGC
CCGAGGTAGACTCAGTACCAGCAGGTAGCCTAGGAGCAGGATTCTCCGGTAGCCTAGCAG
GATTCTCCGGAATCGCGTTGAGCGGCACGGTATCACTTCGCGATGTGATGGATGGGCACG
GAGATGGTGCGAAGCTGATCCGGTGCTCGTCGCGGTCGAATACCACCAAGTGGTGGCGCA
TGAAAGACGCACCCAACGTCAGCGGGGGATCGCCGTAGAAAGTGTAGCACCACTGGTCGG
CGTGCTGGCTCGACAAGTAGCCGCGTGGTGTCCAGATGTACGTTGCCTGACCCATTCTGA
TGTGCAAGTTGGGAAATAAGCTCAGGTCTGCGTCACTTGCCCTCCAACAGGTGGTGGAAG
CAAGAGTTGCGCCCCCACATGCCCCGGCACCACAAGCATTCTGCACGGCGGTGCGCAACG
CCTGCGCTTGCGTCGTAGTAAAATACGTGTAGGTTGTGCCTGAATCGAACTTGTAGTTGC
CGGCAAGCGAGGCCAAAGGTGTAGACTGCCCACTCGAAGTGACCAATGAGGCGCCTGTCA
CTGACACGGAGTAATGTGTCCCATAAGGGACCGACTGCAGAGGGGCCTCCAGTGGTGTCC
AGGATTCGTTGATGCCTCCAAAGGCCATAGCTCCACCTTCGTCGGAAAGACATAACCCAA
GGACTTGCCTCTCCAGTGGCCCGCCAATCAATGCCGTCAAGACATCCCAGTTCTCCTCCC
GGAGGCCCATGATCCCAGAAGGATCCTGGCGCAGGAAGAGGTTGGTCTCCTCTGTGTGGC
AGCCTAGCCACACCGGCGCCCGGGTGTTGTTGCGGTCTGCATGGCCCAGATGCAAAACAT
CCTCAAAGAAGCGCCCACGAATGCTGGAGCCTTCAAGGTAGTGTACACTGTACACGCACT
GGTTGGTGGGCTCGCAACTTCTGCAGTGCGGCTCGCCGCAAGTCACCCAGCGGAAGGTAC
TGGAACGCGATGGGTCAAAAGGCTCATCTATGTGGGTGCCACAGTTTGCACCACATTCCG
TACAAGGGAAGGCAGTCATCCCCGAGCCTGTGTCCACGATGACGGACTGCATCTGCCCTG
GGGTGCCGACAAGCACATCCACATGCCAGGAATGAAGTCTATGAAGGTTCCCGAAAAGTC
GCGCCTCATCAGTTGGCGATAAGCGTCTCCGGTCCCCTGCACCAAGGCCGAAGCGCCTGG
TGAGCGGAACCACGACTGGATCTGCCAGCGCCGGCCAGAGCAGCAACAGCGCCAGGTGGG
CCCAAGGGAGTGGTGTCATCCCTGG
>at00784
TGTAAGGCGCAAGCCTCAGAGCCCACtATTCTGCCTGTGAGCTTTTCCTTCTTCACGGTC

TGCTGCAAAGGACTCCTGCGCTGGCATGAAGAGGCGCTTGATGGCCGTGTTCTTCTTGCG
CTTCCCCACACGCACCTGGCGAGCATCATGCTCAGTGCGTG
>at00785
CCGTTAGCCATTTTGGCTCAAGGGTTGCTCTTAGACAGGCCAAGGGGTCCTTTGATTACG
ACGGTGGGGGGGAGGCTATTCATCGGAACCCACCCTGCATGTAAGTGTCCAGGAGGAGGC
TGACATGAGAGCGGCGCTGGGCGTTGGTCTGGTCACACTGCGAGACTTGTGCCTGTATGC
AGAGAGGGACGAGCAGTACGTTGTTGGCCCAGAGGGTGAGGAAACCGTGGAAAGGCTCGT
GGGCAGCGCCCCTGC
>at00786
CCGTAGCCATTTTGGCTCAAGTTTCTGGGCAGCAGAACATCTGTAGCCCAGCCCCATGGC
CGGTGGACCGGTGCTTGGCTGCACTCGGCGGGACTTCGCTGGGAATGGTGAGAGCGTGTC
TGACACACAGAGCTGCAGGAGTGGTGCCAAGTCCGTCAGTGGTTCCTCATGTGCTGCGAC
AGTGCCTGGGCTGCAGGCATCTGGTCAGCACTCAACGTTGCGAGCGCGGCCAGCTAGTGC
TGGCCCAACGCTCACCAATCGCCGATGCTGCAGCACCCCTGCGGCCGGAAAGCCATCGCA
TGAGGAGCGGCCGCTGAAGACGGCATTCCAGGGCAGTTCCCGTTTCGACATCAACAAGCA
GGGTGAGGGCTGGCGGCCGCAGGGAGGTCCACAAGAGAAGCAGCTGCGCCACGATCCCAT
CTTGCACAAATTGGAGGCCTTTGTACGCAGAGATAGCGGCACTGTGGAGCGCAGCAATGA
CGCATCTCGCGGCACCTCGTGCGCCGTGGGTACCCGCGACCTGCCAGCCACGGCCGCCGA
CGGGGCCTGGCTGAATTCGCAGATCTCTGCAATCCTGGGCGGGGCCGCTGGACCAAGGCA
TGCGGCTGCCTTGGAGCGGAACTGGGGGCTTTCCACGTGCGCCGGACTGATGTGTGCCCC
CGGCAGCCGGATGCCCGCGCCTGTGGCTTGAAAAAAAAGAAAAAAAAAACAA
>at00787
CCAGAACGGGCGCCCGCTACTCCATAAGGTGAAAGAGATCCGTCTGCGTTCACGTCCACG
GAAGTCCTGGACCGTACCATCGACTACAATGAGCCGCCATCAGCTCCTGATGCGTCTCAA
GCAGGACTTCCATTAGAGGATACGGCTCCGGCTCTGGAGGCACCACCGACAGCTCTCGCA
CCTGAGGCTGTACCGCCAGGGCCCTCTCCAGCGGCGCCGGCTCCAGAGGCACCTCCGGCG
GGACCTCCCCCAGCGCAAGAGGAAGCCCCGGTTCTTGCTGAATTGTGTCCTCTTACCAAA
AAAAAAAAAAAAAAAAAANAAAAAAAAAAAAAAAAANAAAAAAAAAAAAAAAN
>at00788
GACCAAGTGTACAGCCATGCGTACTTTCCACAGTACATGAAGAAGGCGAGCAAGGTAACA
AGCAGAGGAGTTCCCAGCACCAAGATCAGCACCAGGCCCTTCAGCAAGTCCTTCACGAAT
GTTGCCACCGTCGTCCTGTTGAAGCCATAGCGCGCCTCAAGGACAAAGGTGGAATAGATG
CTGAAGGGCATGTCTAAGAGCTGGCTTACCAAGCCCAGCGCAAAGATGTACACCGTGCCC
GTGAGTAGGGGCCCCCAGGCCAATCTGCATCACCAGGCCATCCAGGAAGGGGAAGCCCCC
AAGGAGCCAGAACGCAAGGAACGCGACCATCGACATGTTCTCCTGCAGAAGGCCCTGCGT
GGACTTCTCCCGGTTGTACCTCTGGCTCGTTGCGTACTCCTCGGCGTCGTAGATGCCGGC
GAGCTC
>at00789
TTTTTTTTTTTTTTTTTTTNNNNNTGCGAGCAGACTCAAACAGCTACAAGTTGCCATCCG
TCCAAGCGGTCTGATGCGGCACGTGCCCCGCTGCATCTCAGGCGGCAAGATCCGGCAGAG
GCCGCTGCGGCGCTTCGGTTGACCGGCCGGACCAGCCATTGCCTCGTAGGATGGCCTCCG
CCCGGTCACTCTGGCTGCATGTCGTCCGAGGTCTGCTGCTCCCTGCGCTGAAGCCATGAG
CCGCTAGGCAAACCGCCAGGAGCAAGTCCCGGTGGTGGTGCTCACAGGGCACACAGACCA
TGGTCAAGTTGAGAGGCCTCCGGCAGCGTAACAGTTGGAAAGAGCCGGGAGGCACCTCCA
GGCTCGTGCACGTGACAGCCTTGGGTCGCGTCTTGCAGGCCGAGATTACGAACCGCCACT
GACCTTGAACGCCTCGTTGAGCGCCTGAAATATGCGATCGGCCCTGACTTGCTGCTCTGG
GTTCATCTCCTTCGCTTTGTCTGGGTGGCACACCAAGATTGCCTTTCGCCT
>at00790
TTTTTTTTTTTTTTTTTGGGTGTCATGCTGTACACTGAATGCCAAGCTTCGTGTTGAGC
GTAGTACGTCGAATGGCATACGCCTCACGCTACACGTGGAATGTGTGCGCTGTCACATTG
GTTAAACAGAAGATTGAAATCTGGCATCAAACGCCTGATGAGCGTAGCTCCGCTCGACAG
CTGGCATGGAGAGCCGGCCTTCACTGATTAACGTAGTGACTAGGAACATACACGCCCCAC
AACTCCGCCACTCCCTCCGCCCAAGGTATCTTGTACCACATGATCTCTGGCCAGGTTAGG
ATCTGGAAGGATCCTTTGCCCTTCGCGTTCATGGAGTCGTGCACATAGAACAAGCCATTG
CCGTCCGTTCCACCCATGATCATGGCATGACCATTTCCTGCTCCCGCCGACCCTGGCAAT

TTGACATCCCAGACAATCGGATGGCCATCCATCAGCACGTTCTTCACAAGCAACACATTC
ACGGGTGCGTTCACTCGCGTCCAAGGTATCTTTGTGTAGATGTTCAAAGCGTTGACGATG
TCCGCACCAGAATTTGCATGCGAGTTCGAGCAATGATCTTTATTCTTGCAGCAGATATCT
GGAGTCTTGGGGTAATGGAGCTCGCCAACGATTTTACATTCCAGACCCCT
>at00791
GCTGGCTCTGTGGCAACTACACGCTCGACCCCTCTGCAAGCTCTGTGGAGCAGCTCCACT
GGGTTACACCTGATGGGCGTTCCTCGGTCTGGACCCGAGGCAGGCAGTGATGTGCAGCTT
AGTGCTGTGAAGATTTGAAGAGTGTGCGCGGTGCAGCGCAAGAGCGTGCATGGGCTTGAC
TTTTTTGGGCATGCGCGCATGTAGGATTGTTGGCAGATTCTTGACGCAACGGAGCAGGGC
AGCCACCTAGTCTGGGCTCTTTATTGGCTCAAGATGGACTGGAGCGGATTAGACAACTGA
GGA
>at00792
AGGGCTACCAGATCTCGCGCTACTCTGCCGACTTGCCTCTCCTCGGAGGCATGCGACCGC
AACACTGGTGGTACTTGCAACCGCCTTCCGCTGCCACAGATGGAGGGGCAAAGTGGAGTG
CGTGAATCATGGCCTCGTATGGCGCTGCCTCTGCGCCCAGGGCTATTGCGCCGTGGAATG
GCCGGTGCGTCCTCGTGAGACCGAGCAAGGTTGGGCAGGCCACGTCGATCTTGCGAGGCG
CCTTAAGGCTGCCCCCGGAGCCGCT
>at00793
TGCCGACGGTGTCGGTTTTCTTGACCATGATGCTGAAGATCGGAGGGTCCTTCATCCACC
TGATGCTGATCACCTGTGACTGGCCGTCCCCATGCGGGACAAGCAGGGGGCCTTTGACAC
CAAAAACCAGGACCCTCGACAAGTACCCATCCGTTCTCAGCGGGCGACTTCGATATGTCG
ACCTCCGCCCAAGTGCCGCCTGCCGGCCCCTGCCAAGTGCTGCCCGAGCTGTAGATGACC
GCCCCGACTGGGCGCTTGGCCTTCAGGATCTTGGCAGACTCCGGCCTCTCGTCGGGCTTG
ACGAAGATGACGCCCTGGGTGACGGTGTACGCCGTTGGTCCGTCAGTCTGCACAGCTCCG
GTCATCCCGGCCGGCTCGGAGGGCTGCCTCGTCCGCTGGATGTCAAGCGCGCGCCGCGCG
GAGGCACGTCGC
>at00794
TTTTTTTTTTTTTTCTTGCACATCGGCGCTCCAAATAGCATGGCTTCTGCTTCCCTGAA
TTGCAGCGGTGCGAGTGTGGCAGGCTTTCTGCTGGCCTGCTATGCAGTCACTGCCTTTGT
CTGGCTGAGGAAGTCCGTGTGTTCCTGGAAGGGGTGCTTCACGAAGTAGGTTGGTTTCCA
CAAGTCTGGTGTGAGGTAGGAATAAGTCACACGCACTGCTTCGAAGGTTGCCTTCATGAA
GTTGCCCTTGGTGCGCGTGTGTCCACACGTGCATGAAAAGCAGTCGGAAATACCGGCAAA
TTGCATCATCTTCTTCAGCACCGGTGAGCCCACGATACCGCTACCACGCGGGGCAGGGAC
AAGGCGCACTCGGACAGAACCGCACTTGCCGTGAACCTTCATAGGCACGGTGTGGGGCAT
TCCGATCTTGTTACCCCAGTATCCACGGCGCACAGGGATGAGGCTCATCTTGGCCGCGAT
TATGCCACCACGAATGGNCGTGGNCACCTCCTTGGCGCACTTTGAGCCCAAGCCAATGTG
GCCATTCTGATCGCCTACAAGGACGTAACACACAAAGCGATTCCGCTGGGCGGCAGAGGT
CATCTTCTGCACAGGATGGATCTTCATGACCTCATCCTTGAGGGAGCCGGGCGGGA
>at00795
TTTTTTTTTTTTTTTTTCGCACATGAACGCATGTTTGACGGTCCTGCGCGCATGCATGC
CTTGCTGGAACGGCTGCAGCGCTCCTACAATCCTAGACAGGTAGCCGCAACGTCAGCGCA
CAAAGGGGTTCGTCGCCCTCTCTTCGCCGATGGTGGTGGCAGGGCCGTGCCCGCAGATCA
CCTGGGTCTCCGGGTCGAGCGTCAGCAGCTTGCTCTTGATGCTCTGAACAATCTGCACGC
TGTCCGAGGTGCCCTTCAGCGAGGGTATGCCCGCCCAGCTGGTCCTCCCCACGCTGCCTG
AGAAGAGGGTGTCGCCCGGGCAGCAGAGCTTGTGCTGGTCGAAGTAGTAGGTCATGGAGC
CCGGCGTGTGGCCCGGGCAGTGGATGCAGCGCAGCGTCATGCTGGGCGCGAACTGCACCA
CGTCCCCGTCCGCGAGGTAGTCGTCCGGCGGCGCCAGCGGCTCGGCGGGCGCCTGGACGC
GGAAGTCGCGGCACTGCTCGGCCACCCTCTCGTAGAGGAAGAGGTCCTCCTGGTTCATGA
GGATGACCGCGCCGGTCTGCTTCTTCAGCTCCGTGGCCCCCAGGATGTGGTCGAGGTGGC
CGTGCGTCACGAGGATGCGGCTGCAGCTCAAGCCCTGGGCGCTGAGCTTCTTCACGATCT
TGTCCACGTTGCCGCCCGGGTCCACGACCACGGCGTCCTTCGTCTCCGTGTCGCCGATGA
TGCTGCAGTTGCACTGCAGCGGGGGCACGGGGAAGACGTCGATGAAGGGCGCCATCGTCA
CGCAGTGGCGTCTCCTCCTGCTCCCACCCCCCTCCTGTACAGGCCTGAGCCAAACTGGCT
TGAGCCAAAATGGCTACGG
>at00796

TTGTTTTTTTTTTCTTTTTTTTTTTGTAGGACGGAGTTTGTCGCTCCAGCTTGGCCAACC
ATCAGGTTCCTAGCATAACGCGAGTCGTGAGAAGAGGGCGACTTCTACGAACACCCCACC
TTCAGCACTCAACGTTGTCCCGGCGGTCCCATACACCGCCTCCTGCGGTCACGGTCTTCG
TTGTTCGTCTTCTACTTGTCCTTCTTCTTATCCTTCTCGTCCTTCTCTCCCTCCTCCTCC
TCCTCCTCGTCATCCGAAACAAACATGGTGACGACCTGCAAGAGCACGGTCAGGCCCGAA
ACAATGGCGGCGCCGAAAGCCATATCCCGGTTGCCCAAGTCTGGGTGGTACGAGCGCACG
GTGAGACCCAGCGCGAAGTAGGTGTACAGCAGCAGCGAGAGGATCTGCCACTTCGGCAGA
AGGCACCCAAGAATGCCCACGGTGATGTGAGGCACGTGGATGATGGGATTCGGCTCCAAG
ATCAAGCAGTCAATGCCCATGATGACGACCACCCCCAGGCCCCCGAGGATCTGCACGCCG
GCGGTGATGGACTCGAGGCTCACCATGGCTGGCTGGGAGGAAGCCGGAATGATTACTTGG
AGGACGGGGGCAGGACTGCAATCCCAGGACGGCGCTTGAGCCAAGATGGCT
>at00797
TTTTTTTTTTTTTTTTTCCCCCCCTCTCCGCGATCCGCCCGCGCGGAGGTGACGGTGCG
AGGCGGCCGGCCCGCGGTCAGCGCTGGCGGGATCATCTGCCCACGGCCTTGCCTATGAAG
GAGCTGAGGAACTTGGACTTCACGCTCTGCCCCGCAGCCTGGCCTGCATGCTCCGCCTCC
GTTCCCGCCCCGGCGTCCTCGTCTCGCATGTTGATGCGGGCCTGGACCTTCGCTTCCTCT
ACCTCCATGATGAGCCCGTCATCAGTGATCTCGTCCATGAAGAAGTACACGTCAGACAAG
TAATCGACAACAAGCTGCCTCGTCGGGCCATTGGACAAAATGCCCGCGGGGGCAGCTCTC
AGCATCGCGGCCCATATGCACTCCACCACCTGAAGGACCATGAGCTCGTTCTCGCTGCTG
GGAGCGACGGCGCAAACCACGATGTCGTTGGCGGCCTGATAGAGCACGATGAACTCGTCG
ATCATCGTGACGTCGGGCTCGCTCTTCGACGCCTCGCCCCCCCCCTTCGGCAGCTTCGTG
GCCAGCCGCTTCTCGAAGGCCGTCTGCCTCTGGACATCCGGCCACAGTTCCCTCCCGGCT
CGAGGGTACTTCACCGCCAGACGCTGGCCCTCATTGTCAAGGATGAGCACGGCCAAAACC
TGGCACAGGAAGCTAGTGTCCTCCATTGTCCCCAAGAAGCAGGCTGTACAAGGGGACCTT
GAGCCAAAATGGCTACGG
>at00798
TTTTTTTTTTCTTTTTTTTTTGGGCATGACACGCGGAGAAACCAGGTGCCATGATGACCG
CCAGAGCGCTCAGATGATGTCCAAGGAACCAAGAACCCAGAATGTGGTGTAGATGGCCGA
AGGTCCGAAGAGAAGCGCCGCCGCGAGCGGCCAATTGCGAGAGTTGAGGGACTTCTCGTC
ATCGTCGATCTCATCTGCGGGCGAGATGATCACCTTCTTCTCTGGCGTCGTCTCCGGCAC
TGAGAAGCTTGGGAGCTTGAAGCCACCTGCATCCTCTGCGGTGGCCTCAGCCTTGGGCGC
CTTCGGCCTGGAACTCCGCTTGCGGCTCATGCGCATGGGTGCTGTGGGGTCGTCTGGACG
AGTTCCCTCAACAACCTCCTGTGCCTGCACAGACGTGGCTGCCACAGTCACCGCAAAGCC
GCAAGCAGCTGCCAAGGCAACCAAAGGGCTCCAGCTGCTGAGCTGCTCGGGCACTGGGGA
GTCCTCGTGCTGCGCGGACAAGGACTGGTACACATCTGCCGGGCGGCTGCCGCGGAGACC
ACTGGCACGACGGCTCCCGGTGGACACAAATGCGGATGCCGCAAACAGCCACATCGCGAC
GCCACAGGCAAGGGCCACATGGCGAGCGGCGTGGGTCCTGCGCACGGAAGCCATGGCAAA
AATTTGGCCACACCAAACTCGCTCTTTCGCTTGGTGGCGCTGCTGCTTGAGCCAAAATTG
>at00799
GTCGTTCAGCTCGCCCAGACAGTCCACACAGTTGCCCAGGTCCTGCTTCGGCTGGTTGGT
CCTCCAGCGTGAGCCCATCGACGACGTTGACCGCTCCTTGGATCTCTGCCTCGATTTCAC
TCTTGGCTTGGCGTTCGACTTCATTCCGGACGGTCCCGAGACCTCCCCTCGGTTGTCCGT
AGAGTGCTCCACCCTTCCTTCATCATGACGGTCTGCAAGGCCCGCCACATTGGCCTTGTG
GTCGCTCTGGA
>at00800
CCGTAAGCATTTTACGGCTCCACCAGCAGTGGCCTCAGCACCATTGCGCGACAGGCGTCA
TGCCATCCAAGGTTCGCAAGGGGAAGATCAGCAAAGTGGCTGTAGAGACTGCAGTGGCCG
TGGAGGTGGTGGACGATGCGTCTCGAAGTCGGGTGGAGGCCGTTGCGGAGGAGTTGTGCT
TCCTGCCCGAGGTCTTCGTATTCGACCTCGACGATACGGTCTGGCGTGGTGACATCGACA
TGACCACCGGACCTCTCTTCTCCGTTGACGAGGACGCCCAAGGCAAGGTCTCGGCGAAGG
ATGGCGACGGAGTCACGCCCTTCCCGACGTTGCCGATATCTTCGACTGGCTCGAAGCGAG
TGGCCGTCGGCCGCCGTGGCCTCGAGCAC
>at00801
TATGCCTGCCACGTAGTGACTGCTTCGTAGGCTAGAACACCTTTCTCGCCAACCTCACCC
GCTACCAGCACGGTGTCGCACAGCACAGCGTCGGCAGCTATTCGGAGGAGGTCTTGAAGC

TTGTGGACGAGCTGACGAGGGAGGATACAAAGCTCTACAAGGCCGGGGTGGAGCGCTTTG
TGCAGGATGTGCGCATGGCGGAGTCCCAGCATGGCATGAAGATCC
>at00802
CCGTTTAGCAGGGCTGCAACAAGGTGCCATTGCCAACATCTGAGAATGCTAGACATGGAT
GGAAGGATGCTCCGACTTTGTCCTGCGGGCCATTTCTCTGTGAGGGGCCTCGGGGAAGGC
GCAATAGAGGGACTGGGACGTCCGCTTTCGCGGCGGCAGGCGAGGACCCCCGAGCCCGCC
TGGCACGCACGGAGAGGACGGCGTCAACCTCTGGCCCAGCAGCAGAAACAAAGCCAACAA
CATCAGCGTCTGCGCCTGCACCCTATGCACATACCTCGGCTTCATCAGCATCTGCTACTT
CGCCATGGACGCCTATGGCTTCTTCGGCAACAACGGCAACATCTACGGCTCCGTCAGCTA
CGCCTGCA
>at00803
TTTTTTTTTTTTTTTTTGCACGCACACGCAAATCCTTGTCGTGAGGGTTGACGCGTGGA
CAAAGCCTTTACATGGTCCTGTCCAGGACCCCGTGCGTTTTGACTGGCGGGTGAGTATCT
AAACGCTTTACAAAATCCATATGCAAGCGTGCATCCCAGGTCAGTCCCCATTGCAAAGGT
CTGTGGTGCAACACGTGAGGTCTTCGTACGTGGCCGGCATGGTCTTCATGGTGTCGCACG
TGCTCTTGGAGACGGGGACGTGGGCCACCTTGCCCATAAATGTGTAGCTGGCGCAGGCTC
CGTCGGGAACGGCAGTCATGCTGTACTTGATGAGAGCCCCACCGGGGCCATCAGCATAGC
AGACGCTCGGCGACTCCACATCCGACTCTTTCGCAGGCGTGGTCGCGTTGTTGCACAGGT
CTGTGGTGCAACACGTGAGGTCTACGTACGTGGCCGGCATGGTCTTCATGGTGTCACACG
TGATCTTGGAGACGGGGACGTGGACCACCTTGCCCATATAGGTGTAGCTGGCGCAAGCTC
CGTCGGGAACGGCACTCATGCTGTACTTGATGAGGTCCCACCAGGGCCATCAGCATAGCA
GACGCTCGGCGACTCCACATCCGGCTCTTTCGCAGGCGAGGTCGCGTTGTTGCACAGGTC
TTTGGTGCAACACGTGAG
>at00804
CCGTAGCCATTTTGGCTCAAGCACCAATCAAGCTTTGTGCTGCCAACTGATTCGCTGATT
GGGAAATCTGATGGCAGCCATGGTTGCGCCAAGTGCTGACCAGAGCGTCGATGATGGTAT
CCAGCCGGCATGCAGTCTCCTCGCCGCTGGTGGATACCACATGTGCGGGGCTCGCAACAG
ATCCGGGCCATGAGATCTGTTGCAGCCATGATCGGATGCGGCACCTCATCCTGGGTGCTC
GTCGCTGGTGGTTGGAGCAGCTCCCTGGATATTGTGCTTCGTTGCCCTGCACCGTTGAGT
GCTGGATCTGGGCTTGTGCGGGTAACTGCGGATGCGACGGCTTCTGCGCAGCCCGAGTGA
GCGGTCATGTGCAGTGTCGATAGATTGGGTGATTTCAGCCTGTTGCCTGTAAGAGCGGAT
TA
>at00805
TTTTTTTTTTTTTTTTTCACGCATGGACACCCTTGCCTTGAGGGTCAGCGCATGGCCAA
AACCTTCAAATTCAAAATGCCGGGATCGTGCACAAAGGTGGCTCAATAAATGGCTGGAGC
CAGATCATGGCAAGAGCAACGATGCAAGTCAGAAGTGGGGGCCATCATTGATGTGAGGGA
AAATAAGACAGCCAACAAGGCAGATACTTTTTTTTTTTTTTTTTCAGCACATCCCGCTG
CAAATTGTATCATCAATACTGTGATGAATCCAATGAAGCACATGTGGCTTTCACAGCATC
ACCATGCAAAACACAGACGTAGGTGACAGTACACCTTGGCACCATCCCCCACTCACAATG
TACGTCCAGACTTTGAAGACAACGCCCTGTGAGCTCGCCCGCGAGGGGCGAGCAAACTGT
ACATGCATCGTCAGGCCACGTGACACCAATCCAAGCCCTGGAACAATTGAATCTCAAACC
CGGCACGCCCCCCCCCACTCAAGGCTCTGATGACAGCAGAGCAGCCACCACCATTGCCAC
CAGCGCAGATGCTGATGCGCTGATGGACCCAGACATGGAGATTCCCCCCGTCGGTGCTGG
CGTCGGTGCAGGTGTTGGCTCCTCAGTTTCAACACTGATGTGTCCCACAAAAAAACCGAGG
CGGGAATCGAAGTGCATCCTCGATGCTCTCCACCGCCAGAAGGTTGCTTTCCAAAATATC
CCCGAATCCGGAGTCTGTCACGTTCTGCAACCATGCTGTCACGCTTGAGGCCGTGACTGT
GCTTGGAAGAGTGACGACGGACTCCATGAAGATATCAGCTGTTGTGTTGGGGGGAGCGGT
CCGCAGCCGTCGCGCTGGGCCTGGGATGATGGTCGATGAAATGTCATTCCGAGAGACTGT
ACCACACTCAACATCTCATTGGCATTGACCACAATGGCGTCCTCCACGGCACCAATGACG
CCTTGTTTGAGGGTCTCATTTGCAGAC
>at00806
GCCATGATGCGGCCAAAGATCTACTGTCCACGGCCAAGACACGCCAGCAAAAAAAACAAA
GAAGCACCACACCCTACCGGCGCCCCGGAGAAGAAAAATGACACGGCGTCACTCCTTGCA
GATGGAGAATGGCGCCATCACACCCATGTGCCTGTAGGGCCCAAGGCATGCCCCTAATTC
TCAGCGCTTGCAGTCAACACCTGCCACTGTGCTTGAAGCTTCCGCTCGAATACTTTGAGA

GGTGACCGTGGGACCAACTCACCACGAGTACGAACTTGCTCCTCCTCCGAGACCCTGTCA
GGGAAGATTCGCGGGAAGTAGGGGCCACCCAGCGCCTCGCCGTCCTCCAGCTCATGACCC
GAGGCGTCGCCCATAACGCCACTGAGGGTCCGGCGCCAGTAGGTGGCGCCATGCTTCACA
GCCGTGCCGCTGATGACGCGGCGCTGCGCCTCGCCGCCCCAGGGCCGGCCGGTGTGCACC
ACGAGCCGGTGGATGGTCTGGGCGTGCCAGACGAGGAGGTCGCCTGGCTCGACGGAGGGC
TCGGCCAGCTCACATTGCTCCAACATGTTGCCATCACCACCAAGATTGACAAAGCTGGTG
TCCCGTGCAACCTCAGCCGCCTGGCGGTGGGAGCCCAGAAGGAACTCCGGCGCTCCGAGA
CCAGGGCTACAGGGGTCCATAGCAGCCCAGAATCGGAGGGCATCCGAGGGATCGATTGGC
CCGGTCTGGTTGTCCTGGTGCCATCCAAAGCACTTG
>at00807
TTTTTTTTTTTTTTTTGTCGTCGAGTCCGCACAGTTTGCTGCTCTGTACACCGGTGAGGG
CTTCAGCTTCAGCCCCAACAAGTCCAATTCGGCGCAGGGGGATGGCACGCCTATGAATTT
GTCTGGACCAGAACAACCAGCTTGCATGTCTACAGTTGCACGGCACGGCATTGCAAAACG
CATCAGACCTGCGCGGCAAACTGGCCGTGCTCATTAGGCGCATAAAGAGGCAGGTGAGAC
CACAGGCCTATGTTGGAGCAGCTAAAACGTCATCGGGAATAGCTGACCACATGGCCCGAA
GCAGCCCCATTATAAATGAAACAGTTTGGCAATTTGCCTGTGTTGTGACGCAGGGCTCTC
TTGCTGGCGCCCCGGTCTTTGACAAGCAGGAGACCAACTTCGAGTGCAGCACCTGTGTGC
TGCACCGTTGCCTTGTGCATTGGTCAAGCTGTTTCTACCATGAACATGTAACACCCACGA
CATGCAGCCAGCGCATGCCCGACCGACAGCAGCCATCTGCAACAGCCCTTCTGGGGGTTG
ACCCTCGTGCC
>at00808
TTTTTTTTTTTTTTTTTCCACAAAGCCAGCGCTGATGCTCAGGATTCTTGCGCACCCCC
AAAATATCGCGCCATGGATGCTATGACATCACTGGTGGTGCCAGCATTGGCCTACGCGAG
TCATACTGAAAGCATGAGAGAGGTCTCCTGTCACCAAGAGGGAAATTCATGGACTTCTGT
TTTTATGTCTGTACACCTGTCCCCTGTTGAGGCAACTTCTATTGGCAGCATTTTGTTGGA
AAGAATTCAATGGCGGGGCGCTGGCGCCCTTGAGTACCACTCCCAGAGCTCCAATTTGTG
CGAAGCGGTACGCTCAGTCCTCCAGACCCCTCTCAGAACAAGCCGAACTGCTCTCCCGAC
CTGTTTCTCCGATCAACGCCGACGGCGACGCTCACCGCGAGATGGCTTAGCGAAGGCCCA
GTGCACTGAGAGCTTCTGGTCAAGCAGCACTTGCCCGTCCATGCCTTTGATGGCACTTTC
GGCCTCCTGCTTTGTCTCATACTCGATGAAGGCATATCCCTTGACGAAGCCAGTTCGGCG
GTCCAGGTTGAGGTGCAGGTTCTTGATGTCTCCGAACTCGGCAAATGCCTCAAAGACGTC
GTCCTCCTGCGCCTCCTCGTGAACGCCAGAGACGATGATCACCCAACCTTCGATTGACCG
TGCGGGGCCTGCATCGTCGTCATCGTGCTGCAGACTCTCAAACACGCCCGACTTCCCTGT
GTACCTGTCATCCTCTTCCACCTGGCCACGTCCTTTGATCCTGCGGCCGGCAGGCTTGGC
TCCCTCTTCCGGCATCTCCTCATCGTAGTCAGCATCCATGGTGCCGGGAAGGGTCGGCAA
GTCGGGGACGGGGCAAAGACCAGCAGCTTGAGCCAAAATGGCTACGG
>at00809
TTAGTTCCTTATTTTTTTGGTTCTTTCTTAAGTTCCGTTTTTGTACTGGAGCTCCCCTGC
ACACGAGCCCTCGCACAAGCGTGCCGCTGCATCTCGGCAGCCATCGGCCCCCCCGGACCT
GAGACGCTCAAGGTCTGCAAGCCCTGCGCGCCTCTGCGCCAGGCTCCTGTGCGCGCGTCG
CCGGAGGGTCCCAAGGTGAACGGCCAGGACAAGGCCTTCAAGTGCACTACTTGCAACCAG
AGCTTTGACACCCAGCACGCGCTCAACCTTCACGTGAAGTACATCCACCAGTCGAAAGAG
GATTGAAGTGCCGAGGCCAGCCAGGGCGGTGGTGTCGCGGCTAGCAGTCGATCGGCTGAT
ATGGCCCAGTTTTCGGACCCGCGAGCCTTTCAGGACACCATGATGACATTGCAATACGAA
AACGAAAAAAAAAAAAGAAAAAAAAAAAACAA
>at00810
CAGTGGAACAACGCGCGTGTCCCCTGGCTTGAGAACACCCCAGCCACGCCATGTCCCGAC
CTTCGGCGACTCCACTGGAAACTCCAGCTGGTAGATGGCAGGCAAGGGCTCCGATGCAGG
CTCCAGCCCTCCAGATGCCTCCGGAACGAACCAGCGTGTCTCCCCCTCCCAGCGCGTGCC
GAAGTTCTGCTTCATGAGCCTCCACTGCGCAGACCGCTCGGCGGCGCCCACCTGAAAAAA
AGCTGCGCCTTGCAGACCGGATCTCCTTGAACCCTGCTGCAAGCGTGGGCGACGAACAGC
AACAAACAACGTGTAAAGAACGTAGACTCCCCACAGCAACCTTTGCACCTTCCAAGCCTT
CCGACCAAACGCCCGGCCCACATGCGCCCTCATCAAACGCGTCAACCAA
>at00811
TTTTTTTTTTTTTTTTTCGGGGATTGGCATCTTCGCGTCTTTGGTGGGCCGACTGCAGC

TCATCGGTACATGAAGTTGTTGACGCGATCCAAGTAGTATGCTGGGTTTGGCGCCAGGCA
CACCAGGTTCGCCTCATACTTCTTGATGATCGCCTTGACCTGCTCATCCAGATTGGGGAT
GTTGAACACCTCCGAGACCTCCTTGTAGGGCCCATGCGTGGCAATGGCGCCCGCCGCCGT
TGGGTACATGCCGGGGAACTGGCGGTAGGCCTGCACGTTGGCGTTGTTGATGTCCACCTT
GTCGCTCCCTCCGAGATACTGAATACCTTCGATGTCAACCATTGCCGGCGCAGCGGCAGG
CGCGAGCGCCACTGCGGCGGCGGCCGCCGCCGATGCCGCCTGATGGCGGCGGCTGGAGAG
CCACCCGAGCACGGCGCCCACAGCCACGCTGGCGGCAGCCATGGAGAGTGGCCCTGCACT
CGCCTCTGCAGCCTCTTCCGCCGCCATCATGGCAGGCTCACTCAGGAGAGCCTTGGCCTT
CGATACGCGAAGTGCCGGCAGGTCTCCATGTGCAGGTGCCACGTAGGCTGCAGCAGGGGC
GGCGGAGACCACCAACAGGAGCGCAAGCACCGACAAAGGCATCTGTTGGACTGAGCCTAC
AGTTGCCAAAAACTGGCGCAAGGCGTCAAGCGGCTTGAGCTTAAAAAACACTTGAGCCTC
GTGCC
>at00812
TTGATGGCCACCCGCCGGTCCTTGAACATCTTATCTTTGGCGGCGTACACACGGCCATAG
CCTCCAACACCGAGCTTCTGTGAGTCAATCTCGTAGCGGCGGAAAATGCTGGAATCGACC
CTCTCCGGAACAAGGCTGCGCGTATCGAAGCTAAAGGACCGACTTTCACAGTACTTGCGC
ATCAACGTCGCATCACCGACAGCTGGTGACTGCCCTGCGGGCTCAGAGAAGCTCACGCTT
CTCTTAGTCCTCTGGATGGGCGGTGGCGTGCCGGTCTCCGAAGACCCGCCCGTCGGGGCC
CCGTGGCTGCTGGACCGGCGGGCGCCTTCCCGCATCCTGGGACATCGGCAAACCAAGCCC
ATGGCTGCCTGGATCTCGCGCGCCCAAAGCTGCTTGAGCCAAAATGGCTACGG
>at00813
TTCTcTTTACGGGCACTTGAGGGGTCGATGCAGAGCTTGTGGGGGCCCGCAGGCTGCCGC
TACTACCCGCCCCGAATTCCGAGGCCATGCAAGAAGGGGCACCCCCCCCGGGCAGGCTG
CGGACCCCAGCCGCTGCCCTGCCCGAGGGGCACCCTGGCTTTCAGAGcCCCCCGCCACAA
GGGGGTGATTGCCACGGGAGGGTTTCTGGTGCACCCCGAA
>at00814
TGAGCTTGACAGTTCAAGCGTTGGCACCACATTCGTCCGGGCTGTGCCCCCCCGCCGTAG
TGCAATTTAACAAACTCTCCAGTGAACATTATCCGTATGATGGCTGGATGGCGAGTTCGC
CATCGCCACTTGGTCATTGTCATCCTCGGCCTTTCTGGACTCGGATTCTTGAACACCTGG
TACAGTACTTCATCCGCTGTCAATCATCAAAGACGGCGGAAAAGGAAGAGTTGCATTATA
CGCGTGGGATCTTTGCAAATGTCTCCACTGCCCGCTTGAGGACTTTAAATGTTACCGCTT
ATCCGTTAAAACTGGTGCTTGGGATCTTTGCAGTTTCTGGTAGTTGGAGGACAGCAGTCT
AG
>at00815
TTACAGGCACCGGAGAGCTGCAGGACTTCGAGGCGATTGGCGCATGCAATGGCAGCATGG
AGCTGAGGCTGGACCTCCGCTCGGAGGAGGAGCTGAGGTGGCTGAGAGAAGTGCAGCTGG
ATGGCTCTGCGCTGATGGCCGCCCCTCAGCACCTGCGCTCGAGCCGCGACTTTGTGTTGG
CCGCCGTCCTCGCTAACGGCTTGGCCCTTCAGGTGGCTCCTCGCGAGCTGCTCGCTGACC
GTGGCTTCATGCTCAAGGTTGTGACGAAGACCGGCTTTGCGCTGCAGTATGCGGCAGACG
AGCTGCGCAACGACCCCGAGATTGTCGCAGCTGCCGTTCGGCAGGACAGCAGGGCTGCCC
AGCACNCCGCCTGCTCCGTCGCTGGAAGCACGGAGGCATCGCTGCCAGGCTTTGGCGCTA
C
>at00816
CTCAGCTACCTTCCGGTTTCCTTGAAACCTGATGAAGCTCGCTTGACTACAGTTTTACAG
TTTTCAAGAGTGCGAGAATCGTTTTGGTATTTGGGCCGCTGTGCTTCCAAGCTCAACAGA
ACAGAAGTTGGAAGACTAGCGGAGGAAGTGCAGATAGACATGAGCCTTGGCCACTTAACC
TGGAGTGAAAACCCGACTTCTGTGCTGGTCGCGCCAGGCAACGGAGTGCTGTCGTTAACC
GTTGTTGCCTTATGTGTCCTTGCCATGGCGTCCTTGCCTTGGTGTACTCCGTCTGGGAGT
ACCAAGACCACGATGACTGGCATCCCTTTCCAAAGGATGCAATGCCTCCGCTCGAGGATG
CCTATTGGGAGTTTTACTCTTGTCGTCGTGGCTCTACATTTAGCTGGCAGCTGACACACA
TGAGTATGACATCGATTTGAAGGAAATGACCAGAGTGAATTTGGGATC
>at00817
CGGTCTCGTCGTAAGTGGCTGTCAGTGCCAATGCACCGTCAGGCGAGAAAACCGCGGAGG
TCACACTGAGAGTATGCCCCTTCAATGTGCACACACACTCACCAGAGTCCACACTCCAGA
TCTTGGCGGTGCGGTCGTCAGAGGCGGTCAGGGCCAATGCGCCGTCCGACGAGAAGATTG

CCGAGTTCACCTTGGCTGCGTGGCCCTCAAGCATGTGCAAGCGCTCGCCAGAATCTGCAT
TCCAGAGCTCCACACCGGGAACAGTCAGAATGGTCCATGCAGCAGCTCTCTTCACGAGGG
TGAGCTGGCATCCATCGGAAGCCCCCAGGGCCTCCAACGACACTCCAGGGACAAGCACGC
GCTTGCCAAAAAGGATTTTAGACTTTTGAGTTAAGGCACTACCTAAATGCACTATGATAT
CGCGTAGGGTCCAAGAGGGCTCCGCATCGAACGTACCCACCTCCTGGCCTGTCAAGGAAA
GCACAGACACACGCATGTCAAGCGTCGCGGGCCAAATGCAGAACCGCAGGCTGCCGATGG
AGCACACGCATTGATTTAGGCTTAAGCCACAGCGGCTTGAGCCAAAATGGCTACGG
>at00818
GCAGCGACGGGGTCCCCGTCCGCGCCCATGCCTGCGCCTACGCCTGCCGGGATGGTGGGC
TCTGCCGATGAGCCGGGAGACGCGCCGGACTGGGTGCGATATCCCGAAGATATCGTGTTG
GCGCTCTTGGAGGACGCGACCACCATGAGAGCTGCTTTGACCGGATCGAAGCCAGAAGAT
ATTTGGAAACTGCAGCTGAACTTGAATATCTGGACGGGCAGTGGGCGGATTCCCTCACGA
GCCGGACTGCGGGATATCCTCTGGCCCCTAGGCTGGCGCAGCTGATCCCAAATTTGATCC
AGAGGATGGAGGAACACAGCCAGTTCGTGAAGAGCATGGCTTTTCAGAAGCAGCTGCGCT
GGGGCAGGGAGTTG
>at00819
TTTTTTTTTTTTTTTTTGCACCTGTCCATGTCATTCAGGCTCGAAGCTCCCTGATGAGC
CCAGTGCGAAACTAGTCGGAGAGCGTCTCGGCCCGGCCCGAAAAAAACTTTGTCCCTGGA
GTGCAACACGACTAAATGATATGCAGATCGTTTGAGTCGTTCGAATCTCCGTACGGAGAC
TGGCCAAAGGAAAGCTCTTCAAGCCTCGTGCC
>at00820
TTTTTTTTTTTTTTTTTACACATGCACCAGGCGGCCTCTCAGCTCACGTGCGGCTCCGC
GGCCGCGCGGGGGGGCCGGCCACCGCCGCCGAGCCCCTCACCCAGAGACCTCCGGGACTG
GCGGCCTGGGGCGTGGACGGCCTCGAGCCCCCCAGCAGCGTGCGCCTCTGGCTCGCCCTC
TTGGAGGGAGAGCTGGGCGTGCTCGGACGCATCTCTGAGCATCTTGTTCCACTGACCTTC
AGCGAGGACAGCGGCGACTCGGGAACGGCGGCCTCGTCCGCCAGGCTCGTGGGGCGGCGC
GCCTCGCGCGCATCCCAGTTCGCTAAGATGTTGGCCGCTCGTTCAACCTCAGGTGCCTCC
ACCCCAAACTCCTCGGCATCAGCAAGGCACATCTTCAAGGTGTACCGGTCCTTGATGCCG
ATGGCTGCCTCCAGGTCCTTGATTGTTTCCAGCTTCCTCAGGTGCTGCTGAACCTCGCGC
AGGAGCTTCGTGTCTGTGCAATTGTGCTGCTGCGCGCTGGCGATGGCCCACCTCACAGCG
TCGACCTGCTTGACGCCCACGGCGGCTTTCAGCCTACTCCGCACCTCGTGCC
>at00821
TTGTTTTTTTTTTTCTTTTTTTTTTGTTACAGATGGCTTGACGCCTCAGTGCAAGGCCCC
ACACAGCGCAAGAACTGCAGAGGGAGGGCACGCAAGCGGCTGGCCTCATTCCGTGGGCGT
GGGCGGCGTGCCAGCAACAGAGATGATGTTGATGCAGTAGCGGTTGCCCTTGCCGTCCGG
CAGATTGTGCCCAAGATGAGTGCCGTCCACGCTCACGGTCTCCCCATCAGGCAGCACGCG
CACGTTCTCAACGACGAGCTCTTCGTCACGGAAGGAGGGCCAGCCATGGCTGCGGCTCTC
CCGCTCGAAGGCAGCAAAGCTCCGGCCCCGCGGTGCGATGAAGAGGGGCTTGCCCGTGAC
GCTGTCGTAGAAGGTGATCTCACCTGAGCTGGCGTCCATCGTCTTGAGGAAGGAAGTGGA
AAGCCAGTAGCCGGCATACTCGGCCCAGTGCCTGTTGAAGCAGCAGATCTCGTCGGCCTT
GTTGACGTTGGAATTCCACCGCAGTGGGGTCATGGGCGGCCTGGGGCAGGTCCCATGCTC
CTTCTGGCTCATGATCGACTCATCCCCTGCAATGGCGTCGAGCACCTGCGGATGCCCACC
TGCCCCGCAACCAACCAATGCCAGGCACAGACCCATCAGGGAGCCACCCCTTACCACGCT
TGTCATGCTGGGCCGTGGCAAACACGAACTCCCTGCTGCGCCTGTTGAACCCGCGTCTCC
CAGAGATCTTGAGCCAAGATGGCTTGAGCCAAAATGGCTACGG
>at00822
ACTTGAGTCCGGTGACAGCGGCGGTGtCtTGtCtTGGCCGTGGCCACGGTGGTCACCTCG
TTCGCCACCGACTTCCTCATCGACGCCCTGAAGGGCACCCTGGACTCCTGGAAGGTGTCC
CAGGAGTTCGTGGGAATCATCCTGCTGCCCATCATAGGCAATGCTGCAGAGCACTACACT
GCGATCACGGTGGCCATGAGGAACAAGATGGACCTTTCTCGTGGGGTCGCTGCGGCTCCT
CGTGCCAGATCGGCCC
>at00823
CCGTAGCCATTTTGGCTCAAGTAGCCTCTGGGGCCCAAGCATCAAAACTCTTGTTGTGAC
CTTCCTCGGCCTACTCTTCGTGATGGCCCATCAGGCCATGAAAAGGCCTGCGGCATGCCT
GGACGAAGAGCGCATGGATGTGGATGCCAAGATAAACAATCTCAGGGAAAGCCGAGGACA

GGATCCCCTTGAGCTCACACACGCGGAGCAAACGCAACTCGCCAGCAAGTGCAACTACCA
GCTGGACAAGGGTGGTAGCCACTCACTGCTGAACAGTCAGCTCAATGGCCTCGGCAAGGG
ACAGAAGCAGGCTGGATACCAGGCATGTGTGAGGTCCTGG
>at00824
CCGTAGCCATTTTGGCTCAAGCGCGGTGCATGCACTTCCAATAAAGAGCCGTACTGAATC
AACTCCAGCATGCGGTGTCCGTATCTCTACGCTAGTGCGTTCGTGGTGGCGCTAGACGTT
GCATCTGCTCTTTCTCTTGTCGACGAGTGCGAAGGGGAGAATGCAAGCATACCATTGGTC
CGGCCACTGTCGGAGGGCAATCGAGTGAAGTGGTGGCTGGGGCTATCACACAGCGATTCA
AACAGAAGCACGACTCCTTCAAACGTCCTAGCCCATATTTTTCCAGTTGCTTGGCTGCAT
GTACCCAAGAGCGGCACTTCCCTGTTCAATGCAATCATCCACATTCCAGGAGCTTGCCCA
AGCGTGCCCGAGGACTTCGCGGCAACAATACACGGCCTTCACACTGCACTGCTCATGAGT
GCGTTCGAAGATATGTTTGGGGAACTGCCGCATTCTTGCCCTGGAGCCTTTAACCGAAAC
TGGTCTGGTGGACACAGGGGTATTGCCAACAACATTTTGTTTGATACTCAATTCAAAGGG
CACGGCCTGATCATGCTCCGGCAACCTGAGTAACGCATCATATCAGCATGGCTTGACACA
CAGCACTCATGGCCTTATCCGCAAAAGCCATTTGCCAGGAACATCAGCGAGTTTGCAGAG
GGCATACAGGGTTGCGCCACGAAGATGCTGACGAGGGGCGGTCGTTCTTATTTCATGGAC
ACGAGGTCCTTCCACGTAACATACGGGCCTCACTGGGGCGGGCCTTGTGGGGACCCCATG
AAGCCATCCGCCGGTGAGGTCACATTGGCCAAGGCGAGACTCCGCGATTTTGTCTTCTTA
GGTATCACCGATCAGTGGCAAATGTCAATGTGCCTGTTTCATGCCATCATGGGCACCCAC
TGCCAAGGAGCAGACTTCTTAGACTCGCGGACCAAAAATGAGAAGGACCATCCAGACTAC
AACGCAGAGGCGTATCTTGGCGACTTTCATGATCCATACGATGGAGAGCTCTTCGAGTAC
GCTGAGGCATTGTTCACACAGAAACTCGCGGAGTACGGCTTATCACCCCACACTTGTAAA
CCATGCTTCGATGAGGCGGGCGTGAGGTTCACCAACTCTTGAGCGCGATATGCTATTTCC
AGTGTCATGGTTAAGA
>at00825
CCGTAGCCATTTTGGCTCAAGCTCAAGACACGGTCAGCCCAAGACCCCTATCCTGCCGCT
GCGTCTGTTTCACGCGCGCCGAGGCAATGGCGGTCAAGATGGGCATCAACGGCTTCGGCC
GCATCGGCCGCCTTGTCTTCAGGGCTGGAATGGAAAACCGAAGGTCACGATGGTTGGGAT
CAATGACCCGTTCATGGACTTGGACTACATGAAGTATTTGCTGACTTACGACTCCGTGCA
CAAGGGGTTCAAGGGTTCCGTGAGCACCAAGAAGGATAATGGGAAGGAGTACTTGGTGGT
CAATGGTATGCCAGTCCGGGTTTTCCACGAGAAAGACCCCGGTGCAATTGGCTGGGGGGA
GGTTGGTGCCACCTACGTCTGCGAATCGACTGGAGTCTTCACCACTAAAAGAAAAAGCCG
AACTCCACCTCAAGGGTGGCTGCCAAAAGGTCATCATCTCAGCACCTCCCAAAGACAGTG
TCCCCATGTTTGTTATGGGTGTGAACCAGGAAAAGTACACCAAGGACCTGACGGTGGTGT
CCAATGCTTCGTGCACAACCAACTGCCTCGCACCCTTAGCGAAGGTGATCCATGACAATT
TCGGAATTGCGGAGGGCCTCATGACGACTGTTC
>at00826
CCGTAGCCATTGGCTCAAGCTTGTGTTCAAGGATGTTCAAGGGAATTGGCTTGCACCGAG
AACCGAAAGACACCTGTGACATTGAAGTGTGCTTTCGTCCTTCTAGAGGACCCGGGTATG
GAAAGAGTTTGTTTCAGCTCATGTGATGGGACGGCACACTTGCGTGCTCCTTTATGGCTG
GCTGGGTGCGGCTAAACGGCAGTTGCGCCACCACGAGAAGCTTCTCCACAGGCCGGTGGA
CGTGCCCAGAAGAGTGGTATCTCAGGTGTTGCCTGCAGGCACCTTCTTCGGCAGCCAACA
GCATCTGCAGCGCCAGGCAAGCTACCTAGCAGATGCTGCACTGGAAGCCGCCGCCAGTGC
TGAGAAGGTCGGCTTGTTCGCCTTGAGCGGCAACGGCTCCACTGCAGTCGCACTGCTACT
TCAGGAGCTCCGACGGCGAGCTGCAGGCGATCTCTGCGGCTCTGCAGCCGGCACGGTTCC
CGCACAAGTGCTGCTCGAGCGGATAGGTGCCGTGATCTATGATTCTGGCCCCCCGTACATG
TGGCCCCTCCCGTGCTTGCAACTGGCTTTACGACGATGCTGGCTTCAAAGTATGGCCTGG
AGCGGCACCTCGATCTGCAGCGGCTGCTACCCGCCTCAGAGGCATTCTTCCGTGTTCTGA
TGCAGCTGACCGGCCGGAAGGAGCTTGCAGCACAGCTCTACGAGGAGCTGCTGCTTCTTG
TGCCAGTCCCTCACTTGCTCCTGTACTCTGAGGCCGACGCTGTAATCCCCAGCAGCTGTA
TTACAAGTTTTGCGAAGGATCTCCAGGCTCGTGGCGGAAACGCTCATTTACAGGAATTCC
ACGAGGCCCCACATGTGCTTCATCTCAAAACGTATCCAAACAAATACGTTGAAGTTGTCA
ACAGGTTCCTTGACGCACACCTCTGACAGTCCCAGAGCGGCGCAAGCATGCCGCATGGCC
TTGCCCTTCCATCTATTTACGATGCATGTCGGACACATTGTTCGGCGATGAGGGCTTGAG
CTTCACTTGAACGGGTTCGACGAT

>at00827
TCTTCCACGTGGCAATGGAACCATCGCCCCAGTCCTTCCTTGTAGATGCCCTTCACATCG
TTGGTGCCGATCATGATCGACACGTAAGAAGGGCGGCAGGCCGCAACCCACTTCACCCTT
TCCCTAGCAACAGTCCAGGTCAGGATCGAGTTCTGCCCGCAGTTGACCACAGAAAGCTGA
GGGTTGTTCTTCTGGACCATCTCAACAAACTCGCCGGAGAGGCGCTGATGAGTAATGGAA
TCGCCGAGGAAGACTAGACTCGGCTTGCTCGACCTGCCTGACTTGCAGAAGCTGTGCGGA
TCATTGGGAGGTGGCTTGGGTAGATTGCCCGAGCAAGT
>at00828
GCAGCTCAGGTCCTCCTTTCCAGGTCCCCCAGGTTAGCGTTGATTGCGCAAGCATCCTGC
CCTAAACCCAAGCAAAATCCAACCCCTCCAGCGCGCAAGTTCGCACCAGCTAGGCCTGTG
GCAAAATGGAAGCAAAATCGAACCCCTCAAGCGCAAGTCAGCGTCATCTGGGCCTGTATC
AATGGTGGTGGTCGCAATGCTCGATGGTGTGGTTGTCAAGCAAGTTGCGAATCAGCTTGG
CCTTCCGCCAGTCCGTCTCGGGGTTGCCCCCAACTGCCGCGCCCTCCATGGTCTTCTTCT
CACCATCAACTGTGAGCTCGTAGTTGCCAAACTCGCCCCAATTGATCAGGAGCTTGTCTC
CATCCAAAGACCAGTGGGCGTGCGCGGGGAAGTCTTCGCACTTGAAGTGGTTGTAGCCGT
CAGCCTTGAACTGCACGGGGAAGCTCCCGCCAGACCACGCGAAGTCCCACTCCGAGCCCG
CGCCGTCGCCGCAATGGCCGTGTGAAGGACGCCTTCCGCCAATTCTTTTCATCGGCCTCG
TTCTTGGGGATTGCGCTGCCCTCCATGGTCTTCGCCTCTGGGTTGAATTTGAGCTCGTAC
TTGC
>at00829
CGAACACAGGCCCCTATGCACATGGACAGCCCCGCCGCACCCCGCGACCGCCCTGCGGAC
CGCGCGACGACGCTGGAACCCCCCGCGCTGGAACCCCCGACGCAACACAGGATCGCGGAA
CTGACCGCACTACCCAACACCGCGCGCCGCCCCTGCGCACAGACCCGACTAAGCACGGGC
CGCTAGCCTACGACACAAGCGCCGACGAGCGCCGACCTAGCACTGCGCCGACAAACCCCG
CCGACCCCCTGAGAGCCGACACCGGAGCTGCAGCCCCGCACCTGCACGGAACGCACGCGC
TAGCACGCCGCGCCTGGACCGACAGACACAACCGGCGCGAGAACCGAGCTGGCCAGGACC
CGGACCCGCCCGCGCCTTG
>at00830
TTTTTTTTTTTTTTTTTTCAAGACGCATTCCATAGACATGTCAATCGAAAATGGCCACAC
TACAAGCTCTGAGCGCACAAAACTGTAGTAAGGATCCTCATGGCACAAAGCCGATAACAC
AAATTTCACGTCACCCTGAAGTGCAGAAAGCTTCAAACGGTGGTCTGGTAGCCAGCCAGG
CCGCGCATCAGAGCTGTCCACTAAGTTCACGAGTATGCGGACTGTGGCAAGAGGGAGCTG
CTGCCAAACACTGTCAGCCAGAAACCTCAAACCCACAAGTGCAGTACGCACCTGAATTCC
GAATGAGCTGGTCTGAAGTTGCCGGTGTTTGCCAACATGCTGCAGCAAGACAGCGATGCG
CCAAGAATCGAAGTAGTATGGGTGCAACTGGGCATAGGCAAGTGACGCTACTGCCTCATC
ACCAAGTCCTGTGAGGTTGTCCATCACAGCCCATGCCAATCGAATGATCATTTCGTCCCC
CCAATCAGTCTTTGCAGCTGCCAGGATAGCACGGACACTGTTGGGGCCATCGAGAGCACC
AGGGAGAGCAACTTGCCGAGACAATGACCGCCAAAAAGATGCGGGAGGCTGCAAGTTGAG
CTTTGCAAGACCATGCAGAAAGAGGGCCAGGTGTGTCGGTCCACAGTTTTTCACTGCTGC
CAGGATTCCCCTCGTGCC
>at00831
GCTGCAACCTGTACACACGGGTTACCCGGAAGTCCATTCCAGGGCTTTGATCCACTTCAT
CAGAAGATGCCATAAACATCGAGGAATAGGCCTGCTCAGGAAGACATGCTAGGAGGTTTA
ACCAGAAGTAGAAGGACAAGTAATTCTTGGCAATGAGCTTCATGCTGTCTACCTCACGCC
CTCTCGAGTCCTTGTAGGTCATGAAGAAGGTGATGACGAGATCCACCCAAAAGAGCGCAT
CGATTATGTAGTCAATGACTATCCACAGGACATCATCCTCATTGACGGGTTCAAGTCCAT
CCGGCCCAATGTGGAAGGTCCAGAATGCGAAGCGGTACAAGAAGACGGAGGCCACATACA
CAAGGAGGATGCTCACGACCAGGGACCATGCAAGCCGAGCAGGATTCTTCTCAGACATCA
CGAATTGACTCCGAAGTGGTCCATGTCATCGGCATCGTCCAGGTCATCGTACTCTA
>at00832
TTTTTTTTTTTTTTTTTTAGGTGCACGTTGATGCATGAAGTCAAGTCATCGTCCAACAGC
TGGAGTACAACCTAATTGTCTGCCATCCCTACTCACAGGAACTGCGCGAGTGATCTGGGC
AGGGCCATCTGGGGTTCTAGCTTCGATTCTGGTTTGGAATAGATTGAGGCTGTTGTGGCT
TGAGCTTGATCACCCGCGGGACCTCGCCCCAACCGGCTTTCAGGCAACCTTCGGCCTCTG
GCTTGTAAGTTCATTGCAGACCCTGCCCACCCTGCTTCGTTGGAGGCTAGTCCATTTCAT

CCAGGGTGCCTTGCGGCGGCAGCAGCTCAATTCCAGGCAACTGCGGCACACCAGGAGATG
ACCACCCGCAAACGGAGGGCGGCATCTCTGCATTCCCGCTCTGGTGCGACCACGCTTGCG
AATGGACCTTCTCCTGCCTGGCCGGCACCC
>at00833
AAAGAAATCAACCAAGGTGAGGATGTCGATGCTTCGCGCTGCAAGCTCGCACACCTTTGC
CTGCCGTGGCCCACCCTGGGCGTCTGCCTCCAAGTCCATGGGAGGATGCCTGCTCGGACT
CTTCTGCAGGATCTGGACTTGGGGCGTGTTGTGCTGCAGGATCCCAGCCAAGACCATTAA
GCAGATGGCATCCAAGTTGCAGACACACAGTGATGACGACGCATCTGATACGCAAAGCTC
TCCGAGACAAATCTCAAGCCCCCAG
>at00834
CCGTAGCCATTTTGGCTCAAGTGCGCACGGGTGTCAAGGGCTCGGCTCCAGCGTCCCACC
CTGTCTCTGGGGCTCATGGTGGTCTGCCCCAACCCCCGTGTCTTCTTCGACATTGCCATT
GGCCGCCGTGCTGTTGGACGCTTGAGGCTAGAGCTCTTCCACGACGTGCTCCCGATCACT
GCGGAGAACTTCCGGTGCCTCTGCACGGGCGAGACGGGCCTGGGCTACTGGCTGCGGCCG
AGGTGGTACAAGGACACGCGCATGCACAGGATCACGCCAGGCTTCATGTGCCAGGGAGGG
GACTTCAACTTCGGCACTGGGATGATGGGCGAGTCGATCTACGCCCAGAGCTTCCGCGAC
GAGAAGTTCTGCTACAGGCACTCCAAGCGGGGGGTGCTGTCCATGGCCAACGGAGGCATG
CGGAACACGAACAACTCCCAGTTCTTCATCACGTTTGCGCCTTGCCCTTGGCTGGACGGC
AGCCACGTGGTCTTCGGCCACGTGACGGGCGGCTTCGAGGTCCTGGACCTCATCGAGGCC
TGCGGCACGGAGGGAGGAAGGCCCACAAGGAAGGTCTGGGTCCACGACTGCGGCGAGGAG
GACCGCCTCCTGCTTAGGCAAGAGGCCATTGAAGCGCACATGGGCGCCGAAGGCAGCCAG
GAGGCCGGCGTGCTCCCAGACAGCGAGTCCAGCGAGCCTGTCCAGCGGCCGGGCGCGCAT
CCCCACATCAACGAGATCTCGCCGGTGCCCGACGACGTCTGGAAGCGCGCGAAGGCCAGG
CGCGACAACATGCTGTGAGGGGGCTCGGCGGCAGGACTGCGCCGGAGAGTTGCGAAGATT
ATGAGAATTCGAATTTCAAAAAAAAGAAAAAAAACAAA
>at00835
CTCGATCACCCCAGAGCTGAATAGCAATGCTCTGTGCGGCAAGATGGGAGTTGTAGCCGC
AAGCATCATTTGCAATGCTGATGTTGAGCGCAACGAAATCGTGAGACTCCACGAGCAACG
TCTCAGAAGCACCAAGGCTCTCGCCATCTAGTGGTGCGCAGGCTGGCTTTACCGTTTGGT
TGGCACAGCCGGGAAGGCCGTTGCCAGAAGGTGTGCAATCAAAGATGAGCAAGGTGTCCT
CCGGTGCACTTGCTCCCTGCAACGTTATGGGGCCCATGGAACTGTTTATCCATACACGCT
CGCGGTAAACCCCGGGCTCCACCAGGAGCGTCCATCGTTCAAGCTGGTGAGGTGGAATTA
GGGCAACGCCGCCGGCGATGGTGTGCGCATCGGCCCCGGCCGATGTACCCACATGACTCT
CACGTTGTCCAGTGGTCAAGCTTGCAAGCAGGGCGCATACCAGGAACATGGGTGCCCTGA
GCATTGCCATTCCCCAAATCTCGATTTGCCCTGCTACCCTCAATCCATATCCTTGAGCCA
AAATGGCTACGG
>at00836
TTTGTTTTTTTTTTCTTTTTTTTTTGAAGGGATAGCATCACTGTCTCCACTCATGACCCA
GCACAATGCCAGCGTTGCCATGCCAGGGGTCCTACTGACTTCAAGCGCGCACGGCGAACT
GGGACATATAGGCAGAGGCAGCCGCTCGCAAGCGTGTGTTCAAGGCAATCTTCAGCTCTG
TGATCTCCTTGTCGACGTCCTGCATGAAGTCTATGATGAACTCGATCAGCTTCTCCTTGT
GCATCTCCTCGAAATGGGTGTCGGTGATGACAAAGCTGATGTCATACCCGTCAACAGGCC
TCCTGCGAAGGATCACAAAGTGCTCAGCACGCTGCGCGAAGAAGCGCGCAAATTTCCTGC
AAAGCCAGCGCTCTGTGTCGTCCAGCTGCCGGACCTTGATGCTAATCCGCACGGAGTTCA
CGCCAGGCTCAATGAGGCACTTCTCGCCCTCGTCCCTCGCGACGCAGAGAGGCCTCGGGA
TGACCATAGGCGTGTCCTTAAACTCTATCTCGGGACGGTTCTGCCGCTCCGCCACCTGCG
ACGGGTAGCTCCGGAGGCATAAGGCAGCCCGCAGCGTGTGCCGAATGGCATCCAAGTATG
GCTTCAGAGTGTGGGACATGCGCCACCTTGCTCATGCGCAGTGGGCAGGCTATTGGTAAG
ACACGATATGCTTGAGCCAAAATGGCTACGG
>at00837
TTTTTTTTTTTTTTTCTAATACCAGGCGATGTACATCAGGGTCCTCCCGTGATGGACGA
TCAAACAAACTCCGGCCGTCCCAGCCAACGTGGCCCTTTAGGCCATTCTTTGGGGACAAT
GGGCCCAATAATGAGGATGATGATGAAAATCACCAGGATACACCTGACTGCGACAAAGAA
AAGACATAAAAGGTGAAGCCATGTGGGCCAAGGCGGTGCAATGTTGACCAGTCTGCCCTG
GTAAAGCTTCCTGATCACTGATCATGATGGACCCTCAGTTCTCACTCTTCTCCGGCCATG

GCACCCATCAAGCCCCCATTGTCGTCGTTCGAGTTCTCGTTGAAGTAGCGGAAGTCCATG
AAGATGGCAAGCGACATGAGCAACGCTTTGTAGTTGGGATCCTGCACCCCACCAAGGTCC
ACATCAAAGTGATCAACGTCCGGCGCAAAGAGGAACTTGCAACAACCTGGCACCTTCTTC
TTCAAGTGGGCCACCTCCTCTCCGCTGCTTGCATCCTCGATGTTGAAGTTCACCTCCGCA
CAAGGGCCGCAGGGCAGCGGGCAGCAGAGGCCCCACTGGCAACAGCCCCCTTTGCCTTGA
GTGCGTCTTCGCCGTCGGGACCCCGTACTGCAAACGTGAGATCGCAACATGCAAACGGGT
CCTGGATTGACCCGATTTCCTGTCCAGTTGTCACGTCTGTGATGTTGACCACTGGCCTGT
TGAGACAACATATAGTGCACGTGAAGTCCCGCTGCAAATGGAACGCCTGCATGTTGGCGC
CACCCTGGGTGTAGATGATGTCAAGGTTCCACGGCGCACAATCCGGGAGGCACTGCTTCA
TCTGCCTGGAACAACAGTCTGTTTGCTCAACGGCAAAGAAGATCTCCTCGCCGTTTGCCA
AAATCTTGTACTTATTCGCCATCTCGACTTCCTGACCTATGATTGCCGTGACTGCCTCCA
GGATATTAACTTGCTCTTGAATGTCCAGGGAGGTGATGCGCGGGTCTCCAAGGCATTGGA
GATGAGCCATGACTCCACGACAGTTTCAACGGTGCAAAGCTTCTAAAAGCCCCGACCAAA
GCCTCAACAAAACCTTCAGCCAAAATAGCTTGA
>at00838
TTTTTTTTTTTTTTTTTTANTGGGGCTACGCTTGGATAATATCTTTAGTAAGCGCCATCT
TGGAACACTGTGTGTCCCCAGACAAGAGCAAACAAGAACAGCAATTCCAATCTTGATGTG
GATGCTGATCTGGTGCATGGCTGAAGCCATAGCAGGAACAGAACATTCAAGTAACAAGGA
GGCAATCTTGGAAAGACCTTCATCTCGATAGTATGACATCCCAGCAGCCATCATTCACTG
TCCGGCACATGGTACCCCACAAGGTTCCAACCAGATCGGAGATGTCCGTCTCGCTGGTGA
GATGCGCTGCAAACTGCATCTGGATGCCCATGCTATGGCACGAACTTGCAACCTCCACAG
AGTTCAAATCCTCTGAGAGGAGGCGAATGATGCAGGGCCCCGACGTCATGTTTGGCTGCA
CCTTGTCCGTGACGAAAGCAAACCATGCCACGCGCACCTCGCCAGATGCAGCCATGAGCA
AGACGAAGCGAGGCTGCAGCAGTACCGGCTGCAGATGGTGGGTCACCGCGAAGCCCACGC
CTCCGCGGCCCCCAGCAGCGCAACGCAGCGGTCGGGCAGCCCGG
>at00839
CCGTAGCCATTTTGGCTCAAGCTCCTGGAAGCTTTCGAAAAACTGGTCGGTGAAGCCTCC
ACTGATGATCCAAGTGCGGACGATCTGCTGGTGGGCCTGCAACTTCTGGCCCTTGCCGAG
GTTCCCAGTGGCATCCTCGACCAGCGATCGGCACCCTGCCTTGTCCAAGGCATAGTTGAA
TTTCGATGAGAGCTGTTTTTTCNGTTCGAGGGAGAGGCTGCTCAAGACCTCCTCGTCCTG
GCTCTCGCGAAGCTTTGCCATTTTCTCTTCCAATGACATCTGGCCCAACGCTGCGCAAGA
CGGGCGTTTCAACCCTGGCATGGCATGCGAGGGGAGGGAGTgGAGCTCAAAGGTAGGTGC
CACGAAACTGGAAAGGCTCGAAACTGCAAATGAGCTACAGATGAGCTACAGAGATACGAA
AAAT
>at00840
TTTTTTTTTTTTTTTTTTTGACCAGGATAATGACGACCAGGCAACATCCCGGCCGAGTT
CTCACCTTCCAGTACAACCGTGGAACACAGTGCACGGTGCAGGTGAAAAATGAGAAAGTA
AAGGCCCCATCTTTTGCCTATGCACACGCATGCGGAAAAAGTCAGAAGTGGCGTACCTCA
GCCCGCACCCATCAGAAGAAGTTAACAGGTTCTCCACGCATCACCTTTCCGCACTTGTCC
ATCAGCTCGTACATGGTCTCCAGAGTGTATGGGTAAGGGCAATCCGGACGGTCATCGACC
ATGTTGTGCTTCTTGAAGAACCTGGTGTACTCCTCCCACTCCCTGACGCACTCTGCGTGG
TAGGCCTCCAAGGGGTGAGACTCCCACTGCTTCCGAGCCTTGATGCCGTCCGCCTCGTTC
ATCTTCTGGGCCAGGCCCGGGATGTTCCCTCCCAGGGCCATGCCCATGGGCAGCCCGAAC
GTTGCCTGCGTCACGTAGGCTGGCCACTCGCCTGCCTTGTTCTTGGGGAGCTTGTCGTGA
ACCAGCCACCAGTCCTCAGGGTAGTCCAGGAACCAGTTGCACATCTGGACGGAGGGCGCG
AAGCCCGGGCCCACGCTGAAGGAGCC
>at00841
TTGTTTTTTTTCTTTTTTGTCAACACAGTCCACTCAGTGCCCTCAAGGTGTTCCCTGGG
GAATGACCGTGTAGCTGGGGGAACGCACCTTCTCCTCTGGAGACGTCTGCGGCAGCCCAC
TGGCCTCTGCACCGGTTGACTTGTCAGCGCCGCTGTAAAGCGAGGCGAGGTATGAGGACA
CGATAAGCAGCGCTGACTGCAATCCATCAACACGGGAGGCACCATCACCGCAACATGACT
TTGAGCTGCTCACCGAGGCAGTCCAAAGCTGTTGCGTAACTTCGGCCCACCCCAAGCGAA
AGACCAAGTATAGGCGGACCACGAATGACGCCATCGTGATGACCACCATGCAGAAGATGA
ATACGAGCTGCGAGAGCAACTTGCCACCGTCGTACAGCCTTCCCTGGAACTTGTAGGCAA
ACCAGCACACCGGTAAGAAAGTGACCAGAGCCATGGTGTAAAGAGCTGCCATCTTCACGT

AGCTGCAGTAGGCGGCATCGTGCTCAAAGAATATTGCAAGGTTGTCCATCAAGGCGGAGA
CGACAAAGTAGATCACAAGCCCCACGCCCTCGGACATGGTAAACTCCCATCCTGCGCGGA
CAAAGTCTCTGATCCATTCGACAGGGCCGCTACTCTTGCCCTTCTTTTTCCAAACCCTGT
AAGAAATGATCGCCACGAGCACGAGATAACGCAAAATGGTAAAGTGGGCCAGCTTGTGGT
CGCTTTCCACACATTCGTTCATGGCGGCAGGCGTTGTCGCCTCTGAGCGAGAGGCCGGTG
CATAGGAGCACCCCTAGGCCTTGCTTGCTTGAGCCAGCATGGCTTGAGCCAAAATGGCTA
CGG
>at00842
TTCTGCTACCAATGCATGATCTGGAAGTACAGGAACTTCGGGCAGCAACGTCAATTGCAC
GACTGCCCCATCAGACTCTGAGGTTCCCAAAGAGTCTTCTTCCATAGGTGGCGAGCCTGG
TAGCGGGTTGtCGTGGCTGACATCCTGAAGCTCGCTTGGCGAGCCAAAGTGCtTAGTTCT
GAAGTGCTTGATGGCAAGGCGGGCGAGTGGGTGGAGGCACCTCGCAAAGTAGTCTGCGGA
TCTCAGGCCGCCCGCGTCCGGCTGGAAGACGAAGGTGGCGCaAGGGGAGCGCAGACAGTC
AAGGTGTACGCGGATGCTGCAGCCCTgCtGCNNNGGGGGGGCTCGGGAGTGGCCCTTGGA
CGCCCTGGGAGCACACTGACGTGAGAGGATGCTCAAGCCAATCCTTTTTCCTGGGCTCTA
TTGGCTGCAATGCTCGTCACTGTAGGTGACTCAGGGGCCTGAGC
>at00843
TTACACTCATGCTCCCTCCAGGCCATGGCTACAACACGTTCCCGGATGCCCATGATCTGC
TCCTGAATGTGCACTGCCCGCCCACTGCTGTGACTGAGTGCAACCTCTGACACCAACTCC
AAGGCATGACGACAAACGTTTCAGCATCCCGTTTGACCTCCGCCAGTTGCTTCTTGGGGT
GATCCAGTTGACCATGCACTGCTGGAGTCTGCTCCAGCCTCTCCAGAAGAGCCTCTTCGT
GTTGGATTGCCAGCCTCAAGGTTCCAACTGCCCAGTTCGCACCGCCGTGGACAACTGCTC
CTTTTCGTCGACAAGCTCA
>at00844
TAGTTCCTTATTTTTGGCTCAAGGCGATGAGACAGGTCAGCCAACCGACCACCCACAAAC
GGCAGTGCACAGGATGTACGGACACATTGACCTTGGGCGAGTGCGCGGTTCCCGGCTTGC
GGGGATTTCCTGCGCTGTCATCTGGATCGCCTCGCTGAGCTGTGGCCCTGTTTGGGTCGA
GTGGCCCTCTTCTGGACTGCTGCCGAGAAGGCCACTGGTGAGCCGAGCCAGCCATGGATT
GCGGTCTTCACCCTGTGGAGCAGAGTCTCGGCAGCGGCTCAGATGTGCAT
>at00845
CAGGAGCTTCACACAGCTTCTTCTTGATGCATGATGGGAACATCTTGGCATGCGGTTCAA
ATGGCCATGGTGCGTTGGGAATTGGCACACAGATTGATAGCTTCACGCCAGTGCCTGTGC
AGATTGATTGCAAGATGTCCTTTCCAGGTGATAGCTACGTATTTGAAGGAATGGCATCAG
CATCCGAACGCCTGGAGAAAGATACTGTTAGCGCTTCAGTAGTTGACCTGGACAGCGAGC
GGGGAATTCTTGACGTGGGCTCTGGCCAACTCGCAAGTTTCGAGCCTGAGATAATTGCGC
TCAGGAGTGCAGTGGCTGATCTCGGAAGCAAGCTCGAAGCACTCCAGTTGGCAAATATGA
CTCTGCAAGAGAAGCA
>at00846
GACCTGCAAGGCGTAAAGCAATTTGCCGTGCCACCCACTTTGCTCCCGCGACGAAGCTCG
ACATCCCAGGTGGACATGGTAGCTCCAGCAGCCGCAATAGCAACGGTAACACACATATCA
CATTGAACAGGCTGCTGTGTGATTGGCACATGTAGGCTAGAGGCACTTGGTGTTCTGAGC
TTGCAGGTTCCAGATTCATCAGCGCAGGCAATGGCGCAAGCCGCAGTGCACACATCGCCC
AGCCTGAAGGCGGTAGACGCTACATAGCAGCAGTAGCAGCAGGACCGGCAGGTTACATTC
CGAATGCACATGCTGGTCACCATTGTGGGCAAGGCCTGCCTGTTTCCNGTCCTCCGGCAT
TCGTACCCTCGTTTGGATCATATGTTCTCTGCGTTCTGTTCATGCGTCTGCTAGAAAAAC
AAAAAAAAAAAAAAAAANAAAAAAAAAAA
>at00847
AGAAATGAAAGCGAGCAGCCAGCAGAGCCTGGCCGTGGCAGCATGTCACCGAGCCGATTC
GTCTTCTGTATCGCAACCCAGACCTTGGGAAGGTCATTCTTACAGTTGGCTTCCAGATGG
CAGCGTCAGATTTTGCGGGTACGATCGACCTTGGGCCTGACACGAACCAGACCTGGAAAG
ATCAAACCTCAGCCACTGTTGCAATGTTTGCGTTAGCTGCGGGCATTATGTCATACGTGA
CTGGTCCGATATTTGGGCGATTCACCGATTCTGTCGACCGGCGGATAGCTCTTGTGGTCT
ACGGCTTCATCACATTCTTGCCCACGTTCATCTACTGGTTCTTCTTCCCCCTCAACGAGC
GCGTTGTTTGGGCCAGCCACTTCGCGAGCATCCTTGCTGCGATTGGGAGATCCAGCAGCG
TTGACTTCGCCTTGAGCATGGATGTC

>at00848
TTTTTTTTTTTTTTTTTGCGCTGCGCGCGCGGAGGCTCGAGGTCGGCTCGGGCCGCCGC
GCGGCTGAGCTCAGACCAGGCGCGAGCCGCGGTTGTACGCTGGCGTGCCCAGGGGGTCGC
GGGTCCCATTGAAGCCCTCCAGGCTGTCCTTGTGGAAGAGGGATTTGAAGACATCGCTCT
TGGCAGCCTTGGACTTCGATTCCTCCGAGGAGGCCGGCTTCTCCCCTTCCTTGGCCTCAC
TCCCCTTTGCCTCCACCAGCTCATCCGGGGCCTTGCGTTTCCTCTGCGGCAGCTTCGCCT
GCAGAGCCTCCAGCTCCTCCCTGTCCGGAGCCAGTGGTATGAGGTCCTTGTCAGGGTCGA
AGGCCTTGCCTGTGACGGGGCACTCCTTCATCTTGAGCTCCTTCTGCCCCTTCGCCGAGA
CCACGGCCCCGGTCGGCCAGATGAGCACCGCACGCGCGGCGCCGTCGTCCAAGTCTTGGC
GCATGACCGGGCAGACCATGCGGCGCTTGCCGTTCTCGTTCTCGGCCTCCTTCCAGGTCA
GGAGGCACTGCTTCACGTCCTTGAGTGCGCGTATGTGGGACAGCGCTGGGGGTATGCTCT
TGCTGAGGAGGGCGGCGATGACGGCCTCCTTGTTGTAGAGGTTGCCAAGCCTGCAGGCCA
CGATGGGCTCGCGCAGGGGCTCCTGGGAGAGCAGGCAGAAGCTCATCCGCGTGCGCTGGC
GCTCCACGGGCCCCATGCCTCGGTCCTCGCTGATCATCTGCACGTAGTTCGCCATCTCGC
CCAGGGAGTTCGCATAGCGGTCCCCCCCGGACTTCGTGAAGCCCCATCCCTTGGTCTTGA
CCATCGTGCCCCGGTCGATGACCTGGCCGCCATCGCCTCCCATGGCGGGCCACGCTCTGT
GGCCGGCAGGCCGAAGGGGTCTGGGAGAGAGGGGAGAGAGAGAGGGAGACGGCCGGTCTC
CGGCGGGGAGGGAGCCACTTGAGCCAAAATGGCTACGGACTCTGCGTTGAACA
>at00849
AACGCCAAAGCTCGGACAGCAGCTTAAGATCTGACTTGATGTCATTGGCAGACTTAGCCC
TGAAGGCCTTTTGCTCTGCCGTATACATCTCGCAGAAGGAGCACCGGTTCCAAGAGCAGC
CGTTTGTGACCTGGAGTATGATGCTGTTGCCCTCTGCAGGTGGGCGGAACACTGGCTCCA
CGTAGATGTCCGCCTCGTCATCATAAATTTGACTCTCATCCTCCGCCTCCTCCTCCTCCG
CCTCCGAACCAATACGAAAGGCACAGCGCCCAACCAATCGTCGTCCACGATTCGGTACAC
GTAGGCCAGACACAATTGTGACCAACAAGCAAACAGAGTGAGGATAAGGCGTGCA
>at00850
CCGTAGCCATTTTGGCTCAAGGGCATCGTGGCTCCGGCGCATAGCCTGCCCCCTCTCCAG
ACCTCCTCTCCCTTCTCACCATGCCGAAGCAGATCACGGAGATCCGGCAGTTCCTGCAGA
TCGCGCGCAGGAAGGACGCGCGGTCCGTGAAGATCAAGAAGAACGGGACGCAGACCAAGT
TCAAGGTTCGGTGCAGCAGCTACCTCTACACGCTCGTCATGGCCGACAAGGACAAGGCCG
AGAAGCTGAAGCAGTCGCTGCCGCCCTCCCTTCAGAAGAAGGAGATCTGAGGCCCCGAGG
GCGCCCGCAGCGGCGGGGAGAGCCGGCGGAGGGCAAGGTCGAAGTCAATCGGAAAAAAAA
AAAGAAAAAAAAAACAA
>at00851
TTTTTTTTTTTTTTTAATGCACTTTTACTTCACTAAGCACAACCTCCGTGCGAGGCTGCT
CGCATCCTTGCACGGTATTGCTTGCACCCTCTCTCTAGCGTTCGCATCCTGATGTAATCT
ACCACCATCAATGCTTGCCCACTCAGAGATACAGCCCTTCGCTGTGGAGGGATTGCAACG
TCTCCGCTGGGAGCACCTGAGCAGTGGCAGACTCCGCGAAACGCACAGAGACGATGCGAA
TAGTGCAGCTCCGGCCGAACACCTCCGGATGCAGCTCTTCGAGGAGCTGTGGCGGTGTCG
CACCGAGGTCCTGCAGGCGCTGGAAGAAGCGTGCCTCCACCTCGGCACTCCGCACCTCGT
ACGAGAAGACTGCCTGGCCGCAGCTTGGTGCCGCGGCTGCAGCCACTGCAGAAGCAAGCT
TGGCCTGCATGTCCTCATCGTAGATCAAGTCCTCGTGCC
>at00852
TTCTACAGGAAGTCCCACTCGTCCGGCTGCTCATTGCCAAAAAGGAAACAGCCCCGAACC
GCGGCATGCCCATGGCAGTCGAGGTAGAAGAACAGCTCCTCCCGCTCGGCCCACCCAAGC
AGCAGCGCCTTGACGGCTGCGACCCCCTCATGCTGGTCGAACCCTGGGTTCTGATAGCAT
CGATTCAGATCATACCCCAGGGAGTTGGTGCGCGAGTGGCCCCAGGCGACACCGTCGGGG
TTCAGCATGGGCACGATCTTAAAAACAAAACGAGATCTGAGCAGGGCGGCGCGCGCATCG
TCAGAGAGCAGAAACTGCAGAAAACCCAGCACGGCAAACTGGCCTGGCGTCTCGCCCGGA
TGTACTCGCCCCGAGACAAAGCAGATTGGCCTGCCTGGGAAGCGAAGTGGTGGACAGTGT
TGGCAATCCGGCGAGGGCATGCCGCACACTTCCAGGCAACTCGTCATAA
>at00853
GTATCAACGCAGAGTCCGTAGCCATTTTGGCTCAAGCCATTTTGGCTTCCAGTTGTCTGA
GCATTGCTCTGTCGTTGAGCGGGAGCAGCTGTCTCCGGACTCCCAATTCACAGCCAGGAT
GCCGCCGAAGGCAGCTGCGCCCAAGGCTGCAGCACCGAAGGCCGCCGCGCCGAAGGCAAC

CACACCCGCGCCAAAGGCGGCAGCCGCTGAGCCAAAGGCTCCAACGGCGGTGGCAAAGGC
AGCGGCTCCGACAGTCGATCTGGGTTCCTTGGATGACAAGAAGAAATTCATCCTTAAGCA
CGAGACGAAAAAAAAAGAAAAAAAAAACAA
>at00854
CTGTAGGCGTCTGCATCCAGGCGACCTGCCATGTAGAAGAGTATAGCAGCAATAAAGAAC
CGCCACAATAGGTGGAAGTTGAATATCTTCTTGACAGCCCACGATCCCTCTAGGGAAAAC
GAGATTGAAAGTGCGACCACCAGCGAAAGAAGGTGAGTCAACACAAACAAGGAGGAGATG
TTGACCTTTGAATTCCACATCGCTTCGTCCATGACGAATGCGACAGCATCTGTCACCACA
AAGACCACTATGATGGCGATGTCGTCCGGTGCACCAAGGCGGTCCATACACCAAGCATGA
AGCCCGGGATTGCACTGCTGAAGCCCATGGCACCACTCTTCATCAGAGTCCTCGTCCTCC
TCATCCTCGTCGCTGCTTTCTCGTCTGATC
>at00855
CTCCTCATTTCTTGCCAGCGCTCCTTGAGTGTGGCTGCCCCGCCGCCTCCTTCTCGTGAG
CACTTGGCTTCAGCAGCAGTTGCCCTTTTGGAGTTGCCCAGGACGCTTGAGGGATGCGAC
CATTGGCGACAAAAAAAAAAAAAANAAAAAAAAAAANAAAAAAAAAAAANAAAAAAAAAA
ANAAAAAAAAAAANAAAAAAA
>at00856
TTCAGGTGAGGCATTCCTGTGGCAGCTTATTCTCGTCACTCGCCCGTCTTCTGAGACCGG
TTGGAGGGCATGACCGCATATCACATCGCGCCTTGCGAGCGGACGGCGCGCGAGAAGGCA
AGGAGTCAGACGGCGTTCTTCACTTCCTGGTGCCGGTCTCCGCGCCCTTGTCTGCTGTCC
TGGACGGATGTGGAGGGCCCCATGCCGGCTCAATCTACTGCATGGTCCGGTGCTGCGTTG
CCGCGCTGTGGCATCCTGGCGGGGCATACCGCCGGTGTCGAGTCAGCTTTGTCTTCGAAG
ATGGGCGGTGCTGTGGCCTTCTCGGCGCCTCGTCGAGAAGCTCATCCGAGCCGGGCGGTC
TCCCACCGAGTTCCAGATCCTCGAGC
>at00857
TTTTTTTTTTTTTTTTCTCAGCAAGGCAGGCGACAGGTGCACTTTGTGGGTCTGTACTCG
TCACAAGTCGGCAGAATCGTCCCGCTGCCAGATCTGTATGCTGCCGTTCTCCGAGCCAAG
AACGAAAATGCCATCGTGGCCGGCGCAGCACAAGCTCTGGACCTTACATCGTTCCAATGC
CTGCCAATAAGTCTTGAGATTGAACATTGGGAGCGTTATGGAACTCACTTGCCCCTTGGC
ATGCCCGCAGAGAACTCTTGGACCCGATTCCAGGCCTGCTATACAAAGCACCGGGCCAGC
AGCCAAGGGTGGCTGGGTATGTTTTTGATTGCCGCCAGCGTCGAATATGCAGAGCGAGCC
GTCCGAGTAAGCGACTATCATGTGGCCCTCGAACTGTAACAATCCAGCCACAAACTTTTT
CGGCGGCAGCTGGCACGTCGCTTTCAGAGACGTGAGGTCAACCAGGGATACGCCAGATGT
GCCGCCCACCCAGAGGTGGTTTCCCAGCACGGACATACACAAGACATTGCCAGAGATCCC
GTCGCTGATGATGTGCGTGCAGGCGAACGTCTGGGACTCGGGGTTGAGCTGCCAACACCG
AACCGTGCCGTCCATGCTGCCGGAGAGGAGCACGCTCTGGTGGATCAGGAGGCAGCCCAC
GCGCTTCGTGTGCCCGGCCAGGGAGGC
>at00858
TTTTTTTTTTTTTTTTCTGTCCGCGCAGGCAGGAGCCGCTCTGAAGCCTCACGTCATGCC
TCGGCTGCAGACCCCGCGAAAAAGCTATCGTCCGCCAGGCTGGCGCACGGCGGCTCACCC
AATGCCTCCTTGCAGAAGTCACCGTCCTCGCGGATGGCGACGATGAGATCCTCGAACTTG
TCAAACTTCGCCTCTGGACGCACGAAGCCGCAGATGACCAGCCGCAGCTCGCGGTCGTAG
AAGTCCTCGGCGAAGTCGTGCAGGATCCAGGGCTCGATGGTCTTCCTCTTCACGTCCGTG
AAGGTGGGGTTCCAGCCCACGCTCATCGCAGTCTTGTAGACGCCCTTGTCAGGCCCGTCA
GGCACCTGCGCCCAGGCGTAGTAAATGCCAGCCCGGCAGGTCTGCGCAAACTCCAAGATG
GCACGCTCTTNGGGGCTCCAAGGCCTCAGGCTCGTGCTCCTTGTCCCACCGGACCTCCAT
GTTGGCGGTGGGGAAGCCGAGGACCTTGCTGCCGCGGCCAAAGCCGTGCACGACCTTCGC
CCTCAGCTTTGGCATGGGCTCGAGCACCTCCCTGGCACGGTCCACTGCAGAGGAGCTTGC
CCTGAGCGGCTCCGGAGCCGTCTCCGGCGTCTTCTCAGGCGCGGCAGCAGGGCCATCAAC
CTCTGGGACCTCGGCCTGAGTCATGAGGCCACTACGGGCGCAAGCCCAGAACCACAAGGA
AGGGAAGAAGAAGCAAAATTGGGCCTTGAGCCAAAATGGCTACGG
>at00859
TCGTCCTTTTGGAAAAGCTGGCCTCGCAGTGTCGTAGCTCAGCAGATACTGGTAACTCAG
ATCCATTGATCCTTACGCCACCGTTGCGGTACGCTGCAGCTCCCTGCCAGGCGACCGTGC
GTCTGGGCCCGACGACGAGCCTGAGATTTACGATGCCAG

>at00860
GCCATCGCCCAAGAAGGCCGTGGAGGAGACGCCGTTCGTCCCTAAGAAGGCCGCAGAGGA
GACGCCGTCGCCCAAGAAGGCCGTGGAGGAGACGCCGCCGCCCAGGAAGGCCGCAGCGGA
GACGCCGCCGCCCAAGAAGACTGGGGAGGAACTCGCAGCTCATATGAAGCCGTCGTCACC
GGGCCTTGAGCGAGGAGAAGGAAAGATGTGGCTGGTCGTGGGTGGCCCAGGACCCAC
>at00861
TTTTTTTTTTTTTTTTTTAGATGCGCCGGAGCAGCACTGCAGGCGCTTGCAGTTTTTCTA
CGCCTCGCTCTCAGAGTCGCCGCCGACCGCGACAGCGTCGGCCGCTGCCGGGACCTTGCC
CTTCTTCTTTTCCTTGAGGTACGTGCGGATCTTCTTCTGGATGGCCTTCTGCTTCTCCTT
CTTCTCCTCCTTCTTCTTCAGCCACTCCTGGCCCTTCTCCGACTCGAGCCACTCGTTCCG
GAGGTTGTTGATGTAGTGCCTCTTGCCGGAGTTGGTCCTGGAGCCCATCCAAGGCTCGTA
CTCCTTGGGGCCCAAGGTGTTTGGGTTGGTCCAGACGCACTTCTCGCGGACGTACTCCCA
GGGGAGAACGGAGACCTCGCGGTGCCGCCACTCGCCGCGCCACTGCACGATGCCGTCGCG
CAGCGCCACGAGGGAGTAGTCCTTGGCCCGGAGCACGTTCTTGCCGGCGTACCACTTCAT
GCCCTTCTGCTTGAGCAGCCGCGTGCCCGCCTTCACCGCGGCACCTTGGTATCCATGCTT
CGTCAGGCCCCAGAAGTGGGGGAGGAGGTGGTGGCCCTGGTTCTTCGTGCTCGCCGAGGT
CTTCTTGTGCGCGTGCAGAACGAGCGCAGAGCCCCTGCTGGAACTGGTGCGCCAGGATCG
CTTGGAGGAACCTCCGGTGAAGGTGCTCGCATGGGTGCACGGCGCTGCTGTAGCGCCAGC
TGCGCAGGTGCCCGCAGTGGACGCTTCCNGTGCGGGT
>at00862
AGCAGGGCCGTGTACAGCTGATCTGCGCCCTGCTGAGGCGTATGCGGAACGTGCGTATCG
TCGGAGGCAAAGGAGAGGGTGACACTGAGGGGGTGCTCGAAAATGAGGTAGTTGGCATAG
TTGTTCCAGCCAGTGGGATTGGGCCTGACCACTGGAGAGTCCCCCATGGCCGTCTCTATA
GTATATGGACCGTTCTCGAGGAAGAAGCCCCAGAAGCTGGAAGAGCCGGGGCCGCCGTTT
GTCCAGAGGATGGTCGGGCGTTTGTCGTAATCTGCAGCTCCGACAAACCAGTAGAAGAGC
TTTTCTGAACTTGCAGGGTCAGTGGTCGGCGGATCCATGGAGGCATAGCCTGCAAATTGG
GTCGTGCTGACCCGCCCTAGCCCGGGCAACGATTTCACGGTATCCACCTTCATCCCTT
>at00863
GACATTTTGGCTCCGGTTGGCAAGGGCCTTCCCCCAGTCGCCTTGTGTGTTGGGTAGCGC
ATCCGACTTGCAGACATGAGCAAGCTGAACATCGACACCCTGAAGAAGGGCATCAACAGC
GTCCTGGAGGGAGCCAAGGAGAAGCCCCGCAAGTTCCTCGAGACCATCGAGCTCCAGATC
GGCCTCAAGGACTACGACACGCAGCGCGACAAGCGCTTCTCCGGCACCATCAAGCTGCCC
CACGTCCCGCGGCCGCGCATGAAGGTCTGCGTCCTGGGCGACGCCGTGCACTGCGAGCAG
GCGCAGAAGACGGGCATCCCCTTCAAGAGCGTGGATGACTTGAAGAAGCTCAACAAGAAC
AAGAAGCTTGTGAAGAAGCTTGCGCAGTCGTTCGACGGCTTCTTGGCCTCGCAGGTCCTG
ATCCCGCAGATCCCAAGGCTGCTGGGCCCAGGCCTGAACAAGGCTGGTAAGTTCCCCACG
CTGGTCCAGCACTCCGACGTTCTTGAGAACAAGGTTACCGAAATGCGAAGCCAAGTGAAG
TTCCAGCTCAAGAAGGTGCTCTGCATGGGCGTTGCGGTGGGCAACGTTGGCATGACTCCG
GACGAGGTGCGCCAGAACACCCTCATGGCCATCAACTTCTTGGTGTCCCTCCTGAAGAAG
AACTGGAACAACGTGAAGAGGCTCCACATCAAGAGCACCATGGGCAAGCCCTGCACCATC
TACGGCTGAGCGCGCCCCCGCGCCGGGCGGGCGAGTGGGATTTGGCCCTCAGATGCGCGT
GTACGTGCTGCTGAGGCTGTCGAGCAGCTGCGGAGGGCACTGCGCAAAAACAAAA
>at00864
TTTTTTTTTTTTTTTTGTGTCTTCTACAACACCAATCTGCGTGAAGCATGGCCCCCACGC
CCATGAACGATACCTAACAGACAAATAGCCATCACAAAGCGATCACCAGATGCTGCTACA
AGTGGCGCGGGGTCACCTGCAAGGCTGTGTATGGCAACTGGTCATCACTTTGCGTAGCGG
TCAAAGGCCGACTTCCGCTTCGGTTTCGCGCTGCACCTGGTAGCCCATCTTCACAAGCAT
CCGCGCATACTGCGCAAACTCAATCTTCTGCTGCGACGGGCCGATGCAAATGAGGATGGT
CTTTGCCGGGAGCTTGAAGCCAGCAGCAATCATTGCCTTGAGGAAGGCGTCGTACTGGTT
CACGCCGAAGCATGCTACCTCTCCCGTGGACTGCATCTCCACGCCGAGCCTTGGGTCAGA
ACCCTTCAGGCGGCCAAAGGAAAAGACGGGGACCTTTGAAGCAATGCAGTCAGTGTCAAT
CGGCTGGATGGTCACGGGTTTCTGGGGAATGCCCATCATGATGCGCGTGGCCATTTCGAT
GAAGTTTACATTGTAAGTCTTGGAGATGAAGGGCAAGGAGCGGGAAGCCCGGAGATTGCA
CTCGATGACCATGACCTCGTTCTCTTTGCACATGAATTGCACATTGAAGGGCCCGGATAT
GCTCAGTGCACGACAGATCTTCTCCTCGTGCC

>at00865
TTGTTTTTTTTTCTTTTTTTTTCGTTTGCCGCCACCACTGCCGGCAGTCGAATCAATTGC
ATGGCTGCTGCAGGTCAGAGGTCGCCTGGTCAAATCTGAGCCGCCTACTTTGCCCCGAAG
TATGCAGCATGAACGTCCTTGAGGTGCGTTGCGAAGTACGGGTCAATGGTCTTCGTCGAA
GCTGAGCTCAACGGCAATCCCTTGAGTGTCTCTGTCTCAAGGAAACTGACCTTGACCTTC
GAGGCAGCCAGCGCCTCGCCAATCGGCGTAGCAGGAGTAGCCGGGCCCAACAATGCGGAA
GCACCTGAGCCTGGTCGGTAGACCTTCATGGCCTTGAAGACAGCTGCCCGGCCGCCAACT
GCCATACGCTTGAAGACGGTCGTCATGGCTGCCTTGCTTGCAGTGCAAACCACTAGCTGA
AAAGGCTTGAGCCAAAATGGCTTGAGCCAAAATGGCTACGG
>at00866
CAGCCGCTCCAGCTCAGCATTCATTCCAGCACTCATTCACTCTCAATTCAGCGACTTCTC
CTCGGAGTTCTCCACTTATGGCAAGCTTCAGGACTGTCTGCCTGGTGTGCCTCTTGGCCC
TAGNCAGCTCGTCAGGCCCACGCGGCCGTGTGCGCCACAGCGTCGAGACGCTCGAGCGGA
GTGCTTGCCAGAGGGAGGCAGCCGCAAGG
>at00867
CTGGGTCCCTGGCATCAGCGCTGGACTCGCGCGACGGCCCGGTCTCGCCGGCTGCAGGCA
CGCCCTCTCCATCTGAGCGCAGGTCCTCTTGACTGGTGCACGGCTCCGCGGCAGCTCTGG
CAAGTGCTTGCCAGTGTTCCGTCCGCAGACCAAGAGCTGCCCGCTTTGGCGTCTTCGTTG
CGCTCTCCAAGGCTGCAAAGACGCTCAGCAACTGGACTTTGTCGTAGACTGGAGGCTCCG
AGTGCGCTTCCCTGGCCGCTTGCTGGGAGGAGTTGCAGTGGCTCTGGTCTTGCTCC
>at00868
CCAATTTGGTCCATGATATGGCCCTTGGTCCGGTACAATGAAGTCACCCCTTGGGTTGAG
TCCCTGTCCTCCTCTCCGGGAGGCTTGAATGGATCAGCGAACAGGTGTTGCACCAGTTCT
CAGGTGCTTTAACTCTTTGCTGTTACCGAGTTGCCTGGAAAGCACAGCATGCCTGACGTG
GAACGCTTCTCCGAGCCCCTGAAGTTTCTGCCGATTGCGTATATCGCATTCACAACAGCA
GGTCTCTGGACCATCTATGTGGTCCTGCACTGTCTCCCAAGGATGCAAATCGGGGTGGAC
CCAGCATTGGTGGATCAGGGGATGGG
>at00869
GAAGACCACATCGACAACGCCATCCTGCATCTTCCGCACGCGCCGGAGCATCGAGACCGC
CGTGAGCATCCGGCACGGCTGCAGCACGAAGCGCAGCGCCAAGAGCGGCAGGTCCGCGGC
CAACAGGCCCTCCGCTAGCAATGCCCAGCGCGCCAGGCTGGAGCACCCAGTCCAGCAGCG
TCAGCGCTGCCACGCCGGCGTCGAAGATGCACCAGCAGTCTCGCACGAACGCCCTCCGGC
CCGAGAGCCACAGCGGCCAGAGACCGGTCTCGGCGA
>at00870
TTTTTTTTTTTTTTTTTGCTGAATCACAAATTGTCAGTCTTGATGCTCTGATGGCCTCA
TCAGAAGACACATTCTTGGGGGACACAATTGTAATTAAACATTTCCGTGCCCCAGTGTCC
CGCGTGCCTCTCACATGCGCTGAGCCGCCATCAGTGTGGCATCAAAGACGCCTACTGCTG
TGGCGCGAGGGACCGTCCACTGCAGGCGCATCTCGTCCTTGGAGTGGCGGCAGAACATCT
CCATTCGCAGGGGGTCCGAGCGCAAGGCAAAGACCCGCTCCTCCTCAGACTCCTGATTGT
TGATGACCGGACGGTAGGTGATCTCCATTCGCAGGGGGTCCGAGCGCAAGGCAAAGACCC
GCTCCTCCTCAGACTCCTGATTGTTGATGACCGGACGGTAGGTGATCTCCTGAGCGCTCT
CGATCACGCGGATGTTGTCCGTCACAGTCGGACTGCCCGGCTTTTCCACGATGCACATGC
TGCGCTGCATGTAGCCGCTCATGTCACGCACCGTCACGTTGGTCACCTTCAGGCCGCACT
CAGCTGGATTGCGCATGGCGAAGAGCATGGCCTTCCAGAGGGTATCCTGGCTCACTTCAG
TGATGGNCTTGGACGCCAGACCGAGTCCACCACGTCGGATGATCCTTCTCGATGGTGTCT
GCCAGCTGTACAGTCTTTGAGAANGTGTCGCGGGCAATGT
>at00871
TTTTTTTTTTTTTTTTGGGCCTCCCGTACTTTCCTTTCCCACACTGTCTGACAGCCTCA
CTAACCTGCTGGCAGGATTGCCTGGCGCCGAGCACTAGCAAGGCCAGCCATGCTGTCAGG
CGCGAGGGTCAGCCGAAGCCGCCAAGGTTCATGCGCTTCAGACCTGGGGCCGCGGCAAAG
CCGTCGCCAAAGGCCAGCGCGGCGGTGCGCTTGTTGTTGTTGTTCACGGCCTTGGCCGCC
TCCTTCAGCCTCGTGAGCCTCCGTTTCAGCTTGGTCACCTCCCCAAGCACCTTGGGGTTC
ATTGCAACCCGTGGCTTGCCGTTGTTGTCCTCCACCTGAAAGGCGATGACATCCCCGACA
TCAAGCCCCGACTCCAGGAAGTTCTTCTGGCCTATGTAGATGTCGCTCTTGCCCCAGGAG
GCAACGCACTCTGGGCAGATGACAAACCCAAAGCCAAGTTCAGGGTCGTACCGCGCCAAG

GTGCCGACGAAGCTCCCCGAGTCCGCGCTCTCCTTGGCGGCCTTCTCAGCTTCCTTCACT
TCTAGCAACTGTGCCGCGATGCGCTCGCCCAGTGCCTCCTCGGAGAGCTCCAGCAGCGGC
TCGGCGTCGCTGCCGTCCTCCGCCCCAGCAGCGGGCAGGCCCGGATCCATCTGGGGCACC
GCAGCCTGTTGCATCATCGGAAC
>at00872
TGTGGTCATCCTCGGGGCCTGACCTTAGTGGCCCTCATCTTTGTGGTTGGGTTCCAGCTG
ACCATGATGTACCCAGTGTACACTTACTGTGAGGACTTGAAGTGGGACAGTATGGGGCCA
GAGCAGCGAGCCATGGCAGGAAAGCCAAGGTTGCCAACCAGGAGGATGGCCATCGCAGCG
CAGTGGGCAATGCTGCGTGCTGGTCCCCTGAATGAGATACACGGGCGGATGCCGGGCGAG
TACGGCTCCATGTACGAGACGGCGGCCGCCATGGGCGCCGCCCAGCCGCTCACCTCCGCC
TCAGGGGCACAGCTGCCGTACCTCCCGGCTACAAGGCTGGTGTGATCGTGGTGTACTGGA
GGTTCATCTGATCCAAGCAGCCGCGTATAGGAGCTTCCAGAGTTGTGACAGACGTCCTGC
GAACCGAGTCCGCTT
>at00873
TAGTCCTATTTTTGGTCTTCAAGGCTCCCGAGGCAGCTACATCACGTTTTCCGAGGCAAC
GCCCGCAGCCACCTTCACCGGATGCAGCACCTATAAGGTCGCGCGCAAGGGGGCGGCGTA
GCAATGCATCCAAGCCGACTATGCAAGACGACTTTTTTGGCGAGGACTTGCTGGCTGACT
TCTGGGCAAAGACGCTGCCTGCTGATGTATCTGCTGGCAGCTCTGGACCCAGAANAGGCC
ACTTGCAAA
>at00874
AAGCTTCCGCAGGCCGAGTTCTTTGACTCGCCCtTACGGTGCCACTATCGCAGTGGACAA
CCGTCATTTTCCGCTTTCTGCAAATGAGCTGATGATGGAANATGGTTACAGTGACCTGCT
GATCCAGACCNAGGAGGTTGCTCATGTTAGTGGTTGTGACCACGTGTGGGCTGGGGACTT
GGATTCTGAGGCCAAAGCGCTCGTTCGACAGGTGTACGGGCGCGACTTTGAGCTCTTGTG
CAAGCATTTTTGGCTACTGCGACACTCTGGAGGACACATGCATCACTACTGTCCCCGGTA
TGTGCCCTCCGCAGCTCTTCACGTGGAACTCCCAGAGGAAGATGTATTTGAGGAAGTAAA
CCCGAGTGACCTGGCACTGACGACAATACGCGTGAAGGCAATACAAACACA
>at00875
TCCACGAGTGGATGATCGACGTGCACGAGAGGCTCCACCGCTTGCAGAAGCGGAATGCAA
TGGTCCAGGAGAAGGCCAACACCGTCTGGACTGCCGAGGGCGCCAAAGCCCAGCGTGAGC
AGCTGAAGGCTGGCTCCAAAGATCAGAGGGAATCGGCGGACGATCTGGTTGTCCAGGTTG
CAAAGGAGATGATGAGCGAGGCATGGCTGCTTTGTTTTGACGAGTTCCAAGTCACGCACA
TCAGCGACGCCATCATCATGAAGCGCCTGTTCTCCGTGCTCTTCGAACAGGGCGCCGTGG
TCTTGGCGACGTCCAACAGGCCACCAGAAGACCTGTATCTCAATGGGCTCAACCGCCGCT
CTTCTCGCCCTTCACTCCCGATGCT
>at00876
TTTTTTTTTTTTTTTTTCCCCCTTCCCCCCTTCCACACTCCGGCTTCCAGATGGCCGGC
AGCACGCTGCCGGTCCACCCAAACGACCCCGGAACCCGCGCCCGGGCCGAGCCCAAAAAA
CGACAACCCCCAAGCCAGCAACCCGCCGCGCAGAGCCTGCGCGACAGGGGCCCGGGGGGA
AGGCAAAAACCAACAGCGCCAGCCGAGGAGCCTCCACGGCTGCCGGGGCATACCCCAAAA
GGGGAAGACCGCCCCGAGGGCCATGCCCTTCACGGCCCTTGAGAGATCAAAAGGTGGCTG
AGCTGCTTCAGATACCGGGCTGATGCCGCAAGCAAGCACATGCACACAGGCAGGCAAGAC
AAGCACAACAGCACGGAAAAACACCACTGCATGTACAGACGTGTGCAAGTACATGCAGGC
AACATGGGCGCAGAGAGAACATCCCCTCGCAGGTCACCATGGGAGGAATGCTATACAATG
CTTCAGAAACAGGCAACAGACAGAACGCAGAGGCTGTGCATAAAGGACAAAGCAAATCAC
AGCTTCAGAACGCCAGGGAAAAATACTCACGTGCTGTAGCGGCAATAACAAGCGATGCAC
CGCTTCAGATCGCCAGGAAGACACGCATACTCACTCGAAGTAGAGACAACGTCGCGTTCA
CACGAGCAACTGCATCACCGTACCGGCAATGAAATGCAGCAGCTTTCGCAGAGGCGTCCG
CATCTACAGCCAAACGACAATTGTCCACGAGAAGTGGGTGAATTTAGCACCTTCCTGCAC
CTGCGGCAAGCGACAAGTATACCGTCACTGTGCAGCTCTATCTGGTGCGGAGTAATCTCC
GTACTAGTGAGTGCATGACCTGCCTCGCAGCGAGCGTCTGCAACAGGGATGGAGGCAATC
ACCGGCAAAACCTGGCCTGCTGCAAGTGTTGAGCAGGTCCGCAAAGCATCGTCACAAAGG
CCCAACTGGCGCTGGGATACATGACTGCAGGCGGGCAAGAGATCCAAGTCAGCCCGCANA
TCTATAAGCG
>at00877

TTCCCTGCATTCTCCATGGATGAGGCGAGTCTGCAAACTTCTTGCATGCCGGTATGACTG
ATGTCAAAGGCCTTTGTGTCGAAGGATACACAGTGCTGCTGACTGACGTGCTCTGCAAGC
TCCTTCCGATTCCCCACCGTGTAGACATGCTCTTTGCACACAGCACAATAGTCAACACCC
TGGTGATCTGTCGGCTGCAACTGATCGACGGTAATCGGACACACCATATTCCACTGGAAA
CCCGCTGGCAGAGTGGCGTCTCCACCAGCTTGCCCTTGGGCAAATGCAAGGTGGCAGTTC
TCAATGTCAAAAGGCACGACTTTTGTTGTATTGGTGGTGTGCGCTTCTCTGGAACGCCAC
TCCTTGGCCGCTGCAAGTCAAGCTGTGCTG
>at00878
TGGCAGCTGCCTCTGCATGGTCCGCCAGCCGCGCCAACAGCTCTCGGACGGCGATGACAC
AGTCATCTTGCCGCCCAGGCTGCCTGCGAAGCTGCGCACGAAGCCGATGGGTGCGGTCCT
GGACATCCAAGGCCACTGCCGCTTCGCACCGCAAGTCGCGCTCTAAGGCCTGCGCGCGCT
CCTGCCAGTGCT
>at00879
CGGCACGAGCTCGCCGCAGAACGCCGACACGCTCCGCGCGGCCGACGACATNTACATCGA
GGAGATCAGGAGGACCGGCCACTACGACAAGATTGGCCAGGCCTTCGCCGTGCTCCTGCC
GACGGTCCGGTCCCTGGGCGTCATGGGCGACCACCGGACCTACGAGAACGTCTGCGTGCT
CCGCGCTGTCACGACGTCCGACTTCATGACCGCGGACTGGTACGACATGCCCCACGACGT
CCTCGGCAGGATCTCCAACCGGATCATCAACGAGGTCAAGGGCATCAACCGTGTGTGCTA
CGATGTGTCATCAAAGCCGCCAGCGACTATTGAGTGGGAGTAGTCGGGGCTGAGAGCTTG
GACACCCAGGTGTCAAAGACACGACTCTGCAGCAAGCCTTTCTGGTGTGAGTTACTTCTG
TTTGCCTCATATGGCAGGCATTTGGGGTCTGCCCCTGTTTTCAGCTGCACCGTTGAGATG
CGGTAGCTGAACTTTTTTGACCTGTTCTCCTAGTGTTAGTGGGATGCGCAGACGTCTCTG
GACCTGCGAGTGATGACTGTCTCTTGGGGGTGCAACACCAGACCAGTATGGTGTGCGTTC
ACATCAGTCGCGGAGTTGATCTGTCATGCGGAGCGTGCACTGGTTAGCGGCAAAAAAAAA
AAAAAAANAAAAAAAAAAAAG
>at00880
TTTTTTTTTTTTTTTTTCCCCCACAGCTAAGTGATGATGGGCTATATCTGTAAGATAAA
GTGAAGAAGTATCCGATTTGAAACCACCTAAGAATGTTAAGATTGCTGCTTGAGATGAAC
TATTACCTACTGTTGATCCGAAAATATGGTCGTTATCAACACCTAACGAATACAAAACCC
AGTTGCCTGTATAAAATGGATACAAACCTTCTGTAGGGCCTAAATTAATTGCTTGAATGC
CTCTTGAAACTGGTATAGCTACATGTACTAAATGGCCACACCATGCAATAGAGAAAAAGC
CAATCATAACGCCAATGTGATAATTTAATCGAGGGCCACTAACATCAAAAAAACGCAAGAA
ACAACCTAAAAGGGAGGTTAAATATTGGGGCCGAAGCTACTTGCTCATCGGTCTGAAAAG
CGAACCACTGTAACATATCCTCTAAATAAATCAAGTGTACCTTACCTAATAAGAGTGAAA
TTACTGCTACACATTCACTGAGAATTACAAAGTTATATAGGTCATTCACCTGAACAAACC
CAGAAGCGTACAGCCAATTATAAATACCTGAAAAAGATTGAACAACAGCAGAATCACTTC
CGCCAGAAGAATATGCGTCACTTATTGAGAAACCAAAATGTGGATCCCAAATACCATGAG
CTATTGGTATGGTTGCAATTGGATTTTTAATCCACAGCTCATAGTTACCATTCCAACCTA
TGTGAAAAAGATCCCTGATACCCATAGAATAATTATAGACAAGTGACCCCAATGTGACTT
GAAAATTTGGACATTTAATGATGATGTATTATCTATGCCGAAATAAGATTCTATATCATG
AATGC
>at00881
TTTGTTTTTTTTTCTTTTTTTTTTTCGTAGAGGACACGGCAACATCTGCTTCCGAGCACA
CTGTGCTGTCACTACACGTCGGCACGTCGAGCCATGTCACTGGCCGCCGGCGACGGACAA
CTGCGGTCTATGTCTTCGCGATCAACCGAGCGTTGGGATGTCCTCGAGCACTCTGCTCTT
CGAAGTACCTTTCAAAGAGCACTGCGGCACCGAATGCATTCCCAGCCATGAACACGAAAT
TCACGATCGGCACAGTCTCCAAAATCATTGCAACCGCTCCGAAGCTCAAAAACGATCCTT
TGTGCGCCAATGCAAAATCCAGCTGCTGGCGGCACGAGCAGATCATCATTCGGCCAAGCG
ACTCGCTCACCAGGTTCCAAGTGTACATGCCGAAGTAGGCAAACAGCAAAAGCGAAAGCA
GGGCCGCGGCTACAGGCGGCACAAGAACGGTCACACTCGGGAAGCACCCCGACTGCCCAC
AGTATGAGGACCCTCCAGACAGCAAAGCACATGGTTGTACTGCATATTCCGACAACAGCC
AAGCTCTCCCACCTTCTCAGCACCATAAGTCGAGCAAAGCTCATCATACACGCCTCGCTC
TTCAAGGGCAGCTCGGGTTATGAATCGCTGCATTTTCTGGAATACAAAGATCATGACAGA
CTTGTTGATCGCAATAAACTCGAAGATCACTGCACCGATAGCAAGAGGGCCCAAGAATAC
CTGGGCAGCGAGACCAACGGTAAAGATCATCGACGCGACCCAGATTGTGAATACTGCGAA

GATGCACCCCGGGATTACAGCATATTTCCACAGCGTGGGCCGCGAACAGAAGGCGCCAAC
GCCGTAAAATGGCAACAGACAGGCAGGCAAGTGTCTCGCCGCAGGCATATCCAATGAATC
GCCGGTGCACAGCTTTCCCAATCTTCAGCCCAAGTGGCTTGAGCCAAAATGGCTACGG
>at00882
TTGCGGCTCTTCGTTAACCCGGGGGAGTGGGGCTCCGTTGAGCTGGTTTCTTGGATCTGA
ACTTGGGTGCCTTGGCGATCTTCATCAGTTCACACTTTCTCTTGAATGGCCCTTACTATG
CGGATCTCTGGCCCTGGCTTGTCTACCTCACTGCGATCGCATTCCTGGGAGTCCTGGGCG
CGGTCACTGGACGCTCGTTTTCTTGCTGCTCGCTGCTTTAGGCCTGACTATCCTGAGTGG
AAGGCTGGCATGGGGGTCTCGACCTCTCAGCAGCGACATGTCCCTCTGCTCTTCTTCGTG
CGTTTGGCTTTTCTCGGCATGGTGATCATTGGTGCTGGACAGTGGCTGCAGCAGTCCAGG
CG
>at00883
CGCTCCAGCTCTCTCCCGAGCCCCCCCTGCCAGCACTGTAGCTCACTGCCAGTCAGCCCC
CGTTCGCCCGAAGCCATGGCGGCCATGAAGCTCGCTGCATGCGCCCTGCTGGCGCAGATC
CTTTCGGCCTCTGGCGGCAGGGCCTTCCGCAAGGGCCTTGCGTCCTCGGGCGCGCGGCAG
CCGGAGGAGGGGGACGAGTACGAGTACGGGGACCGGGAGTCTCAGGTCAGCCACGGCGAC
GCCTCCGACTTCTCCGTCACCAGTTCCTCGTCTCCGCGACGCCGCG
>at00884
CAGGCTCATGCAGGCGTACATTCCTTGAGCATGTCTTGGAATCCTTGTCCCCAGCACTCA
GCCAACTCCCGCATGTGCCTCATGATGTAGATCAGCACGACACTCACGAAACTCTGTGTA
CCAAAGATGCCGCAAGCCAGCAGGAAGGGGCACGCTGACTCCCCGATCCAAAGGAGCCTC
AGCCCGAAACCCATCTGG
>at00885
CCATTTGGGTTTGAAACATGTAGTGATTCACGCGAAAGGGGTTCCTCCTGGGCAGTACCT
TACAGGCTGAGCTATGGCCCTGGTTCGGGCCCTCAGCTCGCCTGTCGGCCTGGGCCTGGG
GAGCCCTGAGAACAGTGTGGATCGTTCGGCCTCGCGATGTTCTGACGCGAACGAGGTGAA
GGCAAGTGTGTTGTCCTCGGCGTGCAGACCGCACAAGCCCTTGCAGCCACGCATCCAGAT
TCAACGCCCAGGGCTGTTCCATGATCCTCTGGAGGTGCCCTCTCCGGCGTCACTGCACAA
GTCCAGCACTAGCATATGGAGGGGACCTGGTCGTCTGGAGCAGTCGACACTGCCCCCACT
AGCCAAAGGCGCGAGCCGCCATCGGGTGCCGAGCTGGGCAGCGGAGACCCGCGTGGTGGT
GCGGGCTCC
>at00886
AGGGCCAGCGCGATCGCGTGCAGCACGTCCGGGTCGCCCAGGAAGTGCTCGACCTCCTGG
CGTGTGAGCCATGTGGCAGTGCCATTCGTGGCACCGGCACCGGTCCATGTGGTAGTGCTG
GTGGGGTTTGCCAACAGGAAGCTGGTGGTTCTTCTGGCACTAATCGTGGTGGTGACCAGA
GAGCTGGTGGCATTTGCCAACAATTCTCGGGCCGCCATGCATCGCTGAGCCACCTGCAGC
AACCTACGCCTCCACCTCCTTCAATGACACCCCCAGTGCACCCCCTCCTCCACAC
>at00887
TTTTTTTTTTTTTTTTTTCGGGTTGCTGATCTTCTGCCTCGCACTTTGATCGGCCTGTTG
GCTTGCTGGAGGGAAGCTCCCAGGCCAGCTTTCAAAAGCAGACTGTGCAAGGATGGAATT
CAACACAACTCTATCACTGAAAAAAAGAAGCTCTGACCCAACCAATGCAGAGGCGGCCTC
CAGGAGAACTGCTATACGTCAAGGCACCAAGAATGAAACCTCGGACCTTTCCACACAAGC
CTTCACGCCTCGTGCC
>at00888
GCGGCCCCGGCGTGGGCGCCGGGTGCACCCAGGCCCAGCAGGGCGGCGCCCGGCAGCGCC
CCGAGGCGCAGGGCCTCCACCTCCTTGATGCGGGAGATGACCACGGAGGAGGGGTTGCGC
GTGCCCGCCACGGGGCCCCGGTCCATGACCATGCGCTGGAGCTGCGGCGGCAGCGCACGC
AGCCGCACGGCCGCCTCGCCGTCCACGCCGCCCGCCTGGAGGAACTGCTCCAGCTCCGTG
GAGGACGCCAGGGGCACCACGACGGGCGTGCCCGTGAAGATCGCGCTCTGCGGCGTGGGC
ACGCCGCCGGCGTTGGCCGGCTGCAGCGAGCGCTCCCAGGTGAAGCAGGGCGTCTTCGAA
GGCGCGGCGTTGTGGACGGTCGCGCCAGTCGCCAGCGTCTTGGT
>at00889
CCGTTGCCATTTTGGCTCAAGTTGTCCAGAAATCTCCCGGGATAGCAATATCAAAATAGC
GCCGCTTGGTTAGGGCAGCTATGAATACGAAAGGAGCCAGTCAGGACACGGTGGCAGTTG
AGGGGCAGCACAAGTTTAACGAGGCAGCTCTTGCTGAGTATCTGGCACGTGAGTTGCCAG

GCTTTGAGGGTAGTCTGACTGTCCGTAAGTTTGGCTACGGACAGTCTAATCCGACTTACT
TCCTTGAGACAAGTGCAGGACAAAAGTATGTGCTTCGGAAGAAGCCACCAGGCAAGCTAA
TCAAGGGTGCTCATGCGGTTGACCGTGAGTATCGTGTGATGCATGCTCTTGGTCAAGCTG
GCTCCCAGGTCCCACTCATGCACAGGCTTTGTGAAGATGAATCAGTGATCGGCACCACCT
TCTATGTCATGCAATTTGTGAAAGGTCCAATCATGGACAATGCTTTAGCTAAGGCAAGTG
CACCAGACCGGCGAGGCATCATGATGTCGATTGTGCAGACGTTGGCCAAACTGCATCGGT
ATGACCCCGCAGAGTTGGGGCTGCTTGATCCAAGCAGTCCATTTGGGC
>at00890
GACCCGCTTTTCCGGGATGGCCCGGCTCTGCTTGCCGGCCATGACGTAGACGAAGCAGGG
GTCATCCAGGGAGAAGGTGTCGAGCTTGTTCTGCCACTGCGACTTGAAGGAGGCGCACGG
CACTGAGAGCAGAGCCAGCAGCAAGGCCGCGGAGCTGGCCGGCATCACGGGGGACGGCGG
CTAGTAGCATTCGGTGTCGAAAATCGCGCGAGGAGTCGGCTCGAGAGCTTGAGCCAAAAT
GGCTACGG
>at00891
AAGGGACTAGGCCCTGCAAGCAGCCGCAGGCACAGTGGGACAGAGATTGCTTGGATGCAA
CTCTCCGCCGGCCGCCATTGTCGCTGTCAGGTGTGGGTTGCTGGTGCCACTCTGAGGTTT
GCTGCTTTCTCCGGCTGCACCGGCAGCAGTTGCGGGGGTNTGCAGGCCCTTGGGCCTGCA
GTTGGGTTCTTGGG
>at00892
TTTTTTTTTTTTTTTTTACGTGCACAAAGCTCACATCGAACGATTGCTCATTTCATCTC
AGCAGATGGCCAAGAAGCGGCAGGAGGTATCATTTTTGATCACACTGTACAAGCAGAGCC
CTTTACGGCCTACAGCTCCTGGCTCTGGAGTGCCTCACCCTGCTGGAAATGCGGATTAAT
GTGAGATCGGTCCAGTCCGTGCCAGGTCAAGGGGGCAAAATGCACAAAGATGTTCCCAAA
CTCCTCGCCCCGGAACCGCATGGGACGCCCGTGTGTTATCCTCGCGCCCTCATAAAGCAC
CATCTGCCCAGGCTGAAGGTACACCTCCTTGCGCCCCGACTTGTGGGGGTGGATCACCTC
CAGCGGCCAGCCACCGTCTTCGTCGACCTTCTGGGCCACCTGGAGCACTGCGCTCGCTAC
ATGCGTCTCCTGGCGGTCCAGGTGGTTCACCAGCATGGAGCCGCGGCGGTAGACACGCAC
GCCGGCAGTCGCGGAGTGCCGCAGTCGCATCTTGGTCCACCACTCGAGCACCTCCTTCAT
CTCCTGTGAGATCGCACTCCTGACTTCGCGGAAGCTGTCGAGGTTGATGACGTCCATGGA
GATCCTCTCATTGTTGGTGAAGCCGCCGGGGATCTTCCTGTGGCGGGTGAGGGAGTCGGA
CAGGCGCTCCTCGTACCAGGGGTAGAGGGTCCGCTTCAGGCGATCGGTCATCTCCATCTT
GAGGTAGCCACCCTCGTATGAGGTGACGTTGATCAGGTAGCCCTCCTGCCAGGCGTAGAG
GTGGTCAGCGGTCTGGCCAACTTTGTAAGGGGTCCCCGCGAGCTCGTCTGCATGGAAGCC
GTAGCTCGCGCTGTCCCTCTCCCTGACCTCCTCAGGAGAGACGTAGCGCACGCAGCTCCA
GTTGCTCGAGTGCCCGAGGCACGGCGCGGCCGCGTCGTGCACGAGAGCCTCAAACTCCGG
CCACCGGTCCCTGCTCACGCTCACGTTCATGCCGTCGCAGCGCCGGATGGGCACGCTGCC
AGTCGGCTGTTCCTCGGGCACAACATACTCCAGGAGGAGCTGGTCGGAGCCCGAGACCCG
GAAGCGGAAGAGATGTCCGGGATAAGAGTTCTGAGAGATATTTCCGTCGGGCGATATGTG
TCTGCTTTTCTCTCGGCACCTTCGAAGTCCACCCACAGCAGATTCACAGGCACACCGGCA
CGATTCTTGAACCTCACTT
>at00893
TTTTTTTTTTTTTTTTGTGGCGCGTAATTCCCTCCGTGCGGCGGCGTCTGGTCAGCTCAG
CTCGAGGCGGCCTTGGCCTTCTTGGGCTTGGGGAGATTCCTGTTCTTCTTGAAGGAGTCC
TTGATCTTCAGGTACTTCTTCACGGCCAGTTCCTTCAGATCCGGGCGGTAGAAGACACCC
TCGGTTTGCTTCGCGATGACCTCGATGCCCTTCTTGGTCTGCTTCTTGACGCCGGTCCCG
GCAGACATGGACGAAGGCTTCGATGGCTTGCCGTGCTTCGGCTTCTTCGTGAGCATGATG
GTCTGCTTCTTGCCCGTGGTCTGGACAGTCACATCGAGCGGCCTCTGGTTAGCAAGGCCG
CTGTACTTGAAGCTGTTGAGGCCGCAAAGGTTGTTCGGCTCCGCTGTCATGACGGGACAG
TTCCGGCTCTTGCGGATGAAGGAAGAGCTGTCCTTCACGCACTCCCACAGGAGGTCAGCG
GACACCCTCATGGTTGCTGGGTGAGGCAGGTGTGCGAGCTTCGGATGGGAGGGACAAGGC
GGAG
>at00894
AACGGCCAGCCCCACGGCGAACGGCTCTCCGTGCTTGAAGGGCGCTGGAAGCAGTGTGAA
GCCTGGCAGGAGAAGCTGGTCCTCTGGATCGAAGACCCCACTGAATCCAGAAACAACACT
GCTCACAGCCTCCGGTGGGACAGCTGGCACGAGATCCTGCAAGAGGTCGACAGAGGTTTG

GCATTGtTACGTTGCGGTGAGCCGCTCAACAAGGTTTGCTCGGATGATGCCCCCCTcCGT
GCTCATCTTGCACCTTCTCACCTCAGGCACAAAGTCACCTGCaAGCGCCGTCCAGAcGAT
TGAACCTGACAGGCCGCGGCGCTCCAAGGTTCGtCGAAgGAGGGTAAGAGTGCATCACCG
GCCCTTGCCGTCATAGTGATGGCAATTGAGTCTTTTACGAAAAACTAAAAAAAGG
>at00895
TTTGGAAGACATGGGAACCAAGAGCACCAAATACTACACGTTCTCATTGACAGGGAAGTT
CAACGCGAATGGTATGGCAAGCAAGGTCTGCAGCATAGCCGGAGGCTGGAATTTGCCGTG
CGATAATGCAACACGGGCATTAAGCTTCTCATTCAAGCTCTCACCGCGGAAAGTCGCTTA
CGTCATCACGCGCCAAGGGCGAGTGTACCAGTTTGCAACTTTTCTTGGCAGCGTCGGCGG
CTACATCACGATTGTTGGAACAATTTTCGGCTTCTTCTTTATACGCAAGAACGCCAAGGA
GC
>at00896
TTGTTTTTTTTTCTTTTTTTTTCAATTTGCCAAGGCTCTGGCTTCCCACAGTTCCTGCAT
CCAGCGGCCGCCCTACGAGCGCCTGCAGCGCTGGGCACGCGAGCGCGGGTAGCTGCTGCT
ACTTGCGCTTCTGAAGGTAGCGCACGGGCGCGGAGGACGCGGGGCTCTGCGGAGACGCGG
CCCGTGCGGGGGTGACCGGCCTCGCTCGCAACGCTCCTGGTGGCCTTGCCTTGGCCTTTG
GCAGCAGCTGGTTCCTGGCCTTCTTGGCGTGCTTGTTCTGCACAGCGGACATGAAACCGG
AGGCAGCCTGCACCAGCAAATTGGCCAGGTTCTCATTGGTGGCCACGCTTTCCGTATCCT
TGCCCCTCTTCTTCTTCTTGTACTGCACGCTGGGCTCGATGTCCACCGTGGGAGCCTGGC
TGATCATCTCCAGTAAGACGGGGTGCCCCTGCGTGAAGATGCCCCCGAAGTCCTCCCGCG
AGACCTGCCCCAGGCCCTCGACGTCCATCATTCTGATCATCTCGTCAAGCTCCTCGTCCG
TCGGACGCTCGCCCATGGTGCAGAGCAGGTACCGCAGCTCGTTGACGCCGATGTACCCGT
TCCCGTCAAAGTCCATCTCTTGGAATGCCTCGTCTACGTGCTCTATTTGACTCCAGGCA
GCCCCGGCACGCTGGTTGAGGCATTCAGCTCCTCAACTTCGTCGCCTCCTGCAGTATGAT
CGGGAGGCTCCGGCGGCACAAGCGGCAGGTTCGTCATCTCCGGCGCCCCGGGAGGAGGGC
CGGGAGGCAGCCTGTTCACCGCGGGCACGAGGGCCGCTCCAGCCTGGGCGGCCGCCATGG
GCCCACCTCGGTCCTGCAACATCATTGCGCGGCTGGGGGTCTGGGGGGGCTTGGGCGAAG
ATGGCTTGAGCCAAAATGGCTACGG
>at00897
TGTAGTAGCTTGTGCTGCTGTGTGGCGGCTTAAATCTTCTTTTGCATGCTTACAGCGGCA
GGAGCTGCGGTGCGCTTCACTGCGGTCTGCGCACGGCTTCTGCCTGGGATTTTCACCCAG
CAGAAGAGCTATGCTGCGCCATGCGCTTCGTTCCGGTCTGTTGACAGCCGCAGACCTGGG
ATTTTCACGGAGAAAGGAAGCGCTTGACATGACTCATACAGTGCGTTTGTAAAGAAAAAA
AAAAAAAAAAAAAANAAAAAAAAAAAANAAAAAAAAAAAANAAAAAAAAANAAAAAA
>at00898
GTCAATGCACTCACTTCTTGCTGCACCACTCGTATCTCCTGTGAGACTTTGGACTCCTCC
GCCTTCGCATTGAGAAGCCTCGTGCGCAGTACCTCAAGCTCCTTCACCATGGCGCCGAAC
TGTGACGTCAGGAGTGTCCTCCCCTGCTTCATCAGTTTTAACNGGCTCTGCAATGCCAAC
TTCTCCTCCTTCAGGCTGCTGAGTTGGCCTTGCATCAAGGACGTCTCGTCCATCA
>at00899
TCCGTAGCCATTTTGGCTCAAGCTTGACGGTCTTGGCATAACCCAGCCGCCCCGAACCTT
GCGGGTGCAAAGAGGGCAGCCATGGCCGTGGCGGTGCACTCTATGGTTTCTCTTCGATGC
TGGTGATGCTGGGACTTTGCCTCGGGTCGGCTGCTGGCCTGACTTGCCAGGACTTGACAG
ATCAGTACAACACCGATCAGTGCAGCGGAAATGGTGCCACTGAGTTGTCCAGCATCCCAG
GCTGCCCGTACAACTTTGCCTTGCCAGCCTGCGCCAACGCCGAGCCCCAGTCGCCTCGGG
ACCTTAGTACTGGTGCACAGGGCTCCAAGATCCCCAAAGCTGCGACCCTGAACGATGAGC
AGGCAGAGTTTCTTCCGTTGGCCAACGTGCACTTCCACCTTGGTGCAGAGCACAAGAGCA
TGATTATAACCATGGCGAGGACGCAGCGGCCTATGACGCTCAGCACTCATCCCGCCGCCT
CGCAGCAAATCCCCGCCCTGGGTGGATGTGCTCAGGTCCGGGGGAGCTCACCGCAGAACA
GACCGTGTCATACAACTTTCAGTACTGCGAAGGTGTTGAGGTTGGGAAGTCGTACGAGGT
TCACTACGTCCACTCTTCGGCTGGCACTGGCACTATGGGAGACGGCCTGGGTGGCGCCGC
CAACGGCCGTGGCCTCCTGAACCCGATGATCGTGGTGCAGGGCCAAGTCTTCGAGATCCG
CAACGATGGTCCCGATATCGATGACATGCTCCATGGCTGGACCGTTGTAGATCACTCGAA
CTCCGTGATGTATCCTGGGTCAACCACCGGGCCGTCGCATAACAACGAGTACTGCTCTCC
GTACGCCATCACTTGGCATGTGGACTTGACGTGCCACAGCGTCTCCCCTGCCTCCTTTGA

CGGGCTGTGCCAGCAGATGCGTGGGTACAACATGGTCGCGGATGTTGCACCCCACGGCTC
GCGGAAGTTGGTCGATTCCCGCTGGGTCTGCCCGTCTTCGTCTGTTTTGCCGCTGGCATG
AGGTTGATACCAAGGTGGCCTAGCAACTTATGGAGGCAAAGTTGCCCTTCACAGCGGTAG
TGTCCAGCATATTTTTTCTAGGCTTTGAATTCATGTCCCTTGTGTGCTTCATCGCACTTG
TCAGTCAGCGAATTTTGCAGCCGCAAGTAAAGTACTCGCGAAAAGGGTTGCGGGACGAAC
CAGACAGAGCCAGCGACCTCGTGTAGGTAGGAATCCGTTTGACGGTAAGTACTCGAGAAC
ATGCTAATCAAGCCCGAGAGAATCGATTTGGCGTCAGTCAGCAAAGTCCAGTCTCGGCAG
GGTGCAAGTGTGAACGCGCAGCTCAGCAGAGCAGACAGCACTACACGACGGCCGAGCGAG
CGCCAAGCGGAGGCACTTGTCAGGGTAGGGTGGAGCTAGATGCCATCGCCGGAACTCTGG
GGTGGCAAGCTGAGCTGACCAGATGTTGCCGCCCTGTGCGACCCAAAAAAAAGAAAAAAA
AA
>at00900
TATGGACACTGCAAATGAGATGCAGCAATACTTGGCTTTGCATTCTGACCGGCCAGATGA
ATTGACCGTGGAAACGATTGCTGAACAAAGCAAGAGCCATGCAAAGGCAATCTTGTTACC
TCCAGAGGATCCTGAGGAGCACGAGACGAAACGTGCGGACTTGAAGGCCAACGTGGAGAA
GCAGGAAGCAGCTTATCGCGATTACTTCACAAAGCATGACATCCAGGCCATACTTGTGCC
CCCCATGCTTACGGAGCCCTACACACAGAAACAAGAGGAAGCACCTGACTTGAAGACAAT
GTTTAGCGTGTGGACTATCTCCAGTCAGTTGGTCCGGATCAACGTGCCAAGCATCTCACT
GCCGACCAGAGCTAAGCATGTCGTTGGAGGCGGTGCTTTGCCTGTTAGCATGATGCTCTA
TGGTGCGGATGATAGGCAGCTG
>at00901
GACTGGATCCTCCGCCTATGATTCTTGCTCACAGCAGCGGAATCTGACAAGAGCTGATGT
AGAGACAGCAAAGATTCGCTTACAGACTGGCTTCTCCTTCATTGGCATGACAGAACACTG
GGACTTATCGATTTGCCTCTTCAGTGCTATGTTCAAGCAGAGGTGCCGTTCTGATCAATT
CGGGAACTCACGCCCCACCTTGGGAAACGGTGTGACAACCTACGACACCAAATTGCTGCA
TGGTTATCGTGACCCATACGACACCGAGCTATTCGAGATCGGCACAAAGATCTTTCAAGC
AAATCTGAATAAGTACAACGTCAGCGAGTCCTCATGCGAGCCTTGCTGGCGGGAGGCAGG
GTTACTGTGAATGTGGTGTCGTCTTGCCCCCAAAAAAAAAGAAAAAAAAAACAA
>at00902
AAGGTGCCCCCGCTGCCCGTAAGGATGATCTTCTTCACGCCGCCCTGCGGCACGCCCTGC
AGGCATTGGAAGATCGCCGAGTGCTCCGAGTCCACAGGCAGCATGCTGACACCGTACTGC
TCGAACATCGGAGCGATGACGGGCCCGCCTGCGATGAGCGTCTCCTTGTTGGCAAGGGCG
ATGTCCTTGCCGGCCTTCATCGCCTCGATGGTCGGGATGAGCCCCGCGCAGCCCACGATG
CCTGTGACGACCGTCTTGCACTCCTCATGGCATGCCACGGCGCATTGGCCGTCGACGCCG
CTGAGAAGCACCGGGGCGTAGTCTGGGATGCCCAGGGCTGCAACGCCCGCCTTGAGCTCC
TCAAGCTTCGACTCGTCGCACGCGACCATGGTCGGCCGGAACTCGCGCACCTGCTCTAGG
AGCAGCTCAATGTTCTTGCCGGCTGCCAGCGCCACGGGCTCAAAGAGGCCAGCATGCTGC
CGGCAGATGTCCAGCGTCTGTGTGCCGATGCTGCCCGTGGAGCCCAGGATCGAGATCTTC
TTCTTCACCGGCGGCGAGTTCGAGGTCTCGCGCGTGTTGGTCCCGACAAGGTTGCGCCCC
TCGCCCAGCTCGAGCCTGACGACCGTCTTTGCGTGCCCGAGCTTGGCCGCCCTCTCCGGG
CCCTCGCGGAGGCCAAGCAGGCCATCAGAGCTCCGGTGCGTGCAAGCATCGAGCCTGCCC
CATCCAGCAGTGGGCTGGAAGCCTGTCGGCTTCCCGCCTTCGCCGCCTGCACTCGCGCGG
GAGCGGCCGGAACTGCCACTGGTGTGGCAAAGGCGAGCCCATAGCCTCCGCCTGTAGTAG
ACAGGGAAGTCAGCAGACCTGTGGCGGTGTCCATGGCGTCCGCCATTGGCTGTGGACTAA
AAAAGCAAATCAAGACAATTTGTGGTCCTGAGTGTGGAGCCAAACCTGGAGCCAAAATGG
CTACGG
>at00903
CCGTAGCCATTTTGGCTCAAGCCATTTTGGCTAAAGCCACATTGGTTCCAACCAGTCTTC
TGCTTGGTCCCCACTGTTGGGCACTGCTCTGAACCTCGACCATGGCATATCGTCGAAGGA
TCGTGTGTGTGTGTGTGTGTGTGTGTGTGTCTGTGCGTTGTACACCTTGGCGAATGTGATCT
TGCTCAACAAGCACTTGGAGGGTGTCTTGCCGTACAGCAAAACTTCGACCATGGCTGATC
ATGAAGCGAATCTTACAGCTGTGGCCATTTTCGTGCCATCTGCCGGCTGGCATTGGGAAA
TGTGGGGTTTTGCGTTGCAGTTTGCTTTGGATTCGGGCTGCAAGGTTGTGCACTTTCATG
TGAGGAACAGCACCCTGCCTGAACGCCATCGAGTGCGTAATCCTAGATTATGGCAATCGT
GGCATTCCTGGATGGCGTTCTACACCTCTCAAGGTTTGTTTCCTGATTATTTTGCGCTCA

AGATGGATGCGGACTTCGACCTCACGGCATACGATCTAGTGCTTGTGCCGTCAGCGACGG
AACAGTGGCTTGTGCCACATTCTTGGGAACGATTTACATACGCCTCTGAGTATGCCCGAA
TCGCACAGTTTGCGAAAAGCGCAGGCTACATCATCCTACGTTGGGTGTGGGAATGGCGTC
CGAGGAACGTGCTATTTTTGTTCACGGCCCACATAA
>at00904
AAGCAAGCTCTCGCTTCGAGCGCGCCCCTTCCCGAGACCAGTTGGCGAACTCCCCCGGGT
CCGAGGCGCCATGAAGGTCACCGTCAAGTGCGCGAGCGGCGCCTTCGATCTCGAGGCTGC
CGAGTCCGAGACCGTCGCGGAGCTGCTGAAGCGCTCCATGGAGAAGCACAAGTGCCCGCC
CTGGGCGGACGGCGTGCAGCTGAAGCTCGACGGTCAGTCAGAGGACCTCGCAGAGGACGG
CTCGAAGACGCTTGGCAGCGTTGGCATCGCAGGCGGGCGCGACGGTGCAGATGGCCTACT
ACCAGGATGTGCTGCCCTCTGAGGCGAAGAAGCTCAAGCTTACTGGCATCTGCCCCGGCA
CGGCCATGCCTTTCCTCGCGGCCAAGGCGTGAGAGGTCCCGGGTATCAGGCGTGAGTGGA
CACTGCATTCTTCCATCGACACAAAAGAAAAAAAACAAA
>at00905
TTTTTTTTTTATTTTATAATTTCCATTGCCACTCTGCTGTCATGGAGGCAACTTTATGC
TAAAGAAAGGTCGTCCCGACGCGAGGCGTCAGAGTCGGCCCAGTTCCCGCTTCAAACCCT
CAAAGTCAGCCCAAAGGTCCCTCTGCAGCAAACCCTTTTTCAAATGCATATGGTGTGATC
GATGCTGGAAGCGGAAGTACACCTGATATCTGATGTTTGGAGAGCAGTTGTACCCAAAGA
AGTGTGAAAGCTGCTGGTGCATAATGATGACGTCGCCGGCTCTGACCAGCACCTGCTGGC
CTCCTGACAATGCTGGCAGATCAGGGTCCTCTGGCTGAGGGTGCTTTGCTGTGCCACTCT
CGCGCAGGGCGCGCA
>at00906
GCCATTTGGGGCCCAGCTCTGTCAGTTTGCCTTCTCGGTCTTCTCTCTGTGCTGTAAGAA
TGGCGCACCGCGTTTTCACAGTGCAGCGCCACATCCTGGCNGGCCATGCCTCGGTCAGCG
GAGGCGTGCAGATGNGCGGGACAAGCGNTTCTGAGGATGCCGACTTNGCNGCACGTGTGA
AGGAGATGGCCGCATGGATGGGCGGTGAGCGCTTCAAGCACACGACCCGCCCNTACAAGG
TCGAGGATGTCGTCAAGCTGCAGGGCACCATGCCGCNGCACTTCACAGGTGAGAAGGTCT
CCGAGAAGCTNTACGCGATGATGCGCGANCACCAGGCCAAAGGCACTTGCTCCCACACCT
TNGGTGCGCTGGATCCCGTCCAGGTG
>at00907
TTGTTTTTTTTCTTTTTTTTGGTTTATGCATGCATCATATCAACTTGCATATATAAAAAC
ACTGCTCTCACCGAGAAGTGCTTGCTGGTCACTGTACAATCTTATCCCATGGGATAGTGA
TCGGAATCAGTGCTTTAGCCTGGATTCGTGGATTCTGGAGCGTCTTAAACTTCGCCCAGA
GCCGCTGCAGCGCGCCTCTTGTGCAGTTTGGATCGTCTGGTACAGCTTGCCACTTGTCAG
AAAGGGAAGTCTGCTTCATCTCAATGAGAGGTACCAGGCTTGCCATCTTGCCTTGACATT
CTCGCATCCGTGCAATT
>at00908
TTTTTTTTTTTTTTTTTGGGCTGGTCTGGTGCACATTCAATACTGCAGATGGTCACTTC
GTCACAACCCCCGCAGCTTGCTGCGCAATGGGCCACATGTCCCACACTTGGTAGCGCTCG
GCAAACTCCTCCCGCGCGAAGAGAGCCGGGCTGGCGCCCGAGGACCCCTGCAGGCTGCGG
TGGATGAGGCCATCCGTGTCCCGGTGGAGGTGGCAGTTGCCGGCGACGGCCTGCTGCAGG
GGGTCCACGAAGAGCTGCTGGCCCTCCAGCAGGGAGCCGGGCTCGGCCGCCACGTGCTCG
ACGTAGCCGAAGCGGCCGCCTGGCGCCAGGACGCGGCTGACCTCGCGCACCGCCGCCTCA
GGGTCCGCGACGCTGCAAAGGACCTTCGTCATTACCACGGCGTCGAAGCTCGCGTCCTTG
AAAGGCATTCGCTCCGCCTCGCCTTTCACGTACGAAATACGAATTCCAATCTCTTGAGCC
CGTTCGGCAGTCTCTCGCTGCTGCTCAATCTCCGCGGGGTTGGCGTCCAAGCCTACCAGC
TCAGTGCCAGGCGGGTAGAACTCCAGGTTTGGCGCACCAGCGATGCCAACTTCCAGTACT
CTCAGCCCCCCACGGGGAACCGCACGGCCGATGGTGTCCTTGACCCTGGACCAATAGACC
GGATTGAACTCAGCTGCAATGATTTTGTAGAAAAGAAACGCATAGCCTCCTCCCAGCAGC
AAATTGACAGCCTGACCGCGAGATATTGGAGGTCCCACTTCTTCTGGTGTCGCCGGCTGA
GCTGCAGTTGCAACGGCAGCTCCAACGCCCTCCTCGCTGTGACCTTCCGCGCTGTCAGCG
GTTTTGATGCCTTCGCCACTTCCAGGCGCCACAGCCCACCGCCGAACCCAGCGGTCTCGC
CGCAAGGCACGGCCAGAGTGGGGCCAGGTGTGGCCGGCTCGGGGCGTGGGCAGCCAGGCA
GAGGAGGAGGCAGCCCAACAACCGAGGAATGTAAGCAGCACGCCGAGCACGGATCTTCGC
CGCGCTGCTGACATGGGCAAGTGGAGGAAATGCTGCCTGACGGTCCCTGTCTTGAGCCAA

AATGGCTACGG
>at00909
TGGCGCAGGAGTTGGGGGAAGCGGAGCTGGTGGCAGTGGCGCCAACGTAGTGGTCTGCGG
CAAAGGTGCCGGCGGCTCTGGCGCTTCTGGCGCAGGAGTTGGGGGGAGTGGAGCCGGTGG
CAGTGGCGCAGTTGTGGGAGCTGGCGTTGGCTCGGCTGGTGCTGGCGTCGGCGCAGTTGT
GGTGGCCTGAGGC
>at00910
TTTTGGCACGAGGTCATGCACGGAATCGGGCGACGCTGGATGGGTGTGTCATTTTGCTCC
GGTATTGTTGCTTTGGATGGGAGATTTCCTTACAGATCGCAGTCTCGGCTTGGCGGATCT
GGGGGCGTCTGGGCTCTCAGGCCTTCTGGCGGCATTCCTCGGCAGATGCAGGNGGCCCAT
GGTGGCGGTGCACTCACAGTTCCTGGTG
>at00911
GTCTCGACATCTCTCTCATGCTAGCTCCTCATCTCCGATGTGCGAAGGGCAGCCACTTTG
CCCTGCAGCTCTGCCCCATGAATCCTGCCCTTCTCCACGACCAGCAACATATCACATCTG
ATCTTGTCCAGAGGCCAGCACCAAGAGTCCCTCTGGACTGCTGTACTAAGCAGCCAAGGG
AACCTAGCAGGCAGCTCTCTCAACATCGTGTCCAACAATCCCATGGCTGCCTCTTTTGAT
GTGGCCTGCTTCAATGTGCTGCCCAATCGCACCCAGATGCACAAAATCGT
>at00912
GCGCGACTGAAATCAAACACGGCCGCGTCAGCATGCTTGCAACCATGGGCTACATCACGC
CAGAGCTCACCGGCAAGTTCCCTGGCTATCTGTCGCCTTCTCTGGGGTTGAAGTATGAGG
AATCCCCAATGGCCTTGGAGCATCTCCAAGGTTCCGCTTGCTGGTTGGTTCCAGATCGTT
CTCTACTGCCTGTACTGCGAGGCGTCAGGCCTTGGCTTTGGCTACTTCGAAGAGTCCACT
CAGGGTGAGCCGGGGACTTGGGATTCAAGCCGCCACTGCTTGCAACAGTGACCCCGAGCT
GAAGAAGAAGCGGTTGAATGCTGAGCTTGCCAACGGCCGTCTGGCCATGATGGCGATCAT
CGGGATGTTCTTCCAGGATGGTCTCACAGGCAGTGCATGGGGAGACTGGGCCTTGTACAC
TGACTCCCCCTTGAGGGCTTTTGAGGGTGAGCTTGGTGTCCAGCCGCCCTTGGGATTCTG
GGACCCAGCGGGCCTCAGTGCCGATGGCGACGTGGCGTCCTTCACCCGACGCCGCTCCGT
GGAGATCAAGCATGGTCGTATTTGCATGTTGGCAACTATGGGGTACATCACACCGGAAGT
ACAGGCAAATTTCCCGGATATCTGTCTCCCGGCTCGGGTTTGAAGTTTGCCGACGTGCCC
AACGGTTTGGCTGCCATCGGCAAGGTGCCTCTTCTTGGCTGGCTGCAGATCTTCGGTTAT
GCTTTCTTTGTCGAGTACAAGGCAGGCTACGACTCCGATGTTGCTCGCGGCACGCCAGGT
GACTATGGCTGGAAGCCGCCTCTGCTTGCAACTGATGACCCTGAGAAGAGGAAGACGAAG
CTCGCAGCGGAGCTCGCCAACGGAGGCTTGCGATGATGGCAATTATTGGCATGTTCTTTC
AGGACGGTCTCACAGGCAGTGCTTGGGGTGACTGGGCCCTGTACACTGACTCCCCTCTCC
GAGCTCGATCTGTCTGAGGCATGAGCCGTAGTGGTGAACACCTGGCACGCCCACGAGAGT
ACGACACTCACTTGGTTCGAGTGAGCAGAAACTCCGCAACAACAATATACTG
>at00913
GTGGGCTGCCGTGCGGCCGTCGCCGTCCAGGGAGGAGGGGCTGCACCCCAACTCCCTCAG
CGCCCACAGCGTTCCCACGAGGCCTCTCTCGGCGGCGACGTGCAGCGCCAGGCGGCCCTT
GCTCACCAGAAGCTCCTCCGCGCATGCAAAGGAGGCCAGGCGGCGAGCCACCACATCAAC
TTCGACGTCAGCAGCACGGCGTGTACGGAGGAATGTGCGCCACCCACGCGACACGATGTA
CCAAGTCCTTCGCAACAAAGCTCCCTCGACAAAGTCCGTGACGAGAACAAGCCAGGCACT
TTCCCCCGAGCCCACCAGCGCCGATCCCGCCACGCCTACTGTCATGGCTGCCGGGGACAG
TGCTGACAACACCAGTGAGTGCAGCGAAAGAGGGGCCTCCAGCGCTGGCCAATGGACGCT
GACGGC
>at00914
TTTTTTTTTTTTTTTTTGGACTGGACTGCGCGCATCCGCCAACTCCAGCCGCATCGGCT
TGCTCTGTGAAGCACGTTACGGGGAGATAGCGTACATCGTCGAAGACACGCAGCCCCGTG
CTCAGCGATGCACGTCGGGGGAAGGATGTGCGAGTGAATGCCGTGGCCGGCACGAGCTGC
CATCCAGAGCCAGCAGCAAGGTCCCACGGAGCCAGGCGGCGACTGCGGCGGCGGCGGTCT
CTTCACGCCCCTCCGCCAGCGCCATCGTCCCTCCTGCCTCTCTCGTCGTAGCCCTTCACA
CCCTGGGCGCCTGCAAAGTAGTTGTCCTTCTCTCGCTGCATCGCCTCCGCGTGCGCGCGC
CTGCCTGCCGCGCGCTCCTCCTCCGACGCGTCCGTCGGCAGATCCGGCTCGAGGTCGAAC
CCATCATGCTCCGCCTCGCCTCGCACCAGCGTCACCATCTCCCGCGCCTGGCCGGTCTGC
CGCACGGAGGCGGCGATGAAGCGCATGGCGAGGCCGACGCGATGCCC

>at00915
GGCGCACCAGGCTAACCAACTTACTCTACAAGCTCAAATTGAGATGGTGCTGCCACTTCG
CTCCTGGATGGCTTCTTGCAGCTCGGCCATTCCAACTCCGCCGGCCATGGCCATCTTCGA
GAAGTTGCCCAGCTCCTTCTCTTCGGACTTCGGCGACTTGATGTAGGGTTTGCTGGTCTC
CGACATCGGCTGCACTCCCCAGAGCTCTGGGAGAATGCCTGTGAGCAGCGAGTAGTGCTT
GACTTCCTGAAAAACCATGTCCAGGTGGGCAAACTTGAGAGAGAGATGGCGCAAGCTGCC
GTGGTACTCAGCAGCTGGCGCTGAGCAGATGAAGAAGTGCATCGAAGGCCCAGCAAACTC
AGCAAACTCGCGCGGGAACCTCCCAATGGTCGTGAGCTTCGTCTGCCACCGCTGCATGCG
TCCAACGGCTATGTCGTAGCCGATGAAGGAAGAGAGAGAGAGGATCGGAGAGTCGAGCTT
GACCTGAAATGGCACGCGGGGCGCCTCTGGGGTGCCGTAAACCATCCTCATGTCAAGAGT
TTGGAGCTC
>at00916
ATGGAGGCCGCGGTGAAAGCGGGAAAGGCTCGGTCTAACTTGGGCATCTCCAATCTGAAG
TCTTTGAAGGAGCTGAGGCGGATCTACCAGGAGGCAGAGGTGAAGCCCGCAGTGGTACAG
CAGCGGTTCTATGCGGATACCGGCTTCGAGCACGAGATGCGTGCTTGGTGTGTCGAGGAG
GGCATCTACTTTCAGTCCTTTTGGACGTTGACTGCTAATAGaCAAGCggTaAGGtTAgGG
tAGGGGtTAAGGACTTGGTGGTGTcCCCAGTGATGAAGGAGCTGTCGGCAAAGTACGGTG
TTACGCCACAGGTCCTCTTCTTCCGCTTCACCATGGACTTGGGCATCACACCGCTGACAG
GCACGACTAGTGATGAGCACATGGCCCAGGACTTGGGTGCTCGGGCAGTGCCCTTGGaGA
GGGAGGATGCCGAGAGGATTGAGGCCTTGATGCGCCAGGCAGTGTCTGACAGATCTGACT
AGCCATCTCTGCAGCTCCTTCCATTGGCGGCCAATATGTCAGGGAGGGGTGCGGACCTCT
GCTGATCTCTATGAAGCAAGCAACGGAGAGCCAAAG
>at00917
CCGTAGCCATTTTGGCTCAAGCGCCTAGTAGCTGAAGGCTTCCCCTCCTAGTGAACAGCC
CGCTGGTTGCACTGAATATCTCCACGTTCAACCATGTTGCATATGCTGCTCCTCGTGGCC
TTGCTTGGGCTTTCCTCCTCAGAGGACACCTGTTCTGCGGGTACCTCTTGCACTGACGCC
ATCGAAGAGGTCTCCCTGCTCCAAAACCTTGCCGAGCTCCAACACGGCGGGCAGGCAAAG
GAACTGGGCACGGACAGATGCCCCTGCCTCAACTGGAGCGCGCTCTACCATGATAAAGTG
GTCAAGTGCGGCGACGGTGAGGAGCTCTCTGGCCCGGACATTGCTCCGTGGGGAGGCGAC
GGCCTCTGCGAAGTCAAGTCCGGAAACCCTTCTACCCAAGTCAGAACCACCACATCTGCA
TCAAGAAGAGCAGGGTGAGGCAGGAGATGCCGCAGCCCGTGCGAAACGTGTGCTACGTCT
CCGCCCACTGCGGCGCCCTGAATGGCGGAAGGACGGTGAACCAGCGGCGTGAGCTTGAAA
GTGTGTGGCGACGGCGACAGGGCACTGGCCGACTTGTCGCCAAGTGAGCTCTTTGAGATG
GGCGCGAGGGAAGGCGTTGATCCCCAGAACCTTGCCCTCATGGCGTATCCGTGGCAGGGC
CCCCATCCTGCCGAAGAGGAGGGCCAGACCCCAGCTACACTACTCAATCTCGGGCTTCAG
AGTCCTGAAAAGGCCGTGGTCGGCCCGAGCCTGAAGTTCGACACCATTACCGTGCTCTGG
AGGAACCAGGTCTGGGAGATCGAGAGCAAGGCGAAGTGCATCGACGGGTGTCAGTGAGGG
GTTGGACTGCTGGTGAGGTGTGTGCGAAGCCCCGGCCTGGCTCCTCTCCTGGGGCGATTG
CACAGTAGTCCGCACGCATTGATTATTGATTGATGTCGAGTGCTGTGATCCTTGTTAAAA
AAAGAAAAAAAA
>at00918
CCGTAGCCATTTTGGCTCAAGCCATTTGGGCTCTAGCTTCTTCGGCTGCCTGTCTTGGCC
AGCCTCGTTGCCCAACTCCCATAGCTCGCGGGAGAAGCCTTCAATTAGATTGAAGAGTCA
TGCCTGCTGCTGAAGAGGCCCCGGAGAAGCAGGTGGAGGCAGAAGCCGAGCAGGAGGAGG
GCAACTACTTCTACGTCTGCAAGCGTGCTTTTGAGGCAGTGCAGCAGAGGCTGAAGAAGG
GCGAGGAGATCACCCAGGAGGCCGTGAATGAGCTTGCCTTCCCAGATGACCTCCCCGACG
ACGAGATGATGGTGTGCGTGGATATGACCGGCGCCGGGGTGAATACGAGGACGTAGAGCA
GATGGTTGAGAAGC
>at00919
GGGTGGTTCCCAGCGTACTACAACGAAGTCCAGGCGTATGGCAAAAACATGTACGAGCAA
TTCGAAGAGCGTTTCAAGGAGGCCAACAGGAAGAAAAACCGGCTCGTACGGCGTGTTGAG
GTGCATTGGAGGCAAGTTCAAGTTTGATATCATTCACCGTGGGAAGTGGTTCTGGCTTCT
CCGCCCTCCTCGGCTGTGCCACAAGTGCCAGCTTGACAGGCCCGCGACTATCCCCATTGG
TTTATACT
>at00920

GCGCAGCCGGCCCGAAGACTGCGAACTCGCATTGCCGCTTGACATAAACGCTTAGGGGGC
CAGCTGCAGGCAAGAAAGTGAGTGGCACCACCTCTTGCCTCCCCACCATGACTGTCAAAG
CAATGGCCGTCTTGACAGTGTCTTGAACACACATGACAATGAAGACAGTGTTGCCAAAAC
CACAAGGCTGGCAATGCACCCACACACACTGAAACAAGGTCTTCATGAAGAGGCCTGCCT
TGAAACAACGTGTGCGCGTCGCACCGCGGGTCAGAGAAGACAAGACCATGAAGATGAAGG
CGACAAGTAGGGCCGCCGCCCCAGGCCCGCTGTGTCCCCTTTGCCAGGCACACCAGTGAT
GTATATAAGTGGTACGGTACAAGCCAGGACAAGCGTCAACTTCGTCCTCCTCGACTCCTC
GTCAACCCCCTCTCTCGCTGTCAGCCGGGAAGCCATG
>at00921
CCAAGCGGCTTTGCGTAATGTGCAATGGATGCCCCCATGGCAAGTTGGTGATGCGTTGCC
GACAGTGCTGTGGTTGCTTGCATGGGAAGCTCAAGGGCAATTGCAGTAAGTGCACCGGGT
GCCCACATGGAAAGCTCAAGTGGCTTTGCCTGCATGCTGGGGGTNCAAACATGCAAAGGC
GAGAAGAAGTTGCAGCGAATGCTCCGGCTGCGTGCATGGGAAGCTTAAGCACGGATGCAC
GCTGTGCTGTGGGTGCCGCATGGAAGCTGAAGCACACTTGCC
>at00922
GCTGGCGCAGCTACCTGCCGCCCAAGCTCGTCAACTACATCGAGAACTGGAACGCCGTGG
ACATGGAGCTGTACGAGTACGCGCTGCGGCTCTTCAAGGAGCGGTACAACCGGACCTGCA
TGTGATGGCGCGCCTGTCGGAGCAGCGGCCGTCGTCTCGAAGGGGGGCAGCCGAGCAGGC
CACAGCTCGGTCGGCGTTCTTGTCTTGACGTCAGGACAGAGCCGCAGACTGAACTGCGAT
CCACTCCGAGATGTTGTGTCTTGAATGTCGAGATAGAAAAAAAAAAAAAAAAAANAAAAAA
AAAAAAANAAAAAAAAAAAAANAAAAAAAAAAAAANAAAAAAAAAAN
>at00923
TTTGTTTTTTTTTCTGCTTTCCTAAGCCGCCAAGCTTTCCTCTTCTTGTCCAAGCATAAA
AATGGAGCTGAGGGCCATGGACACTTGTCCATGTCTGCGCCTCCTCCTCGGTAATGACTC
GCGCTCACTTGATCAGAGGGTTGGCAAACAGCAACACCAGCGCGATGACAAGGCCGTAGA
TTGTCAGAGACTCCATGAAGGCCAATGAGAGCAGCAGCACACCACGAAGGTCATCAGCCA
CCTCTGGCTGCCGGCTGATGCCGTCAATGCAGCGGCCCGAGGCGATGCCCTGGCCGACGC
CCGAGCCGATCGCTGCCAGGCCAATGGCAAAGCCAGCGCCCACGGCTGACAAGGCTGGGA
TCCAGACACTGCCCTCATCAGCGTGTGCCGCCGTGGGTGCAACGGCTGCCACAGCACTCA
ATGCAGCCGCCGGAAGCCTGGCAATGGGAGCCTCTGCGCTTGCAGCAGGCACCTTGTGCG
CGGCTGGGATGAAGCAGCTGACGGAAGAGATCAATGCCATGCCAGCGAGCACCACGGAGC
CCGTGAGAATCGCGGAACGAGGCCCGGCCATCCTGGTTGAGACTGTGCAAACTCGGCAAC
CGCAGGAGGTATGGGCCT
>at00924
CTCGGCCCCCGGGGCCAAGGATAGGCACCAGACCCAATCTCGATGGCCACGCAACGTCTT
GAGGCATCTGCCTGAGTCCAGGTCCCACAGCTTCACCAGGCAGTCCTTGCCGCTGCTCAC
CGCGCGCCGCTGGCCCCAGTCCACGTCGAGGCAGTAGACCTCTCTCTCGTGGCCCTGGAA
GGTCTCAAGGCAAACACCCGTGGTTGAGGGTCCCAGAGCTTCAGCGTACAGTCTTCGGAG
CAACTGAGCGCACACCTGGAGGCCCAGTCCGCCGCCACGCAGTGTATCAGCTCCGTGTGC
CCCCAGGAGCGTCCTGACGCACGTCCGCTGCCCCAGGTCCCAAAGCTTCAGTGCGCGGTC
GTTGGAGCCGCTGAGGGCGCGCTGCCCGCGCACGCGGGTAGCCTGCAGTGATGCGCTGGT
GCCGCTGGGCAGCTCTGCAAAAGGGCGGCATCATCCCTGACGAGCATCGTGCCG
>at00925
CTCAAGCTGCTCGGCCTCCCCCCCGCCCACCGAGTTGAAGCCGGCCGCCCGGCGACCACA
GCCGCGATGGCGCAGATTCAGATCAACGTGATCAACGCCATGGACCTGCCCAACATgGGA
TTTTCCTGGACAAGACTGACGCCTACGTCAAGGTGACCATGGGTGGCGAAGAGGTCCTCA
ANGaCCAAGGTGATCGACAACAATCTCAAcCCCCGTCTGGGATGCAAAAGGCTGTGGTCA
AGTGGGACGGCATCAACGACCTCATCTTCTCTGTCTgGGACAGCGACCTCCTCACCACTG
ATGACTGGATTGGGCAGTACATCCTGTcCCAAGAcGCCAGATCCAGACGGGCTTCAAGGG
CACAGTGCCGCTCAACGTGGCAGAGTCAAGAGGTGCGGCCAGCAAGCAGCC
>at00926
GTACAGCCAGCAGAACGAGCTGATCGATTCGCGCGAATTCCTCACAATGGTCTGCCTTTT
CAGCTCAGCCACCTTGGTAGAGAAGGGAAAGTTTCTCATCTCCCTCTTCGACGGCACGCA
GACTGGCCGTTGCACCGGTGCCGAAGTAGGGCAGCTTTGTGTTATGATGCTGACTGTTCT
TGCCAAGTGCACCAGTGCATCCGTGAAGCCGAAGGATGTCGTCCCAAGGCTCAAGGCAGA

GTTTGGCGAGATGGTACCTGAG
>at00927
TGCAGACGAGGAGCCGCGCCTGCATGGCCTCCTCGCACAGCACAGTGAGGTCCTCCACGC
AGTACTTGTCCGCGGCTCTGAGCAGCCGGCCCCAGCTCTCGGTGACACAGACCTCAGCAT
CCAAGTTGTACGCTTCCGTCTTGACAACCTCCGTATCGTCTTCCCTGTACTCCCCCTGCA
GCTTTCCGTCCTGCAAGGAAACCGTAATCGTAGCATGGTTGGACAAGTGGGCAGACCAGC
TCATAGGGCCTATTTGCCGCAACTCCCCGTGTATGCGGCCCTTGGCCTCCGTGGTCTCAC
TCCAAGTCAAGATCCCCTTGTGCTCTCGGATCTCGTAGTAGCAACTGTCCACATAGTGCC
AGCGACCAGCTGGATTCCTGATGAGCTCCTGGCCTCCCTCGATTGACCCAGTGTACAAGA
AGCGGAGGAAGCGGGACATGGTCACCGCGTCCAGGTCCGGGATCTCCACGCGCCGCCTCT
CGGCCTCCGACATCCCAG
>at00928
AAGCCTGCAACTGCAACAACACATCGCCCAGCTCGTTAGGCCCGTGCTCCATGTCGATGA
CGAACCAGTCCAAGCCAGAAACGTGCGAGATCATCTCGGCGACCATGGTGCTCCGGAGGC
CCGTCCACAGGCCGATCTGCCGGCGCTGCTCGGGGCCGGGGCGGATGGCCGCCTTGAACA
CGTTCGTCCGAACCCTGGTAGGATCATCGCAGCTTGCTCCTGCTTCCAAGCCCCTGCCCT
CTGCCGTCGACAACCGCCTGGCGCTTGGCGGCACTGTAACACCCCAGCAAGTCTGGAGCC
TCGTCGCCAGTGGCGCCGGCGAGCCGCACGCGAGGAGGCAATGAGCCCTCCGCGGCAGCG
CTGCTGCAGCCTTTGCAGCCCGCTGCATGGCCCAAGAGCTCGCTGTCAGCAGAGAAGGGG
GGAAGGCAGGGGGAGTGGAGGGTGCC
>at00929
GGCGTCGACCTCGCACATGGGCAAGAACTGCACCCGGCCTGAGCCCTTCCAGCTCTCCAG
CACGCGCTGGAAGTAGGCCACAACCTCCACTCCTGAGGCCAGCTCGTTGTCGAAGTCCCT
CCTCGGGGCACTTCTCTCCAGCTTCATGGAGTTCACACCGTAGAAGCAGCTGGGCTGGTG
CAGCCGGACGTACGGGTAGGCATCGTTCCAGTGTCCTCCCGGCAGCCCCCGCTTGTCCAC
CACCAGCACCTTGTAGTCCGACGTCCGCGCCAGGAGCTCGTCGGTGAAGGCCATGCCCAT
>at00930
ACCTCCGCTGAAACCTTTGACCGCCGGGCCCCTGAACGGTCAACAGTTGCCCAGGCAGGA
CCCCAGCCGGACATGTCACCTGCATATACTGCTGCACCGGCACTGGTGCTGCAACAGGCA
TGGCCGTGGCGACCGGCATGGCTGTGGCCTGTGGAATTGTTGTGCCACGTGGATCCACCA
GGGCGGTACCGGAGATCAGGCCTGCTGATGCACCGGCCAAGGCAACCCGCGAACTCCGCT
TCTTGTGCACGAAGGAGACAGCAAAGAGAAGGGCAACGGCTGCGACCAAGAGAAAGATAC
CAAGCAACGGTCTGGCCACAACCCATGCAACTGCCACCGTCACGAGGAACAGACATATGG
CTGCAGTGAGTGCCATCGTGTGAATCACGCACTGTGCCGCTCCGTCGAGGACTGATCCCA
GACCGCAAGTGCAACAGTTCAACATCCTCAAGAGGTCCGCAGCAACTGACAGCGGCTGAA
GGATCAATTCAAATGCGATGACAATCAGCACAAACGCCACCAACCGCCTCAGCCACACGC
TGGCGTTGATTTCACTGTTCCATGCATTGACAAACTCGCTCACGGACAAACGACCTGGCA
AGAGCTTCTCAAGCGGCTTTGTTTCCTTGCCAAACAAGCCAAACCTCTGGGGAGGCCACA
CCCCAAGCCTGTAGTTCATGCTGGGAGTGTCTGCTGTGCTGCTCTGCTTGGCGGCTGCGG
TGGCTGTTGTTGCAGCCCGTCCTTGCAATGAGATGCGCAGATCCCCGACCTGAGGTTGAC
TCGGGTACGTGGTCAGGTACCCACCGTTGACTTGCAGCATTTGAGGAGTCAGACCTCCCC
CCAACCTTTGGTACGACGCTGTCCAGCCCTGTGAGAAATGCAAGAGCCTGCTGGCTAAGT
CTGCAGAAGGCAACTGCTGCTGAAGGCTGGAGTCCAGAGCCAGCCCGTTCTTGTTGGCGC
CGTCGCGCGACAGCACCACGCTGTACTCCGGAGCCACCAGTGCCCCGGACTGCCGGAGGC
CCTGGGGGAAGGTGCGCATGTCGTTGCTCGGGTGCTCGGGGCAGCGGAAGCCGGAGGAGC
TGATCCAAGTGTCGCTCCAGGTGAGGCTGCAAGTGTAGTACTTGGTGCAGACCTGGCCGC
CCTTGTTGTCCTTGTGGCAAGACTGGTGCTCCTCCTCCTTCCACAGGTAGACCTCCGTGC
TCCACCGCAGCTGCGAGCCATTGAACGCCGCGAGGAAGGGGCTCAGCGTCTGAGGCAGGC
CAGGCGCCAGGTTTGGCGCGGAGACGCTGCAGGGCGCCAGCACGAGCTTCCCTGCATCGC
TCGCGCGCGGCACGCAGTCGTCCAGAACGTCGGCGTTCCAGACGACCTTGGCCGTCGCGT
AGCTCACGACCGCGTCGTTCTCGGTCCAGAAGATGAACGCCATCGAGCAGGGCACCAAGA
TCAGTCCCACGCACATCGCACAAAACGACTTTGCCTGCCGAGAGCACCAACTCTCATGTA
TAACCTCCATCTGGCCACAGCTCCCTTCGATAGCAGCTTCACCCTCTGGTGCACTGAAAG
TTTAGTAACTCGCAAGCCCTTGAGCAGATACTTGAGCCAAAATGGCTACGG
>at00931

AGTCTACAGCTTCCAGGCCTTCACGGAGGCATTCTGCAGGCAGTTTATCGAGGAGCTCGA
GAGCTTCTATGCGTCAAAGCTACCGGCACGACGGCCAAACTCGATGAACAACTACGGTGT
AATTGTCAACGACATTGGGATGGAGCACATGATTTCCTCTTTGCAAGAGAGGGTGCTCCA
GCCTTTGATTGGGATGCTCTTCCCTTTAGAAGGGTCAGAGCTCGACGGGCACCATTCGTT
CATTGTGCGATACAAGCAAGGCGAGGACACGCACTTGGACGTGCACCACGATGACAGCGA
CGTCACCTTCAACGTTTGCCTAGGCAGAAACTTCACAGGTGCAGGCCTCGTCCTCTGCGG
CAGGA
>at00932
CCGTAGCCATTTTTGGCTCAAGCCATTTTGGCTCAGTGCCTGCGGCCCCGTCTGCCCCCA
GCCCTGTCTGCCCCAACAGTGACTAGAACTTCGACGCCATGAGCAAGACAGGCACGGTCA
AGTTTTTCAACGACGAGAAGGGCTTCGGCTTCATCGTGCAGGATGACGGCGGCGAAGACC
TCTTTGCCCACAGAACCAAACTTTCGCGGACGGCCAGAACCTCGTCGAGGGCGACGCGGT
GCGCTACGACGAGGCGTGGGACGACCGCAAGGGCAAGAGCCTGGCCACGAACATCACCGG
CGGCAGTGGTGGCGAGGGGAAGGGCGGAGGCAAGGGCTTTGGAGGCGGCAGGGGCTTCGG
AGGCGGCAAGGGCTTAAGGACGGCGGCAAGGGCTATGAAGGCGGCAAGAGCAAGGGCGGC
GGCAAGAGCAAGGGCGGCGGCAAGGGCTTCGGAGGTGGCAAAGGCAAGGGCGGCTACGAC
GGTGGCTTTGACGGAGGTTTTGGCGGTGGGTATGGCTACTAAAGGGAGCTCTGCCATGCT
GCCAGTGGCAATCAGGGAATTGATCTTATGGGGATTGGTGCCGCTTTGCGCTTAACTGTG
CGGCAGGGATCGTCGAGGCGCTGAGGTGCATCGTCGCGGCGCTCCTCTGAGGGGTTTACA
GATATGATCACTTGTGTGTTATAGGCATCATTGTTGCTTCCGCATGCGACTATGATTTGA
GGCTTTTCCTATTTACCGACCAACCAGGcAAAAAAAAA
>at00933
CCGAACTGGCCGAGGACGTCGGTCAGAGGAGTGGTAGGTTCCTGTGTCTTGATAGGCTGC
TCCATCAACAGCGTGCCCTTTGGGAGTGCCAGCTGCCAATTGATGTACGGTGAGCTGTAC
CCGGCGCCGCCAGTGGACTGGTACCATGGCACGTCGGGGTTGCCGCAGCCGAATCCCACT
GGCTTCTCGTACCCCAAGCTGCAGAGCACAGCCTCTGCAGAATTGGAGGGCCTGGCCAGC
AGCCCTGCAAGTCCCAGCAGCGGATCGTTTGCCAGAATGGAGAGATACATGAAGAGCGAG
TATGCACGACCCATGCACGAGTTGCAGTCGGTGTCCTTCTACACAGTTGCCGCAGGCGAG
AGCT
>at00934
CGCGGCGGCCTTCGATGAACATGGACCTCTTGGCAGCTGGAGGCAGTGTTGGTGCGGAGC
GGCCGAATCCCTCTCTCAGTCGCAGCCAGCGGACGGCGACGTCTGGGTCGGTTCGCTGTG
GCTTTGGCTGAACTTGCGGAAACCAGGCATCATCGCTTGGCTGGAAGTCGTCTCCCTGTC
CGTTTGCACCTGCAGAACGGGGCCAAGAAGAAGCTATACATATACTCAGCAAAAGTGCAT
GAATCTGTTCCTCCCACCATGCTTGCCGAAGTCGCTAACATGAGTAAGGCTGCAAAGGTG
CGTAGCCCAGCTCTGCAGGGCTCGACCCAACACAACTTTGACGCAGCAAAGTCTA
>at00935
AAGGAGCAGGTCGAGAGACTTGGCATGGCAGCATCACTCTGTCGCGGAAGTCGTCCCCGT
CCTTATACGTTCCGATCTCGGATGGCAAGCCTGCCAGGTCCAAAAACATGCCAGCGCTCT
TGATAAGGCCAGAGATCGCATATTCGAAGCTGGGCCAGCCCCGCTCCATGTAGGGCTTCA
CCCCCTGTGGAACGACTGTCAGGAGCAGGGAGAAAACGACCTCGTGAGCGTACCACAAGT
TCACGTTGCGTAGAGCTCGACTGAACGACTCTTGCTGCTCGTCAGTCCTGGGAGCCTGGT
ACAGAGAGCACCAGTCAATGAAGGGGCAACCTCATCGACTTGAGTAAGG
>at00936
TTTTTTTTTTTTTTTTTGCCTCTGAGCCGAGCACCTCTGGACCCCTCGCTCGAGTCCCG
GGAAAATGCGCCCTGCCTAGAGTCCGGCCAAGCCGGCAGCATGCAGCGGTCTGCCCTCGC
AGGAGCTCGACGGCCACGCCTCCTCAGGCGCAGCAGCGCCGCCAGCTCAATCCGCTGCAA
GCTTGAGGATCTCGAGCACATCCTCCCAACCGGAGATGGGCCGGGGGTTGGTGTCCACCA
GCCGGTCCTGCATCGTCCGGCGCGCACAATCCTCCAGGAGCCCCCGCCCCACGCCCACGG
NCTCCAAGGAGCCGGGCAGCCCCAGCTCCCGCACGAGGCCCCGCACATGCTCGGCTGCCG
GGAGGCCCTCGGNCTCTCGTGCAGCAGCGTTGCCACCC
>at00937
CCATTTAGGGCTTAGGTCGGTCCCAGGTGCGAGCCAGTTCCTGCCATCTCTGTTTTTGTC
GTGCCATCGTTCTGACTGATAGATGGGCCTCGGCATGAGCAATGGCCGAGTCGTGAATTA
TGGTCAAAGAGAGGCTGTGAATGGCGGCAGCATGGTCATGGGCGGCTCCACCGTGGCGGT

GTCATGATGGCTGCCGGTGCGGGCCAAAAGGATGGGTGGTCGGGTTGGTGCTGCTGGTAG
CATGGTCAGCAATGGAGGCATGATGATGGGCACCGGCGGCCAGAGCATGATCGCtTcCCC
AGGTGGTGCCTCGtTTGTCGGTGGAGGTGGCTACGCCGGCAGCATGGTCGCCGGCGGCAG
TGGCGGCATGGGCTTGGGTGGCGGACGCAGGGTTGTCGTCGCAGGTGACGGTGACACATG
TGGATGCGGCCCGGGCGCCAATGCAGGCGAGTGCGGGGGCGCGTGCTGTGGAGCCGAGAC
AATGTGCTGCGAAGCCGAGGGGGGCGTGGCAAACGTGCAGTGGGCTCAGGTGACTGGCGG
CTCCTACATGCCGGTAATGTCATACCAGTATGTCGGGCCGGGCGCTGGCATGTACGAAAA
GGAGGTGGTGACAAGCTACCACGGATGGAGGGTGCGGAGATGTTGTATTGGCATCCTTGG
GCTCTTGCTCCTTGCCGGCTTATTGTATCTCCTGTCCGGCAT
>at00938
CACTGATCGCAAGTTCAGCGAGTTTCTTCATTCTCATTCTCATCATCAATCCTGTGACCG
CTGCTGGAAAGGGAGTTGGCACAGCGTGAGAGTTCAGCGAGCTTCTCCTTCAACATCACG
TGAGTGACCGCTGCTGGAAAGNGAGTGGGCACTGAGTGTTCAGCAAGCTTCTTCTTCTCA
TTCTCATCACCAACTGCTGCTGCTGCGCTGGCTGCGCTGCAGGCCTGGCAGAGCGGGCAA
GCGACGCCGCAGGCAAAGCCCCTCCCCACATGAACCCGGGGACGAGGAG
>at00939
GTTCCTGGACTTCACGGATGATGAGCAGAAGCGCATGTTGGGCTACAAGGGCCGCGGCTC
GCGCAAGGCGAGGGCGGCCGACCCTGCGGCCGCGTCCCTGAGGAGGCACAGGGCGCTGCC
GAAGTCCTTCAGCGCCGTGGCCGCCATGGCCGAGAAGAAGCGCGCGGCGAGGCTGCTGAA
CATCGTCCGCGACCAGGGCAACTGTGGCTCGTGCTGGGCCGTGGCGGCCACCTCCACGCT
GGAGGGCCAGGATGGAGGTGAAACGCCACGCTCATGGACCAGCTGTCGGCGCTCAAGGNG
ACTGACACCAAGGT
>at00940
GTCGAGCAAGCCGTTTACAGGTTGGCGATGTTGGTGGCGGACGAGGTCGATATTTCTCCT
GTTGGCAGTGGTAAGGAGACTATCCGCGAAACTGTTGGCCCGATCACGATGGAATATGAC
CCTGCGACGATTGGTAGCGGTGTCTCGTTCCCGTGGTGGGATGGCTTGCTGGGCCACTGG
ATTGACTCCGACGGCAACGCAGCGGGTAATTTTGACGTATTCCGGGGGTGATATGAATCC
TGCATTACTGGCGGCTATTATCGCCGCAAATACTAAAACGCCAAACCAGAACCGGAGCCA
GCACCAGAACCAGAACCACCGGTACAGGAGGATGATAATGGCGGGCTTTAATT
>at00941
TGGCACATTACGTGAGGTGCTGAGGAGACCACTGCAGGCACACATGCGTAGGTCTGCGAT
GTTTTCGATGGAGTGCTGGCCTCCAGAGACCGACCATCTGCGTGGAGAAGCCTTTGCCAA
AGAAGGAGGCAGAGCGCATGAGCCGCAACTGGCCGAACTGAATGACCTCCTGCAGCAGTA
CTTCAAGCAGCTCGGCCAGCACATTGACGGCCGCCTGGC
>at00942
TTTTTTTTTTTTTTTTTGCGACGCACACTAAAACGTAGAACTGCTTTGCCAATACACCG
TCTAACCATACGAGTGCAAACAGACTCCGGTGGAAGAGCCCATGTCAACACTGCTCCCTC
AGCTCGAGCGGCCCTCAAACTCGTTTGCGCCAAGGAACTCCTCCATGGCCGACTTCAGTC
CTGCTTCGATCTCCGGCGTGATCTGCTTCTTTTCCATGATCTCATCGAGGAGTGCTGCCC
GTGTGGACCTCATGTGGTCCAGCCAGAGCGCCTCGAAGCGGAGGATCTCCTTGGTAGCGA
CCTTGTCCAGGTAGCCGCGCGTCCCCGACCACAAGGAGGTGATGATCTCCTGGGTCTTCA
TTGGCACAAACTGCTTCTGCTTCAGGAGCTCTGTGAGGATGCCGCCGCGCAAAAGCTGGT
GCTGAGTGGAGGCGTCCAGGTCCGAGCCAAACTGCGCGAAGGCAGCCACCTCGCGGTACT
GCGCCAGCTCGAGCTTCAGGGTGCCAGCCACTTGCTTCATGGCCTTGATCTGCGCAGCGC
TGCCTACGCGGCTCACGGAGAGGCCCACGTTGACTGCAGGGCGGATGCCCTTGTAGAAGA
GCTCCGTCTCCAGGAAGATCTGCCCGTCGGTGATGCTGATCACGTTCGTCGGGATGTATG
CGCTGACGTCGCCTGCCTGCGTCTCGATCACAGGCAGCGCCGTGAGCGAGCCGCCGCCGT
GGGTCACCTCGTTCATCTTTGCAGCCCGCTCCAGGAGTCGCGAGTGCAGGTAGAAGACGT
CACCGGGGTAGGCCTCACGGNCAGGCGGGCGGCGGAGGAGCAGGGACATCTGCGGTACGC
CACGCCTGCTTCGAG
>at00943
TTCTTGATCGAGCGGAAGATGATGTTGGCAAGTGCCACACTCCGCCCTGCACCGCTGGCA
GATCACTTCATCTTGAACCTGGACTTGAGATCGTCGGCGGATAGTGGGGCTCCCTCCTCC
CATGTGTAGAGGTCCTGCAAGTACAGGTTCTCCTTGGTATACATGATGTTCTGCTCGTGC
GGCAGGCTGGAATTGGGGTCCACAGGCACGGCCTCGGCGGACTCAAGGTCAATGCCTGCA

GAGGAGAAGGCATTCAGGACCTTGCGCTCGTCCTTGCTCTCGTAAAACACGTGGACCATG
CTCGAAGCCTCCTCGCCAAGGCCGCTGCTCTTGGAGTACTCCGAGAGGTTCCAGACGCAA
ACGGAGAAGTGGACCTTGCCCTGCTTCTGCGTGGCCTTCTCCAGCATGCTGTCCTTGCAG
ACAATTTCCCGGCGCTTCAGGATCTTCTTCGGCATGGCCTTCAGCAGCCTCATGTACTCG
ACATCGTCAGGGTGAGAACCCCACTCCGCCACCTGGGCGACGCAAGGACCGGCATCTGGG
CGCCACATCTTGGAGGAGTTAGCTGGGCAGAGGGCTGGGAGTGGGGTGCCTGAGGGTGGC
TTCCT
>at00944
TTTTTTTTTTTTTTTTTGCCCTCCTTCACAGCTGAATGCGAGTCCTGCCGCCACCACCC
CTGTCCGGCTCGCGCACGTATGCACACTCCGGGGAGGCAGGAGACCATCAGCCATCCGCT
GGCGTGCCGCTCAGGCCTGCTCCTTGAGCTTTGCAATCTTCTCCGCTGTGTCATCCAGCT
CCTTCAACAGCGGTTCCACTTCCTCGTCCGTGAACCACTTGATGGGCTGATCTTTCCGAA
TCAGGACGACCTCAATGTTCTTGCCGGAGGCCTCCGTGACCTCCAGGAGACCCTTCAATG
CCATCCGCACAGTGGCATCATCGTCAAGGTCCTCCTTGTACTTGGCTTCCAGGAAGTCCA
CGACACTCTTCGAATTCTTGCCAATGGCTGTGGCCTTCCACGCGTAGTAGGTGCCCGAAG
GATCAGTCTGAAAGAGAGTGGGCTGGCCACGGCAATCCATGCCTCCAATGAGCGTTGAGA
TGCCAAACGGCCGCACACCTCCCCGGTGCGTGTACTTCTGCTGAGTGCGCGCAATGAAGC
GCGCCACATAGTCGACCGTAGGCTTGTCCTCCACGTTCAAGCGGTATGACTGGCACTCGA
CACGGGCCTTGTTGACGAGCACGCGCGCGTCCGCGTTCAGGCCCGCGAACGCGAGCGCAA
TGCCGTCGTCGACCGTCAGGATCTTCCGGATGGTCCGGGGGTCCTGCAGCTTCGGCGTGG
CGCGCTTCTCCACGGCCAGGCAGACGGAGTCCTTGCCCCGGACGCCCACGGTCGTGGTGC
CCTTGCGCACGGCCTCCAGCGCGTATTCAACCTGAAAGAGGTGCCCGTCCGGTGAGAAGA
CCGTAATCGCGCGGTCGTAGCCACCCGCCATCCCTTGAAGCAGCTTCCGACGGCTTGGCT
GGCAGTGGTGGACTTGGGGGCGGGGCCTCAGCACTTGAGCCAAAATGGCTACGG
>at00945
TTTTTTTTTTTTTTTTTAAGGCGAGGCACGAAGGCCAATGAGTGTTTCTGCCCTGGTCA
GCCGCCTTCAGCTCAGAGTTTGTGCCGCAACACGCGGTCCCTGAAGGGCTCGGCACCGTC
AGGGAAGAGCTCTGCGAATGGCTTGAAGGCGTCTTCAGGGTGGCAGCGCATAGGCAGCTC
AGGATAGAAGAAGCTGTACTGGTGCCAGAGCCGCATCTTGGCGAGGTCGAATGGCCCCTG
AATCTTGCCACTCGGGTCAACGTACTGCCATCCAGTGTCCGGACAATGAGCGAAACGGCC
CTTCTGGAACACAGGGCTCTCAAAGTCCCCGTTTCTCAGCCGCACACTCCGCCTTGGTGG
CGGCAGCCACCGCCTGGGGAGCCCACCGCACGGCGTCCCACAGGTCCTCGGTGTAGAGGC
CCTCAACCTCCAAGCCGTCCTCGCGCGCGCCCGGCGG
>at00946
TCACAATGCTTCGCGATCAGATCGTTGACGGCTTCCTTGCCTTGCAAGCCGAGGCCGTAG
CTGCCATCTCCAGTCTGCAATGCGGCATGCACCAGAGCCTGGACAAACTTGACTGGGTCT
TCCACCGGGTTGGGCATGCCCTTCATTCCATCCACGATCCCCTCAAGGAGCGAGCAAGAC
CGTCCTTCAAGGGGCAAGATGTCCATGACGTCCACGTTTGCAGCAATGGCCGCCCTGATC
CGAGCCACCGCCTGCGCCGCCAGTTCCAGCTGCGCTGCGCAAGCCGAGAACTTGAAGGCA
GGCTTGTGCAACGGTCTCTCGGCAGCGAAGCGCGCCGCTTCCCAAGTGCTTGCCCACTGC
CTGCGAAGCAAAACGCTGTGCGACTCAATGTCCACCATCGCGAG
>at00947
TTTTTTTTTTTTTTTTTCCCAAGCCACGGATGAATGCAAGGAGTTGAAGCCGCATGCTC
GTTCTGCCTTGAGATCATGCCCCCCTCCCTCTGTCAGAGATGTGCCGCAGCCTCCGGCTC
CCCCTCCGCAGCACGGGAGTCCAGAAACTTAAGAGGGCGTGCCCAGCATCGCGCAGCCAC
ATACTTGGAGCCGTGTGGCCACGCTGTCCGGAAGAGCGGCGGGACAGCAGCCCGCCGTGG
TGCGGGGAGAAAAACAATGCGCGGCTCGGCGAGCGATCGAGCACCAGCTGGCAGTGGAGC
TCAGGTGGCAGCCCCCAGCACCAGTCTGGACCTCGTCAGCCTTTGCTCTCCTCCACGAGG
TGGTCCTCCAAGGCCCGCACGGTCGGGAAGTCGAAGACCAGCGACGGGCTCAGGGCCATC
TGGAACTCCTTGGCCACCATGCTCATGAGCGAGACGCTGGAGAGGCTGTCCATGACCGCC
TCCATGAAGGGGCTGTCCAGCTCGATCTCCTCCTCGCCCGCCACGACGTCCTTGACGAAG
GTCAGGAGCTGCCTCCTGACGAACGCGGGGTCCTCGTGCC
>at00948
TTTTTTTTTTTTTTTCTCATTGCAAGGGTTCACACGCCAACTCCAGCAACACGGGTACT
GCGGCAGGGGTCAAGGGTCAGACGACCGACAACCTGGCCGTGCCCGGCAACCGTGCGTCC

GCGTCGGCGGCGTCGCTCCAGCCCTCGCGGACAGCTGTATGCACGAGTCGGCCGAGGCCG
GGCGCAGCAGCGTCGCGCCAGTGCCGGCTCTCCCCTCGTGCC
>at00949
GCGCGGGAGGACTTCCTGCGGGAAGTCTCCATTCAGGGAGTCGTCCAGAAACACCTCGGT
CGCATCCGGCCTGTGCTTGTTGGCAACGCGAAGCCCTCGCTCACAAAGGCGCACCTGAAA
TTCCTCATCTGAAGCTCCATGCCACGGCGATCCCTTCAGCAATACCCGCAACACCATGGA
CCCCATAGTCTGCTGCCATCGTGCTTTGCCGTGTGAACCGCTCCCTGGCGAAGCCCAGAA
GTCGCGCAAGCGGTGAACCTCCTTCGCAGCAGCGGCAGCTGGGCCCAGCGCAGCGGGCGG
CCCGGCGGTGCTGGGCAGCCCCTGCCCAACCGCCATGGCCCGCTCAGCGGAGGGAGGGCA
TGGAGGGGCGATTCTGTGCAGACGCCTGCGGCTTGAGCCAAAATGGCTACGG
>at00950
TTTTTTTTTTTTTTTTTTGCAGTCCAATGCACGCTGACAGGGACTGAAAGTAGCTCATTG
GCAGATCACGAACTGGTACACTCAGTGCAAGTTGTAAAGCAAGTGGCGGTGCAAGGCGGG
GAGAAGCAGTATGCCTTGCATTCGCAAAGACACGTGGTGGTGGTCGTGCTCGTTGTCTCT
GAGGTGGTCGTGGTCGTGATCGTTGTGGCGCTTATGGTGGTGGTGCTTGTCGTTGACACT
GTTGATGTGTAGGTGGTCGTGGCACTGGTGCTCGTGAATGAGGTGCTTGTGGATGTGGTG
CTACTTGTTGTTGTGAGGGTTGAGGTCGTGGTGGTGGTCGTGGGAGCGCAATGTGGTGGT
GCACCTTGGAACCCCACACACGTGTATGATGTGGATTGACCGATGTAACCGGCTGCACAG
ACAACGCTGCAAGCCTCCCCTGGAAACTTGCCCCAACAATCGCTGGTGTCCACGGGTACG
TCGTCTTCAAACACCATGCAGCTTCCAGGAGGTGGTGTCAGTACCGGGGCTGAAGTTCTT
ATCTCCGTCGTTGGTGTTGCAATCGCCAGCGTGGACGCTGGAGTCGTGGTTGATGTGGGC
GATGA
>at00951
NNNNNNNNNNNNNNNNNNNNNNNNNCCAAAGAAATGAAAGTAATTAGCGGCTGTGTCATT
CTTTTCTTACTGGTTCACGAGTCATTTCCATTTGATTTCTATCTTTTCATCTCTCGTGTT
TGTGAAAAATGAATGGTTTCTTGTCAGCTGTTAATCAATTCTTCTTTCATTTCATCTTTT
TCATCTCGTGATTGAAGAGATAATAGGAACATAATAACAAATCTTAGACCAGGCGTTAAC
AGTCTACTGGCTCTCTCTTCTTTCTCTCCATCACTATCAATTCTTAACGCCTTTACGGTC
TGTCAATCTTTTCTCTCTTGTGAATCTATAATCTCTTGATCTTTGGTTTCATAAACTTTT
CTATCTGTCAAAGGATTCTAATGATATTACCAAGAGTTAAAGGATGTTTCAATAATTATA
CCAAATCTTAAGGTCTTAAACCTGGAATGAAAGAGTGATAATTTCTATTTGGTGAAGTGT
AATCTTTTCCGATCTTGTCTTCTTCTCTTTTTCCTTTAATTTCTATCTTTTTGGAATGCA
TTTTCTAAACATCTCTCTGATTCTCTTTCTTTTACTTGAAAACTTAGAATCGATTCATGA
GCTGTAACACTCTTCATTTAATGGTAATTCTCTTTTCTGTCTGTAATCTTTTTCTCTTTT
GGATAATTATCGTATGGCTCTTGATGGTGACCGCGCGTTCTCCTGTTAATCGATCACAGT
AATTCTTGAAGATTTCTAAAATAATTGGGTGAATTTGTTAGGCTCTTTCCCTTATTCTCT
CTAGTGTATTGGGAATGAATAAAAGTAAAGC
>at00952
CCCGCTCGTACTCTTTGATCATCTGCAGCTTCACAGACCCATCCAGTGCCAGCGTTGCCA
AGGGCACATCGTTCTTCAGTAGCTTGGTGCCACTGAGCAGTTTGATCCTGGATGATGGCT
GCGAGGTATCCGTCGCAACACGCACCTTCAAGTCCAAGGCCTTGTCATCCTTGCTGCAGG
CATAGGTTGTAGCTGCGCCGCCTAGAGCGGCCACCTCCACCGAGATCATCGCTTTCAAAG
CGGCAGGACCCCAAAGGACGCGAATGCAACGGGAGCACAGCTTGAGCACAAC
>at00953
TGCCCAAAGCAGCAGTGCTTTCTTCCAGATTAGGGCAGCAGTGGTTGTGGGATGGCTGCC
CGGGCGGAATGCTCCGGATACATCAGTGCCACCGTTCAAGGTCCCATCGCAGACAGTGAC
AAGGGGACAATTGCCAAAGTGCAGGACAACAAGATCGACTTCTGGCCTTACTGCAGCGCT
ACTCAGGTCACAGTGGAGGACCCTGGAAGTACGGAGTTCAGGACTGCATACAACAAGGCA
CTCGCAGAGATCAAGGAGAAGTACGAGTACGGGCAGGCCGAGTTCAGCGATGACGACATG
ATGAGCCTTCTGACGCCCGAGGAACAGAGGAAGGGCTGGAGTTATCCCCTGACGCGCACG
CAGCAGAAGAAGGAGTTTGGCTGCGTCGTGGTCGACCAGGAGCGCTTCGTCGCGCACCTG
CAGGACGGCGACCTCCGCTTCGTGCAGGAGACTCTGGGCAGCGCCAAGCGCAAGCGCGCC
ATCGACGTCAACCACTTCGACCGCGCGGGCCTCACGCCCCTGCACCGCGCCTGCCTCCTG
GACCACGGTGAGATCGCGCGCGCCCTCCTGGACGCCGACGCCGACCCCTCGCTGCAGGAC
CGCGCGGGCCTCGG

>at00954
CCATTTGGTTATGCCGGGGCCAGCCCCGGGCCGGTCTTCTTCGCGTTGGCCACAAGGCCT
CCGATGCCTGATGGCGATGGCGACCGCAGCCGTTGCACGGCTCAGCGCCGACATGCAGCA
GACGCGGCACACCTCCCAAGCCCGCGCCGTTCCTCGCCTTCGGTGCCGCTGCCGGACCGG
GAGGAAGCGGCCAGCTCTCTGTCCAGGACTTCTGAGCGGGGAGGGAGCAGCGGAGGGCAG
AGCGCAGCAAGAAAGAGGAGTCTGAACACGGGAGGGAACGCGGCAGTTGCAAAGGAAAAA
AAAAAAAAAAAAAA
>at00955
GCTCCAGATGCAATCCACACACAAGACCACAGGGAGTACCTTCTTAGCATGCGCAAAGGA
GAGAGCTCTGAACACACTTCTGCCAGCTGTACTGCCAgGaACATGCTTGCGGCAAACCTC
TAGGGAAGACGTCGAGCGTAAGGAGGTCCAGCCCGCAGGTACGTCCTTTCT
>at00956
CGTAGCCATTTTGGCTCAAGCGCATGGCTCTCAGGCCCTCCTCTCTGATGGCCGCGATCC
CACAGCGGGTGCGCAGTCGGCCCTGCGCGTCGGAGGCCCAGGTGGGGCTGCCTTGAGCCT
ATGGTGCGGCTGCTGGACACTGACTTCGAGGCTGGCCCGGAGCTCTTGCGAGCGGCGGCG
CTGAGCCTGGGTGCGGCCTCTTGAATGGCGGGCAGCGGCTTTCTCTTGCGGCTGCTCGGT
ATTCCCCTGCTCTCGAGGGTGCTCTTCTACTTGGTGGTGGGCTTCCGGCACGCCTTCTTC
ATTGTTTCCTGGAACGGCCGAACGGGACAACGTGAAGCAGCTGACCAATGCCCTGGTCTC
CGCC
>at00957
GAGTGGTGGTGGGGGTGGCGGAGGAGGAGGCCGAGGCTACAACCGTGACCGCGACTGGTC
GACCGCGATCGTGACCGTGACAGGCGCTATTGAAGGTGGCCGTGTTCGTGACCGCGACTA
CGACCGCGATCGTGACCGTGACAGGCGCTATTGAGAGCCTGCCTGGTAGTAAAACAAAAG
TTAACAAAGACAAGTCTAGCGGAGTCTCAGTGCTGAGAGGGCATTCGGGCG
>at00958
TACGACTACGGAGCGCAGGACATCGTGGTCAGCCTGAGGACACGGCGCTGCGCTCGTGGA
GTGGTCGCCCTCAGCGCTCTGGATCTCCCCCCGCGAGAGACGAGGGCGTCATGGTGCACG
CGAACCACTGCGTGCTGGGCTCTACGCTGCAGGACGGGCGGAGCAGGCGCCTGCGCAAGA
TCTTGGAGGAGAGCAGGTGGAGGCAGGCGGAGGTGGAGGACTTCTACGCTGGAGAGCTGC
TGTCTGGGTGCTCCCCGTCCTTGAGCATCCCCCGGATGAAGGCTGCACTCTCGATCACTG
CCGTGCAGAATGACAGCGTGTTGGCAACCATCCTTGTGTCTCCCCGTGAGCGGGCACTGT
ACGTGCGGTTCAGGCTTCAAAGTGAGGGTATGGAATTCGTCGAGAATACAGTCGATTGTG
ACTTCTGGCAGCAGTTTTCT
>at00959
ACTCAAACGAGCAATGCTTCTGCACACATCCGCAGGCCAGGCAGCGTCTGCGACCACAAC
GCCGGCATTGCATGAAAAATTGCCGCGCACCGGCGCGCTGCAAGGACTGCATGCGCGCGC
GCACGCACCGGCGAGCCACGCAACATCGATGGCACATGGGAACATCTCGCCACCAAGGCC
AAGATACTTCTATCCGCGTGCAATCTGTAAATTCTGACGACAAACCTTGGCAAGGGCTCA
CCAGGGGGACTCCACCCCCCTAGGACCCCCGGCCGCAAGAGGAGGGCTACCGGCCACCTA
TGGATCAGGGATTATCTCTAAGAGTGCCCAGGAGGCCTCTCAGAGCAGAGGCGCCATGCA
GCCACTCACTTGTCGCAGAGGGCGGCCACACCGGGAAGGTCCTTGCCCTCCAGGAGCTCA
AGGGACGCGCCGCCGCCAGTGCTGCAGTGCGAGACCTTGTCCTCGGTGTCGTACTTCTTG
CAGGCCGTGGCGGTGTCGCCGCCCCCGATGACGGTGATGGTGCCCGCGCTGGTGGCCGCC
ACGACGGCGTCCATGAGGCTCTTGGTGCCGGTCTCAAACTTGGCCATCTCGAAGACGCCC
ATGGGGCCGTTCCAGATGATGGTCTTCGACTCGTCGACGGCCTTCTTGTTCATCTCCATG
GACTTCTCGCCACAGTCCAGGCCCATGAAGCCATCTGGGATGCCCTCCTCCTTGGTGGCG
GTCTTGATCTCGCCATCCTCGCCAAACTTCGAGGATATCGTAAAGTCGACGGGCAAGATG
ATCTCTACGCCCAGGTCCTTTGCCTTCTTCATGATGTCGGGTACGATCTTGGCCCCCTCC
TCGTCATACAGGGAGGTGCCAATGGCCATGCCGTCGCTGACCTTCAGGAAGGTGTACGCC
ATGCCGCCGCCGATGATCATCTTGTTGACCTTGTCGAGCATGTTCATGATGAGCTGGATC
TTGTCACTGACCTTGGCGCCGCCCAAGATGGCAAGGACGGGCTTGGCGGGGCTGTCCAGG
ACCTTAGCAAAGGCGTCCAGCTCCTTGGACATGAGACCCCCGGAGCACTTCACGTCGAAG
CCTTCGCCCACCATGGAGCTGTGCGCGCGGTGGGCGGTGCCGAAGGCGTCGTTGCAATAC
ACGTCCGCGAGCTTGCGGATGGAGGCGCGGAACTCGGCCACCTTCTCCTTGTCCGCCTTG
AGCTTGTTGCCGTCCTTGTCCACGCCCTTCCCCTCCTCCTCCACGTGGAAGCGAAGGTTC

TCCAGCAGGAAGACCGACCCGGGCGCTGGGTCCGCGCAGGCCGCCTCGACCTCCGCGCCG
CAGCAGTCCGAGAGGAAGGTCACCGGCTTGCCCAGCTTGTCCTCCACGATCTTTGCAACG
GGCCTCAGCGAGAACTTCTCCACCACGCAGCCGTCCGGCCGGCCGAGGTGCGATGCCAGA
ACCACGGACTTGGCACCACGGTCCAGGACCGCCTTGATGGTCGGGAGCGCGCCGTCGATC
CGCTGGGTGTTGGTGATGATGGTGGGGTC
>at00960
CTGCCGTTCCTGCCCAGGGTACCCTCTGCCTGCCAGTGGTGGCATCGACTTCGCGGGTCG
TTCATGATCGATCAAGAGGCCCACACCCTTTCTCGCTCTTTGGGACACGCTCTGCTGCTA
CCTCTTGGGCCTGGTGGGGACGATGCCGGACAACAGGTGCTCCCGGACCTCTTTTCNGTG
GAACGATGCCCTGTCCAAGTTCAGGTGCAGGGCACAGTTGAGGCCTGAGAAGCTCCCTCG
CTGGCTCCCGGGTCTCTCTGTGCCAGAGCAGAACGAGATGGATGATGTCCAGGAA
>at00961
CCGTAGCCATTCGGCTCGAGCGCGCTGCCTCTGGCTCCCAGTCCGGCGCACAGAAGACTG
TTCCGTGCAGGCCAGATGGCAGCCAGTTCCCTCCAGGAGGCCGTGCCCTTTGAGAAGAGG
AGCGCCGAGGCGAGGCGCATCGTGGCAAAGTACCCCGACCGCATCCCGGTGATCTGTGAG
AAGGCTGCAAGGTCTGACCTCCCTGACATCGAGAAGAAGAAGTTTCTGGTGCCGGGTACC
ATGCTGTGCGGTGAGTTCAAGTACATCATCCACAAGCACGTCAACCAGACGGCTGGCGCG
GCCATCGCTGCTGATCAGACTATCTACCTCTTCGTCAACAGCACGTCGCCCAAGACTGGG
GCACTGATGTCAGAAGTCTATGACCAGTACAAGGCCGATGATGGATTCCTGTACATCACC
TACAGCGCTGAGAACACTCTGGGGCTTAGGTAGGAGGGGCGCTCCCTCGGAGCACAGGGA
GTCTGTACATATGGGCGTACAGAAGCCATGGACGGCAGTCCTGTGTGCTGGACAAAAAAA
AAAAAGAAAAAAAAAACAAA
>at00962
TGAAAGGGAAGGGGCAGGTCTTTCGGACCAGTGCCCCGGCGAGAAATCACGACCTCGCCA
CGGCCGTCGTTAAGGCTTGCCCTCTGCTAATGTGGAGCCTGAGGCTGGAGTGGAATCGCT
TTACGTCACGTGGTTGCAAGGGTGACGAAGGTGGCGGCCGCGGCCGCTGAGGTGCAGAGA
CGCTTCTGCGTAGGTTGCTGTGCATGGCCAGACAAACTATCATTGCAACTGCCACTCGAG
CGCGGCACGCCTCTTTGCACATGACACGAGGTCCTTGCAAGGGGCGATGTGGCAAGCATG
TGCTTAGATCCAAGCGGGGGAGGCATAACCTTCCAGCGCAGATGACGCGGTTGATTTGTG
TGCCGCGTGCGTGTGGTGGGACGATGAAAGCTTCCCGCAGGACACCACATGTTTGTTGGC
TGAAGTGTCAGAGACTTGATAGTGACCGGCATCATCCAACCCTGGCAACGCTGGCTTCTC
TTAGCATTG
>at00963
CCGTAGCCATTTTGGCTCAAGGCGAGGCTCGAGTCGTCCGATCTCCCGCCCCCTCTCCTG
CCAAGAGCCTGCTAGGCGCCAAGTAGACATGCCGCCCATCCAGCGCCGCGTGCTCATCAT
TGGTGGCGGCTTCGCGGGGCTCGCCTGTGCCCGCGACCTCCGCGATGACTTCCTGGTGAC
CATCGTGGACGCAAAGGAATTCTTCGAGTACACCCCAGGAGTGCTGCGGGCCTACGTGAA
GCCCAAGCACCTCGACGCTCTGACGTTCACTCTTCAACCAGTCATAGAGGAGAAGATGAG
CTGCAAGTACGTGTGGGGCGAGGTGATGGAGCTGAATGGAAAGGAGAAGACTGCCACGAT
CAAACCCA
>at00964
AATGATTTCAATATGCTTGCTGTTGCTTTGTAAGTCTTGCAAGCCAAAGCTTTCACAGAT
GTCAGGGACGGGCATCTTCGGTTTGGCTCGCCGAGCTTTGCTGCTCGTCTGTGACTTCTC
CGCGCTATTAGTCTTTGCAGCCGGATCAGTGTGTGCCTACGTAAGCTCTGCACGCGGTGT
GTACATAAGCGTGGTTGGAGAACTGCCGAGTGGGCTGCCCCCGTTNACAGTCCCTACATT
GCCGGAGGTCGGTTCATTGCAGGAGTTGGCTCCTCTTGTCTTCTCGATGGCGTTGTTGAT
CTTTGTCATCGCGATGTCCGCGGGCTGCAATCATTGGCGTCTCCGTCCTGGGCTGCTTCA
CGTCCAGGAGTTTGTGGATGGCCTACTTCGTCGCGCCTGGCG
>at00965
CAGCATGGGTTGCTGCACTGTCCCTTTGGCGAGGCGCCACCCTGGCGTGCCTCTCCATTC
CAGATCTCATCCCTTCGCGCGCCCCACTGCCTCGCGAAGGACTTTCCCATGTGCTACGCC
TGCTTCTGCTGCTGCTGCTCCCTCCTTGGTCACGATCGCCATTTGGCTCACAGCCCCGGT
GACCAGTGCTCTCGCATCGTCCATACCCAGTGGATTCACTCGAGCGGCGCAGCCTTTCCC
CAGAGCTGTGATGGCGTTGATGGCCACGTTCTGGGTCGCACTGTTCAGCTTCAGCCCGCT
GAGCTTCCGAGTCTCCTTTCATCAGGTT

>at00966
CCGTAGCCATTTTGGCTCAAGCATGCAGCCACTTCTGACCAAGCGTAGGGGCGCTCCGTC
ATTGAGATGGTCGAGACGTTGATGGACGTGAACGACATCCGCTTCACGCAAGAGCACATC
TACGACGCCTTCAACGCCAATGACGAGCGAGCCAGCGGCCTGGCGGAGTTCATCGAGTCC
ATACTCAGTGGCCACAAGACGCCCTTGGACATGCCCCTGATACGCGTGGCCGTCAAGAAG
GGCGCATATTGGTGCGTCGACAATCGGCGGCTCTTCGTCTACAAGCACTGCCAGCTGGGC
CGCATTCCCGTGCAGGTCTGCGCGTGGAAGGAGAACCGCGAGTTCGAGCTCAAGTGGCGC
AACGGCCGCTCCGTGCGCGCGCAGACCGGCAACGGCGCGA
>at00967
TTATGCAGATCTCTGGGGATCAGGCTGAGCAGCAGCCCATGGACATGTGGCCCTTGGGTG
CTGCTGCTGGGCCATTCTTTGACATGAAGGTCTCCTCTGGCTTGGTGGACGTAGGTGCAG
TTAGCGCCATAGTCCCAGCACTCAAGAGTGCAGCCATGAGGACTGAGCGTTTCAGTTTCT
AGGATGATGAGCTGGCTTGTCATGACGACGGTGTTGAAAACTCTGGGCTAGAGCATGCTG
TCCTTGTGTGCGGCTGTGCTAATGCACATGGCCTGTTTAGGCTGTGGTGCCAATGCGGTG
CTAATGCTGCTTCCCTCTGTCTGCATGCACTGTTAATGCGCCTTCCTTCTGCAAGTACTG
TGGCTGAGCTTGTACTCCTTCTGCCGACGAGGACCA
>at00968
TAGTCCTATTTTNGGTaCTTcCAAGGCCGTTTGGTGCAAGTTCTCTGACTTGGCTCCGCC
CGTGTTTGCCCTACCTGCTCAGCCACCATGGTGATGCGCAACGCCGGCTCCCTCGTGACC
ATTGCCCTCCTGGTGGGCGTGGCTTGCTTCCTGCTGCCCATGCCGGGGTATGAGTCTTTC
GTGGTGCCGGAGGCCAGTCGCTCCTTGCAAAATGCTGGAGCTACTCCTTCCGCATTTGCC
CAGGACTTCCAGCTGCAGGCCCGGTCTTTGCGGGTTGCTGCAGCTGCAGGCCTGCCACCT
CAGCCGGCAGACAACGGACAGCCAGGCAAGATCAACCTGCTCTTCTTGGGGCTGCTGGTC
GGCACTGCTGCGATTGGCTTGATCGCCTTGTTCGCCTATGGTGCATACTCTGGCTCTGGA
AGCTCCCTTTGAGTTTGTAGCGGCATGCTCGGATAGGATGGCGTCATTTGTTCGCCGTAG
TGCACCAGAAATAGAATTGTGTGAGCGGGGTAGG
>at00969
CCGTAGCCATTTTGGCTCAAGCCATTTCGGGCCCAGGCAGGCGCGCTTCGCTCACACACG
GCCCTCGCCTCGCGTGCGAGCTCCCCACCTGCGGGGCAGCACCCTTCGTTGGTCACAGCG
GCTACTCCGGCAGCATGGTCGGCGGCGCTTGGGGCGGT
>at00970
TTTTTTTTTTTTTTTTTTGCCGCCCGCGGCGCCGAACCGACGGCGGCCTCTCCCGCGCCG
CCCGGCCGCCCCGCCCCACCGCCCCGTCACAGCTTGTGCGCGGGCACGGGCGAGCGGGAG
CGCGCGCCGGAGGCGCCGCGCGCGTCGGCCTTGTCCTCCTGCTGCCTCCGCAAGCGGAAA
TACAGGTCGTTTGGGAGCGCGTACTGCCGGACCTGGTGGCCCCCGTCGCAATCCAGCACC
ACGCCGGTGGTGAAGCTGTTCGTCGCGAGGAAGTACAGCGCCTCCCCAGCCTCGGATGGC
CGCCCCACCCTCAGCAGCGGCAGTGACGCGCCCGTGGCCTCCAGCATGTTGGCCCGGATC
TCCGGCTGCATGTGGTCGAAGCGCTCTGTGTCCACGAAACCTGGGGACAGGCAGTTCACG
CGCAACCTTGGCCCCAACTCCAGAGCCAGCGCTTTCGTCAGCGCCTCCACCGCGCCGTTG
GCGGACGCCAGCGGCGTGCACTTCTCCCCCGGCCGGCGGCACAGCACGCCCGAGAGGAAG
ACCACGGCGCCGCCGTCACGCAGCCGAGGGGCGCCGTACTTGGCGCAGTACCAGGGCCCC
CAGAGCTTGCCCTCCATGATCTCNCTGGCCCTGGCCACGTCGAGGTCCAGGAAGTTGCCG
TGCGCCGCTCGACCCAGGGCGGTCACGATGAGCGCGTCGTAGAACCCCGGCTGCACCGCG
TCCTCGTCTTGGNAGAGCGCTTCACCTGCTCCTCGTCCTTGCAGTCCAGCGCCCTCGTCC
GCACGGGCCCCCCACGGCCTCCGA
>at00971
TCCCCGCTGAGCAGCACCTCGCGGATGGATACGCCAGCGACGACGACTACCGGCCGCGGC
GGCCAGCTCGCGGCTTCGGCGCCTTGCCGGGGGATCTCGAGCCTCGGCGGAGGCTGACCC
AGGAGGAGGTGCTCGAGTATGGAATTCGTCAGGCCTCTTCGGGGCCCTCCACATCGAAGG
CCCACTCCTCGACGCGTTCCGCGCTGCCGGCCAGCGCCCTTCGCTGGCGCAGACCAAGCC
GAGGACCGCGATCCAGCGCCACCACGCCCTGCTCCAGTCGCTGCGCTGCCCGGAGCGCAT
GAAGACGGACCTCGACATGCTGCGCGAGAGCCACCGGTTCCTGCGACGGAGGAAGACGAC
GACGGGTCCTGGGAAGCCAGGCTTGCAAAGCGGTACTACGACCGGCTATTCAGGGAGTAC
GTGCTTTGTGATCTTGCAGGCTACCGGAAGGGCAAGGTAGGGTTCCGGTGGCGCACCGAG
TCCGAAGTTGTGCAGGGTAAAGGGCAATTCCATTGTGGGCACAAGCCTTGCGAATCTCGA

TTGGGGTTGAGAAGCTATGAGGTCGATTTCAAATATTGCGAGGCGGGATCCCACAAGCGA
GCCCTCGTCAAGGTGCGGCTGTGTGAGCTTTGTGCGTACAAGCTGCACTACCGGAGCCTG
AAAGCTAGGAGAAAGCGTCAGCGACGCGAGGCAAAGAGGGAGCGGAAGCGTGCCAAGAAG
TCCGACTCTCCTGCGATGCTTGATGTTGACAGTTGTTCAGAGAGTGCTGCAAGCGAAACT
CCGGACAAAGGAGTCCAAGAGCAGCGTTGCAGAAAGCGATAGACATGACCTCGATGATGC
TGTGCAGCCAAGCGAAGAGGACAGACGGCTTCTCGAATCTCTTGCCTGGAGGGGGCCCGA
CCCGAAAGCTCAAACGCGACAAGACGACTTTGACGAGTACTTGCAAGAGCTGTTCATGTA
ATGCATTTCATCTTGTACTAGGGGTGGGTATGGTTACCGGTCCTCCATCTGCAAAGCGCA
GCCCCCTCACTTCTGTCCGGCCTAATACAACCTTGAATCCCACATCGCCCTCCTTTTGGA
GCGACCGCTTTTGGCTGACGCGTACAGTCCTTCCATGAGAAACTCTTATGCCACGTATGG
ATGCCTGGGCACACGTTGGCTTCCTTGAGCTCAATTCATCATGACGA
>at00972
CGTGGCCGACGTCCCTCAACTGGGGCTCGGCACATTCGACCGCGTGGTGGACAACCCAAG
GCCCAAGGACCTTACGACCGATGCGGCCCTGGCTGCTTCGCTGCCAGCCGTGTTGGGTGT
CGGCGGGCACTTGCACCTCTACGGATTTGCGACGGCAGCGGAGTTGGAGCTGGAGCAGAC
TGGTCTCGCGAGAGCGAGCGGGAGCGGGGACTTGGCGCGGCTCCTGGCGCTCACCGGCG
ACGACCGGCCGTTCGAGCTGCTCAGGATCGGCCGCTGCCCACGCGCGCCTATCGGCCGAG
AGGGCGGCCGGTCGCTCTTCAGAGTTGCTCTGGCAT
>at00973
TTTTTTTTTTTTTTTTTACCGGCAAAGTTTGTAAATGACAACGAGGCAAATTCCATGCA
CAGCCGAGCTGCTCCCAATGCCCTCAACCGAGGCTGACAGCCGCACGGCACGCAGCCGCT
CCACCGCATCAGGAGTTGCATTCTTGGAGGCCGAGGAAGCCCGTCGCGGCCCAAGGCACA
TGAGACTTCGTGCCGAAACCGTAGGTGGAGAGCAAGTAGAGGATGGCAAACGGGAAGAAG
GGGCCAGGCATGCAGTGGGTCCCGGGGTGCTCGATGAAGCTCATCCTGCCCGAATCGCAC
ATGGTCCGGATCCCGAACATGTTGTTCTTGTACCACTCGGTCTGCTTGAGTGTCAGGGGT
CTCGGCACCATCCCTGTGCTCATGGGGCCGAAGTGCGCGGACCCAGTCAACTTGTTCGTG
GGCGAGCACATCGGCTCTATGACAGGATCGGCGTACCCAGCAGTCATCACCATGCGCTCG
GTTCGGAGCAGGTGGGACTCCGCAGCAGTCTGCGCGTTCACCTGGTTCAGGAGTATGCTC
TTTGCGAACGGAGAGTACTTCGTGACCTGCCTCGAGGTTGGGGAGTACACGTAGTGGCAC
CACTGCACGAGTTTCGGCATCATGGGGATGAACGCGCAACACTCAGCCCCACCGGGCATA
GGGAAGAACTCGCCTGCGAGCGGGCCGTCGATGGAGATGTACGCCTTTGCGGGTGGGTCG
TTGTAGAGCTGCAGGTAAGACCTTGCGACAAAGTTGCCCTGCGAGAACGCCAGGAGGATG
AACCCATTCGCGTATTTGGGGTCGGCNTTGAGCCACTTTTGCAGTGCCGCCAAGCCGGAC
CTCATGTCGTAGAACACGGAGGCAATCAGCTCGAAGGGGAGAAGGGC
>at00974
CCCCTTGGACTCGCCCTTGGCCTCGTGCACCTTTCCAAATCCCTTCGTGGTGGAGCCCTT
TCCGGTCGTGGCACTGCCGCCTTCCCTCTGCTCCTCTCCTTGCTGACCTGCCGAGGCCTT
GCCGGTCCACTTCTCCTTTCCCTTCCAGCCTCCCTTGCTCTCGCCTGCCTTGCCGCTGCC
CTTGTCCTTGCCTTTGCCTTCGGGCgGGTGCCACCCCTTGCCTTTGCCTTCGGGCTGGTG
CCACTCCTTGCCTTTGCCTTCgGGCTGGTGCCACTCCTTGCCtTTGCCTTNGGGCTGGTG
CCACTCCTTGCC
>at00975
TTTTTTTTTTTTTTTTTTACCGACAGAAACAAAAGCTTGAGATGTCATGATTGCCAGCTC
CATCCGCCCTGAAGCAGCCTGCCAAAGGCCTTTCTTGCGGGAGACTCCCGCCGCTCTCGC
CTTCCTCAAGACCCAGATAAGGAGCGTAACGAAGCAAGCATCGACACTCTCCAGATCACT
TGAGACAGCGGCTCCACCATGGATGAACACACCATCACGCTTGCGTTGCTCCGTCGTGTG
TGGTCAGTCCGTGTGATGTAAGGTAAAGCGCGTCAATGTAGCCTCGCTTCATTCCGGGGG
GGCATCTGAGACTTGTGCTGTCCAAGGTTCGCTAGTTGCAGACAGCCTCATCTCCATCAT
GTATGACTGACAGCCATGACAGGGGTGGTCTTGCACCACCAAGGGCGCTCCAGCAGACGT
CTGCAGCAGCCGGAAGCCGGCAGACTTGTACAAGGAGATGGCCGCCTCGTTCGAGGTCTC
CACGTGCAGGCTAGCCGTCGTGCCGTCCCCACACTGCTCCTGCGCGAGGGACAGTGCGGA
AGACAGCAGCGCGCGGCCCACGCCCTGCAGTCGCTGCGCCTTGTGCACGAACAGCCATGC
GAGGTAGGCGCCATCCTTGGCACACCGCACAGCAACAAAGCCGGCAATGGCCCCGCCCAG
CTCCGCCACTAAGGCTCGACCCTGGGGCTTCCGGACGAACTCCTTCCACCAATGAGGCCC
ATAGAAGACGTGGTGGTCGTCCCCGGCGCACTCTGCGTCGATGCGCATGAGCTCAAAGAC

GTCGGCCGAGCCGTTGCTAGAGTCGAAGGGGCGGATGGAGACCATGGCGGCGGCCTCCAC
CCTTCGGCCTCGGTGTTGAAAGCGGCTTGTGCCCAAACTGGCTTGAGCCAAAATGGCTAC
GG
>at00976
GCGGCCAAACTTGGCCACCAGTTGCGCCAGGCGCCAGCTCACCGTGCAGCCATGAGTGCC
ACTTGTCTTGCCGGCCACGGGCTTGACTTCTATGAGACGCGAGGCGGAATGGCCGAGAAG
TTTCGGTCAGATCAGGTCGCCTCCATTGATGAATATGAGGTGCGTGGTGGCATGGCAGCA
AACTCATCTTCACGTAGT
>at00977
TTTTTTTTTTTTTTTTTTCCCTGAAAACGGCCCAGATCATGCATAGTAGCCCTCATTGCA
CATCGGTCAGGCACGGTCAACGGCCACGGCTGTAATGGTGGCCTTGCCGGCCATGGCGTC
CACCATGCCCATGTACACGATTCATGTTTTCCAGAGCTCTCGGATTGTTCAAAGTGTGCT
GGACGCGAGCTCTCATCTGGTCTTCACTGCGCCCTCCGTGGCGGATGCGAGATACCTCAC
ACCACAAGTGTGAGGTTGCGTCACGTGTCTCCATGCTGGCGGAGACCATCTCCCCAATGC
CTTTGCGCACACTGATGGACACCGAGGTCGAGTGTGGACCGTGCATGTAGATCACTTGCC
CGTGGGGGGTACAATATGCCTTCCAGAGGGTGGCGCATGTCGCCCCAAGAGCAGCTCCGG
TGGTCGGCCACCCGAAGAGCCCACCCACCCATTTCCCCATCGGTGCCTCCCCAACACAGA
TCCTGCACAACGTCCCACATGATCCGCCCAACACCTGGCATCTCAAATATACCGTCAATA
GTATCATACATATCATACCTACCAAGGCCCCCACCAACAAGTCGAGTCTGACTGCCACGC
GCCCCCAAGGTGGTCTTGAGGCTGCGCGGCAAAGCGTTGAGGTCGCGAAATGCGGTGACG
ATCTCGATGTCGTTGCCAGTGCTTGCAAGCTCTGCCAACTTCCGGTAGAATGCGGATGAA
TCCTCGAACTGCAATCTGAGCGCGGGCATTGCTGAACAACTTTCAAGCGTGAGCTATGTG
CAACTTGCGCCAAAGAGG
>at00978
ACCATGTACCTCAACGTGAGCACGTACCGCGGCGTGGACTGCGACGCCGCGGAAGAGCTG
CATTTGGGCCAGGAGGGCGTCCTCCCCTCCTTCACGTCCACATCCGCGGAGCTCTCCGTG
GCGGAGGCCTTCGGCTGCACCGTCCTGCTCTTCTCCGGCGGCGGCTACGACCTGGGGGGA
GTACTCCCAGTTCCCCGGCGAGGCGGAGCTCCTCATGGAGCAGGGCCGCGTCTACCGCGT
GACCCGCCACGAGACCCAGCCGGACACTGGCCTCGTGCACGTGTCCCTGGTCACGGAGCC
CTCCGCCAAGGTCTCGGCTGGCCGCGTGCCCGTCTCAAAGGNGGCTCCGCGGAGGAGCTG
CCAGGGCCTCGGACTCATCGCAGTGCCAGATCTGCCGCTGCTGCGAC
>at00979
TTTTTTTTTTTTTTTTTCAGGGCTAACCAATGTCCAACGCCAACGCTGGGCCAGCACTG
GGGAGTGCCAACGTCACCGATGGGGTTTGTCAGTGTCACAAATGGGAAGTTCTGGAGCTC
AGCGTCACTGCGGTAGCTGGCGTCCCTGGCCCAGCGCACCTCATGGACCAGCCGGGCCTT
TGAACAGCCCAGGCCTGCTCCACAAGCTGCTCAAGTTCAAACATTTCAAGGCCAATCGGA
CGGGAGGCCAGCACCGCTCAGGAAGCTGGCATGCACTTTGTGGACCAGTCGGGCCAGTGG
GCGGCCCGGAACCGCTCCACAAACGCTACGACGTTGGGGCACTGCTCCTTCAGGAAGCGC
TTCTGCGGGTAGTCCAGGGGGATGTCCAGGAACTGCACGAGGTGCGCGAAGAGCATGCAG
TCCGTTGTGGAGGCCCGCTCCCCGTGGAAGTAGGGCTTCTCGCCTAGGTAGGCCGAGATG
GCCGCAAGGTCTTCGTTTGNNNNGTNCCCAAAGCTCCTCGTCGGAGTGCCCCTTGAGGCC
GCGCATGCTCATCTTGTGGTTGGTGATCCGTGGTTGCAAGCCTGCGCATTCCGACGCCCA
CGATGAA
>at00980
TTGTTTTTTTTCTTTTTTTTTTGTAGCTTTTGTCGGCCGCATGAGCCAGGCCCCGTCCTG
CAAGTGTGGCGTTCCTGTGATCGCCAGCCGCCTGCAGAGAGCAGCATCAAATCGGGGCGT
TCTGCCGAAAATTAGACATCCATGACATGGAGGATGGTACCTGTCACCATGAGAGCTGAA
GCTTGCCACACCGAAAGTCTGTTCTCAAGAATGCCTGCGGAGCATACAAACTCAGTTTCC
ATTTTAATTGTCATTCCCAAGCAATGGCTTGGCATCAGGCTTCAAGATGCCACCAAAGAG
CGAGACTGAAGCGAGAGCAATAGCGCCAAGACCGCTCAGCATTGCCACCTTGATCCCAAC
CTCTGCAACTGACTTGACAGCACCAGGAGAGTCGCCCTTGGTCATGCCGAAGCACGCCCA
TGCCGTCGCAGCTGCGTATCCAATGCCAAGGGCAAGCGTTCTGGGTGTGCGGCCCAACAT
CGGAGCAATGCCAGCACTGGCCGCTACGAGCACCCCAAGCAGGACAGCGCCTCCCTGTGG
TTTTGCCAGGGACTTCAAGGCAGGAGTGACCTGTGCAACCAGAGTGAAGCCGATACCAGA
TGCGGCTGCAAGCCATGCCGCGTTGAGGCCCGTGGGAAGCGCAAGAAGGATTGACACCGC

CATCGGGCTGGAAGTCGGCTGTGACATGAGCGCGTGGCGCAACCTGAGAGCTGCTGCCAG
CATAGAGCCATACGCGGCCAGCAGCAAGACCGATGCCGGTGCAAGCACGGCGAGCCGCTG
AGATGGCGTTGCTGATGCTGCCGGAAGCGGCGCGAGCGCAAAGCAAACTGCCAGGCAACT
TCGGCAGCCCAGCCTGCAAGCCATGCTGGCACAACGGCAGCACTTGCTGCAGGTGCACCG
CCGGAGGCTATGAACAATGTGCCACCCATTTGCAACAGAAAGATTGGACCCCAGATCGCA
AAGGTAAACCCGGCCGGTGTCACAGCACTCGGGTATGCAGCTGAGATGACGGCATTGGTG
GGGACTCCAAATGCGCCTGCGCCAAAGATGCCGTTGACGACAAGCAAGCCGGCACTAGCC
CAAATGTGATGCCGGCGCGCATTGCTCCAGACACCTCTCCTGGACCCTGGAGAGACGACA
TGGTTCTTGGAGTCTCTGACTGTGGACAGCTGTGGCCCACTTGCTTGAGCCACACTGGCT
TGAAGACCAAAAAAATAAAAAAAAAGGAAAAAAAAAACT
>at00981
GCATCGAAGAGGCTCCTTGTTCAGCAGGGACTGGATGGACAGCGGCACCGTGGCCAGGGA
CTGGCTTGCGGTCCACTTGGGCCCGTGCCACGTGCCCAGGATGCTGAGGCAGACCTTGCC
GTCCGAGTAGAGCTGCGGGTGGATGCGGAGCGTCCCGCCATCAGTGTTCATGAAGTGCAC
GTGCGGCGGCTTCAGCGGGTACTCTGGCGGGACATTGATCCTGAAGTGGAGCATGGCGCC
GTCGTACGGGGTGTCCTCCGGCCCGACGATGAGCGCATCTATGGTGGTGATGTCGTCGCT
GCGCGGCGCAATGTACACGGTGGGCGAGACTCCCCCCTGCGCCGACACCTGCGTGAGCTC
GCGCTGCA
>at00982
GTCCTGCAAATTGCGCAACGACACTGCAGGTTGGTGGCAAGTGGTTCTGGGAACCAGGCA
CTGGCTTGAAGTCCTTGGGCGAACTTGTGCAGATATACCATGACACTGTAGGAAGGAACT
GCGTCCTGGAGCTGGACTTTGCCATTGACCGTAAAGGCCGGGTCGACCCTTTGCACGCAG
AGCGCTTTCGCAGCCTCGGCGCTTGGATTCGCCAGTGCTATGGAGCGCCTGTAGCAGTTG
GAAGGGCGAAGGCCAGTGGGAAGTTTCAGCAGAATTGAATGCGTCCACATTGGTGGATCG
CATTG
>at00983
TTTTTTTTTTTCTTTTTTTTTTTTGTCTACACCCGCACCATCGCGTGGCCAGTCAGCAT
GTGAAGCAGTGCCTGCGCCTCAGGAGCGGCACCTGTGCACCAGGGCCCCGTCCGCCCTGC
CCAGGACCCCTCACCCAAATCTCTCGAGCAGGGTCATCGGCCGCGCGCTGCTGACCGCCG
GAGCCCCTCCCTGGGCGAGCGGCCTGCTTCGAGCGCTTCTCTGGGAATTGCCGCGCCCAG
CACTTCGGCTGCGGCTCCTGCCCTTGGTGGACCTGCCCTCGGGCACCGCTGCAGCACCAA
AGGTGCCCTCCCTCATCTGGCGGATTGCGGTCCGCACGTGGTCTGCCGGAGTCTTCGAGC
CCTCCAAGACCTCAGCAAGAGATCTCACATCCTCGGTGAGCGTGGCCGATCGCTGCTTCA
TCTGTGCACCCAGCTCCTCCTTGAGCGCTCCATCCAACTTGAAACGCGTCGCCAGCTTGT
CCACCTCCTCCTCCACCATTCTGGCCACCAAGGCAAACAGCGAGCCATCCTCGACATCGG
CCATGGCTCCAGAAGATCCCCGGGACAAGGACAAGCAAGCTCAAGCTGACAAATGCTATC
CTTGCCGCGGACTTGAGCCAAAATGGCTACGG
>at00984
ACTTTCAAATCGCCTTGGCCGACGACATGCCATCCATGCACTTTGtTGAACCtAGAGTGT
GAcCGATCGAGATGCTGAGAcTTGTTTGGNCAGCAGGTCAATGCAACAACGTTAGTGGAT
GACTACCNGGCAGGGGTGTTGCCAAGGCACTACA
>at00985
TTTTTTTTTTTTTTTTTGAGGCGCTCTTTGCAATCATACTTGTCACAGGCTTCCAACTA
TGACCCTGCCACGTGTGTAAGCCAGAGCTTTTGCAGAGTCCATGCCCCGCGCTCGGCCCA
GGCTTGCTGCACATGCCAAGACCGTTCATCCTCGCGGCCCTCTCCGTCAAGCACCGTCAA
GGCAACGGCCGTGCCTCGCCGTGGCAAAACCGCGAGAACTCCATCGGCCTCAGCTGCAGC
TAGCAGCCGCTGCTGCTCCCTGTGGAGGGATGAGGCAGCATCAGAGCCAGGGAAGAAGCC
TGAGCCACCTGGCCCGGCCCAGGGGAATCGGAGCTCGCCCCCTGCTCCTGCTGAGAGCCA
GGCGACCATCTTGCCGCGTACAGCCTGCACCTTGCTGCCGCAAGCGTTGCGCCGCACATA
CTGGCCAGCTCCCTTCTCCTCCCACACGATGCCGAGCTTGGGATCCACAGCATGGGCTTC
CAAGCCCAATGCTGTTGCCATGCGTGCCTCAACTGCTTCCAGAGTCTCGTCGGCAAAGCG
CCCTGTGCAGCGGCACTGCGGTCACTGAGCTTTCTCCAGCAGCTGGCTGCTGAAAGCGGA
GAGCTATTCGGAGGAAGTGATCGCACTCCTCCGTCTGACAAGAAATCTGGAAAAGACCTC
AATCTTGGGAGAGTCGCTCAGCACCTTGGACCCCTTTTGCCAATCCACCGAGCTGCATAC
GTGACCTTTCGGCTGTCAAGTCGAGCGTCGACAATATTCGCCCTGCCCTTCTAACCATGC

CTTGATGTTCTGCGGCGGTGGTACCAAGCGCAACGGCGGCTCTGGCAGGTCGCTCCTTTG
CCACCCAGAGTGATGGCGCAGACTCATCGCCGCACCCTGCCAAGGTCCCGTATACCACTC
AACAGCCACTGCCAGCGGGGCAAATGGCCCCAACTTCTCCCTGAGGAAAGCAGGAGCAAA
GCCTTCTATCTGCACGCCCTTCATCTCTGTCACACCAGTGCAACCTACCCATACTTCTCG
GCGGCGGACCAACCTTAGTCCGTAGGTTGCGGGGGCCTCGTCGCGCATTGCAGAAGCTAT
GACACCCCGAATTCTGCCTACATCGCCAGCAGGAAGGGTGGTGGCAGGTGAACCATGGCA
CCGAGTCCAACACTGGCGGCATGCATCACGAACTGCCTGGCCAGGAGGGGTCCTGGCATC
AGCAATGGTCACCACTTCGTGCACGTCCGTGACTGCACTGAGAGGCCCACTTCTCAAGGC
ATGCAAGGCAGTCTCCTGAGTCATCGAAGCAAACCCGAGGAGCAACATGCGTGGTGTCTC
CTCCAC
>at00986
TTTTTTTTTTTTTTTTTTGGACGGACGCGGCCGTTCGAGGTTCAGGCTTCGAGGAGATAC
TACACCGACCGAGCAGCCGAGAAAGAACCCAAGAAAGGCAGTCTCTAGCTGGCTCGAGCT
GTCTCCCTAGTCAGCCAGCTCCAGTCCGCCGCCCAGCTTCGCTCGCCGCCCGGGCGCGCC
TCGTGCC
>at00987
GGCTGGCTCGCCTTTCCCACCAAAGCTACCCTTTCACTGTCCACCCCGTGCTCCCTTTCG
CACAATGGCGGACCATGGCGAGTCCATCTACAACATCATCCCCCCCAAGCCCGTGGAGCA
GGAGAAGCCGCCCATGTACCGCTCCAAGCACTCGGGCAACGTCCCGCCACATCCTCGACC
TTCCACNCCAAGTCCACGACGCACCCCGGNACCAGCAACCTCTCGGGCGCGCTGCAGACA
AGCCCGTGCCGGACAAGGATGCGCGGACCTTTGGAAAGATCCCCGGCTCCTAACAAGAAC
GACCCA
>at00988
TGAAGGAAGCCCGGGAGGCAGTCGCCGAGGCCAAAGAGGAGTTTGGGGGGCATCTCCGCG
GGTGCTGACGTCGACCTGCAGGCCTGGCTTGGACTCGAGGTCCGGAAGCTGCAGGCCAGC
CTGAGCACATACGATCCTCGGCTGACGAGGGCCTCCAATCTCTCCGTGCGCTTCCGCGAG
GGTGCAAGGAAGAAGGAGGCCGAAGAAGTATATGCGCTGGAAAAGAAGGCCCTTGCCCTC
ATCAAGGGCCATCAGCGAGTCAAAAAGCTTACAAAATGAAGACGCATTTGCAGAGATTGA
TGCCGATAAGGACAGCAAGATCAGTGAGTCTGACTTCCTTGCGTTTTTCACAAAATGCGA
GAGGGAGCCGAAGAAGACAAAAGCCACTGCTGAAGCGCAGA
>at00989
TTTTTTTTTTTTTTTTTTCACCTTCACACCGTGCACAGATACGGATGGCCCCACAACACG
CCAACCGCCAATTGCCAATGACCATACACTTAGTTCAAAACTTGCAAAATCTGCTGGCCC
TCACTTCTGCGGGAAGAGCTGGGCCCTGATGGCAGGCTTGTCCTGCTCCATGACCAGCCG
CAATCGCAGGTCGATCGTGTTGTCAATCGTGATCTTACCCTGCTGGCAGAGCAGCATCAC
GCCGCCGAGGCAGGAGGGCGCCTCGCTGCCCGGCACCGGCGCCGCGGGGAGGTACTTCTG
GCTGTCGACAGACAGCTTGCACGTCTTGGTGGCTCCTGTCTGCGTCTTGATGGCCTTGGC
GTAGTCCGAGGCGGCCTGCTCTAGGCACGAGCTCACCAGGCCCTTGTCGCACTCGCGGCA
GCGCACGACGACCTCGGGCTCCAGGAGCATCAGCAGGCCCTGGACGATCAGCTGCGTGAC
GAAGGCCTTCGACTTGGCCTGGCTCTTCATGGCCTCCGTCAGCTTCTCTCTAACATCGTC
GGAGATCTTGCCGATGACCTCTTGCCTGGCCTTGATCTTCTCCAACCGCTGCTTGTTGAT
TGCCATGGACTTCGCGATGGCATACTGAGCCTCGTGCC
>at00990
ATCTGCCGTGGACCAGCTCTGTCAGCCGCCGCGCCTTCACGCACGGCTGGGCTCAGTCTG
CCGCCCTGTGAAGGTGCCCTCGGCGTTGCCGGAGCTGCCAGCACCACCCCCAGCAGTGGA
GGAGGAGCGGCCACCTCAGGGACTGGTGCTGGGTTCCTCGCCTGGCGAGCTCGACGAGAC
AGCTCGGCGCGTGGACGAGACAGCTCGGCGCGCGGACCTGCTGGCCACCTCGCCGGAAGC
ATTGGTGCAGGCGCTCGGCATGGCCAACGGTGCGGCCGGTGGCGCAAGGACGTTGCAGCC
GCTGATGGACAGGCTCTGGGAGAGCACCTTCGACAGTGCCGCGGCCCACAGTGCAGTTGT
TGCCGGCGCAGTGGACGTGCTCCTTGACGTGACGAGCAATCACGGGGACTCGGCGAGCCT
TGCCAGGGCTGCCTGTGGCACCCTGATCAACGTGACCCTCTGGGGCCCGGGCCTCTCGAC
AATCAGCCGCCCTGAAGTAGTGGAGTTTGCCGTGGGCGCCATGCGGCACCATGCAGGCCA
CCTGGACGTGCTCGAGCTTGGCTGCCAGGTTCTGTACCTCTTGGCCTCTTGTCCGCTCTG
CAAGCACCTGGTCGATGATGCAGAGGGCGGCCATGAGGTGGCAGAGCGCGCAGCTGGTGC
TCCTGCATTCTTCACGCAGCGGTGGGCGGTCGCTTGCGGGCATTGCTCGCTCAGTGAGTC

CAGCAATTGACCCTCTCGGTCCTCCAACAGCCTTGTCCCCGGCAAGATTGCGAGCGGCTT
GTACAGCACGGCCGCCCACCAGCCAGGCACAGGTGCGTTACGTCAAG
>at00991
GCGGAGCAGCTGGGACTCCGGAGCTCCCTCACTAGCAGAAGGCCCTGCGTGCGTAGCCCG
CGCCCGAAGCAACAGACACAGCAGCTGCAAGCCAGAGAAGGCCAAGTCCTCCTTTGTAAG
TCAAATCGGCCCAAGATCGCCCAGCGTGGCCCTCTGCAGCGTCAGCCTCGGCCCGCTCCT
TCTCGCTCTCCGGGGCTTTCTCCAAGGCCACGCCCGCGAGCATGGCCTGGAGCGCCAGCA
ACTGCAGCGCCGCCTTCACCTTCCCGTGCCATGCCACGGCCACGCTAGCCTGCGTCCACT
CGCGCAGGGACGACACTGCCAGCTCCCGACCGAGGATGGCGACCGCCGGCAGCGTCACAG
AGAGCTGCCCGGCATGCTCAACCAGCACGAGAAGGGCTGTGGACACAAGCAGCTTGTCAG
CGAGTGGGTCCAGCAGGGCGCCTAGGGAGGACTGCTGGCCGAGCCGCCGAGCCAGGTAGC
CGTCCAGGTANTCCGTCGCGGCCGCCGCGCAGAAGAGCCCGGCGGCCGCGGCAGGCGCCT
CCGTGTACCAGGACACAAGAAATCCTGGTATGGCCGCGAGCCTGGCTGCTGTCAGCATAT
TTGGCACTGTTGCCCATCTGTCCGATTCCAGAGGCACACTCGCCAAGAGTCTCATTGCCG
GTCGACCACTGTGCGAAGGCAGCAGCAGATCGCATGGCGAGTTGCCTTGTATGCGGAAGT
GTCTTGTAGCCGGGCTCCTGCACGCCAGACGCGCCTGCCCGCAGCGCTTAGCGCCAACCG
TCCACCAGGCCGGCATTATCATTGCTGCCGACACTTAGACAAAGA
>at00992
GTCGCCTCCCACCAGCATGGCCGTACGCATTTGGACCCTACCATCCAGAGGCAGCCGAGC
TTCGAGATCATGGACCCACGCCCACACGGGGAACCATCCAGCGGCCAGAGGCAGACCTGG
CAGCCCCCACACGGCTCCCAACGCGGCTCACGCTCGCAGGGGAGGTCTTCGCACAGCTCG
CGCACACCGATGGGAAGCTCGGCGAGCGGCCCGGCGTCCCAGACTCCGGAAGGCGGAACG
ACCGAGGGGTCAGCGAGCGCTCGGCCCTCCCGGACTGCAGAAGGACGTGACGGGGACGAC
GGCCGTGGCCAGCCGAGCACAGCAGGGCCATCCACGCCCACAGAGCATGACAGCATTTTG
AGCCGGCTCATCGAAGAAGCAGGGTCCGCTGGCGGCGCGGCCTG
>at00993
AGGCAGTTGATGGCATCCCTCTTCCTCCCCCCCTTGCTGGAACAGGGGGGAGTAGCTGCG
GTGTCCGTGAGGCTAGCCCACCTGGGGCCATACCCGGCCCGCCTCCTCCAGCTGTCAGCA
GCATGGCGGGGAGCACCCCTGGTCCTCCGCCTCTGTTTCCAGAGGATGTAGGATTCGCCG
GTCCAAATGCATCACCGCTCACCGCTCCCGGTCTGCAAGGTGCTATACCTGGACCACCAC
CACCCACCACGCCCAGTGTAGCAGGCCCTATACCTGGACCACCACCGCACACCACGTCCA
GTGCAGAAGCTTCTGTACCGCTCACCACACCCGGTGTAGAAGGTTCTGTACCTGG
>at00994
TTTTTTTTTTTTTGTTTCCAATTCAGCTTGCTGATCCCAGCTACCTACCTGCTTGCATG
CCTGCCTGATTGCGCGCCTGCCTGCCAGCTTCTGTGCAGCTCTCGCGGACATCCCGCGAG
GCGCCCGGCCGGCTCGCAGCTAGCGCTCGCCGCGCTGCCCTGAGAGACGTGCGTGGTACT
CCTTCTGGTACTCCTCGGCCTCCTTGTCACCGGACCTCTTGGTGTTGAAGTCCTCGAGCT
GCTGGAATGCCTTTCGCTGCTCGATGAGCTTGCTGAAGGCGGGCGGGTACGCACCCTCGC
AGTACCAGCTACCTGGTGCATCGCACTTCCAGTTTCCGGGGTTGGCCGCCCAGGCCTTGG
ACTCCTCGGAGATATTCAGGACCTTGTATATCCTCACGAGGTTGTACTTTGAGGTGTATG
CCTCCTCAAACATATCTGGGTCCACCTGCACGCCTTCCCTCTGGCCGTGGCCGTGCAGCT
TCCACAAGAGGGACTCCGCCATCATCTCAGTCGGATTGCCCTCGCGGTCCACGGCGAAAC
GGCGGCAGGCGGGGTCTCCCGGGCAAACATCGTTGTACACGGAGTTCGCAATCCGCGCCA
TGTGCGGGGACTTTGCCCTCGTGCC
>at00995
TCAACGTAGGTGGTCAACAAGTTCACGCAGTCCGCATAAGTATGTGAATACCTTGGTCAG
AGTCAACCAGATCGCTGAGCTGATTCACCTGCAAGGCAAAGGCAGCAGCTTCAAAAGCCT
CCCCTAGCTTCTCCGAGCCACGCTTTACCCAGCCCAGGTCCCCTGCTAAATCTGCAGCTT
TGAGGCAGCTCTGGCACTCTGAAAGGTCTCGGCAGCGCTGTGTGAACTTCTTCTGCCCTC
CATCACCGTCTAGCTCCTCCAGGACACCTCGGAGAATCCGCTCAGCCTCCGCGCGCGTCC
GGCGAACCTGCTTATTCCGAACTTTGTCGATCGTTGCCTTGCATTCTCTGTGCTTCAGGA
GGATGTGCCGTAAGCGGACTTGCGCTGGCTGCTCCGTCCTCCGCTTCTTGGCCTGCGGGG
>at00996
TCTCCACGAGGCAACTCCAGCTGGTCAACGCGACATTCTTCGCCTATGTGGTGGACAACT
GGTTTGGTGACCTCGAGTGCATGCGAAGAGCAGCAGACGAGATACCACCAGGTTCCTGGC

GTGGGAGGCAAGTCGGCTACGAAGACCTGAGATGGGCGCTGAGCGTTGTGAAGACCCGCG
GCTTCGCCTTCGAGGGCACGCGCGAGAGCACCATGCTCATTCCTCTGGCGGACTCCCTGA
ACCACAACATGGTGGCGAGCGTGCGGACGGCAACACTGGCAGAGGACGCTTTGAGGTTTG
TCGCCACACGCAACATCATGCCTGGTGAGGAGCTCTGCATTGACTATGCACAGGCTTCGA
ATCTGGAGTTCCTCATCCGATACGGCTTC
>at00997
TAACAGCTACGAGCAGGAACTCCTCATTGTATTGCAACACCAGAAGGGTGTGAGCTTGTT
CGCATGCGATGAGTATGCTGTCTACAGCAATAGGGTCATCCAGCTTGATGAAGGCGTCTT
GACAGTGGTTGTCGACAGCGACCTCAAGTGTGGCAAGGTTGGCGAATTCGGAACTCCGTT
AAACATCGACATCTTCATTGCTGTCTGGACCAAGGTCGTCAGTGACTTCCGCTTCGAACA
ACATGACTGGACGGTGAAGGTTGAACCCGGAACTGTGTGTTCTTCCCTCGGATTCTCAAG
CTAGTCTTGGAGATGCATTCAGAGACGGAGAATGGCGTG
>at00998
TTTTTTTTTTTTTTTTTGGCAACACAAGAGTGCTGAGATGGCGATCAGCTCCGGCGGAA
ATGGCGCATGCCCATCTACGCTCTGTGCCGGCCGGAACGGCCAGGCTGGAGCACAACGAC
ACATGCACTGCCTTGATCACTCGGAGCTCAAAGACCAAGGGACTGGTGGCAGCCTCTGCT
GCTCCAAGTCACTCAGCCCCTCATCTCAAGCCGGAGGCCAAGCTGAAACCGCTGCGTCAG
ATCATTGTGATGCGCCCAAAAGGGGTAGGCAAATGTGAGCTCCAGGTTGCCCATCCCCGG
CAGCGGTATGCCAACTCCAAGCCCAGCCGAGGCGCGCACGCCTTTCCGGAGGCCTTGTAC
TATTGCACTGACCCCCGTGCATGGCTGCTGCGCAAGAGCACCTAGGCCCACAAAGGCGAA
CACACGCCCGTCGATGCCGCCACCCGAGGGACCTTGGGGAATATGGACTGGAGCTGACAG
CGCCGTGAAGGCATTCCACATGCAGTCACCTCCCAGCGCATCGGTCAGACGCTTAGCCGG
CTTGGCTGGATCTCGCACCTGGCACCGTCCAGTTGGTCCCATCCCACGGTTGGCAAATCC
TTTGAAGACCGTCGAAGATCCTGATGCGCCGCCCAGGAAGAACCGGTCTTGCAAACAGGT
CGGCCGCGTGCCTGTTGGAAACAGCACACCGCAAGAGCCAGAAAGGTGCCAGGCCATTCT
CCAAGGAAGTTGAATCGAGGCACTAGCACTGGCTTCGTTGCGGAGAAACTGCACATCGCC
AAGCAGACCAGCAAGCTCCAGGTCGTTGCGCAGGCTGGCAGATGGACCAGGAAGTCCGAG
CAGCTGGCCTGCCCAAGAGAAGGTATATCGGAGCGATGTCTTCACGGACCTCTGCTGCGC
CTGCTGGACGTCGACTGACGGGATCCTGAGTCCATCGGTTGCTGGGTACAGATCACGAAT
GGCTGCTTCAGCAAAGATGCTGTGCACACCAGCCATGTCGGACAACTTCATGACCGCATG
GGTAACGCGCTCACTGTAGCTTGAGGCCTCTCGCTCGTCCGACGATGACCGTGCCACATC
GAAGCTCCAGCTGCAGCGCTGTCCCAGAAATCGCGGCGTGGACAGGCGCAGCATGAACTC
GTGGGCCTGGCTGGCCGTGGAGCCTATGGACCCCATGGCTGATAGAGGACCACCCAGCAG
TGCAGGTTGCGTCAACCGCAAGTCGGCGGAAATCTCACCCTTCCTGTCGACATTGCCGCT
GACGGTGCACTCCCGCTTTCTCTCCTCGACGATGAAGGCGATGTCGACAACCCCGTTCGG
CTCCAGGGAGATCTCCGCATCCAAGGACTTGAGAAGATTGAGCTCAGTCAACCTCCTTCG
GGCATCATGGATAGCATGCTGCACACCTCCCAGCGTCCTGGCGTTTCCAAGTGCTTGGCA
CTCGCCAATCAGTGAGTGGGGTGTGATGCGCTTCAAGCCCTTGAAGCTCACGTACACGCA
GTTGACCGGATGGCTTGCATCGATCTGGACTTCATCTCCCTCATGCGAATCCACCTCAAA
GGCGGCAGCCGCTGGGGCGGTCTCAATGCCGCGAGCACTTGGGCCAACATCTGCCATGGC
TTGATGGGCACGGACGATCGCAACCTGGCGAGGGGTCCACCATGTACTCCCAAAACTTGA
GCCAAAATGGACTAACGG
>at00999
TTGTTTTTTTCTTCATTTTTCATGTTTTGGGGTCGACGCTGGTATAATTGTTTCCATGGT
CATGAGCTCTCATGGCCTCACTGCAGAAGCCTTGTCGCTGGAGATGCTCTTCCCGCTTGT
TGTGCACACTGCCTCCCTAAGAGCTCAAACATCGACTGTGCCACTGGATCTGGCTGGTGC
GCAGCAATGCCAATCAGTAGCACAAAACCTGATCATACATCATGCATGCTGGATCTTGGG
TTGGCAGCCACCACGGCTCAGAGCTCGTCACCTTGAATCCATCTGAGCATCCTTTGCAAG
AAGCTTGGTTCGGGTGGAATGGAATACGGAGCAGTGTCGGCAAACTTGTCTGTGGCAAAC
TCCAAGATGTCCTCCATTTCTCCCAACGGTGGCGCCATGTGGTACATCTTGCCTTGCTTG
AAGAGGACGATGGAAGGGAGCTTGTTCAGAATGCGCCTGAAGCGGACTTTGAGATTTGGC
GTGGCGTCAATGTTGACCTTTGCCACGTTCACATAAAGACTCCCTTCCGCCTTCCGCGTG
GCAACTTCTTGTCCAATCTTCTCCCACACTGGAAGGTACTTTGCACACGTTCCGCACTTC
ACAGCTTTGGTGGCTCCCTTGGTGTAGAACATGACGAACCAATCCCCTGTCGTTGCACCA
GAGGCTGCCTGTGTATCATGCTCAAAACTGTCGTCGTCAAGCTCGATCATGGCACCATAG

GCACCATAAGAAACACCGGCCAGCAAAGCCACCAAAAAGGTGCGTCTGCTCAAGCCCTGC
ATAGTCATGGTGTGGAAGGCCTGCGCACCGCTTGAGCAGACATGGCTTGAGCCAAAATGG
CTACGGAC
>at01000
GTCAGCGCTGAGTAGGTCGGACTCACCGGGTTGTAAATGTGGTGCAAGTTGGTACTGCCT
TCATCCGTGGACAGCACAAGGCTCGCGATGATGGCCGCTGCCCAGTCTGCCGGTGTCAGG
TCGCAAACTGCGTTGGACTCTGCTGCACACCCCAACTGCAAGCAACCACGCACGTACCGG
TCCGGGAAGAACCCTGGTTGCCCATGGCCTGTGCTTCTTGGACCCCATAGCATACCAGGC
CTGTAGATTGCACCAGGGGAGACCCTTTTTCAAACGCCTTGTGCACGTAGGCCTCGGCGG
CCCACTTCGTCAGGGCGTAGGGGCAACCTGTGGGCCGCCGCTCCCCGGCGACTTGGGTTG
CCGCAGCCAAGGACCGACACTGAGGAGATGAAGTGGAAGGGGATGCCTCTGTCCG
>at01001
CCTACAGCGACGACACAAGCGGCGACCAAGCGCTGCCTACGGTGGCTCACACCTTCGGTA
GATGTACACGCTGCAATTCCCTGTTCCTCCAGTGCAATATGCAGCCTGCCGGACGGAAAA
GAAACCTGTCGGTGCTCGATTCGGATCAGCGCAAAGAGGCCGCACGAGGGCGACCACTCG
AGTGCCTTCCGCGACNTCTTGCAGGCCGTCCGCCAAGCgGGAAATGGTGCTCTCATCAAA
GCCAGCGGTGCTGACAAAGATGGTGGTGGCCTGGCGCCACTCATCCTGAACTCCGAAGAT
GTCACCGCAATGCAGGAAGATCCTGGCATGCACACGTCTGGGGTAGTCGCATTGCGGAAG
CCACAGCCTCTTGGTGAAGCTCGGCACACACTTCGATCCCACAAGCTGCACCCTGCGATA
CCAAGAGCACCCAGGCTGCAACCAAACGGCCAAT
>at01002
TCGAAAGCCTAGGCCCAGGTAGCAGAAGTGCTAGGAACTATGATGGGTTATGGCGGGCAG
CAGGCAGGCGGGTACACTGCTGCGCAAATAGCATACTTGCAGCAGCAACAGCAGCAGCAG
GCCCAGCAGAAGTATCAGCAGATGGCAGCCTCGTACT
>at01003
GAGGCGCTTCTGCAACTCCTCTACACGCGCGCCGAGCTCGCCAGGCGAGGAACCCATCAC
CGGTCCCTGAGGTGGCCGCTCCTCCTCCATTGCTGGGGGTGCTGGCAGCTCCGGCAGCGC
CGAGGGCGAGGAGCCCATCACCGTTTCCTGAGGCGGCCGCTCGTCCTCCACTGCTGGAGG
CGCCGGCAGCTCCGGCAACGCCGAGGGCACCTTCATAAAGCGGCGGACTGAGCCCAGCCA
TGCGTGGAGGAGCGGCGGCTGACAGAGCTGGTCCACGGCAGATGGAGCCCGCTGCTCGGT
TGCCGACCGGCTGG
>at01004
TTCATCACACGTGCAGCTGCAGCCAAGACCGCAACCTGCCGACCTCCCTGGCTCATGCCG
CCTGCACATCCTCAGCAGCTTTCTTCTCGCATGCAAACAAGAGCACCGTCTGAACGAGAT
CGTGGGACAGGCGATCCATGTCGAGGTCATGTGGCCGCCGAGAGCTGACCAAGTAGGGAC
GATCGATGCCATGCTGGAGCATGTCCTCACGGATGGATGCCAACGTCTCATGCTCCGGGA
TGTCGTGGTCGTCGAGGTTGTTGTCAATATCGAGATCCACTTTGCTCCGCACCACGAAGT
GAGGTATTTCACAGCTCTGAAGCTTCAACGTGATCGTCTGTTCCGTCCCAGTGTAGGGGC
TCTGCACGATGATGACAACCATGTCGAAGTGGGTCAATCCCATCGATTCAATGTACGTCT
GCGACGCAAACTCCTGGGTGTCAGCACCTGGAATATCCCAAAGCTTAAAATTAG
>at01005
TTTTTTTTTTTTTTAGCTCCAGACACTATCTTCACAACGCAGGAGCTCCAGCGGCAAAAG
TCAAAGCCCCTCCGACGCAACAGGTCGTTGGGCACATCACAGATCCATGCTAGTAGGCTT
GCCTGGATGAGGCCACGCCTCAGCATCATGCAAGTCTATACATGCACCTCCGGCTCGCTC
GTGCTTTCGTACACGGCCACGGAGCTCCGAGGTGCAACTCCAACATCTTGCAAAACGCGT
TGCGCGGCCGAGGGCCAATCACAACAATCTTCTGCTGCAAGGAAAGCTTGGCAAAGTCCG
AATCCATGCCCGCCTGCAGGATACAAGTGCAAGGAGGATGTCACATTTGAGTCTAGCCGC
AGCTTCTTGAAGAACTGCAAGAGGTTCTGCGGATAGGCTATGTCGTCGTCACTGTTGCCT
GCCATGAAAGCCGGTGGCGTTGCTCCACTGACGTTCAACTCAGGAGCAAGCTCCTGTTCC
CGCGGATCATCGTTCAAAAAGAGCCTCCACGGGTATACCAAGAG
>at01006
TCACCACGCCTGGGGCCAGGGTCTGGGGTGGCAAGCCCGGCGAGTATGACGCCAACTTCT
ATGAGATGCCATTCTTTGAGGTTCCTGCAAACTTGCTTTCAAGCCGCAAGGTTGTGCTGC
AGGGCGGGAACGCGTTCGTCCCGGCCAGCGCCCTGAAGCTGATCCTTGCGAAAAAGTTCA
AGGACCGCCTGAATGCGTTGATGGGCGTCGCGTTCCAGGGGTTGCCTGTGGCACTCTCCG

ACCCCCGCATTGGCGGTTTCTTGCGAGACTTGCAGGAGTATGGCATGCAGTTGCTTGTGG
CTCCAAAGTCCTCGGCAAATGATCCTGGCGACAAGCTTTCGTTGGAGAATTTCGAGGAGT
ACTTGGCGCGCTCCTTCCCTCCGTGCATGCGGCGCATTGTGGAGAAGCAGCGCGAGAATA
AGAAGCACTTGAAGCACGCGGGTCGGTTGCAGCTGCGTCCTTCTG
>at01007
CTGCTCATTCAACTCCCGCCAGCGTGGTCTCAGTGCCGAGAATGAGGATAGCCACACCTA
ATACCATCAGGCAGAAGTTGGCTATGATCTGGAGCTTGTGGTTTTGCGCAGCCACGATGC
CACTGTAAAGAGCAGCGATCATGAGCATGCAGGGGATGATGAACATGATGAGGCTGCAGC
AAATGGCGCCCACGAGGCCCACCACTGTGCCGGCATTCTCCAAAAGTATCGCAGCGATGG
TGACAAGGGCCACGAAGAAGATGGAGATGAGGTCCTGGCCAAAAATTACTCTCGATGTAC
AGGATCATGCTGGGCACAACTATCACGCCGAAGGCAACC
>at01008
TGGCAAGACCGTTGACCGCCATGATGAGTGGTTTCGGGAAGTCCCAAAACGCTTTGGTCA
GAGGAGCCTGCACCCAGTCTCCCGGTACTGGGGCCGTGCCACGAGCAGCGTAAGCTTTGA
TTACTTCCCGTGGCACATGAATCTTCATGTCACCCTTGAGGGCGGCACCGGCGTTGAAAG
CTCGCGACCCTGTCCCTGTCCACACTAGAGCTTTAACCTTGGGGTCACGCTTTGCGTGTT
CCAGAACCAGATAGGTCTCCATCACTTGGTTTGGAGCAAGGGCATTCAGGTTCTTCGGCA
TGTTATATGCAGCAATCCCAACACCGGCCTTCTCCAGCAGTCGCCAGGCAATGGAGCGGA
GTTCGTATTTCTCCCCAAGCTCTTCATGTGCTCCGTGGTACACGAGGACAGAGCCATTGG
ATTCTAACCCTGAGTCTGACAAGAAGGCTGATGCGTCTGCAAGCTCCTCCATATTCAGGT
CGTTTGCCAAGGAATCTCCCTGTATCGCCAC
>at01009
GCCGCAGCTCGTTGCGCCACAAGTAGTGCTGGGCGTCCCCCAGCCATATGCTCAAAGAGT
GGAGCGAGTGGAAGATCGCGTCCCTCACAAAGGACGGAGCCGACTGCAGGAAAGGGAGGT
AGACAAAGAGACGGACGGCTGGCGGCCACCTTCCGTGCTGCGCGCGCTCGCCGATTGGCA
TGACGACGGACAGGCGGCCACCTCTCTGCAAGCGTCCCACCACCGGAGGCGTGGCTGGCC
GCTTCGGCACGGACCGCTTCCACAGAAGCTTGCCATCGCTGAGGCTGTAGGCCCCCAGCG
TGCCAGCTGGAGAGCCTGGGGACTCTGGGAGGCTGCTGGTCTGGTACACAATGCCGTTGG
GCCCAGAAACTGGGTGCCGTCAGTCCAGCTCCCTGGAGCTCC
>at01010
CCGTAGCCATTTTGGCTCAAGACCCCAGCAGGTGCTTGATGGCAGCTGCCGCCTCCAATG
ATGCCGCCCATGCGTTCCACTGCGTCCTTGAGGGGAAGCTGTGGGTAGGTCCGGCATCTC
CAGCTGTGGATCGGTCGCAAGACCTTGTAGAGCTTGGCATCTCGCACGTCGTGAATGCGA
CCAAGGACGTGCCTTGCCTGTTCCCGGACCTTTTCAAGTACCTGCGAGTTGCCATCGCAG
ACGACCCAGACGAGCCGATTGCAGTGCATCTCCCTGAAGCCTTTGGCTTCGTCTCCGACG
CTCTGAAGTCCGATGGGCGGATTTACATTCACTGCCATGCCGGAGTCAGTAGGTCGGGCA
CGATTGCCATCTTCTGCTTGATGCGCCTGCAGCGCCTCTCGCTTCGCGAGGCATACACAT
ATGCTGTCGAACGCAGGCCTTGCATCCAGCCCAACCTGGGTTTTTTTGCCGCTCTGGTT
GATGCAGAGGGCAAGGATCCGCCTTCGTACAGTCTCAAGGACTATTGCAACGACACGCTC
AGCGCAGATTTTGCCGGGGGCATCTTCTAAAACTCAGAGTAGGTGGTTGAAGGAAGAAGA
CTGTTTCCTGGGCTTGCGAACCAACCAGAAAAAAAAAGAAAAAAAAAAACAA
>at01011
TTTTTTTTTTTTTTTTTTCATGAGATCGTGGCAAACATATAGGCTGGTTCAGCTGTCTGT
ACATCCTGTACACCAGAAATGGAAGTCCATTTGGATAAAAGAACCGGCAATGATACCCGG
CGTGCGTCTCACTTCTGACAGCTTCCCATGTGTCACCAAAGGTTGTAGTCAGGATAACAG
CGCTTCTTACTGCCAACAGTGTAGGCAATCTCATACTTGGGCAAACACTGGTGTGGGTGG
AAGGCAATGTACTCAGAATTCATTACCGAGAAGCCAGGACGCACCTTGGCGCCCAACCCT
TGGACAAAGAGCATGTCACTCTTCTCCGCAGACTCGTGTGCGTTGGAACTGCTGTCTGCA
CGTCCACGTGCGCGGCATGGCTCGATCATCTTCCCAACTTCGTCCTGCGTTAGCGCACGC
ACGTTTGCCACATCATGCATGGCATCCGCACACTTAAGGGGACCCTCCACCTTGAACGCC
CGTCCCAGAACTGAGCAGACGATCATGCGGTACACTTTCTGCCCGGATCCTGTGACCTCA
GGGTGCGAGACATACCGGTGCGACTTTGGCGCCAAGTCTGCAAGGTAGATCCCGAGACCA
AACATGTGACACTTGTCCCACTCATGCTTCTCAGTGCAGTACGTGCAAGTGTTTGGACAG
AGTGTTGTGCTCAGACCCTTACCACCTGAGACTGGGCATGCGTCTGAGGGCTGGCAATCC
GAGGGAGGCTGCAAGCCATGCTGGAAAACAATGTCATGGATGTTGTTGTTTGTTCCGTGG

TAAAGCTCCGCACCATGGGCTCTCCCGGTGCTTTGTTGCCAAATACCTCGGTATTCATCA
TG
>at01012
AGTTGCACGTCGCAAGCTGGCGAGGAAAGAGAAGTTCATGTCTCTTTTGAACGAGCAGCG
TAATCTGCTGCAGAAGTGTGGCGTATCATGTAGCAATCAGATCGTCGCTGGGGCAACCGA
ACTCCTCATTGCCGAGATGCGATTCCAAGAGCGGCGGATGGAGGCAGAGTTGGGACGTCC
AGTGGCTTGGGAGGTGTTGGAGGAGCTGATGGTGGAGCCCACTTATGCAGAGAGTGGATC
AGAGATGTTCACTTGTGATTGGTTGGGCGCGGGCCGTGGTGCCTGTGAGCATTGCTGGCG
ATGCCCGTGCTACAAGTGGCAAGCCCTCGAGAGCCAGGAGGAGTGGTATTTGACAAGTCC
GCTCGCCCTCCGCTGCGAAGCATGCTTCTGCAAGAACAGCGACCACAGGTACCTTGGTGC
AAAACTGGGCAAATCAGGTTACCTCCCCGCCGTCTGCAAAAGATGATGATCAACAAAAAA
AAAAGAAAAAAAAAACAA
>at01013
GGGCTGGCGAGCTTGGGCCTGGAGAGGATGCCCGGAGGATCGCCGCTCTGCATCCGCTTG
ACAGTGCCGAAGCCGTCTGGAACGCTGGGTCAGGGGGCGAGGTGAGCCAGAGGCGTGCAC
CGGCCTCCTCGACCGCAAAGTTCCTGTTCTCCTCTCCAGTTGTGCTTGCGCTTGCGATGT
CAGGGGTGCTTCGTGCTGCCGGAGCTTGAGTCAGCATCACCGGGCTGGCAGCAACGTGAC
GCGCAGTTCATAAACCTTTGGGATTCATGTAACCAGGCGGGCACTTGTGGTGGGCTAAGA
CCTGCCAGGTCCGCATGTCTGTACCTGCAATCAGGAAACCGGGCACTTAAGGACGGGCTA
TGACCTGCCAGGTCCGCATGTCTGTAACCAGTTAATCAGGTAACCGGGCTCTTGAGGCGG
GCTAAGACCTGACCAAGGTCCGCATGTCTGTACTAAGTTTGCTTGTCAGCCTTATTGCGC
CAGACAGCTAGCCAGCATTCCCACGTGCAGCAA
>at01014
TTTTTTTTTTTTTTTTTTGGCTAGGAATGACATTCCAGTGAGACGTGCTCAAATCATGCT
CAAATCATGTGAAGGTGATCATGCCTTTCCCCCAGGCCCCGAGTGAGGCGTTCAAGTGTG
GCTGCTCAATACTCGGAAAGTCCCGCCCAGTGAAGTACCACACCTGCACAGGCAGGATAG
CCAAGAAACGAACCTCGTCCGTCCCATGAATGTCGCCTACGTCGATGAGCCCTCGGGATA
GCACCTCAAACCCCCGCTTCCTCGCAAAGCCGTCGAAGAAGGGATACGTGCCGTGCGCAG
AAGCTCCAGGTCCGAAGTATGGGCCATCTGAGTCTATGTAGGTAGCGAAAGGCCCGCCCA
GGGCCCAAATGAGCTCGTAAATGCACTGCTTGAGAACCCTCGGCGGTGCATTGAGACTGG
ACATGGTGAACACGTCAAAGCGGATGCGGTCTGCACCTGGCACAAAGTGGTCGTGCAAGA
GCAGGTCCAGCGTCTCTGGCGAATTGCAAGCGTCAAAGTGCAGAACATGCGCGCTGTGGG
CACCGTTCCTCCTGATGTTCCCCTTGGCCCGGATGACCGCCTTGGCGTCTGCGTCGATGC
CCACCGCCCTCGTGCC
>at01015
TTTTTTTTTTTTTTTTTTCCACAGAGCCTGTGCCCTTCGCACCGTGCATCTCGCCTGAAG
TGAAGCGCGCGGCAGCGCCAAACACACGCCAACGATGATGTTGACGTCTCCCAACATGCA
GTTCTGGCCAAGCTGCCGAGGCCCTGCCTCGAGGGTTGGCGCATGGGCGCAGCCTCTAGA
TGAATAGAGGTGCACCATAAGGTGGACGCTGCACCTTGCGCCCAGAAAGGCACGCACAAA
TGGCACTAGGACTGTACATGCCCACCCCTAGCGAGCAGAAGCTAGGCTTCATGAGCGTCG
AAGTACTACACACGCGATCCTGCTTGCTCCTCCTTCTTGTTGCCTTCCGTCGGAGGCGGA
TGTAGGTGGCAGAACTTGCACTTCTCGCTGTTCATGCACCCGCGCTTCGTGTGAAGCCAC
CTGCATGGAGTGCAGGTTCCCATTGCATGCATGGCCGAGCCTTTTGAAGGCAGGCCGCCC
AGCTGCTCGTCCTGCGACAAAGTCGAACTTGGCGTCTTAAAGCTGGACGACTGGTTGCTG
CTGCCGCTGGTAGTAGTATGGCCTTCAGTGTTGCTGCTGCTCTCACTGACGCTCTCACGG
TCTGAGTTGTCGTCCTCGCAGACTGCACTCCAAGTTTTGGGCTCCTCCATCAACCTGGGA
TGCTCTGGCGGCTTGGAGGTCCGCCCCGCGGCCGCTGCCTTCTTCGCCGGCTGCGAGGAG
CTCGCGGTGACTCCCTCAGACCTGCCCTCAGCGCCGGAACTGC
>at01016
TTTTTTTTTTTTTTTTTTAGCACCAGAGGCACAACATTGGCTGGTATCGTCACCTCCCCT
AAGAACTTGCATTGTCATGGCTATCCCCGTCAATAGCCTCCGGCTGAAAGCTCACTTCAG
TGAGGCTGCAACCGGCGACTTGGCACGCTCACGCTGCACCTCCTGGACAAAGATCTGAAT
GCACCTCACGAAGGCCGCGGCGCTCCCGGGCTCCTCCCTGGGCCCCGGCGGGAAGCGCAG
CGCCAGGCAGCGGCCGTCCCGGAAGTCCACGAGCGCGCAGCGGCCGTCCAGGGCCAGGGC
CGCCGCGAGCTCGCGCTGCGCGGCCGCCGCCTCCTCGCCCGCGCGCACGGCCGCGACCTC

ACTGAGATGCACTCGTTGGGAGGCATTCCCGCCCTCTCGCTCGATCTCAAAGGCATCGAC
ATGTCGGCTCAGCCTGAAAACCACAGGATGCGCCTGGCGGTCGTGCCTCAGCGCCTCCAG
GCGGTGCCCACGCACGCTCAGCTCCACGAAGGCCTTGACGGTCCTCCGCAGGAGCTCCGC
GTCCGCGCCCGCGGCCTGTGATGCCCCGGCCGCGGTCAGGAGGCGTCTGTCCTTCGCATG
CAGCGTGGCGCTGGCCGTGTTCGAGACCGCGCTGAAGAAAGAGGATGCCACGGAGCCCCC
TTCCCAACTCGCCTCGCTAATGCGCGAGAGGTCGTCTTGAACCAGCGGGTGCATGATGAC
CCCTCCATCGTCGGCCTTTCCA
>at01017
TTTTTTTTTTTTTTTTTGATCCGATCACATCACAGTTCTGCAGACCCCCCAACCAGTCC
TAGACGAGTCCTACAACAGTCATGGCCAATCGTCCAGTGCCCACGGCGTCAGAGCAACAC
ACCCCCACTGGAGATCGCAGCCTCTTCTGCCCTCCGCTCGCGGAAGTCTGCCAGGTTCTG
CTCACTCGCCACGGTGTCCGGATGCTCGGGGCCGAGAGTCTCTCTGAAGCCCTCCGCGAC
CTTCCTGAACAGAGCCTCCGCTTCGCTGTCCCGACCCTGCAGGGAGAGTAAAACCGCTAG
GTTCTCTGTGCTGCGCAGTGCGTCTGGG
>at01018
TTTTTTTTTTTTTTTTTGACACATACATACACACACAGTTGCTTCCGGAACTTCAAAAA
GTCGCTGCGCTCTAGCAGGATGGCCCACATGAAAAACCATCTTCTGTAACAACACACATG
CTGCACATTAAGCACCAGGCTGTCCGAAGGCTGCTGATTCTCACTTGGGGTTGTACGGGA
ATGGGGAGTTTGCAGAGTAGTAGTTGCCGTATTTGCTCTCCCTCAGAAAGCCACTTTTCT
TCCACTCCGGGATCTTTGCAAGAAGCGCTTCGTTGGGCTTAGGGGGGTAGTTCTTGATTG
GGTAGGTGCCGTTGTCCATGTCAGGGATGGATGAGATCGGGGAGTACTGGTCCATGCCAA
ACTGCTTGTAGCGGCCAGCGCCCGTGGTGGGAATGCTGAACTTCTTGTCCGGTGGGGATG
AGAACTGCCGGTCAGCCCAGATCTTGAACTTTGCCTTGTTCCACGCCTCTTGCTCCGCCT
GCGACTGGCCTGTGCGAATGTCATAGTCTGCTGGTGCGGCGACCTCCTCGGCCTTCGCTG
TGCCTGGCAAGCCAACACCTGCCAGCCCAACCACGAGACCCAAGGCTAAGCCAAGCACAA
GGCCATGTACGGATCGGCCTTCGCCTCGTGCC
>at01019
CCGTAGCCATTTTGGCTCAAGTACCGACTTTTGGCTCAAGCTCACCTATGTTCAATAACA
TGCAGTGGCTCTTTATCCTTGCACGCTGAAGCGTACTTTTGAGGACGTGCGCAAGCCAAC
ACGGCTGAGGTGTCTCAGACAGCTATGACTTGTCAACCGACCTTGAGTGCGGCCGCCAAG
CAGCTGTTCACCCCGACAAGACTGAACTCAGCCGAACCACCTTCGTGGGGTCTTTTCACG
GACTTTTGCAGGCCTTCAGCCGAAAAGGCATTGCAATGCCTGAACAGCTGGGGCATCATT
GTTTCTTGACTTGTCAAGCTGCTGTGGGCAGTGGGAACATCTGGAGCCCCTTGTCCAACA
GATATACGCTGCTGTCA
>at01020
TTTTTTTTTTTTTTTTTCAGCCACTGGTCGCTACATTCACGCGCGACGCACCGACTTGC
ACAAGTAACATGCACAGAGGAGGAGCCATCCTCCAAGCCACTGTACAAGCCTGGCATCGG
CTTGGGCTCACTTTCAAGGAAGAGGATCGATTGCAGCCGCCTGCAAACACCTGTGCTGTG
TTGCACCTGCCTGGCCTGCAAACATCATGTCATCGCAAACAGCTTACGCTGCACGCTCGA
AGACGAAAGGCACGCGCAGCGGAGCAGGCACCTTGAGCTGTGCTTGCAGCCACTCGACAA
GGTCACTGCCCTCAGCTTGCATCCATTCGATCGCCGTCCTTCCGCCATGCTGCATGTCCA
CCCGAGCACCGTGCTTCAGCAACTCCTCCAACAGCGCCTTGCCTGACTGTTTTGTCAGAC
GAATTCTGCCCGGGCATTCGCCTCCTGCAGTGCTGCGCCCCAGCAAAAGAGATCAGAGGG
GAATAGCCGAAGTAACCATAGGGCTCGTCCGTCGAGCGATTCACTGCATGATTGTTCTGG
AAGTTGACCATCTCGGGATACTTTGTGCAGAAGTCCTTCACAATGGTGGTCATTTGGACC
TCGTCGTCTACCCGCTCCATGCACTCGCCGCAGAGCTTGGAAGACACTTGGCGCCATGCT
GCGATTGACTCCTTTTCGTACTCCATGTACTCCGTCGCCTTTCCTTCTAAGTAGTCAACG
AATGCGCTACCGGAGTCCCGCGCCTGGTCGACCAGAAATCCCTTGGCGTGGCGGATGGTG
ACTCTTGCACCACGTTGGATCAGCTCATCGACACATTGCTGCCGCAACTTGGTGCTAGTC
ACAGACTCAACTGCAAAGCTGAGAAGCGGCTTGGCGACCTTCGCATCCACAGTCACAGAC
TGCTGCCAATTGACAAGCTCAGGATAGTTGTCCAAGAAGGTGCGGAGTCTGATGAGCGCC
ACCTCGTCATCTACCTCATGATTCTTCAACTCAGCAGCCACTTCACGCCACTCCTGGAGG
CTCCTCTGCTCGAATCGCTCGAGAGTTGTATGCCGGACCATGTACATTGAGACGGTCTTG
CTCACAGTTGCATCTGAATCGGAACTCATCATGTCCCTGGCAGTCACCTTGCAAACAAGC
ACGTCGTCGTTTAGCACGATGCCGCCTGCGGTGAGCTGGAAGTCAGACGCCTCGAGGCCA

CACTGTACCGCAACTGCTTGCTTGAGAAACTTAATACTCGTGTCTTTTGGCACGCTGACT
CCAAAGCTCTCACCCGACATTGATAGAATGGACACCTGAATCCCTTCATCAGTGGCCTTC
CGATGGTTGACTGAATCAATGCTGCAATCCGGTTGGCATGGCCTCCGAAGCCATTGGAAG
CAGCCGAAGCACCACCTCACCATGCCACAAGAGGACGGTAGAACTGAATGTGTTGTAAAT
AAGCAGCAATGGTGGGACATAGAACGCTTGAGCCAAAATGGCTACGG
>at01021
TTTTTTTTTTTTTTTTTCGACAAGAACAGCTCTTCAAAAACCCGTCCCGAACTCCAGTG
CCGCTTCAACCCAGCACGGCCCATCAGAGCCTCCCAATCGACAGCATGCGTGCAAATGCG
GCGAGGAAGAAGACGAGGAAGAAGATGGCAAATGCGAAGCCAAAGCGGTTGAGTTCCTGC
TCGGTGTACGCAGATGCGAGCTCCGGCGCCACCGCGGCCGCCACTGCGGGTACGGCGGCC
GCCAGGCCTGCCACCGGCCGCTGAGCCGGGGCAGCCACGTCGGCCCGGGGGCTGAGGAAA
GCCTGTGGCAGCAGAGCGCCACGGAGCAGCACCACGCAGAAGACCCCGGCGAGCAGAACT
GGGAGAACCTTCGCCCCGGCCATGGCTGCTGGAGGGGAGTACTGTACTGGGGTGTGCTGT
CTGCTCGGAGGCTTGCCT
>at01022
TTTTTTTTTTTTTTTCTTCTTACTCGTCTTCTCGCTCGTCAAGAATCGGTCACCGTGGCA
CCGCTCACAGCTGCCATCATTCTCTTCAATGTTGAGGCCGCAGCAGGCCCAGCTCCAGCC
AGAACGCCCCCGCGCAGGTCACCAGGACCCGGGAGGGCGACAATGGCATGCACCACGACC
ACAGTCGGCGCCATCGAACTCTGT
>at01023
GTGTGGCCATGGTGTCTTAGCCATGGCTGTGGCTGCGGCATCCTCTACCGGGGGCAAAGC
CCAAGAAGCTCTTGAAGAAGAAGAAGGCCAAGCAAGCGTCCAAGGAGCCCCCCAAGCAGC
TGCAGGAGGCGCCCGCCGAAGCGGCCGGCAGCGGCAAGGTCAAGCGGAAGCGCAAGGAGC
TGCAGGAGGCACCTGCCGGGGCGGCTGAAGGAGGCAAGACCAAGCGGAAGCGGAAGGCGT
CCGAAGATCAGGAGCCTGACGCAACCCGAGGCCGGCCACGGCCAGGCCTGGAAGGCCAAG
AAGAAGAAGCGGAACCGGGAGGCGGCAAAACAGAAGCGGACGGCAGCGGCCGGGAGCAGG
ATTCCTCTAAGCCCGATGCCTCTGGGCTGACGCTCTTCGTCGGCCAGGTCCCGTTCTCGG
TCAAGGGCCCAGAGTTGCAGCGGCACTTTGCGGCAAAGGGGGTCCAAGGGAAGGTGCAGG
TGAGGTTGCTGACGTACAAGGACACAGGGAAATCGAAGGGCAT
>at01024
TTTTTTTTTTTTTTCCTTGGCGCCGCTGCATGTTTGTGACGTGCCATCCATCGCTCGCCT
TAAATCTGTGCAGGTTCCACTCAAAAAATTAACGGCAGCGAATGCTGCTCCGAAAGGTGG
CTAGAGTATCAAGCCTCACGGGAGCCATCCGTCACTGGTGGACGACACAAGGAACGCGTG
GGCGAATCATACTGCCACGAACAAACAGGTCACCAACCGCTGTGGCTGCCTGACAAGCAG
TTGTACCTGTCTATCCTCGTGCC
>at01025
CGGAAGGGATCAGCAGCAGCTCGGCCAGCCAATGCTGAAAGCACACGGCTGTCGAGCCGC
CTGGCGCTCTCCTTCAGGTATTCTACTGCGTTGGCCTGCGCCTGAACCTGCATGTCTGCC
CTGAGGCCAGCCAGCGACGTGCCCCTTTGCAGCATCTGCGGCAGGTACCTCTCTGAGTTG
CCTGTCACTGCCTCGCTTGAGAGGATCTCAATCGCCTTCTGGACTGCCGCAATCTCCTCA
GCACGCAACTCCTGCCGGGATGAGAAGTCAGAGGACTTCTGCTCGCACGTAGCAGTGAGG
TCACTGACGGTACC
>at01026
TTTTTTTTTTTTTTTTGTGCAGACAACACATGATGCACCAACGATGCGCTCAACACTGCA
CAGCGCGACATGTGCGTCCTTTGGGCAAGGTAGAACACCCTGTACACATCGAGGGCTGAA
AGCCTCGACCGGTGTAAAGCAGAGAGCCTCATTGAAGCAGGGGTTCATGAACACGGACAA
AGGCTTCCCAACTGTACACTGGGACTGGGCCTGTGTTCAGCTCCCGCTTGCCTATACTGC
ATCACTTGCCATCAGTGTGGATGTGCACATGTACCGCGCGAGAACTCACTACCGGGGCGC
TCGCCAATGTTGTCGTTGGCCTCCTTTAGCATCTGTGCCTTCTCAGCTGCCTTCTGCCCA
TCGCCATTGCCTTGTGACTCCCCACTTGCTTCCTTTCCAGCCTCCTTGTCAGCTTCATCC
TTCCCACGCTGGTCAATTGCAGCAGCAATGGCACCCACTTTGGCCAGCGCCTCAGCTGCC
GAGCACTTGCGATGCGTCACGAAGAAACTTTGCAGCTGAGCAGCGCTCACCTTGCGCTCA
CCCAACGCCACGAGCAGCGCATCTCGAAAGTCTGAACCGGTAGGAACCTTCTCGAGGGTC
TGGGCCACGCCTTCCAAGGCCTTTGCCTTCTTCAGTGCTGCCAACTTGGCCTCCAACGCT
GCAATATCAGGATCCACATCCTTCGGGGCAATGGTGGTGTCACTCCCTCCGACGTGCTGA

AAGCTGGGTAGAACTTATCAAACATCTTCTCCATCTGCTCTGGGGTGCAGTAGTCGAAAT
GAATCCTGACATCAACCCTCCCGTCGCGAATCAAAGCCGAGTCCAGCTGATCCTTGAAGT
TGGTCGTCAGAACGAAGACCATGCCGTTGTGATTGCCAACACCGTCCAAGGCATTGAGAA
GCCCAGAGAAGGTCAGGGGACTTGACTGGACCTTTTTCTCACGACGCTTGCCGAACAATG
CATCCACATCCTCCAAGATTATGATGGCATTGTCGGGAACCTTCTCAATGGCCGACTTCA
GGCTGTCGTCATCAAGTTCGGGATGGGTGGACTGAGATAGCAAAGATTCCGGCCAAACG
CACCAGCCAACGCCTGAATCATGGAGGTCTTCCCTGATCCAGGCGCCCCATAGAAAAGGT
AAGACCTCTTGTAAGGAATTCCATGGTCATGGTAGAACTGCGCCGTCTCCTCTGACAGGA
ATTCTGACAAGTCTTTGACGAGCATCTCGGATGTATCCATTGGAAGCACTACACTTGAAC
AGGGCGGGCAACAACCCTGCTGACACGCTGCCAGAAGTGGTACTTTACGTGCCACCTGTA
AACCTGGAACGTGTTCTTTGCCGATTTCTCAGACTCAGCCAAGATCTCGGAGAGGAAGCC
CTGGACTGCTCTCGCTGCCTCAGGGCCCTGCCCCTCGCAGAAGAGGACCAGGCTGCTCAT
CACCGAGACTCCGCAGTCCCGTCACCCACAGGCTCCCCGACGTGCTGGTGCAGCGCCTTC
AGCTGGACGCCATTGTGCTCGAAGCGGAAGATGCCGATGCCAAGGGAGGTCGGAGACGTT
GCTCAACGACGGCTCCGGGTCGCAGGTCGCAGCCAATGCATGGCCTCCGCAGCACCGCTG
TTGCCCCTGCCCGGACTTCTCAGCGCGCGCCATCTGGAATGAAATCGCGTCTTCTTCCAC
TATGACACCGGGCTTGCAGAGATGCGCGGTCACCAAGGCAAAGAGAGGGTCGCTGGGCTG
CACCTCAACTCGCACGTGTGCGTCTTGTAATCGAAGGATAGCGAAGACGGGTCAAATGGA
AGTGTCCGGGACTTTGTTGTGGAGTCGCGTCTTGCGGAGATGTCGCTGCCATCAGCGCCC
ATCATTGCCCACTTGTCAGTGCTCATTTCCTTGACTTCTGGTGCAGAAGACGGGAGGGCT
ATGATAGCCGAAGGTGTGATGCTCTGCCAGGGCTTGAGCCAAAATGGCTACGG
>at01027
GCGTCCGCAAGGCCTGGAAACCAATGTTGGCTGGAATCAGAGCCCGGCAGAGGTTGCTAT
ACAGTGCCGCCGCTCGCTCGCTCTGGGGTCTTCGACTCGTTCAAAGTGAATCTGACCTGG
AATGCTCCAATGAAAGAAGCTTCAACGCTCATCATCATTCCAAGCTCTTTAGTAATAATA
ATAACCACGTGAATTCACGCCTCGTGCC
>at01028
NTTTTTTTTTTTTTNTTTTTTTTTTTTTTTTCCTTCTTTTTCGTGGGGCGTTGACAAGCA
AACACAAAGGTCAAGTCTGGAGAGGTTCTTCCTTCCCTCGTGCCAGTGTGTTGCCCCAAG
CCTGTGGCAGGAAGCAGCTTCGCTGCAACTGACATGGCAAATTGAGGCACTACACACAAA
AGTACTCCGCAAATCGCACCGGTGCGCCAATGCTTCACATCATACTGCAACCGCACCCAG
GCTGGCTGTCATAGTTGTTCCCAAATGGACCTTTGTAGGCAGTAACCTCAACCTTCTCCT
CAAGGGGCTTCGACGTGTCTATCTCGTCAGGGAACGAACACCGGTGCATTTTCTTCCCTT
TTTCGATGGCATTGCCAAAGGTGTCGGCGCGCGTCTGACGGCCGCTTTTCCCGCTTAGTA
TACTGTGTCTCGAGGGCGCCCGCTGTATATTCCTATTGGCTGGACTGGATGCAGGGCTGC
CGGCTGGGCTCGAAGACCCGTTCGACTCCAGGGGCATCCGTCTGAAAACCACTGCTGGCT
GCTCCAGTATTTAGGGGCCAGCCTGTTGCATTGCCACTGAAAGGATGGCGCAACGTGGCC
CCCCTTTCATGTGAAGCTTGGGCAACTGCTTGAGCCAAAATGGCTCCGG
>at01029
CAGGTCTTGAAACCAGGTATGTCCTCTTCGCGCATCACCGTCTGCTTGGCAAGCGTGGGG
AAGACAAAGCCTGCAAAATTCTGGCTCGCCAGCCATGTCCCCCTCCAACCAGTCCCGGGT
ACGTCTGGCGACGCGGAGGGATGTTGAAGATGGGGAGAGGCAAGCTCCACGAAGTTTTGC
CTCATAATCCGGGCGCGCAGACCGCTGCCGACGGTAACATCAACGGCCCCGCCGCTGATG
CCGCAAG
>at01030
GACAGTGAAGGACACCACATTGGGCATCTTGGACACGACACTCCCATACTCGATGTCCTC
CTCCTCCTCCTCGCCGGCCTTCCTACTGAAGCTCCCGCCATCCTCTTCGTTGTAGATCGC
ATGGATAAAATCCCACTCAATGGCCTCGTCCTCTGTAGTAAGGACAGCTGGAGGTCTGCA
GTGAGCTTGTCATCCATGTAACCCATCCCACTCAGATCTACGGCAGTAATCCCCGTCGGG
ATCTCCTCCTTAATGTTGTCAAGCTCAGGCTCTCCAGCCATGG
>at01031
GCTTGAATAATTTGGCGCATTTTTGTTTACAACCTGACCGCCTCCCTGGTGTAGTTTTCC
CTGATGGCGGAACATGGCCAGGGCGCTTCAAAGGCAACCCTCAAGTCTCTGCTCCGCAAG
GGCGACTTGCTGACTGCCCCAGGCGTCTTCGACGGCATATCGGTTCGGGTGGCTGAGAAG
GTTGGCTTCAAGGCTTTGTACATGACCGGGTTTGGGGTCGTCGGCTCGCACCTGGGGGTG

CCCGACGCTGGCATCGCCTGCTACCGCGACATGGTGGAGCGGGTCCGAGTGCTCTCGGGC
CTCACTTCTGTCCCCCTGATCTGCGACGGCGACACgGGGCTACggGGGGGCCTCCTGAAT
GTGGAGCACACCGTCAAgGGGCTACGAGGCCGCCGGCGCCAGCGCCATCCaAGcCTGGAG
GACCAGGAATTACCC
>at01032
CCGAAGGCAAGGACAGCGCCCTGCTTGGGTTCCATCTACGGACGTGATAAAGTCGGCTCC
ACGCCATCCAAGTTCCGGAGGAGCTGTCCGGGATGCTCCACGAGATCGAGGGGGGATGTC
GGCTGGGCGGCCGGCACTTGGCCAAGTGGCTGGCGTACGCGTGAGGAGCACGAAGTACGT
CCCGGCTCCCCGTGCTGCA
>at01033
TTTTTTTTTTTTTTTTCTCAGGCGGCAGACTCAAGCGCTGGACTTGCAGACGCAGCCTAC
GTTCACACAACTAGTGTGCTGTCCCGAGCAGTCGCCTTGGCCGGCATCGCGTGCCCTCCC
TCTGCCAGAATCGTGTGGTTTGCGATGGTCTCCTCCAAGAAGGACCAGACCCACTGTCCG
AACTCCGCATCATAGGCAAGACTGTTCGTAAGCCTGCCTTCCAGCTTGACTGAGCTGTGC
CCAATCTGACGCACGCCAAAGTACGTCACCGGGCGCCGCGCGGCGTTAGGAGACGATATG
ATCGTAGCATGCAGCTTGCCGCACTCCACTGGATCCAGCCCAGCACTCTTCAAGGCGAGG
TTTGCTAATGGTATGAGCCACTTGGGGACGAAGCCATGGTCCAGGAGCTCAGTCGCGACA
ACGCCTGGAAACGTGCCGATGTAGTTGACCTCCGGGTGATGCTTGGACATAAGCTGCAGG
TGGAAGCCAAAATGCTCATGACACGTGCGCCGGAGTTCAGTAATGGGAGAACCTCCCTGG
TGACCAGGAAGCGAGAGACCAAATCCAAAGAGATGACCTTGTCCAAGCCATCATCTGTGC
GGTGGATCCTTCCAATCTGGCCACACGCCCACAGTCAGAACAACGTAGTCGAAGCGGGTC
CTGTTTCCCCTCAGCTGCTCGGCTAGGCTCAAGCCTCCCTTCACGGTGAACAAGTCCACC
AGGTACGATTTGGATCTCCGCTCTTGTGGGAACCGGGCTACCGCTGACATGTTGCCCACG
ACGGCCTGGGCCTTCGAGGCCGAGTGGCCAACTACGACGACGCTGGCCCCGGCCTGTGCC
ATGGCAATTGCGGTGCCCCGGCCAATGCCGCCGTGCCACCAACAATCAAGGCAGACTTGC
CGCGCATGCGCTCCGTGAGTGCATCTGCTGTCACTGGCTCGACGTCCCCAACTTGAGGGT
CCAGGGGAAACCACATGTCTGGAAAGAGAGGCTCGCCCGCGACGGCACTGACCAGCCACA
GGGCGGTAACAGCGGCATACTCATGCGCCCTGCCCATCTGTGCCTGTGAAGACAAGCTTC
AGACTTGGGGCTCGGACTGACCGAGGCCGGCAAGCCTGTCGGGGTTGCCAGGATCTGAAC
CCTGACCGACTGCTTGAGCCAAAATGGCTACGG
>at01034
TTTTTTTTTTTTTTTTTTCCAATATGTAGCATGGATTTCAAGAGGTCCACCAACACGAAT
TGTCCGATCTCAAGACTCAAGCACTGGTAGTGATTCAAGTCAAGTGCGAATTATCATCTG
CAGCCACGCGTCACTCGCCACCCTTCACGTTGCGCTTTGCCTTTCCAAGCTCCACGTTCC
GCGCCCGGGAGGCAAAGTTGAGAGAGCTCAGCGTCTCGGTCACGTTCATCTGCGCTGGGG
AGCTCTGCACGATCATGAGCGTCTTCGAGTCCCCGCCCAGGGAATCCTTGAGCATCATCG
TGAGCTTTGAATTCCTGTATGGAACGTGGGGCGACTTGGCCACCAAGGAGCCTATCACAT
CACCCAAGGCGCTGAGGCTTTTGTTGATGTTTTGGGCCTCACGCATGCCTTGTCCCGACA
CCCCGGACTTGTTGACATTCTCGGATCCAGCGAGGTCGATGAGATTGATCTTCCCAGTGT
ACTCGCGTTTGTCCTTCTTGGAGGTGCTCACAATCCTCACCTGCACGATACTGTGAGATC
GGCCCTCGTGCC
>at01035
TTTTTTTTTTTTTTGTTTCCCCCAGCCTTGATGGCTTGTTCAAGTCGCATACTGAGGGAG
GCCATGCCGCTACACACACTACTCCGCAACACCAAGCAAATCCAAAGTTGAAAGCAACGC
AGCGTGAGCCCTCAATTTGACGTCCTCCATCACCCAGCCCTCGTGTGGGCACTGCACCTA
CCCATGCTGTAAAGCCACTAATGTACCCACAGTTGCTAGTGAGCGCCAGCGTCAGCACAT
TCGCCGTCCTCGCCGTCGTCAGAGCACGCATCTATACGATCTCCAACATGAACAGCCAGA
CTGGGCCAGTGCCGCCAGCGCATGCCGTCTTCCCCAAGTGCAGCCTCAGCATCAGCACCC
TGACTCTTGCCCCTGTCATTAGGACACCGTGGCTGCAACACTGCTAGCGGCAAGTAGTAT
GTTTCAAACTTCCAGTAACGCTTGGCAATTCCGCGCCATGTGGAATCGAGAAGAGGTCCA
ACTTCGACGGGAAGGAATCCACACGTGAAGCCCAAAGCTTGCAAATCATCTTGCGCCATT
CGACAATACACTGCGTGAAAGAAGATGTTGTATGATGCCATTCCATTAAAGAAGGAGAAG
CGGTCGCCAGGCCTCAGAAGCCGAGGCAGGTGCGACTCGAAGTCGCGCATGTCGGCGTAG
GACTCTTCATACGTATCGAAGAAGATGGCATCG
>at01036

TTTTTTTTTTTTTTTTTAGTGCTTTAGCTCCGAGACTGTAAGGTCTTGAATCCAGTCAG
AGGCCTGACCCTGTACAAGGGGCTTCACTCAAGCGCGCTCGCCTGTACCAAGCCGGCTTC
GAAGTCCTCGAGATCACGGCTACTCTCGAGCTCAACCGCCGGATGTCGCACGCCCATGCG
GCGCCACGCAGTGCCAGAGCCCCTCATCACCATCATGGCCGCCGCAAGGACCATCACCGC
TGCCAGCAGAGGTGCCACAGTCGTGCCAAGCATCCGTGGGACCGGACGTGCATGCATCTC
CGAAGCTGTCGCATGCATCTCCGGAGCCGTCTGATCGAGCCCAGTGAGACTCTTCATGAG
GGTGCCCGCGCCGTCAACACTGTACAACGACGGGCGCAAGCTGAGGACGGCCTCGCGCAG
CATCTGCCCCAGGTCTTTGCCCACGGCAAACCACTGCAGCTTTGAGAAGTCCGAGACGCA
AACCTTGAGGAGGTCCACTGCTCGATCGACACCCACAGGCACCCCCATGCCTGGCATCTT
CAGCTTCATGTGCAGGTGCTGCAACTCCTGCACGGAGCCCATGAGCATCTGCTCCTGCTC
CGCGTCGACGCCGCACTTCCTGAGGGCACCCGGCAGCCTCATCAGCGTGCCCAGCAGGAA
CGTGGTGCTGGGCACCCCTTGAGATGGCGCAACTCCTCCACGGAGAAGAACTGCATGGCG
TCAGCCATGATCGACTGGAAGAACGGCAAGTTAGCCTTGATGCANCTGTGGAGGTCTATG
TGGA
>at01037
CCGTAGCCATTTTGGCTCAAGCTGCGACGGCTCAAAGACCGGTCCAGGGACTACTCCTCT
CAAAATGGAAGCCGGGGCACCCTCTCCCGACGGCATTCTTGCGGGTAACTACGGCCTGCT
CGGCAAGTGGTGGTTCTACGTGTTGCTCTTCTTCGCCCTCTACACCTTCACAGTTATTGT
GCATGTCTTTTTTGACTATAGGCAGTGGTGCTCGGAGCGCAACCGTGAGGAGATGGAGTC
GAAGGGTCAATAGTTTCAGAGGTACACCTCTTCTACGTTGGCGGCAACTGGTGCTCAGAC
CAAGCCCTTGAGGAGTCATTTGCAGACAAGGCTTCACCGTTCTTTCACATACGGCGACCA
GCACACGATCCTCGGTGAGGTCCATCCAGCTGTACATGCCGCCTTGCAGCTTACCCAACA
ATTGGATTGCCAGTGGCATCTTGGTGGTGTAGCGGGGTTGCTCCTCTGATGCAGACTGCA
GATTTGGGTATCCCATGGCTCGGAGGGGTGGCACAGTCTGTCTCGCAGCTATACAAAAAA
AAAGAAAAAAAAAACAA
>at01038
CCGTAGCCATTTTGGCTCAAGCACACTGGCTGCAAGCATCCGCGTGGGAGCGGGCTTGTG
CCCGGCCATGACGGCTGCCGCGGCAGGGGGTGTGCTTCACGGGCGCCATGACGACGCGCG
CCCGATGTCCAAGATCACGCTCAGGTTCCATGACCGGGGAGTGGAGGCCGCCTACAAAGC
AAAGCTTGAAAAGGAGCTCTTCAAGCCCACGCTGATCTCGTGCACCTTCGTGCCGGTCAT
CTTGTCTGTCCTTGCTCTGAGCACGTACAGTGTCGAGGCAAAGCGGCCGGAGTTGCCCTT
CCTGTGGGGCACTGACGACCCCACGGACAATGTACTTTGCGACGCATCTGGCAATCGTCT
TG
>at01039
CTCTGACCCCTGTCCACGAAGGTCTGCAGCTCGCAGAAGCTGCAACCCCATGTCAGGGCG
GTGGTAGTACAAGGCCAAGTCAAGAGGCGTGCCCAGCTCGCCGAAAGGTCCAAGCTCGTA
GTCGGAGGCTAGCAGCGGTGCTCCACGCCGCAGCCATGCACGGACCTCGCTCATGTCAAG
ACATTCGATTGCTTTGGCCAGGTGGCCTTTCAAAACCTCGGTCGCTGCCTCGGGCGCCTC
TGAGTCGGATGCCTGGACATTGGGTTCGACGGCCCCCTCAGTGGCTTCAACTCCGCCAGC
CTCCATGGGCGTGGGCAATCAATCTATTTGCGCAACTGGTGGTCTGGCGACACCAGCTCA
AACCAAATGACAGATTGAGGGGAAATGGCTATGGC
>at01040
CCGTAGCCATTTTGGCTCAAGGGGACGGGCTCAACAGGATTTGTAACCCTCTTCCCCATG
CCATGAAGGCCATGTTGCAGCTTTGCACGCCTTTGACGTCTCGTCACCATGACTCTGTCC
GGGGCGCTCGCTCCGCAAACAGAAGTCGGCGGCGCTTGAAGGCGCCTCAGTTGGCGATAG
CCTTCCTCTTGGTCGTCATCTTTGCACAGCCCTTCGGTGCGGCCATCCTGGCCGTGGATA
GCTCTGATGGCACGTCCTCTGAGTTGTCCTTGTGGACTGTGTTGAGAACGGCTGCCAGAA
GCGCCTTCCGGAGTGGCTGGGCCGGGTTTGCTGCTGGTATTTGCCAAGTGGTCGCCTTCA
TGTGGCTCCGCTGCATAATGAACCACCAGTACAAGCACGGCGGCAGCTTCGGCGCCACCG
ACGCGTGCGCTC
>at01041
GATCTTCTCCTCTTGCTTCCCCAGCTGGAGGAGAGCATCGACAAGCTCTTGGGCTTGTTT
GGCGGTTTCTTCGGGCCTTGCATTGGTAGCACTGAGCGAGAGGCCCTTGATGAGGCAAGC
AGGCACCTGGGAAATTTGACCTACGTGGCCGGCCACTTGCAGGCAAATGGTGCGGACATT
GTGCGCGAGCTGGGTGATGCCCATGAGGCATTTGAGAAACAGGAGTTCTTGCACTTTGGC

AAAAGCATAGGCAGGGCATGCCGCAAGGTGCTGTTATCAAAGACCACCTCCCGTCTGCCT
GAgGGGCCGCCTTCGCTGTCAGCAATTGCAAACTTGACTGCAGGCGTGTTACGCGGGTTC
TTCGGGCgGGGCTTCGAGCTGGACATCTCTTCAGCTANCACTGACGTGCCGCCGGCATCT
GCGGAGAAGACCACGGTGGACTTCGGATCATTCGTCGTGAACGACGCTGAGTCTGAAGCT
GCATCGCAGGCCCCCGTCAGTTTGCACGTTGACCTTCACAAGTGCGTCAAGGCCAACCTC
AAGTACTTCCAACCTGCCTTGCTCGGCACCTTGGAGTACCTTGCGCGACAAGACTTGGCC
TCAGACGAGGACCCCCGAGGGACGGCCTTTCCTGACCTTGTCATGACCATGATGGACGAT
GGGCCTACAATGCTGAGCAAGTGTGGCCTCGGAAGGAGAGCTGGCAATGTTGGAGGAGGC
GGTGCTGGCCT
>at01042
TTTTTTTTTTTTTTTTTTCACCCCTCCAAAAAAATCCAATGTCTCACAGGTTCTCAATCC
TCTGGGGGCAAGGGCAGCCCTGCACACGCTACGGAAGCCGCACTCCCCGCGGAACGCCCC
CTGGCCGCGCCGCCCCAGGATGGAGCACTCCGCTAGCCCAGGCGGAACAGGCTCAAGCCC
AGCATGCAAACAAGCATGAGCATTGCATCGCTGTGGGGCATGACCAACACGCGCCAAAAG
CTGCTGGGATCGCATTCCTGGTTGAGGCGGAGGACGCTGGCCATCTCCACCGTCAGCGGT
GAGGCCGAGCCTCGTGCC
>at01043
ATTCACACAGATGCTGTCGGAGGTGACTTCTGAGATTCACCCTGAGCATGTCCCCGGGAA
TGGACCAGAGCAGGATTACCTGTCACGCTTCTTCGCGGCTGCTCCGTGGTATCACATCGG
TATCGAGTACAACTTTCAAATCCATCACATCCCATTTGCCTTGGAAAACACGCTCAATCG
CTGCAATTATGCTGCTTCGCAGCCTGACTCCCCAGAGGAGCACCGCCGCTGGCTTGCGCC
GCGCATGCGCATCCCACTGGACGAAATCAGAAATGTTCACTTCTCTGGGGACTTGAAGTT
CTGGGATTACGCCCTGTTTGAAGACGTGGAAGGGATGAGCCACGGACAGGTGGCTGAAGA
CTTCCTGCGGAGAAGCCTAGAAGGATACAAGCGCTGGTTCGAGCTGGCGGAGCCCGAGCT
GTCTACGCGGACTTTGGCTGCCGGCGCCGCGAAGGCGGCCTCGAACTCATCGCAGACAGA
T
>at01044
TTTTTTTTTTTTTTTTTTGCCATCTTCCAGCTGCACTCTCGCGACCAACCGCTGGCTCGG
GTAGCCCGCTGCGGGGCTAGGCACCTCAGCGCTTGCTGGCGGCATCACGTGGCGAGGTGT
CCTGAAGACCTCCATGGACTCGCGGAAGCGCCCAGTGCCTGCGTAGGCGCCTACCAGCAC
GTTGCGCGCGATGTCCGAGCGCGGGATGAGGGAAGGCCGGACCTCCTGCAACAGCCCCAG
CACGTGCCGCCACTGCTGGCTGTGCCCACAGATGCTGATGGCGGTGGTGTAGGTGATCTC
GTTGGGCTCAAGCCTCTGCTCCCGCATCGAGCTGAGGAGCCAGATCCCGTAGCGCCAGTG
CCGGCCCTCGTTGCACGCCCCCATGAGCGCGTTGTGCGTCACAAGATCAGGCCGGACCCG
GCACACGCGCATGTTGCCCAGCAGCTCCAGGCTCAGGTCCCAGCGCGAGCCCCGGCCCAG
GCTCGTGATGCAGGCGTTGTAGCTGAGGCGGTCCGGTTGCACCCCTTGCGTGGGCATCTG
GTCCAGAATCTCCAACGCCTGCTGCCACATCTCCTTTCGGCCACAAGCGCTA
>at01045
TTTTCTTTTTTTTTTTGTTTTTGTTGCCGACCAGGACAGTGCGAGCAGCTTGTCAGTACGC
AAGGCATGCGCCTGCCGCGCAGGTCCACGTAGTGCTGCTGGCGCGCACAAGCCGCAGCGG
GCCGCCGCTTCACGGCACGGCACCGACATTGGCTCCCCTGCACGCGCGCGGAGGTCCTCT
CAGGCTAGGGCCGCAGAGGGGGGCCTGAAGAGCACGCACAGCAGAGCAGAGGGGAGAGAC
AGCCTAGCCTCTTGCAGCATGGCAGCTCTAGGATGTGCTTCAGGGTCGACTGCTGCTGAT
GCTTCTCCTTGAGGACCCAGTCCTTGGTGGGGTAGGCAGCGGGTCCAACTTCAGTGTCTG
GACCTCCGCGCCACCGGCCCGCGTCACGAAGGGCGAGGGCGGCGTTGAAACATGGGACGA
GACCGACCATGGCCGGTTGCGGTCGAAGAAGTTCCTGTCGCGCGAGGGGTCGTAGCCTGG
GAAGTTGGAGATGGACCGAGCGCGAGGCTCTCGCTCGACGACGCCAGGCGCTATGGTTCG
CCTCCAGTATTGTCCACTTGGCTTTCCACCGCGGAGCTCCTTGAGCGAGCGGCTGGCGCC
CCAAGTTCTGAAGTCATAGCTCCCTGTCCGCTGCTGGCTCAAGTAGGAGTTCCTGCCGGT
GCTCTCCCCGAGGCCATCCCGCACCGGCCGCACGGTCTGCGTGCCCGGCAGCGGGAACTC
CTGGAAGACGGGGTGCCACTCCATTCCGCCTACGGCCGGCCGGCGCCCCCTGCTCGCGTT
CCCAGCTTGAGCCAAAATGGCTACGG
>at01046
CCCCCAGCGCACACCTTGAAAGGGGTACTCCCAGGTGAAGGACGCGAGGGGCAAGGGGGG
AGCGGAGCGCAAACCGGCCTCACACAGCCTCCGCTGCTCCATCATGGATCAGGATGTGTG

TTTCCTCGCCCGGCCCCTCGTGGACTATGGCACCCGCCGCTTGTTCCCGACGACTCCGGC
AGTCCTCGGCCTCGGCCTCCCCCT
>at01047
TTTTTTTTTTTTTTTTTTCAAACGCGGCAAACAAGATGCAAATGAAGGCTGCACTATTCC
AAAACAGAGCAAAGCCACGCTACAAGAGGCCTTCGCACGTGGGCTTGACAAAGTACCAAG
CATTGAAGTCATAGCAATCGTTCATCCAAGTGATATGTATGTTTGTCTTGACGTGGCGCG
CAAATCTGTTGACTGCCCTGATCACACCCCACCTGTCACGGAGAACAACATTGTCATTCT
GTGAGGTCTTTTTGCGATATTTGCAGATAGGCTTGATGGCGACTTCTGGACATTAGTGAT
GTGTCCCTGGAGTCAGCATAATCGTCTCCCGACATGAGGCCACCTGGGCGTAGCTTGGGA
TACCAAGCATGAAGGTCACGAATCACAGACTCATAGGTGTGCATCGCGTCAATGTACAAC
CAATCAATGCTGTTGTTGTTGAACCTTTGTGCAGCTTCTGTTGACCAAGCCGTCATCAGG
TGTCTACGATCCGAAGCAAAACTGGTACGATCCTTTGCAAGCTCTGTCGACCATGCATCA
ACCATGTAGTACTGACCTCGCCAATGTCGCAAATTCTTCGCAGCAAACGCTCCAGTCCAC
ACTCCAACCTCGACAGCTACCCCAGTTTTCAAAAAATGCTGCTGAGCTACCCCGGCAAAG
TCATCCCTGCAAGGCACTGGGTCAGTTGGCCACAAATAGTCGTGCAGTTGACACGCTGAG
ACTTTACAGATATTGAAAATACCAAACACATGACGTCAGCCAAGCTGGCAGCTCTTCCCC
AGACCATAATTGCATCGTCCCCAGTTAGTGCGAGCCACGGGGGGTTTGAGCCGAAGTGAC
TTGAGCCAAAATGGCTACGG
>at01048
CCGTAGCCATTTTGGCTCAAGGCATTGTGGCTCAAGGTGCCAACACACACAAAGGGGCAG
CAGGACTGAGATCTGTGGGGTAAGTCAGCCAAGCTGCCAAGATGGGTCACTTCCGCAACC
ATGTGGTCCCCAATGTCCACTTCCGAAAGGTCAATGGCATGATGCATGGCCGCAACAATC
GGGTCATGATGAGGACCTGGCTGGACCAGGCGGGCCGCAAGAAGCGCCGCTTGAACAATC
GCCGGGCCAAGGCCGCGAAGATCGCCCCCCGGCCCACGGGTGGCCTCCTCAAGCCCCTCG
TGCACCCGCCCACGCAGCGCTACAACATGAAGCTCCGGTTGGGCAGGGGCTTCACGCTGG
AGGAGCTGAAGGAGGCGAAGATCCCACGGAAGCTGGCTTACACCATTGGCATCGCGGTGG
ACCACCGCCGGAAGAACCGCTGCATGGAGAGCTTGCAGCTAAACGTTGAGCGCCTGAAGC
TGTACAAGTCCAAACTCCTCGTCTTCCCCAACAAGCACGGCAAGAAGGGTGTCAAGAAGG
GTGACACCCCCAGGTCTGAACTCCAGAACGTCGCGCAGAACACGCTGAAGGAGATTATCC
CGATCCCCAAGCCAGAGGACATCATTGAGGCCCGCGCCATCACCGCGGAGGAGAAGGAGG
CATCGGCCTACAAGACGCTGAGGAAGGCACGCCAGGACAAGAAGTTCTACGGGGCCAGGC
TCAAGAAGGAGAAGGCAAGCGCCGAGGAGTCCTGAGCCAATTCACATATGGACACTGGTC
AGCCGTGCGCCTCTGAGTAAGAAAAAAAAAGAAAAAAAAACAA
>at01049
ATAGCGCCGTCGCGGCCCACCAGCCCCCCGTACCACTTCCACTGGCCTTCAAAGACTGGG
CCAATGGTGGTCACCTGGCCAGTGGAGGGCACTATCTTCAGCACGGACTCGGCATGAGCC
GGAATCCCATAGACGGCGCCATCGGCACCCAGTGCACCGCCATGCCACTTCCAGCGGCCT
GGTGGCACATGGCCAACGGGAATGCCTCGCTAGTTCTTGGCAGGATCTTCAGCACGCAAT
CTGCCCAGGCAGGTATGCAATACACTGCACCGTCACCGGCGAGAACCCTCGCAGCCACTT
GTGCTTGCCTGTGGCAGGAACCAAAAGTTGTCACGTTCGCCCGAGCCAGGGGTTATCTTG
AGCACAGATCGGGCTTGCCCTGG
>at01050
CCGTAGCCATTTTGGCTCAAGGGACTGGCCGAGGCGCCTTGCTTGGTTTGGGGCATCCCA
CCTGCCCTTTTGGGGCCATGCAGATCTTCGTGAAGACCCTCACGGGTAAGACCATCACAC
TAGACGTTGAGGCCTCTGACACAATCGACAATGTCAAGGCCAAAATCCAAGATAAGGAAG
GCATACCACCTGACCAGCAGCGGCTGATCTTCGCCGGAAAGCAGCTCGAGGACGGGCGTA
CCCTCAGCGATTACAACATCCAGAAGGAGTCCACATTGCACCTTGTGCTCCGTCTTCGCG
GGGGCGTCATCGAGCCCTCCTTGGCTGTCCTTGCGCGCAAGTACAACTGCGAAAAGATGA
TCTGCAGGAAGTGCTACGCGCGGCTGCCCGCCCGCGCTGTGAACTGCCGCAAGAAGAAGT
GCGGACACACGAACTTCCTGCGCCCCAAGAAGAAGCTGAAGTGAAGCCTGGGTGGCTAGG
TCGAGGGCCCCAGTTGGAGAGGGAGATGCGTAAAAGCAAAGGTTGCATTATGGAACAAAA
AAAAAAGAAAAAAAAAACAA
>at01051
TCCGGGTGCACGCGAGCAAGCTCGCTGATGCGCGCTCTGATTCGCTTGCTCATTGGCAGG
TCCGTGCACTCGCACTGGCACCAAGCCACACTTGTCTTCCGGCTCGAGGAGAACTTCTGC

GTGTGGCGCCCGTAGGCGTCCATCTCGCCCTTGTCAGATGCCTGCTTCCAGGGTCCCCTC
TGAGTCCTCTCGCTCAGAAGCCACTCGATCTCCTCGTCGCCCAGGAAGTTTGGGAACTTG
ACGATCCAGGGGTCCTGACTGAGGACCTCTGCGGCAAGGCTTGGAATGGAAAGTATCCGT
TGAAACGTGGCGTTGATCGCTCCAGGCTGCAGGGACTCGGGCATATCAGCAAGGTGCCCG
CACCACGCCGTCATGTCACGCAGGTGGCAGGTGTTGCAGGACCGTGCACAGACGTCGATC
ATGAACGAGGGGTTGTCTTCGCACCCAAAGCCGCCATATGCAGACGCAAAGGCAGGGCAA
CTCTTGT
>at01052
GGACAGTTGTATGGACACGCCTGACGAGGACATGCTCGCGGCGTATCTACCGACGTGCAG
CCAGCTTGCCCCAGTCTGCAACTATGCGGAAGACGGGGAGGGAGTCCGGGCCGCCTGCCC
TCAGACGTGCTCTGTGCCGGCGTGCAGCAGCGGGCGCAGGGTTTGCCTGTCGCCTCCGCC
TTCTTCCATGCGCATCTCCTGGGCTCGGCGATGTACCAAACGCTGACGCGGAACGGGACT
ACATGGGACCTGGGCTCTCAGTCTGTGTGGCACTACGATGACCAAGCGACTTTCCCGCTG
ATTGCAGCGAACCTGACCCTCCAGCCTGGAGACACCATCCAAAGTACCTGCATCTTCAAC
TCCATGGGGAGGTCCACGGCGACCCCCATGGGCCCCAACACGGTGGACGAGATGTGTTTC
TGCCAGGTCAGCACGGTGCGCCCCACGCACGGCGGGCACCTGCCCGGGTTCCGATGCACG
GGCAACTTGTGGGCCGGCGAGCTGGGACCCGAGGAGGACGCCAGGAGCGTCGCCGCTCTG
CACCCGCTCGCCAGCGCCGAGGACGTCTGGTACGCCGACTTGCTGGGCGGCGTGGGCCAG
AGGCACCCGTCGGCCACCTCGGCCGCGAAGCTCCTCCTCTCCAGTCCAATTGTGCTTGTG
GTGTCAGGGGTGCTTCATGCTGCCGGAGCTTGATTCAGCATCACCGGGCTGGCGGCACTG
CGACGTGCACTGAGAGAGAGAGCTCAACTGGGGCGGGCTGTGGGATCTCGCTCGGCGCCA
TCGCCTCAGCGGCCAATGTCGTTAGACCATTGGGAGTCAGCAGAGCGTTTGCGGATAAGA
TCCGCAGGTCCGCATACCTGCACCCAGTTTGTCA
>at01053
TTTTTTTTTTTTTTTTTTCCACCCTTCGTTTCATCATTGCACAGTGTTGTCCAAACTGTC
CTTCAAAGCATAAAGTTCCTATGGCGCTATCGAAACGGCCACCAGTCTGGGCCATTCGAC
TCCAAATTCCGCAGCAGTTTCTCCTTGGAGCCATCAAACATGCTCTCAAACGTGAGCTTG
GAGATCCATAGCACAAGCGTCAAGTCGTTGACCGACCGAAGGGTGTAAGGCCACTTAAAG
CCTTGCACACTTGCCTCTTCAATGACCGCTGCAGGCTCGTTGTACACCGCCACCTGCCGC
TCCCCAAAGTCACTAGGCTCCCATGCCTTGACCTCTCCTAGCTGCAAGATGTAAATACGC
TTGATGTCCTCTCCAGCTCTCGCCAAGACATGCCCCGCTTTCAAGACCTGCGGACGCAGG
ATGTTAGCAAGCTGCTTCAATTCCTCCTTAGCAAGACCCTTGAAGAGCTTCACGCCTCGC
AGGAAGTCCACTGTCCGCCGCATCGTTGGTCGCTCGTCAATCTTCGCAACAGCCTTTTGG
AATCTCTCCTCGTGCC
>at01054
TTTTTTTTTTTTTTTCCTGCTTCAAGCCATGGCAATGCGATGTGACTGGTGGTCAGCACT
GCTCATCTCGCCAGTCTCTTCCGCATGCACGGCAGAGCATTCAAATAACAGAAAGAAGGG
TTCGATACTATTCGTCTAAAGTGTCTTTGGCATTCCGTGTTCCTTCAAAAATAGCAGGCC
GTCAGAAGAACGGCCACGCCTTGCCGGTCAAAACTGCCTGTGTGGCGATGCCGCTGAAGG
CAAGCATCGCAAGGCGGCCGTTCTCCAGCTCTTTCAGCCTCATGCTGTTCGCCTGGGCCT
CATCCTTCGGCAGGAAGTTCTTGCCAAGGAAGAAGTCGCCCGCCTCGCGGTTGATCTTTC
CCTCGAAGCCTTCCAAGCACAGCCACCCGCCGTAGATCTCATACGCAGCGATGGTGGCCA
GGAACGGCCCCATGATGCCGGCCTGCACCATCGCGTTGTGCGCCTGGACGGTGGACACAT
TCTGGAAGACCTCGCCTGGGAAGCGGGCCCCGAGGTCGGTGGCGATCCAGCCCACTGTGG
CGAGCATGCAGACGCGTCCGTGCTTCAGCTCGGCCTCTCGGACGAGATACACCGGCAGGG
CCTCCGTGACTTGCAGGGGGTCGAAGCCCTCGTGCCGAATTCTTG
>at01055
GTGGATGGGTGGTTGGGTGTGGAGGGTGTCGGGCTAATGTGTGGCTGGATGGCTGGTTGT
GGTGGGTGACTGAGTGGCTCGCTGGCTTGGTGTTGAGGTAGCGGCAGGTGGCTTGGACGT
CGAGGAAGGATGTGGTAGTCTTCCTCCGCGGCAGCGCAGCTGAGGCCCATGGCGCCGTTA
CGCTGGACGTCCTCTCCGCAGAGCTCCCTTGAGGAAGGCTCGATGGCCGCCGCGTGGCCA
GGGACGGCCTTGCCTTGCGCGGGGTTCCTTGCGAGAAGAGCCCTGAGCGCGGCAGATGCG
GTGTCTGGCCCGTGGGGCACCAGCACGACAGACCCAGGGCGCCTCCCAGAGGGAGGGAGG
GACCCGGGACCGGGCCCGGGCCATGCGGTCGACGTCCAGGCTGT
>at01056

GCCAATCAGCACACTGGCCCCCAAGCAGCTGGACACCCCAGGATGGAGTCCTCCGTCGTC
CCTGGAACCCTGCGCGTGAAGCCAAGGAGCGGAAGCCCGTAAAAGCTCGCGGTGACTTCA
TAGACCCGCAAGGCAAAATGGAGGCACCCGGCATCGATCTGCAGAGGTCTGTCGGAGAAG
ACCAGGCAGCCATTCTGGAGCTCGTGCATTTCCCGCCTTGAAGCCGTCATGCCGTCGTTG
GACAGCATGCAGTGGTCCTGGAGCCGCTCGCGAAAAGAATGCGAATCTGGGCATAGCGGT
GAACGACACGGTGAGGaATGCTGTCGCGTCGAtTGTCCGCGCGTGGTCtTCgGTGCaACg
AcCGCCGCGGAACCCGNNCCTGGCtTCTCATCGAGGAGCAGACGGACTCGGCTTCCGAGT
CGGACGAGCAAAAAGCTCGACGCCGAGCTGGACGCGAAGGCCAATCGCCTGAGTCGACCG
CGAACACTCAAGAAGGCGCGGCGGCNGTTCTCGGATGCCTCAGTCCGCCGTGCTGCGGTG
CAGCGAACC
>at01057
GGCTTGTGGCAACAGGGGAGGTTCCTTGGGTGGCCGTTGGCCTGGCTTATAGGAGCAGTG
TCGATGACCGCGGAGATGGACCTTGGGCTACGATGCTGGCCATATTTCCTCTTCGCCTGT
GTGTGGAGATGGTGATTGAATCCATGGCACTGAATGCTTTGCATACTGTCTGGTTTTGGC
TGCTCCTGCATTCTAGTATCCACGTGCTGCTCTGGGGTTTGCATGTGCAAGaAGATGGTG
GGAAGGCCCTGTTGGTGGCGCTTGCCTGCATTTTCACGGCAAAATCGGGAAATGGATGGT
GCTGATATGTGCTTACAGCTTAGCGATGaCGGaCTCtTTACAGGtTTTGGCGTTACAGTT
GAATTgGCCTTGTGAGCTTGGCTGCTGTATGGCATACAGTGTAGCGAGACACGCTGATTT
CTTGGCCGCAAGGCAAGGCGAGCCACATCCATACCAAAGCTCCAGTGAGGGATGACATGA
AGCATGGAGACCTGGCTGGCAGAGGAAAGGGAAAAGAAAAAAAAAAAAAANAAAAAAAA
>at01058
CCTGGGATCAGAGATGACGGCAAGGGGGCCAAAGGTGCTCACGGGTGCAAGAAGCTTTTA
CGCCGCAGAcTgGGTGGCCGGCCGGATCGCGGCTGCCTCCCATaCTCGGAGCGATAGTGG
CCGGCAGGCTCCCTGCCGCTGCTCGCCGCTTTCTCTACCACAGCTTCCGCGGGTTCCTTA
TCATCGTGCTGACCATCACCTTCGCCAAGTCCTTCAACAACTACCTCAACAATACGAAGG
CGAAGTCGATCCACATCGAGGCCACCGACCCTCAAGCTGCATCTCAAGCCCGTGAGGCGC
CGTATGCAGATCATCCTCGAGCACAGGTGTCTCCGCATCAACCGCAGGGCGCAGGAT
>at01059
ATGCAGGCGGTGGCGTAGCTGCCTAGTAGCCGTCACGGCCAGGGGTGTGTCACCACGGGG
GCCGTTACTACGGAGGACCTGGGCAAGGACCTCCACCTGTGCTTGGCAACTTCCAGCCTT
GGGGTCAATGATGACGAAGTCCCGGCCCTCCACTGGCTCGTCCGGGCACGGAGGGTTCAA
CAGAAGAAACTCACTCCGCACCCCTGCCCGGGCGTTCCATTCCGCGTGTTTCAAGGCCAT
GCTGCAAATCTCGGACCAGCGCGATGCTGGGACGGCCTGAGGCTGCCCACaAAAAAtTTT
cCTCtTcCCAgGCNTGTGCCCGTCTGACTGGCTAGTGGAGTCAGAGTTGTCCAGGAGGAA
GACGCGAAGATCTATGTTTGGACCCTCCTGGGCAAGCATGTTGGCAAATCCCCAGGGCGC
GTCCATGGCGCAGTAGGCCGTCACCCGTTTCTGGCGATGCGCATCTGACTTGGGCCAGCT
TGGGCGCACCGAGGAGCCTCGGCTTCGTCTCCGGCGCTGCTGAGGTCATCG
>at01060
TTTTTCCTTTCACCGGCTCCACCTTAACCTCTGGAACTGCGGGGCACTTCAGGTTTTTGG
CTCGACCCTCTCTACCTTTGCCGTCTTGTTCCTTTTTTTGTTCTTTTCTTTGTTCCTTTC
TTTGTTCTTTTCTTTGTTCCTTTCTTGTTCC
>at01061
TTTTTTTTTTTTTTTTTTCCCAGCGGAACCACCAGAATCCCTGCTGCATCCGATGACTTT
GTCGGATCTGCGGCAAGCCCCTCAAATGGACGACCCAACGGCAGGACTGCTCGAGTCGAT
TAACTCAGATGCCTTCCTCACCTCAATCACGCCTCTTCGCCACCTCGCTCGTCCCGCTGT
CGCATTCACTTTCCGGCGCTTGGCGGCCGGCTCGTGTTTGCTGTCAAGCTGAGAGACATC
CATGTTGACGAGCTCGGGCCTTTCGATGCCCATGTCCATCAGTTTTGCGACGCTGTTCGC
TAACGTCTGCTCGGCTCTCTCAAAAAACTGCGAATCGAACGTGCACTCCGCGCACAAGTA
CTGGACCAGCAGGCTCTGAGTCCCGTCGCGCCCGATTCTGTGCGCACGGTCTTCGGCCTG
ATGAAGCATCCCGGGCGTCGGGTGCAGCTCCGCCATTAACACGACTGAGGCTTTCGTCAG
CGTGTACCCCAGTCCACCGGCGCCAAGCGACATCAATGCTACGCGAGTGTTCGGGTTTTG
CTGGAAACGCTTCACCTCTGCGTCCCTCTTTTCGATCGACACGTCTCCGTCGACACGAAC
AAACAGGTCCGTGGAGGAACAGCGAGACTTTGGTTCCGAGCAAACTGGACGAACGAGACT
GTCCAGTNTGCTCGCTATGGCGTCGAGCATC
>at01062

TTTTTTTTTTTTTTTTTTGACTGCCACTCGCTATGCGGGCTGCAGTGATTTCCGTGCCGA
CGGCCAGGGGGCCGCCTCCGCGCCACGAGCCGCGCAGGGCGTCACAGGGCTTCGGCGGCG
GCCTCAACGGCTGACGGCATCAGAGGCTCGGTCAGGTCTGGCTCCGGTGCCACGGCAGAA
GCCTGGAGGCCAACGGAGCGTAATCTGCCCTCGCTCGTGACAATGCTGCCGGGCGTCGGG
ACCAACGCCTGGCGCATGTCCGCCACCGGGCAGAGGTGCGTCAACGACTTGTGGATGGGC
AGCCTCACGCAGCGGTCCACCGGGAGTTCGGAGAGGACGTACGGGCCGAAGGCGTGGCGC
TGCACCTGGTAGACGTTCATCCTCGCCCTCTCCAGCAGCAGGTCGACCGGCCTCTCAGGA
GTCTCGACAAAGCGCACCCGGAGTCGGGTCGTGGCGCCGTTGCGGCGGGTGATGCTGCAG
AACACCGTGTCCGTAGTCTATGCCGCCCACTCTGGCCCTTTTGCGCCAGCTATGCAAGAG
GTCCACTGGCGGGTCCCCGGCAATCTTCACATCGTAGACTGTCAGTATTCTGCTCTCCTG
TCGCATCAGGGCCTGGGCAAAGAGGCCGTCATTTGTGAGCAGCACCAGGCCATCAGCGCC
TCGTAGGCAAGTCCAGAGACAATGATGAAGTGCTTATCATCGAGGCATTCGTCATCGAGG
CCAGTCGCTTGGGTGGTACCGGTCCATCTCACTTCCTTGTTGCGCCATTCTCGCATTTTG
CTGCGCAGCGTTCTCTCTCCCTCCTTTTCCTGCTCTTGACAGAGCACTTTCCTGGGCTTG
AACATCCCCCAGAGCCTCGGCAGAGGATCCGGTGGGGGGGCATCGAGCCCGTCGACTGTC
ACGGCCGCCTCGCTGAAGACCTTGAAGTTCGAGGTCGCGGTGTCCCCATCCACTTGGACC
CGGCCGGCAGCGATCGCATCCACAGCCTTGTGGCTCGACATCAGCCCGGCAAGGCTGATC
CTGCGCGAGAGCCGCTCGAAGCGCGTGACCAGCATCTGCATCTGCCTCGATGCAGGCATT
GGTGCCACCATGCCACGGCCCCTGGCCGCCCGGACCGAGGAGGCAGCCGCCGCTTGAGCC
AAAATGGCTACGG
>at01063
TTCTAAGCCGGCTTGCATCAaAGTTcGTGTCTaAAACAGAGAGCTGGCTGCCCCTGAAGA
GCCAATGCTGCAGACGCAGTCGGCTTGGCGCCTTGCAGTGACCACCACGCTCCTGACAGc
CCATCtTGCACTGACATTATACACGTGCGCTCGGCAGAAGTGCCTGCTCACTAAGGCCCG
CGATCTCTTGGCCGAGCTTCGAGCCTTCGCATGCCACTACCTCACCGGCTCTGCCAGTCT
AGCAAGTGAGCTTGATGAGTCGGTACTTCAAAGGCTGTCCACCTTCCGAGTTTCAGTCTT
CAGGAAGTCGCTCACAGTGGTCACGATCGTAAGCATTATAACGCTAGTGGACACGA
>at01064
TTTTTTTTTTTTTTTTTTACTCTCATATCCACACAAGCTCAGTCAGCTCATGCCGTCGAG
AGTCAACAGCTCAGCAGGAAAACGCTTCTTGGAGCTGGGCATGCTTGCAGGCGGCCTTTG
TCGAGCACAGCTCGAGACCATCTACTACTTTGAGGTTCCCGACGACCGATCCTCGGAGAA
GCTCAAGACCAGACAACTCTTTCATCAAACACCACAACCCACAGTTGCTGACTTCACCCA
TGCCCATCCTCGCTGTGGATCTCAATCCTTTTAAGATATGCAGGTCAACAACAGCTTCAG
CAGAGGACATTGAAGGACTCGACCGTGACACTGTATGAAGGTTCCCGACTTGCTTTCATT
CCAAAGCTGCCCTCCTGACTGTTTCGTCTTGGTCTCCCTNGNGNCAGAATATCTATTACG
CCCATCGTCCAGCTTGATCTGGTAAGGAGCTGTGCTGCGATTGCCCCATCCTTCCTGACG
CCACCAAATGCGCTGGATGGTACCCCAAGGCCCAGCCATTGTCGATGTTGCACTCAACGC
GGGTACCGACTTGGAAGCGTGGCTCCTCAGCAGTGTCCAGGTCAAGGACTGGATCCACTG
CCAAGCGTGGGTACCACACCTTGCAACTGCACCGTCCAGCGACTTCGACAAGCACCGTGT
CATCCCTCTCG
>at01065
TTTTTTTTTTTTTTTTTTCCAGGCGAGCGTACGTGGCCAAAGTGCCTGTGCGGCCGCAGC
AAGGCTTCCTCCCCAAGGAATTCTCTACAGCTCACTCGTCCCCGCCAATCTTGCGCGTGT
AAATCATCTGCGCGTGGGGCTTGTCACCGACGTGCACAATCAGGCCCTTCTCCTCCAAGT
GCCTGATGGCCTCCCGGGCAACGGAGCCGTTCACCTTGAGCCTCTCGCTGACCACCGAGG
GGGTGATGAGCTTGGCCTTGGGGATCTCCTTCATGAGCTTGTCGTAGGTAGGCTTGTCGA
ACATTACCATGTTGGCGAGCTTCTCCTTGACCTTGCCCTTGCTCCACTTCTTCTTCCTCG
ACTTGCCTCCGGCCATTGCTGCCTTCATCTTCGCCTCCTTGGACTTCTGCACCACTTTGT
TTGCTCCCATGTTTACGACTCCCCAAACGAGTGCTACTGAAAGAGGCACGCGATGGCTCA
CTGGCTGGAGCCTCGTGCC
>at01066
TTTTTTCTTTTTTTCTTTCCACGGGTACCATCCAAACAAAGGCACCATCATCCGGTCGAA
AGCCCTGACAAAGAAGATTCCCCCTCAGGCTCATGCACCACGTTGACATCTGTCCTCCAC
CGCGGGGCCGGAGCCGCAGAGGCCTCAGCAGGAGACCCATCCCTGCTCCTCTGGAGAAGG
AAGAGCGCACCAACTGAGCGCACCAGCTGACGTCACTTGGAACCAGATTCGGATATGCGC

CTGCCCTCCGACCAAACTCCGCACTTGGTGAGTCCGTCCTTCGTCGTGAATTCGCCAACG
CCGTGCTGCCTGCCGTTCTGCCAGAAGCCCTGGTACCTCCGGCCGTCCGGCCACGTGAAG
ACGCCGAAGCCGTGCTTCAGGTCGAGGAAGTAGCCGCCGACATACTGCCGACCGCCTGGC
CAATCGTACCGGCCCTTGCCGTGGATGACGGAGTTCCTCCACTGGCCCTTGAAGCAGCGG
CCGTCGCTGGCAGTGTACATGCCCCCGCCGTGGATGGCGTTGTCCTTCCACTTGCCCTCG
TAGACTGACTCATCCATCCAGTAGTAGCAACCATAGTTGCCTTTCTGCCCATCTCGGAAG
CTGCCCGCGTAGTGCGCTCCCTCGTGACCTACCTCCACACCATAGCCATCCTGGACGTCA
TTGCGCCCACTGGCCCTTGTAGATCGCGACGCCCTTTCTATAGTAGATGCCGTGGCCGCT
GGCCATATTGCCGGTCCCACTGGCCTACGTAGACGTCGCCGTCGGCGTGTATGAAGCGGC
CGTTGCCGGCTGCAGCATTCTCGCTCCAGGCACCCTCGTATGTAGCGCCGTCGGGCCACA
CGTGGCAGCCAAAGCCATGGCGCATGTTGTTCATCCACTGTCCTGTGTATGTGGCCCCAC
TCTTGAACTGGTAGCGGACCCCACGAGCAGCGCCGCCAGAAGACCGTATGCTGAGGAGCT
CCGCAGCTTCCCTATCAAAGTAGCTCATGGACTGATGCATGTCCTGCGCCTTCTCCTCGG
CACAGCCCTCCTGATCTGGGGCATGCTCGCTTGTCGCAGGAGGGGCGCTACTGGGCAGAG
CAGCATTCTTCTCGCTCTCACAGCAGCATTGCTTGTGCGAGCAGTTCTGACCCATCGCGT
CGGCGGGAGCCAGCCAGCATGGCTTGAGCCAAAATGGCTACGG
>at01067
TGTGGCTGCCTCGGAGCTTCGCGACtAAGtTGGttCttAGAAGATGTCAACGACTTCACT
GGGAGACAAGACTCCCGATTGTGCAACCTGCTTGGCGAAGAAGGGAGCACTCATTGCTGG
AAATCTGATGAGCCGCTTCAGTGGCCTCAGGACATCCTGCCAGGTCTGTTCCAGGCTGCC
GTTGGCCAGGGAGTTCAGGCCCTTCTCCTCATGCGGCTCTTCAGCGACCTCCGGGCTGAG
CTCAGGCCGACACTCGCCACCGCCGACTTGCAGCTCTGCCCACCGCTGCAGTGCCGTCCA
GAGCGGATCCTCGTCTACGCAGAGGCCATCGGCGCCAAGGAGGGTAGCTATCGAGACACT
GTGCAGCGCTGCAAACTCATCAGCGAAGAGCAGCCGAGAAGTCTGAAGCCGCGATTGCTC
TCCCAGCATGTCCAGGAAAGTG
>at01068
TTTTTTTTTTTTTATTTGTTTAATTCGTCCAATATGCACTGGGTGACGTTAAGGCAGGC
CTGAACAAGGAAAACCCAGTCGGCTCACTGAGCGCCCTTTCCTTTACTGAGCGCCTTCAC
CTGGCTCCTGGTCCGAGGTCCAGAGCGTCAAGTTGTCTCGCAAGAGCTGCATGATGAGGG
TGGAGTCCTTGTAAGAGTCTTCTGCCACATTATCAAGTTCAGCAATCGCATCCTCGAAAG
CTGTGCGTGCCATCTTGCATGCCTCATCGGGATTTGACAGCACTTCGTATTGGAAGACGG
AGTAGTTCAAGGCAAGGCCAAGGCGAATGGGATGTGTGACGGCCAAGTCCTTCTCGGCGA
CCTTCGATGCGTCCTCATATGCGAGCCTGGCGCTCTCAGCTGCCTTGGACTTCTTGTCGC
CATCACTGAACTCTGCAATGTAACGGTAATAGTCGGCCTTCATCTTCTGGAAGAAGACCT
TTGATTCACCCGTTGAGGCTTTTGTGATGAGGTTGCCGTCGAGCAAGCCAAGGATCGTGT
CGCAGATCTTCTGCAGCTCCGCTTCAACTTTAGCGCAATATTCCCTTGCGTATTGTGCCT
GCTCTTCATTGCCTTTTGTTTTTTCCTTCTGCTCAACGCTCGTGATGATACGCCATGCGG
CACGCCTGCTCCCAACAGCATTTCTTGTAAGCCACTGACAGGAGATTGCGCTCTTCAACT
GAGAGCTCCTCGTTCACCTTGCCGACANGCTCCATGTGATTAGCCATCTCATCATACCTC
TCGGCCTGCTC
>at01069
TTTTTTTTTTTTTTTTTGCTAACTCAACGAGGCAAGCAAGCAACCAACCAACCAGCCCG
TCCATCGACCAGTCGTGCAAAATCGGCCATCGCCTGACGTGGGCAGCTCCGCCCTTCCGC
GGCTCACCCGTGCAGGCGGCCGCGCCGCGCCGCCACAATGCCGGTAGCCGCCAGCGCGAG
CAGGGCCACCCCAGGCGCGAGCAGGAGGAGCGCCTTCGGGCTGGAGGCGAGCATCGGCCC
TCCACCCATCGTGGCCGTAGCGCGACACCCTGACCTCGTCGCCCTCGCACAGCGTGCCGG
CCACGCCGCCGTGCGCGACGTGGATGCCAAACATGGGCCCCTCCCACTCCCTGCCCCACT
CGTCGTCCGTGCACTTCGCCGGGAAGGCTGTCCGGAGCGTCGCCTGCGCCAGCGCCGGCC
TCCCCGCGACCTCCCTGTTGAGGCCGCCGGTCGCCTGGTCGCGCGAGGGCACTGAGCACC
GCGGCGCACGCTTCAGGAAGCGGAAGGGCGTCTCCCCGATGGAGAAGTCTCGCCATGTCT
CCTCCACCCAGGGTGACACAGTGCCCGCCACGACGATGTTGGCCCGGAAGGGCGCTATAG
GGTAGCTGCCAGTGCCCATGCGCAGGACCAGGTCCGCATGCGAGTCCTCGCTCACCAGAT
GGAAAGGCGAGAAGTCGCAGAAGCGGACCGCATCACCAAGTCACACGTCCACCGACTGCA
TC
>at01070

TTGTTTTTTTTTCTTTTTCGTCCATTTCTGGTGCCATGCCCTGAAGGTGCGCAGCGAGCG
CATTGGGGCCAAGGCATTTGGCCAGATCGCCGGCACCTTGTTCGGACAGAACTCCAGCGT
GTTATCATCTACAGTGATGCCCTTCTTGTCCCAGGCCTTCACGGTGAAATTGTCCAAGTC
ACTGGTGCACGTCCTAAGGAGGTCGATGGGGCCCCAGCCTTTGAGGGACATGGCAGCGCT
CTGCGCCGCAGCCATTGGCACCAAGGCGGCGAGGAGCGCCCATCGCATCCCCACCGCAGG
CCGGCCCCGCAAGGGGGGTCGGGGGGAGGCGCTCGGCCGGCGACCTGACACCTTGAGCCA
AAATGGCTACGG
>at01071
CTTGGCTTTTGCAGATGCCTACCGCGACTTGCATAATCCTGGGTTCAGCAGTTAGGCAAT
GGCAGCTGGCGACTTGCAGAAGCATGGGTTTGAGCTCTTGGCGATGATGAGTGCCAACTT
GCAGAATCCTGGATGTGAGCTGTTGGCAAAGGATGCTGCCTACTTGCACAAATCTAGACT
TGAACTGCTGGCAATGCCGATGCCTACTTGCAGAATCCACGATACCAGCTGCTAGGAAGG
AAGATAGCCGTTGCAGAATCTTGGAATTGACTTGTTGGCAAG
>at01072
CCTCTCCAACAAGCCAAGTGATGTCTCAGCAGCTGGGACACTTGAAAAAAAACGCTTGGC
TCGCAATGCCGCACACCTCACAAGAGCTCTGGCTTGTTGAAATAGATCCAGAACAGCACC
GCGGGGCCCAGACCCACGATGAAGATGGCCCAGCCCAGCTGCAGCAGCGCGTAGCTCGCG
GTCGCAGCCTGCGCTGCCTGAGGTGTAGCCGCCAGGGCCACGCCCAGGGCTGCGGCCTGC
ACCGCACGCTCGGCACCGGCTGGGGAGGCCTCCACGGCCCTTGCAGGCTGCGCAACGAAG
GCTGGGGAGGCAAGGCTCGCAAGCAGCCAAACGGCCGCGAGGCAGAGGCTGGCCACGAGG
AGGTGAGAGCAGCGCCTCGCCATGTCGACTGCTGTAAAGCTGCTTTCCTGCACGCCCTCC
GGATGCTGCCGAGCAATAGCTCCTGAGCCTGAGCAGCTTGAGCCAAAATGGCTACGG
>at01073
GATTTTGATCTTTGCGAGAGTGCAGATCCTACATTCGGCGGTAGACCAATCATGAAGCGC
TCACACTGTTGTGCACTCTCATTATCCGGCTTACTACGCCTTGGTGTTGAACACAAACAC
GGTGTCGCAACTTGCAATACCCATGAGTCCTGGTGCCAGTGAGACGCCACCGTTGCTGAA
GGCAGCCTCCGCGTCGTAAGTCGATGCCACCTTGACTCCGGGGCCATCGCTGTCGCGTGT
GAGGTGGTAGATAAAGCCGTCAAGCATGCCGATGTAGTAGTTGCCGTTGCTATCCAGTGT
CGGGTTGCCGACAGGATTAGGGAAGGCAAGGCCCTCGATACGAGAGCAGCC
>at01074
TTTTTTTTTTTTTTTTTTGCGCCCAAAATTATGACATTCAATCGGGCTCTCACACAGTCA
AGACGCTTGAGCCTTGGCCACCGGACGACCCAACTGCCCTCCATCCTGCACGTAGCTGGG
CACTGAGCTTCGATACGGCTTCTGCAAGCGCTGGATCATCATGCACGCTTGCATCCTCAT
CTGGGTCAGCACTACGGTTGTACAACTCTCGGCCTGCAAGAGCGGTCCAGTTCGGCCCAT
GCGTAGCCTTATCGTACTGCACCCATTCTGTGTAGCGGACCTCTGAGCCGTCTTCCAAGC
GTGTGTAGAGGTTGTAGCCCATGTGGCCTTCCATGGGCCATTGTCCGAAAGCTGCCGACT
TGCCAGCGGCGACTTGAGCACCAACGCCAATGAGGGGCTTGAGACTCGAGCCTTCATGAC
AAACTGATGCCGTCTGGCTCATCTCTGGTGACTCGCACCGCTGCGGCACCTCGATGCCAG
CGAGGTCTGCCTCGTGCC
>at01075
GTACATGGTCTCGCCGGCGGCATTTACAATGGGAGCTGCCATGGCGGGCCTCCTGCCCGC
GGACTTCCGCGCCGTGGAGACGTATCCTGAGCAGGGGAGATACAACCGAATGCTGAGCCA
GCACCTGGTCAAGAGGCCCTTCAGGCTGCGCCTTGCAGAGGCCGCCGACCTCCTGAGCCT
CGTTCGTCTCGAGGAGCTGGCGTGGCCCAGGCGGATGCAGGCAGGCCTGGAGGTGCTCCG
CGGGCGACTGGAGACGTCCCCCACACCAACCTCGTCTGCGAGCTGGAGGCAGGGTCTCGC
CGTGCTCTACATGCAGCGGATCGAGAGCCTCGACGTCCTC
>at01076
ACTGACACACAGACCCCAGGCGACAGATGTGGTAGAAGAGCGTCTCAGAGTCGAAGCCGT
AGTTGTTCCTCGCGTCGGTGTACTGAATCGTCCGCTGGCAGAAGCAGATGTTGGAGACCG
CGACCCAGAGCAGCCCAGGCACAGTGAAGTCTGAGGCGCCGAACGAAGAGCAGCAGGCGC
CGAGGACGATGGGCACCAACGTCATCAGCTGGCTGGCGCTGGGGGCCTTCTCCGGCTTCT
GGTACCAGACGAGCGCAGCCGAGACCACAGGCTCCGTGGCCCGAAGCGTCTCCACCAGGG
ACACGTGCATGCACTCGTAGCCCTTGTTCAGCGCCAGCATGCCGAAGGAGAAGGTGGCGC
TGAGCGCCCACATGTCCCGCCGCATGCGCCTGCCGGCCTTGCCCGCCTGAAGGGCCCGCG
GTGGCTTCCCGTGCACCAGCCGCACAACCAGGTTGCCGAGTATTGCAGCGACGACAAACT

GGGCGAGCGAGAGCCAGTAGGCAGAGGGGAAAACGCTTCGGACCAGGACCTTGGACCCAG
CTGTGCAGATCACGGAGGCAGCATACCAGGTGAAGAAGAGTGCACCCAGGGGCACCTGTG
GCTTCTGCCCGCAGTCCATCCGAGGCTGGGATCCGACGCGGAAGCAAGATCCTTGATCTT
GCGGCCTCGGGCCTCGAGACGTACTTGAGCCAAAATGGCTACGG
>at01077
TGCAAATCCCAAGTTTGGTTTGGCGACCAACATCGGACGGGTGCCATTCCTGATGTGTTC
ACGCTTTCTCTTCAATGTGGGGCCTGTTAACTGCTGCTGATCTGCGCTCACTGCCCTATG
CCTACATGCAGTCCTTGCATGTGGCTGCCAACCTAACGAAGGCCGCGCAGTGGGGTGCAT
TACGGAGACCTGCAAGTGTTTAAGTGACTTGAGCAGCCGGACAGCTCATCTGAGCTTTAT
CGTTGCGCGCTGATGTGGCCCGTTATTCTGCGTGGCAGACACTATCCCATCCTTCAACGA
CTGAGTATGTTCCAGCAGCCAGGAAC
>at01078
CCGTAGCCATTTTGGCTCAAGTGCCATTGCTGCTCGAGCCAGTCCCTGTAGAGGCCAGAG
GGCCGAAACTAGTAGGGCCATTGCAGCTTGAGCTTGGGTCATTGGCTTGAGAAAACTTGT
CCCTTGCGTGTTGGCTGACCGAGAACGCCGCAGGACGAGAGCTGAGCCTGCTCGGACCCC
GCGGGACTTCGCCCGCACTGGCTGGTGGCAGCTGGGACCTTGCGAGTTGGTGTGTATGCC
CACGGCACGTGATGGGGAGTCCGCTGGCTGGTGCCCACAATAGCAATGGCAGTGGCGGGG
CTTGCTCTCGCCCGTCGGGACTCGTGTTCGCATGTTGTGGTGCCACTCGTGCATA
>at01079
TTGGGTCGAACCCCCTTACCATTAGCACGAAAGGTCTCCTTGAGGGACTTGACAAACTGC
CCTTGCGAGAACTCGATTGCATGAGACAGTCGATCTCTGAGAGCCTCTGCTTTCTGAACC
GCTGGGAAGCACCTCATGAGGTCTTCTGCCCTGCGGTCGATGCGGAAGTCGTGTGAAAGG
ATGTTCTTCAGGTTCGCTCTGACATGTGGCTCGAGTGCCGAAATCTGCTTCGTGAACTCC
TCATTGTCCCATTGCTTGGAAAAACTTGTCCAGCACATCCGCAACCACCTGCACGTCTTC
ACTCGTCTTCTCAACATGCACCAGCGCACGTTTG
>at01080
TTTCGCAGGGCCCGGCTCAATCCACTGCAGGCAGACGACCAGCGGGCTGTTTGCCGTAGG
GGGTCCTCCCGGGTACTCCGCGGTGCTTGCCGAGATCCCGAGGTTCTTGCACTGCCACCA
GCACTTGTCGACGCGGAAGCCTTTCGCCTCGAGCGCGGCCACACGAGCTGGCTCGGCAGC
CTGCCCTCCAGCGCGCGCGGGGGCTCCCAGGCCGCGAGCGCCTTCATGACGCCGCCGTCG
ACGGCGACCTCGCTGCGGTGGAGGCCTCGCATGGCGCTCGCCATAATCTGGGGATGCGCG
CAGTTCTGCAGAGACTTGCCGACATAGGCGTTCAGCTTGGCCCTCTCCATGGCCACTTGG
CTCTCCGTGATCCTTTTGAACGCACTCGCTGGCAACAGAGAGCAGCTCATGTCTGAGATG
TCACCGAGGAATGCGTCCAAGAGCTTCTTGGCTTCCAGAGGCAGTCGCATGTCGTGCTGG
AAGGTATCGCCGATTCGCGCGGCATCTGCCGCGCAATGATAGTGGCGCTGGAAAGAGCTG
AAATGCTTCATGCGCTGAACGC
>at01081
CCCTGCAGTGTGCCAGTCTGATTCTTTTCCTTATTGGTCATTGGCCAGAGTCCAACAAAC
GCCCCACCCACGCGATGCAAGCGTGCTGGGCAATGGCTGCGTACCTTGGTGCCTGCCACC
TCCATCCTCCTCGCTGTTTTCCCTTGCTCTTACCCCCAGCATTGCCAGTTACTACCCGTG
ACCCAAGGGTCGGCGGATGAGCGCACTTCCACGTCTTTCGGCGATGCCTACGGTGGCCGA
ATCCGGCCTTTCAGGCAGCGCCTTCTGGAGT
>at01082
TGCTTCGTCCGCAGGTTGGTGGGCTTCGAGGGGAGGCCGAGCTTGATGCAGCCCATGCCC
ACTGCGCCCGAGGAGACGAAGACCACATCGAAGCCCAAGCGCCGGAGCTCTGAGATGAGC
TCCACGATCCTGGCGACGTTGGCCAGCCGCACGCGCTGGCCGGTGCTGTCGGAGACCATG
AGCGTGGAGGTGCCTACCTTGAGGACAAGCAAGGGCTTCTCACGGCTAGGGGTGGATGTC
GAGGCTCTCCTCAAAGGCCGCTTCGAAGAAGAGCTGGAGCTCTTGGTCGACGACGTGGGC
ACCGCACCCGCATCGGGCACCGGACCAGCATTGCTTGCAGCAGCAGTGCTTGCTGCCATG
GAACCCTGGATTCGGGAAGCAGGCTGCCTCGGTCCTTTGTGTTCCTGGAACCAAAAT
>at01083
TTTTTTTTTTTTTTTTTTCCACTCATACAGTGTGGTCCTGGGGAAAGCTCGCAGCTCCCC
TGGCATGATATGCATCAAGGAGTGCTTTGACAAAAGGAGGCGTCTTCAGGTGCTGACTGA
CCCGTGCGCCTTTGCTGCGGCGGATGTTTGACGCAAGCTGGGCACCAACTTTTTGAGTGC
AACTCAACTACTCACCATCAAAGCAGGTAGGTCAAAATTTGCCCAAAGACTTGTCAGGA

TCCTGCAACTTGTGCCAAAGTGGCTATCAAGCCATCTCAGCATCATTCAGGAAATCGCAT
TGCATCGGATTGCATGCATGCATACACACACAGAACGCCTATTGACAAATAGGCCCAGTA
GTCCCCGCAGGGTGCGCTATTCAAGACGCCGATTGCCGCGGCCGCGGGACCTTAGACGTC
TCAAACGGGGCGCCCGCCCAATCCTGCCACCTATTGAGGAAGGAGCCGGCGGCAAAGACA
GCGGCCATGAATGGCCCCAGCTCAGTCGTACAAGACCTCTGGGGCCACCGGCACCCGCAA
GAGCGAGAAAGACCCTCGTGCC
>at01084
CGCGCGCTTCGCTTGTTCAGATGTTTTGATCCAGGGGTGCAATGGCCCGTGCAGCGCACT
GACCGCCAACGGCGACAACCTACATTGATCCATTTGTGATCATGTGCCATCCTGCGAGAT
TGTGCGCCGCAAGcCTTGTCGATGACTGTGTGACCGACGTGGAGCGTGTGGTTGCCTTGC
TGGCACGTCCCGTTCAAAATAGAAATTTGGTATTTTGAGGAGCCAGGTCGAGTGTTTTGG
GGNGGCTGGC
>at01085
TTTTTTTTTTTTTTTTTTGCACGCAAAACGCCGCCCGGGCATGCCCACACCAAAATCCCG
GGACCTCTCCAGGCGAAACGACCCATAAGCATGAGAGGCATCACTGCTGGGCCTGCCCGA
AGATTCTTGCTCGCGTCTAGCGAGCGAGAGCCAGCATTCCCCCATGGTGTTTGGGGAAGA
AGGGCAGGCACGAAAACAAGGCTGCCATGGCCCACGGCCCATGGGCAACCGCGACGATCA
ACTCCGGCCCAAAAAGCCGGCGGGGTTCAGCAATGAAACCATAAATCATATCGCACATGA
CCCACACCCACACCCTCGCCATCACCATCATCGTCCCCATCACCGAACCAGGCCTTTGGC
CGATTAGTCTTCGGCGGGGCGGCCACAACTTTGGGCAGTCGGCAAATTGTCAGCATGTAA
AGGGCTATGGGATGAGGCAGTGGCAAACTGAAGTATGCAACACAGAGCTCCTCGGACCTG
TGTAAAGACCTCGCTTCATGTGGTGATTGGCTAGGAAGGTAGTTCAGGACCTTGCCTAGG
GTGATGAGAAGAAAGTCCGACCTCTGAAACACCTCCAACAAGACATCGGAGCCATCATCA
GCCTTTGTGGAGGCGCTGGGCATGTCTTGGTCAGAGCACCGACCAAAGAGGCGAGAGACC
AATGATGTCAGAGTACTCGGCGAGCATGG
>at01086
TTGTTTTTTTTCTTTTTTTGACACATCCATCTTGAAGTACACTGCAGCTATCATCCTGAC
AAACCCCATCAGAGTCACGGGGGAGCTAGGAGGAATTTCCAAGGACTGTTCTGCTTCGAT
GATGCGCAGACCCGGCGGCTTCCTGCCACAGAGGAGCGATACGCCATCTTCGATACCCCA
CCGAATGACCTCCAGCATCTCTGATGCCTCCCAGTAGATTCCGCAAGTGCGGCCGCGGAA
GCGCTCCGTAAGAGAGTCGAACGTGATGACCAGCTGGTGCCTCACTCCATCTCGAAAATG
CGGGGTGTTGTTGGTCAGGCCGCCATCCAGCACCCGCATGCCTCGCCATGACACCCACGG
ACCACGGCAGATGAAGAATGGAACTGCTGCAGTGGCCAAAAGCCCGTCCCCGACTGCGCT
TTGGTCTTCGAAGGAAGAGATGAGGTGGTTGCACCCAGCACTGAGAGTCCAGCAGGGACA
GGAAGTGCGCAAAGGCTCGCTCACACATGCGGCAGTACCAGCACATTGCGGCACTTAGTG
CCACGAGAGGACAGGCTACTGCAACGCAAACCACCGTGCCGGCCTCCATGATCTTGCAGG
AAACACTGCTGCCTCGTGCCGAATTCT
>at01087
TTTTTTTTTTTTTTTTTTGCCGCGAACCACACTCCCTGATTGGTCCGTTTTCAACAGGCC
CTCCCGGCAACCGCGGCCCCCCCCTGCAGTTGCGAATACCCCTGGCCCTCTCCGCCTACC
CCTGGTGACCATGTCGGTGCCTCATCTGCGCTGCGTGGCGTCAGGGCCGCCCTCGGTCAG
GCAGCCTGGCCCTGGTACTCCCTCCACGCTGCCGCGGCCTGCACGGCCGCGCGCCTTGCC
AGAGCTGCCTCCCGCTTCGCCTCGATCGTGTCCTTCAGCTTGGCCGCTCGCCGTGCCAGA
GCGGTCGCCCGATTGGGCTCGATGAGCGCGCGCTGCTCCGCGGTGAGCGCGCCGCCCGGC
GGTCCGCCGGAGGCCGCCTCCGCCCCCGCCTCCGCCTCCGCGAGCCTCCGCTCCTTCTCG
GGCCGGCCCTCCGCGGCGAGCGGGTCGCCGGTGGCGGTGTCCTCCTCCGAGGGAGTGTGA
CTCCGCTTGACCGGCGTCTCCGAGGCTGGGTCACGCACCGCTGGTGGAGGGGTCGAGCAC
ATGCTCTCCTTCTTGT
>at01088
ACCCCGGACACGTCCCTCGCGCACCGGACGGGAAGCCGGAGGTGATGTACTTCTATGACC
AGGCCATCCGGAACGAGGACTGGAGGCGCATTGGCCAGAGGATGCCGCGCGTCTACAGGC
CCGGCGAGGAGTCGCTCGGCAGCTGGCTGCACCACCACGCGGACTACTCCAGCGTGGTAG
ACATGAAGGGCGAGCTCAAGTTCGAGCACGCGGACCTGCCGCCGAGCCAGCGGCGCTCCG
CAGGCCACCACGGACTACCACATGCAGCACTCCCCAGAGGAACCCCCGAGGGATCCAGCG
CATCCGCCTCGAAGGTGGCACACCGCCTGGGGCCCGCGAGATCC

>at01089
CCGTAGCCATTTTGGCTCAAGGACAGACCCATTCCTCGCCGATGGAGAGCCGTGAGCCCC
TTGGTGGCGTATCCTCCGCCAGCGCCGCCCTTCATCTCAGCCTGTGTTCACAGTGATTGC
CGTTCTTGACACTCAAGACGAGGAGCGCATTTGTGCTCTTGCGAAGGATTAAGCCTGACC
CTTCCAACAGAGAGATCTTCATGGCTATCGACAGCGCGCCGCATCACGTCCGCGTCTTTC
ACTGCCTACAGCAAGACAAAGCTGCACGGTTGCATTGGCCCGTACCATGATCAAGGAGC
ACATTGGAAAAAGGGAGTTTGAGCAGAGGGCGGCCATCTTGATGTAGAGTCGATTACC
>at01090
AATGTCACAATGTCCTGTTGGGTCGGCAATGCGGGCACCAAGCACCCTGTCTGATGGTGT
TGAATGTTGCATCCCACTCATGGCCTTCTGCACAGCGCCAGCGAAGATGGGTGTTGCTAT
TGCTGTAACCTATGGACAAACAAAAGCCACCGCGCAAGGCAGCTGCGTGAATGGCCTCAT
TCAGTGTCAGCCTCCTTTTGCCATGGCGCAACGTCGGCACCATGTACCTGCATTTCTGAT
GCTGTCAGAGTTGCACGCCACTCGTGACCTTCTGCGCAGCGCCATGTCAAGTACTCTTTG
ACGCCAAGGTATTCCGTTTGACACACACC
>at01091
TTTTTTTTTTTTTTTTTTCACACCACTCGGCAAGGCCCTCCACAGTCCATCCACAGGGGC
AAGGCTCCCCCCATTCACTCCTCGACCTCCTCCACTTCTGTGCCCGAGCGGAAGTCGTGG
CAGCAGCCCAGGAGCGTGAGGCCGCCCACCAGCAGGTAGGACAGCGCCATGCATGGCCCG
TAGTTCCCCCGCTCCAGCTGCCAGAGGACCCCAAAGAGGAAGTTCAGAAAGACCAGCAGC
AGGGGGTTGCCCCACAGCACCGTCTTCGCCCAGAGCTTCGGCAGGCACTCCGCGCCCTGC
GGCAGCAGCCCGGGGTTCCCCTCCTGGAGCACGTGGCGCCGGACGCCCACGCGGACCCAG
AGGACCCAGGCCACGTTGGCCAAGAGGAAAGCCGCTGGCATAAACTGCACCCGGACGCTC
AAGTGGGAGCTCCGGGATGGTCACGATGAGGGCCGTGGCGGTGCCCAGCATGCTATAGAG
GAGCGCGCCAAAGAGCGCTGCGGCGGCAGACTCCCGTAGGAGCAGGGATCCCACGATCGT
GAAGCCCTGCAGGTACATAGCCACCGCCAGCTCCGGGAAGGCCCCGATGATGAAGCCAAA
TGCGTCCTCGAGCGTCATGACGAGAATGGAGATGCCCACAAAGAAGAGGAATACGACTAT
GTACCAGCAGGGTCTGGTCCGCATGAGGTTGGAATAGTAGTCCAGGCGGTGGTGCT
>at01092
CTCGGCGCGTAACCCTGCACTGCCGGTTGGGTGCCTTGGGTTTCGCAGGGCAATGGCCGC
CAAAACGGCGATGACTCCGGAGGAGCAGCTGGAGGACCTGCTGCGGCGCTTCCAGCTGCT
CGAGGGCGAGCGCAAGGCAACATATGACACGGCAAAGCTGAACATCCAGCAGAACAAGGA
GATCATCAGCCAGATGAAGGAGGAGAACAAGACGTTGCGCAATCAGATCGCAACGCTCAG
GAATGAAAAAGCCGCAGTCAACCGAGAAGCAGTTGGAGAAGACCATGTCTGATGTCCAGG
TGCTGCAGCGCAAAGTGGATGCTCTGAGGTCTGAGAACTCGAAGAAGCGTACCACTCTCG
AGCAGCTCAACATCAAGCTTGGTGAGCTCTC
>at01093
TTTTTTTTTTTTTTTTTTGAGATATGACATCTTGACTTGGAGCTGCACTGTACACACTCA
AGTCCAGCAGAGTCAGCATTGATCTCAGCAGGCTATGCGGATGGCCGGCTATCTTTTGAC
CGGTCTCCCGCAGAACATGCAGGTCCCGAGCAGGGCCTATCCCGCCCTGGGGAACGAAGG
CCCCAAGCAAAGACACTTGCTTTGGCAGAGCCGGAAGACGCGAGGGAGCAACCACTTGAA
TATCGGATGTGAAGGACAGGGCAACAATCCCGGGATGGAGACAGTGATGGAGCAACTGTA
CCCATCCCAAGAACTACCAACATCTTCCACATCAGGTTTGCTCAATTGTGGAGTGTAGTG
CGAGAAAACATCAGGCTTGACTGATGGGCCATCTACTGCAGCAACAGCCAATGCCTTCAC
TCGCTTGCCTGTGAGGCAAGCTCTCCGCATTTCATCTCCGAGACAAAGAACGCCTAGCAT
CAGCCGCCGGAGCTCTTGCCGATTCCAAGGTCCCCAGCCCGCATAATGTTGGGCCCATCA
CCAAGCTTCTGCGCCACGCTGCCCTGAGTCACAGGGCCCTCGGCGTCCTTCTCATGGCGG
TCAGCCTCCATCTGCTTCATGATGGCATCCTTCTCTGCACTCGGGGCCTTGCGAGCGGCC
ATCTGGCGGCGACGTTCATTCTCCCT
>at01094
TTTTTTTTTTTTTTTTTTGGTCCAGCCTCATGGTTGTGGATTTTCGTCACCAGTCACCGC
TAGGTGTGGTAGACACGGAAATCACACTGGGATGCAGCACCTCAGATGTCATCCTCTGAA
TCGATGTTGTATCTCGAGCTCAGCTGCTTGATCATGAGCGAAGACAGCCACATGCTCTGC
CCTGCCTTGCGCATGTCTGCTTTCGTGAAAGCGGCAAACTTGGAGGCAGGGTCCTCACCA
GTGCCACTTGTTGCAACAAAGTCAGGCGAGAACTTGTTGATGAGCTGGACAACCCCTATG
GGCTGCGCGTCGTCAACTTTCTTGGAGAAGACAGGCACACAAAGGGTGTTCTTCGTGTAG

TAGCCTGTGCGCTGATCTACCTTTTGGTTGAAGCGCTCGTCCTGGTAGCAATCTGGGATG
TTCAGAATCTTGCCCTCGCTCAAACAACACCCAGCAATTCCAGCAAATGCATCGACGCAC
ACGTAGGCCGTCTTTCCCGATTCTGTCACGAAGAGCGTCCATAATACGTGCTTGTTCTTG
TCGTAGACCCACAATGTAGCCCGATCAACTCCGCCTCGTGCC
>at01095
TCCGCGGAGAAGTCGAGGCTTGTGACGCGCCAGCCAGCGGCAGCCAGGTCCGCACTGAGG
TTGGAGTCGCCACTGCCGACGACCAAAACTCCGGCGTCCCGCGGCAGCACGCCCTCCAGG
GGCACCCGCAGCTGTTGGAAGCCAAGGCCGTACCACTCGTAGAAGTCACGGCCGTTCTGG
CTCTGGCGACGGTAAGTGCGGTCCCAGAAAACGGGGTTGTCCGCAGACCCGTCATCCAAG
AGCTCCAAGGCATCGTGGCCGCTCCTCCACGAAGCCAAGTGCCCCCCTCGCCCCCACCAG
CGCCACGGCTGCGGCCACGGCTCTCAACCTCGCCAGTGGTTGAGGCCGCGAGGCCGCGCC
GCGTCCCACGCATGCTGGCAGCGTCCGAGGCATGCTGCTAGCGTGCAAGCGCAAGAGCGT
GCGCTCGCTCGCGTTCATGCGGGAAGAGGGGCACCGTGGCC
>at01096
AGCGGCTCAGGCTCCCTCTTCCCCTCCGCAGGCGCGCGCGCGCGCGCTTGCGCGCGCC
ATGGCAACGGAGGGCGCTGGGGATGGAAAGAAGGCCACGGCGAGATTGCAGGACCCCGCG
TCTTGGATAAGCGCCATCGACATTTTCATCTTTGATTGCGACGGTGTCATATGGCGCGGT
GACTCGCTCATCGACGGCGTCCCCCGCACGCTCGACCGGCTGCGCGCCGCCGGCAAGCGC
ATCTTC
>at01097
CGCGATGGAGGATATGAGGCAGCAGTAGGCGCATGCGTGCATGCTGCTACGACGACCTGG
CTCATGCAATGGTGTTCTCAGCTGCAGTGACCGCACACATCGCCAACGTGGATGGCAAGA
TTGGCGTCATGGCCGACGAGTACCGGGACTTGGAGCTCCAGGCTGAGTGTTGGGTGCTTG
TCTGCGGCATGTCAGGGCGCATCCATGGCTACTTCTGCAGGCTGGGCTGCAGAGTTCAGG
ACTTCAAGGGCGCGAAGAAGATGTGGGCCCTGAAGTGGCCACATAACGCCATGGATCCAG
CTTTGTGACGAGCCGGCCTGTGCCTGTCTGGGTTTCTTCCAGGAGATGCAGGTCGTGAGT
GCCAAGGGTCAGTCCCTGCTGAGGCAAGGCAGGCATGGCGACACCTTGGCTCAGAAGGAG
GCTGCTCATGACAAAACGGACATGGGGAAGGAGTTTGCCAGAGCCTTCACAGACTATGAC
CTGCCTGCCTCCCTCATGCATGTTGCTGCCTTCAGGGCCGCATGCGGCATTTGAACACGG
CTGGTCTGCTTAGGCACGCACCTGCCTATGTCTGCCTTCACAAATGACCTCGCCGTTTGC
AGCAAGACCGAGCTAGAGAAGGACGGAGAGAGGCCGATGCCTACATGCATGTGAGCGTGA
GTGCGGTAGACATGAGAAGCGTTCAGCTTGTTTAAAGATGTTGTGAACAGAGAGTGCAGA
TGGCCTTAAGTCTCAGGCAGGATTTTCGCTCCTACGTAGAGTGGCGCCATTCGAGGACTG
ATGTGCCAGCACGAGGC
>at01098
TTTTTGGGTTTTNNNNAACGCAAGGGCGAGAGTcCCCCTCCGCCCTCACGCTGTATAGTT
TCGGATGACGGCGGTGAACCGCTGCCGGCAGAcCCGGGCAGcCGCATCTGCTTCCGCTGC
AACTCGTGGGCGCACGCCATGCAGCAGGCTGTGTGCCCAGAGTCCCCGTGCACGAAGGTC
GCCACGGCCGGTGCCGCTACACAAACGACGCACGTGGCTGCACATGCGTCCATCTCATCA
GTGGGCCCGTTGCCAGATGGAGCACACCTCAGCTCGGTGCCTGTAGCGTCGCCGGGGCCG
GAGGAGCTGCAAGACCCCATGCTGGTCGAAGGCGGTCGGCCTCGGCACCAGCTGTGTCCG
GCTGCTGCGAGGCCTCTGCACTTGGCGGCCATGGG
>at01099
ACAATGACTTCTACCCGGTGGTCATGGCTGCTGCTCAGGTCACAGGCGAGATCGACGGGA
AGAAGGCACTTCGCGCAATGATCTGCCTTGATGAAATCCGGGGTCGGCTGGCCGAGGTCT
TTAGCCTGAAGAGCTACAAGATCGACCACGTTGTGCATGGTGCGATCGGCTCCGCCGCAG
TGTATGGAGCACTGCTAGGGGCCTCAGTGGAACAGATCGAGAGCGCCATCGGCATGTCTG
TGGCGCACTACGTGCCCTTCCGCGCCATCCGCGCAGGCAAGCAGCTCTCAGACTCCAAGG
GCGCGTCTGCGGCCATCTCCACCGAAGCAGCAGTGCTCTCAATGCGCCGCGCGATG
>at01100
TTTTTTTTTTTTTTTGTCTCTGCGGGCAAACAACTCTCTCGGCGCAGGGCCAGCAACTG
CCAGTTTGGAACAAAAACCTCGCGGTTGCATGGGCCATGAAACTTGGAGTGGTCAAAAGT
TGATGTGTGGACAGCTTTCGTGCCACCAAGACTCTTCAAAAGAAACTGCGGGATTAGGCC
GGACCTGGAAGTCCCAGCACAAGGGACAGACTGATGACTAGACATCATCCACGGCCGCAG
TGCACCGACTTGTGAGGTGGCTGCGGGCCTGTTCCACGTCCTGCATGCACTGAGCTGCAC

TGATGCTCTCCTTCGGCAGCGGGTCACGGACATGGCCAAGAACGGTCAAGGACTGAGACG
GCATGAAGGGCACAACCTTTGCAGTTGAGCCCTGGGCGCTGGGATGGCAATCCGCATCAA
CGGGTGGGTGCGACAACATCATCAGCTCACAGGGGTTTGGGCTGGTGGGGTGCGTGTTGC
CGCACACAGGATACACGCCACCGAGCATACCGTAGAAGACGCACGGCGGCAAGACTCCGG
CAACTTCGCACATCATCGTGTCCATGCCGATGCTCTGCATCAATGGCACGCCCTTGGGGC
TCAGGTTGACGAGCTTGACATCCCTGGCCGCCGCGTGCATGGAGTTCTTCTTCGCCTTTG
ACCCCACCTTCGGGGAAA
>at01101
TTTTTTTTTTTTTTTTTGCCACTCGCGTTCATGTATCAGGCAGAGCCGAACAACTCCGG
CACCACGGAACAGTCAAATGGATCCGGGGAAGCTGGCTGAAGCCAGCCAGAGCGCTTCTT
GCATAACAGTTGCAGGCCTTTAGAATCTTGCAGCGTGTGCACCAGGACCAGCCAGGGAAG
AAACTCACACAATCCTGCCCATCCTCCGCTTCCACTTGCGTGACTTGGGGTAGTCGGGTT
CTGAGCCGTAGGCATCCTCGTACTGCATCTTGAGTTCCAAGCGCCGCCGCCGACGCATGA
GCCGGCTCTGCTCCGAGGTGATACCACGGGCGATCTCTCGTTCCCGCCGCAACGTCAAAG
CTCTGGCAATCTGCTTCCTGATTATCTTGAACAAGTGCGTCTTCATCCCCGGTGTCTCCA
CTGCCTTCTGCATCCGAAGCCTCAGCAGTGCCCGCTTCCCAGCTATGACAACGTCCTCAA
GATCTCCTGATCAAAGGCAGATTCTTCGGCTGTCATCTGCCTCCGCAAGCGTTCCAGGGA
GTCTGTGGACTCCTCCACTTGGAGGCGGCCCACGGCCATGTTGGACGTCTCCTGCCACCA
CTTCACCTGCTTTGGCTGGTTCCTCCGGGGAGCTTTTGGCACCACCTGCGGATCGGCGGT
GCTCATGGATGCCTCTCGTGCTGAGAGTGGCCGCCCATCACTGCGGCCAACACGGCCACT
TGCCAGGCGGGCACCAGTGGGGAGGGCAAAGCCTTGTCCAGCCCAGGTGCATGCTGCAAA
GAATGCAACGGTGGCAAAGGCAGATCCCGGATGCTGCCGGAACCTTTGGAAAGAAGCAAG
TGGGCCATGTCTGTAGCTCAAGTGCTTCATCATCGAGGAAGCCTCTCTGCCCTGTCCTGC
AGGCTTCAGCCGCTGTGGCTTGAGCCAAAATGGCTACGG
>at01102
CCGTTAGTTTCCATTTTGGCTCAAGCGCACTGGCTCAGACCTTTGTTATCTCACCACTGC
AATTGCGGCGGTGCGCCCGTGGCACTGGCAGTTGTACGCAGGCCCAAGATCTTGACAATC
AAGTCAAGCAACGCGATTCATCGATCACGTGCACATGGAGTCAGCGTCCAGCACAGCCAG
AGGCGACGGCCCGAGCCATGGGGTTGAGAAGGCGGCCAGCACTGAACAGCCCTGCCTGGA
GGGAGATTCCAACACCTTCCACCAGGCGGCTTGCACTGAACAGCCTTGCCTGCAGGGAGA
TTACAATACCTTC
>at01103
TTTTTTTTTTTTTTTTTAGAACGTCACGCATCCACAATGTGTTGTACCGTTCACAATGG
AGAGAGAGATGCGAGCACCTTTCTGAAAGGCCAGAAAAAGAGCAAGCACGAGAGAGAAGT
CCTGCTCAGGTGGATCCGCAGTGCAGTCAACCTCAGCAGCAACTTCCGAGGGAGCATCGG
CAACGCAGACTTCTCAAGCATTTCTGGCGACAACAATTCACGGCGGGACCGCCCCCCCCC
CCCGACAAAACGCTGTGGACTGGGTGCCAATCACAACCAGTCCAGGCAACAGATTGTCAT
CTAGACTCCATCCCATTGAGTCGCAGCCTGTACACGACCAAAAGCGAGGGCGCGAATGCC
ACATGTCTGAATGCCAGAGAATGCGCTGAGGCATCATGCATCATACTCGGAACTCAGATC
AAGTCAGCAATGTCGTTCGGCATCTCTTCGATCTGTGTGTGGTAGAACCTTGGATCATGC
ATCGCACTTGGAACTCAGATCAGGTCAGCAATGTCGTTCGGCATCTCCTCGATCTGCGTG
TGGTAGAACTCTCCAGATCCTTCATGGTGCGCACGTCGTTGTTCGCCTCGTGCCG
>at01104
TTTTTTTTTTTTTTTTTCACAGGCAAGCATGCGAAAGGGGATCCTCCCGTCGATGGAGC
TCCTCCAGCGGAGCGCCTCCATGAATTCGTGAACGTGCTGAACTGTACACAAGAACCCAG
CCAGTTGCCCAACTACCGAAGACTATGCGCCTCCCTTCCTTCTCCCCAGCTCCTGACTCC
AGTACTGGGGGGTGTCGTCGATGAGACCGTCGATCGCCTCGATCGTGTAGGGGTACGGTA
TCTCCCTCGCGTCGGCCGCGACCTGCCCGTGCTCCTTCATCATCCGGATGTAGGCCTCCC
ACTCCTTGACGCACTCCGCGAGGTGGCCTCCTTGGGGTGGGCCTGGCAGGTCTTCATGG
CCTTCAGCTTGTCCACGGGGTCCAGCTGCCAGCTCAGGCCCGGGAGCTGGCTGATGATCA
TGCCGCAGGCCAGCATGTACGCGGTGTCGACGTAGTAGCCGGGCCTCCCCTTCGCGGGCC
TGTGGACCGGGAGGTCCAGGGACAGCAGGTCCTCGGGGTAGTCGACCACGTGGTTCACCG
CCGTGACCGCGCCGGCGAAAAAGGGCCCCACGCTGAAGCTGCCGAAGTTCTTGGCCTGGA
CGCCCCTCGTGCCGAATTCT
>at01105

CATTGGGAGGTGGCTTGGTAGATTGCCCGAGCAAGTAACGTGCCAAGCAGCTGTAAGCGA
GGAAAAGCCAAACCAGAAGCTCTGCGGCCATAGTCACCTTTGTTATCAGGCTGGCATGAA
CAGAACTAACACCACACAACTTGTAATACTGAAGGCAAGCAAGGATGAGGCCGGCAGCAA
TGATAGACTTGTTCTGCTGCGACAGTGTGGCTTCAAATCCATCTCGGCCTTGGCGTGGTG
GAATGTCCACTGGCGCGGAGAGAACAAAAGGAATGTCCATTGGCGCGGAGAGAACAAACA
CCGACGACGCC
>at01106
TTTTTTTTTTTTTTTTTCATTGGGTCAGAGACTGTACGGTAGTTGGCAAACAATAGGCC
GCCAGGGCCTAGCCTCTTGCGCTAGGAGGCGACCTGGAGATGGCATCTCAGCGCTGCTTG
ACCTGGGGGCCGCCCAGGGTCTTCTCAATGTTTGCCAAGGCCACGTCCCAGTTAATGACA
GAGAAGAGCTCGTCCACGTTGGCCTGCTTGGCCACCAGGACGGCGCGCTTGCCGTAGGGC
ACGGACTGGAAGAGCTCAACGGCCAGGCCACCGGGGCTCATCTCGGCGGCAAGCCAGACC
CAGCCCTTGTTGTCGTTCACGACCTTCTGAGTCTCATCGACGCCGTAGCCGACGCCGCCC
TTGGTCACGAACATGGGGCTCGCCATCTGCGTCTTGAGGCTCGCCACGCCGACGGCGATG
TCCTTCTTGCCGATGTTGCCGGGGGGCGGGCCCTTCCAGAGGCCGGAGTAGCGCTTGAAG
CCCTGCTTCGTGAACTCGCCGTGGAAGAACTTCACGATGAGCTCGGACACCACGGTGCCC
TTCGGGGGGTCGCCGCCCTGGCCGGTGCGGAGCGCCTGGT
>at01107
GGAAGGAGTTGGTGATGTAGGGCTGCAAGCGGGCAAAGTCTGAGTGAGACTCCAAGCACT
TGAGCTGGGAGAAGCTAGGCTTTCGGAGCTTGGTCTTGCTGTCAAACACGACAAGACCTG
TAATGCTGTGATTTCCATCAGTTGGAGACTTGCGCTGTTTCACCAGCCACTTCCAAGCTT
GCCGTATGGATTCTTGGAGTGACCGTCAAAAACTGTTCCAGCTGCGGTCAGCTGCTGCTG
>at01108
CGAGGTGGACCTTGCGCGTGGCCGTCTCCAGCTGCGCGACCAGCTCGGGATCGAGGTCCT
TGCACCAAGGTGCATCTACCAGGTAGATGTAGAGGTCGGTGTCCAAGAGCAGCCCCGTAT
CTCCGGACTCGTCTGGCTTGTGTTCGCCAAAGGGTGGATCCAGAAGCCAGGAGAGGCCAT
GTACAGTGCCAAAGCGGTCCGCTGGCTGGCAGAAGTTGAGGACCATGGCGCTGGAGAGGT
TGCGGCCTTCGGCCAAGGCCTTGCTCACTGCTACAGTGCGGTCCACGCCCACCGAGAGGG
CCACTCCTGGAGGTCCCCGGCGTCAGGCTGCCCTGCAAGAGCGCTGCGTGCATCGTACCG
CACG
>at01109
CCGTAGCCATTTTGGCTCAAGCCGTTTTGGCTCAAACTCTCCTGCAGAAGCCTTGACCAT
TACGATCTCTGAGACTTGCACAGACCAATGGCAAAAATACTCACTCATGCGTTGCTTCTG
GCGCTGCTGAGCCCCTCCCTGGCTTTCATCCAGGCTCCATCTCCGCATGAAGTGCTCGAA
AAGGCACCCTCGACCAACAGGTTCCTAGCACACGGCCCCGTTGAGGCGCCTGAGCTGGAG
TTGCCAGAGGCCGGTGCGCAGCCAAGGTCGTGGTTGACGCCCATCCTCAGCTTCGGCGCC
ATGCTTGGACTCGCCGCGGGGCTCCTCTCGCCCACCCCGACTCGCGCACTCACCGCCGAG
CAGTACAGCCAGCTGACCTACAACCAGGTGAGGGGCTCGGGCCTCGCCAACCGCTGCCCG
ACTGTGGAGAGCCAAGGCAGCTCGGTGCCTGTGACGAGCGGCGCGAAGCTCACGAATCTG
TGCTTCGAGCCCAAGTCCTTTGCCGTCGAGGCGGAGACCGCGAAGGGCAAGGAGTTTGTA
CCACCAAGCTCTTGACACGCCAGACCTACACCCTGGCCTTCATCAACGGCAACCTTGATC
CGAACCCGATCACCTTCAGGGAGTACGATGGCATCCACACTTTGCCCACCACCGTGCAGT
TGCCCAATGGGGAGTACGTGCCCTTCCTCTTCTCCGTCAAGAAGCTTGTCGCGACAGGGG
AGGGCGCTTGAATTCAAGCCGGGCTTTACCTGGGGTGGGGAGTTTGAGGTGCCCTCCTAC
CGCACCGGCGCGTTCCTCGACCCGAAGGGCCGTGGTATGTATTCCGGCTACGATCAGGCC
GTGGCGTTGCCCGCACTGCAAGCCGACGGCGTAGGGGGCCAGGAGGAGCTCTTCAAGGAA
ACGAACAAGGTCTTCGACATCGGCAAAGGTGCCATCGAGATGGAGGTCAACAAGGTGAAT
TCTGAGTTGGGCGAGATTGGCGGCGTCTTCGTCTCCAAGCAGCCGTCGGACACTGACATG
GGAGCCAAGGCCCCCAAAACCATTCTTCTCAAGGGCATCTTCTATGGCAAGGTTGTATGA
TGTGCTCTTCAGATCAGATCGACTCGAGTGAGCCGGCGCGTGCCGCGCACCGTCAGTTAC
GGATGGAATATCTGTGGAATATGATTTGGACATGGGTGGGATTTGAGAGAATAAAAAAAA
AAGAAAAAAAAAACAA
>at01110
TGCAACCAACCAGGTTGAGTTCAAGCGAGAGGTTGCTCGTCGAGCCGAGCAGTGTAACTG
CGAGCCGCCACTGCGCAGCTTTCTCACAAGTGCTGACAACAGCGTTGCAGCTGATGGCGT

TGGGCTCGAGCTGCAAGCTGGTCATGGAGTTGAGCAATGCAAGGGCGAACCGCCACTGTG
CAGCCTTCTCGCAAGCGCTGATGGCTGCACTGTAGCTGATGACATTCGGCTCAAGCCGCA
AGCCTGTCATGCTGCCAAGCAACGTACAAGCAAGCTGCCAGTGTCCAGCCTTCTCGCAGG
CGCTGATGGCGGCGCTGCAACTGATGATGGTCGGCTGAAGTCGCAAGTCAG
>at01111
CTGGATTGAGTCGCTCCAAGTACATATCTCTGGCCTCGTCGACGCATTCGTACACGGCAT
TCTTCAGACTTGCGTCCCGCAACACAACGGTCAAAGTGCTGAGGTTTGCCTCCAGGATGT
CCAGATCGCGATTGGAGTACAGGCCGAAGTTTGCCAGCGCCAAATCCTGGTGGTTCTCAT
AGTCCAGGCACCGAACATCAATGGAGTTGCAGTCCCTCTGGGTGTGCATGTTGCTCACAT
GATCTGCACCATCCTGCAGGACAGTGCGCACATTCGCCATGGAAGGCGACTTCAGGATTG
CCGCCTTCGATTTCAACACCGCCTCGCTCTCTTCCGCGTCCAAAATATGCTGCTTGTGGA
TGCGGAGTTGCAGTGCGAGATTCATGCCGGCGCTTGTGAGGTCCGGGTCCATGTCAGTGA
CGACGAAGACCTGCCTACTTGACGGGACGGAA
>at01112
TTTTTTTTTTTTTTTTTCGCGTGGAGTCCGGTTATGACAATCGAACCCATGCTCGACTT
CACTTGAACCTGCGGTCCGAAATCAGCTCATTCATCACTTAAGATGCTATGCAGGCCACG
CCTTCAACCTGTATGCTGCCGTCGCTGCCTTGCTACTTTAAATCGTGCTGTTTTGGCTCC
TAGGCCCTCACGGATACGGAAGACGTGGAAGCGGACTAAACAGACACCGCAAGAAGCATC
GCGATCCTTTGACAGACTATGACGACGGGTGCGCCCCAAATCGGTCAAAAGCGCGAGTAA
CAAGCAACTTAAATAACCCCATCAGCTGATCTAACGGAAGTGAATTCAGAGCCTCTTGCC
CTCAAATTCCGCTGAATCCGCGGATTCTCTTGCGCACGTCAGCTCGACGTAGTCGCACTC
CGCCGAGCTGTGGCGGCGTCCGGAAGCAGTCTCGTCACGAGGTGTGCAAAACCTCCACAA
CAGCTCTGGGTGTGCCTCAGCGAGTTCAGCCCCTCGTGCC
>at01113
GTCAAGTGCCACATTGAGGTGAATGACTGATTCAAGTGCATCTTCAAGATGACTGGTGTC
AGATATCTTGACAGCCTGCCACGCAATGTAAACAGTCATGGCGGCAAGCGCCAGCATCCT
CACAAGCAGGTGGGGGCCGGATACCACTTCTCAACGCTAAGGTGCTCGGGCCGCACCTCA
AAGTTGTTCTTCACATTGAGGCCCTCAATCGTGCCGCGGAAGGGCATCTCCCAGATGAAG
TCCTTCTGCAGCTTGCCCTTCACCTTGTCGCCTTTCCCATTGCCACCCGAATCTGGAAAG
CAACCTCTCCGTTGCACACGATACCGACCGAAGGGTCTTTCACCACGGACTTGTCGACGG
TCAACTTGATGTCTAGAGGCTTGGCGTCCGAGCCCACGGCGA
>at01114
TTTTTTTTTTTTTTTTTGACCGGAGGAAACATGCGCTCCAACGGCTCTGCGTCAAAACG
CTGCGTCATCCCGGAAGCCTGCCCCTCGCTTTCAAAGTCTACAGTGGCATGCAAAAATGC
TGAACGGGCCGTGCTATGCTTTGACCCTTTGCGGCACACCATCCAAGAGGGAAAGCACAT
CTGCACGCTCAGTTCACTGTGCAGGCAGGCCGAAATCACGATACAGAAACATGCAGAGCC
AGCAAAGACGTCGTGACCCGTCAATTTGACCCTCGTGCC
>at01115
TTTTTTTTTTTTTTTTTTGCGGCAACTTCTATTGTCCAAGAGGCCCCTCCTGAAAGGACA
GCCTTTGGACAAAGTGACTCTTCGAACTGCATCGGTGCTACCTGTGGGTCCGTGAAGTCA
TTCATAGCCGGTAGCCTATTCACCTTGCTGCCGCAGCCCTCACATGCGCAGCTTCAGTCC
TCGTCCTCCTCCTTCGGCTTCTTCTCCGCCTTGCTGAACGCCGGGGCCACCTTGATCGGC
CGGGAGCCGTAGAAGAACTCGCCATCCCAGTCCAACGCCTTCTTCGCGCCGGCCAGCGTC
TTGAAGACGATGAAGGCCGCGCCCCGGAAGTTTCCATCAGGGCCCTTGACCATTCTCACG
GTCTCGACTGGCCCGCAGTCCTCAAAGTACTGACTTGATTTTCTCCATGGCGGTGTCTGG
CGGTATGCTGCCCACGAAGACCGTGAGGTCGGTCTGCTCCTTGCCTTTGCCCTTGCCCCT
CGTGCC
>at01116
CCGTAGCCATTTTGGCTCAAGCCTCTGTTCAAATCCTGGGCATGTGCGCCCCATGAATTG
CCCTGGCTTGCACCTCGGCGTCCACCCAGCAGGCCATGCCGGGACGGCGCAGAGGTGGTG
TGGCAGTTAGAGGATGTTGTCCAACGACTCACCGTCCTCATTGTCACAGATGCCCAAGGG
GAGCCAGCAGATGGTGGTGAGACCTCCCAGAGTGGACTGGTACAGTCCGAGAGCAATGCC
AGCATCCTCACGACTGGTTCAGATGGCGGGCCGCCCGCCCAGCGTCCCCCCCAGACCGCG
CAGTCTGTCTTCCGCCGTGGTGAGGTGTTGCAGCCACATCTCAAGCTGAATGCGCCAACA
GCTCAGACTGCAGCTCCACCCATGCAGATCGTGCGAGCATACCCCACTTTGCACAGGGCG

GAGAACGGCTGCCCGTCTCGGCCGGTTGCGCCATCCGACAGGCCGCGGATTTCACTTCCG
GTTGGCAACCAAGCCAGTCATGTGCAACCTCATGAGCAGGCAATGCCAATGGCCGCTCCG
GTGACGCCAGCTTCGGCACCTCCCATGGTGAACCGTGAACGGGCCGAGCAAAGACGGGTC
TCGGTTCCAGCACCAAAACGGGAGGCCGCACCAACTTGGGTGATTGAGGAAGTCATCGAC
TTTGGCTTGACCGCAGATGTGGGTTCAGATCCTGACCGCGAGGTGTCTGACGCAGCATTC
CACAGAGTGGTGAATGCAAGGCTGATGCATCGAGCAGGCCTCTTTGAAAGTTGCGCTTCA
GACATGTGCGCCGATGAGGAGAGGGACCACAGCACCACGGAAGCGCACTGGCCCCAGTGG
AGGCAAAGCACTCTCGTGCGTGACCTTCTGTGCTTGGACGGATAGCTGTCATGGCTCGGG
GAAATAACAAAAAAAAAAAAGAAAAAAA
>at01117
TTTTTTTTTTTTTTTTTTACGCTCTTGCAAGGGCCCACATGCAAGAGCCATCGTCAGATG
GAGAACTGTGAGATCTACAGGGACCCGCATCGCCTGAAGACTGCTGCAGAAGAAGAGCGC
GAGGAAAGAAGCTCAGACCTGAAGGTGCAGGCAAGCACCCTCAATACATCCATCTCAAGA
GCTCGCGAGCCCCAGGGTGCGTGGGCTCAGGCTTAAAGATGACTCTTCGAATGAGTGCTA
GGGCAAGGTCTGTGCACAGCAGCCAGAAGTCTGGAGAGCCCAGGCCAAGGACCTTCGACA
GTGTGAACAGCGCCACGATCGGCTGAGCAAAGACTCCACAGCAGAGGAAGTCCTGACCAG
TGAGGTACAAGGCCCGCACACCAGATGGGACAATGTTCAAGGTCGGATCAAGAAACCTTG
CCACATTCTGGTCAAGGCCATACGAGCCCAGCCGGCCCAGATAGTAATCGTTCGACAGCG
GCGTGCCAACGTCGACGTACGAAACCTTCGACTGGAGGTGGGGGAAGTGACGGTAAAAGC
CGGCGAGGAGGGCATCCTTGTACCGACTCTTCACCTGCTCGTACTCAGCTCCTCGCTTGC
CATGCGGGCCACGGGTGCCGAAGACCCTCGTGCCGAATTCTTG
>at01118
CAGTCTGCAATAGAAAGTAATGGGCCCGGCTGANGGGGTTTTGACAAAGACCCTATTCTT
CGCCTTTGCCAACCACTGTACAAGACAGGCACTGACTCAGCACCTGCTGTGCTACCAGGG
CTGCACCTATGCCTGTCGAGTGCCACCGAGCGGACAAAAGTATCACCAATCGCCAACCCT
GTTTTTTCCATGCTTACCTTCGTGGTCACCTCCGCCCTTGGCTCTGCCATCCTTGCCTTT
GCTTCGTGGAGACTTTGACCAGGTGGCCCTTGCCTTTGCCTTTACTGCGGCAGCCGCGTC
CACTCGTGCCTTTGCTATGGTTGCCTTTGTAGTGGCCTCTACATTTGCCTTTGCCACCCT
TGCCCCTGCGCGTCTTGTTTTCTGAGCCGTCCCTCTCCGCGCCAACTTTGGCCAGGTGGT
CGGGGTACTTGGTATGGTCTGCGATTGCGGCCATCAAGTTCTCGCCAACAGCACCGAGAC
ACTGGTCGAGGCTCATAACCGATCTGCGGTGCTTCTTTGGCACGTACAAAAGGCGTTCCT
GCTGGAGAAGGCATAGTTGGAGCCCTGGAAGGTCTCTGAAGTCCTTAGTGTCCTCAAGGA
TCTTTCCTGCTGTGCTCAGCCAACGCTTCAATGCTGCCCCGGCGCCCGCAGCCACATCAG
CGCCCAGCAGCCGGGGCACGAAGTCCAGCTCGTTGATGATGGTGTAGAACTTGGACTTTT
CAAGGTGAGGACTTCCATGCTCGTTGAAGCTCCCTGAAGAAACGCGAGGGCCGCCAAAGT
TGACGGCTACAACCTGGCCCTCATGATGTGGGCAGTACTCTTGCA
>at01119
CTGCAGGACTGTAGCAGGGCCCTCTCGCCCAGCCCGCATGTTCGGCCCCGTGGGCAATGC
GGCGGCCGCATCTGCGTCCTCCGCCCGAGACCTGCGCATACGAGGCACCAGTACGGTCAC
GCGCCTGGGCAGCCGAACGAGCCTAGAGGGCGAGGACCTTGCCAGCATCGGAAGAGTGGC
TGGGTTCGAGGAGCAGAGGGATGGTGTGGACAGAAACATCGTCAACGACGGGCTGCACAC
AGAGCCGGCCGAGAGGCAGCTGGAAAGGGCATTCCAGACAGCCGAGCACGAGGAGTGGCG
CTCTGCCCGCTCGCTCCGGCGGACCGAGAGGCAGAGGAGGCGGACC
>at01120
TTTTTTTTTTTTTTTTCTGGGCGCCATTGCACCCCTTCATGTGGTACCACATGCCACGAC
TTTCTCACAATAAGATGCCAGACCGCTCCACGCTGACACAAGGAAATCTGTACACACAAA
CGGTGCAAACCGCACCATGGCTTGACCAGCAAAGGTGTCACTCCAATTGTGACGCTCCAC
GCCATGACGCTCCACGTCATACTTGCTAAGTTGCCACTGCTCAGGAATTTAAGCAGCAGT
GGGAACGCACGTTCTGCGGCTTGTATGCAAGGCACTCCATGCCTCAATTAAGACGTGCTT
GGCTTTGGCAGTCGCCGAGGCACGTTTAAGGCGCCTGTTCACACCACCGTTACGTCCTTT
GAGGCACCTGAATCAGCAGCGTATGCCTTTGGGGGAGAGTGCTCCATGACGTCTGCTCCT
TGCTTTTGGAAATCGTCGCTCAACCGCAACGCGCCCAACATCCGAAGAGGTACGAATGCG
GCCACGCTGAGGGCTGCACTCCACAAGGCGATGAGCAACAGGGCAACAATCTGCACACCC
AGCTGGTCACCGCCGTAGAACACGCCATTGCCACCCATCCCATCGTCAGGATTGCCGAAG
AAGCCGAGAGCCAGGACACCCCAAGCTCCACATGCGCCGTGCACTGGGAAAGCGTCCACC

ACATCATCAATCTTGAGCAGCTTCAGGAGCATCGACGCACCTTGGTAGATCAGGCCCCCG
AACAATCCGATCAATATCGCCTCCCAGGACTTCACGAAGGCGCACCCCGCGGTAATGGCA
ACGAGGCCCGCGAGGATCCCATTGCAAAACCCACCAACGTC
>at01121
CCGGAGAACAATGCAGCTGCGTGCAGACATGTATTATGGTTGGTGCAGAAGCTGCTTCCA
GATTGCCGAGGCCCTGCCCAGACAGTGCGCAGTGGAGGCACCAGTCTTCACAGCTTTTGA
GTGTGAGATGTGCAAGGAAAAGGAGCTTCTTCAAAATGATGAGACCATGGTGAGACAGTC
CAAGACCTGTCCCGCATGCAAGGCGCCGACAGGTGAAGGATCTCAGGCTGTAATCATATC
TCGTGCGTTTGCGGTGCCCACTGGTGCTGGCAGTGTGGCGGCCAGTTCAACGAGTCTGAG
ATCTACGAGCACATGGACAGAGAGCATGGGACCATCGGACTGGATGATGATGCAGGGGAC
TTCTCTGAAGACGAGGACTGAATTGTGGCCTGAGCAGCCCGTGGAGAGGCTACTTGCAGT
GCTTCCA
>at01122
CTGGCACTGATGCTCGCAATGATCATCGCAAACAGATTGCTACCAAAAAGGATAACAGCC
GGAAGTCGCTCTGCAACGACACAGGCAGCCTTGAAAACTAGCCGTTAGGTCAAAAACATG
CAAATGTCAAACACTTCCAAAAACAACGGGCAAGTCAAACCCGTCGTGCCATGTCAAGTC
GACAACATGCAAGTGCACAGAAGGTCCCCACCAGTGCATGACTGTCAGCAGTCGGCGCAT
TTGACGCGCCACCCCCGGCACCCCGGCAAGGTGCCACCTGGTGGCGGATGGTCATGCAAC
ACGCACTCGTGCCTGCACCTCAGCTTCAAGGGATAGCAGGAAGTCCAAGGCCTCTCGTGG
CGTTGGCTTTGAGACAACGATCTCCACAAGCCAAGTCGGTAACCCATCCTTGGGTCCCAC
CACTTCATCTCCTGCACGGACATGAGGGCTGAACGAGATGACACCGTCACGGCCTCTCAG
TGGGTCTAAGAAGCTCATGTCCACAAGCCTGCCCGACCGCAATGTGTTGACGTCGCAGTT
GGCAAACACTCGGTCGGCTGCTGCGGGACATCAGGCCGCGATGGTATGCCCGCCTCGTGC
CGAATTCT
>at01123
AAGTTCCCAGAATTTTGATGCAAATCTGCCAAAGGCCTCTTCTGGCACGGTCATTGCTAC
CACCTCGGCGTGCCCCATGGAGCCATAGTCGGCAACTCCCTGAAAGTTGTGGTAGCAGAC
CAAGCCTCCGGAGCCGACGTGCGTGCCGCCGGCATAGGCAGTTCCTGGCCGCGATGTTGC
TGTCTTGTCGGCAGAGGTCCGAGATCTCGCTCGAGACGAAGCCGTGCTGCACATGCCAGA
AGCAGCCGCATCCGAAGTAGACCTGGACCTCCGGGCCTTGGGGTGGTGGCTCGTCGACGA
TGAGCACGCGGCTGGCAGACTAA
>at01124
TTTTTTTTTTTTTTTTTTCCAAGAAGCTTCACGCATGGCGGCAACGCCGGCATAGGCGGC
ATATTCATCGTGCACAGGGAGCGCGGCAGCCCATCTCTCTCACCCGTACCCTGCCAACGA
AGCAACAGGTCCGCGAGGATCCACGTGGACATTGCCAAGGCCTGCAGCTGGAACTGCTCC
GGCCTCGTGCC
>at01125
GCATCGCCTTCATCTCCCGCTTCTTGTGTGCATTCTGTGAGCTCCAGGAGGGATCCAAAC
CAGTCTTCACTGGTGTCTAACCAAACCGGAGGTCCAGGCGCAACATCGAGTCCCACACTG
CCACTGCCAGTATGTGTTGATCTAGTGTGCCCCACAGAGCTCTTTCTCGCGCGCACCAGA
GCTTTTCCAAGCAGAAACTGGTCATCCGTGAAGACAACCTTCTCCACACTCTCAAACATG
CGCAACAACTGCACCCCGGGACCCATGATTGCCCCACTGTTAGGGTACCGCAGTTGCCCA
TGTTGCAGGCCCTGGCCCCAAA
>at01126
NNNNNNNNNNNNNNNNNNNNNNNGCCGGTTGACCTCAGGACTCCGGTGGCCCAGGGGCCA
GCGCAAGGAAGTCTTTAAATGACTTCAACTCCCTAGCACCCTCGTGACAAACCTCCGGGC
CAGGATTCGAGGCAATGATGGTTTTGAATTGTAGAAGTACCGCCATTCTAATATCATTGC
TCAGTGTATGGGCACCGCACGGAAAACTGAAACACTGTGAGCACCTTATGGCTCTGCCGC
CAGAGTTGTCAGGGCATCCTGCCACTCTTGATTAAGATTTGAAGCAAGATTCAATCTGAT
CGGCCACCTCATCATTGCACATCAAATAGGAGTCAACAGGCGTCGTCTTGAACACCTTTG
CGATGGCACAGGGAAGCTTTGCTGTCCACCCTTTTAACTGAGGGGTATGCTTGCCCCCAG
TCTGGAAAGGCCAAAGCAGTGTTACCTTCCCCTTGAAGCCTTCCATGTTCTTCCCAGGCT
TCCAATCCCCATTTGAAGGGAAGACGCCGTCCCAGATCCACATGAAGTAAGCGCACCCAG
TTGCCTTCATCGCATCCTTTGTCTGCAATTCCTTGAACTCCTTGGGGGCCCAGCTTAGTG
>at01127

TTTCCTTTATTTTGGCTCAAGCATTGATGCTGAGTTGGGTGGGCCCCTGCGCATAGCCCG
AGGGCCATGCTGCAGTGGCTCGATGGCAACGCGCGCGGTCGAGAAGACTCAGAGGCCTTC
TGCTCTGTTACCGAAGGGCTGCAGAGCCTCTATCGTGAGAAGCTGCTGCCAATAGAGAAG
GAGCACTTGTTCCACCAATTTTACTCGCCGGAGCTTTACAGATGCCGACTTTGCATCTGC
ACCTATGGTGCTCCTCATGGGCCAGTACTCTACTGGCAAGAGCACCTTCATCCGGCACTT
GCTTCAACGAGATTACCCTGGGCTCCGGATGGTCCAGAGCCAACGACTGACAAGTTTGTG
TGCGTTTTGCACGGCGATGCAGACTCGACGACTCCAGGGAACGCTCTTGTGGTGGACAAC
TCATTGCCTTTCACGCAGTTGAGCCACTTTGGGAACACCTTCCTCAGCCGACTT
>at01128
GAGGTATGTTTGACTTCATTTACGTGCCGCTGATTTCCCGTAAGCGTAAGAATCGTGCAT
TGGCTTCGTGAACTTCACCAGCAAAATGGTGGCTGATGCTTTCTACCAGCGGTTTCACGG
CACGAAGTCAATGCATCTGACTGATGCCAAGGAGATCTTTGTCACCCCAGCAGATGTGCA
GGGCTTTGAGGATAATGCTGAGCGGTTCACCGCTGTGTTTGCGGTACAGAAACCTGTCTG
CAACCTGCCAATACTCCTGAGATCTTATCCGCGAGCGGGCTCTGCAGCAGCCTTGCCACA
GACCCACCGTCATGGTGCGCATGCGACCACGATATGCACTGTCTGCAACGTGTCACTCCG
CTTCACCTTCAGCTTCTGTCCATTTTGCGGCATGTGCTTGGCATGACCCCTTAACGACTG
GTGACTGGGGACACGCTCGCTGTGCCCATGACTGTCCTTGAGACAGCCAATGTGTATTTG
ACGGATGCCCTTCAGCACCAATTTTCCTTGCGCTGTGATCCTTGCATTGTGATCTTGATC
GTTGTCGATCATGGACTCTTCAGCGCGGGGTACGTGCGAGCCTAGTGATGTCTTCAGACA
ACAAAACCTACACAGCGATAACCTCATTTTCCAGACTGTTGTGTGCCAAAAAAAAAAAAA
AAAAA
>at01129
CGAGGAGCTCCAGAACAGCAAGGCTTCTCAGTCAGGATCTCGAGAAGGCCAGGGCCATGC
TCGACGAAAGGAAGGCTGTCTGGGACAAGGAGCTCAACACCGTGAACGCGACCATGCAGA
GGATTGCGCGGAAGCACAAGGACAAGTCCAAAGCCGTGCTGGACCGCATGCTCGCTGGCC
AGGCATCAGGGCTCATGGGAACTTGCTATGCCGCCTGGGTGACGTTCCTCAAGGCTGGAA
GAACAGCGAGAAGGCCAAGGCCGAGGTGGAGGCGGCGAAAGCCAAGCTCTCAGGCTTCCA
G
>at01130
TTTTTTTTTTTTTTTTTGACACAGACACGCTTGCGACGATGAGGTGCCGGCAACACCTT
GCAAGTGTCCAGCCGTGGACGGAGCGCAAGGCGGGCCGCGCACAACTTCACGTCGCCAAC
CCGTCAGGGAGCTCCCTCAATAGAGGCGCTCGCAGGCAGAGAGGGACACGCCTGCTACAG
TCACGGAACGAAGTGTTTGCACCGCACAGCATTGCAAACGACACCAAATAGACATGACTT
GTGAGGTCTTTCACATGTTGCCACGATGGCACCACGCGGCAAGACTACCGACCTCACTGA
TGAGAAAACAAGCCTAGACCCAAGCTGATGCCTAACACCACCCACATTCAAAGAGGCTGC
CGGAAATCTCGCATGGCTGCGAGCAATTATTGCTCAAGCAGCGCAGCGAGGCTTGTGGAG
GTATTCAGGGGGATGGCGTCTGCAGGCGGTGCCTGGACTTGGAGGCACTACCCCACGCAT
TCATTGGGCGCAGCAGCCACAGACTCATCCGTGGGGGGGGTGAAGTGGTAATGCCTCGGA
CCGCTCCAAAGCTTGTAGACGCATTCGGGGGGATGGCGTCCGTAGGCGGTGCCAGCTGCC
GTAAGGCACACCCCCCATCCATCCGTCGGG
>at01131
TCCGTTAGTCCATTTTTGGCTCAGCAGGGGCTCAATTTAAGCCAGGCTACGGGGTTGCGG
GTGCTTACTGCGAGGCCCCTCCCAGCTCCGGTGCGATGGCGCACAAGTCGAATCCGTACA
AGAAGGCTCGCTCGATGTTCCGCTGGAAGTGGAAGAAGAAGCGCACGCGCCGTCTGCAGA
AGAAGCGCCGCAAGATGAGGCAGCGTTCGAAGTGAGGTCCACACGACACCAAGGAGATGC
CGGTGCCGCTGAACTGAGCAAGGGGGCCGGGAGCGTAGGAAGATGTCAGGCAGACTGTGG
ACGATGGGGCCCCAGTGATGCGCTCGTCTGAAC
>at01132
GTTGATTACACCTGGAAATGGCATGATGTTTTTAGCTGTAAGTGAACCGAGGTGCTTCCG
TCTCTGGGATCAGACCGAAGCCCTTCGGCTCAGGAACAGTACGTTCTCAGACTTATCACA
ACAGACCAGCTTCAGGGTGTTTGCCGCCTGGGAGGTCCTGGCCTGTGTCATCATCGTGGT
GGTTCTCGTCTTGTCTGACTTTGTCCAATCGGAACAGATTATGCGGTCTCTCCGCTTGCG
AAGCTCGGAGGATCATATTCTGGCTGTTTCGCAGATCCTGGACAGACTCTGCGACGCCAC
TTGCCAGCTTTCGCCTGCCTACTGCATCTGCACCCCAGCGCCGAAACTGGATGTCCTCTT
GCACATGTCACCCACTCCCGGAAGCATGGTGGGGATGGAGTTGCGCAGCCTACTCTTATC

TGAGGGAGATTGGCCAAGCATCGAGGGGTCCTTCAACGAGGCTATGAAGCAAGACGCTTC
CTTCAGTGATGCGCTGTACTTAGACTTGAAGTCCACTTCTAACGCCAAGGTCCGCGTGCA
GCTGCTGTTCGCACGATTCGTTGATTTTGCCGGTGAGTATCGAATCCTGTGCGGCTTCAC
TGAACTTCACGCTGACCCGGCAAATGGGAACACACATGGCGCTCGGAGATGGCAGCGAGC
AGTTCGGTCCAGCAGCCAGACGCCACACAAGTCCTCGACGCATGCACTCGACGACAAGGT
CTCGGAGT
>at01133
ATCCTGAGCGATGCTTGCCGGGTTATGCGGATACGGCAGCAGTTGCTCCGTCAGCATCAC
GACCTGCTTCGCGCTGTCGGCATCCACCATCGCGTAGCCCAGTGAACCGCAACAGGCTTT
GCCGGTATAACCGTTAGCATTACCGAATTCATCGCAGGAGGGCACGCCAAGAAATGCAAC
ATCAATATTGAGTTCGCCGCTTTGTACCAGATGTACACGACCGCCGTGGGAGTGAATCTG
AACCGGCTCAGCCAGCAAGCCGCGAGAAATTTCTTCCGCCAGCGGGCCGCGCAGGCCTGA
CGTGTAGATGCGGCTGACCACCCCGTTACGAATGTGTTCAACCAGCGGCGCATGGCAGTC
GCTCAGCGAACTGGAGGCCAGCGTCA
>at01134
ATTTCGACTGGAATGTGGAGCAGCTCAGTCCAGATGTTCCCTTCTATCTGGACATATACG
ATGAGGAGATAGACTTCATTCAGAAACTGAAGCACCTGTTCTATCCACTCTCGAATGTCA
CGATTCGGAACCATACCTTCCCTGCTCCACATGAGGCCAGAATCTTCGTCGAGGCACGGT
ACGGGCCTCACTGGCAGACTCCAGACCACCAATCTCGGGAAGTAGCATCGGCCGGGTATC
CAACGTTGGACGAGGCCGAGAAGTGGTCAAAGGCAGCTGTGCTGTTCGAACAGGTTCGCG
GTAGCAGGTCAGCATCACACCGACTCTCAAGCGC
>at01135
GTCGACTCAACGTCACCATCACTGCCCGTCCGACAGTTCGACAGGCCGAGGTCACACACA
TACAAAGGCGACTCAACAGCACCATCATCCGGGCAGAAAGCATGCATACCGCCCGCCTGC
ATAGGCTTCCTTCCACTTCCTTCACCTCCACAAACACAGAAAGATCCGCCTCCATATTCA
CATTGTCTTCCAGTACTTGTATACCGCCCCGGTTGCCCCGGCGGATACCTCGTTCTTCAA
GAGCTCGCTGTCGCCTTCTTGTCCTTGTAGGCCCTCCACGCAGCAGGACTCTATGGAACT
ACCGTCT
>at01136
TGTTTTCCTTCTTGAACTCATCAATCTACATGTCAACAACTGCATTGTGGAGCACAGGAA
GCAGCGCTACGCCAGTTGCACTGGCAAAGCAACAGGATATGCACCTGCTCACATCATGAC
TTGAAGACAAACACCGAGTCAAAGTTCACAGCAACGAGCATGCCCGGAGCATGAGCGGAT
CCGATGTGGGTGAACACCCCTTTGGTCTTGTAGACGGACACCTCCTTCTCGTTGTCAATG
CGACCGTCGCCGTTCGCGTCACGGATGGCGTAGAAGTCCCCGTTCTGGTTGCCCAGGTAT
ATGGTGCCGTCGGACCCGATCCGCGGGGCACCCCAGGGCGTGGGGCAGCTGATCGGCCGG
ATACCAGCTACGGACCTGGGAATGAAGCCTTCTCTGTCGCCCGGTGCCATTGAGAAATGG
CTGGTCGGGCCCGCCCAGGTCCAGAGAGGCCTGCCATCCTCCGCGTCGAACGCGTGGAGG
TCGTGAGTGACCGTAGCCTTCCCCCACAGCAGCCAGTTGTAGTCATCTCCCAGCATGATG
CTCAGCGTGTGAAAGAAGGGGAATATCAAAGTTTGCTGAACCGAGTCCGGAAGACTTTTG
AGGATTGGGAACCAAAGCCATAAGCGAAATGCTTCCGGCTGGCATCCCCCCGTCTGCTGT
CCAACCGGCATCACCAGGGACAAGCGGCCGCCTTCCACCAAGCGGCCCACGACGGGGGCA
GTGTTTGGGGGCTTTGGCACCGGCTGCTTCCAGACTTCGCTGCCGTCGCTCACGCGGTAC
GCCGCCACGATGCCCGGGCACTCGGCCTCGTGCC
>at01137
GACACCAGACTTGCCAGACATAATATCCCCTCCTCCTGCAGGTGGCTACGCAGTTCCCAA
AGAGAGCCGCCTCAACCACCCAGAAGAGCTTGCGTCGCAGATACTTGTGGCCTCGGGGGA
GGTGTTTGAGCACTACTACACCTTGGGCTTCCAGTCTTGCCACCTGGCAGATGGCACGGT
TGCACAATGCAATGTCCGGCCATTGGGTGCCGGACGCCAGTTGAGCTACTTCAGTGCGAT
GCTGTCCTTCGCTCGCTGGGCATTGCACATTTGCAATGACGGGGGTCCACCGGGTGCTAG
GTGCGCATTGAGATCAAGACTCCCGGGCGATGCCCGGGTGAAAGCCGCTGCTGCATATCC
TCGTCAGCGGGGTTTGACTTCCGCTGGAGCAGCCACAGCTGGAGCAGTGGTTCG
>at01138
CGGAGTCCGCTGCTGAGCCCGGCTGGACACGCGCACAGCACCAACGGCCAGGCCTGCAGC
AGTCATTCCCATCAATGCAAGCGACTTCAGATCCTGAGCACCTGCCGTCTGGTAAGTCGT
CAGCCCATTCTGAGCCTCCGCGCCCTCACCCGACCTCTGGATCACGCCTGAAGACTGTGA

CCGCAAGTTGCCTGGTTGCACAAACGCCTTGCAGGGAGCAAAAGCGCTGCTGCAACAGCA
AGTCCCTTCACCATTGTGCTGAAGAAGCGAAACGGGTGTGGTGAATAGGGTGTGGCGCAA
CAGTGGAGCCAGAATCCGCTTGAGCCAAAATGGCTACGG
>at01139
CAGCGCCGATCATGGGCCAGAAGCAGAGTGGCACTAGTGGGAAGATCCGCAGCGCCAGCC
GAGTTGCTGCTTGGGTCAGGAGGCTGACCCCGTCGCGCTCGTCAGCCATGGCACCAGGTG
ACGGGCGCCGTCATCACGTGACACCCAAATGAGCAGGATGCTTCCGATAATCTTGCTTAC
GAGCAGATGAACTTTTTAGAAAGCCCATGGGGCCATTTCACGAATGCATTCCTGATCGTC
CAAGTTAAGCTTGGATACTACGCGACCATGCACTGTCAGTGTGACAATTCCAGGTTTCAA
TCACAGTTGACGTAAA
>at01140
TTTTGGTGTCTCCCAAATCCTCTCATTGAGTAGCAGCGGCACATCCGCTACGGAAAGCGA
TGACCGGCAAAGTTCCTTTGGCCTCCAGCGAGTCGCGGGTGAACTACGGGCCGCTCCAGC
AGTTCCTGTCTTGGTGGTTTGTGTTCCTGTGCTACCCGTTGCACTGGATCATCGCTTCCA
TTCTTCTCACAGTGTTCTTGTTGTGCTACAGCGCGCGCACGAGGATCTTCATGATCCTGT
A
>at01141
TTTTTTTTTTTTTTTTTTGCTTGAAGCCGGCATCATTGCACAGACGCGAGGATTCCGGCC
CGCCACTCAGCAGGCGCCCGCGCGCACGCCTGCTGCCAGTGTGACGTGCAGAGGCCGGTC
AAAGATGAGGAGGCCACGTGAGTAGGCCTGTGACAGGTCTGCCGACCGACCATCCTCCAG
GCGGTGCAAGCCGGTGGCCTGATCGAAAGCGCGGATGGCCACGCCTGGGATGCTGGTAAA
ATGCCGCAGTTGCATCAACGAGACAAACGCGATGGGTGCGTACTCGGGGACAAGCACGAC
CTTCTCCCCGCAGATCTCCCCGTCCAAAACATCCGGCAACGCAGCGGTGCCATTGTGCCG
CGCCAGCGCTGCATCTCCAGGAACACCACGGGCACGTGCCCATGCTTCAACGTGGAGATG
TGCGCCAGCTGCTGCTCCACGATGGAGAATCCCAGCTTGAAGTACCAGGAGATTGCACGC
GGGTTGCGCTCCACAACGCTCAGACGGAGGTCAGCGGTGGCAGGGCCCTTTCCGAGCAAT
GTCACGAGCTCTTCGAAGAGCAGCCTCGCCACGCCACGGCCATTATGGCTGGGCAAGACT
ACGACGTGGTTGATCTTCACGTGTGGGCCCTCCTCCTGGCCA
>at01142
TTTTCAGCCTCACCGCACAGGGTTGGAAAATAGACTCTCTCTGCCACTTCAGACCTCGAC
GTTCATGGTGCTGCCCCATACCTTCGAATCGATGCGCTTCATCATAGCCTTGATCTGCTT
CATGGGGCCAACCTCGTAAAAATCCGTGACACCGTCCTTGATCATAGCCCTTACAGAGGG
CTCCCAGAGCACAGCACTTGTCAGTTGCTTCTTCATGAGCTCAATGATCTCCTTGGGATT
CGTGCCTGGCCTCACCGGCGCAGCACTGGTGTTCATGTACACTGAGATCTTTGGGGGCTT
CATGCTTGGCAGCATGTTGTCAAGGGCCTTGCTCAACGTGTCCTGTGCAGGTTTCATGAG
TGGGGTGTGGAAGCCGCCAGACGTCTTTAGGACCTTGGCCTGCAAGGCACCAGCCTTATC
TGCCAATTCCTTCACCATATGGATTGCCTCCTCGGAACCCGCCACCGAGAAGCCTTTGGG
GAAGAGCTCATTGGCAATCTGACAGACTGCACCGCCGCCCTGCTTCTTCACAGCCTGCAC
GCAGAGCTCCGCCAGCTTGGGCTTGTCCAGGCCCGCGACCGACAGCATGGCCTGCTTGCT
GGCGGGTGGCCGCGGCCTGCATGGCCTCGCCCCTGATCTTCACCAGCTTCAGGCCCTCCT
CGAAGGTGAAGACGCCTGCAACGCAGAGCGCCGTGTACTCGCCCAGCGACAGGCCTGCTA
CAACCTTTGCGCGGGTGACGGCCTCCTCCCTCTCCGCTCGGAGCTTTTCAATGCCGGCCA
TGCCGGCGATGAACATCGCTGGCTGGCAGTTCTGCGCCTCGTGCC
>at01143
ATGACACAGTCAAGCTTGTCCGCGTCCTGGACCGACGCTTGGGTTTCGTCTACTATGCGG
TCTTCGCTGCTGTGGTTACCTACATTGTTGTTGTCGTGTTCATAATCAAGAAGGAATGGT
TGGATTTTGAGAAGTCCTCGGGTGGATCATGGTCAAGGTCATGAAGCCTCAATTGTCACA
CCTGGGAAGCTCCTGGGATGTCTATGACAGAATCACAAACCCGGGTGAGCAAGGTGCAGT
ATTTATTCCGACGCGTGTCCTTATCACGAGAGGACAAACGCAGGAGGACGAGTACTGTGA
GTCACCTACGCACAATTGCACGCTTGGAGATGACTGCGACATTGGCAATGAGAACCTGCA
GAGGAAGGAATGCGTCAACGGCCACTGCATGCGCCGGCAGTGGTGCCCTGCAGAAGACGA
AAATGCTTCGACGACAGAGACTCACTACTTGGAGTTTGACCAAGTGCAGTTGTGGTTCCA
GAACTACCTTCATTACCACAGGTTTAAGCTTGATGTGTCCACAGCGGATGAAGTTGAACC
AGTTCATTACCCACACCGTCGGGCTAACACCTATCGCTTACGAGACCTCCTGCGCATGAC
AAATACTGATCCCGACGAGTTCAGAGAGAATGGTGCAATCATGATTGTCAACTGTCTCTT

TGACTGTGACCTGGACCACAAGGACTGTGATATGAAGATCGAGACGGCAACGGTTGATCG
GAACTTCTACTACCACATATACA
>at01144
CAAGGATAGAGGTGGTCGTCTACCATAAAACAGGTCCGACTTGTGACCTTGCTGGTCGAA
GGGTCTCCTGACACTGCTGAAGTGCCTGGTGTGCCTTCTGATGGAACATCTAAGCTTGAG
GCCATCGGTTTGCTGTATACTTGCCTTCTAGAGGCTGGTGACCGGATGGATGCGAACCAA
GCAATGCGAAGCTACGACTCACAATGCACAGCATAAATGCTGTAAAGCTAGACCACAAAC
TCCATGTGCTCTGCTGCATTAGACCCGAGGCCACTAGCCTGGTGAGGCGGCCATGTC
>at01145
CCGTAGCCATTTTGGCTCAAGTTAGGTGTTTCTTGCGGGCATGCTGGAGGTAGCCTCAGA
CGCTGGCCGCCCCATGGAAGGTACGAGCAATCGTGCAGAGGCACTGTTGCGGGAGGAGAC
CTCACCGAAGATGATGCGGGCTGCTGCCGCTTTCTACAGCCTGGCAGGTGTAGCCTTCCT
GGTGACCCTGATCGTGACGATGTCGAAGCACCCCCTCTTCCCCTTCAGGCTGGACTCCGC
CGACTGGTCTAGCGCTTGGCTCCTGGCCACCATCGGCGACTACTACGTAACGGCCTTCTG
CCTCTGCGGCATCGTCCTGGCTACGTCGGGCCTCTGGCCTGGAGCCCTTTGGTGTCTGGG
GATCTTGTTCCTGGGCTCATGGGTCTCGTGCCTGTATATGATCTGGCGGTGCCTCTCGCA
TGGCACCTTGGCGCTTTCGTCAAAGCTGAAGCCAGAGTGATCTGGCTGTGGAAGGCAGTT
GCCGATCCAGTTTGGGCCACAGCGGCCTCTGGAATCATTGCTGCCTGGC
>at01146
AAGGCCGCGCTCGCCGTGGCTCAGCATTGGTGAGCACCGCCGCCTGCTCCGCGTGGATGG
TGTGGCGGAGGCACGGGTTCCTGGCGTAGACCCCGGAGGAGACCTCGACGTTCGGGCCGA
AGTAGACGTCCCCACTGGTCCCAAGGGCCACGGCCCCCACGTGGAAGGCCGAGATAACGG
GCGCGGCACACATCTGCGCCAAGATCGTGAGCTTCTGCAGGAGTGCGCCAGTGTCCATCT
CGAACTCGGCCTCCAGCTCTGCCCTCACGTCCCGTGGGATCCTCCACTTCCCCCCACATC
CCCGCCTTCCGGAGCCTCGCAACCACCACGACACAGGATCATCCTCG
>at01147
TTTTTTTTTTTTTTTTTCCAATGTGCGCTGTGGGGCATGGAGGTTCAATGCCTCCTCGA
ACCACCAGCATGACCAGGCCGATTATGTGTCAAGGTTAAATGTTGCACCGTGGCCTTGGG
ATAATACGCCTTCCGCATCATTTCTGCTATTGCCACCGACAAATGGGTTCCCTTTGCACA
ACACATGGACACCATGTTGAGATCCTCACGTTCGATGCGGCCAATGGCTTCCAGCAGCCA
ACTAGCAAAGAAGGTGTTGCGGCAGATTCGCCCCTGAACCACATCGTCCAAGCCATTCAA
CTTCTTGATGTGTTTATTGTTTCGGGTCAGGTAGTGAAACTTCTTGCAACTGCAGTTAAT
GAGCGCCTGTGCCTCGGGAGGCCTGGCCAACTTGAAGAAACCCCACGTGATAAGTGTTAT
TTGGCGGTCTCTTCTCGCTCCCACATCTACGGCCCTTGGTAAGCAGCGGATGTCAAATCC
TTGCGGGTCGCCCTCAGAGTCACTCTCTGCTTCGCTGCCATCCTCTGCATCCGCTTCCGG
CATGCAGTCGCGGCCCTTGCTCCAGTCGTTCTCAACGAGTGCACGGCCAAGCCCGGTGGA
TCCCACCTCAGAAACAGCTTTGGCCACAAACGACAAAATCTCATCTCCGGGGCACCCATC
AACAGAAACTATCGGCAAGGCA
>at01148
ATGCTTCATTTTATTTGTCTGTGCTTTTAGCATCNGCAAGTTCATCAGCTGTATGTCAAT
TTTTATGCGCTTATACTGGATCTGCTTTGTCTGAATTCTTTATGTTCGTAGGTGAAtTTN
CCCTCTTTTCtTATGCTAGATGATTTATCTCGACATGCAGTGGCTTATAGATAAATTTAT
TAAACAGTTTTTAACAACACAACTCGTAACTCGTATGGCAAGTTTTAAGGTAAACCAAGC
TTATAGAAAATGGACTTATAGAGAAATTTATTTATTATTGCGTCGTCCACC
>at01149
GCTCAAGGGAGAGGCAGCTGATGGCGCCGGGTTCTCGTTCTACGCGGCCTCGCATGCCGA
GGTGGGTGGTACGCCGAGCTTGCCGTATGAACGCGTGGAAGGAAGGGTTTCCCGAGTCGT
TGCCGCTTCTGACCACGTGTGGTTGCATGTTCGTCTCCGAGCTCGCTGGGGAATGCAGGC
ATCACGGGGCATGGAACCTGAGCTGTGAGATCAAGTTGCAAGGAAGACTGGGCGTTGCAG
CGCGATATTGGATCTGTCTGGAGGCTCTTTGTCATCATCGCACACAGGC
>at01150
CCCAGTCTGCTGCCGGGCGCCTCCGCGCAGCGGGCCAACCTGCCTACACACGTCGGCCAG
CGGAGGGACGCCAGGGGCAGGCGCCTTCGCGTACATGCTTGGGACTGGCCAAAACAAGTA
TTTTTCACAGCTCAAGAGCTGTACACTTGGGACTGGCTGCCGAGAAGCCTTCAAACACAG
TGCCGAGCAGTACATGCTTGGGACTGGCTCTGCCAAAGTCGCGTCTCGTGCCGGAGTATG

CGACGTCCGCAGTGATAGCTCTTACCACGAGCTTGGCAAGTCTAACTTCTGCTCCTCTCA
GTTCTGCGGGTAGACCACTATTCCGTCCGTAAAGAAGAACGCGAACGGGAGTATGAAGGC
CAGGAGCCGCATCACCCAGGCGGCGCTCATGAAGATGGGGCTGCCGAACTGCTGGCGGTA
CTTCCTGATCTTGTGCGCGACGAGAGCCATCTTGGGCCGCCTCTCAGTTTGGACCTCTGG
ATT
>at01151
GCTTCCTGCTGGAAGTCTGGGCGAGCGCTCCGAGTTCGCGCAGGTACACCACGGACTCTG
TGTACACCTCCCAGTGCGGAACGGCTCTCGTGGCGACAGATGTGCTCTTCCTCCTCGTCT
ATGCTCGGAACTTGAGGGAGTCGCACTCTGCTGAGCCTGACCCTGACAGGCGCTTCTTCT
ACAGAGCTTGGGGCTCGGCGTTCGGGATCTGGTTCCTGGCACTTCCGGCCACGGCCCTGC
TCTCGCGTGCATTGGCTCCCTGGGTCCGCTTCAGAGTCATCCTGGCCGTAGTACCAATGA
CCACGCACGTGGCCGCTCTGGCAGCCTGATGGTGGGGCTGTGGCCGTCCAGGCTCAGGCA
GTCGTACTTCGTGCTCG
>at01152
TTTTTTTTTTTTTTTTTCGTGCGCGAAGTTACGGGAATCTTGCCTGCTCGATACCTCCG
GCACCTCCATCCAGCCTCTGCCGCGCGGGCTTTCCGATTCCGGCCCCCCCGCCACGGCTC
TCAGGCGCAGAGCTCGGCGCAGTAGAGGCGCTTGGCGTGGCCACGGCAGACGTGAAGACA
GGCGCCAGTGGCCGCACACCAGATCCTCGCGGTGCCATCGTCGCTGCACGTGAGGACCGT
GGCCCCATCGGGCGAGAACTGGGCCGAATTCACGCCCGCGGAGTGGCCAACAAGCGTTGC
ATGGCACATGCCATCAGGCATGCACCAAATCTTGGCCGTGCAGTCATCAGAGGCAGAGAC
TGCGAGGGCGCCGTTGGGTGAGAAGGCCACCGTGATGACGGCGTTCTGGTGCCGCACCAA
TGGCCCGACATATGTGCCTTTATCCACGAGCCACAACCGCAGCAGGCCATCCATCGAGCC
TGTCAACGCGAAATGGCCATCGGGCGAGAAGGCCACGGAGAAGACTGGGCCACAGGCGTC
CTTGCTGTCAGCCAAGATGTGCAAGCAAACGCCATCTTCCACGGACCATATGCGAGCGGT
CCGGTCGTCCGAAGCCGTGATTGCGCGCGTATCGTCCTGGGAGAAGACCGCGAAGCTCAC
CACGCTGCTGTGGCCCTCGAGCGTCCGCAGGCAGGCCTCCTGGCCAGCATGCCACAGCGC
CGCCCGAGTGTCCGCTCCGGCGGTCAGCACAAGGCTGCCCTGGTATGAGAACGCCGCGGA
CCTCACGGGCGAGCCGTGGAGCGCGAAGGTCCGGAGGCGGCGACCACTCTCGATGTCCCA
GAGGAGCGCCGTGCCATCATCCGAAGCACTCAGAACTCTGGCACCGTCGGGTGAGAGCCT
CACAACATTGATGGGCCCCGTGTGTCCCTCCAAGCAGCGGCAGGTCTGGTCCGCCACGCG
CCAGAGCCGCACGGTGCCGTCCGCGCCACCTGTCACCGCCAACGAGGCGTCGGCTGAGAG
CACGGCGCAGTGCACGCCGCCGCAGTGGCCATGCAGCGCGCACCTGCAGCGCCCGTCCGC
CACCTCCCAGACGCGGGCCTCCCCGTCCTGGCTGCCCGTGATCGCGGCGCTGCCTGCCTG
TGCCCGGAGGAGCAGCACGGTCGCGTCGCGGACGAGGCCGGCCGAGGCAATGCTCGCGTG
GTCGTCAATGGCCTTCGAGCCAAGAAGCACCCGCACGGACGAAGGGTCCAGTCCAGCACC
TCGTGCAAGCTCGTGGCGTAAGGTTCGACCAGTCTCAGAGCCGCANATCCTCGTGCC
>at01153
TTTTTTTTTTTTTTTTGTCACAAGCCCAACGCCGAGGCATCAAAATGCATCTCCCGCATG
TCGTGCCGGTGAACGATGCCCGGCAGCTTCCACTCCGAGTCGAAGGCGCTCTTAGGCGCG
CAGCGGAAGTCGAGCGACACTCGCGTTCTCCCCGAACTGTTCGGCTGAGTGTGGTGCCGG
CAGAGCGTTCCGTGGAATCGCAGGATTGGCAGCTGCTGGGGTTCACAACGGTCGGCCTCC
GGTGCGTCTTCAGCATACCCCAGCTCTTGCAGCAGCACCGGCTTGAAGTCTCCGCGCCCC
TCGGCGCTCTCCGTCCACATGGTGTTCGCCGCGGCGAGCCTCGTCAGCGGGAGCCAGAAG
TTGACCTCGCCGGTCTGGTGCCCGTACTGCGCGTCGTTGTGCAGCCTCCCCAGCGACTTG
AAGCGAGGGCATGCCTCTTGGGCGACTGCCTCTTGGGCGACTGGCTGTCCGTCCTCATCN
GCGGCAGGCGAATGGCCCTCTTCGACGCCTTTCGGACGCTCCCCAGC
>at01154
TTTTTTTTTTTTTTTTTGTTCTGGTGGCGCACTCTGTGACCTCGCATCTGCAAATGCGC
ACCACTGCAAGCCAACCAGGCTCGCGTCCGCCGTCTCCGTCCATGACCCATGCCCAGCCA
GACCACACGTGTGGAAAACGACTGGTGCGTCCAGCAACGGAGCACGGCCAAGAACGACGC
CGGCCAGCTGCTCAGAGCTTGTACCACGCCTTGATCGCCGGGATCGCATGCATGCGGTCC
ATCCAGGCTGTGACCGTGGAGTAGGGGGTCAGGCAGTCTGCCGGCACGTAGTCCGCGACG
CCCTTCGTGTAGTAGCGGAGCTGCGCCAGGATCTGCAAGTCCGCGATCGTGGGCTTGTCC
CCGCAGAAGAAGGCGCCCGTGGCCTCCAGCTTCTTGGAGAAGAGGCCCATGAACTTGGGC
AGGTGCTCGGCCAAGAAGTTCTCGCGCAGCTTCTTGACGGTGTCGGCCTTCTCTGGCCAC

TCCTCCGGGTGGCCGTAGTTCTTGTGGCGGTCGCCCATGCCCATGTAGAGGGCCGGCTGC
CAGGCCCTCGTCATATCATCAGAGAGCCCGATGACCTCCTCGATCTCGAGCATCGCGTCC
GCATCAGCAGGGTACAGCGAGTTGGTCGCATCGAACTTGCGCGCAACCCAACGCATCATG
GCAACGCTCTGCGCAAACTTCTTGACGGAGCCGTCGTCTCCAGTCACCTCCATAACTGGG
AGCTGGCCGAACGGCGTGGTCTCCTTCAGGGCAGGCCAGTCCTCAAAGTTGATGCGGTTG
TCCTCAAACTCTGTGCCGGTCATGACCAGCGCGAGACGCACCTTCTCTGCCGCGGCCTCG
ATGTTGAAGTACGTGAGCTTGATTGCTGGCATCTCCGGCAAGTTGCAAAGCTACAAGCAC
AGATGCTGGGTGGCCAGCCGAGGCTCCAGTCCAAAACTTGAGCCAAAATGGCTACGG
>at01155
CCGTAGCCATTTTGGCTCAAGGCTCCGAGGCAGCGCTTCGCACGTGCAGGAAAGGGGACT
CTTTGCAGCAATGGCAGCCGTGGACAAGAAGCAGGTCAAGAAGTTTGCCAAGGCCAAGAA
GCAGCCTTGCCGGCTCTACTCCCGGGGAGTCATCCTCGGACCCAAGCGCAGCAAGTCCAA
CGTCTACCTCAATTGCACGCTGGTCAAGATCGAGGGCGTCAAGACTCGGGAAGACACGAA
GTTCTACTGCGGCAAGAGGATTGCATACGTCTACAAGGCCAAGACCGAGAAGCAGGGCAC
CAAGTTCCGCATTATGTGGGGCAGGGTCCGCCGCCCCCATGGCAACTCCGGCGTCGTCCG
CACCAAGTTCAAGAAGAACATCCCCGCGAAGGCCTTCGGCGGCCCGTGCCGCATCATGCT
CTACCCCAGCAGCATCTGAGGGCCTGCAGATACTCTTGTTGGAAGCAGTCGGATGTGGCC
CGAAAAAGAAAAAAAAAGAAAAAAAAAAACAA
>at01156
TTTTTTTTTTTTTTTTTGAAACAATTCTTTTGAAAATGACGCGAGCAGGTGCCAGGAGC
CGGTCGGCACGGGAATTCACACGCACACGACCGCGAAGTCTCCGGCGATGGAGGTTTCCA
GCATTGCACTGCTCAAGCTCCAACGCGCGCGATCACAGCGGTGAGGCTGGAGGAAGGAGA
CAGCTGAAAGCTCAACGGGGTCCACTCCAGGAGTCGGCGGCGCGACCTCCAAGCGGCCGG
CCGCGATCTACGGCAACGGCTTGCCCTCGTTGGACTTGAAGAGCGCGTTGAGCCCGAGGA
AGGTCGCGATGACGACGGCGATGCAGACGATGAGGAGGGTGATGTTGCCGCTGCCCGCGT
CACCGAGGCCTCCTGCCGCCTTCTGGCTGCTGACCATCTTGTACGACAAGGCGTCAGACG
CCCAGGCCGGTGCCGCCCCCAATGCTCCATGCACTGCGATGAGCGCAGCTGCCGGAGCGC
GGTCAAGCTGGATTGACCGTGACGGCTGCGCCACGAACATGTTCGTGCCAGGCTTCAAGG
TTGCAGCCATGGCAAGCAGGCATAGAGTCAGACAGCCCCCGGTACGCCGCGAGGGCGCGC
AGCTCCTTGATGCCATGCTTGAAACCGAGGCTTCCAAGCAAGAGCTGGGCTGAATGACTA
ACGGGCTTGGGCCCACATGGCTTGAGCCAAAATGGCTACGG
>at01157
TTAGTCCTATTTTTGGTCTTCAAGTCAGCGTGGTTGCCGTGGAGGGGCCGGTTGGTGCAG
GGATCCTTCCTTCGGCCATGCCGCCGCGGGCAAAGGCGCGCTCggAGTgGAGCCGTGGTC
GTGGTACGGCGCCACGAAGCGCCAGCGGCGAGAGGTCTCCAAGCCCTGGAGGACGCGGGA
GGGGTTNGGCGTGCGTCGCCGCAGACCAGCACGGCAGCGGGAGCCCCNGTT
>at01158
TTTTTTTTTTTTGTCTTCCCACCATGCTGGAGCCAAACGTCCATGGCACGACACTCCCT
GTGCTCCCAAAAGGAAGTTGAAAAGGCGCGAACTACTGCTTGCTGTACATGATCCAGTAA
AGAGCACGCCGCACACCACGCGGACGGCATGCGGCGCCAGGCGCACCGCGACACGCCCTC
GGCGCCGCAAGCTGCACAAGCCGCCCGCGCTTGGAAAGGGCCTCCTGCACGCGCTAGGAA
TTGCTTGTTGTCCCAAGCTCTTAAGAGCTTGGAAATGTCATTTTCCAAGCTCTTAACAGC
TAATCACGGCCCGTTGCACTGGATGTTGATGTTCGTGATGAGCTTGTAGTCGTCCAACAT
CAGCACTGCCGTTTTGTGGGGGTTGATGATCGGGTCTGCTGCATGGGACGCTAGGTAAAG
CGGCTTGTTCGTCCAAGGAGAGGGGTCGCCCTTCCAAAGCGCATCGCGAAGGTCATGGCC
ATCCACGGGCGGAAGCCCTGCCGCCTCTGACCGCACGTCCTTGACGGGAAGACCAGCCAA
GGCACTGAAG
>at01159
TTTTTTTTTTTTTTTTTGGACTTTTGACCCAAGCAGCCCAACCAGACAGAGTCCCAAGG
TCCTGCACCGAGATACTGCTGGCCATGCTGGAATGGCGAAACAGCAGATGCAGAAGAGGC
CGAACAGACGACGAACTGTCTAGCCCCCGGCTGCATCAACGAACTCTGGAGTAGTCGAGT
AGTTGAAACCGTTCCCGAAAGATTAGGCTACCTAACGCCGCGAGGCGACCTGCCTGGACC
TTCGCCATGCTGATAAAAAATTCCATGCTGCTCGTGTTCCACGAGCAAGACGGTGAAAAA
GCACGGCATTCAAGCATGTACATGAGAAGAGGCGGTCTTGTGGCCCACTCCTCGTCCTGC
TGGCTAGAAGGGTGGCGCTTTGCCAGGTTGCGCCGCGGGGGATATCTGACAACCTGCCCA

CCACCACGTGAATCCCCTCCAGCCAATCAGATGACGGCGTGCTTCACTACCTCCGTGCTG
ATCATCTCGCTGAAGCCGACCTGGGAGCTGCACTTGAGGTGGCAC
>at01160
AAGCATGGTCTCCTGCCTTGGACAGGACCTCACCAAGGCTGAAGAGAACGTTCCCGACCT
CTCGGTGGCCTGGACCGAACTCCCTCTCCCGGATCTCCAAGGAGTGCTGGAAGAAGTCCC
GCGCGCGCGGCAGGTCTCCAAGCCTGTCGTACGCCTCCCCNCCGTGGT
>at01161
GCCAAAAGTTCCTCGTACATGAGGCCGCCGAGCCTGGGGCCACGCGTCTCGCAGAGCTCA
TCATCAACGTCAACGACGACTGGTCGCTCTTGTGGCCCCAAGAGATTCAGCGCATCGCTG
ATCGTGGCGCTGAACTCCGTATCTGTGATGAGCAGCTTGGCGCCCGCATGGCGCACGCAG
AATGCCACGTTCCGAGCATCAAGGCGGAAGTTCAGCGGGTTGAGGACCGCGCCAGCCATG
GCCACGCCATGCAAGCACTCCAAGTGCTCGGGCGTGTTGTTAAGCATGACAGACACGGCA
TCGCCCCGCTTCAGTCCACGTCGGCATAGCGCCGAAGCAAGGCTGCGCGAGCGGGAGAAG
ACCTCACTCCACGTCAGTCGCTTGGGACCGTGTATCACAGCCACACGTTCGGGATGCACT
GTGGCAGAGCGTTCGAGAAATTGCACAGGGGTGAGTGCCGCAT
>at01162
TTTTTTTTTTTTTTTTTTCGCAGTGCCGGCAGATCAACTCGCAAAACCAAGATGGTAGGT
GCGCACAGTGCTAGCGATGGACTGTCTACATTGGCCACTGCTTGCATAGCCGGTGAGGTG
TCACAAGAGCTGGGGATTCCTGCCAGCCTCGCCTGTATGTGCCAAGATCTGGTGACAAAA
CGGTGATGCGACATACAGGCCAGCAAACAGCCCAGGCCTAGCAGCAGAACACTGGCGAGT
ACCCACAAACACAGCGGCTTGAAGCCTCCAAGCTTGCGGCTTCCTGATGCACTTTCCATA
CTGCAGTTAGTAATGGGCACCCTCCAGTATCACGGCACATGTTAAACGAGCCGTTGGAGA
TGCGTTCACTGCCCTTGTCGCCTCGTGCC
>at01163
TCGTCCATCTCTGCAGGCAAGTCCACTGTAGGTAAATACACCGTTCAACAACTCAAAGAG
AATGGACCGACCTAGACCAATTACCTATGTACAANAAGGAGAAAAGGGACCGAAGACAAA
GCCCAAAACATGAGCTTGGCCAGTCAACGCCAGAAGTACAGCACAGCACAGTCCAGTCCA
TGCACTGCAAGATTGCAAGCCGCCCAATCACTCTGGGCCACCCCTCAACCTGTTTCATTT
CTTCGTCTCAGCTGCCTTCTTCTTGAAGTAGTCACGCGACCCTAGCAGCGCTGGCCACTG
GTACAGCTTCATCCGCGCGCTCATGGGGTCCGTCTCGCCCGCAGAGGGGTCCTCCCGG
>at01164
CCTTGTGCAAGGCACGCGCAGCACGTGCTGCCGCAGCAGCGAGGGCCATCAGTGGGGAAG
CTCTGCTGCGTATCGAAGGCAATCACAGGATGACTTTCTGTAGCATGGTGCCAAGGCTCG
ACTCCTTCATGCAGCTTGAGCCAAAATGGCTACGG
>at01165
TGATGACCGAATGACCACCTTGTTCTGCTCGTTGCAGATGGTGCGCACGATGCCAGTCTC
CGGCTCCAACACCTCCAGGGCCTTGCGGAGCCTATCGTGCCTGTCTGAGACCGCAAGGTC
GTCCAGCGAGGTCTGGTGGAGGACTTTGAGGCCGTTGATGGAGATCTGCTCGAGGATGAA
CTCCTTGCTGTGCCGCAGCTCCCAGGCCGCATGGCGCAGGGCGGTCACATCCTTCCGCGT
GGCCTGCATGACGATCTCGCGGTCGGCCTTCAGCTCGTCGGCCGCGTGCTCCAGCGCCTT
GCCGTTCTGCTTCACGGCGGCGCCGACGACCGAGCGGTCGGCCTTCAGGCCTTGGGCTGC
GTGCTCCAGGGCAAGGCCGTCTTGCTGGACGGCCGCCAGGACGATATCGCGGTGGCTTTG
CAGCTCCTCAGGCAGATCCTTGAGTAGTAATCCATTCTGCCCG
>at01166
TGCAGATGACTCTCCTCCAACAGACTTGAGCAGCTGCGCCAGGCAGAATACGTGAGCAGC
CTCATGCCGTCGATCATCGGAATTGAAGCCCAATGTCTCGAAGGCCTGAAAGACTTTGGG
CATGGCCTGCTGACAAGATGCCACTGAGATGTGCAAAGCAGCCGGGATTCGACATAGGAT
CTCACCAGCAG
>at01167
CTGCGTCAGCGCGGTGCAGAATCCAGGGAGGGCCATTGGAATAGCTCTGCCTGCATTTCT
TCTTGTCTCCAATTGCTCGCATGCACGCGCCAAGGTCCTCACGGCACAGCGACGCCATGA
AGTCTTTAGACCACCCCTCCTGCAGAGGCACGCAACCAACGCTGTAGAATGCCGAAACTG
CTTTGCGCGGTCTCACGGCAGTTGTGGACCAAGGACCACGGGTAGACCTCTTCGGGCTCT
TCACCAAGTGCACCGCGCGAAGCCAGTACTCGTCAGGATCCAAGATTCCAATGGCCTCTT
CCTGAGGCGCCACTGCCTGCCACCAGGCAGCTATGGAGGCAGGGCGGTTCGAAGCTTGGA

ACTTGCCATACGGCTTGCCAGTGACTGGGTGAAGCAGTTGCACTCGCGGCGCGTGCCAGA
CGGTGGCGTTGGGTGCAGGAGCTTCGCCGTCTCCGCC
>at01168
TTTTTTTTTTTTTTTTTTGGAGGAAATGATCACATCAACAAACCTGATGGGTCAGCATGT
GCACCTTCATGCCATCCTCTACAGCAGCCGCTGCATGGCAGTCGCCGGAAAATGGGCTCA
AATTAAGCAATTTGGATCCTCACCTGATCCTCAGTGGACCCAGTGCTACGCCCAAGGCCT
CTCCGTAGCATGCCTCGCCAGCCCGCACTCACACTTCAGCTGTCGCCTGCTGCTGACGCT
CAAAGACGGTGAAGTCAAGAAGAATGGTGAGGCCGATAAGTCCCGCCTTGCGCCGCGCGT
CGCAGCCTTCAGGAAAGAAGGTGGCGAAGCTGTCTGCGGACGAGCAGCACTCCTTCTTCA
TGCCTGCCCAGACTTTGGAAATCTGCGGCGTTGTGCCGTCACTGTTCACGACCTTCTCGC
CAGTTGCAGGGTCGCGGAAGTAGAACGGCACCACAAACCTCGACTTGCCTCCGAACTTTG
GCTTGATGCAACAACCGAAGCAGCACGTGTCGGGACTGAGCTTGTACACTGGCTCACCGT
CCTCAAAGTAGGTGAACTTGCCCACGCAATAGTTCTGGTCGCAGTCGTACTGCATCGAGC
TGAACTCGCTGCCCTCAGCATCGCTGCCGACGAAGCTTGGGAGCGCGCAGCAACATGGAA
GGATGCAATTGATCCATGTCACGTCATCGCCGCCCACGGGGACTGGCACATTGACGGTGA
AGTAAGGG
>at01169
GCTACCTCTTGCCAGACGAGTCGGAAGACGCCCGTGTTCAGTGCTGTGCCCCAAGTTCCG
CCCTTGGACACAGAGACGGGCCCGTTGATGAGTAGCACACCTGTGGGAGCCGCCTCCGCA
GCACTCCACGAGGGCGTCGTGTTGCTGAGCACGGCGTAGCCGTCGCAGCCGAAGATGCTC
GAGCCCCTCGCGAACTGAGCCTGCAGCAGGCCAGGCTCGTAGCCAGAGGGCACTGTCAGG
GAGAAGCAGAAGAGCAGCGGGAAGTGCGAGGGCGGCCGATCGCGTCTCCCCTTCTTGGGC
ACCGGTATTGTCGGCAACGCCGCGGCGTGCAGCGACAATGCAAGCAGAG
>at01170
TTTTTTTTTTTTTTTTTTCCTTCAGACTCGCTGGCGGCGGCCCCTTGGGAAGGCACCGCG
GGGTCCCACAAGACGCAAGCAGCGAAAGACCAGCGCAGGAGGCAGGCCGCGCCTGCAGGC
CACCCCCTTGCCGGCACCGCCCGACTGCGGCAGCGCTGCGGCGACGCTCGGCGAGGCATT
GCTCAGCGCTACTGCGGTGGCGCTCTCAGTGGAGCAGCGCGGGCAGCCGCAGACGAAGCC
GTGCCTCTCCACGAAGTACTCCCGCCGCTTGGCCAAGGGCCAGCTCACCAGGTCCTTGGG
GCCGTAGTTGAAGCAGAGGGGGTCCCCGGGCTTGAGCGCGCGCTTGCAGCGGACCACGGC
GCGCCCATCGTCCGGCTGGAAGGTGCCAGGAGCCAACGGACCCCAGTGGAGCTCCATCCC
ACACGATGGATCACAGGAGTGGTTTATCCGCGAGATGCGGCGGAAGACGGCAGAGGCTCC
CAGCTCGAGGCTGCCGCTTTGGTAGTGGAACTGATTCGCCTGCCACCTCGCCAAGACCGA
GGCCACGTGGGTGACGTGCGCACTCCATGTCTGGCTGTCCTTTAATTCCTCGTGCC
>at01171
CATTTCGTCCCATTGAGCTCACGTGGCCGGGAGGGCTAAGCCTGTACGTAGGAATCTGGA
AGATGAGCTGGGTGACGCTGGCGATGTCGATCACTGTCTCCCCCAAAGGGGATTCCGGAA
GCAGAGTGGGAGACGGTTCTGCCGGAAGTTCCATGAGCAAGTCTGGAGTATCACGAAGAT
CCTGTGTCAATTCCGGTCGTCTCGGATATCTATGGCTGTGCATCAAAACTGACGTAGTTG
GTGAGTCAAGGCGAAATTCACGTCGATGCCGCTCCCAGAGTGTCCGTAGCCACGACAAAG
TAATCACCAGAGTGCCAGCAAGTCGCAGTTGTCCTTCAGGAGGTAGAACACCTGGAAGAA
GGACGCTCCTGCGTCACACGGAGCCTGGGAAATCTGACGAACTTCGATCCGCCTCCAGAT
ACTAAGATGA
>at01172
TGTGGTGCATGAAACATGTCAGAAACTTTCACTGCGTGAACCCCGTCATGAGCACGGGCA
GCAGTGCAAGTGCGCACATGTGAGATAGCACAGTGCAGCTTCCATCCCCAGCAGCCGTTT
GCGTGCGACACTTCCAAGGGCACTGTGGGAGGGCAACTCT
>at01173
TTCTGGCAGGGGTCTCCTGTTGCTGCCTCGTGATGTACATGACGGCCCGCCTGGAAAAGG
GCGCACCGACTTGCATGGCCATTGTTGGTGCCTCGTTCTTCCTGGTGGGCAGTGCTGTTG
TGGTCACAGCGGCGCTCATGTACTTTGAGGACCTCAGGCAGGAGCCCCCCGGAAAACTGG
ATTCATGGCCTTGACTTCTTTTTCGACGAGTGCTTCGGCCATCTTCGGCTGGGTGCCCCC
AGTCTTACAGGTTGCTCGCGCCTCTCAAGATTCTGGGGTGCTTTCACGCAGACGCTGCTA
CTTGGCGGCATCTTCCTGATGGTATTCCTCTTGGGTGCTTTTTGCTGTCCGCGTTGCANG
AAAGGCATTTTGCAAATTACCGtTTTTGCCTTGGTGGCCGCTGCTGCCTTGCTGNCGaCA

TCAGTCTACTCTGAAACTGGACTTCCACCGGAGGAGCAAACTTTCTCTGTTGTTGTCTTC
TACTTGACGGCGCTATTCTCAAGGAGCCTCATGCCTATCTTTGCAATGTGGGCAGGTGTC
TCCAAGTGGGGAATCACGCTCTATTCCAGAAGGCGAAGGCGGAGGACCCCTTCACGTAAT
CCTGAGAGGGAATCGCCTTGCACCTCTGGACCTGAAAAGCGTGTTAGAATTTG
>at01174
GTCCAGCGAGAGACGAGTACGTGGCTGCCTAATAGCACGTACCAGCCCATGACGAGCAGA
AATGACAGGACAACAGCAAGGGCAGGAAACAAGCGTCGCGCGCGGCGAAGCCAAAACCAC
CACATCGGCTTCAACAATACACCGTCAGACAGCAATATGCGGGTGATGAGGTAGCCAGAA
ATGACAAAGAAGACNTCGACCCCTGCAAACCCACCTGGGAAGCCCAACTTGAAATGAAAC
AGGACGACAGGAATGACCGCGAATGCTCGCAGCCCATCTATCTCGGCCCGGTATGTCAAA
ATATCGTCGTCGGCTAGTCTTCTCTTGGCCTCCGAATCGGAGACCCCAGATTCCGAGTTG
GTATCATCGCCTATGCATTTAAGTGGTACCGCAGTCTCGGAATCCAAGGATCCGAATTCG
TCGGTGGAGGACCCATCACCTTCACTGCTGTCATCTTCTCCATCAGATGACAGACCGCAG
ATTCCTTCCAGCCACGAGGGCATGGGCATTCTTCAAGG
>at01175
TTTAGGCTGAAGACCGGTAGTTTGCCCCCCCTTCACACATCCCAGCCATGTCCAGTATGG
GCCACAGACTCGCCGCACGGAACCTTGATGCCATCTGCCAAGCCTCATCAGAAAAGCCTT
TGCCACTTCATCCTTCACAGGGGTCGCACGCTGCGACTTGCTCTGAGCAAAGCCTGCATG
ATGTTTGTGAGAAGGTGAAGGTATCGGTCAGCAAAGGAAAGGATTCGCTTGCAGTTCTGG
TGCTGCCAGGTTCTTTCAACCCGTGCATTCCGAGCATGT
>at01176
CCCCGTCGAGCTGGCCGTGCCTTCAGCTACCGCTGGGAGGACCCTCGACATCTGCCGTTT
GTCCGATGGGGGCAACTGCTTGGGGCTTCTGCAGTCGACTCGACGGAGACTCCGGGCATC
TTCTTTGGCAACGGCCCAAATCACTGGGGCACTCCACGCCGTGCCTGTGAACGTGGGCTG
CTTCATGGGAAGGCCATCAGGGAGGCCACAAGGCACACCGCCTTGATGGAAGCGGATGCG
CTGTCAGAGCGGTGGGCCGTTGAAGGTACGAGGACGCTGGACGTGCACCCCGACATCCGC
GATGCAACGCTCAATGCGGTCACAGACATCGTCTTTGGGGGTGCAGATGGGCTGGATCCA
GCCAGCTGTGCGGAGTTGCAATCCCTCTACTGCAG
>at01177
CCGTAGCCATTTTGGCTCAAGGCTGCGGGGGTCGGGGAAGGGGGCCTGGCAGTTCATGCC
GGGGCGGATGTCTGCAAATGGGACCATGCAGCCTGTGTTGTCCTCAGAGAACTTTGAGGG
ATGCCAGCCAGGTGAGCTTCTCTTGAATGACGCGGAGCTCTCCTTGATTGAAGGGCACCT
GAGGCGGTGTGCCCACCGCTGTGGCGGGCTTGGTGGGCTTGCGGCTGCGAAGCTTGTCCA
GGTTCCGGAGGCGCAGCCGGCGCCCACGTCAGGGCTCAAGGCGAGGGTGCCTGGGCATGG
CCTCCGAGTCACACTGAG
>at01178
GAGTGCATGTGAGTCCATGCATCCAGGTCAGCTGAATCTCTACCGGTGGCAGGGAATTGC
GCGTTTCACTCAGCCGTCCGCTTAAAACTTGTCACTTGATGATTCCTGAAGTGACAAGGG
ATGGATGAGATTTTGAGACGCCACCTGTGCAATGCGTGAGTGATTGAACTGTGCTCACAC
GAATGGACTGCAGCAGCTTCTCTGGCATTTCTTGCAAGCCTCTTCTCGTTTGGATGGCCC
TTCACCTCGTAAAACTGTGCATGCCGCAGGTCGAAAGCTCCCTGTACAAGAGTGAA
>at01179
GCCACTGGCCCAAGTACCTGTTCGGCATCTGTGCTTGACTTGTGTTGGTGCTAGGTGCAG
GCATGGGGATCACGCTTTTGCGCATCCTGACATCCCTCGTGCAGTTGGCGCCCCCTCTTG
CAGGTGGGGGCAGATTGGGCGCTCACCGAAAGTGAGCTGAGCGCTGCGCGAGGTGGGGTC
CCCCCGAGCCGGGGGTGGCTCAGCTGACGAAGGNGAATGCTGTGACTCCCTTGATTGATG
GGAAGGCCTTCTTTGGACAGGTGTATCCAGCTATCGAGGCCACGCAGAAGGATGACTTTG
TGCATGTGGCGGCCTTCGCATTGACTGGTGACTTGCTCCTGGTGCCGAATGCAAGCGCAC
CTGACGATGCGAAGGCTACTGGACTGTTGGAGACGCTTCACCGAGCATTGCAGCGCGG
>at01180
GGCCTCGGCTACCAGTACCGTCTCAAGGTAAAGTGCTATCGGCAGTGCAAGCTCAGGTTC
CCACTGGTGAAGCTCACCCTGATTGACATCTGCCGAAAGTGGGAGGCACCAAACCTCGCA
GAAGAAGCGGCGGTCCTTCAGGTGCGGTCTCCATCGGACCTCCATAGCCTCATGCCAGGA
GCAGGCACCGACGGCAGTGCAAAGGCTGTCTGCAACAAGCTCTGCGGTCCAGCACTTGGT
GATAACGACGAGACGGACAAAATGGGACTGCAGGGGGGACTGCGTAGACCGCTATCCGCA

GGTGCTCTCCGTCATCAAACGATCTTGTGGCCGTGAAGTGTGCCCTTGTCCTGAATTAGC
AGGAGCCCAGATGCCACCATGGCAGAAGTCCTGAGCCTATCCCAGTAGACGCATCGAAGC
AGGAGCATCCACTTGTCCCGTGGTTTGCCGCGTTCATGCACGCTG
>at01181
CTCTGCAAGCCAGTGGCCAGCAATGCTGGCATCTCCCCACATCCATCTCAGCTGGCCGCC
ATCGAATGAAGTGTTCTCGCTTGCAATGAATAAGTCCCCGGCCAAAACATGGCTCGAATG
TGCTTCTGCGGGGGGCTGGACACCAATCCCAGCTATCTTGAAAATGCCAAGCCACATGAA
CATCTGCAGAATGGCCACAATGAGCACACAGAGGCGAGATGGCCAAGACTGCCGAACCGC
CGCGACTTCATCGGCTCCGCGATGCATCAAGCAAGCCAGCTCAGCTCGGGGCACAGAGAG
CGAGGTCATGGTGCATCGAGGGGAACTCATTTCGGCTGCTGAGCCAAAATATCTTGAGCC
AAAATGGCTACGG
>at01182
TTTTTTTTTTTTTTTTTGACGGCGCACAAAAAACCCAGAGCTGCAGGAGCAGTGGGACC
CTACGCTCAGTAGCCCATCTCGATGAAGCGCTTCACCAGGGGTGTGATCCTGCCCTTGTT
CTTGAAAGTCTTGTCCTTGATGGGCCGCACGCCGCGCTCGAGGCCGATGGCGAGGTCCCA
GGCGGCGAGGCCGGCCAGGAGCGCGATGCAGAGGCAGAGGGCGACCTGAGCATCAGAGAA
GCCGTCCTCCTCTGGCGAGGGCATCACCAGCTGCTTCTCCTTGGTCCACTTGCCGCCGGG
GATCCGCGCGAGCAGCAGCTGCGCGTCGGCCACCTCTGGGCTGTAGGCCCACGAAGGCAG
AGCCGCACCTCCCGCGGCGACAGCTGCGGCTGCGGCCGCGGGAGCCAAGGCGTTGGGAAG
AGACCTCGGCGCGGGCACGAACGCCGGGAATGCCGCAAGCCGCACTGCAGCAAAAGCCGC
GGCGGCCAGGGCGAAGAGGGCAGTGGCGCGAGCCATGATTGAGAATGGGTTGCTGGGGGC
CGCCGGTCTGGGGATAA
>at01183
TTTTTTTTTTTTTTTTTCCACTGCAGACACCGCTGCAACATCATATGTGATCTGGGCCC
CGTGCAGCCTGCGCCTACTCTCTCCGAGCGTCCACTGCACATGGTCGCCTGGCCGACAAG
AAGCCCCGATCGCCTGGCCGACCGGCTCCGCGAACTCCGCCGCGCGAGGGCAAGCGCCCG
CTGCCGGAGCCTCATCTGACGGGCACGGTCCAGAGGTTCTTGCCCGTCTTGTTGTCGTGG
TAGTAGTACCGCTGCTTGGAGCTTGACCAGTGCTTGGTCCAGCCCGGCGGCAGCCTCTCC
TCCGTGGCCGAGGACCGCTTGTCGGCCGCTCTCCTCTCCGCCTGCCGCCGCTTCTCCTCC
GCCAGCTTCCGCTTCTCGGCCTCCTGGCGCCAGGACTGCAGCACCGTGGCCTTGCCGGCC
GGCGCCTTGCCGGCCGGCTCGTCGCCGGCCTTGACCTCCTCGCGCGAGAGCTGCTTCAGG
AGCTGCCGGGTGCCGCCCTCCTCCTTCGCCTGCCGCCGCATCTCATCTCCCCTTGGAATC
GCGCTGCTGGCTAACGCCCAAT
>at01184
TTTTTTTTTTTTTTTGTCGGGTGAGGGTACTACGAAACATAACTCGTTGAGAGGAAAAC
AGCACGAAGCAGAGCACTAGACCGTCCTACATGGCAGAGTGACAGCTCGGCACATCAGGG
CTCAAACTGCATCACGTCGGAGGTGATGCAAGCGGGAGCCTGGTCCCAGTCCGGGCATTG
GCTTGCACGCAGGACCCGCTGACGTTCCGCCTCTTGGCCGGCAGCCTCCGCCTCGTTGCG
AAGCAGGCGGAGGGTCTGGCATGCGGCAAACCAGGTCCGGACCTTGACCACTTCGATCCT
GCTCTGCACGGCCCAGATTGACGCCTCAATCCTTTTGATGGCCAGGGCCTCAAGTAGGGC
CTTCCGCGTCTCGGCATCCTTCGCCAGGGAGTATAGGATCTCGGAGGCGGCACCGGGCAC
GTTGTACATGTGGTCGTTCAAGCTGCCGTAAACGTTCTGGTTGTCCCAGCTGGCACGGGT
GTCCAGCAGCTGGCCGGAGTGGATTTGCAGCACATCGAGGACGTGACTCACGCCATCCGC
CTCAGCAAGGCTGGCACGGAGCGTCGTGTTGCTGTCGATGAATAGCCTGAGCACCTCTAA
GCCTAGGGCTTGGACCTGTGGGTCTCTCTTCTGCTCCTTGAGGGCGCCTACCAGCAACTC
AAAGAAGCCTTCCTTTACTAGGTCCTCCCTGTTGGATCTCAAGGCACAAACGCCCCTTCAT
GGCCTGCANGATCTCCTCTCTGACCCTATAGCTCTTCGCATGATCCCGAAAGGCGACGTA
GGTATGCCGCATGCCGCCCCAACGCGAAA
>at01185
CATGCTTCTTGGCTCGGATAGAGCTGTTGTGCTTCCTTCACCTCCCTGCAGAGAAGCCTT
GGATCTCCTGAGACAATGCCAAGCCCACTGGACCCAGGGACGTGATCAATTCTACGCTTT
GGCAGTGGGCCCTAGACGCATGTGGACCTCTAACCCCAAAGAGACAAAGCATGCAGAGTC
TTCTCACAGGGATGAGCCCCCTNACAGAGTGAGTTCCAAAGCCATTCACTGAAAG
>at01186
TTTTTTTTTTTTTTTTTCCAGTAGCACGCACACCTGTACAGTGCGTAGCTGAGTCCGAG

TTCCCGGGGCACCGGCGGGTGCTCAGCACCACAGGCGCATACATCACGTCCCTGGGGAAA
GAAGCAGAAAAGAGGCACAGGTCTGCCATCACAAACATACAAACGCATCAAAGTACGTGC
TTTGACGCTTGGAATCCCTTGCGCACAGCAGTTCCGAATTCCGTGCCTAAAGCTGAACAT
TTTTCAGACCTAAAGGATCGATAGGCCATGTTTTCACAGTTTGTATTCTTACTGAAAATG
AAAATCAAGCAAGCTTTTCCCCTTTTGGTCCACATGAGATTTCTGTTCTCATTGAGCTCA
CCTTAGGACACCTGCGTTATCATTTCAGATGTGCCGCCCCAGCCAAACTCCCCACCTGAT
GATGTCTTCCACATAAAACAGATATCCCGAAAAACAACTTTGATTCAAAAAATCACAATC
AAGTAAGCCCCAGCTTCACAGAATAAGTAAAATGACATTAAGAGTAGTGGTATTTCAAGA
TCACCACAGCTCCCACTTATGCTACACCCCCTAAGTCATTTCACCCTCCGTGCC
>at01187
TTTTTTTTTTTTTTTTTTCCTCATGATGCTCGTACTTCAACAACAGTCAATTCCATCCTC
ACGCCCGCCGAGGTCGGGCAGCGTCAGCAGATGACGCAGATGGACGGCACGATGCCGTCG
GCGTTGGGGAAGGCTTGCTGCACCCAGCAGCGCAAGAGGATTTAGCAGTAGACGATGATG
GGCCCGTTCCAGTGGCTCGGCGAGCAGCGTCCTTGCGCAGCAAAGGGTTCCCCTCTTCCT
CAGAGGATCCCGGTGGGAAGAGATCGTTTTCCGTCTCCATCTCACTCTCTTCACTCGAGT
CCACGGCAGGGACAACACGTAAGCCATCGTCTCCCCTGCCGGACTGGAGGGAGCAGCTGA
CAGCGTCCACAGCCCTGGCATTGAAGCACGAGTTCACAGCGCCCTCGACCTTCTCATCCA
CGAGCCTCCGAACACTCGCAAGCATCGAATCTGCGCAGGGTCGGAAGGCCGCATCCGCGG
CAGCCATGGCAGCTGTCTTGACCAGATCGTCAACCTGCGGGGGATCGGCGAGGCGCATCT
CTAGTTCCCGCAACGAGACCACGAAGGACTGCAGGGTCTCAGTCAGCGTGCGCACATGCT
GGTGAAGGTCCGAAAGCTTGTTGCCCAAGGCGCCAACCTGTGCATCGAGGACCGAAAGCC
GCTCCTGCATCGCCGCAATCTGCTGCTGAGAGTCCGAGCCCCCGGCATCAGCAGGCTCCG
CCACCAAAGGCGGCGAGGAGAGGGCAGCACAGAGCAGAGCGATGGAGTCCTGCTGGGCCC
GCACGATGTCCTCGAGGAAGGCAGTGGCGAGCTCGCGCGGCTGCGAGGGAGCAGAGGAGA
GCTCGGCCGCCGCGGGGAC
>at01188
TCTGGGGCGTCGGGTGGTCTGTGCACCTCGGCAGCAATGATGTCGGTGTCGGTACTCAGG
ACTTAGAAGCTTTCCACATGCATGCCAAAGTTGGCCTGGCAGTTGTGGAGCTCCGAATTA
ACAGTGCCGACGCACGGCGCCTCATGGGCACCACGGAACCGCACACAGCTGTCACCCACC
TTGGGCATGGCAGGGCCCCCTGCCACCATGACTCCAACAGATGTGGCATGGCATGGCAAG
GCTCGGTGGCCCCGTCAGCAGGATACAATGTGAGCGCATTATCAAGTTGCCCACGCACTG
CGTAACTTTCTGAGTGGCCAAGTGCCTGGGAAGCGGAAGATGGTGTCAATGTCCCTCAGC
TTGCGCCGGAGCGAGCGGTGCGCCGGAACTCGGCACCCTCAGAAATGCCTTGCCGTGGCT
GGTCCGTGGCCTGGGTGAGGCGGATGAGGCACTTCCGGTGAAGCATGGTGACCAAGCCGT
ACAGCTCGTCCCCATCTCCTGACGGCATGGCAGCAGCAAGCGGAGCCCTACGGGCATGCC
CGAAAGCCTTGAACCAAAATGGCTTGAGCCAAAATGGCTACGG
>at01189
CCCAGGCTCAAGCTTGCCCATTACTCAGGTTTGCTTCTGCTCATTCTTGGCCAAGTGGCA
TGATCAGCTGAGCGCCACTCCACCCCCTTGCAAGCTGCATTTATAGTTGTTAAGTGGACC
TGGCAGGCCCGGGCGATCATCATGTTTTTGGAGACTGTCAACGTGGTGTCAACAAGTTCA
TCCCGGAGCAAGTGTTCGGTGAGGTGGAGCTCTGCAGTGGCATGTCATCGCCACGCAAGG
CATGATCCCATCTCTGTGGCACCAACGGTGCATACATTCAATGGTCATTGGCGTGCAGTT
GGAGGTTCTCTGGTATCGGGTGCAGTTCTTTCCTCCAGAGACGACGTGCTCGTCGACCAG
CGAGATTGGAGGGGCCAGGCCAGACCGCAGTGTGGAGCTGACGT
>at01190
TCTCCATCACGGTGCCGAAGTGGGCTCTCGCGTCCATGGTCGGGCCTCGGCATCGCCGTC
TTCTGGCAGCAGGAGCGGCGAGTGCAGGCGTGCCTCGCAATCATCGAGCAGCATCAGGCA
GGAGTCCTCAGGCTTGCGGAGATGTTCGTGTCCTGCCAGAGCAGTGCCCGGCAGGTTGCC
GAGGCGGCGGCGGCGGACAGGGCGGCAATGGCTGTGCAGCAGACCAGGCAGAGCGGCGTG
CTGGGAGCAGTCCTGGGCATGGGAGCGGCGTCGCTCCTCTTGCTGAGCGATGAGCGGCTG
AAGAGCAACATTCGCCGATTGCCCGGAACTCTGGGGGGCTGCCCTCTGTACTCTTGGAAG
TGGTCCCGGGACGCCGAGGTGAGATTCGGCCTTTCAGGGACGTCTTGCGGCGTGATCGCC
CNGGAGGTGTCAAGGTTGCGCCCGGAGGCTGCCTGCCGGGGGTATGATGGTTACATGCGT
GTGGACTACATGGCGTTGCTGAGGTGAGCGCCCTTCAGTGTGGAGCGTTCGCACTTGTGA
AGGCCAAACAAGTGCCCAACCGAGGCATGGTACAGTGTGAGCCTCACACAGATGGCAACG

CCCATTGCAAGGTGAAAGCTTGCGGGTCCGCATAGCAGCAGATCTGATCTGGGGTTGGCG
CTTTTTAAGAATTCCTGACAGAGTGCATCCCGTGTGCACACCTCCCAAATGGAGACGGAC
ATGTGCGAAAGCTTGTCTGCTTAACC
>at01191
TTTTTTTTTTTTTTTTTACGGCATCAGAATCAGCCATGATGGGGTGTCTTCACGTCTAC
ACCAGGAAGAGTGGACGTAAGCGTCAACAACACTGCTGCGAATGCGGCAGCTTTGCCTCA
GCCTCCGAACAAGAACTCCAGGACATCTGCCTCTCCCCTCAGCACTTTGGTCAAGTTTGG
GCCGGTCTTGTCGATCAGCCCCAAAGAACCGGTCATGAAGGGATACCGCATGTACAGCTC
CCAGTTGCTCATCGCGTCGTCGAGGTGGTCGTCGTTCTGGTAGTAGTGGTCCTCGAGGTA
GAGCAAGTAGCGCTCGAACGTGCGGCGCTGGTGCTTGTGTTTCACCTTGGTGATGTCAGC
GTTCACGTCTGCCACCGACTGCTTGACAAATTTCAGGAAGGTTCGCTGGACTTCCCACCC
TGCAATTTGCTCAGGAGGCATCCCATCAAACACTTTCNGGAGGATGGTATGTAACAGTGA
TCGGGTAGTTCATGTCTTCAGCAAACATGTTCTCAATTTCCGCCCTGTTGCCGACTACTG
TCCGGCCAAGCTTGCTGGTGAAGGCCAACAGCCTGTGCGCGTCGAATGTCGCCGGGTCGA
TGCCGGCTGCGATCATGTTCCAGGTCAACATCTCACCAAGGTGCTCCTTGTCCTCCAATG
CCATCTGAAACTGGAACTTCCCGGACATCGTCACGCCCTGCGGCACGCCCTGGCCCCACG
CCTTGCGGATGCCATGGGGGAAGGGCTCGCCGTTCTCGCCCGTGAGCAGCGCCTCCGGCG
ACAGCGGCACGGCCCCCTTCACGTCGGACTCCTGTGGCACGCCATCCGGCTTCCCCAGAG
ACGCCTCCGAGGGCGCGGCCGCCCCAGCCGACGGCTGCGGCGGGGGAAGGGCAGGCTGCC
GCCCTGGAGACGCACCTCCATGGTGCGCTTGCTGGCCACATTGCTGCGCAACATGCTGGA
GAAGTCGGTCTTGAAGCGCACCCCGTTGAGAGTGTAAAAGACCTCTGCCTTGCCGCCGCC
CTTCTGCAGCTTGAGGTAGGCCTCCTTCAGCGTCGAGTCCTCCTCTGCGCTGGCATCGAC
CCAGGCCTTGCGAAATGGGCACTTGTACTGGAACTTCTGGCCGGCACCAAAGTCCGCCAT
TGCCCCGGCCCCCAGCTAAGGTGGGGCCTGCAATGAGAACAACTTGAGCCAAAATGGCTA
CGG
>at01192
CCGTAGCCATTTTGGCTCAAGCCTCATGTCGCCCCCCACATGACAGTCCGCAGTGTTGCG
ATCCTGGTGCTGGTTTGCGTGCAGAACAGCTTGTACACCCTGCTCAGACGCTATTCCCGG
GGTGTGGTCAAGACGACGGTGCCCTCGTCAGTGGTGCTGTTGGTTTCCGAAGTATCGAAG
TTCCTGGTGTCCGCTGCTTTGTTCGAGCAGTCCGGAGAAAGCGCCTCGCGTTGCGCATCG
ATTCTTGGTGTGCTCAAGAGCGGGCTGCCTATGTTGGTGCCGGCCTTGACTTACCTTGCT
ATGAACTTGATATCCTTCAAGGCCGTCGAGCTCATCGATGCGACGACGTTCTCCATGATC
GCCCAGCTGAAGGTGCTCACGACAGCTCTTTGTTCGCGGTGCGTCATGGGGCGAGTGCTC
TCCATGCCCCAATGGAGGGCGCTCGTGCTCCTAGTTCTCGGGGTCTCCATCATCACCTTC
CAGCGCGGCATGCACGTCGGCGGAAGCGGGATGGCGCAGGCTTCGATGGCTTTCCTGGCA
GGTGTGGGCATGGCATTTTCGGAGATCACGCTGAGTGGATGGATCACTGCGTACTTCGAG
AAGTACCTCAAGGATGGCAGCTTGTCTGTCTGGGGTCGCAACCTGCAGTTGAGCTTCTGG
TCCATTCTTGTCTACTC
>at01193
CTCTCTGGCAACGGAGCTAAGGTACAGCATGGAGCCCAAGAGGGCGATGTTGAAGACCAC
GAAGAAGCAGCGAGCGGCTGGGAGCACCCACGGCACGGCGCAGGTGGCTGCATTGCTGCA
GCCCGTCAGCCGGAACGTCAGTTGGGAGCCAAGGAATATCAGACCATCCACCAGCAGATC
TGCCCCTGTGGGAGCCAAAGTCTGCATCCTCTGCCTGGCCCTTCGCTGTGCCTTCTGCAT
GCCGAGCCGCGCGGGATGTTCTCCGACCCAAGCAAACAAGGGCGCCAAGGACCGCCGTCG
CGCCGGCAAGGCCCACTGCCACGCCGGCCGCCGAGGCTTGGAACGGAGCCATGGAAGGAA
CCATCGCTAGGCTGGCATTCCGTGCCGTGCGCAGGCCCGTTGGTGAGCAGAGTAGCTGCT
TCGACAGTGACGCCGATGGACCTGGGACGAAGCCTGGCGCGGTCCTGAGCACCAGGGCCA
GCACCGCCGCAAGGAGGACAGCAAGGCGCTGGAGAGCGCGGTCCCCACGATTGGTGGAGG
CCATGGGCCCTACAGTAGAGGGGCTCCTGCGGAGGATCTGAGCCAAGAGGGGCTTGAGCC
AAAATGGCTACGG
>at01194
AGCCAGGAAGAGCAGTACTTCCCTCAGGCTCCGAACTTGTATGCTGCGGGGCGGATGGGA
CTCCCTCCACAGATGGCAGACGGTGTGCGCACCATCTGGTCCTTCTTGTCCTTCTTGGGC
GTCTTGTCCTCTGGTGGCTCATTCTCCTGGTTGGCCGAGACGCAAGACAAGGTGTCGAGA
GCATGCCCAGCCCGGCGGCCAACTGCCTCATCCTGCGCACGCTGGAGGCGCTCGAGCTGC

TGCGGAAGCCCAAACTCCTCCGCGTGCACCACCAGCGCGTTCTTGGCCAGGCCTTTTGCC
GAAGCCTGCTCCTGCCCGAACCGCAGCACTGCCTCGCTGGCATCCAAGAGAGCGCAGAGC
AACGGGATGTCAGACTCGGCCTTGAGGGCTTGGGCCAAGACTGACAGCAACCTCCTTGCG
TCGAGCTGAGCAAGCGTGTGGGAGCCGCGCCTGGCAACGTTCGCCACAGCCCAAGCACG
>at01195
TTTTTTTTTTTTTTTTTCCAGGCAGACACACACTGAAAGCAGGCCTTGGCCTTGGAATG
CGTGCAAAAGCGCAAGGAGAGGCCGAGCCGGCAGCCCTCCCCTCACTGAATGACGCACTT
GGCGCACCGCTCTGGCACAGGCTGCCTCGGGGGGCCGAGGTCCATGTCCACCACGACCGG
ATGGCCGTGGAGCTGGTTCCAAACACCCAGGCAGGCTGCATTGATGCCCAGCGTGCCGTC
CATGTCTTCCCACACTCCCTTCAAGGGATCTGCCGGGCGGTGTGAGTGGCCGCAAACGTA
AAGCTTTGGCTTCACTCTCTCTATGAGCCGGGCAAAGGGATTCGGACCAGTGGACGCCTT
GTTCAACATGACGCCCTGAGGCGGGGAGTGAGCAACCATGATGTCCACGCTGCCCTTGCT
GAGGCCCACTTTGTCAATGAAGGCTTCGCCCTCGTCCAGCGCCATCTGGAAGGAGTTGTT
GCCAAACGCTATCGCTCTGCTCCCGGACACCTGGGCCACGAAGGAAAGCCCCGTGCCCCA
GAAGGTGGCGGAGGTCCCGTTCCGGAAGTTGAGCCTGACTGGAGCTCTCTCTGTGTAGAG
GTAGGTCACGCCATACGCCTTGCAGATCTCCAAGGCGCTCTCGATCCCAAGCTGCTCCAG
GACGTAGTCGTGGTTTCCCCCGACGAGGACCTTGTGCTCGAAGCCCAACCGCGAGCCCAG
CCAGCTCAGCGCCTCGTGCAGCTCCAGGCCCGAGGCCTTGAACCACCGCACGAAGTCCGG
GCCGGAGAGCGGCCCGCCGCTCGCCTTGTAGTCCTCGAGGATCTTCGCCTGCGTCGAGCG
CGACTCCTCGTAGCAGAGGTCACCCGCGTGCACCAGGACGTCCCCCCCAGGAGGCAGCTT
TAGGCCCGAGTGGA
>at01196
GGTGTAGGTGCCAGGATTCAGGCCATGGAGGCCAGAACCCCATCACCACANNAgtTGCCG
cCTGCAGCACCAGCAAACTGCCCTCGCAATACAAAGCCTCCTCAGACTCCGCCCGCTCAG
GCACGGTCGGAGAGCAATATGCCACCCGCTCAGCGGTACTTGCTGCCGTACTCGTTCTTG
CCCTTNTTCGGAG
>at01197
TTTTTTTTTTTTTGGCTTGGATACTCCCCGAGAAGTCTGAGACACTCCCCAATGAGTGTC
TTTGCCTCGGCAGGCCCAAGCCAAGAAGCTGCCAGCATGAATACCTGTGCGTGGAGGCCA
AAACCCACTGGACACCCTGGCCCAGCACAGGATTCTGTGAGGCTATCCCTTTGCTGTCTA
AAAAACATCTAGACCCACCAGCAGTCGGGCAGACCGGAGGGACTTTACGAGTACAAGGAT
GCGCATCACGACGAAGCTACAGGGATTGAAATGCCGCGCCGTGGCGGCCCCGGGGGCCGG
CGTGGACGCAAATGCACTCACGCATGGCTGGTGCTAGGGTCTTCCCTCGTGCC
>at01198
ACAGACCAAAACGAACAAGGGAGAGCGATTTGCCACGCTCCTCGGTGATTGGCAGGAAGC
ATCCACTGTTGATGAGCCGACCGTCGAAGCCCTGGAAGAGCCGCGACCTGACAAAATGCT
TTGTGTATATGCCAGCCCACTCGAATACAACGTTCAGTCTCCAGACGTGAAACTTCATCA
AGCAGCATCCACGAGGTCAGCTTCTATTGACACCCTAAGACGTGGCCAAAAAGGTCTCTG
GCTACCAACAGGGCGACTGGCTCATGCTACAACAACCTGCAAAAGGCTGGTTACTCATTG
ACACCAGTGGTCTACGCAATGCGCTGCGGCTGACTCACGAGTACTTGCAGCCGCGAGTCA
TGCAGGCCTTCTCCACCGCTGTCCGCCTGGCCTGGAAGCCGCTGCCGGGTCCGAGGTTCG
TCGACTATGGCCTGCAGTGGGCTCGCTCGCGGGCGAGGTGGTTGACACTCCAACGCCAGC
GACACTCGC
>at01199
CAGAGATGTCACAGGAGCAGCTTACGAGCTACCGTCGCAACCGCAACTCTGCCTTCAAGG
TGTTTGCAGAATGGCTGCAGCGGAACTTGTCTGACTGCAATGGCAATCTGGCACATGGAA
GTCTCAAGCGCCCAAGAGAAGCTGCCGAGTGTCCCGAAGCTGATGAGCGAATGGAAAAGG
CGATGCAACGTCAGCCATCTCTTACACAGATGTGAGTGTCAAATGGCTTTTTGGACCCGA
GCCTCTTTTTGCTTTATTGTCTGTTCCCGAGGTGGGTCCATTTGCCATTACAAGATGCCA
TGACTTATCAGCCGTGGACCTTAAGAGCTTGCAGGCAGACACCAGTGCATCAGCTGTAGC
CACAGGAGCCCGCGGAAGATGACATTGTTGGCGAATCCCAGACAGTCATGAGCTAG
>at01200
CAGTGTTTGTTCAAGAGACTGGTTTTCACGTAGGCACATTTTTTCGTTTATAGGGCCAGA
GGCAGCCTCCTGCCGATTGGCAGCGGACTACCTTGGCGATGAGCATGCACATGCCGGTCT
AGAACGAGTGCATGGAGGGCGAGGCGGCGCGCCATGTGAGTATGCAGGCTTGGCGGAGGA

CCTACCACCAGGGCAGCTGTC
>at01201
CCGTAGCCATTTTGGCTCAAGACCTCAGCGGACTAGCCAAGGTGCTGTGTCCGATGGCGC
CGTGAGCCAATGGCCCTGAAGCCCCGACCTCCAGCGATGCCTGGGCACTTCCGGCTACAA
GGCTCGCACTGCACCTCTGTGGTGGCGGCGAGCCGTCCGGCGATCTGCAAGCATTGTCCG
GGCAGCAGTTCTCTCGGCCTCACAGCCACGGCCTCACCTTCACCCACCCTGATGCTGCAG
AAGTGCTCACCGCCAAGCAGGCAGAGTTCTATGAGACCAACGGCTTCCTTGTCATCCGGG
GCAACATCCCCACGGCTGACCTGGAGCGCTATGTCGGGCGCTTCCGGGATATCTTGGAGG
GCCGAGCACCCATGGTGGAGGGCATGCAAGTGGTGCGCGACATCGGCTGCATCAAGGCAG
GCCGCACCGAAAGGTCGGAGCTGACTGTGAACAAGATAGCGGGCACCGAGTGTGATCCCA
TCCTCTCCGGCTACGCCAGCCACCCCAGCGTGCTCGCTACATCCCGGCATGGACGGGAA
>at01202
AGTCTTCCAAAATCTCTGGAATGCCATCTTCGTCACCAAGATCGAGGCCCTTTAGGAGGT
AGTTCAGCTCATGATAGTGACGCGCCTCCCTCTGCTGCTGCTGATACGCGACTGTCTGCT
GCTGCTGCATCATTAGCAACTGCTATTCCAACAAGGGCACCTCAAGCTGCTGTTGCCTTG
GTGGCTCATGCGGCACCAGTTGATGAGATGTCCGTGCTCTTGGTTGCTGCCTCACGGGAT
TGCTGTCATGCAGCTCCTTCATAAGGACGGCCTGGTCGAGCTGCGCGATCAGGCACCATA
GGCCCATATCCACTACCTCTGTCGACCCCCCCAGCATTGGCAGCGGAAGCGGACCGAGGA
GCCTT
>at01203
NNNNNNNNNNNNNNNNNNNNNCTTGCTGGTCAACCTGCGCCTGTCACCATGCGTGAGTC
CACATGGGTAGCTTCATATCCCGATTACACCCTCGCGCAACCGTTCAGAAGAGCAAAAGA
AATGGCCTGCACCCCAGTCATGGACTTTGCCACTGAGGATACTTACGGCTCGAGTGTGGA
CCCGATGCAGAGGGAACAAGACCTGCAAAGCAAAGCAGGACTCGCATCGAACATCAGCAT
GCCACAACCTCCAACTGCCTGGCATACGCCAATTGTCAGCATCTTGCTGGCCGACTACGC
CGATCTCAAAGGCTTCTTCCAGGTCATCATGACAGCACCGCTCACGATGCCCAGACGGCC
AGCGTTGACAAAGTCTGTGGCGGGATGCACCGCACAGCTCTCGAACCAATCCGAGAAGCC
GGCAGGGAGGAGGCGGATGGACTCACTGGCCTGCACCAGGCGCAAGAGCAGGCTGCTGCT
GAGCACAGCATGGATGCCCGTTCGCTCAAAGCAGGAGTACCACTGGCCCGCGCCGGAGCC
AGGTCCGCAAAGGTCATCCTCCTCAACGGCCACGAGCCCAGCCTTCTTCGCAGCATCTCG
GAAAGCTTGCGCATGCCACAATGGCGGCATGCAGGTGGAGCTGGAGATCTGGTCCACATA
GAACTTCTGCAGCTCGTCGTCAGGCCTGTACTTGTCGGTGACGAGGATCTCATACGAAAG
AAACTTGCCGCCTGGCTTCAGCA
>at01204
TTTTTTTTTTTTTTTTTGCGGCGAGTCGACCTTTCGGGCCGTTCTCCAAGCGTTTCGCT
TGGCGTCCTGGCTAAACTGCGGCTGGCATCATGGCGTGCCGACGATCTTTGGCGTCGCTG
CCAGCGAGAGGTGCAATGGAGTGAATCCAGCGTTCTGCGGGGTTAATTCGGCAGCTTTGG
TCCAATTTGCCTGCGAGTACGACATCGATGCAATCATCCAGACGATCACGAACCAGAAGG
CAGGCCCTCCGGACCTGGCCAACTTAGGGAAGGCTTGGTCCAAGATGATGTAGGGCACGA
GAGATAGCATGTGGAAGATGACACCGAGGAGCAGGGCTGTGGTTCGAGGGGGATCTATCT
GCCGGATGGCACAGTAGGTCCAGTAGACCGCTCCAATGAAGTGCTGGACGTAGTCCGTGA
CGGCAAGGACGATGAAGACGGTGCCAAACGTCCACGCTCCAGAGATGGCAGGCACAAGCA
GATAGGCCTGCGACAGGTATCCAAGTGGCATGGTCCTGGCAGCCCAGTTCTTCCCGAACA
TGTCGAAGATGACCCGCGTTACGTTACACATCGAGTGTAAAGGGAACGCAAGCAGCGTGA
AGATCACAATCATCGTGGCTGGATTCGGCGCTGTGTGGCGCGACATCCCTCCGATGGAGT
C
>at01205
NNNNNNNNNNNNNNNNNNNNNNGTCAAAGGCGGTATGCAGGCAATCCTGGAGGCGCCTC
CAGAGGCAGCCCTCGCCACCGACCACTCTAATTCTCAACAGAACCTACAGGCTCTGCAGC
CGTCGCGCACCCCCAGCGGCCTCGCGCAGCTCCAGCTCAGCCGGGGAACTCCCCGGAGCC
GTCCACCCAGAGCCTCCGGAGTGAGAGCGGCCTTGCCCCACAGCCCTAGCGGCCTCCACT
GGGAGGCCGAGAATGCCATCGGTAACTGCGCTGTCCTCCACTTTCCTCACGGTCTCCCAG
ATGCGCTCGCTGATGTCCTCTGCTCGAGGCCGCCTGTCGAGCTCCTGCCGCGAGCAATCC
TCGATCCAGGGGCGCCAGTCCGCAGTCAAAGGGCAGCTGGCCACAGCGCTGGGCCAGTGC
AGGCAGGGCAGGCTGCCGGACCTGAGGACCTCCAGGATCTGCTCCTTGCTGCGGCCCCCA

AACGGCCTCGTGCC
>at01206
TTTTTTTTTTTTTTTTTTCCATTCGCAAGTCTGGTGGTGAACGTCAAGGCGTCCCGCATT
GTTTGTACAGCCTTTGTGTTTCATGCGCAGGTCCAGGGGTGAACTGCAGGTCTTCCGACA
CTGTTTGTACGGCGTCCTTCTCAGCAGCCCTAAACTACAAGGTTGTGGGTCAGCACAGGG
TCCTCAACAAAGCGGCCTATGAGCACCAGCACGAGCGCTTCCCGGCTTCATCTTGCGGTT
GAGCTTGTTGATGATGCCTTTGCGCTCAGCTGCTGAGGCCCTAGGCGCAGCTACCTCGCC
GCGTGCCGAAGGGTGCTCGCGCTCGGAGTCTGAGCCTGGACTGGGAAAGTCGTCCCGCCC
TCTCCGGACGCCAGACGCCTCGACGTGCAAGCACGTAGGCGACTCGTGGTCCCTCAAGGC
CCGCGCCGCGGCGTCGCCTCGGCGGTTTTTTTGCGGCTCCGACGTGACAACATTTGGCC
ACCCAGTAGCCTTGTCCGATTATCAATCGGTCCTCCAGCTATGCCGCCTCGTGCC
>at01207
ACAGTGAGGAGGCTGATGGCCTGCTCCATGACCAAGATACAGCGCGTGAAGCACAAGTCA
TCAGCCAGAGGGGCTAGGTTTAGCGCAAGATATCGGAGCTGCATCAGCAAGGCCTGCAAT
GCAGTGATCATGAGTTCCTCCGAGGGTGAATCCTTCCATCCCATGTTGATAAGGGACTCA
GAAGCAGCAGCACTGCCCAACCCAATGAACACCATCACCCCACCGACACCCAGCCGAATC
CGCCGCAATGACTTTTGGCCGGCGAGCAGCTTTCGCTCCAGAAGGCGATCCAGATCCGGC
TTTCGCTCCAACATCCCAGCCCTACCGGTGGTTTCAGTCTCACTCATACTTTGTGAGTCA
GTAAGGTCCGCTTGAACTAAGATGCAGAGCTGCACAAATAAGAAGGCGCTGCCATTGTGT
ATGGCTTGAGCCAAAATGG
>at01208
CTTTGGCAGGAGGACCGACATCCTCTTTATCAGACCTGTGTCCCACAAGCATGCAACGTT
TACCACGCAGGGCCAGGTATTCACGACCATGGGTGGCAAGATGCGTTTGTGTAGCTACAA
AGCCACTGATTGCCCCTTCAAGAACGTCTTGCTACAAGGAGGTGATACTTGTCCTTCTCA
CTTCAGTAGCCATGGCCCTGTTCAGCTGTGGAGACCGGTTTGAGACGGTCTATCCCCCGG
GACAATCCCGGATGGAAGCCATTGCATTCTCGCTTCCCAATAGCGTATCAGAGCGAAGAG
TTGATGAGCTTCACGCACTGGCTTCTGGGTATCAGCTCGGGCCGTGGCTGGGTTCAGTCC
CACCAGTGGGTTCCATGCATTCATTTTTGCTGCCATGGCGTGTGACTCTGTGGACCTGTT
CGGTTTTGTTGGAAACGGCACAGCCGATCAGCATCGCATTACCAAGGATCACAACCTTAC
GCTGGAGCATGAGCTGATCCGGAAAATCAGCAAAGGCCAGCTCAAGGACATAATCCGCAC
GAGCCTTCCTTCCTGTGCTCCATGTATGATTCGGAAGATGAATGGCAACTCTTTCCACGT
CGTGTATTGACAGCTTCGACGGCGGAGAGGGGGGTGAAAGCCTCTTCCACAGTCTGGCGA
CGCTGTCGGAACTTGAACTTGAACCGGGCGATGTCGTCGCCTTGGGGGCGGGCACGTGGG
ACCTACGGGACTCCAGCATCGATGACTTCCTGATCGACTACAGGCAACTCTTGAGCAAGC
TGGAAGCGGCATCAATCAGCCATCGAGGTGTCCGCTTCATATTCCGCACTCCACCTTCCT
ACACCTACAAAAGCGGCAACAAGCTCAAAGGCCGGGAATACCGGACCAACGAGAAGTTGC
GTGATGCAGTGGAAGAGATCAAGAGGCTCAGTTTGAACAAGGTTCATTTGTGGGACTCGT
TTTCGATTACCATGCCTCGGTTTGGAGAGCCTGTCGACACACACCATTACCTAGCAGCGC
CGAGCGACGTCGAGGCCGCAGCCCGTGAAGGGACAAGATTGGTGTTGCTGATCTGAACAT
GTTTGTGCACATGGTCTGCAGATGACAAGAAGTCGTCATTCCTGCCATGCTTGCATTTAC
TGGTAGTTCTTGCTCGCAGGCCAACTCGTATTGCCGTGATGACGCC
>at01209
TTTTTTTTTTTTTTTTTTGGTCGATCAAACCTTTGCTGATTTGACACGGTGCCAGTGACA
TCACTCGTCACTCCTAGTTGTAGCCATGGCCGCACCCCGCATGCGTCCGGGCCCGCGGAA
AAATGCTGCTGCCGGAGCCAAACGCTCACCAGCTAGGGGCAGCGGAACGTGCATGGAAAG
CTCTGCTCACGCAGTGCTTGCAGGAAGTGGAACGCGCACGAGCATCTTTCCAGTGTTCGC
GCCGGTAAACACGCCCCGGATGCCATCAGGCAGTCTCTCGAAGCCCTCCACCGCCGTGTG
CCAATCCTGCAGCTTGCCGGCCCTCAGCCACTCGACCATGGTCGCATCACACTCGCTCAG
CTGCTCGGCCGTGAAGCTGCTAGACACGATGAAGCCCTGCAGCTTCAGCCGCTTCGCAAC
GACGTGGAACAAGTTCTTGACTCCGTAGCGCTCCGAAGGCGGCTTGTCGTACTGGGAGAT
GGCGCCACTCATCACGACGCGACCGCCGTCGTTCATCATCTCCAGGCCGTGCTCCAGCGT
CTCACCACCAACATTGTCAAAGAATACGTTCAGGCCCTGGGGGCAGAGACGGCGCAAGCC
CTCANGAGTGGTCTCTTTCTTGTNAGTTGATGCCTCGACGCCGATGCCTTGCAGCANGCG
CACCTTGTCGTCGGATCNGGCGCTGCCCACGACGCGGCAGCCCAGTTCCGCAGTGTCTGG
CAA

>at01210
CCAGCACGCCAGAAGAGCTGCTCACACCGAAGTAAGTGTTTGCCTCGTGTGGCTTGCCAA
CATGCCTGTAAAGGCTTCCAAGTCCAAGCGCCACCGCTTTGCCTCCAAAGCACGGGACCA
TGCGCGTGCTCAGAGCGGTGCTGTTGTTGTAAGCCCTGGTCTTGGCCACAAGGAGTGGGC
AGATCTCGATAACCTCATCACGAGCATGGCGCCGAGTGGCAAAGACCCTCCAGGGCTCCA
GGCCGCAGTCTCCTGAAGGCCGCCGCTCCCGCCAGGCCCGCACCTGCGCCGCGCCCGGAG
CATGAATCGTGGGCGTGAAAGCTCCTGCAAGTCGGCGTGGGTCAACCGTCATGCAACCCA
AGGGTGGTGCGAAGGTGTCCGAACATCGCTCGATGATATTCTGAGTGTAGAGGCCGAGGG
CAAATGGGTGCCCCACCTCCTCCACGGCACTCTCGCCGCTGCTCGGGTACACCGCATCCT
GTGCGGGCCCGCCGCTGCTGGCGATGCCGCGGAAGAGCAGCAGGGCGGCGTTGGCTGCCG
CCTCTGCAGCACTACCCCCATGCTCTCCGACGATTGAATGGAGCCACAGGGAGTCAGA
>at01211
AGTGCAAGTACCACATCACCAAGAACAACTGCAATTCCTTCAGCCTAGCGCTCCTCAAGT
TGGTGACGGGCTCCAGGTGCACCACTCTTCCACGTGGCACAACGAGATTCACTGGAGCTC
TCGATCTCTTACCCCATTCTATCGGGCATGGACTGGTTGCAAGTGCGTACTCAAAGTTGC
TGGCCAGAGCTGACCGCGCTGAGAAAGAGTGCGATGAGTTCAGGGCGACGGGCAAAGGCT
GCAGCTGGACTCAAACATCGAGATGCCCCACTCAGCCCAAAGGGCGTTCCAAAAGTCTTG
CGAAGTCTGACGGGTCAGTGGGCTTCAGGTGCTGCTGCTCGGCAT
>at01212
GGCGACAGGGTGTGGCAGCACACCGTCCCTGGGTCTCCGTCTTCTCGTACCCCTGGACGC
CCTCCTCGAGCTCCCTCACGAGCGAGTCGGTCATCTGCGGCAGGCCGCGCTTGGTGGCAC
CGTAGGCGACGTAGCCCGGGGTGCCTCCGCCCTTCACGCCGCTGCCGACGGTGTTGAAGA
CGTGTCCGGTGACGCCGGCCTGCTGTTCCATCATGCGCATTGCTGCATGCGTGCAGATCA
GGATCCCACCAAGGTTGATCTTGACCACGGCCTCGACAGTCTCTGGAGATATCTCTGTAA
AGCGTCGACGACCTCCGTTGATACCGACATTGTAATCCAATAGTGCACCACACCAATCTT
TTCCTGACAAAGTCGACCAGTTTGCTCACGCTTTCAAGGCTGGACACATCGCAAGCTGTA
CCGTAAATGAGGCCTTCGGCGTCAGGATACTTCTTTTCAA
>at01213
GGAGCGCACGGTGATGCTCCTGCATGCGCTGCCAGTTGACGGCCGCTGCCGCAGTGTACT
CGTCCGCAGCTCCCATCGCCAGCCGTGCGTTCAAGCTACCCTCGCACAAGGCCGTGCCGA
TCGATGGACGCTCGCAGATCCACCAAGCAAGGCGCTCGCCAAGCCGCGTCCAGCAGCGAT
TCGCCTCCCTCTCGTGCTCCGCCTGCTCCCGAGCCATGGCGCCCAGCTCCCCCCACGAGA
GCCTCCACGAGCGAACTCTCAGCAGCCACAAGCCTCTCCCCCCAACAGGCCACAGAGGTA
TCTGCGCAAGCGAGCGCGAGCTGCGAGGCGCTCAGGTCCGTCCAATATGAGCGACTTGGG
GGCCTCTCCAAGAGCGCGTGCCTCCTGTTGGGCGCCTGTGCGACCTCTGTGCAGCTG
>at01214
TTGTTTTTTTCTTTTTTTTGGCTCCCACCTCAGCCGGGACCACAACGAAGAGACCGCCT
GCAGGTCACCGATGCCCGGGTCATCTCTCCTGCGTGGACAGCGCAGGTGAGCAGGCTGCA
CTCAGAGTGACATCTTCATGCTCCTTGTCCGGCTGCTCCCGGCTGGAGGAGGCAGTGCCG
CCGCCCTCAGCACGCAGCGTCTCGGCGAGCGCCCCTAAGCGGTCCTTGAGCTTGATGACG
AAGTTCTTGTTGGACAGTGCAGATGGTGGGAGCGCCGCCTCGTCAAAGACGAAGTTGTAC
GGGTCATTCTGCACCTGGGTCTCCAGCTTCTCGACGAGGTTCTTGAAGCGAAGGCGCTTG
CTCTTGCAAGGGCGGTCGGGCTTGCCTGGCTCTGCGCGACGGTGCAGTGGCCCTCTCCAC
CTCCGGCCCGCTCTTCGTTGCCACCTCCTCGTCCTCCTCGTCCGAGCAGAGGTCTTGCTC
GGCGCCGACGGGACCTTCGGAGCCTCTCGACTCGGAGGGCAGCAGCAGCTTGTCCAGCTG
GGACAGGCTCTCGGACAAGCTGGCGCACGACGTGTTGTCTTGGCTGGAGGACATGGTCGA
GGGCGGAGCCGAGTTCTGCCGCCGCATGAACTGGAATCCATCGTACGGAGCAGTCTCCAC
ATGTAGAAAGGTGTTCTTCGTCGATACTTGGAACGCGTCATCCGGACCGCCACCAATTCG
AGTGCTGCTGCTGCCGGCTCCGCCGAAAGCACTGTGCTGGGCCATCCTTTGGATACCCAA
CCAGTTTAAAAGGGCCCAAGTGGCTTGAGCCAGACTTGAGCCAAAATGGCTACGG
>at01215
GAGGATGGCAACATGCGCAAAGTCACCTTCATCACCTACTGCCAGGAGCTCGTCAACCGT
GATCCCCGGCAGGGCAGCCGGGTCCGGCAAAGACCTCCGTGCCTGAAGCACATGATCAGA
CGGCGAGTAGGTTGGCGTCTTCGAAAGCCTCTCCAGGTAGGCCTCGTTCAGGCATGCCTG
GCGCCCACCCTTCATGAGCACCCCCAAGTAGCGCTTCGAAGGCCTCCCTTCCTCTTGAAC

AGCCTGCCCGGGCTTCGTGGTCGTGGTGTAGAGGTATCCCAGCAGAGTGCGTCCATCATA
TGCCTCAAAGCTGATATCCGCCTTGGCGTAGCCGCGCTCCACGTTGTCCAG
>at01216
TGCAGTGCCCAGACTGCCACCAACAATGCGACTTCAGAGCAAAAAGTATGCCTCGCTTGC
GAACTCCCATGATAGCAGAAGCCAAAGTAGAAACACACAAGGCCAGGCAAAACAAAAGTC
CGAGACCTGCAACTCTGATGCCTTGCGCAAGACCTTCCAACGCATCCCGGGGTCGCGATC
TGGAAAGAACCATTCTGAGTGCAAGTAGAGGGTGCACTCCAGCCCCTGGCGATGACTGAG
GTGTTGGAACTGTCACAATACTTCTGGAAATCTGTGCAGCTTTGTCTTCGATCTCACCAT
CCATACGAGTTCCAGCAAAGCAAGATATCCTCCTGTATGCCCAGTTCTGCACGCGATAAC
CCACAATTGACAAAATGAGGCATGTGCTGCGATTCCAAGGTCGATTCAAGAAGCTGCCTG
AACGATGCAGTCGTCTAGATCGTTGCCTCAATGGGAGACCAAGGTAAGTGGCATGTTGAG
CGGGTCGCGTTGCCAATACGCTCGCTTGCAGCATATGGCTTGATCAGGCATCTACACTGT
CACCACGTCAGCTCAGATTCGAAATCGTATTTCGCAAGTGGGAAGCCGGCCTGAGGTAT
TACGGCCTTGCCTCTTGAGCCAAAATGGCTACGG
>at01217
TGCCCAGCTTTCCAGAGCCAGTGTGCTTTGAATAGTACTAAAGGATGTTCTTCTGGTGGC
AGGCGAGAGCCACATTGATCCTATAGTGCAGGAGCTGCTTCGCTGCAGTGTCACCAAAGG
GCCCGAGGTAGCACGTCAGGATAGCGTTGCAGATGCGACGGGTGAAATGGACATGGGAGC
TGAAGGCCTCGAGGGGCCACTCAACATCTTCAGCAGAGGCACCTTCCCTGTATTCTTGTC
TTCCAAGGGCGTGGACGCAGTGCTCGCTCGTCGCGAGTACTTGCGCGGTATCATCTCGCC
AAAGGAGATTCAGCAATCCTTGCGACAGTTCATGGCCGACCTGGGGTCAGGCCAGCATTT
ACCAGATCTATCGTTGAGATCGCCACGTTTAC
>at01218
CGGCTGCTAGTAAGCCATTTGGGCTCAAGTGATTGGAAGTGACTTCAAAGGCGCTGGTGC
GCAGCCCTAATAGGGAATGCTGACCGATGGGAAACTGCAGCGGTTGGTACTGCCGCTTGT
TCTTGGTCTCGTGTGCCCCACGTCTGCGTCTAAGTATTGTGGCGAGCTGAACTGCTATTC
CGTTCTGTCGGTGGACCCGTCCGCGGACCTTCCCGCCATTAAGAAGTCCTACCGCAAGCT
GTCGTTGCAGTGGCATCCAGACAAGAACCCTGCAAACAAGGAGGAAGCTACCCAGAGGCT
CCAAGAGATTGCCACGGCGTACGAGGTCCTCTCAGACGAGGCAGTTCGGGAAGCCTACGA
CTATTACTTGGCGCATCCAGAGGAGCACCTGTACAACACCATGCGCTACTACAAGGCAAC
CTGCGCGCCCAAGACGCC
>at01219
AAGGCGAGGACGGACACGACAAAGCATGTGAACACCACGACCTTGATGTAGACCTTCACA
GACCCTGGCTGCAGCTGCTCGACGCCACCGATTGGGGCGATGTAACCCATGGGGGCCATG
AGAGCGATGGGGATCGCAGAGGCCGGTATCTGCACGATCTTTGGAAGGAAGCCCTTGAAC
ATGAAGTAGGTGGCTTCGTTACGTTGACCCGTGAGGAACTCGTCATAGTCAATGATGTCG
CTGAGAATGGAGTCTGCAAGGAACTTTGCACCGAGAGGCAGGCCGTTCAAAGCGGCGACA
ACCCACAACCCGATGACCATGCCACTATCCAAAAAAGAGGAACATCACGTTCGAGAGCGC
CATCGAAAGCGACCACAACCACCATGTCTTGACTTTTCCAAGCTTTGCCACTGCCAAGAA
CCAGAGTGGGAGACCAAGGACCGCGGCCAACAAGACTGAGATGCCACAGGCAGCG
>at01220
GAGAACAGGGGTCgGCCATATTTTGTCTGCGGTTCGCACCCCTCTCTGCTGCCAGGTGCT
AGAGGAGGCTGATGGAGGGACTCTGCGCCGGTTTCATGGCCCCGGGCCTTCAAACAGGCA
CATTGGCGTGGCTGTGCTTCGGCTTTNNCGCCACCATCATTGCGCAGGCCTTCGTGAAAG
AAACGCCCAAGATCACAAAGAAAGAGAGCCGCGAGCTCTCCCTTGTGGTTGTCTGGATAT
CGACGATCTGCATGTGGCTCTTCTGGGCGTTTGTCTACATGCACCAGATGGTCCCNCTGA
TCTATCCCAAC
>at01221
TTGTTTTTTTTTCTTTTTTTTTTGTCTTCCTGGCACAAAAACTGAAAATCGAGGGTAGGA
GGCACTGGGCGGCCCAGTTGGAAAATGCAAGAGGCTGCGCCGTCTGCCTCGGACCGCGCA
CACTCAGGCAGAGGGGAGTCCGAGCTTCCGCTCGCCCTCAGCCAGGGGCAGCACGGCCGC
ATCCAGCGCCTCTGCGCTGACGGCCGCAATCTCCGCGGGTTCCCACTTGGCCGCGTTGTC
CTTGTCCACGAGCACCGCACGGAGCCCCTCGACGAAGTCGGGCCGGTACATCACTCGGCA
GGCTGCCGCGAAGTCGCGCTCCAAGGCGGCCCCTGCACGGCCTGGCTCGGCGAGCCCTGC
GGCGGCATCTGCCTCCGCAAGCTCCATGAGCTTGAGGACGACGGCCTGGCTGAAGGGCGC

GCCCTTCTGCATGCCCGGCAGCAGCTCCGAAGCCCAGCCGCCCGCCTCGGCTTCGGCCTT
GAGCATGTCGAAGGCCTCGGAGACGGTCTTCACCTCGGCGAACTTGGCCGGCAACGTGCA
GCCATCGACCAGGAGCTTCGGCGCTGGGGCAGCCTCCGCAAGTCCCTCGAGGCACTTCTT
GACGGCACCGTCCGCATCCCCGCTGAAGTCTGCAGCCTTCAGCGCCTCCGCCACCCCGGC
CAGCTTCTCCATGGGCACGTAGTGCGTGGCCAGGCCCGAGGCGATGACGTCGCCTGCCCC
GATGAAGTGGCAGCCCGTGAGCGCCATCAGCCTCCCCAGGCCCTTGGGCATGTGATTGGC
AGCCAGGTGACAGAAGCCGGCATCAGGGAAAAAGCCGATGTTGTTCTCAGGCATTGCAAA
CCTGGACTTCTCCGTGGCGACGCCGTACTTGGCGCTCGCGCCCAGGCCCAGGCCACAGCC
CATGGTGATGCCGTGGACGAGAGCGACCGAGGGCTTTGTCAGCTGCTGAAGCTCGAAGAT
GGCGTTGTACTCCTGGTACATCTGCTCCTTGGGGACCTCCGTGGCTGCATCTGCCACCAG
CAGTGGGTGCAATGCCTTGACGTCGCCGCCCGAGCAGAACCCCTTCTCACCAGCTCCAAG
GATCAGCACGGCCTTCACGGTGTCATTGGCCTTGCACTTTGCGATGGCCTCCCGGAACTC
GCCCACCATCTTGACGTTGACTGCGTTGAGGGCCTTAGGCCGGTCAATGGTCACCACCGC
AAGGCCATTGGGGCATTCCTCAAAGGTCACTGACTCCGACATGTCTGCCGCCCGAGTAAG
CCTCCACAAGAGGGTCCCCTGCCACCTCGAGCCCAAAATGGCTTGAGCCAAAATGGCTAA
CGG
>at01222
TTGTTTTTTTTTTCTTTTTTTTTGTTGTTCAACAGGGTAGCCTTGCCGATGCGTTCAAGA
GACCTTCCGTGCCGTAAAATGCCAAACTGAGTCCGTACTACGAGCATTCAGCGGAACGCC
GTCATCAAGTCCTTGGGCTTCATGGTCTTCGAGCGGATGGAGAAATGCAAGGGGAGCCAG
AAACCTGGCGAGGGGTAGGGCATGTTGATCTGGATACCGCCCAGCAGCACGAGGTTGCCG
GGGCCGTAGTCCGTGTTAACAATGGCCAACATCTCCTCCTCCACAGCCTCGTACGCCTTC
AAGACGAGGTCGATCATCGGCTGGTCGGAACTGGACACCTCCGGGACGTACTGCTTCAGC
TTGGCGCGCAGCCAGCTCTGCTCCATGTCGGAGGCGTCCGCAGGCATGTTCGCGCCGGAG
GTGAGCTGGCTGTGTGCGGCGATGACCGCCCCGCATGACGTGGAGGGGGCAGACTGACCA
GTGCGCAGGAACTTGCCTGCCTCGCCGTTTATAGTGAAGCCAATGTGAGGGCCAAAAATG
ATCAGGACATGCCCGTTGTCCGGCACATGGTGTGAGAAGGCCATGAAACCAGTCTTTCCG
ACGTAGGGAGCACCACCAATGCCGCCAAGGGGGAAGGTCCTTCCATAGTACCTGGTCAGG
AGTGTGGAAAGGTGCCCGGGGTCGCCGTTGATCTCATCAGAGCAGATAGACTGGCCATAG
ATGGTGTTGCGTCTTCTTGAGGCCGTACGGCTTCACTGCAGAGAGGAGAGTAGACATCAC
AGCAGAACCCTTCATGGCGCCTGGGAAGGCGGCAACTACTGTATTCTGGATGTTCTTGAT
GTCGGAGCCGAGGAATGCGGTGGGAACGAGTGGCTGGGCCGACACCTCCTCAGTTGGGCC
TGCAGTGGGGTTGCTCTTTGTGAGCACGTGGATGCCGAGGATGGCAGAAGCGACCGTCAG
CAACCAGGGAACCCAAGCGTGGGCGGCGCTGCCAGGCCATTGCTGGCCTTGTAAGTATGC
ACTGGCAGATGCCCAGATAGTCCAAGGATCTCGATTCAAAAAAGCTTGAGCCAAAATGGC
TACGGTGAGCCAAAATGGCTACGG
>at01223
AAGGCAGGCACTTCGAATCTCTACAACGCTGTCAACCTGTGCAGACTGCAGGCACTGATG
GAGTGCAAGTCAAGCGTACAGGCGGGCGACTACAAACAGGATCTGTGAACAACGCGGCGC
ATATGCCTAAGTGCTTCGTCACTCACCGGGGAGGCATGGACGGTCCGGTCCCGTGTAAGC
CTCTCTCTCCGGTTTGTCCATAATTAAGCCTCCAGCTAGTGTGGCTTCTAGACAAACCTG
TGGATCCTCTGCTTCGTTCTTACATACATTCAGGAGCGCTAGCGCGGTCTCCTTGTCTCC
AAACACAGCTATCTTTCCCAACGATTCGCACGCTGCCTTTCGAACTCCTGAGCTTGGGTC
TTTCAGCGCAAGCTTGGCAACACCGAGGATGTCTGCATCCCCCTTCACAGATCGACTTGC
CGCAAATGTAAGTGCACAGGCACGAATCTCTGGGCAAAGATCATTCACACAGCCTGCAAG
CGCCGCTGCTGCTTTCATGTCGCCAGGTGAAGCTAGCTTGTGAATGGCACAAACAGCACA
GCGCCGGACCTCTTCATCCTGATCCTTCAAAAGCTGAGTCAGGGCAGCAACAGCCTCGGC
ATCTCCCTTTTCAGCCATCTCACCTACAATGCTTGCGCCATTCTTCCGCACACCGGCATT
TGGACTGGCGAGCTGCACCTTTGCTGCTTGCCACATGCGGCAACAAACCCCAAATTGAAT
CACAACACCAGAAGTGCGCAATCAAGCCATCTTCTTCCGTGAAACGTTTCGGGCCGAAGA
TGGCCCTCGTCAGGCCTCTAGAACCGAAATGGCTACTTGAGCCAAAATGGCTACGG
>at01224
CATCGTGAAAACGGCAAAGTTCACGACAATGAGTGACTGGAACAGGTGCTCTGGGCGAGC
AATGAAGGGTGCAGTGTACGCAGCTAAACCCAGAACAGCGATGGTGTAGAAAATGTCAAT
GGTTCTTCGCGACAGAGTGCTTGGAAACACTGTAATCAAGGTCGCCACAAAGTGCTCTAC

AAGCTGCAGGACGTCCTGTGGGAAAGTGTGGGCGCGGCTCTCGCCCCTCCAAATGTTGAT
CATCAAGTTGGCCGTGAAGGGAGACGCTATGCAGCAAAAGATCTGGCACAACATGTGGAA
CTGATCGAGCTTCTGCTCCCACAGCTTCTCATGCACAATAAGATCCACCTTGGAAGGTTC
TGCCTTAGTCCACAGGCTGATTGTGTTGATGGCTAAGGTCAGGACGGACTTAAACTTCGA
CAAGCACCTGCAAGAA
>at01225
TCTACGCTGGCGACCTCCCTCCACGCTCTACACCCTCGCCGGCACCGTGGGCTGAGAATG
GTGGGCAGCATCAGTCTGTTTGTCGAGTTACACGCAGCATCCTCTTCGAGTTGAGCCTCA
TGACTGCTTGCGCCTGCTTTGGGCCTATGCGCTTCCATTTGAGGACGTCCTTCTTCGAGA
GCCTCTTCTGGGGCTTCGGCATCAGAGTTGACCGGAGGACGAACCTGATGGGCACACCGC
TGATGCGGAACTCCTCCCGCATGGCATTCTGCAGCTGCCGCATGTAGGACCCCGGCATGC
TGCCCGCCGTGGTGTTCGTCCAGAGCACGAACGTTGGGGGCCGCGACCGCGTCTGGGTCG
CGTACTTGACCGTCATCCTCTGGCCGTGCCGCCATGGGGGGGCCAGCGGAGCATCCAAGC
CTGCAGCCAGCTGTTGAGCTCGCTGGTTGGGAGCCGGGCATTCCAGCGCTTG
>at01226
GCCTCGACCTCCAGCCAGAGGCATTCTTTTGCACTGGCCAGCAGCACCCCCGCCTGAGCA
AGCGGAGCCATGGGGTGTGCCTTGGAGCCAGGACGCGGAGTCCGTCGGAATGTTTCTGGG
GGCCGACCTGCACGCAAGCTACTCCTTGCAAAGCTGAtTCTTGCACAGGCATGCTTGCAG
ACTACCTTCATCTCCTTCGGCAGCCCGACTGGCGCGCTCGCGAGCGCTTGATTCTGCGGC
TCGGAACGCGCACGCCCTTGGGGCCGGGCAGGC
>at01227
TAGTTCCTTATTTTTTGGTTCTTCTAAGGTGTCCGAGGCCCTGAGTTCTCGGCAGCAGCC
GGCGATGCTCCGCGCTTGAACCTTTGCCGGAGAAGGGCGCCTCGGCGGCAGCCTCGGCGC
CCTCAAGTGGCTCGAGCCTGTCGCGCGTGAGCGTCGCCTGG
>at01228
GCGATGGACTTGACCTCGGGTAGTTCCTTGGGAATCTCGTGCAGACCCTGCTTAAACCAC
GTCAGGCGCTGCTCTCGTGTATCCTTGACATCGGAGTTGCATGCAAACGCTTCAGCGAAG
GGCGCTGGCAGTGCAGGAATCTCCTCCTCGGGGTTCTCGATGGGCTTATCAGCAATGTAC
TGGCCGTAGAGGTTCACGATAcGGTCCCCGTGCACTGAGATGGAGCCGGGCACGCCGACG
GTGCTGGCGATCGcCTCCCCCTTGGCGCGGAGCGCCCGCCGCTCCGCGTAGACGTCGGCC
TCGGGGTGCTTGGCGAAGACGGCACTGGCCAGGCCCGAGGCGGCCTCTGCATGTATGCAG
CTCACCTGGTGCACGACGAACTGCGCATCACAACTAAGGAGGTCAGCAACGATACACTGC
ACGGGCATGCTAGCGGGGAGGGGCTACGAAACACAGACACTCAAATCATGGCCCGAAATG
C
>at01229
TTTTTTTTTTTTTTTTTACTCGGCTATGCACTCCTGCATGCGGTTTCCAAAAGTTCTGA
TCATCGACCAGTATGCCATCTTATGCGGCATGTGCCACCACAGGAACTTCAAGTCCTGTT
ACCTGTACAAAGGTAATTACCCGGCCCTCCTCAGGAACGGTGCTCGTTCTGTACTGCACG
TCCGATGTAAGAGCCATACTCGAATTGGGGTGCTGAATCACTGCACCTGCACTGCATTGC
ACGACGGCTGGAGGTATAGTCCCTGCCATTGATGACAGCATACGGTTTCAGGCCATCCTT
ATGCCCACGCAAAGAAGGGTGCCGATGTCTGGCTTGGATCAATGCCAATGACGGGTGGAC
ACTCAGTGATGCCGTACTCAGCCAACTGCTGAAGGAGTCTCTGGCAGAATACCTCCTCTG
TGAAGTTTTCTTCAGATAGGAAGTTCTTCGTCACCAAGAATGTGGAGATGGTGCCTGCAA
AATCATCTTTCAACATTTGCTTCAAGATGCGGTTGTCGAGCACGACTTCGGAGTTAATAC
CGTCTGGATGGAGAGGGCTGCCGACCCATTCCTCTGGATGGTCAATACGCTGCTGTACCA
TCTGTCGTAGTTGGTTCTGCCCGTGCCCTCATAGAAGGCTGGTCCCTCCCAATAGCATTG
CCCAACCGACGAGTTCATCATTGACTCCTCTGTGCTCGTACAGTGGCATGTAGAGACGAG
GAAGCTGGAGGGTGTGCTCGTGTGCCCGGTCAGTGGGTCGATGTTCATGCATGCATTCCC
GACGTGGTCCGAAACCTGACTACACGCATCCTCGTGCTGCGAGGCAAAGCGAGGGGCATA
ACACGATAAGAAGTGGCACCCACAGCCATGATTCATCCTGAACCTGCTGCCACTGTCTGA
CGCGTAAGAGCACCGGACCGCTCTCTCTCGAACTGACTCGTTGTAGACCCACCCTACAGT
GGCGTACTCATGGAACAACACCCTGTTACCTTTCACAGAGTAGGCCTGATTCAGTATCAA
TGAGTCCGAACTGCAAGTGTCCCAAGTCTGCCACGCGTCTACGTCGTCGCTGGGAACGGT
GCTGTTGCTGCAATTGTCATCACAGTAGAACACAAACTTGCTACTCAAGCCTTGAGCCAA
AATGGCTACGG

>at01230
CAGCAATGTCCAGCCCCTCTGTGGCCCACATAAGCTGGGCTTCGCACCCACCAGGCATGA
TAACGATCGCAGTGCTCGCATGCGCCTCCACATACCGAGGATCGCCGGGGTGTACCTGAA
AGGGCACCAGCGCGGGCTCCTTGACATTCTGGAAGACTATGCCATTACCACCGCCACACG
CTGACGGATTGGGAGCCTTAGACTGCTGGCCATAAAACTCAGGGCCTGTTGCATACAACT
TCAGAGGTGCTTGCTCAGCCGGATACAGTGGCCAAGGTGCTTCACCTACCGCATCCAAGG
CATCATGGCTATTTCGAGACCCTAAAACAGCTTGTGAGATGGCACGC
>at01231
TTTTTTTTTTTTTTTTTTTTTTTTTTGtTCACACACCACTGTCCAGCTGCAGGTCGAG
GACACCGGAGCACGTGACGCTCTCTAACCCCCGCCCCTGTGGCTGCCGATCAGCATGCTT
GGGTATCGCATGGCCGATCCTCAGGTGGGATCGCTCCGCCAGGCTGAATCTTGATTCCGC
CCTTCTCTTTCCGCTCGGAGTTTGCCCCCCCTCCTTTCGCCGCCTGGTGGTAGACCTCCT
GCAGCAGATGCTCGAAAGTAAGCTTCACGTTGTAGGACGTGACCGCACTGGCCTCCTTAA
AGAGCAACTTGTGCTGTCGCGCAAACTCCACCGCTCCATCGTGGGGGACCTGGCGAGCGG
TGGGATCCTTTTCCACGAGGTCGAGCTTGTTCCCAACTAGCATGATAATTATGTTTGGCT
CTGCATTCTGGCGCAGCTCCTCGAGCCACTTCAGGCAGTTGGAGAATGTGGCTTGCCGCG
TCACATCGTACACGAGCAGCGCTCCGACTGCCCGCCGGTAGTGGGAGCTAGTGATTGCAC
GGTACCGCTCCTGGCCCGCCGTATCCCATATCTGCGCCTTTACAGTTCCGCCAACGGCAA
GGGGTATGGTCCGCGTTGCGAATTCGACGCCGATGGTGGCGGTCGGCGCCTTGGGCAAGG
TGCCCTTCACGTACCGGGACAGCAAGTGTGTCTTCCCGACGGTTGCATCACCAACGAGCA
CCACCTTGTAGAGGTGATCGCAGCTGTCGTCGCCATCATCGTCCGCCGACATTGCCGCTG
AGACCTCCCTCCTCAGGCTTGCAGACCGGCGCGAGCCATGGGATTCCTGGCCAGAAAGGC
TGAGCAACCTTGAGCTAAAGTGCTTGAGCCAAAATGGCTACGG
>at01232
CTCTGTAAGGCAAGGCTGCCGATAGCTGGGCAGGACCTGGGACTCACTGCGCCAGCTAGG
GGCCTGAGGCTGTCTCTGCTTCTGGACCGCCACCCGGGCCTTCATCTCTGAGTGCTCGGC
AATGCCGGGAGACGGTGATCCCTTCAGCTGTTCCTGGGGCTGACGATGGAGCACGATCTG
GATCATCACCGCTGACTCTGTGACGACCATCTGGCGCTCGAGCTGCTTGCCCACTTGGAA
CTCGTTCTCGAAATCCGCAGGGCCCAGGGTGAGGAACTCCTCGGGGAAATGCTGCCAGAT
GTTCTCGGCATAGGAAAGGAGCTCTGGCGATAGAACCATGACCTCGCCGGAATCTAGGAT
GCGTGGTCTGCAGGTGCTACCTTGGCTTCGGTCTTCGGTTCGAGCAAAGGC
>at01233
AACTGCGTCTGGAAAACACTGCCCACCCTGGCAATACTTGTACGATATGTTGAGAGCTGA
CGCCAAGTCCATCATGATACAAGTGGCCAGAGATATACCGGCCTTCGGGGCAGCGAAAGC
TCCATATGTAGCAACACCAGGCTTGTCAACCGAGGTGCCATGGCATTTCGTAGGCGGCTC
TTTCAGATGCGACGCCAGCCACTGGCCCCGCTCGGATGCCGCCAGGTGCAAGTTCATCGC
TAGTCCGCCAGCATGGCGTGCAAGAAGAAGACCGATGATAGGGAAGAAAATGCATAGGTA
AGATGTCCGGTACAAGAGACGCTTCCGTGAGGTTTCCGGCGCAATGAGCTCGAACTCGGA
ACGGCTGGC
>at01234
ACAGTTGTGGAATGACCGCTGCCACTCCCAAGTCTCCATGCAAGACGGCAAGATTGTGGA
GGCCGAGGCGGGCGTTGGTTCCAAGAAGCACAGTGTCTATGGAGGTGTAGAGTTTGGCCT
GGACAGGTTCAGCATTTCCAGGCCCAGCTTGGCATCAGGGGCAATGCTTGCACGTGGTGC
TGCAGCTCAGATGTCGCGAGTCCTTGGAGTTACAAGTGTGAGGCAGTTGGGTCCGCCCAT
TCCGGGGGCAGCCCTTGCGGGCTGGCATCGAAGCGCAGGCACATCTTCAGGGTGTAGACC
TGAACTTGTCAAAGTTTGACTTAACCTCTGCGACTCGAGAGAAGAGTGAGGGCCCCGCAG
GCAGCTGGGGGAGCCACGCCAAGTTATCTTCAGTCTCAGGAGCGGACGCAAAGGAGGCAT
TCTGGACGAACGTTGCAGTCGGCTCAGAGACAGTGTTCAGCGAGGAGGATgGTGAGCCTG
CTCCGCGCCGTTTTCAATCCACATCTTTCAGACAGGCGTGAGGACGGCGACAAGTTTGTC
CCTCCTGACACGACCTTTCGTCATGTGCTGAGCTTGCGCCAGCTGGTGGAGGAGGAGGCC
AAGGCGCGGCAGCGCCGGAAGGAGCATTTCTGTAGCAAGGCGTTTGTGGTAGGCGATGCT
GGTCCGTTGTTTCCCTC
>at01235
TTTTTTTTTTTTTTTTTTCCTGGGAGCACACCAGCCCTCATCAAGCGTGAACGTGCCTGA
GGGTGCCAGTGCGCCGCGCCTTCCGCGAGATCTTTGCTCACCACTGCGCAGACTCCTCGG

GGTTGCTGCGACCACGAAGATGCCGGTGTTGTCCACATGCAAGTCGTGTGAGACCGCGGG
GCGCGTCAGGCGGTGCGCATCAGCGCCCTGCGGCTGGTCCTGGGCGAGTAGACGGTCCTG
CTGCCAGGACCAGCGCTCGTCAAGTAGGACTCCAGCGAGGGCGGCACGTTGCCCCTCATC
GGTCGACCACGATCCTCATGCCACTCCCCTTTCCAGGGCGTAGCAGAACACGATCGTGTC
AGCGATGTTCTTGAGGACGTGCATGAAGGTGGTGCCGATGAGCGCGCCGAGGAGGCCGCC
CAGGACGGTGATGGGCAAGGGGTTCTCGACGTACTACGGCGATGACGGCGAGGTGAATCC
ACCGAAGGTTGTGGTGATGCACCAAGTGAATGCA
>at01236
ACCTCGCGACCGCGAGATTTAGATTTTTGTTTTCGNCCATTCGTNACAAACTCTCTCTCT
CTCTCTCTCTCTCTCTCTCTCTCTCTCTCTCTTGCTCCCTGAACAGTGTAGCACAAAATA
CTCAGCGAGCCAAGGGATGGCTAGAAGACTAGACCTGCACAGAGAGCTCAGCCAGACTCC
GGGCTCCTGCGGAAGTATGAGCCCTTGCCCGGGCCGTCAACCTGCTTTCCAAGAGTGCCA
AGGCCCGTGATATCGTCAGTGGGCTCCTGCTGACCCTCCCACTCCAAGTAGGCCAAGACG
ACAAAGACAAATGGGACCGCGAGTGCCGTAAGCTCAGCTCCTCCAAAGCCATCATCTTTC
AGCTCATTCCCGAATGCAGAGGCAGCCTGTGGTGCTGCTGCAAGGGCCCCTGCCAAGATC
GCCCCGTGTTGCACGTGGCTTCGAGCCGGAGCTTGCGGTGGTGTGAGAAACGCAAGGCCC
GCAAGCTGCAGCAGAGCAATGACGGCAATGCCTCCGACCAAAAGCCGTGATGACTTACGA
GTCATCTCTGGGACGTGCAGTGGACTC
>at01237
TTTTTTTTTTTTTTTTTCATTGGCTCAGCCTTGCCCGGATGAGTTCCGCAATCTCAGCC
CGGCCCTGCATCATTGCCTCGTCCAACGGCGTGCCATATTGGCCACGCAGGCTAAGGTCG
GCCCCATGCTCCACAAGGAGCCGAGCTATTGCCACGTGGCCGTAATAGATCGCCAACGCC
AACGGAGTCTCGCCCTCGCTGTTGAGCGCGTCCACACTGGCCCCATCCCTCAGGAGCTCC
TCCACAACCGCGTGGTGGCCGTTCTCAACAGCCAACAGCAGTGCAGTGGACCCCTTGTCG
CCCTCCGCCGCATCAACACCGACGCCCACGCCCAGCAGCGCTTGTGCGATGTCAGGGTAG
CCGCTCTCTGCAGCGACGTGCAACGCCAAGGCACCACGGCCATCGGGGCGGGAGGCGTCG
GCGCCGGCCCTCAGCAGCACGTCCACAACCTCCTTGTGCCCGTCTTGGGCAGCGATGTGG
AGGGGGGTGTATGAAGTGCCACCTTCCGAGGCCTTGTTCGCATCCGCCGCCGCCTCCAAC
AGCCTCGTGCC
>at01238
TTTTTTTTTTTTTTTTTCCGGAAGGGGCCGCATTCAACACTGCGCCAAGCAACGAATGC
TGCCAACCCTCGTCTTCAAATGCTGCATCTTTGGAGGGACCGTACGTCAGTTGTAGGCGC
GGTGCTTGCACACGCGCATGGGCACGCGGATTCATGGGCATAGCCAGCCTCGTGCC
>at01239
TTGTTTTTTTTCTTTTTTTTTCTTGCAGAAGCAATGCATTACTCCAGTGGGATGGCTCCT
GACCAACTGTACAAGCGTGATTTGACAGAAGCACCTTCAAAGTCATGCACATGCATCATT
AACAGTACTTGGTCCAACTCAGGACAGAAATTTGCATTAAAGACCTACGTGATCTTCCCA
CCAAGGCTCAGCTGACTGTGCAAGAAGCCGCCTGGTGAAAGATTATGTCATTAAGCTTAC
CTAACGTCTACAGCGTTCAGCAACCTGATGGGTTCATGGGAACAAAGGGGGTGCAAATCC
TTCACAGATGTACTGAAGGGTCGGCAGCGCCATGGTTGTCGCAGGAGTGATGCCAGGCGT
GACACCATACATCAGGACGCCTGCAAATGCATTGTACACGCACCCAGTGAAGCCCATGAA
ATTGACCTCGATTGTCAACTTCCCGGAGAGTCCGACGAAAGATGCCGCAGCGCCGAAGCA
GGCCTCTTTTGAGGGAACGGCTGGGATATTCCCGCATCTCTGGCCGGCCACCACCTTGGT
ATAGACAGCAGTTGTGGTCGTGGCAGTTGTGGTCGTGGCAGTCGTGATCGTCATGGTCGT
GGCCGTCATCGTTCCAGTTGTTGTGGTTGTGGTCGTCGGGATGCCACTGCAGACATACTC
AAAGGTGGGCAGCTTCATGTTCGATTCGGAGGACATGCCCAGGGTCGTGCCATACATCAG
CATGTTGGCGGCGGAGTTAAAGACGCAACCGGTAAAACCAAGGTTGTTCAGCTCCATGGT
CATGGTGCCGCCAAGTCCAACCACGCCCATGGCGGCATTGAAGCAGGCCTCCTTCGACGA
AACGTCAGGCACGCCCTCGTCGGAGCACCGAGAGCCAGCGCCCAACTTGGCAAAATTGGC
GCT
>at01240
GGACGAGCCTCCGGAAAGCGCCCGAGGTGGCGCGCTTGATGTGCGAGCTCGGGCTCTCAG
CCAGGACGGGGCAGAGTCGCCCCGCCAGCTCGCGCCGGACTCCCCCACCGAGCCGAGACC
GAAACAGAGGCAGGCGCCGCCGCAGCGGGAGCCGAGGCCTGTGGCCGTGCCCGCGATCCT
CGAGCAGTGCCTGCAGGACGCCAGCAAGGCGGCCGAGCTCAAGAAGGACGCTGAGGCTCA

GGCGGAAGGGAGGCCGCCAGCCCGGAGGTGGTCAAGAGGCTGGAGGACCTGCCCAGCGGT
TGGGAGGCTGCCTGGGATGAGACGCACCAGAGGTACTACTTCTACAACCACGAGCTCGGG
GAGTCCACCTGGGCCTGGCCAGATGGCACGCAGCAGCAGCGAAGAAA
>at01241
TTTTTTTTTTTTTTTTTGGAGCAGTTCCGCGTAAGTTCACGCTGCCGGCAATGCTGCGG
CCCTGCACCCCCTACCAGCCCTCGCCACAGTCCACGATGGCGACCTGCGTCGCGGGCTTG
TCCGGATACTCCGTGTCCAGTTGGTCGATGGCGTCGAGGATCTCCAGGCCGCCCACAACC
TCGCCGAACACCACCTGCTTGCCGTCCAGCTGCGGCAACGACCGCAGGGTGATGACGAAC
TTCGAGTTGTTGGTCCCAGGGCCTGAGCTGGCCATGGACAGCAGGCCGCGCCTGTGGTGG
GCGTCGCTGAAGCCCTCGTCGGGGAAGGTCCGGCCGTAGACGGACTCGCCCCCCGAGCCG
TCGTAGCTCTCCACGTCGCCCGCCTCCAGGAAGTGCCTGGGCACGAGCCTATGGACTCGG
GTCTTCTTGTAGTGCAGAGGCTGGCCGCAAGAGCCCATGCCCAATTTCGCCCGTGCACAG
CGCCCGGAAGTTGTTCGCCGTTCTAGGAACAGTGTCGCTGTAGAGCGCTATCCTCATGCG
CTCCT
>at01242
TTGTTTTTTTTCTTTTTTTTTGTTTCAACAGCCCCAGTCCAGAAGAACGACAGTGTCAT
GCGAGAAGGGTCACACACGGTACTTCAGTTTCCCAAATGGGACTGCTCCTGAGCATTTGC
CTGGAACTTCTGAATGAAGCACGTGAGTGCCTCCACAATTGGATCCTGAGCCCCCATCCG
TTGATCCTTTTCAAGGCTAGTCTCTTTCGAGATGTCGTCCTTAAGCGTCTGAATGTCCTG
CTCTAACTTATCTTGTCTTCTCGATAGATGCTCCTGCTCGTGTTGCCACTCCTTCAGAGG
TTCAGCCACACACTACACAAGACTTGAGCTAAAACAGGCCCCAAAACCTAGCCTCCAGCG
CTAGCCACAGCCCCGTCCATCTTGCTCTGAATCCCCGCGTTCCGTGTCCCCCGGACCTTC
TGCTCCCGATGGAGTTCGGCGGCCAGCTTGTCTATCTGCCCCCGGAGTGGCTCCAGGACC
TCAGTGGCCCCGCGCAGGCGTGAGGCCCGCTCCTCCTCAAGGCCTGCACGGACTGCCTGC
CCCGTGGTCTCAAGTTGCCTCAGTCGAGGCAAGAGGCCATCGATCCGAGCGCGTAAGTCT
CCATGCAGACCGTCGAAGGTGCCCTCTAGCAGGCCGCGGGAACTCTGAATGAAACCGTCC
ATATAAACCGTCAACTCAGCTATGGAGCTGTTGATGTCAACTCCAATCTCGTCAAGACGC
TTCATCTGATCCTTGTGAAGTGCCTCGACAAGGTCACGCTGTTGATCACGTGATCGTGCC
TCCTCGTCAAACACTGTGCCGATCCTGCCCTCAGCGGAAGGCTCTCTCTCGTCTTCCCAG
ATGGTCTCCACAGCAGCAGGAGTCCTCATGGTGGCATCCAAGCCAGTGTCAATGCCGGAG
CCATGCCCACTGGCATGGTGCCCTCCCGATGACAGCTTGAAAGTGGAAATGTCCTTGGAT
CCAGCATTGCTGCGCACGCCATCAGCTCCCTTAAGCGTCATCGAACCAGCGATCTTTAAC
GCCGATGAAGACGGAGTAGCCTTTAGGCTTTTCGAGGCCATGATCAAGAGCCTCAGTGGA
GTGGCGCAGAGTTCGGGGGGCATGCGCGCGTGCGCGCGATCCGTGGGCTCGAGCCGCAGG
TGCCTTGAGCCAAAATGGCTACGG
>at01243
TTGTTTTTTTTCTTTTTTGGTACGTCGCTTGCAGAGTGGCATCAGCCAGAGAGTCACAC
ACTCCACCAACATCTTATCGTTACAATGGCCGTGCATTTGGGAACATGGCTCTCATCTTC
GCGCTCAAATGCCACTGAGCACTCCCTCCTCCCACTCTCCCTCCTCTACGTGGCCTTGTG
CGTCTGTGATGCGGCCAAACCCGTGCAGCTTTCCTTGCTGGAAGATGCCCTCAAGCACGG
CGCCAGATGGCTTCACCTCACGCCCACGGCCATCGGGAAGCCCTGCCACCACGTGGCCTT
CGAAGTGTGATCCGTTTCTCAGGAGGCCCTTGCCGGTGCATGACCACAAGAAGCCGCCTC
GAACTTCACCATCAAAGCCGCAACCGCTGTAGGGGTCGAACACCCGGCCATGGCCAGTGT
CTGGGCGGAGGTCACGCCACTCGCCTTCAAAGCTAGCCTGCTGATCACTCAGCGCCGGAA
GGACTCCGCTGGCATTGTATCGAAGGATGGGAGGACCACCGCCGTAGACCTGGACGAAGA
AGTCCCACATCGGCTGCTCGAGGACGTGGTAGTCGTGCTGGACGCCCTCGCGCAGCCCGG
CTCGCAGGGAACCGTCCAGGTCGAACAAGTTCCATGTTGTTGATGGGTGAAGGAGGCCTG
TCCTTCGTCTCGGCGCTCGGCCGGCCCACGCCCACGAAGCTGCGCCACTCGCGCAGCCAC
TTGGAGCTGATGACGTAGCCGAACCTGCCGCCCATCCACCAGAGCTGCGTGGGCCGCCGC
TTGAGGTCGGCCTCAAGCGCCTCGACCTGGGCCAGCTCCTCCGTGCGCCGCATGTGCAAG
AACGCACGCTGCTCTGATGGCGTCATGCTTGCAGAAGCAATGCACCACTTGTCGAGCTGG
AGCCGCAGGTTCGGGGCCGGCTCCAGCACGCAGCGCCGGGAGTGCTCCGCGACCTCCTCC
GGCCGGACGCGCTCCCCGCAGACGCAACACCTGATGCGGCTGTGCTCCTCGATCTCCTCT
TCCGTGAGTGGTGGCTGCTGCGCCCTGGATGGCGCCTCGCGGCCTGACCCACTGGTGGCG
GCAGCCTGGGTGGATCGCCCGAGAGTTGCCTTGGGGTGTCCGAGCACCACTGTCTGATCC

TTGTCTGCGGCTGCAAGAATCATGGTGCATCCTCCTGCTGCAACCCCGGCGCTCTTTACG
GGCAAGCAAGCCCAGTGTCATCTCACCCCAGCTTGGGCCATGATGCTGCCTTGAGCCAAA
ATGGCTACGG
>at01244
TTTTTTTTTTTTTTTTTAATGATGAGACGCTCCACAGAACCGCCATCCGCGCTTCTACT
ACTTAGGGAGGGTGTAGCTATACAACAGGAGGTGCCGCGACGATCTTGGCAATGCGATGA
CTCAAGTAGTCGAGTGTGCCAGCCAGTTTCTTCACCATGCCCCTCAGCGCGGACACTTCC
TCCCGGTTCTTTCCGAGCCTCGCGTCGTGGTCCTGGGCGGCCAACGTGACCATGGCGATC
AGGTCCTGGTACACCACGTGCTTCGTGCTCCCCACCTCCCGCACCAACTCCGGCAAGGCC
CGCTCCACTTCCTGCGCCACGAAGCCGAAGCGCGGGCTCATGGACTTGGAGTCGCCGCCC
TCGCGGAAGGAGAAGGAGACCGGCCGGAGCTCGCGCAGGACCCAGGCCACGGCCGTGGAG
CGCCGCGCGGCGGGCCCGCCGCCGCTAGCGGCGGGCAGGCGCGGCGCCAGGGCCCGGTGC
AGGGGCACGACGCGCGCCCTGAGCCTCCGGTCGGAGGTCGCGACGCTCGTGTCGGCCACC
CAGACCCCGTGGA
>at01245
GAGCTCAGCATAGGCTAGACTCATGTCAGTAGAGGTTGACTTTGAGTGCACAGGTTGCGG
ACGCATGTTGCGCAGCGCCTGTCCGCTGCAGGCGGCAGTCTTGTGGCCCAAGTCCTATCG
ATGGTCTTGCCGCGGGTGCGGGGCGGGTTGCCGCTACGTCCTCTTTCTGAGACTGCATCG
ATTCTGCTACAGCGTCTGTCGTTGCGCGCCTGATTGGCAAGTGCTCCCTGGCTCCACTCC
AGTGGATGCCGCTTTCCGCACCCCTGCGGGGCCACCAAGGGAGCCCATACCATGCAATGT
CCGCCTGGAGTGGTTCCTGCAGCAGCATGCCGCCGCTGCTGCCCATGTGGTGCCCCGCAT
GCTTGCCTGCCTTCAGCCGGTGGATGAGG
>at01246
CCGGCTGATGCCATCAATGCCAACGGCCGGAGGCAACTGCCCTGGCCGACGCCCGAGCCG
ATCGCTGGCCAAAGGCCAATGGCGAACCGGCCCCGACGGCGGACTAAGGCAGGGATCCAG
ACGCTGCCCCCGTCGGCGTAGGCTGCCGTGGGCGCGANGACTGCCGCAGCACTCAACNcC
ACCNNgGGAAGCCTGGCNACAGGNGCCTCCACGtCTGCANCAGGCAGCCTGTGAGCgACg
GGGGATAAAGGCGCAGCTGGAGATCAGTGCCGCGCCGGCAAGCACCACGGCGCCTGTGAG
GATCATGGAACGAGGCGCGGCCATCGTTGGTGAGAATGTGGAATCACAGTGGAAGCACTT
ATGTGGGGCCGGGAACCAAGCTTGAACTGAAATGGtCTTACNaNNNTaAgGNNCTAAAAT
TGGTCTTACGGTACTTCTTGTTCGTTTGTATTACCtACtTGCTT
>at01247
CTGCAGTTGCTTTCAAGAACTTGACCTTCGTGCAGATCAAAGAATTGACAACATACTTGT
GGCTTTGCTTATCAATCATTGCTTGCCGGAGATGTCCAAGGACGATGTGATAGGTGTCAT
TCCGAAGCACAGGCTTTGCCCAGTCAAGAAAGATGTGCGGGGCACGGTCTCGACAGAGAG
GTGCCTGCCATGTGTTGAAGATATCCTTGATAAAGACGATGTGGGTGAGATCCGCGAGCA
AGTTGAACAGAAGGGCCAGAAGAAACTTCAGTGATTTGCCAACAAGATCCGTGCATGCCG
CAAGAAAAAATGCCCTCAGCATAAAAAAGCCGACCAAAGAAGAGGCCCATAACATCCTGC
CAGAAGTCAAGGGTTGCCACCTCATGAGGAATCCAAAGCGACACATCCCCTCGC
>at01248
CAATCGCTGTCCTCACCAGGATGGACAGGAGGCACTTCCATGTCGGCCCTGCCAATGGCT
CTCAACAGTGTCGCGTCATCGACATCGTTACCTACACACTGACGGTAGCAAGTCTCTATG
CCGGAGCCATGGAAAAGCTGCCGCATTGTTGTGGGCACTGGCGCCACCGGGAAGGAGGGG
CAAAGGCACTTGAGTTGCATGCATGCCCACCTCGCCAATGGTGGGAGCGTTGCCAACTCC
ACGGGCAAGTCCACTCGCAAGTCCCTTGGCAAACCATTGAAGACGCTCGTCAGGGTGGAC
TCAATGATCGTCGATGCGGATGCATGAGCTGCAGAACAACTCATCGATGCACGGCCAAAC
TCTCGCGGTAACAGCAATACGCTAAG
>at01249
TTTTTTTTTTTTTTTTTCCCCGAGTGTCCTGCGAGGCAGAAAGCCTTGGAAGACAATGC
TGAAGACTGTGGTCAAGATGACGGATGCCAGCGGTGGAGGCAAGAGCTTTCCGACCACGG
GTATCCTTGCCCACACGACAGCCGAGGCCATCGCAAACAAGGCCGTAATGGTCATGCTTG
CAATCAGCTCCTTCTCCACCCAAGGGCCGTCACCATGGTAGTGGTACTGGCGCAGCTGGG
CTCGAACCATGACGATGGCGAGCCCATTCACGAACCCGGTCATCACTGAGTGCGGGATCA
TCGTGATGAAGCGCGAAAGCCGCAGCATGGCGGCAATGATCTGCAAGATGCCCACGATGA
AGACGCAGAGCGCCATGGGCCCCCTGCCGAGCGCGGGGTCCAAGGACACGTGCGCGAGGA

CGACGGCTGTGGCTGCCGACGCGCTGGAGATCATCCCGGGACGACCGCCGATGAGTGCGG
CCGTGATGCCCATGATGCAGCCCGCCCAGATTCCGTTCAACGGGGAGACCCCAGTGATGA
AGGAGAAAGATATAGCGTCAGGAAGGGCGGCCATCGCCGTGGAGAAGCCTGCAAGGGCGT
TCTGAAAGAAGGAGCCCTCATCTGAGTCAGACGAGCTTGTCGGCCGCAGGCTGCTGATGG
CACGACTCGCCTTTGGGGCCGTTTCCTCCAAGTTGTACTGCGATCCAAGCCGTTCANTAC
CATTGCTCTGCGTCTCTCCGGACTTCTCGCCCGTCTTGCTGGATATCGCCATCTCTCCAA
CCTTGAGGACGCCAAAGACGACGAAGGGCACGACCAAGCACGCAAACAGGAGGACGACGT
CAACAACCTTGCGTGCNACTGTATGCTCACCCTCANAGTTGATGG
>at01250
GGAGCGGCTTTCGCTGACGCGACCGGGGCTGAGGACTGGCTCAACGAAGGCCTCCCTCAG
CAGCGGCTCGATCGCTCGCAGCGCGTTGCCGCCGGACACGCCGTCCGCTGGCGACAGCCT
GGCGACGGCGTGGCGTCGCTCCGTGAGCTGCTCCAGTCGGCTCAGGGCACGCACGCGCTC
GTGGTCGAGAAGGAGGGGCTCGCGGAGGAGGCGCAGAGGCTCCTCGTGCAGCCGTGCGTT
GACAAAGTTCGGGACCTGATCGGGCCCAAGGTGGGCGACGAGGCGCCATCCGAGCAGGTC
AAGGAGAATGGCGCCACCTGGTTTATGCTCCAGGGCCCGAAGACGCTCGGCATCGCTGTA
GCGCCACCATGTTCCGAACAGCAACAGCCATGTGGCGATGGATGGCGGCAGGAGCAGAAT
CAGGCGCTGGCTGTAGGACGCGCCAAGCGCTGCGAGCTCCTCAGACTGCACCTTTGCACG
TGCGAGGCTGAGCTTCGTGGCCAGCGGAATCCCCGCAGCCGGGCCTTTGGCATGTGAAAG
ATACAGTGTCTTCTTGAAGACGTTCCATGGCCGCAGCCCCATGCTGGCACACACCCCAAG
CGATTGAGCTTGAGCCAAAATGGCTACGG
>at01251
CCGTAGCCATTTTGGCTCAAGCCTGAGCTTTTGGCCCTCGCCCGCTTTCTTGAAGTTGTT
GATGAGCCGGCGCTGAAAGACCTGCTTCTGCAGCGTCTGGGGGTGGCCTGCCTTGAGTGC
TCCTCAAAGGCCTTCTCAAGCTCCTCGCTCGCCTCTGCCCACTTCACGTGAGCTCTGAGG
CTCTTGAGAACTCTCCGCCACACCCAGCAGTCTGGAGCGCCACAGTTGTGGCCCTTCTCC
TGCCCCACTGCACTCACGATATTCCTTAAGCGAGGCTTTGCCACTGAGGTACACAGGATT
TTGTTGGCCTTGGCGGAGAAGGAGACAAAACCCAGAGCCTTGGAGCCGGCTGTGTTCTGC
ATGGTGAGGCGAGCAGTGAGGAGCACCAGTTCACTCACGTCAGCAGCATGGTGAAGTGAG
CAGCGAGGAGCGCCAGTTCACT
>at01252
TTTTTTTTTTTTTTTTTCAGAATGCTGCACAGGGTACCAGACGAGTCAACACATAGCCT
CGCAACGCGATCCTGTAGCTAGGCAAGGCTACGGAGAGGCAAGGACGGTCTGTCTACCAC
GTCTTCCCCCCGCTCAGCCACCACTCCGCGTAGCGCTCCATGGTCAGCGCCTCTGTGCTC
AGCCCGTCGGGGTTCGCGAAGATGCGGCTGAGGTCCTCGTCGCGCTCGAAGATGGCCTGG
CCATGCGTTGTGAGGCCGCGCCGCGCAGCCCGGCCCACGTACTGCAGCATGCCTGCCACG
GAAGTCGTCTCCGCGAAGGACTTGGTCACCACCACCGTCGACGCGGGCACGTTGGCGCCC
CACGTGAACTCCTTCCCCGCGGTCACGCAGGCAAGCTTGTTGTTGGTCATGTTGTCATGC
ACCCACTGCGTGTACACAGGGTTCCCCGGATCGACGTTGAGCTTCGGGTCGAACGACCCT
GCTCCGGACAGTGCTAACATCTGCCATCTCTCATCCATGGGGAGCTCCTTGATGGCAAGG
AACTCCTTGACGGTCGGCAGGAGTCGCACGATGGTGGGCGAGTTCTTCGGCCTGTTCGGG
CACCACCGCTTCAAGAACTGCTCTGACTGAATCACGAGGT
>at01253
TTTTTTTTCTTTTTTTTTTGCAAGGACTTAACAAATCTACTCGCAGGTCTCTCCTCCAACC
TACTTGCCGGAGGCTGCCGATACGGCAGCTCGCAAGCCCAGCAGATAGCTGTCCACCCCA
AAGCCTGCGATCTGGCCCTTGGCGATCTCGGCCAAGACGGAGTGGTGGCGGAACGGCTCT
CTCGCGTGGATGTTCGACATGTGCACCTCGATGATGGGGCACTTGAGGATGGCCAGCGCG
TCTCTGATGGCGTAGGAGTAGTGCGTCCAGGCGCCGGCGTTGATGACGACCGCGGAGACG
GCCTCTTCGTGAGCGCGGTGCACCCGCTCGCACATCTCTCCCTCGAAATTCGTCTGGAAG
CACTCGACCTCGACGCTCAGCTCCTCGCTCAGCGAGCGCAGGCCCGCGTCGATGTCCTCG
AGCGTGGCCGAGCCGTAGTGCGCTGGGTCGCGCTTGCCGAACATGTTGAGGTTCACGCCG
TGCAGGACCAGTACCTTCAAGGCTGGCATCTTGGCCTTCTTGGCCGGCCTCTCCTCGGAG
CCGGCGGCGGGCCCCGGCGCGGCCATGCTGCGGAGTGGTTGGGAGCGGCGCCGGCCGGCT
CGGCAACTGCCTTGAGCCAAAATGGCTACGG
>at01254
TTTTTTTTTTTTTTTTTGCAGAAGCACCACAGGGAACTGCGTTGACTCAGGCGTACATC

TTCTTCCTGCGCGCCTTCGGCTTGGCCGTACTGCCCCCCTGTACCTCACCATCCTCTGCG
GTCTCCGCTCTTGCCCGGAGCCGCTCCGCGAGCCGCGCCTCCAGGGCCTCGGCGTCACTG
CGGAGGGCCTGGAGCTCGTGCAGCAGCCCGGGGCCCGCCAGCACGGCCCCAGGCGCTTCC
GCGGAGTCGCCGGCCTCGGCCCTGCTGCTCGGGCCCTCGTCCTTGCAGTCTCTGAAGGCC
TCGTGAAAGTCCGCGCGCAGCTGAGAGTCCTCATCCGGCTTCTCGCAGGGCCTCGCAGGA
GGGCCGTCGGGCCGGGCAGCACTCGAGGACGCGCCGGGCCGCGGGCTCTCGCCCTCCGCC
GCCGGCGCNTCCGCGCCGCCGCTGCCGGGCCCCGCG
>at01255
TTTTTTTTTTTTTTTTTCCCAAGATTGCAATGGCACAAGTCTGAAATAACTGGAACATC
CAGTTTAAGCCATTGCAGCTTTGCAGAAATCAGCCACAGGAAAAGAATGAACACTGCCAA
GGCGTCTGGACACATTGTCCATACAACCTCTGATGCCTTGGCCTGTCAGCCATGAAATGC
AAAATGGCGCCTCCGCACCGTGCACTGGTGGGCCAAGAGACAATGCACTCTAGGGCACCC
ACCCAGATTGTACCATAGCCTCTGGGAATCCTACGTACCTGCTGTTAAGAAGGCTCCAAA
AGTGCTGGGGCAACGCTGCCTTCTCCGCGCGCCAGTTTCGTGGGCTCCTCGTGCC
>at01256
TACGACGAGGGCCGCATACAGGCAGCGGTTTCCAACATCCTCGAGGACAAGCGCGGCCAC
GAGCATGAAGAGTGGTCCACAACAGCCACAGCCAGCGAGAAGAAGCTGCGCGCACAGGAG
GCCAAGGAGCGCAGGATACAGCGCGAGCGGGAACAGGCTGAGGAGGCAGAGCGCCAGCGT
GCTCAGCGGACGAAGGCAGCCGACGAGAAGATGCGACGCACGATGGAAGAAGCTGAGAAG
CGGCGGTCAGCAGATGCTGTCTCGGGACCACACGGATCACTTCGTGGACCTGGCCGGGGC
TGGGGTGGATGGCGACCTGCGGCACAAGAGAGCACTGCCTCCCTGACCAGAACGATGATC
TCCATGAAGCAGCTTCTGCCACCTGTGCCGATGACAACGCACAGGATCCTGCATCCTCGC
ACGAGG
>at01257
CACGGAAGCTACGCCTGGCTTCCTCCGCTGTGGCCGGCCGGAGTGGCCCATGGAGACAAC
TGATGGCAAGTCGGAGATGGTCTCCAGAACCCCCTTGGCCAGGCGTGTGCGCACGGCGGC
ACGGGCCAGTCGGTGCTGCTTCACCCCTGAGCTCAGCTCCGCAGCGCGGCGCGGTGGCCG
TGAGAAGTCCGACTTGCTGCACTCGAGCTCCGTCCGCTTGATGCGCAGCTGACGGCTCCT
CAGGGACGCCCGCTGTGCCTTCAGCAGGACGGGGTCGACCTCCCACTGACAGCAAGCCAT
GCCGCGAGGAGAAGAGCAACCGGCGGTTCTCGAGTGGAGCTTGATGATGGAATTGGGCCC
AAATGGC
>at01258
TGCCGCCAGTCGATGCGATGAGGGTCACCTGCCAGCGTGCCAAGGAGGGCCTAAACACCA
TAACTGTCACTGGCAATGTCCTCAGGGACTACCTCACAGATCTCTTCCCCATCCTGGAGC
TAGGCACGAGTGCAAAGATGCTGTCCATCGTGCCAATGCTGGCTGGAGGTGGCATGTACG
AGACGGGGGCTGGCGGATCAGCCCCAAAGCACGTGCAGCAATTCCAGAAGGAGGGCCACT
TGCGATGGGACTCGCTGGGGGAGTACTTGGCCTTGCTTGCTCCATCGCCGACCTTGCAAA
GGNGACAA
>at01259
TTAAGAAGGTGCTGTTTAATTAGACTATTGATGACAGTACATCTAGATCATTCTCTTGAT
GAGGTGGTTAATTTTAGACCCCTGCATACCGCAAGCTCCGCCCTCATTGAAGTGAATCAA
CTTCTTCTTGCCACCATAGCCGTGGCGAGGGGGAGTTCAACTTGAAGGGCCACAAGAAGT
TAGCAGCTTGCTTGAAGTTGGGGCCGACAGTGAAGATCTCATGGATGACATCCTCGATGC
AGTTCAGCCGAACTCGCCCAGAGCAGCACCTGATGAC
>at01260
CCATCGGAACTGGCTTCGTGAAGAACTCCTGCAGCCTGTCCGACCAGGCGTACATTGTCA
AGTCCCCGCCCGGGCAGTCCGGTGCCACCGCCTTGCCGACGACGTCCCACTTGCCATCCG
TCATCACCTGTTGTGCGTTTCGCCTGGTTTCATTGAGTATGTTCGTCTGTGGCTCGGCGA
GGACCATGCGTGGACTGTGGGGCTTCAGCAGCTGCATCAACGGACTGTACATCCTCTTGT
CCTCCCAGAAAAGTGTTGGCTCCAATGTGCAAGACCACTGGAGTGGACCCACTCCTTCCG
ATCTCCTCTGCCCGCGCAGCGATAGAACTGTAGAAGTAGGGGGTCCAGAGCCGCGGCATG
AGGGGATGCGTAGGGCAAGAAGAACCAATCTGCGGGCTCTCCATGACATCGCAATTCATA
AAGTCGAGCTTCAGCCGTCCCCCTTGCTGTCCGTTCCA
>at01261
AAAGCGTGCACCCAAGTAGCAATGTTGAAGCCACTGATCTTTCCGCCCCATGCGGCCAAC

AAGGGCGGCGAGATCTCCAGCGGCTGCGAGACTCCGTGGCCGTACACGACAATCTGGGAG
TCCTTGTCGCCGGACTGCAAGCAGTGGATCAGCTGCTCGCAGGAGTCCCGGGTGGCGACG
CCGTCGAGAGCCAGCTTCGGCAGCATCCGCTGGTTGCGGTCCCTCCACCTCAGAAGCGCC
ACGGCGTCGGGGAAGACCTCCTCGGCGCCCATGGTGACGAGCCACTCGCTGATGCGCTCG
TACTGCGCTCGAACTGTCGGAGGCGCCACTGCACGAGAGTCGGCGTCCTGCCAAGCTGCC
TTCGAGCCATACTCGCCCCTGACGATGTTCTTCGGTGC
>at01262
TTTTTTTTTTTTTTTTTCGCAAAACCAAAGCCAGCCCAGCAGCTCCCGCGCCAGGACCC
GCCGGTAAGCGCCCCCTANNNNNNNNNNNNNNNNNNNNNNNTTGCCCGCTTCTCACTTCTT
GCCGGTCTTGCCGCCAATCCTCGGCGCGTAGGTCTGCTGCCACCGGCCTCCCCCGGCCTT
GCCTCCCTGCGGTGGCCGCTGCTGCTGCGCCTGCTGCCACGCCTGCGCCCGCGGCGCCGG
CACGACCCTCGGCCCAACCGCCTCGGCCGCCGGGGCCTGCGGGGCGGCCTCGCCCTTGGG
GGCGCCCTTCGCTTGCCGCGGAGCGGGCGTCCAGCCCCCGCCCTCCTGCTGCTGCTGCAG
CACGCTGAAGGTCAGTCTGGCGGTCTCGTCGCCCGCGTCGATGCGCTGCTGCCGCTTGCG
GCGGATCTCCTCCTGGAGCTTCGCGAAGAAGCTCACCGCCTCGGGCTCCTCCTTCAGCAC
CGAGACCAGCACCTTGGTGACCTCGTCCAGGCGCACGGCTTCCCTGACGAGCTCCACGAC
CTCCTGGAGGGTGTCCTTTCCATAGGACTTCGGGTTCAGCTGCTTCTTGAAGTACTTGCT
GAAGTAGAAGAGCAGGTGGCTCTCGTGAATGCCAATACGCTCAGGGTCGTCGACGTCGTG
CTTGAAGGCCGCCTGCAAGAGGTGGAGCTCGAAGCGCAGCGTCAGCAGCGCCCAGTCTTC
GAAGGTGAAGTTGCTGAAGAGCGGGTCGCCGTCGCCCACGTCGCANATGTCGTCGACGGA
AAAGATGTCGATGTTCATCTGCTGTCATCCTAATCGTCCTCGGTCT
>at01263
GTTTTGTCCAGACCGGAATACAGCACGTTGGTTTGCCTCATCCAACGGAGAGTTGAGCAC
GAGGAGTTTCTTCATCTTCTAAGTCAGCATCAGAGCGTGCTGTCTCCGCCAGGCAGGGGG
ATACGATTGTGGCCGCACCTGGCAGGCGGTTGCAGTCGGCACTGTCCCACTAGTTGTTGA
AGACCCCGTATTCGATCAGCGTCTGCACGTTGGGGTTGGTCCCGCCTTTCTGCCCTCTCC
TGAGATGCTCACCCTGCGAGTCTGGAAATCTTGTTGTCCGAGCTCCGCGACCCTGCGAGC
ACGCAGAGCATCTGGATGTCCGCATTGG
>at01264
TTGTTTTTTTTTCTTTTTTTTTCCTCATGCACAACCACCGGACAGCAGGTATCTACACTG
TCTGTCCATCACAGTGGCTGGTCCACCTCTTGGTAGGGAAAGCGATTGAGCAGCCAGGTC
CAGCTTAAAGTTGGGGATGCTGCTTGCATCTCAGCGCCCAAGGCTTCGACAGCAACATCT
CTGGTCTGGACCGGCATGGCTGACGGCTCGCCAAAAGAGAGTGCACGACCACCTTCTGTG
GATAGAGAGCTGGTCGCTTGGCAAGTTCACGTCCATGAACCATCGCGACACATCTTCTGA
ATTCTCACCAGGCCTTGCAGCCGCATGTGCACCACATGCAGTACTGCTGGTGCATGCTCT
GGGTTCCAACCATTTAAAATCATTCCTCAGTCCAGGCAGACATTGCCCAAGAGCGATGGG
AGGTCGACCCTCCATGGCTGTCTACGGCTCCAGCAGCCTTGTCCGGAGCGGGTTCGACTC
GCTAAACTCGCGCGCGTGGCCCTCTTTGCGGAGTAGAGCTTTCCGGACCTCTTCGAAAAT
ACTAATCCCGACGAAGGCCATCCACGGGTAGATCATCTGCTCCGCGGTGAGGGCAGTGGT
TTTGAAAATATCGGCCACATCCGGCACGTAGATGATAATAGCAGCAAAGATAACCTCGAG
CACGATGCAGATGTTGAGCTTGGCGTTGGGCAGTTTGTACTTTGCAAGGGACTCGACATG
CGTGGTTGTGGAGAGGGCNGCAGCANTCTGCCCGATGACCAACGTGTAGTAGTACAACGT
TGAGGCTACCATGTGGGTGTGAATTCATCGTCTGTGAGATCCTCTTGCCAATGTTTGCAT
GATG
>at01265
TTTTTTTTTTTTTTTTTTACCGTCCACAGGCTTGGAATCACCGATCAAGGCAGGCCTGAC
TTAAAGTACAGGACGAAAAATGCCACTCCTAGGGCTGCATCACTTAGCCTCGCTGGGCGC
AAAACATCCGTGGAGGCCATGCGGAACAGCATGCGGCAACCAAACACGGCATACAGGCCC
GTCCTCGATCCTCGCAGCGTCGAGCACGCAGAGGCACGAGCGATTGCTCACGGCATCAAA
CAGCACTCCCAAAATCCAGCCATCGTCCTCCCCACCAACAGGAGCACCGGGAGTCCACGT
ACCTTGCGGACCACTGCGTGGCACGAAGTGTGGCTCCTCAGTGACACTTCGACTCCCCGC
GTACCACCTTTTCACCTCACCCGTCTGGAGGTCTGCACGTGCCCAGCCGCAAGGAGGAGT
AGACAGACCGCACTCATTGCCAATGACCATGTAGACGTACCGGCATCGAGGATCGCCCGT
GTAAGAGCAATTGATGGTCGGATGCTCCTCGTGCC
>at01266

CTGCACACATTGTTTTGGACGGAGCAAGGTCTGGTACCGGTGTGGATTTCTGGCATGGTG
GACGCACAGGCGGCCTTGGAGGCAGCACCCCCTCTGCCTCCAAAAGATAATGCAGAAGAA
AGCGCATCGCCGGCAACCGCCCTTGCTACCATGGGTCCGGTGACAGGGCGCGCATTTCTG
ACCAGCTACGACGGAACCGCATCTTGCAGGCGGCTCTTCGACGAGCACTCAGAGTAACAA
GCGAAGCCAGTGCTGCAACCAAGGAGCAGGACTCGCAATTGCACCGCTATGTAGA
>at01267
GGGTTGCCTTGCTTGAATGGACGGATCCGCTCTTCACGGGAGGCCACTGGGTTCCTGGAC
AGGTTGAGGGGGCGGGCGGCACCTACATAGGTGCCGCGGCGGGTGAGCGGAGCAGGGTGC
TCGAGCCTGAAGAGCTGACGGCGGCGGCACCCGACTTCATCTTCATCGCTCCGTGCGGCT
TCGACCAGCACCGCGCTGCGCGGGACGCGCTGCAACTTTGGCAGCATGGCTGGTGGCGGG
AGCTACCCGCGGTGCAAGGGGACAATGTCTATGCTTTGGATGCCAACAGCTACTTTGCCA
GGCCAGGGCCGCGCCTCGTCCAAGGCTGTGCCCTTCTAGCCTTTCTCATGCATGGAGTGC
AGACAAGCACCACTCCTGAGGGTGGCTGGATCCGGGTTCAGCCAGGGTGCGCATCCACAC
CTGCATGAGGTTGGTGGGCCGA
>at01268
ACGCCTTCCACTTCCATTGCATCTCACGCTGGCTGAAGACCCGACAGGTGTGCCCATTGG
ACAACGGCGAGTGGGAGTTCCAAAGTACGGCCGCTGATCTTCCGAGGGCAGGGTGTTGCC
CTTGGTGAGTGTGCGGATGATGAGCCCGATTTCGGTAGTCAGGATGGTGTCGGCTGTCTT
GATGGCCGGAGCATGGACAATAAGGGCTTACTCCGACACCCTAATCATTGCAGTCATCAC
TACCCCAGGGACCCAGGCATATGGCATGCGATTGGACTGGACAGACTCGGGGCGTGTTGC
TCGCTCT
>at01269
TGGCCTTTTGGCGCTCAGCACGCGCTACGCCGCGCGAGGTGGCGGCGGGAGCAGCGCAGG
ACTGAGAGGATCCGAAGGGAGCGCGCCGACGGCGACTGCACCCAGGCGGCAAGCAGCTTG
CTGGCTGCGTCAAAAGCTTCACCACTGGCCGTGATGACAGGTGTTGCAGACGATGTGAGC
GCCAGTGCCCTTAGCGGCCCAACACTAAGCCAGGCGCTGTCCTGTCATTCAATGCATTTC
CCCCTTTTTCCTCCGCGAGCAGATGAGTGAATTTGGGCCAATCTGCCCTCTCCTGCTCCG
TGCCATTGTTTGCCATGCCCGGGCATGTAGCCTTGCACGCACAATGATAGCGCACAGGTT
TTTTTTCATTTATCTTGCGCAGTGTGACGAAGGCTGTTGCTGACGTCGGAGCATACAGAG
GAAGAACAAGAAGAACCA
>at01270
TTTTTTTTTTTTTTTTTTCGACGGCCACCGTCCTGCGCGGGCGTGATGGGCAGATCCCCA
AGGCAGCTCCGTCTGCACACTGATCCCCAGGGAGCGAGGCCCTCTGCACTCCGTCCCACG
CCTCAAGGGCGGAGGAGTCGCAGCGGTGACCGCTCACTGCAGGACAGACGATGGCGCCAT
ACCCATCTGGCAGGCGTGCTCGTCCATCATCGGACGTGCGCGTGCTGACCCGAGGACGAG
GGTGGCTGACGCGGATGGCATGCAGACCAGAGATGCACGTGCAGGTGGCAAACGCCCTCG
CGGCAACGAGGAACAGTCCACACGTCACCGGCCACCACCTTGCATTGTGACTCAATGCTA
AGCGGGTGCATCTATCTGATTGCCATGCCGGCGCCATCGAATTCACCACCTAAAGGGCAC
TTCGGGCAAAGAGCAGCAGGCGTACACGCGATGAGACAGATGCCCCTGAGCAGAGCACTC
CCTTGGAATTGCCTCGTGCC
>at01271
TTTTTTTTTTTTTTTTTTGAGCATCAACACTTGGGCAATGCTGAGCTCCACATTCTGCAA
GGAGAAAATCCATTGTGAATGAGTGAATATTCTTGCGACCTGTGCCAAAGCATCGGTGGT
TCCCGGGCACCCCAGCTGAGAGTCAGAACGACAGAGGGCCACTGGGTTCAAACACAGCTG
TACGCGGCCATCCGCTTCTGTCCACAGGTTGCAAGCCGCATGGTAGACGGTCTGCCTTCT
TGCATCAGGGAATCACAGGGGTCTCTCGGGAGATGCCCAAAGCACTACCGTGTTTCACCG
TAGCTGAGGCAACGTCGTGAGACCTGGATTGCGAAGCTTCTGATATACTCTCATCCGGCT
CAAAATTCGTGATAGGTGAAGCCGATATCGTTTCCATGCACAAAGCCGACAAGGCATGCC
GGGTGCGCAAACTCCCGATGCCATCCCCGCTGCCCAGATGATCATGACACCTCTGCCTGA
AGGCGTCATGCTCAGTAGTGGCTCCGAGGCATGTCGGCAGAGTCGTCTGCCCGAGCGCCA
GGTTCTCTGGGACACGACGCCGAATCAAATGGGCCTCCCACCTGCACACGTCGTCGTCAT
AGGTGAGAAAGCTACTCACTGCACAGACAGGCTCCTCCANCGTGTTCCTCGTGCC
>at01272
TTTGTCACACGGCTCAAGAATCGCTTATCCTCGGCCCCCAGGGCGTGTCCCCCTCCCACT
CCCAGAACCTTCATCCAGCATGCGCTGGTTACTTCTGTTCGCCGCTTTTGCGTCTGCAAC

TTCAACTGCAGACAACGAGGGCCATCGGCCAGGACCGGAGTCTGACCAAGTGCTCAGGCA
AAGGCCGAACATCCTCTTCTTGATGGCCGACCAGTTCCGCTACGACGCCTTGGCGCCCAG
CGTGGCCCGAATCTTCTCGCGATGGGGCGCGATGGCGCGATCTTCGAGCGTGCCTACAGC
TCGACGCCTATCTGCGTGCCTGCCCGGCA
>at01273
TTTTTTTTTTTTTTTTTTTTGAATTCTGCCAAGTTGCAATGGGTGATGTCAAGGCAAG
CCTGAACATGGAAGTCCCAGTCGGCTGGCTCACTGAGCGCCCTCACCTTTACTGAGCGCC
CTCACCTGGCTCCTGGTCTGAGGTCCAGAGCGTCAAGTTGTCTCGCAAGAGCTGCATGAT
GAGGGTGGAGTCCTTGTAAGAGTCTTCTGCCACGTTATCAAGCTCAGCAATCGCGTCCTC
AAAAGCTGTGCGTGCCATCTTGCATGCCTCATCAGGATTTGACAGCACTTCGTATTGGAA
GACGGAGTAGTTCAAGGCAAGGCCAAGGCGAATGGGATGTGTGACAGCCAAGTCCTTCTC
CGCGACCTTCGATGCGTCCTCGTACGCGAGCCTGGCGCTCTCAGCTGCCTTGGCTTTCTT
GTCGCCATCACTGAACTCCGCAATGTAACGGTAGTAGTCGGCCTTCATCTTCTGGTAGAA
GACCTTTGATTCACCCGTTGACGCTTTTGTGATGAGGTTGCCATCGAGAAGGCCGAGGAT
TGTGTCGCAGATCTTCTGCAGCTCCGCTTCAACTTTAGCGCAATATTCCTTTGCGTATTT
TGCCTGCTCTTCATTGCCTTTTGTTTTTTCCTTCTGCTCAACGCTTGTGATGATACGCCA
TGCGGCACGCCTGCTCCCAACAGCATTCTTGTAAGCCACTGACAGGAGGTTGCGCTCTTC
AACTGAGAGCTCCTCGGTCGCCTTGCCAACAGCCTCCATATGAGAAGCCATCTCATCGTA
CCTCTCGGCCTGCTCTGCCAATTTCGCGAAATACACCTCCTTGTCGCGCGAGGCCATTTC
TGCGAGCGTGAAGATGCAGAACAGAAGTCACGGGTACAGGACAATGGAATTTGACGCCAA
CTCACTCCTCGTGCC
>at01274
GCCAAAGACTTCCTCCGCGTAAAAGCAGTGCTGGAGATCGCGGGGGAGCGCTTCCAAGTT
CGTAGTACAGTGTGTGCACATGGTGAGAACCACTGGCTTTTCAGGGTCATGGGGAGGAGG
ACTCGCCCCGTGAAAATCGGATCGTGTTCATCGGCCGAGGCATGCAACAACGGCGGCAAG
CCTTGACAGAAGGCTTCAAGGCTTGCATAGCAAAGCCTCTGCGCTTTCAGATTGGAGAGG
AGGTGCAAGTCCAAATCCATGTTGCAGGGCCCTGTAATCATGAGC
>at01275
GGGGAGGCCGCGGACCGCATGGGAGTGAGGCCTGATGCTTGCTGAGGCATCCCACAGCAC
GTTGCTTTTGCATTCCTGCTAATGCAGGCGCAAGCAGCTCGCAGGAGCATGCGCATGCAA
GTGCTGACTGGCTAGATGCTGCAATGCAGGGATCTTGATTTGCCAGGATTGGTGCCCCTT
TCATTCGTTGGATCCTTTTCTCTGAAAGCTGCTTGCCGGAAGGCAGCCCAGCCGATGTCT
CTCTAAGTCCTTTGTACAAAGTGCAAGCGCCATCTGCGAGGCATGTGCCTCGTAGTCTGA
ACGCGCTGGTTCCATCCGGTCCAGGATTGCTGAATGCACATGGCTCGTGCCTGCGCCCCC
CATTTTCCA
>at01276
GCAATGAATTGTGCAAGCAATCGCCGTGCCTTCTGCACGCACCCGCGCCTCGCATGGAAG
GCGTTGAAACAAGCACGATCTGGCAGGATCCGAAGCGCTGCTTACCTACTTTTTGACTTC
CAGGCTGCCGCGAATTGTGCGAGCAACCACCGTGCCTTCTGCACGCATCCGCGCCTCGCG
CGGAAGGTGTCTAGACAAGCATGATCTGGCAGGGTTCAGAGCATCGTTCTCTCTCTCTCC
AGCATGCAACATCA
>at01277
TTGTTTTTTTTTCTTTTTTTCTTCTTCCGCACCCCCCCCTGCACTGGATGAGGGGCCACT
TACCGCCACTCAGGTGGCTGCCCACCCGAGGTCCCTCCGCACATCGTCCTCGAGCTCGTA
GCGAAGCTCGGCCACGGCCTCCCCGCGCAGCTCTTCCTCGAGCTCGTCCGCGACATCAGG
CGCCAGCTCGTTGCACAGCTGCGTCCAGGCCTCTGACTTCATTTCGCGGCGCAACTCCTC
ACGGACTTCCCAAAAGAAGCGTTCCCTCATTTCCGCCTCCGCGCTGTTTGGCACCACGCC
CTCGCTAGCGGCCGCTTCCATGGCAGCGCGCCTCGCCGCCGCCTCTTCACGCCAAGCCTC
TGCAGCAGACACTGGCTCCTCACCCTCGTCCTCCAGCTCTTCCTCGTCCTCGTCCTCCTC
CTCATCCGAGTCCTCGTCCTCGGCGGTCGACCCGTCGTACTCGGCCTGGAGCTCCTCGCG
CAAGTCCTCAAGCACCTCCTCCTCGAGCGCCTCCTTCAGGCCCGACTTGACGGTGGACCG
CAGCTCATCACGCACGGCGTCCCATGCCTCGTCCAGCATGTCATCTTTGAGCTCCTCCAT
GATGAAGGGTCGCATCTTTGCGGCAAGGTCATCCCAAATCTTGGCCTCTTCGTCCTCAAG
GCGGTCGCGCAGGGAGATCTTGGCAGCCCTCTTCGCCACATCTGCATCGAAGTCGCCGCT
CGGCACGCCTTCCTCCTCTTCCGCCGGACTCTTGCCAGCCGGCCGCTGGGGGCTACGAAC

TCAGTGGGGGCGGCCCTAGCAGCCGTAGCACGCCGGCGAGCGCCATGCCGGGCTCCAGCG
AGGCCCCGCCTCTAAGTGCAAAGGGTGAGGTCCTGGAGGCGATGCTCGGCCTCCTGCGGC
CTCGCTGA
>at01278
CCAGCGAAAAGGCTTCCAAACGGTCCTCCCTCCTTCTCTGTAGGAAGGCAGTCTTGCCAT
CTTCGCAGGTGCCCAAGTCCGCAGCGGTTATGGCTCAGCGCATGCATCTGGTGAACGCGA
AGATCTCGATACCCTGCTGAAGTCCGAGGGGGATGGGGTGCTTCGGCAGGCACTCGGCGT
GGCACTTGGCATCGTAGCAGCCGCACTGGTTGATGAGGCAGCGCCAGTAGCCTTGGCGCA
GTAGGGCGTGCGGAGGCAGTGCTCGGAAGGCTGGCTGCAGTTCGTGACCAAGCAGCCGTA
GAGCATGGCGCAGCCCTCTGCAATGTTCTTCTGCCCCTTCTGCTCATGCCTCTGGATCAG
CTGCCCCGACTCGAGGGCCTTCAGAAACTTCCCCTGCTCATACCCCGCGCTCAGCTGCCC
CCTGGAGGAGCGCCTCCGCGTCGTCGATCCTGGGGCTGCCACGTGAGAGCAGGGCTGCGC
AGAGGCACACGGCGCAGCCTCGTGCC
>at01279
GACCTTGTGCGGCGATGCTCTCTCTGGACAATCTGCCCAGAGGTTATCAACCCAATTCAA
CCAGCCAGTTATGTTATACGTGGCTCCAAGCCAAAACAGAAGCCTCGCGTGAATGAGAGG
GCGCTCAATGAACAGGTTCAGATACTCCCGGCCACTCGGCTCAACGCATTGATAAAGCCA
CAACTCGTGGTCTCCGCCCTGCTGTAGCCACCGGCTGGCAATCCTGTCGTCGTAGTAGTC
GTATCTTAGCACCCACGCCGATAGTGGAAGGTCAAGCGGCATTGTCTTGCCCCAATTGAG
CGCTGCCATCGTCCTGACAGCTGGCCACTGCCGGCGCATCTCGCCAAAGAGAGCTCTGAT
TGGCTCTATGCAACTCTGTGGCTTCTCATCAAAACCATATGCATAGAGCTGGTGGAATCC
ATTGGCGGTCATCGTTCGCACCATGGCAGC
>at01280
AGCGGGAACTCGAGGACCTTGGTCTGAGCACTAGGGGTTGCTTGGACAAGGAGTCCCTTC
TGGACTTGCTGGAGAAGCAGGGTCCCGAGGCCTTGAGGTGGTCTGCGCAGAGGGCAGAGG
CCGCTGAGTACCAGCGCAGGGTCTCCAAGGACAAGCAGGCAGAAGCAAGGTGGGAGGCTG
CCGACAGAGTATTTGGCGTTGGCAAAGATGAGGAGTACTACGGTGACGAGGGGCGCGGCG
CGCCGGAGAGAGTCAAGAGGAGGCTGCAGGAGGCGTATCCGGACAAGAAGGTGCGGCGTG
GCTCTATCCACGACCGTCGCCGTCAGCAGTTCAAGTCGTGGAAGTGAGCCCGCGCGCATC
CCCGCCTCTGTGCGGCGGCGGTCTGCGCGGCGGAGGAGGC
>at01281
TTTTTTTTTTTTTTTTTTCATGTCCCCAACGTCGAGGGTCTTGTGGGAGAGTGATGTATG
ACGGTTCAACAGGCTGACTTCATGTCCAGTGGTGGTGCGTGCTTCCTAAACCACTCTCAG
GCGATGTTCGTCAGGTGGATCAACGAAGACAAAGCTGAAGCGGTTGCCCAACTTGCCACC
AATCTTGATGTCGCAGCCTGTGCTGGAGGAAGCCGGGCATTGATGGCCGCCCGACCCCCA
GAAATGATGACCACCGCGCCCTCAGTAGCCGCGCTGGGACAGCATGGAGTAGGTCTCGCC
CACCCCGGCCTCGAAGAAGACCGCGCAGGGGCCGCAGAGCCTCACGAAGCGCGTTGCCGG
TGTTGGCGAGGTCGGCGCCCCGCTCCCTGGAGGCCTCCATGGCGGCGCCGGCGCGGGACT
CTGGCAGCCTGCCTTGCATCGGGCTCTCGTCCTGGAACATCAACTGATGCGTCCTCAGCC
GCTCGTCCAGCGCGATGATGTTCGTGCCCCTCCATTTGAAGCAGTCGCCGCACAGCACGA
GGCCACACGAGCGGCAGTGGTAGCGCCGACCCTCGTGCCG
>at01282
TTGTTTTTTTTTCTTTTTTTTTTGGTATTCGTCAGGGTCAAAAATGAGGCTTGGAGGAGG
AAATGTGCATCCCACTAAAGCTCGCTGGGAGGCTAGCTGACTCCGCAGAGTCTGCACACA
GCAGCCTAGCCCCAAAGCTTCTGTAGCTACTAGTGTAACTGTCAGATTTGGCAAAACGGC
GTGCCACCGATGGACACCGTGGTAAGCACCACGATTGAAAACCTGCCGGACAACCTCCAG
GCAGAAAATCACACTAATCCCTCCACAAGCAGTGCCTCCCCAAGTTCTGCGCGTCATTAG
TGGTGTTCGAGAGACCTAGAACCATGACAACAAAGCTAAGGAGGACGAGGCAGGCCATTG
GCATCGGCGAGTACCAAAAGCTGTATGTAGATGATGAAGGCTTCCTTGTTGGGCTTGCCA
GTGCGCTGTCCCTCGAGATCAATGCCTCCGTGAGGATCTGCAGGGTTCCGCTGCGGGCAC
CGTCTGCAGGCCCTGTGCCATCTCCGGGAGCTGCAGCCATGCCATTCAGCACTTGCATCG
CTATGACTGCTGGAAACAAGCCCACGAAGAAGAAGACGCTCAGAGCCCCGTTCAACACGT
TGATGACCCCCAAGTCGGAAACGTTGAAGCTGGTCACCAGCGCCGCAAGGTTGATCCCCA
TCACTCCCATTGTTCTACGAGACTCCGGTAAAGGCGCGACCATCGGGTACAACATCAACG
GGTAGACGCCCAGGCACACTCCGGCCATTGACAGCCTACTTACGACTGTGTACCACCTAT

CAGAGGGGAGGTTCTTCAGGACGTCGCCATTCGCCGCAGCACCGAACGAGAGGTACCCAG
CCACCGCAAACATACAGAACAGCATGTAGAGAATTGCGAAGGCAACGCCCAGCGCCCGTG
CAAAATTTCTAGGAGACCGCTGCGGTTTGCGGCTACACTTAGTTATGTCGGTGTTCATCT
CCTCATACATTGGCAGGATACAAGGCTGGATCACGATTGCCATCAGAAGGCTGGAAACGA
AGGCCAGGTTGCCTGGGGTAAAGGGCGTGGACAGCACGCAAATATCTGGAGCACTTGCTA
TACCTGCACTTGCAACACCGGCTTCGCTTCCCAGCTGAGCAGCGTGGCTCCTCTCCGACT
CAAGCACCTCGTAGTTGAGCAAAAGGGCAAACACTATGAAGCAGTTGCTGAACGTGCCCA
GGAAAGAGGTGAAGGCAAGTTGCTGCTGATTCAGGAAACAGAGCGGGCAGAGCAGGCGGC
AGAAAGCAGCAGAACCATGATGCGAGCGTTGTTCTCCCCAAAGCTCAGCACATCCAGCGC
TCCTATGGAGTCCAAAGTTGGGATGAGGGAGTCAGCTATGATGATTAGGTAGCCGACAAG
ACAGAAGAAGTAAGTGACCCAGATTGCCGCCTTGCACGAGCGGCCCCAGAGTCTCTTAGC
GTTCAAGTCCC
>at01283
TTTTTTTTTTTTTTTTTGATTCTGCACATAATTTCATGATGCGCGGTAAAATGTTAATC
CAGAGTCCCTTATCAGAGCACAGTGGCTCCCATTGGACGGACTGACCCTGTAGAACGGCA
TCGCCCTTCGAACATCCGAAGTGATACCTTGAAACAGCCTTGTGCTGCGTTGCGAGACCA
TCTAATTCGTCCTCCACTCAAATCACAGACTGGGGGATCGGAGATCACATCTCGACCTTG
CAAGAAGATGCTCAGCAGAGGTTGCCAAATCGGCCCTGGTGCTCAATCTGGAAGCCGCGG
GCCCGGTCGAGCACTCTGCTGAGGGACCGCAAGTGCTGCTCGTCCAAGCAACCAAACTTG
TGGCCTCCGCTGTGGATGCCACACTTGCGCTCGCACTCGTGCTCGCAGTGCCAGAAAGCG
CCGTGACCGCCCCAAAAGTTGTCGTAGTCCTCAGCAGCATATATGACGACGGTCCCACTG
GGGCCACCGTACTGGTAACTACACATGCAACAAGTCTCGCCATCATGGCCGTGGCCCCAG
TCGAGCCACTCAGCTTCCACTTCCCCTTCCCCAGCACCCACCGCCGAGGTGTTGGCCTTT
GCCACACGAGTGCGGACAGCCGCAAGCACCTGGGCCGCCTGCTCGGCATGGCTGGCGCCT
GGGGCAGCGCTGGGGGCCGGCGTTGGGGACGGGGTTGCCGCCGCGTCGGCGCTCCATGGC
ACCGCTCCCGCCTGGCCCGTGGTGCCGCGCAACTGCGGCTGGGTAGGAGAGCTCGACGTT
GGCACTGCGGCTGCTGCTTTCGGATCAACGGCGTCGACGCTCAACGCAACCACGACAACG
ATGAGAAGGATAGTGCGAGACATGGCAACTTGTGGTGGCACCAAAGCCTGCTGAGGAC
>at01284
TCCAGCGAGGCCGCCTGCCGGGTAGCGGTCTCCGTGGGTGGAGTCTTGGACTTGCGATAT
GCCGTGCAGGAGGGGCAGCTCTGCGGCAATGCAGTGCTGAGCGCTTTTGGAGCAGACGCG
CCATACCCCGAAGTGTCCCCAGCAGAGATGCTGCCGCCCTGCCTGGTGAACAGCCTACCA
GCCGCACGTCAGGTTGACGCGTCTGTGCCAAGAACGCGCTTTGGCCTTGTGCATGGAGAG
TTGGACCAGATCGTCCCCCCAGCACAGAGCGTTCGCTTCTTC
>at01285
CAAGGGGCAAGCCTCAAGGAGCAGCTTGTAAAGAGCAGGCGAACCTCGAGGCTCAGGTCA
AAGAATTGAGCGACCAGGGGGGAAAGCCTCAAGGAGCAGCTGAATGAGCAGGAGCAGCGC
GCCAAGGAGCTGGAAGTAGCGCTGAAGGAGGAGACTGAGGCACGTGCACGCCTGGAGAAG
GCGCTCACT
>at01286
TTTTTTTTCTTTTTTTTTTTAACATTGACAACTTCCTCATCAACATAGGTCTTCCAGGT
GATGTGGTTGACATGTCATGCTCAAAATGTCTATGCCATCAAATCCTAATCTACAAGTTC
GTAGCTCTCGAGAAGGGCTTGGAATTCTGAAGTGAGGTACGCCTTGTGCGCTCTCACAAG
GTGCATTGGGACCGGGGAGTGCAGCTTGCCTTCCGCACAATCCTCAAGAATTCTGCCCCA
GTAACTATTGAAAGCGTGCGTGCCTGGGCGTATGTCAGGGAACAACTCTGTGCTGAAGTA
GAGTACTTCGTCGGACAAAGTCTGTGCCGTGGCAGCGTTTGCTTCCAACCAGTCACTGTC
CTCCAGGCGCACCTGGACCCCCTCCAAGGCTTTCACCAGATCGTTGGCGCGATCGGATTG
TCGGGCTGGAAGCAGCAACATCTCGATCACGGCAGACAGGGGATAGCTCGCCCTAATCTC
ATCGTCCCTCTTGTGCTGTGGCTCCCAATGCAGAGCCTGGGTCCTAGAACGACTGATGCG
TCGCAGAGCGATGAATGCGAGCCCCCCAAGCGCACATAGGCCAAGAAACCGAGGCTTGGA
TGGACATGCACCCATCGAGCTGTGGCAGTGTCAGACTCGGCAAGGCACGGCTTCGGGAGA
TGCTACACTTGAGCCAAAACATCTTGAGCCAAAATGGCTACGG
>at01287
TTTTTTTTTTTTTTTTTCGATGACAATGAAAACTAATCCACACGCACCACTGTACAATG
TTGCAGGTCAAATGACGCGGGAACAGCTCCAGAGTTCCTCCCACAACTGCGCAGTGGCGT

GCAATCCAACTCGCTTGGCCATGGCTGGTCCTCCGTCTGCCCTCCATCCGCGCACCCCGT
CCCAGAATAATACTGGGCGGTTGCGGTCGCTATATGGCCGCCAATCACCTGCCATGTCTG
ATGTGTTCAGGTCCCCAGTGCACACAAAGTGCACCCACGAATCTATCCACTTCTGCCGCA
CCTTCAAAGCACCCGGGAGCTCGAGAATCTATTGGTTTTCCTCACCAAGTAGCAGAGGCA
ACTCCGCCGCATGAAAGGCATCCCCACGGCTGTCATAACCGTTGAACTGGTAGCGGAAAA
CTCTCCGACATGTGCCAGGCAA
>at01288
CACGCTCAACACCATGTGGCTCAAGTTTCTGGTACTCTGGCGCGTGGCACGGGCTTGGGC
ACTGGCAGATGGCATCAATCCACCCGAGAATATGAGGCGGTCCTTGAACGATCACTACTC
GGTGGTGTCATTTTGGAAGAGCTGGCATGTTTCCTACAACAAATGGCTCGTGCGTTACAT
CTATGTGCCTGTTGGCGGCAAGCACCGGCGGTTGCTGGCCACAGCAGTGACCTTCCTCTT
TGTATCCTACTGGCATGACATCGAGGCAAAGCTGATGGCTTGGGGTGGACTGAATGCTGG
GTTCATTGCGCTGGAGGTGTGCGTGACCAGTGCTGTTCAAAAAGAGGCTGGAGGCAATGG
GACTGCCTGCCCGACGCCCGTGGCTCTACCGCAGCTCTGCGCGGCA
>at01289
GGTGGCACTTGGCCGAGTACACGCAGACAAAAAGGAAACAATGTCAACTTCCCTCAACGA
GAGCATGCTGAGGCACATGGGCTTCGAATGTCATTCTCGGGCCGCCGCAGGAGAGGGCCC
CCGGTGATTTTTGCCAGCGCGCAGGAGGTGCAGCAGGCCGCGGTGGCCCTGAAGCGTCCA
GGCTGGACGTCCTACAGAGGCTATGATGTGGAcCCCCTACGAGGACGCGCACTCCATGtA
GtCGtCtTCtTGtAACATTGAGGCTTGCTTCCAAGTCGCAGAGGAGAATGGCTTTGGGGG
CTTCTGCACCTGGGAGGGCGAAGCTTACTTCAGGGAGTCGTCCGGGGAGGAgGCTCAAGC
AGCGCCTTCAGCAATCGcCCGGTGCTGGCCTCTATATTTACACACCTGCCCGCAGCTCGG
CTTCGGCGCCTTCGGCTGCTGCTGGCACCGGCGCGGCTGATGACGACGAGAACGACGCCG
ACCTGCAGGAGGCGCTGCGCCTCTCCATGATGAGCTGAGAGCCGCCGGCTGCTCGCTATC
CGCGTCCGCGTGGCGGCCTCGCGCACCGCGCGGTGGAGGGTGGCAC
>at01290
GCCGGCCTCGCGAACGAGATCTGGGGCGCGGTGCAGTGGGAAACCGTGCTCTGCAACAGC
AAGACGAAGAAGATCGATCCAAGCGGCACCGCGATACCCTTTACTTTTGATGACGATGAC
AACTGCGACGGGCTGCATGTGCCCGCGAAGGACGGCTACTACGAGTTTAGCGAGCTGCAC
TACACCGTGTGGCTCTCATACAACAAAGCCTGCGCTGGGCGCGCTGCTGGCCAGTGCCAG
AACCGGGGCTTGGACCGGATGTGGCAGGCGTGGCAAGGTCGCCGGCTTAACCCAGACATC
CACTACCAGGGCTTGCCGCTGCTCAGCCTCTGGTCGTCCTACATCGTGCACCTGCCTTAC
TACGCTTCCCACTCCTTCAACGCAGACCCGACTTGGCAGTCGCTGTTCCGGTCGCACTGG
CAGGCCGACCGGGCCTACTACCAAT
>at01291
CCGTAGCCATTTTGGCTCAAGCACCGGCCCGCTCAGCTCCCGCCATGGCGCGTCTATGAC
TTGACCTGGCCACATGGCAGGGCCCCCATCTCCCGCGGTCACAGGCGATGCCTCAGTGTG
ACACTCAAGGCAGCAATCCAGAAGTTCAGCCAGCCGTCTATCAGCGTTTCACAGAAGGGG
TCAGGTACGGCTCCGACGTTGCATACCGAGTTGTGAGGGCAATACCAAGGCAAACTTGCG
AGGCAATTGAGACGGCTGT
>at01292
GTGATTGCTCAGCCGCATCGGCTGTGGTGTTTGGACTTCACTGGAGCAAGCAAGGCATGC
GTCCTCCAGTGCTCTGCTCGGCAGTTCTTCCATGATAAGTTTAGCATCCTCTGGATGAAC
CTCTTAAGACCTCAGTGTGGCCACTTGGACAATCCATGCAGTGGCCTGGCACCACTGCCC
TGAAGTACCCAGCTTGAGTACCACGGGACATGACGAGCAATCGTATCCCCACGCATCCAC
AGTAGTCCTTGGCCAGGATGCCAGAGCAGACCGTGTGAGCGCGCCTCCCATGAGCCGGCC
CCCAAGGCCCATAGCGGCATTTCCTCCACAGCTTGATTGCCAAGCAACGGCTGCTGTCCA
GCCA
>at01293
TTGTTTTTTTTTCTTTTTTTTTTGAATGCACAGCACTCACTCCTCAGCGTGCCAATCTTC
CGCTCGACCTCTTCTCCCGCTAGCGGACAGGCCCTCAGCTCTCGGTGTCCTTGTCGGAGT
ACTTCCCCTTCTTGGCCTTACCGCCCTTGCGCTCAAGCATGGCCTTACGGTCCTTGTCGA
GCCTGAGCTTCGTGATCACCACCTTCGACGGGTGGATGCCCACGGGGACCGTTTGTCCGT
TGACCTTGTCGCGCGTGACCCTCTCCACATGGATCCGAAACTTCTTGCGGTACACACAGG
TGACCTTGCCCTCGCGGTCATGGAAGTGCCCGCGCTTGATGAGGACCTCGTCGTCCCGGC

GGATTGGTACAGCGCGGACACCGTACTTGGCGCGGAGGTCCTTGCTCAGGGGCGAGCTCA
TGAGCTTCCGGCGCGTGAAGGAGCCCGCCGTGAAGTGCGCCTTGCGGTTCTTCCTGCGCG
AGATGGTGACGCTCGCCTTGACCTTCATGAGTGGAAGTGGTGGTGGACTGAGGGCAAAAG
CAGCAATGAGCTCAGCTCCGGACTTAGGGCCGAGATGCCTTGAGCCAAAATGGCTACG
>at01294
CGGCTCAGCGCTGGCGGACTATCCCTCTGATCCTACAGCATGGGCTTGGCAGTTGGAGAG
CGCTTTCGCCCGAGCCGGGCTAGGATCATCATAGCAAGCTCACAGTGCACGGCAGTTGCT
CAGGTGTGACCCGAGCGAAGCATAGTAGAAGCTTTCCTTGTGTTCAAGCTAATGAGCACC
CATCTTCCACCCGAGCTTCTAGTCGTCCTCTAATCAGAGCAGCGATGCGCTGTAACAGCG
GGAACCGCCGCAGAATCTTGTCCCAGAATCCGGGGTGAGGGAAGCTCATGCCCAGTGCAA
AGTCCGCTGCCACAGGCACCTCTCATAGGAACGCTGCAAATCGCCGACCTCGTACATCTG
CGGCTAGCAGGCCTAGTGATGTTGACCGCATCCTGCCACGTGTCAT
>at01295
CCGTAGCCATTTTGGCTCAAGACATTTCAGCCGAGGTATTGAGGTTGCCATCTGTGGGCA
GACATCCTGGTTGTCGCAAAGCCTTTCCTGGCACCCATGGCAACAGAGATGGCCCAGACC
GTCCGAGTGCTCCACCTCATGGGCTCACCAACGTCCAGCTTTTACTTCGACCTGTCCGTC
ATGTATGCTCGCACAGCTGTAGAGTTTCAGGGGCTGGATCACGCTGGATTCAAGCATGAG
TACGCAGTTGTCTTTGTGAATGGCATGTGGGCGTTCCCGAAGTCTCTGGACAGTGCTGCA
CTGGAGAAGGCTGAGCGGATGCGCTTGCCAGCAGCGCTGGCACACATCGAGGCGTGCAAG
CCAGCGCTGGATGTTGTCATGCCCCACATGTTCTGCCAAGAGGGCATGACGCGGTATCGT
GCCTTGTTTGAGATGGTTGGCATTGAGGTGCTGGGGTGCCCGGCGCACACCTGCACTATC
GCGAATGACAAATTCCTGACCAAACATCTCTGCAAAGCG
>at01296
GTTTTGTAGATCACTCCTTCAACCTCAGGGCCTTTGCTGGCATGATCATGTCATCTCGAA
GGCCTCGCTTTCGGCCACAGTACTTCCCTCCAGTCAAATTGGAGAGACTTCTCAAGGTGT
NNCGCTCCCGAAAAAATGTGTGAGCAGCGAAATAGCTACCTCTTCCTCTCGCCAGGAGCA
AGGAAAAGCTTGATTTCAAGTCATAATCAGCAGCATTCGCTGCTAGGCGCCGAGCAGTGA
AGACATTGGGAATGGTAGCACATCCATGCCAATCCAGCAGTGTGCGCCTAGATGCGCAGA
TGCAAAAATGCACAGGTGGATGAGCATGCTCCGAAGCGATTGCCACAGGGCTCGGAGTTG
GCCCCTCCAATCATCAGAGGATCGAGAAGTCAGATCATCTGCTCACAGACTTGGTTCACG
AAGCGAGGTTTCAGCATGCTGATCCCAACTGCCTTTGACACCGGAGACCCCAGTAACACC
CTCGGACAGCGAGCTGTGCTCCTTGTTCTTGCTGCCTCTGGAGCCCCTGCTCGAGTTGGA
CCCGTTGGACAGCGCGGATTGCGACCGGCGAAAGCTCGCCGGCTGCTCCACCGCCGAGGC
CTCCCCCACCGCCGCTGCTGCGCAGCCGGTAGATGATGATGATGACCACCGCGACGAACG
TCATGAGGAGCTCGAAGACGCCCGTGCCGAGTATGACGAGACTCGCCTCGAGCATCGTGG
ACGCGTCCCATCGGTCCACCCTCCAGCTGCTGACACCAATGAACCCGAACCAGTAGGAGA
TGAAAGCCATTCCGCTGACGACCTTGGGGACGAAAGGCCCGCCGAGCTCCGCACCGAGGA
ATCTTCTCCGAAGGTGCAGTAT
>at01297
TTTTTTTTTTTTTTTTTGCCAGAAAACAAAAGATATCTATCCACACATGTCCAGCTCGT
TGCCGCCATCTCATGGCAGAATGCCGCTGTGCCATCATCGTGATATCAAGTCTGCCTCTG
ATGACCGCGTGGCATGCGCAGGCGGCCACACTCCTACAGCAGCGTGAAACTCCGTCAAAA
AGGGACGTGGCGACGCTGTCCCCTCGGGGAGGCAGGCGGCGGCTTCCGACCGTCGACAGC
GGCGCAGGTCCGTCAAAGACGTGCCGGTGCTCAGAGCGGAAACTTCGCCGCCCCGGCACT
TGCGGCCGGTGCCGGAAACGAAACGGCCGCCGCGAGAGGGGTGTCCCAGTGGGAGGGGGC
GCTGGCGGTGGCGGCGTCTGCGCCGTCGCGCTGCAGCTCGGCGTGCCTCGTCAGCTTCGC
CATGAGCTTGGTGTGCAGCTTGTCGTTTGCTAAAAGGGAGGGCGGAAGCTTGGCCTGGCT
CACGTGGAAGTCTGGGTCGCTCTCGATCTTCTGCTCCAGTCGCGTGAGCAGGTTTGTGTA
CCTCTTGCGCTTGCTCTTGCACGGCCGCTTGGACTTCCGCGCCTTCCGTGGGGCTTCGTC
AGCCTGTGGCTCCATGGCTCCGCCACCAGCAGGCCTTTCCTGCTCTTGGTCAGAGTCCTC
CGTTTCCTCACCATGGTCATCATGTCCTGCGCGCTTCTCGCCTCCGGAGATGAGGAGATT
GTCCATTCCCGCCAACGTTGAGCTGCTGCTTCTTCCAGCAGATGACCTCACACTGGAGCT
TCGGCTTGTGGAGGAGTCCTCTGCAGAAGGCCGCCTTCTCATTCTGAAGCCCATGTCCAA
GAAGTAGGCTAAGGGATGGGGAGTCCCATGTACCTCGCTTATGCTTCCGCTCACGCCTTC
CTTGCTGCCGCTCATGCCCACTTTAAAGCTGCACGCTTCTGGCATACTCAGAACTGAGAC

GGCTTGAGCCAAAATGGCTACGG
>at01298
TTTTTTTTTTTTTTTTTTGGCAGGGGCTGCTGACACTAGAGGCCGGCTGTCCCAAGTTGA
ACAAATCCATGTTGGACGCAAGTGAATGGGGATGATGTTGTGAAAGGTTGTGCTTGCGTG
GGAGCTATTCTACGATGATTAAAATTAGCCTAACTTCGCATACAATTTCCTTGCAAGCCC
TCACATCTCTACTCATGGCATGGGCATCTCTGGCCATGCACCCCAGCAACTCTGGTGGGC
ATCACCACTTTTCCCATGTCGTCAGACCGACTCGCAACTTGTAATCTCGGCAGACAAGGC
GAAACTGGTTGAGGGGTCCAAACACCCCGTAGTAAAGTGTAGCGTGCAAGAGGAGTACGA
CGGTCATGCGAGCAATCGTGTTGCCATAGGTCCTCCAGAAGTCATTCCTGAAGCTTCTGA
ACACAATGCTGGTCCTCTTCATCAGTTCCTTCTTCTCTTCAGACATGTACGACTTAAACA
GAAGCTCGTCAATGCATACAAACCACTGGAGGGCAATGGACTTCAGAACCAAAGTCCCAG
TTGTGTTCGTAAGTATCAAGAACCCTCGTGCC
>at01299
TATGGCGGCAGAAAAAGTGAGGCCCCGCAGCTGAAGTCAAACCCTGAGATGTTGCTCTTC
TTGAATATAGACTCCATCGCTGGAGGGAAGGCGCGGAAGCTCTGGGACACGTCCTACCGC
TTGGGCGTCAACGATCCCCTCGACCCGGACTTGCTGGAGCGGGGGCAGGACCCCGGGGAC
TGCAAGCTGGAGGTCATCACTTTGCGTAGGTTGTTGAGACTCGTGATGCCGACCAAGGAC
TTCTTGGCTGGGCGACGCGTGTTCCAAGGTGCTCCGATCCACTTGGTGTTCCTGAGAACG
AGGGACCTGCTCACCTACATCCAGGTAGATGGTGAGAGCTACAAGCTCTTGAACCCAGAC
ACGTTGACGATTGAGCACAAACAGCAG
>at01300
CCGTAGCCATTTTGGCTCAAGCCGTAGCAGTTCTGCCAAAAGAGTAGAAGCCACCTGGGA
ATTGTGGCGTTCTGGCCACAGCAATGCTGTTTCCGAATCTGCTTACTCTGAGACCGCTAG
GTGTGCCTTGCTTGGCAGTGCTGGCGATTCGTCTTGCCAGCATCCCGGCACAGCTTGGCT
CCTGCAATTTGCATGCTGCCAATCCATCTGACTCCCAATGCCCTGCATCGTCATCGGATG
AGAGGTCATTGCTTCAGACCCTCCATCGAGGACGACTGAATCCATCGGCAATTGATTTCG
ACTCGGAAGGATTTTCTCGGTGGGCAAGTGTTCAGGTGAGGGACCCACCACCTGTTCCAC
CACCTTGGCGAGATTTCATCGAGTGCCACCTGTCGAACTTGACAAAAGCTTGTT
>at01301
GGAAGGATGGCTTCTAGCAAACCATGGCGATGCGGCGAACCTCAGGTTTGCTTTTTGCCA
AGAAAGGCCACTCTCTGCAGTGGATACAGCGCCACGTTTCCGACAAGTATGTCCAGAGGG
CGCAGGAGAATGACTACAGGTCCCGCTCTGCCTTCAAGCTGCGGCAGCTCGACGGTCAAT
TCTCCTTCCTGCGGCCGCGCTCCTCTGTCGTGGACCTCGGCTGCAGCGCCGGGGGCTGGT
CGCAAGTGGTGCTGGAGCGCACGGCGACGCCGGGCGGCCGGAGGACCTCCGTCGTCGTGG
GCGTGGACAAGGTGCACATGGAGCCGCTGGACAACCACCACTTCATCCGCGGTGACGTGC
AAGATGCCGTCACGTTGGCAAAAGTCAAGGAGATCCTGCAGGGTCAGAAGGCGACCATCG
TCCTCTCCGACATGGCCCCCAAGATGACGGGATCCAAGATCGACGACCACGTGGCTTCGG
TCGATCTCTGCAGGGCGGCGGCGAGGTTCGCCGAGCAGATCCTGAAGGAGGGAGGCTGGT
TCGTGACGAAGATCTTTGCGGGCGCGCTGGCGGACCGTCACAgGGGGGGAGCTCGAGCAG
CGCTTCCGCAAGGTGCACAGGTC
>at01302
TTGTTTTTTTTTCTTTTTTTTTGGTCTGACAGTCCTCGAATCTGCTCGGCAACACACATT
CATGTGACGCATGAGCAAGTGCAAGGCCGCTTATCCTGAGCGAGCGTAGCGACAAAGGGA
AGACGCAGCGCCTGCATGCGCAGGAGAAGAACAACTACGACTGGAACATCCCGATTCGCC
GGGTGTAAGCAGCGCTCCAAGTACTCTTCTGAGATCAGGGGAAGGGAGTATCCTTCACCA
TCGTGTCGTCTCCGTATTGGCCTTCGAATCATAGATCATGCCCTCTGTCTTCTCGACCTG
GCCATACTTTCCCACCCACACATCACGTATCCAATCGTCCATTGCCTTATCGGCCCCCAT
CTCTTCCAGGACTCGAACTGTGACGTTGAGGACATGGATGTTCGTCCTCAGGGTGCTCTT
GTTGTCAACGTTGTCGGCAATTCGTCGATCATGTCGGTCCTTTATTGTTGCCCACTCCTT
TGTTGGACATTGCTTCAAGGCGTTTGGTAATGCGGCCACCATGATCATGCAGTTCTGAAG
AGCAGTGACCAGCACTGTGATTAGACAAAGCTCTCTATCCAGCATTGGGCCCCCGTATTC
ATGGTACATCTTGATAAAGGCATCGATATACTCGATCATGTTTTCCTTCACGTTCTCGAA
TTCGGCCATGTTGAGCATCCACCAAATCTTGTGAGGCAAGTTACTGCTGGCAAAACCTCC
AAAGTCAAAAACCCCGCAATCCAACACGTTGTTCTCATCCCGCCAGAAGTAAGCATTGTC
CACGTTCAGGTTCTGATGCCCCAGAGCGCAATAATCTGTGTTTGAGTTTTTAAAGTAGTT

GAGCTCCGCAGTGTATGCATTGACCTTCATCATGGTATCTTTGAACTTTTGCTTGAAAGC
CTCATCCTTCACAAAGTCTGGGAAGACGGCCTCGGCCGTCTCTGACATGAACTTGATCGC
CGAGTCCAATTTGGGAGAGCACGCCCCTGGGTTCTCGCCGGTAGCCCTCACTGGGCTCAC
CCCGTAGGCGGCTGGGTCTGTGGAGGGATCCGAGTAGATGGCGGGCGGCATGCCGCCGAG
CTTCCACTTGGCCGAGATCATTGCCTGCTTCTGCAGCATGACCAGATAGTACTCTTTGTG
CGGTTGCCGGAGGTTGAAGTCCTTGCACTTGTCAAACGGGCCCTCGACCTCATAAGGTGC
CATGCGTGCTTTACGGCCCTGGTGCTCGGCAAAAGGCACCCTCTCTGTGATCAGTATGAA
GTTTGTAGTCTCATTGCTGATGTCTCCGTAGTAAAACCGTGGGGTTGCAAAGGGCAGTGC
CGACTCGAGAAGTCGGTAGGTATTGAGTTCCATGAAATCCATTGGCTGCTTATAAACCGA
GCTGCTGAAACGGTCACTTTGCGTCGGGCCGCTCAATGCAAATGGCACTTTGGCAAACAA
GTGGGTGTGCAAGTCCTGACCCTGCTTTGCATACCTCACTTCAAAGAGGAACTTGCCCGC
ATTGTTTCCGCCTGTCACCTTGATCTTATTCTGCCGGATAATCTTGGTGATGTAATTGGT
CTTTGGCAATGTTCCAGCAGCGTGGAAGGCTTGTGTCATCCACTTGGGACCAAACTCATC
AAGCATCTCCTCATTCCAGGGGAATTCGAGGCCATAGAAGGATCCAGGTGCGCGCTTCGT
ATGTCCTTCTTTGACAGTGTCCAGGGAGACTGGGAACCAACCAATTGGTGGCCGCTCGAG
CTCGTACTTGCATCTTGCTGCAATCCTTTCCTCTGGAGACGCCTCGACAACCTCAGCTGA
GTTGTCGTCCCCCTTTGCACACTGGTACTTGATCACTGTGTGCTCCCAGGGAACGTTGTC
GGTGACAAGTGTCAACAATTGATCCGCAGGTGTGAGCCAGTTCCCCGGCCCCAGCTTGCC
GTCTTGCTGCTGGAGGGCAGAGGACCTCCTGTCTCTGACACACTTCTCTCCCAGCGCGGT
TCGCCCTTGGAAGCTGTACGTCTGCATGGAGGAGCGCATGGTCGTCGGAGGCGAAGTGGA
CCCTGCCCACACAGCTTTCCGGCTGCTACTGCACACCGCCGCTGCCGCAGCAGGCCTGCC
GCTCACCCTCACCAATCACACCGATGGCGCCGCCGCTTGCAAGCGGTTCCAGCCGCGGGA
AGCGCGCCGAGGAAGGAGAACCATTCAGACCATGACGTCTCCGCGGCCGTGGCTGCTTGA
GCCAAAATGGCTACGG
>at01303
CCGTAGCCATTTTGGCTCAAGTCGTTTTTTGTTCAGCTTTGCCTGGCATTTGCAGCCCGC
GCAAGCGCACAGGCAGCAATGTTCCGCTCAGCCCTGGTCTCCGTTGCTCTCCTGGCGGTG
CCTGCAGCTGCTTTCGTCCCGCCGGGAGTGCAGTCAGTGCCAACACCCCGTGGCCTCGCC
GCTGGCACTTTGCGCGCCGAGGCTGCGGCGGGTGAGGCAGCAGCGGCTGCAGATGCGTCG
GCGTCGTGGTCTGCTTGCTTGCTTACCGGTCTCGGTCTGGGCTATGCTGCTGCAGCCACT
GGCGCCTTCGCACGTCAGTCGCGGTCGCGGACATGCTGTGCAGCCGAGGCCAAGCCCAAG
GTTGCAGCGGCAAGGACCCCAGTGGCCTACCCCATCTTCACCTTCCGCTGGCTCGCCGTC
CATGCCATCACCATCCCGACGGTGTTCTTCCTTGGCGCGATCAGCTCGATGCAGTTCATC
CAGCGCTGAGCGGGCGATAGGCTGCTTGTGATGCAGGCAAGGAGGTCTTTTTGATGTGGC
ATTGGACTAGAAAAAAAAAAAAAGAAAAAAAAAACAA
>at01304
TTTTTTTTTTTTTTTTTTCAACATCCAACATCTTGGAGCCCCCAAGCCCCGCACGAACAA
GGCTTCTCACCATCACGAGCAAAGCACCCAGGTCAGTGCACCCACATGGTCACAGCCACA
CGGTCTCGCACATTGGGGAGCACCTCGTGGGGCACGCAGCCGCTCAAGAATACCAGCAGC
CGGCCGGCCAGGGGTTCCACGTCCCGGCTCTCGCCGCCCAGAGCCGGCGGCCATAGTCTG
AGTTCGCCGCCGTGCTCGGGCCGCCAGTCCCCGTTGCAGTAGACGATGGCGGTGAGGACG
CGCTCCCCACGCTGGGCGGGGTTGGCCGGATCCTCGTCTGTGTGCCGGGAGTAGCCTTCG
CTCCCCGCGGGGTACCAGGCCACCTGCATCTCGACCCGCCCGCTCGCCAGCGCCAAGGTG
CAGAGGTCGCCGTTCAGCTCCTCGAGCGCGCTCAAGAGGCCTCGCGGCGCTCCATCCCCT
TTGACGACGAGGGCGTCACCGTCT
>at01305
TTCGGGGATCGGGCTACGGCCGCGGGCCTGGCTCCAAGGTCACGAAGACGGGCAAAGGCT
TCGGCTGGGACGACGTGGGCTCGGGCAGGACCGTGGACGCGGTGCTGAAGCGCTTCACCG
TCGATCCATTCGATGATACGCCTGAGCTTCACACGCTGCAAGGGGGAAGTATATCTCAAA
GCCAAGTGGGACAAGCAAAGAGCGGCTCTCAGTGACATCGTGGCGGACATTTCGGTCTCG
GAGTCCATTCTCTTCTGACTTGTGGCAAGTGTTGCCGCTATTGGATGAGCTCCGCTGGCC
GATCC
>at01306
GCATAATGTCGGCAATGCTTTGTCAAGTGCAGTTCTCGCTCCACCCCTGCTCACCGGGCC
GCCGCGCCGCCGCCCATGGCGCCGCCCCAACTAGACATGAAGTTCTTGACGCAGCTGCCT

CCCATGTTGCAGTAGCCGTCGTTGCAGTGGCACTTGCCCCATCTGCACGAGGCCTGGTCG
TCGTTGCAGCCGATGAAGTAGTTCTGATCCATCTTCGTCTCGCACTTCATCATGCTGCTG
CCGGCGTCCGGGTCCGGCTTGCACTTGCCCTCCCGGCGCACATGCCCTCGGGCAGCGGCA
CGCCCCCATCGCTGCACGTGGCGCCGCGCGA
>at01307
GGTTCTGCATCTATGAACAGCACGGCGCGTTCTGTTTGAAGATCATGCACGCCGAATCGA
GGCTGACCTGGGCCTTTTGCCTCATTCCCGATTGGTGCCCAAGAGCAATCACTGCGAACT
CCCAGATGCGACTGCAGTCTTGCGTTCACACCTCCCTCCATGAGAGCCCTTTCCATGAGT
AGCACAAGCGTTGCAGCAATGCGTTGGATGCCAAGTTGATTGAAACGTACAACCTGGCGT
CTTGCACACGCGGGTTGCCATATTTGGTGCTGGGATCTGGGCTGGCTGCTCTCCCGTAGG
CCCCAAGGACATCCCGCAGGCACGACCCTGCTCTGTTGCCTCTTCTTCCCGACTTCACCT
G
>at01308
TTTTTTTTTTTTTTTTTTGCAACTCTGCGCTTTGCCCACTCTCCCCAAGCCTCTCTGCGC
ATTGGGTAGCCAATGCACAGGCGAGTTGGCGGGCAAGTGCAGAGCGCATTGGCCCGGTGC
ACAGGAGTGGCTGCTTGCCAAGCCCGTGCTTCATAGCTCCACTCAGCAGCGGAGAAAGTC
GATCCAGATCCAGACCCAGACGCCGTGCATGACCACGCCCGGCCAAGCAGACCCTGAGAG
GTGCACAGCGTTCGTGCACGCAATGCCCAGCATGCCAGCCAAGACGAGGAACCGCACGTC
AGTGAAGATGGGCCGCATGAAACCAGAGTGCAGCACGTCCAAATGGTAGACGAGGAAGAT
CACCAGGGCGGCAACCTCCTGGCCACTCCAGGGACGAAGCCAACGACACCTAACCTCCTG
CTGCAGCCGCAGTTGTTGCTTCTGCTGCTGCTGCTGCTGCTGCTGCTGCTGCTGCGGCTG
CTGCACCTGCAGGAACTTCTGAGATTCCGGCTCCTCCGCGGGCGCCGCGGGCCCGCGGCA
CTCCGCGCGGCCGAGCGGCCACGGAGGCGGCCCGGGGGCTGCGGAGGAGGAGCCAGCCGC
TCCGGCGCCTGCTGATCCGGCAATAAGAGGACGCG
>at01309
TTTTTTTTTTTTTTTTTTCACAGTTCATAATGTTTGCATATCCTCCCAGCACAAAAGAGC
AGGCCTTGCGTGCGGTGACCCGGCAAGCCAGTCAGCACACCCCCTCTTGGGATGTCATGT
CTGCGCTCTCTGGCGTATGCGTCGGCATGCGTGAACGCAAAATCCTGCGGAGGGCGCGGT
GGGCGACCAGGCGGTCAGAACCAGAAGTTCACGGAGAAGGAAGGCGTCAGAGCTCTCACC
TGGTGCCACCAGCCCTGTGGGATGAAGAGCGCGTCTCCCGGCGCCAGCTCTGCAACTAAG
CACTGCGCGTTTGCAAATTCAGGGAATCTCTCCAGGTCTGGCGCCTCCACGTCCACCTGT
GAGATGTTCCCTTGCGAGGAGGTGCCGCTGCCAGAGGCCTCCACGTACAGCCGGCCCTTC
TCCTTCGGTGCCAGGAGGACTGCACGCTTGGTGCCCTGGACCTGCGTGAGGAAGTTGTCG
TAGGAGTCCCAGTGGCAGGGGGTGACGGTGCCGTGCGTGCCCAGCCACACGTTGGTCCGG
GTGGGCTTGCCCAACACCCTCTTCCAGTCCTCCGGGAGGCTCATGTCGCTGCGCAGCGCA
GGGCACTGCTCCAGGAGCTCGTGCTGCGCCAGGTAGGCCACCCCGGACTTGCAGCTCTCA
CGCGGCTCCAGCCAGTGGGAGCAGTACTCACAGCTCGGCGCCAGGTAGGCGCTCAGGAAG
G
>at01310
GGTGCAGTCTCAGCACCGGCTGCTGCCGGGCGCGAGAGAGACTCTTGGCCTCTGGACCCG
GGCACCCCTGTGCCGACGCTGGCTCTCTGCTGGGTGGCTGCGTGGGATAGTGGGCAGCTG
GACAACTTCACGGCGGCCAACGTGAGGCACTTCCACAAGGCCTACCCCCAGTTTATGAGC
AACGCCACGCTGTGGGTCGACGAGGCCACGAAGGTGCCCCCGGACGTGCGGGAGAAGGTC
CGGGTCCGGATCCTGCCGCGCGGCTACGACGGCAAGATTGGGAAGACCTACGCCTTGAGG
TATTCTGAGCCTATGGACACCTCACATGTGCTCTTCCTGGACGGAGACACGTGGGTCTGC
CCAGGCTGGATAGACTTCCTCTATTGGCATCTTGTGGACACATCCGTGGACGTGGTGTGG
ACGATCACGCCCATCAAGTGTGGCCGCTACGTAGACTTCAAGTGCGACGTCGCCATGAAC
CCTGCCATCTGGAACAACAGCAGGGAACTGTCGCATTTCAAGAGGTTTCCCGAGCGGAAC
TCCGGCACCGTCTTTGCGGTGAGGCGCTCCGAGCGGACCATGCAGTGGCTCACTGACGCT
GCCGAGCTGTATCCTCGCATGGTCGAGGCTGGGGTGGTGCTTGGGGACCAGCCTGCGCTG
CGCGAGTCCTTCTTCCTGCACAGAGACAGGCTCAAGGAGGTGATCGTTCACGAGAATCAC
GGCTGCAGGGCAAACCTTTGGAAGCTGCCCTTCCATCTGCAATGGAAGGGCCAGTGCCCC
TGCAAATGCTCCTGCTCGACCTGCTTCTTCATACACAACAAGCAGGAGTTCCCTGAGTGC
GCCCGGGACCTCGATGTCTGGCAGCCTCAGCACTGAGCGGCCGGCTCTCCGCCTTCCTTC
GGCCCCTCAGCGCGCCGGCTTCTGGCCACGGGGTG

>at01311
CCTCTTTGCAAACCTGCATAAAAACATAGCCCGCTCCCTCAGCTGTCTTCGCACGGGCCG
CCTGACATGGAACGCACTTTGGGTTCCAAATTCGAGAATGGAGCACCCTGCCGGTAGTTG
CTCCCTGTGCATCTGGCGCCATTGCCATTCCTGAAGGCAAAGNGGCAAGCACGGCTCTGT
ACATTGCCGGCCTAAAGATGGCCACTTCGCTGACGG
>at01312
TGTCATCTTGGTAGGAGATGAAGAAGTTCATGACGAGGTCCATCCAGAAGAGCAGGCTCA
GAATGACGTCCGCGATCACCCACCCCACGTTGTCCTCGCCGAGGGATCTAGACCGTCGGG
GCCGATGTGGAAGCGGAAGAACGCGAAGCGGTAGAGGAAGATGGTTGACGTGTAAAGCAA
GAAGAGCATCACGACAGTAGACCAGATGCGCCGGGCAAGGCTCTCCTCCTTCAGGACGCA
GCAGCTCCGGGCCTGCTCCTGTTCCTCCGCCCGCGCCTCCGACTCCTCCCGGGCCAGCGT
CTTCGCCAGCTTCGAGATCCCGCTTTGTCTCGACACTTCGGTGGAGCTGCGCTTCGGCGC
CCGGCTCGGGCGGTGCCCGACCGACCCGGAAGCGCAGACAACCTCCATGGCGGCCGGGCC
>at01313
GCTTTGTTTCAAGCCGCATATCACAGATATCCGATTGAGAACATCCTGGAGGTAAGGATT
GAAAGCTTTGACGAGAACAACTACGACGACATGACAAGAACCGTCTACGAACACTTCCTT
GGCAAGAACGATACCCGAATCGCTAAGCTTGTGAAGAGCGCCAGCAGGTACGATATCTCC
CGCATGACTCTTTCTCAGAGAGCTGACATATACCATCCAAGTGATGCAAACAATACGGCA
GAAGCGATGATCGGGATGCAGAAATTGTTTGAAGCGGGGGACCCATGCATCAAGGGTATC
GTGAAGATGGACGAGTTCATGGGCTACAGCTCTGGCTTTACATTTTGAGCACACCTCAAT
GCATCATGGCGACATTTTGACGCCGTTGCT
>at01314
CTCCACCCTCTCCAGTGCCTTTGTTGCCAGACTCAAAGTGAAGGAGCCGAGCTCGTGTCC
ACACGTCGTGGACAGCCAGAGTGCATCTTCGCGATCCCCCAACCAGCCAAAGGCGTCAAT
CTGCATGGACCCCGGCGTGAGCTTTGTAGGTGCGGCCGGGCCCCCAGCGTGCAGATCCAC
TAACCAGAGGTCCATATGTTGCGTGATGGGGTAGCTATCAGTGTGATTTGCCTGGTAGAC
GATTCGCGCACCACTGGGTGAGAAGCGGGCAGCATTCCGCCCCACGCGGCCAAGCGGGCC
CTGGGAGATCTTCCGGGGAACGGCCCCTTGACAAGCT
>at01315
TTCTCAACGGCTTGGCAAGGAAGCGGCAAGCAGCCATCGCCATCAAGGTCCTCGAGATCA
TGAAGGATGAGCATGTCGAAGTCAATACCTTCCATTGCAACACTGTCATCAATGCGTGCG
CGAAAAGCGGCCAGTGGCAACTTGCCATCGCTCAGTTTGCCTCCATGCCACGACGGGATT
TTGAACCCAACGTTGTCAGTTTCAACGCTGCCACCAGCGCCTGCGAGAGAGCGCCCAGTG
GCCACGCGCACTCGCCCTCTTTGCCTCAATGCCCCGCATGCGAATTGCGCCAGACCGTCG
TCAGCTGGCGACGCCGCCATCAGCGCCCGCGAGAAGGCCGGGCAGTGGCAGCGCGCGCTC
GCAGTCCTCGCCGCCATGCCTCTCCTGCGGATCTCGCCGGACA
>at01316
TGTGTTTTTTCTTTTTTTTTTGAAACTGCTTTGCACAGAATTGTGTCCTCGATGCAAGCA
CTCTCTACACGCAGGGACGGGCGCCAAGCCTCAGAGCCCATCCAGCAGCGGCCTGCCACC
GCCGTGTCGGCCTGAGGAGAAAAAAGCGGCCGTCAAAGGACGCGAAAGGGCCCCCCTCGC
GCTCGCCGGCGAGGGGGGCAATTATGTCAGCGGCCAGCCTCAGCTGACCACCGCCTCGGC
TCCGGGCTCGTCGTCGCTCGCGTCGGCGCCCTCGGCCTCTCCGCGGCCACGGCCGCTGTC
GCTCTTGTCCTCGGAGGCGTAGAGGCCGTGCTTCGCACGCCTCTCGCCGCGCCCGGCGTC
TGGCTCCGAGCCCACGGCCTCGCCGTCCTCGAGTTCAAATCCGGAGCGCTCGGCCCCGTC
CTCGGAGGGGTAGGCGAGCTCGGCGTGGACCTTCGCCATTGGCTCGATCAGCGGGGGTGG
CAGCAGCGCCGTGGGGTCGTCGATGGTCAGCGGGTCCTCAAGCGCGGAGTCCGTCCCCCG
GCCTCCGCCGCCGTCCTCCAGGCCGGAGGAAGAGGCGTTCGCGGAGGTGGCGTTGCCGCC
GGGGCCGCCGCTGCCGGCTGCGGCCGCAAGGCCCGCGCCAGCGTTGGCGCCTGCACCTTT
GTCGGTGCTGGAACTGGTACCTTTGGTCTTGCTGTGACTGACATCCGGGGCGCTGCTTGC
GTTGTCGCCGCCAAGGNCGGAGTCAAAGCTGTCGCCGCCTACTCTGGCAATCTGGTCACA
GCCGATGTGCCTGTGCAGGCAGCACCAGTCCAGCTCATACGGAGACCAATCCTCGAAAGT
CTCGGGGAAGTTGGCCTTGCAACTGAAGGACGTGGCGTTGGAGCCGCGGCA
>at01317
TTTTTTTTTTTTTTTTTTACACGTGTTGCTTCCATGATATCTCGGGGACTTCTGTTGCCC
AGCACGTCCAGTCCGCAAGCACACCGACTTCACGTTCGACGTCCTCGAACGTGGTCGCTG

CCAGGACCTGGGGGAGGGCTGCACGTCCGTCGATCTCTCTCAGACAGGGCACCCTCCGGC
AGTCGCGACCTCGAACACGTCAGCGCGAGTGCGCAGGTCCTTGGCCGCTCCAGAAGTCCC
GCGGCGACGCCGCGCTTCCTGCGAGCGCCTTCAGCGAAAGACGCCGTGGCTCGCACCTAG
CAAGAACTCGGTGACGTTCCCGTCCGCGACCGAAATCTGCGACTCCTGATGCTGGCCCGG
CACGTACAGCCGCTGCACCTGCGAAGGGAAGTAGGAAGGCCGCCGCCGCGGTCCCCAGGA
GGAACTGGTTGCTCCAGCCGCGCGACTCGAAGCCCTGCTGCGCGTCCATGAACTTGAAGA
AGTCGCGGTAGAGGGGCTCCCTGAAGAACTGGACGTCCCCGCCGAAGAGGCAGCCGTACA
CGACAGTCGCGGCCTCCAAGGCCTCGGGCACCTCGCTCACGGGGATCCCGAGCTTCTCCG
CCTTCTCCTCGAAGCGGCCCCGGGCATAGAAGGCGCGGTTCCGGATGGGAATCCCGCGGT
CCTCCAAGAAGCCGACGGGCGCATGCATGTAGCCTGCCTGGCGCTGCCCCTGCACGTGGA
TCTGCCAGTANGCAAGCCGCGACCCCTCTTGCCTCATCAGCTCGATCGGATCTTGCTCCA
GTGGAGCAGTGAAGTAGAAGTCCGTGTCTATCTTGAGGAAGTGCCGGCACTGGTCCAGTG
CTGGATGCATGAAGAGCGGGCCAGCTGTGTAGCGAGATATCCTCAGGTAGTCAGGAGACC
AGAACTGCGTTGCATTGACCACTCCACGATGAGCAGAGCTCTGCAGCGTGCGGCCTGCTA
CGCAATCCATCCCAGCAATGCAGCTGTAAGATTGCATCTCTCGGGTCAGCTCCTCTGGAG
GGATGACGGCAGGGACCACAGTCGCCGAAGTGAACGCTTGAAATTCTGCCTTCAGTGTCG
GCGCCGTTCTGGCGACAGTGAAGACAATGAGCGGGTATTTGACCCGCAGGGGAGTGAAGA
AGTACTTCTCTAGTGAGCGCAGAGCCGCTGAGGTG
>at01318
CGTCCGAGTATGGTCTTGGGATTCTGCCAATAGCAGGTGGGATCCTGAGACAGTGGAGAC
CCCCGAGGGTCCACAGGTAGCGGTGCGCGATGTCGTGTGGAAGATCTGGGATGGCGTGTG
CGAGATGATCGCGGCCGCCGCCGGGCAGCACGTGATCATCTGGCAGCAGGACCCTGCAGA
CTCCGAAGAGGGGGCCAAGGTGACGTGGAAGGTGACGGGCTTCATGGAGCTGGGCGAGGA
GGTCTGGAAGCTCTCCTTCACGGAGCAGGGCGGCGTCATCCTGGCCTCCATCGGCGAGGA
GGAGCAGCAGAGCATTTTGCTGAAGCAGCGGCTCGATGGCTGCTGGGACGCGGTCCCCAT
AGAGGAGGAGGGAGAGCAACTGATGGCTGTGTGATGGGGGGCCTTGGCGGA
>at01319
CTGCCGAGGCCTTGAGCTACATCAAGCGATACGAGTACTTTGTCTTCCCCGAGGCCGGCC
TCGACTCCGAGATGACGGGGAACCAGGAAGGGCTACTTGCATGGCATGTCCCCGAGGCGC
TCGACGTGGAGGCGATGCGGGTGGCGGCCGCCTCCTTGGAGGGCGAGCATGACTTCCGCG
CCTTCACCGCCAAGCATGGGCACAAGAGGGTGACTCGGCGCCGACTTCTGGAGGTGACTG
TGAACCGCGTCCAGGGCCCATGCAGGCGCGGCCAGCCGCCGACCGAGCTTCTCCAAGTCC
GAGTCGTGGGCGAGAGCTTCCTCTATCGAATGGTGCG
>at01320
AGAACGCTGTTCAGTATGTAAATGGATATGACCATGTAGGGGAAGAAGAACAAAAAGGTC
GCGCGGTGCTCATTATATCCTTTGATCCACATATCTGGATTGTTAGCCGTTGTGAACAAG
ATCCACATTCCAGATAATGCCACCTGCCAGGATTCGAACATTACACCCTCAGCAGTGTTT
GAGAAGATGACGACCCCCATCCATGTAAAAACCAGGATGCAAATCGCCAGCGCAACCAAT
GCACTCGTAAAGCCAGGGATTGACCGTAAAACCCTGGTCGTTGACAGGCGCAAAGGCTTG
TAGCAAGCCATAAATATCAGTGGCCGGCACAAGCGGGCTACCCGAAATCTGCCCCACCAC
GTGACTGACTTGACGCTGAAGACCGCCCTACGCAGTCCAACATCGCGAGGACCTGCACGC
>at01321
GCGAGGCGTTTCATCATGTGGAACGTGGAAAACAAGGCGCCTGCTGACATCAACTACTGG
TACGAGAAGACGGTGAGGTTCGACTCCTCTCTCATAGCCGCTAACTCTTCGGGATGAAGC
ATGCACTCCGGAGGCATGACTCATTAGAAGTCCGCGTTCCCCGTGAGCCTCTCAGCCTGG
CCTCTGCTGCGTCCGACTTTCACCTTTGATTGCCTAAGGTTCATTCTCTGCAGAGGCTGT
GCGTAAGTCAACAGCTCTGCTGGCAGAAGAGCGGAAGGGGTGGGTGAGTGCCAGCTCTGA
GGATTCGTGAGCAGATCAACAGCAACATTAATTGCGACTGCAACAAAAAAAAAAGAAAAA
AAAAACAA
>at01322
CCGTAGCCATTTTGGCTCAAGCCATCATCGTTCCATGAGCAGGCGCTGCTCATGCCATTG
CATCAATATATGTGATGCATGCCCTCTGAGTAGCTCCGTGCGCCCATGGCTCTCCGAACG
CCCGATCGTTCTGGTGCACTGCGGCATTTGACCCCAGCGTGGAAGACCAGGGCTGAGTCG
GGCAAGGCCCCCGATGTGCTGGATGCGGCGTCTCTGCTGGCAGGGGGGACAGGGATGGAT
GTTCGCGTCTCTTGCACTGCACAGTGTGGGGGTGATGACATGCCCATCGAGGATTTTGAC

CTGGCAGAGCTTGAGGCGGCGATCTCACGTGTCTGTGCCATTGGGCAGCCTCAGGAGCGC
TGGCGCTTGACAGCGTGCTGTATCAAGTGCG
>at01323
AGCGGCCGGAACTCCTCGTAGGTGACCTTGCCGTTGCCGTCCCGGTCTATGGCCGCCCCA
CTCCTGCTCCGCCCCCGCCCCCCGCCCAGGCTCCGCAGGGCTCGCTTCATGGTCGCGCCG
CAGAAGCGGGCCTCCAGGCGCCGGGAGAGGACGTTCCTGGAAGCGCCTCAGGAACGCCCC
CATAGTGGCATGAGCCCTCCTGGCCAAACTGCGCCAGGTGCGGCGCCAGCCGCTCCCCAG
GGGGAAGTCCTCGTCGGGCACCACGCAGGCTCGCATGGCGGAGACCCACTCGGCCAGGAG
GCCTTGCCCTTCATCTCGCGGTCGATGCTGTTGAAGTAGCTCAGCAAGGCAGGACGCGCG
CAGAAGATGAGCAGCCAGAGCTGCTCCAGCACCTGCT
>at01324
CATTTGGTTGTAGCTTCAACGCAGCTTGAAGTGGGTGCCCCCTTGGACAACCACTTCCAG
GGATGGCAGCACTGCTGACAGTGTGCATTGCACAAGCTGCTGTGGCACTTGCCAGTGCCA
GCCAAGTCGAAAGCCAGGGTGTGCTGCGGCACTCCAAGCACTCCAAGCATGATGCTGATA
GCGCGGATGGCTTTGTGAGGACGCTGGAATTCAAGCACCGCCTCCGCATCTGCAATGCTT
ACCATATGGTGCTGCAATGGACGTGTA
>at01325
GCGCGGCTACCGGATGGTCCCTCTTCTCGACACGACGCGGTGCAAACAAAACATCGGAGG
CGCCACCCTCACTCCGATAAACGACGAATCCCTCCCTCAGGGGTGTGTGAAGCACAAGGA
CAAATTCTACATGAACGAAGCAAAGAGTAACGCGATAGCTGGCACGCAAGCGGAATCGAA
GTCCAACACTCTTAACGTGATGACTCATACTTCCCAGCTCTGCGAGATGGACCGCAGCTT
GACAGAGTCTGCTGCATTCTATTACTCGCCCTACAAATTGAAAGCAGGTGTGCCTCCGGC
ACGCTCGACTTCGTGCTCTTTCGGCATGATGGCCTACCTCAGCAAATTGGGAGAGACCAA
GAGTTCATGCGCCGCCATTCTCACATACAAGGCACATCCGGACCTCCCAAGTGCAAAAGA
TGCCATCGAGCGGGCCGATGCGCTTGCCAAGTCTGGCAAGACAGAGGCTGCCGAGGCTCT
CCGGGCATACGTCGAAAGCATCCGACAGAAGTCCGTGCTCATTGACACTATCTCAAATCA
TTGGTGCCCTGACACTTGGACGAAAGCGCAGACGAGCGGGAACACGTGCGATGTACAGCG
TCTGTCGACGTGGACAGAAGAGTTCCAGAGCGCAAACCCTCCCAGTGCTATCCCAAGTGC
CTCTACCATGGCCAGCAGGGCAACTACTAAAAAAGGCGGCAACAGGCAGGATGTCACAAG
CCGTCCAAGCAGGCAGAGCGTCAGTGTTACCAGGCAACGCAGACTTGGTCCAGCGTGCCC
GCAAAGCGCTGCTGGCCAACGCCGCTGAGGAAGACAAGAGGGAGACGGTTTCTGGGCTCC
ATGCGATGAGGCATGCGCTGCCTGGCGCTATCAAGCCCTCAAAGCTTGCTCTCATGGAGA
GTGCATGCGATGAATTTGAAGACGAGACCCTTCAATCGATCTTGGATCGTATTGGGGCAA
CACCACCGAAATGGTATTACCAGATCCCCGAGGACGCTGCCGACGTGTCCTGAAAGGGTT
AAGATGTTGGGCTGAGCTTGGCGCAGCAGTTGTCATTCGTCCGGTTAGTGACCATGCTCG
GATAAG
>at01326
NTTTTTNNTNNNTTNNNTNTTCANCCACACGCGGATATCTGGAGCAGANGGAGACGCGCA
ATGNTGTTAAAcAAAATTACGGTCGANGAAACTCATCAACAGCTTNAGGCTCGTCTCCNG
ACTACTTNGNCANAAAAGTTGTNGCNGCTAGCAGNTCCANGNCaACCAGACTCTGCCACT
GGCCCAAACGGCCAATGGCCGTACGCCGGTACTCTCTCTCTCTTTGCCGTCCTTGCGGAG
GGCTCACCGGTCGAAGCCGAAAGGGCACCCGCCCNCCGNGCTTTGGACCTTCCCAGCGAT
GTGCTNGATGGTGTAGTCNAGGTACTCGTCGAAAGTGATGGTGCCACCGCCGTCCTTGTC
CATGCCCTGGAAGAGCTGCNTGCGGGAGGCGAGGCGCTCAGCGTCGCTCTTGAACATGTC
AGAGGACTTGGGCGCCAGGCCGAGGGCGCGCGGGGCGTCTGCAGAGAGCTCCACGAGATA
GTCGAACTGCTCCGCCGTGACGGCTCCCTTGGCCTCCTTGTCCGCCAACACGAAGTTGGT
GAAGAGGAACTGGTACAACTCCGNGANCTcCNCGNGAtNNTCNNNNTGCTCCGCCGTGAC
CGGCTCCCTTGGCCTCCTTGTCCGCCAACACGAAGTTGGTGAAGAGGAACTGGTACAACT
CCGTGAACTCGCGCGACTTCTTGTTGCTCGTCGCCGTCTTCAGGAACGCAATGAAAGTAT
CCTTGCCGCTCTAGGGCAGGTTCGCAAAGTCCACGGTCTCCTTGTGCATGCCCTTGACCT
TCCCTTCGATATGGCGGAAAGAGTACTGGATCCAGTCGTCTATGCCGATGGTGCCCCGCT
TGGAGCTGTCCACCTTCTGGAAGAGCTCGCGCCGGGCGGCCTGGCGCGCCTCCACGGAGC
TGTAGAGATCGCGCCAGCTGGGCGCCAGGCCGAACCTCCTAGGGACAAGACGCAGCTGCC
TTCTCGCACAGG
>at01327

TTTTTTTTTTTTTTTTTTGCAAGGAGCATGTGCAATCCCCCAAGCATCAATGACTAGCCA
GTAGGGATGCACTTAATACACACCACCATTGCTGAACTTCTTCCTCGTCTTGATCAAGCC
CGGTTTGACTCGTCTGCGCTCCCCCACAGGATCGCTGGTCTTTCGGACCTCGTCGACCAC
CAAGATCTTGAGCAGCTCGGAGAGTGACTGCATTGTGCCACGCCTCTGCTTCACCAAGCC
GAGCAGGGAAGACGGCGTCGTGACTAGGATAGAGGGAAGCTCTGCCATGAGCACATCAAA
GCAGAACGAGAGGAGCTGCGTGATGACGCCATGGACCTGCACGATCAGCTCCTCTGCTGG
CAGGGGAGGGGTGTCCTTCGTGGGCACCAGCACCACGCCGCCCAAGGGTGAGGCCGCGCC
CTTGGCCTCTCTCGGTTCCGCTCGAGCGTTGGAGGCCGCTTGAGCCAAAATGGCTACGG
>at01328
TTTTTTTTTTTTTTTTTTGCTGAGGTCAGGGCCCAGAAACTCCGCCGTTCTAGTCGAAGG
CTGTTCTCCGCGAGTGCGGCAGTCAGGTTGGACAAGCACCTGGCTCTGTCTGTACAAGAG
CTTCCGATCTTGGCTTGTTGGTAAAGCCCTTTGGCAGAAGCTACGTGCTCAGCTTCTTGC
ACGAGGCTAAGGCTTCAGACATCTGCTGCGCATGGTCCGACTCAAAGATCAGGCTGCACG
AAGCAATGGCCTTGGACATCTGGTTCACCTGCTCCTGCACGTCCCGGAGCTGCGAGGTCA
CGTGCATCAGCGAGCGGTTGTCCGCAGGCGTCGCCGCCTCATCCAGCTGCAGGATGAACT
TCTCGTAGGCGAGGACCTGGCCGCGGAAGCCGGCAAGTCTCCCCGCACCAGCGGCGTTGG
GCCGCGCGGCGCTCTCGAAGGTGTCCTGCAGCACTCGCCTCGTCAGCGCGGTGGCGTCCA
GCAGCTCCCCTCCCCTCACGGGGTCCTCCAGCAGCGCGCTCCACTCCTGCCATCTCGGCG
AAGCGCCTGCGCGCTGATGAGGCCGCTGCGGTCACGGTCGCAGCCGTGGAAGAGGTCCAG
CAGGCGCTCCGCGCGCAGCTGTCGCTCAGCTTCATGGTGCTGCGCCAAGTCCTCCTCAGC
AGCCTTGGCAGAGGATGTCATCATGTTGTCACTCACCACGGAAGTAAGAATAGCCAGCAC
GGGCCAGTTGGAGAGAACCATGAANGGCGCAAACATGAGCTGCCCTACGGCGGTGTCGGT
GATGGGCTCGAC
>at01329
TTTTTTTTTTTTTTTTTTCCAGCCAAGCGAAGCAGCCAGTCGTCCCTGCACTCCCACGCG
CGCGAATTCGGTGGGCGCGTCCTCGCTCCTCCCCCCCCTACGCGCCCCCACTCCGGCCGT
GCGGCAGCCCCTCACGGGACCCGCCGCTACCGCCAGCAGCACGCGGTGGGCGCCCGGCGG
TCGGCCTTGGGCATGGTGTCCTTGCTCACATGCTCCGAGGACCCTGGCTCGTCCATAAAC
TTCTGCCAAGCCTTGGCTTCGAGCTGTGCCTGCGACACAACCAAAGGCATGACCTCGATC
TTCCAACGGTCAGCTATGACGCAGGGCATGTGGGTCACCCCCTGCATCTTGCCAGCCGCC
CACGGCCCCCAGCCGCCCTCGGCCCAATCGTTGTCGGACTCGGAGGGCACGTCCAAGACG
GCCGCTGGGCGGATGATGAAGCCGCTCCCCCAGTTGGCAACTGCCAAGGCCACGTCTATG
TGGGCCACCGACACGCCCTTGGCGACCGCCGCTTCCATGGCGGCTCGCGAGATCGCGTAC
CAGCGCGTGAAGTAGAAGGAGCCGCGCCGGACCCCCTCAGGCAGCTGCTCTGCATCACCC
TCTCTGGTCAGCAGCACCTCGCCCCCAGAGTTCTGCAGAGAAGCATGCCGCCAAGCAATC
CCCGAGTCCACCAACTTCAGCAAGCTCTCAAAGGCGCGCCCCGCGTGCTCCGAGAAGCGG
ACGTCGTCTTCGCAGACGACGGCGAAGGGCGCGCCCTGCCTCAGTGCCGCCTCGAAGACG
GCGAGGGGGGATTCGAAGCAGCCCACGCGCCCGCTCTCAGGATGCCGCTTCGGCCGGTGC
CAGTGCACGCGCTCGAGCACGCCCATGCGATCGAATTCCTCGGTGATCATGTCATACCGG
TCCGTACGCTCCTCGCAATTGATGCAAGCCAGGAAGGGCACCCGATCCATGGAAAAGCTT
GAGGTGTGGGAAGGGAGGCGCGCATCAAACAAAACATGCATGTGCTTGAGCCAAAATGGC
TACGG
>at01330
TGTTTTTTTCTTTTTTTTTACTACACATCTACAGCCACACGCGGAAGCCCCATGAAGAGG
CATAGGGAAGCTGGCCTCCATTGCAGGCTCCCTATGCATGGCCCAGCACCCCAGCCAGCT
GCCATCATGGTGGCAGCAAGGCTACGCCCAGCGCTTCGCCGCCGCGCGCGCCGACGATGT
CGACTTCCCCGCCTGAAGCAGCTCTTGGAAGGCCGGGGCGCCGCTGAGCGCCTCATGGTC
GCGCAGCTCCCAGGTGAACTCGAAGCCCTTCTTCTTCGGGTCGTGCGCCAGGAGGCCACA
GGCGCCGCAGTAGACATACGGCTCGGTCGCCAGCCGCCGCACGAACATGAGGACCCTCGT
CTGCCTCTCGGCCTTCTCCCCCCGCGAGGTGCTGAGGAGGCGCTGGACAATGGGCGACTG
CTCCGTGGGCCGGGCGCCGCCCACGAACCAGTTGACCTGCCGGCCGCCGCGCTTGAAGGC
GTTGGTGAAGGAGCCGCCCGCGGCGTTGACCCACAGGAAGATCGCGTTCCGCCACTCGAC
CCAGCCGGCGTACTTGTTGAAGCGCGGCGGCGCCGGCACGCCCTCGCCCTTCTTGTCCGC
CGGCGCCACGAGCTGCAGCACGCCCCGCTTCCCGAAGAGCGGGACCGGCCTGCCAAGGGA
ACCCCGCAAGCGGCCCAAGTCAGCTGACAGCGCCTTGGTAGAGGCTCCGGCGGACGGTCT

CTGGAGCACGAAGGCGGCCATGGCCTCGCCCT
>at01331
TTTTTTTTTTTTTTTTTTAGTCGACTGATGCGGTAGCTTAATGGACTGCTTCGCTGGCAC
GTGTTTCCAACCTCACCAAGCAAGAACGCCCTGTACACGCTCTCAACTCGGTGCAGAGGT
GCCCTGTACATGCAGCGCGCGGCATCGCGCTCCAACACAAGTTGGGAAGGTATCAATCCG
GCCAAGGCTGGACGAACCTTCACCCCACTGGGTTCCTCGTTCACATCTGGTTACGATAGA
TCGACTCGATGTTCTGAACGAAAGGCTGCGGGTGCTGCAAGCCTCGGCGCTCGTGGACGT
CGCCTGCTCGAAGGGTCCGAACCGCGCCAGCCTGGCCCTGTGCGGCGTAGACGAGGGTCT
CAGGGGCGTCTGAGCGGAGGCCGCGCTCCAGCGCATCGAGGTCAAGCCCCTCGATGCCAC
CGTTCAGATTCAAGAAACCCAGATGCCCCGCCAATAGCGGGCCCGAAGACATCCCTGCGT
CCGTCAGGTCGTTGTGGCGGCCATCGAAGTAGGCCGTCGACTTGTAGGAGCCAAGCGTCA
GCGCCTGGAGAGCAAGCGACGTCGCGGCCACCTCGAGCAAGAGGGGCACCGACGCGTCGG
ACAGACCGTCCTCGTGCC
>at01332
TTTTTTTTTTTTTTTTTTCCTCGTTGTCACAGTGCACGTAGCTCCGGAAGGCAGCGCAAC
ACGGAGTGCAGCATGGTGCCACACAGGCGGAGCTCCAAGCAAAAGTGGCGTGCAAAGTGG
TCATGCCCGCGTGTAACTCACGATGTAGTTGGGGTAGACCTGGTCCTCGTCAAAGACTGT
GAACTCCCGGAAAGTCCCCCGGCACTTCTTGCGATCTCCCAGCACAGCGTGATACTCACC
TTTCAGGCACGACTCCTCACAGTTGCGTGGGTCCGGTGAGACCTCATCGTTGTAGAGGAG
GTGGCCCAGAGTCACGCGGCACAGCAGCATCGTGAAAAGCCCGGTCCTCCGGTCTGCCGA
GCTGTATTCGTCGGCCTTTGATGAGTTCTCAGCCAAGTAAATACCGCGGCCATATAGGGA
GCCGGTTGACGAACCAGCCAGATCAATCCGAAAGTCATCAGATGTGATGGAGTTGGCAGC
AATCGGCTTGGTCCCATGGAATAACCAGGCCTCGTTCACGGCCGTCTCCAACGGCGGGCC
CGGGAGCTCCGGGGCCAGGGCGGCCTGGAGCCAGAGCTCCGAGCCCGGGGGCTGGTCCGC
GGTGAGCCGGATCCTCACGCACCCATCGCCAACCGGACTCTCCACTTCCAAGAGGTCCCC
CGGTTTGGTGCCTTCTGGTACTGTGCGAGACACCGCCGGAGCAACAGCCGTTGCGGCTGC
CTCAGTGAGGGGCTGCTCGGTGTCGGCGTTGGCAAGGAAGAAACCGGAAGGCACATCCTG
CCCGACCTCGGCTCTGGATCGGAGCTCGGACTTCACGAACTCCCGGCGAGCGATGTAGTC
GACCCACTGGACCTCGTTCTTGGCCGCGCCACGTTGTCGCTGCTCCAGCATTTTTGCGAC
ATCCCAACCTTCTGGCATTTGCCGCACCTCCCTGTACATGTTCACCGCTGGCTCAAACTC
CGGCACGTCTGTTGCGTGCAGGCGCTTGGCGGCCACCAGTGCCCCCTTCAGCCGCCTGGG
ATCACCAGTGGCTGTTGCATCGCGCAGGTTGCGTCCAGCTGTCCGCCGTGCAGCTTGGCG
GAGGGCTGCGGGGAGCCTGTCGTTGGTCAGACCACGTTCGAGTACGGTATCCAGGAGCAG
CTCAACCTTCCGGTCATCACACCGGGTAAGCGCCTCCTCCATCTGGTGCTCCAACTGCTC
GCGGAAACGCGCCGTTGAGCGCTTCGCAGCCTCCACAGCGTGGTCCAGCCACTGCTCCGT
TCCAGTGACTTTCGCGACTTTCCCAACTGCATGGCCAGCGTGCTGCCCTATCGCACTGCC
CACACTGGCCATACGCTGAAACCATTTGCCTGAAGACGATGAAGACGTGGATGTCCGACT
CATTCGTCTTGAGCTTAGATCCAGTGAGTGACCGTCGCTCATGCGTGGAGGATCCCCTGG
CTCAACGTCAAAGCTGCCTCTATGAGCTGAACCCAGAGCCACATGTCCTCATCAGTCGAA
GCACGTAGTTCATAGACCCGCTCCTGCCCACCTGTCAGGCGTGCCCGCAATTTGATTATC
CGTGGTGGGTCTGCTTCGACGTGCAGAACATCCCCAGCTACCAAAGATAACCGTGGGTCC
AGACAGCCATCCTTGAAGTACTGCAGCGACCCGTGGTCTAAGTGGACACGCCGCGGCTGC
CAGAGACCCAACAGCTCAGAGCGCTTCTCCAGAGTTCCCTCGTGTGGAAGGTCCCATGAA
CGGCCAACAGAGTCAAGTCGATCAGGTGGGCTTCCCATGGCAACCTCACATGTTGTTGGG
AAAAGGCACCAAGATTATTTCACTTGAGCCAAAATGGCTACGG
>at01333
TTTTTTTTTTTTTTTTTTCGTGCACTGCTGATGCCTCGACTCCATGAACAGAGGCTCTGG
GAAGGAGCTGGCCGCGCTCAAAGCAAGGCCAGCCAAGAGAAGGCTCACGCAAGGATTTTA
GTCTCTCCGGGCAAGCCAGGGATGGCCTACTCCGACTTGAGCTGAGGCTTCGCCACGCTG
AAGATGGGCATGCTTCCCAGGGCCTTGGGGGCCATGTGCGCCTCCCTGATCCGGAACGGC
AAGCTCGGCCCAGCTCCACCGCCCTGCGCACCGCCCTGCGGCCCCGCCTTGTCAGCCGTC
AGGGCCCCCTCCTCGAAGCGGCTCTGTGCCCTCGCAGCGCCCCACTGCACTGGTGGCTTC
CTCGCGGCGGTGCGGTCGAAGGCGGAGAGGCCCTTGGAGGACTCCTTGATCTCCTGCTCA
AGCAAGTCGAGGCCGCTCATGCTTGTAACACCTCACTGCACGACTTGTCTGGCAGCTGAT
GGCAGCCAGCAGAATGCCGGGTGCCTCACCGAGTCAAGGGGCTTGAGCCAAAATGGCTAC

GG
>at01334
CGTTGCGCACACAACCCCGCCGACAGACCACAGGAAGGCAATCTGCAATGCAGCCCAGGA
GAAGCCAAACAAGTGGAAGAAGCCGATCACCAAGGCAAGACCCAGGGAAGCGACAAAGGC
CGCAACATGCGCCACTCGACTCTCCATGTCGTGCACCTCCTCGAAGCACGCCCGGTAGTC
CGGCGCATGCACGGTCACGCGCGGCAAGGCCGCTGCCGCCCCCGTGCGCGGACCGGACGC
AAGATCCCTCGCCTGCCGCTCACGATGAGGCTGCCAGATCGATGCTGTCGACCTCTGGGC
AAGCATGGTCTTCTGGGGCCCGGTGGGCATCGCAGAAGGCGCGGGAGCACCGGGCAAGGC
CATGGCCGAGGCGGCAAGTGGGAGGTGCTTTCCGCA
>at01335
TTTTTTTTTTTTTTTTTTCAGTGGCACTGGACGGCAGCAGCATCGGCAGCTCATGCGCCA
TGTCTCCCGCAGCTCTTCCGCCGCAGCGCAAGGCTGGGGTGCTCCCGAGTTCCCGCCGCG
GCGCCCTTCAGTTCCTGGAGTACTCGCGGGCGCCCAGGTGGTCGTGCTTCATGATCTCCT
TGACCTTCATGATGTTGGCCTCGTCCACGTACGGCGGCAGCGCGATTGAGGCGGCAAAGA
CCGGCACCATCGGTGCCACTTTAAAGATGAAGAACAAGCAGATGATCGCAGTGATACATG
CACCAAGAAGCGCCTGCACAGCATCCCGTCACAATGCGGGTCGTTTGGATCCAATGCCAA
CACCAGTCTACAACCGTGTTTTGTGGCTAACGGCATCCACGCTGTGCTCCGCACAAGCGC
TTGTGCTCCATGTTGCCAAACTGTGAAGACAAGAAGGCCGGCGAGATTTACATGGTCAAA
TCTTCTGACAGGGGAAAGCCGCCTTCGGCGACCCTCTCACCCTTACTTGGTCCTCGTGCC
>at01336
CCGTAGCCATTTTGGCTCAAGCCGTCCTTCAGCCCATTGTGATTCACGACCTTCGAAGGA
GGACCACTAGGGATGGCAGGTGCCAATGTGCCTCTTGTGCTGTCCGAGGAGATACCCTTC
GAGAAGGAGCACGGCACCGGGGAGCTGGACCTCCTGCGGCCCCTGGGCGGGCCCTTCTGG
TGGAATTCCCTCTATTGGACGAACTTCCCACCGCTTGCCTGCTTTGACCAGAAGTCATGG
GAGGACAATGCTGGGTCCGACTTTGTCATGAGCACAAAAGCAATCAACTCCGACAAGAAG
TTGGGTGAGCAGTACGTTAGCTTCCAAGCCGCCCGAGAGGACCTTTGCACGTCGAGGCCA
GTGCCCAAGGCAAAAACGACCATTTCGGTCGAGTGTATGCAATACGAAGGCGTCAGGGAC
GTCAATTGGAACTTCCCGCTGGCGAATTACATACCAAAGCGAGACAATCACCAGCCGTAT
GATGTACAGGCAAGGCGCTTCTATGACAACAATGTCATTCACCTGGGGCTCAACGCCGAA
GAGTTCTCCCAAAAGTTCAAGCCCGTCGGCGACCTGGACGCGTGCATCACCATCGCCGAC
TACGTCGGAAACCCGAGCTGGGGCTACTACCACGCGCTCCTGCGCGTCGGCGGCTGGCCG
TACAGGATCAAGAAGATCGGGACCTTCGAGGGCCAGGGCGACGGCGGCCACCTGGAGCTC
AACATAGGCCTGCACCACTTCGACGACCAGGTCCTCTGGAACCTGCTCGAGGGGCCGCCG
GCCATGGCGCCCACGGGCGCGAGCGGCGAGATGGAGGCCCAGGGCAACCAGAGGTCGGGG
TACAAGTTCTGGGCGCAGCTCATGGACCTGGACTGGCTCGGCCCCAAGATCGACGAGAAC
AAGTTCCACGACCCCGAGGTCCCGCACGAGAAGGGCTACGTCTGGTTCACCGTCGTGGAG
ACCGTCGCCGTGCCCTCCACGGGCCAGAAGGGCGTGAAGGTCCGGCTCCTGGACGAGGGC
ATTGTGCCTGCTTGGCTGCGACCTCAACTACATGGAGGCGTTTGCGGGCGACATGCCGCT
CGGCTTCTTCAACGAGATGGAGTGCACCGGCCCCCCGGCGGCGTCCACGACCGTCCACCG
GATGCTGGACCCCAACTCCAGGAAGGTCGTGGTGGAGAACGAGGCCAGCAGGCTCGTCTT
CAACGACCCCTTCGGGCTTCAGGTGAACGACTTCTGCGACATGGGCTGGGCCATCATGGA
CTGCGGCTACAGGGGAAGCACCACCCTCAAGCTCGGCCCTACCCCGGGGAAGTGGTCCAT
GGACGTGGGGGACGACGAGGGAATGATCGAGTACAAGGCTTCGGGCCCACCGGCTCTCGC
ACATGGCGAGGGCGAGTACAAGTTCTTCATCCAAGCCCCAAGGTCGGCCTATGGCTTCCA
GCGGCGCTTAGTGATGTGAGGGCCTGTCTGTTGCAAATTCTGCAGACGCCAAATCTGTGT
GCTACTTGCCAGATAAGAAGGGATACTTTGCACCAGACCCAGGATGTTGTGCCCCATCAC
CTGTCCTCCATATGGCGAGTCTTCCGACGCAGCATGAGAAAAACAAAAAAAAAAAAAAAA
AAAA
>at01337
TCCACAAAACGTGTGAACCATGCTGGGACTGCAGCGGGTCCCACGACGGAGTTTCCACTG
AGCGTCAAGAAGAGCCACTGCCAAGCGAAGCGTCGATGCAAGGGACTGGTCTGCTGCGGC
ATCTGGTCCAAACGCCCGACCTTGTACGGGATGCTGAGCTCAACCGGATCATTTCCCAGG
TCTGCAAAGATGATCAGCTCGTTACGCTCATGCAGCAGACCATTTGCGAAGTAGTCATGA
CGGTTGTATGGAATCCCCAAGTACATACCCTCAGCTTCCTTCACGATGCGAGACTCCATG
AGTGGATCCATCAACCGTCCTGTGCTGTAACTACCATGCAGTATCCATGCATATGCCTTC

AAGCTTGGCAAGAACGCAAACNCCAGCATGCCGGCAACCGAGGGCTGGTGCCACGGTCCG
GTAGTACCTGACTGCTGCATGG
>at01338
CGAATCTACgGACAAGATAACAAGATTCCTTTGGTCAtTCACGAACTTCTCAGAGACCCT
GCAGCTCGGCCAGTTCCGAGTCACTGGCAGAAGTCAACAAAGCGTTCTTTGCAGCAACGC
ATCCCTCGCGGAAGAGCTGCAGGAGCTCTACCGAGATGACGTCGACGCAGCCGCGCAGGT
CCTGAAGGAGTCTGGAGAGCCTGTGCCTGCCTCTCTGTGGCATCCGCAGTCGCACTGCTC
GGCTGCCCCGCGCGCGGGGCTGTGCACTGGCAGAACGC
>at01339
TTTTTTTTTCTTTTTGAGCAGCTAGGCATTCCTCCAAACACTGAAGGTCAACTTCTCCCA
TCAAAAAGTTGGGCTCCCGCATGAGGGCCCGCGAACAGCGGCAAGCAGCACACTGCACCG
ATGGTGCCGTGGGCAAGCCCATCAACAAAACCTGATAACCTTTGGAAGATGACCACTCCA
TAGTCGTGTCTCTCCATCCACCACGGAGGCGGCATTGCTTTAATAAATTCCCCAGCCCTG
CGCTGCAGTGAGCCCTGCAGGCAAGAGCATGTTCCCTGTGCGCATGCAACAAGTCAATCG
AAACCCGATCTCGCCCTGGCTATAAAAAGTTAGCGCTCACGATCAGCTACAGACGTGAGC
CTGTCTGCTAGCAGCACGCTTCAAGCACGACGCTTGGGCGAAGCAACGGCCTTCGCAAAC
GGGGGCTCCGCAAGCGCTGGCGCTGCTTCCTATGCAGACGCAGCCGGCGCCGGCCGGGCA
GAGGGCTCCACGCCCCAGATGACCTTGGCGTACTCGGAGATGGTGCGGTCCGTGGAGAAC
TGCGCCATGGAGGTCGCCGCCTGGATGCTGAGCCTCGCCCACGTGGCCTTGTCGGCGTAG
GTCTTGTCCACGCGCTCCTGCGCCTCCACATAGGATGCGAAGTCGTGGACAAGCAAGTAC
CGGTCGCCGTCCCACGTGCCAGCCGCTGTGGCGTTGCAGAGCTTGTCCAACAATCCGCAG
AATTGGGCGTGCGCCTCGTCGTCGCCCAAGGAGAACTTGCCGCTCTTGATGGTGTCAAAG
ACCCTCTGCAAGCGCGGGTCAATGGGATAGTGGCCCTTCCGGGCGCGGGCCTTGATCTCC
GGAACTTCCGTCTCCAAGCAACCGAAGATGAACATCGTGTCCTCACCACCCTCTTCGCGG
ATCTCAATGTTCGCGCCATCCATGGTGCCGATGATCAGACCGCCATTCATCACAAACTTC
ATGTTGGAGGTGCCGCTCGCCTCGGTGCCTGCCGTGGAGATGTGCTCAGAGATGTCCGAG
GCTGGGATGATGGCCTGGGCCGCCGTCACGCTGTAGTTGGGCACGAAGCCCACCTTCAGA
TACGGGGAAACCTCTGGGTCATTGTTCACCACTTCCGAGATGTTGCCCACAAGCTTGATG
ATGGTCTTGGCGTTCACGTAGGCCGGGGCCGCCTTGCCCCCAATCAGCGAGGTCCGCTTC
ATGAACTTGGCACGCTCAGACGGGGAAGCCTGCTTGATCATCAAGTACCTGTGGATGAGG
TACAAGCAATTCAGGAACTGCCGCTTGTACTCGTGGATGCGCTTGAATTGGCAATCAAAC
AGCGAGTCAACGTCCGCGTCCATGTTCATGTGCTCCTTCAGGTATGCCGCCAGCTTCACC
TTAGCCTGGCGCTTCGCGGCCATCCACTCCTTCTGCAGGGCAGGGTCGTCCTTCTTCTTC
TGCATGGCCTTCAGCAACTCCATGTTGGTCAACCACTTCTGCGAGCCCAAGTGCTTCGAG
TAGATGTCGCTCAAGGGCCTGTTGGCGCAATGGATCCACCTGCGGGGGTTACCCCGTTGG
TCATGTTAACGAATTTGACCTTCTCGCCCTTGTCACAGAAGTAGCGGTAGAAGGTGGCAA
ATGTCTCCTTTTTCACGATCTCCGTGTGGAGCGCCGCCACGCCGTTGACCTTGTAGCTGC
CGAGCACTGCGATGTTCCCCATGCGGATAGCCTTCACGCGCCCAGTGTCAGGGTCCGCCT
CCTGGAACAGGCTCAACTCCTGGACCTCTGGGCCATCTCCATACATCTTCCTGAGCTCAT
CCATGAAGCGCCGATTGATCTCCCCTACAATCTGCAAGTGTCGAGGCAGTAACTCCTCCA
TTATCTCCAAGGACCACTTCTCCAGAGCCTCTGGCATCACC
>at01340
TTTTTTTTTTTTTTTTTGCACTTTGGGGACTCTGGTCTGCGTCTCCTGACATCTCCCGC
ATCGTCCGCACTGCTAAAAAAACCTAGCATGACATGTCCAGATCTGGCACTCACATGTGC
CGCCGGGTGTGCGGGCTCTTTAAGGCTGCGAGTGCACCACACGAAGGCCTCGTCTACAGC
GAACCCCAGCAAGACGGGTCATGGTACCACTTTGAAAGCTCTCCATTCTTCTTGAGGATG
CGGGACTGGACGCCTCCCGGGGGCGCGATACCCGCAAAAGCATGCTCAAAGAGGTTTCTG
TGGCCGACGATGACAATTCGCTTCTCCGGCCTTGAAAGGAGAAACTGGAAGAACGACTTG
ATTTCGCCGTCAGCACCACCAAACCACTTCTCTTCGTTGTACTCGGCCCAATTGTTGTTG
ACGTCTCTGTACTTGTCCATCAAATGCCAGGCCAGTTCTCCGGG
>at01341
TTTTTTTTTTTTTTTCTTCAAAAAACCTGCCCGAACTCCAGTGCCTCTTCAACCCGCCAT
GTGAGCAGCCCCAGCGCGGCCCATCAGAGCCTCCCAATCGACAGCATGCGTGCAAATGCG
GCAAAGAAGAAGACGAGGAAGAAGATGGCAAACGCGAAGCCGAAGCGGTTGAGCTCCTGC
TCAGTCGCCGCAGACGCGAGCTCCGGCGCCACCGCGGCCGCCACCGCGGGCACGGCGACT

GCCAGGCCCGCCACTGGCCGCTGAACTGGGGCAGCCACGTCGGCCCGGGGGCTGAGGAAG
GCCTGCGGCAGCAGAGCGCCGCGGAGCAGCACCACGCAGAAGACCCCGGCGAGCAGAGCG
GGGAGAACCTTCGCCCCGGCCATGGCTGGTTGAGGGGAGTACTGGGGTGCGCTGCCTGGT
CGGAGGCCTGCCT
>at01342
CTTTCCTAAGCGGGGCCATGACCAACAGTATGCTACCAACAGGCTCAGGGTTGTGAAAGA
TGAGTCGGGGGAAGTTGTCGTGCGAAGCGGCGCGCCTCTGCCAGCAGTATTTGGAATCGC
TGAACTTGCCTCAAGACTTGTTTGAGCAGAAGCATGACCGATGTTATTGCCATTCATGCG
CTGCTCGTGACAAGCTTCCCGACGTCATGCGCGTGGCCGGTGAGAGATACGAGCTACCAA
TCGGCTTCACAGGATTTGGCCTCAAAGTGCCGCCACGAACCACGATGGTAAACATCTGGG
ACTCCTGGCATGTATCTTTTCATGGCTGCGCTGGCTCTGTGTTGCCATCAATCTTGGCGG
AAGGTGGACTGTTTCTCCCTGGGGACACGCTGCTCGATGGTAGAAAACTTGGTGCAATAC
ACACCAGAGGAGGGGAAC
>at01343
CCGTAGCCATTTTGGCTCAAGAAGCCACGAAAGCCAGGGTGTCAGTCGCACTGGCAATAT
TTGTTTTTGAAGGTAGCTGAGAGCCATGGCGGACAACGCTGCATGGAAGGCTGTGAAGCC
CTTTGCCACTGGCTCGTTGGCCGGCATGTTTGCGACTTGCTGCATCCAGCCTGTGGACAT
GGTCAAGGTGCGCATCCAGTTGCTTGCAGGCACAGCTGAGGCAGCCGGCCCCATGACAAT
TGCGAGCAACATGCTGAAGAACGAAGGTGTGACTGCTTTCTACACCGGCCTCACTGCTGG
CCTCACTCGCCAGGTGATCTACACCGGCGCTCG
>at01344
NTTTTTTTTTTTTTTTGTTCGTGCGCAACCACTGGGAGGACCCCAAATGCTGCTGTGCT
CCTGAACTCGGCAATCCTGCTAAAACTAAGGATTGCTTTGCAGAAGCCCCGACAACCCCT
CACGCCAGCCACATGCAGCCGACCCCGCTCAAGTGTGCTGTGCGCTGGCCATGCTCTCGA
AGGCGGCAATCTTCAGTGTATGCCCCCTCTAGTAAGGACTGAAAGGAGAGAAGCCCACCA
TTCTGCGCCTCCCCTGGAACCCGAGGTCGTTGAGTCTCTCCAATGCATGCAGGAAGCTGC
GGCCGTCTGTCTCCCGAGATGGTGCCTGTGCAGGTTCTCGAGGGTCNTGGGGATGCCTTG
AGTGTCGCCTTAAACTCCGGTTCCACCGTGGCAGATGGTGAATTCAAGCGTTGCTGCTTG
AGCGCTGGCTTCGGTGGAGATGATGCGTGCTTGGCTTCCGCCGCTGCTCGTGGCGGTGGG
GAGCGCATTGCTGTGCACGATGGTGATGGCACATCGTCCGGAACTACAGCCGCAAGTGCA
AGACTCGCTGATGATGCTGGAGGCGCGGCTCCCAACACAAAGCTGTCCAGGAAGCCCAGT
GCGCACTCCTACTGCATAAGCGGAGCAACCTTGTCATCGCCAAGCACTGCCTTTGCGGGC
GTCGGTGTCACAATGGACACAGCAGATTCCACTGCCTCGTTGCTGGTGGGAGTACTTTGG
TTACGTCTTGGAGCAGCCCGCGTGGTCGCCCGGCTTCTGTTCAGCACCGATGCTCTGCAT
GTATTTTCATCTAGCTTCGGCAGCTTCCTCCTCTGTGGAGAACGATCCCAGATACTTGGA
CTTGTAGCTCGCCTGCCACTTGCCCTTGTTCGCGACAATGCCACGATACTTGGAGGTTCC
TTGCTCCAGCTGTTGCCAACTCAAACTGAATTGGACCATGAGAAGGGCCAGGTGCCGGGG
CATCCGCCCTGGCTGCACATGAAGTATACCGAATAGTACGGNCCTAGTGTCCCCTGCAAA
GGTCCCTGCGAAGTTCTTCTCCTCTTCACAGGCTCCTCGGCCTTGGCCTTTCAGAACTCT
TCGATGTCGGGATCTCCTGCTGAGCCTGCCCGGCCACATGAGCATCAGCGTGCCCCAACT
GATCATCAGATCTACTTGCAGAATTCGCGTAGCGCCAGCGAAAAGAGCCAGAGACGCTGT
CCTTTCGGATCGCCTGATAGATGCAGTCCTTACTGACGCCACCGGCGGCCGAGGCAGCCT
CAACACTGGGCAGAGTCTCCAAAACCTCGCGAGTATCCGGGTGGAGCTTCTGCACAGGTT
TGATGACACCCATTGCGAGCGGTGGGGACAGGCGGGTGG
>at01345
GCCTACGGCTCCGCGTAACTCTTATTACGGGTATGAGTGCTGTACAAGCGGACGAAAGCG
GCTCAAGGTGTGTAGGCAACTCCGATCCAGCTCAATCGATCCAACCGTAGTGTCCGACTT
TCTCGCAGTGGAGATCGAAGCTCCGGTGATCGTACGGATCCCCGACGTAGATCCAATCAT
CACAGGGATAGCAGAAGATCGCGGACCCGTCGCGGCGTCTCTCGCCTGCGCATGGACTAC
ACCAGCTGCTGTCCCAGCTGGTACGGCGTCGCTGCACAGTCAGCACAGGGTCGTCCTTTG
CCTTGACGAGTTCGCGAACGGGAGATGCTGCAGGGGGCTGCTTCTTTGGCTCTGCCGGGG
CCTTGCTCTGAGCGGCTTCGTGCTGTCCCCCAGAGTTGCCATCGCCCTGAGCAGCCTGCT
GCTGCGCCTCTG
>at01346
TCTCCGACTCAGGGACCTCTCGTATCCTCCATGGAAGATGGTGGTGCTCCGCGTGAGGCA

GGGCAGGTCAGGCTTCTGGTTCCGGGCGCACCACACCATGAGCGACGGCCTCAGCATGCT
CTTCGCGGTCTGCCCGATGCTGGGCTGCAAGACGGCGCCGACCCCCTCNCCAAGATCCCC
CTCCCGGGGCCCTTGCTCCCGCCCTCGGCGCGGAAGCAGCAGGCGCACCCGCCGGCCGCC
GCGAAACCGAAGAGCTGCTGCGGCTCGTGCGGAGTCGGCTCCTTCTTCCGCGGGGTGGCC
GCGCCCTTGGTGGCCAGGCATGATGCCGAGCTCAGCATCAACCCTCCCCTGGACAAGCGA
CAAACGACCCTTCCTCCGCTTCAACCGCCAGCGCACGT
>at01347
GGCGTGGCCGTGCCAGGCGGCCTTGTGTATGGCGTGTCCAGGCTCTGCAAGTACGGTGCT
GCGGGTGGCGGCAGCATCGGGGGGAGGTCCAGACCCTGGGAGAGCTTGGGCCCGCAGCCG
GGAAGTGGGGCGAGGCCGTGGCTGCGGCAAACGGCGCCATCTACGCGGTGCCGGGGGCCG
AGGCAGGGACGTGCTGCGGATAGCGGCGGCCTCGGGGGAGCTGAAGACGTTCGGCGGGAA
GGACTGCGCGTGGGCGAGGGCGGCGTGGAGGTCCTCTGGCCGGGACTTCCAGTGCAAGGC
GGC
>at01348
CCGTAGCCATTTTGGCTCAAGGCTCAAAAGATGCGTATCTTGCCTTGCTGCGGATGTCGG
CTGATCGGAGTGTGCCTACTTGCAGCGGCCGGACTGGCATCTCTGTTCCTGGCTGTCGCG
TCACTTCAGTCGTATGTTCAAGGGCTGGAGGAGGCATGGCAGTGGTCCAGCGCGCATGCA
ACAGAGGATCATCGGCGGGCGCTGCCGCCAGATAGTGTTCGAAGCAACCTCCCCGCCCGA
GCTTCCTCGGAGCTCCCAAGCAGATCTGGCTTCGCGACTCAAGGGCCTGACACAACTACG
GCAGAGGCGCGCCTGCAGGCGTCAACGTCAGATGGTCCGCACAGCAGTAGCATATCCAGC
TCCGAGCCGCCCTGTGCTGGACCGATGAAGGCTCCGCAGCTGTCTCAGAAGGAACGCGAC
GCG
>at01349
TTTTTTTTTTTTTTTTTCGCTCCTCCTCGAGCTTCTTCTTCAAAGCATCCTGCTTCTTC
TTGGCCAGCTTTGCGGCCTTCTTCCGCTCCTTCTCGAGCTGCTTTTGTCGCTTCTCCAGC
ATCTTCTTGCGCTTCTCTTCGGCCTTCTTGGCCCGGAAGTCAGCATCAGATGCCTCCTGT
TTGGTCTTCAAAGCTAACTCCTGGCCGCGCTTCTTGAATTCTTGCGGGGGCTCTCCCATC
ATGATGGCGGCTGTCCGCTTGAACCCTGAAGACGTCCACTTAGCAAGGAGTTCCTTTCTG
TCCTCCTTCACGAGGTTTGACTTCACCTCCATGACGACGTAGTTCCTCTCCTGGATTGGG
GGCAACAGCCTGGAGGACACGGCGTATGCTGCCATCATCCATCATGGGTATGCCGAAACC
CATCTCTGGCTTGTCATTTGAGGTCTTTGCCGTAGCCTTTGGTTGCGAGAAGCCGCTCTT
CTCGGCCCACGCCAAAATTGCTCGATCACTGAGTTCAGTGTACTGCTTGTTGTCCTGCAG
GTAGTGCCATCCAGCCAATCGAACCGTGCCCTCATCTGGCAAGCAGATGGGGAAGACTGC
CTCCCTGCTTCCCATCCTTCGCTTTTGCTGGACCAGGTGAAACCACAATGTCCTTTGCCA
GTGCATGCTTCAGCATGTGGCAAGTGAAGGGTAGAGGCGCAAGTGGAACGGGGGCAAAGT
TGTAGTGGAAGGTCAGGTTCCTGAAGGTAAACGCAGGGAAGAGCNCGTTTTGCCCCTACT
AGTGCCTCGGGCAGGGACTGCGGCTGGCACGCACGCACGCCATTCTTGAACAGACTGATG
GTGTTTGCGTTGGGACTCTGACTATCAAGATTCACGAGCAGGCCCACGACGTCACCTTGG
CCAAACTTCTGCGAGCACGCCTCCTTCTTCTTGTTGTGAATGAAGGCACCTTCCATGTCG
AAGCAGGCGCTATCCTCACCCAAACCCAGAAACAGCGAGGCCTCTGATGTCGCAATGCCT
ATGCGGAGCTGATTCCGTGGTTGTGGCGCGCGCTGCCGCGCAACAGGGTCCTCCGCGGGG
TTCATGAGCTCCACGACCTTGGCTTCGAAGAGGTAGCGGCCGGCCTTCATCCCAAAATTG
CTGCGTGCTCCCGCAAGAAGAAACTGCAACCCTCCGTCGGTGAGTGGCATGAGGATGTTG
CCATGAGCGGAGAAAACGCAGTTCATGGTGGTGTCATGCGTGTGGAAGGTCACGGGGTCC
TTGACCTTCACTCCCGTAGCTGCCGGCGCATCCGCCTCAAGCTCTGGCGGCGGCCCTTTC
TCCTTGGGCTCCTCCGTGGGCTTC
>at01350
TTTTTTTTTTTTTTTTTGACTGCAAACGCGCTCGCCTCAAGCATGTCCGTCCCGTAGGC
GCGGTGGAGACATCAAAAGCATCTCAGCGCGCGGCCACCCTGCAGCCCTGAGCCTGCGAA
AAAACTCGCGGCAGGGAAGGCCGAGACCAAGGCACGGCCGCCCAGCCGCAACGCCCACGC
GCTCCGAATTCGAAGAGCGATCTCCAGACCATCAGATCCAGAAGCCACGGGAATCCACGG
AAGGACGTGCCGGGGCGCCATGCCGAAAACTGCCTCACGGGTTGAGCCTGCCGGTCTTGT
ACTCCTTCTCGACCTGCTCCGCCGTGACGAAGCCATTCTCGCGGACGCCGATGTACTTTG
CCAACCAAGGCTTTCCGTAGTCGGCCTCGAACCAAGTAACGCCACGCTCCTCCGTCTTGG
GGTCGAGGCCGGACCAGGCGGCCAGGCCACGCAAAAAGCCCTTGGCAGTGCCCTGGGACT

GATCCACGTAGTAAGGAGTCCAGCGCTTGTGCCCATACCTCCTGATCCAGTAGTCGTTCT
CCTCGAGCTTCTTCGGGTCGGTGATGCCCTCCCTGGCGATCTTCCGCTTCAGATCGCGCA
GCGCCTCGTTGTTGCTGGTGCCTCCGAAGGCCTCGCCANACTTGATCTTGACAGGGCCGA
ACTGGAAGAGGAGGTTGTCCTCGTGCCGAATTC
>at01351
CTGCGCTTACTTGCCCAAGGCAAGGCNCCGATTTGGTGACCGATATGGAGCCCTTGGTGA
TTCCATCGAGCAGCAGATGCTCATGTTCGGGCCGGAGCCTGGCGGTGGTGCCGCCATGCA
TGCCCGCAATGTCCTTGCCATGCTGCCTGCGCTTCGCGACCACTTGCCTGATGCTCAGTG
CGCTGGAGATCCTGATTCAGTCAGCGTTGGTCGGTCTGACGTTGATCGGACTGGGCTCCC
CTGAGGCGCAGAGGAACATTCGCGAACAGGGATGGTACTTGGTTGTGCCCGTCATCACCA
ACCTGCTACAACTGCTGATCTGCCACTACGCTCTGCGAGAGTCGGCCACCAGCTCCAGTC
CGCCTCCGGCATGCT
>at01352
TTGTTTTTTTTTTTGTTTTATTTCCAAGCCACGGCAGCTCCAGCCGAGTCACCAAAAGCA
GGATGCCATGATATCAGGTGCCTCGCTTCAGAAGAAGAAGCCAACAATCTTTCCACCCGT
GTTCTTCCTCTTGGCCTCCTCGTGGTCCATGATGCCGTACGGGGTGGTCAGCACCAGGTG
GCCAAACTGGCGCGAGGGCAGGATGTTGCTCGCCAGCTGCTCGATGTCGCCCAGGCAGAC
GTCGAAGCGCGGCGAGATGACGCCGCACTTGTTGATGCGGCCCACGAGGTCCACGACGAC
CTTGCCAGCACGGTGGTCGTCAACAATCTCCACGTCACCAAGGTAACCCTTCTTTTGCAT
CGTCTGCAAAAACTTGATGATCACCTTCGAGTTGGGCCTGCAGAGCACCTGGCGCTTCCC
GCGCTTCTCAGCGTTCTGGATGTTGTTCAAGCAGTCGCGGAGCACGTTGATGCGGACCAT
GTCTGCGAGTAGGTTTGAGTCCGCAAGGGCTTGGGGCTGAGGGGCCGCGGCTAGTGGCCT
GTTCGTTGCAATCCTTGAGCCAAAATGGCTACGG
>at01353
TGGCGACCATCGCCGTGGGCTTCGCTGCCATCTCCAACCTCGCGCAGGGGGAAGAGGAGG
ACTGGAGGAAGGAGCTGGACGAGGAGATGACCAGGCAGGAGTACGAGAGAGGAGGGAGCA
GCGGACCATCGCTCTGCACAAAGTGCGGGAGCATCCTGCTGACGGNCCTCTaACTgGCGA
GaAAgGTGCGNCgGGGCCGTCGGGGGGCGGGGGGggATCCCGGCGGCGGTCCCTGTCCCG
ACCAGATGaAAGGCgGGGGGACGCGTCGGAGGAGGCGTGACGTGCTCGTCGGAGCGtTTT
TTTCTAGTGGCAGTTCTCCTTGTGCTGATACGCTGCGTCCTTGaAAGTCTCTCGTGGTag
GANANTGaACAATAAAAAAAAAAAAAA
>at01354
TTCGATATGGTGCCCAAGGAGACGAAGATGCACTTGCGGCCACTTCTGATCAACTTCTTG
TCGTCGATCTGGGTCACAAGCCGGCGGTCCTTGGATGAATATCCGCGGCCAGTTTGAGCA
GCGATCTCCTGATCGCTGAAGTCACGGCCGCACGCCAGGACGTCCCCTCCCAACCCCAGG
CTCCTCAGGCGGCGGGCTCCATCAAAGCCTTCGTAGATGATGTTCCTGGGCGACCACGTC
ACGCCCCCGGGCGTGAGCCCGTCGATCACGGCGGACAGCTCCTCGAGCGACAAGGTGTGT
CCGCGCTCCGCTGCCTTCTGGGCGTCCGCCAGCAACTGGAGCTTGAGCTGCAGCACAACT
GCTGAGCGTGCCTCGCCGGCGGGCAGGACCCCCACTGGCTCGTGTCCCCTGTCGGCATTA
CAGGCATGGAGCCGATGCTAGGGATGTCGCGCAACAA
>at01355
CCATCNAGGCTCAAGCCTTTCTGGGCGAACGCTCCNGCTCCGAGaCAGTACCTCCACCTC
GGGCTCCATGCTTCTCCAGCTGGCCANgGTTGCCCgAcGCTTCCGTTTNCTTTTCCTGCC
AATNACGGCCATCTCAGGCTTCTCCTTCTCTCAGCCGCAGCGGAGCTCGGCCGTGATCTC
GGGGCAGAGCTCGGCCGTGATNTCGGCCATGGCNTCTGTATCAATCAGNGNACCTCGGAG
TCNGGAGGTGCANACAAGCAAACCGTTGCGGGATGTCGTGTACCATCTTCACATcCCCAA
GGCTGCCGGCNATTCtNTCATGAGNGACGAGCTGGCGGTTCTGGCTCCGCACCACCTCAA
GCAGGTCAGCATGGAGGCTGCTTCGGCTATCAGCACCAGGACTCGAGGGTCTTGGGCACC
>at01356
TTTTTTTTTTTTTTTTTTAGCGCTCGCTCCATGCAGCTGGAAGGATGCGGCCCTCTGCAT
GTCGGATCGAAATCAGGAGAGGCTAATGCTGCCTAGGCACAGCTCTCCTACCGAGGCAAA
AGCATGCATTCCTCCAAATGTGCCGCAGGGCATGGCTCACGTTCCGTCGTAGTTGTATTT
GGCGGACACAAACCCGTGCGGCCGCAGGTCCACGGGGGCGCCCCCCGTGGCGGCGAGCAG
CCGCGCCCGCGCCGCGGCCAGGGCCCGGAGCAGCGCCGCCTCGGCGCCGGGGGCGCCGGC
GGCGAGGCGCAGCGCGGGGGCGCTGCCCGCGAGGAGGCAGGGGTTCTGGGGCGAGCGCTC

CCGGGCCACGAAGGCCACCTCTTCGGCCGCAGGGAAGAGCTGCCGAAATGCGTTGAGCCT
TGGCTGCTGCAGGACGTCCTCGAACAGCTCCGCAGCGCGCACCTGCGGCGCCGTGATGCG
GCTCCCCAGCGCGCCCGCGGTCGTGCGCCAGGCCACGCAGCGCGTGCAGAGCGCGAGGAG
CTCGCGGATGCGGCCGGGGTCGCCGCCGGTCAGCCCGTTGAGGTACGTGTCGCCCGCGTG
AATCGAAGCAAAGGACTCCCGGTCACCGGCGCCGATCTTGTCCTCGTGCCGAATTCT
>at01357
CGAGACAATGCCACCGCTCTTGGTTTTCTTCTAGTAGTACCTATTTGGTTTGTCCTACGT
TCGTCAGCCACGAGGCTTGTTGGAAAAGGTGAGATGGTTGCCCTTGGTGGTTGTGGGTGG
GTCCTTTGAAGGGGACTAGGCCCTGTCAGATGTTCAGCTCATTTGGCTAAGGTAACCAAG
TAACTAAGGACCAAGCAGACAGGCTGTCTGCGGCAAAGCAGCAACTTGGGNGTTCGACAC
TTGTGCATTCCTCATGAAGTAAAAAAAAAAAAAAAA
>at01358
GGTGAAGCCCGGCACAGGCTCCCGCGCGGCCTTCGTGAACTTTGCGTCCGCGCGCGACGC
TTCGTCCGCGGCCAGCGTCTGTGACCAGCTGGAGGTGGAGGACCGTGGCGAGAAGTACCT
CCTGGAGTGCACCGTGAAGCGACGCACGGGCGAGAAGACCTACGGCAAGAGGTACACGCC
CTCCGCCGAGGGCCAAGCCGGCTACGACCGCGCGCGGAGCGAGATGCGCGTGGCCTTCAT
CTCGCGCCTGCCGCTGGACGCGGACGAGTCGAGCTTGAGGCAGCTGTGCAGGCCCTGCGG
CACCGTGGAGGAGGTGCACATGCTGCCCTCCAACAACATCAACCAGGCCTGCTTCGTGAC
CATGGCCGCGGCGAACGAGGCGGCAGTCCTCATCCAGAGCCTCAACGGTGCGGCCTTCTA
TGGCAACGTCCTCGTGGCTTGCTATCCCGCGAGCGAGAGGACAAGAAGCGGAGGCGGCCG
AGAACGAGAACCAGCCGGACG
>at01359
TCATAATTTATCCTCAGAGCGAGCGCAAGAAAGCCTCCGACTCCTCATCGTACTCCGAGC
TGCTGCTCATCGTCGACGACGCAGGTGAGGACTCCTTACTTATGCTTGCTGAGGAAGATG
ACAACAGATACTGCAGCCCCAAGTCGTTCTCTATCCAAGCCATTTCTGCCAGGCTTCTGC
AGCTCAGACCCACAGACACACTGAATGTCAAGAAGGTGACATTGCTGGAGACAAGCTGAA
AGGCTGCGTAGGCATCAACCAGAAGAGTGGGAAGTGCCTCCAGCGAAATCTCCGTGTGCT
TCAGGTAGAAGTATTCGTCTGTGTATATGCCCAGTTTGAAGGAGTCTCTTGCGGCAACG
>at01360
TTTTTTTTTTTTTTTTTTCANCAAGCGCACAAAAGCCCCTTCCGCCATTCCTCGGCACAC
CCCTTTCCGGTGCGGACCTGCCTCAGGCCGACGGCTGGCAAGGGTGGAGCGCCTGGCCGC
GGAGGAGCGAGGAGACCTCCGTGGAGGAACAAGGAGACCTGCCTCAGGCCGGCGGCTGGC
GAGGCGAAAGCGCCTGGCCGCGGAGGAAGAAGGAGACCTCCAGCATGGCGGAGGGCGTGA
GCAGGTGGTCCGAGGAGCCGAAGCGCGGCAGCTGGCTCTTCCTGGAACTGGCCGAAGAGC
TCGCAGGCGCGGACACAGTGTGGCCGCAAGTGGAAGGCCCCTCCGTGAATTTCTCAGTTG
CCAGCAGCGGTCTGCTCCAGGTGTNNNNNNNNNNNNNNNNNNNNNACTCAAGCCCGCGCT
GCCCGACGGTCAACGTGTACGGGCGCGAGATGGAACACGCGTAGAGGCAGCTTGCGGAGA
GGACCAAGACGGCCCCTGCTACGTTCAGAAACGACATGTCAACGTGCAGGTCCAAAAATG
CCGCATTGAGCATCCACTGCGGAACGACCTTGATACCCCCAACGATGCCAACCGT
>at01361
TCCATGGCTGTGGGTCGACTTCGCTGGGCCAAACACCAGCACAAACTGGAGCACCAGCCT
GATCAAGGCCAGGGTGGATTGCATCCAGCTCAGTGCTAACGAGGGCAGGCTCATACTGTT
GCGGTTGTACCCATCGCACAACACGGGGCTAATCTGAGGATTGAAGAAGAGTGCGAGGGG
CTCATACGCCATGAGCATGACGCCATACAGAGCCAAAAAGCTTCTTCGCAGGGCAGAAGA
AGCCCCACTCCCGCCAGAGGACCCCCCAGCCAGGGACCCAGGAAGAGAGTCCCGCAGACC
AAGCTCGCAACTATCGTACTAAACTGCACCACTGCCACGTCGTTCGCAGAGGCCCCCACG
TCTGTCAGCAGGATGGCAGTGCTGAACATGACCGG
>at01362
TTTTTTTTTTTTTTTTTGAGGGACTGCAACTAAGATGAGCATTGCTCATGCTTGCCGTG
GTCTGTTCCCAAAATAGGGCAGCGGTCCTCTGGGCTCACTGGCACGAACAACCCCTTCAT
CGAAGGTCCTCATCAGGCTGCGTCGCCGGAATCAGCAGCTGGATGGTGGTGACGATGACG
AAGTAGAAAATGGCAAGAAGAGGGTAGAAGTAAAGCGGTGCAGCGAGGAAGACAGGGTCG
CACAGCGGGTAGATGAACCACACCAATGGCTTCGACTTGCATACCCTCTGAGCCTGTCAG
TGGGCCGGTGAATGACTTCTGCAGGCAGTGAGGCTGTCAGTGCAGCAGCGGCTCCAAGCG
CGGTCACCTCAATGGGAGCAGGCTGCTTTTCCCGTGGCTGGATGAATGTCAGCTGGGTCT

GTGCCACGAAGCGATGTGCTGCCAAACAGGACACCAAGCCAATGAGGATGATAGTGAGCT
TCGTGCTGCGCATGGCCCGAGCCATTTCTGATGGTTTCTTAGCGTCAAGCGCTCTTCCCT
CAGCCGTGCTCTGCAGCCTCGTGCC
>at01363
TTTTTTTTTTTTTTTTTTCACGCCAGCACCTGAAAGCAATGACAGCTGGCATCATCCCCA
CTTCTGCCACAAGCACAGCCAAAAAACAACTACCGGAAGTGGAAGTGACGGTTCACCAGC
AGCTGCCCAAGAGAAAGGCAACCATTAGCATCCAGTTCCCTCACGACAGTGCGTTAGCGC
TATACATGACAGCGCGTTGTTGCTTGGCCTGCGAATGCCCTGCCAGCCAACACAGCCTCC
CTTGCGGGGTGCAGCAAGCCACCACTGGCAACCAATGCGTCCAGGAGCACTCGTTGCGGG
TATGACTTCATGTCCTCACACCCTACCAGCTTGCAGAACGCGCCAGCAAGGTTTTTCTCT
GCAGCCTCCGCGACGACGGCCATCTCAAGGCGAGTCGTCTGCTCGGCTGGAGCCTTCTTT
GCCTCCATGGGCGTGGGCCCAAAGGCCTGGGAAGCGCGCTTTCGCGGAATCCGCAAAAGC
ATGGAACTGGCTGTTGCTGCCTCCTGAGCCTGAGATGGCTCTGGCTCACACTTCTTGGAC
CGCCTGACCCTTATGATTATCGGCCTCCTCACTGCGGCGCTCCTCACTGCGGCGCTCTTC
TCTTGCCAACTCTGCCCTGGCCTTTGCGGCCTCCATCTGTTTATTGTAC
>at01364
TTTTTTTCTTTTTTTTTTTCCTACGCTCGTCCCAGCAATTGCATCGTCAAGATGCCGTAAG
GCAGACCTATCCGTGTGCAAGAGCACGCCCAGGATTGTTCCATCGCAGGTTGGAAATGCA
CGCGGCAGGCCGATTGACGGCAGGGCCAGACCGGTGCCACCGGCGCAGCGTGCTGCGCAT
GGCGTTGCGCGGGAGGGGGCACCGCGGCGGCGGCGCACGACCTGCACCCGGGAGGGACCT
CCCTCAGTAGTGCCGCACGGCCCGGTCCACGTTCGCCTTGCAGATGGGGCAGTCCGTGCA
GCGGCGCAGCCACTCGTCGATGCAGGTCGCGTGGAAGACGTGGTTGCAGGGCAGGGGCCG
CACCTCCTCGCCGTCTGCAAAGTTGGATAGGCAGACCATGCACTGTGTCTGGGCATGCTC
GTGCTTGACGTCTTCCCACCTGAACTTCTCGACGCGGTCGATGGTCTCGTTCGAAAGGCC
GCGGCGGCGGCTCAGCATCTCCTGCACCTCGATGAGGCGCTCCTCGTAGCCCCCCGCGAG
GGCCTCGGTGTTGAGCAGCAGCATCTGCAGGCCAGGCGGCAAGTGCGAAAGCTGCTCCAG
CAGCTCCGGTGGCAGGAGGTTGCGCANAGATGGATGGAGAACCCCTTCACAGTAAATCGT
CGTGTTCCGTCCGATGCGCGCAGGGCCACAGGCTCGC
>at01365
GGAGCTCCTTCTTCGAGCGCCGGGACAATGTGTACTTCGCTGGTCAACGTCAGCGATGCT
GTTGATTCAGCAATGGAAGCGTTGAACAGCAACCGCAAGATCATTGACGGCATGGGGTCG
GAGGACACCGCGTGGCAAAGGCAGCAGGCGGCCGCATGCGCCGGCGGCTGCCAGCCCTGC
TGCGACGCGGGCAGGAGCGCCCTGCTGCTGCAGGGCGCCAGGTCCCACCGGAAGTGCGAG
GCCTGCATCCAAGACATGCAGCAGTGAACTCCCGGGCGTGCGCACCCGCAGCAGGACCGT
CTTGGGAGCTTGTTGGCCGTTGTCGGATCTGCCTCTTTGAAGGCGAGGGCAGGGCTTGC
>at01366
GATTGTGTGGCTGACATGGTTGCCGCCTATGACGCCAGGCGAAAGGTGCTGCTCGACGGG
CTGGCCGGCATACCCGGCGTCTCGCTGCCCGGCTCCGCACCCACGGGCGCCTTCTACGTG
TTCCCGAACGTGTCGGAATTAGGCCGGTCAGCCGACATTGCCACGAGGCTCCTGGAGGAC
CACCACATAGCCACCATAGATGGCGCGATCTTTGGCTCGGAAGGTGATGGCCACTTGCGC
ATTGCCTACTCCTGCTCTCTGGAGGATTGCCAGAAAGGCGTGGACCGGTTGTCCCAGGCT
CTCAGGGCCATGCGGTGAGCAGCGTCGAGCTGCTTCCAACTAGG
>at01367
TTTTTTTTTTTTTTTTTTGGTTGCGCGACTAAACAACTCTTGATAGCTGCTTTGTCCAGC
CTCGCGATGTATCCGAGTAGCCAGGAATGGAGCGGAGCAGCGTGGACCTGCCACTCTAGC
CAGACCGATGGCGTTGCAGCATCATCTCACGCAGCTGTCCTCCACAACGCGAAAAGGTAG
GCTGCTGCACTTCCTGAGCGATGGCCTGATCCACCGGACTCAAGTGAATGCGTGCGGCAG
CCCTCCAGGGGACACGTCCGCGGCAATGGAGATACCGTCAACATCTGAGAAACTGATGCT
GCGGAGTGCTCGTGCTTGTTGAAGCCAAACTGGGAGATTGACAAGCCAAGCAGAGCCACT
CAACTCCAGGCACTCTTCAAGATCTCCCGCAGTCACGCTAAATCGTACATCGCGTCAGTG
TGCAGAGCCGACGCCCGAGCTCGATGAGCTAAGCAGGGCCGCCTGGTCCGGGGCCAGCGA
GCCACCTGCTCTTCAGGATCTCCCGCAAGCGCGCCGACTCGTACAGCACGTCGGTGCGCA
GGATGTACTTGGCGCCCTCCGTCACGGGGGGAGCCCTCNGTGGAGCAGCGGGACGCCTGT
GTTACCGTGCGGGAAACCGTCGCAAAGCCGGTGACTGGCTTCACTGGGCTTGCCTCCATG
TGGCCTGGCTGGTCGGGCCTCGGCAC

>at01368
AGAGAAGATCATCCAGTGGAGGCCCACCCCCCGACAGAGCCTCGCAAGAGGCTCCAGCCG
GGGGCGAGCTCGACAGCAGACAGCTGCTGCAAGCCGGCCCGCCCTCGGAGCCTGCGCAGG
TGTGCTGACGCTGGCATGCGCTGTCTAACGACGAGCTCCTCAGCTGAAGCAGACTTGCCG
CCGGTGCGGCTTTTTTTGGCTCCTGGGCCTCCCGAGCTGTCCACTGTACGCGCTAATGCG
TGAGGCGAGCTAGGGATGATGTACACCCCGTTTCAAACGATTAGCTGGCTTCTCACAGTA
TGAC
>at01369
GGGCACTACAAGGATGTGTCACCAGGGCTCTTCTGCACGCCCATGCGCCCTGCAGCCGAG
TGCAAGGCCGCACCGTCCGGTTTGGCGAGCAGCTCGCCACGCAGACTGCACACGCAATCC
GATGCTACGAATCCTGACTTAGGGCCCGGTGCATACTCCATGCCAAGCCAGAGAACTCCT
GCACGTTCAGACG
>at01370
TTTTTTTTTTTTTTTTTAGACGCACACCGATCCCGAGCACCAGACCTTCCTGCTCTCTC
TGCCTGGTGCCTGCTTCTGCAGGCATTCGGCTTCAATGGCAGGTGTGGCTTGAGGCTGCA
GTCAAAGAACCGCCGCGCCTGGTGGCAGGGACCCCCCCGCAGCCCCTCCTCCCAGCTCAT
GTGCCGCAGCGATCCCCGAGACTGGCTCCTTGCGCGTGCCGGCTGCGAGCAGCAGGACGC
CTGGTAGCCTGGATGCCCCCGCGAGGACAGCGGCGTCTGCCCATGCTCACTGCAGCAGGG
GCAGTGCGCGCCCTCACTTCGCAGCCACGCGGTCGTCCTTGGTGCGCACCGCTCACTGTC
GGGGCGCAGGGAATCCCAGAAGTGGCAATCGGCCCATGCCTCCAGTTGGGCACCAGATGC
CGGAGCCTCCCCAGCGGCTCGAGGCGAGCGTTGCGGCAGCTCACTCTCACGGCTCAACCA
GCAGGGCTCCGTACAGTCTGCACGTGGGGGGCCGTCAGGCCCCTCGTGCC
>at01371
TTTTTTTTTTTTTTGTTTCGCGTCCAGCTCAGACCTGCCCAGCTTAGGATTGGCGTGCCA
GCAACGGACCCCGCAGCATGCCCAGCATGGAAACCTCCAGAGGAACGGGGCGGAGGTCAC
GAGCTCTTGACGAGCACGACTGCGAATGCGTGTGCCTTGCACGCACAATCTCCGTCTGCA
AGTTCTTGAAACTCTCCACAGGCACATGCTGGCAGAGCGTGAAGGTCTTCTCCTTGGCCT
CTTCATCCTCGTTGCGCTTACGCGACATGCGGACGCGCACTCGGCGAGGCACGTTGCGAA
TGCCCTTGCTCCAGATGAACTTGTTCAGCTTGGCATCAATGCGCACGTCCTTGGTCAGCA
TGACCTTCGCAGCAAACTTCCGGATCTCGCGGATGGCACGCGGAGCACGCCTCTTGAACT
GGATCTTCATCAGAAGCTTATGGAGGTGGATGGTGTGGTCACGGGTAATGGGCTCCTGGC
TGGCGCGGCTGGTCTTCTTGCTGGTCTTCGCCATGGCTGTGGAGTTGTGGGGGGAAGCTG
CCGGAAAGGGCAGTGGCCAGTGTTGCACCTCGTGCC
>at01372
GCCTGCCCGGTGGAGACGCGGACATACAATGTCGTCATAGATGCCGCAGCTAAAAAGGGG
GACCTACCTGCAGCAGAGCGTTGGTTCGATCGTGCAGTTGATGATGGCATCAAACCGGAC
TTGAGGATGTACACGACGGTCATCAACGCAGCAGCTAAGCAGGGTTGCGCTGGGAGCATT
ACCGATGCAGAGCGCTGGCTTGAGAAAGCATACATGGCTGGC
>at01373
AGAAAGTTCTTGAATGATTATACAACTCTCCAAAATACGAACAACCTGATGATTCTCGTT
ATCAACAATTTTTCTGTTAAGCGGGCTTGTGGCAGAAGGCGCAGCTAAAACTTCACGAAC
TATCTCAAACTTTGAGTCAGCTTCTGGAATGTTGGCTTTAAGACTTTGCAATTCAGCATC
AGTCGTGTGAACAGAATCGAACAATAGTGACATAATCTTAAAAAACTTGTAAAGTCAATC
CGCAATTCTTCGGGGAATTAAGCAGACAAGATACCAACAACCGTGACACATTTGTTCACA
AGTCCAGTCACAGCAAGTTGCTCGTCGTACTTCAAACTTAAAAATGATTGCAATTTATTA
TG
>at01374
AGCTGGCCCAGCTCGAGTATGAGAAGTCAGATGGCGGCGGCCACGAAGCTCAAGGACGAG
CTGGTCGCCAAGGAGATCAACCTGGGGGAGATGATCGCCCAGAGGACACTGGAGAAGGGC
GCCGAGGAGGCGGACATGCAGAACAACCAGGGCGACCTCACGAACGAGCAGGACTACAAG
GCGGAGATCACGCCAGACTGCGACTGGATCTTGGGGGCCTTCCGGCACCGCGCCGAGCAC
CGCGCCACGGAGATGGCGGGCCTTGTCAAGGCGAAGGAGTTCCTTGCCGGCTACCAGCCT
GCCCAGGCGGCACTTCTGGACAGGAGCCACGCCGCGCCGTTTGATGACGGGCACTCGCCA
GCCT
>at01375

CCCCCCAAAAAGGCCACACAGGTATCCCCAAACGTGATATTCATTGATGAAGACAGGTAG
ACGGGGAACTTGGCAGTGAGTTTGGTCGTCAAATATCCAATGAGTGCCGTCAGGTACCCA
ACGCGGCGAGCGTGCTGCCGCCATCATGATTGCTCACCTCTCTCCGTTTGCCGTCTGGAG
AAGGCGCAAGCAGGCCTAGGCACCGTCCTCGACGGCCCGCAAGGGCGAATCCGTGTAGAG
TGCCCAATCACCCCAAGCTGAACCGGTCAGGCCATCCTGAAAGAACATCCCAATGATCGC
CATCATAGCCAAGCGGCCATTTGCAAGCTCTGCATTCAGCCGTTTCGTCTTCAGCTCCGC
ATCATCAGTTGCCAGCAGTGGGGGCTTAAAGCCGCAATCCCCAGGTGCCGGGCTGTTCTC
ACCGTAGCCGTTGGAATACTCGCAGAAGAAGCCGTAGGCCACAATCTGGGCCCAGCCAGC
AGCGGGAACCTTGGAGATAGCTGCGAGACCATTGGGGACTCTTCAAACTTGAGGCCCATG
GACGGAGAGAGGTAGCCCGGGAGCTTGCCAGTAAGTTCTGGAGTGATGTATCCCATGGTG
GCCAGCATGCTGACACGGCCATGCTTTATTTCGGTCTCCCTTCGCCGCTTGAAAACTGCA
GCATCTCCGTCACTGGAAAGGCCCAACGGATCCCAGAAGCCCACGGGGGCCTGCACGCCA
AGCTCGCTCTCGAAAGCGGCAAGTGACACCAGCTGCCTGTTGGAACGCTGTGCCTGCGGC
ACCGCACCCGGTCTCCGCTGCAAGGCAACTGCCGTCGCCGCGGCGCCGAGCACCGCCAGG
CCCAGAGCACCCGCAGACGAGGCGGGCGAGTCTGAGCTGGAGGCACCGCTCCTGGGAGCC
TGTACGCTCAAGGCCGAGCTCTGCTTCCTCCCCTCGCGGACCAAGTCTGCAGTGCCACCT
GGAGTGACGAAAGCCTGAGAAGGAGAGAAGCTCACCATTGCCGCAGTGGTCGCCAAGGCC
GCAGAAGAGGTCGCGGGTGCCATGCTGCTTCAAACAATATTTAAACACTTCTAATTAGTA
GACTCACCGTCTGGCTGCTAAACGCAC
>at01376
ACACTTGAACTCCGCAAAGGCAGACTCTCGCAGCAACAGTGAGACCAGAGCCACATGTGA
ACTGTCCATGGACTGGACCTGCAAGCCCTTCTCACTGCAGTCGAAGTTGACGTCCTTGCA
AAGATCTTTCATTGCGTCCACCACCTTCTTCAGAAGGGCTGAGTGCTGGAGGTGTGCCTC
AAGCGCCATGGTGTGCGGGAGCGAATGCAAAATATGGGTTTGAGACTGGTCTGAACCAAA
ATTGCTTGAGCCCAAGAAA
>at01377
GCACGCGTCAGGATGATTGGTGCTTGCTCGCACCGACAGTGCCCTGTCTCAGCACGCCAG
GTCCTAGTCAAGTCCCTACAGGCCTCGGCAGGCCTCTGTACAGTTCAACGATTCCGAGCG
CTGCGAGCGGAGGCCACTCCGCTGATTTCAGTGCGGCCTCGGCTCCGTGGGGTGCCGATC
CGCGGAGTGCCCGTCCGTGACCAAGCCGTGTGCCTGTCTGGCAGATCAGGCTCGTCATCA
CCCGCCAGGCTCGCCAGCACCTTCCAGACCTTGAGGTCGTAGTGCGGCGAGAGCCGCGGC
GCGGGCCAGTCGGAGATCAGGGAGGCGAGCTGCTCCTGCCCAGCGTTGGCAGCCTTGGCA
GCTCTCGCCTCGTCTGGTGTGAGCTCCACCTCGCGT
>at01378
GAGGAGCAATTCAGCGCTTTTGCAAAGGGCGATGTGCGAGGCTCACGGATCAGATCGCTG
ATGTCCAGATTTTGGGGGTTTGCAAAAATACAGCACCAGGCAGCCACCTTTCCTGGTGGC
CAAGAGGCAAGGATCTTGTCAATGAGCTCAAAGGCTCCCTCAGCCCAGCCATGCGTGATG
AATAGGTCACATTTCAAACCTTTGGGATGTCTTAGCAACGCCCAGCTCATTCCACCAGCA
GCCCTCGTCAGCGGCTTGATGAAGTTGTTCACAACGTTGTACATGTTTGGCCCAATCTTG
CCGTCTGCTGGGTCAAAGGGGTCACCCTGCCCAGTCACAGGATCGATTGGGATCGGCTGG
ATCTCCCCTTCAGCACTGCCTCTTGATGCA
>at01379
TTTTTTTTTTTTTTTCCATGTGACTTGCCCGAGAGGACACGTGCCCCCACTTCAATCACA
AGTCACTCCGCTGCTGTGGCACGACCAGATCAGTCCGCCCCAAGTCCACTGCAGCATCCA
GCGGGTTCCAATATCCCAGGGCTGACGCTGTTCCCTCTTCTCCATCGAGGCTGGAAAGCC
TCACCATCTCCTCCTCTGAGAGCGAGAAGTCAAATATCTGCATGTTTTCCTCGATGCGGT
GCTGCCTGACGCTCTTGGGAATGACCTGGAAGCCCATCTGCACGGCCCACCGCAACAGGA
TCTGGGCGGGCGTCTTCCCAGGATGAGCTGCCGTGACTTCCGCTACAACAGCATCACTGA
GCTTCTCCTGGCGACCAAAGAAGACAGACCCGTATGCCTGGACAAGGATCCCATGCTCTT
TGCAGTACTCAAGCAGTGGCAGGCGCCCTCTATACACCAACGGTTGCATCTCTACCTGAT
TCATCACTGGTAGATGCCTGCCGTGGTTGCGTCAGGCCTCGATGTGCAAGACGTTGTAAT
TCGATACTCCGATCGACTTGGCGATCTCCTCCCGCTGGAGCTGGAGTAGCGCATCCCAGG
TCTCTATAAGCTTCGCCCCGATAGGTGATGCACCATCAAGAGGTCCATGTTCCCGATGCC
CATGTCTGCGTTGGCTGCACCGGCC
>at01380

CCTCTTCTTCTTCAAGACCTCCATCTTAGCCCGCTCCTCTTCAGCCCTCTTTTTCTCCTC
CTCGGCCTTCAAGCGTCGCTCCTCCGCCCGTGCCTCGTACTCCTCACGGGCTCTCTGCTC
CTCATGCTCCTTCTCCCGACGCAAGTCATCTTGACGCGCTCTGTCCTCCTTCTCCTCGCG
CTTCTGTCGTTCGGCCTCGGTCTCATCCTTTTCCGGTTTGCCTTTCCCTTTTTTGTCAGT
CTTGATCTTGTGGCCATCCGGACCGAGCTCCTCCGGCTTCCCCGATTTCCCGGTTTGCGG
TGGTCTCTTCCTGCCAGTGGGTACAGACTCCCACGACTCCGCCGCGCGCGCGACCGCGGC
GTACCAGCCTTCACCGCGCTCCCTGGCCTGCCGAAGGACGACGCCTGTTTCCGGCGCAAG
CGCCGAAGAGGGGTTCTTCGTCGGGCGCGCGGGGGCCTCGGAGGACGGGGCACTGACGCT
TGGCCGGCTGCTGCCTCCTCCCGCTCCCCCTTTTGCACGGCGCCGGCTCCTACTCCGAGA
GCGCGAGCGCGAGCTGCGGTCCTTTTTGGCCGCCTCGATCTGCTTCTGTGCCTCGAGCAC
CTCGGCCCGCCAGGGTGTGAGCGGCGGCGGAGCGTTGTTGTATCGCACCATCTGGGAGCG
GCGTGGCCCCTGAGAGCCGGGGATCGGCGCCGAGCGCTCGAACCCGCTCATGGTGTCTGG
CAGCACACAGGACTCTCAGTCAGATGCCGCCTCCTCCAAAACGCATGCAACGGCCCAACC
CCCTGCAGGGGTTGGCCAGTGCCGGGCCCGTCCCAGGAAGATCGAGCTTGAGCCAAAATG
GCTACGG
>at01381
GGTCCtTTCTTGGCAAACTTCTTCGCTGCCAAATGCTTTTTGCGGTCCTCCACGATCTTA
ATGCGGCGCTGCTCCTCAGAAGCCCGCGCCCCTCAcCCTTGTACATCTGCTTGTCCgTCCG
GAGCATCTCTGCGTAAAAATCCGTgGGCCGGGTGCAGGGGGATCCTCATGACTCTGAGTC
GCC
>at01382
TGTGCACTGTGGTGCGTCAATGAAGCTGAGCCCAACGAGTAGCGCCTGGGCCCACGATTC
CCTGCACGTGTGCTGTCTCGCGCCATCAGGGCTTCCATGGCGGCATCACTCGCAGCACGA
AGCTGCTCAACCTCTGCACCGCTCAGCGCTCCACGCAcCCaaACTaGCCCAACCGTTGGC
AGCCACGGCGGCTGCAGCTGCGGTTGCGGCACCCTCCAGCATCATGCTCCCACGTCCGCC
GCAGGAAACGTAGCCTGCGGGC
>at01383
GACGACAGCAGCAGCGGCAGCAGCAGCAGCGGTAGCAGCAGCCCAAGTGAGGAGGACGAG
GAGAGGAGGAAGAAGCGCAAGTTCGAGGAAGCGGCCAAGTCCAGCGCCAAGGGTTGGAAG
ATCTCGAGCTTCCTGCAGGGCGGTGAGGACTCGGACGGCTGACCGCGCGGCAGACGCGGC
TGGCTGCCACAGCTTGAGCCATGGACGGGGTCTTCCGGGCTCTGCTGGCAGCGGGCTTCA
TCTACTCAAGGGCAACTAGCTGCTGGACCACCTGACTTCGAAGGTCAATGCCCTGACTTC
ATGGACAACGGAGCTTCATCGGCAACGGCGATCTGTGTTGCGCATGGTGGCGGATACTGC
CTGACAAGATGGAGTGATGCGTCTCTAGGTTGAGGTGTATTTTTGGTCACAACACCATGG
CTCTTGACGTGCTGTTGACCTTATATATGGAAATGTCGCAGGGAACTCCTCAAGCAACAT
GCACTTTGGCAGAGACT
>at01384
GAAGACGCCAACCCAATCCTTCAGCGGCAAGAACTTCATCTCGCCTGCGTAGGTCTCCTT
GACCTCAGCCTTGGCGCCGCGCTGCATCATGATGGTGGGGAACTTCTTCACCTTGAAGCG
CTGGACCAGCTCCGTGTCCTCCTCCGTGACAAAGCCGAACTTCACCGTGCGCTTGAAGAC
CGTCTCGCTCGAAAGTGCCTTCCAGATCGTGGGCGGGGTCTTCTTGTTGGAGAAGAGGAC
GACCTTGGGCTTCGAGGGGTCCGTGGTGATGAAGGCATCTGCGTTCTCCTTTGTAAGCTT
CGTGGAGAGGTCGACGATGAACCTGGCGATCTTCGCGCTCAGCGCCTTCGCCTCCATCTT
ACCCCTCATACTTGAAGGACGGGACTGGATTCGTGGGGTAGACCATAACGGAGGGCGTCT
CCTTGACGCCGTTCTTCTCGCNGAAGACAGGGAAATCGGTGCAGCTGATGGCGCAGATCT
TGGCCATGCCTTTCAGGTCCACAGCCACCTT
>at01385
TCTACTACACCATGTACCCCCTTCCACCACCTCGCACACCGCACCCGGCCTGGAGGATCA
TTCAGAGGAAGGCCCCTGAGACCGTCACGATGGCGAAGGTCCGGCAGTAACCAGCACTTA
CGTACTAAACAGGCTGACCCCCCACCAGCGCGCGCCAGAGCACCTCAATCACTGAAGCCC
GCCTTCTTCTTCTTCCTCTTCGTCGAAAGAAATTACTGGGGCC
>at01386
TTTTTNTTTTTTTTTTTTTCTATTTTGACAGCCTCTGCACGCTGTTCTGCAGTGCTGGGTA
GAGCGTGCGCCTTTGCTACCGCGCAAACCGTTGGACTACCAACCTGCCAGCATCACGCCG
AAAGAGCAGTCCAGCCCGTCTGCTGAGGCTTACCAGGNACGATGATTCCCATCCTCCTAT

ANGCNACAAGCAATAGCAATGATGCGTNCACGAGAANCGTGTAGAAGACAGGTCTGAGAG
GCCATTGCATCAAGNAGCCGTAAGGAAATTGCACNNTTCCGTCGTCNTTCTTTGAAGCCC
ACGTCTTGACAAAGACTTCCGAGAAGAGGGCCCAAGCTCCGATGAATGCCGGGGCGACAA
GGTAGGGTCTTGCAACGACCATGGAGCACTGCACTCCTCCAAGCATGCTTGCCTGGGCAA
TCAGAGCGCAGGCCAGCAGGCCATGGCACCACAAGTTCTGCATGACGCCCATTCGCCAGT
ACCAAGCATCACCCACTGCCATCCACAACTTGCTGTGCTCTGGGCTGCTTGTCCGCCAGC
GGAGTGGAGGTCCGGACGGAAGGCCCAGCCTCACTATGGAGGCCATGAGCAGGCACAGCA
ACTGCAGAGACTGGTTGGGCAACCTCCTGCTGCAGACCGCTATCAAGGCGTTCAGACTGT
ATATCGCGCA
>at01387
GGATCTTGCGCAGGTTCAGAAGATCCTTTGTCTCCATCGTGGCGTTCGCGGCTCGGTCTC
CGCCCGCGCCTTCTCAAGGTACGACTGATGCTCTGTTTTGTGCCTTGTAGCGAGGGTGTT
CTGCAAGTTGGCTGCGTGCGCCTCAAACTCGGCAACCTTCTGGTCCCAATGCTCATTGAA
CTCCTGCAGCTCCCGCATGTGCGCCTCCTCCACCCCGAGGCGCTCGGCCAGCTGCCTG
>at01388
TGTAGACACCGCGGCACGCAGGGCCGCCAGGGACTTGGCCCGAGCGCCCGCATGCTCCCG
CCTCGGCCAGCCGTGCGGCGAGGCACCGAAACTGCACGCAGTGTTCCTGCGGCCGGCCTT
GCAGAGAGGAGCTCTGCCAGCGCAGTGCCTGCCGGCTGGCCTCTCGCACGCTGTCACCCA
TGCCTCTGGTGCATCCAAGCCCTTGAAGAGCGATCCCAGGGAAACGGCCAGGCCCAGCAA
GGGCAATGTTCGGAGCATGCGACCCCAAGGAGCTATCCTGGCCACCGCGGAGGTCTTGGC
TGCAGCCTTGGCCGCAGAAGTACCCCTGCCATGGGCCTTGCTGCCAGTCAGCATGCGCCT
CACGGTCGAATCGCCGGGAAAAGCGGCCCAGAAAGCTTGGCCACACCGCCCTCCCTCAGA
CTCAGCATGCGCACAAGACATCTTGTGTGTAGCGGCGGAGTGCACGAATTGGCATGCAGG
AAGCTGCACGGAACTCGCAGACTTCCCGGGCTTCTCAAGACTTGAGCCAAAATGGCTA
>at01389
CGTAGCCATTTTGGCTCAAGTGCGGCTCAAGGCCAGCAGACTCGGGCCCAGAAAGCCTTG
TCCACTTGCACCTCTCTCTCGAGACTAGCGAAGCCATGTGGCGCGCGGCGGGTGTCTCCT
ACTTGCGGTACTCCAACGAGATGGCCTCCATCCTCCGCCAGTGCTTGAAGGAGCCCTACA
GAGAGAAGGCCATGGCCAGGGACCAGGTCCATCTCGTGGAGAAGGTCTGGGCGAACGGCG
TCGTGCAGGTCAAGAGCACCATCGAGGACATGAGCAAGGGCTTCGAAGCCGCTGCCGCGG
GCGAGGCCAAGAAATGAGGGGGCCCGATGGCATGCGGCAGCTGAGCTGGGGGGTGCGCCT
GCGGATGCTGGCTGGGGCTGCAGGGGCCACCCTGCTGCATGAACAAAAAAAAAAGAAAAA
AAAACAA
>at01390
TGACCAGGTCCCTTGCAGGTGGAAGGGCCAGAAGAGTGGGCCCCCTGCCCATGTGAAGAT
GGTGTTCATCAACAGCTTCGGGCATAGGCTTGCGTATGATTGCATGCAGTACTTGCAGAC
GGAGTTTCCGAAGGCGCCTTTGTAATGACAAGCTCAAACTGTGCCAGCTGGCCTTCACGT
AGACAGCGCTGACTTGTGCATCGTCCTGCTTGAGAGTTCAGAAAGTGTTTTCGCTAGCAG
AGCAGCGCTACTCCGCCAGAGAGAAGGCTTTGTGGTCTCTCAAGCATGACGAAGCTTCAA
GAGGTGCCCTTCTTCATGATGGATTACGGGCCAATGCACAGAGGGCTCAGGATGGTCGAG
GAGAT
>at01391
TCCCTGAAGACAAAGTTTGCGGCCAGGAATCCAAAGTCCACGAGGAGACTGACGAGGAGG
TATCCGAAGTACATGCGGACATGGCTCTCAAGCCGAGACGACACGCCCCCAAAAGCCAACA
ATGATGAATGGCAAGCCGAGCATTGACCACGCACCATTGAAAGTTTGCGTCATCGGGCCA
CCACTAACACCGAAGGTCGGAATCTTCAAAATGATGTTGCAGAAGGACGTCGCAACATAG
AAGAGGTTCTGTATCAGGTTGAAGATGAGGATGACCGTGACGCCGAAAGTCAAGGGGCAG
CCACAACAGAACATGTTTATCGGCCGCACCATCTTCGGCCAGTGCTGTGAATTCCAGTGT
CCGGCTTGTACTTTGGAACTCAACAGCGCTACATTGCCTGCCTACCTGTCCTTGAGCCTT
GAGCCAAAATGGCTACGG
>at01392
GCGGGCTCAAGCTTGGTTCATAGTCCTCTCGCTCCTCGTGCAAGAAAGTCCAGTTTCCAA
TCCTCGGAAGATGGTGAGCATCCGGGCTGCTTGCGCCTTCTTCCTGCTTGTTGTGTCTGC
CGTGCAGGCCAGCAGCTCCTTCCTGGAGGGCCGCACCCCCTGGGCCCAAAGCCAGTCGGA
GTGCTGCACTACCTGCCACCGCTTCGGCATGAAGGCACTCGCCAAGTGGAACTCCGTCTT

CGCGGACATCACCAGCCCGGTTGCCTGCTGCGACACATGCGCCACGGTCTTTGGNGGCTC
AGCCTCCGCCTCGCCAGCG
>at01393
TTTTTTTTTTTTTTTTTTCGGCCTCCCCACACTGGGTAGGTTCTGTCCAAAATGCCAAAC
GGCAGTGTCTCAATCAAGTCACCTGCAGTCCAGGTGTCAAAACAGCAATGGGAAGCACGC
CTTCGGCTGGAAAAGAGAAGCATGCTCAGTCAAAATCGATGCAATCAATTCTATGTTGCA
CTGAAGCCATTCAACTGCAATGACCAGTGGGCCAGCTCATCTCGAGAACCCTACTCTGCG
GATGAAAAATGCTTTAGCCCACTGCTCCGCGATGCAATTGGCATGGACTCCCCACGAGCT
GGTGTGCACTCAAGTATGCTTTTGTATGGCACCCGCGCGCACGCAGGCCAGGACGTGCTC
AGCTGAGCATCTCAAGCAGTAGTGCCATTCGCTAATTGCAGGGAAAGGTGTCTCGAGCTT
GCGCTCCTCCTGCGCAAAGGGTTCCGATGGAGACCTCTTCTGATGGAGACCTGTGACTCA
AAGGGCCTTTGGAGCCACAGGCCTCTTTCAACGCGAGGCCTCACAGGGCCTTTGCCTGCT
CCGCCACCTGGATGAACTTCTGCGCCGTGCTAGACGAGATGCCGTTGTCTGTGAGATCAT
TCTCCGTGACATGCTTCAGATCCATNNTCNTGNCTTCACGCCAAAGAACTTAAGAACAGT
GCAGAGCCCAACTCCAAGTCCG
>at01394
CCCCAGAAGCAGGAGCACGAACCTCCAACGTCCTTAGGTAGTCACTTCCTGGCCTTCGAA
GTCCTCTATCGGCAGCTCCGCCACATCGCAGCCCATGTAGGAGACGCTGCGGAGGTGTAG
CTCCGCGGCGACTGCAATGACACTGAGGTCCGGACCACTGGTCCTCTCCGCGAGCCCGCT
GTCCACGCTCAGGCTGCCC
>at01395
CGTTCGTGCAGGTGAACGCATGTATGGCCCTGGAAAGGCTCTACCTCAGGGGTGGGGCAC
CTGCCACCGGTATGGAGCAGACGGCTGAATCAGCGATGGCGCTGCATCCATCCAACACCC
AGATCAGACGAGGAGCCAAGAAACTCTTAGAAACACTGCGGAATTTACCTTCGCCACCGC
CTGCGGAGGTGCCTACGCCTAGTGAAGTGCGGCAAACTGCCGCACTTCCATCTGGTGGCA
TTGGGGCCAGTGGAAACATGGCCCCGTCCCTGCCAATCCGCGACTGGCTGATGGAGCTCG
ATGACGTGGGTTTTATGATGGGGTACTACCGGGGTTTGGCGCAGAACTTTGATTCGCTTG
CTCAGGTACTGGAAGCATATGCTCCAGCTGGTGAACTGGATCCACGATTCTTTGATGACT
TAGGAGTGAGCAAGCTCGGACATCGGCGCTTGTTCAG
>at01396
TTGTTTTTTTTCTTTTTTTTTGCTATTTTGCTGCAACTCTCTAAGACAGTGCTGGACACA
GAAATTGCAATCTGGGCCTGTGGCTTGGTCTTTCGCCTCACAGGTCCATGATGAGCTTCA
ATTTCCTGCACGGACTGAGATCAAAGAGGGCAACCGAGCAGCTGCAGGTCCGACGTCTTG
CCGTGCCGCGGCGATGACCACGGTCCTGGTCAGGCGGCCTCTCCGTAGTGCTCCCGAAGG
GTGTCTGCAATGTCGTCGTAGCCGTTGTACATTGCCATCTGCACAATAGTGGGAGGTGTG
AAGAGCTTGGCCGCGGACGTGGACAGACTGGTGTCCGCTTTGGCTCGGGCAAGGACCTTC
ACGACATCCAACGCTCCTCCTCCAACTGCAGACATCATTGCGGTCCTGCCCGGATCATCA
TCGCCATCAGCGCGGGCGTTGATGTCAGCCCCCGACGCAATCAACGCCAGGACTTGCTCA
GCATTGCCTCTGCTCTCGGAAAACACGATGAGTGGAGTCCATCCCCTGGCGTCTCTTCCA
TTCACGTCTGTGAACGAGTTGCCCTGGGGGTCGCGGTAGGAGTCGAAGGCGACCTTGACG
ACGTGCGCTATGATGCCGTCCACGGTCGTCTCTGGGTGCAGTCGGCGCTGCATGACGATG
AGCTCATCGGTTCCCATGACGTCTTTGTACGCAGCCTCGAACCATTGTCTGGCAGAGGCC
CCAGTGCGGTCCAGAGCCTGTTCTGCCTCGGTGTGGTGAGACTGCTTGCAGCACCACATG
GTCAGGCAAATTCGACATCGTGCAAGCGGGCTTGAGCCAAAATGGCTACGG
>at01397
CACCAGCCTTGGTCTGCTGCTTCCCGCTCCAATAGACCGGCGCGTCGATCTCACGCAGAC
CTGGAGGGACCCAGCCATGGCAAAGTACAATGTAGTCCCGGGCCACCTCGCCAGCCCTGA
GCTGCACCAGCAGGTCGTAGAAGGCCTTGTAGGTCTTTGCCAAGAGGACGATGCCGGATG
AGGGCACGTCCAAGCGGTGCAAGAAGCCGAAACTGTGCTCGCGGTCACGCAGGATTGGCC
AACGGTTCGCAGAGATCGCCCGGCAGAACGACAGAAGCTGCCGCCCTACGTGCTGGTCGT
AGACCTCCCAACCCTGAGGCTTGGTCAAGACGAGCCTGTCTGGTAGGTCCAGTACAACTT
GAGGTTCTGACGAGAGCTTCGAGGCTTCAGCAGACGTCC
>at01398
AAACTCCGACTGCCAGCCCCAAACTTCGTTCGCCATGTGGACCCGGTAGCGGAGTGGTCC
GGCAAGCTGCATGTTGCACCCCACCAGGGTGAAGTACCAGAAGCGAGGCCGAAGGTGCTC

TCGTATGCGCACGGGGAAGGTGTTGTTTGTCCTGCCGGGCGTCACAAAGACGGGCACGAC
CTCCGACGCCATGCCGGCCTTCTCGTCGCAGCTGGTGGTCTCCCAGGACCTCTGGGCCTT
CTTCCAGTGCCGCGGCTCGTCGTCGAAGATCAGGAAGAGCAGCCTGCCGGAACCAGGGAG
CTGGCCACTCCTGTTATGTTGATCGATTCTCTGCAAGGTGACCTCAACCGTGCCCACCGA
CTCCTGCCCGGTAGGCAAGAAATCGTAACAGAACTTGGTCACAAAGAGTGCAAAGTTCGA
CTCATGTGGCACTCTCCCCTCAACAACCGTTGCCCTTGCAACTGAGGAGCCACACCAGAA
GACAGCAGCCCACACCCAGAGGTCTCGCGCAACAGCCCACGGTGGAAACAAGGCGGCACC
ATGGGCCATGGGGCTGGCCCCCCAGCAAGTGGCCCCAATTTAAATCAAAGCGGCCGCTGG
GCGCGGCATTGCCGCCCTCAGCCAAAACTGGCTGCGGCTTGAGCCAAAATGGCTA
>at01399
TGGTTCCAGCCAAAGCCCTCCATTCCATTGCGAATGTCACACTTGTTGCCTGAGTATGAC
CCGGTGCATTCTGTCATCATGATGTCCAAGTCCGGGTACTTGCGGTGCGTCCTCTCCTGT
GCATCAGCCATGTTTCCAGCATAGCAGTGATATGCCGTGCCATTGAAGACACCTGGTGCT
GCCTCAATGCCCTCCTCAGGGTACTTAGGGTGATCCCAGTTGTGATCGTAGATCAATATC
TTGGGCGAGCTCCCCAACAATGCCTTCACCTTCTTTCCAAGCTTTGCGTAATTCTGCGCA
GAGAGGTACATGCCAGGGTATGCTGGTGCGAAAAAGGGGCTCGTTCTGCAGAGTCATGTA
>at01400
CCGTTAGTCCTATTTTTGGCTCAAGCACCATTCCGTTTTGGCTCAAGTGAGCTGCTTGTG
AAAGGTCTGACTCCAGCTTGTCTCCCAGTCTGATGGCTGCAGTGGCTGGGGGCTCCGCAG
CATCCAGATTGACTTCATCCGTGAGTGTGACTGCGCTCGATGAACTCTGTGACTCATTGG
AATCCTATATACAGGTGGTGGTGAGTGGCTCGCTGACCAATTCAGTCCACAAGATTTCCG
GGATGCCGCATGGAAGCGTCGCTGCAACATCGTCGAAGACTTCA
>at01401
AGACCCTGGCGTGCAAACTCCTCTAGTTTCACAAGGTGCTCATCCTTGACAGCGCCCGCT
GATGACGCATGTGGTGCCTCAACTGCCGGTGCATCTACAGCAGCTGCGCGCCTGTGCATG
TAGGCCGGCCCACACGCAGAGTGAGAGCCTGGGTGCCGGCACCATGGTTGCCGGGTCTGA
GCTTGGCCAGATGGCATTGTGGCCGCATGCTTGCCTCTGGCACTACGAGTGGACATGCCA
AGACTAAAGGCAACACCATGGATCTCAATCCTGGCAACGAGGTGCAAAGCAACAAACACT
CCGACGACTGACATCGCCCTTCCCACAGTGTGCCGACGGCATGACGTGTTAGGCTGCAGG
GATTGACCTCCAGGAGGATGTTGAGGTGTCCTCACCATGAGCCTGC
>at01402
TTTTTTTTTTTTTTTTTTGCGCGCAACACCAGCTTCATGTCAGATCACCTGCTGTACTTT
GAAACTGAGTGTCCGTTGCGTCAGTGTGCCCGGCGCCGCCTCATGCCCTGCCCAAGCAGC
CCTCAACCAATCTCAGCGTCGGGCTGCGTAGGTGCCAGGAACTGTGCACCTCCTGCAGCC
GTCAGCTGCACGCCCGCGAAGCACGCCACGCGGTTTGCATCTGTCCTCAGGTCCTTAGCC
ACGGTCTCCCAGACCTTCTGCTTCTTCACGGCCTCGGGGGCCAACGGCTGGAAGCTCTGA
GGCACGTCCTTGGGCAGGACGCGCTCGCCCACTGCCAAGCCCTCCGGCACGTCTAGCAGC
TCCACCACCTTCTTCTCGGGGTTCTTGGCGCAGAGCACCATGCCCGAGGAGACGACACCC
ACCAGCTTAGCAGGCTTCATGTTGGCCACAACCAAGAGCTTCCTGCCCTGGAACTCCTCG
AGCTTGTAGTGCTCCCGCAGGCCGCTGACAATCTGTCTAGGACCCGAAGCCTCCCCGATG
TCGATCTCCTCAACAAAGAGCCGTTCGGCTTCCGGGTGATGCCAGGCCTTCACCACGTGC
CCCACACGGACATCCAGCTTCGAGAAAAGTGGCTGCTCCTCCNCCTGGCTGAGCGGCAGG
TGCCGCTTTGGCCTTNNGGCTTGGCTTGTCCTTGTCC
>at01403
GGACAcCACCTgGGGTATGGTCCTGGATTCGCTGGGtAGATGCGGTgGCCGCATgGGCTA
gTCTGCGGATCGGTTGGGCCAATGGCTGGCCGTCCACGGTCAGCGGTGACTGACCACCAC
gGCGTCGCTGGAACTGAAGAGAGATGGTGCCCCTGAAGGGCCTCTACGAGTACGACGATG
GGGACACAGCCTACAGAGCGTTGGGGGCAAGGGGAGCTGGGACTGCATTGGT
>at01404
GCTGGTGCGGCTGCTGGGTCTGGACCCGAAGCCGCAAGGCCGCAGCCGGGCCGCGATCAC
TCCCCACAGGAGGCCTCCAGCTCCCAGGACTGTAACAGCTCGTTGACGGTCTCCAAGGCG
TCTTTCTCGCTGGGCTGCTTCTTGCCGACGATCTTGGAGTCGCCGTCGACGACCACGCGG
ATGCGTCCGATTCTGTCTCTCAGGATGTGGCTGTGCAGATCGCGATGGACCCAATCAGCC
TTCTTCATGGCAGCCCCAAAGGCGCAGAGCTCCTTCTTGAACTCCTCCTTAGTTCTTTGT
ACCGCCACGAGTCATGGGATTCAGGAGGCATGGTGAGCGCGTACCGCAACCCCTCGGTCA

AGAGCTTTCCCGGCCCTTCCACCGCCACCGTTACGAAGCCAAGGTTCGTGTCAAATCTGT
AGACGTCCCCGAATCGTAGTAGTCGACAGTGTGCGGGATCTT
>at01405
ACGGAGTTATTATTCGGAGCAGGAAAGTCAGCGCCGCTGATATGCCCAGATGGAAATCGC
ATCATCAGTGAAGCTCATCAGGCAGGCCTCACCCCGAACATCAAAGGCTTCCAAGAGTGA
TTGCTCGACGGGAAGTTGTCGGGCAGCCAGCAGCACGACTGCCCCGCGCTGGAGCCGCAA
TGCCTGCTGGCCAAGCGCCTTGATCACATcCCTTGGGCCAAGTGACGGCGTTCGCGAAGA
CCACCGAAGCGTCGCTCCAGTCCAGGTCTTGGAGGaAGTCGCCTTCCCTGACGAGCCCCT
TGGGACAGCCTTTGGAGCGCGCCAAGGCCGCCAGGCCATTCCTAGTGTACCNCCTCGCCA
TCTCGCGGGCGGACTTCGCCACGCGCGACTGCAGCTCGCATCCCACGCACCGTCGGAAGG
GGAAGAGCAAGTGCGCCGTCAGGACGGCGAGGCCGGGGCCGCTGCCCAGGTCGAC
>at01406
TTTACTACATTTTCACTTATTATGTTTTAAATATTATATTATTAAATATAATTACTTTTT
AATATTAACTATATTGTCTAATTTTGATTTAATAAAATTTTTTTAATTTTATAACCTATT
ATTAATATATATATATTATTTTCATTTTTGTCATTTTTTATTTTTTTGCAGATTCAATAA
GATTATAAATTATATTATTTATTTTTAATTCTTTATATTCTATACAAATTAAAATATTCA
TTATTTAATAATATTAAATTCTTTTAATTTTTCAAATAATTCTTTTATATTAATAAATTT
ATTTTTTTTTCTTTTCATTTATACTATATTTATTTTTTTAATTTACTAATTTTATCTATA
TTTATATCTTTTATATTTATAATTTCTATATTTTTTTTTTTGTATTAATAATATTAGATA
ATGAT
>at01407
CACATGTGGGCTCGAGGGTCCTCATCACATCTGTGTGGCTCTGTTGCCTCTCTGGCGCGA
CCATGCGTGTTGCTGCGGGGAGAGGCCCTTCCTCGGGCGTGGCAGGGAGCGAGCCAACAT
GTCGGCCCCGCCAGCGAGGGGTTTGCACACTGGTGGCCAGCGTGGCTTGCGGCCTCTTCT
TGCGGTTGCCCTCTGGGCTTGTGCCGTCAAATGGGCCGGCCTGCGGCCCGGCTGCCGAAC
CCATGGCAAGGCATTCTGCGCACCGCTTCTCCGCCGGATGCGCGGCCCGGCGCATCCCCG
GCCGACGGACGCGCTGAGATCTGAG
>at01408
GGTTTTTAGTTCAGTCCAGCAGCGTCATCACTTGATCCGAAGGCCTTAAAAGTGATGAAA
AAGGATGTTCCGAAATGACCCCGATGCCAGTTTCTACCTGCGAGGCGCTTTGCTCCCAGC
GGCTACAATGCACGCACCGTCCTCCCTCCCTCCCCCCTGCCCTCTGCCTCCGCGCGGCTG
CCAGCGCCCGCGCTCATCTCGGCAGCCGGCTTCACCTCCTCCGCCGCCGAGCTGAAGCTG
CTGGAGAGGCTGTCGGAGTCGCTCTCGCCGTCCTGCAGTGGCTCTTCGCAGCGACCAAAA
ATCTGTGGTTGGTTCTGTGGCGCGGTACCTTTGCCCATTTGGCTCTTCCTGGCATGGCCT
TGCTCCNCCTGAGGACCTGGAGGGTG
>at01409
AGACCTCTCGCCTGCCGTTCGGCGCGCTTTGCTTTGCCAACTTCTTGCTGGCTTGGCTTT
CCTTCATGACCAGAGCGTGCTGCATCGTGACCTGTCCCCAACCAACATTCTGGTAGATGA
TGGACGCAGGCTCGCCATCTGTGACCTTGGCTCCGCTCACACGCACTCTGAAGGCGCTTT
GGCGCCACGATGCCGTGGCACGCTCTGGTACCGTGGCCCCGAGTTGCATGGTTCCGGCAA
GCGTGTGCCGCTCTCTTTCACATCTGACATGTGGTCACTGGGGTGTCTTGTGGTAGAGCT
GCTGCTCCGCAAGCCCGCTTTTGCTGGCAACGACATGAATGCCATGGTCAAGCTC
>at01410
CAAGATGCACCTCTCAAGGCTCAAGCTCAGTCTGCGTGCGTGAGGGGACTCACGGCACAG
AGCGCTGCCATGTCCAGCCTCTTGCGATACACTTCAAGGATATTCCGGCCCACCGGCCGT
GCTGTCTTCCAGCGGACACTTGTGGTTCCCGCTGCAACCGCTGCGGTGGGCGCAGCTTTG
CTTGGAGACGGTATTGCAAGGCACCAGAAGCATCCAGTGGTTCATTGTCAAGAGCAAAGT
CCAAAAAAAAAGAAAAAAAAACAA
>at01411
TTTTTTTTTTTTTTTTTTCATTTGGCACCACTGGACCCTGTCCCCAGAAGCAGCTTGGCA
CTGGTGCGTCGTGTGTGTGTGCATACCCTGCCACCCTCCACATCTAATATGCGGGTACCT
CAGCTGCAAGCTCAATCAGGAAATTGCTCGAAGAGGAAGGGGTGAGCCAGTGCGGCGGAA
GCAGAAGGTCGGCGCGAAGCCTCCAAGATCAACAACGAGGAGGCAAACTCCGCCAATGAC
CTGTGCTGTTGAAGCACCAGCTTTGCGAGCGGTCCTTGATTTGAGACTCGGCGCGCCGAA
GAAGGTGAAGATGCCCATTCAGGCCAGTTCAGCCGCCAATGGCCACTTTCAAGATCCTGT

ACCAAGAACTGGTCCTTCCTGATTTCGTTGACCTTTTTGAGCATGGACGCTGAAAACGGC
TCAATCGTCCGCTCCATGAGAGCTAGGTGCTCGAGGCTTTCGTGGGTGCGGAACAACAGT
TCGCCAGTGTAGAGCTCAAGGAGGATACAACCCATGGACCACAGGTCAGAACGCTCATTC
CTCGTGCC
>at01412
TGGGAGCCAACAGAGGCTCGGCCAGGCGAAGAACGAAATATCACCCAGATGCAACTACGC
GCGACGATCCCCCTGAGGGATTACAACTGGAGCAGATTCTCCATGTCCCCGGGAATGCGA
TCTGTCATCATCAATCCACCATTGAGGATGCAGTACGAGGACTGCTTGGGATGGACAGTC
TGCGGTGCTGCCCCGTTTCCATTCGACACACTGCACTTTCGCGACTGCTCCCAGACCACT
GCTATGAATGTGGTGGGCCCGTCCAATGATTTCTGGGCTCGACGGGCCACTGGCCAAGA
GAACGGCCACTCTTCGATTCCGAAGAGCCCCGTGTAGAGAGTACTCTGTCTCTGCCACCC
TTTACGATAGAAAAAAAAAAAAAAAAAAAAAA
>at01413
CATGAGCTCAAGAGCCTTTTCAGAAAGGCTTAGCTTCTAGAGGACCCGTCAAATCCCGAG
GCTTGGCAGCTAGAGAGGGGAACAGCGCACTGCTCGGTTGAGCTTAGCCCAGAGAGGGCA
AAACCAGCCGCTCAATTTGACCTCCGACGGCAAGTGTCGGAGACAGGATATAGCTGTGTC
GTCCGAGTCGCTGCCGCAAGGCCACTCAAACGTTTGTCCCGGCAGCAGCCATGTTCCCTG
TGCTTTGAGAATGCAAACTTGCAACGCCCTCTCATTGAGCGTG
>at01414
TCAGTAGCAAGCAACACCGGAACTGGGTGACTTCATGCGAGCCATTTGCTTTGCCAACAC
CTGCATGGGGAAGGCACTGCCGCGGCTGCTCTGGCTATGGAATCTTTGTCTTTGTGGCAA
CGGTGATGCTGACTTTCTCGCTCTGCAAATATATTGGCCCAAGAGGGAACCTGAACCTTG
CGGAGGCCTTCAAGTGCCTGTGCGACAAGACGGGGTCGCACTGTGTAGAGGCTTTGTTGC
TTATCCAGAAATCCAACACGACACTCTGAGACTGCAGTAAACCGGGCACGCACAAGCATG
CACAGAATATGCATGCGATGGGCACGTTGGACCCCAACGTGACACCTGACCAGGCATGCG
GAGATCTGAGCAACACAAGGAATGACCAAACGGTGGAGGCCAACAGGTCCGTGGACGTAA
GTATTTAAGGGCGTTGCCCAAAAAAAAAAGAAAAAAAAACAA
>at01415
TTGTTTTTTTTTCTTTTTTTTTCTTAACGTAAGCCTCAGGCAGCTCAGTTGAGCCACCAA
AGATCCTGCACTACGAACGCACATGCACATGCGCACGCAGACACGCATACTTGCACGCTG
CACACAAAGCATGCATGGATGTGCATGCGCATGGAGATACTCGCAGCACAACCGCAACAT
GCAGCTACCCTGAACAAGCACACCCCTGCCTTCACAAGCCAAGCATCTCATTCAGGACGC
GGCCCTAAGTCCTTCCCAGCAGCGTCAGCCCACTTCTTCATCTTGTCCCACATGTCGCCT
GGCTTGACCTTGCTATCCTCGTATGCCACCTTGACCGTTTTGGCTGTGATGTCACAGTCG
ACGTCCGTAATGCCATCAAGCTTGCTGAGTATTTTGGTGATGGCTCCGGAGCAGCCCTCA
CAAGTCATCCCAACGCCTTCAAAAGTGTATTCGGACATTGTGGCACGGCCGAGCAGTCCA
AAGATCCCTGTGACAACAGCTCAACTCGGCAGCTTGAGCCAAAATGGCTACGG
>at01416
TTGTTTTTTTTTCTTTTTTTTGATCTCTCCAGCTGTCCACATGTTCGGTCCACGCTCTCC
ATCCTGCTCCGCAACGCGTACGATATGGAGCTCACATCTCCATCTCGCGACATGTCATTC
GGCCTCTATGCGGCGCTTCTTCGTACTTGGCTCGGAGTCCTCGACCTTCTTCCCCTCACC
AGGCTTCCTGTACTCCTCGGGTGTCTCTATCTTCTCCACCTCCCTTGCCCTGGGCTTTCC
TCGGCTGATCTCAACCGAAAAAGAAACCCAGTCACCATTCTTCACTTCTACCTTTGTGCG
TTGCTTGTACAAGTCCGCGACTCCACCAATCTCAGCCTTGTGCAAGAAGACGTCCTTGCC
ATAGACCTTTCTTGCGTCCAGGCAGCTGATGAACCCCCAGCCCTGATCATCGTCCCACTT
GAAAACCGAGCCAGTGAAACGCCGGCTGGGGTCGTGGCTGAGGCTCAGGCCGCCCGGCTG
CCGCGAAAGAGCCGCCAGGGGCATCCCCACGGCGGGCTGACCAGCAGCCTGAGTGGTGGC
CATCGACCCCACGGCTGCAAACTGGAGCTGCAGCGCGGCCACGTACCTAGCGTACTCTGC
CATCTGCTCTTGGCTCGTGCCTGCAAAGCTTTGGGATACAGCCGGCGTGGCGAGCTGTGG
AGCTGCCAGACCGGCCGCAGCCAGACCAGCAGCGGCCAACGTCCCGGGCACTGGCAAACC
TGAGGTGGAAAGCACCCCGGGCACCCCGAAGCTGGGCATCGGCATGACATCTGTGGCGTT
CCCTATCTGCATGGACGATGTGGAGGCACGCTGTGCCTGTGGGGCAAAGGCTGCAGGCAA
CCCTGTGGCAGTCGTGGTGCTGCCATCTGCCTGCATCATGGTGCAGGCAGGAGCCACTAG
CCCTGCTGTGGCTGCAAGTGGCATGGCAGTGGCCTGGGCAACCGCTGCGGGGCCTGCCAG
CAGGAAGTGCTGCGCCTCCGGGAACGACGGCCCGGGCACCATTTCCATCCATCTCTGCGC

CGGGTCCATCCCAGGACCGAGGCGCCAAACTACCGCGTGGGCAGCCACCGCGGCGCGCAC
CCATGCGGCAGGCCGCGCGGCCTCAGGGCAGGGCTTGGGAAAGGATTGCTTGAGCCAAAA
TGGCTACGG
>at01417
TTTTTTTTTTTTTTTTTGCCTCACGCCCCCAATGCCTCTATGCACTCGCCGGCTTTTGA
ACCCTGGCCTGATTCCTAAGGCGCGTCACACAACCTTTCCGTAGAAGATGCCCTTGAGCA
GGATGGTCTTTGGGGCCTTTGCTCCCATGTCTGTGTCCGACGGCTGTTTGGAGACAAAGA
CGCCACCGATCTCACCCAACTCAGCATTGACCTTGTTGACCTCCATCTCGATGGCGCCCT
TACCAATGTCAAAGACCTTGTTTGTCTCCTTGAAGAGCTCCTCCTGGCCCCCAACACCGT
CGGCTTGCAGAGCGGGCAGTGCCACAGCCTGATCGTACCCAGAATACATACCGCGACCTT
TGGGGTCGAGGAAGGCGCCAGTGCGGTAGGAGGGCACCTCAAACTCTCCACCCCAAGTGA
AGCCCGGCTTGAACTCGCCGCCCTCACCCGTCGCGACCAGCTTCTTGACAGAGAACAGGA
ATGGCACATACTCGCCGTTGGGCAACTGCACGGTGGTGGGCAGCGTGTGGATGCCGTCGT
ACTCCCTGAAGGTGATTGGATTTGGATCAAGGTTGCCATTGATGAAGGCCAGTGTGTAGG
TCTGGCGCGTCAAGAGCTTGGTCGTAACAAACGACTTGCCCTTGTTGGTCTCTGCCTCGA
CGGCAAAGGACTTGGGCTCGAAGCACAGGTTTGTGAGCTTCGCGCCACTCTTCACTGGCA
CCGAGCTGCCCTGGCTCTCAACTGTTGGGCAGCGGTTGGCGAGGCCTGAGCCCCTCACCT
GGTTGTAGGTCAGCTGGCTGTACTGCTCAGCGGTGAGCGCGCGAGTCGCCGTGGGTGCAA
GGAGCCCTGCCGCGAGCCCGAGCACGGCACCGAAGCTGAGAATGGATGTCAGCCACGACC
TTGG
>at01418
TCTCAGAGGAGCGGGGGCCTGTTGAGGGAGCGGGCGGCCAAGCGTTAAGAGCTTTCCTGT
TAACCCTCGACAGCACCTATGGCGGCTGGCAGGGGCCCTCCTCATGCACCTGCAACGCCA
TCCATGTCCCCACAGCTTGGCGAAGTGGCCGCAGCCTCATGCGCGATGGCCGCGACCTCT
CTCGGTTTTAACCCATTCGACACCGTTAAGACCAAGATGCAGACGCAGAACCAGCTCTCA
GCGGATGCGACAAAAACGCCTGTACAATGGGGTGGTGCACTGCGTACAGCGGGTTCGGGT
TGAGGACGGCTTCGCACGGGGCCTCTGGCTGCCAGGCCTGGCCGCATCGGTGGGACGGGA
CGTTATCAGCGGCGGCGTCCG
>at01419
CGTACAAGCCACGGTGCCACCATCTCTGCATCCAGTGAGGCCCCGTGGAACACCAGACGC
TGCTCATGGAGCGTCCCACCACGACGCGCTGGCGCCGGCAGGGCAGCGCCGCCGCGGAGC
CGGCGAGGCCCCGTAATCTCAGCGTGTCGATGTAGAAGACCTCGCAGCCCTCACAGCGCT
GGAAGACCTCCTTACGCAGGAGAGCAGCCCTGGCCCGTGCTGTGAGTAATGCCCGCCATC
GGGTACGAGAGACATGGAGCACCACGGCACCCTGCGCTCGCAGCGCCTCCTCCAGCCAGT
CAGAATTGTTGACAAAGGTGGAGGACATAGCCCTTTCCTCCTTTGTCAACTCGTCAGAGT
TGAAGACCGCAGCCAGAAAGTGAAGAGGCGGCTTCGCGCTGCCGAAGTCTGCACCTGGTG
GGCAACTCGCGCCCTCCTCAGAGCACTCGGTGTCGTCCGCGGACGAAGCAGGAGGCCATG
TAGAAG
>at01420
GCGGTTGTTTTTTTTTCTTTTTTTTTTGGACAACCACATCTTTNGAGGGAAAAAACGGCT
GCGATGAGCATGGAGCTTCCAGCGTCAAGTGGGCAATGCGACTTCCTAAAAGGTATGCAA
GGCCTAGAAGCCTGGCACCGGCAGGCTACTGATAGCGCCGGATGCTCCAGATGCCTCAGC
AGCTCAGACTGGGAAGCAGGCCTGCGTAACCAACCCTGTCGTTCACTTCTCAGCCTGCTA
GCAGCTGCTCCGCTGGAGGTCGAGTCCTGAGCCCAAGCTGATGGAACGCATCGCAAGAGT
CAGAGCCCAGCTAGTGAGGAACATTGATCCCAGCTCGCTTCAAGTACTGCTGGTGCTTCG
CAACCTGCAGGAGGATGATCTCGCGCTCGGTCTTTGTCATCTCGTTCCACGGCTTGTCAC
CCCAGAACTGAGCTGTGCCGCTGGCCATGAACCCAACCTTGGGATGCACCGCCTTGTAAC
CATCAGGATTCAACCAGACTGGTCCCCGTTGGGGGTTGATTTCCACGTACTCGTCCCCAT
CTTCAGTATGGACGGTTACCCAGCGCTGGTGTGTCTCGAACTTAGTGCCAAGCGAAGGCC
GCTGATCTTCCAGCTGCAGGCCTCCAGCACCGGCGAATAGGTCAACACTCTCCACATAGT
CTGTAACCGGCCGGGGCGGATTCCTGGCGCGTGGTGGCAAGGGGATGTCATCGATGAGGG
ACATCTTCTCCATGAGGACTCGCACTGCTGCAGGAGGCCTGGAAGTGATCTATGTTTCAG
CTATCTTGAAACTTGAGCTGAAGACAGCTTGAGCCAAAATGGCTACGG
>at01421
CTGTGCCCTCTGCAAAGAGATGTGGGTTGACGTGCTTTGCTCACAGCCCCATTTTTTCTT

AGTTTCCCGATGTCTTCGAAATCGGAGTTGTGCCACATCTTCAGATTGTGCTGGCCTACA
TGCCTCTCCTCGATGTTGCAGTTTGGACTGTGTCTGACCCCCCTCGTCTTTGCCGGTCAC
ATGGGCCGCTTGCCTCTTGCAGCCGCCTCTCTGGGGGGCAGCTGGTTCAACATCATATAC
TACGGCACAGTTGGAGCCACAACGAGCATTGACACGGTCGTTTCGCAGAGTCTTTGGCCA
AGGAGACATCAATAAGTGCAGAGCATGGATGC
>at01422
GGCCCTTTTCCAATCCTGCAACACCACACCAGATGCAAACGCGTCTCTGTACATGCGAAT
CGTCTGGCAGGCCAAAGCTGGGTCCCATGCACTGTCTTAGCACTGCCGTGACCTGGACCC
TCAATTACATCTTGGAGTGTTTCTGTATATGAGTGGGTGGAGCTCAATGCACAAGCTCAC
AATGACCAAGAGACTTAGCACTGTGGTCTCCCTCTCTCTCCAAGGATGTCAGCAACTAGT
TGACGCAAGTTGGTATAAGGCGGTATTCCACCACCCTTATGAAAAGCACATGGCCTCAGT
CATGGGGAAGGTGTTTGGAGCAAGTCATGGCCCTAACCATTCGGCCTGGCAGGGACCTCA
TTCGGGCTGCAGCATTCAGTCCCAGGGCTTTCTTCAGAAGTCTCAAATCCACAGCTGGTG
ACATGCGGCCAAGGCGGCCATTGTAAACAAGAGGAGCAACCTCGACTGCCTCCTTGGCAA
TTCGGTACAACTCCTCCGCGTTGCGAGGGAAGGGTGCGCTCTGCGGTGTTCGGAACAGAA
ACTCCAAAAACTTCGCGATCTCCGGCAGCTTCAGGGCGCGCTCATCCAACAAGTCAAATA
GCTTCTCCAAGGTGCCACCCTCGCGAATCCGATGCATCAGCGGTGTGTACGCAAACCATC
CGTTTCCGCAGTCATACACCTCCTGCGCCTCTCCGCGGAGGTCATCCAAGATGTCCAGCG
GAAGGTCGAGCTCCTCATCAATCTTTGTTGTATAGTCGACGATGGA
>at01423
TACTTGTTGAGGAACTTTCTGTGGAAGTCGCTGCGCACCGCATCGTTGATGAATCGCTGC
TTCTCCTTGGGAGCCATCCTCGCTGGTTCTTGAAGACCGTGTCGTCGTCCCACTTTCTCT
TGAGGGACAGGTTGCCGGACGCCACGATTGGGTTTCCCTTCATGATCTCGTCCCGCCGCG
ACCTGTCGTCCTGCTTCTGCTTCAGCTCATTCTGCCTGTCCTCCTCTGCTTGGCGCTCCT
TCTTGATCTTCGCCAACTCCCGCATGAGCTCCTCCGTGTCCTCGTCGTCGTCGTCGTCGT
CCTGCTGCTCCGACTTCTCCGAATCGCTCGGCGGCGCGGCCTCGTCGGCGTCCTCTGGGA
ACGGATTGTCCTCCAACGCTGGAGCACGCCTCTCCTTCTTCTCCTCGCGCCTCTCCTCCT
TGCCATCCCGCGCGTCCTGCGCCTTCTTCTCCTTGCGCTCGAGCTCCTCACGAAGCTCCT
TGCGCGAGAGACGCTCCTCCGGCATGCCCTGTCCGCTCTGGCGCGTCTTGAGCTTGAGCA
TGCCCGCGGCATCGCGGTTGGCAACCTTCGTCGACTGCGCCATGTACAGGTTCCCGCCCG
GGTTCGTGCCGCTGCCCAGGGCGGACGTCCAGGTAGGTCGGTGAGCAGTCGTCATGGCTG
CGGGCGAGGGGGGCAGAGAGAAAGAGAGAGAGAGAAAGAGAGAGAGGGAGAGGGACACAC
AGAGGCCCCTGAGCTTGAGCCAAAATGGCTACGG
>at01424
TTCTCGGCCGGCTTGAAGTCAATCAATCTCTTGTACGGTTCACAGCACGGAACAACGGTT
CTGCAGCTCAAGCAGTTGCCGGCCACAAAGGCCAAATGCCCTGCCAGCTGGACGCGGAAC
AACACTGGCTATGACGAGAGGGGCTGGTGTATTGTTGAACAGGCAATCTCGAGTATCTAC
AAGAAGTCCGGCTTGCTTGTGAACTTTGCAGAAGCACCTGCATCTCAAGTCCAGTCTGCA
ATGCGTGCCTTTATGGGGCGTCGGATGGATCTCGAGCGGGATGAGAACTGGGAGAAATTG
GGCATCCAGTGCGTCGAGTATTGTGAGGAATTCGAAGGTCTAATTGCGCGCACATCCGGT
TCCTCCATCAGGCCGCCGATCCACCCAGACCGACATGGATGCCATCCTGGGCCAGGCAAA
GTTCACCAATGGCTCGGACGT
>at01425
TTTTTTTTTTTTTTTTTTGACGGGGGGGGCACCTTTGTCGTATGCGTGCCCTAACCCTGC
TCCTCCTTGGGTCGACAGCGTCAGTGCTGGTCACTCCAGAATCAAGAGGATGCATTGAGG
GTAATGGGGTCCATGATGCCATCGCCGTCTTCGCCGTCCTTGTCCTTGTTCAGATCTGGG
GCAGAAAGTCGGTGGCTAGCGCGATTCGGGCAACGGAGTGTAAAATGGCTCCACGCTGTA
CATCGCTGCGACCCCCAGCCGTCAGGAGTAGAGGCCAAGAGTCTCAACAGAAAGCTCTCG
CTCGGCAGCACCAGAGGAGTCGTTGGGCTGGGAAGCAAGCCTGCATCGCGCACGAGGCAG
GCCGTACTGTCGGCGGATGTCCTCTGAAGCCTCTTGGTTCTCCATCTCCCAAGCCACCCA
GCTGCGCAGGCCTCCATCCAAACGACAAACTGAG
>at01426
GCAGTCCTCGTTCAAGGACTTGAATTTGAGAGCAGCATGGGTTTGACACGGTGTCTGATG
CATTGCGGATATAGAACCCTGTAGGTTGCAGTCTCAGCAAACTGGCAACCGTGTATCGAA
CTCGCCTATCATGTATGAGGCGGCCATACACCCACGGATAGGCACGCATGCATGCATGAC

GCCGTTTGAGTGTCGGCTTCTGAATCCCGCAGCATGCCCGAGCNGCGTGGGCTTCTCGCG
ACCCATGGGTCAGGGTGCAATGTGATTTTGATGCCTCTGTGCATTTGCAAATTACTGCAA
GTGCATTTCTGTGGAGGNAATGGCACCATGACAGGAGGGGCCATTTTGCCAGATGTATAT
GTGTGGATATTAGGGCTGAATATGTTTGCCTTTGTGTACAGGAGCTGANATTTATTGTAC
AGGACNGCTCTGCACTGTCATCTaGGTGCTGTGTGCATNGNCTGCNTGCATAAGATATGC
ACGCCNAGTGATTGGtTTCAACNTGcCANCACTTTGTGGTTGGTGCACTTCAGTGCAAGG
TATCTGTTTAGTCTCACGTGGATGGCTGGCTTCGAAGAAAGTTTGCCTCACCAGACACGG
CATTTGGATCATG
>at01427
TTGTTTTTTTTTCTTTTTTTTCTGGTCCCACTTCTCTTCTCTTAAGACAAGGAAGCAGAA
TGTTCAGTCTACAACAGTAGCAAACGCGAAGGAGGCACGCCGAGATCACAGACTAAGGCA
AGGCTAGCTAGATGACAGAGCAGTCGCCACTCACAGCGCTCCTGCTGAGAAGCTCACCCC
CATCGGCCTGTAGAACTGCATGTTGACCAGGGGTCGGCGAGCAAACGGCGAGCACGTGTA
GGTCTTTGTGGCAGCTCTGGATGTCCAGCCGATCTCGTGGCTCGTCGTGACCTGCCGACC
GAACCTCTCGGCAGGAGCCTGCCTGCTCTGCTTGTGCAGGTACGCCGCGCGGCCCTGTCC
CTCTGCGGAGACACCGTAACGAAGAAGCTCATCTGGGGCGGTCTCCGCCGGCGCGGTGGG
CACGTGCGGGTTTTTCACAATGCCGTAGGTCTGGTCCAGCAAGTTCAGCTGCGCCGACGC
CTTCTTCTTCGGCGAGAGGCTGGCAAGCGCCCCGGCAACGGCCGAGGTCGATCCCCCTCG
AGCTTCGCGCTGCTGATCCTTCCATTTCCTGCTGAACTTGGTCTCCTTGTTTATCGTCTC
CTTGTAGATGTTGGCAAGGGTGTGGTCCATCGCCATGCCGACCACAGCAGCAAGACCCGC
GCGAAGCGGTCGCAGGCTCAGGTGGTCGTTCCTTCCCCCCTCCCTCCCCTTGAGCCAAAA
TGGCTACGG
>at01428
CAGGGAGCAAGCACGCTTCTTTGGACACTGCTCTGGGGCGTGTAGTCTCCTCCGCGCAAT
GAAGCCGACgTGTTGCAACGGCAGCCCTGCTCATCTTGGTGCCCAGTGCCCACTCTGCTC
TGTCTGTGGCCACCTACCCCGTTTACTCCCCTGGCGCAGGGTCCATCGACACGTGCGGAG
GCATCAGTTGCAAGCCACTTGAGTGCAAGCCNCCCTTCAAATATCACAGTCCCGAGGAGA
CGGGTCACATGCTGCCCCCTTTGCCTAGCAGAGAGCATCTCGGTGCCTGAGGACAGGTCT
TGGGCCAAGGGGATGTCAgGGCGGCGTTGGCATGGACAACAAACGCGGGACCCCTGGTCC
TGTGCCGCGGCGTCGTCCTGCCCACCGGCCGACTGCCCTGAGTACGAGCAGATGTTTGAC
GGCCGATGCTGCACCAAGTGTCAGTCAGCGGCAAGGGTGACCCCGGCTGACCTTGCACAG
AGCTATCCGCAACAGCCCTAGGAAGTAGGCGGCGCATGGTGAGAGCATCAGCTGAGGCGG
AATAACAAAAAAGAAAAAAAAAACAA
>at01429
AAGTTGAAGCAACGGCCAATGCTTTACTCACGAGCTGCTCAGACTGCTGGTCATAGGCCT
TGATTGCACTTTGCAGCTGCTTGTTCATTCTCTCTAACTCAGGTGTCGTGAAGATGCACT
CCATCTTCTTGATGGAAAGCACTTTGAGCTTTTGGGGCCCCTTGTAATTAGTCACAGCCT
GCTGATGCTTCTTCGTCACTCTCAGGGCCTGACCCTCCGGGCACTCGGTGAGCACTACTG
CCCTCTGGTTTCCTAGTGATGGCAACGCCATAGCGCTGTCGATCGCATGTCGCACCGACT
CCATCTGCTCTCGGACTTGGTCCCTCTGTGCAGCGAGCTGCTTCAAGGATGCGTACAAAA
TTCATGCTGATGCAGTAGTTCCGCTGCTCTGCCTG
>at01430
CATTGGCAGCTTCCAAGCCTGGGTGCTTGCAACTAAGGATGCCTCTCCCGTGACTCCTCC
CATGGAGTCCTGACATCCTGCATGACCCCTGTCCAGTCGAGACCTCGCCTCTATTGCCTT
GTAGAGGTTGGCCTGGCTGACACTGGAGATCTTTGCAAGACCTGTAACAGACGCTTCGAC
AGAAAGCAGAGCCTGGGCCTCATCACTGGTCCTGAATGTTAGGATTGACGCCCGGGAGTT
CCGTGCACAGGGCAGTGCGCTGGCCTTGGCCCTGGCGATACAGAAGCCACTTTGCCAGTG
TCAAGGGTTTCTTCCATGCCATGCTGGTCTGGCACTGCTTACTCAAAGCCAGTTAGAATG
ATACGACCGACAAACAGACAGCA
>at01431
TTTTTTTTTTTTTTTTTAACATGTGGTAACCACTGGCAGCCCTCGCCCCCAGCCCTGAT
GTCAGCGGGCCTGGTCCAGTTACAGGCCTCCTCCGCTCTGAGGAGGCAAAATGACTTTTG
CTGCGAGCACTGCAGGACTACGGTGGCAAGAAGCTGTCCACCACCACACCCCCTCACTCA
CCGGAAAACAGCTGCCAGCTTCTTCATCTTCAGGTAGAAGACGACCGCCGTTGTGATGGA
CAGCACCACCGCGATGCACGCCAGGATGATCTGGTTGGTCTGCAGCCGCAGGCCCAGCGT

CTGCAGGACCCGCTTCGAGAGCCCGAGGCTCTCGTCCACGTCGCTCAGGTGGCCCTTGGT
GCGCAGGATGCTCTCCCTCTGGGTATGGAGGTCGCACATGACGCCCATGCCGATCTCCTC
GGCCTCCAGCGCCGTGCGCCGGGTCTCGTCCAGGCGCCGGGTCGTGGACCTCACGGTGTT
GTAGTGGTCACCGAGGGCCTTCTCCTCAGGGTCGCGCAGGTTGAGGCCGTCGCGTTGCAG
GGCAGCCTGCGCCTCCCCTATTCGCCTCTTGCCCGTGGCCAGCTCGGCCCTGTAGTCCCG
AATCCGCGGCGCCACGGACGCGCCCAGAGAGCCCATGGACTTGACCTCCAGCTCCATCTG
CTGGAGCGCCCGGCCCGCGCGCTCGTGTGCCTCGCTCGCCTCCCGCATGGCGGCCACACG
GCCGGCGCCATCGCCTTTGGCCGACACGGTGGGCCGCCTTCTCGCGCACGGAGCGCAGCA
GGCGGCGAAAGTCCTCCTCATAGGAGTCGAAGACGGTCGAGGTGGTGACCACCTTTGCGC
CCGTAACCGACGAGGAGCTGCCTGGCATTGGGCACCAGCGGCAAGTGGCCACCTGCACGG
CCCTCAAGCTCCATCCATTCTGCCTGAG
>at01432
AGTGACCGCCTCGCCGGCCTTGACTTGCAGAACTTCGCGCAGCGTGTAGCTAGTGCTGGC
TGGATCTACTTGGACCACGCCATGTTGGGTGTCGAGGCTCCTGAAGATTCATCTGAAGCG
ACGATGTACCAGTGTGGTCCAAGACGGCTTCCCACAACTTGGGAAGGTTGTGCATGGGTG
GGCTGACATGGACATGCATCTTGAGCACTCCTGGCCTAGAGGGAGCTTGCAGCTTCTGCC
GGCGATGCTACTGTGCCTGCTGAGGGTCCCATGCGCAACAGCTTGAGTCTCGGATCGTCG
GCCACAGATTACAGGATGGGCCCGGGTCATGATGTAGAGTTCTCCGGCAGGTCCGCGCAC
ACTCAAGCGGTTTTGTGCATGCAGGCGCATGCTTGAGGCTCTGATATTCGTGTGCC
>at01433
CGGCTGTCGCCCGACCTGATTGCCTTCAACGCTGCAACCAGTGCTTGCGAGAGCGGCCGG
CAATGGCAGCTGGTCGGGCAGCTGTTCGAGGACATGAGGCGCGCGAGCGTTCAGGCCGAC
TTGGTGACCTATGTTGCGGCGATGAGGACCTACGGGCGATGCCTGCAGGGGCTAGACAAC
GTAGACTCCATCTCATGACTGAGATGGA
>at01434
TTTTTTTTTTTTTTTTTCCTCCACACGCCGGCTTCAAGACGGCCGGCAGCACGCTGCCG
GTCCACCCAAACGCCCGGGCCACGGACATCGCGGCCCGCCCGGCCGAAGGCCAAAAACAC
ACCTGGCGGCAGCAGGCTCCACGTGCCACGCCTGGAGCCCCCGCCGCCCCCGCCAAACAA
GCACTTCAGGCTCGCAAAAAAGAGCGGCCTGCACAGCATGGCCTGCCTGCACGCACTCCG
CATGCAGGCAGCGACGGAGCAAAAGGGGCGACTGCGCAAGTACACATGAACAAAAATCAT
GGACTTGCACATGTACAAGCGAGAAAGCACGGCGGGCATGAGAACATCCTCACGCACGTC
AACCATGGCACTCGACCTAAAGCCTGCAGTACAAGGGGCCTACGACCAAACAGTTATTTT
TGTGCAGTTTTCTGCAACAAACTTGCAATTTGTCGCTGGACAGAGTGCCTCACACTGCCG
GGAGGCATGCCTCACAAGTGTTTCCTCCGCACTCACAACGAAAAAATATGCCCGAGTGCG
TTCCTCGTGCC
>at01435
CATACCGACAAAGAGTCTCTCTTGTTGTCCAACAGCGCGGACACCAGCGTCACCCACAAT
TCCGATAGCCATACCAGCGGCAAGTCCGCTGAGTCCACAGCACAACCCAGCAGAGAGATG
GGCATAGCCGGTGAAACTGGAGTAGACGGTGGCTCCAGTATTGTCAGGAGTGGTAATTGA
TCCTTGGATGATGACGGCGACGATGAGACCATAAATACCCAATACACCAGCCATGACCAC
AGGAATGATATTCTCAGCACCAACTGAGGGTTCATCACACCCATCGAGGAAATTCCAACA
CCAGACTTGGCGGTCCGTAGGCAGCGCCAATGTTGGCAAAGCACAAAGCAGCAGTCACT
>at01436
CGCGGGGGTACGACGGCACGATGCCTCCCTCCAAGCCGATGCTAGGGTGGGTGAAGATGA
CTTCATAGGTCCGAGCAAGTGGTGCGACGGCGTCCTTGATCGCCATCACTGCCCATGACG
CTTCCCAGACTTTCTTCCCCAGACTGTTTCCCAGCTCACTCAGGCTCTGCACGTCGGCCA
CGAATGGGATCTTGCCCTCCTGCACAAGCCTTGCGCTCAGAAACATTTCAGATCCCCAGG
TTGAATTTCTCAGCACGTAGTCGCTGAGCTTGTTAACTTGGTCGACCATATCCCTGTGAC
AATGCATCTCATGCTCGGTCCTGAAGAACATGTTCCAGTTTTGCAAGACCTTTCCCTCGG
CAGCGCACGCTCGGACCGTAGTAATGTTCTCTGGCATCAACGGCTCAATCCAGTTCTGCT
CCACCTTGGTCATCGTGCGGTCA
>at01437
CCACTTGCGAAGCATTGCCATCGGACGTCGGTCtTTATCTATTTGCCGGACATACGACAC
GATGGGAACTTNGTCAAAGGAGAGTGGGGCGATGCTGGAAAGCAGACAGCTTGGTGCGCT
GTTCAAACTGTGGCCTGCATTTCCATGCCACCAAATCCCCGAATGCACGCAGCGGGAAGT

ACTTCGAGACCGACAGGCGCTGCCCGACTTGCGGGGGACGGGGAGCAAGCAGCAGGTGAT
TGCAATCGGCGCGAACAAAACGCCATGAGCTTTGATGTGGCTGACAGCAATTCAGGCGGT
CATTGAAATTGGCTTGCCATTGATATTGCGGGCCACCTGCAGCAATGAAGGAGCAAGGGT
TAGGCATTGGAAACGCGGAGCTGAAAGCTAACCTGAACAAGGTGTAGAATA
>at01438
TTTTTTTTTTTTTTTTTCCAAACAGCTGCTGACACCCCATCCGTGCTGTATGTCCGTGC
TGTAGCCACGGACATCTCAATTCATGCATCTCAATTCACGCACGCACCCACAGGTCCCAG
TCAGTGTTCTGGAGGCACTCCGAAGTCCGGATGTTCTTGCGCAGCCAGCAATTGCCAGGA
TCCTGATTGCGGAAGACGACAACCCCCTCGCACTCAGGCCTCGTGCC
>at01439
CCGTAGCCATTTTGGCTCAAGCTGCCGTGGCTCAACTTGCACCGTCAATAGAGTGACATC
AACCTTGGCTGCACAGCCCTGAGATGGTTGATCAGAACTTATGCCAATTTGATTGTGAGG
TGCTTAGTGATGAGAACAACAGCGTGCCACCTTCACAGAAGCTTGCACGACACTTGGTGG
GAATATTGCTGATTGTCTTGGTTGCCTGCATTTGGGTTGCTGCATCACAGCTCATCGAGG
GGATTTTCCAAGAGGGTTCATATAACAAGCCCTATTTCCTGACCTACTTCAACACTTGTG
GTTTCGCACTGTGGCTGCTCGTTACAGCGTTTCGAAGACGCTTGCGGCACCAACTTAGCC
AGCCGCTTTCGATCAGTGACCCATCAGTGGAGCCACAGCCGGTGATACAGCGTGGAGTGT
GGGTTTATCTTCGGATTGCCGCATCCGTTGCGCCTGGCTGGATGCTTGCCAATTACTTGT
TCAATCTCTCCTTGGATTACACTTCTGTGGCATCAAACTCAGTTCTTTCCACCACCAGCA
ACCTTTGGACGCTGATCTTCTCGGCGTGCTTTCTCCAGGAGCGTGTGGACCCCATCAAGC
TCATCTCAATAGTTGCCACCGTGTCAGGAGCTGCACTCGTGGCCACTGCGGACACAGGTT
CCAAGCATGGCAGCGAAAGTGGCGGAGGCAGCATCTGGGGAGACCTCCTCGCATTGCTAG
CAGCATGCGCCTATGGTGGGTACAGCGTACTGCTCAAGGTGTGCATGCCAGACACCGCCG
AAGGCCTCTCTGTACTCATTGTGTTTGGATTTGTTGGTCTGTTGGTGCTCCTGTGTGGCT
GGCCGGTGCTCCTGATTTTTGACGTGGTCGGGTGGGAGGAGTTCATGTGGCCTCCAGCTC
AGATCCTGCCGTTCCTACTGCTGAACGCCCCTCATTGGCACCAACCTCAGCGATGCACTCT
GGGCTCAAGCCTTGCAGTTCACCTCTCCACTCATTGCAACCTTGGGCCTCTCGCTCACGA
TTCCTCTTGGGATGGTGAGCGATCTGCTGCTGCACGGCAAGCACTTTGGACCCAAGTATG
CGGGCGGGGCAGTGCTCGTGCTTGCGGGTTTCTTCCTGGGCAGCGTGGCCGAGCATGTGT
GGGACAGGCTTCGATGCAATGGTCAACGAGACGGCTGCTGCAGGCGACGGGGTCAGATGG
TTGAGTGCACTTGAGTGCGCTTACCTCAATAAAAAAAAAAGAAAAAAAAACAA
>at01440
TTTTTTTTTTTTTTTTTGGAAGAGCGGTCGTTCAAACACTCTGACTTCACATTCATGCT
TGCGTCCGCGAGCCTCTTTCCTACGATAAGGGAGAGTCCTGAGCATCCCGGGCTCACTGA
CGCGTAGTCCGGAAGATCTCTACCCAGCTCCTCGTCCAGAACTTGCCCAGCTCCGCGTCC
TTGCAAGATCTCCAGTGTCGAGTTGACTGACCAGTCCGGCCAACTGGGGAGAGAAGCCTC
ACTCTCCTTTGATGATGGCCTCGGACTTGGGCGGCAGCTGCAGCACGCCCGTCCAGAAGC
TTTGGACCCACCCCTCCAGCACCTCGTCCCCCTTGAGCTCCTCGATGATCCGAGCCCCAT
TCAGCATCGCCTGCAGCGTCGTTCTCAGCGTGCTCTTGCCTCCGACGTTGGCGGCGATGC
GGGGGTCGTGGCGGTCCTGGATGGTGGCCCACTCCTTCTCAGGGCACTGCCTCAGGCAGT
TCGGCACCGACTCCACCATGAAGTAGAGGTTCTGGAAGGACGTGAGGACGATGCCTGTCC
GCACGGTGTCGTGGTCCAGCCAGGGCCCCCCACAGCGCTGGTACTCCTCGACAAAGGCGT
TGATGTACTCGTCCAAGTTCTCCGCTATCTCCTCAAACTCCGCACAGTTGAGCTCCCAAT
ACAGCTTGTGGGCGATGCAGTTTGAAGAGAAGCTCCCCAGTCCAGCAGCCCACAGTCCAG
GTTCCCGCTTTCGTCGCGCC
>at01441
TATCCATGACCTCTTTCCTCTTGGGCCGGGCGCCCATCTGCAGCTTCATTGCCTTTGCAA
CCTCAGCGAAGGTCAAGAGGAAGTCACCATCAGCATCAAACTCGCGGAAGCGGGCCTCCG
CCTCTGGCGCCTGGATGGGCCCATGCCCGCCCAGCCCACTGAGCGCGAGCAGCAGAAGGC
ACAAGGGCCCCACCGCGCGCGCGGCCGTCCAGCACCGGCGCCTGCCCCCAGACCGGTTAG
GGCAATGCGTACCGTGCTGCGCGTGGATCGTTTCCAGCTCATCCATCCCAATGGCAGCCG
CAGAAGCGACTGAACGCTTGGCTCTGGACCTCCTGGATTTTTGGCGGGCTCAAGAGGAC
TATCAACGAGAGGCCTCGACAAGTTCCTCAGCCAACCGCGCGCCGCGAGCTTGCCAGACA
CGGACTCCAAGAGCGGTACAGGCAGCTCGCTCGGGCGACCGTCAGGCACGGCCGCCGGCT
GAGGCAGCACCTCGGACACGACGTCTACCGGCGCAACCTCCTCGACGGGAGGCTCATGGT

CCTCCTCCGTCTCCGCAGGCAGCCCCCCAGTGTCCACTTCACCAGTGGATGCTGCCTCGG
CCTGGGGATGCAAGCACTGCCCGCCCCCGCCTCATCACCCTCATACTCCCGAACAGCAGG
GACCTCCACCACGACAAACTGGATCTGACGCACGAGGACAATCCGGGAGAAGCCGCCGTC
GCAGGGCGCCGCAGCCATGGCTTGGAGTGGAAGAAGTGCTCGTACAAGACGAAGTCACCA
AAGACGCCCACGAAGCATGGCACCTGGGGGCAGACAAATTCTGGCCGGACCCGAGCAATG
TCTACGGGGCTCAAGCTCCTTGTCCCCTTCTGCAGGACGACCGCTGCAGAGGGGCATGAA
GGAACTTGAGCCAAAATGGCTACGG
>at01442
CCGTAGCCATTTTGGCTCAAGCCGTGGTGGCCCAAGACAGGCAGTCCTCACTCTGGTCCC
CTTCTCCAGCGGAGTCAGCTCAATGAAGGCCCCGGCAGCTCTCCTCCTCGCAGCTCTCTC
GCTCGCAGCCCGGCCTTTCGGCGCGGCTGCACGCGTGGGCGCCGCGGCGGGTGCGATGCA
GCCGGAGACTGCGACTGCCATGGCAGGGCTGGAGGGGGAGGCCTGCAGCAGCGACGAGCA
CAGGCGGTACCAGACCATTGTCTGCAGGATCCAGACAGCGTGCAGGTGCTCCGACACCGT
GTGCGAGCTGGACTGGTGCAGTGAATATGTCCACAACTGGAAGAAGGAGTTCGGCGCTTG
CATTCTGAGGGGCTGCCCCTAGGCGAGATGCTGAGGCAAGTCATGGAAAAAAAAAAAGAA
AAAAAAACAA
>at01443
TTTTTTTTTTTTTTTTCTCCTTGCATGCCATTTATGCGGGAACATGGTCATGCAAGTGTA
ACTGCCTGTGCCTCCGCGGGTCCTAACTCCAGCCGGAAATGTAAAGAGTAGCAAACCTGC
TGTAGATGGGTTGCCGGTGAGATGACATCGCATCCCCACAAGAGCTGTCAAATGCTTCGC
TTGCTGGCCTTCATGAAGGAGCTCCCTTCAACTGGTGCTTGCAATGCCACGGCTGCCCAA
GAACAGACAAAGTGGCCCCTCCTGTGCTTTCTGGTCCTACATGGCCAACTCCTCACAGGA
GCGCGTTGGGGACGGGCCAGGGCATGCAGCCAGGTCTAGTCGCTCGTCAGCGTTTCAACT
GCTTAAAGCAATCAGCTTCCAAAGCCTGTCGTCCACTTGTCGGGCAGCGACAGCGACAGA
ATCGCCCGTGAAGCCGCCAGTGTCTGGCCACGAGCAAACCAGCCTCAGCCTTCATGAATT
GCCGCCTCGTGCC
>at01444
TTTTTTTTTTTTTTTTTTGGGGCAATTCTCGGTGACTTCTCGCCTGCATGAAGGACTGTA
GATGCCAGCCGGAGGCAGGGCGAAAGGCGAGAGTTGGACACAATGCGATCGTGCTACCTG
TCTGGTCACAGATGTATGCTCGTGACCCGCCTTGACTTCGTGAAGTCAATGCACGGGTCC
CTAAGTATCATGTGAGACCAGACAAGGCACAGGCTGTACAGATATCAGAAGACAAGGTTT
ACCTTGCCCCTCACATAAAAGCAACAGGCGCTGTAGTGCCGGTGCGTCGGTGCTCATCCC
CGAGTTGCGCATGCCCCCAGTGTCAGATCACGGCGATCTCCGATCCCCAAAACTGGACTC
AATGTTATCCACGATGCGTGCGAGCACCGTGCTGCTTTCTGAGCAGTCGTCCCCCATCAA
GTCGTCGGCACACTGGACCTCAGTGTAGTTGTAGCGGCCTCGGACCATCTTCTGGGTCTT
TTGGGCATAATCCCAACCGCACATAATCTCGGTGTCCCCCGTGCCGCAGATGTAGGTCAC
GGGCAAGTCCACCCTCCCCACCGGCTGCCCCTTCCTCTTCTGCTTTTTCCTGAAGGCCTT
TGTCAATTTTGGCGAGAGCTTGAAGGCTCCCTGGAGGAGTAGGCTTCTATCTATGGCAGA
CTTGTACTTGGCTGGGGTGTACCTCTGAAGAGCATGAGCTTCTTAATCGTCAA
>at01445
GAGCCACCTCTCCTCGTGGAGTCTTCAAGGTGGCTGTGCATCTCCTTGAGTGTACCAGTG
GGAAGCTCGGCCATGTGCTTTGGCCACTCCTCTGGCGGGATCTCAGTCAGGGCCATGCAG
AGGATGACACAGTCTCGATGCAAGCAGACGTTGTTGGCCGGCGTTGGCGCCTCTGCTGCG
GACAAGCCGTCCCTGCTGTTAGCGACCGTGAGCTCACGGTCATAAGCGCCGCGCTGAAGC
TCGTCGGAGAGCGTCTCGAAAGCCTCTGCCAAGCTGAGGAAGCCGGCGAACTGGCCCTGC
ATCTTGTCCGGGTGCAGGCTAAGGGCCTTCTGCCGGTAGGCGTGGCGGATCTCGGTCGGA
GTGGCATAGCGGCTCAGACCAAGCATGTCATAACACCTGAGGTCCTTCCGCGGCTGCGAG
CTGACAACTGGCACCGAAGGTGCCCGCCCTGCCCCGGGGTTGGACCGTTTGATGACGCG
GAGGCAGGTGCCATCCCTGGCATTGATGCCGCGCCAGGCAAAGCCAGCATTGCGAGCTGC
ACTCTTGAGAACGCGCCAAATGGAGAGGTCCAACAGACCTCCTTCAGCCTAGCATCACCT
ACGCCCTGTGGTACCTGCCATAGACTCAAGCCGAGACAGCCGCCACTTGAGCCAAAATGG
CTACGG
>at01446
TAGCGTGCGCACCTCTCCAGCGAAACGATCTTGCACTTTGGCGGACGTCCTTGCGACCGC
TCCCGCTCCGCCGCAGCCCCACCCAGGCCTATCCGACCTTGGTGGGCATGCTGGCGCGGA

CGATGCCCACGACACCACGGAGCCTCATGTACAAGCTGTGGCCGTAGAGGATCTCGTTCA
GTGCTGTACAGAAGGCGACGCCCTTGCCGTTCGCCGGGTAGGCGATGGGCCGCGGGATGG
AGGGCTTCAGGAGCGGCAGCCGGTTCTGCACGATGGAGCGCTCGTGGCAGAGGCCCCGCA
GGCCCTCGGGCCCCGCGAAGCGGCCGAAGCCCGAGTCCTTGAAGCCGCCGAAGGGCAGGG
ACTGGCTCGAAGAACTGGCCGCTGGGGAACTCCCGGTTCCTGCCGCCGCCGGCCAGCACG
CGCGCGCCCTTCGCCACGGCGTCGTCCACGAAGCGCTGGTAGCCCTCGAGGCTGGGCGGG
TGCACGCACGCTCCGCAGTCCACGAAGTCGCCGCTCAGGGGCGGGGCCTGCCGCAGGGTC
TTCACGAGCGCGGTCACGTGCTCGCAGAAGCTGTCGTAGACCTTGTCGTAGACGATGAAG
CGCTCGGGCCCGGCGCAGTTCTGGCCCATGTTCTGGAAGGCGCCCGGACCACGAGCTGCG
CGAGGGCTTCGGGTCGGCGTCGTCGCAGACGATGAAGGGGTCCTTG
>at01447
AGAAGGCATTGGCAGTCAAAGGTTCATGCCACCCACGTGTGGATTCGTGCACCGATGACG
GGATCGCGTTGAGTTTTGGACCAGCTGCGGCTTTGAGATGTCGGAGGCAGGGGAGCTGGG
CAAGCTTACTCCGCCGCAAGCAGGCATTGGCTCTCTTGTTGGGACTGGCTTCCTGGGCAG
GCCGTCCAAGGCTTTGCGCAGCATCAGCCAGACCACCGGCATCCGCGCGGCTGGCCCATG
CGCCATCAGCCATAGTTTTCCTTCACGGCAGCGGTGACTCGGGAAAAGGCTTCCAGCGCT
ACCTT
>at01448
GAGGAGAACGGGGATCAAGAGGCAGAGAAGTTCCAGTCCGAGGATGACTACAACATTCTG
AAGATCGCAGCCAAGAGCGGAGTGGATAACTACTGCTTCACTGAGGTGAGCGACCTGGAG
ACGATTGAGAAAGCGCTCCAAGATGCGCTTGGCTGGTTGGACAGGAACCAACTGGCCGAA
AGGGATGATTTTGAGTCTAGGCAGATGGAGTTGGAGGGCCTTGTGAACTCCATCATGATG
ACAGCGAATGCATGCACTACGGATGTCTGATCTGTCCTGCCGTTCATGTACATTTGCTTG
CTTCTGTGCTTTTCCTGCTTTAGGCTAGTCCTAATGTGCCTTCAGCTCAGTGGTGCGGTG
TGGACGCTCCCAGCGTGAGCTGTAAAAATGGAAAAACGACATTGTTCGGTAA
>at01449
GCGGCCGCCACGGTTCGTGACAATGGCCCCGGCCTTCTTCATGATGGGCTCCCAGTCAGG
GTCGGTCATGTCTGCGACCAGAATCTGGCCCTTCTGGAAGGAGGACATCTCGTCGATGCT
CTTCAAAATGTTGACCTTGCCAGAGCCGATCCGCTTGCCCACCGCACGCCCCTCACAGAG
GACATCGGAGCCCTTCTCCAGCATCTTGTAGTCCTCGATCACACCCACCTTCTTCTGCGA
CGCCACAGTCTCGGGCCGAGCCTGCACGATGTAGAGCTTACCGTCATCGCCGTCGCGGGC
CCACTCGATGTCCATGGGCCGGCCATAGTGCTTTTCGATCTTGACGGCCATTTCGGCCAG
GTCCGCAACCTCCTGGTCCGTGGCCGACCAGGAGCTCTGCTCCTCCTCCGACGTAGACAC
CGTCTCGGAGCCGCCCCCCTTGCTGTAGACCATCTTCACGAGCTTCGAGCCCATGGTCCT
CGAGATGATAGCGTTCTTGCCCTCGGCCATTGTGGGCTTGAAGACGTACCACTCGCCTCG
TGCC
>at01450
TCCGCCAGTCCAGCATGCACTGCAGCACATGGATGGCGACAAGTGGGCGCCGCTTCTCCG
CCAAGGTGCTGAGGGCAACTGTGGCCCGCTGGGGGCTGGCCTTCCAGGTGTCCAAGCAAC
CTGCCAATGCCCGCCTCACCTCTGCCAGTGTCGGCGCCAAGTCAAGCGTGAGCAAGGACG
GCAGGCCAGACCCTGGGATGCCAGACTGCCGCTGCGACAACAAAGAGCATGGGCAAACAG
CAGCCGTCGGCTCTGGTCTGGCAAACATGTCGTGGCAGGCAATGGCCTCGACAGCC
>at01451
TTTTTTTTTTTTTTTTTGCAACACGCATGACACGAAGAAACTAGTGATGCGTCAACAGT
TGTGGACCTAGAATGAATCAGGGTGCGAAGACCGCCCGCCATACACTCATATCAAGTCCG
GTAACGCCACATACTTGTCCGTCAGCTCACCTGGCGGCACCATGTTGTAGAAGTTATAGT
AGGCCATCTTCATGCTCTCAGAGTCCGCGCGCCCCGTCCACTTGACGTCGACCACTTTTC
GCCTATCCACGGAGTTCCTGTCGAATGAGATCTCGAACTCAGCACCGGGTTTCAAACAGA
AGAGGAAGAAGACCCGATTCTCCGCTGCCACATCAGGCCCAGCGACGATGGAGTCGCCAG
GGCAGGCGGCGTCTGCCTCTGGAAAGAAGGCAAGCTTGGGGTCTTCGTCCGCCGTTATGT
AGAAGTACTCCTGGCAAGACTCCCCCATTGTGCCTTGATATCTGAATGTGCTGAGAGTCT
CCTCATCGTAGGTCATCTCTGTTTGTCCCCAGTTGTTGAACGTTCCAGAGACGTAGTACT
TGTGGCGCTTCGGTCCCTCAACACTCTGCATGGTGCGAGTACCCATGGAATGGGATAGCA
CAGTCACGGTGATGTGTGCATCAACGACCTGCAACCGGAGTGTCACGATCTCGCCAGACT
TTCCGCCCAAACGCCAGCTCTTATCGGTGCACATCCCGTCAGGGCCGCAGAC

>at01452
AGCATGGTGTCGTCACCGAGCACGGCCTCAACCAGTGCAGTACAGATTCTCGAAGTGCAT
GCGAACACGAAACATTGGGTAAAGGCAGCGCTCGTGCAGCGCATGGTGCAGATGTTGTTG
GACACGATGAAGAAGTGTCGTTTGGACACCGCCCATGCGATGTCCGATTCGACGTCGAGC
AACAGGAGGATGCAGAGAGGACAGCCACGTGGACTGCCCTTCTCTCCAAGCCCCTCTCTG
AACTGTTCAAGGGCAACGCCTACGTCCTGAAGAGAGTGAGACGAAGGCTTGCATCGATAC
AGTCTTGGCAGAGACCCGGGCGCGATGGAGGCCAGA
>at01453
CAAGAGTGGTGGGACCACGCGATGGAACATGGCATCCCCGGGCCGTACGATCAAGGCTAT
GTCGCCGCAGTCTTCTACCCCCAGCATCCGGACGAGGTGGCCGTGCTTGAGGACGACTAT
TTGGCATTTGGCCGCTTCCTCGCCCACAGATGCCGCAAGTGTAATTGGTTTCCGACGAAA
GTACGCCTGTTCGCCGAGGTCACCTGGAGGAACAAAATGCCGCTGGCTGCTGGCCATGCC
AGCATCAAGTTGCAGCAGCTGCAGGGCAACATAGACACGCAGATCAGAGATCAGGCCATT
GGTCATTGAGCAAGTGCCAGATCACGGGCCAGCTTCTGAGTGCTCAGTTGGTCCAGGAGG
TTGGGAGTCTGACCACAAGGAGTCGTGATTTGCTACCAAGCG
>at01454
GAGACCACCTTGGAGGAGGCGAACGAGATGTTGCGATCAGCGCTACGGTCGCAAGGGCTG
GTCAGAGATGGGTAAGGCGTCCGACTTTCCGAACCTGCTGAGAGATGCTAGTGGCATACC
AAAGGATTGTCGAAAGGCTTCAGCAACGCCAGGGCCGCGACGCTGTTCCTCAAGATGGCT
TGCGCCATGGGCAACGTACCATTG
>at01455
GAAAAGGATGCTCGCCTCGTGCATTGCCACTTTCAGCAAGCTGAACGTGCAAAAGCTGAA
CGAGAAGCTGAAGGATGCAAAGGAGAAGGCGCAACGCAGGATGCGGAATCTTATGAACAT
CGTGGAGACTCAGCGGACGCAGCTGAAGAAGCTTGAAAGCACTATCCAAGAAGCTGAGCA
AGAGCGGCAGGCGCAGCAAAAGGAGTTGGAGGGTGTCATCGGCGAGCGTGACATCCTGGG
TGCTCAGTTGATCCGTCGCAACGAGGAGTTGGCACTGCTGTATGAGAAGATCAAGATACA
GCAAAGCACACTGCAAAAAGGCGAGATCCAGTACCAAGAGCGCGTGCAGGAGGTCAACCA
CCTGCGGTCTGAGATCCGGAAGTCCAAGAATGATGTGGCAGCTGCCAAGACGCAGGTCAC
CAACGTGGACAGCCTGAAAC
>at01456
CCGTAGCCATTTTGGCTCAAGCCATCTGGGCACAAGCGGATCCGTTTCTCAGCAGGGTGT
AGAACTTCCTTCAGGTCATCGCCATGAAGCTGAACATCGCGAACCCCGCCACGGGCATGC
AGAAGACCTTCGAGATTGACGACGACAAGAAGCTGCTCCCCTTCTACGACAAGCGCATGG
CGTCCGAGATATCGGGGGACAGCCTC
>at01457
TTTTTTTTTTTTTTTTTGCCGTGAGGTTCGCCTTGGGCTTGTGCTGGCTAGCGTCGCTG
ATGTCCAACAGGCTTGTTCCCTCCGGAATCTCGGTCAACCTCGCCTCTGCCTCCAACAAG
CTTGCCAGCTCTGAAGAGCTGACCTCGTGAATCTCCTTCTGGATCGCCTCGTGCATGGTC
GCCTTCAGCTCGTTCTGGAGCTGCTCGCCGACACCCTTCATGTCTTCCACCGCCTGGTGC
ACAACCTGCCGGGTGCTGCTCTCCAAGCCATCCTTCATGGAGTCAATAGCCAAGTTGATG
TTGCTGATCATGTCCTTGAAGGGGTTGAATGATACCTTGCTCTTCTGCTTCTTCCCAAGC
TGAACACGCGCGACGTTCTCATTCGGGTCCACGTATCCCTCGAACTCATTGCGCTCGTGG
AACTCGGGTGCTGGATCCTTGAGGAACGTCCTGTCGGGATCCTGAACAGCGTGCAGAAGT
TCCCTTCCCCTTTGGATCAACGCAGCAGAGTCATCCAGCTCGCTGAGGCAATAGCCCGTC
AGGCTGGAAGACACCAAGATCGTGGGAATGATTCTGCCGAACAGGACCATTCGTGACACA
AATGAG
>at01458
TTTTTTTTTTTTTTTTTGTGAAGACTTTCTGCTGTTACATCAAGAAGGCAGTTCGTCCA
GTACGAATGTGCGGCTCCACCAGCAGCGCTGATGCTGATGCTCACTTCGGAAGCGCAGGG
GCCTCTGTCAGCCTGAGGGCCTCTTGCTGCACTCTTGCTTCGACCTTCCTCTCGCGATCT
TCGATCTCACGCAGCATTTGCGTACCCTCCTCGTACTCCAATGCCTTCCTTCGGCGCTGG
AAGAAATCGTCGATCTGCGCCATAGACTTGGACAGCCCGAACCTGAGGAACGCCGTGAGA
GAGAAGTTGATGAAGATCTGCGCAATGCCCCAGGGCCATGTCTCGTCAGGCGTCTCATCG
TAAAGCCCGTTGGTGGCGAAGCTGACGACTGCAACAAACTTGATGAAGTTGTTCAGCGCT
GGCCCCGCCAAGTCCTCCAGCGGTCCGCCGATCTGCTCGCCGATGCCGAGTGTGTCGTAC

ACCTCGCTTTCAGGGCCCACCAGCTCACCCTGCTCGTTCCTGAGCATGCCGAAAAGGATG
TACTTCCGCGCCGCGTCCCAGGCCTGGCCGGTGTTGATAAGGAACATGTTGAGCGAGAAG
CACACGGTGTTCCCTCCGCTCACCAGCATCATCAGGCCCTTGAAGCCAAAGAGCTGGCCA
TTCACCACTGGTGCTGCAAAGGTCTGGCACATGGGGGCAAACACGAGCTTGAAGGACTCG
ATGGGCGAGTAGGCACCAGGATCTTCCCTCGTGCCG
>at01459
GACGAAGGTGGGGGCCGGAGACCTCAAAGACTTCAAGGAGCACCGTGTGCCATCCGTCAG
CTGCCTTGAGCTGCTGCGGCGCTTCGGCACCCCGGACGTGCTGAAGATCGACCTGGAGGG
TGGGGACGCCGCGTGCGTCGCCGACCTCGTCGCCGCTGGCTCAGAACGGCCTCGGCTCGT
GGTGGTCGAGCTGCCCAGCAGCTTAGAAGAGGCTGCAGGTCTGTTGGAGTCGCTGAGCGA
TTGGGGCTACAGGCGCTGCAAGTTGTCGAGGCGATCTGACTACCAGGTGCGGTGCTGGCC
GGGAAGCTGGACTGCCACCAGGCGCTGCAGCACGATGGCATTGCGCGCTTCACCGGCACG
GCGGCGCCGGCCGGTGCGCCCGCCGGTGTGCGCGGTGGAGCTCAGAGTGCTCGATGACGA
TGGTC
>at01460
CAAAGTACTCGTAAGCATTGAAACACATGCGCAAGTCTTGGCCGTCACAGGTCGGGTAGG
GCCTCACGTTTGATTCAACCAACCCTGTGGCTGTAGCGGCACTGGCATAGAGTATGATGG
TCAACAACAGCGCTAGCCACCCAAGTGCCCGGCGCGCCCGCGCGATGCCCGCCATGACGA
TCCAGAGCTCTGCGAGGGTCGTCGACCTCTCTGCGATCTTGTAGATCCGACGGAAGCGGA
AAGAATTACGACAGCAGTGATAGTCTCCAGAACCACGTGTACTGGCCTTGGAATACGTCG
ACAAGCATCAACATCAAGATGACTAAGTCCAGCCAGAAGTCCATCATGTGAGTGAGTACT
CGTCGCCAAAAGAACCAGCCTGGACAACCAAAAATTTCACGGTAGATGAGCACGCAAGTA
CACATACAGAGCCGTCGCCAACTGAAATTGGACACTATTCGTAGCAGCATCTCAATCGAG
T
>at01461
TTTTTTTTTTTTTTTTTGACAGCACAGAAAGTGTACTTCATGAATCAGCCTCCCTTCCTC
ACATGCCTCACTTCTGCTCCAGCATTTTGGCCGCAGCCTCCAGCCGCCCAGCCGAGCGGG
CGAGCGAGTCCGCCGCGACCTCGTTCTCCGACACCCTGTCGGCGGCCACGAGCACGCTGG
CCGCGAGGCTCCGGAGCGAGGAGGCCGCGGCAGGCCGGTCGGCAGCAGGGAGGGAAGGAT
CACCGTAGCCTTCTTCATCCGTGAGGAGCGCAATCAGGCCCTCGTACTGCTCCGCCTCGA
AGTACTCCTCGCCTGCGTAGGCCGCCTCCTCCAGCTCTCCCGCGGCGTCGTCCCAGTCAG
ACGCCAGCAGCAGCTCTGCGGTGGTCCTCAGCTTGATGCCCATGGCCCCCATGGCCGCGG
GCGTCTCCCCGCGGTTCGACCAGCCGCTGTCCCCCAGGAAGGAACCGGCGGTGAGGATCT
CGTCGCCTGCGTACCAAAGCTCCACCGCCAATGTGGCCAAGAGCCCATCATGGGTTGCTG
GCGGTGGCACTGGCATCCTATTCTCGCCATCGTCGCAGCGGCGTTGCACGAGTCGCACAC
GAGGCAGCTGGGAGTGTCGGCGTTCAAGCCATGCTCTGGTGTGGCCTCCTCTCCCCACCG
CCGCCCCACACCTCCAAGTCCCCACCGCCGCCCGCGGTGCCGCGAGCACAGCCCTCAGCC
ACAGCGTGCAGGCACAGGCAGCAACCCCGGCTGCGGCGANCCGTCGGCTGACCCCACGGC
ANATGTGGCGGCTTGCGACGGCGGCTGGCAGCCGCATTGCGGCCGGCGCGCAGGTCTCGT
GG
>at01462
GTTTGCAAACAGCCCATTGTCGTGACAGGGCGTGACCACTGCTGAGTAGGTGGGCATGTC
GGGGTGGCAGAGGTCTATGATCTTGGGCACCAGTGTGCCTATCACCGTCTTGCAGGCATA
GCCGCCCACCGACGGCCGCCCGGACTCTCGGGCACACCGCAATGCATAGCGGGCAACGTC
AAGGACTGGAGCTTCACGCCGTTCGGGCGTCACATCCTGCAAGTAGCCCTTGTAAAGCGC
CACCAACTCCGCAACCTTGCCGTCTTTGTCCTTGTTCGGGTCAGGCAGTGCGCTCTTGGC
CAGGCTGACCAGATCCTCGAAGCCGGACGCGGGGAAATGGCCGGACCCCGCCCGCCCAAG
GACCGGCAACGGCCATCAGGCGACCACAGGCTTCATCAAATCCGGTCTGAG
>at01463
CCGTAGCCATTTTGGCTCAAGGCTGATGGCCTCTGCACCAGGCACCGGCAGACATCTCCC
CGCGCCCATGTGCGCGGTAGCCCTTGGCGCCCATGGCGCTGGCTCCAGACGCTGGCATGG
AGCCAGCTTTCGTCACTGCCAGTGCAGCAGCACGGCGCTGGGTGGTTGTGGTCATTCTTC
TGGGGTCAATAGGACTGCCACCGGCTGCGGAGGCGCGAAAACGCATGGGGGAGGTCACCT
GCCCCTGCCTGGATGCGCTGCCCCAGCTGGAGAACCTGACTGTGAACACGGCCACCAACC
AGATGTCCAAGGCCGCCGTGGGCGGCGCCTCCCGGGACCCCGTGGACGACTGCCTCCTGG

CCGAGAGCGTGG
>at01464
TTTTTTTTTTTTTTTTGTGACACCATCCTGTGACACATAGAACAATGCTTTTGACGCGTG
GCACTGCCTGTACATGCCTGCTACGGCCGGGTCTCGTGCCAATCAGCATCAGTGCCCGAT
AAGCACCATGGACTCCAAAGGGCTGTAGCCACTTAGTCAATGTAAACAACAAAGGCAACC
TTGACGGATTTGTCAAGCCAATGCCAAAAATTCGGCGTGCATGCCAACGAATTTGCGTGC
ATGCCAACGAATTGGCTCTCGCGAGGCTAGCGCGGCTAATGTGCGCACAGATCGTGCAGG
AACAATGCATGCGCATGCTTCGCGCCTAGCTTGCTTCTTGCGCAAACAAGCATAAGTCAG
GTTTGCAGGATGTGAACAAGATGCGGCTCCCGAGTGCCGTGCACCTCCTCGTGCC
>at01465
GATGGTTCTCGCGGCGACGTCGGTACACTTTTGGCTTTGGGAAACGCGGGCCTACTTCAA
AAAAGTGTCTCGATTGGAACTCAATGCCACTGTACAGACAAGCATCAAAGCTGGAATCAA
GAGAGAAAAAGCGAGCTTTTCTCTCTCTCTCTCTCGCCACTGTACAGACAAGCATCAGAG
TTGAAATCAAGAGCAAGAGGAAAGAAGTTTTTTCTCTCTCCAAAAAAATGTCTCGATTGG
AACTCACAGCCACTGTACAGACAAGCATCAGAGTGGCTCCAGCGGCAGCCGAGCGCATCT
GCAGCGGGAGCTGCGAAAGTGGCCGATGAGGCCCACTGCA
>at01466
GTCATCACCGGCAAGGGCATTCTACCTGGCACCTTTGTATGTCCGGCTCCCCACGGCGCC
CTCTGCGTACCAAAACACTCCGAGCGTGCCGATCCAGAGTTTGCGCAGGGCTCCAGGCGT
CGCCAGCACACGATCCCCAGCAGAGGTCTTTACACCTGGTGCAGCATTGGAGTTCAAGAT
TTCAACAGGCAAGTTCTGGATCCACTGCACTGTTGAGAGCAAGGGCAAGCTGCCGGGCAC
CTACAATGTCCACATCCCAGGCGGACAGGCGGGGAAGGGCCTCAGGCGACTCACGGNTGT
TCCCGCTGCATGGCTCAG
>at01467
TTTCTTTTTTTTCTTTTTTTTTTGGTATCTCAAGACAGTTCCTTCGGAGCACATCAGATC
AACGCCGCCAGTGGTCCCATCCGGCCGTGAAACACAGGTTGCTTCAGCACGGGGGCTGGT
ACGACTCGTGAGCGCCAACCCTCTCGTACCCCCACTGGGGACCATCGGTGGCATCTGGGC
TGCCATCAGCGTCCAGCAGTCGCGCCTCTTGTGGAGGATGGCGCCAAGTCCACACTGCCA
CAGCACATGCAGCCAGCGTGCAAAGGCAGCAGCAGTACCACAAGGCGCCGAAGGCCATGG
TGCGGGCGGGGCTCAGGATGCGCACACCCGTACAGTGGACGGCCGCGAGCAGGTACGCAG
TTCCACGGCCCAACGGAGTCATGGCAAATCCGAAGTAGTCTTGTACGGCCTGCTGCCGCC
AGTGCAGAAGCTGGTTCTTCAGGCTCTGCTCCCCCTCAAAGCAGGCAAAGCCACCCAGGA
GCAGAACCGCGCTTTGCGTGACGCCAAGCATCAGCGTGGACAGCTCCATGTCCAAGAGGG
ACAGTACAGACAGAGCCACCATGGAACAGGCGCCGGCAGCACCCCCGAAGGCAAGGTACT
CTTGCGTCGAACGTCCCAGCAAACCCGTGCATGCTGACACGATGCTGTCCTTCGAGAGGT
CCATCATTTCTGTGCCGCCTGTTCACCCAAAAGCAATCGGCCGATGGCTGCAAAAGTCTT
GAGCCAAAATGGCTACGG
>at01468
CCGTAGCCATTTTGGCTCAAGTATCGGCCCAAGTCTGCATTGGGCACAGTGAGCACGTCG
GGATGGTCCTTTGCGGTAGATGACATGCCCTGACGAGACCGCGCGGTCGCGCTCTGGCTC
TGCGAAAAAGGCAAGGACGGACGGTCTCGGTCAGGCAGCGATGTAGCCAAAGACCGGTCC
CGCTCCAGGCGGCGGAGGAGGGAGGAGGAGAAGAAGCCGAACAGGAGAAGAAGGGACAGG
TCCTCCCGCTCCAGTGCTTCCTCCCTCGGGCTCCAAGAGGAAGCGCAAAGACAGGGAGAA
GGAACAAGAGGAAGAAGCGGGACAAGGACCGGGACAGGGACAGGGACAGAGACAGGGAAA
GAAAGGAGAAGGACCGGGAGAGGGAGCGCGAGAGGCAGAAGGAGCGGGAGAGGGAGAAGG
ACCGGGAGAAGGGAAAAAAAAAAAGAAAAAAAAAAACAA
>at01469
CCGTAGCCATTTTGGCTCAAGGTTTCACTCTCGTGCGACGCATTTCGGGCGCGATGGGTT
GCTGCAACAGCGCCGCCGGGGATGCTGACGCAGTAGAAGACGTCCCCGCCGTTGGTGGAG
CCATGGCAAGTGGGGCCAAGGGCGACGCAGGCACACCGCTTGGCTTCGAAGCCACTGGCA
AGAGCGGCGCCCCCGCTGCTGGATCAGGGCCGCCAAGCGGGGCCAACAACATCAACGCTG
AGAGCGAAGTTGGCGCTGCACACCAAGGCAACGCAGGCCCGACACTTGACCTTGACTCCG
AAGGCACCATCGAGAGCGGCGGCCCCGCCGCTGGGGCTGGGCCGCCAAAGCGGAGCCAAG
TTGAAACCGAATTACCCTGCCTGGCTCCGTGCAGAACTCGAGCGCGCTCGGGAGCTCTAC
TCCGCGGGAGACGTCGACGGCGCTTGCAAAGTGCTTGGGGCTGCTTTCGCGGATGGGATC

GCTAATCTTGACAACATCAGCCAACAGGACTACAACGAGAGCACCCTGGTTATGCAGCAG
ATACGCGACGAGCAACGTC
>at01470
AGACGTCTAACGGCCCTGCCGCGTCCCACTGAGTGCCCAGCGCCAGCTGCACGACCACCC
GAGCGGACCAGCGACCACGCAAGACTGTTGCCTTACCTTGACGGTGCTCCTTTCGCTCGA
CAGAAAGCCCCAGGACATGCACTCCAGCCCCATGGCCTGAACCGGCCGGAGCCGCGAACC
TTTGCGCTCCAGTCCAGAACCGAACCCCGGTTCACTGAGCAACTGCGGACGCGGTGACAC
GAATGACGGCAGCGATGGGCCGTGAATGTCAGCGTCCAAAAGCCCCACGCGAAGTCCGCG
ATCCCGCAACGCGAGAGCCAACCGCAGGGAAACGCTGCTCTTACCAACACCACCTTTGCA
ACTCGAGACTGCCATCGTGTGAGCCACGCGCAGGCCTTGCATTTTTCAAGCAGCACTCTA
TGCTTGACAAAAAGATGGCTTGAGCCAAAAATGGCTACGG
>at01471
TTTTTTTTTTTTTTTTTAGAAAGGCACAAGATCCAAGGATGCCCAGTCATCAAGATATC
AGACGGCACCAGGCCAACACAAGGCTTGCACCTGTGCGATCTTTCCCAAGAGGCAGCTAG
CTGCTCTCATGCACGCGAAGTGTGCATCCGCGAGCACACTTTGGCCGCCTTGCCTGCAAG
TCCGGAGGCTCGATGCCCTCCTGGTTCGGCAGATTTCCGCCAATGGCAAAGCGGATAGCC
TGCACCGTCACGAACGACAGGCTGAGGCCGGCGACGTCGTTCTCGGACTCCTCCGTCTCG
TCGTCCCAGACCTTCTCGAACTCGTCGACCACGCCGTCGTCGCTGTTTGAGATGTGGTCC
CGGATTCGGTCGTAGCCTTGGTACATGACAAACAAGGCGCAGTAGCCGATGGGCACAGCT
GCCAAGGTCACCAGGGGGAGAGGATTTGAAGATGCTCTGTTGCAAGGAACCCCAGGCGTT
TATGGCAGAGAAGCCGGCAACGTGAGCAAAGAGCACAGCTAGACTACT
>at01472
TTTTTTTTTTTTTTTTTCAAAGTCCAGTCTGGCCAATGAGGAGCTGATACGCCTAAGGC
TGCATCAACAAACCACTGACAAAGACGTGCGAGTCTCTGAATTGCCAAAGCTCACCAAGG
GTGGGCCTCAGCAACGCAAGCAGACTATCTACGTGCCACTGCACGTGGAGAGGAGGTGGG
GGCATTCCCGCACGAACCCCTCGAGATGTATTCTCAGGGATAGCCCTTCTCGATGCTGTT
ATGGCAATGCGCCACCATCTCCCTGCTCAGCACTGTAATGGCTAGGATGGTGGCAACACG
CACGAGCCTAGTAAAAGGACGCCCGCTGCCTGAACCAAGCGGAGGTACGCCACAAAGGTG
GACTCTGCTGTCATCGCCGTTCAGCGAAGGCTTCTCTTCTGGACGTTTCATGGTTGTGCT
CAACGCCATCAAGATCGCCTGTCTGCAACCCCAGCGAACCACACTACACAATCCAGCCCA
CTGCTGCAGGAAATCAGCTGGATACTTGCACTCCTTCATCAAGGCCATCGTCATGCCGAC
AAGCAACTCGCTCACGCTCCAGAAGATAACCCTGTTGTCCTCTGGTCCGCGTTGAGTGCT
TGGCCGNGCCGATGAATCCAAGTGGAGCCCTGACCATCAGTCTGGCTCCAGCCACAGTCT
CCTCGTGCC
>at01473
TTTTTTTTTTTTTTTTTCACCACTCGCGCTCTGTTTAATAAGCATCAGCTAGGCAGCGC
CACACTTCCAGCAGTGCAACCTCATTCGACAAGACGCTCGCATACAGCGTGGCTTTTCCA
CAGCTGCTCGCGAAGCGTGAGCAGAAAAATCCCAGCAGCCAAAGCATGAGCCTGCCGCCG
ATGCCTGTGAATGCATTAGCAGTATGGGGCCAAACAAAACGAAACTGTTGATGCAAATGA
ACCCCACATGGAATCAAATGCATCAATCGCTCTGTCGGTGGCTTGAAGGAGCGACTGCGC
TGCGCGTCTCATTCACCCTGGCCGCGCCGTTCACCGACGTGAGCCTGTCGCCCTTTCGCA
CCTTCTTGAACTCGTCCTCCATCTGGTCATTCCAAGCAGAGATCAAGCTTGGCGCCACCA
CCTCCTCAACCAGCAACTGCTCCAAAAGCGGCTCAACTAGGACCACCAGTCCTATTGTTC
TCCAGTAAGGCCCTGTGCGCGTGATCTCGATCTCCTTCGGTTGCCGCAGTACGCTGGAGA
GGGAAGCTCCAGCTTCCTCGATGACCAGCGATCCGTAACTTTGCGGCGCCTCTGCTATGA
CATCTGCAACCAGGNTCTNTGTGTTTGGGGTGATCCTACCGTCGCAACATAGGCATTCAA
CCATCTGCCTGCCGGCGCATCTCTTGAGCGGAAACGGCC
>at01474
GGGCCAATTCCGCGCCACCTTCACGGTTTACCCGCTGACGGTTTTCTTGCACAAAGTCGC
AGAAGGAGCCAACTTGTCCTAAAATCCCTCGCACCTCAGGAACTTCAGGGAACCTCTTGA
CAGCTGCCATCATTACCTCAATAGCTCCAGCGTCACCACCATGTCTGGCGAGTGGCTCTT
TCCACTGGACGGATGCGGACAACATACTCGAACATGCTACCTGCAACTGCCACTGTTGAG
GGAACCTCTGCATTGCTGAGATNGCCNGCTCTTGAGCCCCGAGGCCTACCATNAGNTCNT
GCAAANAGGTACCNGTTGCCGCAGCAANTTGCANAGTTCGACATGCACTTTGCAGNACGA
CNTGNTTGCTTGGAAAATTCTTCATCTTTGAGATTGTCGTGTTGACCAAATCTGCTTCAG

AACCAGCCACTGATGCCGCGTCTGAACTGCGACGATTCAAGCGAGATCTTTCGCTCAACC
TTCCAAGGCCAAAGAGCCAGATGATTGTAGCAGCGAAACAGAGTCCACAAGCACTTCATC
TCCACCTGCCGTGATAGCTGTGTCCCAGCCGATGGCAGCATGCGCACTGACAGCTAGGAA
AAAGCCATACAGTGAGGATGAA
>at01475
GCCACCCGCTTCACCAGTCAGACATGTGTTCTGTTCGAATGCAAAGCATTGAAACACTAA
CCTTTCAGCAAGCACATAGCGAAACTTACAGATCCAAGAACAGGGAATTGTGCTGAGGAA
TTGTTGCATTGCGCAATGGAAAAAATCCTCAGCGCAGTCTTCGCCATTGAACATTGTTTT
TTTTCTTTTTTTTTCCAGGAACATCATCTCAGCCTTCGTGATTGCATATGAGGCTTGCTT
GCCCAGAACGCTGTACAACGGACCACATGCATGGTTTCAAGGTCTTCGATCCATCAATCC
CCCAACAGCAATCGGCCTCAGAGTCACCCTGGTACCTGCATGGCACTCGCCAGGACCAGT
TGAGCTTGCCAAGGTTCACGATTGAAACAGACATGCACTGCTGAGGTCATGAGACGACGG
TCCATCGTAATTGAGACCTGTCTGGGCTTTGCTCTGTGTAATCCTGAAGATCGGGGAGCC
ATCTGTGTACAAGCACTCAACTGATAGCAGCGCAGCACCGTCACACAGCCAAGGCGCAGG
AGACTTGGGCCATCAGCTTCACAATCACCCTGGAACTTATCTGACACTGATCTGTGCCAA
CTGAGCTTCACAGGTTCACTGGTGAGGATGGCATGCGATTCCCCCATCAATGAGTATGCT
AAGTGCTGCTCCACCGGATTGCTGTGCGAAGAAGGCTGGCAAACGGACAGCCTAGGACGC
AGCAGCTAACCACTCATCAAAGCATGATGGCATCCCAATTTGCATATCACCTTCGAGCTC
AATGAGCTTCCCGTCGAGGTTCACGATCGTCAACGTTGGAACGCCAGGTGGCTGGTACTT
ATCGTTGGTTGCTGTACGCTCTGCAGCACTGTAGGGTATTGCAAGCCAAGGGTCGCCGTC
TGGAAACGACCCCTTCATGGCAGATTGACTTCTGTCCAAGCTGCAAACCACAACTTCAAA
CTGTTTCTCAGATGCATTGACCTCAGCATAGAATGGCTTCAACCTCTCATGAAAGCCTGG
AGATCCGGGTCACCAAGTGGCAGTAAACAAAATACCTAGAACTTTGACGCCTGAGAGTGC
CTCTGCGCAGCCAACCCGACTGCCATCACTCCGCAGCAAGCTCTCGCCAATGATCTTGCT
CATCCCAACGTGTGCTGGGCCAGTAATGTTCTTCTGCTTCTTCTCGCAATCACGTGCGTG
CTGAGAACCCGGCCCCTTACATCTCGTGCAACACGTCTTGTACTT
>at01476
TCCTTCACCCCCTCCAAGCTGATGAGCCCACTGACCGCGGCCTCGACCTTGGCGCCAACC
TCAGCATATGTCTCCCATTGCACAGGACCCTTTGTCCAGAACTGTTTCTCTCCACCATCC
TGCGCTCTTCGACACTGCGCGTCCCATGCACTTTTTGCGGAGAACTTCTTGACGCTCTGC
TTGAAGAGGTTCATTANCGTAGGCTCCTGGTCGGTCGGAAAGCCCTCGGCTTCAAAGCAA
GGTCGCCGCCGTTATTCGTGTAGCACCAGGG
>at01477
TTTTTTTTTTTTTTGTTGCACCGCTCAGCGCTCTGTCTGCCGGGACTGGCAGTCGGACA
AAACAGCAGACTCAGCTCTCCGAGCCCCGGTGACCCTTGGCGAAGCGCTGTGTCGCCTCA
AGCCAGAGCAGAGTTTCTTGGTGCGAGACGTCGTGCTCGCCCTCCACAAGGCCCCAACGC
TCGATCTGGAAGTGCTCCTCTAAGAGGGACCATCGCAGCGCGTCCTCGGCGCTAGCGTGG
CTCTTGTCCATGAGCGCCGCGCCTACGATGAGGGACTTGGCAGTGGAGGTGGCCACCTCC
AGCGCGCAGAGCTCCCACTCATCCCGCGTGCGCAGCTCCTCGCCCACGGACGCCAGCGCG
CCCTCGCCGTGCGCGGGCGAGCCCAGGCCCTGGGCGACGGCCAGGGCCACGCCGAAGCGC
TCCTGGAACCAGTCCCTCAGGGGACCCCACTCCGCGCGCTGCCGCTCGGCCAGCAACTCG
TTGTCGTCCTCGANACAGAGCGTGTCCATGGCGAGGTAAGGCAGCAGCCTCTCCACGCAN
GCCCCGCAGTCCGGNCGCACGATGTCCACGGCGGTGCAGCCCAACGTCGTCAGGGGCATC
TCCTTCGGCTTCAGGTGCTCACCCTGCGCCCCCCAACTCGTCGCGACACGCTGGGCAACG
CCTCTGGCGGGCAGCTCCC
>at01478
TTTTTTTTTTTTTTTTCTGATCCAAACTCTTGAGGACCATGCCGCTCCGAAAGAGCAGTG
GACTGTACCACTGCACCCTGCCTATGCACCACCGCAGACACGTTGCCCACGTGCTTTACA
CAAGGAAGACGCCTGTATACGACCACCTCTCCCTAAGGGAACGCGGGCTGGAGACACGCC
AGTGGCGCGGGGCTGCGCGCTGCGTGAGAGCCTCTGCCCACGAGCGCCCTCGGACGTCTG
CGCTCCTCGGTGGGCCTCAGACCTTGACGCTGAGGAACTGGCTGCCCTGCTTGAAGTCCC
GGTCGCGCTTCTTGTACTGCTCGAAGATCTCCGCCAGGGAGCAGGAGGCGGAGGCCTCCA
CGAAGTGCTCGCTGAGCATCTTCTGGAGGCGCTCCGCGCAGAGCTTCGCCATGCGGAGGA
CGTAGTCCGCCCCCTCCTGGTAGAGCTGCTGCGCCTGGTAGGGGTTGTCCGCCGTCACGA
TGCAGTGCGCGTGGGGCCAGACCTCGCGAGTCACCTTCAGGAGCTGCGCGTTGGTCGTGC

CCTGCAGCAGCGAGTCGGGGATCGTAGAGATCACGATCCGCGCCTCGCCGTGGTGGGCGT
GCTCCAGCACGTCGGGCTCCTTGTAGT
>at01479
GCAAGGCGCACGTATCACCTCACCTCTCCAGGATAGCCATGACAGATGCAAATGGAACCT
GCATAAGATAGAATAGTATGGCGCTGCTGCTCCGCAAGGCCAGACAATGGCATAAGTGAC
CAAGGCCACTGGAATCCAGCCAATCTGGAATATGACAAAAGGTACATATGTGAGGGCGGC
ACCACAGGTAATGATAATTTCACGCCCCCCGCCAAACGGAGCGATGAGGAGAAAAGCCAA
AGGGGCCCAAACCACTATCGTGAGGCTGCACACTGCGAACAGAAGCAGGGAGAGTGCCAC
CATAGGCATCATGAGCCGCTTGAGGCACCGGAGAAGCCGGTTCTCGCCGCCGCGCTCGCC
GCTCTGGAACTGCTGGCAGCTGCCCGGGTTGCCGGGGTTGTAGTGCCAAGAAACATTGTC
TAAGGTAGCCGAGCACAACCAACACCAATGTCGCGAGCATGCGCTACAAGTCATGTGGTT
GCAGCCATTCAGCTTCTCGGTCGCAATCCCGCAGTTCGGGCAAGGCTTCGTCCCTCTGAG
GGCCCCATCTATGATGAGGCGCTCCTCCTTTGCCACTTGGCGCTGGTACTCTTCGCAGGA
CTTGCCAACATGAGCATTGGAGTGGTAGTAGCAGAACTGTGCTCCACAGGCACTACAGCT
CATCTCCGCAAGAATGTTGCTCTCCTCGTCCACGTTTGGCTTACACAAGACTCCACACCG
AGGGCATTGCCTCAATGTTGCGTCCTGCCGCATCTGCCTGAAGCGCTCGTACTTCGCAAA
TGTCTCAGAGCTCGTGAAGCTCTTGACCTCGTCCGCCGTGATGACCGCATGACACTCATT
GGGAACAAACAACGAATGTCGTTCACACGGGCGTCGCACACAA
>at01480
TCCTTGTTATGCCAACGATTCTGGCACTCCTCTCTCCGGCTGAGAGGAAATAGTGTGTCA
CCACCAGCCTCAACATCACTAAGGTAGATTAAGAGGGTTGCAGGGCGTTCGACGTCATGC
GAGGGGTCGGAGTCAACGTGTGATTCATACTTCTGCCCGGGAGTATACCGCCCGATCTGC
AGTGCCTCTGCATCCGTGTCCGGGAGGTATGCCACTCGATGAAGACGTTTCAAAACGTGC
TTGAGCACTGGGATCGTGCTTTCTTCCTGTGGGGTAATCCAGATTTGTTCATTATTCCGC
GATTGCGCTTGAATCGTGTGCTTGTCCTCCGTTTGCGTCACGACACCGGCAGGCCGGAGG
CGACCACTCACCAGCCTGATGAGAAACTCGCACTCTGCGGGGCTCAGCAGGTTCTCGAAC
AGGTAGATGCGAGGCGAC
>at01481
GCGTCTCAGAGGCAGCCAAAGAGCGTCACACATATCAAGAACACATTTCCAACAATGCAA
ACACGTTCGTGGCCGCAAGCAGACCACGCCCACGGCCGCAAGCGGCACAAGACTTCGCCT
CTCCTCTGGCACGGCCTACTCTTCCACCTTGGCCTTCTTCGTCTGGGTCTCCTGCTTGTT
TCCGCGAGCCCCAGTCTGGGGGGGCTTGCGCTTCATCCCGCGGCCGTCTTTCTTCACCAC
CGAGATGAGGTAGGCGCTGGGGTTCTTCACCTCGTCGGCCTTGCCCTCCAGCTCCTTGAA
GATCTCCATGGCGCGGGGCACGTCCACCTCCAGCATGACGTCCACGGCGTCGCTGTCGAT
GGGGTTGGCGGCGAAGGAGTGCTTGTTGAGCCAGGCCACGCGCTTCTGCACCCGCTGGGC
GTCCT
>at01482
TTTACTTTTTTTGGTAGTCAACGTCAACAGAACCTTTGCAGCTCAACACATGCGCGTCCA
GATCGCGCCAGGACCACATCCCCCCTcCAGCGGTACTTGCTGCCATACTCGTTCTTACCN
TTCTTGGAGTCCGTGCCAAGCTCGAAGTGCTTTGTCCGCTTGATGGGCTTCATGCGCTTG
GCCTTGCACTTCGTGCACTCAAACTTCAGCACGATCTTCTTCGTCGTCTTGGCCTTCTTC
TTGAAGACGGGCTTCGTCTGTCCGCCGTAGCCCGCCTGCTTCTTGTCGTAACGCCGCTTG
CCCTGCGCACTCAGAGACGCCTTACCAGTCTTGTACTGCGAGACCTTGTGGGTGGTATGC
TTCTTGCACTTGCGGCAGTAGGTGTTCCTGGTCTTCGGGATGTTGACCATGGCTGCAGTG
GGTCCAGTTGGCGTTAACTACTCCAACCTTGCCTCGTGCC
>at01483
TTGTTTTTTTTTCTTTTTCTTGATGCGGCACATGATCGCCACCAGCTCTGCTCCAGACAC
AAACACACCTATGTACTACCCACGGAGGGCCCATCAGCCAGGAGAGAAGCGACACAACTT
CCAATGCCGACGCCTACACCGGGCGCCTCCTGCAGACATTGCTGCACGAGACAATACAGC
TCACCTCCCCTTGAGGAGTACTGAAGAAGGACCCGAAAAGCCGAAGAAGAGGCTGGCGTC
CTCGAGGTCGTCCAGGGCGCCGTAGCGCTGGTAGAGCACCAGGTCCTCGGGCAGGAGCTC
CAGGCTGCTCTTGAGGCCGCCCAGGATTTCCGACTCCACATCAATCTTCTGCTGCATGGC
CTGAGCGTGGGCCTCCTTTGTAGTCACGGTGTAGTTTGCCTGCCCATCGGCCATGATGAC
CCTCACCTGCTTGTCGAATTCAGCCTCGATGACACTGGTGTGAGCACGGATCTGTGTTGA
CTGCTGCTCTTGCTCTTTGATAAGCATGTTCTGCTTCTGGATCTGTGTAACGACGATGCG

GTGCTCAAAGACGTCCGGTAAATCAATGAGCATCAACTGTAGGCCCCAACACTCCGCATA
AGCCTTTCTGAGCTCCCTGTCAACCATCTGCTGCATTCTATCTCCGAACTGCTCACGGTC
CTCCCACAGCTGTGTGGCCTCGTATTCGGAGGCTGCCCTGATGAGAGTGTCTCGCACGGA
GCTGGTGATGACCGGCTCGTAGTTCATGTTGAACTCGCTATACAGGTCCCCGAGGGTGTC
CTTGGAGAGTCTGTACTGAAGCGAGACCTGCAAGTGAAGTCCCAGGCCGTCCTTCGTCCG
CGTGTGCAACGGCTCATACCTCACTCCCGTGCTCGGCAACCCCATCTCGTCCGAAAACTG
GATTGTCTGAACTGTGGAAGGGAAGAGCATGAACTTGTTGAAAGGGCCAAGAAAGTAACG
GCCTGGTCCATAAACAGTTCCTGTATCGGCTGCCTTGGTGAAGCCGTTGTAGCGGATGCC
CATGCTGAGCGGAGGGACCGTGCCCAGGGACATCACGGCGACCACGCCGGTGAGCAACAA
GCAGCCCAGGGAGCCCGCCAGCAATGCAGGGTTGTCCACGACATTCAGGGGGTCGCAGTC
GCAGTCGAAGCCCTCGACGACGTTCCCAGGTGCTGGGTCATAGTTTCCACCCCCAAGGCG
GTGACCCCGATCCCCAAACATTTCTGGCGTACACCTATGGGATTGGAGCCACAAATGGCC
TTGAGCCAAAATGGCTACGG
>at01484
CCACTTTGGCCTAAGCAACCACTGTTCACCTGCGAATAGGATTATACACGAGATACTTCT
TCAGACTGCGAGCAATGCTGGAGCCATCGCACGCAGCGAGGTGCTTGAGCAGTCTCGGTG
CTTATGTAAGCTGCGGGTGCGTGCTTCTCTGTCTCCAGATTAACGT
>at01485
TTTTTTTTTTTTTTTTGTCATGCCAGGTCTCTCCTATCAGGCTGGACGGCTGCAATGCAG
TTCACCCAAGTTGTCAAGTCCATGAAGCCGGCCATGGGCCGGGTCACACATCGGAGAAGA
GGGGCAAGGCACCTTGCGGAAGCTCCTCAGGACCAGTAACACGGGATCCATGGGCGTTGC
CTAGGGCAATGGTCGCCCACGCAAACTCGCAGAGCGTCAAGGCAGGCCGACGTGCGCCAC
CAGGCGCCGCAGCGAGGTCGGCCGCAGGAGGGCCACGTGCAGGCTCGCGCCGACCCGCCA
GAGCGTCAGGGGCACGGCGGAGGCGTCGCCCGCGGAGAGCAGGGCCGCGACAAGCGGGCAG
AGGCCGGCGAGCAGCGCCAGAGCCCGCGGCGGAGGGTGTCGACGCGCGGCCCCGCTGCAC
CTCACGTCACAGCCGAAGAGTTGTGGGTTCCCCGCTTCGCACTTCTGGATGAATTGCGTC
TCGCGGCACGTGTTGAAGGACACTCGGGCGCAATAGGCGTGGCATTTCTCGATCTCACAG
CGCTTTTCCTGGGTGATGCCAAATGTGTACATGTCAGCCCTCGAGTCTAAGAGGTACTCG
TAGCGTTCCCGGCCCCGCAGCGTCATGCATTGCGTTCGATCACCACCCCAGACTTCGCAA
CCATGTGTACAGCAGAGGTAGGCCTCGCAATCTGCATCCCAGGTGCTCATCGCGGGTGTC
TCTCGGCAATACT
>at01486
AGCACCAGCTCCTCGGCCTCGTCCGCCCCCTATTAGTCCTAGCTGACCGTCAGGGCCGAC
ATCCATGCCGCCGTCCACCAGCGCCCTCCAGACGGCCTGCAGCTCCGGAAGCAAGCGTCG
CGTGTCATCATCAGCGTCTGCAAGCTCTTCCGTGAGTGCTTTGCGGTGCTCGGCGGCCGC
TTCATGATCACCCACGGCCCCACAACTGGCCGTGCCGTACGCCAACATTGAGAGGTCCTC
GAGCCGACCGCGGGAAGCTGCGCCCGCGCCGCCGTGTACGGCGTCCaACAATCGGCGCAT
CGCTTGCACTAGGGGATGTTTGTTGGGGTCCTCCTCCTCATCGGGCTGGCCACTACAGGG
GTCCATCCCTTaACTGATGGGTTGCGGCGAAGAGGGCAACGCACCAACAGAAGGCCAGGA
CGCGACATGGAAGTGCGGCAGCCCAAT
>at01487
TTTTTTTTTTTTTTTTCTGCATTGACCACTTCTGGATCGTCGTCAACAGTACGAGACTCA
CAGGATCGCGAAGCTCCGCGGCGCTCCACTGAACTCAGAAGCTGCAAACTCTCAGGGCAT
GGCGAGGGCAAGGTCAAGGAATGGGCAACAGGTGAGCAACCCCGTCGACAAGCAGCGCGG
CATGGCAGTCCTTGTACAGCAGGTCAGAGGTCACCACTTGATCTCAGCGCCAGCAGAGGC
GCTGCCACAAGTGCTGGAGCACCGAGGGACCCTCTCCTCGACTGAGCCGCAGAGGTCCCA
ACCGGTGACGACGTCAGCGCCTCCACGTTCCATGAGAAGCTGCGCGGCGATCTGTCCGGG
TAGAGGCTGTAGCAGCGGCGGTTCGTGATGACATACGTCCGGCGAAAACCACCAGAGCAG
ACGTCGTTCCCAGGGTAATCCACCTGCGTGATATTGGAGTATGAGGCATCAACCTGGAAG
GTCTGTGTCCCATTGCTCCACCGTAAGCAGGCATTCTGCAGGGGGTATTTGAACGTCTGC
ACAGGCAAGCCGTCACAGCCAGCGTCGGAGAAGAAGTTCTGCGTTGCGACGTT
>at01488
TTCTTCGGTTGGGCAGCTGCCGTTCTCCATTGGTTGCCACTGCCAGCGTCAGAGGACGAG
ACCAGCTCCAGGTCAAGTGTCAGCCCATGGAGTGCCGACGCACAACATCCCGCGCCAAAG
GTGGCACGTCCCAACAATGCGCCCTCGCCACCCTCCCTCAGCTCCGCTGCTGAGAATGCT

GGCGTTGATGCCAGCAAGGCGCTGCTCTCCCGGGCCTTGACCTTCTGCTCCTTCACGGCT
TCCTCTCCACTTCCTGGTGCTTGCCTGCTGCCGTTGCTTGCTATCAGAACTGCATCGGCG
GAGACGTGCTTGCATCAAGTATTG
>at01489
CCGTAGCCATTTTGGCTCAAGCGTTTGGGGGAAGTCCCAGAACTTGGAGTTGGTGTGCTG
TCCCCTCTCCTCTGCAGGAGGCACCACACGGATGCAATGGAGTGTGGGAGGGAGGATGGC
ATTGAGGGTGATTCTGGAAAGTTGCAGGGGATTCGCCGTCAGCAGTGGAGGCCGTCACAG
CTGGCTGGCTTGGCCGTGTCGGGCTCTCGTGGCGGCTCGTCCTCTAGCACGGCAGGGTGC
ACGGCTTGCCATGATGCCACGCCACTCGTCCAGCTGCTCTGGGGAGGTACATGAGGATCT
CCGTGCCTACCTGCTCTTGTTTGTGGAGGTCGCATGCGTGGGCAATAGTGCCGCGGCATG
CCAGCAGTTACATTCTTGCCTCTGGTCTGATAGCGACTTCTGGCGGACGTATGCTGGTCC
TTGCCTGTTTGCCGAACCATCAGCAAGCTCTGCGGCACACATGAGGAACACATTCCGCAA
GTGGCTATTTCGCTTGGAAGGTCGCTGGGCTGAAGACTTTGGACACTTTGTGGAGAGGTC
ACGCCAGTCTGAGTTTGGTGCAGACTTCATGCAGCTGTTGGCAGACGCTAGGTATATCGC
GTCTGGTCTCATGCCGTGCGATGACAGAGGTTGTGTGGATGAGTTCAGCGAGATTGTCAC
CAGTCTCCTCAATGAGTATAATCCGTCGCAGCTTGACGAGAGGAGCATTGCAGAGGCACT
GGTCTCGCACATCGAGTTCCGAGAGGATGTCTTCACGGCATCCCAGATGGCAAGTGTTGC
CTCTGCCTTTGATCAGTCTGTTGAGCGTGCCATCCTGGATCGTGGCCTGGAGGACCGGCT
TGAAAGCCACGATCACTTCATGGGTCCTCTGGTTGATGAGTCTGAGGAGGAGGGCTTCCC
CAGTGAGCCTGGTTGGGAGCCACCGGCACGGTCTCGTTTGCGGGAGTATGATCTTGATGC
TGACCTTGACAGCCCGCGAGAAGTGGATGAAGAGGCTAGCCTCAGCTTCTGGCGAACTGC
TGCTATCGAAGAACCGACATGATTGGACGGCCACTGGCCAAGTTTGCAAAGCTGAGCCCT
AATGCTGTGCTTGCCCA
>at01490
TTTTTTTTTTTTTTTTTTCCTCCAGGTGTGGCCGCTGTTGCCCGTGGTTCCCAAGGCCAT
GCCGCGGCAGCCCTCAGCCGAAGAGGTCAAAGTCCATGTCCTCCTCTTCCTCCTCCTCCT
CCTCCTTCTTCTCCTCCTTTGCCTCTGCTGCCCCACCAGCGCCGCCGGCAGCTGCAGCAC
CCCCACCAGCGCCGCCCGCAGCGGCGCCGCCGCCGCCGCCGCCGCCGCCGCCGGCGCCGC
TGCCCATGCGGATGAGCGCTTCCATGCCGACGCTGTTGATCATCTTTGCCATGAGCATGG
GCCAGTAGCCCTCCACCGTGACGCCAGCGGCCTTGATAAGGTTTGCCATGGCCGTGGCGG
AGATCTCCGCACCATCGTCATGCAGGATGAGTGCCGAGTAAGTGCAGGCCAGCTCATCGA
TCTGATCCTTGGAGAGCTCACCTGTGGCGACACCGGCCATGTTTGGCAAGGGGGGTGGCT
GGGGCGAGGATAAGCGTAGGGGCTTGGTGGCGCCACCTCCCTCGTGCC
>at01491
TTTTTTTTTTTTTTTTTTCGCACGCGTAATCAGCAAAGCGAGAACAGTCACTGGAACATA
CTGTCAGGCAGCGGGCCCCGAGGCGTCGGCGGCCCACCCTTGCCGCCCGAACCGCCAGCG
TAGGGGCCCGCGGGGTGGCGGTGCAGCCCGGGACCAACGTCCCATGGAGGAGGAGCGCCG
CACCACGGGCCCGGCGGCCCTCGCCATCCCGGCGGTGGCGCCCCAGGCCACCCGTGCGGC
GGCGGCGGGCCCAGCGGCCAGCCAGGCCGAGGCGGGCCGTGGAGGTGCGGCGGCAGCGGC
TGCATACCAGGCAGACCCCAACGATCCGGCGGCATCATTCCAGGCGGAGGGCCTTGATCG
CGATAATCGTGCCTCTGGAACTGCCCCGATCGGTGGCTGTCCTTAGACTTGGCGTCCTTT
ATCTTCTCCGCCATGTGCTGCTGGACTTCGGAGGCCCGTTCCACCGACACCTTCACATCA
AGGCGCATGCCGTCCTCAAACCGCTGTGACAGCGACTCTCCAGTAATCGTCCACGCTGAT
TGACCATCTTCCTCGTGCC
>at01492
TTGTTTTTTTTCTTTTTTTTTCTTGCGAAAGAGGAGCTGAACCACAGAACAGCTCACTCG
GTCAAGGCTGCTGGCAACCACCACTATCAAAAGCATGGCCGAACTGTGGGCCGTATGGTC
GGTATGCACGAGCCTTCAAACACACCCCTCATGTGAGAACCTGCAACACGCACTGTACAC
TGCAGGCCACATCTGGCAAGTGCCCATCTTCAAGTGGCACGAGCAGAGTCTAACCTCAAA
GACGAGATCGCAAATAAAGCTCAAACTCATGATCAGGCCTGCGCATGACGCTCAGCCCGA
GATCAAAAATAATACTTAAACCCTCAAGCCAGCCTGCGCATTACGCTCAGGCCGCGATCA
AAGAGGGACGCATCACCGCCGTTGCCCCCACCGCTGCCCACCACCCAAGACCGAGGCTGA
CGCCGAAGGCCTCAGACGCTTCAATGTTGGTGGGTAGCGCCACCGTTGTGGTGGTCACGG
CGTCGCGGGTGCACATCTCGTGGTTCTGCGCGCTGTCTGTCTCGTCATCATAAACTACGT
ATGACATGCTCAGCGGGCAACGCCTGCAAGCGGTCTCGGCCTGCAAGAAGTACGGGCTGA

AAGAACCCCACCCCTGACCTCCTGCGTGGCGGTGTGAGACCCGGTGCGCACGTGCAGAGC
TGCGACTGCGAGGTCGAGCGCCGCCGGCCCGTTGCGAGGGCTGAGGGTGGTGGCGCTGAA
AGGGATAGAGATGAAGCGAGCGGCCCGGAGTACCTCCAACTGGCAGAAAGACGGCGTCGT
CGTAGAGATACCTGATCTTGTCGATGGCCGTATCCCACGGCGGCGTCAACGACGACGATG
ACGTCAGGCTGGGCGAGTGGCGATTTCGCTCACGCTGACGCAGACCCAATTGCCAGACTT
GTCGGCAAGATGTTGGTAAGGCCAGCCTCCTGCATGAGCATG
>at01493
TTTTTTTTTTTTTTTTTTCAGCTTGCTTTAGCCTCTGCGGTTCTGCCATCCGGCCGCACA
TGTCAGTCGTCTGAATTCGCCGCAGCCTCATCGAAGTGTGCTCGGCCCAACAGCAGCATC
TGGGTCAGCTTTGCACTACAACTGTCAAAGTCCTCGTATGGTGGCAAGTCCAATGTGCAT
ATGCAAGCATGCGCCATCGGCAAGGACTGCGGCGTGAGGTGACTGCCCTTTGACAGTGTG
AAAGGGTGCTTCGCTCCATTGAAGCCAACCAACTCGCTGAAACCTCCGACTGGAACTCGG
GCACTGCCTGTCACAAACTGCAGGAGCTGTGGCAACCGGACGCTCTCCTCGGGTGAGGCT
GCCCACGCCTGGACGGTTCGCCAAAACCAGACAACCACATCACTGTCGCGAGTGAGACCT
TGTGCGTAAGCGGTGTGCTGCTCCCAGTCGTCAATGTTGATCTCACCCAAGCCGCTGAGT
AGCTGTGCGATCTCTGCTGGCTTCAGCAGACTGCGCAGCTCCTGAAGCATCGACTCTGGA
ACCACCTTGCGCAAGCCAGTTCTGATAGCATCTACCTGCAGCTCAATGGATCCAAACAAC
CGCCACTCCACGAGCGCCTTGGGGAAATCCTCGTGCC
>at01494
CCGTAGCCATTTTGGCTCAAGCGATGTTGGCTCAAGCCCGATATTTGGGCAGCAGTTTTC
ACTTGTTGTTGGCGATGGAGCAGTTGTCGGCTTCATGCAATGCCACCGATACTGGACTTG
AGTACATCAGTCTTGAGCGATGCCGGCGGCTCCGATCGGGGTTGTTCCGGGTTGTTGGAG
GCCGAGCGGCCGGGCAAATTCTTCCGAGTCTCAACCTGATTGCACCAACATCGGTTGTCA
TCATCAGCATCAGTTACAGAAGTGTCAAGAGCGAGTGGAGGTAAATTTACCTTCTCTCTG
GCCGCACGCGCGAGGAAGCTGGACAAGCAAGTGCGCCGCTTTGAGGGCGCCGCTCGATCT
CGATCAACCAGGT
>at01495
GCGCTGCGGCCGAGGCTTCGGCGTGGCCGTCGCGAATGGCACGGCCGCGCTGGACATCGC
GGTGAAGGCCCTGGGCCTGGGTGAGGGCGATGAGGTCATCATGCCGACTTTTTGCATCAT
CTCCTGCATCACGCAGATTGTCCGCGGAGGCTCAGTACCCGTCTTGGTTGACAGTGACTC
AACCTTCAACATGGATGTGTCAAAGGTTGAGGCAGCCATCACACCCAGGACGAAGGCCAT
CCTTTGCGTGCACACCTACCACTTTCCCGTAGACATGGAGCCCATACTGCGCTTGGCCAA
GGAGCGGGGCCTGCGGGTCATCGAGGATGCTGCGGAGATGATCGGCCAAACGTGCATGGG
AAAGCCTTGTGGGTCGCTTGGTGACATCAGCACCATGAGCTTCTACCCAAACAAGCACAT
TACCACTGGCGAAGGCGGCATGGTCCTCGTGGACACTCCCAAGCTGGCCGAGAGGTGCA
>at01496
CGCAATCCCGCAGACAGCCGCGGGGCTGCTGTGTCCATAGTCACGGTGAGGTGGGACCGA
ACTGTGGTTCAGGGCCAGCTCGCGGACGGCTCCAAGCATGGTTTCACGCTTCACTTGGAC
TGGGCTGCAGATGATCCTGCTGCTGCGCTCGTGGGCCGTCAGTTGACGGATTACTCGTGG
GGCAGGACCGTGGTCAAGGGCCAGCTGCAGACTTGCTACGGAGAGGGCTTCACTCTGCAA
TACAATATTCTGGATCCTGAAGCTGCTCGGGCGCGCTTGCGCGAGCCGTACTTTTTGGCG
TAAGCCGGGCGAGGCAGGAATCTCCTCGTGGGTGTGGGGTGGTCATGGCTTGAAGGCATG
ACGATTGCTTAC
>at01497
CGTTAGGCGGCCTCGGCTCCAGTGCCCCCGTAACGCGCGGCAGCCGCCCGGGACTGTCAA
GGCTCGCAGAAGTGGGCTACAGGCAACAGGAGTGCATGGCCAGGCTCGCCTCGGAGGCGC
TCGCGCCCATCGGCCGCAGGCACGGCTTCGCCCTCCGCTGGTGCGTCTGCCAGACGCCGG
GCGCCGCCTCCTGGTGCTGCGTTCTTCTCCAGGCGTGGCCGGCTGCACCCTGCAGTAGTG
ACCTGTTGGAAAAGTAGTCCCTTTGCATCTTGTGGCAGTGCTCATTGATGAGGTGGATGC
CGTGGTGGAAGCGGGGGTTCCATGATCGGACCTCGCAGGAGCGGTCCAGCATGACGCCGT
CCGGCCGGTGCCCCCGGGCATCTCCAGCGAAACCCTGGGGTCCGCAGCGGCGCTGATCAC
GGTACCAGGCGGAGTCGTGCCAGGCTCTTGCAGTTCCGTGGTCGTCCTCGGCCCACAGAA
GAACTCGCGCTTGGACTTCACAAAGTCCAGGTTGCTGTGGGGGTTGCCCGACTTCGTGTA
GAGTATCTGGTGGTTCTTGTCCCAGGCCTGCCTCGGCGCGGCGGCCTGCCTGCCGCAGCC
GAAGGGCCCCCGGGCCTTGCCCCAGTACGGCGTCCTTGCCCCAGACTGCCTGCTTGTGGG

TGCTCTCGAGGGCGCGGAGGACGAGGCGCGCAACTCGGGCGCAGAGGCGCTGTCAGTGAG
GGAAGGGAAGGCCTCCGGCGCAGGCTCCGC
>at01498
CCTCAGCTCCCTTCTCCCTCCGCCCAGCCCCTCGGGCGCCCCCCTCTCGCGCGCCGCCTC
CCCTCACCCTCCCCTTTTTGCGCGCGAGAGGGGGTGCCTTTGCTGGCGCCTTGCTGCCTG
CCTCGACGCAGGCCCACCCCATGGCGCAGCCCCTTGCAGCTTCCCCAGCCGCTTCCGAGG
GTGGCCTCAATTTGCAGCTTGTGGCGACACCGCCTGA
>at01499
TCTGTGCGGCGGGGATTCTCGTGGCCATCGACCAGGTTGCTTTGGTACGTGGACATAGTG
GGTTTACTTTGTCACGGGCAAGATGCCGGtTGAAGGTCTGCGGCTACCTGTTCTGGCCCT
CGACGTCCCTCGCGCGGCGAGTCACCGCGCTGCACCATGTGCTGTTCGAGCCGGCGGTCA
TCATGCTCTGCGCGCGGGGCGAGGGCATTCCCCCGTGGGCCCGCGGCTTCCTCGTATCGG
TGGTGCCAGACGGTCGCCTGCCAGGCCGTCTGCCGCTTCATGACGCCCCTCGAGGTGCAG
CACGCTCGGG
>at01500
GGCGGCTGCCAGGAGAACGGATACAAGCTGCTGCTGACGAAGCTCGACCCGTGCTTTAAA
GGTACGACAATGTGGAAGTATGACCTTGACCCTGAGAAGAACATCCTTTTCAAGCCAAGG
TACCAGCAGTGGGGTATCAAGAACTACAACAAGCTCGTCGAGAACACCCGCTTCAATGTT
CGCAAGCACTGCTTCGGCGAAAGCGGCGTGAATTTGGGGGGCCTTCCAGACCATGCTGCG
ACCCTCCTTCACAACACTCTCGGGCAGTAGGGCCCAGACCCCTGACGCGGAGCGGATGAG
CTGTGTCTGAAA
>at01501
CCGTTAGCCATTTTGGCTCAAGCATCAATAAGGACGCCGCCTGAGAGAGATATTGCCTCC
TCTGTGCGCCACCCCCTCCACGCTTGGCTTCCAGAAACGCCATGGAGGCATCCAGCTTGC
TTGGGAGGGACGCCACGGATGTTGAGAGCGCCACCGGCGGCAAGACGCCCATGAAAAAGC
CCATGCTGAGAACGCAAGCACGCGGAAGGCTCAGGCGCGTGGCATGCCTGGGTTCCGCAT
TGATCGGGCTGTGTGTGGTCGCTCTCTTCGTAGGAAGCGGCGACAAGCATGGTAACGGTC
TTGCCACATTGCTCACGCTTGAGCAAGAGCCGGAGGCGAAGTCGCCGAGTTTATGCTATG
CCGATATTGGTGGGGCCCTCTTCAAGTACGGCATGACTGCTGTTCCCGACGGAGTTTGCG
CCAGCTACAAGTTTATGGGCACGCTGAATCATGTAGCCATTCCCAAGAGCACATGCGACA
TCATGAAGACCATGCCGTCCACATACG
>at01502
CCGTAGCCATTTTGGCTCAAGCCATCTTGGCTCAAGCCTTGCTGGATTCCAAACCCATTC
CTTAAGCAGCTGGCCATGGAATCTCAAGGCATGGAGGCAGACGCTCATGCCTGACGTGCC
GTACCCGGCTCACCACGCAGACCGGTCGTCAACGGGGGGCAGTGGTAGTGGGCAGTCCGG
CAGAAGCCTGCTGCGGAGGAGCAGCACGATCGGTCGTTCTGCCAAGATCCGAGAGGCGGT
CGGGCGGTTGCGCGTGCGCTGCGTCAGCGCCAGCCAGCTGGCAACCAACACGGACGTGAG
CCTCCTGTTGCGGCCTTGGAGCAGCCGCAACCCC
>at01503
CCGTAGCCATTTTGGCTCAAGCGTCTCTTGGCTCGAGCAGCCAATGTGAGCATCCTGATC
ATGCGATGGCCGCAGTCCGCTTAGTCGGAGTACATGCCGGCGCGTCCCGGTCTCGTGCCT
CAAAGCGACTTGGGCAGGCCAGGCAGACACGCGAGATTGCGATAGAACAGGCTACCTCAG
CCCCGTCTACAGCCAAAGGCTCTCACTCACTCGATCTGCAGCAGCGGTCAGCGAACGAGG
AGCCTTTGCGGTACCTCTGGAGATTTGGCTACAATCGCGCCGCAGGGGCACCAAACCCAA
GGCTGCAGATAGCACTGGAGGGCCACGAGGAGGATGATGGTCACACCTGGTACGTCATCA
ACAGCTCGGTGCATCGCAGCGACGTCAAGGTGGCAGCT
>at01504
TTTTTTTTTTTTTTTTTCAACCGCTTCGTCACAGCATACAAACCCTCAAGTCCCTTGTA
CAGTCACCTGACACCTCTGTGGCAAGTTGGACCCCACAAGCTGCGGCGCCCACAAGCGCG
TCAAGGGTATCCTAGTCAAAGTAACTCCTGGATCTTGGAGGTCTTGCGGACGTTGCAGCA
AGCGGCTGCTGGCATGGACGTCTCCGGGGCTTCCCTGGAGGAGTCCTGCCCGCTCGCCGG
TGAGAGCGCCCCAGAGTGATTTGCCTAGGCAGGCAGTTCCTGGCTGCCGGCGCGGTCCGA
GAGCTGGGGCCCCAACTGTGCCACGACGTCCCTCAACACCGAAACCACTTCATTGAGATC
CTCCATGTCTTTGTTCTGCACCTGCAGAGTCTTTGCGAACCTGGGCGGCAGCAGGCTGTC
CTGGTCTTCTGCAGGTATGGGTCCCTGCTGCATGTCCTGAAGTGCTTTGTTGCACTGGGA

GACGTTCAGCGAAATGATGCGCAAGATCTCGGCCATGCCCTCCATGAAGCCTTCCACTTG
GACCATGCGTGCTAGCTGCGTGTCCTGACCACCCTCGGTCTCTTTCNCTCGTGCC
>at01505
TTTTTTTTTTTTTTTTTTAGTTTCATGAACGCATCAAGACAGGCCCAAGGCCAGAGGCCC
AAGCCTCTGGGTCCAAGTCTGCTGTGGTCAAGACTCAAGCCACTCTGGACCCTTCACGAC
TAGCCTGGGCCCAACACACCCTGAAGGCCGTTGCCTCACATCTGCCCGCAAGGCGTTAGC
TGTTCACGCCCAGTATGGGCCGCCTTCGGCATTGGAGAACTCAGCGATCGCGGAGCCAAA
GACCAAGCTCAGGATGGCCGTGAGCTTCATCACAATATTCAGCGCTGGGCCTGAGGTGTC
CTTCAGCGGGTCGCCGACAGTGTCGCCCGTCACTGAGTTCTTGTGGGCCTCAGAGCCCTT
CGCGTATTCCTTGCCCAAGCCACCGGCCGAGATATACTTCTTGGAGTTATCCCAGGCACC
TCCGGTGTTGCTCATGGAGATAGCCAACTGCACGCTGGAGACCAGAGCGCCTGACAGAAG
CCCCGCGCAGCAGTTCTTCCCAAAGCCCAGGCCGGCGACGAGCGGGCTGAGGATTACAAG
GGCACCGGGTGCCAACATCTCCCGAAGCGAGGCCTCTGTGGAGATCCTGATGCATCTCTC
GTAATCAGGGGTCATCTTCTCGGGGCCCATGATCTTTGGGAACTGCGCGAGGCACTCCTT
GACCATGTCGTTTGCCGCTTTGCCCACTGACTTCATCGTCCAAGCGGCAAACGCATACGG
CATCATCGCACCAAAGAGGAGACCCGTGAAAGACC
>at01506
CCGTAGCCATTTTGGCTCAAGCCATTTTGGCTCAAGCTCACTTTGGGCCATTTGCGCCTC
TTGTCGTGACTTGCACCTTGACTTTGCCTGCCATGGCGGCCTCCTCCAGAACTGAAGAAG
TGCAACAGGCGCAGGAGGAGTCCGAGCAGCTGGAGGATCAAGTCAGAGATGCAGCGGAGA
CGCCAGAGGCAAGAGAGGCTGAACCTTTGCCAGAGCAAGCGGAGCTGGAAAGAAGTGGAA
GAGACGCAAGGTCAGCTTTCAGCTTGTTTCAAGGAGACTTCCGCCAGAGCAGGTGGTGGA
GGAGATTGGCTCAGACGGCACATCGCTAAGAGTTGCGCCAGCTTTGGCTGCCAAGTGGAG
TGGAAAGACCTGAAGAGACCAAGGCAGGCTAAAGGCAAAATACGAGGAGCAGGTCCGCAA
GCAGCTAAGAGCCCGAGATGACTTTCAGAGAACATTGCCAACGGAGCGGTTCGAGCAGTT
GCCGCTAGGAAGAAACGCCAAGCTGCGGCAGCAGGAGAGAGCGAGCAGCGCGGAAGACTA
TCCGCACGCCCGAGTGCGCGGGACTGAGCCCCAAGAACAGAGCCAAAGAAAGAGCCGAAG
ACCGTCCAGTTACCCTTAGGAAGAACGCCAGACTGCGCCCCAGGAGAGAGCCACAGAGCT
CGTAGCTCCCACTGGCTCTGCGAAAACAGGACTGGAGCGTCATTGACAGGCTCGTTGCCG
AGGGTAACAGCAAACCCAGCTTTGCAGACATCGGTAGAGCCGCTTCCAAGACGTGGCTGG
CGTTGGCGGAGGAGGCAGAAGAAACCATACGTGGAGCGGTGTAGCCTCCTGATGAAGAAG
TTCAAAGAGCTCAACAGAGCTTGCAAGGAGTACAAGCAGGAACAGATGAAGGCCGCAATG
GCCAGGGCAAAGTTGGCAAAGAAGAGCGCCGCAGTAGAGGAGGCCGACAACCAGAAAGGC
CAGACAGTCCAAGAAGTTTGAGCCAGAGCCATCCCAGGCTCAAGAGGAGGCAGCAACAGC
CAGCTCCGCGCATGCGTTGACGCCGCGGAAGCGCGGCTCCCAGGCCTCTAGGCCCACGCC
CCGTGAAGTCAAAGAAGGCACCAGCCAAGCAGACGACCTACCTTGATCTGGCCCTCGTCG
CGGAGGCTGCAGAGAAAAACCTTGCTGGCGCGTTCTGCAAGCTGGTAGAGAGCGAGGACA
TGAAGTCATACCCGCAGCGAGTGCTCCTGGACGCATTGGTTGCCAGTGGTGGCTTGCTGC
ACCCCACAAGGGAGGCCGTGTTGGCTCGGCATTCGAAGGCCTAGCAACAACGCGCTTTCA
TGTATAGCGCTAATGCACTGCCGTGAGGGAACTGGATGCTGATGGGTGCGCTGCTGGTGA
ACCGTCACCTCCACTTCCGGTAGTTGTTTTGGCTGCGCTTC
>at01507
TTTTTTTTTTTTTTTTTTGACATGCAAGTGGCTTCAGAGTGCATCAGTGTCCCACCAGTT
GCACTCCCTTGTNTGGGGCGCGCCGCCCCGCCGGTGAGAAGGGATGGCGGGACGGCCACG
CGCTGTCAAGACTGCAGACACATCCGCTCAAGCGCAAAAGTGAAGGCGGCGCTGCGGCTG
TGTAACGGGGAGCCTCGACACCGCAGACACGGACGACGAGGACGACGTCGACGGCAACAG
CGACGGCGGCGGCAACCACGACTGCTGCAAGGCATGCTCCATGGGCCTCAGTGCAAGCCT
CAGAAAGTCATGGCAAGTCCTCAAGAAGCCGAAGCCGCTGTGCCTCCACGCCAGAGCTGC
GGCGAGCCTTTGACCACGCAAGCACACCACTGGGAGATGGTGCCACCAAACAGGGAGCCG
GCTCTGGAAGAGTGGCTGGCTCTGCTGCTGCTGCTGCTGCTGCTGCTGCTGCCGCTGCCT
TGGCACTGCTGCTGCTGGCCGTTGCATCCTCCAAGGCTGGGCACTTGCCAGCAAGAGCGC
CGCTCAGTGCAGTGGTGGTGCACAGGTGACTGCCGGCTCCAGCCGCTGCGTCGGCGGCGT
CAGGAGGCTTTGCCGGCAGCGGGGCAAGGTTGCAGCGCTTGAACTTCTTCGTGGTGTGTC
CCTCGTCGAACTTGACGCAGTAGCGCTCTTCCATGGCCGCCCCTTGAGACTCAACGAAGC
CGGTCACTGTGCCTGTC

>at01508
TTTTCTACCGCGCGCTTTGCAATGCCTGTGGCGCAAGCATTGTGATCAAGGTGTGCTTCA
ACAAATATGTTGCAGGACTTTCCCGCAACCGCAAACACCTGCCACAACAGAAAAATGACA
CCATGATCTGTACAATGATCTGCTGGAAGGCCTGCCGGCCACCTCTGGAGTCATGTCGTC
GCACCTACTCAGAATCGACCGTAGCCATGTGCTTAATCAGATCCACGACCCTTGAACTGT
AACCCCACTCGTTGTCGTACCAGGCAACAAGCTTCACGAAGGTCGGATCAATCATGATAC
CTGCCTTGGAGTCGAAAGTGCTGGAGATACTGCTGGTCTCAAAATCCGTGGAGACAAGGT
CCTCATCGCAATATCCCAAGAAGCCCTTCATGTCGCCTTCCGCTCTCAGCTTGACCTCTG
CGCAGATCTCCTCGTACGACGTCGGCTTCTCCAACTCGCACGTCAGGTCGACGACCGACA
CGTCGATGGTCGGCACTCGGAAGGCCATGCCGGTGAGCTTGCCCGCAACGGCTGGGATCA
CCTTGGCCACAGCCTTGGCAGCGCCCGTCGACGAAGGGATGATGTTTCCCGACGCCGCGC
GGCCACCGCGCCAGTCCTTCTTCGAGGAGCCGTCCACCGTTGGCTGCGATGCCGTCATGG
CGTGGATCGTAGTCATCAGGCCCCTCTTGATGCCGAACTTGTCGTTGATCGCCTTCAACA
GTGGAGCCAGCCCGTTCGTGGTGCAAGAGGCGCAAGACACGCACTCCATGGAGGAGTCGT
ACGTCTCCTGGTTCACGCCCATCACGATGGTGTGCGAGTCGTCTTG
>at01509
CGGGTATGGCCAGCAACGTCCGGGTCTTCGAGTTTGTCGTTGCGGCTGCAGTGGGCATAG
GCAGCGGTATGTACATCTTCAGCCCCGTGATACAGGAGTTCAACAAGCGCCAGGCCAGCG
TCATGGCTGAGAGAGAGCGCAACGCAGCGCAGGCCTCTACCACGCCGCCCGCACTGCCGG
AGGGGCAGGCGCCGCCAGCCCGGGACGGCGCCAGCCGATGAGCGCAGCGCGGGGTCTGTG
AGGAGCTGTGGTGGCTGCGAAGGCAGGGCTGCAGGGAGTCGGTGTGCCCAGTGCTGCAGA
CTCGGGCGATCATGGCGTTTCCGTTAGAAAAAAAAAAAAGAAAAAAAAAACAA
>at01510
ACATGGAAACAGGCCAGCAGAGGTGGCGGCAAGAGCTGCAGATCTTCGTTTACTGGTGCG
CCAGNTCCCTCCTGGTCTACAATGACACCATAGTCTTCCCGTTTTCGAGACCACTTCGTG
GCCAGGCCAGTCCCTGCCGGTGATGATGGCAACAACGCAGTCGGGGGCCATGAACGTGTC
>at01511
CCTGCACGAGCCGCTCTCGCAGGGCTTCGTCGCATAGGTCGAAGCCCGAGACGTGCGTGG
CCTCCTTTGTGGTCCCAAGAGCACTTGCCCGTGTTGCCGCTGATGTGGGCTCCTGTCGGG
GGTAGTCGCTGGCCAGGTCGATGCCGCCAGCCTTCTCCACATACTGGTAGGCGGTGACCG
TGTCCCCGCCATCGCAGCCATCNTCCTTCTTGTCGCAGGAAATGACTTGCTGCGTGGAGA
GCTGCTTCAGCTGCCCCGTGCTCATGAACAGGGCCGACTCGATNTCCTCTGTCGCGCTGA
ATGCCCAGCAGCTGCCACATTCTCCCTGATCCTTCACTGCCGTCACGGCACCACGCTGGC
GCCAATCAACGGCACTCACTGCGGAGAGATTGGCCTCGAGCGCAAGCACCGGAGCGCGTG
GTCGCACCACGGCGCGGCGATAGCCCAGCCGTGTGGCGGGAACTCATCCTGGAACATGTC
CGCGAACTTGGTGATGCCAAAGACGCAGTCGACGTCGTCAAAGAGGTCCGCGCAGTCGAT
GCCCTGCTCCGAGTTGAGCGCATACGCCCGCGCGACGTTGCGCTTGAAGGCTGCAAACTT
CCGGGCCTCATGGACCTCGTCGACGTCCGGGTGGCGGAACTCGTGCCGGAAGCGCTTGTA
CATGAGTTCAAGTTCAGCCTCTGAGTGCTTTGGAGGAGCCCCCAGGGCACAGACAGCAAG
GAGGCTTGCAAGGAGCGCAGCGCCTGCAACTGCCGCCATGGTAACAGAGCTAAGGGCCAG
AGGGAGTGAGGGCAGAACACGTCAGCTGGAGAGGGATGGTGGGAGCAGCTCGCTCGCACT
GGGGATGGCTGGACCCAAAATGGCTTGAGCCAAAATGGCTACGG
>at01512
TTTTTTTTTTTTTTTTGTCACAGTGGCGCAAGGCTACCTGCAATCTTTGAACATGAAGCG
GAAGCGAACGTGTTCCTGCAGGTCGCAATAGCAGCACTTGCAGCTCGCTAGCGATGACGG
AAGGCTGCAGAAAGTGCTCATATGGTGATATTTCAAGGAGCAGCTTGAATCGCCCCTTCAC
TTCTATGAGCTTGCTTCCTTTGCACGCAGCCTCTCCCGTGGGCCGCTCTATTTTAGTACC
AAAGAGTTTTGGAATAATGCAAAGCGGCATGGCAGGCCACAGTTTTTTTATCCCGGGTCC
AATCGTTGGTCAAGATCCGATTTGCCACGTCTGCCTTGGCTGCGAGCTTCAGAAGGTCCT
TGGCATCATCAAGAAGCTCCGCCGCGGTGGCGCGCTGCGCGTCGTCGGACACCAAGACCC
GCTCCAGGATCTCCACAACCTTGCTTGCCACGGGGGTGTTCTTCTTCAAGTACTTCGAGA
TCTCTTCGTACTGCCTCACCTTGAAAGCCTTCACCTTGTTTCGCAAGTCCGTGTCTGAGC
CACTGAGGAACGATGGCTTGCTCTCCACAAACAGCCGCCACAAGAGGGACCCCATGCTCC
AAACATCGTTGGCTGGCGCGGCAGTGCGCTCGTCCCAGACTTCCGGGGCCCGGTACCCCG
TGTCCTTCGGTGCGGGACAGCTCCGCT

>at01513
TTTTTTCTTTTTTTTATTTTTCATTGCCGGTTTGCTAGGGCGGTACAACCTGTGCTCCAT
CCTCCTTTGCAACCTTTGGGGGTGTCTTCCGACACACCCTTCCGAGGATCTTTGTACTCA
GAGGACCACTGGTCAGGCTCTTGGTCCCACTCAGCCCACCAAGCATCTGTCTTTGCCTTC
TCCTCTGACGTGTCTACCTTGATGGGATCTGAATCGAATCGCCACTGCCGCTCCTCGGGG
ACTTGATTCCTGAGGAAGAAGGATGCCATGCCCCACTGTGCGTGGGGGTACGTACGGAGC
CACAAGCAGTGGTTGTCAGGCTCAGAAAACAGAGGGCAATCAGTGAGCGGCCCCGTGACC
CACCACAGCTCCAACGTGGCACCTTTCGGTTTCCAACGCCATTCCCTGACTCGGACCTTG
CACTTTTCGCCTCCAATGTTGAAGCCCGGCATCTCAATGTTTCTAGACTTCTCCTGCAAG
CCAGCGGCAATGAAACCTACAGCCAAAACAGGACTACCAGCACGGCTGTCGATGTCGATC
TGGTATGCCCTCATGGACAGACTCGACAAATTCTCCAGTACACTTGGTGCTGGTCTCAAA
GCATCACCTGGCCACGTGGTGCCAGGCCAATCACACCGCCCAGAAACTGGCGCGACCGCA
GCAAGCATTTTACCGTACTTTATTGCAAGGTTCCATGTGCCTGTTGCCCCAAGGGATGTC
CCAGTGACGTACAAACGTGCTGGGTCCACCTTTTCTGGCCCAAGACGGCGGAGGACTTCA
GTGAAGAGAGCCCAAACTGCATCTTCAGACCACCGTCTTTGCCATCCCTTACGGATGTAT
GTTACAAGACCGCTAGTGGTTGTCGGTTTGGGCACGACGGTGATGAATTTGCGCCTGATC
TGTTCATTCCTCAGTAGAACATCAAGTCCCATCCCTCCAGTCATCTTATCTCGGTCATTC
ACATGCCCGTTGCCAAGAAGGAAGAGGAGAACTGGACTCTTCTCGGAAGTCTCCTCCTCT
GGCATGACGACCAAGGCCTTTGCGTCATCCTCGTATGCTGAATCAGCGTGGAAAATAATT
TCATGCCCAAATTTCTTCACATGGTCCTCACAATTGAAAGTCAATGCCTGCAGGATTGCT
TCCATAACGGCGACTGAAGTGGGAAAACCGGAAAATCAACGTGAGCGTTGCAGAAGAAGT
CGCAGGATGCAAAACCCTGACATGGAAGAGGACTGCGCGACTTGACCTT
>at01514
CCGTAGCCATTTTGGCTCAAGCCAGAATCGCTCCAACGCTTTGCCGGCGCCGGCAGCCCC
GTTGTACGTTTTTCGGGAACCTTTGAGTCGGACGAAATGGCGACGACAAAGGAAGAGGAC
GTTCAGATGATGCTTGTGTGCAAGACACATCTCGGCACCCGCAATTGTGATTACCGGATG
AAGCGTTACGTCTTCCGCCGCACAGTCGACGGAATCCACATCATGCACCTCGGCAAGACA
TGGGGAGAAGATCATGGTCGCCCCTTCGCTTGTGCTTACAGTCAGCAGCGTCGGGCTTTG
CCCTTGGGCCGGCTTGGCGAACCCGATTGCGGAGTTTTGNGCATCAGCCCCCCCA
>at01515
TAAGGACACGCGGCTGCCCCATTGCAATGGTGTACATTATCTTGATTGTGAGTGGGAAAG
TAAATAGGGCGCAAGAACCTTGACGCAGCCGCCTGAAGCCGAGGAGCTTGCCCTCGTTTA
TCCTCTCTTTCAGCCGGACCTCAAGCTCGTCGGTCTCTGTCTCTGTCCTCCATGACCGCA
ACACTTCTGCAAGCGACTTGAGCTTGGAGTGCATCCGCGAGAATAGACCATAGACGACTG
CAGCCCGAAACTGGTGCACAATGATCCCAAGCAGAATGATACCTTCCAGAGTGAAGACCA
CTACGTTCCAGGTGTGGCTAGTCATCAAGTCCATGCTGCAAGCGCTTGCTGGCACGCGCA
G
>at01516
TTCAGTAGTTCGTGGAAATATGGATGATAAGACTGAGTATGCTGAGATAATCGCAGTAAA
ACCTAATTAAATATTCAAAGCTGTGTTGTTTAGCACACTAATTGGGCATCATAATGTGTT
TGATTCAAAAGTGCAAACATTGAAAAACATGTTTCCTTCAAAGCTCATATATTTTCCTGC
ACTTTAAAGTAGAATGATGTTTAACTAGTTGAATAGCACACTTATAATGGCAGTAAGTAA
ATAAATGTATCAATATTATGACTAGAGCTATTTCTATGATTAGTCATTTTGAAATACCT
>at01517
CTTCAGCGCCTTCTCGGGGCTGTCCGAAGGTCTTCCGAGCTTCTCCCCATGGACGGCCTC
GACGATATGCCTGTGGTAGAAGCATTTCCCCTCTGGGCTGATGTAGCATCTGAGCTTCGA
TGCACACGTAGTCTTGACTCCTTCACCCCACCCCTCTGGCAGCCAATACACAGGGCGGCC
AATGTGAACGCGGACCCCCGGTGGCCAATGCGCCGGAGGCTTGGGCCAGCCCCCAGGTGG
CGGCCGCTCTTTCTTGCGGCGCTCCTCCCAAGCCTTCTGATCGGTGTACTTCCGATTCAG
TGCGGTCAGACCTTCCTCATTAATCGTCTTCTTGCCATCGAAGATCTTCTTCTTGACTTT
GGCACTGGCTTTTGCCAGTGAAACCGCCTTCCGCTTCACGACCATTCTTTTCACCGTGAC
TGCTTCTGTGATCAAGGCTGCTCAAATTAGAGGGCTTGAGCCCACATGGCTTGAGCCAAA
ATGGCTACGG
>at01518
TTCTCTTAAATGTTCTGGACAACAGTGTGTGCTCGGCTATGGAATGTAAACTTGAGGGTG

GCGAGGAAGACAAAGCAGATGGCGGTCTTGCTGCTTGGTCCAAGAGGCTGCGGCATCAGC
AGATCCAGTCGCTGGCAGCAGTTGATGAAGAAATGATGGAAGCGTTTGCCGAGTTCGATG
GCGAGGTGCCGCGGATACTCATATGGTCGGCTCTCCGCCGAGCAGCGGCCGCCGGCAAAA
ATCATGCCGCTAATTGCTGGCAGTGCGAAGAGCGGTCTGGGTATTGATGCCTTGCAGGAT
GCTGTGCGCACGTTCGTTCCGGCACTGGACTGGTC
>at01519
TTTTTTTTTTTTTTTTTACTGGCCGACACACATTCCTGCGAGGGGGCGTGGGTCCTGGA
TTCGGTGGCAGAGAGCCACGCCGCAATGCTCGTGTAGATGATGCAGATGATGCGCTGGTC
CCTACAGGCAAAGTTGCGCCCACGCAAACGTCCTCCAGCACGCGTCCTGCTCACTTCGCC
AGTTTGCCCTGGATGCTTGCGATGCACTCGCTCTCCTCGGGCATCGAGATGTGCGAGGGC
ATGTTGCTGCCCATCCCGCCCTTGACCATGAGGAGCCTCGCGGTCCAGAAGCCGCCGTTG
TCCAGCGTCTCCTCCAGCGCCGTCCTGTTGAAGTGGCCGCCGCGCTCGTTCACGTCCTGG
CCGTTCTCCAAGTACTCCCACAGCCTGTCGATGTCCTCCGCCAGGGCTGCACCGAACCAA
GTGTACTGCATCCCCTGGCTCTTCAAGTATGATGCAATGTCCATTGGGGCGGCGCTGCCG
TCAGGCATGACCGGCGGCTTGTCCGGGTGGTGGCAGTGGCAGGCCGCCGCGTAGTCCAGC
GCGTTGTAGCCGCCGTAATCGACAAGCTCCAAGTCCANCNTGNCTTCCTCCACGAGCCAC
TGGACCATCGCCCAATTGCACTGCTCGATGGCCAGGAAGAGCCCGGTGCCGCCGTAGCCG
TCGAGGCGCGCGTTCACGTCAACGCCCTTCTTGATGCACTCCTTGGCCCAGGGAAAGTTC
CCGAGGCCCATGGGTGTTGCAATGGCGTGGTACCACTCCTGGACCAACTCAGGCATAGTG
TCCACAACATCGTTGCCATAGATTCGGATACGCGGGTAGTTGGTCTTCGACATGGCTGCC
ACTGGCGGGTGGCAG
>at01520
TTGTTTTTTTCTTTTTTTTTTTCTCACCTCNACCCGAGAAATGACGCGAAGGAGCTATG
AGAACGCCACCGATAGCACCTGACCGCAAGCTCCTGTTCGGGCCAGGCGCGGAGCTTCTT
CCTTTCATGCCTCGGCGGGCCGGGCAGGGAGCCAGCGTTCAGAGGGACATCTTCTCGATC
CTCGGCGGCTGTTCTTGGCTGGAGGAGGCGGCGGTGCCGCCCTCGGCGCGCAGCTTCTCC
GCAAGCGCCCCCAAGCGGTCCTTGAGCTGCGTGCGAAAGCCCGCGTTGGCAAGTGCAGAT
GGCGGGAGCGTCGCCTCATCCAGCACAAAATTGTACGGGTCTTTCTGCACCTCGGCCTCC
AGCTTCTCCTCGAGCTTCTTGAAGCGAAGGCGCTTGCTCTTGCTCAGGCGCTGGGACTGC
CTGAGTTTTCGCGACGATAGGGCTGGCCCTCCCGCCGTCGCCGCGGTGTTCGGGACCACT
TCCTCGTCCTCCTTATCTGACGAATCACACTGCTCAACGTCTTCAGTCTTCGGGGCCAGG
ACCTTCCCCCTTTTCTCTCCTGGCAGCAGCAACTCATCCATCTGGGTCAAGCTATCGGTG
GATCTTCTGCTCGACGCACAGCTGGAGGACTCACGGCTGGAGGACGACATGGAGGGCGGA
GCCGAGGTTTGCCGCCGCATGGCCAAGAACCCATCGTACAGTGACGGTCCTACAGGCTGC
ACATGTAGAAAGGAGTTCTTAAGTGAAAGCTGGAGAGTGTCGTCCGAAGCGCGGCTCCTG
GCTCCAACGGAGTCATTGTGCTGAGCCATCTGTGGGCCGTACCCTGACGAAAACTGAGCC
TTGAGCCAAAATGGCTACGG
>at01521
GTCTTGGAACGAACTCGGAACGTAGGATCCAGAGGCGACAGAGCCCCTGCACTATTCGCG
CGTCAGTAGAGGCGTGCCTTTAAAAGTCAGACTCACCCAACCGCGGCCAGGACCATCCCC
TGAGACCTTGCGGAAGGACATGCGGTTTCCGTCCACCTTCACCTGGGCAACACGGGCACC
CTTCATGAGCAGCTCCCCAAGCTGTGGCGACCGCAGCTCCTGGCTTTCGCGCACCAGGAG
GCCTCCCTTGCTGAGGCCCTCAGCGGTGACCTTCCAGATAGGCGGGCAGGACACACCGCT
GCCTTCGTCCGACAGCAGGCCAGGCGGGCTTGCATGTCGCCTGGTGAGTCGGAAGATC
>at01522
CGCTTGAAGAAAGGGCAGCTCCTTGGTCGGTACTTCGTCTACGTACGTTTATGGAGTCCG
TCCTTGCCCAACCAGAGATTTTGAAAGAGAGGGACTGCGCAGGCAGGACCTTGCTGATCA
AGGCAGTCGCGAAGAGTGGCTCTCCGGAGCTCGTGCAGGCTTTGCTCGCGGCTCGATCGG
ACCCCAACG
>at01523
TTTTTTTGTATTAACACCACCTCTCTGCACGTGTCGATGGGCCGTGAGCCCTTCACTGAA
CCCACTACAGCTCAGAGTGCTCAAATGAAAGCGGAGGCGTCTTCACgTCGAAGGGNTGGT
TNGGGAGAcCCAATGCACGCCTTGCCCGCTTCTCGGACCAGTCTTGtCAAGCGTCTCATT
GCCTCCTTCACACACGCATCATGCGGTGGCTTATAGTGGCATGGGTCGACATACCAGCCT
TGCAGTGTGAAACGCCTGGCATGGCCCTTCTCCAAAACAGGTGCCACTGCATGATAGCTA

CTCCGGAAGACGGGGAACACAACTGCCGTGTTGAAACCTGGGGCCAAGGTCGTGCCGTTG
CGAGAGACGTTGAACGTCTTGTGGAAGTGTGGCCTGTCCGGAGACTCGACAAGGACGTCA
TCGGTGCCGTCAGGCCCGCACCAGACGAACTCGCCGCCTCCTTCAGCTGGCCAGTCCTTT
GCAAGGTGGATTGTCACGGCCAAGTACCTCATCTGGGCGTCGTCTGCATGCAGTCCGTAG
AAGTCCCCAGGCCGGAGCCACGACCACGAGGAGTCGTACGCCGAGGCGTGTCCGGGCAAG
TCG
>at01524
GAGATATATTTGCCCTGGACGTCAAGACTGGAGCTCAGAAGTGGAAGGTCAAGATGAAGC
TTGGCGTGTCCAATCTTATGCCAACCTTTGCTGACAACTTGGTGGTCTTCCAGTCAATCA
GTGGGGAGGTTGCCTGCCACGGCCTTGCAGATGGTGCAAGCAAGTGGATGACAAAGCCAG
CTGGCCAAGTGTCCACTGGTTCTGCCACCATCTGTAAAGAGACCGGCCTAGTTTATTCCA
CCGGTAATCTCAGAGGGAAGAAGGAGTGGAACGCCCCGAGAGGAGGTAAAGGAGTTCTGC
GTGCATTCAGCCTGAAAACTGGTGAACTGGCATGGCAGAAGGAGCAAGAACTTGAGTGCA
GCAACGCTCCCATCGTTGCACCTGT
>at01525
TTTTTTTTTTTTGATTTCTTCCCGGTGTGAACGGAGCATCACTGCCGTGCTCGCCAAAT
TCCAGCGCCAATGTGAGGCCACAAGGGCGCTGTAAGATCTACGTTTCAACCATGCAGGGT
CGGCGTCAGCTCACACCATGCAAGGGCGGCTCTGACTCAGGAAAACAAACTCACCCCTCG
ATCACTTGAGCTCTACCAACTGGCACAAACTAGTGCTCGTCTGACATGCTTAACCTGAAG
CTCTGCAAAACCACCGGCTTCTGTACAAGATGGACGCAGGCCCAGAAGCACCTCATGGAA
GTGACCGCACATACAAACCTTACAAAGCACAACCTGTTCATGACTGCACCGTCATCATGG
CAACGCTTCTCTGGTGCCTTGTGCACTAAGCCGTTCACGTCATGAGGAAACAACTTCCCC
TTCATGAAGAGCATGTCCGCGACGTTCACGGCGCAAACTTGAGATGTGTCACGGATAATG
TGCGAATTCTCGCCGCAATGAGTTAGCTCCCACAAGTTCACGTGCTCATGATTCACATAA
TGGGTGCCGCAGAGGTTGTATGAAACGATCGCGCGACCTACGATGTTGTCCTGGTGCCAT
CTGCTGCAGTAATTATCTCCGAAGACCTCCAACTTCGCGATCAGCTTCTCCGCCGTTGGG
AACATCTCGGCCAAGGCCATCCCCCAGTTGCTCAATGTCGTTGCGGATCTGGTGGCCGAC
ATGCTCAGGCAGGCCTGCGTCCACGATCTTTCCAGCAAGCTTGCGCGCCTTCTCAGACAC
ATGAGCTGCGAGCCACGGCTTCGAGCCCTCTGCCCTTGCAACCTCAAGCTCCTCCTGCAT
CGAGCC
>at01526
TTCACCATCAGGCAATTTCTCACAAGCAACACGTACCAGCGCAGCAAAACTGTGATAGCC
AGGAATGCCCTCAGAATGCTTCCCCTTCTTTCCATTCACAAGAGCAGCATACTCCTGCCC
CCGCGCTGCCGGCAGCAGCCCCTTCCGAGTTCATCTTGTCAGCCACCTTCTGCAGCTTCT
CTATGAGCTCCTCCTTCGCTCTCTCCGCATCTCCTGCCGGANCCA
>at01527
TTTTTTTTTTTTTTTTTGCATTTAGATGCGCATCGCAAGGGAAACACCACTCAGTCAA
GTGGCCAATCACTTCATCTTGAACCTTGACTTGAGGTCGTCCGCTGACAGAGGAGCTCCC
TCCTCCCAAGTGTAGAGGTCTTGCAGGTAGAGGTTCTCCTTGGTGTACATGATATTCTGC
TCATGAGGCAGGCTGGAGTTCGGGTCGACCGGCACAGCCTCGGCCGACTCGAGGTCGACG
CCCGCCGACGAGAAGGCGTTCAGGACCTTGCGCTCGTCCTTGCTCTCGTAGAAAACATGG
ACCATGCTCGAGGCCTCCTCGCCAAGGCCGCTGCTCTTGGAGTACTCGGAGAGGTTCCAG
ACGCAGACGGAGAAGTGGACCTTGCCCTGCTTCTGCGTGGCCTTCTCTAAGGCGCTGTCC
TTGCACACGATCTCCCTGCGCTTCAGGATCTTCTTGGGCATGGCCTTCAGCAGCCGCATG
TACTCGACGTCGTCCGGGTGGGAGCCCCACTCCGCCACCTGCGCCACGCACGGGCCTGCA
TCCGGGCGCCACATGGCCGGGAGACGGCTGGCTGCGCTCCGCGGGCGGAGCGGGGGCGGG
TGGGGCGCCTGGGGCCTCTGCCTCGTGCCGAATTCTTG
>at01528
TTGTTTTTTTTTCTTTTTTTTTTTGTTTCTTGTGCACTACTGGGACGTTCGTGGTTCGA
CGCTGGTCTGAGGGCAACAGCTTCGGGTGGCAGATGCCAACGTGCAACACCGCCCGAGGC
TCTGTGGCCTCGGCGTCCACCTGAACAAAGTGGTCATAGGAGTCGTCGAAGATCGCGGAC
ACCTTGCCGCTTTGCCATTCCAGGTAAGCGACCTCCGAGTCTTGCTGCACCACGGCAAGG
TATGATCCAGGCGAGAGCCCTGTCAGGCCTAAGTGCAGGTTGAGCCTCGTGGGCTCGCCG
CTGTGAACCACGACGTGGGCCCCTGGCGGCAGCAGGCTGATTGCCACCTCCTCATAGCCA
TCGAACACCCCGCGCTGTAGTCCCGGCAGCTCTCTCTGCATGAGGTTGCACAGCCTCGGC

ATGGGCTCGCACAAGCTCATGCTCCAGCCGGAGGCGGTGGTGTAGAACCGCCAGCGGTAC
CAGGAAAAGGCTCGAGGTGTTGATGAGGTACTCCGGATAGTCCAGATGCTCCTCGCTATA
GATCCAAGGCAAGGTGCGGAAAGACCGCCATTCATCCAGGATGCCATAAGCGTGAGCCTC
AAGCACCTGCGTGAGTCTACTGAGAACTGGGTCGTCTGCGGGCTGCCATAGCGCTGGAGA
CGCTCGTATTTCAGGCATGGGGAGGTAAATGTCAAAAGTCTTATGCACATGAGGCCATTC
CAAAACTCCCATGACCTTCATGGATACCAAGTAGTCATAAAACTCGGTTGCGAGGGCCCA
GTCGTAGCTCTGCTTGACTGCCCACATTGCTCCGAAGGCCTTGTGAGCAATGCAATACCT
CTCCAAAGGGGCGCGATCTTCCCCCGACACTCAGGATCTAACCAAGCTCGCCTCACAGTC
CACCAAGCATAGAGCCAAGTCGCTTCCCGCCTTCGCGAGGGATCCACATGCTGGATTAGG
TAGTTAAAATGGTGAGACCCTGCCAAATTCTTGAAATGCTGCAGCCATGAGTCGAAGACA
TATAGCAAGTCTATGAATGATCCGCAAGCCTGCACTTGGAACTCAACTGAGCACGTCTCC
ACTTCACGGAAGCTTTCCCAGAGCCTTCTGACCTTATCTAAGTGTGACAACACGCGAGCA
TCCCAAGGTTTGAAGTCGTCCAGAAGTCTGTCTGCTTCAGGCACCGATGCGCAGAACTTG
CACAGTGCTCTGATCATCTCCTCGT
>at01529
GCGCCACTCTCGAGCCCTTGAACTTGAAAATACAGTGTCTGATAATGGAGAGCAACCCAG
CCGACGCCTCCTATGCATCCCTGCGCTACGAGCACACAGAATGACGGTATCCGGAGATAA
TCAATGAGACGCCTCACCTCCGCGAGACAGCTCTTGCTGCCTGATGCTCGATTAGAGCTG
TGACGCGGGGGCTCCGTCATCACAAGGGCCACGATGGCGCCCACAATCACGGCAAAGCTG
CCCGTGCAGAGGAAGACGGCGCGCCAACCTTGGTGACCAGCCACTTGGTGGGTAGACAAC
GGTGTCGCAAACAGAACTCCGCACACATACCCATTATGCAGAAGAGCTGGGACCAGCCGA
ACATCTTGCCTCGATCTTGCTCTGAAGCCACATCGGCAATGATGCCACTTACTATTGGCA
GCAAGCTCGCCAGCATGGCGCCGTTGCTAAATTCGGAGCAGGAG
>at01530
TCTTTTCGTCCTCGCCATCCCACCTGCGGCGGCGCGGTAGCAAACCGCGGCTCGGGCTGC
GGCTCGGGGGCAGACCGCCGGCTTCTGCTCCTGGGAGAAGCACTGCCTGCCGGCGTCTGC
GCCTGGAAGGCGCAGCCCAACTTTCTGCTCCTGGCAGGCGCAGCCCGCCAAGGATGAGTG
CACGTCACTGCTGGCGCGTGGTGCAGTGCTTGGGCCCAGGCGCCCCCCGAAGCTCCACGC
ACCCGCACATGGCCCAGTCCATGAGCACGTGAAGAAGATCGGAAGGACCGGGATGAGCAC
CGGGGCCCACAAGACCATGCCATCAATCTGGCACCCAATGTCCATGCGGTCAGCCGCTGG
TGGCCGATGCGCTGAGGCCGCA
>at01531
TTTTTTTTTTTTTTTTTCCCCGCGCGCGTGGAGCCCGTGAAGCTGATCATGTCCACGGA
GGGGTGGGACGCCAAGGTCTCGCCCACGCCCATGCCGGTTCCATTGATCATGTTGAAGAC
GCCTGGGGGGACGCCAGCCTCGTGGATGGCCTCCGCCAGCAGGAACGCATTGATGGGTGT
GACCTCGCTCGGCTTCAGCACGACCGTGCAGCCCACGAGCATGGCGGGGCAGATCTTGGC
GGCGATCTGGTTGAGGGGGTAGTTCCAGGGCGTGATGCAGCCCACGACGCCGATGGGCTC
CTTCACCACGACGCCTCCCATGGCCGTCTTCTCCTCGAACTTGACCTGGTCGATGAGGCC
AATGGCCGTGCCAAAGTGCATGTCCACAAGGTTTGCCTGCACGCGCTTCGCGAACTTCTC
GGTGCAGCCCAGCTCCTTCTGAAGCCACGCAATGGCCTCCTTCTTCTTCCCCTGCCACTT
TTCCAGGATGCGCTGCATGGCTGCCTTCCGCTCTGCCAGAGGTGTCTGTGACCAGGCAGG
GAAGGCGTTCTTGGCGGCCTGGATGGCCTTGGCCATGTCCTCCGCGGAGCCGTCAGGGAT
CTTGGCGCAGACCGTGGCGGTGCTTGAGTCGATGACATCCAAAAACTTCCCCGCGTTCGT
GCTCTTCACCCACTCCCCGTTGATGTAGTGGGAGTCGTANGTGCGCAACGACATGTTGGC
GAGGGGCTGTGGAAAAT
>at01532
GGCTTCCGCATGGATCGCTCGCGCGGCAAACCTGTGGATGTTGCAGCCTTGCTGGAGCTC
TCGATGGCTGTGAGGGAGTGGGCAGCTGAGGCTGCCAAGCTGCGAGGGAGCTGCCCAACC
ATCGGGGACGTCATGCCCCTGAAATCGCCTCCCGAACTGCGTTTCGATCGCAGGCCAAGT
GCAC
>at01533
CTATTCCCATGCCACAAGATGCGAAATACAGAATGACCAGACAGCTCGATCTTAGCTATA
CAAGATCAAACTGTGCATCTGAAAACGACACAGCATGACTTGAGGGGTCAGGCATGTAGA
AGGTCTCCATGCCAACCTTCGTGACCCTCCTTTTCTGACGCAGCAACGTCATCACGGCGA
CGAAGGCGttTTTTTGTTCGCCACTCTGGACACACTTTGTCGAGCTCGCCCTCCTCCAAG

TCAAAATGCGATGCATCGATGACCGGTGGGTGCCAGACGTACCAGCACTTGGGCTCCCGG
GCGTTCCGCACCATGCGCTCCAGCTGCTCCGGCCGCGCGGCGCGCAGGAGCTCCGCCAGG
GCGTCCGTCACGTTGCGCATGGGCCAGCGGATCAGCCAGTTGCTTTTGGCTTGGAACAGG
CTCAAGAAGGGTAACTCATAGTCGTCGTTCAAGATCACAGGCACGCACTTGGCAAACAGC
GTGCGAAAGAGGCGGCTTGACCAAGCACTCTTGCCACGAGGGATGAAGCAAAACCTTGAA
GATATGCTTCCTTTGAGTGCCTCATCGTAGGGCACATCTTGACGGATGTGCATGTCTTCA
TACTTTGCTGCCCACTTGA
>at01534
TTTTTTTTTTTTTTTTTACCACTCGACGTCATGCCTTCCTACGTCAAAATCAAGCTGTC
ATGGTTTGCAGCCCACATGAACCACTACATGGCGCAGCCCCCCTTTGTTGCAAACGCAGG
GGCGCGTGCACCACCTGTACATGAGGCACCCTCTCCAGCGAAGGAACCTCTTGTGCCTCA
AGAGCCCTCGACAGCAAACTCCCCTGCCAAGGACTGCAGCAGGAACAGCCGCACCGTTCG
GGCCAGTTCCTGCTCCTCGCCTGGGAAGGCGTGCTTGCCAGGCAAGACTGCCAGCCGCGC
CGGCCGAGACCTGCCGCGGTGGAGGTCCCTGTCCACGATCTGCTGGCAGTACTCCGGCCG
CGTGAGCCGGTCCTCAGTGCCGCAGACGAAGAGCACGGGAGCCTCGAACCGACTCGTGAA
CTTGCCAACGAGCTCGACGTCCTGCCGGTGAAGCCGCTCACCGCCCTCCTTGGCCAGGTC
ATCGTCGGCCAGGAGGTACCCCAGGGGGGGGATGCCCCAGTTGACGTCCACGTAGGCGGC
GACCCTCTCTCTCAGCACCTCCGGCGGCAAGGCGCAGAGGGCCCGCACGATGCGCGTGTT
GCCACCAGAGTGCGCAAGGATGACAAGGCGATCCCCGCACTCCGCAAGCGCGCGACCCAA
CGCTGCGGCCGCGTCCTCCAGCATGGCCTGCATGCTCGGGGTGCCCTGTGCCACGGCCGA
AGCCTCGGTAGTCAAAGCGCACCGTAGGGATGCCCAGGCCCCACGCACCAATGCGCACAA
GCTCCGTGTACATGGGGTCGGTCATGCGCGCGCCTCCCGTGCCCTCCAATGGCGTGCCAG
GCACCACGAGCACGCAGAGAGGCGCGCGCCCTGGCCTCTCATGCCTTGCCAGTGCCACTC
CAAGGCTGCCTCTGCCGCCGGCGACTTCCCAGAGCAGCTCCTCCTCGGCCAAAACCTCCT
TTTGGAAGAGCCTTGACGGCAGCCCGTGCTCAATGGAGTCTGCCAGCTCGGGCATGCCAC
GGTTGGCTCCCCCCAGGTCCTCCGGGATCTCGTCGAAGTCAAACATCCCAGGCAAGGCAG
TCGGGCA
>at01535
CCGTAGCCATTTTGGCTCAAGGGCCAGGTCGGCTCGAGATCAATAGCTGGTTGACTGGCA
CGCACGGGCAATACGGCTCAGTCTCTTCACATACCATGTCCTCTAACCCTGCGCATGTGG
AGCCGCGCTTCAAGGTACTAGCAGTTGCAGTCTTCGTTGCACTCCACCTGGCTCTTGCGA
TCTATTTCTTGGTAGGCACCTACAACATATACTTCAATGACGTGCTGCCAACACACGCTG
GTGTTGAGTCCAAATGGGGTCAAATGCCAGACATATCCCTGTGTGAGGAAAAAAATACAC
CCATTTTTGTTGCCGCAGATTCCTATTTGCAAAGCGATGATCGACCACACCAAGCAGGAT
ACCGTCTTGCCAACTCCAGCGGGAAAAGGGCTTCGGCAAAGGATCTCGGGACGCCCGATG
AGCTTAGTGCACCCACTGAATCCTGCATGCCCTACACAGTACTGGATCGATGCGGCCAGC
AAGCATGACACTTGTGACGCCGACCTTTACGTCGTCGACATCTTCGTTGATATTTCCCAG
GAGGTAGTGCCAATTGCGGAATTTCAAGGAAGAGCTCTCCAGCTGTCTGTCCTCATCTCA
AAAGTTGGTGGTTTGATGTCAATTGTCGCGCTCATCTACAGCCTCCTGTTTGTGAAAAAG
AACCCAAACTCGGAGATCACGCGTGTCTACGAAGAGACAACTTTGGTCGGGCATGAAACC
CCCGTCAAGGATGATGACAGTACCTCGACGGAATGAATCCCCCGTGCGGATGAGGACTCG
GCCGCGAAGCGATGAAGTCTGCAAGTGATACCGGCCGCATGG
>at01536
TCTTCGCAACCGGATCAAACGCTCTGCCCTTCTTGTCCGTGCCCAGGTTGAAGAGAGAGT
CCAGGAGAGCAGTCTTCTGCATCGACTTCTCCTTGGCATCCTTCTCTTGTCTCTTCTACT
CTTCAATCCCACCTTTCGGGGCCCCTTGGGCATTGCTGTGGATCGACTTGATGTACTTCT
GTACCACCTTGCTCTTATTTTTGTTCTTGAGTCCGAAGGTCTTGTCGATGGCAATCTTCT
CTTTCTCTTGTTGTCTCTTTTTGTCGACCGCCTTCGGTGGCATGTTGGCTCAGGCGACTG
AACCCCTCTGTGAGAAGGCCTTGCCAGGGGCTCAAGCGCCAGATCGCTGGCGATGCCCCG
CGCGCCAACCCCTGCCAAGGTGGCGTTGCGGCGCGCCCTTGCACCCCTTCAGGCAGCTCC
GACTCTTGAGCCAAAATGGCTACGG
>at01537
CCGTAGCCATTTTGGCTCAAGCCACTTCAAGTCAAGCCCAGCGTTCCAGACAATGGCGGG
CGGCACCGGTGCGCCACGCGCCGTGACGTCAAACCCCGCCCATGGCTGGATGCGGACTAC
GCGGCAGGCTGTCCTGCTCCTGACGTTGCAGCTTTGGCACTGCCTGGGGCTCGTAGAAGA

GCCTGCGCTGGAAGAGGCTTTGCGCGGTGACGATCAGTGCAATGCTACAGGGATTGGCGG
CACATGCCAGGTCGAGATGATGCAGCTGAGGGCTGCCACGATGCAGTGCCCTCAGACACA
GGATGGCGAGATCTGGACTGGAGAGGCAAACGTGAAGAAGGGCTCGAAAAGGTGCCTCCC
TGCGTGGTTCAAGTCCATGCAGGAGGCGGACAAGTACTTCAAAGGGAAGGGTTTCAGGGT
GGAGCTGTTCAAGCTCGCCGAGTTCAACGCGGTTTACAGCTTCGCCACAAAGAACGGCGG
CTTTGACCACGCAGCGAACCAGGTCGCCAAGTACGAGGAGTTCTACGCGGTCAACGGCGT
CCATCCCTACGTGGATGCCATGGTGCTGCCAAATCTTCCGGGTGCGTACCGCCTGTATTC
GTCCCGGAATGAGATTTACAAATCCTACAACGTGTTCTGGAAGTCGTACTACAAGTTCAT
GGTCCGCTGGTCCAAGGTGATGGTCTTCTTCATCACCAAGGAGTGGCTGGAGAGCAAGAA
CACCTTGGAGGAACTGGACTGGGCTCTCGCCATGTTCACGGGAGAGCGGATCATCGTCAC
ATGGATCGGACCGGAAGTCCGGAAACGGGTAGGCAAGTGGACGGATATGTTGAACTGGAA
CAGCCCCTTCTGGATTAGGAGGAGGCTTCTCTGCAGATTTGGTTCGAAGGTGGTCCAGTT
CGTGGAGCAGAACAGCCTGGACGTGAGCTTCAACAATCCGACCAAGGGAGCCATCGAGGC
TTGGCTTCGTGAGAGGGGCCGCCCTCGACTCCCTCGAGG
>at01538
TTTTTTTTTTTTTTTTTGGCAACTTGTGGACCCAGGGACGGTGCACACCGACATGGTCG
GACCAACGNGAGCAGCACATGGTCACCACCTCGAGAACGAGGCCAGCCCTGCAAGAGCGC
CAGTCTGTGCTCAAAAGCTCCGGCCTGTACAAGGCAGCATCGGAAAAACTCTCGCAGACG
ACGTGGGCACAGCCCGAAGAGCGGTCATTGCTGCTGGTCACCGTCAGGTGCAGTCATCAA
CACCCGCCGGGGACCTGGCAGCAGCATCCCACGGACCCTGGCGAGCCACACCTCCAGAGG
AATGCACTCGGTGTCCTGGAGGCGGATTCCCAAAAGCTGGAGGAAGCGCTTCTGTGCTCT
GAAGCGCGTGCGGTCGGACGCCCATCCATTTCGGCCGTCCCAAGAGAATACCCAGATACG
ACCGTGCCATTGCGGCGCAAATGCGGAGTCCATATCACAGTGAACGTGCGCGTCGCCCGC
GCCGCTAGCCGTGGCGTATCGGTGCCGGGCTGGCTGCATATCGCGGGAGGGCTGCTCCGC
AAGGTCGTGAGACGCCGGCAGCGGATGAGTCACGAGGCAGGACGCCTTGCCCTCGTGCC
>at01539
AAGCCGTGCGTATTCTTTCACAAGGAGGGTTGCGTGAACGGCTGGACTTGTCTCTACTGT
CACTTGTGCAAGCCTGGGGAGAAGAACCGCCGTATTCAGGAGAGGAGGAAGAGACAGAAG
CTCGTACGCAATTTACACCGGGCCTGGAAACAATCGTGCAGCCACAGCAGCGACTGACCA
GGATTTGAGCATTGTTGTCTCTGGGGTCGCGTCTTGAGCGCCAGGCGCCAGTCCCCAGCT
TCACCAAGCACAGCGGTAAAGCTTGCGTGTTGCCAGACGCCCTCGTTGGCATAGGATGCG
GCTGATTTCTGCAGTGCACGGAGACAATTGTCCCAATTTTTCGGCGACTTAGGAACGTCT
AGGCGTATGTTGAAAACAGTCTCGGCGAAGCTGTGGCCCCCCGTGTCACAGCGCCAGCAG
TAGTATGGCCAGGACATG
>at01540
TTTTTTTTTTTTTTTGTTCATGTAACTCGAATCGACGCATCTTCATCTGCCGCAATGTCA
GTATGTTGACAAGCCATGACCCTGGGCCAGCCATTGTCCTGCACAATCGCCGGCGGCTGC
AAGCTCGTCACTCCAAGCCCAAGATCCTCTACCAGCCGCTCACCTGCGCTTGCAGCAGCA
CGCTTCTGGGCGAGAATATCCTTTGCGGCTGTCTGATTCGCTTCGTTGAGGTGCGTTGCA
TTCCTCACGGCAAAAAATGCCGACCTCCCGCAGCATTGGCAGCTCATGGTCTGAGTAGCA
ATGCGATAACAGCACACGGAGGCCCCCCGCCAATCGCAGAAAGTCCTGGGCTCGTGGTTG
CTCAAAGAGGATGTTGCCACACAAACGTATCTGATCCACAAGGAGGCATGCGCGCGCAGC
TTCAGCAGCTCTTGGTGAAGCTTCTAAGCGCTGTGGTGCCTCGGACTGCACCTCTCCAAA
CCGCAATCGATGGAGCGCATCTATGAATGCAATGTTGCCTGTCAGGTACACCCCTATTAG
TCGTGCAGGAAAGGCAAACCCGTCAACTCGAGGCACTGCAGCAAGTTCTACTGCTGCCTC
CAGCACTTCTTGGAATAGCCGCTTGGGAGGAGCATCACTTTGAACATCATTGGCTCCAGG
TCCATCACATGGCTCGGAACCCTCGTCATTCGCCTCCCCCGGCGTCTCTCGAGGTTTTTC
AATCACCTGATCTTCATCACGCATAGCCGCAAGAGAAAGTAAGTCCAACTCACTGGCTGC
GACACCAAAGCGCAGCACAAGCCTCGGCAGATGCTGGTTGAGGGTTCCACTCAAGACCCC
ATTCATTTGCAAGCAGTGCGAGAGACTGAGCCATCTCTGCCGCGGCCAGCTCCACAAATT
CTTCATCTCTGAGGAGGCACTGGGCAAGTTCTACAGATGCAGTCCGGGAGCCTGAGTCTT
CGCCACCTGGA
>at01541
TTTTTTTTTTTTTTTTTCAACCCAACGGCTGCTGCGCTGCTATGCTCAACGGGAGCCTC
CTGTGCCCCTCTAAAATGCAAAACCGAGTCCGGACTGTGAGCGGTCAGCGGAACGCCGAC

ATCAAGTCCTTGGGCTTCATGGCCTTCGAGCGCACCGAGAAATGCAACGGGAGCCAGTAG
CCTGGCGAGGGGTATGGCATGTTGATCTGGATGCCTCCCAGCAGCACGAGGTTGCCGGGG
CCGTAGTCCGTGTTGACAATGGACAACATCTCCTCCTCCACAGCCTGGTACGCCTTCATG
ACGAGGTCGACCATGACCTGGTCGGAACTGGACACCTCCGGGATGTACGGCTTCAGCTTC
TCGCGCAGCCAGCTCTGCTCCATGTCGGAGGGGTCCGCGCGCATGTTCGCGCCGGAGGTG
ATCTGGTTGTACGCGGCTATGACCGCCCCGCATGACGTGGAGGCGTCGGCCTGACCAGTG
CGCAGGAACTTGCCTGCCTCGCCTTCTNTTGGTGAAGCCGATGTGAGGGCCAAAAATGAT
GAGGACATGCCCATTGTCCGGCACGTGGTGCGAGAAGGCCATGAAACCGGTCTTCCCGAC
ATAGGGAGCACCGCCGATGCCACCAAGGGGGAAGGTCCTTCCATAGTACTTTGTCAGGAG
CGTGGAAAGGTGCCCAGGGTCGCCGTTGATCTCATCAGAGCAGATTGACTGGCCATAGAT
GGTGTTGCTCTTCTTGAGGCCGTACCGCTTCACTGTAGAGAAGAGAGTAGACATCACATC
AGAACCCCTCATGGCGCCCGGGAAGGCAGCGACTACTGTATTCTGGACTGTTCCTGATAT
CGGAGCCGAGGAAGGCGGTGGGAACAAGTGGCTTGGGCCGCCGCATCCTCAGCTGGGCCT
GCAGTGGGGCTGCTCTTTGTAAGCAGGTGGATGCACCAGGGTGGCGGAAGCGACCGCAAG
CAGCAGGGAGCCCAGGCGTGCGCGGTGCGCTGGCTCGCTTCGCTGCTGCCGGGCCATTGC
TGGTTTGCAAGAATGCACTGGCAAATGCCCACAAAACCCAAGGATGTCGATCCAAAAATG
CTTGAGCCAAAATGGCTACGG
>at01542
CTGCGTGGCGGCCTTCGCCATTGCGGCTCTCTGCTTGCTGCATGTCGCTACCCCAGACGC
ACCACAGCAGGACCTCTTTGTGGTTGGATCCACACCGATGCTCCGTGCAACAAGCGCCAA
GGGGGCATTTGCAGGCACGCTACAGGCAGAGGTTGTGCCAGCTGTCTCCATGCAGGCACT
CCCAGAGCCACGGCCGAACGACGCGATGCTGCCTGTGGACCTGAACCGCAGCTCCCTCTA
CTGGGCTCTGCTCTGCATCCTCGTGCTGTCGGTCCTCTTCTCCAGCTACTTCTTCAATTG
AGCCGATGGCCTAGAACTGCTGAAGCAGCAGTGGAGTCAGGGTTGTGCAGTGGCTGCCTC
CCATGTCGGGGGTGGTGTCTTGCCGTGTAGGCGCACATTTTCGTCACGACCATGACTCAG
CGATGGGATCTGTGCATCCCTaGAAANAAAANAANNAAAAAAAAAAAAAAAAAAAAAAAA
AN
>at01543
CCGTAGCCATTTTGGCTCAAGATCGTCCATGGCGGCTGCCACGGCGGGGGGTGTGCTTCA
CGGGCGCCATGACGGCGCGCGCGCGATGTCCAAGATCACGCTCAGGTTCCATGACGGGGA
AGTGGAGGCCGCCTACAAAGCAAAGCTTGAAAAGGAGCTGTTTAAGCCCATGCTGTTCTC
CTGCAGCTTTCTGCCGGTCATCACGTTGATCTTTGGTGTGAACAACTACGGTGTCGAGAC
AACGCGGCCGGAGTTGCCCTTCGTGTGGGGCACCGACGACCCACGGACAATGTACTTTGC
CACGCATGTAGCAAACATTTCGA
>at01544
GGCACGAGGCGCACGCGGACATGTCAGGCTCCCACTGCCTGCCGGGCCGGCGCGCGCGCT
CGAGCTCTNNNNNNNNNNNNNNNNNNNNNNNNNNNNAGGCGGTCCTGTGCTTCCGTTTGGCT
CATCGACATCGACAGCACCCGCTGCTGCTCCCACTGCCCGCCGAGCGAGCCCACGAGCGC
GCTGCCTGCGAGCTGCCGGGCGTCCTGCCCTCCTGGGTGGCTGGCGCACTACCAGCGCGA
GGCCGGCAGGGTGCTGGTTCTCGTGCTCGTCGTGCTGCTCCTGCGCTGGCAGGGGGACGT
GGAGCACTTCGAGTCGCACTGCGTCTTCCGCGAGCTGCTCCCACCGCCTTTCTTCAGCGA
CCAGCGGATCCGCAACGTGGTACAGGGCCTCCTCTCGCACCTACGTGCCGCGTACTTCGA
GGACTGGGTCTCCCTGCCCAATGCCTCCGGCGCGCTGATGGACGCCTTCCTCGCAGTCCG
TGACGGCCACGGCGCCTGGCCCCCCAGGATCCTGTCCTTCATGCAGGAGGCTCGCGAGGT
GCTGAGCTGAGGGCGGCCCCCGTGAGGCAGTGAGGGCAGAGGCACCGGCACTGGAGGTCC
AGCTCGAGAATGCAAAAAAAAAAAAAAAAAA
>at01545
CGTATTGCTGAGGACCACGAGGTGGTCAAGCAATGCTCATGGCACCTGCCATAAAGCTGG
ATCTGTAGTCCAAAAGCAGATGTGGCCTGGCCCTGAGTAGTTCACACCAATCCGGACACC
CACGTCATACTCCCACAATTCGAGGCCCCAGGGTGCATACACTATCaAGAACCAGTACAT
CGAACTCATGCAGCTTCACCCTGTTAAAGCTGAGCTTCCAACGCACAGTGGACCTATCCC
ATCTGATTCGCGAAGACTTGGCCTCGACGCGGAGCCAAGTCTCATTGCATTTACGCTGCC
AGTCATGGGCCTCAGAGTGTCGTCCACGCCAATTTCCATTCATGTCCTTACCTGGAGCCG
GGTCGCCAGTGAGGTATGCTGTGATCTGGTCATCATGACGCCTTGCGATCTTCTCAAACA
TCTTGCCTCGTCGTGG

>at01546
GCGCGGCCCCAGCGAGGACCGTGGCTCCGAGGCAGGGACCCCGGAGGTGCAACGCTCCGC
CGAGCAGCAGGCAGCAGCACCCCATGAGGAGAAGCTGCAGGAGGCCAGCCCCGTCCAGAC
CCAGGAGCAGCGGCTGCTGGGCCTTGAAGAGCAGATGGCCGAGAGGAATGCAGAGCTTCA
CCGGAAGCTCAGAGAGTTGGAGGACCTCCGTCACTGGATGACAGAACAGAGGGCACTCGC
GTCGACGCCACAGCGCCATGAGGACAGCGTTCATCAGCCATCCGCCTCACCAGCATCTCC
TCCGCCATCAGGGGCCCCGAGGAGCCCGGTCACGCCCCCCTCGACGCCCCCAGCGGATGT
GCCCAGGCAGGA
>at01547
CGCGAAGGGCGAGCGCTCCATGATGGCATACCAGTCCCAGGGCACCGAGCCGGTGCTGGG
ATGCAACAGCGACGGCGTGCTCTTTGTGGCAAAGGGCGGGAAAGTCTTGCGCAACGAGGG
GCCACCCAGGCCACAGGCCCCCCAAGATGTTGCTGGCGAAAGCGGCGCGCCAGTGAAGGC
CGCGATCGAGCAGCCACGCAGTAGAGAAAGCGGCATGGCGGAACTTGGTTGCCTTCCCAG
GGCAGGAGCGTGCATCTGGGGCGCTCTCTTTGGTCTTCGGAGAGCAAAGGTTCTCGGCGC
AGACCACTCCTTCCATTGAGCTTTTCGGGCTGCCCATGCTCGCAAATCCCTGTCAGCGTT
TTCCCCTCCCTTTTTTCGCCACTCCGGGCCTTGCTGAGGTTTGCGAGCTCTGCGCCCCCA
CCCCCAGTGGTCTTGTGGGTCATCTCCTAAGCTCATGAGTTGGATGGGTGACGACAATT
>at01548
CCGTTAGCCTATTTTTGGCTCAAGGCTGTGCTCTCGCAGTCTGTTTACTCAACTCTGGCA
CATGCCTCGTTTCAAAGTCTTGATGCAGAGTGAGGCACTGCTAGTCTGACAGTTCAGGCA
CCTGGTGGGATTCAGGCACCTGGTGGGAAAGCGAAGACGCTCTGAGCAGCTGACTAGACC
ACGCTGATGGCAGAACTTGCAGCAGGTGGTGATCTGGTAAGATTTACTGTTGGTCAGTGC
TGG
>at01549
GTTCGAGCGCAGGGGACTGCGCGGGGGGTGGTGCCGAGCCCGGCGGCGCAGCTTTTGGGC
AAGGCCAAGGGCGGTGCGCCCTTCTCGCGATGGGCAGGAGTGGCTGGAGGCTCGAGAGGT
CATGGCCCTGAAGCCCGGGCCCAGCCGCTGGGTCGACATGCGGGAGGCGGGGGGTGCAGC
GCCGATCCTGGAGCCCAGCGCCGCGGCCTGCGCGCCTCGGGAGTTCGACATCTCGGGCGG
CGACTGCGCAGGTGAGGGCTTTTACTCGAGGAACATGGGGG
>at01550
TTTTTTTTTTTTTTTTGTCAACCAAGGCGCTGGATAGTGGACTCCCCGGCCAGAAACATG
CACTTGTGTGTCAACCCAGACGCTGGATTATGTGGACCCCCCTGCTGGAAGTCACGATGT
CACTGAACTCGTTCATGTCCAGGGCGAAGGCGGCCTCGTCCAGGCTCGCCTCCTGCTCGC
CCTTGGCCACCCAGCCCAGGTGCCCCGTCATGTTGCCCGGCTGGTCCGCGCTCTGGCAGT
CGGAGAGCTCCCGGCAGAGCTTGGAGAAGGAGCTCGGGGCGGCCTGCAGCTTCTCGAGCG
CGGCGAGTGCCGCCGCCTCCGCCTCCCCCGGCCCCCTGGCCGCACCCTCGCGCCGCGCCG
CGGGGTCGGCGCCCCTGAGCTGCGGGTGCCTGAAGAGGATGTGCCGCAGGAAGATGGAGC
CCTCCTGCTGCTTGCCGCCCGCCGCGGTCCTCTGCCTCTTCGCCGCCGGCCCCTGCTCCT
CCTTCCTCCTCTTGGCCTCGAAGCCCACGCAGACGGCGCCGACGGAGGGCGCCGAGGCGT
CCGCGTCCACGCACCAGCGCAGGGCGGCGGCCTTGGGGCAGCGCTTCAGCTTGATGGTGG
TGGCGCCGAGCCTCTCGTTCGGGAGCGAGGTGGTCGCGCCGCTCAGCATCGCGAGGCGAC
AGTTGGACGTCCGAGGCACGTGCCAGGAAGTGCTCTCCGGCTCCATGCGCACTATGGACG
>at01551
CCGTAGCCATTTTGGCTCAAGCCACTTCGGCCGAAGCAAGCCGCACAACCCATCCAAGCA
GGCAGCCGCGCGCCGTCCGGCCCCAGCCATGACGACTCACCTCGGCATCCCCCGCGTCCC
GCCTCCCGGCTTCCGGGACCGGGGCGAGGTCATGCGGCACCCGGACTCCAAGCCTGCGCG
CCGCGGTGGGCCG
>at01552
CCGTAGCCATTTTGGCTCAAGCCTCAGGCCCAGGTCAGGCACATCCTGTGTGCATCGCAC
ACCCATCATCGTGTAGGGCCAGCCCCCCATGTCCCAGACAGAGAGCTACAAAGTCACCTG
TGCAGTCGCCGTCTCCCTCCTTGCGGCCCAGCTCCTGCTTTTACNGCACACAGCTGTCAG
GCAGAGGTGCTTGCTCACCACGGCCCGCGCCGCCGTGCAAAAGTTCCGCGCAGCAGGCTG
CTTCTTCTTTGGTGCCTCAGTCACGGGGGCCGCCAGCTTATCCAGCGAGATCGAAGAAGC
AGTCCAAGCAAAGGTGCTGGCCAGCCAGCTTTCAGTC
>at01553

TTTTTTTTTTTTTTTTTTACATACACGCGTGCTTCAGTACCCAACAGGCTCCCATCAGGG
AAGCAGCCGTGAGCGGAGACCACTCCAGAACTCGTCGCGGCTTCCTTTGTGACCACGCCT
CTTCCGGCGACGACGGTAGTACTCAGCAGCCATGTGTGGTGCCAGGAGGACGCTGTGCCC
CACAGGCCCCAAAATTGAGGTGTAAGCGAGAGCAACACCCAACAAGGCGGCCAGCTGCAC
AAGCCCATGGAACGGCAGCTGCTCGAGCCGGCGGGCAAGCTCGGCCCACCAGCCCTGGTT
CCAGTAGAAGTGGAAGACGTAGGTTCCGAGTGCAGTAGAGCCCCACCACGAGAGGTCGTA
GGGCAACCATGCCATTGCAAGTGGAAGGAGCACAGCCAGTGCAAGGTTCACTCCGAGCTC
CACCGGGAGCCAGCCCCAGGAGTAGTCCCTGTCATTCGGCCCGTAGTTCAGAGCGATGTC
CGGATAATGAAAGGCTGCCATGGCACAGTTCAGCAGCACGTAACCAGCGAAGGCCGCGAT
AGCCCAAGCTGGACCAAAGCCAGAGCTGATTGAAGTGCGGCGGAAGTGGCGCAAGCACGG
GCGACAACAGCCTCGTGCCAATTC
>at01554
TTTTTTTTTTTTTTTTTTACCTTCCCAAAGAGGAAGGGATCCAGACGATAACCTCAGTCC
GTTCACATGCATACACAGCCTGAGCCTGCTCTGGGTCAGTGGCCTCATTGTGAGACACAT
CCGCCGGCCTCATTGTGAGCACGCGTCCTTTCAAATCTGCAAAATCCACAAAAATCCTGA
GGCAAACGCATGGGCAAAGCCTTCGACTTAGCTGTCCGGATGAAAACGCACGTCTTGTGG
CTTCGTGGCTACCAGGACGGGACCTTCAGAGGCTGCTATCACGGCCCGGATCGCGGTTGT
AGGGCACCCTTTCTTGTGGGCAATCATCTCCAATGCTTCCTCAAATTGCTTCTGCGAGAT
GTGACGGCTGCCTCGAGGGCACACGCGTGCAAAGATAGTGTCGACATCGTGACCACCGAG
CTTGTCGTCATGAAGCCCACAATCTCTGCACATTCGTGCAAACTCTGAACCCTCCAGAGT
GTCATTCCTCCCAGCATACTTCTCATACGGGCCACGCAACTGCTTCCCGGAGACCTCAGC
TTCCTCTGCAACATGATCCCAC
>at01555
GCGCTGCGCTTCGCCGCGCCCTGGCCGCTTCCGCGCGGTGGCCCAGAGATCGGAGCTCCG
ATTCGTGACGCACACCGTCGAGGACCAGCAGCCTTGCGCAGACGTCGTCGTAGGCCTCCG
CCGCAGCCTCGAGAGCAAAGGCCCCCTTGGCCGCGCTGCACATGCCATCCACTTCGAGCC
GGCGCTCATGGATGGCGATGAAGGCACGGAGGAGGCCCTCCTCCGACCGCGCGCCCCCCA
CCAGCGCGTCCCCACTTTCGGGGCTGCCCTGGCCACCTTCATCTTCCTCGCCACTGCTGG
ATGCGAGCTCAACAGACTTGAACTTGCTGTAGTCAATGACCATAGCACGCCATCCAACCC
TGCGTCGTCCTGCGGCGACCCCCTTGCACCCCCAGCGAAAGTGCGGGGCCAGGGTTGGTC
TTGAGGCTTCGGCTTGAGCCAAAATGGCTACGG
>at01556
NTTTTTTTTNTTTTTTTTNTTTTTTTTTTTTTTTTACGTNCGNTCGACATCTCGTTGCAT
GTACACAGATACAAGTCCTGTCTGATGTGCCTACGTGCTGGCCAGTGGCCAGTCCTTGCA
TGCACGCAACGAGCTTGCGCATTTATCCTACGAACAAGTCCAAAGTAGATCAGCGACAGG
TAAAGGCAAAGTTCGTTTGGCCACATCATGGCTTCATAATTGGCCTGTATGAGAATCTAC
CCCGCTGACTTGCCTCTGGGTCGAAGCGTGGACCCCTCCTGCTGTCGGTGGGGTCATACG
GCAGTGCAGCAAGCTGGCTCTTCGTCATCTGTCTCATAGCAATGGAATCGTCAACCTTCA
GGACTTGCCTAAGGGTTGGAACCGCCACGTTCATCTCGTCTTTGTAGCGAGTGATGCTCT
CTGCGTTCATCCATTTGGAAAGCTCGTTGGCCTGCTGCCCTACAAACTTCGGCATTGAGT
CAAACGTGACAGCCTTGCCCTGCTTGTTCATGCCCAGCCACTGGTTTCCAGCCCTGCTGA
TGGCGTTGGTGTCACCGAGGTCCCGGCTCTGCTTCAGGGCATCCTCGAGGCACTGCCTGC
CGTTCCGCTGGCGGTCCTTGGCGGCAGTCATGCGTTTACGGATGTGAATCTCCCCCGGCA
TGCCAATGTTGTCATCGGGCATCCCACCCATTCCGCTCTGCATGCTGAACGTTGGGCTCG
AGCTCGCTCTGACCAGCGCATTGGTCCACATCGGGGCTGTACACAAATGCCCGAGTGCAT
CAACTGTTTTCAGGGAGCAACATGAGCCTCGAGCCTGGACTTGAGCCAAAATGGCTACGG
A
>at01557
GTCTGtAGGATCTCACCAACCTTGCCCGCAATCTGCTTCGGGAGCTGGTCCACGTACTGC
GGCTTGATCATCGCCTTCAGGCGGTCCTCAAGCAGGCCTTTGATGTACGACTGCAGCTCG
CTGCCGCGGAAGTATGCCAAGATCTCCTGCCAGTCGGGCGGCGCGTCAAAcCTGCCCAAG
TTGGGCTTCAGCTTGCCGGCGAGGACCTTCAGGGCCGGTGGACTTGCCGATGCCGTTCGT
GCCCACGAGACCCAGGACCCTGACCCTGGCCGGGGAGT
>at01558
GGCTGGTTCAAATTTCCCAGGAATCGTTGGCACCGGAATTCACGGAGGTGCCTGAGCTCA

TTGACACTGGCTAGTACCACAACTCCATGGGTGCGGCCCAAGTGATCATTGACAGCTTGG
CAGCACAGGACTTTCACGATGCTTTGACAAAACATGACGGTCTGAGGGCCCATGGGCATG
CCTGCCTCAGCCGCATGACTTGAGCGATCCTGCAGGTGCCAAATGCCGACGTCATCGCGC
AACTCATGAGTATCATGACCGCGGCTGTGGACGAGGGCGTCCGTGCCGACGACAGCACAA
AGATTCTGGAGAAGAGTGGGCGCATCAGGGAACGCGGCGGGTATGCAAGCGCTGATCCGG
CCGCTTGCAGTGGAGAGTCCGGCTTGCAGCTATGCGTGTTTTGATGA
>at01559
TTAGGAGTGCGGAGAGAAGCGAGCTGGGTCGTAAGTATTGACGAAGAAGAGGAGGACGGC
TCGGACGAGGAGGACGAGGCCGCGGAGCCGGCCAAGAAGAAGTCCAAGACGTCCTGAGCC
GGAGGCCGTCGCGACGCCGGGGTGAGCGCGCGGCTGCCGGAGTGGGCGGTTTTGGGGGGA
GGCGATCCCACGGGCGCATGCATGCACTGCTGCTGCTGCTGCTCATGGCATACATGGCAT
CAATGCGTTGCCAGGGAGGGTCCTCT
>at01560
GTCTGTGGACGTCGCTGGCGTCCACTTGCTGCAAATATCTCGCGCTCCATCTCCTCAGGC
AAAGTGACCGGCGGATCCCAAAAGTTTGTCATGAGAAATGTCGGCCAATGCTGCCCAACC
ACAGAGAGCTGGCGGGGAGCCTCGGCATCACGGAGCACGAAGCCGCAAGCCGTGTACCGT
TGCATGATCGCTGGTTCATGGAAGGGTTGGCTTTGATCTGCACCATTGGCCTCGAGCAGG
ACCTTTTGGCACTCATCCGCCGTCATGTTCTCTGAGAAGACGGCGGCATCCGCAGTGCCA
TCCCACCGAGGTCCTCGCGGCACTCCAAGATCTCTGCATGCCAGCCGGGCCGCTCTGAAA
ACCTGCGTGGGGCTCGGCCAACGAGGTAGCAGTCGTCGCAGCCGTCACCCAGAGG
>at01561
TTGTTTTTTTTTCTTTTTTTTTTTTTTGTCTGCGCGAACTTCCGCAGGCCCTGCTGCCACT
CGCGCTCGCCCAGGCCCGACGGCGTCCGCACGGTCACCACGCCCCGGCCGCTCTCCTCGC
GGCACGCCGCCGCGACCTTGCCGCAGTGGTCTCCCCAGAGGCAGTCGTCGAAGAAGAGCA
TCTGGTCGTAGGCCACGCCTGTGGCTTCCTTGAGGAGCGGAAAGTGCGTCGCCGCCTTGT
TGGAGGACAGCGGCGGCGACCGGCCGATCTGGTTCACGTCCCGGCCGTCCCAGTCCCGCA
GCAGCAGCTCCCAGACGGTCAGCCCAGGCAGCACCTCGAGCATCGCCAGCGCTGCTCGGC
CCACCTTCACCGCGAAGGGAGTGTCGGCGCTGCTCGCCACCGC
>at01562
GGATGACAGCCAGCTCTGTGCGCTTCATGCTTCTCCAAGTGGCCGCCTTCGAAGTTTGGC
ACGCCGGCAAGCTCCTGTACTCGGGGCTGGAGTCATGGCACCCGCCGTACATTCGCGACG
TTGAGGCCATGCTGCAAGAGCTTGAGCAGCGTGGAGTAGCAGTGAGCAAGCTTGCAAAGG
CGCAGGCCTGCATGGCGAAGGGCGGTGGCTGATGTTAAGGGGCGCTGCCGGCTGAAGGGC
CGAGGGGCGAAGGCCCGCACGGAGGACGGCCAGTGGATGAGGGCACTCCCTTCAGTGTGC
TCGCCACGGCCTGAGTTGTGACGGGCTCACCAGTAGCCACCTCGAGGCACACAGCCATTT
TAAAGGCT
>at01563
TTTTTTTTTTTTTGTTTTCACACGTCGAAGGACCTGCCTCTGCCGCAAAAAATATGCAAG
TGCAATTGCAGGATCTCACAAGGACATGCTTTCCCACTGAGGGAAAGTCTGCCACCGAAG
GTCAGCCAAGCTGGTTATGGATCTGAAAAGGCAGCTGTAATTCCTCCTCTGGGTAAGATG
CACAAGCTGCAGTGCACATACTGCCAGACAAGTGCACAAAGTTAAGCGACGTGTCCAAGT
AAGAAAAATACACACAAAACGCTTCGTTGCGCCCCTGTGCTGCTTCGCTGCGATGTGGTC
ACTCATCGGTCTGGTCACCCACGCTTGGAATGAGCGTCCCACCTGTAACAGCGCGCATTC
CTTCCCTCATGCCCCGAAGCACCGTGCCCGGCATTGCTCGGCACTTGTAGCCCACGCACC
CGGCTGCGCCACCGCCCGCAAAACCGACTGTCGTGCCAGCAGCCCCGCCTATGCACACCC
CGGCACTGCTGCCAACTACTGCACCAACTGGGATCGAGTAGCCCCCTAAAAGGGTTGAAC
AGCGCCGGCACCAGGCCGATCAGCACCCCGGTGGCACCACCAACCAAGGCGCCGCTGGCT
GCTCCTGCAGTGCCGAGAGCCATGGCTCCGGCTCCAGCGCTGGCTGCAGTCACCTGCACA
CACTTGTCCGCCGCAACGTCAGTGACCAC
>at01564
CACATGTGCTAGCTCAAGTTGTGCACTGTGCATGCCCCATCGCTAGGAGTTTACTTGACC
GCCCCATGCTTGGTCCCTCTCTGAGCTGGGATCGCAGGCATTGCGGGCAGCAGAACCGCA
TAGGCTTGCGCGCTACCTGCAGGGCGACTGCGACCCCATCCACGCCCTCTGATCAATGGC
AAGAATGCAGTGCTCCTCCGCTTGCCGCGAAGCGCTCTCCTGCTCAGCG
>at01565

TTTTTTTTTTTTTTTTTTGCGCCCATGGCGAAGCGGCAGACCAGGACAGCCACTCTACAT
CTCGCGCCAGCCATTCCGGAGCCTGCCCTTACCAGCAGAGCTCCCCGGGGCCGTTGCTTT
GCGCGGGGCGTCTGGCAAAATCCCTTCAAGTCGCATCTATATTGCCTCCCATTGAACGCG
AATAGGTTTCCACATGGGCAGGTGAACGTTCTTCAAGTGCTGATCGGAACGGAATGCATC
GTAACAGTAGGAAAGAGCACGGAGGAGGTCGAGTTCCTGGAACGTCAGCAGCAAAATGTG
CACCACGAGGGCTCCCTTGCCTTCCTTGATCTCAGCTTCGCACAAGTCCCGGGACGTCCT
GCGCGCCGCGCGCTTGCCATGAGAGCATGCCCAGGTGCTGATAGCCACCTCAATCCGCAA
TCGCCTGGGACCCT
>at01566
CCCANANGAGGTTGTTTTTTCTTTTTTTTTTTGTTCTGCGGTGAATGTACAGCAGTGTTG
TCAATGCTCGTGCTTGCCAGCTGAGATCAAGTTTTCAGCCAAGAAGCAAGGCATGGGGCC
GCATCAGGAACGCAACTCCGCCCCCACTCCCTCTCACAAACGCCTGCACCAAGCTCATTC
GAAGTCATCCAGCTGGTCGTCATCCATCTCCGCAACGTAGATGTGCAGGTGCGTGGTCTT
CTTGACGTACATGTGCGCCTGACCCTTGGACACAGGCCTCATCCGCTTCATCTTGGGGCC
CTCCAGGGCGTAGCACCGGGAGACGTAGAGGCGCGACTTGTCCATGTTCATGTGGTTCTG
CGCGTTCGCCGCGGCGGACTGCAGGGCCTTGAGCGTGGGCTTGCAGGCCCTCCAGGGCAG
GAACTCCAGCAGCATGAGCGCCTCGCGGTAGGACCTGCCGCCCT
>at01567
TTTTTTTTTTTTTTTTTTGCTGCGAAGCAGGAATTGGTTGGTCACCTTTGTGCTCCCGGA
AGACAATGAGCTCTTGTCAGGCGTCAAGCAAAGCCGCCGCTTCCGCAGCTGCAAAGGGGA
ATGACCCAACCATGGTGATTATGGTTAGGATGCCAATGATAATGCGGGCGATGATCCTGG
TACGCAGCAACCCGCCAAGCCAGGGTGCCATGCATTCGGCGGCAGGCACTCAGACCTACT
TGAGGGCCCACGACGGCTTTGGCTGGTTGGGGGGGGAGTTGCGCACACGGCAGAAGACCT
GGCCAGTCTTCACGGGCTGGATGTAGATGTCGTCGACCATGAACGGCAGCTTGGNNNNNN
NNNNNNNNNNNNNNNNNNNNNNCGCGCGGTCAGCCACGGCGTCGTGCCGCTCGAAGCGGG
TCGCGTCTTCCTCATGGCGCCGTGAGGTCTCCGCCGCTGACCAGTTCATGGGGGCCTTGC
CGCTGCGGGGTGGCGCCGTTGGCTCCCGTGCGCCGCGGTAACCGCCGTAGCTCGACATAG
CAACAGTGGTGGGCAGGACAGGGCCAATTGACCTGTGTTCTGTGGTGCGGCTCCTCGTGC
CGAATTCTTG
>at01568
TTTTTTTTTTTTTTTTTTGCCCTCTGCTTGAGCTGACACGTTGCCTGCGTTTCGCCAAGG
AGTCAGCTGCGGGAGCGCGCGACTGCCTCCCGCTCAGCTTGGAGAGAGAGCAAGGGCTTC
TGCAGCTGGGGTGGGCACCTCAGGTTGTAGAGCTTGATCTGAGCGTTGATGGCCTGGCAC
TCGGCCTCCGCACCCTCGCGACCGTCGCGAAGCCACGTCCGCAGCGCCTCCATGCGCGCG
CGCAGGCGCTGCTCCTCCTCGAGCCACTCCGGCAGCACGCCGGCGTTCTTCATGACCTTC
GCGGCCAAGGCCTCCTCGCCCGTGGAGAGACGGCCGAGCGTCCAGGTCGATGGGCCGGCC
TCGGCCAGCCAGGTTCTCGAAGGCCCCCTCCTGGGCGGCCTGCTGGATGGCGCGCTCAGC
CATGGCCTCGAACGCGGAGGTCTCATTGAGGCCGGCGATGCCGGCGTCCAGGCCTCCGGT
CTGGGAGGACATGATGTTCGAGTCGCTGAGGTCGTCCGCCATCATCTCCAACGGTGTGCG
CGGCCTCGGCCTCGGGGGCCGCGAGCCCGAGGAGTGGTCGCTGGATGCCAAGCATCTGCG
AACAATCTCCACGTACGGCCTGCAGACCTGGGTCCTGCGCAAGAGGAGGCGGTGCAAGGC
CGGGGCGGCCTCGTGCC
>at01569
TTTTTTTTTTTTTTCTTTGTTTCGCACGCCTCAGCGTGCATGCACATCGGCCCTCTGAAC
AGCATGGCTTCTGCTTCCCTGAATCGAAGCGGCGGGAGTGTGGCTGGCCTACTATGCAGT
CACTGCCTTTGTCTGGCTGAGGAAGTCCGTGTGTTCCTGGAATGGGTGCCTGACGAAGTA
GGTTGGCTTCCACAAGTCTGGCGTGAGGTAGGCGTAGGTCACGCGCACCGCTTCGAAGGT
CGCCTTCATGAAGTTGCCCTTGGTGCGCGTGTGCCCGCACGTGCAGGAGAAGCAGTCGGA
GATGCCGGCAAACTGCATCATCTTCTTGAGCACAGGCGAGCCCACGATGCCGCTTCCACG
CGGTGCAGGGACAAGGCGCACTCGCACAGAGCCGCACTTGCCGTGAACCTTCATGGGCAC
GGTGTGGGGCATCCCGATCTTGTTGCCCCAGTAGCCACGGCGCACGGGGATGAGGGCCAT
CTTGGCCGCGATGATGCCGCCGCGGATGGCCGTGGCCACCTCCTTAGCGCACTTCGAGCC
CAGGCCGATGTGGCCGTTCTGGTCGCCCACGAGGACGTAGCACACGAAGCGGTTCCGCTG
GCCGGCGGAGGTCA
>at01570

CTGCACGTGGCACCACGTGCTTTGAGGAGTTGCAGCACCGCCAAGCAACCAGCACGACGG
CCTTGAGTAGGAGACTTCACGGCCCACCACTCCGCCTCGTCACGAGGCTGTTCGCCCTGC
TTGTCAGCTACGTTGGGATCGATGCCCAAGGACAGAAGCAGCTTCACGGCGGACATTGCC
CCTGCCCTGGATGCCCAATGCAATGCCGTCTTACCGCCACGGTCAGTATCATGAAGCTGA
GCACGCCTGGTAAGCAGTTTTTGCATGCGCGGCACATCGTCATCTTCAGCGGCGTGCCGC
AATGTTCGCGACAAGTCGTCT
>at01571
TTGTTTTTTTTCTTTTTTTTTTTTCTTTGCTACTGGCGGCCTGCGCATCGCCCCCCGCCT
CCGCGGCCTCCTCGTCCTCGTCCAGCTCCTCGACCTCGTCCTTGACCTGTTCTTCTGCCA
TGACTGCTTTGAGTTGCGCTTTTCTGCTGTGGTCACCTTGGAGTCCTTTCAGCAGCCGGC
AAGGCTCATGTCTATACCCGCCGTCGGTCCGGGCGCTGGGGCTCGGGGGGTGGGCGCGGG
GCCTGCGCCTTGAGCCAAAATGGCTACGG
>at01572
TTTTTTTTTTTTTTTTTTGATAACACTGCAAACATACACCAGCCATGGCAGTGGCTCCAC
AATCCCCCTACGTGTCTCCCGCATCTGAGTGCGTAGAAATGTAGAAACAGGACTAGATGA
AGAGAATGGTGGGGAAGCCATCCCCGTCCGCATCGCATAGCTCCACAAACGTGAAGCTGC
ACGACTGACCAAGCGCATCCACGTCGACGCTTTGCCACCTGTACACATTTGCGGCACCCT
TGCTCGTTCATGCCCTTGAACCTGCCAGGCCGTCTCCACTCAAGCGCCGGTGACACTGGT
GAATGAGATGGTGCCGCCATGGGGACGAAGATGCAGCCACGAGAGGTCAAGCGGAGCTCG
ATGAGATCAGGCAAGGCGTCGTCCCCGAGAAGCAGCCGAACAGGTTCCAAGTGCGATGAA
ATGGGAGCTTCAGTGTACAAGCAGGCCGTTCTTATCTTTATATCAAGATAACAAGATCGA
CTGTTTGCGGTTTACAAGAGAGTTCAGATGAGATCGGCTGATCCATCCCAGTCCAGGCCC
CTGCAGGTCCGTCCAGCGAGCCTTTGGAAGCCCACGGATGGATGTCCGACTGCTCTGGTC
CTTGGAGTGCAGCTCGCCATCGGAGAGGGAGCAAGGCAGACGCAACTCTGCAGTCTGCCA
CAGCCGTTGGGGGTTGCTTCANGAGATGCGCTGGCTGGCAGATGCAAGCCGG
>at01573
TCTCGACAGGTGGCTGGAGATCTCAGGGTGTTCACAGCGGGATGGGCTATGGAATATGGC
CGAGTCCATTATGCCGACAGAGGCACGCTAGCCGGTCATTGGGACCACCGGCTCGCTCGC
GCAATTCTTGATGCCTCAGCACGCGGGACGAAGGCCTTGCACGCACAGCATTGGAGCAGG
GGGCGAGCCCGNTCGCGGCCACGGCCGAGGGTGTCTCTGCGCTGCACGTCGCCGCGGCCA
GCGGCAGTGCCGGCGTGCTGCGTCTCTT
>at01574
TTTTTTTTTTTTTTTTTTATAGCCCACACTACATCGAGTTTCTCGTGTCAGAGCAAAAGC
TAATGCGCGGAAGGATCTCACAGGATGCCTTCTCCTTCACTGGAAGTATCAGTAGTGCTG
TCATGGACCTCTTCCCTCGAAGAGCGTCGCTTTGCGCGACCTTTGGCCACCTTGCGGCGC
ATTGAGCACATGACCCAGACCATGGTCGTGGAACTGACAACGGAACACAGCCACACCAGG
GGCACGCTGCGCAGGCCCAGGCTGAGAAACAGTGAGATTAGCACGGGATGGAACAGATAG
AGCAGTAGCCCCATGTGTGCCAAGGTTCGGGGCAGTGGATCACATGCCACCCCACGGCTG
CACCTGTCCACTATGAAGGAAATGACTAGAGCACCGCCAACGTAGAGACAGCGGTCTGTG
GCATGACGAAAGAAGGGATAGGTGAGCATTCCAGCCAAGTCCCAGTTGGTTGAGGGAGCA
AAGACGGTGCCCAGGAGCAGCATGCACGGCCAAGCGCGGCTGAGTGCTGGCTTTGCCTCA
TCTCCTTCTGGCCCATTCCGTGCACGAGTCATCAGCCTCGTGCC
>at01575
TGGTGCGCCCCGCGGTCCACCCAGTCGCCATTGAAGACATAAGCAGGCCCATGCTGGTGA
GGGAAGCCGAAGTCCTGGAGCCAGAGGAGGAGGTCGCGGAACTGGCCGTGCAGATCGCCG
AAAATACGCGTTGGCACCGGCGCATGCTGCAGGGTCGGCTCCTCTTCCAGGAGCTCGTGG
ACGCCGTCGGCCAAGGAGACGATCGCCTCCGGGATGTCCCCATCAGCGTCCTGGAACAAG
GTCTGCAAGGGAGACTTCTTCATGTTGCTGCTGGGTCCAGGCCAGTCCCTGTCCAGCACG
TCGACTTTCTCCCGAGCTACCCGCGCAGCCAGGCCCATGGCTGCATCGACGACGCTCTGC
GCCAGGGATCTCGGTGGCCGGC
>at01576
CCATTTGAGGACCAAAGCTAGGCCCTTTCTCGGGAGAGAGAACAGTTTGCCCCCCCCCCT
TCCAGGCGCTTAATGGTCACCATCCGACCTGCGGACGTAGGTCCATCGCCGAGGACGTGA
TCCAGCACTTCCTCATAGGTGGCAAGACTTCGAAACCCGACTACGGCAGGCTTGGGTGGC
AGCCCCAGGCCCCACGGCTCTGCAGGAGATGGACATCACCACTTTCAACTTGGAGCTTGA

ACGGCTCGTGCAGGACGAAATGCCGGTGGGGATTGCATCGCCGCCAAGCCCCACCTCGGC
TCCGC
>at01577
TTGTTTTTTTTTTCTTTTTTTTTTAGCAAAAGTTCTGATTCACAGCGTGTATCTGAGTTG
CGGATGTGCCTGGACCTGATCATGCAACTCCCGTTCAGGAGTTGGGCTAGGCCTGGCAGC
ATCTTGCGAAGCTGACAAATTCAATTCTCGGATTTGGGAAATCTTGAGCATGACGTGAAG
GTCCCCGTCTTCGTCCTCACACACGTTCCAAAGTGTAAATTGACCGATCCATTTGCACGG
TGGCTCGTCTGGAGCTGGCTGCCTGAAGACCAGGTACCCAAGGTCAGCCGACGTCTCGGT
GACCGCATCAGTCGACAACCTCACACAGGCTTGGCGCATTGATGCCATCAAGTCGCCGGG
TCTCTCCATCCAATGAAGTAGGTTCCTGTGCCCCTGGAATTGGAACATGAGGTGAGCGTG
CTTCGTGCCGCCTCTTGGGACAACTTCACGCACTTGGCCATCCGAGAACGACACCCACAT
CATCAAGCATTCTTCATTGTCGTCGGACTGCAGCGTGTAAGCGGTGCCCTGGTTTGCACT
TCCTGCTCCCGCCATGAGCTTCTTGAGGCACTCATGGATTGACTGACGTTTGCAATGCAA
TTGCAGGTCGTGAAGGGCAAGGCGCATCGGAGCGGTGGAAGAGGAGGCCGCCGGTAGGTC
TTGTGTCAGATCGACGGACAGCTTGCATGTGTACATCCAGCCAGCCCCAGGAGGCCGCAT
CTGCACAGAGCAGGAGCTACGGACAGGCTGATCTGCCAGCCAAGTCCGGACTTTGGCCAA
GTCCGAGGCGTTCAAATACTCGGCGAGGGAGGCCCTGACACTTCCTGGCCTGGCAAGCAT
CGCAAAGTCGCTGGAGCTTTCCAACACCTGGAGGCTTCTGCTGCCCCCGTCGACCAGGAT
GTACGGAGCCTCGCCGTCATCTCCACTGCACTCTGAGGGTGCCCACGCTTGAAGAGAGTA
TGCTGTGCTCTGCCCTGAAGCGCTCTGGCAGGGACGCAAGCTGGGTTTGCTCGGTGCTGG
CCAGCTCCATTCCGTCCGTCGCCTCCGGGTAGCATGGCTGGATCTCATTGTCCTCCCGAA
TGCCGACGAGGTAGCTTGGCTGGCCCTCTGCAGTCGGCACGCAACTGTAGAAGAGCTCGT
CCGGCAGCAGTGAGTTCATGGAGTCCTTCAAGTGTATGTGGCAAAGACCCGCAAATGGCA
ACCATAGTCCCTCTGCATGGGCATCAGGGTGAGTGATTTGCGAGGTCAGTTTGCAGGCAC
TCATGAAGGCGTTGTGCGTCCTCTGACGGCATCAGGTCTGTGAACTGCGAGCCCAATGGA
AGTGGGACTTGGCGCAACACCAGCGAAGACAACTTTCCGGGAGGGCTCGGCCAAGCACAG
CTTTCCATCCATCCTCACGACAGCGTCACACATGCGAGATAGTAGTTGGTCGACAAGAGC
TAAGCCATGGTCAGAACGCTGTGCGCGGACCTTGGCCCTGACCTCCGCAAGCAGATTGCT
GCTCCAAGACCCAGGCCAAGATGCACATCATCGGAGTCACATCCGACTTCCCGGAATGTG
TGTTGCCAGCCCGTTTTCGCTGTGTCGGGATGATACTGAACGACTGAGGGTGGTGCTGGC
AGAAACGATGATCGTTGAGGAGAATGGATAGGTCGTGCGTTGCCCAATATCAAGGCCTGG
AAGGTGCGGCAAGCCTGAACCCACATAATGGTGTAGTCGAACCAGCCGCGACCGGTGACA
GCGAGCTTCCATGACCACAGGACGGACATCAACACATTTAGGAGATCTAGGGCACCAGGG
GTCATGAGTGCGGGAACCCTGACTAAGAGACTAGAGACAACCGCCACGCACAGGAATGGT
ACAGCCTCAGAATCGTAGAGATACCAGTAAACTTCGGTGGGGCTGGCTTCGGCTGGCGTA
TTGAAGGCAGCAATACTGTTGAACAACAGGACTGGAACCATGATCCAAATGAGTTTATGC
AGGTTGATTTGGGCTTGGCGCAAGCGCAGCTTTTCAGCGGATCGCCGGATCGTTGCGGCC
TCCGAATCCTCCATTTCCCTGTGGGACAGAAGACGGATCCACTGCCAAAGCGCCTTGCAA
GGCCTGACAACTAAGGCCCAGATCTTCCTGGTTGTAAGCTGACCGCGGTAGCATAACCCA
AACACCGAAAGGTTCTGCCAGATGAACATAAGGATTTGCACCGAGGCAAGGCACGCATTG
GCAGAGCCACCACCAAGGAGCCGATGTGCATGCCACGCAAGCCCCCAACGATCATAACCT
CATCATGGCACGTACTGGGGCGATCTCCAACAATGCTTGTCCAATTGCACCCACACGGTC
CAAGCTCCGTGGAACGTCAGGGCCACGAAGAATGCAACCGCCAGTAGCAATAACATGCAA
CGCATGGCTGCATGCTTCATCAAAGCACAGGAAAAGACCCCCCACATGAGCAAGAATGCA
CGGGTCGAGTCGGGAAGGCTCGAGCCAAAATGGCTTGAGCCAAAATGGCTACGG
>at01578
TCTGGCTCTGGGGCGTAAGTTCGTCACCTCCTAACCCTCTTCATCAGAAGCTACACTACC
TTCCTGTGGGAGTATCTTATCCAAGAGCTCCAGAGTCTCATCGCTTACTCCGTTGCCGCC
AACGTCAATGTATGTGAGCTTGCTGGTACCAATGTGCGGGATCAAGGCTTGCACGCCAAC
ATCTGAATCTTGTATTATCCAGGTGCAGCTCAGTGAGCTTCACGTCT
>at01579
CTCCATCAATTGTTCTCGGCTTGCACGCCCATCTTGACGTATGGAATCAAAACATTGTCC
CGCGAAAAACATACCTCAGCGTCAAGCACCACAAACCACACTCTGGCTCTCCAGGGTGTG
ATCAATCCGTCTTTACAGATGGACTCTAGGCAGCTCTTCATGCCAGGCACTGGCTGCTCA
AATTGACCCATGGCCCAATTCCTTTGAGATAGTAGTCGGCTTGAAATAGCACTTCACCCA

TCGCTTGTTTCTGCCAATGACTTTGGCTCTATCCGCTTAACGAGCATTGACGACGGA
>at01580
AGCTGTACACAAAAAACGCATATGTACAGTGCTGCCACAGGCTTACGAGGTGGAGTCCCG
GTTCGCAGCCTCACGATTTCTGCCGCGCGGAATGACTGGCGGAAGCGGCTGCTCGGGTCT
AGGGAATGGTTCTTCCTCTCCAGAGTCGTTGAAGAGGAGTGGCATCCACTCAGGATGGTC
GACCAACTGATTCATCACTGGGCTGTTGCGAAGGCGTCGCACATTCTCCTGTAGGCCTTG
AACTCTTGCACGTGTCACCTCCACCACCTTTTTCGAAATCAGGCCGGGGAGGCATTTGCG
AACGTCCACACCATGGAAGTCATTTACGAACTTCTCGCATGCCTCTGCCGTGCGGAAATT
TATGAAGGCATACCCCACGTTGCACTTGTTCTTGAAGTCGATAGGCAAGTACATGAAGTC
GAACTTGCCGTGGAAGTCGACGCAGAGCTGCTTGACGAGCATCTCCCGAGAGTACTTGTT
TGGGATGTTCCTGAGCATGACCGTGGTCTCGGCGGAGGGTGCCTCCTGCCCGCTCTCCTT
ACTCTCGGCCACTGCCGGACCACGCTCGCCGCTGGCAACTGCCAA
>at01581
GGCCGCGGTGCGGGATGCAATGGTGCACTCATTGGGGCGCCTACGTGAAGTACGCGTGGG
GCCACGACGAGCTGCGGCCCGTGTCCCGGTCGTACGAGGACTTGCAGTGCTCCACCGCCT
CCGGGCCGTGTCCCCAGCATTCCCTCGGGCTCACAATCCTGGATGCTATGGACACACTTT
GGATCATGGACATGAAGGCGGAGTGGCAGCAGGCCCTCGACTTCGTGCGGTCAGAGCTGG
ACTTCAAGCG
>at01582
GCTGCAGGGCGGCGAGAGCCGCTACCCCTTCTTGTTGCGGACATGATGTTCTCCGGGCAC
GCCTACTTCTACGCGATCCTCTCCTTGGGCCTTTACGATCTCGTCCGGAAGCACACGGCG
CCACTTGGACTGCTCGCACGCCCGCTCTGCCGCTTCCTTGCGGGGGCGGTGCTGCTGGTG
TTGATGTGTTGCGACGTGGAGTCCGCCATTGCAAATCGGTTCCATTACACCATGGACATT
GTCATTGCGCTGGTCCTGTCTTTGCTTGTTTTTTCGAGCCCTGCAATAGCAGCTGCAACG
GAGTTTTGGGCAGTGGACCTGGATGCAGAGCATAACTTTTTGGAGAGCGTCTGCCCAGGA
>at01583
CGAGCAGCTCCGGAGCAGTTCCCCACCCACAAGCTTTTCCAGCACCTCCACCTCCAATCT
TCAAGATGGCCCGGTCCAACGTTGCGTCGCGGATGGTGTGCGCCCTTGCTGGCATCTTCC
TTGTGCGCCAAGGCGGCCTTCTCTTCTCTGGGGTCAGCACCAGCCCTTCGCTCAGGCAGC
CATCCCTCCGCCAAAGCCTGACTGCCCGTCAGGCCGAGGAGGGCTCCACATCCACCTCGG
ACCGCGTGCAGCTCAAGGTCTTGTCCCCAGAGGGCCTTGGCATCACGGAGGCGGTGTCGG
AGGTCGTGCTGCCAAGCGCCAGCGGCCAGCTGGGCGTTTTGGCAAACCACGCGCCCATGA
TGACTGCACTGGACATCGGCGTGCTCCGCTACAAGCAGGACGGCCAGTGGAAGCCCCTGG
TCGTCATGGGCGGCTTTGCCAGCGTGGATTCCAACCAGCTTTCCATCCTCGTCAACGACT
TCGAGAAGGTCGATGACATCGACTTGGACGAGGCCAAGAAGGACATGGAGGGGGCGACCG
CGGCGCTCGAGAAGGCCGATTCGAAGAAGGCAAAGCTGGACGCCACGCAGAGCGTCAAGA
AGGCCGCGGCGCGGGTGCAGGCGGCCATGTTTGCGGCGAAGAAGTGAAGCTCGAGATGCA
TCCAGAGCAGAATGAGTACTGCAGACGCACGGAGCCGAGACGATTTTTTGAGGCCTGAGC
AAAACATCAAAAAAAAAAAAAAAAAA
>at01584
TGGCCGAGCGCGCTTGCCATGGCCGCGGACAGCAAGTCGAGCAGTAAGCCTCCGCGCCTG
AGCGAGGTTGAGACCGATGCTCTGGGACCGTTgGCTCGAGGCGTGCGAGAGGTACTGCTC
GGTCCACAGCGAGAACCGAGCGCCGGTTCgGGCAGTGAAGTACGCGCTGGAGCAGCGGGA
GTTCCTGGCGCTGCTGGCTGATGCAGTCGCTGCTGCGGTGGCAGCCGCCAGTCCAC
>at01585
TTTTTTTTTTTTTTTTTTACTGCTGCTACTTTTAATTGCTACAGCCATAGGAGCGTGGCA
GCTCTGGAGGCAACGCCTCCGCAAAACAATCCACATCGAGTCCCCAGCTTGGCAACAAAT
CTGCGCCAGGTGAGACGCCAGGAATGCCTCCAGGAGGGAGTGCGAGCAGCGAGGAAGGCA
AATACGACCGTGCAGCGGTCATCTCGCATCCCAGCTGCAGACCCCAAGCGCCAAGCCTCA
GCACAGCATTGCGCCATCCTCCGCGACGCCTTTTCAGTCCCGAGCCACTCAGCCCTAGCA
GTCCTTCACGGGCCCTCCCCGCCTCGGCGTGGCCCGAGCCCCTGCCCACGCGTAGAGTCC
CACCCCCGACGCCCCAACACAGAGCGCCAGGAGGTCGCCTCGTGCC
>at01586
TTGTTTTTTTTTTCTTTTTTTTTTCGTATTCAGAAAGGCGATCAACGCGCTCACAATACC
CAGATGAATTCACCCAGTCCGTCCGCGATCGCATTGGAGATTGCGCAATCAGAAGATGCT

GTGCGGAAATTCTGACCACAATGCCAACGGCATCTTTAAGCAACATGCTCTTTGGCTCTG
CCGATGGCCAAGGCAGGAGCGTAGCCCTTCTCTGAATCTTGATCTGAATTTCAATCCGAC
TTCCTCGACTCTACCTTAACCTGCGCACGCTCTGCTGCATCTTGGAGTGCCTGCAGGCCC
GCGGCTGTCCATTTGTCAGTGCACTTAGGGTTCTTCCAGATCTCCAAGTACTTCAGTGTA
GGCACACACCCGTCCGTGATGACACCGGTGAGAGCGGCGAGGGCTTCGTCACCATAGTCA
TTCCCAGACAGCCTGAGACTCTCGATGCACGGGAGTCGCTTACGACGGCACGCACCAGCA
AGCGCCACAATGGCGTCGTCCGTGACCATAGCGTACATCAAAAGGAGCTCGCGGAGGTGC
TGCCCCGTCTGCGCCGAGAAGTGCTCGAAGAAATCAGCATACTCCTCAGTTGGCATCCCC
TGGCCCCATAAGACGAGCCGCTCGAGGCTTCGGCAGAGGTCGAGCGTCTCGGCAACGCGA
CCACCATCCCCTACTTGAGAAGTATCACTTTCGATGCGCAATTCTTTGACACCGGTGAAG
ACGGCGGTCGCCGTCCTTATGTACAACTTTGTCACCAGGTGATGGTCTGTGCCGTTCGTA
AACTTCTTGGTCGCGAGCAGACCCTCAAACTCCTTCGGCGAGACAGGGAGACGGCGCGAG
CCATCATGCCCTCCATGTATGCCTCCCATGTCGATGATCATGTTCCACCCCCACGGCGCT
GATTGTCTTGGCTTGATCAGCTCTGAGACTGAGCGCTCATAGGTGGTCCAGCCGCGGTCG
TCGTACTGCGCGCAGGTTGTGCCGGGCGGCAATGGCACGATCAGGACATGGACAGCCGCA
TGGCAGTACAACAAGTCCATGGTGCGGAGCGCGTGGTCGAAGACAGTGACCTCATGCTCG
GTGCGCGCTCCGTGCTCGTCCTTTTGATACAAGCTCGGAAAGTCCCAGAAGACACCGATG
TCCTCGTAAAACTCTGACCAACCCTTGCTATTTGTGCCTATACCCCCACCGCTGAATTGC
TTGCCAAGCTCTTCGGCGATTCTGCGCAGTTGAAGCCCATCAGGGTCCGGATGTGTGGCG
GTCAACCAGCAGTATGAGATGGAGACGATTT
>at01587
TAGTTTCCTTATTTTTGGCTCAAGACTAAGTGTTAATCCATTCCCCATTGCTCCTCAAGC
ACAGGGGCCATTCACAGTTGGGGGTCAAAGACTTGCTTGAGTACTCCGGGACCGTTTCCA
AAACGAGGCATGTCGTTCCTGAAGCGAGATCCAGATGTTGTGGTCAAGGGAGGCAGGCTG
GGCGCCTTCGTGGACTGCCTCTCGACAAAGTCTGGC
>at01588
TTTTTTTTTTTTTTTTTCATCAAAACCCCCTGAATNAGAGGCCTCACGTCAGACGAGCC
ATTCTCCATATCCATCCCTTGACAAGGCAGCCAGCNCTGCACTTCACTTCAGAGGCACGC
GGAGCAGAACACCAAACTCGGCGGCCCAGCTGCATGAGACCCTCGCATGCACGCGCCCCT
GGCGGGTGAGGAAGCGCCTTGGCCGTAACTCTGCATGCTCGGGGGCCACGTACACGCCAT
TGTGCCACGTGCCTGGGTGCAGGTAGACGCCGAGCCCCTCAGGCACGAGGAAGGCACGCA
TGTCCTCCGGCCGGATGCCGTCACCTCTAGAGGCTGGACCCAGGCAGACGACAAAGGGCA
CCGGCTCCCGCGGCCAGAAGAGCTGGCCGCCGTCCGGGTGGTAGTCGCTCATCCACAGGA
AGATCGCATCAGCACTGGCAGGTCCCTGCCCCGAGGCCTGCTCGGGCAGGGCGCCCAGGC
CCTCGACGTATTTGTTGTTCTCCGTGGCCACGGCCAGGTTTTCCGCGTGGTAGAAGTCGC
CGCGCCACTGCACCTCGAAGTCTCCCTCGGTGGTGCCGGCCTCGTCGCCTGTGCCTGGGT
CCAGCGGCCGCCAGCCGGGCTGGGGCCAGCGGGTGATCTCGAAGGTGCGGCGGGCGACGG
TGAAGTCTTCAGGGTTGCGGACCAAGAGACCGAGGCCCTTGAGCGCTTCGTCGGTCGCCA
ACTCCCGCTGCACCTCGCGCACCGTGAGGCCTACGGGCAACTTCGGGGCGCGGAAGTTCT
CTGGGGTGAAGAGGTGGGAGGGCGTGGCCCGCCTTGCAGACTCCAGACTGCACGTGCCAG
AGGCAGCTGCGCGCGGGAGATCTAGAGGCAACAAACGTGCGCTCACCCGAGGGTGCCCAA
ACCGGCGTAATGCGTACATTTCAAGGCCGGCTGGGGAGATGTCGCAGCCCTCCCATGCCG
CTTGAGCCAGGTCGCCATGCTTGAGCCAAAATGGCTACGG
>at01589
TTTTTTTTTTTTTTTTTGCCAAACATGCTCTGTTCCGTCTCCGCGTGTCAAAACTCCCA
ATGTCATGAATATCGTCTGCAACCCATGTCTCTGTACAGTATGCGGGCAGGGCTAGTTCA
GCCAAGATACCCATCATCGCCAAAAGCTTATGTGGCATACAAAAAGTGCATGGTCTGATG
AGCGCTGCAGTGAGCAGCTCAGCCAAGCAATGGATGCTCAACGTCAAGAGTGTGCTGACA
GGAGCGGAAGGCAAGCACGCCCCATTGTGTCCCATAACAAGATAGAGCTGCTTGTGGTTA
CGTAGACAGGTGGAGCCGTGCCACCCCTCATGGCTCGGCAAGCAAGTTCACGTATCCCTC
AGGTTCCATCGCCTGACGACGACGACCCTTGCCGACGTAGGCACCGAGCAGGGCAACCAG
CACTGCCAATGCAGCGATGCTTGCGACGGATCTGCCCACGCGAGTCTCCCTAACAAGCTC
CGCTGACCCTTCAACAGCCAGCTCCACGGTGGATACATTGGTGGCCAGAAGAGGCGGTGT
AGCCGTGGCCTGGTCGGCTGAGCCCTGGTCAGTCTTGTTGTTGGCGGAAGCAACAGCCGC
AGTCGTCNCATTGCTCCTCTCGTCAGCAGGTGCAGCCGAGCCTTGCTGTGCCGCCTTGCT

AGCTTTCTTGTCTGCAGGTGCAGCGGAGCTTTCCTGAGCTGACTTGCTAGCCTCCTCATC
TGCANGGCGTTGGGCCTGAATGTC
>at01590
TTTTTTTTTTTTTTTTTTCCCAAGGGTTAGGCGCAGACGCAAAGCCTGTCCAGCTCATCA
AAAAACCGTTTCCAGACTAGAGCTGCTCGCTCTCTCCCAGCCATGGCTAAGCCAGCTGAT
TGGTAACGTCTGCGAAGCCTCAGCCGCTCGCGCTCCCCCACACTTGTCTCCGAANAAGAA
GTACGGCAGGAAGTTTGCAACGTACTCGTCAAACTCGTCCTCGGGCGGCACAAGCACGGA
CTGGTTGTTGATGAGGAGCAGCAGCAAGTAGAAGAGGAAGAGGAGTACGATGCCCGAAAT
GAGCACGATGTAGGTCGGGATGCTGTCCTCCTTGGCGGTGACGCCATACTCGCTGTTCAA
AGGCTTCAAGAGGTCGCTGAGGCCGGTGCGGATGCCATAGCCCTTGGGAAAAGGGGCCCG
CTCGCCGTACACTGGATTGGAGCTCAACGCTGTNGCGGACGCATGCGCGGAGCCCTTGGG
TGCCGCACCGGTCACGAAGGCACGCGGTACAGCCAGCAGGCA
>at01591
CGCTGTGCCACCCACCATGCCCCACTCAATGGGTTTTCAACGATTCATATTTTCTTCTCA
TGACGAGCTGTTCAGCAACTGTCCTGGGAGGTATGGAAGGCAGCCCAGCAGACGGAACTC
AGCCATGCAGCTGGAATGCCACCTGGCCACAACGAGCCAGCAAGGNGAGCACGAGGACGC
GA
>at01592
TTGTTTTTTTTTCTTTTTTTTGTTCCCAGCCACAACTCGGCACTCACACATGGCCTGAAG
CTATTGAGGGACTTACTGCTCTGTACATGGGGATGTCCAACAAAACAAGCTAGAAGCACA
CTACCCCCAGGCATCAGAGGGGAACGTGAGTGAGACAAGCCATGCAGTGACTCCGCTGCG
GCACCCTGAATGATCAGTAGTGGCCTGGACCCTGCGACCGGAAGCCCGCAGCAGACCTCG
GCTGCGCGTCCCCGCCAAGCACGTTCCGCTCCGAAGCCGAGAGCTCGCCGAGGCTGCGGC
CGCCGTTGCCGCGAGCACATTGGTGCATTGCGAAGACGACGGGTATTCCGACAATGGTGT
AGATGAAGATGGTCACGGTCAAGAACGGAGGGGCTTCCGTCCC
>at01593
TTTTTTTTTTTTTTTTGTCAGATCACCTCAGAGATTTACATGGTTTTCCATGCCGGAGAA
GGCGAATGGTCAGGCACCCAAGAGAGCCACCACCCCCAAGGCCAGGCGAGGTGAGACAAG
TGCCTCCCGGCGTTTCTCCAAGTCCACTGAGGGCCTGTCCGTCTCAGGCAGTGCCACTGG
CACTTCCTTGCTTGCATCGTCTTCTGGAGGGCCAGTTGGATTTTGCGGGATGGCCGCTTG
CTGCCGAGGCGCTCCAGGTCCAACACCAGGCTCCACAAGCTCACCGCGCTCCATAAGGGG
CGGGGCTCTGCTCGAAGAAGACGGAGTCGCAGCTGCAGATGCCTGAGGACGGGACTCGCC
AAGAGCTCGGCCGATGGCCTGGTGTTCCGCTACCTTCCAGCAGATGCTGCTTGCGAGGCA
CCACGAGCCAGTTGGTCTGCTGATTGATCTGCCCTGAGGACGGGACTCGACAAGAGCTCG
GCCGATGGCCTGGTGTTCCGCTACCTTCCAGCAGATGCTGCTTGCGAGGCACCACGAGCC
AGTTGGTCTGCTGATTGATCTGCTCTGAGGGTCTGCCGAGTCGCAAGTGCCCTGCCACTT
GATCTCGCTCATGGCACTGCCCAGGCCAAGCTCACCAAGCTGCTTCACAAGCCGCATGCG
GATGCGGCGGGCAACCTGATTATGCAGGGGCAAGTGGTTAACTGGCATAACAGCTCTAGC
CATGTCAGTGACATCCTGCTGAACTGCGACAAGCACCCAGATGTCCTCGCCAGATCTTGT
GTTTTTGGCAATGATCCTCGTGCC
>at01594
TGCGCCCAAAGACACCATGGGTTCAGGCAGGGCCGGTGGGTGCAGATGGAACGCTTGTAC
ATCCAGTTTGACCCATATCTACGTGCAAGTAGCACCTGGTCGAGGTGCAAGTGGGCGTCA
CTGAACGTGAAGATGGCTCCCTTCAGCTGCGGCGGCAGTGCAACAGGACCGCAGCGGAGA
TCTAACTTGCCATGGGCGTCCCAACACTGGTGGCTGGCCTTCTCGCACGTCAAATCCTCG
AGCACGTACCACGTGCATAGGGAAGTCTTGTAGCCAGGCAAGCCCTGGAATGCCTTGGGA
GTCCGTAGCTCCTCCCTGCCATGTGAATCGAAACAATCCTCTGCAGAACAGTGGCCGGAC
GTCAGAAACCTCTCGCACATAGTCATCTTGTAGCCTGACTCATTCGTGACAGGCTCATGG
CTGAAGTAGCATGCATCATCGTACCAACAACAGCCAGACTGGTAGAACGTACACAACTCT
AGAGAAGTCTTGGCGTGCCCAGCTTCCAGTTCCGTTGCCGGAGGCGCCACAAGCAGCTGC
TGCCTCTTCTCTGGCAAACGCCGGGTCTCTGCTCTTCGCTTGCTTCGAAGAGACCGACCT
GCACCGTCGCTGTCAGTGACCCAAGTGCCATGGACAGGAACGGGAGGAGGTTCTATATCC
AAACTCGCGTCCTCCATGAGCCTTGAGCCAAAATGGCTACGG
>at01595
GTCCTGGCTCGCGCACCTATCACATACTGTACCACAGTCTCTCTCGCGTGAGCCTGATGC

TGCTCCGGATGACTGAAGCACTGGATGACGTTTGTCCGCACGGTGCCAACCTCCCTCCAC
ACCTGATACTGCCCTGGCTGTCCTTGCCGTGCAGGGGGGCGGCAAGGACCGTTGGTGCGA
ACTGCAAGTGCAAATGGATGCATGCACGCACTGCACGTGCAGTCTACTGGTTTAAGGCCC
CCTTACTAACTGACTTGACTGCTGGCTGACGGCCGTCATTGCAGCTGGCGGCTCTCTGTA
ACC
>at01596
AGAGGAGTGCCCGCATCGCGGAGGATTTCCATGGGCACTACTTCGTGCGAGGTACCACGG
CGTCGTATATCTCTTGGCTCGCCACGTCCACCCTTCCGATCAAACAAGTGCAGAGCTTCC
TTCAGTATAAGCAGTTGAACCCTGCCAAAGATACCGTAGTGCTTTGGATGGGCTCTGCGA
TCTGGGGGTGGTTCGGCGGCGATGTGCGATGGGTCCAGCCAGAGACGCGGGAGGCCATGC
TGATGGATGCCCTGACGAAGCGAGCTCGGCACTTCCAGGGTGCGCCTCATCTGGGACTCG
CCGAGCTACATCGACTTGCCGCTCAT
>at01597
CTTGAGGATGGGGTCGATGTTGCCAAGGACGGGCGGGAGGTTCATGTCCAGGCCTCGCAC
GACCACCAGATTCAGCTCTGGGGACGCCTTGCCCTGCAAGTACATCTGGGTCCTCTGGGT
CGGGTAGGAGATGGTCGCCCGAAGCTCCAGGTCGTTCTCGCAGACGCACACGCTTCCGTG
CTCTGCCAGCAGGTTGTAGAAGTCCTGATTGTAGAGCGCGTGCTCCTTCTCCGTCTCGGC
GACCAGGTTGTCCTCGATGGCCCACTTGTGGTTGTTGAAGATGTACAAGGTGACGAGGGA
CTGCGCGTGGTAGCCCTGACGAGGTGCAGCGCCAGCTCCACGCCGCCGCTCAGACGGCAG
GCGCCGTCGCCGATGAGAGTGTAGACGCCGCTCCTCGCACAGGGCTTCGACGGCCACGAC
CTCGGTTCAGCTTCGAGATGATGT
>at01598
GCGGTACGAACTCCGCGGGGCCGCTCCATCGTGCCACCTTGCCTCCCAACACAGGCTCAT
GGCTGCACAGCGCGCGGGGAGGCCTTTCCCTCAAATGTGGGGCCCTCTACGGCCTTCTTG
GCCTCTTCGCGTCTGTAAGGGGGTGGCCTGCGTGGCCGGCACCGCGAGCGCCCGCGCGGC
CACCTCGTCGGTCACAGCTGAAA
>at01599
CACGCTGCGGGGATGAGCTGGCTGGGTCACTCCGTGCCCTTGCGCCGCAAGGGAACGTCG
CAGACCGTGGAGGGAGCAGCCGCCCCAGACTGTGCTCAAGCTCTGGTGGCACTGCGCGCC
CTGCCGGAGTGTGCCGTTGCCTGCCGCCGCCAGGGCGCGCCCTGCAAGCCAGACTAGTCC
TGCCAAGCGCCACAACTGCCAGAATGCCTTCTCCGGCGATCCCTGCGTGAGCATCCGAGC
TCATGCCTAGATGCCGGGAGGTGCAAGGCCTTCGTTCTCCAGTGAGCCCCAACTTGCGCC
AAACGTGTCTCCATCAGATGGCGATGCAACAACCACGTGGAGCCAGTGGCGGAGCTGGGG
GTGCTCTGTTCCTCCGCTGATACAC
>at01600
CCGTAGCCATTTTGGCTCAAGACATGTTGGCTCAAGCCATTACGTGCCCAGGATCATCGG
CATTGTCACCCCAGCAATCCGAATGGGCAGCGCTGCTTCTGCGTTGCTGAGACAGTTCTT
GGACGAGTGACCGCATGTTTGACAAGGCCGCACACGAGTTTGCCTCTGTGAACTCAAGCA
TGTCTGACTCTGACGAAGAAGCTACCATGACCAGCTTAAGCATGCTGCTGCCTCAAAGNG
GCGTTCGAG
>at01601
CCCTTGTGACAGCCCTCTGCTCCGGCTCCCCTCCACAGCAGCGATGGCCTTATCAGTGGC
GCAGTGCGAGCAGAGCCGCAGCCGGTCGCCTCAGCAGCGAGTTGGCTCATGCAATGGTCA
TGGCAAAGTCAGTCTGGCGTGGCAACACACAGCGGTCCAGTGGAAGACTCGTGAGGGCGC
TTGCGGCTGGGTCCGCCTCGCGCACCGCTGGCACGAGCAGCCATCTGAGGAGTGCCTTCA
CTCCGAGCCGTTGACGCTGGTCTGCATCCACGGCTCCCTCTGCGATGGCACTTGGTTCGA
CGCCCTGGAGCCACGCGCAGACTGCAGTGACCCACCGCTGCCCGGCTGCCATTGCCTCGT
TGTGGACCTGCCCGGCCATGGAGCTTCAGAGCGTGTCTC
>at01602
CTCCCTTGGCTGAGGCTTGCACCACGTATTTGTCAGTCTGTGTGTCCGGACAGAATGTTG
GAAAGCTGCTGCAGGTTGCGGAGCAGCATGGGCTGGTCAGTCTCAAGTTGGCCTTGGTTT
ACTACGCCACATCAACAAGCAAACGCCTTGATACTTTGACAGACTCGGATGAGTATGCCT
CATTCTCGGCCGAGCTAATTCGCGAAATAATGTGTCATCGAGTGAGGCCGATTCCAAGCG
AATCTGGACAAAAGCGGTGCTTTTCCACTCTGGCTTCAATGGGGCAATGTTCAACGTGAG
CTTCCTGATGATACCAAGTTTGAGTTACTTTCAGCAGACACTTTGCGAAGGGCGTGCTTT

GAA
>at01603
CCCGCTCCTCGCACGTCCAACTGCTGGGCCACCCGAAATCGATGTAGCCTTGCGGCCAAA
CTTCTTTGCCGTAGTTGCAGTTCACGTGATCTGCCACAATGCGCTCCCTTCGCTTCGCTT
CGCAGCATACGGTGGTCCCCGCCGCCTTCTTGATGTGGACCCACACCACCGGCTTTCGGC
CAGGAAGGGGCGTGTACGTCGCGTCGGTGGTGGGGTTGAGCAATT
>at01604
TCNTACTCAGTACGACTTCATGCACGGCACTTGCTCTACTCGGCTCCTCTGTCCACAGAC
CTGCTGGAGCAGGTATCCACGGAGCTGATCTTCTCCAGGTGGCTGCTTCAGCTCTCTGCA
ACTGTCGGGACCTTCGTCTTGGGTGTGGCTCTTGGCCGCGTCTACGAGCGGAAGAGATCG
GCACCCTCTACGACCGGCTACATCACGCTTGCATGAGGCAGGATTGCTGAGCTCTCGCGG
AGTGATCGATCACTGATGACGTGCCATGACATGTCTTGCTTTTGCGCTTTTGTTTGCATG
TTTGTCGTTTCTTGTCTGGTGCATGGTGAGTTCCGGTAGCAACGATCATCAGTTGGTACA
CCAAGCTCCTGAACTCTTGGCTTGCGTGAGGCAAGCCTGGAGATTTACCCCACCTCTGTG
CCACATCGTGCTCTTGCAGCCGTTTTCCAGCCGGAGTGCAGACAAGTCTAGGTTGCCATG
TGCTTATCGCCCAGCCGGAGTGCAGACAAGTCTAGGTTGCCATGTGCTTATCGCGCATCC
TTTGTCCCGCATGCTTGCGGTTGAGACTAGAATCTTACTCCTCGTGTTCAGTGCACGCTA
AAAAAAAAAAAAAAAAA
>at01605
GAGCAATGCCACTGTGGTCGCGAAGGAGTTCGGTCTAAAAAGTTCCCATTTGACCATACA
CGCCATGCAGGTTCCCAGTGGGAGCGTGTGATAGTACTCCGACTGCAACCGCGCCAGTGG
CTCCCGAAGAAAGACCACAAACTTCGGCATCTTGCCCCCAGCCTCGCCGTGGAACTGCCG
GATCAGCTTTGCAGTATCGAAGGTCTCCGGATGGTCGCCTTGCGCTGCATACCCCAGGTC
CTGGGACCAAGCAAAGTCCGAGGGCAGTGGAATGGTAAAACAGGTTGACGACTGAGAAGT
CGCCCATCACGGCGGGCATAACCCCACAGTCCGGCAGGCTCTCCAGCCACATGGCCTGTT
TTTTCGCGGCTGGCAATTCGTTGTACTCTTTGAAGAACATCC
>at01606
GAACCTGTCACAATGGACGCACCATCCAAGGAAAAGGATGCTGACATCACGGCGCTCTCA
TGGCCTGCAAGGTCAGCACGCACTCCGCACTCTTCGCGTTCCAGATCTTTGCGACGCCGT
CGTCGGAGGCAGTGGCAACAAGGCTCCCATCAGGCGAGACGGTAGATGCGTTCACTCGGT
GAAGCTCCTTGATTGTCAGGATGCACTGGCCGCTATCCACGTCCCACACCTTTGCAAGCC
GGTCGTGCGCACTGCTGATGACCAGACCGCCCCGCCGGCACATGACATAGACGATTGCAC
CGTCCTGGATCCCAGCTGAGGCGAGTGTCTCCTTGGCCTGCAGAGTCTGCGCACCCAGCA
GCAGCACGATGGCCGTAGTGGACATTCCCGCTGCTCCGCTGCAGCCTGTTGCAGCTGGCC
GACAGTGTCAGACTGTCGGCTCTTCACCTTGG
>at01607
TGACACTCAATGTTGCTGCGCACAACAGCCTCATGAGCGCCTGCAGGGATAACCAGGAAT
GGCAGCGTACCTTGGTGCTTGTCTCAGAAATGCAGCATGATGCTCTCCGCCCAGACGGTA
TCACATGTCTCTTGGCCATCAGTGCCTGTGACGCGTCCCAGCAATGGATGAAGGCCGTAA
GCTTAATGATGACAACGCACGGCAGCCTGCTCAAGTCGAACCCGATCTTGCGCAACACGC
TGATGAGTGCTTGCGTGACGAGCGGGCAGTGGGAGAAGGCCTTCCTACTCACAGGATTGC
AATCTTTTCAGAATCTCCGGCCAGACGCCATCATGTACAACGTAATGGTCGACGCTTGTG
ATCGAGGAAGACTATGGAGGCCTGCTTGCAA
>at01608
TTTTTTTTTTTTTTTTTGCCAAGCGGACGGCTGGTCAACCTCGCAACCGTCCCCGGACG
TCAAGGAGCGGCCGCCTCTCCGCGGGCCGCCCGCGCGAGCCTCGAGGCCTCACAAGGCGA
AGGCGCCGGGGCAGGGCGCGACGGCGAAAAACTCGCGCTCCAGGCCGCCGCCCTTCTCCG
GGAACTCGGCCTCGTACGCCTGCCGCCAGGCGCGGCAGAAGGCCTCCGCCTCCGCCGCGC
GCACGAGCGCCCAGCAGGAGCCCCCGAAGCCGGCGCCGAAGGCCGAGGCGGCCAGCGCCG
TGGTGCCCGGTTCCCGGGGCAGGCCGGCAAGCGCGGGGCCGCCGATGCTCCCGCGCCGCG
CCCAGCGCGGAAGCCAGGCCGTCTCCGGAACCGTGTTCCCCAGCTCCAGGACCGTGGCCT
CGTGGCTGCTGTCCGCCAGGCGCCCGAGCGCCGCGTAGTCGCGCGCCGCGAAGGCGGTCG
CCATGCCGGGCACGATGGCCTCCGACTCCTCGAAGAACTGCGCGAAGCGCGCCTTGAGTG
TGTCCATGGTGATCTGCTCAGCGTCAAGACCATCCAAGCCT
>at01609

TTCTGGGGCTCTGCGGCTTCATCGCCGCCAGTTCCGTCGTTGCCAGAGTCGCGCTGCGGC
TCGGCACCGTGGACGCTCTCTACTTCAGCATCGTGACGCTCACGACCCTCGGGCTGGGGG
ACATCGTCCCCCGCACGGCCGGCGAGAAGTGGGTCATCTCCGCCCTGTGCCTCGTTGGGG
TGCCAACTCTTCGGAAGCGCTCTGGCTAGCCTTGTCAGCCTGGCCGGCAGCCACCACCAC
ATCCATCGCATCCACGGCGGGCTCACGGCAAGGAAGCTCATACGCATGCGGCAGTTCGCC
GAGGCCATGTACAAGCGGGGCCTGTACCCGAAGGCCCAAGAGGTCTCCGACGCGCTGGCG
GGCGAGCGGAAGATTACGCCCTTCGAGTTCTGCTGCTTCCTCCTCCTGCAGAACCGCGTC
ATCAGCATGGAGGACATGGACGTGATGTTGCGGAACTTCCGTGAGCTTGACCTGGTAGGA
GATGGAATAAACTTAGTACGTACCAAGGACCTGACATGCGTGGGAGGAGAAGCGGCATCG
AGAGGTGGATGGGCCGTGAGGCTGCGCGGCCGCGAGCCTCTCGACTGCGATCGCGTGCGC
AGCGCTGGCATGCATGGCTGCCTTGTTGGACAAAAAAAAAAAAAAAAAAA
>at01610
TTTTTTTTTTTTTTTTTCGCGGGCAAGGCGCGCCTCGACAAGCGCTCCTCCCATCCCCG
CAAGGGGGTCCCGAGCGTCGAGCGCGGCGGCGAGAGCAAGCGTCGGCGCTGCCGGCGCTC
ACAGCACCTTGCTCTCGCTCTCGCGCTCCAGCTGCCTCATCTGCTCCTCGCGCTGGACCT
TCTTCTCCTCGAACTTAGCGGCGTCCTCCGCCTTCCGGGCCTTGCGCTCCGCCTTGCGCT
TGGCCTGCTTGTCCGCGTTTTCTTTCTTCATGGCCACCTCCATCCTGTACTGTTTCCTCT
CCTTGTGACCCCACTCTTCCACGGCCTCCTTCCGCATCTTCCTCCAGGACTCCTGCAGGT
CATCGCCCATCTTCAGCATGTGGTCGGTCATCGTCCAACGGAAGGCTCATCCAAGTGTCT
TTGGTCGGGGTTGTCGCAGTAGGCTTCTCTCGGGACTGGGTCGTTCGCGGGCGACTGCTG
CAGGAGGCACACAGCATCGTTGACCGCATGCGCGAAGGCGGCAAAGTAGCTGATGCAGCA
AGCACTGAGGAATTTCATCGCCGGTCCTCCTGCGTGCAAACTGCTTGGCCCTGCCAGGCA
AGGGAGTTGNNNTAAGGTATGGGGATGGACGCAGAAGGCAACA
>at01611
CCATGTTCGCCTTCGCCACGTTGGAGGCGGTGACGCAGCCCGCCCTGCCCAGCACCCGGC
CCCACACGTCAGAGGCCGTCCTCGTCTTCCACGCGCAAAAGTCGCACTTCTCTTCGCCGT
GCGTGGCCGAGTAGAGCTCCTCCAGCTCTGCCTGCGTGGCCTCTACGCATCCTGCACTGG
TCAGAGGGTGCGGCCGCGAGACCCTCAGGGAGTTGAAGCGAACCTCTTCGCAGGACTGCT
TGTTGAACACCGAAAGCACCCGCTGCTGCTGGACGCGCTCTAGCGCACTGGCTTCAGGAT
CTGCATCTGACTCCTTGCCAAACCATTTCTTCAGCTTCTCCGTGAACTCCGGTGGTACGG
AGCACCCGCCAACCGAGAGGCTCAACTCATACAGCTCCAGCCAACGCTGGCGGTCTGGCT
CACACAATCGGTCAACTAATGCAGGGAGCTCTATCACGCTTT
>at01612
GTCTGCCTTCATGGAGAGCAGGACCTCGTCCCCCTGCTTGGGGTTCTTCCAGCCATTGCC
CTCCTTCAGCTCCGCCTTGATGACGCCGTCGTCTCCGAAGAGGTCGTCCTTGGACACCCA
GCTGATCAGCTCCACCTCGAAGACCAGCGTGGCCTTGGCCGGGGATCGAGGGC
>at01613
TCAGTGATGTAGGTACTCAGACTTTCTGGCTTGTAGGCAGGGTCCCGGTCCGGATGTTGG
CCACGATTGTCCATCCTAGAGTTGTTGTCAACCGATTCAGACACCCAGCGCCACAAGAGC
GTTCGAATTCACGCCCTGATGCATGTGTGCTGCGAGATTGGATCCAGTGCCCCTCAAGGT
GCGGTTAACAAATGGACAAAAAAAAAAAAANAAAAAAAANAAAAAA
>at01614
TTTTTTTTTTTTTTTGTTGTTTATCTGCGCCAATAAAAAAATCTCGGGCTCTAAATCCCA
GCATCCCTATTCCGAGACATGAGTTCAGCCGGCCAGGTGTGTCTGTACATTGACTGGAAT
AAATTCGTTTTCACCTTGTGTGCGATGCCAATGGAGACGTTCTTGTCGAAAGCAGTGCAA
GAGCTTTGGCATACCGGTTCAGCTTTCTGCGGAGCAGCGTACAGTCAGCGGCTTCCGTGA
AATGCGGGATGACACGCTTATTGGAGGGCCCAGGCCTCCGTACTCCTGTAGTGCTGATCC
TCCGCTCGCGATCAATACTGCAACGCATAGCGTCGACATCATACCCTCGCAATTGCAGCC
GCCGAAGGAAGAACCTTTTTCTGCCTCTCTGCGTCGCCGACTCTGCAGCAGGTGGCTTCG
ATCCTGTATAACTCCGCTTCATGAAGCGTAGGGTCCACTCCCCACCCAGAAGGTCTCACA
ATTTTACGTGCAAGGATCCGCCAGGACGTCCCTCAAACTCCTGGCGACACGCCTCTGCCA
CTGACCGCCGAAGCTCTTCCGGGAAGACCATCCAAATAGTCTTGGACCTTGTACTGCGCA
ACCTCAAGGCACAGACCCTCGTGCCGAAT
>at01615
GAGGCTGGAGACCATCACCCTGCTGTTCTCTTACAAGGTGAAGTACTCCGAGAACTTCTT

CCTGCTGCGCGGAAACCACGAGTGCGCCTCCATCACGAGGATCTACGGCTTCTACGATGA
GTGCAAGCGCCGTTACAACATCAAGCTTTGGAAGCAGTTCTGCGACGTCTTCAACTGCTT
GCCCGTCTGCGCGCTGGTGGACGAGAAGATCATTTGCATGCACGGCGGCCTGTCCCCGGA
GATCACCAACATGGACCAGGTCCGGCGCCTCGTGCGGCCCACGGACGTGCCCGACACGGG
CCTCATCTGCGACCTCCTCTGGGCAGATCCCGACAAGGACATTGCCGGCTGGGCCGAGAA
CGACCGCGGCGTCTCCTTCATCTTCGGCCCCGACGTCGTCACCACCTTCCTTCAAAAGCA
GGACATGGACCTGGTCTGCCGCGCGCACCAGGTCGTCGAGGACGGCTACGAGTTCTTCGC
GAAGCGGCAGCTCATCACCATCTTCAGCGCGCCCAACTACTGCGGCGAGTTCGACAACGC
GGGCGCCATGATGACCATCGACGAGACACTGATGTGCAGCTTCAAGGTCCTCAAGCCTGT
GAAGGGAAAGAAGTGAGGCGCGGCAAATTTCTCGCCCCGACGCCGGAAAAGCCGCGGGCC
CTGGCTAAAAAAAAAAAAAAAAAA
>at01616
TTTTTTTTTTTTTTTTTTCCGAGCCAATGCTCATCAGATGTGATAAACTGCATAGTCCCG
TCATGGATGCTGTCCAGTCATCGCTGGCATGCATCGCTGCTGAGCGGGGAAGTACATCCA
AGGCTCTGACCGCCACACTTGGGCGTCAAGCGCTCGTTAAGAATGCACTCCGTCTGTACA
ACCGATACCGAGCTCAGTTGTGAAAACAGCGCAAATTACGCATTACACGTGTAAGAACTG
CAGGAATGTCCGGCCGGTCAACTTAGAGACACGGATCCGAAGCGCTGGCCAATCCGGACC
ACCTACAGACCCGGAGACCACGCGGCTCAAACTAGCGCCTGCCGCCTCTTCAGCACCATG
CACTCTGCCCATAGCATGCAGCGAAGAAGGGCATCTTCTGTGCCTCTGTCTTGCCGCCCT
CTGTCGGCACCGCTTTCTCTTCGATGACTGGAGAATCGGCTTTAGAGTCCTCGGCCGCGC
TTCTCGTGGAGGCTGTGCTTGCCGCAAATGCGGCTGCTGCGTCCGCCACGACGAGGGCAT
CTTCTGGGACAGCTTTGGGCAAAGGGGCCGCTGTCACCTCCTCTCCGGGCTTCGTGGTGC
CCTGGGCCGCGGGCTCTGCCGCACACTCGTCCGACGACTTGCCCCCCGGTGCGGATGCTG
CAACCTCGGCTGACGAGGCAGGCGCATCTGCCGTGGCCACCTCCTCCTGTGGAGCTTCTC
CTGTANGCTCGACAGGAACGGTGGNCTCTGC
>at01617
TTTTTTTTTTTTTTTTTTGGTGCAATGCTCTAACACTCAATGGCAGCTGCATGTCGTAGC
AAATCCTGATGGTGCACGAGGAAACCTCTCTTCGACCGATCCCGAATCAAAAACCTGCCA
TCCCGCAGCACGCGTTGCTTCACGCGAGCCCACCTGCTCAACGCTGGATGAACTGCATCG
AGCTGATTGCGCCAAGGAAGAAGACCGTTGGGATCGTGATTGCGTGCACGGCAAGCCAGC
GGAAGGTGAAGATTGGATAGGCCACAGGTGTCCTGGCCGCGGCAACCTTGGGCTTTGCCT
CCGCAGCACAGGCTGTGCGCGACTGGCGCGTGAAGGCGCCAGCGGCCGCAGCAGCGTAGC
CGAGGCCAAGACCTGCAAGCAGGCAACCAGGCCACGGCATCCTCGTGCC
>at01618
TTGTTTTTTTTTCTTTTTTTTTTCTCACGGGAACAAGCACCTTCCTTTTCGGAAAGCTGT
GCGCTCTGCATGGACAATTGTCAGTTGTTCTCTGCAAGAGGTGAAAGGCAGAGCAAGAAG
AAATTGCTGGAACCGAATGTCAATCCCCAGCTCTGGACATCTCGTGCTCCCCTCTGCAAG
TGCTCCCTGCAGAGTCAGTACATGTGTTTTCATGCACTGGGCCTCGAGCCGTTATGAAAA
TGGTGCGTGCAGGGACTACCTCTGCTGGGCCCCGCCCAGCCACTTCCTCTCCAGGCCGAT
CTTAAGGTTACGAGCGTTCTCCACGATCCTCTCAAACTTCTGAGACACGAGTTCGAGCTG
CTTCAAGAGCTCCTCTGGCGATGGGTTCTCCCTGAACTTGATGGTTTTGATGAGCGCCTG
GCTGAACTCCTTGCCCATCACGTGCACGGCGTCCATCTCGAAGTTCGAGTCGCCCAGTGA
GATGAGGTTCGTAATGATCTCCGAGTCGAGCTGCCGCTGCACCTCGAGGAACGCCTCGAC
CTTCCACTTGCTGACCTCCCCGGGGAACTGTGGCTCGTACTTAGTGCGAGCAGAGATGAC
GCGCACCTTTCTGCAGCACAGGGAGCAGCTCGGGGACCCACTTTGCAGCGCTGTACTCCA
CCCAGCCATTCATGGCGTTGGTGATGATGAAGGTGTGCCCCACCCGCATGGCCAACTCCA
ACAACTTCCGACCAGCGTGCTGAATCTCTTGCAAGTGCCGCTCAACGACTGGCGGCAGCG
TCTGCTCCAAGCGCAAGTTCAGGAACGATGTGCACAGCAGGGTGTCGTCCCAGTCGAAAA
TTATGACCGTCTGATGCTTGGGAGGTCGCTGCGCCTGCGGCACCCAAACCCCCGAGTACG
AGAGCTTGCGGAGGTAGTGCACGCGGAAATCGTCTCGGCTCTTCTCGTCCAGGGTCTTCA
TTTCGAAGATCTGCTCCTCGGGCGGGCGATGGTTCTCCTCCGGAACTTGTAGCCTGTCCG
GCGCCTCCACGACCCCCGTGGGGGCAGCGGAAAAGAACGCCTTGTCCTTCTGGATGCCAA
GACGGTTAAGGTAGCCCTGGCGTCTCATTCTGGTGACTTCTGCTTCTGGCGGCGTTACTG
GCGAATCCAGTCCCTCGGCATTGCTCTCAGCTTGGCCCATGGTGCACAGAATCAAACTTG
CCACTGCGGCGCGGATTGAGCCACGAAGGCTTGAGCCAAAATGGCTACGG

>at01619
TGTGCATCGCCTCGACACACTTCTTTGCCTGCTCGGTCGTCTCATAGTTGACAAAGGCAA
ACTTTCGGCCCTTGTTGTCAGCCCGAAGCGCCATGGACGTGATGGTGCCGAATTCACCAA
ATTCAGAGTTGATCTTGGCGTCGTCCCACTCATCTGGGAAGTTCTTGACGTACAAATTGG
TGAAATTTGTAAGGTCTGGCTTCTCACGATCTCCGCGCTTCACGAATTGGGTCACCTCCA
CTGTCTTCTCTCCAATCTGCATGCCATTGACGCGCTCGATGGACTGCTTCGCAGCTTCCT
CTGTCTCGTAGTGGACAAAGCCGTAGCCGTAGGACTTGCCTGTGGAGTCCGCAGCAACCT
TGCAAGACAAAATGTTCCCGAAGAGGCTGAAGGTGTCATACAAGGCCTTGTTGTCAATGC
TCTTGTCCAAGTTCTTCACGTAGATGTTACCAGCGCCGCTCTTCCGCAAGCTTGGGTCCC
GCTGGCTCCACATGATACGGCATGAGCGACCTTTAATGCTGGAGTAGTTCAGAGTGTCCA
ATGCCCTTTCCGCGTCAGACACACTGTGGAAGTTGATGTAACCATAACCCAGAGAACGAC
GTGTAACGCTGTCGCGGCACACGCGGATGGAGGCCACAGGCCCTACAGAGTTGAAGATCT
CGTAGAGCATGGCCTCAGTAACGTCCGGATGGAGGTCACCGACATAGAGCGAGGCAAACT
GAGGGACGCCACCTGCCGCCATCGCTGCTTCCTTGCCTGTACACAAAATACCACTCAAAT
CTGGTCTTTGTGAAGGCCCAGACCAGCCCACAAGCGAGAGTTCTTTAAAGGTTTTGCTTT
TGAGATGTTTAGTTTGCTCTTTTGAGCCACGTTGGCTTGAGCCAAAATGGCTACGG
>at01620
GCACAACCCAGGAACAAGATAGGACGAGTCGGTATTGTACGTCTTGTAAGAAGACACTTT
GGGGGGTGAGGGAGGTCTGGCCAGTTTGCCCACGCCTGTTCACCGATGAGTCTATCATCA
GTGTACGACCTCAGGGGTGCAGGTAGTGTGGCTCTTGGCAACCACGGGGCAGGACATCTG
CCTCTTCTGAGTGATATGCCGATACGTCTCCTAGGATTCGTTGATGGGA
>at01621
CCTGAGTCAGACACGTTCTCCACGTCCTGGTCTCCTCAGGGCTACAGGTTCTTCGTGTAC
GTCTGCGACCGCTCCCGGCCGCCCCAGATCACCGCGGAGAGCCGCAACGAGACCTACACG
GAGTGGCAGGCGATCTGGGACGATGCCAGCCAGTCGTACTACTACCACAACCAGGAGTCG
GAGCACGTCGCAGTGGGAGGTCCCCCCAGCCGCGGGCTGCTGGCGGGCCGAGTGGTCGCA
GGAGCACGGCGCCTACTTCTTCTGGCACGCGCCCTCGGGCCACTCGCAGTGGGAGGTGCC
CAAGTGCCTCGCCGACCTCGGGTGGGGCACAGGCACGGATAGCGGCTCCGCCGGCTGAGG
GGTTGGGGGGGACGC
>at01622
TTTTTTTTTTTTTTTTTAAACGGCAGCTTCATAACCAAGCCATCCGACGCGTGCGAAAA
CGGGCCGGGCCTCCGCGCGGGAACTGCGGCACCTGCGAAGGCGGTGCGGCGGCACCGCCA
GGAGCTCTCTTTGGGCTCGGCGCGTCCCGGGAGGGCCGCGGCGGCCCCCCCTCCGCGCGT
CCCCGCCTCAGGGCGAGAGGCTCGAGGCTCGAGGCGAGCTCCGCCGCGCGCCAGCGAGGA
ACCGGGCGCCGCGCGCGCGCGCGGGGCCGAGTGAGACCGGGTGCCGGCGAGGAGGCCGGG
CTCGCCCGCCGAGCCCTCGGCATGCCAGCGCCAGTCGCGCTCCTCGGCGCCAATCTTGCC
CTTCAGATCAAACGGGATGCGGTTCCCCCCGCGTGTGGGGCTCCGCGACCACGCAGAAGT
GCATTGCCGCTGCATCGCTGTCGCAGCCTCTGTGGCTTGAGCAGCAGGCAGCGTCCTGCA
CGGGGCGTGCCTCGTGCC
>at01623
CCGTTGTGGTCAACCGTGCTCCAAGCACGTTTCCCAGCCCACCCGATTTACGATCTATCC
AGCCGTTGCGCTTCATTGAGATGGCATTCATGTACCGAGCGTTCTTCGTGGCCGCATCAT
CCACCGTTGTGGCAGCGTCCGCAGACTTGGCGGACGTGACGTCTCTCATTCAGCTAGGGC
CTGCCGTACAGGTACGCCCTTCGAGCAGAGTGCTGACGTCGGCGAAGCGCACCCTCTGGC
TGAGTTCCGCGATGCAGTAGTGAAGCGGCCAACCC
>at01624
CCGTAGCCATTTTGGCTCAAGTGGCCAGTTTGGCTCAAGCCCTATCACTCACCTAACTTT
TCAGTCAGGATCCCTCTCTCTCCTTCAGGAGCCAATCGCGAGTAGGAGAAGAGTTTCCAT
TTCCACAATGATTGGCCTCGTGATGCGCGCCGTAGTGGGCGAGGCCATGGGGCCGCTTTC
AATGATCATTGGCCCGCTTATTGCCGTCTGTGCAATCTGTGGCATTATGCACCTCTGCCG
GTGCCGTCTTCGTGATTGCGGCTGCTGCAAGCGATTCTTGCGTGCAACTGGGACGGACCC
CTTCGACGACTTTGAGATGATGATCTTGGTGCACAAGGCAAGCTTCACTGCATCCACAAA
AACATCTGTCTCTATACGGATAAGAGCAGGGGAGCGACGAGGTGGCCACAGATTCAAACA
AGGAGGAATTTCCAGCAAGCTCTCGCTCTCTTTGTGGAGCAGGGCACTGAAGTCCTCAAG
TTTGAGCTGGTGAACTCCTCTGACCAGGTCCTTGCAGAGCTGCAGATGAACATCATGAAA

GATGTACTCCAAGATGATAAGTTATGCGTACCTCGGCCTGTGAAAGAAAAGATGTTTGTG
ATGAAGCAGAAGAACAAGAACATCCTGGACCCGCGAGTCGTCCTCACCTTCGCCGCGGAG
GTTCCCGGCGATGAAGAGCAGGCCTTGCTGGCCGGCCTCAATGCCAGCTCCGAGACGGAA
TGGATGTTGCAGCAGCAACTTCAGAAGGTCCACGGGCAGGACGAGAGAGGGGTGGCGCGG
ACCAGTCCTGGTCAGAAGAAGCCACCCGAGCAGATATCAGAGATCGAGCTCCTTGCGCGA
GGCTGCGTTGGGCCGCTGCACAAGTTCGGCACCCTCGGCGCCAAGAGTGTCACCTACGTC
GGTGTCCTGGGGCCACCCCGAAGGAAGAGGTTCGCTCTGCACATGTGGGACAGCGAGTCT
GACTACCGTAGTGATAAGCCTGCCAGGGAGGAGATAGAACTGCTCCGCGTCTCCGCTGTG
GCG
>at01625
TTTTTTTTTTTTTTTTTTCCTGCGGGCCGCCGAGCGTGGTGCGGTGTGTTGCCTGCCGGT
GCGCCACAGCCCCGCGCTGTACAGAATGGCTAACCCTTGTGAGGCCAATTCGCCAACCCT
GTTTAGGGCAAGGCGCCGGAGAGCGTTATTAGAACCCTTGCTCGACAAATGCAATCTGGC
TCTATGACTTACAGGGGATGCTTCCAGCGATCCAAGCCACGCATCCAACCGCTCTGACGG
AAAAGTACAGTGGAGACAACCATCACAAACCCGAATGACATTGCTGCATCTGGAGTGCCT
GCACGAAACAATGGCAGTTTCTGCCCTTCTGGCTGTCCTCAGATCCCCCTCAGGTGCATG
ATGCGCGGCCTGTCGCAAGTGCTGAAGAAGTCACTTGAGCCGGTGCAGGTGGTGAAGTCA
CTTGAGCCGGTGCTGGTGGACGCCTGCGAGGAGTAGTCCTGGTGCTTCAGCCTCTCCACA
AAGGAGACGCCAGCCATCGAGTGCATCGGCATACTTTGGCTGNNAGCCATCGGGCATT
>at01626
AACCCGTTCCACCTTCTTTCCACTGAACTGGATGATGAGGTGGCCAGCCTCCACCTGCTG
CAAGCTGAACGTCAGCTGAGTACGTGCGGCCGTGCTGCTGAGGCTGCATGGGTATGGGCA
TGTGCGATGCTGGGTCTCTCGTGGCGACGTGCGGGCATGCTGCAGTGGCTGTATGTGTAC
TGTCACGAGTGAGGGGCTGTGAGGAGCTTGCAGTGGTCTGTGGGGTTTCCTTGGCATGTG
TGCTGTCTGCCTGATGTGCATTGGTCAAGCAGGTTATGGTTTCCCCCTTGCGTGTGTGTT
GTCTGTTTGATGAGCACTGGTCATGCAGGGTAGTGTGACGTAGCCATCCTTGCCTAGGAA
CAAAAAAAAAAAAA
>at01627
AGCAGGCGGGGCAGTGGAAGCAGCGGTGCGTCCAAGCACACACGTGCAAGCGGGGGCAGT
GGAAGCCGCGGCGGCCGCTCCGCCCGCTCGGAAGGTGCGCGCGAGGGGGCCCGGCCCCGT
CCTCCACGCGGCTCGGCCGCGGGTTATCACGAGAGGCTCGCCCCCTTCCTGGCCTTGTGC
GATCGGAGGAGCCAGCGGACCTCGTCCTTCTTCGCGAAGTCCAGGGGAGTGGCGCCGGCG
GCGTTCTTGGCGTTGGCGTCG
>at01628
CCCAGATTTTCTCCTCGCTTGGTCAAGCCGGTGGCTTTCTGCATGGAATCGTTTTTTCTT
GTCACGGCCGGCGGCCAGTCGACCGAATTCTGTGGCGAGCTGACGAAAGTCACTGTGCTA
GATGTGAAGCTGAAGTACAACGAAGTGGCGAATGTGCCGGTGTGGCAGCAGCGTTTAGTG
CTTGGTACTGACGTCTTGGAAGCGATCTCGTCTTGGCTTCTTGCGGAGTTGGCGCGGGCA
CGAAGCTGCAGCTGGTCTTGACCCAGAATTTGTCGGACTTGCCGGGACTGGGGACTGGCT
GGGTGCTCTACATGTTC
>at01629
GTACATGGCCAGACTAGGTCTCAAGCTGCTGATGCAGACCCCGCCTCTGTATGACCGCCT
CTGTATGAAACGTCAGGGCCTGTTTGGCAGAGATGCCAACAACATCTTGGCAATCATGCA
GCCAGTGTCGGATGTCCATCCATGCTTGCTCCAGGGGCCGTCGGTGGCAGCAAGCCCTGT
CGCTTCTCACAGCCGCAATCGCAGCTGGTTACGTGCCTGACTTGGTTGCACTTAGCTCAG
CGATCACTGCTTGCACCGTCAGATGGGCCATTGCCTTGAATCTATTGGCTACAAGTTGCG
AGATGCGCCTCGCTCTGGACTTGGTGGCATACGGCGCTGCAGTCAATGTGTGCTCCAAGG
GCCAACAGTGGCGGGCGGCGTTGGGTTTGCTTGGCGAGGCGTATCAGAGGCCCTTAGGTT
GGATGTGGCCATGTACACCTCCGCAGCATCTAGCTGCAGGCAAGCACAGCAGTGGCAGCG
TGCGCTGGCCTTGTTTGCTGACGCCAGTGCCAAGCGTCTGAGACTGAATGTTGCGATCTG
CGGCGCCACTATCAGCGCGTGCGACAGGAGCTTGCAGTCGGCAGCGTTCCTTGCATCTTA
TCGCCGAGGTGCTCTCAAATTCCATC
>at01630
CCGAAACCATTAGTGAATCAAACCACATGACAATTCACACGAGAGCCTGTGCCGAGCGCA
CCGGCTCGTGCGTTCTGCAAGACAGGTGCACCAAAGCTGGGCACTGAAGTGGTCCGAGGC

CAAGAAGGATGTGTTTGTGGGAAATGTTGATGTGCAATCCTTCCATTCAACATCAGAGGA
CCAGCGCGAGACGCAGCTGCCGCCTCAGTATGGGGAGTAGCCTTTACCACCTTGGCGGCC
GTAGCCGCCACCGTAGCCACCATAGCCGCCGTCACGGCCGCCACCACTGCTTCCCCAGCT
GTCACGGCCGCCGCCCGCCACCCCAGCTGCCACCACCGCCACCGCCACCGCCACCACCAG
TCTTGAAGCCAGTGCATGAGGAGGCATTGTACTTCTGCTTGCGGTCGTCCCACTCTGTCT
CATACGTCACCTGGTCACCCTCTTCAAGATATGCGCTCCGATCGTTGCCGTCGCCAAAGA
CCCTGCGATGGACAAAGACGTCCTCGCCGCCATCGTCTGGGGTGATGAAGCCGAAACCTT
TCTCCTCGAAGAACTTCTTAACCGTTCCGGTCGGCATGTCGGGCAAATGCGGGTGGGCGC
TGGGGGCAGAAGTTTGTGCTTCAGCCACACTGGCTTGAGCCAAAATGGCTACGG
>at01631
AGAAACTCCGCAGCTGCACCCGTCAGCGCGCGCGCTCGATAAGCAGGTCCACTGGGAAGT
CCTGCACGAAGCCACCGATCACGACGCGCCGTCGGGAGAGCTCCAGGACAAGTGGGGCGA
GGCCTGCCTGCCAGGCAGCCATCGCGCCTTCAGTGGCGCTCCCCTCCTCCGTGCTGGTGC
CTCCCGCGGTGGCAGGTGCCCCTGCCGGTTCCTCCTCCTCCTCTTCGCCCCTGCGCTTTT
GGTAATTTGCCTCGCACTCCTGCTGCGTGAGGTGCCGCAGGCGCTCCACGAAGCCAAGGA
GCATGGGGTTCAGCACCTCGCAGAGCCTCCGGGGACCGAAGGTGCCGGCATCAACCTCCA
TGCAGAGGAGCTTGAAGGGTTGGAGGGCGTGCCACGACATCTCCAAGAAGTGCTCGTGCC
GCCGCAAGTTCTCGAAGCACCGGTCTGCCACCTGCTCCTTCAGCGACA
>at01632
AACAACCACCACAGCGAACCCCTATGTTAGCCTCTACAGCTGCTTCACTCACTCAGACCC
ACGCTTGGAGCGACGCTTCCAGTTCCTTTACCGAGCAAGGCCTGCCCGATCAGGGACCCA
ATGTATGTTTGGCGTGACACCCCAGGATGAGGGTAGCCACTGCGTGATGGACGATGGCCG
ATACGGCTCATTTGGCTGGTGCTACACCAAGGCGGACCGTAGTGAATGGGGCAGCTGCTC
AGAGTCGTGTCCTCTGGAAGGAAGCACTGAAGTGCTCGCCCAACGCATCGAGATGCTCAC
CACGAAGGTGCAGTCTGCGTTGCAAAAGCTGAACATGCTTCACTGCCTGCCGGGCGGCTC
TAGGTGAGATGCTCAATGATCATCTCGCATCCCTGAATGCGACA
>at01633
ATGTAATCAACTGGTCTGTGCAGATTGGCATGTTGAGTGATTATGGCCACACTGGTGTGG
CTGGCGATCTCACGGCTACGTCGGATCTGTCCCTCAAGATCGTAGAGTGTGCGTCAGGCA
GATCGGATGACCTTAGATCTTTTAATGGCATTCATCCCTGCGCTATCAGATTTACTCACC
AGGCATCCACCTGCAAATACCAACGGCAGGGAGCCCAAGTGGTTGCACTAGACAGGGGCA
CGGAGAGCAGCGCCTCGAATAGTTTACATTCAGCGATGTACACAACAGTGCAGTCGTGTT
GCAGACTGCGCCCGACGCCATTCAGCTTCCTCTGTGCTTGCCGATTCTGTACCA
>at01634
ATACGGATACGCTCGCAGGTGCACGTTTGAACCAAAGCACCTCATCACCGCCTGGCAAGA
TGATGCGAAAACCCCTGCCGGCCGACAGTTGAGGTTCGATCATCTCTATGGGCCATCGAC
TGAGCACTGGTTGCATCCGGTCGGCGGCGCCAGCACATCCAAGCCCGCATGCTTGCCAAT
GCGCTGGGCCGATTCAAGTGAGCACTCCTGTAACCCCNCGAGGTCAGG
>at01635
GTCATCGGTGGGGTCTCAGGTCTCGCAAGTGAGTCCTTCCTGTACACTGCGGTTGGCTCG
CACGCGAAGGCTGTGTCCGTGGTGCTGCTGCCATCCTTTGTCTCGCCCCTCGTGGTGCAC
TGCTGCCTCCCGGAGACGGCCGGCAAGGACCTGGACGAGATCGCGCCGGAGCGCGCGGAG
GAGGGCGGAGGCAAGGAGGACGTCGAGTTGCTCGAAGCCCCAAAGGGGACGGCGGTGCCG
AGAAAGTCCAACAGTGGGGTCATGGTGGTTTGACATCCTAGCTGGTGGCTGCATGATCGG
CGGTGAGGGCCGGGGGGGGCTCTGGACAAGACCTCCGGTCCACCAACACATAGTTGCAGT
TGCTGAATAAAAAAAAAAAAAAAAAAA
>at01636
GCGATGCCGAATTTAACTGTCACGGCGAACCGATTGGTGTACTTGAACAGTAGTATCAAT
GAAGTCTTGGCACCTGATGGGTTATTTTACAACATCACTGCACTCATGGACAGCTTGGAC
CTGGTGGTCCAGCGACCAATCCCAATTCTCCACGACATATCGGTCTCTCTGTCGACCTTT
GAGGACACTTTAGACCTCTTGTCTGACGAGATGGCTGACACTGTCGCCACCCTGAAAGTG
CTGATTCCCTTGATTGAAACGCTTCTTCCTAAATTTGAGGAGTTGGAGGGTGCTGTATAT
GATATGTATGCATCTGAAGACCTTATTCCCGTTATTAACATCCTCATTGGCCAGATTGAG
AGCGTAAATGAAACTTTGTTCAGTCATACTATGCTGGATGATGTTGTGGAATCAATGCTT
GAATTGAATGCTACAGTTTACGACTTCCTGTATAACGGCACCCTGAGCGATATATTGGAG

CAAGTGAGCG
>at01637
CTGATGCTCGCCCAGATCCCATGAAATGTTCACCACATCCGCTGAAACAATCATCGCCCT
ACATGCAAACTGTTAGTACACATGCAATAAGCAATTGCCACTCATTGTTGGCATGAATTT
GCCCAGGTCACTCACTTCTCGCTCTCAGGAGCAGCGTACCACTCATCAGCAGATGCCCCT
GTACATGAGCTGCGTCTGCCAAATGCCCTCAATATCAAAGTGCTCACGGACAGCAGTCTC
AAAATCTGCAGATCCGCCTTGCGGCTCACCAT
>at01638
TGCCAGCACCATGCCAACAGTGCACGCCCACCACGAAGGCGCCCGCGACAAGGAAGACAA
CCACAAAGTAGCGGCGAGCCCAGCCGACCACCAGAGCGCCAGCACAAACGCCGCGAAGTA
CCCCCAGAGGCCCGCCGTAGCTGCAGCAGCACCCAGGGCTTGCACCAAATTCACCTGCAA
CTGCCGTGCCAGTAGAGCAGGCCGAGCATGTAGAAGACCCCCCACAGGACTCTCCCGAGG
TCCGTGTCACAGAGTACGCCAGTAAGAGGAGCGGCCGGCTGCCAGGTGCCCACGAGCTCG
AGGAACTGGGTCAAGTAGACGCTGCAGTAGCCCCACAGCGACAAGCAGCAGCCCGGCCCT
GCCGACAAGTGCCCGTGGAACAGCCCACAGCCGCGGCCGCCTCGGTAGCGGCGTG
>at01639
GCAAAAGCGCGTCGCAGACCATTACACAAGTGTTGAAGAGCATGCAAGGCCAAGGCAACT
TCCCATTCAGACACCAGGTGTACCACCGGTGCAATTCAAATTCCGAAGGGCCGTGTTGCA
ATGCCAAATTCAAAGTAGCATGATGCATAGGCAGCTCCCCGCAAAACAATCCCTGCTGGC
AAAGTGCCACGCAAAGTCTCAAAAGAGGTTACCATGCAGTCACTTGAGGGACCGAACCCT
AGCCCTGATCTCATGTAAAGAGTCTAGGACAGTGATGAAGTGCTCTTGCATGTAGCGGAC
CTCGCACTGCATAATTTTCATGTCCAAGCTACGGGACTCGCCCTGAAGGCGGTAGCAGCC
AGTCAGGAACTCATCGATACTGACTGCCTCTGAGCGGTCGTAGTCGAGAAGCCGAAAGAG
CACTCGAGCATCACTGACATCTAGCTTCAGGGCATTGAAGTCCTCGTGCC
>at01640
TTTTTTTTTTTTTTTTTGAACCTACAAAAGACGACTTCAGGCCATGGGACGGGAGGCGT
TGCCTCCCTCTGTATCCACTTCCACGCGCCGGCCGCATTCCCCTCAGCATGCGCGGAGCT
CAGCGCCGCGAGCGCCTCTGCTCTCCAAAGAGCCGGAGGAGGAACATGAAGAGCCGAACG
AGGTCCATGTAGATGGAGAGCGCCGCGAGCACGTAGTCGTCGATGCTGAAGCGCTTCTCC
TGGTGCTTGCCGCCGACGACGAGCTGCGTGTCGTACACGAGGTAGCCCGAGAAGAGGATG
GCTCCGCCGACCGAGTAGGCAGCCTGCAGGAGGGGGACGCGGAGGAATATGCCCGCCAAG
CCCAAGACGAAGAGCGAGAGCGACATCGCCCGGAGGTAACCGCCGAGGCCCGTGATGTCC
ACCTTGGGGGTAGCAGGGGACCAGGCTCAGGGCCCCGGCGATGGTGGCCGTGGCGCCCAA
GCACACGACGATGGACTGCACTTCGAACATCGCGCAAAATAAG
>at01641
CCATTTCGGCGTAAGTCCCCAAAGCATGGCTCACAGAAAACCCTGGCCGGTTCTACGCAT
GTCGTATGACCCCTTGGGTCCGGCTTGTCCTTGCCCTCTTGCTTGCTGCCTCCTGCATTG
CCCTGTGCGTCAAGCTGCGCTGGCGGGCCGTGAAGCTGCACGACGTTGNCGAGCTGGGCG
CCTTCCCCGAGGCTGGCGACGTCTTCGTCTGGAAAGACTGGGTGGACAGCTTCAGCGGCC
TTCCGCCCTTCAGCAACCTGCCCTTCACGGCCACAATCTCGTGTGAGCCTCTCTTGTCCC
CGCTGCTGAAGCGTTACGACTGGCTGGTCACCCCGTTGTCGCCCTTCACTGCCaAGaACG
GTTGCGAGGAGATAGATCCGACTACTTGGGAGGAGATCACCTTCAAGGGGGACCCACGGA
CCGTGTGGGCAGCCCCGATGGCGGTAGACAGGCTATTGGATCTCGCCGAGCGCCACTGTG
ACCCCTCGCACAGCGATCGCGTGGTCCACTTTGCTGGCACGGAGGATCCCCTGGCGCACG
CCTTTGGCCGGACGTGGGCCATGCG
>at01642
GATTATGCGAGTCTTGATCATGAGGTAACAACAAACTCAACTTCCTTCGCAGGCCTGGGT
CACATTCCAGAGCGTGGCGTGGCCCTGCGAGTCCCCCACGAGCGCAAACCCGCTCCCACC
ACCGGGGGCGTACACACAAGCACCGGGCCGCCGCGGAGGCTTTGCTCAGCTGCAGCTGCG
ACCACGGGTNTGCCAG
>at01643
TTTGTTTTTTTTTCTTTTTTTTTTTGGTTTGTTCACACGTTGGCATGATCCATACAGCTC
GACACAGATGCACAGCTCCCGCCAGCACTGCGCCCCCGCCCCTCAGCGGTACTTGCTGCC
GTACTCGTTCTTGCCCTTCTTGGAGTCGGTACCCA
>at01644

GAGTGATGCCATTCGAAGGAAGAAAGCTCTGATACTAGATCTTGAGGCAAAGCTGCACGA
GGTGAAGCATGACATAGACAACAAGGTCCAGGCCGTCGGCCTTGATGAGATGTGCCTCCG
ATCCACCAACGCCAGCTTCAACACGGTGCTGATGCACACGGAGCGCGCGCCTTCGGCTGG
ACGAAGTATAAAGTTCTCCAAGTGGAGAAGGCCAGATGAGCATCCGGAAGTGCGTGACAG
CAGGAACAACGAGCTGGTACGCGAGCGCAGGGTGGCATACCTCATCGGGACCACTGAGAA
G
>at01645
TTTTTTTTTTTTTTTTTCGCGCACGCGCGTGCGACGGGCCCTCTGGCTGCTCACCGCAG
GCCCTCCATACGGCCCCCTCAGTTACTCTCGGTGCCCGAGCTACCCGAGGTGCCGAAACT
CGAGGACTTGCGGTGCCGGTGCTTCTTCACGGTGCGGGTAGAGGCGGTCCAGGAGAGAGT
CCTCTCCCTCAGGACGATGCTCACGGAGCGGAGGAAGCGGCAGCCCTTGCGGAAGGAGCG
GAGGGACTCCTCGGTAAGCAGAGGGCACCCTCGTAGATCGACTGTCTGCACGTCATGCAA
CAACTCTGCCAGAGCATCGCCGGACCTGTCCGACAGCTCCGCGGCGCCCCCCAGCGTGAG
CGACGTGACCGATCTCAGCGCCAGTGCGCAGGGGCTGCGCGGGTGCTGCCGGCGCCGGCT
GATGCGCGGCGGATCCGGTCGCGGCGCCGGCGCGGGAGCCAGCGGCCCCATGCCGCCGGC
TGGCCCGCCGAACTGCAGCGAGGACATCAGCGCCAGGTCCAGCTGCTCCAGGACCTCGGC
CTCGTCGTGGCTCTCGCCTCGGTTGCACCACTTGGGGA
>at01646
TTTTTTTTTTTTTTTATTCAGCCACGCGCATAAGAACATCTACGCCACGGGTGTTGTTAG
CAAGGTGTGCCGAAAGAAAGCTGCAGGCGTTTCCTGCAACTGCAAACACTTGCCACAACA
GTAAAAAACAGCCTCAAGATCTGTACACCATGATCTGCTGAAGGCGTGCATACCAACTCG
CCATCGCACCTACTCAGAATCGACCTTGGCCATGTGCTTGATCAGATCCACGACCCTGGA
ACTGTAACCCCACTCATTGTCATACCAGGCAACAAGCTTCACGAAGGTCGGATCAATCAT
AATGCCTGCCTTGGAGTCAAATGTGCTGGAGATACTGCTGGTCTCAAAATCCGTGGAGAC
AAGGTCCTCATCGCAATATCCCAAGAAACCCTTCATGTCGCCTTCCGCTCTCAGCTTGAC
CTCGGCACAAATCTCCTCGTACGACGTCGGCTTCTCCAACTCGCACGTCAAGTCAACGAC
CGACACGTCGATATTCGGCACGCGGTAAGCCATACCGGTGAGTTTGCCATCAACATCTGG
AATCACCTTGGGCACGGTCTTGGCAGCACCCGTCTACGACCGGATGATGTTGC
>at01647
GAGAAAGCCACTGTCCCAGTGGAGACTGCGTGCTCACCGTGCTGCTCAAGGATATTAATG
CTTCGCAAAGCGCTGACGTGACAGGTAACCAATCACAGTGGTACACGTTGCTGATGTCAT
GGTCTTCAATATCAAGAGAAACAACCTGAACTGAGGCCGAGCCCGCACACGGCAGGAGGT
CAGCTTGGTAAATCTCCTTGGGGCTGGTGCTGCAGGAGGAGGCAGCCGCAAGAGGCGCCC
TTCGCCCAGCGACAGCACTGCAAAGCTCCTCATACATCAAGTCGCACTTCACAGAGTTGA
GGTGCCTCGAGATGGCAAAGGCCGTGTCCAGATTGACTCATTTCTTGCACAGGGAGTTGC
CGATGAGTCCATTGATACGAGCTCGCTGGGCTGCGATACTGTCCCTGGAAGTCTTCACCC
AGTGCCTGACAAAGGATACGATTGCACTTGCTGCGCATCGCACTAAGTTCTCCAAGGAAG
CCAGCATCTCCAGTTGTCGGCATCAAGTCAAAACTGCAAATTCCTAATTCGGCCCACCGG
CAGTCCGTAACCCTTGAGCCAAAATGGCTTGAGCCAAAATGGCTACGG
>at01648
TTTTTTTTTTTTTTGTTGCCGGTTCAAGCTGATGGATTACTGTACAAGATTCAGTGGAG
GTTACAAACAAATAATGTGAGCAGGAAAAAGGCCCTGTTAGCCATGTTCATGATGCACTA
TACGACAACACAAGCAGTTTGTGCCATTCAAAATCGTGCCTGCCGTCCTTCGACAGCAGC
TTCACAATGACACAGGAGTCTTCACTGCAAGTTCTGCAACTTCCTCGCCTTCTCCAGGCA
TGCCGAGGCATCCAAGTCCTCCAGCGCGTCGAAGAGCGAGCCCTTCTTGCCGCTCTCGAG
GGTCACCTGCGGGTCCACGGACCAGCTCTTGATCATGGCCTCCTTCACACCCGCGCGCAG
CATGGCCTTGCAGTAGGCGTCCTTGCAGCATGGCGTCTCAAGCCAGTTCATTCGCTCTGC
GAGGCCCTCGAAGGGCGAGGCCGAGGCATGAACGCCGTTGTCGCCCACGTTGGGCTCGGA
CTTCAGGCCAAGCTCCCTCCAGTCAGAAAGGATCTTGCCACGAAGGGAGTCCTTCGGTGC
ATCCTTCGGGTCCGTAGGCCCCAGAACCTTGCCGCGGAAGTCCTCCCAAGACAGCTTGCT
CTCGTCNCACTCCACCGTGTAGTANNTGGATGCTCCGGCCGGGCTCTGTGAACTGGCTCC
GCATGGTCATGANGAAGCCGTTGAAGACGTACAGCGGCGTTTG
>at01649
CGCCGTTCTCTCGGATCTCCGTTGGCCCCCGGTTACTTGGCCCTTGAGCACCTCACGCCG
CAGGGGCGCCTTCTCGCTGCGCCGGCCCGGTGCATCCTGACGGATGACGACGACCTTTGG

CCTGAGAACCTCCTCGACGGCGTCTGCGATAGGCCACAGAAGGGCATCGAGGCCAGCCGC
TGAGGGTTGCAGGCAGACGTGGTCTCCGTAGCGGTCTATTACCAGCCCCCGGAAGGCCGT
CGGCCTCCCCGTGCACGAGCCTGTAGAAGGGTTCTGGAAACAGGCGCTCTCGGTAGGTCA
GACACTGGCGGAGCCTCTCGGCGAAGAAGGCCGTGTCCACCTGCGTGTAGGCGGCTTGGC
TCAGCATCCTCGCCGTGATCGTGCCATGGCGGTTGTAGAGCGCCACGCCGAGGGGCTCCC
CGTCCGACGCCTCCACGTTGACGAGGCAGGGC
>at01650
GAGACAGTCGCCCTCCACTGGCAGGCGATGCACCGCACGTGCAGTTGCTCTAGAAGGCTG
GTCTGACTAGTAACGCACCTCACACGACCTTGCCATAGAAGATACCTTTGAGCAGAATTG
TCTTCGGAGCCTTTGCTCCCATGTCCGTGTCAGATGGCTGCTTGGAGACAAAAACGCCAC
CAATCTCGCCGAGCTCAGAATTCACCTTGTTGACCTCCATCTCGATGGCGCCCTTGCCAA
TATCAAAGACCTTGTTTGTCTCCTTGAAGAGCTCCTCCTGGCCCCCCACACCATCGGCCT
GCAGTGCAGGCAGCGCAACGGCCTGGTCATAGCCAGAATACATGCCGCGGCCCTTGGGGT
CAAGGAAGGCACCGGTGCGGTAAGAAGGCACCTCGAACTCGCCACCCCAAGTGAAACCTG
GCTTGAACTCGCTGCCCTCACCCGTTGCAACAAGCTTTTTCACAGAGAACAAGAAGGGCA
CGTACTCGCCATTAGGGAGTTGCACGGTGGTGGGCAAAGTGTGGATGCCATCATACTCCC
TGAGCGTGATCGGGTTGGGGCTCAAATCACCGTTGATGAACGCCAACGTGTATGTCTGGC
GAGTCAAAAGCTTGGTGGTCACAAATTCCTTCCCCTTGTTAGTTTCTGCCTCGACGGCAA
AGGACTTGGGCTCAAAGCACAGGTTCGTGAGCTTCGCCCCTTCCTTCACTGGGACCGTGC
TGCCCTGGCTCTCCACGGTCGGGCAGCGGTTGGCAAGGCCAGAGCCCCTCACCTGAATGT
AGGTCAGCTGGCTGTACTGCTCAGCGGTGAGTGCACGAGTTGGCGCAGGCGAAAGGAGCC
CGGCCGCCAGCCCAAGAACAACACCGAAGCTGAGAATAGATGACAGCCATGACCTCGGCT
GTGCTTCAGCCTCTGGCACCTCTACCTGAGCTGCCTCATTCAGGCCGCGTGATAGGAACT
GGTTGGCTGAGGGTGTCTGCTGCGGCAGTGCCGGAGGAGCTGGACCTTGGACATAAGCAA
GGGGGGCGCCAGGTAGCACCAGAAGCAAAGCAAGAGAGAGCCTGGCTGGCATTGGTGAAC
GTGCGTCTAAAAAATCGCTACAGCAGCAGTCACGGCCACCTTCAGCCCAAATGACTCTTG
AGCCAAAATGGCTACGGA
>at01651
GCCACGGACTcCGGACCGAGGATTCGACCGCACACGGCGGCACGCCCCATCAGCCCCCGC
GCGGGCTCATGGTGTGGGCGTGCAGGCGGCAAGCGCTTGAACTGCGGAAGCATGGGAGGC
ATGCGCCTCTCAGTGCTCAGCTACAACCTCCTCCACTCCTTTGGCGCCTCCCGGCTCTTC
AAGGCACACAGGGGAT
>at01652
TTTTTTTTTTTTTTTTTCCCCTACTACATAGTTTGCATGCAGCACCGTTGGATGGCTGG
ACTTGTGATTTCCCGGCGGGAACTCGTCCTCTGGGAGCGCAACGGCAAAGTCTGGAAAAC
ACCTGCTGATCTCGTGGACGGCCTGGGTGAACTTGACTTGGTTCCTCTGTACCTTACCCT
CCATCAGGAACTCCCATCGGAGAATCAGAGCCTTCGCGCTCGAGGTGGGGTGCATGTACA
AGAAGCACGAGCACAGATTTTTTGACGTCAATCCGTGGTTACTACCCGAATCGTCGGTGA
TGTACATGTCGAACTTGCCCGCACCATCGATGCTGCTGAAGGGATTCTTGAGCCAAGCCG
TGTCAATGTCAGCGTACAAGACGCTGCAGCCCTGCTCCAAGAAGTGCCTTATCTGACGAG
GGCGCGTGCTGACGATCTTGTTGTAAGCTTTGGAATCGAATGGGTGGATGTCCTGGATAG
GCATCTCGTAGACGTCTTGCAAGTAGGACCGGTCAGCGGCTGCCGCTGGCATGTTCCACG
CGATCTGCGTTTCGTTGGCGAGAATTGCTATTTTGAAGGGAGTCCGTGCGCCCTCGTGCA
GGAAGCTGCGCAGCATGGGGACGACTTTGTCGTCTTCAGCAAAGACCAACGACTGCTCGG
AGGAG
>at01653
CAGCCGTGCGACGGTTCCAACGACAACCTTCAGCTTCCCAGCCGACCGCGCCAAGAAGTA
GAAGTCGCTCGTCGGGAACATCACCTTCGACGGTGAGAAGAAGAGCTGCTCCAGCTCTTG
GTCGCCATAGCCAATCAAGCTGTACACACTTCGCAGGGCGTGAAGCACAGCTGTGGCTGG
TACATGGCGATCGACGTTCCGGTCGACGAAGAAGCCCGCCGCCCCGACTCATCACAACCTG
GTCATGCCGCGCAAGCATTACAACCGAGGCAGCGCCGCATTCCAAAGCATGGCGGGCATT
CTCGATTGCAAACGAGCCATGGCCTGCAACGACCACGCTCTTGCCTGCAAATGGACAGCC
CTGCAGATCCTGTCCATTACCCCAAGCCACGACACCGCGGAAGCTTGGCTCTCCTGCAAA
ATGTAGCTGTCGCCGCTTG
>at01654

TTTACCACTGCATCTACGTGGACACGGAAGGTCTTTTGGGGGTTTCGTACTTCAACGAAC
AGCGAGCTGCGACATGCAGATATTTTCGCTCTGCATACTGATGTCATCGTTTTTTATATA
CAACTCCATCGGAGCTATTGATGAGCAGGCCATTGACGAATTGCACCTTGTGCTGCATCT
TGCCAAGCACGTCCACCTTCGGTCTGAGCAGGGGGANCGAAGAGACGCCAAAGTCGGAGC
TTGCCGAGTACTTCCCTAGCTTCCTTTGGGCACTGCGAGACTTTCAT
>at01655
TTTTTTTTTTTTTTTTTGATCTCAGGCTCGCATTCACAGGAGCAAGTCCAGCGGTGTAC
AAGACTAGGACAGTCACAAAACAGGGCAATTCTGACACGACAAGAGCTAGACAGATTGGT
GAGCGAAGTGAATGTGAACACCCTGCACCAATCTCTTCCACACCTTNGGGTCCTTCAAAA
GCCTGCCGCTGAAAAGCAAAGCTGTAATAGCCCACAGCGCAGGCAGCCAAAAATTAGATG
CCATACACAGAGATCTAAGCCTGTGACACCTTATATGCAGTCGGTGCCCCAGGAACTGGA
TGGTACATCCATG
>at01656
CCGTAGCCATTTTGGCTCAAGTCACCGTCTGCTAAGGAACCCATGCCGCAATGGCAGACC
CCTCAGGAGCACCCGAGGCCCAGCGGCTGTGCCCTCCACAGAACGGCGGCACAGAGGCCG
CTTATGGGCAGTACGACCCAGAGCTGGCACAACACGGCGGGGAGTGCGAGAAGAAGTTTG
CTATCGGGCAGGTGACAGTCCGGCTGCCTTTGGACTGGAGGCCCCTGCCCTTGCTCGCCC
TTGCGCTGAGCTTCCTCCCGTGGCTCATCCCTGCCGCTCTCGTAGCGGACATAGTCTGCA
CCCGGCGCCAGTCAAGCATAGTCCTGCTGGTCCTGCTCGTCACGTGCTCCGCCATGACAG
>at01657
TTTTTTTTTTTTTTTTCTCTTTACCGCGCACAAGGGCGTGCATCAGAAGTCCTGAGGAGA
CAGATTCACCTTCGATGGGTTTTAACAGCCAATACCAACAAAAACGCCCAACGCCAAGAC
CTACATGGTATTATTCTGACAATGCCGATGGGCGCGTTGGCCAATGCGGCTTGCCTGTCA
GAGTCAGACGGCTGGTTGACTCCTTGTTCTGGAGGGTAGAGCGTTGGAGTCATGAATGGT
TTGCTTGGGCATGCTTTGGGATGTTGCCGTCATGCTGCGTGCGGCATTGATACTGCTGTA
CTTCAGTGGTGGTGTGGGCTAACTGGCAGATTCTGCTCAGGATACAACGGGTGGAAGCGG
AAGCATCAGAAGTCTTGGCGAGTCTGCCAGGCTGTGATCTTTGGGTGGATGCATGCCCAG
GTGGGGCGCATGGATGCCCGGGGATAAACTCCACAGCTCATCATGAGACCCTGCGATCTC
GTGATGGTGCTGTACTCGG
>at01658
TTTTTTTTTTTTTTTTTTCCCGAGGTGACAAGCGTGTCACACAGACCAGGGTGTATAGCC
CCTCAATAGGACGGACTACCCAAGACGTCCTCAGCACGGCCAGGATCATCCACGCCTGTA
GCATTCTGTGCATCAGACGAGGTGCCCCTCAGCAGGAAGTCCCAGGGCATGCCGAGCCCA
GATCGCCGCCAAAGCCAGGCGAGTACCACCGGTGTTGCCAGGGTCACCCCCCAGAGCACA
ATGCGCATGCCTCGCTGGTATTTGGCTAACTTCTCGGCTCGGGCGAGGGCTGCCTTGTGC
GCCATCGCCCGGTCGCTGGTCTCCCCGAACCTGGGAAGCGGCCGCCGGCGCGCGCCGCCA
AGAGGCGCCGCCACGCAGTCCATTCTCGAGGCCAGTTCTACCGCCGGCTTCCAGGCCTCG
GCAGCCTCAGACCATCGCTTCTGGAGTTGCAGGTTCTCCGAAAGGCTGCAGAACGACCGG
TAGGTGTCGTGCACGCCCTTCTCCTCCTGCTCGATGGCCCTCTGCAAGCAGTCGCGGAAG
AGGGCCTCGCCCTGCAAGAGCTTCATGGTGGTGCCTTGCTCTGTCAGCACCGTCCCTAAG
CCGAACATCGTGGCATAGCTCAACTGGCCCGCCTTCTGCTGAGCATCAAACGCTTTGTTG
AACATCTCTTCGGCCTCCGCCCAGTAGCACTGCTCCGTC
>at01659
AGGCTACACTGTGCCCTGTGCCGTGTCATTCCGCATGGCCCTGTTCCCCACGGCTGGAGG
CATGCAGGCACAGCGCGCATTGTTGGTTACCGTTGCGGCCACCATGACTCCCTTCATGGT
CTCAGGGGCATCCATGGACGAGGACTGGATGGGGAAGACCCGCAGGACCTTCCTCCTCGA
CCATATGACCAGTGGTGTGTACACACACTCCGCCCACTCGGCTGCCAGCAAGTCCAAGGT
GGCGGCAGCGTTGGCAGCTGGGCCTGCCAGGTGGCGACGGGCGTGCAGCGAAGTGAGACC
AGAGGTTGCCGTGGATTCTGTAACTGGAAAAATATATGTGTGGCTGGCCAGCACTTC
>at01660
TTTTTTTTTTTTTTTTTTGCATGCACACACACGCCGAGCTTGCGAATGACGGAGTCCACG
CTCTGCATCCGCCTGGGCACATGCCGCGCGACTCCGCGGCCGGAGGCATTGCCAAACTCG
CCTTTCATCGATAAAAACTGGGCTTCGCCCAGATCCGGTCAAGAGCACGCCGAAGACGAG
ACTTGCATTCCGAGCCTGGGGTCGCAAAAACAAAGAGGAACATGCACACGCACGCTATGC
ACAGCATCAGCCCCAGCATGCACAGCAGGCTCATGGGAAGGTGACCGACCGCATGGCACA

TGGGAAAAGCCGTGCATGACCTCGGGCATGGACGAAGAGGAAGTCTCAGAATACGATCAT
CACCGCATGAATTAGCTGACTGGCATTCTGAGCTCACGGCCGGCAGCTGCATGTACAGTG
GGCCGAGGGACCGTCTGCACTTGAGCAGCAAATCCGGTTCCGAGAGTCGTCAGGCTGTCA
GTTCCCTTGCCGCCTTGTCCTGCAGGCCTCATGCATGCGCTGAGCCACTAC
>at01661
TTGTTTTTTTTTCTTTTTTTTTTTGTTTTCTTACTTTGTTTCAGCGCTTCCTTCGGCT
GGAGGCGCAGAACTCTTGTTAGGCTGGCGGTCCCTTTTGAGCAGCTTACGCTCTTGTTTG
CTCAGTCGCTTCTTGTGGTTGGAGCTGTCCATCTCCGCAGTGCAGGAATACAATATCTTA
ATCGGGTCGAGACTCCAGCAGCCGTCGGCCAGGCACCTGTACAATCACATTCCCCACGGA
TGCAGCCCTTCTCCCGACCACTTCCGCCCTATCCCCTTCAAGCACCCGTAGCTTGAGCCA
AAATGGCTACGG
>at01662
CCGTAGCCATTTTGGCTCAAGCCATTGTTTTAAAGAAGTCGGCTTTGGGAGGTGCCTCTA
CCCTCCAACCCACTGTTGCAACGCCAGCTCAGGCATGCTGGAACCGAGCCTTGTGAAGCT
GGTCTTCGGAGTCTCCCTCTTGACAACGCTCTCGATAGCCTACAAGGGCCGGTCCAAGGA
GGGAGCGAGGGGCTTGGGGAAGACTTCGAAGGATGAGCACTTCCGCGCCTTCCAGCGGAC
ATACCTCCTGGTTTACCTGCCTGCCATGATGGCGGACTGGCTCCAGGGGCCCTACGTTTA
CGCCCTCTACGAGTCGTATGGCTTCACGCAGGGCGACAACGCGGTGCTCTTCACGGCGGG
CTTTGGCGCCTCTGCTGTGCTCGGACCCATCGTCGGCTCGCTGGCCGACAGGATGGGCAG
ACGGCGCTTTGCCGCGCTCTACTGCTTCCTCTACGTTACCTCGTGCCTCACGAAGCACGC
AAATGACTTCTCATGCCTGTTGGTTGGCAGGATTACGGGGGGTGCTGCGACGTCCTTGCT
TTTCAGCGTCTTCGACAGTTGGATGGTCTCGGAGCACAACGCCCGCGACTTCGACCCGGA
GCTGCTGGGGGCAACGTTCTCGCTGGCCGTCTTCTCCAACAGCATCGTAGCCATTCTTGC
CGGAGTGGTGGGACAGATGGCGGCTGATGCGATGGAGCTCACGCCCCTAGCCGGCAGCCT
GAACTACGGCGGCTACTGCTCGCCGTTCGACGTAGCCATTGTGTTCCTGGTGCTGTGCCT
CGTGATGATGAGCGGCCTGTGGCCCGAGAACTACGGCCAGAAGGGCTCGGGCGCCGGCGG
CAGCTCGCTGGCGGAGAGCATCTGCTCAGCGGTGCGCGCCATCCGGACGCAGCCCCGGGT
GCTGGACTGCGGCGTGGTGTGCGCCTTCTTCGAGTCCTCCATGTTCATTTTCGTCTTCAT
GTGGACGCCGGCCCTGACGGAGGAGGGTGCGGCAAAGCCCCCGTATGGCACAATATTTGC
GTCCTTCATGGTGATGGCCATGCTGGGAAGCCAGATCTTCTCCATGCTGGTTGTGAAGCG
ATCCGTGGAGGAGATTGGTCGCTTGACACTCCTGGTCGCTGCCGTGAGCCACGCGATGAT
CATCTTGAGCTCTGACA
>at01663
CCGTAGCCATTTTGGCTCAAGGGATATTGTCAACCCAACGGCGGCTGTTCCTGAGCTCGA
CCACTTGTGTTTGGCGTCGCCGGGGACTAGGCTCCCCCAGCTGGGTGGTGGTTTAGCTTT
CATCAAAGTCGGAAAAGTTGGTGGGAGTACATGGGGTGGTGTCATGCGGCGAATTGGTCA
TCGCCACGGCCTTCATCGCACCAATGATGGTCCGGGCTGGATAGATCTGGAAGAGAAGCC
ACAGGCGTCAGTCTGGGCGAATCATGGGACGCGCCGCGAAGTGGACAAGCGCGTGTCTGA
ACTGATGCCGAATGCCATCTGGGTAACATTGCTCCGTGAACCAGTTGCAAGAAGCATGTC
CGAATTCTATCACTTCGAAGTGAGTCGC
>at01664
CACTTTTTGGCTCCAGCTTTCAGCTGTTGGATGGCAGGCTAGTTTATGCCGCAGTGCCTC
TTTGAGTTGGACGGCGGTCTGCTGCCAAGACTGGACTTTGCGCAGATTCTATCACTGCGG
GCGTGTCTCAGGAAGTCTTCTTGGATCATGACTGCGACCGCCCGCTGGCAGGAAGCCGGG
CTCAGGACTTGACCTTTGGAGGGGCTTCCTGGATCAACGCTGCTTGCTGCGCATCTTCGT
CAGCTCGATGCAATGCTGGAGGCTCGGGGCAATGCGGTTGAGTCTGATCCCAGGCGAGCA
AAGATGCAGCATGGATTGAGCAAGTG
>at01665
TTTTTTTTTTTTTTTTTTGAATCTCCACCAACATTGCGCGTGGATGGTCCCTTGGCCAGA
ACTCCGAGGCAAATCGCTCGGCCTGTACAGCTCGCACACATGCAACTTGTCTCATTGAAA
TATTGAAAATCGCGGCCTTAAACGGCACAGGGAGCGGAAGGACAATAAAGCCCAAGTCCA
GGCTCTTAGCCTTTTTCAAAATTCGGTGTACTCTTCAAAGGCTTCAGGCTTCACAGCCCT
GACCGACTGCTGTATCGCCAGTCATCGAGGTCCGGCCATGTCATATCCCGTTCGATACTG
CCCTAGACTATCATCATCCAGAGGAGTCCGAGAAATGTTCCTCACATATCAGGAATCCGG
AGACGAGCCGCGGCAGCAGTCTCCTCATTAAAAAATGCTGCTCTCATGGTGTCCGCCGAC

CCTCTGGGCCAAGCAGGCGCCTCGTGCC
>at01666
GGGAGCTTGGAAAGCTTTTGCTGTCCGCTCCGACGTCTCATAACGTTTGAACTGGACGCG
TATAAGAGTCTGACCACTGTGGCTGACATAAGCATCTGCAGGTGAACCGGGCTTTGACAT
AGCGGCCTTGACCGTCAATGCTTCCACACCGCGGCCAAACCCTCCAGCGATCGCATTCTT
ATTGAAACTCTGAACATGCCACTGCTCCTGCACATGGCCAGCGCACTGAGGAAGGCCAAG
GTTTGCATACTGCTCAGCTTTTGCCTTGATTGCTGGATTTCCAACCTGCTCAATGTATGT
GTTTGCAAAGACCTGATTAGTTATGTTTGCAAATGCCCATGACGATGCTGTGAGTGGACA
GTTGGAACTGACGGAAGCCCGAAAACGCCCGGAGCACGGATGGGCTCTTTGACTTTCTGC
T
>at01667
CAGCTCAAGTGGTGCGGGCCATGGCGTGGTGCAGAAACCGCGACGATGCGCTTGGGGATG
CCGTGTGCCGCAGGAGGCAGCGCTGCTGACCAGGCACGGATTCCTACGTATGGAGCTCAT
CAGATGTGCCCAGATGCAGCCGCCTTGCATGATGTGATGAGTCGAAGTTCAATGCGCTCG
GGAGCCAGCCCTGGATCGCCACTTGGCTCTGGAATTGGTGGCCAGGGATGGTCTGGCATT
GGAAAGACGAACTAGACCGGAAGGCCTGGCAAGCCGACAGGTCCATTGTCCTGGCTGCTA
CCGGAATCGACCGGCGTGCACTGCAAGTGG
>at01668
ACGGCGGCCGCTTCCCCGGCCGCCCGCCGAGCGCCGGAGAGAGCTCAAGCGCAAGCACTT
CCAGCTCGAGAGGAGGGATGTTGCGGACCCCAGGGAGAGGCAGAAGAAGAGGAAGAGCGG
AATCTCCCGCGGGCTCACTTGGGTGGCTCACCGGCCTCTGCCAGCCAAAGAGGCGTCTAC
CCTCATGCGCAAGCACTTGCAGCTCGAGAGGAGGGATGTTGCGGACCCCGAGGGAGAGGC
AGAAGAAGAGGGAGAGCCGCGCCGTGGGCATCTGCTGGAAACTCCCGCGGGCTGACTTGG
GTGGCTCACACGTACGTGAAGGGCAGGCAAATCCAGAAGGTGTTCCGACCGAAGGACATG
TCGGAGGCGTCAGTTGAGGCCGCCCGCCTCGAGGCCGTGCAGCACCGCCTCACGTAGGGA
GCGCTGCCGTTAGGCCGGCAGCCTCTGAGGGAACTGCGTCATTCCTCAAAAAAACAAGTT
TCAGAAAAAAAAAAAAAAAA
>at01669
TCAGGGTCCACCGCAAGCCCCGCAACGCTGCTCACGGTGATGATTCGACCGCTCCGCCTA
GGGATCATGTCACGAGCCACGCGCTGGCAAACCAGAATATTCGCCTGCAGGTTGTCGTCC
ATGATGGAACGCGCGTCATCGTCCTTGATGAAGACAGCGTCGTTGACCTCTAGTGCGGGT
CCTCCGCCAGCATTGTGTACCAAGATGTCAATCCTGCCGAAATGCTGTACCACCTCTGCA
ATCGCTTGGTCGACCTGTGAAGGCACCGAGAGGTCGGCAAGAACAGGGAGGGAAGGCACG
CCAAAGCGAGCCCCTATCTCCCTGGCCACATCGGTGAGAGTAGTCCCTTCCCCAAACTCT
GCTGGGCCGTGCTCCCTTCGGCCATGGACGGCAACACTCGCACCCAGTGCAGCAAGGCGC
TCGGCAAATGCGCGGCCAAGACCTCGCCCTGCCCCCGTGATGAGAGCAACCTTTCCTGAC
AGTTCACCCATGGCCGCTGACAAGATGCCTGCTGAAAGCCACGTGAGCTTGAGCCAAATC
GGCCACTTGAGCCAAAATGGCTACGG
>at01670
TTTTTTTTTTTTTTTTTGGACGTGCGCACCCACATGAGACAGCAGAGGAGAAGGACGTC
GCTTGCTCCTTTCGAAACTGGTGAGCTGCCGGGTCTCAAAAATGCCCTGCTCAGGCTGCC
AGACGTCACACGCCCTGTGGCCCTTCACGCCTCCATCACGATGTCCGGCAGCTTCGACAT
CTCCTCCGGCGCAACGGGCGTCTCCGGCACGCCGCCCCCCTCGATCTCCCTCATGAAGTC
GCTCCCGACGTCGTCCTCGGTCTCCAGGACGCCCAGCTGCGGGCCGGCGTCGCTGCCAGA
GTCCTCAAGGCAGAGCGCGCCGGCGCTGATCTCGCAGATCTCCTGCGTAATCGCAGCCTG
GCGCTTCTTGTTGAAAAGTGTGGTGAGCTTCTTCGCGAGGTCTTCAGCATTGTCAGTGGC
GGCCTTCATGGCCGTCATGCGCGCGGACAGCTCCGAAGCCTGCGAGTCAAAGAGCAGCGA
CAGGAGCTGCGAGTTCAAGTACAGCGGGAGCATGGTGTTGAGGATGGTCTCGGGCGGCTG
GTCGAAGATGACGTCGGGCTCGATCTCCTTGGCCTTGGCTGCCTTCACCTTGGTCTTCTC
GACTTTGAGCTTCCCGTCCTCGGTCGTCATCTTGAACGTCTCGTCATCGGGATCCTCGAT
GCCCGTGGCTGACAAGGGCAACACAGTGCGGAGGGTGGCTTCGTTCTTCAGCAGATTGAA
GAACTTCCCGTAGACACTTCGACCTTGTCCACCTCGCCTGAAAGGAAGAGCGACCGCAGC
TCGCTACCGATTTCTTCTGCAGCCTTGGCAGTGACGGTATCAGGCATTCTGAAGTAGGCT
CCGGTATAGTTGACTTCCACTTTGCTCAGTCGCGTATTCAGCGCATTCAAAGCCTTCTTC
CCAACAAGGACAAGCTTGGGAACAACGCCCTCGGCATTGAGATCTGCAATACGGCTCGCT

GCCTTCTTCAGCGCGAATGTGTTGTATGCTCCGCAGAGTCCACGGTCCCCAGTGATGATT
AGGAGGGCGACATTCTTCACCTTTTCAGGAACTCGCAACATGGGGAGCTCAGCCTCAAGA
CCACTGCCCTTGAGCTTCTTCACGAGACCCTTGATC
>at01671
CCGTAGCCATTTTGGCTCAAGGTGCTCAATGGCAAGCCACAACGCCAGCATGGAGTTATG
GCTAGATTGCCGGGACTGTACATCTCTAGGCCCCTTGCCAACAGAATGCGACAGATTCCT
ACTTCGAGAAGGCGCCTCACGGATGAGCGAAAGGGACTTGACATGGGACGAAGGGCGTGA
TCACCTCTTGGACTACGGTGGGCGGGTTGTTGGCGGAGTGTATTCAGGGAAGGCTGCAGA
GGCACAGATCCGCGCACTTGCTGCAGTAGGCTCTGCTGAATGGATCGTGCTTGATGCATC
TGACGGGGCCGTCTTTGTTCCCGCAGAAAATCTAATCGCTGCTGGCCGCCAAGCCGGCAC
ACGCGTGGCTGTCATTGCAAGAAATGAGAAGCAGGTGACTGGCTTGGCTGGAGCACTGCA
ATCAGGAGTTGATGCCCTAATCATACCTGCCACAGCTGATGGAGCTGAACCTGAAGAAGA
CCTTTGGCAGGCCGCCGCAGAGGCAAAACGGACACGCCAACATGCCACTAGAACACATGT
TGCAGAAAATACTCCACAGGAAGATTCTGAAACCACACAGTGCCTCAGTTTGGTACAAGT
AGAATCAGTTGAGCCTGTCATGGGCTTGGCGGATCGTGTTTGCCTTGACCTGGTCCAATT
GATGAATGAAGGTGAAGGAGCACTTGTTGGCTCTACAGCCAAAGCTCTCTGCTTAGTGCA
AGCTGAAACTGCCCACACTGGTTTGGTGCCTCCACGTCCTTTCCGCATCAATGCTGGGCC
GGTCCATGCATACGTGGCTCTTCAAGGAGGCCGCATGAAATACCTGTCAGAAGTTGCCGC
CGGTGATCAGATTCTCGTCGTTGATGCGTCCACCAGCGTCCCTGGCAAGCCTTTCAATAG
TCGGAGCATTGCCGTTGGACGTTGCAAGATTGAACCACGGCCTGTTGTGTGCGTCCGCTT
CAGCGATGGCAAGAGACATGGTCAGATTTTTCTCCAGCAAGCTGAGACAGTAAGGGTGCC
TGCCCGTCTGGGAAGCGGAAGAAGCTCAGACTGCAGTCCGTGCTCCGTCACAAGTTTGGC
ACCAGGGGATGAGATCTGCGCACTCTTGGCTGATATGGGCACTCACGTTGGACAACGGAT
TGCTTCTTCTGTTTTCGAGAAGTAGTGACGCTCGCAAAGG
>at01672
CATCAGCCACCTGTTCAACCGCCATCCTCGCCAGACTTGCAGCCGCTCGACTACCACTAT
TGGGATTTGATCCAGTCTGGCGTCCCCAAGTGCGACACACGCATCCAGCTTGTGGCGGCC
ATCAAGCAGAAGGTGGAGATGGTGAACCGCAACTGGGACGCCCAACAAAGAAACGGACCA
ATCACGGCCGGTTTANGCATCGATTGCGTAAGTGCATCAGTGCAGAGGGAGCATCTTCGA
GAACTGATGCTTCAGAGCAGAGGAGTGCTGGGCCACAGAGGCTTTCAGCGTG
>at01673
TAGCACTTCTTCTCGCAAGCGTGGCATGCATGCCACGGTCAAAAGAAATACTACCCCAAG
CATGGCTGGCCCTATGCCCCAGGTGCTTTCAGACATTGCTCAGCAGCCGCAAGCCGCGAT
TCTCAAACGACCGNGAGGTCCGCGGCTTGCGGACGGAGCCGCAGAAGG
>at01674
TTTTTTTTTTTTTTTGTTCCACAGCACAACTCGGTCATGCGTCGTCCCGCTGGTAGAACA
GCAAGGCAACGCGCGCACCGCACGCAGGCTCTGATGCCTCGAGTGCCATCGGAGTGCCCT
CAGCCTGGCTCGGTTGCGTCACCTTGTCATTGTCGACACGCAACCAGTCCCCGCCGCAGA
GCACATAGGCAAAGTAGTGCCCAGATCGCTCAGTGGCTCCTCGATGAAACACGGCACCAA
TCAAGTGGTAGCGCATGGAGCAAACCTCAAGGTTTCGGTCCAATGGCTGGCCAAAGTGAG
CCACCAGCTTCAGCCGCCCCAAGTCCAAGCACGACTCAAATTCGAGCGGAACCTCGCACC
GGCTGCCGTCTTGCCGAACTCGATTCACATGAAGCACAAGCGTGGCAGGAAGCCTGAGGA
ACCGCGCGGTGCGGGCGCTGCAGCCCACGTGCTCGCACTGGGGGTTGGGGCAGCGGTAGC
CCTCGGGCGCGGGGCTCTCACAGCTCTCGCGCAGCAGCTCCCCCAGCGAGGCCCGCGCGG
GCGGCGCCGGCGCGCTGCCGAACCAGGCGCCCAGGCGGGCGAGCCAGCCGCCGGGCTCCT
GGTCGTGCTCGCCCACCTTGACCTCCTCCACCACGCAGGGCTCCGCGCCGCTGGCGCCGT
ACCTGGCGCAGCTCTGGCAGCGCGT
>at01675
AGCTTCGCTGTTCTTCGCAGTGTCTGGTGCCGCCTCAGCATGCGCCGGCGAGGTACACCC
GTCGCACCTGTCGAAGTAGAGGTTGTTGCGCGCCGAGTTGACGTTCCAGTCGCTTGCCCG
CAGCATGTGCAGGTCCGAGAAGTCCGGGCCAGAGTAGCCAACGCCGTAGGACTTCGGCCA
TAAGTGCTCCCGGTTCCACCCAGACGTCTGCCCGGACAGGCTGGCATCCACAGGGCTTTG
GGTGTAGATGGGGATGACCTTGCTCGCATCTGTGGGGTCTGCATCGAGCACTTTGAGCGC
GTCCCAAGCATCTGTTCCGCTCGAGGACGTGTATGGGATGACGACGTGCGGCGAGGAAAT
GCGGGCTTGCAGGGCTGCCTGCAGGGCTGCCCCTTCCTCCGAGAGGTTCACGCCTGCATA

GTACGCGTCAGAGTCGCAGCTGAAGGTGGAGGCCGCCAGGCGGACTGAGAGCAGCGAGAA
CAAAGCCCCANACGCATGCCTGGCGGCGCAGCACCT
>at01676
GGGAGCGGAAGCAGGAGCGGGAGCGGGAACGGGAACAGGAGCAGAAGCAGGAGCAAGACG
ACGAGATCGTCATTGCTACCCCAAAGGTCCCAGTAACCTCCATGGAGCAAACCATTCCGG
TGGGGCTGCTGTTCTCGGCCCAGGCCTCTGGACATGCGCCGTTGATGATGATGCAGTTGG
TGCTGGACCTCCCGTTGTTCAACACAATGCGCGAGAAAGCCAAGGCCATCCTGGGTTGGG
ATGTGCTTGATATTTGCCTCTCGGGGCGGAAAATATGAGCCCAAGTTCTGCGAACCGGTT
GCGTTCATCATTGGCCTGGCGGTCATGGAACGTCTCCACCAGTGTCATAAGGAGGCTGCC
GAGCGCTACCAAGCCACTGCAGGCTTTGGCACGGGCGAGTACATAGCGCTCTGTGTTGCA
GGCGTCTTCAGCTTCGAGGACGGACTGAAGCTGGTTGATATCAGAAGCCAGGCCATGATG
GAAGCTGG
>at01677
CCGACGGCAGCACTCGTCGCATTGGCATGTGCCAGGCAAGGAGCGACTTGGTGGAGTGGA
CAGTGCCTCGGTGCGCGCCGGCTTCGTGAGGAAGGTGTACGCTATCTTGATGTCACAACT
CTTGGTCACATGTGCCGTGTGTGCGGCAGGAATGTACGTTGCGCCCTTGCGGGGGCTCCT
CGTAAGCATTTGCGGTATATCAGACACGTGGTGGTTCCGGCTGGGGCTGATGATTCCCAC
CTTCGTGGCAATGCTCGTGGCCCAGTCATCGAAGACCAAATACCCTTGCAACCACCTCTC
CTTATTGATCTTCACCTTGCTCATGTCCTTCAATGTTGCGCTTGTTTGCGCAGTGTGCTA
TGAGGTGGGCCTTGGCATACTGATCCTCGTATCAGCAGGCCTCACGGCATTTCTGTTTGC
CTTGCTGTCTGTGTATGCGACCGTCTCAGGCCAGAACTTCTCTCGCACGGGTGCTTACCT
TTCCTGCGGCTTGTCGATCTTGTGCCTCTGGGGCTTGGCACAGTGCTTCCTTGGCATCGG
TCGCTGGGTATTGTATCCGAGTGTCAGGAGCGTTGCTGCTTTGCGGATACATTGTGTACG
ACACTCGGCGCATCATGCAGGTTTTTGGATGCGACGACTACATTGCGGCAGCGATTGAGT
TATACCTAGACATCTTGAACCTGTTCCTGTACATTCTGCAGATATTGCTGAGCCTGTTGA
CGGCAGGGAAGAACGATTAGGTTTGGCTCCTGTAGAAATCAATCTGCCAAGCGCGAGTGT
TTGGCAAACATGCTACATTTCAACTTCTGGAGT
>at01678
TTTTTTTTTTTTTTTTTAGAACTTATTCTTGGGACGTGAAAGTCCCAGGGCCCCGCTGG
AGAAACGACAATCCCCACCAGTCACGCAATGTAGCCTATCATGTAGTTGCATGATGGGGC
TACCATGAAGCCAGGCCCAATTGCAGGATTGCCATTGGAACCGTAACCATGATCATAGCT
GGAATACTCTTGAGCGTCCATCGGGTCGATCTTCCCATCGCTGTTTGAGTCCCGCAGCGC
AAGAAACTTGCCTCCAGACCAGCTGATGTAAACTGTCCCGTCAGCACCAATCGACGGATT
TCCAAACATATCAGGTGCGCAACCGTAAGACGGGAATCGCAGATTCAGGTTTGGCATAGC
ATACATCTTTCTAGAGAGGTCAAGCGACCACACAGTCTCCCCAGTCTCGGCGTCGAGTGC
AACGACTTGGTTGAAGTCCTCACCATCCTTTGCGAGGATGTACACGTCCAAGTAGTACAG
CAAGTAGTGGAGCGGCGTGAGCAGCTGCCAAGACGTGACCACGAGCGACTCCAGACCTAG
CCTTCGCACCAGCGGCCAGAACCAGATGAGCTGGGGGTAGAGCTGCGTATTGTTGCCAGG
GGCCGCGATGACGGTCAGCGGACCTCCAGGCCTCAAGGGTCCCACAGCCAGGCCCACGTT
CATGGCAACAGGAAAGCTGTGCTCCCAGACAACC
>at01679
TTGTTTTTTTTTCTTTTTTTTTTTTACTTCCAGCCACTTGGGACCACCCCTTCAAATCAG
CGCGACAAGTCTCCACTGGCTCACCGAGCAGGCTCGTATGGCCTTCTTCCAGCGAACTCA
CGAGCCGCCAACGATGATGTTGTTGATGGGAATGAAGACCAGCTTCACGGTGTAGCCAAT
GAATCCCATGATGGCAAAACCAATGGCGCACGCCACGGCGATCTTCTTGAACTCCTTGGC
ATCGGGCTTCGTGCATCGCTGGATGAGCCGGCGGGAGTCCACGCTAAACTCATAGGCGCC
CTGCACGACGTAGCCGACCGGGTTGGTCTTGTCCGTGAGGAAGGGCATCGCCGACAGTCG
AGACGCCATGGCTCGAAGTGGCTATGTCAAGGCTGCTGTAGGGGTAAATCCAAAATCCCT
CTTGAGGCAAATGCCTTGAGCCAAAATGGCTACGG
>at01680
CCGTCTCGGCCCAAGGACCCATGCTGGCTCGAGCGACTCAGCATGCCCTGTGCAGGCTTA
CTCGCCTGTAGTACCAGcCCCCAGCCTGGATCATGGCGCGCTTCCTCCTGGCCGCCACCT
CGCCACTGGGGCTCCGCCCGGAGAGCCGACAGCATTCAACCTGCCCGTTCCAGGCAACGA
TGACTTCGAGAATGCGATTAGTGGCTTGAAGGACGTGAAGGAGAAGCTGGCGGATGTCTG
TGAGCGGTCAAGGTGTACAGGCTTGCCAAGCTGGAGCCGGGGCATGCGTTCACGACTGTT

CTGAGAAGCATCGAAGACCACCAGACTCCGCTGCGTGGCAGGCTGTGGACAACGCGAGGA
TCGCGAAGGA
>at01681
TTTTTTTTTTTTTTTTTTCCACAGATTCTCAACAAGTGAAGGCTTCCACAGATCCTGACA
GATGCATGACGGGCAGAGGAATGCCACGCATGCCTGTACAGTGAGTCTCCGAAGAGGCTG
TCGTTGACAAAGATGCACCAGCACGCGTGCTCACTGCTGAAACAATCTCCGGTGCGTCAC
ACTGTACATGGCAAGCCAAAACGATGCATGCTCGTGCAGTTGCCGCACGAGGATTCAAGG
AAGCGTGATTCTCCGATGACCCTGGGTCTTCCAGGTGTGTCGGAGACCTTGAGACTGGAC
CAAGAGATGTGTTGCCTCTCCGTTGGAGACAAACACACAGACACTTAAGCACACAGACAC
AGACACACGCTCGTGAGCGCGCGCACCGACACACACCTGCATACTCAGAGATATGCAGAC
ATGGGCACAGGCGGCTCCCATGGTCGGAGCGTGGGCGAAGCCTGTCGATTCAATCTCCTA
CTGCGTGACCCCTGACAGTTCACGAATTCATCGTGGCAAGCTTGCTGTCCATGTAGGTGC
CCAACACCTCCACGGCATAGGCCACTCCCTCCTCCGATCGCAGAGCCTCCGCCACTTTCG
CGCAGCACTGGGCCATGGCCTCCGACTCGCACTCGCAGAGCTTGTGCACAAGTTGCGAAG
GTGTGACACNCGCAAGGAGGCCCGTGCGCACACCCGCATGCTTTGACACGACCTTGTC
>at01682
TTGTTTTTTTTCTTTTTTTTTGTTCAAAATGCGGCGGCGCCGGCGTCCCCTCGGCAGGAT
GCAGCCCGGGCTTGCCCGCCCCCAGCAAAGCGCGCCTTCATGGACCGGCACCCAGCCAGT
GCCAGTGCCAGGACGGCGCCGCTCGGCCCACTCAG
>at01683
CTGACCCAATCGGGCAGTTGTTTTGCCGCTGCAGTCTCTGCAGTCTTCAGTCGGCATTGG
CATATCCGAGGCAGTTTGTTGGAGACTTTGGTGAGTTTACCAGGTCCGCTGACAAATGAA
TATACATCACTCGCCAAGCATGCGACTCCCGGAATCTCCCATCAAACGCGTGCTTTGTGT
TCTCAACTTCAAACAGATTCCGTCCGTTGAGACATTGATGCAGAGGACTGACCGAGCTCG
AATTGAGGCCTATATTGCGACTGGTT
>at01684
TTTTTTTTTTTTTTTTTTCATAAGCACCAGCCCAACACCACTTGGGAAGACTGGGTATTA
GGACCATGCTAGAGCTCTTCTCCGGCCTAGACGGCTGTGACCACCCACAGTGGGTCGCCG
TGCCGCGGGCTGACATCCGCACTGGAGAGCTGAACCCATCCTCCGCGAGGACCGTGCTGA
AAGTACCTGCATATGAGGTGTGCGTGTCCTTCACCGTCATTGATCTCCTTCACCCACTCG
TGCACGCACTTCTCAATGAAGCAGCGATGGGAGAAAGCCACCAGAGCCATTCCTCCAGGT
CTCAGCACACGGTGCATCTCGGAGAAGACTGCGCGGGGATCTGTCAAGTACTGCACGCTG
AGTGCCAACGTCACAAAGTCAAAACTGCCGTCCGGCCATGGCAGGCGACGGTCGCCATCG
AGGTCCTGCACGTGGCGCTCCGTGGCCTGCCTGTTGGCATCCCCTCGTGCC
>at01685
TTGTTTTTTTTCTTTTTTTTTGGAAGACGACCTCAATATTTACACTCTGATCATCTCAG
TGGCCAGCGTGGCTCCAATCCGGCGGGGAAAAAAGGCCTTACTACGAACATCAAGGCATA
CAGAGATACACCTAAGTCCAAGCAACCTCAGCTCTCAGCCGATAGACACCATGCCAGCAG
GCCCGTAGAAGTTTGCACCCCCGTAGCCAGCCGGGGCAGCCTGGTAGCTGATGTTTCCCG
CCACCGGGTTGGCAACCATGCCCCTCCCAGAAAGTGATGCCTTGTAGCCACCGAGCGCCT
TCCCGTTCACGTAGTAGCTCAGCCAGCCGTCCATGAGAACTTCGTCGGCGCCACTGTTGA
GGAGTGCTGTTGACATGGTAGAGACTGCCACTGGATCACGGCACGATGGAGACTTCTCCA
CCAGGCAGACGCACGTCTGCCGCAGGCCCCGCGCCCGGAGCTCCTTGACATACCGCTGGG
CGCGGCCAATTCTCTTTGGCTCGACAATGACAAGGACGCCCTGCATGCCCGGGCCCACGG
CGTGCGGCTCCGGCTCGGGGCCCTCCGCGCCTTTTGGTCGAAGCACCCTCCACTCCTCCC
CTGCTCGAACGACCTGCCAGCGCGGGTTCGCGACGAGCTCCTGCAGCGCCTCCTTCTCGG
CGGCCTGCTCCGCGTCGCCAGACTTCTTGATAAGCTCAGTGCACTGCGGGATGTCTTTGT
GTTGCCTCGATGGTGGATGTGAAATCTTGGGCGGAACGTAGTAGCGGATCAGAGTTTCAG
GTTCCGCCTCCTGGGTTGTCACTTGCTCATGCTTCTTCTTGTCCTCGTCTGTGTTCACAA
TGCCCGTGGTACCCCAACCATACTCGATGTCAGAACCGTGAGGCCCTGTGATGAATTGGT
CGGTCTTGCCGTCCCAGCCCTGTTGCCCGTCCCAGGCATCCGGTGGTCGGTATACTTCTG
GTGGGAGGTAGCCGTAGTCCAGGTCACTGCCCGCCATGGCTTCTACGGAAGATAAAGAGA
ACGGCACTTGAGCCAAAATGGCTACGG
>at01686
TTTTTTTTTTTTTTTTTTCCGAGACGCGACAGCGACAGCGCCATCCTAGCCCACCCGGCT

CATGATGCCCTTTGATGGGCCAAGGGAGTGGCACGCGGCCGCTGGGCCCCTGTGCTGCAA
GCTCAGCGGCGGCTCAGCCGGCACGGCGGCACGGTAAAGCCCTCATCAGTGTACTCGTTG
GTGGGCAAGACACGCGGAGGCATAAAGCACATTGGGCACTCCGCGTTCCGCACCTCGCTG
GAGCGGCACCACAAGATGAGGTTCTGCCGCCGGCCGGTCTCCACAGGCTCCGCCTGGTGC
AGTTCTCTCCCCGCGTGCAGCACGCCGTGTCCGCGCTCCAGCGCCACTGAGAGGGGCGCC
TCACCCTCGTAACCGACGAGGCCGAAGAAGGTAACCTGGCCACCTGTCCACTGCCCACCC
ACGTTTATGTTCAGCGTGACCTCCGCGTTGTCGTAGTGCTGCGCCAGCTCCCTGTCGCCG
TCGTCGGAGGCCTCGTACTTGACCGTGAAGGCACGGTAGCTGTCAAGGCCGTCGGCGCAC
GTGGGCAGGAGCTTGGCGGCCACGGGGTCCACAAAGTCCCTGACCAGTGGGTCCAGGAGC
CCGCCGCAGAGGCCCAGCTCTCTTAGGATCACGCCGTAGCGGTTCATGGTGTTGGGCCGG
GCCTTGGGCAGGTCGGAGGCCTCGAAGTGCGCGACTTCGGCCTCGCAGAACTTGCGTCCG
CAGGCCACCCAGCACGCCATGCCTCCACAAAGCACACGCCGCCGCAGCAGGGGGAGCTGC
GGGGAGGGGTGCCGGGAGAGGGCGGGCGGCGTGCCTTGAGCCAAAATGGCTACGG
>at01687
CCCAGATGCTCTTCGAATCGAACTAACAGAGTTCTTGCTGTACACTGCTAGGGAAGACAG
GCTGGGCACATACTGGAATGATGCCAGGCCTGAACACTATGTCATCGCAACAGATACTGT
TGGCTCTCAGCCAAGACAAGGAGTCCGACCAAGGTCACCGATGACATGGGCAATGGTCAA
GGGCCATGCTGTGAAGCCCTACACTTAAACAAAGTTTGAGCAATGCTGTGAGCTCAACCA
AGCCTTCACTCGAAAGACACCTTAAGTCGGAAGCTCCACTCCTCGACTACTCTAAGTCCT
CGAGCGGCAAGTACACCTTCTTGCCGACATCTGCAAACTTGCTGCTCATCTCTGCCATGC
CCTCCTCGATAGCCTTCTCCCTCGTGCC
>at01688
TTGTTTTTTTTTCTTTTTTTTTTGGTTCTTCCCCACACTCTGGCAGTAACGAACACACAC
CTTCCATGTTCATCCTTCAGAATCCAGCACGCTCATGCGTGTTCGTGTGCTCTGCCTCTG
GGGGTGCATCCGCAACCTGCCACAGCTCGTGCTCTGTCTCGCTTACGCCTTCGTATGGCC
GCAATGCAGCCTCAAGCCGCCACGGGCTCGGCAGCCTCATACCCACCCCCATAGCCGCCC
TGCTCCTGCGCCATGACCGGGGCTTCCTCCTTGGGCTCGTGCACCACGACCTTGTCCGGC
ATTGGCCAAGAGCAGCCCATCTTGCCCTCTGGGTCATAACCGAGCATGATCTTGACCTTG
ACGCCGAGCATGCCCTGGCGCATCGCGACGTGCCGCACCGCCTCGCTGATGTAGTGCTTC
TTGGGCTCGCCCGTGGAGATGAGGTAGCCATCCTTGAACTTCATGGCCTTCGCACGCTGC
GCGCGCAGCTTGCCGCTGATGATGACCTCGCAGCCCTGCGCACCGTTCTCCATGATGAAG
CGCACGACGCCGTAGCAGGCACGGCGCACGGCGAGGCCGCCGAGGAGCTTGTACCGCAGG
GACTCCGCCTGGGCCATGGCGCAGGCCGCGCGGTTCTCCACGCGCTCCGCGAAGAGCTCC
ACGCTGTTCTCGGGGAAGCCGAAGCGCTTCTGCACCACGGCCGTGAGCTCGCGGATGCGG
CGGCCCTTCTCGCCCAGGACCTCGCGAGTGCGAGTAGCACGGATGATGATTTCAGTGCGG
ATCGGAGTGACGCGCACCTCGACGCCCGCGTAGCCGTCCTCTGCCAGCGTCCGCATCAGG
AACTCGTTCAGCTCGGCGTAGAAGACGCCATCCGCTACGAACTTGCGCTTCTTGGAGATC
TGGCCCTGGTGCGACATTGCCTCTTCGGGTCCTGGTGTCTGTCAAGGGAGTGGGGCTGGG
AGCAAAGCAGCCTTGAGCCAAGAACACCTTGAGCCAAAATGGCTACGG
>at01689
GTGGCANNGGCAAAAAANGTCCGCTGCTCGGTTTGCAAGCAAGTAGGCTTGCTGTAGGAG
CCGCGCGTGCATTCATTCGAGACAGTGCGGTCTGACCTGCCGTGCAGAAACCCTGAGAGT
GCACGAGGCCAACAGTACTCACAGATGCCTCACAACCCCAGCAGCACGCACCAAGCACCG
AGCGCAAGGACTCTCGCCCCAGGGGTGGGACAGTCGGCAAGCCAGTGTGGCCAGCAGAAG
GTCACGACGCCGCGACAGGCCTTCGCACCTTGTCCGCCGCAGCGCCTCTCTTGCAATTGA
GGTCATACTTGATGACTCCGTCCTTTTCGTGCTTCGCCTCCACGGAGGCGTCGACCCACC
GGTTCTTGGTGTCGCTCCAATACTGTACCGAGTCGCCAACCTTAAACGTGGCTGGGGCAG
GCTTGTACGTGGGAGACGAAGACACGGATGCAGCCTGGGGCTGCACGGCGGCAGCCGGGG
CCGGCGCCCCGATCCTGAAGGCTTCGTTCAAGCCGCCCTTCGCGGCCGGCAAAGGCACGT
CGGCGGCTGCAGGATCCTGGTCGACCTCGTCGTCGTAGTCGCCGTCGTAGTCCTCGTCGT
AGTCCCCGTCCGTGACCATCGCGAGCTCTCTGAGCACCTGCTCGATGGTC
>at01690
GGGCCGCGGCTTCGAGATCGCGCAAGGCCGCCTGGGCCCGGGCCGGATCCACCACTGCAT
GAGGTCCCTAGGCGTGGCCGAGCAGGCGCTCGCTGCCATGGTGGATCGCGCACACCGCCG
GAAGGCCTTCGGCCAGCTGCTGGCCGAGAAGGACGGCGTGAGGCAGATGATCGCGGAGGC

CCGCATGGAGATCACCATGTGCCAGCAGCTCTGCTACCTTGCCGCATGCGTGGCCGACGA
GAAGGGCTTCAAGGCGGCCAAGAGCCACATAGCCATGATCAAGGTCGCTGGCATACGCGC
CGTCATCAAGATCATCGACGACGCTATACAGCTCCATGGGGCGCACGGCGTGTCCCAGGA
TTCGAAGCTGGAAGAGATGTACAGGAGCTACCGCTACCTGCGTGT
>at01691
AGCCACGCGCATCCAGATTAACTTTCACAAGATCTCCAGCAATGTCAACCTGGTTCTCCC
CCATGGAGTCCACCGTGTCAACAGAGACTTCGTCGCAACGCAGGTGTGGGAAAGTGATGT
TCAAGCCGATGGGCAGCTTTTGATTCAGAGTGGTGTCAACAATGATGTGATCTACAACCT
CAACTGTGAGATAGTTGTTGAGCTCTGAGATAAACAAAACTGTCATGATAACAATTGAGA
CAAGTGATATGAGCCCTCCACTTTTCGTCTTGACCCTGTAGTCATCATGGACTTTGGAGT
ACACGTCGAACTTTTTAAGCTGCGTCAAGAAGTTCCCATTTTCCTTTGTCCGTTGCCGCA
TCGCACGAGGCGAAAGAAGCCCAGGTTATCTCATGGCGTGCGGTCACAGGCTGCTGGTTC
TATACCAGCACCTCAGCCGTAAAAC
>at01692
TTTTTTTTTTTTTTTTTTGACAGTGGAAGGAAGTCACGAGGCAGGCCGCCGCTCACCAGA
CGCTTCGCCCGGCCGATCACTTCATCTTGAACCTCGACTTGAGATCGTCCGCAGACAGAG
GCGCTCCCTCCTCCCACGTGTAGAGATCCTGCAAGTAGAGGTTCTCCTTGGTGTACATGA
TGTTCTGCTCGTGCGGCAGGCTGGAGTTGGGGTCCACGGGTACGGCCTCGGCCGACTCGA
GGTCGATGCCCGCCGAGGAGAACGCGTTGAGGACCTTCCGCTCGTCCTTGCTCTCGTAGA
AGACGTGGGCCATGCTGGCGGCCTCGTCGCCAAGGCCGCCGCTCTTGGAGTACTCGGAGA
GGTTCCAGACGCAGATGGAGAAGTGGACCTTACCCTGCTTCTGCGTGGCCTTCTCCAGGG
CGCTATCCTTGCAGACAATCTCCCTGCGCTTCAAGATCTTCTTGGGCATGGCCTTCAACA
GGCGCATGTACTCGACATCATCTGGGTGAGAGCCCCACTCGGCCACCTGGGCCACGCAGG
GGCCTGCATCCGGACGCCACATTGCTGATCAAACGAGGAGAAGGCTGCTGTGGCGTTGGG
CGGAAATGGCTTGAGCCAAAATGGCTACGG
>at01693
CCGTAGCCATTTTGGCTCAAGTGCCTGTCAAAGCGTGAAGCGGCAGACCACTGGAACTGG
CAGCCATGGCCGCCCTCGGCTTCAGCCGCGCACTCCTCGCGGCCGTGGCGCTGCTGGCGG
CGGCGGAGCCGGAGCCGTCACGCACCGGTGCGCTTTGTGAACAGCTGCTCAGGGGTGTTG
GTGAGGCTGGGCGCTCAGACTTCGAATTGGCGGCACAGTGCCGCGCCCGCTTGCCGGAGG
ACGCCTGCCGCAACGCGCGTCACCTCCTCGGCAGCCAACCATGGAAGCCAGAGAACATGA
GGGTGGCTTGCGGCCATGGGAGGTAGAGCACAATGCCTACGTGCGGCAGCTGCAGCAGAC
TCAGCCGTATTCGAAAT
>at01694
TCTCGGCTCCAAAAGGGCACCGCAGGCTTGTCCCCTGCATCCCCAGACCGCCCTGTGTcC
CCCTGGATcCCCCAGGACGCCCTGCTGCTCATGGTAGAGGtTTCCACtTGGACAGACAGC
AAGAGCTTCTCGCTCTGCTGGATGCCAAGTTCGACCCCGACGGCGCTGTTTCGACCGCCG
ACGCCGACCGCTTCGCGGCGGTTTATGTGCCAGGAGGCACACTGTCCATGCATCCGCCCC
TGGGGGGATGGCAAGGCGAGGCCTGTGGAGGTGCATGCTCGGGGCATGAAGAAGGGCATG
AAGCCTGGCCAGCTGGTCTATTCGAACAGGCGCGTGACCTTTCACCCCGACGGCTTGCGA
GTGGCCGAGCTGCATACAGTGCGCACGCAGACGCCCAAGAGGACCTCCGACGTTATAATC
GTCTTCCTC
>at01695
TTTTTTTTTTTTTTTTTTGGAACATGTGCATACTTAATGGTACATGATGCCTGCATCAAA
AAAAACACCGTCATGAGCGTGATGTCAAAATAGCCGCTAACTTCATAACAAGGCCCTGTA
CAGTATCCAGTTTGGACTGACTCCACATGGGAACGGTGCTAAAAGTCAAAATCATGACCT
CTTGCCTTCAACTGATCAAAGACATGCCTGAGGCCAGTGTCTGCGTTGACCCGTTCTCCC
ACTTGCTGTAGGGTGGCTGTCACATCTTCGCTCGACTGGAGCTGCTTCACAAGGCTGATG
GCTTCGAGCCCACTGGGGTGCTCTGCCAAGTCAAGGTCCTGGGTAATGTCGTCCATGGTC
TCAGGTTCGTCGGCCCAATATCCTGCTCGGAGGAGTCGTCGCAACAAGCGGCACAGGTCC
GCATCATCCAAGTCTGAAAGTCCCAAGTCTCCTGACTGCATCAGCTCGCGGAGCTTCCCA
GTGGCAGGCTCGCCTTCGAGGATTGCCTGCAACTCGGCTTGGTCTCGCACAAGTTTCAGC
AGGTTCTCTGGCTTCAGCAGCTTCTGGCTGCCTTCGCGGAGGATGGTGACGGCGTACCTG
CCGCTGGCCTCGTCATACTTGACAATTCGGCCAGCGGCGCCGTTCAAGTGCTTGGCGCCG
ACCAAGCCCTGGAGCATGACACGCTGTCCTGGTGACAGTGCTGCTGCATCTTGCTCCTCA

GGTGTGGGCTCAGGCACCATCGGGCCACAACGTGAATCAACTCAAAGAAAATGCAAAACC
CTCCTTGAGCCAAAATGGCTACGG
>at01696
CCGTAGCCATTTTGGCTCAAGTACAAGTATTGAGGCTTAAGTTGATTCCGGGTCTAGAAG
AGTAGCGCATCAGCTGCGGATGGTGGCCTCCTTGATGCCATTGGCCCCATGCGGTCTTGG
ATACCACGGTTGCCACGGGTATGGCAAGCACGCCAGAATTGACAGCAAGACCATCGACGT
TGGTGATTTCCTCTGGGCAGGCAGCGCCATTTCGAAGATGCAGCGACGGCAAGCAGGGCT
CTATTGTGCTTGGGTTGTTATCGTCATTGGGGGAGAAGCCCTCTCGTTCAGATGTGCAAG
TGGCCCTCACTCCATCAATAGATGCTTGGAGGGAAAACCCACGCTCTCCTACACTTGT
>at01697
CCGTAGCCATTTTGGCTCAAGGCCGCCGTTTAGAACGCTCAAGCCGTTCTCGAGTCAGCC
TCACCCTTCTCCAGAAGAAGCAATGGACGCAGCGACGGAGTGCAAGTGGTGCCTGAAGGG
CGAGTGTTGGAGCCATGGGCAGATCGAGAAGCCCGAGAAGAAGCCCCGGAGAGAGGGCAG
GGCAGACGGCAAGGGCAAGGGCAAGGGCAAGGGCAAGTTCTCCGGCGGCAAGCCCGGCTG
CAAGTGGTGCCAGGCCGGTGAGTGCTGGTCGCACATGGGCAACGACATGATGGGCGTCTT
CATGCAGTTTCTCAAGAAAGGAGGCTGCGGCAAGGGCGCCTTCGGTTACGGCAAGGGCGG
CGGCGGCGGCAAGGGCGGCGGCGGTGGCGAGAAGCCCAAGAAGTTTGTGGTCGACAAGTC
TGGCGGGGAGCTCGGGGAGTTCACCGGCACCATCAAGAGCTTCGGCCCCAGCAACTACGG
CTTCATAGAATGTCCAGACCTCGCCGGGGTCTACGGAGACGTCTTCCTGCACGGCGACAT
GATCAAAGGCTACAAGAAGGGCCACACAGTGAAGTTCTCAGCCGTGATCACCAAGGACGG
CAAGGTAAACGCCATCGACTTGAAGTCCGGCCTCAAGTGATCCACGGCTGATGCTTGGCG
GGGCTCACAAGAGGTTTTATAGACTGGTCTTGGTCCGTCTGTCTGACGTGATTGCCTTGA
TGTCTTAGCTACCAAAAAAAAAGAAAAAAAAAACAA
>at01698
TTTTTTTTTTTTTTTTTTGGTTCTTAACACAACTCCTCACACAGGTCTCTGCCAGCGCTC
TTGCCCGTCCACCGCAGACATACTTGTGCGCAGCATGGAACTGAGGCTGCGGTGACGAAT
GCGCTGTCGTCTCAGCAAGCGTGGCACTGGACATTCCTGGTGGTGGAGCTTCGTTATCCG
CTCCGACGCCCAGTGCTTCGTGGGCAGATACCTCTCCCCGCTGGAGCATTCTGCCCGGAA
CGCGCGCAGGATCCAGCGCTCGGCACTCTACTGCCCGTCGACGTCGATGACGTCGTCCGG
CTTCTTGTCGCCGCCCGGA
>at01699
TTGTGTGGCTTGACATGGAGAAGACGCGAGAGGAGCTGTGTCCATCGAGATTTTCTTCCC
GGAAGCCCTGAGTGACCGTGTCAAAGTTGCAGGTGACGAGCGCGAGCTTGGCAAGAGTGA
GGGGTAGTGCATCTGCTACAGTGTGCACAGTGTCCTGCACAGATCACAGTTTGAAGTGGT
GACCTTCCCAAAGGAGACTTAAGACATGTCCAGGGCATGCGTTGAGGATGAACAACTCAG
ACGTGACATGACGCTGGTTGTTCTTACTTGTGGCTGGGCTTGCGGGCTTGCGGCGATGAG
GGCGTGTTTACCAGCATGATTGCAAGAGCCACCAAGTCAATGCGTCAGTGGAGTTTGCTC
TACATTCAAATTGATGCCGCGGCGTC
>at01700
GCATTTCGGCACAAGACCAGTAGGCTGCCACTGCACGGACCGGCCTGGTGCATGGCCCGA
CTAGGTGCCCATGCATTCCGCGGTGTGCTGCTCCAGTTTCTGACGGCTCAGGGGCTCAGC
GCTGCTGGAGCACCCCTAGCACCCCTGCNGGTGGTGCTTGACTTGGACGAGACCCTGGTT
CATGCCACGCTGTCGAGCGGCAGCCCAACGGCCAGAGGCCAACCCATGCCCATGCCCTcG
cCCCCGCTGCCTCGCTCGCTCCTGCGCCGACTCACGCAGCCCTCCTCTGACTTCACTGCC
CGGGGCCAAGGGCTCATCCTTGGAGCTGGGGAGGACGTGcCCCTCGAGGTCTCGCTACGC
CCAGGTGCAAAGAGCTTTGTTGAATGGCTCCTCCGGCAGCCATGGATCGAGGTTGCTAGT
ACTAACAACGGCAGGTACAAGGGCTTATGCGGGGCAGGT
>at01701
TTGTTTTTTTTTCTTTTTTTTTGGGGGGGGTCATGGACAGCTCTGGGTTCAGCTTCAGCA
TTTGGACTCCAGGCTGGAGCATTCAGGGAAAAGCCGGTCCCTGTCACTACACTTGCCCTC
CGGAAGGGCCGTGAAGCTTGTAAGCCATCTCAAAGCCGTCACAGTCAGTGTAAAAATTGT
GCAGCCTGGCTTCTTGCCTGAAGCCGAATCGCCTGTAGAGACGCTGCCCCGATAAGTTGT
CAGCCCTGACCGTTAATCTCAGAGTCCTAACTTCCTTCCACTCGTCCGAGCCCCGCAGGA
CCACGTTCCGCAACAACTGCTCGCCAATACGCTTGCCGCGCCAGTTGGCTGCAACAGCCA
AACTGCAGACATTTAAATATGGAGCGAGATAGGCGCTCCGTGGGACCACGTGAGCCCAAG

CTATGTAGCCTACAATTGAGGATTCTTCGGAGACTGCAACGTCAATGAAGGTCTTGGGAT
CTGCACCCTCCCGAGCGCACCGGATCCACCGTCGTAGCGACCCATCCTCTCTGAAGCAGT
CCTGTTCAAGAGCAACCAGTGCCTCCTCATCCGAATGGCGGAAAGGGCGGACCATGCACG
TAGACACGTGCTTTCCATGACCTTCACTGACAACATCAGACTCCCTTGGGGGGGAGTTTT
CAAGTATCATGTGCCTGCTGCGTTCACCCCCTTGCCCTCGGGGGCCGAAGGGAGGTGCTG
GAGTTGACATCTGCAAGGGTCTCTCCAGGGCGCAAGTTCTACCGCGGTCATCTGTACTAT
CGTGTTCTTCCACTGGTACGGTCACCCTCACCAGGAGAGCTTTCATCTTTAGAAGTGAAT
CGACACCTGCATCACGCAAGTAGAGTGTCACCCATCCAGACTCAGGCCCGTTGCCCCTAA
TCTTGTAGTAGTGGAGCCGTCCATCCTCCAATGCCAATTCTGTAACAACAGCACCTACCT
CCAGGAGATCTGCCTCCGCTGGCGAGGATAACGATTTGCCTTTTCGCACAATGATACCAC
CTGTCGTCCCACCACCAACAACCTCCCAAGATCTAGCTTTGCTAATCTGAGCCATCGAAG
TTGGGTCATGCTCTGGGTCTTTGCCAGCAGTGCTCTCTCAGGCGCAGCCCGCCAACAACC
ACCACCATCACTCTGTCATAGCGGACCTGA
>at01702
GGCCCCTCTGCCAAGCTCCCGTCCCGCCGGTAGCCACGGAAGCGGTATGGAGGAGCGCCC
AGCGGTGCCTCTTCGGCGAGCCGTCGTCGCTGTCGCCGCTGATGTCCACGACTTCGGCAA
CGTCATCCCCGCTGTCTTGGGTCACGTCCACCACTCCTTCCTTGAGCATCACCCAGGGAG
TTGCGCTGGCGCGCCTCAGTCTCAGGTGCCCAAGGGGTGAGCGTGAACAACCCGCAGCTC
TACGACTGCATGGCCACCACACGGTGGGACGGAGCACATCTTGGAGGGCTTGAGCCAAAA
TGGCTACGG
>at01703
TTGTTCTTGGTGCGACGCTGCTCCGCCAGGTCAAGAACGTACCCTGCAAAGGCGCCTCAA
GAAAAATGTTTGAAGCCAATGTGAGAGGGGCTCTGAAAACATGCCAATGAATAGTTCAAG
GCACTTGCTGCCAATCATTGGAAAATGAAGGCTCCGAGAATTCGGCCTGAAAGTCCTTGC
TCTTGCCGGCAACGCGCTCGGCTCCTCTTGCCAGCCGCTTTCAGAGGCCGAACTGCTGGA
TCGCATCCTTTCCAGTGGCCAGCTCGGCTGCAAGGATCCCAGTCACGGCAATCATGGCAA
AGCGCCCGTTGTTGATCTCCCTGTTTCGCATCTCCACGTCGTACATGCCAAGGCCAAAAG
GGTCGCCAAAGTTGCCGGGCTCCTTGCCGGGCTCAGTCCTTCCAGACCCCCTCCAACACG
CCGCTCCACGCGAAAATGGCGACGAAGCCGACCTGTCCGGCGACAGTGCTCAAGGCCCCG
ATGCCAGCAGGGACGCTCTCGGCTCCAGGCAGCTTGAGGAAGTGCTGCGCCACAAGACCG
ATGGACGCCATCATGGCAACCCGTCCAGTGCTTCAGCTCGGCCATCCGCAAGATCCGGAA
ACCCGCCGGGTCGTCGGCCTTTGCGAAGCCGAGCGGATCGAAGAACCCGAGCGGTGCCAT
GGCACCAACCTCAGCCGCCGGGTCGAATGCCGGACGCGCAATGGCACTCCGCCGAGCTCT
CTNGGCGACCACCGGTATTCGCGGCGAGGCTCCTCGTGCCGAGCGCGGCAGCACCGGCTC
CAAGCGCGGCGACTGCAGCCAGTGGGACGCCTGGAGACCTCCATGGACGACCTGCGTCAC
CGCTGACCTGCTCGCTCGCCTGCACAGCAGCATTGCCCCTCCTCAGCAGGCTGGGACCCT
GCACGCTGCCACTCCCAGAGGGCAGCACGAAGGCGTCGGCCGCTGCGAGGCCCGCCATGG
CAAGTGCGACGCCGCTGCTCATCGCGCTTGCAAGCCCCGCGTCCGAAAATCTGGTCCAGA
AATGCTTGAGCCAAAATGGCTACGG
>at01704
GTCTTCATAGTGGCCCGGGACGCATGCCTCGGCGTGCAAATGTAGTGGCCAGCCTGGAGG
GCTAGGCAGGATGCAGGCTGAACAGCGGCCAGCAAACACCCAGATGAGGCGGTCTGCTTC
GGCCACCCTTGTCGCGTCCCCAATCTCTGAACTGACGATGACCAACCGCCGGGCGAGCTT
CGCAAAAAGTCAGAGGGGACAAGGGCATCCTGCTCTTGGAGTCATGCAGTTCTATCAGAC
CCCCTTTGAGGCAGCTTACTGCGATCTAGCGAAGCAGCGCAACCTCCGGAAACTAGAGGC
TCTTTTCCTCGAGGCGGACGCCG
>at01705
GGTGTTTTTCCTTTTTTTTTTTTTTTCCCCACACTACAAAATGGGTGCATCAAAGATGAT
CCTTTGCTAAGAGGCGGCCCCAACACCCTCCACACATGAAACCTAGGCACTTCAAGAACT
AAGTCTTGGGCACCGTTGCGTCTAGAGGAGTGTGGCGACCAAAACAGGGAGGAACCCCCC
TCAGGCCTTCAGCTTAAACCTCAGGTCGCCCTTCTCAAAGAAGCCCACGATCTGATTTGC
AGCTGCAACACCAGCATTGTTGTTTGCCTCCAGGGTCTGGGCACCCATCTTCTTCTTCGT
GAAGATGACCCGCTTCATGTACTTGTCCCCCACCAATGCCTTGATCTCTTCTGCGTTCTT
TGGGGCAACATCGCAAAGGTAGCAGAAATCCGGGCGCTCCTTGAACACCTCCAGGAGTTC
CGCTTCATGCACAACCTCATGCCGGGCCGTGTTGATCAGGGTCCCACCCTTGGGCATCTT

GGAGAGCAGAGCCTTGTTGATGGACTCCTTTGTCTCGTCTGTCAGAGGCACATGCAGTGA
CACGTACTGGTAATTGAAGAGATCCGCGACGGTGTCAACTGGTGTCGCGCCCATCTCCGC
AATCTTGTCCTTGGGGATGTAAGGGTCATAAGCGTACATCTTCATACCAAAGGCCACTGC
CAGCTTCCCAACTGCCCTGGCCACAGCACCAAAGCCATAAAAGGCGATGGAGCGGTTGAC
GAGTTCGAAGCCACTGGAGCCATCATAGTTGTTCCGTGCACTCGTCAGCATCATGCCGAA
GACGAGCTCCGCCACGGCATTGGCGTTCTGCCCTGGCGTGTTCATTGCCACAACACCCTT
CTTGTCACATGTCACCAGGTCGATGTTGTCATAACCTGCACCTGCACGCACACAGATCTT
CAGCTTTGCTGCCTTGTCCAGGACGGCTGCATCCACAATGTCGCTACGGATGATCATTGC
ATCAACTGAGGCTACCGCTGCGAGGAGCTCGTCCTTGCTCTTGTAGTTCTCCAGCTTCTG
CAGCGTGTACTTGGCGTCCGCGAAGACGCCCTCGATCTTGGTGACGGCGTCCTTTGCAAA
GGCCTTCTCTGTGGCCAGGAGCACGGTCCTGGGCTTCCCTGCTTCTCCTGCCATCGTTTG
TCAAATGTGGCTGATGGGGGCGTGAAGACGACGGGAGCCCAATGCTCTTGAGCCAAAATG
GCTACGG
>at01706
GCTCTGCTGGGTGCACTTGCCAGCATGCATGACTTGCGACCACACTGTCCCAACATAGTC
TACTACAAGATGAGCCCATACTACGGGAACCTTTGCTACTACTTATGTCTCAGTGACATC
TTGGCCCTGGGTTGCGCGTACATTTACGTAAAGGTGCGGATGAACGCACAACTGATTGGC
AAGTGGAGATATCGAGCCTGGAAGACATTGTGGTTAATCTCTGCAGTCTTTTTCGACTTT
GTTGCCATTTGGGCTGCCTTTCGGACATTTTGGCGGCCTTGGCTCTGTCTTGAAAGCCAT
ACTCTCCAGCTTCTGTACACTCTTCTTCAGGTTCGGCCTGTCATTTCAGGTCAACCTGGA
CTTGCTGAGGATTATGACTTGTACCATATTCTTATTCGAGGGTCTGGAGTTAGTTTCAAA
TGGTCGCTCTCATACTGGGGCCGAAAGTGGCTCCAAGATCTTTCAGAAGCTCCAGCTCTT
AGGACGCG
>at01707
TTCACAGTTTCTCGTACAAGGACCTTAGATCCCTCCTTGCGTGCACTCTGGGAGCTGAGC
CTCTTCAGTGCGGGCCTTCTCGGGCCCGAGCGCATGCAGGGTGGATGGAGCATTGGCTTG
CCCTCCCCGCTCATGGTCTCATACATTGGGCAGGCTGTCAAGCGCGGCGCCGCCGAGGCG
ACACTTTAAAATCATGTTTCTTGCGAGGCATGTTGATTTTGAGTGGAGGTACAGTTCTTG
CCTCGCGCGATCGCACGTGGTTGGCACGCGAGTGGCAGCAACGCGAAGCTCAACAAAAGC
ACAGCAATTGTTGGCTCGCGCACGGCTCAATGCCTCAAGGTTGCCAACCGTGGCGTATCA
AGTTTGGCTCTTCTTTATCCTTGGCCGCACTTGAAGGGGCCGGCATGCGCAAGTGTTTGA
TGGTGGTTGGTTTGCT
>at01708
GTGCCGTTCTTGCTTAATAGGTTCGGTGCCTTTAGCACGTGTGTGGTGGCCTGCCTGCTT
GTAGCTGGAATGTGCTCAGCTCTCGTCGCTTTGACGGCAGTTAAGATCATGCCCTACGTA
TATGCAGCAACAGTTGGACTCTTTGGCCCCATGTACGGCATAGGATATGCGCACCTTGTG
CGGCAAAAATGCGAGGCGAAGAAGCTCGGGAGCTTATTTGGCCTCCAGAGCCTCTTCAAC
GGTGCAGCCGGCACCTTAGGCCCGGGTGTTGGCGGGGCTATCTATAACCGCGCCCAGCTG
TTGCCCACCATCATCACTGCAGGTGCGTCTGCGATCACA
>at01709
TTTTTTTTTCTTTTTTTTTGGTACTCGTGATTCTTTTCTTGTACTGCTGAAGTCCTCGTC
CTCCTTGTTATGCATGGCGAGCTCATTGCACGGATGCAAAGTGAAGAACAAGCGGGTAGC
ATCCCAAGTTTGTTACGAGAGGCCACCCCGAGTGGTAGCAGGCGTCAAGCTGAGCGCATG
CCACTCAGCGTCATTGCATCCCAGGCGCTTGTTAACTATCAAATCCGTCTGTTTCTCGAC
CTTCAGCTGGAGGTATCGCTCCTTGAAGCTTGTCAAAGAAGCAACATCCATCACGCCAGG
TGTATCAAGGGCCTGCTGCAGCACTTCTTCTGCCTCACCATCTGCCTGCTGCGAAGTGGA
TGCCAGGTTCGCAATGCAAGACTTCTGAAGTCTGCTATTTTCATCTCTCACCTCCTTGCT
CCTATCAACGAGGACACGGGCTTGAGGCAACGTCAAGATAAATGCATCCAAAGCACTTCG
GCTTGCTTTGAATTTGCCAAGTTCTGACTCGCTAACTTGCATCAACTCCGGAAATGCTGC
AGGCACCAGAGGGGCAGCTAGTGATGCGTGAGTGGGAAGTCCTGACATGGTCTCCACAGC
AGCTGCATGCTGTCCCAGCTGGTTGGACGCTAAAGAGGCTCCCACGGCCCCTACGCGGCG
GATGCGCCTCCGCTTTCCGCTGAGCTTGTTTATCTGTTCCATGGTATCGAAGTCAACTAT
GTAAGTCTGCCGCCTAGCCTGATACTCCACTCGGTGCTGGCCACTGGCATCTGCCTCTGC
GAGCATCTTGGCAATCCTGGGCTCGAAGGGAACCCATCCCCCCTCCAGCTCAATCTCGAA
GACTCGAAAAACCCCCAGTGGTCCCAACGTCTTGTCGGCGCCTGGCGCCACTGCGGCTTC

GGCGGGGGAAGGCGGCTCAGGGCGTGCGCATGCCGTACCCATCGGAGCGGACAGTCACGG
CCCTGCAGGCAGCACGCCAGGGGCACAAGAGCACGACCGAGTGCTGCTCTCGCTCAATAC
GAGTTCACGTAATACCCGGCAACAAACACGAATTTGAGCGTGGGACTGGAAATCAAACGA
ATTGGTATGCAACAACCGTTGGAGCCCAGACGCCCATCTTGAGCCAAAATGGCTACGG
>at01710
TTTTTTTTTTTTTTTTGTCCTGCGAGCATGCGCAAGAGCGCTCCCAGCTCTCCTCAGTCC
CCCAGCCGCCCTCAGCACTGGCGCGGGCGGGCGTCTCAGCAGATGGCGGGCGCGCAGGAG
GCGATCTTCGGCCAGAGCTCGGCGCACTCCTTGGCCACAGGGCCCTGGATGGCTGAGCCC
TTCATCTCGCCCTTGTCATTGACGATGACGCCGGCGTTGTCCTCGAAGTACACGAAGATC
CCGTCCTTGCGGCGCCAGCACTTCTTCTGGCGAATGACCACGCCCTTGAGCACCTTCTTG
CGCAACTCCGGCTTGCCCTTCTTGCAGGTGCAGAGGACCATGTCGCCGGGGCCGCACTTC
GGGATCTTGTTCAGGCGGCCGCCGACGCCCTTCACGGCCATGATGTAGAGGTTCTTCGCG
CCACTGTTGTCCGCGCAGTTCATCAGTGTGGCCACAGCACACCCAACCGACATCTTCAGC
TTGGCCACCGAGCTCGAGCAACTCAAGCCCCTCGTGCC
>at01711
TTTTTTTTTTTTTTTTTCCGAGAGCAGGCCATCACTTCCTGCCGAGGATCGCGTCATTC
ACCTTCTAGTCCCGCTGGTCGGAAACATCTTGACACCACCACGTGGGAGTCCTTGTGCCT
GTACAATGGCCTTTGCACAACGACTGGGTTGGTCTTGAGCATCCCGACCCATATGAGACA
CCGCGGATTGATGTCGCTGCTCAGCGTGACGAAGCTCTTCTCCAATCTGGTGGAACTGCG
TGCCCCCGCTCTCACGTAACCATTGTTACCAAGGATGCCCGTCCGCGCGATGGCCGGGCT
GCACTGACCGAGGATTGACTTGCAGCACTAGTCGCAGATGCGGAAATGGCCTCCTGTTTC
TCTTCGTCAACACGCATCAAGAGTTGCCCAAGTCCTGCTGCCACGTGCGCGCAAAGCCGC
TGCCGTCTGAGAAGCGCAACCAGCCATCGACGGGCTTCGCATCCGCCTTCACCTCGGCAC
CAGCCTTCAAGATTCTCACCACAGGCGCCTTTTCTGACATGCTGCCGCGAACGAGCAGAA
GCGAATGAACACAACGCCAGGTTGCACTTGGCTCGGGCGAGCCTCGTGCC
>at01712
TTTTTTTTTTTTTTTTTCCGCGAGCGCGCTGCAGAGGAGAGCCCGGCAGGGCTAGAGCA
TCTTGCTCGCTGTCGGCAGGTCCAGGGGCGAGCCGCCGCTCTGGGACCTTCTCAGGGGCA
CCTTGAGCTTGCCGTGCCTCACCTGCACCCTCTGGTACGCGTGGGAGGCGATGGAGTCGG
CCTCGCTGAAGAGGTAGTACCAGCCCAGGTCCAGGAGGACCTGCGCCAGGCCAAAGGCTG
CAAAGCCAAGCAGAACCTTTTCAAACAGCTCGAAGAAGCTGAAGTATCCCACACTCCCTG
TGCCCACGGCCACCAATCTCAGCCCATGCTGCAAAGCCATGGGTTCAATGCCGTGGTAGA
ACCAATGCAGCGGCGTGAACTGGTCTCGGAGCACCTCGAACCGCAGAGTACACTCCAGGT
CGCTTGCAAGGGCATAGCCAAATGGGTTCCACCGCGTGAGCGGCCAGCGGAAGGGCGTGT
GATAGTTGCGCAGATCGAACTTTGCAACAATCTCAAGGCCTGTAAGGATTAGTGGACCCT
CAGAGCCGGTCCCACTCAAGTTGCGACCGGCCGCTTTCAGGATGTCGTGAACAGACAGAA
TCAGGTAGCCTTCACCATAGTCATGCTCAGGGAATAGCTTATCGTATCGGGACTTGATGC
CATTTCCGTCGGGGTGC
>at01713
TTTTTTTTTTTTTTTTTCATCACGACCTAACCCACTAAGTTCTCGCACTTGCCACCAGC
AAGGCACTGAACGGCTTAGGGGGCAGTGAGTCCTCTAATCCGCAAGACCTCAGGTCTTTA
GAGGCTCCTTCTTATCTTTGACCTTCTTGGGATCTACGGCCGTGTTGCTGCCGGCGACCG
TGGTGCCGGTCTTTTTGCCATGGCCCTTCCGCGCCCGCTCGGCTTGTGTCAACTTCTGGA
TCTGCATGACATCCTCGTCCTGCAGCTCATGCTTCGCGCCACAGACCTGCGGGGACAGGC
GGACGCTGCTGCCCCAGACCATGGCGCTCTTGAACTCTTCTAGAAGGCTCTTGTGGAGCA
TCCCTACGGCGTTCTCTATGGTGCAAGTCTTGTTGCCTCTCTGCGGCGTGATGATCATCG
GGTCTTTGAAGTCGGGGAACATTCCCTTCTTCTTCGTGTAGATGCGGACGATCTCCAGCT
CCTGCCAGATCCTCTCCAGGAGGCCGTCCAGGTTCAGCTTCTTGTTCACGGAGATGACCA
CGGAGTAGGGCTGCCGCGCGATCTCGTCCACTTGCGCCAAAGAAAGCATGTCTACCTTGT
TGTACACGTAGAGGCACTTGACGTACTTCCGTGGCGCCGTGCCATTCTCCTCTAGGATGT
CTATGAAGTCATCGATCGAGGCATCCTCGTGGACAACGACATCCACGTTGAAGATCTTGT
ATTGCTGCAGGATCGAATGACACATCTCACGACTAAGGACTGTAGTGGGAACAGTGGCAT
TGAACTTCAGCCCTCCAGTCTTCGTCCTGGTGACGGAGATGTTTGGGCGTGTCTTGTTCA
GCCGGATGCCGACATCCTCCAGCTCCTTGGTCAGCATCTGCCGCTGGGCGTCGTCTTTCG
TGGCGTCGAGGACCATGATGATCATGTCGCTGGAGCGCGCCGTGGCGATGACCTGCCGGC

CCCGCCCCTTGCCCTGGGCTGCGCCGTTGATGATCCCCGGCAAGTCCAGGAGCTGGATCT
TGGCATCGTTGTACAGAATGACGCCAGGAATGCACGTCAAGGTCGTGAACTCGTAAGCAG
CGGCCTCTGACTGGACACCTGTCAGGGCTGTCAGAAGGGAGGACTTGCCAACTGATGGGA
AGCCAATGAGCGCTACACGCGAGTCCCCATAACGCTGCACCTCGAAGCCCGGCACCTTGC
CGCCACCGGCTTTTGATGGCTCCAGGAGTTCTCTCCGCAGCGCACACAGCTTGGCCTTCA
GGGAGCCCAAGTGGGTTGTTGTGGCCTTGTTCTTCTGCGTCCTGGACATTTCCAGCTCGA
TCGCTGCGATCTTCTCCAGAATTCCTGGCATGGCTGCGACAAGTCAGCGCCCGCAACGGC
GCGCGCAAGCTCAGGGCACCCTCTACCTTCAAAATAGAGCATCACATGCTCTTTGTGCAA
GAGATGCTTGAGCCAAAATGGCTACGG
>at01714
CCATCGGCAAAGCACTGGCGGAGGTCTTCCGCCGCGTGCGCCTCGCTTGGGTCCGAGACG
ATGACGTGTAGCGCATAGTCCGCTGCCGACTTGGTCGCCGTCTGCCGATAGGCGGTGACC
ACATCGCGGATTCGCTGGCCACGGTGCTGGCATGCGAAGGGGCGATGGTTGTGGTGCCGC
CGGCAAGCGCGCTCACGCTGGCCGTGTAGAAGTCGTCGACGGGCGTGAGGCCTGTCGACG
TCTTCTGCTCAATGTGGCAGTGCGAGTCCACACCACCTGGCAGCACGAATAGGCCGGTGG
CGTCGATGTCCTCTCGGCCAGGTGGCAACGTTGGCTCAATGGCAGCGATGCGCCCGTCGG
AATCAATGCCCAAGTCAACGCGGCGTGCTCCCTCTGCCGTCACGAGCGTGCCGCCGCGAA
CCGTCACATCATGGGGCCTGCC
>at01715
TTTTTTTCTTTTTTTTTTAATGCTCCAAGCATGTCACAGCACGAGCTTTTCTGTGCTTCT
GGCATCTCGCAGTATGCAAGCTACTGCATGGAGGCATGTCCTGGCCATACTACTGCCGGC
GCTGTGACAGGGGAGAAAAGAGCTTAGCCGAGACTGTTTTCAACATATGCCTAGACATTC
CTAACTTGACGGAAAACAAACTTGTCACCAAGCAAGGCAGAACTCAGCCGCATCCGATGC
CAACGAGGGCGTCTGTCAGCATGTAGGCTTTGAAGCTGTGCTTGGTGAAGATTCTGGATG
TGAAATGCTAGGGTCTGGCGCCTGGCGCTCAAGACGCGACCCTAGAGACAACGATGCTCA
AATCCTGATCAGTCGCTGCTGTTGCTGCACGACTCTTTCCAGGCCCGGTGTAAATTGAGT
ATGAGCTTCTTTCTCTGCCGCTTCTCCTGCTTACGGCTCTGCCTCTCCCCAGGCTTGCAC
AAGTGACAGTAGAGACAACTCCAGCCGTTCACGCAACCCTCCTTGTGAAAGAATCCGCAC
GGCTTGCACCACCCTTGAATGTGGCGGGCCGAGCCGATACTCGGCAGCTCTGTTGAGCCA
AGTGCCAGACTGTCTGGTGCAGCTTGAGGCGGTTGCATGCTCCGTACTCCCGCAGAGACG
TCAACGCTTGAGTGGGAATGAACCGACCATTCCGAAGGCGGCCAACTGGCAGTGACATCA
GAAGATGGTGAGACCCCAGTCCAAATGAGGTTGTTGCCGATTTGAGCTCCAGTCCCACAA
AGGAAACCGTCCGTGAAACCTTCAGCATGTTCCTGATAGCAGCCCAGTGTCTGCACTGGT
GACGGCGGTGGGCCCATTGCATTGTTGAACGGGAGGACGAGGGGCGCGCTGCCCGGAGGG
GCATCGACAGCGCTACACGCCTCCGGCAGTTTGACTGACAACAGTGTACAATCCGCATCC
GCATCCTGGAACAGGCTCCACTTGCCTTCGTCACTGTGGGTTGGCGAGCAGCTGGCCACC
TCGCTCTCCTGGTCAGCCTCGCTCTGGCGACGATGAGAGCTCTGAGGATGTGTCGATGCT
CGTGCCTCGGATGCGAAGCTTCACCGGCTGCTCCTTGGGCATCGTGATCGTTTGAGGTGA
GGTCTTGCCCCTCACCTTCACAGGGCAGGGGCTGGGCACGTTCCTCAGAGAGGGGAGGAG
AGCACTCGCTTCTTCGCAAGCCACTGTGCAGCTGATGTGCATTGCCATGATCGGCGATGG
AAGGACCCGTGCTCCACACAGAGACCTTGAAGGACCTCGTGCTCCACATCGGAGACCTGA
ATGGCCCGTGCTCCACAGAGTCCTTCGAACCTTGAGCCAAAATGGCTACTTGAGCCAAAA
TGGACTACGG
>at01716
TTTTTTTTTTTTTTTTTTGAGCTCACACGCATCACCATGTGCAACGCAGTGCACAGACGC
CCATGGCCTCAAAAACATGGCCACAATTCAAACGTGTGACACGCGTGCACCAGCATGCGC
GCTCGCACACGCGAGTTTGCATGTGTCCGCGCGCACACGGCTGCGTGAACCGGCACAATC
AGGCTCAGCGGAAGCCACGGTACACCAAGCCTGTTGCGAAAAAGTTGAGTTGCATTGCAG
CTGCCGGGGCCTATGAGCCCACCGCATCATCTCTCCCTCCGCAAGCGGGTCTGATCGGCA
GCTCCAGGCACAATCTGGACATCACCGCTTCGAGAACCTCAGGGGGCCGCACCCGCCGCA
GCGAGAAACTTGTCCATGGCCGCCAGCCCCTTCACCACCTCCTGCTTGGCCTCTTTCAGG
AACACCTGCTGGCAATCCCCGCCACCACACCTCTTTGAAGCGTTCATGCTGGAGAAGTTC
GCGAAGTTCGACGAGTATGCCAGGCTGTCGGCCTCCCGCAGGCGCCCGCTGGCCTGCTCG
GCATACTCCAGCAGATCCAAGTCACCGGTGCTTTCCGCCAGCGCTAGGTATGCGTCCTGT
AGCGCCCCTTTCATCCCTGGTCCTGGCGTCAATGGTGAATCAGAGCCCACTGTCCCCAGG

TACCGTCGGACCTCATCCCCACCTTTCTTGGCGGCTGCCTCATAGTTTGCGACCAGGTCG
GCCACAGTTTGACGGCAGTCTTTCACCACCATCAGTGGATCGCCACCTGTCTTTCTGATT
GCCCATGCCGCTCGTGGTGCCAGGGCGCCAGCTATCGGCACAAGGAGCAGCAGTGGCAGC
AGCCGTGAGGCTCCCCGCCTGCTGCAGCCTTCGGGCTCGGCTCTGCCCAACGCTGGACGC
AGCACCACCGGCTGCTGCCCACGCTGCTCCAGAGCCTTGGGCGCCTGCACTGTCGCTGAG
CCAGTACCGCACTGTAGCGACAGGAATGCTGCGCTGGCAAGGCCTATGCTTGCAGCAGCT
ATCGCAGCCACTGCCAGCAGCAGTGTGGCCTTGACGCCGGCGCACCCCAACCGCCGCCGG
TGGCGTGCGCGGACGACGGCCATGGCGCCACAACGAGCCGGCTTTCAAACTTGGTGGTCC
TAGACGGAACTTGAGCCAAAATGGCTACGG
>at01717
CCGTAGCCATTTTGGCTCAAGCCATGCTGGCTCAAGCAGATTCACCAGTCATAGTGCCCC
ATCCCAAAGATGTCGCTGTCACACACAAGTTACAATGATTTTCTTGCACCTGCTGGGGTT
GCTGAATCGGCTGCAACCACGCAAGACTTGCACCATGACTTCCTGTTTGGTTCCCCTTCA
GAGCCTATGGCTGTGCCTCACCACATGGTTGCCAGCAGCTTTGCTGAGGTAGCACATGCG
ACACCACAGCCGGGGGTATTCGATGGCGCAGGCGCAGTGCCCCAGAGCCTCCCAGATGCA
GTGCCATTGGAGCACTCACGTTTGGTTGAGTCCAGCATCGGCCTGGCCTCAAGCTTGCAT
ACCCTGAGGACAGGCTTCATGGTTGCTGGCAGCATGTGTAGCAAAATGTTTGGCCTTGGT
ATGGTGGGATTCCTGGCGTTCCAGTCAATCAAGGCTAACATGGCAGAGAGCCAGCAGCGG
CAGCAGCAAGAGCAACAGCAGCAGCAGCAGATGCAGCTGCTGCAGGGAGGGGCAGCCGCG
ATACATGAGGGCGCCTCCTTGCACAGCCCAAGGCAGCAGTCCATGCGGTTGTTCTGAGTA
GGAGAGCAGCATCTCAGAGGATCTGCACTCCAATTGCCAAGGCATCACTCGAACGCAAGT
GCTTGGAGCTTGGAGGCATCACCGGAATGCAAGGGCTTTGACTCAGAAGTTTGACATCCT
TGAGCTTATTCTGACTTCAGACGTTGCCACGTCTGACTGGCGCGACTCTGGCATGAGGCA
GCCATCATTGCTGCTCTGAGATGCCCACTTGACGTAGCAGATGCCTAGTCAGTGGCCAGG
GCTCAGGCGCTGGGATCAGCGCGGAGAGGGTGGCAAGTGCGCCGGGAGGGTAAAAAAAAA
AAAAAAAAA
>at01718
TTTTTTTTTTTTTTGTTCAACTTCGATTGGCACCACTGAGTTCAAAACGGCCTCAGCAA
CCATCGGAAACACAGGAAACAAGACAAACTTGAGGAAGGTGTGTGCCTCCTGCACTACTG
GATTGCGATGCAATCCCGCAGCAACAAGCAGTGAGACTGCAATCCCATCAGAGCTCTTCG
CTTGAGGGTTGTGCATCCAAGAGAGTAAGATGCTGCATCATCGAGCTTTCTTCTGCATCC
TCTGCATGCTTGCGGCGCGCAAGAAATGAGGCACCGTCTGCCCGCCTACCAACATTCGAA
TGCCGTTTCACCGAAGAAGTTCTGGAAGGCGTTGTTGCTGAGAGCTGCTGCAAGGACTCC
CCGCACCACGCATAGCTGAAGGAATCGCACCACGACTTTGGCTGCGTATAGCAGACTCCA
TTGCTGGCCAAGCAGGATGTGCAGACGTTACCCTGATAGTCCAAGCATGCAGCGGGAGCA
GTTGTAGGCACAGGAGTTGGCGCGAGTGTGGGCGGAGGAGTGGGCAAAGGAGTGGGCAAA
GGGGTGGGTGGAGCTTGAGGCGGAGATGTTGCAGAAGGAGTGGTCGTGGAGTCTGGCGTC
TCATCGCAGCAGTTGTCTCCTGCATAGCCGTGGNCACTCTGGAAACAAGAAAGTTGACAG
TACTTTTGCTTCTGCCACGCATCATTTGCATTGCAGTTGTTCTGCAGACCCCAAGTCCTC
GTGCCGAATTCTT
>at01719
AGGAGTCATGCTTCCTTCATGCCGGGTTTCAAATCCATCAACTGTAGATACAACACGTTG
GCTGGCAATTATATGAACCTGTTGCATGCTAGGTAAGCTGGGTTGCATGGGCATTTGCAC
GAACACTTGCATGTGAAATAGCCACATTTCAAGATTTATGCTAGAGTCGGTATGGGAATC
ACAAGCCCGTGGCATGCATCAAAATACATTGATTCACATCTATTACCCACATCTGGTATG
CTGTCAAGCTGGACATGTCATTGTAACTGCAAGCATGTCTCAAAGCACTACTCTCCATAG
GATTGCTGTCAAGTGGCATCTCCTGA
>at01720
TTGTTTTTTTTTTCTTTTTTTTTGTATATCTTCACTGCGCTGATGCTTGTGATATCCACT
AACGTCAACACAAGTCAGAACTTATCCAATCAACCGGTTCCGACTCAGAAGCAGCAGACG
TGGGAAGTGACAAGCTCGTCGATGGCGAGGCCGCATTCGCTCACGCTGTCCAGCCGCTTG
CACAACTCGTCCACGCGCTGCGGTGATCACGGAGCCCACCGCCCCATGATTCAGCGAGCC
CCCTCTCTCACGCGTGTCGTGAGGCGCCGCATGTCCGCGACTGCAGGTCTTCCAGCAGGG
CCTTGCCGCTTGAACCCAAGCGGTCT
>at01721

GCCCTCCAAAGGACAGCGCGTAGCTCACAGCAGAGGCTCCTGCGTCACTGAACAGAGCCA
GGCCCAGGCAGGCGAAAATCACTTCAATGTAGTGGACCCAGAAAACGGGTGCCAAGGCTG
GTCTTGCAGGTGAACCGTGGGAAAGTAAAGGCTTCCCCAACATCTTCAGAAGGTCGGGCA
GGACGGACCAGACAGCCAAGACAAGACCCAGTCTTGCATCTTCAAGCCAGCCATAGCGCC
TGGTGAGGACATAGAAACCCGTGGCCACAGCACACGGAATAGCACCACATGTCATGCCTC
CCAAGTACAGCAGGCTCGCTTTCAGACTTTGGTGTTTACCGTACAAGCAGCGCCCGACTC
GGT
>at01722
GAAGGCAACGGTTGATGATGGCACTAGATTCTGGCGTGTGCCGCCTATGGTTGCCTTTGT
GAATCCTGCTTCTCTGGCACGTGCAGGCATTGGCACGGGTCTCCTTGAAGGCAATGATAA
CCCCTTCCTCGTGGTTGGCAACTCCTCCCGTTTCCGTGGGCCGGAAGCAGCAATGTCAGA
AGTGGATGCGGAGGGTTCAAGAGCTGTTCTGACGGCACTGCGCAGGGACATGCGCACGAG
AGGCGGCAGTTTG
>at01723
TTTTTTTTTTTTTTTTTCACCGCACGGCCCTCCACTTGCGACGGTGGCCTGTGCCGTCC
GCGTCTGCTCTGCCTGGATCGCTTTCCATGCGGTTTTCTTCTGTACGCGCACTGCTCCAG
CGCCGCCTCTGACTGGCCTGCGCAGCCTCGGCCGTGCCGCCCGCGGGGCCGCCCCTGCCA
GAGCCCTGGGCTTCGGCGGCCGCGGCACCGGCGGTCCGAGGGCCTCTGGAGGCGCTGGCG
TAGTACCCCTTGCGAGGGTTGTGGTTGACAGCTACAGGCCTGGTGCGAATGTCCTCGCGC
GTGCTGTAGAATATGTTCAGCGAGGCACGCGGGCCCTCTGTGCGCCCACGTGGAAGGCCA
TGGCGTTCCCCGTCCAGTATGACGACGTCGCCGTGCCGCATGGTTTGTGTCCCGTCGCCC
ACCAGAAACTCCCGGGGAGCCCCAAGGGACAGCGTCAGCTGTCGGCAGTCGTGCACATGC
GTGGGACAGGAGTCCTCGCCAGTCTCGTAGAGACATGCAAAGCACTGGAAGGGGACAATC
GGGTCACCCGGGCACTCCGCCTGCATGGCGTCCAGCAGCCCCCTCATCAGCGCGGCCAGG
AANCGGAGGTGCGGCGGCGGCGGCG
>at01724
TTCGCCAGGGCCTGCACCGTGGAGGACACCTCCTGGGGCTTGAAGGTGCCCAGCCTCATG
GTGCATAAGGGCGCAAATCTGAGAACGAGCCCCTCCATCGCGTTGTGCTTTGGCATGACC
TTGGCGCACGCCCATAGGATGATGGCGAGGTGCTGCGAACCCAAGTCCATGCTCATCGCG
GCCTGGATGCTCATGAGGAACACTTCTTCATTCCAGTAGCCCAGCGTGGCGAAGCCCCAC
AGCATGTTGGAAATCTCCTGGGCCTTGAAGCTACTCATCAGGGGCACTGCTGCCTGCGCC
ATGGACGTCAGGAGCTTCCGGTCCCGGTGCGCGGCAGACGCGAAGGCCCAGACGGTGTTG
CCGATCTCTTGGCAGTTGGCAGTGCGCGCGGTGCGGCGCATCTCGCTGGCCAGGCCCGAG
AAGAGCCGCGAGTCGCGCTGCCGCGCCGTGGCAAACGCCCAGGTGATCATGGCCAGGCTG
CGGAAGGAGAACTGGCCGAGGTTCGCAGCGATGCGACCGCTGGCGGCCTGGAAGAGCCCC
TGCACGGTGACTGATATGCTGTCTGCCACGCCAAGCTTGGCAAATCCCCAAAGGAGCATG
GACAGTTCAAAGGGCTTAAACCCGTCCATGTGCTGAGAGGAAAGCGAGGCCAACAGCACC
AATAATTCAGAGTCTCCGGCATGGAGGTGCGCCAGCGACCACGTGACGTTGCTCAGGCTC
TGGTGCAAGCATGACAGGCCTTGCGAGTGGAGTAGCTTCGAATAGATCGCGTTCAAGAGC
TCCTCAAAGACGGGACTCTGCGTCACAATCAAGCACACTTCGGGATCCTTATCCGCCCCT
TTTGCCAACCGGTGGATGGAGGTCGACGCGTTCACCAAATTCATACGGTGGATGTACTGC
CCGATCACGGAAAGGAGGCCGAGCGTGTTGCCATCATTTGACGCGGCAATGACTTTCTGG
TTGACCTCACTTGGCTTGAGGGGTTGGCCGGGAAGCGACTTTTGCATATCTTTGAACCCT
TTCCTTCCCAGGTTCTGTGCCTTGAACGATACCATTGCTCGGGCCGCCCCGCCTAGTCCG
GATGCTCCTGTTCCCCCTCAAAGCATCCACGAAGCCACCTTGAGCCAAAATGGCTACGG
>at01725
TGCTTGCCAATCCGCCCACTGAGCTGATATCCAGTGGCTGAAGTATCTCTGGGACACTTC
TAGCTGGAGTGCGTCCTGGAACTGCCTAAGGTCCCAGAAGAGCAGCTGGCCGTCCACCAT
TGCACCACACCGCTTGGCGGCCATGCGCACCGTGGAGTCCTTGGCCTGGAAGATTGGGAA
CCGGCTGACAAAGCTTTTGACCAGGTCAGTGAGCCAAGCCCTCGTCCCATCTGGCAGCTT
CATAAAGATGCCCTTGCTGCCCTCCATCGCCTTGATCTGGAACTGGGTCTTCATCCAGAC
CCTGCTCTGCCCTCCTGGGCATATGCCCAGCCGTCCTCGTCAAAGATGAGCTCTCCAGAG
ATGCGCCTCATCATAT
>at01726
TCCGTGGTGGACCTCCAGAAGGGAGGCGAGTTTGCGCGGTCTGCCTGGCAGAAGATCTGC

GACGTCAGCCGCATGGCCTTCAACAGGATCTACGACCGCCTGGGCATCACCGTGGAGGAG
CGCGGTGAGTCCTTCTACAACTCCCTGATCCCTCCTATCGTGGAAGATCTGCAGGCGCGG
GGCCTCTGCGTTGAGAGCAACGGGGCCATGTGCCTGTTCACGCCCAGGGTGGCCGAGATC
CCGCTCATGGCCGTCAAGTCGGACGGCGGCTTTGGGTACGACTCTACCGACTTGGCCACG
ATCTACCACCGCCTCTTCCTGATGCGTGCAGACTGGGTAGTGTACATTACGGACTTGGGC
CAAGAGCTCCACTTCCACATGATCTTCGACGCAGCCAAGCAAGCTGGGTGGCACAGGCCG
CCGTG
>at01727
GAGGCTGCAACAACACAGAACACGACTTCAAGTTTGGTACCGACTTCTGCAATTATGAGG
ATCCCTTTTTCTCCAGCCAGGGCCTTGCCCACATCATTGGCTTCTGTCCGGCGACGTGCT
GCAGAGGTTTGCCTTCTTCTGCAGATTCCGCTTGCCCTTCCGCTTGTTAACTCCAGTGAG
TGGTGAACCACCGTGAGGGCTCCGCCAAAGATTCAGGCTGGATCTTCAGCACCAAATGTG
TGCGAAGGCATG
>at01728
AACGTGAGACAGGTGGTCTCCATCATTCTATCTCGTACATCCAGTTTCACCACAACATCA
CGGGATGGCAGGTGCTTGGTCTCGCAGTTGTCTTCCTGGCGCTCTTCTACAAGAGCTTCA
GCAGCCTTCGCGAGTCATTGACTTCCAAGCAGGAAAAGGAGCCCTTGGTCCCCCAACAAG
GACCCCAGAGGCAGTGGAGGACAACAGGGCAGTGGATAACAAGGAGCCTCTTAAGAGCAA
TTGAGATGCAGCATCGCAGCTTCCCGTTGCTCAGGCTCGTGGTCAAGCTGTCTTTGTAGC
CCGGCGCA
>at01729
TTTTTTTTTTTTTTTTTTGGCGTGCAACGAAATTAATTACAGATCTGTCACCGGACTCGC
TGTTAAAGCCTGCGCTCACCTTGAGAGGTAAGGCACGCTGTCACTTGCACATGCACTGGC
AGCCTTCGTGACAAATTCGCTACGCGCGAGAGCTCAGCAGCTATCACGAGAGGTACTGTG
CCTGCCCATGATGAGCACCTCCATTGCTCATCTGCATGGCAAACATCTGCATAGCCGCCT
GCTGACTTGCTGCCCAGCTCTCGAAGCAAGCCTGTGGCTGTCCCGGAGTGCTGTAGCACT
GAACCTCCTGCATCGAGGGGCCCGGCAGGGGAAACCCCGGATGATGCATGGGCGCGGCGA
ACCAGCCGCCTCCGGGAGCCTCCCCTGGCACCGGGGTCTCGCCGCCACTGCTCAGCGCAC
CTCCAGGGCCTGCACTCATGGTCGGCGACGTTACTGGTGCTGGCCCCGCATGCGCCATGG
GCGGTGCCGAGCCAGGGGCGCACGTGACCGGCCACGGCGGCAGCTGCGGCATGGAGCCCG
GAGGCGGCACCGGCGCCCCAGCATGCCGAACATCGGGTGCAT
>at01730
TTTTTTTTTTTTTTTTTTGCGAGGCACTGCGCGCCCGCACCTGTACTGATGATGCCAATG
CCCGGCATGGGCCTTGACCGAAGATCTGCGTGCTGCCGCACAAGCAAAGGGCAGCGCTGC
GACTAGACGTCGCTTTGACCTTGCGCCTCTCTTGCGAAGGTACTCGAGGAAGGAAGTCCC
GGGCGCCAAGCCCTCGGTGCCAAGCGGCAACCGCCGATGGGCGGCTGCTCAGCAGCATGC
TGTCCGTCAGGAACAACCCGACAGTCGTACGAATGCACATACGCCATCCCCCGGCCAATC
CCAGCAAGGCGCCGACCAATGCACACGGCAGCATGGACGTCAAGCAAACGGCGCCGCCGG
GAAGCATGGCTCCCCCAAGAAAGCCTGCGAATGCCGCGTCCTTGAAGTCGGTAGTCACCC
AACGCAGCTCCT
>at01731
CCGTAGCCATTTTGGCTCAAGGCGTTTTCGTCACTACTTTGGTTATCCTCCTCACAGCGC
TGTAGAGTCTGCTTCGTTGTGAGGCACATTGCAAATGGAGGAGGGTGTCTGTACAGGAAT
TGAGCTCCATCGCAACATTGACAAAAGCAAATGGTGTGTGACGAAGAAGGACGTTGCTGA
CTTCGGCCGCCTGGTTCGCACGGCCATACAGGAGGGTCGAATTCAGCCGTCCGAGCAGGA
TCCATTTGATCCAAATGATGCTCAGATAGGACCAAGCATCTATACAGTCAACGAGCAGTA
CATCAAGCCAGTCACGAGAGCTGCAGGTATGATGAGTTGGGCCTTGATGCTTCACCCATG
CGGATTGCAGTGTGACCTATTTATTACACACGCGTGGGCTGAAGGTGTCTTTGAGTTCAT
TGACAAA
>at01732
TTTTTTTTTTTTTTTTTTGATGTTGCGCGCGACTCGATCACCAAGTCCACAAACTGTGCA
CCCCTCGGGATAGAACAAACGATCACATCAATCTGCCCTTCGTTTGTCGCCGAGGACTCG
AGCACGAACATCTTTGATTACGCGCTGTAAGGCAATGACGAGTGGTGCAAGACGATCACC
AACTTGCCGTCGGGAGCTTTGAAGTAGCCAAATGTCTTGTCCACAGTGGTGACATCGCCG
TATTTGTCGGTGAAAATGGCGTTGCCCTGCACCATGGCATAGTCACCTTGCTTCCAGATG

GCCGTGTTGACGAATTTTGCCTTGCGCCAACCCTTGATTCCGAAACCATGGTCCTGTGGA
TACCAACTGTCGCCACCTACAAAATAAGCCAACGCGCCTCTTTTCGTCGTGCGGAAGGTC
TGCTTCCCACTGGCGAGCGTGGGCTTGAAGAGCACTGGCCCGTACTTATATCCGTAGGCA
GAGTCGAGGACTGCTTCCGCCAACTCCTTTGCCGCTTTGAAGCCCTCATTCTCGTAGGTC
TTGGAGATCTTGACTAGGGCGTCTCCCCAAGCCTTCTGGGCAGCCCGTACTTCTCCGTTC
GTGATGGTTGAAGTCGACTTGACCCCCCGGGGCCCTGTGTCTGTGA
>at01733
TTTTTTTTTTTTTTTTTGGGATGTCAAACATTGTCCGAGCCCCGGGTCTTCTCACATGG
GGGCGAGCGGGCCAGCAGCGGCCCCTCACTCGCCTTCCTGCCGCGTGGCGCTCGCCGCCT
TCCTGGCTGCCTCCTCTTCAGGGCTCAGCTTCCTCATCACCATGTTCCCCAGTTGGGTAA
CCTGGTCCAGCGCCTTGCCTCCTCCGAGCTCCCTGATCTTCTGCTCGAGCTTGGGGTCAT
TCCCCGCCAGGGCGTGGAGAGCCCTCGAAGCTCCGACCGCCGCGTTGGGGTCGCGGCCGC
TGGCAAGCTTCACGAGCGCCTCGATGCCTCCGGCGTCGGCTATGCGAGACTTGCTTTCAG
GGTAGCTCTCCGCCATGCCGCGCAGCGCCATGGCCGCCCACACCTTGCCCTGGGCACTGC
CGGTGTCCAGGAGGCCCACGAGGAAGTCCAGGCCGCCAGCCTCCTCGATGCGCTGCCGGG
TCTCAGCGCTGCCAGAGGCAATGTTGCCGATGAGCGCGGCTGCAATGTCGCGCACCTTGG
GGCCTTGAGCAGACCCCACGAGCACTTGGATGCCACCAGCCTCCACAAAGCGCGCCTTGA
CGTCGGAGCCCACGTTCACGATGTTGCAGAGGGCCTTCACGACCTCGAGCTTCGCTTGGT
CACGCCCCGTCTTCAAGAGGCCGACGAGCAGCTGCAAGCCTTCAGCCTCAACTATCCGCC
TCTGGTTGTCTGCGTGGTTGAAGGTCACGCTGCTGAGGGGCTTCACGGTCAGCTCCTTCA
GCCGGGGGTCCTCCGCCTGGCTGCTGGCTGCAGCGGGGTCCAACGCCGTCTGCAGGCGGG
CAGCAGCTGTTGCGGTAATGCTGGGGT
>at01734
GCACCTCGGGACAGCATGCTGTAAACCCGTCTACTTTTGTTGTCTTGATCCGCCTTGGAT
GAATCATTGGTGCTTGATTCCCTTTTGAAGTTGTGCTATGTGACTGCCCAGGTGATATCC
GCTAAGATTCAAGGTGACAGGCGGCTGCTAACTATCGAATCCGCTGCGTTGTGCGAAACG
GCAAGAATGTGCATTCTGGCACCTTCCCTTGAATTGCTTGTTTTGGTTATTGCAGAGGTC
CGTTTGGCTTGAGTAGTTATGCATTGATGCATGCGATTTCGCACGTTGCCAGGTCTTCAA
GGGCAGCATCAGCAGGACGTTGGCGCGAATGACTCCCTTCGGAATTCATCATACTGTGTT
CAAAGATGT
>at01735
TATGCAGCCATTATATCTCAGAGGCCGGCCGGACAGGAGTGGATTCGGCCAGCATGCGCT
ATGCGGTCCCCCCTGCTGGCCCGCTTGGCCCTTGCGGGCAGCTGGGCGGAGCCTCTGTCG
GGAACAGACGCGGCTTTGGAGTGCGGCATGCGCAAGCTGGCCTTGCAGCAGGCGAAGCGG
CTGCAGCCTCAATGTGACCTGAGGCGCGGACCTG
>at01736
CCGTAGCCATTTTTGGCTCATGCCCTGCGTGCCCATGCCCGCGCCGTCCGCGCGCGCGTG
ATGGGTGGCTTGTGCCCAGCCTCGGCACCCGCATCTGAGGACTCCGGCAACTTGAAGGCT
CGGATGGACAGCGCGTTGGGCACGAGCATGAAGGTGGGGGCGGCCTGGAGATGCTCAGGA
AGAGGTACCTGGCAGGCTTCAAGGACGCGCACCGGAGCACCTACCCAACGATCCCGATGC
TCGAGGAGTCCGCGGGGCCCACGATCGATGGGACCATCGACGATGTGGCCAAGGCGCACC
CCGAGCTGGTCCCTGTACTATACAACGACGTGAAGGTCTGCACAAATTGCAGCAAGCCTT
GCGCCTTCACGCTTACGGTGTGCAACGCCTGCGGCACGAGCC
>at01737
CCCTCGCACATAGTAGACTGTACAAACCATGCGTTGCGAGTGGGATATATCACGGGACAC
AAGAGTGCTAGTTGGCAAATCCGGGCCGGAAGTCAATTTTTGATGCCCATTCAGGATGAT
CCACGAAGGGGTTTCGATTACCCTGATGCGCAAATCCGACTGCGTTGCGATGCTTCTCCC
AAAGTGTCACAGGCTCCCGCGCAGCAGCTTGAACCAGCCATACCAAAGATGCATCTGGTA
CGCGACCCCTGTCCAATGCTCCGGGGTAGGCTGCAAGGACGTACAGTGCGGCTCGTGCCA
CAGCGCCTCGGTTGATGTGTGGCAAGAAAAGTGTCTCATTGCAC
>at01738
TTTTTTTTTTTTTTTTCTGGACAAGCACATTCGTGAGGCTCAATAGTGGTTCTCATGCAC
AAGGTTTGCGTACTCGGGCTGCTGCCTCCTGTCCGTCTTCCCTTTCTTAGGAGCCAGAAT
CGAGCGGCCAGAAGTGGGAAGCGCGAAGCCGAATTTCGAATAAACCTCGCGCAAAGCCTT
TACGAGCACCTCCTCGTTGTCTGTTCCCCAGGGCGTGACGGTTATCAACTCCAGTACCTC

CCCAAGCACGCTGGCTGCCACTACTCCCCCCGGTGGAGAAGGCTCAGCGTGAGCTGGCGT
CGGAGTGAGCACCTCACTTCCAGCTTGGGGTGCGTCGACCTGAGGAGTGCTTTGAGGTGC
TTCCTGTACATCGCCTGTGAGCCTCGTGCC
>at01739
TTTTTTTTTTTTTTTTGTATGCGACCACACTCTTTGCATGCTTCGCAGATGATAGACCTA
AGTAGGAGGTGGGGGTCTTGGGCTCAGTCCCGATTGTCCATCGAGTAAAGCTCTGTCCTA
TTGCTCCACGATCCTGACACTGACTTGTTGAGCAAGTACGCAAACTTGTCAAGCATGCCT
GTGAAGGCAAAGATGCCGCCCAAGATTGCGCAACACTCCGTCAGGAAGTGCCAGAGGCTT
TTCCGGCTCTTTGTCACCTTCACCTCAAAGGGCGTGAAGTCGTAATTGACCCACAGTCCC
AAGTTGGAGGTGGCCTCGTGAGCCAGTCTGGCGTCCACCGTCCTCCGGTGCTCGGTCACG
CTGTACTGATGGCTGGAGATCCCCGGGAGGCCGTCCTCCTCATAGCGCGTGGGGATGACG
TGGACATCGTACAGGTAGTATTCTGAAGTTCCAGGTGTGTGGTTGCTCTTCTGCCGCCCA
TCCAAGACGTAGATGTTGCCAGGGAAGTCTGGGCCGAAGGCTAGGTTCTGGATGGTGTGC
GAGAAGTCCAGCGCCTGCATGTACTCCTGGACCTCGTCGTAGGAGTTGGAGACCGGCAGC
TGCTCAGGCTTGAAGCTCCTCGAGACGCCGATGTGGAGCGTCGCGGGCACCTTGCGCACG
TGGAGCTCCGCCTGCAGACGGCAGCCCTCGTGCTGGAGCGGGGGCGCCTTGGCGTAGTCT
TCCTCCGCGCACTGCCCGAAGACGTAGTCCGAAGGCCTGTCGGCCCAGCCTCGGGCGCGG
AACTCCTTCCGCACGTCCTCGCAGGTGGCGCAGCAGTCGTCCTCGTCGGTGTGGCTCTGG
AAGCAGGAGTGGCACCGCAAGTGCGTCGTGGCATGGGTCGTCCGCACACTGGTGGTGCTT
CTTGAGTCCGTGACTTCGCCTGGGCTCTGGGCCACGTTGAGGAGACTATCACGGACCTTG
ACTCCCAGCACTTGGCCCTGCGGCGAAAGCCGAGTCTTGTAGATCTCATGCCCTACGTGC
ATGAGGTTTGCCTTCTTCGGATCAATGAGGTTGATGCCAAGTACTGCGCAAGGCATGTCT
GCAAACGTGATGTCAACGCTAATGTTGAGGTATTTCTGGTCCTGGTTCTGGTCCACGACC
ATCTCGTCCTTCGACTCCAGCTCCAAGAAGTACTGGAGCTCCTGGACGAAGAGCGTGGCC
ACAAGGCACAGAGGCACACACCGATAGGTAACCTCCGCACGTTGCTTCACCTGGAACTCC
TGCACGGGCCTCGCGAAGGCGTCAAAGGACTTCATCGCCGGCAGCGGGGCCCCGCAAGGG
GACGGCCCAACGGCCGTGCCACGCGGCCCAACGCCTGCGCCCACTGCCTGGGCTCCGGCC
TTGAGCCAAAATGGCTACG
>at01740
AGCCATTTTGGCTCAAGTCCCGAGCAAGGCTCAAGCCATGTTCAGCTTCTTGCATGATGG
ATGCGTACAAAAAGTGTTGTGTTGTCTCTCGTTCCCTGAGCTCCTTGCAGCCACCCGCCT
GAGTTGACGAGCACACCAGGCTTTGCATGGGACATCCTTTGCCAATAGACATGTGCAAAG
TGCCCTTGTGGTTTTGACACAGTCGGAGGATCCTGCTGAGCCTTGGCGACTATGGATGCT
GAAAGAGAGTGCCTCCTCTTCTTGGGAGAACCTACCTCTTCCTTCTGAATGCCAACGGGA
AGGGTCTTCGTTTGTCCTTCTTCCAGGCGACGAAACTTGGCCCTTAATGCGTATTTTGGC
TGTGAACGAGAATCGTCAATTGGTGAGTGGTGACATTGCCAAGATGTATTGTTTCGAATA
TTCGGCTCTCAGGTGGATTTCCATGCAGAATCTGGACTGCGTGCGTTCGTCCCTACCCTA
TTCGAATATTGTGACAAGTGCTTCAACAGCCAGACGCTCTTTCGTGCACATGCTCAATCT
TTCTTGTTCGGATCAGGAATCTTTCGATCCGTGGGGATGTGAGCCAATCAGTCAATACGT
GGGCATACTACCAGT
>at01741
TACGGCGTGACCGCCTTCCGAGTCGACTGACCTCCCGCACTATGCAGAGGCTGGCATCGA
AGGCATCTTCTACGAGCAGCTCGACGTGTTGAGCCCTGACTTTGCGGAAAGGATTCAACT
CATCACACGAGGTGCAGGAGATAGGCAGACAGCCCTCATTGGAATGCACCTTTGCGGACG
ACTCAGTGAATGCGCCATAGAAGCATTCGTGAGTGTCGAGGGGATTAACATATGTGTCTT
GTCTCCATGTTGCTTGCCTGCTTTGGAGGATGCCCCAAGTGTGTTGAGACCACTCTATGG
AACCAGGAGTCGTAACTCGCAGGACAGTTCGCAGCGTGGGAAGCACACCTGGAGTCACAG
>at01742
TTTTTTTTTTTTTTTTTGCCGGGCAAGATGAGAGTGCTCAGAACCGACTTGCTGGCAAC
CTAGGCGAAGAAGCAGCAGATGGTCAGGTGAGGTTGAAAAAAAAGCCTTCTCACGGCAGC
CTTATTTACATGGATTGAAATGATGATGACTCTTGCCATGCCATGGCAAGACTACAGTGG
CACTTCACCGCCTCTGCTCTGTCATCATGTCATCCTCACTCGGCACTTCTGAGCACCCTC
CTCTCTTGCTGCAATCGATCGGAGTGGCGCTGGAGACATTGCGACAGGAAGAGAACTCCA
CCCAAGACATAGAATCCTTGAGCCAACTTCAGCGCCTTTTGGGAGTTCAGACGCGTCCAG
TGAGTCATCACTGCAAAGAGCACAGAGCCGGAGAGGTAGAGGAAGCTGCCAGCAAAGGTG

GGGTCGGACACTGCTGCTTGCCACAGGCTAACGGCGAAGCTGCTATGTGCTCTCTTGTAG
AAGCGCTGGAACTCCTCCAGAGTGATGAAGTCATCACTGGCTGGGTCAATGTCCTTCCAG
ATGGCAGCAAAGCCGGTCATATCCATGATGCCACACCTCAGCATGTGCCGCCGCAGCTCC
AGCCTGTCCACGACGCCGTCTCCGTTCGCGCCTCGTGCC
>at01743
GCCGGTGAGCCAGGTCCGGGCGCTCCTGGACGCCTGCCCGGAGCTCAGGCCGCCGCTGGA
GCGGGTCTTCATGGACACCCTCGCGGCCAAGTGTGCAGCCATGGACGGGCAGGTCAAGAG
GCACAAGTACCAGGCCGCGCTGGAGGTGGCGTGCACGTTGGAGGATCGCGCCGGCATCGC
CTCGAGTCTGGTGAGATTTCGGAGGCAGAACGGCATCTCCGACGAGCTCCACGTCAGGCT
GATGGGAGACATACAGCAGTGTGCTCACAGGCCTTTCCCTCCAGCCACACTCGCCACTGT
GGATCCATCATCGGCATCGTAAGAAAAAAAAAAANAAAAAAANAAAAAAAAG
>at01744
CTTGGCTTATCATGGTTGTGGATAAGGGGGTGTGAGCCTGGGACTCCTGGGGTAGGTTGG
TATGGCCGACGGTTCTTGCTGCGGTGCCAAGGGGTTTGACCCGACCTTTCTACGCGTGCA
GGGTGCTGATTGCCACGGAGGGAATGATGGCAGACGCAGCAGCACCGACTCCTACCGAGG
ATCAACTTGCGGCTTTCTTCGCGGCAACTGGTGTGCCAAACAGCCGCCTCGGATTCGAGG
CTTGGCAGATCCTTGAGCAACACCCAGACCTCACTCGCAAAATCCGGGAGCGGTTGCAGA
CATTGGCGAAGATCCATCAGCGTCTTCGGGGGCTTGAGCGCCAGGCGCGGCCGGGCAGAC
CCGAAA
>at01745
AGCACTTTGTGAACTGCCTCTCCTACTCACTTGGCCTATTTTGCGTTGGTGCTGTCCTCT
ATCGCGTGTATGACACTCTGGTATTTGGTGGCGCCCACATCTGGCTGCGCTGCCTCATGG
GGTTTGCGTACATGGCAGCGTTGCCACTCCCCTTATTCTCCTACCTCCACTCGAGGACGG
ACGTGTATCATCATCACCTGGAGCAGTTGTATTGCTTGCACTCAAAGTTCAAGGCACACA
GGGGATAGG
>at01746
TTTTTTTTTTTTTTTTTGCTTTGGCCACGCCAAACTGCACACCACACTGTACTTCAGTG
CGCAAGAGCTCATCCGCCTCGACACATAATAGCCGACGTGGCATTCTATGATGGGGGGCA
CCCTACTATAATATCATCCCATGACTGTCAGGCTCTATCACTCACGTCTGCTACGGCCTC
ACAATGCCAATCACGACTAACAGAAGGTGCCAGTGCAGCCAGGCATCAGTGGAGTTGTTG
CACCAGTGCTCTCCCAACGGCGTTCAGCGGTTGGCCTTGGCCACGCGCTCAATCTCATCC
TTCTTCTTGATGGCATAGCTGTTGGAGGACTCCTTGGCAGCGTTCATGATCTCATCAGCC
AGGCACTCCGCGATGGACTTGATGTTGCGGAACGCACTGTTCCTCGTGCCTGTGGTGATG
AGGTAGATGGCCTGGTTGACGCGGCGCAAGGGGGAAACATCCACGGCCTGGCGGCGCACC
ACTCCGGCGGAGCCGACACGCGTAGAGTCTTCTCGGGGACCCCCATTTTTCACCGCATCG
ACGAACACCTGGATAGGGTTCTTGTCCGTCAGCAGATGAATAATCTCAAACGCATGCTTC
ACAATGCGTACGGCCATGAGCTTCTTGCCGTTGTTGCGGCCGTGCATCATCATGGAGCAG
ACCAGACGCTCCACGATCGGGCAGAGGGCCTTGCGGAATCGCTTCTGTTGGTAGCGGCCT
TGCGTGTGTGGCAGGAAGGTCTGGGATGGCTTTGCACTCACCGCAATGTAGTCAAACAAG
CTGAGGTCTGAGACCATAACATCATCGTAGGCCCACCGGCCGAAGAGCTTCACCTCGCAG
GCCGACATCTCGAATGCAACACTCTCTACTTGGCGGAGGCCGATTGCAGGCAAGTGGGCG
CAACTGATCCTTGAGCCAAAATGGCTTACGGACTCTG
>at01747
GGCAGGAGGGCCCGCAGGAGGATGCGGAAGGCAGCTGGGGAGGCTCTTGCGCCTCAGGCG
CCGTGGCGCCAGATGTCGATGCAGGCATGCTTCAAGCACTGGCTGCCACTTGTGAGGAGT
TCCCCGAGGGCACCCTGACATGGAATACACCCTGACGTGCGAGCTGCCCATCCCAAGGTT
NCGCCTTGATTGGCAAGAGGGTNATCACATCCGAGAGGTTCGCAAGATGACCGGCGCAAA
GATTCACTTTGACGAGGATCCCGAAGGACAAGATGCGGA
>at01748
CTGCAATGTCCCAGCCCCGCTTCCCAGGCAAGGGCAGAGGAGCAGGGAAGGGGGCCGGCA
ACAGCAGCAGCAGCAGTGCCAACAGAGAGCTGAACAAGGGCGGTGAAAAGGGCGGAGGTC
GAGGAAAAGGTGGCAAGGGCGGCGGCGGCGGCGGCATGCCGCGAGTCCCGGACATGATGC
AACGCGGCTCAGACAGCAATGCAATGATGCAAGGGCCAATGACTGGATGGCAACTGCTGG
AATGGGATCCGGCTGGCCCTCAATGCCAGGAGCACCGGCAGCGCAGCAGCGGCACTGCCC
GGCATGTGGCAAGGAGTGCGGACCGACCCACGGTTCTGCGCCTTCTGTGGGGCGGACCAG

ATGGCCATGCAGGGCCCCATACCCGGCATGCGAGCGGATGCGCCGTCCTTCACGCCCGTG
A
>at01749
TTTTTTTTTTTTTTTTTTCCGCCCAGGTGGAACTACACATGCTGTACATGCTGTACAAGG
AGATCGTCCGTGCCCCTATTGAAGGTCAGCAGATGCCAGTGGCGTCTTTGCTCTGGGATT
CTTCAAACTGCAACCCAGTCATCGTCTAGCTGAAGCTCGTGATCCCTGGGTTTGGAGCCC
AGCAGCTTCTGGAGCGCTTCGTACGACACATCCGTCACAGAAAGCCCGAAACGCTGGCGC
AAGCGCACGACGAAGCCCAACCCAAACGACCGAAGCTGGAACTTCACGACAGTCGGCAAC
GTGGCTGGAGCCGAGTCCAGCTCTCCCTCGGAGCACGCCAATGCAGCGCCTGCAGGGATT
GGCTGCGACCCTGGAAGCTGAAGCTCTCCGCAAAGATGCCGCTTGTCCTCTCCCAACGAG
TCAGTTAGCATGTCCACATCCGCCTGCGACTCCACGAGCATAAACCGGATGTTGCTCACC
AAGTGAAACACCAGAATGTCAGTGCTCCTCTGAGGCCGGATGGACCAAATGACCTTGGAG
AGAGACGCAGCTTCACGAAGTGGGCTTCCGAAGGTCAGAACAAGCTTTGCACCGATGGCC
TGGGGCGGAATGTCCACGTCGCAAGGCTGAGGCAGATCCGCAGATGCTGGCCCATCAGGG
CCT
>at01750
TGCTCGATCACTTGATCGAGCAGGACGACTCGGAGGACAACGCAAAGTACAGCCGCGCAG
AGCTTCTAGCGCTACTGCAGCTGCATGCCCAACGGCCTGAAGAGGCGGAGGACAGGCCTG
ACAGCCGCGATTACGACGACCAGACCCGCCCCGTCATACAGCGGGACGACGACAGCGTCA
TGTCTGGGCCTCGCCGACCCGGCAGCTCGTCTGCTGCTCGGAGGAGGCATTGGACTCTGA
TGCGCCGCATGAGTGCCAGGAAATGGCCGAGGACTCCCCACTGCCTCACGTACTCAGAGC
TGCAGTTCATC
>at01751
TTTTTTTTTTTTTTTTTTCACCATTAAGCGTTCACTTGCAAGGGCACGGAAAATCCACGT
TCCCGATGCCTCCGGGCCCTCACCTCGGAATCTCCAGATGCTGCCTGCCCCTCAGACATG
GATCTGAAGCTGCCTCGCCTGCATGCTCAAAGCCTGCAACTGCCAGTGTGCAGGGCTGAA
TGAGGGAAAGTCCACTCACCCCTGCACATACTGAATGGTGGCTGGCTGGCCGTAGGTGGT
TGGCAAGACTGCTTGCTGAGACTCCTGACGAGAGATCACGCCATCATGGTTGCGATCCAC
TGCATCAAGGGCACTCTGCCCCAACACTGCCCCCGCCGAGCCGAGGCTCGCAATCGAGGC
G
>at01752
TTTTTTTTTTTTTTTTTTGCATGAAATCGACAAACTTGCTCAGTGCAACTTCAACAACCC
CTCTGTACACCCCTCTGGGCTCAAACATTGCCGAGCTTCACAAACAATTCTTTGCGGTTC
CAGAACGCGTCCCTGCCTCGCCATCTAGTCAAAGCGTCAAAAGCGTCAAATGACTGGCGC
CATCACGCTGGCAGCAAAAACTGGCGGTCAGGGACAACATAAACGTCAATTCGTAAAGCC
TGCCAGTGTCGGCAGAGCAGTGCCACTGTACATGAACACCTGTCGACAGTTTGATTACCC
CTTTCATAATCGAGCTGTGCTTGAACCTGAGCTTGTTTGCCACGGGACCTCCCCGGATGC
ATCTTGACTGTCAACTGCACGAGCATATACCAGTCCAGTCATGTTCACTTTGCGATGCAC
TTCATGAAACCGGACGTGAGCTCCTCCCTGTTCAAATTCTTTCCGATGAAGACCATCTTG
CAGCGCCGCTCCTCTCCAGGCTTCCAGGCTCCTTCTTGGGAGTTTGCAAAGAGCATGTGA
ATCGCCTGGAAGACGAATTTCTGCTTCATGCCCTTCACCGCAAGGACTCCTTTGGAACGG
AACAAGTCAGTCCCCTTTTCCTTCAGAAGCCAAGAAATCCAATCGTTCAGCTTTTGCTGC
ACCACCCCCACTGGAACGTCAATTCCAACGGAAGACACTCGAGTATCGTGCTCATGATCA
TGGCTGTCCTCCAGGAAAGCGTCCTCGT
>at01753
TTTTTTTTTTTTTTTTTATGAGCACATCGAGCCAGTAGGTTCGTGTGTGTGAGGTACAACA
GTCAATGAACCTACTGTTAGCCTAGATTCAAATCAAAGTTCCCCCTCACTGGGCTTGTAC
TGTGAGATTGCGACTCTATCGGGATATGGAGCCAGGATATCCTCAAATCGCGTTTCAAAA
GGTCCTTCGCCTTGTACATAGCTGAGGTACTTGGCCAGAATGCGGGCGTCGCGGCCAGCG
TTGTCCGCATGAACTATCCCATTGGGAGCAACCGCTCCTATGCTCACCAAGCCTCTCAAG
TCTTTCAGGTAGTGCGTGTGGTCATGATCAAGGAGTACGACGTTGAAGTGCTTCAGCTGC
AAATGATCCAGCACTGCTTGGGCAGCCTTCACCACGGCGCCCGGCCTGACGACCAGCGCC
TCGTGCCGCANNNNNNNNNNNNNNNNNNNNNNNNNNNNACGGGCGTGGTGCACGAGCGCCCGG
CAGCCCGAGGCCCAGGCCTTGTCGCCCTCGAAGCTGACGACGGTCCTCGAATGTTCACCT
TGAAAGCTGGCAGTGACTGCATGCCCCTGCAACGCGCGTATGATTCGCAATGTTCCATCA

CCGAAATGAGCGCCAACCTCCAGCACTGCAAATGGGCTTGAACCATTACCATGCCGCGCA
NACGCATCCTGAACTGTTGCCTCAATAACGTCGCCCTTTTCTGCCCCCAAATGCATACCA
AGGTCATGACTCTCACTGTAAGCATCAAAGCCAGTGATGGCAGAGTTAATGTCACCCTCC
TCCGTCACACCCATGACATTTTTCANGA
>at01754
TAGTCCTATTTTTGGCTTCAAGTAGCCATGTTGGCTTCAAGCACGGTTGCCTCCACCAAT
CTGCGCATCTCATGGCGGAGAGTGGCACAGACGACAACTTCACGCTCATCTGGAAGGGCT
CGCGATCAGGGAAGGAACACCATCTACATGCCCGTAcCTCATCAGACGATCCAGCAGTTC
AAGCAATTGGTGTACAGTGAATTCCATGGGCCGGAACTACCACCATTCGAGCAAGACTGG
ACATCCAAGCACCATATCAAAGTGATTCCCGGCGGTCTCCTCATGAGCGATGAGACCAAG
TCGTTCAGTGACTACGGCATT
>at01755
CCGTAGCCATTTTGGCTCAAGCCGCCGCCCCATTTGCAGGTTCAGGGCGGCTGCGACCGC
GGGTAGCGCCAGATCTTCGTTCCCATGTTGGCGCAGGCATTTGATGGGCTCATGGATGTG
CAGGGCCTCCGAGCACAGATGCGTTTAGCGTTGGAGGAGGCGTGCAGGAGTGGGCTCCTC
ACGACGCTCATCGCGGAGGCCGTAGGCTGGGATGGCGAGGCCAAGGACGGCGGCTCCACC
GCCACGGTGTACATACAGCAGCCCCTGCAGCTGCACTTGGAACTCGACCAGAAGGTGCGC
GAGGAGAGGGCTTC
>at01756
TTTTTTTTTTTTTTTTTGACCACAAGGTCCCCTTGATCCTGGTGTAACCGTACCTCGAG
GTGGCGTTACAGAACACTGAACTGCCAAGACTGTTCGCTTGGCCGTGTCTTACCAAAAGC
CGGCCCACACGCCGGCCGCCAACCAAAAAGCGTTTGCAGGAAAAAGCGTTGACATGTTCC
AAAGGGCAACTCCTAAGGACCGAACGGCGCCTCGCTGATCACTTGACCTTCCAAACTCCA
GGCGCTTGCAGCAAGGCCTCCTGCACGCCGACGAGCTGCTTGCTGATCGACGCCAGCAAG
AGGAGAATGGAGCTCACCGTGCACACGTCATCGTGGAGCAGGGTCTCCGCCACCTTCCCG
CCCGAGCCGTGCGCCGCGAGGTGCGCCGCCGCCTCCTCGAAGATCGCGGACTGGCTCTTG
GGGGGGGCCTCCCTCATCAGCCTGCGGTCGCCCTTGAAGCTGGCCGGCAGCGGGCCGGCC
ACCGGCGCCTGAGACGTGTGGCTCATGACCTCCACCGCGAACGA
>at01757
TTTTTTTTTTTTTTTTTCCCACTCACATAACTCAGCATGACTGACCTCTGGCGAGCGAA
AAACAGTCATCTAAACCGCCACTCATTGGTGTTGGTGCTGGAGAAGAGCCCCGGCCAAGA
CTTGCCTCGCGACGGCCGCCTTGGCGGAGCCGCGGCCGGGGCCCTGGCCGTCCATTGGCT
TCAGGCGGGTGACGAGCTCCCGGCCGTGGAACCAGTAGCCCGGCTTCTGGTCGACCTGAA
CATAACCCGTTGCTTCGTCCACCAGTGTGACTCTGCACGGAATCCACGTATTAAAAGTAG
TGGACCAGAGTAGCACCTCCTGGCCCTTCCTGTAGGACGGCGCCTGCTGCTCTGACCGCC
TCGCGACCATCGCGTCGGTGCGAAGTCTCAACGAGTGGAGGTCCTCGCGCAGCAGCCAGC
AGTCCGGCTTGCAGCTGACCTGTACAGCACCATTCGCTTGGTCGATGGCCGTGATTGTAC
AGTCAATCCACGCTCCATGCGACCTGGACCAATATTGCGCCTCCTGCCCCACTTGATAAG
GTACTGAAGTACGTCGCTGGCTTTGCAATCTCTCGCCCTGGACTGCTCGCCGAAACTCCT
CGAACGAAACCTGCCCATCCCTGTTCTGGTCCATCAGCTGCATCAGGGCCTCCGGGGAAC
>at01758
GTTCTTCTTGTCGGTGTCAATGACGCGGAAGAGCTGGCTGCGGCTCTTCTGTCGGGTGGC
CACGTCGCCGTAGAGCGCCTTCCAGCTGGGCGCCAAACCAAAGCGGCGCGGGAGGGCAGC
GGCCTTCTCCACCATGAAGTCGAACTCGTCCGCATTGACGAAGCCGTCGCGGTCGGTGTC
TGCGTCCTTGAAGCACTCGGTGAGGAACGTGTACAGCTCAGCCCGCTCCACTCCGTTCTG
CGAGACGGCGTCCGTCACCCAGCGCTTGAAAGCAACCGACTCGGTTGCCCAAGGGCACGC
GCCCACGGCGACCGCAGGCTTCTTCGCAGGGCGCGCCGCGCCACCGCAGATGCCGGCAAA
ACACCTCATTGCTTCTGGAAACGCCTAGAGATGTACTTGGGTACAAAATGCCTTGAGCCA
AAATGGCTACGG
>at01759
TTTTTTTTTTTTTTTTTGAAGTCTGATCACTTCTGCGGCCACGAGGTGCGGAGAAGCTA
GGTGCCAACATGGCCGCCAGAGCGCTCAGATGATGTCCAAGGAGCCAAGGACCCAGAAGG
TGGTGTAGATGGCTGAGGGTCCGAAGAGGAGCGCCGCCGCAAGCGGCCAGTTGCGTGAGT
TGAGGGACTTCTCGTCGTCGTCAATCTCATCTGCGGGTGAGATGATCACCTTCTTCTCAG
GTGTCGTCTCCGGCACGGAGAAGCTTGGGAGCTTGAAACCACCCGCGTCCGCCTCAGCCT

TGGTGGCCTCGGCCTTCGGCACCTTCGGCAACTTCCTCTTCTTGCTCATGCGCATGGGTG
CTGCGGGGTCGTCTGGGCGAGGTCCCTCGACCACCTCCTGTGCCTGCACAGGTGTGGCCG
CCACGCTCACCGCAAGGCCGCAGGCAGCTGCTAAGCCAATGAAGGCGGCCCAGTTGCTCG
CGTGCTCAGACATCGCGGAGTCCTCTGGGCTGTGCAGCCATTGGCTGGTACACATCTGCA
GGGCGACTGCGCCGCGGAGACCACTGGCACGTGGGCTCCCAGTGGACACAAATGCTGTGG
CTGCAAAGAGCCACATCGCGACACCGCAGAGCAGGGCCACATGGCTAGCCGCGCGGGTCC
GGCGCCCTGAAGCCATGGCAGGTAAATCTGGCCAAACCAAACTCACTCTTCCGCTTGGTG
GCACTGCCGCTGGCTTGAGCCAAAATGGCTACGGACTCTGCGTT
>at01760
GCATTTTTGTTCCCAATGCGATGCAGCTGTTGTCTGTTGATGGTTGTCATGGTCGGGGGG
CGTTTACGATACGGAGTTTGATGAGAGAGGTCAACGACAATTGATCACGCAAAGGTTGTA
ATTTGTAGTTGCACGGATTCCCCACACGCGTGTCATCTTTATCGAAACTTTGGCTTTTGC
CGGCCTTGCTGAAGCCTGAGAGATGGAGTCATCATGGAGTCCAAGCCACTACTTGCTGTG
CCTGCTTCTCTTGTCGGACTTGGCGAGCGGTTTCATCTACAGCGAAGGGTTCCATCTTGC
CTCGGCCAAGAAGCGCCTGTACGAGAAGCGCCACCCACTCTGCCGATTTGGAGAACATGC
ACTACCTGGCTAGTAGTGTCA
>at01761
AGCGCTCCCGCAGCTCCCTCCTGATCATTGCGGCCCTGGCTGCCACGGCACTGTGGGCCA
CGGCCCCTTCCGCCTTCGTGCCGAGCCAGGTGGCAGGCCAGGTGCCTCCAGCGGCGCTGG
CTGCTGCAGCTGCCAGCGCGCTGCCCACAGCAGCGTGGGCAGACGCCGTCGACGCACCCG
CGGACTTCTTGGACGTGGGCTCATCTATGAATCTTGCAGGCCTGACAGAGGTCCTCATGT
GTGGCATCGTCATGGGCGTCGTGCCCACCACAATCCTCGGCCTCCTGGTTGCTGCTTGGC
TGCAGTTCAAGAAGGGCCCCACACTCGGCCTGTGAGCGGGGGGGGCTCCAGTCAGCTCTT
GCACGTTTGGCGGAGAACGGGCTGAGGGCCAGCCGAGCTAGTACCTTCCACTATCACTNG
TGAGTTTTCCAAGACCTTACGAAAGGCCTTTGAGCTTGCGAATGTCTTCCCTCTACACAC
GCGTATTCAGGGGGCATCGGGATGTAATCTGGCCGAAGCAAGCTGCTTGAAATTAGCATA
CTCACATGCTCGTGTCGAGCCGACTGCCCCTCTCCGCTCAGAGAGGCTGGACAATTTTTG
GTTTCAGCCCGCTCTCATGGTAGCGGTTGTTTGTCCCGAGCATAC
>at01762
GGCATCTGGCGCAGGCTCCAGCAGCATCGCCCAGATCATCATAGGCTTGGTGCTTATCCT
GGTCTCTCAGATAGTCCTTGCATACCAGATTGTATTGGAAGAGCGCATGATGACGGAGGA
TGGGGGTCACCCCGTTCCAGCTGGCTGGCTACGAGGGCCTGTGGGGCCTGCTCTTCTTCG
TTGTGCTCATCCCCATGCTCACCCTGACGcCTGGCAACCCAAAcCCAGACCAGAAGCCCA
GCCCCATGCTGGCTGtTTATCATGAGGACTNCCGCGACTCACTGGTGAAGCTCCACAACT
CACCGCAGGTgGTTCTCTGGGTTGTGGTGTACTTTGTTGTCATCGCCATCTACaACGTGT
CTGGCCAGGCgGTCACGAANCNNCTGAGCGCAGTCCTGCGCTCCATTCTGGAGGCCTGCC
GTACCCTTGGTGTTTGGATCGCGGACCTTCTGCTCTTTACGCCATCGGCAGGAAGCAGGA
CGGTGAGGCCTGGACAGTCTGGAGCTGGGTGGAACTGGCAGGTTTCCTGCTGCTTGTCTA
TGGGACCATGTCATACAAGGGGCTGCTGCCGATCCCCTGCGCAAAGAGGCCCATGGCTGC
TGTCGCAGATGAGATTCCTCGGGGATGTTCGTCGTTGTCTGCAACCGGTTCGTGGGTTGC
TTAAGCATCGGGCTCTGCTGTATCGAGGTTCTTGCACACTTGCCAACCACCGATGTTTGA
CGGCGTTTGGCTTCACCGGAACTGGACGATTGCACGGGGCAATCGCATGGCCAGATGCCA
AGCTTTCTGCGTTACCGCCAGATTTTCCTTTTGCATCTGGCAGAGAGCAATTATCCTTTG
ATTTTGGAACCTGCCTGAGGGGGCGTGTTCCAAAGCAGAGCCAACCATCACCATGCTGAC
TCAGGTCGCATGTTGCGTGCTTTCCGATGGTGAGAGTAACTTTTTTTCTTCTTCGAGCTG
CCGCCAGCTCGAAGAAAGAAAAAAAAAGAAAAAAAAAACAA
>at01763
TTTTTTTTTTTTTTTTTGACAACAGCCGCTCGAAAAGAGTTCGGTTCAATGGCAGTATC
ACCGACCCGGGAGCAGGGGGCGGGACGAGGAGGCAACTCTAGCCGCGGCACTGCGGACCG
CATTACCTACCGGCAGCCTGAAGTAACCGTCAGTGCAGTCAAAGCCTGTGCCAGTGCCCC
CCGGGGGCCAAGGATACTATGAAGCAGCATCAGCTCGAGCATGCTCCTGTTCAAGCACCA
CTTCGAGGGCCTTGCCAGCCCAGCCGGCGCTGTCCTCTTCGGAGAGCCTGAAACAGCGAT
CCCGCCTTTCAAGAAGGACCGCCGCAGTGGAGACGGCCCTGTTGAAGAACTCCTCAAGGG
CGCCAAGCTCGGCGACGAGGTGCACAGCACTGTCTGCCTCGGCCTCTCCGCCAGCCCCGG
GCAGCCTGGAGCTCGCCGTCGCGGCCTCGGCCTCACATGCAGATCCGGCAGGTTTGGATG

ATGCACTGGGTGTGGCTGGATCGCAACTTGGTCCAGGGCACTCAGCTGCTGCTGCTGCCG
GATCGGTTAGGCGACCAACGTACCACCGTGCCAGGTGCGTCACCGAGGCACCGGTGCTGC
CCACGGTTGGTGGCTCCCAGTCCGGGATCACACCATCTCGTCCTCGTGCCGCCTCGTGCC
GAATTCT
>at01764
TTTTTTTTTTTTTTTCCTGGAACAATCCCTCCCAGTCTCCACGTCACAATGGTGGAATAT
AAGTGGTCGGGCATGCATGGGCCCTCAGCCACGTCAGAGCTCCCTCGGACTCGTGGCCTT
CTGGGATCACGAGCTGGCCCTCGACTTCTCTGCCCCAGCAAACCACGGGGTTGTCCCCGA
CCCGCTGGCCGCAAGTGAAGTAAACGCCAGCCACCAAGCTTCGAAAGTGCTGATCTTCCG
GAGGGCTTGCCTGCCCGTCACCGTTGAGGCCCCAGCAAGCGACGGTACCATCCTTCCGGA
GTCCGCAGGAATGGTCACCGCCAGCTACTATGTAGTTGAAGCGCTCATTGGGGGGAACAT
CCGACTGCCCGTAGTGGTTCAAGCCCCAGCACACGCCCGAGCCGTCGACCTTGCGCACGC
CGCAGGTGTGGTATTCCCCGGCCGAAATGCTGGAAAACCGCTCGTGCGCGGGGGCCGTGG
CCTGTGGGTCCAGCCCCCAACAAACGGAAAAGCCGTCCTCTCGGAGGCCGCAGGTCTGCG
CGGAGCTCGCCACGAGCGCCTCGTGCC
>at01765
CTATGCGAGCTTCACTCAGCATGCTGGATCAGTAAGACGGACTGCAGGGACGAGGGAGTG
TCCAATGCCTCGCACTGAAACACTAAACAAACAGTTGCAGCGGGAATCGTCAATCCGCCT
CAAAAAGTTGTGGCAGATACCTTGAATCCACCTGGTCCGCGCGCATATGCATGGCTGTGT
GGGGGCCAACAGCCGCACCAGCACAGACACGCACGCTGGCATGTGACCCTCATGGCCTCC
ACAGGAGACAAGATCTCGATGTCGAGGCCGGATGTGTCGAACTCAGGCAGGTAGGCCTTG
ACCTTCTGCATGAGGTTCACGTCATGGGAGCGGCAAGCGTCAAGCCAGAAGATGGCCTTG
CAGGGCTTGTCATTCCTGGGGAAGTTGTTTGCACGGCACCTCGTGACCGCCAGCTTCACC
CAATCCTTGATTGGAGCGTCCTTCGTGCCTCGTGCC
>at01766
TTTTTTTTTTTTTTTTTCGTTTTCACATTCGGCGCATGGGTCAAGCCTTATAACAAGAA
CAAAACACCATCAACTTCAAACCTCAGACTGCACGCTCTCGATCAGCAACCAAACTTGCC
ACCTGCACAATCTGGGCTGGACACGAAGTCCCGAATCAATTCTTGAGATTGAAGGATCTG
CCAGGCACAGATGTTGCTCACGAAGCTGACTGTCGCAGGGTCCCAATCAACTATATATGT
ATTGACACGGGGGAAGGTTGGAACCTTCTCGTCAAGGTATGCCTTCGAGCTCTCAGGGAG
TCTCTCCCACCACTCTGCTGGCCTTCCGTTGAACACCCATACCGTGTCCACTTCGTAGCC
GCCCTCTATGCCCTGTTCAGGATTCGGAAGCACCTGAAGCTGTTTACGGATGACCTGGGG
CTGACCCTCTGCTATTTGCTGCCGCAGCTGTCCAAAGAGGTCGTCCCACGCGGCCCGCTC
GAAGACTTGCGCTGATCTGTTCCACGCCTCCGCCGTGACCTCGAGCTTGCTGTACTTGAA
ACTCTTGCCCTCGGGGTAGGCGCCGAAGTAGCCTGAGATGTCGAACTCCTGCTCTGCCCA
GTCATCGCAGACCTCAACAGCCATGGCTGTGCACACCACCAACTTCCGAACGCCCCGTCT
CAATGCTGGGTAGATGGCGCAGTTGTCCGAGCCGCCTCCATCTGTCATCTCCGCTGTGCA
ACCGGAGTGATCCAGCCCAT
>at01767
CCGGGCCCGAAGCTGCCCCCCACCCAGGAAGACCCAGGGCGCCATCCAGCGGCGGCTCCC
GCCCGGCCTTGCCGACGGCCAGCCGCCCCGCCGGCTCACCCTGTTCACAGGAGCGAAGCG
TGCCAGCCAATTCTCCGACGTGGATTCTGCTGATGAGCCTGCGGGGTGAGTGAGGGAAAA
TGTACCGCGCCTCTTCGCGAGAGGAGGTGGTGAGGTGATCCACAGAAAAG
>at01768
AGGAAGCTGGCGCTCTCGTCCCTCGAGAGCAAGGAGCAGCAGATCAGGGCAGACATCGAG
AGGGTGCGGGAGCGCAACGCCCAGTCTCCACAGCTGGCTCTGGACGACCTGCGGCTCATG
GGCCTGAGCACAGAGTAGCCCGGGCGAGCAGAGTCACTGCGACTCCTCATTGCGACGATC
CGACTAGGATCAGGATCTGGTTCAGGGCCTGGGTCTGATTCGGGATCGGATTTGATTGGC
TTTCTTGCTGCACATTTCAACAAAGACCGCGCGCTGGCTGGTGCCTGTGAGCCTCTTTGG
CCGCAAGGCAGCTCGTGTTCACGGAGTAGTTCAAGGCCTGAGCTCCGGATTTGCCCACGT
CATCCAAGGTGCTGTCATTCCCATCGTGCTTGTTCAAACCACGAGGACAGACTTGCCGAA
AGTGCTTGTTGCTAGGCATCTGAGCAATGAAGACT
>at01769
ATCCAGAGAGCCCTTCAAGTAAGTTTATGCGCATTGCCAAGCGTTTGGGTCCCTCTTTCC
GGCAGAACTCCGCTAACCTATCAGGAGGAATGTCGAGGATTTGCCGCATGTTTATTGCCT

GAACTGGTGCAGAAGAGAGGTACGCCACCTCCGCCCTGACGAGCTCATCTAAGCGCTCAG
GGGACACCCGTACGAGACGGCCACCCGAGCTGCAAAAGTCTCTCTGCTGTGGTGGCAGAT
GTGAAGCTCTGGGTCCACGCTCGGCTTTCGGCACCTTCCGGGCCACCCCGGAAGGGGGAT
GCCTGCAATCCGTGCGAAGACCATGGCACGCAGTCCAAACAATTGCCAGGCCGCAAGTGG
GGAACCGTCCAAAGGGAGCC
>at01770
TTTTTTTTTTTTTTTTTTACCTTANGCGCCATCAATCCATCTGCTCCAGCTGGAACGAGG
TCATTGAGCAGCTGCCTGTGGCAGGTTCAGCGCTCAGCCTTGGCCCGCAGTTTTCTGTGG
CCGCCTGCCCGCTGCCCCTGTCCTCATCGTCCTGCTGTCTTGGCAACTCCGCCACAGTGT
CTGCTGGCAGCCGCTCAGCAAAGGCCACAGGTTCCGCTCCAAGCCTCTCCATCGCCTCCT
GGCTACCCCTTCGGCCCACGCCATGAAGTCGGTCGGCAAAAAACTCCATGACGACGCGCA
TCTCCTCTTCATTACTGCCTCGCAGCATCTCCCCACCACGCTTGGCAAATGCCTTCAGCT
GCACGCTGCCCTTCCCATCAGCGAGGGCCTTCCGCAGGAGCCCCGCGGACGCCTCTGCTG
AGGACAGTGGCACTAACGCGTCCCTGGAGCCATTGATCAGCAAGACGCCGGTGCCCTCCA
CGCCGCAGTCAGATACAGCTCCACCAGTGGACGGAGTTCGCGCGAGGTCTCCGAGCTGCC
ACTGGCGCTCTGGCAAGACCTCCGAGGCGACTGCAACCACTCCGCCGAGGCGACCAGCCC
AGGACTTGGCTCCCCTAGTGCCTTCAGGCGGCAAGAGCAAGTCCAGAGCCAGTGTACCGC
CTTGGCCATAGCCAAGCAGGAATATCTCGTCAGTTTGCCAGGCACACTCGGTCCACGAAG
GGCATCAATTGCCTCTGACAGAATCTGCCGGCTTCTCTGCAACGAGACAAGGCGGCGAAG
CTCCCCTGCACAGGCTTGATGAAGTCCACCGCCTCCTCATCCAAAA
>at01771
TTTTTTTTTTTTTTTTTTAACTGTTCGGTCATCTTCCACGCGGTGCCCACGCTACTCCAA
CGACCCTTCAGCCACCCGCCCGCCAGCTGCCCACTCAGTACGGCGTCGAGCGGAAAGCGT
CGCTCAAACTGGCGCCCAGCGCACCGTAGCTGAGCAGGCCGCCGGCGCCAGAGTCCAGTG
AGCTCCCGCCGCGCGAGGCATGCTTGGCGTCCCGGATGCGGCCCATGACGGCGGAGCTGG
GGTTCATGGTCACCGACAGGGAGCCGCGGTCCATCACCGCGGCCTGCACGTCTGGGGTCA
CGGACAGGAGTGCGCGCGCGGCGCTGTCGTCCAACCGGTTGTCCAAAATGAACTGCTCGA
CCTCCGACGATGTCACAGAGCCGCTCCTGGGGCCTGACGACAGGGAGCTGGTGCTGGCGG
AGGCCGAGGAGGAATTGAGGATGGAGTAGGAGCTGCCGGTGCCAGTGCTGTTCTTGCGGG
CGGCCTCCTTCTTCACCTTCCCGTCGCGGATCCGTCNCATGACCGCGGCGCTGGCGTTGA
TGCAGTCCATCAGGGAGCCCCTCTCGAGGACCTCCGCCTGCACCTCGGGGCTCTCGGTCC
GAAGTGCCCGCGAGGCGTTGTCGTCGACGCGGTTCTCCCTGATGAAGTCTTCCACTTGGC
TCCGCGTCGGCAAGAGCATGCCGCTGGTCG
>at01772
TCGCTCCAGCACAGGTCGGGGCTTGAGAAGCGCCCAGTGGGCGCGAACCGTCGCCATGGC
TTGACTTGGATTCTCTGTACATTGTGTTCATCGTGAGCTCAGAGAGTGCAGTGTCGAATC
TGCACTTCGGGCATTTCCTTCCGCATCAGTTTGCCAAGTTGGCAACTCCC
>at01773
CCGTAGCCATTTTGGCTCAAGCACTCAGCGAGGGTGCCCCTTTTTTGTCATTGCAGTCAC
GAATGGCATGATGGCGCTTGGTGCTCCATGTGCATAGGTCGCACGGCCTTTGTCGCTGTT
GCTGTGTTGCAGTTGCACTACTGCGACCTGATATGGCTGAGACGGAGTGCCAGTCCTTCA
TTTATGTGCTTAGAATTTAAAGGGAAAGCACGCTCATTCCAGTGATTGGCCAACTGAGCC
GTGTTTTCAAGGCAAGGGATGTGTGCAGAGACCAGAAGAGGACAAGACCTCTGTTCCCGT
GTTCATGCCTCCTTACGCATGCAAGGCATGCACCTTTGCTCCAAGCTTACTGAGTGGGTA
AGAGATACCTGTCTGCAGTACGCATTGCA
>at01774
TTTTTTTTTTTTTTTTTTCCAACTGATGGGCATCGTGACAAAGCTGCAACCACCACCGGC
CAGGCTCGTCAGCGCGCAATGGGAGAGCCATCATACCGCCTCGAGGCTCAGGCCAGGGGT
CGAGCGCCGGAGACAAAGCCAGCATGGTTGATGCGGTACTCCCGTGTGGCCTTAATCCAG
CAGAGGTCCTTGTGGGTTTCCTCCCGTGACCACGTCCAGAAACGGCAAAGTTCGTTGCTT
CTGCAGAGTTCGCAACACGCAGCAGCTGTCGCCACGTTGTTGAACATCTGCACGTCATTG
CCCGCATAGATCCAGCCATCCTCTGGGGCGCAACTGGGTGCTATATCAAAGGCCCTGTCA
TCCCCAGCAACTCCGCCCGCTGCTTCCAGCGGTGGTTTGCGGACAAGGACTAGGATAGAC
TCTGCCAAGTGAGCCACCTGCGAGGCGGCACGGAGCTGAGCTGTCAGGTCCCGATCGAGA
TGTAGCCCTGTATTCTGGGCCACGAGTCTCAACACCTCCGTCTCGCTCTGAGGGTTGGTG

CTGCCGAGCCCTTGGAGCCCAGGGCGAGCCCAAGTCAGGATGAGACCTTCGCCCGTATGC
GCATCGAGGTTGCGGAGGAACACCCCGGTCAAGTCCGGCGGGATGTGCTCAGCCACCTCC
ACGCAAAGCACCCAGTCAAACTTCCTCCACAATGCCAAAGGGTCGACCCAGGTCAGCACT
GAGCACCGTGCCCTTCGTGACGAGCTCGATGTCCGGCGAGCCATCGAAGGCATACGCTGT
GACGAGGCCGGTGTCATTCAGCCAGGTGGAGTAGTGGCCGGAGCCGGCGCCGAAGTCGGC
GACCACTGCGTCTATTGGGAGGATGTGTCGGATGAGTCCCTGAAGGAGGCCCTTGTCCAG
CTGCCACTCGTCGAAGGTGTCGCGGCGCAGGGCCTCGAGCGTGCTGAAGCGGCCGTCCGC
CTCGTGGCTGTAGAAGCGGGCCTCGTCCATGGAGGGTGGCCGCCAAGTGCGCAGCACGGC
CCTCAGGGTTCGCAGGTCCGCGTCCAGCAGCCCTGCGGCCTCTGCTAGCCACTCTCGTAA
GTGCTTGGAGTTGGCTGCAGCTGATTCCTCAGGTCCATCCTGGGCAGCCAACCACAATTG
CTTCAGGTGCCCTGTGGTCAGGGACTTCATTCGTCGGAGGTTCACGATCAGCAGCAGCCG
CATGTGCTCTGAGAAGCACTCGGATCGCTGTGAGGAAATTGCAGCAATGACGTGGGTGTG
TGCGGATAGGAAGGAGGACCCTCTTGAAAGAGTATACAATATATACCTCCATGGCAGTGG
GTCGTCCTTCTCATCGCCGGAAGCAGTGCGCAGCTTTTCCATGCGCTCCCACACCGTCTG
ACGCAAGATGTACACAAACTCCATGAGCTTGGCACAGTCCATGGCATTTGTGCTAATCTC
TCTTTCCACGTTTGCGGCCTCTGCAGGGCTGACCGGCATAGAGCAGAAACCCTCCTCGAA
GATGGCCGGGTTGACCCCTGCAGCGCCCAGGCCCGCCAGCATGTCTTCACTCGTCGGCGC
TGCCAGTGGCTGGGCTCTGGGGCCGATGCCGGCAGCCATGGTGAGCAGGAACAATGACCG
GGACGCCTGAGCACCCAGCAGCACTGAAGGCG
>at01775
ACATGTTCCGGGGCTGCACTCCTCCCGCTCGCCATCTTTGCTCCAGCTGCGGCCTCCGGG
CCGCTGGTCATGGCCCTCAGCTGTCTACTTGCCCTTTGCTGCCTTCCCCTTCTTCTTGTC
AACCTTCTTGGAGCTCTCAGCCTCGTCAACCTTCTCAGCCTCCTCAGCCTTGGCGGACTC
CTCCTTCTTGGCAGACTCCTCCTTCCGAGCCTTCTTCGACACGGAACCTCCGTAGAACTT
GGCTTTCTCTTCGCTCGTCTGGAAGCGTCCATGTCCCATCTTTGAAGACGTGTCGATGAA
CTTCAGATCTATCTTCTCAGTGGCTCGGCGGCTCACCTGTGCAAGCAAGGACTTGCGGAG
GGTGATGACGCGCTTCTTGGGGCCCATGACTGTGCCCTTGATCATGACCCAGTCTTGCGT
GATCTCCCCGTAGTGCATGAAGCCGCCCATCGGGGTGATGCCCTTCTCCGTAAGGTCAGC
CTCCGTCATAGCTCCATAGGCATCCTCCTTCACCGCCTTGCCTACACGGTAAATCTTCTT
GTTGATTTCGGTCCTGTGGCCGTAGCCCTTCTGCCCAAATCGCGGCACCTGGAAGCTGAC
ACGTGCAGGGTGCCATGAGCCAATGCATGCGACCTTCCGCAGCCCGCGGTGAGACTTCCG
CACGAGGCGAGTCACGCCCCAGCGCGACACGACACCCTTGAAGCCGTGGCCTTTGCTGAC
GCCGATGACGTCAATCATCTCATCCTGGCTGAAAACGTCGGCAATCTTCACCTCTTGCTC
AAGGAGTCCCATGCAGAAGTCCACCTTTTGCTCTGTGGTGCCGCCGTTCACCTGGATCTC
CTTGATGTGAGCCTTCTTTGCCCTGATCTTGATCTTGCCGATCTGTGTGTGGCAGATGGC
ACGGATCACCTGGCAGTACTTTTTAGCGCGGGCAATCTCCGCAGCCATGGGTGCTTCAGA
ACCCTTGCTGGCCTCGGACCAGCGCTTCTGGTACTTCGTGAAGGCCTTCTTCTTGCACTT
GTACCAGTTCTTGTAAAATCGCTTCTTGCACTCGTCGCTCAAGTGCCCAGCCCAAACAGT
TGTCAGTGCCCGAAGGCCTTTCGGGGTCTCCACGTAGCCGACAAAACCCGCAACAATCAT
GGGGGGTGCCTCAAGGATCGACACACCCTCCGCAACCTCCTTCTTGTGCAGCTTGGAGCC
GGGCTTGTCGACATCACGGACGATGTGCGTCATGCCGGCCTTGTAGGACATGAAGGCAGT
AAGGTGCGGAGGCTTGGATGCGTCATCTTTAGGGAAACTCTTGATCTTGCCAAAGTGGTG
CTTGGTACGCTTGCGGGGCAAGAAGCCCAGGGAGCCATGGCGGGGACGCTCAAACTTGCG
GTGGGACATTCTGGCTAGCGGGGAGACACCGTCTACGCCTCTGGCGCGAAAACGTGCCGC
ACCGTGAAGGACCGCTTGAGCCAAAATGGCTACGG
>at01776
GTCGCAGGCTCTCCTCCAGCAGCAGGTAGCGCCTCAAGAGCTCCGAGCAGTCCCTCGCCC
GGTACAGCGCGCCGGCGGCCGCCTCCGGCGGAGCCCCGGCCCAGCCCATCCAGCCACCAG
ACTCAGCTGCTTGGGCACCAGACTCGGTTGCTTGGGCATTCACTGCGAAAGGAACAGGCA
ACGGTACAGCCTCGGTGTCATAAAGCGGTATGGCTACCCGACCATGTTCAGGTCGAACGG
GGCAAAGTGCCGTGAAGTGGCCACGAGTGTATGCCACCACGAGTGGATTTGTGGAGCACT
CCGCGGGGTCCCACATAAAAGGGGTAAGTAGACCCCCACTCATGCGGAAAGGCACCTCCA
AGATGTGCCGTGCTTCGCC
>at01777
TTTTTTTTTTTTTTTTTGCGTGCGCAACTGCCAGTACTTTTTTTTTTTTGGTTTCGATC

TCCGATCTAAAACAATCCATCAGAATCACATAAGCCGTCGGCGTCTCTTGCGCCAATTCG
AGGTCAAGTCCAGAAGCCATGGTGCTCGGGATCAATCCGCTTGTCGAAAACTGCTCCCAG
CATCTGGCTCACCCGATTCCAGAATTTGCTGCATGCCCTCGGCTGGGCGTGGCATCGTGA
AGGCTCAGGCCGCTGCTCGAGGCTCGGCCATTACCAGCCATGTGCCCACTGAAGGCCTCA
ACTTGAAGTGGCAAGCAGCTGGTTCTGGCACTGCTGATGTCATCGGTGGAGCCATGACCG
GCGCTGACAGGCCACTCGCACCCGGTGAGGCCGCCGGTGAAGCCTTGGCCCCGGCCAGGG
TCAGCCAGGTGCCCACTGATGGCTGAAGCCCGAAGCCACCAACAGCCGTTGAAGCCACAG
TCGGTACTGTCACTCGGTGCTCACCGCCGGCGCCGCAGCAGGTGCGGGTGCCATGGGTGA
CGAGGGTCCGTCCGGCTTCTTGTTGCCCGACGCCAGCCACGTGGCCACAGAGGGCTTGAG
CCGGAAAGTTGACGTGGCCAGCAGCCGCGGCATGTCAGCCGCTCCACGTGCGGACGGTGG
AGGCAAGGAGACCTTTGCACAGCCCTGGAGCGAACTGGCCACAGGGGGCTTCAGTTCCCG
GTCTGGTACTGGCTCCAAGGCCTCCTGCTTGGACCGGGCCAAGCAGGGGAAGTCGTCGAC
GCTGCCAACCGCACCCATGGAGAACGAGGGCAGCCCACACCGCTCCGGGAGAGGGGCTGG
TCACACGGGTTGAGCACAGGTCGAGACTGCCTTGAGCCAAGATGGCTTGAGCCAAAATGG
CTACGG
>at01778
CCGGCGCGTGAGGGGCTGGTGGCTGCGCGCCTCTTAGGCGCAAGGTCTCGCTGGCCACGG
ACCCCCGGGAAATCAGTCTCTATGGCAGCGCTGGTTCATCAACCGTGTCCTGGATATTTG
CCGTCACTCTGCAGGATCGACAGCCCAATGCTGCTCGCTTGCCGGACTACCTCTTCCGCT
GTAGCTATTGCATCAAGTTCATTGCGAAGCTCGCCGGTCTACATGCGGCATGACAACTGC
TATTGC
>at01779
AGCGGTGGGTTTCAGCGAGTCCTTGCAGTCTATTTAGTGCATGGGCATGCAGATCTATCA
AACTCAGAAAGCTTCATGCCCGGCGCATGCACTGCGACAAAAAGGGNGGTATTTGATCTT
CTCCATGCGTCACAGCTTGAGCTCAgGGATGAACGCTCTGTTCGATTCCACTGGACGTCG
CGCCAGCTCAAGTCCTCACATCGACACGAGCGATCACAGTTGGGAACCGCCTTTGCTTCA
GGTTGAGGATGCCGACAAGTATTCTCGAGGTCGGTATCTGCTAGAATACAACGCTTGGCA
TAACATCGGTGGCCACC
>at01780
TTTTTTTTTTTTTTTTTCAAGCGGCCAGCAGTGGATCTTGAATCAAAACCAGTGGTGCC
CCACACCTGCTCCGCATAATGGCTCCACACAATGACGTGCACTTCACTCAGAAGAAGTAG
CGGTGCATAGTACCCCACTTGAACTTGTGCTTCTGCCGCAGCACAGCGAGGCCGTGGCCA
ACGGTCTGCTCAATCTTTGGTTTGAAGCTCGGGTGCTTTGCCAGGAGCTTGTCGTAAGCC
TCGGCGCAGAGCTTGGCATGCTCCTCCATGTTCTCGACCTTCTCGTCAGCGGCATAGCCG
GTTCCCATGAAACCCGAGGCACGAGTGGCAAGCTCTGCAAGCTCGTTGATGTGATCGCGC
ACATCCTCGCACTGGTAGAGCACGTCCATCATTTCCTGGACCTCGGCAAATTTTGCCCCC
ACTGCAGGTTCATGCAAGTAGGGCTGGACAATTGATGTGAGCTTCTCAAGCTGCGCCTCC
CAGAAGGGGCCGCTGTAGACCAAGTTCAGGTAGTCCGCCCAATTGGCGCTCAAAACAGTG
GAATGGTACTCGTCTAACTGCTCCAGCACGTCATCACGCGTGTACTTGGCAGTGGTGATG
TATGGCCTGAAAGCCGCTCCCATGAAGGCCGGTGACGGTCGGCCAACAGGCATACCACGG
GAAAAAGGCGTTGGCAACACTGGCTGCAGACCTGCAACAGCCCTTGCGGTTGTTTGCAGC
CTTGAAGAAGCTATGCGACGGGCGCACGACAAGGCCATGGCTCCAAACAGCTGCCAACCC
CAGATTGGACGATGGAGGGCAAGCCAAGACTGCACAAAATAAGC
>at01781
TGAGCGACACGGCGAAGAAGGCCACCCCCCTGAACCCCGCCCGGACATGGCACGGCGTGG
ATGAGCTCATCTTTGACGAGTGCGTGATCCCGCTGGACACGATGCACTACGGCATGGACG
CGGAGAAGGAGTCGGCCAAGGAGGAGAGGCTCATGCAAAAGGACCCGGCCAGTGAAGATG
TGGTTGGGACTGCGACGGCAGAAGACCACGAGCATGAGCCTTGCCATGAACGTCTCATGA
AGAAGCAGCTCACTCATCTCGTGGTGAAAGGCGCGGAGGAGCACGTTGCGCGCAGGCGCA
CTTCGCGCAGTCAGACATGGCATAGCAGCAAGTCGACCACATTGTAGCACATCCGTGCTG
AGCAGAGGACAAGTCGGCGTGCTTTGATGGTACAGTGAGGTTGGTGCTG
>at01782
CCGTAGCCATTTTGGCTCAAGCCATGCGGATCAAGGACCCTGCAGGCACAGAGTAGAAAG
AAACACATGGTGTTGTGCCTCTACTGCTAGGCTCTTCGATGACACGCGGCCCACATGCAG
CAGAAGATCGCACCTGCTTGCAGTGTGACGCACCTGCCAAGCTTCAGTGCGGGCGTTGCC

GCTTGGTATGGTTTTGCTCTGGAGCCTGCCAGCGGGCAGCATGGAAGTCACACAAGGAGT
CTTGCCGCTTGCCAGCACAGCCGTGGCACAAACTGCCAGCTGCTGAATGCTCGCGGATAC
TTGATGCTGCCCAGGTGGGCCTTCAAGAGCAGGGCCTGGATGCTGTTGTGATTGTGGCCC
GGGAAGCGGACGTGCTCTTAAGGATGTCATCGGCAGAGGGCGCTGAAGGTCCAAACGGCG
TGGAGGAGACACCAC
>at01783
CCGTAGCCATTTTGGCTCAAGCCATATTTTGTTGCCTTGCCCATCCGTGCTTCGTGCTTT
CTCCCACCTTATGGCAGAGAAGCAGGCTGCGCCCGTGGACTGGGCCAGCTTCAATCGGCT
GCGGCGCGAGCATAGCCGGCTTCGCGAAGACTTGATTCGTCTCATCGACTGCTTGGGCCA
CGGACATACGTTTTCCCAGATCTCTTGGCAGTGCCACAGGGAGAAGTTCAGGGCAACTTG
CCTCCGCAGCCCCCTGTACCCCATCAAGACCTCGCTGGCAGACGCCATGACGATCAAGCC
CTTGCGCCTCGTTCAGCGGCTCTGCGGGGGCAGGAAGGAGATGCTGTCCCTGAGGGAGAC
GGCCACCCGTGCCAAGGCATGCGCCGACAGCATCTTCACGGGCTAC
>at01784
TTTTTTTTTTTTTTTTTGCACCGAGCGTTGCCCTGAAACAGGTCCCTCCAGTTGATCAC
GGGGCTGTGCACAGGGGTGCATGGCCGAAAAAGGCCCCAGGCTTGTGCTGGCGCTGGACT
GAATTCCTCGGATGCATCAGCGCATGTGTGCATGTCAGTGGCGTGCACGTGTGTACATGT
GGGCGGGCAGTCCTCAAAACGCTGAGTCACACGACGTGCAGCTCATCCCTCGATGCACCT
TGCAGCCATGGATGGGCCAATCCACTCTGTAGCTCTATCTCAGCCTCCAAGCCTGTCAAG
TTCATCGGGTAGTCATGCACAAACTGGGAGTCGGGGGCGCCTCCGTCACCTACCTCGTTC
GCTTGCAAAAATCCGACCGGCTCCAAGCCGGAGCGCCAACGCATCAAGGCAAAAAAAAGC
TGGCTCTTGACGTTGCTGTGGCCCGGATCGTCAAAGAGGTTCAGCAGCTCGTCTGGATCA
GACCTTCGGTCGAAGAGCTGTCCACGGCCATCGTCGAGGTAAAAGGTGAACTTCCAATTG
CGCGTGACGACC
>at01785
GAGTCTCTGCGCTACAAGCTCCTGGGCGGCCTCGCTGTGCGCCGGGCGTGCTACGGCGTC
GTGCGCTTCATCATGGAGAACGGCGCGCAGGGCTGCGAGGTCGTCATCAGCGGCAAGCTG
CGCGCGCAGCGTGCCAAGGCCATGAAGTTCAAGGACGGCTTCCTGATCTCCACAGGCGAG
CCCAAGAAGCACTACATCAGCGAGGCAGTACGGCACGTTGCGATGCGGCAGGGTATGCTG
GGCGTCAAGGTCAAGATCATGCTCGGTTACGATCCAGAGGGCAAGATGGGCTGTTCGATG
CCGATGCCGGACAAGGTCGTTGTGCATGAGCCCAAGGAGGAAACCCCAGTCTTGGCGCAG
GAGCAGGGTGGCTACGGCGGCGGCTACGAGGGCACTGCCGAGCCGGTTGGGGCTTGAGAT
ACACGGCAAGAATGTGAGGTTGTGAACGCGGCAGCCGTTTGGAGGCAGAGGGCCCAGGCT
AGTCAGAGCACATTGACTCGTGAAGCATGAAGCATGGAAGGCGTGTGCTCGTCATTGCTG
GAGCGTAGAA
>at01786
CCTTCTGCCCACGCTGAACCACGTGATGCAGAACTACGACCAGTTTTCCAACGAGTGCCC
TGTGGCCTGGCTCGCTACTCTGCTCTTGAAGCTTGAGTCCAACCTCGACAGGGGGGGTTC
AGGGCCACACCTGCGCCAGGGTCCACGCTGCCGGCTGCTTGCGTGACGCGAGGGGCTACT
GGGGGGCATACGCCGCAGCAAGGTCAGCGCTGCCCTCAGAGTCTGACTCGAAGGCGGAGG
AGGAGCCAGGCGAAAGCGTGGCGGAGGTCTTGGCCCAGGGCGAGGCTCGCGTCATCAGCA
GGTT
>at01787
TTTTTTTTTTTTTTTTTGAGCCCATTTTGACAATCCACCGAGCGGGGCACATGCCAGCG
AACGCCCAGGCTCACTCAGCATGCAAGGGCATGCAAAACATGCACAGGCGTGCAATGCAC
GCGCAGCGGAGCAAGAATCAAGCCCTCCTTCTTGCCTCCACCCAGCAGGCAAAACAATCG
ACTTCACGAGCTGTGAGGTCAGGCTCATCCTCGACAGCCTCACGAGACCATTTCCAATTG
CGCTGGTAGCTGCTCTCCAGAAGACACTGGCTGGAAATAGCATCCACTTGCCAACCACGG
ACTGCCAACAATTCTGCAACTCTATCAAGTACTGTGGCCAATTCTGTCGGCACTGCATCG
CCACTGGAGGAGTACGCCACAGTCAGCTTCCCTGTATCAATTTCTGGCAATTGACTCAGT
TCACCATAGATTGCGAATCGCCACGTTGTTCTTATACCAGCAAGCGCAGCCCGTGCAAGT
GCATCAGCCACANGTACCACGTGCCCAAACAAAAACACGCANGCACCGAGCAGCTCAACC
GCCTCGTGCCG
>at01788
TTTTAATCACAGGGAGCTTGCGTCTTCAACGCCACCGCCCCGATTCCGCAGTGTGGTTAG

GAGCATCCCAGCATGCAACTTTGAAGGCTTTGCCTTGAAGAGCGTGCCATTTCTTTCCCA
ACAGGCACGCTAAGCACGAGAGCCTTTCCTCCATCGGGCATCCGCGTAGGTGAAGAACGC
TCCACCAGACCAAAGACAATGCGCCCAAGCAGATACGCCGACACACCACCAAGCCGCACT
GCTACAGATGGTCCACCTCATGACAACGTCGTAAGATGGGTGCACAGCAGTGCCGAAGAA
GGAAACTCCGCAGACAGCCCACAGGATCACGA
>at01789
TCGCTTTGGCTCCAGGCATCCGCCTCTCCGCCTTCCTGCTCTTCTGCTGCCCTGCCACCC
CACCTCATGGCCTTGATGACGGGATGTGTCTGCTGAGGCGATCCAGCGCGTATTCTGGCG
GCGTCTTCGACCTCTCGGACTACTGCTTCTGGGTGCTGGCGGCGGACATGCCCAAAGCGA
CGAACCCTGCCCTCCCATAGTTCGTGCTCTGACGGCTTTACGCAGGTCTCCGACTTCCCC
ATCGCCGACGGCTTCTCCAGGGACTCCTACAAGCTGCCGGCGACCTGCCCTGCGCGGTCC
CCCCAAATCTGCGCGTGCCTACGGACAGCTTGGGCTCCGAGCTGACGCCTTTGCATTGGA
TGGAGGCTGAGCTCACCTCCTCGTGGCCTTACAGCAGGCTGCTTTGGCCAGTTCCTTCTG
GCCAAGGTTTTGTGTCTGCGAGCTG
>at01790
CCGTAGCCATTTTGGCTCAAGCTGCTCCGGCTCAAGTCCGAAGTGCGTGTTCAAAGCCAG
GGCGGGGACGCTCCGATGGCAGACCAAGCTGCAAAATGGCAGCAGGAGTTTCAAAAGACG
CGAGCTGCCACGGCACAGGTGCAGGGCGAGCTCCAGCAGTGGGAGCAGCTGGCCGGCCAG
AGCGCACCTGCCGGCGAGCGAGCGAAGCAAGGCGCGTGCATCAGGGCCAAGGTCATGCAG
CTGAAGTTGGACCTGGAGCGCCACCAGCGTGCGCTGGACGGCCTCGGCGCGCAGGCCGCC
GCGGACGAGGCCGTGC
>at01791
TTTTTTTTTTTTTTTTTGAAGAATCGGGCTTTTGACATGAGATCGCACGCCTCTCCGCG
CGGTGCAACCGCCACACTGATCCAGCCATCAAAAGCGGCTGCCCAGCGTTTTGCTGGTCT
TGTTGCAAGCCTCCCTCCAGATGCGCTGGTGACAAAATGGCGACTGCACGGAATGTACAT
GTCTACCAAAATGGTTCAAGCCGTAGGGTTAAGTGTTTTCAAAAGGTTATCTACAAACAA
CTTAAAAAAGGCAGCGTGAAGACCATCAGAATGCAATCTGCATGCGATCTGAAACCAAGT
CTTGGAGCATCGAACCGATTGAACGGTCCCTGAGCGGTCCGAAACATGCACTGCACACGC
AAGGCCAGTCAAGCTCTCATCTTGTGCGCGAGCTGACTCGAGTCCTGAATTGGGTTGCGC
AGGGC
>at01792
TATGTGATGCTGCTGTGCTCGGCAAGGTCAAACTGCGGAGACAAGTCATCCGCCTCATCA
CAGTTTGGGTCTGCTCCAGCCTGGGCTCGCTTGTGCTGCACGCTCTGCGGGTAGACCTCG
AAGCCGACCACGCGGAAGCCCTCGAACTCGTCCGGGTCCGCATGGTACTGCAGCCCGATG
CGGACGTGGTTGTTGACGTAGTACTTCCCGTCGCGCTTAAGGCCGACAGGGAAGCCGTTA
GTGTACGAGTAGCCGTGGTCCCCCCTCCGGTAGTAGCGCATGGCGGCTGGGAGGTTGTCG
ACTAGCCAGTTCACCTGATACTCGTGATCGATCATTCTCCTGAACTGTGCCTTCTCTTCG
TTGCCCAGCTGCATCTCGCAGAGTTGCGAGCACGAAGCGTTGACGTTCATCCTGATGTCG
TACACGGAGTTCTCGATGGAGTCGCCGAGCAAGATCTCCCCCAGGTTCTCGGCGGAGACA
TGTATCTGGGTCGGCTTGCAGTAGGGGAGGACGTAGTAGCGGTAGGGGAGCAGCGTCTTG
ACGGAAGTCAGCTTGTTCACTTTGAGCTCGACGTGGCTGCCCTCATGGTACTCAGTTGGT
GCAACGCCCGGCAAGTAGAAGCTATCTGCCCTGAAAGGGATCGAGAGCAGCAGCGCCCAC
GGAACACCGCATGGTCTATGTACCATGTCGTGACTTGCCTGAGGTAGGACACCGTCAGGG
CCCGCTAAATCCCCTCCCCCGCGCCACCTTGCCCATGGGGGCTTGAGCCAAAAATGGCTA
CGG
>at01793
CCGTAGCCATTTTGGCTCAAGCCCTTTCTTTGTGCAGCATGACAAAGATTTCCCTTCCTG
TAGATGGGTGGCATGTCAAGGGTGTGGCTCACATCGGTGTGGCCCATCCAACAGGTGCAC
CAAGGACTGGGTCTTTGTCAAGAAGGGCAAGGAGCTGCAACCTACCTGGTCTCAGCAGAA
GGGTGGGAGGTGGCATCCTAGAATTTGGATTGCTGGCAAGCTTGTGTATGCAAGCAGGGT
CGTTTGCTTTGCTTATCCTCAAGGGACACAAGTTGGCTGGCATGAAGTACTGAGGACTGG
CAGTGACTGGCAGGCTGCACACCTTAACCTTGATGAACTTGACTTTAGAAGGTCTAACCT
AAAGTGGTTGACCAAAGCTGCTAACCTTAGCCAGTACAGGGCTGAGGCTAAAGACAAGCA
TGGCAAAGTGTACAGAGGTCAGTGAACCCAGCGCCATGACAAAAAAAAAGAAAAAAAAAC
AA

>at01794
CCGGTGCCGCTGAAGAGCTGACTCAGATCGACACTCGCCTCGTGCGTAGTCAATCCTTCA
GACTTGGACGGGCGCACATGCCTCATGTACGAACTGTGGTTGAAACCCTCCTTGCCCCAC
CGGAAGCCAACAACATGAGACTGCCGGCTTGCAATCTGAGCACATGTTGCCAGGTCCAGA
TGAGTGCCATTCAACTGCCAACCAGGTGGCTCAAGCCCTTTCGAGTCTACACAGCGCCTG
CCACCGTTGATCACTCGCAGCGATGCCTCACGGTTACCAGCGAGCGAAAACCtatCNCNN
NTGTGCCTTGGGAGGGTGGCgTGGGATGAGGTGACGTcCCTGTTCAGGTCGTACGCTGTG
ACTTGCTTGCGGATCACTGTGTTGTCGTCCGGGTGCTGCCACCTCTTCTTTGCTTAGTCG
CTGCCTTAGTGCCACACCCTCTCCGCTCACCTCACCAAGAGCTTGGGTTGGCGTTGTCCC
CCAAACCTGAGACCCAAAGGGAGGGCCAATCGC
>at01795
TTTTTTTTTTTTTTTTTANAGCATACTCGCGACAGGAGCCTCACTTGTTCTCGAAGACG
TAGAGCGTGTCGCAGCTGGCAGCGAGAAGCTTGCCCGGCACGATGGTGGGTCCGCCGGTC
GGAAAGGCGCTGCCGGAGTCGAAGCTGCAGAGCTCGGAGTCGCCCTGGATGCGGCCGTCG
CCGTCCCAGTCCTTGATCCGGTAGAGCCGGCCGTCCAAGTGCCCGATGTACACGATGCCC
TGGGAGTCGAGCGTCGGGTAACTGCTCGCGAGGGGCAAGCAGACAGGCCTCCTGCCGCCG
AGGAGCCGTCCGAGGAAGCCCTCGCTGTCGCCGGCGGAGCTGTAGTAGGGCCATGTCGGC
AGGTCCACGCTCCACTGCCTCTCGCCGGTCTCGGCGTCGAACGCCCAGACCCTGTTGTCG
TGCGCCCGCGTGCCCGGGAGCGCGCAGTGGCATCCCACGGCCGTCCCCAGGATCGCGCCC
AGGAGCGCGTGCTTGGCCAGCATGCCGGCCAGGCCGCACCTCGTCGCTCTCCTGCACCGG
CGGCCGCCGCAGCAGCAGCAGAGGCAGGCCAGGGCGAGCCCGGCCAGGGCTCCAGGCACG
CCCGCCCCCAGAACCATCATGCCCGCGATGGCAGCCACGGNGAAGAACCCAGCGAGCCCT
CGCCGGTCACCACCGAGAGGGCGGGGCCGTCGCCGAGCCGGNCGACCACGGGATACGTGT
AGATGCCGCGCTTAAAGTCCGTGCGCTCCCAGAGCTGCTGCCCCGTCGCTGGCCTGAAGG
CGCGGAGGGCACCTCCGGTGCTGGCGGGGGCCCGCATCTGCGGGTCCTTG
>at01796
CAGCGGTACAGCCTTAGCCCCGATGGTGATCATAGCCCACTGAGCCTTGCCGGAAACGCC
GCCGTCCTTGTCTCGCAAAGCAGTCATCACCTGATCCACAACGCTGGACGCCTTGGGGCC
TATCTTTGCCAGGGCGTCAGTGACCACGTATCTCACCAGAACCTCGTCGTCGCTCAGCGC
CTTGCCCAACAAAGGCACCGCCTCCTCCGCCTCGGCGCCCAGGGATCCCAGCGCCATGGC
CGCAGCGATGCGCACGACGGGGAGCTCGTCCTGCAGCGCCTTGGAGAGCCGGGTCACGGC
CTACGGGTCCCCGATGTTGCCCAGGGCCTCGGCCGCCCCGCAGCGGAGGCCGTAGTCCTC
CTCGCCCAGCGCCTGCAGCAGCGCCGGTACGCTCCT
>at01797
ACATCACGGTCGGCTCGCTTGGCGGCGCAAAGAAGGTCTGGAAAGAGGGGGCGTTCAAGG
GCGTTCCCGACTCCAAGGAGCCTCTTAAGAACCTCGTGGAGATGTTGGAGAAGAAGCCCA
AGGCATGACCAGCCGGCCATGCAGCAGCAGGCCACAGCATTTGCACGCGAGTGGACGTGC
AGTTCCAGGCATTGCGAGCAAAGAAAAAAAAAAGAAAAAAAAACAA
>at01798
CGGTTGAGTGCCCTGAGGGAAGGCTTGTGAGGCAGTTGCGGCATGGGTGCTCACACGGAA
GAGAAAAGTATGATTGCAAGGCTTGCAGCGGAGGATGCTTGCACGGAAATGTGAGACGAC
ATTGCCGCTTGTGCAATGGATGCCCANATGGGAANCGGAAATCCAATGCAAGTTGTGCAG
TGGCTGTCCACATGGAAGGTGGAAGTCGATTGCGGTAAATGCAATGCATGCAGCCATGGA
C
>at01799
ACTTCAGCCAGCTGCTGATCAGAAGATGGCGGCCCGGGCAATGGCTGCAACTTCAAAGAA
GCCTTATGACGACTACTTGGCATTCATGGAGCGTGCTGTGGACAAGGTAGCATGCCCAAC
GATGGTTGTCATGGGCGAGTTCGATCCAAACCTTGTCGTAAAGGAGGGAGGAGCGCGGAC
GGTCGTGGACCGATTGCAAAGCCGCTTGCCACGGGCTGAACTGCAGGTTCTGGAGGCCAC
CGGCCACGCCTCGGTGCGGACCAGCCCTCTGCAGTGCGCTCGCGTCGCTGTAGACTTCCT
GCGGCGGATCGGCGTGGCTGCTCCGGCGGCGGCCCTGTAGACGCGAGGGCTGAGGCTGCC
GCGCACACGCGCGGGCTGAGGCCTCTGCAGGCT
>at01800
CCGTAGCCATTTTGGCTCAGCCATTTTGGCTCAAGGTCCCTTAGCAATTTTGTCCAAAGC
ACAACACCACAGCATCGAGCATATTTCGTCCCACCCTGGGCAAGATGGCACGCCGCAGCT

CCCCGTTGAGCCTTCTGATGCTTGCCTTTGGCGCGGTGAGCTGCTTCCGCCTGGCCCCTT
TTCAGCTTCTGCCTTCACCTCGGGCACCGCTGCCCGCCGCGAGATGCTCGGGGCCTGTGA
CTGCAGGGATGGTCGCGCTGAACGCGCCGCAGGCAGCACTGGCGGACTGGCAGGGCGACG
CCGTCAAGGCCTTGAACATGTACGGCAAGCAGGTGCTGATGCTGGAGCCCGCGGTGGAGA
GCGGCGACCTGGAGGCCATCAAGAAGAAGTACCGGAAGCTTGAGCTCCTGTCCACGGCCT
ACCGCTACAAGCAGGACATGGCCAACAAGGTTACCGGCACCGTGGACAACCTGATCGAAG
CCTGCGAGAAGGGCGACAAGGCCACCGTGAAGAAGGAGTACGGCGCGCTGCTGCAGTTGA
CTAACATCAAGGACCTCTTGAAGGAACGTCCTGTGCCCAGGGGCGCCAGGATTGTCGATG
TTGACAGCTCGGTCTCAGGCTCCACGATTCAGGCCCTCTCGAAGGATTAGGAGTGAGAGG
GCCAGTCGCTTGCGTAAATTCATCCACCACCTGCAGGTTCCCAGAAGAGTTGTTTTTAAT
CCAAAGACACGTACTTGCGAGCAGCATGCCAGTGTGCTCTTGAGGTTCTGGCCACATCGG
CGGCCCAAGCAGTAGACGCCAAGCCCTGGATGTGTTCAGTGATGTGCACCCAAGGGCTGG
GAAGATCCATTGTGAAAAACCAAAAAAAAAAAAAAAAAAAAAAAANAAAAAAAAAAAAAA
AAAAAAANAAAAAAAAAAAAAAAAAAAANAAAAAAAAAAAAAAAAANAAAAAAAAAAAAA
>at01801
TTGTTTTTTTTCTTTTTTTTGTTTTCTAATGGGTGTTATGTTGCACTGTCCGCCAGCCAT
TGAGTGATGAGTCGCATGCGAGCTGTGTTAGCAGGCTTGCCTAGGCTTGTGAATTCTGAC
TGCCCACATGCCCCCTGTGGTGTGGGGCCTGACCATTCTCCCGGCTCTGCGCATAGTGGC
CATGCGGGCACGCCTTATGGACTTGCTTCTCCAGCATCCTGCCTATGCGCAGACCTCACA
AGCTTCTGGAATCACTTCTCCGCGTTGGCGCTGCTGCTCCCCCAGAAGAAAGCCACCATG
CAGGTGTCGCTGCAAGTTGGGTCTGCCAACATCAGGTCTCTCAGTGACCCGATGGGGCAC
TCCTCGGTACGACAGGGCCAAGCTCAAGTCGGCCCTTTTAGATGTCGGGCAGAGCCGACA
TGTTTCGGCATCGGTTCCACGATGGGGGCTACCAGGTGGTCGGCATTCAGGAGTTTCGCA
AGGCCAATGCCACTTGAGCCAAAATGGCTACGG
>at01802
CAAAGCCTGAACCAGCACCTGCCTCATCGATGGGTGCATGATCTCGAAGCTCTCGCTCAA
AGCACCTATCAGGGCAGTTGGCAGCGCCGCTCCCTTGACAGGGAAACTGGGGGCTACGTG
GGCAAGGAACATCACCTGCTCGCCGAAAGAGGCGTGCGGCTTCTGTGGCTTCAGCTTGAA
GATCTCCATCATGCTCTCAAAGTGAGACCACTGCTGGTCGAACTCCGGGCCGTAGGCCTC
AGGATCCTTCTTCATGAGGCTCTGCAGCTGGGGCAGCGAGAGAATTCGCTGCTGCCCCTC
CAACTCCATGGCGTCCGGCTGAGTGGCCCTTGGCTCCGGAGCTCAAGTTGGGAGGGGCGG
CAGGCCGCTGCCGCGGGTTGCCGTGAGGGTTCGAGTGAAATGGCTTGAGCCAAAATGGCT
ACGG
>at01803
TGCGCACCGTGTTGGTTCCAATCATTGGAGTCATGAGGTTTTCCATTCCATTTATTGGAA
TTGCAATTTCGCGTTGGCCCATAGCACCTGCATACAAAGCGGCAGTGGTGGAGCTGCCCT
CGCAGTCCGGGGAGTCAGGTGCGCAGAGATTGCGGGAAAATGCGGAGGTGTCGGTGAACT
ATGCGCGCGACGCAGCTCTGGCTGGAGCTCAGATCATAGTACTGCCGGAGTATGGCTTGA
CCGGGTTTGCCGCAAGCTCCCGGGATGCTTACCTGCCGCTGCTGGAGCAGATACCTGAGC
CTCATGGTGATGTTCCATGCGACAACCCTGTGCGCTACGCGGATGCACCCACAGTCGTCA
CACTCAGCTGCGGTGCGAAGGCAACGGCATTGCAATTGTTGCCT
>at01804
GAAGTGCTCGTGCTGGTTGGACACCAACCACAAGATAGAGAGCATTGCTCCATATGTTTG
CTGGCGCTTGTCTTCAGCGTTGGCATCGAAGTAGGAGAGCTCTGGGAAGACTCGGCCCAG
TCTCCCCCTTGAACTGCTTCTCCGAGAGCTGCCCTTTGAGCACATCAGCTGCCAGTTCCT
TCCCTTGTAAGCTCAGTGCGTCCAAGACTGCCTT
>at01805
GAGGTCCGGCCGTCAGATGTCCCGCCGCCGTCCCCGGCGGGCCGCGCTGCCCCCGCTGCG
GAGGAGCGAGGGCAGGCCGTTGCAGCCATGGGGATGTTCGGAGGCGACAACGAGCTTGGC
AACATCCTGCAGGAGGCTGTCAAGCTCAAGTCTGCCCAGATGGGCCAGAAACGGAAGCAG
TACGAGACCTGGCCATACTTCGTCCAGCACACCCTCTTCCATGGCGAGAAGGACGACTTC
AAGGCGTGGCGCCAGCTGCCCTTCTGCGAGAAGATCAAGATCTCGGAGAAGATCGAAGGA
GGAGGGCAAGTGAGCTCTACGCCAAGGGTCAGGTCGGACCGAC
>at01806
CCCATTGCTTGCCGAATGGCAGAGAGGTTGCTAAGGAGAGCCCATCTGATCGCAGCCACT

TCTGCGACTCCGAAGGCTGGGTGCAGCAAGGGTTCGCGGAGGTGATGCAGCGTGCCGGGT
TGCCGCTGCTGCCGGTTCACGCGATCATGGCACAGATGCGCTTCTTGCACTACCTCGAAG
AAGGAGGAGGCCTGCCACCCCACGTGGACCTTGCGCGGACGGACTTGGATGGCCAGCGAT
CCTCCCACACGTTCATCCTCTACCTGACCGACTGCTTGTCAGGAGGTGAGACGGTTTTGC
TCGAAAGGATGGAGGACACTCAGGATGCAGTGCTTGC
>at01807
GGCAGGCCCGCCAGCCTGTCGTCCCGGCCTTCTGGGGCGATGGCCGCTATCCCGGGGATG
GTGGCGCTGCTCTCGAGCGCCTGTGGCGGGCACTGCCGTGGCGGGAGATCCGCAACTGTC
CGGGCCGCTACACGACGCGGGCGTTGATGCTCGTTCGCAGACTCCAGAGGCACTCTTGGA
GCGCTGCGGCTTGGAGGCAGAGGGCCTGGCACCCTGCGAGGTCCGCTGCCCCGGCAAGGA
CCCCATGGCCCTGCAGCGCCTGGCGGGGGGCGGCGGCCTCCTCTGCTACTGCAAGCCGGA
C
>at01808
CCGTAGCCATTTTGGCTCAAGCCATGTTGGCTTAAGGCTTCTGGGTGTCATCAGACAAGG
CACGGGGCTACCGTTCGAATGTTGAAGGACAGTCCCCAATGCTAGCTGTTCCTGGTAATG
GCAAACTCTCAACGCTATCACACGCCATCAGGCACCCGCAATGGTCATTCAGCGGCGCGC
CGCGTTTCCGACATCAAGTCCAGAGCATATCAGGGCCAAAGCTGCGGATCGCCTTTCTCA
ATCCTGCCCTTCATTGGTGACCGCCGAAGGCGCACCAGCGGAAGCAGGGGATGGCACCAG
CGCGGAGGCTAGAGC
>at01809
TTTTTTTTTTTTTTTTTGATTTCCTGGGGCAAATTCTCGGTGGCTTCTCTCCTGCATGA
AGGACTGTAGTTGCCAGCCTGAGGCAGGGTGAAAAGGGGAGAGTTGGCCCAGATGCGATT
GTGCTACCTGTCTGGTCACAGATGTCTGTCCGTGACCCGTGTTGACTTTCGTTAAGTCAG
TGCACGGGTCTTTAAGTATCATGGCAAATATCATGTAAGACCAGACAGGGCACAGGCTGT
ACAGACATCATAAGACAAGGCTTGCCTTGAACCTCTCATAAAAGCAACAGGCACTATAGT
GCCGGTGCGCCGGTGCTCATCCCGGAGTTGCGCATGCCCCCAGTGTCAGATCACGGCGAT
CTCCGATCCCAAAAACTGGACTCAATATTATCCACGATGCGGGCGAGCACCATGCTGCTT
TCTGAGCAGCCATCCCCCATCAAGTCGTCGGCACACTGGACCTCAGTGTAGTTGTAGCGG
CCTCGGACCATCTTCTCGGTCTTTTGGGCATAATCCCAACCGCACATAATCTCGGTGTCC
CCCGTGCCGCAGATGTAGGTCACGGGCAAGACCACGCTCCCCACCGGCTGCCCCTTCCCC
TTCTGCTTCTTCCTAAAGACCTTTGTCAATTTTGGCGAGAGCTTGAAGGCTCCCTGGAGG
AGGATGCTTCTATCTATGGCAGACTTGAACTTGGCTGGCGTGTAGCTCTGGAGAACCATA
AGCTTCTTAACCGTCAACACGCGAGGAAACGGCATGAGACCGCAGTCCACAGCACTGACG
TACCAATATAGGGCGTTCTGCAGGGATCCGGGACTCGCGAAGCCCAAGCTCCTCGCGTTG
AGTGTCCTGTAGATCAAAGCGTCGTGATCCCAGGCACAGTTGGGTTGGAGGAAGATGGGG
ATGTCCTGGCAGAGAGCTTGCTGCAGCTGATACTTCTTTTTGTGTAGCGTTTTCTGCAAC
GCAACTGGATGCGGCATCGATAGCGAAGTGTAGGAGAGGATCTGGCCACGGCTGCTCTCT
TGGCAAATCTTCCATCCGAACACGAAGCAAGCCCAGAGGCGACCAAGTGGAAGCTCCGCC
ACCCCAATTTGGCAGCAAGCTGGAAGACGTCTGAGCTAAGCTCNTTGCAANTATANTTCT
CCTTACCCTGCCAGGTGCNCCNTTCTTNAATGAGTAACCGCGCTGGCTGCATGCTATGGA
GGCATACCCTTTGTCCGCTAACCGCAGCATCAACTTTATCCAGTCATAGATAGATTGAAG
AAGCCATGCATGAAGATAATACCCTCCTTGCCTTGCTTCCCTTNTTGTCCAACCCCCGCC
TCAAGCAGCTGAACTTGTACCTGCCAACCNCGATTGCGGTGTACAATTTCAAGCCCTTCG
TGATCTTCTTGCCTTCGTAGATCACATCTTCAGCTTCATCCTCATCCTCTCCTTCCACTT
CCTCCAACGGGATGGTCCCGTTGAACCAGCTGCTGTTCCCTCCCACGTTTGGCACAATCT
CCCTGGACGCGTTCTCCAAAGGGGCTCCCGTGTCGCGCGCTGAAGCGCCATCCGCCTCTG
GCTCCTGCGCAGGAGCTGCCGTCACCTCCACTGGAGCCTGCGTTGCCTCTGCCGGTTCCT
CGNTGCACTAGGCTTTCAGCCACAGACCTGGGCCTCAGTCCCGTGCGGGCATGGAGCAGA
GTGCTTTCCTCGTTTTCTACATCGCAACACAGTCCTTCTGCTGGGCATGCATCGCCACGA
ACTGCGGAGCCAAGGACCAACAGTGGCTGTGCAACGCCATGATCAATGGCAATGAGAGCC
TGGAGGCCACCATCCCTTCCAAACTGCACAAATGGGAGACCGAAAGATGCG
>at01810
TTTTTTTTTTTTTTTTTTAGNCANGCAACGTCCGCATCACACCTCGATCCAGTCCACTCC
ATCAAAGGACGTCCTGCTATGCCCGCAGGGTATATCCTACAAGGGGTACCGGCGGGTCTA
CTGGTGGCGCGTCCATCACTAAGGCAAGTCCAGCATGTCCGTGATCTCCTTGGAGAGCGA

CCTCCGCCGCTGTCAGCCAGTGGGCGCTTCCAGCATGGCCAGCGTGGTGTTGGAGAAGGG
CTTCAGGCCCCATATCTCGGGGAGGATGCCCTCACACGTCTCGTCCTGCTGCTTCAGGTG
CATGACAAAGTCCAGGTGCGCGTACCGCGCAGCCAAGGACTCGTCCTCACCCTCTTCGAC
TGGGCCCGCCACAGCCATGATGACCACGCGCGCCGACCAGCCATCAATCACCATCATCTC
CACCTGGGCCTCGCCGATGTAGAAGTCCGGCACCCTCATGAAGGCGAAGACGACGTTGTT
CCACGCATACGCGTACGAGTCAGGCGTAGGAATGGCCTTCAGCGGCAAGCCCAGCCAATC
CAGAGTGATGTTGAGTGTGGAGAAGTGGTCATCAGGCACCTCCCCCCTGCTTGAGGCCCT
CGAGGTCGCTCCTGATCGCGACGCTCGCTACAGCCTCTCCATCCAAGCCTTCCAGCACCA
AGCGCTTGATCCACTGGTCGGGGCCCAGGTTGAAGGCCGCGCCCAGCACCCTGAACTCAC
TCGTGGCAAAGAGCTCGATCAGCGCGCCGTCGGGGAGGTAGAACTTCTGGGCGACGCCGC
CGTAGTACGTCAGCGGGTCGTCGCCTACAAACTTGGCCATCCACCAGGGCGTGTAGGTCG
TCGTGAAGGTGGTGTACGTGGACGTGGACGAGGTGGCGGTGGTGTAGGTCATGGTCGTGA
AGGTGCTGGTTGTGGACGTCGTCCG
>at01811
TTTTTTTTTTTTTTTTTGCCACGTGACGATCCACTAGCAGAGCGCCGGTGGGCAGCAAG
GCAGTACATGCCAATGCCAAAAGGTGCCTCTACAGCACTGGCATGAGAGACCACCTAGTA
TGATGAGGGCCCCGCTCACGCCTCTGTTGAGTTCAAGACGACAGCGAAACGAGTCCTCAA
GATGTCGCAGACCTTCTCAAGATCGTCAGTCTTGCCAGAGAGCTTTGCACAAACCTTCTC
GAAGAGGGCTTTGTCGACACCAATGGCGACCAGACCCTGCATCATCTCAGTGAGCTTCTG
CCGGGGGTCCTTTGGGGCATCGGCGTCCAATCTCACTGGGGCGCTGTCTCCGCGAAGGTC
GGCTCCGCCAAGGAACTTCTTCATGTCCTTCTGACCTTCGGGAGTGGCACTGCAGAAGCC
AGTCTGACAACCAGGTATCCTCATGAACTCCTCCCAATCGTAGGCCTTGCGGTCAGGGCA
GCAAGACCACCACTTGGCCGTCTCG
>at01812
GGCCTCACCTACTTCTTTGCCACCTTGATGCACACGCTCCAGCTCGACGGCCAAATCCTG
AGCATCGACCGGAACCCCCCGCAAGCCGTCTTCGATGCCCACTGGTTCTGCCCGGTCTGC
GAGGAATGCGTCCGGCCGTGGGACACGGAGCCGTGGCAGAGGCACGTCAACTATTTGCAA
GGCTGGGCGGACCAAAAGGACCTTTATGACGCTGTGCGGCAGGAGGCTGCGCGGTCGACA
GGCCCTGTCATGGTCAACCTGGATGCCTGCCATGAGTATGATTGTGTCTATGCTGAGATC
GTGCTGTACTCTCGGCTGGTCACAGTTGACTCCTATCTAGTTGTGCAAGACGCAAGCTCG
ATAA
>at01813
GCAGTCGCGGCACCGTCTGTCCGACTTTCGCCGCAACTGGCTGCTGCTAAAGTCAGCCCT
GGCCCTGTCTTTGCCGCAGACTCCGCAGAGCGGCATGACTGAAACAACGATGGAAGTGGC
ACGGGTCCTGGAGCCCCAAAGTATTGTTCAAGAGCTTAGGCAGCAGGTTGCAGACCAATG
GGGAGATACCAGCAGAGTGCCAAAAAGTTAGTTGCAGGCTCCTCTGTGCTGAAGGNTGGT
CAGACCATTGCTTCCTGCTGCCAAGACAATGATACACAGCTTGCACTGACATTGCTTATC
TCCCTGGAAGAACATGCCTGGCGTTGCGGGTGCGGAGAGCACGGTGAAACGGGCCGCTGC
CTTGGAGGT
>at01814
GCTGCCTTCAGGCACTGCCATGGACTTCCTCCAGAGACGGTTCCACTGCCGACATCAAAC
GAAGTCTGCTACCTTTCAGTACGTTGAGCCATTGCCATATGAAGCTTTGCCCCTTGGATT
GGCGGTTGGAGCCACTCTTGCATCATGGTCCTCATCTTAGGAGGCGCGGTGTTGGGGCAC
ACCTGCGGTGAAAGAGGAATGCTCCGCAGATGTATGCCTCGGGGCGAACAGGCTTTTTTA
CATATTCACAACTTGATTTATGCTTTTGCAGACACATGCACTTCTACCATGCAGGACATC
CTGGCTGAACATTGCAGATTAAATTGAGAACCACACACACACACACACCATCACA
>at01815
ATGTCTTTGATTCAACCCTTTCTCCGAGTCATCAGGAATTAGGGCGTTGCACCTGCACGC
GTTGCAAAGGGCAAGTGCCCAGCGGCTTGGTCGCATTCCCTTCGCGCAATTCCCTGGCTT
ACCCTCGGCTGATCAGCCTTTGCATTTATTGCGCCCTGTGTCTTTCCCTCAGTCCGCGTT
GAGATGTCTTCCATTTTTCTTAAGGCGCTTTTTACAAAGCTGGGCTTCTCGTGTGACCAA
CATCGTGACACTCCAGCGAAATAGATGTGCGATGAAAAAAAAAAAAAAAAAAAAAAAANAA
AAAAAAAAAAAAAAAAAN
>at01816
GTCTTCATCCGCAAGAGGACTCCTGACATGATCGCGCCTCCAGAAGGACGACCCCTCGCG

CCCTCCATGCGGGAGCCGCCACGGACGCTGAGCGTTGCAAGAAGCCGGAGTGGCTGATCT
GCATTGGGGTCTGCACCACCTTGGCTGCATCCCGCAGCCGGACGCCGGGAACTACGGCGG
CTACTTCTGCCCCTGCCACGGCTCGCACTACGACCACTCCGGCCGCATCCGCCAGGGCCC
GCGCCGAAGAACCTGGAGCTGCCCCCCTACAGCTTCATCGAC
>at01817
CCGTAGCCATTTTGGCTCAAGAGCAGTTGGGCTCAGACCAGGACCAGATTGACAGCATAG
CATTAGGGTTATTGGGACTGGAGCGATGATGGCGTAGTGGTCAGTGGGCGCCGCAGCAGC
AAAGCCTGTCTGCTGTGCAGCACAAGATGGCCACGTGGAGGAGGTCCGGACCTTCTTGCA
AAGCCGGTGCGATCCCAACGAGCGAGATGAGCCGGGTACCTTCTCAGAGAGACACCTGCC
CCGGACCTTGCTGATGTATGCGGCGCAGCACGGGCACGCGGAGGTGGTTGCCTGCTACTG
CACTACGGCGCCGACGTCTACCTCAGGAATGAGGACGGCGCCACGGCCGGCCACCTCGCT
GCGGCCAACGGGCACGTCGAGCCGCTGGCGATCCTGGCCGGGCAAGTGCCGGAGAGCCTG
GGGCTGAAGGACGGCAACGGCAGCACGCTGCTGCACGTGGCTGCGAGCCACGACAATGAG
GAGCTCATATCCTTCCTCATAGGCATTGGCGCAGACTGCTCTGTGCCGGACGACTAGGGC
AACTTGCCGCTGCACTGCGCGCAGGACCTCAAGGTCCTTGACGCTCTGCTGGCGGCCTGG
CCCGGCAGCATCGACCGGCAGAACGGGGACGGCTACACGGTCCTCCACCTCTCCGCCAAG
GAGGGGAAGCACCAGCTCGTGGAGTCGCTGCTGGAGAGGCGGGCCGACCCCTCGTCGCGC
ACGAGCATCGGCCGCACGGCCCTGCGCTTGGCCTGCAGCTCCGCAGACAGGGGCGACGAG
AGGCGATCACACCTGCAATGCGCTGCCCTTCTCCTGGGCTATGGTGCAGCCGTGGAGCCG
GGGGACGCACTGGCCGCCGGGAGCCACCGTCCGCTGGACCTGATGCTGCAAGCCTGGGCG
AAGCGGCAGCCGCAGCCTCCCCCGGCGCCGCCACGGACGGAGACGGAGAAGGACGCGGAG
ATAGCGAGGCTGACACAGGCCTACGAAGCCATGGAGCGAGCCAAGGATGACGAGGCCGGC
CGCATCCGCTGCTGCATCTGCATGGCCTCGCAGAGGCAGGTGCTGCTGCTGCCCTGCGCC
CACATCGTGTGCTGCAAGCAGTGCGCGGACTGCATCTCGGAGTGCCCTGTGGACCGCTCG
CCCATCGAGCAGCGCGTGCGCTTCACCCTGGCCTGAGCCTGGGGAGCCCGCGCCGGGGCC
GAGGGCCCCGGACCTCCGGGCGTTTCGTCAGCGCTGCCGTCGTACGACAAAAAAAAAAGA
AAAAAAAAACAA
>at01818
GCGGCCGCCTCTTCAACAGTGGATGGATAGTCCATGAATGTTGTCAGCTGCTTTGCCAGC
ACTATGCGCCACAAATCGAGTGGTTTCCGTCGCCTCGGATTCAGTCCGGTGGGAAATGGA
TATATACGGAGTTCCGACTTTACCCCGCGACCAGGAAAGCCGCGACATCGCATGTCCTCC
AAAATCCTGAACATAGTCGGGGTCATCTCCAACGAATGTGATGCAAAAGGAACTGGGAGA
GGTCTTCCTTCAACGCCTGGCCAGCCCTTCGTATCCTTCAGCTTACTTGCGAGTTGAAAA
GCAGGCCTACCGCTGCAGACCACTGAAGATTCGGAATTGATGATATTTGCCGCAGATCTC
AGACAACTGTATTGCAGGTATGCGTTAGGCCGTCCTCGCAGAGCAAACATGCTGGACTGC
TTCAGCTGGGCATTGTTGTCCAGTGACATATGCCGGAGCCCTGAGGCCTGTTGCTCGTAG
AGTCCTTGCCGGAACACTGCCCAAACAGCCTTGATGGCAAAGCGGACGAATGACAGGCAA
GCAGCACTTGCCAAGCCCTGGCTGTGTCCCACTGCACACGAAGAGTATGCTGCAAGTGAC
TTGGCCTTGACCAGCTGTGTCATGCACATCTCGTTGACGAGCGTTGTTAGGAAAATGATG
GTGCACGAGACCCTCGTGCC
>at01819
TTTTTTTTTTTTTTTTTGGCACCAAAGAATTGATGAGCCGACATGTAGGTGCCATCAGA
ATCCTTTCATAGGAGCTCTTGGGATTCACGATTGTCTGTCATTTGATAATTCTCTAAGAA
AAAGAAGAGTTGACAGACAGTTGACCAAGAGCGATTCCTTAAGACCTGTATTTGATGGTG
ACCGCGCGTTCTCCTGTTAATCGATCACACCTTTGGACTCACCGCTTGTTTCTATCTCTT
GATCAAATGTAATTCTATATTCTGAAGAGATATAACTTAAAGAAAGAGTGATAATTTCTA
TTTGGTGAAGTGTAATCTTTTCCGATCTTGTCTTCTTCTCTTTTTCCTTTAATTTCTATC
TTTTTGGAATGGTGTTATCCCGAAGCCGTTTCATGCTATCATTTCACTCTTCTTTTTGGG
ATCTTTCAATCGGTTAAGGGATTCTTGGTTTATGATATGCTGAAAA
>at01820
TTTTTTTTGTTTTTTTTTCAGCCGCAAGGCCCAGCATATCCTGCAAGAACAGTTGCNGGA
GGAAATGACAAGGACGCAACATGGGCTAAAGTAGACCAGCACGTTGAATCCACAAGTGCC
CTGGTCACTGTCTCCGCCCCAACCCTTCACTGCCCCTTACAAGGACATAAGTATTTTGGA
TGATGCAGAGGCCTCGGCATTCAGTCTCGCCGCCATCTTTGCACGCTTCCGCTTGCCTGG
GCGCTTTCGCCGTACTGCTGTGTGGAAGTGGCAAAACGTGCACGTCGAACCGTTTCGGCA

CGTGTTTCCCGATCTGAAGAAATGGCAAGGCTTACATTTGCCAAGGTGGTGCCATTGGCC
GCCCGGAGTCATCAAGCTGTCAGGCGTCGGATCCTCGCTGGACGCATCATCGTCATCGCC
GCTGAGAGGACCGCTCTCATGGTGCCTTTCGGCGCCCAGGTGCTCCTTGTGCACATCCTC
GGAACTATCCGTGATGGAAATGAAGGAAATCTTGTCACCTATTGTTGCCACCTTTCGGTT
CAAGCTTTCGGAGTCGCATGACGAGTTTACCGGCTCGATTTGACCCCATACCCTGTGGCT
GTGAGGCGACTTTTCTCCTCCAGACGGTTGGGGCAGTGGCTCACCACGATCGCCATTTGG
CGCACCGCTCATGGCTTTAGCTAGCCTATGCCCTGGCCGGCTCGCAGTGCTGCGGGCGAC
ATGCTTGAGCCAAAATGGCTACGG
>at01821
TTTTTTTTTTTTTTTTTTCAAGCATGGACCCCGCCAACAAGAATTCAGACGGGAGTGCTA
GCCTCTGCGTCAGCCCGCAGAATAAAAAGTTGGGCCGGCGCGTTCCCCAAGCGCGGCTGC
CTCTTCTGTGCTCCCAAAGCAAAGCACAAGGGCAGTATTGGCACTGAGAAGTGCAAGCGA
CTCCTGCCTAATCTCTCTAAAACTGGGCACCTGCCTCCGGTGCGATTTTATGCTATCAAA
ATGGCCAGGCGCCAAATCCAACAGGCGCCAAATCCAATCCCGCAGCATGCTCGCTCACCA
CCACTACAGGACGATGCCATCCTGTCCATGGGTCCGTCATCGCATCTTGGCCACGCATAG
AACTGCAGACGCCTGGAAGGCACCGACGCTACTTGCTGCAGCGGTCTATCTTTAACTCAA
TCACGGCACAACGG
>at01822
TTTTTTTCTTTTTTTTGGTCCTCAACACCAGGCATAAGATGCTGTGCATTGGTCCACCAC
AGGCATGGAATTAACACCTGGTGTGTCAAGCTTGTGCAACGTTGCCCGAGCCTTCACGGC
CCTTGCATCACAGGCCCACTGACCGCCACTGCCGGAATGTCAGGGGACACCCGGCGGACC
GAGCCAACCCGTTTCCGCGGAATGCCGTGCAGCCACTCCCACCGCGCTGGCCCTGCCAGG
ACATAGCAGGAGCGAGGCTCCAACAACAGGGCTCCACTGCCTACGGAAGCAGCGCAGCTG
TTCGGCGGAGGCCGGCAAGCAGGCCGGAATTCCATGGTCACAGGCGCTCCCAGGCTGATG
CCGACAATCAGGGCCCCATACATGGGCCGGTCCTTGTGCATGGTGATCCCTCGACGGCTG
CCCGGAACTGAGCGGCTGTAGGCCTGGACGAGAACGGTGTCGAACTCGTGG
>at01823
TCTGCTGGTTGGAGGCAAAAAGTTTGATCTGCGTCTTTACGTTCTCGTGACATCGTACCG
TCCGCTGAAGGTCTATTTGTACAGATCTGGCTTCTGCCGCTTCTGTGTCGAGCAGTACAC
TCAAGATGTGGCAGAATTCGATAACATTTTCGTTCATCTGACGAATGTTGCCATCCAGAA
GCGAGGCTGGAGGACTAACAATGACAGGCGA
>at01824
TTTTTTTTTTTTTTTTTGCATCCTGCGATGCCAGCCTCATCCTGATGCTTGAATCAAGC
AAACTCCGTGATAAATCGCGATTTATCACACCAAGGGCTGCCCCACACGGAGCTGCACAC
AGCCCTTCATCTTCAGAAGATAAATCAAGATTTATCGCACTCGATGGTCAGCATTTTGGC
AAACTGGTTGAGGTCGGGCTGGGACGAGTCCGACTCGTCGATGTGCTCAAGGTCGGCGGG
CTGCTCAATGTCGGCGGGCTGCTCAAGGTCGGCGGGCTGCTCAAGGTCGGGCTGCTCAAG
GTCGGCGGGCTGCTCAATGGCGGGCTGCTCAATGGCGGGCTGCTCAAGGTCGGGCTGCTC
AAGGTCGAACTCCCCCNGGTGCGACTCCGTAGGACATGAGCTGCTGGTCAATGATGTTCA
TGGTGGCCCGCTTCTGCTCCTCGATGGGCTTCCCGTGCGACGGCACGGCCTTCTTCTTGG
CGGGCGCCTGCAGCAGCACGACGTGGCGCGGCCGCATTCGTGGAGGAGGCACGCCAGCAT
CCGTGAGCTCGCCATTGAGCCACTTCTGCACGGGTAGGTTGATCAACTTCTTGCTGTTCG
TGAGGTTCGCCTGTGCGCGGCACCAGTTGTGCTCCATGGTCCAGTCGGAGTCGACGAGGT
CACGTGTGATGATCCAGTCATGCGGGAGCTGGTAGATGTCGTCCGAGCCGTTGACCTTGA
TGCCAATGATGGGGATCTTGCCGGAACTGCTGCACGCCTCGCCGCCGGGGACCAAGAGGA
AGCAGCCGATGTCCAGCGACCAAGACCACGAGCCAGCAGTCAGGCCGGCCAGCGGACTGC
CCGCGTCGCCGTGGATGGACATGTACCAGTTGCCAAAGCTCTCCACGGGCAGGCACAAGG
ATGGGACCTTCATTTTCTCATCGCCCCCGCCACCCCATACATAGATTCTGAACGTCTTCT
CAGCATCCAACGATTTCCACCAATGCAGGCCCACGGCAGAAGCTTTCGTGAGGCAATAAT
ACTGCAGCCAATCTGGGGTACTCTTAAGGTTTCTGACACCACGCGGAACTTTTGGGCTAT
CCTTCATGACAGACCGGTTTGCCTTAAACTTTTTGATGGCCCCGATGACCACATCGATGA
GCTCTTCATCGGCATCAAGCTCTCCCAAAATCCATGCGTGAGCCTTGCCCACCCGCTCGT
GCTTGTTGTCCTGCCCGAACTTGGACAGCACGGCGCAGCCCCCACGGCCTTTGCGGCGCT
TGCCAGGCTGCGCGCCCTCGCCCTCGGAGTCCTCCTCCTTGACGGCCGGCTCCGCCTCCA
GCATCGCCGCCAAGGCGAAGGCCCGCGCCATGGCGCCACGGCGCCCGCGAAGGCTCCCGC

TAGGGAGAGGACCCTGGAGAGAACTGGAACCAAATGCTTGAGCCAAAATGGCTACGG
>at01825
CCGTAGCCATTTTGGCTCAAGCCATGCAGGTTCAGACGCACCTTACACTCTCTACCACGG
GAAGCAACGAGGATGGTTCCGCCATCTCGATGAGGTGCTGAACCGGCGCCTCTCAGCAAT
CTCAGCACTGGCTGAGGGACAAGAGGCGGCCCCCGGCACAACAGCACGTCAATGGAGGAG
AAGCAGGAGGACGTGGAGGAGAAAGACAACATGACCTGGGCCGTTATCTTGATTGTCTTG
GGTGCAACTTTGGTATTTCAGATGATACTGCTCATGGCATGCCAGCGCCGCCCGGACAAT
CAGGTCACCGCAATCAGCGGCATCGACAATGGCGCCGCGCCGCACGCGGAGTACATTGAT
GGCGAC
>at01826
TTTTTTTTTTTTTTTTTTCAATTTTGCGACTCGTGCAGCGGCCCTCCATTGGCCGCCGAA
ACGACCCGTGCAGCGGCCACTGAAGCTGTCCGCCCGGCGTCACAGCAACGTCGCCAGGCT
TTGCTCAGCAGCGCTCTCAATCTGGCCCGGCAGCACCGCGAAGGCCTTCCTTCCGCTCCC
CTCAGGGGGGCTTCCACCTGGCGGCCTCGGCGGCAAGGAGCAGCTCGAAGACGGCGGCCA
GTGCTCGGGCGCGAGGGGAGGGAAGGCTCCCGCCGTCTCTGCGCGCGCCGTGCTGACCTC
TGACGACGCCTTCGCCTTGCCTGCGGCGTCCTTGCCAGAGTGCGCCGCCACCAGCCGAGC
GTTCATGAGGAGGTTTCCCTTGGCATCTACAAGGCCAAGTGACTCTGGGAGCCCAATGAG
CTTCTCATGGACCTGTGTCATGCTGGGGCGATCCGTCTCCTTGCCTCGGATACAGGAGTG
CACCAGATCTTTGCAGTCTGTCTCAAAGAAGCATGTCTGTGGCCAGGCTGGCAACGGAGG
CGGCCCGTAAGTCAGTACCTCCCTCAGCTCGTCGCTGCGAAACTCCGACAGGGGTGAGAT
GCCTGTGGCTAGAAACGCAAGTAGGCGGCAATAGGAGTATACATCCGCACTGC
>at01827
TTTTTTGTCGCCGCCGTTGCCTCCGCGGATCGGCCGCTCCTCCCGCCCGGCACCGTCCA
AGCCGCCGCCGGCCGCCGCTCAGAAGTAGTTCTCGACGAAGGGCAGGGGCGCGAGCACCT
CGACGTCGCCCTCGTTGGCCTTCACGATCTTCATGGGCTTCGGGCCCTCCGGCTGGCGCT
TGCAGATCTCGACCTTCTCGCCCAGGAGCTTGGCCTCCAGCCTCCTCTTCTCGTTCTCCC
GGAGGCGCTTCTTGAAGTCCTCGTTGCAGCGGGACTTGCGGACGTGCTCCACGCGCACGT
GGATGCGCTTCCGGAGCTGCCGGTTGCCCACGATCTTGGTCATCTCCACGCCCAGGGCGT
TCCTGTTGACGTTGAAGACGACGCCGGTCCTGCCGTGGTAGAAGGAGTAGGGCATACCCT
TCTGGACGGAGGAGTCCACCACGATGTCCACGTAGTCGCCGCGCTTGAAGGTCACCAGGG
ACCGCGAGACGCCGGGGAGGCCCTTGTTCCTGTACGCCTTGGCGTACTTGTCGCGCGTGC
CGTGGCGGTAGCCCTTGGAGATGGTGTGCAGCTTCCCCATGGTAAGGCCCTGGCTTGAGG
CAAGGGCTGCACGCCCTGTGGCAGATCAGCGCGCGGGCTCGAGCCAACCTCCTTGAGCCA
AAATGGCTACGG
>at01828
AACGCAGAGTCCGTAGCCATTTTGGCTCAAGCCTTCTGCCCACAGCTCAGCTCAGTAGGC
CTCGCCCCCCAGACCGTGCCGCCATGGCCACGATGCGCTGCCGCCACCTCCTCATCAAGT
TCCAGGGCTCCCGCAACCCAGTCTCCCGCCGCACTGGCCAGAGCACCTCGGACGTCTCGG
CGGCCGCCGCGGAGGCAGAGCTCCAGCAGTACATCGACCAGATCAACAAGGAGGGCGCCA
CGGAGGAGGCCTTTGCCAAGTACGCCATGCAGCGCAGCGACTGTGGCTCCTACCGCGAGG
GCGGCGACTTGGGCGAGTTCGGGCCCGGGCAGATGCAGAAGCAGTTCGAGGACGGCACGG
CGGCCACGGCGGTGGGCAAGATGTCGGGCATCGTGCTGAGCGACAGCGGCTACCACATCA
TCTTCCGGACCAAGTGAGCCGGAAGGGGGCCTTAGGGGGCCTTAGGGAAAGCGCTGCATC
CTGGAAGGACCCGA
>at01829
CCGTCTGCTGAGCAACTCCAGTCCATCTCATCCGGCCTTGTGCCCTCCATTTGcccttCC
CTcCCCCCGCTGACCAGcCCCCCCCCATCNCGAGATGACGACGCCAGCCGcCaAAAGACA
GTCGTGCTCGcCCTCCTGTTCCTCCAAGCCACGCCCGGCTTGACGTGGCGCGGCGAGACC
GAGGAAgGGGGAGATGGCCAGNAgGCCtTGGCCGAGACTGCGGACCCGTGtCCCAGAAGG
GCTC
>at01830
TTTTTTTTTTTTTTTTCTGCGCATGACGGCGACAAGGAGCCACTCGCGGCACCGTCTGCT
TTCAATCAGGCCCCCGAATGCTTTCGATCAAGCGTGGGAACGGACCAAGTTCACTGCAA
TCTTGCGGAGGTCGCAGTTCAGCTGGCCAGGGAAGCGAAGGCACGTCGTGACGCCACTCA
TAGCGGCGCTCACCAGGTGGTTCAGGTCCCCGTATGTTGGCGTCGTGAGCTTCAGCGTGC

GGAAACAGATGTCGTACAATGCCTCGTTATCGAGCAAGAAGCACTCGTCGGCGTTCTCGA
CAAGCTGGTGAAAAGAGAGCACGGCATTGTAGGGTTCCACGACTGTGTCAGAGACCTTTG
GGGATGGGATCACCGAGAACGTCTCCATGATGCGATCTGGATACTCTTCGCGAACCTTGG
AAATGAGGAGCGTGCCCATTCCCGAGCCCGTGCCTCCACCCAAGGAGTGGCACATCTGGA
AACCTTGGAGGCAGTCGCAGCCCTCTGCCTCCTTCCGCACAACATCCAACACGGAGTCAA
TGCCTCGTGCC
>at01831
GCATCCGATCAGTAAGTGACGGATTGTCTCGCAAGCGCTGCACGTGCGACGACGTGTCAA
CGCAAAGGCGCGTTAAATTCCGATTCGAAGACGGTGTCGCAGAGATTTCGCCGATAGCGT
GGATCGAACGCTTCAGCATACCCTGCACGGAACTCTGCCACGGACCGTGCTGCAAATTTG
CGGTGGACTCTGCCACCGTCTATCCACAAGTCAAAGTAACCGCTGTACCCATGCAGCACG
ACATTCCCAACAAGCCAATCGAACTCGCCGCTGACATCCCAAAGCTCGATGAGCCATTTC
TTTACCCGATGTTGCGCAAATGATGCGGTAGCTCCACGCAGCGCCACCAGCACAGAGTTC
TCAATGTCAACCTTTGCTATCGCAACCTGCGTCATCTGATTGAAGACGCCTTCATCCGTA
AAGATGCGATCGATCGTCTTCACCGGCACTGCAGGAGTGAATGAACGAGCGTACGTGTGA
GATGTAATTCCGGAAGCATGGTCCGCCAAGTACAGCTTTTCCACATGCAATTTTTGTGGG
TCATTAAATTTGCCACTACCCCAAGGATTTGCATTCATGGCCACGGACGCGCGGAAGAGG
TGCAACATCTGCGCGTCGGCATCGACAGACAATACCCGGCAACCACAGGCAAGGGCGTAG
AGCGAAAAGAATCCAATGTTACTGCCAAAATCTGCGAACTGCACTCCAGACGCATGGCAA
CCATCTCGCTCCATGAACGCCAGATGCAGATGCGCATACGTATGAGGCTCCCAAACTCCT
TTAGCATGCAAGCTCCGATGCGAGTGCCCACGGTAAGGCTGCCGTCGAATAAGGAATGAC
GGATCTGT
>at01832
TTTGGCATCATGACAAACGAGCCTCGCTTCGAATGGCAGCTCGAGAATGTGAAGCACTTG
GAGTGGAAGCGCACGCTTGCCCGCAGTTCAGTCACCGTGCCCGGCAATTGGTATCCGGAC
GAGCGATTTCTCCGAATCCACATTGTCAAGCAGGCGATCGAGAAGACCAAGCAGCCAGCT
ACCTACAGGGAGGCTGTGGCGAACACAGTCGCTGTGCTCAACACAATCACGGTGCCGATG
GGGGACGTTGCGGGTACTGATTCATCACCTCATGGCGGGGAAGGCATGGGTGATCACACG
CAATGGGGCCTCATTCACGATCACTTGAACAGAAAGGTCTACATCCGCACTGAAACCAAC
CCATCATTCCGCCGCATCTGCCTTGCGGAACTTGAGCTGGAAGAGGGACACCCAATAGCG
ACCATGGAGTTGGAGGAGGACCCGTGGTCTTGGACGCTTCAAAAGCATTCAGACCAGCTG
CCACTCTGGTGTG
>at01833
TTGTTGTCGTTGTTGCTGTTGTTGTCGTTGTTGCTGTTGTTGTTTGTCGTTGTTGCTGTG
GTTGCTGTTGTTGTCGTTGTTGCTGTGGTTGATGTTGTTGTCGTTGTTGTCGTTGTTTGC
TGTTGTCGTCGTTGTCGTTGTTGTTGTTGTTGCTGTTGTTGTCGTCGTTGATGTTGTTGC
AGTCACTGTGAAGGTCGTTGTCGTCGCTGTCTCCGTCAATGTTTCCGTGGTGACAGCGGT
GGTGGGAGGCGACTCGCACGCCACTCCATTCCAATACCCGCCACCACCACAGTTCGCGTC
CTGGCAGCACACATAAGGCCCCATTTCTGATGGGCCTCTGCAGCTATGGCAAAGAACGTT
TCCAGTGAGTGAATCGTGCCTCGGCACGCCATCAGACCAGATCCAACCTTCACTACTGAC
GCTGATCGTCGCTACTGCTCCCAAGTGAGAGTTTAAGAAATGCCTGGCATCGTCATACCT
TCCAAGTTCTGCGCCAACGGATGTGCAGGCTGCTTCAGCACCGGTAAAAGACGTGGACGT
GCTATAAGCTGTACACTTCTTTGTAGGTCGGACCTCACAGTAAGGCCAACTCGTCCCTTC
CCCCACACCATAGTATCCGGATGGGCACTCAGTCACGCAAGTGCCTAGGTGGTACCATGG
GTTCGCCCCCCCAACAGTGCATACCGGGTCTTTCAAACAATACTGTCTGCTTCCCCCGGT
GGTCCATGAATTGGTGGGACCAAACAACTGACAGTTGAGGATGGAAGCTGAATTCCATTC
GTATGCTGCGCAATAGGTGTGAGAGGAGCAAAGAGCCTCGCAATCACTTTGGGCAGTTGT
GTAATATTGAAGACCAACCTTCGCTGATTGCCCCGCTCCTGCTTGACAGGTGTACGTTCC
GATAAAAGTTGATGTCACAGTGGATACAAAAGCCGCGACACAGATCGCCAAGATTGCCAG
CGTCATGTGCAGCAGTTCTGTGCGGTAATGAGATGAAAGGGCCACGAGCTGCACGAACAC
GTTGCACAGGCTAAGCGATTCAGCTTGGCGTTTGAGCAAAGGTGCTGCCTGGAGCCAAAA
TGGCTACGG
>at01834
CCGTAGCCATTTTGGCTCAAGCCATCCGCTCAAGAGACCTATCCAGTCAGTTTTCGCTCC
GCAGAGTCTGCGTATGCTTCGTCTGGGCTGAGTACTGGACTCTGCTGGACTCCGTCAATT

TCGGGAGCTAGGCCACATAATCATTACCAGGTGACAAGTGCCTGCCCCATTTGAAGCAGT
CATGGAAGAGGAGGAGGCGTTCCAATTCAAGTCCAAGGACCCAGAGTTGCAGAAGTGGTT
GATGGCGGTGTTGGCAGAGCAGCAGTTGGAGAACAAGAGACACATCAGCCAAGCTCACCA
GCAGATGAGACACTTCCTTGAGGTGAAGTTCAACGGGATCACCAAGGCCAAGGGCTCGAA
CGAGTCCCTGGAGCAGCCCCGTTCCAAACCAGTCGGAGGTTCCAACCAAATGGGCTT
>at01835
CCCAAGAGATGTTTTTTTTTCTTTTTTTTCCCCTTGAATGTACTCAACAGGCACACTCGC
CTCCGTGAGCACCTTGGCATACAGCTCCGCATTCTCCTTGATCGGCGTGTAGTTGTCCAT
GGAAAAACCAACTAGGAATGTTGGGGGCGTGCTCCCCAGCGGAGAAGCCTATGATGCCCA
CGCGCTCGGGGTCGCAGCCCCAGGAGGCGGCGGAGGCGCCCACCGCGCGCGCGCAAGCCC
GCCAGTCCTCCAGCTGCGCTGGCCAGTAGTGGCCCCTGGACACGCAGCGGTACTGCAGGA
CCACGGCCGTGACGCCGTGGCCCGCGAGCCACTGGGCCACCGGCTGGCCCTCACCGCAGT
AAACGTCCGACTCGTCGTAGTTCCCCCCAGGCACCACGATGATAATGGCCCCCGTCTTCC
GCCCAGGCGCGGGGTCGTACCACACAGACATCACCTGGTCCACGCGCGGGTCCGCGCCCG
GCCGCAGGACGCAATGCACGGGCCTGGGCGGAGGCCCCACGGTGCGGGCGCCGGGCTCGA
TCGCACCGGAGCGCAGCAGCCGGCGGAACGCCGCGGAGGCGTAAGGCCACAAGATCTCGG
GCCGAGGCACACCTGCTACAGCAACAGTGGACCTCTCGA
>at01836
AGCCATTTTGGCTCAAGGCGTATGGCCCGAAACCGGTCGCACTTTGTATGAACTAGGGCT
GTCATCGACCGTGTCCGCTGAGTTGAGAACACCATCATTCGCTTCATGCCAAGAATCTGG
ACCCCTCCTTGAGACCGCTCATCTCTTGCCACTTGGGACAAGCACGTGCTTGCCAATGGA
GTTCAGAGCCTGGATCGTGTTCGGGAGGCTCTACGGCGTTGAGTCTTACACGGTGTGCGG
CTCTGTCGATTCTCCGTTCTGGATGTATGCGGACGGCCGTGGTGGTTGGATAAGCTGTGG
CCCCTTGAGCGTGGACCCCGTGTATCATGAGAGCTGCGACTTGTCCGTGGATGACCAGCG
ACGTTGCAGGACTG
>at01837
GATCCCTTCTCGGATGTGACTCTCTTCAAGATGAACCCAGCACTTCTACAGGAGCTCGGT
GTGGAGCCACGGACTATGGCAGATGGTGATGTCGCGCCTGAAGATGAGGAGAGGGTGGAG
GACCTCTTTGACAGCTACCATGCGGATGCAGTGCCAGGAGGCAGAGTGCATGGTTGGGTT
CGCATTCCCCGCAAGGAGCGGCATGCCCTTCGACGGTTGCTCTCAGTCATGCATGACGTA
GCTGCAAAGCGAGCTCCGGTGCTGGATCGCCTGGGGGTCAAGCTAGACCAGGCGAAGAAC
CCAAAGCAGGATGACGACGATGATGACGACAGCGACTGGAGCGACGATGAGAGCTGCTAC
GGACCCAGGCCCCACT
>at01838
TTTTTTTTTTTTTTTTTCGCCATTTAGGCACCAGGCGCTGCTGCTGCACAGCAGCAGCA
GCCGCAGTACCAGCACAGCGGCAGTGCCTTCGACCGCGGTACAAAAGCGGAATGGCCATC
CCGTCAAGCCTACACGCTCCGGTATCATTCAAAGCGAGAGGGGGGGCCTCAGAGCTTGCT
CCGAGCAGCGGCCCCGCTGCCAGGCCAGTGCCCGGGCACCTCTCTCGCCAGCCAGGAGAG
CAGCGCGGCGTTCACCTCCCGGGGCCTCTCCTGCGCGGCCCAGTGCCCCGCCTCCACCGT
GGCCACGCTGAGGCGCCGGCAGAGCTTCTCCATAGGGGCCTTGAGGTCCCGCCAGGCGCA
GGTGGCGTCGTACTCCGCGCCGATGAAGAGCACGGGCATCTCCAGCACCCCGCCCTTCAC
GCTCCGCCTCGCGTACTCCGCGTTCGCCCGGTGGTTCATGTACCAGGCGTCGGGGCCGAA
GAAGCCGTTCCGCCGGAGGGAGGAGGCGTANGTGGCCAGGTCCTGCTCCGAGAGGACCCT
GGCGTCNCGGGGCAGGTCTGGCGCCGCCGCGCCCTGGGCCCCGAACCAGCCGCCGCTGAT
GCGGACGAAGGCCGTCCTGCTTGGCTGCGAGATGGCCTCCGGGGTGCCGGCCCGGAAGAG
GGCCTTCACGGTGTTGTCGACGTTCGCCTCCATCTGCGCCACGGNCTGCTCGAAGTGCTC
CTCGT
>at01839
AGCCAAGAACGAAATGGACGAGGCTCGCGACGATGCCCAGCATGCTCACGAGAAGGAGGA
ACAGTTGGGAAGCGACATTGGCGGCGCCGTGGACGAGGTCAAGGAGAAGATGAAGCACCT
CGAAGATTGCCAGAAGGAGCTTGCAGACGCAAAGGCAAGATTGCAGCGCCTGATGAAGGC
AAAGGAGGAGCGCGCAAAGAATGTCACAGAGGCAATGGAAGCCGCCGAGAAAGCTGCAGC
TGGTGAGAAAGCCGAGGTGGACGAGCACAACAAAAAGGCAGACTCAGAGTTAGACGAAGC
CATGGCCAACGGCACAGCCAGGATTGAGGACGCCAAGGCAAAAGCAAAGAGTGAAGATGA
GAAGCTGGATGAGGAGGAGCGCAATTTGCAGAAGGAGATCGCAGAGGCTGAGGCAGCGCA

CGAGTCTGCCTTGGCGGCATACAATGAGGAGAAGGAGCAGATGGACGACGCACATGCTCG
TTTGAAGGCAGCTGCGGCAAAGTTGCGGACGTTCCGGGACGATGTGGATGAGGAGGGCGG
CATCTACAGGAACAAGCCA
>at01840
TTCACCCCAAGTGCGCACTACTCCCTCCGGCTGCGGCCGGGCCGACCAGCGGATGGTACG
AGAAGTACAACAGGGACTGGGGGCGTCAAGTTTGGCAAGGACTGCTGCGCACCCGACTCG
GTCTCCTTCCACTACATGAAGCCGCCATCCCAGATGCGGCACCTGTACGCTTTGCTGTAC
ACCTGCGGCGTCTGATCGCCATCCGGAGATGATTATAGAGCCATGCATCACGCCTCCTGC
ATGTTTGGGCATGGTGCATGCTTTCTTCATCTGTTGCTGGGGCTTGAGACACAGCGATTG
TCATTGCGTGACCGCCA
>at01841
CTGTTCATCAACAATCCAACCTTCGGGTTTGTATTGTATGCTGAGGCTACTCCTGCTCTT
CAAGAAGTTGACCTCACCCAGGAGCAGGTGACGGATGGCACTCCCATTCCTCTGAAACTC
GTGAAATTCCGCAAGGTTCACTCTCTCCAGATATTTGTTGAAGAGAACCATGGTGCAGAG
GTCACTCGAATCAGCCGTCTAGATTTCCTGGGTGAGAAGGTCGAAGACCAGAACTCTGAG
GCTGTGGGTGGTTTTGGACTGCCCATTCCGACTGGGTTCAAGCTCGACACCACGAACACA
CCAAGCTACTTCTCGTGACGAGTGTCAAGTCAGAGGTGTCCTTGATTGCCCGTACTCAGC
TGGCCACTTCCTTCATTGCACTTGAAGGGCCGAGCCTGATTGGCCGGCGCATTCCGGCCA
CTC
>at01842
CCCACCCTTCTCGCACTGTTTCTGCAAGAAGGATGCCCTCTGGAGTATACGCTTTGGTTC
AAGAACCCTCCTCGCGACTGGCTGAGACTGAGCCATTTGAGGTCCTGTTGCCAAAGCTGG
CCTATGAGGAGGCTGTCAGAGACCTTGCCAACAGCGAAACATGGATAGGTAAAGCCGTGC
CCATGTCATTGTGTGTAGGTCCTGCGATCAAATGGGCACAACTCAACACGCTGCTGCTAT
GGGCGTGTACGCTTGGGAACTGTTTTCTGACCTATTGTATGACGGAGTGTACTCATGATG
CAGTGAGGACCAGCAAATCTTCAGTCATGTGGGTCTTGTTGGGAAGTCTTCAGGCTATCA
TCTTCCTAGCATGTACATGCCTGGAGTTGATCTGCCTTCGATTCACGACGGTGCCCTTTT
TACAAATTGCGCGGCAATTTAAGGTCTTGGGGCTGGAAGTATCATTTCGGGTTTGGCTTG
CAGTAGCGTTCCTCCAGTCTACTCTCATGAAAGCAGATATGGTCACAGATGGGAGGTTTG
TGGCAATATTGCTACGGCAGGAAGAGTGTCCTGGACAGAAGATCAGCTATCTGTGGAACG
CTGTCACAGCACACTCGGTCGTGCCTGGACTCGCAAGTGTCAAGCTGTCCCACCTCGCCC
TGATCGTCTTCCTCCTCACTCTCGGGCAGTGCATCTGGCCGCAGTGCAGAGCAC
>at01843
AGTGTGCAACCGCGGCTTTCTCCATCTGCTGACATGTTGGTGCGATGGAGGAGCGTCGCT
GCAGCTGCATTGTTCCTGGGCTCAGTTGGAGTTGCCTCTGAGGAGGCCGCCCAGACGGCA
CCTGCACGCCACAAGTTCTTCAAGATCAATGAAGTAGCGCAGGTGATCCCGTTGCCGCCT
TCGAGCTATCCAGCCGCACTGCAGGGCCTCATTTGGATGGACCAGTCAGGCTTCTATGCC
CATAGCGATGTGCCTTTCTCAGCCCCTGACCTTGCCCT
>at01844
TTTTTTTTTTTTTTTTTGGAGCTCTCGAGTCCGGCATTACGGAAACACACCCCAGCATC
AAAGCTCGCGTTACCGTCAACCTAACAAGCTGTGGACTCCATCCACCAAAGCTTGACGTC
AAAATCTGCGTTCCGCGAGCTGCTCAACTGAGGAAGGTCCATGCATATTGCAGGAACAAT
CTAGTACAATGGCGTGGGAAGTGGCTGCGGATTGGTCATGACGCCGACGTTAAAAAAAGA
TCGCACCTTTTGGCATGAGACTGTTGACGTCCTATAGAAACCTGCGAAATGGCTCTCATG
CCACCAGGCGCGCACTGTGGGAGTATGTTGCCGGATGGCACTCCTGGACTCCAGACCTCA
TCCCTGGACTGGATTCAAGACCTTGTCTCCTTAGAACACAAAAAAACCGAAGCGCAAGCAA
GAGCAAAGACACCCGACGAATGAGGGCTCACCGCGCCTGAATCCAGTCAACTGACGATAG
AGTACTTAGCAGCTCCCGGCTCCACCTAGGTGAGCATGCCAGCCACCCTCTGTGCATGTG
TCATTACTGGCGTCTCAGAACATGGTCAGCAGCGCTCTCCACCGACGTCGTGACGAGGGC
ATCATGCATCAGCCTGACGCTCGCGCCTCGTGCCGAATTCT
>at01845
CAGACGCACTCAACCATAGAGACCCAGGAGAAGAAAGTCATCCAATCAGGGGCAGGGATG
TCAGAGAGATGGAGTGCCAGAAGCAACGCAGCTCGCCCCCAAATACCGATGTCTCACCAT
TCACATGCACGAGACTCTCATGTCCGCGCTGGCCCACCCCTGGGGAGACTTCGGCATGGG
CAAGCACTGTTCGGCCATCACGGGCACTCCATGGACCCCGCAAGCCACTGCCTCTGACTC

TGCAGACAGACCAAGGCCTCACCACACGCCCGCCTCGTCAAGCGGAGCAACCAGGGCAGC
AGGAGCAACCAGGGCAGCATCCGCTGGCCGACATGGCCGACAGCGAGCTGAAGGCAGTGC
AATGCTGGCTGATCTTGTTGTTGATGAGGTTGTGTAGGGCCACCGTCGAACGGAGCATGC
TGCCCAGGTAAAGGGCGAGCATCATGTCGTTTGTCTCCATAGCGAAGGACCGCACCAGTT
CCTCGGAGTCCTGGTCTGGCATCAAGTTGAAGATCTCCTGCAGCTGATAGATGATCTCCT
GATTCATAGGCAGCTTGCCCGAAACAACCTGGCTAAGGTAGTTCGAGATCTCCAAGAGCC
GCGTGCTGAGGCCCTTGAGGGCTGCAATCTTGTCACCCACGCGCACAGCGAGCGTCGAGG
GCGACGCATTCTTGATGTCACGCAGCAGGTGCTCGACGCCTACCTCTTCAGCTTTCAATG
CACCAACGGTGGACTGCACATGC
>at01846
TCTAACGTCTAGTAGTTTTGTTTTTTTTTCNTTTTTTTTNNTTTTTTTTTTTTGTTTTTT
TTTCTTTTTTTTTNTNgTccACgCTCCATGTGAAAGAGAATCAAGTCCTGGTCCGGAAAC
TGTGCACAGATCTGCCTGCAAAGGTGAGGTGGGGGCCTAACAAGACTGTACCGGCCTCAC
TTCTTCTTCTCCGAGCCCAGGAGCTGCACGAAGAAGTCCAGCTTCTTCTGTGCCTCCGTG
AAGGTGCCCGGTGCCCTTGAGTCACAGATCACCACCTGGTGTCTGGTAGGCTCCTTCACC
TTGGGCTCCGCCTTCTCCTCCTTCTCGGGCATGGCTGGCTGGTGGGCGGGaAggctggAc
ttgggAgcGGcaCTGGACcttTTtGGcaacttGAgccgGCGGCAGCTCGGGGCCTTTTTG
GCAACTTGAGCCGGGCTTGAGCCAAAATGGCTACGGACTCTGCGTTG
>at01847
NNNNNNNNNNNNNNNNNNNNNNCGGCTCCCGCATGAGCGCTCGAGGTCGCACAGCTCAGT
CTGCGACCACCTGTCTGCTTGAGCGCCTCTGGCCAACCGTGTCGCAGAGTTCCAAGTCGT
CTTCCCCTTCACATGCCTTCGACACCGCCTGCCACTGCACAGCCGCCGACTTCACACATC
GGGAAGCCACGAAGCGGTAGACCCCCAGGGCAGCAAGAGTTGCAGCGGCCCCCGAGACGA
CCAAGAGCCATGCATCAGCAGGTTCACGCCTTCCACCCCGCACCAGGCCCAACTCCTTGC
AGAGATCCGGCACGAGCCGTGCGCCAGTGCCTGCACCTCTGCGCAGGTGCTCAGCAAGCG
CTGCACGGCCAAAGTCCAGCATGTGGCCAAGCGGCTCCTGAGGAACGAAGGCCAAGAATC
TCCGCGGATGGAAATCGGAAGCAGGCCTCGCGCATGTCCGACATGAACTCTGCATCGTAG
AAGACGTCGAAGAGGAAAAGCAGCCGCTGCTTCAGCTGGTCCGGCTGCTCTGGCGAGGGG
AAGGTTGCCGGGCGCTCCGTCTGCAAAGGATTCAGCAAGACTGCCAGCACACGCTCCGCA
CCCCCTCTCCAGAGCATGTAGGATCGGCAAGTTCGCTCTGACGCCCCCGTCCATGAACCG
CTCGCCCGCAATCACCACTGGCGCAACGATGGGCACCAGTGTTCCTGATGCCACAAGGGC
CTTGAGAAGCACCTCTGGGGCCGCTGTCTCGTTGAAGTACTGTGCATTGCCCTGTCTCCA
GGGATACCGCTGGCACCCCCAATTCCATGCCAGATGAGCGGATCTTGCCCGCGTTGACGA
GCTGCGAATAGAGCCTCAATGCAGGAGTCATCTCACAAGTAAAGCCACCTGTGTTCTGGT
ACTGTAGATCACCTAAAATCCAGATCCCAGAATTGAGCTCACACGGAGAAGTGTCGCCAG
TTCGCACCATCAATTCAGTGCTTGAGTTGATGTTGCGCCATTCCCGCTCCAAGGCCGGCA
TTGCATGCGAGCATTGCTCAGAAGGTCCGTACTGTGCTAAATATGCAGCATTCAGCGCCC
CAATACTTGTCCCAGCCAACAAGGTCCATGAGTCTGCGTAAGCGGTGTTACAGATCGCAC
GGAGGACGCCAGCCTGGAATGCACCTTTGGCGCCACCACCTTGCAACACCAATGCAGTTT
TCAGCGAAACAACATTACCTGCTGATGTAAGCAAGTAGAGTCCGACTGTGGTGATGCTCC
ATGCCCAGTTTGAACAGTTGGGCATTAGTCTGCCGAAGAATTGCCAACTGGCCACTTGAG
CCAAAATGGCTACGG
>at01848
CCGGTACTTGCGCAGCAACCTCCTGAGCACGCGCTGGCGGCGCATCCAGAGGATCTTCGA
GGGCAGCCGGGCCTCCCTCGTGCCCTTGCGCTTGCCGATGCCCATGTGCCGCCCCTTCCT
CTTGGCCTCGAGGTTCTTCCGCACCCGCGCGCGCGAGTGCATCTTCACCGCCTTGCGGAT
GATGAGCCCGTCCTTGATCAGCTTCCTGATGTTCATACGCGAGTTCGCCATGGAGATCTC
GCTCACCTCATTGGGGTCGAGCCAGATGCGACCCTTGCCGCACTTCTTCACGGACGCCGC
CAGGCGCTTCTGCAGCTTCATCTTCCTGCGGCGGATGAGGTGCGAGGGGGAGGGCAGAGG
CCGGACCGATCCCCAGGGCGGAGGACTTGAGCCAAAATGGCTACGG
>at01849
TACTATGACGCGCACAACATCAGCTTCGACACGGGCTTCCCCTTCGGCCACGGTCTCTCC
TACACCTCGTGGCAGTACTCGTCCCTGGAGGCGTCGGGCCGCACTGTGAAGTTCAACGTG
ACGAACTCCGGGCCCGTGCCCGGCAGGGAGATCGTCCAGATGTACCTGACCTTCCCCGCC
GCGGCGGGCGAGCCGCCCAAGCAGCTGAAGGGCTTCAAGAAGACGGCGCTGCTCGGGGCC

GGCGAGAGCGCGGCCATGGAGATGACCCTCGCAGATCGCGACACCTCGATCTGGGATGTT
GCGCAGCACAAGTGGGCCCCCGTNAGCGGCACCTTTGGCGTTCACGTCGGCACGTCCTCG
CGCGACATCC
>at01850
TTGTTTTTTTTTCTTTTTTTTTCTTTTTTTGTCTCACTCCTCTGCCTGGGGCGACACTAT
CGCAGACTGTCATGTTCAGCACCCGAGCTCACCACTCGCATCCCCATCAAGACCATTGCC
TATCAAGACTCAGATGTTGCCTTCACGTGTCTGTTACCCTGAAGGGAGTGTGCTTCAAAA
ATCGGCTTCCAAACGCCAGGGCGCGTCCGCTCAGGCTGGATGGAATCATCGGAAACCCAC
AGAGGCCTGCCAGAGGAACGCCAGGAGCAAGAAGAAGATCGGGAGGGACGGGAGCACGTC
GACAATCGGGGCAAACATGACAAACTCCTCCGGCAGCCGTGCCTGTGCCACCTCGGGTGC
CGCGATGATGGCGCCCGCTGCTGCCCACCCTGTAAGCTCCGGCCTGGTTGCCACCTTCGC
CTCCGCCCCGCGCAAGACGTGCTGGCTGCCGGCACCCGCTGGCTCGACGAAGACCGTGGC
CAGGCCCCCAGAAGGCGCAGGCCAGGGCGACGCAGGGCAGCAAGACGCTGGAGCGGCGGG
CAAACATCTTGCTACGTCGAAGGCAACTGTTTTCACTCCTCAAGTCCGACTGGGCCGAAA
CTTGAGCCAAAATGGCTACGG
>at01851
TTTAAGCATGTCTACCAGAGGCTGAATTACAGGCCAGTCCAAGAATTGCATAGCCCTTCT
AAAGCCATCACTTTTTGAGCGAAAGCCTGCTTGGTGAGGTGCCTGTCTTTTTCTGCTCTG
AAGAACATGGCCAAGTAGGGCCAAGTGGGCAACCACATGGTTGGTAAATGCATTGACGAC
GTTAGGGGTCTGCTCAGGGTCGCACAGCAGCTCACCTTGGCTCATCTGCATCTCAAGGAG
CTCAGACAAAGTGGCCATGCCAGTTAGTGCCACACCTCTGATGTCCTTGGCCATGCGAGG
CCCGATCTTGGCCTCAATCAGCAAGACGGGCATGCATGACCTCTGTGCTGAGGAGTGGAT
TGGGCTTGGAACACGCATGAGGACCATCCCCAATGACTGCTGAGGGAGGCTTGGAAGGTC
CACT
>at01852
GTGTATGCTTGCTACACTCATCAACAAGTCGGCTGTACAACACACCTGGCGAGCAAAGAG
CTACAAGTCATGGCATTTTTAACACTGCTACGATCACATGCGCATGCAAATGCAAACGTC
TGGGCATTTGGTAAACNGAGCAACGGTCCATATGACGGGCTCGCCGACGGGCGCGGACAG
TA
>at01853
TTTTTTTTTTTTTTTTTTGCTTANCCAACCAGAAGCGATACTTTCCGGACGCTCCTTCGT
GGCAAGTCTCTGAGCGCACCAGACTTGAGCAAGCCGGTGAAGTGCAACCTTTCGGTAGCA
AGCAAGGACGTCCGCTCTGTGAATGGAGTTCTCCACCATCACGTTCAGAAGTTCCAAGCA
AGAAACTGAGGACGCGCTGGGGGCCTGTTCCCCAGACACCGCCGCAGGAAGAGCACTTTG
TAGCCCAACGTCAAGAGGCAGGTCATTGCAGCGGTGAATGAGGTCTTGGATCAGGAAGCA
TCATAAAAATTGGGACTTGAATCATGCATCGCCAAGCTTGAAAGGTGTTGCGTCAAAGAT
TGTCATGGCAACCGTTTTTCGCGCTGCTCTGCAAGTGCGTGTCAGAGCCCCCCCTCGGTAA
CGTAGGCAGCCGCTTAAGAGTTCCAAAGGCCATTACAGATCTGACTCCGCATCCAAGTCC
TCACCGCCGAGCTCGTCCTCCGTGTCGTCTTCCGATGCTTTCGGCTTCTCGCACGTCAGC
ATGCGCTTCTTGTATGCCGTGAAATCGTCAACGTACGGGTCCGTGATCAGCTCCGTGCCG
TGCTTGTAGAGGTAGTGCCT
>at01854
ATATCAATACTTAGCGCTTGAGTCGAAGGTGACGCTTACAATGAGCTATTGCCGTTTGAG
CAACGACCTCTGGCGGATCGTTCGCATCCAGGTGTAGAGCCCCCCGGTGCATGTTTCAGC
TGCAAGTCCCGGTACATCAAGAAGTGGGACCAAACAATGTCACGATACCATTCCGAGTAG
AGCTCGAAGTCCGTGCCGCGGGCCTGGCGAGaCTCGGGCTCgGGACGGGAGCGCCgGCGG
GGCGCGGGGCCgGCAGCGTGATCTAGAAGTGTCCCCACGACTCGAGCTCCAGATGCCGGA
TGTGTTGCGCATCGGTCACTGCAGCCTTGAACTCACACTGAACCAATGCAGAGCGTCCGT
CCACGATGGATAAGCCACGGTGGACAATCAAGGGCTCATCAGCTAGGAAGTACTTTCGCC
CCCTCTGCTTGAGACCAAAAAATAGGAACTA
>at01855
TTTTTTTTTTTTTTTTCTGGATACAATGCTGGCAACAACATGAGCACTCCTGGACTTTGC
ACGCTCCTGGGGCGGGCCACCTCACAGCTCAGCCCAGAATATGTTGGTGGGCCAATGGCC
TCGGCCGCCTTACTCCGCCATGCGCTCCAAAAAGCGCTGCAGGAAGGCGAAGGGGTGCCT
GCTTGGGTCGCAAGTGCCACCGCCCTCCTGGTTGCAAAAGTCCCTCCAGGCGTCCGTGTT

CTGCTTGGACTCCCTCTGCAGCCGCTTGATCTGCTCGACCAAGTCCTGCTTGGTCTCCTG
AGGCCGGGCTTCCTGCACCTCTTCTCTCCGTGGCCTCGTGCGCCTCTGCCAGCGTGGCGG
ATCATCGTCTTCATCCAAGTCAGCCTTCGCCCGTCGGTCTCGCCGCCATTGGCCGGGAAG
GCTCGCGAGGAAGGAGCGCAAAAAGTCGGCGTCGTGCCTGCTGGGATCGAGAGTGCCGCC
CCTCGTGCC
>at01856
GCACGAGCGTACCACTGCCGTCGGAGAGAAGCCAGGGCTCACAGGGCCAAGGACAGCGGC
TTCAGTGTTGGTTTGGTCGGCTTCTGCTTCATGCTGTGTCTTCGCATCCTCATCCTCGCA
GCCTGTTGCAATGTAGAAGCGCTCCCGGAGGCCTTCGATCTTTCCCCCCGGGCCCAATTC
TTCCCACCACTTGCCTGCCTCTTCGTTGCATATATTTACTAAAGCCTCTGCATCTGCCAA
GGGGATGCAGCATTGCAGCACGTATCGCGCGAACATAAAGACGGGTCTTACTATACCCTT
GTCATCAAATAGGCACTGCTTGGCCTCAGCCAGTAAGGACTCACGCTCCAGCCCCGCAGC
CTTCTGGAGAAGGTTCGAGAGATCTGGGCAACGTCGTGCATCAAAATGTAGGTAGATCGA
GGCTCCATTGCAGAACATGTGGAACTGGCCAAGGAGGTCCAAAGACCTCTGTTCACACAA
CTCCTGCAAGAGCGCCACGAGAAAACGTTCGCTTAAGACTGAAATGTTATTCCCAGTGAT
TGCAGCCAGCTTAAACCCGAGCCTGGCAAGTCTGATTAGCGTCCTGCCGACACCCTCACG
GAACGTCCGCAACTCATTAGGATCACCTTGATGCAGGACTGTCCTATCCACATCAAAGGC
TACTAATGACCTTT
>at01857
TTTTTTTTTTTTTTTTTCCCCGTCTTTCTTTGAGCCAGAGGCCAGTCAGGTATCTTCCG
CTCTCACGCGTCCGATTTGTACACCTGCTTCACAAACAAAAAATAGCACTCCACCTGCTC
ACCTACTCACAGGGAGCTTCCCAGAGCCCGGAGTAGGCGCCGTAGGCGAACAAGGCAATC
AGGCCGATGGCAGCAAAGCCGACGAGCAAGCCCACTGACCTCCTTGAGCCCGCCACGGCG
CACAGGGCGCTGCTGCCTACTCACAGAGAGCTTCCAGAGCCGGAGTAGGCGCCGTAGGCG
AACAGGGCGATCAGGCCGATCGCGGCGGTGCCCACGAGCAGGCCCAGGAAAAGCAGGTTG
ATCTTGCCCGGCTGGCCGTTGTCCGCCCGGGCTGAGGCGGCAGGCCAGCGGCTGCGGCAA
CGCGCAGGCTCCGGGCCTGCAGCTGGAAGTCCTGGGCCAATGCGGAGGGAGCAGCGCCGG
CAGACTGCCGAGAAGCCAACGGCTTCCGGCGCCACGAAAGACTCGTAGCCCTGGCGTGGG
CAGCAGCAAGCAAGCCACGCCCACCAACAGGGCAACAGTCAAGAGAGAGCCAGCGTTGCG
CATCGCCATCGTGACGGAGCGGGCAGGACGGAGACTGCTGGATGGATGCGAGAACGCTCC
CTCGTGCCGAATTCTTG
>at01858
CCTCAGATTTGGCATTAGAAAAGTTGGCCAAAGCCTACTCTAGGGTCTGTGTATTCTTCG
GAGCTTTCGGAAGTAAAGAGAAGAAGGAGGAGGAACATATGTCCCCGGATTCTCCCCTGT
AGCTCCGTGGCTGAAGCCACACAGGAGAGCAGCATGCCCGTACTGATTCGTCACGGCAGC
TTTGAACAATGATGTTGTTTTTTTGTCTGACTCTACACTCATGCTAAAAGTTCCTTTGTA
CGACACTCCAGGTGCATGTCTGATGTATGCTGGATCAGCCGGTCAGAGCTTTGCCGTGGC
ATCCCGCTTCGGATCGCTTTTCGCAGCGCTGGGCCTCGAGTGGCATCGTGCAAACCAACT
GGGCAGCCATTGGAGCGTCTCACCGAGCTCCTTGATGAGGGGCTGGCCTTGCGCATTGAA
GAAGTCCAGAGAGCAGAAGAGCTGCAAGTCTGCAATGCTGAAGAACTTGCCGTTGATCCA
GTCTGGCGATCCTGACTTCACCATGAGGTCATCCAACCACTTCAAGTTCGTCTTGGCCGT
CTCCCGAAGCTTGGCTGCAGCTTCTGGGATCACTGGCATTCGGGACTCAAAGAACTTCAC
TGCCGGGCCGAACCTGAACGCCGAGAACTGGTTGAGGACGATGAGCTGCTCTATGCGTCT
CTGCCACATGCAGCTGTCAGCGCGCTCACGCGCAGTGCAGCCAAAGAGGGCGGGCTCCGG
GACAACATCTTCCATCAACTCGCAGATCGCCACGGTCTCTGCAAGGACGCCCCCGTCTGC
AAACTCCATATAGGGCAGGGATCCCACCGGATTGCGCCTCAGCGACTCTGCCGCGCGAGA
GTCGTCGTCCCTGCCTTGCCCTATGGTGAGAGTCTTCGCTATGGAAGCCAAGTCGATGCC
CTTCTCAGCAGCAAACATGTCCACGACTCGGGGGTTGGGTGCCCCGGGTGAGGTGTAGTA
AAACGTGGCTCCGGGAATCTGCTTCACCATGGGGAAGGCAACCAAAATATTGTCCTGACT
GCAAATGAAAGGGTGTGCGGCCAGCGCACGTGGAGTGTGGACAAAAACCTGGACTTCAAC
AAGCTGAACCAAAATGGCTACGGCTTGAGCCAAAATGGCTACGG
>at01859
ATACAAGCTGGCAGGGCTGGCGGCCGAAGAGCGCGTCCTCGAGCGCCTGCAGCTTGCACC
AGGTCATGGGCAGCTGCCGGACGCGCGACGACTGGGCCTCCTGGATCGCATGCCGGCAGC
CCGTCTCCGAGAAGTTGTAGAAGAGGAAGCGGTAGCCGTGGCGCCGAGCATAGAGCGCAT

TCGCAGCCAAGGCAAGCGAGACGTAAGAGGTGTTCTCCAGGTCTGCCGCGCTCAACGGCG
GCTTCGCCGGGCGGTTGTCAAACATCACCACGCACTGCTTCGACGCGGCAGAAGGCCTCG
TGAACGTCGGCTGAACTGCTTCTTCTGCTCGTGGCAATGGAGGTGGGAACTGACAGTGCA
GCGACAGCACACCCAGCCCGCAGAGTGCCACGGCTGCCAAGGCGCACACTGCCTGAGGCC
>at01860
TTTTTTTTTTTTTTTTTCACTTTTTTTTTTTTTTTTTGGCGTCGACGTTCCTGAGAGC
TGCTCCCGGGCGGATGGGAGTGGTCTACCTGCTGGTGGCGATGCCCGCACCGGCAGTGCC
GACCTTCAGCCGTGCGGTCTGCCGTGTGGCTGCGGGCGCAGCAGGAGGCGCGGTGTCGCG
CGAGACCAGGCCGCCGGCCGCAATTCCGATCTGCCGCGCTCACGACGATGAGCACCGCGC
GGGCCCCCGCGAAGACGGCCGCGCAAGGCATGCCCGGCAACCAGCGGGCAGCGCTTGGCG
CAGGGCGGCCTCTCGCTCACTCGCAGCGCTCCGCCGGCTGCGCCCGTCGTCATCCCAGAC
ACACGTGCGTGCGAATGGTGTCGACCGTCTCGGGTTTGCTCTCAAGCCTCGCAAAGACAA
TGAAGGTAAAGAGGGTGCAGAGCTGGATGAAGCTAAGGACCTCATGGATAAGACGGGCGC
GGAGAAGCAGCCGCCGCCGCTGCTCTGCGTGCAGAGGCCTAGGCCATGGAAGAGCAGTGG
TCGCTGCGGCCTTTTGGCCACAAAGCCGCCAGTACCCAGATGCTCCAGGGTCGCTCCAAA
GCCAAAGGGCACGAGTTGGTCATTCGACTCGGGCATGCCGCAAATGCTGGCAAGTGAAGC
GGACCCACCCCTTGCACCTACTTGAGCCTCGTGCCGAATTCT
>at01861
CCGTAGCCATTTTGGCTCAAGCTTCTATTGCTGCTCAAGTTTTGAGACTTCTTGTGCAGT
GATGGCAGTCGCTGAGGGAGCAAGACCAGCTTTGGACGAGTCTTGTCAGGAGCGTGCTGA
TGCCAGACCTAGTTCCGGACCTGATCCTGCACCGGCAACTACAGCTTCTGCTGCAATCCG
ACGATCGATTATCCCGTTCATAATTCCTGGTATGCTGCAAGGCAAAGCTGGCTCAGTAGT
GACACCCATGCTGCCAATCTTTGTGCACGTAGATCTTCATGGGTCTACCAGTGTCGTTGG
CGTCATTACTTCTGCGTACGCGTTGGCACAGCTTTGCAGCTCTGCTCCTT
>at01862
TTTTTTTTTTTTTTCCTCGACATCAAATTTGTGGGAGAGGAAGTTGCACTCGCCCGGCC
TCATTTTACGAGTCCTTTTGAATCACTGCTTGAGTCACTGTCGCCGTCCTCCTCCTCAGA
GTTGTCATCCTGGTCAGGAGCCAAGGGGGAGTTCATCCAATCGCGAATGCATCCACCATG
ACAGCTGCAGCCACCAGTTGGTTCAAGGCACGCCATGCAAGGAATGAATCAGCACTCAAT
ATAAATGACACCTGCAGCGTCGCGCTTTGGCCCGCGTCCATGAGGATGCATTGAATAACG
CTGGCAGTTGCCATCAGTTGCGCATTGCCCTTGATGGTTTCAGGGACCAGAGCACCTGCT
CCGCACTTGAAGATTGACAGTCCGACTCCAACGAGGACCCACGAAGCAGCTTCGAACTCC
AACATCCAGTGCTCAGGGGAGGAGGATAAGGCGATGACGCCAAGTGAGAAGCCCAAGACG
TGCGCTATTCCTGCAAAGACGAAGAGGGGCCCGCGAAGACGCTCGCCTTGCAAACAAGTT
CCCGCACACTGGAGCGAGGAGCAGGGAACAGATCGGCACGAGGCCCATGATCCGGTTGGC
CGTCTGCTCACTCAGGCCGCCGTACTTCTGCAATAGG
>at01863
TTTGTTTTTTTTTTTCTTTTTTTTTTTTTGGTTTTGCCATATTCCTCCTCACACACAGCC
TGCTCACTTGTGAGGCTCGCCAGCCGGTGAGCCAACGAGTGCCCACTGTCACTAAGGCTC
GTGGCAGACAGCATACGCTTTGCCGCCGACCGGTGCTGGATAGCCGTTCCCATAGTGGCT
GCAGATCTGACATCGCCAACCGCCTGGGCGGGGATAGTTCTCACGGATGTAAGCGTCGTG
TCGGCCGACGATGTCTTCGACCGACGTGGAAGGCACGTCAAAGTCACAGCCACCACCTGT
CGTAGTCCATGCATCAGGGCAGTCAACGTAGATGCGCAAACACCCATAA
>at01864
TTTTTTTTTTTTTTTTTGAGCAAAATGACAACTTTCAAAGACCGCCTATGCGGCGCCAG
TGCTGGCCCACCGCAAGTTCGTACACGCGCGGAAGGAGGCGGTGCAGGACCCCCCAGGGC
TGCACTCAGTCCACGGCCTTATACTCGTTGGGCTGCATAGCGTGCTTCCAGTAGGACTTC
ACATCGGCCTCGGAGTAAACGTCCTTGTACGTCTCGATCAGCTGCGCCAGCGTGTAGCGC
TTCCCGTCGTACGGGTCGACCCGCTTCTCCCTGACTTCAAATGGCTCCCGGCCATCTGGG
AAGCGCTCCAGCTTCTCCTTCCGGATGTTCTCGTCCGCAATCTCCTTCGGCATGACGTCG
AGCTGGTCCCAAGCCTTGCAGCGCCAGGCCGGGTCCATCCACTGCGCCACGATGGAGGCG
GACCATGGCTCCAGCGGCTCGAGCTTGTCCAGCGCCCCGCGCAAGTCGTCCGGCAAGGGG
GCAACCGCCTCGCGGGGGATGTCGTCCAAGTCGCGGAAGGCCAGGCGGTACCGGTGCAGG
AAGTTGCGCGGGCACCACTGCAGGTCGCGGAGGAAGGCCTCGCGCGAGATGGCCCTGCTC
ATCTGCTCGGGCAGGTCATCCAGCTGCACGACGATGCCCCGGGAGAGGTCCTCCTCAGTT

AGGTCGGGATCCAACCGCAAGGCCTCCTTCAGGAGCTGCAGTCGCAACGGCTCCGGCAGG
TACTTTGCATCAAACGGCATGGCAGCCTGCTCTCACTACCCGCGGCCGACGCCACCTCAG
CAGCAGCCATTGGACGGGTTGATGCAGTCCTGCGACGAGCGCACGCTCGACACCCAGCAA
GCGAGCCCCGCGCCGGGCTGTCTTGAGCCAAAATGGCTAACGG
>at01865
TTTGGGCTCGAGCCACTGATGCACACCTACCATCTTGATAGCGATATGCTCGGCATCGCC
GCCAAGGCCTGCGAGGCAGGATCTCCTCCGGTGTTGTGGATCAGACGATGAAGCTCATCC
TTTCCAGCGCCCCTGAAGTGCCAAAGGCATGCCCTTCACGGAGGACTTCCGGAAGCGTGT
TGGCCATATTTCCGAACAGGCGGAGAGAGTGCACATCGAGTTCATGCAGGCCAAAGCCTC
GGCTGTGGCTCCAAATGCTTTAATTTGTCACAGTGGCACTCGCGAGCACAGGTTTGCAAC
AAAGCATGCAGGCATTGGGACTGGAATTCAAGGGCTCGCATGCTCATCGCTCACCTCCAA
GTGCAGTCGGGTGGTTCTGGCAGCTCGGCTGCAGGAGG
>at01866
ACCCTGCCAAGCGCTGCCAGCGCCAGCTTCTTGCAGATGGGTGTGAACCTCAAGGAAGTC
CGCGCACGCGCCTTGACCGCGCTTCAGGAGGCACGCGGGGTGGCCGATCGCTCGAATAAG
CCGAGCCTGGAAATCTTGATGTTGGCGTTGTCGGGCAAGAGGGCGATGGGCAAGGGGACC
TTCGACAAGGTCATCAAGATGGTGGACGAGATGGTGCAGGTCTTGAAGCAGGAACAGTTA
GACGACGACCACAAGAAGGAGTACTGCGCCGCAGAGTTCGACCTCAGCGATGACAAGAAG
AAGTCCCTGGAGCGGACCGTGGCCGACGAGGAGAACGCTATTTCCGCTGCAAAGGATGGC
ATCAAGACCTTGGCCGAGGAGATCTCCGCCTTAGAGGCCGGAATCAAGGCTTTAGACAAG
CAAGTGGCGGAGGCCACAGAGCAGAGAAAGG
>at01867
CCCACCACATTACCAGCCCCTCTGCAAGCGGTGCTAGGCTTCCGCACAGGCTTCCGCACA
GGCTTCCTCCCTGCGCCACATGGGCTGTTGAGTCACTGAAGCTCTGCTAGGAATGAACTT
CGTCAAGGCTGAGACGCTGGTCCAAGAGATTACGGCAGATGGTAAGGTTAGGGACATCGT
GACCACGCGGGTGAAAGACCCAGGCGGAGAAGGAGGCAGAGCAGAGGGAAGAAATCATCC
GCAACCTCCCAGAGTTCCAACGAAACAGA
>at01868
TTTTTTTTTTTTTTTTTGGCATTTCGTGTTGTCGCAGAAGGCGGGGTCACACTTCTGCG
GTCTATACTGGCATGCTTACTTGAAAAGGAGCTCTGACCCACCTAGTAACATGCAGTGCC
AAGTCTAGCTGTACAAAATCTTGGCATGCGCAAGAAACGCGACATTCTGAGCATGGCCGG
GTAAAGGTCACACTCACAACTCATCATGAGCCTCCTCTTCGTCCTCATTGTTGCCCACAC
CACCCGCACCACCACTGTAGTACTTGGAAACTATCGGCGCGCAGATGTCCTCTACCTCCT
TGTGCTTCTCCTTGATCTCCTCTGCACCCGCCCCAGGATTGGAGTCTAGCCATGACTGGC
CGTCCTTGAGAGCGTCCATGATCTTCTCCTTCTCACCTGCGGTCATCTTCTCACTGAGGC
CCTTGTCGTCGCCAGAACCCACGGTGGCAGACCGCATGGAATAGATGTAATTATCGAAGG
CATTCTTGGCATCGGCGCGCTCCTTTGCCCTCCTGTCCTCATCCGCGAAGTCCTCCGCCT
CCTTGACCCTCTGATTGATCTCCTCCTCGGTCAGGCGGNCTCTGTCATTTGGTATGGTGA
TCGGTTTGCTCTTTCGAGTGGGTTTGTCCTCTGCTCCCAC
>at01869
ATCCGCTTCCTCCAGTGGCGCTGTGCGGACGCGGGGCTCGTGGCCCGCTTCTACGAGGAC
GCCCTCGGCTGCCGCGTGCAGCACAGCGAGGGCTCCGAGGCCGTGGTCTGCGTGGGCCCC
TCCGTGCACCTCATCTTCAGCACGGCTCCCGGCCCCCGCGACCAGGAGGCTCTCAGCCGT
GCCAGCGGCGTGCACGTCTGCGTGTACCTCTGCGAGTTCAAGCGAGCCTACGACCGTCTG
AGGGCGAAGGGCCTCGTCTGGACCAACCCTCGCTTCGCGTACCTGGACACGTGCGACAGC
TACGAAGAGGCGGCCGCGGGCAGGCAGTTCCGCTTCCGGAGCCTGCGGGACTCTCGGGAG
GGAGCTGGCGGAGCTGGAGCATGAGGTGCGCTCGCTCCGCCACGTGCGGTTCATGAAGCA
GGTCTACTACGTGGCTAGGTGAGTAGACCAGGGACCAAGGGGCACGTGGGGC
>at01870
CACTGCCGTGCTGGCGCTCCTGTCTCTGCTGCAGGGATTGGGTCTTGTATGGCGACTTCG
TGGAGGCTGCTGTTTTGGTGGGCTGGGAGACGAGAGGCGGTGCAGATGCTCGTGTACGGC
TTAGCGCTGCGGGTACTGGGGTGCTGGCGCGTCTTCGGCTTCCTGGGCGCGGTCTCCACT
GCGAAGTCTGGCTCCTGGCGTGAGGGGGTACGGGGGTCGTCCCCTCCCCGGCACTAGGTC
CTGTAGTTGCCGTGTTGCTTCGCCTTGGTAGGATGTGGTACAGGATGCCGTGCATGCTTT
TTCAGGGGTCTGTCAGGCATGCATGATGTGGCAAAATCTGTGGTTTTCCTAGGAAAGTTT

TGATTTTTGTCATATGAAGGGAAGCGGATGCCCCTAATCGTGTCAAAGAA
>at01871
TTTTTTTTTTTTTTTTTTGGAAAGGCCGACACTGAAATGGGAAGTCACTGCGTTCAAAAC
ACATCCCTGTACCGATGCCTGCAAGGGTGCTTGCCTCGCCACTCCAGTACCCCCTAACAC
CATCAGATCTGCTGCCTCCTCTGCTCCCCAAATGCCATCACTGCCTGCCCTCAGACGCTC
TGGCTGTAGATCAGGCGGCCGCCATACTGCAGCTTCCTGAACCAGAGGTACATGTAGTCC
GCCGACAACAGGGTGTGGCAGATATCCGCGAAGAGGAGCTGCAGGAAGTGCTCACCCAGG
AAGTACATCTTGCCCCAGAAGAGCATGCGGATGAAGCGGGCGACGACGATGAGCCCCACG
TAGTGCGACGTCAGCGCCTCCACCTCGTGCATCTTCCGCATCAGCCACAGCTGCGGGAGC
AGGCCCATGGCCTCTTGAAACATGG
>at01872
CTTGCGTCGCACACGATGACGGGCAGCTCGCGCGGCGTTGCGTGGTGGCAGATGCTCGGC
AGCCTCAGAAACTTCGTGTCCATCCCGTGGCGGCCCTTCAGGTACATCCCCTCCGTCCCT
TCCAGAGCGAGGAAGCAGACGGGCACCTGGTAGTGCTGCATCACTGACAGAGTCCATCGA
GAGATGTCGGTCTGAGTGATCGACCGAATTGTAACGTCAGGAAGCGACTCGTTCGTCTTG
GTCGCGAAGGTGACAGGTGGAGCGTCTTCTCTACGATCATTCTGCGCATCCGAGCCTCCA
GCTAATGCTTCGCATTGGTCGGAGGCGCCTGTGCTCGAGGCGGCGGCCATCGGCTGAAGC
AGGAGAAGCTACACTACAAGTCCGCAGCCTGAA
>at01873
CCGTAGCCATTTTGGCTCAAGCATGGTGAAGAGCAGCCTGGGCTTGTGGGTGCCAGGAGC
ATGGCGTCCAAGCAGGGTGTGTTGCCGTGCAAGCGCATGGTGAAGAGCAGCCTGGGCTCG
TGGGTGAATGCGGTGCCGGCCAAGGCAGCAGATGCCTCTCGTCCGTTCTCCTGCAAGGTG
TGCGATGCGCGTTTCAAGACTCGCCAGGCACTCGCAGGGCACGAACAGCCCCGCGCCCAC
AAAGCCAAGGTGGAGGCAGCTGCCAGGGTGCTTGTGCAGGTGCTTGTCTCTACTGCGGCA
AGCTCCAGCATGGCGGCTGGCCCTACTCCGACCTGCAACTGGTGGATGCTGCCAAGCATG
GTGGCCAACTGCGTCCACAAGGGCATCCGTGCTTCATTGCGCGTGAGCAGAA
>at01874
GTAGAATTGAGTCCCGTGCTGAGACTGCGCTGATTCGTCTGTATCGGGCCATGGCCTCGG
AGGTGCAAGACTCCACCAGGGCACGCGCAGAAGCTCGGCGCCAACGGATCTTGGCAGGGG
CCAGCAAACGCCTTGCACGGCTTGGGCCAGGCCTGGATTCTACAGAAGAGAGCAACAGAC
TTGAGTCTGCAGCAGTGACGCCCACAGCCGGGCCCCATCACGCAGCCCCCAGAACATTAC
AGGTTTCCCCTTGGTGAGCCGGAAAAGCCTGATGCCACAGCGCGACAGGAGGAAGCGCAT
CGCGCAGGCGAAGCCTGGGTCCAGAGCATAGTAGCTCGGAGGCGAGCTCGCATTATCCGC
CTCCTTGGGGGCACACTACTGGGAGCAGTCTTTGGCATAGGAGTTCCTCAAAGTATGGTC
TGGTGCCCTCCGCTGGCTGCATTTGTGGCGCTGTTTTTGGTGACTGAGGTGGCTCTGCTG
AGATCAACGCGGACCAACATGGCCACACGAGATGGCGACCAGATCGACGTTGCGATGTTG
CAGCTGCTGGGGGGGGTCTGTGGAGCACAAGGTCTGGACAAATTTAGCTTCTTCTTGGAC
TTGGGTGCATCGGCGCTAAGGTTCCTGGAAGATGTTTTGTGGTTCATGTTTGTGATGCTG
TTCACTCATTCCGCTGTGCTCGGCGTCCTACAATGACACTGACCCTCAGCGCGAAGCCAG
CGGGAGGTGCGCAATGTGTGCGAGTACAGGAAAAACC
>at01875
GGCGACAGCCGAAGACGTTGCAGGCGAAGCTCTACTAGAAGCCTACAGGCGGGACCCCGG
TGCCTTCAAGGGCCTGGACGGCTGGGGACGGGACCAGGAGACGGGCAAGCTCGCGAAGCT
CGACCCTGCACGATGCGAGGCGCACGTCGCCGCCGCCATCGCAAAGCTCGCGGCGGCCCC
AGAGCGGCGGATCGTGCTGGTGGCGCATGGCCTGCTGTTGCACCAGGTGACGGCGCGCCT
GGGCCTGCGCGAGGATGGCGCGGAGGAGCTGGGGATGGGAGGTTCCCCAATGCGAGATCC
GCGTGGTTAGGCGTGGAGCTCTCGGA
>at01876
TTTTTTTTTTTTTTTTTCCCCTCACCCCGCTGGCCCCTCAGCTCGACGGCCCCTCCGCC
ACCAGCCGGCCCTTCACCTCAGCGCAGCGACCTCTGCCGCGGCGAAGTCCACAGGCCCCG
GCAGGCACTGGTGCCCCGGCCGCGGCGCGCAGTCACGCGAGGCGCAGTCGGCCGGGAACG
GCGCCAGCGGCTGGACTGTGAAGTTGAAGCTCCTCTGTGCCAAGACTTCCCGATCGTGCT
CCTCATTTGCCACAGGCTCGCCAAACATGCCACCCAGCAGCAAGGTCCCTCTGAAACCCA
GCTCCTTGAGCGCGTGCGCGAAGTCCGGCAGCGTGAACGGCCGGAGCTCCACGAGCTCCA
GGGCCTCGCTCGTCCAGCAGAGCGGCATCGCCCTCTCCGCCACGAACTTGCTGACCGCCC

AGTCGTAGGAGTTCCAAGGGCCGACCACGATGGTGTCCAGCGCAGTGGGGTCAAGCCCCA
GCCATTGAAATGCCCGCTTCAGACCCGGGATGCCCTTGTAGATCCAAGGGTGGTTGACGG
CGATGGAAACGCTCCCACCACCATGCATCTGCGCGCGCGAGAGTTCGTCCCAGCAAGGCT
CTTTCTTGGAGCCGCCGTCGCACTTCTTGGAATATGGCGACGCCTCCAGGCAGCCAAACC
TGTTGAGGCGGGCCCACAAAGTGATCATGCTCTGGCGCTGGCACGTGAATGACTCAGCCA
CCTCACGGATGTGCGAATTGCCAATGAAGAGGATGCTTTGATTGAAGGGCTGCTCTGCAG
CTCTCGCGTAGATAGACTGCANCGCATCACCGTGTGCCTCCAAGTATGAGTCGTACGCTT
TGCCGTGACATTTGTAATTGANGCGGCGTGCTCCACAGCGATGC
>at01877
TTTTTTTTTTTTTTTTTAGGGTTGCAGCCGGCCAGTGGAAGTGGAGTCGGCGGCCCGGT
CAGCCGCACGGGGGGCACGCGTCATCTGTGCAACCACCATGAGCTTGTGGTCATGGCCCC
CCCCCTCCCTTCGGCGCGGCGCGCCCCCGGCGATCGAGGTCTCTGGTGGGCTCCGTTCCG
CCGCCCGAAGCTCCGGAGGGCCGCCGCGGGCAAGCGTCGACCTAGATGGCGTCGATGTCC
ACGCCGGCCTGCTCGTTGAACTCGACCCCGTCGTCCGAGAGCTCGCCGTCCACGATCTCC
GTCTCGTTGATCTTCACGTTGTCCGGCAGCTCGCCGTAGGTCTTGAGGTTCCTCGCCTCG
TCCGAGGTGTACTTGACGATGATGTCGCCCTTCTCGTCCTGGAAGTCGCGGAGGGAGACG
AGCACGATGTCGCCTTGGTTGACCCAGACCTTCTTCCTCATCTTGCCGCGGATATGGCAC
AGGCGCTTTGTCNCGTCGAAGCACGTCACCTCACACCGGNCGTTGCCGAGCATCCGCATG
ACCTGCCCGTACTCCTGACCGTCCTCTTTNGACTCGAGCTCCTTCTTNGGCTTGGTCGCG
TCGCCCTTGGCTCGCTTGGNCTTGTTGCCCACAAGCTTGTTCTTGNNGCATCCTGCTGAG
GTTGGGTNNGCGAGCGCTGGCGGGCTTATACAAGGTCTGGCACGACCTGATCCTCGT
>at01878
TGCCAGTCCACGGAGCGGAGGGCGCCCTGGGCGTCGAGGGCAGCGACGAGGACGACATGC
GATCCCCGCTTGATCTGCTCCTCCGGAAATTTACCAATCTGCCAGTTCAGGCCGTAACCC
TCGTCATTGGGTCCGAGGATTGTGAAGCCCTCAAAGGGGCGTGCATCTTCGGCATCCGGG
TAGAAGGCGCCCTCCTCGCTGCCATTCTTCAGGAGGTGGGAGCGCTCTGTGCCCCACTCC
GT
>at01879
TTTTTTTTTTTTTTTTTACTTCATCCACGAGAGTGACTTGATCTCTCAAACTTCACTGC
ATCCCAAGCAGCGCCCCGCTGCATGCTGCCGTGCGCAGTGAAGCGCTTGAGTCAAGCGGG
CAGCCACAGTCTGCCCAGTTAGAAATGGGTCAAACGAGTCATGTGGCCTGCCGGGCAGGC
ATCGCCAACGGCGATACACGCGTCTCTGCTCACTCTCTGTAGAATTAGCTCGCCGCAACG
TCCTGAGAGACTCCTTTTTGAAAGGAAATATCTGTAGCTCCTCATCATCCTGCTCTGAGA
ACTTGGACATTGCTCGTGCTCTGAACTTCACTTGCCACTCCTCAAAACTCGGAGAATCGA
AGTGCAGCACAACTGCCGCATCATCGTTCAAACAGCAGCTTGGGTGGGTGCAGCCGCTAC
CATCATCGTGGAGCCCGTATTCAGGTAGAGGCGGCTCAAAACTCCGATCATATTGACAGA
AATGGTGCACTCCGCTGCAGAAGACGTCACCAGGTGCAGTGAGGTTCGCTGCGCTCTTCC
CATTGCAATATAGCACGTGCGCCTGCGTTCGGAAACATGTGCAGGGCCGTGAGAATGGCT
GGTCAGTGGCCTGATCGTATGGGAAGACCGCCTCGAGGT
>at01880
ATATGCCGCCTTCATAGGCGAGTCCTTCAACTTGGCGCTGGCATCATCGCCGTCTGGACC
GTGGCGCGTGGGCGCATCTTACGGCCATGCCATGCATACGGCACTGTCAAACTACAACGA
AAATCCTGTGGTCACAGAGTTCAGACGGCCAGACGGGTCGATCATCTATGCAGCAGTGTT
TGACACCGTGTACAATGAAGCAAGAGGTTCGGAATCACCTGGAGTGAAGACGGTGTTGCT
TGGACACATGGGATGGACATCCCAATGCCTGGCACAATTGGCGTCCGTACACCACTTGGA
TTGATCGACCAGGAAACGGTACGGCCGCAATG
>at01881
TTTTTTTTTTTTTTTTTGACTGATGGCTCATTTTCTGGCCGTCTACTAGAGCACGGTCA
GCTGTACAATGCGCGTCTCAGAATGCACTCGAAATGTGCGTCTCTGGATATCCTGATGGC
TGTGCCGTGAGCCTCAACCACTCCGGGGCCTCCTGATCCAAGATGTCGTCCGGGGTCAGC
TGCTCCAAGATGCGTTGCGACTGGGGATTGATGGCAGAGGCGGGCCGCTCCGGGCTAGGA
CTGGCGATCTCGGGCTTGATGGCAAAGGCGAGCCGCTCCGGGGAAGGACTGGCGATCTCG
AGCTTGATGGCAGAGGCGGGCTGCCGCCTGTTGGTCCGCTGCAATTCTGCCAATGCTCTC
GATGTAATCGATGCTCTCGCCGCGCAATGTGGACATCGGGCTGGTGCTCAAAACCCAGAA
GATGTGCTCGTAAAGCTTTGGGGCGCGTGCCAAGGGCCAGGACTTGCGCGAAGGCTTCGG

CTCTCCAGGGCCCATGAGCCGCTTCCATTCGGCCTCTGGAGTGGTGTCAACACACTTGCA
TGGCGGCACCAGCGCGCTGGCGCAGGCGTCCCCGGTAGTGCTTGGCGACACCACCACGCC
CTCCCATAAGTACCTGAGTGCCAGCCCTCGACACCCGGACCTGGTACTCCAGCTGCGACG
CCACGCGGCGCGGCGGCCTGAACTCCTGCAAAACCTCCGAGCTCTGCGANNNNNNNNNNN
NNNNNNNNNNNTCCAGCTCCGGCTGCGAGGCCAGGCGGCGCGGCAGGCAAAAGTCCTGAA
GCGCCTCCGGCCCCTGAGAAGGCCCAAGTGGGCCTGCTGACTCCTTGGTGGCTCCGCTGC
GGGCTGGCAAGCGCGGGCACCTGCCGGATGGCC
>at01882
GTTGGCTCCCTTGGTCGGTGTCAAGATGTCGCAGCCACCGCTTGCGATCTCTTCGTCGTC
CTCGCACTTCACGCCAATGGAGGTGCATCCATTGCTTGATTGGAGGACGCTCGAGTACGT
CTTGCAAAGCATCAAGTTAGCATCAGACTCGCCCTCTGCCTCATTGGCCGAGCGGGCGGA
GGCGTCCGCGTCGTGCCGCAGCAGGGACCGCTGGGTCAGGGCTTCCGCGTCCTCGAGGCA
GACGGCCCCTTTGCAGGCGTCCTCTGGGACGGCTGCGCAGCCGCAGGCCTCGAGGCACAG
GACGAAGCAGATGGCCGAGAAGCTGCGGAAGGCCATTGCGGACAGCGAAGGAGCGCAACT
ACGAAGGCCGGTAGGTTCAATCCAAT
>at01883
TTGGTCAGCAACATCTTACTTCAACCGACGTGCCCTACCAGTGGGTGGTGAAGAGGCACA
ATCTGGGTGCCGAGGCAGGCCGGAGGCCGGCCCCTCATCATCTTTCTGGGCCTGGGCTTC
CTGCTGGGCCGTCGCGTGTCCCTGGACGCCCTGGGGCGCAGGGCCGGCTTCTCGGAGTTG
GGCTGAGGGGCGGCGGGGGCGAGGTGGCGGGCAGGGAGGGGAGGACCACCCAAGGGCTAT
TCTCCACGTG
>at01884
GGAGACAAAACAGGAACGAAAAACAGAGGAAACAGAAGGAAGAGAAGAAAGAAGGACGGA
GAAAGGAACAAAAGACGGAAAAAGGAAGAAGGAAGACGAGCGAAGAAAAGAGACAACAAG
AGAAAAACAGTAAAAGTGGAAAAGAAAGAAGGAAAGACAAAGGAAAAAGAGGAGAAAGGG
GGGAGGAGCAAGAAGAAGAAGAAAAAGCAAG
>at01885
CCTCCGGGTCGTGCACGGCTGCGGCTTCGTCCACTGCGACATCTCGCCCCTCAACATCCT
CCTCGGCCGGGTGCCCGGCGGCGGCCCTGCAGACATCGCGCCCTACCTCATCGACTTCGG
GATCGCAGAGAAGTTCCCAGGGGGCAAGCCGCACGGGCCGCGCTATGGAACCATGGAGTT
CACCTCCGCTCGCACAGCGGCGGGCGGCAAGCGCGTGCGTGAGGATGACCTCGAGGCGCT
GGGCTGGACCATGGTCTTTCTGCTCTTCGGTGAGCTTCCATGGATGGAGCGGCTGAAAGN
GGACTTGAGCAAGCATTCCATCTTCGAGTACGAGAGGAAGCGCCAgGGAAGTTgGTCGAG
ACGGTGAgGAGCAGAGAAGTGTCAACTTCTTGAAGATGGTTGGAATTCGtTgGGGcCTcC
ATgGGACAAATTTGCCGAGATGCCTGAGGGGTTGGATTCTTTCATGCAAGCCTGCAGGTC
GGAGACCAAACCATCCGAGAAGCTGGATTACGCGTCGCTTGCAAGATTCTTGGGAGAGAA
GAATAGACGGACCTAAAGCCAAGACGAGGTTGAGTGCGAAGATCGCAGGGAATTCCAAGA
TCATGTGCTCCCATTG
>at01886
CCGTAGCCATTTTGGCTCAAGGTGCTCTTTTGCTGAGCCTGTGACTTTTTGGGCGTGACC
TCCAGCGCCCAGCGCTCTCAATAGACCTCAAATTCGATGATGTTGCAGCAAGCTCTCCGA
CCTGTACAAAAGTCAGCCTCGAGGCTATTGCTGGCAGGAGCAAATGCTGCAGCGGACTTC
AAGGATGTGCCGCCTGAGGTACAGTCCACGGCAACAGTACCAGGACTGGCACACACAGAG
TCAGGCTTTACTCCAGTGGTGCCGAGCCGG
>at01887
TTTTTTTTTTTTTTTTTTCGCGCTGCGCGCGCTTCCGGCGGCTCCTCAGCGTCTCCGGCC
GCTCCGGCCAAGACGGAGGCCCCTGCTCAGCGCGCTCTCCTGGCTCTTCCTGCCTCTGCC
CTTACCTCCGCGCCCCGTGTGCGCTGGCGGTGGCGGCGCCGCCGCCACTTGCGGGCTGGC
GGCGGCGGCTGGGCTGCCCAGCATGGCGGCGTCCTGGCTCCTGCCGCCCCTGCCCTTCCC
TCCGCGCCCCACGCGCGCTGGCGGCGGCGGCGCTGCCGCCGAGCTGGCGGTGGCCACTGG
GCTGTCCGGCATGGCGGCGTCCTGGTCATCGTCGGCCTTCTCATCAAGGATGTCCTCCTC
GTCCGACAAATTCTCTTCTTCAGAAGACGAACCGTAGTCGAAGTTCTCATCCCAATACAG
CACGGACTCCCGCACTTCGCGGTCGCCGTGCCGAAACTTCCACTCGCAGATGTGGTGGCT
GCGGTCGTCTGACAACACCAGACTCACATGCACCGGCACGCCCCGGATCGGTAGGCCGTC
CGGCCTCACCAAGTCGTGCTGAATGCGAGAGAACGGCGTGGCGTGGACGAGCTGCCTCAC

ATCGTGGTGGATCTCACGGATAGCCACCTCGCCCATGACCTCGTGCCCCGTGAGCAGGAG
GGCCTCCAAGTTCTTGAGCACATAGACGAGGTGGTCCACCCGCAGCGGCGTGGCCCAGAG
CGCCGCGTTCTTCGAGAA
>at01888
TTTTTTTTTTTTTTTTTAACCGCGCCGATCGCCTTCGCACGTGCCCGCAGGCCATTCAC
TGCAGAGCTCAGGCCATCCCGTTCAGGCTCACTTCTTCTTCAGGATCTTCTTGATGGCCC
TGGAGCGTGTCTGCTTTGCCTTCATGAGCTTGAAGCGCTCAAAGTCGGTCATATTTGCCT
TGGTCTCCTTGGCCTTGAGCTTCTTTGCCCAGTTGGTTGCCGCCCACTTTGCCATGATGT
CACCCTTCTCCAGCTGCTGCTTCAGGGCCTTCTCGCGGGCGCCCCGCACGATGCCCGACT
TGAAGTCCGTGAGGCTCAGGCGCTTGATCGGCATCATGTGCCTCTTCACCCTCGTGCC
>at01889
CCATGCATTTCTCAAGAGACACTCCAGGACCATGGCAAGCTCTTAGGGGCAGTAGGTTGG
AGTGATGGCATGGTGCGGGAGCAGCCGCCAGGGACCTGGGCACAGCGAGTGCCAGCTGCC
TTGGCCACGGGCTGGCAAGCAGCTTGAGGATGGCAAGACCTTGTCCGACCACGGCATCCA
GAAGGAGTACCACGCTCGACCTCACGTGCTCCGCCTCGCGCGCGGCATCGACGAGT
>at01890
TAGTTTTCCTTTTTTATTTTTTTTTTGGTTTTTTCTTTTTTCTTTTAAGTTACTCCCCTT
TCCAGTGACGTCTGTGAGACCAGTGCCTTTCTCAACCAGTAGCCATGGAGTCCAACTCGA
TGCCCGTGCGCATTGCGCATGGCTGGGCCATAGCGTGGCTGGACTTCTACCAGACGCCAG
CCATCGTGAGCGAGGGCGTGGTCTCGGAGGTGAAAGGCTTCTTCAGGGGANATGGTCCGG
CCCAAA
>at01891
ACAGGTCACATGCTGAAGCTGCCCATACCCTCAATGCGCGGGAATGCATCATTGAACACG
ATGCACCATGTCAGCAGGCTTGCCAAGGCAGCCACAGGCATCAAGCTGGCTGTCGTCAGG
CCTCAGCAAGACCCTCTCAAGCCGAATCAGGCTTCGCCTAGCCATGGAAGTGCCTTCTCG
ACCTGGACGCACTTGTCCTGCACTTCATTTAGGCGGCAAGCCAACTCATAGTAGTTGCCG
TACAAAACCCGGATAACTGTGGGGCTCGTGTTAGAGAGCACGCCGTTGAATGCAAGAGCA
AACTTGCCTGGCTCCCAAGTGTCGTGCAAAAAGTGGGACGTTGCCGGGTGGTATGAGTTG
AACTCTGACTGCGGGGCCAGCCGCACCTCAGCAGGGTAGATGTCCTGCATGTCGTCGGCA
TCGCCACCTGATGATGCAGCCGTGTCTTCACCCCGGAACTTCTGTGAGTTGTCCTTCAAG
AAGTTCCAAAGGATGGCTGCATTCTCCCACCATGGATGATCTGTCCACACCGAGTCGACA
GGACCCCAGACTTTGCGCAAGAATTCTCGGCTCCAATCAGATGAGCGGAGGAAGAAGGCG
CCTGTGTTCAACATGGCATGATCCTCCGCGACCAACATGTGTATATCCGGATCCAAGCCA
CCATCCCTGCCAACACTGCCGTACTTGTACAACAGCGAGTCCATTGTTACTGTCATGTTC
ATGAAGTAGGTGTCGCAGTCGAACCAAAGCCACCAATCCACGGAGGGGTCTTCGAGGACA
TCGTACATCAGCTTCACTTTGCCCCATGCCGGCGGCCTGCTTGGGTCCAGGCGTTCCCGG
CCAACCCTGTACTGGTAACCGTGACGGTCTGCATAGATCTGATGGTTCGACGTCGCATAC
CGCGGCAGCACGTGCTCCGGAGGGTAGGCGCAGAGGCTCACTATTCCAAGCTTCAAACGA
GGATCACGGAAGTCGTGCGGCACGTCTGGCCACCGAGGCTGATGACCTGCGGGACGGCCG
TCGTCTGCAATCGCGTTCTGCACTGCCGTCATCTCTGCGTTTATCTCCTGCAGGGAGATC
GGCCATGCTGAGTTGTCGATGTATTCCGTGTCATAATGCGCCATCCCTGACCCTGGTGCC
TCGCGCCTTGGCAAACTTCTCCCAGGCATGCGTAATCCATACCATGGCAAGGTGTTGGAA
CCGCAGCGCAACCTGCTGCGCCCGACGGAACGCCCAAGTGCCCCGCACGAGCCCACCATC
GGCACTGAAAGCCAGTGCCAGCACCCGGTAAGCTGCTGCAACTCCCAGCGAGCAGGCAAA
GCCCGCACCCGGGTCTGATGCCAGCCCATCGGTTATTGTGAAGAGCGCGTCAGCCAGCGT
GGCCCTAGCACTCACGAAGTCCCCCTGACTAACTGCATTTAGCCCAAAACTCACATGTGA
GAGGACTTGCTGCCAGAATAGCTCAGCATCCTGGCCCGGCAAAGGCAGCCCTACAGCGCC
GGGGGAGCCAGGAGCATTGGCAGCACTGTGAGGACAGTTCAGAGGGCCTACCTGCCCATA
GAAGTTCTGCATCACAATGAAATATGGCTACTTGAGCCAAAATGGCTACGG
>at01892
TTTTTTTTTTTTTTTTTGAAGCAACTTGTACATGCGGATCAACATCAAAAAGTCACTGC
GCGAGAGCAGAGGCGCATATACATTGAGCAGCATGCGCTACTCCGCCCCTCCCCTCTCAC
CACACGAGGACAATCCAAGGGCACAGCAGCCAATTCTCACCAGAACGTGTCCGGGAAGAA
CCTCTGAATCTCGATCAGGCCGCCGGCAGTCATCACCATCATGATGCCGAAGAAGACCAC
GTTGAGAGACAGGTAGCCCTTGATGCCCTCAGAATCGACCTCCGGCGTGATGATTGAAAC

CTCGTTCCGTGCGCCCGTGCTGAACTCGAAGGCGCCGGTGGCCTCCTGGAGCTTGTCGCC
CGTGGCTGGCGTGGCCGCCATGATGTCCTCCTTCGCCCTGAGTGCGGTCCGACCACGGAG
CCCCCTCTCACCCGCAGCCGCGGGAGCAACGAAGAGCTGGCCGGCAGAGCGCAGAGCCAC
GAGCGCGAGGGCCGCAAGCAGCAGGAGAGGCAGAGCACGTGCCATGGCTGGTCCGAAAGA
AGGACAGTGGAGCCACAACAAAGGCCT
>at01893
CCGTAGCCATTTTGGCTCAAGTCGGCAAGCAGATTGGCTCAAGCAGTTGAGGCGAGCCGC
GCGAGCAACGCCATCATGCGCGCTGATACTGTACCCTTGACAATGTGCCTGCGTGCCAGA
CTGACCATCAGACCTTCTTTGCTGCTGGCCATCGGGTCCATCCTCGGCCCGGCGCGCTGC
CAGAAGCCAGTGGATGAATCTCGCGCCGCTTACGCAGTGAACCCCGATGCTTCAGTGTTG
CTGCAAGGCAAC
>at01894
ATCGTGTACGGCACATGTGAATGTGGAACCATCGTCAGGCTCGCCAAGTGCATTATTCGA
TGCCTGAACTGACAGAGCTAGCAAGGAGATGACTCCAGTTGTTCCAATCCGTGCTGGCGC
TGGCTCCAACAAGCAGACACCCGAAGGAGGGGACGAGGCCGATCTCGGACCTCTCGAGGC
TGATGCCACGGAAAGGCTGATGAAGCAGCGGATGTTTCTGTACCTCGTTGAGTCGACGAA
CTTGGCAGATTTCGTCGTCATCTTGTCGATCACCTGCGTTTTCCTCGTCGCCAAGATCAA
CCCCTTGGAGGGGGCATCGTCTCCTTGGCCGCTTGCGAAAACGCTTGCAACAGGTGCCAT
CGCCTTGCTCTTTGAGTTCATGACAGACGCAACTGTTGCAGTCATCGTCGCCGCAACGAT
GCGTGACAGGAAGGAGA
>at01895
TTTTTTTTTTTTTTTTTGATGGCGACCTGTTCTCTATGTACGAATCCAACCACGTGTGC
TCAAAACGAGTGCCACACATCAGCTCCACTGACGTGGGTGCCATTTCAGGAGCTCCACCC
CACCGGTGCCAGTGCCATGCATCTTCGATGCAACGGCAAATCACGCTACACACAGGCGCC
CACTATCGAGGCAATGACCACAATCCGAAGCCTGAATTCAACAATTAAGATGCGCAAAGC
GCTGCATGCAAAGAGCCACAACATCTGGAAAAGGCCGATGTGGCGTCGCACGTCCTCCAC
ACAATCTCCAAGTCACATTGAGGTGCATGTGCAAGCGCTGCTGCCGGGGTGGACCGTCGA
CCAGATGCTCCACAAGAGCGCCAGCCTCAGGTCTCTTCGCAGGCACGCAGTCCCAAGCAG
ACTCCATGACCGGGCGGAAGACCGCAGGACACTCAGAAGCCTTCGGCCTTGGCTCCATCC
CGCGTGAGAACTCGTCCAGCACTTGCACGGCCTCGTGCC
>at01896
TTTTTTTTTTTTTTTTCTGACGTACATTTTTGTTGGGGGGACACACAAAAGGTGCTCCGC
AATTGCTCACTCAGTTGTTCGACTCCTTCGCTGCACACATGTCACACAGTCCGATCTCAA
AGCGCTCTGCAAGCATGCCGCATCTCGGACATTGCCAAGTGTACGAATGATAGTTCTGGA
TCTCATCGCAGTTCCTCCCTCGTAATTCTGTCAAAGTGTGCATGCCTGCCTTCACAGTGG
CATGATATGAGCAGCAGTCCTTCCGGCTCAGTTTAACGTTCCAAGAGGACATCAGATCAA
GCGCAGAAACTCCTCGTGCTTCGGGATCCGTTAGCTTCGGATAACTCATGGTAGTGCGTC
TAGTAATCGTACGAAGCGTGGGGTCCCCCTGGCTGGACTCCACATCTGACAAGTCGTCAG
AGGGGGCCTGTGGAGGCGTACCTTGTGCACTCGAGCTGTGTTCAATCACCGGCAGACGCG
GAGGCTTCAAGCTCAAGGTCACTGTCGTCGTCTGCCCGGCAACCTCAAAAGTGCCAGAGA
CTTCTGCTTGGTAGAGAATGGTGGCACCATGCATGGGGCAGGTCGGCAAGATCATGCAAC
CATACTCGGACGACACTGGTCGCCGCTGAGCTCTAGTGAGGCAGTACTGCAACCACTTCA
GGAGGTCACCGCGCTTTTTCCCGTTCACCCT
>at01897
CGAATGATTCGATCAGGTAGCCATCCCGCAGGCCATTGACAAGCTTAGCACAGAGGAATT
GGCACTGTCTTCCACGGGCCGTTTCCAATTAGCCAGACCTCCTCGCCATTGCCGGAGCAC
ACTCGCAGGTGGTCCTCATAAACTCACTGGAGTCAAGACTTTCTCTCTCTATGCCTGGCT
GCACTCACGGCATGCCTGGCTGCACTTACAGCTCACGGTGATGGGGGCCAGGCCGACGTC
CACGGGGACACCCACCAGTCATTAACTGGGGGGTGGTTCTCTGGCAGTGAGCAGTCAAGC
TGTGCCTGTTGGTCCATGTAGGCAGAGCTGCTGGCCATGCAGTGGCAGCCCGGGACGCTT
GTCAGAACGTCCACGTTCACGCCGATGCCACGCGACGAGAAGTATGCCTCGAAGCCGCCG
TTTTTGAAGAGCTTGTCCTCCACGACCACAGACTGCTGAACGCCGGCATCGGTGGATAAC
TCCGTCCGGGTCCAGATTCGCATGGGCGGGAAGCAGTGGTCTTCCGGAGGGAACATCACC
GGGAAGCCAAGGGAGGCGCAGCTCGTGGCCGAGACTGTGTCGTGGATGTGCATCGGCAGC
TGCGCTGAGCCCTTGAGGACCCCCATCGCCAAGGTCGCCAGCACGAATGGGCCCTCATCG

CACATCAGCTCCGCGCCAGTGTCCGGGTCGTGGTGGACCCAGGCGCCGGGCTGGCTGCTC
AGCGCGTCACACTGGCTCCCCCGGGCGGCAACGAGCGCGCTGCTGGTGTGGCAGGTGCAG
T
>at01898
TGGGCACCTGCAGGACCAGCACCTGAAGCAAATGCTTCTTGACAAGTGCACGGACCTCCG
CGGTGCATCCGGCACGCTACATGACACGAATGATCAGCGTGTACCGTGGTCGTTCGACGT
TCTCATGCACGGATTGGTGTACTTCTGGATCATAGTGATGCCTTTCACTGAGTTGGCCTC
GACTCGTGACCGCCAGGTCATCGAGAGCACTTCCACCATCCTTCCCACCATCATTTGCAT
GCTCGATGCCTGGTTCTACTACTCGATGCTGAATCTCCTGGAAGCGTTCAAGGACCCCTT
CGGAACTCACATGGACGCATTGAACGTGCAGGCCATCTTCCTGGTGACAGAGACGACCGT
TGTGGACTTCCTTACAAGCCTGCTTCGCGTGCCTTGCGGCCCTTGTGCAAGGAGGAGCTC
CACGACGGCAGGCGGATGAGGTATGCAGCGCTGATGCAAGCATCG
>at01899
CAAGGACAACCTCTACATGTGGTCCCTGTGGATCCCCGGAGACCTCCTGGTCTATGCCTG
CCCCATCTGGATGCGCCTGCCACTGAACCACGGCATCAGCCTGGTCTGGACCATGATCTT
GAGCTCCGTCCGGGGCAGCGAGAAGTAGAGAGGGAAGGTGAGGAAGGCGGCGTATGCATC
CTTCCTCTCGCGAGCGTACCTCACAGTGGCTGGCACAGTCAGCTCGGCGCTCTCTAGCCT
CTCCGCTGTTCAAACGTGCCCGGCATCGCCCTCCGGCGGTCGACGTTGGCAAGTAACGAC
TTGCTCTGCCTCGGTTGGTTTTCCTTTTCGAGCTTCTGGACGTGCGCGGCGTCCATTGCC
AGTTGCATTGCGATTGCCATGGAGGCGGTCGATTTGGCTTGCTAG
>at01900
CCATCTTGGGTCAAGGATCCGGCCCGCACTGGTGCGACCAGTGGCGAGCCCCCCTTGCTC
CACCTCCGCCTCCGCGCGACGATGGCTGGGCAGCGTTGCAGCGAGTCATGAGCAGCTCGT
TTGTGGCGCTGGCGAAGAGAGGTTGAAATCATGAGTCATACATGTACCATTTTCCACATG
ATCTGGAGGCAGTGCTGAATACCCGAGCTAGGGCCAGTCTGATGGAACGATTGCCTGTTT
ACATCGTGGTCGAGGTACCGGTACCGGGCACTGCTGCAACAGCAAGCGCACCTGACAGTT
TCCTGCGTACCCGN
>at01901
CCGTAGCCATTTTGGCTCAAGCTATTTTGTTTGACGCGCATCCCCATTGCCACCCCTGTC
GCACCATGGTTGCTGGGCGGAAGTCGCCATTGCTGCGCCAGGTGCTCGGCCTCGCCTTGG
GTACGGCTGTGGTGTGGCAGCTGGCAGCTGTGCCAGGCTTTTGTGGAGGCATCAGCAGCG
CGGCCCCACGTGCAGCTTCCCCCGTAGCTCAGAATGGCTGGCGTTTGGACATGATGGAGG
TAGGCCCCAAGGGCATTGGACAGCTCGTATCCATGAGGGCTACTTCCTCGGTGAGAAGGC
ATTGGTGCAGATCATGAGCAAGAACGGGTATCGCTACCGCATGCGTGCCACTGACGATGA
GACGGCGCAGGGGATCGAAGTGCCTCCGATCACGCAGATTGGCCCGGTCAAGTTCAGGCT
CTACGAGATGTTTGGGGGGTCCTGCCTCAAGCCAGGCCTGAAACGGCCCGAGGGCTACCG
TGGTTCCGCTGGCGTCGCGGGCTGGGACACAGACGTCATTGTCAAGGGAGCCGGCTTTGC
ATTGTCAAAGTGAGCATGGTCTGAGGGTGGCGCTTTAGTATGTTGCGGTGGTGATGTTGC
G
>at01902
CCATACTTCTCACTTTGCAGCGGGATGTATTGTGAAAGCGCGTTGCTCTCAAATCGCACC
AAACCCTCCCCACTCTCCGTGCTTTGCTTCACCTTCAGCACATACAGGCCTCCTGCCACC
ACGTGAGCATGAGGAATCAAGCGACCGTGAGTCCATGCAGCCTTTAAGCTGGACCAAGCC
CTACTCTCCAAATCGAATATCTCCAATCTGTCAACCGGAGAAAGGTTGACGTCGGCACCA
CCAAGTACGTATAATCGACGGCCTAGCGGCCCAGCTGCACATCCCCATCGTGGTGTTGGA
ATAGGTGGTGACAAGGCCCACGAAGCATCAACAACATGAAGCACTCGACGACGTCAAGTG
GAGGGACAGCGACCTGCATGATCTGTAGGCGTGCCGGTCTCTGTGTGCCGCCGACAACAT
ACATGGAGGAACCGACAAGTGCTGCTCCAAAGTCCCTCCGTGGGGTCGGCATGGAAATGA
TGCCGCTCCAAGTATTTGCTCTGGGATCGAAGCGTTCAACTGAACGCAGCACACTGCCAT
CTGTGCGCGAGCCTCCGACAACATAAAGGCTCCCCGAGGCATTCAAAGCTGCACACTTGA
CCCTTGATGTTGGCATTGGTGCAAGCCTCGCCCATGTTTGCATCTTTAGACTGAAGCGCT
CAAGAACTCCACAGGCTTCGTCTGGGAAGGGGTGTCCCTGACAACCTCCGACAACATAAA
GGCTATCATCCAACACAGCTGCCGCACATGAAAACCGAGCTGTGGGCATAGGCGGCATGG
GAAGCCATGAGGATGCCACAGGGTCAAATGCCTCAACAACGCTCAAAATACGGCCATCAA
TTTGGCCCCCGACAATCACCATCAATTGATCGAAGCTTTTAATGATGTCACAGAGGTGCC

GCATAAGTTGCTCTGGTGTCCGAGCCGTTTGACACAAAGCTCTGACAGCAGCAATACCCA
CTCTTTGCCCAACGAGCAAGGCCAACTCCTGGGCATTCAACACACGTTCCAGCGTCATCG
CGTTGATTCCAATGATTGTGACCACCTTGAGCCAAAAATGGCTAACGG
>at01903
GATGCTGCTTGGCCGAGGTGCATTGGGCTCAACTCGTGTACTGTCCGAAGCAAGCGTGCA
GCTGTTGTGTGCCAACACCCTTCCGGGTGACGCTGAGCTTGTTGGCCCCTTTGCGGTGGA
TGCAGCTGCAGCCGCCGCGCCCGATGGCACTCTGCTTACGAATGCTTCTGTTGGGAGGAC
TGATGAATTGAGTGCAGCCTTGTCTCATTATACCAAGTCCAAGACGCCCGTTGGGGGGAG
ATTCGGAGTGGATCCTTATGAGGTCAAAGCTTGCCGACCCGCCAACAGTTGTAAAGGGCA
TGGATTTGGGCTTGGGCTCGCAGTGGTCACGGATCCAAGGAGGGCAGGCCTGCATCCCAA
GGCAAAAAGGCACATGCTGGTGGCAAGGGATCATG
>at01904
CCGTAGCCATTTTGGCTCAAGCTTAGACTCAATCCCAAAGTCAAGGTTAGGAGCATGGAT
GAACATGCCGAAAGCGCCGGCACATCTGGGCAGGCATCATCTGCTGTAGTTTTTTTCTGG
ATATGCCACATTGCTTGCCAAATGCTCGAGCAGAGGCCCCAGCCTGGCATGGAAGCCTGC
TTCGTGCAGATAACGCAACATTGAGGGATCACATGGAAGGAAGTACAGATGATTGTGCGG
CAGCCTTCAGATTCCAGGGCACGTTCTGTGCGTTCTCCCTCTGGGCAATGATCCTGTTCA
CAGCGGACATAGTCTGGGATAGAAACGCACCCAAGTCATTCTGGGTACCTATCTTTGCAG
CGCATGCACTGTTTGCTGCACTCGTGTGTCTGATGACTGTTCAAAAGACTGACATACAGC
CATTGCCCATTCTGGGCCGGCTATCACCACAAGTGCTCTCTTGGCAGAGTCGGATTGCTG
GCCGCTGCCCTGCTTTGAAGGGCCAGGTGTTCTGCACCGTCGCCTCCATGTACTTCGGTC
TGGGCGCTTTCACGCGAATTCCAGCCTCGGGGCTGCCGCATCCGTGGTTGTCCGTGGCCA
TCCTGAACTCCCTCGTCCACCCCGTGAATCTCCTCTGCATCCTGTACAAGCTGTGCACGA
CCTGTGGCTCGCACTCCTCCGACGGAGGCAAAGCCAGCGCAGAGAAGAAGGACGACTGAG
ATGCAAAGTCGAGAGCGTGCGGCTGCCCTGCAATGGACCGAGTGGTCAGAGGTGAGGGGA
GAAGCAGTACCACTGATGACTCCATGCCAGCACCTGCCTGACG
>at01905
TTTTTTTTTTTTTTTTTCGAGCGGACTCCTGAGCCGCGGTGCGCTCAGGACTCCCGGAC
CACCGGCGGCACGGCCGCCTGCTCCAGCACGAAGCCGATGATCTTGTCGATGAGCTCGTC
CTGGTCTGCGGGAGTGCTGGGCAGCGGCCGGTGGCCGTCGGGGTGCGTGAAGGCCTCGGC
CGCCTCGAAGAGCTTGACCACGTGCTCGGAGACGACCTCCTCGCCCTGCGTGTCGATCTC
TTTGCCCTTCAGGGCCTTCTTCACGCCCTCGTCGTACTCCTCCTTCTCGCCACGGACGAT
GAGCGCCGGCACCGGCAGCTTGGCCTCGAACAGCTCAGGCAGCTGTGCGACGTAGCCGGG
TGCATTGGGGCAGATGGGGACGACGAAGGAGAGTGGCTTGCCGGACCCTGTGTAGGACTG
CGCCGCCAACATCACCGCAAAGTTCGCGCCCTGCGAGAAGCCAAGAACGCCATCGAACGG
GCCGTGCTTCTTGAGCTGGGCCTGCAGCCATGTCAACGTGTCCCTCACCTTCTTGTAGCA
GCGCCAGTTCCGCTCGTCAAACTGCAGCTCGTCGTACATCATCATCGGCTTGCCCTTGAA
GAACTGGGACATCGTGTCAATGGCCTCCTGAAAGTTGCACTGCTTGGGCCCCTCT
>at01906
GGCCGGCAGCCGACATGGTGGCGGGCAGCGAGAGATGGCCAGCAGCCTGGCGGATCTTCC
AGACCATGCCGCCCATGAGCGGCATGGACGGGTGGATGCCGTGCAACTCGGAGACCCGCA
ACCAGACATGCCCAAAGCACTCGGCTGGGCAGGCGGAGGGCGGCAGTTGGTGCAGGATGG
AGCGGACATCGTCGAGCTCGCCGAGGTCCTGCTTGCCTCCGAACTTCGGACTCTGCAGCT
GCATAACGAACCAATCAACCTTGGCACCCTCAGCAAACTTCACTGGCGGGTACATGAGTC
CTCCAGTGAGCCCTGTCTCCTCGACGGCCTCGCGCATGCCCGAGTAAGGCTGTCTGGGTC
GGTCCGGTCTCTGCAGCCCCCCACCCGTCGCCAGTGCCAAGGCATGACTGGCATCACTTT
GAGCGC
>at01907
TTTTTTTTTTTTTTTTATGCATAGCACGTTCATGGCGTGCTGGGAGTCATGCCGTGCCAC
CCTGCTCTCTGCCCGTGTCCCGAGCTCCTCAGACGTCGGCGATCGTCACCTCAACCTCCA
CACCAGGCTCGATTGAAATGGACGTGATCTGCTTGACCACATCCGACGGGGAGTGCAAGT
CAATGAGGCGCTTGTGGATGCGCATCTCCCAGCGGTCGAAGGTGTTCGTGCCCTCGCCGC
AGGGCGACTTGCGCACGCAGAGCCGCAGGGTCCGCGTGGGCAGCCGCACGGGGCCCGCGA
CCTGGAGCTCCTTCTCGCGCGCGCCCACAATGAGGTCCGCGCAGACCTTCTCCAAGGACT
TCACATTACGGCTTGTCAAGGTGATGCGGATGCGGTGGAGGCGCTGCTCCTCAGACTCAA

GGGACTTCACAGGCTTCGTCGCCATGTTGCAGGCTGACTGAGTGGCCTATGTCTCAACCA
GATGCGGCCACCTTGTATACCTGAGCCAACAGCTTGAGCCAAAATGGCTACGGA
>at01908
GCGTGCATGAAGACCATGGTGACCTTGGTGGCCATCTTTGCCCTTTCCCTTTGTGGTGAA
GCAGGCCACGGCCGGACATGCAATTTAAAGGCATTGGGAACCAGGACTTTCATCGACCCA
GTTTACCAAAAAAAAAAAAAAAAAAAAAAAAG
>at01909
TGTGGCAGGTGCTCAGTGCAGTCACCACAGTGCCATTTCAGTTCTAGAGAACTGCGTGTG
CTCATGACCCGCTGGATGGCTCTTGCACACCAGAGTCATCGAACCTGCCAGCACCGATGA
ACTGCACGGTAGATGTCAGCGACGAACTCTGGGTGTCCGGCCTCGATGAAGACCAGGCGG
TGTTGGCAAGCTGCTGTGGGGTAAACAGCTGAGGTTCTGCCAGCAGGGCCATAAAGAACG
CCTCCACTGAAGGGTTGCCTCCACTGCACAAGTGGCGTGAGGCCACACAAGGCCAGAAAG
CTCCTGAGCACGAAAGGAGAGTGTCCCTGTCACTGTGGCACTCCAGACGGGCCCAGGAAA
CCGAGAAAATCCGCATATTCCAGTTTGGCAAATTGCCAAGCGCTGCTGGCGAGGCCCTGG
>at01910
TGCGTGGAGACATCTTGGAACAGGTTTGTCTTAGGCGGTTTTGACGAGAGTCGACTAGGT
TCACTTGGAGCCACATGCGGCGGATGGACTCACGGAGGACGGCAGCGGCAGAGGTTGGGG
AAGCACCGTCAGCCTTGTGATTGGATCCTGAGCAATGGTCAACGTGTGGAGGCCAAGTCG
GCACAGTTCAGCTGGAATGGGAAGCGTTGGCTATTTGACTTCAAAGCGGTGAAGCGTGAC
TTGTTTGATGAATGCCGCATAGTTATGTACTCACTCGTGGCCTTCACATTGTGGACTGGA
ATGGATTGAGGTGGTC
>at01911
CCGTAGCCATTTTGGCTCAAGGTTGGATACTCTTGCACTCATGCTCGCATGCCGTGGTGG
CAACCTCCACTCTTTGCCCGCACCTCTTCGTTGTGGCACACCGCCAAGGGTGGGCAGAAG
GAAAGTGGCAAGAGCCCTTTGTGCAGCTCTGGCGGCAGGCTCGACATCTTCGTGCTGCAT
CCCAAACATGCAGACGCTCGGCATTGGTCTGCCTCGCGACAAGGCAACGCAGGACCAGTT
GTTGGGCGTCGCCCGGGGAGCACACCTGCTGGCACAGTTGGCGAGCTCCACCAGAAGCAC
TTGCTGGCTCTCAATTTGCGGCATGCACCAGGGCAGAGGGTTGTGCCGCGCGC
>at01912
CGAGTGTCGGTTCAGTGTAACATCATGTCGTGACCGCTGCGCAGCAGTAGATGGAGGTTA
NCTTTGTCGGCCTCATTGGCGGCTTCATGCCTGGCCTCTGCTTCGCCGTGCGCCATCTGC
GCTCGCTTCTACGTCTCAGCAGCAGCGCCTGCGTCTGCACAGCCGGCTGGCACCAGGATA
GGCTGGGCATTCCNAACCGTCGCTGCTGTTGNGGTGCCAGTTAGCGCCGGCTTCTTTACA
GGCCACTCCCTG
>at01913
GCTGCCTCCGCTGCTTCTCCACCCCAGGCACCGCGGAACCGTCCGCAGCACCCACCGCGG
AGCCCCTCACGCGCCGCCGGCCTTGACGCCCTCCTCCTCCAGCTCGACGACGAAGCCCGC
CTTCCGCAGCTGCGCGATCGCGCTCTCCGCCGCCTCGGACGTGAGGGACCTCAGCACCGG
AGACCTCCCGCTGGAGCCCCGGATGCGCGCGTAGCAGGCGTTCAGCTCCGCCTCGTCCGG
CGCGAATCTCTTGGCAGCATCTGAGGCCATCTCCACGTAGGCCCGGAACTTCATCTCTGC
GTGGCGGCCGCCTTCGAAGACGGCGTGGTCGCTCTTGACTATGACGATGGTCTTGGAGGC
GCCGGAGGACTCGGCCTCGGCCTCTTGGCGGCTCTGCTCCGCAATCTCCTTCAACTCAGG
AGGCAGCTCCTTCCAGTCCCGCCGCCTCCAGGAACCCTCGCCGCCCTGCGCCGCAGCCTC
CACCTTTCTCGCCTCCGCCGCGAGGCCGTATTCCGGAGCTCCTTGACGTATCCTTCACCA
CGTAAAGCGGGG
>at01914
GCCCCGCCGGAGCGCCAGCAGCTTGGCGGGGCCGGTTGGCGTCCCTGGGAGAGTGCCAGT
GCGAGGGCGAGGTATCCAAGCCACGCATGAGCCACCCTCGTCCAATGCTCCCATCTATCG
TGCTTGAAGTCGTAGCCGAAAAACACCGTCCGAGAGTAATGTGCCATGAAGATGGCAAGA
TAGCCAACAATAGCTACCAATACAGCCACGGCCATGATTGCTGCGTGTATTTGCCGCCTT
GCTAGCTTGCCCACGTTCCACCATGGGTCCAAATTGTAAGACCAGCGACCCACTATCATC
AGGCCAAGGAAGGCAACTGACATCAGCACGGGATGCCAAGGGACCATGGCACTGAAGAAT
ACAACAAGATCCAATGCCACCGCCATGGCAGGTCGGATAGATTGCNGACCACCTGGAGCA
GAAATG
>at01915

TTCCTGGCCAGGGCCATCATATTCTTCTTCAGAGCAATGATGGGATTGGCTGCTCCAGCC
GCTCGCAGAAGGCTGCATACCATGAGACCCTGCCACCACCCCAAAGCATTGTGCTTTGTA
CGACGCCTGGGCCATCTCACCTTGATTGCTGGGTGCCTCTGCTGCTGTCTCGATTGCTTT
TGTTGTGCGTCGAACCCTGCTTCCAGCCTGTGTCCGCGCACTGGCTTTGCCCTCATGCGG
AGGGCACGCTGGGCACTCGCAGGGCGAGGTGATGACTGGAATGCATCACCGGGCCCTCTG
GCACGAGAGGCGCTTGGGGACTTCCGTGTCGAGGCCATGAACCTGTTCTCGAATCTTCAA
AGGAGTGCGACG
>at01916
CTCTTCTTTCGGCTCAAGGAAGCGTGCTGGGCGAACGTGATCCCACTACGCGTGGCGGCC
GTTCCTGCCTGGATGGCCGACCCTTGGGATGCATATCCTGAGGAGGACGAGTTGCGCGGC
CTCCTTTCAGAGGAGCGCGAGTCAGGCCTTTCCCACAATTCAGATCACGTAGCAGCAGCC
AATGATTCTGAGGACCCTCCCGCAGATGCATGGCTTGCTGTTGAGCAGCCCAGCAGGATG
CCTCACCAGCTCCATGCGATCAGACCCCACGATCTGCAGTCGCTGCTGCGTCACGAGCAG
AGCCTGCTGCGCAGTGAGCGCGCCTCCGAGGCGCGCGAGGCCGGTCGGATGTTGACAATG
GAGACCTCCCgTGGAGGAAAGACCCGGTACGCTGATCCTGGACCCCTGCTTGGGCCGCTG
GGAGCTCCTACGAGCCAAGGCGCTGTTGGGAGCGTTTGTTCTGCTCGCCGGGATTGGACT
GGCTGTGTTCGTTGGTCATGCTGGCCATCGCTGGCTTGATCGATGCCTGCATGCTGGTGG
AATCCCGCCCTTGGAGGCAACGCTCCTGATTGGTGTGGCGATGCCTCTGGTGTTGGCCGC
GTTCCTCTGCGTCTTGGAGCACTCCGAGTGGCTCTACGTGCGTCGGGTCTCGCCGCAGTC
TCAGCTCTCGTACACGCCGTTCCTGCATGATCTGTCTCGACGCATGCAAAGTGAGGATCA
CGTTCCAGAAAGCCTGGGCTGCCTTCCAGCAGGATGCAAACNTTCGCTTGAAACGTCACA
GATGCTGTGTC
>at01917
TTTTTTTTTTTTTTTTATCCTGGAATGGTTGGAACGCCAGGACTCGATCAGCTGCAGCGA
GGGGACAGGCTTCAGCAAAGGCACCCCACCAAGCCCCTCAAGATCATGGCCTGTAGTCCA
TCCCGCAGAAGCCGGCCAGCTCTCGGAAGACCCGGTCCCGCATCAGCTTGTGGTTCTTGA
TGGACATGTGGTCAGTCTCAAGCTCGTGGTAGTAGAAGTCCTCCGTGGTCCAGTCCTTCC
ACTTCTTGAACGCGCTCCTGTCCCAATTCACGCTGGTGTCTGGGGGTGAGCCAGTGACCT
TCTTCTTGGCCTCCATCTCCTTCTTCATCTGCGAGTTTCCACTCGCCTTGATGAAGTCCT
CGACGTCCACGGTGTGCCTTGCGACGAGCACGTGCAAGTCGCAATTGAACTTGTGGAAGA
CGGGCTCGGCCCTGGCCTGGCACCGCTGGGCGTGCTCCTCGAAGAGCCGGAGGCCCCGGG
ACCACTTCTGCAGCAGGGCCTGGCCGGGCTTGGAGTCCTTGCCCCCCAGCTTCTCCACCT
GCCTGATGGTGTCCGGCTGGAACGCGACGTAAAACTTGTAGATGTCGCTGCAAAGCATGC
TGTAGCCCTCCGGCGAGAGCGTGGACACGTTCGCCGGCGGGCCGTCGTTGGCGAAGACAA
TGAGGGG
>at01918
TTTTTTTTTTTTTTTTTTGCTGTTCGACGCCCTCGCTCAACTCATCCATGGCTGCGCGCT
GTTCGGGCTTGCGCGAGAAGCGCGCAAGCGACGCAGCGTATGCCACGCACGCGCTGCACG
CAATGAACGCCAGAATCTGGCGTCGGCGGACCTTGGACTGGCCAAGGTCTCGGGCACGCT
CCTCTAACCACTGCGTGTCGCGGTGGTCGGGGCCGTGGCACGTGCGGCTGGCCTCACACG
CCAGCTGTAGCCAGGCCGCGGCGGCTTCGACACGGCCACCGGCAAGGGCGGCCTCGAAGG
CCGAGCGGCAAAGGACCTTGAGGTGGCAGTGAAGGTTGGAGTCCCTCTCGCTCATAGAGC
TTCAGGGCCTCCTCCGATGCCCGCAGTCGGGCAGGCAACGGTTGGGCCGTCGAGCCGCCC
TCGGCACGCTTGAGAACCTCCCGCAGGCGCCTTCGCCGTCTGTCGCTCTCCGCTGCATGA
CCCCTGCAAG
>at01919
TTTTTTTTTTTTTTTTTTGGGGTGTCGCCTCATAACTCTCGTCGCTGGAACTTTCCTGTG
CTGGAACCACTGCCCGCTGGCTCGTGGCCAGGAGGGCGCGGCGCATCGATATCGCACCAA
AGGTGTTGGTGGCCACGAGCAGCAGCAGGCCTCGAGCTCCCCTGCCGAAGGCAACGATTC
CAACCAGCACCGCCGCAAGAGCCAGCCGACCGCTGACAGTTGCCCACAGGAAGCCGTCCG
GCCCAGGGAGAGCGGCACCGCGATAGTACCATGCCAGGAGGGCGCATAGGGAGCCGAACA
CGCGAATCCACGCTGCGGACATGTCGCCGGCATCGAATAAGAGAGAGAAGGTCCGCTGTG
GGAATAGCAGCATATCGAGGCCGATGACGCCCATGTAGCCCGCAGTCAGTCTGGCAGCAC
TCTGAGCTGTAGTGCTCTGCGCTGCCT
>at01920

TTTTTTTTTTTTTTTTTTAAGACGAGACAGCCACACCCCCCTTAGAAATTCAGCCTCACA
GCTCAAACCTGGGTCAATCCTAGAAGGCAAAGATTGCCACGCAGTGAGACCAACTCTCCC
AGGGCATCAGCATTCTGAGATCAACAAGCTAAGGCTGAAAAGACAATGGCAGCTGCTTGT
GCACCAGGATCTGTGGTTCCTCTCCAAACACTTTCTGGCACATTCTCTGAGCGGCCAGCT
CTTGGTACCATCAGTTTGGTTGCCTCAGCGCCAGCTGCGGCTGCTTCCGTCGCGCCTGCC
AGGCCCTTGCCATCCCAAGAGTGCCTGGGCCGCTGGTACCAACGCATCAAGCATGGTGCG
ATCTCCCTGTGACGCCCCTCCTGCATCCATCATTGCCTGTAGCCCGTCCATGAATTGCCC
GTGCTCCGCCCTCTGACCAGGTCTTCGTCTCAGACTGCTCAAAAGAGCCGGCCATTCCCA
TGAACATGATGCTCATGAGAACTCCGCTGGAGCCGCCCATAGTTTTCCCAAGAAGGGCTG
ACAGGCAAGCACAGGTAGCCTTAGGGTCCGAAAGCGGCAAGGCATCCTTCATCTCGAACA
CTTTCATGGCCGCAGAGGCCATGGTTGAGCCGCAGTCAGCATCGTGCCGACCTTGCCGTC
AAGCTCGTCCAGCTGCTGCTTGGCACCAACGAGCGCTTGACACGCCTTCTCCAGCAGCTG
GCCAGCCATCTCGTCCTTGGACGGTGCCACGCCCTCCATGGGGTCGAGGCCTTTGATGCC
CTCCACAGGCTTTGGGAACGATGGCACCACGGCCCTTGGCCACGCCGCTGCGTCAGTGGG
AGCCGTGAGGTATGCTTCCAAGTCCGGCGCCAGCCGCAGAAGGGAGAGCGACACGCCGTT
CATATCGAGGGAGGTACACAGCGCAGCCGGGCCAACAACGAGCCTGAGACTGGATGCCCA
CTTGGACTTCATGAGTGCGCCGGCAACGAGGCACATCTCTTGCGGAGGCACACCACCTAA
GTTATTGATAAGGACGGCATATCCGTTTGGAGCCTCAACCTTCATTCGCTTGCCCTTGAG
AATGCCTTCCATGATGATGTCGACGACCTTACTTGCTGGTGCCAGATCGACCGTCTCGGC
ACCAGGTTCCCCATGTATGCCCAAGCCGACCTCCATCTTCGCTGCTTCGATCATCTCTGG
CTTCAAGCGTCGGACTGAGGAGAACGATACACCAGGCTTGCCGTTGCTTCAATGACAGCA
CTGGCACAGGCCTTCACTTCCTCAAGTGACTTACCGGCCTCGGCCGCAGCTCCTGCCACT
TTATGCACGAAGAGAGTGCCTGCGATACCACGCTCAGCAGTGGTTGCTACATCATCGCAT
GTGATCACAATCTCAACCTTGAGTCCAAACTGTGTCCGGGCACGCTGCGCCGCCAAGGAG
AAGTTCAGCCTGTCCCCGGTGTAGTTTTTGATTACCAGGAGGCAACCCTCCGGACCTGTA
ACTTGAACAATGGCTGTTAGCACCGCATCTATAGTCGGAGATGCGAATGTCTCACCTGAT
ACTGCTGCTGTGAGCATGCCCTTTCCTACAAAACCTCCATGCATGGGTTCGTGACCTGCG
CCACCACCGGATATCACGGCAACGCGGTCTTTCTGCCAATCTGTACGAAAAACAACTTTG
ACGTCGGGGTACGTATCCAGACGACCAAATTTGGTGTCGTCCAAATTAGGCCATCGAGCG
TCTCCTGAACAATATCGCCTTCTCCATTGACAAATCGTTTGGGGCAGGCTGCCATAGTGC
CAAGGCTGGGAGCTGACAACTCGAGGATGCTTGAGCCAAAATGGCTACGG
>at01921
TTTTTTTTTTTTTTTTGTGATGCAAGCTGCTTGATGCATGAACTCCAGGGCCTCATCTGG
CCGTAGTGACAGCAAGCTCAGCGTGCTCCTCCTGCTGCACTGTGTCTGCAGCCGAAGGCA
GCGCGCCGGTGTGAGAAGGCCGGCCCGCTTCGCGCCGCTGCTCTCTCCGGAGCCTCGCCG
CAGCGACCACGGCGCGGAGGCCCAGGGCCAGCGACGCGCAGAGGACCTGAGCCGTGCCCA
GCGTCGCGCGCAAGGGCAGTCGCCAGGGCGGCCAGAGCATGCGCCGCAGCTGCCGCCGCA
GCACCAGCCGCCAGGCCTGCCTCGAGGTCTTGAGCGCCGCAGGGGGCAGGACGTAGGGAT
CGTCCCGGCGCCACTTGCCCCACGGCTTGGCCGCCGCCTTGCAGCGCGGGCCCCACCGCC
GCTTGCTGCTGGCGCTCGGCCGGGCAGCTCGCCGCGTGGCCCAGCGCTCGGCCAGGGCAT
CGGCGGCCTTGCGCCTGAGGGGCCTGGCGTCGCGGGAGACCAGGAAGCTGCGGAGCTCCT
CGAAGAGGTCGATGTCGTAGAGGAAGCGCCTCAGTGCTGCGCCCTCGGAAGTGCGCAGAA
GCCGCCGCTGGACTCCCTCGGTCGCCCCATAGCCGTCTCGCTCCTGGTCATGCGCATCAC
CGTCCGTGTCGCCCTCTTCGCCCAGGAGGTCCATGAGGGCTTTCCAGTTCAGGCTGTTGG
CGGCATGTGCGCGCCCCTCGCGCACACGGTCTCTTGCGCCTGACTCCTCTTTGAGAAGAG
CAGCGCGCAAGGCAGCTTGTCCCTTGCGAGTCTTTGGGGATA
>at01922
TTGTTTTTTTTTCTTTTTTTTTTGAATCGACCGCAGGACCTGAGAACAACAACATCGTCG
GACGAAAATCAAGAAGAGCTGGTCCGATGTCTGCGAGAGCTCAGCCTGGCTCAAGGACAA
AATACCACCTCACCCAAACCAGCAAGAGCCACACCGACCCTTGCACGTGCCTCCACACTG
GAACACCTTGTCCACGACCGCAGGTCTGAACACCACAGCGGATTCATAGTCGATTTCATC
AGGGCAGAGATCTTTGTTGGCCTTGAATCCCTCTATGGATTTGGCAATCATGAAGGGGTA
CTTCTCCACGACGGCGCGCAACGAGCCATCGTTGCGGTTTGCTATTGTTTTGGCCTCAAG
CCAAGCCAAGGCATTCTTGACGCGCTTGGGCGCCGGGATCCGGGGCATTCCCGCAAACTG
CAAGTCTTTTCCGTTCGCAGCCAGTGCCACGCAAACTCCGGCGGCCATCTCAGAGCACCC

CAGCTCCTCCATGAGCAAGCTGCGGATGGTTGTAAATCTGCGAGCCATCTGCTCCTGCAA
CAGTCCCAGACGTGCGGGTAACCCGGGGTCTGCACCCATTATCTGCGCAAGGAGGTCCAA
CTCCGGCGGAATCTGGCGCTTTCCCAGTTTCATTGGGCAAAACTTCTTGATGGCGTATTC
CGTGGTGTCAACCTCACCAGTCCCTACCTTGGAAGTCGCCGCTCCCGCCCATTGAGCTCT
CACGTCAACAGGGGCTCCAGCCCTGTGAGCTGCCAAGCGGATGCGCGTGATACGCGACAC
CTTCAAACCCACAATTGACAGTCCCACTGCATTTGCAAGTCTTGCACCAACACTGGCTCT
CCAACGGCTGGGACCTTCCGGCAACGCTTTCAAGTCTCTTTGCCTTGCAATCTGTGTCCC
TGGCATGGTATTCCAAGTGCGTTGCACATTGGCAAATCCAGGTGGGGGTGCGGCACTGGC
AGCCACAATGTCCATCAGAAGAATTGCACAAATCAGTCCTCGAGCCCAAACGGCTTGAGC
CAAAATGGCTACG
>at01923
GCGTGTGACGAATCCGCCCCCTCGATGTAGCACGTCACGTCGAGGTCAGAGCCCCGCGTC
GCGAAGCCGTTCGGAATGCCTCCGAAGGGCTTCACGCGCCACTGGGGCCCCAGCACGAGC
GCCGTGAGCCGGAGCTCGCTGATGCACTGCTTGACGAAAGCGAATTCCGAGTCGGCCAGG
GCAGCTCGGGCGAGGCTTGCACTCAATGCCCGGCCGAGCTCGTGGACCTCCAGTCCGAGC
ATGCTCGCGGCATCGCCAAACCGGCGGGCTCTCGTGCAGGCGGTTGAACTCCTGCACGGG
GCCCGGCCAGGCGCCGATCGGCAAGCGGTCATTGACATCTCGATTCCCTGACTTTACGGT
GAAGTGCTGCATCCCGTGCCCCCATGGGTCAGGACGGGATGCCGCGGTAGACGTCCAGTT
GAGTGCCATGGCCACGCGGCACTCAACAAGCCACGAAGTGGCTTGATCCTGAGCCAGATT
GGCC
>at01924
GGCGACCACCCTGTATACTCATCACTTGTTCCTTGCATTAGGCACTGCTACTGGACAGCC
TCCAAGCTAATCCTCGACGATATACTCCTCCATGATAGAAGTCCTCTTGTAGTATTTGGC
CTGGAGCCGTGCGTGCACCGTGCGCACTTTCTTTGCAGGCTATCCCGGCAAGGAGCTCCA
AAGCTGGCACAGCAGGTCTAGCTTGCAGCACGGATGAGCTCCAATTGACATTATACTGCT
ACTGGACAGCCT
>at01925
TTTTGAACTGGGCCGCTGGCATCGGGAATCTCGTCGGAAGTGAAGCACCATGACGCTACG
TACTCATGCCAGGGTACCACATCCTCAGGGACGCTGTCGCACGCAGACCAGAAGCGGCGC
TTCGCAAGAGCGGGACGGGCGACTGTTCCACCTCTGCTCTGTAACGCCACCGGAATATCA
GTGCTCCGAAAGCAATCCTGTGAGCTCGAACCGCCGCGGCAACATAGATGCTAGCCCCCT
CTTTGTCTAGCATGAACTTGCAGAGGGCGGCGTGCGTCGCATACATGCAGAGCGCTTCGT
CGAAAGGGGGGCAGTGGTGGCAAGTGAAGGCGGCGAGGCGGCGCCAAACAGCGAGCGCCG
GTGCAAACCGCTGCAGTTCCATGAGGGCGTGTGCCTGCACCACCCGCAACTGCTGCGCAT
CCGACCCCAACAACATTGCCGGTGCCGAGTCTGTCTCTGTCTCCTCGCTGTCTGCGGACT
CGTCGTCTTCGCTGCCCACCTCCCCATTGAGGGCTGCTTCTGCAACCTTGACGCGCCTGG
CGGCAGGCAAGTGCGGAAGCCTCTCTTGTAAACGTTCGTCGAGTCGACGGAACCGACACG
GAGGTGGCGGCCTTCCTTCCAACAAGCTGGCACTGCATAGCTGGCACCCGCAAGTGAAGT
CNCGGTTAAGAAATTCTGCCGCATCGGTGTTGGCGTGCACAGGAGCTCCGTTTCGATGTA
GTTGATGCAGAGCTCTTCGCCGCCCCGGACGCACGGTTCGTCACGAACGCACAATATCGC
CGAGGGCG
>at01926
CGAGCTGCCGGACTCGCACTTTGCAGAGGCCCTTGGGAGCAGTCGGAAGTGCCTCGGATG
GCCAAAGCCATCCAGACTTGCGGCATCCTCTCATTGGTTGGTTCGAGCAGCACGTGAGGC
AGGAGTTGCAGTCTATGAACCAGAAGCTTGACGCGCTAGTGGAGCAGGCAAGCCCCCGGG
AGAGGGGACTTGAAGAGCAGGGGTGACTTGAGGAGCAGGCTCCACGTGCCAGCGCGAAGG
GACAGGAGGGCGCAGACTGGGTCCTCCGTCGTCGGGCCGCCGACAGTATTGTCCCAGACC
TCGGCTTGCACTTGAAGGACTCTGGACAGAAGGCCCCCTGCGGAGCACAACAAGTGCTTC
CTTGCGGACCAACGGAAAGTCGAGCGGCAAAGAGTCGAGCCCCATGTGTGGACTGGAGCT
GGTCCTGTCGCCCCCAAACCCCA
>at01927
TTTTTTTTTTTTTTTTTGAGATGTCCTCGAGATGCAATCCAGTCTCAAGAACCACTTGT
CATGTTAACAGCTCCCAAACTCGGCATTCCAAACAGAAGCCCAGCCCGTCGTTCAGCCAA
GCACGCGCACCAGTTCCCAGTAAATCAGTTGCCCTTGCCCTTGCCCCAGCCCTTGGGTGA
GTTTTTGACGTACACGCGGAGGGTGCTGCCGTCCGCGAGCATGGTCCCATTCAGAGCCTC

CGCGGCCTTTGCCGCCTGGGCCTCCTCCACAAAGTCGACCCAACCAATGCCTGTGCACTG
GCCCTCGGGCGTCAAGGTGGCCTTCACCCCCCTGGGCGGGATGCCGCCAAAAGGGCCGAA
GATCTCGTGGAGGTCGGCGTCAGTGGTGTCCGCAGGGAGGCCCTTCACGTAGAGCTGCTG
TGCGTCGCTGCGGCCCTTGCTGCTCTTGCCACCAGGCAAGATGTTCATGTTCATGAGGCT
CGTCTTCAGCATCTGGATGGAACCACCCTTGCCACCGCTGTCGCCGCCCTTGCCACCGTA
AGGGCTGGACCGCTCGCCCTTCCAGCCACCTCCTTGGCCACCCTTCCCACCCCAGCCTCC
CCTCGGTGGATTGGCAAACTTCACGGCGATGGGCTCCTTGATACCTTCAGGCATATTACC
ATCCAAGTTGTCTACCACCCACTTTGCCTCGTCAACAGATGCAAATGTTACTATTGCAAC
GCCAGGGCTGGTCACCTTGACCTCCTTGATAGTGCCATAAGCCCCTAGTGTCTCCTTGAG
TTTGTCGGAGTCAATGTCTTTGGGTAAGCCCGTAATGTACGCGCGTTCAGAAGGAGGTGC
CTCTCCCATTGCTTCCGTGAAAGCTTCCGTGAAAGTTGGCAGCGGAGGCAGCTCTGGGCC
AAAACTTGAGCCAAAATGGCTACGG
>at01928
TGCGACAAAGGGAGCATGGACGTGGAGGCGATCCCCGATGACGATCGACAGGCGCGGATC
GCGGAGTTGGCGAGGCGGCGGCGCGAGGGGCGGAGCACAAGAAGGATGGCCCCGACGAGT
TGTGGTCTGCGTGGGCGGCGCATGGCTGTCTGGGCCACAGTCTGAGGCATCCGCCGCAGG
GGCAGCTGCTCGCGCCGCGAGGAAGCGGCGGCTGGCTGAACTCGGGAGCGCCGAGGCTGT
GGCGGCAGCGGAGATGGAGAAGGAGAGGAAGAAGGAGAAGAAGGAGAAGAAGGGGAAGAA
GAAGAGTAAGAAGGATAAGTCCAAGAAAAAGGCCAAGAAGGCTTCTTCATCCTCTTCCTC
GGAGAGTGCCACCCCTTCGGCAGAGAAGGAGAAGAAGGAGACCAAAGAAGCGGTGGAGCT
CGCGCAAGCTCTCGCGCAGGATGCGGCGCAGTCGAAAGCAAGGAAGAAGCTGGAGCAGGA
CATTCTTAACAGCTACGACCGCATCACCAAGACAGAGGCCGAAAAGGTCCGGCTCGTCTG
GCCCAAGTGCGGCTGAGCTCTGCTGCTCGGTGAGCGCTCCCAGGACCCCTAGTTCCAAAA
AGCCTTGGCATGCTGCTAGG
>at01929
TTTTTTTTTTTTTTTTTACGATCTCTGCGTACATGTGCCTAGGTTTGCTGTTGAGCGCT
ACACTAGGAATCAGGCTGATCTTCGACTTGGCTCCGAACTAAAGGCCAAAGTCGTTGTTA
GCCTCGCTGTACAGATGCTGCTTGCGGTGTGATCAGAGGCAAGGCCTCAAGACTGCTACC
CACAACAGCTGCGTCCCAGCCGTCTGCGCTCAAAGACGAGTACGTAAGCAGAGGAGCTGC
GAATGCAACACGTTGCAGAGCCATGGCGTGCGTCACAGAAAAGTTGTCAGACTGGCTTCA
GTGGATCATTCCCTGGGGCAAACGCATCGAACTGCTGCCTGGCACTGCGCGATTGCCGCT
GCATGCATCTATCCCAACACCAAACTCCATCCGCCCCCAGCGCGCCTTGCTTGTGCAAGC
GTCTTCTCCATGATAAGATCAATGAGCAAAGGCTGGTCTGCAACCGCCATGTGACCCGCA
TTAAGCACTTTGACCCAACTCAGCTGTCCGTCACTACTCTCCTGAAGGAAGCCAAGCACG
TGATCAGTACCACCTTTCCATTGCACTGGATCAGCTGCATGGAAGCTGCCAGCTGTCTCC
CCTGTCAGCAAATCAAGCCAGGCCTGCGTACCCAAGAAATTGCAGTCTTTCGCATCATTC
AATCCTGAAATGACCAGGGCCTTCAGACCGCGATCCAGAAGCTCCTGTACAGTCTCCTGG
TAGTTATCGTTCACAGTGCTCCTGTACAACTGTGCAATGACGTCCGACGACCAAGATTCC
ATGGGCGGACCACTTTTTACATGGATCGCCCTTCTCACGTCCTCACGATTCAAGTACTTC
TGGACGAGATTCCACTGACGGTCAT
>at01930
TGGAGGCCTGGCGCTCGAACTTGAGACGTTGCGCGATGGCATCATGCGCTTGTTAGACGC
TCCGAGCGAGGCTGCGGCCGGAGACGCGGTGATACTCCACTCGCCGAAGCTCATCGAGGC
CCTTCACGTCGCCTCTGCAATGTCTCAGAACACAGACCTACTGGAGGGCGACCGAGGCCT
CCCTGCTAGCCTTCCACCGGACTGCGACCTGAGCTGGGATGACGTCACCATCATGATGCT
CCAGTGGCTCCGCTGCGCCATCGCGTGGCAGAGGAGCCGGACACTTATCAGGAGCAGGCC
CGGCAGCGGTCCAAGA
>at01931
TTTTTTTTTTTTTTTTTGCGCCCAGAAGGCCATGTGCATCATCTATCTCTGCTCAGGAC
AAGCCGCTGGTACTGCGACAATTGCAGGTCTGCGCTGACAGGCCGCACAAAGAGGAAGGA
GGAGGTCCTAGCGCCCGCATGGCAGTGGGCCCCAGCCGCGTGATTTCACGACCTAGCGGC
CGGCCGCGGCGGACCTCTGACTCACGGTCTGCCTGGATGTCTGCCAAAGTGCTCTTCGAG
AGCGGCGTGATCCCATGGGAGAGGTCGAGCGTCGGGCAGCGGCTCTGTACCGGGGGCCTC
TCCCCAGTTCGCGGAAGGCCAAAGACCGTGGCGAAGACCGCAAACTCGTCAGCGCAGCGG
TCGCCATGTGGGCCGGGGCCCACCATCACGCTGCTCCACCCGACGGCCCAACGCTTGCCT

CCCAACCGTGCTGTCCACTCAGCCTCCTCGTTCACTGGAACCCAGTCCTCTACGAAGCGC
TTGGCATGGTCACGGTTGAGCACCACCCACTGGGAGTGCTTGACCAAGTCGACGCGCACG
CCATTGGCTTTGATCGAGTCCCACTCGCTAGGTTCCCGGAGGCAGAAGTCAGACTCAGGC
GACGTGACCAGCGCTGCGCGTAGCACCGTGAAAGGCTTTGCCGGCAGGGTCGCCTCATCG
ACAA
>at01932
TCAAGCCAATTTGGCTCAAGCTTCTATTCCAGCGGTCTACGTCACGCACTTGTGCTTTAT
TTTGAGGTTTCCTCATCCTGTACGTGCATGTCCATCTGGGCTTGGGCCGGTCACAATGCT
TTACGCATCGAAGCTGCTTCTCTTTGTGGCCAATGCCTTTGCTATCTGTGGTGCGAGCCC
ATCAAGTGCCCAGCGGAGCACTTGTGCCGCGGAGGGCGGGATTTTTGGCACATCATTCAT
CCAGAAGCCGCTTGTCAAGGATGTAAGTCTCATGAACACTGAGAAATGGCCATGCCCAGG
GCGTTCCCTGAAATGCAAACGCAAAGACTTGTGTGAAAAAGTGGCCCTCACCGATACTGA
TCCTGAAGCCAG
>at01933
GACACACGGCCAGCTGACCTTATGATTGGGAGGGGCAGCCTAATTCTGCCTTTCCAGAAT
TGTAGAAACAGTTGATTTTCCAGTCTTTTACCAAGCTGCTTTCCGAGAGCTGGTACGACC
AGCGGGAAGCAAGAACGCTGTATAAGCTTACATCAAAATCACCCCTCCAAGCTGCCGTGC
GACTTCCGTCTCAGAGGCAGCTGGTGTGACTAGCGAGGAAGAAGAACGCTGTACAAGGAC
TGTATATTCAGTCACCTCCTCAACAATCCGTAGAAGACACGGAGGCGGCCAATAACACCG
CGACAGCATGTCAGAGAAAAGGTTACCCCCCACCCATCGTCACAGTGCGCTCAGAAAGGC
ATCTGCCACAGAAGCGCTCCTGTGCTGGTTACTCAATAAGGCCCCGCAGCCTGTTCCACA
TTGCCCTCAGTGCCCACGCTAGAACAGCTACCAGACACATACACTCCCATCACGGCTTCG
GTGTTCGCACTCGCGCGGGCCTTCAGGGCCNNCTGCGCCGCCTCCNTGTTCTCGNCCTTG
CCCATCCAAGTGACGATGCACGTCTTCTGCAGNGCCTTGCCGTACGAGAAGGACAGGTGC
CAAGGCAACTTGCCCTGGTAGAGCTTATGCATTGTCATCAAGTTGATAGTCGCTGTCTCC
TCATTGTCCTCNTCNTTCGCCGTCTCGNCTGAGAGGAANAAGATGCCNGGCATTNCAGGN
GGCACNGTGCGGAGCAAGGCCTGCACtGTGTAGGCCGCCACCGTCTNNTGGNCCNCCCTG
GGcCCCNGTCAGGNCNTTCTTGACCATGTTCGGcTTCAGGACGGCAcCCTCCAAGTAGAN
NNTGTGGAGGGCCAgNCGCCtGNA
>at01934
CCGTAGCCATTTTGGCTCAAGCCCAAGATGCTCCCGCCCGACACCTGCACGCAGCCATGT
CTCCTGATGCCAAAGGCATCTGTTCCACCCTTTGACAGTACCTGGCAGCGCCCTCAGCCA
CAACCCATCGAGCTGCGGGGCTGCTGGCAGAGATGAGCGCTTCGACCGGACGGGAGGCTG
CTTCTCATCGGTAGCCTCGGGAAGGTGAGAGGCCCCAGTGCTGAGATCGCGAAGTGCTAC
TGCAAGAGGAGCGGCAGCGTCAGTGGCTAAACCCAGGACGAGCGGTATCAAAGCAACATC
GTCACAAAAGAGGTCTGCATGCGTGCCGTTCTGTTGGGCCAAAGTGGGCTGCGAAGTCCT
CTTGGCATCTCTTGACAAGATCTCCAGCCGTAACGCCATCCGGAACAAGAAGCTGTGATG
CGCTTACGCAACCTC
>at01935
GCAGCGCGCCGTGCACGACTACCACAGTCGACCTGACCAGCGGAAGGTGGCGCCAGGCTG
GCCGCGAGGTCTCGCGCGGAGCGGCCTCTCCTCAGCCAAAGGGGGGAGGCCTGCTGGCGG
TCTTCGTGAACTCCTGGCTCGACGAGCAGCTGTCCATGGCGGCGCCCGCGTTCGGAGACT
ACGCGGTCGCGCCCTTCCTGGGGGGCGCCTCGTACCTCGCTGACCGCGCTACCCTGGAGA
CGACCGGCAATCCTTGGGCGAGGGCGCTGGAAGCAAATCCCATGGCCTCACCGCACGACG
CCCATGTGTTCTGGCTCG
>at01936
TTTTTTTTTTTTTTTTTTGCGTGCAAGTCATGGCACTTGTCCCCTGAGCTTCAGGGCACT
TGTCCCCTGAGCTGAACCTCGTACCTACCCACCGAGGCCGTGGGACAAGTCAAGCGATAC
CCTCGACTGTAGAGGCCGGGGGCATGGATAAGGATGTCAGTTGCCAATTTGATGAACTCG
GCCAAACTGATCAGTGCAAACCATGTCGTTCGGTGCGTTCTGTGCCTGCGCACAGGCGTA
CTGGAGCTCGTTCATGAGCTGCAAGAACGACGTATCCTGTCCGTTGGAAGAGAAGTAGGC
AACATTCTCAGTCCAGATGATGGGCTTGAACCGCCAGATCGCCTGTCGCGCACCGGCGAT
GACCTCCTTCTCCATACCCTCGACGTCGATCTTGATGAGGCGCAAGTCCTCAACCGGAGG
AACGCCAAGCAAAGCTGTGCTTTGGGTGGGCGAGATGAGGAGCTCGTCAAGACTCACAAC
GGTGATGAGCTCCGGCGATGAATCCCAATCGTACAGCTGCATGGCCTCCTCCGTCTTCAG

GCCGTTCTGCTGGTTCTGCAGCTTCATCCCGCCGGGAGAGGAGAAGAAGCGCAGCTGCGG
GGGGCGGGCCTCGAACTGAGAGGTCTGCACGCCCAGTCCAAGAGGCAACACCCACACATT
GCTGAGGCCATTGATTGCCACATTCCCTGACACTATCTGCTGCAGCCATCGGAAAGGCTC
GAAGGCGAGCACCTTGCCGCGTGTTCCAACTCGCTCAGCAAAAGGCACTGTGTAGCAACC
AAGGTTTGCGCCCACGTCCACAACGACATCCCCCAAGCGCAGCAATGACTGCATTTCGTA
AGCCTGCAAGGGGTTCATGGACCCATGCTGCTTCATCCAGTGGCCAATGTTGG
>at01937
GTGTTCTCGCGCAGCTCCGCGAGCCACTTGTCGAGGCTCTGGAACGACTGCTCGTTGGCG
ACGTCGAAGACCAGCAGGGCGCCGTCGGCCGCCCGGTAGTGGTGCGTGGTCACGGACTGG
AACTTCTCCTGGCCCGCGGTGTCCCAGATCTGCAGGGCGTACTCCTCGCCGGTGTCCTCA
TCCACCAAGTGCTGTTTGAAGAAGGCAATGCCGACTGTCGCTTTTGACTCGTTCAACAGC
TTGTTGTGGATGAAGCGGTAGCACAGGGCAGACTTTCCAACCGAAGAGTTTCCAATGAGG
ACGATTTTCATGTCACAAGCTGCAGAAGGGAACTGAATCGGGGACCAC
>at01938
TTTTTTTTTTTTTTTTTGAACGGATCACACCTGCACAACTGTCGTGCTGACCGTGCGCG
CAGCCCTGCGCACACCAGTAGGATACGAAGAGTCAGCCAGGATACCGCACATGCCACAGA
CAGTGATGCTCTTCGGGCAGGCGCACCAGCCAGTCTTGCCGCAATAACCATCAGTTGGCT
GGGTGTCTACAGCGCACGGCTCCTTCTGCCCAGGCGCAGCTCGCAACAGGCGCATGTAGC
CCTGCTCGCCCCACTTTGGGCCATAGGAGTTGCGAACGAGCCAATAGTTCGTGCCCTTGT
CTGCACCGTAGCCCACAAGGCTCACCTGGTGGTTCAGGTCAGCGCCGCAGCCCATGCCCA
ACTCTCCGTCGAAGATGCCACTGTCATAGAGCATCCATGACGTCGCAGCGACGGTCACTG
CAAGGGGCCCCTGTTGGACCAGGGCAGACAAGAGCGACTGATCATCGTTTGACGGCAACC
TCACGAAGCCCCCAACAGCTGCAAAAGGTGTGCGCACGAGCGCTTGGTCGCAGGTGTGGC
GGCTGAAGGGACCGTGCTTGTATGGGTAGGCCGCCTCAGTGGTCACGCCACTCTTCACCG
ACAAGTTGAACGCCTGCTCCGCCGTGGCACCACCGCAGCCGCCACTCCCCCCACAGTG
>at01939
TTGTTTTTTTTTTCTTTTTTTTTGGCCATGGGCAAAGCTATGTACACCAGACGCGCGACG
AAGCTGCCGCCCTTACTACAGCTGTTAGCATCATGTCTCCCAAGAGACCCCCTGTCAGCA
GCAGCAGCCGCCAGTCTTCGAGTGCTCGACGGCCACAACTGCAGAGCACAGATGCCCAAG
CAGAGGACAGGCCATGTCACTTGTCGAAGCGTGCAAGGCTCCACACCTGGCAGCACACCG
TCCTGTTGGAAGCAGCGCCGACAACGGGTTGTTTAGGCAGCCTGCCCTTGCAACACCATC
TGAGTCTGCCGCAGGCTTGGAGTCACTGATCGAGGTGGGAACTTCAGGTCCCGGTGGAGT
TAGGGGGAGCTCTGCCTCGTCCTTCTTCCGCGATTCAGCGCAGCCGAAAAAAGCCCCCAA
GCGGCTGAAGAAGCCGTCCTGATACGCCTTGTTGCCAGCATATGCAGCGCTGCCAGCAGC
CACCACTGGCAGCGCGACTATGCACACGGGTCAGGCCATCCTTTTACCCAACGCGCGCTG
TCAGTAGGGGTCGTGAGCTGAAACAAGCCTTGAGCCAAAATGGCTACGG
>at01940
GCTCCTCTTTGTGCTTCTTGGCCGGGCGCTGAACTCCTCCGTAGCCTGCTTTGCCTGCGA
CGCGCAGGACAGGCTGGAGGGCACGAGGAACGTGCAGTGCAACACACCATCAGTTCGCGT
GTCTGCTGGGAGTTCTGCAGGACCAGCTTCGCCATAACTGGCAGCACCTTCAGCACAGCG
TCTTGCACCTTCTTGGACTGGGTGGACTTCCTGCCCTTGCTGCTGCTGCCTGCGTTGCTT
TCGCTCTTGGACTGCTTGGGACCAGTCCTGCCAAGATCTATAGAGTCGCCTGTATCTGCC
TTCCCCCTN
>at01941
TTTTTTTTTTTTTTTTTCGCAGTCGCCAGGCACCAACCGAGCGGTTTGGGGCAGTTCTC
GGCCCTAGCTCGCCAGCCCCTCCGCCGCCACGCGGCTGGCCCCGCCTGTGTGGCCGGCTC
TCTCCACGACGCGGGCGACCGCAAGAGTGGCCATAGTGAGCAGCAGGGCTCTCTGAAAGA
TGACTGGCGGCCAAGCGCTCGGCATGGATGGCCTCCTCTAGGAGGCGGAGTCGCCGGCCA
GCAGACTCTTGGCTCTCGAGGAGGGCCGCGGCCTGGCCCTCCAGGGAGGCGTGGGACTTC
CTGAGCGTCTCGATCAAGGTGCCTTGGGCTTCTCTCTCCGACGCTCCTGCACAAGCCGGT
CATTCAGCTGGTCCAGAGTGCCGCGAGCCATCGAAAAGCCCTGATCGTTCAACGATGCCA
AGGCACGAAGCTCCCGCTCGAGGACGTCGAGCCGGTCCACCAAGCCCGTGGCCCGGGAGT
CGGCGGACTCGGCCCGGCCCTTGCGGCGGCCGGGAGAGAGGTCCTCGCAGTTCTCCAGCA
CACGCTCCACTCTCACCCTGAACTTTGACATGTCGCTCAGCTCCCCTGAGAGACTGTCGC
AGCGCCGGGCGAGGCTCAAGCTGCGAGCACTGATGTCGTCGGCGTGGCTCTG

>at01942
CTCTTCCGAATGATCCACTCCACCGAGGCCCGAAACATGGTGAATGCCTCTTCCACGGGC
CACAACCACGTCTGCCAGCCTGTTGCAACCTCCAGAGGTTGAACCTTGCCTTTGTCTGCA
CGCAAGGCAGTCCTTTGACAATGCAAGGCCGGACCTAATCTTGCACCTGCGCAAAATGCC
AAGCTAGCTACATTGGTCTGGTCTGTTCGTCGTGCTGCATGCGAGTATGTGGAGGAGGCG
CAGAACGTACGTCTCTTTGCCGTCTGAAGAAGAAGAATACAAATCACCAGGCACAGCCAC
TCTCGCAAGCCCGAGGAAGCTTCAGCCATGCTCCCCGTAAACGGATCCTGTGGCAAAGTG
CGAGCCTATACGGCCTTGAGCCAAAATGGCTACG
>at01943
TTTTTTTTTTTTATTTTTGACCGCTCCCGCCGCAGGGCTTCTCACACTNNNNNNNNNNNN
NNNNNNNNNNAGCAACTCGTCGCTGAGGAAGCCCTTCCTCTCCTCCTCGCTGTAGAAGGG
CCAGGTGTCCGAGCTCATTATCAGCGCGATCTCCTCTGGAGTGTAGGTGAAGTTCTGCCG
GAGATTCTTATAGAACGGCAGGTACCAGCGCTGCTCCGCCATCCGCTCTCTGGCGATCTG
GAAGTCGGGGATCAGCTCGCCGATGATGCGGCCCCAGTGGTCGATGTCCTCATAATGCAG
GAGCAAAATGTTCAAGTCGATGCCAAACACAGTTGAACGTGTCTGGATGAAAAGGCGGCC
TGACTTCCGGTCGAATTCTTCCGCATGTGCCGACAAGTTGAAATGCACACCTTCCCTGTT
GAAGTGGCGATCGAACCAGTTGTAGGGCACATCGTACAAACGGTGAAATTCGTGGAAGTC
TTCAAACAGCGTTGCTGTGTCCCATTTCAGAATGTCTTCCTTCTTGGCGCCCTTCAGCTT
CAGCACCTGCTCGAAGTACATGGACATTGGCTGAACGAAAGGGTTGCGCGTGACGCAAAT
GATCCAACACCGCCGGCCTTTTGGCACATGAGCCAAAAAGTCCCTCGTGCC
>at01944
ATGCCCGGGCATGAGCAGGTGGCGGACCAGACGGAGGACGCCAACAAGAAAGAGAAGCAG
CCGAGGCAGGAGAGAGCGCCCAAGGAAACGGCACAACGGCAAGATaGTaGGNACAgAAGA
AGGAAAAGGCGCCAAAGCAGGAGAGTGCAGTCAAGAAGGATAGTGCGGCGAAGCAGCAGC
ACCATGCAAAGAATGATACCACGACAAAGAAGGAGAGCGAGACCGAGAAGGATCATTCGA
CGAAGAAGGCTGATGCAAAGAAGAACGAGAGCGCGACAAACACGGCTGATGCGACGAAGA
AGGAGAGCACGACGAAGAAGGATAACACGACAGAGAAGGCTAA
>at01945
CCACTTTCGGAGCGGATAGCTGCTCTCGAACTCCTGATCGAGTTGCCGGTGGCTACTCAG
ATCTCTAGCAGCACAGGCTCCGCTCCTGTAGCTCCACTCCCGCTGTCTCGCGTGCAGGTG
CTGGTCGGACAGAACCTTCGCCCGTCACATCGCTTCAAGCTGGCCAGTCTAGCCAAGCCT
GACAAACCAGCTGTCAGTGCAGTATTTTTAAGGCAGGACCTTTATCCTGTGATTGTATTA
ATTTTGAGAAGCGTCCGGCTTCCCCTGTACACACTGTGCAGGAAGGCTTTAGGCAACCA
>at01946
TTTTTTTTTTTTTTTTTCACCGCTAATGTGCTCGACAATCTAGCTCTGTTACCATGCCC
TCGGTGGTGAACAACTGCAGTGCTGGCCAGATGGCCGCCTAGCTTGCAAGCATGCTTGGA
ATGCTCACCCCGCCCACCTGTATGTGACCCTATGTACCTCTGTGGGCACGCTTCGGCCCT
GGCTAGCAAAAGATCGTGGGGCGATGGACCTATCATCCAAAGCACAAAATAAAGGAAAAA
GTCAGCACATCCAGCTTTACCCAGGCAGTTGCAAGCACCCTCCAAGGCAACTGTGCTCCT
CCCTTCCAGTTCCAATGAGGCCAAGTCACTCAGTGCCACAATGCTAATGGAGTGCCCATT
CGTCCATTTCGCATGTCGCAGCATTCAAGGTGCCCGAGCAACCGCGCCATCCTCCATGCA
TTTTGCATGTCAAGAGATCGCCAAGGTGATTCAAAATGCCATCGTCGACTTGATGGCATC
ATAGACCCGCAGGGGCGTTGGTAGATAGAACTCCTCCCAGGCGAGGGGGAACGGCGTGTC
CCAGCCGCACACGCGCTGCACCGGCGCCTCCAGCTGCAGGAAGCACTGCTCCTGGACCGC
CGCGGCGAGCTCCGCCCCGAAGCCGCTCGTCCTCGGGGCCTCGTGCGCCACGATGCAGCG
GCCGGTCTTCCGGACCGAGGCCATGACGCAGTCCTCGTCCCAGGGGGCGATGGACTGGAG
GTCCACGACCTCGCAGGACAGGCCGTCCTTCGCCGCCATCTCCGTCGCCTTCAGCAGGCG
GTGCACCTGATTGCCCC
>at01947
ACGTATACGGTGCATCCACGTGCACGTAGACGTCGTCGAAGCATGGCCGCCCCCTGCCGG
CGCGCTCAGAAGGGCTCCCCGGCGTACTGCTCGCTCCGCGGGGGCGCGTCCAGGCCGCGC
CTGCGAGCAACCTCGGCGTAGAAGCGGCCGGACGCCTTGATGGTGCGCTTCTGGCTGGGG
TAGTCCACGCGCACGATGCCGAAGCGGAGCCGGTAGCCCTCGGCCCACTCGAAGTTGTCC
AGGAGGGACCAGACGAAGATGCCTCGGACGTCCGCGCCCTGCTCAAGTGCGGCGTGCACC
GCGCTGAGGTGTGCCTGCAAGAAGCGTACCCGCTTTGGGTCATCCAAGGTCTCATTCTCG

AAATCCTCCGGCTCCGAAGCTTCCACATTGTATGGCTTTGGAACGAGGGACTCTCCCAGC
TTATCCTCCGGCTCGAGCGCGCAGCCGCACTCGAGTGATGAAGATGCCGGCGGGAGGCGC
GTAGCGCCGCTGGATGTAGAGGAGGACCTCCCTGAGGCCCCAGGGCACGATCGGCCAGTG
GCAGTCAGTGCGCTCCCAGGCCGGGTCCTCCTTCACTGCGACCGACACATCAACTGCGAA
GCCACCTATGTGCTCTCGAATGTAGTCCTGGTGCTCTAAGCAGGAGACACACAAGCCAGT
GCTGTAGCTATTC
>at01948
TTTTTTTTTTTTTTTTTTACAAATTTACATGTAAAGTGGAGATCGGATCAGCGCTCCTCC
CTGGCAGCGCTGCTGTCTCCTCAGGCTCAGTACCGCGGCAACGGCGCGAAGCGCGAATCC
AGGCCCCCATAACCGCTGTCTCGAACTCCCCGAGAGACAATGGGCTGGAGCTGCCGGATC
CGGTCCTGGAAGAGCTGGAAGTCCTCGTTCCGCGGCTGCGCGAGCGACCTGTCGACTGCA
TAGACGGAGAGGGTGCCCTCGCTGTCGCCTGTGACGAGCTTCCGGCCATCGATGGACCAA
TGGCAGCGGTTGAGCGCCAACTTCCTCTGGTTCGGATTCTCGCAGCGGACGATGGGCGTC
TCCAAGTTGGCGTTGAGGTTCCACAAGTCCACGTGGCCCTCGCCATCAACGGTGGAGAAC
GCCGCGGGGTGCGTCGGGTGCCACCTCACGTCGTACACATAATCCTCGAAGGAGTCCAAA
GACAGGATTGGAGATTGGTGCTGCTTGACATTCCAGAGCCTCACACTCCAATCGAAGGAG
CAAGTGAGTGCTAAGTCGTGGGAGGAGTCCACCTCGTGCTGGGTCAAGTGACTGTGATTG
GGCCGGATGTCCATGCCAGCGACAGGCCCCTCGTGGCCGTCGTAGAGCTCTGTAACACCG
ACCTTGGAGCCGTGTATATGGACCTGGCAGACAGACCCATCCNTCAGNNCCNACGTACAG
CGTGTTGGTCTCGTTCTCGGGGAAGGCCAGGGTCGTCACCGCGAGGTCGCGCATCTTCTT
CGTCTCATTTTTGAGATCGACCGTCTCTTGCAATGTCACATTCAACATCGCCAGAGACCA
GACGCAGAGGCGGCCGTCGTTCGAGGCCGTGACCAAGTTCGTGGCGTTCTGCGTGCCCAC
CTGCTGCATGGCCTGCACGGGTGCGAGTGGCCTTGCCGCTGAGGGCGTGCGAAGCA
>at01949
TTTTTTTTTTTTTTTTTTGGCGCGCACAGCCGCTTAAGGCCAACCTCGGAGTGCGTGTTG
CAAGGCACGGCCGGCTCAGCTGAGGGCATCGGGCTGTGACTACCCGAAAAGGTCAAAATC
CATATCCTCCTCTTCTTCCTCCTCTTCGACCTTCTCCTCCTTCTTCTCCTCCTTCGGCTT
GTCCGCAGCAGCAGCGCCTCCGCCAGCCGCAGCACCGGGCGCTCCGCCGCCGCCACCGCC
CCCGAATCCCTTGAGCTTCTCCTTGCCTGCGGCGATGACCTCGTGCACTGCCTTGCCCTC
CAACTCTGAGACCAGCTTGCCGGCGACGTCTGCGTCAAAGTCTGCACCCACAGACTCCAG
GATCTTCTGGATGTCATCCGCAGTGGGGCTCTCCGTGCCGCCCAGCACGGACATGGCGTA
GGCTGCGGCGTACTTCAAACCCATGATGGCTAAGATACTGAGCTGCCCTCCCCTTGGTTG
CTCCCGCCCGCCGACCCTCGTGCC
>at01950
ACCTGCTGACTTCGTGGAGCAGCTTGAAGACCGCCGCCGCTTAGCAGCGGAGCGAGGCTG
TCCACTGTTCACAAACGGCAAGGATAACTCGTTTGTCCCGAAGAAGTACGAGGACGTGTT
CCAGGTCATTCTGAACGCGCGGTCCCGCAACTATAGCGGCGCGAGGCTCGACGACAAAGC
GGTGCAACTGCTCTGCAAGGTCATCTGCAGCTCCAAGGTCGAGGTCCTGAATTTCGGACA
CAACGAAATTGGCCGCGAAGGCCTCGGCGCAATCCTCGAGATGCCTTGCTCCGGGAGCAC
TCTGCGGACCCTGCTCTTGTACGAGAATCGCCTTGGCGAAGACTGCGTCCAGCTGATTGC
AGACGCCCTCCCGCGCCTCCGGTCGTTGTGCAAGCTTGGACTGGAAGCCACCCCACTGAG
CGGCTCTGCCGAAGCCAGAGCAGCACTGCGGAGCGCTTGGGCACTTGCTGGAAAGGCGGA
GCGCGGGCTGGTGCTTGACTTCCCTTACTACCCGCCTCCTGAGCCGGACGCGACGTTTAC
GCCTCCTGGTAGGTCGGGGAGGGTCAAGCCTGCGAAGCAGGCGCAGCGGTCGGTCATGTA
TGGACCCAGAATAAAAAAAAAGAAAAAAAAAACAA
>at01951
TTGTTTTTTTTTTCTTTTTTTTTTCTATTACTGTCCGTGTTTTTCCAAGATTGTCACTTC
TCTAAGTGCCTAAGAAAACTGGCAGCTAGCATCTGCCCATTCCACCAAATGCAGTGAGAA
GTTCACATCCGAACTCATCAAACTGCCTCGCTCAAAGCTCATCTTCGCAATGCGTTTTCA
TGAGTCACGTCCGAGGTTGCGTATGTGACGCAGGTGAGGCAGTAGCCATCGCCCATCTGG
TCATCGTCCAGGAAGGCCTGGTCGGACTGGTCGATCGAGCCGGACAAGACCTTGCCGGCG
CAGCTGGAGCAAGAGCCGGCACGGCAGGAGTACGGCAGCTCCAAGTCCCTAACCTACCTC
TGCTTGATCCAGGATGTACACGTCCTCAGGGCACTCGAACTCCTGTGCGCCATCTGGGGT
ACTACCAAACGTCACCTTGAAGTGCGCCGCGACGCCTCGGCGGGCCGGCACTCCACTGAC
AATGACAGGCAGAACCCCTGCATGGTGTCCTGCAGCTAGTGCACCGGAGGTTTGCACATG

CTGGCAGTTGCGGTGCACCGTCTGCGACGTGACGTGTGGTGTGGTGAAAGCAGCGCCAGT
GCTGCCGTTGGTTGCAAGCAAGCTGTGCAGGAGGAGTGCACAAGCCGCTGCAGCCAGGAT
CAGTGGCAACACTTTCATCTTTGAGGCCATGGCGCGGGGTCCNGATCGCAGAANNtTGcc
CTgGGGCTGCCTTGGTCGACAACANTGATTACTTGAAGC
>at01952
TCCTCCCAAGGGTTGAACTGTGCCATGCCGGGCGAGGACCACGTCGGGCGTGTCTGGGCA
TCACTGCAGCTTGGCCTCACGTAGCCCTCTGGCAATGCATGCGAAGCGAAGCCAGGGTGG
GTTGGGCTTATGCCTGCCATGTAAGCGGAGGCAGCGTCACCCTCTCGCCAAACGCAGCAA
GGATCCCCGAGCTCCTCACCATTAGTGAAGCCGTCACCATCGCTGTCTGCGCTGCATAGG
GC
>at01953
GGCCTCAAGCGGCAACGGAACGTCTGCGTCGGCGTTCGGTGCCATTTGCCAAGACTCACC
TGGCCTGCGCGGCGCCGCGTCCACCACTCGCACGCGAAGCCCGGCCCTTGCAGCCGCAAG
ACCGGCGCCAAGCCCTGCCAGGCCGCTCCCCACGACGACAATGCTCGCTTTGCCCTCGGT
TTTGGCAGCTGTTGCTGTCCGACCACTCGCTCTGCACCGAGAACGGCAGCGCCGGTGGCG
GTGGTGGGTCCTGCCAGCACAGACACCCACGAGCATCACCGCGCAAAGCGGACGATCACC
ACAGACAGAATGTCGTGAAGAGAGCTGGGACTTTGCCAGCAACAGCCCTGCTGGCAGCGA
GGACCGCGACTGCCCAAAGCCAGCATGCGTCACATGAGTGCGGCGAGGTGCTGTCTGCCA
GTCCAAGGCAAGCGCAAGCAACATCGCGGGCCACCGCACGTGACGTCAAGGCTGGATAC
>at01954
AAGGTCTACTGGACCGACAATGGGAACCTGAACATCAGGCGCTACAGTCTAGACGGATCT
GGAAGTCCGGAGGAGGTTCTTGGCAGCATCGGCGACTCTTGCGCCATGGCATCCTATGAA
TTTACTTCCACAACTTGGACAACGACCACTTCAAGTACCACTACTACTACATCCATGACC
ACGGTCACCACGTCAGTGACATCCGTGACTGCAACAACAATAACCCTGACAACGACGAGC
ACCTCCACCACATCCTTCACATCCAGCACGACCAGCACCACAAGTACAACTAGCACAACC
AGTACCAGCAGCATTACAACCATCACTAGCACCAGCACCT
>at01955
TTTTTTTTTTTTTTTTTCCACGGCAGGGGATGGCCAGCGAAAGGTAACTAGCGCAGAGG
ATGTAGCGGATACTCCCGCATGCAAGTAGTGCCCTCAGAGGTTGAGCACTGCTGGTACCA
GCGCCGCGAAGCCACCCGGCGCGCCACAGTCGAAGCCTCGGCAGTCGTCGCGCAGGAGCT
CCGCGCCCAGTTCCGGGGCCGAGAGCCAGCGGTCCAGAAGCTCCAGCAAGGTGAAACCGC
CAACGCGGACCCAGAACACAAGGTTGAACAGCGTGGTGCAGTGGAAGTAGCAAGCGGAGG
AGAAGACCGCTGAGCTCGAATTGGCCGACAGGATGGGCTGGATGTAGTCAATCACCTTGT
GACGATAGCCCTCCGCCCAGGCTTGCAATGATTCGTTGACCCTGCTCTTGGGCAACCACC
AACTGCTGTGCACGTTCCAGTTGATTGCGAACTTGTCGTACTGAGAGTGGCTGAGGACGT
ACGGCGTGTGGACAAGCGGCAGTCGGTAGGCCGGGAAGATGCACTTCCAGCGCTGAGCGC
CAGGGTGCAGCTGGAGACACTCCTAGCTGAAGAGCTCCGGGGCGGTGTAGAAGATCGCCG
CCTCTTTCACCTGCTCCTCAAAAGGGGTCACGGTGGGGCCGTAGGGCTTCAGAGGCACCC
AGAGTGGCGAGTCCAGGAGCCCAACTACACGCGCACGGCCGACCAAGGAGGCGGCCACGC
GATCCAGGTGGATGAGGGCTCCTCGTGCAACAGAGCTGCAGCCACCGAAAACCACAAGGT
CGTCAGCCCTCTCCCCCAGGCCCGTGCGCCGCTGGAGGTCCCCGAACACCGCGTCCACGA
TGCGCTTGCCACGGTAGTGTGTCCTCAGGCCCGTAGTGTACAAGGACGGTGAAGTGTCAC
CCAGGAAAGCGTCGCTGCTGCAGCTGACCACGTAGGCCGTGTTGAATCCCGCCAAGGAAC
TTCTCTCCAGGTCGAAAATGCCCCTCATCTTCAACGCCCACCCACGGAGCCTCTTTCCTT
CCCGCGGAAGCAACTTGGACGTGAGTTCACGCAATGGCCTGGAAGAGCAAGATTGAACGT
CCCAGCACCACATGCCGCCTTGGAGGAAGACAAGCCACCTCCTCGCTCCGGGGCCTCCAG
GCCGGTAGTAGTAGGCAGCGGGCGAACCGTCGCCGCAGACGGCCAGGGGGTAGTCCTCCA
GAAAGCGTAACTCCCATCGCTGGTCTTGTTTGGGACTGGCAGCCAAGGCCTGCAACGCCC
ACACTGCTTTGAGTACTACTCCTCCCGGCATGCTGCCGCCACAGGTCCACCTGTCATCTC
GCCATCCTCTCCAAGTGCCAGTCTTTCATCTCTGGCATGGCAGACCTCGACCGATAAGTG
TGCGAGAGGAACTGAAGACAGCAAGCCGTCAGGGGAGCCATGAGCCAAGAGCTTGAGCCA
AATGGCTACGG
>at01956
GGCCAACAGCCGCTGCGTTCACCTCTGCGTCAGCCTCCACAAGTGCGTGGACCAGTTCCG
CGCTGTCTCTGTGCGCAGCGAAGTGGAGAGCACAGAAGCCACGGGTGTCCCTCGCGTTGG

CATCGGCGCCAATCCGCAGGAGCATCTGTGCAGCTTCTGTGTGTCCACCACGGCAGACTG
CGTGCAGCAAAAAGAGCGAGTCCATGGCTTCCATGTCTGCCCCCAGCAATGCCAGAATCC
ATCGGCGCTGACCTTGCTCAGCGGCACCTGCAATAAAACGCCGGAGATGCTGGTTAGTGT
GAAACCATTCGAGAGGCAGAACTTTGAGCAAGGCTTCGTCGCTGGCTCGCACCACCCTCA
GCACCAGCGCACTCACATTGCCCGTAAGACGCCATTCCCAAGGCCAAAGTCCACTCAGAA
TCCTGCAGAGAAGGTCCTGGCGAAGCCTTGCAAAGTTGGCTTCACAAGAGGTAGTGACAA
CAGCCATAATGCGGCTGTGCTCGGATCCAACTTCTAAAACCTGGCTTGAGCCAAAATGGC
TACGG
>at01957
TTTTTTTTTTTTTTTTCTGTCCACATCTGCACAAACAATGAACAAAGTCATGTGCCACGG
AACCAAAGGATGTCTTCAAAAAAATCATGCAGATTAAGTTGCAGCATATGCAACCTGCCC
GTTTGAACCTGCGTGGCTACTCACAAATATGTTCTGGAGTCCGTAATGAAAACCTTACAG
TGCTGCAATGGCTGCATCCATGAACTTGGACTTCTGCTTCTCCGAAAGATTTGAAAGCTC
GCTGAGCCCTCTTTTCACGACCTCCTCCTGGATGTATGCCTCCACCTTGTTGTTGGCACC
TTCCTGCAGGTCGAGAAGGGTCTTTTCAGCGGCAGCTGCCGCTTTCTTGAGGGTTACCTC
CGTTTGCGCCTTAATGGTCGCCTCAAACTCTGCCTTGTCGCTGTCAATGCTCGCGTTGAT
TTCGGCAATGACCGAATTGGCCTGGGCCTGTGCCTTCTCAGCTTCTGCAAGACGTTCCGT
GGCCTCGTTGAACTTGGTGATGGCGTCCTCAATGTCCGTCTGGATCTCCTTCTCCCGTGC
TGACATTGACTCACTGAGCAGAGGGGCCAGGAGGTAGATCAGGCCGGCGATGAGCACTAC
AATGTTGATGATGCCTGTCTCAGGGATGTCCAGGTTGAGTGCAAAGCCGCCTCCCAAGGG
AACCTTCCCAAAATTCAAGAGACCTTCCTCTGCAGATGCGGGCGAGGCACCTGTCAAGGG
CGAAGAGACCGCAGCGGCCACAACCCNAGCAGGAGCAGCCCTGGCTGTGTGACTCTGTGC
TG
>at01958
AGGGGCGTGAACACCACGAACCTGGCCGGGTGCATAAAATCGTTCCCGCACCATGAAGGC
CTCTCGGAGCCATGCTTAGCGGCCTGGACTGGTTTGCTTCACAACGTCGTTAAGGGCAGG
AGCGTGGACACCTTTCGCCTGTGCCTGGACATCTGCGACGGCGTGGACACATGCGTTGGC
ATGGAGAAGTGCGTCAAGAAGGCGGCTGAATGCTCACGTTGCATCGAGCCAGCACTCTAC
AAGTTCAAGCACCAGTTTGGAGGTCATCTTGAAGTTCAGCCTGCCGTCGTGCAGCCCGAC
CTTTTCATGCGGGTGGTCATGGGCTTTGAGAAGAGATGGGACAAGGATTGATGGAAACCG
TGGCAGGGCCGGCAGTCTTATTGGACTTGGAGCAAGATGATGTGATCCAGTTGGTTTTCT
GGTCCAAAAAAAAAAGAAAAAAAAAAACAA
>at01959
CCCCCGCACCCATGCGCAAGATGCTCCGAGAGGCGGCCACCCGCGGCAGCTGCCCGTTGC
TACCTGGCCTCCGGAAAGGCAAGCAGCAGCTGCGCCTCACTTGAAAGGAGAGCCCTCATA
GCGGGGCGCGGGAACATCCGCGCGATGAAGCGCACCGTGACGTAGGGTCCCTCGGACACT
CCCCGCCACGCCGTCCCCAGCTTCGCGCCCATGCGCGCGAAGAGCTCCAGGTCCCGGCCG
AGCAGCCGCGCAAGGCTGGGCCCCCCGAGCAGCTCTA
>at01960
CCGTCTAACTCCTCGAACACCCAAGTCCCTGGTAAAGCCAGCGCCGCGGCCGAGAATCAA
TAGCTGTCCCAGCGAGGATTCGGCTTGGCGTGCCGAATATCTATTTTGTCCTTCAGTGCC
GCAACGGCTGCGCCTGCCGCGGCAGGCTCAGCAAAGCGGATGATGGCCCCTGTTCGGCCC
ACGCCAGGGCGCCCAGGAATGGACCTTCAGGCCCAACACCTCACCGT
>at01961
TNTGTNTNTNTNTNTTNTTNTTTTNTTTTTNTTTTTTTTTNTTTTTTTTTNTTTTTTTTTNTT
TTTTTTNTTTTTTTTTTTTTGTTGCTGCAGCTTTTACTAAACCAGGCAAGTGTTGGTCGCC
AGGACTCTCGGGCTGTACACAGGTCCCGGTCAGGCACAGAGCATCTCTGCGTCAAATGTC
AAAAGATGAGTGCGCATCATCTTGCTGAAATTGCAGTGGCCATGTTACATCGGCGTGGCC
CCCAGCTTTCTTGTGCAACCTCAGACGTATGTCAAAGGTTCCCCAACCTGTACATTGGAG
CTGGAAGGGCAAGGTCTCCAGGCTAAAGTGGTTCTGCTGAAATGTCGGATGGAGCCAGAC
CTCCACTCTGCTCAATTGATGGGCTCCATCGCCAGCCACATAGAGTCGCCATGCATGCTT
GCCATCTCCAAGCAGTTCGTGGGCATTGCCGACGACGACTCTCGCCTCCGAAGTCTCCAG
AACCGTCTCGACAACATCTCGTGGGCGCGAGTGGCTCCTGCTGGTGTTGGTCTTCGCTCT
GCCTCGGAGCACTGAGCGCGATGGGCATCGTCGCCGGGGTGCAGCCTGCATGGCGTCGGA
ATCGTCCGGCTCTGCTACCGCCAGGCTGAGTGCAAGCAGTTCACCAGCAGCCTCCCTTTC

CCGCTGAGTCGCCACTTCCTCGAGCCTCTTGGCTGCATCGCGGTACGGCTTCGAGACGTA
GGCGGGAATCTCGCTCAGTGGCAAGCCATCGCGAGGCCGCCAGACTGGAACCGGCATCGG
CTCAAGGCCAAGACGTGCCAGCAAGCCAGCCGAGACCGGATCGCACTGGGCCCAAATGCG
AAGGGTGCACTTGCCGTCGTCGGGCGTCTTCTGCAGATTCACGATCACTGTCCTCTTAGC
CTGGAAGGGAAAGTGAGTGATGTGGCGCAGAGACATTGAGGTCCCCATGACAATGCAAAG
ATCGGCTTTGGAGCAATGCAACTCTGCCTCCTTCCAATCCCTCTCGTCGAGGAACTCCCC
AAAGTTGATATGCGTGTCCTTGGTTCCAACATTCGCATCACGGGT
>at01962
CCGTAGCCATTTTGGCTCAAGCCATTATGGCTCAAGCCATTGGTTCAGGAGCACCATGTC
TATGACTCAGGTCAATTGCGACCCCCATGCTCGCCCAGATGGAGCCACCACCACCACAGG
CGAGCTCATCACCGAGATCTTCCAGACCGCGTCGGAGCCACAGAAGAGCATCGAGATCCT
TCTCAAGCTCGTCACAAACGCCATGGCAAACCCCAAGGAGCCGAAGTTCCAGGAGGTGCG
GAAGGCCAACAACGTCGTGCGGACCAACATCGTCGGCGTCAGCCCCTGTGCCACGCTCCT
GCGGCGATGCGCTTCGTGG
>at01963
TTTTTTTTTTTTTTTCTCTCCCACGCGCGAGGCGCCCATCCACCAGATTCCCTCGGCCG
GCCACCTAGCTGCCGGCGCGGCCGCCGGAGCCGATCTTGGAGCCGAGGTCATCGCCAGAG
ATCGGCGGCAGGCCCTCTGGATCCTCGCCACGGGCCGCGCGCGCTGCCGCCTTCAGGGGC
GCCTGGATCTCGCCCTCGAGCCTGCCCAGCCGCACGGCCGAGAGCTCGTCCAGCAGCCTC
GCCGTGATGTTGTCCTCGATGCCCGCCTCGCCGTCCCGCTTCTGCTTCGCCTCGAGCATC
TTGATCTTCTGGTCGAGCAGCGCCAGCTTCTCCTCCCTCGAGAAGTGGCCGTAGAGCTGC
GCGGCGCGCTGCCTGTGGCTGGGGCCCTCCGCTGGCAAGGGCTCGAAGCGCTCCTCGTCC
TCGGGCAGGACGTCGTAGGAGAGCTCCTGCACCTCCCGGTGCACGTTCTCCACGTGCGGG
ATGCGCCGGGACTTCTGCTGGGGCCAGCCCTTGGCCCCGCCCAGCTGCCGCAGCAGGAAG
GGCACCGAGACGTTCCTGCTCAGGAAGAGCCCCGAGGCCGTGATCGGCTCCGAGGCGCCA
TCCCTGGCCGCGGGGCTCGGCGCTGAGCCCACGCCGGCGGGTGGCGCAAGGGCCTCCTCA
AAGTTGTCTGCGGAGGCGATGAGCTTGGACAGATTCTCCATTCGGCCGAAACAGTAGAGC
TTCCGGCGCTTCAGCTTGGAGCGCCGCGCCATCTCGATGGGCTCGTTGACCTTGGNCTCG
ATGCGCTTGCGGTCCTTC
>at01964
CTATCCACAACAGGAGTAGTTCTGAATATAAGGATCTGCTCGCTAGTAGCAACTCCCAGT
TGCTCCTCCACAGCCACCTTTATTTCACGTACTGCCCAAGACGCATCAGCCATGATGGTG
CAACTGACTCCCGTCAGCGTAGTCACCCCCACTTCCAGCAATGCCATGATGAGCACCCAC
AGGAGACTACATTGCCTGGTTTAGACTAGTTGAACATGTGTGGCCCGTCACATCCGTGTC
GGAGTCAATGCCAACCTGCAGCCAGGTGGATGACGGTTGCAGGCAGGAGAGCAAGCAAAC
ATTTGAAATCGCGGGAGGTGTTACAAACACTTTTCCCTCAAGCCAAGGC
>at01965
TTTTTTTTTTTTTTTGTAGCCGACACATGCATTCAAGTTTCACCCGTACAATTCATGCG
GCGTTGCGCAGTACACATGCTCTGTACATCGGAAAAGGAGACAGTCTCGATGCTGGAAAG
CCAACAAACTCACTGAGAAGCCTCAGGCTTGAACAGGAATGAACTCAGCTCCCAGAGTGC
CACTTTGCCATCGCTGTCCACGTCGCACAATCTCAGAGCATCTTGGAGCTCTCCAGGCCC
GAGAGTATCTTGGAAAACAGCAGTGAGCTCTGCCAGATCGACATCTCCATTCCCATCGGC
ATCGAGCTGGTCAAGGAGCTTGCCCAGGCCGCAGCGCAACTCTGCCGGCTCCATAGAGCG
CCAGTCAAAGGTGGACGCCACAAACTCAGTGAAGCTCAGCTGCCCACAACGGTTGATGTC
AGCCGACTGGAAAGCGGCACGGGCTGCTTCCGAAGTCAGCCCCAGCACCTTCGCACTGGC
AACAAACTCCTTGAGGTTCACATGGCCCGAGAGATCCCTGTCCAGCTCCTGGAAGACAGC
CCAGCAGCTCGCACAGTGGTCCCGCTGCAGCCTCGTGCC
>at01966
TTTTTTTTTTTTTTTTTGCTTCCACTCAACGACAATCACTGCCTATCTGAAGCGGCTTC
GTAACCGGGGAGAATCACCACTAGCAACCTGTACACTGTACACTGTACAACTTGGCCAGA
TGGGAATCTCCCGGGAAGACCATTGTGGCGTGGACCACGCTCAGGCATTGTCGACGTCGA
AACTGCTCTCCAAATATCCCCGGTGGCGGCGCACTGCCCCCCCAGACAACGCATACACGC
TAGGAGAACTTGTCCAATTCCTTGCCCAAATAGTCGTTGGCCTCCGGGGCCTTCTGCTTC
TCGGGCTTTGGGCGCAGCTTCCAAAGAGGAATGTATCCCTTCTTGACGGAGTCCTGCAGG
CTCATGAAAAGCAGCTGCGCCAGCTCCCTCCCCTTGAGGGGCTTAATGTTCAGGTTGGTG

AAGTACTCGGCCCAGCTGTTCAGCCCATCACCGTGCATCATGCTTCTCATCTGCTCCTGC
AGCATGTCTACCTCGCTGGGCTTCGGTATGCCCTTCCGATCCTCGTACTCCTCCCTCGTG
CC
>at01967
TCAGGCATACCGAGTCTCAACAGCGTCCGTGCATCTTCCTGGGTCCGCCTCCGGGCAAGC
TCCTTCACCTAGGGCCTGCCGTCAAGAGCCGGCCCCCCTCACGCTCCGCGAACCGCGGCT
GCCACGGCGACCGTGAGCTGCAGGATTGGCCTCCCAGAGAACCATCTCGATGTGTTGCAC
GGCCTGACTCGTCCCANAAATGGCAAGGTCCTTCCGACGGCCTTGAGCAAGAAGCCTTGG
TTCCTCGAGCTCCG
>at01968
TGAGATGCCCCAGATCACCGCAAAGCTTTCCCTAAAACCCTAAACTGTGAAGTGGGAAGA
ACCACCAGCAGCCAGCCTAGCACCCCAGCGCTGACAACAATGCACTGCAGTTGCCAACGA
ACGGCAAACGTGGCGACCGCATAAGCGAGCCCGTAGAGTGCACCGTACAGCCAAGTGTGC
GCCTGCGGATAAGCCGGCCGCAGAACTGCCACGAAGATCCAGCCACTCGAGATGCCGCCC
AACACCTTCAAACGGTCCCCAGGAGCAGAGCCTGGAGATCCCAACATTGACAGTACAGCC
TGAGTGCTCGCTCACAGCCGAAGAGAATGGCAAACCCCAAATAGGCAAGCAGAAGCTCGC
GCGAGGCAAACTGGAAGACATACTGGCAGCTCTCGGACGAGGTCGACTTGGGCGTTGAAG
TGCTCGGGGCCTTTGCCATCCATGGCTCAGGACCCAC
>at01969
TTTTTTTTTTTTTTTTTGCTCTGAATTCTCGTTGCTGACACCGGAATCTTGAAGTCGCA
TGTGGTGCACCAGTCAGCTACTGAGCTCGGTCCCGATCTACGAGTTCTGGGGGACGACTG
GGGTGCCACGTAGATCGCCTGAAGATTGGCGGCCGCTGCTGCCGTGCCCTTGCGTAATGC
TGTAGAACAGCACCTGCACATGCTCGAGGCTGTTAGTGTTCTCTGTTTCGCCGTCCTCAT
TTTCGTAGGCCTGCCCCGCTAGCACCCTGCAGCATGCCCTGATCTTTCCCTGCGGCCTCT
TGAGAACTACGCGGCAATCTGGTGGGGCCGGCTCGCCATTCCGCTGAGCATTCAGAGCTA
GCTGCAACCAGGTCATGAACTTCTCATGGTGCCGCCTCCCCAAGAGGTCTGCTAAGGGGG
TACCTGGGGCCATGCGGCCAATGCGCATGCCAAACCCAGGAGAACAGGATTCGAAGGGAA
AGCCTGTCTGAGTGCAGTCCAGGACAGCCACAGCAGCGGGTTGCTCTAACGAGTCGATAC
TGTCCGATGATGAATCTGCCAGGTCAGGACCCTGGACGCTTGGGAAGCTGTCCACAGCCT
GTGCCACGAAAGAGACCATCCCTGCTTGCCGGTGATGCCGCTGG
>at01970
CCAGATTGCCACGCGAAATGCAATGGACTACACTTGCAAAGCCCAAGGTTTGTTGTTCCA
AACCTTATCCGGCGCCTGAGCAGGTCTAACAACTGCTAGGACATGTTCTCACAGGCTTTG
TAGTGCATGCCCACTCTGAGCCCCTGATGGACCTCCCCAGCCACAGGACTCTCGGAGGTT
CTTTGCCAGTGCCTGAGAAAGTGGAACAAAAAATCTCCACCGGCCTGATAGCCTGCAATG
GGAGGCCCCATACTGCTTCCACCAACTTTCGCTGTCGCCTTGTCTAAAGTCTCCGAAATG
CGCCGGCTGATGTCATCCATGCGCGGCCGCCTGTCAGCGGCCTGtAAGAGAACAGCTCTC
AATCCATGAGCGCCAATCCGAAATAAGAGAGTTGCAGGTCATGGGCCATGGCAGGGGAGG
CAAAGTGCCGGACCTGATCGTCTGAAGGATCAGCTGCTTGCTGGAGGTGCTAAAAGGGAA
GCGGCCCGTAAGAACGAAGAATGAGAGACGTCCGAACGAGAAGACGTCGGCGTGGACCTG
GGCCGGTGTGCCTGGTTTGAGGACCACTTCCGGCGCCACCCACATAGCAGTGCCGCTCAG
GGGTCTGGCGTGCTTTGTGAGGACCCGCGAAAGGCCGAAGTCCAAGAGCTTTGCTCGGAA
GGAGCCAGGATGATTAGGGTTATGCTCAATCATGATGTTTGTCCCCTTCAAGTCCCCGTG
GACGATTTGTGGCTGTCGTGAGTGCAGGTAGCACACTGCTAAGCAGGTGTCCATGACGGC
TTTGAATTGGTCTGAAACTCGCAACGGGCAATTGGGCATGCCGCGTTGAAGAACGAACTT
TTCCAGACTGGGACCGTGGACCCACTCCAGCACCAGAGCAACACAGCTGCCTTGGAGGTC
AAAAGATGCCCATAGAACGAACAGACG
>at01971
TTGTTTTTTTTTCTTTTTTTTTTGTTGTCATCGCCCGGCACTGCTCCTCATAGCTCGTCT
CCCCGGCCCGCCCGACGCAACATGTCAGCGATGTGCTTCCGGCTTTCGATCTTGGCCACC
ATGTCCAGCGGAGTCTCGCCATCCGTGTTCTTCGCATTCAAGTCCGGCTTCTGCCGGAGC
AGGAGGCGGACTTCTTCTTCTCCGCACGGGTTCATGTTCACGTACCAGTGCAAGGGGGTC
ATCGACATGGTCTCGCCCGGCTGAGTCTTTGCGTTCACGTCTGCGCCCGCCTTCAGCAAG
GCATCAATCGTCCCGTGGACGCACTGGATCCCTGCTACATGTAGCGGCGTCTCCCCAGCG
ACCGTGCGCGCATTGACATCTGCCCTGCCGGCAAGAAGCCTTTCGACCTCTTGCGTGTCT

CCTTGGCCCGCGGCATCAATGAGCATCTCGTCGTCTCCTGTGGCAGAGACTTGCTCAGCT
GCAGAGGCCGCGCAAAAGCAGCAGAGCAGCAGCAGTGACCGAAGGAAGCGCATGTCAAGT
TGATCTCGCCTGTGCAGTGGACACCCTTCTGAAGGGCGGAGTGGCCGGGTGACATTGGCG
AGAGCGCTCGAGCCCACATGGCTTGAGCCAAAATGGCTACGG
>at01972
TAGTCCTATTTTTGGTCTCAAGGTCCCTCCCAGCCGGAGGCCTGGACTAGCCGAGTCAGA
AGTTGAATACTGTGCCTCCCCTTGCTCCGAGCAATGGCAGAGCATCGTTGGAGAGGCTAT
GCTCACGCTGACACTGGCGATGGGCGCCTTGCTAGCTCCCACGCGCATGGGAAGGCGGAA
GCTCTCGGACTGGACGGGGAACCCTTGGATGCGACTCAGTTGACTGCTGCTCAGACGGCA
CATAACCCCGATGACACATGAGGCGGATCGGGTTCTCGTGCGATCCGCTTGAAGCCTTGG
CACCGAGTGACATGAACTGCCAT
>at01973
CGGACCAGCAGACGGCCGGGGTGCGAAGCGGCCTTTCAGCTTGTCGAGCATCCAGTGGGA
GGGGGCAAACATGCGCTTGAGGTGCTTCTTAGGTCCGCGCGCCATGGTGAAGAGGGGACT
GCCTCAAGTGACGGCGGCACCTGCCTGGAGCTGACAAGAGCTTGGCTCTAGGAAATGCCC
TTGAGCCAAAATGGCTACGGACTCTGCGTTGA
>at01974
GCGGCCGCCGCGGTGGGGCCCCTTGGCCGCGGCGGCAGCCTACTGCGGCGGGCCCTGGGG
CCGGCCCGCGAACATGACGATGGAGGTGGAGGGGCCTCCACTCCAAATCCAAGCCCATCA
TCTGGCTGCAGATCTACTCTCAGGTGCCTCCCATGTTCACCCGCGACATCGCAATTTCGT
TGGATGCCCTCGCAATGAACCCCATGTACGAGGCCACCTTCGCCGCCTGGCGGTCCGAGG
GCACGTGGAAGCCTGGGCCCTTGGGCTGGGCGACCTGGCCGGAGCGGAGCTCCTTCATCA
TCATGTTCCTGTCCTCAGATGCGACGAGCTTGGTTGCCATTCTCGACCAACAACTGCAGG
ACAAACTGCCAGGAGCTGCGGGAGGCCTGGCGCAGCCAGCAAGTGGGCGGTCGGGCCTCG
TGCC
>at01975
GGATTTCCAAGTCCGCTCACGGTACACCAGGCATCCTTGACACTGTTGTGTGTGTTTGTG
TGGGAGATGGCCCTCGCGGGAATTGAGGCAACACGCTCGGAACGCCTCCGCTCACTTGGC
GTCCGCGAAGGTGCAATCGCAGCCTCTGCGATCACCGTGCCGATGGCAAGGAGGCCAGTG
AGTCCCACATTCGGCTGCTCCAATGGAACATCCTGGCAGATGGGCTTGCAGATGATGGCT
TCTGGTGGCTGACGTG
>at01976
TTTTTTTTTTTTTTTTTGCGTGCATCCACTGCCTGTGAGGAGAAGACCCATGGTGCCCC
ACCCGCGGATGAATCAATTCCAGCAGAATCGCCGGGCGTCTGCTGCTGAAGCTGGTTCCA
GCCATGCCCACCTTCCCAACTGACCTGTAAAAACTACGGCAGTGCACTGCCACGATGCGT
CGCGTGTGGAACACACGAAAGGCAAGGGAGATGCAAGGGGGACAGTGTCCCACTCGAGAC
GTCTCCGCTCGTTGTGAGTGTGGCTGGCCAAGCCATGGCACCGACATCAACCATTCGCTC
CAGCATCTCCCACGGGGCCGGCTCCTGCGCCAGTTCTCGGCAATACTATACAGGTGTGAA
AATCGATACGCTCGGGCGGGCGCCCCTGGCTCACGCGCTGAGCAGCTTGTCCATGAGACC
CGCTGCTGCCTCCTTGCCCAGGACCCCGGTGCGCTCCGCGCCCTGGATGGCCTCGATCCG
GTCGACGATCCTGGCCGCCGCCGGCGAGAGCGCCTGGGCCTTGCTCTTGTCGAGCACGGC
CAAGGCAGTGGTGCCCATGAGGTTGCCGAGGTAGGTGGCCTCCATGAGGTCCGGCACGGG
GATGTCGAAGAAGCCGGCGTTCGGCTTGTCCTTGGTGCAGTAGGCCGGCAGGCACTTGGA
GATGCCCACGCAAGAGCAGTTGAACTCGCCCACGATCCATTTGTCGATGGTGCAGGGCGT
CCCTGGCTTGCCAAGGTCCGAATTGATGAAGTCGCCCGTCCACCAGAGCGGCAGCGGCTC
GCCTGCGAGGTCCAAGTTAGACATCAGAGTGGGGAGGTCCTTCTTTGTGAAGTCCGCCAT
GAGCGTGGCAAACTCTGGGGCGTCG
>at01977
TTTTTTTTTTTTTTTTTGCCCGAACAGCAACGAGGCTTCTACTCAGATTGGCCATGCTG
AGAATTTCGAACTGTCGTCCGGAAGACTTGCTGCAACTCGAGAAGACGCGTCACGTCTGC
ATCTTCCAGTCCAAGCTCCTCGTTCTGCCAGTCTTCCTCGGAGGCTTGGCAGAAGAGGTC
GGCGTCGACTTTGGCGACCTCAATGGACTCGAGGCCATCGTCGCTGATGCCCCCCTGCAA
CCGCAGCCACCGTGTCATGCTGTCGTGCCCCCACTCCTCCATCGGCTCAGGGTCCTGCTC
TGGAGGCACCCCCGGGACAGGGCGTTCCGGTGAGACTGATACGGTGCCGCCCGTACTGCT
CGGCCTTCCACTCAGGTGTGCTGACGCCTCCTGAGCTTTCTGGGCAATTCCATCTAGCAG

CTCTTGGCGGTCCGCAT
>at01978
TTTTTTTTTTTTTTTTTTCAAAGCCCGGAGGCTTCAGCCGCGCGAGAGTCAAAGCGTCCC
ACGCCCTGTCCCGGCCGTCCGCGCGCGCGTCAGCCAGGCGACTCAGCGCCTGTACATGTA
CCCCTTCATCCAAGCCTCATCCTCCCCCTCCTCGCCCCAGCCGTCGCCGTCCCCGGCGCC
GCCCTCCTTGTCCCGGCCGCGCGGCTCGTGCTCCGCCTTCCAGATGAGCTTCTCGATATA
GGTCTTTGCTCGATCCACTCCGTCTCTCTCCCCGACGATCACCACGTTCTTGTTGGCAGA
GTGCTCTCTGGGGATGTTCATCTTGACCTTGAAATTGTTCTGGATGTGCCGCAATTCCGA
GCCTGCCTTTCCGATGATGAAGTCTGAAGCACCTTTCGGCCACCTCCATCTCCTCGTAGA
CCATGTCAGGGTGAGTGATCGGATGGCAGTAGTACATCACGATGTCATTGATCACTTCCC
TGGCCTTGTCGACAGAACCGGGTCTTCCCTGCAAGCAAGACCTTGTACTTCTTCCCTGCA
GCAGCATTCTTGGGAGCGTCTGGGATGGTGACGATGACGCCCAGCTCATCCCTGATCTTC
CGTATCACGAACGCCGACCCCTACCCGATGATCTCCGGAAAGCACGAGGGGGTGGTACCG
CCACGAAGTTCTCGGCAAAGTCCTCGAACTCCAGGGCGGTGTAGCCTTGTCNATGAGCTC
TGCGACCGCGCTCTCTGCNACTTTAACGCCTTCTGGTGGGCCGGTGATG
>at01979
TCTATGTCAGGTCCTCCTTCTGCAAACAGAGCCATGTTCAAAGGACTTCACGGAcCCCCG
AGAAAGGGAGTCCTTGTTGGACGGACTTCCCTTGGGGTAAACGGCCCATCAACTGGCGCG
GAATCCATGACTGCATGCAACTNCACATCTAATGCAGATCCCACCAAGAAGGCTCACTCC
CTGCGCGCCCCTTTTCAAAGGCGCTGGACCCTTCCCGCCATGGCTTCAGATGGCAACTTG
CCTATGTGCTGGTTGGCAGCATGCCTCGGCGTGCCCAGCCTCTGGCTGTCGCCGCTGaAG
tTGCTGCCACCTCCGCTcGCCCTcCCTcCCCCCTGcCTCCTcCCGCCAcCgTAcCgGGGG
ACtCGCTCCGCGTCCTGGACAGCAGACCACCGGCAA
>at01980
TTTTTTTTTTTTCTTTTTACTTGGTTGTGAATTTTAACGACATGTCAGAGGACCTAGGGA
TCGGCCGTCTGAGGAACAATGACAGGTGCAGTGGCAATCCCGCACGTGTTCTTCCCATAT
TGCAGGTAAATGTAGCCTCCAGTTGGCTGGTCGGCTGGCCCACAAGATGCTGCGCAGACC
GACATCACTGTCTGGCCAGTTGTGAGCATTCCAGAGCAGCCGGCTTTGGTCGGTCCCGCC
AATGTGGCACAGTTGGAGTACTCGTCTGTAGGTGCCGGCGATCCATCTAAAGCTACACCC
CAGTCTAGTCCCCACGAGTTTTGCACGATCCAGGCACTACGACTCGCATCAAGGCCAATC
GCCACCACGGCATGGTCAACGGCAGTGCCACAAGAAGCAGCACTCATTACGCCGCCAGTG
TATGTTTGCCACTGGGAAGCATCGACCAGGATGGAGACGGACCCCGTGTTGCAAACATAT
GTAGTCATGAACTCTTCGTTGTCGTCTTCCACAGGCGCACCACGCAAGGTTACAGCCTTC
GGGATGTCTGTTTTGCAAGGGAACGATGTGGTAGGAGCATTGCCGCTGTAGGTGACCCCG
TCCCCCGAATGCGACACGGGACCAACTTGGGGAGATGGACCGGTCTGGGGATCCGGGCTT
TGAGACGCCCCCTGTGGTGTGACACCGTAGTAGTCACCATACGCTTCTTGCGTGGGGAGG
CCACCCTGTTTCTCCAGCCACTGCATTGCTTGGGCAGGGTTGCCTCCACAGCACCCGTTC
ACCCCACCCTGGCAAGTTTGTCCTGCCATCATGCAGTCGACCAAGTACTGTGTGCTGAGT
GGGCCCGGATCGAAACCACGCTGCTGGATGGATGCCGCACGCACAGTTTCTGCCGTCGAA
AACGCCCAACAATTGCCACACGCTCCCTGGTTGCGAACTTGGCCACGCGAAGCCCAGTTC
TTCGTGCAAGGTGCTGGCGATGATATCGCCCCCACACTCGTTGTGGTCGATGTTTGATTC
TTCAGTCTGCCCCCAAGCAAAGCCTGAAACTCCCCTGGTGTCCAATCAGCAAACTTCGTG
ATGCCATGTGCTCCTCGGTTTGGGAGCGTTGCATTCTCCTTGGCGTTCCTCTCGGCAATC
CTTTGCAGATTGTCATAGAAGATCAGCAAGCGAGCTTTGTACTCTGACACGTTGAGCCCC
TCTAAGTACGCACGGTTGTACTTGAGCAGGAAGTTATCAAATTCATTTGCCACGCCTTGC
GCACCTGCTGGCATGTCCTTGATATGTTCTGAGAAGAACTGCTGCCTTTCGACAAGACAA
TTCGAAAACGCAGTTGCGTTTCCTCCATGCCTGCTGCAGTTAAACTTCTCGACGAGCTCA
GCTATGGACGTCGGCCCTCCGCTTCTGAGACCGTTCCACCAGAACAGCAGGGCCGCGCAT
CCGGCGAGGAAGGCGCAACCAGCGCAGATCCCCAATGCAAGCTTGAAGGTTCGCGCATCG
CCATCTTCGCTCGAGTTGTCGCTGGCTGACCCTTCCTCCCCACTGCTCTCTTCCCCGAAG
CCCGAAGCATGGCCGCGACGGCCGACCAGGTGCCGGGTCTCCTCGAAGGACATCGAGGTC
GGGCACGCTGCAGGGAGGGCTCGAGGGCTC
>at01981
TTTTTTTTTTTTTTTTTTCCAAACATGCCGCACTGAGTCCCGGGCGGTGCGCCTACCACC
CGTCTGGCTCAAACATTGATCAGATCAGGTCACCAGCGAGAAGACTCAAAATCATCAAGC

TGCCCTCCCATCTGCAACGTCATCCGTTGCTCGTTGCTGAGGCCATCCCAGGTACTGCGC
AGTCCGTACACGCCCTGACAAAGCTTGACGAGGTCACCCTGGGATCTTATCCACCCCATG
GCCTTGAACTCAGTCGTGCCGTCTTCGCGCAAGAACGTGTACTCCTGGTGTGGTCTGTCC
TCGAAACGCTCACCGCCTCTGAACGCGCGATGGCCATAACCATCGTCATTCATTCCAAAC
AGGCCATCCCCGTTGGAGTCCTCGGAAAGCTCACTGCCTCCAAACGCGAAATGGCCATCC
CCGTCAACGCCCCCAGAAAACGCGCCGAATTCAAACGCGCGATGATCGTAATAATCGGCA
TGCATTCTGAACAGGCCCCGGTCACCGTGGTACAACTCCTCCTGCACCTCCTCCTCCT
>at01982
TTCTGGGGCGAAGCGCGACCCTGTGAATGCAGGAATCGCGTTGGCAGGTGCAGAAGAGAC
GGCCATGAGCAGCCCTGCTAGCAGGCCTGCGCCCAGCCATTTCAGCAGGGACGGCGCTTC
CTTGCTCTTTGCCTCTGGCAAAAATGGAGCAAGTGGCTGAGAGTCCACGGCCTTCAGGGC
CGCCAGGGCATCCAGTTGCTGATGGGGATGGGGCGCGGAGTGCACGACGTTTGGCAGTGT
GACGAAGGCGGAGGCAGCACTTGCTGCGGGCACCAGTGCACACACTGCCAATGTCTTCAT
GACGCTCTGGAGCGACACGCCTGGTAACAGCAAGGGATGTTCTGCCGGAGCGCCTTCAGC
CAAAATGGCTTGAGCCAAAATGGCTACGG
>at01983
CACGACAGATGCGCGAGTGGGCAAGCGGACTCTTGCAGTCCGGTTGGGTGCCAGATTCGC
ACGAGCTGAGTATGCCACGCTAGTTCTGAGTGCATTCGGAGCCATCTGGGCATGGCCACT
TGGCTTTTCTGGCCGCTATGGTGGATGGACGGTGTTACTGCCAATGGCAGCCTTCCCTGT
AGGCATGAAGTGTGTCAAGGCGATGCAGCAGAATGATGGCCCAGAGCTTAACGCGCTGAT
TGGGGGCACTGCCCGGCTGCAACTGCTGTTCTGTATGCTGTTGGCCATTGGTGCTGTGCT
CTGAGCATTCTCAAGAGCAGCGCTATGCCTTTATGCAGGCTGCGCACTTCAAAAGACAGA
GCTGTTCGACAACGCTTCGGAATCGTTGTGCCATACAAGAAAAAAAAAAGAAAAAAAA
>at01984
CCGTAGCCATTTTGGCTCAAGCCCTAGCCAGCGTAGCCAAGAGGCCCGCGATCCCACGAA
ACGAACAGGTGGCGATGGCGGCCCGCCTCCACGGTACGTTGCGGCATGCAGTCTCAAGTG
TTGGCAGAGCGCGACGTGCAGCTTCAAGTGTCGGCAGAGCCACTCGACCAGGAGTGTGCT
CCGGGAGGCCATTCGTTCAGATCACCGCCGCACGCATGGAGGAGTTTGTGAAAGCGGAGC
TCAACGTCTTCGCTCGCAGGGTGGGGCCGCCGCAGCCGGTGACGCTGCTAGAGATCATGC
GGGCCTCTTCGTCGTTGGAGAGGGTGGCTGAGCTCGTGCACGAGGAGCTTCCCGTGCGCT
TCGCGAGGCGCGTGAAGCTCATCGAGCGCGTCGAGGGCTGGAGCGAGATCGCCGAGCTCG
CGCTCCTGCACGACATGCACGTGCAGTCCTTTCGCGAGCTCCGGCTCTCGGATCCGCGGG
ACGGATGCGG
>at01985
TTTTTTTTTTTTTTTCTCCTCAACTGTCCGAACTTCGCAAGAGACCATCAAGGCAACAC
ACCGAACTGAAAAAGTCAGCTTGCCCAAGCCTGCATTCGAATTCGAGTGCATGCAAAGGT
CAGTCTGTCCTCTTTAACCACATGCCAGAGCCTTCGAGCGGTGCCCTGAAACTCTGGATG
CATGCCGCCTCACGTCTCCGGAGATGTGCTTGGCCCGCGTTGCGTACTTCAGCGCACCCA
CTGTCTCGTCAAGGTCGGCAGTCGCAGGCGAGCAGTTCACGATCATCAGCGTTTTGGACG
AGCCGCCTAATGCATCCTGCATGAGCTGAGTTAGCTTGTGATTCTTGTACGGGATGTGCT
TCTCGCCCTTGGTAAGGGCCTCAATCACGTCACCAAGTGCTGTGAGGGACCTGTTGATCT
CGATGGCCTCCTTTTGTGCGTCCCCGCTGACCTCGGAACGTTTCAGCCGCTCAGAGCCAG
CCAAGTCGCAAAGGAGGATCTTTCCCCGAAGCTGCTCTTGGCTGTCCTTGTTTGTGCTCA
GAATGTTGATCATCATGAGGAGGTGCGAGACGCGAACTCTGGCTGTTCATGGCTGTGGCT
GTGACGGTCCTCTGGCGGATGCCCTCTCAAGCAGCTGCAAGAGCTCTGCTGGGTCTTGAC
ATTCTTCCTCCGTGAGATGGTCGACGTGCACCGCCTTGTC
>at01986
TTTTTTTTTTTTTTTTTGCAGCGACCGCATGCGTAGGAGGAGTCTCCATTCGACTCCCG
CCCAGCCCCCTGCCTCACGCTCATGCCAGGTGTTTCTTGATGATTGAGACTGCTGCAGCG
GCCAGGAATCCAGCCAGTGTTGAGAGGAGCTTGGTGCGGAGCGCCGCGGTCACCTTCATG
CCGAGGATCTGCTGCGGTTCCTGCAGCTGAAGCCTCTCACGCGTGATGTCGATGAGCGCG
CGGACCTCCATGGGCCCGTGGAAGCCAGTGAGGGGGTGCTGGGTCCCGGTTGGCGAGCCG
TCCCCGGGCGGGCCCAGGCGCTTCTCCTCGCCGTGCATCCAGACCTTGGCCACCGTCTCC
CAGGGCGTCCGCGCGTGGTCCTTCCACATGAGGAGGACCTGGTCGGAGTACTCGAAGAGG
AGCTCGTTCATCGCCACGCAGTAGCTGCAGGCGATGCCTATGTAGATGAGCATGGCCCCG

ACGTCTATCAGCGCCACAAACTGCGTCAGGGAGACGTGCGGGGCGCCGATGTGCTCGAAG
AACACCGGGACGAGCGCCACGCCCATCAGCGTCACCAGCATGCTGACGAGCCTCGTGCC
>at01987
CCGTAGCCATTTTGGCTCAAGCCTTGGCTCTAGACTTGAGGCAGGGGCGCCATGTAAGGT
GATGCGGGTTGTTGGGATGGTTCCAGTTTCTTGCATTGGGCTAATTGCTCTTGGCCTTCA
TCCTGTGCGTGCTGCAACTGGAAGCAGTCATGCTGAGGAGACGTGTGTGAATCCACAGTC
ACCAGGATGCCAGATAGCCTCACGACCCCATTCTGGCCGTTCAAGAACAGGGACTTGAAC
CGCACAGGGCTCGCACCACATGTGGCGCCCTTTGACCTATCGAAGCCTGCCTGGTTGTTT
CAGCAGCCACAGGACTCTGGGGTCTTCCATTCTGGTCCGATAGTAGAT
>at01988
TTTTTTTTTTTTTTTTTTGACAACTTCAGCAAAAGGCCCTGGTCAGCAGAAGGCCCCTGT
CAGCAGAAGGCCGTTGCGGGGCCCCGGCAGTCCGTGACCGCCGTGCCCGGCTCGCAGGCC
CCGGACCCCTCTGCGGACTCGTACCACTGCGCCGCAGCGTCGGGCCCCGCTGGCCCCAAG
CCGG
>at01989
AAACATAAAGGCGATCAAGCAGAAGCTGACAGATCTCAAGGCCTCAAACAAGCAGGCGAC
AGCAGAATTTGCAGATGCAAAGCAACAGAAAGAATATGAGGAGTCGCAGGTGGCAAACTT
GAAGGGCATCATGATCCGGGCTCAATCCACTGGCATCTGGAAGGAGCCAAGCAAAGGCTA
TCTAATGAAGGTGGCAGCCAAGCGCCAGTTGCAGAATCAGTTGATTGCACTTGGGCAGAC
AGACGTGGTTGTCGATCAGCAGTATATCATGACAATGAGGGATCTCTTGCATGACTTTAA
AGGATAGTCAACCAGCAATCCCACACCATCTCAATGTTCTCAGAACTTATTTGGCGCCTT
GCCTACA
>at01990
TTTTTTTTTTTTTTTTTTCGCCCGGGCTGGCTCGGATTGCAAAATGGGCAAATCCAGCAA
CTGCTGCAAGGCCAGCTGAATCGGCCAAGTTTGAAAGCCAGGTTCGTTGAGAACGTGGCA
GAATCAACTTCTGAGTAGTGGCCACCGCTGGGACGCACGGCGGACTCCAGCAAGCAAGTT
GCGAAGCGCGGCGGACTCCGGCTCACCCGCGCGTCTTGCGCTTCCTTATCCTTTCAAAGC
GGGCGCAGAAGAAGCCGTTCGTCAGGTCCTCATCATGGCTCGCGCGGCAGCAGAGTGGAC
CCGCCGGCCCATGGGACAACCTGCCGCGACTTTCCCACTTGGGCAAGCACTGGACTGCCT
GGAACTTGGAGGTCTTCTGAAGGATCTTCGCCACCACGGACTCGTTCTCAGCCTCGTGAA
CCGAGCACGTCGAGTAGGTCACTGCGACTGCGGCCGGAAAGGACATGCAGTGCAGCACGA
TAGCCACCTGCGCGTCTGCCAAGGCCTCCAAGTCCTCGGTGCTGAGCTGCCCCGCAGCTC
TGCTCTCTTCGGCTTCGGCATACTGGTTGGCGAGCTGGCCGCTGCCACTGCACGTGGGAT
CGCACAGGAAGTGAGTGACATTGGAGAACCTCTCGTCGTTGGGGTCGACATGCATGAAGT
CCATGCAGAAGACTTCGATGCCGTTCACGCCAAACTTATCCATCTGCCTCCGCAGCGTCT
CGCAACGCTTGGGGTTCCGCTCAAACGCGAAGAGCTTGCCCGTTCCTTCCAGCAGTGCTG
CTACGTGGGAAGTCTTATTGCCAGGCGCGGCACAGCAATCAATGACATGTGACCCCTTCG
GTGGCGCAAGGCCACTGCAGGAATGCAAGAGGAGCGGTCCTGAATTATGAGAGAACCGGT
CTCCACCAACGCATCACCATGAAAGGATGTCCGAGGTGGGAAGCCCAGCACGTTTTCCAA
GGCAGGATCCTCGGTATAACTCCAGCGTCCTGGCACTCCCTTCGGAACCCATCCCAGCGT
CACCAAATGAGTGATGGCATCCTCGTAAGACACACGCAGCGTGTTTACACGGAGATACCT
TGGCAGCTCCTTCTTTTTCTGGTGCTCGGCGGTCACACCTTGTTGCCTACGGGCCGCCAG
AGTCTTCAGCAGAGGGGCCTTCCATTCCTTCACCAGGTCAAACAAACCTCCATCGCCTCG
GATGCGTCGGGTGCCGAGCAAGCACTGGTAGAGCAGGATCAACAACAAATTTGTGTCGGC
GCTCTTGTCCACATGCTCGGCCAAGCCAGATTCCTTGGAAACCTCATGCAGTAATGCCTC
ATGCTTGCGGGCTCCCAGAACAAGAGCTTGCACCTCTGCCATCATCCAGGGCTCTACCCC
CGAATTGTACAGCACATCGCGGTTCCAAGAAGCAGCACCATGGCCGAAGACGTCGGCTGC
CTTGCGGCATACCCGCTCAGCTGCTTTGGCCATACTGAATGAGCCAGACTGATAGCTTGA
GCCAAAATGGCTACGG
>at01991
GGAGAACAACAAGAACCGCGAGTCCTGGTCCTGCACCGCGCCCTGCTACGAGCCAGGGGC
ACCCGCCTTCTCCTCCGAGTACTACACCTCGCCTCGGGGCCAGCAGCACTTTGCGGCGGC
CCCGGACCCGCAGGTGCCATGGCCGGACGAGGCCTGCGTGCCCGGCTTTCGCCACGCGAT
CTGTGAGTACCACGAGGAAGTGGAGAAGCTGGGGAAGGTCCTCTTGCGGATCTTCGCCCCG
CGTGCTCAAGTGCGAGGAGGAGAAGCTGCTTGCCATGGCCGACAAGCACGTGAGCGCCAT

GAACCTCTTCTCCTTCACCCATGGGAGAGGGGGGGCGAGGTCCTGCCTGCGCACGGCGAC
GTCAGCGCCTTCACGGTGCTCTCCCACGA
>at01992
CCGTAGCCATTTTGGCTCAAGGGAAGGGCTCAGGTTGCAGCTCATACAGCGAGCGGGAGA
AAGAGCGACTGCAACGTCAGCGCCAAGTCTGTGCATAAACTGCTCACATGGGCTGCCTCC
AAAGCTCAAACAAGGCTGAACGACCGGTGTCGATTTCCCCGCAGCAAGACCAGGCGGACA
TGAGCCAGATCTGCACTCACGGATCACAGCCATCGACGAGTGCATCGGAATCGCAATCCG
ACGCACGGCCATGCCAGTTCATCGCTTTGGAACCGCCCTTCATGCGCACGGCAGCTCCAA
ACGGCTCGCCGTGGTCGCAGTACGTCATCTCGAGCCCCACCTTCGCCGACGTGCGAACTG
GCTTGCTGATAGGCCCTTGGGCCCGAGTGCCTGTCGGCGGCCGTGTGCTACGAGCACTAC
GGTG
>at01993
TTTTTTTTTCTTTTTTTTTTCCTTTGTCAAACGGCCACAAGTGTTCTCGTGCCAGTCTCA
ACATCGTAATTGTCTGCAGTGCCTATTACCCTGTTGCTGCAGTCGCCCAGACTCAGTCAT
CTTTCTTGGGTACTGCGGCAATGATCAAGTCTATCATTCTGCTGTCAGGTGTGCTCTGCA
GCCACACACTGCCAGTGCCCCGAAGAGTTGTTACGAACAAGTTCCCTCCGATGAGCGCAT
TTCTCAACCCAGGCTGCGCAATGTCATAGTCAATTCCCAAGGTGAAGGCCATCAAGCACC
CTGTCTCAACAGTCAGTGCCTCGTTGTTCAACTCCTTCCGCACAATAGTGCCTCCACCAT
GGATGAACAAGTCGCCTGCAGAGCCGCTCTCGGCGACCAGCCGCTGCAAGATGAAACCCT
CCCCACCAAAAAGTCCAGCGCCAAACTTCTTTACGAGCTCCACTGAAAAGCGCACCCCCG
GTGAGGAACAAAGAAAAGCCCCCGACTGTGCAATGAGGGTCCCCTCGGGAAAGTCATTCA
ACTTCACGTGAACTATTGTGCCCATCTTTGGGGCAGCAAGCATGAGGGTACGCGGCGCGC
CGGTATTGTTGGTAAACCACGTGTAGAACAGACTTTCATTCGAGACAACCCTTTGGAAGC
CAGCCTTGATTGCAGACCACCATCCCTGATCCTGAGGCATGATGCTACCATTTCCCAAGC
GGCACTCGAAGCTCATACCGTCATCCATACACATCATACTACCTCCCTCGCCAATCACCG
TCTGCTGAGGCTGGAGCGTGAATTTAATTGCCTGCAAGTTGCTACCCAGAATCTCATACT
GAAGAGCCATTGGATCTCAGAATCTACGGCGTACAGAAGTGATGCTGCCGCTGCCAAGAG
CCTCAGAACGGCTTGAGCCAAAATGGCTACGG
>at01994
TTTGTTTTTTTTTTCTTTTTTTTTTGTACCGTCCATGCCTCCAGATCACTGTCCCCTACC
CGTAGTCCACTTAAGAATGCTACGAGGGCACGTGATCCACATCTCAGAACAGCTTCAGTG
CATTGTCTGGCCGATCAGTGTCAGAAATGCCGTTGTTGATGAGGTTGCGGAGTGCAGGAT
TCTCCAGGGCCGCGGCCACTCGCTCCTTGTCGGCGAGCTGCTTGTTGACCTGGTCCTCCT
GGTTGTGCGTGCCCGGGGTGATCTCGACGTCCACCTTGAACCTCCTCGGCAAGGAGCGCT
GCAGCTTTGCCTTGATGGTGAGGCCGATGAGAGTTGCGACGCTACAGGTCGGCACCGTTG
GCGTGAAGCGAACGGCCACGCACTTGTCGCTGACCCAGACATGCCCCGCCTCGACGACCT
TGAGCTGTTCAAGAGATAGGTTCGGGTGTTCTGGATCCTGCAAGTAGCGCAAGCTCTCAT
AGACCTCATGCGAGGTAAGGGGGTCTTCACCACCCTCGTCGCAATCAGGCGAGAGCATCT
CCTCAAGACTCCGATGCTTCAGCGCCGAGCCACCACCTCCGGCCTCATAGATAAGCGGGT
TTGGATTGTCCGCCGACATCAATCTCCTTGCTGGCACGGACCCCACGAGGGGGGTTCTGA
CAGCTAGGGGGTGTGCGGCAACACCCCCCCTCCGCTGCTTGAGCCAAAATGGCTACGG
>at01995
TTTTTTTTTTTTTTTTTGACAATTTTGCAAGTGCTGACGGATATCTCTGAGTGGAAAGT
GGGGGTCTCCTCTGCCGCAACGTCCTGCTACGGCCGAGAGCAGAGGTACCGCATAACAGA
AGCAGCCTGCAAGGCCGGCATCTCAGCTCGCGCCCGAGGCCATCAGTTCAAGCGTTCGGT
GCGTACTTTTCCGTGTTCGCCGCGCTCAGCCAGCACCTCTCCTCCTTGCGCATGCCGCCC
TGCGACTCCAGGTACTCCACCATGGCGATGTACGGCTTGTTGCCGTTCTTCTCGCTGAAG
ACGCCGGCCTCGCCGTACAGTCCGGAGTCCAAGCAGTAGTCCAGCGGGGTCAGGCCGTTG
CACTCCTCGATGTTCACGTCGTGGCCCCGCTCGACCAGCAGCTCGGCGATGTCCAGCTTG
AACTTGGACGCGGCCATGTGCAGTCCCGAGAAGCCGTTGGCGTTAGCCTGGTGGATGTCG
ATGCCGGTGTCCAGCATCGCCTTCACCTGGTCGACGTCCGTCCAGCAAGCCCGAAGGAAC
TCTCGGGTCTGCGTGGAGATGTTCTTGTTGAAGATCGCGTCGGGGTAGTGGTAGAACTTC
GACGGCTTGCCGTCGGCAGGCAGACCATAGTCAGGCGTCAGCAAGTCGTTCGGAGCCTCT
GCGAGCTTCGATGGGTGTGTTGGCCAGAACGACGGTGGCATGGCATCGGGACTGTCTGTG
GGAGCCTCGAAGGGGATCAACGACGCCATGGCAGCAACGGGGCAAGAGGGACTACTGGCG

GTAACCAAGAACTTCGAGATTGAGCTTGAGCCAAAATGGCTACGGACTCTGCGTTGATAC
CACTGCTTAAGCAGTGGTATCAACGCAGAGTCCGGTAGCCATTTTGGCTCAAGGCATTGC
TTGGTAAGCGCCAGCAGGGCGCGAAACGCGCCGCATGTACGCCTGATTGCAGAGCCTTGA
GCTTCCACGGTGAGTCTTGAGA
>at01996
TGGCAGGGGGCCACCAGAGCTCTCGTTAGATCCGGGTCGATGGGTGGGAGGACCCCGGCC
AACCTTCGGGAAATGTGGCGGCGGGCCGCGTCGAAGGTCCGCGCGGCCAACGCCTTCTGG
TGGGAGGTCACCTTCTTCTCTGTCGGTTTGTCGTGCTCGGCCTGATGGTGCTGCTCACAT
GCCTCTATGTGAAGGCCCACTCGGCGCTGCCGCTGCATGCTGTGCTCGCGGAGGCCGCGG
TCGGCCTCGGCTGGGCCTTCGTTTTGGTAGGCCCATCTTCGACTTGCTCATATCTCACCG
ATCCTCGGACAAGCACACGGGCGTGAAGGCGGAGTGGTTCATGGGCTTCCTGCATCGCTG
GTGCAAGGAGCGAGACGAATACATCAAGAGGTCCACGGGCTGAGCTGTTGCAGCAGTTTC
TGTGGATGGCCGCGAGGTTGCTTGGG
>at01997
GCGGCCGCTCCCCTCACAGGAGTCGCCAGAGACCTTGCGGTATGCGTCCGCATAGAAGAA
GTCTGATGATGTACATGTCAATGGTGTAGCGATAGCACCATCGTCCACATACTTGCAGTC
CACTGATCCCACCTTTCGCGTGAAGCCCATCTCACACTCGAAGTTCTCCTCGGTGCAAGC
GCAATTCTTCTTGACCACGGGCCGCTCGAACTTCTCACCGTTGAAACATTCCGACGTTGA
TTTTCGCCGGGTGTATGTCATCTGCTTCCCCAGCAGGCACTTCTCTGAGCTGCGCCCATC
AGACGGAATCCAAGTCTCGTAATCAGAGGACACGGAGTCAGCAGCCCAAACTCCCTTGCA
CAGAGGCTGGCCCAAGGGATCGAAATCCAAGTGATACAGCACTCCCGCCTCTCCACGGGT
CCCATACAGCAGAAACTTCGTGGATGTGGAGTTGGGCTCCGTGACAATATTGTCAACCTC
GATCGGATCCTTCCCAAGCTCAAAGTCATACCAGCTCTTGCCCTCGTTCCATGAGAAGAC
AACCTGCTTGGTCTTCCTGATGTCATCGGCCATGAGAATCAAGCCGCCGTGGTCGCCAAA
CTCATAAATGTATGCACCCTTGTGCGCTTCGACCCACGTTAGCCCACCATCGCGGGACAG
GAAGGTGTTGGTCTGGTCGGTCTCGAACCGCAGGTATGGTCCAATGTTGCCAGTGCCCAT
CATGATGCCCACTGCAGCCTCAT
>at01998
TTTTTTTTTTTTTTTTTGGNTGCGACATCTTCTGCCCCCCAGCCCGCGTTCTGGTCGCA
TCCGCAAAGGCTCTCAGGACAGCCCGTCTTGCAGGAGCTCGCGCAAGCGGAGGTCCACAA
CAAGAGGCTCCACGCGAAACTCCTCCAGCCGCCGCCGCCTTCGGAGATCCAGGGGCATGG
GCTGCTGGTCCTGCGATTGCCAACCAAGGCTGGGCGGCACCAGCAGCCGCCGCAGGCCGC
CGGACCGCATGCCGACGACGCCCTGGCGGATGCCCTCCACGGTGTCGTCCCGCATGCCCA
AACCTCGGATGCCCTCCACCAGCTTCAGCGGCACGAACTGCATGGTCGGCGGCCGGCTGC
TCGCGGCTGGCTGGCCCTGGGACTCGCTCGCAGGCACAGACTGGTTGAAGCCCAGCGACT
CCTCGATGCGGGCCCCATTCAAGCGGCGGATTCTCCAGTCCACTCTGACGGCAGCGCCCT
CCGCGCAGCACTCGCCCGCCCTGGCGGGCTCGAGGTCGAGGAACCGGAGGGCGCTGGGCG
TCTCGGCGAAGTCGGCCGTGGGGCCGCCAAAGTAGTTTGC
>at01999
TTGTTTTTTTTTCTTTTTTTCTCCGTCTGGCGCTCTGTTTGAGACGGCATCAGACGTGGG
TACCCGACAACTAAGGGCTCTCCATAGCCAGACATCTCCTCTGAGCGACTGTAGGGCCGC
AAAGGACGCCGACAACGGAGCCTCAGTCACAACGGATGTCGTTAGAACGTGATCCTCATA
AACTTCCTCTTCGAGAACTGAAACCACCACTTGTTAGGCTGTCCGTCAGGGTACAGGATG
GGCAGCAGTCTCTGCAGGGTCTCTTGCCGCAGCTGCTTGAAGTAGTCCTTCGCGGTCTGA
TAGTCCTCGGGAGAGCCCTCTGAGAACAGCGGAGTCAGGACAAACTCCTGGTCACCAGGG
CCCGGCATCTTCTCCTCTGAGGCCATTGCCTCGAGCTGCCGTGCAGCATCGGGCAGGCCG
CTGGCCTGTGAGGCCACGGTAAGGCACTTCTGAATCCAGACAGTCACGTAGACGAGCACT
CTGTCAGCGCCTCCGAAGAGCTCGAACTCACGGAATAGGATATTCCATCGGAATGCCTTG
ATCGCCTCGTCTATTATGTCCTCGAGGCCCGCGCCCTCTGCCGCCGCCTGCGCCGGCCCC
CGGAAGCGTGTCCTCACCGGTAGCAGCGAGCAGCCGCAGACGCTTCTCGCGGTGGCGCCA
TTGAACTTTGAGTGGTATGGCGGCATTGGTCACACCCCCTCCCCAGCTTGAGCCAAAATG
GCTACG
>at02000
TTGTTTTTTTTTCTTTTTTTTTCGTATGCTGAAGCCCGAGGTGCTCTCACAGACCGGACA
GCTTCCAACATGCTCCCCAGAGACGCAATACTACGGTGCAGAGCTTTGCAGCACCGTGCA

AAGGCGCAAAATATGAGTGAAGCCCCCGTGAGTTTGCATGAATTGCCATCAGCAAGTACG
GCAAACTTGCATAATGCAAATCCCAAGTGACATGGGTTTGACATCATGGCCGAGTGCAAC
TGTGGATCGAGCAAGTGTTCCCGCATGACGCGGAGCCTTCTGCTTTACATGAGGCTCAAG
TACACTCGCACAGAGGATAACAGCATGACCGAGTGCAACTGTGAATTGCATGAGTGTTCC
CGCATGATGCGGAGCCTGCCTCCAGCTTTGCACGCGGCGATGACCACTCATGCACACATG
AACATAGAGCTGAGACAAACTCAAGCCAAGTACAGATGACGACTCTTGAGGCCATGTTGC
GTCCGTCTGAAAGCTATTTACCTGGGGTCCTGCCCGACCCCTCACTCCACCTGCCCGGGC
ATGTTCGCACAGCACAGCGTCACTCCGCAACACACGACTCCAGGAGTGTATCCTGCAGGG
ATGGAGCAGTCGATCACGCAGCAGCAGCTCTTGCCGCTCAGCTCGATCCAGCCGTCATCG
CAGCAGCTGGCTGTTTCGGTATTCACCCAAAGGCAGCAGAGCTTGCCCTCGTTCTCGCAC
ATAGGGGACATGTCATAGGTGAAGCCTTGGAAGCAGCAGTACAGAGACCAGCACCAGAGA
GTCTCCTGGTACATGTCCAGCTCATTCTTCGCCGCAACGCTGGTGCAATCGTCGACGGTC
CTCCCGTCAAGGTTGCCCATACACCGCGCAGGACCACAGCCAATGCCAGGAGTGTTGTCC
GGTGGGCATTCGAAGCCGCATAGGTTGCAGCAGCACTTGGAAGAGAAAGCGACGCATCCG
TCCTGGTCATAGCAGCTTGTGATCTTTGTGGTTCCACCGCAACAGCAGCACTCTCCTGTG
GTGAAGCAGCATGGAAGGTCCAGGCCATCAATGCCGCAGCCGCCGTAGCAACAATAGATG
CACCATGTCGTTTGTGTCAAGACCTTATCCTGAAGTGCATCACGAGCTCCCTTGAGCTCG
CCACCCCTCAGCTTGCCGGACTGTGGGTCGGGACCAGGTCTGCCATGGCGATGGT
>at02001
TTTTTTTTTTTTTTTTTCCAGGCAACACAATATGCTATCGTCGCTGAGAAATCGAGTAC
GTTGTCACTGCTCTGTGAATGAGATCGACGCAGTAGCCGGCCCGGACCAGAAACCAGATT
TGCTCAGGCACATACAGAGAACACCAGTAGCCTCGGCGACAAGACCTCCCTGGTGGAGCA
GCCCGGCAGCAACGGATTCCTCTGTAATTGTCGGAGATGTAGTGGCACTAGCATCTTCTG
CTATGAAGCAATGTGAAACGTCTATGGGAGAGCCAGAGACAGAGATGGTCCAGTTCAATG
CTTGACCCTTGGTGAGCCATCGCACAGCATATCGTTCAGAACCCCTGCCGATGGTCAAGT
TCTCCATCTTTGAGGCGCCATGCACAATGTCCATCCAAGAGAAGATTGCTCTGGCACTCA
ATCGAGTGGACATGTTAGTGAACGTCTCCCCACTTGGAAATACCTGTAGCTTTGCCTGCA
TACGGTCGTTCAGTAAAGAATTGAGAATGGATAGGACCTTCGCAATCAGTTTCGGAGCGT
TGAAGAAGAAGGCTTGATGAACGATCTCTGGATAGTAGTCCTGGATGACTCCCAGGACTT
CTTTGAGCTTTGCTAGGAAGGCCGGAGCTCGAGTTAAGATGGAGACGAGCGGCGCACCAC
TGAAATCGCGGAGGTCCACGAACTTCACCATCCTCCTTTGCTG
>at02002
CCGTCTCAGTTCAAGAGCCGGTGGACACGATCGATCTGCCTTCAAATCCCTGAAGCAGCG
TGCACATTGCACCAAGTCTGTCCAGCGAATGTCCATGAACTTGCGCATCCCCCTCATCTT
GCTTGGCCTCCTGTGCGTCGCAGCTGCTACCGCAGGGTTCAACAACACTCAGGTCGCAGG
GCGGCAGGCCGCCTGCTGATGCGCAGCGGCGGCGCGCTCGCCGCCGCCGAGAAGCAGACG
TGATGGGCACGCGCTGC
>at02003
CCGTAGCCATTTTGGCTCAAGCGGCGTAGTGCCATTGTGGGACCCAAATTGCAAACCCTT
GGGCCTGCCGCCCGCCCCATCTGCTGGGGCCATGCAGATCTTCGTGAAGACGCTAACAGG
GAAGACCATCACGCTGGACGTCGAGGCCTCGGACACGATCGATAACGTCAAGGCCAAAAT
CCAGGAT
>at02004
GCGGCCGCTCCAACATCAGAATGGTAACACCTCGTGCCGAATTCGGCACGAGGTTTTTTT
TTTTTTTTTCCACTGGCGTGTCCAATTCAATCCAGATCCGCCTCATTCCAAAGTGCACA
AAGAAGCACGTACATATGAATGAAATTCAGCCCCTGATGTGCTTGATAGTGGACTGCACA
CCAAAATATGCGCAGTTCACACTGCGCTGTGCAGTTCACTCTACATGTGGAGGCTCGTTG
CAGCACTCGCACCTACTGAAAGAAAGAGCAGAGAATGCTAGGCCTACCTTCAGTCAAGCT
CTGGCGTCAATCTCTGGCTGAAAGTAGGCCACATGAGCCTTAGCGCACGGCTTGCAGATG
GAGAAGCCGCTACCCTCCTTTTGGATGCCCTGCTGCATCCAGGTCAGCTTCGTCCCAATG
ACCATCGCCACGGGCCTGTCATGGCCGCAGCCTGAACAGGTTCTGTGGTACACATGCTTC
TGGTAGCACACATGGCAAAGCGGATAGCCGTTGTTGCTCTTGACCGCCCCAGGTTTCATC
CACTTCTCCTGGGTACGTACGCTGGCTACCAAAGCCTTGTCCTTGTTGCAACCCGAACAC
TTTCCGGTTGGCACGTGGACGCGGTAACAGTTCTTGCAGACTGGGTAACCCACATCGCTG
GGGGCTGAAGTCTTGTCACCAGCGTCCAGCTGCATCCACTTCTCNCGCTTCGCCAGGCGC

GAGGCCACCACCCTCCTTTGTGGCGCAGCCTGCACATGGG
>at02005
AGACGTCTTCAACTTCAGTCCCACTGTCTTGGCAGCCAGAGGCGGTACGTGAGATGCCAT
CTCCTCTGCGACTCCACGACACAACTCGCGAAGGCGACGGCGCAACTCATCAGGATCCGC
CTCGGTTGAAAAGGTTCGTTCACTGGAGAGGGACTTGCGGCCAACATCCCCAATGATCTC
GCCCTCCTCGTCTGCCACTTCACCACCTGACAGGCCCAAGGAAATCCGCATCAAAAACTC
GCTGGTCTTGGGCCTGCTTGCAAAGGCACGGCGCACGGCGGCAGCCGCCGCACGGAGCTC
GCCACAGTGACAACATTGAGCATGACCTTGATCTGCCGCTCCAAGACC
>at02006
TTTTTTCTTTTTTTCTCTGCCCGCAGCACCGCATCAGATTCCATGGACTTTACAAAATTG
ACCGTGCCATGCACCTGGAGCTAAAGCGCTGCTCTGAACGTGTTCCAGGTTGCGATGCCA
TGCACCAAAGCGCGCAGTGCCACGCACTATGCGTGGTTGCCAATCTTCGACGAAGGCATT
GGCCGCAAAAGGTCTGCAAAACCGAGAGGCCGAGGTAGGACAAGCAGGCACGTCCTGTAA
ACCCTGCAGTGCTACTGGATGCGCGTCATGGAGCCCCTGGAGAAGGCACAGCTCCGAGCC
AGCCACTAGGCATAGTCAGAATGGCCCTGGGCAGCTCTCACGACCTCTTCTTCTCGGTGC
GCCAGCTTGTAGTTCAGCACCGTCTCCGCGAGGCCAGGGGTCAGCCTGGTTGTCCCTGAG
CCGAAGACGGCTGCCTGGTAAGCCAGCACGAGCTTGGCAAGCTGTCGGCCCGAGAAGCCT
TCCGTCCTCTTGGCTACGCTGTCAAGGAAGGTGTCGTCCAAGCCCGGGTCGACCTCGATG
ACTTTGCCTCTCCTCGTCGGCTTGTGCACATGCTCCTCCATGAACATCTCCAGCATCCGC
CGCCGCTCCCGTTCGCTCGGCAAGGGGAACTCGAAACTCTCATCTACGCGGTCGAGCACA
GCTCGGTCGAGGATTTCACGGATGTTCGTGGCAAGGACGACGACAAACTTGTCGCTCTCG
GTGCCGGTGTGATGCAGAAAGGCTGAGAGGACGTTGCGCGCCTCCTCGCTCATGGCTCCC
GACTCAGATCCGCGTCCAGTGCGCAGGAAGGCATCGGCCTCGTCAATGAAGAGAATCAGG
CCACGCTTCGAGGAGTTCGCCCACGAGAAGAGCTTGTTGAGCTCTTGCACCGCTTCCTTG
CCCAGCGGGCCGACATCACCGCCAGACATGATAGCGTAATCCAGGCCCGACTGCTTCGAC
AAAGTGCGCGCGAAGAGCGTCTTGCCGGTACCTGGCGGGCCATGGAGAAGCAGGTGCCTG
AAAGGAGTCCCATTCCGCTTCGCGTTCACTAGCGAGTTGGTCGTCCAGCTCAATCGCTCA
GAGAGCTCATCCTCCAAGACGATGTTCTCCATGAGATTCATCTCGCGGCGCTTCCATGGC
GTCCACGTGAAGTTGTCTCGATTCCAAGTGAAGCGAGAGGTCTCCCGGACCAGAGGCGGC
CGCATGAGTCGCTTCTCGACCAGGTTGCCAGCAACTCTGGCCGAGGCACGCGCACCGTAG
ACACCCAAGGCCAGTGCTGTCAGGCCCCCACCAACGCCGTCATCTTCGTGCGATCCTCCA
GCAATGTTCTGGATCCTTGGCCAAGCCCCGCAAAGACTGCTGTGATGGCCTCAAGGCGGG
TCTTTCGCTCCTCCGCCATTTTTGCTCGCATCTCCCGCAAGCGGACCTCAACGTTCTCGC
GCTCCTGCTCGATCCTTCCCTGCGACTCGGCCTTAGCCCGTGCCATGGCCGTTTCACGAT
CGAGCCTGGCCGCCTCTTGCATGGTCCTCCTCTTCTCATCCTCGAGCTCTAGGTTGTTCT
TCTTGCGCATCTCCTCCTGCCGAAGGAACTGATCGTGCTGCATCTGCAGCTGCTGATCGA
TCTGCTTCTGCTGATCCTCGAGCTTGCTCTGGTAGAGCTCGTTGTCCAAGCGCGCCTTGT
ACTCCGCGGTCCTCCTCTCTTGCTCGTTCTGGTGCGAGATCGTCTTCCGCTTCTCCTCGG
CGTCCATCTGGTTCCTCTGCAACTGCGCCTGAGCCCGCACGGTCTGCTGCTGCTCGATCT
CGGCCTGGATCTCCTTCTGCTTTGTTACCTCCGCCAGCTTCGTCAGCTCGAAGGCCTTGG
TAGCGTTCGGAGAGGTGTCGAGATCTTTGAGCGCCTTCGCGCCCCGCTCGAGTGCCGACG
GATCGAACTTGCCCGTGATGTTGTCATCCTTCTTCTCGTCCTTCCCGCCGGAGCCACTCT
TGCCAGTCGACGGGTAGTACATGGCCGGCGGGCGCGGCGCGGCAATGGGGGCAGCACGCC
ACCCGAGACCGCGCCTCCACGCACACGGGCCGGCTGCGAGCCGACTGGGCTTGGGCCAAA
ATGGCTACGG
>at02007
AGCATCAACAGCAGTGGATGTCCTAAAGGTACTGAGCTTTTGGAAGCGCCGACCCCCAAG
GGTCTGAGCTTCGGAAGTGTTGACACTTGTGACACCGAGCCGCGTGCACAGAGGGTCGCA
TCGCCTGTGCTTCGGGGTAGGAGGGAGGCAGGCACGGCCAAAGTCCGCAATGATCGCACC
TCCGCATGCGCACCTTCTGCCTCAGGCCCTCTCGTCGCCCTCAACGCCTGGCGCATCCAT
CTCGCACAACGTGCTGCTGAACGCGCCATCCGAAAAGGCATGCTGCCAGTCCGAGCCCCT
CCCTTCAGGTGGCGCGTCTGCTGTTGGTGC
>at02008
TTTTTTTTTTTTTTTTTTGCTGAATCACAAATTGTCAGTCTTGATGCTCTGATGGCCTCA
TCAGAAGACCACATTCTTGGGGGACACAATTGTAATTAAACATTTCCGTGCCCCAGTGTC

CCGCGTGCCTCTCACATGCGCTGAGCCGCCATCAGTGTGGCATCAAAGACGCCTACTGCT
GTGGCGCGAGGGACCGTCCACTGCAGGCGCATCTCGTCCTTGGAGTGGCGGCAGAACATC
TCCATTCGCAGGGGGTCCGAGCGCAAGGCAAAGACCCGCTCCTCCTCAGACTCCTGATTG
TTGATGACCGGACGGTAGGTGATCTCCTGCTTGGTCCCTGCATCCACAAGGCGATAGATC
ATCTCACCCTTGCGCTCGCTGGCATAAATGTGCTCCTCCACACGCGCTTTCGTGGCATTG
ATTGTCATGGAACGTGCAATGTATCCGGGGCGGTCAGCCACGACGACGTCAGACACATCC
ATGTTGAACTTGGATGGGTTGCGTGCCTTGCGGACAAGAGCCTCCCAGAGCCCATCAAGG
GACGCCGTGGTGATCTCAGGCGATGTCCAGCCCATGCCCACGACTGGTGCGCCGACGGCA
GCCGGGGCCTTCTTCACGATTGGCGTCACGGCCTGGTCTTGCGCCAACTTCCATGCTGCA
CGCAAGCGGGCTGTCTGAAGCCTGCTCGCCGCATCTTGGAGGCCTGGGAGCTTCTGGATG
ATGTCGCTCTGGACGCCCTTCTCGTACTCTGCGGCTGTCCAAAAATTGGCAAAGTCAGAG
ATGGTCTCAAGCTTGAGGGTCTGGGTCATGTACGACACAATCTTGGCATCAACCCTGGCA
TCGCTTGACAGGAACTGAGCAAGCGAGGACATGGCGACAACTGATGANGGC
>at02009
ATTTTTTCATCCCTGCTCCTGCTCGACGTAGTTCACAGCCTGCCAGTCTGTGCAATGGCA
GAGGTGATAGCAGAGGGGCGAGACGGGAGATGCACTGTGACCGATGATTGACATGCGCCT
CACAAGCAAAAAGGCGAGCATCGGCAGCAGACTGCTCAGTAAAGTGTACTAGTAACCAAG
GGATTCTCAAAGCTAGCTTCGCGGGCCACGCAGATGAGCCGGGCAGGCCCGGCAAGTGCT
TTGGACACCCGTACGTCTATC
>at02010
TCCTGGCTGAACATATTCCAGATCTTGAGCCAGTCCACCTGCACATCCTGGCACTTCGTC
GTCAGGGCGGCCTTCCGCGCGGCTGCCTGGGACGGCAGGCCGCCGCACTTGGTGGTGCAC
ATGGAGGCAAACCAGCACTGGAACTCCTGCTCGAAGGTGGCGTTGTCCATGGTGTGGTAG
GGGTGCTTCCACAGCTCCACGGCCGCGGTGGCGTAGTAGTCCTGCGAGCCCTTGACCAAG
TTCTCAAAGCCCTTCCCACACGCGGTGTCCGGCTCCTGGCCCGAGCGCAGCACGCGGAGC
CTGTTCCCCTCCGCCCCCGAGGCCAGCGCCAGCGCCACCGCGGCCAGGAGCTTTCGCTGC
ATTCTCTACGGAGGAAAGGAGAAGCGGCT
>at02011
TTTTTTTTTTTTTTTTTGGCACGAGAATGCGCCGATAAGCGGAACAACCTCTGTGTGAC
CAACACTGTCACAAAAGGTTGGGTCTGTGGGCAGAGCCGTCCATAGGTACTCTTGAGGAC
AATGCCGAGGAGCGACTGGAACTGAAGCTGCAGCTACCAACACATCTGCGGAGCAGATCA
CCGGGCATTCAAATCCGCTTTCACCTACAGAGACGACAGTGCTGGTGAAATGCAGGTGGA
AGCGCGTGACCTATGCAGGGCGAAGCCAAGGCCAACTGCAATGGCGACTCGCCTGATGCC
AAGCTCGCATGCTAGACGTGCGCGCAGCAGAATGCCGTGGCTCCTTGCTCTGACACTGCC
AACCCTCGCTTGCGGCTCGGCCTTCACCTCCGGGCGAGCCCACTCGCGCAGTCAACCATC
CGCGACTGGAACTTGCCGGCCTACACCTTCAATCTGAAGTGGCAGTCAGGTCAGGAGGGC
CAACCGGAGCGTCCTCTTCAAAGGCGCGATCCCAGATGAGCGGGCTGACTCCGTCTGTGC
CGTCCACGCTGATGCCAGCATTCTGCTTTCGCAAGAATACGAAGAGCTCCGCCTGTGCGT
CTCCCTCGAGGCTCCACGTGCTCTCTGCAGGCTTCACCGCTCTGAGGAGCTGAGCATCGC
ACACAGTTTCATAGTCGTCACGCCCTCCATCACCCACTGCATCAGGTTTTCGCAACATCA
CCCACACNCGCTGAAGCCTATTTGCAC
>at02012
TTTTTTTTTTTTTTGTTTCTCTCTGTCACTGATGATCCAAGGTCACGCAGCTTAAAACAG
TCCGCCTCTCTGATGGCACTGTACATCTCGATCATGTTTGAAGTGGCCACACGCCCTGCA
AAGCGGTCCTGTCACGAGGTGGAGTGATCGACATCCTCTTGCAAGACAATCCAGTCATCA
TCATACAGTTGTTCGAGGTCTGTCTCGACACCGCATTCTTCTTCGCCACTGTCGTCAGCC
CGTGCTGCACATGGTGTCTCAATACGCGCCCTCATTGCCACGCTCGCTG
>at02013
CTCTGTCNGTNCNNAGttCtTGtTCCATGCTCGGTCCAACATTTGCTTCAAGCATGCTCG
CGCTGCAATGCGCACACTCAGTTCCCACTGAAGCTCATGGTTCCGTTCACtACACAAGTG
CCAATCCAAGTTGGCCTGCGAAGTGTCACCACCTCGTCAGCGTCCAGAGGAAGAGCCTTC
TGCCAAGTCCTCGACATTCTCAACCACCTCCTCGAGCTCTGCC
>at02014
TCGAAGACGGACCGGACGTCCTCGTAGAGCGTGTCGGAGACGTCGCCGCTGACGACGTGG
GCTCGCAGGCGGTCCACGGACTCCACCCAGTCACTCTGCTTCGGCAGGAGCACAGGGCAC

GAACTCTTCGCATCGCGCACCGCCGCCTGCCTGGGAGTTGTCGCTGCCCATGGGCTCGTC
GCACCGTCTCCAGAGTGGCAACTGCGGCTGCTGGTGCTCGCAGTGCTTCTGCTGGTGTAG
AGGTTGTGGTCGAAGGAGGCGCCGCCAAACTCGTTGCCGACGTCAATCAGGATTCGTCCA
GACAGGTGCCCCAGCAAATCGGGAAGACCGTCGGGGAGGTCAGACAGGCGCCCCTTTAAG
ACCTCAAACGCCAACATCCGCTGCATGATGCCCAGTTGAACCACCCCGAGGAGCGCCCCG
CCTTGCCGGCAAGTGAGCGTGCGGCCCTCCAGAGCCTGGGCAGCAACCAGGCGCTGGTCG
GTGCCAAGCTTTTGGCTCTTCATCTGGGCCAAGACGGCGTCAACCTCAAAGTCAGTCATG
GCAGTCAGCACTTGTGGCACTGCATCCACCGCCCCGGTCTCCATAATTGCC
>at02015
CATGTTTTTGCAATTTGCAAGGTGACAAGGCACACTCTTCAACACCCTTTGCCGCCCGCG
AAAGTGTCTCACGCCTCCACTACGCCATGGGTGCTGACGTAGATGCCGTCCAGGAACTTT
CGGATATCCTTGTTTTTCACCAGACATGACTGCCGAATAAGAGCAGCTGAGCGTGATGTC
AAATCGATGTCGGTGCCGCTGACGACCAGCTCATCCTTGGTCTGAGCACTCTTCTCGACC
TTCACGCCTGGCAGCATGTTCACGGTGCGGACAATCTTCTCCCCCAGGAAATTCCGGATC
TCAATGGTAGAGCCGCCGTTGGTGATGTTGGCGTTGATGGGGAAGTGGGCGTACACGAGC
CTCATCCGGTACTCGAATCTGTGCTGGACACCGTCGAAGAGATTACT
>at02016
CCGTAGCCATTTTGGCTCAAGTACTTTGGAGCTAGGCATATCTCAGGTTTGCAGAGGCCG
CAGGCCCGCCACGCCATCATAGGTGGCTGCAAAGAAAAGAAGCAACAGCCACGCCATGGC
TGCACAAATTCAGTTGAACAGAGAGAATTGTTCTTGAAGCGGACCGAAGCATGGAGGCGC
TTCTGAAGCTCGTGCAGTTGCATTGCCAGGAAATGAGCCTCGTGAATGCCAGCACGGCCT
GGCATTGCATTGCACGAGTTGGGTCCGACATCAAGCATCATGAGGAGTCAGACACATGCA
TGGCTATTCTCCAGAAGCAGACCAAGCTGCTGCTCTCTCGCTTCCTGGCTGCAGAGCATG
ACAGGGACCCTCAACCCCGTTGTGTGGCTACGATCACTTGGTCCTGCGC
>at02017
TTTTTTTTTTTTTTTTTGGCTCTCATGAAACGCTTCGCCTGACAGCATGCAGCATCTTG
GCCAGAGCTCGAGCGGGCGCATGCTCTCGCATGAAACGGAGCAACCCCGCAGACTCACGC
AAGGTGCGGACTGGGACTTGGGCTGCCTCGTGGGCCCAGACGAGGGCGCTCACTGCCGAT
CCGTCGGAGTCTGGGACCTCGTGCTGCTGCATCTGCCCAACGACGCCAATTGCGGCCTCC
CAGTTCGCTGCACTGCCGCAAGCGCGGACCACGGAGCGGTACCCGATGATGTTTGGTGGG
CAACGCGCATCTCCACGATTCTTCCAGGATGGACAAGCACAAAGACCAGGAGCCACGACG
TTCGCACCCAGATATTGCTGTGGTGTAGCTGAACAAGTCTCGCGCCGACATCAGCCGCAG
CAGCCGCAATGCGCGGGCCCACTCGTGACACCTTGCCTGTGCACTCATTGTTGCGTTGAA
CACCGAAGTGTCGGGCATCCCGTGCTCGAGCGCGCTTGCGAGCAGGGAAAGAGAGACTCC
CA
>at02018
GGATATTGCCATGTCGATCCCCGAGGGACGTCCACGTGAGTCGCTGGTCAAGTACGACGC
CCCCCTGGAGCTGGGGCCGGCGCCCGCCGACGCGGGCTCGCGCAAGGGCAAGGCTGGCAA
GCCGGGCTCCTTGAAGGCGCAGCTGTCTAAGAAGGTGCAGTTGCCCCCAGTGGAGTCCAA
GCCAAACACGGATGATGTGCTGCATGCCCTTCTGCCACCACGTGAGTGGATCGAGGACAA
CAAGCACTACACGCAGTACGTGAGCAACCAGCCTGCCACTCGCTTGGACGTGATCCAGTT
GCAGGAGGCTTTGGACCAGCGCTTGATGGAGCGTCAGGCGCGCGAGACGGGCATCTGCCC
GTGCGCGAGGAACTCTACTCGCAGTGCTTCGACGAGCTCATCCGGCAGGTCACGATCAAC
TGCCCTGAGCGGGGTCTTTTGCTGCTGAGGGTGCGTGATGAGATCCGCATGTCCATTGCA
GCGTACCAAACTCTCTACCAGAGCAGTGTGACTTTCGGCACAAGGAAGCAACTCCAGTCC
GAGCAGGGCAAGGCCGGGGACGAGCAGATGATTGCCGAGCACGAGGAG
>at02019
GCCACGGCATCAGACTTGAAGCAACTGATGTTCAAGCAAGACCCTGCCTTGGGCGTATCG
TCTCCTGACCAGTTGCGGCTGCTCTCCGGGATCGTTGAGATCAGAGACGAGGACGTCATT
GCCATTGCTTCGCCAAGCCTCTTGACGGAACCCCTCACCGTTGCCACGGACGGCCGCCCC
ATGATGGCAAGCATCCTGCGAGCATACGACCCCTGTTCGATGGGCCTCCGTGGTGATCGG
CCTCTCCTCAAGCGGATGGCCGAGCTGACCGAGCGGATCGTGGTGCCAGGCAGCTTGGAG
GAGTTTTGAGACGGAGTTTAAGAGGATTTTCCATGCTGTTGTCGGGGAGCGCTGGGATGC
ACTCGAAGGCCAAGAGTCGGTGTATGTTCCAGAGCTGAGCAATCATCGCGGGATGTCTAC
AGGCAGGGTCTCC

>at02020
GCCCACCCCCCCCCCTGCTCCGGCCGGCGGCCCCGTTCCCCAGCGCGGCGATGCAGGTGG
AGAGCTCGACGCTCGAGGATGCCGCGTCTTGTGGCTTGGACTTAGACCGCAACGCCTTCA
TCTTGGTTCACAGCTGGACCGCTGGCAACTTTCTGCTGAATGTTGTGCAGAAGGCGAAGG
GGGCCCTCGCGGAGAGGCCCAGGACGGCCGGGGAGCTTGCCGCGGCCGCCGGGCTGCACG
AGGGCCCGCTGGCCGTCGCCCTGAGGGTCTGCAGCATCATGGGATACATGGTCTTCGATC
CGGACC
>at02021
CCGTAGCCATTTTGGCTCAAGGCCGGCATGCCGGCCTGAGCCACCCCCCGGGCGCCAGGG
GCGCGGCGGGGAGCGCCGCCGGCCATGAAGCGGGGGGCGGAAGGTGGGGATAGCTACGGG
TCCTCCGCCAAGAGATATGGCAAGGGCAAGGGCGGCAGCTCTGGGGCCGGTGAGAAGGGA
GTGCCGAGGACGCGGCACGCCTTCAAGGTTTTGCTCACCGACAACCTCACTGCCGGCCTG
CTGGGCACCCGTGGAGCCATGAAGGACGAGATCCAAAAATGAGACGAACACAAAGCTCGT
CTTCAGCAGCCGGAACGACT
>at02022
AATGCGCGATGGAGACGCAATGTAGAAGCGACCCTCTTACGAGGGGTCGCCCCTCTCCTC
TACTCTTCTTCAGCTTCAGCGGTCGACCATGTAGGCCAAGATTAGCCGGAGCTGCAAGGA
AACAGCAAACTGCAGGATGTGGAACCGATAGGCTACAAGGCACGAGAAAGGGATCACAGG
AAAATTTGCCAGAAGTCTGGCAGGTCGACGTGCGAGGCACGGGCATGAAAAGGCTAGGCT
CTGCAGGACCGGCCGGCAGAGCCTCCTGGTCCGGCAGAAACTCAAAAAAGGAGGCAGCTA
GGCATCGCGGCGCCGGATGATGGACATACCGTCCCTGATCGGCAACACGACCTGCTCGAG
CTGCGGATGGTCGCACACCCACTGGTTGAACTCGCGGATCGCGTCGCCAAAGTGCCGGCG
GGCTGGCTGGGCGTCGAAGTGCTGATGGACGTATGGGTAGCCGTTGTAGAGCACATTGTC
GCAGACAATGACTCCAGTGGGGGAGAGGAGGTTGCGCTCGAGGATCAGCTCGACGTAACG
GTGGTACTCCGCCTTGTTAGCATCGACAAAGACGAGGTCAAACTCGCCTGTGAGCTTCGG
CAGGGAGTCGAGGGCGGGCCCGACCACCACCTCATGGCGACCCGCGATGTCCGGGAAGCC
AGTCAGGCACTCGCTGAGCCAGCCCTTGAGGAAAGCATCAATCTCCAAGGACACACCCTC
GTTGAAGGAGCACCCTCGAGCATGGCAACAGAGCCGTACCCTACGAACATGCCGATCTCA
AGCACGCGGCGAGGAGTGGCCATGAAGGTCAACTGCTTCAGGAGCATTGCCTCCAGGGGA
TCCGTAGACATTTCCTCACCATACGAGAACATGGTCCTATTCTCGGCCCACTCCTTGGCC
CAGTCTGTGGCAAGCATCTTCTCACGCACAGCACACATCGCCGGTCCCTCAGCTTTGGAC
TGGGATACACAGTAGGCATCCTGCTTTCCAACAACCTGCGACAGCTCTGCAACCAGACCC
TCAACTCGGCTTAGAGAGAACGCTCCATTTGGTGCTTAGTTCGGGAGGTATGTTGGGTCA
CCCTCCTGGCTGGGGAAGCAGGCG
>at02023
TCGCTGAGTCCCCAGGCTCCAAGCCAGACGCAGTTGCCCCTGCTGCATATGTGCCAGTGC
GTGTCCTGTCCCAGAGCGACTTGGGCAAACCGCTGCAGGAGGTGCTGGAAAGCATCCAGA
GTATGGATTCGGTAATGCCTGCAGAGGGTGACGATGCGCGCTTACGGCGACCATTGTTGC
TTTTCAGCGGCTGGCCTCCCGAGAAGATGCTCTACGCCGTGAGGAAATTTCGGGATTTGG
CTGCCCTGCGGCGACTTCAACAAGAGCCAATGGCTGCGATGGCGGTACCGCgGAGCGGCC
AAGAAGTCGATGAGTCAGCTGGTGGAGGAGATAGAGGGCGACTTCGAGCTAAACCGCACT
TGAAGCCGTGGTTGCGAAACCTAGCGGACAAGCTTATCCGGCGTTTCATTCTGATCCTTC
ACAGTTTCTGCATTGA
>at02024
CCGTAGCCATTTTGGCTCAAGCCGTTCGGCCCGACAAGGCGCCCCTGCCTCCACTGGCCT
GAGCGTCCCCGCGAAGCCGCAGCATGGACTTCCCAGCATCCCCGGGTCGCGGACCGCTAC
GTCACGGCCAACAAGGGCCGCGTGCCGGTGCTGTTCGCCAAGCGCGACGGCGCCGGCGAG
TGGAACCTCGCTGCGCCGAGCGCGGCGCTTGCGGCGGGTCCATCTGTGACAAAGAGGACA
GCGGAGATGATGAAGCGTGACCTCCTGGGGGCCATGT
>at02025
TTTTTTTTTTTTTTTTTGCAGGGCCAAGCAACCTCCCAAGCCAACACTGCATTGCTGGA
ACATGGGCTGATCACAGTGCCAACCAAAGACTCAAGTTCAACAGACAAGTGTCATGCGAC
AGCATGCTCCAACATGATAGGATCGGTGGCCCGATCAGCGCATGAGCAAGCAGAAGCTGT
TTGATTCCCATAAGCTTGTTGCCATGGAAATCTTTCCGTTTGGTACAGCATTCCCTCCAC
GGAGCGAGGTGAAGTTCACTCTTGAGCTCTGCTCCACAGCTTGAGCGTTGGGAGGACGCC

ACAGGTGGTTGTGCCCGCCAAGCCTGCTCGTTGAACTCAACACCATCATCGCTGAGCTCA
CCGTCCACAATCTCTGTCTCGTTGATCTTCACGTTGTCGGGCAGCTCGCCGTACGTCTTG
AGGTTCCTTGCCTCATCTGAAGTGTACTTGACAATGATGTCACCCTTCTCATCTTGGAAG
TCGCGCAACGACACAAGCACGATGTCACCTTGGTTGACCCAGACCTTCTTCCTCATCTTG
CCGCGGATGTGGCACAAACGCTTGGTCCCATCAAAGCATTGGACCTCGCAACGGCCGTTG
CCCAACATCCGCATCACCTGTCCGTACTCCTGCCCGTCCTCCTTGAACTCCAGCTCCTTC
TTGGCCTTG
>at02026
ACGCTTCAGAACAGCGCTAAGCCTCGCAAGTCCCATCTGGATGTCTAGGAGCTCATCCGC
GTCCAACCCAGCATCTATTCTTTCATTCAAAAGAGTCCGCTGCTTTAGCAGTTATTGCCT
CCTCTCTCGTGCTTTGACTTGCCAGTCAGGCACCTCATCCTTCTTCATGTTTTTGAAGTG
CTTTAAAGCTACATCTCGCAACGTCGAAGCGAACAAATGCCATGCCTCATTCAGCCGACA
AGCAGCTCTCAAGTTGATCCATGTCTCTTCGATGCCACTGAGCCTGATTTCAAGTTAATC
GAAAACATTATCGTCCCTGTCCTCCGACAACCGCTCTCATCAATTTATCCATGTCAAACA
CATCATGAGCTGCAGCAAAGGACGCCGTCAGCTCGTATTCACAAGAGAACAT
>at02027
ATCGTCAATCGATACGCCCAGCGCCTGCGCTTTTCGTTGGTCAATATCAATTTGCAGTTG
CGGGCTGTCGTCCAGGCCGTTGTGGCGCACGCGGGTCAAGGAACTGTTTTTCCCGGCCAG
CTCAATGAGTTGATCTCGTGCGGCCATCAGCGCGTCATGGCCTGCTCCGGCGTGATCCTG
TAATTCCATATCAAAACCGGCGGAGCTGCCCAGACCGCTAATTGCCGGCGGGCTGCTGGC
GAAGACGCGAGCTTCTTTAATCTGGTTAAATGCTTTGTCGCCGCTCATAATGGCGAACGA
GGAGCCGGTGGTGGGATCGCGCGCGTCCCAGTCTTTCAAGCGAACAAACATGCGCGCGAC
GTTTTGCCCATTCCCGCCAGGGCCGGAACCTACCGTCGAGAAG
>at02028
CGACACACGCCACTTCACCAGCGGCCAGGCAACAAGGAAACAAACCGGGTAGATGGTGGC
CTGTGCCAAGGCAACCCGCCGAACGGGATGGCTTGCAACCATGGTGGGGGTCACTGCTCG
GTGAGGCTGAAAGCGCCTCAGTGGCCACCACCTCGGCGAGGCCAAGTCTACCGCAACCAA
GAGCAAGCCAGCTCCCCAGTACACTGCCAGGAACAAGACATAGGCCAGTGCAAATAGGGC
CCGGTCTGAGAAGCTCTCCCTCATAGTTGCCCAGAGGCACCAGGAACCGTCTTGTGCGCT
CCCGCACAAGATCCAGGCATCATCAGGTCGCTGGTCCATGGCACACGCGCACCGCGGCAA
GGGCGGTTGCAACAAGGGCCGCCTTCAAAATCT
>at02029
TTTTTTTTTTTTTTTTTTGCCAACAGAGCAAACAGAGCGCAGCACATGTCCAGGCCTCGT
CCGCGGGACCGCCACAGCCTACGCCGCGCTGTCCGCACTCGCCGGGCGGGGCCGATCACT
TCTTCATCTCCTCTGGGAAGGTCTGGATGTCGGTGTCACGGGAGACCTCCGTGGGCTTGC
TGAAGGCCAGGAACTCGCCCGGGCACAGGCAGTAGCAGGCGCCAGCTTCCACCGCGAGGA
TGCCCGCCACGGCGTCCACCTGGGCCTTCAGCGGCACGCACTCACTGAGGAAGAGCTCGG
CCCCGGCCTTGCTGATCACGTAGCACCAAAGGCCCGCCAACCAGCCGCCCTCTTCAGACA
TCTTGAAGGGGCCCTCCGGGTGCCTGCCTGGTGCCAGCAACCGCTGCTCAGGGCCCTCCG
GCGAGTGGAAGCCGAGATAGCAGATCAGCCAGCCCTTCGGGAGCCTGCCAAGAGCAATAC
CAAGATCTTCGTCAAAGTTATCGCAGATACCAACCCTCGTGCC
>at02030
TTTTTTTTTTTTTTTTTTGCCAAAATGAATGAGATTGTACTGTACCATCAGCCCGTTCAA
ATGAACTGCACACGGAAAATATGCACTACTCCTTGCTGGTCGGACTCACTTGAGCTCGAA
GAGCTTGTAGGCGAACTCGCTCAACTGCAGCAATCAGTGGGAATACTCCTTGAATCGCAA
CGTGTCCCTGCTGACTCCCAAGATCTTGAGGATGCGCTGCTGCTGACCAACTGACATCTA
GTTGGAAACGTTACATGCTTGTGCTGCGAGCCGCTTGAAGAAGTCACCCTGCTGCTGGAT
CGCCTCGACCGCTTCCGCGAGAGGCTTGGCGTCAGAGCCGCCACACCCTTCGGAGTAGAG
AGTGAAGGAGTGCCACATTCGGGGCCACACAGTCACCTCCACAGGCGATCCCGCGGCCAC
GGCCTTCTTCCCAAACTCAACGGAGTCACTGAGAAGGACCTCTGCATCGCCCACATGGAT
CTGTGTGGGTGGGAGGCCGTGCCCTCGTGCC
>at02031
CCGTAGCCATTTTGGCTCAAGCCATTCTGCCCCCGGGTCCGAAGTACACAAGCTCGCGAC
GGGCACCCACACTTGCGTTGCAGTGACTAGAAGACCATGGCAAGGCGTTGCGTCCTTACC
GCTATGCTGTTGGGTGTTGCCACTGCCGACTTTCGTGTCAAATTCGACGTGGAGACAACA

GCTGGCCCTGAGTCTTTCGTCGTATTAGTCCACGAAGATTGGGCTCCGATAGGGGCAGTC
CGTTTCAAAGAGTTGGTGGAGGCCAAGTTCTACGATGACACTCGCTTCTTCCGTGTCATC
CCAAGCTTTATGGTCCAGTTCGGGCTGAGCGGCGATCCCTCAGTATCTGCAGCATGGCGC
GCCAAGACCATCAAGGACGAGAAAGTCATCAAGAGCAACAAGCCGGGCTATGTCACATTT
GCAAAGACCGGAGCACCGGATTCGCGCACATCACAGCTGTTCATAAATTACGTCGACAAT
GCTCGACTCGATGGCATGGGCTTTGCCCCCCTTTGGAGAAGTGGAGGGCGATGGCATGAG
TGTGGTGAAGAAGATCTACAATTGCGGGGAGTCGCCCAACCAGGGTCAGATTCAATCACA
GGGAAACAAGTACCTCGACAGTTCGTTCCCCAAGCTGTCTAAGATAGTGCAGGCCCGAGT
CATCGTTGAGGCTGAGTTGTGAGCTTGCGTAAAGGCTCGTGCAAGTGTGCTTCGTCAGCG
TGCACGAGGCCAGGAGATCGATTGCAACCC
>at02032
CCTTTGCCCTGCCAGCGCATGCAGTATTGGCGTGGTGCCAAGATTGCATTCCTGGCATCT
TGGAGTTCGACCGTCGAGGGCGCACTGAGTCTGATAGCTCCAGTGACGTGACATGAGCTC
CACTACATATCCTGGAAGGTGGGTGGGCCTCCCAAGCTTCCGGACCCCTTCGTACAGCTG
TGAGGCCTGCCCTGCACCGTTCCTGGTTTTCCAAGTGGTGCTGTACAATACAGGGATGGC
GCGCAAGGCTGCCGTGCAATCGGCTCGCTCATCCGGCACCCCATGAGTTCACTGTGGCTC
ATGCTGTTCTGTATGTATCTTGATATATGCACGCCCCCTTAGGGATCGTCTCATCACTG
>at02033
TTTTTTTTTTTTTTTTTACCCAAACGAGCAATGCTTCTGCAACACATCCGCAGGCCAGG
CAGCGTCTGTGACCACAACGCCGGCACTGTATGAAAAATTGCCGCGCACCGGCGTGTTGC
CAGGACTGCATGCGCGGGCGCATGCACCGGCGAGCCACGCGACATCGATGGCGCATGGGA
ACATCTCGCCGCCAAGGCCCAGGTACTTCTATCCACGTGCAATCTGTAAATTCTGACGAC
AAACCTGGCAAGGGCTCACCAGNGGGGACTCCACCCCCCTAAGGACCCCCGGCCGCAAGA
GGAGGGCTACCGGCCACCTAGGTTTAGGGTCTACCTCTAAGAGTGCCCAGGAGGCCCGTC
AAGAGCAGAGGCCCCAGCAGCCACTCACTTGTCGCAGAGGGCGGCCACACCGGGGAAGGT
CCTTGCCCTCCAGGAGCTCAAGGGACGCACCGCCGCCGGTGCTGCAGTGCGAGACCTTAT
CTTCGGTGTCGTACTTCTTGCAGGCCGTGGCGGTGTCGCCGCCTCCAATGACGGTGATGG
TGCCCACGCTGGTGGCTGCCACGACAGCATCCATGAGGCTCTTGGTGCCGGTCTCAAACT
TGGCCATCTCGAAGACGCCCATGGGGCCGTTCCAGATGATGGTCTTCGACTCGTCGACGG
GCTTCTTGTTCATCTCCATGGACTTCTCGCCACAGTCCAAGCCCATGAAGCCATCTGGGA
TGCCCTCCTCCCTCGTGCC
>at02034
TTTTTTTTTTTTTTTTTCCTTTCATCATATTTGCGAAGCCTCAGTGCACGAAATGTGTC
TTCTCAAACTGTACACGAGCAAACAGACTAGCTATGATGAGTGCCAATCTGTTGGTTCAG
GCGGGGCCACTCTCTGACCAATGCCCAATCGCCCATGCGGGTACATGCGCCGCTGATAGG
CCTCATCTGCTGGCCAGCGCAATGCTGCATCACCCATAGGTCCATCTTGCACCCACAAGT
CCACAATGAAGCGGTGCATACGTGGCCTCAAGGAGACCTCAAGCCACTGATGGCGCTCCG
GGCTGTCACCCACTTCCCCATCGAAGACAGTAGCATCATCTGGGTTCACGGAGCTAAAGG
AAAAGCCTCCACGATAGCAGAGGTCAAGCCCTAAAAGTGGAGTCCATGGGTAGAGGAAAG
CGCACATCACAGATGAAACCGTGTGACAGTGACCCTGACCATGCACCGCAAAACGGGCAG
CATCGTCGCACATGGAACAGCAGCGGCCTCTGTCCAAACCGCGCTGGAAGGAAATCATAT
CAAGGCAGTCTCGAATACATTGGATGCAAGCCAACTGAACTTCTTCAGCTGCAGCCACAT
CTGCTGCAAAGTTGAGCCGATCGACTAAGGGGGCCAAGCGCATCGCAATCCCTCGTGCC
>at02035
CTCGCTGCTCGCCTGCCGCCACGTCAATGACGTGCTGCTCAGTGCTCCGTGGAAGGTCTC
GCAGGACATGCTCACGACCATGAACATCTCCGTGGTCTGCCACGGCAGCACCGACTTCTG
GGACATAGACCTCAGTAGCCAGGGAGATCCATTCGAGCTCCCGCGGCAGCtTGGGtCATG
CTNCAGCGCCTGGACACGGGCTGCGAGGTGACGGTGCACTTGATCGCGCACCGAATATGG
CTGCACTCAGAGGAGTACGCTATTCGGCAGCAGAAGAAGGAGTCTGAGGAGAAGAAGTAT
CTCGCAAGCAAGGCCTTTGNGGACGAGGCGTGAGTGGAGGGTCGTCTTCTTGTTGCCATG
GAGCTTCTCTGAGAGGGTCGGAGCTTGCAGGGTTGCATGCCTCGGGGCTGAAATCAGATC
TGTGCCTTTGCACACACCTGTACAGACAAAAAAAAAAAAAAAAAAAAAAAANAAAAAAAA
AAAAANAAAAAAAAAAAAAN
>at02036
TTGTTTTTTTTCTTTTTTTTTCCCACAACGGACAACACGATGGACGCAGGGAGCCTTTGC

GTCAACACCCAAGTGCTCCGGCCGGAGCGTCTTCGCCTTGGGTGACGCAAAATTTGCCGG
CCTCCCAATCCACCGAGTACTCGCAGCTCCTGTACCAGTCGTCGTCCCATGGCATCTCCG
GCCGCTTGCTGCATCGGTCCTTGTACACGGCCTCTACTATTGCGAGCCGACGGCAATATG
CCTCGTACTTCTCCTCCGGCATCCCCGACATCTTGGTCATCTCCCGCATGTATTCCCAGG
CGCTGCTGCTCAGGAAGTGAAGGTCCTGCGGCCTGTCTCCAACAAAGGACCGCCGCGCAT
CGACCCACGCCTCCAATTCCGCCACGGTCGAGCTGCTGCT
>at02037
TTTTTTTTTTTTTTTTTTCCTTTCCGCTCTCTTCTGAGCCTCTTTCTTGAGCGCTGCCAA
CTGATCGTCTCGTGCCTTCTTCATGGCCTTGACCCGTTGGGCGCCTTCCTCCATCCTCTT
CCCAGCATTGTGGAGCGTGAGCTTGGCCTCGAGCTCCTCCAGGACGATCTTCTGCACGTC
CTTCTTGGCCTTCAGCGCCATCCTGTCGAAATTTCGTTGCTCGGCCGCGAGAACCTTGGC
CATGAAGGCGCTGTTGGGATTACCTTTTGGGGCCTCCTCGCTATTATAGAACACCTGCAG
CGCGTCTGGGCTCCTGGAGGCCATGTCGCCGACCTGCCCCATGAGAATCCTACGCTTGTT
CTCAAAAAGCTCGTAACGTGGCTCCATGCCGCCATGCTCCTCGAGCGTCGTCTTATCCAG
CTCCTGTGGCAAGATGCCCAGCACGTTCATGGCCGCCTGCACACGTGGGCTGTCGCCATC
TGGCACGGGTGGCTCAGGGATGAGCGTCATGCCCGGGGATGCCGCGGCCGCCGCCATCGG
CGCTCTCCACTGGGGTGGGCTGAGACAGGGCTATGACCCTGCCCCACCCCACTCCACGCC
GCGTTTGCGCCT
>at02038
TTTTTTTTCTTTTTTTTTGGGAAGCCATGGTCACTCTTGGTGGGTCCGAGCGCGGCCAGG
AAACTACCGTCAGCCACTCGGGGGATTGGGAGCTGCCTCCAGCTGGCTCCATGAGTGACT
CATCAGCAACAATGACTAAACTGCCCGAGCTGACTGACTGCGCGACTGCAAGACTGCATG
ACTTCGCCGGCCAGTCTGCTGCTTGGGGCACTGGACCGGTGTCCTAGTTCCGAGCATCAC
AAAGCTTCGCTCATTGCATAGAGTCAGACTATTGAACAACTGCCGGGAATGCAGGTGCCA
ATGGCCTCACCTTCACCGCCCAGAAATATTACATTTTAAGAAAAATAAATCAGACAATGT
CCGATGTCGCCTGCCTCATGCAAGCGTCCGCATCTTATCTGGCTCTTTCATGTTGTTCTT
CCACAGCGAGCTGAGCGTCTTCCCAAGCAGCTTCACATCACCTGTCGGGTCGTTGCCCTG
GACTGTCTTGTACAGGTAGGAGTCGAAGGTCACCTTCCTGGACATACTCCTCCTCGCACA
GCTCCACGAAGCTCTCACGAGCAGCGTCAGAGGTGTAGAACAGCTTCTGCAGAGCATCGA
GCACAAAGTACGTGGGTCCGTACTTGGCATCCCACTCCCTTATGTACTCCATCAGGTCGT
ACTCGTCAATCATGCGAGCGCCATTCTCCGAGCGCTTGGCAATGGTCTCAGCCACATGCG
GCCACTCTTGGCGGCAAAGTGATACCCTCACCTGAGCACTTGGTCACGTAGCCAGCGGCA
TCGCCAATGAGCGTGGCGCGCCCTCGAGTCCTCCAGGGCCTTGGTGTTCAGGGATGGGGT
GCGCCTCCACGCGAAGGATCTCTCCGCCCTCGATCCGCTTCGCAGCCCGCTCGCGGATGC
CTTGCTGGTACCTCTGAATGCCCTTCTTGTCGATGACCGTGCCGGTGCCCACGGCCACAT
GGTCGCACTTGGGGAAGACCCAGGCATAGAAGTCCGGCGACACGTCCTCACCAACGTACA
TCTCAGCACGGTCCTTGTAGTACTCCATCTTCTCCTCGTCGACCTTCATGCGCTCCTGGA
AAGCGATTGCGTACTCGTAATCCCCGGCCTTGATGTCCTTGGCTCGCGGCTGTTGGCACC
ATCTCCACCGATAACGACATCCACCTCC
>at02039
TTTTTTTTTTTTCTTTTGGGGGGCAGCATGTTCGAGTCTGTTCGGGTCTCGTGCGGCCT
CAAAACCGAACGCCGTCCGGTCTATGCTGCCCGCTGCAAGCGCCATACGGCAGAGCGGTA
CCAGGTAAGCGCTGATGCACAACCAACCGCAAAGAGCATCACGGGGAAGGAGGTGACCAG
CCACAAGGGAGGGGCAGTCTGGGCCTTGGTGAAGCGCTGCAGGTTCCAGGCCACTGGTGC
CAGCGCACAGATGTGAGTGCGTAGCAGGGCCACCCTCCAACGAATCCAAGTCCGCTGAGT
GGGCGTCAGCTCTGGCGGCACGGCAGGAGGCGAGGGCGTGTCGCCGAGCAGGGCATACTT
CAGCATGATGGCCAGACGCCTGTTCTCTGGGAACCCCAGGGATTCCAGCAGACCTCGCGT
GTACCCCAGCGAGTGCAGCACACCGATCAAATTGGCACCGCCCTCGCGCGTCGCCACCAC
CCTCGTGCC
>at02040
TTACCAACTCCTCGGACTCCGTCACCCACCGCTGTGTGGATGCTGGATTGGGCTTCCGAG
AACAACCAGGCTCTTCGCAGCAAGGCGACGAGCCAACTGAACAGCCGTCACAAGGGCACT
CGGTCTTCCGACAAGACAGTCTTCCGTTTCGCCGACGTCGCCAACAGGACTGAGGTGCTT
CAGGGCAAAGCTGTGCAAAGGCCCATGCAGGTGCCTCTCTGCGGCCGCAAGGAGGTCAAG
AACTGGCCAACGTACAGCTGCCAGCGTTGGCAGGGCCAAGCGGGGCGGGAGCATTCCAAG

TGCCAGAAGCCCTTGCAAGGCATGCACAGCCTCCAGGTAGGGACCCCTGAGCTTCGCGAG
GGCAGCAACTGCCAGACCAGCCAAAACATCGGCATGGGCTTCTGCAAGAGTGGCTACAAG
GAATGCGGCCACAACTGCTGCAACGCCCAGC
>at02041
CTTCGGCTGAAGTTGCCTTTTGTTCAGTGTTGCGACGGAGTCGTCCATAGGGTGGAGCAG
CCATGGACCCAGCCATGCTGAAGGACACGAACACCGAGGTCTTCGGCGATGGCCTGCCCT
TCGGAGATCCATCTTGGTATCAGGCTTACAACTCCCCCTACTACAATGACACGCATCGGC
AGATCCGCAAGCACTTTCGCGCCTTCATCGAGGAGGACATCATGTCAAACGT
>at02042
TTTGTTTTTTTTTCTTTTTTTTTTGTCACGCTACAGGGCTGGAAATGGTGGCCACCTCAT
GGCAGCTCTCCAAACGGCGCCAGTGCCCGACGCCGCACGTAGCAAAGCCCTCAGAAGACT
ACTGGCTCTCCAGGCCCTTCAGCCCCTGGAGCCCGCCAAGGCCGGTGGAGAAGCCCATCT
GCAGCTCCATCTTTCCGAAGAGCTCGTCGCGCGGGTTCTTCTTCTCCCGGAGCGCCTCGG
GCAGAGGACGCCTGGGCTCGGCCTGCTCGGCAGGGCTCATGAGCACCGGCCGGGCGGAGG
CGGGGACCTTGAACGAGGCCGAGACAAGGCGAGGCAGGTAGGGGTCCTTGCCCAGGAACA
TGACCTCCGACTCCGCCATGAAGGCATCCGACTCAAGCAGTTACCGAACGTGGGCCTTGT
TGCAAGCGACCTCCGCCTGGGTCGTGCCAATCTGAGCCATAGCTGCTGTTGGTGGAAGGG
CGCTTCGAGAGGGGAGTCAGAGTCGGACTCTCTGCTTCTGCAGCTTGAGCTTGAGCCAAA
ATGGCTACGGACTCTGC
>at02043
TTTTTTTTTTTTTTTATCTCGAAATTTTAGCATGAAATGTCCGCTACATCTCCACGTGG
CGCCCGAGCTCATAGAGGCATATCGCCGCAGCGCTGGCTACGCTCAGAGACCCTTCCCCC
CGCTGCGGCACGCAGACGCGGACATCGCTCAGGCGCCTGGACGCGGCGGAGACACCAGAG
TCGTCGCTCCCCAGGATCAGAGCCCAGCGCTTATCCCTTGCTGGCTGGTGCGGCTGCACC
GGCGCGTCGGAATGGCTCTCCGCTACGTACGTGCGGACGCCGAGACCCTGCAACTCTCTG
AGGGTGTCCTCCAAGGACGTGGCCAGGTGGAAGTCCATGTTGTAGAGAGCCCCCATGGAA
CTGCGCGCTGCGGGGCCGTTGAGGTAGCTCCAGGAGGTTGGGGTGGCAACGACGGAGGTC
ACGCCGAGGTGAGCCGCTGTCCGGAGGATTTGGCCGATGCTCTGCGGACTCTCCAGGCCG
TCGAGGACGACGAGAGGAGGCCGCAGCTTCGCCAGAGGCTTGGACACAGGGAAGGCAACC
ACGAAGCTGCAGGCCAGCGCTGGCAGCCCAGCTAGTCTGGACTGGAAGTGCGTTGGAATC
AGCTGCGGCTCCACGGCGTAGACTTCGGGCGTGACCTCTGCAGCGGCCTCCAGGAGGTCA
TCGCGGGCCAGGGAGGAGACGAGCACGGAGTGCACGAGCACGCTGCGGGCCTCGCCGCCC
GCGCCTTGCGCAGCTCCTGCAGGCACTCCCAGCTGGAGTGGAGTCCGATGTGCACGCCCA
GCTCCTCGGGGT
>at02044
CTTTTTTTTTTTTNTTTTTTTTTTTTTTTGTTTCTTGCGCTGCCTGCTTGCCCTCTCCTTC
TCCAGGGTGCATGGCCGCTGTAAAATTTCAGCCAAGTGGCTGAGGATCCTTGAGGTAGCT
GTGCTCATGGTGGCCGCAACGGCCAGCGAAAAGTGAATTGATACTGGTGCTTGCACCTTT
GCCACAAGCCACACAGATGAGCCTATGCACATGACCCCCAAGATGACTTGCGCGTCAACT
CATCTGAAATTGAGCAGATTTACTGGCGTGAGCAACCTGAGCAGGGGCTCTTGCCGATGG
CCCTATTGCGTGATGGTGAAGTCAAGGCCAAACTGGGACCAGGCCGTCTTGCCTGTCAGG
AGCTCGGCAACGAGGATGCCAATGGTAGCAAACATGGCAAAACGACCGTTGTTCAGCTCC
CGCTGCTTGAAGCCATCAACCCACTCAGCCTTGCGCTTGGGGTCAGGGTCCTTGTAGAAG
ACGCCCCAGCCCGCCGGGTCGCCAAAGTCGCCGACCTCCTTCTCCGGATCCTGCTTCCAG
ACAACGAGCTCCAGGACGCCAGAGGCAATGACGATCAGAATGAGGCCATAGAGGCCAGGG
GGAGTGTAGGCCGCCCCGATCCCCCGTGGCATCNTCNTGCNAGGAAGCTACCGGGCACGA
CGCTCTGCACCAGCAAGCCCAGTGAGGCCATCATGGCGACGCGGCCATGCTTCATCTCCG
CCTCGCGCAGCCGTCGG
>at02045
TTTTGTTTTTTTTCTTTTGATTATCATGGTCATCATGATTCGTTCTTTTTGGTGGAAAAT
TGATGCATGAAAACTCCAGACCTATGTACAGCAATGGATGACACCTCAACCCTTGGGATC
TCGCCCACTACTCACTACGCCAGGCTGGCGATGGCCGCTGTAGCTGCGGCTACGCCGCAT
AGGGCAGCGACGAGTGGTGCGCAACGATCCGCAGCTTGCGGTCCCTGCCCTTGAAGAAGG
CCCAAGTCTTGTCCACCTTGGTGACAGCGCCGGACTTGTCAGTTATGAGGACGTTGCCCA
CCGTGACGGCGATNTTGCCTTCCCTGAANATGGCGCTGTTGACNATCTCCACCTTGCGCC

ANCCCTTNAGCGCGAAGCCCGNGTCCTNNGGGAAGGNCGNATCGCCGCCCACAAAATAAG
CCAACGCGCCTCTTTTCGTCGTGCGGAAGGTCTGCTTCCCACTGGCGAGCGTGGGCTTGA
AGAGCACTGGCCCGTACTTATATCCGTAGGCAGAGTCGAGGACCTGCTTCCGCCAACTGC
CTTGGCCGCTTCGAAGCCCTGACGCCTCGTAGGTCATGGAGATTGCTCACCAGGGCATCC
CCCCCAACCCTTCTGGGCAGCCAGCACCTCCTCCTCCGTGATCTTGGAACGCAGCTTGAC
GTTTTTCACCGTTCAAGAAGGTCCAACTCTTGTCCACCACTGTTGAGGTCAGAAACAGAA
CTGCCAAGCACGCAATCTTGGTGGAGCGCATGCTGCAAGTGGGGGAGACTCCAAGCTGAT
GCACTTACGTCGCGAATGTGTGGTCTTGAGCCAAAATGGCTTGAGCCAAAATGGCTACG
>at02046
ATTCGGCACGAGGCGCGAATGTCGTGCTTTCAGGCGGCACAACAATGTTCGCGGGCATCG
GAGAGCGCATGACCAAGGAGCTCTCAGCTATGGCACCATCGACTGTGAAGATCAAGATCG
TCGCACCCCCTGAACGGAAGTACTCGGTGTGGATCGGCGGATCCATCCTGTCGTCTCTGA
GCACGTTCCAGCAGATGTGGATCTCCAGGGCCGAGTACGATGAGTCCGGTCCGACCATCG
TTCACCGGAAGTGCATTTGAGCTCTCAAGGTGTACAGTGCATTGTTGGGTCTGATCAAGT
GGCCTCTGATCGTGATCCTCTGCAGACTCCCTGCGATGTTGCCAGATGATTCAAGCAGGC
AGACCTTGTGGTCTGTTGTACAGGCACCGTTTCCAGACATGCAAGCAGTCCATGCTCGTC
CCCACTGTTTTGCGGCATGTTCCCCAGCCTCAGCTGTGCCATGTGGTGGCAAGTTGTCCC
AGAATTCACAGCTGTTCAGGCGATGAACTTGTCAAGGGGATGAGGACAGCCGCTTGTCTC
GCAGATGAGTGTTTGCCGCAAAAAAAAAAAAAAAAAAAAAGAAAAAAAAAAACAA
>at02047
GACCACTGTGCCGTTGTGCCGTTGGGCGACATCATGAATAGCCAAGAGCTTGCACCAAGA
AGGATGCCGTTGCACCTGCTTCCCATTCTGGTGCGTGGAGCAATTGCCTGTGTACAAGGC
AAACTCGAAACGGATAGCCATGCAGCTTTGCATACTGCATGTTCAGGGCAGCCGCGAGTG
CTACCCAGGAGGCATTTGATGGCTCCGTGGCATTCCATGGGGAAAGGCGAGCGGCTATCT
GCCATCAGAATGGTGATGTCTGACCCTGCCACTCTCGACCTGAAGTGCCGCTGGGTCTTC
TGAACGAGTGCAGGACAATGGGCGCCCTCTTGCTTGACCCCCCCTTGACTTGTGAAGTGC
AGGTGGCGTGCCCCGCAGCTCTGAGCAGCACGAAACAGTTGAACAACACTCGGCTGAAGA
ATACCATACCGAAGAGTGCAGCGTT
>at02048
TGGTCTTTTGCAACCGCTGCCTCGGTCTACCTTTTGGCAAGTTCTGTTCATATCATCATA
CCCGATGCTTCCAGTTCTTGTCTGGACCGCGTCCCACTGCACCGTCTACACACGTCGTAG
TAGCACGTGCGCGTGCCCCTGGCACCCTTGTAGCGACAACCCCACATTGTAGAACCCTGT
GCTGACTTTGTATTGCATACTTGGCATTGCACACCAGCTGGGGCAATGAATCTCTGGAGG
GAATGGCTCTTGGGGCAGACAAAGTCCTCCACCGCCTTCTCTTTTCCTGCAGGGGGTCCC
TTC
>at02049
TTTGTACGTACAATGATGATCTTGCCCGTCAAACCCTGTGGCAAGCACTAAAAGGCTGCG
ACAGGTGGCACCAACGAGCCTCGAATGTCAAGAAATGCACCTTCAAAAGTAGTGGAAGGA
TGAGAGCGTGTAGAACAACATCAAGACTCAAATCCAGCATTAAAACAGAGTAGGGATCCC
CCATCCAATAGGACCAAAAACACGGAACTATATCGAGCTGAGGCTATCGTCGCTTAAGGT
CCTCATCCTTTCGACCCAGGCTGTACGTCCGTCGTGGTACTAGCGTTGCGAATTTGGGAA
CCAGGCACCATGTTGTGCACGTGGCAGCTTACATGGGCACGTACTCATCCATCTCC
>at02050
TTTTTTTTTTTTTTTTTTCGGGTTCGAAATGTCTGATGAGTGTGACCAGGCACGCACTTC
ATGCACAGAAGTGCAAATGCCAGTACCGGGCAAAGCGTCGCTCTATCACAAGCTTCCCTC
TTGTGTCCATTCACATGTTTGGAATGGCCTTACAAAAGCTACTTCCCCGGACGTCAACAG
AGAGAACCCAATACTGCTCCCCACTCTCACTCGTCAATCTTCGGAGCAAGGTAGAACTGC
ATGTGTCCATTGTCAGTCTTCTCAAGCTCGTACTTTACCAGGAGTGGAGCATCAGGCCCA
AGTCCCAACTCCACTGCCCCACAGAGAGGCGCTGCCTTGGCAAAGTTGACCAGATACCGA
AGTGCAAAGGTTGCTGTGACAGGCTCATGGACCGTTAATGAAACCTTTTCCTCGGGCCTC
TCTGCCTCCCTTGGCTTCAGCATTACATTGCCTGCTCCGACATCTCCCTGCACACTGAAC
GTGATACCCTCCTTGCTTGCCTTCACCTGCATCGTCTCACCAAACTCCTTGAGATCTCTG
CAGATCTTCTGGAACTCCGCTGAAGGAAGCTTCGCGGTGACCTTGTAATGCTGCTCAGGG
ATTTCCATGTGTTCTGACTCGATTTGCATCAGCTTCAAGTCGAAGTCTGCGATGCGATCC
TCCTCGCCACTTTCGCACTGGAAGTTCACTGTGTCAGCATCAGCTCTCCACTTCAGCTTG

AGGGAGT
>at02051
TTTTTTTTTTTTTTTTATGCGCCCCGTGCCTCAGGTCAGGCGTGCCTCCATGATGCTCAG
GCCGCCCCCTTACTCTACGAGTCCACCAGCGGCGGCTCCGAGATCGGCATGTTGACGATC
GCGGAGATGATGGGCACGTTGGAGCTCTGGCTGCAGAGGAAGAGGCGCACGCAGTCCGTG
CCACCGTTCTCGTTGCCGCACGTGATAGGAATCTCGACGAGGTACACCTGCTGCGGCACC
AGAATGGGCAAGTAGAAGCAAGGCTTGGGCATGCGGTAGAGGGCCGGGTTGGTGGTGCAC
ATGAGCGGCACGTCGTACAGGGCGTGTACGCCCGCGTTCTGCACCCGCACAACCACCTTG
AACTCGGGCCCCATGCCCACGACCTGCGCGTTCAGCCGTACTTGGGCGCCGCCAGCGGAA
GACATCGGCCCATGACCAGCCCCCAGAAGGTTCACGTATGCCCGAGCGGTGGACAGCCGC
AGCTTGCACAAGTCCCGCTGGAAGGTCCGGTGCATGTCGATGGCCTGCTCGCGCTCCCGG
CTGGTCTGCTCCACGTAGAGCTTGGTCTTCTTTGGCACGTCGAGTGGGATGTCTTGCTCA
GGCGGCGGGCCGGGCGTGGTANAGGAGACGTCGAGGTTTG
>at02052
AGCAAGGCGAGCTTGAAGCACGTACACTGCCGCAAGGAGCACCAATTGGGATGCAGGCAG
AGGGCTTAGATGGGCATGTGTTTCCGACTGATGCGGATGGAGGACTAGCATGGCGCAGAA
GTGTGTCGCAGGTGTACTTTACGGTGCCAGCGACACCAAGGCTGCTTGCTTCCAAAGCTC
AGCTTCCTTCCGCGCAAGCCAACGCTGAACTGATCTCTGCCCCGAAAGCTTCTCTCCAGC
AGCTCATTACCCAATGGCCGCTGCCTGCAGACGAGCCTCAAAAGTGAGAGAGCTGGCACA
GGTATGTAGAGGATTGCCCGGAGGAAGGGACACCACCATGGAAGAGCACCAAAACTGAGA
GTGCGGCATTCAGCCTGTGCCATGCAGATTTAAAATTGCAGTATTTAACATGCTA
>at02053
TTTTTTTTTTTTTTTTTACAAGCATACGCTTTGCAGACCTTCCAAACTCAACTGAAGCT
TGACACAAACGTACAGGCAGCGTGGGCTGCAGAGACCAAAAGGCCAGTCGTAACAGGTTG
AAATGCCAGCCTCACGACCCATGCTGCATGCCCTGAACACAAACAGCAGCAGCAGCAGCT
GCCACAGCAAGAATTGTTGCCCCAGACATGGCCGCAGGGACCTTTCCCACGACCCGCTGG
CAGCAGGACAGGATTAGGACGGTGCAGAGCACGGCAGCCGCGAGGATTGCAAAGACTACC
ACCTGCGCCAAGACTCGCAGGCGTCGCCTTCTCGCCTGCACAAGCACCCTCCCGCATCTT
AGTCTGCCATCGGCACCTCTTGCATCGAGCAACTGAGCGGCAGGACCTGCAAGCGCCTCG
TCGTTGCCTCTCGCCTGCTGGCACAGTTCAAGCTCTGCAGCTGACAGCTCAAGCTCTGAT
TGGGCCTCGGCGGCCATCCAATGATTCAGCCCCCACCTGTCCTCATTGCCCAAGCAGTAC
AAGACTCCAGCGCACACAGCAAGCCATAAGAGCATGAACATTGGGCTACAACGAGACCTT
GCAGGGGTCTCAGAGGTTCCANTCCCANTGCAGACATCTTCCTCTGCACNGCATGCTTCA
GCTTGGGGAG
>at02054
TTTTTTTTTTTTTTTTCTATCTCCAGCTCGGGCTCTGGCTCCGGCTCCGTCGCTCTCCGC
GGGGAAGCGCAATCCTCATGGTCGTTTGTTACTGAGACGGCAAGTGCATCTGCATTTCCG
CTCAGCTGACTTCCTTTCAAGGAGTGGCAGCCCGACGAGTCTCACCATGTTATGTTCAGC
CTCTGGAGCCCCAGCATGTACTCTGCAAGGCCACCACGAGCCTTTGGTGGGCCACTTGCT
GCGGCGGCGGCGAAGGCACCGTCTTGCGAGAAGTTCTGCACGTGCGTCTGAAGAAGCGGC
ACAGCGGCCGCTGCGATGTTTGCCCAGAAGCGCAGGACGCGCTGCTCGCCCAACACCAGC
CGCAATGCGTTGTACTCGGGGCTACCCTGCTTCTCAAGCGCCAATCTTGCGCTGATCGCT
GCTTCTGTCCACCTGTAGCGCTTCAGGGCTGCCTCACACAACCGACCAAGCTCGGACATG
ACGCTGGCCTCATTCTCTCTGCTCATTGCCTGTG
>at02055
TTTTTTTTTTTTTTTTTTCAACCTTTCGAAAATTCGAAAGTTCGAAGACCTCCGAGGCTC
AGCGAAATCACTGCACACTGGAATCTGTCCTCACTCTCAAGGAAGCGAACAGACAGCCGC
GCGCTCAGGCTTCAAAGACGTGCATCCCGTCGCAAGGCGTGGCGACGAGCAGCCCGGGGG
CGAGGCCAGGAGAGCCGCGGTATCCGCGGCCGCCAGGGTAGGAGGAGACCTCCGCCCCCC
GCAGAACGCCGTCGCCGTCCGCGTCGCGGATCGCGAAGAGCTGGCCGCTCATGTGGCCGA
GGTAAACGGCGCCGTCCCCGCCCACCGCGGGGCTCGACCAGGCGTGCGGCATGCAGAGCT
TGAAGGGGAAGGCGTCGCCCGCGGCCGCCTGCCTCGCGTAGGCGGGGGGCGCGAAGGCCC
ATCCCGTGGGCAGGCCCGTGGCGGAGTCCACCGCGTACACGCGGGTTGCCAGCGCCAGGG
GCGACCCGTC
>at02056

TTTTTTTTTTTTTTTTTGGGCGAGATGAGTCTGCCAAGACTCCAGGTGCTTCTGCTTGC
TGCCCGTCACTTCAGCGCACTTCCCGTCGCGCTCTTGCCTGCCACAAGACTGCGCCAGAT
CTCCGCACCGAGTGATTGGACTGGCTGTCGCCAAAGACAAGCTCGTACTTACCAGGGATC
AAGCCTTCGTTTTCTGTGTCACCATCGTCTTCCTCCTGAGGCATCTCAAAGGCAACCTCA
GGCCGTCCCTCGAGCCAAGCCACTTGCAAGGATGGCTGCAGCTCTGCGGGCAATGCCACC
CAGCGGGGAGACCGGCCCCTCTTGGACCAGACCTCCCACACCGAGGTCAGTAGCTGACCG
TCAGCCGCAAGCTCCACCAACAGCGGAGGTCTATGGTCTGGAGTGCAGCCGGAAGGTGAG
TCCGACAGCTCGCGGTGCTCGTGGTGCGGCACTANACACAACATCACGGGCACCTGTGCC
GGGAGCGCCACCTGCAACCTCATGGCTGTGATCGCTGACGCCAGCAGCGCTGAGCCAGGC
AGTGGACCTTGACGCTGGTAGTGGCAGACGACGTCCACTGCAGCAATGGCATGGNTCGCG
GCTGCAGTGGCCGGGCAGGCAAGTGCCCAACACTCCAGAGCAGCAGTGTGTCCAAGCAGC
>at02057
AATAGTGGCCGGCCCACTCTGCCTGACAGCGCCCTCACTGGCGTGTCGCTGGGGACGGGG
CCTTTCAGGAGTGTGCGTCGAGCCATTCGAAGAGCAGGCGGTCGACCCAGCGCTCGAGGC
GCCAGCTCGTGTAGCACGCGAGGTGGGAGCCGTGCTTGAACATTGCCAGCGCAGCGTTGT
CGAGGCCATCCAGCGTCTTCATCCAAGAAGCGGGAGGCCCTCCGGGCATGACGGGATCGT
CCTCCGTCAAAACCCACAGCAGGGGCACGCGGCAGGGCTCCAAGACCCCCGCTCCACCCG
CAAAGGGGTTCAGCCGGGCCTCAGCCTCCTTGGGGCAGTCC
>at02058
TCAAGACCTTCAAGGCCGGTTTTGTCACATACTGAGATCACAAGGCAGGGCTACTCCGAC
CTTGAGGAGCCGATCGATCAGGAGAAGGGGACGGGAAAGGGACACTGGAGGCATGTCATG
TTCATATGGTACTGGGTCGAGCAATTCACCACTGAGTTTGCAGCGCAAACTCTGCACAAG
CTCGCCAGCAGCCTGCGGATATTCTCTGTGACGACGATAAGATGTCGAACGCTGGGAACT
GCCACGGTGGCCCTGTCCATGCTGCACCATAGGATGATGGACCTCGCAGAATCCAACGAC
GGCCTTTGAAGAAAAGAAAACGGGAAAGACGAGCAGGGGCAAGGGCAGACGTGTGTGGAA
GCCCCGCTTTGAAAGAGTCTCATGAGACTGCGTTGGGTGCC
>at02059
GGTACTGCGGACTCAATATTCTTCTACTGGGACCTCAAGTGCTAGGCCACGATGTGGTGT
CTCGTGAGCTCAGGCTTCAGCGAGACCGCTCGCCAGTAGGAAGTGCAGTTCCCAACCCGT
TTTCCGCCAGTGCTCGGAAGCAGACAGATGTGCATCTTTCAAATCTGGAGTGGTCCCTCT
CCATTGAAACTGTAAGCGTCGAGGCCGCAGAGTCACACGATCACCTGCTCCAGTTGTTCC
GTTGCAACAACACCGAGCGCACAACCCTCGTCGCTGACGTTGTGGCTGATCTGAAGGCAG
CTGCCTGGTCCCAGTGGAGCTTGCCGGCCGGTGCCTCCGAGCGGCTCTGCGCCCACGCCT
GCTTGCAGCATCATGCAGC
>at02060
TTTTTTTTTTTTTTTTTATGCGATCTCTGACTTGCGATCCCCGCACTCCATTCCTCAAA
GCATCTCGCCGTCCTCAAACCCGCCACGGAGCCTGTCTTGCCTCGCGGCGAGCTGAGCTG
TACACAACGCGCTGCAAGGATGGGCAAAGGACTGCTTGGGCACCTGCGCACCCTCCAACC
TCACTGTCCAACAATGCCCCAGAGCATGGGGGCAGCGGGCTCTTGCACCCGCGGCTGTGC
GAGTGGCGGCGTCGGCGCCCTGGACAAACCGCTGAGCGCTGACTCCAGGAATGCCTCCAA
GTCGTCAGCGGACTGCAGGGGAGCCACAGGAGCTGGGGCCTGTGCTGGTGGCTGCTCCTG
TCGCTCCAACTCGCCGAAGAGGTCCTCGAAAACTGCATCGGCGATCTCCGCCCTCCTTGC
CAGTGGCGCTCCGTGCCTGGCCTGAGGCGGCGCAGCCTGCGGCTGCTGTGCAGCCGAAGA
TGATGAACGCGGCTGCAAGGTGGCCGGGCAGGTCTGCGCTGGTGCCGAGGCTTCGGAGCT
TCGCACACGCGGAGTCGCGTGAGCAGTCGCTGCGGCATCGCGCTCCCGGTACTTCCGGAG
CACGAACCGCTGCTTCTCCTCCTTGCTTGCGCCCGAGGCCACCTTCTCGGCAACGTACAT
TGCATCTGCCCAGCTGTTTCCTCTGGTCTGCATCAGTTCGATCTCGTCGGGTCCCCAGAG
GTAGGTGCCCGTGCAGCCCTTCACCTTGCTCACATGCGTCCCGACAGACCGATGCACGTC
GGAGCAAGTCACACAGATGAAGACGCCATGGGAGACAGAAGCCCAGGAGCTGTCCTGCAC
GCCACAATCAGCACAGAGGTGGTTGCCACAAGCTTTCAAGCCGCGCAGGCGTTTGAGCTC
ACGCTGGGTTGCAGCATCCTGACGCAGAATCTCGTCCTTTGGGTACTTGTTTCCCAGCTT
GCTTGTCCACATGGCTGTGGCCATTGCGCGAGCTGGGGAGACA
>at02061
AGATAGGAGCGAAGATACTCGATGTtCCTTTTCGTTCGTCAGGTAGTAGTAATAATGCTG
CCAGTTGTAAGTCTCTCTGCAATATCCCTTGGAAGTAAAAGACTTCATCAAGCTGAGTAC

gTTCCAAATTGGGGGAtTTTGGATTTCATCAGAATGTTGAGCTTGCCAGTAATCCTTCTT
GGCAACGAGAGTTCCTTCCTTGAACAGGTAGGAGTAAACCGCAATGCGGTTGGGCTTGGT
ATAACATATTGATAAAAGTGTTTGGGGCTTGTTTCACGGCTCTTCGTCGCAAAAC
>at02062
TTCCGAAGGGCCTGCACCACGTTCCTCACATGTCCTCGCTGCATGGCATTGCTCGAGTGG
GGGGTTGGTCAGCAGTTTTGCATGGAGGGCTTGGCAGCTCTGGCGTCATGGCCGACGTCT
GGCTCCTGCAGCAGCCTCCGGGCCTGGCACGAGATGCGGACGAGTGGCGGCAACGTCGCC
CGCGCGCACCACTGCGGTGCTGTCTGCAGGGACCGACTCCTTGGTGCACTCC
>at02063
ACACTGGGCCCTCCGTCCTCTCCCCTCTTCCGACCTAGTTGTTGCTTGAGCTCCGCCTGC
GGAGACTGAGCCTGCTGCAACGGAAGCGGCCTCGGTGGAGACTGCAAGGCTGCTGCCGCC
GTGACGAGAGCGCAGGTTGGCCTCAACCTCTTGGAGTAGCATCCGAATCATGCTTCGCAT
GACCGGCCGTCGTAAGACCTCGCCTATGGACGGTCGGCTCCGCGGGTCCTTTGTCAGCAT
TTCGGGAATCAGGGCTCGGAGTGCCGCCGAATATGTC
>at02064
CCGTAGCCATTTTGGCTCAAGCTTGCCGTTCATTCCCACCCCACCCACGGCTTGATGAAT
TCTCTGCCCGTAGTCCATGCTTGCTCAAGGCTTGCCTTAGTGACGCTTGCACTCGGCGAC
CTGGCATGGTGCCCTCTGTCTGAGTCGTTGGGCCTCAGCGGTGACGGACTACTGCGGAGG
GTTGTCGATTACGAGAGCTACAGGGCCTACTTGTACGAGCCGAAGGGAGCTGCTCCTCCG
GATGGGTGGCCACTGCTTCTTTTCTTGCACGGTGCAGGAGAAGTTGGAGATGACTTGAAG
AAGACGATCGCGGAGGCGGCCGACGGACCCATGCTGGATGTCTCCAAAGGCCAATGCATT
CAAGAGCTTTGCGAGCACTTCGTCGTGGCTGCCCCCCAGGCATCCAGGCAAGAGCGCGGC
TTCAAGACTGACAAGATCACTAAGCTCTTGGACTTCC
>at02065
TTTTTTTTTTTTTTTTTTCGGCCCCAGGGGCCCAAGCAATCCTTCCAGCCTGCCTGCCGG
CCTGATTGCCTGCCTGCCTGATTGCCTGCCTCTATGGAAAACTCGACGCGCAGCCCCCCC
GGACTGTCGTCTTGCTCAGCTCCTGCCGCGCTTCAGCTGCACATGTCGGTACACCGCCAG
GAAGAGGAGCTGAAGCCCCAGCAAAATATGCCCCAGGAGCCAGTGGAGGAGCTTCCAGTT
GCACCAGTGCACCAGAAGATAGACCGGCGTGAGCATAGCAAAGGCCGGCAAGAAGTCGAG
CTCATGGTTGGCCAGATTGATGGCACGAGGAATGCGCCCGCTCTTCTTGCACGTCTCCAA
CTCCAGAAAGTGGCAGAGATCCTCGTAGGTAGTCTTTCGAGGGTCGACTTCCAGGCGCCT
CTCCGGCGGCACGTCCCGCTTTATCCGCTCAAAGAAAGCTGTGTGGGTTTCCTCCGAATT
ATTCTCCCACTCGCTGAGGCCCATGAACCAGTGGCCGACGTAGCGGACGTTCGCCATGTG
TCTCTGCCACAACATCAAGTAGGGGGACTGGACCTGATTCAAAGGAGGGCTACCTTCTTC
CAAGAGCTTGTGCATAGGCCATCCGCAGAGAGGCTCCAGGGCAAGGAAGATGAGGGCCCA
AGGAAACACGTGGAGGCCGAGATCAAGATTGCCCATCATGATGAGCCAAGGGCCGAAATA
CATTATGAACTGCTCCATGGACTCTTTTGCCTTGGCATAGGTGCGCCAGTTCAGGACAAG
AACCTTCGCAGTGGGGCTAACTTCCATGATCGGCCAGAAGAGCTTTTCCGCACCATCCAG
GGCCACAGCATCTGTACGACAGCGAGACGTACCAGAAGCAATCAGCTCAGGCGCCGTGTT
GTTCAACACTTTCATGTCGTCCGTCGCCCCGAATTTTCCCAGCGAGCCTATCTTTGGCAC
CACGTGGCCACCGTTCTCAGGCTTCGCCAAGTAGTCGTAGTAGATGGGGTGCCAGACGT
>at02066
TGCCGTCACTTGCCCTGGAGGAGCCTGTCCGAGAAAGCCAGCCTCCTGCAGAAATCAGGG
GGAATGGAGCACCGGAACTCCCGCCCAGTCTCGCAAGATGTGCCCCTACGCCGAGTCAGA
CGAAGCTGTTGATGCTTGTGGGCACCCTGGCACATTGGAACATCCCGCCGCAAGCCACAA
GGTGTTGTGCCTTTCACACTGCCGTGGATGACGTGTTCATTGCCTTCCAGGCGCTGCGGG
ACCTTCCGTGTAGAGCCAATTTGTTCGACCCCGAGCCGGCACTACGTGCAGTGTCCCGCG
TGCCTTGCAGTGAGCCCAGCAGATGAGGATGGGGAGTTCGAGTTGCGGCTTCTGCAACAT
GCACAACGCTCACCTTCCCATGTTGATGGTCCTTGCAGTGAGC
>at02067
GCAGCTCGGGCGACAGAGGAGTGTGCTTCCCAGCGTGGTAGAAGGCAGCCACCTCGCTCC
AAGTCTCCGTGGAGTAGGGGTACGACATCACCGGGATGAGTGTCGGGTCCTTCATCCACC
CGCTCTTCTGGATGAGCTTCACCATCTTGCCTGGCGCGACCGTTCCGAAGTCAGTGTCCT
GCTCTGGGCTGCAGACCTTCCAGCTCACTTGCGAGTTGACCCGGCGGCCACCGCGAAGGC
TGGCGCATTCCTGCGAGACATAGCACCAGGTGAAGTCGGAAATGGTGTGGCTGGAATAGT

TGAACTTCACGCAGTAGTTCTCCTGCAGGTCAGGGTAGGCCGCATACGAGCCGTGTGGCA
AGACTCCTTTGCATCGCCCAGCCTTGCCATCCCAGTGCGGTAGACCTCCGCCCAGCCCAG
G
>at02068
CGGCCCGCTCTTTGTTCGCTTCGCTTGGCATTGCACCGGCACGTTCCGTGCGACCGACTA
CCGCGGTGGTTGTGATGGGGCACGCATCCTGCACTCCCCCGAGAAGGACTGGCCCGACAA
CGACGGCTTGGTGGTCGGCGCTGACGAGCCTCAGTCAGAGCTTGCGGTGGATTTGCTGCA
GCCTATCAAAGACCATTTCGGCACTGAGTTGTCTTGGGCGGACCTGATCATCATCGCAGG
CACTGCTGCATTGGAAGACATGGGTGGCAGGGAAGTGCGGATCTGCCCCGGCCGATCTGA
CCTGAGCGCCCAGGACGCCGCAGTGGGCTCCACGTACTTGGATCCGACGATTTACCTCAA
CCCCGAGTTTG
>at02069
GTCCAGGAAGCAGTCCGGAAGAGGAGGCCTCGTTTCAGCCGGCTTCCGCCCACTCGCCTT
GGCCGCGAAGGGTCCACGCCGGCAACGTCGCGGAGGTTGGCGACATAGTCCATGCCGTAG
GCGTTCAGCCAGCCCTCGGCCTGCCCCTCGGGCAGCGGCGGCAGCCAGGACGCGTCGGAG
TCCGCCGGCGGCGGGAAGTCTTCGGCGCCGGCTGGCGGCTTCGGCGCCGGGGGAGGGTCC
AGAAGCAGGCCGATCTTGAAGCCGAGCTGGCCGATCTCGGGGTCCTTGTTGCTCCAGTGC
GTCATGATCTGCCCCATCAAGAAGACGCCCCTTCGGGCTGGCCTCGAAGCCGAACTTGGGT
ATCACGGCAAGCTGCACCGAGCTGTACAGCTTGGAGCGGTCCATATTGAACTGCCTACCG
CCTTCGGGGTACTTGGCCTTGAGCGCCTTCACTCCGCCTGGAA
>at02070
TTTTTTTTTTTTTTTTCTCCCGGAGATGACTGAGGTCAACATAGATGGCTGTGGGGCACG
GTGGTCGAGTCCCTGGGCTGGTATTTGTCACCAGGTCTACGGAACCCGTTGACGTGAGGC
TCATGGCTGGGGCCAACGACTTTGGCGATGGCTCTCTTTGTTGGAGAGGTGCGTTAATGG
ATGTCAGTGCCTGCAAAGTTGTCTGCATTGTTTCTGGCACTGCCATTTCTGTCGCAATGC
TTGGTACAGGTCCTGCAATAGCACCCACTGGCATGGCAACTGGCACTCCAGCTGCCACAG
GCACCGGCATGGTCATTCCGCTAGTTCGTGTCGGCATAGCCACATGAGCAGGGATTGCTG
CCATTTGCAGGCCATTGGCGCCAATAGTGCCTGCAGGGCAGAGGTAAACTTGTGGAGCAA
ACCCAGGTGTGCCAGGCCAAGTGCTGAAACCCATGGGGCCAACAGGTACTATGGATGCTG
CGCCCATGGGCGCATTCCCATGTGAAGGCACTCCTTGCATCAGCATGCAACCGGAAGGCT
GTGCTGAGCTGCTTGGGCAATTAGGTCCCAAGAAGTTTGCCGCCTGCGGCCTTTCGAAAA
ATCTGTCCATGGGTGACTGGCAAGTCCTTGGATCGAGAAAATCGTAGGTCGACTGGCAGC
CAGGGACGTTGGTCGCTGGTGTTCCCTCGTGAAGATGGAGCCTCCCTGGGGCTCATTCTC
CGCAACTGGCTTTTGCAGTAGCAGACT
>at02071
GCGGGTGCAGGGTGCGGTAAGTGTCCCACAATGAGAATGTTCCATACTGCGCTCCACTCT
TTGCCCGCCGAATCGGCATGGTGTCGTCGAGATCCGCCAAATGGTAGCCGTGGCCCCCTC
CCCACCAGAAGTATGGGTCTCCCTTCTCTGGTGCCGATAGTTTGCCTGGCGGTAACCTTT
CAATGCGGAAGGACCCGTCGACGTCACTCACCAGGTTTGGTGCAAGCATGGTGTGATAGA
GGGCAGAGTAGAACATCCGTGTTCTGTTTAGTGAATTGAGCTTTACCTGAAGAGTACTCA
GCTCGCGATCCCACATCATCCGTGCAGCAGCCTTCACCTCGTCCAGGGACCTGCCAGCCA
CCTCGGCTCCAAAGTTCGCACGAGCATGCTCGATGCTTGTCCGGCTGACGCCAACACGGC
AACCAATGAAGCATTG
>at02072
AATCGCAAAGCCGTACGTCATGGCAAGCATGATCACGGCCGCGGTCAAAACAGCGCGGAT
CCCAGTCAGCATGCTAGACATCATTGTCTCAAGCTCGGGCATGAAGCGGCAGATCTTGAG
CATCCTGGCAACGCGCAAGATCCGGAGGATTCGCAAGGTCGCCATCACATTCATCCCACC
AAGGTTGTGGTTCCCAGTGACTGCGATGGCGAGGGTCAGCACCCAGCTGTCTAGGATCAT
TAGGATAACAATGGACGAGTCCAGCAGAAACCACCTGTCAGAAAGTGCGATCCTCTTCCG
CTCAAACGCCAGGAGACGTACCACGATCTCCGCGGAGAACATCGTCACGAAGAAGTGTTC
CGCGACTTGGAACACAGCTGAGGACTCCAATAGCGCTTTGCCATTGAAGTCCAAGTCAAT
GGCCATCCACAAAGTGTTGAAGACTACAAGACCAAGAGTGGtCtAtTACtTCAAAGGCTG
TGCTCCTCACTATGCGCTGTGCAACTCCGCTTCCCTTGTAGTGGGGCCGAGCAGAGTCTC
TGCGATACGCTACTGAGAGTGCCCTGAGTGGAACATTGCCAGTGCCACCCAGCTGCTGCA
GGGCTGACTTTCTGGTGCCCCATGGCTCCACGGTTTCGACACGCTCGGGTAGTTCATTCC

TCAAAGTCTCGCATTCTCCTCCGCTCCACTCACGGTGCCAGTCAAGCTGACGGCTCAGAC
CGTGTAGTCGATGCTCGTGCGTCTTCCCATCACCCTCTTCGGGGGAGGGCTCCGCAACCT
GCCTCCTGAGCAGCTCATTCTCAGCTTGAAGACGCACTACGGCCTCGTG
>at02073
TTTTTTTTTTTTTTCTTTGAGAGCAGGCTCACTGCGCGCCACAGCTCCTGAAGAAATCTG
CCAGATGAGCGATCAAAGGGTAGCCAAGGATGCTCACCGTGTGCCACGGTCCAGGAAGAA
CTCTGTCAGATGAGTGATCGAAGGGTAGTCGAACATCATGGTGAATGGCAAACTCGGCCC
TGGGAACTCCTTGGTAAGGGCGTTTCTGAGCAGCACCGCCGACTGACTGGTCAGGCCCGT
CTGCATCAGTGGGGTCTCGTCCTGGATATCGTCCAAGCCCACGATGTCGTTGACAACACT
ACGCACCGACTGCTTCAAGACATTTGCGTTCACGCTGGATTGTTGAGCAACAGCCTGCGA
GGACTGGAAGCGGGCCTGTGTGACTTCGCCGTAGCCTTCAAGCATTTGATCCTCCAAGTC
AGGAGGGTTGTCAATGACGTCAGTGATGTGCAGTGCCATGTCCTTGCCCCGCGTGTCTTG
GACCTCTGCGAAGATCGCCAACGCCTTCCCAGCTGCCTCCCTGGCCTTCTCCTGCTTGCC
TGAGGTGAGGAGCCCGCTGGCGTTCAGGAGCAAGGCATAGGCCTCACCGTGTTTGTCCCT
>at02074
TTTTTTTTTTTTTTTTTCGCATGGACACTGACGAAGCGACCCACTTCAATCACCTACTA
CCCACACGATCGCCACAGGACTCCTTGCAAACATTGGTAGAAGTTTGAATGATATGTCAG
GTCAAGCCATCGATCAAAGCTCCATCTTGTCCGTCTTCTGAAGGGTGTATATTGTCGCTA
TGCCTGTACCTGCGTGGAAGCTGCCGTCGGGCTTCCGCCAGTCCTGATGGTCTCTGATGG
CCTCTTCTAGTAATAAACAGTACGCCTCCTTCAGCTGAGCCTCAGTCTTGCCGTTCTCTT
CCGCCACCTTCGCGAACGGGAACCCCAGGACAGTTGTGGCTGCACATATGTCCTGCATCG
TGCCTAAGTTAAAATCGTAGCTCAAAATCTCGCTGGCCTTCACAGTAAGTCCAGGCACTG
CAGAAACCCCGTCTTCCACAGAGCTGATGCCATACTCCGTCGGACTCAAGGACAAGGGGT
TGATGGAGAAGGCCGGAGGCGTCCCTGTGAAACCGAACCTCTCGTACATCTTGCTGAGAG
CCTGACGAGGAAGAGTGTAGAAGGGCATCTCCTTCCATACAGAAACAAAAGCGAGACCCC
CGGGCTTAAGCACGCGGTGGAGCTCCTTGAGAGCCTTGACCTGGTCTGAGAACATCAGGA
CATAGCATGCCGTGGCCGCATCGAAGCTCTCGTCGGCAAAAAGGGGAGAAGTCCTCTGCT
GGCATGACGTGGGCCATCACGTTGCTGAGTCCTTCCGTCCGCTTTTTCGCCTTCTCAACC
ATCGGCTCCTGCTTGTCGGTGAGGACGAAACTGTGCTTCGGCATGCGTCTCGCCATTGTG
GAGCTCGGTTCCCCTGGTCCAGTACCGATGTCAAGCACCCAGCTCCCCTCAGGTATGTTT
GCCTGCACAAGGTCTGCCATCTTCCTCCAAGCAGGCTGGTTCACTTCGAAGATCTTTGCA
TAGATGTTCGGATCGATGCTCTGTGCCCCAGCACGTGCCAAGGCCAAAAGCCAGAAGAGG
GGAGCAGATCCGACAGGCCGCATCACTGAATGGATCGGCCCACCTTGTCGTATTTGCAAC
GGAGTGAAACTGCTTGAGCCAAAATGGCTACGG
>at02075
AGGGCTGCTTCATTCGACATCAGGtTAGTTCGTCCAGCaATGAATCaAGCAGAGCCTGTG
CACAACGTTTGGCGTGTTGTGCTCGGCAATGGCCGGGCAAGCACAgGcCATGCGAGTATC
CACGCTATCCTGAAGCCGCTGTTGCTGCCCCTCGCTGCTGCCGAAACCGAGTGTGCTGTC
AGTGGAGGTATCCGCAACATTGCAAGCTTCTTTGAGGGTCTGAAGGCGCGCTTGATCCAG
GCTTGCCCGAATTGCCGGAGCTGCTTCGAGGTTTGCATGGGGCTGTAAGAAAGTATCCGG
CCAGCAGT
>at02076
TTTTTTTTTTTTTTTTTCATAGACTGCAGACGAGTGTTGCAGAGCGCAATCCTCCTTTG
TGCCGGGTCCCACAAAAGGCTGTACACAAGCATCAATGGAATGTGGCACTCTTACAATTG
CATGCATTGCGGAAAAGCGAGGCGCCTAACCCTTGTCGTCCCACACAAAGTTCCCTTTGG
GTACTGATTCGCAGCCAGGCATGCTGACATCACCAATGCACACCTTGACAGCGTCCCTCT
CTGGCCCTCTGCTGACTTCCAGCTCACTCTGCCCATTCGCGAAGTGAAGCTCCGAGTCCC
AAGTGATGTCCCACTCAAACTCAGGCTCATCATGGAGCTGCGGATCCGCCTCCTCCTCAC
TGGCAGGGCCCTCTGCTTTGACCTGTAAGTCCTCGTCCCAGTTCCAAGTGATGTCCCAAG
GGGAGCCCTCATCCTCCTCATCCTCACACATGGCATTACTGGGACTGTAGGTGGCCACAT
CACTCCAAGAATAACCACCACGCAACTTCTGAATTTCCACGTCATGGTTGCAAGCGAAGC
GTGCCGGGAAAAGCGCACCAGGCTCGGATGGCACGCGCAATCTCAGAGAGAAACGCGGGT
CCACTTACTGCACCTGCCGCATCTTCTCCTCGGCATACGTTGCCACTTCAGAGGGAACAC
AGAAAGGAAGCCAGCAGAAGATGACGAAGTCCTCAGGCACAAAGTGAGATGCATGGAGCC
ATTGCTGGGACTTCACAACCTTCACAAGCACGGTGTGCGCGAGCGTCTGCTGCATGTCTG

CGAGGCTCAGCTCAGCCACAAGGGGAACTCTGACAACCTGTACACAGGTCAGTGCGCCAT
CCCAAGTTTCAAACATGCCGTCCGGCGTGCCGGGGAAGTGCCGACCGTCGAACGAGCGGA
CCATCTCCCGCTCAGACCAACTCCTTCTGACCTCCAAACCCCTCGTGCAGTCCTCTAGTG
CAAGCTCCTCCGCGTGCACGTTGAAGGGGTCTGCGTTCAGGCTCTGCGTGCAGACACTGT
TGCAAAGAGTGAGGTCTGGGGCGCCGGCCAAAGGCTGGCAACCCCCAGTACCCTGCACAT
CCATCTCCAGGATGACAGGAGGAGGAGGAGGCAAGTGTGCCTCCGGTGGTGGTGCTGCCA
CTGAGATCACCGATGCCGATGAAGCCAACGTCGCCGTTGCCGCTATCAGCGCCGCACTTG
TCGCCTATTCCGCCCTCACTGGAGGAAGTGTGGCCCACCGCCGCGCCTCCACTAAGTTGT
GCCATGGCTTATGGGAGTCTGGCCACAGCTACCTTACGCAGCTCACTTGTGCAACTGCTC
CGAGCTGGGATGGCTTGAGCTCTTGAGCCAAAATGGCTACGG
>at02077
TCTCCGACTCAGCCATGGCGAAGCTCTGGCCATCGAGCGCCTCCGTGCGGAAGCTGCCCA
TGGGGGGCCTGGAGTCGGCAGAGAAGGAGTCTGCNNNCGGCAGCGtTCNGAAGCTCGCCC
TcGTCGGGCgGCAAGCGTGTTGAATTTCTCACCATCTTGCGGCAGCGTTCTGAAGCTCGC
CCTGTCgGGCGCAAGCGTGTTGAATTTCTCACCATCTTGCGGCAGCGTTGAGAAGCTCGC
CGTACGTGGCACGAGGGTGTTGAAGCTTTCTCTGTCGGACACGCCTCGGTCCGCCTCAGC
CCCGGAGTTCCCTACGATCGGTGCTTCTTCTGACGGCTCGCTAGCGTTCCCGC
>at02078
CCGTAGCCATTTTGGCTCAAGGCGTTTCAACCACATCTGTTGAGGGTTACTGCACGATCT
CGCTTCAGCCAATGGGCGCCTCATCGAGCATACTCGCCTCCGACCAATGTGAAGCATTGG
CTGGAGGCCCAGATGCACAGCGTGACAACGAAGCACAAGAGGCAACGCCCGCCACTTTTG
CTTCAAAGACAAACGAGTCGCTTCCTGACATTACGATTCGGTCAATTTCGCATTCTGACA
TTGGGGGATGGACGCTCTCGGTGATGCAGCACTACAAGGTACCAATTTGCTTCCTCGCGC
TGGAAGGGACCGACGGGATGTACCTGAAGGGTCGTCACGGCCTCGACACGAAGTTCCTGA
AGCTGCCGAGCATATGCCACCATGCAACGCCGCGCGAGCTGCCCGTCATCGTGTGCGACG
CGGTCCATGAGATCATGTACCGCTACGATCAGCTGGTCGTGGGGCCGCCTCGTGGCCGCT
TCTTCGTGGGGGCTCCCTTGCTGCTCCCCGGTCATCGATGCATTGGGACGCTCTGCATCA
TGGACCCAAAGCCGCGAGATGCCTTCAGCCTGAAGGACTGCTCATTTCTGGTCCGCGTCG
CCGAGAAGATGGCGGTCCGCCTGTGCGCGGAGGCAAAGCGGTTCCTCTGCCCAACAGCGG
AGAGCCTCGCCAGCTGGCCCAGCGGCAATCTGGGCAGCGCGGGCTCGGGCTCGGGCTCGG
ACGAGAATGGGACACTGGAGAGCCTGCCGTCGGTGCAGCAGCTGAGGAGCGGCACGCAGG
AGAGCCTGGCCAGCTTCCCCAGCCCGACAACGGAAAGCCTCCCAGTGATCCCAGAGTCCT
TAGAGGACGCGCAGAGGCAGGGAGCTTCCGCCGAGGACCTCGGCAGCCACCTGGAGGAGC
CGGGTGGAGCCGGAGAT
>at02079
TTTTTTTTTTTTTTTTTGGGACCCGGCGCGCGTAACTAAGTGAACAGAAGAAAGAAACA
TCCTCAGCGAGAGATGCAGGTGAAGTCCACAGGACTGGAAGACGGCAGCATATTCAGCGC
AGGCAAACATGTGGGCAGTCGTGGCGGTACTGGGTTCAGAAGAGGGGTTGCCGCACATGA
TCTCCAGACCTCCTGCCCACTCGTCCCACTACAAAAAGTTCCTTAGTTCGTTCTACGTGC
CAGTTCTCCAGCTCGGACACTGTCTCTTTGGATACTTAGAAGCACTGAAACCGCCATGCT
TGATCTTCATGCCCAGTGTCGGCCAACCAAAACTGCTGATGGGAACGGCCTCACCCCTGC
ACGCACTTACGGACTGTTTGCCAGGTGGATCATCTTCTCCATCCGTGCTTCCATGGCCTG
TATCTCCCGTGACAGGCACTTTCTGATGTCTATGACGTCCTTGACGGTTGCCTGATTCTC
GTCGCGGAACCGCAGCACTGTCTCGATGAACTCGTCAAGGGCGAGGGTCGGCTTGTCGCA
CAAGATGAAGTCGGCATAGTCGACGAGCGCCAGGACGTCCACGCCGATGTCGTCCAAGAT
CTTGCACGCCTGTGGCTCCTTG
>at02080
TTTTGTTTTTTTTTTTTTAGCCCGCACCTACAATTCGCATGTCCAATCATTGTCGTCCCT
CGCCCCGATGCGCGCCCTGTGTCCGGTGAAAACAATGTGGCATGCAGGGCGCTTCCCCTC
ACCTCGCCGTGCCGGCCTCACCGGAACTCGCCGATCGCGACTGCCGCACTCACAGGTGGG
CTTGCCATGGGGGAAGGCTGCCGGGCAGCTGGTGCAGCCTCCTGGCCAGAGGCCCGCGGT
GCTGCTGCTGGCGACTGTGTTCGGCGGTTCACGGGCTCCGGCGTGCNNNNNNNNNNNNNN
NNNNNNNNNNNNNNNNGCGCAGGCCCAGCAGGCCGTGGTGTTCTGATCCGCGTTGACGTCG
ATCCGGCTGTGGGCCTCGTCGGCGCACTCGCACGCCTCCTCCAGGCAGTTCGGCGGGTCT
GTTGGGCAGAAGATTGCTGCCCGGCTGTCCCACACGGGCCCACGCCCTGGAGCATAGACG

ACGGCGCAGACCGGAGCGCTGGTTGCCTTCACCGGGGCCGGCATCAAGGCAGCAGCGGTT
GGCCAGACGCCGAGCACGCGCAAGGCCTGGTCGGCTCCCGCGATGCGATGGCAGACGTCG
AGCTTGCGCAAGGTGACGTCCTCCTGCTCCTCAGCCTCCAGCCGTGGCACCAGGCGGTCC
GGCAAGAGGCTGGCGT
>at02081
CCATATTTAAAGGCTTACCGAACGTTGCATCAATTGTCCACGTGGCAGCCCGCACAGCAG
AATTGACTGTAGCTTCGTACTCGAACCAGCTCCAGTTTTGCGATGAAATTTGTCATCAAG
TGCCTCACAGGCCAGATCGATGTGCTTGATGTTGCAGTCTCAAATAGCAGAACCGACGAC
ATAACGGCCGAGATTTTCGAGAAGTCTGGCACTGCGGCGCAGACATGTCTTTTCTTCGAG
GACACGAGATTGGCAGATGGCTCCAACTTGTCCGAACACATTGTTGATCTCTGCAAGGGC
ACGGACATCAATGGCAAAGAATGCTCCGCTGTAAGCGATGGTGAGGACACAGCTGCTCGA
TCGGCAGAC
>at02082
TTTTTTTTTTTTTTTTTACTCTCATCTGACTCGAGGCGATACTTGAAAGATTCGAAGGC
AGCCCCAGTTGCACTGCAACGGGAGGTGAAGCCTTGGATCAGACTACATGGTGCACATCT
GTACAGGCGCGCGCACCTTCAGAAGCCCAGCCGCGGCGGCAAAACACTACTGAGCAATCC
TTCACTGCCTGTCATGGTGCCTGACGATCGTCTGGCAGACGTCCAAGGAACCAATGCCAG
GCTCATCCTTGGACCCAGCGGAAGCCATGGCCTTGTGCGCAGGGCCGGCAGCTCTCTGCC
CTGTAGCTCCGAAATCACGCTTCGAGGTCTGCAACGCGGCGTCGCGGGCGCGCAGCAGGT
CCTCCCCCCTGGGCCTGCTTGTGCTCATCCTCCCGTTCTTGCAAGCCGAGCAGCACGCTG
GCAGCGGGGTGGCGCTCGGCTGCCGCCTGAAGTCCAGTGATGAAGGCCGTGGTGGACTCC
TTCACGGTCTGCCTGGTCTTCTCCAGCAGCGCCCCTGGCCCCGAGTCCACACCCAGCGTG
GCCTCCAGGTCCTCCTCAGAGACCTCGCAGATGCGGGAGATCTGATCGGCAACGGTCTCC
TCCAGACCCCTCAAGCCCCTCTCGCACGCCGACTGGTAGATGTCCCAGACCGCCTCGCAG
CACTGCGAGATGGAGGAGACGGCCTCCGGCATCACCTGGGCGCAGCCGCATGCCGTGGCC
TTGTTTGCGCAAGTTTCCAGGCAGGACACAGGGTCCGTGAAGCAGTTTGCGCAGTCATAG
GGGCAGGTCTCGGAGCCCAGGCGGAAGCCTGCGAGGAAGACGTCCAACCTTTCGGCGACG
CAGTCCTTGATCTCGCTCACGCTCCATTGCTCCTCGGCGAAAAGCTGCTCCGCGCCTGCA
CCGCCGCAGAGCGCCGCCACGACGGCCAAGGTGCAGAGTCCGGCGCAGCACTGCATGGGC
TGGAAGTGGAGCAACGGCCTGGGAGGGGGGACCGGAGGGCACGCTTGAGCCAAAATGGCT
ACGG
>at02083
CCGTAGCCATTTTGGCTCAAGATATCCTCACTCAAGCCGCGGCTGCAGATCCCTCCTGAG
ACCCTGATGGCTCGCGCACCCTGCTTCTGGGTGGCCGCGTACAGGCTGTCACACTTCAGC
TTCTTTCGCGTGGTGGTTTTACCACACCACATGCTCTCAGGGGAGTATTTCTCATCGGTG
GAGAGAGTGCGCGTAGGGGTGAGCACGTGGACCTCCTTCTGCTTGAAAGCCTGGGGCTTC
TGGGCACTCCAAAAAGATAGAGCTAACGAGCTCTTGGATGGCAGCTAAGAAAGCTGCCGG
>at02084
CCGCGGTTGGAGAACCTGTACCTGGGACGCCTGTAACTAGCGACTGCGAGCCGGCAATGC
AGGTGGGCCTGCACACCTGCGGCAGTGGGCAGTTGTATGGCCTCAGCGGGAACGTGGCAA
AGTGGATTGTGGCAAATTTCGGGCCAGCGGTTGGCGTAGAGGACATCGTGGTGTGTATGT
GGATGAAGCGCTTCGAGGACACGCATGGGCTGGTTGATCGCTGCGGATTGCTGGACCCGC
CAGACTACTTCGGCTCATGGGTGCACCCGTTGAAAGACCCAGAGCGCTACTTACATTGCT
TCCACAATAGGACCCATGGCTGCAGTGTATCAGAGTGGAAGCCATGCGAGGAGAAGA
>at02085
CCGTAGCCATTTTGGCTCAAGCCCTCGTGCTGCTGCGGCTTTTCCTCCATCTTCTCCGGC
GGCGGCGGGATGGCCTCCGGAGGTCAAGACGCCGGCCCCCGCGTGACCGAGGTCGGCTAC
ATCACGGACCTGGAGGGGAACATGGTTCCCTTCGAGGACTACCTCCGAAGCCCGGAGACC
GTGCTCTACATGGACGAGGAGGGCCGCCTGCAGTTCCACCGCGACGACACGGCCTTCGTC
TACGGCGGGGACGTCTTCGACCGGGGCAACGCCGAGCTGCGGATCGCGCGGCTCCTCGTG
GACTTGAAGAAGCGCCACCCCGACCGGGTCTTCCTGATCCTCGGCAACCGCGACATCATC
AAGATGCGCCTGACCTCGGAGCTCGACGAGGAGTACATCCGCACGAGCAGCCCCAGGGAC
GCCTTCTACTGCTTCTGGGA
>at02086
CCCTTTTTTCAAGGCCGCCGCACCACCATGGAAGCACCACACCGCCCACGCTGCGATTGC

GGATGTGTCCCCCCCTGTGCCTTCACTGTTTGAGATGGCCCAGCTTCGCGCTGCCAGGAG
GAAGCGAAAGGNGGACAGGGCGAAGCAATCGGAGGCTCAGGCGCATCTAGACAAGcCTGC
TCAcCCGTTCGAGAAGATCTCAGCCGCCAATTGGAGGCAGAGCGGGGCTGTGCGCTCTGC
GTTCCTTTCACTACACGAACCTGGCGCCGCAGAGGCAGAGCGTTGCGTCGAGATGATGCT
ACCGCCACCTCATGGCAGAGTGGAGTTTGTCATTGCCCAGGACTCGAGCCTAGCACATGG
AGGGCTCCTCTGGGAGTCAGGCATTGCCCTTGCTGCACACCTCGCACAAGATGCCAGTTC
TGGAGCTTCAGGCTCAATGAGGATGCTCGAACTGCCAAGTGGGAGGCCCATGCGCATTCT
GG
>at02087
TTTTTTTTTTTTTTTCTTGTGGAATTTGGGGCTGATCTAAGATGTGCAGAGACCCTGCAC
GTCAACGCAGCCGAAGTGGGGCCATTGGCCTCACTACCCACCAGAGCAGGGCACACTAAA
TGACTTCTGTACTCTCGCCCTCCTCAAATTCAGGCCTTGTAGTACTTGGTCGGCACAAAT
GGCATCTTGACAGTCACCGCCGGATACTTCTTGCCGCGCACAACCACCTGCAGCTGGGTG
CCCTGCTTGTTGAAGGGCTTGTCAATGTACCCCATCGCAATGTTCTTCCGCAGGCACGGC
GACATGGTGCCGCTTGTGACCTTGCCCACGGGCGTGCCGTCGGGCGCTTCAATCACAGCT
CCCTCCCTCGCCGGTGGTCCAGCTGATGGCATCAGTCCCGCACGCAACTGGGTGTATTTG
CTCTTGTCAGCAAGCTGCCCCAGCACCCGCTCGCTGCCGATGAAGGCGTTCTTCGCACCA
GGGGTCCGGCGCGACTTCCCCACCACCCAGGCAAGGCCAGCCTCCACGGGCGTGATGGTC
TCGTCCAGGTCGTGGCCGTAGAGGCAAAGTCCTGCCTCAAGGCGCAGCGCGTCACGCGCA
CCGAGGGCCGCGAGCCGCACCTCGGGTTCCGAGCGAAGCCGATTCCACACCTTCTCTGCC
GCGCTGCCCGGGACGANGACCTCGACGCCGTCCTCGCCCGTGTAGCCGCACCT
>at02088
CCGCGCACGCCTCCGCAGCCGCCCAGCAGGAGGCCACGGCGAGATGCCTGAGGGCAACGG
GAGCGTCCCAGTCGTGGAAGGAGAGGTACTGCGCGCGGGAGCGGCAGAACGCTCCCTGCG
CTCTGGAGGAGATCGAGTCCGCGAAGAACTGCGCGTTCGCAACCTCTGGCAAGGCCGTGA
GCTACCACAACATGGACGGGCGGCTCAGGTACGGGCGCAACAACGTGGGGGACATGTTCT
TTGATCCGCGGCTGGGATGCATGGTGGCGGACGGACAGTAGGCGTAGGATGCATGGCAGT
GCAGCTGCCGCGACCATGACGCAGGATGCCAGACGGTACCATATGCTGGCTTCTG
>at02089
AGTCCGAGGTGAGCAATGAGATGAAGCCTGGACTGATGACAGAAGAACTTGAGAACGATG
TCATGCAAGAGCTTCCAGGTGAAGTCTTGAAGAAGCTCGAAGAGGAGTTCGAGACCGCTG
TGAAAGAGGACAGGAGAGAATCTGGCACGACTTCCAAAGCCAAACAGAGGATCCGTTGCA
AGCAAAGCTTGGCAAAGAGGGAATGCAAGCAGGAGCCTCAAGACCCACAAGCTCAGTGGT
CTTGGACGAAGGCAGACCCAAGCACGACCTTCGCAGCACGCCTCCTTGGTTCATCGCGCA
ATGTCGCGCCTTCCAGGTTGTCAGAAGAACATGGTCTCCTGCACGGCCCTGCTGCTT
>at02090
GTTTGTGAAGTGGCACATGCCGGATGCCATGAAGGAGATCTGGGTCTCCGATCCCACTTG
GGGCAACCACAACACCATCTTTGCCAAGGCAGGCTTGACGGTCAAGACCTACCCATACTA
CAAGGCAGGCACCAAGTCCTTGGACTTTGA
>at02091
TTTTTTTTTTTTTTTTTCATTGGATGGACGCTATCACAAAACTCTGCACAGTGCTCTTC
ACCAGAGGCATTACATGCCGTTGCGGGAACGCTTCAACTTCCTCGGTGTTGACTCCCCTT
CGTCGAGGCGTCTCCGCCCGCCATTTATGAGCATCCCCATAGCTGCTGGGTCTTTCATGA
TCTGGGTCCAGCCCTCAGTTTGCATCACAGCTGCCAGGTTGCGCTTGATGATCGATTCGC
ATGCTTGCACCAACTCTTCTGCCGAGTATCGAACTGCAAGGGAAAAGCGCTCAACGACAT
TGTCTACTGAGAGCGTACATAACATCTGCTCCTTGACGAGAGCCACAAGTCGAGGTACCT
CGTACTGCGTACCAAGCTCAAACAATTCACAACCAATGAGATCGACAGCCCCCTGATTTA
TTTCATCTGTGTAGATGCAACTGAGGAGCTCCCGCACGCCAGCAGACGATGCGTTGATGC
TGGCAATGCCTGACTGTGCCTCCCGCATCTGCCCGTAGAACATTGCACGGAAGACTGGTG
AGCGCATCGCCAGCAGCTGCCGGTGAGCTGCGAACGTGTTGCCGTCCTCCGACTTGAGCG
TGACATCAGCAAAATCTCCGGATTCCAAGGCGCACTGCAAGTTGCTTGCAAGCGACAAAG
GTGGGCGGACTTCCAGCCCTCTCTCCAGTGGCGTCTCCACAAGCTCGCCCTGGGCAGAGA
CAGTCGTAATCTGGACAATTGCGGAGAAGCCATTACACTCCCGGATCGCGCACCCAAGCG
GGCTCTCCTTCTCCACGGAGAGGAGCCTCGTTTCACCATCACGCAAAGGCCCCAGGCCAT
TCGATTTCACTGCAGTAGCCTTCTCTTCGTGCCCACCACAGCAAAGCTTGGCATCCACTG

AAAGTATGTAATACTCAAGTGAGGCAAGCTCCAGAATATAATGTTGTCGTTTCCACACAA
ACCTCAATCTTGCTCGCTTGGGCTGATCAGGAAGAGCAAAACCAAGAATATTGTTTGCTC
CTTCGGTGCCGTGCACAGCAAAATCAGGACCCCAAATCCTCTCGACCTCCGCTTGGGTCA
TTTGCTCCTTCAGAGACCGAAGCTCCCACTCAAACTCGGTTGTGACATTACGCCTGAGCG
GCCGCTGCTCAAGTGAGCTCACAACCTTGGAGCGATGCACCTCGGCCATCTGACACAGTC
AGCCAAGACCGCTGGACAACCTGATTGCTCGTATGCTTGCACCAATTCTGAGCCCAAACT
AGTGACACCTTGAG
>at02092
TTTTTTTTTTTTTTTTTCCTCATGACAACCGCCAGCACGGTCAGATGGCTGTACATTGA
AGGGGACAAAAAGTGCACTTGTGTATGTGTGGCCAGTGATGAGGATGCCACGGCGACACA
ATCCCAGAAGCACTTCCCTCAAATGCTACTCTTCTTCAACAACTACCGGTTGCCACAACC
TACGCTGGGGCAGTCGGGTAGACTGACTGTGAAGAAAAACAACGCAGAAGTCTGTACAAG
TGATGAGCTCACCACTGCGGTGTCATGGTCTCCTGTGATCCACACACTGCTTCAAAAGTT
CGAAAGACGCGGGCCCTAGCCTGACTGACACGGCTTGTCAGCGGCACTGGAAAACCAGGG
CCTTGTACCGCCAAGCTCTGTCGCCTCATGCCGAAGGGAAGTCATCATCGCCGAAGACGT
AGTCGTCGTCATCGTCATCGTTGGAGTCCGGGAAAAGGCCTTTTCCCTTCAAGTAGTTAA
TGGACTCGGTGATCGCGGAGTCCTTCAGCTTCAAGACAAACAAGCCCTTGTCAGTATCGT
TCTTGATCTCCGCCTTTGCGGTGGTCGCATCACCGAAAAGACCTCTCCTCCGCCAAGCTT
GGCCAAGATGTCCTTCATCCACTGCTCGGCCGTGAGCTCCGCCTGCTTGGCGGGAGGGCA
ATACGCGACCATGGCCAATTTGGAGTCCTCCTGGTTCTTGGAGATGATCATCTTGCCAAT
GCGCCCGCTGCCACCCTTGCGCTCCTCCTCGTCAGGGTTGCTCTTGGCGTTCATGGCTTT
CATGCTCTCGTAGAGCATGTCCACGTCGCCGTTTGGTTCGTCCATGGTCGTGCAGAGAAC
TGCAAGCCTCCCATGTCGGCCGCGCCCTCGATCTCGACGCCGCGCTTGCCACCCTCCTTG
GTGACCTTCCTCATCTTCTTCTCCATCTTCTCGGTGGTGAGGCGCTCGGGAGTGGCCGCG
CCGTAGGCATCTCCGGATAAGGCTCGCTTCCTCTTCGGCAGCTGGAGCCGGATCCG
>at02093
GGCGAGATCATGCTGCCGTGNAAGACgGTGGAAGGCGTGAGGCTGAAGAATGGCAGGCGC
CTGCTNTACCNGATGAANGGcCTGATGTGCTTTGGAATCAGCAANGTGGCCGTGGTGGTC
CTGAGCTACTTCGGCATTATCAAGCCGTACTACGTCNTCTTGAACATGGGCGCNCTGCTC
ACGGAGGCCGTCATCACGAGCTTCGCGATGGCANTCTGGCTCTACATTGACTTCGGGCTG
CTGTGGCANCGCCACGTGAACGATCCGGAGTTTGAGGAGGATCACGGTGTGTTCTCGGTG
GGCGAGTGCTTCAACGACTGGTTCATGGGaCGTaaGGTaaGaCGGaAACCCACGGCTCTT
CCACAGCTGCCTGAAGGTGCCCTTCGACCTCAAGCGATTCTGGAACGCCCGCCCGGCCTC
ACGGGCTGGGTCATCCTTAACATCTCTTACCTCTCGGGCATGTACTACAACTGCAAGCTC
CCTCGGCGTACAGCAGCGGTGACTCC
>at02094
GCACGCACATTGTTTTTTTTTCTTTTTTTTTGACGCGTGGGCAAAGCCTTTACATGGTCC
TGTCCAGGACCCCGTGCGTTTTGACCGGCTGGTGAGTATCTAAACGCTTTACAAAATCCA
TATGCAAGCGTGCATCCCAGGTCAGTCCCCATTGCACAGGTCTGTGGTGCAACACGTGAG
GTCTTCGTACGTGGCCGGCATGGTCTTCATGGTGTCACACGTGATCTTGGAGACGGGGAC
GTGGGCCACCTTGCCCATATAGGTGTAGCTGGCGCAAGCTCCGTCGGGAACGGCACTCAT
GCTGTACTTGATGAGGTCCCCACCAGGGCCATCAGCATAGCAGACGCTCGGCGAACTCCA
AGAATCCGGCCCTTTCGCACGTGAGGTCGCGTTGTTGCACAGGTCTGTGGTGCAACACGT
GAGGTCTACGTACGTGGCCGGCATGGTCTTGCACGTGCTCTTGGAGACGGGGACGTGGGC
CACTTTGCCCATAAATGTGTAGCTGGCGCAGACTCCGTCAGGAACGGCAGTCATGCTGTA
CTTGATGAGAGCCCCACCGGGGCCGTCGGCATAGCACTCGCTCGGCGACTCCACATCCGG
CTCTTTCGCAGGCGAGGTCGCGTTGTTGCACAGGTCTTTGGTGCAACACGTGAGGTCTTC
GTACGTGGCCGGCATGGTCTTCATAGTGTCGCAGGTGCTCTTGGAAACGGGCACGTGGGC
CAGCTTGCCCATAAATGTGTAGCTGGCGCAGACTCCGTCAGGAACGGCACTCATGCTGTA
CTTGATGAGGTCCCCACCGGGGCCGTCGGCATAGCAGACGGTCGGCGCCTCCGCCTCCGA
CTTTTGCTCAAAAGAGGACGGGCTGACGAACGAATGAAGATGCTTGCCGCTGCTTCCCAT
GACCAGAGCGACCACCAGCAGCCCAACCAGCGCGGAGCCCAGGCAGGCCACGCGCCGCAG
CCCTCCCCGCCCCTGCGCGTTCAGCGCCTCACCGTCAGTGGCGCTCTCGACGTCCGTGGC
GGCCGTCCTGAGCAACCCCAGCGAATCCATGGCTTGTCTGGAGGCGGCGCGCGGCGGAGG
CGGCGCGCTGAGGAGGCAGCAGCTCTGTCAGGCGATGCTTGAGCCAAAATGGCTACGG

>at02095
TTTTTTTTTTTTTTTTTTGGAATGGACCACCGCTGGAATAGGCAGACGCCATGCTGCTGC
CGGTCTATCGGTCCACATACGAAAGCTATGCGCATGCCCTGCTAAAATGGGCATTGCCAA
GATGTTCACCGCATCACGAACGTGCCAGCAAACACACACACACACACACACACAAACAAA
AACACTATAGCTGAGGAACAAATGGATTCCCGATTCCTACACACCTGCTCGGAGCACCGG
CGGCTAAGCAATCACATTCAAGGATAGGGCGCTTGACAAGCACCGGGCAGCCATGAACTC
ATCCTCAGCCTGCACCAAACACAGCCCGATAGACAGCGCCCTGGTGCCCGAGGAGTTCCA
GTACCGATTCACCAGACGCTGCATAGAACAGCCGCGCTTTCCGGTCGTCGCAGGCGGTCA
GGACGTGGCGCCCGTCGGGCGAGAAGACGGCGGAGTTGACGCTCCGCGCGTGCTCGAACT
TGCGCCTGCAGACGGCCATGGCCGCGTCGAAGAGCAGCACGTTGCCGTCGTCCGAGGAGG
TGAGCAGCTGGCGGCCGTCTGCCGAGAACTCCGCGCAGTTCACCGCCTTGCGGTGCTCCG
TGAACTGGTGGTGGACACGCCGGTCGCAGCGTCAAACAGCCTCGCCGTGCCGTCGTCCGA
GGAGAGGGCACCTTC
>at02096
TTCTCTGCATACGGCTCTACCTGCATGGAGAGGGACAAGGGTGGCTTCGATCACGGATTG
GAGTGCTGCGGCAAGCTTGTTTATCGCAACACCTCGCAGAGGGCTCTCGCAGCCAGCTTC
GGACTCAGGGCGCTCTCTGACGCTGCTCTGGGCCTGCCGTACGAAGAGCAGCACTGGGCC
CCGCAGGTCCAAGTGCTCTGGAATTGTGGCTGGGAGCGCTTCAGTCCCGACAAGTTGGAC
TTCAGAGGCAGCATGTCCGCAGGT
>at02097
TTTTTTTTTTTTCTTTTTAATTGGCACGCTGGGTGCCCGCTGAGGTGATGACGCTGAGCA
AGGGGGAGCCCACAGCCTGCGCGAGTTGAGTTGACTCTCAGTGCGAGTCACGCTGCCTCT
GGCCCAATGCTGCCGACTCAGGCTCCGGCAGCATGGTTCACAGCTGACATCGCAAGCACA
ACTGGAGTGACGAAGAGGTACTTTGCGGTGCCCGCTGCAAAGGACACCGTGGTGTCATCG
GAGTTGCTCCTCCCATCAGCGTCGCAGCAGCGGTAAAAGTGAGCCGTGTTGCCCAAGCCG
TCCTCCCCGCAGACACGCTCAGTGATCTTCACTTTTCCATCCGCGCACGAGGCGCTGGCG
ACAGAGGCGCAGCAGTCATCGTCGAATTCGCCGTCTTTGCGGCAGGCTGAGGGCTCACAC
CCGGCATTCCGCCTCTGGCCCGCGGTGCCGCCGAGCCCAGCGTGCCAGACAGCATCTGCG
CTGGCGAGGGGGTGCAGAACGGCAACGTTCCTGGCGTCCTCCTCAGGCCCCAGCTCGCCG
GCCCAGATGCTGCCCACGCATCGGAACCCCGGCTTCTGCCCGCCGTGCGTGGGGCGCACC
GTGTTGACCTGGCAGAAGCACATCTCGTCCACCGTGTTCCGGCCCATGGGGGTCGCTGTG
GACCTCCCCATGGAGTTGAAGATGCAGGTACTCTGGATGACATCTCCCATCTGGAGGGTC
AGGTTTGATGCGAGCAGTGAGAAAACCGCTTGGTCGTCGTAGTGCACACAGACTGGGAGC
CAGGGTCCATGTTTGTCCGATCCGCGTGAGCGTCTGGTACATCTCCGAGCCAGGAGATGC
GCATG
>at02098
TTGTTTTTTTTTCTTTTTTTTTGTCGTTGCGCACATTTGCGGGTCAGGCAGCAAGAACGT
GCCGCTCCAGCCAACCGGTGCTGTCCTGGAAGTGGCCGTGCAAGTCAGAAGCAGGCAAGT
CTGCACTCTCTGCCCTGCGCTACAAGTCGCGTAGCAAGCACGTGCTCAAGTGCATGCACG
CCAACAAGCCAATCAGCCTGTGCTTGCACTGCATCTGCACGCACTCCACCTTCATCAGCC
ACTGCTGGTCTACCAGACCTGCTGCGAGCCAGGCAGCTCGTCAGCGCCGAAGACCTGCAC
TGCCTCTGCCTGGTTCTCCCTCTGCTGATGGGCAGCCATCGTAGACA
>at02099
GGTTCCTACCATACTTGCTGAGCTCGAGAGCCCGGGAGCCGTTGCAGGGACTGTGCTGCC
TGGACCTTCCTCCACAGCTGAAGCCGCACCGGTGGCTTTCGCGGGTTGGCCACTATGGCA
TGGCTGTGGGTCCTGATGCCCCACTGGAAGGTGCTGAAGTACCGCCCTGCGCTGCAGGTG
CTTTGGTCCCAGCTGCAACNGCACTGGCATTTTGGCTGCCTCTGCATCTGCACCAATCTC
TCCGCTTGCTTC
>at02100
CGTGTTGGCTCCAATCAAACATCGCCAAAGCCTCGGATGTTAGAGGTGTGGGGGCACATG
CCACAACGACCTCACAAAGGTCGTGATCGTGGGGGGCCGCAATGCAAAACTCCAGCGTGC
AGCTTGTCTTGCAGTCCTTGCTGTTGGCGCAGTGTGGGCGGCCCAGGCCTTCGTATTGGG
CTCATCGAGACGCGCTGCAGCTGATCTCCAGACCACCTCCTCGTTGCGAACCTCGGTGGC
CTCTTCATCTTCACCGGTCACTCGCCACGCAAAGCTTGGTCACCGACAGGGGTGAGCCTC
ATGGCCGTGCCCCAC

>at02101
TTTTTTTTTTTTTTTTGTACGAATGCGTCCAGAGAAGGCTCCTGAAGAGTTCAATCCACC
CTCTCGATCGGCGCCTGCGCACTAGGAGGCCATGCGATGCTTCAGATCAGAAGAGTGTTC
AGGCAGCGCACCCTTGCCTGTGCTGCCCGAGCAGCTTGGTGCACGAGCAGGGCGTCCCGG
GCCTCCAGGGACCAGCCCGCCGCCCTCTCGCCGCACGGCCCAGTGAGGCTCTCCAAGGTG
GCCAGCGCGCCTCGGAGTTTGCCCTGCTCGATGCACGCGGCGGCCCGGGAGAGCGCCACC
AAGTTCCGCTCGGCAGCGGCCGTGGGCGAGTTGAAGGGGATGCCTGGATCGAGCTCTGCG
GCCGGCAGGTGATAGAGAGAGCCCAGCAGTCTCCCCACGATTGCCGCGCCGAGCTTGCCA
ACCAGGCCTTCAGCGGGCGGTGCGAGCGCAGCAGTCGCCATCTCAGGTAGCTGCGACCTG
AAGCTGCGCTTGAGCTGCGGCTCCGTCAAGACCTGAACGCTGCTCCGAGCTAGCGTATCT
TCGGGCAGGTGCGCCAGGATGCGATGTATGAAGCCATCAGACGTCTCAAGGTCCCGCAAG
GCGGCCAATTCCGAGGTCGGAGACCGCCCCTCCATGAAGGCGCCGTCCAGTGACAGCAGT
GCTGCCGAGAGCATGCCGCTTGCCTGCGAGCGCTGCTTCA
>at02102
TACAGCTGATCACCTCCCGGCACCCCCAGCCGACAGCGGGCGGACATCGGAGGCCCAGGG
GCATCTCCTGGAGGACCTCCTGGTCCCACTCGCCGTTGGCCTCGTCCCAGGAGTAGCTGT
CGGCCTCCACGCTCCCCACTGTGGCAGGGCTCTGCGCCGCGGTGGGCCCATGCCACGTGC
GCCCTGCCTCCGCGTCTTGCCACGTGTCCCCCCAGCGGTCTTGCCAGGCCTCATCCTCTG
CCTGGCCCTCCGCTGTCGGCGTCTCGTCCCTCTGCCTCACGTATCTCCTCGCCTCACGCT
CCTCCTGCAGCCCCTCCACCGCTCGCCGGCGCCTATCCTCTCGGCCTCCGGCTGCATGCG
GCTGGCCGCGGGGGC
>at02103
ATTCAAGTCGGTATACCGATTGTCGTCAGCATCTGCTGCAACCGTGCTGTTCCATTGAAC
ACAAACATCAGCCGAACAACACATCACGTTCCTTCCTAACCAGCTCAGTCCGTCAACCAC
TCAAGGCAATTATCCAGACGGCCACATCGGATAAGCAGTCAACTGGGAAACACGATCAAT
TGGTCAACCAACCAATCAGGCCCAACAAGCCATCTGCCCCACGAAACTGATAAGGTCAAC
CAGTCAACCATGCCAAGCTATCATCCAACTCTGTCAGAGACTGCAAAGTCCTCCTCTCAT
CTATGGGGCTTNGNTTTTAAGtCGtTCCGGCATTAGTCACGAAGCTCCTCCATACTGGCA
GCAGACTGCGTGCAGTCATCGCCAAACCTGCTTTGACCCCTCACTTCACTGGATCCAAGC
ATGCTAGACAAGCCAATGTAGATGTCGAATCACCATGCTCCCGTCACGGACCATCTTCAA
GCCGCCCTCTCATCTGACGAGCTATCTGAAGATGACGAGTGGCGCCTTTTC
>at02104
TTTTTTTTTTTTTTTTTAACAACGTACAGCAAATGAGGGCACGTCGTGGGCATCCAGTA
ACTACGGAGATACCAGTGGACTCAAGTCGGGAGCTACTGCCAGAAGGGAAGGGCTCATGG
CTCTAGCCCGCCGGAACTGGATCGAAGAGCTCTTTGCGATCCTGATGCAGGCATTGGCCA
GCCCTGGATCTTGTGCTGTCATCCTCACTCAAGGCCTCTGGGGGCAAGTCCTCTGCTTCG
GGCGGGGCAACAGCCTGCAAGAGTCTTTGATCGTTCCTCCTGCAGCGCCACGGCAACTCT
GGGCAGAGCAGTGGCTCCGTGAGTTCCGGCAAGAAATAATGCGAGCGAACTTCAGAGATG
GGCAAGATTGACCCCTCGTCAAACCCTGCAGTATCCTCATCGCAACTTCCATCGGTCGTT
CTGCCGTCATGCACATGTGTGGTCGAAGGTGGCGTAAACACGCTGACACCTGGAGCCAGG
CGGTGTGCCATCAGTGTTTCGTGTGCATTAGGCAC
>at02105
TTTTTTTTTTTTTTTTTTCGTAAGCTGCTTGCAGCCGGGCACCCTCCGAGGCTCACACC
TGCCTGCTCTCCCACAGCTGCCTCTCCCTGAAGCTGGCGATCATCCACTGCACCATGGGC
AGCATGGCGTCCACCGCGCGCGACGCGTCCCGCAAGATGGCCGCGGACAGTCTTGCAGGC
TCGTCCTGCACTGCGGGGCAGAGCGCTCCCAGGCTGCAGAGGCCCTCGCGGCTGGGCATG
GCGGTGAGGGCCGCCTTCATCCCCAAGGATGTCATCCATCCGTGATACTGCTGGAGAGTC
TGCACATAAGCATTGCTGGCGGCGGACTTGTCTCCGTCGACCATAAGCCTCAGCATCGTG
AAGAAGAACTCCAAGCCTCTGAGGAGCCATTGCAGCATGCACGCGCCACTGGGATCCCGA
AGTTGGCACGGGCTTCTGCTGTGGATGTTGGCATCGAGCTCCGCTTGGAGGAAGACCCGC
AGCGTCCTGCGCCCCGTCAAGTCCGCCTCCACGACAGACTTGACATAGTTGTAATTCTTC
TCGAAGTCCCCCAGCACAAGCTTCACGGCGGT
>at02106
CCTGGAGTTTTGCAGGGCAGCGCCGGCTTGCTCCACCTAGGCCCGCAATGCGGCAGCTGC
TGCTGCTGGCGCTCTGCCCGGCCCTGGCCGGTGGTGCGCTGGACGAATGCAGCAGCACGC

GCCCCGGCGGGCGCGCCGCGCGCAACTACCGGGTCCTGCGCTATGCGACGACACCGCAGT
TGGCCTCGGCTCCCACAGGCTTCCGGAGCAACTGCCGCTGACCAGCTG
>at02107
CCGTAGCCATTTTGGCTCAAGCTCGGATGCCGGCATAGCCTTGTCTCGCATTCAACACAG
GGACTTGGGCTGAAGTATCGCCTCGCGGCTGAGGCAGACCACGGAATAGTCCGTGGTGGC
GCGGCATGGAGCACGTAGACTCCCCACAAGAAGGCTGGTCTGCCTGTTCTCCTTTGCCTC
CTCCTCCTACTGCTCCCCCTCCTCCTCCTCCTCCCGCAGCTTCCTGTGTAGCAACAGTAC
CCGCTGCAGGATCAGCCGCCACAGCTGCTGGAGGATCTGCTGGCCATGAGGTATTGGCAT
CTACATCGACGTCTGTGCCTTCAGCTGAAGGGCCGGATGTCTGGTGTCGATGTTGGATGA
TTACAGCCGCGACTACTATTGGAA
>at02108
TACGCCATGCTTGCCGGCGTGGCTTTATGTGCTCTGGCCTTGGCGGCAGCGAGCGGCGAC
ACATTCGGCGCGGATTCGAAGGAGCCGTCGTGCAGCCAGGACCTTGCCATGATGGCCCTT
GCCAAAGGGGACATCAGCCAGGCAAGGGCGGAGCTTGCGATCTACAAGGACGGGTCCAGG
GTGTCACGCGAGTGCGCAAGCGGAGTCTGGAAGCAGGGCATCGCGGCATTGCCCCTCCCA
CGGGCTTGGGAGGTGGGAGCGGTCGGGGGAAGCAGGGTGTTGGAGCGCTTCGGCAGCAAG
GGTTGTGAGGCGAGAGCAGAGGACGGCACGTGGTTGGACATCCCACGCTTGCAGGAGGTG
CTGGTCTGCATCCCAGCTGGCGAGCGCATGCTCGTCCTGGAGCAGGACGGCTTCTTGAGG
GCGTTTGACATGCCCACCGTGCTCTG
>at02109
GTTTCTCTGAGGCGCCATGTGTTGAGGTGCCTGAGGCACCACTGCGAGAACAGTGACGCT
CCCCAGAGCTCAAGGCCAGAAGACCAACAGCTTTGTCTGCATGGACGCACAACATCATGC
ATCATCCGCAAGGCCAAAGCTCTTCAAAGCCCGAAGGCAGCTCAGTCCAAGAGGAGGGCC
AAATGATGCAATCATCCCAGGGTCCATGCAAGACCTGCTCTTCAGTCAGACATCGCTCGC
AGCTTGTCCCCTTGAACAGCCTTCACCTGGCCGTCAGAGTGAAAGCGCACCTCGAATCTC
TCCTTCTCTGCGGCGTATCCGAGCACCTCAGCTCTCTGCCCATTCAGGTGTGCCGCTTTC
AGGCCGTCCAAGACGACCACATCGCCCGGCAGGAGGGCGCGGACGACGGTCAGGTTGTCT
CCCCGGAGCGCCTTCAGAGCGCCCTCGTCGCCCATGAGACGGACCTCGAAGCGGCCCTTG
TCTCCCCGATACCTGACTACCTGGCCCTTCTGTCCGTTGAGCTCCGCAACGGAGAGGCCT
CCAAGCTCTACTTCTGCCCCCGGGGGGATCGCCTTGACAACCTCGATCTTGTCCTTCGAG
GCGCGAATGGCATCAGCAGTGCTGGCAGCATCCCGTGGCTTGACAAGAAAGCAGCCCCGA
TCGCCATCTTCGCCTTGAAATCCCACGACGTCGCCCCTCAAGCTTGTCATTCACACGGACC
CGGCTCCCAAGCGGAATGTCCGCTACCTGCAGGAGCTGCCAGATGACGCCCCTGAAAGAGC
TCAAGGCTCTGTGCACCGCTGAACCGCGAGGGTTCGGCTGCGCCAACTCGAATCGTGAAG
TGTGGCACAGATGTGATGCCAGATTGGAGTGCGGTGTTGTGAAAAGCCTGCACACTGGAG
CGGTAGGTGCCAGATTCGAGGCACTGCTTGGCTCCGCTCACGCCAACCTTCTCGGCGAGT
CTGCATAAGACGCCAGTGTCGTGAATGTCTTCCC
>at02110
CCAGGTCGGGGGCTCGTACACATTGAATTTTGACGACCACGCTTGGGCGAGCCATTTCCT
GCTGCAAGTGACCGCTCCCGGTGCTGTAGCCATCTTCGCACAGCACTTGCCCACTGAATT
TGAGAAGGACTTCCACTACTTCAAGACCTCAGCTGGCGAGGACGTGGAGCCGGCCATGGA
AGTGTCCGTAGCGATGTGCTCGGATGGGGACGGGAGCGAGGCTACAGAGCAGAGCAACAC
GGGGCTTTGGGGTGCGGTGTTGGCAGGATCCTTTCTGACAACTGTGCCCGCCCTCTTTGG
GGTNTTCTTTGTCGTGACAAGACCTAGGCAAGCCAGGCCAAGAGCGCCTGCAGATTGCCT
CTCC
>at02111
CCATCATTACCACTTTTCCTGGAACCACAAGATGCTGAGCGAGAGCTACATGGCACAGCA
CTGCTGCCCGCAACGCTGCAGCTCCGCGCACGACTTCTTGAAGATCGCGATGTGGCAAAC
AAGTAGTGGAGAGAGGCAGGCAGAACGGCAGCGCGGACATTTCCAGTTCTATGTGGAGCG
ACAGGTTATGGCTTTGGGCCTGCAGCATACTCCTCACTCGTGGTGGGCTGGGCCTTTCCC
TTGCCGGCAGCGAGTATGGCCTTAATA
>at02112
TTGTTTTTTTTTCTTTTTTTCCGCGTGAGCAAAACTCCTGGTTTGCTCGTGACTAAGCCG
CAAAGTAACCTACGCCAACAAGGGCCCTGGTCTGCTCCATCAGCACACCGACCAAGGTGA
AGTGGGGCGCCAAACGCGCCGCGGACGGCGTCCAACCGCGGAAAAAAACGCCAGGCCCGT

CTTTCCTGGCCATATCCAAAAAGCAATCCGTGAGGCCTAGGTACTGCTGGGCAATATCGG
ACATCAGGCGTGTCTTGATGATGTCAACGGGCGCGGAGGCTGTTGCAAAAGCCATGCCCG
AAATGAAGGACCCCAGCATGTGCAGGGGCACCCCCTCACGCATGCCCGCGTGGCGCTTCA
ACAGCCACTTGGAGTGGTCGTAGCACGAAAGCTGCGCCCCGCGGCCGCAGGCCGCCCGCA
ACATTGTGGCGCTGCAGCCCCGGAACATGCCGCCGAAGCCGAGCTCACGGCCCATGACCC
CGAAGGCGTGCAACGAGTTGGCGTGGCTCGGCCGCTGGCCCGCCCGCAGGCCCGTCTCCA
GCAAGCCGTTGGTCCCTAGCCTGCCGGCCTCCGCCTGCACACGAATCTTTACAAAGTCCG
TGGGGTTCACAAAGCCGCTGAAGAGGGCGCCAGTGACAACCCCCGTGGCGAACTTTCGTA
CAAACCCAATGTCGGCTGACCCACCTCCGCTGCCAAACAGCTTTTCCTTCATGAAGGGGT
ACATGCCGATGGACAGCCCGCTGTAGGACAGGTCCCGCAGGCAGCTCGCCACGATACCAG
GCAGAAGCAGGCCTCGAAGTCCCTCCTCGCGCAGGATCAGGCGAACTGTTGGCCCGAAGC
CCTGGTAGCGAGCAGCCGTGCCGCCAGCTGGCACCTGCATTCGGACCTTGGCTACATCCG
TGAAGTTGAAGAGGCAAGACACCCACACGGTGGTGCAGCCCGCAGCCGCCACCTCACGGA
AGACGTAGGCAGGTGGTGGCTTAGAGCCATCCCCCCGGGTGGCCACGAGTGCCGCAATGC
CGGTCCCCGTAGAAGCCAGGAAACCAACGAGGCACTGCCCCTCTGAGAGCGCCGCGATCA
TTGCACCGCCAGGCCGCCAGCCTATCGGTCTCCCACCTAGAAGGCCATCCTGGCCACTAC
CCCGTCCTCACCATCCTCGCGCTCCGAAACGAAGCTTGAGCCAAAATGGCTACGG
>at02113
AGGAGATGGATGTCGACTATGTGCGAAAGAGATATACGCTGCTGCATGGCGGGCGCTGGG
TGCCATCGGCCCTTCCCGCTGCAACATTGGAGGAGTTGAAGGCGCAAGGCGCTTTGGCAC
CAGCCGTGTCAGTCTCGGACAACCGCGTCATGGAGTGCTACTTTGCCGATTCATCCAGGC
CGCGCGTCATTGGCGCACTCAAGTTCAGCCGGGCTGCGGCCGGGCCGCCCGGCCGTGCAC
ACGGGGGAGTAGTCGCTGCTGCGTTGGATGAGGCCTTCGTGTGGGCAGGCTTGCATATGG
GTATTGGGGCCCCCA
>at02114
CCGTAGCCATTTTGGCTCAAGTCGTGGCTCAAGAGGAAGCTTTGCTTCCCGACTCAGGTC
TGAATAGTCACAGTAGCCATGGCTGAGGATACTGGCATCGTTATTGCGGTGCCGGAGGAC
ATTGCTTCCCAGATCCTGCACGAGGGCTACCGCTGTTCCAACCGAAGGCGGGTCCCAGCA
TCGCGTAATCGAGAAGCAGCGCTGCGTGCCTACCGACGCTTTCAGCGCTCACGACCACCC
GTGCTGCTTGAAGTTGTGTCTCTGCCCGTTGGAGTGCACACGACTCCACACAAAGATGGG
ATCAAGTTGTCAACACCGCACCTTCCAGGGTGGNGCCTTGCACAAGCTGCTCGCCCTCAC
AGCATCCCATCATTTACAGCAGGGTCACACAGCTTCCGGCCATCAGATCGGACATGCTCC
CTCCGTCCAAGGCTGACGGCCCAAACCATCAATCAAAGAGCAGCTCCCCGAGCAGGATGC
AGCTTGAATGGCCTAGACAACGTGCGAACATTATACCATGCAACTTGTGAATCTAACGCC
CATTTGATCTGCTCTCAGAGGTCCTTCCACGCTGGCCACCACGGATTCTCG
>at02115
TTTTTTTCTTTTTTTTTTGAGGAGACACACCTGCCTGTACAAGCGTCGAGAAGCCGGTGG
TGTCAAGCATGCATGAATCGGACTCGAGCCAGTTGTGCAAGCGGCCATCGCAGCACTCAT
CCTGTACTAAGTGCTTCGATGGCTCCATTCTGCAGTGCCCGCATTCTATGGCCCACTGAC
TCGCTTCTGGGTCAGAGGCTTCGAAAAGTCCTGCACCTTCGATTGCTCAGCAGCAAGGAA
AGGGCCACCAGTGAACATCAGCATGAATATGACTATAACCCGGCTGTCACCCATGAAAGA
GACACACCTGCCTGTACAAGCATAGAGAAGCCGGTCGTGTCATGCATGCGTGAAACGGAC
TCGAGCCAGCTGTGCAGCCGGCCATCACAGCCTGCAGTATGTGCTTCTATGGCTTCACTC
TGGAGCGCATGAAGTCCATGGGCTGCTCACCTGCTTCTGGGCCACTGTCTTGCTTATGAG
CCAGTGGCTTCCAAGATTCCTGCACCTTTGCTTGCTTAGCATCGCATTTGAGTGGCCTGG
CTCCTCGCCGCTGAACCTACGAGAACGCGACAGCTCACGCGCCGCCAGCACTCGAGCGTC
GCAGCATGGTGACAAACTCATCAAAGCACAACTCTCGAGCCTCCTCGTCCTTCCCCGGCT
TTGCCGGCATGCCCGCAGAGTCCCTCGACACACCGCAGCGCACTAGTTCCTGGACTTCGG
CCTCTGAGATGGAGCCGCTGCCATCCTTGTCGCAAACACGGAAGGCCTCCCAACAGGCGT
GGGCTGGGCAAGCTGTCCTTGCTGATGGCGGCACTGAGGAACTCGGAGTACTCGATGGAG
CCGCTTCCGTCCGTGTCGAGCTGCCGGAAAAGTTCCTGCGCCTCCAGGCTTGAGAGGCCG
AGCTCAGGGCAGCCGCCGTCGAGCTCCTCCAAGGAAAGCTGACCATCGCCATTTTTGTCC
AGGCGCTTGAAGGCAGTCCTCTGCTCCGCAACATGCGGGTCGTCGAGGCGGTAGGCCGCG
ACGCGCAAAGCTGCCCTCTGGAAGTCGCTGAGCGCGGAGAATGCCCGGAGGTTCTCCGCG
ATGGCATGCCGGCAGGAGGGCCCACAGGACCGGGAGGTGGCGCCGTGGCTCTCAACCACA

ACGCTGCTGAGCCAAGGGGTCTGGAGGGCCTGCTCGGCAGAGAGACGATCGTCTGGGTTC
CGAGTGAGCAAGCCCAGCACCAAGGCCTTGGCTTCTTCAGACGCTGCATCCCACTCGTCA
CCTTTCAGCTGAACAGGACGTGATGCGGCCATCCTGAGAATCTTCATGGGCGAGGAGCTG
TCGAACGGTACGCGACCACTGAACGCCTCGTGGAGCAGCACGCCACAGCTCCAGAGGTCG
CACTTCTCGCTGTAAGGCCCGGAAAGTATCTCCGGAGCCATGTACACCGGGGTGCCGCAG
GCGTCGGTCAGGCTCCTCTCGCCTGGCGTGAAGCCCTGCGCGAAGCCAAAGTCGATCAGC
TTCACGACGTTGTCCTGCATGGGCAGGCCTTGGAACTTCAGGAGCAGGTTCTCCATCTTC
AGATCCCTGTGCGCAACATTCTTCACAGCATGCAGGTAGAAGACAGCTTGGAGGACCTGC
TGTATCATGATCCTCGCATCTGTCTCCTTCAACTTGCCACCCTGGCCGTGCACAAAATCA
AAGAGCTCACCCCCTTCACAAGCCTCCATCGCGATGTGGACATGGTCAGGTTGTTCGAAG
GTCTCATAAATTCTG
>at02116
TTTTTTTTTTTTTTTTTCCAGGTGACTTGCCTCACAGGCCTCCTCTTCCCACTCTGCAC
TTGTCCCGCCAACTTGACACGGCAGCTTGGCTGCTTGGCACGCCTCCGTTCCAATCTCCG
CTGCGTCCTCGCTCCCAACCTTGGCCTGCACACCTCGCGGCACAGGACACTGCGCTGCAA
GCAGTGCGATGGCTTGTTTGGCATGCTGCTGTCGCAGGCCCTCATGGCTGAGTGTCTGCA
CCACATGGCCTTCAAAGGCATGCCTGAGCTGGCAGCACTCCCTGCCCTCAAGCAGGGGCT
CGTCGTCCAACGCAACCCAGGCATGCCTTGCTCCGCCGGCCTCCCTCTCCTGCAACCATC
TCCAGATCTCGTGCTGCCGCACGCCGTGGTAACCAGGGTCTGTGACCAGAGGAAGTTCCA
GGACCTGCCCCAGAGGGCCACCATGCAGGGCTGCAAAGCGCTGGAACTCAGCCAGTAGCT
CCTCCATGGCCTGCTCGCTCGCACGCCACGTTGACGAGAGCACAATCAAGGCGCCGCTGG
CACTGAGGATCTCCGAGAGTGCGTGAAGGCAGCAGTCCGGGAACAAAGCCGGCCTGGCAA
CTTGTGGCGCACCGTAACCGAAGGGTAGCATCACGCCGTCGATGTCAAGGAAGACGAGTG
GCTTAGACTGCACCCTGCTTGAAACTGGTGGAGAGGACTCCTCAACGGCAGCACGACCCG
AGGTCCTAGGCACGGATGAAATGTCCATGGCCGAGGAGTCTGCGTGGAACAGTGGTCAGC
GCTGTCATGACCCACCATGCTTGATGCCAAAAATGCGCTACGG
>at02117
TAGTCATTGCAGAGGCCGCTGCCCATCAAGAGGCGTGGCAAATGCCACTCTCTCTGACCT
GCCGCTGGCCAAGGAGGCGCCGCCCCCCACCCGGCAGCGGCCTCACTCTGCCTCTTCGCC
GCCAATGGGCAGCGTGGAGTTGATGAGGTTCTTGAAGAAGCTGGGCTCGGGCGCCCCGTC
GATCATCTTGAAGGTGAACGCCCCCCTTGGCCCACCATCCGCTCGTAGAAGCCGAGGTGC
ACCATGTGCCACGTGGGCGTCCCCCAGCCACCCCGCAGCTCCAG
>at02118
ATGAAGCTCTTCTTCCTCTACGATCGCAACTTTGCGCCATTGCGTGACCAGATGGTTGCA
TCACTGGAAGAGTGCGGGGAGCAGCTGCGATTAGTCTCCAGGAGGACTTGCTGGACGATC
TAAAGGGTAACAACCGCGCAGGCGGCGGACTGCCGACGTACTTGTATAAGTCTAGCAAGA
TTAGAAAAGCGCTTGAAGCCGTGGAGAAAGATGAGGTGTTCTTGTTCACGGACGTCGACA
TCCAATTCTTTCAGCCAGTGCATTCCATTGTCGAGGAGTGCATGGCGTCGGGCGTGGACA
TGATCTTGCAGAAGGAGTTCGAAGGCATCGGAGTCAACATCGGCTTCATGGCCATCCGAA
ACACGCCTGCCTGCCAAGCCTTCTGGGAGCACGTGCACACCGAGATCAGCCGAACGCAGG
CCTTGGACCAGCGAGTGGTGAACAACTCGCTCTACTCGGGCCACGCGGCGCGCGACTTCG
GCCTGCGTTGGGACCGCTGGCCCAGCTGCATCTGGGCCTCCTCCATGGCCTTCTCCGGGC
CGCTGCCGGAGCCGCTGGTGGTCCACCACGCCAACTTCCTCGTGGAAAAGGCCCCGGCCG
CGGACCCGCAGCCCAAGGTGGAGCAGCTGAGGCAGCTCCGGGAGGCAGTCCTGCGCGGCG
AGGGCGGTAGCGACCCGGCCTGGTTGGAATTCATGGACGCTGCCAGGAGCAGCCCCGCCA
TGCTCGACTACCGGGACCGGCATTTTGGAGCTCGGCGTCCAGGGCCGGAGTGGTCCACGC
TGCCGGAGGGGCACGTGGCCCGGCCCGGTGGCTACAGCGAGAAGGCGGCGAAGAAGGCGG
CGGCAGCTGCTGCGAAGGCGGCAGCCGACGCCGCG
>at02119
TTTTTTTTTTTTTTCTTTCGTCGTCGATTTTGTAGTCTATCGATTGCACGTGCCGACTGC
CACCCTTCTCCATCATCCGCCCAAGCGAGAAGCTCGCCACTCTGCCGCTTCCCCAGCCGG
CAACGCCATGCTGTGGTGACGGCGGCCTGAAGCGTGGCGGTCAGGCAGGGCCGCGGAGCA
GGGTACCACCCAAGGAGTTTGATGCTCACGACCTCCGCGCGCTGGGCGTCCACACGCTGC
AGGAGCTGTTCCAAGCCACCAGCTCGTTCCACATCCTCAGCGTCTCGGAGTGCCGACTGC
AGCACAGCCCCCGAAGAAGCTGACGACTCTTCGGCACCGCCGCCTGCCTCCGGGCAGAGG

GAGACCGTCTCCCGGACCTCATCCACGAGCAGTCGCTCGCTGCAGGCAAAGTGGTTCACG
CCCTCGAACACTATGCGCGGTTGGGGGTCGCAGCCCTCCCTGACCT
>at02120
TTTTTTTTTTTTTTTTCTCGCTGTAGTCATTGCCACAACGCGCTGCTGAAGGTGCCATCA
CATAAGCCAGCGAGCCGGTCACTCATCCAGGGGTATGGCGGGCTTTCCAGTAGTTGGGGC
CGTAGGAGATGAACAGTTCCTCGCCCTCCTGAATATCTCGCGTAGCCCGCATCTCGACGA
TCTCGAAACCCTCGATCCAGCCCCATTCCATGTTGTAGCCCGGGTTCTTGTGGTTGTACA
TAGCCCCGTGGCCAAGCGCAATCACGCTAATCGGGTTCGCCACATCCCGATTAGACGAGG
GATGCTCGTACAAGAGCATGGCAAAGTCCTGGAAGACCCCCTGGGAGCCCTCGTCCAAGA
CAAGGCCGGGGCAGAGCTCCACCACGTCGCCCTCCCTGAAGGAGGTGGCGGCGAAGACGC
CGCGGCCGTGCACGCTGGACTGGCCCAAACAGGCCGCGCCAGCTGTCAAGCCCACACTTC
CGGCGGCCCAATCCAAGTCATCTTGGGACCGCTCGTACGGCCGCGCCCGCCGCGCAAGGG
CCCTGGCGCGGTCGCTGC
>at02121
TTTTTTTTTTTTTTTTTAGTCAAGCAAGCGCTCGCTTACAAGTGGTTGAATCTCCGAAT
CCGTACAGGGATGCCGCTACAAACGGCCATGCGCGCATGCCCTGAATACGCAAGGCCTGC
AGCATAACCTCCCTGTCGCTCCGCAGCTCGGCACTGGCAAACCTCAGCGGGTTGCACCAG
TCGGATGCGAAACGCGACTTGGACAAGGCCGCCAGCACGACCTCCTTGTCTGCGTGCAGC
GATGGGTCTGCCCATCTCAAAGCGCTGCTGTCAATTCTAACCACCTCCATCACGGCATAC
TTGTCCTTGCGCAGCGTCTCGTGAGCGCGAGCGAAGCAGCTGGTGTCCCGAGAGAGCATG
AGACGCACGACCTCCCAGTCAGCCATCCAAGAGGAGGGGACCTCGTTGATGGGAAAGGCA
TCCTCGCTGAAGTCTTGCAATGCTTTCAGCAGGATGCCCCTGTCCGCCAGGAAGGACTCG
TGCGCGAAGCGCAGCGCGCGGCCGCAGCTGCCGACGGCAGCCAGGATGACGTCTCGATCT
GCCCTCAAGGAAGGGTGCGCGGAGAGGAGCGCCTCCGTCTCCCACTTAAAGAGGGGTGTG
TGCTGGTTGCTGACAGCTGCAAGTACAACCTCTCTGTCGGCCTTCAGCGATGAGCTTGCA
TACTTCAGAGAACTGCCAAAGTGGGACACTGCCGCCAGGACGACGGCCCTGTCTGCGCGC
AGCGACTCGTGCGCGAACCGCAGCTTGTCGCCGTCAAAGCGGCAGCCGGGAACCGTACTC
ACGGGCGCGAGCACGACCTCTCTGTCTGCTGTC
>at02122
TTTTTTTTTTTTTTTTTCACCATGCCATATGGCTCTCCCGGGCACCTGTCGTCCGTGTC
TCCCGCGCACTCACTTCGGGGGGCCGCCCGCGCCGCCGCCCGCGCCGCCGCCCTTCCCGT
CGCCCTTCCCGCCGCCAAAGGTCGGGCGCACGTAAATGCGGCCCTCGGGGCTTGACCGGA
TCTTCTTGTTGCGGACGCGGACGATGCGGCCGTGGATGGCACAGGACACGCAGTACTGCA
TCTTCATGTAAAGCTTCGGCAGCATGAACGTTGAGTACACGCTGGCCTCGCGGATGTCGC
GCTGCGATGAGGCGTCAACCATATTGCGGACTTGGAAGCGCTTGATCGCCTTGTCCTTCG
GAACGCAGCGCGAGCAGTTCGAGCACCTAATGATGGCAGTGTGGCCGCGGCCGTGCTTGG
AGCGCCCGTTGTTCCTCCCCTCGTGCC
>at02123
TTCCCGGCAGCATGCCCTTCACAGTATCCAGTCGGCGTCCCATCCACAGATGTGGGACTG
ATCTTCAGTGTGCCCTGCCACATGAGCCTGCCATGTCTGCGCTGTTGCACTGTTGCCACC
CTGCTCTCCCCGGNTTTCGACTTAGTCTTGAAGGCTTGGCCAAGAGCGTCTTCAACGCAG
GACTGCAGCACTTCGAACAGTGACCCATCAGAGGCAGTTCCACGCGACACTGTG
>at02124
TTCTTGNTTGCATGCTCNGTCGACAAGGAAGCTTTGCCATGATGCGCAgGGGAAGATCAC
CATGATCTTTGTCAGCAGGAAAAANNAAGCATGCAGACAtTTTAAGTGCAGAAGGCTACT
GCCTGCTGCCNNTACAACCTCGCCGGCgANAAGCNGNAGGAtTGCAACGtTGAANGACAA
CTCCACATGNTGNGCCAAAGACGATTTATCATCGTGTNCgGGTGCGCAGAGTTGGCAACA
cgcCtGCCGGCAGGCCACATACATGCATCGGAGCTGATCAAAGTTGTTaaAAAAAATCGG
GGACGGtTTNGTGCACTgccgaGCGTCATGCGGCGACTGTGTCCGCNTTGCCAGTGCTTC
TGCCCCCGGAAGTGCTTCAAGCANGTTgCNTNGTGTNCTGAGCGATCANGCTGCACCTGA
CTCCACCNCNGCCGGCGCGGCTCGCGATGAGCGCANNGAGGACGCCCACGCTGCAAGCCG
GCTCGCTCTGCGGAAATATCCGCTCCGCAGGGGGTGGAGGCGGCGGCCACCACCGCTGCC
GCCGCCATCGC
>at02125
TTTTTTTTTTTTTTTGTGCCCACATGCTCAAGTCCTGAAGACGTAAACCCCAGCACACG

TAGCATGGGTCATCATGCCAGGTGCGAAGGCCTGGCCTTGGTGCAGCGACGCATCACCTA
CATCTAAGACGGAGACCTCAGAATCGTCAATGGCACCGTCGGAGTTGGCGTCCCGGATGG
CATAGAGCTTGCCGTTCATCGCCCCAGCGTAGGCGGTGCCGTCGCCGCTGATGGTGACCG
CACTCCAGGACGCAGGCCCGCAGACCGTGCGAGCGGGCCAGCGAAACACTCTGGTTGCGA
AGTGCGCCTCGTCCCCCTTACAGGAGAAACGCGGCCAGGGCTCTATCCTCTCGTAGGACC
ACCGGTAGGTCCCTGTCTTCGCGTCCAAGGCCAGAAAGAGCAGTGGGGCGCTGGACCTGT
GGAAGAAGGCCCGTCCCCCAGCCAATTTTCGAACCTCAGCATCAGCCTTCTGACAACGTC
AGGCAGCCACAGCGTCTCCAAGTAGAACGGGACGGGCGACTTGAGGCCCCCGACAGGGAC
CACCACGTCGCGGCCGTCGCTCGCCGGCCACGTCACGCACGGCTGCGGGAGGGGCACCTG
CCAGAGCAGCCGCAGGTCCTCGTGCCGAATTCT
>at02126
GCAAAGTCTTTCGGGGAATTGTGGTCAAAGACCTTCGGGTCGCGGAGTGCTTGGGTGGCT
GATGTTTCAGTACTCTTGAACAACAGCTTGGCCTGCCTCGCTTACACTGTTCTCATTGGC
GACTTCCTGTCAAAGTCTCTGGCGGGGCTTCTACCTGGGATGGTGGCACTCCATGCTCGA
GGAACTGACCTATTGTTGGTGGGTGCATTCCTGCTCGTGCCATTGTCACTGCTGAAGGAC
TTGTCCCCTCTCCGCTTCAGCTCCATAGCGGGGCTTGGGGCGACACTGTATGGCTTCCTG
CTTGTCTCCAGCGATTGCGCAACCCAGGCTTCAATTGGCGATCCAGCGGGGCCCGTGTTG
CAGAACATGCTGCCGATGCGTGTGGACTTCTTCT
>at02127
TCACCACGCTCGGCCTGGGCGCGGGAGACATCGGGTCGCGCTTCGGCAAGTTCATCGATG
CCTGCAAGAAGAGCATGGGCGTGGAGCCCGACGAGCCGCCCCCCGTCCGGAAGTGCCACA
CCTGCGAGGAGAACTACAAGAAGCAGACCTCCGACATGAGCGACGCCAACGGCTGCATGC
TGCACTGCGTGCCGACCGTCGCGAGCTGCGCCGGGGGCATCACCGACGCCTGCCTCCGGA
AGGCCCACCCGTGCCTGCGCTGCCACAAGCGGAAGCTGGCCGCGTTGGACACCTGCCTCG
GCACGGACGCGCACGCAGGCATGATCGAGATGATCGGCCAGCTCTCCGCAGCCACGAAGA
CGGCGACCGTGGAGCAGGACGGCGTGGACGCCCTGATGGACAGCTTCCTCGACGCCATGA
AGGTCTGATGACTGCTCGGAGGCCGCCTGAGGCACCTCTGCGCTGCAGCTCTGAACGATG
TCGCCCGCGAA
>at02128
AGCGGACAGTGATTTATCAATATCTCCCGGTAGACTTCTTTTGCCTTTTGTGGATCTGTC
CAGAGGGCCTCAGGGCTTTCAAAGGACCATGGTTCTGCAGAACGTCTCTCTGGGAGCTTT
GCATGACCTCGGGCATGGGACCGAGGTCACGGGCTTGCCAACGCTTAGCTGCTCCACCAA
CTCCATGGCACGCAGGGCAACCCAACGCCATCCTCTTGCACTTCGAGGTGCCCAGGTTCC
TTCAGCCGCATTGGTCTTGATGCCAAAAGTGCCAGCCGACCGTGCAACCATGCTCAGCAT
CGCATCGTGGATTACTGCGTTTGTCTTCGGCGCGATGTGCGACATCAATCAATTGCGCAT
GTTGTTCGCAGGGCTGCTGCGCAAAATAGCAATCATCTTGTCTCCGGATCCCAAGCCCGC
GGAGCCGGTAATTGCACCTCGCACTCCGGATCCCAAGCCCGCGGAGCCGGTAATTGCACC
TCGCACTCCAGTGGCGGAGTCCGAAGGGGGAGATGCCTTTGTTTTTCCAGGCCGCTACAT
GGTTGGCAGCTCCTCGCTCACAGCCATATCGGCTGCTGCCGTCATGCGCCGCGCCTACCT
CCGCTCCAAGGATCGAAGGCATCGGCGGACCTCGAGGACGCAGACCGCAGGCAGCAGAAA
CAGCATTGGACAGCCAGAACTGCACAGGGAGGTGTCGGAGACGCTGTCACGGCAGGTCTC
CGTTCAGGTCGCAACAGACCCCGTGGCTCTGGCCGCTGTGAGGTCCAAAAGCAAGGGCGC
CCGCATTGCCGCCACCCAGGAGATTGCACCAGCGGGTGG
>at02129
TTTTTTTTTTTTTTTTTTCCGCTCCAAACTCATCAAACCCTTGACTCACCACCCTTTTCA
AACATGCAAAGCAACACGGTCCTGCTAATTGGGCGCTATGTGGCTGCCCTTGCCGTGACG
TCAGGCAAGCGGTCACGGATGCAAGCATGTATCGCAAAGGTGGGCGCACTCACGTGCGCA
CACCGGCGACCATGCGCATGCACATGCAAACAGGCATGCCGCAGTTGCACAGCGTGCGCG
TCCGCACGTGTTCGCAACTCTACGTACTTCTATCAACAGTGTGTCACTGCAGCTGCAGCC
TGAATTGATCCACATGCACCTGCTCAGATCCGGCTCATGCCGCCTTGCCATCAGCCTTGA
CTGCGCCAATCGTTCCGGTCAGGCGCTTCCGGATGAGGAAGCCCGCTCCGCCAAGCATCG
ACGCTGCAGCTGCGGTTGAGCCAACCACAACTGCCGAAGGGTGCCGAGCGACAGCATCCA
TGCTGTCTTGGAGCATGCTGCTGGGGAGCCCTGGGATGTAGATGGCGGCAGCCGCGACAA
GGACAGTCAGCAGGGCAGCAGCCACCAGTGCCATCACAGCTCGACGCCCTGGCAGTGCCG
CGAGCCGGCTCTTGATGGAGGTCCTGGAAGCCAACTGCGCAGCAGCAGTGCTTCGCTCGG

GCTTTGCCGCTTCTTTGACCACCTCTGTCTTCAGGTTGGCGTGCGCATTCTCCTTGCATG
CCGCATCATCANGGCTGACCTTTCCGACAGCGGCATTGCGCGGTGGCGGAGCCT
>at02130
GGGTCATCGAGGACAGGATGCTGCTTGTTGGGACAAGACAGCATCCGGGACCAACGCCGT
GGTCTAACGGACATCCGTTAAAGCCGCTGCGCCGTTGGATCTCCTTTCCTGCCCTTGTGC
GTTGATTGGAGGCAGCCTGCCTGTGGTTGCATGACGAAGCGGTGCGGGATTTCCTGCGCA
CTGCATTTTGAGTGTTCTTGCAGAGGTCCGCGTGCTTGCACCTGGACTTTGTGAGGTGCT
TGCATGCATCCCGATTGACTGGGCCTCTTCAAGGAACACCCAACGAAGGTTTGCGGAGTG
AGAGCCCGAGACACTCTGCCACTTTCTGTGTACATCTAGCCTTGCGGATGCACGAGAACT
CGCATTGCCTGTGGCAAGTGCTTGAGGCGATGGAGGCTTGAATGCCTCTCGGGCCTAGTC
TGCGGAAGCTAATCGTGCGATTCACAGACCTTCTTGAGAGCCTGTTTAGACAAAAAAAAA
AGAAAAAAAAAA
>at02131
ACGTTCTGCGAGAAAGTTGAGCGGGCCGCTAGTAGGTCTTTCTCAGCGCTGCTCTGGCGG
CGAGTTCATCATCTGTGCTCGCACCGATGCTGCCGGTGTCCCCGAGGAGGGCATGGAGCG
GGCTGTGCAGCGTGCCTCGGCATATGTCAATGCCGGTGCGGAGATGATTTTCCCAGAAGG
CCTCCATTCCAAGGAGGACTTCGCAGTGTTTGCGGAGGCCATGCGCAAGTTGCCAGGCCC
CGGCCCCCGAGGGGGGCCGGGCCCCTTCATGCTGGCCAACATGACGGAGTTCGGAGACGC
CTACTTGACGCTGGATGAGTATGCCAGCCGTTGGCCTACC
>at02132
TTTTTTTTTTTTTCTTTTCGGCGCTGTGCATGGGAGCTGTGTGCCAGGTCATCCCTACGT
ATGTCGAGCCAACCACCGCACTGGAGCGCGCATCTATAGGACCTATTATTGCAAGCTGAC
CGGTCAGGACAGCCAGCCAAAACATGCCGGCATGCTCTACACCTCTGAAACCAGCCGGAA
AATGCGGCAGACTTTGAGCCGCACTAAGAAGCCAGCGACGCCATCGCGCAGGTTGCCTCC
GCCGGAGCGAGTGGCCCGGAAGAGACGGCGGAAGCATGGCAACGAGGAAGCGATGAGCGT
GGGCTGGAACTACTGCGAGCAGGCAGTGGCTGCGCTGCTGCTACCACCGCCGGCAGGAGG
TCTGCGCTCTCGACGTACAGGTGCCGCCCTCTCAGCAGGCTCGTCTGCGCGGGAACTCCA
TGCGAAGGTAGTGGCTCAGGAAGGGGTCCACCCTCCTGGCGTTGGCAAGGCTGCGGACTG
CCCCAACGCCCGGAGCAAGAGGGTCCGGAGCAGGGTCTTCCACTGGAGCAGGTCCTTCCT
CGTCGGTGGATGAGTCGGCCAGGAAGCCAGCGTGACCTGTGAACTTGGCCAAGGGCGGCT
GGGTCCTGTCCAGATCATCCAGGAACGCTTTGTACTTGCTTGCCTC
>at02133
CACGGCGCCCGTGGGACGGCTCCCATGCGGCCGTGCGTCCAACTCGTAGACCGAGTCCAC
CAAGTCCTTTCGAAGGTCTTCGATGATCTGCGAGATGCCTACGAGTCCACGGCGCCGCTC
CAGCGCCTCTCTCCTGCGGCGTTGCGCGTCGAAGAGGCTCATGACCGGCACGGCCGCTGA
TCGCGGAGCTCCCGGCGACGGGGCCCCCGGCCGCCTATCTACCCTTATTGGGCTGGTGGG
CGTGCCATGATGCCCCGGATGTCCCCAACATCGGCCTGGCCATGCCATGGCCGGCAAGGC
ATCC
>at02134
TTTTTTTTTTTTTTTTTACGGCAGACACACCGACATTGCGCACATGCAAGTCAGGAGTC
ATCATCCGTATCTATGTCTATGGGAGCGCACGGGGCTGTGCGCCGAAGATTGGACCAAGA
ACCAAGCCGAACTGGCTCGCTCTGAGCATTCACGTTAATTCCCGAAAAAACCTCCTCAGC
CTGAGCTCGCAGCTCTGCTGTTGGCAATTTGTCCATGCCAGCAAGCCACTGCAAAGTCCA
TTCCGGCATCTTCTTGACTAGAGGCGGCAGCAGTTCTCTGGAGCTCTGCAGATTGATCCA
CGACAGTCCGCCAGCTCCTGTGGTGCAGTAGGCAAAGCGCAGGTGTCCCTTGACAAAGGC
GTCGCCAGAGTGCTTGCACTTGCCCCGGCTGCTGGGGCACACCTGAACTTGCAGATTGCG
AGCGAAGCATTCGAGATGATGGTGCGCCGTGATTTGACCACGCGACCAGCCACGCGCCTT
GCATGCCACACTGACCCTGCAAGTGCCCTTGGCAATCGCCTCGCCACACCCCCGACATCT
GGAATGGC
>at02135
TTTTTTTTTTTTTTTTTGCTCTACTTTCGATGAGGCGCCTCTAGGAAATCCACGGTAGG
CGCTGATGCTGCCAGAAGCATGCTACAAACCAACACCTTTGCGTTGGCAGGCAGTGAATA
CTCACTTCAGGCGAGCCTACCCCCCAGCATGCCTGCAGGCAGACCTGCACTACAGAGTTA
AAAAAGACCTTGATACAGTCACGGACTTGAAAACACAGCTGGCCCAGCGGTTTCAGCCTG
AGATGCAGACTCGGAGGTCAACATGTGCGCGGGTTGCCAAAACTCGGCTGAGGGAATCAC

GCCAACCGCACCAGGCTGCATGAAAACACCGAAGCTGTCGTCGTTGAGGGCCGAAACCTC
TGGGGCCCACGGCAGCAGGTCATCTCCCCGCTGAAGACGTCTTCGCAGATTCCTCCTGGC
CGGCGAGGTACGTGGGCCGGCGGCCGGAGACCACGACCTGGCCGGCGCCGACCTCCTTGC
CGTGCGCCTGCGCCGCGAGCTCGCGCTCCTGCTCCCGGCGGATCTCCTCGCGCGTCTTCG
CCACGCCGCTGAAGACCTTGCGGGCCCAGCCGGCGCTGCGCACGT
>at02136
TTTTTTTTTTTTTTTTTGGCGCGCCGCTTCCTCGCCGCCCGCCTCCGCGCTCGGGTGCT
GCCCCCAGTCATGCCCCGGCCCGCCCTCCCCGGGTCTCATCGCGGCGTCGGCTTCCGCCG
CCTTCAGGAGGCTCTCCGCGGCGACCTGGGGCAGGAGGCTCCGCAGGTAGTCGATGGACT
GGAGGTGGCGCTGGACCTTGATGTGGCCCAGGAGCTCGGGGTAGCCGAACATGAGGGCGA
CGATGTCGTCGGCGTGGCCGCAGGTGCCGGCGAGGCCGCAGCGGGCGACGGCCTCACGCT
CCACCGCCTTCACGACAGTCCCGACGGTCTCATAGAACTTGGCCTCGTCGCCGTTGTGAA
TCTTACCTATGTATGCCCACGTGAGCTCCTCCTGGACCTCCTTCGTCTGGAGGCCGAGGA
GCGCCTCCTCGAGGAGATGGAGGGCGTCCTCGATGGCCAGCTGCTTCTCCCCATTGCCGG
CATCCGGTGCCTGGGCTGGCTCCGGGGCGGAGTGCCCCGCAGACTGCGAGATGCCCAGGG
CCTCCGCCACCTGCTCCTGCTCGAAGACCCGAGCCTTGTGCTTGGCCCGCAACTGAGGCA
GGTCCTGCGCAAGGAAGTTGGTGAAGGCACTGAACTTCATGACCTCTGGGATCTTCATCT
CGGGCTTCTGGAAGTACAGGACACTCCTGTGTACACCTGCAGTGTTGGCTTCAAATCCGT
ACTTGGGCAAGATATACTGCTGGATGCTGAAGCACAAGGCCTGCCGTCCCTTGGCCAGCG
CGGGCATGTTGTTGTCCACCATAAACCTTGCGAAGCAATCCTCCATGTTGGCCTGGAAGC
TGGGATCCAAGTACGCATCGCAGAGCTCTCTCAGCATGGCGTAAGCTTCTTCTGGAGAGA
GAACTGGATCCCGACTTTGCAGCGCTTTGGGCTCTTTCTTCTCCTTTTTGGGTGGCGGCG
GTGCGGGCTCACGGGTGCCAATCGGGGCGAAGTCAGAGTCCTCCGCCAGCTTCGTGCGCA
CACTCAGCATGTTGG
>at02137
TTGTTTTTTTTTCTTTTTTTTTTTCAATTCAATTCAATTCACCTCAGGTCCATCCATGAG
AACACCGAGAAGAAAAAAACCTGTGAAAGTGTGCATTTGCACATATCCTCAGCATGCTGA
TGCACGCACTCAATGTCTCTTGCACCAGGTGCAGCCAAACGCTGTGTATTGTCAACCTAA
GCCCTAACGCGGCTGGAGGCATCTGGACGGGCCTCACGCGTCTGGACCAGTGCAAGCATG
CTACTGCTCAAAAGCACTTTTCAAGCATGGCGAATGCATCTTCAGGACATGCACAAGTAC
GGCAGGTCTGAATTATGAGATTCACACTTGGATCAGCAGGGAAGGCTTGGGAGAGGCCGC
CTAGGAAGGACTCAAGCTCAGCGGCTGCCGCTGTAGAGTCTGCCGTTGGTGCCTGAATGA
CCGGGGTACGCAGTGACGGTGCCGAGGGCGTGGAAGACGCAGAATGCGCCCGTGCGGCGG
GAGCCACTGCAGAGCTTGCCGTTGGTGCCTGTGTGACAGAGGTCCGCGGTGAGTGAGCCG
AGGGCGTGGAGGACGCAGAGTGCGCCCGTACGGCGGATGCCGCTGCAGTGCTTGCCGTTG
GTGCCTGTGTGACCGGGGTCCGCGGTGAAAGAGCCGAGGGCGTGGAGGACGCAAAGTGTG
CCCGTGCGGCGGATGCCGCTGTAGTGCTTGCCGTTAGTGCTGGAGTGACCGGGGTGCACG
TTGACGGTGCCGAGGAAGAAATGGATGCAAAGTGCGCCCGTGGCGGCGAATGCCGCTGCC
GAACCTGCCGTTTGGTGCCTGTGTGACCGGAGTCCGCGGTGATGGAGCCGAGGGCAAAGT
GGATGCAGAGTGCGCTTGTGCGGCAGATGCCGCTGAAGTGCTTGCCGTTGGTGCCTGAGT
GACCAAGGTCCGCCGTGGTGAATCCAGTGCGGCCGGCGGCGGTAGGGCCCCCTCCGGCCA
CCATCTAGGTCGTAGAGCCGCCCCCGCCTCTCTGCAGTGCTGCTCGGGAGGCGCCGCTCC
AGCCGGCCAGTAGCGCACGATCCGTGCCAGCGCTGCCACAGTGCAGCAACGGCTGCGACC
AGACATGCCAGCATGGCCGAGGCGCGAAGGAGGTGCGCCCGACGATGCGTCCAGTGCCCA
TCAACGCAACTCACGAGCTAAAAATGGCTGTGGTCCCGTCTTGCAGCCTGGTCTGCATGC
CACATGGAGGTGCACTTGAGCATCCTTGGCTTGAGCCAAAAATGGCTAACGG
>at02138
TTTTTTTTTTTTTTTTTAACTCACCACAGTGACGCCCAGTTCTGATCAGCCTAGCTCCG
TCGGACCCACTCGATGCGCACGGGCATCTCATCTCTGATGTGTAACCTGATGACGAAGCG
GGACCCAGTGCGAACAGTTTCATCTTGGCCGCCGACCATCCAGTTGATGCCGTCGCACTG
GCTCGAGTCGCTGGGGCCCATGATTTAGGGTGGCTCGGAATCTGACACCTCCGGGTGCAA
CACCAGCCTCCAGTCATTGTCCATGAGGATCTGGAACATTTCCCTTCCCTCTGGTCCAAT
GGCTATCTGGAACTGGAAGTAGCCACCCTCAGGGTTCCAGGTCATCTCCTGAGCTCTCCA
CCGGTTCCAGGAACCCACAATGAAGTAGCGCACTGCAGCAGCCTCCACGAGCTGCTCATC
AGTAAGCTCTTGATGCCCTATGCGCCTGAAGGAGACGGCCTGGCGCACGGCGCCGTTCTC

CCGCGCCAGCTGGTACTCCACGCGGAAGAGGTCCCCGGGCTTGCCGTCCAGGAACCAGTT
CATACCACGCGTGGCAGGAGCAGCGTCAGGCCCATTTACGGGGTGCGCCAGGCTGCCAGC
T
>at02139
TTTTTTTTTTTTTTTTTGCGCAATGATCGAGACTCAATGTGCAAGAGCTCCGACCATCG
CGCTCTAGCGAGGCTGCGTCACAGAGCACTTGCACCACGTCCTTATGCCCGTGACTTTCG
GCAGCGAGCAGTGGGGTCACACCATCCCGCCTTGCCCACTCCGCATTGGCCCCAGCCTCG
CACAGTACCCTGACTGTCTCCGCATGGCCGTTGGTGGCCGCCGCCAGCAGTGGCGTGGCC
CCATCGTCGATCGCCCGATCCAGGTCCGCCCGAGCGCCGCACAGGACAGCCACGCACTCC
GCATCGCCCCTGGNNGGCGCCGCGAAGAGAGGCGTCCGCCCGTCGGGCTTCGCCTTGCCG
ACGTCG
>at02140
TTTTTTTTTTTTTTTTTGGGGGGGGAGCGCGCGTCCCTCGCCATCGCGCCATCGCGCCT
CGCGCGGTCTCCTCGCGCCGCACGGCTGCCAAAGCGCCCACCTGCGAAGCAGGAGCCGCG
AGGCACCGCAGAGCAAACACAGTTCAGCAGAGCACAGAGCAAAGATGACATCCTAAATGT
CACGATTCCAACGCATGGGCACCAAGACAGAGAACAGTCCGCGTTCCGGCGGTCGTTCGT
TTTCGTCCAAGGCAGAGAGAACCTTGGTCAGAAGGCCTTTCTATGCAGGATTGGGAATCC
ATCCTCCTCGAACTCTTCCTTTGAAACCCACATCTTCTTGAACGTGGCCAGTGAGGCAAG
GATGGAGCCGCCTATCCAGGTCGAAAACTTCCGCTCCGGAGGTGCAGAGATGCGGATCTT
GATGTCCTTCGGAGCCAGCTTTCGGACCTCGTTCAGCAGGCGGTCGCCGAACTTCTCGAA
CATGGTGGAGCCGCCCGACAGGACGATCTGCGAGAAGAGCGTCATCCGCAGGTCCAGGTC
GGCCCGGTTGATGGAGTGCGCCAGCAGCTCGTGGATGCCCGGGTACTCCAGGCCCACGAC
ACTGGGGTGGAAGAGGATCTCCGGCGCGCGGAAGCGCTCGGCCCCGAGGGTGATCATGGT
GCCGTCCGGCAGCCTGTAGTGGTGCTGCGCCGAGGCCTTGTCATGCTCGTTCTGCTCCTC
CTTCTGCGGGTTCAGGGCGACGTAGCACTCCCTCTCCTTGATCTGGCGGACACCTCCCGC
TCGCCTCGTGCC
>at02141
TGCAGTTCCGTTTGTTATCCTCCAATGCTGGCATTTTCAAACAGCGTCTTATGCTCTGGG
CAGCCCGAGCACCAGATCTGTCCTTGTGGCGGATTTTCCCTCCTGCCCAGCCACGTTTGC
GCGGGTCCACGCTTGTGCAGAGGAAGGCAGGGAGCTATGAAGAGGCAGTACGGAAGAGCT
GGACTTCTTCGTTG
>at02142
TTGTTTTTTTTTCTTTTTTTTTGGAGAAAACTGAATTCATCCTTCGCCTGTTCCTCACGG
GAGTTCCGGTCCCTTGGAAAAGTGTTGTCATTGAAATCTGTGTGAAATGGTGCGCACAAC
CGCGCAGTGACACATCCAAATGCCATCACCTTGGGTGAGACTGATCTATACTGAAAACTG
AACAGATAGGTTTAAAGCCAAGTTGGAGCGCTGGGTCCCCAATGCAAGACTCTGGAGGCG
AGCTTGAAGCTCTGTCGACAGCAAGCCCTCGCCCCTTCTTCAGAGCTGGGGGGTCTGGCA
AGCGAGGAAAACTGCCCAGCAGCATAAGTAGAGCGGCAAGAGAACAAACCCAGACAGCCA
TGATGGAATGATCTGCGTGGGGCCGTGCATCAGCCAAGAGATCTGGAGCGCAAGAAAGCC
CAACAGTGCTGACGAGATCATGGCAGGTAGGCAGAGCCACACCAGGAGTGCACCGAGCGA
AACCCCAAGCACTAGCGAAACTGCCACGACAAGATCATGCACTCCCCCGTTGTTGTCCTC
CGATGCATCCTTCGCCTCGAACTCTTCCTCGTTGTCGCTCTCCGCCAATTTCTCACTGCA
GGCCGGCTCGACTGTGCTCGCGTCACTGGCGCCGCGTTCCCTGGACTTGTCTTCGACGGC
ACTATCCAACATTCCCTCAGCCATGACGCTCACAGGCGCAGCCATTCAAGCAGGAGGGAC
TCTACCAAGGCCCTTGGCAATGCAAGGAGTCGGAACTCGAGCGGAGTCAGGACTTGAGAT
CTTGAGCCAAAATGGCTACGG
>at02143
TTTTTTTTTTTTTCTTTCACGAGATAGTATTGGTGATAGCCGGGGGCAATGCTTTTCTC
GCACACTGCCTCAAGCTTCTGTTGCCAGAAGACGAACGTACCTGCTATCCGTGCGTGCAT
CCCACGATACGTCGGACACCTTTTGGACGGTTGAGTGGGTTCGCTGCCATGCAGGGCAAA
CCCCCATTGGACCAGGAAGGGCCATACTTCAACTCCACGGCGTCCGCTACTACCAACCAT
TCAAAATAAATTTGAGGACCTCCAAGCGAATCACAGCTGCATTGCACATGGCAGAGCGGC
CTGCTGGGTCTCGTGCATGGGGCGCCAGAACTGAGTAACTTGCTGGCGTGCAGCGGTCAA
TCGGCCGGATCCTTCGGCTCACCAGGCTCTTCCGCTATGAGCTCATAATTTACTGGGCAG
TTTATGCCGTTGCATTGCACCTTGGTCTTTCCAGAGTTCATGCTGAAGTAGTACGGGTGT

GTGTCTTTCGTGATCTGGGGTGCCGTCCACCCCTTGTCCTGCAGCTGCGCGGCCAGATAG
TTCAATCGTTTGGCGTTGGACCCGAAATCTGCCTTCATGCCAAAGCGACCAGCAGACCGC
ACCTGCACGGCGCCGTCGCTCCCGAGCTTGAACTCGACGTCGTCCACCTTGCCCTTCTTC
AGGCTCTCGAACTGCGCGTAGAGGTAGCCCTCCGCGGCGGAGGCGCTCTTGACCTGGAAA
GCCGCCCCGTCCACGAAGTTCTGGCCGGGGGGGTANGAGCGGACGGGCTGCTCCAGCTGC
GCG
>at02144
TTGTTTTTTTTCTTTTTTTTCCGCTGCTGATTCCGCTGACGCTGCCAGCATCCGTCCGCT
AGCCGCAAAGTATGGATCGTCATCACACTGCTGTGCAATCTAAGCGCGCTTGTGGCCTTA
GATTATGCAACATGATCGGTCGCAATGATTGCCCGCAACACTTCAATGCATGGCGCGAAC
CTGCACAGATTGCCTCATTGGGCAAGTCCGCGCAGACCTGTCTGTATAATCGCCTAGCAT
TGCAAGGCAGAGCCACCATGCTCACATCGATTGGCGTCTCGCAGTGCAGCTTCTGTGCTC
AAGCGCCATGTCATCGCTTATTCTTGCACAGCCTCCGCTGCACGAGCTCGGGGCACGGCC
TCCCACCAGGCAGCTCCGGATTGACGACGACGCGCACGCGCTTGCTGATAGAGGCCCGCA
ACCCGTCGTGCTCCTGCATGCAAGCCAGCCAGTCGCTCCAAGGGCTCACCTTGCAATCGA
GCTTGGCAACATCGCGGAGCTCCCTCATTCTTGCCATCTCCTCCCTAGTCGGCTTCGGCA
CCGGCATCAGAGTCGGGCCTGCGCGAACGAGCCCGGAGCGCTCCTGCAGCAGAAGCAGGT
CAGTGGGCTTCCCGTGGCACGACCGGAAACTCCCGGTGTCGACAACTTCCGAGCTGGCGA
TGTTCAGTATGTTTGCACCTACCATGAAAGGCAGGAAGTGCAGTCCAGGGATGCTGGCCA
GAGGGCCTCTCATGGCAAGAGGGGGCGCCGATGGACCCAAGACATGATCATGGACGATGC
CATTGAGCCGAGGCATCCACTTGAGCCAAAATGGCTACGG
>at02145
TTTTTTTTTTTTTTTTTTCCCCCCCTGAGCGCATTTCATGCTCCCAGGTCAAATGTCCGC
TTCAGCTAACAGCTGCATGCCAGGAAAGGCAGGCACCAAAACCAATAGCCGTGATTCACG
CTCCGCACTTGCGGATACCGGAACGAGCAAGCAACACTGGGGGGAACTGCTTTCAAACAC
TGCCACCTGAAATGTCCACCAAAGGTCGTGCCAATCTGGGAAAAGCACGTCAGCTCCTTG
CCCTGAAACGTGAAGTCCTGCAGCTCGTCAAGTGTGTCAAACTCTGGTACAAGTGCATCG
TGGTGGGGCAAATTACACGCAGGACTTCCTGTGCGTTGCTGCATGCCAGACAAAACCAAT
GGCCAAAAGGCAAATGAGGAACAACAATCTTCAACACTCCAACAGCACCTTGAAGCCACG
TCCAGGCGTTAGCCAGGACTGCACCAACATGAAATACAGCCAAGGCAGCACTTGTGTTGC
GCCGGTCTCGGTGTGCGTAAGACCTCGCCGCACAACAAGCCCACCAAGGAGGGCCAGTGC
AAGCAATGCTGCAGCATGATG
>at02146
CCCGCAGCCGCCCCTCCCCAGCCCCTCCGACCTGCTCCAAAGGGGCGCTCGGCCGGGCCC
GCCTTCGGGGACCCATGGGGCGCGCGCCGCTGCCGACCTGCCGGTGATCTCGGCCGGAGG
CCGCGTTTCGCCGAGGACTGCCATGTACTTGCTGCTGCTCCACGTGCTGTCGCTGACGCT
GGCAGCCGATGGGAGCTATGTCTTCTGGGCGGAGACTGCCACCCAGAAGATCAGGCGAGT
GGACACAGGGACGTCTGCGGTACAAGACGTGCAGACGTGCTCGGCCCCACCCCTCAGCAT
CGCCGTGGACTCCATCCGCAGCAAGATCTACTGGCTC
>at02147
TTGTTTTTTTTCTTTTTTGCTGAGGGACAAATAGTCTTGCCCACATTGATCTTACAAAAT
TCATGCTCAGCCACTGAAGTGCCAAATCACAACTTCCGCATCATCCCCGGCCGACGCAAG
CAAAGCAGAACCATTCGGCAGAGGCTTTGGGCACCATGCGACGCAATTTGCGTCACCAAA
GTGCGCCTCCACTTCTGCGACGCACGTCCAACCCTCCAAGGTCGCCTCATTCTGTGGCTG
GAGCACGCGAACGCAGTAGTCGCCAGATGCAGTTGCAATGGAGGCGGAAGACTTTGCGGT
GCCAAACGGCAGCCAATCAACCGAGTAGATTGGTCGCGTATGATGGTCCTCAATGACCGC
CGCTGTTTGCCACTCGCACGCTGCATCCGGAGGCGCCCGCCGGGGCTGTGGACTGCAATG
GCTGCCTGATGCAGGTGCGGTCACTGCTGCCGAAGTGGCAGCGGCACTTGCGCGGAACAA
TGGAGTCACATAGGCCGCTGCAGCGACGCTGACACCCCTGGATGTGGGGGCAGTGACCGC
AGGGACCTCCTTATCGGGCTTTGCGGCCTCGTTCTCAGGCGCCCAGATTCGCAAAGTCTT
GTCACCCGAACACGATACAAGGCGGGAGCCTTCGGGATCAAAGCCGAGGCACCAAACCGT
GTTGGCGTGGCCTGAGAGCGTCTGCTTGCAGGCAAAGTCATCGCCATCAGGGCCCCAGAT
CTTGATGGTGTCGTCGTACGAGCAAGAGAACAGAATGTCTTGCCCCGGGTGCCACGTCAC
CATCTTGACGTCCTGCGTGTGCGATTGAAGTACTGCTATGCACTCATACTCGAAGTTCTT
CGCTGTCTCATAGATCCAAACGGACTTATCCCGGCTGCAGGTTGCCAGGTAATCTCCAGA

TGGGCTGAATGCGGCCGACTTGACCTCGTTCTCGTGGCCAGTCAGCAGACCTACACACTC
AAAACAAAGACCGTTGCTCGACTCATCATCATTGACGGCCTCCTGCCAAAGGGATGTGCT
GGCGTCAAACGACGTAACTGCCAACAGGTCACCGCCTGGGTTCCATGACAGTGATCGTAA
GGTTCGGCTGTGGCGGTCGGCTGCGTCGACCTCTGCACGAAGCACCCAGCTGTCCTCTGC
ATCTGCAGCAGCTTGCGCCCGCTGTGCCCACACACGAATGGTGCGGTCAGCGCCACACGA
AGCAAGCTGTGGAGGATCTGCATGAGGGCGCCAAGCGATGCACCATACACGCTCTTCGTG
GCCCTGTAATCGGCACACGCGAGTCAGAGGTGCCATCGCAAGATTGTAGCTGGGGTTGGC
TGAGTACCTCTCAACTCATGAGCCTATCTCCTTGAGCCAAAATGGCTACGG
>at02148
TTTTTTTTTTTTTTTTTTGGCTTGCTGTCGCACATCGAAGACATGTGGACGCCATCACAC
CCCTGTACAACCCGACAGCTGTTCCTCCCGTGCAGCCTGGTAGCGCGCACGACTGCTGAC
CCACCAGGAACAGATTCCTGAAACCAGTTTACAGACCCAGCGGTGCATCCGAAGAGCAGC
GTCACACCTCGAAAAAGACCCAATGGATGGCAGTCCCTCCTGTGCCACATGCAACACAGC
TCAGTGCTTACACTGGCATACCAGCACCAAGCAAATGAGAGACAGGAGTCTGCCAAACCC
AACACAACATCAGAAGACACCCCTCACATGCAAGAACCTCAGCTCTCGAAATCGATGGCA
TGCGGAGGTTCCGCACGGACGCAGGACTCAAGCCTCCGTCTCCAAAAAACACCCCTCATG
TGCGAGAACTCGCAGAGTTTGCTGGGCCCCCCTCGTGCC
>at02149
ACCCTTTTCACACGCGCTGATGACAGAATTGATGCTGAAAACATTGGGGATGATCCGCAA
CACTGCCATGGTGTCCAACGTGGCCAATGCAAGCTCCCAACGCCCGCCCTTTTCGCATGC
ACTGATGACAGCACTACAGGTGATGACATTGGGCATGATCCGGAGCACGGTCATAGTGTC
AAGTACGGCAAACGCTAGCTGCCAACGGCCTGCCTTGTGGCAGGCACTGATTGTGGCGCT
GCAGCTGATAACGTCTGGCATGACCTGCAACCTCATCATGGTGTCAAGCATGACAAACGC
AAGCTGCCAGAAGCAGCATTTCTCAAAAGCACTGATGGCAGAATTGTAGCTGATGACGTC
GGGCAGAGTCTGTCTCACAGTCATGATGTCGAGAATGGCAGGGCACACTGCCAGC
>at02150
GGACGAACGGCGGGACCGACCAGGCAGCGATGCGCATGTGGCGAATCATGCTCTCATAGT
TGAGGCGATTCTCTGTCACTAGCGAGGCGGACGGGGTGAAGTTGCCGTGCCAACCAGAAT
CCTGAAGCCGCCTCCATTCTGTCAGCCAGCCTTCGAAAGTCGCCGGCATGCCAGGGGACC
CCGGCTGCCGCACTACTCTCTGGTACCTGCTGATAAAGTCTTCCATACAGAAATGGTACA
TTGACAAAATATGCTCCCGGGGATTCCGAAGCAAGACAGCCGTGCCCAGTATCTGTGGAT
TGCTGTCCTGGAAGCTGAAACAACCTCCTGGCTGACCTGTTGGAGACCGTGCCCCTTCAA
GATCGACATCTCGTCATTCATCCAAGAGAGACCGGCTGCCTTGGGAATATGGAGATGATA
CACGAGGTTCCGTTGACGGTTATCCGCATGCACAGCTGCGCTGGCTGCAGAGTGG
>at02151
TTGTTTTTTTTTCTTTTTTTTTTTATGGATTGGCCCGTGAGGAGGTCTGGGCACTGCTGC
ACGGCAGCTGTTCCGTTTGTTTTGCGTCACGTCTCACATCCAGCCTGCTTTGAAATGACT
CCTGCTTTCTGCAGTGTGTCTTTGAGTCCGTGGTAGCCTTTCGAGTACCTTTCGGACATG
TCATCGTGGTATTGGTGGTAGAGCTCAGCGGGGTAGAATGGGAACTCAGAGCTATCATAC
ACGTAAACAATGCCCTTGCCGAGCGTATCTGGATCCCCACCCTTTCCAGGAAGAAGCTGC
AGTCTGCCGGCGTTGGCCTGCTCTACTATCTTTCCGGCGGGGGACGCCAAGCCACCGGGC
AAGCCAATGACGCTGCGGTACGCGCTTCCTGTGTCTTGAGGATCATGCCGGCCAGCAACC
GCTTCTGGGGAACCAGGGTACATGAAGCCACCTGGCAGCGTAGGTCCCTTGCTTGCCTCA
TCAAAGTAAAGCTTGGACATCTGCTCCAGGGCAGCAGTGGGCAGCTCCAGGCCCACCACC
TCTGCATGGCCCAAGTCATCGTAAGACTTGCCTTCGTAGGCGTTGTAGCACACCTGGCCA
TTTGGATTCTTGCCACGGCCCCCGGCGTACCCCGCGATTGCAGGCGAGAACGAGCCCTCT
GGGAAGGCGCCTTGTGCTTCCCGGTTGATCTTGAGGCTCCTCAGTGTAAGTTCATGCTGC
ACGTGCCAGAAGCAGCCCTGTCCGAAATACACCCTGACTGTATCCTCGGCGCTTGCAAAC
TGGGTTCCGAAGGAAGGTATCAAAAGCCCTAGCGATGAAAACAATGCCCGCCTACGGGGC
TGCTCATGCAGCTGAGGCCGTTCCGTCTGCTTTGGCTGCGCATCCATCATGTCCCCGGAC
CTGCTGGACTGGGGTTTGGAAGCCTGGAGTTCTGATCGAGCAATGCCTGCAAAGCAACCA
GGGAGCGACCAAGCGCTAAGAGCACCGAGTGAAAGGCAAAGCAGGGCTCCCAGTCGGCAT
TGCCGCAAAGCCTGGCCTGACGCAGGCCGTTTCGCTCGTCGAGTCCCCATCAGCCCAATC
GGGAGGTCCGAGGCATTAGCCGCAATGGCTTGAGCCAAAATGGCTACGG
>at02152

TTGTTTTTTTTTTTTTTCTATCGACGGTGCGCCACACTGTACTGCTCCTCTGGAGCGCGC
GCCATAGCAATGATGGAAATGCGTGGACCCACGGCTGCGTCCTCAGGCCTCATCGGGTGT
ATGCCATGCTCAAACGCACTGTTGACGCTCTCTCTGAATGCAAACACATCTCCATTCCAC
TGGGGGAAATCAAATGAACGGCCACGGTCGCTGGCGTGGCGGAAAGTGAGACTGCGCATG
GCGCCGAAGGAGGCGCCAATGGTGATGTTACGCCCTCGATCGCCAGCAGAGCAATCATGG
TGGAAGCTGCGGCCAGCGCGACCGTCTTCGTAGAGGTTGACCCACCACACAAGGCACTGG
GCATCAAAGCGTTTACACAACTGCCTCAGGACAGAGGTGCATGCCGGCAGTTCCCGGCCC
TCAAGATCCTCAAGCACAGACCCTTGTGGCGGCAGGCCCTGGCCGTTCTTCCCGGGCAAT
GACCACTTTCTAGCGGCCTTACCTGTCGTGCCGCGGCTCCAAGACCCCTCGCCGCGGCCA
AGCTCCTGCCGCAGAGCCTCGAAGAGACATAAGTCTGTTGCCTCGCAGAAGGCACCCCGC
AAGTGGTGGGCTCCGCCGCCGAGAACAGCTCCTTCGCAGTCCCTTGCCAAGTGGTCCTCC
AGCAGCAACTGCCATGGCGACGTCAAGCGGCTCCGGGGATAGAGCCCCTCCTTCGCTGCA
CGCGCAGCCCTCTGACGGTCATCACCTGAACCGCCTACGGCAGTTGAAGACTCTCTGGGA
GCCACGATGATTGCATCCAATGGGCTGGCCAGAAGCACTTTGATAGGATCGTTGCCATGG
TTTACAGCAGGGGAGCTGGTCTCCAAGGCCTCTTGGGCAACAGAGCTGCTCGGATTGGCT
GTGCCTGGTAGAAGTTTGCGGCGGTGCTGCCAGCGTGACTTCTGCACGTGCGCCGGCAGC
ACTTCCTGCCCAAGCAATGCAGGGTCTGGACAATTTCCAGTTGCTGAAAAATCTTCCTGT
AACCTGCTCTTGTCATGGCCCCACCTCCGCATCTTTCGAATTGGGAAGACCTTGAGCCAA
AATGGCTACGG
>at02153
ATCGATGAGCTGGATGAGTTTATACCATTCCTGGCAGAGAAGAGGCTGCCTCGGGTGGCC
CAGGACATGAATGCGCAGAATGTAGCAAACGTCTTCTGGGCAGTGGGGAAGCTCGAGTTG
GACTCATCGCTTGCAGACCACGTATTACGGACGACGCTGCCTCGTTTGCGGGAGGTCCTA
AGCACATCTGATCCTCTAACGATGGTCTCTATATCTATTGGCATGGCCTCATGCGGGTGC
AAGGACACGCCACTTCTCAAGTCTATAGCTGCACTGGTAGTGGATGTCGCGCCAACTTGG
CCTGGCGAG
>at02154
AACCTCAGCAGGGCAGGCAGGTTTGCAGCCTTGGACATCTCCGAAGTGTAGACAAACTGC
CAAAGGCATTCAATGTCAAATGCGTCGGCATCCGGCAGGCTCATCTCATTGGCACCTGCC
TCTTGAAAGCTTCCATGCATCATTGCCTTCAAGACAGGTGATGATAAAAGCACCGCTCTG
TGCACCTTCACTGCACCATCTCTGCACACAAAGGTGAAATCCCGGTGATCGTGCTCGTTC
CACAAATGGAATCCAAGATGCCATGATGGCTCCCGCTCTACAAGCTCTCGAGGGCAGATC
TCCAGCACGTCGCTCAGAGATCTGGGGAGGTCATTGCAACTCAGACTAGGAAGCAACATG
CCATGCTTTGA
>at02155
TTTTTTTTTTTTTTTTTTCAGCCCAGAAAGCGTTCGAGAAGCTTCCAGAGATACCCCGTC
GCAAGCCGTTTTCAGGACAATGCATCTGTGCCTGTCTCGAGGCGCGGTCCTGCACACTTG
TCAGGTTACGCGTCGAGCCAGAATGACGGCAGCATTCGGGCCCCCTGGAAGAGCGGACGC
GACGGCCGCAATCGGCAGATCGCAGTGCATACCCCAGTGCCGACGCCCAGCTCAGCCTGC
ACGAAAACTGGCGTGCGCACACCAATGCCGTGCCAAGGCCGGCGGGCGGTGCGACCGCCC
AAACACTGAATCAAAAGGCACTCCAACCCGTGCCGCCAGACCCCGAATTCAGCCGGAAAA
CTCGTGGAGAACCCCACGCGGTCCACGCCCGAGCGCGTGCATGCTCTTGGCACGTCGCTG
TTGGCAGTCTTACAAGCGCATCGATCATCTGCATGTCCAAGTGGTTCGTGGGCTCGTCCA
ACAGGAGCACATGGGGGTTCACCAAGGAGATGAGGAGCATCACCACCCGCGTACGCATGC
CATCGGACATGGTCTTCATCTTGCGCGTCTGGAACTCGCCCGTGATGCCAAAGGAGCCCA
GCTTGCCGCGCCACTCAGGTTCCTCGAGCTTCTTCATCCCCGAGGTGGTCACCACACCTT
CCGGGTAGAGGTGCTTGAGGAAGTTCAGCGGCGTCATCTCCAGGTCCAGCACGTCCTCGC
TGTGCTGGTTGTAGTGCCCGATGCGCAGCCCTCGTGCC
>at02156
TTTTTTTTTTTTTTTTTTACTTTCACTCATTCAGCCGCCAATGTCCACAACCAGGTGCTG
CTTCTGCGATGGAGAAACCCACCAGCATCACGCCCCGCGTCAGAACGTTGTCAGCCTCCT
GATCATGATGGACGCTCACGAGAGGCTGCTGTTGCATGGATGCTACTGACAAGTCATCGC
AGTTGGCCCTTCTCTAATCCGCAGCTCGTAGCTGGAATGATCCGACAATCACACCCAATG
CCAGACAGAGGGTCAAGGTGCACCTTATCATCCTCCTGCATCGATATGTCAATAGATCTC
CTTCGCTTGTCTCTTTCCCGCACGAAGTCCAGCCTCCTGTTGAACTCGAGAGTGAATGCG

TCATTGGCGTCCTGCGCCACTTCGTGGACGTCAGCCTGCCATGCCGCCTCTACGACAGCC
TCAACTGCGTCCGGTGCCCGTGACACCGCTTGTGCCCCGATCCCGATTGGCGGGAGCAAC
GATGAAAAATCGGGGGCAGAGAGCAAAGCCGGCGAGTCGTCCGTTTGGCCAAGCCTCCGA
ACCAGGTTGTCGGTGTTCTCCTCCTCCGAAATTGAGTCATCACCAACCACAAGGATGCTG
TCCTGCGTCTCCCTGACCCAGATAGGTGGAGCTGCTGTCTGGCCCGGAGTTGCCTGCTCC
TGCATGTTCTCGACGTCAACGGGCCCACTACGCTGCTGTGTTATGAGCGTCCTGCTGGCA
AGTCCTGCCTCAAGACAGACGCAATCCTGTGTGTCATGGTCGTTGACCTCAATTGTCTCC
GACTGACTTGCTTGACCTGGCAGGAAGCTCTCCATCTCCAACACAGACTGCTCCATGTCC
TGGCCCATGCGCTGGCCCACTCCGACGCAACAGACGCCTTTTCTAGACCTTTCTGTCTGC
GAGTGCCTCGACGCTGTCAGGCGGGTATGTGGCACTGCCAACGGCTCCCTGAATGTGCTC
GTAGCCGCGTCAGTGCCGGGCACTGTCTCAGCATCACTGGGACCATCCTCAGGAAGATGT
GTACGCAGTCTTGGCACCAACTCAGCTGGGGTTGCTGGCACACAATTGTGAGAAGTAGCA
GCACTGGGCGTAGCACAGGCCTCCAGCAGGTCTGCAGCTCTGCCCAATGATTCGTCATCT
TGACACAACAGCCTGCGCGTGTAGACCTCCGAATCGGTCATATCCGAGGCCGCAAAGCCT
CGAGCGCAGACCACACCTCTCCGCCCGACCAGCCGCGACGCCTCCGGAGAGCAGCACGCC
AGATGCCTGCATCAGGTCGACGGCGTGAATGGGCGGTCGCCATTCGGCGTGTGGCCAAGA
GCCCGCAGGCATTGCAGGGCCAAACGCCTCCGCTCCGAGCCTTGAGCCAAAATGGCTACG
G
>at02157
GCGAGCAGGGGTGCGACCAGTGCCATAAGACCTTCCTCCCGAGCGATTCCTTCCCCGACC
CTTGCCTCTTCTGGGTCCTCCCGTGCCACCTTCGGCAGCCTGACCTCTTCCGCCCATGCT
TGCAGCAAACTTCGTCAGAGAGAATGCCGATGCTCGAGGCCGGAGGAATGACAGCGTCAG
CATCAAGGTGCAGCTGACGCCAACGGCAACTCGTCGGCGGGTGGACGCCCGCATATGGCA
GCTGTCAGGCCACCTGCGTGTGCAATTCACGCCAGGACTGAAGCCAGATAGATCTAGTGA
AGCCACACAACCCCTCTACAAGTACCTGTACGCCAACACCTTCGCACCGACGGAAAGCAT
GAACTATGCTCTACACATGCCTCGTCCAGAAGCCTTGAGCCAAAATGGCTACGG
>at02158
TGTCGTCAACCCTTGGCCAGTCAGAGGGTAGCCCGCTGCGGCTCACAGGCGGCTGAGCAT
CCAGGGCTTCCGCTTCAGCGAGACCTTCGAAAAGTCCGGCGTCTTCTTGGCCATGAACGC
GGCAGCTGCAATGCCCATCTCAGGGTTGGCGCCGGCCTTCGCATGCCGCTCAGACTCGAC
GTTCAGCTGCTCACTGAAGGAGTTGGTCTCGGAGTCGTTGAGGTTCGCCTTGATCCTCTG
GAGTGCCACAGGCGCCCTCTCAGCCAGCTTCTGCGCCACTTGGCCCACGTGCGACATCAG
GGCCTCCCTGTCTTCAGCACCTTGGCCACAGCCCCCCAGCGCTCCATGTCTTCAGCAGTG
TGCACGTCAGAGAGGAGGTAGAGCTCCCTGGCCTTCCCAGCACCCACGATGCGTGGCAGG
GTCCAGGTGCCGCCAAAGTCGCCAGTGAGCCCGACGTTCACGAAGGCCGTGGTGAACTTC
GCCCCAGAGACGGCGTAGCGCAGGTCGCACGCGCAGGCCCAGCTCAGGCCAGCCCCGGCG
CAGGGTCCGTTGATGGCGGCGATGGTGACCTTGTTCATGTTGTGCAGGAGCTCCGAGGAG
TAGCTGTCAGCTCGCAGCCCCCAACCGCGGCGTCCATCGGCGCGGGCCGCGGCTTCCGCT
TCGCCTTGGGCCGCTGGCCCGCGAAGCCCTTGACGTCGCCGCCCGCGCAGAAGGCCTTGC
CAGCCCCGGTGAGAATGACGACGCGGAGATCCGGATCGCTGGCTGCCATGGCAATGGCTG
CGCATGCGCCCTGGATAATGTCAGGAGTCATCGAGTTCATCTTCTCAGGGGTGTTGAGCG
TGATGGTGCAGATCTTGCCGCTTTCGTCCACCTCGTAGAGCACCGACTTGCCGATCAGGT
CGAGGCCATATCTGCTTGCTCCGAAGCCAGGCTCTGAGGCGATCTCGTCTTCCTCTTCAG
CCATGGCTGCGCTGGAGCCCCCTGGAAAAAGGATCTTGAGCCAAAATGGCTACGG
>at02159
CCGTCCTGGCTGAAACCTATTTGCCTGGAAGCTCGTTCTGCCTGCAAAGTCAGCCCGCCG
CAAGCAATGGTTCCAAGCTCCCTTCTTAGCCGGTCTTCCATGGCTGGGGCCATGCCCTTG
AGGCGCGTGCTTGGTGCAGCACGAGGCATGAAGATCCTGAGTGCGCACCCCGTNAACGAC
GGCAAGCAGCTCGACATTGaAATTTGTGGACAGGACCACTTACCGATTCCACGTGGGGTG
GATGAAGGGCGCGAGCCCCAGTCTTGTGGGCAGTGATTACCACCACAAGTGGGCAAAGCA
CTTGTTCGACACCAGCAAGTATGTTGTCACCAAGGCGCGGCCAGCCGATGACGGGTCCAA
GCTCATCGTTCA
>at02160
ATTTGCATACTTTGATCTCCCTTGCCATGGCTGGACACCCACCCTCCTGCTTCACATGCG
AAATGTATTGCTCCAGCTTCTCTTTGTCCGCCTGAGTGGCCTCCTCCAAGGCCGCTTTGA

TCAGCTTGTTCCATGCCATCACGTGAGGGCTGGTCAAGAGCTTTGCCCTTTCCCCCGCCT
CGATCTGTTCTGCCGCTTTGTGCCACGCACCGGTGGCTTCTTTTANCGCTGCAATGGGTG
GCGAGCCCTGCTTGC
>at02161
TTTTTTTTTTTTTTTTTTGAACTCGCATGCTTCCGCTCCTCTCCGACACCATCCTCCTCC
CCTCCTCACCAGCTTTACCTGTCGGTTGCGGCCCCTTGCCGCCACCAGCTCCGCCTCACC
CGCCGCCCATACGGCAGCGGTGCGGAACTTGGCCTCAGGCGCTGCCCAAAAGTCCCGCCG
CGGCCCGCAGCGCCTCCGCGGGATCTTTGCCCACGCCCTCGCCGTAGAGCTTGACCTTCG
GCTCCGTGCCGCTCGGCCGGATCTGCACGCGGAGGCCGGTGCCGCCCTCCTCGCCGAGCC
ATATCCGAAGGAGGTTGGCTTCAGGGAATTCACGCACGGCGGCGACCTTCATACCAGCGA
TCTCCTCCGGAGGGTTTGACCTCAAGCGCTGCACGGCGGCGGCGCCGGCCACGGGGTCCA
TCCTCACACTTGTCTCAGCGACCATGTGCTTTCCGTACACCTCGTCGATCTCCTCGAGGC
GCCGCTCCAGGCTCGACCCCTGCGAGTCGAGCAAGGCTGCGAGCTCCGCCATGAGGACGG
CTGCCGTGATGCCGTCCTTGTCGAGGGGTCTGTTGGTGAC
>at02162
CCGTAGCCATTTTGGCTCAAGATTGCGGCTCACGAGGGCAGCTTGCTTCGTTGCATCTCC
TCTTGCTTCTCCTATGTGCCATGCAATCGTGATGCCGTCCGTGCCCATGCTCGGTGTTGC
TTGGTTGGCCTTTGCCACCACCGACGCTGACGCCCTCAGCGAGCCTGCTTATGCCTTCGC
TGATTACGTCCGTGACTTTCGCCGTTCCTACGCACCCGCGGAGGTCCCCCAGCGCCAGCG
AGCCGTTGAGGATGTGCTGCGTCGCATTGCAGCGCACAACGCAATGCCCAGCAGGCGTTG
GAAGGCTGGCGTGAACGAATTTACCGACCGGACAGCAGAGGAGCTGCGCATGCTCGGGGG
ATACGACAAGGTCCAAGGCTTCAGTCACTTGGGCGGGCTGCAGAGCCACCGGCACGTCTG
GCACTGGATGCATTGCCCAGCAGG
>at02163
TTTTTTTTTTTTTTTTTTACATGAGACTGAGAGTGAAGAAAGAGGAAGCACCTCCAAGAC
TCCGTCGCTGTCTGTAGCTGATGGCCTCCCAAGTGGAAGTCATCGGAAAGAACGGCGAAC
ACTGGATGCACGCCAATCGTTAACTGCCATACCTGTTCAAAAGCTGCTGCGATTCAGCAA
GGAAAAAATCGCAGCCGCGCCGTAGGATCAGAAAGTCACCTAGACTATCAAAGAAGAGGC
CGCCCTGGGCTTGACGCACTTGGCCTCCACAATCTGATTTGGTGTCACACCTCTGAGTTC
GTTCTGGGTCTCCTTGGTGATCCATGCTCCGCCACACCTGACCTCAATGTCAAATGTCTT
CACGCCACGTGCCACGGAAGTGGCGAGACCGAAATATGCATCGTATCCATTCTGTGAGCA
GAAAGCGCTGCCGCCATCAATGTTCAAGGGTGCAGCCGCCAAGGTGCGCGTGCCTGCCTC
GTACACGCGCACCTCCTGGCCGAGGAACGCGGCATGGCCACTCTGATCGACTGGACGCAC
CTTCAGCCATGCCCCAATCTTGGCTTCATTTATGAACAACTTGTTTTCTTGAGATGGCCA
GCTCAAAGGCGGCGTTCCGAAGGTGTCCACGTACATGTCGAGGTCACCATCGCCATCGAC
ATCACCAAAGGCAACGCCCTGGCCGAACAGATGGTAATTGACCCCAGCCTCAGCAGTGAC
GTCCTTGAAGTGACCCTTGCCGTCATTCTCGTACAAAGTGCCTGACTCTACGACGCCGCT
AACGAAGATGTCGAGGTCACCGTCACCGTCTATGTCGGCAATGTTAAATCCTTGAGCAAC
TCCCGCGTGGCCCACACCAGCCTGACTCCGTGCCATCAGTGAAGTGCCCAGTGCCATCAC
CAAAGTACAACTTGTTTGCTGCGAGAGGGCCAC
>at02164
GCCCAAGCTCTGGTCGTAGCGCGGCTGCCCCCAGCCGTCGACGATGGCGTCCACAGGGCA
GGATGCCGTGGGAGCCAGGAGGTACGGGTCCTTCTGCCAGTCCGTGGGCACCTTCCCGGC
AGCCACCTGCTTCTGTATGGCTTTGCCGCTTGCGATCGTGCCGCCGCTGATGCCCCCGCG
CATGCCAGTCACGACGGCCCTGACGGTGACGTTACTCGCCAGCTTGCCCCCAGCAGCCTG
GGCGTCCAGCGCTCTCAGGGCCACGGCCGCTCCGACGTCGAAGGGCGCGCTGTCGTAGCC
GGCGGCCACCACGACCTTGGCGCCGCTGAACTGCGCCTTCGCCGCGTGGCTGTCGATCAT
CTTGCGCATCCAGAAGAACTCGCCGCTGACGTCCACGTAGTGCGTGCCCTCCTCCGCGCA
CACCTGCAGAAGCTTGGAGCCATGCTTCGTGTAGGGGCCCGCGGCCGCCAAGACCACGCG
CGCCTT
>at02165
TCAGAGCCTCCTCCACAGAGTTACAGTAGGCCAGTATGCGGCGGTAGTGTCCGGCCTCCC
GCAGCAGCATGACTGCCAATCCTCCAAGCGCATCGCCCGCGTCGACGACTGGCACGGTGA
GGTCGTAGTCGCGCAGCACACCAGCTTCTATGGCCTCGTCCAGGCGGTATCTGAAGTTGG
GCGGGGTACGTTGCGTCGCGGAAAAGAGGTAGGTGTTGTTGGCGGTCGGACAGCCAGGTG

GCAGGGGATGATGTGCCTCATCCACATATAGGTCCGCAAAGGTCATGTTTGCCAGCAAGT
GCGCAGACTTGTACACGGATATGTAGCCCGGGGCATCCCAGTCGATCCGAGTGTTGTAGC
CCATGCCAACCGGGCAGAACTCCGGGAACACGCTCATAAGCTGCCTCAGCAGAATGCGCG
AGGGGACTATCACAAGTACGCGGCCCTCTGCCTGATCTGCCAGCTCTCGCATCACGCGTG
>at02166
GTTGTAAACGTACAAGCAGCGGATATACTTTGTCGGTTTGTCCTTTCTAATTGACGTCGA
TCAACTGGTCCACTGAGATGTCTTCTCGAATAAGGACTTCTGCGTTATGGACGCGGTACT
GATGCAGCACACGATTCACCAGCTCGCCCGGGTTATCGCCGAGGCGTGTGAGGGGCACAG
TCGAGTTAAACTTGACACCTCCACCAGCCTTCTTCTTAAAGGTCACATCAGGGGGTCTCT
GGTTCAGTCTAATGCCCAACGTTTCCATTTCTCGCTCGAGAATCTTGCGTGTTGATTGCC
CGTTTC
>at02167
TTTTTTTTTTTTTTTTTGAGGTATTTTTTCGCTTCCGCACAAGGTACGCAATGTCAGAC
CATGCAACTAGCTACTTTCTTCGTAGTCATCAGCGGTGCTGGCCGCATCTACTTCACTTT
GGGCACCTGCGTCAGCCTTACTAGTTTCCTCAGTGAGGAAGAAGTAGGCGCCGACTCCAG
AGAGTGCCAATATAATTGGGGTTCCAACTCCAGCGAGAAGATAACCTACGATGGGCTTTT
TCTTCTCGGGAAGGCCCTGCCCAGCGATCCCATGCTCGCCCTCCTCACCCGCGGGGCCGC
CTGCCGCGGTGCCAGCCTCACCCCCGGCATTGCCAGCATCGCCCCCCGGCACCGGCTGCG
CCGGACCGCCCGACGACTGTGGCTGCACAGGCGGGTGCCCCGGCGGCTGCGCCGGCGGCT
GCGCCGGCGGCAGTGACTCTGGCTGCTGGAACTGGGAGAAGGTGGCACGACCAGGCCCGT
TGCGCCTCGTTGTGCCGAGGAATCCAGCAGCAGTGGCCCGCCGTCTGGCAAGTGGTAGCT
CATCCAGAGGCGCAGTGTCAATGCGCGTAACGCTGGTCCGCAACCGCGATGGATGCGCAT
GAGCTATGGTGCCGTCTGA
>at02168
GCGAGTTCGTCAAGTATGTCGAGGAGGCAACTGCGACGACCATAGACATCGAGGGCGAAG
ACCAAGCAGAGCCAGACGCACCTAGGCCAACGACCATCGTGGGCCAGCTGCTGAATTGCT
ACACCGCGCACGCGATGCTGCTCATGAAGCTGAGGACAGTGGAGGCCCAGGAGCAGGAGG
CCGAGAGGCGCAGAGCCGAGGAGCAGAGCGAGGATCCAGCGGTCCTCAAGGCCAGGATCC
AGGAGCTTCAGGCCCAGCTGGCCAAGGTCTCCAACCAGCTCCAAGGCATCTGACGCGCTG
GCTCGCTGACGCGCCTCGCCCCCTGCCAGCGCGCCCGCGGGCCTTCCCCC
>at02169
TTTTTTTTTTTTTTTTTTCCTCATGCACAACCACCAGACAGCAGGTATCTGCACTGTCTG
TCCATCACAGTGGCTGGTTCACCTCTTGGTAGGGAAAACGATTGAGCGGCCAAGTCCAGT
ACCAATTACGGATGTTGCTTGCGTATCAGTGCCCAAGGCTTTGACAGCAACATCTCTGAC
CTGGACCAGCATGGCTTGAGGGCTCGCCAAAAGAGAGTGCACGACCATCTTCTGTGGATT
GCGAGCTGGTCGCCCGGCATGTTCACGTCCATGAACCATCGTGACACATCTTCTGAATTC
TCACCAGGCCTTGCAGCCGCATGTGCACCACGTGCAGGACTGCAGGTGCATGCTCTGGGT
TCAAACCACTTAAAAGCATTCCTCACTCCAGGCCACGGTGCCCAAGAACGATGGGAGGTC
AACCCTCCATGGCTGTCTACGGATCGAGCAGCCTTGTCCGGAGCGGGTTCGACTCGCTGA
ACTCGCGCGCGTGGCCCTCTTTGCGGAGCAGAGCCTTCCGGACCTCTTCGAAAATACTAA
TCCCGATGAAGGCCATCCATGGGTAGATCATCTGCTCTGTGGTGAGGGCAGTGGTTTTGA
AAATATCGGCCACATCTGGCACGTAGATGATGATAGCAGCGAAGATAACTCGAGCACGAT
GCAGATGTTGAGCTTGGCGTTGGGCAGTCTGTACTTTGCAAGGGACT
>at02170
TCTCCGAGCCCATCGCGAGTGTGCGAGGGCCCAGCTAAAATATCCCTTCTCTCATTTCAC
TTCAACCCTTTGCCCCACAACCCCCCGCTTGGAATTGCACTGCTGTGATGCAGTCCGTCC
CACTGTCACCGTTTGGTGCCAAGCGCCTTGTCCCATCTGCTTCTGTCATTGGCATAGAGG
ACCCCCAGGCGACCCCCACTGGGTCAGCTGTGGTGGAAGGCCCGAAGTTCCGGGCAAGTC
CATCCCAGGGGCCGGAGCAGAATGGGCCTGCAATTGGAAAATTGCAGGCACCGGTGGTGG
CAGATGAGGAGGTCGAGGCCCACAgtAGGGCAGTGGGGTAAAGAGGCCTCACGTGGAAGC
AGGCTCGACCTTCGACAAGAGAGCCCGTACGGCTCAGGTTGAGGCGCCTCCCTCTCAAAC
ATC
>at02171
CTACATAGATCAGGTGGACCAGGTGTACCAGGTCATGAAGAAGAGGGGCAGGGCCCCTGC
AGATGCCCCCAGCAGGATCAAGAAGGAGCTCTCTGCTGCGATCAGGCAGGTGAAGAGGGG

CCCAGAGGCCACTGAGCAAGACAGGATCCTCTTCATTGGGTGCACCTCCAAGCCATACCT
CGACACCGTGGACTCCAAGGAGCTCATTGCCGCCTTCGACGAGAAGGTTTGGGTGAGCTA
CCCTGAGTACGGCAGCCGCGTCGTGCTCTGGCAGCAGTTCATGGAGATGCACAGCGCCTC
CATGTCCCTCGCCGACGTCTCCACGCTGGCGAAGGTCAGCGAGGGCTACTCCGCCGGCTC
CATCAAGCAGACGGTGGACCGGGTGCTCACCGCCAGGCGCGTGCAGCAGCTCAAGAACCG
GCCCTGAAGGTGCAGGAGTTCATCGGCCCTGTCGCGCACGGCGTACTG
>at02172
CCGTAGCCATTTTGGCTCAAGCCACTTGGGCTCAGGCACCATGAGGCAAGGCGGGGGCCT
TGGGCGATGGTGGTCGGCACTTTGCTCGCCATGCTGCCCTCTGTGTCACCCTGATGGCA
CAATGTTGCAGCGGTAACCACCTACCAAGACTATTGATCGGCAGTACGCATGGCGACGAT
GAAACTTCCACGACACTATTGCCGCAACTGGAGCCACAATGACAACCACAACCCTGACAC
AGAGCACAACAGCTGCAAACAGCACTTGGGCTCTCTCGACCACGTTCACTGATCCCATAC
CAAGCAGTTCTATCGGCAGAAACACAACAGCAGTGAATTGGACTGCAGCCGTCACGACCT
CTCTGGCACCACCCATAACCAAAA
>at02173
CCGTAGCCATTTTGGCTCAAGAGCACGGACCGAGCCAACAGTCCCGTCTGACGGGGAAGC
TTCGTGCCTGCAAGAAGTGCACGCCCCCAGCTCGATGGGCCCTGTCACGGCAAGCCACCA
CGGCAGTGTTGTGGCTAGCGCTTACTCCGCAGCAGTCGGTGCCGCCATCGGCACAGAAGC
CGAGGCTTGGAGCCTGGAGGTGGACGTGGACACCAGCGCCCCGGGCTCGCAGAGGCGGTG
GAGGTGGTTGTGGACAAAGAGTCCAGTGCGAAGAGAGCGCTGCGACCTTGAGGTTGCTGG
GGGCACGAGCCCTGTAGGACCGAGCGTGCACAACTTGCGGTGGTGACTGCGGCCGTAGTG
G
>at02174
TTTTTTTTTTTTTTTTTCCCACCCGAGCCAACTCAATGAGTGTCCCACGCGCGTTGCCG
GAGTCCAGTCATAACATATTATCATCACCAAAGCAAGTGACCCTCACTCAAGTCGGCTTT
GCCCACTGGGTCTTGCCCAGTCTGGCATGAAACCAGTACCGGAGCCCAGGGTGGCTGCGG
CTCTCGACCAGAGTCCACCCGCTCACTTCAGGGGGTTCCTGCGGCTCCATGTCAGCAATT
ACCAAGCCTGCCTTCTCGAGCTCCTCCCAGCAGCTGCCGCAAAAGATGCCACCCCCTTCC
TCGTCAACGCAGAGCTCGTCCTCTGGCTTTCCGTGACACGCCTGTGCCGGCCAGCGCTGA
CACCCGGCCCGGCCCGGCAGCGGCGGCATCTTCTGGACCGGCTGCTGCTCGGAGCTCGCG
GTGGCGCGCGCAGCCGAGGCCTCGAACTCCTCCCAGCAGCGCCCGCAGTAGACGCGGCCC
ACGCAGCTGGGGCCGAAGCGGATGAGCTCAGCCCGGCGGCCAGGGGCGTCGTCGCGCCAC
AGGCCCAGGAAGGGACCGCCGAGGCAGACGCCTGCGATGGCCCCCGGCAGCCCGCGCTCG
CAGCGGAAGCCCCTCGCGGCGCGCTCGCTGCCGGGGCGGGCTGGAGCGGGTGACGTGGGT
GGGCCATTGGTACCATCGCTCGGCTCAACCTTGACTCTGATGGTGATGCCCCTTGCTCTG
TCTTCCTTAACAGCGCCCCGGTGATTGCTTCCATTCACGATCTTGGGCTGTATGCCTGCA
TGCGTCTCAGGGTCACTCTTGATGTTTGCCGATGGCCCCGGAGTTGGTTGCCGCTCCG
>at02175
TTTTTTTTTTTTTTTTATCACACATCTATCTGCATGCGCTCCGCGAGCCTTCCTGCCAGG
CCGAGCGCCGCCAGCTGCGCTCAGCCCGCGCCGGGCGCGAACGTCTCGGAGAACCACTTC
TCGAAGATCCGGCGGTGGCCGAGCTTCTTGATGCCAGCGTCCTCGAAGAAGGCCTTCTGG
ATCTCGCCGTTCTGGCAGTAGAGATCAACGATCTGCTCCAGGGACTCAAAGTTCTCGGCA
ATCTGGTCGTGGTACTGGCAGAGGAACCGCGACTGGTCCAAGGACTCCAGCCAGACCCTG
AAAGGCATCCTCTTCGCCTCCTCGGCCTTTGCGCTCTTTGAGCCCTCCGGCTTCTCTGCC
CGGCCCTTGCCAGAGGCAGGCGCTCTGGGCTCCTCCTCTGCAGTGGCGCCCTTGCCGAGA
TTCCGCGCTACGGCAGGTCCAGTGGCTACAGGGTTGGCGTAGAGCTCCTCCAGCCTAGGT
GCCCCGGCCCGGTAGGTTGGTCGGTCTGCAACCTTCCGGCCCGAGAGCTCCAGGCAGCGG
GCGAAGGGCATGGCGCGCTCTGCGCTGACACCTGCCAGCGGCCGCTCCTTCGCCACGACG
TCCGCCACCCAGTCAGGGTACGCGCGCGGGGCGTACGGGGGCGGCTTCTCGTCGTGCGGG
CGCTTGGCGTCGACCCAGGGCTTGCACTCGATGCCGTAGCGCAAGTAGTTCCGCTTGACG
TACTGCGCAAAAGCGGCCCAGTCAGCTACTCGAATCTGGTCCTCCTCCGAGCGCTTCCGC
ATGGTGTCCGTCTGCCAGGCCAACCGCTTCAGCTCCTCCGGGCATTCATCGCAGACAGCT
TGCCGGTAAGGCATCATCGAGCGGTGCTTGTTGGGGTACTCCTTTGAGCACCCGATGTCG
ATGTACCAGTTGTCGTCGTCGTTGATCAGTATCCGGTAAGAGAGCGGAGTAGGGGGCCGG
AGCAGCGAGAACGCGTCCCGGTTATTCAGGAGCACACGATCGCCGGCGCTCAGCTCCTTG

TCTCCACAATACACAGGCTGCACTCCAGATAGAGCTTCCAACCACCAACTTCTGCCAAGC
TTCATCACCTGGCAAAGTCGGCGTGGAACGCGCTTGTCCAGCTGCCGGATGTGACAGTCG
TTGCCGGAGCCAATGATGAGGACGGGGGGCGCCTTGGTCCTGGTGTGCTCGTGGCCCTCC
TCGAAGTCGAACTCAGGCACAGCCTGCCCCTCTGGCGGAGGGTGGATCCACGAGCCCTCG
TACCAGGGGAAGCCGGGATGCTCAACCCAGAGGCCATCCTCGTTGTCGGTCGGCTTTTCG
AGACCGGCAGGCAGCAGCCTCATCTCCAGCAGCCCCTATGTCCACCAGGCGCCGCAGGCC
GGTCCGCGAGGCGGCACTGCCCACGGCGCTGGAGCAGCATCAGTCACATCCCTGGTGGGC
TCGGGTGCTGTGCAAGCGGGGCAAGCCTGCAGACAGCTTGAGCCAAAATGGCTACGG
>at02176
AGCCGCTCTCTCCAAGAGCTCCTCCAAAGAGAAGCACTGCCCCTTCTCCCCGGCCTTCAC
CAGCAGCATGATGGTGAAGTAGCAGATTCCAAGTATCAGGAGTGTCCAGCAGAGGCCGTT
CAGGATGCCTGCCCCAGCCAAGGTCCACGGCAGAGACAGAATGCCCGCCCCGAGCCCGCA
TAACACCAAGTTCACCACAAGCGTGGTGTCAAGAGGTGCTGCGGGCTGCATGGGGTCTGT
CCCTGGGTCAGTGCTGCTGGCGCTCTCATCGCAGGGCATCTGCTGTGGGAGTGTTGGTCG
CGGTGCAGGAGACGGTCGTTTGGGCCTCTCAAC
>at02177
CCGTAGCCATTTTGGCTCAAGGAGGCGCCACAGCACTCAAGGGGCAAAGGGACTAGGATC
GTTGAAGAAATCAGTTGAGCCGTCAAGATGGGTCACTTCCGCAACCACGTGGTGCCCAAT
GTCCACTTCCGCAAGGTCAATGGCATGATGCATGGCCGCAACAACCGGGTCATGATGAGG
ACGTGGCTCGACCAGGCGGGCCGTAAGAAGCGGCGCCTGAACACGAGGAGGGCGAAGGCC
GCGAAGATCGCGCCCCGGCCAACCGGGGGCCTGCTGCGGCCGCTCGTACATCCGCCCACG
CAGCGCTACAACATGAAGCTCAGGCTGGGCAAGGGCTTCACCCTGGAGGAGCTCAAGGAG
GCCAAGATCCCCAGGAAGCTCGCCTTCACCATCGGCATCGCGGTGGACCACCGTCGGAAG
AACAGGTGCATGGAGAGCCTCCAGCTCAACGTCGAGCGCCTCAAGCTGTACAAGTCCAAG
CTCCTCATCTTCCCGAACAAGCACGGGAAGAAGGGTGTCAAGAAAGGAGACACACCGAGG
TCCGAGCTCCAGAATGTCGCGCAGAACACTCTGAAGGAGATCATCCCGATCCCCAAGCCG
GAGGACATCATCGAGGCCCGCGCAATCACTGCCGAGGAGAAGGAGGCGTCCGCATACAAG
ACGCTGAGGAAGGCAAGGCAGGACAAGAAGTTCTACGGCGCGAGGCTGAAGAAGGAGAAG
GCGAGTGCCGAGGAGTCCTGAGCGAAGCCCTACAGGGGCGTTGGCCGGCCGTAACCCGCC
CGTGGAAGTGGACTCGTGCTCCTAGGAGGTGTTTGGTCTTTTGCCTG
>at02178
TCACGGTCAAGGCCAAGAAGGCCGCTCATCTGCCAATACGGATCGAAGCGCCGGTGAAGC
GAAGCGTCAATGGTGCCAAGCTGGAAACCACTGACCAAGGGATTCGCCGCGTGCCACTTC
ATCACCGTCGTGTACATCTCCGCAGATCGGGGCAAGTTCTGCTTCCGCGCCCTCAGGAAC
CGCAGCAAGCTCATGGCATCCTTAGGCCCGCTGACGCCTTGCACAAGCTCCGCAAGCTCC
TTGCAGCGCTCGCGCTCGCCATCTGCACGCGCAGACCGGCCCAGTCGTCCAGCAGCGCCG
>at02179
TTTTTTTTTTTTTTTATCATGATGATTCTGCTCTTCATGGCCAGAGCGCGAAGCTAGGC
CGAAAACCGCTGTACACATGAAGTTCTGTAAACTTAAAAGCAAGCCAGTGAGCATGCGAT
GCATTGCAGCGCAGTCGGCTATGAGGAACGCGGTCACACCTAAGCACTATCAACCTTTGC
CATGTGCTTGATGAGGTCCACAACGCGGCATGAGTAGCCCCACCTTGGCACGCGCAGGTC
ATTGCTAAATAGGAAGTGGCAGTTCACCCTGAGAAAGGAAGGGGGTTGAGGTCCCGCCCC
CCACTCATTGTCGTACCACGCCACGAGCTTCACGAAAGTCGGATCAATCATGATGCCTGC
CTTGGAGTCGAAGGTGCTGGAGATGGTGCAGGTCTCAAAGTCAGTGGAGACAAGGGACTC
GTCGGTGTAGCCCAAGAAACCCTTCATGTCTCCCTCTGAGCGCTTCTTGATCTCTGCGCA
GATCTCCTCATATGTCGTCGGAGTGTCCAACTCGCATGTCAGATCGACCACAGACACATC
GATAGTCGGCACACGGAAAGCCATGCCGGTGAGCTTGCCCTTGACTGCTGGAATCACCTT
GGCCACGGCCTTGGCAGCGCCCGTTGAAGAAGGAATGATGTTGCCCGAGGCCGCGCGGCA
CCTCGCCAGTCCTTCTTGGATGAGCATCCACTGTCGGCTGCGATGCAGTCATTGCATGGA
TGGTCGTCATCAGGCCCCTCTTGATCCCAACTTGTC
>at02180
AAGGAGACCAGCTTCGCCTGGATGAAGGCGCGGTGGCCGAAGTCCTGCGCCCCGCAGGAG
CCGGCCTCGTGGCCGAAGGACCGCGCTGTGGCGGCGCCGCGGAAGCCCAGGGAGTTGGAG
AAGCGCTGGAGCAGGGAGGGCCACTGGAGGAGCGAGACCTCGGTATTGGCCTTGACGCAT
GCAACGAAGTTGTCGTTGGGGCACGGCGTTGACGGGTCCTGGCAGTACTTCACCGTGCAC

ATGCGCGTGTCGGCTTCAGAGGCAGGCTCACCCTCAAAGCCGGGGCACTGGCAGTTGGGG
TACTGGCCCGTCTTCTTGCACATCTCCTCACACGCGATCCCAAAGGTCTCCAGCTTCTCC
TGCAACAGCGCCCGGCGGCTTGCGTCCATGGCCAGGC
>at02181
GTTTTTTTTTCTTTTTTTTCATCAATGCACTCTGTACATCACAGTAAGTCTCTGCAAAAG
CTCTGGTCCGGAATGCCCCTCTGCGCGGCGACCTCGCAAGCCCGCTCCAGCCTAAAGCAG
TCATTGGCTTTGCATGGGGTGGAGCCTTGATGATTTGACGAAGCACATGAAACAAAAACC
CGCACCTCCTGCAAGGCAACCGTGCCTCTGAGGNNNNNNNNNNNNNNNNNNNNNNNNNCA
AGCACAAGCACAAGCACGAGCACAAGCAATGCATGCGCACGTGTACGAAGCCCAGTCAGG
ACATCTGAAAGGATGCTCAGCAGCATGGCCGCGGCCCTAAAGCAGCTTCTCAGCATCCGC
CTCGCTGGGCGTAGGCGAGGTCTCGGTCTTCGCCCCAACAATGCCATGGACTTCTGAGTG
CAGCGGCAGCTCCTCCACGCCCGGCGGGTCGTGGCGGTTGCAGGGGGTCCTGAGCGCCAG
GGGCCCCAGGCAGTGGGTCCTTGCCCCTCGACAGGTCAGAAACGAGTACAACTCAGCTTT
TGCAGTGTTCCAGGATAGCCCTCTTCGTAAGGGCCCAGCCTCGCTGTGCAAATCCGTATC
GGAAAGCATCAGTGCACCATACGAGATGCCGGCTCCTGTGAGCGTTGGAGCTCCGACCGT
GGGGCTGAGAGCCGGAAATGGACAGCAACAGAAGAACACGAGAATAGCATATGGCCGCCA
CATGTTGAACCAGATACTGGCGCTTCTCCAACAAC
>at02182
TTCGGCTACACTGCCTACACGGCCACTCGATACCCGGACAGTCGGATGGAGGTCTGCAAG
GGAGGCGCTGGTTGCTGCCAGGTTAGCCTGGCCGAAGAAGATACTTACGCCATTACTGTC
GCCCCATTGATTGATCCGATCTTGATGGTCAGCAGCATCATGGCCATGGAGTCCCTTAAG
GGGAAGACGGCCTGAAGGCTGCGTGGAAGCGCAGCCGTTTACGTAGTCATGAACGGCCCA
GTCCGCGCGTGAATGCAATGCTGGTGGACGCCTGGGTTCCTCCCAGTGCATCAGCGGAAC
AGTGCCGCAAGTGCGAGGGCGCAGCTTCG
>at02183
TTTTTTTTTTTTTTTTTGATGCGGTGTCCCTTTTGCCCTTAGATGCTCTGGCAGGATCT
GCTACGCCTCTGCTGCTGCACCGCTGCCAAGCATTCTGAATTGCACTAGGGCGGGATGCT
GCTGGCACCAGGAGTATGGGGTTGCACACAGGAGTGCCAAACCAAATGGTGGCATTGGAT
CGAAGGCCTGCACTTCGCTCCTGGTCACCATGAATGGTACAATCACACAGGGAAAATTGA
CGTGACACAACGAGCGTCAATGGTATTATTCCAATGGAAATGGACACCTATAGGTGTCAG
GTGAGCATACCGCTTGTCAAAAGACGTCTGCACTCTATACATTTGCTTCACAGAAGCTTC
AAGGACATGCAGATGGCTTCCATGGCTTAGAGCCCTGGGTACCCAAAGCCGCTCGCCCAG
GCCTCGACTTCACCTTTCAACGCCTTGATCTCAGGGTTCTCTGCAAGACCAGCCTCGAAG
TCCTTGAGCTTCTTGCCCTTCTCTTCCTGGACTCTGAGTGCTATCTGGCAGCAGCGGTCC
AAGAAACTGGCAATCTTGCCGAAGTCCTCTGTGGTGCATCCTCGGGTCGTCATTGCTGGC
GAGCCGATGCGGACACCACCCGGGGAGAGGGCTGAGGCATCACCATGCACTGCATTGCGG
>at02184
TTTTTTTTTTTTTTTTTCAACGGCAGGTTCCCCAGCTATAACCGTGTAACACCTTGCCC
ATGAGGTCGACTTCAAACCATACTTCTTCTTGAAGTGGCGGGTCCCTTCTCGGAATCAGC
AGCGGGGCCTCGTCTGCCATGTCCTCGCCATTTGTGGAGTTCATATCCACGGCGAGGGTG
TCCTTGCTGAACTCACGGAGGCCGATCAAGTGGCGAGAGCGACCGGCTGGATCCTTGAAC
TGAAAGGAGAATACCTCCACCTCACGGCTGGTACCCTCCGTTGAGCCCAT
>at02185
CCGTAGCCATTTTGGCTCAAGCAGCAGCAGCAGTGCTCAAGCATGTTCGGTTGGTGGCTT
TCGCAGTTGGTGTGTGTTGCGGCAGCGCCATGAACATTCGCCTCGAAGGCGAGGGCAAGT
TCTGGGAGATTGTGCAGCAGGGCCAGAAAACGATCTCGGCTTTTGGAAAGCTTGGCGGCA
CGGGCAAAACCGACAGCCAGATCCACTATGAGCTTGCGGCGGCAGACAGGTTCTGCGAGC
AGAAGGTGAAGGAGAAGCTGAGGAAAGGTTACAAAGAGTGCGGCAGAGCCGCGGTGTGTC
CCAACTTCTTGTTGCCGAAGCATTCCGCGGACTTTGCGGAAGGCACCATCACCAACGCGA
AGAACGACACCCCGAGAGGCATGGTGTACGAATGCGAAGCGCAGGTTGCTGCGCTGGCAC
GGAAGGATTGCATGGGCTACTTTGTGCACAAAGGCTATAGCCTTGGCGAAACGTATCATC
TCTTGGTGCCAGGGGATGAGACTTGGAAGGAAGGCTCGCACGGGTCCCTGGTTAAGTCAG
TGAAGGTGAAGAAGGCCGGTGCTGCCGTTGCGACAGGAAAGAAGATCGGTACGAAGGCTG
GCGCGAAGGCCGCCGTGGCAATGAACAAGTCGGGCGGAGATGTTGGTGCTAAGTTGAAGC
CGGCACGCGTGATGAAGATTATGAAGGAGGCCAAGCCGGCGATGAAGGTCATGAAGGAGA

AGCCGAAGAAGCGGTGAACCGCGCCGCTCCCGGTGTGTGCGGTTCGGCGACGATGCATCT
GGCAGTATCTGGCATCATGGG
>at02186
TTTTTTTTTTTTTTTTTTCCAACTGCCGTATCTTTTCATGAGGTCTTTGGGGTCGAGAAC
GACGCTATACAAACCAATGATCTTGCTATCGCTGAACATGCACGGCTGTGATGCTGCCGC
CACTGGACTCCTCACTGGACAGCATCCGAGCGCGGCCGCTTTCCGCCCCGGGCATCTGCC
GCCAGTACCTGTGTCCGCATTTCCTCCACCAACTCCATCGTGGCGATGGACTCTGCGTGC
GTCCAGTCCGCGCACTCTTTGAGCCCCTGCCGGAGCGCCTGGACGACCGCCGCTGCTTCA
TAGAGGAAGCCCTGCGAGCCAGGGTAGTTCCAAGGCAGGATGGCGCCCTCCGGCGTTGCC
GGCAGCGGGAAGTCGAAGGTCTGCTCAGTGGACTCCGTCCGGCTACCGTACGCCTNGTGN
NCGTCAAGCGCGTGGGCGTGTGCGCGGAGCGATGGNTGCGCNGGGTCCCCTTCGTGCCCG
TGTACACGGCCTCCTCCGGGGTGCTTGCTCGAAGCGTGACGAGGACCGAGGCCGTGAGCC
CTGGGTGCGCCGGGGGCGCAAAGCCGGTCAGTGTCGCGGCCGCGGACCAATCGACTCGAG
CGCCAGCAGGGCCCGTGGTCACGCCACCGGCGGAGACCTCAGAGGGACGGGCTCCCCGAG
ACCCAAGCAGCACATGCCCCAGGGGGTACATGGCGATGTCCATGCTGGCGCCGCCGCTCT
TGGGGCTGAACAGCCGCGCATGCGGGCCTTTGGGGTCGTCCACGGCGGGCCAGCCAAAGT
CCGCCGTGATGGCAATGACGTCACCGATGGCCCCGTCTTGAAGCAGCTCGCGCGCCTTCT
TCGCTGCCGGGAAACAGCGGGTCCACATCCCCTCCATGAGAAACACTCCCTTGGCTTCCG
CAGCTGCCACAACGCGGCGGCACCTGCAG
>at02187
GCGACAAGTGGGAAGTGGCTGTTGAGTTGCTCCAGAGCCCGTTGACAGATGCCGTGACCT
TCAATGCGGCAATGTCGGCATGCGCCAAGCGAGTATCAGTGGGCTGAAGCACTTAGACTG
CTTGGCATGATGGGCAAGGTCCGCGCACAGGCCTTGGAGCGGGATCTGCTCAGCTACAAC
ACGGCCAGCTCGGCGTGCGAATGGGCAGGTGCATGGTCCCACTGCCTCCATCTCTTCGGT
GCAATGTGGGCTTTGCGTCTACAGCCAGACCA
>at02188
CCGTAGCCATTTTGGCTCAAGAGCGGGGATCGGTATTGCTGCAAGGCCGTACCATTGATG
TGCTACCCAGGAGAAGTGGGAGGATGTGCCCCTTGAGATGTCCATCCGGCCAGCCCTGGT
CATCAGTCTCACGGGCAGAGGATGCACACTGCTTGCAACCTTCGGATGCCAGCGGAGCAG
CTTGTGATGGATGCGGCTTGGACTTGAGCGCAGTGGCATGACGCCAGAGCCTCAAGTGCA
GGTGACTGTGTGGCATGCTCAGGGCCACAGCCGGTCTGTCCTTGCCGTTCCCGCGACCGC
CACGATCTTACATTTGAAGGAGGCCTTGGTCGAGAAGGTGGGACGTGGAGTTGTGACCAA
GGTGCAGCTGGCAAAGAAGGTTGGGAACTCATTCAAGCATCACGAGGACACTGAGGAGCT
TGAAGGCAGGCGAGAATTTCATGCACTTGGACTACCGAGTGAAGGGTTGGCTGCAATACC
GTCTATGTCAACGTTTACGCCGAGTCCGATGATGGGGACCATGCCGGCCTACGACCCTGG
ATCTATCCTCGCGGCAGTGGCCTCCGTCCCCCATGCGCGAGGGCAGCTCACTCGACAGCG
CATTCATGAAGCGTCTGGCCGCGGAGAGTCTTGTCGAGGTCCTCTCCAGCTGCCAGGGCG
AGACGGGCTTGCGCCTCCGCGTCCGCGACGCGAGCAAGCAGCAGGGCTGGGTCAGCGCAG
TCGGCAGCGACGGCGCCGAGCTTTGGGCAAACCTGCCGCCCTGCCTCCGCGCAGCACACG
GGGGAGCTCCCTGCGTGCGGCCCTACTGGCCGACTGACCGTGGCCAGCTTGATGAGGTCG
ACCGAGAATGCTTCGGGACTGGCGAGCAGCTAGGGGCAGACTAGGTTCGCCTTGCGGGGA
AGATGCCTCAGCGCTGCTTCGTCAGCGTTGCAACGGCTGCTCAAGGAGGCGTGGTGGGGT
ACTGCGCCTGGGGGCTTGAGCGACCAAAGCGAGATGACAGACTTGGGACACGCCTCTATG
TGGCAAGTCTTGCAGTACACCCAAGCAGTCGCGGCAAAGGAATAGGGACGCAGCTGCTCA
AGCACGTGGAGGCCATGGGAGACCACAGGTTCAAGGAGGCTGAGTTCGTGGCACTCCACG
TCCGTGTGGATAACTCGAGTGCGAAGCGGCTGTACGAGAAGCTCGGCTTCAATCCCGTTG
CGGTCATCTGCAAATACAACGAAGGCGCGGACGGCGTGGAGATGGTCCGGGAGCTGAAGC
TCATGCGCCAGCGGGATGTCGACTCTTCCTTGGCAAAGGAGCTGCAGGCCATGGGTTTTG
CCAAGGCTGACGTCAAACGTGCGCTTCACTGGGCGGTGGGCAACCGCCAGTTGGCCCTGC
AGCTGCTGCAAAACACCTGCCCATCATCCGCGGCGCATCGGTGGCCATGCCCTGGATCGA
CGAAAAGCCTTGAGGGTTGCATTCGATTCGGCAGTTAGATTGGCATTTGCGCTTAG
>at02189
CCGTAGCCATTTTGGCTCAAGGTACTCCATGTGAGTATTTGAAGGCCGAAGGACTGGCGA
TCACATTCTGTGGCCAGAAATGGGCTCGCCAGCCACATGGAGAGCAGGGTTGCTGTGCAG
ATCGCGTTCGACGCCCATTGTTTTGGACGACGCTCCCTGCACAGAGGAGGCCAGAACAGT

TGCGAATGGGACCTTGCGGAGCCAGACCTTGGCCGTCAGTGGCCATCAAGGCCTTGCGTG
GGAAAAGGTGGGAACCGGTTCTTCGCCTTCCAGCAGATGCGAGGAAGAGCTGAAGTGCAG
CACTACCCTCCAGGTCTCCTGCACACTGGGAGTTTCTGATGAAGGTGAGGTTCCTGCGCT
TCCATCCGGGAAGAAATCAAAAGAGAGCGCGAAAGTCCAAGCTTGCCCACGCAAGAAGCC
CCGGTTGCGTGGTGATGGGCCTGCTGGCAATCCCGAAGCTGTTGATGCTAGCACCATGCG
CTCTTGCCTTCCTGAAGTCAGTGATCACCTGAGGGCGCCCAGTTTGCGAGCTGTCCGCAG
ATTCCGTAACTTCCAAGAGGCCCATGAGCAGTACGGTTATCCATCAAGCCACAGAATCGG
CACGATCCGTGATCCTGGCACGGGTTGCGTGATTCGTAGTTTCATCAATCCGCTTCATTG
CAAGGATGTCGTCGAGGACGAAGGAGAGAGAATACTGTACAGGCTGAAAAATGCCACAAT
CAGGGGCTGGTTCCTCAAAAGCAAGGAGAGTTCTGCATCTTTGCGTGTCTTCTGGAGAAG
AGAGGAGGGAGTCCATGACCTGGGCTTGTACTCCGTCATCGACTTCGTGACCAGATCGGA
AACTGATTATGTGCAACTTAGCAGAAGCCAGACTGTGAGATGAGTGCAATTTTCCAACTG
TGGAACAGGCATGTCTTTCCATACGCCCGACAAGGTTTGAGCACGTCAAGCCTGTCCAGA
GT
>at02190
TTTTTTTTTTTTTGATCGTCCACACTTGGCCTTGCATCACAGGCACCCGCGGCTTCAGC
GCGAGCGCAATTTCCTACCAGAGGCATCGATCCGAGCCACTCAGTGGAAGAGGCCTCGAA
CCGGGGAAGAGGCCTCACCGCAGCGACAACTCGTCCGAGAGCTCACATTCGCCCTGGCTT
CCGCCGTCGTCCGTCTCCACGCAGGGGGGCGGCGGCGCCCGGCGCGGCTTGCGGACCTTG
AACTTGCCTTCCTCTCCGCTCCAGTGACGGTGTCGGCAAACACCTGCATCATGACGTGGT
CCTTTCCGATCTTCTTCTTCTTCTGTGGCAAGTGCTTGGAAGCAGCTCTTGCAGGTTGGG
CCTTGCGCTCGGCGCCCTGGTTGGCAGCAGTGCCGACCTGCAGCAGGGCCGCGCTCAGGG
CACAGAGGCCCAGGAGCCGAAGGCCCAGCGCCATGCTTGGCCA
>at02191
GCCTCAGATTCCCTGTGTTTCCCCCGCACTCAAAGGTGCCCGGAGCTGCGGGGCCGAATC
CCATGCATCTTACCAGCAGCCCAATTTCGCAGGTCTTACGCAGAGTCTTGCTGCGGATTG
ATCACGAAGTTTCGCACGATGTAGGCGTGGGCTGCATGGATGTCGCCGATGGTTCCTGAA
ATCATCAGAAGATTCTGCTCACTGTTAGCGGTCACACGCACAGGCCAATTGTGTCGGACA
GAATTGATGTTCTTCCCTCCCTTGCCAATGACCCTTGCCATCTGTTCATGCGTAAACTTG
CAGTAGATCTCAGTGTAGGAGTCAAGCTCACTTGATTGATACGGTCCGGACAGATTGTCC
CTCACATCCTTCTCAAAGTTTGCTAGTTTGGTGAGCAGCCATGCAGCCACTTCTTCCACC
TCTTCTTTGGCACCCTTCACAATAACTTGGGCTTCGGACATTGTTCGTATCTCCTTGACG
TGGATTCCCGCTGAGGTCTTCTCCGCAATCTTGTTGATCTCCTCGCCACCAGAACCTATA
AGTTCACCTTTTGCGGACCTTGGCACGAGAAAGATGATCGAACCCCTCTCGGGCCAGCGG
CTCATCAACTGCGATAGTGCTGCAAGGACGTTGCCCTCCTCGCCTGAGATGGCGACGACC
TTCATGCTCTTGCCATTATGAGGGTACCAATCCCCCTTCTTGGACATCATGATGCTTGCG
CCTGTCTCTTTTGCGAATGCTCGCGCACCCTTGAAGACCATGGCATCGTTGCCAAAGTCC
ATAAATGCAGGTTCAAGCAATACTTTCAGATACTTGGGGCCCTTGAAAAAACCGTCATCT
CCTGAAGCCGCGACCAGATCCGGGCGCGGAGTGCTGAGGCCCATGGCTTGGAAGCAGGCC
GAGGGACAAGAGCCAAGCAGATGAGATGCCGCACAGGCAGCTGCCATTGCAAACAGACAC
GCCTTCCTGGCCATGCGGTGGGTTGGAACGGACGCCCGCAAAGCCATGGCTGATGATGGG
CGAGCTCGCGCAGTGGCAAAAGCTGGAACG
>at02192
CACAGAAGTCACTCTCAGACCAGAAATGCCCAGGTAGTCCTGGGCCCTTGACAGCAGCAC
CTGAGCTAGTGTAAATCAGCCGCTTGACGGAACCGCTCAACTCAATTGCATCAAGTACAT
TCTTTGTGCCATCAATGATACCGTTGTACATCTTCTTACCGTCACCCCAGAAGTTCTCAT
AACCGCCATGTCCAAGCTCCGTGGCCACATGGAAGACAGCGCTGCAGCCCTTGAACACCT
CATTATAGACGCCTGGATTCTTCATGTCGGCCTCGACAATCTCCACCCTGCCCACACCGT
GGCCATTCATGGCAATGAGGTGGTCAGTCTTGTCCTTGCGGCCCTTCTCGCGCACGCACG
CGCGCACGACGTAGCCATGATCCACAAGGTTCTTCACGACATATGAGCCAATGTAGCCCG
ATGCGCCTGTGACAGCCACGGGACCGTCAGCGCTGG
>at02193
TTCCATGACTCGCCGTTTGCTCCACCACTGGTCCACCGCCCAGTGTTGGCCCCCTCTCCC
AGTGCCATGGCAAGAGACGGCGTACTCGTATGACCCACAGAATGTCCATGATGTGGCGCC
TGCTCCACCACTGGCCCACCTCCCAGTCTTCGCCCTCTCTCCGCTGCCTGGGCA

>at02194
TTGTTTTTTTTTCTTTTTTTTTGCATTTGCCAGATTCTTGGGCGCGAGGTCCGCTTCGTG
CGCAAATTCGCTGTGGTTCCGGCGGCACGCTCACGGCGACTCGTGCCCCCGGCTCCATGC
CGCGCCGTTCGGCACCTCCAGTGCATGGAGGGCCAGGCCCGGCGGCAATGCCCTCGAGAA
CCTGGCGGTGCTCGACTTTCTGCTCTCGCCTCCAGCACCTCATCAGCACGGCCTCGCTGA
CAAAGTCGCCGACGAGGCGCTCAGCAAAGGGCTGGTCCTTCGAAGAGGTGCTCCGCTCGA
GCCGGGCAAGAAGCTGCGCCCACACGGAGCCCGGCTGCGGTGCCTCCACGGAGAGCGTGG
ACCCGAGCCCCGCGATGGCCGCCAGCTCGGACCATCGCAGCTCGCGGCCCAGGTCCTTTT
TGAACGTTGACACGACCCCGAAGAGCCGCTCGTTCGCCTTGAACTGGTCCCAATGGCCAG
TGCCCTTGTGGCTGCCACTGAGCTCCTCAAGTGTCCCGTGGAACCGAGGTGGCACTGCAT
CCTCATCCTCCCAGCGCTTGAGATCTGACTGCCGGCCCCCTGCTCTCGATGCACTGATCT
CGGTATCAGTACACCATGACCGGGAGCTTGAAGCAGCACTGGCCCCCGTGCCCCAGGCAG
CTGAATGCCCGTCATTGCTAGGCCTCACCTTCCCACCGGCTTCAGCCTCGTGCCGA
>at02195
TTTTTTTTTTTTTTTTTCCCCATCGTCAGCAAGCAGCACTGTACACAAGAGTCTGTCAA
GGGTGGCATGCTCAATGCACAGCAATACGCAAGGCAGTTCGAGCGCCGCCACTATCTACC
AAGGGCGTGGCGAAAGCGCCACCCCCCTGCAGCCCATGCGTCCGCCCGAGGAGCGCAACG
TGGCGGCGCACCGCTGCCGCTGTACACGGATGCGGGGAGGCCAGCAGCGACAGCCCACCA
CCGCTCACGACACCCTCGTGACAAGCTTTGCTGGCATGTAAGACGTTGCAGTCTGTATCG
GCTCGAAGCCAATTTCCTCCGGATCGATGCCGAGGTTGGGCAGCTTGCTCTCGTACACGC
GGATGGTGTCGTTGTACGCCTTCGTGCTCTGGTGCACCTGGTACTGCAGGTCCTTGATCT
GCCTGTTCTTGGCACCCAGGACCTGCTCCAGCTTCTTTGTGACGCTCGCCACGACCGTGG
GGTCCAGGTGTGTGGCCGTGAGCACCTCCGTCAGCTGCGCCTGCTTCTCCCTCGTGCCGA
ATTCTTGGC
>at02196
GCAGCAGCGACGACGGCGGCAGCGGTGGCGGCCGCCGCTTCCACTCACTGCGGAGCGGAT
GTTTCCGCAGAGCGAGCCGGCTCGCAGCGTGGGCGTCCTCACTGCGCTCATCGCGAGCCG
TGCCGGCTGGGGTGGAGTCAGGTACGGCATGATCGTTCAGCAAACGACGAACGTGCTTGC
AGCACTTTTAGGTGCAGAAACACACACAATCGCCACGTGACACTCAGCGGTGCACGAACC
CCCCCGATTTTTGTTCACTTTGATCGGCTCCGACGCAGTGTGTGGCCTGCCGGCATGTTG
CAAACTCTACGCATGTAGAGCTGTCTGT
>at02197
TTTTTTTTTTTTTTTTTGCGTGGCTTTGCATCAAGAATCGTGATGGATCAGAAGAAGGT
GCGCAGCTTTCAGGGCCGCGGTGGATGCAGGTGCGTCAACACCTACAGGTGTTGTTACTG
CGTGTAAATCAGCAGCATTGTCCGGGAAGTCAAATGCCGGGAATGTTGGTGAAGAGTAAA
CAGAACGCCAAACTGGCAACCAGATATGACAGTTGGAGTGCTCGCACGTGTCGCTGGCCG
CATGCTCCGCTGCTGTGGGTCACTGAGCGTCCACTGCCATTTGCAGCATCTGGCACCTAA
CGAGAGGCTCGATTTACCGCGCAGAGGCATGGCCAAGCAGAGTGTTATTTGCACCATCAG
CGCGCACTCCAACGACGCCTTCTTCACCGCCAAGCCTAGAATTAGACTCGCGCCCAGGCG
GCCCGGAAAGGTCCGCACGGCACACAGGACATCCGGTGGCTTGCCAGAGCCATGGACCGA
GGCAAGCGCTATGGAAGTAGTGGCCGCAAGTTGTCCGTCGAATCTCCTCTTCTGGCTGGA
ACTCTTCCAGGCAGATGTAACATTCGCGCGGTGCGCTCTCACCGTTCAAGATGGCGGCGT
CGTAAGGCACCAGCTGTTGAGCCTCTATACAGCCAAGCGCTGTTGGCGGTGGCATCCGCA
AGAGGCCTGCCCAGAAGAGTAGTCTGAGAGTCAGCGCTGGCAAAACGCACACAAGCCAAG
ACACGACGATTGTCATGCACGCAGTGACTGTAAGCTTACTACTGTAGAAGAGATCTGGCG
CCGTTGTGGGGCAATCTTTGGCCAAGGCCAC
>at02198
CCCANANGAGGTTTTTTTTTCTTTTTTTTAGCTTTGGACAGACTAATCAATCCCGGGTCA
TGAATTGAGTCCGACCCTCCCTCTACAGCTGCTGGCCATGACGTACCCGGTGGCCGCTCA
ATCCGCCGTATCGTCCTGGACTTCCTTGACAGCTGCTGGCCAAGGTGTGCCCCCCGTTGC
CGCGTAATCAGCCATATCATCCGGCGGCACCAGAAAGCCCACGGGCACCTCCTGGAAGGG
CGGGCTCTCCTTGACAAGTTCCTGCTTCCAGACGTGGTTTGCGAAGGTCGCCGTGGGCCG
CTGCAGCGTGCGGGTGAACTCGCCCTGCGCGTCGACCACGTCGGTGGTGGGACCCCTAGC
CACTGTGACCCAGTGGTTCTCGCCGAGCCTTTGCTGAAAACATCGCCACATGGGTGCGGA
TATCCCATCGATGCTGATGGTGGCCGGGTAGGTCAGGTGGTTCTGCATATGCTCGAACTT

GAACTCTGCTCGGTTAAGCTCGAAACCAAGGCCGTCGCCGCGCGCCTTCACGAAGGCCTC
TTTGGCTGACCAGAAGCACTGGAACGCCTGGTAGCCCTCGGGCTGGTTCAGGGGCCGGAC
GGTGTTCGAGTGGTCGGAGGACAGGCCGGTGCCCTCCAAGTCCTCCACCTCCAGTGCCCA
GTCCTGGCTTACCTTGGCGGGTGCATCCTTTGCCATGCGCTTGGCCACTGCAGTAGCGGC
CTCGCGCTCCACGACGTNGCACTCCTCCTCCGTCAGCTGTTCTTTAAACGTCTCGAAGAC
ATCGAACTCCTTGCCCCTCCGGTCGTGCGGGGGCGCTACA
>at02199
CTCAAGGGTAGGGACCCTGCATTTTATTCCGTTTACCTTACCTCCGGGTTGCCGCCGGCG
CTCGCCTCCAATGCCCGTCTTGGGTGTCCTCCTCGCCGGCGGCCTTCTGGCGGCTGGCCT
GCCGGCGGCGGGGGGCCTTGCGGGCGAAGCGCGACGGGGACGACTGCGAGTGCCTGAGCT
GGTCGGGCGCCTTCACGCGGAACGGCGCGCTGTGCGGCGACGGACACGAGTTCTCCTTCG
CGTACAGGTTGGGCGACTCCTTCGTGGCTGGCCCACACTTTGTACTACCATGAGCTCTGC
AGAGAGTTCCTCCAC
>at02200
CTTTGGACCAAGCTTCATTTTGTCTCGGTACCTCACCACTCGCCGATCCACGCCCTGCGC
TGCCGACTCTCGGCAGCTTTTTCGTCGCTGCAGGCAGCCATGCTGCGGTTTCCGNTCCTG
CTGCTCACGCTGAGGGCTGTGGCCAATGGGGAGGAGATGGGGGGACNCTGCCGCGCTGGT
GCAGCGGCGCCTGGCCGTGGAGG
>at02201
CTCAGCCATCGGCGCCTCAGTCTGAATTCATTCTCCACCAGGCATTTGTGCCCCGAAGGA
TGGTGAAGGCGTAAGCCAACAGTGAGGTCCTAAGCCCGCACCCCTTCACATGGCGATGTC
GAGCCTTGACGCCGGGATGCAAAATGGTGTGTTTTTCGATTCC
>at02202
CCCACAAGAGGGTGTTTTTTCTTTTTTTTTTTTGTCTGCCTCGCAACTCTCTGGCTGACT
GGCTACCTGGGTGCTGTGTCCTTCTGTTGGCAAGTGGGCGCTGATGCAATGTTCTGTACA
CTGTACAAGTGTGCACACTGCACAACGTCGACTCCACGGCAGAACAAACCTTTAAAATCG
CTGTCTCAAAGGCCTGTACGGCACGCTTGGCAAGGAAACCTGCTTCTGACGCTCATGTGT
GCGTAGGCGTGTAAGTCACTAGCCACAGGGGGTAGGCGCAATCCTGCCCCACAACCCTCA
TCGTCTGATTGTCTCGCTTCACCGCCGTTCGTGAGACTACGCAGATGAACAAGTGCCTGA
CACCAGATGGTTCTCGGTAGACAAAACCACTGTCACTGTAGGATGCCGCGTAGGCGCACC
AGATGCCATAATCAGTTCCGCCGCTGACACACTTGGAAATCTGGAAGCCTTGCCGCAATA
TGCTGGCCTCGTTCTCGCTTGAGCGGCATCCGTGAAACATGATGGTCAAGGATGGCTCAC
CCCTTCCTTTGAAACGGGAATACACCTCCTCATTCTGAACACGATCCAGGCTCAGGATGT
TTGGCAACCAACCCAAGGTGCCGCGAAAGTATCCGGTCACTGCGTGGAACTCGTCAACTG
CCGGCCTCCAC
>at02203
TTTTTTTTTTTTTTTTTGGGAGAAAACAGCGATCAAGTGCAAGCGCCTGTCGTTTGAAT
TGGAAAGGATGCCATCCAGTGGCTCCTAGGCCCTCCTTGCCACCAGGTCACGTTTCGAGG
CTTCTGCTGTAAACTGCCTCGGCCCACTCCTTCATCGAACATCCTGCAGGACCCCCAGCG
CCCGGCTTCAGCATTTCACGGCGCATCCTGGAGAAAGATTGGTGGTCAGCGTCAGAGACA
GGAACGAAGGCGCGACATGTAGCCGACGACGCCTCGAACACAGAGCGATGCTGGCTGGGC
GAGATCATCGTGTCGTCCAGTGGGCTCACCACTGCTATGGGTACGTCTGCAGGAAGCGAA
GCCACCGCCCCGGCCGTATCCCAGTCGTCCAAGTGGAAGGTCCACGTCCAGGGGAAGAGG
GCCACGTACGCAAGCATCCCAAGCGTGCCAAGAGAGCCCTGGATGACGTATCGAGTCTCT
TCAGAGACGCTCGCGAAGGGCGCGAGCAGCAGGACCCCCGCCACGCGGTCTGGCTCCCTG
GCCGCGAGCTGCGCGGCCGCCGCGGTGCCCAGGGACCAGCCCATCAGCAGGGCCCTCCGG
CCTCCGGCGGAGCGCAACGCGTGGTCCAGGAGGTCGGCCGCGTCCGCGGCCACAGAGGCG
CGGGAAGTCCAGCCGGCGTTCGGGGGGTAGCCGCGGTAGGACGTGCTGAAGCAGCCCACG
TCCAGGGCCTTCTCGTCCAGAAGCTTGGCAAGCGGAGGTATCANGTCGAAGTGGCACATG
TCCCACGAGTCGTACATGTCCATGGCGTTGCTCCAAAGGCCAAGGCCCTTANCGTCGAGA
GCTCA
>at02204
GCTTACTTGATCGACGGCTGCGTCGGAGTTGGCTTGGAGAAGAAGCTGCCCAGCATAACG
GCAGAAGAGCTTGCAGACGCCATCGTCCGTGCGGAGAGCATGGCTCCGGCAGCTGGGGAG
CTTGGCGAGCGCATGCGGATGGATGGGGGCCTTTCCCAAGCAGCTGATGTGATTGAGCGC

TTCTTGCGAGAAGAGGTGCGACCGGGAACGTGGAGATCGCAGTGGCTGAATGAGCGGAGG
GTGCTGGGTGAGGCAGCCTCCTCGCATCTTTCAAGCTAGTGGCTATTGGTGCGGAGAGTT
GTTGGATTGGCGTGGACTTGGGTCCGCGGGGCCCCGTGCATTTGCAGCGCCATTCTCAGA
CGTTGGTGGCTACCGTGACCT
>at02205
GCACGCTGCAGGAAGACAGGCCTGTACATGTCTGCAGACATGCGCCCTGCCAGATGGACG
GAAGCGCGACTGAATGGAAAACTACGGAAGACCCGAGGTCAGGTGCCCAGGGGACAGCAT
GAGCCTGACTAACATCAGACGTCCTTCTTGCCCTCACCTGACGGCCCAGCGGCGCCTGAC
GCAACCGCCTGGGCCACAGCAGCTGCTTCGGTCACGGCCACCCAGTCCGCTGTGGATGCT
GGCCCCGGCGGAGCACCCCCAGCTGCTGCGTCAGCGCTGCCGGCCGCAGCTTCGACGCTG
TCAGCAACAGATCGATCGTGCGGCACTGCCGGCTCGACTGAGGCCTCCACTGGCAGCTGG
GCCTCCGCCCACGCCGTGCCATCCTGGTCTGGCACAGGAGTATGGGCTGCTGAGGAATAA
GCATGCTCCTCCCAAGCCGCATGCCCACTCCAGCCTCTTCAGGGCCTCTTTCGATGCCTT
CCAGGGGTCGAAGAGGCCCAGCCACTGCTCCTTGCTGAAGACGCCTGCCCCGCCAGTGCG
AGCCCACTCTAAGCCCAGCAATTCGTCCAACTTGTAGAACTGCCACTTCTCATAACTGAT
CCAGCCGTCTCCCGCAGAAACCATTTGCCACATGACTGTGGCCTTCTCTGCTTGCTCAGT
GGCCGCCCCAATGAAGTGCTCATGCACTCCATGTGAGTAAGCGATTGCCTGGTCAAGCTT
CTTGAGAGTTGCATGGCCCTGCAGAGGTGTGACGTTCAGTGTCTCTATCAGACTAACCCA
GTGATGGATGCCANAAACACCTGCCCCA
>at02206
TTGTTTTTTTTTCTTTTTTTTTTGCTTTTGTGTCTTGGACTTGCACGACCCCGCAAGTGG
GTCTCTGATGTGTGGAAGCTGCACCGAGAATTGCAGTCCCCTCTCCTGAAACGGCCTCAG
CTGCTTGCAGCCAGGGCTCTTCAGACGTGGACACACTAAACAAGTTGACCTGGGTCGCCA
GTGAGCGGAGCACGCTGTGGGCCCATCAGCACCACACTGCCTTCAATGCAGGCTCATGTG
CCCCGCCTGGACTCCAGGTGAGCGCTCACAGCTCCACCGTCGTGTTCTCGTACGTGCCGG
CGGCCACCCCTTTTGCCCCACCGCTTGCGTTCTTGATGGTCACTTCTCCCTTGATCACGA
CGTCCTTGGCAAACGTCACCTCGCCCTCGATGACGAGCTTCTTGCAGTCGATGAGCGAGG
GGGGGCCCTCAGGGATCAGCTTCTCCATGGCGTCGGTGAACTTGTACATGCCGTCGAGCT
TGATGTTGGGGGGCACGCCGCCGCGGGACTCGGCCAGGACGATGCGGCAGTCCGGCGTCA
GGACGTAAGCGTCGGAGCGGAGGGCGAAGAGGTCGTTGGTGGTCTTCACGGGCGCGAAGC
GCGAGCGCGGGATGACGAGCGCGGTGGCCCCGGGGAAGCACTCGATGGCCGCGCCCATGG
CAGTCTCGAGCTGCACGACCTTGGTGGACGCCTTGTCGCGGGGGTCGACGGTCTTCGAGT
TCTTCATGACCGGCAGGGGCAGGGCGCCGTCGTTCTTCGCGAACTGGTCCTTGAGCGCCT
CGAGGTCAACCCACAGGTTGTTCGTGTTGAAGAACTTGTACTTCGTGGTGTCCTGGAAGG
CCTTCTCGTCCTCGTCGGGGCACTGCGCGGACTCCCTGAGCAGGAGGCCTCCCCCCTCCT
TCTTCTTGGCCAGGTGCCCGCCCTTCTTGTCGGCGTCCGTACGGTCAGCCACCTCCATCA
TGAAGGGCGCACCGGTCGCCACGAAGTGCGCCAGGATCTTCAGGTCCATGGTCGCTCCCA
GGTTGTCCGAGTTTGACACGAACATGTACTTGAAGCCCTTCTCCAGGAGCTTCTCAAGAG
TGCCGCTGCCCAGCATTGCAGGATAGAGGTCTCCGTGGCCTGGAGGGCACCACTCGTGTC
CAGGCTCCTTCTCCCAGGATGCCGGGGTGAGGTCCGAGGCCGTGACCTTGGGCGCCTTGT
TCTGGACAAACTCGAGGTCCGAGCCAGAGCCGAGGTCGGGGTATTTGGCCAGGGCCTCCA
GGGTGTCGGCGGAGGTCGAGAAGGAGTTCATGAGCATGAAGGACAGGTCGGTCTTGTAGC
TGTCCTTCATAGCAGCCACCTGCTTGGCAATGAGGTCCAGGAAGCTGTTGCCGACCGGTA
ACCGGAAGAAGGGACTTGGCCTTGTCCAGACCCATGCCGTGCCCAAACCACCGTTCAGCT
TCAGAATGACAGTCTTCTTGAGCAGAGACGGATCTGGCTCGACCGTCAACTCCTCCAACC
TGGGCAGAGCTTCGACACTGTCGAGCGTGCTATCAGGGATCATCACGTTGGCGCCCGAAA
CAAGCACCGAGTAGGTGTACTTGAAGGCGGCAATGGCTGCCTCATTGCAGCCTTCCTTCT
CCATCTTCGCCTGGTAGGCGGGCCAGTTCTCCTCCATGGACGGCATCTTTAAAGACGGCA
AGCGACGGCAGAGAATGCAAAGAAAATGATTGCCAACACTTGGGTCGCTTCTTGAGCCAA
AATGGCTACGG
>at02207
TTTTTTTTTTTTTTTTTGCCCACTCTTGACTGACTGCAACGCACATTCCTTTGTGCCTC
TGTACATCTCTATGCCTCCTCATCTCTCGGCCGGGTACCCAGGCTGGTGGCCAGCTCACA
CCGTCTGCCAGGCGGTCAGGCTGACCGAGCCACAGCCACGATGTCGTGGTTGTGCCTGTG
GATGTCGTAGCCGCGGGCGTGCACCGCGCGCACCAGCTGCAGCAGGTCGCCGAGCTTTGA

GGCGTTGTTGGCGTGCAGGTGCCACTCAAACATAATGCTGGCAGGAGCGAAGCCGTCCAT
GGCCAGGAACATGTGCATAAGCTCTGTGTCATAGCCCTCCGCATCCAGGACCAGCACGTC
CACATCACGTGGCTCCAGCTGCGCCTCGGCCAGCAAGCTCGCGGGCGTGTGGCACTGCAC
GTGCACGGCCTCAATGTACGGCAACCAGTCTTCCACTGACTCGGGGAAGTGCTTTGGGAA
AATATGTAAGGCCTTGAATAGCCAGACCTTCTGGTTGGAATTGAAGAACTGCGTCTCGTC
TATCAGGTGCTGCTTGTCCAAGCTAGCCCAATACCGGATGTAGAAGGCATAGTCATTGAA
GCGCCTGAAGAAGCTGTCGGATATCCTATAGAAGGTCACACTGTCACCGAGTGTGGGGCA
AATTGCCGTGGGCAGAATGGTTACCGAGCTACGGTCATGGATCCCATAGATCCGTTCACG
CAAGCGACTCTGCTGTCCCGGATGTGGTTCCACCAGGACGATGCGGCCGCCATCTTCCAT
CAGTGGTTTAATCCTCTGGTACATAATATAGTCATCTTCCATGAGGTCTGCGGGGCCAAT
GTGGACGATCTTCAGCCCCCGCTTCTCCGAGCGCTTCTCACGACATCTCTGTTCCCAGAG
CGAGTGGATCCTGCCAACCTCCTCCATGGACAGATCCGGTGAGAAGACAGCGTGCCGTTC
AATCTCTTCCTCTGACAGTAAGAAGCTCTGCTCCGCCACCTTGCACTCCTCGCGCGCTCT
GCATAGCAGCGTCTCGTGATTGCCCTTCAGCTTCTCTCTGATGATAAAGGCAAGGAAGGG
CCACATGAACTTGGCGGCTGCCCACATGGCGTAGGCGCCCAGGGCAAGGCCCGCTGCGCA
GAAGGACCCTCTCATGGCGACCGCTTGCGCGAGGCGGTGGCAGACGGCGCGCTCGGCTCG
GAACCAAATGGCTTGAGCCAAAATGGCTACGG
>at02208
TTGTTTTTTTTTCCTTTTTTTTTTGTCAGCCATCTGCATTGTGGTGTCTCATCAGCAGGA
TATCCAGCGGACTGCGGTGTCAGAGTTACCAGGTCCTACAACGCAAACGATTTGCCCAAC
GGCATACAAGCACTTGTGTCAAGAAACAGTACCAAGATGAGGATCCGGAGCGCGGTTCAG
CCTACAGAAGCAGCTGCCTCACCCTCCAGTGCCAACAACTCAAAAACTTCGTGCCTGCAC
AAACAGGGAGAGGTGACCTCAAGCCCCCTGCGGAGTTGCCTCCAACTGGCTTTGCAAGAT
GCAACTGGCTGGCGCCCCTCAACGCCAACCGACTGCCATGATGCTGAACTGCTTCAGCAT
TTGCTGATGACTCCTTATTGATTTCCTGCTATTGCAGCTGCCAGCGCTGTACAGCACCCA
TGCAGGGGCGGCCAGGGCACTCACTCCGCAAAGCTGTTGCAGGCGGCGTACTCGAGCCCG
GCATCCACCTCCTCCTTGAGGGTGCCGATCATCTCGTCCAGCCTCGCCTGCTCATGCTCA
CTGAGCTTGCCCAGAGGGTGGACTTTCTGAACGCCGTCCTTGCCAAAGGTCACTTTGGAA
GTGAAGTACGGGACCTTGGGGTGCACCGAGGACTTCACGAAGGCGCACTCAGTGGTTTCC
ACGCCGGCGAGCCCCAGGAGCACCGCCTTGCCCAGGCGCGCACCCGCGTATGCCATGGAC
AGGG
>at02209
GCGGTCGCACAGCTTCGCGTCCACCTGCTCCATGTGCTCCCGGCTGGGGGCGGCAACTCC
CCAAGAAGCACACTGGGCAGCACTGGGAGGCCGATTGACCGTGCATACCAACAGGCCCCA
CGTGTTCCCGAAGGGGGGTGGACTTCCAGATGGGTCCGATCGCTGCGGGCCGTACTCCAC
CCCTACGAGAAAGACCGACTTCTCCATGTCGGCGGCAAAGTCTTCGAAGGCCCTTTCGAT
GCTGCCCGGGACCCTCGTGCC
>at02210
TTTTTTTTTTTTGCTTTTCATGTGTAACGTCTCCCAGTATCGGCGCATACATCCGCCCGT
CTGAGGTGCCACTGTGGGTCGACCGCTGAGGATTAGCTCAGCGCTGCGACTGAGAATGTG
AGCACCTCCGAAGTCCAGTGCTGAGGATTAGCTCAGCACTGCAACTCAAAAAGTCAGCAC
CTCTGAAGTCCACCGCTGAGGATTAGCTCAGCGCTGAACAACTGAGTGGCCGATGGAAGT
GCCCCTCAGAAGCCATAGCAGGAACGAGTGGCCGAAGGACCACGCACCTCGTACGCGCGG
GCATACACAAGTGAAAAACGGGTGGAGCGTGAGGGGACCGTGGCGCCAGCCCCAACTCAG
CTCCAGAAACGGAAGATAAACCACTCGGGGTGCCCCATGGCAATATGTACACGTAGGTGA
AAGCTCCATCGCTTTTGCTGAGTGTAGTGTGTCCCTAACGCCGTACTGAACTCACCGGCA
ATGCTTGGGGTTCCTGGTCATGGTCTGTACATTTAAATGGGACTCTGGGCTGCCGCGTCG
ATCTGCGCGCAGTGCATCCCGCCTTCCCTCTCTTCTCCACCGCAACTGTTGGGCGGATCA
GCAATGCTGCATTATGGCCCTGGGAGCAGTGATTGTAGCAGGCAAGCACTGAACGAATTC
GGACGGCCGGCCCTGGCACTTGCCCGAGCGGCCCAACTGTTGTTCGTCNCAAGCCTCCCA
GCTCACGAAGATCGGGTCTCACCACCGGCAGAGTTGGGGGCCAGCTGGGGGCCATGCACC
ATGCAGTCCTTGGGCCTGGTGGGCTCAGGTTCCATGCGCCTCGT
>at02211
TTTTTTTTTTTTTTTTTTGGTGATTACCACGCCTATGATCAATCATGTGCCGGCGGAACC
AGCGCTTGCTTGCTAGGAACACGTGCAACGTGTCGCACCCTCAAGCACCAAGGCCTTCAT

ACTACGACCGTAAACGAACCGAATGCATCACGCAAGCTTTGCTTGCCAAGCAACTTGCAA
CCAAGCCTCTGCTCGACTGTAGCCGAACTGCGGCTAAGGAGCAAACCAAGGAGAGGTTGC
TGTACAATGATGCACTTGGCACTAGCTGCCACAAAAATCATTCTTGAAAACAGCATGAGC
CTGATGACCTTGCTGAGGCCTCGTGCC
>at02212
TTTTTTTTTTTTTTTTTGGTGGCGCAACTAGCCCCTCGACTAAAGTTGCCATCATCACT
TGACCTGAGCTGTACAACTGTGGCTGCTTCGCCAAAACGTCCAATTAGCTGGGCCACGAC
GGTGTATCACTCATGGGGAATTCGAAGATGTTGACATAAGACTATTCTTTGATGGGCGCC
CCTCATTGCAGAGCTCGCATGCTGCTGCGTAGTCGCCAAGACCAAGGCCGCCACATATTA
CAATATTACGATTTGTTATGCGGCGGTCGATCGTCTGCAACCGCTCTGCTCTATAGAGCC
CAGCANNTACTCCACAGTTGAGCGTGTGCGTGACGTCATCCTCGTCCTTTCAGTGCACTC
CGACTCGGCAAGGG
>at02213
TTTTTTTTTTTTTTGCGTCGGGGCTCCGCTTACCGTCTCTCCGCCAATCAAGCCCTGCCT
CAAATGCCACAGACCTCGGATGCCTCAGACATTCGGTGCTCCACCAACCTTGAGGCGGGG
GATCGCCAGTTGCCTCTTGGGGAACCTCTTTGGCTTGGCCTCCTCCTTGGGCTTCTCCAC
CGGCTCCTCCTTCTCAAACAAGATCATCTGCACGTGGCACGGAGAGGACATGTATGCGTT
GATGCGGCCATGGGCACGGTATGTGCGGCGCCGTCCTTGCTGCGCGCAATTGACATGGAT
GTGCTCGATGAAAAGGTTGTCCGTGTCGAGGTTCTTGAACTCGGCATTGCTTTCAGCGTT
CCTCAACAGGTCGATGAGGATCTTGGCTGACTTGATGGGCCAACGGCCCTGTGTGGTCTT
GAAGGCCTTGGCCTGAGCCGTGCGGCCGATGCCGCCGGTGTACTTCCTGAAGGGCACGCA
CCGCCTCTTTTCGCACACGTCCTCAAGGTACTGCTGTGCATCCCTCAGCTTCATGCCCTT
GATTGCGTTGGCGGTCTCGTAGGTGTTCTTGTAATGCACACGAAGGTCAACACCCATTGC
CTTGCAGGACTTGCTGGGATTCTTGGGCTCCTTGC
>at02214
TTTTTTTTTTTTTTTTTACGCAAGACGCAAGACGATCCCCGTCCGCCACTCCAGGCATG
GGTCTGCACCGCATCTGTGTGCATCTACGGTGCTACATCTCTACTGTATCCCACGTGCCC
ATTCATGCCAGAGGCCCTCTAAGCCTTCATCCTCGATCACTGCTTCTGCAATCAATGCTA
CTTGAATGTTGTCTATCAAGTTCTTCATGTAGTGCGTCGCGGCCTCCGGGAGCTTGTCCT
TCCTCCGAGCCGCATTCTCTTCCTCCGTGAATTCGCTTTTGACGTCCTCGACTCTGTGCA
AAATCATCGTGGACGCAGTATGCAACTGGTTCGTCAGGACCTGAGGAATGGGAAGTTCGA
TAATCTCGCACACAGGCGGGATGCCCTGCTTCGCTGTTTCCTCCATCCCGAACTTGGCGC
CGCAGGAGCAACTAGCGACCTCCCGCCCCTTCTGGTGCGGCTCCGCATCGTCATTCTGCA
CGACCCACTTCTCCATCAACGGCGAGCTCTGCAAGGACAGCTCGCCCCTGCGCTGCGCCA
CGTTGATGTTCATCGTGCCGCCTCGTGCC
>at02215
ATTGGAGCGTTCTTTGTGCACAGCCTCATTGGCCTGCCCAGACAAGCCGCTCCCCCTcGG
AGACAACGCAcGAGAACTCCGACGGCGATGCCAATGGCCAGAGCGCCGCCGATAATTATC
GCGATTTTGCTGCCACCTCGTCCATNCTCGGCCGGCATGCTGGGCAGGCCTCACGCTGCG
CTGCCGGAGCAAATTGCGCAAGGATGCGGCACTGGTGCCTGGGCTCGATTGGACGGTTTT
GGCCAAAATGGC
>at02216
TTTTTTTTTTTTTTTCTTAGGACTAATTCCTTGCGGCAGTCTCACTGTAATTCACCCGAG
CCCAAATCTCACCACTCGCACGGTCATCGCCATCGCTGTGGTGGCACTGCGGGTGGGCCA
TGCATCCCGGCCATCGCTGCACCTCCGTGCATCTGGCTGAAGCTCTGGCCAAAGCTGTGA
CTGATGCCCAGTGGTGCGGAGCCACCAGCAAACAGATTTGGCGGCGTTAGAGCTGGCACT
TTCCCAGATGCTGGTGCCAGTGGCATGCCAGGCTGGCTCTGTGGCCACAATCCACCCGGC
GCCTGCGGCATTCCTGCCGGCCGTGGTTGAGAGCCACCAGCAAAGAACCCGAAGAGGCCA
AACTCAGAAGGGTTGGTCGCCCCTGTATGCTCGAATCCAGTGCCTGCGAGCTTTGCATCC
TGAGGAGGCTCGCGTGCTAACTCTTGGCCACTCCCTTGCTGGGGTCTTGGAGGGGGGTAG
CCTATGCCAGCTTGCCCTGTAGGCTGAGACGTCCCTCGTGCC
>at02217
CGTAGCCATTTTGGCTCAAGACCTCAGTGGCTCCTCGGGAAGCGGCCGGGGCGAGGCCGG
GCAGCAGCCATGCACCTCTTCGTCCGCGACCTCGCGGGGCGGACCTTCCCTGTCGCAGTC
GACGGGGGCGCGGATGTCGCGTGCCTTCAGCAGCAGATTGCTGCGGTGGCCGGCGTGCCA

GAGGATCAGCAGCGCTTGATCTACGGTGGACAGTCACTCGCACCCAGTGCGCTTCTTGGT
GCGTGCGGACTTGAAGATGAATCAACGGTCTTCCTCTCGCTCGAGCTCAGCGGCGGTGCC
AAGAAGCGCAAGAAGAAGACCTACACCAAGCCCAAGAAGCCAAAGCACAAGCGCAAGAAG
GTCAAGCTCGCAGTCCTCAAGTTCTACAAAGTTGACTCCAATGACAAGGTCTCCCGTCTT
CGGCGCGAGTGCCCCCACGAGCTCTGCGGCCCGGGCGTCTTCATGGCCATGCACCACAAC
AGGTACTACTGCGGCAAGTGCCACCTCACCTACCTCATCAAGAAGGGCGACGACAAGTGA
GCGCGAGGGCGGCCCCAGTCGGAGGCAGACCGACCGCTCCGGCCCAGGCGCAGAGGTGC
CGCAAAAAAAAAAGAAAAAAAAAAACAA
>at02218
CGGGGTCGCGGCTCGCTGAGGAGGGCAGGCTGGTGCCCATCGCCTGCCTTGCCAGTGCGC
TCTCCTGTCGCTTGCACTCGCCAGGGTCTTGATGTCGCCTCTGCCAGTAAAGGGCGAGCC
TGGGATCCCGCAGATCCGTCAGGAACGCCACTCCAGTGTGCTTTTGCAGTGACTCTGGAA
GGTCTCCCACCTGCCACTGCGAGGATACNGATTCCTCCTTCCGCGCACTGCGGGAgGACG
TGTCATCCTCCTCTCGCATTGACTGCTCTGGCCTTCTCCGCCTGGACTTTCTGTGCATTG
GTGGTTTCTGTCCACTCCCTTCTTCCACCTTGGGCGCTTCACCAAATATTGTGTAGGGTG
TCCGGATGGAGCTCGCAGCGCGAAACGGCCAAACACAAAAACGATGATAGTGTGGAAGGA
ACCGCAAAGGACGAATCACCCAGCAACTCGGTGAAGAACGTATGGCACGTCAATCCCACT
ATGCTTCTACATCTGGAAACAATCC
>at02219
TTAGTTCCTTTTTTTATTTTTGGCTCAAGATTTCCTGTCTAGAAGGCCTCAGGCCAAGCA
AGTGAAGATGGCAGTGCTGTCGTTTTCCTTGTGAGCTCAGCAATGGTCACAGCCTATGCT
CATTCCGAAGACCGTGCACAGCAGCAGTGGAGGACCCCGAGCAGCAGGAACGCTCTCCGT
CTGGTACGGTGT
>at02220
CCTTCCACTTTCCGTGCGGACACCCACTGCAGCCTGGACAATTCCGCTTTGCCTTTCCGT
GCGGGCACCGCTGGCACACGGGGCAATTGCGCTTCGGCTTCCCGTGAGCGCATGTATTGC
ACTGGCCGCACTCATACTTCAGCCTTCCATGCTGACATCCGCTGCACACCTTGCAGTTGG
ACCTCCGACTCCCATGCAGACAACCATTGCACAGGCGGCAGAACCGTCTCACCTTTCCAT
GTTTGCATCCGCTGCACACCTTGCAGTCGTACTTCTCTCTTCCATGTGAGCACCCATGCT
GCAGCTTTTGGCAAGGCGATTTTCGGATCGCCCAAGCGCTGCCGGCAAAGATGCAGTCTC
CTGGGATGGGGAGGTTGTTCCTCTGGGGCGGAGACCCCTTGAGAGAAAAGCCAAT
>at02221
TTTTTTTTTTTTTTTTTGGGAGCATGCGCCCACATTTGGGCACCAGTGTCTTGACACTT
GTGTGATGTGCTGCCTTTCCCTCCGCTGCGCTTCAAGAACCGTCGCGCAAATGCTCGAGC
TCGGTCGGGGATTCACTCTTTAGCATCACATTGAAGAAGCCGGCTGGGAGGTCCCCCGAC
CTGCAGAGCCGCCAGTGGAGAAGGTAGGCACCCAACAGGACGGGCGTTGCGAACATCTCG
GTGTACGGCTGCTGGCCAAGTGCGATGAACAAGGAGCTGACCCAACTCTCTCGCCAAACG
ATGCTGGCGCGGACGGTCGGCATACGGCATGTCCTCGACGGATCGCACACGACTGCCTGC
AAGCCAAAGCACGAGTACTCGCTGCAATGCCGGTCGGCCAAGAGGCAAGGGGATCCCCTG
CCGGGGCTCCGGTCTCTGAGGTTCGCCAACACCCTCAGGTCTTCTTCACGCCAGTTGATC
ATCACGCCGACGTTGTCAATGCCGACCTCGCCCGTGACGGAGATATCGTGTGTTATCAGC
CGGGGGAAGTCGTCTTTGCCTCGTGC
>at02222
TTTTTTTTTTTTTTTTTTCCGCCGCCAGTCCGTGGGGTCTTCGATCGCGCCGGGGGGGCC
GAGCGCGGCAGCGCCTCACGCCGCGGCCCAGGCGAGGGCGTTCCGCACCAGCCGCTCGAA
GGCCGGGTTGGCCCAGGAGGCCTTGTAGCCGGGCCGGCCTGGGAAGCCCACGGTCGAGTG
GCCCACGCCGACGTAGAGCACGCCGCCCCGGCCCACGGAGCGCTCGAAGGCCAGGACGCG
GTTGTTGCGGTGGCCCACGGAGGGGTGCTGGGCGTTCCTCTCACCAGGGTCGCGCGTGAG
CATCATTTCGGACTGCCGCGGCGGCGCGTGGCTTCGGAAGAGCTCCTGCAGCTCGCCGAT
CTTGCGCGTGGGGTCCCAAGGGTAGCCCTTGGTGTACGCAAAGTCAGATCTCTCCATGTC
TACGCCCAGGATGTCGTACCCTCGTGCC
>at02223
TTTTTTTTTTTTTTTTTTGCGCAGGCATGTCATAGCTAACCACATGAATCAGAAATCGTG
GCCCACCACAAATCGTGATCTGGAACCACACACCACGGTGACGCACAGCATCCCAGCATT
TCAGCATGTAAGCTGAGCCTCTCTGTACAGACAACTAAGTGCATGGGCACTGAGTTGCAG

CCACGCCCAATTTCGCATGGCCACAACCACTCCCCACTAACAACAGATCAACGCAATGCC
GTGATTGGCACAAGAAGTGCTGGATCTCGGCCGCCAGGTTGGATCCACCAGTCCTCTGGT
CTGTCCAGAGTGCCCTCTTGGCGCCTGAGCATGGGGTAACATGGAAGTCCCGAAAGCACA
CACGTGGCTCTTGCATCCTCTCGAGCCCAGCCGTGGGCTTCCCAGACATTCGGATGACCT
TCCCACGCCTTATGCCCAACAGGCCAGACCTCGGCGGGGGAGATTCTGGGCGTCACCTCT
GCAGCAGATAGGTATGCGGACCGGCGCTGCGCTCTGAGGTTTGAAGCCTCCCCCGCAAAG
CAGATAGCACAGCGCCAGAAGGTCAGCCTTCCGTTGCCAGGGCCTGCTCCCGAGCCCACC
TGTGAGGCATGCCGAAGTTCGGGCGACCACAGCAGCCACGCGTGCCCGCTGCTCCCCCGC
CTATGGCCTGCAGCTCCAGCTGACTCTGCTCCAGAGCGCTGAAGCTGAGCGAATGCCGCA
ACAGCTGA
>at02224
TTTTTTTTTTTTTTTTTCCGGAGCTTCCCAATTTCTTCCGTCCTGGGAGCTACGAGTCC
TCCGCCAGTTCACGGGCTCGTCTCCCAGCCCGCTTCAGCAGTCCTGCCTCCATCTCCCGC
TGCTGCCGCTCTGCGTTCGCCTGCCGCAGCTGTGAACGCTTGGCGCCAAAACTCTGCCGC
GTCGAGCTCCTCCTGGCGCCGGTCCTGCCACGAGGAGTAGGCACTGAGGCCCACCGGCCA
AGGCCGCGAGGCCCACGAGGTACCTCGGCTAAGCCTTCTGCAGGGCCGTGAAGTCCGAGC
CGGCCGCCTGCTGCGCCGTGGCGGACCTGGCCCGGAACCCCTCTCCCCATGCAGGGTCGC
TGGAGGGCATCTGCCCCCAAGCGCTTGCGCCGTTCCCCTGCGCAGAGGCTCCGGGATGCC
CGCCTGGCGAGGAGGCGCCGGCGGTCTGAGAGCCTCGCAGCTCGCCCAGCAGCTTGCGGT
GGGCCTCCTCGAGCTCCTGAAAGCGTGCAACCGCAGCTTCCCGATCCTCCGGAGGCTGAA
CGTCAGGATGCAGTCTCTTCGCCTTCTCGCGGAAGGCGGCCTTCACATCCTCCTCTGTGC
ACTGCCAGCTCACACCAAGCACGTCCCTGGGGCACCTTCTTGAGGAGTGCGCTGCCGACG
TGGAGGACAC
>at02225
TTGTTGGCTGCAATGCAACATGCGTCTTGCGAAACAACCTTGCAAGGTGAAGCTTTGCTG
ATCTGGCTTCAGGCATCAGCTTGGCTGACGTGCAAAGGACCGTGGCAAAGGTGCAGAGGC
ATTGAGTAATTGACTATGCTGTCATTGCGTTTGTTGAGTGGAGCTCATAGGACCTGCAAG
TTGCCAACACGCTGACACTCTTCGCGTATCCCCGTGATGACCTTGCGACGACATGAGCGT
ACCACCCGCAGAACTGCCACTGTGCAGCAGGGCCGGAACGTGATGCTCGCAAGTGCCATG
CGTTTGGGCTTCATGATTCAGTGTTTAGCAGGGACGCGTAAA
>at02226
GAGTGCTGGACCTCGAGGACCATCGGACGGCGCCCGCCCTGGCTCTCTTGAGGGAGCTCA
TCGCCCGCTTCGAGGCTCAGAAGAGCGCTGACGTGTCCCTGGCGCGGGGCGGCAAGCAGT
GGGGGCAGCTGAAGGCACTGTCCGACAGCAGCAGCGATCCAACTTCGCTCAGCACAATGC
TGCTTGAGACCAAGGATCCACCCTTGGAGAGGTCTGCTGCCCCTGGCAACACCAGTTCGG
AGCCTTGCCGTCACGGCAAGATGAACTGCTCGAAGTGCCGAGGATGCCCCCACGGCAAGC
TGAAGCAGAACTGCCCCCTGTGCTCCGGATGCCCCCATGGCAAGCTGAGGTGGCTATG
>at02227
ATACCTGCCGAAATGTTGAAATCCGGGGCGGCCGAATTCCAGAACTGTTCCTTCAGCATA
CAAGGGTCCTCGAGCAGCAGCACACGCAAGCCGGCGTACAAGTCTGGCTCGCTGAAATTG
GCCCTCGCCAGTTCCGCCAGGCCCCGCCTGGCCGCTCGCTCTTTTCGGGCAGCCCCGCCC
TCGGCCCAAGCAGCATTGGGCAGGCGAAGCACGAGCTCTCGAAGGGCATTGCGCAGCTCG
CTGTTGGGCACGCCGGTGCTCGTCAAGAGCACGAGGCTGCNGCCATCGGCCTTGGCGTCC
AAACGGCAGTCCACGCTCTCGCGCGGCTGCGGGAATGGCGCGGCAGTGACCGTGCCGGGC
TCTGCAGCAAGTGTGGCTGaAGAGAGAAGAGCTACTGCNGCAGACCACGCAAGGAGCATG
AGTGCAGGGCGCCAAAGCTGACAGGCAGGAGGGGTG
>at02228
TTTTTTTTTTTTTTTTTCCTTGCACAGCACGGCTGAGAACTCAAGTGTGTCAAAAACAT
TGCAGGCGTCTTCACCGCATGTGTAACCAGCTTGTGCCAAGGCATCGGTCCTAGACTGAC
GCATCTCAACTGCATGGCGACATCGAGCCCAGACGGGGAGTGCCGACGGCCACCAAGCAG
ACATCGGTGTTCGCGCCGGCAATGTGCCTGTAGTCAAGGGAGATGAAGGGAAAACTGAAG
ATGAAGCATCTCGCCGTCCTTGAGTCTGATCAACCCCAGTGAGGCTCGCGTGCTCCAAAC
TAAAGGCCGATAGATGCAAGCACCTCGTCTGGTGGATGAGGCAATTCCTGCGTTGGCTTA
GGCAACGTCAGTGGGTCCAACACCCCATCGATGAGCTTGGTGCATGCAGCAAGGTCTCGC
TGGTCAATCGCCTCGAAAGCATTCACATTATTCATGTTCAGCACCGCACTGTGGAGGAAG

TTCCATATGTGTGTCGCTTCCGGGTTGAGGACGAGCGAAGAAAGGTAAAATCGTGCTGCA
TCTGCATGCTGACCAAGATTAGCATGCGCAATGGCGAGATTTGACCAGCTTCGGGCATAG
TTGGGCTTCAACTGTAGACCTTTGGTGGTACGCAACCACTGCCTGCTCGCTCTTGCCGGA
GTTNGGCAGCGTTGCGCCGAGCTTGTTCCAGACCGTGTGGTCGTTCGGTCGCANGCGGCA
GGCCATGGCGAGCGCCTGAATGGCAGCATCGNTATTGCGATTGATGTTCTCCATCACTCC
AAGTGCGATGACACATTTGGCGTCAGCGGGTTCGATC
>at02229
TTTTTTTTTTTTTTTTTGCAACGCTGCGCTCTGTACTCGCACGCACTCCACCAGTGCGC
GTCCTCTCCGACAATACTCCTCCATAGATCTAGACCGCCCGAAGCGCTCACTCGACATCC
GGGGGGATTCCCATCCTCTGCCGAAGCAGAGCCGCCCGCCAGTCCCAGTAGTTCGAGCGG
ATCTTGTCAATCTCTCTCAGCAGCGCGTACTGCCGCAAGGCCTCTTGCGTCTCCTTTCGC
GCTTCATGCACAAGCGCTAGGCCCTCACAGGCAAAGCGGCAGGGCTTCTGCGGGTCCGCT
GCGTGCGCCACAACCTCTCGGCAGAAGGCCTCCACAGACGGCGTGGAGTCCCAGGGGACC
TTGCCCTCGCCCTCACCAAGGAAAGCGGAGAGGTAGTTCCAAGCACTCTCGTTGTTTGCA
CAACTGCGTAGTGCCTTCAAGGTGAAGTCGATCTCCTCCTTCCTTCCTTGCTCGGTGAGG
GGCCACTTGGTGTGCCGCACGGTGGTGTACCTATGGTTCCACGCAGAGTTGTTCCTGATG
TCGTCCCTCAGCAAGTTCTCGACGAAGTCCATCTCCCCTCCCCACAGGTCGAACTTGTCC
ACGATGAACTGCCGGTGGGACCAGCCGTTGTAATTCTTGAAGTCATCGTTGACCTGCATG
GTGTCATTGTGGTAATCCAATTCTCGTTCACCAAGGGCGCGAATGTCCTCCGCCAGCTCC
TCGGCTTCTTGGCCTTCCAGCTGCGCCTGCCGCGCGCGGAGCTGCTCGGAGAGCTCTGAG
ATGAGCCAGCGCCTATGATACCAAACCTGGTAGTTCTTGGGGCAATCCCGGGACCACTTA
TCGGTGAAGATGAGCTCGCTGTGCAAGTCAGAGCCCAGCTCCTTCAAGCAGCGGCGCCGG
TAGTACCAGGCCGTGTAGTTCGCGGAGTTGTGCTCGATGACGTCAGCCGTGAGGTCCAGC
GCCCTCTGGCTGAACTCGTTCATCTGCTGGATGGCGCGGAAGTAGCTGTGGACCTCCTCG
AAGCCGGGCGGGTAGCGGATCACCGCCAGCGGCTTGGGCCCATCGTCTTGCGGCACTGGC
GTCACGTCGCTCCAGTCCGGCCTCTTTGAATACGGCAGGGCCTGCTCATCCCCGCTGAGC
TCCCCCGCCATGGCGGCCCGAGCGGCGGCCGGGGGGAGGCGTGGCAGCGGAGGCTTGAGC
CAAAATGGCTACGG
>at02230
GCGGCCGCAGCCACTAGCCTCTGCGAGGTCGCCAGGCTAGTGAAGAAGCCACGGACGAGG
TCGCGGTCAGAGGGGTTGGCAAAGGCCTTGGAAGGGAGAGCCTGCTCGAAAGCCTCCGTC
AACGGACTAATGTCCCCACTCTGCCGGAAGTGGGCGGACATCTCCCGGAAGGCAGCGATC
TCGTCCTTCACCCCCTTGTCAACCCAGGCTAAGTCACGGGGACTGGTGTTGATGGCGGTC
ATGATGCATTCGAAGTAGCACCACCCTCTGTCCAAGTATGGGATGGCAGACTCGGCTTCT
TTGGGAACCTCGTCGATGATGATCACCCGAGTCGTCAAGTAGATCCGACCCATCAGATCG
CCCGATAACGCAGACGCAAGGCGCTGCTTCTCTGCCGGCGTCCTGTCATCAACTCCAGAG
GTGGTCATCTGCGGAAGAGACATGTAGTCTACGAAAACGACGTCGTCCTGGTCAAGCATC
ATCTGTTTCTTGAGCTTCTTGAGCTTGGCCCGCAGCGTCTCCATTTGGATCCCTTGCGGA
TCCGGATGGAAGCGACCCAACCATGGGTGGGTCACGCCAAAAACGCGCACGGCCTTGCTC
AGTTGGCCGAAGGCCTTCGGAGGCAAGTCCTGGCATCGTGGCATTGGCTTGCCGTCTTCC
AGGAAAAGAAGGATGTANTGTGTGCCTTGACCCAGCGTACCCCGGGATACTCCGCTTCAT
CATTGGGCTCCTCCTCTTCCATCGCCCGAACAAGGAAAACTAAGGCTGCAGACACGGAGG
TGTTACTGAGAGCAAGTG
>at02231
CCGTAGCCATTTTGGCTCAAGCCTGGCGCGCCTCGGAGCCGGAGTTCCTCTGCCCGCGCC
ATGCATGAGCTGCCGCGCGGCCTTGCCGAGGCATGACGTGAATGGCAGCACTTGGCAGCG
ACACCGGGGAGCCAGCCTTGAAGCTCTTCCTGGCAGAGCTCCGATACACCGGGGAGGACC
GCAACGGGCAGCTCGTGATCACGCCTTCGCGGTTCCGCCTGAAGCGGGTCTGGGTACAGG
GATACGTGGTCTGCAGGGACGGAGACGACGTGGTAGACATAGATGACGGATCCGACGTGA
TGAGCTTCGATGTGGCGGACCTGCTGCAGCGGAATCCTGAGGTCAACGACACGTTGCAAG
CCGGGAGGTATGTGTCCTGCGTGTGCGAGTTGGAGATGCACCCGGGGGGAGTGCCTGACC
TTCACATGGAGAGCGTGTGTGCACTCGATGGCGCTGGCGATGCGCTGGCAGAGCCATTCT
GGTGGCTAGAGGTGGCAGAGGCTCACGCAATCTGGTCTTCATCCTTGCTTCAGCACCAAG
CTCCCTCAGGTACAGAGGGTGTTGCGGGCATCCAAAGTCTTGGCTAGTGCTGCAATATTC
CCTTCATGCAGTGTGACATTCCGGATCCATTTTTTTGGATCGCGCAGTATCCAGCGTGCA

TTGTGTCTTGAA
>at02232
CCGTAGCCATTTTGGCTCAAGCCATTTTGGCTCAAGCTGCTGTTCGAACGCGGGCAGCCA
ACGCTTTGAGCACTCGACGTCTGTACCTGCTTGGCAGTTTCTGCAGATCCCATGAGCCTG
GTTGGTGAAACCACCGGTGAGGATGCAGTTTTCTTTTCAGCCGCCCTCCTGCTGCTGACA
GTGCTCATCGGAGCCATTCAAATTGCACATTCTTGCAGGCCCTTTGCAATACTCAAGGCC
ATGGTGAGTTCAGTCATGAAAGCGATCAGCAACTGGACTGAGGCGGTCCCTTCTGAGGTG
GAGCTCATTGTGCATGAAAAGCTGCGGGAGCAGAAGCTTGATCAGTTCCACAGATTGTGC
CAGATCTTCTGCTTCATAGCGTCTCCCTTCACGGTCAACTTGATGATCAACATTTGGAAG
GGTGAGCGTCGCGGCCACACCATAACACAGGACGTTGTGCAGCTTGTGGAGCACTTCGTG
GCGGCATTGATCACAGTGTTTCCTGGCATTCTGTCGTGGAGGACCATGGACGTCTTCTAC
ACCATCGTGGTTCTGGGCTTAGCTGCGTACATCGCACCCTTCATCTCTCAGCCGGCGCTC
TTGTTCCAGTCGCTCCTTATTGTGAACTTTGTCGTCATCACCATGACAATGGTGAGGCGG
ACGACTACCCCCATGGTTCCCCTCCTCAACCTTGTGGTGATCACGGTCGCATGCAGTTCC
ATCTGGATGCATGGAGGGACCGTGTTCGCGCAGCCGCAGTTGATGTTGGTTGGCCAGTTT
CTGGTGGGCGTCGTCTCGACCATTGTTGCACACATCTTTGAGAAGGGTTCCCAGGCAGTG
ATGCGGCAATGCTTAGAGATGCAGACAACCAAGGAGCTGCTGTCCGCCGCTTGCGCATTG
CTGAGGAGTTGCTGTGACGTAGTCGTGGAGCTTGACCGGCAAGGCGTCATTATGTGCCCC
GCCGTTGACTTGGGAAGCTTCTTGTTGCGAGGGCCTGGCCAAAAGATGCAGGGCTGGCGG
CTTGAGGATCTGATGTCAGACGCAGAAGACAGGCGGGCATTCTCGAGCAGATTGGGCGCA
CCAAGGCTGGAGGGTATGGGTATGGCGGAGGCCATGCACGTCTCCATGAGGGATGGCAAT
GGCAACAGCTTGCACCTGGAGCTCCTCTGGTTCCAGTTCTCCTGCCTCTCCGGTGAGCCG
CGGTACATGGTTGGCATTCGCGAGTTCTGCGACCTGACCGTAAGTCGCCAGCAGCAGCAG
CAGCAAGACCTCATCGAGCCCACCGGCAACAACAAAGCGCCGCTCCCTGAGCACGAGGAA
GGCTTCTCCCTACCAGAAGCATCCTTGGTCAACACCGACTCCCTTGCGGAACCACAGGCC
GTTGCCGTCTTGGACTGCTCAAGAAAGGGATTCCTCATCAAGAGCGTGTCCTCTGGATTT
AGTTTGCGTGTTGGGCGACTGCCAAGCAATACTCGCCTCATCCCTATTGTGAAGCACGGG
GATCGCTTGCGTGCCTGGATGCAGGAAGCATTGTACGCTTGGTCAATACATGCTGCGCCT
CCAGTCGACTGCCAGGTCATCCTCCGCATGCCGCAAGGGGAAATGAAGGCCACCTGCAGT
ATCTTGAGAGATGATATTGCTGCCTCCGAGAGCCTAGATAATGTGGAGGAGGAGAAGGAG
GACCACCAGAACAGTACGGATTGTGCTGACATTGAGAATGTCCGGCTACTCTTCTTCAAT
ATTCGACCATCTCACAGCCAAGGTGCACGCCATTGCAATGCTCATGCTCACGGACCAAGT
CAATTCAAAGGAACTCCAATTGCTGTCATGAGCCTCTAGATCAGGCTCCCCAGCACGCTG
CTTGATGTGCTTGTGCACAGGAAGCAAGTAAACGGAGGGAAACAAAAAAAAGAAAAAAA
>at02233
TTTTTTTTTTTTTTTTTGCCGCGATCCAACGCACCAGCCCACAGAGCGCGCTCACAGCA
AGTCCACCGAGCTGCCCGCGTCAGTCACGGCAACGATAGCCAGCCGCCCTGCACAATCGC
CAGCGGCCACGAGTGGCAGAGCTGCTGCGCCAGTGCCTGCGGCATGGGCGCCCCAGTTCA
GGGTCTCAATGCTTCATC
>at02234
CCGTAGCCATTTTGGCTCAAGATAAAAGACCATCCACGCAAAACGATGGCCTGATCGCGA
TCCTCGGCAAGGTCTCGTGCAGGCCATCGAATTGCAATGGCGGACACTGAGGAGGTTGAG
AAGCCCGCCAAGAAAGCGAAGACAGAGGCTGAGGTCGAGGAGTTCGAAGATCCTGCCCTC
GACAACGACGACGTGGAGCCGACTGCCCACAAGGTCTCGGTCTCGGGCCTGGACTTCGGC
ACGAAGCGGAAGGCCCTGCGCAAGGTCTTCAAGAAGTTCGGGACCCTCGTCTCCGTGCGG
CGCTACGTGCGCGGCGCGCCTCGGAGGGCCGACCTCTCCTACTCGTCGGAGGCCGAGATG
AACGCGGCCATCGAAGGCATGAACGGTCAGGAAATCGAGGGGCGCACGGTGAAGGTGCGG
CTTACACCACCAGATGAAGAGGCTGCCCAGCCTGCCAAGAAAGGAAAGGGCAAGGGTAAA
GGCAAGGGCAAGGGCAAAGGCAAAGGCAAAGGCAAGAGCAAAGGGACCGGACGAGGCTTC
TGAGGCCTTCCTAACGCTGTGGAGTGTTAGGCCGCTGTGAGCTTCGGACGGCTTGGAGAG
AACGTGGAAGTGGCGATGAAAGAAAAAAAAAAGAAAAAAAAAACAAA
>at02235
TTTTTTTTTTTTTTTTTTCCTCCTGTGCCGAGTAGGGCTTTGCTGGCTGTTGCCATGCTC
TGGGCCAAAGCATGCCTTGTGCGATGCTCACTCCATGGTGGCAGATGGTGGCCGATAAAC
TCGAGCTGATCAACTCACTTGTTCTTGCTTTGAGCAGATGAGACCAAGCGGCATAACTTC

TCAAGCTTTGCCTCGTCGTCAAGCTCAATGAACTCCACACCTGTAGACAAAATGAGCACA
CCTACACCGGGCGTGCACATATCTCTGCAGCCATCGCTGAGCAAGCCCAGTGGTCCTGCA
TCAACCATTGCCTGCAACTTGCCGTCTACAATCTGGCCGTTCAAGTCTGCGAATGCTGTT
GTGACAACAATATCGCACACATCACTTGCTAGAGCGGCCCAGTGGTGCAGAAGCCCCTTG
CAGGCCTTGCTCATGCGATCCGTGCTCTCATCTTGCTGGTGGTGTATTTTTCTACCGTGC
TTTCCAGCGACTGTGCCCAAGACCAAACCAAGCCAGCCGCAATCTTGCCGAGCACGCTTT
ACAGCCTCAAAGTCTGGCTGGTCTTCAACTCCGAGCCACTTCTCCAAGCGCACGGCAATA
TCTTGTGCGAGGCGACCAAAGCCAAGAGTTTCCCACTNCGATGTCGGCAGGACTGCAGGG
CAAATCCTCAAGCCACTCTGCATCCGCAGTCAGACAGTGTTGAGNCNCNNNCCTGAACAC
CTGAGCTGCACCGAGTTCAGGAACACTCAGGCCAATCCAAGAATCAGGAAATGCTTTTCG
CACTGCGCAAAAGCATTCCGCGAGAGCGAGCAACTGTGTCCACCCAAGTTCATTGGAGGG
TGCACGTGGCTCATCCAAGTTTACATGCTGAGGCACAGCCTTGAGTGGCGCAATCTTTGC
ATCCTGTTGCAAAGACGGCAGTGTGCTTGTGGGACCCCCGGGGGTNAGTAGCACACCGCA
TGCACCAGCTGAAAGCGCGAGGCAAACATCACTTACAGCTTGTGCTGGTGACCATGCGCC
GACGATTACAACGGAGTACGGCGGGACTGTGTCGGCTTGCCG
>at02236
TTGGAGCACACCCACCGGGAGTTGCGAAGAGCGATGTTCAGGCACGAGAAGCTGATCCGC
GGCCAGGTGCAGATCATTGACGTCGGTGACTACGGGAAGCACGGTGTTCCGCAGTGGGCT
GCGCGGATGTGGGCCACCTTCCAGCATGGGCCGGAGCTCTACAGGGTCTTGGACTTGAAC
TATCCAGAGACGGTGCGGACAGTGTTCTTCGTCCGGACCGGCCCCGTCACGAACTCCATC
TGGCGCCTCGTGCTGCCCCTCATCCCGCCGCGAACGAAGAAGAAGTTGAAGCTCTACGGG
CCACAAGCAAAGGACTGGACTGCCGACCTTCTGAAGGAGCTTCCGCAGGACCCGNAGGTG
CCTCGCTTCtTGCTGAGCGATAGCGAGGAGGCCTTCGCTGAAGCGACGCCGAAGGGCGGC
CTCCTTCCTACCGGAATCTCTGGCCCAGACTTTGGGAATTGGAGCGTGTCGAGTCCGCCA
TCCCCCAGAGGCATTCGTGGCAGAGCCGGGAAGGCCAGCGCGGCCAAGGTGCAGCCCCTG
GCGGCGAAAGGCTTCTTGCCGCTGCTGCTGGCCGTGCTGGCCGTGCTCTTCGCCGCGAGC
CTGGGA
>at02237
ACTGGAGATGCCTGGGCGGGAGATTTCAACCTTCCTTCGCTGCTACCGTTACCTGCCGGG
AGATCTGAGCAAGCCACATTTGACCGCTCTTTTTGCTTGTGTGAGCACACCCCAAATGTG
CTCTCCAGCTTCTCTGCATTTTCGCTGCTGCTTTATGTCAACGATGAGTTCGAAGGAGGT
GAGACTACATTCTTTGAGCCGGATGCCACGATCCCAGTCTCTAAGAAGGGCCTCACTCCC
CAGTGTGAGCGCAGCACTCTGCTTGTTGCATCCAGTGTTAAGCCACGTGCTGGGGATGCT
CTGATCTTTCCACATGGGTTACATCCAGGGTGCCACCCAAGTCCGTTGCACGAGGGATCA
CTTGTGACAAGTGGCGAAAAAAGTGCTCATAAGGACCGACATCATGTTCTCTGCT
>at02238
TTTTTTTTTTTTTTTTTGCAGCTGCACTCCTCTTCAGCTGTACCACGCTGTGCAAGCCC
CTGAACAAGACCTACAAACACGCACAAGAGGGGGAGCTGGCAGCAGGCTGTTCTCACGTA
CGTATCAGACTGGAATCGCACTTCTTGTGTTACTATGTTCCGCCATTGGAAGCTCCAACA
TCCTGGCCCCTAAGTGATAGGCCGCCAAGGAAGGCTCAGCTGGTCAGCCGGCGCGCCAGG
AGCCCGTAGGCAGCAAAGAACCCGCCCAGGCCTGCTAAATAGCCAGCGGTGAGTCCTGGA
GGCTTGAGCCCCTTCTGGGTGCCCCGCGTGATCGCAGGAACTTGCTCAGCAATGGACACC
ATGAGGCATGCAGTAGCCAGATTGAGCATCTGGTAAAAGCAGCTTGTCAACGCCTTGTAT
GTAGTCACGTCCTTCACCCAGGGTGGGATGTCCTCAGATTTTCCAAAATGAAGCAGAGGC
CCAGCACCGAGAAAGAGAGTGGTGGCAGGGTATACCAAGAAGGCGTGTGGAGAACCCAAA
CGCCAGGATGTCTCACCCCATAGTCCAAACTGCATTGGGATTTCGCACTGATCTGGGAGA
GAGGCGTACAACTGCAAGCCACGAAGGAACCCAAGCGCACCCACCGATACGTTCCCAACT
GTGAGCAGCTGGAGACTGATCTGCAGCCCCATAGCTCTGCAGGAGCAGAGGAGTGGACCT
GCAGCCAGATGTCTGTCTACCTGTCTTGAACCAACATGGCTTGAGCCAAAATGGCTACGG
>at02239
CTATCACGCTGACTTCACTAGCCAGGCCATGCCAGAGCCACACCTTGGGAACCTGCTGTT
GAGCAGCCATGTTCCTGCCGTGCCGCTGCCAGCGTGTCCTGCGGCGCTGCTTGGCGCGCA
GCTGAGCCGCCAGCCACCTGGCCCCAGCGGTGTCGCTCCTTT
>at02240
TTTTTTTTTTTTTTTTTGAGCATGAAGGCAACTCACCTGGTCCAAATGCAACAGTTCAA

GGTGACCTCGCGCGCTGGGCCGCAAAGAACGCAGCCGTCAGTTCGTGCGAAGCTTCCCGG
CCAGCGCTCGATGAAGCTTCCCCGTTCATCCTCAGCAGTTCCGCAAACATGTCTTTCATG
TCGTCATCAGCACATAATTCGTTGATGAAAGCCTGAAGCTGCTCGGGGTCGTCTCTGCGA
ACGAACGTAACCTTCTTGCGAGTCTTGGCCGGCATCAGCGGCAAGATCAGCTTGATCACC
CAGACGAGTACTCGCGGCACGTCGATGATCACGATACGATGGAAGCGTTCTGCAAAGTAC
GAATCGATGCTGCGGGCGACATCGAGGTATGGCATGATGTTCAGGTTCAGCAGCGGCTGG
TAGCCGTGGGCGTCCAGCACGTAGTCTAGCTGAGCAGTGGCCCCTGCCGCTTGCAGTGCC
TGGTCCCAAACGCAGGCAAGCAGCTGGCCGGCTACCTCGCCTTTGCGCTGGTTTACACAG
CCTGAGTAGACCAGTGGCCTCGAGTCGCGACCGATGGCAATGACACGGGTCTCTGTCGTT
GGCAGGATGTGGCTCTGCGCCTCGAGCCACCTGTTGAGGGTAGCCTTCTTCCAGAGGACA
GCCTCCTTCAGCTTCGCAACAGCCAAGCGCTGATTGCCCTCTGTGGCAACTAGCATGCGC
TGCACGTGCCCTGCTGAGATCCAGTCTGCAGCTCCGTCCGGGAAGGCATTGACAACTTCT
GAAGCCAGTTGCGTCTCCACACTCCCGCTTTCTTCTGCTTGGCATTCCAGGGGTCCTGCT
GCGTGAAGTATTCTCCAACTTCTGGCCGAGGCTACCCATCGACGGCGCCTGGTCATCTCA
GAAGCAGCTGCAATCGAAGCAGCAGTGGCGCCAACAACAACCCATGGTGAGAAGTGCTTT
GGAGAAGGCCTTGCCCTGAACAGGGACTGGCAGCACATGCGCGTCCAGCTGTGAGCATCA
ACTGCTCGATGTATTGGCCGAGCATGACCCAGCTTGGGCAACATTGTAGTCAGGAGCAGT
CAACAACCTGAGGAAAATAGCTTGAGCCAAAATGGCTACGG
>at02241
GCTGTGTTTGCATCCCAGTTTTCAAGGTGCACTGACATGGTTCTGTTGGATGACTTGATG
CCCTGTAGCTGCCGATCTAGCCGAGCGCTTCAGACTGTACACCTGCCTGCCCCACACGCG
CAAGTGACATCTGAATTTGATAAGTGAGTCATGTCTTGCATGGTGTGAGAGGGCTCACAC
TCACCTCGTAATTAGGGGGCAGGCCCCGCAACTGCAACATCGCAGCCGTTCTGATGAACT
TCTCCTGTACAATATCCCAGAACTGCCGAACAATGCAGAGCAATACTGGGAGACAGAGGC
AACCTCTAACCCGTAACCGTCTCCTTCGCTTTCTTGACAGCGGTTTTCTTCACCGTCTTC
AGCGCCTTCACCGTCTTCACCGCCTTGGCCTTCTCCCTTTCTTTCTGAACCTTGCGCTTG
GCCTTTTCCTTCTCCTTCAGTGCCTTCACCTTGAGCTTCTCCTTCTCGGCCTCTGCCTTC
GCCTTCAGGCGCTCCTTCACTGCCATTGCCTTCTTCTTTTCCTTGTCCATGCGCGCCTGG
AGCTTCTCCTTTTCGAGCCGCTTCCGCTCTGCTTCCTCGTCTTCCAATGCTTTGATGCCA
ACCTTCTCCTTCCACGCCTCTTCGCTGCCAGAGGCCTTGTATTTTACCAGGTCCTTGTCA
TACTTTGCTTTATTCTTTCTCATACTGCGTCACAAAAGGCTTCTTTTTGCCAGCCGGAAG
ATTCTTCCAAGCCTCCGCTGCCTCCGCACCCGGTTTGTATTTGTTCTTTGTCAGGAACAG
CAAAAACGCAGTCAACGGCCGTCTTGGCCGATTTGGATCGCGTTTCCACATCTCTGCCCT
GCCCGAGTCTACGTACTTCTTCCAAGCCTCGTCAAACGCAGCCTTGGCCTTTGAAGCCTT
GGCCTCGTATGGTTTCTTCTTGGCTTTGGTCATACCGTTCCACTCCTTGCTTGCCTCCTT
TACAAGCTCTGTTGGCGCCAAGTCTCTCTTTTTGCTCCGCGAGTCGTTGAGGAAGAGAAA
GAAAGCCGACATTGGGCGCTTGGGCCTCTCAGGGTCTCTGCCCCTCTTGCGAAGTATGCT
GGCAGCCACCGCTGCGCAGGCCACCAACCAGCCACGACCCTCGCTCTGGGCCTGCGCAGC
TTGAGGGAAGAGCTTCGGCGCTTGAGGGAAGAGCTTCGGCGCAGTCACCCTGCAGGCTCC
TGCAAGCCGTGGTGGCCTCACCTGAGGAAGCTGAGGCTGACAGCCCAGGAAAGCCAAGGA
GCTCAACATAGCAACCACCAACACGCTGGAACTCCTCCAGGCACTTGAACGAAAATGACT
TGAGCCAAAATGGCTACGG
>at02242
ATGTTCCCCGGCCATTCAGCGCGCTGCCTTGGTGAACCTGGGGGAGCTGACGACGTATGA
CACTGCGAAGAGCTACTTTGTGACGGTCTTTGGCGATAGCTTGAAGTGCCACATCTCCTC
TGCCATCGCAGCGGCTTCTTCGCCTCCCTCTGCAGCACGCCTGCGGACGTGGCCAAGTCC
CGGATCATGAATCAGGCCAAGAACGAGGATGGCTCCCTCATGTACAGAGGCACTGTCGAC
TGCTGGGTCAAGACCGTGCGCAGTGAGGGGTTCATGGCACTCTACAAGGGATTCCTGCCT
GGTTGGTTGCGGCTTGGCCCTGGCAGCTGGTGTTCTGGGTCACGTACGAGCAGCTGCGCC
TCGCCTCCGGCATGGGCGGCTTCAAGTGAGGCGTGCACGGCCTCCGGCGCGGCGGCCC
>at02243
TTTTTTTTTTTTTTTTTGCCAACAGAAGCAAGCCACAGTCAGTGCCTGTCAAGGAAACG
ACGACAAGAGAAGGATATGTCGTCAGCAGCAAGCATGGCTCCTTGCTTGCCAAAAAACAG
GACAGAATGAAGTAGGGCGAAAAAGGAAACGGACTACACCAGTTGTGTCCTTCTTCCTAA
GTGACAGTTTGGCTTTGAAGGGGCGACTCCCCTTCTCCAGTAGGGCCCACGGTGAGGTCG

GCAGTTGAGGACACATCCTTCGGAGAGTAGCCCGCTTCCCTTCGCCGGCATTGCCACTCA
AAGACCCGCCTTCGGCGCTCGGCCTCTGGCACAGCATGCAGCCAGTGTCTGTAGTGACGC
TGGATGTTTATCACCTGTGCCAGCAAACGAAATCGCCGATTCCGCACTATGAAACCACGA
GCAGCTGCCTGTATCTGCACAGTCCGCTTACTCACGACCTGGTTCCACAGCTTGCACCTC
ATCCGGTGTCCGCGATACACGCGCTGGATCATGACAGCGGCATCCACCTTCCGAGCACGC
ACAGCTGCAATCCTCTTCCGGGCCAGGTGCCCGCGCACCATTGCTTGGATCTTCTGCGCC
CCCTTAAACCGCCTTCTTGCCTTTTCAAACTCAGCTCGCGCCATCTTCTTGCGCAACCGA
TGGACTCTGGTGCGCGCAAGCCAACCGCGGACATATCGCTGCAGCCGATTTGCGGCCTCC
TCTCGGCTGTGCACTGCCTCGTACTCGAGCTCCCATCTCAAGCCGCCACGGTAACCGCGC
CAGCAGCGCTGTATCTCCACAGCAGCCCACAGCTCCTCTTCAGCGCGGATGGCGTCGCGC
CACATCCTCAAGCGCACAACATACCCACGAACATAGCGCTGGATGGTGATGATGGAATTC
TCATGAGTCAGGAACTCCTTCCGCAACTCCAGGAAGCGCCGGCGGTGCAAGTGCCGAAGC
CAGTGCTTACG
>at02244
TTTTTTTTTTTTTTTTTGCCACTGGGTCTATATCCATTTGTCATGGTCTACATCCATTT
GTACATCCCAATCATACGCTGTACCTTTGACACACTGAGACACCAAAAGGAGTTCCGGCA
ACCTTCACGGATGTCCCTGCTCACACCAGGCATACTGCTCCCAGGCCCACTGCCGCGGAG
CTAGGCAGCCGAAGCCTTGCCCGCGCCAAAGCCAGCCAAGAAGCCCAGACTTCAATCCTT
GCCTCGAGCCGGCAGGCAAGGCCTCTTTGCAGCGGGGGCACCGCAACCCTTCCAGTGCAA
GAAAGCCAAACTCGCCCGAGGCCCTTCGTCTTCCTTCATACATTGATCGCCAGGTGGAAC
GCCTTGTCTCTGCAGCTGCAGAGAGCCGCTGTGCTTCGATTTCCACTTCTCCTCACGTGC
TAGAAGCCGGTCCAATTGTGATAAGAGGTTGTCCTCCTCGTGCC
>at02245
TTTTTTTTTTTTTTTTTAACCACATCGTGCACATTGCACAAAGTGTCCACTTCAACTCC
ACTTAAAAAGGAGCACTGTAGTCAAGCTCTTTTGGCTTTCGATGGGCAAACGCAGCAACA
ATTCCGCAGGGTGGTAGAGCAGGGGAGCCGACCAAAGTCGGCCAGCGAATCGAACCACTG
AGCGCGCGCCAATCCTAGCCTTAAAAAAATGTCGGTGACTCAGCTCATGCAGATCTTGCC
CTTGATCGCAACTGGCAATTTCTGATCTCTTCTTCTTCAAAATGGTCGAACGTCACCCAG
TGCAATCCGGTTGGCGCCGCGAAGTCTCGAGGGCCATCTTCAGGAACTTGCTGCACATCC
GCCGGGGGAATCCGAGGCGGGGCTGCATCGATGAGCCTGCCGTCAGCTACATCGATCGCT
CCATCATCTAGCTCCTCTCCAAGGGCGTGGACCTCAGTCCTCCGCCGCAAGGCCGGCAAG
GCTGTGCGCAGGCGGCGGAGCTGACGCTGAGCGAAGTTGAGTGTCCTGGTCAAGGCAGCC
GCCATCGCTGCAACNNTCGTGCNGAGTAGTCACTAGGGCAACTGGAAGCCACCCCAGATG
GCGA
>at02246
AAGGCCCGGGCTTTCGAGGACGTCATTGACAAATTTTACTCGCGGTACCAGCATCAGTCC
TGGAAGAACATCATTAGCATCGGCGATGCGCCGCACGAGCGCGAGGCACTGAGCCGAGTG
GTCAGATGGGCGCCATGTAGCCTTGGGAAGAGATGCAGGTCAAAGTCGGTGAAGTTTGTA
CTGCGTCCCTCAATTGAGCAACTCACTCGCGAAATTCAAATGCTGAGGGAAAGTCTCAAA
GAGATCGTGTGGCATGATGACGACTTGGACTTGCACTTTTCGGCCGAGTCGCTTGCCGAG
CCGCTCATTGCCTCGCCTTCATCGGAGTCCCCTCCGTGAGGATGAGTTCTGAGAGCCTGG
GCACCTGGATGCCTTGTGCCCGGACTTCTAGGAGACTATGCGTGGGTGCACCCTGGGCAG
CTCATTCATGCACATGCACCGGAATTTCGCACCTGCATGCACTTGACCTTGAGCATTTTG
CAAAGGTGCGCGGCTTTTAGACTGGTCTTGACCTCGAACCAGGGGCAGACTACCCTGACA
CTTGCACCAGCAAGTTTTCTGGAGCTTGATGCATAAGAATCCCTTAGCCGAAA
>at02247
CTTTGCAGTCTGGCTCAGGCATACTCCAAGAAATTTGTGTTGGATGCCGGCTTGCAGCAG
CGCGCGTGCACTAACAGCGCTGCCATGCGGCTAAATGAGCATGTGAGGCTCATGGGAGAG
CGATGCATCCTGGTTCCCTACCAGGCTGCCATGGTCCCGACCTATCACCAGTGGATGCTC
GACCCTGAACTGCTCGAGCTGACGGGGTCGGAGCCGTTGAGCCTTGAGGAGGAGCTGCAG
ATGCAGGCCAACTGGCGCGAAGACGCGGACAAGCTGAC
>at02248
TGCGACGACTGTGAAGATGTTGCAGGATGTCAGCTGCTCGCTGTCTGGGAGCAGGCAGCG
ACTCAGCTCACGCCGTCGCCCGTCTCGCGCCAGGACAAGGGTGCTCCGGTGGGTTTTGTG
TTCTTCCTGAGCTTGCTCGCGGGGAGCGCCCCGCTTCATACCGATGGTGGGCCATTTCCT

GCTCCGGTCATCAGCTCCGAGCGTGAGCTGATGATGAAGTTCGATGGCAACGGTGACGGC
AGGGGTCTCATCTGTAGGTGGTATCGGCGGCTCGGGCAGCCGGGCTCCCGAACGCAGATC
ACAGCCACTGTCCAGCACTTCCGCCTGGACCACAGTGGGCGCTGGGAGATTGATGAGATC
CCTGGAATTGTTGAGTGGCTCAGCGGTGACGTCAGCGGCGGCTGATG
>at02249
TTGGCCCGAGGCCCGCCCGAGGCCAGACGTGCCCGGCCCAGGCTCTCGAGCGGCCGCCGG
GCCTGAAGCACATAATGCAAGGACCAAGCGAAGGCCCGCGCGAAGCCGCTGACGGAAGGA
GAGCCTCTCTTCGTCGCGGCCGCTGACACTTACTTCGAAGGCGAGCCCGGAGTCATTGCC
GCGTTTGACTTCAACTACGATGACATCGTTGACTTCGAGAC
>at02250
TTCCGTACGTCAAAGTCGCAGAATGATCCTAACGGTCGTCCTAGTCAGCATTCGCACAGC
CTCGCGATCTGGAAAGTTCTGCATGGCTGTAACCATCATTTCCAGGGCCCCAGCATCAGC
CAGTGCTCGTGCCAAGACCCAGTTCCACTGCACCATGTTGGACA
>at02251
CAACATGGCGCAGAAGGAAGGGTGTGTGGAGTGCTATGCTGCCCAGGACTGGGTGGACTG
GCATTCGACGCAGCCAGCCATGGTCTTGCACGAGCTGTGCCATGCCTATCACAAGCACTC
GCCTCCAGAGGTCGATGCCATCATCGCATCTGCTTACNCAAAGTGCATGGCGTCTGGGAC
GTTTACGACCAC
>at02252
TTTTTTTTTTTTTTTTTCCTCGCTTGCAATCACTATCAAGCCTTAGCAATGGCCTACCC
TCAGCCATCGCCTCATCAAAAAACTCCTGGCTCAGAGCAGAAAATGTCTTACACGCCCAC
CCACTCAACGCTGGATGAATTGCATCGAGCTGATCGCACCGAGGAAGAAGACCGTCGGGA
TGGTGATGGCATGGACGGCAAGCCAGCGGAATGTGAAGATGGGGTAGGCCACGGGTGTCC
TTGCCGCTGCAACCTTGGGCTTTGCCTCTGCCTTGCAGGTCGTTCGCGATTGGCGCGTGA
ATGCACCAGCGGCCGCTGCGGCATATCCCAGGCCAAGCCCAGCCAGCAAGCAAGCAGACC
ATGGAGCCTGCGCATCTGCAGGGGCCACGCTTGCAAGAGCTGCTTCAGCTGGCAAGTTGG
CGCGTCCACTGAATCCCTCAATGCCACGTGGTGTGCTCACCGGCTGCGCGCCAGGCGGCA
CGAATGCAGACACGGAAGCCACGGCAAGAGCCACGAAGATCACGGCCGTGCGGAACATTG
CTGCTGTTGTGCACAGGGCCAATCTCGCAACGCGGGCCACAGCTTGTAGGGACTGCTCGA
GCCAAAGTGGCTTGAGCCAAAATGGCTACGGACT
>at02253
TAGCCGCACTGCACCTGGTCGGCGCGATGGTGGCTGCGAGGAGAGACTCGATTGCAGGAG
GCTCCCTGGAGAAGGAGGAGCTATTGCTTGCCAAGAGCGCTGGCAGGCCGTCGTCAACTG
CACGCTATGATGCGGCCGTGATCCGGCGCGATGAGAAGTACGTCCTGCGCAAGATCGGGG
AGCGGATTCAGGCAGCCGAGGCTGAGCTGGACGCACGCGGCAGGGCTCCGTCGAAGAGCG
GGAGGAGGAGCAAACGCCGGAAGCAAAGGGGGCACGTCGCTGGTGGCGGAAGCTTTGGAG
AGCTGTAATCACTTCATGGAGAGGAGCCGGAGCTTTGAACCATTGCTGGTACCGCAGTGG
CCATCTTGGCAGTCGGTAGCAGTAACAAAGGGCAACGGGTGAGCTCCATTGTGGACTGTG
ACAAGTCTGACTACGCTGGAGCCCTGA
>at02254
TTTTTTTTTTTTTTTTTCCCTCATGCCCTCCAGTCTACCCCTGGCATTGCCACTCTTCA
ATGCTGATGCTCCTCAGGCACACATCTTGCAAAGATGAACCGAAAGAGGCTCGGACCAAT
GGAGAAAAGTTGGCAAGTCTGATTCCAGACAAATATCCTAGCATCCAAGCGCGTCGATGC
ACGAGGCTTGAAAGACGCAGGTCCCCTGCACATTGAAAGCAAGGTATTGCAACAGAGACG
GTGGAGCACGCGCGACATGCGCGCGCAAAAGCATGCATGCACAGAATGAGGACGATGTCC
CGTGTGGTACGTAGTCAATGCTGTGTGGCCAGCGGCCACGGCAAGCATGGAGGCACTGTG
CCCTCACATGCGCTATACTCATCTACTCAGCTACACAAGCAGACAAACACATCAGAGATA
CATGCTCCGGAACAGCTCACTGCTTCTTCTTGCCTCTAGACATGTGCTTTGGAGATTTGC
TGCCCTTGGCCTCAGCTGGGCCTCGTGCC
>at02255
TTGTTTTTTTTTCTTTTTTTTTTACCGGACAGTCTCGGCTTGCACATGCTGTCTTGCAAC
AAGGGATGGAGGGTAAGGTCGCTAACGAGTGGGAGCACGCGTCAGCATGCGTGCAACATC
CAGAAAGGTAGCCCAGGCCGGTCTCAGGAGTAAAATGCCTCAGCTTTGCCAGCAAGGTGG
TCATGAGTAATTCTCGTCGTCATACCCATCCTCATTTGCACCAGCAGGACCGCCAGAATA
CAGTCCATGCTTCGTGCGGTCCTCCTGTGGACGTTGCCGCAGCTTGGCCTGCAGCAAAGT

GTAGTTCACCACACCGATCTTGACTTCCAGCTGCCGCGTCTCCTGCCGCAGCTTCTTGAA
GGCATCCTTCATCTTGACGATCGGAGCCGTGTCTGTCACAGTGGAGGAACGCTCCTCCAT
GTCGTTCTTGACAATCTCAAGCTCGTCGGAAACGTCCTTCAACTCAATCTGGAGTTTCAT
CACGACTTCGTTGCTGTTGGTGTATTGACCCTGCACCTGCTGGAGCTCCTCCTGCTGCTG
CCTGTAGTCCAGAGCGCGGTGGTCGAACTGCGTGTTGATGAAGGCCTCCTTTGACTTGAT
CCTCTCGAGTGCACTAGAAAGGCTCGATGAGAGCTTTTCAAGCTGCCCCTTTGCTGGCGG
GAACTGAGTGTCGATGACCTGCTTGTAGCCCCTGGTTTGCTGCAAGTGTGTCCTCCACTC
CTTGGAGTCATTGGGCATAACAATCTTGAGCTTGGGGGCAACTCGCTCCACCTCAATGAG
CCATTCCTTGGCGTCGATGTTTCCCTCCAAGACCTGATGGTCCCCGTCATCGCTATCCTC
GCCTTTCTTTTTGATCTCATCCTCCTGGTACATCAGGTCATCCTCCTCGCCATCAGCTGG
CATGTCCTCCTCGCACGTTGAGTGGATCTCAGCATCGCTATCAACCTCTGCTTCCTCGGC
CATGCCCTCGTCGGGATAGACCGGCGCTGCAAACTCAAACTTGGTGGCCCGCAGCACCTC
CTTGAGCAGGCTGTGGAGCACTTGGCAGACACCCTCACCGCATCCAGTCTTCAACTTGGC
CGGGACAGCGTTGACCTGGATACCACAATCCTTCAAGGTCACAAGGATGTTTGTGATCAA
GGTGTTTGGGTCATCATACTCGTCCCATTCCGCCTCCCATCGAGCATCCCGCTGCTTCAG
GCACCAGGGCACCAGCTGTGTGAAATATCTGAACTGGTAAGCCGTGTTTGCAGCAGGGTA
CGTGAAGAATGTCCGCGAGATCGGCTTCAGGTCACTGTTGCAAAACCTCGTCTCGTAGTT
GAGAAGCATCAGCATGTCAACTATGTCATCATTGAGCTGGAGCGCCTCAGCGTTGTAGAG
CGCGCTATCTGAGCCACCCGCCGTGGCGGCCATGGCCCTGGCCCCACTAATGGATGGGTC
CAAGGGTGCTAGTGGGGCTCCAGCATTAACTGAAGCTTGAGCCAAAATGGCTACGG
>at02256
CCATGTGCATGATCTTCTTGCCGCCCCGGTCCTCGCACGCCTCCTTCCACTGGCCTTCCT
CCTTGAAGAGGCACATGCATAGAGGCAGTCCGCAGCAGCAGAAACATTGGTAGATGCAGC
TCTCACCGCACGGACACATGCGGTACAAGCAGACGCCACAGCACGCGTACCAGCAGCCTG
TGTAGTCCGACGATTTCTCGCCGAGCTTGTGAGCCGGCGGAGCGGAGCCGTTTGCGGCGG
CGCCGTCCAGTGCCTTGCCCATCGTCTGCTGATCAGGAGCGCTCATTGTATCTACGAGTT
GAAAAATCACAGATGCTAATGAAAGACTGGAGCCTTGAGCTCAAATGGCTTGAGCCAAAA
TGGCTACGG
>at02257
CTTACAAGCCTGCTTCCAGTCCCGCTCTTCCGGCAATGCCAGCAGCATATTGTCCAAGAG
ACTCAGCGTTCCTGGCCTTGATCCTGGCGGCGCCCGGAAGGCGTACATCGGGCAACCCAA
GCATGGCTGCCGGCATCGCCTCGAAGCTGGCTGTCGTTGCCCGAAGGGACTTGGGCATGT
CACCGGGCAAGCTGGCAGCGCAGGTCGGGCATGCGGTCCATGAGGCCGTCACCAAGGGCC
CAGGGGGGAAGCGCCGCCTGGCTGCATGGGAAGGCGAC
>at02258
CCGTGCTGCGATTCTACGAACTCGGTCTGCCTCGTCAACAATGTGATAAAAACACCCAAA
CATCCAGGGAATGACACTAAGAAACGCCCAATTGAGGTCCGCATATCTCCAGGCAAAATG
CGGGTCTGGCAGTGCGGCGTAGTAGGCAATTACAAACGCACCACAGTAGTTGTTGAGGCG
CTTCCAGCAGCTGGTATCCAAGAACAGACTGATGACTAAAGGCCCAAATATTCCGAACCA
AGAGAAGTATGCCTCGACTTTGGTCAGTTGCAGACCGAAAGCAAGGCGCCGGGTCACCCC
AGCGGCTGCGCCGCCCATCCCGGCAAGTGCCACCATGGTACACGCGCTGCTGATTTTTGC
AGCGCTGTCAGGG
>at02259
TTTTTTTTTTTTTTTTTCAGGCTTCAGATGCCTTTGGCCCTAGCCAGAAACGATGCCAA
GCAAATGAGCTCGCGAGAACCCTAGCATGGTTCCACATACTGCCCTTCGTCCAACTGAAG
AAGGTGTTGTAAACTGTACATAATCCCGCTCCTCATTCCTACAGCCGCTCCAAAACCGTG
AGCATCAAAGCGGATGTGGCAAGCAACCACTCCAAGAAGTGCCGAAATACTGAAATCTTA
AAACAGTTGGCTCCTCACTTCTTGCCGCCTTTGGACCCCTTCTCCTGCTTGGGCTTCTCA
GGCTCCTCCAGCTTGTTCACAAAGGGGAGGAACTCCTGACCGAGCAGGAACGGCACCAAG
ACGCGCGGGACGCGGACTCCGTCCGCCTCCTGGTAGTTCTCGACGATGCAGCAGAGGGCG
CGCTCCGTTGCGCAGAGCGTGCTGTTCAGCATGTGGCAGAATGGGTCCTCGGGCCTGAAA
CCGCACTTGGTTCGCATCGACCGCGCCTGGTAGTCCAGGCAGTTGGAGCAAGACACCAGC
TCTCGGTACTTGCCCTTTACCATCGTTGTCGCCGGGGAACCAGCCCTCCAGATCGTACTT
CTTAGCTGCGGCATTGTTGAGCTCTCCTGAGACGATGGCAACGGTCCTGTATGGGATGCC
AAGGGACTGATAGAACTCTTCAGCCATGTTGATCATCTGATCGTGCTCCTCCCAGGACTT

CTCCGGCTCGACCAGTGTGAACTGCTCGATCTTCTCAAACTGATGCACGCGGAAAATCCC
GCGGATGTCGCGCCCTGAGCTGCCGGCCTCACGCCGGAAGCAGGAGGACACACCAACGTA
CCGAAGAGGAAACTTCGCCCTCTCAATGTTCTGCCCCTTGTGGTATGCAGAGATCGGCTG
CTCAGATGTTGCGATCAGGTACTTGTCAAGCTGGGGCTGGTCCTTGTCCTCAATGATCTT
GTAAAGCACATCATCGTAATCTGCAAGCTCAGCAGTCTTAGCCATGAGGTCCTTCTTCAT
CATGAAGGGCGGCTGGAGCGGTGTGTAGCCGCGCATGCTCAGGAAGGCAAGGCCGTAGTT
GATGAGCGCCTGGTTCAGCAGCACGCCTGGCCCCGACAGGAAGTAGCCGCGGCTGCCAAA
GACTTCATGCCCAGCCGCAAAGTTGACAGCGCCGATCATCTCCAAAAGCTCAAAGTGAGG
CCGGAAGC
>at02260
CTCTCTATCCGTCGCTCTCCCTCATGCATCCTTGCCAAAGCCGCTCTCACTGCTCAAGAG
CGAGGCGCAGGCAGCAGAGGCTTCGGCAGGCGGGGCATCTGGGAGAATGTAGCCGCACCG
CATCAGTGCCTCGGCAGGAGAAATAGCCAGTCCCCCCGAGAAGACCATGACCTTGGCAAC
GCCAGCTGCCTTGACTCGCCCGCCCGAGACGAAGCGGTGCTCGA
>at02261
AGGATGACTTTACGCAAATGTACATGACATGCACGCAGTATTGAGCTGAATTCCGCGAGA
AGCAATGGGCTCCCGTCTCTGAGAACAAGCACGGGGCCACACCAATGCCCCGTATGCCTG
GCAGCCAACTCCGCTGTCTGGTTTGCGACTGCCCCTCTCCAAGGGTCTAGCTGTCATCGC
AGTCGACCACGTGCCTCTCCATGCGGGAGCAACTCCACCATGCTGTTCCAGCGGGAGCGT
GCCATTGATGGGAGCCGTGGCCGCGGCAATGCAAAGGATCGCGCCAATCAAAATCGCAGC
AGCCATTCCGTCCATGACAGGAGGGCTGGTCAAGATTGTGCTTGAGCCAAAATGGCTACG
G
>at02262
TATCAACAGGCTTGCAAAGAAGCTGCTGTGAGACCTGCTTTCAGACATGCACGCTGACAT
GCTAGGTCATCCGGTTGTCATGGCTGACCGTCCTGTCTTCTGGGCAGAGTTGGGCTTGGT
ATCGTCTCCCGCACTGCTTGCGCGCATTGGCGGCTACCTGCAAGCGTGCGCTGCGAGAGA
TGAGGAGCTGAGCAAGGCCATGCACCTAGCTGCGAGGCATGTCCAGCAGGAGTGCTTGGT
TTGGGCTCCCTACATGGCCGGAGTCCCACACGATGCAGCAGCAGTTCCCAAAGACGTGCC
CGTGAAAGCTCTCAGGACAGCAATGGCAACCCCGAGAGTCACAAGTGATGCCACTGCTCA
ACAACGTGAAGAGCCAGCTCGAGCCGA
>at02263
CCGTAGCCATTTTGGCTCAAGCGTCGTGGCAGCCCTGTGCAGCTGCCCTGCCGCCCTGGC
GGCCATGGCTGCCTCGAGGCAAGCTAGGGGCCCACTGATCAGTGGGCCACTGCGGTGGCG
GATGCAGCGGCTGGGCGGCAGAGAGGCCGCCACTGAATCCTGCCTTGGCAGTCTTAGGAA
CTCTCGGGCATGGCGCTTGCTTGGTGCTGCCATGCTCGTTCCCCTGGTGCTGTGGACCTC
AGCATGGCCGGAGGCCTTTGCGCGGAAGGTCTACATGCCCCTGCCAGAGGCCTGGCGAAC
TCTTGGCGTGGGCCCTGACAGCACAAAAGGTGAGATCAAGAGTGCTTTCAGGAAAAGGAT
CAAGGAAGTGCACCCCGATGTCACGGGCGACGACGGCACCATGCTGCAGCGCGTGCGCGA
CGCTTTCGCGGTCGTCGAGAGCCTGAAGAATCCCACGGCCTGGGACGCCAAGGGCGTGGA
GGACGGCCTCCCCGCCTGGGCCTCGGGCCTTCTGCGGGGGGTGCAGTGGTCCAAGGACTG
CCCCAGCTACGCGGCCTTCCTGGGCAAGCCCGACCAGAAGGCCTTGGCCGTGGGCGAGTT
CAGCGAGAAGACGGGCCTTCGGCCCTGGGCCGCTGTCTGGGGGAAGTACTTACAGCAGGA
GGCGAACGCAGAGGCCTTACGCATCTGCCGACAGCACGGCTCGAAGTGCAAGCTCATCTA
CGTGGGCAGTGGTACGGGCCGGGTGAGGCAGGCACCGGACCCCACTGCCGGCAGGGACGA
GCGCGAGTGGTGGGGCGAAATGTTCAAGGGCTCGGGTGACATGCCCGGCTTCGGCTGGAT
GCCCATGATCAACCCTGATAAGGAGAAGCTTGTGGGCTACAAGACCATAGTAGGCGGCGA
CCGCTTCGGCGCCGACCAGCGCGTGCGCGTGCCCGTCTTCCGGCTGAAGTCGGGCGGCCT
GCCCTACTACTATTCCCCGTTGAGGCCGAGAGAAAAGATCCACATGCAGAAGAGCAACTT
CAAGCATACGGCGCCAGCCAGGAAGAAGTTCAACATTAAGAAAGACCCGAGGCTGAAAGA
CCTGCGCGAGATGTCCGAGTGGCACAACATGTGGTGATCCTGGCGGAGAGCCATGCCGCC
GCAGACCTGAGAAAGATGCCATCGACGTCCGCGTGCGTCGATTGGAACTACCAAAAAAAA
AGAAAAAAAA
>at02264
TTTTTTTTTTTTTTTTGTGAAAATTCGAAAAATTCGGCATTAGCTGGGTCTTGGCCATCG
GCTGCGCCTCACTACTCAGACTCAACATCGGGAAGGTCATGCATCAAGTGATGCAAGTTG

AGCCGCTCTTACATGTCACTGTCCGTGTCATCCTCGGACTCCTCTGACCAGATGTCATCG
CAACTCTCATCGTCACTCCCCTCCTCTTCGGCGTCCCCGGCTGCATCTATCTCGTCGTGC
GTGTCAAGGGTGTCGAAAGTGGAGACCATGGGGCGGAACTGCGCGTCCAGCTCCTTGGCG
TTCAAGAACCTTTGGCGGCTGCTGCTCGATGACTCCGACATGTCGTTACCACTGCGTGGT
GGGAGGTCGCCGCAAGTGCCCGCTTTGGCCAGCAAGGGTAGGTCCCGCATGCCGTCGCCG
CTGCCGCACGCCGGGAGGCTGCTGCACGTGG
>at02265
TTGGATCCCAAGCTGCTCTTCTGCAGCCAGGCGGCTGGCGCACCCGCTCCCAGCGATCGG
TGTGACTCTACTCCCGCTCCTCCCAAGCCAAGCACATGCCGCAGCTTCTGAGCAACTTTC
TAGCTCCGGACGGTGTTGGATCTTGTGAGAGCGTCGCGGGGGTCGGCCGATCAGACGTAA
ATCATACGTCATACATCATAAGCCCACTCGCTTATTCCCACACTCACTTGCTGAGCCGTG
CCAGGCAGCTCTCCGCCAGCATTGGCTTGTCAAGCACTGAGCACTTGCTTTGGAACTTTG
AGAGCCGGAT
>at02266
TTTTTTTTTTTTTTTTTTTTTTTTTTTCAAACGCACGAAGCAGAGGCACTGCCTGAATG
CTGGCTCAAACGACTTTGCATCGTGAACGGCCAGGGCTGTAGGGAGGCCAAGAGTCATGC
AAGCACAGCTGGCAAGTCTATCTCTGTACAGTGGCAGACTTACGCGAGGAGGCCTGCCGT
GGCAACGTGCGCGCTCGACGCTGCCATCACCATGACTACGGACAAGACACCCTCCACTAG
GCGACACTTCATGAGGTGACCGGCGGCCGCATTCTCAGGCAACTGTAAGGTCACATCCTC
TCCAAATACGTTGACCTTGACCCGGCCACCGGGGACGACGCCTTGCGGGAGTGTGAATTC
TGCATGCTTGCCATGGAGCAGCCTCGGCAAGGCCGGGATGCTCCTGCTTTTCGCTGCCAG
TCTATAGCCCTTGAAATACCGCGGACTGAAGAGGTCGTTGGCAGGGTCGCCTCCGGACCA
CTGCTCGCATGCAGCTGCAATCTGCGCGAGGGTTGGATACACTGCCACCGCTCCGCGGGC
GGCCAGCCCGCCAGACCAGTACCTGATAGCCGAGGCAACAGCTGCAGCATATGCTCTGGC
ACGAAACTGGTAATCAGACATGGAGCCAAGACTGATGTTACTGTAGACAGACTTCTCGGT
CAGGAAGTGAATCAGCAGGCCTTCGCCCGCCGCACCCTGTACTTGCATCAGCCGGTCCTC
TTGCTGCAGGTCAACAATCAAGAGCAAGTAGACTGGAGCGCAAGGGAACGCGCGCACCAG
CGCGTCCTTCAGCGCAAAGGCCAAGTCCAACTCCCTGGTCGAGTTCAGCAGCCTGACGAA
GACTCGGGGCTTGGATGCCGGGACCTCCCCGAAGCCCAGCAGGCGATCCGCGCGCGCCAC
GAAGTCGCGCTGCACCTGGGCGTCCTCCGGGTCGTGGTGCCAGAAGCTGCCGCCCCACCG
CGCCCCCGCGTAGACCGTCAGGTCGTGCTGCTGCCGCACCTCCAGGTAGGTCAGGAAGTC
CTCGAAGTGCGACTCGAGGCAGTGGATGACCCCCTCCATGGGCGACCTGACCCAGTCGAA
CGGGTAGGCGTGGCGCCTGAGGCCCATGTACAGCAGCGCCTCGGTCAGGGCGCAGTAGCA
GCCCAGGCTCACGAACTCGCAGGCCTGGGCGAAGCCCTCCTCGGGTGCCCCGCAGCCCAG
CGCCAGCCTCCGCACCCGCGCCTCGGAGTCGCTCGGCCTGGCGCTCTCCCGAGCTGGCGC
CTGCTCGGCGGCGGCGCGGGACATCGCTGCGGGGTTGCACGCCGCCGTCGGACCTCGAAC
>at02267
TTTTTTTTTTTTGTTTTCTCGTAGAATGGACTCTTGAAACGTTGCGATCCTTGCACACC
TCGATGCTGTCCCATGCTCACAGCTTTGCGGTCATCTTCCCAACCAGCATGCCGGGGCTC
ATGATGCCAGTGGGGTCGTGGACAGCCTTGGATGCAGCGAGGGAGGCTCCCCAAAGTGCA
CCCGACTCGGCCTCGTAATGCTCCTGATGCAGTCGTCCGACTGCATGATGATGCGTCGAA
GTGCCCCCATGCCTGATGATGGCGTTCATCACATCCTGCTTGATCGCCAACCACATCCGG
ATGCGGGGGTCGTCTTTGGACCCGGCTATGCCCTGTTCCGACAGGCCTCGGACGAAAATG
GTGTAGTACGGTGCCGGTCCATCAGGGTACACGTGGGTAATGCGGCAGGTCACCTCCCCG
TCAACGCCACACTCACGCCGGACTGCATCTCGCGCTGTCCGCAGAACAGCCGCATGCAAA
GTGGGGAAGGCGGCCCACGTACAAGCTGTCTCGATCGTGTTCACGAGCATGGATGGAAGC
AGGCAGCTTATGGAGGACACATAACCCCCTCGCATGAAACCTTTTCCCCAGGAGCCTGCA
ATGCCCTCACGCTCACCACCTGCAGCATCCCCCTGGGCAGCCAAGCGCTTTGAGANCACG
ATTCCGCGATGCACATCGGACTCTGCCATGCCGAGGAGCAAAGACATCTGGGAGTTTTCA
GACA
>at02268
GCAATGGGTGCTGCTACTGCCGCCGGGCAAGGTATTGATGAGCACTGCCGGGGCAGTAGG
CGGTGCAGCGGCCGGCGCAGCGGGGTCCACCATGAGCGCGGCGCAAGGCATGGCATCTGG
CGCTGGGGAGGCGGCCGCGGGGGCCATGGGGCACCGCCGCAAAACACGATGGCTGGCACC
A

>at02269
GCAATAATAATCGAAACAACCAGAGACTTGTTGTGCTTTGTCGCAAGGAGGTGAGGTAGA
GCGCAGCATTGCAGCCACAGCCCGCACCAGACAAATCCGTCGTGTATCTCAAGGTCTTGC
CCAAGAAGGGGAGAACGGCATACTGGCTTGGGTTGTAGGTCCCACTGCAAGACTCGGCAA
AGTAGGTCCGCCCTTTCATGCCTGGTATCACTACTCCCTTGCTCCCTTGGCCCTCTACGC
GTACTGGCCCAGCTGGTTCGCCTGGGACATTCCACTCCGCATTC
>at02270
TTGCCTGTGGACTGCTGAAGGCCGGTGCCTATTACTCGGGACCCACAGGCCCATCCGGTC
GCGTCTTCCTACTCATCACGGACGCCAAGTTTCCTGCACGGACACCTGAGATGGCAGATG
ATGCGGTGCGCCTTGTCCGCTGCATCGGCCAGGCATCTGAGCTTGGATGGATGCGTGAGG
CTGGGCCTGCTGTGGACGCATTTCTGAAGAAGTTTGGGGCAAAGCTGCAAGGAGATGGCA
CCTACGAGTTGCCTGGTGGAAGTCTGCTGAAAGTGGAGATATCACCTGAGGGGCGTTTGC
AAACGATCGACTCTACTCTCAAGGCATGTGACGCATGATTTGCCATCTTCACTGCTTGAC
TTGATTTTGCTTCTCCT
>at02271
CTGGCTGTGTGTACAAGAATTTGACCTTGCCAGGATGCTCCTTGCAATGCCGCTCCAACG
CATCCAGGTCGAGGCCATCATCCTGCATGGGAACTCCGAGGAGGTTCATGCCACGGTCGC
GGATCATAGTGAAGGAGAGGTAGTACGTGGGTTCCTCCACGATTGCATAGTCGCCAACAG
AGCAATGCATGCGGCAGGCGAGGTCGGTGCTCATAGATGAACCAGCCGTGGACATGATCG
TTTGCCAATCTACGGGCTGACCATACTGCTTCTCCAGGAAGGTTGCCAGCGCTTTGCGGA
AGGCAGGGTGACCGAGCATGTAGGCCCCGTTCACCA
>at02272
CCGTAGCCATTTTGGCTCAAGCCATTGACGGTCAGGAGGCAAGGGCCACACCTAAGACAT
TTTTCAGCTGTCTTGGAGGTAGTTGCCAGCCATGTCTTCCACAGAAGAGTACCATGTGCG
TGCTGACCACCACAAGAGCACTTCCGTGCGTATCTCCCGCAAGATGTCTATGAAGGGCGA
GCCGAATCGCCGCCTCTCCAAACGGGTGGAAGCAGGCCAGGTGGATGCGGCGCTCGCGGT
GGAGAAggcGCCAAAGCAGNCGTCCCGCAAGTCCTCGNTGtTNGTTGCAAGTGCTGCGGC
CTTGAAGCAACGCCAGCTTGcCCTCTACGCNGCCGGCAAGGACCCAGGTcGATAAGGACT
ACCACACTGGAGTGCACAGNACNGAAATCNNGGCCTTGGNGCAGTCGCAGAACAGCATGC
TCGGCAATGATGCGCAATGTAAGGAGGGGCCAGCCGGGATTGACGTGAAGGAGGTGAGTG
CGCTCCAGCAGGTGGTCGGAAAGAACAAGATTACCCCCGA
>at02273
TTTTTTTTTTTTTTTTTGCCTTTTGAACAGAGCCCTCAGCAACATCATGACAGTTGTGC
ACGCGCCTGTCACAAGTGGAAAAGCAGTGGCAAGGACAGCCCCTGCAACCCTAGTTGGAA
AATCGCAGCGACGAGGACATTGTGACCTTCCCTAAGAGGACCTCTCCTTCCGAGCACACA
GAAGGAGATGCAGAGGTGCGGAACCCCTCGGCAGCGAACTCATGCATTGCTCCGCCGCAT
CCCGAAAATGCGGCGTGACAGGACGAAAGTCTGGTCCAATTCTTCAAAGACTCACACGCA
CGGTTCTTCCCTGGATGGCTTCCTGACGGTGCTCCTGCACAAGCGCAGCTCTATCGTCAT
TGATGCCAGGTTCCTCCTGCGCAAGCGTAGCTCCGCCGTCATTGACGCGAGGCTCCGTCT
CCGGAGGCGACTCGAGCGCTGCCGGCTCTGGGCAGTCAGTTGCGCAGCCCTCCGCGCCGG
CGTCGGAGATGCGCGTCCGGCAGGTAGGGCACCTCGCATACCGGAAGGTTGGGCATTGAC
ATATCCACCTGTCGGCGCAAGCGTAGTGAAAAGCATGCCGGCACGGCAACACCACGACAT
CACTGCCGTCCTTGAGCTCGTCGAGGCAGATGGCGCACTCTTCGCTGTTGCAGGCACTTG
CAAGATCCCCGCCAAGGCTCTCCAGGAAGTTGTCCGGCAACAGGTGGTCAAGAGTCACGT
TCGGTGGCGGGGGCATGGGGGCCATCATGGGGGGCGTATTGCGGCGTCGGGAGAGGTCTC
TGTGCACGACGAATCTGACTGTGCTCTTCCTGAACCTCTGCAGCGTCGCGCACCAAGCAG
TCCAAGAGCTGTCCGCTGTGACGCCG
>at02274
ATGCGTGTGCGTTCTTTTCCCTGCAATCTCCAGGAGAAAATGCTCCATCCATGCCGGGTC
AAGGGATTCCCTTGCGGCTAACAGGCAAGCTCGTGTTCGAGCATTGAATGCCACACCACG
GAGAGATTTCGTGGCTTCAAAACAAAAACCAAGACTACCTAGTCACTACCCACTCAATGC
TTTCCCTGGCTACCATCCGACAGGATCCCAGTCTCTCCCTTCATGCACTTCTCAGCACAA
GCGACGACGTTTGGTACATTGAAACCAAACTTGTCCATACAAGTGCCTCCTGGGGCAGAG
GCGCCAAACACGTTGATGCCGACATGTGCATCAGCCCACTCCTGCCATCCCATGGTCACC
GCAGCCTCAACACTCATTTTCGGCACGCTCTTCGGCAAGAGTGCCTCCCGGTAGCTGTCA

GGCTGGGACCGGAAGAGCTCCCAGGACGGCATGGAAACCACGCGAACCTTCTTGCCACTC
ATCTCCTTGGCAGCGTCCCAGCACAACGACACCTCCGACCCCGTCCCGATGAGGATGAGC
TCAGGGTCCTCGCACTCCACCACGGCATACGCGCCCTTGGCCACGTTTCGAGGGAGGAGT
TGGGAAGATTTGGCAAGGCTTGGCGGGTGAAGGCCAAGAAGGTGGGCATGGACTCGAGCT
TTGACTTCTCTACTGCAACCTTGTAAGCGCCAGCTGTCTCGTTACAATCCGCCGGGCGAA
TCACAGTGAGGTCGGGGATCATACGCAAGGAAGGCACGGTCTCAATTGGCTGGTGTGTTG
GACCATCCTCTCCAACGGCAATGGAATCGTGCGTCGTCACGAAAATTGTCCCGCACTGCG
CCAACGCAGACAGTCGAATGGCGTTGCGCATGTAGTCCTGAAGATGGTGAAGGTCGCGCA
GTACGGCACCAATCCGGTCTTGTGGAGCGCGATCGCATTGCACACGGCTCCCATGCCGAA
CTCGCGAATGCCAAAGCGCATGTTCCTGCCCTCATACGACTCTTTGAGGAAATCTCCAGT
GCACTTCATCAGGGTCATGTTTGACGGGGCAAGGTCTGCAGAGCCACCAATGAGCTCCGG
CATGACGTTGGCAATCGCGTTCAGGCAGTCCTGCGAGTGAAGGCGAGTAGCCTTGCCCTT
GTCCTCCGGAGTGGCCTTGGGCAAGGCCTCCACCCAGCCATCGGGCAACTTCTTGTCCAT
CGCGGACCGCTGGAACTGGGCAGCAAGCTCGGGCTCCTTCTTCTGGTACTCCGCCCACAG
CTCGCTCCACTCCTCCTGCTTCTTCGCGCCCTCAGCCGCATGAGCCCTGTAGACGTCATA
CACACTTGATGGCACCTCGAACTCGCCGTACTTCCAGCCCAAGTTCTCGCGTGTTGCTGT
GGCCTCCTCAGAGCCCAGAGGGGCACCATGCGCGTCGTGGCTGTCAGCTTTGTTCGGTGA
ACCATATCCAATCACGGTCTTCACCTTGATGAGTGTAGGCTTGTCTGTGCATGCTTTGGC
CTCTGCAATGGCTTTCCGGAGCCCATCCACATCCGTGTTGCCTTCCTCGACAGTGAGCAC
CTGCCAGCCATAAGCCTCATAGCGCTTGCCAACGTCTTCAGTGAAGGAGAGCTCAGTGTG
GCCATCAATGGTGATGTTGTTGTCGTCGTAGAAGGCGATGAGCTTCCCCAGGCCAAGGTG
ACCAGCGTAAGCACAGGATTCATGCGAGATGCCCTCCTGCATGCAGCCATCGCCAAGGAT
GCAGTAGGTGTAGTGGTCGACGAGGGGCAAGCCGGGTTTGTTGTAGACTGCAGCCAGATG
GGCCTCCGCGGCAGCGAGGCCCACGGCGTTGGAGATGCCCATGCCCAGTGGCCCCGTGGT
CACCTCAATGCCGGCGGTCTCGAAGTTCTCCGGGTGGCCCGGCGTCTTGGAGCCCCACTG
GCGGAACTGTTTGAGGTCATCCATGGACACACTGTCGTAACCTGCCAGATGCAACAGGGA
GTACTGCAGCATGCAGCCATGGCCGGAGGAGAGTACAAAGCGGTCACGGTTCACCCAACT
GGGGTCCTGTGGTTGTACTTCATCTCCTCAGCAAAGAGCAAGTAGCCTATGGGAGCCTGG
CCCATCGGAGCGCCTGGATGTCCGCTGTTGGCCTTGTTGACAGCATCAACCGCTAGGAAA
CGCACGGCGTTGATGCAGTCCGTGTCCACTTCTGTGCTCACTGCGGTGGCACGAGCCACA
ACCTTCTGTGTCCTACGGCGGCGTGCGCCTGCTGCGCCGCGGCCACTGCGGCCGTCAAAC
AAGCCACGCCAGGCATTGCATTGGATGTGCTAGAGGAGGTAGAAGACGTGGCAGTGGGCT
GCTCCAAGTCGCCACCAGCGCAGGCTGCTGCAAAGGCGAGCTCGCAAGCAAGCTGCGCGT
GGGCGCCAGGCCGGTGAAGCCGCTTCCTGCGGGTGACCCCAGCGCTACCAATGTGGCTGC
CCCGCCAGCTGCAAGGAGCTTTGTGCGCTTGTCGCGGCATGCATCAGTCTTTGCGAAGCG
GGAGAGGACCGGCATCTGAAGTTTGATGACTGGACAGATGGTTTCCAAGATGCAGAAAAC
TACGCAAACGCGTGTGGTGGGGTGACGGCTGGAGCCAAAGGCTTGAGCCAAAATGGCTAC
GG
>at02275
TTTTTTTTTTTTTTTTTGGCAGCCAGCACAGGCCCACCCCATGTGACGGAGTGAATGCT
CCCACTCCCCACACTGTACCAGCTACCATCCCAATGCCGATTGCCGGGCATCACACTGCC
TCCACCATCTCCCTGGCGGTGACACGCAACCGACGTCCCAGCTCAAAACCTTCCGCGCTT
GGCGCTGCCACCCGCGGTCCCTGGGCCCTTGCGCTTGCCCACCCCCGCCTTCGCCGCCGC
GACCTTGCGCTTTGTCGGATCAAACTTATCGCTATCCTTGATCTCTACCTTTGCGGCACC
GAAGAAGCTGTCCAACGGCCGCTGCTTCGAGCGAGACTTGGCAGTCTTCAAACGCTCAAG
GTACCGATCGACGCGCTCCTTGCTGAAGGAGCAGCCCTCAACAAGGAACTTCGTCAGCCC
CTCGCAATCAGGGTCCTCGAACTTGAAGTCCACTTTGGAGGTGTCGACCGCCTCGCACTC
CTTGAAGAACTCTCGCGCCGCCTGATACCTGAAGTTCTGCGGGACCTTATCTGGGCCTAG
CACTTCGAGAACCTTCTCCAAGCTTCCGTGCTGCACCAGCATCTTGATTGCCGTCGATGG
GCCCACACCCTTGAGTGTGTCGCAGTAGTCGCATCCAGAAAGGATGCAGAAGTCTATGAA
CTGGTCCATGCTGATATTAAGCTGCTCCAGCGCTCGCTCCAAGCTCACTTCCAAAATTTG
CTTCTTCTGCGATTCAGCAGCCATGAGGTTCCTGACCAGAATCTTGGTTCCGAAGGTCAG
GCAGTCAGCGTCCTCTGTCGCAGCAGCATAGACCTTCCCATCGCGGCAAAGTGCCGCGCA
TGTCGCTTCTGCCTCGCTTGGCGCGTCCACTACTGGCACTCCCATAGCCTCAACAGCTGC
TTTGTCTCCTCGTTTTGCTCCTTGGTCACTCTGACCGTGGCCTTGGTGCTTTTCAGGACC

TGCTCACTGTCGCCCGCCTCCATGGCGGCCTGTAGGTTGGCAGTGGCTTCAGTCGCTTTT
GTCGCCGCGCATCCAACTCATGCAACTTCAGCTCTGGGGGCTTCCCGTCAAAGACGTACA
CCGGTTTGATCCCAGACTCCATGAGCTTGATGGTACGCGTGAGCATGCCGATGATGTGGG
AGGTGACGTGCCCATCTTCGTTGGTCAAATTGTTGTACGTACCTGACCGGTTTTCCCGGA
TCATGATGAGAAACTGGTAGAGGCACATGCTTGCATCAATGGCAACAATGCGACCAGTGT
AGGCCTGCTGGCTGTCAATCTCACGGACGGACTTGGGCGCAGCCTCCTGCAGAAACTTCA
TCAGGCCTTTGATCCCCATGGCTATGTGTTTGCAGTGAGAGCGGCAAGTGGAGGTATGGC
GCCTCGGTCCTTGAGCCGAAGTGGCTTGAGCCAAAATGGCTACGG
>at02276
CCGTAGCCATTTTGGCTCAAGCCATTATTTCTCAAGTCAAGAAGCAGATGATGGCAATGA
CTGGTGCCAGGAACCCAACTTTCATTTCAGCATGGAGAGCACAGGATTGCGACTGGTAGA
ATGGCCAGCCTCGGCCAGCAAGGGCTGTGGCTCCGGATCCCGGCGTTGCTCTCGGAGGCT
GCCCTCACCGCCCGCTGTATCGCGGCACGCACCCCGTTGGCAGGTGCTACTGCTACGTCC
TCAACCCTGGTTTCGCGGACTTCACTGGCAAGCAGTACATCCTCAACCACGGTGTCACAG
GTTCCAACGGCAAGCACTACATCCTCAACCCTGGTGTCACTGACTCCCACGGCAAGCACT
ACATCCTCAACCACGGTTTCACAAGCCCCAACGGCAAGCATTACTTCCTCAACCCTGGTG
TCAAAGACCAACGACAGGCACTACATCCTCAACCACGGT
>at02277
TTTTTTTTTTTTTTTTTGATGTGCCAAACGCAATAAGCTCGACCAACCAATGCCCCCAT
CACTCCGTTGGGGAGGTGCTTGCTTCTGCGTTCACATGGCGAGAGTGGCGATGAGATGGG
CTTTCTGTGGGCACCTGGCGTGGCAGCTGGTGCTGCACTTGTCGAACTCTGCAGTCCGCT
GGAGAACCCAGCTCTGCATTGCCACGCGAGCCATTGCACATCTTCGAATTAGAGCGACCG
TCAGGATGCATAACTCTGGAGTAGGAAAGACTGGGTATGCGACCCGACGGCCCATTCAAG
AGATCAAGCATGCAACATGACAACCTCCTCATCTGGGATGTTGGCAGCAGCCGACGTAAG
ATCCAGCACCAAAGTGCCACAATTCCACAGGCATGGCACGGGCCAGCACCATGAGGTGCT
CTCCTTGACACCCCCTCCTACACTACTTGGCGACTTCAACTAGCTCCAGCAATCTCAGAG
GAACTTGGTCCGATCAAAGCCAACAAGCGTGCGCAATGCCTCCCTGGTCTGCAAGCATCC
GCGTGCCGTGCTTGTCGAAGCCAGGCCCGACTCGACGAGGAAGTTGAGATCGACACTGAG
GACGACGCCCGCAAAGGCTGCAGTGATCACGGGCACCAAGACGGCCAGGAGAGTGGCAAC
AGAGATGGGTGCCGCTTCATGCCCTCGCCTCCTCTCCTGAAGGACGTCGCAGTCCAAGAC
CTGGCACCTCTGCCGCGAGTACTGCACGTGGGAACTTCTCGTCAAC
>at02278
CCGTAGCCATTTTGGCTCAAGCCATTCTGACTCGAGCAGCGATTTAGCTCTGAATTTGGC
TTGCCCCTTAGAATGGACCTTTATTTCGTGAATTGTCGTCTATCTTCATCCGTCGCGAA
AGTGGGATTTGGGAGGAAGTCATACTACTTGCCAAGGGCACCTCCTTACAATGGGCGGTT
CTTGACCACTTGGAGGGAGTTCACGTCGCAGACTTTGCCTTGCTAAAGTGTCGCCCCATA
CGGCCGCAGGTAGGAAGTGTGCATTCAGTCGGTGAGCGTCCGGCATCGGCGGAGATGAAG
ATTTTAGTGGACTTTGGCAGGGAACTCGTCAGAAGCAAAACGCATGTTCATCACACTGCG
TTGGATGCCAACATCTTGCCGCCAGCAGAACCATCTTCCGACGTCGACTTTGGCAAAGCA
ACCAGCCACAGCCGTGACTCCGGCAACGTATTCACGCAGCAGCGTCTGGCTCTGCAACCG
CAGGTGCCCGGTGGTGGAGGTCGGGGTCGCTCTGCTTCACATGTTCGAACGGTGCGCGTG
CGAGCGGTGAGCGTGCCAGCGAAC
>at02279
AGGGCGGCCACGAGCTCCGCCACGTAGGCGTCCACAGCCTCCTGACCCTCCATGCCGCGA
CCCTCCAGGGCTCCCTCAGCCTCGCCACGCTGCAACGCCTCGGCTCGCGCGACCAGCTCC
TTGTCGGCCACCTCAGCATCCAGGTACTCGATGAGGAGGCTCAGCTCAACCGGCCCGGTC
GAATCGGCACCAGCATGCAGCGCCTCGGGGGGCTCGGCCGCCTCGAGCGAGAGCAGGGCA
GCGAGGGGAGCATGCTCTGCTCCTCTGGACTGCTCTAAGTAGCCAGGTTGGCGAGGCTCA
GCAGTGACCACCTCCAAGAGGTGGTTGAACTCATCTTCTCCCCATGTCATGGCCGGCCCT
TCACTCTGATGCTGAGCACTTGAGCCAAAATGGCTACGG
>at02280
CCTCGTTGTTGCCGTCACCGACTCCACGGCTTCTTCCTCGCGATGATGAACCACGCCGTC
GCCAGAGGTAGCGCTGGCGCTCTTCTTCACACACAACACTGGGCCGCGGATGCCAAACCA
ACTTCCCAGGGTGACGGCGCCATGCGTGTCATGGACAAGCCAATTGCTTGGCGGGTGTCC
GTCATGGCGCTCAAAGAAGTATGCCCTATGGCCGCCATAGAAGCTCTGGCCCTCGCTGCT

CACGAAGAAGTTGGCTGCGTTGGAA
>at02281
TCAGAAGGTAGACTGCAGCCGGTATCGCGAAGGGGGCACTGTGCCGAGCGCCACACCACA
GAGGTGCCGGCATACCCAATGCACGGCGTCCAGCAAGGACAGAGCTGCACAAGATGAGCT
TAAGGCTCTCAGCTGCCAATTGGAATTGCTCTGGCGGCATGGGCACTCCATCCGACGCCC
GCGTCATGTGCAAGAGTAACGGCTTCAGCGCCATCATTAACGTCGCTACTGCAAACAGTG
CAATCGTATCAGTGTGCTTCATCATCGCCACTGCAGACAACTCTTCACACTGTGCAATTA
GAAAATCGATCACATGCTGAGAAGCTTGAGCCAAAATGG
>at02282
GCAAACTCCAAGTTGCCTCCGCACAGCTTGCTCCTTCACAACCACCATCTGCTGCATGTT
GCGCCCAATGAGGAGCGTGCTGCTCAAGATGACAATGAAGATGAGGGCGACCAAGGGCAC
GACAGCCTCCAAGCTTCCACGCAGACGCCTGTATCTCACTGGCTCCAAGTTCTGCACAAA
TGTGTGGACAACGGTGGGGACCAGAGTCTCCCATAAGATTTCTGGGAAGTCGGTGACAAA
GCCAAGGGCAACCGCATTCAGGATCAGGTCAGTGATGGACGTCGTCGCCGCCAACCAAAA
GACTCCTGTGTAGAGCAGCATGATGGCAACGACAGCACGGGCAATGCTCGCAGCCAAGAA
GATGGCGAATCGACCAGGTGAAAGTGCCACCAGGCAACCGTTCTCCA
>at02283
TTTTTTTTTTTTTTTTTTGAGATCCAAGACAATTGTTTCCGTACACCACCGTAACTATGA
AACCCTGAGAACCCCTCACAGCTCTAGCTGTTTGAGGAACAACGTGAAAGAAAAAACACT
GTACACCTCCTGGGAAACAATAAGGTGACACACTGGTCGTCACTCCTGCCTTTGGCAGAA
GCCGCTCACGTGAAGAGTGTCCACTGCAGCTGTGACTATTGGTCCCTGCACATGGAACAA
CTCGACCTCTGTGCATGATTGGTCACTAAAGTGACAGCATGTCAACAACACCACTGAATG
CATCCCACTGACAAGCACACAAGGTGGCCAAGTGCAGCACTGAAGTGGTAGATTGTCCCG
AACAATCCCAAATCAGTGCTTCCACCAGCATGTGCTGTCACTAGCATTCCTGCTGAAGCA
CACTGCACTGCCAAACCTGCACCTCGATGACTGGCGAGGCCTGGAATTCAGCCGATGTTG
ACCACACTGCCAGACTCCAGGACCATGATCTTGTTCTGCGCCTCCAGGCTGTCCAGGCCT
GCGCTGAACTCGTCCTCATTGAAGGCCGGCTCGCCCTCGGCCAGGCCCTCATTCACGCTC
ATCAGCAAGTCTCCCCTGGTGGACCTCCTGCTGTGCCTGCCGCGCGAGGCTGCGAGCAAC
GAGCGTCGTGAGCACCGCGAGCCTGCCCGCCTCGTGCCGAT
>at02284
AAAGGCTCTGGTGGCAGTGGTTGCCCTTGGAATTACAGTCTGCTCTCAAGATCGGAAACC
ACCACCAGGAGTGGGCTGCGTACCTTGCAGGCACTGATGGGCAGTTGGAAATGCCGTTCC
AGCGTTTCGATTACCGCCCCTACTACCGTGAAGAGGTTGACAATCCGCTGGGGACAACGT
ATGTCAAGCACTTCTCGGTGCAAGAGNGTATCGAGATGTTTGACAATAAAGTCTTCGAGA
TCTCGAACGCTGAAGGAGAGGCGCTGGACCCAATGTTCCGGCAGATTATGGAGGTGGGCT
ACTTGGCTGTGCACCAGCTGGGAATCACAAAGAAGTACTGCAACACCAACCCAATCCATG
CCTCGGTCTCAGTGGGATGCGACAAGCAGGAGTGGCTGAACATGCCGGGCATCCCGCGGA
GCGTGGCGACCAACAACCAGCTGGCCATCTGCGCGAACCGCTTCAACTACGTCTTCAACC
TCAAGGGCGGAGCTACGTCTGCGACACGGCCTGCTCCTCGACCCTCGTGTCCATGCACCT
CGGGAAGATGAACCTCATGGAGCAGCGCTGGGACCCGCTGGAGTGGCACCTGGCCCTGGG
CACGAGCCTGACCCTCACCGTCGGCTCCTTCATCGGCAGCTGCGCCTCGC
>at02285
CCGTAGCCATTTTGGCTCAAGAGCCAACTAGGATCAAGACGTACCCTAGCCGGCACCTAG
ACCAGATGCACATGTTTCTGAGGTCCGCAATGGTCCAGGATGTGTGCGCCCAGGCGGCGC
GCCAATGATCTGGCGCACCATGCAGCTCCTGCGCCTGCTGCGGCTGGGAGCGAAGAAGTG
GAGGCCGTACGACTCAGGCGAACGATGGTGGCCCGCGGGCGATCTGCCGCATGCAGGTCG
ATGGTGGCCCGCTGGCGCGCCTGCGCCACACTCTTCCTCGCTCCCTACGCGCGATGCCAG
CGCACTTGGTGCTAAAACGCCTATACCTTCCTCTGCCTCTCCTTCGACACGCGCAGCCTT
CGCACATGGTACTTCATCTCCTACACCTTCGTCTGCGCCACCCTCTCCTTTGACGCGCGC
AGCTTGCGCACATGGACTTCATCGCCTACACCTTCGTCTGCGCCACCCTCGCCTTTGACA
CGCGCTGACTGCGCACAGGGAGCTACAACGTCTACACCTACTTCTGCGCCGCCCTCTCCT
TTG
>at02286
GTCCTTTCTCCGCCTTCTCCTTGCTGGCCTTGCTGGCCTTGTTGGCCTTGCTGGCCTTCT
TGGGGGTCTCTGGCTTGCCCAAACTTTCTCCGGACTTTGTCTTGTCCAAACTTTCTCCTG

ACTTTGCCTTCTTGCCCTTCTTGCCGCTGCCGGACTTCGGGCTGGGTTNCGCTTTTGATT
TGTCCAAGGAGTTTGCCTGCTCGCTCCTCCCTTGGGACTTCCCAGAGGTGCCCGGTTTGC
CCCTGACCT
>at02287
TTTTTTTTTTTTTTTGTTCAAACGCAACCCTGGAATCGGAATCCCTTCGAGGTCCGTTCC
CAAGGCTGTCCGGACATGTAGCTTGCGGATCTGCCAGGGGCGCATCTTCAGGGCTGACCT
GCCGCGCGAGCTCCATCCTGGGCCACTGCGGCACAGGCCATTCCGCTCCTAGGGCGCACG
GCCCTTGGTACTTCAAGAGCTGCAAGGTGGCGTGGGTAGGGGCATCGGAGGGTTTGACAA
GCTTCACCCTGCAGAGTGCCTGGTACTTCTGGGATGTCTCTCTGTCAGAGTAGCGCTTCA
GAATCATGCTCACCTCTCGTTCATGCTGAGCATGAGGACCGTCTGAGCGCATGCCCTCCG
AGAGGCAATTCACCCTCGTGCC
>at02288
TTTTTTTTTTTTTTTTTGGTTGCAAACGCACCAACCAGCTCACAATGGCCGATGGATAC
CAGGTCCAAGGAGTCCTCTCACTTTGATGCCCTCAATGGAGAGTCAGTGTAGAGGGCCCA
GTCACCCCAGGCTGCTCCTGTAAGACCGTTTTGAAAGAACATGCCGATGATGGCCATCAT
TGCAAGGCGGCCATTGTTGATCTCCGCCAGCAGGCTCTTCTCCTTCTTCTCCCTGTCATC
AATGCTCCCTCCTCCGGGAAGGCCGAAGGCACCGTAACCTTCGTAATCACCAGCATACTC
GCCAGGCTCCGACTGCAGGTTGAAGACCTCAATGGTGCCGACAAAGGCGACAATCTGAGC
CCAGCCGATGAGAGGGACCTTGGCAGCAGCACCGAGGCCATTGGGGATGTCCTGAAATTG
AATGCCTTCGGAAGGGGAAAGGTAGCCGGGCCACTTGAAGTATTCGGGTACGATGAAGCC
AATGCAGGCCAGCATGGACACGCGTCCGTGCTGGATCTCAATGATCCTGCGGCGCTTGAA
CTGAGAGACTGCCACGGCAGGGTCGTCGAAGTTGCTCAGGTCCAGCGGATCCCAGAAGCC
GAGCGGCGGGCAAGCACCGAGCTCCTGGGAGAAGTCCGGAGCTGCGGCCCGCAGGGGCGA
GTCCGTGTAAAGGGCCCAGTCACCCCAGGCTGCTCCAGTAAGACATTTTGGAAGAACATG
CGATGATGGCATCATCGCGAG
>at02289
CGAATAGCCGGTGTCGCTGCACTGCGTCGTGCAAGGCCAGGCGTAGCCAGAAATGGTCAT
GTAGTCACGCTTGTCGCCACCGGGTGGCGTCAGCGCCTTCTGCGTTGGACTCCATGGACC
TGCGGGGACACCCGTTCGGAGGCCAGCAGGAAGCTCCTTGACCCTGTCAGCGTCCCGCAG
CAGCCTGCTCTGCGCAGCCAACGCCCAAGCTGGGGCAGTGTCCGGCTGCATCAAGACCTC
CTGGACTCCGCTGAGCGTAGGGCCATCCCAGGAGAGCACAGAGGGAGGCGGCGCGCCTTC
GACTCGCGCCTGAAGCTTGCGCCT
>at02290
TTTTTTTTTNTTTTTTTTTTTTTTNNCAGTATCAGCGGACCACTTAGCAGGGTGCACTC
AGCCGCAAGGAAGCTTTGGAGCTGGTCTGCGTCGGCTGCCCATAGCGCAAGTTGGCAGTC
CTGCTATGCACCTCAACATGCAAGTTCGAGCGGGCTTCACTCTTCCAGCTGATCGGCGGG
AGCCCACCTACGTAGTTGCTGTGCATCGAGGGCTTCGGCGGCTCCAGGGGAGACGTGAGC
TTGAAGTTGAACGCTGCCTCCCGCTGCGCACCTTTGCGGTTCATTTCCTCGCGAGCCAGG
GCGGTCTCGATGTCCTGCATGCGAGCAGCCCGCCTCTCGCTCTTCAGCCTCTCCTCCTTG
GCCAGGACTTTCGGAGCCTGGCTCCTCTTCTTCGGAACTGCTGGCTTGGCATTGCCCCCG
TAGTCGGGGTACATCCTGCTGTACGTCGTCTGGAAGAGCTGCCGCTGCACGTCCTGCATC
AGCGTCCGCACCTCCTCTGACGAGAGGGGATGGTCAGGGGCGTCGGGAGCTTTGGCAGCA
AGGCGCGGCTTCGAGGCACTGGCACCCTGCTCGTGGTACTCGTGGTCCGCCATGTAGCTG
GTCTTCACACATTGGGCCCGCAGACGGGCAAACGCATCGTCGAGGGACGGCGAGCTCTGT
GAACGCTGTCCGGCTAGCATGGAGCAGCCACTGCCTCCTTTGGAGCGCAGGCCATCGACT
GCAGGCCAAGCCCTTGAGCCAAAATGGCTACGG
>at02291
TTTTTTTTTTTTTTTTTCACCAAGTGACTTCGCAGCAAGCGCCTCAGCCTCAGCGCGCC
GACGCCACGCGCCGCCCCCGCCGCCCGGCCGCTCAGTCCAGGTACGCGCCGCCGCCAGCC
TCCAGCGCCGCCGCCGAGCGGGCGCCGGGCGGCGGCGGCTCCGGCGGCGGCTCGGGCGGC
AGCGCGTCGCAGGAGAGGGCGTACTGCGCCGGAGCACGATTGTTGGAGACAGCAGGGTAG
ACCGTGTCCGGGAGGTACGAGAAGACATCATTGTGACGCCTTCCGTCCACGAAGGCCGTG
GCCGTGCGCTGCTGGCCCTCTGCGCCAGCTCCAGGAGGAAGCCCACGGAGCTGCCCTCGC
CGAAGCCCTGGGGCAGGCGGGCAACCTCCTCCCCGCAGGCCAGGACCGCGCCGCTAGAGG
CGATGCCCCAGGACTCGGGCGCTTGGCCGACCGGGGCGTGCACGGCAGTCCAGTCGGCGG

AGACCAGGCCGACGTAGGAGACGTCCGCCTCGTGCC
>at02292
TTGTTTTTTTTTTCTTTTTTTTTTTGTTTCACATCCTGAGCAATGAGCACTCTGCGTCTT
GTGAGCGATGCGTCGGAAGACCGCTAGCAGGAGCCAGCAGTGCGCTCCTCTACGCTTCAA
AGTGTGCCATGCGCACTGGCGCCAGCAATGCATGAATGCATAGAATCTGTCATCAAAATG
GCCGCGATGCACCGTTCAAGGCATGTAGGCCAGCTGAGCACTGGAAGAGTCGGCCATGAT
GTAGCCACGCCCCTACACCCTCTCCTCATCTGATTCATCCCAGCGGCCGGGTGCGTTGCG
GATCTT
>at02293
CGGCGCCGCCATCTCCACGACCTCCCTGCCAGCCAGTCGGTCCGCGTCCGCGGACGGGCC
CTCCCTAGCCCCTTCCGCGCGCCGGCAGCCTCATCGGCCCGGCCGGCGCCTCAGCGGTAC
TTGACGAAGCCAATCTCGGTGGCCTTCTCGCGGAACTCCTGGCGCTTCATCATGATGCCG
TAGCAGCGGATCAGCCCCATGCCGCCCAGGCGGCCGGACATGCGCGAGGGGCGGTTGCCC
TTGGAGCCCGGGAGGCCGCGCACGGGCGTGACAAAGGGGTTGCGCTGCTGCAACGCTGGC
TTGGAGCCTCCCTTGAGTGCAGTGTGGCCAGCCGTGAAGGTCATGCGAAGGGGCTCGGCA
GCGTCGAAGCTCTTCAAGGCAGCCAGGGTGTCCGCGTCCTTCAGGACCTGGTCCTTGTAA
ATGAGCCGCATGTGCTCGGGCTCAATGTTGCAGTGCTCCTTTGCGAGCTTCTTCACGTCC
CTGATCGCCATGGTGGGCTCCAGCTCCAAATCGAAGGAGGGCCCGCCCGTTACCTTGACC
TGCATTGTCAGGCAGTCGCGGCTCGGCTCCTCCTGCTGCTCCTCCTCCGGTCCCTCCAGG
CTGATGGCGCCAAAGGCGTCCACGAGCTCGGCCGACATTCTGTTCTGGGGCTCTCTCTCG
GTGTTCTGGTGTCTCGCTGGCTGGACAGACGCTCTCTCGCCACCCTTGAGCCAANANNTN
NANCTAACGG
>at02294
GAAAAAAGGTTCGTCAAGAGTGTGGTGAAGTTGACGCTTGAAATGTTCCGTCCGTCTGTC
CAGCAAGTGGCCCTCCAAAGGTCGCCACACGGGTAGTCTTTAAAGCTGTAAGTCTCTGCC
GGAGCGACCTCATACAAAAAACGTAGTCCCTTGCAGAGTACACCCACGGCTGCCCGTCCC
GACATCTCCCCAcTAGNGTCCCGTCGTGGCGTTTGACTTCTTGACGACGTGGAGCGGCAC
GTCCGTAACATCGATTGGCGCTTCCAGCCCGAACTCCGCAGAGcCCCCAGAGGCAGCTAG
GAATGGCTATGTAGCCGGGCTCGTCGAAGCGTGTGCCGGTGGTGGCTGGGTGCCCGGAGC
CTCCGTAGATGGGCTGCGACCGGCAGAGAAGTCTGCCGACAGCCGCGTTGCAGTCGGCTA
GTGCGGTGCCCTTGGCGCATGCGTAGACCTCCACAGACAGGCAGCTGGCCGCATGGCAAT
GGTTGTTCAGGGCGACAAACGTATGCTTACCCAGCATGCTGCCCTC
>at02295
GCTCCACTGGAGGGCCCAGATGGTGCCGTGGATGCCGCCCAGAGTGTTGATGAGCGTTCC
GGAGGCGCCGGAGCCGGAGCCGGAGAGCTCCCAGACGCGGAGCTTCTGGTCCTCGCCGCC
CGCCAGAAGGAGGTCGTGGCCCCCGCTCTGGGCCGAGGGTATCCAGGCCAAGGCGCGGAA
GTAGCCGGAGGCCTGGGGGCTGAGGGTTGCCACGGCGTTGAGGTTGTCGGCCAAGCAGAA
GGCCTGTGCAGTGGGGAGCCACGAGCCGAGGAGGAGGCACTGCAGTAGGAAGCCCATGCC
AGGCTCCAGCCGTGCCGCAGTTGGCCGCGGAGGCCCCCGGCGGGCCATGGCGGCAAGCGG
CGCTGCCCCCCGGGGT
>at02296
TTCCGGTGGCCGACTCCAAGTACCTATTGTTGTGGGCATGGTGAGTGGCAATGGTGGTGA
TGGAAGCGAGCTGGATTGTTATTGGCGGCGCGGACCGTGGCGGCATCCTTGTCCGCGAAG
GGCGTGACACAGCCTCGCCACAGGCAAGCGAGCGTCTATCCACTGGCGCTTTGGTGCGTC
AGCTGGAGCTGGACGGCGAGCGTTTGCGCTACAAGAGGCTCACTGGCGCTGGACCAGCAG
GAGGGGCTGGGGTGAGTATCCGGGGTACCTGGGCAAGGAACTCATGGTGCCTGCTGAGGG
CGTCTGCGAGGTTGTTGGCGGCGGCGAGCGCGGCGGCATCGTCGTGCGCAGCGGTTGCGA
CCTCTCGTCGCCGTTGGCGCTGGAGCGCCTCTCGA
>at02297
CCGTAGCCATTTTGGCTCAAGCGCAAACCTGCTCAGGCGCGCGCCTCTTTTCTGCCAGGC
CCTTTCCGCCACTGCACCATTCTCTGCCTTCCGCTTGGCGCCGCATCCGGCGTCGAAGGC
GGCAGCCGTCCCTTCCGGATGGCCTCCAGGCCCGAGGGCACCGCCCTTCGCTGAGCTTCG
CGATGGGCTCGGGGGCGCACCTGCTGGTTTGCGCTCTGTCCTCGCTCGCTGCTTGGCGTG
CCTCTGGATATTTGGATGATGAGGTGGGACTGCTGCAGGAGGGCTCCGCGCGGACGCGCC
AGCAGTCGCAGC

>at02298
GACCGAGGAGGTTGCCCTTGCGGAAAGCATGCTTAGAAAAGGTCCATAGAGCAGGAGAAG
GAAGCTGCGAGGAATGCACTGTGCCAACGTATTCAGGTTGGAGCAGGGAAGGAGAAGGAG
CTTTTGGCGGTCAAGTCTGAGGCCGAAGCTGCTGGATTCCATGATGTTGTGGCTTTGGCC
GAGAAAGCCATTGGGGA
>at02299
GGAAGCCGTAGCCCATGTTCTTCTTGGTTCCGAAGCCTGTTGGCAGGTAGAAGAAGTCAA
ACGCACCCTCAAAGCCCTTCTGCAGCATCTCTTCAAGCAACTCTTCGGGCGTGTAGCGGT
TTGGGATGTTTCGGAGCATCACCGAAGTTGTGCCTTCAGGCCACTTGCACCCTCCCTCGG
TTGTGCCTTCAAGCCACTTGCGCCCTCCCTCACCAGGGGTGTCTTCTAGCACCTGCTGTG
ATAGG
>at02300
CACGGCAACGAACTCCTTGCAGTCGTCCTTGTCAAAGGGATGGGGATGCCGAGCTTCCTG
CACATGGCGATCACAATTTCAACCACTGAATACTCACCATGCCTGTTGATGATCACAGCC
CGCACGCTCAAGTCTGCCTGTGCAGCAGGCTGTAAGTCATCCTGAGGCTGGTACAGAAGC
AGGTCGTGCCGGCGAAGCTAGACCCAGGGGGGCACCAGTCTCAGCTTGCGCCACTCAAGC
ATACAGCCAACAACACCACAAACGGAGCGGCACATGCTGCTAACGTCATACGCATCCGCA
TCCGTTGCGAGGTGCTTGAGACCTTGTTGAGCTCCAGAAGGTCCACACTGTCCAAATTTA
GATTGTCGCATGCTCTTGCGCAGTGGCTGCGGCAATAACACAAACGCCTTGGTACTGCTT
TCAGAAGTCCCAAGATGCGGCACTAGGAAGAGAGGCAGCGA
>at02301
GCGGTTCACGTCCTCGCGGCCGCGCGAGGGGGGCCGGAGGCTGCTCTTCCAGGTGGACCC
GCAGTCCGCGCTGAACGGCGTGGCTCCCGGGAGCAACGCCACCGTCGTCGCCGAGATGGT
CTTGGCGGCGCTGCCTGGCCAGGACACCCAGAAGGCGAAGATCATCCACGTGATCTCCTG
CTAGCTGCTTGGGGGTCTGCTGCCGGCCGTCTGCCTTACAGGGCAGGTAGGGCATGGAGC
ACCCTTGCCGTTTTCCTTAGGAGGGCAGACAGGGC
>at02302
CCGTAGCCATTTTGGCTCAAGCCATTTCGGCCCAAGCCGCTGCCGTATCTTCCACTCCCA
GAAGCCATGAAGGTCGTCGTCAAGGCCGCCAGCGGCCCGCTGGACTTGGAGGTCGGGGAC
TCCGACACCGTCTCCGACCTGCTGAAGAAGGCCATGGAGAAGCACAAGTGCCCGCCGTGG
GCGGACGGCGTGCAGCTGCAGGTGGAGGGCCAGGACGAGGACCTGGCAGCGGAGTGCTCC
AGGACCCTCTCAAGCGCAGGCATCTCCGGCGGCGCCGCGCTGAAGATGGCCTACTACCAG
GACGTCCTCCCCGCCGACGCCAAGAAGCTCAAGCTCTCCGGCATCGCTCCGGGCACGGCG
GGGCCCTTCCTCGCGAAGAAGGCCTGAGACAGCGCGCCGCGGAGGCCGCTGGCGAGTCGG
GCGCACTTCCAGGATGAGGGCAGGCTGCGCTAG
>at02303
TCAGTCCAAATTGGACCCAACTCTCTTGGTACCGAACTCTGGCGATTGCCCCGCCTTCGA
GTTTGAGGCAGGCAGCATGCCTGAGCTGAGGTGGCTTATCCATCGCCACTGTCTGCGACC
GGAGGAGTTGGTCTTTGGGGGTGTGCTTGTGGCGACTTGAGTGCCCTCGCAGATAATGCT
CGGAGGCAGCAGCGGCGCAGGCAGCTCCAAAGGCCAAGATAGTTACGGCCGCAAAGACGG
CAAGGAGGAAGAACAGGAAGGGAGGCCGGGGGCAAAAGGGGAGAGGAAGACCACGTAGCA
GAGACAAGGGCCCTGGGAAAGCTCCGGGAACTCACGTTGCGCAACATGCAATTGATGCGA
GC
>at02304
CAGGAACGACCGCGGAATCAGCGGCTGCTTCAATTCCATGGACATTGCCGGCCGTGTGTC
CGTTGCCGACCCCTGCTAATGTGGCCTGGGCTTTCATAATATCGACCATGAGGCCTTCCA
GAAGATCTATGACAGGTGTGCCGGCTTTGTGTCTGCAGCCGCTTCGGAGTTTTAATGACC
TGCGCTCTCACGAAATCGACCTGGAACTACTACCAGCTTTCCGGTCACATGCCCGTTGCC
GATCCGGGGTTTCGTAACGCAGACTTTCGCGAGATCGTCCATGAGGCCGCCCAGCAGACC
ATGACCACTTCTCCCGCCGTGTGTCCACTGCCACTTTTGGGTTCAGTGACCTGGAC
>at02305
CCCTCCTCAAAGCCATCGAGCGCGTTGGTTATTTTTTGTTTAGTCGGCCGTTGCATGGCC
CGGCCACCACCTGGGAATGACACGGGTTGGTCCGGCCGCTCCAACGGGATGAGCTCGAGG
CCTGCCGGCTGCCTGTCGGCGTGTGTCCGCACAGCCACGATGGCATCCTCGCGGGCAAAG
CGCAGACCATTGACGGGGCTCACGCTCACATGCTCGCCGACGTGTACATAGCCACCCTTG

TGCACAGTGTGGTTGACAGCATCCCCAGTCCAAGCAGTCCTTCCGGTAGAACCGATCCTG
CCATCATCGCTGCCAAGGAAGAGGAGGTCACGGCCAAGGT
>at02306
GCCACCACAATTCCGAGCACTGCAAGATAAAACTCCAGGATAAGCCTCTTGCGGTCGCCC
AGAGCCTGCAACCAACCCAGGAATCATGAAGTGCGCTGAGCAGAACTGCATTCGTGAAGT
CGATCTGGATGTGACAATCGGAACGGCACTCGTAAGAAACACCCACGTTGCGACAGATTC
CGATCAAACAAAACTGTGGCAATTACCACTACCCTCAAGCTGTTGGTGCCACGCCTTCGC
AGGGTACGAGTCCTGCATCATGCCAGCTCACCTGCTTCCGGCCCAGCTTGATGGTGTTGT
GGGCATGGCAGGCCACTGGCCGAATCCATCAATGAAATCCTCGACACAGCCATCGGTTGA
AGGCTGCATCCATCTGCAATCAGTCATCA
>at02307
ACATCGAGGAGCTGCGCTTCGAGTGCATCACCGCAGCAGACCTCCTGGCGGAGACGGGCT
TCGCCGCCCGGGACGTCGCCGCCATGGTGATCGACGCCGAGGGCCTCGACAACAAGATCT
TGGACCAGTTCCTGCGCATGCCGGGCTTCCAGCCCGCCCTCGTGATGCACGAGACTCCAG
TGCAGGATCGCACCTTCCAGCAGCTGCTTTGGCGCCCGCTCAAGAAGCGCGGCTACATCG
TGGGCGCCACCATGAACCGCATGAACGTGGTCTCGGTGCACGAGAGGCTGTTCCGGTGGC
CTTGGAGGGAGTGGCTGCTGCGCTGGCTTCGGCCTTTCGTCCCCTGTGATGCACAGACCT
GCGAGCCGGCGCGGCGCATCTTCAGCTTCAAATAAGAAAAAAAAAGAAAAAAAAACAA
>at02308
TTTTTTTTTTTTTTTTTGCAGCATTCTTGCGTCTTCGAACCGCGGTCCCCATTGATTCG
CATGCCTGCTTGCATTGTGATCTCTAGGCACGGCGTCGCGGCCCATCTGTAGCGAGCCTC
TCCGCAACCCCCTCCCTCACTGCCCCGCAGCTGCGCGGCATCAGCCCAGCTTGTGCACCA
ACAGGAAGCCGTCGAGGCCGACAGACGCAAAGGAGCCCTCACCGCACCCAGCCACTCCCG
TGACGCCACCCTTGTGCGCCTCGGTCACCTGCACTTTCTTGTCCGGCGAGTCCACGTCGA
AGACGTAAAGGTGCCGGTCGAGAGATCCGCTGACAAGGCGGCGGCCTCCTGGGAGCCAGT
CAAGGCTGGTGACCCGTGCGGTGTGGAAGGACCAGCTGGAGATGTGCAGCGTGGCAGTGC
CATCCCCCAGCTTCCAGACCAAGATCTTGTTGCCTGCATCGGCCGACGCGAGG
>at02309
TTGCGTGCTACGGAGCCGATTCCCAGGGGCCCATCTGAGACTGTGCTTCTGCAGCCATGA
CCTCCCGCTTCAAGAAGAACAGGCACCTCCGCGGCCACATCTCCGCGGGCAAGGGCCGCA
TCGGCAAGCATCCGCAAGCACCCAGGAGGCTGCGGCAACGCTGGCGGCCAGCACCACCAC
CGAACCTGGTTTGACAAGTACCACCCAGGCTACTTCGGAAAGGTCGGCATGCGGAACTTT
CACCTCATCAAGCACAGCAGGGTCATCCCGACCATCAACGTGGAGAGGCTCGTGGTCTCT
GGTGCCGGAGTCGTGTCCGCTTGGAGTACAAGGACAAGACCGACAAGGCGCCTGTGATCG
ACGTGGTGAAGAAGGGATACATGAAGGTCATGGGCAAGGGCCACATCTCCTCAACAGCCA
GTGATTGTCAAGGCCAGGTACTTCACAAAAAGAGGCGGAGTCCAAGATCAAGGAGGCCGG
TGGCGTCTGCCATCCTCCGCGCCTGAGATGGCGAAGGATCTGGCCGGTCATTGATGGGAT
GGCTTGGAGCGGAGGACTGATGTCTTACAACATAATAACAAAAAAAAAAAAAANAAAAAAA
AANAAAAAAAAAAAAGAAAAAAAAAAAAAGAAAAAAAAAACAAAAAAAAACTAAAACTAAAC
GTAACGTTAACGTAAGTAACCTAAACCTACGTACTTACTTACGTA
>at02310
TTTTTTTTTTTTTTTTTCAACCGGCGATCAAACCCACAGCCATTACATCCGGAGTATCC
CTTCAACTGCAATGAATCACGGATTGCATGCACAATGTCCCATCCATGATTCCCACCAAT
GTCACCAAGTGCGTAAAAATCACCAAGTGCAAACTCCGTGTAGATCGGTTCATAGCCGAG
TCCTCCCAAGGTGTTCAACGTCACAATGCCAAATGCAGTCAACATCATTACAAGCAACAC
GCACCTCTCATCCTGGTCACCATCCTGCACTGCATCACCCCTCATGGTGCGATCGCATCA
TAACATACAAGCTTGCGCCTGGCAGCACAATGGCATTCTACGATTGACCAGCTATGCGTA
ACATCCAAAATGATTACTCAATCATGATGCTGCCACCTCCAATCGTCAAAGGCACAAGAA
CTGAAATAATCAAATATACAAAGCAATCGCCAATCGCATACACAAACAGCTACCCAACAT
CCCAAGTAGCTTTTAACAACAAGTGCTCATTCCAATGCTGAACTTTTTCCGAAAGGGGCA
ATCAAGACGATCAGGGAAAGCTGCGGTACATGTGCAGCACGTGCAAATTGCCAGCGACTC
GACTTGGCGTTTGGCTAAACATGTTTTCGTTCCGACTCTATGCTGAATGCGTGTCANAAG
CTTGCTGATATGAATCGATGCGCTCACATGGGTTGAGGTCGTGTTCGTATC
>at02311
CCGTAGCCATTTTGGCTCAAGAGGAAGACATTGCCCTCTTCAGACCGGATGCTATAGAAC

AGCGCCCATTTCTTGTCTCTCCGCTCAGACCTCGTCTCTTTGGGGGATAGTCCACCTCAT
TTCCCAGTTCATTGCTCAGTTGCACGAGTACACGGCTTGGAGAACTTGAACACAGCGGAT
TCTGACGATCTGAATCTGCCAGCAGCCTCCTCGCGTTGCCTTGGATTCTGCATACCCCTA
CAGCTCCTGATCGGTGCCGCCTCGCCCGTCCTGTTCCTCAACTTTGGCCCCAGCGAGCTC
ATCTTCTGAAAGCTGCTGCCCCGGCCTAGCATCATTGATCTCATCGTAGCTCTGCGGACG
CGGT
>at02312
CACGGACCTTCGCGCACTTGCTGCCTTGGCTCACAAGCACGGGGTCTTGCTGAGCGTCGA
CAGCACCATGATGCCACCGGTTATCTGCAAGCCCCTCACGCTTGGAGTGGACATTGTCGT
ACACTCCGCAACGAAGTTCTTCTCAGGCCATGCAGATTGCACAGGAGGGCTCGTCTGCGT
GCGGGACCCGGTCCTGGCCAAGCGCATTGCCTTCCTCCAGAACGCCGAGGGCACGGCGCT
AGCGCCGTTCGAGTGCTTCCTCTTCCTGCGGGGTATCAAGACAATGTTCTTGCGCGTGGA
GAAGGCACAGGACAGCGCCCAGCACGTCGCGGCATTCCTCATGAAGCGGCGCCCGCACAT
CCAACGGGTTTTCTTCCCgGGCAAGAGCAGCGGCTGTGACGCAAAGAGCCTCCGGATCCA
TAGCTCCCAGTGCCAGGGCCCCGGGTGTATCATGTCCTTTACGACGGGGTCTGTGGACTT
CTCTCGCCGGATGCTTGACGCATGCCGGATTTTCAAAACCACCGTGTCCTTCGGCAGTGT
CAACAGCCTCTGCGAGATGCCTTGCACCATGAGCCACGCATCCACTCCCCGCAGAGAAGA
GGACGCTCGCCCGACG
>at02313
ACGTCCATCATGGTAAAACTCATGCGGGATGGCGTCTCTGAGTTCAGTCTTACGAACTGG
CCAGTTGACACTGGACATTGTGCAGGCTCATCCGGAGGAAGGTATGTGCGGACATCTGGC
AATTGGTGTGCTCTCAGCTCAGGCATCCGCGTGACACTGACCACAGGGGACAGGTGCAAG
CATGCAGCTGGGCACTGGGGGCACAGGCAGGTCATTGATCTCCAACAGCAGCTGTTTTTT
GCCCGGCAAGATGATGAGCTTGTGGCGTTGGAATCGCCCACACCTGAGCATCTGTCAACA
TGTCTTGCTGTGCATGTAGCAGTGGGAACTCGATTCCAGCAAG
>at02314
TCGCATGTTTACtTTGGtCtTCACTTCaAACAGACCCGAGTACCGTCGATGGATCCAGTT
GATACGAAGGCCCAACCTAGTgGGGGCAGGTGTCtTTTCGCTGAACTTGCTCCGCAAATT
GATCGAATGCAAGCTCCAGATGACCACGACCTGCATGTAGTGGGCAAGCTGGTGCGAGAT
TGTGGtTTTTGATGTGGAATGTCTGGTGaAAGACAGGCTGGAACTTGACGAGCAGCCAGG
CAGAGACATTCATTGCtTgGTTGGGAAGAGcCGCCCACGGACCAATCTCAAACCGCATGT
CATGGTACCAGACAATCGAG
>at02315
TTTAAATTATTTTAATATAATATATAATAATTGATATAATACCAATAGTATTGAAAATAA
TTTATTTTCTAATTTAATATATTATATTTTTTTATAAAAATAAATTATTAATTAAAAAAA
AAGTTTATCAATTTTTTGATAATAATAAAAATATTTATTTAACAGATAGATTTATAAAAG
GTATATGTCCTAAATGTTTATTTAAAAAATCAATATGGCGATTTTTTGTGAAAAATTGTA
ATATTAATTATGATTCAATATATATAAAAAAATCCAAATTTT
>at02316
TCCTATTTTTTGGTCTCAAGCCGCTCTTCAGTTGCACGCCGCGCAAGGAAGCAGCAGCAT
GGCGGGTGTTGTCGAGAAGCGTCTGGCCGAGCtTCGGCTTGtAACATCCCCAAGGCGGTC
GCGCCCGCCGCCAACTACATCCCGTGGTGcCCGCACgGGGGAGTCTCGTCTTCATCTCAG
GCCAGATACCGAAGACTGAGGACAACCAGCTCCTGAAGGGCAAGCTTGGTGCGAACTACA
GCCTGGAGGAGGGCAAGGCGGCGGCGAGGCTGGTGGGCA
>at02317
CTTACGAGATAGCCGCCGCAGGAAACGTTCAAAGCAATGATTGTCTACCTAGATTGCATC
TCCATCACCGCGGCGAAGTTGTGACCGCAGAAACTTCTCCATCATGATCACGGTTGGCGT
AATCAACACGTTGCCGCCATTCTTGTATACCAATTCCTTCACTAGCTTGGAATACCCACC
ACCACTTGGAACAAATGTTTCGCTGTTGACCATGAAGACAACATCATCATCTGGCGCTTG
GCCCGCCCGCACTTGCACGGGGTGCCCCCTCTCTTTCATGTGCGCAGCGACTGCGTATGT
GTACACGCACGACCTTTTGAAAGGCTGCGTCCATGTTGCGTAATCTGGTTGGTGAGGTGA
CCCAACAAGAGTGACCGGCTGGCCAAGTAGAAGCCTAGCAGCGACCTTGTCGTAGTGCTT
CAAGGGCATTACATATCTGTTCGAACGAGCATTACCTTTACTCGTCCAATACCAGGTTTG
ATGTCTGAGCATGTCTACGACAGAGAAATTGGCATTATCGACCACGTCGCCCATCCGGAT
GTGTACCGTGACACCCTGGTTCTTGGTCTTCTTGCCGATCCTCCTCCGGACAATTGGGAA

GAGGTGGGTCAAGGCCTCCTCTGTACGGTCTCTTTTTGAGGGAATGCGCGCTTGTATTCG
CAGTACAAGGAATCAGGCCAAAATCCTGCAAACGACCCATTGCCACACGCGTAGCTCGAA
GCGTTACGAGTCCCATTAATGCCATAGAATCTACCCTTTTCCTTTATGAAATCGTTCAAT
GCGTAATGGCCCCAATAGCGATCCATCCGGAAAGCTCCAAGTACATCAGGATGTTCACAC
GCGGCTGTCGCGTTCTTCGACGAGCGCTTGAACATCTTTCGTGGGCGGAACCGCTGAGCC
TTAAAGAACGAGCCGTGCTTGAGCCAAAATGGCTACGGCAAATGAATGAAGTACTGTACA
GGAGGGGGGGGCATGTCAGCAGATACGGCCCAAGAATGTCTACGCGTGACTTTGAGCAT
AAATGACTTGAGCCAAAATGGCTACGG
>at02318
CCGTAGCCATTTTGGCTCAAGCGTCCAGCATGGCTCAGCTTCTGAACCATCTGGGCTTGT
GAGTTTGAGGAATACTACTTCTATGTTTCGAGCATGCCCATTCGGGCTTCCATGGTGGAC
ACTCCTTACCGCGGGTACCGTCCTGGTTTCAGGACAAGGACCTCAGCATTGAGACCTGTC
TCGACCTCATCAATCAAATAGGTTCCGCAGATCAATTCCTGGGCATGATGCCATGTGGGG
CAAATCTGCTCATCATTGTCGAGCTTGCCTGTCAATGCCAGAGTCGTCGGAGAAGGAATG
TGAGTCTTACCAGCAAAT
>at02319
GGCACGAGGAAGGGCAGTCAGTGGCAGAGTGCACTGAGACTCTTCACTGATATGAGGAGT
GCTGAGATGGTCCCTGATTTNGTCNNTTACAATNCCGNGCTTGAGGCAACGTTTGATCAA
GCGGTTGGCTGTGAGCTGTTNTGCACAGCACTCAATGACAAGGTCTTCAATGGACTACTG
AGATGGAAGGGTCCTATTTTTGACTTGCACNNNCTTTCCACAGGTGCAGCGATGCTNGCN
GTGCAGTGGCGTTTATGCCAAGCTCCACCCGGtCGaGNTNTTTGGCAGGCGAGCCAGTCG
CATATTGCGGATCATCACAGGCTGGGGCAAGTCGCGGAAATCGTGGGGCACGTCAGACAT
TCGAGCTGCTGTGCAGGAACTTCTTCAGAGTCTTGGATTGCCTGTTATAAATCCTGAGTG
TAATCGAGGCGTATTGGACGTGGACATACGGCACTTGGATGACCCATTTGCTCATTGATG
CGTGCCAACGCGATAGGTTACGGTGATTCATGCATGATTGAGAAAGTCCGATGAC
>at02320
GACTGTATCCGTTGTGGCTCCAGTCATTCAGAAGAGAATCGGAGCCCCCTAATTCAGTTG
TGGTAGGTGATGCTAGAGCGTGCTCCGCCTGGCGACCCTGGGACAgGCTATaAAATTGGC
ACCGGcCTCCCTTGGCAGTTGATGGTGTTGAAGGCAGTAAAGTaGGCCGTTCtAGTAATG
ACTCTGCAGCTGGTTGtCAGAGTCTTGCGGAAGCCGATCAGCTATCTATCCCTTTGCCCG
CCGCGCAT
>at02321
CATCAGCACATGCCACGCAACAATGAAGCCAATCTACATGTTGTAGTCGTGCAGAGCATT
GACGCACGTCAGCTAAGCCTCTTGTAAACTAATTACTTGTCGGTACGTAGGAGCACTATC
CCTGCACCTCCGGTGCGCAGCTTGTGCCGTGGCTCCGCAGACTCGATGAACCACTTGTGG
TGCTATCAAGATNCCGGGAACTGTTCCATCCCATCTCCTTTGCAATGGGCTTTGAACTAC
CGTGCAACTCCGAGGCTGGTCCGTGTTGCCAATGGGTCCTCACAGAAGCTCTCGTCGCGC
CCGCTGGTGCCGAACGCGTCGACGCTGGCTCCGTTTGAGTGCCCGTGATGAACCACTGaC
CTCTGACTTCCTCTCCGGCGTTGGGCACATGCAGCATGCCCAGAAGCCACGGAGCTTGTT
CTGCGCACGCTGCTCCTCAAGCTCCGCCCGCAGCCTGCTCACCTCCTCGAGCTCCTCCTG
CAATGCAGCCTGCGAGTCTGCGAAGGAGGCACGCTCGGCCTCGAACCTCTCCTGCTCCGC
GCGCAGCTCGTGCTGCTCACTCGACAGCCTCTCCAGCAGCTGCTGCGTCTCCTCCTTCTT
CCTGTCGATGGCCTGCACTGCGCGCATCATCTCATTGTGCAGAGTAGTCTTCAACTCCTC
GAGGGTGTCCTCCATGCTCGAAGCCGAGGCATGTACAGAATGTAC
>at02322
GGGGGGGGTAAAGCCGGGCAGATCAGCATGGGGAAGATGTTTGGAAAGACCCGCACGCTC
CAGTTGCTGCCAGGGTCGTCCGGTCAGCTCCTCCACGCCATTGCAGTCGAAGCAGAGGAC
GTCTCGGTGGCGTCTTGGCTTCGCTGCCGGGGCAGAATGGGCACCTATCGCTGTGT
>at02323
GCCTTGCCGATGTCGTCATCCTGCCTTGAAAAGTAGGATGCTGCCGCGAAGGCTGCTGCG
CCCACCCAGATGCCGCCGAGCAACAGGCCGGCCAGGCCTCCCGCAAGACTCACTGTCGTG
ATCGTGTTCTCCTCCGTGATCTTCACGAGGGCCGTGGACTCGGATGCGCTGGGTGCCGGG
GCCGGCGCTGACTCACCCTCTGCCCGCAGAAGCTGTGAGTGCCGTGCTGGCGCCGCACCC
AGCCCAGACACGAAGTTCACCGCCGGCACCAACGCCGCCCCAAGCAGGGCAACGCCCAAT
GCGAAAATGCTCAGACGAGCCAGGCTGCTGCGGCGCTTGCTCGCGCCGCGCGCAGCAGAG

GATTGGAGGAGGCGGCGTCCAGCGAGGGCCATGGCGTCCAGGGCAGCTACTACCTTGCGT
CCGCAGTCTTTGATCTTGAGCCAAAATGGCTACGG
>at02324
TTTTTTTTTTTTTTTTTTGACGGCATGGGACCTTCGTCCGAACCGGCAAATGGCGCGTGC
CCAGCCTCTGCAATCCAAGGCCTCCAAACCAGGGCACCCGTCACCATTGGCCAAAGACCT
TGGTCACTATCGCAAGCAGCAGGTTGGCGTTTGCCCTGAAGGCGCCAATGGGTGGCACGT
CGCAGCTGTGGCCGTCGCCACCTGGCGTGGAGCCAGCCTGCTCTGCAAGTATCAGTTTCT
GGCAGGTCCGTCTGAGTCGCTGGAATTGGTACTGCATCTCGTCAAACAGTGGGAGGTCGA
CATATGAGAGAGCGGCAAACGCCCACTCAGCAAGGTGGTTCAGCCTCTCTGAGTGCACTT
CGAAGGGGCTAGCGCCATCCGAACTCGCCAAGGCAGCACTCCCGTCTTCAAAGGAATTGT
GCACTTCAACGATCGCATCCACGACTGCAACAACGAGGTGGTTGATGCTGAGGAAGTCCT
GCGCCGCCAGCGTCGCGGTCGACGGCCGCGAGCCCCCAAAGTGCTCCGGCCACGCCTCGC
CCTCGAGGTGCTCCTGCGGCGCCCGGGCGGCTGCCAGGCGCCGAGCCTCCTTGCAGCGCT
CCCGCAGCTCTCGGAAGGCCTCTGCCACGTCCTCGGCCCACTCTGGACAGTAGCCGAGCT
CCTCTGGCACCTCCGGGACGTCGAGCTGCGCCAGCAGGCTGCGGTGCTTGTCGCCTTGAC
GCTTTCGCCGCCTCCTCGGCTTCTCCACGACCTCTACGTCAACGACCTCCGGGACGTGCA
TGCGCTCCCACTGGACCTGCCGGAGATACTCGTCCGCGTCCTTTGGAGGCTCGTCACTCG
GCGGCAGCGGCGAACCGGAGACAGGCAAGCATGCCTGCTGTAGCATCTCTTGGTCCCGGT
CAAGCTCTTGCTCATCGACCTCCTCTCCCTTTACAAGAACCTCCGCTGAAGCCTGGCCAT
CTCTTTTGGGCTTCTCTTCTGCC
>at02325
TTTTTTTTTTTTTTTTTTGCCCTCGTCGCCCCTCTTGTCACACCGGAGCATGCTGTGGAG
GACGCAGAGCCACATGTGGACCTACCTGCCTCTCCACTCTGAACCACAGGCCTCACTCAG
GCCAGCTGGCAGCGCACCCCTAGGCCGGCCGGCCTAAGCTGCTGAGTTCGCCCCGAGTGC
CTCCGCGCCTCCGGCAGCATCGCCACGAGCGGCTCTTCCGCGATGCCGAGGCAGGAGATT
TCGGCAAAGACGGGCGAGTGCACCGCCTCCTGCACTTTGTTCGCCGGCAGCCATGCCGGA
ACATGGTAGGCCACGGCGCAGGCGTAGGGGTCGTGGAACACCAACCGAAGCGGCAGCATG
ACGTGGAGCTGACGCCGCAGCAGCTCGGCGGTCCAGAAATACCGGCATCCAGAGGGAACT
TGAAAAGCAGTTGTGAGCATGTGCCTCCCACGATGCCGGTCGGCACCAGCTGCACTGAGC
GCACGACCGCTTGGGCTTCCCATGCGCGCACCCTGAACATTGAGAGCAGTTCTGCTTGAT
CTTACCATGTGGGCAGCCGCTGCACTGCACACAGGCAAACCTCACCTTCNCGTGAGGGCA
ACCATGGCANATCTTGCAAGCACTCTTGAGCTTCCCGTGGGGGCACGCATTGCAGCGTGG
GCAAATGCGTGCCACTCTGCCGTGTGGGCACGCGATGCACTGTCTGCAATCTCG
>at02326
CAACAGCCTCTTCTCGTGGTTCGCGTCGCGAATGTGTTGGTGCCGCACGTGCTTCAGAGG
CTGTAGGCCCTCCCGCAGAATCCTGTGAACTGTCGACCGTGACAGTGGCCGCGCAAAGCG
CGTGGAGAGCTCCTTCGTGACCTCGGACACTGAAGTTTCCGGGTTCTCGTCCACGAAAGC
CCGGAGCTCCTG
>at02327
TTGTTTTTTTTTCTTTTTGAAGCGCGCTGACAGCAATGCGCATGCCGAACCAGTGCCAGT
TCAAACACGTCCCTCAGGGACCGTCTGCGAAGGCTAGGATCATCAGCGTTGTACATGCTT
CTGCCGGATCAAGGCCTCGCGATGCAAGTAAGTCTCGTCGTAAGGCCTGCAGTTGCGGGA
CTCCTTGCCGCTCCCAAACTCTTTGACCTTGTCCACAGAGCGGGGGAAGAAGGCCACCTG
GCTCTCCGTGAGGAAGACGCCCGGGCGCTGGCGGTCCCAGTGGAAGTCCGGCGCCTTGGC
CTCGAACCCTTCGCTCAACCGCAGGGCCGACTCGCTGCGCGACCGGGCGAGGTGCTGCTC
CTGCTCGGTGATCTCGGCCTCAAGAACTTCTTGGCGGAAGACATGTGGATTGGGAAAGGT
CATAGGATCCTGCGGCATCTGGAAGGGGCGCACAGTGGTGGGCTTCCTCCTTGGAGCGCT
CTCGGCAGAAGACTTCGGCTCTGAGACGCTCGAAGCGGACAAGGATCTTCTTTGCTGTGG
CAGCTTCGGGATGACGAATATGGATCTTGGCTCCGGCTCCGGGGGCGCTCTGTGGAGAAA
CTGGAAGCGCGTCTCCTCGTCCCTGAGTACATTCCTGAACTCCTCCCGAAGGTCCAAGCC
CTGGGTCCAGCGGTGGATCAGGTGCTGCTCCTTGGGGATGGCTTCAACCACCCGCGGCTT
AGAGTTGCAGTGACGGCCACTCTTGCCAGGGTTCCCCACGCTACCATGGGAACGCCCTGA
GCCCCAAAGGAGGCTCTGATGCGAGAGTGCCATGGCTTGCCGTCGGTCCCATCCCCTCCG
CCCGCCCGCCCTGCCTCTTTGTGAGCTTGAGCCAAAATGGCTACGG
>at02328

CCGTAGCCATTTTGGCTCAAGTCCTCCTAGGCCCCTCCACCCTCGGCCCGCCCTCCATGG
CGGCCGCCGTGGCGGAGGCGCCAGAGGGCGAGGAAGAGTCCGCCGGGCCGCCGGCCGGAG
GCTGGGCAACCTTCGAGGACCGGGCAACGGCCCTCGAGAAGGCGGAACCGCGGAAGCCCT
CGCTGGAGCGCATCGAGGGCCTGCTACGGATGCATGTGCTGGCAGCCGAGCCGGAGAACA
GGGGAAGACCTGTGGGACGATGACCAGGTCCGGGCCGCGATCCTCGCGGGGGCCACTCCG
CCGGACCCTGATGCCGGAGGGGACAAGTACGCCGAGGACTACAAGGAGCCATCGCCGGAA
GACGACATCGTGCGAGTGGTAGCCTTCGGCACGCTGGTCTTCCTGCTCACGTCCCCGGTC
ACCTGCCGGCTGAATGCTG
>at02329
CCATAGACGGACGGCCCTTGGCGCCGCCAACTGCCGCCATGGAGCTGGTGTGCACGTAGT
GCTTGACCTTGAACTTGTTCACTGAGCTCAGGATGTTGAGCGTCCCTTCGGTCGAGGGCT
GCACCATGTCCTTCCACGGGTCCTTGACCACCCCGGGATCGACCTGGGCTGCTGCGTGAA
TGACCGCATCCGCGTTGGATACTGCATCGTCGTAGGAGCCCTTGGTGACCAGATCGGCCG
AGAAGAGATGAAGCTCGCCCTTGCCGACTTCCTTAGCCATGTCCAGCAGGAACTGGTTCT
TGGGGTCGCTTTTGTTGCGCACGCAGGCGTTCACTGTGTAGCCCCTCTCCAAGCATTCGC
GGACAATGTGTGCCCCGATGAATCC
>at02330
TTTTTTTTTTTTTTTTTTAAACCCTTCTCTTTTTGTTGTTGTTGCGTTGGAGAAGGAATG
ACCGTCTGCGCGCTTACCAAAGCCACAGTCTCCGCTCTTATCAAAGACTCACGAAGGCGA
ATCTCCCAGCTGGCGTAGGAAAATGTGGGCTGCGATCCTGCCCCTCCTGCAGAAGTGGCG
CCCCCTCGTGGCCCAGGAGAAGCTTGGCCGGCACGTTCTCGGCCGAAGATGCCAGACTGC
AGCCCAGGTCCGAAGGCCACGCGCCTTGCCATGGCGAGCGGTGGCAGCGGGGTGTAGTTT
CCGCTCTTTTCCTCGTCGAAAAAAAAGGAGGCTCGATGTGCCACGCTGCCTCCGAGCAAT
CCTTGCTTAGGACTCCTCCTCGAGGTTATGCTTGGCGCTCTTCGCTTCCATGAGCCCAAG
CATTTTGCCCCAGCACTCTGGGTGCTTCAGAATCTCAACGGACTTCGTGGGCACGCCCTC
TTGGATGTACTTGTACTCACTGTCCATCCAGTTCTTCCACTCGGCGTACGATGCAAGGTC
GTAGTCCAGGGTACTGGAACTGCTGGAGCCCATGTACTTCATGCGCTTGGGGGGCGTGGG
TACAACGCAA
>at02331
AGCACATCCTGACGCTGATCGACCTGGGGTACACGCGCTTCAAACTTGTGTGCCAGGACG
ACGGTGGGGCGAGAGATGACTGGGGGCACACCAGCGGGCCCTTCGGCGAGTTCGGCTTGG
ATGCGAGCTGGGGTGGAAATGGCGCGTCTTCAACGGGTCCAAGCCCAACGCGGACGCGAC
GGGTTTTGCCGCGGACTTATTGCGTTCCCGCGAGCTGTGCAAATGGTGGGACATCCACGC
GGCTAAGCCGGTAGGCTAGGCTGTCTTGTCTCGGCGGCAGGCGGTCACACCGGCATT
>at02332
NNNNNNNNNNNNNNNNNNNNNNNCGAAGCGGCGCTCCTCGGCACTCGCTGCTCCGAGGCG
CCCTCAGGCGCAGTACTCGATCCGGCCGGTGATCATGCCCCAGTAGCCGTCCACCGTGAG
GCAGTTCTTCCTGAAGCTCTCTAGCCTGGGAGCGAGCTTGCGCCACCAGCGATCGGTCAT
CGCCTTGGTGATCTCGGACCATTTTTCGACCATGACGAAGGGGAAGCCCAGCGCCACGAG
CTCGTCGTGCGCGGGGAAGACGGTTCGCTGGATCACGGGCACGGTCAGCACAAGCAGCGC
CTCCAGGCTCTTGGCCGTCTGGATGGCCGAGCCCAGGGGGTTGAGGAGGAACCTGTACCT
CCCCAGCTCCGTGAACCACTCCGCCTGCGAGAACTTCTTCATCTCCACACCCACGGCCTC
CGCCTCTGGCGTCGCCGCCCAAGTCCGCATCGCCAGCCGGCTGTAGTAGGCAAAGATGAC
GGCCTTGCAGGAGGTGTTGCTCGGAGGGTAGAGCACCCCTGGGAGTTCAGCTTCTTGGCC
TTCTTCACGGTCGCCAGCAAGTCGAGCCAGCCGGCCCACACGCTCCGCACCGCCAGCATG
TTCTTAGTCTTGCTCTCCACCCTGTTGGAAATCATCGCCTCGAAGTCCATGAACTTGTCC
GCGGCCTCGTGCC
>at02333
GCTCTGCCACATGGAATGCAAGCATGTTCATGCAATTTGCCCGTCTCATCCGAGACACAC
CATTGTTTTTTTTTCTTTTTTGCGATCTGGGCCCTCATTTCTGTACAGCTGTACACTGGC
GCAGGTAGCGCCATGAGATGCCCAACTGTACTCACGCACACATGAACGGAGACGTCCATG
ACGGCAGTGCGGGTGCCATGCCGCCAATCGACCAGCTAGTCATCACTTGTAGCCACCCCC
CTTGCCGCTCTTCCCCTTGCCACCCTTGCTGCTGCCCTTCCCGCCGCCATACTTGGGGCC
CTTCTGCGCCGCCCTCTTCACGCAATCGTTGATCCAGCCGCCGAGGGAgGGGAACTTCGG
CTGAGAGGCTCCAATGCGCTCAAAGTAGAGCTCCCAACTGTCGGTGGAGGCCTTCTGCTC

ACGCCACAGCTTCTGCAANCGCTCCAATCGCNGAGGGACCTTGCAATTNGTGGCCTCCAT
CTCCNCGAGCGTCTCNTGTGNNTTNCCGATGACNANNTTNATGACCATGCANTGGAACAT
GCAGATCTCNCGNGANATCTTNGTCTCCTGGAAGACNGGCGCGGGCTTCGGCTCGATGCC
CGCTCTCTGCCACCACATCACACATCGCACGAAGTTCTCATCCGCGTAGGCGTCGATGAA
CTCCTGCTGCGAGGGGCAGCCCTCCAGCCCCTGNTANACNGNGTACAGNGCCAAGGCCAT
GCCNAGGTCGGGCGTCGCGTCCTTGTGCCGGAGAGCTTCGCTCTTGACGAATCCTTCAAG
CTGGCGGCGGGTGCGCTCACGGAGGCTTGGTCGCGTCTCCACGAGCCACCGCAGAGTTCG
CCAGAAGTTGCACAAGGCCTCGAAGGTCGCAATCGCCTCAGTCTTTTCCTGATCTGGGCG
TATCATTTCCACGATCATCTGATTTATCAGCCGCGCGAAGACTTCAACGATAGCATCGTC
CTCGCTTTTGACCTCCCGCAGCTTGTCTTGGTTCTTCTT
>at02334
CTTCTGGTTCAATCCCTGCTTGGAGACAGCTGTCGTCTGTCGCCATGCCTCTCATTGGCC
AGAACGCCAGGGATGACGTGGTCTTCATTGCCTTCGGTACCATTTTGCTTGTTGTTCTCC
TTGGGTTGCTCCGCGCTGTATGTTCTCGTCCGGGCTTCTCGCAAGTGGTGGCCCACCTTA
GGGCTGCGATCAAGGCCGTCAGAGCTTGGGATGGCATGGCAGTCGAGGAGGTTGACCTCA
TCGTGTGGGACAAGCTGACGAAGCAGAAGGTAGACAATTTCCGGAAGATGTTCCAGATCT
TGAGCATCATCCTGGTTCCTTTCACCCTTAACATAATGATCAACATCCTGAACCACAACC
TTCGCACCTACTCTCTAACGCAGG
>at02335
CCTCTCCATGATCAGCTGCGACAGCGCCTCTCTCTGGTGGCTTTCGATCGTTCGCCGGCC
TATGGCTGAGGCAAGCACCAGACTCGAGTACAGAATCATTGTGCCCCACGCTTGAGTCCC
CTCTGGCCCACCAATGATGAGTCCGAATGCTGCGTACACCGCGACGGAGAGCACTTCCAG
TGGAAAGAGGACGACCCATCGAATTGGCAGTGTCAAATGGCTTGCCGTCATCAACACGGA
CAATATAAAGTACCGGCGGGGTGTCCGAATGCACCACATCCGAGGACCAAATGTCCCGCG
GGTTGTAACTTAGGATCGTTGCGCTGGTAGAAGGGAGAAAATCAGGAAGGGAGCGACAGC
GAGGAACATGATTACCAGCACTGTCACAATC
>at02336
ACAAAGGCaCGaNGCTCATCCGCATTGGtTTTGGAAGAGATGATCACCTTCATCCGCCAC
TTNTATCCAAACGTCAAGGACAGCtACCtTTCNTGGAGAGTTGTGGGGTGGCAGACCTCA
TCACCACCTGCTTTGGCGGCCGCAACCGAAAGTGTGNTGAGGCCTTCGTCCGGGCCAACG
GCACAAAGTCTTGGGAGGTGATTGAGAGTGAGCTGTTGGACGGTCAGAAGCTCCaAGGGC
ACACTCACAGCAGAGGAAGTNTGGCCAGTGATGAAGGAGCATGGcCTNTGCGAGCGCTTG
CCGCTGTTCACGACAATNTATCAGATTGCATTTGAGTCAAAGCCGGCGGCCTCCATTGTC
CAGCTTCCGCAGCCAGTTGTGTCATGAACACCCTTCTTGTCACACTGGTGATGTGCACAT
AGCGAGCCGCTTCATACAGCTGCCTGCGGCGCTTGCTGCACTCGTCTTTTGAACCCGTAG
GGAGAACTCAGTTTGTATCATCGGGACTTTT
>at02337
GGCGAGAGGGTGCCTCGCGTCCGGAGCCCAGCCACGCTGCTCAGAGAGCTGCGCGACTGC
GGCCTGAACTTAATGCCGGAAGTCGCTGACGCAGAGTTCCTTGACGGCTACACGCCCAAG
GACCCTGAGACACAGGCGCGGGCCTACTCCGACCTCAGCGAGATCGCTGGCTACTACGAC
ATTGCTTCCTCGAGACACAACAAGAAGTTGCCGAAGGAGCAGGCCTTGGTGCGGGTGAGG
ATGAACCCCCTCTGCGAGCACTTCGACCCCCTGGACCCCGACTGCGACACGGACTACCAG
GCCATCATGTTCTATCCCGACAAGGCCTGCTTCAGCCCGTCCTTCGAGCTCCAACCCGTC
AGGACCGAGGTGATGCCCGGCCACACGACGCATGCCAGCCTCTACCTCT
>at02338
TTTTTTTTTTTTTTGCTTATACTCTGTTGTTGCATCTCGGCCTCAGCTGCTGCTCAATCC
GCGCTCACACTGGATTGGCTGGAGCCTCTGACTGCGCTGAGACCAGCGTCTTACACCACA
CAAGCATGCAAGGGGAACAGACTTTAGACAGCCTCAATTGACCGGCACCAAGTGCTATTA
CAGTGCACATGCTGGCCTGAGTGCTGCAAAAGGCCTCAAAAGAGAACAAAACCCTGCCGA
AACGGCAACTGCCCCTCAGGCAGAATGGAGAAAGCCTCCCGGCAGCATACCCATCCGGAG
TGCGGTGCCAACTGGGAGTTCAGAGTACAGATAGGAAAAGAGCAAAGTGAAACCTGCTGA
AGTAATCAGCGAAACATGCTGCATGCACACGGCACATGAGGCCCTTTGACCTGCTCGAAT
TCCTAATCTCTGGCAGAAGAAGGATGAGCATGCATCAAGCCTGACGCTTTGCACCATTCT
GCAGCTTCTCCACGGTGATGTTGCCATCCTCCAGGTGCCACTTTTCGTTGCAGATCCTGG
CGACAAAGTCGGACTTGGGCTCAATCATCACGATGCCCCCCCGGAAGGTCTGCAGTGCCT

TGGCCAACGCCTCCACAGTCTCAACGTCGAGGTAGTTGGTGGGCTCGTCAACTGCAATCA
AGTGGGGCTTGGTCCAAAACATGGCCGCCAAGGAGAGCCGGACCTTCTGGCCAGCGGAGA
AGCCTCGGATCTGCCTG
>at02339
GTAGTCAGACATTTGGCGTTCGGCATTGGATCAATTTTTGTATGCTTTGTAGGCATTGAT
GACAATTCAACAAGGAATGGAGGCGTTCCATCGCCCAACAAGCTGTGCTTTTTGACGGCC
TTTTCCAGCCGGCAGCTTTTCCAGTGCGGCATCCTGTGCACAGGCCGGTTGTTGCCACGT
CAAGAGTGCACTGTCTTTGGTGTTCAACACCTTTTGCTTGCGACAGGGTCCTCCATTTGC
CATCCTTTTGCCAACCTGTACCCAGCATTGCTCCTAAGGCTCGGTA
>at02340
CTCGTCGCTTGTCAAGGAGTGGCGCACAATGTACAGGGCCGAGACCGGAACTCCAGAGCC
GGCCATGTCCGCGGCAGTCCGGTCCCGAAAGTCCCGGCGCATCTCCATGGACCTTGGGGA
GAGCGCCTACGGCCGGCACCAGAAGGTCGCCCACTACATGGAGGTCGTCGACTGGACGTG
CAAGATCATTATTAGTGATAATGAGGTCAGCAATGGATTGTTGGCGAGGACTCTAGCAAG
CAAGGAGCTCCTGGCCCGGTCTGCTGAGGAGTTGAGGCAGCAGAAGGCATGCCGGAAGCG
TCAGCTTCCTTCTGGCTGAGTCCACGCCGTCAACCCAGTGATGGCATTCTGCATGGGCAT
GTAGCACGTAGCACGTAGCACGTGTATGCTGATGTAT
>at02341
CCCGGCCTCTCCACCGCGCCGAGCCGACCCCGCGCGCCGCATGGCCGCTGCTCTGCCTGC
GGTGGTCGTGGTCGACACCGACAACCACCGGGTGGTGCGGTGGGACCCCGGCGCTGCGGA
GGGTGTGGTGGTTGCTGGAGGCAACGGCGAGGGAGACGGGCTCCATCAGCTGTGCGCTCC
CTGCGGGGTGGCTGTCGATGTGGATGGTAGCATCCTGGTAGCCGATTGCGCCAACGATCG
AGTGGTCCGATGGAGGAGGGGCGCCAAGGAGGGGATATTAGTGGCAGGCGGCAATGGGAC
GGGGGCAGGGCTGAATCAGCTGGACAGCCCTTGCGGCATCGCACTTACGAAGGACCGCGC
GGTGATCATTGGGGATTGTGCCAACCATCGAGTTGTGTCCTGGGCCCTTGGTGCAAAGCA
AGGGGAGCTCATCGCTGGAGGCACTCGTGGGGCAGAGCTGGATCAGCTGTCTAGCCCGAA
TGGTGTTGCCGTCGCAGCAGACGGTTCCGTGCTCGTGGCCGATATCGAGAACAACCGCGT
CGTGCGCTGGGCCCCTGGAGCCACGCGTGGCGAGCTGGCAGCTGGTGGGCAAGGGCCTGG
CCCAGAGCTGAACCAGCTGGACGGCCCCTGCGGTGTTTCAGTGGCGCAGAATGGTGACAT
CATCATCAGCGAGCGGGGCAGCCAGGTCCGGAACTACCGCATCACCCGCTGGCGGCAGGG
CGAGCCACTCGGCGCCGTGGTGGCCGGGGGCAACGGCCACGGTAGCGGCCTGGAGCAGCT
GGATCTGCCCTGGGGTGTGCTGGAGGCGCCGGACGGAGGATTCATCGTCGTCGACAGCGG
CAACCATCGGGCAGTCCATTGGTCGCGCGGCGCTTCAGAGGGCCGCGTGGTTGCAGGTGG
CAACGGTCGTGGTACGGGCGTCCACCAGCTGAAGAGCCCATGTGGGTTGGCCTTGGTGGA
GTGACGGAGCTGCCGTTCGTATAGCTGTACTGGTGTTATTCAGGCCGCCCTCAGCAAAAA
AAAAGAAAAAAAAA
>at02342
CCATTTTGGGCTAGCCTTGATGCTGCGCTACTCCTCCGCACGCGCAAGACTGATGGATCT
CTTCCAGAGTCCCGACTAATACAAGCTGTACATGAACATTGGCGCGAAAACGCATATGAT
GCCAGGGTTGTCTCCTCTGGGGCTCAGCCAAATCCAGTGGGGAGCCCCATCTGCTTTAGC
ATCTTCATCTCCTCGGTCTCCTCCTCTTCCTCTTCCTCGTCCTGGCCAGCCTCGGTCTTC
CGGCTCTTCTTCGCAGGCTCGTCTCCGTCCTCCTCGTCCTGCTCGCCCTGAGCGCCTCCC
TTCTTCTTTTTCTTCTCCTTCTTTCGGCGTTTTCGCTCCTCCTCAGCCTCCTCGGCAGCC
TGAATCTTGGCAGCCACGTCCTCCTTCTCTGGCTGCTGCTGCGACTTCTTCCTCATGGCT
TCCAGCTTCTCCTTGACACGATCCACGCCGATCTTCTCCACTTTCATGTTCATCCCCAGG
TTTCTGTTGTGCCTCCTGCCGTTGACGTGCTCCAGGTAGGCGGACGAGTCCTTGATCAGG
CACTCGCACACGCTGCACCAGTAGCCGCCCTGCATCGGCTTGATGGTGTGCTGCGTCACC
ACTTTGGACTGGCCCAGCGTCTTGTCCAGCTGCAGGTCCACCGACCGCTGCTTCAGGAAC
GTGCGCTGCGAGGAGGGCAGCGGGATCACGGGGTCCCGCCTCTTGCCCACCTTCTGGGGG
CCCATCATGATGTTGTCCATGGCCTTCTGGGAGTAGAAGTCCTTGTCCCAGACCTTCCTT
CCCATGGCGTCCACCGCGTCATCTTCCGGAGCATCTCCACTGCTGCTGGCCGACTTCCCC
TTCTTCTCAGCCATGGCTTGCGGCGCCAGAAGTGGCTTGGGGTGGGCCAGGGCTGGGGTC
CCAGCTTGCCTTGAGCCAAAATGGCTACGG
>at02343
CATTCAAGGGACCAAGGCGTCGCACACGGTTCGTCCATCAGTAGGCATGGCATTGAAGGT

GGACTGCACACTGGCTTCAGAGGGGCTGCCTACTCTTCTCACCTCTCGGCTACACGTGCC
CAGCAGCGGCAAGAGCCCTGCAGGCGGAGGGAGAGAGGTTNAGCCTCTGGTGAAGTTGCC
CGAGGAGTATCCAGTGAAGATCCGCATCCGAAAACACCTTCATCGACACTTCTGCGGAGC
GCTCGCCATCGCTGGAGCGTTTCTACCCAGGCCAGCGGGAGTGGCGACATGCCCGTGGCC
CACATCGGGCG
>at02344
TCAATCGGAAACCACCATGATTGCCCCCTCACTTGCTGCCACCATCATCATTGTCACCCT
TGTCAGCCTTGTCAGCGCCCTCGGTGGGTTTGACAGCTTCTTCCACTGCTTCCTTCGCTT
CCTTTTCTGAGAAGCCCACGTCGACCGAAAATGCTGTCAAGTCCCGGCGGGGTCGAGGGA
TGCAGACGCTGGCAAGGCTGCCTGCGGCCGAGGCATTCCACAGCTCGTGATGGCCCGACA
GAGCAGTGGATCCCGGTTGCTGGTGTCCCAGCACTTCGGCGACTGCCGCGTGCAAGACCC
CACTGGGCATTGCTGCGACTCACAAGGGCGAGATGGCAGCAGAATGGGGACAAACATGTA
TTCTTGGATCTTCAAACATTGCCTTGAGCCAAAAATGGCTACGG
>at02345
CCGTAGCCATTTTGGCTCAAGCAAGGTGCAGGTTTGTCACAATTGTTTGACCGTTCTTCC
CCGCCTCCGTCACCAGCCACCATGTCTGCCTCGTTCAACGCCTTCGACCGCAATCATGAT
GGCGTGATCACCCGCGCGGAGTTCAACAGCGTCATGGCGGGCGCCACAGCGCCGGCGATG
GCAGCCCCGATGATGGCAGCCCAGGCTCCCGGGGTCGTGTCTTACGGCGCCCCGGGCGTC
CAGGCCTATGCGGCCGCACCGGCCATGATGACGGCAGCACCCGCAGTCACGACCTATGCT
GCGCCTCC
>at02346
TTTTTTTTTTTTTTTTTGACTTGCACAGATCCAAGACAAACTTCGGACCGCATCGTAGT
TAAGCCCATCATGGAGAAATGCAATGCCAGTGCTGCGGCAGTCGACGAGACTGCACACAG
CAGAGGGCACTACCCACCGCGCAGCGGCAGACTTAAATGAGAATTGGACGCGGCCAGCAG
TCAGTCACTGATCACTCCTCTCCGTCCAGAAGGCAACTTCTCGAAGCGCCTCCACTCAAC
CGCATCTCCTTACTGCCTCTTCGCCTTCAGGTCCGCGAGCTCCTTCTGCAAGGCGTCAAT
CTGTGACTTCTGGGCATCCATCTGCCTCTCGTCGCGACGTTGCTGGTCCTGCAGCATCTG
GTCCGACATGCTCGAGCCCATGGATGGCACAGGCACTGGGACCATCGGAGGGCCAAACAG
ACCCATGGGAGGCCCGAAGAAGGGCGATCCAAACATCGGCGGGGCGAACAGCGGCGGTGC
GATCATAGGGCTGACGCCAACGGAAATGTTGGGGCCAGTCGAGAAGCGCGGCCCGGCTTG
TGCCTGCCGGTCGGGCCTCTGCTGCGGTGCACTGCGCATGCCGCCGCCCATGCCGCCCAT
GCGGCCGCCGCTGCGTGCCGCCTGTGCGTCGCCCATGGGCACGAGCGCGACAAGCAGCGC
GGCGCACGCGGCCAGAGCCATTCGCCAAGAGCTGCTGATGGCTGCCGTANCAGGCTCTGC
TGGCATGTCCACCCCGTGGGCAACTTCGGGAGCATGCTTCCATGTGGTANNGCACGGCTG
GTCAGACTTGGGCAACG
>at02347
TTCACCAAGGAAGATTTCCCTTGAGTGCGCTGTCCAACAATGGGGACAGGAAACTTTGAA
CACACATCCCGTGAGCGCTTACACACACGCTGAAAGAAAGCCCGATCGAGTCCCAGTACG
CGGGTGCCTGCGAACGCCTCCTGAAGATGCACACCCTGCAAGATCTGTTCATTTGTAAAT
TTGTTTTGTGCCTCTCTGAACACCAAGGCGTTGATGTAATGGTCTAGGCGCCTTCGTCTC
TGGGCCTTCTTCTGCCTCCGGGCGACATCCTCAAAAGCACCTCTTCGCCTTGAAGTACAA
GTCTATAGATATGTCGGCTCCTACCAGTGCTGATTGCCAGCTTCCCATGACTACGAAGCG
GGAGTGAGAGTGCAATGGAATAAGTGCCTCAGCC
>at02348
CTGGTAATGGGACACACGCGTCTGCAAGGTTGGACCAGGCACCGCCGCAGATGTGGCCAC
ATCAATTTGTTGCAGACCATCAATGCTGCTTGATACTGCAGTAGCCCTCAGGGGAAGGCA
ACCGTATTTGACTGCAACAGGAAGGCGCTGTCCGCTGCACAGACCACATTTCTGAGCACC
AAACATTTCAATGGTTGTTCGCTTGGCACAACCATGTCACCGTCGGGTTATTTGAAATGA
TTAGACACGACCAGCACCACTTG
>at02349
TTGTTTTTTTTTCTTTTTTTTTTCTGTTCATAATGCGAAGCAGACAGAATCTTCACTATG
TGACGTATTCGCCCTGCAGGCTGGGATTTCGCCTAGAGAAGGAGTTCCAGCATAGCTCAC
AATAGATGTCCCCAGCCTCTCCGTTTAGTATGTGCTGGACGAGTTGGCTGTCCGGGGTGC
CGATGCAGTCCTCCTCAGATGTACACCTGGGGTGCCCACCGCTCTTGCCTTCTGCATCCT
CAGCATCTCCACCCTCGACATCGCTCTCCCCGTCCAGCTCGTCGCCTTCCTCGACCCCCA

CCATTGAAGCCCCTGAACTTCCTCGGGTTGGCCTTCGCACTCAGATGCTGAGAGGAATTC
GGAGTGGGCACCCGCACCTTGGCTCTGTTCTTTGACTTGGCCTTCGGATGCTGTTCAACT
GCAGCCTCCTCTGCAGCCTCCTCGGCAGCCTCCTCTGCAGCCTCCTCCTCCCCGTCGTCA
TTCCCCCGTCGGCGCTTCTTCGCATGGCTTCGAGCCGCGCTGTCGGCCCAGGAGCTGAGA
AGCTCCTCGACAGCCTGGGCAATGAACGGCCAGTCTGAGGGCGGCCGACCTATAGTCGGA
GAGAGGGCTCCAGGTATGCAAGTGAAGGGATCGTCGTCGCCANACGGGGCTATGAGGGCC
TCGAACAGCTCGACGATGATCTCCCCAGTCAATTCTGCTCCAANCTGCTCGACAGCCCCC
ACGCACGATTCATAGCTTCAGTG
>at02350
CCATGGCGCTGGGCCCCCCTCTTCCTGCTGATGTCCTGCGTCTGCGATGGCTACATCGGA
GGGCGGCAGAAGGCGCTGAAGGCCAGCGTTCGGGGCGGAGCGCCGAGCTCGAGGGCTGCA
GCCGGAGGCCCTGGGGCCCCTGGAGATGCAGCTCTTCACCAACCTCTACATGCTGGCCCA
CCGCCTCGATCTTCGCTGTGGCCCTGGGCGCTTCTGGCCGAGCGTGCGGNTGCTTGTAGA
GGGTCCCCGCCTTGCTGCGGAACGTG
>at02351
TTTTTTTTTTTTTTTTTGCTCCGCATGCCAGACGTCCAGGACAACCTCTGTTGCGCCCC
CAGCCGGCAGGCGTGCCTAAGTCACTGGCTCACATGCCCACCGGGCCCATGCCCACTGGG
CCCTTGCCCAAGTGACGTCCAAGTCTCTGCTGAAGATCACTGCCAAGGCGCTAAAAGACG
ACTGTCTGTTCGGGTGCTGCTTGAACGCAAGCTCAGGAGGTGCGCTGCCATCCACTGCCG
CTTCGAAGCATCGCTCCTGTGGGTCAAGAAAGAGCACGGATGATGGCTGTAGACAATACT
GCAAGGAGGGCAAACCACATGGCGGTCAGCACCAGCGGCCAGGCAAGATGCAGTTCCTCC
TCGTCCCCCGCCTGTGCTGGCGTCGGAGCAGGTGTCGGACCACCGCTGGCCGGCACGGCT
GTAGCTGGCGCGACTGACTTGCCATTTGCCTTCCGGGGCTCCAGCGGCTTCTCAGCAGGC
CCCTGGCCAGGCCAGCCTACGACCTCGAACGCCTCCACTGGTGAGGGGTTGCACTTTGCG
AAGTCCACGGTGGTGCGCCCATCCGCCGTGAGCCGCAAGTTGTCGCCCCCCCGCGCACAGG
AGCGCACGCAAGACGTACTTCGACCCGTGGTGCGCGGCCAGGTGCAGCGGCGTGACGCCG
TTGGCGTCGCCGGNGTTCGCGTCCGCGCGCCGCGCGCAGAGGTGCCTCACCAGCTGCAGC
TGCTCGCCCGCGGCCGGGGCGGAGAGCGCGCATTGCAGGGCCGTCCGGGCCTGGTCGTCC
AGGGGCCCGCACGTCCAGCCTCTGCGTCTTCGGGGGAGACGGGGCCGAGCATGTCGCGCA
CGCTGCCGAGCGACCGCTCCGC
>at02352
TTTTTTTTTTTTTTTTTAAGACGCCCTGAGATGCCACCAACCACTTCGGCACCCTCTGG
CACCCAGCTGTTTACGTAGTGCCAGGGCCAAGTCTTGCAAGGAAACAGCCTACCACGCAG
CTCCTACACCCAAAGCTACCACTGGGAGTGTAGGCTTGCCCGGTCATGTGCAGGCATGCT
CACTCAGACAAAATCTGAAAATCCTAAAAGTGAAAAAATGCAAGTCTTCGCCCGGAGCCA
CATCGCAACACTTTGGCTCCATGACTGTACCACGGAAAGTGGACCTCAGACTGTTCCAGT
CTACGCTCTAGACTCCGTTTGCCCGGCCGCTCCTCTACTGGATCGGCCGCAGGCATGCCC
CTATCAGGCGTTCCAGCGCATGAGCGAGGGGAACGTCAGGCCATCCCCCGTGGGCCCGGG
AGCCAGCCAGGAGAGCAACCAGCGGAAGCGGCTTATCGGGCTGGGCCCGAAGACCTGCTG
GAAGTTCCGACTCCGGCCCAGGTCGTACGGATTCCGGAAGTATCCCCGTTCTTCCGAGCC
TCCTTGCGCCGCATGATGTTTGTCTGGAATTCTATGGTCGACTGGTTGCTGAGTACCAGG
AACACGTGAAAGCCCCCAAGCACGCAGAGCGCGACGAGGATCGAGCAGCAGATCATGAAG
GATGTCATGATGCACTGGCGCGCCGAATGGGGACCTCGTTTCCGTGAGAG
>at02353
TTTTTTTTTTTTTTTTTCCCCCTTGCTCTTGAAGGTGCCCCTGTCGTGGACGTTGGTGT
TGGGCCAGCGGTAGCCGTTGCAGAGGAGCCTGTAAGCATAAGCAAAGCCCGAGAAGTCGC
CAAAGTACGGGTTGCCGTCCATCCTCGCTTTTCGGATCAGGCCAGTCATCCAGTCGAACG
TCTCGACCCACGGATAGACAATGTTGCTCTTGAGCAAGACTTTCTGGACGTAGTCCCATC
TGATCTCCTTCTGGCGGCTGGCTTTCGTGCTCTTCTGCCAGGAAGGGAAGGGGCGCCAGG
TCATGACGAACTTGGNCTTCGGAGCCAAGTGCAGCACAAGAGGCAGCATGTCTGTCGCCG
GTTCCAGGGAGATCGCCTCCATCCGGCATCGCGTGGCGTACTCTGCCCAGATGGNCGGGT
TCACCTTGTCGANCATGATCGCCTTGGCGAGCGTTG
>at02354
CCGTAGCCATTTTGGCTCAAGCTGTCGGGCTCAAGAGCGTCCCTCCATTCCAGGAAGTCT
GCAAGAAAGTTCCAATCTGAACCAACTCGTTGCGTTGCAAAATGGCCCTGCGTGTCTCGG

CTCGCCTTGCTGCCAGGCTCGGTGTTCTCGTGGTCCTTTACTCATGCATGCTCGCGGAAG
CTGCTGCCACTGAGCAGTGCCCGAAAGTCTCCGGCTACTCTAAGTGCCGCGCCCACAAGG
ACCTCT
>at02355
GGGCCGCCCGCGTGTTCATCTCCTCGCCCGGGTGCCCGTCGCCGAAGCCGGAGGTGAAGC
TCAGGAGCATCCGGAAGGTCACGGTCGACCGGAGGTCCTTGGGATCTTGTGTCCACCAAG
GCAGGTACTTGGTAGACCGGGTCATCCAGTGACGACACTGAGCCATCATTCACGAGCCCC
GCAAACATCATCGCTGACGGCCACTTGCTGGTGCTTCCCGTCGGGATCGGCGTGCCCAGC
TTGAAGTTGCCGCTCTCGTAGGAGAAGAGCCTTCCATGCTTGGCGTCCCCTACCGTGATG
GCATACTCCGTGGTGAACTCCCAGCCCGCCAGCAGCTGGCGCAGGGGGTCCCAC
>at02356
GGAGTTCGCTCTTTCTTGAAGGGACGTCTGCTGCGCAGCGGCAAGGCCCCGCGCAGCCGC
CCGGCACCCGCTCCTAATGCCTCTCCGCGTCGCCACTCTTCGAGGGGTTGAAGCTGTCGA
CCTTGGGGAAGGGCTTTGCAGGCGTCCTGACCTGCAGGAAGTCGCACAGGGGCCCCCAGC
CGTCGGTGGGGGTGAAGATCAAGAGCTGCTCCGGCGGAACCAAGTCCCTGATGCGCTGGG
TCTCGTTGACGTACTCCTGGGCGCAGGCCAGCTTCTGCTTCGTGGTCATGTTGGCGGGGG
TCTTCACGAGCTCCCTCATGCAGGGGCTGAGCTTGGAATCAAAGT
>at02357
CCGTAGCCATTTTGGCTCAAGCTCGAGCTTCCTGCCCCGCTCCCCAGAAGCGGAGACAGC
ACTGCGGGGCAGCTCGATTTGTGTCAAGCGCCATGTCCTCAGACCCCAACCAGCCGGCCG
CGACCCAGCAGGTCATCGGCGCGGCGGCGAACGATCCGAAGGTGCAGACGGCGGCCATGG
AGGTTGCTAGGGAGGCAGCGCACGACCCCAGGATACAGTCCGCTGCTTGGAACGCGGCGA
GGGCTCGCGCGGAGAGTGCGGTCCAGGAGGGCGCCACCCAGGCGAGGGCTGGCTTCCTCG
AGGTGAGGACGTACGTGCAGGAGTCCAACTACGGCGTCCGCGCATACTGCTTCCTTGCAG
CAGTTGCGCTGCTTGTCTCCTCCATTCTAGGCATCTTCAACGTCTTCAACGCCATGTTCA
GACCGTTCCAGTACTTGTGGGCAGTCTACAACGCGGTCTTTGCCGCCGTTATCATCATCA
TCGACGGGAAGCCGGAGTGGTTCACCAGGTGCTGGGACGTGCAGGCGAAGCTCTACAGCA
ATGCAAACTTCCTGGCCACGTGGTCGGGGCGCGCCATGCTCTACTTCTACGTGGGGTCGA
TCAACCTCTTCTTGCTGCCCGACACGTGGCTTTGGAAGGTGGTGTACATCTGCATCGGCG
GTGTGCTCTGCTCCATTGCCGGCTTGATGATGATGCAAAGCTGTGGATGCAGGTGCTGCC
AGCCCCCCAGCAGCCGCCACGAGGGCCCCTGAGGGGCGCCCTGGCCGCCCGTGTACAGTG
TGGCGTGTCGTGGCGGGGCACAGGCTGGGCAGGCGCGCCCCCAGTGACGTTGCTGAGGGC
CCGCGCGATGGTGAGAGTAGGGTGATGGGCATGTGAGCGTGGCCATCTCTGTGGATGGCG
GCATCCATGTAGGTGTGCATGTTCTAAAGTACAGGCTCCGGCACGTCTGCGCAGATTTCA
TGCAAGAGACATGAGACCAAGCATCTTGTCGGACAAACGGAAAAAAAAAAAAGAAAAAAA
AAACAAAA
>at02358
TTTTTTTTTTTTTTTTTGGCCACGAAGGGTGGACCGATGGTTCAATCAGCCAAGCCATC
GACGCATCGGCGGGAAAATGAAGGCTCCGCCGCCGGCAGCCCTGCTCTCATAAGGCCTCG
CCCTTCTCCGGGCTCCCTCCCCAGGTACGCCCCCGCAGCAGGCACAGAATGGCGACGAGG
CCCTGGAGCAGGGCCACCGCGAGCCGGGCGGCGAAGCTGTAGACGCGCATGTTGATGTTG
TGCAAGAGGAAGTAGAAGGGGATCCGCGCTAAGAAGACGACAGGATAGTCTCGCTCGAAG
TTGAAGACGTTGATCGCACGCTCCAGCTTGCCAGTCCGAGGGCATGGGTCAACCTCCAGG
AAGTTGCAGAGGTCCTCCCAGTTGTTCTTCCTCATGCTCCACTCGAAGAGCCTATCCTTG
GGGACGGTATTCCGCACTTGCCGGTAGAATCCGTCGTAGATCTCCTTGTTGAAGGGGACC
ACCGGCAGGCCGCTGTACCAGTGGCTCATGATGCGGCGGCCGCCCACTAAGGTGTGGTGG
ATGGCCATCCACGGCTTGTAGTCCTGGCACACGGCTGGCCGTCCCTCGCGCAGCATCTTC
GCCATCGGCCCGCCGATCAAGGCGTCTATCTTGTCGAAGACCCAGACCCAAGGGAGCCCG
TGCTGGGCCGAGAATATGAAGCC
>at02359
TTTTTTTTTTTTTTTTTGCAAGGAGTGTTGAAACGAATAAGACATGCGCGCAAACTAAA
CAAGTGCGCGGTGGGGATCCTTGCTGCTATACATCAATGTGCGTATCCGTGCTCTGATAA
GCATGCAAGCCGCAATGCATGCATGGCAATTCTTGCCACTCGTCTGTACAATCAAGAGGC
CTGCACGCAGCCCTTTGAAAATACCTGACAGTGCAGGTCTCCACTGGCACGGCTATGTCA
CAAAGGTAGACCAACCAAGCGCTGATGACACAATTGCTCGGGGCTCGACCGTGACATAAC

AGGCTGTCCTGTATCCACCCTCGTGCC
>at02360
TGCTGCAGCTGGCGGGAAGATCTTGAGAACAAGGAAGACGACGCAGAAAGAACAGCTCTG
TGTCGACCAAGCTGTGCACTGCAGAGCACAGCGATCACCGTTGATGAGCTCGCAAGTCCC
GATCAATAGGTTGACGTTCGCAGTCACATGGTGCATCTATTGCCTTAGCTTTCTTGGTTA
TTCCATGCGTTGCCATTGGGTCCTGCGTTGCTAAAGCGACAGATCGATTTAGGAGCCTTC
ACGTCATCCTTCTTCCTCGGGCAGCTCTTGGGTAACGTGAGCT
>at02361
GGTCGAAGTGACCGGGCTCGGGGATGAAGTAGTGCAGCCCCCGCTGGCCAGCAATGACTG
GGTGACTGCTGAGTGCCTGGCATGCCACAAGGACACCATGGCCAGTGGGGTGAACGAATC
CATCACGTCATTGTTGGAGTCGATGTCGCTCTTCACCAAGAGGACGTTGAAGCACTGACT
GGGGANCGGCGGGCNCCGCGTAGGAGCCTGCCGGAGCCGCCTGTGA
>at02362
TGGAATCAATCTAAAACTTGCTTTGTCAACTTGGTCAATTACTCTGAAAGGGTAACCAAT
GGGCGATTTCACAGGGGATACGTGATTTGCCCAGGTGAATTGTGGTAACGTATGAGTTTG
ATCAAGGCAGAAGAAGCTCTGGGGGCCGCTGTTTGCCGAGCCACGCTCGACCTCGCGTTT
TGTCTTCACTGAGGATGATGAGTGGTCTTTACGTGCGACCGGCTACCTCAAGGAGTCGTT
GCCGGAGCTCACGCACGTAGCCATCAAGATGTCTTAAACTGTCCTCGTTACTGTGTGCAA
GCACATGGGGGCTAGAGTCTCTTAGCAACTTGGCTGCCTTCTCGTAGGTCTGCAGCGGCA
TGGCGCACCTCACCATGGAGATGCTCAAGAATGGCAAGATTGCCTTCTGCGCAGACATAT
TTTGTGAGTTTTCAAGACCTAAAAAAAAAAAAAAAA
>at02363
CCATGGAGGCTGGAACCTGTCTAAAGGCGCGACAGTCCCAGCCATGCCGGGAGTCCTCGT
GCGGCCGCTGAGCGCCCGCGGCTGCACGCTCGGCCGGAGAAGCGCGGCGGCAAAGCTTGG
AGAGAGGAAGCTGAGCCTCCAGGTCCGAATCAAAAAAAAAAAAAAAAAAAAAANAAAAAA
AAAAAAAAANAAAAAAAA
>at02364
NNNNNNNNNNNNNNNNNNNNNNNCGAAAAGCATCCTCCCTCCCGCAGGGTCAGTCACAG
ACAACAAACGCTGTCTGCAGCACACACAAGCCCCTCTGAGATTGCGTCTACGCGACCTTC
AGTCACGGACACTTGCTTCTTGGGAAAATCCGACGGACGGACAGTGCGGGTCTCAGCAGG
TGTCGTACTGTGCAACTCCGCAATCAAGGCCTCGACCTCGTCATTCCGGACAACCGCTGT
ACTGAATTTGGCTGCCAGTGCCATGAAGACCATCCAGGGGGTGCATGCGTCAGGACCCAG
GCATCCAAGATCTCAGCCAGAGGCAGGCGCTGTCAAACCAGAAGCATGCGTACCACATAT
TCAGTGCCTTCTCAAAGCTTGCTTGCAATGAGCTTCTGGCATGCAGGCATGATCTCAGCA
AGCAAGTTGCGACTCGTAGTCTCGGGCCGGAGCACACGTTCAAGTTCACCGATCACAACG
GCAGTGCCACGCTCGAACGCGCCAACGTGGTTCGCACGGGCTTCCGCCGTANGCGCCTCG
GATGCGTCGGCGCCGACGGAGAC
>at02365
TTTTTTTTTTTTTTTTTTACCCAACGCACAGGAGCCTTTTTCAGCTGCTACCGCGCTCTT
CCACGGGCTCTGCAGCCACTCCAGGCTCTTGCAACAATTCGCCTGCCTCGGGAAAATCAC
CTAGATGGAGCTCGTCATCCGAATCGGGCTCAGACGCCGCCAAGGCTTCAGTCCTCGCGA
GTTCGCCAAGCTCCGGGCGCGCCGCCCAGACCTTGGCCCGCACGGCCTGCAGCTCGGCCC
GGAGCGTGGCCACCTGCGCTCCCCGCACGTCCGTCTGGAGCCGGTCCACGCCCCGCTCGA
GCTCGGGGACGTCCCACTCCCGCTCGGGGGCCTGGCGCTCGTCCATGGAGGCCATCTCCG
CGGCCAGGAGCTCTGCGGCGAGGATGCTGCGGAAGCTCGAGCCGGGCGCCGGGGAGGGCG
GTCCAGCCCAGGGCTCGTCCGCAGGCCGCACCCTGGCGATTCCATGCTTGGCGGGCGACC
GGTGCAAGTCAGCCGCCCCTATCTGTTTCCCAAGCAGCTCCATGTCGGACGCGATGAAGT
GCGACGCATCACTGGCCTGTCTGTCCCAGTCCTCGTCCGCGCTGTCCATCTGGTAGACCT
CCGGGCCCAGATTGAAGACCAGCGTCCGCGGGGTCACCGCCTGGCCGGCCTCCGCGGCCG
CGTGCGCCCCCGCAGTGCAGCCGTTTCTGAAG
>at02366
CTCGAGAACGAGTTTGTGCTGGCGACTTCTGTTGCGGAGTCTATCAAACCCAAGCCGGGC
TATGATCAGATCGACTTGAAAAAGAACGGCAGCCAGGGCAAGGGCGACCAGAGCGACATC
CAAGCAGACGTCACCAATGCTTCTGGCAAGACTACGGATGCTCATGTGAGACCCAGGTGC
TCGGCTCCATCCCAAGAGCGTGATGGTGGCCAGTACTTCGACAAGCTCGAGCCGTTCACT

GTCGATGCTGGGTGCATCGAGTGTGACACGGTCCACACCCTCCTGTCACTGGTGAGTAGG
GCGCAAGGCAGCAAGGAAAAGATGTCGAAAATGGGCCGACTCTGGAAGACGCCACATCCC
ATTCTCCTAGCAGCAATTTTGA
>at02367
CACGTTCACCGGCCGCGGGCTGCGGACGTACTCAATCGGGGCACCAATCCAGCCGAGTGA
CCAGTTGTAGGCAAAAGCGGTACCATTGCGGATGAAATCACGCCCAGCCAACACCAGGGC
ATAGGCACGTAGGGCCGCCTCGCTGGGGATGAGCTTCTGGATCTCCTCTACACTCATTTT
GCGGAGGCTATCCAGGACCTCGTCGACAGACTTGCCCTGACGGGAAGCGATCGCAAGCAA
TTGCTCCTTGGCCCACTTGTCGACAGGCTTCCCGTGGAGCCTCTCCATGACGAAAACGTT
CTTCGTCACAAGGCCCCTGCCAAAGAGGGCCCGGCACCGTGGGTGTGCATCGTCGAAGGG
CTCAGGCACCTTCACATTGGGCACATTCCCGAGGATGCGGCAGGCCTCGCGCTGGAGCTG
CGCCTCGCGGCAGTAATCAAACTCGTTGGCGAAGCTCCGCTGAAGCTGCCTCAGGATGTC
GACGTAGTAGGGTGCCAGCAC
>at02368
TTTTTTTTTTTTTTTTTTGCCTACACCCTCTCAACACGCACGACAGCTTCAATCTACACC
GCTCGCATTTGCCGTCCATCAAGTGGCAGCGCTGGTCAGCAATTCCCGAACGCGAGCTAC
CAGATGCGCAACCATGGTGATCCGATCCCTCTTCATCTCCTTGAAGTTGATGCCACCCTC
CGACTTTCCCAATGTCTCTTCGACGGCCTGCGCAAGCTCAGCCGATAGGCTGCCATCATT
CCCAACAGCCTGTTCCTTGTCCAGCATCCCGAGCTCAAAATCGATCTGCTTCGAGACGCC
TGCCAACATGCCATCAAGGTCGACACCCGGGCCGCCCTTCCGCAACGCCTCCCGTATGAA
GGGGCCGGCAGGTGGTTGTGCTAGTGTAACCAGCCGTGCCAAATTGAGGGTGGCAGTCAG
CGTGTCCATACCATCGACGACCTGTACGTCGCCAGAGAGTGTGGCCCTGAAGACCTTTGT
CAATCGGCCCCTTTCTTCGCTCATGCTTTGCCCAGACTCAGCCAGGGAACGCACCTGTGC
CCACCAATCGTCCTTGAACATGGTGACTACCGCTCCGATGATGGAGTCAACACGGCACTT
CGATATGATGGTGAGGTAAAGCTCGAAACGGCAGGGCCATGCAAATGCCCTGAACACATC
GCCGACTTTCCTGAAGAGCGTGCCCCGGACCTCACGCTCTATGTCAGGTGTCGAGGCCAG
CGCCTCAAGCAAGGGCTGAAGGCAACGTGCTGGATACCAACGGCAGAGTGGGCCACTGAG
CGAGGTGTACGCCCTCGGAGTCTGAACTGCTGCAAGTTTGCACAGCGGGTGCACGCAGGC
TGCCTGCGAAGAGTGCAA
>at02369
AGGCGATGCGCGAGCAGGCTGTGACCGTACAGGGCATGGTTGTCAACTACGCCGCCATCG
CCGTGCAGTGCCCGGAGATGTTTGGCGCTGCGCAAGGTGGGGACGTTGGGACAGCCTTGT
CGCAGGCCGTGGAGGACGGGAAGGTGGGCAGCCTTCTGCTCACAAAGCTCGCAGACAGGC
TGGCTGAGGAGGACCAGGAGGCCTTCACGTCCATCATGACCCCCGTGCTTTCCCGCATGG
CCACGGCCATGGCGGGGAGAGACCTCCAGGACATGAAGGTGCGGGAGACTGGCTTATTCT
GCTCCGTGCTTGCGAGACAGGAAAGGGCTCACCGCTGACTCAGCTCTTGGTAACGCTGCC
CGTTTTT
>at02370
CTCACCTGtTTTGTTGTGAGCGTGGCAGTTCCTTGGAGCGCATTTGCGCCGGTGCCCACC
TAGAGCCATGGCTGAGCCGGATTACTACGAGTTGATCGGCGTCGAGAAGAACGNGACCCT
TCATGAGATCCGATCGAAGTTCNGCGCGAAGGTTTTGGCTGAGCACCCAGACAAAGGTGG
CGATCCCAAGAAGTTCCAACTGCTCAACAAGGCATACaANGATCNTGcACCGNANCgCAG
GAGAAGCGCAGGCGTTACGACTCCACAGGCCGTGCGGAGAAGTCTGCCGAGGAGGAGTTT
GTCGAGGGCTTGGCGGA
>at02371
TTTTTTTTTTTTTTTTTTCCGCATGAAAGAAAAAGGGGGAAGCAATCAACCCGCAAGGAA
CTCAGAAAGGAAGAAGGGGAAAGTGGCTACACAGGCAGCGGCAATCTTCAGTTCGCATTG
CCAAGCCCCTCGAAACGCCTTGGCCGGGCCTCACGTCCAACCTATCTGCTCTTAGTGATC
ATGCTGCTGCCTTTGCTGCTGCCTTTGCTGCTGCCTTTGATGCTGCCTTTGCTGCTGCCT
TTGCTGCTGCCTTTGCTGCTGCCTTTGCTGCTGCCTTTGCTGCTGCCTTTGCTGATGCTG
CTGCTGCTGCTCAGGAGACGGCGCTGGGCAGCACGGCCACTGCCGGAACGTCCACTGCGA
GCGTCTGCGGATCGTCAAGCTGTGCCACGACGATGTGCTTCGGCACCACTCCGACGAAGG
TGTCTTCCGCAAGCACCGTGGGTGCATTGTTCGTGCCATCGGTGGCGGCGATGACCCGGT
TCCCTCGGAACCGGTGGCACCTCAGAAGCCACGTGCCAAGCAAACTACCATGATCATGAC
CAAAACGGCCGTCACGCAGGCCCAGACCTCACCTTCAGAGATGCCCGCCTCCTGCTCCGG

GTCCTCCTCCGCCGC
>at02372
CGCTCACCCTTGGCATCCCTCACGTTCCAGCAGGATGGTGCTCCAAGCCATGTCAGTCGC
TCCACCTTGCAGTATCTGGCACAAGAGGCCCCTGGCATGACATTGACGAACTGGCCAAGC
AACAGTCCAGACTTGAACCCGCTGGATTGGTTTGCCGGGGAAAACTTTCAGATGCCATCC
CAGAGCGACATGGCACAGAGCTGGAGCTGCGGGCAAGTCTCCAGCAGTCGTGGCTGCAGG
TGCAGGAGGTGGGCCACATGAAGGTTTGGGCAGAGCTGGAGCGCAGACTCTGGGCTGTCA
TCGCTGCAAATGACGATGTCTTCGAGTGATTGGAGAGAGGGGGCAATGAGTAACAGTGGA
TGGGGTCAA
>at02373
TTTTTTTTTTTTTTTTTTAGGCACGAAACGCTTCACTTCAATTAGGCAAGCCATGCATCT
TGAGAAGGCCTAATCATGGAACGGGGTGTACATACGTGAGCTGGGTACCTCATAGTAGTA
GAACTTGCAACTCGTTCAAAAGCTCCATGCCCGAGCGCATGGAAAGTGTCGTTCATGGTA
TGCGGAACTTCACCAAAAGCTCTGGGTAGATCTGGGTGTCTCCCCGTGGGACCACAAACT
CTGTGTGCACTTGCCTCTCCTGGCCTTTGCCCTTGCCTTTCCCTTTTGGGATGCCTGGGC
GAGCAACGATGGAGTCATGAAGGACATCAAGTCCAGGAACACAGGGTGGGCGCTCGTGAG
TAGGCAGAGGCCCTTCAGCAAGGAAGGGGTGGCCAAGCGACACACGCGCAATGATGATGC
AGCCAGAAGAATCACTGCCTATGTATTGTGCAGCTTTGCAAGCATCAGTTGTGAAGTAAA
CTCCGCGGCCATACAGGTGGCGGTGCGCCAGGCGCTCGTCAAATCCCTGTTGAACGATAT
GCTGCGCACTCTCATTGGTAGTGCCGTGCATCAGCAACACCTCGTTGGCGAGCGGATGCA
GCGTGATGTGCTCCAGGATTCCCTGGATATTCTTGATGCCACGAGTGATGCCACCAGCCC
ACGGAC
>at02374
TTTTTTTTTTTTTTTTGTCTGGCCACTCGACTTGCCGCTCGAAATAGTGTCCGCCCTAAT
CCGGAAACTCACAGATCCGTTGCCTCAGCTCTGCCCATCAAAAGGGCGTGCCCTCCCGCG
TCGCTGCACTGGATCCCTCTTAAATGCCACCAAGGGCTTCCCATGCTGCGTCGCGGTCTT
TCTGAGAGATCCTCTTCAGTGGCTTGCTGCCTGCGGCAACGCTGCTGCTTGCATTGGGCT
TTGACTTCCCTTGGGGCCTTGGCTTCGCAGTACTCTCCAATGGCTTGGCTGTCCTTTCCA
ATCGGCCGGGGCCTTTCGTCCCAGGGCTTGCCCTCCGAACTCCGTCAACTCCAACAACTG
TCCGACCGTATTCGTCACACCAACCCACGCCCTCGAGGAAGAGGGAGGCCTCCGCGGAGG
GGTCCGCCGGCCCCGGAGCCGTCGCTGTCGGGGCCGCCGAGGNCACGGGGGGCGCCCGTG
GCAG
>at02375
CGTGTGCAAATGCCACATCAGATATGAGTTGACCCCAGCAGCGGGTGTGGCAAGGGTGTG
ACTGGAGATGTTTATTGTGGCCCCGTACATTGGCATCCACAGACAGAAGTTTTGACCGTT
GCTTCGGGAAGCTAGTCCTTGTGAACATGGGGGCCCAGGCGTCGCAGCTGGACCCGACGG
CGGTCTCGCTGAAGGCCAACGNGTTCGCCAGGCAGGTCCAGGAGGCGTGCGATCGGGAGG
ACCTCGCGCCAGTTCAA
>at02376
TTTTTTTTTTTTTTTTTTCGGGCGGAAACAACAATGAACCAGAGCTTCAAGTCAGCATAG
CATGAAAGGCCACCTCTGCTCTCTGACAGGTGTGTTCAGACACGCTTTCCAACCTCTGGT
GCCTGGGAAAACTGCTGTCTCGACTTCATGCCGATGAAGCGCAGCATTTCGCAACAGCAA
GGGCTGATAAAATCCGCAGTGTGAGAACATGCGTGGAAGAAAATGTGGCATAATATCGTC
CCAAAGGTGACAGAAGGCTGGGCCTAGATCCATACACATGGTTGCCTGGGCTCTAGGGCC
GCGCCATGTAATCGCCCAGAACTATGTGAAAGTCATCCGGGCGTCTCTTCTCCGGATGGA
AGCCATTCCCTGCAGTCGACCTCCCATCATCGTACGTCTTTGCTTTGAGAGGCAGCCGGT
ACTCTTTCTTTGTGAAAACTCTCATGAGTTGACCATCATGGGCAAGTGTGGAACCTTTTG
GGAGTACGATGAGATCGTAGCCAACATCGTCATTTCGGTCTCTCCTCCGCGCCCTCTGGT
CGCGGATCGCCGTTCGGCTCGCCAGCGCAGCGGCCTCTAGATCTTGGACAAGGCGTTCCG
AGTAGGCGCCCTGAGCAACGTGCTTCTGCAGACGAGCGGGCGCTTCCCATGGACAGTGAA
TGAAGCGTGCGGGCAGCGACCTCAACTCCGGGCACCAACGACGAACATAGTCACCTGTGG
GGTCCATGCGCCTCGTGCCGAATTCTTG
>at02377
AGTAATTCAAGAACGTCTACCATGTTTTCCTTCCTTGCTCTCCTTTCCACTGCCGCGCTC
GGTGTAGTCAGTGCTACTGGCGGAGTTGACGTCAGCCAGCGCACTTACCAAAGCACTTGG

TCATGACAATGAAAGTCGAATGGCGAAATCTTTCGGCAATTGTTCGTGTCTATCGTTCCA
GTGGTTCGCCCGATCCTAATGGCCCCGNAACTATCAACGATGCCTGGGCTGGTGGAATGT
CCCACGTTGATGGCTACATCTTCCCTTGCTATTCCTGCGGCAATCCTGCGAAGCAGATGG
ATGATACCA
>at02378
CCGTAGCCATTTTGGCTCAAGGATTGCTCCACCTCCGTATACCGTGCCTCTGCTCGCCCA
TCACTTCCTGGTGCCGCGATGGTGAAGACCCAGCTCTTCATCCTTTCTGGCCTTGCCCAT
TTGGCAGCATCAGGTGCAGTGGCTGACGTTTCCTCCCTCATCCAAAGAGACGCCCAGCTC
CACACAGGATCGGCGGTCACATCGCACAACACCAGCTGCGAGTGCTTGGGGTGGAAGGAT
GCATACACCAAGCACGGTGCCAGGTGCGGCGATGGCCACGAGCTGGACCTTGCAAACATC
CCAGGCCGCATCGCCAGATTTGTCCCGCAGCTTGCGTTCGAGTTTTGCAAGATGTACTTC
GAGAACCTGCCCAACGACAACCTGTGCATGAACCAGAAGTGGCTGGAGAAGCCCACCGAG
TGGTGCTACGTTTCCGCAGGCTGTGCCGGCGCCGCC
>at02379
TTTTTTTTTTTTTTTTTCCCCCCTCAGATCTTTTGATGCCCTTGACCAACCGAGTCAGC
TCAGACTCCAGGCTTTTCACCTTTCTGAGCTTCTCCTTGAAGTGCTGGGCTGCGATCTGC
TTTTTCGCCGAGAGCCCCTGGATCTTTCTGTCAATGTCGCCTACCTCTCTCTTGGCCTTC
GCCACCTTCTGCTCATCTGCTGAGTAGGTCTTGCAGGACTGCGCACCCGAGTTGATTGCC
TCTTGCTCCGGGTTTTGCGTCTTGACCCGGCTCGCCATGCCGCCAAACCAGCTTGCTTTC
TTCCTCAACCATGCACAGTCTTCCGGCTCACTCCCGTGCGCTTGCGGCGTCTGGCTCCCA
GAAGCGGGCGCCGTGCCTGTGATTGGGACTGGGGGGACCAGCAACGTTGGGGTGGGTGGC
TGGAAGGGCCACTTTCTGTGGAGTGTCCTGTCGAGTGCATACTGGATGGAAGCTGCCGTG
GAGAACTCCGGCCAAGCCAAGAAGATACAGCAGGTCAGCCACCGTCGGACTTGAAGCCAA
GCCATGGCCAGTGATGTGCGATCTGGATTTCGGCAAAAGACGGGGTCGAGCCTTTTGCCG
ACCTTGAGCCAAAATGGCTACGG
>at02380
TTGTTTTTTTTTCTTTTTTTTTTTCTTCTTGAACCAGGAAATGGAAAATGTGGCACGTCC
AGTACGCGCACGCAGGAGCCCATGCAATGGCCCTTCTCGCTGGCAGCCATCCTCGAGCAA
TGGTACAATATCTGAGATCCTGCGTGTGCGCCGACTAACCAACTCACCTGGAGTCCCTGC
GTCCAGGGCGGCCGCCACCGCCCTTGCCAGCGTCGATGAAGTACTCGTACGGCAGGAAGC
TCCGCAGCATCGTGATGTTGGCGTCGATGCCGGCCTTGGCGTCCTTCAGCATCCTGAAGC
TCTCGTGGCCGGCGCTGCAGCTGTCCAACGCCTGCAAGACGTCTGACTGGCACTCCGCAA
ACTTCTCGATGGCCTGGCGGATGTTGCCCGCCTTCATCAGCTCCTGCGCCTGGCCCTCCT
TCTCCGCAGCCGCCTGCACGGCCATCTCCAGCGGCCCGCCCCTGCGCGCCGGCTTGGAGT
CGGGGTGGCGCATGACCTCGCCCCGGTCCCGGAAGCCGGGGGGGGCACGCGCGGGATGC
CGAGGTGCGTGGTCATGGCGGCGGGTGGGCTGGGGGGGGTGCAGGGGGCTCGGCGGTGCC
TCGTGCC
>at02381
TTTTTTTTTTTTTTTTCTCCAACGCCCATCTTCAGCAAGCAACAACTGTGAGCTCGCCAA
AAGTTGTCAAGACTAGATTTTCCCACGCCCACCTTTCAGCAAGCAACAACTGTGAGCTCG
CCAAAAGTTGTCAAGACTAGCTGTCAAGTGGTTCTGATGACGATACCACCAAAACGGGCT
TCCCCGGCAGAAATGCCTATCACATGGAACTTGCAAGTCCTCCGCTCAAACACCAAGACA
TAATTATGAGAAATGCTATCCGGAGGAACATCCTTTCTGCCGTTCCCTTCTGCATCAACA
AATACGCGCCTGAACCCTCCACACATATCAATATTTCCGAATTCAACACGTTGATTCTCC
TGGCACTCGGCTGTTGCATAATACTTCAAGCCCTCCATGTATGTAAGCGTCTTTGGGTAG
CGCCCTTTCTGGCAAGTCCAGACGTCCTTGATGTTGTGGATCTCAGGGGCGTTCCCTTTG
CGTAGTGCAGACACGGCCATTCCATCGGTGATGCCTGCCTCGGCTACTGTTTGAGAGACC
TCAAGCACGTTATCTGCCAGTATGAGCTGGTATATGCACGGCTCCTCCCTTTGCTCAGCA
ATCGCCCGTGACAAAGCCAATACCGTGTCTGAAGCACAGCAGGATACAATGCCAAGCGAT
TCGCCTGAGACGCCGTTCACAACCTGTACGTTGACAGGATTCTCATGCAGAGGAGGGTCT
GAGGAATTGCGGCTCCAGCTGCTCCCCATGAAGAAGGGACCTTGCCGTTAAGCACGAGAT
CCTTGAAACACTGGGGTGTGTGTGCTTGATGGACATGAGGTTTGAGTCACAATGGCCAC
>at02382
CCTCCGCACAGCAATCGCGGCCTGATACGACACCTTCGGCAAAGGCATTCCAGAACGCTG
CACAGACTTGCGACTTCCAGAACGTCATCGAGATTGGCAAGCATGAGCAGTGGCTGATCG

ACCGCAAGGAGGTGAAGCTAATGGCCCAGCAGGTGCTTGGGAAGGGAACGTTTGGCATGG
TGGTTGCAGGACGCTACGCTGGTGCCCCAGTCGCCGTCAAGCTTATGCCAGAAGCCAGTT
CCAGACTTGAGGAATGCAAGGACTTTCTGGTGTCCATCATCCACGAGATCCGGGTCCTGA
GGCGCTTACGCCATCCTGGGCTTGTACAATTTTATGGAGCATGCATTGACATTGAGAATG
GCAGTGCGTCCTTGGTGCTCGAATGGCTTCAAGGCCTGTGTCTCGAGCGCTTCATTCTTC
AGGGCGGCGTGCCGGACTGCCCATTGACAGCAGCTATCCGGCTCAAGGCCATCCTCGATA
CTTGTTGGGCGGTGTGGTACCTTCACTCGCGGCAGCCGTCCATTGTCCACGGAGATTTGA
AGGGGACGAACATCATCATGGAGAAGGACCCCCTCAGCTTCAACTCCTTCCGAGCAAAGC
TCTTGGACTTCGGCCTTTCTCGAGTCCTGACGAAGCACGTCGGAGGCATGGGCGGCACCG
CCATTTGGATCGCTCCTGAAGTGGTTCGAGGTCAGACGGCCCACTGCGAGGCCGACGCCT
TCTCGTTCGGCCGCCTCTCCTTCTTCGTCCTCACAGGCATCTTTCCGCTGGGCGACTTCA
GCAGGAAGCAGATCCTCCGCTTCCTCAAGTCTGGCAGCCTTCCGCGCCTCCCCTGGCCCA
CCGCCAGCTGCAGGCTGGTCGAGGAGTGCCGCTGGTGGGTAGAGGCCTGC
>at02383
TTTTTTTTTTTTTTTTTGGTGGCTAAGCTCCGATCAATATCCGCCGCTGCGGTTGCTTC
ATCGTGCCAGAACTTACGCTGGCATGCGCGAGCGGAACGCCCGTCCCACAGTGGAGGCAG
TGCTGCGGTCCATCGCAAGATGTCCGGTCAATCGCTCGCAAGATGTAACACCTGCACATG
CCAGTGATGCTGTTAAGATCCCGCAATCTAAGTTCCACCTGTGGGCTTGCATCCCTAGGC
ATAGAGACCCAAGGGTCTGGGGCCGAAGCACGCAAAAAGCCCCGGACTAGTGGCGGTATC
CACTAGATCCTTTGACCTTGAAGGTGAAGCGACATCAAGGGTTGAGAGGGTTGGAAAACA
GAGAACACGCCCGAGAGCTTCCAGAACTTCCATGAATCTTTGAGCGGACATCGACTGGGC
GCCCTCTGAGGAGGGCGGAACTCTTCCCAGCCACGATGCAAGCTCTGCCACTCATCCTTC
AAAGCATCTCCAGCTTCGAACCCTCGAAGTACCCACCCAGCTTCTTTGTGTCCCACAAGC
GGAAGATGGAAGACTTCTTCAGCTGAGAAAGGGCATGGTACTCCTCGGTCTGCCATCGGT
CGCTGTANAAGCTGTAGTTGGCGGCGGGCGCGTCTCAGACCCCTTGTACTTTGGGATCAT
GAAG
>at02384
TTTTTTTTTTTTTCCTTGTCCGAGCAATGCCCGTCACCAAGTAATCCCGAGAGATCCAT
GCACATGCTTGGCTGAATCGAAAAGCTTGTCCACAAGGACGATTTGTTGGATCTGAATAT
CCCAGTCAAGCAAGCTGATTCCCATGACGCGGCATGGGCATGTCTGTGTGGAAAACGGCA
AACGCCACAGAACATGCACACTGCGTTGACCTCCGAGAGGGTCACAGGACTTCGGGCCGA
CATCAAGGAAAAGTCTAGGAGGCCAGCTTCGGACAGAAGTGTTACCTGCCGATCGAGGTC
AAAGTAGCTGGACTGAGGCCAAGTCGGCTGACGTGTCTTGCGAAGCCCGCCTGTCTTTTC
CCTCTCAGACGTCACGCCAGGCGCTCAGCCAGCTCTGGTACTTCGTCAGCGCTTGCCCTG
GAGAGCCTCTGTGCTCTGGTGCGCCGTCTGCGGCGTGGGCTTCCTGAACTTGCTGAGGTC
GAGGCCGGCAAAGTACTTGATGGTCTTCTCTAGGCCCTCGCGGAGCTGGATCTTGGGCTC
CCAGCCCAGGACCTCCCTCGCGAGCGTGAGGTCCGGCCGGCGCTGCTTCGGGTCGTCGCC
GGGGAGCGGCATCTCCACGATCTTGCTCTTCGAGCCTGTCAGCTCGATGCAGAGCTCCGC
AAGCTGGCGGATCGTGAACTCGTTTGGGTTGCCAATGTTGATGGGGCCTGGATGCGTGGG
GTTGTTCATCATCCGGATGAGGCCTTCGATCTCGTCCTCCACGAAGCAGAAGGACCGCGT
CTGGGAGCCATTGCCGTAGACCGTGATGTCCTCACCCTCCAGTGCCTGCATGATGAAGTT
CGACACGACGCGCCCATCATACGGATGCATGCCCGGCCCGTAGGTGTTGAAGATGCGGGC
GCAGCGGGTGTCGCTGCCCTGACGGTGGTACTCGAAAACGAGCGTCTCTGCCACGCGCTT
GCCCTCGTCGTAGCAGGAGCGCACACCGATGGGGTTGACGTTGCCCCAGTAGGTCTCTGG
CTGAGGCGAGATCGTGGGGTCGCCGTAGCACTCCGATGTAGAAGCCTGGAGGAACCTTGC
ACCGCATCGCTTCGCAAGCCCAAGTGTGTTGTATGTGCCCAAGAAGCAGGTCTTGATCGT
CTTCACCGGGTTGAACTGGTAGTGCGGCGGGGAGGCGGGGCACGCCAAGTTGTAGATCTG
ATCCACCTCGACTTTGAACGGCTCGATGACATCGTGGCGGATGAGTTCAAAGTTGGGCTT
CCCAATGAGGTCTGAGATGTTGTGCTTGCTGCCTGTGTAGTAATTGTCGAGGCAGATCAC
GTCGTGTCCCTCATTCACCAACCGCCGGCAGAGGTTTGATCCCAAGAAGCCGGCACCGCC
AGTCACAAGGATCCGAAGGTAGGGGCTGGCTGGTTTTGGCTGCGCAACAAGCTTGGTCTC
CTGCTCCTCAGCGGTCTGCTCTGTTGCAGGTGCCATGGCTTCCAGCGGGATGGGAAGGGC
TTGTGACTTCTGTAAGATCTATCCTGAGACACTGCTTGAGCCAAAAATGGCTACG
>at02385
TTCAGTGTTCTGCTGCCGGCATTGTACAGTCCAGTGCTGGCGCTCTACTGATAGGCCTTC

CTGCAGGACCGAGACGGCATCTCCAGCTCCGGGCTCCTCGGCTGCTGGACCTGGAAGCCC
TTCATCGACGGCAAGCGCCTCATGGCGGCGCCCTTCAACGTCCCCAAGGGCCGCAGGCTC
GGGGAGGTCATGGAGGCGCAGCTGCAGTGGCGCCTGGAGTgGCCCACAGCTCACGGAGGA
GGAGTGTGCTGCTCGGGTGCAAGAGCtTGTGAACTCATGACAGTTGACGCGAGACGCGGC
TTTTGCACAGTGGGCGCAGGGGCCCATCTTGCGGCAATACAGGCTTGCTGATACGTGCGG
TGTTGTTTGTGGACTGGCCATGAGCGG
>at02386
TTTTTTTTTTTTTTTCGTCCAGAGCCGCCACGCAGCGCAGTACCGCGCGGATCCAAAATC
CTCTGCGTGCAGCGGCGCCGGCCGGCGACCCGCGGGTTCAGGCCGCGGCCCGCCAGAGGC
GCCAGGTGAAGGCAGCGCCGCGGCGGCGCGATGCGGCCCCACTCTAACACAGCGCGGGGG
CTTCCGCGGACGCCGCCCCAAGGCAGCTAGAGCCCCATTCAGAGAACGCCTTCCTCTTGA
AGATGTCTTTTCAGTTCCGAGGCTGAGCATCGTCTGGCCTCGAGGCCCGTTCAGAGAACG
CCTTCTTCTTGAAGATGTCTTTTCAGTTCCGAGGCGGAGCATCTTCTAGCCTTGCACAGG
ATCTCGGCGAAGTCGGAGATGTCGAGGGCCTTGCGAACCGCGTCCAGGCCCTCGCGTTCC
CGGAGCTTCCGCAACGCCCCCCGCAGCAGGCCCCAGACGAAGGCGCAGCCGTCGGGAGAG
TTGCTGAGGGAGTGGAGCGGCCGGAGCACCGTGGAGTTGAAGCCTCGCCCGGCGGCGACA
AGCAGCCGGGAGAAGAGAGAGTCCTCGTGCC
>at02387
GAGCATCCTTTACGTCCTCTCCGCTCTCCATCTTGTAGAAGAGGACAAGCCACGGGTTTT
TGGCCTTCTCGTTGGGGTAGTGCTCGTCGCACAGGGCGATGGCGGCATCTTTGGCCTCGC
TTTTGAAGAGGCCCTTCTCGCACTTCAGACTTCCGCCCATCTTCTTCTTCGCCGAGGCCA
GTGCTGCCAACAGCAGCAGCCCGCCTGCGAGCCTCCAGCCCGGCGCTGCCATCTTGGGGG
GCTGCCCGGAGCCGGCGCGTGCTGCTAAT
>at02388
TTTTTTTTTTTTTTTTTACACGCAGGACATGCCTTATCGAACAATGTTCGTTTGCACAT
TTGCAGCTGTACGACTCGCCACAGGGAGCGTCCATGACAGCATAGGGATCTTGTGCACCC
TCATGACAGCACGCATGACAATGCACTGAACAACAGATGCCACACAGATTTAAACAATTT
TTAATCACCGGGAAAAGCGTCAGCTACATTACAGAAGACAGGTGCTCTCTTTCAGCTTAG
ACTCAAGCTCATCCGTTTCTGCCAGTTGGTTCTGTCCAACCAGTCACGTGCATCTTGGAC
CACTAGAACTCACGTTCATCATGATGGCGTTCACAAAGCCCTCCAGCTCCTTCTGCTTAG
CCTCAAGCTCATCTGTTTCTGCCAGTTGGTTCTTGTCCAACCAGTCACTCGTATCAAGGA
GCGCTTTCTCAATCGTCTCCAGGTCGCCCCCTTCGAGCTCAAGCATGCCCATCATGCCCT
CCTCATTGAGTGTGTGACGCCTGGTGAAGCAGTAGTTCTCCAATCCGTTATTGGCCAAGG
TCTTCAGCTTGTTGTCGTCATCCTCGGACTGGAACTTCTCCGCCTCATGATCCATGCTCT
CCTCGTCGAGTGTGTGACGCATGGTGGTGCAGTAGTTCTCCACTCCGCCCTC
>at02389
GGCACNAgNCGGAGGTTCAGAAGCCAGGAGGATGGCGGAGTCCCATGTATTGTACCATGG
tTTTTCGGCGCCGCCATGTCCATGTCCTGCATTTCAGGCAAGCTGGCATTTGGGCNTANG
TGTANCAtTCGAGGACGGATTAGCCGGACGGTGGCACTTAGTCGGCNAAAGGCNGGTCAA
TTGAGGCAGCTGGGCGTGGCNTGGATTGGCCACAAGCNGAGCGNGGCAGTGAGAGTNAAG
GCCCTTTTCACTTGCAGTCAGCGATGCCGGTCTTTGCTTCGAGCTGTCAAGGGCGGCTGG
CTGCGGGTGAGGTGGGCCTGAATACAGAGTGCTTGGTGTGAGTGGATTCCAGCAGCCCCA
GCAGAGCTTGCGCGCAGTGGAGTGCCGGGCAACCGGGGCACTTTAAACATACTGGAAGGC
ATTGACGGTGAGGGGATTATGGGCTTGGACTTTGTCTACGACTCAGACATCGTGCTCTGC
GTGTGATCTGCCCAAACTGCCCGTGAGGGCAGGTGCCGTGGCCATTTAGGGGTTCCCTGT
ACAGAGCTGGTCGCTATCACAGTTGGGAACGTCTAGTGGAGTTGCATTGCATTGC
>at02390
TCGAGGTTGACTCGCTGCACGATGGCAACGACTTCAGCCTTCGATTGTCGAGGGCCAAGT
TTGAGGAGCTGAACATGGACTACTTCAAGAAGGCCATGGAGCCTGTGACCCAGTGCCTGA
CAGACAGCGGCTTTGCCAAGACCAAGATCAGTGAGGTGGTACTGGTCGGCGGCTCTACGC
GCATCCCGAAGGTCCAGCAGATGATCCAGGCCTTCTTCGATGGCAAGGAGCTCTGTAAGG
ACATCAACCCTGACGAGGCAGTGGCCTACGGTGCCGCCGTGCAGGCCGCGATTGTCTCTG
GGGTCGGCACGGAGGAGGTGCAGAACATGTTACTGCTCGACGTCGCCCCGCTGTCACTGG
G
>at02391

GATGCATGTCTTAATTAGCGATGAGGTGATATAGGGCAGCACGTGCAACATAAAACATAG
AAAATATCACCGCACACCACATGAATCACGATGATGAAGTGCGCACTTCGTCACAGCAAT
AATAAGCTTATATCAAGATCTTAGCGAACACAGAAAAAAAGGAGATGACACCATTACATA
AACAAAATGAAATGAAACTTATCAAACCGACAATAAAATGGGTTTTAACGATTAGCAACA
AAATTTTGACACAAGGTCAGTATAACGCTTGCCTCATGAAGCAATAGCTATT
>at02392
TTGTTTTTTTTTCTTTTTTTTTTGTGTTTTCGGACGCTCTCTTGTGAGTGGACGACTGCA
GACCCCCCCAAGCGAGGGAGCTGGCACTTCTCAGGCTGTGGATCTCAGCTGGAACCGTGT
AACGGTATGCTGAACAGCGATCGGTAGTAGCCGTCGTCACCCATCTCATCCAGGCACGAC
AGCTGCAGCTCTTGCAGCTTCAGGTCGCCAAAGTTCACCTGACCGAATCGCTCCATGAGG
GCACTTGCATCGAACGTCTCCCGCTCGCCCCGCGAGCCATCCTCTCGCCAATTGGTCCGA
CTGTACTTGGTGTTCATCAGGGTGGCATGCAATTTCACCTCTGCGTGAGCACCGTCAGAC
GAGAGCAGCCTCTGCGACATCAAGCTCTGTGCCGAGACCAAGCCGCGGGATTTCAGGAGG
TCAAAGATCATGTCTGCGACGTTGTTCATGCGATTTTGCAGCGCGTGTGACCGATCCGTC
GTGAACACGACACCTACGTTGCTCGGGTCGTCGGTCATGATGTGAAGGCCCTTCAGCGAC
GCAACGATTGGCCTGTTGAAGTCGGCAACGGCCGAGATGCGAGCAGACAGGTCCTTCAGT
GCCTCCTTCATCTCGTCCACTTGCGCGTGCGAGTGGAGTTTCAGCATGCAGACCGTGAAG
TGCATGCGCCTGGACGGCATGAAGATGGAGGCATCGATTCCTGGAAGGCGCTGCAGCACG
ACGTCCTCGCGGAATTGGTCAACCAATCGAAGGAATCTGGCATCGTGTGCCAAGGGCACC
GACAGGAAGTGCGTGTATTCCAGCTTCGCCTCCTCCTTCTCGCACAAGAGCTCGATCTGA
GCCTTACAGCTATAGATCTGCTGCTTCTGTCGCGCTCTCAACCAAATGACATCCTCTTGA
TCCTCTCTGTGCGGCACCACGATACGCGCTCCTGACTCCATCTCGAGCTTTTGCTTATTG
CGAGCCCGTGACCCCATGATGAACTTATGGAATACAGCGGGGATGGGCAACTTCAGCACC
CACTCGTCATTCTCAAATTCCATTTGGTCATCCAACTCGCGCTCCTCATCGTCCTCCTCA
TCATCAACGTACTGGCGACCTTCGCACTTGAAGCGAAGGTCCTCAGGAATGATAAGCTTC
CGGTAGGAGCACGAGCCGATCTTGATGAGACTGAACTTTGGGTCTTCGAGGTCTTGGGAA
AGTGCCATGGCCGCCAACTTTGCTATCCAACAATGCTGCCGACCATTGTTGGAGTGACAC
TGTACTTGAGCAAAGCTTGAGCCAAAATGGCTACGG
>at02393
CCTTCTCTATGTTCCAGACGTGATCTGCGAGAGTGCACTTGTTCCCCGCCACTGTGTAGT
ACCCGCAATGGTTGACCAATCGCAGGGGCCTGGGAGGAAGGTCCAGGGACGGCAGGAGCT
GATCCTGCCTCTGGTGCGAGAAGAGGACGT
>at02394
TACACTCCTTGAGTGAGCTCGCCCGTGACCAAATACCGGCGCTTCGCAAAGTCCTCCGTT
ACAATGCGGACCAACTCTGGGCCGGAAACCGACATCTTGCCAACAGGCACCGCCGCCTGC
GACGGCGCAACGCCAGCGTGCAGAGCTGCCGCCACCACGGCGGCGGACGCCCATGCAGAA
GCCCTGGCCAGAAACGGCTGAGTCGAGGGCAGCCGAGGCATGACTGCTGGTGGCACCGCT
GACTGCCCCGGGAACATGAGAGCAAAAAGGGCAAGGAAGATGACGGTGGCCCAGCGTCGA
GCAGTAGGATGTCCACTTCGGCGCTGCCAGCCCCACTTGTGGCGCTCGATGCGCCCACCA
GTCGCAGCTCCCATCGCACTCAAACCGTGCTCGGTCACAGCCAGCGGTGCATGGAAGATG
CTAGCAGCTGGGTGCTCAGGGTGCACACCACTGGCGTGATCTCCAGGCTTGAGCCAAAAT
GGCTACGG
>at02395
CCCGATCCGGAAGTGAGGTGGGCGGCCGTGGTACGTTATTTGGGGTAAGCTTGGCAGTGC
CGGGTGACGAGCAAGTTGTAGCAGCGCTCAATGAGAGAAGGAAAGATGAAGACGGCACGG
TAAGGCAAGCGGCAGTTGAGGCCCTGGTGCATGTTGTTGGGATGGTGGACGAGCGGGCCG
TAACTGCGGTGAGAGGTGCATTCTTCGAAGATCCTGATGCTCAGGTGCGTGTAGCGACCT
TGGGCCTGTTGCAGGTGCAAACAAGGGCACTCGTTCAGAAGAATCATCTTCACCGAAAGC
GGCATTAGTCAATCTTGCTGTGTCTTTATTTCCCAATGACCCGCTCATGGTAGCAGAGGT
GA
>at02396
CCCAAAAGATGTTTTTTTTCCTTTTTTTTTTTTTTGCTGAATCACAATTGTCAGTCTTGA
TGCTCTGATGGCCTCATCAGATTGACACATTCTCCGGGGACACAATTGTAATTAAACATT
TCAATGCCCCAGTGTCCCGCGTGCCTCTCACATGCGCTGAGCCGCCATCAGTGTGGCATC
AAAGACGCCTACTGCTGTGGCGCGAGGGACCGTCCACTGCAGGCGCATCTCGTCCTTGGA

GTGGCGGCAGAACATCTCCAGTCGCATGGGGTCCGAGCGCCCCGCCCAGACCCGCTCCTC
CTCAGACTCCTAAAAGTTGNTGACCGGACGGTAGGTGATCTCCTGAGCGCTCTCGATCAC
GCGGATGTTGTCCGTCACAGTCGGACTGCCCGGCTTTTCCACGATGCACATGCTGCGCTG
CATGTAGCCGCTCATGTCACGCACCGTCACGTTGGTCACCTTCAGGCCGCACTCAGCTGG
ATTGCGCATGGCGAAAAGCATGGCCTTCCAGAGGGTATCCTGGCTCACTTCAGTGATGGC
CTTGGACGCCAGACCGAGTCCAACCACGTCGGATGATCCCTTCTCGATGGTGTTTGCCAG
CTGTACAGTCTTTGAGAAGGTGTCGCGGGCAATGTCATAAGGCGCTTTCCAGTCAACACG
CAAGCCGCTGGGGGCACTACGCTC
>at02397
CCATGGATCGCTTGGAAGATGCTCGTGTCCAGCGCTGTTGTGGCGAGGGTCGGCCACCAG
CACTTGTTTGCATCTGCGTCGCCACACCTCTCACGGAGCGTGAGGTTCATGAGCGGTAGG
GATGCCGTGGACGTTGCAGTGCGTATGCGATGTTCCGTTTCAAGCACCATGGCCTTCTGC
AGGAGGTGCATCGGTTTCTGCCGTCGATGGGCCTCTCGCACCGCGACATCTGCGAGCTGC
GGGCAGCATACGACCGCCTTGGCTTTGACGTTCCATTGTTGGGGGAAATCCATGAACTCC
AGAGGGCTGGAATGTCGTTGGACGACAGGTTCAGATGACAAGCATGTTTGGGTCC
>at02398
CCGTAGCCATTTTGGCTCAAGCCATTTTGGCTCGAACCATCGGCAATATTTGGAGTTCAC
GGTCCCACATCATACCTTGTGTACTTTGTCTGAGCCATGTTGTCATTGGTGCCATTAACA
ATCATCGGCCTTGCATGTGTGACATCCGCCTCACCTGATACAGCAAGCGCTGTCACAGCT
CAGTTGGATGCACAACGAGCTCGTGTAGAGGAAAGTGATCATGCAGGCAGCAATGGCAAC
TCAACAACCTCAACAACTACACTGCCAACTGCCTCCTCTGCATCCAGACACGGGCATACA
AATCTGAGGGGTGCTAGGCACAGTGTGCACGGTGCAGAGGTTGCAACTGCGGCACAGACA
GAGGCGCCGCCATGCGAAGCGCAGCCAGACTTCCAGGAGGCAAGGAGGTCCTGGTGTGGT
GATCAATTGGAGCCCTTCGCCTGCTTCAAGCCTCCAAGCAGTCCCAGTCGGATATGGGAC
TGCCACTTGCAGCCAGGCCAAGGATGCTCCAAAGGAGGGGAGCTGTGCACTTGGCCGGTC
TCGGGCCCACAGCCCACGGCAGTTCCGGCACCGTTGCCGCCAACCGGCGCAACACGTTTT
GTGACCTGGAACCTCTATGTCTTCACACTTGCGGCACGCATTGGTCCTGTAGCGGAGGAG
ATCGTGAGGATGCAGCCTGAGATCGCGGCCTTACCGGAAATGTGGCACGAAAAGGCTGCC
ATATTGAGCCGCCTGAACGAGATCTCTGGACCTGTTTGGGCATTTGCCGATGGCGGGCTC
ACCGAGCGCTTCAATGACGCAGACATCTTGTATCGATCCGACATTTGGGAGCATCTGGAC
AGTGACTTGGTTTCCTACTCTGCTGATCGAGCCTTGAACTGGGCCGTC
>at02399
CAACGCAGAGTCCGTAAGCCATTTTGGCTCGAGTCCAGAAGCAAGGGGTTGAGGACTGCA
GCAAGGAGGCGAGGCGGCAAAGTCAGAGACCAAGCGAAGCATGGGAAACGTTCATCAAGA
CCGGCCGCATTGTCGTGCTCCTCCAGGGGCCGCTTACACCTGGCAAGAAGGCCATTGTCG
TGAAGGTGTTCGATGACGGCACCAAGGCGCGGCCCCTTCGGCCACTGCCTTGTGGCTGGC
GTGGACCGGCCGCCCAGGAAGGTCCCACAAGAGAATGTCGAAGAAGAAGATCACCAAGCG
CACCCGCGTGAAGCCCTTCCTGAAGTACATCAACTACAACCACTATCATGCCGAACGCGC
TACCAGGTGCCGGCGGACGCTTGGTGCGCAGTCGCTGGCAACCGACCAGCAGATGGAGAC
CCCTGACGGCAGAGTCGAGGCAAAGAAGTTTCTCAAGAACTTGTTCCAGGAGAAGTTTGT
GAACCCACCTGCAGACAAGACTGGCCGGCCATCCAAGGACGTGATCTACCTTCGGAAGAA
GCTCCGCTTCTGAGCGTGCAGACGATTGGACGGAGGAAGATGGAAGCCCTGGTGATACAG
CGAGAAGAAGGTAGCGCAAAAAAAAAAAAAAAAAAAAA
>at02400
CCCCCTCTCGAGGCTCCTCGCCCTCTCGAGGCTCCTCGCCCTCTCGAGGCACCTCGCCCC
CTCCAAGCTCCCCGCAGAACTGTTCCTTCTCTCGAGGCTTCTCGCCCTCTGGAGGCATCT
CCCCCCTTCGAGGCATCTCCCCCTCTCGAGGCTTTTCTTTCTCTCGCGCGGTCGCCTCGA
AGCTCGCCGGCGGCGGCGGCCTCGGGCCGAAGACCTTGGGTCTCGATCTTCGCGGCAGGT
CTGCCGGCTGTGGGGGGCCGTCTTGCCACTCGTCGATGAGGCCGGAGCTGAGGTTGGACT
GGACCCGCCAGGTGCACTGGGGGCAGACCCGCAGGCCTCCAGCCTCCGTCTCGATGCGCT
CCCTGCACCCCCAGCAGAAGGGCCCGTGGCCGCAGGGCGCGGCGTAGGAGCCCCTGACGC
CCAGCTCGCCCCTGCACCGGCAGCA
>at02401
TTTTTTTTTTTTTTTTTCTCCCACGCTAATTGCAAAACTCACATATGGCCCGCAAGTGGA
GGCAGCAGCGCCCGATCTCACATCATGCCCATGATTTCCGGCTTGAGCACCTGGAAGGAG

CAGATCAGATTCTCGTCAATGGTCAAAACTGAAGCTGCGTTGTCAAACTCCCCACAGTAA
TTCGGCGCAGAGAAGATTGTTACAAGCTGGCGGTCCGCAAAGAATTCGTAACCGTCCTCC
ACGACCTGGTGCGCCCGCACCACGAGGTCGAGGTCCTCCTTGTGGAGGAAGGCCCGGACG
ATGTCGGGGCCGAAGGACACGGAGACGCCGCGGTCGTTCGCCCCGAAGCCCATGACCTCC
GCGGGGTCCGACCACAGCAGGTCGCAGAGGAAGCCGGAGTCCGGGATGTCGGCGGGCCGG
CCTATGCTGGACACCATCCGGCGGACGTCTGCGTCAGGCCGCAGCATGTCCTGGGAGAGG
CCTCCGTGCATGCAGATCACCCTGTCGTCGATGATCGCGCACGCCGGGAGGCAGTTGAAA
ACATCACAGAATGTCTTCCACAACTTCACACTGTAGCGCTGCTTGCACTCGTCGTAGAAG
CCGTAGATCCGGCAGATCGATGGCGACTCGTGGTTTCCGCGCAGCAGAAGAGTTCTCAGG
GAACTTGATCTTGTACGCGAATAGCAAGCAGATGGTCTCCAGGCTCCTCTTCCCTCTGTC
ACGTAGTCCCCAG
>at02402
CCGCTGAACGCGGAGGCTCGCGAGCTTTTTCGTGAACGTACCCGGCCGGATTTGCAGACC
GCGCTGCGGAGTGCGCAAGAGGCAGCTTACAGAAAGCCTACGAGAACCCTTCTGGAACGC
CCCTGCTGTTCACCAAGGGCCCTGCGGAGGCTCACGACAAGATACTGGAGAATCTGCACG
CCCTCGACACGTCGTACAGTATGGAGGACCGCAAGACGATGTTCGTCCAGTGGTTTTGCG
ATCACTACGGGCACGAGCA
>at02403
CGAGCTGGACATGTACCAGAAGACCCTGTGGTGCTGGTCATGCTACTGCTGCTTCCAGGG
CTTCACCTACGACATGACGCCGATGTGCCAGAACGAGGGCAAGCTCTGCTGCCTCTGGAT
GAACACCGAGACCGCGAGCTGCTGCGACGATGGCTGGATTGAGCTGAGCGGCAAGTGCTG
CTGCACCGTCATCGACTGCTCCATCCCCGCCGGTTACACTCCAGGAGTGGTCTGCTGCGG
TGTCACCATCTGCTGCGCCAACTTGCCGGAGACTCCCGTGGAGTGAGCCGGAGAAGCCCA
TGGAAGCCAGGGCCAAGACAATTCGCTTGATACCGTCCAGTGACCA
>at02404
TTGTTTTTTTTCTTTTTTTTTCCGTCTCAGTGGGCGAATCAGCCAGTTCTCATCACTGCA
TGGCAACTCTCCGCGCGAGGCAACATCCGCGCTAAGTGAGTGTGCTGCCACGAGAGCAGG
GACAGCTTGGGATCCCAACCCGACGATCAAGGTCAAGCACTCGAGTTTCCATCAAGAGCT
TTTTGTGAGGTGTAGATGCAAACCGTTCGAGACCTCTTCGAAGAAGGCCTCTTCCTCAGA
GGAAGCAAAGGTGACCATGTTGCCGCGAACTCGAGAGTGATCCATGTGAGCAGCCCGGTT
CAAATACGTCTGGCTGGAGTCAGGCACGTCGTAGCCAAACACTAGGTTTACACGGTCGGC
ACTTGCTATCCTCTGCCCGTCAGTGGTGACCAAGATCCGCTTCTGAAAGTTCCTGAAGGA
CTCGTACCTGATGGAGCGGTCCTTCTCCTCCAGAGCCATGTGCACTGCAATGGCGGGGAA
GCCACCCTCCGCCAAAACCTTCTCCAAGCTTGCAGCCTTCTCCGTAGACTTCACAAAGAT
GACAACCTGATCAAAGTCCAGTGTATCCAGAGACTGGCATAGCTTCGAGTCTCTGTCCTC
TGCAGCCACCCTCACGCAGTGATGGGCAAGCCCACTGAGGGCGAATACCCTCTCCTGGTT
GCGCGTTCTGCAACGCTTCTGCGCCTCCGAATCTGAAGGTCCAAAAGGATCTGCCTCCTG
CTTTCGCTTCCGTGCTTCTGCGGAAACCTTCTTCAGGGAGCACTCGATGTTTGCTGTGTT
CCCTGGCTCTGAGCACCCTGTTGGGACATCCAACAACAGCTGCGTGGATTCACCGAGCTG
CAGGTCCCCTTGGCGTGCCAACGACTGAATACGGCCGGGTGTCCCCAGCAAGATGTGCGG
ACGTGAGTTCCTGAGCATCTCTTGATTCTTCGCCAATGGCATCCCGGCATAGACGACACC
TGTCCTCCTGCCCTGGACCTGACGGCAAGCTCGATCGATCTCCTGCTTGATGGGGTACAA
TCGATGTACGTGGCAGACGATCAACACCTGAACCGCTGTCGATGTTACGTCCACGTGCCG
CAGGAATCCTCGGCTGGACTCCGAAACCTGGTCAAGGATGTTGCCCCTGATCACTGGCGG
CGCTTCGGCAACAGCCATTGCGCAGGAGAGGGTCGCCCCAGTGTGAACACAAACGGGGCG
GGCTGCTGCTTGAGCCAAAATGGCTACGG
>at02405
GGCGAGATAGCATTATTACCAAGCGACTATTTGTACCATCAAGCTGGGCGACATCGGTGA
GACCTGGAGCCTCTACGACAACTTCTCCGAGGAAAGCGCATTCCTTAAAACAAACATCTA
CACATGCCAGAAGCTCGAGTGCGCTGAGTGGTTCGACAAGCAGTTGGGCAAGGACACCTC
CGGCGACTTCGACTTGGCCCAGGAGACCCCCAAGAAGAAGCGCAACTTCACCAGGGGCAA
GTCTTCTGTATCTCTGAGTCCTAGCACCCCCAAGCCGTCAGATTCCCACTTTTCAAGCCC
TCACCAGCAGCTTCCTCTCCCATTGCAACATCAGGTGCTAAGACATTCAGTA
>at02406
TTTTTTTTTTTTTTTTTGACTTGCAACTCCATGCTCATGATCTTGGGATATCTGTCAAC

ACTTGCGGCTGCAGGAGGGTCTTGCAACTCAATGGCATAATTGCTGAGGCGCTCTCAACC
GCACGCCGCATCGTCGAAGGTGATGCTTTTCCACCACCCTCGCGCAGTTTGAAACCTCCG
GGCAAGATCTATGAGCCAGTTTGCCTCGCGAGACTGCGCGCAAAGCCTAGAACAGGCAAA
ACCTCCTGCATGCCCCGAGCATCAGCCCTCTGGTGCACGAGGCTAGCATGAGGAAAAGGG
CGAGTAAGTGTCCAAACACTCACAGGCAAAGCTTTTGGGCCCTCATTGTGAGAAGCCCGA
ACAAAGCCGCTGCTGGAAGATCCTCGTGATCCACCGCATCAGCGGAGGTGACTTCAAAAA
GTGAACGTGCACTTGAATTCTATGGTCCAGCTGCTCCCTTCCAGCTGGAACAAAGCGCAT
GCCTTAGCCAGGACGTCCTCTGCCTTGCTAGGACCTCCAGCCAAGGGTCAACTCTCCCCG
AAATCCCTGCCTGGAATCCTCCGCGATGTTGCCAGCTCGTCTGTCTGGCCTTCTTGGGGG
AGAGGGCTTGCAAGCAGCAGCTTGCAAGCAAAGCCATTGGCTCTTGGTGGCCGAGGGCAA
TGTCTCGATCCACGTTGGCTGCCAACAGCACTGCCACACTCGGACCATCCCGGACTCAGG
GAAACAAGTGTACGCTGGCCAATCCAGCATTGGCATCTCATGCCANCATCCTGGCAGGAG
GGCAGTGCCTCCCGCATTGGGAGATGCCGGGACAACACTCATGAACAGGCANGGGCACCA
GT
>at02407
CCGTGGAGCCGGGGGACGCACTGGCCGCCAGGAGCGGGGAAGGAAGGCGCGCCGCGGGGA
GGGTCCGCTGCCGCGCCCTGCGCGAGGTACGACGCTCCCATGGAGATCACCGGCGGCAAG
AAGATAGGCTCCTTCAACCGGACCATCGTGGAGGGCGCTCCGACCAGCGGGAGGCCCAGG
GGCCTGGGTTTCGAGACGCAGGCGAGGCGCTTCAAGCTGCGCTCAACGAAGGAGACGCCC
GGCCCCGGCGCCTACAACACCGAGCCGGACTGGATCACGAAGACCCACAACGTCCACTTT
GGCGACGTGGGATGACGCTCGCCCGCTGGGTTTTGATGGGGGCGAGCGGCGTCGGCCTTG
CGTTGCTCTGCGCGCTGGTCGCGGAGTGGAACTGAGGTGCTTCGCCAGCGCTGTGGCGAA
A
>at02408
GACACTTGGGCTCGAGTCTTGTACGATCAGGTGCTTGGTGAAACCATGAGTATGCTAGAT
GCCCTCCGCGAGAGGTCAGCTGTGAAATACCTGTTCAAGGTTGTTGCAGAGGAGGCGATC
TTGTATGACAAGCCAGACCTGACCGCTTCCCGCATCGGTTCACTCGGGTATGGACAGGTT
GTCCTTCTTTGTGATGTTTCCGATGATGGTTGGGCTAGGCTGCACAGTGATCAGGTTTGG
GCCATGAGCAGCAGCCTTGAAGGAATGGGAGTACTCCGAGGCTGTGAGGTG
>at02409
GCTCCTTGCCCCAGTCGGCAATCACATCCTTCAGCCCACTGCTGAGCTTGTTCTTCAACA
CCTTGGCCTTGTCCAGGACTTCCTTGGCCTTCTTCTCCTCCGCGCCGGCCTGCTGCAGCT
GCGCCTCGGCGGCGGCCACCCGCTCGGGGAACTGCTCCTTCAGCTTGTCCACACTCTTGC
AAGCTATCTCAGGCGTCACATCGAAATCTGGAGGCACCCACCGCATCTTGTCTTCAATCT
TCCTCACCAGCGGCGCGCACAGCTCGTCCATCGACTTGGCTGAGGACACCTTCATGTTTT
TCTTGCAATACTTGGAGTAGAGCCCGCCCACTTCCGCCGGGGTTATCTGCACGCCTTTAG
GGCGCTTCAGCACGACCTCGTACACATCAGTGCAGCCATCTGCGGCTGCACAAACTATAC
AGGCTGTGAAGCACAGCAGTATCCGCCATGAGCTTGCTCCCTGCCCGC
>at02410
TCCTATTTTTTGGTCTTCTAAGTCCNATTTTCTTGGTCTCAGGTGAGGTCAGCTCAAGTT
GTCGAGGTTCATCCTTCTCAACTGGTGAGCTGGTGGTGACCATGGCCGACGTTGGTTTTG
TCATTCCAACCGGTGCCCCTTGGTCACAGTCGGTTGGCGGCGCACTGCGCGGTGCGAGCC
ACGTGCATTCCAGCACTGCTCCACACACGCACGTGGCAAGTGTGGAAGGTCCATCTGCTG
TGGGCTACCTGGGGGGCTTTAAGCAGTGCAGCTCTGGCCATCGCAGTTGGGGTCCGTGCA
AGCCGTCAGCGTGCACCACGCATGCGCAAGGCATCAGTTGTGCCACTCCAGGCCCAGGTC
GGTGAAAACCATCCCGAACATCGGGCTAGACAAGGGCTTCCCACCAGAGAAGGTGCCCCT
TGCCGATTACTGCAAGGGCAAGAAGGTGGTCTTGGTAGGCCTGCCTGGCGCTTTCACGCC
CACCTGATCCAGTACGCAGGTGCCAGGCTACCTGGACAAACAAGATGAGCTGAAGGCCAA
GGGAGTCTCTGATGTGCTGGTCTACTGCGTGAACGATGGTGCCGTCATGACGGGTTGGGC
AGCGAACCAGGGCGTGGAGGGCTCCATCATCACCTTCCTTGGTGACCCACGTAGCGAGCT
CACAAAAGCGCTTGACTTGGTGCTGGACGCTCCTGGTCCCATGTCAGTCCTGGGGAACCC
CCGGTGCAAGCGCTTCTCGATGCTCATTGACGACGGGGTCATCAAGACCATCAACGTGGC
CGCCTCCGAGGACGACCCAGCGGGTGACGACAACCCAACCGTGTCCCTTGTGGAAAAGAT
GTTGGAGGACCTGTAGGCATCTGCCAGCAGGGTATTTAGTTCCAGCCCAGACTTTGAGGG
CGTGTACAGTCGAGCCAAGTGCTACACACTTAGTTATGGGTTGGAGCCTAGCTGAAGGAC

AACCTGAGAGCAATGGTGTCAAGGCAATGAGCCTTTGATGATAGATCCAAGCATTGAGAT
GCACTTGTGTATCGAGTGCTCGGAGGCTTGAATGAAGACTTGTGGGCCTAGGCTTTGTGC
TTTCCGGGAACTATTGTCACCTTCAGTTACCAAGCTCTGCACCTTGAAGCCAACAAAAAA
AAGAAAAAAAAACAAAAAAGAAAAAAAAAAAGAAAAAAAAAAACAA
>at02411
CCGTAGCCATTTTGGCTCAAGTCAGGAGGTTCTCTAGATGCCGTGCTTGCTCTACCCAAC
ACAAGGAGGGCCCTGCCCTCATATCACTGGGGTGGATGAGCGGGATCGACGAGGTTTTG
CTCCTGTCTTGGGCAGAGGCTCACGGCACATATGTGAAGCCTGGTGAAGGCGCGGACGTC
GAGGTCCGTGGGCGGCTTGATGCCGAGCCGCGGACCGCTCGCGCCCAGGCTGCCAAGGCC
AAGCTCTGGGTCTGCCACTTCTGCGGGGCGGAGCTGACCGTCAGCGGGTCTGGACTGAAG
CGCATCCGGGAGCATGCGGTGGTGGCCACTCATACCGGCACCAAGAAAGTTGGATGCGCG
CAGGTGACAGAGGAGGCCCTGCAGGAGCTGCTGTGCG
>at02412
ACCTGTGCATCGATGGCTTCAACCAGTGCATCTTGCCTGGTCAGGTCTTCATTGCGCCTC
TCGAGCATCATGCTGCCACCACAGACGTTGACGAGGACGTCCAGCGCGAGATCCGCAACT
ACCAAAAAAGCCTCGTGCGCTTCTTTGAGGAGGAGGAGCCTCCCCGGGCGGTGATCTTCG
TCGAGTCAGCTGTGCACCGGGTGTCGCGCGACAAGGCGCTCCTGGGCGCGGGGCCGCACA
CTGCCATCGTGGCCTACCCCGTGGAGCCGGGCCTGCTGCAGGAGGCGCGCGCGTACTGGC
GCAAAGCCTTCGACGAGGCGGAAGTGCGAGTTCACGGTTCAGCA
>at02413
TCTTCACATCTAGGTTGTTTGGCGCAGAAATCCGCATGGCATACTGGGACCTCCGACTAG
ATAAGTTGTCAGATCCTGTGGAGACGTTGCTTCTGACACATGGCGAGCCATCTTGGAGCT
ACTTGTACCGACGCATGGTGAAGCCCTTTCTTGCCAAGGGCTATCGTGTAATTCTCTTCG
ACCAGGTGGGATTTGGTTGGAGTGATAAGCCAGCAGACAAGGCCGACTATTCATATTCAC
GCCATGTGGCATGGAACGAGGACCTCTTGATCAATCACCTCCATCTCACAAAGATCACGG
GCGTGTTCCAGGATTGGGGCGGCTTAATTGGCTTGCGCGTCGTAGCACGCAACCCCGATT
GTTTGATCGTCTCGTGCTTGCAAACACAGCGCTCCC
>at02414
GCCGGTGAGCCTGCTTGCAGGCGCGCGTCTGCGCCACCAGCTGTATGTACATGTTCATAG
TCCTAACCTGATCCTAACGGCTCAGTCTACACAAGCCCTCAGCATCCAGGTGCTCACGTC
AAACAGGCTTCCAGCGACCCCCGCCAGCGTTGAAGCACTCCACCCAAATTCGAATCCACC
AATCGCAGAGCCGCAAAGCCAGTGGCAATCATCTTGTCGGCAATGTCCTCTGCCTCCAGC
CCCCTGCCTCACAGCGTCCGTGCCCCGCTGCCCTTCTGCAATACCTCCGCCTCGGGCGCC
TCCGGCACGCCCGCGGGCACCACGAAGGCCCCCACAAGCTCCCTCGTGTTCAGCCACGAC
AT
>at02415
CCCAAGGAAGCGCCTCGTTTTGGACTCCCCGGCGCCATGTCGCGGCCGCTGCAGCCTCCC
GCCGGCCGGATCTCCCTGGCCCAGCCGCCCGAAAATGGCGACGCGCCCGCGCCGAGATGG
CGGAAGGCGGCTGAGGCCGTGACGTCCCACTGCTGCTACGCGGAGAACGACGGCAGGCCC
ACGAGGTGGTGGAGCATCATCGGCGAGTACGCCTACATGCGGCCGATCCCGAAGGTCGAG
AGCAAGCTCATGGGCATCATGCAGCGGGGCGGCCTGCTGCAGACCTTCCAGGGCGAGGTC
AAGGCGAGCAGGGCCACGCCTGGGTGGAGCTGACTCCGCTCATGAAGTGGCGCCTCCACA
TCACCGCGGAGCAGGCCGCCCACGTGTACACGCTGATCGATGG
>at02416
CTCAACAGCAGCAGAAATCTGTAAGAGCGGGACATTCTTCACAGCAGCCTGTCGCCGGAG
CTCCTTCACAGACAACCTGTTGAGGCGTGCGGTCTCGTCGGACGTGTCCGCGATCATCAT
CTCGGCGGGGCTACCGCCATGGAGGCGGCCCCGCGCGGGACGGGCGGGGGAGGACGCTTG
AGCCAAAATGGCTACGG
>at02417
TTTTTTTTTTTTTTTTTTGATCGCGTCACGTTGAGCTCAAACTACGAAGGCCTGGAGGTC
ATTGTGTCCGCATGACTTGTCAGAAGGACTAGACGAGTTTCCTCCACCCCATCCCAGAAT
GCAAGCGCCGAAGGTCTCCTCTGGAACCTTACGGTTTCCAAAGTACGCGCCCGTATTGCA
GTAGCATGAGTAGTGCCAATAGGTGTGGGAGATCCCGGCCATCTGGAAGGCCCTCCACTG
TTGACAACCCCAGAATTCCGTAGCAAACAATGGCACGTTCCAGCTGTCGCGAAAACCCGA
GGCTCTCTGAACGAGCTCTTCCGGATTACTGAAGTCGTAGCCGTGAAATGCATAGAAGAC

AGTTTGGGATCTCTTGATCCATTCCAAGGTCCATGGATCGATGCCAACACCTTCGCCCAG
AGTCTCGTACACCCAAGCTGGAAAGACCGGCTCCTGCCCCGTGTCTGACAGTGACACACT
CATGTCCGCCTCGTGCC
>at02418
AAGAGCGCGTCTATGTCGAGCCGCGGCGCCAGGGTCGAGACGACCTCCGCCGTCTCCTCC
GCGGCCTTCGCGGTGGCGGCACTCTCTTCCGCTGGGGGAAGGACATAGTACCGGAACTTG
CCAGTGGTCCAGTCCGAGATCACAGACCTCGCAGCTGAGGGCAAGTCAAGTCCACTGCCA
CGCTTCAACTTCCCTCGCGCCTCGTGCC
>at02419
GCGGCCGCTGGCCTGGAGCTCGGTAAGACCACAAAGGACGCGAGGGTGGCCATGGATGTT
TGTGAACCGTGCACACAACAACTGCATCCGAACTCTCATGCTTGAGGAAGATTTCAGTCC
CAAGCAGAGCGCGTCCTTGAAGCCATCTTTGATCCCATTGTAAATGAACGCCGCAAAGCT
TGCACGGTCCTCTTCCTGCGCGATGATGCTGCTAAAGTCGATGCCGTCCAGGGAACGCTG
TCCTGCGGCTGACATGTGCAGGATCGCGCCCAGACTTGGTGAATGGCCACATATGCGGAA
ATCGGATGAGAGCTGGCAAGTTGCGTCGCAGAGTCTGTCCAAGATCTGCGACACTGCCAG
AAGCTGGTCTTCTGAGCTTTGCAGCTGGAGAGACTTCAGGATCTCCTGGGACATGAGTGT
GTGAAGAAAAACAAGACACACCATTACAATTGTAAGGAGTAAAATCTCTGCGAGAAGAAA
GTCATGGAAACTGGTCTGCTTCTTCAGGAGCTCGTCAAGCAAGGCCATCAAGCTCTCCGC
GGTGCTCACGTCGGTGACAGTGGACAACTGTCGTAGGTTTCCTCGTGCCGAATTCT
>at02420
TTGTTTTTTTCTTTTTTGTTCTCCTCATGCCTTTCCCGTCCACGTCTGCGCTCACAACG
GAAAAAGCCAAGGGCGACGGAACACAAGTATGCCTTCAAAAACGGCACGCCCATTGAGTT
GCAGCAAATGCAACTTGCCCGTTTGAACATGTTTGGCGGCTCAACGTTGCATGCTGGGCT
TCGCAATGAAGGCCTCACAGTGCCGCAATGGCTGCATCCATGAACTTGGACTTCTGCTTC
TCCGAAAGATTTGAAAGCTCGTCGAGGCCTCTCTTCACAGACTCCTCCTGGATGTATGCC
TCCACCTTGTTGTCGGCACCTTCCTGCAGGCCGGAAAGTGTTTTCTCAGCCGCGGCTGCT
GCTCTCTTGAGGGTTACCTCCGTCTGTGCCTTAATGGTTGCCTCAAATTCTGCCTTGTCT
TTGTCAATGCTTGCATTGATTTCGGCAATGACCGAGTTGGCCTGGGCCTGTGCTTTCTCA
GCTTCTGCAAGACGCTCCGTGGCTTCGTTGAACTTGGCGATGGCGTCCTCAATGTCCGTC
TGGATCTCCTTCTCCCGTGATGCCATTGACTCACTGAGCAAAGGGGCCAAGAGGTAGATC
AGGCCGGCGATGAGCACTACAATGTTGATGATGCCCGTCTCAGGGATGTCAAGGTTAAGT
GCAAAGCCGCCTCCCAAAGGGACCTTCCCAAAATTCAAGAGACCTTCCTCCGCAAATGCA
GGTGAGGCACCCGTCAAGGCCGCAGAGGCTGCAACTGCCACAGCCCCAGCAGGAGCAGCC
CTAGCTGTGTGACTCTGTGATGGCGAAAGGGAAAGGAAGGCCGCACTTGATGCCCTTAGG
AGGTACACGGAGGCGGCTCCGGCCAGAAGCCCAGCCAGGAAGCCGCATGGCCGGGCAGAA
GAGCTTGCCATTGCTGCGCAAACGATTTGCCAAGTGGTCTAAGCGACAGCTGCTGCACTT
GAGCCAAAATGGACTACGG
>at02421
GCGTGGCTCCTGACAGCGCTGTCGCGTTTGCTTGATGGTCGTGATCTGGCCATTGGGGAG
CACGGCGCGGTAGGGGGCCTTGGCCTCCAGCTTCGAGCTGATGTAGAAGTCCATGCCCAA
GACCGGCCTTCCCTGCTGATCCATCCCGTTCTTGAAGTTGTGCCCAACGAACAACGCCGC
GATGAATGTGGCGGGGTCCCTGTTGCGAAGTGCTGCCTCGAGGTCCATCGGGGGGTCGAC
GCGGAAGGGAATGCCGCTGTTAGTATTCTTCATCATTGCCTTCATCCAATCCCACAACAA
GTCCATATTTGTGTGGCAATGCTGATGACGCTCCTTGGGCCATGGAGGTACCTGGCAATT
TGGACTGCTGCTGGCGCTGAGACGCGGACGCTCCACAGCGTCTCAGCTTGGGCCGCGTTG
ACGATGTTGCCGCGGTGA
>at02422
TTTTTTTTTTTTTTTTTGAATACCGAAGCAGCATCAACGGCGACTCAAAAGCACTTCTT
CCTGGATGCCAGTAGGGCCGCCTAAGGAGCCAAGTCACTCAGCAGCACAACCCATGTTCA
TGCTGCTGCAGCGTGGGCAGGGGATGCACTCTGAGCGTTCGCAGACACATCCTCCAAGAA
GCAGAGGAGCTGGAACAGGGTGAAGAGCTCTGGAGCCTTCTCGACACCAATGACCTCTCC
AACTTGCTTCTGCAGTTCCTCTGTGCTGGTCTCGCCCGAGGCGCGCACCAGCGAGTACGC
CCTCAGGAACTGCGCTTCGGGCATCTGGCTCCGGAGATAGCTCCGCAGCGAGTCCGCCTT
GGCTCCGATCCGGCCGCCTTCAGGGAGCGTCTGCGACGGCGCGGAGGGCGTGGTCACGGT
GCTGTCCTCCTCCGCCACGGGCTCCAGCTTCTGCTTGTGGGCCCCCTTGCCGAGCTTGAG

GTTGGCCACGCTCTGCTGCAGCTGGCTGGGCGGNNTCCNCCTCGCCGTCCTCGGACCCCG
AGTAGTCCTCGAACTCCTCCTCGTAGTCGCTGTCGTGG
>at02423
CTGCCAGCAGCTGCGTGAAGCTCCGCATGTCGACGAGAGCATTGACAACCATGTTGTAGA
TGGTACCCAGGAAGCTGAGAGGGGTGAACACATTCATGATATACTGGCTGACGGCCACGA
CATCCCCTATCTGGCCATGCTCCTGGAGAATTTGAGCTGTTGCGAGGAGCATCCCACCTG
CCAATGCAAAGTTGATGATTATCTGCTGCAAGACATTGAGAACGGACAGCGACGCCTGCG
TCGCCATGGAGAACTTTTGGAACTTCTCCACCAAGCTGCGGTACTCCCTCCGCTCGTAGT
CCTCAGCTGTGAAGCATTTCACAGTCTCGAAATTGACCAGCGAGTCAGTGAGCCGGTCAT
GCAGCTCATTA
>at02424
TTTTTTTTTTTTTTTTTGAGGCCACAAGGCCGATGCGCTGTAAGATCCACGTTTCAACC
ATGCAGGCGTCAGCTCACACCACGCAAGGGCGGCTCTGACTCGGGAAAACAAACTCACCC
CTCGATTAGTTGAGCTCTGCCAACTGACAGAAAACAGTGCTCGTCTGACTTGCTTAATTT
GAAGCTCTGCAGAACCACCAGCTTCTGTACAAGATGGATGCAGGCTCAGAGGCACCTCAT
GGAAGCGACCGCACATCGATCTTCAAGCACAACCTGTTCATGACAGCACCGTTAGTGTGG
TAACGCTTCTCAGGTGACTTGTGCACTAAGCCGTTCACGGCATGAGGAAACAACTTCCCC
TTCATGAATAGCATGTCCGCGACGTTTACGGCGCAGACTTGAGAGGTGTCACGGATAATC
TGCGCCTCGTGCC
>at02425
TTTTTTTTTTTTTTTATTCACTCAGGCCTCGCACAAACACGGGTGCCTCTTGGCATCATG
CGGTCTGTTCACCGATGCCGCAAGCACTCACTGCTTACTGGCCGCGTTGCTCCCAAAGAT
CAAGCGCCCAAGAAGCCTGTACTTCTTCACGTCGATTTCGGCGAGCGCGTTCAGCGCCTG
GGGGGGCAGCGCGGAAAGCAACAGCCCCAGGCCCAGGAACTTGATGTCCGCCAGGACGGC
CCGCAAACCACTGCTTGGAGGGACCTTCAGCATCTTTGACTGGTAGCCCGATTGCCTCTC
GATCGTACCACGCTCAAACTCGTCTGTGAAATTGTTGGCCTTTCCAAAGCCCACTTGCAT
GAAAATTCGGCCAGGCCCAAAGGGGTCATAAAATGTGTTGCCTGCGTGAGATATTCGCTG
GTCGATGATTACGGCATCACCGAAGCGAGGATGCAGCGCCGCATACCCCCTCTCCAGGCT
TATGTCTCGCAGTACGTGAGAGCCAGGTATCACCTTCACTGCTCGCGCATCCTGCTCGTG
GTCCTGCAAGTATAGGAGGACCTTGTAGATCTCATGCCGCTCGCCTGCCTCGTCGGGCTC
CCAGAGGTCGCGGACCTGGTGCTTCCGCTGCGGCCCCCGGAGGACGTCCTTGTGCCAGAC
GCCCACGAAGTCGCAGCCAATGTCGTTGTGTGATGCAAAGCGGTACTCAGCCCCATTGAA
CATTGCACCCAGGACCTTGTGTAGCCTTGAGTCATCCAGAAGCCAGCGTGCGCTGGCAAA
TTCAGGCAGATCCAGAAAGGCAGGCACCGTGTAGCCTCCCCAAGAAAGAGGCAACGCCTT
CCTTTTCTCACCGTAGCAGAAGTCCTCTGCAAGTCGTCGGAGGCTCCGAGCATCGGACTC
GTTCAGCAACTGCCGCACGTGCGCATAACCATG
>at02426
TTGTTTTTTTTTTCTTTTTTTTTGTTAGGAAGCAAGCGATGATACTGGCTTCGTCTTTGC
TCTCGCAGCCACGCTTGTGTTGGGCTGCTCAAAGCTCCTCCTTCATCTTGCCGAGCAGCT
TGATGCGGGCGTTCAACCATGAGAGCTTGTCCTCCTTCATGGCGTTACCCCGCATACCTT
CCAGGCGTTTCAGCTCGGACGCAACCTTTTCCTTGCCCTTTGCCTTCTGCTTCTCCACAT
ACTCCTTCTCCTTGTCGGTGCACTTCTCCAGCGTTGTCACGTCGCAGCGGCTCGCGGGCT
TCTTGTCGCCCTTGATCTTGATGATCTCCATGGTGAAGACCAGCACGTCGCCACCGCCGA
TCTTGGGAGGGCTGCCCGAGTCGCCGTAGCCGAGCTCGGAAGGGATGTACATCTCCCACT
TGTCGCCCTCAACCATGAGCTGCATGGCCTCCGTCCAGCCCTTGATGACCTGGTTTGGGG
CGAAGGTCGTGGGCGAGCCGCGGTCGTAGGAGCTGTCGAATTTGTCGCCGCTCGGCCAGT
CCTTCGCCGTGCGGCCCTCGTAGTGGCAGTCGCAGGGCGAGTCCGCGGTAGGGTGGTCAG
TGCCCTCGCCAGCGCGGAGGACCTTGTACTGCAGGCCTGAGGGCAGGGTGATCACGCCCT
CCTTCTCCTTGTTCTCCTCCAGGAACTTCTTCCCAAACTCGTTCGTGCCTGCGGCAGCTA
GATGCAGCGCACTGGCGAGTGCCACTGTGGCCAGGAGTGCCGCGCTGGCAGGCATGGCCG
CCAAGCTGATGATGCGAAGGGGGGCTGGGGTGGGCCTCCTGTCGGCTTGAAGCAAAACTG
ACTTGAGCCAAAATGGCTACGG
>at02427
CCTTTCCGCTCAGAAGCACAACACCGGCAGAAGTGGACGAGACCTGAACGGGGTACAGGT
CTACAGAGAGCTGGAACACTGCTATGAAGCCGAAGGTGGCGCAAACAGCAGGGCTGTTCT

GGGCCGCGATGTAGCAAAGCTCCACAACCCCTGTGCGATGCAGAATGCCACCGCCCCAAG
CAAAGAGAACCATTCCCGCAATGCCGAGAACCAGAGCAAGGGCAATCAAAGCCCTCCGAC
TGATCAACACGCTTACAGGAATAGCGACAATGTTGATGGCAAGGCCAACCAAA
>at02428
TTGTTTTTTTTTCTTTTTTTTTTTAGTTGATTCTGCGCTCACCACCAACTCACAGAAAAG
AGCTGCTTTGACGCGTCTGTACGACGCCAGAAGCAGCTTTGAAAGCCATGCGAAATGGGC
GATGAAAGGTGCCATCGCCACTGCTGTACACTCAATCGAAACAGAGCGGAGTCCATGGTC
TCAGGGCAGGGGCATCACCAGCACGCACACGCCATGCGGCAAGTCCATGCCGTTCATGCA
ACAAATTGATAGTGCGCTGCTTGGCACCTTCAAGAATTAAGCCGGCTCGTGCGGCTCACG
ACTGTCTGTGGCATGCTCGCATGTGTACTGGGTCATGCGATGGACTGCAGTTATGATAGC
ACTTACAGTCTTGACATCGGGAATATCATCAATACGTCTGCAGAGGCGTCTGACCATCTC
TGTCCCATCCAGCACCGCCTGAGCTCCAGGCCGCGCTCAGGACTCAAGCAGGTTCTTGCG
ATAAGGCCGGCAGCAGCATAGGCAGCAGGGGTACAACACGACCAAGATGCAGATCACCAG
CAGCACGGCGGACGCACCGACCCAGGCGGAGAGCGCCGAGGCAAGGGCCGAGAAGACGAC
ATTCCAGAACCGTGGGAAGGCAACCTCGATGTGGTCCAACACCTCCGCCACCGTTGGATC
ACCCCTAAGCTGGGCAATCCTGACTTCGCTCGCGCCAACCTTCCAAGCCGCCCGCCAGCG
ATGACGGCGATCATCAGAGTGAAGGCCGCGAGCCAAAAG
>at02429
AGCTGATCGAGGAGTCCCTGCCGCGGCTTCTGGAGGAGAAAAGCCAGCTTGAGACGCTCC
GCGGCGATGTGGCGAAGCTCCCATTGGCGGGCCGTACGAGCTGCCGAAGAACGCGGACGT
CTCTGCCTGCCGGCCCATCCCTGGCCAGAAGGACGTTTCTGGCACGCTTGCAGAGCTGGC
TAACATCAAGTACCCCGGCATGCTGGAGGAGGTCAATGCAGACGTCAAGCAGATCTCAGG
CAAGAAGTGAGATGCCAGCAGCACTTTCTTGGGGTGATTCATGCCAGTGGATGGAGAGGC
CAGCGTGGCAAAAGTGCGCCTCGTTTTGATGCAACCGTCCAATGGATGGCGCAATTGCTT
GAGCCGAAATTGCATCATGAGATTGATCCAAATCAGTGCTACTATCGCGTACGCCGTCTT
GTCATCTGACCTGATCTTCTTGGCTATTAGGGTGTAGCTTGCTGCACCTGTACAGCCACA
CAGAAGTAGGGATCCCAATGCTCATGGACGTCG
>at02430
GGCACGAGGCCGAGGAGCGCAGTTGGCGGGACAGAGAGGTAAGTGCTCGTGACGCTAAGA
CCCCAAGCACCAATGGCTCGGGCCCTGCGCAGCACGAAGCTCACCATCACCCTCATTGGC
TTGGTGTTCTGCTTTGCAGCACATCGGTTCGTGGCACAAACCCAGCTGACGTTCATCCAG
CCACGGGAAAAGACGCCTGCTGCCAGTGAGGTGACCGCGCTTGGAGCCGCTGCTGCACTG
ACAGCCTCGCTGCCTGCAGAAGCATTCACCGGCCCGCTGACAGGCTCGGAGGTCTGCAAG
TCAAAGCCACTGGTGTGGTTCATCTACCCGCTTTGCGACCCTGTCTTCCTGGCTGCACCA
CTGTACTTCTACCCCCTTCTCGCCATTTTCTACTTTGTCATCGTAACTACTATCCAGTTG
CTGATTCCAGCAACGCAGCCTGACGAGGACCTTCGATGAAGGGGTTGCCGGTGCCAGTGA
GCCGAGAGGGTCGCTGCCCTATTTTGCGAAGGGACCACGACAAGCATGAGCAATGCTCAT
CTTAGTTGCAGTCCCTCATAGAGTGAAGAGGAGCCGCGAAAAAAAAAAAAAAAAAA
>at02431
CCTTCTGCATGGTCTTCGAGAATCtTTACTCAACAAGCCAAGTGCCAACGGCCACTCGCT
GCACTTTCCGCAAACACTGACTGCAGCGGTACATGTCATTGCGTCCGCCCTCACCATGGC
CGAcCCCCATTGTGTTCAGGAGGAGTAGTCCATTTCTCCAGCTTCCGTCTTTTTGACATG
CACTGAGTACTCTTCCATAATCGGAAACTTGGACCTCAAGGCACACAGGGTACTTAGGAC
TCTGCAAAACCTTCAGTGCTTGTGCGGTTTGCCTTGCCCTTGCATGCGCACTGAGGA
>at02432
TTTTTTTTTTTTTTTTGTCGTGGAGCCCACACAGTTTGCTGCTCTGTACACCGGTGAGGG
CTTCAGCCCAACAAATCCAATTCGGCGCAGGGGATGGCGTGCCTGTGAGTTAGTCTGGAC
CAGAAAACTCAGCTTGCATATCTATAGATGCAAGCCATTGCACAACGCATCAGACCTGAG
CGGCAAACTGGTCTTGATCATTAGGCGCAGAAAGAGGCACATGACATCGCAGTACCTATG
TTAAAACACCCAAGGCACATGAGACCTCAGGGCCTAAGTTGGAGCAACTAAAACGTCATC
AGGAACAACTGGCCACCCGGCCCGAAGCAGTCCCCATGATACATGGCTCAGTTTGGCAGT
TTGCCAGTGTTGTGACACGGGGCTCTCTTGTTGGCGCCTCCGTCTTGGACAAGCAGGAGA
CCAGCTGCCAGTGCAGCACTTGTGTGCTACACCGTCGCTTTGCGCACTGGTCAAGCTGTT
TGGGTCTTAAAAGGGCAACACCCATCACATGCAGCAAGCGCATGCCCGGCCGACATCAGC
CATCTGCAACAGCCCGTCTGGGGGTC

>at02433
GACTGGGCGATACTCGTGACACAGCGTCTCCAAGAAATTAGCGAGACTGGCAGGCATATC
AGAGCGGGAGTGCATGGACCGAGTGTCTGTCATCAGTGCCCGAGGTGGTCTCGTCATGGA
GGCATTTCCATGGTGGTGAAGTTCCAGAGAAAGACGACTTTCCATTCATCCTGGTGTTTG
GACCCGTCACCGTGGAGGTCATGGGCCCTACGCACACAAGCGTTCAAAGGCTGGTTCAAG
AGGATGAGCGCGGCTTCACAGTCTGTTGGAATACATCGGGGGGCGGCAGGCCTCTCTGTG
CCCGTGGAGGCCGGCGGTG
>at02434
TTTTTTTTTTTTTTTTTGCGCGGGAAAGCTCTCCATTGTATCCCTGGTGAGACCACACA
GCACCCCAGATCTCAGCAAGCTGCGCTGGTGTCTGCGCCGGCTTCCATTCTTTGAAGAGC
AGAGAGGTTGCTTATGAACCGTGAAACAAATGCAAGCTGCGCGTCCAGCCCGCCAAGTCC
TTGGAGGTTTGTGAGGTGAGCGGCAAAGTCCCCAGGCTGCCACTGACTACGCTGTGGGCG
GACTAGGGACTGCAGAGCGCGGTGCTCCACGAGCGCAAACAGACCCGCGCCGTTGTCTCG
TATGTAGGCTGCTATTGCATCCTGGTCCCACCAGCCCGATGTCCGCGGCAGTCGCCATAC
GCCGCCGAAGAGCTGTGGCCTCTTGCTGATGCGGAAGAGGTCCTCAATGAGGCGAATGGT
CGCCTCATCCCTCTTGACTGCAAGCAGGCCACTGTTTAAGGGCACGCGCTCCCATGTGAC
CTCGTCACGCGACGCAAGCACTAGTTGCTTACAAGCCGGGCACATCAAGTGCTTCGTTAC
AGCCTCGTGAAGGGCATCGCGCTCAGGGCTGGTGATCACAATGTCATCGTCAACCCTCGT
GCC
>at02435
CCTTGACAAACGAAGAAGCAGACGGATCGGCCGTCACCTCCGCGTCGAGGTTGTGCTGAT
TGTTGGCATAGGCGCAAACCCAGTACGCGATACCCACATCGACTTCGAACCCTCCGTCAG
CTCCGCGCTTTGTGTGGTCCGCAGCATGCTGCTCCAGGCAGCGAAGGAAGAGACTGACCG
CTTCGCCCCCACCAATGCGACACGAACCACGCCGGTCGCTGCGGTCCGGTTGCAACGAGC
TCCACGTACGAGCAGCGTCGCTCAGCGGTTGCCGGGATGGAGAAAGTGTTTAGAAGCGTC
ATACAGGTTGATACACTCGGGAGCCAGCGGCTTACCGTATCGATCAGTCCACCTTTCGAT
CGCACACTTTGGCTTCATCTCCGACTCCACCGTCCGCAACT
>at02436
GAGCGACCAGCTGGAGAGTTGCCCCTCGCTAGACCGCGGTAAAGAGTGGGGCGAGGCCCA
GCACGAGGCTGCCCCCATCTCAGGAGGAGCCAGGGGTGAAGATCCTGCAGTTGGACCTAG
AGATCCCCGAGCAGGTGGGTGGCCTTGTCTGGCGCGTTGGCACCGACAAGCGTGGCGCCT
GGCTTGCGGGCGTCAAGCGCGGCACGCCTCTGCCTGAGGACAACGAGCTTGAGGTTGGCG
ACCAACTCGTCGCTGTCAACGGGGAGCCAGTCCAGGGACTCACAGCAGAGGACATCAGGC
AGCGCTGGGAGGAGAGCCtAGGAGCAGGTGTGGNAGGCCAGCGAG
>at02437
TTTGTTTTTTTTTCTTTTTTTTTAACTACTGGCAAAACGCCAACAGCGGCAGGAAGATGC
ATCCTTGGTCCACTCCCCTGCACCTAGTTGACAGCTGCAGCTCCAACCTTCTCCGCCTCA
CAGCGCTCACGCATGCAGGCATTCATCTTGGCGACCTGGGTGATCAAGACTTCCTTCTCG
GTGGCGGTAAATTGGCGCCATGTCTTGTACTGCTCACCCGAGTTGGTGCTGTCGGAACCC
TTGTAGAATACGTTCTGTCTTCCGGCATTCGTGAAGGACATGCCACCGTCGAGATTGAGC
CTGAGTGGGCCGCGCTCCGGATCAAGCTGCACCACCTCCTCTTTACCCCCACCCAGATCC
ACGGTGACTTTCCTCAAGGCGTCAAGGTCTTTCCCATACAGGAACGATGACATTGCACCT
AAGATGTGGACTGCGTCCTGCGGACTTTGCAGCAAGGCCTCGTGCC
>at02438
TTTTTTTTTTTTTTTTTTCCAAGCGGACTCGATCTCTGCAACCCTCACGTTGGGCAGTCG
TTGAGGAAACTGGGGAAGTCATCATGCCTGCGTCCTGCTCCGAACCCGCGGGTGCCCGCG
GTGCTGGCGCTGCTGCTGGCGCTGCTGCAGCTGGGGCTGACCATGGCGGTCTGGGCCGAG
CTGGCAGTGGCATGTGTGGCGTCGGGTGCTCGGCGTCCACCACCCAGACGGCGAGGCCCT
TCACGGCCGCCACGGCCTCCGCCTTGCCTCATCTCGCCGCTGGCCAGCCGGCGCTCGAGC
TCCGGGGGCACCGGCAGCGCCATGCGCCTGAAGATCTCCACGATGCTCCGCGCAGCTCCA
CCATCGTCGTTGGTCAGCAATGTGATCGCTTTCCCCTTCAGTCCCGCGCGACCAGTGCGC
CCGACACGATGCACGTAGTCTTCCTCCCGGTTG
>at02439
TTAGTTTTCCTTTATTTTTTGGCTCAAGACCGACAGCCTTCGGGAGTGAAGCCCCCAGCA
TCCGAGAGCGCTACCCCGCTTGCCTCTGCTTCGGCTCCCGTCATGGCAGCCCAACTACGA

TCTGTCCTTCTCCTTGGCCTCCTGGCCCTGCAGATTGCGCCCGCTGGCACTGCGGAGGCG
GAGGCCGAGGGCCTGAGCAGCATGAAGGCCGGCGCAAGCCTCCGCTCCCACAGCTCCGCA
CTCCGCGCGGAGATGCAGAGCATGATGAGGGACGAGGACGACGACGTGGACTTCGGCAGG
CTCTCGCGCGAGGGCGAGGACGACTCCGGCCGCGGGCACCTGCAGCGCAGCGGGCCTTCG
G
>at02440
TTTTTTTTTTTTTTTTTTGCTCGCCATCAGTACGGCAATCTGCCGGCCCCGCTTGATCCC
ACAAGACTGTCCGCCCTCACAGCCAGCCAAAGCAGTATGCAGGCAGCCAGGACCCGGCTC
AGTCTCACCTGCCACCCAAGCGCCTCAGCATCGTGAAGGCAACCCTCGCGAAGAAGCTTG
CAAAGAGGTAGATGCCGACCGAATACCAGAGGGAGAAGCCTAGCACAAAGTAGGAGCCCA
GGCCAGCACCCCAAGCGAAGCCAACCCGCAAGCGGTTCTGA
>at02441
CCAACAGCGGCTACCATTTTTGCTCCACTAGGCTTCCCAGATTCGCATATCACAATGCTG
GAGGACGTGCACCTCATCGTTGCACTTGCCGGGCTCGAGCCAATCCGAGACAGACTTTCA
TTGCTTTCCCACTTGAATTGACGATTGTCCTGCAATGCAGGGGCTCTTGCAGCGCACTGC
AATTCCCTATGTCTGCTTTGTGCAGTTCATGTGGGGAAGCTGGGTCTGCTGGCCAACACT
CCTTTCCCCGGTCACTCTTTCAAGACCATCACCATCCAAGACCAGAGCACCATGTGCTGT
GTCAACCCATCCATCGTCCAAAGACGTAAGGACCCGAGCAGTTGAAAGCCCAGCCGCAGC
ATGCTCAGCCT
>at02442
GGTACTGTATCAACAACAGTGCTAAGGTGGCGGCCTCTTCCATACATGTCCTGAAGCTTG
CCGCCACGAGCAGCCTGCCAGATAGCGCTGGCGACAAGTTGTGTTACCTCAACATTCTGC
GAGCTGTCGGTCACAGGATCCTCCCAACCACGTCGGCCTCGGTTCCTGAAGCCACCGCAG
CGGAAGGGTGTGCAACGCAAGCCCGAAAGGACTCTTGCATCATGGCATTCCAAGTCGATT
TCTGATACCTGCGCAGCATCGACAACTGTATGGCTCCCACCAAGCTTGCCCATGGACTCT
GGTGAAAGAGCATCGACAGCTGTAAGGATCCCACCAAGCTTGCCCGCGTACTCTGGTGCG
AGTGTCACAATCCTGACC
>at02443
TTTTTTTTTTTTTTTTTTCCCCTTGCGCAGGGTAGGATGGTGGTTCCGGTTCTTGTTCAG
CCTTCTCTCCAGTCGCTTCTGGCGCATCCTGCTGTGCATTTCGTCGAGCATGCCCTCTGT
CTTCGCATACTCTGTCAAGACCGCATCGTCGCCGCGCACCCTGGCCTCCTCCTCGAGCAG
CAGCTCCTCCCGCTCCAGCTCCTGGAGTCGTCTGTCGATGTCCGGGTCGACGAAGTCGGC
GATGTTCTTGCCATCCATGATCTCAGGAACGACATCATACTTCCAGGAGCTTTCCTCAAG
GATAGTCTTCCTCCAGAGGTCGACAGAGTAGACGCCAGCTCCACCCAACTCCTCCATCCG
CTCACGCTCGAGCTTCTCCTCCTTTTGCTCCTCATCGACCGCCATATCCTCTCCAGCACG
CTTCTGGAGGATGCTCTGAGGGATGCACGGTGGTCGATGAGGAGAAAAAAAAAAAAAAAA
AACCTCATATGGGCGGCCGCCTCGTGCC
>at02444
TTGTTTTTTTTTTCTTTTTTTTTTGTGCCACCACCTTCCTTTACCTGCCCCATGCCAGCTG
TGGAGTGCGCAAGTCGGAAGACGCAAACTCTTGCCCACGTCCCTTCCTGCGCCGTCCAGT
CCGCCTCACTTGATCAACGGGTTTGCGAACAGCAGCACCAGCGCGATGACGAGGCCGTAG
ATTGTGAGTGACTCCATGAAGGCCAGCGACAGCAGCAGCACGCCCCGGAGGTCGTCGGCC
ACCTCNGGCTGNCGGCTGATGCCATCAATGCAGCGGCCTGANGCNATGCCNNGGCCAACG
CCCGAGCCGATTGCCGCGAGGCCGATGGC
>at02445
TTGTTTTTTTTTCTTTTTTTTTTGTTAGTCGCAAGTCCGCTCACACCATCAACTCGAACC
AACGAATTCCGCCAAGACACCCTCTCGAGCTGGCACGGGGGCGGCTGAGCTACATGCGTG
CGGCTCAGTGCGGATAGAGGCTCTTGATCTTTGAGATATACTTGCTGAACGGAAATGACT
CTATCCACACAATGCCCTTGGTCTCTCCAGGCGGCAAGCTGATAATCGCATGGAAGATGC
CCTCCCCTCCGAACATCATGCTCTTGAGCCCGCCGGCCAGAGCAATGTCGTACGTTAATC
GACTTGTGAAGGCAACAAGGCAACCAGTGTCAACTCGAATCGTTGGCCGCTCCGGGGAGA
GCTCCTCTTTGACCACAACGCCACCGCCTTGCAGCAAGACCGCGCCCTCTCCAGAGATCT
TCTGCAGGATGAAGCTCTCACCGCCAAAGAAGGCCGCACCCAATCTCTGCCTGAAGCAAG
ATTCGACATGCAGTCCATCCGACCCTGCGAGATACACTCCACCGGCACCAATGATCTCTC
CACCGTACTCTCCAAGGTTCACAGGTACGACGTTTCCAGGGACCACAGTCCCAAACCGCA

GTGTCTTTGGCTCTTCTGTTTCGTTCGTGAACCGCGCCATCATGAAGGATTCACCGGAGA
ACATTCGGGAGGCTGCTTTCTTCATGTTGCTCAGCAGCCCGGCTTCCGTGCCATCACCCA
TCACAGTATGAAAGTGCACCCCCTCTGGCAGACCGATCATCCGCCCTGGTTCTGCACACA
TGCTGCATCCAGGCTGCACCACAACCTCGACCAAGGGGAAATCCTGGGTACCCAGCAAGG
TGAATGTCACTTCGTCGGAGCTCGCAGACAGAAGTGGCACTTTGGTGATGTCGTTGTCAC
CAATCTCATTGTCAGCCATCTGTAAGTCACGATTAGGCCCACCTTTTGTGGAACCCTCGA
GCAGCAAACACTTGAGCCAAAATGGCTACGG
>at02446
TTTTTTTTTTTTTTTTTGCTGACGCAGGTAATTTAAAACTCTGGCCCAAGTCGGTGCTA
AGCACCCCTTGCCTCCAGCATATCACAACTCGATGGCGCAAGCCAAACTACAAAAATTCA
ACCACAAGTCTGTACAAATCCATGCGGCCAACTGCGGACGCTTTGAGGTGATTAGACTCA
CCTCGGAACAGCATGATGTGAGGTGCTTGCTAGTATCTGATCACAAGATGCTGCCAACAT
GCAGAGATGTTGCAGTGGAAGGCAAGTCTCCAACTGCCCTTGCCACTGACTCACGTGTGA
CACTTCAGAGGACCACAGAGGCAAGCTGGCAACACGCCCTTACAAATCCATACATCACCT
GGGCTCTTGACAAGGCGGCCTCTGACAAATTGAAGTTATCAAGTGCACGAGACTGTTCAG
TGCACATGAGTCAAGCATGTACAAGTCTTGAGGGAGAGTCAAGAAACACTGACTCTGACA
GAAATGAAGCCCACCCAACGAGTCCTCCTGCTGCGTTGTGAGGTGTAGCATGCTCTGCGT
TGGCATGCGCATGGCTTTGCCGCACCGTCGCGCCATGCTTCAAGAATCCACTCTGCCGCC
TCGTGCC
>at02447
CCGTAGCCATTTGGCTCAAGCAATTTGGGTTCAAGGGAGAGGGCCAAGCCCAGTCCGTTC
AGAGGCACTGCTCCGCGTCGGGAAGGAAGATGGCTCTCCGGTTGGCGGCGCGCCGCCTGT
TTGCAAGCCTGTGCCCCTGGGGCTCGAGAGTAAGCAGGTGGCATCCTTCAAGGCCTCTCT
CAAGTCGTTCATGGGCGAGGTGCAAGGCTTCTCCTTCAGCAAATACTTCGAGGAGAAGTA
CTTCTGGGAGAAGGCCAACGTGGGCCCCTTCTTCCTCCTCCTCTTCTTCACCCCGAGCAT
CTACCGGTCGGTGAAGGACTTCTACTGGACTCGCCAGCTGAAGAAGTTGAACACGGAGGA
GATCATCAGCGACCGCTACGAGTGGCTGCGCCTCTCCATGCTCCAGGACGAGGTGGAGGC
CGCCCTGGTCAAGCAGCCGCCCGTGGCGCCCCTGGAGCTGGGGCCCTCGACGCCCCAGTG
AGGGGCCGGCCCCGGGCGGGCGGGGCGGAGGCATGGATTGACCGCGAAAAAAAAAAAAAA
AGAAAAAAAAAANAAAAAAAAAAAAAAAAANAAAAAAAAAAAAAGAAAAAAAACAAAAAC
TACTAG
>at02448
TTGTTTTTTTTTCTTTTTTTTTTTCCTTCCTCGATCGGCGGAGCACCTGCACGCTCCGGG
GCCGCCCTTCCGCCGCCGCTCAGAAGCGCAGCTTCTTGCGCAGGTAGATGACGTCCTTGG
AAGGCTTGCCGGACTTGTCGGCAGGCGGGTTCACAAACTTCTCCTGGAACAGGTTCTTGA
CAAACTTCTTGGCCTCCACCCGCCCGTCCGGCGTCTCCATCTGCTGGTCCGTGGACCAGG
GACTGCGCGCCCAGCGTCCGCAGGCACCTGGTAGCGTGTTGGCATTATGTGATTGTAGTT
GATGTACTTGAGGAAGGGCTTCACGCGCGTGCGCTTGGTGATCTTCTTCTTCGACATCCT
CTTGTGCACCTTCCTGGGCGGCCGGTCCACTCCAGCAACCAGGCAGTGACCAAAGGGCCG
TGCCTTGGTGCCATCATCGAATGCCTTCACAACAATGGCCTTCTTTCCTGTGTAGCGGTT
CCCTGGAGGAGCACCACAATGCGGCCGGTCTTGATGAACTTGCCCATTGCAAAGAGGCGG
TGGTGTGGATGTGGCGGTGCGGATGCTCAGCACTTCCGAGCCAACAGAGCTTCAGCCAGC
CAAA
>at02449
GCATTTCAGGGGTCAAACTGTTCGGCTTTCGAGTGTCAAGCTTCAATGGGTGGCAGTTGC
TTTGAACAGTTGGGCGAGCAGCCAGCTGATAGTGACCCGGAACATGCAGAACTTGACAAC
TTTTGGCCAATTTGCGACATGCCGCAGTCCAAGCGGAGAGGCCGGCCGGGTGATGGCGCG
TTCGAACTGGATGGCAAGATATGGCCGACTTGCGAGCATTACTTCCAGGCCGCAAAGTTC
CCCAAGGATCCAGACTACCAAGAGATAATTCGCACCAGTGAGACGTGTATGGGTCCAGAG
GGGTGCTTCTCATTGGGTCGTAATGGGAGCATGAGCATGAGCGTCAGGGACTGGGACAGC
ATGAAACTCGAGGTCATGTA
>at02450
GGAGACAGGCTTCCGGAACCCATGATGGTCGAAAATGTGAATGGCTGATTGGACCAAGAG
ACTAGAGCGGGTGCAAGCTATATGATCAACCCTGGACACGTACCAGGGATGTCATTGAAG
GAACCTGCTCAGCCACTCATTCACATAGTCTCAACGATGAGGCCCTGGCCAATAGTCTTC

CCACCCTCGCGCATGGCGAATCGCATGCCCTTCTCGATAGGTGTGCCAGCGATGAGCTCA
CAATTGATGGTCACATCGTCACCCGGCATCGTTCCTCCGGCGCGTTGTCGATCTCCTCGT
AGGTCTTCTGCTTCGTGTCTTGCGTCGTGCTGAACTGTGCGCAGACAAGGGAGATGGCAG
CTGAGAGAGTGGTCTTGCCATGGTCAACATGCCCGACAGTGCCAAGATTTACGTGGTCCT
TGGCCTGCCTCACAATGACAGCAGCACGTGTGGACCGAGGCCTCCTTGCAGCAGCACTGA
AGGCAATTGCACCAGCTGCCATCAAGCCAAAGCTGCCGATCTGCAGCAGAGATGATGCTG
GAGGCGCACTTGAGACCCTTGGAACCCCATCCCGGAGGAGCACTGTTGCCTCGTGCC
>at02451
TTTTTTTTTTTTTTTTTCCACGCGGTGAGTGGACCCTCGAGGAGCATCCGCACCTCGCG
TGCACGTTGGAACCCCTGTACACGCCGAGGTGCTGCCCACGCCCCAGGTTGCCCCTCCAG
GGCTTGCACCTGGCTTGCACGTTCAGTAGTCCATCTCCTCCTCGAGCCCCCTGCGCTCGC
GACGCTGGTCGTTGCCGTAGAAGGACCTTGCGTTCTCTGGTTGCACCCGCTTCGCCGCGA
TCTTCCTCACCTCCTCCGCTTTCTTGGGCTTCAAGCGGCGCCCCTGCTGCTGAAGCTTTG
AGTTGTCGAAAAAGCGGAAGGCTGGATGATCAGCCTTCTTCGACGTCTTGTCCCCAGTCT
TCGACTTGTAGCCCTTTGGTTGCCGGAGGAGGTCATAAGGATCCACGTCCAGAGCAGGGT
ATGGCAACCCCAGGGCATGTAGCTTCTTCCGGTCGTCGGCACTCACGGACGCATGGCAGC
CCACGGTGAAGTAAACGATGCTGTACCGGGACCCCTTGAATGGCTCCACAGAGTGGGCGC
AGTTCCCGTTGAAGAGCGCCAGGCCGCTCGTGAGGTCGAAGCTCATCTTCCCGCTCTTTG
GAAGTCCCTGCAGGCTTCCGCCATTGTCCTCTGGCCAGTAGTTCAGCGTGCCGCCCGAGA
AGTCGCCGAAGGCCTTGATGAAGCTCGGCCCAAAGTTGCCGTTGTCACGGTGCATCGCGG
CCGCATAGTTGCAGTTCACGTTCATGGACGTGAAGGTGAAGTTCTTCGTCTCCTCTGGGA
GGCGGTCCGTCAGCCATCGCGCGAGGAGCTCCGCCACCTGGGGGTACGGCGCGTCGGANC
GGTGATGTGCACGT
>at02452
TTTTTTTTTTTTTTTTTAGCCGCGACCATGCTGCGAGCGAAGCTCCACAAGTGCCATGG
TGCCATCACCACCGGGCACTGCAAAGGCCCGGCTCTCCGCATCCCGAACAGCGCAGGCAA
GGGAGCCCGGTGGCGCCGACGCGCACCTAGGGCCGCCTGCGGCGTCCCCGGCCCGCCAGC
GCCGGTCCAGGGAGCCGAAGCGGCGGAGGAACCAGGGCTCGTCGGTGAAGAGGAAGGGCC
TCAAGCCGTGCCGCCGCCACGCCGCCCTGCGTGGGCCGCGCTTGAAGCTGCGCATGTCGT
GGATCGCCGCGAAGCGGACGAGCCCCCCGCGAGCAGACAGCAGCCTGCGGGCCAGCGCCA
GCGCGGCCTCGTGCTTGGGGCCGTCGATGACCACGGCCACGGAGCGGGCACCGGCCGCCG
CCGCCGCGGACAGGGCTCGCGGAAGGGCATCGCGGGCGTCCTCCTCCAGGAGCGAGACAG
TGGGCGAGGGGCCTGCCAGGAACGGCGCCAGGCGCTCTCGCGCCTCGGGCCGGAGGACGC
GGTCCGCCGCGACGACGCGGGGGCCTGCGCCGCCGGGGGCGGAGCTGTGCAGCCACCGGG
CCCAGATCTCCGTGGAGAAGGCCAAGTTCACCCCAGACTCCAGGATTACGTCCACCTCAA
ACGCCTCGCAAAAAGCACCACTGCCAGCGCTTCGCTCACGCGGACTGAGGTGGAATCCAG
GGCTGGCGCCAGAT
>at02453
CGAAGCATCCCTCGCCGCTGACATTGCCTTCTTCTCAAAGAGGTCCTTGAGCTGACTTGT
TGCACCGGCAGACGCAGTCTCCGGAGGAGACATGGGGACCATGGGTGGACATGGCGACTC
GCGGCTGCTGCCTTGCCCCTCGATCTTTGCACCCTCAAAGTGCGAAGGCCGCCGAGGCAA
CCCCGACGATGCCAAGCCAGCGCCATTCCTCGCACGGGAGGGTGTCGGAGCGTCCGCTGG
ACTTGGAGCCGATGGCTTTGTGACCTTGGCTGCCACTCCCTCTGCCGCCGCTTCCCCCGC
CC
>at02454
TTTTTTTTTTTTTTTTTGGGTTACCTTCGGGCCCTACTGGAACAGTCACTCTCACTGCT
CCACCTGTACAGCGGGTTTCCGGCCGACTCCGGCCAGCGACACAGGTGCCCCGTGAAAAG
CACCCGGTTGAAAAATCCTCCAGGCGCATCCAAACCGATCCCGCCGGAGGATCAGAGCTT
GTCGAGGTAGTCGCCGAGCGCGGGCATCTCGACCTTGATGTTCTTCCGCTTCCGCACGGC
CAAGACGAGCTCCTGCATCTTGGTCCCGGCCTCCATGGCATTGCCTGGCATGTTGTCCCA
GTGATCAAAGACGCACTGCGGGAAGGCCTGGCCGCTCGTCTGCTGGCGCAGAGCTGAGAC
GAAGCCGAAGGACTCTGCAACCGGCAAGTGCGCCTTGACCTGAATGAGCGGAGTGCCCTC
GCGCGGGTTCTCCTCGAAGACGCAGCCGCGTCGCAAGTTCATGCAGGCGTACACACCGCT
CATGGCCTCCTGCGGGCAGGTGATCTCGACAAGGAACACAGGCTCCTGCAGCGTTGGCTT
GCCCGTCATCTCTGCTGCAAAGCAGCAGCGGCGCGTGGGTGGCATGATCTGCCCCGCGCC

GCGGTGGATCGCATCGGTGTGCAGTCCTCGTGCC
>at02455
TTTTTTTTTTTTTTTTTTGAAGCGCCCAAGAAAATCAAAAGTGCACGGGGCTAAACAGTC
AGATACCTGAGCCGCAGCAGTGTCCCCGGCCATGCACTGAGCTCACGGTCCATTCTTGAG
CGTCCCGTCGCCCTTCCCCAGCGAAAGCTTCCTGAGCTCCGCCTCCATCTTGGAATTCTG
CGCGGGGATGCGCTCTGCCCTGGGATTTTAGGGATCACGACCTTGTCCGCTTTCTTGCT
TGTCGTGGGGGCCGGCGCCGCGGAAGGCGCCGGCGCCGCGGAAGGCGCCGGTGCCGGAGC
AGGCTCGGCCGCGGCTGGCGCAACGGCCGGAGCGGCCGCTTCCTCAGCCAAGGGTGGCAC
ACCACTGAACGCCACAACCAAGCCGAAGGCAACGGCCGCCATGAAGGCCCCGGCGCCGCT
GGGGGAGCCGCTGCCTTCCTCAAGCGGGGC
>at02456
ACAACGCCGTACTTCCATGCCACTGCCATTGCCTAAACGTGCCTGCCACGAACCTCGTGC
AGATCGGCATCGGCGGCTGGCAGGTGCCCCGGGAGGCAGTGCCCAACATACGGGCGCGGC
AGACGAACATCGTCACCATGAACGACATTGAGCGCCTGGGTCTTGAGAAGGTGGCAGAGA
TGGCGCTTGATGCAGCATGGAAGGACGCTGATGCTGTGTGGCTCAGCTTCGACATTGACT
CCATTGACTGCGCCTTCGTTCCTGGTACGGGCTGGCCGGAGCCGGGTGGCCTGCTGCCGC
GCG
>at02457
GGAGATCGCCTGCGGATTCCATGCTTGGTTCCCGATCCTCCGGATGAGGGCGCTCGCCAT
GGGGAGGTGGGAGCTGATGAGTCCACTGCGCAGGCTTGCTCTGACAACTATCGGAGTGCC
ACTGGCAATGGCCATGGAGGCCAGTTTCACCTGTTGCCATGGTCGCTTCCTATGCCCTCA
GAGAAGCGCGTGCCGAGCTCGCTCATCCAAAGTGCCTGCTCGATGCCGCGCTGCGGATCC
AGCGGCACGGAGCGAGGCAG
>at02458
GTTTTTTTTTCTTTTTTTTTATTCATTCTCGGAGTGGAAGACGAGCATTGATGCCGGAAT
GTGGCCAGCTTCAGTGCGAGAAGCTGGCAGTCGCATCTGTGTGTTAGTCTATCACCAGAT
CACCTCACATCTCAAAGCTGCCAGTCTTGGGGTCGTATGTGGCCTCAACAAGCCTCCCAT
CCTCATACTTCAAGACATCATACTTGCCAGTCCTCAGGTGAGCCGCAACCGTGTAAGAGC
CGCCCTCGCTCAGGTCATATGAGCGTACATCAGAGGCTGGTCCTCCCTTGACCGAGCCGT
TTGGCTTGATGACAGGATCGTCCCCGAAGGCATCAGCGCAGACAGCACCGCTGTCCTCGA
GGACCTTTTTGGTCTCGTAGAAGGGCTTCAGGTCCGGGCGCACTGCCAAAGACGTGTCAT
CAAGCCGATTGACCTGGTCGAAGTCATTGAGCCGGGAGTCGCGCTCCTCCTCGTAGGCGA
TTTTGGCGAGGACAATTTCCAGCGGCGTTACCAAACCGAGGGTCGCGGGCAC
>at02459
CCGTAGCCATTTTGGCTCAAGTTGGCTCAAGCAGGTTGGGCCCAGGCACTTCCCGATGAG
CTGACGGAGTTGCCTGGTGCTTTCAGCTAAGTGTACCTCATGGAGTCGTCTGCCTCAATG
TCTATCGGCGCTTTAGCCGCTGTAGCGATCGCTGTTCTGGTTCTCTCTGGCCTTACCCAT
CAGATGCGACTCCCCATCAACGAGCTTCTGGCTATCATTCGCATGGGCATGAGTCTACAC
GGTGCAGAGAGTCTGCACAGTGCAGACGAGCAGGTCCGAGGCTGCCGAGCCTTGATGGAG
AGGAACTGGGCAGATCCAGACAGCGTAGATTTCCTCGTATGGGCCGGTGGGATGG
>at02460
GGCACGAGGGGCCGCTACCTCGAGATTTTTTTTTTTTTTTTTCGATAACCATGACAGTG
ATGCAAGTCCACAGAAAGTCTGCAGTCCTAAACAGCAGCAACAGGGGCTCGAATTAAAGA
GTTCTGATCTCCAGGCCAAACGCGTTTGGGATGACAAGCACAATCAAACATTGCGGAAAC
GGAACAAGCCAGTCAAATTGAAACGCCTCTTGACCTGGCATGAGAAAGTCTGGCCAGCCA
GGAGGCAGCTGGGTGAAACGGCCCCGGCCTCACTGTTGCACCTTGAACTCGGACCGCTCC
CCTGCAACTTCATCCAAGCACTCCTGTGCAGCAGTTCCAATGGCGATCTTGCGAAGGTCA
AGATGCCTGAACTTCGTGTTCTTCCTCGCGGCGTCCGCCAAGAGCTTGATGCTGGAGTCG
CTGAGGGCGGTGTTCTCGGCGAAGCGAATCAGCTCGAGTGGGCACCTTTCAGCTCGGATC
GCGGCCGCCAGTAGCGCCGCACCGACATCGGTGATCTTGTTGCCCCGAAGGCTCAGCTCG
CGCAATTTGGCACCATTCGCCCTGCAACGCGTCAGCCGAAGAGCCCGGCGCCAACATCGG
TGATCCCGTTCTCCGCGACGGAAGATTTTCTCCAATCCCGTCTCATGCAGCACAGATACC
AGGTGCGCGACGCCCTCGTCCCCGATGTTACAGCCCTGCAAGTCGAGCGTCTTGAGGGTT
GGGTACAGCTTCAACGCCTTGGCAATGTGCTTGACGCCCTCCATGCTGATGGCGGAGTAG
GTCACGTCAAGAAGCCCCAAGCTGCGGCACCAGGGTAGGGAGGACGCGAGATCCTTCCCG

TGCTCGGCGTTCCAAGCGAGACGCGTCAGCTCGAAGGACTGGGCAGATGCCATCACCTCC
TGGAAGGTGCTGAGATACTTCTTCATC
>at02461
TTGTTTTTTTTTCTTTTTTTTTGGGGGGGAAGGCGTGAACACAGGATGTACTCTGTCACC
AAATTCAAAAGGCATACCCTCATAGGATCTGTAGCTATGCGGGTCCGCAGGCGTTCACCT
TGCAGTGGCCATTGCCATCTGTGTGAGGCTCACACTGTACCATGCCTCTTTAGGCACTGT
TCTGGCCTTCACACGTGTGAACGCTCCACAATGAAGGACGCTCACCTCAGCAACATCATG
TAATCCACACGTATGTAACCATCAGACCCCCGTTGGACAGCCTCCGGGCGCAAGCTTGAC
ACCTCTTGGGCGATCACGCCGCAAGACGTCCCTGAAAGGCCGAATCTCACCTCGGCGTCC
CGGGACCACTTCCAAGAGTACAGAGGGCAGCCGCCCAGAGTTCCGGGCAATCGGCAAATG
TTCTTCTTCAGCCGCTCATCGCTCAGCAAGAGGAGCGACGCAGCTCCCATGCCAAGGACT
GTTCCCATCACGCCGTTTGTGTGCGCCCTGTCGAGTCCTGACTGATGCTGCTCGAGGATC
TTGAGGAACAGCCGGGCTTGCCGCTCCTGCTGCCCGAAGACGGTCACGCCGAGGCCGACT
ACGAACACGAGAGCCCATTTCGGGACCGTGATGGAGACCGGCGCCTGCAAGGCCTCCACG
ATGCCCTCGTGGTGCAAGTGCCGCTGTGCAGCCGCGGCGGCACCGCCCACCTGGCTCCAC
ACGCGGCTCCAGATGTCAGCTGCATCGCCAACCTCACCCGCAGGTTTGTCGTTCTCGTCG
AGCTGGTTCGCTTCGGGCGGCTCCTGTATGATC
>at02462
TTTTTTTTTTTTTTTTTTGCAAACGCCAACCCATGCGCATGCTGGCCCAGCGCGATCACG
CCACACTGCGGATCATGGCCGCACAGGCATGCATCAGGCCATCACCTACGCCTCCTCAAG
GCCATACAGCCGCGATGCGGTGCCCGCGAGGATGTTCGCCCGGGCATCCTCGCTCAGGGC
CTCCCAGGCAGGCCACTGCTCCAGCACACGTGTCGCTGGCCCATACTCTGTGTGCTCAGT
GACATATGGGAAGTCGGAGCCCCAGAGCAGACGCTCTGAGCCGAAGGCCTTGAGCAGCTG
CTCCAGGCGACTCGAGAGCGACGTGAAGGGGAAGGGGTCCTGCGCCACACGGAAGAGTGC
CGAGAGCTTCACGCAGACCTGCGGATAGGAAGCCAGCTTGAGCAGCCCCTCCCAGCTCTT
CTCATCGATGCTGCGGTCATCACCGTGGCCAGTGGCAGGCTGCAAGAAGAAACCCCAATG
GTCAATCACGACCTTCGTGGACGGGGAGCTCTCAAGCAGCGCCTCAATCTCCACCATGTG
GCGGCTCAGGCCTTTGAAGGGCCTCGTGCC
>at02463
TTTTTTTTTTTTTTTTTTGGCCTTGGGGCGCTCTCGCAGCGACCGAGCGTCTTCCGGCCG
CAGGCGGCTGCCCGGAGGGTTGGCGATGGGAAGGCCGGGCCGCGGAGCTGTCGGGCGGCA
GGCCTCCCCTCTGTAGCCAAGCCTTCTTAAATCGCGGGGCGTCTTCTTGTCCGGGTCAGG
ACGCTCTGTACAGGATCCTGGTGTGATGAGCCAGGCTTCAAACTGAATGGGTGGTCCTTG
TAAAAAGCAGTGGTCCAAAGGCATACTATGGAGCGCGTAAGCAGATTGAAACGTGCAAAC
AGTGGGGGTAGGGGCAAGTTTGCCTTTTGAAAAACAGGCCATGGAAATCCCACGTTGTGG
CCTGACTGTCTCCAGGCATGTTGGGACTCAAGGCTGTGACCGTGGCCTAGCAATGCCAAG
CATCTCAGAAGCTGGTGTCAGCCTCCTCGTTCCTTGGCCCGCCGCACGGNCTCCTCTCCT
CTCTCAGCCTGGGCTGCCTCTGGGCCTCTATCCGAGCAGTGTGGGATCGGAGCCGA
>at02464
ACGAGAGAGGGAGCCCATGGAGGAATCGAGCGGCGTGGTGTCATAGATCTGCATTCCCCA
CTGCCCGAGTTTTCTGGCAACCTCCCGAAGCCCCTCCGGCTCCAGGCGCCAGGACGACGA
CAAGACGATGGCCGCGCCGGACTCCACGGTGATGCGGCGAAGCCTCTGCATGCAGCTTTC
CAAGAACAAGTCCTCCAAGGCCACACAACTCGCCTCCGCCCCTGAGGCCAGCGACGGCGT
ATCCGCTGGATGAAGCACTCCGTCAACGTCGAGGAAGACAATCCGAAGACTCTTGGTGTC
TGTCATCTGTTAACAGAGCTCAGCTTCTTGCCGTCCTCCTTCTCAGGTCATGCTCCACCG
AAGGGTAGCTGTGTCACGCAACAGACGATGCGGCTCTGCTGCGGATCCAACAGCTCTTGT
AGGTCATCCAAGCATAATCCGGGAGGAGCATGCACACGCAAGGATTAATTCAAGCTGTGT
AG
>at02465
TCCATTGACAGCATCTTCCCGTTGGGCAGTTGGGCCTCGCAGATGGTCCTCGACGTCGCG
AGGTAGAACTCCATGTTCTCATGGGGCCGCATCTGAGCCATGGCGATGAGCTCTTCGACA
CTGATCGCGTCTGCTGGCCGCTCGTCCCATACCCGAAGCTCTGGAGGGAGAAGCAGCATG
CTGCGGCCCTCGCAGCACTCCATGCGCGTAAAGAGCCAGTCGAAACAGGCACTTCGCCCC
TGCGGCAGCAATTGGCAACAAGCTCTCTCACGTGCTGCAGCACTACCCTCCCAGCACGTA
ACTCGCAGCTCGGCCGGGACATGCCACTCTGGCGCAGACGGAAGACCGCCCGAGAGGCGC

AGGCCAACGCTGGACAAGAGAAACAGCACTTGAG
>at02466
CTCTCTCGCTCCACGAAGGATACCCTCGAGAGCGCCTCCACAACGAAGTGGTAGTACGCA
GTCGGCCACATCTGGGTGAGGACCACCAGCGTGCCCTCGATCTTCTCATCCCAAGTGTCG
CCACACGAATGCCCCGAGTACCAGTGCGGGCAGCCGTAGTAGGCGGCCTCGGCGCCGAGG
ACCCTGCCCGTGCAGGTGACCCACACGTCGCTGAAGGTCTTCAGGACGTCCGGATCTCGG
GGGGGGCAGCGGAACTCCTCGCGGGGATCCTTGGGCACCGGAGTCGGCGGATAGGAGGGC
ACCTCGGGCCTCCCAGCAGGCTCAAGCCTTGCGCCTCACTGTCGTTTCTGTCCTGGCACT
TGGCGCAATGAACCGCTCTTTGAATAAGAGCTGACGACTGAAGGCCTTGGCTCCGAGGCG
CACTGGTCCGCACTTGCCTGGAC
>at02467
TTGTTTTTTTTTCTTTTTTTTTTGGTCTAGCAGCCTGAGTGCAAGCATGCCGGCCTGGGT
CCTTCCGAGCAGTCGCGCCGCCGAGCGCCATCACTTCTTGCCCCCGGAGGCCCGGGCCTT
GGACTTCTCCTTGCCGCGCGTCTTCTTCATGCGGTTCTTTGGTGTCCTTCTTGGCCCTGC
GGCCGGCGCCGGTCTTGGCACCTCCCAGGCCCGCGCGCTTCAACCGGTACTTTGGCTCGA
ACTTCTTCATCTTGTCAACGTTCTCGTAGATCAGCCCGAAGCCGGTGCTTCGTCCGCCTC
CGAAGTGGGTCTTGAAGCCGAAGAGCTGGATGCATTGGGTGTCGTGCACCTTGTACATGG
ACGCCAGTTTCGTGGCCAGGTCCTTCTTCGAAACTCCGCCCAAGTTCGGGTGGATGACGT
CCAGGACGAACTGCTTCCGGCCGAGCAGGGGGTTTGTCTTGAACTTGCGCGTGCGGATGG
TGAACTCCATGGCTGCTGCAACGCTATTGCCGTGGGGGAAGCTCCTCGCACTGGCCCGGA
CCGACTCGCCTTGAGCCAAAATGGCTACGG
>at02468
TTTTTTTTTTTTTTTTTTCGCAATCAACACACAGTTTTCTGGGTCCTCGGCGTGGCGCAA
GGAACCAGAGGTCTCTAGGACTTACCAGCGCCGCGAGACTTTCGAAGGTTGGCCAGCTGC
TGTTGCAGTTTTCTGTTCTCCTTTTGCAGGTTGTCGCAGAGTCGATCCAGAGCATCACGC
TGCTTCTGAGCAACCTCCTGCTCCTTCGTCAGCTGCGAGAGCTGCGCGCTGTTCCTAAGC
TCCTGGTTCTCCTTCTCCAGGTCCTGCAACCGGGACTGGAGCGAGTCGATCTCCACCTTG
CACTCCCCGTGCCGCTGGTTGGAGCCCGTGACCGCCTCGTGGAACTCGTCGAACTTCCCA
AGGATCAGCTGCAGCTCGTCCCGCAGCACTGTCTGCGACTTCCGGAGGGCCTCGTTCTGT
TTCTCTAGGGACGCGGTCTTGGTCTTGGACTCGCTGCACATGGTCTCGTGCTCCTTGAGT
CGCTCCTGAGCGGCCTCCATCTCGCGCTCGCGCGCCTCGCGCTGCTCGGCGAGCTGCCGC
TCCTGGGCCTCATATGTCTCCGTGAACTTGAGGAGCTTGCCCCGCAGCTCCTCATTCTCA
CGAAGGAAATGCTGACGGACCTCAAGCTCAGCGCCTCGTGCC
>at02469
CCTCGCATGACAGCCACCGGCTGCGCACGGCCCGGCCAGCCCTAGCGGCCGGCGGGCTAT
CAGGAGCAGTGCCTGAAGGCAGTGCTGGCCAAGAAGCCCACGGTTGCCCGTGGTGGCATG
CAGCCACATCAAAGGTTGCAGGCGGAACTTCAGGCGGCTGCACGGTCACAGAAGCAATTC
TGCAGGTGACTGAGGCACTTGAAAGACGCTCTTTGTGTGCATGCTGCGGACAAGCGCAAC
CTTGCCGAGGCCAGGAAGGAGCTCGAGGAGGCGAGGCGCATTGCCACATGGACAGCAGAT
GTAAGATGCCCAGTCCCTTGCGGCCTACGCCAGGGCTCTTGCGGCAGAGCTTTTGCCATT
TCCATGAGGCTGAGACTGCGTCTCTG
>at02470
TTGTTTTTTTTTCTTTTTTTTTTTATTTCCCttTTGGACAACtTGATAGCACCTcCTGTC
AACGTCGCTTAGGTGTTGCTGACTGCAGCCCTCACTCCGCCAAACCCCACAAAGAGATGT
GCTTGATGTCCATGCCTTTGTGCAGCGAATGCGGAAACTCAATCATGAAGATATCGCCTC
TGATCCCAAGCTTCGTTCCTGACGGTTTGGAGCTCGTTCCTCAAAACCATAAGAGGCCCT
ATCCGGATCGTGTGGAAGCCGAGGGTGGTTTGGACAGCACCTGCGGCGGCAGCTGCTCTC
GGCGCTTAGCGTCTCCCGCGCGACATGGCCGCCCAGGCTGCGGCTGGCAGCCCCTCCTCC
TCAAAGTTCAGCCCAGTGTGCGCAGACACACTTAGCCACTGGGCTGCGCTCATGCCAAGC
TGCGTTGGGAAGGCAATGAATTTGGATTTGCAAGGACACAATCATTGGGAAAAGGCTGGA
ACTCTGAGCCCATTCGCAAGCTTGAGCCAAAAT
>at02471
TTTTTTTTTTTTTTTTTTGCTGAGATGCGCCAACTGCATCACAGTCTTGGGTACGTGCTT
GTCAACGTACTGGACAACCGGCACCGGCACCTGCCGCACCACTTCGCGCACCTCGATCTC
CGGGACCTCCACGATGCGCTCCTCGTAGATAACCTGCGGCACCTCGACAATCTTCTCGAC

CCAGTTGATGGTGGGCTTGGCCACCTGCTTCTCGATATACTGCACCTCCACCTTTGGGAC
CTGTTTTACAAACTCACGAATCTCCACTTGCGGCACCTCGACCACCTGCTCTTGTATCTC
AACATGTGGAACCTCAACGATGCGCTCGACCAGCTGCACCTCAGGCTTGGCCACCTGCTT
GTCCACTTGCTGGATCTCGGGCTTTGCCACCTGCTTGAAGAGCTCCACGCACTCTGGCCG
CGGCACCTCAAGGATCTGCTCGCGCACAGTGACCTGCGGCACTTCCACGATCCGCTCGCG
AAACTCCACGACCGGTTTGGCAACATGCTTCTGCACGACCTGGCCGACCGGCTTCGGCAC
CTCTTTGACGCAGTCGACAACTTGTGGCTGTGGCACCTCCACAAGGCGCTCTTGAACAAG
GACATGAGGCACTTCAACGATGCGCTCGCAAGCCTGAGCTACAGGCCTTTCCACCTGCCC
CTCGTGCCC
>at02472
CCTTAAGCTCATTCAGCTTATCTTCCATCTCCCACATCTCCTCATCCGTCAAGGTCTTCT
TCTTCTTGCCGTCCTTGAGCTTCTTCTCGATGTCTTTGATGACCTTCTTTGTATCCTTGG
CACTGAGCGCAATATTCTTTTTCACATCAATCTTGTTTCCAGCACCGTCATAAACCTCAT
CCGGGGCCTGGTTTCCGTCCGTCTTGTCCTCATCCTTTTCCACTGACTCACCTTCAATGC
GCAGGTAGCCCCCCTTCATGATCCACTTCTCCGTGGCCACATTATCACAGAACTCCTTGT
TGTGTGAGATGATCAGGACACCTCCCTTGTAATCCTTGATGGCAAGGACAAGGGCACCGA
GACCCTCACGATCCAAGTAGTTGGTTGGCTCGTCAAGGATCAGGATGTGTGGATTCTGCC
ACATGGATGCAGCCAGCACGATCTTGACCTTCATGCCTCCACTCAACTGGTTGATCTGCG
TGTGGCTTGCAGACTCAGGGTCCACGCCAAAGTCGCCAAGGTGCTTTTCGACCGACGGTG
CGTCAACTGCTTGGTCATGAGACCAGCCATGGCGGCCTGCCTCTCGTCTTCCCGCTGCAC
GAGCTTGAGGTAGCCCATCTTGATAAGGACCTCCTTTTCCACCCAAACGTTGCTCTCCAT
GGGCTTGAACTGCCATTTCACCTCGTACTCGTATGTCTTCTCCTTCTTCTTCTGCCGGCG
GTTCAGAATGGCATCAGGGACCACAGCCGCAGCCTCGTCCTGCTTTGCCTTCTTGGCATC
CTCTTTGGGGTCAATGCACCGGCGACAGCTGCCGCTGACGGCATCAATGCACCATTTCGT
CGCGCGAGCCTTTTCCTCGTCAACAGAGAGCTCCGTGCTCTTGAACTCGATGCTCTCCTT
GTCGTCATTGCCTGCAAACCGCCACATGATGTACTCTGTGGGCGTCTCTTGCATGTGCTT
CTCCAAGTGGTGGAAAGCGTGCTGAGCAACGTAAGCCATCCGCAGGCCTGCAGCCTTCCA
GATGGTTCCCTCAGTTGGCTTCTGCTCCCCGACCAGGACCTTGATGGCCGTGGACTTGCC
GGCCCCGTTCGCGCCAATGACTGCTACGCGGCTCACCTGTGAGACAGTGAGGGTCACGTC
CATGATCGTTGGCTTGTCCTTGGTAGGAGTACTGGAATGTCACGTTGGTCATGCGCAGGA
CCACCTTGCTGCGGCTCTTCACGCCCTCCATGGCCCCTGGCTCAGGGAAGGTGAATTTCA
TGATCTCGTTGGAAAGTTCGAAGTACGCCTTCTTCTCGGGATACTTAGCCACAAACTGGG
TCAGCGTCTGGCCCTTCTCACCCTTGAAGGTCTTCAGCTTCCGGTCTTGGAAATCAATGA
TGTGCGTGCACATCTTATCAAGGAAAGGAGAGAAGTGCGAGGTGCAAATGATGCTTCCAG
AGAAAGACTCCAACCAGTCCTCGAGCCATTTGATGTTGTCCACATCCAAGTGCCCCGTGG
GCTCGTCAAGCATGAGGACATCGGCATTCATGAGCTGCGCAGCACACAGCTGCATCTTCA
TTTTCCAACCACCAGAGTAGGAGGTCACAGGGTTATCCAAGTTGGCCGCTCGGTCTGGGC
CACCTGGATAGCCGAAACCAGTTGTCTTCATCAGCTCCTTCACTGTGTCCTCTGACACTG
GGTTGTCAATCTTGTAGACATCATTGCATGTGTGCTGCACCCACCACCAACCAGGCTTGT
CCACACTGAGGATGGGAAATCCATCATCCTGCACTCCCACCTCCTCGTCTTCAATTTCAT
GCTCCACAAAAACGCTCTTGAGCTCATCGCGCTTCGGAAATCCTTCCAACTGTTCGTTGG
CAATGGCGCGCATGAGTGTCGTCTTCCCGCACTGGTTTGGACCCAGCAGGCCATAGAAGC
GGTTGCGCTTCAGGTGCATCTTTGTATCACGGAGCAGCGTCAGAGTGCCATATGCCAGGG
AGAAGGAGCCCTTGTACAGGTCCACCCCCTCGTCATCCTCGTCCTCGATCTCCTCGACTG
GCTTAGATGCAACCTCCATCTTTGCTCGGATGTCCTCGATGACGTCTGCAAACGGCTCAA
CCAAGCCTACACCAGTTTTCCACGCCTCGGCTTCGAAACTACGCATGTTAGCGGCCATTG
CTGCCAAGGCTGCTACGTAGTTGAGTGTGACCTGAGCAGCATCGTCGTCTCCAAGCTTAT
CACCGACAGCAGCCTTCAGCGTCTCTGTGGCTTCAGCCTTCGTCACAGTCTTGCTCTCGG
CGCCTTCAGCCGACTTTGTCAAGGTCTTGTAGGCTCGCTCCGCCACAGCTCTGGCCTCCG
GGTCGGAGATCTGTTCAACAGCACCCTTCACCAGCGGCTCAAGCAAACTCATAACCGGCA
GCACTGCAGCTGGGTCCTCGACCACCTTGCACATGTTGTCAACAATCAGGCAGCAAGTTC
GCTTCACCTTCTCTGCCTTGTCATGCAAGCCTCGAGTGAGCACCGGAAGCATGATGGCCA
GCGCAGGCGTCTCGACGTTCTGTACGAAGACACAGCCAGCCAGCCGCTCGACG
>at02473
GATATCCCTTGCGCTGGAGACTCGCAATGGTCCGCACGGCTCGGCCGATCCAGGCCATCG

CCGTCCTAGCGGTGGCGGCGTGCCTCTTTGGGCAGCTCCTGGCCTTTGTTGCCCCAGCGG
CACCCACCACGCCGCCGGCCCAGCATGGCCGCGGCCAGGTCTCCCTCAGGGCGCGAGGTG
GCGGCGAGTACGACGTCTCGGACGCCGACATCGAGGCCTTCTACCAGGCGCTCCGCACCG
GCCAGGGCGGCGACCCCGCAAAGGGCTCCGTGGTGTCTGAGCTCATCGTGAAGTTTTTCC
ACGGCGAGTTCACGAAACAGGGCTTCAAGCGCTACTCTGGCCTCTGGAAGGGCCCGCCCC
CCGGCAGCATTGGCAAGAAGGACATTGCCGTCGGCATGGAGAGCCTCAAGACGCAGATGG
CGAACCCCATGTTCGTGACCAAGGGCGGCGTCGGCTACGGCGTCGATGAGACTCAGAAAG
TCGTGAACGACAACAAGGGCTGGGTCTGGCTTGCCGCCGAGATGAGCCCCGGTGGCCTGG
CCGTGGAGCTCTTCCAGTCCGTGCCCTACGGGAAGCGCGCGGTCCTGGTGGCGAAGCAGG
CCAACGTGGACGAGCTCTTCTCTGTCATTAACTGGGACGTGGCCCTGGCAAACATTGACA
AGACCCTGGGTGGCCCCCAGGTCAAGCAGCGCTGAGATGCCGTCTCAAGGTCGCCTCCTA
GCGCAAGAGGCTAGGTTTGGCGGCCTATTTTAGCCAACTCTTGTATAGTCTCTGGCCATC
TGAAAAAAAAAAAAAAAAA
>at02474
TTGTTTTTTTTTTCTTTTTTTTTTTGTCAATGCAACCATCGCCAAGCGGGGTAATGTAAC
AATCAGCGGCCATCGGCCTTCAAAAAGAGACAGGCAAGCAGCTCAAAATAGACGCATCCC
CCCGTCAGACGAGGCTCATCTTCAGTGCCTGCTTCTGTGTGTCCGAGAGCTCAAGCGGCA
CTTCCAGGACCTTGTTGACGATGTACTCAATGGTTTGTGCCTTGTTGGCGTTGTAGTCCT
GGGTCACCAAGTCAATCTCTGCCTTGGTGGAATCCTTCAACTCTGCCGTGGGGTCAGCAG
CTGCCTTGGAGCCTGTCTCCCTTTGGAACTTGGCCTCCTGCTCTTCACGGAAGGCCTTCA
GGTCCTCCTCAGCCTTCTCCTTTGCCTGGCGCAGCTTGGCAAGCCGGTTCTTCTTCGCCG
TTGAGATGAGGTCATCACCTTGCTTCTCAGCCTGCAGCAGCTGCTGAATGAGCTCCTGGG
ACTTGTTTGCGGCAGCCATTGCTGTTGAAGGTGCTTAACAAGTGGGAGGGAGATGCTGAG
TGGGCTCACCTGTGCCTGCTTGAGCCAAAATGGCTACGG
>at02475
TGCGCTCGTGAACGTGCGTGTCCTTATCTATGCCATTGTCTTTGTTGCCTGGTTCGTCCT
CAACAGGTGCATTCTGATGCGTAGGCCTAAAGGTAGCGCTGTTCGAGGTGTGTCGCTTGG
CTGTACTGCCGGATCGATTGCTGGGAACATGTTCTGCGTCAAGGCGATGATGGAGCTGAT
CCAGAGGTCCATCAATTACGAGGAGGGCGAGATATGGTTACATTGGCTTCCGTATGTGCT
GCTGGTGGGTGCAATCTTCTTCGCTTTGTCCAACGTGGTGTATATGACGCAGGGCCTGCT
CGAGTACGAAGCGCTCTTCATGGTCACCGTCTACGAGGGGTCTATGATAATCAGTGGGAG
CTTATCCGGTGCCGTAGTTCTGATGGACCTGAAGGGTCTACAGACTTGGCGAGTGTTGAC
GTACTCCGTCGGCATCCTCATTATCATCATGGGGATGTACGTCATCTTCAGCCAAGAAGC
CATGGGCACCAGCTCGCTCATGGATGGCACTGCCAGCATCAAGCCATCGCCCTTG
>at02476
TTTTTTTCTTTTTTTTTTGTGCCTTGCTCACTTTGCATGGCCTTGCTCGAGGAGCTATTT
CTTCGAGAATGCGTGCCATGTTTTTGTAACTGATCAAAAAAATTGCCGAATTGCCGCCTA
TGACGGTCTATGTTGACGGCAAATTGTGGACGCTGCCAGATGCTCCCAAGCTCCACTCAA
GGTACACTTTCAGGCAAGTGCCACATGGAGCTGCCACAACTCGAGATGCCCGGCTCAAAG
CTTGAGTCAAGACCCTCCCGCGGGACATGCTGTTCCAGAAATTCCTAATTCCTACGCGCT
GTACACTATGGGACTGCTGAGAGGTATTGGCCATGAGCCAAACTCCCAGAACCTGTGCCA
CAATTGCTCAAGGATCAACGCCCCCCCAGAGTTCGCCGCGCTTCGCACTGGCGAGCTCCA
GTGGGCCCCAGTCGCTCAGGCGACCAGCACGTTGTCCTGCTCATACCCAAGCTGCTGAAG
TTGTTCAGCGGCTGAGCGGGTCATGGGCTCAGGGCCGCACATCAGGGAGATGGTGTCGTC
GGCCGCAGGGAAGAGCTGCGCCTCCAGCATCGCCTGGGTCAAGAAGCTGACGTCGTANGA
CCAGTTGGTCAGGTCCACAGTGTACCAGAGCTTGAAGCGGCCGGGGTGGGCGGCTGCCAG
CGCATCCAACTCCTGGCGCAGCAGGATGTCCTCGGGCGTGCGGTTGGCGTAAAGGAGCCG
GACCTCCGTGGCATCGTGTGGGTCCCGCAACACCGCGGTCAGTACCTGGTAGCAGGGGGT
CAGGCCCGTCCCGCAGCGACGAGTGAGATCCTGCGGCACTGCCGGGCCTTGCCCTTCC
>at02477
GTACACCACGAGCTGGAGCAGCGAACCTCGCGCAGGCAGATCCTCCACGCGGAAGCTGCC
GATCGGCAGCCCGCCGCCCAGCCTGAAGCTCAGCAGGCTGGCCCGGGGCCAAGGCCCTCC
ACGGCCGGCGGAGGTCGGCGCAGCGCTTGTAGGGTAAGGGTCCCGACGCGCGCGTGAGCC
TCGAGGGCTCAGGCCGGGGCCGGGAGATTGGCGGCAGGGGGCTGTGGAAGGCGTCCGGAC
TTGAAGCCATAGGCGTTGCAGATGCGTAAGTTCCAGGACTTCTCCGTGGATGCCGCCAAC

TGTTGTGCAGTGAGTCCAGCTTGCCGCGAAGCCAATTGCCGTATGCCTCCACCACCAATG
TTGACGTC
>at02478
GTCGAATGCCGCTCGCCTCTTCTCAATTGCCCATTCTCATTGTTGCGGGCTCCTCAAGCA
GGAGTGCCTAGATTTCATGAAGGACAACATTTCAGACGTCATGAAGTCCGAGGACTGGGC
TACACACCTGGCTGGCAACGCTGAGCTTCCAACGAAGTGCTTGCTCACATCGCTGGCGTG
AACCCTGTGTCTGCCCCCAGTGGGTCAGCAAAGCGCCAGAGATTGGAATGAAGCGCATCG
CTCACTGCGCGATCTCTGCAGTGTCAAAGCGTGAGCATGGCCAGCATCGAAGTGCATGGG
ATGCTCGAGACGTGTCCGCCCGAAGCTTGATTCTACTGTAGCTTGCACC
>at02479
TTTTTTTTTTTTTTCTTTGCACATGAAAGGGCTGGAAATGGTGGCTGTTGTCCAGCAGCT
CTCCCAACGGCTCCGGTGCCCGGCGCCGTGCTCCGCGAAGCCCTCCGAAGACTACTGGCC
TCCAGGGCTTGTGGAGAAGCCCCTCGTGCC
>at02480
CAAGAACCTGTAATGTGGGCCCAGTTTCTTTGGTACTTGCAGGGGTGCGCTGCGGAGGCA
AAGCCATACTGATACATCTGTAGTACCAGCGACCATCAGCATCGTAGTCATGCAGCGTTG
CTTTCAGTTCTGGGCAACAGCTCCTGCGGCTGCCGTGCGAAAATGGGTCCTCACCCTCAG
CTGCACAATTGGCCGCAAACTTCCCTTGCAGAGTCACTTCTGACGCAAGGAATGGCTCCA
GGGACAGGTGGCCAGACCTGCACGACACCCATATCAATATGCCACTCAAAAAAAGGCCAC
TCAACAAGAGCAGCGAAAAACGCAATGTACGCGGCGGGCACCGTGTTGGCACCTCAAGCA
ACCGGTTCCCTGATAGCAACAATTCGGCGGAGGCCTCATTGTCCTCCTCAGCCGGCAAGT
CTGAGGGCATCGCCAGCTGACTCATTGCGCCATCTGCTAGCATCTGCAGACAAGTGAAGA
GCTACCTGAAAACACATGCCTGGCGTCTTGAGCCAAAATGGCTACGG
>at02481
TTTTTTTTTTTTTTTTCTGCTGTCATATGAGAAACACTTCCTGAACACCTCCTAGAGTCT
GTTGCAGCCGCTACCGAACCACCAGTCTCTTAGATCTCAACCCTGGCCTTCCTGGTCAGG
GACGGAAAAGCATGAGGGCTGATTAGACCAATGTAAATGGGAAGAGGCTAACCGAGGCCT
GCCCCCTCAAGTGCAGGTCAGCACAAGCACGCAGGCTTCCATGACCTCCAATGGAAGTCG
GCCAGTGCGTCTGATGAAAGAGGCTCACAGAAACTGGCAGCAAACACATCAGTGGAGCCT
CGTGACGAATATGCATGTCAGGGATGGCGATGCTGGCACTTGAGGCGGGTAGAGTCTCAG
ACCACAGACCAAGACTCACTCTGCGCACGGTTGCTCAATGTGCTCAGATCCCCCTCCTCC
CCCTGACCTCAATTGTTTGGACGACGACCTCGGAGGAAGCACTCCGAGCACGCCTCCGCG
TCTCCGCAACTGCCTCCGCTGGCTCTGTCAGGTGACCCACGTANAAAGCGCCTCGAGAGC
GCCTGGCCCTCGTGCC
>at02482
TTGTTTTTTTTTTTTTTTTTTTTCCAAATACTCTGTACAATATCAAGAAGATTTGCTAT
GCAACCCAAATGGCATGCTAAGGGGTACCCTTGCAGCCAACTGAAGCAAGCTCTCGTTTG
AGCTTGACGGCCACATCATAAATAGACTTCGTGGCTCGCCACCGAAGCAATACAAACTAA
TCGCTGCTCCCCTCAGTGCTTTCCCTGGCTACCATCTGAGAGGATACCAGTCTCGCCCTT
CAGGCACTTCTCAGCACAGGCAACTACGTTGGGCACATTGAAGCCAAACTTGTCCATGCA
AGTGCCTCCTGGGGCGGAGGCACCAAACACATTGATGCCAACATGTGCATCAGCCCACTC
CTGCCATCCCATGGTAACCGCAGCCTCGACACTCATCTTCGGCACACTCTCGGGCAACAA
TGCCTGCTTGTAGTTGTCAGGCTGGGAGCGGAAGAGCTCCCACGATGGCATGGAAACCAC
GCGAACCTTCTTGCCGCTCATCTCCTTGGCAGCATCCACGCACAGCGAGACCTCCGAACC
TGTGCCGATGAGGACGACTTCCGGGTCGTCGCACTCCACCACCGCGTAAGCGCCCTTGGC
AGTGTTCTCGATGGATGAGTTCGGGAGGTTCGGCAAGGCCTGGCGAGTGAATGCCAGGAA
TGTGGGCATGGACTCCAGCTTCGACTTCTCTACCGCAATCTTGTAAGCGCCAGCTGTCTC
ATTGCAATCTGCCGGGCGGATCACAGTGAGATCTGGAATCATACGCAATGACGGTACAGT
CTCGATCGGCTGGTGCGTTGGACCATCCTCTCCAACAGCAATGGAATCATGTGTGGTGAC
AAAAATTGTCCCAGCCTGTGCTAGGGCTGACAGTCGGATGGCATTGCGCATGTAATCACT
GAAGATAGTGAAGGTGGCGCAGTATGGCACCAGCCCAGTCTTGTGGAGAGAAACGCATTG
CAGACAGCTCCCATTCCGAACTCACGGATACCGAAGCGCATGTTCCTGCCTTCATATGAC
TCTTTGAGGAAGTCTCCAGTACACTTCATGAGCGTCATGTTTGATGGGGCAAGGTCTGCG
GAGCCACCAACAAGCTCCGGCATGACATTGGCTAGCGCGTTCAAGCAATCCTGTGAGTGG
AGGCGAGTAGCCTTGCCCTTGTCCTCTGGAGTGGCACTGGGCAATGCTTCCACCCAACCA

TCAGGAAGCTTCTTGTCAAGGACGACCCGTTGGAACTGAGCAGCCAGCTCCGGCTCCTTC
TTCTGGTACTCCGCCCACAGGT
>at02483
CTACACGGGCCTGTTGGTCGTCACTGGGGGGACGCCCAGGTGCGGGGCGGTCCCACTGCC
CATGAGGAACGTCGGATAAATCATTCTGAGCCTGAGTGCAACGTGCAAGAAGAATGCCCC
GTTTGGACAAAGTAAAGAATCACTGCACCTTTTGATTCGCCTGTGCGTGGTGGTTTGGGG
TCTCAACCCAGCGATGCTGAGGGGTTTGCTGCTGAAATCTGTGCTGGGGTCAATGGTCGA
GGAGGTGTGAGACTGCAAGCATCGCCGCCACTGCTGCAACTGCTGCTGCTTCAGCTGCGC
GTAACCATCGCATGGCCCTCACTCCCTCGTTCCCGCCTGCCTCATGGCGTCAAGTCCGAA
TGAAC
>at02484
TTTTTTTTTTTTTTTTTGACAACGCACCCTGTCTGGCGAAGGTCTAACGTCGTAGTGTA
CGCCTCGCAACCAATCCCCGAGGCAGGGAGCCCCCCAACGTCGCCCTATGTACTTAGCAC
AGCCCAGGCCGCCCGCCCAGCCCAGGCCATGCGGGATGGGGCAGCCACGCTGCCAGAGCA
GTCTGCTGGCAGGACGTGGCTGCCCTTGGGTGGAGGCTCCGCGAGCTCAGTGCTTCGCCC
CCGAGGTGTGGTGGGGCTTCGTCTCGCCTCTCGGCGCCTGGCCCTGGCTCCCGTCCAGGC
GCGTGGCCATGCACTTCTCAACGTTGCGGCGGAGCGTGTTCACGGCCATGGTGTCCTCGT
AGTTCGTGAACCAGACCTCCAAGCGGATGACGTTCGCGGCGCGCGGGCCTGAGAGCTTGT
CCACGAGCCGCGCCCCCGTGATCATGTTTGCGGGCTCGATGGTGGCGCCGATCATGCCGA
GGATGAGGTTGTTCCAGAACTCGTCGATCTGGCCGCCGCCGGTGTTTGGCTTCAGCTGGT
ACTGGAAGTGGCCACCCTGGGCGTTCATCTTGTCCTCCCACTCCGGCCGGATGTTGTCCC
GAAAGATCATGATGGCGTCGATGACGTGTAGACCATCCGGCTGCTCGCGCACCATGCGCT
TCTGCTCCAAGAGCTCGGAGGGCTGCGGCATGTGGTTCCAGAGCTTCCAGAACTCCTGCA
CGGAGCTGAAGGAGGAGACCTTGTGCGTTGCGTCGGAGTACTGCGAATTCTTGCCGTCGC
TCGACTGCATGATCTGCTCCCAGATTGCCCATGTGTAGCGCAGCGGCAAGTCCTTCTTGA
GCATCTCCCCGCTCTCCTCGGAGTCCTCAGTTGTAAACTTCAGGTTCGAGTTAAAGTTCT
CGTTGAAGCTGAGGTACGCCATCTGTGGAGCGTGAGGATCGGGGGCGACTGGTAGATGGC
ACTTCGCCGCTGCGGACTGCGAGTTTCGCAACAGCTGTGTGGCGAGCTTCCTTGAGCCCT
TTTTGAGCCAAAATGGCTGAGCCAAAATGGCTACGG
>at02485
TTTTTTTTTTTTTTTTTTGCCTGTTTAGCCGGCCCTTGGACAAAGTAACACATTGAAAG
TCTGAACTTAAATGATGGAAAAGGCCCAAACTTGTGCCCACTCACAAATGGCCTTTCCTT
GCAGGGAGAGTGGCGTCACGGACACAACATGCACATGAAGCTTGGCAAATCAAGTCTGCA
ATCACACACTGCACCTGCAATGCTTACGAAGGGTGCTCAGGCCAAACAGAAGCTCACACG
AGGATGCGCCCATGAACGGTCCGAGCTTGAAGTCGTGCACAACGAATGCCCCATTGCACT
CGCCTGGAGTGGCATCTTCTCACTTCCCCTGGAGTGCCTTGGCTTTGGCATACAGGGCCT
TGCCTTCAGGTCCCTTGTTGATGAGCACAAAGCCGGTGATACCCAAGGCCTTACGCGCAG
CTGCAATGGCCTTTGCCCAGGCATTGTTCTTTGCGCGCGCTGAGGCCTTCTTGGACACAA
ACTTGCCATACTTGTTCTTTGTGATGTCCGAAGCCTTCAGGCCACCTACAGTCTTCTCCC
TTGCGCCTTTGTAGACCATAGCCTTGGAAAGCCTCCCCTTTGCAATCTTTGACTTCCGAG
CCTTCATGGCCTTCATGGCCTTCATGGCTTTCATGGCACCTGGCTTCTTCATGACAGCCT
TCATGGCCTTCATCG
>at02486
AACACCCGGCCATCATCAATGACCCCTTCATAAGTGCAGTCTGAGGTTGAGGCAAGGAAA
ACTCCAAGCACAGGGTCGAAGCGTGTTGCACTCTTCCCCTGTCCGCCAACCCCCCACCAC
CGCCGGGGTGGCTCGGACGGCGGCGCGATGCTGGAGCGCGTCAAGTGGCCGAAGTAGCCG
GCCACCTGGAAGCGAGCGTGGTCTTCGACCTGCGGCCCAGCAGCGACGGT
>at02487
TTTTTTTTTTTTTGTGTCTCCATTCTTTCTGCAATTGACTCCAGCTCTGACTCCGAGGA
ATGTGTTTCCCAGATGCCTTACACCACTCCTATCTCCACACAGCAAGTGCTCTGGTAGCG
CTCAGAAGATGTTTAACGCCTCACTGCACCCACAGCCTTGCACCACTCCAACAGCTCACC
AGAGTCCTGCTTCTCGAAGATTTCATCAAGGCCGCCAATCCCAATGCCACCTTTGATGTG
CACCTGAGGCAGTTCTCCAGTGCCAGTCTTGCTCTCCATGTAGTCCTTGTATGGACGGGC
TGGGGGAATGGAATCCAGTCCGACCTCATTCTCGAGCTCCATGGTAGCAAACTTGATGCC
CAGGGAATTCAAGGTCTCCTCTGCCATTTCACTGCGCTGACAGCATGCCTTCGTGAACAG

CACGACCTTGGCTTGATCGACCCCGCCCTCAAAGTCTTCCTTCACACCTTGCCGGGACAT
TGGGCTCCGGGCACGATGCTGGTGTACCCTACTGCTTCGCACTCCGGCGAGTACCCCAAA
AGCCATTGACATTGCAGCAAGTGCCGCAGACGTGCTTGTTGCTCTGAGTGTCGCAAAATC
TGTCATGATGCTGTGAAGAGCGAGGGGCTTGGTATGCTGACCCTGCCAGACTGCCGCTCG
TGCTCCCGCTGCTGGTGTTGTGAAGACGGTGGCTTCGTACAGCAGGGCGCTAATGGAACT
TAGCGCAATAAGCACACACACGACCACCATCTTTGTCGACGGCGGCACGGCTCGCAATCC
AGTCCTTAGGTGCACCATGGCTACTGAACAACTTGAAACTCTTCAGCCAAGATGGCTTGA
GCCAAAATGGCTACGGA
>at02488
TCCACATCCACTCTAGAAAAATGTCCACAAAGGCAAAGAACTTTCAATGGCTCTCGACAA
GAGTCTGTGCAGCGGCAAGGCGGGCGAGTGGAGCAGCTTCATGCCAAAACCTTACAGTGG
TATCGGTGCCGGCGAGCCAAGGCGGCATACCTCACTGGCGATTACCCCAATGTCTTTACC
GAGGTACTGTCCGATTTCGGGAAAGCCTTGACCTGTGTGGGCGTTAGTGTCTACAGCATC
TCCTGCTACGGGTCTCAAGGGTGGGCCATCCTATGGTCGCAGGGATGCTCGCT
>at02489
TTAGTTCTAACGTCTAGTAGTTTGTTTTTCTTTTTTNTTTTTTTTTTTTTTTTTTCCTTT
TTTGTAAAGCCAATACCGATGACAATCTCCACTGGGGTGGGACACGGAGAGGTGGAGAGA
GTCAATGGACCTGCAAGACGAACAAGAGAACCATGTGCCAATGTGGCAGAGGTGCTCTTG
GACTCAACAATTGGACACTTCAAAGTAAATTCCACATGGGAATGATGAATTGGATAGCCA
AACCTTCTCCGAGCATGGCTGGAGGTGCCTTTACAAGAATAGCGGATCCCAGAGCAGCCT
TTGCTCTGAGGTGTATATGACTTGCACTGAAGTGACCTGAATGTGGCGGGCTTTGGGAGA
GAAAATCGGTAGAAGGGAAAATCGGTAGAAGGGAAAATCGGTAGAAGGGAAAATCGGTAG
AAGGGAAATCGGCGTGCATGTCGGAGCCAGGCCCTGCCTTGATGTTGCCGGACTGCGGCC
AGCCTTGCCCCTGCATG
>at02490
TTTTTTTTTTTTTTGTTTGGGAAAATCTGCACACCCTCAGTTGTTCACTGTCCAGAGATG
CATAGACATCCCTGATGGTTGTGCGTCGGGCGGGTAATGATACTAAAAGCGCCCCCATGC
AACGAATCACCGCGCTTCTGGTCCAAGTGTGAAAACAAAAGCATGACAGATAGCACCGGA
ATGGCAATCGACGAGAGTCATGCCTCCAAACAGCCTTAAAACTCGCGTTGACGGCAAGTT
TCTAGGGAAGATCTGAAGCCGTGGCCCTAAGCTCCTCATAGCCTCTCCGGNAAGCGGGTC
GAACGCCTGCGCAAGTATTCGCAAGACNCTCCTCGCTAGACATGTCAGTCACATTCGCGC
CGTTGATTTCCAGCAAGCTCCACTGGGGCATGATCATCATCTCCTCACCCACACTGCCAG
GTTTGATAGCCTTGACGGAAATCGGAACCGTTGTCGTGAAGCCCACGCCCAGTGGCCTGA
ACGCCACCACCACCAGCTTCTGCTCCCCGTCCGGCAGCTGAAAGAGCAGGTCGACCTGCA
GATCCTCCAATGGGGTCTCCTCCTCGGGCGTGGGCTCCTCTGACACAATCTCCTCTGACG
TGGTCTCCTCCAACGTGGTCTCCTTCAAGGCGGTCTCCTTCAACGCGGTCTCCTCCAACG
CGGTCTGCTCANACGCAGTCTCCCCTCGTGCCG
>at02491
TTTTTTTTTTTTTTTTTTGCCACCACGCCACACACAGGACTGGCTCACTACCTCCGCCGG
CCATCAGTGCCCCAGGCTTGTGCGCACACTGCACCCCCGTCGAAGATCAGCACGAGGGGC
GCGTCTGCGTGGACCTCCCTCTCGCTGTTGGCCGCGGGTGCATGGACGTAGCGGACGCCT
TCCTGGACCTCGGAGCACGGGCAATGACTGTGGCCATAAACATGCAGCAAGCTGCCAGCA
GCACGCACCTGCTCATCTAACATCTCACAGCCCATGGTCTTGGCTGATGCAGGGTTGAAT
GGAAAACTCCTCCGCGGCAAGAAGTGAGAGAAAGTCACAACTGTCCCGTGGTACGGCAGC
TTCAAGTGCTCCTCGTTGAGCTTCAGCATGTACTTCCACACCTGCATGTCGGCATCCATT
GGCCACTTGCAACGCGCGTCCGGCTGCTCGCTGGAGTCAGGCCCCTCGTGCC
>at02492
TTTTTTTTTTTTTTTTCTCGGAACTCCAGATGCCCTCCCCGAGATCATCTGCCACAGCTC
CCTATGCACGCAGGAAGGAACGAGCGGCTCACAGTCGGGAGCGAGCGGCCTACGGCCGCA
TGGATCATTTGCACGTCGATGGGCGTCGACTTTGCCGACGCTGCTCCGCTAGTCTGATTA
CTGGGCTCTCCGCTGCTCGGCACTCTGCTGCTCGATGCCGGAATGGATGTCGATGGTGAG
CCCACAATTAATGGCGCACTCCCGTTTGTTGAGAACCCGTTGCCTTCAGGGATGTCTGGG
CCTTCCTTCTTCGCAGGCATCTCAAGCACCTGAGGCTTGGTCACTGCGCATGCGTCGTGC
GGACGCTTCGACCCCTGGCATGGCTGGTGCGGCTGACTGGATCCGCTGTCTTTGACCGGC
GGCGGTGTGTGATACACCACAGACGCCCTCGACCTGCCGTTGACGTACTTGTGGCTCTTG

TCACTGACCTTGATGACGGCGACACCTTTGGTCACCTCAGGAAAGACTGGTGAATAGAGA
TCGGTCGCGTCCGACATCACGACGAGGAGGTCCTCATGCACCGCTGGGTCGTAGGTAAAC
TCTGGGGGCCGGTGCGCCTTCTTTGCGAGCAGTGTGCAGATGCTATTGTTCCAATCCTGC
ATCATCCTGTCCACGGGCAAGTCATTGGCTTTGCTTCCGAATGGAAACTCTAAGTCCAAG
GCATTGAAATGGATGCAC
>at02493
CGTTGCCGCCTGCTGTGAGGCCGGCGCCTATCCCGAGGACTGCCGCTGCTGGAGCGTGCC
GGTTGCAACTGTTGCCAGAGTCGACCCAGAGGAGGCCTGCATCCTGCATTACCACAATTG
TCAGGGTGTCGACGGCCTAATGCAGAAGTACAGAGCGCGCACTGAGGAGCGGTGGAACAA
CACCAAGTTTCACAGCTTGTGCCAGCGCCTGTACACTGTTGGGAGCACGGCTTTGGGCAG
CATGTTGAGAAGTGGACTGGTTCTCAGTGACGGTGACCATGAGGAAGTGGCAAAGCAGCT
TCGAGCCGGGGTGCTGATTCGCATGACGGACGTCCGCGATGCGTGCAAGGAGATCACCAC
TTCAGACCAATTGCAACGTCTACTTATGCAAACTCGAAGCAC
>at02494
CACTCGTGAACCTGTTGATCGTCTTGTCTTGAAATGCCGACGTCAGAGAATTGAGGCCAC
CTTTGCTAGGCAGCTCACCATACATCTGCGTTGCTGGGAAGTCATTCACTGCATCATTGC
CATGACCGCAGGCTTCTCCAACACCACTGTCTGGAGCACACCAGGGTTCCGGGTATGCTT
GCGTCGACAGGAAATCAACCTCTGCAGCGCTCGCCGCCAGGCTCCTCTCGCAGGGCTGTT
CCAAGGCCGGCGCGGCGCCATCCTGCTCTATCGGTTCGGCTGCCTGGCGCTGCTTCTTCA
CAGGCACCTGGAGCGCTGGGTCCTCGGGCATATCCTCGGGGTACTCCTGCGTGGAGGCGG
GGCTGTCCTCGATGCCATCCGGCAGCTCCTGGTCGCGCGCTCCCTTCGGGTCGGGCTCGC
CGCCTCAGCAGCTTC
>at02495
AAGCTGTTCACCAGGACCTCCACTAGCGAGGCGATCGAGAACACACCCCCTATGATCGCG
CAGATCGAGGTGAAGATGTAAGACCATGGCTTCATCTTCTCGGTCCTCTGCATGAGAAAC
GGGCTGAAGTCATACACGATGAAGACGCCCGGGAGGCCGGAGAGCTCCCCGGTGCGGACC
TGCACGTTCCGCGCGCTGTCCGTCGCCGAGTACTGGTTGGTGTAAGCCTCGTTACCCCAG
CGGTCGGTGTACACCGTGGGAACTAGCTTGATGTAGTAGTGGAACATGAACGCCCCGTGT
CTCACTATCTTCGTGGTCCCGTCCAGGGGGGAGAGCACGCCAGGCACGCGCTCGCCGAAG
GAGATGGAGTTGATCTCGTGGGAGGTGTTGAAGCCATCACCCACGTCCTGCATATTGAAC
TCATGCACGTGCTTGCCGCCTCTCACCGCAGAGCGGCCCAGCGCGACGTGCACGTTCCCA
CTAACCTTGTTCACCGTGACGCTGCCCACGATGCGGCAGCCGAGGGATCTCCTGCACTGC
TCCGCCACGTCCAAGACGTGGAAGTAGGGCAATTCCTTGGCAGAGTAGGCGTCCTTCAAC
TCCTGGCACGTGTTGCAGCACTTCCTCTCCTCGTCCGCGGCCTCCATGCAGGACAGGCAC
TCCCCCTCCCTGGGTGCCCTGTCGCCCTCCACGACCTGGCCCTGGGCGTCCAGGGCCACC
TTCTCGAGATTCCCGTGCACGTCGACCTGGTTGTCGCCCGCGGAGTCGACGGTGTCGACG
GAGACCTCGTCGCACCGAAGGTGCGGGAAGGTGACATCCAGGCTAATGGGCAGCTTCTGG
TTTATCCTGGTGTCGACGGAGATGCTATGCACCACCTCGACGCTGCAGAACTCCAGCAGC
TCGGCCCCGAAGAGCAGCGCGAGGATGCAGGGAGGTGAGGAGCGTGACCACC
>at02496
TTTTTTTTTTTTTTTTTGCTGCACCTCTGCACACTGCACGCGCCTCTGCGCAATCGTAC
GGGAGCCGTTCCCTATGCCGCCTACTGTTGCCTCTTGCTCCGCTTGACTGCCCGCTCGTA
CAGCTCAGGGCAAGGCCTCAATGCGAGCGGCTCAGTGTTGTCGAAGCCCATGGCGACATC
ATCGTTGTCCACGAAGTCCTGCATCTTCACTTCCTGGAACGGTGAAAAAGAGCCAGTGCC
TTGACCACTGGGCTGGCTCTTAGAGGGCAGCTCGCCGACAAGCACATCGTTGCACAGCCT
GTACACCTCCTGCGACCTGTCATCCACATGCCAGCAGCTACCGTCGCACGAGCTTGCCGT
GCAAGTGAATGTGACTCCTGCCTCCTCAGCCACCCTGCGCAGCAAGACTCTTGGGCGGAC
GGGCAGTTGAAAATTGCGTTCTGGAAAGGCCACCTGAATAAAGCCGGTCCGTTCCCGGGA
AAACTTCAAGCGGACTCGACTGGGGTCCTTTGCACCAGATATGAGCTCCACTGCGGCCTC
CTTTGAATTGCAAGGCATTCCATTCACCATCATCAGCCGGTCTCCCTGCTGCACCCAGTT
TTGAGCGATTGAGCTGTCCGTCTCGAACATGTACTTCTTGTTGTGCTCCGCGGCGCTGCC
CCCGTCCAGCACCTGAGTGACCCCGACGGCATCCCCCATCGACGAGTTGTCGAGCGCGAT
GCCCAGCGGCAAGCTCAGAGTGACATCGACCCAGACGCTGTTGCCGACCCTCTTGCCGCC
CCGCGGGTTGGGGTTCTG
>at02497

CCAAAGACAGCAAGTGCGTGCCTCAAATGCCAAGCGCAACAACATGGCAATGAGCTGACA
CATTCCAAGAATCTAAACCAGCATACGTTGCATGCATCATTGGACTCCCTCACGGGCGTC
AAAAGGTCTGTACACTGCCCAGCATTTTATCTTCAGACCAAATGATCATTAATAGGGGTC
AATCTCCAGGCAAGTGCAATTGAACTAAGCTCTAGCAAGCACTTGTATGCCTCACGCTGA
TGCTTCTTCCTCAAGGAAGCAGCGCAGGTGTGTTTCTGGTTTGGATCCTCCTACCAAGTG
CACTACGGTCATAAGGGTCCGGCGCACTCGGCAAGAAGGACGGTCTGGCCTCGCTACATC
AGGTCATGGCCAAGTTGCTCTAGGTCTCGTACTCACTATCGGATTCCGACTCCGTCTCAA
AGTCTGACTCGTACTCCTCGTCTCCTTCGCTGTCGCTCTGACTGGCATCGCTCCCACCAA
AAATGCTCGAAGCGAGAGGGAGCGGCTGATCACCGTCGCTCACACTGCAGTGAGTGCTCC
TGCTGCTAACGCTCTCTCCCCCCGCAAAGGCGGTGCAGCAACGCGACGTGTCTACAGGTC
TCCTTGGTGGGACCTGGAGTGTTCCCACGGGAGGACAGGGCGCACTTTGAATTTCCCCTC
GTGCCTAATTCT
>at02498
TTTTTTTTTTTTTTTTTGCAGCACTCAGACATTATCGCTGTGAGCCGAGATGCGCGCGC
AGTTCTGTAGGGTTTTCAAAATCAAAGATCCAAATGTGCTTGCAGTCAGAGGACAAGATG
GCCGAAGCTCAGCCATCAAGCATCCTGCTTGGCCATGTAGATGGCCAAGTCGACGAGACG
ATTGGAGTAGCCCCACTCATTGTCATAAGGCCCTCGATGAGGCCGAACTTTGTGTGCACA
ACCTTCGTGAGCGGCGCCAGGCAGTTTGTGGTGCATGATGCATTCGAGACGACGGTGTCT
GTGGTCTTGTACTCGAGGTGGTTGACGCCAACCACATAAATGGGCACGGCATCCTTGGGA
GGCGCAGAGATGATGACCTTCTTTGCTCCACCCTTCAAGTGCAACTCTGCCTTTTCCTTG
GCCGTGAAGACCCCGGTGCTTTCGCAGACGTACCCGGCACCGCTGTCACCCCACGGGATC
GACGCGGGGTCCTTCTCGTGGAAGACAGCAATGTCGGCTCCGTTGACGACGAGGAACTCC
TTGCCGCCCTCCTCCTTTGTGGCGATGGCATCTTTGCGGGCAGTGTGGAACAGAAAGGAC
GGCGGGGAGAATCGATCC
>at02499
TTTTTTTTTTTTTTTTTGCTCATTTTATCAAAGAGAAGCGCCTGTCCAACTCGGCGCGG
CCGCCCACGCTGAGGCCTCTCACTGAGACTGCCTGCTTACGCTCAATGGTCTGACTCGAT
GCCTGCGTCGTTCTGGACCTTGTTGTAGGCGGGGTTCACCTCGTGCCTGCGGACGTTGTT
GAAGTACACACCCGTGCACTGGACGAAGGCAAACATGAAGGGCAAGCACATGCCCAGGAA
GGGCTTGTAGCCCATGTAGGTGCAGATCTTGAAGCCGTCGCGCACGAGGCCGAAGACCAT
TGCGGCTTGGGCGAGGGCCGGGCGGCTGGCAGGCGGGAAGGGCTCCTGGGAGCGTAGGGG
CGGGCGCCGGAAGAGGATCTTCGCTTGAGCCAAAATGGCTACGG
>at02500
CGCACGCCGTTTGAGGCCAGGTACTCAGCCACGTCCGGATCCAGGGGCGCGCCATTGCCC
CGCACCAGGAAACCATCTGTCACCACGTGGCAGCCGGCCAAGCTGGGCATCATGAGGGGC
TGCCCGCCTCGCCACCGCTGTCCAGCCACTTCCTTGTCTTTCTGAAAGTGGGGGTACGCC
TCGAAGAGCCGCAGCTGCCGCACCTTCCAGACCAATTACAAGCTACCGCAGCCCAGTCCT
CCAACGGCGTGGACTTTGGCAGCCGAAGCGGCGGCGGCTGGCCCTCCAGCGCGCCGTCGC
GGCAGTCCTCGGCAGGGAGAGCAGCAGCCCGCGCTTAGATCGCGCCGTGGATGAAGACGC
AA
>at02501
TGAGACCGCACAGGATTCGAGGCTGGCCAAGAAGATCTGTGAACAATGCGCACAACAGTT
GCATGCGGATTCTGACGTTCGAGGCTCCTTTCAGATCTAAGTACAGCGCATCGCTGAAGC
CTTCCCGCACACCCTGGTCTATGAATTATTCGAAGTGTACATGGTCTTCTTCGGGTGGTA
TGAAGGTACGAAAATCCCTACCTTCCATGGAGAATGTCCCTGACATGGTGAGGAGCACTT
GCAATTTTGCCGAGTGCTCACACAAGTGGAAGCCGCGCGAGAGCTGGCAGGTTGCGTCGC
AGAGTCTGTCAAATATCTGCGACACTGCAAGCAGCTGCTCTTCCGAACTCTGCAACCGCA
AGGATTTCAAGATCCTCC
>at02502
NNNNNNNNNNNNNNNNNNNNNNNNCAGTGCTGGGCGCAGCACTACGAGCATGCGAGCAGC
ATGTGCCACATGCTGAGAGCTCTTGATGAGCTCCTCACAAAAGAAGCGCAACGTCTGCAA
GCTTGAAACTTTGTGATGGGCTTATCGCCACAAAGTCACAAAGAGCGTCTTCTACTAGTG
CATGGCTTCTGCAGCTTTGAGAATGCTCTTTGCCAGGAGCAGAAGTCAGCCTCCTCAGCT
CTAAAGCGCGCCGGGGCACTCAAGGCGGCCCAGGCTTCCGCGTGGGCGGAGGCGGCGGTG
CAGGTGGGACTGGTGGCAACAATGTAGAACCCCTGGATCCCCGTGTGCTCGTCTTGTCCA

GGGGGACGAAGGGTGGCAGCAGGGTCCCTGAGCGGAACGGTGCCCCTGGTGGCGGGGGCC
GTGGCGGTGGCCGCAACTCCCAAGGTGGCGATGTCCCCGGTGGTGGCTCGCGTGGCGGCG
CCGGCCCCTTCCAAGACTTGATCGGTGGCGGTTGCGTAGGTGGAGGCGGCTGGTCCTTCG
GGAAAGTGCTCTCAACCTGAGCAGCAGCCCTCAGAGCGTCGCCCAAGCCGTCCCCTTCCG
CGGGCAGCGTATCCAATCCGAGGTCGGCGGTTGCATCTGCCTGGATGGCGCCGCCGCCGA
CCTGCTCAGGCAGAAGCGTGCCAGTGTCCATCTGTGGGTCCGGGTCCGGGTCCGGGACCT
CCGGCAGGACCTGCCTCGTCAAGTTCCGCAGCGCCTTCGGCGAGAGGAACTCCCGGACCT
TGCCGTCCACCGAGCAGGAGAAGATCCTGTTCATAGGACCGAAGTGCACTTGGCGCACCC
AGGAGTCATGTGCGGGCAACATCGACACCAAGATGGGGGCGTGCAAGTTCGTCACTAACC
AGACCCGAATGACGCCATCAGAGGATCCGGAAGCCATGAGGCAACCGTCCTGTGCAAACG
CGACGGACCAGACAGGCCCTTTGTGGCCTCGCAACTGATTGCCTGTATTCTGGGGTCCTC
CCTCTGAGCTAGGGTTGAAGTGCCACAGCCAGACGGCTCCATCCTGGTTGCCAGCAGCGA
CGACGCCAAGCTCCGTGGGGTGGCTCGCCACGCAGAGCACACCGCCACCCTCACCGCCTG
CAAATCGGGTAATGAGCCCATTCTCTCCGTCCATGACATTCCACAGTGCAATGGAACCAT
CTGCTCCGACAGAGAAGGCTGCAAAGGCGTCCGCTTGCGAAAACTTGACGCCATGCACAT
CACCCTCATGAGCCTTCCATTTCTGTACTGGCTCCGGATTATCGAACTCCCACAGGCATA
ACAGAGAG
>at02503
TTTTTTTTTTTTTTTTTGCACCATCCTCGAATCCTTGAATCCACGCGAGTCCTACTGCG
GTCTCTGATCCGAGTCGCGATGACGACGACCCCTCGCTGGCCCCCGCTGCCTCACATCAT
GCCCATGGGGTCCTCCGGCACTGCGGAGGCGGGGTCCGCCCACTTCCGGAGGCAGCGGCT
CACGTCCCTGCGGACGTCCGCGAAGACGGTGAGGGCGAGCAGCTTCGTGCCCCGCTCGTT
GGGGTGCACGCCGTCCTCCGCCATGTAGCTGGGCCGCTTGAGCTCGGCGCCGCCCAGGGC
GTTGAAGGCGTCGATGACCGCCGTGTGCGCGAGGACTTCCTCGGGAACTGGCTTCCGCGT
CCTCTTGGCCATCTCCTCCATGGGCGCGTTCATCGTCCGCTCCGCCGCGTGCGCCACCCT
GGCCACCGCCTCCTGGAGCTCCTTGTTCACGACCTCCTGCTGTATGTCGTAGGCGCCCGG
CTTGTACACGGGAGGGGGCGCGACGAGGAAGACAAACGGCCGCGGCGACATGCGCTCGAG
GAACTCGAGGCAGAGCTCCGCGTAGTCCTTCTCGAAGGCCGCCATGTCCCACTGGCTCTC
CTTGGCGTCGTTGTGGCCGAAGGTCGCAATGACGAAGTGGGGCCGCCAGAGCCTCGCGGG
CTCCATCCGCCTCTCGTCCCAGTAGGGGCACTCCGAGAA
>at02504
CACTGCGCCTCAAGGACGGTCTGCTGCCCATTTGAGGTGTTATTGCATGGTGGGGCCAGT
TGCCATGGCATTTTCACTTGGGTGCCGTTTGGCACACAGATCTCAGCACATTGCCACAAA
GGGATTGCCATGCGGAGTTGTGTGAGATGGCAGGCACACGCGAGATTGATGAGAAGGCAA
ACTTGGACACTGCGCTATCAGTGGCGCGA
>at02505
TTTTTTTTTTTTTTTTTCAGCGGAATGATCAGAGCTCTACAGTGCAATAAACAGATCCT
CTGCGAAGTCCGGGTGAGAATCAAAAGGGGTGAAACGCGTTTGGGCTCAAAGCTCTCGCA
GCCTAAAAAAAAAAAAGTTGCACTTGGCTGCTGAAGTGCTGCAAAGGCTTGGACTGATGA
AAATACTGTCTGACGGTTCCCATCGAAAACGTCTTCAGGAGAGGCCCAGGCTCAAACACC
CAGGCGAAGGTCACTCTGATCTCGAGCTTGATCACAGCTGCTCCTCATCCTCCAGGCGAT
AGGGCACGCCGTGCGCCAAATACAGGCCGCCGGGGACCGGTTGCACTCATCGGCACCTGC
GGGGGACGCGGCATGATCCCGTGGACCCGAAACGTGCTTCCCGCCGTCGCGGCGGCGGCG
TAGGCGGCCGCGGCCTCCGCCTGGCGCTCCCTGTCTATGCCGAGCAGCTGCGGCTCCGGG
TCGTCCTCTTCCTCCTCCTCGTCGTCGGTGACGTCCTCGAAGGAGCTCGGCATCCGCCTG
AAA
>at02506
CCTAAACTGCTTCCTTCCATACATGGTAACCAACCAGACCCAATATACAAAAAAAAGACC
AATGCTGTGCAGTTGCAGCCATGGCAGCGCTTGCCTAGTTATCACAGTGACCACTGCGAT
GATGCTGGTTGCCAATTCAGTCCTCCATGCAGTAGAGGGGGTCAAGGTTGCCATCTCTTC
CATTCCCTCCATGCCCATTGTCGGCCAAGGACCATGACGTTGTCAAGGAGAGATGGCAGC
CGGCGGCACCCTGCAGTGTTGTCAGAGAGACCGCCGCTCCTCCATGAGCTTGCCACCGCT
CCGTATGTAATGAGGTTCGGCTGCACAATGAACTGACGCATGTCCTCGAGAACCAAGG
>at02507
TCAATCTCCTCTGTGCGTTGAATGGCCTCTTGCTGGCAAAGAGCCGCAACAGCAGGTGCC

AGGCGCCTGCGTGCCAAACGCATCCTCTCGTTCACAGGACCTTGAACTTCTGCCCTTGCG
GCATGCAAAGCCTGTTCACGCTCAGCGAGCTGGCTCTGCAGCCTGGAAATGAGAGACTTT
GCAGCCGAAAGTCCAGACTCAAACAAGCCGAGGCCGGTATACGCCGGGTCAGGCTGTGCT
TGCAGATCAGCCATCGGACCGAAATCGCACGCGGGACAGATCCGGCCTCGTGGTGGGGTT
GTGCCAACTTGACGCTTGATAATCCTTGAGCCAAAATGGCTACGG
>at02508
TCGAAGAGGACCAAGGATGCGTATACAGAGGCCTTGGCGTCACCGTCGCAGTCGAAGAGC
TGGTTAGCAGACAAGCAGATGTCCTGGCTGCCGCAGTGCCGCACAGGAACAACGACGTAG
CGAGTGTCTGCGCGGAGCCCGCCGCGCGGCACCAGCTGAGGCGGCCGCCACGACAGGAAA
TCATCCCGCGGAAGTTGCGTGGCCCGCAGCTGTGCCTGCTGCCGGCAGCGGCGGTGCAAA
GCCGACACTGTCGTGGCGGCCGGCACAATAGCCGATGAACTCCGTGCCTCAGCATCTCGC
CCAAAGTCTTCCGCTTCCATCTGGCCGGCTTCTCTTGCATGCATGCCAATGTTATTTCTT
CCCCAGATCCAGGGCCGCCTGGGCTGCTGCCAAACCTCGGCCATATCATGATGTGTGGTC
CTATCCATGGAGCTGGCCCCCGGAGTTCGGGACCGGGG
>at02509
TTTTTTTTTTTTTTTTTGCTCCCATTGCATTCCTAACACCTTCCCTACAGCCCCGGCGG
CCTCAAGCGAAGAAGGCGGAAAGAGGCTCATCCCTCTCCAGGTTCAAGTAGATGTTCGCC
TGCTGCTCCGCGCTCTCGAGGCTCTCCCACGGGCGGGGCAGCGGCCTCCTCCCAGCGCTC
GCATCCCTCCTACGGCGGCCGGCCCGCGAGCGCGACCTCGACCTGTCCGGCGAGGCGGCT
CTGAGCCTCGCTCCCTCGATGGTCGCGGCGACGCTGCGGCCGAAGGCGGCGGCAGCGTCC
TCGGCGACGGAAGTGCTCGGTGGCGCCCAGTCGCCGTGGTCGAACCTGCAGAGCGCGCCG
TTGTCGCAGCGGCCTTGCAGGAAGAACTTGCAGGGTACCTTCCTCAGGTTCTCCCCCCCG
GTGGCTGGGAGCATCTCCTCCGCTGAGTGCACGAAGGGGCACTCCCCACCGTAGTACAGA
TGGGTGTTGCAGCGCCCCTGCATGAAGAACTGCAGACGACA
>at02510
TTTTTTTTTTTTTTTTTGAGAACGCAATGGTCTGTTCCAGTGACAAAACAATGTGCCGA
CGTGTTGTGTTGCCAGTCTTGCAACACGGCCTCTGTGAGGCACATTCCTGTAGCGCCAGA
TATGCCGAAGCAGCTTACAACTGAGAAATCTTGAACAAGGCACGGCCCTTCGTGGCCGTG
TCCTCAACATCGGACGGGCAATTAAATTCAATTGTAGGCTGTACACTCGCAGGTGGAGCA
AACCCTCAAGATTAGCATACGTCCTATGCTGTAACCCTCACATAACCGGCTCCACTTGCA
GCAAGTGCCCTCATCTTTGTCGCGACATGCTGTGGCATCACGCCGAAGTGCATGAGGATG
TAGAGCGCTCCCAGACAGCATAAGTAAAGGGCCAGGATCTTCTGAGGTATGCCAGTGAAG
GAGCTGAGCACCCACCAGATTGAGCCCTGGAAGACATAGAAGAGGCCGCGGCCCAGGCTC
AGAGCAAGGAACCTCATGTTCAGCATCAGCATGTTGTGGTAGTCGTTGATTAACTCAACC
TGGCTGATCCACTCCATCCTGGCCTCAAACAGCATGGTCGTAAGGCTGAATACGACCTGG
TAGAGCGAGACTACGTAGGTAAATCCGGCCCCAACAACCGTGAACGGGTTCAGCACCTGC
A
>at02511
CTGACCCGTTCAGATGCGAAACGCATGGCCGAGCCGGGAAATTCTTGGGCATGCATGGCC
GCTAAGAGCATGCCAGGATCATCCCGGAGCTGAGAGGAAGCATGTTCCAACGCGTTTCCA
TTCAGGGCAAGGGCTGCAAGTACAACTTCGCGGTCAGCTCGCAATCTTCGGGAGGCCCCC
CGCATTAGTCCCGGGACGGCATAGACACTTTTCTCGGAAAGCGCCGCCATCAATGCGTCT
CGGTCATCCTCGCCACCTTCACAGATGCTGGGGGTGCAGTTAGAAAGCGCCACACCCATT
GCAAGAGGAGTTCAATGAATGTTCCGCAAAGACTGCTCTGCCGAGATGCTCGATTCCGGA
TCAAGCGCGAGCCGTGACGACTGAGCCAACAATGGCTTGAGCCAAAATGGCTACGG
>at02512
TTTTTTTTTTTTTTTTTGCCCGATACGTGGCGAGTAGAAAGGCAACAGCTCCATGCAGG
CGTCAGTGGCAACTCCGCCCTGGTCGCTAGCTTGCCGTGGCCATCCGCGGCTCCACCGGC
TGCCCTCCAGGAGTCTGCTTCTGCGCAGGCTCCGTGTCTTCCGGCGCCGGCAGCTGATCG
GAGCAAGCCGCCCAGTGCCGCCACATGTCCTCGCGACACGGGGGGTCGGTTTCCTCCCGG
TAAAGCCTGGTGTCCTTGAACCACTCGTGTTGGCCGCTCAGCTCCTCCTCCTCCTCAGCA
GCCAGCAGGCCGCTGTCGCCCTCTGCAGCCTCCAGTTGGAAGTCCGCGAAGACCGCGAAG
CGGATCGTCTCGGCGCTGGTGTGGACGCCGGCGGAATGCAGGGCCCGGCCGTGCCAGAAG
ATGACGTCGCCAGCCTCGCCGCAGAGCTCCACCGGCACGACTGCCTCCAAGCAGCGCCGC
AAGCCCTCGCCGAAGGCCCCGGGTGCAGGGCTGAAGTTGCTCTCGAGCTCCATGGTCTGG

TACAGCAGGCGGTGCGAGCCGGGCCAGACTGTGAAGCCGCCACATCGGGGGGGCACGTCC
TCTANGTACGCGCACAC
>at02513
GTACCGTTAATAATACACTTACAGAGGTCCCCTTTCTGAAGAAAGGAACAGATCACACCT
CCTAAATGCCCACCAGCCGCCTACTACACCCTCACGCCGTGTCACTCATGGGCCGGGGCC
GCCCTGGCTGAACTGCTCAATCATCTTCTTCGTCTCCTCCTCGATCGCTCGGCACTTCTC
CGCGGTCTCCACTGCCTTCTAGCGCGACTTCTTTGGCCTTGCCCGCAGACTCCTCGACCT
CGCGGAGGCACGTCTCAAGCTCTTCTGGTGCTGCTC
>at02514
GCCTCAAGCGGTCCTTCCACGTGCCCGCGATGGCTTACGCCAAAGCTTCCACGGAGGCCG
TGTTCGGTGCGCACGAGGACTCCTGGCACTGCTCGCCCGACTCGGAGCACGCACTGGAGG
CGAGTCGCTCCACAGCACGCGCACTCATGGTCCAAGGCTGGCTCCAGGTGCTAGGTGGCT
GCCTTGCCTGTGCCGTGCTCCAGCTGAAGGCGATCACGCCCGCCTTGGGCGCAGTGCATA
CGATTGGCTGTGTTCAGGGAGCCCTGCTGGTGGGTGCCAGCGGCAGCTGGCACATGCTCA
GGCTGTCGACGGCGAACCACCGGCTCGCCTTCTTGTGCCTCTTTTGGTGCGTTTGGCCGA
ACTTCTGGGGCCTTCGTCGCTGCCGCGG
>at02515
GCAGCGTTGAGGCTGATGACGTCAGGCGCGACGCGAGCGAGTGGCATGGAGCTGAGCATT
GCCACTGCGCGCTGCCACTGACCACCCTTCTCGCATGCGCTGATGGCAGCGTTGAAGCTG
AAGTCGTTGGGTGCAGTGCTAGACGCTGGCATGGAGACGAGCAGCCCCAAGGCGCGATGG
CCATTGGCCGCCCTTGTGGCAAGCACTGATGGCGACGCTGAAACTGAACACATCTGGTCT
GATCCAAAGTCCCGGGATGGAGGCAAGTAGCGTTACAGCATGTTCCCACAGGCTGTGCGC
TCTCACAGCCATCACAGGGCGCCGTACTTGTGCCGAGCTGCTGAAAGCCGTAGTCGCCAA
CCTTGAGCTGTCCGTGCGAGCAGTCATTTGCGT
>at02516
TGTTAANTATCAATCAGtCGACTTCACTGAAGAAGATGTGTGCATGTCAAGAGAGAAAGT
GTATGGCCTCCTGCCCGCCGCTGaAACAGTGACTACTGTCGCTGCCTCCCTCAGCCTGCG
CGCTCAACGTGGAGCTGCGCCACGAGCTAGCACACTGAATTGCCAATTACGGGGTCAGGG
>at02517
TTGTTTTTTTTTCTTTTTTTTTTGCTGCTCGCATGATGGCAGAATCATCCTGGCTGTCTC
TTTGCCACAAAGAGTTCATACATGCCAATGAAGAGCTCGGGCATGCTCTGTTCCAGTTCG
TGCCACGCTCCCAGGTCCAAGGGATCACGGCCCGGCTTCGCCTTCCGGAAGAGGTCCAAC
AAGTCTGGATCCACGTCCAGGGCCAGCAGTCGAAGACCAAGCCGGTCCAGCATCTGCTGC
ACCTCTGGCCAGGTATACTCGTGCTCCAGGGGATGGAAGAAGGTGTCCCGCAGCCGCGCC
AAGCTGTGCACGTCCGGAGATCGCGCCAACTGCAGCAGCTCGGAGGTCCAGGTCTTCGCG
AGCAGCGCCTGGCGGAACTGCCTCAGGGCCTCGTCGTCGAGGACCGGCGTGCTACTGCTG
CCTTGGGCCCAGCCAGGGAGGACCATGGGCAGCAGGTCCTCCCGCAAGGCCTTCAGCGGC
CTCCTGCCGATGCGGCTGTAAGAGCGACACCAGCATCACGCCGTCTGGCTCAGCATGGAG
ACCAGCCGCGCCCAGGCAGCCTCTGGCTCCTCAAGATGGTGCAGCACGCCGCCGCACTCG
ATGAAGTCGAAGCTCCGCTCCGGCAACCACGTGCGCAGATCGCCTTGCTCGAGCTCGAGG
TTCCCCAGCGCGAAGCGCGCCTTGCGCTCCCGCGCGAAGCTGATGGAGGCCTCGGAGACG
TCCAGGCCCAGGACAGATGCGCCTGGAAAGCTGGCTGCGAAGTGGCAAGCCCAGCGACCA
GTTCCACAACCTGCCACCAAGACGGACTTCCTAGCTGCGCCCGCTGCTTGGATTGGGCCT
GCCAGCAGCTCAGGTCGTTCCCACGGAGTCAAAGTCTCCCAGCGCGGAAAGACGTGCCTC
TCATAGAAGCATCGCACCGCATTTCTATCATCCTTGGATGGTGCAGCCCCGTGTCATG
>at02518
TTTTTTTTTTTTTTTTTGCCTGCCTCTGGCTCGGAGAAATGATGACAATGCTGTTGTCA
AAGGCACAGACCGGTAGCCACACCTTGGCACCATATGTTAAATTCAACCACTTTCGGGGC
AGCCCACCACGGCGCCATGCTCACCACTTGGTGGCCTGTTCGTACACGTAATTTGCCACA
TCCTCGATGTCATCGGGTCCGAGGCGCTCGCCGAAGGCAGGCATGGATCCCTTGCCGTTT
GTGACCTGATACTTGATGGCCTCGACGTTGTAGCCACCGGTGAGGTACTTCTCGAGCGCC
TCCTTCTTGAGCTTCTTCTCCGCGACGATGCTGTTGTTGCCTCCGGCATGGCAAGCCGCA
CAGTTGCCCGCAAAGACGCCCTCGCCGTTCTCGAGATCTGCAGCCAAGGCCGGGCGGCCA
CCAACCACGGCGGCCAGGAGGCCCAGGGCCACGCCAAACGCCAGGGGGCTCCAAGCGGCA
CTAGCCGACGCGGCTGCCGCTCCAGAGGGCTGGGCAGCCTCCGCAGCGACCGCTGCCGAC

CGTGCCTGGGTCTCTGGCAGCCTGTGTGGTGCGGAGGGCGCGACGAAGCAAGAGGCAGCG
TAGAGCGCCAGTGAAGCTGCCGACACAAGGCACGCAGTGCGTGGCATTGCAGCTCTTGCT
TGAGACACACTGATGGGATGCGGCTGCAACTGGAGCTTGGGCCAAGCCGCCCTGAGCCAA
AATCCTCGTGCCGAATTC
>at02519
GTTGCCTTCATGTCTCTCTTTTTCTGTGCCCTCCCTGGTCCAGGGCTGTGAGGCGACTGC
ATGGACACACTATTTAGGCGAAGCCACTCGGGCGACTCCGGCAGGTAACACAGGACGACT
GGCCAGATTGCAAAGCTGACGGCAGCCTGCGTGAGAATCGGCACATTCAAACCGAGAACA
GAGAGACCTGCTCCGATGGAGGGCCCAAAGAGGGTGGCTGCCTGTGTCGAAGAGAGGAAG
TAACTCATGTAGGTGGGCCTCAGCTGTTCAGGTACCACATCTGCTATGTACACCTGGCAG
ACACTGCTGACTCCCGCCCAGATGCCGCTGAAGGTGCGAGCTGCCAGCAAGGAGGAATAC
CCCTCCCGCAACGCCTTGCCAGAAGGATCCACATGTAGTGCCAAGCAGGGAGAACACCAA
CACCGTCCTCCGTCCGAATTTATCAGAAAAACAAGCAAGAAGAGGCAAAGCCAAGGTCTG
CGCCAAAGCAAAAGCAGAGAACAACTGGCCAACCTCTTGATAACTTGCACCAAACAACTT
ACTGTAGAATGGCAAGATGGGCGTACTAATGCTGCCACCCAGAGAGTCCATGAAATTCAC
AAGACACACCAACACCACCGAACTCAGGTTAACCCCCAGCGAATCTCCAGACTCACTTGC
ATGGTAGACGCGCCGGCCGCAGCTCGCCAGGTCTGGTGAGCCTCTGGGTGGCCGTTTCGC
TGGTGCACCCTCCTCATCACTCACGTCCGGCTCTGGCGCAGAACTTCGCCGCATTGTCAC
TGCCAGGCCGCGGGACTCAGAAGCTCCCCCCGTCAGGGATGTGTTTAACCAAATATGGCT
TGAGCCAAAATGGCTACGG
>at02520
GGTCTGCCAGTCGGAGCTGCACTGGCCTCGTCAAGGCTCTCTCCAGCAGTGGGATCCAGT
GGAGGCATGGGTGGTGCCGGGGGCGCCGGCTTTGCCATGACGAGAACATCCGTGGAGCCA
AAAAGCGCCTCTGTACCCACGGACCCATTAGATCCAAGGCTCAAGGTAGGAGAGGACGGG
CCAGACGGCAGCTTCTCTTGAGGTCCTGTGCAGTGCCGCTTTGCGGGCCTCTCAGCACCG
GTGGCTGCAATACCGTCCTGCAACCACCTATGGCTGCTGACATCCTGCAAACACAGCCTG
GCTGTTGGCTCCAAGCGCAAGAGCCCCTGCACAACAGACCTTGCCGTCTCGCTGACCTTC
AAGCTTGCCACAACACGGTCAATATCTCCCTGTTCGGCGCTGATCTTCTCGAAGGGAACT
GCCCAGCGAGCAATACATACAACAAGACA
>at02521
GTCGTGGTGAAGACGCCGTTACAGACGTACCGCCAAGTAGTTTAATTCTTTCACATTCTG
GCTAGAGATGCCAAACATAAGGAGGTCAGCATCAACGTTGTACAGGCAGCCGGTGAAGCC
TGCGCCGTCCAGTCTGAAGGTCGTCTTCGAGGAGAGACCGACGGCGGCCGCCGCCACGTT
GAAGCACTGCGTCTCCGTGGCCACATCAGGGATGCCGTAGTCCGCGCAGTCGTTGCCAGG
GGTGACCAACGAGTACTGGTTCGACGCCCCAGCCAAACTGGCCAGCGGCAGCAGTGCAAG
CCACTCCACTGCCCCAGCCCCGGGCAGCCTACGGCGCCCCCATGGAGGCATCTTGGCTAC
CGGCGCGCATGGGCGGACT
>at02522
TTTTTTTTTTTTTTGTTTAGCAACCAACCGGATGCCCGCAGTGCCTTCCGCGGAGGCTGC
TTGCTGCATTCCCGCTTTCTAGGCCTGCGGAGAGGCTTGCACTCCCACCCGCGGCCCCCA
GACGGTGAGTGTGCCGGGGCTCTGCCACGCCGCCCTCGCTCTACCTTGCCGAGGGCAGCG
CGGGCAGAGGGCAGCGCACGCAGTGCCTGGCGGCTCTCCGATTCACAGCACGCACTGAGG
GCAACATGCTCTCCCGGGCCCTGCGAGAGACTTGCCCCCT
>at02523
GCAGTGTCGTGCATCTCTGTGGCTTCTCTCTAGCCCGCTTACGGCCTGTGCAGAATCACA
TGGCTGGAGTGTTGTCCTCAAGGGGCGGTGCCACTCCGAGGAGTGATACTGTCGCCGAGC
GGCACAGAGCGGCGTGGaAAGAAAAAAAAAAANAAAAAAAAAAAAAAAAAAAAAAAAAAA
AG
>at02524
ACCTTCACGTCGTTCGTAAGTGATGAGCCAGGCCGGTCAATGAGGCCCTCGTTGACAAGC
TCTCTCTCCAAAGCCGTGTACTCGGAAGAGCTCAAGTCACGCTTTGGGAGCCGCGATGGC
CCCACGTCCAGCCCCGTCATTCTCATGATCCCCTTCTGTACATTGACGTCAGACTGATAC
GCACCAAACTTGGCACACAACTTTGCATTCTCGAGCTGTGCAGCCCGGGCCGATGATTCG
TCGTTGTGCCTGTACGACGACACGACCGTACGGAGAGTCGGAGAGAACTGCACGGTCGAC
GACACAAGCCCATCGCATCCCATCTGAAGAGCGGACAGGACCTGCTCGTCCCGGCCATAG

AGCATGTTGTACTTCCGGGTGCCGCCCACCTGCATGCACAACTGGAAGTCCATAAGATTG
TAGTCTGTGTACTTGATGCCCTCGAAGTTTGGGATCTTCCCGGACTCCTCGACCATCTCC
AAGAGCTTGTGGGCCTCTCCCGGCAACACGCCACTGATGCCAGGAAAGTGGTAGTACCAG
AAAGGCAGCTCTGGCGCCGCAGCAGCGACCACTGCAAGGAAGTCGTGCAACGTCGTCAGC
GTCGGCTTGAAGAAGACTGGAGTCATGCAGACGATGCCAGAGACTCCTGAGGTGCTTGCA
GCATGCTTTGCCAACTCTACGGCGTCAGCGAGGCTCTCAGCGCCAACATGCACGTATACG
CGGATGCCGAGGGGCGGCCCTGCACGTGCCCATGCCTCTGCAAGGGCTTTGCGCTCCACA
ACACTCAAGGAGAGGGATTCTCCATTAGTCCCTGCAGACAGGATGCTCGTAATGTTGACC
CTGACAAGGTGCTCGGCATACTTTGAAATGCTGTCCAGGTCCAGCTCAAGACTGTTCGAC
TTCATGGGCGTGTATACGGCTGCTGTCGGCTCAGTGATTCTGTGTTTGTCAATGTTGAAG
GCAGCGGCAGCTTCCATCAGGACTGAAAACAATGCAAACCAAAAGAATCAAATCTGTCCA
AAAAGCATGTGCTGCAACGCCAAGCGTCCTTGAGCCAAAATGGCTACGG
>at02525
CCGTAGTCCATTTTGGCTCAAGCTCAAATTCCAGTGGCCTTGCCAAGCTGCCAATCTCGT
GTTTCGAACACATCATGATGAGTCCTTCTTCGACAGTAGCCTGTGCTGCCTCGAGGTCAT
TGCAATCGAAGTGGCTTAGAGGTGTGATGCAGGCTTCGCTGAGACCAGCAGCCAGGAGGC
CCGTGCTTTCACGGGGGGATATGATCCGCAGGTCAAGGCATTTTTCAACCCTGTCACTGC
AGGAGCTACCAAAAAATGTTGTTGACTGCGAGTATGCAGTGCGTGGTGCCGTCTTGCTGC
GATCTGAGGACCTTGCGAGGAAGCTTGAGGAGAAACAGAGACCCGACAGTCCTCTGTCTG
CTGAGGATGAGGAGCCTCTTCCCTTTGACAAGCTAGTG
>at02526
GCCCCCCTTCTGCACAGGCTCTCCTGGGCTGCTTCCCAGAGGAAGACCATTTTCGTCCTC
CGAGTCTGAGTGTCGGCCCATCGCGCTTTGGGATTGGGAGGCTCACTTGGCACCGTTGGC
GCCGCTGGGGCTACCCATGCGGGTGCGGGCATGGCGGAACTGCTTGCGGCTTCCACCTCA
TCCTCAGAGTCCGGCACGTCAGCCCATTGCATCCGGAGGCCTTCCGGGGGGCCGCTCTGA
GACGGAGGCCGTGGTGGAGGCATCCGATTCATCACCGTCCTCCGCTTCTGTTGCAGCACA
AGCT
>at02527
GAGGTCCATGACAAAGACGCGGTGAAGTTTGAGCAGAGCGGCGGCGATGCCGCGTCGTCA
TCGCGCGGGCTATGGACGACTGGCTGCGAGGCATCAACCGACGTGAAAGGACTTCGGAGG
AAGGCGGGCTTTCCACCCAGACATGAATGTCTCCGGCGCTACGACGAACCTGATGCTCTG
GCAGGAAGGGCCACAGAGGATCCGCACTCACATCCCACCGAGTCGTTGACCATCCTGATA
CGAGGAGTCATGCGCGTCCAG
>at02528
GGGCCGCGCTTCGCGGGTCCTCGCGCAGCTGGAACGCCTCGGCGTGCCGCGCAGCCGTCT
GGAGATGGCGGCCTACGCCGAGAAGGCGCTGCTGGCTCCAGAGGCCAAACTCCGAGGGGA
ACGCGGCGAATCGTCGGGCGGAGCTGCGCGCGCTGCTGCGAGAGACTGCGGAGAACCTGC
GGTCGATGCAGCAGTCGGAGAGCGCTCTGTGCCAAGTTGCAGCGCTTGCAGCGGGTGCAG
TGCTCCCCTTGAGCCTGGGCCTCCGCTGCGTCGCTGCCGAAGTTTTGGACTCCTGGGGTG
TTC
>at02529
TTTTTTTTTTTTTTTTTTAGCCATGATCATGCTACTGTCCTCAGCATTAGTGTCATCAGT
GCTATTGCCAGCATCATGATTGATCCAACGTCCGGCGACGCCTAGACAGACAATGCCAAA
TACCCATCAGGGACTGCCTGAAAAAGTCTGAGGAGATCATGCCAGCCTCACTTCTACGCA
GTTTGCACGTCATGGGCGCTCCGGTCAACAGGCAATTCAAAGGGGCTTGTCGATGAACTC
TTCGGCTGCACTGCTTTTCAGCCTGCTGGATGCACGATGACCTTGCCTTGGGCGACATTG
AGGCGGCGTCCACCATCATGCCCATACTTTGTGCTGATCGACATTGAAAATGCGTCCACT
GTGGCTCGGACCTCCTGCCAAACGGATGCCCATGCCCGCAGAACGACCCACGCCAACAAG
GCGTCCGAGCCGGGGCAAGCTGCCCAGTTGACACGCTCCATGCGCTCGGTGTGCCGGCGC
CGCACCACTCGAGCGCTCCACAGACTGAAGACCCAACTCATGGCATGGAGGGCTCCGATG
ATCAAAGCTTTTGCAGGCAATCCTTTCATGTAGGACTGCTCTTGCGAAGATAACCGCCAG
GCCCATAGGATAACGCGCAGACGAGCACCGGCCGCAAGAAACACACCGTGAAGGGCCGCA
GGTGACTCTTGCCCGCCCTGACCTCNTGCCATCTCCGGTACTGCTCGCAGCCGATTTCGA
CTGGCATCCCGAAAGATGCGTT
>at02530

TTTTTTTTTTTTTTTTTANGCAAGACATGATCGAAGGCACCGGGTCCCTGCATGATTCC
GTGATCACTGCACTGAAGGCTTCAAAGTGTGGCCTCTGGACAATGCTGGCATGGCTCACA
ACCCTGGAAAGTAGAGCATCAATGCGGCCATGGAAATATCACCGGAAGGAAAGACCTCAA
CACATCGAACTGCCTGCCCTGACTCTTGATGGTCGTCCTGGAGCCCTGCCTCAAACCCGA
CAATGAAATAGAGCGAATGCCCACAACCTTTGGCAACCAGTGGAAGAGGTCAACATCCTT
GGTGTGCAATGACTTCTTTGCAGGAATGACATGGCCATGATGTCTGCACGTTTCAAAAAA
GCGTGGTGTCTTCATGGGCCAATTGCAGCTCCCAAGTTTTGATGTCAAAGGTTGAATTGT
CCTGGAAGCAAGCAAGGCAGCTGCTCTGCAAACCCATGCGATCATCAAGATACCACACAT
TGCGTCCACTGCGGCGGCCATTATTGAATCGAGATCCGGAAGCATCCTCATGCAAAGATT
TGGTAATCCAGAGATTTTGCATTCCCAACAAGCCACCCCAGTCTGATCACCGGAATAAAT
CAATGCATAACCCGCATGAAAGCTGCGGCTCCGGAGAGGGCCAGCAGAAGGCAGCCAACA
TTTTTTCGCCAGTTCCTTGATGAGAATCACTGAGCATCGCCACTGGTGG
>at02531
CCGTAGCCATTTTGGCTCAAGTCAGGCCGCCCCACGACCTCCTGATGCGTGGTTGCCGAT
GGCCCGTCCGGTGGTTCCCTTCAGCCTCCAAGACTTGCTGGATGCAACCTGCGAGATCTT
ACTGGAAACCTGATTCAGTTGCGACGCTTGACCACTGAGCGGGGTACCAATGACATTAGT
CGTTTCAAAATCACTGTCACGGAGGTCTGGAATCTTTGGCACTGCCGTGGACTCCAAGCG
GCGCAGATAAGCTGGAGCGTAGTCAGGCG
>at02532
CCGTAGCCATTTTGGCTCAAGTTTATCAAGTCTAGACGAAGTCCAGCGGTTCGGGTCGGA
GGCGCCCGGCGGCGCATCCTCCATGACAGCCGGGAGTTGCTCCATGGCGGGACTCTGGCC
GACATCGGAGTCGGAGAGGGTGCGCGGTTCACTCTTGTATACACAGCTGGTGGACAGGTG
GTCACTGCTGCTGGCAATGCGGCGCAGATCTGGGACGCAGAGACGGGCGAGTGCCTGCGC
TCCCTCCAGGGGCACGATGGTACTGTGGGATACGCGGCAGTTTCGCCGGACACCACGCTG
GTGATCACCTCTTCGTGGGACGGCACGGCGAGGATCTGGGACGCGGCCTCCGGCGAGAGC
CTGCGCTCCCTCCAGGGGCACGGCGGCCCTTTG
>at02533
TTTTTTTTTTTTTTTTTGATGCCTGGCAAAGCAAAGGGCTGTGCCACACCTGTCTGGCA
TGCAAAATTGTACACAAAAAGACATAAAAAATCATGGCGTGTGCAACACTTAGGTTGACA
GTCCTACTGAATGTAACACACAGACACACAGATGGACACACCGAGAGGCACACACACATA
TGCACAAATTTGCACCTCCATTCAAGCCGTGAGCATCGAGAGAAGCACTTCCAACCGCTC
ATTCGCGTCCTTCAGCGCCTGCCGGGACATCTTATCCTTATCCGCCAGCTGCGCGGTCAA
GCTCTTCAGCTCCTGCTGAATCTTGGCCTGGTCGGCGCTTGAATGACCGGAAGCTTGCGG
CGCAGGTTGCTCTGCCGGTGAGTCTGGACGGAGACGGCCGCCTCTGAGCATCCACTTCTC
AGTGGCAATCGCTTCGAAGCGCTCTTGGTACTTGCCGTGGCACGTGATCACAACGCCGCC
CTTGAAGCCTGCCGCAGCCTGGGACAGCGCCTCCATGGCCGCACAGTCGAGCCAACTTGC
AGGGTCATCCAGGACCAGAAGTTGCGGCCGCGGCCAGAATGCCGCTGCCAGCAGGACGCC
GGCCTTGGAGGCCGCACTTAAGGTCGCGACCTTTGCACGACTCATCCACCCAGGGGTCGA
GCCC
>at02534
CACCAACGGCCGGCACCTCCACAGCAGTGCTGGTTATCCGATGCACGAAGACTTGTTCGC
TGTCACTGGGAGGTGGCGAGGGTGCCTGAGCGTTGAAACTGAATGTGGCGAATCCGCGGT
TCATCCCGTGGTAGTGCAGCTCGGCATTGTCCTGCTTTGCCCACGCCATGATCCCGCCCA
CGGGTGCATCCGCATCGAGCAGCTGCACATCTTCTTCAATCAAGCAGGTCTCACGCGTTC
GCGTGATGACGCACCACACGCCGACGTTGTTTTCACTTCCACCCTCAACCAATGTCCAGT
CCTGCACACGATCTTCAGCAGGTGTTCCATAGCTCCGAGCGTGATAATCCTTAACCACCA
CCTCAGTGCTACCTCGATGACATGCTTACG
>at02535
CCGTAGCCATTTTGGCTCAAGGCCGTGTCAAGTTCAAAGCACCGTAGCCCATATCCTCAT
CCCAGCCTGCAGCTAGTGAAGGATGTCTGCACCAAGGATCATGCGAGCACTTTTCGGTGC
ATCCGTGGCGCTGCCTCTCTTTGCCGAGCCGGTGCTCCCTGCAGACATCTGCACGGGCGA
AGGCTGTGCCTCCGAAGCTGATGATGCAGCCCTGATTCAGGTGCCTATTCAGAAGCTTGA
TACCGATGATGAGTTCTTGCCTAGACCATTTTGGAAGCTATTCAATCAAACTGGAGCCAA
GATTTGCAAGAATAGCGACGGACTCTTTTTTCAGTGCTCACCGGGATCTACGTGCTGTGG
TGATGTCTGCGTTGCCCCCGGTGGAACATGCTGCAAGAACACCCATGGCAACAATTTTGC

CTGCGCTGCTGGGAACACATGCTGCGGCAGTACATGTGCGGCTCCAGGGAACAAGTGCTG
CACCAATACGTTGGGCGACAAGTATCCCGTGGCTCCAGGCACAGAGTGTGCAACTGATAT
CGACAAGAGTGTTCAGTGCAAGAACCGTCATGGTGTCCCGTTTGTGTGCGGTGAAGGCTC
CTCATTGCTTGTGGAGACATCTGCGTCGCACCGGGCGGCACGTGCTGTGAAAACACCCTG
GGAAACAACTTTGTATGCGCAAAGGGTAATACGTGCTGTGGGAACACCTGCGCCGCACAC
AACGGCAAGTGCTCCTGTCTTCAAACAGCGCGGGCTACCAGTATGCTGTAACTGCGGCCA
CTCTCTGCCCAGATGAGATGCCTGATAGCCATGTGTGCACAAACCGCCATGGAGCAAAAT
TCCAGTGCAGCCCTGACTCTTCGTGCTGCGGAGACATCTGCGTCGGAGCGGGTGGCACTT
GCTGCAAGAACCGATATGGCAATAACTTTGCCTGCAGCAAAAGAAGCTCGTGCTAGACGG
TAGAAGAGTGTACCGCTCCAGGCAGCAAGTGCTGCGTGAACAAGGCCGGCACGCACTATC
CGGTGACCCAAGCCACAAAGTGCGTGGCATGAATTAGCTAACACACTGTACAGATTGTAG
TGCCACTGCAAGCCGGTGGCATGTGAGATTGATGGATTTCACTGCCACCATATTTATCTC
GTATAGGAGATTGGAAAGCTTTGCGATGTCAAAGCATGATTGTTCACCCAAGCAAGGGCA
GCATATGCTGCTTGATGCATTACAGTGGTCCGGAAGGCGGATATATCTGCAGTCGGCGCA
AAANTGCCCCGATCACGCGTCTCCATCCATCCTGGTCTTGCGTGTCTGTGTGGCAACGAC
ATTCCGGTGTTCAGCTTTTCCTGGAGTCAGCATGCACATGCTTGTCAACAGAGACCAGTC
TGCCTGGTAGCAAGACTGTCTACGC
>at02536
GGCCACGCCGACCTCTCCCTGACAACCCACTTCGGCGCCGGAAATAGAGGCGTTCATCTT
ATACAGCGAGCCAATCGCGCTGGCGACCAGCATATAGCGAGCAAGCGAGTTAGCATTTAC
TTCGCGGATAAACTTGTCATAGTACGCCAGC
>at02537
TTTTTTTTTTTTTTTTTCGCGGCCCGTCTTTCTCATGAAGCCTCCCCACCCCGCCAGGG
AGTCCAACGTGCCATCAGCTTACTGAAGGTGATTCCCGCCTCCACCTTGGGAGTTTCCGT
CCAAGTCGCGCGACAATAACGCGCGAAGAGAGGAAGTGCCAGTGCACATCCGTAGGATGA
TTCAGAAGTTGCCTCGTGCC
>at02538
CCGTAGCCATTTTGGCTCAAGAGGGCTCTGTTTGACCAGATCCCTTCGTCATGTACTCGC
GGGCGGATGTCTGCGTAGGCTTCCTGAAGGAAGATGATAGAATATGAGGAGGAATGGATC
ATCAAGCTCTTGTTCCGAACGGAGTGCAAGGTCGCTGTGAGGGCACTGATTTTTGCCTTC
CCTGCAACGATCTTTGCAGTGCTCCTGAACATGGGCCACATCCCATCTCGAAGACATTAT
AGAGTCGAGCGGCCTGCCCACCGTCTCGGACGATTCGCTTGTGAAAA
>at02539
CCGTAGCCATTTTGGCTCAAGGGCTCGAGCAGCCAACCCGGCACAGCCTGGCGGCCGTCT
CCGTGCTATGGTAGCTCCGTAGCGCGGTGGCTGAGGCGGACCATGGCCTGGGAGCCGGGG
GCGGAGGAGGAGGATCAGTCCGCCGCCGCAGTGCGCTGGTACTGGGAGGCGGTGACGCGC
CAAATCGAGCAGAGCCAGAGAGAGCTGCAGAGGCTTCACAGCCCTCCTGCCGACGGCGGC
CCCCACCCCGTTGACGAGGCAGCGTTGCGCCGCTGGCGGCGCTGGCGCGAGTCCGAGGCG
AGTCGGGAGCCGGTCCAGGAGAGCGCCAAGGCAGAGGAGGACCCCGCGACGAGGTCGCCG
GAAGCAGAGGAGGCTCAGGAGCCTCGAGGGACGGCAGCCGAGGAGACCGCAACAGCGGTG
GAGAATGCGCAGGAGGAGGAGGAGGCTGCAAGCGGAGCGGCTGTTGCGAAGACAGAGGAG
CCTGAGGCAAGTCAGCGTGCGGCGGAGGAGCCTGCGGGACGAGCAAAGGAGGAGGAGGAG
AAGCCAGCAGAGCCTCCAGTTTCGAGCATAGCGGCGCCAGAGGAGAGGCCTTCAAGCATA
GCGGCGCCAGAGGAGAAGCCTCCAGAGGAGGCCGCGGCGCAAAGGGCAGCTGACGCGAGG
AAGACGGCAGAGGAGAGCGTTGCCGGAGCAGGGGAGCAGAAGGGAGCAGGCCAGGCGAGC
GCGACTGCTGGCGCAGCCTCCGGGGCGGAGAAGTCCTCTCCTGGACAGGCGGCGCCCGCC
AAGAGCCAGCTG
>at02540
GGCATACGGACGTCCAGCAGCAGTGAGACCGCTGGCGCACTTCCTGTGCGGATCTGGATG
GGGGGCGCATGCTGGCAAGCAGTGCCCGCGCCTTTGATGAAAGGCCGCGCCTTCATGAGA
AGGTCGCTGAGCTGGAAGATGAGCTCGCCTTGGCGGTTTTCTGCCAAGACCAGCGCTACA
GCGCGGAGCGCGAGCTGAAGGACGCCGGGGTCCCCTCACCGCGGGTTGAGGCTATCTCGC
TCAAGGCCAAGCGTGTGGGTGTGGCCGCCGGCCATGGCTTCACTGCAGGGCCGGACATGG
ACTTCGACCAGTTCAGCTGCCATGCTGACATCTCTCTGCGCGGGTAACACTCATCACAAG
GGCACTGATTGCCACCCCCCGA

>at02541
TTTTTTTTTTTTTTTTGTGCAAAGGACGACACATGCTTGCAAATATTCAGCTCCAGCCTC
TGCAGCACTGCTGGGGCTTGTGCCACGAGGAGTGTGTCCCTCCCCCCATCCGAAGCTCTC
CTCTGTACTATGAGGCTGTCGGATGGATCAACAGAGCCCAGCAATCTGACCCTTTAAGTC
AAATTTTGAGGGCCGCTGCCTGAGAGACTCAACGGGTCTGAGGGAAAATCGTGAAGTCTG
GGCCAGGAGTGTCATGCGTGGACTTGGCGGCTCTGCAGTCCCTCAGTGCAGGATCGAGCG
TCAAGCCGTCGAACGTGGCACTGCCTCCATCGGCGTACCGATCGGACTTCGTTATGATCG
TGCCGAACCATCCGTCGATGATGTAGAAGAGCACCTCGGAGCTCGCCAGCTTGACCTTGC
CGCCTGAGAGCTGGTCCTTCATCTTCCCGAAGCGGTCAAAGTGCTGCAGAGCCACAGAGC
CGATCGGGCATCTTGGCCAGGCGGAAGTGATCGAGCTCGCGGTCCACGTCGAGACGGCGC
ATAGAGCTCGGGTCAAACGGCGCCGCAAAGCCGGAGCAGTCTTGAGAGAGCTTGTTCTCA
GTTCCCGCTCCACTGCACGTCGAAGACGCGCGTTGGCGGGGATCTTGCCGCAGGTGTCGA
TGAG
>at02542
CCAGGTCAGGGGTCAAGCCCCTAGTGGATTCGCCTTGTTCAGAGGCCGTTGGATGCTCTC
AGAACATGAACCCACGGAGCCTCCGTCCATGGCACCACAGATCAAGACCTGTGTTTGCAA
AGGGATCAGGCGATGCGCAATTTGTGCACCTGAGAGCCTCAGATCTTGCCCAGCGCAAGG
GCCAGGGCAAGAATGTTCGAGGCAGTGGGACGCAACGGCCTGCTGCCGCGCTCTACCTGT
>at02543
TTTTTTTTTTTTTTTATTGATCGTTGGTGTGAACACCTGAAGCTCTAACCAAAAAGAGCA
GTGGCAAACTGCCGCTAGACTAAGAGGGCATGCCTTTGGCTCGACTCATGCCTCTTGTAG
GCAAGTGGCACGGAGATGGCCCCATCCTGCCTTACCCAGATCAGTGCCAAGAACACCCAG
CACCGATGTCGCTGCAGCAGCACCCCAGGTAACACATCTTGCTGTGGGCATTCCTTGCAG
CCAGGCAGCCAGATAGCCGCCGGCGAAGAAGTCACCGGCACCGGTGGAGTCCACTGCCTG
GGCAGCGGCGACGTGGTACCGCTCGGGCTGCGCACCATGGGCTGTTGTGACCCAGAGGCC
GCGCTCGCCCATTGTCACCACAACCATTGGGCAGTGCACCGCGGAGGCGGCAC
>at02544
GCTGCGTATGCGGCAAGCGAGGGCTGCAGCACCGACGCTGCGGAGGCTCAGGCAGTTCAG
ACCTACGGGGAGCAGTGGCTGCACTTCCTGCACACCACATTCTTGCCCGAGTTTGCAGGT
CCTGCCCGAGATGAGCGCTACACAGTGCGGCGCATCTGCTTTGCGGGAGGCGTTGCTGAG
CACAACTGGCCACTCCTGAGTAAGGCAGTCATGGAGCCGGGCGGACAGCTGAGAGCTGTA
CCGGCCACGCTGGCCACACCCAGGCCCAAAGTCCGCCCACTCAGCACCCACA
>at02545
CCACCATCACTGTCCATACAAATGCATGAGGGCTGTCTCAATGCGACTGACAGCATAGCA
TGTCGCAACATCGCAATTCATCGCGCTCCGAAGCAAGGCTCAGCCCGCGAAGGCCTCCTG
GTCgGGCCTGGTCCCGCTGCAGCCACGCCAGCAAGGACTCAGGCATGtCTTCCCTCTTCA
TGACGGTCGTGGGGAGGAGCTTGCACGCCACAgGTCCTgGcCCCCGCAGGGGCAGGTCCA
CGGGGATGTACACGTCGTTCTGCCCGTCAAGGACCACGCGGCTCTGCGGCCAGCGCTCCG
CGTTCTTCTCGACCGTCCAGTGCTGCTTGACCTTGTTGACGGTGTACATGGCAGGGCGGG
TGGGACGGAGACTACCAAGCCCCAACAGCTCCGGCTAGAGCAGAACAGAGG
>at02546
CACCCTCATCGAGCAGCGGGAGGACGGCCTCTTCAAGGCCAACGTGGCTGTCCCACAAGG
TTCCGGCGGTGATAAGACCGTGGTGTACCCAGCGGTCAATCCAGCGAACATCCGCCACGC
GCGAGGCTCCAAAGATGCGCTGGTGGTGCCCAGGAGGGCCATCGTCTTGACGGTGCCGAA
GTCGGACCCCATGCTTGCGACGCTCTCCATTGACGGCAGCGAGCTGGTCACGCACTTCTT
TGCCAGGCCCACTCCGGCGCCGGCGGCCAAGGGGCAGCTCTTCGGCAGAATGCAGAGGAC
GAATGTCGACTTTCAGGTGTCAAAGGACCGGAAGGTGGTGTCCTGTTCCGTGGGGCACAG
CGTGCTGGCGTGGTTCCTCAGTGGCGAGGCACGGACGGTGCACGAGGAGTGCGAGAAGCT
GAGGCATTCGTGGACGGTACGCGTCGGTCCTTTCGCCACGCACACGATCGTGCTGGAGAA
GAAGTACAAGTCAAGCAAGGTAGTCACCCTCACCGTCGACAACAGCGTACTTGCGGAAGG
CTCGCTGAGGACCTGGAGAGCCCCGAGGGGTACTGGTTTTGCTCCTTCCGCTT
>at02547
TTTTTTTTTTTTTTTTTTGCTTGTTTTGACGAATCCTTAAGGGACAAAGTGAAAGTGACT
GAGGGAACAAAGTCATGATCTGCATGGGCACCCGACCAGCCACACGAGACAAGCGGAAAA
CACCACAGCAAACCTCCAGGGCCCCAGAAAGACGGAAAAGATTCTCAAGTCTACCCAGCC

GCGCGTGTCACTAGCAGGAAGGACAAGTCGAAGATGCTCCCCATCTGAGCCAAGCTTCAG
CGGCCAGTCTGGTTGCAGTTCAAGTGACCCTCCTCCAGCAAACCCTCTTTGGCTTGCCGT
CAGACCGTATGGTCAGCTTCACGTCATACTGTGCGCCGGTGGCACCCTGGTCCTCCGGGG
TGGGTTGCCAATGGTCCAGTGCAGACCCCGGCACTTACGCCGATCATCAGGGCCCTCTAT
TGTATAGTCAGTGCCAGGGGCACCATTGACCTCTTCCTCTGGGTAGCAAAGCAACTCTGC
GAGGAGCCCTCATGCAGAATCTGGAAGCTCTCGACGCGGTACGTGAGTCTCAAGCGGAAC
GTGTAGGACCGGCTGCTCCGGTCCCAAGTCATCTCCTGAGGCTCACCCCAGTTGCTCCAT
GAGCCGAGGACATGGAACTTTTCGCGCTGCTCCTTCTCTCGACTGGTCCGCTGTG
>at02548
TGCAGGGCGACAGCGCCAACGTCAGCGGCCTGGTGACCTGGCCAGTGTGCCTGGACCAGA
ATGGCACTGTCATTGGAGTGGGCGAGAAGATCGTCCAGTCCTTGGCAGGCGTAGACCAGA
TCAACTTCAAAGATGACTCCTGGTGGCGCCTCCGGCCCTCCGACGGCTCCATCTCGGTGA
CTCTGCGGACGGTGGGCGGCGTCTCGCGGCGCCTGGAGGACCTGAGGCCGCACGAGACGC
TGGGTGCGATCCGCATGCGTGCGGAAGAGGAGCTGAAAGAGGAGCTCACGAGGTCTGCTG
CGGGCATGGCCATACACTGCGAGGGGCGCGTCCTCCTCCCTTGCGAAGATCTCCGGACGC
TCACCGAGCTCGGCATCCGAGACGGGGCCACGGCTGCTCTGCGCGCGTTGAGCGTGGA
>at02549
TCACGATGGCAAAGGTGGTCCCACTGCTCCGGATAACAATGGCGGCGGTACCAGCATGCT
TTGGCTTTGCCCTCCAGCTGACGCGGCAGCACGAGAATTAGGCCTGCAGCTGACGCGGCA
GCACAAGAATTAGGCCGTGATTGGGCTTTGCTCGGTGGACTTGAAGGCTTTGCCGAGACA
CCCGCCC
>at02550
CCCAACCAACAGACAATGACATTTTCTCTGGCCCATCAAGCTTCAGCCCAACAACGCCCG
AGATCCTCAAAGCACCAACCATCGGCGCCGCGTGCAACTCCGGAGCGCACAGGCGTCAGA
CGCCCTCCGTCTTGACGCCCCGCCTCCGCGGCACGAGGCGCTCACGGTCGCTTGGCGTGC
ACCACCAGAGCTCGTCAGGCCGGAAGTCGAAGAGAGGGGTGTTCGTGGCCTTCTCCTCGT
TCCGGAGGGCGTCGAAAAAGCGGTGCATGAGCACGTGCACGGGCGGGAGACGGGAGGCCG
GGTCATACCTGAGCATCAGGTCTGCGAGCTCGTTGGCCTCCCTCGACGTCCAGCCGCGGA
AGACCTTCTCCCAGGGGTGGGCCGAGATCCGGGGCGTGAACTCGTACTCGGGGTAGTTCG
GATTCATCGCGCGCAGCTCCTGCGGGGTTGGCGTGCCCAGGACCTTGATGATCTCTACGA
GCTGGTTGATGCCGTCCTTGCCCGTGAAGAGGGGCTGCCCGAGGAGCATCTCAGCGAAGA
CGCAGCCGGCCGACCAGAGGTCTACAGAGGTGGTGTAGCTCGTGGAGCCCAGGATCAGCT
CCGGCGCTCGATAGTATCGCGAGCAGACATAAGGCCGGTTCTGCTCACCAAAGACCATGC
GCTTTGCAGTGCCAAAGTCACAGATCTTCAGTGTCTGCGTTCGCCCATCGAGCAGCAAGT
TCTGCGGCTTGATGTCGCAATGGACGATGCCCCGGCCATGGATGAAGGCGAGTCCGCGCA
CGAGCTGATAGAGGTAGAGCTTCACGTAGTAGTGCTCCATGACCTTGTGCAGCTGGTTGT
AGTGCTTGATGACGCGGTGCAGGGTGTCAGACAAGAACTCCAGGACCAAGTTCAGTCTTT
GCTCTGGCGAGCCAGCCTCAGTGCTGAGGAAAGCGCCGCGGAGGCTGACTATATTTGGGT
GGCCGTCAAGCTCTTTGAGAATTTGAACCTCACGGTCCTTCTCCTGCAGTCTGATAGACT
TAATGGCGACAATCTCGCCTGTCTCTATCACCTGCGCCTGGTAGACTACCCCAAAGGATC
CTTTGCCAAGAATCCGCTGAGCTGTGTAGGTGCGTGATTCGAGCTCGGGTAAATCCTCAC
CATCGATCTTTCGCTTTTTGTTCGGTGTGGCCTTGCTGGCTGCATCCATTCTTGCCCAAA
TCCAGCACCCCAACACGCCTGGACCAAAATGGCTTGAGCCAAAATGGCTACGG
>at02551
TCGTCCGCCTTGCCCATCAGCAGCGCCGCCAGGCCTCGCAGCGCAGTGTCGGCCTCTTCC
TCCGCCAGCGAGCCCTGCTTGACGGCGTCGCGGACGATGGCGGCGCGGTTCCTCACCACT
CTGGCCGCTGCCTCCTCTTCCGGAGTGGAGGCGACGTGTGGCCTTCCGGCCGCGAAGGAG
TCCCAGTCCCCTTCGCTGTGGTCGTCCCCGACTGTGGTTCCATCATCGCCCACCAGGACA
GGCGCAATGCCTGAGTTCCCGTACCCGGCCATGGACAGCACCTTCCGCCAGGAGACGCTG
TCCCGCGCACCCGCGCGCCGTAGGCGGGCACCTGCCGCATCATCGCCGCGTACCA
>at02552
TTTTTTTTTTTTTTTTTTGCTTGTTGAGCGCAGAGTAGCAATCGGTCTGGGCAGCTGCTC
GTGTCTCCCTCTTCTCATGAGCACGCAGACTGATACGCACGGAGCCGCCGCCCTGGTCTC
AGAGCTTGGCATTCTGCGAAGCTGCCTGCTCCACAGCAGGAGAGGTCGTGTCAAGCATGA
AGTTCTTCCTGTATCGCCAAACTGTCCCCACCGTAGGCCGCGCAAGGTGCCTCAGAGCCT

GGCATTCGACGAAGCTGCCTGCTCCACAGCAGGAGAGGTCGTGTCAAGCATGAAGTTCTT
CCTGTATCGCCGCACTGTCCCCACCGTAGGCCGCGCAAAGTGCGTGCCAAAGACCAATGT
CGGAGTGCCGCAGAACTCGTTTAGGAGCTCCTCACGTGTCCGTGTGGCCTGAGCGCTGTC
CGTGTCCGCGCGCGAGCCCACGGCAGTCTCTGTGAACTGAATGGGGTGGTGGAAGCAGTC
TCCAGTGACTGACTGCCTTGGAGCCACACGACTCGACCATGAGTGACACATGGCCTGGCG
TGTGACCAAGCGTGGGCCGCAGGTAAACCCGTGTGTCTCCGGTGTCCATCAGAACGTGGT
GGAACTCCACCAGCTCCACGAGACCGGCATCCATGACCGGAGTGATGGAGTCGGCCAGTT
CCTCCTCGATGCCGCCTTCAGCACCTCNCTCACCAGATCTCCCACCGCCGGACCAGTATT
CAAACTCCTGCTTCCCGAACAGGTACTTGGCGTTCGGGAACGTGGGCACCCACTTCCCGT
TCTCCATCCGCGTGTTCCCAGCGATGTGGTCTGTGTG
>at02553
CTCTGAGCAATGGTTGGCCTGTGCCGTGTGTCTCAGCTGAAACCATTCGGCCTCTCCGCG
ATCGCGTCAAAGTTCTGTGTGAAGTCTGCTGCATATCTGACAGTGGATGATCCGGCCTTG
TTGCTGGGACCTCGTTGCCAGCACCAGCGCCGTCTTCTGAGGCTGCAACACCCATACTGA
CAAGAGGCACTATGACGCCGTCCTCGGAGAGGCAGACTTCGGCTGCGAGCACGACAAATG
CGATCCGGCCCTGCCAGCCCCTCTTGCGCCACCTTGGCCCCAGCCAGCTCCCAACAACTC
TTCATGCCTGGCACTGGCTGCTCCAATTCGACCCAGGGTAA
>at02554
TCCTCGGCGACCGGGCAAGTGAGCAGGTGGACCCAATGAGCTCGCTACGATCTGACGTGT
TGTATGTTTTCCTTGAACGGAAATTGTCAAAGCTCCAGCGCTCGGAAGAGGTGGACTTGG
CAGCAATTCAGAGCCAGCCCAAGCTTCTGCGCCAGGCCACNATTGTCAAGACTGTTGGGG
GCTCGGTTCACGGCTATATCCACTGTTCGTTGAGCCGCCTGGCCATAGGGCCAGGTCTCA
CGGAGCGCGAGGTGGATATGAAGATGCTTCAGCGCATCTGCAGAcTCCTGGACTGCGAAG
CTTGCTGGCCGTCGCATGGCCGTGCGGGGATTCAAGCTTACTGAGATGACTGGCCTGGGA
GCTGGCGGGAACAAGAACCCTTTCGACCATGCAAAGTGGTCCCAGGATATTCGGCTGAAC
ACAAAATCTGGGGCTTTGGAAGCGAAGAGCTGCGACGGTTTCGAGTGTCATGCGG
>at02555
TTTTTTTTTTTTTTTTTGGACACATTCCTCCTCACTCTTGCGCCGCTGATGCGTGCGTC
TTCCCCACAGATCTTGGGCAACATGTGGCATTTGAAAGGGAACCACACTGATGCTTTGCT
GGCGCGGATCAGTCCGCTGCGTGCTTGTAGCCGGTGCTGCAAGTTGCAGCATGCCCAACG
ACCGGCAGGATCGGTGTAGCACGTTCCGCAGCCAGCCAGTCCTTTTTTATGATCCAATAC
ACGGCGCTGAGTGGCGCATAAAACTGGCCATACACGATCAGCTCCTCCCCGAAGTGTTCA
GGCAAGCCAAGCTTGGTGAGGATGGAGGCAATGATGAAAAACGCGCAGGAGAAGCCCATG
ACAAGAAAGGGAAACACGGGCAGCACCAACCGCACGGGGGCAATCAGCTCCTGTCCGTAT
GCAGCGAGACAGTATGGCTGCTGCCGTAGTCGCCGAACATCTATGTACGCGGTGGCTGCA
AAGCCAATGGCCACTGCGAGGAATCTCAGCACACCACGTACAATTGATGGTAGCTCTAAC
AAAGGTGCAACAACGCCCGCCTGCGGCGAGGGGGTGCTGGTGCCGGCCTGCGGGCCTGGC
GCT
>at02556
TTTTTTTTTTTTTTTTTCAAACGAGCAATGCTGCTCCAACAAATCGACGGGCCAGGCAG
CGTCTGTGACCACGACGCCAGCACTGCATTGAAAACTGCTGCGCAACGGCACGCTGCCAC
AATAGCGCATCTGAAGATCTCCCCGCCGAGGCCCTTCAGAGCAAAGGCCCATGCGGCCAC
TCACTTGTCGCAGAGAGCGGCAACGCCGGGAAGGTCCTTGCCTTCCAAGAGCTCCAAGGA
CGCGCCGCCGCCGGTGCTGCAGTGCGAGACCTTGTCCTCGGTGTCGTACTTCTTGCAGGC
CGTGGCGGTGTCGCCGCCCCCGATGACGGTGATGGTGCCCGCGCTGGTGGCCGCCACGAC
GGCGTCCATGAGGCTCTTGGTGCCGGTCTCGAACTTGGCCATCTCGAAGACGCCCATGGG
GCCGTTCCAGATGATGGTCTTCGACTCGTCCACGGCCTTCTTGTTCATCTCCATGGACTT
CTCGCCGCAGTCCAGGCCCATGAAGCCATCTGGGATGCCCTCCTCCTTGGTGGCAGTCTT
GATCTCGCCGTCCTCGCCGAACTTCGAGGAGATCGTGAAGTCGACGGGCAGGATGATCTC
GACGCCCAGGTCCTTCGCCTTCTTCATGATGTCCGGCACGATCTTGGCCCCCTCCTCGTC
GTACAGGGAGGTGCCGATGGNCATGCCGTCGCTGACCTTCAGGAAGGTGTACGCCATGCC
GCCGCCGATGATCATCTTGTTGACCTT
>at02557
TTGTTTTTTTTTCTTTTTTTTTTGCTGTGCACATGCTTCAGGCTGCAACCCGCGCTTGTC
CGTGCTCAAACTCGGACTGAGCGCCTTGGAGGGACCTCGGGTATTGCGGCGAAAAGGGGA

GCCGCCTCAGCGCAAAACGATGCGAACCGTGGCGGCAGTCATGGCAGAGGATCCGTCACA
CTGACGCCGCGAGCCTTCGCAACGCCTGCTCACTGTAGCCCGAGTCCTGATCACGCTTTG
CGCGATATTCGCCTGCCTTCTGCACTGCCTCCGACTTTCTCTCCAAGGGCTGCAGCTCAA
AGCTGCGGAGCCTGCCGGAACCCAGGAGAGAGGGACTGACGCCCTCAGCCCAAGGAGCTT
GAGGACCAAAGGACTGGGCCTCGGCTTCATCGCGCAGGCGCAGGCCACGTCTGAGGGAGT
ACTTGATCACCTGCGTGCAGTACTTCAGGTAGTGCCACTTCTTGTGGTAAACAGAGTCGA
TCAGGTCTTTCACTCGCGCGTCGCAAGGGAACTCGCGCCGCTGGATCCTGAAGACCCTCT
TGAGCGTGCCGTTCTGACGGCCTATCCTCTTGATCGCGTTCAGCTGGCGGACGGCGTCGT
TGGGACGGTCGGCACGAACGACAGCGGAGGGCTGCAAGAGATTCCTCGCGCATCCGGCTT
TAAAGCCATTGAGCCGGGCTCCGATCGCACGGAGGGCCATAGTGCTGCTGGGGCTGTGCG
CACGCCGCGAGAGGGCGGCGATCCGCTTGAGCCAAAATGGCTACGG
>at02558
TTTTTTTTTTTTTTATTCGCTGACCCTGTACAATGTCCCTTCTTGCTTCAGCTTGGGAC
TCTGGAAGGATGCCTCACAGAAAAGGCCGCAGAGAACCCTCAGGCCGCAGCCGCTCCCTG
GGTGTCCCCGGGCAGGTCCCGCAGGCAGCGCGCCGGCGGCAGCTGCAGGCCAGCGTCGCA
GCGTGCCCCAGAGGGGACTGGGCAGGCCACTGTCAGGCAAGGGTGTATCAGGACGCTCGC
CTCGACGGTCTGGCCGACGACCATCTTGTTGATCTTGCCCTTGGCCTTCTCGAGCATGTT
GCTCACCGACTGGATATCCTCGGCGATGCCGGACTTGCCGGAGTCGCCTATCAAAGCGCT
GAACCTTGTCACAAGCGGTCACCAGCTCGGAGCTCTCCTTGGTCACGGAGTTGACGAGCG
CGGCCACGGAGCTGTCCATCTTCTGCCGCGCGTCGGCGTCCCCGAGCCTCTCGGGCCTGG
GGCCTGCGACGGCCTCCTCTAGGCGCTGGCAGGACCAGAGGTAGTCCTTGACCTTGCCCA
GGCGGTCCTTTTGGCCGTAGAGGCTGTCCCAAGCGTTAGAGAGGGTATCTGACACTGCGG
CCTTTCCTTTCGGACCGCGCCGAAGGAGCTGAAAGTCTTTGTGAAAAGGTCTCCAAGTCC
TTCAGAGTCTCCTGCAGCACCGCCACCTGCGGGCTGGAGGTTGCCGCGTCCTCGAACGCC
ACTGGCATGCCGCCGCTCCACAATCCGGGCATTGGCCCCCACCCAAGGCAGCGGACGCCT
GGATAGGC
>at02559
CCGTAGCCATTTTGGCTCAAGGCATTTTGGCTTAAGACCACAAGCCAGAGACACAGAGCC
AGCGATGTCCGCGACATCATGTGATGAGCTCCTACTTCAGGCTGAGGCCCTGCAAAAGGA
GGGCCAGCACTTTGCTGGCGTACAGAAGATCAACGCTTGCATGGAGCTATTTGAGACTGA
ACTTGGTGGCTCGGACTCCAAGATCCTGAAGGAGCCAAGTTCATTCCTTGCTGAGTTCAT
GCCCAAGATGTCGCCTGAACAGAAGCTGAAGTCGCTGAAGATGTTCTCGGTCCGTGGCGA
CTTGATGGTTGGCCTGGGGGCTTCCAAGCGGGCTCTCGTTGAGCTTGAGTGCGCAGCAAC
ACTGGCGCCTGAGAACGAGGAAGTCAAGGCAAAGCGCGCCAAGCTTGAATCTCCAGCTCC
TTCGCTTGCAAAAGATTCAGACAAGAAGGTGCC
>at02560
CCGTAGCCATTTTGGCTCAAGTGCTGCCTACGGCCTGAAGCGTGAAGCATTCATGGCAAA
CCCCACGGCCCATTTTGAGATTTCCGAGGGCAGCTTCGAAGCTGAGATCTATCTGGACCG
TGTTCCCATCACGGCATCCAACTTCATCAACCTTTGCAGAGTGGGGTTTTATGACGGGCT
CCTTTCCATCGTGTCATTGACGGTTTCATGAACCAATTTGGATGCCCACACTCCAAAGAC
CCCACTCCCCCATGGCAGGCAAAGGCGGCCCTCCCGAGGTATCTTTTCAAAATCTCAGAA
CTGGCGCACTGATTTGCCGTGGATACGGAGGCACCATCAGAGATGAGCATACGTCGCCTG
ACAGCAATGTGCCTGGCTCGCTTTCGATGGCCAACGTCGGCGAGCCGCACACAGGCGGCT
CACAATTCTTTCTGAATGTAGCCCACAATCACTTCCTTGATTGGTTCACACCTGGATCTT
CCAGACATCCTGTCTTCGGCATGGTCAGGAAGGGCATGGACGTCGTGATGAAGATATCAA
AGGTCCAGACTCAGAACGAAAGGCCAGTGCAACCCATCAGGATGATCAAGATCTCTGTCA
CGGGCGCACCAGAGGAAGCGAAGCCACAGCTGAAGAGGAAGGCCTCGTCAACGTCGTCGT
CTTCCTCCTCCAGTACTCGCGATCGGAGG
>at02561
GCTGCACCCTATTAGCAAGGCCAGCTCATAAAGAATGCTCCCTCGAGCGCTAGCCGGTTC
GTCTCCCAAGAGAACAGCGTCCAGCGCGCAACCGCATCCAGCGGGGAAGCGACGCAACCA
CGAACCGATGCCAGAACGGATTGCAGACAGCCTCTCATGCGGATGCCTTCCGCAAGTCGC
CTAGCAGCTCCTCTGGGCAGGGATGGTCCGGCCTGTTCACGGTGGAGGCATACTCGCACA
GGTCGTGCACGAAGGCTACCGGCAGCACGCCATCTCCCATGTACGCAGGGTGCTGGCGCA
GGCGCTCCCGCAGGTAGGAGGCCGGCGTCGGCAGCGAGCCCTCAGCGCGGCGCTCGAAAA

GCTGCATGTACCTCTCCAGCCTCTCCTCGGACTCTGCCGAGCAGGCCCCTGCTTGCCGGC
GCCCTCGTGCC
>at02562
TTTTTTTTTTTTTTTTTTGANAATGCACACCCCTTCTGCGCACAAAAGTGATGTGCACCG
CTAGATGCACACATCTGCTGCGCAGACAAGTGCTTTCCACCGCTACAGCCCCGCAGTCAC
TGGCCGCACCCAGGGTGTGGGATCCACCTGCGCCCGCGCCGCACGGAAAGAGTCTCCGCC
CGCGTCCGCGATCCAGGAGGCCACGGCCACACCGGCGCCCAGGCCCTGCATGAAGCCCGA
GCCGCCGAAGCCCGTGCAGAGCCAGAGGCCCGGGCGGAAGAGCAGCTCGCCGACGAGGGG
CCCGCCGTGTAGCGTGGGCCCAACGACACCAGCCCAGGCCTGCCTGCTGCCCTCGGCGAG
GCCGAGCGCAGGCAATAGTTCGTCCACGTGCGCCAGATTCTCCTCGGCAGCGGCCTTGTC
GACTTCGAAGTCATCGCGGCCGTGGGTCAGCGGCAAGCGGTCGCCGCCAACCCAGACGCA
GCCGGCGTCGGGGGCCGCACGCCCGTAGACGTGGTGGCAGAGGCGTGCCCCGCGTGAGTC
GTGCGTGACGGCTTGCGGCACGCCAGCGGCGGCTTCCAGAGTGGAGTGGGCATCCCAGAA
GGAGTGGCTGCCAGCAGAGTAGATCACGCTGGAGAGTGATGAGCACTGTCCAGCAGCCTC
TGCCACCATCATCTGTGCCTTGACCTTGACGACAGGAATCTCCCATCCAAACCTCCGGCT
GACTTCGTTTGCCCAGGCGCCGGTGCAGATGACGACATGCCGGCAAGCTATCGTTCCCC
>at02563
TCTACGCCAAGTGCCAACCCTCCCCTGGTCAAGACCGAGGCCCCATAATGCACAGTACGG
CTCCATGGGAAGCCACGTGAACAACCAGGGCGTTGCGAATGCCTTGTCATTTGTAAATTT
CGTATACCAGGAGCTTGTGGTGAACCTCGAAGTCCAAGATACATGATCCCTTGAGGAGGC
CATGGC
>at02564
TTTTTTTTTTTTTTTTTTCAGCACCCGCGTGCATCCCAGAAAGCTGTCCTTGCCTCCCCG
GGTCCCACTGCGTTGGCACTAGAGCTGTGCTCCTGTAATGGGAAACGTGACCAAAGGTGT
CTCATGCACTGTCTGCAGAGTGTCCAGGCCACGCCCATGTGGAATCCTGCTTTAACCGAC
TGTAGTGGCAGGAAGTGGCAGATGCTGTTTATGCGCTGCAATCATGCAGAGCCTTACTGC
CAGAGCCATGGAGATCTGTCCACCAAGATACATGCACGTGCACAGCCCAGTGCCTGACAT
TGAAAAAGATCAGGCTGCTTTCACGACACTGTTCGTGTCACCTGATCTAGCTTTGGCAAG
ATGGGCAGTCCACCCTCCGCGAGGATATCACTCCAGGCTCGGGAAGAGTGCCAACAACTG
CAACTTACTGCTGTGCCTCGAACTGAGCCTCTGTGACCCCGGCACTCATGATACCGGTGG
AAGAAGTACCGCCTGTGATGATGTTGCCGTATGCGTCGACGAGCTCTGACCCGCTCCACT
TCTGGTGCTTGATGGCTTCCACGCCATGCTCACGCTCCACGCGCTGGATGAACTGGACAT
AGGCCGCGGCACCATGACTTGCATAGTCCTTTGCAAAGTCGTCAATGCTCAGTGCGTCGC
AGTGGAAGCCGGCCAGAGTGATAAACTGCCAGCAGTAGCCGAGCTTCCCCAAGTCCC
>at02565
TTTTTTTTTTTTTTTTGTGGGACAGGTCCACGTCTTCTCAAGCACCTGGGTCTCTGCTCT
GGCTTGAACATGTCAAGCCATCATCCACAAAGCCGCCTGTACAGCCCGCCGGCAGGAAAA
GCACCATTACCTCTGCAACGATGGATGGTTTACAGCTCGAGGGAACCTGCCCAGCCTCTA
CATGAATTGCATTCAAATTCAATCTAAACAGCACTTGGATGCATCGGATAATTCAGGCCA
TGGGAAAAAGCAGATCACGAGTCACGCCTCGTGAAGAAGGATGCCTCACAAGAGGGATGC
AAGCCATGACCTGAGAGCAGCACCTGCCGTGGAAAAACAAGAACTCCGGGGGTGGCCACT
GACCGTTCAACGCGGTGCCACCTTACAGAACTGCTGCTTGCAAGTCCGGACGTTACACGG
GGGACGGCGTACGCACCCGCTGCTTGCTGTCTGAGGCATGATGTGCACTTCCATGCGGCA
GAGCAGCAATGGACTTGTCAGTGGCAGAGTTGCCATGATCGGCTGCAAGGCTCATTTTGG
TGTCCTGCATGCTTTCTCCCAAGGCTGGGGCCAGTTGGGCGATGCCCGTTGTGACCACGA
CGTCGAAGGGCAATCCTTTTACCTCCGCGCTTGTGACCCACTCTGCTGCCTTTGCCCTCT
GGCGCTTCAACACCTTCGCACTCTGCCCGCTCGAGATGACAACGACCATGCGGCCAATAG
CCACATTGGTCGTGCTGCTTGCCATGCCATCCATTCCGCCCTCGCTGCCTCTGCACACCG
TGAGCTGTGGCCTTTTTGCATCCGGGCGAAGGTGCTCCACATGTCCGCTGCGCATTGCAA
GGACACCTGCCGCGCATGGCTTTGCCTCACGCCAACCCGTCACAAGGATGATGTCATCAT
GATGTCGGCCGAAGAGCCACCTTGTGAGTTCCTGTGCCGAAGAAAAGTTCAAGACGTCTC
CATACCCCAGTTTGCTGATGGCCGGCTGTAACTGATCGCATGCACGTGACTCTTGGCAGA
CAACGACCTT
>at02566
TTTTTTTTTTTTTTTTCTGCTCCATCGCGGTGCGGAGGCAGGGGTACCAAACAATTTGTC

GAACCTCCTTCCGCCGCTGCGGACTGCCCTCCCTCTTCCCCAGTGCTCCCTAGACGGAGG
AGAGGGTGGCAGCGGGCCGCCCCTCAAACGGTCAGCCCCTCCACAGCGGCATGCAGGAGA
CCCCACAAAGGCCACTGCGTGCCCAATGCAATGTCAAGCTCCGGTGCGCTCCCGATAACC
TGCTTGAAGGCCACCTGCAAGCCCTGCAAGATCGCCAACCGCTCTTGCACCGGAGCAGTG
CCGGCCGCCTGTGCCGCGAGGGCCGTGAGCGGGCCCAGGGCGCTGCCCTGCACGTCCTTG
GCCAGGAAGGCCGCGGAGGCCTTCTCCATGGCGGCGAAGTCCGAGGCTGCGATCGCCGCA
ATCAATTCAGCCTGGAAGGCCTTCGCCACCGGGTCGTTCAGCCCGTCCGCCTCGTCGTTG
TTCAGCGCCCCGGGGAAGTGGCCCCTGCGCAGGTTCAGCTGGGCCAGGAGGCCGAAGAAG
GGCCAGCCAGCGTTCACCACGCCCACCCACGGGATGTCGAGCAGCAGCGTGAGGACGGGC
GAGGCCAGCTGCTCGAAGCCCGAGAACAGCCTCGTGCC
>at02567
ACTCCATGCTGTTGCATGGAAGGGTGCAAGAGCGAGATAAGCTGCAGAAGCCGCACGGTC
AAAGCAGTCCCCTGCGACTCGTGCTCCGCCGAGAACTGCTGGCCGGGAGAGGGGAGGGTG
TGCAAGCACGGCTTCGTTGAAGGGGACTTGTGCAGCTGGTGCAAAAACCGCCGGCGTGTG
AGCCGCGGGGTCGAGGCGCCGCCTGAAGCATAGCGCTGAGGAGGGGGCCGAGGGACTCGC
GTTCAATGGGCGTCTGGAGCAGACCCACTGAGAAAAAAAAAAAGAAAAAAAAAAACAA
>at02568
TTTTTTTTTTTTTTTGTTTTCACCTTTTTTGTTTGCTCATTCCGCCTGGATCTCGGAACA
CTGTTTGCCAGGTTGCCCTGTGCCACAGAGCAAAACCAGCTCATGTTGCACTAGGTTTGG
TGCGCATACGCACCAAAGAAGCATTCCTCAACCAAGTATGTACAACGGACCTTGGTTCGT
GGACAAATCGGGGAAAACTGTCCGCACAATCGCGGCATCAAGCAAGTTTGGGTTTTGCGC
AAATATACCAATGCACAATGTTGGGTGCATTATCGATGGCAGTCCGTGCAAGAACCACGG
GCCAAATCTCTGCTGCCTACTGCAGAGGACCTACCTGGGTACTCGGCGTTGATCACGATA
CTGGTGAAGATGGATCACACCCTTCAGTGATTGCCACTCTTCTCAGCCGCAGCGCAGCTC
TGGAAGCATTGGAGCTGCCAAGCCCAAGCGATACTCCACGCCACCGCCCAGCGGATGCCT
CACTTTCGCATGGAAGAAGGAGAGCAAGGCGGCGCCGATGATAGTGACAGTGACAACAGA
TATGAGCAATTACTCCTGGGGATATGAGAGGTGCCATAAGCCATGG
>at02569
ATCAGAAGAAGCACGCCGAGTTGCTAGCGATGAAGCAGTTGCTGGACATGGCCAGACGGC
TTGACAAGGACAATGATGGGTTGCTCTCACATGATGAGATGATGACTGCCTTTGACGACG
ACGCGATCCGCGAGACGTTGGAAGTCCTGGGCATCGACAGGGAAGACTTCAGCCAGATGT
TCACCTTGATGGACGTCGATGGGTCGGCGACCTCGACTACGACGAGTTCATCGAATCCTT
CCT
>at02570
TTTTTTTTTTTTTTTTTCACAATGCATCAATGGCTTACGTGCATTGCTCGAGACACATT
GCGGCATGTCGCTTGAGCCGCTCGGAAAAGTGTTGTAAATGCAGTGCTTCGCCCCGATGC
GGCAAAGGAAAAAAGCTACAGCACTGGAAAACAAGTTCTTCCATCTCAAGGCACTGTAAC
GGACAAATGCACGAGGAAGATCATCTGCTCGGCGACCTACTGCCCAAAACCGTTGGCTGC
ACATCAACAGGCTGCACACTATCAGCCTTCACAGCCTTGAAGACTACTGCCTGCCTCTTC
TGCAGTTCCTCCAGGGCCGCTCCGCGGATGGGGAACCGCCACGTGTGGAGGGCACAGATC
AAGTCCAAAGACCGGGACTACGAAGGTGAGGACAGCTTTGATGTAGTCAGGCACAGCACC
GGGCTGGCTTGAGATGTCTTGGCGGTCGCAGTCCCTCACGCAGCTGTCACGGTCTTCCGA
GCCCTCGCACACGAGCTCGCAGTTCTGGGTCTTCAGGCCTGCCCAGCTGAGCCCGAGGAA
GACCGCAAAGGCCAGAGCCCGGCCCTCGTCCTCGAACAGCTTCGTGACGCCCGCGTGCAC
CGCCTCCCGGCGGAGCCCTTGGCCCCGATGGCAGTCGTCATCCACAGCCCAGCAGAAGGA
GCTGGTCCGGAAGAAGGCCTGCGGCGAGTAGCAGAGGCGCACCGCCAGGTTGTACGCGAC
CCAC
>at02571
TTTTTTTTTTTTTTGTTGCTCCCTCTCTGGCAGTTTCCGCTCGAACGGAGAATGCAGAC
ATACTGAGCAGTGGGGAAACTTATCCCACTGAGTATGAAAAACTGGAACAGCACTCAACT
AGCCGGCAAAGATGGGAAACCCCAAATGCGGCTCGAACACAGCAGAGATGCTGAGTTCGG
TCGGCGACAAATGCAAGGGCTGTATTGGAACCCCGAATCAGCCCAGCTGTTGCGGCGCCC
TTCTGGGATCAAGCTTTCCCTTGCGCACCATGCTTCAAATGAACTAGGCACTTGCGGGTT
TGCTCAACATCTGAGACATCTCACTTGGATCCCGTCTCCCCTTGTCCATCGCAAGTGATG
CGTCCAAGCCCTGGAGCTGCTCGTTGAGCTTCTTCCAGGCAGTGAAGTAAGCATCCGAGG

CGAGTAACCGGGTGAGGTCACAGCTCCTCGCCCCTCGAGGCTCCTCAGCGCTTCCAGAAG
TGCACCCTGGCTCATGAAGAGCGTACTCCTCCAAGATGATGAACTCTGAGCTGGTGCTGT
GGTCCGCTTCCCGCCAGCACAGGCTCTCGTCATCTGGAGTCTCAAGAGGCATGAGGTGGC
ACAACGAGCTCTGGGTGGACAGCTGTGCAAGGATTGCCTGGCTGCCCGACTTCATGGCAG
CGTGTTCCTGCACCCGTGGCTTGTCCTTGAGGTGCCTGCGCANGAACTCCGGCGGACAGT
ACATCGCCACGGCTTCTTGGGAGCTGATTCGCTGACCGTTGTGAATCACCGC
>at02572
TTTTTTTTTTTTTCTCTCTCAGCGGAGCTTGTGGAGAGCATTGGCAAGCGTCCGGGGTG
TGCGATGCTCCATCACACCTCCTGCTGAGCACTGAAGTCGAAGTGCCTGGGCGCCGGCGG
GGACGGCGTGGCCGCCAGCGCGCACCAGCCGCCGCCGCCCGCCGGCCGCCGCGCGAGCGG
GCAGGCGGCGCGGCAGCCGCCCGGCGAGGCGAGGGGCGTCCGCGGCGTCGGCGGGCCGCT
GCCGCGGGGGGACTCCCGGGGCGAGGGCGCGCCCTCCTCCGGGGGCTGGGCGGCCGCGGC
GCAGCGCCCGGCGGGCCTCCCCGTCAAGGCTTCGAAAGCGGCAGGAGCAGGAGAGTGCCG
ACCTTGAGCGCCGGGCTGTGGCCGAGAGGCACAGCAGCGCCAAGCATCGAATAAGGGCTG
GAGGCGGACTGGATGGGCCTGCTGCGGGAACATCACCACAGGCCTTGCCACATGCCATTG
GCACGGTTGAAGCACTGGCCTCTGGATGCTCTCAACCTTGACCGGTATTCTTGGTGACTT
GGGCTGCGAGCACGACTTCAGCTCAGAGATGGCGGCCTCGTGCCGAAT
>at02573
ATTCGAGCTGTTTGGAACTAGAGGCAGCCATTGCAGCGCGGTGCCGGCAGTTGGAGCAAT
GTACGCTGGGCCAACACTGGCCAGTCAGAACAGCTATGTGGCTGGCAACACCGCATTCGC
ATCTGGCAATATGATAGCTTCCGGTGCTGGCGGCTCAGGTGCGTATGCTCTTGCCAGCCA
GCAGCCAGCAGGGACGTGTTGCGGTGGCCTCAACAATGCTGGAGAGTGTGCTGGTGCATG
CGGTGGCATGGAGGCACCTGGCGCTTAGAAAGTCTGTAGAAGGACTGTTGAGCACGGGAC
TGCCCCAAATGGCAAG
>at02574
GTGCACTCTACTTTTAAAAGGGGGCACGTTCCATGGCGTGGAGCCAGAGGCCTCTGGAGC
GCAAAAGGCGGGCCTGTGCTGAGCAGCGCGGCCGACGGGTCCCACTTCTTGTGGTGACAG
TGCTGGCGGTGCTGCTGGCAGCTGGGGCGCTGGCCACATTGAGGGGGACTGGAGGCGGTG
GTTGTGGCACCAGGGGAGGTACAAGACGCTCACGCGGATCCGCCTGCGTGAGTTTCCGAG
CGTGGACTCCGCTCCCACAGGAGAACGCCTTGAGAAAGGCGAGGTCTTCGAAGTTGATGC
TGCTGTGCCTCCCGCCTCGACCCCTGGCCTCAGTTACCTCCGCGTCTCCGGCCGTGGTGG
TTGGGCCTTCGACCACGGGGAGTAGGCTGGCGCCTGGGTCGGCAAAGACATCATCGAGCG
CC
>at02575
CTGGGAATGATCCCTGGCGCCTTCAACTCGACGCGCCTGCGCTCTTTAGTCTGGATTGGC
CCCTGGCCATCAATGGGGGTCCCGAGCGCGTCGACGACGCGGCCAAGAAGGCCATCGCCG
ATGGGCACGTCCACAATGGTGCCTGTGCACTTGACACTGTCACCCTCAACGATGGCACGG
TCATCGCCAAACACGACAACACCGACATTGTCTGTCTCCAAATTGAGGGCCATGCCCTTG
AGGCCGCTGGAAAACTCCACCATCTCGCCTGCCTTGACACCCTTCAGGCCGTAGATGCGC
GCAATGCCGTCGCCAACGCTCAGGACGCGCCCCACCTCCTGCACATCCTGCTGGTCCTGA
AAGTTGGCAATGCGCTCGCTCAAGATCTTGGTGAGCTCAGCTGGGCTGACCTTTGCTGCC
AGGGCAGCTGCGGAGGTGTGGAGTGGACGGCTCAGTGGAGCAGAGAGGTGCTGGACGGCC
GGCTGTGCAACAGCACTCCGCGCGCTGCCGTTGAGCAAGCGGCGTGCGAAGGATCGCGAG
GCCATTGCAAGGTGTGGTCTGCCGGCAGGCTGAGGGGGGTGGTGTCCTTGCAGAAGCTGC
CCTTGAGCCAAAATGGCTACGG
>at02576
TTGTTTTTTTTTTCTTTTTTTTTTTGTCACAGACGCACACAATCACACGTGGGGAAGAGT
GGCCTCATCCACCTCCATACCACCCTCACATCTCGCCGCCCTTGGACTTGAGGAGGTCGA
TGGCATCCTTCTGCTTGTTCTTGGTGGCCAAAGCCAAAGGCGTCTGGCCCTGCGTGGTCT
TCGCGTTCACAGCTGCACCGCCCTTGATCAGGAACTCCAGGAGCTCCTTACGCCCGTAGG
CTGCAGCGTAGTGCACGGCGGTTCCGCCGCTGGAGTCGCATGCTGAGGGGTCGGCCTTCT
CAGAGAGCAGCAGCTTCACGACGGCAATGCGGTTGGCACCGACGGCGTAGCCCAGGCAGC
TGATGCCCTTGGAGTCCTTGGCCTCCAGGTCCATCTTCCCGGCACCCCTTGCTCGCCTTGA
TGTAGTCCTCGACGGCCTTCAGGTCACCCTTCATACAGGCCTCCTGCAAGGTAATGGAGC
TGGCGTGCACCTTGGCCAGGGCGTCCTTCACTTCTTCAGGGATGCCACCCACCGCCCGGT

TGACCTTCATCATCAGAGGCTCGTTGTATGAGTGCTGCAGCGCCGCCTGCGTGCCGCCGC
GCTTCACGTCCTCAAATATGTGCGCGAACTCCGGCTGCTTATCAAGCCCCAACAGCTTGT
TCGTGATGGTCTCGTCACCGGCACTCAACTTGGACTCGTAGTACTCGGTGATCGCCTGCT
GCTGCATCCAGGCCTTGACTGCCTTGAAGTCCCTAGTGAAGGACTCCCACCTACGGGCCT
CTTCAGGGCTCTCTTCCAGCATCTTCTTCGTGTCCTTCCACTCCTCAGCACTCATGCCAG
GCGTCTTCGAGACTGTCACAGTGGTGCCATCGCTGAGGCTCAGGGTCTCAGCTTCCGAGA
TCGCCATCGTCATGGCTACACAGCAGGACAATGTTGCAAGCAGAGGAGGAGTGCTTGACC
CAATAATACTTGAGCCAAAATGGCTACGGACTC
>at02577
TTNNNNttNtNNTNNNNNgctNATGTTGGGCAGAGCTGCCAATGCCGTACATCCGCGGCa
CcTCAaGAGGGAATTGgCCAGCATGCTCGAGCACTTCCGAATGCgAcTGCTCAAtTCCTC
CtTCCGGAGaCGcGGGTAGAtCGTATgGGGTTGTgGAGCGTAGAGCTTCCCCTGGTTCAC
cGTAGTTCCAGAtTGTcGGTCCCACATaGCGGCTCCAGTAGTAGcCCACCGACGATGCCG
CCGCAGACCATGAAGCCGTAGAAGCGTGGGGAACTCTTCAAAAACACCTCGTAGAGCTTC
AGCGAGTTGAAGCCACGGAAGAACCAGTCCAGTGAGTCCTTGCCTTGGCGAGTGTTCTTG
TTCCAGTTCTCAATCCACAGTGGCTCATGATCCCGTGGCGAACCGAAACCACCCCCACGG
CGTGGCCGCGAGAGATTGACTGCCCTCAGCACCCGAGAGCGGAAGGCCATATTTGAGCAG
TGAGTAGGGAAGCTTCTTGGCACCTGCTGAGTCCAGTGCTGATCTTGAACCAAAATGGCT
TGAGCCAAAATGGCTACGGTAGAAGACCAAAAAATAAGGAACTA
>at02578
TAAGAGATCCCTGCCACTGGTCAACCCCAGTGATGATACATGCACGGCCCTATGCGAATA
CTTCTCGTCAGTCACTGTGGGGTGGCCCTGCCACTGCGAGTGCACTCGGATCTGATGCCG
GCGGCCAGTGTGGATGGCTATGCCGACTAAGCCCAAATGCCAGATCGCCCAGGAGTGCTC
ACGGCGTGCAACATGGAAAGCAGCCTTCAGCCACGTCTCCGCAGGCCTCCCCTCCTCCTC
CACGAAGCTCTGCCCTGGCAGGCAGTCTTGCACGGGGGCGTTGATCTTGCGCAAGGCGCT
TGGCACCAAACCGCTCACGAGCACGGA
>at02579
TCCAGGCTGCCGCCACATCCCGGACGCGGTCTGTCCTGGCAAAGCTTCGCGCGTGCTGCA
GGGCTATCGACGTGTTCCGGGCTCTCTGGAAGTCGTCCATCAGCAGGGAGCTGTAACGCT
GCTTCACCTCCCAGTCCCTGGGCTCTCCGCAGTTAAGAAAAGCAGTTTGAAGCGAGCTTT
GCGCGATGGCGGTCAAAATCCTCCGGCCATCCGCAGTCCAGCGGCAGCTCCGCGCTGCCA
TAGACCCAAACCTCTCCAGACCTGTTATGCAGCAACTCCTCGATCGCGCACAGTCCATGC
GCCTCGCCCTCCCATCCTAACGTCTGCGTTGCATTCCAGTTCGCTACGTAATCGGTTCCG
CTGACATGCTCGTGCTCTGCGGAGAAATCTGCCAGAGCGGATTCACCAAGAGCGCGGACA
TGGCGAAGTCCTTCCAGCCTGCTGAGGGCATGTGCTG
>at02580
CCTGTTCTTCGCAGGACACCTGCTTCACTCGTGGAAAGTGGTCTACGTGAAGAAGGGCAG
CGCGTACCTTGCCTGTAGGCGCATCCTTCTTGCCAAAGGTGTTGAGATCATAAATGTTCT
AGTTGCTCTTGCGCAGATCTCGCTCTGGAGCATCTACGTATCCAGTGTGCGACTCCTGTC
GCAGAGGGTGGAAGCCTTGCAAAGACCCAGCGCTGCGCCACTGCACAACAGCTCCGATGC
CCATGTGTGGGAGGAATACCACCATGAGATTGCTTACATAGAGCAGATTGCAGAGTCGGG
CATCCGGCTCATGAGAGCCATTCGCGTGCTTGCTGCGGTTATACTGTCTTGCTCTTTGAT
GAGGTACTTTCAGGTCTGGAAGGGATCCTGCCTTTGCAGTAGTGACCAAAAC
>at02581
TCTTTGCTTCTCCCATGCCCGTTGATGCCGGGCCGGAGGCAGCTGTCCCGTCATTCCTCT
CCTGATGAGCCAGTGCCGCTGCTAGCGCGACGTTCCCGACATTTGCGGCGAGCGTCGGAT
CGGTGCTTCCCTGGGAGTTCTCAGTGCACGTTGGCTCGACGACACCGACATTTGCGGTAG
ACGTCACCATGGGGACTACCTGAAGATCCTGGAATCTTCGTTCTGATGTGATCGCATCAA
CGTGAACCTCCACGTCAGCGACCTCCATAGACACGCCAGAGCGCCAGTGCTCGGACGCGT
CCGCCCACGGACTGCTGTGGCTGAGGCTGCCCTGGTCGGTGCCGGTGCCTTCTGGCCAGC
GCGGAATGCGGCCAGTGGGGATGGAGTGAGACCGTACTCGCGAGGCTTGCGCAGTGCGGG
GCTCCTCAACAAAGTGCAAGAACGTGTTCTTCATAAGCAAACGGCCCGCCGGATTGCCCG
GCACATCCTGCTGCCACATGACACCGCGGCCAGCTCTCAGCCCAAGCCTTCGACTTGCCC
ACAGCCTTGAGCCAAAATGGTTACGG
>at02582

CGCTGAGGCTGTTGGAGACCTGGCTGATGGACTTTCTGGCGCTGCTGCGGTTGGAAGCGA
CGAGCAGGACCACGGCCTCTCGTGAGTCTCCACGGCTCGAGGGTACCACAGTGTACACGA
GAGAGCTCTGCTGCCCAGCCGGAGTGGTGAAGTTTTTGCGGTGTGAGATGAGCTGAGCCT
TTGCCAAGGCCTTGTCCAATGCTTCCTCCACACTGTGCTCGCTCTCGATTTGCTCCCTCA
CTGACCGCAGCCGCTGAGACCACTCGCCGCCGAAGCCGCTGTTGGCATAGCCGGAATCAG
CCCATGCTCCTCGATCTCAGTTCGGCGGTCAAAGCTCGGGAATATGCACGACCACAATGA
ACGCCG
>at02583
TTGTTTTTTTTTCTTTTTTTTTCCCATCTACGCTTATGGTCTCCAAGTACTCACCCATAC
TCGGGTAGCTGTGCCAGCCTGACACATCACTAGCTCTAAGGTCGAGCCTTCCTAGCGTCG
CAGTCTAAAGCACGCGACCACTGCCTGGGTCGATGCAGCAGGCTGCCAGTACATTGAGCG
AGGCCACTCGAATCCAGAATCGAGTTGCGTTTCAAATGATCCAAGCATTTGGACCAAGCT
AGAAGAACAGATTCGTCACGTGCAGGCGATTTGTGATCTAAGGATTGTGACCGCGTCAGC
TTACTCCAGAGCATTTGGAGCCCGGCCGAAAAACGCCTATCGCCATTCTTTCCTGGAAGA
GGGAATGGCTCAGAGGATGTACAACTGTGAGCCTCAGCATCCTGTGTAGATTCAAACCGC
TCACTGTGCCGAAAGCTCCAAGGAGGCCGACCCATTTAGGTCGCCTACAGCATTTGCGCA
CAATTCCTCAGGACTGTGAGCAAACCAGCAAGCAGCACCAAATGTGCAATGCTGCTGGTC
TGACAGGTGGCGGCACATCTGCGTCTTGTTCTTCCGCTTCAGCAGGTCCCGGTTCCTCGC
TTTCCTGTAAGTGTTCTGGACTGTGCTCTTGGAAGACGTGACCACATCAATGTCCTTCGC
CTCCACTGTGTCCTTCGCCCCCACTGTGCTGATGGAGCCATTCACGGTTGCCTTGTCCAT
GACCTTAGGACCAATCAGCTCGAATTCGCTGTGAGCAAACCAGCAGGCATCGCCGAACTT
GCAACTCCCATCTTCTGTCAAATGCCTGCACATCTGCGTCTTGCTCTTCTGCTTCAGCAG
CTCCTGATTTCGCTTCTGGCGCCTTGGGTTCCGCACAACGTGCTGCTCACACAAGGGTGT
AGTCGCGAGCTGTGTGGGCAGCTGCAACTCTGCAGCATTCACATCCTTTGATGCATCCAA
ATCCTCTGCCAGCTCGCTGTCCTCTTCATCGGTATCTACCATGTCAGCCCACCGTGGGGC
GATGCGCTTAGAAGCAATCCCTTCTTCGGTCAATGACATCTGAGGAGGCGTTGGTGCAAG
CTTGTAGCTCTGGCCTGTGAACCACTGCATGGAGGTAACTTCATCGCCAAGCTTGTGTGT
TGCTCCCGTATATGGCAAGGGCAAGAGCCTGCCCAACATCAGGACTGGCGAGCTACTTTG
GGATCCGCTTGATCCCACGATCGTGCTGTCTCCAGCAAAGTCTTCCGTACACTTGCAGTC
GCTCTCTTGTTCAAAGTCTTTGGCTGCCTTCGACGTACTTGCGCTGGGACTCTGCGCGGC
ACCCGCCGAAGGGTCCGAATCCAGGACTGCTGCGCTGGATTTGGACTGTGCTGGTTTCCA
CACAGGGCGGTCTTCGTGGGCTTCTCTGACCCAGCCAGAATTGTCAACCAGCTTGAGCCA
CCCAGGATGGGCGGAATCCCTCACCGCGCTGATCCGGTCCTCATGGTGGAATACATCGGT
CACTGCTGCATCCACATACGGCTCCGCTCGGAGACGCCATGAGAACAAGTCGGTACTGGT
CAAGTCCAGAACGAAGAACTCCGGGGCATCTGGGTTAGAGTCCGACGCCATCTGTAATCA
GCTGTCGCAGACTACTCGCAAGTCTACCTCGTAACTGGTGTGCGAATTGATGTGCGGGGT
GAGCAACTGCTGAACCGCGTCGGCTTGAGCCAAAATGGCTACGG
>at02584
TTTTTTTTTTTTTTTTTTCCCGCCAGGCGCGCGCGCGCGCGTTTCCAGCAGCGACGGGCG
TATTTGCAGTATCGCACTCGTCGGCAGGAGGCGCGGCGCGGGGACAAGCGGCTGGCGAAG
CCGGCTCCTCGCCAACGCCGCGGTCTCTTCTGCCGCGCGCGCGCCGGAAAGTGCCCGGCC
CCGCCCGCGCGGGGGGCGCGCGGGGGGGCCCGCAACGAATTGCGTCAGGCGGCCCTGACT
CCGATGCCCGCGGGGCCGTACCTCATGTGGCCG
>at02585
ACAGGGGAGGCTGCAGAGGCTCTGTCCGGTCACAGTGGCCCCCTCTTGGCTGTCCCAGGG
ACTTCGCCCTGGCAGGTGGGCAGGACCAGTGGGTGATCGTCGGCAACCAGGACTCTGACA
CCATACTCATCCTTGACCGCTCGGTCGACAATGGGAAACTGACGTCAACTGGCACGTGTG
TGTCGTGCCCTGCCCCGATCTGCGTGTTGCCACTGCTTTGAGGCACAGTGAGTCTTGCGG
ATCCTGGCTGGACTTCGCTGAGAATGCCACAATGGCTAGCGCGGACTTGTTAGGGATTGG
CTGTCTGCGTGATTTTGAGATGAGTTGCTTTCATGCCAAACTTTGTGCTTTAGGCATTGT
CTGTCATCTGTCACCGGCACTCCTAGGTGCAAGCGCTCTGCAGCCCATCGATCATCAATG
CCTTTTTGCTTCTTGACCACGCGCAGCCGAAGTTCACGGCCTTGACGATCATAATGGTTG
GCAAGTTACAAAAAAAAAAAAAAAAAAAAA
>at02586
CCGTAGCCATTTTGGCTCAAGATCCAGCATCTTTGGTCAAAGTGGTTCCAGCATCAGCAC

TTCCTAGCAGCAATGGCTTTGGCTTTGCAGGATGTCTTGGTCGTGGACCTCACGCATGTT
GTTGCAGGCCCTATGGCCACGAAAACGTTGCGGGACTTGGGCGCGCGCGTGATTAAGGTC
GAGAACCCAAACAAGAGGAATCCAAAGCCTGCCCGGCGACGTCACGCGCTACTTCGAGCC
ACTCATGGAGCAGCCGGATGGCCAGCGCCGGCGCTCCGCATACTTCGCGATGCTGAACGG
GGGCAAAGAGTCAATCATGCTACAGCTGGACCCCAAGGCTGAAGATGCCCAGGCTGATCG
CACGGTC
>at02587
GATGTCGGCATATTCCTCCTGGGAGAAGTTCTAATGTGCTCCAGCAGCGTGCCTGGCAGC
TTCTGCTTCAAAATTACGTCCAATGCCTCAGACCAGTCAAGAACACCACACGGTCCACGT
ACTCTTACAGAGCCGCCACTCGTCTTTGTGCGCTTTCCATTGGGCGAAAAGCAAGTCATG
TCCCACTGCACAAGGTCAAGTCCGCGTGGGCTGTAGAATACAAGGATGGCAAGGTCAAAA
TTGTCCACTTTAGTATTTACGGAAAAAATTTAAAATTCCCCATCGACGGGTGGCTTTGCC
ATACTGAAGCTGTGCGGACTTGACTTCAACACGCAAACCGCCCGCCAGAAAGTCACAAGT
GGAGCTTCCGGCTGGCAACAGGTATCGCTGATCATGCGACATTGAGCGAGGCGCATCAGC
AACTTTGAAA
>at02588
CCGTAGCCATTTTGGCTCAAGCCCCTTTGCAGCGCTCCCGCGCCGTTTCCTGCACCCGCT
CTCGAGTGGGCTTCAAACTCCGCCATGGGCGCTTCCGGCTCTCAGCTATTGCACGACGGC
CTCTCGACGGTCAGCCCTTGGACCACGAGGGTTCGGATCGAGAACCAGTGCTCCTCGCAG
GTTCTCATATATTTAGCACGCAATGCCGCGGAGCCCAGTGCTGCTGATGCCATCGAGCAC
ATCGTGCCACCGAGCAGTACGTACGGGTTCAACTCAGGCTGGCTGCACGAACCTCGAGCT
ACTCTGTTGATCAGAACTGGAGTCCACGAAGCCAAAGTCATCAGGATCTCAGACACTGGG
CGCCTGATGATATCACTTACTCCGCACGGTCTGAAGGTAGAGTCACCAGACAATATTGCC
ATTGAGGACTTTGCAGATCCTGCCTCGGTACCCGGTGCTGACACGATTCCAATGGTAATG
CATGGGGAGACTTTTGCCATCGACACGCCGGAGCCTGTGGCTCGTACAGGTAGCGAGGAA
AGGGATGCCGCCTCCAGACACGCAGCAGAACGAGCACAGACAGAGGACGATGTGGAAGAT
GTTGAAGCAGCGGTCGTGCACAGAGGCGCTGATGAATTACAGCCAGCGACCTTGCATACA
GGCATAGAGGCTAGGGAAGTGGCTGCCACACATGCAGTCAGCGAGGCAGCACCTGCGAAG
GCAGAAGGGCAGGTGGAAGGCGTTGAGGTGGCGGCGCCACCCAACGGGCCAAGCGCCGAG
GAAAGTGGTGCAGTTGGCAGCCATGGCCCGTAGCAATAGGAGGCACTTTCTGGCCTGCAT
CAGCCAGGCAGAAGTGTCGAGGCAGGTCCACACAGAGGCCTTGGCCTTGAGGGGAACAGT
ATTTTCAAGTGAGGATGCCATATGTGCACTCCTAAGCTGTTGGGGACCTTTGCCGTTGAC
TGGCTGTGCACAGGTGAATGCAAGCAGCCATAGCA
>at02589
CCGTAGCCATTTTGGCTCAAGCCATGCGGCTCAAGAATGTGACTATCAGACCCTTCCATG
AAGTCGCAGCTGAGGCTTGGGATCATGCGCCGCATCTGACTTGTGCCTTGAAGTGAAGCC
TCTGGAAGTCCGGCTATCGGCCACTGGCTTGGGGCTGCTCCACATTGACATCCCACAGCA
TGCAATGGTTGACCTGGACTGTTCCGGCACACGCTTCGGGCATGGGGTTTGGTGCTATC
CTGGCCACCGGTTCGTGGAAGATTTTCGCAGGAGTGTGTCTTCGAGATGGTGAGCGAGGC
TCCTCT
>at02590
TCCGTAGCCATTTTGGCTCAAGCCATTTCGGCTCCAGGCTTATGCCTCTGACCTCTGGGT
CCCTTGTGGCGCGCGGCGTGGCATGAGTGGAGTCTGGCTTGGGTGCAATGGAGGAGATTG
CCGCAGATGAGACTCCTAAAATGCTGATATGCGGGCAACACCAAGCTGGGAAGACTTCCA
TCCTGCAGGTGATCCTGAAGAGGCTGCCTCCGCATGAGACGCGGCATATCGGGCCCACTC
CAAAGCTGGAGGTTACGTCAGTTGCCCGGAACGCGCTCGTGAACTTCAGGGTGCTGGACC
TCCCTGGCTCCACCAACGGAATAGAAGACACTACGGACAGCAGGCTGCTACTCAGCAAGA
CTAGTGTAGTGGTCTATGTGATCAACGCACAGGAGCCATACCTTGATGCGGTGACGGCAG
CCAAGAAGGTTATCGAGTTTGCCTACAAGGTAAATCCCAAGATCGCCTTTGACATCATGA
TCCACAAGATTGACGGAGACCAGTTCTTCTCCGACGAGCGCAAGACAGAGATCACGCGGG
ACATTCAGTCCAAGCTCTCGGAGGAGGTCCACGACAAGGTGGATTCGCAGATCTCGTTCC
ACTGCACGACAATCTACGACGTCTCCATCTTCGAGGCCTTCAGCAGGATCATCCAGAAGC
TGATCCCGCAGCTCCCCCTGATCGAGCAATGTCTGGACCTGCTCATCACGTACTCGCGCA
TGGAGAAGGCCTACCTCTTCGACGTCCTCTCCAAGGCCTACATTGCCACAGCTCCCCACC
CAGTCGACCTGCAGTCCTACGAGCTCTGCGCAGACATGCTCGACGTGGTCGTCGACACCT

CGTGCATCTACGGGCAGTCTGAGGACTACGCCCTCAACTGCATCTCGCGGGACTCCAAGA
GCGTCATCCACATGGAGAACGGCACCCTGCTGTACCTCAGGGGTGTGGACCGCGGGCTCG
CGCTCATCTGCATCCTCCGGGAGGAGAACTACGACAGGCAGCACCTGATCGACTACAATT
TCAAGGTCTTCCAGGAGACTCTGGCTGAGCTCCACAGTGTCGTGCAGCAGCGCCCGGCCA
GCTTCATGGCCGCCGGCTCGTGACACGCAGGGAAGTGGGCTGCTCCCTGCTCGGCATGGG
AGCCGAGCCGAGAGCAGTTTGGCATCGAAAAAGCGCTTGAAAAAGAAAAAAAAAAAGAAAA
AAAACAA
>at02591
TTGTTTTTTTTCTTTTTTTTTGGTTTTCGTTCGTACCCTTGCTCACCTCTCTGGTGGAGG
CAGGAGGACGCCTGTTCGCTGGCTGTACAAGCCGCCATCTTGGGCACGTCACATCATGAA
GTACATGGGTGTGCTGCTTGTGTCTGTGCGAGGAGACACCGGACTGAATTTGGGCACAAA
CGCCTTGGCCTATGCCTCCGCTGCAAACTGGAGAGTTGGTCAGGCCACAGTTGCCCAGCT
GTCACCTGACTCGATTAAAGTTTCAAGAGCGCATCGCTGCGACAAGAGAGCCAGTCATCA
TGTAGAAGATGATCCAATAACGGGGCACTGTGGAGGCAAACATGATTGGGCTTGGATGCA
TGGCGGGGTAAATCGCCGTCAAGAAGGAGTAAGCAAAGCCAATGTATGCACCGACATTTG
GGCTCACATAGAGCGAGTACAACAGAAAGCTGGCCAAGAACATTACGGATTGCTCCTGCA
TGTTCAAGAAGCTTCGATCAATGTTGCGGAACCAAGCCTCGATAGCAGGCTCAATGGCAT
CTGGCGACTTGAGAAGCTCAGCATTGACAATGTTTTTAGATGCTCCCTGTCTTGCCATGA
TGAAGTACCACACCATGAAGTACACTGTCAAACTGTACACTGCCAGCTTGATCTCCGGAC
CATCATGCAATCTTAGCCCCTGTGTCTGCGTGAACCAATTCAGTGCCAGTACTGGGGCAG
ATAGGATAAGGGTAATGACCGCCGCGTGGCCAAGCGACGACTTGGGAGTTGGACCATTGG
CGAGTGGCGGCTGCATTGCAGCCTCCAAGCAGTCAGTCGTAATTTCTGAGTCACCTACAG
GAACTTGAGCCAAAATGGCTACGG
>at02592
CAGCTCGAAGCGCATGCTCCCCACCAGGCCGGCCAGGGACGTGCATCTCCGCGCTCCCGG
AGGGCCTCCTCCTGGAACTTCCTGGAGAGGCTCCTGCTCCTGACGATCTGCTCCTGCAAG
GTCTCGCGGCTCCTGAGGGCGTTCCAGGAGGGCGGCTGGGCAGAGTCGGTCGGGCGAGGT
GGTGGACACTGCACGCTGCTGCCGGCAGAGCGTCCTTGAACCAAAAAAAAAAAAAAAAAA
AANAAAAAAAAAAAAAAAANAAAAAAAAAAAAAA
>at02593
TTTTTTTTTTTTTTTTTTCAAGAGTACTTTGTGGCCTGCTCATCGGAACCACCATTTACA
TGCTGGGACAGATCACCTGCATGATAGCATACTGGCTCTCATGCCCAAAAAAACTGCACC
TGTGCTGTGATGCAGAGGCTGCTGCAAAAAACACGCTTGAAAAGCACGCCCTACAAGTCA
CCTGGTTGGGACTGTTCTGTGCACCACTCTTGAATTCTGTGATGGACCATCTCATCGCAT
GCCAAAAAGCTGCTTTGAGATCTTGAAGACATTTGTCACACGCCCTCACCAATAATGGCT
TCAGCTCGCTACGCCTATACATCGATGCACATGCAAGCATCAGCCACTAAGAATACCATC
AATGGTTACATCAATGGCATGCGAAGGTCTGAATTGTTGGCGCTCCCATGCCTTTCAAAA
CACCAGATAGAGATCCTGCCCAAGCTGCCATGACTGAGCACAATCGCTATTAGGTAGCAC
TTCACAACCCTTAGCAGCAGCAGCTTCTCTTGATGTCCATGCCCCACTTCCTCTCAGTGA
TGAGCTCGTTCTCCTGGGGAACCTCATCAACAGGGCTGTCGGCGACCACTACCTGGGCTT
CGTCAGTGTCCATGGGGAACGTGTCTGTGGCGACTGCAGCTGTCTCAAGTATTTCGAGCT
CGTTCTCCTGNGGAACCTTATCAACAGGGCTGTCAACGACCACTACCTGNGCTTCGTCAG
TGTCCACGGGGAACCCGTCTGCAGCGCCTCGTGCCGAA
>at02594
GTCCAGCACTTTTGAGTCGATTGCTCGTCCGCATGTCAACACATACCAGCTCCTGTGCTG
AGTCACCAATCAAGTGTATTCTCAGCAAAGGGAAAAGCTAGCAAGTGTACAGACACAAAA
TTGACAAGACAAGCGCTGCAAGGAGGAACGTGCCGAGCACTTGCTCCCTGGACAAAGTGC
GTGCTCACGCACAGTATACTTATGACAAGAAGAGTCAGTTGATAACATTGGGCTCGGCAC
AGTGGCTCTCAGACTTTCGAAGTCATGCTGCCAAGAATGGCTCC
>at02595
TTTTTTTTTTTTTTTTTTGGAACAAGCGCGCCCGCGCCGGACGCAGCCCACGCGCTCCAC
ACCAGTCCTCTGACACCAGCCCTGGCCTCACAGACGTGACGGCGTCCCGGCCAGGCGCTC
GATGTGCCGAGCCACGAGCTCGGGGTGCGTCATGGGGATGAAGTGCGAGGCCCCGTCCAG
CAGCACGTCCTCCACCTCCCCCCCAAGGCGCTGGGCCAGCGCGGGGTCCACGACGCCACC
CGTGAAGGGCCGGT

>at02596
CCGTAGCCATTTTGGCTCAAGCCTAGGTTGAAGCAGTGCTTCATGCGATGAGCGCAGCAC
CCGCAGCCATGCCGGAGCTTCGTCATAGGATCAGCATGAGATTCAAGAACGGCAGGACCG
AGGCACACTTCGTGAAGGAGCCACTCTAAGCGTCTCCTGGACAATTTTTACTTGCTTGCG
GTGGTGGCCGTTCTGACACTGGCATGCGTGCTGTTCATCGTGCTTTCAGTCCCTTCTCCC
GCTCCCGATTTACCTGAAGTGAAGAGGGTAAGGATGCTCTCAGCTGCTTCGAGCGTTGCA
TGCTTGGTTGTCGCTTTTCTGGCAGGCATCGCCACCAAAGTTCCAAGCTTCGACAGGTGT
GTCAGCCCCGAGATGAAGGAGATCTTGGCAGTGATCGTGATCATGTTCCTCACGCTCATG
TGCTTCCTGATTGATCTGTGGTACGGCGCGAAGCTCTGGCGGTATGATCCCATGGACGTT
GTGGGTGCCGCACTACGTCGTTTCAGCGACACCAGGCCAGTACTTGCCTTGTCTGTGCAG
ATGACCGGCAGCCACATGGCACTGCCGATACGATGGATCGTCCTCCTTCCGCTGGAAATC
TTCTCCATCATGGTGTATGTGACGTTTGCATTCATCGTTGGCGGCCCTGACGGATTGCAG
CGCTTGGACCACCGTGGTGCTGTACACCGCAGCTCGTGATCTGCACGGCCATTGGGCGAC
GGACCATCGAGAACTACGAGAGGGAGACGCTGTCACTGCTGATCACAGGAGAGGAGCCTG
CGGGCCAGAGACTGAGTTCAGGCTGACCAAGCTGGAGGAGACCGGCCCCAAGCACCCTTC
GCAGCGCGCCGGCAGGACGACCAGGCCTCCACCCGGGAGTCGCGGCCCGAGACGACGGCC
TCCGCCGAGGCATTTGTGAGGGCTTCACAGGGCGGTGGCTTTGAAGAAATCGTTGAGATT
GGCAAGCATGAGCAATGGTTGGTTGACCCTGAGGAGGTGGATCTAATCGAAGATCAAGTG
CTGGGGAGAGGGGGCTTCGGCAAAGTGGTTGCCGGAAGCTACTGCTGGCACACCAGTCGC
CGTCAAGATGATGCCCCACGAGCAGGCATTCAGGGACCCAAGAAGGATCACTATCTGGCG
TCATTCCTCAATGAGGTACGGATTCTCAGACGCTTGCGCCATCCGAACATTGTCATGTTT
TTCGGTTCATGCCTCCGCAGTGAAGACAGCAACATCCTCTTGATTCTGGAGTGGGTCCAC
GGACCGTGCCTGGAGAAATTCATTCTTGC
>at02597
TTTTTTTTTTTTTTCTGTGGGCCATGTCTGTGCCAGGAGCCATTGGTGCAGAACACCAAC
AATGTTGGACGTGACAAGACGTGCTTGCAGTTAAAGCTCTGCAGCCACCAGCCTGTACAC
TGGTAACTCAGACCTGGATGGCATTAGAAAAGCATGCGTTGCGCATTGCACCCCTGTGGA
TCTCAGGCCTGCTGATGGGTAGGAACAGCAGATGCGATCATGGTGCGGTGCGCACGCAGA
TGGGCCACTAAAAATTATGAAAATGAGAATGCCTGGCACTCCAATGCAAGGACGGCTGTA
GTCACTGTACAAAGGCATGCAAGAAGCAACCAGGCAAACCAACATCCTGTGTATTGGTGG
GTATTGAGCACGCAGAAGAAGCGTGCAAGCAAACCGACTCAAATGAGCTTGTAGAAATTA
GTGCCCGTCTATCTTCACACTCAAGGACACCTGACCGGATTCCCATCAGAGCTGCCCAAC
CGCCACGTCCAATTGCCAAAGCGCATCAGCTTCACCTTGATCGACTGCGGTGTGATGGTC
TCCGAAAGACCCATTGCCTTTCTGACAGGGCTTCCTGGCTGGAAGTCCATTGGAACCTGG
TGCGTCNCAAGCACTGGGCTGGACTGGCGACGGAAGGCACCATGGGCAAGTCCGGGTCAT
GTGCCAGCTTCTTGTCTTGGCTGCCCCTGGCGATGCTGGAGCG
>at02598
GGATCGTTCCAGTGCCCGATCACGCAGGACCTCATGCAGGAACCGGTTCTCGCCGCTGAT
GGCCAGGCGTACGAACGCAGTGCAGTCCGGGAGTGGATTGAGCGGGGCAAGACGACGAGC
CCGCTCACAGGCGCACCGTTGGAACATCTGCGTCTCAAGCCAGATCGAGCGCTCCATCGT
GCGATCCAGGAGTACGAAGACGTCCGCCGCACGCAGCAGCATCTCTTCGACAGCCTGCAC
CAGATGGCGATGAGTCTGCAGCAAGCAGCTGTGCCAAGCAGGTCGCAAGTGGAGTTGCAC
GGCTTGCACCGAGAGGAATTCAACGGCCATCGGGGCGAAGTTGTGCAGTTTCATCCCGAG
CAGAAGCGTTTTGAGGTCTGTTTAGAATCTGGTGGCATGACAAAGCTTTTCCGTCCTGCG
AACATTCGCGTCGTCTCCTCGCCTCTGCAGTCGGCTCTCAAGCAAGAGGCCTTGGAGGTG
GCTCGTCAGCTGAAAGATGCATGCTCTACAGGCACGCGCCCAGTTTCGCTTCCGAAGTGC
GCCGAGGACACGTCTGGTGGTTTCAGTTTTCATATCAGTGTTTCATGGAAGAGCGATTGC
CCAGGAAACCGCCCGCCCACGCTTGCATCCTTTCATTGTCCCATCACACGGAAAGTCATG
AAGGAGCCAGCCATTGTTGCTGATGGTCAAGCGTACGAGCTCAGCGAGATCACAAAGTGG
TTATCTCGAGGGTATCAGACCAGTCCACTGACTGGTGCTCCTTTGAAGAATTGCCATGTC
AGGCCAAATGTTGCGCTCCGTCGTGCGATTGAAGAGTACGAAGGTGCCCGGGACGCACAG
GAGCATCTCTTTGCCAATCTTTGCCAACTGGCACAGCGATTGGAGGCGGGAGCCTCAGCT
GAAACTCTACTGGAGGACGTGAGCGAAGTCGTCCCCGTGCTGGAATCGCTGTGCTCGACC
GACCAGTGTCGTATTGCGGTTCCATCGCATGTGGAATACGACAATGACATAAACGGCTTC
AACGTCGAGGTAGATTCTGCCAATGGTTGTACGTTACAGTAATGCGCAGCACAGCGTATG

AGAAGGGGAATGGCACTGCCAGATGAAAAAAAAAAGAAAAAAAA
>at02599
CCGTAGCCATTTTGGCTCAAGCACATTCAAGCTCAGCTGGTGTTCTGCTTGTGGAGGTGG
TTAATGTGGCTGCGTCGAACGTGAGCTTCACCTCACCGAGCAACGAACTGTTGTGTACAT
ACCGTCTTTGGCCAGAAGCGCAACCCTTGCCACA
>at02600
TTTTTTTTTTTTTTTTTCATCGCACCCCGCATGGCATAGATAGGCCTGTTGCCCGCAGC
CAACGCCTACCTTGACTTTGCAAACGCCTGGGACATAAAGTCGCACAGATGAGGACTCGC
TTGCCACATATGCCCTGAAGGGTTCAGCTGAAGAGTGTCCACACAAGGAGGACCAGCACG
ATGAAAACAGCCGCCAGCAGCAAGTAGACCTTCATCTGCTGGTTCCCCCTGGCCACAGAC
ACGGCTGCCTCTGCAGCAGAAGTTCCGAGCACGAGAGATTCCATGTTGATCTGTGGGTCC
TCGTACTGCGTCAGTAATCGCGCCATCTGCGGCTGCAGCTGCTGATTGAGGGAGTGCTCA
GTCGCATTCTCTACACCGGTGAGCTTCTGGACTTCCGCCATCAGATCGTGGAGGAGCTTG
TAGGCCGGCCGCTCGGGGTAGGCCAGGCTGGAGGTCAGCACGCAGTAGAGGGTCGCGCCC
TCCTCGTCCATGAGGCAGCAGACGCTGCCATCGTTCCACTGAAGCCTCGTTCTTTCGCCT
CGTTGAAGCTTATTCTGCGCAGCTTGCAGAAGCTTTCGGAAAACCTCCATGGTCTGCTCC
CTCTGCTCATCTGTGGAAGTGGCAAAGTGGTAGGCCAAGCACACCGGTTCCGGTCTCACC
CTGCCCACCGCCAAGAACGGCAGCTTCTTGTCACCCCAACCTCCCATGGCCTGAAGAGCA
TTCCCTAGGACAGATCTTGAGCCAA
>at02601
TATTCCAGGTGTCGCTGCAGAACGCAACTCAAGTGGCAGCGCCATATCTGCTCGGCATCG
CCGATCTCGGCTCCGAAAGAACGGGAGACATGACATATGCACACGATGGCGACAACTACT
TGGATGTCTGCATATCTGACCCACGTCAAGAGATCGTGGCCTCCATCGCGCAGCTGCGTA
TGCCTTGTGCTGAGACAAAGTTATACTATCCCAGGGGTGCGAAGCATTGGAGTGGCTGCA
AGGACCACGCCATCAAAGTGGACAGCGAGATCTCCTACGAATTCCATGTCTAATGCGGCA
GTGGCAGCAACAGCACTGACAGCGTCTGGATCTGCGCCGTGAATGGTGAGTTGGGTACGC
TGGCGGCGGTGCATGATTCAGAGTGAAGGCTGCTACGACAACTAGAGCAGAACATGAGCG
CCAAAAAAAAAAGAAAAAAAAAACAA
>at02602
TTTTTTTTTTTTTTTGTTCCGCCAGTACGCACATGCGCATGCTTAGGCCCCTGTCTGCAT
GCCTGTGTCTGCGTGCTGGCGTGCCTATCTACGTACAGCGCAGCAGGCCTCACTGCCCTG
CAGCGTAAAAATATATCTGTAGGCTGACGATGCAAGGATGCACCCTTGAATGTTCATCCG
TCGCGCCCTGGGTGGAATAACAAGGGCCTCTTACCATGGTTGGGTTCCTACAATGCAAGG
CAGAGCCAAGACAGAGCCATGGAGGCCAGACACCTTCAAAAGGAAGAAATACAAGCAGAC
CTGCCAAGCCTTCCGTTGCATAAGACTGTACGACTGCAGCTGTACAACTGTGACAGCCCA
AGCTTATGCTGCGCGCCTGTTGCCCAGGAGTCGGAGCCAGTGTCGTCTGGCTACAAAAAT
GGAGCAATCCACAAATATCCATGGTGTCATTGGCATCTCCAGGGTCCGCCTCTTCATAGT
ATGTACAGGACAAGAGGCCTTCCGTCCTTCCCTCCCGGCACTGCTGCCCAGCCTAGGGTC
CAACCCGAGCACGGCACAGCGGACAGCGAACATGCTTCCGGGCGCGGCCTCTGTGCCCAT
CTCTGCGCGACCTTGAGGAGGCCACGAAGTGCATCTCGGCGCACTCCTTGTGGAGCGCAT
GGAAGCAAAGCAGCGTTCGGGTGAGCTGCCCATGCTCCATGGGCTCGAGGCACACCGGGC
AGATGTCCTCGCGCTCATCCTGGCAAGGAGCCAAGACTGAGTTCTCCTGTGCCTCTCGGA
GAACCACGTCCTCCCGCCGCTCCAAAAGGGCCGGGTCCATCCAGGCAAACTGTCCGGTGA
CCTGCACCGACCCATCGACCTGAGTCACGGCCAAGGGCTGGCCGGGGACAGCTCGCGCAA
CCCACTGGCCGTTGATGTAGACGTCGTCGTCGACGACTTCGGTGGCTGCATCTTCGCTGT
GAATGACCCAGTTGCCGATCTGCTGGTTGAGCTCGCGGATGTTGCCCTCGACGTGCAGCT
GCACCTCAGCTGCAAACTCCTCCACGTGCTGCGCATGCCCAAACATGAGACTCTGTACAA
CATGAACGTGTCCTGGACCTGCAGTGATCAGCACCTGAACACTTGCCATGCTGGGGGGCA
GGTGGGAATCCGCAGCTCCTAAGGAATCACCTCAGCCAATGCATGCTTGAGCCAAAATGG
CTACGG
>at02603
TTTTTTTTTTTTTTTTTTACATGGAGAAAGATGACACGAATATCCTTGCAACCAACCACT
TGACTGCATGCCCTCCCTGTGTGCACCACCTATCAATTTGGCTGTGTGAGACCAGCATTC
CGGGCAGCTTGGCTCCACCCATCTCCCAAGCTGGCCCAGCAGCCACCACATGTCACTTTC
CAGAGTCCACACGCCAACATCACCGCATCCTTGTTCGTGCATTCAAAGCGGTCTTGCTCC

AAGAATATTGAAAAAGTCATGTGCCTTGCAGGGAGCTGAAGAGGCCATGTGGATCTGGCA
CCCGCTGTGTTTTGGCCCAAAGCCAGACGAAGCAAGCACAGCGCTGACTAAAGAAATTAG
CTTCACCAACGTTCAAGGCAAGACCGCACAAAAGGACTTCAAAGAACGTCCCACGCCACC
ATGACTTCACAGCTCCCAGAAGGTGCGAACGTTTGCGCTGGCCTCTACACATGATAGTCA
TTCGTGGGACGGGTGATAACCACTCTGTCAATTCCTTCTTTGCCATGGGTCCATCATTGG
CAAGACCAGCTCTCTCTTAAATCTCGCTGTAAAGCTATGTCACAGAGGAGTCCACGTTGG
AGTCAGCTACCTCGCTCGTTTTGGCATACCGCTCCAGACTCTCGTCCGATGCTGAGCTAC
GCCTTGAACACAACCATGACAACCGTCGTGTCATCCTCCAACTCTCCTTCCTGCTTCTCC
CGAGCTGCATCGATCAGCGCTCTTGGCGCTGCCTGTGGTGTCTCGATGTATCTTGCGGCG
ATGGACACCGCTTCTTCGTTGGTCAGCGCATCCCAAAGACCATCAGAGGCGACGATGGCA
AATCTGTCATCTGACCGAAGCCTCGTGCC
>at02604
AAGCACCAGCCCCCCACTGATGTCGGACTGGTTTTGGAAGTCGTGCTATGCTCGGCAGAT
CGAGGAGTACCTCAAGTACTTTGATGCCAAGCAGTTTTTCTTTGCCCCCGACAAGGAGTA
CTACGCCGTGGACCCTCCGAAGTTCAGCGCCGACCTCCTCGCCTGGCTCCACATCCCGTC
CCACACCGTGGCACAGGGCCCACCACGCAAACTCGCAAGAGCATCCGGAGTTGGACGAGG
ATCTACCGTCCACCGCGCCCGCTCGCAAGGCCTACGAGGCCTTCATGGCGCCAGAGAACG
AGCGCCTGGTCCGCACTCTGGCCCAGGCCCACACCCAGGGCGCGGTCCTTGCGGGCTACG
CAGGGTTGCCCGGCGACGTCGCCGCCGTCCGGGACTGGCTTGTGCAGAACTGGTAATACA
GGGGGCAGGGCTGGAGGAGGGGGA
>at02605
CTTCTGGGCTCTTCCGGATCTCACACGAAGTCCTGACCTCGGGCTTTCTGCCGTATCTCA
TGTTGGTCTCGGTTGCCTTCTGACCTGTTGCTGACATGCTGGGAGTCGACGAGCTTCAAA
AGCATGGATGAAGAGTTTTCTGCAACCGAGTGGTTTGAAGGCTTGATCTCTGCTCTTACA
CCAGACAGTCCCCCAAGCTGGTCGAAAGGCATCGACATCGGAGCTTCAAGTGTTGTTGTG
GCCATTGCCGACCACATCTGTGTGGATGATTTGATTGTGGACGCAGGACTCGATCGATTG
GTGGTGCGCTGCAGAGATCACGCGGAGGCGTTGGTCATAGGAGGGGTTTGCATGGAGGAG
GCCACGACGGTGGCAAGATGGAATGTCAAACGTCGAACTC
>at02606
GCCTTGGCTCTCAGTATCTCCTCCTCCTTCTCCATGAGGATGTGCCGCAGCACGCAGCTC
CTCCCTGCTTCGGGAGCGCGACGCCCAAGACGAGATACGAAGACAAGAAGTGCAAGAACT
GCTGCCGCAACGGCCACACCGCATTCAAACACTGTCACTTCGTAGCCCGCAGCTGCAAAG
ACCACACGATCCACAAAGGAAGCAATAATGCTGTGGGGCCGACGGCGGTTTCCAGAAGCC
AGGGGGCTGCTTCTTGTTCCACGCCTGAACCTGCTTTGCCTCGGAGAGTCCCTGGTCCCC
GAACGGCCTTGACTTGCTTGTTCTTTTTCTCCTCGGCCATGTCTATCTGACCCAAGACAC
GCCACTGGGTGTCAGCGAATGCCTGCTCAGCCTTCTCGATCGCCTTT
>at02607
ATCTCCGGCGTGCAGGTTTGGCCATCGTTCTCGGCTGTAAGGTTGCACGATCGTGGCACA
GAGAGTAGTAGCTCGCCAGCGCTGATGGCCTCGTGCGCGAAGAGGCCGCGCTCGGAGCCC
TGCCCCTGGCGGACCTCCAGCTTCTCAGAGACGGTGGCCCCGCGCGCGCGCAGGAGCTCC
CTCAGGGCCGCCAGGCCGGCGTGGGCATCCGCGCCGGGCCTGTTTTCAGCGACATGTCCG
GGATGCCCACCCAAGCAAGGCACAAGATGTCGGCGGCGCCGTGGCCAAGTAGAGCAACCA
GCAAAGCGAGACTTGGTTATGCACCAGGAGCTGGGATCAGCAGGGTGCCGGGCAGCGGCA
CGGAGCGCAAGCCATCAGCTGCGAGG
>at02608
ATGCCCAGTCTGTTGGGCACTGCGCATGAAAACTGGTGATGGGCTGAAGGTCATTGCACC
CTCGGTGATGTAGACGTTGCGCTCCTCTCGGCACTTCCTCTCAGATGTCGATGTCCCAGG
AGATCTCTGAATAGGCCAAGGACGTCAGGTCATCAGGCTGCACGGCTGCATCTTCTGCCT
GTCCCAGTCCAGCAGCGAAGGTCGCGCTGCTTGAACTGGAACCCCCGACGCGAGTGCTGG
CGGATGTCAAGAACGGCAAGGGGGTGCATCCCGGACTTGACCGCTGCGCCTCCAGCGCGC
TGGTTGCTGGGCGTGCCGACGCCTGGGCCTCGGGGTCTGAGCCGAGTGAGGTTGGCAGGT
CACGGACTCCACCGCTCAACTCGAGGTCGCCTTCGGGGAGGATGCCACGCAAGGCTCCGT
CCGTGGCGCGGACCTCGTCTCTTGATGGGGCCGCGAGGCCTTGCCCGTAGGTCGTGGGCG
AGATGGCCACGTCGGCGGCCTACATCGGCTGCGGCCTGTCTGCGACTCTGAAGCCCCGCC
TTGGGCAACAGAGCATCCTTCGCAGGACTCCGCAAGCTGACCTCACGGCATCGCAGTCCA

CGTAGCCCACGCCAGCAGCGCCCAGGAGCATGGCCAGCAGGATTGTCATGGCCACGTTCA
CCCACTTCAATGCATCCGTGCGCTCCTCCGACAATCGGCGCAGCTCGTGCAGTGGGAAAG
CGTAGAACATGAGGCGCTCGCAGCCGCAGGCACCGGCGCACAACGCTGTAAACAGAACCG
CAATTCGCACCAGGCCACTCCCGACCATAGTCTTCACAATAACCGCTTGCTGGGCCAGGC
ATAGAGCAAACACGAGGAGAAGAGATCTGCCACTGAAGCACCAGTTGTAGAAAGCAATGG
CCGCTAAGACTTCGCCGGCAATAATTCCAACCCAGAAGTACGACTTCCGCCGAGCTCCAC
GAGTCAAGGCCGCCGCCATCATGAACATGAATGTGGCAGCAGCAGATGCATACCATACCG
GCGCGGTAGCTCTCGACGGAGCGCATGGCCATGCTGTCGCCGCTACGGGTGCAGAAAAGG
CCAGGACCAAAAGGACAACCGTCCCCCCGGAAAACATGCTGGGCTCCACATTATCCTGTT
TGGAAGCCATCCTGCTGGTTGAAACATGTTTCCGCAGTGGGCTTGAGCCAAAATGGCTAC
GG
>at02609
CAGCAGCAGCAGCAGCTCTGGAGGGGCAGAtTTTTGGGCGCTCCTTGGGCGGCCGCGCGG
CCAGCGGGCGCAGGGCGAGATGGCCGGCGAGGACCGCCGCGTGCCCGTGACCGTGCTCAC
CGGCTTCCTCGGCAGCGGCAAGACGACGCTGCTCAACCGCATCCTGACGGCGACGCACGG
CAAGAGATAGCGGTCATCGAGAACGAGTTCGGGAGGTGGGCATCGACGACGAGCTGATCA
A
>at02610
TTTTTTTTTTTTTTCCTTGAGAGGGATCTTGCTCTTCAGACACTGCAGGCAGGCGCTCAC
TTGGCCTCTTGCGTCGCTCCCTGCTGCGCCTTCCTCAGGCGTGCAACCGTGGCCTTCACC
GTGGCGGCGGCGCCCGTCGTGAGGAGCCAGCAGCCGCCTGCGATCGTCCTCTTGCACTGC
TTGGACTTGCACTGCCAGATGCCGCCGGCCACGCGCTTCACGTTCTCCTTGCCGCAGAAA
GTGCACATGTACTTGGCGTGCTGCTGGATCTCGTACTTCTTCGAGATCTTCCTCAAGGAT
GCTCCGTAGCGCGTGCCGTACTTGCCTGTGATGCCGACCCTCTTCGTGTGCTTCGCCATG
TTGCTGCTACTTGTTTAGATGTGGGCCTCGTGCC
>at02611
CGGCTCGAGCATGGGCCGCCTCCTGTTGTCCTGCACTGTCACGTAGCCTCGTGCCAACTT
GGCAGCTGGTGTCGCCGCGCTTGTGCAGGCGTGGCGGCAGGAGCTGCTTCCNGTTGGCAG
GGTGGACAACCAGCTGGTCAGGCGCGGGACGCCGGGCCGCTCGTGAAGCGGCGGGCGGCG
TCCTCGCGGGCTCGGTTGAGCGGGGAGAAGCTGACCAGGATGGGAGGACAAAGGCAAAGC
GGCCTTCAGATCGACAGCAGCCGATGGGATGCTGGATTGCGAGGCGGCTTTT
>at02612
GTTTTTTTTTTTTTTTTTTTAACCACACTTTCAATCAAGACTTGCCCGTCAGAACTTGTA
CACCATGTCCGAAGTGTATCCCAACTCCTTCAGTATGCCCTCAACCTCGCCCTGGGTCGG
TGGCTTTCCCTTCTCAAACTTCTTGTTGCCCGCCACGGCGATAAGCATGGGCGGCGGCCC
GCACACGTACACGAGCGAGTCAGGAGAGGGAGCCGGCAGGGCCGCTGACAGAAAGCCTGC
AGTCACATAGCCAACGTGCGTGATGGCAGGGTCCTTGGCCTCGTTCTTGTCCACTACGTA
GATCACCTTGAGCTGACCTTTCGAAGAGGCAGCCAGCTTGTCCAGCTCCTTGCGGAGGAG
GATGTCCGCAGGGGTCTGGTTGCAGAAGACCAGCGTGACCTCCGTCTTGTCCTCAGGGCT
CTTCAGGATCTCCTTGATCACCTGCAGCATCGGGGTGATGCCGGAGCCCCCGGCCACCAT
GCCGATCTTCTTCTTCATGTTGGCCACGTAGGGCAGCTTGGGGAACGGGCCCTTGACCTC
GACCGAGTCCCCCGGCTTCAGCGAGCAGAGGTACTTGGACACCACGCCGGTGGGGTAGCC
CTTGACCAAGAGCTCGAAGTACCCTGCCTGGTCGTCCGTGGTTGATGGGAGTGTATGGGC
GGGCAGGGGCCTTCCCGTCCTCCCCCTTTTGCCCGTTGACCATGACCATGGAGCTCACTT
TCATGCCCATGACGTGCTGGCTGGAGGGGAGCTGGCACTTGAGGACCTTGCTCCCCGGGG
CGGCGTCTCGCACCTCCAGCACCGGGAAGGACCGGAACTCCGACCTCGAGAAGGAGACAT
CGTGCCCCGCGTGGCACCGCGCGTAGCCCTTGAAGCCGGAGGAGTCGAAGGCGCCGAAGC
CCGCCAGACCCGCCAGAGTGCCGGTGATTGCGGGCGTGAGACGCGGGACCTTGCAGACCA
CATGG
>at02613
CCTTGAGTCAGATTCGCCCGTATCATCGTCAGCGATCGTCAGCAGGGTTGGCTTGTCTGG
TTGCCAGATTCTGGATAGCGTGCTTGAGATTGAAGGCAAGGCCGTCGAAGCGATGTACTC
ATAGATGCACAGGGTATCTTCAGGTGCTGAATGACTGATTCTTAACGCGCCAGGTTGACC
GACCGATCAAGCTCTGCCCCCGGCTTCCTGCAAAACACGGGAGCCCAGAGCGCCATGGAC
ATTTTGTTCTGAAGCTGACAGCAGCTCGGCGGTCGAGGAAACAGCGTCACAGGTGCACGG

GCTACCACAACATTTTGATAAATGAGCCCATCCCAACCCCAGGTAGCAAGCATCAATAGG
CATTGCCTGAAAGAAAAAAAAAAAAAANAAAAAAA
>at02614
TTGTTTTTTTTTTCTTTTTTTTTTTCCCTTCCACACCAGCATACACGTGAGTCCTTGCGC
TGGCTCCTACAACCGGTGGTGAGACGTGCTGCGGAAAGCGGTGCTGGCCATGAGCATACT
AGCCATCACGGCAGAAGTCATACATGTACACTGCATTGTCGCGGCCATCTGTGACCATGA
TGCCTTTGTCGTCCCGTGTACAGATAGAACCCAGCCAGACTTTGTGACCTTTGATCACGA
AGTGCTGCGTGCCGCTGTCGACCCACCATGTCCGGACCGTGCCATCCAGAGCACCCGTAA
CCAGCGTTTCACCACCGCCTCTCTGAATGGCAACGACCCTACCTTCATGTTGAGCGCGCC
AGATAGTCTGCCAATTCCCGTTTGCGTCGACTTGCCAGCGCGCAAGCTGTCCCGTAGCAT
CACCCGTAATCAGCCCTTCATCAAGTACACACACAGCTGAAACTGGGCTGAGGTGCGCTG
CATGCCTGGCAGTCAATTTCAAGTCGGCGTTACGAAGCTCTACAGACCCACCTCGTGCC
>at02615
TTTTTTTTTTGATTTGTTACTTGGCCTTGTCACCAAGATTCTGAATTCTCTGTGGCACAG
GAAAATGAGTGCACAAGAGGATTCCCGCTTCTTCTGAGCTGGCAACTCCTCGACACATGC
TTCTGTGTAGAATCACCGCGGTGCCTCAGACGCATCCTGTCGCGTCGCATTCCCCGTCTG
ATTTGCGGCACCGCCGCCAGCACTGCAACAGTCCAACATGACACCGGTGGGAGTGGGGCA
GCAGGCTCCCTCAGTAAGGTTCGCTGCAGCACAAGCGGGGAAGGCCTTGCACTGTGCACT
TTGCGGCGCTCTGCCTGCACAACAGTCGAGCATGCTGCCCTCCGCAGTTGGGCAGCACGC
CCCAGTCTTGAGACCGAGTTCATGGCAGGCCTGGAATGCGGAGCAGGCCGTCGCCCCACT
GCTGCAGCAATCCAGCTTGGAGCCTTCGGCACAGCACGTTCCTGTCATGAGCCCTATGGC
GACACACCCGGGGTATGCTGAACAAGCCGCGGGTCCACCTTCGCAACACTCGAGCATCTG
GCCATCTGGTCCATCACCCGGGCAGCAGGCGCCACTTGTGAGGCCCAGTGCCACGCACTT
GGGGTAGGCTGAGCAAGCTGTGGCCATGGGAATGGCACTGGTCGAGTTGCCTGCCGAGGA
TGTGGTCTTAATAGTCGATTGCAGAAATGCGCTGGACGTGGGACTTGAGCTGTTGACGGC
ATNGCATGCAAGAGCCTCGTGCC
>at02616
GAGGGCATCTACGAAGGCATCTGCAAGCTCGACGCCCCGCCGCCTCCGGCGGCCGCGAAG
CAGGGAGCGGCCCAGCACGCACCAGCGCCAGCGCCAAAGACTCCGGCGGAAGCGGCAGAA
GGGTCGACCGAGCACACGCCACTTCCAGCGCCAGAGACTCCGACGCCAACGGGGAGTCCG
GtTGCCGGAGCCAGCGCCAAGTCCAGCACCGAGCTCGACCACAGAGCCGGATTCGGGGCC
AAGCTCGCCCAAGGTGCCTGGGGCGGCAAGCAGCATCGGGACGTGGCAGGCCTGGACGGG
CCTTGTCTTGGCAGTCCTTGCCTGGACCGAGCGCATGCAGCCGTGAGCGGCCGGGACGTG
GCCATGCTGACTTCCGTGCCATCCCCTN
>at02617
TCCGACCTTCTGCCCACAAAGCCCACACCAGGCTTTCTGCACGGAACGCATGCTGGGAGG
GCACAACCGCAGTTAGCGCTGATGCCGCCGACTGCAGCGGAGGCGGTGGCAGTTCTCCCG
ACATGGCAGTGGCAAGGCGGAGCTGCGACGGCAGCAGCTGTGGATACACAAACGTCCTGC
CCTGTCGGCACAGGCATCCGTCAAAGGTCCCATCTGCGTTCACCTGCTGAAGGGTGGCGA
GGAGCCAGCCGTCTGGGAGCTTTGCCTCATACCGCCATGGCACACCCGACATCCGCTGCA
AGTTGCTCCTCGAACTCCATCTCGCTACGGCGCCTCAAGAGGAGCATTTGCAACTGCTGA
TCATGCGCTTGCTGGGCTTCTTCGTCCATGAAATAGAGCACCTGACTCTCTGGGGGGTTC
AT
>at02618
GTCCTGATCGATGCTCCAATTTCCGCCATATCAAACATTCCCGCCAGTCTATGCATGCCA
GGGCTCACGTTTCGTCGTCAGGGGGTCCAGGGGGAGTCCACGAGGAGATGTGGCGGCCTT
TGCTCGCCTTGCGCAGCGTGCGTTTTTGAAAGCGTGCCACTCTTGCGGGGAGAGATGCGG
CGGCCGCTGCCCGAACTTCCTCCGCAATAGTATGTCGCGACGAGAAAGGTGGCCAGGCCT
TGGTCCTGGCCCCCACGTCTCCCAGTGCCACTTCTCGTCTTGCGTCATGTTCTTCATTTT
TGCCCTATTCCTTTTCTTTTTTGCCTCCGCTCTGTCCTTCTTACTTCTCGCTCTGTCCTT
CTTACTTCTCCCTTTTGCCTGTGCCTCTGCCTCTGATTGCAGACCGTGGGTCCAAGCCAT
ATACAAGTCTTGGCCGTCCATAGCAGGATCGACACCATTGTCCAAAGCCCACTCAATCAT
CTCGTCTGCCATCGAGCGCAGCAGCGTTTGCGGCTCTGCAGGCCGACCGGGCCGGAAGAA
TGGCGCGGGGGGCAACCTTCCCCAGCAGTGAGGGACCATGGCAAGAACGTCTCCGATGCG
TACGTCCACCAGCGCACCATAGAGGCAGACAACAACCACCCATCTCCAGTGCGGAGAGCG

GGCCATAATGGAAGGGAACGTCAAAGCAAGCACATCGGAAACGAGCTTGAGCCAAAATGG
CTACGG
>at02619
CCATTCCGGCTAAAGTAATTTTGTTTAAGCTCAGCACGCCTGGCATCATAGGGCGTGATG
GGCTCTCACGTGAGCCAAACGGGCTGCAAGACCAACTTCGTGACCATGGAGTTCACGTCC
AGTCTTGCAAACTTGCCAGAGGATGCGGGAAATTTGCTTCAGTTCCGGATCCACAAGATG
GATGGCCAAGAGTTCAAGCTAACAGTTCGGCCAACGGACAGGATATCCGACATCATCGAG
CGGCTGAGCGAGCAGAGCGGCATCGAGTCGTACCGCATGGAGCTGGCGCAGGGCGCCCGG
AAGCTCGAGGCCTCGGCCTCCGTGCAGGAGGTCGGCCTC
>at02620
TTGTTTTTTTTTCTTTTTTTTTTGTCACACTTTTGCTGCCTGCAAAACGGAGCCGCCCTT
GACCACTCTTCCTGTTCCGTCGGGCAGCTCAGCGCTTGCCCACTGCGCATCCGACCCCTC
AATCGGACTGCATCTCCGTCGCTGCGCCTCGTCAGGTTGTCCTCTTCCCCTCCTACTTTT
CGTCCTCGTCGTCCTCGTCCACCTTCAGATCTTTTGTCTTATCCATGAGATCTTTCAAGG
CTGCTTCGTCAAGGTCACCCATGGTTTCATCCTGAGGTTCCTCGGAGCCGTCGTTGCTCT
CCGACCCAGATTCGCTGCCGCTTTCCCGGGCATCCTCGGGCTTCTCATCCTGTCCGATGA
GCACCGGTTTGGACTTCTCCTCGAAGACCCCGCCCTTCTCCTTGATGGCGTCCTCGACCT
TCTTCATGGCCTCCTCGATGGCAGCTATGCCCTGAACCTTGTCGCGGCAGGTGCACGTGA
CCACGAAGAGGGGATGCGCCACGAGGTTGATCTTCAGCTCGCAGTCCTCCTTGCTGACCT
CGAGCCCTTTGAGCAGCGCCTCGCGGACGGCGTCGATGCCCTCGTACTCGGAGCAGGAGA
CCTCGATCTTGGCCCGGAGCCGCAGCGTCTGCTGCACCAGGCGGCGCTTCAGGTGCACGT
CGATGTCCGCCTTGATTTTCTCCGCCTTCTCGCTCAGGTCCGTGCCGGGCTGGCTGAAGT
CGAGGCTGTCCCAAAGGTTGATCTCTCCGTTGATGTGCTGCTTGAGTGCGTCGTGGGCGT
CTCCGTACTTCTTGTTGGCGTACAGGGGCCAGGAGACCTTGTTGCACAGCTCCTCCACCT
CTACCTCATTCATTGCCGCTACGTGCCGCATGATGCCGTGGACGGCCTTCGCCTTCGCGA
AGGACTCCTCCTTCCGCGTCGCGTCCTCGGGCTCCACGCGCCTCTTCGACAGGTCTATGT
AGCCCTTGTCCTTGTCCACGCGCAGGATCATGCAGATCTCTGTCCGGCCGACGCGGAGGA
GCTTGGAGACCGATCGGATTCTTCGCTTTGAGAGCTCGCTGAGCAGCACCATGCCCTCTT
TCCCGTTGTACTCGAGGAGGCTGACGTACGCGCCCATGTCCACGATCCGCTTCACCTGCA
CCACGACGACATCCTCCAGCTCGGGGAACTCTTCCTCGTAGAATCTGCCCTCCGGAAAGC
CCGACATTGCTCGCTGGAGCTCCTGGGCAGGACGATGGGTCGGTGGCTGCTGGCGGCTTC
GAGCGCTTGAGCCAAAATGGCTACGG
>at02621
TTTTTTTTTTTTTTTTTACTTGGAACGTGGCGCTCTGCCAAGCAGCATTCGCGACTAAC
AAACATCGCCAAGTCTCAAAGATAAAGAGCAGCACCAGGACGTGCAGGGCAAAAAGTTCA
GGAGCGCACCGTCCCCCCCGCACCGGGAGGTGCAAGGCAAAAAAATTCAAGTGTCTGCTC
TTCCTGCACCTTTCGGAGCGCCCAGCGAGATGAGACCGCGCAGTGCCTTGTCAGGAAGTC
CTCCACGAGGGCCGCGGGAAGTGCCTGGGGTGTCCTCTGGCCGTAGCACTTCTTCGTGTG
GCCGACGTACCAGTCCCAGGCCTTCTTGACCTTTTTGCTCTTCATCTTCTCCTCCAACGT
GCGGAGCGTCTCCTCGCTGGGCCGCTGCACCCTGAAGCGGCCCTGCTTCCACTCCTCCAG
GAACCTCCGCACGATGCGGTTTGGCAGCTGGCTGGGCGCGCGCACGCCTTGCGCCTCCTT
CTCGCAGTAGCGGGCCCAGGGCCAACGCGCGGCCGGGTCGCGTCGCTGGAGGCTGTTGAC
TCTTTGCACAAGGTCCATGCTCGCAAGCTCGGCGGGCTTCTCGATCCATTCAGGCAAGCT
GCCTGGGCAGTGGTACTCCAGGAAGTGCTCTACGACATCCGCTGGCAGGGCGGCGGGATC
CTAGACACCGTAGCAGCGCTTCTTGTGGGCGCAGTACCAGTTCCATCTCTTCTGGACCTT
TGCACAGTGCTTGTGCATGTTCAGCTTGTCCAGCGTGTCCTGGGAGGCGTGTCTAAAGTT
CAGCTCCC
>at02622
GAGGCCTCCTGCTCGAGCTCTCGGCACCTGGAAGGTGGCTTGCAAGCGCCGTCGCAAGGA
CGCGAGTTCAGAGTCGTACCTCGCCCTCAGGTCCTTGCGCTCCCGCCTCGCGTGCTGCAG
CTCTGCCTCCAGGCTGGCAGATCCTTGGCAGCTGTCCGCGAGTTGCCGCTCCAGGCTGTC
GGCCTCCTCCGCTAGCGTGTCGCACCTCGCGACTGCGCCAGCAGCAGCCTCCTGGGCCTC
AGAGCACTCCAGGACAACCTGCTGGAGGCGGCCTTCCAGCAACAAGACCTCGGCGTACAG
ATGTGCGAAGTGAGCCTCCTCGCACATTTCCATCGGGTACTACCACCCAGCTGTCTGCCC
GTGCGGAGTTCTAACCCTGAGGCCAACCACTTGAGCCAAAATGGCTACGGA

>at02623
GTCAGGCCCAAGCTTGGTCAATGCCGACATACTCGAGACGTAGATGTTTTTCTGGCTGTC
AATCAAACTTGACCACAGCATATCATCAAACGCTTTGCTCCATGCGAGCGAAGCAGTCAG
ATTCTCTGCCTGGAAGGGGAAACGGGTGATCGAAAGCTGGTTCGCAGGACCTCGAACAGT
TGGCCAGTAGTGTGGTTGAGCCGCTGTGGCTTCAATGAGCTCTGGACTCTGGTGGCTGAA
TGAGAGATCAATCTGCCAGAAGGTCATAACCAGTGCAAGCGCATCACAGCCAGTGCAAGC
GCACAGCAATGCACAGAATCTCAACAAGGCCATTCCCATCTTGTCCAATGACGTTAAGTG
GTCAGCGTATGAAGGTGCGTGCAAGATATGAATGTGGGTTTCACTTGATCAGTTCGTGCT
GGA
>at02624
AGCAACACGTGCAGCGTGGTGCCGGAGGAGGCCGAGACGCCCTGGGCGCCCAGCGTCAGC
CCGTCGGAGAGTTCCACCCCGCCGAAGATCAGGCACTGCTGACTCGCCGGAGCGCCCAGC
TGCTCGCAGAGCTTCGTCTTGACGCTGCCGACTGTCTCGGACTCCTCGGACTCCACGTCG
AGGCCGC
>at02625
TTTTTTTTTTTTTTTTTGCGTTATTTCAATAACTTTGGACTCTCCCGTGCACGTTTGTG
TGCACACATGGACTCAGGGCCCGCGCAAGCGATCGGCCGCGCCCTCTCAGCGAGCTCTCT
GGCTTTGCCAGTGAGTGGAAAAATCCTGCGCGGATAGCGAGTTGTCAACAATCAGCGCTA
GATGATGAGGGCATCTTGCAGCAGCTGCTCAACGACCCCTGAAAATTCCAGCTTCTCATC
AAGCACTGTGGCTATGCCAACGAGTGCATCACTTCCGAGCGGCGGTCGAGTCGTCAACAA
TCAGACACAGGCGAGAGTCCGCGCCACACCGGCAGGCCTCCCATCAGGGCCCACATCCCG
TTCTCAACCCTGCGCCTGCCCTCCTCGATGGAAGTGCTCAGCATAACTTCTGAGCGGCGG
TCGAGTCGTCAACAATCAGACACAGAGGACAGTCCGCGCAACATCGGCAGACCTCGCTTC
AGGTTCCGCCTCCCCAGCCTCGGCCCTGCGCTTGCCCTCCTCTATGGAAGCGCTCACGAA
TTGGGAGAGCGACTTGTCCACGACACGTCGGCTTGAGTCCCACCCACGACGTCCGTCTTG
ATAGCAT
>at02626
TTGTTTTTTTTTTCTTTTTTTTTTGGGTCTAGAAATGGCTTTCGGGGACTGTGCCGAGCA
AGGCTCAATGACATACTGCATGAATTCCGTATGGTACCCACAACAAAGTTCTTCTTTCTG
GTCTCGGCATAAGGAGTCTTCGGATCAATGGGGAAATCATCCTTTGCAACTGTAAGATAA
TCTTTATTCAAGACCATGTAAAGAAGTTGGGTGAGGTATGTCCCTGCAGTCTTGCTGATT
CGCCTGAGTACAAAGCAAGGCTTCTGCGGGCAAAT
>at02627
GACGCCGCAGCAGCTGCTGAAGTCGTGGTACATCTTCGTCTTCCAGCTCCCGGCGCTGCC
GGAGTTAGTGACCTCTGCCGGCGGGGCCTCGGTCGTCCGCGGCGTCTTCACTTACGACCC
CGACGAGCCGCTGGAGGCGTGGGAGCTGGACCGCTACGCGGATGCAGCTGCAGGGCGCGG
AGGGATGCAGGGTGCCATCAACTACTACCGCGCCGCCATGTCTGGCCTTTGGCCGGCCTA
CCTTCCTGACCTGCCGCCGCCGCTCATAGGCCTCATCCAGCGTCTCCAGGGAGTGACCGG
GGCAGGGACCAGGGGCGCGAGCGGCAGGGAGCGTGGAGCGGTCTGAGACCA
>at02628
CCGTAGCCATTTTGGCTCAAGGGTTTTGCCGTGGAAGTTGCGATGTGCCTTCCGTGCCGG
GCCTCGCCCAGACTGGGTCTTCGCACGCCCGCCTGTGGAAGAGAGAAGCGAAACCAAGCA
GTCAAAAGCCACCGCTGGGTTGTGGCGCAGGAGCATCACCAGTTTCGTTGTTTGCCTTCC
TGCTCAAGTGGCCCTGCTCCGGTACCCGGCCACTGAGCACAAGGTGCAGGGCTGCCGTGT
CAGTCGCGGCCATTGCAAGTGTGCAGGCCAGGAATGTCTGGGTATGACGTGTGAAGGGAC
TTAGCTGGGCTTCCGCGTCTATGAAAAATCTGCAGCCGTCGGTGA
>at02629
CGTGTGCGGCGCTGCGTGCACACGTTTGAGCATCAGTACCAGATCTTGGCTGTTTCCTTT
GACGACACCGCCGAGCGCATCTTTTCAGGGTCTCTTGACAACACCGTACTCATCTATGAC
ATGCGGATGCGCGATGCCGATCCTGAGGTGCTTGAAGGTCACACCGATAGTATCACCGGC
ATTGACGTCTCCAAGGACGGAAACTTCCTGGCGTCCAACTCCATGGACAACACCGTGCGG
ATCTGGGACGTGCGGCCCTTCGCGGGGGCAAGGAACCGATGCCTCCGCGTCCTGCGGGGC
GCGACGCACAACTTCGAGAAGAACCTCCTGAGGGTGCGGTGGTCCCAGAAGGACGCGATG
CTGGCAGCAGGCTCCGCGGACCAAAACGTGAACATCTGGGACA
>at02630

GTTGTATAAGCTGTGGAGTGGCCTCCACGGAACTCGCAAAGCATCCCTGGATCTATGTGG
CTCTGAGGATCGACACCAACACTCAAAAGACCAGGGCAGAGCCGAGCCCCTCTGAGCAGA
GCGAGGCAAAGAGGTCAAGGCACTGCGGAATTCGTTCAATGTTGCCTCGTCGTTGTACAC
TCTGTGCAGGCTTCAGCGTGCCGCAATTCTGTGAAAACAATATTGAAATATCAGGGTCAC
CTCTGTACTTGACATGGAGCAGCTGAGCTTGAATTTTGCCCTTCGCTCTGGCTTGGAAGC
AAGAAGCCAATGTCCAGGGAAgTCCAGGTGCGACAGTGATACAACCAGTGAGCACTTACA
CAGGCTGAGTGTCACACCTCTTATTGCGCTCTCTGTTGGAGGACAGCACAATaGTAATCG
TCCTCGTCATCAAATGACACTATGGACAAAGTAGGTGCACTTAGTTCCAGAAGCCTCTTC
AGCTCCCCAAGTCCGGGCAATGGATGCCGAACCATGCGTGGATCCTGAGATCGGAGCTGC
TCAAGCCATTGCCGGCCCAGTGGGTGGCTGATCACCACAAGGCCCCCTTCAGAAATGATC
GACGAGGCAACATGCTGCAAGAGTAGCCCCTGGTCCCAAAAGTTCCCAAAGCAGGCATTA
AAGATCACTCGGTC
>at02631
TTTTTTTTTTTTTTTTTCAATTTCGAAACTCTAAATCATCCATGTTGAAACGGGTGTTT
TACAGTGCACTGGGGATGTAGTGCACACTAGCATAGCCCGCAATCCTGTAAGCAGTCTAA
TGGCTAAGGACGACTCCGCAGGAGCCCAACGCTGTACAACTCCCTCACAGCTCGTCCCTT
GGCTGGATCATCTTCTCCGGACGTNACGCAGCATCAAAGGCCTCTGACGAGAGGTAACCG
CGG
>at02632
CCGTAGCCATTTTGGCTCAAGTTCTCGCTCAAGCCACTTCAGCGCGAGCGTGCTCACGTG
GCCATTTCGACCCTCGGCGGCTCACCTTGCCCTCGACATATGCAGCGTCGACCTCATGCT
GAACTTCTGGATGACATGCTGTGCACAGAAGGGCGAGTCCCTTCAAGAGACGGCCGAGGC
TGCGCCCCATGAAGAGACGGCCGAGGCCACGCAACCGAAGCAGAGCGGAGGCGAGAGCGG
GTCCCATCACGAGAAGGAGTACTACGAGCGCTACGTGGCAAAGGACGTCATACTCGAAGC
GCTCGAGGGAGAGGCGCTGCGGCTGCTCTCTCTGAAATGGTTGCTGGGCTTCGCCAGCAC
AGGCGGTGTGCTCAAGCGCCGCCAGGACTTACCAGCCGAGGCGTTCATCTCAAGCGCTGA
GATCCGCAGGATGCACGAGAAGCTCAGTGCCACCGACT
>at02633
CAGGAGCTGCCAGCCCTCCTGGTGGAAGAACGTCGCGACGTCCGCAGTGGCGTGGACCAT
GTCCTTCGCCTTCCAGCGGTACTTCTTGTGGCTGAAGGCCTCGAACTTCGGCGGCAGCGC
CACCTCCTGGCAGCCCCAGCCGGAGGCTCCCCGGAGGAAGGCGTCCAGCCGGGCGTAGAT
GTCGTCCGCGTCCTCGCCGTTGACCTCGACGAAGCCGGAGCCGCGCAGCTCCACCATCAG
GTGCGAGCTCGCCCCATCGGGTGGTTATCCGAGGCGGAAGAAGAGCTGCTGCTCGCGGTG
GGTGCCGCTCTCGTCCGTAGTTCCCCCCGTTNATCGTCACCAGGCTCCAGGGCTTCCTGC
>at02634
CTTGGGCTGTCAGCCATGAACCTTGCTTAATTTACATGCTCTGTTCATGCTTCCTTCCGT
TGATTCATCATGGGTAAACAGACAGCCCCTACTGCTACTCTGGAAAACGCAAGGAAGACG
GTTGCTTCTCCAGTGAAGGGGACCTAGGCTACGGGAGACGGTCCGAAGTGCAGTGGAAAG
TTGCCGATGGAACATCATCCGGTTCATGCTGACGTCCAAGCAGCACCGAGAGACAGTACG
TATTCTCAGGCAACTCAATGGCAGTACATCCATCGCAGATGCGCTCACCTCCGAGATGTA
CGACTCCCAGCCCGACTCCATTCACGAGGATACTCCGCTGGACTCGCAGTGACTTCTCGC
TACTTCACAGGTGGAGCATGAGTCGCCTCACCCGGCTTTGGCGCGACTTGCAGCGACATT
GGAGATATCG
>at02635
TAGTTCCTATTTTTGGTCTTCTAAGNCCCTTCCCCAGGTCTCCTCCGGCTCTTCCTAGCA
CCGCCCCACCCCTCTCATCATGGGCCCGGCTCCGCTCCTCAGCCTCCTGGCCCTCGCGCT
TGGCGgGGGCCTGGGCAGGGCCGCTGGCGCAGAGGTGTCGGCCACGGCTGGCCGCCTcGC
AGCCCTGGCAGTGTTCGTGCACGGGGGGANCCGGCGCAGGAGGACGAGGAAGACGGCACT
GAGGAGG
>at02636
TTTTTTTTTTTTTTTTTTACGACACGCCTATCCAACTGCAGCCTTGCAAGAGATGCCAGG
AGAACATCCCTGAAGGTGCAAAGGCATTGCAATCTGGCCCCCCACCCGGGAGCGGGCCAT
CTTTGCAACCCTCTCTGGCAGACAACACCCGCTATGTACATCTGTTGCCTGCTCGAGGGT
TAGCAGCCCCGTATAGACTACCCACTCAAACACTAACTAGATCCTACTGCACAAGCTGCA
CCCAGCGCACTGACTATTGCCCCAAACAGCTACTCTGAATTACATGCAACCCAGCATCAA

GCTCCATGCAATAAGAACAATATGGTCAATCAACCAGGCTCCAGATGGCATCCATCACGA
CAGGGCAAGCACCCGCTGGCAACACTTCCCAATCATCCTCGAAAGCCACTATCTCCGAGC
TGAAGACAGTGTCAAGGCTGTCTTCCGTCGCCGCCGCGGCGCAAGAGGCACAAGAGCGTC
TAACCAAATCGTCGACCAAAGAGGTCTCAGCCTCTGCCAAGTACTCACGCCAGTTATGCT
CCACGGCAAGATCTCGTACATCAAAGTCGCCAAGAAACTCCTCTGCA
>at02637
TTTTTTTTTTTTTTTTTTCGTCGTTGTTCAGCTGGACCTGCCGCGTGGGGCCTCCGCCGC
CCGCCCTCTACATGGCGCTGCAGCCAAGTATGCAGCTGCGGCTGGCGAAGTTCCTGGGGT
TGATGTCGAAGACCACGGGCTCCCGCGCGGGCACGCAGAGCGGCGTCGAGAGGCTGGCGT
TGACGCACGCGCACCTGCCCTGGAAGTCCACGCCGTAGCTCTCCCGCACGTGGCCGAAGA
GGTGGACCTTGGCGGAGCCGCTCCCCTCCACGGCCTCCGCCAGCTCGGCGTCGCCCCGGC
CGGCGTCGCAGCGGCCCCTGGGCGGGCCGTGGGTCAGGAGGATGTCCGGCATGTGCCGGA
CGAGCGCCTCCGCCGCCCGCCTCCTCACCTCGGGCCCGTCGCCGTCCAGCTGGAAGGCGG
TGGTCTCGCTGCCCGCGCTCGAGGGGGAGACGCCGCTGCCGGCGATGGTGCAGCCCGCGT
CGAAGGACGTGGGCCGCACGAGGCTCAGGTCCGGGAGCAGCTTCACCGCCCCGTCCAGCA
CGACGTTGCGCACCTCCTGGTCCCGCGCCGCGCAGGTGCTTGGCAGGGCCGGGCTGAGGT
CGGCGGGCGCCTGCAGGAGGACGAGGTTCGGTGGCAGCAGCCTGCAGAGCCTGTCCGGGT
CTTTGGCCCACAGCTCGTGGAGGGCCTTGTCGTGGTTGCCTCCGACGAGGAAGATCACTC
GAACTTCGAGTAGCTCGGCTGGTGGAGGATCTCG
>at02638
GCCAAGGTCCTGGGCAGGGGATTTGCACCAGGTGCCCACCATTCGTGAAGGCACTTGCAG
GTGCTCCCGCTGGGGCGCACTGCCAGAAGCTCTTGGTGCTCTCTTTCGNGCGGCCATGTG
CAGAAGCTGCCCTGCGAGGCTGGTGGCGGAGAG
>at02639
TTTTTTTTTTTTTTTTTTGCCATCGCTCACCAATAGCCCATCCGGCTGTCTGGTCTACGC
GTACGCGCGCGGCGCGGCCACTACGCGCCGAAGCCCCTCAGAAGGGCGGCCACAGCGCGT
CCGCTACCGCCCTCACGGGGCGGGCTGGGCCGCACCGGGCTGGCACCGGCTCCTCGCGGC
GCCCAGCCTTGGATGCCTTGCACCGGGCGGCTGAACGAGTGCCGCCTGACCTCTGTGCTG
GCGTAGCAGGCGCAGCCAACCTACAGGCAACGCGCCTTGCATCTGTACCAACGTAGCAGG
CCCAGCCAGCCTCCGCCTTGCTCGACGCCGAAGTCCGCCACCACGCTGCG
>at02640
TTTTTTTTTTTTTTTTTTAGAACACGCGCACACATTGCAAATGGTCTGTCATGGCGATGC
CATGCAAGAGCTACACATTGCTGTGTTAAAATCTCTGCCTGCACGCACTCTGCAGCCATG
AGAGTTATTCGAGAATGAGACACTAAAGTCGTGTCCATCATCGCAATGACAGCAAGGCTA
GACAGAGCCATCAACACAAAGGGCGTGCTCAGACTAAGCTCAGTCTGAAGCGTAAGCTCA
TCAGATGCGAATGCTCGTTCTGGAATCCGAAGGAGGCCCGACCATCCGGGTGGAGGAAGA
CCTCCGTTATGCAGGCATTTTGCAGCTTGTGCGTGATCTCGCCGTATGACCACTCGTCGG
CCCGCCCATACAGGAGTAAGTGGCACAACAGATCTGCGACTGTCTGGTGCGTTGTGAGTA
GCAACGTCCTTGCCAGTCCACCTTCTTGCACTGCAGCCTCGCGCTCTGCCTCTGCCTCTT
GCCTCAACCACTGGACGACCCGGGTGGCCCGGTCGCGGCCTCGTGCCGAATTCTTGGCCT
CGAGGGCCAAATTCCCTATAGTGAGTCGTATTAAATTCGTAATCATGTCATAGCTGTTTC
CTGTGTGAAATTGTTATCCGCTCACAATTCCACACAACATACGAGCCGGAAGCATAAAGT
GGTAAGCCTGGGGTGCTAATGAGTGAGCTAATCACATTATTGCGTTGCGCTCATGCCGC
TTTCAGTCGGGAACTGTCGTGCAGCTGCATATGATCGGCACGCGCGGGAAGCGGTTGCGT
ATGGCGCCTCCGCTCTCGCCATGATCCTGC
>at02641
CCATCTGGCTCAGCTTCGCCAGCAGATCCTGCCGGGCTTCGTTCGTGCGCAAGAGAGGGC
CACTCCAGATCCAGGATGTCCGGTTACACGCTCCATCCACGGCGCTGCCTGACGATGAGA
TTTCGGAACGCTGACACAGAGCGCCATTTCGTCGAGATGCATTCCGACATGCTCGCACGC
AACGTGTTCCGGGGAAGCCTGGCTTGCGCGGCTGCGTCTCTCATCGTCATGGCATCCCTG
GGTTCACGCATGGAGTACGTTCATGCTCATCCAAGGCCTCATCGTCTGCGCGGCCATAGG
CCGGCGGACCATCGAAGACCACCGGAGGCTCACCTCTGCACGATCATCAACGAGAGGAAC
ATGCGA
>at02642
TTTTTGTTTTTTTTTCTTACCTTGTGCACGCTGATACACATCTTCAAATGCCGACCGTGT

GCCAAACCTATCTTCAAATGCTGCCTCTTCACCAAATCTGATTGCCAGCGTCTGTGGTGG
TTCACCGCACTTGCCTGGTACAGAAATTGCTAACCTCGAGGCTGCCAGGCGTGAGAGTCT
CTGCCCAGAGTTGTTCAAAGCTTGGTCTCAATGAGGTCAGCGACATCACCCACGTTCTTG
AGTGCCGTCGTCTCGTCATCTGGAAGCTCCACATCAAATGCCTCTTCCAAGGCCATCACT
GATTCAACGATGTCCAGTGAATCTGCCCCAAGCTCGGTGAGAGTGGCCGTGCGCACAACC
TTGTCCTTCTCGACACCAAGCTGGTCAGCAATGACATCGGCAACCTTCGCCATTGTGTCA
TCAGACGCAGCAAAAAGGACCATTGGCGACCTGTGACGGATGGCTGAGCCTCGCAATGTG
GGCGATGCCGAGGTGCCCACCACGAACACATTTGCAGGCTGATGATCCGCCCCTGACAGA
GTGATGCATCTCAGAGCGAGGCAGAACAACGCTGTGAGGAGAACGGCGCCGAGTGGGCGT
GTGGACGGTGCACGAGCCGCCATCCCCTTGATGCCTTAGCCTCACGTCAGGCAGCCAGGA
CTCAGCACAGTTGCCTCGTGCC
>at02643
GTCTCTGGGCTTATCCGCCCATCACCTGTGTACGACCTCGTAAAGCACGAGCAATTCTTC
ATTGCCCCACGAAGCTCAGAGCTGAGCCGATTGGCGATTTCGATACCAACNACAAGATGN
TGATGAGTGAGCTNTCTGCGCCCCTCGACANNGATGGCGTAAGCTNCCACACTGAGGGCG
GTGGCGAGCGCAGCTGGGGNACCAGCGGGGATTTCGGCNCCCCTCGATTGCANGGANCGC
GCGTCGTTCTGGATCCCTTGCGANGCGTCTGCGGACTTCTTCTGGAGCGACTGTGTCATG
GAGCCTTCGGACGTGGACCCCGGCGTGTGCTTGAAGCTTGAGCCCACACCCGGGACCCAG
GGCACCGATCCCTCAGANTCAATGGGCNGCAAGTGCAGCTCAGACGTGAGGTCTGCCAGC
CNGGAGGCNTCCACTTCCACAGAGGTCAAGCCGTGCGACGTCTGGCTGCTCAGCGATGCC
ACGTTGGAGACGTGCAGCTTGACAGTCGGCGAGCAGGAGGTGCCGAGTGTCTCGCAACAT
CGCAAGGTCCGCGCGCCGCCCGGGAGAGTGGTGAGGCTGTCGGCTGATGGCCGCCCACCT
CGCCTCCGGGCAGAGGCGGGCATGCATGATGGGGGACACACGCCGCGGCCCACTTGCTGA
GGCAGACTTCTTCAACGCGGCGCAGTGAAGCTTGGACGTGTCAACTGTTTGCAGCAGTCA
GCAATCCTCAACTCCATGGTTGCTCCACCTGATGTCAACACGAGTTTTTCCNCATCTTCT
GATGGGGTCGGCATTGTCTGCTCTTTGCCAGATAGATTGTGGTGGACACAGGCAGCAACT
GCGGAAGTCTGATTTTCCTAGCAGGTGCCGTCTCCGCGCCGGCTGACACGAACAGGAGTG
TTGAGTTTTTGACAAGAGAC
>at02644
TCTTTTTTTTTTTTTTTTCCGGCCCATCACCCGCCCAAGCACAACGGCAGATACTCACGA
CGCCGATGCCAATGGTAATTGCTGGGGAGCGACTGTAAGTCACAGGAAGCAAGTGCTTCT
TGCATGCAGGGGCCATCCCTCGGACCTGAGCCCTGTCAATCGGTCACACATGGTGCAGAA
GCAAGAGGGACTGCATGTTATGAGAGCTTTGCAACCACCAGTTGACCTTGCACGACATGA
CTGGCATGTGCCTAGCTCGCGTGCTACATCCAGCGCCCTGCTTCAGCTGAAGAGCATGTC
GAAGTCATCCTGCTTCGCCTTGGCCACCGGGGCGGCCGCCGCCTGCCTGGGCCGGTTCGC
CACACCGCCTTCTATGGGCGAGGGG
>at02645
TTTTTTTTTTTTTTTTTTGAAAGATCTTTGCACTCGCTTGAAATGAAGTCGCTTGAAAGA
GGCACGGGACAAGCCAGACCAGATACTCTGTACAGCGGGTCTGCCGCGGACACTCTCACT
GTTGGAGGGTCCTCCACCAATCAATCTGCCCCTGTGCTCTGCCTTGGCAGCTATGTACAT
AAACCCCGTCAGGCAATGCCAAGCACGTTCAAGAAGGTGGAGACTATCTGGGCTGCCTTC
ACCTCCAGCCTCGCCAGTGCCACGTCCACCGCCCGCGGCGGCGCCGGCGCCCACGGCAGC
GGGGAAGGTGGCGGCGCGGACGCCTGCGCCGGCGGCAAGACCATCGGCAGTCCTGGGCCT
CGCCGCCTGAGCTCGTTCAGGGCTGTGCGCTGGAGCTGCTTTCGTGCGAGCTGCGGCGCG
AGGCACCGTGACTTCAAGATCGTCACCTGCATTGGCGGCAGCATCCCTTGAAACTCAGCA
GCAATCCCGCTGCTCTGGGACCACAAGTGCTGCTATTTAAATGGCTTGAGCCCTCGTGCC
>at02646
CACAGCGCGCTGGCATACATGAGGCCGCTCATCGGCGAGGCGGTGCAACACAGCGACCGC
ATACGCTGTGCCAGGCTCCCCGCCCTGTCGCAGTGCCTTAGCCAGGAGGGAGCAGTGCCA
CTCCGAGTCGGCGATGAGGCCTGCGATTGCCTTCGCGTGCACGGCACCTTGCCAGCCCAT
CTGTACCAATGCCTTTGCAGCGGCTGTCCGAACAAAGGCTTGGGAATCGCCGAGGAGGTG
GGCGACCGCATCTGCCTGCTGGGCCGCAGGCTCGCCCATGGAGCCGAGCAGTTCCGCAGC
TTCTTCGCGCACGTCTGGATCTTCGTGCGCGAGGAACCTGCTAAGCTATTCACAGCGCTT
GCGATGTCAGCATCAGCCTTGAC
>at02647

ACAGTCCTGCTCCGCAACAGCGACGATCTCTTCGACCTCCCGGAGCCACGGAAGATTCGC
GCGAACCGCTTGGTGGGTGCTTGCAAGCAGCACCCCTTCATGAAGAACGGAGACACGCAA
GTGGCAAGCACAAACCTCCTGGTGGTCAAACCTCACCTAGCAGACTTCCATGCGATGAAG
TATCAGTTCTTCCTGGCGAGGCAGAATTATCAGCCCGGGCTGGAGAGCACTGCAGTGCTC
AACC
>at02648
GCTCCCTGCATGAGGCCCGGGCACTGAGGAGCCCCATTGCTCGCCGTGCAGAGGAGGGCT
CTGGCTCCGCGTCTGACCGCGTGCAGCTCAAGGTGTTGTCCCCGGAGGGCCTTGGTATCA
CTGAGGCCGTGTCGGAGGTTGTGCTGCCGAGCGCCAGCGGCCAGCTGGGCGTGCTGGCGA
ACCACGCGCCCATGATGACTGCGCTGGACATTGGCGTGCTGCGCTTCAAGCAGGCCGGCC
AGTGGAAGCCCCTTGTCGTCATGGGCGGCTTTGCCAGTGTCGACTCCAACCAGCTCTCAA
TCCTGGTGAACGACTTTGAGAAGGTCGAGGACATCGAGCTGGACGGGGCCAAGAAGGAAA
TGGAAGAGGCGACGGCCGCGCTGGAGAAGGCCGACTCAAAGAAGGCCAAGCTGGACGCCA
CACAGAGCGTCAAGAAGGCAGCCGCGCGGGTGCAGGCGGCCATGTTTGCAACAAAGAAGT
GAGGCATGCCTCCGTGGTTTTGCGCATACCAAGGTATGTGTTGTAAGGCAGGGACATGCT
TCAGTGCGCTTTCTCACAAGAATAGAGTGGTAAAAGGAAAAAAAAAAAAAAAAAA
>at02649
TTTTTTTTTTTTTTTTTTAAGCATACTCGCACGCAGCACAAGGTGCAGTCTGCGGTCAAA
ATTCCAGGACTGCAGCCCAGGCGACGCGCCGGCGAATCCCCTTTATGCGCAGTGGGCGAG
TCGCCATCTGTACACAGCACCATGCACCTGTTCCCAGATGAAGATGCTGAACTCTCTTGC
TGGCAAGTCGCATGTCGCAGGCCAGCCAGAGATCTCACCATCTTCGACGCAAAGGTCGCA
ATCGCTGGAGTTGACCTCTGCACGAGAGGGTGGGTGAAGGCTAGCAAGCACAGAGGCGTT
GGCAGAGCAGTCAAGAAGGTCGATGTGCATGTACAGGAATTTGCCAAATTTCCTCCCACA
GAGGAGAAATCCGTGGTCGTAGTGCACCAGCAGTGGGTGGTGGTGGTTCGTTATTGCATT
GAAGGGTGGCTGCGGTGCCTCAAGTGCTGGCCACTGCTTCTGGGTCTCATGACGAAGCCA
GGCCGTTGCAAGTGTGTAGCCAACCATCTCTGCGATCCAAGGTGCCGGAAGATTCCCGTC
ATGCACCAAAGAAGTCAGGGAAGTGGACCTTGAAATGGCCTTTGTGATCTCGTAATACGG
GCGCGCGAGTCTTGTCAAGGACTCCTTTCTCAGCAGCATGGGGACACCTATGGATTGCAA
CTTCTCAACATCCGTGATGCCGAAGTATCGCGCAAGCGCCCAGCTTTGGGCTTGCAGGCC
GTTGACTATGTATTCANAATCGCTGCCGATGGCGTGTGTAGCTCCAGAATGCACACGCTC
GATCATTGGAGTCAGGACTTCAAGGGGGGGCCCTGTAGAAGACCATGCCGGGGTCCACAA
TCATGATCACTGCGTTGGCCGGCAGCTTTCGGCCTCTGCCTTCTTTCAACCAGGACAAGA
AGCCCCAAGGCTTGTTCAAGGGCAAGTAGTCAGAGTCAGCTGTGTTGTGGAAGTGTGTCT
CAAAGCCTGGAAGGTCAGAAAATGTCTTGCTTTGGGATTGTCTATCTGGTGAGCACGCCA
CGATCCGCACAAGCTTACCAGGCAAGCCGGCTTGCCTCCACGAGTAGTACAGTGTCACCG
ATTGCCAATCGTGGTAGGGCTTGCACTCAACTGAGAAGGCAGTGACAAACTTGATGTCT
>at02650
GCTTCCTGGACCTGGCATCAGCAGTGCCAGTAGCAAGACGTGTGCTGAGTCCGCAGTTGG
CTCGCCATCCCAAGGCGTTGAGCTTTTGTGCACACGTCTGCAAGATGCGCGCAGTCCCGT
AGCCAACAGACTGCGCAGCGCCCGCCGCGGGCACGGCGGGAGGCGTGCAGGAACCGCCCT
CGATGGGCGTATCTGCAGCGGGGAGCTCGTCTCCAAGGCAC
>at02651
TTTTTTTTTTTTTTTTTTGCGCAAGCAGCCGGAGAAAACTCCGCACAGGATACGCTCCAG
CTTGCCCCGCGGGCCTTCGGGCCCCTGCTCATTTCATCCCGTTGATGTACGCGATGAGGT
CAGCGCGCTCCTTGTCTTTCTTGATGCCCGCAAAAACCATCTTTGTCCCCGGCATGTACT
TCTTGGGGTTGATGAGGTATTCGAACAAGTGCTTGTCCGACCACACGATGCCGGAGTTCT
TGTTTGCCTCATAGTAGGCAAAGCTGTCGTAGCTGCCCGACTGTCGGCCGATCAGGCCGA
AGAGGGGAGGCCCCTGCTTGGCATTGCCGCCCTTCTCGATGGTGTGGCACTGCGCGCACT
TGGCCTTGATGAGCTTTGCGCCCTTCTTGACGTCTCCATCCGGAACATCTACCTGCGGCT
CCGCAATGACCATGGCTCCGATTGGCACTGAAACTCCCCTCGCCACGCACGCGAGCCC
>at02652
GCCGAGGTGCCGCAAATGCCACCGTGTTGCACGCCCTGCTGTGTTAATGTTTGGCGATGG
GTGCTACTGCTCCCCACGCAGCGCGGAGCAGGAGCAGCACTACTTGGCCTTCCTGGCACG
CCTGGAGCATACAAAGCGCGCCGTGGTCCTGGAGCTGGGTGCGGGGACGGCAGTGCCAAC
AGTGAGGAGACATTGCGAGCGCGCCGCCAACCGCCCTGACGTAACCCTTGTGCGCATAAA

CCTTGAGGATGCAGACGTGCCGGAGGGTGGGATCTCCCTGGCACTCGGTGCCCTGGAGGC
ATTGCAGCTCGTGGACATGGAGCTGAAGGCCCTGGGGCACTGATGGCTGTGGCAGGACGA
TGTGCCTTTGTGCCTCAGTGGCT
>at02653
CAGCTGCTTCGGCGCCCGCCACACAGCGACGCCGCAGATGCGCTGATCCCGGCCTGGCTG
CGCAGTCCCACAGCCTGGaaCTaGaCGCAGTcCCCGATgGGGTGGCAAGCTTGTTCTGGG
CCCAAAGCCGAGGCACTCATCCGCTTACAGCATCACGCAGGGCATGAGGGCAGGCTCCTC
CGCGTGGGCGTGCGTCCTCATTCTTGAGGAGGCGCAGTGCTCCGTTC
>at02654
GGCCTTGCTCAAACCCCTGTTATCGCATCGCTTGCGAGGGTTGGGGATTGTGCACAGGGT
CAAGCTTAGGCGAGTCCAGCNACTTGCTCATTGACCCCGTGCACATGGAGCCCGCGTCCA
GCACTTCCAAAGGCGaGGGCCAAAGCCATGGGGGTGAGCCCGCGTCCAGCACCGCCGAAG
GCGAgGGCAACAGCCNTGGGGGTGAGTCCTCATGCAGCACCACCCAAGGCAGGGCCAAAG
CCATGGGTGTGAGCCCATGTCCGGCACCGCCAAAGGCGAGGGCCAAAGCCATGGGTTTCA
GAAGGCA
>at02655
TTTTTTTTTTTTTTTTTTCCGTGAGCAAAAACCAATGGCACCAGTGTTGCTTGTCGAGTT
ACACCGCCTCCTCTGCTCTTTCTGCACACTGAGCCCACTCCGGCAACGCTGCTTCAAAAC
GGAGGTTCAGCCTCTTGAAGCTGTATGAATGCGCATGCAGGCATATTGCTCCCGAAAACA
TCACAGGCCGCGGCGACTGGCCAGTGATTGCCGAGGCCACCCGTGCACATTCCGGGCACC
AGGGCCTGAAACAACGGTTGAAGGCGCGGCGCAAGATGGGAGAGCTGGTGTAAACCTCTC
TCGATTCCCTCTCGGTAACGCCATACATGAAGTCATTGACTATGGGCCAGCCGAGTTTGG
ATAGGTGGGCGCGGATCTGGTGCCTCCGCCCCGTCCGCGGCCAGCATTCAACCAGCGACT
GTGCCACAGAGGCGTTCCCACCACGCAGCTGCCGCAAGCAGCGCACCCTTGTCTGTGCAA
ACTTTCCAGATGGTATTTCGACTGCTCGGTCATCTTGAAAGCCCAGGTTACCCTCAACGG
ACACATCGGTGCCCTCTGGCACTTGCCCAAGTACCCGCGCAATGTACTGTTTCTGAACGC
TGTCGTGCCTTGAACTCCTCAACCAACACTCGTCTTGTCCTTTTATTCTTTGCACAGACA
ACCAGACCGCTGACACCGGCATCAAGACGGTGCACTGGTGAGGCGCCGGCATGGTACTGA
CTGGCAATACCTGCAAGCGTGTTCTTATGCAGACCAGGACCCGACATCGCAATACCACCT
AGGCTTTGAACAGGACTAGCATGTCAGTGTCTT
>at02656
TTTTTTTTTTTTTTTTTTGCGTTCTTCGAACACTGGGCGCATCTCACTGAGAACCTAAAT
GTCAGGCCACTAAGCATGCTCGCATGTCATTTCTTCCAGTGCAACCCTGCATCACGTTTC
GCTGCCCGTATCATTGCCTGTGGCAGCAGGCTTGCTGCCGGCGTTCTTGTACTTGTCCAC
CAAAGGCAACTTCAGCACTGGGATGGTCTTGGTGTCCGATATCACTTTCGCAGTCTCCCT
CTCTTCGTTGGGAATGACCTCCAGAAGCTGGTCTATGGCGTCAACGAGTGCTTCAAGATT
CCGCTTTGTGGTCCGGATCGCCGATGGCCCCACAAGTCCTTCGGTCCTGGAGATGAGCAG
GCACTGGGTGTCTTCCTCATCGAGGGCCCTCTTCAGACGCTCGACCTCGAACTCTGCTGC
ATTGGACTTCCCCGTCTTCTCTCGCATCCACACTCTTCGTGCGAAACGTCTCCTTCCGGA
TGAGGCGAATCGGATCCACGCGATTCTGCTGGCGCAAGACCTCGTAGAATTCGGGGTCGG
ACTTGTTCACGCGAGGGTTGTCCATCTGCTCCAGGATGTCCTCGAACGCGCGCTTCGTCA
AGAACACCGTCGGCAGAAAGCTCTGCAAGTCGCTTGAATTGTAGTTCTGCGTCGGAGTCT
TGTAGGCGTTGAAGATGGACTTGCTCGCATCCTCCTTCGCCTTGCGCCGCGACTCTGCTA
TGATCGCCTGGATGGTGGACTCAGCGAGAAGCAGGACCGCCAGACGCAGCCGCGGCGA
>at02657
CGACGCAAAAGTCAGCTGCGGAAGAGCATTCTCCCTTTGCACAAAGACGCCTTATTGGTA
CCGTCTTCACCAACTGCTGCGCATCATAGTGCTCGCCTGCTGTTATCTTTTCCTCGATCG
ACCTCATAGCACGGGCTGCCATGATGGTGTTAAACCACTGCAGCTGGCCACTCTGTAGTG
CAGTGCGGTGGATATGCAACCCAGTCTTGAATCCTCATCTTGAGCCTTGAGCCAAAATGG
CTACGG
>at02658
GGCCTTCCTCTCCTGGGAGGAGCCAGTGGGCGCCTGAAAGCCCTTCCAGGGGCAACGGAA
CTCGCTGATGGCCCGCTCGTAGGGATCCCGCACCGCCGTGAACACCTCGAAGCCGTCGTA
CGGACTCTGCAGGAAGAGGGAGGGTGGCAGGTGCCACGGCTCACTCATGCAGTTCTCGTG
CGGTGCCACAAGGCCCTGGGACCAAGCTTCCTTGATCCTCTTCCAGTGCCGGCCCCAGGC

ATAGCCGCGCTCGAAGCCCCAATCCTCGAGCGCCTTTCCTGCACACTTGGTGATGTGAAT
GAGCTTGGGCTCGTGGATTCCTTGCAAAGGCGTACGTCGGGAACTGCGACTTCGGTCCCT
TGGCATGGCTGCCAGGGCTGCGACCCATCCATGCTTCAACGGCTGGATGAC
>at02659
TTGTTTTTTTTTCTTTTTTTTTCGTTTCAATGCCGTGGCGATATCTGATTGAAAAGCCCC
CGCCCGACCCTCAGAACCAGGAAATGCAAGGTCGAATCCTCCCTCCGCCACATGCGATGG
CTCATACTCTCGCAAGTGTTCTTCTGTGATGGCTACAAGGTCGCCGTCAAAGGTCTCCAC
GATGTATTTCTTCTGCTCTTCAACCCAAGCGACTGTCTGGCCCGTTTTCCCACTAACATC
GATAGGCTCCGCATTTGGATCCCTTTCGAGGGCTTCTGGGGGCGGTGGCAATGGCGGCTG
GTCAACGATCTCAACGAGCTTGTACTCTAAGGGTCTGCCATTATCCATGTTCACCAC
>at02660
TTTTTTTTTTTTTTTTTCAACGCACAGGCACAGCAGAAGCGGCCAGTATCGCTGAAAAAG
GCTGCAGTCGCCCGCTCCGCCGTCGCTGGCAGCAGTCAGAGCTTCTTCACATGCGCTGCA
AGAAGGCATTAAAATTCGCACCAGAGAGGATTGCCTTTGCTTGCTTCAAGGCCTCAATCT
CCTCGTCACGGGCAGTCTGGCGGACCTCAAAATTCTTCATGACAAAGTCGCAGCTCTGGT
GCAGCTCCGCATTGTAGTTGGAAAGCTGCTCCAGCTCAAGCATGACTGCCTCCTTCGACT
CCTTGGCCTGTACCAAGTCTGCTTCAGCAGTTGCTTTCTCTTCAGACTTGTTCACGATCT
CCCGGCTCTTGGCCTCAATGGAAGCGTTTGTCTCCTTGACAAAGTCCTCATATGCCTTCT
GTGCATCCTCCTCAGAACGAATTGCTTCTGCCTCCATGGCCTTGGCATCACCGATGATTT
GCTTGATCAGCCCCAAGACGCCACCGCTCGCAGAATTCTTCTTGTAGGCCTCGAAACCTG
GAGGTGGCGGAGGGCCCACAGGTTCTTGCCTCTGCAAGAACGAGGCACCCTGCTTACCAT
AGAAATCCTCCAGGAAGGTTAGAGCTGCCGTGAGCAGCTTCTGCGTCTCGCGCTGGTCTG
CAACCGTGGTCTGGAACTCCTTGTTCTCCGTCTCACGGTCCTCACCAGCCCGCTTCATCT
GGACCTGCATCTCCGCTATCTGACTCTTCAGGGAATCAATCTCATCAGTCAACGTCCTGA
TGGTCATCTCCAGGTCCTCAATGTGCGCGATCAGGTCCTGCTTCTCGCGCTCTTTCTTCT
CAGTCTGCAACTGATTCTTGTTGAACTCGTCAACGCAGAAGTCCTTGTGCTTGATTTCGT
CCTCTTTTTCTTCCAGCAACTGGGCAACCATGTCATCGATTGCCTTTTTCACGCGGGTGA
ACGCATCCAAACGGACCTTGTATGCCAGCGTCGCCAGCCTGGGCTTGTGCAACTTCTGCG
CCACAGCGTTAAGCAGCTTGGCTGCCTTGGCCCGACGCTCGGAGTACATACTGCTCTCTT
TCTGAATGAAGGCTGGGTTGAAGGTGCGCGTGAACAGGTCGTGTGCATCGTCTCCACTCA
ACACAGAAAGCGCCTTGGAAACTGCCTCCATTTCGAGCTGCCGCGTTTTCTGACGCTCCT
CCCACTCCTTATCGGTCATCTGGCATTTTTCCTTCAGGCTCATCAGAAACTGTTCGTCGG
CAGAGAGCGAGCTCTGTGTGTCATCCTTGTCAACCTTTGCCTGGGCATTCTTCTCATCAG
TTGTAGCAAGTTCGCCCGTCTTCATCTCAATCTGCTCCTGCCCTGCAGAGATCTCTGACT
CTTTGGCCGCCTTCAAGTCTTCATAAGCTTTCTGATTTGCCATCTCTTCCTTCTGTGACT
CAGACAGGTTTGACTCGAAAGTCTCCTTCATCTGCTTGAGGATGCCAAAGATTTCTCCAG
ACTGAGGAGCATATGACTGTTTGAAGGTCGGCGTGGCGTCAAAGTAGTCCTCTGGTGCCT
GGATGAAAGAACTCGCCGCCCTGCGCTCTGACGGACTGATGACGCCTGCCAGTAGCCCGG
CGTGCTTCTGCATCTCGTGCTGCAGCGTCGTGGCGACGCTGAGCATGTGGCTTCGGGGCA
TCTGCAGCAGCGAGCCGCCCTGATGCTTGGAAAGGACAGTGATCGCCGCCTTGAGTGCGG
ATATGGACTCCAGGAGGTCCTTCTCCTCTGCATTGAATTCTGCGAGCTGCTTTTGGCGCA
ACGCCGTGGCCTTGTCGAGGGAGGCCTGGTTCTCGGCCACCTCCTTCTCATGGTTCTTGA
CCTCGGTATTGAGGCGCGCACTGCCTGCGGTGAGCTCCTCAATCTTGTGGTCAAGTCGCC
AATCTTGATTTCCGCATCTTTGATAGCCTTCGTCTTTTTCTTTGTCGTTGGTTGTGCACC
AACAAGCCAGCTTGTCGTAAACTTCTTCGTCCTCCGCCGCCTCCTTCTCCAACTGCTTCA
TCATGTCCTTCAGCAGCGTGATGACCTTGGACA
>at02661
GAAAAAGTGGAAGTGGCAACTACGCTTGGCCCAACGGGTCTTACTTCGAGGGTCAATTCG
TGGACAACGACATCCACGGCCTTGGGATTTACGTGTGGAATGATGGCTGTAAGTATGAAG
GACAGTGGGTGGAGCAGCTTGGTTGACCTCGAACACCTGAGTCACACAGGTGTGTAGCCA
TGACATGCCGGACGTAGGGCGCGAGCAGACGGGGCTCTGTGGAATTACTGCCATGTAGCG
GCACGAAGCTAAGGTCGAGAGGGTAGCGGCAACAGCTACGTGCCTGGGCGGTTCAGGGTA
ACTTTTGGAGAGACATAATCTAAATGGTGAGCACTGTTGGTGACTTAAAACAAAAAAAAA
AAAAAAAAAAAAAANAAAAAAAAAAAAAAAAAAAAAAAANAAAAAAAAAAAAAAAAAAAANAAA
AAAAAAAAAAAAAN

>at02662
GAAGTAAATTGTCGCGCAAGCCAAACAGTGAAGCTGACTCCCAGGCCTATCCAAAGCGTC
CACCAGGCAGTGGACTCGCTGACTGCATCCGAGGTAATGGACGCCACAAATACAGTCACC
ACGGGAGAGCACCAGATAACCTGCATCTCCTGAATGTACAGGAAGAGCTGGCTGCTTGAG
GACATGATCACCGGCATCCAGGCGAGCGAGGCCGCCGCGAGGATCAGCGTGAAGACTCGA
CCCACCCTCACCAGCTCCGCCTCCGAGTGCCCCGGGGCCAACTGTGCGTAGACATCAAGG
GTGAAGAGCGTGCTGCAGGAGTTGAAGCATGAGGGGAGAGCGGCCGGCGGCGGGGCGCCG
ATGTGGCGATGGGGAGGCGAGAAAAGTTGAACCACTCCTTGAGCCAAAATGGCTACGG
>at02663
TTTTTTTTTTTTTTTTTAGCTCCAGCACTCGCGCGAGCGTGGGCCCGCACGCATACTCC
GAGGATCCAATCAGCATCCGCGCTTGATGTGGTGGGCAGCGTCCAGGACCCAGATGCAGC
AATTGACGCACCCAAGCCGTATTGGCTGCTTGGTTTCGCCCACTCCTATCTCTGTACACA
AGAGGGGTACCACGTGGTTCTATACAGAGTCTTGCAGGCACGCATGATCTTTGTCAAAGC
TCCAGGTCTCCCTCGTGCC
>at02664
CCGTAGCCATTTTGGCTCAAGGCGCCGCTCCCCTCCCCCGCCCCAGCGCGCCCCGCGGCC
GCGATGGAGAGCGAGCCGCTGCACCTCGCGCTCGTGGGGCCGAAGGGCGGGGGCTCGGAG
GTGCTGAAGCTGGACCGGGGCAGCAGCGGCTTGGAGCTGAAGCTGCGCGTGCAGGAGAAG
CTGGACGTTGACGCCGCCTCGCAGCTCCTCTTCTTCCAGAACGGCTCGCGCAACGCCGCC
AAGGTGGCCGTGGATGACGCAAAGACCTTGGAAGATCAGGGTATCCTGGACGATGCAATC
ATCACGGTGAAAGTCAACCAGTCTGAGGTCAAGCCCGAGAACTCCGTGCTCAGGCAGTCG
ATCGTCAAGAACGGCGGGTCTAGCTACTACTACGCCCACGCGAATGAGAAGGAGCTCCCG
CCCGAGCTGCGCTATGTGTATGGGGGCGCGCCCGCGAAGCTGGGGGAGGCCGAGCCCGAG
CAGGCCCAGGCGGAGGCCCCCCCGGCCCAGGCCATCTCCCGCTACTCCTGGGCCGACGAG
GGCGACTTCGTCTGCGTCTACGTCGCCGCGGACGGCGAGGCCGAGGCGGTGGAGGCGGCC
GGGGACGGCAAGGGCAGCGAGGTGCAGGTTGCTTTCGAGGCAAAGGCCGTGGAGCTGAGG
ATCACAAAGCAAGACAAGGAGTTTGCCCTCGTCCTCAGGAATCTGGAGAACGAAATCGAC
CCAGACGGCTCCAAACATCGAGTCAGTGCCGGCAAGCGGGTGACCCTCAAGCTCAAGAAG
AAGAGGCAGGCAACATGGACACGCCTCATCAGGCCGCAGAGGTGAGCCTCAGGCGCGCGC
CTTTTTGCGCTTTCTGCTCCCTGCGCTGTTCCCTTCTCCACGTTGCGACAAAGACCAGGG
CCAGCAGTGCCATGTACAGTGAAAGGCTCTGCGAGTGACAAAAAAAAAGAAAAAAAAACAA
>at02665
GCAACACCGTGACTTGGTGGGGAGTGTCCAGCTGCACCGCTGACATCAACGTCGCGAAGA
ACTTCGCGAAAGGTTGCGGTGGCAAGTGCACCATTATCACCGTAGAGTCAGAGACAGCCA
GCGACATCTCTCAGATCACTTTCTACAGCAACGAGAAGGAAAGTCTGCTTTGTCCGGGCA
CACAGTTGCAGGTCAAGAGCAACAAGATGAACGGCAATGTGTGCGAGATCACACTCAAAG
AGATTGGTCGTGTCATTGCCTGAGGCGATGAACGGAGGTCGGGATTGTGTCATCAACGGT
GCATT
>at02666
GTTTTTGAACACCAAGTACAGCCAGCGGTCACAAATTGGGTTGCGCTGGTTTTGCTGATC
TAAACGTCAGCTTAACGCGCAAAAGGAAAAATCTGGTCATGCTAATGGGCCGTGGCCTGT
GCCAACTCAAGACATCGAGCACTGCTGCTGGTCTCATCCGCCAATAAGGGTGCAGAATGG
CGCCGCCACAAGCGTGCGGTGCCCGAGCATGGGAAGCACTTACGGTCCTCATCACCCTGT
TGCGTCAGAGACCTGTCTCCAGCTCCGCAGAGGCCAAAGCATGGGCCAGATGCAAAGCCG
AGAATTTGGCAGAAGAAATTCGTGAAGTGGTGGCAGTTCCTCCTCCATGGGTCGTAGGTC
CCTCCCAGCCACTTCTGCCTGAGTGCAGGCATGAGCTCCTCAAGGTCCTCGCGGGACAGC
AACGCGTCGCCCATGGTTATGGACCTAGTGTACCTGTGCACAGGATGCGACTTCGGCTCC
GGCATGCAGTACACGCCGTGGGCTGCCGGATCCTCCGTGCCACAGAAGTACCACTCGCGG
CCGTACACCTCCACGCCTGTGTGGAAGACCCCAAAACGTGTGGAGCCAGTGTTAAGCATC
CTTGGCAAGCACGAGCGCGAGCCCCACAGGTCGTAGACATGCACGAGCACCACGCTGTCA
CCAGTGGGGCAGGCCAGCTCCTCTGGAGTTGGCACGTGCTGCAACTTGAGGACCAGCGCG
CTGGCTGTCGTGCATTCCGCGAACATGCGGTCCACATCACCGCAGATGCCATTCACCTCG
ATGATGTGGTCGCCCACCCTCACGACAGCGTCCCCTCGCCTGCGGTTCCAGTCTTCGATC
AGGCCTGCCTTCATTCTCATGATGATCAGGCTGACCCCGTTGCTGTCATCAATATCCACG
CCAAGCTTCGCACCGCTCGAGCGGTTGATCCGGATGGTGAAGTACCCGTCGTCGCTGGGG

CCCGAGCTCAGGGCCAGGCCCGCCGCCGCGTCGCCGTCAGAGCCCATGAGCCTACTGATC
AAAAGGGGGACTTCATACCTAAGGGAAGGGGCAGGACCGCTCGAGACAGGCGCTGCGGCC
TCTTGAGCCAAAATGGCTACGG
>at02667
TTNTTNTTTNTTTTNTTTTTTNTTTTTTTNTTTTTTTTNTTTTTTTTNTTTTTTTTTNTTT
TTTTTTTTTNTTTTTTTTTTTTTCTTTTACGGCTCTCTCATCGATGGCACTGCTCATCAA
ACCATAACCACTGAACATGAACAGCTCCAGTGACACACAATCGATGCACCAGAGGCTCCC
AGAAGAGCTCCCAGGTCCGCGATCAACTCCAAACCGGCGACAACCATGCTCGAGGCACCC
TCCTGCCCGTGGGCATCACCAGCTGGAAGATGCCAGAGCGCTCCCTCGGGTTTAGAGTCG
GATCCGCAGATGGGCATCGGAAGTACGTGCAATGGAGCACGGGCAAAATCACTCGTGGCT
TTGAGCCTGAGAGCTTGTTGTATGCCAGCCCTCTACCCGGACTTGAAATGACTGACTTAA
AATGGGGAGGCCCGTCTGTGCGCATATGCGCAAGCGCGTTGCATGCCAGGCGCATGTGTG
CGCCCATGTGCAGGCGCACATGTGATGCACTCACTTTCCCACCTTTGCAAATCCGCGCAG
CTACAACTTCACTGGGTTCTCTTTTTGAGCAACTTCTGGAATCTCTTTGCCTGCTCCTCA
TCCATCAGGCCGCCTGCTGAAACTGCGTCCGAGTTTCCCATGCGCTTCTGCAGCAGCGCA
TTCTGCGCCTTCAGCAGGTCCCGCTGCTGCTTTGCCAAAAAGCGCTCTTCATCCTCCAAC
TTGCACACGTTCCTTGCCTGAGGCTCTCCCTTCGCATTGCGGACAATGGTGAAGCTCACA
TCGTCTCCGACTTCACATCGCTGCATCCTGGTCTGGCGTGGACGTACACGTCATNCTCNT
CGCCAGTCGGCCTCCTGTGACGGCCGCAGCACCTGGCCAAGCTCCTCCACGCACTCGGCC
TCGCGCGCTGGGGGCCCAGGCAAGCCCGGGAGTGCAGGGAGCCCCGGCAGGAGGGACTGC
GAGAAGACTGCTCCCAGCACGCCGGACAGCGCCCACGCGAGTGGGGACGGGCTCGCGGCT
GGAGGTGAGCCTCCGAAATCCATCAGCAGGCCATCCCGACTGCTCCACCTAGAGCAACAG
AATGG
>at02668
TTTTTTTTTTTTTTGTTTGATCTCGCAGGTTCATGCACAATCACAGGGTATCCAGACAGA
AGGACTTCGCCCCAGAGTCCAGGAGTCTTGTCTCCGAGGCGATGCCAGGCAAGGCAAGGC
TGCGCGCCAGTCGGACGGGGACCTCCGCTGCCGTCTTGGTGGGCGGTCGCTCTCCACCTG
CCGGCTGCCAAGGGCAACCGCAGGCACACTCGGTTCTCCGAGGACCGTCGGCGACCTCGG
CCAGTGAGACTCTTTGTCGGGCTACAGCCTTCAGGCGGGCGAAGGCTGCGCGTCAAGCTT
GCTGACCTCCTGTAAGAGCTCGTAGGCGTTCAGGCCACCCACAGGCCCACTGGTGGTCGA
GGTGAGGTTCTCTTCACCCTTCTCCTTGTTCCAGACACGCTGCATGATGCCCTTCCAGTC
TTCGAAGCTGATCTTGCCGTCGCCATCGTTGTCAAACAGCGCCACAAGCTCCTGGCTGAC
CTTCTTGCAGACATCCGGGCTCCCAGACGTCCGCATGTCGGCCAGGTCGAGAATCCGCTG
CAGCTCCC
>at02669
TGGCTCTTTCGGCTCGAGTCCGTGCAAGCAGTGTTGCGCAGCTGACGCGCGCTGCATACC
GGTCATGTTGAAGTTGATGGGGCATGCCATGTACATGCACTACGGAGGCAGCACCCCCAA
GACCGACGACTTCTGCAGCATCCACGTGGGGCCAATCCAGGAGGGGCGTATGGTGGTTCT
CCACTGCGGCCACAAGTTCNGCCTGAAGCACTGCTTTCCGCAGCTGCTCAAGTATGACCA
CAACCAAAAGTCGTACCCGT
>at02670
CGAAGTTGATGCCTTCTCGTCATCGCGGCACTGGAACTCCAGGCTCCAGCTGTAAGTGCC
GTTCGCCTCGTGGCCAACGTCCACCACCGTGTTGCGGATGCTTGCAAGGCCAGGGTTGAA
GATCGACGTGGAGCGCCAGTGGCCCATGAACGCCCCTCGCTGGTTGGTGATATTGAACTC
AAGATCGCAGATGGCGTCAA
>at02671
GCATCCGGTGGGCCGTGGACAGCTCCAGGCAGATAGCCAGACGCTCCCGGTCTAGCGCCG
CCGCTCTTCCCTTTGCCCGCGATGCCGCGCGTGGCGGCATGGCGGCTGCTACGCTGCCTC
GTCGTGGGCCTCGCTGGCTTGTCGGGGGTGGCTGGGCAGGACCAATGGGCCGTGCTCATT
GCGGGGTCCAAGAGCTACTTCAACTACCGCCACCAGGCCGACGTGTGCCACGCGTACAAG
GTCCTCACGGACCGTGGCTTCCACCCAGACAAGATCATCACGATGATCTACGACGACATC
GCGAACTCGACGATGAACCCTTTTCCGGGGAAGCTCTTCAACCGTCCCG
>at02672
TTTTTTTTTTTTTTTTTNTGGCTGAATCATAATACAATTTGATAGATCAAGCTGACAAGA
CAGGTTGGCGTGAATGCCTGTGTGCGCCTCCGGGTGGCGCCTTTTCAGGACATCTCCTGT

TGCTCGGGCTTTCCGAGGCGCTTGGCGATGTCGATGACGTCCGGGGCATTCGATATTTCC
TTATACTGCTGATGCAAGTAGCAGATGCCGCAACACCATGTCTTCAGTACGGCAATGGGA
ATGGGCTCCGCTTCGCCCTTCTTCTTTATGCCGTAGTAAGCAATCTGGCACCCTCCAGTG
CCGCAAAAGGCCAAGAGGCACGCAAGTTGGTCCTTCCCCTCCCCGAAAAGGGGGCTGCCA
TTGTCCTTGTGTATGGCTGCAAGGGCACAGGGAGCGCAGCAATAGTGCTTCAAGCAATCG
GGGCACTTGATGTCAAAGAGGCCGTTGCTCCACTGCCCCTTCGTGAACGCCTCCGCCATC
CAGGGTCACCGGCCGGAGAGGTGCACAAGCAGATCACTATCACTCGAGCCGCTAGCTTGA
GCCAAAATGGCTACGG
>at02673
TAGTCTGAGTCTCCGTGGCCGACAGTTCTTGCAAGTCATTTGGATTTGCGCTGCATCCGA
GTTGGGTTGGCATGCGCTCTGATCTGCAGGCGAGTTGGAGTTGGTTGCCAGCACTACTCC
TTGTTGACCGCCTGTCATGACCATTAGCGCGGGAGTCCCAGTCTCAAGCCGCAAGGCTTG
CCTTTTCCTTGGGCTCCAAAAGGGCCAAGCGCCAAGTGCATCGGCTTGTAAACTTGCCTG
AGGCATCTCCCTTCAGCTTGCAAGGATCCGTTGCGCAGTGCAATTCAAAGGCCGAAGAA
>at02674
TTTTTTTTTTTTTTTTTCAGACAGCAGAAGCCTTGACAACTCAAGTCGCCGGGTCTCAG
ATCCTGACTCAACCATCCGTCTTGATTGCCAGCTGAGGGAAGTCAGACTCCAAGATCTTG
ACAGCAGGAGTCATAGTTGCTGCTATACTCACCTGCGTCCAGTACTCCGGCTTGGCACCC
TTGGGCGCCTTGTCTGCAAGGCCCTGCAACAGTGCCGTCAAGTTCTCAACGAGCTTCTCT
CTCCCGAAAGAGGCTTTGCCAAAGACAGTGTTGATCAATGACTTCTTTGACGTCCTCAGC
TCAATGACACCACCGGAGAATTCCTTGATTCCCTTTGCATAGTCCTGCAACACTGTACCT
GACTTGGGCGATGGCATAAGACGCCTCGGACCTAAGATCTTGCCAAGCTTGGCAAGCCGT
GGCATCATCTGCGGCTTTGCAAGGAGGACGTCAAAGTCTATGATCTCTTTCTCGATATCT
TCGGCAAGCTTCTGGCCGCATTTGTAGGCGCCCAATGCCATGACTTCCTCTTCTTCCTCC
ATTGGACAGAAGACGGCAACCCGGACTTTCTTGCCTGTGCCATGTGGGAGATTGACTGCA
CACCTGACCTGCTGATCTGGGAACTTCTGATCTAGGTTCATCACCATCATGCACTCCATG
GTCGGGTCGAATCTCCCTCG
>at02675
TTTTTTTTTTTTTTTTCTCCACTGAGCATCTGTACAAGCTCATGGTCGATCCGCGGCCAC
GCTCGCCGGTGCCAGCCCCCCAACACCACGGAGCCAGCGGCAAAGCTCCTTTGCTGACAG
CGCATCCCTAGCAGCCAGCTGTCGCAGCGCTGCTGTCAGCTCCGGGGGCAGAGGCGCAAG
CACATCACGCCAGCCGCGGGCCCCATCGCGGAAGGCAAGGTGGTAGCGATGCAGAGCATT
CCTCGGGCACCACGCACGGTCAGAGTTCACCGTTGCCTGCGAGGTGTAGCGCCCGTCGCA
GGCCGTGGGGTGGCCGAGGTGGGCGGTGTGCGCGCGGATCTGGTGCTGCCGTCCCGTGTC
TATCTGGACATCAACGAGGCTGAAACCTCTTCGTCTCAGATGGGCATGCCCAAGGACCTT
GAGTCGCGTTCGAGCAGGTTTTCCTTGTGGACTGACCACACTTGAGGCCTCGTGCC
>at02676
TTTTTTTTTTTTTTTTTTACTAGCATGCTTGATTGGCACCGGCGCGATGCGATGCAAATT
CTGGGCATGCCCGATCCGCCCGGCTGCGGCTCACACATTCCAGCTGCAGCCCCGCCCTGG
GTCGCTGGAGACCGTGCCGACACCGCAGGACAGGCCCCAGTTGATCTGGTTGGGGCATGA
GTTGTCGTCGCACGCGATGCACTGGAGGATCGAGGCCGAGCAGCCGTGCTGGCCGCATTC
CTGGCAGCAGCAGCTCACCTTGCCTTCCCCAGCCTGGTCGTCCCCTGCCTCAGTGAGCCG
CGCGTCGCCCCGCAGCTGCCGCAGCGACAGCTCACACTCCTGCGCGGTGCTGCCGGCGGC
GCAGGCGTCGTCGGCGGCGAGGGCCGTCTCCAGCGGGCTCGCGCCGGCAAGGGAGCAGAG
CAGCGCCGCCGGGAAGAGCAGCAGCGTTGGGGGCATGGCGAGATTGGCGCGGACACGATG
CGGGAGGTCACTGCAAGCACAGAAGAGGGAGATCGCAGTTGATTCANCAAGCCTTGCGCC
TCGT
>at02677
TTTTTTTTTTTTTTTTTCGCTGCAACCGAAGCATACGCCGGCTCGAGCCTCGTGGCAAT
GTTGCATTGCCTCAGCACAGCAGGTTGGCTGCAGCAATCAGGAGCCAGAGACGGACTCCC
TGCTTGAGAATTGAAGTAGAAATGAATGCCCTCGTGGAGTGCCCCAGACGGCCTCCCTCA
ACCTCCCTCAGTCGATCTTCGCTGCAGCTTTGAACGCCTCCAGCGTGGTGATGCCCACGC
CCTGCGGCGCCCTCGTCTTGTCGATGTCCTTGCCGCACATCTTGCCGTCCTTCATGATCT
CCAGCTCGATGCAGCCCTGCGCCCACGTCATGACCTTGTCGGTCATCGGCGTGGACACCT
CGACGATCTCGGCCAAGCCCTTCATGAAGCAGAGGCCTGTGGGGACGTCCTCGGCGAGGT

ACCGGTACTTCAGGTCCGGCATGAACTTCCCGTCTACCTCCTTGCACGGATGCGTGAGGC
CGTCGTAACCCTTGTTCGTGTTCATGCACGCCTTCAGGGAGGAGGTGTCGGACATCTGGC
CGTCGTAGCAGTCCAAGTACCACTGCTTCAAGGTGCAGGCGTCCTTCAAGCTGAGGCCGG
GGCAGAGCGCCTCCATCTTGCGGCAGACGGCCTGCGTCTCCTCCGTCATCTTCACAAGGA
CGTCTTCCGTGTAGTCGTCCACGCCCTGGTAGAAGAGCGGCTTCGAGTCCTTTGGCGCGC
CGTCCCAGACTTCGGGGTTCCAGCGGCCAAACATGACGCCGGGGTGCACAACCATCCCTG
GGTTGCGCAGGCTGATGCCAAGGTTGTTCGGGCTCTCCTGCAGCCAGGGTGTGACCCCGA
CAAGGCCCTGCAGGGGTTGCCGAGCGCGTAGGTCGTCTTCGACGGCGGGGTCACCGCCCC
GATGATGCTGTTCTTGATGCCCAAGATCGTCGCCTTCTTGCCCCACTCCGTGAAGCGGCA
AGCCCACGGCAGTGTCTCGAAGCCGCAGAAGATCATGTCCTTGGCTTTGTCGCCGAGCTT
TGTGTTGAAGAGGATGTCGCCGCCCGACCGTGCCGGCATCGTCGCCACGATAGTGCCGGG
CTTCATGTACGGGGCAAACTTCTCGAAGTACTCGCCATGAGCAAACGACGGAACGACCAA
AATGATCGCATCTGCTTGGCTGGCAACCGAGGGGTCGTTGGTGATGTCGTGAGGCTTTGC
CTTGATGACCTTTGGCGTGGCGCCGTTCTCAAGGGGTGTGATCGTGAGCTCGAAGTCATC
GTCCCCAAGCGCTGCCTTCCACTTGGCGGCCTCGTCTGCGAAGAAGGACACGGCAATGGT
CTTGTAGCGGTGGCTGAACAGTCCCGTAGCAACCTGCGCTGCGTTGCCACCGCCGCAGAT
GAGGACCGTGAAGTCGGTCATGGTTCAAGAGCCCGGACCAGCAAGGGGTAGTGAGCCAAT
GAGGGGCACGGCCGTGGGATTTGGCTTGGTCTCCTTTGGGCCAAAATCGCTTGAGCCAAA
ATGGCTACGG
>at02678
TTTTTTTTTTTTTTGTTGACAAAGGCTGAAACCAGAGACTTTGATCGCACTGATGCGGT
CCAGATAACTGACCACACGTAACAGGCACTCACAACATCGTGAGGGTTGTGCAAAAGACA
CAGTCGGTCACTTTACAGAGAACTCCACAGTAATTCCTGTGGATGGAATGCTGCTGGGGA
GTCTGTGTGCAAAGGTGCAAGCAACCCCGCCAAGTTGCTCACACTTCTGTTGCGACAGTG
TGCATTCCAAATACAGGCGGGGCCTTCCACCGCAGCAAAGGTTCTGCAATGGCTCACTTC
TGGGCATCGCACGCTGCAGCGCTGAATTTAGAGGGCTATACAAAAAAGGATTTACTCCTT
ATGTCATCCATGTGCCTCTCTAAACCGCCCTGATGCGCGGAGTCTTGTGTTCAGCGCTAC
CAATGGCGGAAACAACACGATGGAGGAGACTTCTGAGGGCAAGCACTTATGGCCTTCAGA
ACAAGGTTGGTGACGTGCGCGCTGCAGGATGAGCTGTACAATGCACAGCATAAGTACACC
CCGT
>at02679
TTTCGGCTCCAGCAAACCAGCAGCTTTAGGCATCCGGGGCAGCGCGCAGAGGCTCGTCGG
GATGTCCAACACCATGGAGATGAAGGTCAGCATGAAGAAGGGCATCGGCTTCTACATCCG
CGCGGCCAGCTCCTTCCTCAAGGGCGTGGAGGCGAAGGAGGCTGCGGAGGGCAAGGAGGC
GGTTGAGGCGAAGGCGCCCGTGGACGAGCTCAAGATCTCGGGGCTTGGGCGAGGCGATCA
ACACGGCCGTGACCGCAGCCACGCGA
>at02680
TTGTTTTTTTTTTCTTTTTTTTTTGTATTTTTGAACGTGGTCATGGCAGACTCGCTGGTC
AAACTTACATCCGCTAAAAGGTCATTGTCGAAAGATGGCGCCGCTTCATCTACTGCACGC
TCCCCTCGCATCTCCGGCCTTTCAGCACCTCGTTTTCGATCGACCACTCAGGAAATCACC
GTTCTCCAAATCTTCGTCAGAGCTCCACTTTAGCTCTTGCCTCAAGCTTCTGGTTCCTGG
GGGTATCCGATGCCTCGGCCATA
>at02681
GTCAGCCTGACTCTCCCCTCCATTGCTCGAGCCGGAGCTTTGATGGGGGAAGGTTGCCTG
CCGCTGGGAATGCCTTGCGTAGCAACTTGGAAGACTGTGAGCTGACCATTCCAGATCGGG
ACAAGCACGCATTCGGGAAGCTTCATGTTTAGGCCGGCCGCACTTGCGAATGACCAAAAA
CCAGAAGGACTCCCGAAAGTCGGCATAGATCGTGGTCATTATGTCCATTCTCGAGCCCAC
CGCGATGAGCAGTCTGGCCACAGGTTCTATGATGAATGGAAAGCCAGACCGGACGCCGGG
CAGCCATGCCTGATTCCACTCAAGGAGGGCACTGAGTTGCCAAGCCTGCTGCTCATCAAG
ATAGCGGTGACTGCATTGGAATACAA
>at02682
CCCTTGAGGCTCATAGAGCCGTACAAACTCGCTGCGGCATGCAGTTTCCAAAGCGTGTGA
ACCTCGTGTACGGAAACAGGTGGACGCTGACCAAGCGCGGCGGCGGCGCTGGCAAGGTGG
ATTCAGCGCCGGTGGCCGAGGCTGAGAAGACGACAACTTCAGGTATGCTGCGATTGGCGC
AACTGCGACTACCGCTGGCTCCTTGAGCCTGTGAGT

>at02683
GGTTTTTTTCCTTTTTTTTTTTTTTTCCCGCGAGAATCCTTTCCGACAGCGAGCGGTGCC
ACTGCCTCCAATGCAAAGCAGGAGTGTAGAAGGAGACAGCACCCCTCTTGACACTGGAGT
CGCGCACAAGTGCAGGGAGACAGATTGTTGGCTCTGGCCACGATATTTTGCTATTCTTGG
TTTACTGCAAGAAGGCGCATTCGGGATGACGCTGAAGATTCGATTGCAAGCCCTGGATGT
CTGCTGGGGCTACCTGAAGTGGTTTCGTGGAAGGGTACATCCGAAGATTCCTTCCATGGA
ACCGGTGGTAGAAAAGTTCCGCGGTCGCTGCTCCGTGGAAGTTCACAAAGCACATGCCAC
GGTTGAAGGTTCTCCGTGACTGCAGCGGCAGGTAGACAAAGTCAAAAAACCCTGCGAAAC
CGCAGTCGCAGAGCTCTTGCAGGAAGGCCTCCCTCGTGACGTTCTGCGGGAGATGTCGGA
CTACGACAGTCGAGCATCCACTCAGACTGCCGTCTGCCAAGGCACGGTCGCGAGCTTTCT
GTTGATGGCCCAGACCTGCCAAAGGCAAGGCTTGATGTCGCTGGGGCTCATATTCAAAAT
CAGGTCGAGGCACTTGTGGTGGTCCACATGTGTGAACACCTTCAGCTTGAGACCCTCCCG
CTCCGAAGCTTGCAGTGGAGGCAATTAGTGGTTCCAGCCGCCAAGGTTTGTGTGGTGCTC
GCTGCCCTGGCTCACCGAGCACAAACCCCCCGTCGCCGGCAGAGATGCCGGCTCCGCTTT
CGGACACCAACCGCTCGATTCCCCGAGTTCCGGTGAGGATGGCATATCGTTCAACGTCAC
ATCCTCGAAGAGGTCCGTGATGTCGGCATGCTCGAAGCCTGAGAACTCCCTTGCCCCTGG
AGAGGGCTCGATGGGAAATCTGCACAGTGCCGCCTTCAAGCAGTTCATGTTCTTCATATC
GCAATGGCTGGGATGACGGAGAGTGAGGCAGGGCTGCTGCACCTTGAGCCAAAATGGCTA
GAAGACCAAAA
>at02684
TTTTTTTTTTTTTTTCTCTGTCAATCTCTTCACTCTGAAGCAGCACGTCTTGATCGGTG
TGCCCTCCGGCGCCCACCCGCCCCGCCGCCCTGGGCGCCGCCCGGCCACGTCAGGCCCCG
CGCGCGAGGAGCGAGGCGCCGGGCGCCATGGCCGCGAGGAGGGCCTCGTCGCGCTCCCGG
AGCTCCGCCGCCCCGGCGCCCGTGCGGCGGACCGCCCAGATCGCACTGACCACGGAATCC
GGCTGGGCCCCGTTGGCTCCGACAGAGACGAGGGCAGTCTGCAAGGGCGAGATAGTGCCC
ACACCTCCGCGCACGCCAATGATCGACCCGGTGTATAGCGAGCCTGTGCTCGTCCTCACC
GCGCATCCCACGAGCGGCTGCTCGTCCGGCGCCGCGGGCTCCTGTCCACCTTCACTATGT
GGCTGGTAT
>at02685
TAGTAGTTTTGTTTTTTTCTTTTTTTTTTGTTTTTTTTTTTTTTTTTTTCACCTCCAT
CGCGGTGCGCAGCGCATCTGCAGGTCTCTTGCTGTGGCTCTCCTCCGCTTCCGATCTGCG
CGTCTCTACCCCCACTCCACAATCACGTACATCCCCCCCCGCGAGCCCTGAGCCGTGTCC
CACACAAGAGCGGCCGAGCGCCCTCAGTTGTTCTTCGCCCTCTTGGCCTCGCGCTCCTCC
AACTCGCCGTCCCCCTCGGCGTCAGGGTCCTCCTCCTCCTCGTCCTCACCGAGCTCCTCC
TCCTCACCATCCTCCATCCAGCCCTCCTCGTCATCTTCGCCAGCGTCCCAGTTCTCCCTC
CACTCCTTGGCAGTCATAGGCTTCGGCCGCTCTGACTCAGCCTCCCTGTGGCCGTTGGCC
AAGAAGAGCCTCTGGGCCTTCACCATGGCCTCCGCGGCGCCCTTGGCCCCCAGCTTCTGC
ACGAGGCGCTCGACGTCCAGGCGCATGTCCATGTCGACCAGGTCGTCGTCTGGGAAGTCC
TTGTCCAAGCAGCTGAGGTCGACGGGCACGAGGACCTCGTCGTCGGGGCAGTCGCCGGGG
AAGGCGAAGGTGGTGAGGAGCTTGCTGTCGATCTCCTCGTTCCTCTCCAGCTTGATCTTC
AGGAGCAGGAGTGCCTTCTGTGACGCGTAGAAGATGTCCTCCTCACCGCCCTCCAGCTCC
TCCTGCACAACCTCCGCGCCCATGGCACTCGAGACCACGGCTCCGGCCCACAGGAGGAGG
GCGGGAGGAAGGAGGGGATCCAGCTGCTTGAGCCAAAAAGGCTTGAGCCAAAATGGCTAC
GG
>at02686
TTCCGTTTTTTTAGTTTTTCCTTTTATTTTTTGGCTCAAGCGCCGGCAAACAGTTAAGCT
CCCCGACAGCCGCCTCAGCTCCCATACTCAACCGTAAACCATGGACTCCCTGGTGTACCG
TGGAAGCCTCATCGGCCACCGTGGCTGGGTCACCTCGATCGCGACGACGTATGAGCAGAG
CAACATCGTCGTCTCTTCATCGCGTGACAAGAAGCTGATGATCTGGGAGCTCACTCCCAA
CACTGACGCGGTCGGCTATGCACGACGGTCCCTCTCTGGCCACAGTGAGCCAGTGTCATG
CGTTGTGCTGTCCTCAGACGGTCAGTTTGCGCTCTCTGGCTCCTGGGACAAGACGATGCG
GCTGTGGGACTTGAACACCGGTGCTACGGTCCGCACGTTCCAGGGCCACAACAAGGATGT
GAACAGCGTGGCTTTCTCTGGAGACAACCGGCAGATCGTGTCTGGGAGCCGCGACAAAAC
AATCCGATTGTGGAACACATTGGCCGAGTGCAAGTACCAGATCACAGAGGACATGCACAC
AGACTGGGTGAGCTGCGTTGTATTTTCGCCGTCAGCGAAGATGCCCTTGATCGTTTCTGC

GGGTTGGGACAAGCTCGTGAAGGTCTGGAACTTGAGCAACTGCAAGCTCCGCACCAACCT
AGTTGGGCACGCGAACGTTGTCTACACAACCACCGTGTCACCTGATGGCTCTCTTTGCGC
CTCGGGTGGGAAGGACGGCACTGCCATGCTCTGGGACGTGAATGACGGCAAGCACCTCTA
CTCCCTCGATGCGGGTAGCACAATCAACGCGCTCACCTTCAGCCCAAAGAACTACTGGCT
GTGCGCAGCGACCGACACATCCATCAAGGTCTGGGATCTGGAGAACAAGAACATCTTGGA
CGAGCTCAGCTCGACCTCGCCACCGAAGAGCGGCATTCCATGGTGCGTGTCTTTGACGTG
GAGTGCCGATGGCAACACTCTCTTCGCTGGCTCCACGGATGGAAACATCTACGTGTACGA
GGTCGGGCAGGCATGAACCTGTCCAGAGTATCGCCGGACACCAGGAAGGCCTCGAGAGGA
AGGGTGAGCAGGGCTTGGGCCATCCCGCTGCCGACTGCCAGCTGCAACCTGCAGCTTGGA
GGAGGGACCTAGGCATTGGGACAGATTTTGATGTGGCACGGCGAGT
>at02687
TTTTTGTCTTGGTCTGGAAGATCAGCATTGATGCCAGGGATGTGGCCGGCTTCGGCGTGA
CAGGCTGGCAGTCGCATCCGTGCAGCCAGCCTGTCACCAGATCAGCTCACATTTCAAAGC
TGCCGGTCTTGGGGTCGTACGTGGCCTCGACGAGCCTTCCATCCTCATACTTCAGGACGT
CGTACTTGCCGGTCCTGAGGTGTGCCGCAACCGTGTAAGAGCCACCCTCGCTCAGGTCAT
AAGAGCGTACGTCGGAAGCTGGTCCTCCCTTGACCGAGCCGTTTGGCTTGATGACAGGAT
CATCACCGAAGGCATCAGCGCAGACAGCGCCGCTGTTCTCGAGGACCTTCTTGGTCTCGT
AGAAGGGCTTCAGGTCCGGGCGCACTGCTAAAGACGTGTCGTCAAGCCGGTTGACCTGGT
CAAAGTCATTGAGCCGGGAGTCGCGCTCCTCCTCGTAGGCAATCTTGGCGAGGACAATTT
CCAAAGCGCGTCACCAAACCGAGGGTCGCCGGCACGTTGGGGTCGGCCTTGGCGTCAATC
AGCTTCTTGCAAAACTCCGCGGACCCGCACATGGCGGCGGCGTGGAGCGGCGTCATTCCA
TCGTCGCTCTTGTAGTTCACGTTTGGCTTCTTCTTTAGCACACGGTCGAGCCGGCGAGTG
CACTTGCCCCAGCTGCTGATGGTCATGAGCTCGGCATCCGCGGACATCTTCTGCCCCGGC
TTCAGGTCTGCTGCCTCGTCCTTTGCCATGACGACCGGCATCGTCAACGCAGAGTAGCGC
CTGACCAAAGGGGAGAGCAGGAATTTTGCAAAGGCAAGGTTCGCTTCAGCCAGACTTCGG
CCGAAACGGCTTGAGCCAAAATGGCTACGGACTCTGCG
>at02688
TCTTGCGAGCGTCAACGTGAGCGTAAGCGCGAGCGTGACACCGTACAGTGCCCTCCGGCT
GCACCGGCGCTCCTAATGGCAAAGAATCGGGAGGTCTGCATGGTCTGTTTCGGAGCAGCC
CACTGCTTTCAGTGCCATATCTCTTGGCAGGATCCGAGCAGCACTATCCTCGGAGCCCAT
GCCCAGATTCGCGTAGGCCAGACGCAGCACCCGCGACACGTGGTACTCTCCTCCTCTGGA
GCCGTAGGGTGACCACATGTAGTCCTCCAAGCTTTCGATGGGCGTGAACGACCCCATGGC
GCCGTAGCTGACTTCATTCAGCCGGAGCCCATCGGGCCCACCCACGAACCAGTCCGTCCG
CAGACAGTCCAAGCCAACGCTGCGAGCAAGCTGTTCGGACCATCTCTGGATCTCTGGCAC
GTGCGGCTGCAGTGCTGTCCTGCATAGCCGCTGATCAGCGACGGACAACTCACAATTCTC
GTAATACACGGGGTCTACGCTCAAGGGGCCACACTCTGGCCAACCACCGCGGCCGTCTGC
ATACATCAAGAACGTATTGCTCCATGAGCCGCACATCGTGNTAGACTCGATGGCGTACAG
TCTGCCAAAGAGCACCCAAGCTCTGAACTCCATCGGCAAGCANGAGCTTGCGAGTCCGGA
CGATT
>at02689
TTTGTTTTTTTTCTTTTTTTTTTCTTGGAAATGCGCGCTTCAAATGTAGTCCCGCAAAG
CTGTCACTCTCGCAGAGATTGCACATGCTCTCCGTGAAAGCACGCGGGGTTGAAAAGAGC
GGCAGGACTGCCGCCAGACCGGGTCTGGGGCACGTCAGCCGACAACTCCACTGCCCTGGC
CGGCATCAGCACTTCAGTCCTCCTTCGACTGGTGGATGTACTTCACGTGAAGGTTGAGCG
CGTGCTGGGTGTCAAAGCTCTGGTTGCAAGTCTGGCACTTGAAGGCCTTGTCCCCGCCCT
TCACCATGGGACCCTCTGGCGCCGCTTGCGTGGGGCGGGGCGCAGAGGCGCACAGGGCTT
GCAGACCTTGGGGGCAAGCTTCTCGGATGCCGGGGGAGCCATGGCCGCCTCAAAGCCGTG
ACACGCTCGCGGGAGGGCTTGTGCGGAGAGGCAATCGGGTAAAGTGGCTTGAGCCAAAAT
GGCTACGG
>at02690
CACCCGAAGGAACTCAATCCCTGGATCTGCCCTTCCTAGCCGTGCAGCCATCCCCCCCGC
ACATAATGAAGTCAGCCTTCTGTGTGGTCTTTGCGTTCTGCCTTTCGCTGCTTCCAGCAT
TTGAGGCGCGCAGAGCTGGGTCAGCAGTGACGCCCGTGCAGAAGGTCGTGGAGCTCATGG
AGGCATGCTCGAGAAGGGCAAGTCCGAGAAGCAGGCCGAGCAGGTCCAGTTCGCCGCCTT
CAAGCAGTTCTGCGAGGACACGGCCGGCGCGAAGCAGGCCGCCATCGCCAAGGCCGCCGA

CCTCATCGAGAAGCTGCAGGCAGACAGTCGAGGTCTACGTCGCGGAC
>at02691
TTTTTTTTTTTTTTTTTTGAGCCTCACAGACTTTCAATCAAACTCAATTGGAGCTTGACA
CAAACGCACCAACCACACGAGCCAGTCAAAAGAGTGAAATGCCAGCCTCACGGCCCAAAG
TGTGTGCCCTGGGCACAGGCGGCAGCAGCAGCAGCTGCCAGAGTGAAAATTGTTGCCCCG
GAGATGGCCGCAGGTACCGTTCCGACGACCCGCTGGCAGCAAGACAGGATCAGCATGGTG
CAGAGCGCCGCGGCCGCGAGGATCGCGAAGACTACGGCCCGAGCCAGGACTCGCAGGCGC
TGCCTCCTCGCCTGCACGAGCGCCTTCCCACATCTCAGTCTGCCCTCGGGCAGTCGGGAA
TCGACCAGTTGAGCGGCGAGCGCCTCGTCACTGCCTCTCGCCTGCTGGCACAGCTCGAGC
TCCGCAGCCGTCAGCTCCAGCTCTGAGTGGGCCTCGGCGGCTGTCCAGTGACTCAGCCCC
CCCCAATCCTCATCGCCCAGGCAATACAGGATGCCAGCGCACACAGCAAGCCACATGAGC
CAGAACATCGGCTTGCAACGAGACCTGCCAGTGGGTCCAGTGGTTGCAAGCCCAGCGCAG
GCCTCTTCCTCCGCGCGGCAAGCCTGGCCCTGGGGAGGCGGCTGCGCGGCGCAACCCTCG
GCCGGCGCCGCCGAGGCTGCCTCGCCGCCCTTATCTTCAGACTCTGCGTGCGGCAATCTT
GCTGCCTCAAAGCACGCCTGCAGCTCCGGCGTGGCGCTGTCCCAGCTCGTGGGCGCGCGG
GCCTCCGTGGAGCGCCGCGG
>at02692
TTTTTTTTTTTTTTTTTTGACCATCAACCAGCAAGACTGGAGCATCAAGATCCCTCGATG
GTTCGCATCTTCACACGGTGCCTATTGACACTCAACACCGAAAGGATCCCGCACAGAACA
TTCACTGGACAAGCCCCGGAAAATGAAGGGCCCGACCTCAATGTTATAGTGGCGCATCAC
TAAGGGATTGTAGCCCCAGCAAACACGGTACGTACCGACCTGTCCACTGTTGGAGCGTCC
TAGGCGGAAGGTAGCCTCCGCAACGCCATTAGTCACCTCTCCAACAGGCACAGGGTTGGA
CAGGCCATCAAAGTATGCAACAGGAGGGCTGTCTGTCCCGCCAAGACCCACTGCCCTTCG
ATGATGAGTATCCCGTTGCCGCGCCTGAACTCTGCCCCGTAAAGCCGGAATGCGCACACA
AGCCCGAGCATACACTCGATGGAGTACGCTACGAAAGGGCCGCTCATTGTGAAGCTGCCG
ACTTCCACGACGTGATTGTGCTCGCTACTCACGCCCGCNGTGGATGCCGTGTGCCCAACA
GAGTCTGTAGTGACTGCCAATGCAGGACGCAACTGGNGGTGGGCACCCGGCATGTGGAGT
AAGCACCGCCGACGACTACCATGCCCATGCTGTACGAGTTGTCGTACAGGTCGTCCGTGA
CAAGGCGTGGATTCTCAATCCCCCGGCAAGAGCGCCTGCGTGATATCCGAAGAGCCGCAA
GCCTCGTCACCCGCCACGATCATGAGCC
>at02693
TAGTCCTATTTTTGGTCTTCAAGCCGGGAAGTGGGCTCCCCCAGCTCCTTCTCCTTCCCG
CCACTTGGGCCGCCATGCACGTAAGCGCCCTGCCAGGCCTTGGCTGCGGCGGCGGAGGCG
CGCTGCACTTCCTCCCTCCGGCAAGCCCCAAGGATGCCGCGTTTGGGGGTCCTGGCCTCT
TTGGCCCTGGC
>at02694
TGCTGAGTTTCGGGGCGCTGGCCCATGCTGTCAGTGTCTACCTCCAGAGGCCCTGGGAGG
CTCGTGAAGTGGGCGGACAGCGCCGCCATGACGCTGGCCATTGTCGAGCCTCTGCAGTTT
GGCGCCAAGATCGCTGTGGGGTTCGACTAGGCCTGACAGAGCAGGCCCACTTGCTGCGTG
GCTTTGCGCGGCAGGCGGACTCGGAGAGCTCTCCCCGCGGAGTGAGGCTTGCGTCGAGCT
CCAGGCCGCGGCCAAGAGGGAGTTGGGCCTTTTCAAAAGGCTGGGAATGGCCATTCCTGT
CAACGCAGCAGCAAAGGCAGCATCAAACGCAGCAGGCAGAAGCAACCATCCAGCAGCAAC
CAGCAGCAGCCAGCAGCAGCTGCCTTGTCAATCTTCTTGCGGACGCGGCCGACTGCTGTA
AGAGAGCTATGATGCTTTGACCAGCTGGGA
>at02695
CCGTAGCCATTTTGGCTCAAGGCCTTGGGCTCAAGAACTGTTCCATGAGCATTTTGGGCT
CTGCCGCTGCTCCGCCATGATTCGTGCTGCCTTGGCTTTGAGCATTCCCTTGGCGGCTTG
TGGGCTGAATTCGAGCTGCGTGTCCTTCGACGGCGAGGTCGCGCATGGAAGCTTCCCCTC
GCTCACGTTGTCGCATCCAGGCCTTAAGCTCGAAAGCAACTCCTACGGAGTAGGCGGCCG
GAACACTTCCTTACATCAGTGCGTGATCGAGTACGGCAACCAGGGTGCCTTCGGTTGGAA
CTGGACTAGGGGCCACACCAGGCCTCAGTGCGTCACCTCCTGTAAGGCCCCAGCCTGCTT
T
>at02696
GTTTTTTTTCTTTTTTTTTCTCTGTCGCTCCCCAAGCGACCGGCCACGCCGTGCGGATGC
TGATGCTGCGCGCGGTCGTCAGCGACCAGCCATCCACCCCTCAGTCGTCGTTGTGCAAGG

CTACCATCTCCTGGTGAGTGCTCGACTCCATCACGATCCTCATGGCGCCTTGCAAGGACA
CCTGGGCCTCTGTCCCAGTATGCCGGCGGAGGAGGAATACGCCGAGGATGATGGCCGTGG
CGAGGATCTCGTAGTAGGCAATCAGGGAAAAGGACGATAGCAGCGCCAACATCCAAGATG
GCCGCTCTTGCCTCTTCACCGAGACGACGCTGTTCTGGCAGGAGTACTGCGTGCAGTCCA
CTGCGCCCTTGAAGGCGCGGTACTCATCGCACGAGACACCGTGCAACCTACACGGAAACT
CCATGCTGAGACTCGCAGCGCGTTGGCGGAGGTCCTGCTGCATCTGCCGGAACCGCTCAT
CAATGTGGCTGGTGATCACCGCCGGCGGCAACTGGACAGTATGATTGAAATGCAGCCTGT
AGACAGTGTCAGTGCACTCATAGGCATACGCCCCGGCCCAGTCAGTGCATGGGGAGTCAA
ACCACCTCGGAAGCCCATCTCCCACGGACTCCCCGGTGGAGATCATCATCTTGGTGAAAC
AGACCCCGTCGGGATGCCCCAACACCTCGAGCTGCGTCGCATTGACCGCACGTGGCAAGG
GGACTGATGTCCACAAGTATGGGCTCCAACGCACTTGCGCTGCCT
>at02697
TTTTTTTTTTTTTTTTTGGGACGAATCGCATCACCGCTCCAGCACCTACGTCGCAGTTC
ACGCCTATCGGCAGGGGCCCCCTCGCGACTGTTGCGAGCGGGCGAGCCCCCCGCCGCGCG
TCCGTGCAAGCCGGGCCCTCCGAATTGCCGCGGCTCCGACGCCGGGGCGCGGCCCGGAGT
CCGCGGCGGCCCTCTCCCGGGCGCCGACGGAGCGGCCCTCTCAGTCGGGCCGCCTCACGT
GCCAGCCGTGCTGCCCCAGCACGATGCCGCGGGCGGAGCAGCTGCCCTCGGCCGGCACGT
AGCGCCGCGGGAACTGCTCGCCGAGCCCTCGGCTGGCGGGCCTGTCCGGCGCGAGGGCCT
GGAACGCCGAGGCGTACGTGGAGCAGAAGCGCTCGTCGGGGTGGAGGTCA
>at02698
TTTTTTTTTTTTTTTTTGCCAAATAATCGTTGACTTCAAGCATGTGGTTCCTGGTACCA
TCGCACGAGGATGAGTTGAGGCCTTCATAAATGGACAAGAATAGGCCGTTTGCCGCCCTG
AAACCTTGGGCCGCTCAACTGGAACAAGCCACGCATCAAGTACTACGTTAAGGGGACGCC
AGCTGGCATCACCATCTCCAATCGCTTTGGAGATTGCAAGAGTAGGCAGACTTAAATAGT
ATCCATGAGGAGTGCACGCCGATCCCGGCGCCAAGCGACACTGCCACCTGCCTTGCTGCA
TCTCTTCTGCCACTCCATACGCGGGCGCTCAGCGGTTCTTGCCCTTGCCTGGACCCTTGG
CATGCCGGTGCCTCCCGCCTTTCGCGGAGCCAGCTGAGCCTCCAAAATCCAGCCACGTGC
CCTTGCCGTCCACCTCTTCGCTGTGGAAGGTCAGTGTCTCCTCGTCGCCCTGCAGCCTGC
GCGGCGTGCAGGAACACGTGCAAGGGCTCCACGTCGGCGGGCGGCAAGTGGCCTCTGAAG
AGGCAGTCGAAGAGTCTGAAGATCGACTCCGAAAGCGAGTGCGCTGCTTCCGGCCCTCCG
CGTGAATGACGACGCGGTCCACTCTCCGCAGCCGATGCTGCAGGGCGTTGATCCTCCGCA
GCTCCTTGCGGTCACCCTTCCGCAGCGCTGCTGGC
>at02699
CCGTAGCCATTTTGGCTCAAGCAATGTGGGCTTGAAGTCATTTTGGCTCAAGTTCAATTT
GCGTACCCCCATATCTTGGGGCCGGTGGTATTCAAACACGAAGACCAAGCAAGCAAACAA
AAAAGTGTGTCCTGACATGGAGCGCCCGGTTCAGCAGGAGATGGCGCCTGCGCTGGCGCC
CCCGGTCTCGGAGCCCTTCAATATCGCCAAGCGCATGGAGCAGCTAGGCATGAAGAGCGA
CCCAGATCCCTTTGGTTGCTGCAGGTTTGGCAAGTACATTGTAGTCCCCGACAACCACCA
GGCCGCGCTGTTCTTCTGCGGACGGTACACAGGCACTGTGAAGAAGTCTGGCTGCTCTTG
CATTGGCTTCTTTGAGGAGGTCAAGATTATCAGCATGAAGAGATACACGCTCAACTGCAA
CAAGGTGAAGGTTCTCGACCTGAAGGGCAACCCGGTTCTGATCTCCGGGGTGGCCACGTA
CCATGCGACCAGCGCGAAGAAGCCGATCACCGATGTCTCCAACCCGTGGCCTGGCAACTT
GTGCGAGCCAGACACGCAGTCCTTCATAGAGCTGCAGGCCCAGGCGGTACTCAAGAAGGT
GGCCGCACAATACCCCTACGAGGCCCCGGAGGGTCAGGCGTGCCTCTTGAGGGACACGAG
TGGGATTGCCGCCCAGTTCCGGGAGGAGCTCCAGCAGCATGTCATTGAGACCGGGTGCGT
CATCGTCTCCTTCGACCTCACAGACCTGTCCTATGCCCCGGAGATCGCCCAGGCGATGTT
GCAACGCCAGCAAGCTGAGGCCACAGTGGCAGCCCGGAAGCTCGTGGTTCAGGCCGCGGT
GCAGATCGCGAACGACGCCGTTGTCGAAATCGGGAAACTCGGCCGCACTTTCGAGAAGGA
TGTCGAGGCCGGCATCATCCGCAGCCTCCTCACCACGATCGTCTCAACCCAGACCGCGAC
GCCCACGATGCCCGTGTGAGAGAGATAGATCGATAGATCGATCGATGGGGACCCTTGCCC
TGAGCTGAGAGAACATTCTGGATTCAATCGTCGCCTTGGGGCTGGCATGCAGATGTTTCA
GGCTGCCATCCCCTGCAGATGCAGTGGATGGAGTTGTTCCCCCCAGAAAGTGCTTGAACT
GAGGG
>at02700
GTTCCGGTTGCCCACATGGCAAGGTCAAGCAGGACTGTTCTACGTGCAAGGGCTGTGTGC

ACGGCCGTGCGAAGCGCAGCTGCAAGTTCTGCAATGGCTGCCCGCATGGAAAGCTCAGGA
AGAACTGCGTGAAGTGTATCGGCTGCCCCCATGGAAGGTTCCGCACGAGTTGCGCCAGTT
GCTGTCCATGCCCTCATGGACAGGTAAGGCGGAAATGCGTGACTTGCACGGGCTGCATCC
ATGGGAAGATCAAGTGGCATTGCAAGGAGTGCAAAGGATGCCCGCACGAGAAGTTCAAGC
ACAAGTGCTCTTTGTGCAGTGGCTGTCCTCATGGCAAGCTGA
>at02701
TTTTTTNTTTTTTTTTTTTTCTATTCCCAACATCGTCCTGACGCTGCGCCTGGGTGTAAA
ATCATCAGGACTGCTCCAGCTATGGAGCAGGCTCCACCCTTTGGGGTACAACGCTTAAGC
ATCGAAAGGGGCGATACAAACCTGGTGGCATCTCTGTCCAGGTCGCAGAGGGAGCCAGAA
ATGCACAAGCAGGCGCAGGCTTGTGGCGGGAANCATTA
>at02702
GAGGGAGCCGACTTCGTGCGCGAGATGATTGTAAGAGGCCGCGCCCCGGTCCTGGTGCAC
TGCTCTGCGGGGAAGAGCAGATCCACGGCAGTGGTGGTGGAGTACCTGATGAAGTACCAG
GACATGTCTCTTCGACAGGCCATGCTCCACGTGCGAGGGAGGCGGCCCAGGGCGTACCCC
ATTCTTAACTTTTGGCTGGAGCTGATCGACCGAGAGGTGGAGCTTCGAGGTTCTACCTCC
CTGGACCGAGATGAGGTCAAGAGGCTGCATAGCGAAACTCGGAACCAGGAGCTGGCTGAA
GAAGGCAGCCTTGCCTACATCCTTTCCCAGCTCTTGGCCATGGGAGCTAATGAAGCCGAT
GCTCTGGCAGGTATCAGTAGGCATGGAGGCAACCCGCAGCTCTGCGTGGATGAACTGT
>at02703
TCGGCGAGTTTGGGGACCAGTGGGCGCCGCTTCGTGACGTAAACTAAACGTGCGGCCGCC
CTGCCGCGTGTGCAACGGTGAGCTTGCAGCTGTGACCGATCGGCCTTGGTGGACTGCATA
GCGGTGCGCCATGGCACTTCCACAATGCTGCCTTTGTGGAGGTCCTTCACGGCTCCAAGC
ACTTCGCGCTGCTGCCCCCGGGGGACCCCGCCATAGACGACATCAAGGATGCGATCCCAA
ACCTGTCGCAGCTGCACTGGCACCTGGAGGAGCGGCCAGCATTGGAGAGCGCTGGGCGGT
TGTCCAACTTGCAGGAGTGCGTGGTCCGGCGTGGGGAGCTGCTGTACTTCCCCGACAGTG
GCACCATGGCGCCGTGAACCTGGCCCCCTACACCGCCTTCGTCAG
>at02704
TTGTTTTTTTTTCTTTCATTTTGTCCGCGCTCGTGCGAAGCCCCACGTCCAGCCTTCATT
CCACATGCAGCCCCCTCTGTGGTTGCATGAATTGCAGAATGCACGCTCAGATTGCGCCCG
TGACTTGCGAGGCAATAGCGGAATGCGCTGCATGACTTCTGGATGGGTGCCAGCAATCAG
AAGTGAATTTCCCCGCACACAGGCCACAGGCTCCAAGAGCAGCCACCGAAAACTTAAACC
CTGAGCCTCGCGGCTCCACGCATGCACATGCAAATGCCGGTGTGCCCCCGAAGGCTGTCC
CGGAGAGGACAAGTCCCTGGAGGACAGCACAGTCACGCAAGGCAGCCCTAGTGTGCAAGC
CATTGCACAACCAATTAGAAGCAGGTGTGGTGGCTGCCTCAAAGCTGAGAAGCAAGTAAT
CAGTTGACGCCGGTGCCCACCGACTCTCAGAGGATGCCGCCATAGTGCCTCTGGAGCCGC
TCTGCGTTCTTGCCCCCGCAGTTGAGGGCCACCCATGGGTGATCCAGGACCTGCTCCAAG
GTGAGGCGCTCATGGGGGTTCTTGTTGAGGAGCTTGGAGATGAGGTCCTTGGCCTCCTTC
GTCAGCTGCGGAGGGAAGACTGGGGTGCCGGTCTTGATCTTCTTGTAGGTGCCCTTCTCG
CTCGTGTCCTCGAAGGGCGGCTTGCCGACCAGGAACTCGTAGAGCAGCACGCCCAGGCCC
CAGATGTCAACGCGGCTGTGGTACCGCTTGTTGTGCACCATCTCGGGAGGCAGGTAGTCC
AGGGTGCCGCAGAAAGTCTCGCGCCGGGAGGAGGGCGCGTGCACTGCCCAGCCAAA
>at02705
CCGTAGCCATTTTGGCTCAAGTTCAAGCCGTTTCGGACATCGTTCTGCCCAGGACCCCCA
ACCCCCACTTCTGCAGCTATGGCGTTCTCCATGGGCCCTGCCTTTGTACCAAGCTCCACG
GGAACCCATGCGGAGGGGGTGCCACTGCTCAGGTCATCCTCCATGCAGCAGCCGCCAGCC
TCAGTCACCAGCCAGTCGCCCGGACTGGGGGCCGTCGCGACCATTGCCACGGCGGCAATC
GGCCTGGGACTCGCGAAGCGCGCACGCCGCGCATCCCAGCCACAGGTCGCGCGAAATGCG
GCCCGCCGCGATCTTGCCATTCGCCTACGAGGACTCCTGGCATTGACCTCATGGACAACG
GCAAGTTCGCTCAGGGCCTCGTTGGCTCCGAGGGCGCTTGGGGCCCATACGAGTTCGACC
CAGTCGGCTTCTCCAAGTACACTGGAGCTGGTGCCCTTCTTCCGCGAGGCCGAGCTGAAG
CACGGGCGCATCGCCATGCTTGCCTGGGTGGGCCTCGCCGTCCCCGACTTTGTGCGGATC
CCAGGGGAGCGCTACTCCTTCGAGGCGTGCCCCGAGATGGGCAGAAGCTACTCCTTCGAG
GCCTGTCCGGTGACCATCGATCGCGCACGACAAGCTCAACGGTGCCCGATGGGCGTCAAC
TTCCAGAGTGCCCGGTGACTACTCTCGGATTGCGCTCCCTGGACGTTCTGCTGCGCCAAG
AAGGTCTTGGCAGAGCTGGAACTCGCCTTGGAGTGCGCATGGCGGCATCTACCCAGGCTT

TGGGCATGACCTTCTTCCCCAAGGACGAGGAGGGCCAGACGGAGGACTGCGCGCTGGCAG
AGCTCAAGAACGGCCGCCTCGCCATGGTTGCCTTCGAGCGCGTCAGCCATCACGCATGAT
GCTGTCATCACTCGCCACCCCTTCCCCTGGCTGTTCTGAGCTGCGGTGCCACGGGGTAGC
CGCATGGGAGGCCTGTCGAGGTTGGATTCAGGACGTGGTTTTTAAATGACTCCCGATGGC
AAAAGAAAAAAAAAACAA
>at02706
TTTTTTTTTTTTTTTTTTGCAGCCGCCCTGGACTTGGGTCCCTTGCGGCAGTGCCGTCGC
CGCGTCGTCAAACCCTTCGCATGGCAACGGTCGCGCAATGGCGCTTGCCGGTCGGCAAGC
GAAAGACCAGGCTCCCAGGGCAGCAGCGGTGCATGATTGACTTCAAACGCTACGTCGTCG
GGGTCACCAGGCCAGTCCTGCGTCGAATCACCAAGACAGACCGGCTTCGACGCCAAGAGC
GGTTTCAAAGCTTCTTTGTAAAAGGCCATGAAGTCGTCGCGGGCGATGCAATTGGTCCAA
ACAAGCGTCACGAAACTTTGGGCATGCTCATGGCAAAACTCTCGGGGAGCGTCTGGTGCG
GCTAGGCCACCTGCACGGTGTGTGACTCGTAAACGGCGTTGCCGCTGGCGGCCACTTGGG
TGGGTGCCACTGATGCCGTGGTCCATGATGGCAAGATCGCCATGCGAAAGAATTGTGGAG
ATTTTGCCTGTAGGACCGCCGGCCGGCTCCCACCANAGTGTCAAGAAGCCATCGCCGCCG
>at02707
TTTTTTTTTTTTCTTTTTCAACTGGTTCGGGTGTTTCGAAGCGGGGTAATTCTCGAGCAC
TGAGCCCTGGCTTGCAACAGCTGTTCAATAGAATTCGTTGTACTGCGCAGCCTTCTGCAG
GCCCTCGTCTGCGCGAAACTCGCGCAGGCACTGCCTGGCAATCTCGACGGGGGAGCGCCA
CTTCTCGTCGCCCTCGTGCTTCACCACGAGCTGGCGCATCTCCTCGAGGATCTCTGACTC
CTTGAGTTCGAGGGCGGCGGCCTCATCCTGAGTGGCGACGTTCCTCATGGTCTGCAGCAC
GGAGCGGACCACGTTGGGCCTCTCCTGGTACCGCGCCATGACCAGCTGCCCCACCGCCAG
CACGCGCACGTCAGCATTGTTCAGCCGGGAGGCTATGTGCGGCTTCCGCCCT
>at02708
CCGTAGCCATTTTGGCTCAAGGCGGCTCGAGATCCACGCCCTTGGGGCCTGGACGGTGCA
ATGGTGCCCGGAGTGTGAGTGCCAGGTGCGTGCCTGGTGCAGCAGGCGCAGCCTATGGGG
AACTGCAGCTCTTGGCTGAGGCCTGCGCGAGGCTTCGCGAGGCTGGACGAGGAGCGGGCC
CAGGAGCGGTGCGAGTTCCTCGCGGAGGGCGAGCGGCTGCGGAGGCGCTGGGAGGCGGAC
GCCTGCGCCAGGGAGCTGGCCGTCTCCATGCTGGGAGGCCAGTCTTGGAACATCCGCGTG
AGCTCCGACGCCACCGGTGCCGAGCTGCGGGCCGCGTCGCGACGGTCACGAG
>at02709
TTTTTTTTTTTTTTTTTTCCGTGACAACAGAGCCGCAGCGCCCCTCCTGTGCACGACTTC
AGGCATCAGAGTCGCCAACCCCGAGGGCAATTTGGGATCGCAATGAAGTATTTCACTCCA
AAGTAGGCTCAGCTTGTTGGGAACAGAACAAGAAAGTACGAGGCAGGCAAACGGCTTCGG
CGATTAGCACGAATGCGGGAAAAAGGACGTGAGGAGCATCTCTGCACTCCAGAGTCTACC
GAGCAAGTCCCGGGGTACCAACAGGCTTTGGCTGGATTGGCAACCTAGCTCGTGAGCTGC
CCAGGACATCGCAAGAACATACTCCGCGTTGATCAGACTTCAACGCGTCGTGCCCTCGCT
TGGAGTCCTTGCACAGGAGCAGCATGCTCGCAGCCACTGGCTTGAATTCCCGTGCAGCCA
CCCGAGCAAGTCACCAGAAGTTTGCCCGGCCGCTGCTGCCTTGGGCTGGTTGCCTGGCAA
AGACCTGCTCCGACGAGCACGCGTGGAAGCACCCGCTGGTGAACCACCTCCCGCCGCCCA
GCCTGCTGCGCGAGGGCGCAGGTATTCTCCTGTCT
>at02710
CCGTAGCCATTTTGGCTCAAGCCATTTTTGCTGAAGACTTTCTGATCAGGCGTCGGCGCT
TCTGGCAATGACACACTCCACACCCAACCTGATGCGGAGGCAGCATCACCGCGGGGAGTG
CGCTGCTGCTTGCATAGTTGGTGCCTTCGTGGCTCTTGGCTGTGTTGGTTGTGGCGGAGA
TGACTCCACGACGACTGTCACTACAACGACGACATCGACAACGACTACGCACTTCCCCGT
GCACAGTCCTTGTGTGGTCGAGAATGACGCTGCAAAGTGCGTGGACTACCTGAACAAGCA
GTATATGGGTTACGACCCGGCCAACGAGTCCAGCCCCCTAGGCCTGGCCATCCGTGGACA
GCCGCGCTTCAACGATCCTTGGTTCTTCTGCACCCAGAAGTGCTTCCGTGGGCAGCCGGA
TTGCTACACGGCTGCCTCAATGATGAACCACATGGTCATGATCAAGAAGGACGCGAAGAC
CATTGCCATGCCCATGAACTACCCCGTGGGCATCATCTTCCAGCCCGATGCTGTAGAGCA
CCACCTCACCAAGTGTGCTTGGCAGTTCGACGGTGCTGCGTTCAACCGCATGAATGGCGG
CTGCGGCCCCGGACGGCCCAGCCACGATTGCAGCCAACCG
>at02711
CCGTAGCCATTTTGGCTCAAGCCGCAGCTGCCGCAGCTGACCACTGCTCCGTGCCCAGTT

CCAGCGGCCTGCTAGGAAACCATGTCGGAGGCCAAGGCCCCCTCTGCTGAGGAGCTCGCT
TCCAAGGCGGAGGCCCTGAAGAAGGTCGAGACGAAGGCAGAGGGCTCGGCTTTTGACGAG
GCCCCGTACAAGGCTGCCTGGGACGGAGCGGGGGGCGACGCCGCCAAGGTCGCGGAGACC
CTTGGCTTGAAGAAGGTGCCCGCAGACTACGCAGAGTACATCAACATGTGCAAGCTTGGA
AAGTTCAAGGACGCCTGAGCGGCCCGGCTTCCTGCGGCATCGCGGGCAGCTTTTAACTGA
CATGGATTTCAGAGACTGGAGTTAGTGGGGGGGGCGGAGGGAGGAGTACTAAAAAAAAAA
GAAAAAAAAACAA
>at02712
CCATGCGCTCGGCATGAGGCCATTGCCCATCGTACTGCCGCCCTGGGGGTTGGCTATGAG
TCGCTCTTGGCCCTAAGGACGCGGCAGTCGGCCTCGCCCTCGGCGGCCGCGCCTCCTTCG
CATGCTGCGGCACGGGCTCCGGGCGGCCGCGGCGTTCTTGATTGTCGTGGTTTCCCTGAG
CATCAAGAAGTACAAGTTGGACAGGCCGGAGCAATGGCTCGAGGAGCGCAAGGACATCGT
CCTTGTGGACCATCCAGCTCAAGGACAAGACCTGAGCTTCATGTGGTACTTCATGAACTC
GACTCCTATCGAGATCAAGCAGCCCTTCTTCGCAAAGTGGGCAGAAGATGCTGCTT
>at02713
GGTGACTCGAGCATTCTGCGGTCCGACACCCTCCGGTACCGAACGCTCCCGGCATGCCGA
CCATCATGTCATACGTGTGCTTCCTCCTGGCGGCTTCTCTGCCAGCGCGTGCCCTGCAGG
TGCCAGAGCTCCGGCAGCCAGGCCCCACCGCGGCCAGGGAGAGCTACTGCGGCCACGGAA
AGCTGCTGCCCGAGCTCTACGTGCTGGGCCCGATGAGCACAGCAACGAGCTCTTTGTGGC
TGGACATCAGAAAGGCAGGGTTCATCACGGCTCCTGACAACACGACCAAGGAGTGGAACT
TCTTCTGGAGCTCCGCGGGCACGCCAGTCCAGGCGATGTACGAGAACTGGTTCTCCAGTC
TGGCGCCCTGCCCGCA
>at02714
TCATCCATTGTGGGCTTGAGCCCAACACTGCGCAGCGTCTGATCTACAGCAGGCAACCGG
AGCGCTTCTAACCATGTGAAAAGCGGCACCAGCTGGCCAAATGGAAGTGCGGCAAGGAAG
ACAACACAACGCACGATGGGAGCCCGATGCCCAGCAAGGTGGCTGCAAGNGGAATGCATA
TGCACACTGAGCCAAGGCGAAATCGCCAA
>at02715
TTTTTTTTTTTTTGTTTTGCAATGCTTTGCTCAAGTAACAAGTCTTTGGTGGCCTGTGTC
AGGGAAAAAGACATGGACTTCTCATGATAGAAGATTACATCCTTCGCGTCCTCCTCGGTC
TGAGCACCAGTGCACTTGACATGCGGGAAAATGCACAGCTTCATCAGGCCCTTTCCTCAA
CCATCATGGCTTGACAGCATATGCTTCGCGCACGGTCACGTACCCAGTCGTACCCTTCAC
GATGCTCTTGATCGGAGTCGATTGGCAGAGGAGGGAAGAAGTCGGAGAGAACGTTCTTGA
GGGCTTTGTTGTAGGTGATCCACCTGTAATGGCGCTTCGTCAGCAATGCTGCCACATACT
CCCCCCAAGTCCCCGTATCTCCTCCGGTGTCCCATACTCGTCCTGCCAGCCTTCGGAGTC
TAGCAAGGCCGATTGCATGCCCACGGCTCGGCGGCCTCTGAGGAGCTGGCGAAGACTAGC
TGTGGTATGCAGGGAGAGTAGCTGCGTCATGGCAGCAAGCAGTGCCATGATACTTGACGC
ATAGAAGGGAAATGTATCGTCCATGCCAGCGAGCCTGCCAGAGATGACCGGAGCAAGGAC
TGCCCCAAGG
>at02716
TTTGTTTTTTTTTCTTTTTTTTTTGCATCCAACACTCCAAGGGCTCGTTTGGAGCTGTG
AGCCCCCACCCCTCCTCTGCCCCCATGGCACGGCAAAGCCTCACATGTATGGGTGCAGCG
CCTTGATCCCAGCGACCACCTGCTCGTATGTCTTGGAGTACGTCGAGTCGCTCGGCGGCT
CGAGAACCATGACCAAACTGATTGGGCGCTTCGTCTGGCAAGCACCGCCCAGGACATGGC
GCGAGCTGACGTAGCAATAATACACGCCCTTCTCCTCGCAGAGGACGGGCACGTGCGCAA
AGACGTCCATGGGGAAGATGTCCCCGGCCAGGAAGACGATGCCCTTCTGGCCCTTGCGGA
GCGCCTTCGTGCACTCGGGCACTCCGCGCTTGGTCTTCTTCTCCGAGACCGCCTTCTTCA
GGAGCTTGAGCCCACGGGCGAGCAACTTGTCCGTGACAAGGGGCTGTGCCAGAGGCGAGA
GGTACAGGGACTTGGGCTCCTCCTCCTTGTCCCCCTCTGCATCGGACATCTTGATAGATA
ACTTTACCGGGGAGGCTGCCACCACCTAGCCGGCAATAGACGAAATCAGCTGCAACACTT
GAGCCAAGATGGCTCAATACTTGAGCCT
>at02717
TCTCCCCCTGGTCCCTTCGTGACAGTGGATGGCCACCTCACAGCGTTGCCTCCTCGGGCC
GCAGTAGTGTCGGCAGCCCCCACGGGAAGCCGGAAGTCTTCCATCAACCTCCAGCTGGCA
ATAGAGAGCTGCTGGGTACGGCAGAGGAGGTGTGAACGACTTGCGAATTGGAGGAGGAGC

TCGTCGACGACCCCTAGGAGCCGCATCAGGCCAGGTCCAGCTCCATGGCCAGACAGAGGT
GGCTGCTCTTGGAGGACATGCTGGCCTTCGGACACACGTGGACAAAGGATCTAGGATGCT
CGGTGCTGGTCAA
>at02718
CCGTAGCCATTTTGGCTCAAGCCATCGCGCATGAGCTGAAATGGCCGCGGCCTGCGGTGC
CATGGCCTGGGGGGCAGCGGCCGGGCACCTGCAGCGGCCGAGTCCTCCTGGGCCACGAGA
GGAACTGGACTGTGCGGTGAGTACGCCTTCGGGGTCAGTACCCGCAGCGGTAAGTACGCC
TCTGGGGTCAGTACTCGCGCCGGGCGCAGCAGCTGCGGTGGGGCGGACTCGCTTCGGCTC
CTGCGACGCCGGCCTCGTGTTCAGCTTCTCGAGGGCAAGCAGCTCGAGGACGGCGGGGAC
CTCTTCGGCTTCGCCCTGCAGGAGACCTCGTTGCTCGCGCTGCTTGCCTTCGCTGCCGCG
CAGACTCTCCTCACGAA
>at02719
ATGGAAAGCGCGCGCAATTACCTCCTCAGTTCTTCCTCCACATCGCCACAGGGCTCCAGC
CAGCCGCCCAGAGTCCCAGAAATGGCACCAGCAGACCACCCCACCATACAAATGGTGCCA
TTGTCCAATTGGGAAGATGACTCCTGGCACCTGCAGGGANCACGAGGTTGTCCGCAAGAA
AGTAGAGGAGCCTGTTCTCTTGCTCCGGAGTGTCAGCCAGAGCACGAATCTCAAGATCGA
CGTGCAGCCACCCTCCTGGTCCTTCATCCACGCTACGCAACGCCCAATCGCCGCCTGGGC
CTCTCCCTCGCTG
>at02720
TCGCCTTGTGATGTGCCACTCCGCTCATTTGCGGAGTCCTTTGCCTCCTTTGCACCTGCT
TTCTGATCAGAAAGCAAAGGCCCTAGATGGAATCCAGTGTGGCTTTCTGAGTCTTGACGA
GCACTCTGCTCTGTCAGTCAGGCGCAAGTCCGAATGGACTACACCCACGATCATGTGCTC
ACGCATGGCTGC
>at02721
TTTTTTTTTTTTTTTGTTCAGGCATCGGGCCATTCTGCTGAAACAATATTCATCCAAGCT
CAGCTTGCTACACCATGCCCACGCATCACGCCTGTAAAGGACTGCTAAGTGCGACTCCAT
CTGCATCGCCTCAGGACACCTATCAAGGTTGCGGGCGATCATAAGGCCCTAGCTTGTCCG
TTGCCGCTTCGCATCGGGCATCGCGCCGTTCCAGGTGAACTCCGCGGTGTCTGTGTCAGC
AAGGCCCGAGTCCATGGACACGATGCGGCCTACGCACCACTTGCCCGGATTCCGAGCATC
CGGCTCTAGGGTCACTCGCAGCTGCCACCCGATGGTGAGGCCGCCACTCTGGCCGAAGAC
CGAGCGGTGGATGTACGCCCGCCTCCCGTCGCAGAGCTCTATGAAGCCGTAGCCCGAGGC
CTCGTGCCACTCGGTGACGAAGCCCTCCCCTCGTGCC
>at02722
AAGCTCCACTTCTCCAAGTCTGATGACAACGGTCGCCGCATGGGACAGGCCTTCCTCGAG
TTCAGCTCTGCAGCTGCCCGGCGGCGTGCCCACACCAAGAACGAAAGCTACGTGGCGGAT
TGCCAGCTGCAGCTCCACTACACTTCTCAGCACAGCCTTGCCGAGGCCGTTGGACAGAGC
CACTTCTCATCGTACGAGATGTCCTCGTACAAGATGCAGCAGCAGCTGTTGGCAGCAAGG
CAGATGCCCATGCTACAGCAGCAGCAGCCGCCGCATCCACAGCATCACTCCGATATGGGT
CGGGCACTCGCGCCGCTTCCATACCAAGACGGCGATGGCGTGATGATGCCGTGCCCTACC
CACAGTCACTGATGGGGGGTGATGCCAATCAGTATCCATTCCACTTCGGCATGATGCAGA
GGCCTTCGAGGCAGCAGCAGGT
>at02723
GCCGATGCTTTGACTCGTGCACTTCCGGTAAAGAAACCATACTCTACGGTCGATCGGCTT
GCGCAGATGCAGCCATGCATCAGTCGGTTCATGGTCCGAGTGCTCGGCATGCTTCTGGGC
ATGCCATGCGCGGCTCTCCTCCTTAAGCCGGACTTCCTTGATGGCGACATGATGCTGGCT
GGCGCGTGCATGGCAGACTCCGACGTAGGGGGTATCACAGCATCGAGCATGAACTTCTCG
CGAATCGCCGCCCACCGCAAGGCCGCCTTTGCCCATCCACAGCCTTGCTCTTCCGAGGAA
GACCACATCCGTGGCAACTGGGTTGCGCGCAAAGTCCGAGCAGACCAGATGACGTTCACC
TCGG
>at02724
AGGATGAGGAGGTAGCCGAGGTACACGTGGATCACGCGCTTATACTCTGCCCACACTTTG
TTTTTGAAGTCATAGCCGAAGAACTTGGCCTGCGGCAGGTGCGCCTCAAAGATGGCAAGG
TAACCCAAGAGCATGGCCAGCGAGCCCAACCCCATCAGGATGCCATGCAGCGTGCGCTGC
GATGCGAGCGGCCTGTCATCTCCCGTGAGGTAGGACCAGCGCCCCATGGTCATGAGGCAG
GGGAAGGCCAGAGTCATGAGCACAGGGTGCCACCCGAACCAGATCTTTGGATCCATGCCG

CCGCCCATGATGGGGGCGAACATCGCGATCGCCATGAGAACCACACCCCCCAAGCACGTG
AAGCACACAAAGAGGTTGAGCATCAAGCCGTTGATCTCGACCATTGCCTGAAGCGTGG
>at02725
GCTTCGGGCGCGGAAGAGGATCTCCATGCTTACCGCGTACCTGGACTTGGTAATGGGCAT
AGTCATAACCCTCAATCTCTTTACACTCTTCGCTGAGGTCGAGTGGCAGAGTTTGCCGGT
CGCGTACATGTTGGGCTTACGCAGCGACGACGGCGATTGGGAGGTCGCAGAGGATGTCTT
CGGTGTCCTGGAGAAGGTCTTCAACGCAGTGTACTTGCTGGAGATGCTAGTGCGCATCGC
GATGATGAAGTGTGACTTCTTCCGCGACTGGTTCAACGTGTGTGACGGCGTCGTGATCAT
TGTCTGCTGCGTCGAGAGCTTCGTGCTCTCGAGCCTGCCCAATGTACGGGGCCAGGCAGC
TCCTGGTTGGCGCGGATGCTGC
>at02726
TAGCCTCTTATAACCGCGGTCCAATTTGCTCCAGTCGTCTTCTATTCATGGCCTCTCTGC
TCCTACCATCCTACTGTCTGGGCCCTTGCCGCACCTACTCCTTGACCAAGTCTGCAATTG
ATACCAGCGTCGTTGCGGCATTACAAGACTCTGCCTCGAGTTGGAGGGACTGATTCCAAT
GCCGGGCCATTCACTGACCGCTCACACTTGGAGAACACACCCACATGCGTCATCCCAGCT
GGAAGGGGACATGAAGCCTTGCACAGAGCACAGAGCCAGCTCTCGTTGAACCGTCACGCA
TGGCGGCACGGGCTGGATGG
>at02727
TTTTTTTTTTCTTTTTTTTTTTTGGAGGCCAACGCTGTTCATTCCTTCATTGACACCCCA
CATCACAAAGGTACAGGCACCCTAAACAGCCGGAGGGGTACGAATGTGGCATGCGTGCGC
GGCATCCTGCCTCATCGGGTGGAAATAGCTGCAGCTTTGGCAGCTGCTCAAGCGATCCCT
CCGGTGCTCGAACCGCGCTGGGCATTCTGGAAGATCGAGGGAGGGCCACCCGAGGCTACG
GTGACAAGCAAGATCGCGTCCTGCTGCTCTTGGTTGTTGTTAGTCTGGCCGCCCCTGCAG
CCAGACGCCGTCCTTGCGCCTCCCCAGCTGGCGGGATCTCGAAGCTGGCCGCAGGAGCGG
CAAGCGGCACGCCAAGCCGGATGGCAGCCCGTCCCACTGGATGTATGTACTGCTCGTCGT
CACTCACCAGGTCGTTGATATCAGGCCGTATGACCTCGAGCACACTCAGGAAGCCATGTA
GCATCACCGCCAGCCGCCGGGGGTAGGGCAAGTGCGCAAAGCTGTGGGCGAGGAAGCTCG
AGGCAGGGCTGCCGTCGCCGCCGTCAGCCACAAAGAAGTTCGACGTCAGCGTCAGCGGCG
AGTAGAGGCCGGAGCCGGCACCCGCGCTGATGGACTGGACCGTGGCAGGGCCCTTCGCAG
TCACCACGGCGTCGCCTGGCTTGAGGCCTGTGGCAAAGGTGTAGGCGCCATCCTCGCCGC
TGGCAATGCTATGGAATGGCGAGGCGATGAAGTCGCCAGCATCGGTGCGGATCCTCACCA
TGGCCACCTCTGAAGCTGTGTCGCGGTGCAACCAGGCACGGACGGGAGAGAAGGTGACCA
GGCCCGTGCGGTGGTCCAGGCCCATGAGCTCCTCCCCAAGTGCCACTTTGGACATTGGCT
TCGGCCCTCCGCGAGTGATGACCGTCGCGTTCGCGGGGAAGCAGGCTGGGGCCGAAGACA
TGGTGCCCCTCTGAGTCCAGCCTTTGCAGACCTTGTCAGTGGCGTCGTACGCGCTGCACG
AGTACGTGATCTCTAGCTCGTAGTTCGTGCAGCCGTGAAGGAGTACCGTCAGCAGCGCGA
GCGCGGCCGCCGCAAAGACCCTCTCCGCGCCTTTTGACATTGGGCGGTTGCGCAGGAGTG
GGCAGGTGGATGAGGTTGGGAGTTGCTTGGAAGCTGGAGCCGAAATTGCTTGAGCCAAAA
TGGCTACGG
>at02728
TTTTTTTTTTTTTTTTTCCTCTTTCTCCGCACTGGCCGCCCCTAGGCGGCAGTGCCCGT
CCCCTGCCGGCCCCATGGCCGATAGGTGTACAGCAAGCGGCCGTCCTCATCGTCATCACC
CGAGAGGCCATAGCCATAGGGCGCGAGGGAGGTGGCTTCCACATCGAAGCGCTCGTTGAC
TCGGTCAAAGAACTCCATGTCGTCGACCATGCCGTCGCGGCACTCCACCACGAACAAGCA
GAGGCCGCCCACGCCCGCGAGGCGCACCATGGTCTGCACGAGCGCCTCGTAGACCTCCGC
GCCCAGGCGGTAGATCAGCTCGCAGCCGATGACCACGTCGAAGTCTGCCAGCTCGGAGCC
TGGGCCCTCCTGGGCACCCCAGCGGAGCTCCANNNNNNNNNNNNNNNNNNNNNAGACCTC
GGCGTTGAGCTCCACGTTCCGCTGCAGCAGCGGCAGGGTGCCACGGAGGTCCGTCAGGAC
CACCTCCTTCGCGCCCAGCGCCGCCGCCGCCAGTCCTGCAAGCCCCGCCCCCGCCCCCAG
CTCCGCGACGCGGTGCCCCTTCACAAGCTCCGGGTGAGCGGCGAGGTACGCCGAGAGCGC
TCGGGCGGTCGGCCAGACGAAGAAGCCCGTGGAGTCGTCGCCCTCCGAGCAGCTCCGTGC
AGCACCGCCCTTTGCAC
>at02729
TTGTTTTTTTTCCTTTTTTTAGACACCAACAGAACACCAAGGTCCACGTTGCCAAGAGCA
AAGCACGACCATCACCAGTCCAAGGTCTGTCCTGCAATTCAAAATTACCCCAGTCATCGC

TTCAGGACTCGCGCGCCGCGTCCGCGGCCCTTGCCGCCGCCCATTCCCATTCCCTTCCCC
TTGCCTTTGCCTTTGCCTCCCATGCCTTGCGGCTGCTCACTCTGGGGGTTCTTGATGGTC
TCGTCGATGGACAGTATCAGGCATGCGGCTTCCGTGGCGGCACTCAGGGCGCTCTCCTTG
ACGCTAACGGGCTCCCAGATGTACTCAGCAAAGCAGTCGGTGATCGTGCAGTTGATGCAG
TCCACCCCGAACCAGCGCCCAACAGGGCCTGTGACGGCATGCTTCTGCCGCAGCTGGTTC
AGGATCAGGGTGCCGTCGGCACCGCTGTTCTGTGCCAGCGTCAGAGGGATGACCTCCAAC
GCGCGGGCAAAGTAGTTGATCACCAGCTGCTGCTTGCCGGAGATGGACCGCGAGTACTCC
CTCAAGTAGCGGCTCAGCTCCATCTCGATGGCCCCCCCGCCGCCGACGACCTTGGTATTC
CTCATGGCGGACTTCACGATCAGCAGCGCGTCCTGCAGCGACCGCTCCGCCTCGTCGATG
AACTGCTGGGCCCCGCCCCGGATGAGGATGGTCGAGGACTTCGTGCTGGAGCACTTCATG
AAGAAGTTGTAGCGCTCCGCCCCGATCTGCTTCTCCTCGAACTCGCCGCAGGTGCCGAGC
ACGCTCGGCGTGATGCCCTGGACGGTGGTCTGCACCTCGCCGCCGGTCGCCCGCCTCGTG
CGCTCCAGGTCGTCCTGGTCCACCCGGCCGGCGCAGAAGATGTTGCGGTCGGCGAAATAC
TGGGTGGCCAGGTCGCCGACGGCTAGGCGGCTGAGCACCACCTGCGCCCCGGACTTGGCG
ATGAGGTCCAGCTTCTCGTAGATGATGTTCCACTCGGCGTCGACGATGGACTGGTACTGG
TCCGGGTCCGTGATGCGGACCTCGGCGTTGTCCTTCTCCGCCTTGAGCTCGAGCTCAAGG
TTGAGCAGCAGGATCTTGGGGTTCGTGAACTTCTTGGGCTGCTGCTCGAAGCCCGCGTAA
GAGAAGGTCTTCTTGAAGGCCACGCCCTCGACCAGCATTGAGTCTGTGACCGAGCCCCCA
GTCACCTTCTTGATGCCGACCGCCGAGATGCTCAGGTCGTCCCCGAGCATGGAGATGGCG
TCGACCGACATCTCTGCGAAGAACTTCTTGTAGTCGGCCACGAGCTTCGAGTTCAGGGTC
GTCTCCGCGCACTTGACGAGCATCGCGCGCTTCTCCTCCGGGCCCTTGGACTGCAGGTCC
ACCGCCACCTCGCTCAGCTTCTGCAGCGACAGGTTGCAGGCGGTCCGGAAGCCCTTGATG
ATCTGAGGGCCTACGAGGCCGTCCTCGAGGAAGTTCTTCGCCTCCTTCAGCATCTCCCCG
GCGAGGACCACGACGCTGGTGGTGCCGTCGCCCACCTCCGAGTCCTGGGCCTTGGCGATG
TCCACCAGGATCCTGGCCGCCGGGTGCACGATGTTGAGCTTCTTCATGACGGTGGCGCCG
TCGTTCGTGATGGTCGTCTCGCCGTTGTTCTCGATGAGCTTGTCCATGCCCCTGGGCCCG
AGCGTGGTCTTGATGATGCTGACGATGGCCTGGCATGCGTTGATGTTGCTGATGATCTGA
GCCTTGCCCTGTGAGGTGTCAGTGCCATCCTTGAGCAGAATGATGCCCGGCTTCATGAAT
TGCGACATGGCCGCAGTGCTACTCCTGCCCCTGCGCTGCAACCACACTGAGCAGTTCCCT
TGGGCCAAAATGACTTGAGCCAAAATGGCTACGG
>at02730
TNNACGTGCaAAAAGCAATCACAGCTGCAAAGTCAAATGTCAGTCGGGCAGCTCGGACTC
AGAGCGACCGGCCGGCTCAACGACATCCGTGGTCATCATGTCATCTGTTGTCTgGGGAGC
AGcCGAGCCACAGGCACGCCGAGGGGAACGTTCCGAGAGTCGCCACTCCNAGGAGGCGCA
GGACaAGAACAATGAGGAAGCACGCAAACACTCTTGTCCGGTGGCGGTGATGCGGACTCC
GTATCGGCCGTTAATGACATCCGAGACCACGACACCCCTAGAGCCATTGAGTAAAAGGCG
>at02731
CGCGCCGAGCCTATCTGGCGCTGACCtACGtCtAGGTGGACGAGCAGATCGGCCACATCC
TGGCCGCGTTGGAGGGAGTGGGCGTGCTGAACAAGACTTTCATCCTCTTCGTCTCTGACC
ACGGGGATGAGCAGATGGACCACTACCTGTGGAAGAAGGGCTATCCATACCAGGGCTCCA
CGCACGTCCCGATGATCATCCGTTGGCCGGAGTCCATGGATCCTGAGATGTCAACTCCCC
CGTGGCCTCCA
>at02732
TTTTTTTTTTTTTTTGTCTTCCTCTGGCAATTACTTCACGGCCTGCTGTTCAACCTCCG
TGAACAAGCCAGCTAGAACAGACACAAGACGGGCTGCTGGGAAACCCTTCTAATGTCAGG
GCGTCATAAAAAAGCGCGCACACAAACACCCACACTGACACCACTCGGAGTCTTTAAGCT
GTGCCTATGGCGCAGCAGCGCAAGCGTCCCATCAGGGCGGGAAGTAACAGCCCAGCAACT
TGGTTGCCTCCTCGCGCGCTGCAGCCAGAGCGGACTTCGGCCCAGCCATGCGCAGCTCGC
GGCGGTCAGGCGAGACGGTGAGGTCGACCTGGTAGTCGGAAGAGAATTGCTCGACGTCGC
TTTGCAAAGTGGCCCAGTTGACGTTTGCTCCGGACGATGGCACAGCTCCGCAGGTTTCAC
AGTGTGGCTCAAGCACCACCTCCACACCGTCGCCTAGATGTTCAACGAAGGTCGGCTCGC
AATCCTCCAACTCAACCAGCGGCACGCCGTTGATCGAGATGATACAGTCACCCGCTCGGA
ATCCTGCCTCGTGCC
>at02733
CCGTAGCCATTTTGGCTCAAGCCATTTGGGCTCAAGCCTGGGGCCGGGCCTGTGGGGCAC

CGTTTGCCTGGCCGTGAGACGCCCACCACAGGCCCGCGGGATGCGCCTCACTCAGCACTT
ATTTGGCACAAGCTGCACCAGTGCTCTGCTGTGACTGGCCTTGTTGCGGAGTGACTGATG
GCGGTGAGCTCTGCCTCCTCTTGTTCAGTTCCCGTGCCTAGGAGAAGGAGGGAATCGCCT
TCGCCTTCAGACTCGCCGGTGCGCGAGAAAAAGAAGAAGCACAAGAAATCAAAGCCGAAG
CACGAGCGCGGGGAATCGGAGGAAAAGGTCAAGCGCGAGGACAGGGACAAGAAGGACCGC
AAGGCCCGCAAGAAGCGCCAATGTCAGAGTCGGAGCAGGAGTCGAAGAAGGGACCGCGGC
AAGAGCGCAGGGCGGCGGAGACCTAAGCCAAAGGAGAGCGGGGAGACCGATGAAGATGTG
GAGGTGCCGGCCTCCGGCCGACAGCAGAGGTCCTCACCCTCGCCCTCGCCGCCGCCTCGG
CAGCAGCAGACCTCGAGGGCCAACTTGCCCGGTGGTGGTGACACCGACACCAGGATGTTG
CACAGATCGAGGAGTCGGGACGACCCTGTTCCTGGAGCGTTTGGTTTCGACGACCTCGGA
TCGGGCAAGGGCAAAGGAAAGGGTAAGGGCAAGGGCAAGGGTGAGGAGGAGGAGGCCGCC
CCCAAGGAGCAGCCCAATTTCGAGCCCTCAGGGTTGTTGGCTGTGGAGGACAACGCGAAG
AACGGGATTCCTTTGAAGTTTACCCAACCACCGGAAGCACGAATGCCGACAATGAAGTGG
CGCTTGTACATCTTCACAAAGCAGAGTGAGGCGCCGAAAACCATGCACCTGCACCGCCGC
GTAGGCTACCTCTTTGGCAAGGACCGGCGTGTTGCAGACGTGCCCACAGACCACCCCACC
TGCAGCAAGCAACATGCTGTGCTGTACTATCGCCTGGCACCTGGTGGTGAGGTAAAGCCA
TACATCATGGACCTCGAGAGCACCAATGGTACCTTTCTGAATGGTGAACGCCTGGACTCT
GCGCGGTACTACCAGATGAAGGAGCGTGACGTCCTGAAGTTTGGCATGAGCTCGCGCGAG
TTCGTGCTCCTCCACGCAGGGTCTGCAGACGACCAGTACATCGACCCGAGGCAGCTCCAA
TCTGATCCAGAGAATTGATGCCCGAAGGTTTGGGGTGACCGGCCGCAGAGCTTCGCAACA
AGGGACCTCCCTACGGCCCACTATGATTCA
>at02734
GGCCGGATCACAAGTCTGGAGTTTTGTGGGTGTGCTGCAAGTGTGGCTACCGCAGCGAGG
CCGTGGTTGAAGCAGGAGCGCCTGCTGGCCCAGTCGTTGCCAAGTTCCTGACGCGCAGGA
AGAAGCTGTGGAACGCCATCGAGAGGCAGCGCAAGAAACTGCTGGCTGACCAGAGCCTGC
ATGTCAACATGAAGGTGCGCACCTTTCGGCAGACGAGGTTTGCCAGTGCTCACGCGCTCT
GGAAGAGCCTCAACATCAACTGGGGCTTCATTGAGACGATGTTCAATTCCGGCAGGGCAT
CGCAGTACGTCGCCATGACCAACAAAGACGAAGCTGCCCGATACGAAAAGGAGAAGCATT
ACGTACTGTCAGTTGCCCGGCGCCGCCGCAGCCAGAGGGCCGAGCTCATCCTGGAGCCGG
TGACGAAGTTCATCCGCTTGTCTGCGTCCAGTGACGCGCCCTTCAGCTCCGGCGTCGTGC
TGGTGCACGGCCTTGAGGAGGAGTTAGCCACTGCGCTGAAACAGCATGACTGTGTGGCCA
ATGAGAGCCATGAGATCGCCGAGGTCAAGAACTTCCAGCAGCAGATTGTGCTTGCCGATG
TTGGCCAAAGGTTGCGGGCGTCTTTCCTCACAGCGGAGCACCATGTGGCCTACATGATGG
ACCCCAGGGTCAGATGGTTGATGAAGCAGCCTGACCATGGCATGGCAGTTGTCGACTTTC
TTGTGTATGAGATGTGGCAGACTGCGAAGACCTACATTGAGCCCATGTTCCGCAAGTTCT
TCACTGAGCTTGCCAAGTTTGAGCGGGCCATGCGCGAGATGGACCGGTGGTTGTTGTTGG
AGGGCCCGTTTGTTGCAGGCGTATGCTCCCTACCGGAATGGGGCCGTGATGGTTCTGCGA
GGCTGGACATTGACCGCTTCTGGAGGTTGCGGGCAGGGACTGCGCACCTC
>at02735
TTTTTTTTTTTTTTTTTAACGGCCACTGAACTCACCAGGCCTCTATCCGGCCACAAGGC
TATTGGTATGAGCAAGAGCCTCAGTGAAGGATGCACACACCATCAAGAGATGGTCCCCAT
GCCCAGGCTTGGCTTTTCTCTGCTCGCACTCCATGCGAGCACAATATGCATGACAGAAGT
ACTCCTCAGGAGACCACTGCGCAGTGGAAAGAGAGCAAAGCTCAAGAAAGCACGACCTGC
TGCTGTGTATGCAAGCCTTGATCCCTTCAAACGTACTTACCACAGCACACCCGCCGGGAT
CCCTACGCCCAGTGACCCGTTTGGCCACGTGGGTGCAGGGGCGGAGCCGTGAACAAGATG
CGGTGCACATGACTGGCTGCAGGTC
>at02736
TCTAGCCGCAATGTGTTTGGCCCTTTTGACTCATACGCACTCATGATCAGCAGCCCGGCT
AAGCCAATGCTGTTAATCCCAGGCGTTGTACTTGTCATCTTGACCTCCTTGCCTCCAACT
GGACTGGGTTACCCATCCTACCGCAACCATGTGCCCAATGGGCTTCGCGTCGCATGCCCT
CTGATGCTCAGGGTTNCCGACCCGGG
>at02737
CGCGGACAAGCCCGTAGGGTTGCCTCTTCTCTTTCGGCATGGCTGTGGGCTACAAGGCAG
ATCGGTCTTAACGGACCTCGTCACCACCTTGCACCGGCAGTGTCTTGTGTTGCTTTCGGC
GCATGCTGGACGGCCTCTCCAGGGCCTTTTCACAGGGCGCATGTATCTTGCGTAAGCCGC

AGCACCGCGCCTTGCGTCGCAAGATCCGCAACATCGACATCCTCTTTGCGTACTCGCTCC
AC
>at02738
TTTTTTTTTTTTTTTTTTGCTTGGCCTGACGAGGTGGACTGTCCACCGTACTGTTGCCGA
TACGATGTGTCATAGTTTGCACCGTGCTGGCCGGGACCATCCTGCGGTGGATACCGGTCA
TAAGGTTGCCTGTAGTATGGGTCATACTGTGGGTCGTAGTGTGCCCCTTTTGAGCGGGTT
GGCACATGAGCGTACGGGTCGGAAGAAACTTTTGGTGGTTGTCTCGGAGGGTTTCCTGGC
TCAGACTCGTCACCAGATGCGGAAGAGGAGGAACTCCCTGATGATGGGCAATCTCCTGGT
TGTGTCCACTGCTGCGCCGTCTCTGTTCGGCTGTAAGGTGCACTGCGCATTTCGTGGCGG
CCATCACGCAAGGCAGCAGGTCCATAAGCCGGCTGTGCTTCCATGTTCTCCTCGTGCC
>at02739
TTTTTTTTTTTTTTTTTTGCGGCAATGTACGCCTTTGGACAGAGCTGTGCAAGGTGTGTG
TGCAAAACCGCAAGGGTGGAACTAGCAGGCAGGGGACGCGACGTCCTCAGAGCTCACTCG
GGAAGGTTGACGTCGTCGGTGTCGAGATCCTGCAGTGCTTCAGAGAACGACTGGTTCATC
TTACTCACGACCAAGCTCAGGTCCCCCTGCTTGGCTGCTAAGCGGCCCCATGCACGATCA
AAAGCTCCCTCGTCTACTCCAATGCTGACAAGTCCTGCTCGCAGCTTGTCCAGCTTCTTC
CTGGGGTCGATTGGGATGTCGTCATCGAGCCGTTTCGGAGCATTCTCGTTCCGAAGGTCT
GTGCCTCCCAGGAACTTCTTCTTTGGGTCGACCACACTGCAATGACCCCTCTGGCAGCCG
GGGATCTTCATGAACTCGTCCCAGTCGTAGGCCTTGGAGTTGGGGCAGCAGGCCCAGTAC
TTCGCCGTCTCGTGGAAGACCGGGGGTGACTTGTGGTGGACGCACGTCGTCAGCTCCGGC
TTGGTGGGGTCGAAGCGCTTGTTGCAGCCAAAGTTCTTGCAACGTACATGCTCCGGCGGT
CGCGGCACGACATCACCTGCCGCCGCCATGGCACTTGCACTGGCCAGCAGCCACCGGGCC
AACAGATTGCACAAACCCAACCCCTTGGGTATCGATGTCCTTGAGCCAAAATGGCTTGAG
CCAAAATGGCTACGG
>at02740
CCGTAGCCATTTTTGGCTCAGAAACACCTCATTGCAGGTCTGGCCCCACTGAAACTTTTC
ACCAGGGGTGGCTACTGGTGCCATGGTGCGGAGCCTCCCAGGGAAGGCGAGAAAGGAGAC
CAACAGCGAGAAGCGGCTCAGGCGAGAGAGCAACTCCCAGGCACAGCAGTACGCGCCCTA
CGTTCTGGGAGGGTTGGCCGGGATGTTTGTCCTGCTCTTATTCTGGCTGCTGTGGGTGAC
CAGGTGAGATGCAGCAGCGCCGGCCCGACTGTGCAGACTTAAAGTGGCCAAAGACCATAC
TTTAGCTGGAGGTGCATGCCCAAGGCGGCGGCCAAGGTGCAGTAAAGCGGACATGCAGTG
GAAAGACTCACTGAAAGTATCGGACATGCATGAACAAAAAAAAAAAAAAAAAANAAAAAA
AAGAAAAAAAAACAAAACTACTAGACGTT
>at02741
GGCACGAGGCCCTGACTCTGCAGTGGCCCACGGCCCTCTTCGCAGGTGCCTTTGAACGTC
GCCTCCTGACAAGCCATGGCCGAGGAGGAGGGACAAGAGGTCATCTTCACAGAGAACACC
GCGCCGGATCGCGAGATCTGGGACGAGAAGGGTGGCCTCATCAAGAAGGGCGCAGCGGAG
ATCGCAGAGCGCGATCAGGACCATGTCCCGGAGGTGAAGGTGCTGACGACGCTGGGCATG
TTTGCAGTTCCCGTCACTGCTGAGACCACAGTGGGAGAGATCCGGCAGAAGGTGGCTTTG
TGGATCAAGGAGAAGAACGAGGAGACTGGAGAATGGGAGAGCACATTCTGGAAGATGCTG
GAGGAGTCGAAGTGCGATGATTGGAGCTTCCAGGGCCACACCATCTGAGGATTTGCTGCC
TGGTGTGAGGCCACCGTGCTCATGCTCCAAGGCACAGGCGGCTGAGTGGTTACATCTTCC
AGGTTGCGCTGTATAAAAAAAAAAAAAAAAA
>at02742
CTCAGGAAGCGGCCGCCAACGTTTGGGAACGTGACCGTCGCGTCCGTCGCGAGGTCCAGG
ACCAGCTCGGAGTACAAGGTGTCGCGGTTCATGCGGATGACGTCCTGGCCGTGCTTTGGG
GCACTCTCGGGGGAGGGGTCAACTCTTGTGACCTCTCGGGTGTGGACAAACTTGCCGAGC
GCACCCAGGCCCACAAACTTCGCCATGTAGTTGTCGCTCTCGGCTCGGAGGAAGTTGTCG
ATGGTGACGCGCTCAGAGGCCCAGATGCAGAGCGGCAAGCCGATCAGCGCCGCGACGTGC
GTCATTGTGTGGCGCATCATGGTGGCGGTCCTCCCAGTCCCTTGGGAGCGCGACAAGTGC
TGCCAAGGTGCTCTTGACCCTGACGTCTTGAGCCAAAATGGCTAC
>at02743
TTTTTTTTTTTTTTTTTTCCGCCAATTTCGATGCATTCTGCCACGGAACAATCGCAAGGC
CCTCCCTGCTGCAGAATGGAAAATGAATGCTCATCCTAAGGCACGGAAGACGCCCACATA
TACGCTGAGGACCATGCAATAACTGAGGCATCCCCACCAAGGCCGCGGGCTCAAAATAAA

TGTAGAAGTGCATGCCAGCAGGGTCCAACACAAGAAGGTAGAAAAAGAAGGATACAGCAA
ACCCAGCAAAATCGACAGCCACAGGTCCCATAGAACCAACGGCCTGTAGGTGCTGTAGTG
GTCATACAATGCCAGGGGCGAGTTCAAGAGGTGGACGTTCACCAGGAAGAAGCTGAAGTT
GAACTTGATGAAGCGTCGACGACTTTTCTCATCGCCGGGCATGCAGACGAGCATGACCGG
CATGATCACCAG
>at02744
TACATGGGAGGGGTTGCTCCGCGCGTAATCCTGCACCGCAGCTTTCCATCCTCGTGCCGT
GTCGCTGTCCACCACGCCGCGGACAATGACAACGCCACGCTTCTTGATCTCGATCTCAGC
CTCCTGCGTTAGATGCCCGCCGCTCCGTCGGATCTCGGAGAAGTTCACAACTGGAATGGC
ATCTTGCTGCAAACGAGCAATCTCAGCAAGCTCAGTGGTCAGAGCACGGTCAAGGCGTTG
CCATGCATTGCGCAAGAGCTTGGCATCGAGGGCGCAGTCGCTCCTTCAGCACTGCAAAGT
CTGCGAGGCCACGCATAGGTCGTCCCAATCGCGCTCACAACAGATGAGATCGTCCCAGCA
GGCGC
>at02745
GCCAAACCATCAACATGCTGTGGTGGAGCCACTTGCTGTGTCATCAAGCGCTCCAAGAAA
CCTAGATGGATGCGAACAGCATCAGCAGCCTGCCTCTCAGAGTCAAGCTGTGGGTTGCTG
GCCTGGCCTCTCAACTTCACAAGTTCGGTTCGAGTCCTCACCAAAGCACTCAGCAATACA
GCCACATCAAGAAGCTCTTCTCGCTGCTGAGGTTCAGGGTGCAACGCAGCCTCTTCAAGC
CATGCTGTCAAAGCACGTACGAGACTCAGATCTTGCACGACGGGGACTACCTGCGCAACA
GATGAGACACCTGGAGGCAAACCTCCTCCAGAGTCATCGCTGCTCAGAGACTGTGTCTCC
AGCAAGGGATGGTCTTCATCCAAACCATGAACACTACTAAACGAACATGGCAATCCTTTC
ACCAGCATCGTAAACACTGCACTCAAGGATCG
>at02746
CCGTAGCCATTTTGGCTCAAGTCTCGGTACAAAGTCCTCCGCGCGCTAGCCTGGTGGGCG
GAAGGGGTGCTGTTAGAGGTGCGCATACGGCACATCTTGCTGAAGCACCGGGACTGCAAG
GCGCCCGTGGACAAGGTCCGGGGCCGGACTGTCACGCGCTCGAGGGCCGAGGCTGAGAGG
CAGCTTCGCAAGGTGCTAGAAGAGTGCTGCGAAGCTGATCCCCAACAGCGCGGGGCACTC
TTCACACAGCACTGCCGCGAGCTCTCCGAGTGTCAGAGCAGCCGCCGAGGCGGCGACTTG
ACAGGGGACCTTGCGTGGGTGCCCAAGGGCAAGATGGGTGCTGGCTTCGATGAGGCCGCC
TTCGGACTACAGGTGGGCCAGCTCAGCGACCTCGTCGAGAGCGACGCCGGCAT
>at02747
TTTTTTTTTTTTTTTTTTGCCTCGACACCCACAGTTACCACAATGTACAAAAGGTGGATC
GCGGCATGCTCACGACTCGACCTGCAGGCTGCATGGAATGGTGGCGGTCCAACTTCGAAT
GGCGTAGCACCCTGGATCTGCCAATGCGCATACCACGCCGCAAAATCGAATGTTCAATCG
CACAAGCATAATTCTGCACTCGTCCATGTGCCTTGCTGTCCGATGGTGGTGTCCCTTCCT
CTGAGCAGTCCTCTGCGTGCCGGCGCCAACTCTGACGCTATTGAGGTTGGTCTGCTGGCA
ACAGGAATTGCGCAAGATGCTGTCTAAGAGAAGCTTTTGGCGCAAGCATCGCTTCACCAT
TAACTTGAGCGGAAGGAAGCGGAGCTTCGGAAGTCAACCTTGGCGTCTCTGGCTGTCCAA
AGTACCCCTGCGGCACACTGCTGAACCATGAGAGGTCTCCGTCTCCAGCAACAGGAAACA
CATTCGCAGCTACGCCCTCCGCCTTCGAATCTGGCCAAGGGGCCCGATTCGAGATAGGTG
CTCCTTGCAGCAGCGTAAGTGCTTCAAACTCACCTTGCCGAGATGCTTGACTGTGTCCAC
CGACACGTCGATAACCCTGAGGAGGCCCATCTTCTGGCTTTTCTTCTGTAACTTTCCAAC
GAATCTGCTCGGGCCGATGTGCAAATGTGCACGAGAGGTCACCGCATACACCGTCTTTCA
TCAGCTTCCTACACATCCTTGTTCTGCGCAGATCAGGGCGAGGTTTGAGATCAGCGCTAG
AGTGGGCAAACGGACAGTCCAATCCTTTTGTACATGCACCTCGATTGTGAAATGCGCAAA
GGGCTGTTTTGAAGTATATGGCGTTCGACTTACCAGTTCCGCTTCCAGATTTGACAGATG
CCTCAGATGTGGCATTCTGTGCCATCCTTCTGGCGTGGCCGGTGCGTCTGACTTTGAGCC
ACCTGGCTTGAGCCAAAATGGCTACGG
>at02748
CCGCGCGCGGTTGACAGTCAGCGATGGCGGGCGCAGGGACCAAATCGCATGCATGGCAAG
ATGGGCCATGCAGCGACGACGGTGGCCACTCAGCCTCTGGCCACTGGTCGTCTGGAGCGC
TCGCGGGGAGAAGCTGGTGCAGGTGAACGGCGACACACGCAGATTGCACGTGGAGCTGGC
ATCCTTGTTGCGTGACCCGCGTCTGCAAGCAGTGTGCCCCCGGTGCGAGGCGCTGCCGCG
CGACTTCCCAGAGGCCTGCGCGGTGCTCGGCATCTCCAGCGGCTTCCTGCCGTGGcCAGA
TCTCCGCGATGCAGGCACCAGTGAGGtttTTTTTGGaAACTACCTGTCCAACGTCTTCTT

CGCATATGCAGCTGCGCAAGCCCGTGGTCTTGCCTTCCTCGTCGTCGTGCCCTTCCGGGA
AACGAGAGCCTGGGGCGAGTACTTCCCGAATCTTTGGCGTTTCTTCGAGCGTCTGCCGTT
ACACGGGTTGGGCGGTGCGATGTCCGTGCCATCTGTTGATTGCCAGCGATGTCCGGGCCG
CTTCCCCCACGAGTGCAGCAACCACAGGGATCTCTGGGAGAAGTCCGACCTTACCCTTTG
CTGCAATCTTGCGGGATGCTGTC
>at02749
GGTCATGCAATGCGTTCACCCAGGACAATGCCAATGTCACCGCTGCTGTTAAGGACACTG
TCAGTGTCAATGGCACGGGAGAAAAGAAGGCATGGCTTGGCAGGAGTTGTACAGACTTGG
GGCCCAAAAGAAGGCAGTAACGCTTCTGCAGCAAAGATTCTACAGCTGATACCAAGACAC
TCATCTACCGAAGCAAGCCACTGCCGTGACTGCAGCGGT
>at02750
TGGATCCCTCGGCGTCGCAGGGCCCATCCGTCCTGCTCGGGACGACGCTCTCGACCAACC
GCATCTGGGAGGGGCGGCTGAGCGCGGAGTGGGCGCGGAACTGGACAAGCTACTGGACGT
TGGGCAAGGGGGTCTTCGTTGCCTCGCTCATGGAAGATGTGGGCGTTGCCATGGCCTTGA
GTGGCTTGGCCCGTGCTGGGCGTGCGAGGGGTCCTGGGAGTGGATTGCTGGTGCTCCGCA
CCGCAAGCAACTTGGTGGAGCCGCCTCCTGGCGTGTCAGCCGCGGACTTCATCATGAAGA
CGGATGGCGTTGAGCTTAGTTCCCGCAAGTTTGAAGCAGCGCTCGAGGCCGCCTTCCTCG
TCGGCGCTCCTGTGGCCTTCGCTCTGGCAGGACGGCCGAGGGAGAGGAAGCTCGAGCCGG
TGGTCCTATTCATGTGAGCAGAATGCGCGGGCTGCGCTGCTCGCCAGCATCGCGCGCGAG
GGCTGTACAGTTGTCATGCTCCGTAACAAAAAAAAAAGAAAAAAAAAAACAA
>at02751
TTTTTTTTTTTTTTTTTCCTTTTCACTACCTTTGGCTGCTCCCAGGCACTTTGCGTAGG
CCTCAAATACGGGGAGCCTCCCTCAGCACCACTGCCAGCACGAGTGTGGTGAGCGTGGTG
AAGAGGATGAAGACGTAGAAGAGCGCCTCCTCCCCGCCGGCGACCGTCATGCTGCTCTCC
ATCAGCCTCTCGCTGCCGGCGGCCCCTGCTGCGAACGGCGTGGTGCTCGCCGCCGCAGCT
GCCACGCCTGCAGCCTGCCGGCGCCTGGTGTTGAGCCAGCCAAGGATCGCACCGATGCTG
CAGCTGGCGGCCACCAAGCCCATGGCCCCGGAGCTGGGCTCCTCGGCAGCTGCTTCCTCG
TACGCTGCTGAGATGGTGGCTGGCAGGGGTGCCTGCACCATGCTGCCATGGGCGATGCGG
GCAGTGGGGACTGCGCCTGGAAGGGGCTGCACGAAGGCGAGGGCAGGGGCTGTGAGGCAG
GCGGCCATGATCAGGAGAGACGAGGAAGCCATGGTCGGAAAAAGGCTGCCGGTGCTAAAA
AAAAACCAAGGATCACAGGCCTGTCAAACTCCTCGTGCC
>at02752
ATATTTCTATATGACACCTTCACTTATTGCATGCTATGTACGTTTGCCCTGCTCTCCGCA
ATCATTGGCGCGGCCTTTTGGTGCCGACACCGCACCTGCTTGTGGAGCAGACCTTGTGAC
AAGACACTTACTGATACCATCCTCATGCGCGCGGAAGAGCTGAGCGCTCGGATTCAGGGT
GGCATGGGCAGTGGGCGCCGGTTGTCAATGCTTGTCTGGCTTAATGTCATCCAGATGTAC
GCGGGCATGGCTTTGGATGTTTGGAACTTGTATGAGCTCATCGATGGTCTGCATTGGAAA
GTGGCTGCAGCTAAGGCGTTTTTCGTCTTATCGAGCACTCGTGCGTCG
>at02753
TAGGTTCAAAGAGGCCCCTTGGACTCACGCACCTTGACATCGTGTCCATAGCCGTGGGCG
GCAAGCAAGTGCAGCGGCTGGTGCAAGTCTCCTAGCAGTGCCACCATCCATCTCAGCTCT
GTGGCGTTTGTGGCGACCATCGCCTCCAAGTGGCGATCCCTGATGTATCCCCTGGAGGAT
CTCGGCGTACTTCGTCTCAGACCTCCCAGAGTGGTTCCTTGGCTTTCGGAAACTCACGCA
GCAGGGCATCGTGGGCATACCTTCGGAAAAGCGAGCCCAGCGCGCAGAGCACGGAGTCGT
CAGCAGCCTCCTTGCTGTTGCAGCGCAGCTTGCCA
>at02754
TTTTTTTTTTTTTTTTTGGGTCCGCGCATGGGCAGGCCAATCATTCTTCCGGTCTCGCC
AGCACTGGTCTTGCTACTCTGCCCTCAAACTGAGGCTTGCCGCTCACATCCTGATCAGGC
GGCGATCTTGGTGGCTGCGTCATTGACCGCAGTCAAGACCGAGTGTCTGTCAAAAAGCGC
AAGCTCCGACACAGCCAGGATCATCGGAAGCCGACCGATGCCTGCCACAGGAACGCGAGC
AGCAGGAAGAAGATAGGCAGGGATGGGAGCACGTCCACGATCGGCGCAAACATGACGAAC
TCCTCCGGCAGCCGCGCCTCTGCCACCTCAGGCGCAGCGATGACTGCCCCAAGTCCTGCC
CAAGCTGCAAGCTCTGGCTGGGTCACCGCCGCCTGCTGCCTCTCGGCGCCCCGCAGCGCA
TGCTGGCCGCCGGCTGGCTGGACGAAGACTGCGGTGAAGCCCAGAAGGGCGCAGGCCAGG
GCAACGCAAGGCAGCAGCATGCTGGAGCGGCGGGCAGCCATTGCGTCTAGAGATGAAGAG

GTGGTGGTCCTTTGCGCCCTCGTGCC
>at02755
CCGGAAGTCTGCCGCGCGCACGTGTAGTACAGTACTCCACCGCCCAATGGGGCGGCGTCG
CCATCTACCCATGTGGTGACTGCAGCAAGAAAGATGGATGCATGGGGGCAGCTGCTGAGG
CTGCTGAGCACTGCAGGTGGACATGCAAAGATGCGTCCTGCCCTTTCCATCTTTGCAGAG
TAAGCGTGTTGTCGTCCTCACTAGCTCCAAGGCTTACAAAGGTATGTCT
>at02756
TTTTTTTTTTTTTTTTTTCCTTCCATCCAGCGCCACGCCAATCACGGTTTGAGAAACGAG
AGCCTGCCATCGCAAGAGTAAACGGCCAGAATGTTTCTCAAAGGGTGATGGCAGAGACCA
ACCACGTCCTTTTCAGCAACCTTGACGGTCTGCTCCAGGTCACCTGTGGCAGTGGAGAAG
CAGTACAGCTGGTGGTCCTCGCCACAAGCATATAACCACTCCCCCTTTGGAGAAGGGGCC
ATTGCAACGAAGTCACCTTGCTCGCGCCTGCCAGAACAGAAGCTTTTCAATAGCTGCCCG
CTGAGATTCATGAGCAAGATCGTATTGGTGCGGCAGCACACAAATATGAGGTCATCATGG
TCTGACTGCAGAGCTGGCAGCTTCGGGGCCAGCACGACTGCATTCACAGCGTACTGCATC
GTCGAGCTTGCATGTACTGGCGGGGGCGGCGAGATGGTGTTGATGATCTCTGTTGTCTTG
GCGTCAAACACTATGACCTGGCCATCTGAGGATCCCGTGATGACCTTCGAGTTATCTCGG
GTGTAGATGGCCGAGTTCACGAAGGAGGTGTGGCCCCTGTACTCCTTGAGAGTCTTTCCG
GACTTCAGGCCGTGGACGCGGGCGGTCTGATCGAAGGACGCCTCGTGCC
>at02757
TTTTTTTTTTTTTTTTTTGCCGGGAAGCTGGCAAACCCACATGTCCCAGAACTGAAGGAG
GTCATGGCAACATCGCCCAACAAGGAACGACCGCCACAGCGACCTGTCAGCGAGCGGGTC
AGAGAGTAAACAGCCGACATGGTCGGCAAGGGTTGAGGAAAAAGCTACCGTCCGGCGCCA
ATTCGACGAAATGACGGAAGGCAAATCTGGACAACCGACATGGTAACAGTTTAGAGAGTA
GCTGCCTTACAGCGTCCATTCCAAACGGCCGGAAGGCAAATCTGGACAACCGACATGGTC
GGTATAGGTCAAAAGCTGCCTTCCAGCGTTAATTCGGAAGGGCCGGAAGGCAGATCTGGG
ATCTTCTAAACTCCAAGAGTCCATTCTACGACGTTTTTGACCAACCTTGATTTCAAAATT
GGCTACCGGAGCTAATCTTGCTCGCCGTCTGCGCTCCCTCCAACTGAAGCAACGTAAACG
CAAAGGAGAGGAGTCTT
>at02758
CGCCATGGCTTCATCCTGCCTGAGCCGAGGTCAGTGCCATCTCATTTGGGTGCATCATCC
TCCCGATGTGCACCCGTGCCTGCCCGAGAAGAGCCCGATTGGTCAAGGATCACCCAGCCA
GCACAGGTCAGCAAGGGCGGAGGCCGCAATGTCCCATGCCGGAACGGCCAAGGTGAAGGC
AAGGGACGGCGAAAATGGGGGACTTAATTTTCTATGGGGCTGTACACTGCTGTCCACCAC
GCCCGGATCCACATGCTCTCCCTCGGATGCCTAACACGGTGTCTGCTGGACTCCTCGCAC
TTCTTCAAAGGACTTCCGGAGCACGACGAGGCTTCTTACACGTCGAGCATGGCTGTGCTG
CTGCCACTGAAGCACGGCATGGCGGTGCAGTTGCCGGTGAAGCCCTCACGGCGCTCGAGG
ACGAGCCCTC
>at02759
GGGGTTTTGTGGCTCGAGGAATGCTCGGCTTGGTATTAGTGACAGCAATCCTCGCACCAA
GCGCTGAGGCGCAGCTGAGTCTTCCTCCGTCTCCAGAGACGTGCTGGCTTGCAGGCATCA
CACCTGAACAGTGCTGTGACATCCGCAAGAGTCCACGTGGACGACCCGGCATGGTTGGGA
TGCAACTTTCACTTTCGAAACTTGCTGTCCGACTGAGGCAAGCTTGGTAGCAACTGCGTT
TGAGCAGAACTGCGTGGGTTGG
>at02760
ACCCTGCTGTCCTTGGTGCCCCCTGCCCAGCGTTGCTGTGAACTACTTCAAGGCATGTCA
CAAGACGTCCAGCGGTGGCTTTTATGGCAGTCCCAGGCATCTACAGCTGGGAAGCGTGGC
ACTGAGGCGCCACGGCGACTCCAGAAGAGGCACCACAGAGGCAATCGTTTGCATGGGCAC
AAAGCATCGCGTGCTGCAGCAGCACCTGAAGTACCGAAGGGCCGCTCGATGCCATCAGCC
GAGGAAGGTGAACTATTCTCAAAGTGCTTGTTCAGCAACGGTGAGGCAGTTTGGATGTTC
CACGCCATGTTGCTATGCGCTCTAGTTGCCTGCCTGATGGTCTTCTGGTGTTTGCTTGAG
CAGCCGCGTGCATCCTTTTCAAGTCCGTCCAGGAGAAGCGTTGCGTGAAG
>at02761
TTTTTTTTTTTTTTTTTTGGCAAGAAGGGATGGCAAAGCATACTGACCGTGCCAGGGTTC
GAGCGTGAAAGTGAGCATTGGAAAAGGCTCGTACCTCAAAGCTGCTTCTCTCCAGCAGTA
TCAAGTGCAGGTGCACCCTCGTCCTCACATGCTGATGGCAGTGCCGAAAGCGCCAGTGAT

GCGCTTGTTGAGCCCAGGGTCCACAAGCAGAGTCTTCGGCACATGCATGGCATCGGAGCA
CTTGGCAATGGAGGTCTCGATCTCCTCCACCTGCCTTAGCATGCTTGGCTCCATCGCTAA
GCTCCTTTGCAGACTTCATTCCATCCATGTCGTTGACGCTGGCGAAGGTGCCCATCGCGG
CATCGCCAAGCTTCTGTGCCTTGTCATAGTCATCGTTCTTCATATACACATCACCCAGCT
TCAGCATCGCCCGGCCCTCCTCGGCAGTGTCTCCCCGCTGCTTGGAACATGAGCACGCGG
TCCTTGCCCACCTTGACGGCCTGGCTGTACAGCCCGCCGTCCACCAACATGCCCATGATA
GTCTCCAAGACCATACCCTGCCCGCGCACGTCGCCCGCCTCCGCGAAGAGATCCTTGGCC
TTGTGCAGGCACTCCGCCGCCTTCTTGACGTCAGCCTTCTCAACAATCGCGGCGGCGTGC
AGGCAGCCAGCCTCCTTCGCCTTGTCGCCGAGATCACGGTAGATGTCGGCGGCCTTCTGG
ACCTGCTTCGCAGCCAAAAAGGTCTCTGGTGGCTGCTTCACGAGGTACGCGGTCTTCACG
GTGTTGTAGGTCTCCGCCATTGCGCGCTTCTCGCCCAGCTCCGCAAAGATAGCGCCATGC
TGCGGGCGTGCCTTAGCCATGCTGGTAGATGGCGGAGAGTGTCGCCTTCTTCGGCAGGCC
TTGCGTAGCCACGCCGGAAGAGTTCAGTCGTCAAAGAGCCATGGCCTCCTCGAGCTCCTC
CTCGTCCTTGCCCAGCTTGGCCGCGTGCATCTTGGCCATGAGCGTTGGTGGCCGCAGCTC
GTGCCCTCCTCGAAGCTTCATCCTCGCTGCACATCCCCATGGCCTCCATGACATATTGCC
CATGGCGTCATCGGGCCGGCCCATCTGCACCAGGGCGTCGCCAGCCTTGATCAGGCAGAG
GGCCCTGCCGCGCTCATCGCCCGTGGCCCTCGCCGCATCGCATCTTCTGATGGGCAACTG
CCACAAGCGCCTCCTCAAGGCTCTCATCGATGCACAAGGCCCTGCCACGCTCCATCGCCA
TGGCCCTCGCCGCGTCCATCTTCTGAAGGGTGCAAGTAGCAAACGCCTCCTCAAGGCTCT
CATCGATGACGAAGACCTGGTCAAGCTCCATGGCCATGGCTGACTTGGATTGGTGCTGGA
AGGTGCAAGTAGTAAATGT
>at02762
CCGTAGCCATTTTGGCTCAGCCATTTTGGCTCAAGCCATTTTGACCGAAGCCCGTTTAAT
GGATCAGAACCCCCACTGAACCAAGGGAAATCCGGGTCTCAAGTTTCATGACCGTTGGAG
GTGCCATCGGGGGCATACTTGCATGCCTCATCGGTGCATGGGCGTCCGATGACGATCTCC
CATCTGACGCAATGTCCCTCCTGCAGACAAGTTGAAGCACGCATTGCTGAGCCCTGCTGA
TCGTGCTCGAGATGCGGGTGCATGGCCCAACCCAGCAGATCCAAATCTTCAGCCAAGTCA
AGGCCAGAGGAAGCTATTCGATTGCTGAAGGCATTTCCTCACAATACTGCCCTCGTGAAC
AGGTCCCTGTCTTCCATTGCCCTCACTCCTGTCTGGTATGTTGTTGAGAATGGCGGCTAT
GACATCTTGTTCCGAGCACTGCATGATTACGCCAACGACTCCACTGTACAAATGAATGCG
TGGCGGGGTTTGTCAGACCAAAGTCACACCAAAGTTGGTTCGAATTACATCGCCAATTAC
GGCGGTCCGAACAAAGGCATCGAGTACATGGTCAAGCAATTGCTTGCCCATCCAGGGACC
CGTGATGCAGAGTGTGCTGACCACTTGACATACCAATATGAAGTTCTACAATGCATCTCG
GGACTTTTGGAATGGGACGTCAATGGAACACGCGGTGTGGCGGCTGTGGCCACCGGTTTG
TTGCCAGCAGTGATCCACACAATGCGGGTTGAAGGTGACCTTCGCCCTTCGGTGGATGTG
TGCTGCAAGACTATACGGTCACTCTTGACACAAAACACCTCTTATGTGGAGCCTCTTCAG
AAGCTCGGAGTTGTGGAGTTGATCAACACCGCCATCGCGAACTACAGTGCCTATGATCCA
ACGCCCTTCTACTTTGGTTCCACTTACGGTGACAGTTACCCAGTTGTTCCATCGTGCTCC
GCTTCTCTTACCATGATCCAGGCACACCTGTCATCAACTGAGGCAGCATCATTTCTGCAG
AAATAGGCATTAGGATGCATGGACACCAGTGACTGAAGAGAATGAAGGATGACGACATCA
CCAAAAAAAAAGAAAAAAAAACAA
>at02763
CCCAGGGCCTCAAGTCCAGACCGCCATccCCCCCCCCCCCCCTcGCCGCCGCGATGTCGG
GCTGCTTCACCTGCGGCGGGGACCACCTTGCCCGCGACTGCCCTGAcgGGTGGCAAGGGC
AAGGGCgGCaAGGGCAAGGGCgGCAAGGGCGCCGTCGACTGCTTCAACTGCGGCgGACCT
CACTTCGCCCGCGACTGCCCCG
>at02764
TTGTTTTTTTCTTTCCACTCCGCAGCGGCACAACTGCGGTGGCCGTCACAACGACCGCC
ACCCAGCGACTGCAAGCTCGCCACGCGCCCGCTCGCCCGCCCTCACAAGCAAGCTGCCCA
CGTCTCGGCGCTGCAGTCGGCCTCTCAGAAGTGCCCCCCGCGCATGCCGAGGTCAATGGC
CGAGAACAGCACTGCACTGATGGCCGAGTACTTTGCGACTGGCTGCCAAGAGCGGCCCGC
CGACTTGTAGAGGCCGCCCGCGAGGAGGCCTGCAGAGGCGGCGTTGCCTACATCATCAGC
TCCACGGATCCAGGAGATCAAATTGTTGAAGGACACGTAGTACATTGTGATGATGGCTGA
CTGGTTGCCCAGCGCCGGTCCATAGCGGCTGCAGGAATTCAGCACCGAGTTCACGAAGAG
CTTGGAGGTGGCGCCGCCCTGCCTCAGGCCGAGCAGGGCGCCGTAGGTGCCGCCGCAGAG

CAAACCCAGTCCATACGTCAGCCCAACGCTGTAAGTCATCTTCTCTCCCCACGCGCGGCC
GTAGCCCTCCAAGAAGAGCTCATCACGCGAACTCGGCGCAACTTCCCGCCGCGGGGTGGC
CACGAGGATCTCGGGGGACCGGATCCCGCCAGTGGACAGATAGTCGCTCATCGGCTCTGC
TAAGGGAGGGCGCCTGGCCCGTCCCTGCCGTGGGGGGTGGGGCCGCCGCCAAAAGCACCA
ACGCGCCTTGAGCCAAAATGGCTACGG
>at02765
GCGATGAGAATTCGATGGGTTGTGATCAGTACCCTTTCACAACAACTGTGCAGACGGGGA
ACTGCGCTCTGTGCGGAGTAATCATTGTATGCACCTTTTTTTTCATACTGCAACGACATG
AGCTTTTGGCCACTGGCAATGTCTATCTACCAGGAGTGCTGCCACGCGTTGCGGTTTCTG
CTGCAACATTCGGAGCACGGGCCCATGCGTACAGAAGAGCACCTTCAGGAGACGCCAAGG
AAAGACATTTATTATTCACGGGCAGCTCGCAGGACCGAAATGGGATTTGCAGCTGAGGAA
TGGCTTTTGGGTGTCGATGCGCACCTGGTCCACATCCTTGAACCTCGCAGCCTTCGAGGT
GCTGCCTTTGTCCTGAAGGATGCGGCGATGAGAAACGCATCTTCAGACTGAACAGCGATG
AACAAGTTCAACTTGCCCGTGCTGGGCCTGATCTTCAAGAGTTAAGGAGCTCTGCCCAGA
ACATCCTTGGAGACGCAGCTAGGTGGTCCCCGCAGCCCCGGCCATACTGGTGAGTTGCCG
TGGGCGTGCTCGAGACTCGATGCTGCAGGCCTTGGCC
>at02766
AACGGCCTTTCGTGAGGTCTTGAAAACTCACAAGCCATGATGCTTGACGGTCTCCCGAAG
GCAACTTGAGACTTACAAGGCGGGCTCGGGTACTTCCGGCCATGCGCCCATGATGCTCGC
TCCAGCTTCCGTCAAGTACCTCTCAGCAAGGCGCCGCCCTGCGCTCTTCTTTGCGCGAAG
ATAGACGTCGGCCGCCCACTCCGGCACCTGGTCAACGTTCGACCTGGTGTGCCCAAGTTC
CGACGCGGACGAAGCATCTGGTGCTCCCAGGCTCCACTCAGGGTCAGGCTCTAGGGTCTT
TGTGTACGTTGCCTCATCCACGATGGTCGATTCGTAGCCCAAATCTCGAGCGTGGACCAC
CGTTTTCAAGACGCAACGCTCAAACCGCTGGCCCACGAAGACGAGAGCTGCAGTCTCAGG
ACCGCATCCTAGGCCCGACAACACCTCCCCAAGCCTGCTGCAACCTGTGCAGCCTTCTGG
ACGCCCTCCCTCTAAGCAAAAGGAATCGTCACTGTTCTTCTTGAAGCAGATGTCCACATC
CTTGTCCACCTCGAGCTCATCCAGTAGCTCCGCACCTACCGTGTTCTCCATGCAGAATGG
CGTTCGTCCCGCCAGTAGTTCCGCTTCAAGCCAGCCCATTGTGAATACAACCTTGTCCCA
CTTTATGGACCTCCGAATCTGATTGATCTTCGGCACGAGTGGGCTGATATTGTTGGCGTA
GGGCGACCGCACCTCTCCGTACAGCT
>at02767
CCGCTGTTTTTTTTTCCGAGTGGCAGAGGGTCTGTACATATTTCCGGTCAGTAGCGCCCG
ATGGCAAGAAGCACGGCATGGTTTCTTGCTGCCTACTTCCTCTGTGCTCTTACAGCCTGG
AGCAACATGTTGCCCGTGCCCACAGAGCCTGACCTTGTCTTCCTCATCAACGGACTTCAC
GCGTCGAACACAAGTAAGTGGTATGAAGTGGACTGGGGGTCGCTGGATGAAGGCAGCTCG
AAGTGGGTACCTTACTTTGCGCTGCAGCAGTTTGC
>at02768
TTGGAAAGACCGTGGTGGTCGCGCTCTTCTGGTACTTCTTCCAGCCTGGATCGATCAGCA
CGCTCGTGGACCGGATGCCCGATCAGCAGACACAGGTTGTCTTTGCCGTTCTTGTCCATG
GCGTTTTGGACTTGATCCAGAGCATGGTGACCATTTTCACGGTCTGGCTCGGCCGCTTGG
GGGATCACTTCCTGAAGGGCTAGCGCTGAGATTGGCACTGAATGCGAATAATTATACCTC
CAGCCTCCATGTGGCATGGCTTTTTTGCTAATGTTTCCCTTCCCGCGATGCTTGGATTAG
CAGCCACGGCGTCAGGATTAGGCGGCAGTAGGGCATGTCATCAATCCTGCCCGTGTCATA
GCATCCCTCAATTCAGGCAGTCGAACGGCTCTTGACGCTTGCCCAGTCAGCAGAACAAAC
AAAAAAAAA
>at02769
GAACCCTCAAGAATACGCACTCTTGTTACTCACTGGCGGATAGTGAATGTCCAAAAGCCC
ACTCGTCTCAGGCGAGGTCAGCAGGCACGCTGCAGGGCAAATTGAATCCGCCAGACTAAA
ACTTGAATTTGGATTCCTGAAACAGCTGCTTGGCCAAAATCCAAGCCAGGGATCAGTCTC
CAAACCCAGAACGCAAACAAGTTCTTAAGACAACACAGATGCCGCGCATCTGTTTGGAGC
CTTTGTTTGGaAAAACGAGTCCCGCCTGTCGCGNCGGCCTCAGACAAGGATGGNTGTGCT
GGNGNCgGGCATCCTgccGCCAAGTAGCATGCACNGGGAAGGGCACTCGCAAGCCCAAGC
TCAtTTTTTGCCAGCCGCTNGAGAGTCCGNGCGTCGACCACCTCNGCAAANGTCTCGTTC
NCAANTCCGTCAAAGACAAGACCCACNAGCGCCCCGTCATCCTCGGCATCAcGCCCCCGG
CCGGGGAATGAAGGCATGCTCGGAGACGTAGAcgCCCCTCCTCATACCGCGTGACGCTCT

TGCCTGTGCAGAGGTCGCGCTTCACAAGTGCGGTGGATGCGAAGCCTcCGGCCCCCTGTA
CTGAACTGCACGCCCCACATGATGCAGTGCTTCTTGCCAAACAGTTGCGGGTGGATAGTA
GGCAGTTCGATGTCGTCATCCGGCTCCTGTGG
>at02770
CCGTAGCCATTTTGGCTCAAGCCGTTGCAATCTGGGCTCCAGAGCTCTGCTCTCAGGTGG
GCAGGGTTGCCGTTGATGTGTTGAACAGGACATGGCAGCATCCATTGCTCTCGGTATTTC
GCGAGGCGCTGCTGGTACAGCGGCCAAGCAACTTCTGGGCCGGCTGCCTTCTCGTGGCTG
CGCGGGCCTCACCGCACAATCCTTTGCCAATGAGCAGCAGCTGACACTCGCCAAGGAGAG
GCCTTTCAGTCACCGTGGACTTCCTACGTGGGCATCTCCTGGAGGCTGCATTGATGTGGC
GTCCAGGCCCTCACTCCCAGATGATACCTTCCTTATTCGCCTGCTCGAGACGGGCAACAA
CAGAGAGTCTCCTCGTTTCAAAGACGGCACCTCTCTTCCCGAGCTTGCAGAGGGGGATGC
TATGCCTGTGCCGTCCTTGTGGCCATGGCAGCCTCCTGCCCTCTGGGCATTGAACAAGAC
GAATCGCCTGGCTCGAAAGAAGAAGCGGAAGCGCATGGGTGAGCGAGTGAGCTTGCGGAT
GCGCTGAGGACGTCGTCGAACTGGCCATGCATTACTGGCTTCCTGTCCAAGAAGAAGTGC
GCAGCATGCGGATGCTGAGAGAACGGAGATTCAGGGAGGTAGACTGGCATCATCCTTGGC
ATGGTGGGTTGCAAAAAAAAAAAAAGAAAAAAAAAACAA
>at02771
TTTTTTTTTTTTTTTTTAGGAACACAGGCATTACCACTCGAATGTGTGTCTGTACACCG
CCGTGCACGGATGCGGAAACATCCACACTGATGGTGGACATCAGCAGCCATTGTGTGCCC
CAGCTCTCAAGCACTCACGAGGGCCTCGATCACCCGCTCGAGGCGGCCCGCGTACTTGCG
CAACAAGGTTCGGTTGCGCTCCTCGTCCGCGAAACCCATGGCGGCCAGCTCTCGGAGCTT
CTCCTCCACGCCAGCAGGGGCCAGGGCATCGCCGGCCTCTTCGCCACTGGGAGGCGAAGG
TGCTGGATTGGCCTGTGAACCCGCCTCCGCTGGCGCCGCGCCGGCTTCGGCGCCGCCCTC
GGGCCGCGCGGGCGCCGCCGCGGGCGGCGGCATGGGGGCCTCCGCGGCCTCGGCCGCGCG
CTCGCTCAGCAGGTTCGCCACGCGCAGCAGCCGGCCGCCGTACTTGTCCAGCAATTCCAC
TGCCTCAATCCAGTTCTCGAAACCCATTTCAGCCAACTCCTTCAGCTGCGCCGCGTACTT
CTTTGCGGCTATGGCTGCCTCGGGTGGCCGCATGCCCAGGGCCTTCAGGCGGTCGAAGAC
TGCCACGAGCTCCTGGCCACGGATCTCGCCCAGTGCTGGCTCACTCCGTGCCGCAGCCGT
AGGCGTGCCCTCTCGCCTCGTGCCGAATTCT
>at02772
TGTGCGAGGAGTGCCTTCGCAAGCAGCACACGCGAGGGCAATGGCGTCGCAAGAGCAGAG
GCAAGTCTGGCTCCTGACCGAACGCCCAGCCTCAAGGCCTTCCTCGTGGAACCAGAGCTC
TTTGTTGAGTCGATGAAATCCTGCGTGGAGGCCTTGAGCTCAGACGAGCTCAGAACGTCG
TCGCCGCGAGAGACCGTCATCAATTTCAAGGCACTCTATCGAAACGGCAAGGAGGTCTGC
TCTCGAACCCGCACGGCCGTCTGCAGTGTGGAGCTTTGCAGGTCCCCTGGCGCCTCTAGC
CACGTCAAGCTGCGGCTCGTCACGTTCAGCCGGGCCCACGAGCTCTGAGGGGCCACTTAC
TGCGCGAATTCAAAGGATTTGCACCCGAGGGTCTCAGTGCA
>at02773
CTGTTGGAACAGCCACCAAGCGGAGTCCAGCCCATCCCCCCCTCCTGTGTCTACCAGACG
CGATGGCTGATACGGAGGACAGGGGCACGAAGAGGCAGAAGACAGCTGGTTCAGCTGTTG
ATGGCGTGACTGACTTCCAAAAAGGCCAGCGGGACTTCTCGCAGGCGCAGGGTGCAGAGT
CCAAGGCCATAAGGATGGACTTTGTGAGCGAGTCCAAGGNGTACACCAACGGTGTCGTAG
CCAAAAACCGTTGGAGGAGCCACACTGGACCACTCACCGGGGACTAATCCGCATCCCCGA
CGTGGACTCCGGCTACCCCAAGAGCGTCTTTCCGCTGAAGTGGGAGGAGGGCGGTACGTA
TCCTGCAGCAG
>at02774
GCGGCCGTCCTCGAGCTGTCTTNCNAcGNNAAAGATGAGGCGCTGCTGATCGAGGGGGAT
GCCTTTTTGTCCTGGATCTTGGCTTTGACGTTGTCAATGGTGTCGGAGGCCTCGACGTC
AAGTGTGATGGTCTTTCCTGTCAGCGTCTTGACAAAGATCTGCATNGTCAGACGTCTGAA
TCAAGTGCAGCAAGCACTATCAGACCTATCGGAGATCTTACGTCCTGCA
>at02775
TACTGTACAAGACAAGGGAACAGTCGCCTGAGCACTGTGGTGAACAGAGGCACGCTGAAG
GTGGCAAGTCTCAGAACGTGAAGCTGGCGGAGAAGAAGGTGGAGAGTGAGAGCGAGCGGG
AGAGGGACAGCGGGACGGTGAGGGCTGTAGAGAGAGAATACTGCGTGTAGGAGACCGCCA
GACCGACAGACTGACAGAGTGTATGATGCATGTAGCTACAAGTACAGCTGCACGACTCTT

TGTTGTGGTATTTAGGAATATAGTAGGAGCAGCAGCAGTGAGTGGCGTGGTTGCTTGTTG
TTATTATTTGTAAGGACTCACTTAGTTGTTGCGTAATGTGTAAGAGTAATGATTGATGTA
TGTGATATGTTGGTTATCAGTGTGATTTCTGACTAATGAAAATAAACCTGATGTATTGGT
>at02776
TACCTTGGGGGGCTTCACTTGGAAAGGCTACTTTGCTGAGCAAACATCGAAGACGGTGGC
TTACGAACCCTTCGTCTTGGCCTTGGCATCGAAATACGACCTTGTTGTGGACGCGATCGC
GATCACACCAAAAAGGCGCCAGATGGGAATGACATTCGGTTACCCGATTGTTGATCAGAG
TCCAGTGTTGGTCGCCCGCGTCCAAATCGAAAGCCCGAGCTTTTTTCAGATGTTGATGCA
GGTTTTCCGGCCGTTCGACCAGAAGACGTGGGTGGCGTTGCTGGCATCTTTCCTATTCAC
GGCGTTCGTGTACATGGTGATTGAGTGGCCTGGTGACGATATCAAGGATTACACAGCACG
CTTCTTTCATGCATTGGGACAAGCAATGTACCTGACCGTGGGTTCCTTCACCGGAGGGGC
CGGTTTTCACCCCCGGACGACACGCCCCAGGGAAATGCATCTCTATCGGCCGGACATACG
CAGTG
>at02777
GTCGCCAACCCAAAGGTTCACAGCTTCTGGCTCGTTGCCAAAAGCGCTGACACCAAGAGG
CACCGCATCTGGAATGTCTTTGACCAATTCGGGCAGCTCATCGCGGAGCGAGTCATTCTG
ACGCGAGAGGTATGGCACGCCGGGGCGCTTTGCAGCATCCCCTTGCAACAGCCAATCCCA
AAAATCGCGGAAAGGCATCCGCTCCTCGCAAGGCTTCACAAAGCGTCCGCTGCTATCTAC
CGAGTCCCCTCGGCCACTGGGAGTGGCATTGACAGACACCTCCACATCACCCACCGTGTC
GAGCAAGTATGCATCGTCGCGCCAGAGCTTTAGACATGGCCAGTCCTGTTTGCTCAGACC
TGTCAGAACAAGGGGCTGCGAAGCAGCGACAAAATCACGAACAAACTCCAAAGGAGTCAC
ATCCCTGGCAGGCTTCCTTGTAACAGAGCGCGGCACCCAGAAGCTCCGGGAGTTCTTCGA
AAGGCGTGCAAGACTGCGCTGTCTTCTCCACCTCTCCTCTTTCAGGAGACCTTCAACATG
TGCGAGCTTGGCCTCTAGGTCTGCCCGAGAAGAGCTCTCTGAAACAGCATCGCTGGAAGC
CAAAATGG
>at02778
TCCATGGCAGCACCTCTCATTGTCGATTCTCTTTCGTTAATAGCAAAAGTAATGTTGTCA
GAGTGCTCAGACATGCGGCTAGTCTGCTCTTGCGGCGTTTCCAACAAATCTTCTCGCAAA
AAACACTGGACTGCTTACTGATATTTTGACGATCCGCAGCAAAACACATGCTTAATTTTC
GCCAACACAGCAGCAAAACTGAGCTCCACCATGACCAATAGGATCAGGTAAACAACACCC
ATATCAAGAGACTTTGCACGAACAATCCCGCCACAGCGACATCCAATACACTGGCATCAT
TGTTCAGAGTACGCGCACCGAA
>at02779
TTTTTTTTTTTTTTTTTGCGGCAATAAGCAATCGACTGTATGGCAATGCCCCAAAAAGT
TAGGAGGCGTGCAAGCCAGCTTCGGATTCAGCGCACCACTTGACCTCACCAGCTTACGTA
GGCATCATGATGCCTCTGGCCAGGCATGGACTGTATCGCCATCCGTATCCCGCAGGCTGC
TGCTCGCTGCTGCAGCGAGTGGCTCAGTTGCCGGTTCGGCTGCTCTTCCCTCCACGGACT
CCCCCCAACTGAATACCCGGTGAGCATGCGCTGTCTGTCTCCGCGCGGGGGATGCCATCG
TTGGGATCCACGGAGGACCAGCAGCATCGGTCGGTCCAGAACCAATGGTCATGAGGCCGA
CGATCCAAGCTGTTTGGCTCGTACACGCTCGCAAAGTTGATGTCCGTGAACTCCGGGTGC
GCCACATCGTCGTCGCAGAAGATCGGCGAGAGGCAGTCCTTGCAGGAGCGGCGGTAGCCG
TTCTTCGACGAGTAGAACTTGTGGAGCTTGTCCTCTCCCTGCAATATCTTTACCTGCGAC
TTGTTGACCGCAACCCATGAAACATAGGAAGCACCCGAGATTCGGCGGCAGACCGAGCAG
TGGCAGTGGCCAGCAAAGATTGTGGGGAGCGAGAATCATATTTCACCGCACCACAGAAGC
AGCCGCCTGCAACTTTGCCGCTTGTCGGAAGCCGTGGCTGCGCCTCCTTTTTCGGGCGGG
TCAAGTAATAGACAAAGCAGCTCCGGCTGCAGCTGCTGGCACGCGA
>at02780
TTGTTTTTTTTCTTTTTTTTTTCATATTTGTACTTGTATTAAATATTTAATGTGTAGAA
TAAAATATTAACTTAAGGAACGCAAATGCACCCCAAATCATTCTTATGCTATTCTACACA
ACATTGATGCCAACGCAATCCATTCACATAAAAATGTAGCCAATACATGCAATAACAAAT
AAAATAATGTATATGGTGACAAATGACCGATATACAAATCTTGGGTCACATGATTGCATT
GTACACAAGTGAAAGAAAAGAAGCTATAAATGTAAATAAACGCACTCAAGTAACACAAAG
TTCATGGCTAAGCTACAATGAAGATAAAACAATAACC
>at02781
AGTCCACGACCTCCTCTGTGTTCCCCTGGCCCTGGACCTTCCTGCTGTGCTTCGTCTCCT

TGCACTGGTACATGGCCACCTCCTGCCTGACCTTCACAGCCTCCACACTGAACGCACCGG
AGAGCCACTCAGCACCAAGGCTCTGCGGAGTCCACGTGGGCACGCTCTCCCTCGCGATGG
GGCAGGAGAAGTAGATCGGCCTCCCTTGGTCGCCGGCCTGCCGGGGGCCGTCGCACTCGA
CGCTGAGGTAGTTGGCGCGCGCCGCTGCAAGGGCCTTGGCGTGGCACATCGTGGACCTCT
CCCAGATCGCGACCGCAGCCAGCGCTCCAACATACAGGCCACCACCCCCCACCAGGCCTC
TGGCAGAGCGCACCGCGGACC
>at02782
TTTTTTTTTTTTTTTTTTACCTCGCGGACCGATCACTTCGGATCAGCCCAGATCACTGAT
CACAGGCCGATCCGCTATCAAGGGCCATCAAGGGTCTCAGGCTGTACATCAGGTTCAGGA
CCTCACAACGGATGGCACTGTACACCCCAACCGGTTTAGCTCTGCCGACTGGCCTGCCAC
CCGCTGTTCTGGCTTGCCCTGCCGTGTCGGGCGTGGCTTTGCCAGCTCGCCCAGCTTTGC
ATCTCGCTGTATCCTGGGCGCCAAGCATTGCCACACCTGCTGGACAGGGCTCTCGTCAGC
GCCCGCCAGTCCAGTCGCCACTGCTCGGCAGCCGCACGAGGCAGAGCGTGACGTTGTCCC
CGCCGTGCACGACCAGCTCCAACGCCCTCTCCACGTGCCCGCGCACGAGCGCCTCCACGC
GCTCGGGCTGCATGTCCGGCACGATGCGCATGGAGAACTTGCCCACAACGCAGGCGGGGA
TCACGGTCTTGGAGCCGGGTCCGTCGAAGGCGCCCTCGATGCCGTGCAGCGAGAGCGTGG
GATAGCGCCAGCGGTGCATGAGCACCTCCTCCTTGGTTTCGTGGAGCAGCTTGTCGGTGA
CCCCATCCACGCCAGCGTCCTTCTTGNTATCCTCCACGTCGAAGTCCACCCGCGCGTACA
TCTCCCTCTCGCGCTCCGTCAGCGGNGCACGTCTTCGTAGATGCGTCCACG
>at02783
TTTTTTCTTTTTTTTTTTTCCACAGCAGATCCGACACTGGTCAAAGGGTGACCCCGCAGCC
ACCAAACGTGTCCCTCAGTCACACTGGCAACCCAGAATGGCACACCTCATCTGGCCAACG
ATAGCCTGCAAAGGGCCGCCACGACGACTCCCCTTGTGGAGCACTCCGCGGAATGCACCA
CTCCATGTGCCCAGAACTGGCTGACCATCTCCTCTGGAACTTTGCCGGGAAGCATTGTCA
AGCTCCTTTATCACCATCGTCGGCTCTTGCTGGCGCTTCAAAGGGCTCCGGATGTAAACT
GTTCCCCGGCACTTGTAGATCTTCAGAGCAACTTTGGCAAATGACATCAAACACATTGGC
ACTTGCTACCGGGTCATCTGGCAGAGCGTCAGCATCTTCCCTGATGCCATTGGCATCCAG
CTCTTCCATACTATGCTCAGGTGATTCTTGCACTCTGAATTGACTCGAGCAAACTATGGG
CATCGGCTCAACGCATTCCAGTGACTGGGCCTTCACACCGACCTCCAGCTGTGCAGGAGT
ATCTTCAGAGGCAGATGAAAGAGGTAGTGCACGCGTCGCGTCCATCAAATGGGCAACCTC
CGGTGAGGCTGCTCCAGGAGGCACAGCATCCATCCCCAAAACCAACAAGGACTCCCTCTG
ACGAGATTACCGAAAGCTTGAGCCAAAAATGGCTAACGG
>at02784
CCGTAGCCATTTTGGCTCAAGCCACTTGGGCTCAAGAGCACTGCTGATGGGGGCCCTGTT
TGGGTGCACCGCCACCGCGCACTCGCGTGGCCGTGCCCAAGCAACGCTGTGCTGGCTCTG
CCGCAGCGTGGCTGGCCTTGGAGGACAGGGGCACGATGGCAGGCATTGACGCCAGCCGCT
TCAGGAGGTTGACCCCACTTCTAAGCCTGGCTATGCTGATCCAAGGTACTGGGTAGCCCG
CTACCAGGCCGACAGCGAGCCTTTTGACTGGTACCTCCGTTGGGATTTGTTACGGGACCT
GCTTCTGCCATTGCTTTCGGCGGATGCCGAGATCCTGGTCCTCGGGTCTGGTACTAGCCT
GCTAAGTGAGCAACTCTATACAGAGGGGTTCATGAACATAACTAATGTGGACCGCTGTGA
GCTCGCCATCTCAGCCATGCGCGCC
>at02785
TTTTTTTTTTTTTTTTTTGGGGTTCAAAGACATTCAACACAAGTCAAATTTCAGCAAACG
CGACAAATCTCATCTGAGACCTGCGTTCTCTGAGCAGTTTAAACAGATCACACGGGGGAT
GTCAACGCTGATCCGCGGCCACCACAGAAGTCCGTGGCTCAGCCAGGCAGTGCCGTCAGG
AAGCTCAACCGGAGAGAAGCCCAGAAGTGCGGGCATTCCCCAGCCAGAGAAGGCACAACA
TAGCACAAGATGAAGCTGCGCCGGGAAGACCGCGAGGAAGTGCGCCCACAGCGGCAGCGC
CTCCGCGCTCTCGGGCTGGTTCTCGAAGGCGAAGATCTCCTGCTCGGCCCTGGCGGCGGC
CCTGCGCTCCTCGGAGGCGTCCTGGGGGCACGGAGCGACACCGCTGAGAGCCTCAGGTGC
CTCCGGCTTGGCAAACCGGGGGAAGGCGTCCATGCCCAAGCGGCAAGCGAGATAGACAGC
GGCATCCTCGCACATCTCCTGCACGAGAAAGGCGCCAGCCACCCAGAAGTTCTCGCAAGG
AACCATTTCATGGCCCAGAGAGACCCTTGCAAGCAGGAC
>at02786
TTTTTTTTTTTTTTTTTTGGTCTTCCTCTGCTCTGCACTACGGCCCAAACTTGCTGAAAA
GAATATCCCATGGCTCCATGCCATCACTGGGCCTTGGATGCCGCAACATAGGCCTGGGCT

GCCTTGGCAGCGCGGCCGTCATCGAGGGCCTCGAGTGCCAAGGACGTGCCCTCCTTGAAG
GTCAGCCGCCTTCCCCGCCACGTACAAACCGGCGCCGGCACTTGAGGGCAACCGCCGCAC
GCACCGCCTTGCGGCCCTTGCCACTGAAGAGCTCTCTCATCTCGGCCGCGTTCTCCGCTG
CGGTCCCGCCTGCGATCTCGCACAGCGGATGGGCCTCTATCCCGAAGTCGGCAGGGGCGA
CCTCGTAGTGAGAGATCGCTCCGCCGTCTGCCTTGAGCTCCCAAACCTTCGAAGGCCCTG
CAATGGAGAGCTCGTCGAGCCCTTCGCAGCCGTGAACGACCATGGCGCGCTGCATCCCCA
GCATCCTGAAGACCTCCGCAAAGAGCTGCCCGAGGGTGGGAGAATAAACGCCGCAGACGA
TGCCGTCCGGCTTGGCAGGGTTCGTCAGTGGTCCCAGGATGTTGAACACAGTCCGAATCC
CGAGTTCCAGTCGTATCGGCCCCACATGACGCATTGCCGGATGAAATGACCGCGCAAAGA
CAAAGGAGAATCCACTCGAAGACAAAACGCTAGAAACCTCTCCTGCTGACAATTCAAGCC
GGGCGCCCATAGCCTCTAGCACATCAGCTGCGCCACTCTTGGACGATGCTGCACGGTTTC
CATGCTTTACCACCGAAGCCCCAGCACCTGCCATGACGAGGGCCGCGGCAGTTGAGATGT
TGAAGGTGTCCTGTCCATCGCCGCCAGTGCCGACGATGTCGGTGATGAACCCTTTGTCAC
GTGCCAGATCTACAGGTATAGCATTTGCCCGCATGACGTCTGCAAGAGCGTGAACAATAC
CAGAATGGCAACGGTCCAAGCTCAGTAAGGCGAGGAATGCCCCAACTTGTGCCGGTGTTG
CCTCGCCTGCCATCAATTCACGCATAGTTTCACTTGCAGCTGCTGGCACCAGTTCGCGTC
CAGCTGCCAAGGTCTTCACCGCATCTTTGATTGCACCTCCCGAAACCTTGAGCTTTTTGG
CATCCGACTCCACACTCATGGCTGTACGAAGGATCTTGAACCTGTAAGCGGACCGAAAGT
GTAGCCGACATGTCTTGAGCCAAAATGGCTACGG
>at02787
TTGTTTTTTTTCTTTTTTTTTGCTTCCCGGGCGTTCTGTCGCCCTTGGTGCCCGCATGTC
TCCACACACCAGCACCCAGCTCCACTGCGGTCGCTCTCAGTCCTCGGCCTTTTCCTCCTC
CTCCTTGGCCTCCTCCTTGGCCTCCTCCTCCTTCTCCTCCTTCTCCTCGGCCTTCTCCTC
CTCGGCCTTCTCCTCCTCGTCTACGCCCTCGAGGATCTTGCGCTTCATCTCAGCGCGGGT
CTCCTCCCACTTGGCCACCTTGCTCTTGTACTCCTCCAACTGTGCCTCCAACTTCTCCAG
CGTGGAGGGAATGTCGTCTGTGGTGATGGGAGCATCGAGCTTCAGCTGGTCGAAGAGACG
GAATGTCTCCGCGTTGTGCTTGATGGGCTTGGAGCTGCCGCCCTCCTTGCCGCCCTTCTG
CTTGCTCTTGCCCTTCTTGGCCTTTGGTGGGCGCAAAGTAGTACTCCTCCTCCTTCTTCA
GCGGGACCAGATCGCCCTCTTTTGAGCTGAGCACCACCTCCTTGACCTCTTTCTCCTCCT
TCTCCTCTTTCCCCTTAGTCTGCGTCTAGGCCCTTGCAGAAAAGGATGGTCTTGCTCAAC
GAGCGTCATCTCTGCCACGTATGAGCTGCTCATCTGAGCTTCATCAGCCTTCTTCAGCTT
CCGGATCTGATCCTGTTCCGCCTGCCACTTCTGGCGCTCCTCCTGCTGGCGCTCCTGGCG
CATCCTGCGCTGCTCGGCCTGCCAGGCATAGTAGGCCTGCTCTTCAGCGCGGAAGTCGTC
GCGGATCTGATTGCGCTCCTTGATCTTCTCCTGGATCTGCTTGCCGATCTCCGTGCGCTC
CTCCAAGATCTGTGGCATGTCGCCGAGCTGATCCTTGCGAGAATCCATGAGCTCAGTGAG
CTTTGCGGACACTGCTTTCTTCTGCTCGCGGTAGATGGCCATCTCCTCGTTGATCTTAGT
CACCTGCTCCTTCATCGAAATACCCTCAGTGAAGCTGGCCAAGCCCTCCTCCATCTTGTT
GACCTGTGAGACCTTGGGCCGGTTGCGCTTCAGATCCTGGATCTCCTGAAGCATCTTCTT
TTCATCCTTGATGGAGTGGGACTCCGTCCACAGCTTGAACTCAATCGTGGCGATGCGATC
ATCTATTTGTTCCTCGGAGTTGTAGCCAATAGTCTTCTTCATCTTGTTCACCTGGGAGCG
CATCTCCCGCCCCTCCTCCCTCTTGTCACCAATGACCTTGTTGATCTCCTCCTTCCGTGC
CTGGAGAGCGTTCATCTTGGTACTCAGCTCAGAGATCTGTTCGTGGATCGCGGACTTCTG
GGCAAAGAACTCATTTTTGCCGCCCGAACGCTCATTGATCTTTTCCGTAAGCTTTGCCTG
CTCCTTGTTGAGCTTGTCGATCTGAGCACCGATGGCGGCGAGCTTCTCATTGTGCGCCTC
CCGGTCCGGCTGGGGTACCTTGTTCTCTGGCTCCTCGGTCTTCTCCACCTTCTTCTTCTT
GGGCTCGGCTTTGGCCTTGGGCTCAGCCTTTGCCGCCGGGGCTGCTGCAGCAGCCTTCGC
CTTTGGTGCCATCGGGGCAAAGAGGGACGAGGAGCGGAGGTGGGAGACCTGGGTGTGGGC
GTTTCGGACTCCTGTCTTGACCTAGAAAGGCTGAGCCAAAATGGCTACGG
>at02788
TTTTTTTTTTTTTTTTTTCATGATGCACACAATGTGTGGTCCATCACTGTGGCGCGTCCG
CTGACAACGGTATTGGCGCACCGGCACGCCATGGGGGCCGCATCCCTGTACCGCGGCACA
CATTCGTTCCATGACGGATCCTACCAGTTGGGTGTGCGTGGCAAGGTATTCGCACGTGCG
AGTGTGGAGGCAAATCAATGCAAAGTTGCAGGCACCTGTCCACGTCACGCCGCGCACACA
GGGCTTTAAAAAAGTAACTCACCGGTGCCCACCGGCGAGTGCAAGGTCTTGTAGTATCAG
GCGAGTCGGGGGTTCTGCGCGACGTCCTTGCGCCACTCACTTGGTCGACTTAGCGTCCCA

GACGCCCACGTCTCCAGAGGCCGCGTTGCAGTCGACACCGCCGCCATACTCTGAGGAGCC
CTTCTCGTAAAAGTCCTCGCCGACCTTCTTGTAAACGGAGGAGACGTTGTTGGATCCCAG
GGGGAAGAGGGTCTTGTAGGTGCCGTTCTTCAGCTGCTGCTGGTAGTCACGCTGGGCCTT
CTCCACCAGAGGGTTCATGACGGCAAAGAACAGCGGGGGGAACCACGAGAGCACGAACAT
CCCGGCGTAGCCGGTTGGATACGTCGGTGACTCCTTGTATGTGCGAAGGATCTGGTAGGG
GCGGCCCGCGTTGACGTGGTGGTCGGGGTGGCGCCTCGTGCCGAATTCTTG
>at02789
CCTGGGCCCTCCAATCACGCGATGGGCAGTGACAGCTGGAGGGCCTCATCACGGGCAGGA
CGTGTACAGCATCATGCAGTTTTGTGGCATCCTCTTCATGATCGCCGTTGCCAAGATGAT
TGTGCTCCCTGCATACAGCAAGCTGGTTGAAGAGTCGTCCAAGGAGGTCAAGCTTGATGA
GGGGACAGGAGAGCATGAGTTGTTGATTGGGTCCTGCAAGGTGAAGGACGAGTACGGCGA
GTATTGGAAGTCGTTGCCGGATAAGCTCGAGCATCACAAGCTCACCGAGGGGCCGTTCAG
CCCTTGGGATCAGGACTCCGGTGGC
>at02790
GCGCCGTCCTCCCCTCTGCAACTCCCTACGGAGCTCGAGCGCGAGTGGAGAGGAGCAGAC
AAGAGAGAGTCACACCACATGTAAAAAGGGTTAAATGAGTCTAGGCATGGGATGGGATAG
GAGAGGATGGCATGGGCCAATGCCATCACATGCTGAAGGACATGCCCAGCTTCACAAGCG
CCTCATCGCTACCCGCCTGCCGCCCAGCCGCCTGGAAACCCAGCCCCCGCGCGGTGCCCG
CCAAGGGTTCCATGGGTGTCCTATCAGCCAGGCCAACGCTCATTTTCCAACTCCCCGCCG
CCATCTCCGCGCCACTGCAGTACTCCACGCCCTCAGCTGCCACAGTGCCTGGCACGGGAG
CCCCTGAGGGCATCCTGCTGGGCAGACATCACATCCCAGATAATGAACTCCATCTCATCC
TCTCCGTCTGGG
>at02791
TGTAAGGACTCGAAGCGCCTCGGGCGAGAACGCATGCCAGTCGTCGGACATGATGAACAT
CGAGCTGATGGGTCCCTTGGTGCCCTCCAGCCACGGCAGCAACGCCCCCGCGAAGGTCCC
CAGCGAGTCGCTCAGGTTGCCGGCGTCAAAGGCCTTCCGCTCCTCCGCAGACTGTTTGCC
ACCAAGAAAATCCCTGTACTTGTCAGTTCTCCGGATGTGGATCAGAGTGACTGGGCCGTT
AGATGCCACTTCCGGCAGCTGCGTCCGGCGCTCGACGCCCTCGATGGCCTCCCGGAGAGC
TCTGTTCGGGCGGAAGGCCACCTTGAGAAGGAGCGCACGGAACATCCCAGCCGCTGCGCG
GCTTCAAACTGCATGGGCCCGGGTTGAACGTGGGACTCAGCGAGCTCGAGGAGGGACGCG
GAC
>at02792
TTTTTTTTTTTTTTTGTTGGACTGACTGTTGCCAAGACAATTGTGAGTCGTAGGTCCAAG
CCAACCTTGACAGCAACTGCTGCACTACAATCAAGGTGGTGCACTCAAGAGTGTGCGGCG
CCGAGATCGCACACGGCCGACGAGAACACCGATTGAAAAGATATCGCCAGTGTCTGGTAC
CACTGGTGGACTCCAAACGGACTCAAGAACAAGAAGGCAAACCAGTCCGGCAGATCACAA
GTGTGGATGTTGCGGTACCCTAGTGCGAAGATCCTCTTTGGGAACACCGGACCAGTGCAG
CTGCCTCTCAAAAGATCCCATGAATGAGGTCAGCCCACCTACGAGTTGCTCGATTCTGCT
CTCATCTTCACCCAACTCTTTCATGAGAGCAATGAAGGCCCCTGCAGTCTGCACCCGCTC
TCCGTCTCCGTTCTTGTACTTGCGAGTGTTGCCATATTGGGCCCGCGTAGCGGTGGACAC
GCAGCATCGGGTCACGCGGGAATCAATGGACAGGTTCATCTTGCGGGTCTCCTTCCAGCC
ACCATCCAGGACAATAATGCAGTCCGGCGCTGTGAAAGCGTCCTTGGCTGCCTCCGCAAG
GTCCACGGCCTGCTTAGCACCGGGGGCGGGGA
>at02793
CGCTGCAATTGAAGGAGGCACCTGCCGCCGCCAGCCTCTCGTGCCACAGCGCGAAGTCAC
TTTGCCTTTGCATGCCGTGGTTGAAGAGCGTGGCCTTCGGGAGATCCGACTCGGTGCACG
TGGTGTGATCGCCAAGGCACTGGTGAGTGTAGCGCCAGGCGGGTATGAACTTTGCACCGT
GGCGCCAAGCCCAAAGGCCGACTGTAGTGTCGTACGACCCTCCGGTCGTGCTGCACAAAA
CAGAGGTCCCACCTTGATGGGCTGAGGGAAGCAGTCGCTCCATCAGCCCCTTCGAGATGA
GAGTGTAGAAGAATCCCTGCGGAATGAGATCGTTGCATTCGTTGAGCGCGTGGTTCCAGT
AGCGGACTTTGGGACCGTTTTTATCTCCATCTGCATTTACAATTAATGGCATGGACG
>at02794
TTTGTTTGGTTGGCTACCCTTGCTTTGTCGCACCGGACGCTTCGCACCTATCGAAACACG
GCGATGCCGGTGCTTAAGCCGAGCCTTTGAACACCTAACTAGCCAATCAAGCGCGGCCCT
CGAGATCGGCGGTCCTGGAAGACTTCAAGACCAACCGCGGATCAGTCCTTCACCTTCTCC

CGTGCCGCGAAGTCGCTGCGAGGCATGCTCATCATCATGAGCTGTGGAATACCAGCTCCG
ATGAAGGCTGCGATGAACCAGGCCGTGCCCTCGTAGATGCCCCGCTCCTTGCAGCGCGCG
TACCACATTCCTACGAAGACCGTGTCCAGTGACTGCAGCAGCGCTCGCATGTTGTTCGGC
CCACGGCCGAGAACTCGCCGTTGCCGTAGCCC
>at02795
TGTACTTCCGCACGAGGCCTGCGAAGCCCTGGCAGTTGCTGTAGTGGAGGATGCATCCGT
CCTTTGTGCTGAGGCGATGCTTCGCCTCAGGAGGCACGTTCCAGAACTGGCTCTTCTGGG
AAAGGCGCCCAGCTTGCGGCCCTCGCGGACGCGCACGGCAGACTTGCCCATCCAGTAGGC
GGAGAAGTACATGTCGTCGCTGCCGACTCCGCGCAGGCACTGCTGGTCCCCGGTCCTGGC
CACGACCACTTTCCTGCCATCGGTCATTATCTTGGACCTCCAGAAGCCAGTGGCCTTCCT
CTGGGCAGAGCTCAGGCAGTACGGGTTCTTCTTGAACAGTGTGCAGGTGATGAAGAAGTC
GCTCGGCTCAGTAACCTCGGGAACGGCCTCGTGATTGAGGATCCACAACTCCAAGACATT
GGAGGGCGTCTTCGCAAAGACCTTGCCAACTCTGTGGGCGGACATGAAGGCCTCGTCTGC
ATCGAGGTTGCAAACCAGCCAGTCCATGCCGATTTCGTCAGCAAGTATCAGGCAAC
>at02796
TTTTTTTTTTTTTTTATCTTTCATGCACACACACAACATCAGTCCGTCTGCACTTGGGG
CCAAGTGTCTGCACGGGCAGCCCTGAGGAGCTCACTTTGCAGGAAAGGCCCACCCCACCA
CGGCCCCAAGTGCAAGCCCCGGACTGCAGACCAGCCCGAGCCGCTCAACAGGCAGACTGG
GCGCGACCCTTGCGGGGTGCCTCCGGTGCGAGCGCCTCCCCCGGCAATCCGCGCGGGGGG
AGCTCGGCGAGCCTCCCGGCGCTGCTGCCGCCGCACTGGTGGCCAAGGCGGCAGCAGGGA
CGTCGCCCTGCACGGCCGAGCTCACTTGCCATCGAGGTCCCCGGGGCCGGCAACGGGCGC
GGGCGCGGCCGGCTGGCTGGCTGCAACGTCTGCGTCCGCCTCCGTCCACTCCAGGCGGGG
AGAGACCTGCGGCGTCGTCCAATCCTGCTCGTAGAGTTCCTCCACTTGTCGGTGTCGAAC
TGCCCCAAGTACGGTGTCCATGGCGCCCTTGACCTCCACGGCCCGGCGTGCCTTGTTCCA
GTTCCCAATCGCCCGCGGAGGTGGACTGCAAGGGCGTGTACGCAGTCTCGTCGGCAGTGT
CGTCGGCCTGCAATGTGCT
>at02797
ACTTGTTCAACATGCTCAGATGGCCTCAATAGGTTTATCCGCTTGCTGGCAAGCGGACAT
TGACGCAGTCGACAAGCTGCAGGTGATGACCACAGAGGAGATTCACTCCTGGCTGGAGCA
GAGGTTGGCCACTGAACGGGTGGCCCTCTTCGCTGAGCTAAATGCCAAGCTGGATCAGCT
CTTCGTCAACCACCACAGGGCATTGCTGGATGGATTCCAGGATTGCTTGAGTTCAACCCT
TGCTGGACATCGCTCCTCGCGCCCAGAAGGGACTTACTTGGAGGTGTCACAGCCAGGACC
AGAGCCGCCTCTCGACAACGGGGTCAAGTTGGACACCCCCACTGGAAACGAGCAAAGGGT
AGATCAGGTGGCCGCTCCTTaACaACCTCaACaAGaAAGTCCGGaACaAGaCCTGaAGCT
CAAGAGGAGCCGCTCATCAGACCAGTACAAGACCAAGCAGTTGGCGGCCCTAGCATCTCA
AAGTCATTCGAGGCACTCTTTGGGCGAAGATGCTCCATGTGTCAAAAAGGTTGGTGGCGA
GCAACACGTTTGAGGTTCTGTGCTCGGTGGTCATCATTCTCAACACTGTAGGCTTGGCTG
CCGAAGTGCAATTCACCGGGTGCGAGGTCGCGCACAGCATCGGCCATCCAGAATGTGCAA
GTCCAGCTGACGAGATTTGGCCTGCGGCGGCGACTGCCTTCAGGCTCATGCCGCTGTG
>at02798
CCATTGGCTCGCCCAAGGGACATGACGAAGTGGACGACTCCCATCGTGCTGGACTCCTGC
CCCGGTGAGAGGCCAGCGCCGGTGGGGCAGCCGACGCTGGCTGACCAGCTGGCAGCTGCC
AAGCAGCTGATGCTGGCCTTGCCTCGGAGGGACGAGCTGACCACCGTGGAGCAGCGTGAT
GACCGCCTCGCGCCGAAACAAAAAAAAAAAAAAAAAAAAAAANAAAAAAAAAAAAAGAAAAA
>at02799
TTGTTTTTTTTTCTTTTTTTTTTCTTTTTACCTGCAACCTACAGCCGTTTGTCAGTTGAC
CCACCAGGCTTAGCCCACCGCCAAAGTCCCTGCTTTCCAAGTCTTGCTGAGACTCGCTGC
CAGAGTTGCCACCGGACGTAACACCACCCCAGCTCCACAAAGGTGACGGCGAAGAGCCAT
TGCAGCGCCCACTGCCGCAAAACTGAGAGCCGCTTGCCGCTCTCAACGTAGAGAAAGAGC
AGCGCTCCAATGCCGCCACAGAACGCGAAGACCACGACTTGTAACGGCACCCAGGCTGCC
CCGTTGGACAGCCGCACAGCGGCGACCATTGTGCGCCGGGTGATGAACTTTGTCGCCTTG
GACGCTGCTAGCGTAAAGATCCAACCCTGAACCTGAAACCGACCGCTGGCACTGGCAGCG
GAGTCGCCGCCCTGTGGCCCAGCAAGTCGCCAGAGCAGCATCACGAGGCCGCCGACCCCT
AATGCCATGGCCCGCACCCGTTGGTCGCGCTGCGTCAGTAGGCCCAAAAGCTCATGGCGC
CTGCGCACGAAGGTGCCAAGGCTGCCGTCAATGGCGGCTGTGGCTGTGGTGCCGGGTAGT

GGCAGGGAGGCAACCCCTGTCTCATCCTGACGGGCACCCCTGTGCATCTCGAAGGCTGCA
TATCCCGAGACGGCCAGCACAAAGAGCAAGTCACCCAGGGGCAAGGGTGTCATGCGGCTG
AGCAGCCAGCACAAGCCCAGGGAGGCGATGGAGGCCGAAGCCACAATTGGCACGCGGACA
TCCCAGCCCTCCAATGCCTGCACGTCGGCATGAGTCAGCTTTGCCATCGCCCAGCGAAAC
TTAAAATGTAAAGAAAGGGCCTAGCTTCTCAGCTGTCATGGTACAAGTTTCCTGCCGCTG
CCCTTGTCTTGAGCCAAAATGGCTACGG
>at02800
TTGTTTTTTTTTTCTTTTTTTTTTTGCACGCCGGCGCGAGTGCCTCGGCTGCAGTGCGCG
CGCGCCCAGGTCGAGGCCGCGCTCAGAGGTCCGCCGCCTTCTCCTGTGTCTTCAACACCC
CGAAGCGCTTCTTGCACTTAATGCGCTGCTCCGAGTACTTA
>at02801
TTTTTTTTTTTTTTTTTAACACACCATGGGCCAATGGCCCGGCGAACTTCTGCTAGGTG
CGATGCGGTGCGGCGCAGGGTGCATGCACAAGGTGCAGGCTCTGATCTCCCTAGCCGGCG
AGGGGCATCACTTTACAACGCGGACGCACCAAGCAAGGACAACGAGCAGCTCCTCAGCAT
GCCCTGCAGCCGGCTTCTCAGTTCAGCTCCTTGATCTTCCACAGCCCGTCGTTGAGGAAG
TGCGTCTGCTCGAGGCACTGGCCCTTGTTCGTGTCCTTCACCTCTTTGTGGACATCGTC
ATGACGCCAACAGCGCAGGCATGGACCGTGCCCTCCGCCAGCACCTGCACGGGCGCTCCG
GCCTCGACGTCCTCCATGCGGCCGCCGGGGCTGGTGAGGCCGGGGCACATGATGCTGGCG
CCGCGGAGCACGAACTTGATCGCGCCCTTGTCGACCTGCATCTTGGGCATCATGGAGGGG
TACTTGTGCGCCAGCCGGAGCGTGGGCAGCCAGGGCCCGTCGCGGTGCTGCCAGAAGAGG
GGGACCTTGTCCACCACGACCATGGTGATGTGGTCCTTGCACTTCGCGATCACCATGCTG
CCATCCTTGGGCCACACGTCCTTGAAGACAGGCTCCAGCCGCGGGTATGTCTCCAAGATC
TTCTGCTTGATCCCTCTCTGCACGCTGCTCTTCACCTGGTTCTGCCCCGACACATCCTCG
GGCCGTGAAGCCCTTGAACATCTACCTGGAGTCTTCCCTCTCCTCCAGAGGCACTGACAG
GTTGCAGATGCCACACTGAGCCAAAATTGTCTTGAGCCAAAATGGCTACGG
>at02802
TTTTTTTTTTTTTTTTTCATGCAACTTGTCGCTCTTTCAAGGTTGCTCCGAGTGCAGCA
GCAATGCCGGCCCCACGCCTAGGACTCGGCCTTGTCAGAAGCAGCAGCGGCCCCACGCTT
GGCAGCAGCCTGGCGTCCGGTCTTCATGTTTGCGACGGTCAGGCCCTTGTGCCACGGTAC
GGAGATCTCGCGGTCCCTTGAGGCAAGCGAGGCCGTCTGCGAAAGCACTCGACCCTGCCC
TCGGGCTGCTGAGGCGCCAGCCCTAGCGTTGCCACTGACCCCGGAGGCGCTGCCTGTGCC
CTGGCGGCCTTCACTTGCCTCCACACTGAACTGGGGCCTCTCCTCTGGTCGCCCGCATGG
CTTGGCCAGGTACTTGGACGCATCGACGCGCTGGCTCAAGTCAGGCGAGGAGGCAGCTTT
CTCCATGCCCGACGAAGCCCTGGTCCTGGCGTCCGCTAGCTGAGCAGGCGTCACCTGCAT
GGTGGCCGCTGAAGGGGCGGGCTTGCAGCCTTCGCTCCTTGCCTGCTGCTGCTTCTTCGC
CATCACCTCGACCAGGTCTCTCTGCAGCTGCAGCTCGTCTCTTCCCTCGTGCC
>at02803
CCGTAGCCATTTTGGCTCAAGCATTTGGGTTCATAATGCCACCAGAGCCCCTCCCGGAGT
AGCTCTTGCTGTCTCCTCAGCGCCATTGGCGCTCGCACAATGGCAGCTCAGAGTGACAAG
AAGGAGAAGATCAAAGAGAAGGACAAGCCAAAGGAGAAAGAAAAGGAGTCAGACAAGGGC
AAGAAGAAGGAGGAGCCCAGGAAGAAGGAAGACTCCAAAAAGAAGGAGGAGACCAAGAAG
AAGGATGACGCAAAAGAAAAAAAAAGAAAAAAAACAAACAGACGAGGGAAAGAAAAAAGA
TGATGCGaAAAAAGCCGGAAGACGNCAAAAAAAAAA
>at02804
CACACCCGGCTCAGCTCCAGTCCCGTCGACAAGCATGCAGGGTACGCCTCTCAAGTGAAT
ACGGCATTGAGGAGAAAACGTGTGGATATTGCGAAGGTGATCGCTTCACCGCGTGACAAA
AAAAAAAAAAAAAAAAAANAAAAAAAAAAAAAAAAN
>at02805
TTTTTTTTTTTTTCGTTCGAGAGGCCGGACTGCCTGGTGCACATCAGAGTCGCATGCAT
TTGCCAGGGCTCGGCACATGCATGCGCAGGTGAACAAGAACGAAGCAAAGAAACAAAGCA
TGTACATGTACAGAATGGTCGACGCAGTGGAAGAGATATCTGTACAGAAGGTCGACGCAG
CGGATGAGATATCGAGGCCGGATCGGCGAGCTCTCTATCCGCAAGGCGATCCTTGCAGCC
ATGTCTACAACAGCTGAGCTGGCAAGGACATTGCCACTGCATGTAAGCCAAGCTGAACAC
CATAAGGCGATGGAAAATGCATGGCCAATGCGTGGCGACTGATGCAAAGGTGTCCGAACT
TTGCGAGGCGGCGGGAGGATGTCTCAGCCACAAAGACATCAGACCTCCTGTCCTTTGCTT

CCGATGACAGTGCCATCTCCGCCGCTTGAGAAGCCAAATGCGTCCTGACAGCATGCACTG
GACCCCAATAGACTTTGATGCCCTTCTTTGCAAAGATGTCCTCATTCAGCTTCTTGACTG
CGGCAGGAAGCAAGCAGAACCTCAGCAGATGGAGCGTGTTTGGCCAAGCCCATACATCCC
CATAATCCCTGGATTTTCGTGGCAGACGAGATTCCGGCAATGACGCCACCCGCAATCCCA
AATGAGATGCCGAGCAACATCCCTATCGTACCCTCAATCTTGTTTCCGGCGCGGATGACT
GCTTGGAACTCCTCCTCCGTGACGCCCTGTGCCTTCAGCTCCTCAGGCATCGGCTGATCC
CACGGTTGGCAGCACCTGCACTTTCTCTTGACCACGACGTCGTTTGGTCCTGCACGCTTG
GGGCAGCCAAACGGCATGGCGATTGGCTTGCAACTAGGGCCGCAACTTGTGTGGATCCAG
AGGCGCACTTGCACTTGAGCTTGATCTGAGCGGCACTTGAGCCAAAATGGCTACGG
>at02806
TTTTTTTTTTTTTTTTTTGCCTCCCAACCAGGGATGCCCCCAATCATCCTTCCCACATCA
AGGCCTAAGGGCCTTCTTCTCCGGCGCTATCCCCAGGCTCTCAGCACTCTTTTCCGACCA
GTCGCCACCACCAGGCAGGCCCACGCACGCTGCCCATGGCTTTGGTCGGCCACCACCGGC
CCAAGTGGCAAGTGCGGCCGCTGAACGGGGCCCCGCGGGCACGAGGTGTGTAAGGGCATC
ACGGAGGTCCTCGGGGAGGGGAGCGACCGCCTCCTGTGGCTCACCCCCGACATCCTGGAA
GCCCAAGCGGTAGCGGTGAAGGAAATTCCGGCCACACCACTGCCGATCGCGAACGAACGT
CTCTCGGGGCATGTACTTTCCGTCACAGACCGTGGGATGGCCGACGTGCGCCAAGTGCGT
GCGGATCTGGTGACGGCGGCCGGTGAGGATCTGGATCACCACGATGCTGAACTGCTCATC
AGGGTGCTCGCGCAGGAAGTAGTGGCCCACTGCGGTGACCCTGGTTTGCGAGGGCTTGCC
CTGCTCAAGGACCTGACTCTCCTTGCCGCCCTCCGCGTGCGAGTGGAAGACCTTGGCGTC
GATGAGCCGGAGGTCCG
>at02807
CCAGCGTCCTTGTCCTCCAGGCCTGCGTTGACCGCACGGATGGAGCGGTCGCAGCCCTGC
ACCGCGGACGAGCCCAGTTCCTCGAACGCCTTCCTGCGCTCCTGCGCGTCGGCGTCCTCG
AGGCGCGCGAAGAGCGCCGCCTCCTCGCTCTGCTTGGCCGCCGCCTCGGCGCTCTTCTTC
TGCTCCGTCTGCTTGTCCATCTCCTTGGCCATCTGCAGCAGCAGGAAGTCGGACGAGTGG
CCGATGTCGATCTGTGGCTTCACGGTCTCCACGCTGGTGCTCCCGGGAGCCACGTTGACG
ACCTTGTAGTGGCCCTTGGGGTGGTAGTGGTGCATGGGGGGAGCCTCGCCATCGCCGCCA
CCCAGGAGCTCCGCAATGAGCCAGGCGCCGTCGCAGTAGGGGGCAGCCATCGCTTGTAGC
CTGTAGTGTCTGCAAACAAGTTGTTGCTGAGTCTGGTCCCGAAAACCTTCCGGCTCGAAA
GCTTGAGCCAAAATGGCTACGG
>at02808
GGGCGAGGCGAAGTGCGAGTCCGCCTTCCTGTAGGCGATGACGTACTTCTCGCGGTAGCG
GCAGAGCGTGACGTTGTTGTTCGCCTGCAGTGCGGGCGTCAGAGTGGACGCGCGGTAGGA
GCGACCGAGCAGCACATCCAGTGAGCCTTGGTACCGTGGCAGGGCGCTCTTGCCGCGGCG
GTATGAGGCCAGCAATGCAAACAGCGCCCCCAGAATGACCAGACCGGTCGCAAATGGAGA
AACAAAGAAGGCCGCAACAGCTACCGTAGCCCAGAAACAAGCCACGTGACTGCTGTGCAG
CAAGAACTCCGCAGGAAAACTCAGAACGGAAAATATGACAATTTGGATGTCCACCATGCT
GTCGCCATGCACTACTGCTGCTTGGAGCACAAATGGC
>at02809
GTCCAAGTTTTAGGTTACGTGGAGGCCTCCGGCCGTGGCCGACCCCAACCGACGACTACT
TCGTTGGTCGTTCGTTTAGGTACGTTTCGTTTCGAACCTTAACGTTCCGGGACCGGGAAG
TAGTCCGACCGACGAACTAACTAACGAACGTCCTACCGTAACCGTTTACCTTAACCAAAC
TACGTACGGGACCGGGCCCGGGCCGGACGAAGGAAAGGGTAAGGGTCGGTCCGTTCCGTC
CGGCCGTCGGTTCGGTACTAGTACCGGGACCCGGACCAACGAAGGTTTAGGTTTAGGGAA
CGGAAACCCGAACCCG
>at02810
TTTTTTTTTTTTTTTTTAACCACTTGGGCACAATTTCAATAGTTTGGCATGCATCCTAA
AAGACTTGATCATTCGGCCTCCTGTTCCCTGTGCAATAAAAAAAGTTCCTGCCACTGTGC
AGCATGCTCGTACAGCAATGCCTCAGGTTTGGTGGCCCAGGAGTGCCGTTGGGCGTGGCA
GGCCGCTCTCGTCGTTGCCCAGCAGAGCCTCCTGGACGACGGCCTCCTGCTCTGCCAAGT
GCAGCTTCCCGATGCCTGTGGCAGGCGAGGCTGCGGCGCGGCGGCCGACGTAACGCACGA
CGAGGTCCACGTCCAGGGCCTCGCGGGCGACCGTGACAATCCGCGGCCGGACGTACGCCC
AGGCGCCCATGTTCTTGGGCTCTTCCTGGCACCAAATGATGTCGCCAGGGTGCACGCCGT
CGCCCCTCTTGACGTTGGGGTAGAGAGCCATCTGCTCCTTTGCCAAGTCAAAGGGGAAGG

GGGCCACCATCTCCACCGTCGCAATCGCCACATTCTTGAGGCCAAGCTCATCACGCTTGG
CCACAAGCTCGTAATAGATCTTTCCAGTGCANAAGACGAGGCGCGTAACCTGCGACGGAT
CCCCAGCAATCTCGGGGTTGCGCTCCGGAATGAGGCGCTGAAACTTTGAGTCGGCCCCA
TGTCCTCCAGAGAGCTGACACACATCTTTGTGGCGCAAGAGGGCCTTTGGTGCGGCGACA
ATGAGGGGCTTGCGGGAGTCGCGCTTCAACTGCTGCCGCAATGCATGGAAGTAGTTGGCA
GGCGTGCTGAGGTTCATGACCTGCCAGTTCGACTTCTGGACCTGCTTCTCGTTGGACTCC
TCGTAGTCAGGGACGTCGTCTTCGTCGTCGTCGCAGAGCTGCAGAAAGCGCTCGATCCGG
CAGGAGGAGTGCTCGGCACCCTGTCCATCGTAGCCGTGAGGCAAGAGCATGGTCAGCGCC
GACTGCTGCAGCCACTTGTGCTCGCCTGCGCAGATGAACTGGTCGAACATGACCTGTGCC
GTGTTGGCAA
>at02811
TGCGTGCGTGTCCCTGGGCATCAGCGAGGGCGCTTGTTGTCCCACCGCTGATGGGACGAT
GTTGGGGTGCTGCAATAACGATGTGGTCCCCAACTGAACGACAGTGCAGCCAGAGCCAGA
GAGCGAGTGCAAGAAGTTTGATGCATGTGTCGCTGCCAACCTCACTCAGGGCTTCTGCTG
CCCGACAGTCACTGGCTTCATGCTTGACTGCTGTGGCGACTCCCCTCCTGCCCAGGCAAG
TCTGCCACCAGCGCCCGAGCCAGCCTCGGTGCCAGCTCCGACAAGTGTGGCCCCGGTGCC
TGaACCAGCGCCCAGTGACGACTCGGACCAGGATGCNATGCCACCCGCGCCAGTGCCGGA
CCTCCAACCCGCGTCGGTTCCTNCGACTCCNCCTGCGGCGGCaACaACTCCtTGCTGGAT
CCCAgGGCTNGCNGNNATCGGCTTTGTCAACTGCGGGAAGTGCGTCTGCGTGCACTACAC
GTCCTGCCCGGTCTGCATGATGCGCTAGTTGCCGAGATAATGGTGAAGGTCTTCGAGGCC
ACCCCTCTTGCTAGCATTGCAGGATCCTCCAGTGTTAGTTTCAGAGGTGGGTCTCGGTGC
ACTCGAAGAACGGTGCAGTCTGCATGTCAAAA
>at02812
GCTGCCAGCCTTCATGCCCGCAATCAATGTCCTCTCAAGTCCACGCCCGACGCATGGATA
CCACCACATGCCAAGCAGGGACATGCTGCCCCTCCTGTGACGGGCCACGGCGTGGCCAGC
CGGTGGAGACCACACTACGCGTCCCTGCCGCGGCAAGGAGCCCGCATACGCGGCAAGTAT
ACTCCCATCACAGCTCCCAAAACGTGCCCTCCCTGGCGCGGAGCTGCGCCAGGGTTGCAG
TGAAGGTCCTCGACTCCAGCTCCTGCTAGTGGCCCGCCCGCCGCGGTCCTCCTCAGCCCA
AACCGCAGGTGGCTCTGTGACCGCCCTCAGACTTGGAACGCCAAGCCGCGCAGTCGCGGC
CGCGTCTGCGCCTGAGCGGCTGCTCACTTGCCCTTGCCCTTGCTGTCGTTCCAAGACTGC
CGGTAGGAGGGCGGGCGCACCGTCCTCGGAGCCACAGTCCACGGCGCCGGCTGCTGCCTC
GGAGGAGCGTAATGCTGCTGCTGCTGGAAGCCGCCGCCCGCGCCAAAGCTCGGCTTGCCG
CCCTTGCTGGCCCCCTTCGAGTCGCCGTACCAGCCGCCCTTGGGACCTCTCTTGCCCGCT
CTCTGGCCCTGCGCCACCGGCGCGAACTGCATTGGCTGTGTGACCTTGAGCCTGGCCGAC
TCCTCGCCCAGTGCCAGTCGGAGGGTCCGGTACCTCCGGGCCTCCTCGGTGAGCTTGGCG
AAAATCTGCAGGCACTCCATCTCGGCGTCGAGCTGGGACTCCAGGACCTGCTTCTTCGTC
AC
>at02813
CCGTAGCCATTTTGGCTCAAGCCATGCTGGATGAAGTTTGTCAGTGAATTACTTGACTCT
GAAGAGAATGCCAGCGCCTACTGTGCTGAATGTATGTTACATACCGTTTCGTCGACTCCA
GCTGCTGGCAGTGCGCATCTGGCAGGCTTGCCTCGGCTGCCGTGCCGAAGGCCGGCGGGG
CCTTCCCAGCGTTGTGATAGAGGTCCCTCCACCGTTTGTCCAATGCCAACAGGGCGACAC
TTCCCGGCACGTGCATTTGCTATGCAGCGCGGCGAAGAATGGAGACTTTGCAGCGTTGCT
TGGCATCGTTGACTTGACTGCACCTGAGTGGTTCCTTGCGAAGCCAGTGGCGGCCGGCCA
ACAACAATGGCCAGCTTTGGATCAGATCTGGCTTTACTGGAGCCAGCCCGAGCACCACGC
CAAGCTGCTGCCAGAGAGGAGGCAGGATCTGCGGGAGTTGAAAGACCGCCTCCGGGCAAA
GGCGGTGCCGGTGAGGGTCTGCAAGATGTCTGGAGAGAGCCGGGAGTACACGATCTCTCT
CGCCGCCACGGCATCAGACTTGAAGCGACTGATGTTCAAGCAAGATCCTTCCTTGGGCGT
>at02814
TATGGAAGGCGGCATCTGGAGACTCTTGTCCTAAACTATGGGATGCCTGGGCTTGGCTGC
GGAGTTCAAGCGCTGGGCCAGTGTGACACCAGAGGCGAGCGGCTGCAACTTCCACACAGC
CAACCATTATCTCTGCACGCAGGAGGCGGCTCTCGACTTGAGCACCACGCCCACGTCCAC
TGGCGTGCACACGCCGCGGTCCGCCGACCTGGACTCCATCTCCGAGTCGGGCCTGCCCGC
CCGGCCGATGAGCAGCCGCGTGCTTCAGTTTGAGCTGTTGCTGATGAACGAGCAGAGGAA
GACGCAGGAGCAGCAAGCCAGATAGTCAGCTGGAACGCCAGCTTCGGAGCAGACCATGCG

CAAGAGGACGGAGCAGGAGACCTTGCAGGAGAGT
>at02815
TGTGAGCGCTGCCACAGCAGGGTGGCGCACCCACTCCCACACCTCCCGGTTGGTCAAGTG
CAAGTTGTGTACCTGCGCAGGCAGCCGCCCGCCTGGCTGTTCGGCCTCGAGCTTGTCGAA
GATCGCACGTAGAGTTGCAGCCTCACCTGGTGAAAACACTTTTATAGGCGCTGCAAAGCC
TCGGGCAGAATAGAGCTCCGCATGCCGCTCGATCGGGATCCCCTGCGCACAGCCCTGATT
GAAGAATGTGAAAAAGGACGCTGCAAGCATACAGAGCAAATATAACACAAGAGTCGTGGG
GCAACCCAATCAGCGCACCCATTGCGTGCAGGTAGCAGGTAAAGTGCTTGGACCAAACTG
ACCAC
>at02816
TTTTTTTTTTTTTTTTGTCAGAAAGAATGGTGAGCAACCCTGGAGTCATGAAGTGCACAG
CATGCCCTCCCTAATAAAGCGCTTCATAAAGCTGGGAGCTCCCCCTTCGAAAAAGATCCA
GGTCTTCAAAACGGATGGGAATATTACATCATTCGGTTCGGTGTCGCTGTTCCGTGCACT
GGTGCAACATGCACAGAGAACCAGCAGAAATCAGAGCTCTTCGTTCTTGAGCTTCGTACG
CTCGACCTTGCACACCCTCCGCAAGATGGAGGGGACGGTTTCGAGGTACGGGTTGTTTGC
GTTTGGCTCTTCTCGCGGCACGACTACCTCCTGCAATGTTGACTGGATCCTGGACCGAGC
ATTCTGGATGATGTGATCCGGGGTGTTCACCTCCACTCGCATTGACCGGAGCAGCTTGAC
ATAGTCTCCTGTGCCAACAATCTCCAAGAGGTGCTGCAAAGAGCACTTTGTGCTGACATC
TTGGTGGATCAGCGCGCAATAGTCCTGATCATTATGACTTGGCATGGTGAACATCGTGAT
CTGGCAGACAAACACGTCCCCCGAAGCATTTCGGGCGGGGAGCAAGACGCAACCAACCTT
GTCCGACGCGGCCTCGACAATCTTCTTCAGTTCCAACAGAGCGTAGCAATGGCAGTAAGC
TGATTTCATCTGTAGTGTCGTGAGCGGGTAATCAACAGCATCAGTCTCGCGGAACAGCTG
GAGTTCTTCATCAGGTACCGCTTGCAGTCTCGAGGTCGCGCAGACTTGTTCACGGCCGTA
GAGGTCGTCGAAGCCGGGGCTGGAGTACACAACGTCCGTGGCGGTGTGGCAGGGCGTCAG
TGTGAATGCAAGTCTGTTTTGCATGATCCTCTTAGCAACGGGCCAGGAAGATTGGGAAGC
CTGCAGGTGGCCGTCAGCTTCGAGCCAAGCTTGAGCCAAAATGGCTACGG
>at02817
TCTGACCACCCACTTTTTTCACATCCCTGCTGCATGCTTTGGCGATGGAGTCAGCTGCGT
TGCAGTGCGACACGAGCACCCTTCAACGGCAGGAGGTGTCTCGCACGCACCGGCGACCAC
TGGACGGGCTGCTGAGATATGGGCAGTTGTTGATGCATTAAGCGAGGCTGGCCACCAGTT
GCAGCAGCGCTTCAAGCCACCACGCTTGAACGCGGGTATGCAGACGCTTCCTGAGCCAGG
AGGTTGCCCTTTCGGTGCATTCCAGCCAACGCACAACCCATCGAAAAGCCTCTCTTTCGA
ACTTTGCCTCTCTTCCGAGC
>at02818
CCGTAGCCATTTTGGCTCAAGCCATTTCAACTCAATCAGTCATTGAGCCCTCCGGCTTCT
CTAGTACCATCCCAGTCCTTGGTGGTCATGGCCTTCCTCAAGAATGCCATGCCGATAATC
CGGGGGAAGTGCGACGTCTGCGGCGACTCGAGAAACTTGTGGGTCGCAGAGATGGGAGCG
GAGCCAGTCCCCTGCAGCCACCGCGACCGCGGCTGCCTGGGCCATTATCGGGTTGCCTAC
ACGGGGCCAAAGGCCCTCCTCCCCAGGGAGCTCGAGAACGTCCAGTGCACCTGCGGCCGC
CAACCCTGTGCATCCGACGGGACTTTGGCAGCCTTGCTGGACATGGGCTTCCCTGAGGCC
ACGGCCCGTGAGGCCGCTGCGCG
>at02819
TCGATCCCTTGAAAGAGCACGCGTGCAAAGGCCAGGAATGCCTGAGCAGCATCATCAAGT
CGGAAGTCCTGGCTGCGAGGCTGCCCCTGACTGTCAAGTAAATAGTGGCTCACTGTGATC
TGCGCCAGGGAAACACCCCAGGCCCGCTCGGTGAATGCGGGATCGTTGGTGATGGCCAAC
ATGTCTGTGACACCTCGCCGACCAACACGAGCGAGCATCATGACAGTGGCTGCCGTTACA
TCCTTCCCCACTGCATACGGCAAGTGGAGAGCACGTCGCAAGCCTCGAGGCCATAGCGCT
TTGAGGGTGACCGTGCCTGCATGCCATGCACGACACACGCGCGCCAGTTGGCATGCCTGG
CGGACCACGTGGAAGATCCTGTGCGCCACGTCTGCGGCAGTCAGGCCGCG
>at02820
ACACAATACAGGCATGCTGATGTCCAATCCCCCGTCCAAGTCACCAAGTCCTCGAAGCTG
TGAGCAAGTGGACTTCGAATGCCCAACTGTTGTCATTACTGATACAAGCAGGCCACCTTC
ACCGACACCACCTCCAGAAGACGCCTTGGCATGTCCAGTGGTGGGCTCTACTCACACAAG
CAGGCCGCCTTCACCAGCGCCAACTCAAGTTGACATCGCAGACAGTGCATCAGGAAGCGA
ATACCGGGCGTCTCCGGCAGTACTTCAGCAACAAGAGAGGCAAGACGCGGCAGCCTGTCT

TCCAACTCAGCCTGGCCCATTTCTCGCTTCACAGTCCTTCAGGGAGCACTCCTCGGCAAC
ACTGCCCCCACCAGAGAAGCCTACGCTCCTCACCTCAAAGCATTTTGAGAGGCAAGTTCT
TGAGACTCCAAAGGTGTATGACCAGCCCGGGCCAGA
>at02821
ACTGTTCAACACAGTTGTAGACAATCATGTTGAGTGGCCATGGAATGAACACAGGCATTT
CCACTCAAGTAAGCGACGATGTCAGAATCGAGAATAATCTGATATCAAGTGTCAACGATT
GGGGTGGCTATGCCCTGGCAACGTCCAACAGCGCaAACATCCAGCTCAAGCACAACATTG
TGCAGGGAGAGTTTCGTAAGCCAACGGAGGATCAACTCCGCGAGACAATCTTTGGAGACC
CTTCCTTTGCTGACCGAGACGTTCATGACTATCGGCCTAACTCAGACCTCGCTCAGACAT
GCTTGTATGATCCCGCTGTCAGCTTCGACGCCTACGGGCAGGTTCGCCCGCAAGCCAAGC
ACACCACCTGCGGTGCGATACTCCATCAGAACTAATGTTGGCTGGGCTATTGCCTGCCCT
GCATGACCGCATTGACTGAAGGATTTCA
>at02822
TTGTTTTTTTTTCTTTTTTTTTTGGGAGAAGCGCTCTCGGCTGTGTCCCGACATCTTGGC
GAGCTGCCCTGACGCGCGAATCAGGACACGGTAAAGTCTGGCCCGATCGGTGGGGCAACG
TATGGGCGAGGCCGCCCACAACAATGCTAGACGAAATTGAAGATATCGGCATCCCGCGGC
AGCCTCTGCCTCAGCTGTGGCCCGCTGAAGTCACCGGCAGCCGGCTTCGCACTTTTCACC
GCGCCAGTCCCAGTAGACCCTCCATCGCGAATTACCCTGTTGCACGACAGCCTCGTCCCT
CTTCAGCTCCTTCCAGTAGGTCGCGCGGAGCCTGGAATCCTTCAGACCTGGAACTAGCTC
GAGGTCCTGCTCCCACGTCG
>at02823
TTTTTTTTTTTTTTTTCTGCTTCACACTCATCAAACCTCTCGACTGGTCACCATTTCCAA
ACATGCAACGCAACACGGTCCCACTAATTGAGCGCTGTGTGGTTGCGGAGAGAGAGCTGC
AGGAACCAGTAGGTGAAGCGCCAGCCAATGAACGCGAAGAAGCCCGTCAGCCTGCTCAAG
CCCGGCAACTTGGACATAATGGCGTTGTTGCTACCGAGGTACGCCATCACACCCTTGGAC
CTGAATTTGAAGGGGTTCATTGTTCGACTGTTGAGCTCCTTGGCGAGATGCTGCCCCTGC
TGGTTGGCCACCTGCGCAGTCTGGGGCAGCCAGTAGCCATCAATGGTTGCGCAATCACCC
AGGGCATAGATGTCCTCTTCGCCCTTGACGCGGAGACTCTCGTCAACCAAAATCTGAGTG
CCCCTCTCATTCATCTGGAAGCCATAGTCCTTCACCCTCGTGCC
>at02824
TTTTTTTTTTTTTTTTTTCAGGCTCAAAGTGCTCAAGGAAGCAGCAATTCAAGCCCAGGC
CGCGGGCCACTGCTTATCTCGCTACAGCCGCGTCCGGGGCCTCTCTGCCATGAGTGAAAA
GTCGCCTAGCCGTGCCCGTACGCACCAGGATCTCCTGGGGACTCTGCGGAATCAAGAGGC
CGAGGCTCCTCGGCCGGCGCAACCGTTGCCGCTGCAAGCGGTTTTCCCAGGAGCTTGCCC
AGGCAGAGGCCCGGCAAGCTGCGATAGTCGACTGGCACCCGGACATCCGCGCCAGAGTCG
TCCGCCGCAAGTCTGCAGGAGCTCCATGTTCGAGGCGCTCAACGCCTGGACCTTGCCCTC
CAGCTTCTCCGTCTCCCGCAGGAGCAGAGCCGCATCGCGCGAGGCCCGTTGGCCCGTGGC
TGGAAGTGCCGCTGGCAGCTTGGCGGCCTGGAGGCGGCCGCTGAATTCCTCGATCTGCGC
GTGCCGCGATGGCTCCTCTACCGAGCGGCCCGCCAGCCGGGCCTGCAGCGCCGCGGCTGC
AGTGCGGCTCTCACACAGTTGCTGCCGGCGCTCGGCCACCGCCGCGCGCAGCAGGCGGTT
CACCTCCAGCGCGGNTGCCAGTCTGCGGTCTGCCTCCGAACCGGAGCCGCCTTTCTTCTC
CGGGGGCCACAT
>at02825
TTTTTTTTTTTTTTTTTCTCTGCAGTCCCGTGTCTTGTAGGAGCCCAATACACCCATGAT
GGCTTTGGAGGTGGGAGAGATACGAAGACACGGGACTGCAGCGCAGCCATCTTCGGCACA
TTCAGCCTTCTTCGCCATCATTGCCTCCAGACATGCATGCCTACTACGTTGGTGCAGGTG
GAGGTTAGGAAGGCAAGGTTGTTGCATTGTGGAGCCACGACAACCGAGCACTCCTTGAAG
GTGTGCTGCGTGAAGAATGCCCTAGCGGCAAGGTTGTAGGTGGTGAGGTCTCAGACTTGT
GTGACGTGTGGGGTCTGGGCAAACACAGCCACAATGAGCAGCACCTTGGTTCCCAAGCCA
TCTCTCGAATGACATGGCAGCCCGACAGTAGCTCAGCAAGAAGCAACCGTTTAGTTTCAA
TGAGTTTACTTGATTGCAGCGTGTTCGAGCTGTTGTGGGTAGGCGAAGTACAGTGGACCG
CCCAAACTTTAAAATTGTACAGGGTGTCTTCTTGGACTCGTGCACAGAGACATTGAGATG
AGCATTGGGGCTGGCATTCTGATGACACGCAGCGGCCTTACAGTGGACAATGAGAGACGG
GCGCAGAGAAAAAAAAAAAAAAAAAATCTCGAGGTAGCGGCCCTCGTGCC
>at02826

TTTTTTTTTTTTTGTTTTCAGCGCTTCCTCCCTTCGCCAAACACTGGCTCACAGGCACTG
GGTCCATCAGCGACTGTACAGCTCCATGCCGGATGCTTGAAAATGCCTGAAAAGCATAAG
AGACGGCCAATGGCTTGAATTCATCTGGTGCCGTTCCCAGCAACCTGTCAATCGAATTCA
TTACAGCTGGGTTGCAAGCACCAAGTTCAGGGCATGAACGCGTCGAGGAATTGAGAGCAG
TGCATCGATGCGAATAGCGTTGCGTATCTGCCCACGACAAAGGCGATGATCACGTCAAAG
GTGAAGGAGGACCGCGTGCACAGAGCCAGCACGACCTGGAACAGGACGTTCAGGTAGCCC
AGCACTGCGATGGCGCTCCTCAGCACCCTTCGGCCACTGAGCGTGAAGCGAAGTGCGTCG
AGTCCGTTCTGGCTGTCCGGCATGATTGCCCGAAGAATCTCTGTGAGGGCGATGACAGAC
ATACATACCCGAAGGCTGAAGAACGCGGTCGGGT
>at02827
TTTTTTTTTTTTTTTTTTGCGCTTTTGGGTTAGCACTGGCAGCAGTCAGTGAGATGAGGC
CTTCTTCATCTACAGAACAACTATTGAATCTACAAGGCCGCTCAGTGCTAGAACAGCACA
AAGTTCTCACATTTCAATGCCAGGAGTGTGCAAACCACTTTGAAAGTCCTTGTGCATGAC
GACAAACCTCGGTGCGTAGGGGGGGCGCTGAGATTCATCTGGAACGCATGGCTCTGCCTC
CTCCCCGTTCATGTCAGCCTCCAGCCGACAGGAAAAGTGGGGCATGCGCGGGCATCAGCT
TCCGGGACTGCCTGACGATTCCAGCCAGCAGTGGAGGCAAGATCCTCCGGCAGCTTCAAG
ACGGCGCGAGACCCTCAAATTTCATCTCTCCAAGCTCCTCGTCAGAGGTCCCAAAGACGG
TCTCCCTGTCCTGCAGCAAGATCATCTCATCTAGGGCCTTTCCAGGAGCCACCATGGGCA
GACAGATGTCGGGCACAACTTTGAACTCCATCAGCATAGGTCCATCATGGGACAGCCACT
TCTCAACCATTCCAGGCAGCTCCTCTTCTGTTGAGCACCGCTCTGCTTCGATCCCATGAG
CCCGTGCCAGCTGGACAAAGTCCGGATTGACGTTGTCCACACTCATGTAGCGGCCATCGA
AGAACAGCCGCTCCT
>at02828
TTTTTTTTTTTTTTTTGTCTTTCAACCACAGCAGAGAACTGTATCGAGGGAGGTCGTGTG
CATGGCGTCTTCTGCGCACTGTAGCCACGGCTGTCGTGCTCGACGGCATCCTTGGTGATT
GTCGCAACAACATGAAAAGCACGGACGGTGTTGGCCAAGCTCCATATAGACATGGTAAAT
TCCCTCACCTAACCTGATGATCCAAAGCGGAGGCCCTCTGCAGGGCATCGTGCATGCCAT
GCTGTACAAACAGAGTCCTGCCTCCTGGATCCTTGCTGTGCCATGAAAAGAATCAGAGCA
TGTCCCGGGAGAAAAGCGAGCATTATAGCGCACAAGGCTTACTTGTGCCAGGCTCCGAAA
GCTGCTGTTCGTCACAAAAGCTAGCCTTCCGGCGAGACAGCGCTACGGTGGCACTCAAAA
CTGCTGCCTGTGGCGTCGCACCCATGGGACCCTTCAAATCTGCCTGGCTGGCACATCGCA
CTAAACTTGAATCGACAGGCTCAGGGCACTGTCAGGGCAGAGACATCGACCCCACGTGCT
TCCTGCACCTTCGGTGT
>at02829
TTTTTTCTTTTTTTTTCCTTCGGAGGACTGCTGGGGACAAATGGCGTCATTGCCCACCC
AAAGCCACTACAGCCAAGGCTGCCTCAAGCCCTCTCCATCGTTGCAACGAAGAATCCATC
CATGCCTTTGCTCTGAGGCAGAGCATGCAACGGAGGGCGGGACAAGTATAGGCCAAACTT
CTCGCAGAAGAAGCGGACCTGTTGCGCATTCTCCTCCTCCAATGTGCTGCATGTCGCATA
CACGATCTTGCCACCATCGTTCAAGTACTTCAAGGCCATCTCAAATATCTCCCGCTGCTG
CGCAACCCAGAGCCACAGCCTGTCGTCGGAGTAGGTCCACTTCATGTCCGGGTTCCGGCG
CAGCGAGCCCGTCTGGGAGCAGGGCGCGTCCACGAGCACCCAGTCCATCTTCCCCCGCAG
CTTCGGGAGGAGAGGATGGCCGGGAGGCAGCAGGGTGTAGTTCTTCACGCTCGCCTTCCT
CATTCTCTTCCTGGCTTCAAACAGCTTCAAGTCCCTGTTGTCATGCAAGTACACATGGCC
TTGTTGAGCATCGGTGGCGTGAAGCAGAGTGCCTTCCCACCACTTCCAGCGCAGTAGTCG
AGGACGTTGTCGCCGGGCTTAGCGTCCACCTGCTGTGCAATCAGGTGGGAGGACTCGTCC
TGGATCTCCACATAGCCAAGCTTGTACTCAGGCATCTCTAAAAGTTTCTGTTTCTCGGCC
ATGTACAGGCCCACCTTCGAGTTGACCGTCTTTTCCACGGGCACCCCTCGGCTTGTCAGG
AAGGTGTGCACCTTGCCACGGGTGATCCGGCCGACATTGACCCGCAAGAAGGTCACCGGC
GGCTCATTCAGAACGTCGCAGATCTCGCAAGCTACCTTTGTCCCGTACTGCTTCTCAAAG
CGAGCAAAGAGCGGCTCGGGAAAGGAGCACCTGACGTGATGCGGCAGCTTCTTGTTCTGA
GTCTGCGAGCGCCAGCGGTTGCTTGTGAAGAACGTTCTCATGCGGTTGACCCAGTTGTGC
GGCGAGTGCGTGAGGTGGTCGATCAAGCCGCGCCATCTGTGGAGCTCATATGTCTTGTCG
GCAATCCATGCCCGGTCCTTCGCGTCCAAGAACTTGTGCGCCTGGAAGTACCGCCGGAGG
AATAAGTCCAGCGGCAAGTACGGCGTCTCGTTCTTTCGCGTCTTATCGAAGGCGTTCAGG
GCATACTCCAGGTGCCGGACGCGAGTGGCGTTCATCCATGGGTGCTGGAGTCAAGAGCTG

GGCGGCTGGGCATTGAAGGTAACTGGGTCTTGAGCCAAAATGGCTACGG
>at02830
TTTTTTTTTTTTTTTTTTGGCCCCAGCGATGCTGTGTTGCATGCACGTTTTCCAAACTGA
GCACTGTAAACGGTGCGGGCTCCACCAATTGGGGAAGTTAACCATGCAGCTTGAAATCGG
ATGTGGAATCTTCAAAAGGCAAGGCTCGGTAACCCACAACACGTGAATGGAGGGCCTCAG
GGCTTGTGAAGTTGATTCGCAGCAGGCCGTCGAACAATGAGCCCAACAAAAAGTTCCACT
CAGCAAACTTGGAAACAAGTCGACAAATGGGTCAGCACAGTGGAAAACATGATCTGGCTG
ATAATGCGTGCCTAGACCTCATTTGCGGTGCGCACCCGGACGTTCATCCCCCAGCCCCAG
GGGTACGCCCGCGTGTCCGCTCTCTCGATGGTGACAGATTCGTCATCCACGTGGTGTCGT
CCATCCCTCTCCTCGTGCC
>at02831
CCGTAGCCATTTTGGCTCAAGTGTTCCACTGCCAGTCCACACCCCCCCTCTTCGAACTCT
GGACGCCATGGTGGCTGCGCGGGCAGCATTTGTCTTGGCTGCGGTCGTGTCGGCGGTCTG
CTTGCCAGTGGCGTTGTCAGAGGAGCCCGAGGCGGTTGAGAAGGCCTTTGCCATGGACGA
CGCCTGCGCAGCGCAAGGCGGCGAGAATTGTGAGCTCTCCCTGCGTCAGCTGCGCGCAGG
CCAGGCTTCTGCCACAGAGCAGAATGCCACTGTGTCGAAGCATGAGCATGGAGGTGAGGG
CCCCTGCGGCGCACGCAGCTCCAATGCACGCCAACGTGACGAAAGCCGCCGGCTGCACTG
CGGCCGACGCGGCGGCGATGGCAAAGCTCGGGGGCGGTAATTCGCGGGGCAGCTTCCCAG
AGATCAGCGCTACGTGTGGCAGCGACGCCTATTCCTGGTTCAGCTTCCACCCTGACCGCA
TGAGCTCCTGCCTGCAAGAGAGAGCAGGCGTCAGCTCTTCCTGTGCCGCATGCTTCGTGA
AGGCCGGCCAGTACGGCGTCGATCACTGCAAGCTCCAGTGCCTCTTTGGCAGCTGGTGCT
CCCATGGGTGTCTCAATTGCAACAGCAACGGCAACGATGAGGCACAGGCTTGTGCGGGGG
TGGCAACCCCTGCAACCTCGTATTGCTGAGGGTTGGCAGAGGGAAAGTCTGACTGTTGGT
GTTATACGTTTCTACTCTTAGATGCCAACGACACAGGCTCGGCCATGCACGCGAAGACGT
GCATCTGTGACCAGTAACGAGGTGAATCGCGGAGGGGAAAAAAAAAAGAAAAAAAAAACAA
>at02832
ACAAGCTGATGCTGCTGCTTGGTTGAAAGGTACGCGGCAGACGTCCACGCGCTGGCCACC
GTGCTCTGCTACCAGCCGGCGCCACTGGGGTGGTCCCGGATAGACTACAGGAGGCTCCTC
CGATTTGTCACCGGAGCTCCCGCTGCTGCCAGCGGAACCCTGATCGCTTTCTTCGTCATC
ATCATCGTCGTCGTCAATCAAGAAGCAAGGCTTTGGCACGTCTACCAGAGCTAAGGCAGC
CATGTGAAAGGTGCCATTGCTGCTGAGGCATGCATCCCAAATGTCCTCGTCCTCGTCATC
CATCTTGCCCCACTCTTTGGGAAAGCTACCAAAGCTGCGGGCAACTTTGGCACGAGGCTT
GTCCCGTCTCTTCCGGTCAATTTCCACG
>at02833
TTGTTTTTTTTTCTTTTTTTTTCTTATCCTTTTTCTTCTTCTTGTCCTTCTCCTTCTTCT
TCTCCTTCTTCTTCTTCTTCTCCTTCTTCTTCCTCTCCTTGGCTGCCTGGCTGCCCGTGG
GCAGGTCTACCGTGCTCTTCTTCTTCAGCACCTCCTGCACAGGGTTCAAGCCAGTGGCCT
TCGCCAGCTTGAAGAAGTCCTCTGCGCAATGCCCCGACTACGACAACGCCTCGCTGGTGG
ATGCACCCACGAGGAAGGCCAAGGCCATGAGATTACCCCGGGGCCCTCGCTCCGCTGCTG
GAGCTTTAGAGGGGCTTGAGCCAAAATGGCTACGG
>at02834
TTTTTCCTTTTTTTTTTTGCTCTTTTGCTTGATCACCGAGGCTGGAATCCATGGGCCATG
TGAAGCGATTGTAGCGAGAACAGCATTCACAGAAAGTTGCGATTGCCGGACTTCACGGCG
TCAGTGCCTTGAAGGCCCGCGGCAATGATTCCTCGTGCTTGATCCTTGTCTCTTTGCGGC
TCAAGCGCCCCCTGCAGCACTACAGAGCTTCAATGCCATCAGGTCCCAGAACGTAAACCA
AACCGAAAGACTCTGCAGAGGGACTACGACGACAAGGTCGCCGTGCTGCAAGACTCGCTG
ATCACCTGGCTTGAGAGCATGTGCACACTCGATAGAACCCTGATCGGAGCCTTTGCACAC
CAAGACTGGGTGTGCGGTGCTGCAGTTTCGAACTGCGACCTGATGCATATCCCCTCCCTG
GTCGCCGGCGTGTGCTGCCTCAAAGGCGACGTGCTCTGAAGCCATTGAAGACAAAGCCTC
CTGAGTGAGTAGGCGCTGCCAAAATGAAGCCGCATGGCCCGTGCCAAGGATGAGTGGACC
GAAGGATCCCCATTTCTCCAGGCCTATCCCTGCTGGAGGTCCGTGGGCAATGAGTCGGCG
AGACTCTCCACCTCTGCAAGCAACCTCTGCCGCCACCTCCAGCAGAAAGCGTGGCACACA
TTGCCTGCGAGAGAGGTCCCCTCCGCATGTCGGTCAGAGCTGGCGGCACCGATGCTGGCA
CACTGCTGCTGAGATATCCTCGTGCCGAT
>at02835

CAACGAGTTGGAACACCGCCCTCCTTACGCACATTCTTTCAACGCGTGCAGATTCCCCGG
TCCCGCAGCGCGATGCCTAACAGGCAGATGGCATGCGGAGAAGCAAAATAGGAGGTTCTC
CTATAGCCGCGTCGGGTCCGGCATGCTGAAGCTACCCGCGCGGTGGACGAACTTTTCGGG
TGGCTTAGCCTTGCCCAGGGCATGCACTTCGAAGCCCATCTTCTGCAGGCCAGAGTGGTC
CAGCATGTTTCTGCCGTCGAAGATGAAGGCGGGCTTCATCATCTTCTGGTAGAACTTGGA
GTAGTCATACCCCTTGAACTCGTCCCACTCAGTGAGGACGACGATCGCATGTGCACCGTC
CGTAGCATCGTCTGGCGAGGACACGAAGTGGAACTGACTCCTGTCAACCTCAACGTCGTG
GTACTTGAACTCCGTGATGGCATCCTCGATGTTCACCTTGGGGTCATAGACGTAGAGGTC
CGCCCCATCCTGCATGAGCATGTTGCAGACGGTGAGTGCCGGGGTCTCGCGAACGTCCCC
TGTATCCTTTTTGAAAGCGCAAATGGTGTCTCGCGAACATCGCCTGTGTCGAGCTTGGAT
GATGCGGGACGTGAAGCTCGTCTTCTGGTAGTCATTCATGTCGACAACCTGCTGCCAGTA
AGCAGCCACCTCGCTCAAGCCAAATTGCTCACAGATGTAAACGAGGTTGAGCGATGTCCT
TCTGGAAGTCATGAACCGCCAAATCCTATGCGAGGCAATATGCAGGAACCTTGGGGCTCA
ATGCGCGAGTCTGCTCGCCGATGGCTCTAGAGACTTCTCCAACATCAGCACCTGTGCGCT
CGCAGAGTCTGGCAATGGAGTTGATGCTGCTCACGCGCTGTGCCAGCATGGCGTTGGCCA
CGAGCTTGGAGAGCTCGGAACTCCAGAGGTTGGTGGTTAGGATGGCCCTGGAGACTTCCC
CGACATCAGCACCCGTTCGCTCGCACAGCCTGGCGATAGAGTTGATGCTGCTCACGCGCT
GTGCCAGCATGGCGTTGGCCACGAGCTTGGAGAGCTCAGAGCTCCAGAGGTTGGTGGTGA
GGATCTTCTCCCGCGGGACCCAATGGGCATAGATCTCCACAAGCACGCTGATGGCTTCCT
CCACCTGGCCACCGATGAGCACACGATCGGGCTTGTCCAAGTCCTTCATGGCTGTGCCCT
CTGCGAGAAACTCCGGGTTCGAGAGGATCCAAAAGTGCCTTCCCTGCCCACTGGCCGCCT
CGTTCGCTGAAAGCACACGATCCAGTGCCTGGGCCGTCTTCACGGGCACTGTCGACTTCT
CGATGACAATCTTGTCTCTGTTGGAATGCTCCGCGATCGTGCGTCCAACGCTCTCGATGA
ACCTCAGGTCAGCCGCGCGGCCCGCGCCGATCCCCCTCACCTTGGTCGGCGTGTTCACGG
AGGCAAAGATGATGTCTGCATCCTCGATGCCCTTCTTCACGTCCGTCGAGAAGAAGAGGT
TCTTGCCACGGCACTGCTTAACCACTTCCTCAAGCCCTGGCTCGTAGATTGGGAGGCTGT
CGGAGTTCCAAGCATCGATGCGGGCTTGGTTCATGTCCACAATGGCAACTTCAATATCAG
GGCACTTCAAAGCGATGGTTGCCATCGTGGGGCCTCCGACATAGCCTGCACCAATGCAAC
AGATCTTGTTTGTGCGGATGCCGAGCTTGGGGTGCGCTGGCACGGCCATAGCTTTTGTCG
CGCGGAGCAGGGTTGTAGGGCGCAGCACCTTGTGGCCAAAATGGCTTGAGCCAAAATGGC
TACGGAAGCTCGCTTTTGGTCGGCTTGTTCCAATTCTTCGAGGTTCGTGCGACGTTGCTT
CTCACCTGTCATGGGCGCAGGCGGAGCCAAAGGCGAGGCCTACAATGTACAGCCTGGCAT
GGATGAGCTGTCAATGTCTGACCCCGAGTGGGCTCGTGTCGCCGACCTGCTTCAGAAGGG
CTACTCGTTCAAAGTGACAAAGGTGTGGCGAGTCCGCCCCTGTGCCAGATTTAAGGAGTA
CGCGTCCAAAGCAGCGGCCCTGGGGAATCCCACACAGCTCTTCCACGGAACAAGTCATGC
GGCAGCACGTAAGATTGCCAAGGAAGGCTTCAGGCTGCCACGTCGCTCCGGCCTTTATGG
ACCTGGGCTATACTTTGCACATGACCCTCGCAAGTCGGCAAGGCTACGCACCTGAACGTT
CTTGGTCACACCTTCTTTCGACGCTGGGCGGCTCAAGGGTACAAGGCAGCATTGTGCGGA
GAGAAGGAACATGGGCAGATGCTTGTCTGCGACGTTTACCTTGGAAGTTACAAGCGAGCG
GGTGTGTCATGCATCAACGATCCTGTAAAGGACCTCCAGGGAGGCTGGTTTCGGTCGCTA
CTGGGATTGGGCGACTACGACAGTGTCTACGCGGCCGGATGGTTGTCCCACTCGGAGTAT
GTTGTCTACCAAGAGCACCAGGCAGTGCCGCAATACCTGATCGAGTTTGACTATGTGCGG
ACCTGATCTTCATTTTTCGCACAACTTGCATCACCGCCCTTTTGATGTCAGCAGAGCTCC
TTACAATGTACAGAAGGGGTGCTATGTCACTGCTGCTTGGTGTTGGGCTCTGTCTAACAA
AAAAAAAGAAAAAAAAAACAA
>at02836
AAGCCGTGAGATGTACTCCGGACGAACTCTTGGGTTGTCCATGTAAGCTTCAAGAGAGTA
AGAGAGACGCACGCAGTTGAAACCCAATGCAGCTATATTGGAAACAACATTCTGAAGAGT
ACTCTTTGAAAGACCATTCTGCGTGAAAGTCTGCGAGTGTGCGCCATACCAGTTTGTGCA
AGCCAGTTTCGCTCGCACCCCATCTCTGTCCACAACATACCGCCCCTGAGTATGCAAAGG
CACCTTTGGAGCGTCCAGTGCAGATGCCAAACCGGATATTCCTCCGAAGAAACAATAATC
CAGAATGAGTGCCCCAAGAAAATGCATGATCCC
>at02837
TTTTTTTTTTTTTTTTTTGGGTTGCCTCACGGCAGAAGCGCCAGAACAATCCGAGAGTGT
GCACGGCAGTGTCCAGCGGCCTCAACTGTACACTGTCAGGAACGGCTTGCCTTTGCCGTT

GAGCCACTCGTCGAAAGTGGCCTTCAGCGTTCCCTTCCTGATGTAGTAGGTGAATGCATT
GATCGTCGGCTGGGAGCCGCATCTGGTGTAAAACCGCTCCTGATACTCGGGGTCGAGGGG
GCCGGTGAAGGTCAGGAACTTCTCCTTGGGCGACTCCTCGAACACGTGCTGCTCCACAAA
TCTGAAGAGGTCGTACATCGCCGTCAGGTACGCCAGGTGGTACCTCAGCAGGACCTCCTC
CACAGAGACCTTCGGGCCCCCGCAGCGGTGGTACTCGTCCACGTAGGCCTGGCAGATGCC
CTCGATGTGCCCCAAGAGGATCTCGGCGGGGGCCCCGCTGAGGCAGCCCGTGAACCTCAT
TGCGAATGGGGCTCTGGCAAGGCCTCCCCAATCCAGTACTCCACAACTGAGGTCACCATA
CTCATCGCGCCAAAACCATGCATTGTCAGCTTGCAAGTTGGCGTGATTCGCGGCAATGTA
GTCACTGTTTGCATTCTGGTACGAGCCGCCCATGTCGCCGAAGTAAGGGCACATCTCCAG
GATTTCCGACTTCACTCGCTGCAAGGTCGCCTCATTGCGCACCTCANGTGGCGCCAGCTT
CTCGCA
>at02838
TTTTTTTTTTCTTTTTTGCCTTGTGTGACATGGAAGTCATTGGTGGGCAGCAGACGATT
CATGCGAGAAGGTCAAGGCATCTAGGCTGTTGAGCCACCAAAGTCCCGCGCGATCGCCTT
CACCATCGTATCCACAGACTGCTCGCCTTGGCACAAGTACCAGTCGCACAGCCACGGGCC
TGCGGGACCTGGTGCAAGGCCACTCGAGATCTCCTCCACGCGCGTCGGCGTGCCCTCACC
GCATCCCGTGAGCTCCAGCCACGGATCTACCTCGGCGCGCGGGATCAGAGGGTCCTCCGC
GCCGCGGAAGACCACGATGGGGCAGGCCAGCTTCTCGTGCGGAGGCGGAGCCGCTCCGTA
CGCCTCCTCCATGAGCATGTCGGCCCTGATGACTGGCAGGTACATCTTCTTCATGTCCGG
ATTGCTGAGGATCACGTCCGTGCCGCCCTTGTCCTTCAACACCTTCTCGAACGCCTCGTC
CGCTGTGTCCTTGGTCGGACCGCCGTCCGCCCCCACCTCCCTTCGCACGTCCCTCCCAAG
CCGGATGCTCGGCTGAGGCAACCACCAGACCGTGAGGCGCCATGCCCTTCTGGGCGAGAA
GCGCCGCGGTTTCGTAGGCGATGATCGCTCCGAAGGAGAAGCCGAACAGGACCGTCGGCA
TTGGAGGCCCTGCCTTCTTGATGGAGTCGACGGCTGCGCGGGCGATATCGCCCGCGGCCG
TCGCATTCGGCTCCTTCATGCGAGATCCACGGCCGGGATAGCGCACTTCACACACCTGCC
ACGTCTCAGCCGGGCTGATCTCGTTCAGCATCTTGGGCCACTTTGTGAAGTTGTGAGCAG
AGCCTGCACCGCCACGGTTGCCAGTCCAGGAGAAGATCACCAGCCGCATCCGCGACTTTG
GAGGTGCCTTTCCAACAGAGCCCATCCAAGGAGCTGCCTCCTTGAGCGTGCCCTCAGAGG
GCGCTGCTGCCAGCGCGGCCTGGTGCTTTGCAGGCCACTCGGCGGGGTCTGCGGGCGCCG
CGGGCTCCTCGTAGGGCACGAGGAGGTCCTTGCCTGAGAGCTTGACGCTGACCCAACCTG
TCTCGGGCCCAGTGCCGCTGGTCACCTTGTAGTGGAGGCGGTCGCCGACCCTCTCCAGCT
CTTCCACAATGGCCCCCGTCGACAGGCGATCGCTGGTCGCCGGGGACTTCAAGTCCTGGC
CTTCGCGCACGAGGATCCCGCCCTTGTCTGCCCCGCCGACGACTTCCCACTTCGGCATCT
TGGAAAACGACTGGCTCTTATGTAAATAAAACGAGCTCTGGAAAACGCGTGTTGGCTGCT
AGATCCAAGACGGCTTGAGCCAAAATGGCTACGG
>at02839
CCTGTNCCCCATCGAGCCCATGGCGAATACNTGTCCAAACCCACGCTATGGCTTCCACAG
TGATGGAGCTGNGACGGTTGTCAGAGGAGAGCAGGGATGCATTGAAGTGGGTGGTCGTcG
ATCATGACGATCCTGACTGCCATGATCTTTGCCGNGGCTGCCGGAGGTTACTTGAACTGG
GATGCCGTGAGGGCAGCTTGCGGGATCCgTGCCGAGGATGCTCTGCTGCCGAAGGCAGGA
CCGCAGAGTCGCAGACGCGCCGCCGCCCCCCCAGGCCGCCGCGGTGGACATCCTGCCGAC
GGCCTATGGGCAAGGCC
>at02840
GAACTACACCAAGTTTGATACCTTTGGCTACGCTTACGACGGGAAGCCTCACTTGCAGAA
CTGGTGCCGCCTTCCTGTCTATATGCCTTACGCGTCCAAGTGCGTGGCCGGCGAGCTCAT
TGAAGCTGCGCACAGCCACTTCTTCCTGACTGAGGCAGGCTTCTACGGTAACTATACACC
TGAGTTTGATGGCAGCTACTGCTTCATCGAAGGCCATTGCGTGAACATGGCTGTCACCGA
GAACACGACCCTTGAGGAGGTGTCGCCGCTGTGCGACGAGCGCTTCGGGCGCGAAGCTTG
GACACGCTGGGGCGCGtAGtCtAGtCtAtTGCCGGAGGAGGACAAGCTTGGCTACGGCCT
CAATGAGTCTTCGGcCCCCGAGAGCGGCTTCAGCAGCCCTAATCAGACCAGGCCCTTCTC
GCTGGCTTCCTGTGCTATGGGGAACTTCCACTGTGACGTCATGTACTGCCGAGAGACCTA
CTGCAAAAAGGAATACTACGTGGAGAAGTACGGCCACTACCTCCAGAAGTTTTGGTTGGG
TGACGTGACCGCAACTCTGCAGAGCCATGCGTCGCTCCCGCGCTCGGCAAAACTTGGCGC
CCTTGCAAACACCATATGCTTTTTTTTTGCACCAGGGTGTAGCTCCAGGATTACCCAGCA
TCAGGATGACCCGAGGACAAGGTCATGAGTACTAGAACGTTCAGACATGAAGACTGGC

>at02841
CCGTAGCCATTTTGGCTCAAGAATTTGTGCACGCCCTGAGGCAGCCTTGCTACCATGGAG
GTGCTTTCCTATGCTTAAAACACAGACTTGAGCACGTCGGAGGCATTGGCTTTGCCTCAG
TGGTGCAAGAAGTACGAATAGATCAACCTGGGTAATCTCAATCAGCATGGAGGCATCGGT
GATTATAACAATGGGATTCATCACTGATTTGGAGGTTTGGCGGCTTGCTCAAATAACGTG
TTCAATGCAACACAGCGTAGCAGCGGTCTGTGGCCACGCTCTTGGCCCTGTTGTTTTCTC
TGACGCTGGGATTGCAGGGCTTTGGCGCTATTTGCCATTGAGCTCATTGCCGACATGTGT
GCGCAGATTGTCTGCGATTAGATTTCGTGGTGACCACGTCTGTGGGTGCTGGTCAGACGT
TGCAGCATTTCAAGAGATTGCTACTGAGGTGACCGCACATGCTTACGGGTCTGCGGTCTT
CAGTAAGTTCCGCTTTGATCCCTCTGAGGTCCGGTGTGCGA
>at02842
TCTTCGCGGTTTGAGGACCACGGAAGGTACCGCTACCTCATGTCCACGGATGGCTCCACG
ATTGACGACACGCGCGTCTACTGGATGCTCTCCACGGGCCCCCTTGTCTTCAAGCAGGTC
ACGCCCTTGCTGCCCTTCGGCGTGCCGGCTTTGGAGCCCTGGAGGCACTTCATCCCAGTC
CGTGAGGATCTTGGCGACCTGCTAGACAAGGTGCGCTGGGCAAGGGCCCAGACCAAGAGT
GCAAGGCCATGGCCCAGCGGTCGAGGGCCTTCGCGGCGCAGTTCTCACGCAGGAGCAGCT
CCTTCACTACCTTCAGCGGGC
>at02843
GCTCCATgGGAACGCgTGCTATCGCTACTGTGACACAGCATCTTCCTGTTGTGCTTCCAT
CGATGGGATTCGCGCCACAAGCTCGGGGCTGTGTAAGTGTCAGTTTCCTGGGGTGTCTTC
AGCTGAGACTCCTTCTCAGTGAGAGTGCCGTTTCAAGGTACTTTCNGTTTGATTACCTTC
CTGCAGCACATCCACTGTGCTTCGCATGAGGCTTGCAGTTTCCGAGGATGGTATCTTGCT
ACACCTGATTCCTGTGTAGGTTGTGTGCAGCTTTGCACCCTGGCCGTATCGTTGTCGTAT
GTCGATGTGTCGGTAGTGACGTGTA
>at02844
TTTTTTTTTTTTTTTTTGCACCGGACGGCATGGACAGCACTGCACCTTTCGACCACGCG
CGCGTTGGCTCTACTCCCGGGGCTGCATGCCGGGCTGCACTCTGAACGCCGCCGAGGGCC
TGGCTTGCTGCAGGCCCTGGCCGGGCACGCCACCTGCGAATGCAGCCTGGGCTACAATAC
CAGGGGCTGGCCGAATCACTGGGGCAACTGCAGTCTGAACTGGGACCACATAGGGCATCA
CCGCTGGAGCGGCAGCCGATGAGGAGGCCGCCGGGACCATCGGAGCCGGACTCGCTGGAG
CTGCCGTCTGGCTCGCCTGGCCCATCTCTATCGAGGCCGGCGCAACCCCCATCGCCTGGG
CGAACTCGGATTGGGTGATCACGCCGTCGTGGTTTGCGTCGATGAGGTCGAAAAGGCTCG
ACTCCTGCTCCCCTGCCACCAAGGGAGTTTCCACAGATGCAGCTGAGCCGGGCGCTTCAG
AATCCTCCAGTGAGGCAGTCTCTCTGCGAGTCGTTTGGCGAGTCGTTTTGCGCCTCTTCT
TCTTCTCCTCCTTGGAGCCCTTGGTTCCTCCCTCGTGCC
>at02845
ATGGTTACAGCCCAACGGATCGCCACGACAGAGTTCTACACCATCCCCAGACTTCAGCGG
TGTGGCTTAGCGCACTACTTTTGCAGAGCTGACCCAGTTGATGCAAGCAAGGCAAATGCC
GAGATGCTGATGTACCACAAGAATCCTGCCGGGGGTGCAATCTACACTGGCCCGGAGGCC
TGGTGTAAAATGCCCCTCATCGGCAGGATCATTGCGGGTGAGAACTGTTGGAGGCTCCGT
TTCCTGCCCTGTAATGTGCTCTTTGTCGATGACGCGTTGGCAAACTTCGAAGGCCATGAC
TCCCGAGGGATTTCAGTCCATCACGTCCAGCAAGATGGGATGGTAGAAAGCGACATGGCT
GTGGTCGAAGGCTTTGCCACGGCTGGCAGGGTCTTG
>at02846
GCAAAGCAAGCAGACCGCATCTGGCATGCCCGCCAAGTCCGGCCTCATAGTCAAGTGCCT
CACAAGCTACGCCCAGCTCAGTCCTCCCTTCTCATGAGCTCAACATCCTTGTGCTTGACG
CTCACCCAGCCGAAGTCCGGCCCGTCACCCTGCAGCTTCCTGTAGTGCACCCTGTCGCGC
GTGATGCCCACCTCGAGCACTCTTGCCCCTGTGCTCAAGCGGCCGATCTCCCTGGAGTGG
AGGCTCACGGCGGCGCGCACCACGATGCCGCCGCTCGCGCCGCCGCCC
>at02847
CTCAATGCCTTTATTTCTTGGCTGGCTTCGATGGAGCCTACACCTACTTTGCTGCAGACG
GATTCACACCTGGGTCACATCCGAGGAAGTGGCCAAAGATAAAGCAGGAGTTGGACAGAA
GAGGGAAGTCCTTTATTCCTGCAGTTGGCCCTGGCTACGACGATACACTCATCCGCCCTG
GAATGCCCACAACGTCCGTGATCGGCAGCATGGCGGCTACTACGATGCTATGTGGAAGCA
GCGACCGAACTGCAGCCGCATGCTGTGTCGATCACTTCGTATAATGAATGGGGCGAAGGA

ACGCAGATCGAGCCAGCTG
>at02848
TTTTTTTTTTTTCTTTTTTTTTGCACATGCACCCACTTTCAATGCAAGATCTTCTGTTCT
GATCAAACGTGCCCTGCCCGAGGGTTCTCACGGCCCGTACGGCAGCTTGTTGCCCTCGAT
CGCCGTCTCCCGCACGCCGTCCTTGGTGATCACCACGGTGCGGATCATGCCGCCCGAGCT
GCCGTCCCGGGCCATCGCATGGGCCACGGAGCGCTGCACGAACTCGACGCACTCGGCCTC
CGTCATGCCGGGCTTGTACTTCTCGTCCAGGAGGCCAGCAATGTAGCCGGATCCGCTCCC
GTCGGCCGCGTAGGGCACGCGCAAGAGGGCGCCACCGAGCGGGATCGAGTAGACCTGGCC
GCCCCTCGTCTCATCCCAGCCGGCAACGATGAGTCCGGCCATGAGGCGGTCCTTGTTGTT
GTAAGCAATCATCTTGAAGAGGCTGGCTGCCGTGTTCACTGTCGGCAGCCTGCCAAGTTC
GACGGCATGCTGCCCAAGGTAGTTCTGCACGAACCCAGTCAGCGCTTGTGTGTCCGCCGC
GCTTCCACAACGACAGACGAAGATCCGGTCATGTACCTTGGAGATCTTCCGTGAGGCGCG
ATTTGCGACGTAGTCACCTGTGGAGGTCCGTGAGTCCGCCCCCATGACCACGCCTCCATC
AAAGGCGACTGCCATGATCGTCGTGCCGGTGGAAACCGCACCTTCAGGAGACATCACATC
ATCTTGACGGTATGGGCAGCCAGGCTCCACGTGCCGGTGAAGAGGCTTCTCCGGCTCCAT
CACCCGTGGCGCTATGACCTCCATGGCCAAGGCGATGTGAACAGTGCACCCAGTTAAGAG
TAGGCCACGTTGGAAAGGGTTTCGGGTCTGCGGCAAAGCAGCACTTGAGCCAAAATGGCT
ACGG
>at02849
TTTTTTTTTTTTTTTTTCCCCGCGCGCACGGCTCGCACTCGGCGGCCCTCCTCCCAGTC
GCACCCGCCCCGCAGCAGCGGGCACCCTGCTCACGAGGGCGCCGCGGAGGCGTTGACGTG
GGAGCCCACTCCGGTGCGGCGGCTCCTCCGGCCCTCGCGCGGCCGTGCCAGGCTCCCGCA
CGGCCGAGCGAGGGCCCTTGCCACGCTGAAGTTCGTGCCGGGACTGGCCAACACCAAGAC
GCGCTCGTCATTGCCGTGGGACTTCCAGTGGACGAGGATGCCAGCTCCCCGCGCGGCCGC
CTCATNNNNNNNNNNNNNNNNNNNNNNNNNNTCCTCGCCGACGGCCGGCGGCTCGTCGCCGT
CCCAGCCCTCGTCATCCCTCCGCCGCTTGTTCGGAGGAGCCAACTCGGGGGGTGGCCGCT
TCGTGCCGATCTCCTGGTCCCCGTGGGCGTTGTTGCAGTTGGCCCCATAAATACACCTTC
CCGCCTCCCAATTACGACAAAGCTGCGTCTTCACCTTGCCGCACACCGCAAGGCCAACCG
TGTTCAGCTCGTCTTCGCCATGCGCGAAGGAGCACACCTTACCCAAGGAGCAGTATCCAC
GCGCAAAGCGGCCACAGAGCTCCTTCTTCTTCTTGAGGCGCAAATCCGGGAGCGCGTTCG
GATCGGAGCTGATGTTGTTGGCTGCCGGATTCCCCTGCTCGGCAAATGCAGCGGCTAGTA
CCTTCTCTTCGCCAGGCTTTGGGAGGTCTGCGGATGACACATGC
>at02850
CCGTAGCCATTTTGGCTCAAGAAGGGGGGCCGCTCCCTCCCGCCAGGACGCAGAGTCCTC
CACAGCCGGGGCCCAGCCCGGCGCCTGCCCGCTGCAGCGCCTGCTCTTCCGCAGGCCGCG
CCCGCGGAGCCATGGCCCCTAAGGACGACAAGGAGAAAAAGGAGAAGAAGGAGGACAAGG
AGAAGAAGGAGAAAAAGGAAGACAAAGGAAAGAAGAAGGAGAAAGAAAGGAGAAGAAAGA
GGACAAGGATGACAAGGAGAAGAAGGAGAAGAAGGAGAAGAAGGAGAAGAAGGAAAAGGA
GGGCGGCGGCGGCTTCGATTTGTGGACCTCGGCCGAGACCGCCGACACGGGCAACCCCTT
TGCTGGACAAGTCAAGGGACCTCAGGACAAGCGAGAGAAGAAGGTCGCAGCGGAGGAATC
CAAGAAGCAGCATGAGGGCCAGGTCCTGATGAAGGGCGTCGATGCAGTCCAGATGCTTCT
GGAGAAGAAGAAGGAAGCAACGAAGGAGAAGGCAAAAGAAAGGAGGAAGAGGAGAAGGCG
AAGAAGAACAAAGCCGCCAAGATTGCCATGGAGAAGGAGGAGGAGGCAGACGAGACATTC
CAGATTATGGACAAGTCGGGCCTCGGCAACTTCATGGACAGGATGGAGGTCCAGAACATC
AAAAAAAAAGAAAAAAAAACAA
>at02851
TTTTTTTTTTTTTTTTTTCCTGTCCACCAAACACTTAGTGGCCGTCTTTTGACACGCAGA
GCAAAGATGATGGCCCAATCAGCGGTCATTCATCAAGCTGGACGTCCTGTGCAGTGGCCT
TGCCAGGCGGCAATTGCCTCGTCAGAAGAGGAGGATGTCTCGCCGTGGAACTCCTTGCCA
CGTGCCGTCTCATTGCTCTCCGGACTGATGTATGACACGCTGGGATCGTCAGAAGGGTCT
GGCGCTTGCTCTGTCATCTCGTGATACAGACCAGGGTGCTCCCGTTGAAGACAGTGTCGA
AAATGTCGAGCCAGCACTGGATGAGTAGCTGCAGTGCAACGCCAGACATCCCTCAAGTTC
CAGGAACTGCAAAAGTTGTGTGTGACAGCAATGGTGTCCTCGAGATTGAGCACGGTGTGC
CACCAGCGTGCAGGCACGAACACCACCTCTCCGGCGCGCTGGACGAACTCGATGGGCTGG
AACTCTGCAGGCCACTGCCTCGTCTGTGCCCTGACTTCACGCCACCAGAGCAAAAACCAG

CAAGCCGCTGAATCTTCACCGTGAGTCGGCCTATGTTCGGGACACAGCCATGCAGTGGGT
GTATGAGGCGGGAACAAGACCCAGAGCTTTGTGCCTTTCATCAGCGCCTCGTGCC
>at02852
CCGTAGCCATTTTGGCTCAAGGGATCGTGCCCAGCCTCGAAGTCCGGCGACGCCCCGCCC
ACCTGGCTCTTGCTACAGGTTGGCAGCTGTAGGCCCCTCCCTCAAGATGCCGGCCCAGCA
GCCCCTGCGTCTGCTCTTTCAGCTGGCTCTGGCCGCTTGCCTGGCGGCAGTTGCGCAAGC
GGCCTCAGTGGAGGGCAAGAGCTTCTTCGATGTGGACTGGAGCGACCCCACGAAGGCCGC
GCTCGAGCTGCTGTGGCGCTCCACGAAGATCTGCCTGGCGCTCTCGCCCCTCATCGCCAT
CGCGCTGGTCTTCCGCTGCACCTCCGACGATCCGGGCGAGGAGGAGCTCCGCCGGAAGAA
GAAGTTCGACTTCAGCCTCGCAGGGGCCGAGTGAGGGCGCTA
>at02853
TTTTTTTTTTTTTTTTTCACGCCCAGTCGGCAGTGAATTCGAAGCAGGGTCCGCGGCTA
CATGCGCCTGTTCACAGCAAGGCGGATCTGCTCGTAGCTCACGAAGAAGGTGGCGCACCA
GATCAGCTTGCGCACGCAGATGGGCAGAAATCCCTTGTACAACGCCATCGGACCCTCGTC
CAGAAAAATGCGATAGCCCGCATGTAGCATGCCGCCGTACCGCTGCTCGCCCCCCGCGGC
GTTCATGAGCCGCGTCTTCACGACGTCGGCGGGCGTGGAGACGCAGGCGGAGGTGAAGCC
CGCGCAGCCCGAGGCACCTACGTGCGCCGCGAGGCCTTCGCCGAGGTAGGGCACCAGTCG
CGACTTGGCCTCATCGTAGGTCCCCAACTCAGCAGCATTTACCAAGAAGGTCCTGGCGAT
GTTTGGCCGCACGCCTGCCCAAAATCCCAGAATGCCATCCTTGGCCCACACGCGCGAGAT
GACATCGCGCATGGGAATACTGTCGCCGTGGTGAGATTGCACCTGCGTCTTCACCACCTC
CGCAGGATTGAACACCGAGATGGCCGCAGCGCCAGCGGTGCCCCCTGCAGCGAGGCGCTG
CATGTAGGAGGGGCCCGCCGAGCCGGGGCTGCCGGAGGGCTGGAGCAGGCTCCCGTAGAA
GTCCCGGATCGGCTCGTAGATCACGAGCGAGAGGGAGGTGTAGGACACCTGGCGCACGAG
CGCGGGCGCCAGGCCCTTCCAGAGCGCCAGTGGCCCCTCTCCCGCGCTGTGCGCACAAGG
CAGTCCCCGAAGCCCGTGTAGCGGGGGGCGCCACCCGCGGAGACCTGGACCTGCAACGGA
CCTTCGCCACGTCTGTAGGCAGCGTGACGGTCTCCGCCACGAGTGCGCCAG
>at02854
GCGAATGTGACCAACGGTTGCATGCAGTATGTCAGGGGCGGCCATCGAGCTCACTCTGGT
GTGATGAGACACCGCTTCGCCAACAACGGGACCTGGTACTTGAAAATGGAGGAGGACGAT
TGGACCAAATACTTAGCGGACAAAGAAGTTGTCACACTGGAAATCCCTCAGGGCTCATTC
ATACTTTTCAACAATTGGGTGCCTCACCGATCTCTCCAGAACACGACCGCGGACACAGTG
CGATGGTCACTTGACATCCGTTGGCAGGACCCCAGACTGCCAGCTGGCATGGGACCGAAT
GGCAAGCTCATGCATCTACGCAGTGCCGCTAGCGATTTCCATCCACAGTGGACTCCTGTG
GTCGGGGCGCTTGGAATCATGAT
>at02855
CCGTAGCCATTTTGGCTCAAGCAACTGCTCCACCCATCTGAGCCCCGGCTTGCGCGACCG
CCGCACGTTGCGCGCGCCATGGGCGATGACGGTGAGGCACTATACGAGTTTTCGAGGCGT
GGCAAGCTCGCCAGTGTACGAGAGCTCCTTGATCGCCAAGTGCGGCCCGACCAGTTCTTG
GCCTACGATGGCAGCTCACCGCTGGTCATAGCTGCTCGTGGCGGCCATGGGCAGGTCGTG
AAGCTGCTGCTGGAGCGGAGGGCGGCGCTCGACGTGCACACTGATGATGGCAGCTCCGCG
TTGATCCACGCAGTCAGTGGAGGCAGTGCAGAGGCAGTTGCCGCAGTGCTTGAGGCCAAA
GCTGATCCGAATGAGGAGAATGAGGACGGCCTAACGCCCCTCATCCTTGCGGGGGAGAAC
GGGGGGCTGGACGTGGCGAGGTTGCTCCTAGATGCAAAGGCAGACGTGACGGCCACGGCA
GACGACTGGGGCTCTGCCCTCGAT
>at02856
TTGTTTTTTTTTTCTTTTTTTTTTCTTAGGCCCACTGACTTCGCACCGCCAGCCACTCAA
ACTAACGGATCCAGGAGAGCTCTGGAGCACCTCAATCCTCCTCGTTGTCCTCGTTGATGT
TGAAGTAGCGCAACTCGTAAGATGTCTTGTTTGGCGCAACAACACGGAGGAAGTCACGAA
GCTGCTGCATCTTGAGGTACTTCTTGGACAAGTACTTCAGGTACCGCTTCGAGAAAGGGG
CCTCGGCCCTAACCTGGATCTTCTGCTTCTCCCGCGACACCTCAATCTTCTCACCCAAGT
TGCCTGTCTTGCCATCCACCTTGATGCGATTCGTCAGGAAGTGCTCAAGATCCTTGGTCT
CAATGATGTTGTCGTCTGCAGGCTGCTGGCAATCAATGGAGAACTTCAGCATCTGCTTCT
TGCCCTTGGTAACAGCCTTCTTGGTCAGGCCCTTGACGACCATCTCTCTCGGAACTGCTG
CCGGCTTGCCGGCTCTGCACGGGCGGGGGGCTTTGCACGGCCAGGTCTTGGAGCTGTCGA
TCCCTTGAGCCAAAATGGCTACGG

>at02857
ATCATCCCGGATGCCAAGCACGCAGCCGAAGAGCTCAGCTAGGTCGGAAATAACAACGGT
GATAATGCCAATGCCTGCGAGGGAAGAGTAGAAGCATGCCCAGCCCCCCCAGAGGGTCGT
GGGTGGGACCAACGTGAACAAGAGCTTCCATGGCAGTGAAATGATGTGAAGTGCCCAGTC
CACCATTGTGGCCTCCTTCTGTTCCTCAGGGCTTCCACAGCAATACATGCAGG
>at02858
GCGAGCACCTGCTTCCTGGAGGGCCACTGGAAGACGCGGCCGTGACGGAGAACACGACCG
CGGAGCAGATGACGGAGCTCTGCGACGAGCGCTTCGGCCGCGAGGCGTGGGCCAAGTTTG
GCAGCCTCGCCGCGCCGGAGGCGGACAAGCCAGGATATGCCATGGTGGAGGGGATGGACC
CCCCGGATGGGTTCACCAGCAGGGAGCAGACGCGGCCCTTCACGCTGGCGGAGTGCGCCA
TGGGCTCATTCCACTGCAATGTGCTCTACTGCCGTGAGACGTACTGCAAGGCGAGTACTA
CATCAGAAAGTATGGCCATACCTACAGCACCGGG
>at02859
TTTTTTTTTTTTTTTTTGAAGCGCACGCCATGAAGAGGCCATTGCTGAGACGTTAGAGA
CTGCTAGCGGTGCTTCAAGCCCTGCCCCCCACGACTGTCAGGGCAATGGCGGACTGGTGC
CACACCCTCCGCACCACACTCCGCCTTGCCTGTCAAAGCCGTGAATTAGTAACTAGTGCA
TGCTCCACCTCAGGCGGGCAACGCCAGCAAGCCGTTGCCGAGGTGAAGCGCCTGTGCGCC
GCCAAGTGGCCTGTGCTCCAGGGCCACGACCAAGGGAAGGTGGTCAGTCGGCACAGCCTG
GCGGCAGACAAGGCCCTGAGGATCCCTCCAGGGCCCGCGCGAGTGACCGGCAAGCGCTGC
GAGCGGATCCAAGGAGGAGGAACCTCCGCTCAGCCAGATGCCGTCCGTGGCCCGGAGCCG
GACCTCTCTCCCCAGCGTGCCCCTGCCCTCGATAGCCGTCCATGGCAGTTCCTGACCGAG
CACATCCGGGTGCGAGCTCCGAAAGCCCTCAAGGCCCTCCTCGCGGAGAGTCCTGCCCAC
CTCCGCGGCTTCTTCACCGAAGCTCCCACACAGGACCGCGGGAAGCTGCACGTGACTCTT
CAAGCTCTGGATCTGCTTCCGGAGATCCTCCTTCCCAGCACAAGCCAAGCAGGAGATGCC
GATCTGCCACGTCAAGCCACCCTGTGGTTGGAGTGCCACGTGGAGTTCACCATCCTCTCC
AGCGTCCCAAGACAGCGCTGCCATCTGGCCCGGCGCACANGATCATGTTTGCCC
>at02860
AAGTCACAGCACGGTGGATGTGGTCACATTCAAGGTAATCACTGGCCAAATTCTAAGGTC
CTTTGCAGTGGTGACACCTATCATGACAAGGTACCACCAAGCACTTCGCGGAGCACCGCA
GGGGTGCGCACGCTAGAATGAGGGTTGTCCTTTGAAGATACAAACACAACTCTCTTGAAG
TGCGATGCCATCGGAGCATCTGTGCCAAGTGCTCCCCTGTCCTGAAGAACGTCACGCCAG
ATCTCTGCCACCTCATGGAGAGGATGTCCGAAGAGTTCACTGCCAGCAATTCCCTCTGTG
CATCCAACACAGACGCCGTCAAGGTGCAAAAGGCAGCAAACCTTAAGAACTGCCGCAACC
TGCTGGCGGTACTCCAGCCGATCAGCGCGGGACATGTAAGTGCCGTTCATTGGTCGAGGC
AAGAAGCAGACGGCTGCCGGAAGCTCCTTAGCTGTGCGGACAAGGTCTCCATCGAGCTGA
TTCTGCCCCTCCTTGCGGAGCACGGCAATGTCCTCCGCAAACAAACATGTAGGGCTGCCA
GCCTCAAGACACGGTGCCGGTGCAACAGTTCTCTCCTCCCTGATGTCCGCGACACTGCCC
TCCCCGCTCAGGGATGCACAAGTCGATGGATCTACAAAGGCCGTTGACCAATTTGTTCGC
GCGAGGAGCTCTCTCTCCTCGTAGCAGCAGAGTGCTGCCTTGGCGTCGGTGGGGTGCCCA
GCAAGTATGAGCCCGGGCTTCATCAAGTGTGCTGCCTCTTGCAGATCCAATGCAGCGTCC
AGAGCAGAAAGGTTGTAATTCACCTCCACCAAGCACTGCCTTTCTGCTGTGCACACTGCA
CCCCTTGGCTGAGAAGCTCTCTGGAGCTGAAGCGGTGGGGCCATTCGTAAAGCAGCTTCT
AATGTCCGATTCCTCAGTGCATACTCAGTGTAGGCGAGGCGGAAGTCGACGTCCCGAAAG
TGTGGCGAGTGGGCTTTCAACCGACAAAGCACGGCTCCGAGATGCTCCTGGGTTACATCT
GCTGTTTCACGCGGACCACGTGGTCCGCAATCCGCAGACGTGTACTCATGGGGCCTCCGT
CCATAGTGGTGCCTCAAGGACGGAGCTGGAGTCTGCACAGGCAATGGTCTGTGCATAGTT
GCTATGAGCGTTGGCCAGCAGGGCAGATCAGACACAGAGCTCTAGCTGGAGCCAGCACGG
CTTGAGCCAAAATGGCTACGG
>at02861
TCGCAGTCTCCTCCTCGTTCCAAGAGACCGTTCGGTCCCAAGTCTTGAAGTAGCGTATTG
AGTACTGGGCTCCACTATGATGTACGTGCATAACCTTTGTGTAGTTCACTACTGCCTGTT
CGAACGAAGCCTGTGCTCTGCTTAGTCCGATCCTGGAGCCACTCTCTAGAACCGGAGGGA
TATCCGGTGGCCACAAAGTGGAGTAGTTGTTGGAGCAGCACTCCTCGGCATCGTAGGGAG
GAGCAAAACAGTCGTAGGTTGTTTGCTTCTTGTTGCAACAGAGTTCACGGAAAGCTGCGT
TGTGCTCACCGGTACGATGGTCTTGAAAACACGGCCCTCGTTGCACCTCGAGGGCCGAGG

TAACCACATTCACAACGCTGACGGCAATGCAAAGACGGCATTGCCTTGCCATTGAGCTGG
TAGAAGGAACAGCGGCATTGCTGTACTACTGAGCTACGGAAGCCCTCTCGCTCCACCACC
TCCCCCTTGAGCCAAAATGGCTACGG
>at02862
CCGTAGCCATTTTGGCTCAAGGGGGATGACAGCAATACCATAAGGCCTGCCATGGGTGAT
GGCATTGGCTGCCATGGTGTTGCTACACCTGCCTGGAGCCCGAATATGCTGCGGGCATCT
GCTCGGCCAGTGAAGCGTACTGGCTTCAAGGCTCGTGCGGTACTGGTGCTGCTTGCATTT
GCAAGTCTGAGGGCGCTCTACACTGGCTGCTTTTGTGGAGGGCGGGGACGTGTCCTTGGC
ACTGGTTCTCTGCGGCGATGCTCTACAGATTGCCGGAGTGCCGAAGGAGATCTTACGGCT
GCTGTGCCAGCGCTCATGAGTTGGCTCCGGAGACTTGGAGCATTCGGCTTGGATGATGTA
CGCATCGGTCAGAGTCGTGATGGGCCTGAGGCTGGGCTTGGCGTGTTTGCAGCGCGACAC
TTCTGCGAGGGGGAGGTGGTTCTGGGGGTGCCGACCACAGCGTGCCTTGGCGGTGAGACA
GCAGCAGATGAAAGCAGCGATGACGATCACGCGGCAGCGCT
>at02863
TCACAGCATCAACAGCATCCCGATCTCCCTTCCTGCAGCTGCGCAAAGCTTCTCTGCGGC
AACACGCCGGATGTGTTCATCTGAGCTCTGCACCAGGTGCAAGAGTCGAGCCACCGAGCC
AGGGGCTTCTGGCAAGGCTGTATGATCCTTGTTACCTGTGAGGTCTATGGTGGGAACCGG
CCTCTGGCATATCATATCGCCTCCACGAGTTGGTTTCAAGTCTGCATGTAGGCGTAGAGG
TCCTT
>at02864
TTTTTTTTTTTTTGTTTCGTGACGCAAGCACATGGTCAAATGGATTGGCACTTCAGAAC
AACTTACGGAGAACAGCTTCAGCGACAAGAAAAGCTCGCACGACTGTCACACAGGGAAGA
TGAAAGTATGTTCCACAGAGGTGGAGCCGAGCGTTGGACGCTTGCCTAGGACTCGGCTTT
TACTTCTTGACCTTTGCGAAAACTTCCGCGTCCCATATCCAATCAATGTCGTCAGCGCAC
TCCGCCGCTGAGTCTTCACTGCAATTGGCACCCGGGATGCCATGAGTCGCAATTTGGCCA
GGCAACAGTGAAAACGCCTTCGGGGCGGCATCCTCCATGCAGTCTGACCCAAAACCATAC
CTCAAACCCATGTCGCGGACCTGGGCTGCCAGGTCGGGGACTTTGTCAAACAACTCCGCC
CAAGTCACAGCCTTGTGGGCCCGGTGATTAGCGGGGGCGCGGGGCAGCAGCGGGGAGTCA
CAGGCGGTCTGGTTCACCTGCTACAAGATCGAATGGGCAAAGTATGCCTCGTGCC
>at02865
CCGTAGCCATTTTGGCTCAAGCCTTTTCCCCAAGCCAGCAAGTCCAGAGAGTTGCAGCAA
GTTGACCAATCCACCGGAGTCTGGAACATGGAGGCCGCAGTGCTTGCTCCACTAGCATGT
GCTGCACTGGACCAGCGTTGGATGCTTCAGGTGGAGCTATGCCGATGCTTGCTTGTGGAT
CTGGAAGAAAGCTGAAGACGGCGCTTACGCGCAGCTTAAAGCCAAGCCAGTTTTTGCCAG
CAAAACCTGCGCTTGGCAGGAATCATCGCATTACCCCATCTTCCATGAGTGCTGGCGTGC
ATGTGCAAAGCCCATTCCAGCAAGCTGTCTTTTGCCGCGACTTCAGCAGACTGAGCGACA
ACCACCTGTCAGACATTACGCGGCCTCTCTCCACACATCCATCTGCAGTGTCGAATCCTC
GAAGTAGTGAAGCTTGCTCAATGGGCACGGCAACATGGTTGTGTGACCCTGGCAGCCTGC
TGGCCCCTGGCAATGCGTCTTTGATTGAAGACATGCTTGACCACGTCAATGCCAGTC
>at02866
CGTCCACGTCGTCATCATCAAAGTCACCACCTCCCACCGGGAAGGCGGCTGAGACCCCGT
TGGTGGCACGGATGAGCGCCCCGTTGGACGAATGGTAGCTGCTTCGACCCAAGTAGTTGA
GCACGAAGTCTTTGGCCAGACCGGTCATGCCCTTGCTGATGCCCAGCACGCTGAGCCAGT
CGTCGCTCCACGCCTCCAGCTGCGCCGCGTCGGCCTCGGAGCCCTGCGCCCTGAGCTCCG
CCGCCGCGGCGCCGAAGAGCTCCAGGGCGCCGTCCCGGTAGCTCTTGAGGTCGGCGAGGA
TCTCGAAGGTGGAGTCGGCCCCGGCGCCGTGGAGGGCCGCCGCGAGGAGCAGCAGGCCGC
CTGCGAAGCGTCTCCCTGGGAGCATGATCAGCCGAGAGTGGACGCAGGTCCAGCTGAGCA
CTGTC
>at02867
CCGTAGCCATTTTGGCTCAAGGCTCAACTCATAGATTGCATCAGCATAGGCCGTCAACAC
ACAAAGGTTTGCTCGGGCAATGACATTGGATGCAAGAAGTTTGGCTCAGACAGTTACGCC
GCTGGAATGGAAAGATGCGGTGGTGGAGCCCGTTGACAAGCCGTTGACAAACTTGTTCTG
CTATATTGTTTATGGCATCGCTCTGGCGGCCTACGGTGTCTTCGCCGTCGTGATGTGGGC
CACAGCTCCGGACGTTGTGACTTACAAGTCCCAAGAAGTAGTTGACTTTGGGCCTGTTGC
CATCACCATATCCGTGCACTGCCCATCATGCAATGTCCACGCGTCTGGGGACCTCTGGAG

GGTGGTCCAGGAACTACACTGGTTTCGCGCACTGCGCGGAGGCTGGGCTGGCCCCCGTTG
TGCAGAGAGATCTCAGCGACACTGTGGCGCAGCTATGCCGGACGACAGACAACATTGTTG
ACAAGTCTGGGGTGAGGGTGTGGGTGGGCAACATGACCCTACATGCGAAGAGGGCCTCCC
TCGTCATCTCGGACTCATCAGCAGCGCTGGAGGTGACTACACCCCTGGAGGCAGGCATGA
GAAGACCCTGATCTTGGGGATGACCGTCAAGCGGGACAAAGAGGACTGCAC
>at02868
CAATCGCTCGTACTTGATACGGTCACCCTCCGCCTCCGTTTCTCGGACAAGCGCTCCTGT
GGACATGCGATTGGTGCATTGCGGGGAGGAGACATCGACCCCAGTGCGAACCACGATGCC
GCCCTTCGTCCTGCACCGCCGACTACGTTCCCGATACGTCGTCAGCTGGCA
>at02869
TTTTTTTTTTTTTTTTTTAACCAACAATTTCACGCGGCTCACATGCCACCGGTGGGGTTC
GCACTGAGGAATCTTGAGCTGTACCATGGTGAGAGATGCCTCAAATACATCGGAGTGCAT
AGGGCCTGCTAATTTGATGAACGCCAAGCTGCAAGGCAACCGTGCTGCAGGCTGAATGGC
AAGTGAATGTCGCAGCAAACACTGACAAACATTCTGAACTGTACTAGCACACTCGTTCGC
GTCAATGAACATGCTCGTGAGCCCGGCTAACTTCCGCAAACAATTCCGGCTCGGCTCACC
GCGACGCCGGGGTGCCTTCCGGCGTCCGACACACACTGTACGTCCATCGGACCTGCTGGC
ACTGCCACGAGCTACGTCTCTTCAGCACTCCGTCCCACCACTGCTCCACCACTGAAGTCC
CAGCTGGCTCACGAGCACCTCCTGCTCCGCTTCCGGCAGCAGCCTTCCATTCAGCATCTC
CTGGTGGCAGTATCTCCATGGGCCCTGCAAAAAACTGTGAGGACATGGCCATGGACGACA
AAGGCCACCATTGCCGTCACCCTCGTGCCGAATTCT
>at02870
TTTTTTTTTTTTTTTTTTCGCACCCTGCAACTGATCGATCTCTGTACACAGAAGCTCCAC
CTCTCTCGGAGTGGTGCACATTCAAGGTAGTCGATCGCGGATAAGAAAGAAAGGGCTCCA
GCGCAAAGCTCTGAAGCGCAAGCTTTTATCCGGCAACTTAAGATGGCGAATTGCACACGG
TGCTTGCCACGATCACGACTGCAGGCTCGTGCTCTAGTGATTCAGCACCCTCAGCGTCAC
CCATGTCTGAGATCGATTTCACTGCTGCCAGCACCTCTGGGGCCTTCACGTCATTGATTG
TGTCTTCGTCGAACAAGGCAAACCAGGGGAATCCACAACTGGTGTATTGAGCTGCTGTGA
CGGGTGACGGCGGTGGCAGGCGGCCAGTGACCGCTGTGTACATTGCCGGGCTCGCCAGAT
TGACATTCACTGTCTGTCCACCTCTGGCATCCCAAAAGCGAGTGCCTTGTGGATCTGGGT
ACACATCCTGCCGCATTTGCCCACCAACAGCCAGGGCCATCTCATTGCTCTCGTCACCCG
ACCCACC
>at02871
TTTTTTTTTTTTTTTTTTCCGCCAACCAAAAGCTTTCGAGCCACCTCCTTGGCGCGTGTT
CCCGGGCAGCCAGCCCTGCTGAGGCGGCGGCGATGCCCAGGGTCCACGCCTCCCACCCCA
AGGAGGCGGCGCGCGAGCCGAGTCTCTCTCGCGGGCGCGGGTGCCGTGCGCCCAGTCAGT
CAGTCGAGCAGATCGGCCACCTCCTCCACCTTGCGCTTCTTCGCCGCGCTAGACGCGCTG
GGGATCCGCTCGACGATCGCGGCGCTCGTGTTCGCGTTGAAGCACACGCAGTTTGCCAGG
GGCGCGTCGCGCTTCGTGATGGACTTGATGACTTCCTGAGCCAGGAGGCCGCCCACGATG
GAGGCGACGTGGACGAGCGGCTCGAGGAAGAAGAGACGGTAGGCCTCTGCAAGGTCGGTG
ATGCCCTCCGCGGCCGGCGGGCAGCCGGCAGTGTCCCGGCAGTAGGCCTGGAAGGCCGCG
CCCTGGTCCGGGCACAGGCGCGCGGAGTGGCTGCGGGCAAAGGCGAGGAAGAGGGCTACG
AGGATGACGGACGGGTCCACCTTGCCCTTCTGCAAGTCTGCAGCGGGGCACCCGAGCCAT
TCTTTGAAAGACGGGAACTTTATGATCTCTGGTTCTGCAGTTGTCCCCGATGGTGTTGAA
CCAGCGCCGCCCTGTGCAGAGCTATGTTCCTGCACAGTGTGATCGCCAAGATCCGAAAAG
AAGAACGCGATCTCGCCACAGCTGAGAGTGAGGGCAAAGCCTACATTTGCCTCCCTGCAG
ACAGCGTCAATGTTGCAGATCCGCTCCATGTTCCATTCGACAATGCCCGGAGTTGCGAGC
ACAAAGTCGTACCCCTCAAGCACCTTCCGCAGGCCCCCGAGGCTGTCCGCCTCCTCGACA
GCAGTCCTGCTTGTCGAAACTGAGCACAAGGGGTTCATTTCCTGCACCCTTCTGGCAGCA
CACTCTGCGCGGTTGGAACCGATGTCCTCGCTGCGCAAAAAGTAGTTGAAGCCGACGTCG
GCAGTCTCCACGTTGCGGTGGTCACATACTACCACTGAAACGCCAGCTAAAACGAGGTTC
TTGCACGCTTCGATGTTGCTTCCTTCAAGACCCCAGATCAAAACCTTGGCCTTGAGTAGC
CGCTGCTGTGCCTGCACACCCCAAAGCCGCAACTGTCGGTCATAAACAGCAACCTCTCCT
TCGCTGATTGCCGCCATTCTGAAACCTTGAACAGCTACAAACCTGCAGCGCTATTCCATG
GACTCAGGCGGCCTCCGGGCTCGAGCCTTGAGCCAAAATGGCTACGG
>at02872

AATTCAATTCGCTCGGCCGCGGTGCGCTCTGGCACGGGGGCATCACAGATCTCGTCGGCA
CGGCGGCACCATGCATAGAGAGCGCTGACGGCCCGCTGCTCCTCCTCAGGAAGCAGCGCC
GAGGCCAGCTGCAGCATCGGATCTCGGTTGCCAAAAGAGGCGTCTTCCTCCTCTGCCCGT
AAACTGGCCGCTCTCTCTGTCATGGCTGCCTCACTACCTGCAGTTGCATGGCGAGGCATA
CCAGCGGTCCGTCGTCCCGGATGCGTCGACAGCAGGCTTGGCCCCAGCGGGAGGAGTGGC
AGCGCACAGCACAACAGCAAGAGGATAGCGTTGCCAGAGCGCCATGGCCGCCGAATCAAA
CGGCCCATTCCAGTAGAAGGTCCACAGGACCTCCGTGATGTGTGGCCAAATCTGCGCTTC
CTCCGCGCCC
>at02873
TACTCTTGGCGCGGAACTTCAGGCTTTGAAGGAGCGCTGCGACCGCTACGCGGGCGCGGG
CGAGCTCGCGGAGGCAAAGACACTTGCGGAACGGGTGAAGCAGCTGGAGGATCAGCGGTT
GAGCATTGCAGCCCTGTATGAAACAGACTTTTGGCTTGAGCAAATGGTTCTGCCACAAGG
TGATACTGGTGGCAGAGTCTCTTGACTCGGCACTCCCTGCATGGCGTTGCCACCAGCCAG
ATGAACTTCCACGACAGCAGCAACCAGGTGGTGCTTCTGTGCTCGCTCTCTTCGTGA
>at02874
GATAGAGAGCCTCGGGCAGTGGGCCGAGGGTGGTTGCGATGCCGCGGGGAGGCTTTACAG
CGGCACTGCTGGAGGGCAAGTCGGATGTGAAGGCTGAGCACGCCCCCCTCGATGTCGAGG
AGCAGGCCGAGCTGCAGTGGCTGCGCAACCGGGTCGCCACTCTGAGCAACCGGGTCATCA
CTCTGGGCAGCCGGGTCGCCACTCTGGAAGCCCAGCTCAGGATACGAGGCATAGAGCCGG
TGTCTGCTGCAGACGAGCTGCCGGCAGGCGCTCTGAACCTGGGCCCTGAGCGGAGGATGG
AGGCCAAGCTCCTGCAGATTCTCTCGCAGTGTGGACTCGACCACTACGTGAACGCTTTCT
CGAAGCTCGGCTTCTGCGACCTAGACACGGTGCGTGGC
>at02875
GCCCGCAAGAACTGTCCGTGGGCGCATGAGGTTGCCCCAGTGGTGGAGGATCTTAAGCAT
GCATTTCCATACATAAGCTTGGATGAATCTGCCAACGAAGTTCTGCTCTTGCACGGCACA
TCGAGAGAGACAGCCTCCAGAATAGTGCGCGAAGGCTTTGACGACCGGCTGAATGAGCGT
GCAATGTACGGAAATGGGCTTTACTTCTCCACTGATCCGTGCAAGATCATGCAGTACTGC
GGGAAGAGGTTCACTGGCTGTGCAATTCTAGCCAGGGTTGTGCTCGGACATCTGTATTTG
GCCACGGGG
>at02876
GGTTTCGCTAAAAGGAGTGAGGTGGTTCGGGTCTCAGCGCCCTGAACCACCAGCTTGCTC
GCTTCCCTTCCAGTTGCTATGAAGCGGCTTCTTTCGAAGAGATACGTGCCGACGAACGCC
GGGGGCTCATGGAGCGCGAGCGACAGCGCGACAGCCTGGATGTCCAAGCTTTTGCCGGAA
GCCTGGCCAGCCGCAGCTGGATTCTCGTGGCGTCGACAGTGACAGCCAAACCGGCACGCC
TGATTGCGTCTTCGGCATG
>at02877
TTCGTTGCAAAGATCAGGAGAATTTCAGAAGAATAGAGTAAAACTGATTTCAAAGTTTAA
GTTTGAATGAAAATTATTTGCAAAGTTTTTATAAAAAGTACGAGATTTCGTTTAAGTTTG
AACTAAATTTTACTTTATTGAACTTGATTGTGCGATTTCCAACTTTAGAAAATCATTATC
CATCATTTCAAAATAAATTTCATGCGGCGTTATTGTCGCTAATAAAAATTGCAACCAAAT
TTCAAAACAAGTAAACAATAAAGTACAAGATCTCGTTTAAGTTTGTAAAATTTTATCACA
TGTGTGTGCACATATATAGGCACACCGAAGGTGTGCCATATAAAATAAGATAATATATTT
TTAACTAATTTAGAATCGATGACACC
>at02878
GTCCGTAGCCATTTTGGCTCAAGCCATTTTGGCTCAAGCTCCGGCAAGCACACTTTCTGG
GCTCCCCACGTCCTGCAAAGATGGCTCGTAAACGGAAATCGTCTGCGGCTGGACGGCTTC
TGGTCGGAGGCCTTGTGGCCATGGCCCTGCTGCAGTGCATGGGGCTCGTGTTTGTCACGC
CGCCCCAAGCACCTCTTCGGAGTGACGTGCAGCAGGGTGTTGCCAGTGCTGCTATCCTCG
CAGGGTCCATTCCGCAAGCTGCCTCGGCCTTCGGGAACGAGCTGAAGGACGATGGCTTCG
GAGGAGCCGAGCTCACAGCCCTTGCAGTCCCCTTCGTCTTTGTCGTTCTGGCCTACCTGG
AGTGGGAGGGCCAGCAGGAGCCCACTGACGACATCACAGGTCTTGGCACACTCGGCAAGC
AGGTCGACGGCCCCGGCAAGGGGCTCATACTTCCGCCGGAGCCCGGAGTCCGGCTGAGCT
GTCCGGATGCAGTTGGTTTTTAATCTCAAGGACATGGTGAGTCATCGAGCTGATTGTGCC
GTGCTGGTNCATAGAAAAAAAAAAAAAAAAAAAA
>at02879

TTTTTTTTTTTTTTTTTTGCATTCTCGTGCTCGCAAATTCAACCGCCAAAACCAGTCAGG
CCAGGTCAGAGGGACTCACTGCGCTTTCTTCTTCTCCTCCTTGACCTTTTTACTGCTCGA
GTACTTCTCCTCCAGCTCTCCCAAAATTCCACCGCACGCCATCGCCACTCCAAGCCACCC
CTGTGCGTTCATGTGGTGGCCCTTCATCAGGATTGAGTACAAGACCGAAAATACCTTCCT
GGTAGTTGTGACGGTCGTGCAAACAAGCGGATCAAAGCTTGAGATTGTGAAGAATATGAA
CGCCTGACCAACAGCGCTGCATGCAGCAAAGATGAGGACAGTTCGGAAGATCTCCGGGTT
CTCTATGAAGAAGCGGTAGCCGGGCCCCATCTCACCCATGGCCGCAGCGAACACCACAGC
CGTCAGCGCCATGTAGAGGTTGGTGAAGAACTGCATCTCGAAGTTCTTCTCCTTCATGCC
CATCCCTGCCAGCACCCGCCTCGTGCC
>at02880
CCGGAGGGAATCTTTGTGACTAGCCAGCCCAACAGCTCTTGAATATCTTTACTACATGGA
GAACCAGTGCCCGGTGAACCGGCAGATCATGGATGATGGCGGCTTCGCCGATTTGCCGCA
CTTGCCGCGGTCGTCAGTGGTTCTTGAAGCTCAGGTTCGCTGCAAGCACTGCCAAAGCCT
ACACGAGGACGTTGGTGACTTCTTCGGCGAGACCGAGCACAAGAAGCACCTCTGCGGTCA
GTGTGGCCGCGACATCTTCAACAAGGGAAGGGTCGGAAATATCCGGGGAAACCCAGTTTG
GTTGCCCC
>at02881
GAACACCAGGAAGACGGAGTAGGAGAAGGTCTCATTGCCGTATGGTATGGTCATGATGCG
CTCTTGCAAGACCCCGTAGATCACGAGCATGCCAATGATGCCAGAGGCATAAAATACACA
CCAGAGGCAAGTCTGGATGTAGCTGGCCTGGGACACTGCGGGCTGTTGTCGCTGGTG
>at02882
TTTTTTTTTTTTTTTTTTGCAGCCTCAGACCACCATGCAAGGTCAGGAACAGCATTACCT
TCAAGATCATTGACGCCTAGGCACCACTCACGAGGCAAAGGCTTCCGGCACACCGAGCCC
GGAGCTGAATGATCGTCGGGCTTCCGGTCTGAGTTGCTCAAAGGCAGCCGTCTTCTGGGC
CCGCCACAGCTGAGGCTCCCCAGGAGCTGCAACTCCGAGCCCTAGCTGCCGTTGTCCTCA
TCCGAGGTGTACTGCGACTCGCCGGGGGTGAGCATGCGGTAGTCCAGGATGTTGTAGGCG
TGGCCGCTCCGCCGCTGCTCCTGCAGGTACTGGTCGCGGGTCTGCGGATGGTACCGGAGC
GCCTCGAGGAAGATCTCCGTCCGGAGCGCCGCCATGTCGATCTTCCGGCGCTCGAACTCG
AAGTCCGCCACGTCCAGCGGCGCCCTGGTGGGCTCGTCCTCCGGGCAGTGCAGCTGCGCG
AGGTAGGGCTGCTCCAGCGCCTCCCCCACGGTGACGCGGCGCTCGGGGTTGAGGCGCAGC
GTCCGGAACAAGACGTCCAGGGCCTCCTGGGAGGCGGTCTTGAACATCTCCTCCAGCGGC
ATGCCGCCGGTGGCGGGCAGGGACTCGATGAACTTCCGGCACTTCTCGTTGGGGATCCTC
CGGAGGGCGCTCTTCTCGGGCGTCCCCAGGCAGCCGATGATCAGCTGGAGCTGGTGCTGC
GTGTTCTTGCCGGGGAAGAGGGGCTTGCGCTTCAGCATCTCGGCGAAGATGCACCCGACC
GACCAGATGTCGATGGACTTCGTGTAGTCGGTCCAAGAGCAGAGCACCTCGGGTGCCCGG
TACCATCTAGTGCA
>at02883
NNNNNNNNNNNNNNNNNNNNNCAGTATTGCTATCGAACTAGACCAGGCGAGGAAAGCATC
TCAGCGCCCCCCCTCAGCGCTCAAAGACGAGAGCTTTCAGCTTAGTATCGTTGTTGACAA
AGTAAGCCAGGTGCAGGACTCTTTAACAGGCCACCAGAGAGCTTCTCGAGGGTCTCCCGA
GCTGCAGCAGAGATGGAATGTAAGTCTGACACGCGCAACTGGAGCACATGCCAAGCATCG
CCTGATAGAGATTCTACAAGAGGCTCTTCACTCTGATGGACACTTCATTTTCATTGATAG
ATCAGTTAGGGGCGAATGTATGAGCAAACCCCACCCGGTCGAAGCCATCAAGCTTTGAAA
AGATCCGCACTGAGCGGCTGCCAAGGGATACTGTATGTCTTGCCGCTGCCGTCTTTGAGA
TCCTTGAGGCACGTGGCCACATCCACAAAATTCGGCGGACAATCTACGATAAGTACGCGG
TCGCCGTTCTGGCGGAAACTCGTGCTGCTCACGAAGGGAAAGCCTTTCCTGTGGCAGCGT
GGCTCTTGACAGTCGCAAGTGCCCTTCAGGAAGAAGCGCCAAGCGCGGCCGTCCCACAGA
AAGCCCCC
>at02884
CCGTAGCCATTTTGGCTCAAGCCGCACTGCCTGAACGTCAGCATGGTAGCTCCGCCCGTG
GCTCATTCTAGTGTCCTGCCATTGAAGCAGCAGGGTGACAAGGCCTATGCTGCTCGCGAC
TATGCCTCGGCAGTGGAGCGCTACTCGGAGGCCCTGGAGAACCTAGAGGACGCGGTTGTC
CTCTCCAACCGCTCTGCCACCTATGCACAGCGGCGGCGCTTCGACAAGGCGCTCGCGGAT
GCAGACAGGGCCCTGAAACTGCAGCCGAGCTGGCCGCGCTTGCACCATCGGCGCGGCCAC
GCCCTCTTCCACTTGGGGCAGTACACTGAGGCGATCCGAGCCTTCGAGGCTGGCCTCAAG

CTGGACGCGGAGGACTCCACCCTGCGCGATGCCTTGCGCAAGGCCAAGGAGTACACGGAG
CCAGACCCCACTGCCCCGCCGCCGGCTCCCCGACCGCGGAAAGCGCCTAGCAATGCATCA
GCGCCGCAGGCAGGAGGCGCAGCAGCCGGAGGCGAGGCTCCTTCGGCAAGTGCGGAGGAG
CTACGCCAGAAGGGCAACACCTTCTTCCGATCTGGCAAGTACTCCGGGGCAGTCAAGGCG
TACACCGAGGCTTTGGACTTGACCCCGAAGGACCCCAAGATCCTGGCCAACCGAGCAGCG
GCGCAGATGGAGCTGCTGAAGGAGTTCGGCAAAGGGCAGGCGCCCGCCAGATGAAGTCCA
ACCCGTACTACCAGAACTCGATGAGCGACC
>at02885
GTCGGAATGCAACTCCCCTCTCACCTCGACCATTGTGCCCAAGACGATAGCCGAGACACA
CCACATGGCAGCTCGTGTTGGCAACTTCACACCCACGGCGCGCATCACGATAATGATGAT
GAGAGGGGTGAAAGCCTTCAGCATCTGAATGAAGCCGAAGCTCAAGTGAAGGTACACGGC
GTTGCCTGAGGCCATGGTGAGAGCCTTCATGAGCCCGATCGGAAAAGCAACACGGAACCA
TCCAAAGCCTGACACGAGCTTCCGGGACTCCTCACGGATCTCGCCCAAACCGGAGAGCAC
CGCCATGTGGCCTGACGAAGCTGGAGCTCACGAGGCCTAAGCTGC
>at02886
ACGTCGCTGGTTGAGGCGTTGATGCCGAGCACCCGGAGGTTGCCTCCATCGTATTTGTCG
CTTCTGTCGACGGCGGCAAGAAGGATACCTTGATGTGCATCCACGTAGTGCGTATCACCA
CCGGTCGAGTCCGAGTACTTTCTGCTCCAGAGCTcCCCCGCCCGTCTGCAGATNGANGGC
GTANGTNGTGGAGTCCATCGAGCCCCCCGTANACGACGTCGTCCATGATGCATGGCACAA
CGTTCATGGGCGCTNGTGTCTTCGCCACNTAGNNCCAAAGGANGCGACCCTCNGGGCTGA
GTTTGAAGAGGCCGTNCTCCGTCAGAATaACNACGTTNCTTTCGGAGTCAATGAGCGGGT
TCCCGCGCATCGTCGTGCGGAACCTGCCCTCTGGNTGNTGGAACTTCCANGANAANGCNG
ANGAGAGGTTTCGTGGCAACGTCTCTGCCGTCAGAAAATACTCGCCGACGTGCCCACGGC
CAGCCGGCCAGTAATGCGCCTTGGGCCTCTGGGCTTGCGCAG
>at02887
GCAGCAGCCTCCTTTTTTGAAGATGGTCTGCAGAAGCCAGAGCATGAAGTGCATCACATC
GTTCTGCTGGCTGACGACGTCCGCAACCTTGCTGCTCTTAGGTGCGTCCAGTGCGCTGCC
AATGATGATGAGGTCCCAGACCTTCGTCCCCAGCATGGCGTCAATGTCCTCCAAGGTGAG
CATGCCCACAGGCTTTGTCACGACCTTCTTTGTACCCACGCGGTCCCCCGGGGCAGCATC
GACAACCTTGTTGCAAAGGCCACCCTGGTCCGCAAAGATAAGGATGTCCTCCTTCTTGGG
AGCTTCCGCGGGTTGGGGAGTGGGTGGCACGCTGTCTCGGTAGCAGGCGTTGCAGAGAGA
CTTCCGGATATGTGCATCAAGTGAGGTTCCATCCTTGTCCTCCAATGGCAGATTTTCTTG
AAACCAGTCCACAACAAATGTGCAATGCCCGGTCATGTCCTGGGCTGCGACCGGCATATC
TTTCTGAAGAACGTAGTTGAACGCATTGCTAACATAGTAGGTCTCTTCTACAAGATCCTT
GTACATGTCGCTCATATAAGGCATCTGATTGGACATGCTGAGTACTGTAGCAGAAAGCGA
CCCGGCTTCTCAGAGAGCAGGCTTATCCCTGCAGAGCACGCTGGAACTTGGGCCGAAGGG
CTTGAGCCAAAATGGCTACGG
>at02888
CCGTAGCCATTTTGGCTCAAGCCTGCAGCCTTCTTGGCTCAGATGACGTCCACCTGCTTC
CAGTTTCGTCGGAAGGCCTCCCAGCCTAAGGCTGCTCATGAGGCAAATGAGGGGAAGAAG
AGCCGCCAGAGGGCCCAGCAGGAAGCCTTGATCGCCATGGTCTACGGGAAGAGGCGATTG
CACATCAGTGAGGACGATGACCTTGAGGAGAAGGTGCAGCAGATCCTCGACCGGGACCTC
GGCAACTTCTGCTGGTCCCTGGACTTCGGGGAGGACTTCATCCTCCGACTCTTTAGCCAC
GGCTTTCTGCCCATCTGCACCTTGGTGGAGGCCTCGTGGGGCGGCCAGGAGATATTCGTG
CTTCTCCCAAAGTTGCATCGAACAAGGTCTGTCTTGAGGAACATGCACATGCTTCATGTG
GACCGGGGCGCTCGGAAGAGGGCCCGGCAGTACCGCCTCACTCTGGACGCCGCCTTTGAG
AAGGTCGTGGAGGGCTGCGTCGAACAGCACGGGGAGAGCTGGCTCTACCCGCCCATGCGC
CAGGCCTTACTTGCAGCTTTTCAAATGCACCTGGATGCCTCGAAGGTGGAGGCCGAAGCC
TCTCTCCACAGCGTAGAACTCTGGGCCGAGGGCTGCGAGAATCCAGTCGCAGGGGAGCTC
GGCTACATGTGCGGCGCCATGTACACGTCATTGACAGGCTTCCGCCGTGGGGATGGCACC
GGCACTGTGCAGATGCTTGCGCTGGGCGGCCTGCTGCTCCGCTCCGGCGTGCGGTGCTGG
GACCTGGGTATGCACATGGACTACAAGAGGGGCCTCGGCGCCGAGGACATCGACAGGCCG
GACTTTGTGGCATTGCAGCGGTCGCTTCGAGAAG
>at02889
TTTTTTTTTTTTTTCTTGTAGAGCAGCACCCTGCATGGACCATCGCCTCTGCCCTCCAG

CCGCGCGCCGGCCAACGGCCCCGCCCCTGCCGGCGGCTGCCGCCACCAGCCCCGCCGCGG
GTCCTCAGAAGAGCGCGACGTTCGCCAGTGCCATGACGCTCTGCTGGTCGAGCGCGAGGT
GGTGCGAGTGCGCCGCCAGAAAGCCCTGCAGCTGCCTCACGCGAGCTCGCAGCGCTGCTG
TGTTCACGGCCCCCTGTGAGGCCGCGTGGTGAAGGATCTGCACGTAGTCTCCCACGATCC
GGGGGGCCTCCCCCAAGAGCTCCCGGTCCACCTCGGAGCTCATGCTGTCCTCCGCGCCCA
GCGGGAGCAACTGCTCGCGGTCAAAGACGTCCAGCGCGGCTTCGGGCCGGCCTTGGTCGC
AGAGCATGTGGAACATCCTCGCAGCATAGAGCTTTCGCGCCACTGCCCACGCGTGCGAGG
ACAGCGCGTAGCGGTCCAGGTTGCGCTCGTAGATGCTGAAGAAGCGCTGGATGTCCTGGC
TCAGCAGCGCCGCCTCGTCCGCCGCCGCCGCCGCCGGCTCGCGCCAGACGGGGAGCCGGA
GGCAGCGGCAGAGGACCTGCAGCACTTCGCTGTGGCACTTGCCTAAGTGGAAGAGGCGGA
TAGCCTCCCTGTACTGGGCGCGCTCGCAGGCCGCCCGCCCCGCGCGAGTGCAG
>at02890
TTTTTTTTTTTTTTTTTTCTTGGAAGGATGCACAAGCGCTAGGAATGGTGAACATGCTC
CCTGATGCCGAGGAGGCGTTCAGATGCCAATGACTGTGCGCGCTCACTTGGCCCAGTCAG
ATGTTGGCTTGATCTGGCCACGTTCCTTGCCCACGCCCGTGGCCCGGCAGTACGTCATGG
GCACGCCCACGGGCAGCATGGCCGCTTGCATGCCGCCACCGCCTCCGCCCACCTGGGAGG
GGTCCATGCCGGCAGAGATGGCCGCAATAAGTTCGGCACCGGAATTGACAATGTAGGGGC
GCTCAAAGAGCGTCTCCTTGATGCTCATGTGCCAGTCGTTGGCCAGGCCGCCGGGGCTGG
GGAAGCCGATGGAGCCTGTGGCGGCCATGAGCGAGACGTGCGACACACCGGGCAGCACAA
CCGCGCCCATGACCCTGTGGTCCGTATACATCCGCATGGATGTGGGCGAGATCACCGTCT
CCGTGAAGAGGCCGCCCTCGGGCATGCCGCCCACGCCACCCACAAGAGCGTGATAAATTT
TGCGCCACTCGAAGCTCTTCTGCCCGTCCTTGCTCCATGCAAACTTCTGTGACTTCATCA
TGCCACCCTCACCAGCACGCTCGAGTGGGCTCTTCTTCGCCTCGATGCGTGCCAAACGCT
GCTCCGCCTCATACTGCCACTGCTGCTTGTAAGCCTCCTTCAACTCCAACTCACTGTCCT
TCCTAGAGGCAACGTAATCAGCCGATGTCATGAGGGCCACCTGGCCATTGAGGACTTGCA
TCTGCTTTTCGTTCATGGCATCAGAGACGCCGAGGCCGAGCATGTCCATGCATCCTCCGC
CAGGGACACGGTGGCTGACAC
>at02891
TGGAGTTTGTTTTTCAGGCATCAATGCATGTGCCCGTTTGTCNCACTGGCTTGTCGTCGT
TGCCCGCCCAGTCAGTCCCTTGGCACTCCGCTCAGTTGTCCACGTCCTTGTCGGAGTACT
TGCCCTTCTTGGTCTTGCCGCCCTTGCGCTCCAAGAGAGCCTTGCGGTCCTTGTCGATCC
GAAGCTTCGTGATGACGACTTTGGACGGGTGGATGCCCACGGGCACCGTCTGGCCGTTGA
CCTTGTCGCGCGTGACACGCTCCACGTGAATGCGGAACTTCTTGCGGTAAACGCAGGTCA
CCTTGCCCTCGCGGTCATGGTAGTGCCCACGCTTGATCATCACCTCGTCATCGCGGCGGA
TTGGCACCGCACGCACGTTGTACTTCAAGCGGAGGTCCTTGCTCAAAGGTGCGCTCATGA
GCTTCCTGCGAGTGCAGGACCCAGCCGTGAAGTGCGCCTTGCGGTTCTTCCTGCGGGAGA
TGGTGACAGACGCCTTGCGCTTCATCTTTGCGTGCAGTGGCAAGCTTTCGGAGGAATGGG
CACACTTGTGCTGGATACCTTGAACC
>at02892
TTTTTTTTCTTTTTTTTAAAATCACAACGACTTCACGGCCAATCACAATGTTCACGCCCC
CAAATGGTGTCCCTGGGCACTGCGGCAGCAACGCAGGCGCCGCGCCCACTCTACACTGAT
GCAGGGCCGCCATTTGTCCCACTCATTGTTGATCTCTCGCACTGCACTCCTCAAGGCAGC
CGCCAGGCATTCCCTGCTTGGCTGCGTGCCTACCCTGCTGGCTATGGCTGCCCACAGACA
GTAGTCCAAACGCATGAGGTCCGGGCTGCTTGGCGCCTGATTGCGCATTGATATGCCATG
CTCCTTGCAATCGTGCTCAATGTATCCACCATGGTTCTGGCAAGTTGCGATGGCGCATTG
TCGTGGGTAAACGTGCCGCCATATGGAGTTGGCCAA
>at02893
CCGTAGCCATTTTGGCTCAGCCATTTTGGCTCAAGCTTGGTCCAACGCTCAAAGCATTCA
CCCGAGCCAGCAGGCTTGTACAGCAGCCTCTCTCCTGCTCTTGCACCGGGCCGATGGCGA
TCGCAACGGAGCCCTGCCAGCACGCTGCCTCGGATGGCGCCGCTGCCCAAGGTGCCAAGC
GCATTGCCTTCCTCACGATCTCCCTCAGCGGGCATCGGCGGCTGGCCTGTCTCCCTTGCC
ACGGAGATGTCGGACCGGGGCTTCGAGGTCGACTTCCTCGTGAGCGCCGCCGGCGTCACC
GAAGAGCTGTGCGCGCTCGAGCACGAGGCTCCGAACTTCACGCTGCACTCCATCACTAGG
GGCATGGACCTGATCTCGCGCATGGACTGGCAGAGGGTGGCCGCCAGTCACGGCAGGCTC
GGTGGCAGCAAGCTCGCGCTGTTCCAATGCATCGACGAGATCTGTGAAGACATTGAGGGG

TCAGT
>at02894
CCGTAGCCATTTTGGCTCAAGCGTGCTCAGTTCCGAAGGTTGTTTCGGCATGGCGATGGA
TGAGGCGCCGCCTCCATGTGGGTACAAGAAAATGATCGCAGATGTCGTTGCCTCCTGTCC
GGTGACGTCTCTCATTGGGACTTCAGGCAGCAAGGCGAGCAGCCAAGCAGGGACAACTCT
GGAGGATAGCGTCTTGAATATCCGCGAGATCGAGGCCGTCACACTTGCCGGTCGCATGAA
GAAGTTTGATGAGTTCGATATAGAACTTGATTCAGCAATGTCACATGCTGCTATTGAAGG
TTGGCAAATGCTCATTCTTGAGATCTGGAACGGCGAGGTTTGGGTTGTTGGCCCACTGGG
CCCACGGTACAAGATGAACAATGTCATACCATGGATGACGTCACAGGGTACGTCCAGAGC
TGAAACGGTCCTCTTTCAACATTTGAAGGGACTTACGGGTTTCAGGCTCGACACAGTTGG
CTCCTCAAAGTACCAATTATCATGGCGGATGCCCGGGCAAGTCTGGAGGGGTGTCGACGC
TGAAGGAAAACAGAGTCCAGGTCAATCCACGGAGACAATTCACAAGAAGTACCGAAGCTT
TGTTTCGGAGGTTATCATGTCGAACCAAGTGAGAGCTTTATTTCGCCTTCGCTTGACCGG
TCAAGGTGTCAGGGACCTACCAGAGGCAGTTTGGAAGAATCTCCGCAACTTTTGTTTCGA
CGCCGTCAAGGATAGCGTCCTAAATGTAGCGGAGCTCTCAGCTTTGACTGAAGATTGTCG
TCTCTTGTCTTACCCGAAGTTGGATGCTGAGCTAAATTGTCGTGCGATGGAGGCTGCATC
TGACGGGTGGGGCTCCCTCAAGCTCAAGGTGCACAAGTGGCGAGATGCACGCCCAGCCTC
CTGGTAGCTCTGGCACAATCAAGATTCAGAGTTTCAGGTCATGGATGGCGGGTTCTCTGC
GTTCTTATCTCAGGAGCAGAGGCTTTCAAGATTCCGTCCTTGAGGTCTGCGGTAACGCGA
AAGCATACGAATTGTCCTGGAAGTCAGGGTGAGGTTGCCCTGTGCAGAGGGCCATTGGCA
GTAGTGCCATTGGACTGAGGGGGGCTCGCAGCCTGCATGGCTGAGAAGGGGGGGCAGCCA
CTGTCAGCAGTGCTGTTGGGAGAAGGCAGGGAGAAGGGAGGTGTGCGGCATGCGTGGCTG
CGAAGCGCGGGAACTGTCATGGAAGGGGAAAAAAAAAGAAAAAAAAAAAAA
>at02895
GCCTTGGTGCGGAGGGCAGGGGCAGAGCCTAGTGAGGGAACGGGAGGCCAAGCGTTAAGA
GCTGTCAACGCTAATCCCCGACGGCATCCATGGCGGCACAGGTCAACGAAGTGCCTGCGG
CCCCATGGGTGAAGGTTGCTTCTGGCCTTCCACGTGCACCTGCAGCGACTTCCATGTCCC
CCACAGCTCGGCGAAGTGGCCGCGGCCTCGTGCGCGATGGCCATGACGTCCCTCCCTTTC
AACCCATTCGACACCGTCAAGACCAAGATGCAGACACAGAATCAGCTCTCAGCGGATGTG
AACAAGCGCCTCTACACCGGGGTNAAGCACTGCGTGCAACGGGTCCGAG
>at02896
TTTTTTTTTTTTTTTTCTCTTTGTTCAGCAGCTTGGCCGTCGAATGCGGAGCGCAGTCGT
GCCTCGCGCGCCCGCCGCGGCGCGGCAGCCCGCCCTCGGCCTCACTCCTCCTCGTCCTTC
TCCACTCTCGGCTGGAACGGGGTCCAATTGGGGTTGTTGAACGGAGGCTGGCGGTACTTG
GTGTGGATCTCCGGCTTGATGCCGTAGTGTTCATAACCCAACTTGTAGTCGTCGAAGGTG
AACTCCGGAATCTGTGGGAGGCCTGCGTCCGGCTTCCTCAGGTGCACCGGCACGGGCAAG
TGATGAACAGTCGAGTTGATCAGCTCCATGTAGCCTTTGAAGCCGTTGGCGTTGTTGGCG
ATGTAGAGCTCTGTCTCCTTGTCGGCCCAGGTGATGAAGCCGGGGTACTTCTCGGCCCAC
TCCTTCCACGGGATGGGGTCCTCCTCCATCTCGTAGACGTCCTCTCCGCAGTTGCTGTTC
TGCTCCCTGATCTTCGCCGCGTAGAATTGGGGGTCGGGCGCCATGTCGCCCTTGGGGATC
CAGTCGGCCGAGATCCTCCTGCCGGCGGAGNATGCCGCCCGCGGGCGGCTCGGACAAGCT
GAGGGTTACCCGGGCAGCCTGCTTGTCCACGCTGGCCACATACATCTCCAGCTCTGTGTT
GGCCGTGTACTGATCCGGCTCCTTCCCGTCGCCCATAAGAGAAGCGGGCAGAAGCGCATC
ACGGCGGCCCAAGCCAATATCCATGAAGAGGCCGTAGTCCTTGACCTGGCGGACGACACC
TGAGATCTTGTCGCCGACCTTCAAGTCGTCCATTGAAATCCGACCGCAGGATCATCTGCT
CCTGCGGCGTCC
>at02897
CATTGCAGCCTTTGGAACTGTGAAGTTCTAAATTCTTCCCTTCGCATACTTGAGAACCTC
TTTTTTGATTGAGCATGCCATCCTTGTCAACATCATACTTCGTAAAGGTGGCAGTTACTT
CCTGCATCTTCTTCCGTGCTTCAGCCTTTTTGGACGGCCTTGCTCTTCAACTCCTTCGAA
GCACATATGGAGCTGTCGATATTGCGTGTGCACTCGTTGACCCTCTGCAAGAGCTTTGCC
ATGGTCTGTTTGGCTCGGCTGGCGGCTGTCCAGGAC
>at02898
GAGCATCATCCGCAAAGTTGAAGAATGGACTTTCAATTGCGTTGATTACATACTTTGCGA
TATTGTTCAAGGGATTCTCGGCCTTACAAAGTCCGCAGCCTGGAGCAGTTCGATGGCCCA

CATGCGGCTCCGCCGAGTGGGCGCGAGCGTACCAGCTGCTTCGGGTCGCACCCACGGACT
CCGAGCCCCTCCCGGCGGGATGGTGGCAACGGTCTGGGTCTTTGAAGTCGCGCTTGGACG
ACTGGCGGGTGGTGATCTTCTTCTGCTTGATCATGACGCGTGCCGTGGCCTCAGAATAGG
GTGGTGATCTTCTCCTCCTCCTCGTAGAGGCATCGGGGCCTTGGCAGGCCCCTTCTCCTG
CTTGATCTTCAGCGCCATGGAAGATGGGTCCCAG
>at02899
GAACGCGGACAAGAGGGATGTGACGGGTGTGGTAAGGGAGAAGGAGGAGGACACAAAGGC
CAAGCCTGACTTGACGGGGAAGTGGAAGCACCTTGTCAAGGGCAAGGAAACCTTCGAGCA
GATCGTTGGCAACAAGGTCGTCTTCTCCAACGGCAAGCACGACCAGGCGACGTTCGCGTG
GACCACCGGGGACGAGATCGCTCTCAAGCTTGGGAACTCGAGCTACACCGGCATGGTCCG
GGTCGAGGGCAACGGCACCAGGATCTTCTGGACCGACGCCTCCGACCCTGTCTGGACGAA
GGTGGAGGCCTGAGCTGGCTTGTTGCGCTCGCCTGGGCTGCTGATGCTGGCAGCGGCATG
GGGACTGGAAAATTGCAATGTCCTGCCAGGATTGCAAGTTGAAGTCAAT
>at02900
TCGTAGCCATTTTGGCTCAAGCTGCTCGAGAAGGAGAGCTCGCACCGGTCACATGATGCG
AGACCATCGAAGCTCTCCAGAATATGGCACGTGCCTTGCACGAGGGTTGTGCCTCAAGAA
GCAGACGAACTCCTTGAGCTTGTCGAGCATGCACACATGGTACTCTTTATCTCCATGGTG
TTGTACTTTGTGTTCCTTGCGATTGTGCTTTCCCGCTTGCTGCGCACTTTCCACCAGTAC
AAGAGTTCAAAGCCTCGCATGGACAGGTTTCCACTCGTGCGTCTGAGCAGAGGATTTTTG
GAGCACGTAGATTGCTCACGGACTTATTTGCTGCGGCGTGTGCA
>at02901
CCGTAGCCATTTTGGCTCAAGGAGGTCCAAGGGGCAGGGCTCCCGAGCCCCAGATGAGAG
TCCGATGTGTTGCGTTTCCAGCTCCAATAAGAAAAGGGCACGGACGAGGACAAACGGCGT
CCTCGTCTTCGATGGAGTGGGCGGCTCCACGGTGGGTTGCTGAGCTGCCTCACTCTTGCG
GGGAGATCAGCGTCTGGGCTCAACAGCAAGCAGCACATGCCCGTGCGCGTGCATGATTGC
CTCGATATCGCTTGTCTGCTACAAAAAAAAAGAAAAAAAAACAA
>at02902
TTGTTTTTTTTTCTTTTTTTTTTTAAAAGTACAACGCGCAGCCTCAGCCTCCCCAGGC
AGCCAAGCGCATCCCGCTCGCGCCCGCCCGCCGAGGCCCCCCTCACCGCAAGTCCTCGT
CGGGCTGCGTCGCCGGGATGAGCAGCTGGATCGTGGTGACGATCACAAAGTAGAAGATGG
CAAGCAGAGGGTAGAAGTAGAGGGGCGCGGCCAAGAAGACGGGGTCGCACAGCGGGTAGA
TGAACCACACGAGCGGCTTCGACTTGCAGACCTCAGAGCCCGTCAGGGGCCCCGTGAACG
CCTCTGCCGGCAGCGAGGCGGTCATCGCAGCGACGGCGCCCAGCGCGGCCACCTCCTCGC
TGCTGGCCTGCGCCTTGCCGGTCTTGGGCTGGACGAAGGAGGGGCTGGCGAGCAGGTGCG
CCGCCAGCCAGCAGGAGGCCAAAGCAACAAAGACAACCGTCAGCTTAGCGCTGCGCGCGG
ACTTGGCCATTGCTGCTGCTGCGGAAGTGGAAGTGCAAAGGGCTGTTCCTTTACCTCCTC
TCTGCTGAGTGCTTGAGCCGGTCTTGAGCCAAAATGGCTACGGACTCTGC
>at02903
CAAGGAGAAGGATGCCGAGCTCAACAGCGAGCTGCAGACGATGAAGGACAAGATCGACGT
CCTGGGCAAGCGTCTGCAGGATGACAAGGCCGAGACGATAGCAAAGGACGGGCACGTGAA
GGTGCTGTCCGAGCAGAACAAGCAGATGCTTGAGATGTTAGAGACGGAGGAGACAAAGTC
CAAGGACTATGCGGAGAAGATTCGAGATCTAGAGGCCAAGAACCGGAAGCTGCAGGTGGT
CGCCGACGCGTTCGACGCCGCGAAGGCGGACATCGAGAAGCAGGTGGCGGAGGCGAAGAC
CAAATGCGCCGAGATCGTAGCCAATGTGAAAGGCGTGCGCGGCTTGAACGAGACCCTTAG
GGCGAACATCCAGAACACGGAGGCGAAGACGCGCGTGGAGATCGAGGCCCTGGGTCAGGC
GCTTCAAGTGGTGGACCAGAAGAACCTTGAGTACATGACGCGCATCAGCAAGCAGGAGAC
GAGGGAGCAGCAGCTGAAGGCAGAGACTGCGGACCTCAACGAGGAGGTGGAGAAGGTGAG
AATAGAGATCGACAATCTGAGGAAGCAGCTGGAAGGCGACGACGAAGGCCGGATCAACTT
CGAGCGCTCGAGGAGCCAGATGGAGAAGCAGATCGAGGCGCTGGAGGTTCAGGCGGACAC
GCTGAAGAAGGCCCTCAGCACTGCAGAGCGCGCCAACGAGCAGCTGCAGGAGGAGAACCG
GACCACCTCAGACAGGTGCCGCGAGACCGCGGACAAGGTCTACGCGCTGATGGACTCCCT
GCGCCTGAATCAGGTGG
>at02904
CGTACGGTACTTTGTACTTACCGCTCTTCGCGCCGCCAGTTGCTTTCCCTTTATCAGAAG
CACCTTCATCGTAGTCTCTCCAATCATTGCGACTGCTGTAACCATCTTTCCCACCACTGC

CTTTGCCCTTGCCATGGCCCCCCTTTCCCATGCCTGTACTAATTCATCAACCTCGCATTC
TAATACTTGGATTGAGCCTGAC
>at02905
CAGTAGCCCGGGCAACTCCCTTTCCATTTGCATCCGAAGATGCTAGTTTCGCATTCGGAC
GCATCCGTGTAGCTTGTGCAGGAAGATCCGCCTTCGCAATAGCCATTGCGCTGGCGGCGG
GGCCGCTGACACTTCCAAGTGCACCCAAAGATAGAGTTGGAGCAATCAGCTTGCGACTTA
CCGCTGCACAGGCTGGATCCGGTGCAGTAGCCATGGCAGCTGCGACTCCAATGACAGCTG
NAAAATGCTGGTCTCACAACTTGAGGGGTCGGAGTAATCGGAGCACAACGACCCTCCGGT
GCAAACACCTCCGGGCTGCCGGCTTGCCCCCAGCAGGGCTGCTGCCACAGTCGCATTCGC
ATGCACAGGCCGTCCCAGTCCCGCAGTGGTATGCGCGGCTGGCACGTCACTGCCTGACAT
TACCCCACTGGCAGCAGCAACCGGGCCAGGGGTTTGGCCATTCTTTGGTTCGACCCTTCG
CTGAAGGGCATGCAGTGCACATGCACTTGGCTCGTTGGCCAAGCACTCTGCGTCATTGAG
CAATGCAGTCTCCACCTCAAGAGGCTTTGCATCTTCGATGGCGGCACTGCATGGCACAAG
GCACACAGGGACTAAGGGCAGACAAC
>at02906
TTTTTTTTTTTTTTTTTCCGTCCCATTCTCTCACCTTCAATCGGACGGTCCAATTTCAG
AAAGGACGATCAAACCCAACGCGTCAGCCAAAGCACCATTGGTGCTCCAAAGCCGCTCAG
AAACGAACACGGCTTGGCGTGACTTCAAGTTGCCGAGCTGGTTGCATGGACTCCGGGCTC
AGGACTGGCCGCCGTGCGCATTGTCCCTGATGGTCCCGTGCGCAACGATCTGGCTCAGCA
CTTCCGCCAATTCGGTGTGACCGTGATCTCGTGCCTCGCTGAGGGCGGTGTGCCCCCAGC
GGTCCTTTTTGTTGACGTCAGCGCCGTTCTGCACCAAGTGCACGGCAATGCTCATGTTGC
CCTCCGCACATGCCAGGGGGGGTCCGGAGGTCGTAGTCCGACGCGTCAACGTCGAGGTCG
CAGAAGGCCAGGAGATTGTTCAAGTAGGAGATCTGGTTCGCGAAGGCGGCCTGGCAGGGC
TCCTGACCGGCGGGGATGCTGTTGGCGTCCGTGCCTCGTGCC
>at02907
TTTTTTTTTTTTTTTTTGAGTTCACGTGGTCATTGACCATCTCCCTAGGAAAGGGTTCG
CAAAATCCCGGGTACTCACTCAACACGCCTCGATGCCATGCCTTCTGGCACGGATGGCCA
CCCACATGGGCCCCACGAGCGACATCATTCGCATGGCCCCGCAGCAGCACCTGACCGGCA
GAGTCGTGCAGGCGATCTCGCTTGCTCGATTCTCTCAGCGGCCATAGCAAGTGTCCGTCA
GCGCATTCAGAGGCATCCGCTTCAGCAGAAAAGCCAGGCCTCCCCCGGCGGGCCGAAGAG
GGCCCGCCGGGGGCGGGGGGACAGGGAGGCCTGTTCCCCCGCTCCCTGCCCCCGTCGCAG
CCGCTGGCGGCATTCCCCCCGGGGGGCCGGGACGGCACCCCCCGGAGGTCGCCGCTGCCA
CCAGCGGAGGCCCGCACGGCGGGCACTGCGAAGGCGGCTCTGGAGAGCAGCGACCTGCAG
TGCGCCTGTTGCCGGCTGCCAGCTTCCGGCACTGGGCACGAGGCTCTGTCAATAACGCAG
CTGCCTCCANCTTTCNCGAGCTGGGGCGGTCCAGGTATGCAGCAAGCANGAACTGGGCTG
GCGCCGAGCCCCCAGAAATACTGCGGCTGATCCCACTGCTGTTCGCACGCACTGCTGTGG
CACATCTCATTTCGCAGCATTCAGCACTGGNCTTGTGAACCGGCACCTC
>at02908
TCGTCTTCGGGGTTCGACACCCGCAAGAAGGCAATGTGGCAGTTCCACGCTTGCAAAGTC
AATGGCTTGGACTGGTCGAATGGGTATACAGAAGTGCTGCGGCCGCACTGGGCCCACAAG
ACGGGCCAGGCCGAGAACGTTGCAAGGNAAGGAGAAGACACGTCAGATAGTTGCCCAGCA
GACGGGAAATGAAACCGAGTCGGCGGTGGAAAAGCCAGCGGAGGCCCATTGTACCCGACG
GCAGGCTTCACCAACTTGGCATCCTCGCGCGCGTCCCCGGCGCCAGCCTCCGCACCGCTG
GGATCTGCTCTTTGCCCCCGGAGCCAGTGCTCCCTGAAGTCCTTGCGTCCGTCTTGNTCG
CGCGCTTGCCGAGNGTTCGTCCTCGCGCTCCGGAGCCTCGCCCCTCGGA
>at02909
CCGTAGCCATTTTGGCTCAGCTGAAGCCAGGCGTTGCCTGCCTGCGTATTATTGCCTCTC
ACGGGCCATCCCAGCACCGAACCATGGCTGAGGAGGAGGGCCAGGAGAGTCATCTTCACC
GAGAGACACCGCTCCGGACCGTGAGATCTGGGATGAGAAGGGCGGCCTTATCAAGAAGGG
TGCGGCAGAGATCGCAGAGCGTGATCAGGACCACGTCCCAGAGGTGAAGGTGCTGACAAC
CCTTGGTATGTTTGCAGTCCCTGTGACTGCNGAAACAACAGTGGGAGAAATTCGCCAGAA
GGTGCAGGAGTGGAAGCCGGAATATTCGTTGGAGCAGATTGAACTTGTGTGCAAGGACAA
GGAATGGCGTGGGGGCCCGGCTCTTCAACAAGACGTCNCGCCCGCGTGATGACGACGAGA
CTGTTGAGTCTCTGTGGAGTGACTGGGACATCCCGCGCCGCATGATGCAGGTGGCCCTTT
GGATCAAGGAGAAGAACGAGGAGACTGGAGAGTGGGAGAGCACATTCTGGAAGATGCTGG

AGGAGTCGAAGTGCGATGATTGGAGCTTCCAGGGGCCACACCATCTGAGCNNNGGCCAGG
GAGAGGTGCCTNTTTCACGTGATTCACANTTCCNCATCACCAGAANAGTCTTANTNCGCC
AGTTGTCGAGCAGGGTCCAAATCCCAGGCACAGGACCTCCCATGCAGGGCGCGTCGCCGA
ATTGGGTGCTGCACCGCCCCTGTGCGTACCGTTTCGGCCGCCAGCCAGCAGGAAGTCAGG
GAACTCCTTGCGAACTCTGCGATTAG
>at02910
TTTTTTTTTTTTTTTTTGGTCCTGCGTTGCATGCATGACTCTATCGGAGTCCCCCCAAG
AAGCGGTCAACAAGGTTCGTGTCATTCACTTTCCAGAGACTGTACAACGTGAATGGAGAC
CTTGGAAGGTAGCTCGAAGAAGTGAACCCACCTTCTTGAACATGCACCCACATGCCAGGC
CCCAACACCTGAACAATGTCCACTCAGAAATCAACGTCCAATGCAAAGCCCTTGATCTCC
TCCTGGCCGCCTTCCTTGTGACTCACAGAAGACATGACCCCTGCCTTCTGGTACTCGCCA
ACGCGCTTCTCAAAGAAGTTTGTCTTCCCCTGCAATGAGATCAACTCCATCCAGTCGAAC
GGGTTGCAAGCTCCGAAAAGCTTTGAATGGCCAAGTGCGGTCAACAGCCGGTCGGCCACA
AATTCGATATACTTTGTCATCAGCTCACTGTTCATGCCGATGAGATCACACGACAACGCC
TCACAAATGAAGCTGCGCTCCACCTCCACGGCAGTCCTCACGATGCCATGGACCACATCC
TCTGGGAGCTTGTTCCCTCGTGCC
>at02911
TGATGCTGCAGGGTGGGCAGGCGCCTCAAATGGTGGAGTCGGCCCTCGCTGTCGCTGATT
CCGTCAAGTGCGACCGTGGCACGATTGTCCACCTTGCTCTGACCACAAACAAGGATATGG
CACCCCTTGATGAGGAGAATGTTCGTTGCAGCAGCTTGGTCTGGATGGGAACCCCTGCAA
GGGCTGCGCAGGCCATGCAGGCCGGTTATTTTGATCAGTGGCCAAGACTTGCTACTGAAG
CTGATGAGCAATGGCTAGTGTCAGAGATTTCTTTCTACAGGGCTGTGCAGCAGAATTGCA
ACAGCTCCTACCTGGAAATCAGCCGGAATGCAGATGGGGACCTTCTAGAGCGGCTTCGCA
GAGACGGGACCGAGCTCTCTCAGCTAAGTTTACTAGTCCGCAGTGGCGAGCTGGAGGCGT
ACCGGCACTGTGCAGAGTGGGCTGACGAGAGGGCGTCAGTGCTGGTACCCCG
>at02912
TTTTTTTTTTTTTTTTTACAACACAACATCTGCATTATCTCTGGAACTCGTCGCCCTCC
GCCCTTTGGGAGTGCGCGGGGGCTCAGCTGAAGCGGATGTGAACGCACCGCCTGGCGGCG
CCCCGGATCTTTGCCTTGCTCCCCTTGTGGAAGTGGTTCTGCCGGAGGTCCAGGGCGCGC
AGGGAGGCCAGCGAGGGCAGCGCGGCGGCGATCGGCTCCGCGCAGGCGTCCCCCAGGCAG
TTGTCCCGCAGGTCCAGCTCCTCCAGGCGGGCCATGGAAGGGAGCACGGCGAGGATCGCC
TTGGCGCCCCCTGTGCCGAGGTTGTTGTGGCGAAGGTCGAGCCAGGTCAGCTTCTCCAGC
CTTGGCAGCGCCTCGGCGAGCGCCTCCGCACCTCGCTCGCCCATCCGGACGTCGTTGAGG
ACGAGGCGGCGCAGGCCCTGCTGAGCCGCAGAGCAGTCGAGCACGGCCTCCGCAAGCCTG
CGCGCACCGTGCTCACCTTGGATCAAGCGGTACATCTGCT
>at02913
TTTTTTTTTTTCTTTCTGTCCGCCCTGTGAGCTGCTTATCTTCACGTCACACAGCGCCG
CGGAGCGCAGCCGGCTCAGCTGGCTGCACCGCCTAAGTCCTTCACGGCCTTGCGGAAGCG
CACAGCGTGGCCGAGCTTCATGCCGGCGAGGCTGGTGAGCTCCTCCAGCCTCGAGGCCGG
GACCCCTTGGAGGTCCTCCAGCTGCTCGAAGCCGTGCTCCACGACGGCGTCGTGGTAGGC
CTCCAGGCGGATGCTCTTGAGCCAGGCCAGCAGGGCGGACTCGGCTGGCAGGGCTTCCTC
ATTGGGTGGAGGATCCACGGCGCCTTCGGCACGCTCTGGCTGCGGATCGGCCATGGCTCC
GCCACCTGAAGCAACACTCGCCTCCTGCCCCTGTTCCTGCAGCTGGCAGATGTCCAGTGA
AGCATGCTGCTCGCTTAATACGGTGTCGTCCGGCAGCATCAGGGCCTCTCGGCCAGCATC
CGCCGNTGGACAGAGCAAGGCCAAAGCCCGATGCCGTGGTGCTTCCTGTGGGGGTCGTCC
GCTGCCAAGCGCTCCTTCAGCTTCAGCACGGTGGTGCCCCTCGGGACGAGCACTCCGTTG
AGAAGCCCAGCGCTCGATCCACTGTGATGGTCACTCTGAGCTCATTACCACCCGCTGCTG
CCAAGTCAGGCTCCAGGGCTGTGTCTTGATCCGC
>at02914
GGCCGACGAGGACTGGAGTGCAGTGCTGGAGGCTCTGGACGTCCGGGAGCGCCATGGTTA
CATCCGCACCGTGGCCACCCTGCTGCCCTCGGGCGTTGCCTCGACGGATGGCCACGCCAG
CCGGGCCATTGTCTATCATGCCTACAAGCCTCATGCAAATGCCGCCTACGCTGGCCCAGA
GGCCGTGACGGAGACGGCAAAGGTGATCGCGATGGCGCAGGGCCCTTCTGGTCGCAACGA
CGACTACCTTTTCACGCTGAAGGCAGCGCTGGACGAATGGGCTTTGCCTCCGGATCCCTA
CTTGGAGGAGCTGGTCGAGGC

>at02915
TTTTTTTTTTTTTTTGTTGCTGCCTTCCAATGCCAAGTGAACAACGCAACGAACTGTCAT
TTAAAAACGTGCTCAGAAGAATGATGTTAAAAAAGAATTTGCAGCTTGGAGGGAACTCAT
TAAAATGCCACGTCGTTACTACGATATGACTTAGTAGCCATGGCCTACGACTGGTGTAAG
CCTCCGCGAGAGACTTGCAGATAGCGAAACGCCTGCATGTCTCTTCTCACTCGACTCTCG
CAGCCCGCTTCCAGGTACTCTCATCGAGTGTTCACTGACCCTCAGGCAGTATAAGTGTGC
GCTCCCGTGCCCGTCCATTTAATCCAGCGAGCACCATCTTGGTCGTACTCTAAGTCCGGC
TCCTCGTTATCCGACTCATCGTCCACAAACCGGCCGAAACGCCACTCGTAAAGCTCCTTT
ACCTCTTCAAGCCGATCTTCGAGAACCGAGATGGACAGAGCACCAGTGCCGACCGTCTTG
ATCCATGGCGTGCCGTCCGCATCACAGTAAACCTGTTCATTCTCTGACTCGGCGTCCATG
TCAAAGCCAAAGTGTTCAGTGTACAAGGCCTTCACTTCCGCGATCAT
>at02916
TTTTTTTTTTTTTTTTTGACGGTCACTACAGAGTCGGGGAACTTTGGCAAAACAATCGA
AACACCGTGCAGCGAATCAAAGGGCTGCTTCCGACAGCAGAGGCTTGAACCTCAAATTGC
TGCTCAAGAATGAAATCAGGGGGAGCGGGAACATCTCTAAGAAAAAGGACTCTTGGAAGT
AGTGTTCTTCCATGGGTCCAGAAACTAGCCTCCCAACGTCCAGAATCCATGCTAGAACGC
TATCTCAGCACGGAGCAACACCCTGGACTGTTCCCTGCTCAGTGCCAGTGTGGCTCAGGA
TGGCTTCCCTCGCTCGTCTACGGTGAGGCTGAACTCATTGACGGGGCAACCTTGCATGGC
GTTCTCTTCATTGATGTCTTGATCGTCCGTAGCACATCTGCAAGGCGCCGGCGCTTCTGC
GGAACGTCCTCGGTCCACGCCTCTTGAGCTTCTTCCCTGTGTACAGGTAAGGGAAAGGCC
CAAGCTGTGGAAGGCATAGGAGCCCGTATTGCCAGGCCTGTGTGCGCCTTCTGGCTACCT
CCGGCAAAGCTCACCTTCGAGGAGCTCTCAAAGCCCAGAGCGAACGTCATGCGATGGCCG
GTGTCTCGGTGGTTCAGCCCCCATGTCGTGTCGTCCAAGGCTGGGAATGCAATGCCCGCA
ATGCTCGGGATAGCCGCCTGGTCCTGCAAGCCTGGGTCAGCTCTTTTGGACGACCAGTCC
ACTGACTTGTCGAGGGAAGCTTCAAGTTGCTGAGCTGCAGCGTGAAGAGCACCACGAACA
GCACCCATTGAGATCCGTCGTAGCCTCCTGTCCACCTCTGATACGGACGGATGTGGAATA
GTGGTGATCAGCTGCATCTCCTTGTGACTGCTGAGCTCGGCAAGCATTGGCATTGCAGGG
GGTGGCCAGGGTTGCAGTTGCAGTGCCTCGAGAGGGCTTGCGCCGTCTTGAGCCAAAATG
GACTACGG
>at02917
GGACAACACCTTTGCCTACGGCAGCGGTGAGGTGCTCTTCAAGCCTCCCGACGTCGAGGA
GGTGCCCTTCCGAACCACGCGCTACGGCATCTACTCCTACTTTGCCGGCGCCAGCGACGA
CTACCCGCGCGAGTATTTCGAGGACCGAGGCTTCGCGACCGAGGGCTGGAGCACTGGGGA
CTTCCACAATGCGGCGGTGGTCCTCGATGGCGACCAGGCGTGGGCCATGGGCATCTACAG
CTTCGGTAAAAAGGGACGAGGAGGAGCAGTTCCACGCAGAGTACTCCATCGGCTACGTCC
GCGACAGCGAGGGCCGGCTCCGCATAAACCTCTTCTCCGCGTCCATGCCCTCCGAGAAGG
AGAAAGAGAGGCACAAGAAAGAAGCGAAGGAGGACTGCTGGGGTGCAAAGGTCAGCGCTT
CGGCCATCGAAGAGTTGCAGAGGGATTGGGCGAAGGGTATTGTCGACATTGGCTCC
>at02918
CGTGCATACACAGGCGCTGATCAGAGCCGCCGCCTCCGGCTGCGAGCCTACTGTGCAATG
GTGGCTGGTGGGCACACTATCCAAGCAGTGACTGGGGATGTGGCAGACAAGGTGAAGGTG
AAGACCGCGTTGGTCTCGGTCTTCgGACAAGACAGGCCTCGAGGACCTCGGCAAGTTCCT
CGCCAAGGAGGCGTGCACATCCTGTCCACAGGCGGTACCGCGGCGAAGCTGAGGGAGCTG
GGTTGCACCGTGCAGGACGTGGCCGAGTACACAGGCTCACCCGAGATCCTGGACGGCCGC
GTGAAGACGCTGCACCCCAAGGTCCACGGCGGTATCCTCGCCGCGCGCGGCAACGCGGCG
CACGAGGCGCAGATGACGGAGCACAGCATCCTCGGGATCGGACCTCGTCATAGTCAACCT
CTACCCTTCGCCCAGGCCGTCG
>at02919
CCATCCCTGTACATTGTGGACGGGAGTATGCTGGTTAGACTCACAAGGATGACAGCAGCA
AGCCTGCCTTTCATGGGCCTGGTTCACTTGAGGTGGAGGTGGTGTCGCCAGAAGCCGAGC
ATGCCTCCGTGGAAACAGAGTCGTCTGCCGAGGCTGCACGGAGCAACATCGTGCTATCAC
CCATGCACCGGAATCCCAAGCAGCGGCCCTTCGCACCAGCTGCCCTACCTCGGCGGTAGC
T
>at02920
TTTTTTTCTTTTTTTTTAGTCAGCGAGCACAATCTAGGCAAGTCTGGTGTAAATTGTC

ATGGGTAGATGAGCCGGACCTCTGAGGACGATCCTGAGGCAGCCGCGCACGTCAGAAAGG
CCTCAAGCAGTTGCCACCATCCAGGATGCTCTCGACCTCCACGGACATTTCTGGGCTGAG
CTGAGGTCCTACGGACTGCCAGAGATCCCGTGCAACAAACTTCCACCTCATCTCTTGTCT
TCCCTTCTCAACAACAGCTTGCCACACTGCCAGTGTGCTGCGATCATCCCTGCACGATGC
CAGATAGCCCTCGATGACCTTAGCAAGATCTTGGCAGTTGTGCCGGATGAGTAGCAGGCA
CCACCGGAACAAGACCTCCCCGTCTTCTGATTTGTTGAAACCATAAGCGTTGCCCATTCG
CTGGATGCTCTCGCGGTACACGGCTTGTGTGGCCAGCAAGGAGTCCAGGAAGTGCAGTCT
CTGCTCGCAGCTCCACTGCAGCCTCACATGCGGCGCTGTGCGGACTATCAGCTTGGACAC
CTTGTCTTCGCTGGCCCCTACGGTTTCAGTGGGGACGACTGCCTCCCATGCGCGCAAGAT
CTTGTCCACCTGCTCCATGGCAGGGTCCGGCATTGCCACCAGGGCTCAGCGCGTGCTGCT
CGCACCAATCAGACGGCTTGAGCCAAAATGGCTACGG
>at02921
TTTTTTTTTTTTTTTTTACTTTCCAGGGGTAGCTGCTTCTATCCCAGCTAAACGACCAT
GCCACAACACTTCCTCAGGTACCGACACCAGAGCAGCATGATACTTCGGAGCTCACGCGA
CTGCTAAGATGCGCTCCCCCGCAAGCTGTGCCACGTGCTTTGAAAATGCGTGCGCGTTGG
CGTAGAGCGCACGGAGCGACATGGCCATGCCAACGGCAATGGCGAAGCGGCCGAGTTGAG
GACTCGTGAAGCTGGCCAGGGAGGCCATCAGCATCGCCCCGTGCTGATCGATGCGGAAGC
CGCCCTCTGGACTCCGGAGCTCCTGCCAGATGTACAGGAGGAAGCCTGGAACGGCCAACG
CGAGAACACCGCTGAACGCTATTCGCGCCAGAGCCGCCGTGGCAGCATGTGCGGCAGCTC
GCTGCCACACCGGAGCATTCAAGACCTTGAGCAAAGTCCAGAGGAGCATGTTCCCCCCGG
CCTCCTCTGGCACTGGTACATGTACGTGCAACGCGCCGAGCAACAGCCCAGGTAGGTTTG
CATGCAGCTCCGGGAACGCCAAGCTAGCCACAAGACCCAGCAGAGCTGCGGCCGACAGAA
ATGCTGCAGCAACGAGGCACATGGGCGCCCGCGAGGATAGCATCAGTTGAGCAAGTTGCG
TCCTCTGGACTCGATACTCGTAGTGGCAGCTGTCGCAGCGATAGTGCGCCATGGAGTTCG
CTGCAGAGCCTCGTGCCGAAT
>at02922
TTTTTTTTTTTTTTTTTCCGCAAGTGCACTCGCGGACAGAAGCTGCAGTGCCCCCGCAA
ACACCACCACCTGCGTCAGGTAAAATGTACGGAACCGCGTATGGTAGTCAAGCATGTGCT
GCTCAACTTCAGCAGCAGGCAATCACTGTGAGAAGAAAAATCTATTAGGTCCACTCTTAT
GCAATGCTTCAAAGTGCACGAATTGAGTCGGAGCCGTTGCATTCTGTCTTCACGGCCAAA
CTTCTCAAAGCCCAAAGTGCTTTGCGTAAAAGCCCTTCTCGTTCACCGCCCTTTGGATGG
GCTCCATGTCGAAGCCCGTTCTCTTGATCCACTCATCATCCATCATCTTGCTCATGTAAT
TTCTGGAAGAGTCTGCGAAAAGCACCGCCACGCGCTTGTCCTTCCCGATGTTGTGCTCCT
TGATGAAGCGGTATGCGCCGGCCATGGTAAATCCACAGCTGCTCCCGATCAATAAGCCCT
CATGGCGGACGATGGATCGCATCGCGGCAAATGCCTCGTCATCGTCGGTCTTGACCCAAT
AGTCGACGATGTCTCTGTCAAGTACTGTGGGGATGAAGTCATAGCCAATGCCTTCAACCT
GGTACGCCGTCAGTCTCTTCTGTCCGGTGCGCTTGGACCCGTCATTGACGCTGTCGGGCT
TTGCAAGAATGCTGCCATAGGGATCAACTGCCACGATCTTG
>at02923
TTGTTTTTTTTTCTTTTTTTTAAGTGCCCGCGCGCTGCTGCAGCCGCAGAGCCCAGTGCG
GCGCCTCAGCGCCAGCATCACGGTCTTGTAGCCGATGGTGACGCCCATGCCGATGATCTT
CCAGAGGCCCGAGGCCTGCATGGAGAAGACGCAGCCGCACTGGCAGCCCTCGTGCTCCTC
CCACGTGATTCCGTAGTTCGAGCGCCACTCCGGACGGCCTAGAGCGATGAAGCAGCGGAC
GGACAAGGTTAGGCAGAGGGTCGTCATACAGATGCAGAGCACAAAGATCTGCAGG
>at02924
GCGGGCGCCGGCGTCTACCTCTGGAGCGACGGCCGCAGGTACGAGGGCCAGTGGTCGGCG
AACCAGATGCACGGCCACGGCCTCTCCACGTGGCCTGACGGCAGCGTCTACGACGGCGAG
TACGTCAACGACGTGAAGCACGGCCACGGCACCTTCAAGTGGGGGGATGGCCGCGAGTAC
GAGGGGCAGTGGTGCAACGGCAAGCAGCACGGGAAGGGGCAGCTGTCCACGTCGAAAGGC
CTCAAGCGAGCAGGAGTGTGGGAGGAAGGCAAGCACAAAGGTGTGGGTGGAGTAAGTGCA
ACTGGCCCCTGCTGGCATTGTCTGCCGTGGGCAACCCACTCTGTCACACGAGATTCATCA
ACAAGAGCGCCGCCACTTGGTGCACCCTCGTCGGAGACGAGGCTGTGGTACCCTGTCGCG
GCCATCATCTTGTTAGGTTCCGCTTGCGTGCGAGCACATTCCATATACTCGAGTGTGACT
GGTTGACAGCCAAAGCAAGGGCC
>at02925

TTTTTTTTTTTTTTTTTTCCGACGCAAGGTAGACAGTACAGAGCAGCTTGCGCATTTGTG
CCGTGCAGTGCACCTGCATTGACGCATGCTGCCTTCAGCAGCCGTGAACCAGTCTCAAGG
ACGAAACTAGTTGGCAAAAGCTGACCGCATTTGAGAAGTCCAGCCCCCGTGTGAGCACTT
CTCGCTGCGGACGGAAACGTGCAGGTGGGTGGCCGCGGAATGCTTAAGTTTAAAAACAAA
ATAAGCGCTTAAATTTGTTTTAAGCGCTTAAAAAATTTTAAGCGCTTGAAAAAAAAAAAG
CGCTTAAAAAATGCTTTAAGCGCTTAAAAAGAGAGGCTGCGCAAAGGACACCATGGCCTC
TGCTGCCTTGGCAGCGGCAACACTCGAGCACTTCTCGCTGCGGACGGAAACGTGCAGGCG
GGTGGCCGCGGAATCACACTAGGGAATGGACACTCAAGCAAGGAACAATAGTGGTACTTT
TTGCCCACAAGAAAGCTTTTCGCGCACTTGGCGCTTAATCGTCACTCGAGCCAACTCAGG
CAACGGCACAGCACGGTGCAAGTGACCGTGACGTGCAGCATGCCTTCACATGGCGGGAAT
ACTTACCTAGACAGCTGAAAGAAGGAAGAACTGCGCTGGGATTTCCT
>at02926
CATTGTTCGGGCGGCTGCCTACCTTCGGCACCTCGGTAGTGCCGGGGACGCAGGAGAAGT
CGTGGCCGATTACGGGGGCCATCGCTGCGAAGTTCACCGGCGAGGAGCTCGCCGCCGCGT
TCTCGGAGGCCTGCCTCAAGGGCGCGCCGGTCAAGTACGTGCCCCTCTCCATCGAGGATT
GGATCAAGAACGGCATTGCGGCCGGGATGCCTGAGCCGGTTGTCAAGGACTTTGGGGCCA
TGTTTGGTCTTTTCCAGACGCAGACGTACATTGACATCCGCGAGCAGGCATTGAAAGATT
GCACAGACAAGGGCATGACCGATTTTAAGACTTGGCTTAGGAAACGGTGAAAAGGCATGC
TCTATCCCGATGCATAGTTTCTTGACGATCCGCACTACCAGACCAGACGACACCCACCAC
ACCGTCAAAAGGCGCATGTACAGAAAAGCTTGTCCGTTCCCGGAGGGAAGGAGGGCAGAG
CCAAGGGAGCAGCGCAGCGTGCG
>at02927
TTTTTTTTTTTTTTTTTTGCACGCGGCGCTGCGCTGGCGGTCCTCCGGCGCTGCGGCCCC
GGCTCTCCGGCCGCCCTCGGGGCCCGCCTCAGAGCGGCCGAAGCTCGCCCGCAGCCGCGC
GCTGGAGTCGCGGTGCTTCCGGCCAGTCCCTTGAAACGCTGGGATCTTGGTCGAGGTACC
ACTGCGTGCAGAACTGTTATCGTTGGGACAAAGCCCTCGATGCAAGAGCTCCTGGCCGGG
CACCGGCCGAGGCGCTGCTGACCCTTCCGGTGCAGCGCGCGCCTCACGCTGGCGGGCGGA
CCTGGCACACTCTGCCAGCGAGGGGTGCCGTGGGCGCTCCTCGCGGCGCTGCCGGCCGGC
GGCCGGCCGCCCGGCGGCGGCCGCCGCGGGGAGGGCCGCCGGCGCGTGGCCGGGCGCGGC
GGGGAGCATCTGCGGGCGGGGCGCGGGCGCCATGGCCGCANGCAACTCCGTGGGGATCAA
ATCCCTCGTGCC
>at02928
TTTTTTTTTTTTTTTTTTAACTCTGACAGGCTCCATTTCAGGGCGCAGCACGTCTCCGGC
TCTGGCGGTGCAAGTTGCCACCGAGCCGGCTTTCCGAAGAGGGACGCCGCCGCGGCCGTG
GCCCGGTCAGGAAGAGCGGCCGGCTCGTCTTGCGACCTCGCACAACTTTTCGTCTGCCTG
CAGATGCCTTGCCGCCCAGGCCAGGGCCTGCGCCCCCGCAGAGCGCGTGGCGGTCATCAC
GACCTCCCGGTCTGCCTTCAGGCGGGGCGAGGCGTAGCGCAGCGTCGCGCCGAAGCGCTG
GACGGCCGCAAGCACGACCTCGTGGTCGTCCGCCACTCCAGGCCCCCCCATCGCCAGCGC
CCGCGCCACGTGGACTTGCGCCGCCGTCTCCTGCTGCACTGCCGCGAGTACAATATCCCG
GTCTCCCTTCAGCTCGTCCGAGGCGGCCAGCAGTACCGTGCCGTTTTGCCGCACGGCGGC
CAACATGATCGCCTTGTCCTCCTTGGCAAAGTCCGCGGCAAAGTCCAGTGCGTGGCCGTT
CTCTGTCACGGCCGCGAGCACGATGTCGAAGTCCGCCTGCAGGTGCTCGGACGCATAGCG
CAATGCCCGGCCGGTGACACTGACTGCGGTCATGGCGACCTCGAAGTCCGCGCGCAGCTC
AGAGGAC
>at02929
CTTCCCTTGCCCTCGAAGTGAGCACATTCTGAGAGCTCGACACAATAGCGCAGCGCTGCG
GCGCACCTCTCACGGCAGACATGCTGCCAGTGGGCAAAGACATGTTGCCAGTGGTGAAGT
TCGATTTGTTCCCCCCAAGCTGCCGGAAGCGAACTTTGACGCGCTTCAGTTGCTTCTTGA
TGCGTGCAATGTCAGCCTCAGAATAAAACGCGTTCAAGTTCTGGAAATCTTCCATTGCTG
TCGTTGCAGTCGTCCTTCTGCTGTAGACAACGCCTTCATC
>at02930
CGGATGGATGTTGCTCAAGCCGTCAGAACAGCTCCAGCCATCAGCCATGGCAACATTTCT
GCGCCAGAGTTTGATGGAGGAGGCAGCGGTTGATGGCCGCAGACGTCATACATGGTTTGC
TCGCGTTGTTGTAATTTGTGTCTTGTCTGCTGTGAAGCAGATATCTTTTGGCTTCATCAT
CTCCGGCCACGCTGTGACCCCGCCAGCAGTGTGTAGAGGTCATTTTGCTGGCCTTCACTG

CAGGCAGTTGAGAGCTTCTCACCAATCAGAGTTTTTGAGAAGCCATTCCACCGTGCCAGC
GGCAGCTGCTCAGACAGCCGCCGATGCTGCTGCAGATACGGCTTCATTAGTGCCCAGTAC
TAGCTTCCTTCCAAAGTAGGCGCCGTGGCTCTGCTGCCGTACGCCTTGCG
>at02931
GTTCGGTTCCGGTGCTGCAGTGCACTGTAGTGCGGATTGGAGTCGCAATACGCAACCAAG
TCTTTGACGTGTGGCGCAATGGCCTCCATCTGCTGCTGCAAGCGCGCTGCATAGGTGGGT
GTTCTGGTTTCTTCTGGGAACCAATGGGGCACCGTCTCCTGGATAAACTCAATCACTGGT
GCGATATGGCTCTTCACCACCGCCGGAGATGCGCTGAACTTAGCAACAGGTGTCCACTGG
ACCTTCATTGCATGAGGGCCAAGACTCCCACGCTTGTGCAACGCTGCAACGACTCCTTGC
TTCCGAACCATAGAGAGGTAGCACAGGTGTGGGCTGACC
>at02932
TCAACATCCGTACTTCCAACCCCCTCCCATTTCTGGCAGCGACCATGGCTGATTCTGCCT
GAGGCGTTGTCTCAACAGCCGCTTCCGAGCATCCGCAATCCTTCTTGTGGCAGTTTCTCG
TTGAGAAATAATATCTTGGCTCCACATGTTTCGAATCCGCTCAGCCTCAGCAATATCATC
CTCGTCCAGAGTCCCATAAGATCTGGCAGCCTTGTACTTTTTGTTGACTTTGAAGCCTCT
TCGCGTCTCCCCACGAGGTTGTCCGGACTGCTTGCCAGTGTTTGGCACTGCTCTCCGTCT
GGGACCAACAGTGCGTCTATCATATCGACGTCGATTATGTTTGGGCAAGAGCCCAGTAAG
AAGAACTGTGTGCGAAAGGGCCAATGCTTCACACCAATGTCTGCCTTGACTTGCACGCTC
ACCGACAACTGCTTGCACAGGCG
>at02933
CGCCCACTGTGCAAGCTCAGCGGTCTTGTCTTCGCTGAAGCCAGCCAAGATGCACGATCG
AACTCTTGCCAGGTCAAGTCGCCGGGATCGCCCGGCACCGGTGAGCCTGGTGCAGGCGCC
CATCTTCTGAGAGCTGCGGCTGCCTGGGCATACTCTGGACGTGTGCCGGCTTGTGAGNGG
AACTCCAGCTCTTTCAG
>at02934
TTTTTTTTTTTTTTTTTCCAAGCATGAAAGAGTCAAGGGGACTGCTGGAGCAACGCACG
ACTTGCGTGTCTTTCCTTCGGAAAAGGTTCAACGATACATACTGTACAGGTTAGCAGGTC
ACAATATTGTGCATCTGGAATTTCTACGAAGCTGCTCCTTGTGCTGGAAGCTCCAGGGAA
TACGTGGGGTCGGGGCATTCAGAAGCGGCACCCGTCTTGAACAGTCCTTGCTCGACAACC
CTGGCTAATTTGCCGATCTCCTGACGGATGCACTTGCGCAGGTCAACAATGTCTTTGACA
GTGGCCTTGTTCGAGCCGCGCAACGCGAGGACCATTTCCATGAAGGCAGGGAATTCGAGC
TGCGGCGAATTCTCGAAGACGTACTCAGTGAGATCCACCAAGCCCACAAAGTCCACACCC
AACTCCTGGAGTGCCCTGACAGCCTCAGGAATTTGCAGCAGCGCGGCGAACTCGGCCTTG
GAGATCACGTTGTCGCCGTTTGCGTCAAGATTCGTTGTCTGCATCATGTTCAGGAGCTTG
TCCTTTGTGAAGGACACCGACAGCTGTTCGCGCTCGAACATAGACACCACGTGGACAGTC
TCAACCATCACACCGATCAGCATGTTCACCACAAGGAAGGATCCGATGAGAACGAACAGC
AGAAAGATGGCGCCGTACAAAACGCCCTCGTGCCGAATTTCTTGGCTCGAGG
>at02935
CCGAGGTGGCTCAATGCACGGAATACTGCGGACCGACGATACTGGAACCACACTTCCTCC
TGTCCTGGGGCCACACTCCATACCAACGACCTGAAGACATGGTTGGCAGCGCATGCATGG
CCAATGTTCTCTCCCCTGCCCGACGCTCTCCGCTCTTGCTGCTGTTGTTGCTGGTGCATG
ATTGCCGTGGGGCAGGGCCAGTTTGCGAATTGACCGCAGCACAGCCATCTGCCACCGGCC
ACTGCCTCCTCCAGAAGGTTTCCAGCGCCAACAAGATCAGCAGCCTCATGCCGGGGCATG
CGATAAAAGAAAGCCGACAACGCGACGGCCAATGTCAGCTCAATTGCGCTGGCTGAGGTG
GGCGGCCAGCAATGACCGCCTCAGCTGCAGGTCTCGCCATCTTGCCTTAGCCTCTGGC
>at02936
TTTTTTTTTTTTTTTTTGGTTGGCACCCCGCAATCCAGATCTTCACCTAAGTCGCGGCA
GACTACACTTCAATGGCGCCGGCATGCATGCCATCAGCCCTACAAGCTCCACAAGTTGAA
GCTTGCAACGAAGGTATGCCTGATCCCCTCGGTCACGGGCCGACCCGCATGCTTCACGTC
GCCGTTGAAAAGCACGGCCGTGCCCTGCCGCGGCCGCACGACCACGGCCCCCTGCCCGTT
GGCCTCCTCCGGGGGAATCCTGCGGCCAGAAGACCGTGCCGCCGCCAGAGAACGCGCCTG
GCTCGGAGAGCAGCACGTTCAGCGTGACGGACTGCTTGTCCGTGTGAGGGGCAATACCGC
CACCCACCATGTATCTGTTCACAGCAGGCTCTCCGGGAGAGAAGCAAAACTTCATTTCCC
TGAGGCGCTCGCTCTGCCCGAAGAGGTCGAGGGCCACCTCCGGCATGTGCTGCTCGATGA
AGCCAAGCAGCCTGTCCGAGACCACGGAGGACGACAGCATCTGGGCCTCCAGGTCCAGCT

TGCGGACAGGGAGTCTGTGCATCTGGCCTTGGTAAGCGTACAGATTCTTTGCAGTGGAGC
CGCTTCTAGCAGAGCGATCGGCCGCATCCATGAACACATGGCATTCGTCCTTTGAGAGGA
AGTCGGGGATCGTTATAACACTGTTCCATGAGAATAGCTCGCGATCGCCCCCAATGCAGA
CCTCATGATAGGACCTCGGCGACTGCTCTTCATCAGCGAGGAGGGCCTGCATCAGCAAGG
CATCCCTTCCCGCCTTGCTCTCAAACAGCTCTGAGAGGGACTGAGCACAAGGCGTCAGCG
GCTGCCCCTCGCAACCTCTAAATCTCAGAGCCTCGGGGAGCACATTTGTGCCCAGGGATC
GTGTATCCACTACATAAAAAACAACCGCGCACGATGTGCCCTGCTCCACCCCAGCGCACT
GAAAGCTCTGCCAAGAGCCCAGACTGGCGCATCCGGCC
>at02937
GGGCAGGCCGTGACGCTCGGCGGCGGCTTGCAGTTGANCAGGTCTACCGCGAAGCCATGC
AGCGCGANGTCTTGGTNATCGGAGGCACTTGCGCGACGGTCGGCCATGTCGGCTTCACGC
TTGGCGGGGGTTACGGCAGCTACAGCCGCATGTACGGCTCGGGCGCGACGAACCTCCTCG
AGGCGGAGGTCGTGCTCGCGGACGGTCGGATAGTCACGGCCAACGCCTGCGGAGAGCACG
CGGACCTCTTCCGCGCGCTGCGCGGGGGCGGAGGAGCCTTCGGAGTCGTGACGAAGGCCA
CGTACCGGACGTACCCGTTCCCTGGGGGTCGGTCATGCGCGGGGCGTGCTGCAGGGCNGC
ATGCAGGCGGGCCTGGCCAGGTTCCTCGCGTGGTACGCCGACATNGTCCGCCGCGGCTTG
GCCNAGCANTTNGGCGGCGTCGTGCAGCTNGGCGGCCGNTGGGGCGTCAAGGGCAAGGCC
AGCNTGaNAGTTGCANTACGTCGGCATCNACGANAGTCAGTGCTCGGAGCTCCTCTCCCA
GCTGGGCGAGGTGAGCTGCAAGGCGGCTGATTCCCAGACATGGCCCCCCGGCCCATACCG
ACGTGGGCCAGCTTCCTGACGGCATCTACCACAACGGAACGGCGTTGCGGTACTTCACCC
TGGAGGACATCGAGAATGTCAGCTTTGCCGCGAGAGCGGTGCAGATCGCGGATGCGTGCT
CGAACGGCCTTGCGCTGTCTCTGAACTACGTTCTCGGCCACGGCTCGGACATTGCACTCG
CCTCTGCAAGCGAGACCACAGTTCACCCGCAGGTCTACAAGGCCATCGGTGTGCTTAAG
>at02938
TTTTTTTTTTTTTTTTTTGCATCGAGACACATGGTGCCGGCAGTCATACACCACACAGCA
GTCGCGCGGTGACGTTGCGGAGGGGGCGTCGGTCTGATGAAGACCTGTACAGACCCACGC
CACGCTACTGTGCTACAATGCTCCGCTGCTAAATACACCGATGAGTGCATCAGCCCTGGG
ACGAGTCGTGGGCTTTAGAGGTGTCTCCAACTGTCTTCAGGAGCTCCCATGTCTGTGCAA
GCCCTGCGGCGCCCGGCAACAAAGCCCCAGCGACTGATGCCTCACCACCAAACACATTCA
GGCGGACGTCCCGGATCGGCAACTTCTCGCACGCCTGCGCGGCTTTCTCCAGGATCATGC
GCTTCAAGGAGGCAGCTGGCATGTCACGGTCAGCGTCAAACTGCACTTTGGCCCGGTCCG
CTTCAGCCTCCTGCTCGATCTTTGCCACTGCTGCGTCTGCCATTGCTGCGGCCTGGCGCA
CCCGCGCGTCCTTGGCCACCTTGGCAAGTTCAATCTCCTCCTCCATCTTCAGGCGTGCCT
TTGCTGCCTCCGCCTGCTTTGCGAAGAGCTCCGCCTCCTCCCGCTCTGCCCGGAGGCGCT
GCTGCGCTGCCATCAGCCGCTTCTCGGTCTCAGCTTCAGCTTGGGCCTTGGCCATGTGCA
ACTCGTGTGCAAGTGCCTTCTCCTTGATGTCCCGCTCAGTCTGCAAGCGCGCTTCCTCTG
CCTGCCTGCGGACCTTATGCCGGTAGTCGGCCTGC
>at02939
TCCAACCACTTCCGTTGCCAGACCTCGCGTTTACGCTCAACGCGAAGTTCATAGGGTGCT
TTCTTCTCGCAGATTTTGCCACATGCTGCTACTTTGCCTTCGTCCAGTCCAATGGCAGCA
GACCAAGGCTCTTTTCATGGAAGAAAACGTTGCAGCACATGGTTTATGGCACGTTCAACG
CCAAGACCTACCTCTTGCTCGTGTTCCTGTTCTGTCGGGGCATGCGGTTCAACTTGGTGT
GGTGGCTGCTGGATGCCGTCTTCGGCCTCGGGGCGCTGATTAACAATCTGGTTCAGCAGA
GTTGCCTCGCCTGGGAGTGCATGTTCTACAACGCCCACCGCCTTGGCCACCTTCGTGTGG
TCTACGAGCACGCCCACAAGGCCCACCACCGCCTCCACGATGCCATGGCCTTCGACGGAC
ACGTCTACGGAGGCTCTGGCTTTCCAGAGGAGTGGTTCCTGATCTTCTACGAGATAGCCA
TGATGAAGCTGAT
>at02940
TTTTTTTTTTTTTCTTGTATTTGCGGTATCGACATCCTGGGCAGCAGAGGGCTGCTGCTC
CATGACCAGGGGAGGTGGGTCCGCACAAAGATGTGGGCGGTCACCTGACAGTTCAAAAGA
AGGCCGTATTAGAACTCACTCATGGTTTAAGAATGGCCCTCCGCCGGAACGATGCAGGAA
TCTGCTTGCCTAAGTCTCAATTGCCATGGCGTGATTGCCACCACCATTGGCTTCCCATCA
GTGACTCCAATCGGCCAGGAACCCTTTCACGCGGAGGGTCCTCATGATGATCATTCTGGA
CTTCATGTTGGAAAACGCGTCTAACCTGCCTACCTGATGGCACCGCTGACTCTGGACTGC
CACTGGTGTGCTCTCTGTTGTCGTTCTGACCTCTGCTACGCCTTTGATGGTGGTGGTGCT

GCTGCTGCTGCTGCTGCTGCCGCACAGTCTGAGGCACATCTTCAAAGTGGGACAAGTCTG
TGTTTCCAATGATCCGCGCCTCTTGCCATTGAGCGGCCTCCTGCGCATTCGTTGGCGCCG
CAGCTGATGCAGATCCGCGAGTCCTTTGCGACCGGAATCGGCCATGGCCACTGCCACGTC
CTTGTGCAGGCTGGGAAGCTGGAGCGCTTGCCACAGCGACTGGTGTGCTTCGGGGCGTGG
ATCTTGGCGAACTCTTCGTCGAACTCGCGCAACGTTGCATCACGGGAATTGGCCGTGTCA
CATCGCGAGGAGTGTGCCGCCCTTGATGGGAGTGGAGATCACTTGCCGGCGTTCCACGCG
AGCTTTCACTGTGCCCGACTGGGGGAGAAGATCCCCGCGAAGCGTTGGAGTTGTTCAAGT
TTGGCCATTCCGTTCCCGGGACCATGGCGGCCTGCTTTGCCCCACTGCTTGACTGCATTG
GCCTCTGGTCACTCCGGGGAAGCCCGATAAACTTTTCCAACCGGTCTATGCGCTTGTGGA
GGCAATACTCAATGTCGGCGATTCTTCTGAAGATGTCCCAGCTCGTGTCACTGAGGACAG
TGCGCACGTTGTTTAGGATGAGCTGCTGCAAGACGGGGTCCTGCATCATCTCAATCGTTG
CGGCATCTGAGCTTGCTGCCTCCATAACAAGGTTCGCGTCCAGGCAGGGGATGGCGGAAG
AAAAGTAGCCCTCCTTGAATGACTTGAGCCAAAATGGCTACGG
>at02941
TTTTTTTTTTTTTTTTTTCAGATGCAGCCACCTTGGAGCGCAATGGTAGTATATAGGAGA
GGGGCCGGCAGGATGTCCGCGAATGTGCAGCGACTGCCGGCTTTCGTCGCCAGCCGCCCA
CAGTCATGGAAGGTTGCTGCCTTCAGGTCCTGCTTGGTCGCAAAGAAACTTGGCCTCTAA
GGGATCATCCTCTACGAGGGACATCTCCCTGAACCAGGCCTGAACCTGGAAAGGGCCGTG
TGAAGACGCATAGGGGGAGTGACTTGCCTCAGTCGCCAGAAAATGCGGTCAGGCAAAAAT
CTGACAAGGGCAGCAAGCCCTTCGTGCCTATCAGGAAGCAGAATGATTGATCTGACAGCA
CTCAAGAGCGTGCCACGCGAGCTACCTCCTCTGAGCACTGTCGCTCACGACCCTCAAAAG
GCAGAGACTATGCTTGCCTGAGGCTGGCTTAGCCGGTGAGCTCTCTATCTATGACGACTG
CATGTCCATTTCCACCTTCAGTGGGTCATGGATCCAAACTTGTCGATGTGGC
>at02942
TTTTTTTTTTTTTTTTTTGATCCAAGGCCTTTGCACAAATGGATCATTTCGACCTTGCCA
GAAAAAAAAACATACTCCAACACTCTCTCTCACAGGTATGCCCTTCTTACTGCCCTCTGC
CTTGCTGGGCATGGGTGGCCTTGCAGCCATGCCATGCACTGGGAAAAAAGTCTGCTTTGC
AGTTGGCCTGTCGACCCGAGAGTCGAATCTACGCAGATTCATGGCTCGGAGTCTCATGGC
TGCTCCTGCCGCTTCCGGATGCGCAGGAACGTCGGCTTCCTTGCTATCAGGATGAGCAAG
GCCAAGCAGTTGAACACCACGAACTCCACCGCCAGCGCCAGAGTGCACTTTCGTATGGAG
ACGTCCTCAAATCCCAACTGCCTGAGCCAGTCATTGCCCTGGATGCAGAGAGGGCCGAAT
ATCTTCTCCTTGCAGGTGAAGTTCTTATTATCCCGGAGCTCGTTGACGGCGAGCGCTTGG
AAGGCGCCTCGTGCC
>at02943
CCGTAGCCATTTTGGCTCAAGCCTGCTCTGGCTCATGTAATCAAGGCCCTCTTCATGTTC
TGGTGCGGCGCAATGATGGATGTTCTGCACACTTCGTTGTTGTGCATGGCTTGTGATCAG
CGGGCTCCTCCGGCTTATCTGCACATTAGTGAATGCACACGCATTCCGAGACTGGAATTG
CCCACTTGCAGCGGACCCATCAGGACATAATGATACAGGCCTCGAAGTCATTGTCCCGAG
CCTTCCAACTTCGGGCTCGACCACTATGCAGGTTGCCTTGGAGACATTGGGCCTTCGCGC
ATACAAGGGAGATGAGCTGCTATTCTACAGCCCTCTGATCCTCGACTATGACTTGCAGCC
AGCAAAGCTGGTCGCACCCTTCCAGACATGTGGGGTGAAGGCATTCGTGGGCGATGACAC
ATTTGGCCTCGTGCCTAAGCTGCTGGAGGTTAGCCCTGGCACGAAGGTCATCCTTAACCG
TAGAAGAAGTTGGCGCGAGTGGAGGGAGGCATTT
>at02944
GGTTTTTTTCTTTTTTTTTTTGCTCGGCGCGGTATGCGAAGAAAGACGGGGTACGCAACA
CCATCGCATGCGTCCATGCCCTACACTTCTACGCCCAATAGGGCTGCCCCTCACCACCAG
TAGTGGTAGCCTCTCTGGACCACGGCCTCCTTCAGCACCTTCACGTCCTCCTTGTCCAGC
TTCAGCGTGCAGTTGAAGCCCTCCTTCTCCTCCGGCTCCTCCTGAGGCTTGGGGGCCCTC
CTTCTCCCGCCTCCTGGCGGCCATGACCTGCGTCTGGCGGTGGTACACTATCTTGTCCAC
CTCTTTGTGCGTGAGGTAGTCTACCTCCCCAGTCCTCCACGGGCAAGGGCGCTGCACCCA
TGTGGCTGAAGCAGTCAGGCCACGGCGGCGCGGGCCTTGGCGCGCTCGTCGGCGTCGTAG
CGCCACTCGTACTGCTTCACCCAGTCGTCGAAGCAGAGCTCCTTCTGCTTGTCGTAGCGC
CGCTGCTCGCAGTCGGAGAGGTATCGCTGGTAGCGCTCGTCCTGCATGGCGATGCGCTCG
TCCTCGNCTCNTNNCGGTGATCATGCCTCCCCAACTTCCTGTCCTTCTGGGTCTCGGCCT
TCGCC

>at02945
TGGGCGCACGTAGATACGCCCCTCTGGGCTGGACCGGATCTTCTTGTTGCGCACGCGGAC
AATGCGGCCGTGGATGGCGCAGGAGACGCAGTACTGCATCTTCATGTAGAGCTTCGGGAG
CATGAAGGTCGAGTACACGCTGGCCTCGCGGATGTCTCGCTGCGAGGACGCGTCCACCAT
GTTGCGGACCTGGAAGCGCTTGATCGCCTTGTCCTTTGGCACAGTGCGTGAGCAGTTCGA
GCACCGCACGATAGCAGTGTGGCCACGGCCATGCTTTGAGCGCCCGTTGTTGCGGCGCTT
CGAGGGCATGGCTGCCAACGGGTCTGGGATGAGAGTCTGGGGGAGGTAAGCTTCGGGCCT
CGGACCTTGAGCCAAAATGGCTACGGA
>at02946
TGAAGTACCCATTCAAGTAGGCCGCCGCAAAGCCGAACGTCAAGTTCGTGAAGGAGAGGC
ACCAGATCTCTGGAGCGCGCCACAGCTCCATGGCCGCCAGCAGCTTCCCTGCGCAGGCTG
GCTTCGCGGCCCCAGACTGGGCGCCGAGATCGAGCAGTCCAAACACGCCCAAGGCAGACA
GCAGGGCGAAGGCGGTGTAGACGGCGAAGGTGATCCCTGGTTTGACATACTTGAAGGACA
TGGAGCAGAGGAGCTTCAAGACAACCTCCAGTAGCAAGTAGTACACTGCAAAGACTGAGG
CGTGCTGCGCGGTGACTGCCTCGCGGTCCAGGTCGGTGGCCTCCACGATTCCGGTCACGG
AGCGGTCCAGATAGGCGCCCTGCGCGGTCCACATGATGCTCGCGGCGGCACCACCGAAGG
TCGACCCCGCCAGGAAGCTCACCCACTGCCCGCTGCTTCCTCCCGGCAGAAACGCCGCCC
GGGCGAAGAGGCCGACGAAGGCGCTGTAGAGCGCCATGGCCAGGAAGAGGCTGCTGCGCG
GGCCCTGGGAGATGACCACGGGGGCGCCGAGGAAGAGGGAGCAGAGGCA
>at02947
GGATGCGGGAGTCTCCGGCATGTCAGGAGCCTTATCGACCTCTATTACCGCTTCGTCCTG
ATGAGATGACACAATCTGCTCTGCGGCCTCAATCATATGCCTTGCTTCGGCATACGCCTG
CTGTGCAGCACGGTCATGCGCGGACACGGCTCGGTACAATGCGTGCTCTTCTGCTGCAAG
TTCCTGGTGCAATCGAGAGGCAAGCTGCTCTTCGCTGCGAAGCGCCTGCTCCATTGATCT
GAGATCTGGTGATGTCATGTCCAGCTTCTTGGAGTCAGCCGCCAGGCTTTGCAAGGCCGA
CTCAACCTGGCCCTTTCCTGTATCGAAACCTGCCGATCTGATTT
>at02948
TTGATATCGGGACTGGGCCCAATGGTGTGGATTTGTTCTTGGATTCTCGTCTCAGAGAAC
TACGCTCCTCAGCTGGACCATTTCGTCACAGCGCTCCGTGAGGCGGGTGACCGCGCACGT
TTGCACGTGCAGTGGATTCCGCAGCTACGTGATGCACTCCAGAGCCGGTTGGCA
>at02949
GGGAGGTAGCACCGGTGCAGGCCCTGCTCTACTCGTCGGAAATACAGTTGCACGAGCAAC
ATCAGCACCTCTCATTTATGTCTACGAGTATGTCATTGATGATGAAGATGCCAAAGGCGA
GTACTACAACTGGTTCGGCGAGTCAAGGAGGTTGCTTGGGTTGCCTCGTGTCATCTTTGC
ATTGTCAAGTGCGGCGGCCGTCTCCTTTCTTGTCATGCCAGCCAAGGCAGCAAGGTCtAA
AGtGT
>at02950
TTTTTTTTTTTTTTTTCTCGAGGAGCGCTCCGCAGCGCGGCTCCGCGGCGACTCCGGCCG
CAGCGGTCTCCTCGGCAGCGGCCGCATGCTGAGCCGCAGCCGGCCGCTCCGCGGCTCGGG
CCGCAGCACGCGGACGCGCACCTCCTCACCGGCCGGGAAGGCAGAGCAAGTGCTGTCCTC
CGTCGGCAGCTCGGTGAGGTGCACGAGACCCCACCTCCTTGCGCCCCCGCCGGGCGGCGC
GAGGCTCACGTACACGCCGTAGGAGGCGGCGTGGTGCACGACGCCGCCGAGCCAAGTGGA
GGGCGGTACTTCCACGAATGGCGAGATGTCCTGCGTCTCTGCCAGAGGCCCCGCCGCCGC
CAGCGGCGGCGGCTGCGCGTCATCGCCCCCCTCGCCGTCGCTGTAGTCCAGGACCTCCTC
CTCTGCGCGGGCAGCGCCTCCCTCGCCGTCGCTGTAGTCGAGGACCTCCTCCTCAGCGCA
GCCTCGGGCCGCGCCGGCCGCGGCCATGGCTCCGCGGGCGCGGGCGGCCGCGGCCTCGTG
CC
>at02951
AGGCCTCGTCCCAGGCCGTCCCCAGGCAGGTGCGCAGGGCATGTCGCCTAGCGTCCAACT
CCTTCGCGCTCTTGGCCTCCAGCACTGCGGCGCGCAGCCGCTGGACGTCCTCAGGCCGCA
GCAGCCCCCGCAAGGCCACGTGGCCGTCACGCTGGAGGCTCTGGACCTCACACGCTTGCA
GCGCCCGTGGCAGCAACAACCCCAGGAGGCTCCACAATGCCAAGGCAGCGGCGAACAGGA
GGGCCAGCAGGCCTCCGTGGGCCGCACGGCTCCCG
>at02952
CGCCCTTTTTGACCCAAGGCCTTACCCGCTAATTGAACATTCCAATTTGGATGGGGACAT

GGACAGTGAATTGATTGTTGCATACATCGTCCTCTTCTTCTCATTGCTGGGAATACCGGT
CTGCTATCTGATCCACCGCTTGGTTGCGAAAGCACGCCAAGAGCAGCTGGACGAGGGTGT
GCCACCTCCGCCACCGCCGCCATCGCGGCCTTGGGAGCTCACCTGGCTAGCATTGCTCGC
GGCATGTACCGTAGCATTGCTCATTGTGCAGATCGTCCTAGGCGTCTGCGATCATTCACT
GCTACTGCTGGCAGATTCCACCCATGGTGCCGCCGACGCGGCAAGCTATGGCCTTGCAGC
CTTTGTCGAGCACACAAAGCACGAGTTTGGACGCCAGTCAGTGAACCAGCAGGTGCGTGA
TTACGG
>at02953
CGAGTGGCAAGGCCTGTACGCGATGGCTGCTGCCACCTCCTGCTTGCAGGAGGGTATCTG
AGGAAAATACAACATCATGCAGTCCCTTTCAGAGGTGCGCAGAAGTGTGCTGGAATGCCA
AGGAGGCGTTCGCAACTCTGACGTCGTATCTCTGAAGACCGCCCACCCAATGGATGTGCT
CATCTGCGCCGAGTCCTCAAGGCAGCAACGGCACTAGGGGCACCGCCTTGAGCGAAACCG
GCAGGGAGAGCGGTCCAACCCATTCGGGCATGCGTGAGGCTCGGCCGGCTGGATACGCTC
TTGCACACGACGCACGTGCTCCAGGGCCGTCTTCACCCGGGAGTGTACAAACTTTGAGAG
GTCGGATCGCCCTATGCCAGTACCTCCG
>at02954
AGCGCGCCCTGGGCCAAGCAGCACAGCATGCCTTCAGCATGGACCTGTCTGCACACAGGG
TGAAGGGGACTGCGGCGGGGCCCACCAAGGATCTCACTTTTGTTCCCGAGGACATCGTGC
AGCGGGCCCTGCAGCTTGCGGACGACGCGCTTGCACTGTTTGGCCGTCGACtACtTGGtA
tTAtTGGCAGTCAGGCCTGCTCtTTGCCATTGATTTGGATATCTCCCTTCTCAGATTCGC
AGCACCACAATGCAGCGATTAcCTCCAAGTATGTCGTGACAGTAGTTTCCAGCCTCCTGG
ACATGTGGGACTGGCTTTCGACCGTGAGTCTCACACAGGATCCTGCATGTTGGCTGTATA
CCCGTGCAGAAAAGTTGCTTGCCTTGTTGCCGAGGACGCTGGACCCAACTTTGAGCCGCT
GCTGCGATGAACTGCGGCGTCGAATCTCGGACATTGATGTAGCCGCGGACACGTTGGGGA
GCCGAGCTTTCCTGCCGAAAGGTATGGCTTCCACGTTGGCCAACTAGTGTTGTGACGCGC
CTGAGCAGGGTNTGGCCAGTGGTCCCCAGT
>at02955
CCATTGACAGCGCCCGCCGCTGTACGAAGAGTGCAAAGGCTGCTGCCCTCGCAAGCAAAC
CACCAAAAGTTTAAATCCCCATCACACCCCATTTTCGGCTGTGCCTTGGTGTGGAAATGA
CCAATGGGATACTAGCAAGCGCCAGGGGTAACCCAAAGCGCGAGAGAGGCAATCAATCAA
TTGCCCACTTGAGGAGCACTGACTGTGGCCTTGAATTGAGTGCAGGCCTTTTCAAACGGC
CAACAATCTACGAAATCCCTGAAAGAGCCGCAAAAGTAGCGTTGGTTTGGAGTTCACCGC
AGCGCCCCCCTACCCTGGGTTCGGGGTTCAAACGTGGATGCGGATGGATGAAGAGCGAAA
GCAAGAATGTAAGCATCTTGGGCAAAGATAGCCCAAAGCATGCAGACGATCGTGGCGCAA
AATCAAACTTGTCGCCAGGAAGCTGCAGCTGGGCCTTTGACCTCTCCGTTCAAAAGCTGT
CATTGTCGGCCGTTCAAAAGCTGTCGTTGTCGGCGTACCGGAAGGCGGCCTGGCGCTTGA
AGAGGAGCTGCTCGGCTTGGGAGAGCTCGTAGCTGAGCACCGAGCCT
>at02956
TTTTTTTTTTTTTTTCTTCCTAATTTCAAACTACCCGTCAGAGTGCATTTGGAGAATCCG
AGCACACTCACACTGGGGAAGGCTATTGAGCCAGCTCTCTAGCAAGGTGCAAACCATGGA
AAGATGCTATAAGGTCACGAGGAGCATCCCTAGGATTCTTCTTTAGCTTTCCCAGGCACT
CTTCGTGCTTTGCGTTCAGAAACTCTTTGATGGCGGCGCTGAACTCTGAATTCTTCTGGG
ACTTCTCCTCATGTTCCATTATCTCTTTCGCTAGACTGTGAGTCACCAGGCCCCAACCCT
CCTGACCTTGAACATTGTGCGAATCATCCTCCTCATCGTCATCGTCCGGCTCGATGTGTC
CCCCATGTATCTTGAGCCAGCATTCTTCGATGCTTTCCAAGTCCATCTGATTTTTGTGGA
GTGTGAATGCTACATACGCGGTGTAGAAGACGGCTGACCAGCACACTAGCTGCACCACAA
CTGCAAACACAGCGACTGCAAAGTTCTGTTCCAGCGTGAAACCGATAACGGTTCCAATGC
AGCCTACCACCCAGGCAGTGAAGACGAGCATCAGGGGACCACCGATGCAACGAGGCATGG
AGAGCACGTACTTCGTCATCGCAATCTCTTTGAGCCCAGCCGAGGCTGTCCTGTGGACTA
TGCACGAGTACGCTGAAGACGCATAGCACGCGGCAGCTGTGATTGTCGTGCTGTAATAGG
CCGCCACCGTCCAAGGGATATCTGCCTTGTACTTGTCGAGAGCTGCAGTTTGAGATTGAG
CGATAGTGTGGGCCAAGAAAGAGGGTCGAGACACGGACAGCTGTCCCCANTGATTCGAGA
TCGCCCCAGCAACGAGCTGACTGACT
>at02957
TAAGTTTGGATCATGGGGCAAAGTCCAGCTTCGGGCTGGATCCAATCGCGAGCACAATGC

AGTACCCCATGCACTCATATTTCAATCTTGGTCGTGGCGTTCCCGACCTTTGGGTCCGTA
AACTGAATCAGTGTTTTGATAGAACTGCGGCCCAAAGCATAGAAACCAGTGACACCAGAC
TTGATCATTGCCCGCACGGAGGACTCCCATTTCCCAGGGTTGGTGAGCTGGAGCTTGAGC
AAATCCACCACCACTCTGGGATTGGTTGCCCGGCTGTATCGGCTCTGCTGCGATGTCCAT
GTAAACTGTGTGTCTGGGGGGCTTC
>at02958
TTTTTTTTTTTTTTTTTCGCACGAAAGGACAGCCCAAGGCTCGCCGCACCTAGCCCGGG
CCCACTGCCCTCAGGCCTCAGTTGCGCCATTGTTGGTGTCCGCAGCGTCGGCCGGGTAGA
TGGGCCGCAGCCTCTCCACCCCCCGCAGCCTCTCCGCAAAGTGCCGCTTGAAGCGCTCGC
CGTAGGCGGCGGGCGGGATGGTGGCCTTGTTGAACTCGAGCGACTTTATACACCTGGCAA
CCTTCTTCGCAGCTGTCCACGGCTGCAGGAAGTCGATGATGCTCAAGGTGACCAACACAG
CAGTCTCCCCGTCGACCATGGCCCATTGTCTCACCGCAGAGCTGGCTCCGTTGGCCATGG
AGGCTGGTTCCAGCAGCTGCTGCGCCTGGCTCTTTGGCACACGGCGGATGGCAACGAAGA
GGGAGTAGTCCATGAGGCCCTGATCTATGAGCCACTGCGCGTCCCCCTGCACGAGCTGAA
CGATCTCGTCCCGCTGTTTTTTTGGAAAATCAAAGTGGAAAGCGCGGGCTCTGACCTTCC
CCTCGTAGTAGATGTGCCGCTTGGGTGTCCAGTTGCATCTGCACCACATGTGGGGGCTGT
AGAAGCGCTTGCGCACCGGCTCGATGAACTTGCCATTTCTCATGACCGTCTTGTCATCAG
CACAGCCCTTCAAGTCGTACTTCTCATCCCACGCGACGTCTTCGGACGTCAGGTTCCTCA
TGGCGATGTACGCCTTGTCCCTACCAATGACTCTCGAAGTGCAAGTAGATTGGCACCAAT
AGGCTCTTACCCTGGACCAGTCTCTCCGCGTATGCCTCACCGCGCAGCATCAATGACTTG
TGGTCATCCCCAGAGACGGATTTGACGATGTATTTGGAGTCGCGCGAGTTGTACTGCATC
TCCCCGCCCTTGCCCAGCTTTGTCTCACTTCTGGCATTCGTCAGGTCCACAGAGTTCTCC
TTCTGCATGTTCAGGAAGTCGTCCGGCACCCCGTGGCAGCGGCGGAGCCTGCTGAAGGCC
TCGTCGTTCCACACCTCGATCTTTTGACCAGAGACCTGCACGGTCTGACTCTCCACTGGG
AGAATCTGGTCGGGCCGCCTCCGCGGCTTTGCGGCGAGCAGGGGCTCCCGCGTTGGCTGG
CTGGCGACCTCCATCTGATGGAGACGCTGGCCGACCGAGGACGACGGCTCCCAGACGGTG
AGCATGCCACCGCGGGCCCGTTAAACATATGGGGGGGCCCACCTGCCCTTGAGCCAAAAA
TGGCTACGG
>at02959
TTGTTTTTTTTCTTTTTTTTTGTTTCTTCATACCATGCGGCGGCAAACGTCAAGAATCAA
TGTAGGGACGGCCAAATATGTCTTGCTCAAGACATGGCCAACTCGTACTCCTTGCTGTCG
ACAAGGCGCTACTTGACCATGAGCTGCTTGTCAAAAGCCATGCGCTGCTCGGTGTCAACG
GTGTGGGTGCGGCCGTTGGATCTCTGGCGGTAAATGATCCTGTGCCACAACCGGAGGGTA
CCAATACTTGAGCTCCACCACATAGACAAACTGCTGCGATGCGTCATACAAATGGAGTAT
GGTATGCCTCGGCGAAGACGCGATGACACGCAAGACAAGCATATGACTTCCCTGCCACGG
CAGGCGGATGTCCATGGCAACTGCAAGATGAGCTTGGCATGGCGCCAAAATTGATACAGA
GCCTCTGTCTTGAGCCAAAATGGCTACGG
>at02960
TTGTTTTTTTTTTCTTTTTTTTTTTACGTGTTACAGAGGCAAGGACGCGGAGCAGCACTG
TTCCAGTGGAGCAGTGCATGACCGACAGCCCCTACTCAACGAAGGCGCCGTGTGCGACCG
GCCTTTCCGAGCGAATGTTGGGCTGCCAACGTTCAGGGCCTCCTGTCGGCGTTTCAAGGC
TATGTTCTCAGGCACTGCTCCTGCTACGTGAAGGAGGTCACAAGCCACGCAATCCATGTC
GTACCCCTCACGGCTCACCTGCAGGCCGGCTGCATAAGGGTGTCCCCCACCCCCCACGTA
CTTGGCAGGATCCTGGTCATGAAGCTGCCTCGCGAGCGAGCCTAGTGGAGTGACATCCTC
GCTCGTACAAGAGAGTCGGATCTCCACAAAGTCAGAGCTTTTGTACAGGTTGGCGCGGAG
CACGAGCGCATTGGCATATCTCTGGCAGAGCTGAGCAAAATGCCGCGTGTCGTAGGCCAG
TGCCTCCGACTGCTCCACGCCAATCACCAGCAGCTGCATAGGGTGGTCGGCCTCTGCGAT
CACCTTCACCTGCTCGAAGAGCTGCAGCGCATTCTGCTGCAGAATCTGAACTGCCTCCGC
GCCCTTGGCCAGGAAGCCCGGGAGGTCGGAGAGGAACTCGTCGATCATGGCCAGGGTCAT
GCGCGGCGCCAGCTGCGTGCGTGCGACGAAGGCCGCCGCCTCCGCGGCCCACTCGCTGGG
GATCTGGTACTGCCAGACGTCCTTCTTTCGAAGGATGGCAATCTTCTTCTCAATATCGGC
CATGTCCTGCAGAAGGTGGCCAATCTCTTTGCCTACAAGGCTGGCAGCACAGTCATCACC
GCCCGTCTTGGACAGATCCACCATCGAGGGCATCTCAGCAACCAGCGCTGACATGCGATC
ACCCACTGACGCATGGTGATCCACTACAAGCACCCTCCGCGCCGATTTCAGCAATGGAAG
GTCCTCTGCATAGGGGCTGATGTCAACATAAAGCACGGCAGCCTCTGGCCCAATCAAGTC

AGCCCGCTTGCTGGCACGATCTGTGTGGTCCAGCTCCACAATGTCTGACCTGCTCTGCCC
GAGCCGCTCGAGTGCCATGCACGCCAGGCGCGCCGCACCGAGGCCGTCAGGGCAGCCCCC
GTGGCAGAAGACGACGCAGCGGCTGCAGCCCTCGAGGAAAGCCTCCCAATCGAACGGCGG
TCGTTCCCTGGCGTCCATGGCAGAAAAAAATGAGCTCGTGCACGAAATAGCCTGAGGCCT
GGTTGCCAGGCCGTGCCTCCGTGAAAAGTGAAGCAGCTCTGGTGGAGTTAAGCCAAAAAG
GCTTGAGCCAAAATGGCTACGG
>at02961
GAGGAGGCCGACGAGATGGACGGCATCTTACCCCGTCGTCCCGTGCGCCTGGCGCCCCAG
GGCGAGTTCAACTCCTACCACCCCGTGGCGTCGCGCCTGGTGGCGGACACCTGGCTGCCG
GGGAAGTTCGTCATCGCCTTCAACGGCGTGGTCTCGAGCTCCAGCCCCAACGTTGCCTCA
GAGCTCTACGGCCGCTACTACGAGCTCTCGTGCGAGCTGAATGAC
>at02962
TTTTTTTTTTTTTTTTTCCCAAAGCCTGCGGATGCTCTCTAGACTCTGTACAAGCGCGC
CGCTATCTTGATGCACTGTACATAACTGCTGCGCGGCACCCACTGCTGACAGGTGTGCAA
GGCCACCTGGCTCGCCTCCAAGACCTAGCCAGTCGCAATGCGCAGGGCGCATTCCATGCA
CTCGCGCAAGCGATCACGACATCTGCCCTCCCTGCCTCTCCTCCTCCCCTCAACTCACAA
ACGTCCACCGAACTTTGCCTGGGGCCGTGGAGGCCGCTGGCCGTCAGAGGTGGCAGCCTC
GGCAACCCATGCCCCGTTGCGCACGACGAAGAACTTCTGAGGCGCCGCCTGGCACTTGCG
GCGCCTTGGCCACTGATGCTGCCGGCACTGGTGCTGCGACCTGGGGCGCAGGTTGCTGTG
GCAGCTTGTACGTGAGGAGCCACAAGGGGTAGGCGCAGCCTTGGCCCACGACACGCATCA
CGCAGTCGTCCTTCACCTCGTGCC
>at02963
TAAAATTTCTAAAATAAAATAGAATTTCTTCACAAAATTGTAAAAATTCTAACATATTTT
AAGTTTTTAAAACATGTTTTTTACCTTTGTTTTAAATTTTGAATAAAATTTTAGTTTAAA
ATTTTAACTTTTGTTTAAATTTTATAAAAATATTTTTACTTTTTGTTTAAATTTTTATAA
ATTCTTTTGATTTTTAAAT
>at02964
CGGGCCTCAGCCACGAGGAGTGGAGGAGTGGTGGACATACAGCGCACCACATGTATCCCA
ATCTGGCCCAAGATCCCGACACAGACTCGATGGAACCCTTCGTTCACTGGGGTCCGCCAA
CGAGCGAGTTCAGCATGCTCCGTCGTTTTATCTCCCCACTCGGTTTTCACATTGTTGTTG
CTCTGGCAGTTCTGACTATGCAGGTGCAGCATCTCCGTCCTGGATCTTTTGGGCGCTCTC
ACAGCAATTCTGGGCTC
>at02965
CCGTAGCCATTTTGGCTCAAGGCAGCTCGAAGCGAGAAACCAGGGCTCCACTTGGCTCAA
TTGATTTGTCCGGTGAGCAATGCGCATTGACAACCCTGAAGCGCGGGGGCAGCCTCTGAG
TCCACAGCGATGCCACACTCAGAGCGCTCCCGAAGCCCGCTTCGGCAGACTCCCTTCTCC
GAGAGGATCTCGCGACAGATAGCTGCATTCGGGCGCTATCCCGACAGGCGGCCACAGGGC
CTGCATGTCGACGGAAATGGCGCGCTTCGCCTCGAAGACCTGATGCGTGTGTGGGGTCGC
CGAGAGAACCTGAAGGAGTGTGATGTCTTAGATGCTGTCCGCAGGCATATGTTCCATGAC
AGTGGAGGAGGCGGCAGTGCGTTGCGCTTCGCTATCGACACTGACGCGTCAGGGCGCATC
ATCATCCGAGTGATGCCCAAGAAGCCCACAAGTGAGCACGGCGCGCACAAGCAGCCTCCC
CCTGGCAGAGAGAGGGAGGCAGCATCGCGGCCGCCACAAGGCCAGCTGACGGTTCGGTCA
TTGCATGGCAGCGTCCTGCACGAGGTGGCAAGGCAGAGCGAGACAGTGCCCAAGGCTGCT
CCCGAGGCCCTGAAGTCAGGGTTCATTCCGGTGAAGACGCAGGAGTCGCCGCAGCGCCCG
CAGGCTGCGCACACTAGCCCGATGGACGTGGATGCTGGG
>at02966
TCAAGAGATGGTTCGAGACTTGGTAGTGGATGAATGGAGCCCAACTCCTCCTGCTTGGCG
ATGGAGTCCAATGGCAAGCTCACAGCACGCAGCCATGGTGTGCCCAGGTGGGCATTGGCG
TGCTGCTTCAGGCTAAACATGAAGAGAAGGTCCAGTCCGATGACCCGGAGTTGGTGCGGC
ACGTGGCTGAGGTCTTGGGGCCCGAGGTCTTTGCGGGCGAGACGGCGCGCGTGCCACGTG
CGCCGGCCCCTGAGGGCGGCACTGACAGGGCCGCGCGCCGCACTGCGTCACACGCACTGC
GCCACACGCCTGAACGAGAAGTTTCAGGCAATGTGGAC
>at02967
CCGTAGCCATTTTGGCTCAGCATTTCATGCCCCGAGAGAGAGGCAAAGTCTGGATTCATG
AAGTTTTCTGGGCAACAGGCTGGCAGACTAAAGGCCAAGTTCCGGGCTCTGGATCAGGAC

CGCGATGGCATGTTGGACTGTGAGGAGTTGTTGACTTTCCTTCAGAAAGGAGATCCTACT
TTTTCGGTTCAGGCCGCCCAGACCCTCTTCGAGCAGTGTGATGCCAACACGGATGGCCGC
ATTAACTTCGATGAATTCGTGGACCTGGTTTTCTCACGTACCGAGACCAGTTTCGACGTG
GTCACAGACCATTACACCGACAAGAGCTCTATCAGGGAGGCTTTGCGTGGAGAAAACGTC
GTTCTGCTGCGGGACACCTGGTTATTGCAGCTTTACCATGCAGGGGGAGTCTTACCACGG
CGCCAGGATTTGCCAGACGGTGCGATTTGGAGTGTGCATGAAAGAATCTGGGAGCGGGAA
GACTTGTCCATCATTTCCATCTCTTACTGTTGGTTCACAAAGCATCACCCAGACCCATAT
GGAAAGCAGCTTCATACATTCATTGGGCCGTTACTGGAGCTTTTCCTCCGGGAGACCCGC
AGAGCAGCAGTCTTCATTGACTGGTGTAGTCTTCACCAGTATCCACGAAGCCGCCGCGAG
ACACGGATGTTCAATGCAGCACTGAAAGACATCAATCTTTGGTACGCTCACAAGCAAACA
ATAGTTTGGATGCTGACATCCACGCCGCCGGAGGTGACAGCATACATGAATCGTGGTTGG
CCCTGCTTCGAGCGTGCAATCAGCTCCATGATTTGTGACTCTGACAAAGTGCTCGACTTG
GGCAAAGCTGGCTGGGATGCCGAAGAGCTCTACTCCGATGTCAGAGACATGTGTATTTCT
GCACGGCCGCCACCAATAGCACCTGAGAAGTTTGAGCAAATGCTTCGTACCAAGCACTTC
ACAAATGGTGCTGACAAGGATTTTGTGGTTGCCAAGTATGCGCAAACTTTCTATGACGTT
ATGGGCTGTGCGGAGGATTTGAACTACTCGAAACTCCAGTGGTATGACGCAGACATGCCT
GATCTTTGTGAAGCCCTGCCCTTCTGCCATCGCTTGCAGCACATGTCCCTTGCCAGGAAC
CACATCAGGGATGATACTCTGCTGAGCCAATACTTAGCCACGGGTGCCGGCTGTTTGCAG
GCACTCAACCTCGCTGGCAATGAGCTCGCAGGAATCAGTACCTCTCTCTCTCAGGCGCTT
AACTCAAGTTCGGTCCTGGTTAGGCTCAACCTCAGTGCAAATGGCATTTGCAACATAGAT
CCCTTGGGCGAAACACTGGCAGTCAACAACATCCTGGAGGACTTGGACCTCTCATCCAAT
CGCATCGCAGGACTTTCAGCTCTTGGTCAAGGGCTTGCAAAAAACACTGGGTTGAAGCAG
CTGCACCTGGAGCTCAATTGGATTGAGAATATCGGCCCACTGGCGCAAGCGCTCAAGCAG
AATCGATCATTGACAAGCCTTGATCTTCGTGCAAATTACATCAGTGATGAGAGTCAAGCA
TGCATGCAGTCCCTTTGGCGCGACGCTGGGAACGATGAGGCCATGCTTGCCTTGTAGTCT
ATTTAGAGGCGCTCTGTCTCTGATGACCCAAGTGTCAGAATGACTTTGTAGTAGCCTCGC
GGACGTTTTCCATTCTAGTTGTCCAGTCCTCCGTCCTGCAATGCGTGAAATGAACTCGTG
ACCTTCACCATGGGCATCGCTGTCATGTTCCCAAAAAAAAAGAAAAAA
>at02968
AGAGCTCCGTTCCGGCGCAAAGGCCGTGCGGGGCGTTTCTCCGCGACGACGTCCTCCAAT
CCAGAGAGCATCCGCATCAGGCGGCCGTCTTCCGAGATCAGGCTCTGCCGCCACCGGATC
CTCCGCTCCGAGTTGTGCTCTTGCAATGTGCTCGCAATGAGGTCCGTCGCATCGGACAGC
CCGCCGATGACCTCGTCAACCTTGGCCGCCAACCAGGGGTCGGATGGTAAAACCCCGTGA
GCTTTCCGACGTAAATTGCAATCGAGTGGGTCTGCCCTAGCACTTGGCCATTCACCTCCA
GAACGGGAAATGTTCCGAAGCGCATCTTCCCGGACTGGCTGAGCTCGTCCCAGTTCGCTC
GCCGGTCGTCAAAGGGTATTCCTGCGCTGAAGAGGCACAATCTCACGCA
>at02969
TAGAgGGGGGACTAGTATCTCTCAGAACCCTAAGCTACGACTTAAATGTGGCtTTTAtTC
GttCCtTCtTGAAGCCCGGATCCTGCATCTGCACGTACACGTACTCCCCTGAAGACGTGA
TACACCCTGAGAACACTCGACACAACCTTGACGGATCGTTGTCGTTTTCTATTGTGGTAT
GGAGAGGCACCGGGTCTGCTATTAACGTGATGGCTGGCAAGATTGACGCGGATAACATCA
AGCATAAGCTGATGACACTGGGCGTGTGGAGACCCCAAGATTTCCAACAAGGACATG
>at02970
TACGCATTTTCCATTTCAAGGGTCGGACATTCGCAATGCCCCCCATTACACCAGCAGATG
GTCCTGTGGCGGTGACCGGTGCAAGTGGCTATATTGGAGCGCATGTGGTCCAGTGTTTGC
TGAAGCGCGGCTATCAGGTGCATGCGTGTGTGCGCAACAAGGATGATCAGGACAAGACTT
CGCATTTGCTGGCGATGAGTCGTGCTGGCCATCCGGGGAGCCTCAAGCTCTTCTCAGCTG
ACGTATTGGAGGCAGGCAGCTATGATGTCCCATTCAAGGGTTGCAGCGCAGTCATGCACG
TTGGAACGCCAATGGGCTATGCTGGCAATAATGGTCCGAGAGCTATCTACGATGGGGGTG
TCAAAGGGACCAAGAATGTCATAGAATCCGTTAAGAAGAGTGGCTCTGTC
>at02971
TTGTTTTTTTTTTCTTTTTTTTTTGCTCCAGTCAAGGGCTGACCTTGCTCATCGAGGGAA
CCTGCAAAGTATGTTTGCGTGCATATTCACACGCCCAAATCACCAGCTCAATTCGCGGGC
TCAAACATCAGGTGCAACTCGGTTCCGTCAGGGCCCCAGGGCTTCAAGCCATTGTCGCCC
ATCTTGCCCTTGATCGGGGTGCTCTCGTCTTCTTTCAAGTAGAGCTTGAAGTCCGAGTCA

GGGATCCAGCCCTGATCGAAGCCGAGGAGCCTGCGGGAATCCGTGATGATGGACTCGACT
GTGGTGTCATCCATTACGCTCATGCGCGTGCGCTCGCCGACTGAGGGATTTGTCACGAAG
ATCTCGCAGATCCTTGGGCCCGCCGGAACCGGAGCCGGTGCTGGCACTGGCACCGGTGCC
GGAACCGGTGCTGGAGCCGCAACTGCAGGCGCTGGTGCCGGTACGGGCGCAGAGGCCTCG
CTCACGACGAAGCGGAAGCGGCCGTTCTCGTCCACGGTGGTCACGCGGCCGGAGCCAGCA
CCGTCAACGACGAAGCGGAAGAGGCCGTTCTCGTCCACGGTGGTCACCCGAGCATGTGCA
GCGGTGCGGGCCGCCACGCCCCGGGAGGGCCGGCAGCCGGCAAAGGCTGGGCTGAGAGCC
GCTTGCAGCAGCCAAGTGCCCACGGCGAGGACGGAGACGGCGAGGAACGGCCGGTGGACA
GCCATGGCTGGACTGGCTCAGGGCAGCAGAGAGCCCAAGGAACGCCCGGCAGGCCCGAGT
TCAAAATCTTAAGCCAAAATGGCTTGAGCCAAAATGGCTACGG
>at02972
TTTTTTTTTTTTTTTTTTGCACGCCCATACATCACCCTCGTTCGCACTTCCCGACCTCAA
CGCAGGCAGCCCAGCCACGCGACGATCAGAGGAAGTGATAGGCGTGAGCAGTGCCATGCT
CACCGCAGGGTTCAGCCACAGTTGTCCTTGAACCACTTCTCAAACAGCCGCTTGTGGCCC
AGCTTCTTGACTCCGGCATCCGCGAAGAACTGCTTGTCCACCTCGCCGGCCTGGACGTAC
ACGTCCACGATCTGCTCGAGGCTGTCGAGCTTCGCCGCAATGCTGTCGTGGTACTGCACG
AGGAAGCCGGAGTCGTCCAGCGTCATGAGCCAGTCCTTGATGCACATGTTGAACCTCTTG
GCCTTTGGCTCCTCCTTTCGCGGTGCCTGCACCGCCTCCGGCTGAGGCCTCGGCGCCTGC
CATGCCTGCGCCGCCTCTGACTCCGGCCTCGGCGCCTGCCGTGCCTCTGCCGCCTCCGGC
CGCAGCCTCGGTGCCTGCTCCGGCGCCGGGGCCCTGGCAGGCTGTGGCCTGGCCTCGGCA
GATGGCTGGGCGCTGGCGCCGCTGGCCGAGCTCTGCGCCGGCTCCGGCTCGGCGGCCGAT
CGGAGGGGCCCTATGCCGGGGCGGGCGTGCGGCGCCGCGCTGAGGCGCGGCTCCTGGTTG
AGGCCCCTGACCCGCTCCTGCCTCCGCAGCTCCTTGAGCCATTCCGGGAGGGGGCTGGCG
ACGCCCAGCGACCAGAGGAGCTCCTCGGCAGCAGGCCCCTCGGGGTTGCCG
>at02973
GCCAGATTTCCAGAGCAAGTTCTTCCCAGTGTGCGTCGGAGCCGAAGCGGGCGGGCAAGT
TCGATGGGCACAGCTATGAAACGCTTCCTCGGCTGATGAAGGGCGCTGCCAACAATGCGT
CGGTGCTCGTAAAGATGGACATCGAGGAATCCGAGTATGATGTTCTCGGCGCGCTGACGG
CCGATGACTTCAAGCGATTGTCCGGCCTACATGTGGAATACCACTTCAAACTAATAGAAG
TAGTACCTAGGACCAGCCATGGTAGAGAAGGCTTTACGCGTGGCGCGGCACGTGAGGAAC
AATCTTGCAGTGATCGATTCGGCGGCAGGTTACTACGGTGCGGATTTCAAGAGTCCATTC
CGATTGCAGTGA
>at02974
CGCCGCAGAGCATGCAGAGGGCGAAGAACTTGAGACCACAACGCGACATCACGAAGATGC
CCATCGGCACCATGAAGAGGCTCTGCAACGTCCCCGGGTTGTACTGCAGAGCGAGCATGG
CGGGCAAGAGGTGGCCACCGAGGCCGTTGACCGTGGCCGTGCCCCAATTGAAAAGGCCCG
CGAGCCTGTAGCGCCCCCCGAGGGCGTGGGCCGCCACCATGGTCAGCGAGAAGCCGACCC
AGACCAAGAGAACATTGATGTACGTCGTCCTCCGCACGTCCAGGGGACAGGTGAGGTGGG
AAGTCTTGTCGCAGATGGGGAACAAGAGGCTCCCGGTGCCGTGGTTGAAGTGGGGCACAA
ACGCATACCTCCAGCCCCGAATGTCATACGCATGCTCCTCGACCTGATGAAGACAGTACA
CCGGACGGTGAGCCAGCCGATGAACAAGGGTTCTCGAACTTCTCCTTCAAGGAGCCCTTG
AAGAGCAAGAGCAGGGGGCACAGGAAGAGTGGACACAGAACTCCGCTCACAAACATCCAG
TTCGCGTCGAACCAGTCCGCGCCCTCGAGAAGCAGCGGACCGCTGTTGCGGGTCGGAATT
GGATTTGGCGCCATGCGAGGGCGGCCGCACTTAGGCAGCGCTGGTCTGCACTTGGCAATG
ACACCCTTGAGCCAAAATGGCTACGG
>at02975
TTTTTTTTTTTTTTTTTTCCCCCGCTTGACTACTTCAAATCAATCACGCACTGCTCTCGG
AAGTCAGGACAGCAGTCTTGGTAGGTGCTGCAACCCTCGTTACATTGGCATGCATGCGAC
GGCTCGTAGTGGACGCAGCCGTATGACTCGCAGGTGTGCCGTGCCTGTCCCAAGCTCGAG
GCGCCGCAAGTCTCGGCGTAGTCGCTGCAACAGTTGTCGTACTCCTTGCAGCGCTTGTTG
CACTGGCACGTGTTGGCCGTGTCATAGCCCATGCAGCCATATGCTTCACAGCTGCCCTTG
TGCTGCGTAGAGCTGTTCATTGGCTTTTGTGTACCAGGCTCCTCCATCTTCTCCCTTTCG
CCTCTGGTCACACTGCTGCCGTTTGGTTGAAGTGAAACCCCAGCCTTCAGCGACTGATTT
GTTGTCACTTGATACCTCCGGGCTTTTGCGTCTTCATGAGAGCTGGAGTTCGCATCAGCA
NGACTTGAAATATTTTCGCCATCAGGGCCCATGGCTTTCNTGTCTTCATGAAAACTCGAG

TTTGCATCAC
>at02976
TCGTGCACGAGGACAGTCCCGTGAGCCTCCTGGCCTGCACCCAGGCTCGGCCAGAGGCTC
ACGCAAGGCAGGACCATTGGCTCCCCGCCGAACGCGGCTGCGGGGCCTCCCTAGCTGGAG
GCTCGGCGTCAGTGGTTCAGCGGGCCCTTGACTTCAGGGTCTCAGCTTTCTTGGCCAGCG
GCTGCCAATCGATGTGATCCTGAAGGTCTTGCCAAAATCTGCCCTCGTTCTTCGCCTGAA
AGTCGCGCTCTACCTTCCTGATCTCTGCGAGGTTCTCCGCGGGTCTTTGTTTTTGCTGAG
CGTGTCCTCCATGCGATCCAGCAGCTCCGAGTAGGTGCTAATTGCAGCGCGCTGCGCCTT
CTTCTTTCGGACATGGCGGCGGTGCCCAAGCGCGAGGCTGTGGAAAG
>at02977
CAAATTCGTCCTCAGACCTCTGCTGCACTCCCTCGCATGCATCGAGCCGCCCCTCAAGAG
CTTTGTTGCACGATACCAAGCTGTCGTAGCTCTCCGCCCAGTCGACACCGCCAGCCTTGA
GATCAATGCTCTCGTCAAAAGGCACAGCTGTCTGGCCTCGGCCCTGCAGCTGCTCACAAA
GCAAACGGTTGCGCTCCAGCTCGATGTTGTAGGCTTCCTTCCAAAGGCCTCCCTCGTCGG
GCTCTGCCGCAAGTGACAAGCGGGCAGCACGATGACCAAGAGCCCATGGGCTTGCCAGCT
GTTGGCTAAACGAAGGAAGAGACGGCGACAGAGGCACCAGTCCGGGCTCGGGCACCTTGG
GCGTCGAAAGTTGCAGTACGAGCACAGCAGCTGTGGCCGCCAGAGAGGTCAGCAGGTGTG
GCCGCAAGCCTTGCGTTGTTCCTCGTGGCCGCGGAATGCCCATGCTGGTACTCAGAGCGA
GCTTTCCAGGATGCGGCAAGTTGAGACTGAAGCCAGGGGCT
>at02978
GAGACAACGCTCATTTCAAATTTGCGGTGCATATCCCTCGGGAGCGGCAAAGGCAAGGTC
TCCTTCCCAGTGCACTTGCCGATCACACCTCTGCGGTACACATACCTGGGGGCCCCAAGG
GCAAGGACTCCTTCCCGGTGCACTTCTCGAACACACTGTTGAAGACGGGGCCAACATGCC
CATTCATAACCTCGTAGCTGGCCC
>at02979
GGAATAGGTATGCGTGAGCTTCAGCTGAAAGACGAGGTAAGCGCCGTAGAGGCCGAGAAG
GAAGACCGACGTCCATCGGGACACAAGAACCATGTGCTCCCACTCGGACTCCTGTCTCAG
TATGTTCGCGTAGATCGTTGGAAGGCCCACCGCGAAAACCGACATTGCGAGCAGAGAGGT
CTGCACTGCAGCACCGGCCTGATTGTACTTCTGCACGGGGTAGAAGAGGCCCCCCGCAAG
GAAGGCGCAGCCCATGACGAGAAGCAGATTGCTGAGGATGGACCCCAGGAGCGTGCACTG
AACGACTGTTATCTCGTCGTTCCTGATACCAGCGATGCAGAGCAGCATCTCAACCACGTT
GCCGAAGGTCGCGTTGAGAAGCCCCCCTGCCGTCTCACCCAGCTTGGCAGAAACATCCTC
TGTGGCCTTCCCGATCAGCCACGCCAAGGGGATGATCGCCACAAAGTTGAACAGGAAGAT
GTACTGCGGCTGCATCTCCATGAAATGTGCCGCATAGCCCATGGGCACTGCCAGAAGCAT
GCTAGTCAAGATTCGGTTGCCCCCAACGATGTTGGCGAAGCCCTCGAGGTCGTTCTGGGG
GCCTCTCTCTGCGGCCTGCTCACTCTCCTCCTCCGGGCTCGCCGTCGGCACCGGCGCGTA
GTCCCGGGGCGTGACCTGGCCCAGCTTGGAGCCGGTTCGGGCCATGGCGCCGGCGGGCCA
TGACACCGACGGCCGCGGAGGAGCAGCGGCGGCCTCTGCGCTGGAGCCGCAATGTCCCAC
TTGAGCCAAAATGGCTACGG
>at02980
TTTTTTTTTTTTTTTTTCGTCACAGGCGCAGTCTTGCACGTCAAACATTGTGGGTCTCC
AAGAACCTCGATCAAGCACGGCATGCTTGGCGTGACCTGGCTGCAAAGTCAAGGTACATG
TGACTCCTGTTTGCTTGAAAGACTGCCGGAAAAGCGGGCAAAGAACAAATGTTGAAGTGA
GCCTTCGCGAGCGTCGTAGTCCTGGCTGCTAGCTGAACCAAGACCGCGCGCTCCTTGCGC
ATAGGCGAAACAGTTCACACAGAAAAGCGGGCAAGGACAACATTTGATATAAACACCCTC
GCGCGCATCATGGTCCTGGCTGCTTGGTGAGCCAAGACCGCGTGGTGCTTGCGCACATGC
GGGACAGTTCACACAGAAAAGCGGGCAAGGACAACGTTCTGAATTGAACCCTCGCTGGCA
TTTGAGTCCTGGCTGCTTGCTGAACCAGAACTGCGCGGTGCTTGCGCACAGGCGGAACAA
TTCACACAGAAAAGCGGGCAAGGACAACATTTTGAATTAAATCCCTCACGACCATCGCGT
GCATCCTTCGTCTTGCCTGGCTGGGAGGCGCCCTGGACATCAGTGTACTCCTCTTGACTT
CTGCACCTTCTCT
>at02981
TCACTTGTTCGCGTCCAGGGACACCCTCTCCGACACCGCGGCCCGCTCGAAGATGGACTC
CCTGAGGCCCCGCGTCCAGCGGCCCAGGCCCAGGCGCTGGCCCGCGGAGATGTAGCCGGC
CGGCCGGCGCTGGGGCTGGAGCCGCAGCCAGCTCTCCAGGCCCTGCAGGCAGGCCCTGAG

GCTGACGTCCTCCTCGTAGCCGTCGAACTTGATGACCCAGAGGCACCGGGTGTCGTCGTC
GATGGGCGAGCAGTCCACCTTGACGGCCACGAGGTCCTCCAGCAGCCGGTCACTCTCGGG
ATGGAACGAGATGAGTGCCTTCACTGCGTCCTCGTCGTCTTCAAAGCGAAGATGCATGCC
CTCCTTGTGGTCCAACATGATCTGGACCATGTCTTCCTGGAGTTTGGCAAGCGACTGGTA
TCTGCGCCCGGCCACACGCAGGGCCTCACAGTCTGGAGGCCGCTCCGCCAATGCAGCGAC
TGCCAGCTGCGGCGACATCGGTGCTAGCATCACTTGTTCGGGGAGGTCTGTCGGATTGTT
CCGATTCAAGTCGAAGAGAGCAAATCGTCACCCTCGCTTGGGCCAATCAAGGAGCCGGAG
TTGAACGACTCCAGTACGCCCTGATCGCCTGCAGCTCAGCGCCCTCGGACGCCTCTGCCT
CGACACGTCTCGGGCACTCGCGTCTGA
>at02982
TTTTTTTTTTTTTTTTTTGCATGCTGCGTGACGCCAATAGCTCCCCCCTGGGTACGGCAG
CCGCCAGCGCGAGCTTATGGACGAGAAGCGACGGCTCAGATTTGAAGCATACTTAAGAGC
ATGGCCTCTGTAGCCAAGCGAGGCCAGCAGTATCGACCTCTCCGCGAGCGAGAGCTGCCG
TCCCTTCGGAAGGCTTCTGCCCAAGTCCGCCACCTCGTTCAGAGGGCAGGGCTCCGGCAG
GTGGAGGCACCAGTGAAAGAAGTGGTTCTTGCGGCCCAGCTGACCCCAGAAGAGCCGCAC
CATGCCCTCCCAGGCTCACCAGCGGACTCCCCCGCAGGACCTCCCCACCAAGTCGTTGCG
GGACAAACCAACAGGTTGTGCTCGCAGACGGTGCAGGGAGCCGTGATGTCCATCACCTCC
ACAGCCACGAGAGAGTAGCTGTAGTCTCCAGATAGGATTGCATAGAGGGCGTCGCAAGAT
GCTCCATATACCCCACTGGCTGCTGGAGTGTGGTTGGCGTCAGACAATTCGTGGTATTCC
AAGTACCATTAAAAAGCAAAGGAGGCGGAATGTCAGGGTTCATCTCTACCATGACGACCT
TCGGGCGGTAGCCAGCTTCCAGGACTGCCTTCAGGATGGGTGCATCGAAGGAGTCGATGT
CGATCTTCAAGGCATCGAAATCGCGGGGCACATCGTACCTGCTCATCAGAGGCTTCACGC
CACTGGGGCTCACTGCCCCAATGCTGATCTGGATCTTCCCGCTGCTGTTGACCTCCCCCA
GGTTTTTCCGCAGCTCGGGCTCCAATTCAGCAGAGGC
>at02983
CACGACGAGTTCGAACAAAAGGCTCAAGATGCATACAAAGTGGTCAACACCCTCACGGAT
GCTTTGAAGACGGGAAACGAGAAAGTCTCGAAGGAGGTGGCACTTGGCACCCTACCGGGG
GCTGGCAGTGCCTTGGAACATGGAAAGCAATAGATGTGTCACCATCAGGGCATACTAGAT
CTCAACTGTCGCGACTCAAGCCCTGCTTCTTGGTCACATGGTGTACAGAAGTGATTGGGG
GAGCTGCCAGGAATCACATGCTTGAATGCATCAAGCAGTAAACTGTCAACAGATTCCGTG
AGAAAGCCTGGAGAG
>at02984
GGTGCAGATGCATACAATGCCtAAGttACtACtAGAGGTGTTgGACGAAGCTGATCAGGC
AAAGGCAACGCTTGTGTTGACTCGAGAGGTCATCGGGAACAGCGCCCTGGACGGGGGAGC
GTGCAGCGGCAGCTTGATCTTCGAGCTGGCGGACGAGTGGTGGAAGGACAGCAAGGGCAG
CCCCAGTCGGCACAACCATGGTGGCGTTgGCTCCCGGTGGTGGCCCATTTCCGGACAGCG
TGTTCAATGAGGAATATTGGGCCTTGTGTCCATAGATGGGACCCGCGCCAGGCCTACCAT
GCCTATGCGGCGATCCAGGCGCCAGGAGCTCGCACTGGCCGCCCGGCCGTTCGCGTGGGG
CAACATCACGGCAGCGTACAGG
>at02985
GTGAGCTTGAGCGCTTCAGCTATGGCGCCAGGGAGTGAGTCTTGAGCGGCGGCAGGGGCG
CCCACGTCATGCCGGACATGGGCGGGAGCACCTGTGAGAAATACTTGCCGTCAGCCAGTG
GCTACGAGGGCTTCTTCCCCTACCTGGTCTACTACTTCGTCCCCATCGTGCCGGTGGTGA
TGGTGCCACTCCAGCCTTTTCATGAGGCTGGAGTTGGCGAGAGCAATTCCTCCTCCGCTG
AGCCCGTCCAGCCTGTTGGTGAAGGAGTGGCCAATGGTGGCAATGGCCACAAAGAGGAAG
TGATTGACGACTTCTACGAAGGAGCTATTGGCGCGGGGTGGTGTTGGCCGGCGTGGTGGG
CCC
>at02986
CATCTCGAATCTCCTCCTGTTCAACCATTTTGCCCATCCTCGGAGCCGTCATCTACCTCA
ATGCCAAACCTGGCCCGGAACAACTCCTTCACCTCTGCCAACTTGCCCTCCAGGGTTTGT
TCCAATCCCTCTCCGGCTGAAGGCATCGGGGGAAGAGGCTGCTCATCAAAGTCGTCAGAG
ACGTAGTCTTCATCGTCATCCTCAGGCGCCAGGAGTACCTCTGGTGGCTCTGTCGAGGCA
CCAGAAACAGTACGGACaAAATTTCAAGGCTGGNGAGCACTGTGGTGTTGATGGGGTGCC
GCGAGCAACCTTCACAACGGAAGCACCACGAAGAACGGACTTGTCAAGAATGGAGGTCTC
GTTCGTGGCAACTCCAGTAAACGAGGGTGCACCTACAAGGCTGTGGCCTGCAAGTGCGCC

CTCGCTCANCATTCCAGACATCTTGCCTAAGTGGTGTTGCGAGTAAGTGTCCTCGGAGGA
CTTAACAAGGAACAGCTTGAGGTAGACTcTGAG
>at02987
TTTTTTTTTTTTTGTTTTCAACCATGCTGTCCCCAATGTTGCGAGCAATTCACAGTGTAG
CCTCCACCTATTTTTGGATTATAGGTATACAATGGAAGAAGATGGGTATCAGTTCGAAGC
CTGTCTGGCCATCAGCAAGATACGTCTCGAGCCAAAATGGCAGAGGGCACGTACGCATGC
AACAGTGTACATGCGTGTGTAGACAGTTGATTGCCACCTGTACAAGCGTGCCTGAATGCA
CCTTAGGCAATTGCAGGCAACATACTTCTCACGTAGACTATACAGAGGCTAGTGGTGACT
CTTCAGGGGGAATCTCGCAAGGAACTTATGCCTTCGTCCCAGTCCACCAAGACTTCAGCC
CCACCCAATGTCATGCCTGCATCAAGTACACCGAAGGGCGATGAACTGACATGGAGCTTC
AACCAAGCACTGCTGTCGGAACTGGGACTGATTGCTTGGTTGGTTGCT
>at02988
GGCGACTTCAACGAATTCAATCTGATGATAGACTCCGACGAGAAGGGACGCTCATAGACT
TTCCGCAGATCATCCACTTGTCGCACGAGAACGCGAAGGAGGTCTTCGACAGGGACGTGA
GATCGATAGTCGAGTTCTTCCGGAAGAGGCTCAACATCCAAGTAGAGGAGTGGCCCCAGT
TCGATGAGGTCATGGGGGAGCTCGCCGCGTCCGGCGAGGCGCCCGCTTTGGCCAAGCAGC
CGGCTGTGGAGGGCCTTGGCCGCCGGGAGGACGCCCTGCTCGTGGCGGCGCACGAGGAGG
ACA
>at02989
TGGAGGCCCTCGTGAAGGCATTCGAGGATTACCTGCAGGGCGAAGGGGGTGCGAAACTTC
CGCTGCCGGAGGTCGAATTGCCTAAATCTGCGGCTGCGGAAGAAGGAGCGGCTGCTGAAG
AAGGAAGGGCATGATACTCGCACTTTTGATTTCGCGGCGCTGCAAGTCAAGTGGTACAGT
CTTGACCTGTGCTGCGAAAGGGGTCGGCGATAGCAAATACATGAGGAGTACGAGGATGTT
TGTATATCAACGTT
>at02990
GCTGCAGCAGTGCCGGGAAGACTCCGCCCAATCGTAATGCTGACTACATGTTTTGGAGCA
GTATTTTTGCTGTTATCGCTTCACGTAAGGCCTGGTCACGCCAAGTACTTCTGGCCCGAA
GCACGAGGCCGTCCTGATTCCTACGGCGTCTCGGCGTTCTCGGCGCCACGCAACCTCACA
AAGAGGACGCTTGCCTGGACCTGGTCAATTGGCGGCCCGTGGGAGAAGTACTCTCAGCAA
CCCCTTTCAGCTGTCCTGATAGATGACAAGCAGGACCTGTACCTCTCCCCCTACGGTGGC
ATCTACAAGCTCAGCAGTGTGGGCGAGCTCCTGTGGTCTGTGCCAATGCCGACGTGGCGC
GCCCCACCATCTTGGACGGCAGGCTCTACATCACGGACAGTGGCGGTATGGCCCGCGCCT
TTAGCATGGAGACGGGTGAGCAGCTCTGGGCC
>at02991
TTTTTTTTTTTTTTTTTGACCTGGAAGTCATGCTTTGCGCAAAAATGGTTTCTTACACT
TGCGGTCAAGCGAAGATCGGAAGGCGCCCGGCACTAGCGGCAGATGACGACAACGACAGC
AACCATCAAGATCACTGAACGTCAACGAAGCCATGGATCAGAAGTGCTTCAAATCGGCAC
ATGGCCAATTCGCAGCCCTCCGCTGCCGAGACCTCCATAGCACCACGCAGCCATTCCGGC
TCAGTAGCCAATGCTTTTGAGGTACTCAGCAAAGGCCAAGGCCGTCTTCTTGGCGTTTCC
GGCTGTCTGGCTCTTCTCCTCGTCGTAGAAGGCAGCCACGACCTGCGAGCCCGTACTGAC
CAAATGCACTCCTTTCTTGGGCCTTGCGGCAGAGCACACCACGAGCGTGGCGTCGCCGCT
CTCGAAGTTCGGGTCGTACTGAACGACGCTGTACTTCAAGCCGCCGAGCCACAGACCTCC
ATCCGGCGCCTTGCCGGTGTTGACTACCGACATGATGCACGCCGGCTCGTTGATGGTCAT
CTTCTTCTTGCTGTCGTCGTCCTGGGTGATGTCCTCCTCGTGATCCTCCTTGAAGACGTA
ACCCCAGCCCTCCTCGTTTGCCTGAGGCGCGGCCGCGTANAAGGCCCCGTCTTCAAGCTG
GGCAAGTGCACCGGCATAGCAGTAGCCCTCAGAGAGTACCCATTCCTCGATGGTCT
>at02992
TTGTTTTTTTTCTTTTTTTTTTGCAGGCAACCACACCTCAAGTCACGGCAGCTTTCGCT
TCTTGCAATGAACGGAGAGTCCCGCCCCTTCTGACTCGCCAGGTCATGCGTGCAGCAGCC
AGGCCGTCGGCGAAGGGATCGTCAGCGTGGTAGTTCTCAGAGAGGGCCACTTGTGGTTCG
GAGAAGTACCTGAACCTCTCTACTGGCGCAGGCTTCTCGGCAGTCGGGAAGAAGCCTTCG
GGCAGTTCCCGAACGAGGTCTCCTGCTGGCGGCTTCGCGTTTTTCGGCAGGCGCGAACCA
TGGTAGTGAAACTTCGACACCGGGAACGTGGGGCATGAAAGGGCCGGTGAAAAGAGGTCG
CTCGACGTTGCTGCCGGCCCGTTCGCAACGGCGGTGGGCGCGGCGGCTGCAGCGTTCAGT
ATCTTGGCCTGCACTGCTTCGCGCTTGGAGGACGCGGGGCACAGGACGTCGTTCACGCAG

GGAGACGAGTGGCTCCGCTCCCAGCGCTTCAGGACGGCGTTCTGCGGCCGCGCTGCCGGG
GGGCCCGTGGCCTTCTGCAGCTCCCCGAAGCAGGTCAGGGAGGTCCTGGAGGGCCGCAGG
GAGGCGGTCGGGTGCTCGACCTTCGCGCGGCTGGCCTTCCGAAGTGCCCTGTCCCGCAAG
ACCTCACTGGGCGCTCCACCTCTTGGCAGCTCCCAGGCAGATCTGCCCTTCCGGAACTCA
GCTGAGCAGTAGATGCCGTACTCGCGGCCATCCAGACTGGCAGGCTTGAGGTTGTTGATC
AGTCTCAGTCCGTGCGCCATCAGAGGCCTCCGCTGATCAAGATGGGGCCAACGAGATGCC
AAAATGGCACGAAGATGAGGCCTTGAGCCAAAATGGCTACGG
>at02993
CCGTAGCCATTTTGGCCCAAGCGCGGCTGTACTGGCTCATCTCAAGCTCCCCAGGAATGA
AGCACCAGCGTTGCAAGCCTCTACCTTGCACCGTCATGGCAGCAGCCAGCCCATGCCGTG
ACTTTCTGGTCTGCAGCACCCCGTGTGCGTGGAGCTGTATTCTGCTGGTCGTTCCAATCT
GCCATCTTGGATTTGAGCCATTAGCAGGACCTCCTCCCTGATGATTCTGTAAGACCACCA
TAGGCAGCGACCCATAGCTCCTAG
>at02994
TTTTTTTTTTTTTTTTTTCCAGATCCGTATCCCTGAGCCCCGGCTCCTCTCGCCAACGCC
GCCTGACTTCGCTCCGCAATTGCACGGCCCGGCCCCTTCTGCCGCTCACTCCGTGGGGCA
CTCGAACTCCTCGTTGTCAATGGCCTCCTGGAGCTCCGTGGGCTTCATGCCCTCCACAGT
GCAGCCCACCGCGTTGCAGGAGCCCAGGACCTCCTTCACGGTGCCGCTGAAGGCCTTGGC
CAAGCTCTTGGACCGCATCTGCCGGGCCACGGAGAAGAGCTGCGCCTTCGTGAGGTTGCC
GCTGTGCTTGATGTTCTTCGTCTTCTTGCGGTCGCGGAGGGGCTCCTTCAACTCCTTGAT
GACGAGGCTTGTCGCATTAGGCTCCACATCGACCTTGGCCTGCCTGTTCTGGATCGTCAG
CCTCACCGTCACGCGGATGCCCTTCCAGGCGCTGGTGCCCTTGACGATGTCGTCACCGAT
CTTCTTGGGCGACATGCCGAGAGGCCCGACCTTGGGGGCAAGCACGGATGAGGGGGCCTG
CTCCCCGCCATACTGGCGCAGCTTCACGATCTTGACCTCGTTGGGGTCGAACTTCGGCGC
CATTGCCGCTCGGCGCGTGCGGGCCCGCCTCGTGCCGAATTC
>at02995
GCCTTGAGGACTATTTACTGGCTCCGGCGCGCCGGGGCAAAGGTCATCGTCATCAGCTAC
GCCCAACTACTGCTGGATTTGGATAGGACAGTCGAGCGCCTAGAGGCATTCCTACCCGAG
CTGAGGCCGCTCAGGTCCGATTTTGTACCGCAGCTCAACCGGGACATCTTCCCGACGAAC
AGATGGAAAGTCACCGGCAGCCTGAAATCATACTCGTCAGTCTTCAAGCAGAAGCTCATC
CAGCACGGCCTGCACGCGAACAACATCCTGCGCCAGCCCCAAAACTGCAGATGGGAGTCG
CCCCATGGCGAGTTGCATGCCTCGCCAGACCTCGCGAGGCGGCTGAGGCGCGCACAGATG
CTCCTCCAGAAGGAGTCCTCACAAGACGTTGACTGAGCAGAAGTGCTGAGGTATCTGAAA
GGCAAC
>at02996
TTGTTTTTTTTTCTTTTTCTAAAAACTGCGCCCAAACTCCAGTCCCGCTTCAACCTGCCA
TGTGATCAGCCCAGCGCGGCCCATCAGAGCCTCCCAATCGACAGCATGCGCGCAAATGCG
GCAAGGAAGAAGACGAGGAAGAAGATGGCAAACGCAAAGCCAAAGCGGTTGAGCTCCTGC
TCGGTGTACGCAGACGCGAGCTCCGGCGTCACCGCGGCCGCCACTGCGGGCACGGCCGCT
GCCAGGCTTGCCACAGGCCGCTGAGCCGGGGCAGCCACGTCGGCCCGGGGGGCTGAGGAA
GGCCTGCGGCTGCAGAGCGCCACGGAGCAGCACCACGCAGAAGACCCCGGCGAGCAGAGC
AGGGAGAACCTTCGCCCCGGCCATGGCTGCTGGAGGGGAGTCTGGAGGGGAGAACTGGGG
CGCGTTGCCTGCTCGGAGCCCTGGAGACCCAGATGGCTTGAGCCAAAATGGCTACGG
>at02997
TTTTTTTTTTTTTTTTTTGGGAGACGGGCATCTTCTGCAATGTGTCCCGCATCCTTGGCT
GATGTCCGAAATGCCCAAACGTAGCAAACCCATGGACTGGTGCCATGAGCAAGCGGGCAT
GGCCTACTACATGTCCACCTCCATGCCAGTCTCACAAGGGAGGGCGCACAGACGGGCCTT
TGGCAAAATCCATTTGGGCAGGCACCCAGAAGTGTTGCAGCGCATGCGGCTGCCTCCATA
GCCCTATGGATGCCAGGGACCTGCCTAAACACGCCAATGCTGCCACTACCTTCCGGATCG
CGCTTCCTTTCATGGAGCTCGCCTGAGGCAAACTCCATCGCTCACGCGTGGATGCTGCCT
GAGCAGTGCAGTAGACAATGCACTCTGTGTTGTGGCTGGCCGCAGTACAGCCCCCAGCAG
CCGTCACGGCCGAACACAGCACTGCTGTCCGAGGGCACATCCCAATGTGCAGCACGAAGA
ACGGCGAGGGACGTAGGAGGGTGCGGGACAAGGCGACATGACCGCAAAGATGTCTTCAGT
CTCGCTGCGTGTATGCTCGCGCGAGGCGAGAGCGCTTCCGGACCTCAGCGTAGCCATGCT
CCGACCGCCTTTGCTTCTTCCTCGTGCGCTTCAGGCGACCTCGGTTGCCAGTGCGGGTTA

GCAGCTCCCCATCAAGGCCCATTGACGGCGCCTTCAAG
>at02998
TTTTTTTTTTTTTTTTTTGCAGGGTCCTGAGCCCCGCCGAGGGATGACTCCCCCCTTCCC
GCTCCAAGTCACCTTCCCTCTCCCACCCCCTCCCACAACAGCCGAGGCGCTCAGTAGGGG
CGCTGGCGGTAGGCAGCGCGGGGGCCCATGTAGGGGCCTGGGCCGGGGTAGGAAGGGTAG
CCGTAGGGGTTGTACCCCATGCCGTATTGCGGGGGCGGAGCCCCGTACGGCGAGTACAGC
GCGGGAGGCGGCCCGGAGTAGCCGGTCCTGCCTGCCCACAGCGGCTGCGGCTGTGCGCCG
TAACCCTTGCCATCGCATCCGTCGCCTCCGTTGCCGCCG
>at02999
TCTTCCGGGACGGAACGGTGTACCAGAAATTCAACGATAGCTCTGGTGCTTGGCATGGCA
GTGAAGGGTTGGATCTCACCACCAAGCTGCTGCTCAGGGGAGACCTTCCGGAGAAGCTGA
AGGTGCTGGCGTTGCTGAGCAGGCACCAGAGGACGCACGTCCTTCCCAGCGACCACACCC
TCTTAGGGATCGACGCCGATGCAGAGACTGCTTTGTTGGAGGGCCTGACGCGAAGAGTGG
TGGTGAGCAGCAAGGAGTGGGTAGGGCACGTCCGCGAGCGGTACCTCCAGTTCCAGGCCT
GCATGAACGTGCACCTCTTCTCCAACCCTCCTGGCCCAAGCTCGGCCCCATGGACGCTGC
GGAGTGGCTGGAGAAGCTGCCCTGCCTGGCGAGGGACGCCGCGGATGGCGCGGCGTGGCT
GCAGATGCGGCCGCCGGACGCTGCCTGAGGAGGCCCGGGGGCCC
>at03000
TTTTTTTTTTTTTTGATTCGAAAATCCTAGTTCTGTCCTCGGCGCGTCCATTCCCAGGCA
TTGCCATGGCCCCAGACGGCCGTTGCAGCTACAGCGCCATCGTCGCAGCCGCCGGCAAGT
CGCGCTCCTCCCTTCCGCGATGCTCGTGGGTCGAGCGCGACTTGCAGAAATCGCAGACGA
AGCCCCCCTTCCTCTTCGGGCAGAGTGCGAGGCACACAGGGCACTGCATGCAGCCTAGCT
TCGCGTGGACGCTCGCCGCCTCGCACGGAAGAGACTGCATCTTATGGACCATGGTCATGA
TGTCCTCCGCCGCCCCGTGGAACGCGCAGCAGCAGGCGCCCCGGGCCGGGCAGTGCCACT
GCTCCATCAGCTCCACGAGCATCGGCAGCTTCGTCGCCGCCGGCGTGGGGCGCCACCCGG
GGCCGGCCGGCTGGGCCGCCGCGGCCTCGCGCTGTGCGGCCGGGCGCGGCGGGTCCGCCT
GGTGAGCCTCGCCGCCGGCGGGTGGGGGCGGGTCCTGGAAGGCGTCTCTCCCCGAGGCGT
TCAGGAGCGCTATTGCCA
>at03001
TCCAGCAGAAGCTCCCTCAGCCTCCCAGCCACCTTCAGCGAGGGCAGCTCGTAGTCTGGG
TGCCTCTCGAACGGCACGTCCGAGCACCGCCAGTGCAACACTGGATGTTCAACGGGGACA
GCTGCCTTGCTGTACCAGCGCATCACAAGCTCAGTGTGGTTCTTCACGCAAGGAGACACC
CAGTACTGAGCGAAGCTCGAAGTCTTCTTCCAGGGCCTTCTCCATCTGTGGTGCTCCTCC
AGGTAGATCTGTTCGAAAGGGTGCCAAGAGGCTCCGTCGGGATGAGGGCAGATTGCTATG
ATGTGCCAGAGCAGCACGTTGCCGAGTCCGCCGCCGACCAGGAACGTCACCGTCTGAGCC
CACAGCATGTGAACGTAGTTCGCGGTGCATACCTGAGGCTTGTAGACGGAGCAGGTTGCC
ACTGCAACCGCCAAGGCAGCATTGGCCCCTGCAGGCGCTGAGGCGATGAAGCGTGCGGCC
GCCACGAGCGCAGCCGCAGCGCCGCCCAGGAGAGGAGATGGCCCAGACAAGCGCAGCGGC
CATGGCTGGCGTGGGGGCCGTGGCGTGGAGGAGGACGGCCCCGGCCGGTCCCGTTTCGG
>at03002
TTTTTTTTTTTTTTGTTGCCCATTGAATGATAGATACCCTCACTGCTAAAGAAGCAACT
GTGCACAGTCTGTGTGGTATCACCTGCGAGACAAGGGCGGCAAAAAGATTCGACTACCAA
AAGCATCCGGTCTTACGGAGGCGACTGAATATAGCATGCATCGCACTCAAGCAATCAGCT
CAAAGCTTCTCCAGCAGCTGCACTCTCCCCATGCTTCAGACACCCCGGCTTTACCGCACG
GGGGAAGTGGTACTCGAATGAGAAGTGACCGATGCCGAGGACAATGCGCATGCCGACACC
GCTGGCGTGCCACCCGGCAAACACCTCGCCTACCATGGACAGCCCCGCATCGGCCTTTCC
GCCAGCGCCAACCATTCGGTACCCCTGCGTGTCGCACCAGCCAAGGCACATCTGGGCACT
GACGGCCAGTCCGGCAGACGCGACAACGCGCAACGACATCAGCTCGGCGTCTAGGGCAGC
ATTCGACCCCTCTGTCTTCGCCGACTCAGATGCCCCACTCAAGACGTGGCTGAGGTCGGC
GACGTTCAGGCCCAGGGACTGCGCGATCGAGCCAATTCTGGAACCTGCCTCAGAGGCAGA
GTCGCTGAGGAGGCTCTGTGCTCGCTCAAGCGCAAGCCCAGCTCCTGACCAGATGGCCTC
TGCCTCCCGAGACCCGCAAGTGCCAAGCTTCTCCGCGCAAGAGGACTTGTCCAAGCCAAG
CGTCTGCAGCGCAGACGTGATGGCAGAGGCTGCCCCAGAGCCTGCCGCCGCAGCTTCATT
GCTGATCTTCAGCCTGATGTTGTCATGCAGCTCCTCCAGGGAGTGGNCAGTGCTCTTCGC
GTCTGC

>at03003
CCGTAGCCATTTTGGCTCAAGGATCGCCATCGAGTTGGAGTCTTGCTCCTGCCTGGCCTC
ACATCTCCAGCGCCCCGATGCGTGGCGGAGCCGCCATCGCCGACATACTGATAGGCGTCC
GCTCATCGCCCGCCGAGCAGAGGCACCTGGCAGTCGTGAGGGCCATGGGCCGGGGAGTGC
CCCGGCCGCTTCGGGGCCTGCTGCTCTGCGCTGCGGTCCTGCAGCTCCTCCGGGGCGCCG
CCCACCTCCACGCTGGAGCTGCCTGCTTCGTGGGAGCTGGCTCGGCCTCGTGGAGGCTGC
TGGGAGGCCCCGAGCGTGCTGGAACCGAAGGTGGTCACCGGCCGCGTGTGGCGGTGGA
>at03004
GCATTTGGCCGATGCAGATCCCGTGCATGAAAGGAGCTCAGAGGCTCCGATGGCAGATGN
AGAGCTGTCGCCCAAGAAGNGCAACCCCTATGCAGGCTACCAGCCAATAGGCGCCTcCGC
CAGCCAGAGCAGCAGTCCGCAGCGCGGCAGCCTGCGCGAGCGACTCTCGCAGGCCGATGC
TTCTGAAGGCGACGTCTTTGGCATCTTACTTGCGGAGTTGGTCGTCGTGCCGGAGGAGAC
GGAGTGGCTCACCGTCTGGGACGTCTGGGTTGTCACACTGCTCTTCGTCACGGTGTACTA
CGAGCCCTTCGCCGTTGCGGTGCTTGCGGAGGACCCTGGATGGGCGTACGTGCTGAA
>at03005
TTTTTTTTTTTTTTTGTCCGAACAAAGGATATCCTGTACAAAAAATCATCCGTGACAGC
TTGCATGCATTGCATGAGCAAGAGAGCGCACCGAGAAATACTGGCATGATCCCTTGATCT
GCCTGTGAAAATCACGCGAGATCCCATGATCGCCACGTGAGGTGCTTTGAGTCTCTACTT
ATGCCCATGGTGATCTGATGGTGCACAGTTGCGCTGCACAAGGAGCTTCCGCAGCAGCCT
GCTGACAAGCTGTGCGGCTGAGCTGCTCAAAATCGTGCATACAGGTCCTCTTCGAGAGGG
TTCCCTCGCAAGCCTCGCTGCAGGCCAACCCGATATGCAGCGGTGCCAACAATTGCCGAG
AGAGCCACTGCCAACAGGAAAGGCGCAACACTGGACAGGCTCTGAGGCACCACCGCACTG
AGGGACCATGGCACGTCAACCACGACGCCATGGAACGGGCCTCGCGGAGTCCAGGTAGCA
AAGCCCACCTCAGAACTCAACACCCACCATGCAGGGTAGCGCTGGAGCACCTCACCTGTT
GCTGACTCGTTGTACGTGACTGTGTAGCGCCCGAACTTCGCGAAGAGAGAAAAGGCAAAC
TTCCACCACTCACGTACCTTGTCATCGGCANAGGCGTTGCTTCTCGTTGTCAACAGATCC
AGAGCAGTCGCCTCGTCCGCG
>at03006
TTTTTTTTTTTTTTTTTCCACTTCATGTGTCACCGCAACGCTTCCGAAGCCACACTCTC
ACGAGCTCGTCTGACCTCTCTGCGCTTCCTCCGCGTGAGTGGCCCTCGAAGAGCGCTCGG
ACCGCAGCCGCCTGCGAGCTCTGCCTCCTCTGCTGAGGCAGCTCCTCCAGCGCGTCTGCC
TCCTTGAAGCCGCAGCGCTCATAGAAGCATCGCTGCTGCTCCTCGCCCTCCGTCCAGACG
TAGAGGAAGGCGAAGCCCCGGACGCTCGCTTCCTCCTGGGCTCGCGCCAAGAGGAAGCGG
CCGATGCCCTGCCCACGCTGCTCCCGCCTCACGACGACGCTGCACAGCACTGCGCAAGGT
TGCTGGGTGCCCTGGCCGGCAGCAGAGGTGGTGGTCTCTCGCATGGCGCAGTGGCCAACG
ACCACCGGCGGCGAGTGCGCGCCGCAGCTGCTGCCGCTGCTGGAGCTCCTTGGCGAAGCG
TTTTCGGAAGCCTCGCTCTGCAGGCCAGCCGCGTCGGAGGACCTGAGGACCACGAAGTGG
GCAGGCAGTGCTTCGCAGGAGCGCCTGAAGAGCGCCAGGCGCCCGGATATCCCCGCGGCC
CGAGGCCATTGACTCTCGAGCAGCGCGGCGGCTTGCGTGGCGATGCAGTCCGCGGCATCG
TGCTGCTGGTGAGCCACCGGCTGCCCGGCTGCGTTTGCATGCAGCGCCTGCCACTGCAAG
GCCGTGGGCGCACAAGAGGACCGGAGGGCCGACGCCATGCTCAATCAAAATTGGCTTGAG
CCAAAAGCCGACAACGATACACCAACAATACTTGAGCCAAAATGG
>at03007
TCGCAGCGTCCTGCTCACAGTGCGACATGAAGGATGCCCCGGTCTGGCCAAGCCGCTGGA
AAGCATCACGTCGGAGTTCCGCTCAGGATCACAAAGGCGTCTCACTTGTTCTCTTGATCA
GTGATTCCCACCGGTCTTCACCTGTAGACCTTGTTGCAGTGGACATTGGCTCACTGGCAA
GGGCTTGTGGAAGGCAGCCGGCGTGAAATGTGTTCCCTGCAGGCTGGATCATCTGGGCGT
TGGAGCAGAGTCCAAAGGTGCAGTGAGGCTGGCGGATTGGTTTGCAGCGTTGAAGCGGGN
AAAGTCTTGGTGTGGCCGTTCATCGAGGCATTGCGCAAGTCACGTTGCAGCAG
>at03008
GGAGGATCCTTTCGCGATTTTGGGAGCGCGGACCATCCTTCCTCCCCTAGAAAGGACAGC
ACAAGGGAACAACCCTCAGGAAAGTACTTGGTGAACGTAATCGGATCATAGTCGGTGAGC
ACTGGCGCGACAAGTGGTGTGAACAAGCGCGGCCGACGCCAGGGGGGTCAGCACGCCGCG
ACCAGCTCAGCCAGTCATCATCGAAGCTGGCGTCCGAGTCTTCCTCTGAGTCCGACGCCT
GCTGCAGAGGAGGCTCCTGATGATGATCGCATCTAGCTTCAACTTGAGAAGCGGCAAACA

CCTTGATATTTC
>at03009
CATCAATGCACTCTGTCCATCACAGTAAGTCTCTGCAAAAGCTCTGGTCCGGAATGCCCC
TCTGCGGGGCGACCTCGCAAGCCCGCTCCAGCCTAAAGCAGTCATTGGTGTCCACGTAGT
TCGGGCACGAGCACTTCTCAGGATAGGCGCCAACCTCCTTGCACATGTCGATACAAACGC
CGGCAAGCTTGTTCTGTACCAATTCCCGGTGCTTCGCATCCTCCGCCATGCAGGCCTTGC
CGACCTGAACGACCCGCACCGCTTTCTTTGTCCACTGAAGCTGTGATGCTGCTTGCTTCT
TCCACTCCTTCAACGGTCTCCTCACCCCATGAGCTCAAGTCATCCATGTATTTAAGCAAC
TCGTCCCAAGTCATGACACCTGGTGTCTTGTCAGTCGTATCCACGTAGTTGGGGCAGGTG
CAATGCTCTGGGTATGCCCCTGACCTCTTTGCACATGTCAATGCAAATGTCTGCGAACTT
ATTCTGCAGTCTCATGCGCGCAGCAAGGTCCTCAGCTGTGCAAGCAGAGCTGCGTGTGTC
CATTCTGCGCGCATCAATGGCAGTCGGCAGGATTGCCAACAATGCGAAGGACTTCATTTG
GAGCAGAGGAAAAGCTTTACCTGTTGCACCAGAGCCTACACCTCACAGTGCtTccTcGAG
CCAAAATGGCTACGG
>at03010
TTTTTTTTTTTTTTTTTCGATGCTCGGACAATCGTAGCGGCCACTCCTTTCCTAGTAGT
GTCGCGCCTTTGGCGGTCAGTCGAGCCACTCCCTGGACTGAAGAAGCTGGCCGGGCGCCA
TGGGGAGCAGGTGGCCGTACTTCCTGACGTAATAAGGGTTCTTGCAGTAGGTCTCCTTAC
AATAGACCACATCGCAGTGATAGTTTCCCATGGCGCACGCCGCCTTCAGGAACAGGGTGG
TGTGCCTTGGATTCTTGAAGCCCTGATGCAAGTCAGCTGGCATGCCCTGCAGGTCCTGCA
ACATCTCGAGCGCATGGGTCACCTTCCCAAGCTCGCTCCATCCCTTACGACCATAACGCT
CGTCGCACATCTGGTTGGCCTCCTCCAGGGTGGTCTCGTTGGTGACCGCCGTGTTGGAGC
AGTGGCCCTCGATGAAGCAGTAGCTGCCGTCAGGAACAGGGTGGTGTGCCTTGGATTCTT
GAAGCCCTGATGCAAGTCAGCTGGCATGCCCTGCAGGTCCTGCAACATCTCGAGCGCATG
GGTCACCTTCCCAAGCTCGCCCTCGTGCC
>at03011
GGCACCGCCGGACTGTGACACAGCCTATCACGGGATGGTCCCAGCCGACGAGAGGAAGTC
TCGGCGCAGCGAGCTGGGCAAGCTTGCGCAAAGAATCCTGGACAGCTGCAGTCGGGATGG
TCGGGCGGTCGTCGATGCCCGCGCTGCGCTGCCCATGGATGACAGTCACTCGGAGTTGTG
GGACGATGCTATTCACATGTCCCCGCTTGGCTCGGACTGCCTTGGCGATGCCGTTTTTGC
TGCGATTCAAAAGCATGGTCTTTGACGACA
>at03012
GAGTCAAGTCGTGGTCATCTCAGTTCCACCCGTTCGCCTNGCAGATGCCCATGGCGGATC
CGGGCGGTAAAGCCGAAGTGCCCTTCCAGCGGTCATTCTATCAGGCTCCGCAGGCGGTGA
CTGCCCAAGACGTTGAAGAGCTCCGCCGCAGCNTgGNGGATCCGAATAGACGAAGATCCG
GGCGGAGCATGCCCACCACCAATTGCTAGtTTTCGAGGAGCTCGGTGTGCTGCCAGACTA
TGCGCTTCGCGGCTTGAGCTACCAgGGGAATCACCAAGCCCANGCCGATACAGGCGCAGG
CGCTGCCATTGGTTCTTTCTGGCCATGACGTGATTGGCTTGGCGCAGACGGGTAGCGGCA
AGACGCTTGCCTTTCTACTGCCTGCAGTCACGCACATGGAGGCGCAGCGCTCGGTCGAG
>at03013
CCCTTAGGAAAGTAGCGCCTTGCAAGTCCATTCAGGCGTGCCGTCTCGCGGGGCTCGATC
CCGAAGGCCTGTTCCGCCTGCCGCATGAGATGCATGCAGACTGGAAGTCGTTCCAAGGGG
ACCAGGTTGTGCAGACGGGGTCCAGAGTCCCTTGGCAGCTTGATACCACGTCGAGCTGCG
CCATGGCGGGCTTTAACGGCGTAGAAACTGGTTCCCAACTCCCCCGTCCGTGCCTGAGGA
GGCAAAACCAGGGTATCTTCCAACTCTGCCAACGCCGCAGCAGCTGCCTCAGCGGGCAAC
TGGCAACTTCGAAAGCCAGAAGGCATGCCAGGTCGAGCCGGAACATCAGCAATCAACATC
GCGAGGCCATGTGCACCAATTGACAGTCCAAGAAATGCAATTAGACGAGTTGGCAAACGC
CGGTGCCATGGCCGTTTCCTAAACAGCTTTGGCAACATTAATGCCGCATCCTGGCGACAG
CG
>at03014
TTTTTTTTTTTTTGTTCTCCTGCAAACTACAACTCAGTCGAGAGCGCAGATCCCAGACCG
TGTCCGGTGGCAAGATGGTGTGCATCTAGGTGCCATGCACGCGTAAAATTGGTGCACAAG
CTGCCAATCGGAACGCACGCAACATGCGACCTGAGTCAGCATGGCGATGGTTGGCTCTGC
TTTGGAACATGCCCCCTCAGGCAGGTTCCCTAATCCAAAAGATCATTGCCNNNNNNNNNN
NNNNNNNNNNGACAGATGCAGGAGGAAAAGCTGGCAGTAACGCAATCAGCTTGGCAGCTT

GCCATGTGCCTTCCCCCTGCAATCGTCTAGTTCCGGTGAATTCAAACGCCGTCAAACAAC
TTCGGCGGTTGGCAGACGTGCAATAGCCCCGATACAGCAGGAGAGCCTGATGCTCAAGCA
ACCCACGAACCAGTTGCAGACACTGATGAACATCCCCGAGGAATCTCATCTGCCACAGCA
GCCATGGGCCTCTTCGCGCAGGGGATCGGCAGCAGCCCCTTGTACGACATGGTCCCGTAG
ACGAGCAGCAGGAAACCGGCCAGCTCCACCCAGCTCCAGAGTGTCCAGGCCTCACCGTCT
TGCTTCCTGCCGATAGCGTANAAGAGCAGAAGGTCCGCGATCCAGACACCAAGGGTACGG
CAGGCCTCCAGGATGGAGCGCAGGACTGCGCTCAGGTGCTTCGTGNACGCCCTGGCAGAG
ACGTTGTANATGGCGATGACCACAAAGTACACCACGACCCAGAGAAGCACCTGCGGCGAG
TTG
>at03015
CGCTCTGATTGGCCAATCAATGGTCAGCCCCCATGGAgTGATGGtTTgGGAGCGATGCCT
cCATCGGAATCTGCCgGTTcCCCtTcGCAAcCTGGTGGTTCTGCCAGGGGAACCCATCCA
CCATTCCGGCTGGACTTTGGATTCCTGCTCAGAGCCCCAGGGCCGCCGGAGGCAGCGCAT
GCCGGCAATGAAGGTNCCAATGACCCTATTGTCAAATTCATTGAAGATGCGTCGCCACTG
TAGCGTGCTAG
>at03016
GAgGGCAGCGCgGGCGGCGGCGGCATCTAGtTTGGttAAGGtAAGGGCAGGCCGGCCGGC
TGGGCCCAGACCTGGCCAAGGCCCAgGGGATCGGGGCCTCCCCGTCTTCCAGTTTTTGGT
TGAGCTCCTTCTCTTGGCGCAGGCTCTCTGCCTTGTCCTTCTTCCGCGCTCGCATCTCTC
CTTCCGGGCAGAGGTGGCAATGGCGGCACTCCTGACCATTTTTCGGCACCCTTGCGGCTT
CCAGAACCACGCGACATGGTCTGCACTCGCCGGTGCCGTGGGTGGCTGAGCCAACGGATG
GGAGGGCCAATTTTGGTTCCAGTGAAAGCGCCTGCGCGTCCATGTCGGCGACCTTCAGCG
ATGCCGGCTCTGCCAAGCCTGGAGGGGG
>at03017
CTCGTGGGAGGTATTACCGTGTCTCCGCGCCCATCGGCATCTGGAGCCCACGTGTGTGTT
GGGAGGCTCATCATCCTCAGCGGTTACCGGCGCTTCAGCAATGCCGCCTCCTTCAAACCC
CTGGGTACCACGTGTCGCATCATGACACCAGTCCTTCGCAGAAGAGGAGCTACTTGCGGG
GGGTCTGAGCCTCTGAGGCAGCTCGAAACTGGCAGGGTGACATGCAGTCTCCAGGTCAGA
GGTGGCACTGCTTCCCGAGGCGCAAGCCTCAGGTGAATTACCTTTGCGCCAGCCGCTGTT
AGAGCAGATGCCACACACTGCCGAATGTCATCCTCTCTCCAATCGATTGCGTTG
>at03018
AGGACAGAGGCCTCCACTGCAGCCGAGAGCGGCCAGGATCCAAGAGCACATTTCCAGGCA
GTGGTTGATGCCCGCCTGATGGGGATTTTCACCGTCAGCGTCCGCACTGACGAGATGGGC
GACGCTGCAGTCTTTGTCACAGGGCTGCTGGCAGCCGTGGAGGAGTACGCCCCCAGCACT
GCCAGCAGGGCCAACGCTGGACGGCCCTGGCATGTCCGCCGTATCCATAAGTGGACCTTC
AAGCGTCAGCTCCCCTCTGAGGACGGTGAGGAGCCAGGGGACTGGCAGGTTGTCGCTATG
AACAAGCGCCGCTGGCAGCCACCTGCCAACGTGGATGCCTAGGGCAGGGCAG
>at03019
CCGTTCCGGCTGAAAGGGTGAGATCCACGAGTTCGCTGAATGGCCACTTGGCCTTCCCAG
CCCCCAGGCAGCAATGTCGGACCAAAACGGGGATGCCAGTAAGCAGCTTCTTCAGGCTAC
CATGGCTCTGCAGAGGCGCATCGATGAGNTCGAGCAACAGCAGCAGAAAGACACTGGAGA
TGG
>at03020
TTGTTTTTTTTTCTTTTTTTTCCTACTCTTCCTTCGCTTGACGAGATGCACCTTCCTGCC
TGCCACAAGATGGAGCAATGATCAAGAGGACTCGAAAGTCAGCAACACGGTAGTTGATCA
ATCAGCTTGAAAGAAATTCCTTGGAATCCTTGAGGAAGGCCTTCGGGCCGAGCCATCTAT
CACTTGCCACATACTGCAGACTGGCACGGCATTGGTTACTGAGTCTGTGCAAGGGCATGT
CAGGCTCCTTCAACTCGATGTCGTTCTCGTCGAGAACGCGGACATCACCGAGCTGCTTTG
CACGCATCACTTCAGCACGGTGACGTACATCTGCCACTGTCGCGTCCGGCTTCTCCAGAT
GCACGTAGAAGCGCTCTTTGGCGCGGTCGATCCCAAAGTCCAGCCCGCTGGAGATGCGCA
CAAGAACCTGGAATTCTTTGCATCGCTGCTCCTGTTCACGATCACCCCGCCCGTACTTTG
GGCACGTACCAGAAACCTTCTCCAATTCTTGCCTGCCCTTCACTATCAAATCCATAGAAT
CAGCTCAGGCTGTAAACAGCCCGGGATCACGATGACACCGTTCGAGGGTGGACTGATCAC
CATGCGTCTCTGTGCAAGGGATGGCTTGAGCCAAAATGGCTACGG
>at03021

TTTTTTTTTTTTTTTTTTCCAGTTCAACTCATCATTGTCAGTAGGGTCTGGTTGCTGGTC
TGCCCACTGCTGGCTGCCACCAGAAGGGACATCACGCCGGACGTGCGCACAGAACCTTGG
ACCAGATGCTTGCAGACCCCTGGCCAGCATTGAGCTGGAATTGCGTCAACCCGCAGCTCA
AGCCTGTACAACCGTTGCTTGAAAGTCAACGTTTGCCACTAAAAATCTTGGCGATTCCGA
TCGCCGTTCGAATTCGAAGGCCACACCTCGTGCC
>at03022
TTTTTTTTTTTTTTTTTTGGTCATCTTCTTCCTCTGCCAGCATTCCGCCACACGACTTAT
TTCGTAAAAGCAGGCAGCATTCCGTACAGGACGTGCTGCGCCGTCAGCTGGCAATGCGAT
GCCTGTCTGAACTCACATCTGCCCCGACAATTTCTGGTCCTGGATTCATGCGGGGCCAGT
CTCAGACCATACTGCTTCTGTCGGGAACATACGTTCAACCTCCTGGATACGGCTGGGTGA
CATTCCTTTTGCCATCATCTCTCTTGGACCTACTTGCATGACTCGGTTGATCTTCAAAGG
TCCATAACCGCCTGCAATGATCTCCTCCAGATTGTAGGCACTGGTGATGGTCTCGAGCGA
CATGTTCGCCTTGTAAGCGTCATGCGCTGTGAAGCCAACACTTTTGAGCGGGTTGGCGTC
GAAACCGGCCTTGTAGAGAGCACTCATGTCAAATCCAACAGTTTTCAAGTCTTGTAGGCT
GAATCCAGCATCTCTCAGCGCGCTGGCCTGGAAGCCAGCTTCTTTCAACTGAAGCCCATT
GAAGCCGATGGCTTTCAGACTCGCAGGTCCTGCCCGCCGAAGCTGCTTCGCATCAAGGCC
GCCTCTCTCAAGGTCTTGAACGGTAAAGGTGCCCGACGTCAACTCCGTTACTGTACACCG
CTGGCCCCTGAAGACAACGCGGCTTGGACCACCGCCAAGAAATTCAAAGCAAATACAATT
CATGGAAGGCGGCCTGGGA
>at03023
TCTGCCAGATCACCATGCGCTCCGTGCCAGGCAACGATGGTCAGGACTATTGCGCACTGA
TCCACGAAGATGTCAGCGCGAAGTGCTCCCTGCAGCACCTCTTGGAGATTGTCGGCACAG
GAGACTATGTCAATCTGCTTCGGTCGATACGGATGAACGCGAACACCCCGGATCACATCA
TCAAGAATGCTCGGTCCATGATCCAGTTCATGCTGCAGGAGGAGCCATGCCGCGAGAGCA
GTCAAGCTCAAGCAACCCTTATCTCGAAGCCGTCTCTTCCAACTTGCGGAGGATGGGCGG
>at03024
GAATTGTACATGATCTCATATTGTTGTTGGCCGTTGCGTCAGGCACCTGCGGGCATCGGG
GGGTCCCTCGGGGGACCGCGCTGCCTCGGGAGGTGCACGAGCAAACACATCTCACTTAGG
CCTGAGTACTTGCTGTTTGGCGGCTCTCTGCCAGGACTTGCTCTCCAAAGTCCTTCCTGT
CGCAATGGGCCTCCCCGAGGACCGCACTCTGCAGCACCGAGATGGCCTCCACCGCATTGG
CGGGGTGGACTCCGCAGAGTGTTGCCGGCACCGCCGACCGCAGCTGCGGCGAGTCCGCGG
CAACGCCACCGATGGAGATGATGACGTCGGAGCCATCCCACGTCTCAGA
>at03025
AGCTCTGCGGACGACCTTGAAGGACCCCTCCAGCTGCTCGACGAGCCCCATGATCCGCTC
CAGCAGGGGGCCCCAGAGGTACCGAAGCCGCGGTTGGAGGCTGCCGCGAGTCGCAGCAGC
TCGCGCTTCCAGGCATCGCGCGCGGGGTCCTCCTCCATGCCAGGCCATGAATCCTTCTGC
TGCCCCAGGCCCGGCCCGAAAGGCTCGGCAACGTCGGTCGCACCCTCTGCGGCATCGCTT
GCAACAGGCTCCGCCGCGGCCGGCTGGCTTGCCGTCGTGTCAGAGGCGGGCGTG
>at03026
TCTTCCACTTCCTGGCCGACGGGAGGCGAACGCTCTCGCGGGAGCACGCGGTTCTTGGCG
AGTCGAACGCGGCTATCTTCAGCATCCCACTTCGCAAGTGCATTCTCGGCCACGACCTGC
AGCTTGCGCAGACCCTCCAGAGCGGCGCTCCGTGGGAGCGAGCAGTCAGCACCCCCGACT
CTTCGCTGCTGCCGCACGTGGCAGGCGGGATGACTCGGCGGCACAGCGCCCACAATCTCG
CCTCAATGGCAGAGGGCGGGAGCGAGGACGACGCCGGCCTGCACGGAGTTGTTCCTGATG
CTGACCCACTGCCAGACGAGGTGTTCAAGGGCCTTCTGAGAAGAGAATTGCAGTTGCAAC
TTTGTGGCTACAGGATCCCTGAGGAGCCACCAATATTCCCCCAAGGCTGTCCAAGGCGTC
TGCGGCTCAGGTCTGGTGCCCACCAGCTCAAGAAAAGAGGCCCTGCAAAGTGCGAAG
>at03027
GTCTGTCTAGCAGGACCTCAGTCACCTAAGTACTTATTAAATTAAACCGTATTCTACCAG
ACTGGACAACGCGCACGAGGGAGGCGCCTTCCAGGAAGAGTCTGTGTCTTCCGAAGGACG
CCTGCCCGTCATCTTCATACGTCAACTCCCACTCCCTGTCCCAGCCGTCCGTCAGAGTGT
TGCGATAGATCAGCAGTGACGCAAACCGGTCGTTTACCCACCACGAGTCAGGATGGCCCT
GTCCGCTCTCGAGGGTCCACGTACCCTGGAACGGTAGCAGGCCTGTCTGAGCTGCCAACT
GTCCCGCTGACCATAGTGAGACTGATGATGCCTCACGTGACACGGTGCCATCAGAATGGT
CGTTCTCAACGTCCGCAGAGCCGGAAGAACCCTCTGGAATCTCCATCCCGGTACGTTGCT

GCAACNCACTCAGAATGTCCGAGAACTGCAATG
>at03028
TTTTTTTTTTTTTTTTTTCGAGCGCAAGAGCTTCCCCCGCCCTCCCCGTCTCACCACAGG
CCTCAGGATGCCCTGCCGCAGCCGCGGCCACACCTCTTCTCTGCTGCAACTGCCCGGTGC
CAGCTGCCAGCTGCCCGGTGCCAGAGCTCAGCCGCTCAGCCCGCGATCCTCTGCCGCTGC
TCTTCGCTGATGACCCGGCAGGTGATCTTGACGCCCGAGAACGTGGAGCCGTCGAGCGCC
CGAGCCACCTCGTCGGCCTCCTTCTGGCTGCCGATCTGGGCCAGGGCGCAGGCGGGGCGG
CGCTGCTGGTAGACGCGGCCGCCCAAGATCTGGCTGCCGTGCTGGGTGACGAGGCGCCGC
ATGGAGCTCCAGTTCGACTTCGGGGGCAGGTCGCCGATCTCGATCCACGCGGTCTCTTCG
CTGCCCTTGTCCTTGTCGCTCCGCGCGGGCGGCGACCGGCTGCGGCGGGGCGACCGGCTG
CGGCGGTGCTCGCGCCTCGGGCTGCGAGAGGCGCTGCGTGAGCGGCGGGCACGCCTCGGC
CCGGGGCTGCGGGAATGGCTCCGGTCCGAGATCGGCATCGTCAAGGACAGCGGGGAGCGA
GTACCCGGTGCTTCCACTGTGCTGCAGCGGCTATCTTGGGAGGGGAGCTGCCAAGTAGCG
GAACAAATTTGACATCCGGGGGACGTCCAACACGTCTGCAAGCTCCT
>at03029
TTTTTTTTTTTTTTTTTTGACTCGCCACCACCCTGCATCCTCCGGCCCACTCCGCCGAGG
CAAGAGGCAGCCCGCAGCGAGCCGACCGAGAGCGGCCCGCCAACTGCGGCGACCTCACGC
CCAGCTCCGCCGCCCGCGGCCGCTCGCCGCGCCGAGGTCGAGCCGGCTCGCCCCCCCGGC
GCCGCGCCGCCCCTCCTCCGCCGCCCTCAGCGGCGGCAGCCTCGGCGGTGGGCGCGCCAC
CAGGGGGGTCCCCC
>at03030
AAGCCGGCGACAGACGGGGCGCATGCCACCCTCCTGGCCGCCAGGCCCAACCCAAATAGT
CCTTGCCTTTGGTCGTCCACGGCACTGCTCTGCATCGACTTCCAGAAGGACTTCCTCGAC
GAGGGTGGCTTCGGCCACGCCCTGGGCAACGACGTCTCCAAGCTCCACGAAGACTGCTTC
CCCGGCGGGGCGGTGCTGCTGGAGGCCGCTCGGAACTGCGGCCTGACTATCGTCCACACC
AAGGAGGCACACCGTGAAGACCTCCTAGATTGCCCGGCCTCGAAGATGTGCGGGCCCCGG
GCACCGCCGGAGGGCAAGCGCGTGGGGGAGGTGTTGGTGGAGGGCATGGGCAGGCTGTTG
GTCCATGGCTCGCCTGGCAACGACTTCATCGACGATGCAAGCCCCTTGAAGGTGAGAAGG
TCGTGTGCAAGCCTGGAAGGGCGCCTTCTTCATGACAGACCTCCACGAGTTCCTGTACTC
CAAGGGCATCAG
>at03031
CACTCCTGGTCGCGCTTGGGGGCCCCGCCGTAGGCGCACGCCGCAGTGATCTGCACCGGC
TTGCCGAACTTCTCCGCGTCGGTGTAGATCTGGTGGCAGAGCTCGCGCGTGGGGGTCATC
GTCAGCAAGGCGGGGCCCAGCGCCGTGTCCACCTGGTTGCTCTTCTTCAGCTTCCTGAGC
TTGATGAAGCCCGGGAGCAGGAAGGCGAGGGTCTTGCCGGAGCCCGTCTTGGCCACGCCC
ACCAGGTCCTGGCCGGACTGCAGCACGGGCCAGGAGTAGGCCTGGACGATCGTGGGGTTC
TGGAAGCCGCAGCCATCGCTGTCCGTGCTCGTCTGCAAGAACGCTATCAGGCCGCCAGGG
AAGCCCTTCTGCTCCTTCACCTTCTCCCAAGTGTCCCACGCATCTACCGTGCCACTGATG
TCCTTGTCCCAAGCATCCCAAACCCGCACAAGATCAGTGGGCATGGCAA
>at03032
TCTCTGGATTCTGCAGGCCCTCGCGGAGAACGCGCGAGAGCTGGCGGACATCAGAGCGCG
AGAGAGCGCCAGCCCAGTGCCTGGCGTCCCGTGGTACGTTGACGCGGCCGTGCAGGACAG
CAGCATTCCCACCCTCCATAGCGTGGACAACATGGAGCATGCCCACACAACCGCACACAA
GGTGGCAGAGTGCGCCACAGAGCTCGGCATCTCCAGGCATCTTCAGATTCACGTGGCCGA
CGCTTTTGACATCTTTGATGCGTCCACCGCGGCCGGACAGACTCTGGCAAGCTTGCAGTG
GGACATACTTTGGTTTGACGGGATCAGTGCCTGCTCAGAATGGCCGGCCTACTTCCGTGA
AGCGTGGTGCAGACTCAAAGACCGAGGCTTGGCGTTGGTGCACTCCTCTCTGACCAACAC
AACTACGCGGTCGTGGCTGTCTGCTCTCATGGAGGAGGCCGCCGTCCAGGGACCCTGGGT
TTCCTTCGAGGTCGCTCCAGCTCCTGGCTCTGAAGTTTCCTTCGGTGAGCTCGCGGCGCG
CATCAAAGAGTCTCTGCCGGCATACACGTGGAGGCACAAGGTGGAGGCGGGAAGGCTGGT
CTTGCAGGTGGCGTTGGCCCCGGGCTGCAGTGCATTCGACGAGGGTCTTGCACTTGCGGA
TGAAGTCGCTGGGGTTGATGGCGTGGGCGCCGTCACGAACGTGCTGCCGGCGAAGAGGTG
GCTCGACGCGCTGCACCACGTGGGGCTTCTTGAGCCGCACAAGAGGATGCAGAACAGCGT
TTCGATACTGCAGAAGCGCGCAGCTGGTCATGAACTGTCAG
>at03033

TTTTTTTTTTTCTTTTTTGTCAGTTCTTTGGACGTTGGGAGGGTGGAGCCAAAGCTGGCG
TTGGCAGGCGCCGCGCCCCGAAGACAGCGGAGGCTAGGAGCTCACTGCTGCCGGCAGCCC
CCCTCCTCAGCCCGCTCTCTCGCTAAGGCTGCGTCACGTGAGGAGAGCGCACGAGGTGCG
TGCTCCCTCTCGTAGTAGGGCCACATCACATCCTGCGGCGAGGCACTGTGGACTAAGCCC
ACAAAGTGGCCCACCGCATGCAGCAACACAGGCTGAAGGCGAACTGCTTGCGGTCTCCGC
AATGGAATTTCATCCCTTTGCAGCAGCCAATGCTCTCCTGCGTCCAGTGTGATCTTGCCA
TCCGTCACCTCTGACAGCTGACCCCCTTGCACGGCAAGGCCACCGTGGAAGTCCGCACTG
CTTGCCGACAGGTCCGCAAACCTGAGGACAACGTCAGTTCGCGGCCAGCTACCGTCTGCG
AGCTCAAACTTCACCCCTAAGGCCTCGCCCCACTGCGCGAAGATCTGCGAGATCTCGGCC
AAGGCAAGCTCCCTAT
>at03034
CTCACGCGACGAATGAAGCTCCACTGCAGGCCCCTCGCGGGGTGCACCAGGCGTCCCATC
TCCGTGCGAGAGGAAGTACCGTCAGCATGGCTCACTGCAGTGGTCACTTTCACTTGACCC
TCCTCCTCCATGCCGTGCTGTGCCACAGAGCTTGCATGGCAACCAGGAGCAGCGTCCGAG
GACTCCATGGGCAGCGATGGCGGCAAGGCGTATCCAACCTGAGCAGTGCTGTCCCGCTCC
AGTCTGAGCACCATTCATGGTCGCTCTCGACGCCGAGTCCGAAAACGNGAGGCGGCGCAG
GCATCTGGCCGGCATGGACGCGTCCAGCCGCGCTT
>at03035
CTACGCCTGCGTCCTGCTCCTCGGCAGCGGCGTTGGCCCGGGCGTTCGGGGTGAAGGCCG
GGCTGCTGGAGGCCGAGCGGCGGGGCGGCTGCCGACTCATCGCCTTCCCGCACCCCTCTG
GCGTGAGCCACTACTGGAACGAGCCGGCTAACGTGCAGCGCGCTGCCAAGGTCCTCAAGT
CTTCCCTTGCTGGATGCGCCGGGGTCGCGATGGCGGTCGCGCGAAGGGTCGTAGCGCACG
CACGCTGCTGCCG
>at03036
CTTTTCAAGTGTGACCGTGAGTTCGCCGACCATGGCATCTATCATCGTCTTGCCAGACTC
GCGGCAAGCCACTGTCACGATGGTCTCGGGATTCTCAAGGATGAAGCGCTGCAAGACGCG
CAAAAGCTGGCGGCGCTGCGCAAAGGAAGACTCCGACCAGGCCTGCTGCGCTCTGCGCGC
TCGGGCGACGATCTGCTGCACGTCTTGCTCTGAATGTGCCGGCATCTCCCCCCAGGAAAT
CCATCGTGGACGGGTCCCAGCAGGGAATGGCCGATTTGGACTTCCCCCACTCGCTTGCGT
AGTCAAACTTTCGGCGGCCACCCAAGTCGCGTGAACGCTCCTCCTCTTCGGCCTGCACGA
TAACTCGAGGTGCCGACCCAAATTCGGTCAATAGGTCTTGTCCCCATGATCGCCACGATC
TGACTGCAAGAAACGCCAACAGGAGCAGCACCAAAGCATCGCATCCCAAATACGTGGCGC
AGAAAGAGTCCAAGCAGGACGCTGCCCAGGCAAGCCACCCCATCCGGTCAGCTTGTGGCT
CAAGGTGAGGATGGCGAATTGAGCTTGAGCCAAAATGGCTACGG
>at03037
GTCACCGTGCGAAGACGTCGCTGGCGTCTGTGTTGCTCATCAGGAGAGGGGGACACACTG
TCGGAGAGTGCCGACTCCTCAGCATACTTGCGAAGATGCGCATCGAAAGATGGTAGCACG
AAGTGCGAGCACAGGATATCGAAGACGTCAAGATCCGAGGCCTCTTCGAAGGAGAGCTTC
CTCCCGTAGTAGTAATTAGTAACGGAAACCGTACGTTCGGGTACGGGTAGGTTTACGTTA
ACGACCGACCTAGTCGTCGTAAGT
>at03038
TCGTACCGCAGCAAGACAGTTTGTTCGGATGGCAAAGAAGAAGCAGCAGCAGATAAGACT
CTGCGCAGGCTTTGCGCAAGGCAGTTGCCGTTTTGGGGACAAGTGCTGGTTTTCCCATGA
GGCAGGTCGGGACGTGCCGCCGCCGCATGCGGAGCCAGCAGCTGGAAATCAAGACGGCGG
CATCAGAGATGGCGCCAAGGAGGCCACGATCCAGGAGCTCAATGCAAGACTGATGGCTGC
AGTTGCCGAGCGCGAGGCAGAGCTCTCAGCCAGCCTGCAAAGAGCCAACGAAGAGCTGTC
AAGACTTCGAGCGCAGAAGGCGGACTCTGAGAAGGCGTCGAGTCCCGGATAGCGGAGCTG
TCTTCCCAGAACGACGAGCTGACGAGCATCAATTATCAGCTCGCGCAGAAGGTGGTGCAG
CTGAAGGCCCGCATCGCCCACCTGGAGGACGACGCGCGGTTGAAGGAGGGCCGCCAGCGG
CGAACAGCCTTGGCGCCCTCCCCACAGGCTCGAGCGGCGCCGAGTGGCAGTTCGAGAGCT
GCCCGGGCGACTGGGTGGGCCTGCCGGCGGAGCGGAGCTCGGAGCTCATGCAGCGTTACG
CGGAATATGCAGATGGTGGTGCGGCGACAGTCCGCATG
>at03039
CCAGTAGAGCATGAAGAGCACAGGCTGCGCCACTACGATGCCTGCCGCGAGAGATGGGAG
GAACGCAAGCGGAGCCAGGACTGCGCCAGTGTAAGGCTCAACACCAATTGCAGGGCAGCC

GTTCTGAAAGCAGTCCATCGAGACCATCAAGGAGCCGTTGCAGACACCACAGCAGCAAGC
GGAGAGGAAGCCAAAGATGACAGTG
>at03040
TTTTTTTTTTTTTTTTTTGAAGCACAAATCCGCAATGAACTGTCACCGTTTGCGGGCCGC
AAGTTGATGGACATGAATGCAGTCGTCCATGCCACTCTCAATCAGAGTGACAACGATCAA
TGCAAGATCGGGAAAAAGCTGCACCTGTAAGAGGCAGCACGCCTGAAGTGCAGCTCAAGA
GCAAAAGGTTTAGAAATCCTGATGAAACTGTTTCAAAAGGATTGTGCACACCATGGAAGA
AGGGGGCAAAAGAACCGCTGCAGCCAGGTCAGCTGATTCATCAGAGGTAGCCCACAGGAG
GACCGAAAACTCGTGCACAGGTTTCTCATCAACGAAAGCAGCCTTCCCCTGCAAGCCGAG
CCTTGCCTCGACAGGTTAAGGACGCAGCTCGCTCAACTGGAGGGGCGCGGTGGGGGAGGC
GGCGCAGCTCCCCACGCGCTGCCTGCAAACTTCTTACCACGGAAGATTGGCCTGTGGTGG
GAATAGTCGTCAAAGGCTGCTTTCCCCCAGATGCTGTAACCGTAACCTGAGTCGCCGACG
CCGCCGAAGCCGATGTTGAAATTGCTTTGCATGCGCTGCGGCCCACAATTGACGGCGAGG
TT
>at03041
ACTGGTGGAGCCGGCGCATCCGGCGACGAGGCACATCTGGGAGGAGAAGCTGGGCTACAC
TGTCTGCGCAATGGCGTCGCTCGCGGACTTGACCGACAAAGCGGGAGCCAAGCCGTTCGC
AGGGCTGAACAGGTCCGAGCAACTTGCAATCTGCGAGAAGGCCATCAACCGCTCAGGCTT
CTGGTATTCCGCGTGGGCCACGCCTCTCATCGTTCCTGCAGCCTTGCTTCCCTGCCGCCC
TGTCTTCTTCTATCGCTACCCGGTGCTCACAGAAGCCCCGGAGGCCAAGTCGTGACGCGA
AGTCGCGCTGCAGCTCGTTTCACGCCTTGCTATTGCTTGAAGGG
>at03042
TTGTTTTTTTTTCTTTTTTCACTCGTCGCACTCTGCAAGAGGCCCTGGGCTGGTGCTGGA
GCCTTCGAAGCCCCCGCGCCCCCCCCTTGCAGCTCTCGCCTCACAACTCGCCGACCAACC
AGCTGGGAGCTACCCCGCCACCCAGGACAAGCAGGAAGAGGAGCACCGCCACGAACGCCA
GCCCCAGAAGCCCACGATCCTGAAGGTTGCCTTCGTCTGCCCCGACATGGGTTCGCTGGT
GGGCTCCGGAGACTCGTCATCGCCGGTGCCAATTGCAGGCCGCATGTGCCGATGGAGGTC
ACCCACTCTGGTTGTATCTGGCCGTCTGGACGAGCCAATGCCAGGCCGCGATGTCGTTGC
GGCCTGGTGGCCGAGATTCTGCCGGGGGAGCCGAACAGCGCCAGCCCAGCCGAGCACAGC
ATTCACTGGCAGCAGCAGGCCGGCGGCCAGGCAGACGCAGCAGAACACCGAGCCATGAGA
GGCTCGGACGCTCCGGCGGCCGTCATGGGCCTGGTGCCGCCTCGCCATGATAACCTTCTC
GCCACTGGACAGCCAGCTGGACGCAGCGGCTGCCATGGCAGCTCTA
>at03043
TTTGTTTTTTTCTTTTTTTTTTGCCATGCCTAGCCTTCAAAAAGGTTAGGAATTGCGCT
CTCTGCTGCCGGCTCAGAGCCCCCCGGCCCAGGCGAGCTCAGCGGTTGGCCTTGGCCACG
CGCTCGATCTCGTCCTTCTTCTTGATGGCGTAGCTGTTGGATGACTCCTTTGCAGCATTC
ATGACCTCGTCAGCCAGGCACTCAGCGATTGACTTGATGTTGCGGAAAGCGCTGTTCCGG
GTGCCTGTGGTGGATGAGGTAAGGANATCGCCTGGTTGACGCGACGCAGCGGGGACACAT
CCACGGCTTGGCGCCGCACGACTCCAGCGGAGCCAACTCGCGTCGAGTCTTCGCGAGGTC
CGCCATTCTTGACGGCGTCAACATAAACCTGGATGGGGTTCTTGTCGGTCAGGAGGTGGA
TGATCTCGAAGGCGTGCTTGACAATGCGCACCGCCATGAGCTTCTTGCCGTTGTTCCGGC
CGTGCATCATCATCGAGCAAACCAGGCGCTCCACGATGGGGCAGAGGGCCTTGCGGAAGC
GCTTCTGCTGGTAGCGGCCCTGTGTGTGTGGCAGGAAGGTCTGGGATGGCTTCGCACTCA
CTGCGATGTAGTCAAACAAGCTGAGGTCTGAGACCATGACGTCATCGTAGGACCAGCGGC
CGAAGAGCTTCACCTCGCAGCTCGACATCTGGACTGATCCGCTGCTGCACTTGAAGGAGC
AGATGCCTCCAAAAGCGTCCGAAGCCGGTGACTTGCTTGAGCCAAAATGGCTTGAGCCAA
AATGGCTACGG
>at03044
CGATTCGCATCTGAAGGGCTTCTCAGTGACTCAGAGTTCATTCTTCAAGCAGTCACTGAA
AATTGGGGTCGTGATTCCTTCAAACATGAGGTCATCAACAGTGCGCGCGCCTTCGAGTGC
GTTCTTTTAGACGCACCGCCCTCATGGTTTTTGCAGGAGGGCTTCTGCAGTGCAATGGGC
GAAGGTGGTGGGCATCACCACCTACAGTTTCTAAGCTGGTTTGACTTGGTAGTGAGGGCA
GATGGCAGTGGAGACCTAGAGGAAGGATTCGTACGCTTATGCTTGCACGCTGCAACCAGA
CATGAATGTATTCTTGAGCACTTGCTTCGGCAGGGTCCGACAAGCTTGAAGAAAGAGCTC
CATTTGGCCGAAATCAAGCCGGATGCTCCGGACCTGCAGAGTGCGATAGAAGACGACTCG

AACGAGCCTGTCCTCGATTGCGGTGATCCACGTTCACCTGCATTGGCAATGCCGTTTGAA
ACACGCGGCCCGGGCTGCATCGATGCGTCGTTTGGCGAATCTGATCCACAAATCCGACTT
GGCAGTTCGCGTACCACAGGCCAGGAGACTGGACTCCCATTTGTCGGACACCACACGGAT
ACAAGCCGTTTTTGGAGAAGCGCATGACACGCCTATTCTCGCGCTTCTTTTTCCCCCAGA
GGGGCACCACCAAAAAGCTACGGATATCGAGGAGGACATCCATACTTCAAGCCAAGTGGT
TGGCAACGCTTTTCAGTGAGTAGTGAAGCAGCCATACTTCGATCA
>at03045
TTTTTTTTTTTTTTTTTTGGCTTTCCTTGGGAACATTCAGGGATGCCTGCCGTTGTCATG
CCGCCGCATGATGTCCCACGCTGGCTGCATACGCCCCGCAAGCGCTCTCAGGGCAAGGTC
GTAGTGGGGATGGTCTCATGATCTATGTGACAGGAGCGATAATGTCGGCATTCGTTTGCA
GCAAGGCCAGAACAAGGAGATGAAAGTGCTGGGAGGGAGGCAGAGCTGATCACTGCGAGG
GATCTACGCGGCGAATCTCCCGCACCGTCTTCGAGTCCTCGTTCTTCTGGGTCATCTTAG
AAAAGTCGACGGTGTACACGCTGCCGTAGTCCTTGTTGAACGTGAACTCTGTGGTCTTGA
ACTTCGAAGCTCGGCCTTTCGACTTGAAGGCCACCTCCAGCATCATGGAGTCTTCTTTGA
CATACCGACACCCAGCCGGCCTTGTCCGCCTTGAACTCCCAATAGACAGCATTCTTGCCC
CAGACCTTGGCCGTCCTCGCGATCTTCTTAGGAGCCATGACGGCCCAACCGCACAGGGAC
AAGGAGCACGATCAGCGATACAAGCCAGGGGCTAGGCTGCCATGGCTTGAGCCAAAATGG
ACTTGAGCCAAAATGGCTACGG
>at03046
TGGTTTCTTTCGTTCTCTGTTGTGAGTTGATTTCATCACTAATCAAAAAATTGTTCGATT
TCTGTTGTTGTGCATCTTTTATTCAATTCAGATGCCAAGAAACTATAATAATATTTATTG
TTTATGAACGCGTACACTGTGCACTTGTAATTAATGTATACGAATTTTAAAAAAAAAAAA
NAAAAAACAAAAAAAANAAAAAAAANAAAAAAAAANAAAAAAAANAAAAAAAANAAAAAA
ANAAAAAANAAACAA
>at03047
ACTCAAAAAATGCCCAGATGTTTGGAGCACATGGGGTCTGGCGGCCGTTGCACACGGAAA
CAAAATGTCACAAAGAGACCAGTAAGTTTCTATTAACCATGATCTTCCTGTGACCCGCTT
GCATTTCCAGCCGCCGTGCACTGTACAACCTCCTGCTCATGCCAGAGGGCCTGTTTGCCA
TTGGCAAACCGCATCTTGATAAGGTGCCATATACCTTATTCTGAAACCGTTTGTAAGCAT
CCCACTTAGCGCCGGGACTCCTCCAGGACCTTCTTGATGCGCTCCGGGCCGGAGGTGTCG
CGGTCCAGGCGCGCCTGCTCCTGCTTGCCCCACTGCTCCATCTCGGCCTTCATCTCCACG
CGCGCCTTCTCGTACTCCGCGTTCCAGTCCGCGAAGGCCTTCTGCGCCTCGTCCTTGCTC
TCGAAGACACCCAGAAGGTACTTCATGCCAAACGCATCGATGGCCTCAGACTTGTAAAGC
ACGTCGGCCGTGCCCCAGCTGTGCCTGTACTCGCCCTCAACGTTCGCGGGGATCTCCGCG
AGCTCCGCGAGGACCTCCTCGTCCAGGGGGGGCAGGGGCTTCTTGGTCCTCTTGGGCAGC
TCCTCCCAGATGCCCTTGAGCACGTCCGCCTTGGTCTTGCCCGGCATGTCCGCCTGGGCG
CGGTAGAAGGCCTTCATGTTGCTCGCCTCCGTCTCCACCTGGTCGCGCGCCTCCACGTAC
TTGTCGATCAGGTTCTTCATGTGGTGCGCCGCGGCCGCCGGCAGCTTGTCCTTCCTCCAG
GCCGCAATCTCTTCCTCCGAGAGCTCGTTTTTGGCGTCCTCGACGCTGTCCGCGATCATC
ATGGACATGGTGTGCAGCTGGTTCGCCAGGACCTGGGGAATGGGAAGCTCGATGTTCTCG
CCCACGGGCGGGATGCCCTGCTTCACAGTCTCCTCCATCACGGACTTGGCGCCGGGGGGG
CAGCTGGGGTCCGCGATGGCCTTCTTGATGAACTCCAGGACGTCCAGCGCCTTCTGGTGC
GACTCCTCGTCGTCGTTCTGCACGACCCACTTCTCCGTCAGCGGCGAGCTCTGCGGGAAC
AGCTCGCCCTTGCGCTGCGCCGCGCGGATGTTCATCGTGGCGAAGTGGTAGCGGCGGTCG
GCCTCGCGGTTCACCTCGACGACCCTGTTCTTGAGGCTGAAGTCCATCTTCTTCTCCGAC
GCGCGCTTCGTGAGGCCGTCGTAGTCAGGCGTCCGGGCGGCGACCTCCTTCTTGTAGTCC
TCGGCCTCCGCGTCGAAGAACTCGTCGGACTTGCGGTACTTCTTGTCGAACTCGTTCTTC
GCCTGGTTCTGCACCATGGCCCTCTCCTCGGGGCTCAGGGAGGAGCGCCACGCCTCGAAG
TCCTCCTTGAAGCCCCGCAGGGCTGTGCCGGCGCGCGAGCGCAGTGCCGGGGCTGATGGC
ACGCCTGTCACAAAGGCCGCAGTGTTTGCAACCCCGGAACACAGCTGCCACGCACCCAGC
CCCACGAGCAGGGCTGGCAGCAAAGCCGATGTGCGCTGTGCTGTTGCCATGTCGCCTGCA
CGGCTGGCGTGTAGGATGTCCACACGCGCGCGGAGGTGGGCGAGGTGCTCTTGAGCTGAA
ATGGCAAAACTTGAGCCAAAATGGCTACGG
>at03048
CCGTAGCCATTTTGGCTCAAGAACAGAGGGGGCAGGGCATTCCCCCCAGTCTTGGTGTTG

TTTGCACCAGGTTCTGTTCATGGGGAATGCGCCTGCTGCTGCGCCAACCACAAAGGGCAA
GTTCAAGCGCGGCTTCTGCCTCATTCCAGACAGGCCGGATATCTACAGCAACCAGCACCG
CTATGACAAGTTCCGAGACCTGCCCGAGGCAACATACCCTGTGAACGCAGTGGCTGTGGC
CGAGGTTGCAGCCATCCTTGCCGCGGCCACTCTGGGGACAAGCGACGAGGTTCACTTGTG
GGATCTGATCACCAAGGAGCTCAAGGTGTCGCTGCACGCCCACAGCTCACATGTCTGGGA
CTTGGAGTTCTCTCCCAACGAGACGACGTTGGCAACTGCCAGCACGACCACACCGTCCGA
TTATGGGACACTTACACTGGTGAGCCTATAGGTATT
>at03049
CCGTAGCCATTTTGGCTCAAGCCATCTTTCGAGGCATTTTTAATCCCGATCTGCTGAGAT
TGGCAAGGACCTATGGCAAGGAAGGTGTCTCTTGCGTTGCTTCTGGCCGTGCCGAAGGCT
TCCCTAGCATTTGTCCAGGGCCCGGCTCCACGTGACGTGCCCCAGCAGGTGCCCTCAGCT
GGCCAGTTCCTGTCGCGCAGCCTCCTTGAGGCGCCTGATGTGGAGGCGCCAGAGGTTGAC
CTGCAGCCGAGGTCAT
>at03050
TTTTTTTTTTTTTTTTTGCAGTGCACCTTGTCATTGCAAGAGCGCATCGGCCAGAGTGC
ATCAAGTGCATCGCCAATCACGATGCACGTCGCAAAACGGACCTCATAGCTAAACAAAAG
CTCTCAGAGAAGGAAGGGGATAAACGCCCTCCTTTGCTTCGGGTAGGTGTCCTTGAACTT
CTGCAAGTACCACCTGTGGTGCTCGATGGCCCTGGGCCCAACGTTGCAGGCAGTGCAGAA
GGCGAAGGTCACCCCGGGGAGGGCCCCGCCCATCGCCACGGCGAAGCCGGCCCACTCCAG
GGTCTCCCCGAAGAGGTTGGCGCCGGAGACGTACTCGAACATGCCTCCGCGCGGGATCTT
GTACCCTGTCTCGCCAGGTTTCCGCAGGTTCCCGGCAGCAACTTCACCTGCTGCGCGTCG
ATGCGCCAATGCTCCTTGATCCCCATCAGCTCGACCTGCTCCAGCACCTGTCCAAGAGCG
GGAGCCTTATTTTTTGATAGGGAGAATGGACCTCCAGTGCTTTGAACATTCANAAAAATA
GCCCAAAAAAGCGTCAAAAAAGCGTCATCCTGAGGTCTGCACACTCTGTGCAGGAGTCAA
AGATCTGTCCAGTTGCTGTTGTCGCGGGGAGGGATTCAGGGACAGACTCCGGTTGGGTCG
GTCTGCTCTGGGAGCCNGCGCTCCGAGATGACAGATTGAACTC
>at03051
TTTTTTTTTTTTTTTTTCAAGGGACATCCTGATGGCATTCTCACGTCCACTGCCCGAGC
GTCAATAGATCCAGGGAGTGCCTTGCGATTTGTACAAAGGAATGTGGCCCCTACAATCCT
ACAAATAAACGATCGCCGAAAAATGAACCATGCTGCCAACCGATGTGCTGCATCAACACG
TGCATGGGCATCACCGGAAACCTCCTAAGTTTGTCGCGTCGGCAGTCTTGCTACCGCCCT
ATCTCTCTCACCGTGGACTTCAAGAAGACCCTGGCTGCGCCGCATGGTTCCGACTGCTTG
GCTGCTCAATCATGCTTTCCAAGCTGGCCTCAATTGCATCAAATGCTGCATCAAATGCTG
CTGCCTCTGCGTGGGCCACCTCGTTCGACTCGCCATCATCACTGCTCTCTGCATCCTCCT
CGTCGATCTCCTCTTCACAATCAACATCATCATTGGAAAGCTCCGGCTGCACAGGCACGT
TCATCCCTCCCTCCTGGCTCTCAACGTCGCTGTCAGGCTCATCGTCTTCCTCCTCAGCAG
ATTCGTCGTCGTGAAACCGCATCAGCGCATGGCGACCAATATGTCCACGATTTCCAAGGA
TTCTTCGGAGTAAG
>at03052
TTTTTTTTTTTTTTTTTCCATCGTTAGCGCTAGCCAGCCGCCCAGCCCTCGGGCTCGCC
CTCCCCGGCCGCCGCAGCCTCCACCGCGCAGGGGCCGCCCCCCGCCGCCCCCGCGGCCTG
TGCGTCAGCGGGGGGCCGGGGTGCCTGGCCTACGAGGGGGCCTACCCTCTCAAGGAGAAC
GTCCCGCACCATGACCCGGATCTCGCGCGAGTTCACGACGGTCAGCTCGCTCAGCAGGGG
GAAGAGCTCCTGGGCGTGCCTTGCGAACTGCCCGGAAGGCAGGTCCTTGAGGCCTCTGAG
GAGCACTTCCGAGATGATGCGCTCAAGCCCCGAGATCTCGCGCTCCATCTCCACGTCTCT
GTCCTTGGCTGGCGCGTCGGCCGGCTGCGGGTCTTGCGCATGCCTCGCATCAGTCAGCTC
CTGGAGGTGCTGCTCCTTGCTCACGTAGTTACGGAGCACCGAGGTAGAGAGGTGCATCAG
CCGCTTCACAGCTTGATCCGCATGCTCGCTACCGTGCATTCGTACGTCGCCCTGGATCCG
AAAGAGGACCTTCAGGGAGCAGCTCAGCGCCTCCCGCTCCTGCTTCAGCAACCCTGGCAG
CTGCCTCATCTCCCGCATGAAGCCTAAACGCTTCAGTGTCTGCCTCAGTTCANTCTGCTG
GTTGAATTCCTGAGCGAACTCAAAAGACCGCTGGAGAGCATCCA
>at03053
CGTCCAAGGGGACTGGGGCTGAGGAGCTGCACCAGTCCAACGCCCAGCAGAGGATGGAGT
TCTTCGAGAAAGCCGACACGAACGGCGACGGCCTGCTCACGAAGGCGGAGCTGAAGGAGG
CGATGACGAGGGGCAAGGTCTTCGACTTGAAGCTGCCAGGAGAAAGTCCAACGGGAGAGA

CGCCTGCGAAGGACGGCAGCGCAGGCCATGACACCGACGAGCTGTGAAGGGGGGNGGGGC
TGTCGCG
>at03054
CCTTCCGGCTCAATGCCACCTGAGGAGTTGCCTGATGCAACGGAACTGAATTGAGCCTCG
TCCATCATTTGGATAGCACGCCAAGAAGGTGGGCCCGGCCATGCGGCTACGGGAGCTGTC
GCTCACCTGCGTTCCCGCAGGCTCCTACCTCTTCGCAGTGGTTGCGCTGGCAGTCGTGGC
TGCCACGGCACTTCTGGTTGGCAGGGAGCGGCCTGCCGCGGAGGCCACAGGCAGGCGAGA
GGCCCATCTGGATAACGCAAAGTTCCTGGCATCGGTGCTCGTTGTCACTGGGCACCTTGC
CAGCATGATCGAGAGCAGTGGAAAGGAGGAGCACAGCCGGGTCCTCAGCCCCAGCACTGC
CGTGCAGAAGGCCATCTTCTTCCACGTTCCCTCTTCTGCCTT
>at03055
GCGAAGAACAGGAAGCTCGTGAACGTGGTTGACCCACACATCAAGAAGGATGACAAGTTC
CACGTTTACACGGAGGTGCGGGACCAGGGGTTCTTCGTGAAGAAGAAAGACTGGAGGATG
ATGGACGACCCCACGGACGCGAAGCCCGAGGACTGGGTGGAGGTGGAGAAGATCCCCCGA
CCCCACGGCCACGCCGCCGGACGTCTGGAACGAGGAGGAGGACGGGAAGTGGACCCCGCC
GATCATCGACAATCCAGAATACAAGGGTTCGTGGAAGGCGCGCCAGATCACCGACCCGAA
CTCGCCAATCTCGGACTTCGAGGTTGGTGCTGGCCAGGGACTTCCCTCTACCCCGACTTC
ACTGATCCCAAGATGCGTGAGTATTGGGCTCGCAGTTTGCTCTCGACAGTACAAAGGCAC
CAATGCGGACACGTACG
>at03056
TTTTTTTTTTTTTCTTTACACAGAGAACCATTGGCCCTTACAGCAATGCCAACTTCGGTC
ATGCGGCCCTTGGCGATCACTAGATCTGGCGCTCGATACCACGCCTGGTGGCGGGCCGTG
GCGACAAGACAAAATGCCAGAGCTGGTTGATCCGCAACTCGAAGAGCGCCTCGCACATCT
TCAAGTAGTAGTCCCACATGCGCTGGAACTCGGGCGTGTATGCCGTTGGATTCTCCCGGA
AGAACGCCTCCGAATTCACAGCCCAAGCCGCCAAGGTCTTGGCATAGTCATACCCAAAGT
TGTGGAAGTCTTCCAGGATGAAGTCATTCTCTATGGCAGCTGAGACTGAAGACAGCGCGG
GAATGACAGCACCAGGAAATATGTACTTGTCGAGGAATGGGTCACTGTTCGGCACGAAGT
CAGGCTCGCCAATGGTGTGCACGACTGCAAGGCCATCTGGCTTCAGCAAGCGCTTTATGC
ATTTGAACAGTGTGGCGTGATTCTTGAAGCCGACATGCGCCAGCATGCCAATGCTGTAGA
CGCGATCATATCTGCCCACATTGGCTGGGTTCTCACAATGGTCGCGGTAGTCTTCCCACA
CAAACTTCAGCCCTGTCTTTGCAAGGTTTGCCTGTGCCCACTTCACTTGCTCCTCGCTGA
GGGAGACGCCAGTGACCTCGACACGTCCATGCTTGTGCACGTAGGCAGCTGCGGTGCCCC
AGCCCATGCCAAGGTCTGCCACCTTCATGCCATCCTGCAACTCAAGCTTTCGCCGTATCA
AGTCAAACTTGTTGGTCTGAGCCTCGTCCAGCGTCGTCGCGTTGCGCCAGTAGGCGCAGG
TGTACGACATCGAGGGCCCCAGCATCCTCTCGTACAGGTCGTTGCCCGCGTCATAATGCT
GCTTCGTCACGCGCGTAGACCGCTCCCGAGTCTGCTGGTTGAGAAGGCGCCCGGCCAAGG
CAGTGGGCAGGTTGATCATGTCCAAGAAAAAGACCATGTAGTCCGAACCCTCCTTCCGAC
GCGTCCCAAGAGCAGTGCCGTTCAACAGAGTCAGGAAGAAGGGAAGCGGGTCCACCTTGA
TCTTGCCGTTCATGTAGCTCTCGGCAACCCCCAAATTGCCCTTCGTGATGATGTCATACC
AAGCAGTTGGGTCGCGGAACTCGACATGGTCATACAATGAGTCGGGTATGCCGCCAACCT
TCACAATGTACCTTGCGAGGGCCTCGGCAATGTGAGGCTGCGTCAAGGCAGCCTGCAGCA
AACCCGCCGCGGCCGCCAGTAGCAGCACCGGCACCCCACAGAGCCAGAGCCGCCGGCCCA
TCTGTCCGAGGTGTCTGCGCTCCGGCCGGAGCGCCTGCCTACGCCAAAACTCCTTGAGCC
AAAATGGCTACGG
>at03057
TGGGAGGAGCAGCGCGTTGCTGAGATGCTCATCGACAACTTCACCATCCCTGCAAGAAGG
ACTACGTATGTGGACATTGCCTGGAACTTCCCCGACGACTCCATGGACCTCTTCCACGCC
GTCTTCGCGCAAGCCATCGTCAGATCCCCCAAATACCTCCACCACTATGTCGTGAGGGGC
TGCTCGAAGAAGTGGCCGGCAAGTAGAGTTGGCAAGAGAGTCAGCAGGGGTGAGGCTGCA
GTTTGTGATAAAGAGATTGGTAATTGGGCGCCCGGCAGGCCGATCATCTCCACGCCATCG
TGGCTTGGCGTGCCTATAGGTCGCAGTGTCGGCATTGTTGCCTTTGTGGCGAACGTCCAC
TTTGACAACCCAACACAAAAAAGTGGGTGTGGCCTCTACCGATGGCATGCGCATGTTCTA
CACCCCGACCTTGCGCACCGAAACCTTGAAGACTTTCCCCATAACGAAGCTGTCGATGGA
TTCAATAATGGGCATCCCCAAAGGCAAATGTCGATATTTTATCACAAGGTCCTGCCGTGT
CACAGTCCTTGACCCTCAGGGGCAGCCGGCTCAGTTCCACGCAACGTCGGTGGCCTACCA

TGCACACCGTCTCGGGCGTGAGATGTACGCTGAGCTGACGCGAGGCAGCGAGACCAGTGA
TCTGGGCTCCCACCGCATCTGGCACTTTGATGACCAATACGAAAAGACATCATTCCGATG
AACATCACCATCCA
>at03058
GCAGGCTGCGCTCAAGGCAGCCGACTGTTCAATGGTATTCCCAGGTGTTTTGGGCAATGG
ATGTCACAGGACCGCAGCTCCTCTCTTCTCGTCTTGTGGCTTGCGGCGATCCAGAGCGCC
CTAGCTGCGCCTTCGGCTCCGGAGGCCGGTGCCTGGCAGGCCTATGAGGCGCGATTACAA
CAAGAACAGCATGGGTGGCAGTCCTTTATAGGTAGCTTGGCAGCCGCACAGAGTCCTCCG
GTCAGTGGCCGAGGCGTCGTGATCGTCGGCGGCCGGGATACAGCCAAGCCGTCCAGTGGC
CCTGAGCGCACGGGCGGCACAGGCGGCAAAgGCCTACTACACGCTGGaACGCCCTCCTCA
GCCTCGCCGGNCTTCGATGGGCCGGCTGCCGTCTGCCCGTGGACGC
>at03059
GCGGCCGCGCTGGCGCTGCCCACGGTGGACTCAGACACCGTCGTGTCCCCGTCGTTCGCC
TGGTGTTGACCGGACGCCGCTTGCAAGTGCGGGGCTGGTCTTGGCACCCGCCGTTGCATA
GCACCCCNGGTTTTTCTTAGCACGGGGCATGAATGCAAATGGGCTGGCACCTTGATGCAT
GGCCGCCATGCATGGAGCCTCCGGCCTGCTTCCGAGCCCCGCAGCCTCCTGAGGAGAGCT
ACAACCAGCACCATCCGCAGGTGCAGGTGAGGCCGTGAAGGTTGCACGACCGAAAACAAA
AACTCCTCTCCCATCCGTGCCCTCCTCTTGCTTCGACGTCGATGTCACTGGCTGGCTTGC
GCCAAATTGAAAGACGCCCGCATCCCCACTCTTGGGATCCGAGGTCTGCCCAGGGAAGGG
AGTGCTGTGAAAGCCACTCTGTACGCCAGCGGTAAATGTTGAGTACGTGGTGTGGGCAGG
CTGCTGCTCATGCGAGGATGTCTGCCCAAAGACGAAAAGAGCTGGTGCGGGAGACCCTTC
AGCGGTATCTTGCTTCCTTGGTGAGGTCTCTGTGGGAAGTCCAGTGGTGCCGGCAACATC
TTGGCCGCATGACGCCGCTTGGCATCCAAACTCGAAGATAGGCCGACAAGCGGATGGCAT
CCGTTCCCACAAACGGCCCT
>at03060
TTTTTTTTTTTTTTTGTCCGCACTTTGGACGTTGGATCACCCAGCAAGCTCATGCCAGC
TGGCGCTCAAAATCGGCCTGGTCCGTGACAAAGAGGATATCGCCGACGCCGTCGCCGATG
CGGGCCGTCTTGTCCACCAGCTGAACCGACGGCTTGGCCCGCGTGCCGCTCAGGAACTTC
TTGAGGCTCGGGGCCTTCTCTCCAGCAGCCTTGGCCACCAAGCGCTGGCGCTTCAGGTCC
TCCTCCTTGGCAGCCTCCTCTTCAGCTCCCAGGGTGATGGCGGCCTCGGCGGCTTTCGCA
ACCTGCGAGGCGCCGGAAGACAAGAGCAGGGCCCCATCGAACTCGTCCAGCTTGCGTTCC
TTCAGGCGACCGTCGGGAACAGGACTGAGCAGCAGGTTGGGGCCGCCAACGTCGCTTCCC
AGGAAGACCGAGAGCTTCTGAACGGGAGGCTCGGCATCGCTGTATTTCAGCTCCGTGAAG
CCGAAGGCCCGCTGGTAGAACTTCGACACGGCATCAAACGCGGGCACCTCGATATTGAAT
GAAACTAATTCCACGCCAAGCTGGGCGTCGCGCGGAACAAGGCGCACTTGCACTCCACGC
GGAGATTCGGCGTCCAGGTAGGATGCATCGCCGATGAGCGAGGAGCCGCCTGCCTGCTGC
ACAGCACCGAAAATGTCGATGATCCTGCCCTTGACCCCAATCTGCACAAAGCTCAGCGAG
TTGACAGTCGGCTGCATGACATCATAGTTCAGCAACCTTGGCCTTCTCCT
>at03061
CTGATGAGTCCTCTTGTGGATGCCACTGCCGCGCACATTGATATCCATCGCAGCGCTGGG
TGTCGCGTGTTGGTGACCTCATGGGCGTCACCCACGGACCCCCGCTGCTGACCGGCGCAG
CTGGCCACTGCCTCCGCGGCGCTGGCAACACCAGCCGCAGCTCGGCCTTCCCGTGCGCGA
AGGGGCACTCCCGGTCCGCGCACGTGCCCATCTTGAAGTCTCTGCAGAGCGAGGTCCTTG
TGAGGTCGACCCTCTCCCGCAGCTCGCCGGGCTCGTGCGCAAAGGCGCAGGTGCTGCCGT
ACTGGCAGCACCCCCGCGCGTGGAACTTGCACATCTGTGTCTTGGGACAGTTCCCCAGCC
ACGATGTCGGACTCGAATCGCCTCCCGAAGCGATCATGGAGCTGGTGCGCCGGACTGGGT
CTGCTCATCCTTGTGCAGTCAGGCGGGAGC
>at03062
CACTGACTGCGTCATGCTCTCGGGCGAAACCGCGGCGGGAGGATATCCTGTCGAAGCTGT
GACCATCATGCGCAAGGTCTGCCAGACAACCGAGAAGATCTTGGACTACGGCAGTCTGTA
CCTCTCGACTCGCATGCAGGTTGTTGATCACGGCGCCATGAACCCCGTCGAGTCTGTCTG
CTCCTCTGCCGTGAAGGCAGCAATCGACGCACGGTGCAAGCTCATCGTCGCACTGACCGA
GACGGGCTCCACGGCCCTCAAGCTTGCCAAGTACCGCCCGCAGGCACCCATCCTCGCGAT
CACAGCGAGCGAGAGCACGGTGAGGCACCTGCAGGTCGTGCGGGGAGTCATCCCCATGCT
CACCGCATCCTTCGTGGGCACTGACTCCGTCATCGCCAAGGCCGTGGCCAAGGCCAAGGA

AGACGGCCTTGTGGCCTCTGGCGACCACGTCGTGGGCGTGCACGGGACGAAGGAGGAGAC
CCCAGGGAACTCCAACTTGGTGAAGATGATCGTGGTGCCTTGAGTCGGCGACCTTCCCGC
TTGAGCGCAGTGGCTCCTCCCGCGTCGGCATTATGAGCTGTTTTGGGAGTTCTGCGCCAG
TTCCCCAGGAGGAGGAATACATCATTTTTCAGATAGTGTCTGCAGCACATCCTGGGTGCC
TACAGACGCATTCCAGTCGATGCTATTGTGCTGCATTCAAACAGAACCTAGTCTTCTGAC
TTGCATCGCGGCGTGCTCAGTCGCTTGACAGGATTGGCATCCATTTCAAACAGTAAAAAA
AAAAGAAAAAAAAAACAA
>at03063
TTTTTTTTTTTTTTTGTCCATCCACGCTCATGCAAACGCGATGGCATTCCATAACTTGA
CCAGTGGACCTTTGTGGATTCTAGGCATTACCTGTGTCAATGCCACCTACAAAGACTTTA
CAAAGCAAGGGGGGACAAAATCCAGGGATGCAGGAGGAAAAGATGACACAGCCCGAATCA
CAAGAAGAACATCATGTGGAAGTAATTTGCGTTCTGTGGATCGATTGTGAAATTGGGGTT
TCCGTTTGGCTCCAATGCAATCCGCTCTGCAGCAGCATATCCGTCTCCGAGCACTTCTTC
ATCAAACATTAATCCGTGCTGCAACTTCTTTTCAAGGTCGTCGGTTTCATTCTTCATCTT
CTCGTAAGCCATCCTGTGCCGCGGCATGGGTGGTGTCTTTGGCCACATCTCGCCACTCTC
CCGTTGAACCATCTCTGTACCTGGCCAGACAAACTGTGGCCATGCCTGCGTGTAGCCAAT
ATCAAGTTCGATGGGCTCGTCCTTTACGACATCCTTTTGGTAGTCGCCCTGGAAGTAACA
CACTGCTTGCCGGAGCGGGGCAGGTCGATGTAAGTCCTTCTCCAGCATCGAGAAGTCCGC
CGGCGGAATGGACTCTGGCCAGTTGGTGAGCACTATGTCGCCCTCTTCGTCCACCTCGTG
CC
>at03064
TTGTTTTTTTTTCTTTTTTTTTTTTGGCAGGCGCTCCATGCTCCAAGTCGGAACCGGCG
GCCGGCCTGACTTCATAGGCGCTCCTTGTCCAAAGCCGGAACGGGCGGCGTGCCGCCGTG
CCTGCAGCCCTAAAGCGGCGGGCGGGAGCTCAGCGCCTGCCCGGCTTCTTGTTGTCCTTG
CCACCGCCCTTCAACTTCTCAAGCACTTCCTTGAACTTCCTGATGGGCTTCGTGAGGGTT
GCCCTCTTGCCCTTCCTGCACCTGTTCATGACAGTCTCGTCCTTGTACGCGCCTCCGAAG
TCCAAGCTGGACTCCGCCTCCACTCCTTGGAGGAAGTCGGCGAGCAGCTGCAGCTCCGCC
TCCGCGGTCTTCACGCTTGGGCGCCGGAACGCGAGCTCCAGCTCCTCGACGGCCCTGTCC
ACCGCCACGCCCGAGCACGCCATGCGCGGTGCCACCTCTGGCACGGGTACCGGGCCGCCA
ACGAAGGCCGCAAGGGGGATCCGAGCAATGGAGCACACCGCTGCCAGGAGCAGCGCCCAG
GCCGCCAGCGGACGAGCGGCCCTCGCCATGATTCGGCCAAGGTGGTGGGTGGTGAAGGAG
CTCGGGAGCGGAAGCTGGCTTGAGCCAAAATGGCTGAGCCAAAATGGCTACGG
>at03065
TTTTTTTTTTTTTTTGTCTCAAAAGGCCGCACTGTATCCCAGGACCGAGGAGAAAGGCC
TCAGCATTCGGAGTGATGCAGGTTCAGGACACGCAAAAGCCTCTTTGGGACTGAGATTCT
TTGGGTCTGAGCTGCATTCAAAGCCTCAGCCATGCTGTCCCGCAAGTTGCTCCCCAATAA
AACATACCTGTATGGGCACCTGTCAACCCAACTGAAAAGAGGCAACCGTGAACAAGGGTG
CGCATGACCACTTGTGATTAGTTGCAGGCAGCCACTGGAACCAAATTACTGCATTACTGC
ACTGTTCAGCAATGGCAGCTCAGTAGCACGGAGCCTCTTTGGACCTCTGACAAATCAGAG
GGCACAGGCGTCCGAGCGTCCTGGAGCGTAAGACCTGCACACACCATGAAGGAAAAGACA
ACATTGGTGCACAGTCAACGCTCAGGTGCCGAATTCAGGAGAAAAGCCGCGCTCTTGCTG
GCCCAAGTCAAAAAAGCCACAGCTCGCCTTCCTTTGACGGATTCACACTGACTGGCCTGA
TATA
>at03066
TTGTTTTTTTCTTGTAAATTTTAAATTTGAATCTAAATTTTATATATTTATTATAATGGC
CCACCGAAGGTGGGCCTATATGTGTACATATATAAGGTATGTGTGTACGTTATATATAAG
GTTTTTAGGATGTGTTAACAATCACTAGGTTTTGCTACGCAAAACCAGAAATAAGTGAAA
AAGGTGAGTTATCGATTCTAAATTTAGAAGAATAAGTTATTTTAAACTAATTTAGAATTA
TTTGACTTTTGTTTTTACCTTTGAATTGAAACTTGGAATATGTTAGAATTTTATAATTTG
TGAAGATATTTTATTTTATATAGAAATTTTCTGAGGAAAATTTCATAAAACAAAGTAAAA
TTTATATGAAAATTTACAAAAAGAGAAATTTAACATTGAGATTTCGTATGGATTCAGGTC
ATTAACATTGAAAATGCCAATAAAAATTGTCCGAGGGTCCCCTATGGCCCGAAAAAACCC
AAAAGAGAAAGTAAAATCAAAAATCAATCGCACAAGAAAGTACGAGATCTCGTTTAGGAA
GAA
>at03067

TTTTTTTTTTTTTTTCTTCCGAGCTCCCATCGCGCACGGACGGGCTCTGTCGTGAGGAAA
AGGGCCCCATGGTGCCTGGAGCCCGGACAGGACGGCGGAGGCCCGCCGGCGGCTGCCCGA
GCGGCAGCCCGCGGCCGCCGCGGCCCCCCCTTCGAGCCCCGGGCGCGCTCACTGGCCCTC
GAGGCCCTTGAGGCCCTGGAGGCCGCCCAGGCCCGTGGAGAAGCCCATCTGCAGCTCCAT
CTTCCCGAAAAGCTCGTCGCGCGGGTTCTTCTTCTCACGGAGCGCCTCGGGCAGCGGCCT
CCTGGGCTCGGCCTGCTCCGCGGGGCTCATGATCACGGGCCGCGCGGAGGCGGGGACCTT
GAAGGACGCGGAGACAAGGCGGGGCAGGTAGGGGTCCTTGCCCAGGAACATGACCTCCTG
CTCTGCCATGAAGGCGTCGGACTCCAGCAGCTCCCGCACGTGCGCCTTGTTGCATGCCAC
CTCTGCCTGGGTGGTGCCGATCTGCGCCATGAGTGCTGTCAGAGGGAGGGTATCGAATCT
CTACTTTGGCGGCGTTCACTGGCTTCCTCGTGCC
>at03068
GCTTCCTGCAGAGTGTCCTCGCCTTTGCATCCCTCGTATTGGCTGTTGGTTTCTTCCGGG
TGGGCAGGTTCCTTTGTTCTTGCTCCCAAACCGAAGCTGGTGTGCGCTGTTGCAGTTGTA
TCTGCACACCAGTCCAAGCTTGTCCTCTTCGAGATCCCCCCCCTCAAGCCTCCCAAACGA
CATCTCCCAAGTCTTCCCGCTGCAGAGCGCTACGTGGAAC
>at03069
TGGCAACATCaAAAATGTGGGATCGCGAGAGTAGACGTCAAACGATGTGTTGTGGCGGGG
NAAAAACGGCAGAGACCTCCTGCCGATGGACTCGTCTTGCTCAAACTGTGTGTAGCCGAT
CTGTGCGCAAGTGCCCTCCTCAAAGCCGCCAAATGCGACATGCAAGAAATAGTCTGGGCT
GAGAGCACTCTTGTACACACGCCGCTGTCCACAAGCGGAGCGCTCTCCAACACGTACTTG
CGGGTAGCCAACCTTGCGGATAATCACCGTCTTTCCTTCAAGAAGCTCTTCTTGCGCGAA
CGATCCATGAACCAGCACTGCACATGCAAGAAGGGCCCACATGAGCTGAACGGAGAAGGA
CAAAGCAAAATGGTTGCGGCTGCCTGTGTTTGACACACC
>at03070
TGAAAGGTAAGAGCCAACGCAAGAGCGATTGGATGATGTCATATTTTGACAAGCACAGAC
AAGTTGATGGAAAAAGCCCCACATATCCGCATCCAGTGAATGACTTTGCTGCCCTCTTTC
TGAAGGCAGATGAATGGGATGATGGACTCGAAAAGGCAATGGCCTTTGACCTCCTCGGTG
CGCACAAGGTGGAAGTTGTGGAGAATGCTCCAACCATTGGGGGGGATCCCTGCCGTTCCT
ACTCTCCACGAATGCCTTCAGCAAGATCGAGGTTCGACCGCACTTCGGGAAGTATGGTGC
GGACATGTACTTCACAGCCGAGGGCATTCCTGCCCCTCATTCAGACGCCAGATGG
>at03071
TTTTTTTTTTTTTTTTTTCCCCCTCAAAAGGCACTACGCACCCCCTCCAAAGGCCGGCTT
CTCGGCAGATGCACGTATCTCCTTGGCACCTCTTGGGCAGCAGGCGGAAGAATCGGACAG
ATCTGGATCTGCATCTGCACCTCCAGCAATGATGTGACTTTGTAGCTGCTTGAGACTGTC
CATGATCTTGCAGCGAATATGGTCGTTTGGGATCAAGTGCCTCGGAAACTTTAAGTCGTC
CATGTTGAATGCAAGAGGGTCCACCCGAATTTGTTCCTCCAACTTCTGCAAGAGATTCTT
AAGCCCCAAGCGCTTGCTCTTGCAAAGCCTTTTAGCACCCTTCTTCTTCCTGCCATCCTT
TGATTCACTCACAGTCTCCTCACTGATGACCTCCCCAAACTCATCACCTTTACCTTCTGG
AAGATTCATGCTTATGGGGGACTCGGTGATGGCATCACTCCAGAAGGCAGCCGGGGCGGA
GACCTGCCTGTGGAAGGCAGGTCCGCTGTCCAGCGACGGCCCCTCAACCTGCACGGCGTG
CAAGAAGGTGTTCTTCTCGATGATGACAAAGACGCCGGACAACAGTGGGACACATCCTGT
GGCACAAGTACAACAACCTCCGTGGTTGGCCATCACTGCCAAGGTGCTAGATCAGCGGAA
CCTGCCCT
>at03072
GTGCGAGGAGGGTCATCACATGGGCGATGCGTAAAGTCCACCCCCCTGCATTTGAAGGGC
GATAAAGGCCTCGACGAAGGAGGTGGAGAGATTGAGCTGGCCGACAGATTCCATCGAGAT
GTCGGCACCGCCCACAAGGCAGAAGCACTTCCGGAGGTACTCCAACAAATTCTCCAAGGT
GGATGACAGCACGGCTTGAACGAGCAGCTTCTTGATGTTGTTGATCTCACAGTAGACCAC
CACCCGGGCCGGCTGCGAGTCTTCCCCGGGCTGCCCTTGGGCGTCAGCTGCGAGAAGCGG
AGGCTGGGCCTGTGAACTCCGTGATGAACGCCGCGGTGAACATCAGATCGCCCATGGCTT
TGACTGCGCTCGCTGTCATGATGTGGACGGCCGCGTACTTCTTCCC
>at03073
TTTTTTTTTTTTTTTTTTCAGGCACAAACGCAATTACATGTCTGTCCACATGCCAGAGCG
TATGCTGTTACAGGCGCGGCCAAGTCAGGAGAAGAGGCACAGCACTGAGGCTTGTGACTC
TTACCGGCAGAGGCCATCGATAAAGGCCTCAGCGCAACCCCGGAGCTGCTCACGGACATC

GGCACGCCGCCGTCGGAGGTGCAGGTCAGCGCAGTAGCAGCGCTTCACATCCTCAAAACT
GAGGGTAAGCGTGAAAAGCGAGCTGCCGCGTTGCTCCTCCGTGGCCAGCCGGAAGTGTGA
CTCAGCAACCATTGGATCACAAGGAAAGGCCTCACTGGGGCATGCAGCTCCCCTTGCCAA
GCGAATTCCCAACCGCAACGTCCGCGGGTCAGTACGGTCGATTTGGGAATCGACTTGGCG
AACAGGTGCCAGTGTCCTGACTACAGCCCAGCCGGGGTTTTGGAAGTCTGGCTGAACCAG
CAACAGGAGGAAATCATGCAGCACTAGGTATCGTGTTTGACGGCCCTCCGGAGTCACGAC
ACCGCAGACGATGCGGTCCTGGCGGTGAAGCTCGAAGGCACGTCCTTCCTGGAACCCGTC
AGAGGCCTCCTCTGCCACTTGCAGTGGGTAGCCGTTAAGATCGCTGGACGAGCCTACAGC
TGGTGCAACTCCAACTGAATCTGCCATCAGTGGAAACCCACAGGTGGTATCGGTTGAAGC
TCGTGCCGATTC
>at03074
TTTTTTTTTTTTTTTTTACATGCAAGCCCAGGGACAATGAGCAAGAATGGTCATTGGCA
ACCGCGATACCGCTTCCATGACGTGCCGAACCGCACTCCTCTTGGTCCAAGCTGGTAAAG
CTATTGCCACTACCTTCGCCGTCGCTCGCGCAGCCCCGGCTGCGGCCCGAAGAGCGGGCG
TCAGCTCCTGACCACAAGGGTACCGGAGGTGCTCTCGTTGCACCAGCCGGGGGGGTGTCA
CCAGTGGCGTCCCCGTATTCCTCATCTTGTGGGAGGGGCTCCGGCAAGAACTTCGGCCGC
CTCAGATGCTTGAGCGGCAACCCTCTGGGAGCGGCGCCTCTGGGCTCTGCGGCGGAACCT
GTGGCAGGTGTTCCTCCCTGCTGCTCAGCTGCACCAAGGGTGCGTTCCAGCCTGGTGAAA
CGCCTTGCCTGCGGGCCGCTGCGGTGCCGCGAGGGGCGCGTGCCTTCCCCGGGG
>at03075
CGCCGCCCGGATCTTCCTGGCTGGGAAAGACTAGTCATGGCCAATGTTTATCTCGTGGAA
GAGACAGACCCCATGACCCGGGGTTGGGTTATGGGCACATACAGCTCTGTGGCGGTGACT
GGTGGCGGCTTCGCCGTGATCCTTTTTGGTGCGATTGGCGGTCTCCACGACGGCTGGAGG
TATCTGTATGGCCTGGCTGCCATCCAGTTGGTGTTCCTTGCGCCACTGTGGCACCGCCTG
CCCGAGAAGACGCGGGC
>at03076
TTTTTTTTTTTTTTGTTGTCGCATCGCCGCGGCGCGAGGACGTCGTCTGGCCACGAGCC
GCGCTGTACACGCAGGCGGCGCCGGCAGAGGTTCCGAGGCGCGCGCCCTGGCGGCGCTCA
CGGGATCAGGCGGCTGGGGTCGCGCGGGAACATGGAGGTCTTGCGGATGTTGTCGAGGCA
GAGGAAGAGCATGACCACCCGCTCCATGCCGATGCCGGCGCCGCCATGGGGGTACGAGCC
GTACTTCAGGTCTGCAAACGGGTACTGTCTCCTGACAGACTCGATCTCTGTCGAGAAACG
CTTGTTCAGACCTTCAAAGATGTGATGGAACAGCCCGTCAAGCACGTCCAAGACTTCGTG
GTAGTGATCCTTGAAGGTCATCTCGATATCAAGTCCAGTGAACTCTGTCATGTGCCTGTG
CGTGAAGGACTTCTCCGACCGGAAGATGGGCCCGATCTCAAAGACTCGCGGCATGTCCGT
CATCAGTGCCATCTGCTTGTAGAGCTGCGGCGACTGCGCGAGGAAGGCCTTGCCGTCAAA
ATACTTGACCTCAAAGACATCCGCGCCACCCTCGGAAGCGCCGCCGANTAGCTTGGGCGT
GTGGATCTCTTGGNAGTCAATTTCAGCAGGTACTCCCTGAACAGAGCGCATACGCCGGAC
TGCATCCTGCCTCGTGCCGAAT
>at03077
CCATTCTGGAAGCGAGAAGCTTGGGTCATGAATCTAGCCCACACCAAAGCTGTCTTCTGT
TGGGCTGCCTTCACACTCATTTGGCTTGTAGAAGCCGTCATACATGTCCGATGCGGTTTT
GACGCCAAGTTGTTCGAAGCTGAGGCCAAGTGCATCAACCAACCCAGCTGGGGGTACTTG
GTGGCAGTCAGCGAGGGCGTGTGCAGAAGCGGGATGGTGGTGATAATGGCCGGCATTGTT
TGGGAAGTCGTTGCGCTGGCGTTTGCTAGTGAGCTTACAGATCGTATTCTGGGACTTGTT
GAGGGGACTGGCAAGCTCGCCTTTCCGGCAATAGCTCTGTCCTCGTTTGTCGTGGGTCTT
GGCCTGTGGAGCATTGTTGACGAGGAGGACATGCCGTTCTGGACACTTGGCGTCTGCCAT
GTATGTGACATAGGATGCCATTCGATCATGGCGGCGGCGTTGGTTGGTTCCATCGTTGAC
TTCTTAGTAGCGACCATTGAAGGTCGGGCTTACCACTTCATACTCCAGTTCATTCGCTGT
ACCGACTCGA
>at03078
TGCTTCTTCCTCTGCTGCTCCTTCTCCCTGGCCTGCTTCTCCTTCTTGAAGTCGTCCGCG
CTCTTGAGGTAGGCCGCCTGGCCGGTGGCGCTCTTGGACTCGCCCGGCTTGGCCAGCTCC
GCGGTCTGCCGGAAGGAGGAGGCCGAGGGCAGGGGCTTGACGATCTTCAGCTGGTAGCGG
CCGGCGTCGCTGTCGCTGTCGTCGCTCCAGCCGACCATGCCGCCCTGCTTCCCGCCCTTG
CTGCGGCTGCGGCTCTTGTTGGTGGCCGACCTGAGGTTCCGGTCCGAGGAGCCGCCAAAT

GCGGGCGCCACGGGGACGGAGGGGCCTCTCGCCGGGCTTCGGCTGGCCCTCGGGCACGCT
GCTCTCGCTGCTGGAGGAGCTGTCACGGCGCCGGCGCTTCTCCTTGTCCTTCTCCCAGAG
TCGGGCAT
>at03079
CCCTCCATTATCCGCTTACCGAACTCCGTACAGGCTGCTTCCAATGACTGATGGAGGCTT
CCAGGCTACAGAGATCCCTCCTTTCTCGCTGGCCTTGCAGGTCCTGGTGACCAGCTTGAG
CTTGTTCAACTTTGGATACAACGCCGCCAGCATCAGTGGCGTTCTACTTTACATACAGCT
CTCAGACACGAACGACTGCAGGTTGAAGCCCGTCTGCCTGACCAGCGCCCGTGAGAAGGG
ACTCGTGGTGGCCTCCTGCCTCCTGGGCGCGGTCGTGGGGCGCTGCTGGCCGGCGTCCTG
GCC
>at03080
TTGTTTTTTTTTCTTTTTTTTTCTTGAAACAGATTGCGGGTGTGCAAATCAAGAGTTTCA
GTTTGTTTCGAAAATGTGGACATGTGCCACATCACGGTCAGTATGCCGCAAGCAGACAGC
GTGAGGCAGCCGTGCTGTTGGCGAGCAGCGGCAACCTGATTGAAATCGCCTGGGCTTTAC
CTTAGTGTCCAGAGGTCTGGGGTTGTCTGAAGTTCTACAGCGCTGAACCTAGAGCACCAA
CCCGTAGGGACAACAAGGGCCTCATCCAGGAAACTTGGAGATATGGACATACGCAGAGCT
GAGAAGCCTCCACCAAGCCCGTCAGAGCTGCTGTCAATCAGTCTGCTCAGGACAAGTTGT
CCTTCAATTCGCACATCATCTGCAAGAACTCATCAAACTCGAGTGTGCCTGAGTTGTCCT
GGTCATACCGTCGGCCCAGTGCGACCAAGTTCTCAACTGGCATGGGTAGCCCACTCGCCG
CAAATGCGTTGGAGAGTTCTGTAAGGCCGATGGTCCCGTTGCGGCTTCGATCAACGGCCA
TGAACTTTGCCTTCTGTGCCTTCAGGAACTCCATCAGCATGCAGAACTGCTCAAAGCTGA
GCAGACCACTACCCCCGCTGAACTTGCGTATGAGCAGTTCAGCCGTGCGCAGCTGGAAGG
GCCGCTGAACAGTGAACATGTTCTTGTAGATGAGGGCGCCTAAGGTGCCTGCAGAACTGA
AGCCAGCCAGCCCAGCCATGCAAGGGTGCACTGCCAGCGCGCCCATCGGCTCTAAGGACC
GCTTCACTGCGTCCAAGACCTTCAGCAGCTCGTTGGGGCTGATGGCAATAGCTCCCGCTG
GCACGTGAGCAGCCCACGCGTCCAGGTAGCAATCCCACTCCAGCCGCAGCTGCACAAACT
CGTCGAAGGAGAGCAAGCCGTTGTGATCGGCATCGTAGGCCCTGCCGATGCGCTTGGCCA
CCATGAGGCTGAGGGGGGTCTCCACCACCAGGCAGTCCTGTAGAACGACTAGAAAGCCAG
AGAGGTTCAGCGCGCGGCTGAGCTCGTTGACGCTGAGGTCGCCCGTCCCGTCAGTGTCGA
TCTGCGCAAAGATCGCCTTCAAGCCTTCCAGGTACTGCATCATCTCCGCAAACTGCTGGA
AGAAGATGGCCCCCCACTCCGGAGCGGAAAACCTGGCGGCCAGCCAGAGCACTGTTTTTG
AATTGAAGGGCGCCGGGAACAAGGCGGTCATCGAGTGGCTCTTCACACTCCGCTGGACAG
CCTGCAAGGCGTTGTTGAGCTCACAGATGTCGATGGTCCCG
>at03081
AGCGCCAGTCGCAGTTGCTGGGCGCGCGCCACTGCCTCCTCGGCCTTTTCATTACGTCCT
CCGGCCGGACGTGGACCCACGGGTCGATCCGTCCATCTTGCTGCGCTTCGACCCGCCGGA
GGGGCTCCGCTCGGGTGCGTCGATGGTCGTCTGCCCCGGTGGTAACTACGAGTTTGTCAG
CATGTCAAGCGAGGGACACCATGTGGCCGTCTGGCTCAACAGCCTTGGCATCACCGCGTT
CGTGCTGCGGTACAGGTGCCTGACGGAAGGCCACTACTGGCCGGCGCCGCTGGAGGACCT
GGAGGTTGCCATGCGGCTGATCAGGGGCAATGCGGCTGCTTGGAAGCTCGACAGTCAGCG
CATTGGGGTCCTTGGGTTCTCCGCTGGCGGCCACCTCGCCGGCACCGCGGCGGTCACCCG
CGACGCGTCTATGAGGCCAAACCT
>at03082
GCTGCACAAAGGGGGCAGAGGTGTTTCCTGGCCAGATTCCTGAGCCACTGGTCAAGCGGC
CTCCCAAGTCAGCATTTGCCGCCAGGAAATTTACACCGTACGCACGTGCAAAGCCATCCT
GGCTGCCCACCGCTGTAAACCATGGGAAGGAACTGATCCAGAATGTAGAAAAAACAAAGT
CACGCACACCGGACTGAAGCAGTGCCCTTGCTGGCCCACCAAAGTTGATGTCATTGCAAA
TAAGCATCCCAAACTTGACACCAAAGTGTGAGTCGAACGTCACGACATCCGGGGTGTCAA
CCTCTGCCCACTGGGGCTCATACTCCACGCCGACCCCAGGCCAAAGTCTTGCCATGTGGC
TCTTGTGGTAAACAGCCAGAAGTGACCCATCGTGTCCGAATGCCGCTTGAGAATTGAACT
GCGTGGTGTTGGTTGTCCT
>at03083
TTGTTTTTTTTCTCTTTTTTTTTTTTNTTTTTTTCCTCCTCCAATCAGAGTAGATCAGTCC
CGCCAGAACAAGTTCATCAAGGTGCAGGCAGATCAATCAGAGACTAAGAGGCAGCATTCG
AAACAAGCGGGCCAACCCCTCACATCATGACGACGGTCGATAGAGAAGGTGTTCCAGTGC

TAAGGACTGGCCGGCATCAACTTCGTGAAGCACTGAGCTGGACCAGCTTTCAGCTCTGTG
CTGGAACCGCGTCAATAGTCCTGGAAGACCGACACTCCCGAGCGATGCTGCTTGCCTTTC
GTGATGTAGGTGGCCTGGCGGTAGAGCCGGCCCTTGCGCGCTGCGAGGTCTGCCCTGACC
TTGACCATGGCGCCCTCGCCCATCGGCAGGGACAGGGCGCTGTCGGAGGTGCTGTGGAGG
AGCGTCCGCCCCCGGCCCTTGCTGTCGGCCAGGGGGTCCAAACAGGTCCCGGCGGCCCTC
ATGGCGCTAGGGTCTTGACGGGGCGGTGGCCTCCGAACGCCCTCGCCGCCAAAGAAGTCT
CCCGTAGTCGTGTTGAAGAAGCGCTGCTCGTGGTTGTCGCCGTGCCTGCTGATGGCGGCG
CCGAAGCCGCTTCTGGGTCCAGGGAGGGGCCGGCGCTCCGGGGGGCACACGGGCGCGTTC
TCGGTCGTGATCATGACGGGGGCCGACACGTGGCCTTTGAAGAACGCGGCGTGGCTCATG
GGATGAGCAAAGTCCACCGAAGATGTCCGCAGCTTCTCGTTGAAGCCGTCATCCGGGGTT
GCAAAGGACGGCGGCTGTGTCTTGCGGGCTATCCGTGACAGCACGTCATACCTCTCTCCG
ATGTAGGCCAAGTCTGTCTCATAGGGACGGGCAACCTTCTCCTCGGGCATTGACATCTCC
AATGAGGAGACTGGCGCGACTAGCCAGCTCAGGGAGCATGCAATTCTGCCCTTGGTCCCT
ACGGGCTTGAGCCAAAATGGCTACGG
>at03084
GGTCCTGAACACCCGCGCGCACCCTTGCATGATCTCCTGCTCCAAGACCAACCTGGGCCA
CTTGGAGGGCGGCGCAGGGATGTCTGCCTTCTGCAAGTGCGTCATGGCAGTCATGCACTC
CGAGGTGGCCCCCAACCAGCATCTGCGCGAACAGAACCCTCACCTCGACATCGAGGGCTG
GCCAGCCTACCTCCTCCAGGAGGCCGAGCCCATGATCTCGGACGCATCTTACGTCGGCGT
CAGCGGTTTCGGCTACGGCGGCACCAACAGCCACGCACTGGCTTATGGCAGGAACATGGT
CACATCGGCGGGGAGCGAGTCAGAAGTACG
>at03085
TTTTTTTTTTTTTTTTTGCACCCTCCCGCAGACCAGCCAGGCCAGCAGCCGCAGCCGCT
CCACGGCTGCTGTGAACCCAGGCCGGGACGTGGCGTGCGCCCCCCCTTGCTCTCTCCCGC
GCGCTCGTCAGATGAGGCTGGTCAGCTTGAGGGCGGCTGGCTGCTGGCCGTTGCTGGTTC
CGGGCGCCTTGGCCTCCGCCGGCTCGATGGCGCCCATGCGCAATGCGGCAACCTTGGCCT
TCTTCCGGTTCTTGAGCTCGCCGTCGGG
>at03086
GTCGCTGAACTTGCCGCCGTAGATGGACTCGCCACCCCGTCCATCGCCCAGCTCGAAGTC
TCCGCCCTGCGCCATGAAGCCGGGGATGACGCGGTGGAACTTCGCGCCCTGGAAGTGCAG
CGGCATCTTCGTGATGAGGCCCGTGCCACGTTCGCCGTTGCATAGACACCGGAAGTTCTC
CGCCGTCTTCGGGCAGATGCCGTTGTAGAGCTTGAAGATCATCCGGCCAATGTTCACATT
GCTGATCTCCACCTCCAGGTAGACGTAGATGTTGCCGTCG
>at03087
CCGTAGCCATTTTGGCTCAAGCCACTTGGGATCGAGGGGAAAGGCTGCACGTGGCGCCAC
CCCTTCCATGGTATCCGAGCCACGCCCGTCCCGGTGGGGTCGCAGTGCACGCAGTGCCAG
TGTAAGTTGCTCAGCAGTAGCACGCTGTCCCATTTGCTTGGAAGATGTTGTTCACAATGC
GGGCCTTCCTTGTGGCCATTCGGTCTGCAGTGCTTGCGTTGAGCAATATGTGAGTAGTCT
GGTCGACGAAGGCAGGGTTTCGCCTGCAGATCTGTGCTGTTGCTTGCCCCAGTGCAGGAT
GCCAATACCAGAGGGCCTCCTTGCTGACTTGCTGTCCACTAGTCCTGAGGGCATAGCGCT
GCACAGGAGGCTGCTGGACTTTCAGGCAAGCCGTTTCGTGCCCGAGCAGGACGCCGATGA
GCAGCTTGTGGACTGTCCCACGCCTGGCTGCGGCAAGGTCCTCATAGCGAACGAGCTGGC
AGCAGCCAAGGCGGAGGTTCGCTGCCCTCTCTGTGCCTGTTCCTTCTGTGCGAGCTGTGG
CAAGCCTGCCCATGCCGGAACCAGCTGCGAGGCCGCTGAGCTTGAACGCATGGATCCCGA
GCTCCGCAGACTCATCGCCAGGGAGAATCTGAAACGTNCCAGTGTGCCTTGCCTGTGTG
AGAGAGAGTCTGGCTGCAACTTCATGACTTGCCCGTCGGAGCGCTGCCAGCGGAAGACAT
ACTTCTGCTACTTGTGCGGGGAGCTGCTGACGGCAGCGGACCATGCGGCACACTACGAAG
GTTTTGAGGGAGCCATGGGCCGCGCGGGACCGTTTGGCTCCATCTGTGCAAACAAGCGGA
AGGTGGACATGTCCCTTCCCACCCAGCCACCGCCGCCAAGGTTGACTGTGGTGTCAGGTG
AGGTACAGGGAAGCATCGCTCTACGCATAACTTGGGCTTCGCACCGCAGCGAGCCACCCA
CCATCTATTACCGAGTGCGCTTGTCCACTCCCGGCACAGACGAGGTACGGCACCTCAGCC
TGCAAGCTGGTGAGCCTCACCATGACCTTCACAGAGGGCTTCAGAAGTATATTCGATATC
AAGCTGTTGTGGTTCCCGTCAACGTGAACGGGGCTGGACCCCCGAGTGAGGCAAGCGAGG
TTGTGCATTTCCATCCGCGGGAGTTGGAGGAGATCAAAGCGCGCGAGCGTGAGGCGGCAT
CAAGTACAGTTGCAAAGAGCAAACGCTGGAACGCTCGCTAGTGGAGGGTGTAGACGAGGC

TCGGACACCAGTTGAACCAGCAGATGCCACTCCAAGAAAAAAAAAAGAAAAAAAAAAACAA
>at03088
TTTTTTTTCTTTTTTTTTTACTGTTTGACAGGGGTTTTTTCACAGCGGATCCGACAATGA
TGACAAGGAGGACGTCTGAAGCCGGACAACTGCAGAAGCTCAGAGGCTCCAGCGGCTGAT
CCAAGAATCTGGCAAGGCGGTGATCAGTCCAGCGTCGGCCATGGCCTTGAGTCTTTCCAG
ATGAGGCCTGTAGAAGCGATAGAGTCTCTCCAGAGCCGTGCTGTTAGCAATCTGGAGAGT
GGCAACAGTGCTCCAGCCGTAGTGACCCGCCCCGTTGTACTTCCGCCGGATAACGTCGCC
GGGGTAGGTGAAGTTCTGGAGCCCCAAGTGGGCATGAATCGCCCGGACCACTGCCTCGGG
CTCTGCCGCAAGTTGCTCCAAGTATGTCACGAGGATGTCCCTGGGCTGGAAGTTCTCGAA
CC
>at03089
TGAGGAGGCCAAGAAGAGGGGCCTGGATTCTGAGCTTGCGAATGGCCGCTTGGCAATGGT
GGCAATCTCAGGCATGATGTTCCAAACTCCACAGAGGTCTGGAAATGCGGCTACCAGGCA
GTGCTTTCGAGAACGAGATGGGTGTGCAGGCGCCCGTGGGCTTCCTTGACTTGCTTGGTT
CACCAAGGATGGCGATGAGAAGAAGCTTGCGAGGCGTCTCGCCTCGGAGCGCAAGCACGG
TCGAGTGGCCATGCTCGAGCGATAATATTCCGTTTCCCGACTTCTGCTCGACCACCCAGT
ACCTTGAAGTTCACGGACATCCCAAACGGCCTTGGCGT
>at03090
AACCGGTCTCTGCTGTCAACATGTGGTCTAGTATTTGTTTGTTGGAGGCCGTCAGCATCT
CTGCTGCTGCCTGAGGGCCTGCAAAGGAGGCCGTTACGGTCAAGACCTTCGCCGTGCCAA
CCAGGGCACGTGCGCCGAGGCTGATGGCCTTCTCCGAGTCTGAGGAGAGGCCCGAATCAG
AGCGTAAGGTCATGCCAGACTCCGAGCACAAGGAGCGCAAGGATTGCGCGGCCTCCGGAG
TCTTACCCTGGCCATTGAAGAAGTCCTGGCCAGAGCTGGCGCTGCCACAACTTGCGAGGC
TGTCCGCATGCGGCAGGGCAGGTCCTTCACCTGGAGCCGTCGTGCCATCCTCCATCACAG
CTGGCTCTGCAAGCACCTCCAACCCCTCG
>at03091
GACTCCCACATCATTCAGATGGGCGTGAACCAGGACACGTTTGACTCGACTCTGGAGGCC
GACTCGTTTGCCTCTTGCACCCCAGGGCGGCTGGATTCGATCAAAGGAAAGTTTGGCATT
ACGCGAGGCTTGACGAGTACTTTACGCTTTGTTTTGACACCTGGCACCATTAGTCATCGA
GGCAGGGGTGACGCAGAATACAGCAATTCATACGATTTCCGTTTTCCCTCCCAA
>at03092
GTGCCTGCTGGGCCAAATCTGCCTCTTCAACGAGTACGCTTCAAGGAAGGCCGGGAAGTC
TGGCGTGGTGGAGGCGACCTTGGCCTACTTCAAGGGCAACCCTGCAGATCGGAATGTCCA
GGTGCAGCTCGGTCAGCTCGGCTGCTTCCTCGACTACGACGCCGAGAACCGCCAGATGAT
GGCCGACAGCGGAGGCTTCGCGCACTGCTTCGAGATGTGCCGGCTGCACCGCGAAGACGC
GGAGGTGTGCTTCAACGCGCTCTGCGCGGCCTCCAACCCCCTGGAGTTCTATCCCCAGTG
CGCTGCGGCTGGGCGGCCGAGGTCTTGACCGGCGTGCTCTGGGACTTCCGCGGGAGCGAC
AAGGTGAAGTCTGAAGCTGGAATGGTCGCGAAGGGCTTGCTGCGGAGCGAGCACGCTTAC
GAGTGCTCCGTCAGCTTCGGGCAGGCGGGAAT
>at03093
TTCTTCACCAGCAGAGGAGGCACCATCTCCTGAATGACCTCGAACGCCCTCTTCTGCCCG
TCGTGGCCCACTCCGCACGGCTGGCCGATCAGCTCGCGCAGCTCCTCAAACGTCTCAGGC
TTGCGCACTCCGTGACCCGAGTGCGCGTCTCCCTTGACCAGGTACTCATGCTGCGGGCCC
TTGCATGGTAGCCACGGCGGGCTGTGCGTCATCCAAGTGCGTGGGCTCCAAGTCAGGTTC
AGCGCTGCAATGGTCTTCTTGTGCCCCAAGGCGAGATCGTAATGGAAGCTCCCCCGTACA
CGCTGTCCGCTGAGCGTCGCGCAGGTAGGGCCCGGCGCCAGTTGCACGGAATCCGAGAGG
ACTGCCGTGTGTTCGTTGCTTGCCCGTCGCCAGCCGCGCCGTTCCAGTCCAGGCGCCATG
TAAGCCTTGTCTCTGACCTGGAAGGTGAAGGGCTCGCAATCGACCTGGTCCGCTGAAGCG
TTGCCGCTGCGCCGCTCCTGCCAATCATCAGCAAGCTCGTTCTTCAGAAAGCTGTCTTCG
TTGCCGGCAATTGTGAGGTTCACACACGCGGGATCAATAGCCTTATTAGTGGTCATGCAC
TTCACTGATAGCACAGATCGCCTCATGAATTCTTGAAGTTTGGCATCGGAGCTCACGGGA
GCGGCAATCGCAGGTCTTCTCCTGGCCCGTGCCCTGGGGCGACTCCAGACCAGCGCCAAC
CCACCAGAGCTAAAACAGCTAGGGCACCAAGGTAGCGCAATCCTCTCAGCAAGCGGTAGG
GTATGCGCAGATGTTTCCACGAAGCCCAAGCGGTCCGCATGAGATCGGATTGTTTCGGCC
AGCTGCATAGGGATCCGCTCCCGCTCTCTCTCTCGTGCAATGTTGCCACCAGTCAAAGTG

TGCAACCACATGAGTATTTAATTCCCGTATAAGCGTGTCGGGTAAGTCATGACGTATCA
GACTAAGTCATGACACGGGCC
>at03094
TTTTTTTTTTTTTTTTTTGGNGACAGGCCTTACTCAGAAGCTTTGGGCTGAGGAGCTTCC
GCGCCGTCCCCGTCCTTGCCCTCAGCCTTGGCCTTGCCGGGTGCGTACAACCCCTTGGGA
GCTCTCGTCCTCACCTTGGCTGCCGCCTTTGGCTCAGCCGTGGGGGACGCGACAACGTAC
AGAGCTGGATCGGTGATGTCCGCCACTCCCATCTTCGTGGCGTTCTCCTGAGTGATCTCG
GGCGGTCGGCCCTCTTGGTCCAGAGCGCCGATCGACGCCGCAAAGCGCTTCGCCTCACGG
AGGATCTCCACTTTAGCGATGTCCTCAAAGTTCCGCATCACATGGTTGATTCGCGACGTA
GTACGCCTGCGCCTTGACGTTCTTGGCGATGTCCATAGAGGCAAAGTCGGGCACTGCAGA
GCCGTCGCCCCCAAGCGCGACCACGGTCAGAGGCAGGTCCTCCAGCTGTGACAGGAAGCT
CTGCTCGAACTCGTGCAGGAGCAGCAGCCCCCTGCCCCTGTTGCCGGCGCGGCCCGTGCG
CCCCAGCCGGTGGATGTACCTCGTGCC
>at03095
TCGCATGCATCGTGGACCTCGTCTCCATGTCTTGCTTCTTTGGCGGTGCGTACATGTCCG
ACCCGGGACTCTTCAGCTTCAAGGAGCTGCCAAGCATACTAAGGCTCGCGAGGGACGACT
TTGGCTCGTACTGCACCGGAGAAGAGGGGTGGCATGCATTAGCTTGGATCAACCTGGCTG
CGCTGGGCGAGTGGTTGCTCGTTGCATTCCTGGCCATAAGCGTCGTAGCTTCTGCCTCTG
CTGATGTGCACTCCAATCGTGTTTTGCAGCGTAACACCGCAGCTCGTGTCCGGCCATTTC
TGGATCATGACACCAACGACATCGCAAGGTGCCGGCTTAAAAGA
>at03096
TGGCGGGCCTGGTCCTCGTGGCGCCCTTCCTGTCCATCAGGGGGGCCCTGAAGGACGTCG
TGGGTGTCATTTCCTACGCTGTGACTGAGAGGTTTCCAAGCAAGGACCGGATTCATCTTG
TGCAGTCGCCGACGCTCATCATCCATGGGCAGAAGGACGATCTCATCCCTTGCCGGCACG
GCGCCGCTTTGTACACCCTCTGCCGCTGCCGGAAGCAATTTGTGTGTCCTGAGGATATGC
ATCACAACTCGAACCTTCTCGCGGACACCAGTTACCTTGCGGTTCCCATGCTTCAGTTCT
TTTCCCTTCCCGACTACTCCTCCGAGGAGCTCCGCGTGCCACCTTGGGCGTATCGCCCAG
ACGTTTCCAACCCCAGTTCTCCGCCAACTTGTGGCAGTTTCTCTTGACGGGAAGCGAAGA
ACTCGCAGGTCACAGAGGACATCATCACCGAGGAGACCGCCAGTAGCATCATGGACGAAA
AGGATCAGCGTGCATT
>at03097
CCCCCCCTGGAGCTCGAGGGGCGGAGCCCCCGGCGGCGCGGTTCGGGCGCCATGAGCCGC
GGGGCCTGGCGGCTCCTGCGCTGTGCTGGTCCGGTGGTCGCCTCTGCCGCTCCATTTCTC
CATCGCTATCAGTGGAGGGGTGCCAACTGCGACGTTCATAGGACCGCACATGGGGCTGCT
GCTGGAGACACATCAAATGGCGCTACGTCCAAGAAGGCAGAGATATTGGAGCAGGCGCGG
TTGCTTCGCAGCACCTACAGGCCGGCCTACGCCTTCCCTCGCTGGGTTTGCGACAGATAC
CTGGCAGAGCGGTTTGAGCACTACGAAATCGACGCCAATGCTTGCTCCGTCTTCTTCGAC
GAAGGTGATTCATTGGGTACAGTTTTATTGCTGGCAACCCAGCGAGAGCCGTGAACATCG
GGATGCGCCGAGTGTAT
>at03098
GGAACCACAGATGGAGCCGGCGCCAAGGCGCGCCCGCGGATGGTCGCACGCACCGGCCTC
GGTTGGCGATGCGGCTCAAGCCGCGCCACCACTCCTCGGTGACCGAGTGGAAGTGGAGTG
GCCTTGCGAGGCTGAGAACGGCAGCGGCGAAGTGCGAAGAATCTGGTTCCCCGCTGCGGT
TGTGGAAGTGCAGGAGGGCCGGAGGACCAAGGTACCTCTTGCGCTTTGAGGATGGGGGCG
AATCATGGAGCTCTTGCGCCAAATGGCATGGCGCCGTGTCGAAGAGGAGCACCATGATGA
GGCCGGCTTTGTGCACCTCTTCACTTTGGAGGACGAGGCTGCTGAGATTCTGCGATGACT
TGCATGAGCAGCGTGAGG
>at03099
TACGCGTACGCCTGCACCGGCTTCTTCCTTTTCATACTGGTCCTCAAGCTCCTCACCGTT
AAGCTGATCTTCCTTCCGAGCCTGACCCTTCAGATGCGACACCTCAGTGGCTTGAAGTAG
GTTGCACGGCAGTGGACCGAGCGGCCTCAACGACTTCGTCTCCATGGCCAACGATCGCCG
CTTCCAGTACAGAGCGGAACGAGAAGGAGGAGCAGGCGGCGTGGAAGAGGTTGGTCCAGG
CCGATGAGGACGCACTTGCTGCTCACTGGATTACCTCATTGGACTTCCCGCTTTCCACGC
TCCGTGAAGGACTTCTGAGGCATTGCGATCGGTACCTCTAGATGACCTTGGCATCGCAGC
CATAAGAGACCGCTTCCTGAGTGGCGAGCAAAGTAGAAAAAAAAAGAAAAACAAA

>at03100
TCCCCTGTACGAGAGCCGCCGCGCACCCCGCCCCTGTACGCAACTCTGTCTCCTCACCCC
CACCGCCCCCTGGCGGCGGGTCCCTTAGGTGGCGCGGCCGGCGCCGCCGCAGCGCTCGCG
CCGCACTGTGGCTGGCCGCCCCGGGCCTCCGGAACCCCGGTCTTGCCGAGATTGAACGCT
CTTGGCATTGCATCGCTGCTGCAGCGTGGCGAAAGGCATTGCGGCACTCACTGCAGTGGG
CCAACATCACGCAAAGACGGGTAGACAACCGCAGGCTCTGATGGATCGCAGCATGCTGCA
TCCTCCAAAGGTCCTCCACTCCCCGATGTGGCTCCAGGTGTGTATGACATTGGGAAGACC
TCGACGGAAACAGTGAGGCTCTTGTCTGGGTCCAGCTGCTTGAGCCAGTCACCGAAGTCA
CCGCATTCGCTGACAGTGCTCTCCGCAAAGTTGTGAGTGAACGGTCCCCTGCGCACCGAG
CCCAAAGTGAGGTAGTACTGGAGCACGCATGTGCCGGGGCAACTCGGTATCTTGAGCTTC
AGGAGGCATCGAGCGGCCCCTCAGTGATTCTGCGCGTGTAGAGCTCCTTCT
>at03101
ATGACGACCATCAACTGTCTGTACACTCAAATACAGTGCTATCTCAAAGCATCACAGACC
GTCCCTTCACAGTGCCAACCACGTTTCTCTATGCAGCGCACCGAGATGCCGCTGTTGTGC
TTCTGGATCAAGTGCTTCAAATAAGAGATGAGGATGAGCAAGGCGTTATCTGAAGCATCA
TGCTGCTCCCCATCTCCATCGTAGGTGTTGATGTATATCGCATTCCCATAATTGCAGCGA
CAAACTTCGGGCGTGTTGTCGATGATGATGGTGGAACGACGAGTGTAACCTCTGGCGCGA
AGAGACCCGTTCTTCCAGATCTTGCTCAACCGTTTCTCTCTCGGGCGCACAGGAAAGAAG
TGGTCAGTGGAGAAATCGAGACGACCAAGGCTCAGCCTTCCGCTCCATGCAAACGCCCAA
GGTCGCTGTCCATGCGGATCTATGCTTGTCGTGAACAGCTGGAGCCACTCCCTGGAAGCC
GCGGTCCAAATGCCCACTGCGCAGAAGCTCTCGAAGGCAAAGTCAAGAAATTCCTGAAGG
TGTGGACGCAAGCACCTGCGATACTGGCTGGCGGTGGCTGCCAGCGGGGCAGACTCGGAG
AGGAGGGTGCCGTCGATATCAAGAACAAGGCACAGCGCGCTGCGATGTCTCCCGGAATCC
AGCGAGTTGCTTCTTGGACATGTCGGCTCTCTCGGCGGCTCGTAGGCCTCATCTAGGCAG
AGTGGGCACGGCGCCCCGAGTAGAAGGCCGGTGCCATCGCAAACAGGACAAAGTGTGGCC
ATAGCTCGGTGATGCCCAGAAGCATCTATGGCGAAGAAATCCTGTTTTGGGCACCTTGAG
CCAAAATGGCTACGG
>at03102
TTTTTTTTTTTTTTTTTTCGACGGCGACAATTATGGACTCAGGCGATGCAAGATGTTCTC
TCCGTGGACACCCTTATGACATGCACGAAAATGGGATGCTTGTGCCCTTCAAATCACTTC
TTGAGGTGTCGTCCGGTCACAAGCTCGGGCAATTCGCAACTGGCATAATCTCCGGTCATC
ATCTTCTTGCTGAAGTCCTTGGACTGTTCGTCAACCTTCTTCAGCTGCGCTTCAAACTCC
TCCTCTGACAACATCCCTTCTTTCAGGGTCCATGTGGTAGTGACGCCGGGGCCCATCGCC
CAGCTTTGATCGGCCAAACGCTCCCGCCAGGTTGTTCCGTGGAAAGCGAAGGAGGGCGTG
ACCACGCCACCGCGCTCTGATGCCCTTACCTTGTCTTGCTCCTCCACAAAGACGAGGGCA
CCGTTCACGGCCGCTTGGGCTGTGAATTCGTAGCCCTCGGGACCGTCAAACTTGACATTA
GCCCATTTGCCATCGACACTCTCTGCAATGGCAAAACACTTGCTGTGTGACAGAAGTCGG
GTCTCACGAGGTGCACCGTAGCCTGGCCGGCCACCCGCGCCTTTCATCAGACCGTCTTCC
ATTGCCTGCGCGTTCGACATGATCTTCTTGATATCCTCGGGGGGCTTTGCCATCTGTGCA
TTCAGCTTGGCCTGTCCGGCGCTGAAGGTGCAATCGCAAGAGCTGATAACGATCTCCTCG
CCGAAGTCTATGGCATCCACGGAATTCTCAAACAGTTCACACGAACGTCGGCAGACGCGC
ACACCAGGATGTCCTGCAAAACCAGTGGAGCACAATACGTTCGGGAAAAACTCAGACTTG
TAGGCCTCCACAGCGTCCTTGTCAGTGTCCCGCTTTCCGCACTTCCTGTGACCGCCTAAG
CTGAATGGATCAACGAGCAGAGGAGGCTCGTCCGTTCTCATGTGCTCGAACTGCGACCAG
CCGCTGAGAAAGGAACCACCACCACGGACTGCAGCTGTGCACATGATGTACTCATGGAAT
CTCTTCAATGGACCAAGCTCCTGCTGCAGGACATACAAATTATAGTCACTGAGAGCACAC
ATGAAGCCCACCATGATCACGATCATGATGCCCTTCTCCTTGGCCTCATCATGATACTTG
TCGATGCACCTCCGGTTGTACACGATCTCACCAGTGACATCGATGTAGTGCGTGCTGTGC
TTCACGCATGCCTCGACCACCGCCTGGCCGATAAGCAAGTACGGGCCGGCGGTTGCAATG
ATAACAGTGGACACTTCGGCTATCCAATCCAAGTCCTCCTGGGTCTCTGCCTTGATGACA
TTGGGGTTCGTTTTGCAGTTCTTCGCGACCTTCTTCAGCTTCTCCTCGTTGCGGCCCGCT
ATCGCCCACTTCTTGTCGGTCTTTCCTGCAGCTATGAGCTCATCCAAGTGCTCCGCCACC
ATTGCACCAGTGATGCCAGTGGCACCGTACACCACAATGTCATACAACCGACAGCCAGGC
TGTGCATCCAGATCGTCGAAGTCCATGGCTCCGGGGAAGAGTCTGGTGGGAAAGGAAAAT
TAAGCCCACACACTTGGAGCCAAAATGGCTACGGA

>at03103
GGCGACGGCTTGGAGGCAGCAGAGGTTCCTGGGCATGCTAGGCAAGCCAAGGTTGACAAG
GCTGCATGCAGTAGCCGCTCAGCAGCTGCCGTGAAACTGACGCCCGAGCAAACTGATTTT
GGCAAGACCGCAAAGCAGGCGAGCAAGAACGACCAAAGTTCCGGTGAGGTGATAGCGATT
GCCGCTTGCAGTACCACTTGCAATTTCAGCTTCGACCCCGAGTTTGATTGCCCACCGGAC
GAGCCCCCTGCCGTGTCGAAGAGCGTGCTTTTCCGACTTGTAAGCATTTGATTTGTTCAT
GTCCATGGGTTGCTGCTTGCGTCATTGGAGGTGACAGTAACAGCTGCATGGATGAGGGGT
TGATGCTTCCGATGGTGCGGCCTGAAGTTTGCATGAGAGCGCACTGCCAGTTACCAA
>at03104
GGTACAAATAAATCGTACGCATCAACGGGTCCACGGGTCCGCAGGTCCGCGGGTCCGCAG
GTCCGCGGGTCCGCTGGTCCCCGCGTCCACGGGGCCACAGGGCCACAAGCTACCAGCTAC
CCAACGGAAAACCGAGGGCCGGATACCCGTTCCTTAAGCAATATTGGGGACTTAAAGCAA
TTTTGGGGACCCTTACAGCCAGAGCTGTTCAGGGAAAACCGCGAGCCATCAGAGTCTGCA
GAAGGCACTTGTGCGCGGTGAGCCGTCAACGGGCTCTGGAACCTCTTCCTGGATGCTGGC
AAGGCTGGATGCGTGGCCGGCAGCCCCGCCGCAGATGGGGCAGGTCATGTGCGCTTCGAA
GTCCAACATGCCACAGTTGCTGCACTGATACTCGCTGTAGGGCGCAAAGTTGAGCTTGCA
GCTGGTCGTCATCAGCAGCTGGCTCATCCTCTGCAGCTCGGCGACGGCGGCGTGATACTT
GCAGCACGAGTCCGGCGGGCTGGGGTAGTTCCACTGCCGCAGGATGTCCAAGATCATGAA
CGACTTCGAGCTGTTCTGCGTTTCCAGCAGCGCGGGCAATGCCATGGGCAGGGGCAGCTC
CGGCCGCCCCCCCTGGGCTGCCTCCGTCCCGGCGGGCGCGGGCACCTGCCGCTCGGCTCC
GCCTGCTGTCGGCGGAGTGTCAGCCACCGCGGCCCCTGGGCT
>at03105
TTTTTTTTTTTTTTTTTCCTTTCCACCACAATCATCTTTCCAACCTACTATGCTCAAAC
CCCCTTTGAGCCAAACGCCCCCCATGCATTACACTCCACCTTCTCAGGAGGTAATGAGCA
ACTCTGTCTCGTACTGAGGAAGGCCGCGTGCCTCTTTGATGGCTGAGGTGTTTCCAGGCA
TGCGAAGCCATTCTTCCTGGTTGATTTGGTAGATCATCGCGCCCATCACGTAGTCAATGA
TGCTTCCGTTGAAGTGCTGCCTGAAGTCAATGTTGCGAGTCTTGTGCGAGAAGCCAAATC
CGTATGTTAGAAGAACCCAAATGTACGCGGCATGAAAGCAGAGGTGTACTCGGTTGTAGT
GATCCTTGACTCTGGCAAAGACTATGTTCAACTGCTCCAGTGACAGCGAGCAAACGTACT
CAGCAGCGACCCGCAGGTCAGAGATCTCCGCTTGCGCTGGCAGCGCCAGGAAGTCAGCAA
TCTGCGAAAAGTGAGAGAAGGCAATGAATTTGTTGTTGTAGAGGTGTGGCTGGTAGACCC
CGTAGAATGAGCACTTGGGCACTAGGCAGTCTCCTTCCTTGTGGAAGATCCTCCGGATGA
GGTTCTGACAACTTTCAAAATCTGCAGACCCAGACATCTTGATGTCAATTGGAAAGCTGC
GGTTGCCGGAGTAGCCAAACCTTGGGTGGTAGGTGTATCCAACTGAATAGCATGGGTGGT
AGAGCGCATCTACAGACTGCACTTTGAGCAAAGCATCCGAAATCATGAGGCTTGAAGTTC
TGTGCGCTGCCTCTTTCTGGCCGAAGTGCAGAAAGGAGGTCGAGTAAACGCGCAGATTGT
GGGGACCAAGCGTGAGTGGAAAAGCGTGAGACATCACTGACTTTTCAGAAATGAAGACAA
TCTGGGCTGATGCTCCTCCCAGGTCCAGCGCGCCAACACTGCTTGAAGCTGTGATCTTCC
TGTCAGAGCGCAGATTGCCAATCAAATAGTTCACAGCAAGCCACCCATATATTCCTTCCT
CTTCACCAGTAGCCACTACTGCAAAGCTATCTTCAAAGAGAAAGGGGCTTTGTAGCAATC
GTTTCCGGATGCTATCAAGCACCGCCTCACGCTCATCGGCAGTCAACAGACGCATGCCGG
CTGTGGCTCGCAGGTAGAGGGGAGCGATGCTCCACTGCTCCCTGACATCCTTCAGAGCTT
CCTTGGCATACTCGATCAGAGGGTCCAGCGAGGGTCCCGCTCCATCTGGCTCCAAGGCAA
ATGAAGCCACACCCACTGTAGTAGCCTTGCTGCTCAGGGCCTCAGGGAAGACAATCCCCG
GTGTCGGATGAGGCAGCCATCTGAAGATATAGATCCGCGAACCCTCACTGCCAGCATCAA
TCATCATACCATATCGATGCGACATTTCATGCCCATTACACCTGCAACCCTGTATCGCCA
GGACGGCCAACCACCACAGATGCATGGCATTCACTTGCATATGCCGCAAAAGGTAAACCT
TGAGCCAAAATGGCTACGG
>at03106
TGCGGCGCCCGCACGCTGCCCATCTAGTCACCGTGTCCCCAGACCCGTGAGAATGGGCCA
GGCCGGCATTCTCGGCCTGGGAAGCGCCTGCAGCGGTGGGCAGCGGCATCTGTGCTGCCG
CTGGCGGCTCCTTGCGCTGCAGGAACGGAGAAAGGTCCACACCGAGGTTGTAGTTTCTAT
TTGATTCCTGCATGGCTGCATGCAAGAAGGGGTTCTCGAGGATGCCGCGAATGCTTGGTC
TCATCCCGGCGTCTTTGGACAGCATGCATCGGACTAGGGAGACCAAATCCTTGGTATACA
TCTCATCTGGTGGGGGATACTGATCCTTCAGGATCTTGCTAGCCAAGGTCACCAAGGAAT

CTGCATCGAAGGGATGCTTCAATGTTGTCATTTCGTACAGGACAACTCCAAGACTCCAGA
TGTCACTCTTGAAGTTGTACGGGCGGTCTTCGATGATCTCAGGGCTCAAGTAATACGGTG
TGCCAACCATCGTCTTAGCATAGTCCTTTGTGGCGTCAAGGACTCGTGCAATGCCGAAGT
CGCCAAGCTTAATTTGACCTGTGGCCATTAAGAAAATGTTTTGGGTCTTGAGGTCGCGGT
GCAGAACCCTTCGGTCATGCACGTGCTTCAAAGCGAAGCAGGTCTGTACAAACCATTCCA
GGATCCGTGCCTCTGGTATGAGCTTACCTTCGCGTCGCTTTATCTCCATGTGCACGTCCC
CTCCATCTGCGTAGTCCATGACTATGCAAAGACGGCCCTTGCGCGTCATGAAGACCTCCT
GGAAGCGCACAATGTTCTTGTGATCCATCCGCTTCAAGAGCATCGCCTCCTTGACTGCCT
CATTACGCTCTTTCGGGTCCATCATGGAGGTCTCCATCTGCTTGACTACGCAGAGCTCAT
CGGCCTCTGTGTTCTTCACGAGATACGCCTTTCCAAAGGAGCCCTTTCCCAAAACTTTGA
TCCTCTGGTACTTCTCATCCATGGCGCTAGAACCAGACCCATCCACTTGACCCAGGAATT
ACGGTTCGACTTGGGCTTGAGCCAAAATGGCTACGG
>at03107
ACGGAGGCCTCAACCCCAGTCTTCCTGTATACAAGGAACAGCACAGAACTGGCCGCGCGA
TCTTCTCCTTTGGATTTCCTGCCCGGCGCAGTGCGAGGAACACGGAACAGTATCTCCTTC
TGAACAGCTTCCAACCATGCAGGATCTAGATGAACTGTCCAGCGCCTTCGCCAGAGTCCC
AGAGCTCCTATTGCAACGACAACCGAAAGTCCCCAACGCATGACCAGTTACTAGCAGCAA
GCACACTCGGGAGTGACAGCCGAAAGACTTGCCAGTTGGTCCATGAAGAGCATGCCGCGT
GTATAGTTCGGAATATCGTTGTGCCATCAGTGTGCCGGCTCCTTCATACCATCGCTGTGC
TAATTTGGGACACGGGAGGTGAACCAAGCATGCACTGCTACAGTGCAAACCGCTTGA
>at03108
GCAACCCCAAGATGACACAGTCTGTATACTCTTTGCCATCACACTGCAACTATGAGCAAA
CTCCGGGTCTCCCGACTCCTTTGCTTCCTCTGCATCCTGCTCAAGCTTGGCACTGAGTCG
CGCAAGCCCATACATCAAGGGTATCAACACCATCGAAGCTACTGCTGCGCAGGCCCACAG
AGCTCCTGCGCTTGCAGTGGTTGGCTCTGAAGAGAAGCTCTCAGCAGCAATGACGCCAGT
CAGCGCCAACCATGCCAAGCCCATCGCAAAGTTTATCGCATTTGCGGTGGCCGCATATGC
ACGGCCGGACACCTCAGCAGATGCAGCAGCAAATACCCTCCCAACTCTGGGTATTCCGTC
GCCGGGCTCTGGTGACACCTGGGGCGCCATTTGGGGCAGGTTTGTCCGGCACTGGAGCCT
CGAGGGCAAACAGAAGCAATGGCCTGAATTCACAGCTTGGGCCAAAATGGCTACGG
>at03109
TTGTTGTCGAATCAGGTTGTCGCACTCCATTTCCGGTCCTTCACCCCACCTGAAGCGAGG
CATGGCTGACCCTGCTCACGGACCCAGTCCTTGCCCCTCAAAGGTCGCTGAAGCAACGCT
CGCCATTGTCAGATCGGGTCATTACCGCGTGATTTCTGACGATGAGGCTGTTGCGACTGT
GAAGCTCGAGCCCTTCTCGCGGCTCCAGCAAGCGGGCAAGACCGTGACCACGCACTGCGC
ACCGCACTCGGCGCTCCCGCCTCCACAGCAAGGAAATGAAGCACCTCGGATGACGCTGTT
CCAGGGCGACAGCTTTGATGCGGCAAGCTGGCTGATGGACTCCGCGCCGAATGATGCGAA
GGCTGGAGAAGCATGCGTTCTGGACTTTGCCAGCGATTCGGAGCCGGGTGGCGGAT
>at03110
CCGTAGCCATTTTGGCTCAAGCCACTAAGGCTCAAGGGTCAGGCCAAGGCTTTTGCGTTC
TGTTCGAGCCATTCAAACTCTGGAGTTTGCTGCCCAGTTCTGGCCTGATGTCGCATGAGG
TGGCATTCGTAGCACCGCCAGAGCCTGGAGTTGTGCCGCGGTCGGTATTGCGAGGTGCGG
CTTCTATCTTGCCAGAGGAGTCAGTCGCTAATGCTAGACCCATACCAGAGACCAGTGCTA
TGACCACGTCCTTGCAGTTTGTTGGACTCG
>at03111
NTGCATGCGGGGCTCTGCGTCTCCGTCTCTGACATTGTGTACAGCGAGAAGGTCACGCCA
TTCATTTCGGCGGCTGCTTcCAACCCAAAGGCACTCTTAAAAACAGAGGAATGGCCACTT
GAAACTTCATGGCTGGCAGAcGACACTTCCGGCTTGCAGGACCACCTGGTGGGTGGTGAA
ACGGGGCCCCCCCGAACAGGATGG
>at03112
TGAGAAGCACGGGGTGCAGGGTTACCCCACGATCAAGTATGGCGACCCGGACAGCCTTCA
AGCCTATGAAGGGGAGCGCGAGTACGAGGCCCTCAAGAAGTTTGCGGACGAGAACCTAGG
CCCCGTCTGTGGACCCGAGCAACTGGAGTTGTGCGGCGAGGAGGACAAGGCACTTCTTGG
AGAGGTTTCCTCGCCATGCCCAAGGCGGATCTTGAGGCCAAGATAGCATCAACGCGGCAG
GCCGAAGAAAAGAAGATGCTGGCCTACAGGAAGAGGTTTAGGAAGTTCGATGCCAAGTAC
AAGGACTTCAAGTCGGAGGAGAAGGAGGAGCGCGAGCAACTCTCCATACAGAAGGCGGAG

AAGGCCAAG
>at03113
CAGACGTGTGGGGCCCAAAGGCTGGCCTTTGGAGGATCAACACACCACTGGTGGTGAGTT
TGAGCATGGCAAGCGTTTGCACTTTTGTGCCAGTCCGATTCGAACTGGTGGTGGTGCTGG
CAGTGCTGAGCGTACTCGCGGTGACGGTGGACTACGCGTTGACGTGCCGATCTGCAATCG
GAGGATTGAATACTATTCTGCTCCTGTGCTGTGTCGTCATCTGCTTACTGAAAGGTGCAT
GGTCCAACGAGCAGCGCCTTCGGGCAGACTGGGAGACGAGCAGGCTGGCAAAAGCAAAGT
TGCAGTCTGCAAAGCGCGTGTCACAAGATACCCATCACAAGGTTCAGCAGAAAGAGCAGC
GGAAAGAGGTCCAGGAGCGCATGCATGGAGTCTTGGTTGGACCTCCCGGCAGTCTC
>at03114
TTAGGACATTCCCCGATCTGAAACCAGATGCACCAGCTCAAATCTGTCAAGGAGCCTGCT
ATGATGAAGTTAGCTGAGAAGAGCGTCTGCCAGTGCAGTGTCTTGCATCCGCAAGGCCAA
CGCATGCATCGCTGCAACTTCAGGGAGTGCAGCGTTGCAGAGCAACAGCAGGACAAATGT
GAAGAGGCCAGGTGCCCTNACTCTGCA
>at03115
TTTTTTTTTTTTTTTTTGCATCTGGGTCACAGCTCATCTCTCTACATCACTGATGCTTC
TCTTCCACATGCATCGCACACACGTGCTGCCCAGCTGGAAGAGGCCATCCAAAAAGTACC
ACGACCAGGCAAAAAGTGAAGCCAAGGCCGTGTAGTGCAAGGGGCTGCACTCGTCAACGG
CAGTCTCGGGGTGGGCACTGGAGTTGGGCTGGGAGTAAGCCCGCCAGCGACACTGGCCGT
CGACGTCGGGCAACGAGGCCAAAAGGCCTGTGAAAGCAGAGTGTCCTAGCTGGAGGAGGC
TACCCACAAAAGCCACGACCAGGTCAAGAGTGAAGCTGAAGTCGTGGAATGCAAGGATCT
GCAGCCGTTGACGGTATGCAAGGTGGGCGCCGGGGTAGGCATTGGAGTTGGGCTTGGAGT
AATCCTGTCAGCGACACTGGCCGTTGGCGTCGGTAACGAAGAGACCGAAAGGCTTGTGAA
AGGAGTCTCGGGGTCCAGCTGTTGCAGCTGCGTGACGATCTCCGAGGCCAGCGCGTCTGT
TGTGATGCTTGTGATTGAGTTCACAAGCTCGGTCTTCCTCTGGTTGGAGGCATCCGCACC
TGCAACTTCAACAGTGATGGTGTACTCCCCT
>at03116
CACCTCGGCACCGTCTGTAGGCCGTACCCCTGCTCCATCGTCCTCGAGCGTGAATGGGGA
CGGCTCCAGCACAAATTTGAcCCAAGCTCTGATCCTTCGCGTGGCTGATGTCCGTCCTGT
CTGACTCAAACCCCTTGTTCACGTCCTCCTCTGGCGCCTCCTGCATCTCTCCAGCCGCCT
CCTGGGCGTTGGAAAAGTCACAGCAGTCGGCGGGGACCGCATTGGCAGCTGCGGAACTCG
TTGATGGCACCGCTGGCGCGTCCGAGGCGCTGGCAGCCATGCCCGCATCAACACCAGCCT
CCACCTGCACGGTGGAGCTGGCCAGCCCGTCACTGCATTGCGTCTGCGTATGTGTACTGC
TGGAGGCTGCTTCAGTGGAAGCCTGCTCTACTGCTGCGGGTCCAATGACTGC
>at03117
GCAGCCTGGGCCCtAGGTAGAGCCCAACAGCGGGCGAAACGCGTCGGCCATGAGAGCCTG
CGCCTGTCCGTGACCGTGGGGTTCTCCATCCTGCGGCGGCGCGGCAGAGAGGCTGCATTG
GGAAGAAAAAAAAAAAAAAAAAAAAAAAAAA
>at03118
CCGTAGCCATTTTGGCTCAAGGCCATTCGGGCTTGGAGGGCAGGGTGTTTCTCTTGGAGC
TCCTGAAGGTGGGGTCATGGCAACCAAGCCATATATCTTTGCTGCGGAGCTTGCCAAGTC
TGGTGCCGCTGGGTGCAAAGGTTGCAAGGAGAAAATAGCAAAGGGTGCATTGCGTGTTGG
AGTCATCACTGTCTCCAAAGACTGTTTGGAATTTAGCAACTTTGGCCAACGTTATGGTGA
CAATGCCGGCCCTGTCGCTTTGGTTTCCCAAGAGGACATGGACGAATGCGAAAGCATATT
TGATCAAATGGGCTGGCTTTGGCGTTGGCATCACGTGGATTGCTTCCACTTTGACAAGTA
CCCTGCACTGAAGGACATGTCGTGCTTCTGCTCCAAGTCCCCAATTGGCACACTCTCAGC
CGCCGATCAGAAGACCTTGGAAAGTAAGATCGGTGGAAAGGCTGATGGCAGTGCCAAGGG
TAAGGCCAAGGCCAAGGG
>at03119
GAGGAAGAGCGATGAAAGCGCCTTGAATGTCCATCCGCCCCTCCGTCCCTTGATCTTAGA
TGGCTGGGCCTCCGTGACAGCAAGCTGGTGCACTTACGCAAGGTTGCCGAGAATGGAGGC
TGGCGCGTGCAGGAGGTTGCCTACGAGCAGTCGATCGTCATCCTGGAATTGACCCGGTGC
TGACAGGGCCTTTGGAGCCGCGGTTCTGGGCTGGGAGTGTGGAGCCGGTGCAAAGGCCAA
TGGGCGCAAGCGGACTTGGAGGCCAGCAGGCTCGCGG
>at03120

CCATCTCGCTCGCCACTCGCCGCCGCGCCCGCTCACTTCTTCTTGTCCAATGCCATCTTG
GTCGCCATGATGGCGTCAACGGCCTTGTCCCGCTGCGCCTCGAACTCGCCGAGGCTCTTC
GCCACCGCGGCCGCGCTCGCGACCGGCGCGCCGCCCTCGACCGCCACGAGCTGCCGCTGT
CGAGCT
>at03121
TTTTTTTTTTTTTTTTTACTCTTGATCTCTGTACATCGGTCCTGGCTGATCATGTGCGC
CGAGCAAAGCTAGACTGTACAAGGCTTGCAAGGCCACGCCTGGCTACCGATATGGCTTGA
GAGTTGACCAATGGAACTCATGCCGGCACCGACAAGTCATATGATCGCAACCGCCGTTTT
TCTCAATGTACTCATTGCAACTAGGGCATCGCTTCACCTTGACACCCATGACCTCTTCCA
CGCCTATGTCTGAGGGGCTTTGTGCCGGGCTCCTCTGGAAGCCATTGATGCTCACAAATG
AAGCACATGACGGTGTCGAAGCCCAAGTAGCCCAATCCCCAACAATCAGGGCGGGGACAA
TCAACCTGCATTCCAGCGGGGAAGAGGGTGTTTGCGCGCAGCCGACTTCGCCGGGCAACC
ATCGGAAGCTTAGACTAGAAACTCTGAACCTGCTCCGACTCTGTGCAGCAATGCTGCCAA
AGGGCGTGCGACATCGCGTCTAGGCATCGTGGGAAGAAGCAGCGCGCTACATCCCGCCGC
ATGGAATGGCATCGAGGTATCTCCGATTCAGTCCAGAGCCGCCAGCAACCCTCACAAGCA
CCATGGTGGCAGGGAGTGTTCTGCAGCAAGGCCAAAGCAGGCTCGAGGCAGATAGAGCAG
ATGGGGCCGGGACATGATGCGCGAGCCGCCTGTACAACCCGCACGCCTGTGGAAGCAGTT
TTGCGATGCCGCGACATCTCAGATTCAACGCGGCAGTGGAAGGCCGCGACCACGTGAGAC
CGTGCGCGGNAGTGGCTCCACAGAGGCTCGAGATCTCGTGCGGACAGCCAGGGCGAGACA
CCTGGACGCGCTGGCACTTGTCCGGCACTGCGG
>at03122
TTTTTTTTCTTTTTTTTTGCACACCGCCAGTTCCATAATGTCATAATGTGAGTTGACTAC
GCATCTATGTACGGTACAGAGGACCTAATATGATTCTTCCTGGTCTCACCCTGGCTCAAC
CCTTCACCACAGCAATCGGTTTCAGCTTGATAGCCTTCTTTGATATTCCAGCATTATGGC
AGGTCTCGGCAACCTGTATGACGTCCTTGTAGCTCTCTGGAGCTTCCTCCATCACGAGCT
TTGGGGACGCAACCCGGATGGATATTCCCATCTCTTCCAGACGTGACAAAACATCCTTGT
AATCTAAGTTATTCCGAGACTTGTTGCGGCTAAGCGCACGCCCTGCGCCATGGCACGTGC
TGCCAAAAGTGTCCCTCATACCAACGTTGGTGCCCGTCAGTACGTATGAGCAGGTCCCCA
TGGTCCCGCCCACGATCACAGGCTGACCAGTCAGCTGGTAGTCCACCGGAATCAGCGGGT
GGTGCGGAGGGAACGCTCGCGTGGCGCCCTTCCTGTGCACAAGGAGCTGCATCTCCTTGC
CATCCACCATGTGTTCCTCGATCTTGGCAATATTGTGCGAGACGTCGTAGATGAGGAACA
TGTCCAGGTCCTCGGGAGACTGGTTGAACACCTTGGAGAATGCCTGCCGCGTGAGGAAGG
TCATCGAGGAGCGGTTCACCCAAGCGTAGTTCGCAGCGGCAGCCATCGCGGCCAGGTAGT
CCTGACCCTCCTTGGAGTGGATGCGAGCGCACGCCAGCTGCCTGTCGTTCACCTGGATGT
TGTCCCGCGCCATGGCGCGCTCCATCTCGACCAGGGCGTCTGTGGCAACCTGGTGCCCCA
GGCCACGGCTGCCCGAGTGGATCATGACGCACACCTGGCCCTTCTGGTCTATGCCCATTT
TCTTCGCGACGAAGTCGTCGTAGATCTCCTCGACCACCTGGATCTCCGCGTAGTGGTTCC
CGGCCCCCAGCGTGCCCATCTGCGGAAGCCCGCGCTTCTTGGCGCGGGCGGACACCTTCG
AGGAGTCGGCGTTCAGCATCCGCCCGAACTCTTCACAGTGCTCCTTGTCCTCGGGCCAGG
CGTAGCCCTCGCGGAGGGACCAGTCGACGCCCAGCTGAAGTACCTCCTCCAGGTCGGAGT
TCTTGGTCGGGATGATGCCCTGGGAGCCGACGCCCACCGGGATGTGGTCAAAGAGTGACT
GCGCCAGGCGCTCTTTGACCGGCTCGACATCCGAGAAGAGCAGATTCGTCCTCAGCAGCC
GCACACCACAATTGATGTCAAAGCCTACGCCGCCTGGCGAGACAACCGACTGTGGATCGT
TCATGTCGAAGGCCGCAGCATTCCCGATGGCAAACCCGTATCCACTGTGCACGTCCGGCA
TGCCTACTGAATGCTTTACGATCCCTGGCAGGGCAGCTACGTTGGCGATCTGCTTCACTG
CAGGCAGGAAGCCGCCTACATTCTGGGACTGTCTGCTGAAGCTCTTCAGCTCCTCTATCA
TGAGCGCCTCCAGAGCTGAATTCACGTAGAATTTGCCCGGGACATTCATGCCTGGAACAA
ACCCGGGCTTGATCGAGAACATGGAGTCAGTCTCGCGTTTGAGGAACGCCATCTCATCGT
CAAAAGTCCGCTTCGTCTCCATTATGTCAGGCACAAGACCAAAGTCTAAATCTGTGTGCT
TCCGACAAAACTGCTTGAGCCACTCCTTGAGCCAAAATGGCTACGG
>at03123
CCCCGTCAAACCGCCAAATGTTGCAATCTGCAAGCTTCAATGCAGTAGCTCGGCTTTGAG
CGATGGTCCAGAAAAATGTGCACGCAAAGCTGCATGGAAAAACGCTCTTGGGTGTGGTGGC
ACTGAGCCACTTGGTGAGCTCGTTGTGTGAATGTAATCGGCCACTCTGCAAAGAGCGGCC
GACGTGCTAGCTGCATCGCTTGCTAAGGTGCTCCGGACCTGAGGAGCTGTTCTCCAAGAT

CAACTGGCTCTGAGCCGTTGATGTATGACCACCAGCGGTTTGGCATAGGGATCAGAGGAC
TGGTTGTGCGCTGATACGCTCGATACAA
>at03124
CGCTTCGACCACGGCAAGCCCCTCTGCGTCGGCGCCTTCGAGCGGGTGGACCGGCAGCTG
CTCTCCTACTGGTCCGGCATCGTCTGCAGCAACGCCGCGGTGCGGCTGCTGGACCACCAC
TTGCCGGGCGCCACGCGGGACGTCGTGCAGAGCCGGCTGGGCTACTCAGCCGGGGACTTC
ATCATCAGCAAGTGCCTCCGGGACCTCAAAGTGCCTTTGGTCGACCTGCCTGGCTTCTTC
GGCGGCAACTGGCCTTTGGCAGAGGACGCTGAACTCTCTCCAATTGCGGCGACCTTCCAC
CGGGGCCAGAAGCCGCCAGATCTTTGAGACGATCGACCGTCGGTTGCATAGAAGCTTACC
GTCAAAGAAAAAAAAAAAAAAAAAANAAAAAAAAAAAAAAN
>at03125
GCCGGGGAGCAAGGAGTATGGCGTGCTGGTCCGTCCACTCTGCAGCTGTATACACGCCCG
CGCAGAGCTTTCACTGTACCTCCAACTGCTTTCTATCCCAGACCCAAGGTGAAGTCTGCC
ATAGTAGAGCTTGACTTTCTGAGCGAGCAGGACGCGCCCAAGATCGACATCTCGTGTGTG
CGGGAGGTCGTCTCGACGAGCTTTCAGCAGCGCCGCAAAGTGCTGCGGCACAGCCTTGCA
CGGCTGCTCCAACGCGAGCGTGCGGGCTTACCATCAAAGTGGGCAGGTCTGCGAGCTGAG
AATATGAGGCCGCAGGGACTTTGTTAGCGTTGCGCAAGCAATCTTCCCTA
>at03126
CAAATTGCCGCGACTCGCTCACAATCAGTGGTCCAATCAGCACTATGAGCACGTATGGGA
TGCTGAACAGGAGCACCTCGGTACGCCAGCGAAGATTGCTTGCAAGCGTACCAGCAACAA
CTGCCACAAGCACGCCAATGCAACTGTAGCAAGTGTTGAGAGCCACGATGGCATTGGTCC
ATAGTCGTTTGGACAAGCTCTCTTGCAAGAGCAGGTATCCCACCTGACCAGAGGCTTTCA
CGAAGAATCCAATAAAAAAGGCTGTGACCGCATACAGGACGGCTGATTGCGCACGGGTGC
>at03127
TTTTTTTTTTTTTTTTCTGGGCCCCAGTCCACTACTTCATCTGGTGCCACATTCCAGTAC
TTTCTCACAAGAAGATGCCAGACCGCTCCACGCTGACACAAGGAAAGCTGTACAAACAAC
CAGAGACCACCACACCATGGCTTAACTACCATGGCTTGACCAATCCAATTTGTGACGCTC
CACGTCATGACGCTCCACGTCTTACTTTCGAATTTGCCACTGCACAGGGCTAAAGCAGCA
TCGGGCACGCAAGTTTGAGGTACTCTGCGGCTTGTATGCACGGCACTCCATGCCTCAATT
AAGATGTGCTTGGCTTTGGCACAGTTGCCAAGACGCGTTTCGGACTCCTGTTCACACCTC
CGTCGTTTCGTTCTTGGAGGCACCTGAATCAGCGTAGGCCTTTGGGGGAGAGTGCTCCAT
GACGTCTGCTCCTTGTTTTTGGAAGTCGTCGCTCAACCGCAAGGCACCCAACATCCGAAG
AGGTACGAATGCAGCCACGCTGAGGGCTGCACCTCGTGCC
>at03128
AATGTCCTCCGCGAGCGGCACGAGCTTCCGCAGTGCATCCTGCCGGCCGAGATCTTGCGC
TCGTGACATTGACTCGCCGTGTTGCCGGCGATAGTTCGCAAGGTCGGCTTGCAATAACAA
GAAGCCGTCCAATGCCTTCTTGACATCCCCCTTGGCAGCATCCCTCTCCTCCATCAAGCT
CATTTCTGCCTTAGATGGCTGTCGCGACCCGCATTTGTGGCACTCGTCAACCTGTGGGAA
GTTCTTGGCACCGCAGCTGCTACACACCCATTTCTGTGGCTTCTTTTCCTCTGGCGGCGT
GGCCTTTGCCTCTGCTTTTGCCTCTGCCTCACTCTCCTCACCCTCGGCAGGTGGCTCTTC
GTCGCTGGGCGCCTCCGCCTCGTCCGCAGCCTTGTCCTCCGCCACTGCTTCGCCCTCAGC
CTCTTCCTTCGCCTTGCCTTGTTCCTCCTCACCCACTGCCTTCTCAGGTGCCTCAGCCTC
GGCGGCGGCCTTTTCGTCACTCTCCGCCTTACGTGCCAGCCCGCCGCCCGGTCGCCGCCG
GGAACCAGCGGCGAGCAAAGCAGCCGCTGAGGCCACCAAGATGCTGCCGGTGGACAATTC
TGTGGGGATGGCTGCACCTTCGTTCACTGCCGCGGCACCACTAACCGCCCCGGGGAAGGC
CACTGGCAACGGTGCCCGGCTGGTGCGGCTGGTGGCCGGTCCTCGGGCCACGCGGTGCAA
GGGTGCAGTAAAGGCCTCTGCCGCACCCCCAACACGAGCGCCGGCAAAGGCTGCAAGGTT
GCCCCCGCCGCGAGCGGCGCCGCCCGCCCCCGTCGGGGGATGGCGGCGACTGGCATGTGT
GGGTCTGCGGGGGAGCGGCCCCGCACGTCGGAGCCAGGAGAATTGAAATTCAAAATCTTG
AGCCAAAATGGCTACGG
>at03129
TTTTTTTTTTTTTTTTTTGCACGGCACAGCTCGGCCAGAACTCAATTCAGCACTGTCACA
TGATCTATCCTACATCAAGAGACTGCCCTAATTAGTGCACTATCTTTGCACTCTGGTCCT
TGATGCCCGCAAAGTTGGTGTCCAGCAGGTGCTTCATGAGGAGCAAGAAGTCAGCAAAGT
CAGCATCCCAGTTGTCCCCGCTCTTATCACCCGTGCCATTCGTCACAACCTGCTGCCAGA

CCCGCGTGAGTTCGTCCACACGCTTGTCACCAAGGGGTGCGATGCGGTGGATGAGCTGGC
GGACCTTCTTGAAGGATAGGCAGTCTCGTTCCTCGACGTCTGCTGCGATGAATACCTCGC
GGAACTCCCTCACCTGCGCCGCGGTGAAGTCGCAAGCCAGCATGGCACGCGTCTCCTTTG
CCCTCCGGCCCTTCTTCCGAATCTCCACAAACTCACGCATCAATCCCTCGTGCC
>at03130
TTTTTTTTTTTTTTTTTGATCATGTGCTGTTGTGTGCGGCGCACGTCGAGCCAACCGCT
CGCCGAGCATGATACCCTACACAGACAAGCTCGGAGGAGGCCCTAGGCATCACTCTGCCT
TCTCCCTGAGTTCCACGCCTCTGCCAGAAGGCTCGGCTCCGCTGCCGGAAGGCCCGGTCG
TGCCCGCAGCGAGCCGATCAGTCTCGTGGAGCACGAAAACTATGGAGCCTATCAGGCCTA
TGACTAAGAGGATGAAGCCTCCGTAGAGGAGGGCCAACTGTGTCCGGCCGAGCATCACCT
CGCGGTAAACCAGGCCACCGCCCATGAGCAGCAGGAACGAGAGCACGATTCCCAGGGCCT
TGGTCAGGGACGCGCGGTCCAGGCCCACGAGCTCGTCGAGAATCTCCCAGATGCTCGGCT
GCTCCGCCGAGTCCTCTCCGGACCCATTGGCGACCGGCTCAGCAGCATCCGCTGCGCGCT
CTGGCGCCATGCTGCGGATCTGGGCCACCCGGGGGCGCGTGCCCCCCCTCTGAGCCAAAA
TGGCTACGG
>at03131
CCGTAGCCATTTTGGCTCAAGGGCTCAAGTAAGCAGAAGCCACTCAATGCAGCTCTCCAC
CTCTACTTGCAGAATGAAGGCCGCGATCGCACTCCTGCTGGCGCTCGTCGTGCGCGCGAG
CCCCTCGTGTGATGAGCAGGACCGCTTTGACATCCTCATGTGCAACTCTCACATGTGCAC
CAACTGCACCTTGGAGTGGTGCATGGAGACTTGTCAGAAGGTGCAAACCGACTTCCCCAC
CTGCCGCTGCGCGGGCTGGCCCTCCACGCGCGAGACCTACAGCAACGGCACCTTCGCGGG
CAGGGGCTTGTATGGGGACGTGGGCGATTATGGGCGCTGAGCTCTGGCTGCTTC
>at03132
ACGATCACTTTGGGCTCGCCCGGCAGCTTGCGCGAGACCGGTACCTTCTTCCAAGTGACG
CCGGCGTCCTTGGAGTGCAGGAGCAGCGGGCGGCTTGCCCACAATCCAGACGTCATTGCC
GTTCACCGAGACGGTCTGGAAGCGGTAGCTGATGTCCTCGCCTTTGCTCAGGTTTGCGAA
GGACCTGGACACCCACCGCTTCCCACCGTCCTTGGTCTCGTAGAAGAGGCCGCGCGCACC
TACGACGAATCCGTGATTGGGGTCCTGCTTGTCAAAGTCAACATCATAGAGGGTCTCCCC
TGTGTTGAGATCATACAGCACCCAAGTGGGATCGACCTCAGCGGAAGCGCTCGTGGCAGC
CGAGCTAAGCCCCGCCAGGCTCGTGCACTGCATCAGCTGCCGGCGTCGGACGGCGTCCGC
G
>at03133
CCGTAGCCATTTTGGCTCAAGCGAGACTTGCATGTACATAGGGACCGGAGACATTGCACC
TGCGAGCAATACTTCTTTGCAGCCATGAGGATTGAGAAGTGCTGGTTCTGCTCGAGCAAC
GTCTACCCTGGGCACGGGATCCAGTTTGTGAGGAATGACTGCAAGATCTTCCGTTTCTGC
CGAGCAAAGTGCCACAGGCACTTCAAGATGAAGCACAACCCCCGCAAGATGAAGTGGACC
AAGGCCTGGCGCCGCGCGGCCGGCAAGGAGATGACAATGGACAGCACCTTCAACTTTGAG
AAGAAGCGCCTGACACCTACTCGCTACAACCGAAACCTCATGGTGAAGACAGTGAGGGCC
ATGCAAATCGTGGACAGGATCAAGAATGTTCGGAAGGAACGGTTCGCCAAGCAGAGGCTT
GCTGTGCAGCTTAAGAATCGCCAGACGTCTGCGCAGAAGGAGGTTGCAAGGAGTGCAAAG
CTCCTGGAGGGACCAAACAAGTTGCAGGCCCTCGAGTGGCAGAAGAAGTTCGAGGAGGAG
AAGGAAACCAAGGCGAAGAACAAGGCCAAGGCCCGGTCCAAGGTCAAGGTCACGGCGAGC
GGCAGCGGCGGCACCGCGGACATGGAGGATTGAAGTGCCGCATTTGGCGTTACCGTCACC
ATGGCTGCGATTTGGTAGCCGCCAGTGGTGTTTTTGCCCTCCGCAGAGTCAGCCGCTCAT
TGCAGTGCTGGCATGGAGGCATGCATTGGGTGACGATTGTGTGCTCTTGCTGGTCGGCAC
AAGGTGTTGTACAGTACCCATTGAAAAGCCCGGCTGTGTTGTGTTTCCTGTCTGGTACAG
TGTGGGATTCGTTTTGCTAATTTGTGTTGTGGCATCTGAATGCACTCATGAAGCTTGACG
GCTCATTGCAGTGAAGGCCAAGGCAAGTCACCTGCATCTGGTCACTTTTCACATGACCCG
CAGCCCGCATAGCGGCAGTCATTTTCATCCACGGAGGGGGCGGCGACGCGGTGGTTCTCC
ATAAACCCGCCTTGACATGCGCAGCCCCCAGCGGACGTGCCGAGCGGGGGGAAAACACAA
AAAAAAAGAAAAAAAAAACAA
>at03134
TTTTTTTTTTTTTTTTTTGCAGTTGCACCATGAGCTGGATGGTACATGCTGCTCCAGCTG
ACATGCTTGCCAAGGAGGCCACAGCGAGCTGCTGGCCATGATTTCGACACGTTGGTCAAC
GTCAAGTCCAAAGGTGCCAAACATGCAAGTAACGTCGATCGTGTCTGCAATCATGTGCCA

TCTTGTGACTTGCCACAAGGCAGCCATAATTATTGGCCATTAGAGCATCAGTAAATGAGG
TGAGGCATCCCATAAGCTTGCTGTCATGACTGCAGGCTGGATGAACGAGCAGCACACCTG
CTGCTACTTCTGCATCCCATGGGGAGGCAGCGTGCACGGGGCATCTTGATGGAAGGCGGG
TCACTCGTGCGCCCTGGCGCTGTGGTCTTTGTCCTTGTTTGGGAACCACTCTGGTACGAG
CTTCTTTCGCAAGTCCGCATCCCACAAGGCCTGTTCCTCTTTTGAGAACACAAACAAGCA
CTCTNGGCAGGGCCTTGGAGCTGCTGATGCGCATGTTTTGGTCGCGGAGATCGTCGAACA
GGGGGTGTGTACAACACCAAAGTCCAGACGGTCGTGTCTTGGGGTCGTACACCAAGAGCT
TGGAAACGTAATCAACAGCCTCCGACGAAGTTCTTGAGCGGAAGACCTTGTGCCATGGAT
GGGGCTTGATCTGTGGGAACTTGAACTCCGTGTAATTTGGGTTCATAGCCATGAGCTCTT
CGCGAGTTGGAGTGCCAAGCACCTTGATGATCTCAACAAGTTGGTCGACTCCACTCTCCC
CTGGGAAGATCGGCTGCCCAAGAATGAGCTCC
>at03135
CCGTTTCTGGCACAGGCTAACAGCGATCCATCCTCACGCGCACCACTCGCCGGCCCTCTC
CACGGCAAAACGATGCAGAAGCCTGGCGAGCTAGATGACTCAGACCATCCAGCGGCAGTC
CATCGGTGCAGCCGTTCTCAAGGNTCCGTGCGCGC
>at03136
TTTTTTTTTTTTTTTTGTGCTCGCAGCCGCGACACAGCTCAAGCCAGCTGCACCCTATCA
GACATGCTCCTGTGAGTCCAGGCCATGAGGAGGAATACCTCCTCCGGCCGGGGACTACCC
TTGCTGAACCAGCGTCGATAAGTGCTCGCGCGACACCAGATTGTCTTTGTTCAGCGGGCG
GAGGCGACCGATGAGGCCCCCGAGTCATCTGCCGCTGGAAGTGCTTCGGAAGGCCCCGAT
CTTGAAGCTCACCGTGGACACCCAGCTGTGACGACTCCGGGCGGCCTTTTCCCATCGTCG
AGGCTCCTCCCTCAAGGCTCCCCGAGCCTCCCGACGGGCCAGGCCAGCTCCAACTCTAAG
ACGTCGTGGAAGTACTCGCCTGTGAAGTACCGGGATCCGCCGAAGACGACGACGGAGCGC
CGACCAGGCACGAAGGCGGCTGCGAGATGGGACCGGAGAAGCACCTCCGTGATGTAGTCC
TCGCGCCGTGCGCCGTCGGAGCTGCCGCTCTCGCGCTGGCGTCGTGCCTGCTGTCTTGGC
CGCTGGAGAGGGGGAACGTTCACGGCAGCTCGTCGCCGGGTCCACGTCTTCGTGCCAAGC
CTCGTGCC
>at03137
CCCAGCTGGCCGACAGCGTGTTGCAATGCTCGGCATGTAATCGCAATTGTCAGCCGGCCA
CCTTGGCGACCAGCAGGTGCCTCCATGATGGCTCAGTGTTGTCAGAGACGAAACCTCTGC
AAGACACTGCGTCTCAATTTGCAACTGCTGATGGTTGCCCTTGGCGCCTCTGCTTTGGGA
CTGGTTGCTCTGAGCCTGCCAAGCTCTCGCGTGGTTTTGTGGACCAGTGGCACGCTTCTG
GAGCGGCGCGGCGCTGATGCTGATTTGCCAATTGGGCAGAATTTGATCAAGATCGGCCCC
TTATCCCGGACAGCTGCCCAACTGGCACAGGTCAGCGACATGCTGCCATCTGCCGTCTTT
CTCGGGTTGCGCGCACACTGCATTGCATGCGTATCAGCCCTCGGCTATGGCATCGTCGCC
TACCTAGCTTTGGAGAAACACGAGGTCGTGCTGCGCCGCGTTGTTCTCGTCATCACGTCG
TTGCTGTATGTTCTCGTATTTTCTACTTTCATGGGATTCGGCGCAGCATACATGACTGGA
CTGCTCACAGGACCAAAGACTGCAAGGATGGCCGCTCTGCTTGCAGGCGCCACTTGGGCC
CCCGGTGTGACCTTGCTATTCGTCATCCTTTTCCGCGCCCGAAGAGGTGCGCCGCCATCC
TCCGTTTGGTGCTGGTGACCATTGCCACAAGT
>at03138
TCCTCTTCACAATGGGGTTTGGCTTCGACAAGCACCGGGTCTCCGCTCTCAGCTCAGCCA
GCTCCTGATCGCTGATACCGCCTGCTGCGCCACCGGCTCTGGGCCCTGCCGCGATGCCGC
GGTGCCCTCGACGCCGACGGCCTCGAGGACGCAGGGGTTCTCAAGGCGGTCGCGGACATT
GCGGACGTGGGTCCCCGATCCGCAGATCCACGTGAGGAACTCGTTGTAGTCGATCCAGCC
GTCACTGTTGGCGTCCGCGCTCTCGAAGATGACCTGGACGTGGTCAGCAGGCACTCCCAG
CCTCAAGAGCACAGCCCGCAAGTCTTCGACGCCGATGCGGCCTTCGCCACTGGCATCCCA
GCTCTGAAAGATCGCTGCGATCCTCCTCTCTGCAGCCTCGCTGCAGAGGCCGCCGTCACG
GCAGCCTCGGCCTCGGCCATGGCACGGGTCTGCGCACGCTGC
>at03139
TTTTTTTTTTTTGTTTTGGGGTACCCATTGACACGCCACAGAATTATGATTTCAGCCGC
AACACCCAAGGCATGGAACCCAATGATGGGAGGCATCCACAAAAAATATGTGCACTCTCA
AGGAAACAGCCGAGCTGTACTGGTGCCACCTGCACCTCCTGGTGGACAATCACCAATGAT
TTCAAAACGTACTGCATAGGACAACCGGGCCGCGCACCAACACTCACATGGATTCGTGCA
CAAGCCTCGTGCTGCTCAGGTGAACCTCACGCAGTGAAGTCCACACCCTCAATCTTGTCT

TCGCACTCTTGGTACACCTGCCTCAGGCGGTCCAAGTTGTCCTCACTGGTCTCCCAATAC
CCCTTCGAGTTTGCCTCAAGGAAGGTGGTGACCATGTCTCGAAGCGCCTCCGGGTTCTTG
TCCATCATCTGCTTCCTGATGTCCTCGTCTGAGATGTACGTCTCATTGGCATCCTCGTAA
ACGAAGTTGTCCACAGCGTCAGCCGTAGTGCTCCAGCCGAACGTGTAGCGCATGCGTTTG
GAGATCTCGCGGACGCCTTCGTAGCCGCCCTTGAGCGAGGCCTCGCCTCGTGCC
>at03140
TGGATAAGTTTGGCATCAGAAAGGGCTTGGCCAAGTTAATCCACGGCATGCTGTTTTCTG
ACATGGCGTGTGAGCTGGCGAATGCGACGTCGTACAAGGGGATTTGTGACGAGGTCAAGC
AGAGAACTGAGGCCTCTGTGGAAGGTTTCGTGGGACCTTGTGGTGAGGTCCTCGTTTCCA
CGGACTCTGAGCCTGGCAGTATTTCCGGCCCCCTTGACGCCATGGCTGGCAGTACGAATG
ACCCGGCTTTCGTGAAGCCTGCTGCCTGGCATGACAATGAGCGGGATTCCTCTTCCACTC
TTGGTGGGCGGAGAAAGGTCAGCTTTTCTCTGCAGGACGATTCTGATCCATGGCAGTGGC
CATGCGGCTCATGTGGCTTCCCATCTAGATGTGATTGCGGTCAGTGTTTCTCTTGCCAAA
CTGCCAATGTCCAGACTAAGTTTAATTCGTTGCATGTCATGAGGTGTCACTTGTTCTGAA
TGCCCAGCCCACA
>at03141
CAGTCTGGTGCGTTTGTCTCCTTTGACGACAAATCCCGCCGCATCCTTGAATTGGATAAG
ATCCTGGATGCCAATGCGGAGTCTCACGATGTGGGTAGTGCGTGCGATTTGCAACCTGAA
TCTGGCACGCCGTACACGCCGTGTCCTCCTGCCGATGTTTGCTGCGTTGCTGACCGTGGT
ATCGAGTGGTGCACGTCGGAGGAAATTGTGAACCGCATGCATGATTGTGCCAGTGATGAA
GTGGCTGAGCGAGTGCATGCCTCGGTGGTGGTTCTTGCCTCCTCACGTGAGGACCATTTC
CAGCCCATTGGTTTCATTGGGGATGGCGGTTCTGCCTTGCATGAAACCTGACGCTGAAGC
AAGCGATTCTGTCTAATGACAGGCCGTCTT
>at03142
TTTTTTTTTTTTTTTTTTCACATCAAATCCTCGCTCACATCGATACCTTGGATGATCTGG
CTAGGACTCTTGAAATCGACACCGATAATCTCGCCAACGAGTATGAACATGGAGCCGATG
ACAAATAAGCCAAGCATGTTGTTGAAGAGAATGACTCCCTGGAGCACCAGGCTTGAATCG
CCTTCAGCGATACCCAAATCCTTTGCAAATTCAACAACGGCTTTCTTGGGCGAGAAGTTC
TCCCTGACTGTACTGCCACTTTCAAAAGCCTCTAAGCTTGAATCGGCATCGTTCGTGAGA
GCTTTGTAGGCCACGGTTATGTCTGCAAAGCGCGAGGCGGCTGCATCATCGCCAGGCCAT
TTGTCAGGATGCTCAGACGCGACCAATTGCTTGCAACGTGCCTTGATCTCCTCTTTCGAC
GCTCCTGGTGATAAGCCAAGAACTGCCCAAGGTCTGACATACTCGTCCTGAGTGGCTCGC
AGGATGTGCGCGCCATGTGACCGACGTCGATACTGCGTTCGAGTCATCCGTGGAGAGATC
CAGATATAAGCATATCCGCTCTCCGATGCTTTGCAGCTCATGCAGGTGATATGGCATACC
GCGCAAACGAAGAGCAGTCCAACAGAACAAGGCGCCAATAATCCGACTTTGTTGGTTCGC
GATGAGGGTCTCCCAAGAGGAAGGCGCGCTGATTCGCAGCTGGAAGGCATCCAAGACTCA
ATGAAATGTCACAGGCAGCAGGTAAACCTTGAGTCGCAACGGCTTGAGCCAAAATGGCTA
CGG
>at03143
TTTTTTTTTTTTTTTTTTTTTTTTTTGTCTTTTCTGATCCACATCCTTGGCCCTCAGAG
CCCTGGTGGCATCTAAAAATCCGTGAGCTCCGCTCAGCTGCCGAAGACAGCGGTAGAAGA
GTAGCATCCAAGCCATCGCCGTCTGCCGGCCCTGAAGTTTATCCGTTTGCAATGTGCCCG
TAGGGCCTGATGGCATGTACAGATGGGGCTGCATCGCCAGGGGCCGGCCCTCGGACGAAG
AGTCAATGTGCTCATGTTTTGCTCAGCTGCCAAAGACTGTGATGAGAGCATGAGCAAAGC
CAGCGCCGCCGATGCCCCCCAGGGCATGCCAGTTGATCAACTCCACAAAGCCGGCGCCCG
GCGCGTCGCCCTTCTTGTCAAAGAGCTTCGGCTTGATGGTGGAGCCGAAGATGAGACCAC
CCACGGAGACGATGCCTACCACCGCGCAGCCGCAAAGCAGGCCGACCGTAGCCGCCTCGG
GTGTGTTGCGCAGCGGCCCCAGGGCGACGAACGGCCCGATGAGGAAGTAACCATGGGCCA
TGCCGATCTCCAGGCCGCGCTGCCATGGCTTCAGGCCTTCGCGGTGGAAGGCCATGGCGT
TCACATACTGACTTAGGACAAAAAGAAGGCCGCTCGTCACGGGCGTCTGCA
>at03144
CCAGAGTGCGGGCTTACGTCTAGTCTTAGATGTGGCTTACGTCTGGTCTCAGGTCTGATC
AGCTTGCATAGCCGTGCATTCGAGATGGATCTGTCAATGATTGCGGGTGAGGCGCCAGCG
CGTATGTCTAGGGCATCACTAATGCTGGCTGTCCTCACCTTCTTCTCTCCATTCATGCTC
CTCATCGACTGGCCTTGCAGTCAGGCGGCGGCAGCCGCCATGGTGTATCGGTTCAACCAT

CTGTGTACGATGTGCCTTAAATGCGATGGCCTTCTTGACCCACTGTGAGAAGCGG
>at03145
TTTTTTTTTTTTTTTTTTGACAAGCATGCAACAACACACCACCGGCTTCAAGGGTCAAAT
CATGGCGTGGCTGGCAATGCTGCTCAGTACAGCTACGACTTGCTGCACTGGACTGCAGAA
GTAACTACAATCATACTCATCATCTGCAAAGCGCGGGTCTGATAGGTTCCCGCCATGCCA
TCAAAAGATCAAAAGAGCTTTGCACCGGACCTAAATAGTGAAACATCACTGATTACAGCT
TGCTGGCAGGTGGACCAGCCATCACTCCCTTGTACTTCTCGCGAAGCCCCTTCTTGTCAA
TCTTGCCAGTGGCACCCTTGGGTAAGGCCTCGTCGAGCAGGAAGATGTGCTCCTGCAGCG
GCACCTTGAACTTCGCGAGCTTCCCCGCCGCGGCGTGCGACGAGAGCTCCGCGGCGGTGA
CCGGCCCCTGCGGGAAGACGGCCACGCCCACGACCTCGCCCAGCCGCTCGTCTGGCAGGC
CGAACACCGAGCACTCCCTCACCGCAGGGTGCGAGTAGAGCACCGCTTCAACCTCCGAGC
AGTCGATGTTCTCGCCGCCCCGGATGATGATGTCCTTCAATCGGTCCAAGATGTACACGA
AGCCACCCTCGATCTTGGCCACATCCCCAGTGTGGAAGTAACCTTCTGAGTCGATGGCTT
TCGCCGTGTCCGCCTCCCGATTGTTGTAGCACTTCATGTTGAAGACCGACTTGATGCACA
ACTCCCCGCGAGACCCGTCAGGCATAATCTTGCCAGTCGCAGGATCCTTTATCTTGGCGT
CAACCATGAGTGGGATGGGCTTCCCACAGTCNNCNTGCCGATGCTTCAAGTAGTCAACGC
CAGAATTCACAATGACACCTCCCATAGTTTCCGTCGGACCATACCCCTGCGCTGCTTCTG
TGCCTCTGGACTGCTTGCGGAGCTGAGCCACGAGAGAGGGAGGCACTGGTGCACCACCTG
CTGCAATGCCCTTCATGGTGGCAAAGGCCTCCGGCTTGAAGTTTGGGTGCTCCAGCATGT
CTCTGACCATTGTCGGAACGCCTGTGAAGCGCGTGGCCTTCTCCTCCTCGATGATCCTCA
GCGCCTCGCCCGC
>at03146
TTGTTTTTTTTTCTTTTTTTTTAGTGCATACCCCTTGATGTCCGTGCAATCCATGGTTTC
ATGTGGGCTGTACACATCTGGCAATGAGTGCCCAGGCAGCCTTGCCCCATCAACGGACGT
CATAAAATGGTGCAGAGGCGGCCAGCACGCCTCGACTCGGGATCTACGAAGCCATAAAGC
TGACGCTTGGCGAGGCTGTACAAGTTGCTGATGCCCGGGATCCTCCCGAGGACTGGGCTC
AACATAGCATCTATCTTCCTGAAGGACGCTTGCGTCCATGAGCTCATCATGTAGCGCATC
ATCATGAAGTTCCAAAAGAGGAGGACAGATATCGGCACAGCCCGACCGGTGAGCATGCCG
CCAATTAGAACGAAGCCCAGCAAGACCTCAATGTTGGCGCGGCCCTGCATCATCTGATAG
CGTGCTGAGATCTCTGCCAATGGTGAGAAGAAGTTCCTCACTGCAGCCGGGATCCTCTCG
TGAGCCTTGCAGATCTTTGCAAAGCTCATCAGGGCAGTGAGGAATGCCGGCGCGAGTGCG
AGCGTGCGGATGACATTATGCATACCGGGCGTAAGTAGTGTCAGTGCCATGATAGGCATA
ACTCCCACATCGTCCAGCACGACCGTTTGCACGTACTTGGAGTTGAACTTCACGCCGTAC
TTTGCGAGAAGCTCAAGGACGAAGGAGCTAACCAGCAATACAAGAGCCTGGTACGCGGGG
AAGACACCTGCAACAGCCCCGACAGACATTGTTAGCGCTACCGCATAGACCCCAAAACAG
ATCGTTGCCTTAGTCCCCATGACGGCCCATCGGCTGCCATCATCATGCATTGCGTTGGTG
GTTGGCGATGCTCCTGAATGTACCTTGCTATCACCAGTGCTACCGCCAGTGCTTCCACCA
GTGCTTGAGCTGCTTGAGGTGGACGCTTCAGGCTCATACGTGTCGTCATAGTCTGAGTCA
ACGTTTTTCTTGTACCACTTCTTCTTGAACCGCAGGACTTGCTTGTGGTTAGGGGGTGGG
TAGAGACCGCTGAGGTAAGTCTGCCACCGTTCATCTGACCAGTCAAATTCAAGGAACTTT
TGGTAGCCCTCGGCCATGAACTCCTCGTGTAGCCGCAAGTCTGGAGCTACAGCAACTTTG
TGTGGCCCAGCAGCGCCCCTTCCCTTGTCTCTGCTTCCAAGGCCTTGAGCCAAAATGGCT
ACGG
>at03147
CCCTCTTGTCTACCATTCAAGCATTTTTGTCAATCATGCAAAACGAATTCGGACAACCTA
TTATTGAAGTTTTCCTGCCCACCTTTCAGATGGCGCCGGCTGAAGATGAAACGTTCTACC
CCATCCCAAGCAAAGAAAATTGCTCAAGAGATTGTAGAAGCTGAGCTCAATGGCCAA
>at03148
CAGTTATGGATCAAGCCATGGATCAAGCCATGGAGGCAGTGACGTGGGTATCTCAGATGA
CGTAGTTGTACCTTGAGGTCACCACGACGTGTGGGGATCATGCGTTGGCGGTGGTTGTTA
CAGCAGGTGCTGGGGGGCGTTCGACGCCTTCCTGGTGCAGAGCTGCGTGTCCTTGAGAGC
GGGGGCTTGCAGATGGGCAATGCAACCTTGGCGTTACGGCGGATTGCATCCAGGATCTTT
GCAGTGGCAAGGCGCCTGTACTTGTTTGCCGTGGTGCCT
>at03149
CGGGTGGCACTGACGCTGTTGATGCGGCCAAGCCTGCAACCCCCTGATTCCGCGCCATTT

GGAGATGGCTGCTCGTGGTCGCTGTTGCTGCTCTCGTCCTCTGATTCGTCCTCCTCCTCC
ACTTCTGAAACCTTTGGAGGCAGAACCCTCTGTGAGGCAGTGGTTGTTGTGCTTCCTGGT
GACGTGATCCGGCTGTACAGAACCTGCTTTGAGGGCTCTGGCTTTGAGAACATGCACTTC
GCTGCTGCATCATTGATGACAACAGCCTTCGACGTAAGCAAGGTTGGTGCCAAGCCCTGC
AGTGGGACGTTTGCCCTTGGCTGCAGGCCGTCAGATGCCATCAACTTCGCTGCCAGTTGG
TCCTCATTATTCCTGGGGCTGCTGTTCTCGAC
>at03150
TTTTTTTTTTTTTTTTTTGCTTGCGACGGGAGCAAAATGTCAGAAGTCGCCCAAGTCAAA
CCGAGGAAAACTCGTGTACGCAATTCGTAACACGTTATTCCTGTCAAGAGATTTGCCTTC
CCTCCATCACACAACGATGGGAACGTCGGCACCGCCCATGCTGTTCACCTTGAGCTGGAA
GGGATATGCAGGGAAGCCCTCTTGGTTGTAGATGGCACACTGCGGGAAGGTTGCCGAGGC
TGTGTAGCGCAAGATGGACGGCACTGCGGGCAAATGCACTGTGAAGCGGATCTTTGAGCC
ATTCACAATCTTGGCCGCAGTGCCGTTGGCCCACACCTGGCCGTTCGCGGACGCATCGAA
GTCCGAGCCCACCTTCGTGCAGCACTCCGTGCAATTCCGTGTCGGTCTGAAGGCCAGCGG
CGAGGACGCGCCCGCCACGAACTGCACCTCCACCTCGTAGCCCCCGGCCCCAGTGGCCTT
CGCCGTCGCCGTGAGCGCGCGGGGGGGCTCCGGCAGGGCCTCGCCGTACAGGAGCTTGCG
GAGCATCCTGCCCGCTCGGAGGCCGACGTCCTGCTTGTCGGTGTTGTGCACGGAGCCCTT
GGGGCAGTGCGGGCAGCTCAGGTCGTAGCTGGGGACCAGGGCCGCCCGCTCCACGCCCTC
CAGGCCCTGCGCCTGCGCGAGGCGCATCGCGAAGACGCTGCTCGAGGCCGCCACCCCGCC
GGTGTAGCCGGGCAGCTGCACCGCGACAAAAGCCGGCGCCGGTCCCGCGGCGTGCCGGGC
GCTGAGCGCAGTGCGCCAGGTCGTGACGAGCTCCTTCTCGAGGCAGGGGTAGAACCTCAC
GTGGGAGCACTGGGCGTCACGTCGGGTTCTGC
>at03151
AGGCTTCGTCGCTGCCGGCAGCTGCTTCGCATGGCTGCAGAGCTGCGCAGCAGCAGGCTG
CTGCGCCGCTTGCGCAAGAGCCTGGCCCGTGCTATTGGCTGTCTGCTTCCTCGGCATCTG
TCTGACCTCCAGTCTGACGTTGCATTGTGCATGCGGGTGGCTTTGAGTTGGTGGTCTCGC
TCATGGTCAGTGATGAAGACCAGCCGCACAGTTGCCATAGATTGGTTTCGAGCAGCATGG
AGAAGACGCTCCTCGATGGCGCCGCAGCAGTGCATGACCCTCGGGTTTTTACTTCAGCCA
CTTGTATACTTGACTTGGCTGTCAGGAGTTTTTGAAAAT
>at03152
CCGGGTTCATGCTCTGTCCTATGACGACGCCGCTGCGCAGTTCCGACGACCTCCTGGACA
GCACATGCAGAGGAGGTGATGACTCCGGCCTGATCGCCGCATCCAAGACGCGCTGGTGGA
TCCTGTTGACGTTCGCGCTGTTCTCGCAGCTGCAGGCTGCCACCTGGAACTTCTACGGCC
CCATCTCCGAGCAGATCGAGGCAATCCAGGGCTGGTCTCTGCCGGAGATTGCGTGGCTGG
CAAACACGGCTAAATGTGGCCTCTAGCGCCCTCATCGGTCCCCCCTTCGTTTC
>at03153
CTAGATTAATGGACTCGAACATCCGCCAGTATACAACTCCAAAAATCATCTGCTCACGGA
CTCGACGACCTGTGACTCATAGCAAGATGCGACGGTCTGTCGTGCAAGAAGGCACCTCGG
CAAGCGCACGGCGCGATCAGGTCATCCACGTCGTCCCCGAAGCAATTCCGACAAGAATTC
TCTCTTCAGCCAGTTCTGGCGCTGCGGATCCCTGCCTCCTCCTCCTCCTCCTCCTCTTCT
GCCTTCCCCCTTTCACGGTCCCCTCGTCCCGCATGCT
>at03154
CAGCGGAGATGTCGAGCCAGATGTTCGTTGTCAAGCGCAATGGAGAGCATCAGGAAGTAA
AGTTGACAGCATCACAAAACGCATCCGGCAGCTGTGCAGCAATCTCGATCCGAAGTATGT
TGACCCCGTGCCCATCACTCAGAAGGTCATCGAGGGATTCTTCAACGGCATCTCCACTTC
AGAGATCGACACGCTCGCTGCGGAAACATGCGCTTACATGTCACAGAAGCATCCTGACTT
CTCCACACTTGCAGCCAGGATTGCCGTTTCCAACTTGCACAAGAATACGTCGGACTCTTT
CGCGGAGACCTGTCGTGTTCTGCATGACTTCCGAGACAAGCAAGGCCGACGGGCGGCGCT
GATCTCAGATGAGGTTTCAGCGTTTGTTCTTGAAAATGCAGCGGAGCTTGACAAGGCTGT
GGATTATGGCCGCGATTTTTCATACGACTACTTCGGT
>at03155
GGTCCACAACTCCGACGTGTAGGGCCCACGGTCTGTCTCAGCCCACATGCGCATGCTACG
CATCGTCATCGGGGCGGGCAAGCAAGTGCTCTGCATTGTTGGATGCGACTGCCGATCTCA
TGCAACACCTCAGCAGCAGTACGCCACCATGAATTATCCTCCACGCCAATGTCTGCGCCC
TGAATGCCCTTGCCAAGCCAGCGACCGCACTCGAAACACTGAATCCCGATCTTCTCCAAG

AAGACGGTGGGGTCGAACACATTCGTGTCCGTCGCCATCATTGCAAACGAGGGCCAACCC
CCCGGCAAGCACGGCGCTGGAGTGCTGGAGCCAAAAACGGC
>at03156
GTTACCGAGGCTGCCAACGCTTTACTCGCGACTGGCAAGACTTGCCAGAGATAGGACTCG
CAGACGCCGCACAGGCACGACTTCGATGAGGAGGATGAGGATGAGCATCACCTTGTCGAA
AACTACTGCAGAAATCCGAACGGCAGAGAAACCATTTGGTGCTTCACGACAGATCCCAGC
AAAAGATGGGAGTACTGTGACCCGGTCGCGAGCATGTGGGCGGTGGACTCTGGACCTTGT
CAAGTTGAGGAGGGGACCGGGTGCTTGCTGAGCCCCAACTACCCGGCAAACT
>at03157
TTTTTTTTTTTTTTTTTTGCAACATAGGGAGGTTCAGAAGAATACACCATCCACACAGCA
ATTTCACTCATATGACTCAGACCGGAGCTCAGAGACAACATACACAAATTATAAATTCAC
AGGTCTTCCTTGAGCCTGAAAACTGTGACTCTGCGGAACTCCCTCCGGAACGAGCGGTCC
TAAGCTAGACACCAAAACAATTCCAAGTCCTTCAAAAGAGGGTCACTCAGGCAGGCCTGC
ACGTGAGAAGAGGGCGCGTCCACTTCACTCCACATGCTTGACGCTCACGCGCGGCCAAGC
GTTGTTGTTGATACCACGCAAGTTTCATATGTGCTCAATGGACTCTGGCTGGGTGTTGTA
GGCCACGTCTATGGCCTTTGCACAGATCTCCACCATGGCACCGCTTTCCAGACCCTCTGG
CTTTGGCACCTCGCATTCCCAGAACGTCCACGCCCAGGCCTTTGTGGGATGCTGGTCTGA
GCCTTCCTTCAGCTCTGCCGTGTGCCAGGTCTTGCCCCCATCCGCGGAGACATCGACGCG
CACGATTCCACGACCACCACCACTCCACGCGAAGCCGCGGACCGTGATGTCGTCAAGCTC
GCTGGTGGCACCATCCTTT
>at03158
TTTTTTTTTTTTTTTTTTAGTGCTGGCTGCTGGGATGACTCCATGCAAAGTGCCGCCACA
AACAGGCAGGTCTGAAAGGAAAGGTGTGGGGGTAGCTCCCAAGTCATTAAGGCAGGGGGG
CCTGCTGGGGCCATCTGTGGCGGCCTCACTTCGCCAAGATTGCCTGAATCATCGAGCAGT
GCTTTGCTGCCTCAGGCGGTATCAGGCTGGTCGACCTGGATGCCGTAGAGTGCCCGCCGG
CTCCTGACGAAGCGGATGAGCCAGCCCATGCAAACGAGCCATGCCACGAGGAGCAGAGCA
ACGCCGCCCAGGAGCGCCGTGCCCCACTTGTCCCAGAAGTTCCTGCGCCGCCTCGTGCC
>at03159
TTGTTTTTTTTTCTTTTTTTTTTTCGGCCAGAGCCAGCACGAGGCACGGTCTCATACAGC
GCAGGGATCAATTGCCTGAAGGCAATGGAAGGCCAAGGCAAAGCCAACAGTCTCATTGAC
GAGGAACACTGAGGTGAGCTCATGCAGGGCGAGGGGATCCTCGTCGTCATCAGTACTTGA
CCACGGTGCGGGTTTTCGGAAGTCTGCCGTGTCGCTGAAGAGATCATCGATACTCTTCGG
CCGAGGTGCGGATGGTGGTGGCTTGCCCCCTGCTCCAGAGCCTGAGGATTCAGGCCCTTC
CGGAGGCAGAAGCTCAGCGCTAAGGATGCGTGCTGAGCCGCATCCGGAAGGCAGCAATCG
CGCGAGCACCTCCTCCTCCAGCACCTCACGCTTCACGTACTGTCGGAGAAAGGCAACCGA
GCCAAGCCGGGGCGCATCCTGGTCGCCCACGCCGTGTTCGGACTCGAGGACTTCCTCCAA
CAGTTCTTGCCGCGAGACGACGCCCTCATCTGCGAGCCGCAGGCGCGATGCAGGCACCGG
TGCCTCAGCGCAAGGAGCCGTCTCCGCCAGCGCAGACATCG
>at03160
CTGCTCAGTCGTTCCATCTACGTGGCTGGGGGCTATGCGGGCAGCCGCGTGCTCCGCTCG
GTGGAGCGATTGGACACCTCCACCTGCACTTGGGAGCCGGTGGCGTGGATGGCGCTGGCA
CGCCGAAGCTGCGCCGTGGTGGCCCTGGGCGGGGTGCTCTACGTGCTGGGGGGCTCGGCC
GGCGGCGAGGCAGTCGCTGCAGCCGAGCGTTGGGAACCCTCCATGAAGATTTGGGAGAGC
TTGGAGCCGCTCTCGCAGGGGCGCGAGAGCTGCGCCGTCGTGGCGCTTGGCGGCGGGCTC
CTCGTCGCCGGCGGCTACGGGGGTGGCGGCATATCTTCCTCTGCGGAGTACTTCGACCCC
GCGCTGGGGAAATGGAAGGCTCTGCCTTCGATGCATGAAGCACGGACGAAATGTACGGCA
GCAGTCCTCTAAGCAGCGGCCATGGGGCGTGGGAGGTCGAGGCGCATACAAGGTGGAGCC
GG
>at03161
CTATGGAACTGTCGGTTTCACTCGCAGTCAGCGATGCCGGTCTTTGCTTCGTGCTGTCAA
GGGCGGCTGGCTGCGGGTGCGGACGGGCAGTGAGGGTGCCGGTGAGCCGCCTCTGCATAT
GTGTGACCTCGGCCTGTAGATATTTTGGATTGGAGGAAACAGTAGTGGATTGGCTCGCCT
CGTAGAATGGTAATCCTGTTAGTCTGGCGGCGTAGAAGGAGTAATACGGGCATGTAGAAT
AGGAGCGTCCGACGGTTTCTCCTGCAGTGCAGTGCTTGGGAGAGGCTATATGCAGTGCTG
GACCTGACCGTTTTGATAGGCGTCATGCAGGTAGAAGATTGCAGAGAGATGAGGCCGTGA

GTACAGGATAGAGGCTATGACCTCTAGAGCGGCAGACCTTGGGCGAGG
>at03162
GTTTTTTTTTCTTTTTTTTGACTATGCCCAGGACTATGTCCGTTGCCGGACGTGGTTCGG
TAACGGATAACGGCGAGATGATGTCCGAACAAGCATCACGCAAGGCAAAACTGCGAGAGA
GGGCTGCCACCTGCATGGCACGTATTCACGGTTCCAAAAGTAGAGCCTGCAAACTCTCCC
GTGAAGACATGCAGGTGCCTTATATAGCTCTGCTAGCAAGGTGCCGGGAACTTCTCAGCA
TGGTAGCAGAGCGCAGCAGCTAGTGCGCCCCGATGGACCGCCGTGACACATTCGCCATGC
CTCACACCCGGGAGGAAAACAGCCCAACCCCACAGGATAGGAGGAGGAGTGCAAGGCAAA
TGTCGAGACCTGTGCGCCTACTTTTGACCCGCATTGCGGCCCCCGAAGCCTGTGGCCCTC
TCAGACCCCTGTGATGCCTGCCGAGGGTTCAAACTTGCTGCCGAGCTCTGAGAGCTTCTG
GTGGCTCTGAGCGTAGGCGGCGAAGAAGTCGGCCTGGCTTGCCTTGAAGGCCTCGAAGTG
CGGCTTGAAGCCGGGGTCAGTCATGAGAACCTTGTCGGTTGGGAAGGTGACGACCTGCGG
GTCAGCCTTCTCCATGTCGGTGAAATAGGAGTTGTCGAACTTCAACCAGTTCTTCGTCCA
CGACCTACCGCCTGCTGTCTTCGACTTCCCGAGGAGAGGGCAGCCTCGGACCGTGTACGC
CGTGCCACCACGGTATCCCTCTTGCACAGTGCCCGAGCGGTCGCTGAAGGCCCGGCCGAT
GGTGTGAGCACCGCTGAGAGCGACAATATCCTTGTCATCCATGCCATACTTGTA
>at03163
TTTTTTTTTTTTTTTTTTGGATTGCAGAGCGCACCATCAGGTCCGCAAAGAGGATCACTC
AGGTGCAGCGCAGAGAAAAGCTCACTGCTTCGAGAATGAATTGGGCCGGGGCCTTTGGTC
CAGCCACATGGCGCAGGGCACTGCGCACATTCCCTTGCCGCTTGTCTTCGTCACACCTCT
GCAAAAAGCCGGGGTTGTTGACCCACACCGGCAAAGGCTGGACCGATGGACATTGCCAGC
GTCCCGGGGCCCTCGGGGTCATGTTGGAGAGAAGACGTCGGGCAAAATGCACACGTAGCC
TCGCAGGGCGGACCTGTTCCTCGGCCCTTGCAGTGCTACCTCAACTCGAGGGGTTGCACC
TGCAAAGGATGGTGCACCAACATGCTCAGAAGGATCTGCATAGTCTGCAAAAACTAATTG
CTTGCCGTGCTGGAGCCAGCTCGCGCCCATGGCCCAGTGTCAACATCAAGCCAGACACTG
GCTGAACAGAAATGCTCAGTAGAAAAAAAACACTTTTCCTCGTGCC
>at03164
CTCCACAGCCCGACGAGGACGACCTCGTGGATCAGACGCTCCTGTCATGCCGGCCATGGC
CTCTGAGCTCAGGCATGCCACCTGCCCTGTGTTGCCCGACATGGACTTGACCATGGACTC
GCTGAGAGACTACGAGCAGCAGCACAGCTGTGAGTCGCTCCCAGAAATGCAGATGATACA
GATGGTTCCCCAGCTCGCACAGCTCCGCCGCGTCGCGGAGATGGACCAGAGGAGCATTTC
CT
>at03165
TGGCCGGCGTCACTTGTTGGGGTGGTGTCGTGTGGTGGATAGTCAGAGGCCTTCTGAAGC
CACAGCACGGTGTCATCCACATGACTTCTGGTAAGTCCATCGCCAATTCAGGATTGTCAG
GCAAGGAGTCTTCTGTGAGGAGGGGTGCCTCGTGCACGACGCCGTTGTACAGAGGCTGAA
GGTTTGGGGAATGGAGAGAGTTTGGAGAGACTGCCCCTCAAAGGACCTGATGATGCCAAG
CAATGACGCACTTCTTTGTGTAGAATGCTGCCATGGCATCCAGACCTTGACTGAATCCAG
AACGGTTCCTTGCTGACAGAACTTTTGCTGGCTTGCGTCTGGGATGCGCCCTTCTCTGAG
CTTGCCGC
>at03166
TTTTTTTTTTTTTTTTTTGGCTGCAAGCACGACGAGTTGCGATGGCCCCACCACTGTACA
GAGTGAAGCATTCATCGACAATCGCAGGCAGCGAACGAGGCGTTGTGCAGCAGCGTCACT
CTGGCCTCTAAAAGCTTGCTAAAGAAACGACATCCTGACGGCCCGATTGTTGAGCCAATC
AATGAAAATACTGCTGTGATCTTGTACTGTAGCAAAATATTCGAGGACGATCTAGCCAGA
TCACCCTATGCTGTAGGTTCAGAGCGAGTCCGGATCTGCTGTATCAGTTCATGGACAAAC
ACTCGCACAAGAACTCANGGTTCAAGGATATGATGATCCAGGCTACGGNCANGATAGGGA
GCAGGGTTGTGGCGACTCTGCTGTTCGTTCCAGATTGGTTCGGCACTGCGCGTCTCTGTA
TGATGGACAGGCACATCGGGCC
>at03167
GCCGAGAAAGGAAAGAGCAGCAGCAGCAGCGGCAGCAGCAGCAGCAACAGCAGCGACAAA
TTATGCGCTATCAGCAACAGCCATGCCTTTGATCCACGACTTGCTCGCACAATTGGTCTA
CCAGGAGCGCGGCTGGAGCAGAAGAGGAAACAACTGGACAAATGCGCCGAGCATCACCGC
GCCATTCTACATCGACACCAGCT
>at03168

TTTTTTTTTTTTTTTTTTCAAAAATTGATTGAGTCCCGGTGGCCCAGAGTCTTGGAGCCA
AGCTTGCCGCGTCCGTGGTGGCACCTGTACAGCAGAGCACCTTTTGTCAGCGGGCGCACG
CCTGGAAGGGATGTCGATCCAGGGCACTTCAAAAAGAAACGCCTGGCCAGTGCAGTCCGA
CTTCGGAATGCCTGCCATGAACGCTGTTGCTTGTCCCTCGTGCC
>at03169
TTTTTTTTTTTTTTTTTGTTTTCCTTACGCAAGGAATCGCACGGAAACTCCAACGCAAC
AGGAAAATTGGGGAACCCTGCGTTGAGGAAGGGCTCAGAAGGCAAGACGGTGGAGGGCTT
CGAACCTGGAGGGACGGAGGGGGAGGGGAGCGAGGGTAGGCAGGAAGGGGTAGGAGGCCA
TGGAAGGCTCAGAGCTTGGCCTTACCAGGACCGGGCGCCGTCGCCGAGGCAAAAGCCTGC
ATCGGCCCCAGCATGGCCTCATAGGGATAGGACACCGCATGCCGGTCCCCGCCGGCAAGT
GCTGCGCAAGCCTCCTCCCAGTCCGCTTTATTGCCGAGGAGGACGTCCAGCATCCCGCGC
TCCCACTTGGCCGTCCGCCGGCCGTAGGCCTCCCGGACGGCCTGGGCGCGCCGGGCGACG
GCCGGCACGCTGTCCATGATGAGGCGGAGGCCGTGCACCATGCACTCGTAGTTGTCCTCG
TAGATCGGGGTCTGCGGCAGCCGCTCCATGATCGCGCGCAGCTCCTCCGGGCGCTCTCGG
AAGTGCATGAAGATCTCCATGTACAAGTCGCTAATACAATAGAGGCTCCCGGAGAGATTG
ACTCCGCCACTGGGCACAAGCCTCCCGACCTTGGGGTTGCGCTCCCCCTTCATCNAGCAG
ACCCGCTCCGTGATGAATAGCGGGCTTCGGATGTGCTCCCTGCCGA
>at03170
AGGGGGAGCGTGCCACCAGCAGCGGAGGCGCCGCACGAGGTCGGCGTGCCACACAAAGGA
GGCGAAGAGAGCCCCGGCTACTAGGGACAATCCTGGACTGGGTATGCCAAAGTTGAACTT
GAGCACTGAGCGGCACCTACGCCGGCAGGGACGTCAGGCTCGGTCCACACGTGCTTGGCG
AGGTTAGGCGCGGTCGTGCGTGTGCATGGCGTTGCGATGCTCCTGCGGGTCGATGAACGC
CTGCGCGGCTCGCGTGGCAGCACGAGCCATAGGGATAGGCTGACCAGCGATGGCAATGTG
CTCGGACTGGATGGCGAGCGAGTCAGAGATGGCCTTGACACGCGGCGCAACGTCGATGGG
GACCTGCAGCAACTTCTGGCTGGATCCGAGGGGCCTGGGCTGCAGATATCCCCTCCTCCG
CGCTCTCTCACGG
>at03171
TTTTTTTTTTTTTCTTTGGCATTGCGTTGCATGCATGACTCTATCGGAGTCCCCCAAGA
AGCGCTCAACAAGGTTCGTGTCATTCACTTTGCAGAGGCTGTACGACGTGAATGGAAACC
TTCGAAGGCAGCTCGAATAATTAAATCCCATCTTGCTGAACATGCACGCACATGCCAGGC
CCCCGCACTTTAACACTGTCCACTCAGAAGTCGACGTCCAATGCAAAGCCTTTGACCTCC
TCCTGGCCACCTTCCTTGTGACTCACTGAAGACATGACCCCTGCCTTCTGGTACTCGCCC
ACACGCTTCTCAAAGAAGTTTGTCTTCCCCTGCAACGAGATCAACTCCATCCAATCAAAA
GGGTTGCAAGCTCCAAAGAGCTTCGAGTGGCCAAGCGCGGTCAACAGCCGGTCGGCCACA
AATTCGATGTACTTCGTCATCAGCTCGCTGTTCATGCCGATGAGATCGCACGACAACGCC
TCGCAGATGAAGCTTCGCTCGACTTCCACAGCGGTTTTCACAATGCTGTGGACCACATCC
TCTGGGAGCTTGTTTTCCAGCATTCCGTAGATGAGGCATGCAAACTCCGCATGCAGACCT
TCATCACGAGAGATCAGCTCATTGGAGAAGGTCAGGCCAGGCATGAGACCACGCTTCTTC
AGCCAGTAGATGGCACAGAAGGATCCGCTGAAGAGGATGCCCTCGACACACGCAAAGGCA
ACGACGCCTCGTGCC
>at03172
CCGTAGCCATTTTGGCTCAAGCCATCTATGCTCAAATGATGCAGACTCTGATGGCCCCCT
TTTTCGATGCAGATCTAGTGCGATGGGGTACGCACGCTATTTCTGAAGCACTGCAGTGCG
GAAGAAGTCAGAGCTTTGGAGGCTCGACCGACGTGGCTTGAGGGGGCTCGGGCTCTGGAG
CCTCACAGCAGCCATGTGGAGGAAAGGCGGACTTTGCGGCAGGCATGCCGCTCGCTGCAG
CGGCCATGCGGACCGCCCTCCACGAGCCGCCAGGCCTGGCTAGGCCACCGGGGCTTGGAA
G
>at03173
CATCAAGGCAAGCACAAGGTGTGGGGGTGTGGTGCGGACGATCAAGTTCTGACACGGGAG
TGTACGTATACAGGCACGTTGCACGTGCATGCATGCGCGTGCGCGTGCATGCAAGCCGGC
ACAAGTGCATCAGCTATGTCAGTTAGGCGGAACGTCAGAAGTGGTCGACGCTGAATTTCC
AAACTCCAGTGGTGCAATCGTAGTCGATGAACGTGGCCTGCTTCTCCTCGGTCATTTGCT
GGATCTTCCGCTTGTAGCGCTCTTGGCCCCTTGGGTCCTGCAACAGCCTGCTGTTGCTTG
GCGGCCAGCACTGATACATGGTCACCGTGGCCGGCTTGTTCAAGCCAATGCCGATCGGCG
GCTTCATGCCAGGCTCGGGGTAGACCAGCACCTCGCCAATCTCCAACCGCACAATGTGCT

CAAAGTCGAGACCTGTGCAATCAGTCTCGCCATGGAAGGTGATCTTGCCCACGGCAGGCC
TCGAAATGGTCAGGTCGCGCACCTGCTGAAGCCTCGTGCC
>at03174
TTTTTTTTTTTTTTTTTTCAGCATGGACCAATTGCATGCCCGGACCATCCGTTTGGAAGG
AATTGGGCACGTTCTATACGTCCCGAGGCGCGGGCAAGTTCACAACTTCCACCGCTGTCA
GCAACTCACGAAGTCCTCGGGCCAGTCGGCCTCCTCGTCCTCGCACGCAACCTCGCGGAG
GGGCTCCACATTGGCGGTCTTGGCCGTGGCCAGGAACTGGTCCTCAGTGGTGATGGTCCC
CGTCGGGGCGGGCATCGTCTTGTCCTGCAGCTTCCGCCAGTCGAAGTCGTGGTACCAAGG
CTGGTCCTTGAGGTTCTGCACTCCGAGGTTCCCCATGGTGATCCTTTCCTCAGGCTTCTT
CTTGCACAGCCCAAGGATGAGATTCACGCACGCCGGCGGCATGTTCTCCGGGAAGCTGAC
CTTTGAGAAGCCCTTGATGATCTTCTTATATGTAGTCATGGCGTCGGGCGCGTCGAACGG
CGCTCTGCCGCTCATCATAATGTAGATCATGACACCGAGTGCCCACCAGTCCACGGCGCG
GTTGTGGCCGGTCTGGCGCAAGGTCTCGGGTGCAAAATAGTCTGTGGTGCCACAGATGGT
GTAGGTCTTGCCCACAAGGACCTTTGCCAAGCCCATGTCAGTGAGCTTTACGTACCCGGA
GCCAGTGAGCANACAGTTCTCAAGCTTCAAGTCTCTGTAGACAATCCGCCTGTCGTGCAT
GTGCTCCAGGGGCAGCGCCACGCAGGCCGCGAAGAACCGCGCGTGCGATGACGTTCCGAA
GAGGTCGGCGTTCTTGTCATAGATATCANAGAGCTCGCCACCATGGCACACCTCAAGC
>at03175
CCGTTAGTTTCCATTTTGGCTCAAGCTGCTCAAGGGTCAGTGCACGCGGGAAGTTGTGCG
CTCTGCTCGACGGGAGGAGCGGATTCTTCAAGACAGGGCTGATGTCCGAAAAGGAAAATG
CGGCGCTGGCCAAACTGAAGGCTGCAGCCGGGGTCATGGACAAGCCGCAGGTGGAGAGGC
TGGACTGGATGTACGAGCAGAGCGCCGCCCAGAGCGCGCAGAACGACGACGAGCGGATGA
ACGCGCCAGTGGCCGATCAGAAGGACCAGGACCTCGAGGACATGAGGAAGCTGGGCGAGA
GCACCGCAGGCTCCCTCTTCCTGCGCAGCGCGACCAAGACCTCCGAGGACATGCTGAGGA
GGCTGGAGGGAGGACCCCCTCTTCCAGATAAGAAGGCAGGAGCAGGCCGCGCGGGAGTCG
ATGATGGCCAATCC
>at03176
TTTTTTTTTTTTTTTTTTANGCCGCAACACACTTGGATGGACAAGCATCACCATTGCACC
TCAATCCAACTCCGAGTGGCGCGCTTGTCATCCCTGTCTGTCATGCACTCACTGCCTGCC
TTTAAGAAGCCAGGTGTGTCCTAATTTGTGGGCATCAGAACTCCCCGAGCTGTCCTGGAT
TCCACCGCGGCGTGCACCAGAAGGACAGGCAGTTGCTGACGATGTCCTTGTCGAAGGAGT
TCCTGTTCTTGTCGTACTCGAAGGCCTCGAAGTGATCGTTGAATTCGTCGTCTGTAAGAT
CGTTCACCGGCACAGACTTGCCGGTCCGCGCGTTGGTGATCACGTAGAAGTCGTTCCTTT
TCCACTCGTTCGCCAGCTCATTCCTCGAAATGAAGCCGATGTGGAGGCGGAAGATGGGCC
CGGCCAGCGACACCAGGACCACGGAGTACGCGAGGTGCAGGGCCAGGAGCAGCGCCAGGG
GCCAGCGCCCGCCCTCGCGGGCCGCCGCGACGACGAGGCCGAAGTCCAGCAGCGCGCCGC
AGAGGCCGATGAGCACGAGGCCCGCGACCATAACGATGAATTCGCGATGGTTCAGCAACC
CAATGCAGTTCGTGAGCCATCGGCAATAGTGGTCGTAGCGCCGGATGGCCAAAGGGTATT
GCCACGTCTTGTGGCAGCGCTTCGGCAGCGGCGGCTCCTCGAGGGACCCCTCCCCGCCGC
AGCCTTCGGGCGGTGGGCCTCCGACCGCTTGCTGCAGCCTGGCGTAGGCCTTGCGCTGCT
CCTCGCGGTTCAGCTGGGCGGGCTCGCACTTCCAGCAGTANGCC
>at03177
TTGTTTTTTTTTCTTTTTTTTTCCTTGAGAAATTGCGCATCCGTGATACCACACGCAAGC
AGGTTCATTGAATAAAACAAATAGATCATCGTCCGGGTTATTGCCCTCAATGGGACACGT
CCTGCTTTGGCACATCTGATCTACAGAGATGCAAATGCCTTGGGACTTACCGTCCTGGCC
ACAAGGGCTCAGCAGCATGCGCCGCCCTTCTTCTTCTTCTTGGACGCCAACTTCTTTGAG
GAGGACGCCTTCTTGGATGACTTCTTGTCCTTCTTAGATGCCAACGTCGTTGTGGCCGGC
ACCGTCGTAGTAGGTGCCGTCATGGGAGCAGCCGGAGTTACGGGTGAGATAGTGGACGCA
GTCGTTGGAGGCGCGGTCGTGGAGGCAGGCATTGAAGGAGTCAGTGCTGGCTCCACTGCT
GGTACTGTAGTCATGCTCTGCATCGGGTACGCAACCATGGACGGCACGGACTGCAACGGC
GCAGCTGAACTCGTCATCGTCGTAATTGGGGCTGCTTGGTACGTGGGCGCCGCCGAGTAA
ACTGTGGGCATTGCCGTAGGAGCCATAACCGTTGTTGGAGCTGTAAACATCGTAGGTGCC
GCAACCGTGGTAGGAGCCGCGACCATGGTCGGAGCCGAGAATGAGGCAGAAGCCCCGACT
GTGGTTGGAGCCGCCACCGTGGTAGGAGCCATCACCGTGGTAGGAGCCATCACCGCGGCA
GGTGCCTGTGCCGTGTAGTAAGTTGGTGCTGGGCTTGCTGCAGGCGCCGCTGCTGTGAGG

TAGGTGCTGCTCACAGGTGCAGCGGCCGGGAAGGATGTCGGAGCTGCGTAGGTTGTCGGC
GCTGCCGCGGCCGAGTAGGTCACAGGCGCCGCAGACGCGGTGGTGTAGGTCACAGGCGCA
GCGGTCTGGTAAGTCGTGGGTGCCGACGAGTACATCACTGTTGGTGCTGGGGCCGGAGCA
CTGTAGGTCATGGGGACGGCCGTCGGCGCAGCCGCGAAGGTCATGGCTGGTGCGGCAGCC
ATCGTCGAAACAGACGCTGCAGCGGGGGCAGTAAGTGCCGTCATGGGCTGCGCCATGACC
ATTGACGCGGACATCCCTTCAGTGGCAGGCGGACGTGGGGCAAGGCTGGGTGCGGGAGTG
GAACCGCTGGAGCGGATCTTGCCCTTGAGCCAAAATGGCTACGG
>at03178
GACGCTGAGTCACCACCACACTTGTACGCAACAAAGGATACCGCTGAGCAGTACCGGCTC
TGGGCGCGTTACCTTGCAGACCAGCTGCACAGTGCGGCGCCCGATGGGCGTGTGACATTC
GAGGCGCTTCGTCGGCTCTGCCCTCTTGAGTAGCTCAGCCGGTTGCTGCAATGCAGGTGC
TGCCAGACGTGCTGTCGCCGAGGAATGTTGTGCTGTGCGCAACCAAGGCTCAGTGTGGCA
TGACGTGTTAGGGCACGTTTTGAACCGCTCGGCTGACTTGTCAGTTGCCCTGGTGGTTGC
TCAGATGAAG
>at03179
TTGTTTTTTTTTCTTTTTTTTTTGGTAAGGGTAAACAGTCACGCCAAACTTGAAGGTCAT
GACCTGCTGGTGTGTGTTCGGATCGAAGCCCACATCGATGCCCTGAGCCTGCGCTGCGAA
GGCCAGGTCGATCTTCCCGATGTGGATCATAGCTGCCTGACTAGTCACAGTGGTGCCCGA
ACAAGTCCAAACTGGCTGATCCTGGAAAGGTGAGATCAACAAGCGCCAGCAAGCGAAGGG
AAGGTGGCATCAAGTGGGCAAAAAAGGCCTTGAGCCAAAATGGCTACGG
>at03180
GCAGCATGAACTTACCGCCCATGCATGGGCCGCCGCCTGGCAAGTCCGGGCAGGCGCAGG
ACGTGCTCGCCAGCCCCAGGCAGCTGGCCCCGACGAGAGTGCAAGGGTCTTGTGGACAGT
CCGGGGGGTTTACAACCTGGCGGCCTCACTCTACCTCCAGATCGAGTCACGTCTGCACAA
CATCTTGGTCAACGGCCTGGCTCTGGAGAGCCTGCCATTCTGCAGCGAGCAGGCGGCCTT
GCTCGTCCCCCTTTGCCAGCGTCAGCTCCAGTGACGCCGATGCAAGTCTCACGGCAGCAT
CGACCAGGGGCGTCACCAGTGCAGATCGTTTTGCCGATGTCGGGAGGCACTCACTCCTTC
CCACCTGGTGCTATGCTTGTGCACTGAGCTTGCGCAGCGGGCGTCGGTA
>at03181
CCGTAGCCATTTTGGCTCAAGTGCCTGAGGCGGTGGCTGCTCAATGCTGGGCTGGCCAGG
GGCCTCCGCCAGCGATGCATCCTATCCCCATCCCAGCGGACAGCAATGTGTGGTCTGAGC
ACCGGCCCACCTCGCTGGCACCTGGCCAACAAGTCTCAATCTGCCAGGAGCTCACCCACC
CCAGCCACAAGCTGCGTGGAGTGGCCATGTGGCCAGACGTTCCGAGCCCATGGCACCAGG
AACTCCTTTTGGCATAGGGCCACAGGCCCTCCCTCAGCTTGAGCCGGTCTTCATACTCAG
CGCGTGACTGAGTAGTTTTGGCCGAGCAGTTGAGGTCAGCGGCTCCTCAAGTGATCAGAC
TTGGGGGGCCCATCAAAAGTGCAACGGTGCAAAAAAAAAGAAAAAAAAAACAA
>at03182
TTGTTGCCTTGGCATTTCTCACCGAAATTGACAACATCCTGATTTCAAGCCGAACTGTAC
GCAACTTTGTTGAGGTTGAACACGAGATCCAAGTTCGTCATGCCAGGAAGGCTAATCATG
ACGAAAAGACGCCCTGGACGGTTGGAATGGCTGTCTGCACCGACGCCTTAGTGATGATTG
GCCTTTACGTTTCCCAGCATGCTCGTGACACCGTTGCAGAAGCCCTTGGTGGAGTGCAAG
ACCCTGGTGGTCAGATTGAGGGGCCGCTGAGCGCTCTCGTTGCACTCCTGGTTCTGAACA
TAGCGATGTGGGTTGGTAGCAGGGGCATCACGACCTTGAGATGTGGGGCCCTGCTGGCCA
TACTCTGCATCGCCTTTGTCGCCGTGGACACTCGATTCTTGTACGTGAACCACTTCAGAA
TGACGCCCTTCCTGATGCCCGTGGTGCCCTTCTGGTTCGCCCTGCTGGCCATGGTGTCGG
GCAACGCCGT
>at03183
ACGCCATCAAGCGATCAAGCGATCCAACCCACCGCGATCAATCATCAAAGGCCGATCCGC
GATCAAGGGCTGTCTAGGGACTCCACGAGCCTCACGCTGAGAGATGCACAGGCAACAGCT
GCACGTCAGTATCTGTAGCGTTCTCAGCTGTGCTCAGCTGATGACCACTTTAGAAGTGCC
CCTCATGACCATGGCTGCGTGGACTCCGAAGCATCTGAGTAGTTGGGATAGCCTGGCCTT
GCAATCGTGTCCGGCACCTGCCTGCCGGTCCTTCCTCTCGCCCCTCGTCGTCGAAGCGGA
GCGTCGTTAGGCC
>at03184
TTTTTTTTTTTTTTTTTAGCGAACTGAGCCCTGCCAGCGTCAACTCCCTTGGCTCAGCC

AGCCCCCCCGCCGCCCCACCGCCCAGCCGCTCAGCCCAGGCGGGGCCTCTTCGCGCCCGG
GCCCCAGCCGCCGGCGGGCGGCCGCAGGGGCGGCCGCCAGTTGCCGAGCTGCTGTTGGAG
GGGCGTCCAGCTGCCGTTGGCGGCCTCACGAGACTGCAGCTTGCACTTCTTGATGTAGGC
GGTCACCGCCGCCGAGTAGTCCGAGTCGCCCTCCGAACGCGGCCGGAAGTCGCGAAGCAC
CTGAGACTGCACATCCGCGGGGCAAGTCTGGAAGTAGTCCTGTGCCCTGGCGTCGCAGGG
GTACCGGTCGAAGAACTTCACCATGGCCTCGTGCC
>at03185
GTCCAGGATCACATCGGATGCTTGGATTTACCCAAGGGCGAGTCTCCACAACAGAGAGTT
GCATGAGCCTACAGGCGTATACCAGCGGAGCACAGTCAGACCAATGCTCACGGGCTCCCT
CGTCCGCCTTTGTCGCCTGTAGCCCTTGCTTGTCGGTCTCCCCAATCCATAACCGTTCCA
G
>at03186
GTTTTTTTTTCCTTTTTTTTTTGCCACTCTTTGCATCTCCTTTNGATACGATCTCACTCC
TCGGACGCATTAACACATCGCGAGCAGGTACCGGCAAGACCCTCAGCACAAGCCGGGCTT
TCATCAGCGCCGCCACCCATGACCTTGGCCCTGAGGCGGATGATGGCCATAGCCCTGCGG
CGGCTGCTGGCCGTAGCCCTGCGGGGGGTGCTGACCGTAGCCCTGCGGGGGGTGCTGGCC
GTAGCCCTGCGGGGGGTGCTGGCCGTAGCCCTGCGGGGGGTGCTGACCATAGCCCTGCGA
GGGGTGCTGGCCGTAGCCCTGGGGGGGCAACTGGCCATAGCCCTGGGGGGGGTGCTGACC
GAAGCCCTGAGGGGGGTGCTGGCCGTAGCCCGGCTGACTGGCATGGCTCTGCGGGTGGGC
ATGCTGCTGCTGCACGGCCTGCTCGTTCAGGCGACGCAGGAGCACGTTCACGCAGATGCT
GACATCAAACGACGGAGAGCTCTGCTTGATCAAGGCCAGAGCATCCTCCGCTCCTGCTGT
CTTGATGCGCTGCCGGAAATTCTCGTTTGGCATGGTCAGGAACTTCGAGAGAGCAAAGCA
AGCGTTCTCCTGAACCCCCGGGTGGCTCGCATGTCGCTTGAGTGCAGCCACGACAAACTC
AATGCCGTTGAGCTGAGCAATCTCAACCATGTTGTGTTGGTGGATGCCCAAATTGGGCAA
GGCNACGCACGCCTGCTCCTGCACCCCGGC
>at03187
TTTTTTTTTTTTTTTGTCCGCAACTCTCTGCATTATCCGTCATACCTCAGCATGCGCGA
GGTAAATGGGCGGAGGTGAACCGATTCCATGCGGGAGCGGCCGAGCCCGATGAGGGAAAA
ATGCGTCCCGCCGTGTCTCGGCGAGCAGCTCAAATGGCTAGGGAGGGTCCGCACTGCTTC
AGGGCCACCACTGGATCCCTTGACCAGCGGCCACTCCGCCCGTCCCCGTGTGTCCGGACA
GCGTTTGCACGGGGCACGGTCTGCTAAACTTCCTTGAATTTGTGAATTCCTGACCAGGGC
TGGTTGCTGATGCTGCCATCAGAAGCATTCTTCGACGTAATCGCCTACCCCACCAGGTTG
TGCCGCTTCAAAGCCCGGCTCGGCGATGTGGTCGTCGTCCCT
>at03188
GATGAAGGTTTTGCTCGAGGGCGGGCGGAGGGCGCGCTAGGCCGGCTTCCCCCAGAAGGT
CGAGAGGAGGCATGGCGCCGCGGAGGAAGCCCAGGAGGATCGCCGCGCCGAAGAGGAAGG
CACCCGCACCTTGCAAGAAGGAGGTGAGGGGAGCAGAGCTCAGAGAGCAGTTCCTACAG
>at03189
GGTGCAGGCGCTGGCGTGCCTGCCGTATGCGCTGCCCGTGCTCGTGCACCTCGAGGCGCC
TGCCACTGGAGACGCTTTGAACGTGCGAGCATCTTCCTTCGCGCACGACTACGCGCTCCT
TGCGGAGGCGTACTTGGCCCTCCGGGAGGTGGGGAGCTCTCTTTTCCACTTCTGGTGGGT
CCAGCCGCTGCTCCTCTACGCAATCGTTCAGCTGGCCGGGGCAGAGCAGCTCCTACCGCG
ATGGGTGCGCTTCAACATGCTGCAGGCTGCCGTTCTTGACGTCGCTTGCTTCTTGCCCGG
GCAGCTGGCTGTGGCAACTGTAGCCCTAGGCGTTGGCGATGTGTACTTTGACCAGAAGCC
CCTAATCTTCTACCTGCTCCTCTTGTGCGTCGCGTACTGCGTCTTCTGCA
>at03190
CCGTAGCCATTTTGGCTCAAGCCATATGGGACCAAGGGCCAACCTCTCCCCCTTCAGACT
GGGATGGTCATTCCCCCTTCGCCCCGCTCAACCCCACAGCCTCAGCCAATGCAAGGCCCC
CCGCAATCGCACGTGCGGAGCCGCGTCTTGTTGGTGTTCTGAATGCCGAGTCTCTGGCGA
GGTTCCGGGACGAGGCTGCCGGCCCGGGTTGGGAGAGGCAACTGGCAAGGCTCGTGCTGC
GGAGCAAGGTCTGGTCTGTACGTCGAAAGTGGGAAGAACTGGGAAGCTACAGCACTGAAA
AGAGCGAGGCGGTCTTGGCAAGCTTCACGTTGCCACCCATGGTCGCTGCACAGAGCGGCT
TGCCGGAGCGAGGAGGGTTTCACTTGGCTCGAT
>at03191
TTTTTTTTTTTTTTTTTGCTACTTCAGAACATACTGAATTCTGAGCCAAGCATGAATTC

GGGCAACGCAGATTTAAGCACTCATACTCCCTTCACTACGCCCATGAAGCCAAGGATGTT
TGAAGCACAAACAAGAGGAGCTCTCCCTGAGAAAGCTCCACCTCACAGAGTGGTGCTCAT
ACCCAGTGTGCGTCCATCAGCTCTGCTTTGGCGTGTACCACAGCTATCCACCACCGCGGC
GTATATTCATCTTGCGGGCAAGTCCCGGAGCAGGCGTTAGATTGCACCGCTTATCCAGCT
CAGCGGAATGCCCGCAGTCCTCAGCCTTGACAGTGCCTGTTGCAAGTTGTCTGATCTCTT
TGTCATAGCTGCTGCGCTTGAAGGGCCCTGTGTTGCAGTCTGCCACGCCCATTCCGTCCC
AGTGGCTGCACCACCTCTGCAACGCCAGCCTGGGCAAGCTCTCCGGAGCGGACAGTCGGA
GAAGTGCCAAAGAAAGAAATGCAACGCATGCAGTTCCAAGGTCAAACAACAAGGCAACGA
TCATTGAGGCTCCGAATATCAAAGCAATTTGCAGGCATCCCGAATTAATGCGGCAAGAGG
TCGCTGAAGCAGCGTCGCAGCAGATGGCCTCCCCACCTCCATCGTCGGCGCACTCGTCAG
CGGCATCCCCATGAATTACTTGCAAGAGCAGGTCCCGATGCATGCCCCTTGCAGATGGGC
TCACCTCACCCGCCACTGGATAGTTCGTGGGGGCAGCCCAGTCCACACTCCTGGCGACGG
GCTTCCAGCAGAGTGTCCTTCGCGCGTCCTTGGCGGGAGCTGCGGCCTTTTGTGCNGCAC
TGGCAGCACTCAANAGCTGGGCAGGCG
>at03192
TTTTTTTTTTTTTTTTTGCTATCCAACACGCTCCCCTGCGCACCCACATGTCCTCGTCT
CCCATTGCTGGCCAACAGTCTGCGCGTTTGTACAAGAGGCCGTGCAAGGACTCAGAGGCA
CCGCCGGCGCCTCTTCCCAGATGGTTGCGCTGCCTCAGAGGTCGCCGAGGCGCTGCCCGC
CGCCGGCCGCAGGCGCAGCTCCTTCAGCACCACTGCTGGCAAGCTATCCCTCATTGCAAG
CAGCTTCGCCATGTTTGCCTCGTCCACTGCGAACCGCTTGCACGCAGCACGGAGCGCCGG
CAGCTCCTTGAGCACGGCCGCCTCGCAAATCTCGCCCAGAGACTCGAGGCTCACCAAGTT
GATCAGGGCTCTCTCTGCCATGTCCACGACATGAGCGACCTGCCAGCGGTGGGCCAGGTC
GATGACGCCTAGCATGACACAAGCCTTGGGCTCGTCGCAGATGGTACCTGTGTAGATCAA
CTGCAGGACCTGCTTTGCAGCCTCGAAGCTCACACCGTCAACGTGGATCACGCCAGTCTT
GCCCTCCTGCATAGGAGAGCCTAACAGCGCGTCCAGCACGCAGGATGCCCGGTGCAGGAC
CGCTCTGTGCGCCCTCAGCACGCCGTCGCCGCTGACCACAAGCCTCACGTCGCAGGAGGC
CTCGTCCTCCAGGATGCTCTCCCACATGTCCACAACTGCCTCTGGGACTGACACCTTGTT
CCCCTCATTCTTGGGCACAAAACCGGCAAACATCTTGAGCATCTTTTCACAGTTGGCAAT
CTGCGCAGTGTACTGCTTCTGATGCTTCTGGCAGGCCGCCATGGTCGCGAGAACCAGGCT
GATCGCAGAGTGGCCCTCGTGCTTAACAGCCTCGATCTTCGGGAACTGTTCGTTCCCACC
CCAGCCTATCGAGCCGGAGACCTGCTTGGCCTCCACTCGAGGGTCAGCACCCGCAGCCAG
GCATGTCCTCAGGAGTCGCCAGACGGGTGCGCTGTCCTCAGAGTTCTCGTTAAGGCGGAT
GCCCTCTTTGACGATGGCCATGAGAGGCGTGCCGTAAACGGGATGAATCTGATTCCAGTC
CCAGTCCACGGGGACCATCTGGGAGGTCACCAAGTCGACCTTGAGATTTCGGAGAGCCTC
CCAGAGCCATCGGCAGTTTTCCGATGACTGTGCCCGGGCACCGCTGCCTCCGCCACTTCG
GTCGGAGACAGACATGGCTGCCCGAACTGCCGGCTGGGCCGGCACAAGTGGGAAGTGTGT
TGGCGAGGACTTTGAGAGGCAGCTTTTGGCTGCTGCTTGAGCCAAAATGGCTACGG
>at03193
TTTGTTTTTTTTTTCTTTTTTTTTTGTTCCACTCCTCATTCTCATCACCATGTNTGGCGG
CCTCTTGGTGCCCGCCAGAACACGACCTGATGAGCGCCCCACTGTACTGCGGCACTTGCC
AGGGCCACGTGGCAGCCATTCCATGATCTGTAATCACCAAGGCTCCTGCTTGATGCCATG
GCACGGCCTGCACAGCCACCGCCAACCAGCTGGTCGGCGTGGCGGCCCATGTTAAAAGAC
AGAGAGAGGGAAGCTACTTCCCCTTTCCGCAGCACACCCTAAAAGGACAGCAGATGATCG
CCTTGACGAGCTCAAACCCGAGCTTGATGACGCAGCCGACCAAGCACAGACCGCAGACTA
CCACACCAATGACGATGAGGGGCCAGGCCCACCATGGAAGGTCACGCGCAATATTCTCAA
ACTGGGCCGTGCAGCAATCGATCTCCTCCTGTGTGGCGTTCTGACGCATACCTTCCGGCA
GATGCTCACGCATCCATGCCGTACACCGCGAGAAGTCAAACCCCTCGCACTGGAGGTTGC
TGGCCTGGAGACTGTCGAGGTCAACCTCAAGCTCGACTGGCTGCCATGACTCTGAGCTGT
TGCCCCTTGCCAGGAAGGCAGTGTCGTTGGCACCCTCAACCGATAGTGGTGCAAGCATGA
AGAGTGCCAGAAAGCGTGCAAGCTTCAGGGGCAGAACCATTGCGCAATGCAGAACGATGC
GCGTGCTTTGCCTGCTCGCCTGTCCAGTAGTCTTGAGCCCCAAACTTGAGCCAAAATGGC
TACGG
>at03194
TCCGTAGCCATTTTGGCTCAAGGTTGCCAGCACGCAGGCAGTAACCCCCTCCACAGCCCC
ATATCGTCTCCCTGCCCGCCAAGATGCCGAAGCAGATCACCGAGATCAGGCAGTTCCTCC

AGATCGCGCGGCGCAAGGATGCCCGGTCCGTGAAGATCAAGAAGAACGGCACACAGACCA
AGTTCAAGGTGCGGTGCAGCAGCTACCTCTACACGCTTGTCATGGCGGATAAGGATAAGG
CTGAGAAACTGAAACAGTCGCTGCCTCCATCTCTGCAGAAGAAGGAGATCTGAGGGCCTT
GGATTAATGACAGCTGGACGTGGGCAAAGGGCAAGTATGCG
>at03195
TTTTTTTTTTTTTTTTTGCACCACAAGGCCACATCAAAGGATTCATGGCTGTCTACGGT
ATCCTCACTTCAGGCAAGCCAACCCCGGCAATGCAGCCTCATGCCTTTGCTGTCTTGACA
GCCACCTGCAGAGTGGCCCCGTCGTTCAATGTCAGCCCGTTCAGCGATGCAATCGCCCCC
TGAGCCTGGTCCTCGCTGGCGTAGTGCACGAAACCGATGACATAGTTGTCCTTCGGGAGC
GCCTTGGCGCTGAGCACACAGCCAAAGGGTGAGAAGGCCTTGTAGAGGTAGAGGTCATCC
GCGCCCATGGGCAGGCCCTTGACATAGAGCGTGGCGTTCGGGCCGCTCTGCTGCATCATC
AGCTTCATGTTGGGTATGAGGGAGTCGGAGCCAGGGCCGCCGCCCTTCTGCGCGATAGGC
ATGACTGTGGGCGGCAGCGCCGGCAGCGTGCCATAGCTGCCGGTGTGCACCGGGGGGCCG
CCCTTGCCGTAAGGCGAGTATCTCTGGGGGACCGCGGTGGCCTGCTGCTGCTGCTGCCTC
TGGCTTGACGCAAAAGTGATCTCGACGGGCTGTGTCAAGCCCTGGGGGATGTTGCCATGG
ACGTTGTCCACCAACCAAGCAGCCTCGGTAATGGTGCCCATCCTGATGATCGCCCACGGA
CTGGCCTTGACCGTCGGAGGACTTGCCGTTGGCGACCAACACCTTGGTGTCCGTGACAGT
GCCGTAGGCGGCGAAGATCTCGCGCACGCTCTCGTCCGACATGCCTGGCGGCAAGCCTTT
CACC
>at03196
AGTGCCCTTATTTGGATCACTTGCCATGCTGGCTGATGCTCAACAGGATGTGCAGCAGGC
TGAAGAAGACTTGCGTCACGAGGCCGACCAAGCGACATCGCCGATGGACGTTGGCTTCCA
AGCGTTCCTCGCTAACTCACTAGCAGAGAAACCCGAGCATAAACCACTCTGTAAGCACGG
CATGGTTGCCGTGGATCTGATGAGCACAGGAAGAGAAGAGGATTCGCGGTGTCTGCTGCA
TTTCTTATTTGCAAAGTGTCAGGTGTGGATGGAGGACTTTGCCAACCTTCCACCAAAGCG
AAGAGACTGGGTTAGACGCTTCATGGCACTCGCCAGCGAACTGATTCGCCAGTGTGAAGA
GCAGGAGGTGCGCGGCATGACTTTGCAGAACAAGCATCTTCACATGCTCA
>at03197
TTTTTTTTTTTTTTTTTGGGCAAGCTCACGTCGAGCCTCTGCCAGGGGGTCATAACAAG
AACTGCTTCCGCTTCGCGCCCTTGCCTTCAGTAGGCGGCTGCTGCCAAAGCGCTTGGAGC
TCAAATAATACCTCAGGGGGACAGCTCGCCCCGATTGAACGCTTCGTGCACCGGCCAGCT
CTGCTCCGCAAAGTGAAGACACGCCATGCGGAGTGGTCCCCTTCGTCCAAGGTCTTGGCC
AATGCCGAAGCTCGCCACCTTGTCGAAGTTGGCGGCGGAAAGCTGCGCAACGATCCGATC
CACCATCTGCGGCAGAAACCCCTGCACCGCGTACTGCTTCGAGAGCTGGGCAGCGCCGAA
CAGCAGCTCCAGGGGCGGATGCAGCTCTCCTCCCTTGCCCTGCAGAGCTGCTCCCTTGCC
CTTCGCCACCCTCTTCGGNGGCCGCGGAAGGTCGGAGGAAGAGTCGTCGGCGGAGTCGTC
ATCGACAACGAAAGGATGGACTTTGCTCTTGCACTTTCCCGGTTTTCCCTTGCCCTTTGC
CGCCTTTCCTTTNNGCCGCTGCTCGTCGCCGTCCGGTGTGGGCCACTCCTCAGGCTCCCA
CTGGCCAGTGTAC
>at03198
TTTTTTTTTTTTTTTTTAGCTCCAGGACTGTACACACCAGCATGTACCGTCCTGACTTC
CATTTTGGAATCACCCCTCTACCACGCATGCTTGCAGGGAGCGATCACCCCAAGTCGAAA
CTGGAACCGGAAACTCGGAGTATCAGGCACTGCGGCATACCACGCATCCTGGAAGAAGCT
ATGTACACAGGCTGCTGGGACGAGCTGCTCAGTGCCGACCAGCGGAGCAGGCATTACGCT
GCTGCTTCAACGACAAGCCAACCAACAAAGCTTCTGGGGTATGCCTGCGCACTCCGCTTG
CGGCAATTGGCTCCGAAGTTGTGGCGAGCATCCACAATGTCGTACTGGAGCTGAACGTTG
GTCCAGAGCATGTACGCGCCCGGCTCGCAGGGCGCCTGGAGCCGGAGCTCCTTGTTGCTC
CTCTTCCCGCGGCCCGCCACGCCGTTGCAGAGCTCGGCGGCCAGCGTCGCGTCGAGGCCC
AGCAGGCCCTGCTGGATGCAGGTGTCGCCGCCGTGGTTGTGCGTCCAGACGTCCGCGCGG
CAGTGGACCGTCTCGCCGGGCCGGGCCTTGAGCGAGTTGGCCGGCTCTCCCGTGGGGAGG
AAGAGCTTCGCGTGGGTCTCCCACTCCTTGAGCTCGGTCGTCCGGCGCTCACGGTCCGTC
AGCTCCTCCTCGAGCTTCCTCCAGACGGCCTCGTCGGTCTGCAGCCACTCCGGCTCCTCG
AGCTGGTGCCTGGCGAACCACTCCCTCAGGAGCGCCGGGTGCTCGCTGTTGAGGCCGAGC
GCCCGGGCGCACTCCGGGCAGGGGACCCG
>at03199

GCTCGTGGCAAAAAGCGTGCTCACAGATGAAAGCCAGGACAGACTCGTGCAGTCTCTGCT
TCCCAAGGCTGCCAACAACCAAGCTGCCAGGTTGACCCAGCAACTTGCAGTGGATGTGTA
AAGAACAGGCATCGCCAGCAGAGGGTGGGCCAGGAGACGGGGCCAGAGGGCAGCAAGGGC
TGGTGGACCCCCGGNGCTCGAGGGCGGTCCAGATATGCCACATCTCGAGGACTGGCCTGC
CCCCACCTCAAAAGCAACCTCTCGAGAACCACTTT
>at03200
TTTTTTTTTTTTTTTTTGCGCATCACGCCAGACTTTCCGGCCAAGTGGCGTTGTCTGTG
TGGCTTCGCTGTGGCACGGGCTCCTCACTATCCTCCAGTGGAGGGCAGGAGAAGTTTCGC
ACGCAGCGCTCAGCGCACTGCCGGCAGCCGGCGCCCTGCCAGAAGAACTTTGGGTACATC
CTGGCGAGCTGCAGCGCCACGGCGCCAAGATGCTGGGCTGGGCACAGCACCTTAGGGTTG
ACCGCGCACTGGCTCAGGTTCTGGTAGTAGTAGTCTGTCACGTGCTCGCGCTCCTTCATG
CCATGGTGCATCAATGGACCAATAGAAGGACTCCCCAACTTCTGGAGGTACACACCATCC
ACGGCGAAAAATTGCGCGCGAGGCCAGACGGTCAGACGACTGCCGTTGCCGGCCTTCCAG
TTTCGCGGCCTCGTGCC
>at03201
ACTCCTCAAGCACTTGGGGATTGACAGAGTTGTTGTAGTGGGGATGGCCTGGGGGTGCTC
GACCTGCGCTACTCTTCGCTGCAAGCCACCCCGAGCTCTCCGAGGCTTGTGTGCTCTATG
ACATTTCGCTGGGCAATTCGCTGACCCCTGAGCATTCTGGAGCTCAGAAGTACTCGTTGC
TGCTGGCTCGAGACAAGATGATGCGACTGGGCGGTCTTGGGGAGCCG
>at03202
TTGTTTTTTTTCTTTTTTTTGTTTGGAATGATGCATGCATGCAGAATCAGCAACCCAGAC
TGGTGCAGGCTGTACAAGTGGGGAGGGGCACCATCCAGGTTGACGCGGGGAAAGCACCAT
CACAGTTGACATCGGATCTAGACTTCTGAAACAATATTACCGGACAGCCCACCAAGCTTT
GCGGAGAGCCTGTCAGCTGGTGCAACAACCTCACACCAGCCCACCAATACCTGACCAAGA
ACCATGCCCCAGACTCCCAAGGGGAACTCCTTAAATGCCATCAAATGGACCATTGCAAAA
GCCAGCGTGACCTGGCCCTGACGAGTGCAAGCACGTGCCTAGATCTCAGTCGCACTGCAC
ACGCGGACCTTCATCCCCAAACCGCATGGGTATGCCCGCCTCTCGGCTCTCTCGATGGAT
ACAGATACGTCGTCCACGACATGCCGACCATCCCTCTGTATCTGGCCCCAAAGGTGCATC
ACCGCGTTGTGCTCGCCCTCCAAGACCTGCCAGACGGTGCGCAAGGTGGCGTCATAGGAC
ATGAAGCCCGTAATGCTCTCCGTCATGCTGTGGCCAGCAACACTTTCTGCAATCTGCCGG
ATGGTCCTGCCCAGGTCCTCGCCGTCGCAGTCGCCCTTCATCGTTGACATGTAGGAGATG
CGGACGAGGGGCTGCTCGGGGGTGGAGGCTGCCACTCAGCGTCAGCCACCGTGCCGCGGC
TTCCATTCCCCTATGGGGGGCACAGGCAGCGCCAGTGCTCAGCAACGGAGCTTTGGGAGA
GAGAGGCAGCTTGGCCGGCCACAGCCACACGCTGTGCTTGGCACGAAACTGCTCGGGCTG
GCCGGCGGGGAGCTGCGGGCTAGGGAGGCGCTTAGGCGCGAGC
>at03203
TTTTCTTTTTTTTTTTTCTTTTGTTAGTTATTGAGGCCTCCTTTTTTATGAGCATCAGGG
TCTCTGACTTAATTCCAAGTCATCATCAACCACATCATGGCTCTTTGGCCGCTGTGTGCG
CGGGCTTGCAGAGTCTTCATCCCAGATGGATGGATAGTAGCGAGAATACCAGAATGCTGC
CCAGGCAGAACCGATCACGGCTCCTCCAACGACGTCAGCTGGGTGGTGGTCGTTCTCACG
GGACGCGCGAGGAGGCAATCCAGAAAGGCAATCGCCATCGGGGACAGCGCCAGAAGCAAG
AAAACGTTGGTGAAGTCGATGTCTCCGATCAATGGCAATCTGAAGGGCTTCGAGACAGCA
GCGTTCACCTTTCCAAGTGCATAGAGGGACAGGAAAAGCATCCCGTCAAATGACATGCTA
GAATGACCAGACGGGAAGGAGCGGTGGGCATCTGCCTTGTCGTGCGTACACCTCCCCGTC
GACTCATCAACACCGCACTCGCCGTAGAAGTATGGCCTCAAGTAGCCAACGTAGTTCTTC
ACGAGGTCCACGCAGGCAATAGTACATGCCATGGCGAGGAGGAAGCCCTTCATGAACGCA
GCAGCATCCCCGATCTGCCGGCACAGGAGGATGCCAATCAACAAACCTCCCAAGGGCAGG
CCAAGACTCAATGCAACCAACTCCACGCCAGGGACAATCTGCTCATCATCGGGCACATAC
ACTTGATCCCACACCGCGCCTTTCACAAAGACAGTCCCCCCACCTCCCTCTTCAATCCTT
TGTCCAGGGACCGGACGGTCGTATGTCGCCAACACCCCGGGCAACTCGAGCGCAAAGCAG
CAAGCACTAAAGACCAGAACAAAAAACATGTCCCACGAGATCAGCGTTTGCATCTCTGCA
CACAGCATGTCTGCCTACTTCCTGACGGGCAGCTCGTGTTGCAAGAGAATTGGACTATCA
TGCATCGAACAAAAACGGCTTGAGCCAAAATGGCTACGG
>at03204
TTTTTTTTTTTTTTTTTCCATATTGAGAATGGGATTACGCACGGCTCACAATGGTCTCA

TTCTTGCGTGTCACCTGTACATAGGAATGCACGCTGCGCCTGATAGGAGCGAGCCCAAGC
TCGTCCCCCACCAAGATGACGCCCATTTCAGAAATGAGCGTTCAGGCATAGCGCCTGATA
TGCAACACTCGAGCCCAGCATAGAAGGGCGCGCACAAGGTACAAAAAAGAGGCTACCTGG
CGGCAGATGAAGAAAGCCGCGATGCGCAGCCGTGAAAAGCTCCAGCCGTATATGCGTGTG
GCCGCAGGCCCATGCCGATGCACTCACACTTGCAAGCTGAAACTGGCTGGAGGTCGGGGC
GTTGCAAAAATGAAGCGGATCATCAGAGATCAGACCGCGAATTGAAACACGATGTCTTCA
AACATCGCAAAACGGTAAGCCTGTGTGAGACTGGCAACGGCGGGCGCTCAGTCCGATGCC
TCAGTCCCACCAGTGCCACTCCGGCCCACAAGCCGTGACTTGGCAACGGATGCCTGCTCG
GCCATGTAGCCTGCTGAAGCGTAAGCCCGTGCCTTTGTGAAGTCTGCTGCCTCCAGAGCG
CGGCCCAAGGCAGCCGTGGACCTGCCTTTCACAAAGCCAGTGGTCTCTGTGGCCTTGCTC
TTCACAAAGTCTGTGGTCTCTGTGGGCTTGCTCTTCACGTAGTCCGCACACTGGCCAATC
TCCTCCCTCTTTGAGTAGGCACCATANGCCGGCGCGCCTCCCCCCACAGCACCGAAGGTG
CCTCCGACCGCAGTGCCGACCATGAGGCCTGCGCCACTGCCGATGGCCGCGCC
>at03205
TTTTTTTTTTTTTTTTTGGCATGTCAGAGTCCTTCATGACACAGCCGTACACGCGCCCC
TGAAGGCGTAGCAATGTCATCGCACACGCGCCCCTGAAGGCGTATCAATGATTGGGCGCC
TTGCTCCCAACCCGAAGGTCCGTTGAGCAGAACGCTCCACTTGCTGCCCAAAGCGCTGCC
CCAAGCCGGGCGTCTGCGCTGGCATGCTCATGCCTCGTGCC
>at03206
CTGCATCTGTGTAGTCTTTGGGATAATCATCCTGAACCAGGTCTTCAAGCCGAAGAAGTC
ATCCCACGGACATGGCCACGGCGCCCAGAAGGGCTCCTCCAGCCCGCTGTCCAACTTGGC
GAGGAGGAAGGAGCTGGCGAACACCAAGCGCTTCTCCAGGGACAACTTCTCCTGGATGGA
GACCTCCAGCTCTGCAGCCTCCGCCTCCGGTGACTCGGACGCTGACGGCGTCTTGGACGA
GGCCAATGCGGAGTCGCCGATGGTGCAGCTCGGCCGGCTGGATGAGATCCAGGAGCACAA
CCCGGAGGGCAAGAAAAATGACTGAGGACTCCCTCGCCTCCCGGGCTGGATGTGGAGACA
ACGCCGGCGCTTGGCAGCCACCAAAGCAGATAGTGACGACGACAATGGTGATGCCATTTC
GATTATAGTCATGCAGCGTATGCATGC
>at03207
CCGTAGCCATTTTGGCTCAAGCTGTTCTGGCTTCAGCGCCCCGCACCTCACCCCGCATCC
CAAGCACGGGTTGTGGCCCACTGGATTGGCTCTCCGCCTCCTCGTAGTCATGCCCGCAAA
GAAGCAGAGCGTCAGCAGCAAGCCGGCGGCTAGGTTAACCAGCGCCGGCCTCCCGTGGAA
AGAGCGTAGTGTTGATGTGGAAGGCCTTGCAGCAGCAAAGGCAGCAACGAAGATGGCAAG
TCAGCAGTGGAAGAAGCTACAAGTAGGCATGAAGGAGCAGTGGCCGCAGCCGGAGTACAT
GAAGAACTCACACCCCGAGGGAAAGGGGCACACGTGCTACCGCGACGAGACCTTTGAGGT
CGTCACGCGCTTCATGGAGAGCACCAAAATCTCCTACCGACCTCATGCGAAGGCTCCCGG
GACGAAGAGCCATGTGCGCTATGAGAGGTACAGCAAGGCAAAGACCGTGGGAGAGGCCCT
CAGGAAGGGGTCTTGGCCAGTCGATTGGTGCTGGGACATCGAGCGTGGCTACATCAAGGT
TCACGGTCCTGTTCGCGAGGAGCCTCTAGATCTTTCCCAAATTCAGCATGACTCTGAGCT
CACAGACGTGGACAATGCCG
>at03208
CGACTGAGTTGGCCGCGTCGACGGGTGTGTTGATGTCCTGGACGGTGTCCGGCGAGGCAG
AGCCCGCGCTGCTAGTTCCCCTGCCTGCGCGAGGTGGAGTGGCCATGTTGTCTGTAGCTG
GGCACTTTGGGACCATTCGGGATATGGATCAAAACTGTGGTCAAGACGACCGTTGTTTCC
AAGCAACCTCGGTACCGACGAGCCCCAGGGAAGCTGTCCAGTGTCCGGTGTAGAAGGATA
AATATGGAGGGCAGGGTATTAGGCCAGATGCCACCCCATGGCGGACCAGGGAAGGGCTTA
ACTGCTAGCAAGAATACGAAGAGAAGTTTCACCGCAGCTCGCGACCTCATGGT
>at03209
GATGTCCAAGTGGACCACGCCCTGGTCGTGCAGATCTCGCATGCCCTTCACAAGCTGCCA
GAAGTAGATAGCTTTCTGAATTAGCGATTCCCTGTCGACACCGTAAACCCTACGGTCACG
GAGTGTCACTTCCCGGCCGAGCCACTGGACTCTATCCTTGTCGCTGAAGCCCTCCACGAG
CGCGTAGCCCTCCTTCTCGAAGACGGCGGGATCCCCAATGAGCGAAGCCACATACGGGCT
CTTCCCCCTCAGCTGCTTCAGCAGCTCGAAGTTCTGCGTGGCGATCTCCAGCCTTGTCCG
AGAGTCGTAGTGTGAAGGACCGGAGGGTGCGCACAACACGTCCCCATATTCAGACACCTT
CCATCTGATAATGGCCGGCACCTTGGGAACCACATCCCTGTGCACGGAGTCTTCC
>at03210

TTTTTTTTTTTTTTTTTTGCCTCGATGCGACACGCTGTAGCGCCATCGAGAGACAAACGC
GCGCAACGACCGCAATCCAGTGGATCAGTGCCAGAATGCTCCAATAGGCTGCGGAGGCG
GACTCAAGCACCGGGCGCCAGGGAGACGGCCTTCGCGATCGCCGTCTGGGGCAGCGACTC
GACCTCCACGTGGGCCATGCACGTGTCGGACTTGGTGCGCCACTCCGGGTCGTTCTTGAA
CTCGAAGCCGGGGTGCCAGAAGTTCGCGAGCAGCACGACGCGGTCCCCCGGGTAGGCCTC
GTCGGCCCCGGGCTCCTGGTACACGACCTCGTGCTCGAAGGAGTCGTCGAAGACGATGCA
GCGCCCCTCCTCCCAGCCGCGCGGCGCGTCGGCGGCCACGCGCACCCAGCAGCCCTGCTC
CAGCGTGCGGGGCACGCGCACGCCCAGGTGGCACGTGAGGCGCGAGTTGCTGGGCCCGCA
GTGGGGCCGCAGCCGCGCGCCGGGGCCTCGTGCC
>at03211
TTTTTTTTTTTTTTTTTTCACGAATCGGAGTGTAAACGCAGCTGCACCGGTGAGGCGCTG
TGCTAAGGCTCCCTCGGAGCCCTGTTGCACCACAGGATTTTCCGCACTTGATGCCATTCC
GCCAAGACCTAGACCTGCCCAGACTGCTCAACGGTGCTGCCCAAAGCAATAAGGGACGTT
GCTCGAATAGACACAAGTTGGAGCTACAGGTGCCCGAACATGTCTGCCAATCCCTCTTCA
AAGCAGATCTGAGAGATAGGTGACCAGGGAGGGAGGGCCTGCAGATCTGCAATGCTTGTA
TGGCCGTACCAATCAAGCGACTGCTGCACGTGAGCATTCACCAAGGATGAACTTATCTGC
GGGCACGTTTTGCCGGGGGTTCAATGTCTGCCTGTGGAAGGCGCGCATAGTCAGCAGGCA
CCACACTTTCAGACCCATCTTCGTCCCAAGTGATTGTCAGAGAGCCGTCATCATGCGCGA
GCTTGATGACAGCTGGCAACCAAACA
>at03212
TTTTTTTTTTTTTTTTTTGCTTCACACTCATCAAACCCAGTGAAGTGACTCTTCACCCTT
TCCAGACATGCAACACAACACGGTCCCGCTAATTGAGCGCTGTGTGGCTGCGCTCGCCGT
GACTCCAGGCAAACGTCCACACATGCAAGCATGCATCGCTCCTGTGCGCTCACTCAAGTG
CGCACACCAACGACCGTGCGCGTGCTCATGCGTTTGCACTTGTGTCGGCACCCCTGCTTC
TATCAGCTGGGTGTCACTGCAGCTGCAGCCCGAATTGATCCACATGCACCTGCTCGTGAC
TCAATCATGCAGCCTTGCCATCAGCCTTGACTTCGCGAATCGTTCTGGTCAGGCGCTTCC
GGATAAGGAAGCCTGCTCCACCAAGCAACGATGCTGCAGCTGCGGTCGAGCCCACCACAA
CTGCCGTAGGGTGCCGAGTGACAGCATCCATGCTGTCTTGGAGCATGCTGCTGGGGAGCC
CCGGGATGTAGATGGCGGCAGCCACGACAAGGACAGCCAGCAGGGCAGCAGCCACCAGTG
CCATCACCACTCGACGGCCTGCCAGTGCCGCAAGCTGGCTCTTGATGGAAGTCCGGGCAG
GCGACTGCGCAACAGTGTTTCGCTCAGTCTTCGCCGCCTCTTTGACCGCCTCTGTC
>at03213
TTGTTTTTTTTTCTTTTTTTTTTGCTAGGCACTGGGACCAAGCAGCAAAAATAAAAATCC
ATGGTATCCTCGTCGGGCAAACACGGAAGCCAACTTAGGATTTCCGTGTTCTTCTCCCTC
GCCAGCCCGCTCATGGTAGTGTTGCTGGTGGTTAGGGGCACCACCTTGCATGAAGACCCA
GGGGTTCTGAGGCGTGGCATCCGGGGAGTGGGCGTTTCAGCTGGGCAGCCGACTCCCCCC
GAAGGGTGTAAATGACACGCACTGCCGATCGGAGCCCTTGCCGTGCGAATGCTGGGAGTG
CCAAGCTAGCCGGGGATTATCCCGCAGAGGCCATCGCCGATGTAGTACCCAATGTGTTCG
TCTCCACGTATCCGTCCTCAGCCTTGACG
>at03214
CATCCTCGCCGCGGAGCGCCGCATCCCCGTTCTGTTTGCCAAGCGCCTGCAGGATGAGCC
CTCTGGCCAGGGCGCACCGAAAGATGCTGGCCAGAGCTACTTGCCGATCAAGCTGAACGC
CTCCGGTGTCTTGCCATTGATCTTTGCGAGCTCGTTGCTGTCGCTGCCAGCGCTTGCGGC
CAACTACACGCNNCTgCCgCCaNGGTTGCAAgGGGTAgGCCAAgGTCCCTCTTGCCCGGC
TCCTCGGCCTACATTCCAATCAGCATGGTCCTGATTGGCGTTTTCTCCTACGTCTCGACC
TTTCAGGTGCTTGACCCGAAGGACATGGCGACGAATCTTCGAAAGCAGGCGGCCGCGATC
CCGCAGGTCCGCCCCGGCAAGGAGACCGAGGAGTACTTGCGCACCTCCCTGGAGCGTGCC
TCTGTCTTCGGTGCCGTGATCTTGGCTGTTCTCTTCGTGCTGCCCAACGTGATCGAGCTC
CTGACGAAGCTGAGCACCCCCAAcTCCTTCGGCGGGACCTCGCTGCTCATCCTGGTGGGC
gTGGCgGCAGaCACGTGGCGGCAGCTCCAAGCGGAGCTGCTCATGCAGCAGTACGCCACG
GACGTCGACCAGTTCTACAAGGGCAAGAAGCTGTGAGCCTCTTCATCCGCGCGGCCGGCG
CGCGCAGCGGTGCGGAGGGCGGCAGACGGCGGACGGC
>at03215
GTTTGGCTCCATGAACATGATATTGAGGGCTTGGTTCGCCCTTTGGTTTCCCCATCAGAT
TCCTGCACTGGCACCTCCACTGGCCCTTCACAGGATCTGATGGCCTCGGTCTACCAAGA

GGAGATCGAAGAGGAGCACTTAAAGCTGATCTCTTGACAGGGGGTCGCAGACTTCAGGGC
CACTGCACCATGCTGGACCTGCGGCATGCACTGTGTCCTGCGCAGCTGTGGTGGACGGCT
TGGGCTTATCCTGAAAC
>at03216
TTTTTTTTTTTTTTTTTCAGACAATCAAACATTGAAAACAGGGAACTGTCTTGCCAGGA
AATCTCTGCACCCGGGTCGGAGTGCCATCTCAGTCCATGACTGAAAGGAGCATCCCATGG
CTCGACCTAGCGATGGCCTCCTCCTCGCAAGTGGCTGGCAAGTCATACCACTCCATGGCG
TGTGCCTCCACAGATGAGCAGTCGTTGCTCTCGAACAGCAGCTTGCTCGGTGCGATCTCG
TCGTCCAAGATTGGCTCGCACTCTGACTCGTCCTTGCTGGCTGGGCCGTCGCAGATGTCT
TCCAGGCTGAAGTAGCACGCCGGCCGGTGGTGCTGCTGGTGCTGGTGCGGCTGCTGCCCG
TGCCTGGCAGGATGCCTCGGGGTATCCGCCGGCCTCCGCGCCTGCGCCTGGGAGCCGCTC
TCGTGCACCTGGTTGTGCTTCAGCTGCAGCTCCGGCACCCGCGCCGGCAGGAGCAGCGGC
TCCCGCAGCGGGAGCTCCGCATGCCGCTTCTCCGGCCCCGCAATGCCAATCACGACTAAC
AGAAGGTGCCCCTCGTGCCGAAT
>at03217
TTTTTTTTTTTTTTTTTAAGGTGCACACTCTTGAGTCCAATCCTGTACTGAGTGTTCAA
GCCGCGGACTGATCTGTACAAAAGCTAGTCCAAAATCTCCGAAGATCCAAGTGCAAGCCT
CTCATTCAAAGAGGCACAAGTCCTTCATGTCATCCTTGGGCCGTTTCTGGTAGAGGCGCC
TTTTCAGTGCCGCCTCGAAGTCGACATGCCGTCGAGGCTCTACAAGGCCAGGCCCGATGG
AGCCTGCAGTAAAGCTGAAGGTGCTCTGGCTGGAGATCAGGATCTCCACGCTGCTCAACA
TTACAAGCGCAGTCCACGTCTCGCGAGTCTGGTGGGAGACCAAGGTTCGTCTCTCATGCG
TTGGTCTGGCACTGCTTGTCCAACATGTGCAGGGCCCACTTTCTTTGCATGACTTCTTGG
CTGCCTTCCTTGAGCCGTCATTGTCAGACTCGAATATGATCGTGCAGGGTGGCTGGAGGC
CAACCTGCCGTGCAGTCTGCTGGGCGCATTCCACAATCTGTCTGGCTGCCCTCTCGGGCG
TGCCCAGGCCCCACTGCTTGTCGAAGATCACGGGCCGGCGGTCCGTGCTCTCCTTCGACC
AGGAGACGTCGCCCACCCGCAGGTGCACGCCTATCCTCGGGCCGGCCGGCACGGTC
>at03218
TTTTTTTTTTTTTTTTTGAGGCGGCGCGCACGATCATAGGCACCTTCGTCCAACGGCGC
AACGGCTACCTGACGCGTTATTTGAAAAGTAATGACCCCCTAGGAGCTCTGACTGTGCAC
AAGTGACTGGTGCACTCTGTCATATCGGATTATTGGCATACACCGCCAAGCCCAGCCCAT
CACGAGCTGCCACTCATCATGGCCTCCGAGCTCAGGGCGCAGCGTCCGGCCACGCCTCGC
AAGACTATCCCGCCACCTCGTCGGCGCCCAGGCCCTTGCCGTCGCTGAAGACGTGGAACC
AGCAGGAGGTGCCTGGCATCAACCTTGAAACTCCATCTGCGAGGTCGCTCTTGCTCACGA
AGAGCCTTCCGCCGTTCTTGTATGCCAGAGGGTGGTCTATGGCTGTGTAGGGCTGGACCC
AGCCATACTTGCCCTTCCACTCTAAGACCTCGCCCTCCATCGGCTCCTCGGTGATCCTCG
ACCGCTGCGGCTCGTCCCAGAGCTCGTCGCCGCCTCGGGACCTTCTGCTTCCTCTGCCCG
ATCTCGTAGGCGCCGAG
>at03219
CGACATTCGGGCTTCCACAAAGCGTACCCCTGCACCCCCCACTTGTCCCCCCTGCCTCGT
GACTGAATTGCTGTCATGGACGCAGCATACGAAGCCGATGCAAAGAGGAGCGAGGACACG
AGGGTGGATCCGGCATCCCTCAAGGCACACAGGGGATAGG
>at03220
TTGTTTTTTTTCTTTTTTTTTTTTGTCACGCGAGCTTTGCTGCGAGCATGTCTGATCTGC
CCCTGTGAAACAGACTATTGCCGTCGTGCACCCCTGCCAACAGGACCCGAAACATGCACG
GCAGCTGGCGCTCCACACCAGCTGCCGTCAAGAGTTCTAAGCAGTGGGTAGTCTGCAGTG
GTCATCGAACAACTCTAACTCGATACTCCTACTCCTCTTGGAGCTGGCCACTCACTGGCG
GTTCTCGTCCATGAAGGACTTCATGAACGCACCAAGCTTCTCCACACCCTCCATTGGCAT
GCCGTTGTACAACGATGCGCGGCATCCTCCGACCGAGCGGTGACCGGCGAGGGTGTAGAG
CTTCTCCTTCTTGGCACCGTCAAGGAACTTCTTCTCCAGCGCCTCATTGCCGCCCATGAT
TGTGAAGGGGATGTTCATCCGGGAGCGAGCTGGCTTCTCGATGGGGCATTGGTAGAAGCT
GTCGGAGCCATCGATGGCATCGTAGATGAGGCTGCTCTTCTTTGCCGACTGCTCCGCCCA
GTGTGCCACACCGCCAACCTTCTTGGTGTACTTCAGATACTCGCCCATCACGTACATCGT
GAAGCAGGCCGGTGTGTTGTACATGGAGCCGTTCTCAGCGGCAACCTTCCAGCTGCAGTA
CGTCGGGCAGATCTTCAGCTCATGCTTGCCAGTGGCAAACTCTGGGCTGATGAAGCCGAC
GGAGATGCCCGCTGGGCCCAGATTCTTCTGGAGGCCTGCATAAATGTACGCATGCTTGCT

CACCTCGATTGGCTTGGAGCAGAAGTTGGAGGACATGTCGGCGACAAGCGGGACGTTCCC
AACATCTGGAGTATAGCTGAACTCGACGCCGTTCACTGTCTCGTTGGCACAGTAGTGAAC
GTAAGCGGCCTCCGGGTCCAGCTTCCAAGTGGACTTGTCGGGGATGCTGGTGAATTTGGT
CGACTTCGTGTTGCAAGAGACCTGGCTGGTGCAGTACTTGGCGGCCTCCTTGTGCGCCTT
CTCGCCCCACTGCCCAGTGATGAGGTAGTCTGCCTTCGTCTTCGTCCCGATCATGTTCAG
CGGCACAGAAGAGAACTGGAGAGTGGCGCCACCCTGCGCCAAGATGACCTTGTAGTCGTC
CGGCACGCCAAAGACGTCGCGCAAGTTCTTCTCGGCCTCGGCCGCTATCTGCATGAACTC
CTTGGAACGGTGCGACATCTCCATCACACTCATGCCGCTGCCCTTGTAATTGATCATCTC
AGTCTGAACCTCCTCCAAGACCTCAAGCGGCATCATGCTCGGGCCGGCAGAGAAGTTGTA
CACACGTCCTGAGGCCATTTTGGGTGGGGGAAATCAAAACGCGGTGAGTGGGATACCCG
AAGCTTGAAAACTTAAGCCAAACTTGAGCCAAAATGGCTACGG
>at03221
TTTTTTTTTTTTTCTTTTTTTTTCCTTTGATGCGCAGACTGACGGATCAGCGGAACTGT
GTACAGCCTCTCACCCACGCGCGGCGGTTCAGCACTCAAGCCGGAATCACCTTCTGCGTG
TGGTCCATCCTGTAATCGCCGACGACGTGCATGAGCACCGTCTTGCTGCTGTACATCATC
GTCTGCTCGTGGGCGAGCTCGCTCTCCGGATCCACGTCCTCGGCCTCCCGCTCCGAGAGG
TCGACGCCGATGGTCCTCAGAGCCTTCAGCAGCGTCTCCTCATCGTCCGGCTCGTAGTAG
ACCCAGACGACATAGCCGCGGTCATCGCCGTCCCCGGAAAACATGCTATCGGCTCGGAGT
GCGCACACATTCAAGGAGTAGTTGAACTGCTCCATGTAGCTTGTGTCGCTCAGCTTCCTC
ACCTCTGGCGCCCGGAGGTGCACCCAGCGCTTGCAGAGGACCTTCTTCGGGAGAGGCTCA
TCGAGATAAGTCAAGAGGTCCTCGTTCAGCGAGTAGTCCTCTGGGTAGAACTCTGGGGCC
ATCATGACTGGCCGCTCCCGAGCCACGGGGGCGGTCCCGGCCTGCAAAGCCGCGAAGATC
GGATTGGCGCTGCTTGAGCCAAAATGGCTACGGACTCGCGTTGATAACACTGCTT
>at03222
CTCAACGCAAAATGCCGCAGAGAtTGGtAtTTGCTGCTCTCTTGCACGCTTGCTTGGAGG
TGCTACAGTCTCCCAGCTCAACGGTGCTGCCTGCATCGACCTCACTGCAGACGGTGCTGC
TGTAGAGATTGCAGAAGAcCCAGAGCGCAGCAGAACTGGGCGACGCCCTCCTCTCGCCGA
TGGGATTCACGGTCGAGGCATGGGTGCTTGCGGATTCAAGCAAACTCAACATGGAAAGCG
CACAGAAATCCTGTGGCTTCCAGGCAGTGGGCCTGCATCTGGGTGGAGCTTCGGCTTT
>at03223
GGTTGGCAAGCAGACCATGGATGTCTCCAAGTTCCTGCAAGCTGATGGGCTTGACTACTT
TGGCTTCAAGGGCAAGTTGGATGGCACTCAGATTACCTGGAACAACGGCGTGGTCTGGAC
GAAGGTGGGCTGAGGCCAGCCATGTGGGGCGCCTCGCCGCTTTTCGGCGAGCGGGGTAGT
AGACCGGCACGCAAGGGGACGCGTGCTGCTTAGACCGGCCCTGTGTGTGCTGTTGCATGC
GTGCC
>at03224
CCGTAGCCATTTTGGCTCAAGAGTGGCTTGAGGCCACTGCGGCCGCCTCTTGTGTTGCCG
CGGCCGCTTGCTGGGCCACGGGGGCAATGGTGCCACAGCCCGCGGGCGGCCCGCAGGTCG
TGGCGCTGAGTCTGGGATCCTTGGAGGCAGCTGATGAGGCAGGCGCACGGGAGCTGGACG
AGCTGCTGGACCAGGCCTTCGGCGCCGACGGCCTGGGCATCGTCTGCGTCACCGGCGACG
AGAAGTTCCAGGAGAAGGTGCGACAGATCCGGGAGGAGCTGCTTCCCCTGGCTCTCGCGC
TGGAGCGGCTGCCCCAGGCGGACAAGGAGGCCATCACGGAGCGGGGCACCCTCAACGTGA
ACAACTACTCGCGCGGCGTGGACGGGAACCGCTCCGGCCTCTACTTCCACCCAGCTGCTG
GCAGGCCCGGCGAGTGCCTGCCGCCCGGCGTG
>at03225
CCGTAGCCATTTTGGCTCAAGACCGCCGCCACCAAGTCCGTGCTGCACTGGCAGCACCGC
CTGCTCCACGGCTTGCAATGCAGTGGTACCACATGGCGTCACTCCCTGCGCTGTTCCGCT
CACCTTGAGACTGGCAAGCGCCTGTGCCTGTGGCTTTGGAGGCACCACCCGCAGTGGCGC
CGCCCCGTGGTCGCGGTTCGGTCTTGAGGGAGGGAATGCAGCTGTCCACCGAGGCACCTC
GCCATCATGACCTTCTTGCAAGCAGTGCGTGGCCTTCTGCAACGGCTGGCAGGTGAACGG
CGCGGCACAATGGTGCGTTGGACGACATGACGACCGGACTACACTGACAGGCCTGGGAGC
TCGGCGCCGCAGCGGTGCACTGCCTAGACAAGTTTGCGCAGCTCGTCTAGTGGAGCCCTG
ATCGCCAGCGGCACGTGGCCCACACGGCAGAGGACGGGACCGGGCAGAGTGAGGCGGAGA
CAGGTCTAACGGGGAGCTCTGTGAGTCATTTCCAAAAAAAAAAGAAAAAAAAAA
>at03226

TTGTTTTTTTTTCTTTTTTTTCCATCGACGTATGGCTCAAAACGCGTCCTCACCGTGATT
TGAACCAGAGCGGCGAAACCTGTCATGCACCAACAAGAGGATCGGACCATTAGAATGAGT
CCGAGCCATGCAGGAAACACATCGAGCTCACGCTTCTCGAACTGGCATGCAGAAAATCAA
TGGAAAATCAATGGATCCATGATCATGTGCGCAGCCTCACTTCTCGTCCACTTCGCCGCC
GAGTGACACGACCTTGCTCGACAAGGCCTTGTTCGCAGCCTCCAAGGTCTCCATCCTCTT
CAGAAGATCGGAAATGTTCTCCTTGTTCACGTCCACCTGCTCCTTGTTCGCATCGATCCT
GCGCGTGAACACCTTCCTGGAGAAGCCCGAGGCGATGAGGAAGCCGGCAAGGTCCAGGGA
GAGCACTATGCCGATCACAGCAAAGAGGTTGCCGCCGTTCACGGCTGCGTGAACTCCTCG
GACGAACCGCCCCACGATCGGAGTCAGGTAGTAGCCTGTGGCAACGGAGACGCCCATGTT
GAGTGCACCCAGGCTGGTGGAGCGGATGAAGTTCACTGGCTTGAAGAACTCCTTGAAGCT
GAGCTTTTTGGGGCCTGACTGGAGGAGGAACCCCCGGATCTCGTTCAGTGGCGCGGAGAG
CACGCAGGCGCCGATCACCACAGGGAACATCAGGGCTGGGGAATTCGGGGCAAGGCCTGA
GAACTGCGCGAGCAGGAGCACCGTCAGCAAATTGCGGCCGATGGCGTAGATCATGTCCCG
GACGACCGTTGGCAAGTACGCCTGGTAGAGCAGCTTCGGGTTCAACGCCTCGGCCACCGG
CGTCTGGATGGACATGCGGTATCGGAAGTTCGTGATCGGCAGCGTTGCCGTCGTGAAAAT
GAAGCCCACGATCAGGCCGCGCACGACGTTGCTCATGTTCGGCAAGAGGCTCAGGAACAT
AGACACCGCCTCGAAGAGCGGGAACTTGAGCAGGTTCGTTGACATGGTTGTTGTGAAAAA
GCTCAGGATAAGCTTCGGCGACATCTCGGACATGGCCTTGCCGATGCTCATGCGCTTCAC
GAGCACGCGGTTCATGACGGGCTCGGTGACAGCGGCCAGCGCGGCCGAGACCACGGAGGC
CACCAGGCCGGCCGACATCGCACTGAGCACCTTTGCCCCGATGTCCACGGCCTCCATTGC
CACTTTGGGGTGGCCGCTGGGTTGTTCGTAAATTGCCGGTTCTGGGCCGACGAAGAGCGA
CGGCGCTGAGGTGGCCTTCGTTGCATGCCTTCCTGTCACTGGGGGGAGCGCGAAGCTGAG
CGAGGCGTGGACTAGAGCACAAAGTCCACCGAGCATCGCCAGGCGCGGCAGTGCACTGCG
GCTCCGCTGCGAGATCCTGGATCCTTGGAACTGGCGAGCCATGGTGGCAGAGGACGCACG
GAAGAAGATGCCCTAAAACAGGAGGATGGGCTGAAGAGCCAGGACTCACAGGTGCAGCGT
GCGTCAGAGGGGCTTGAACCAAGCTGGCTATGGCTTGAGCCAAAATGGCTACGG
>at03227
TAGGATCGGCAAGGCATCCAGTTGACTTGCCATGGTGCCATCCTCAAGCTTGACGACTTT
GCGCTCATCGAAGCGCACGTAACCCCCAATCGGGAGCAAGCGGAGGGTGAACACGGTATC
TGGAGGATTGGCCACTTCATCGGCCGCCGCTTCAGCAGCCTTCTGCAAAATTGCCTCTAC
CTCATCCGTCGCTTCATCCGGCACCTGGCACGTCCCTTGCCCTTCGCAGTCAAGTGTTGC
ACTTGAGCGGTCAAATGTTGGAACACCCCTGGTAATGACTGTGCCGTTGACGATCACGGC
AATCTCGCCAGAGTATCTTTCCAAGCGCACTTTGTCATCTTCCGCATCTTTGAACTCGCA
AGTATCATCCAGGGGTGCAGCAGATGCCTCTGCATTGCTGAGCAATTCTGGCCCAAAGCC
AAGCGAGAACTCGGCAGCTGGAAGGTTGACTGTCCGAGCACACATGAAGTGACCGGTTTC
GTGGCAGAAGATTGCTGCGGCCAAGGTGGCCACGGTGGCACCGGAATTCAATCCGAACGC
TGCGCTGCAGGCAAGGGATGTCTGCACAAGGTCTAGATCACCCACACTCTCACTCCCGAT
GGCAGAACGTGCCGTAGTCTGTGACTTTTGCGGCCAGGACACCGGACGACCGCGAAGGTT
CTTGTCCGGGTAGCGGCTGCACTGACTCAGTCCACCAACAAATCTAGCCTTCCCAGCCTG
GACTCCACCTACAGAGGACACTGATATGCAATTCACCCTACATCGTCTGCGCCTCCTCGC
GACTGCAAGCGCATTCACGCCCAGTGCAGCTGCAACTGCAGCTGTGCCAAGCACTGAGTG
TGACACAACTGGTCGTTCAGGCAGCCAGCACTGTTGCGGCACCAGCCTTCCCTGCAAATG
CGGCTGGCACTCTGGCCGCAGCTGTGGACGTGCAGAAGTTTCAAGAATCGGAATGGCCAA
AGCCATGCCAAGTGGGTTACACAACCTCACTCTCACATGCAGTGCATCTTGAGCCAAAAT
GGCTACGG
>at03228
TTGTTTTTTTTTCTTTTTTTTTTGGTTTTTGTCATGGGCCTTCCACGGGAGCATGTACA
CAGTGGTTCCGCTGTCGTCAACTCCACACAATGAGCAACTGTACATTCGGCCTGAAATGC
CGAGCGGTGCAAACAGGCACTGTTGACAGACTAAACAGATCCCTTATGCCAGGTACCGCA
TGCTGCCCACCCGAAGCATGCCTTGCGGCAGCCTGCGCAGATGAATCCGACGCAGGAAAG
CGTGCACCTGAAGAACAGCAAGTGTCCTGTCGGATGAAATGAGTCACAAGCCGAGCGAGC
AACGCTTCACTCACTTCTTCTTCTCTCCCGATCCCATGGCCATCTTGGCCATAGCCGTCA
CGGCTCCACCAAGAGCACCCAAAGCCGCTCCAGGCGCCGCCCCGACTCCACCGCCAGTGA
CCGCACCAACGGCGGCGCCACCTGCCGCGCCTGCAGCAGACTCCTTTGCAACCTCTTTTG
CCGCCTTGCAGGCTGCATTGCGGTTTGCCTGGTGCTGAGCGCGAAGGCTGTGGTTGATGG

AGCCATCGGCGTTTCGGCCCATCCTTGCAAGGGCACACTTTGCACTGGGCATCTTGTGGA
AGCCTCGCTGAACCTGGAGTTGCTGTACGCCGGTCTTTGCCCCTGATACCTCCGGCACGC
TGCTGGTGCTGGCAGTCGGGGTCCAAATGGCTTCAGCCGAAATCGCTTGAGCCAAAATGG
CTACGG
>at03229
CCTCATGNCAACCGCCAGCACGGTCAGATGGCTGTACATTgGANGGGGACaAAAAAGTGC
ACTTGTGTATGTGTGGCCAGTGATGAGGATGCCACGGCNACACAANCCCANNAGCACTTC
CCTCNAATGCTACTCTTCTTCAACAACTACCGGTCGCCGCAACCTACGCTGGGGCAGTCG
GGTAGACTGACTGTGAAGAANAACAACGCAGAAGTCTGTACAAGTGATGAGCTGACCGCT
GCNGTGTCATGGTCTCCNGTGATCCACACACTGCTTCaAAAAGTTCGAAAGACGCGGGCC
CTAGCNTGACTGACACGGCNTGTCAGCAGCACTaGGAAAACCAGGGCCTTGTACCGCCAA
GCTCTGTCGCCTCATGCCGAAGGGAATGGAGTCCGGGAAGAGGCCCTTTCCCTTCAAGTA
GTTGATGGACTCCGTGATCGCGGAGTCCTTCAGCTTCAAGACAAACAAGCCCTTGTGCTG
TATCGTTCTTGATCTCCTCGTGCC
>at03230
TCCATGTGTAAGGTGCCCACCACTCGGCAAGTCTAGGCCCATCATCCGAGCGTGTGGTGG
CAACAGTGCGGTGTAGACAGCAAAGTTGGCAGGGCTTCCGGAGTAAGGCTGCACGTTCAC
GCCCCACTTCTGCTCGTCGAGTCCAAAGGCTTCCAAGGCGCGCTTCGTTGCAAAGATTCT
CGACCTTGTCGATCACCTCATTGCCTCCGTAATATCGGCTCCCTGGCTGCCCCTCTGAAT
ATTTGTTGGTGAGTGCCGAACCCAGGCACTCCATGACTGCCCTAGGAGGTGAAGTTTTCG
GATGCAATGAGCTCGATGGAGCGGATTTGCCTGGCCTTCTCCTCCTCGACCAAGCGCCGC
CACTGTGGGGTCCACCTCGCACAAGGGCTTGTTTCCCGGCAGCTGAGGACGGCCAGCCAT
CGCACCAGCTCACACGGGACACTTCACGTCAAGCAGGACAAACCAGAAGATGCCTTAAGC
CGAAAGGG
>at03231
TTTTTTTTTTTTTTTTTGGAAACATCTGAAAATCTTTAAGTCTGGAATAAAACTCAAGT
CCTCAAGTCAGAAATGACCTTGGGCAACAAGAAAGTGGTCCTCCACGCTGACCACACCCT
TCCTGTCCCAAGAGTTGACCTAGGAAAAGGGTCCACCCAGAGTGCCGTGGCCCTCGTATG
TACACGGGCAGGAAGGGCTCATGCCACTCTGCTACTCCAGGTGACATGACAGGAAGTGGC
TATTTATCCTGTCGGTGCTTGGCATTCCCATGTCACAACTCGCTTGCCATGGTGCCGCTC
TTGAGGCATGCTGACGCAGCTCCGGCGATGTGCAGAGCTGAAGCCGCTCACCCACCGGCT
GCCATGGCCTGGAGCGCACGGCTGAAGAGCTGATCCAGCACGTGGCGCGCCGCAGGGCGA
GCCTCCAGGAAACCAACGATCTCGAGATGCTGCCCTCTGCGCGCCATGTCGATCGGCGTG
CGCCCGTCGAGTGCTTGCGCTGAGATGCTGGCACCCGCGGCCACCAAGTGCTGCACAACG
ACCAGATGGCCCTTCAGAGCGGCCATGTGGCAGGGCGTGCGGCCACTGTTGTCCACGGCC
GCCACACCCATGGGGTCGTGGCGGAGCACAGCTCCACAATGGGCAGGCGGCCTTCGAGGC
ACCAAGTGAATCGGNGCGGCACCATGGCTGTCACGGCGTGTCGAGTCAGCCCGCATCGAG
AGCAGGAGCTTGCAGGCGTCCTTGT
>at03232
TTTTTTTTTTTTTGTTTACCCTTTCGAAGGCTAAGAGAGACAGCTCCGAATGAATCTGT
GGATGCCCACTCCACAAGGCATTAGACAAGCATGGCAAGACACGGGCTTGTTGGACCCTG
ACTGTAATGCCTCTTTGCCATGACTGTCCTCCTTTCTTGCCGCACCTCTAGCAGTCTCCT
TTAGGCTGCGGATGTGCTCTGGTGCTGGCAGATTCTGATTGGACCAGCCAGCAGCTTCAA
AGACCACTACCTTTCTGGATTGCAGATGCCGCTTCTCACGTCCACAGCCGGCTTGAGCA
CGTATGTCGACAACAGCATGGGCGGGGGGCCGTTGAAAGCATCCAGGCTCAAACCACCTA
CGAAGGGTATGCTGGCATCCACTCGCCACCTTGCCAAGTCAAAAGCCCGTGCTGGAGCCC
AAAATCCGAAGGTGTGAGTCTCACCAGCTTCAAAGACCATCTCACGCAGGTGCCTCCTGT
GCTTGACGGGATCCCGAACCTTCAATATGCCAAGGCTGGGAAAGGCTTCGTCAAGTAGGT
CTGGAGGCCCCCCCGGGGACGGAGCTATGTACTGGTCGGCTTCGGAGATTGGTATGCTGG
TTATTGGCTGCTCGACGTCAGACTCTGCGCGCCCTTCCGGATCGTCGCCTGGGCTGTTGT
AGAGACCGTGCAAGCTCGGTCCCATCATCGCTAACATCCAACGGTGCACCGCGACCTGAC
CCGGTCCCAGCTTCTTTCGAACGGTCGGCGAACAGCCGATCCGCAAGGAAGAGGCACTCA
CTCGTTGCTTGAAGCAGCACTGGAACCTTACCTCCCAGAGCCGCCTCTTCCCGTGAAAGT
AGCTTCTGCTTTGGTGAGCATCCGCCCCATGCTCAGCGTCTGCAGCGTCAGAGCCAAGCT
CCCATCTTCGATGCAAAAGCAAGTAAAAACCGCTGGCAACCTCACTATCGAAGCACATTG

GAGCCGACGGGTTGCTTCTGGTCATGCGTCCAGTTGTCATGTCCAAGATTTTTACTTCTG
CATAATCTTCTTTCTCTGGTGTCTCTTCCATCTTTGGCTCATCCTGGATGATCCGAGCAC
GCGAAGATTGCTCACAATATTGCTCGCGCCTGGCAATGAAGACAACGGTCACCCAAACGG
AAACAAGCAGCAAGGTCGTAAGCAGAGGGAGCTCCAGCACGGTATGGAAAACGAGGTCGA
GCGGGCTGGACGCAAACTGCCTGAGAAGCCAGACAATTGCTGGCAGAAGCAGCAACAGCA
GAGACTTCAGCTCTGTTCCCATCGTTGGAAAATTGAAAGGCAGTTCGGGTCTCCATCTTG
AGCCAAAATGGCTACGG
>at03233
CTTCTGCAGCAATCCACGGATACCAGGGCCCCTTCAGGTGATGCACCAAAGGCGGTGCTC
TCTGAGTCGCAGCGGCAGCTCCGGGCAATCCGCAAGAAACTGCGTGAGGTTGAGGACTTA
GAAGCGCGGAGCATAGACTTGCTCACAGAGGTGCAGCGGGGCAAGGTCTCACGGAAGGCT
GAGTTGCAGGAGCAGGAGGCCGACGCTCTGGCACAGATCGCTCAAGAGGAGCGAGAAGCG
AAACGGGCGAC
>at03234
CGCCTCAGGTCCTTGAGATCTCGAAGCTGGCTCCCGGAACTCGGTGCAGGTGGTAGGAGC
ACCGGAGCTAACGGTGAGGTCGCCCACCCCTTGGGCGAGGACCCTGGCAGCAAGTTGCTG
TCACTGCCTCCGTTGGCCGCAGCGTCTGCGTGCTGCGTGAGCCAGTTGTCCACCACGCTA
TGGGACAGCTGCTGCTTCGCCAGGGACGCCAGAGGCGGCATCGAGGACGAGCTCGAGGCC
GGCCCCTTGCCCCTCCAGATGCTGCCGCGAGCGGTCTCCACAGACATGGCCATCGGGGCG
AGGCGCAGAGCCTAGGGCTCCCCTCCCCGACGGCCGCCATACGGGAGGGCGGCCGC
>at03235
CCCCCGTCTGCCGCCTGTGCGCCTCGCCCAGCACCTCCACACCCCGTCTCAGTTGCTCAT
CCAGGTCTCTGGCGTACTTCTCCTTCTCCTTTTGGACCGTGTCGGGATCGGCCAGCTCTT
GCAGGTGGCCGAGGCGCGTGGCTGGGGGCATGGCGAGGCTGCCGGGCTGCGGCATCGTCT
GGGCCACCGCCGGGGGCAGCATGACGCTGCCGGCTCCCGTGAAGGCCGCCACGTTCGGAG
GAAGGGCGAGGCTGCCTCCGCCGCCAGGAACGCCAATGCAGCCAGGGCGTGGGCCAGGTC
TGGGCCCG
>at03236
TTGAGACCATCAGCTGCTTATGCTCTAAGGCCTTTGGCAGCTGGAAGAGGCCTACCAACA
GCTCAAGAAGTGTCAGCCTCAATATCTTCCAGCTCGGCAACCTCAGAGTCATTGATATTT
TCCACAGTCCTTGACATGAGCTCCTCTGAGACCATACGCGCCATCATCTCCATAACTCGG
GCGACCTTGTCCTCAATCCACGCAACGGCCGCGAACTCCTCTTTGAGATGCTCCATCAGG
GAGTGCGTCAAAGGTATGACAAACCTATCGTTCAGGTACTTCCCCAGCGCCTTTGCTTTC
TCTTTCATGCTCATGGCCTGGTCCATGAAGTACTGGAACAAGTCGATGATGGGGTTGTCC
AGGAAAGTCTCCAGGGCCGCTTTCATGATCTTGTTCAATCTGACGACTTGGGGCTTGATC
ATTTCGGCCTCCGTCTCCTCGGAGACTCCCCAGATGCCAATGACGTCGTTGATGCGGTCA
ACGATGTCATCGATCTGGTCCTCGGGAAAGAAGCCTTCTTCCAGGCTCTTGGGCGGCGGG
AGCGGCTCGTCGTCCTTGGCGCCCTCCGTGCCCTCACTGCTCTCGGCGGCCGTCGTCCTT
GCGCCACAGAAGCCGCAGAACGGGGGGGCGCCGTGGACCGCCTCTTCCTGGATCTGGGGG
ACCGGCCGCTCGTCCGCCCGCTCGTCCTTCACCTTATCGACCTGGCGGATCCATGTGGCC
CCGTTCTGGAAGTGGATACGGTCGTACTTGACGTTCGCCTTGCCCTTGCAGACGAAGCCA
GAGTCTGCTTCAGTGAGCGTCACGAAGCGCTCGACTCTTGAGCCCGCGATGTCATCCTTG
ACCTTCTTCTTTTCGACCCGCATTCTTAAGTGACCTCTTGCTGTGATGTAGCAATGGACA
GCCTCCGGCGTGCCCTCGTTCATCTCATCGTCATCTTTCCACTCGATCGGAGTC
>at03237
CCGTAGCCATTTTGGCTCAAGCGGGGCTTGCAGGGCCTCGCGGTGCCGATGGCACCTCGA
CGATCCTAGCATTGATGTCCCGGACTGCCTCTGGAACGCAGAGGGGGCTTCGGAGAGGAG
GGCTCCATCGGTTTCTGCATTCCGTCGGACACTTCTGGTGACTGCAGCCGACCATGCGGC
TCGCTGCCCACAACCTCCTCTTCCGCGGCCGGCGTGTCATGGAGACCATGAAGGTGCCGC
TCTGGCGCGCTGCGTGGGGCGAGGTGCAGAAGAAGTGTGGGGGTCCTGACAAGTTTCCAT
CGTATGACGAGTTCTTGGGCTCGGCGACGGACATCTACAAGTACTTGCAGGGTGAGCTCT
TGGCCGCAGAGAACACACGTCACTTGCAGGAGCTGTGCACGGAGGAGCTGTGGCCCGTCG
TCAGGGCGACGGGCGCGGACTACGAGAGGGTGAGGAGACACTTCGGCGCCGAGCAGTGCC
GCATCGTCTGGGCCGGCACGGACGCCTTCGTCTGCTCCCTGGGGCCGCCGGAGGACCAGG
CGCAGAGCGAGGAGGGGCCCGTGGTCATCGCTGGGGCGCGCTTCTACTCGGTCATGGGCA

TGGCCTTCGCTCCGAACGAGCCGCCCAGGCATTCCATTGATGACGTCATCTTCAAGTCTT
CATGGAGCGCTGATCAGGGCGTGGTACAGTGGAAGATCGCCTACATTGGCGGGCCTGTCA
CACCTGTGGAGTGATGACCGCCGTCGGCGGGGGGCTTGCCTGTACAGGCACACGTTGTGC
TTGCATGCGTGTTTCGCATTGGCGGATGCGGATGTATGAAACTGGTAGCCTCACGGATAC
AAAAAAAAAAGAAAAAAAACA
>at03238
TCCTCCAGGACCCGCAACAGCATCAATCCTGCATCACGGTGCATGATCCCAAATCCGGAG
ACAGCGTCTGGCTCCTCACCTGCACTCCGGCCACACCCCGTGTAGTCAAATCGCACGGTG
GGCAACCCAAGTGACCAGCAACGTCGAACAAGTGCGGTCATCAACGGGTCCATCATCCGC
GAGCTGCCAAAGACCTTCTGTGGCATCCCAGGAACAAGCAGTGCGCAGAGGGGCGCTTTG
CCAGCCGGCCTTGGCTGCAGACGCGCCACTGCTACTCCAAGCTTGGCTTCTACTGAATCG
CTGGCATCACCTTCAGCCGGGGCAGAGATTGTCCACAGCTCCTCCACCTCCTCCCCCACC
GCGGTGCCGAGAGGCGTGTCTGGCGCCGGCA
>at03239
GCTGGCCGCAGNCCTGCTGGCAGGGCTGCTCATGCCGTTCTCTCCAGCACCTGCCAGCGC
GATTCCTGCATTCACGGGCTCACGCTTCGCGCCGGAGAAGTTCACCATCAAGGCGGCGAA
GCACCTGGAGCTCTGCAAGGACAACAAGAAGTATCACAAGAAGATCAAGGACCAGATCTA
CAAGATCACGCAGCGCCAGAAGAAATACCCTTGAAGGCTCTGTTGTCTACAACCGGTTTG
TCAAAAAGATCGCGGGCGTTAAGGCGCGCGAGGAGGCCTACGGCAACAAGTATTGCGGCA
AGAAGGACGGGCGCCCGCGCGTCATTGCCACCGGCGAGAACGTGCCCGGCGGCGTGGTGA
TCCCTTCGCTCATGTTCCTCTACACTGCAGGCTGGATCGGCTGGGCCGGCCGATCTTACT
TGATCCGGACGCAGGATGAGAAGAAGGAGCTCAACATCGATGTTCCGCTCGCTCTCACCT
GCATGGCCAGTGGCTTCTCATGGCCCGTTGCCTGCTGGCAGGACATCGTGAACGGCGAGT
TGGTGGTGAAAGGACACGGACTTGCACCGCAGCTTTTTCTGAGGCACTCGGAGCCTGCTT
CTTCTGAGGCCACGGAGATCAGTGTTCGACAAGGGTTTTTGACGGATGGCTGGTGGACAA
TGTATACGTCGCTGGTTCGCAAAAAAAAAAAAAAAAAA
>at03240
TTTTTTTTTTTTTTTCTTCCAAACGGACCTCAGGATCGGATCTGATTCAAGACATGGTCC
ACAAGAGCGGGGCTGACTGCCTGGCCAGTTCGTCACGCTCTCGAGGAAAAGGCATGGCGG
CGATCCGGACGTCAAACACAACTAGGCTGCGGGCACTGCTGCCTAGGAGCTCTGATCACT
GGTTGGGGAAGCAGCGTGCGGGGATGTAGGCGCGAAGAGAGCCTCCACAGCCTTTGGCTG
CTGCAGCTCATGCGCCTCCCGGCTGAATTCATAGAGAGCCCACTCCATGGGTGCGTCCTT
GCCCGGGCCAAAGTAGGGCTCTGGCAGTGGGCGGCTTCCGAGGTCGCTGGGGAAGAGCCG
AGCGTCGGCAGGGCCTTCGAGCTGGAGCCGCGGGACGATGGGGGGCTGCAGGAGGTGGAG
CCTCTCAAAGTCCACGCCGCTGAAGAAGGCCTCCCGCCGGCACTGCTCCGCCGTGAGCCT
CTCGGCCACGCAGCAGATGAGCCCCAGCAGCACGTGCTCCGCTTCTGGCCCCACGTAGCC
CTTCGCCTTGCCCCTCTCGAGGCGCTTGGCGAAGTGCTCCTTGTGCTTTTGGACTTGCTG
CAAGACCCTCTGTCGTTGCCT
>at03241
CAGTCGTCAGTCAGGTACTTGCCCTGCATCCGCTCGGACAAGTCACGCACAAGCTCTGGC
TCCTTGTTCTTGTATTCTGTGTAATAGCGCAGCAGCATCTCCTCCCAATCGTCAGCCAAC
GCATCTCCGCGTGCTCCGTGGTCTCGGAATCGTTTCATCACCTGCTGGGGCACATGGAAT
TTAGGCAGAGTGCTCTCAGCTTCATTCGCGTTCTCAGGGCTTGCTACAGTTAACGACAAG
CCATCCAACTCCCGCTGCACCAATGAAGCCACCTGTGCATCAATTGCGTCGCCACTGCCG
CGCAAACCCTTGGGTGAACTGCCACCTCTCGCGAATCTGCTCGAGGTTCGCAAATGGGGC
GCCA
>at03242
AGCGTATTCGCGACATTGAGGACTTCGTAGGTCCGTTTCATGTTGCAGCCGTTCAGAGAG
GGGAGTTGAATTACAACGACCCGTCCACAGGCTACTCGGTCTTCACGGAGCTGTACATGC
TGCGCGGGGTTATTGCTGCGGCTCCGGCTGTCGACATTGCCCTTACGGGCATTTCAACTT
GAAGGGCCGGCCGCGAGTAAAC
>at03243
GAGCGATGGCTGCACGGCAGCTGAGTCTTCTTTGCTCGGCCGTGCGAGTGTCCTCTACCG
CTGTAGAATGAAGCTTGTTCAGGGGACTGTAGGCCTCGTTCTGACCGAACTGCCGGGTGG
CAGCCAAGTGGTTCAAGTCTGGCATCAGCCTCTGGAGCATGCCTGCGCAACACATCTCAT

ACGAGAAGAGTGGAGGATGTCCAGCTCGCTCGCCTGCAAGGGCCTCGGCCATCTCCAGAT
CTTCCGAAGAGCGATGGCCGATCCTCGCCAAGACGTCCGCAGGCACAGGGTATGCACGGC
CGCCGCTATCCAACAAGTGAGAGTCATGCCCACGAGCAGCGCGGCTCCAGCATGACTCGT
TGCCGCGGGGTCTTGCCGCACG
>at03244
TTTTTTTTTTTTTTTTTCCGCCACCAGGCTTCGTCGGATGTCCGCTTCCTGCGCACCCC
TCGCGATGCATTGCACTTTGGCTTTGGGACGCTTAGATCCAAGCGCTTTCCGTTCGGCAG
CCTGCTGAGGTGGGCGGGGGTCACCTCTCACGGCGTCTACACCTCACCGCTGCAGTACAA
ATTGCCGAAGCTGGTCGAGAACACGCACGACCCCGGCGGGCATACGCACTTTGCCTTCGA
GCAGTTCGCCTCGCGCCACGGGTTGCAAGAAAACCACTTGCAAGTGGTCCCCGTGTCTTT
GTTACACTTGACGTGCCACTCAGGCGGCAATGGCATTGGAAGGTAATTGAGTGCAGTGAC
GTACGGAAACTGAACAGTCACCTTCCCAAACATGTTTGGGCCTGCCCTCTGGTCAATGTA
GTAGAAATGCTTCGAGCGACCGAGGAAGGATGCCAGGTCCCCGACCAGGTCTGTCAGTGG
GGTGTCCTTGATTTTGTCGAGGGCATACTTGAGCGAGAGCTTCTTATAGAAGGCGGCCAC
GTCCGGATGTGAGCCGTTCATGAGAACAGCCGGAGATCCATCTGTGTAGACCTTGAAAAA
GTTGCGGCGAAGGATCGGGCCATCCGTCTTCAAGTCAGCCCCGACTGCGGCGATCTTCCA
GATGCTCCACTGGCTGAAGCTGTCGCATGAATTGTCGATGTTGTGTCGCTTCTTCGCAAC
AGTCAGGCGGCCTTTGGGAGCCAGGCTCCAGAACATGTAGTACTTGTGGGCACCGCTGGC
CAACCACTCAGAGACGACGAGGTCGAAATACTCGTTGAGCTGGCCGCCCGTGAGCGTGGC
GACCTTTGTGCTCAGTGATCCTTGCCAGTACTCTTTGATATCGCTGGTCATTTTGGGGGT
GAAGACGATGGACGACCACGAGTTGTTGAAGATTGGGCCGTCCGGCTCGTAACTCGTTGG
CATCAGGCTCTGCACCATGCCGACATCCGCCCACTGCTCAATCGCCATGTCGTACTTCGA
GGATCCGTCCTGGACCTTGAGCATGATGCCAGAGTGGTACTGCGACAGGGCGGACATGAT
CGAAGAACCCATAAGTTTCACTTCGCCGTAGTAAGCCTCTACTCGGGCATCGGGGCTCAT
TGTTGGCAGGTGCTCCCGCGTCCTTACCCAAGTCTGGCCCTCAAACATCTGCCAGGAGGG
GTCGCGCAGCCGCCCCATCCCTGGTCGGCCCAGACGCCGGGACACCTCGGCCGAGCTC
>at03245
TTTCTGGGTTTCGCTGCAGGACGGAGTTCACATGCGTCTGCAGGACTGTGGCGCACTGCC
CAACGGCTCACGCGCGGCAGTGGGCTGGAGTGTGCGCAACAGTGGCGCGGTGCTCTGGCC
TCCTGGCCTATGGCAGCTGGTGCAAGTTGCTGGCCGCGAGCTGAGCCATGCTACGACGGA
GCTGCCGGACTTCGAAGTCGTGGTCGGCAGGACCGTCACTGTAGTCTTGCACTTCACTGT
TCCGGCGAGCCCTGGCGAGCACGTGTCGACTTGGCGCCTGGTCCAAAGCGAGACGTACGA
CGTGTTCGGGCCGACTTCTCCGTCGCTCTGCGGTCCTGCCGCAGCGCAAATGGG
>at03246
TTTTTTTTTTTTCTTTTGGTAATGGTCAAAGTCCCATCCCTGTGCATATTGTAATTCAT
TTCTTGCGTGGCTCTCAGCACGGCACTGCCATCCTCCCACTCGGAGGAGCATAATCAAGG
CGAAATCGTATCACTGACCGTGGTCTGCTGCTCTTGTTCTCGCTTGAACTATGCAAGCAG
CGAGGGTCCCACAAGGAAATGTCTCCTGGCATGCCATTAAGCTCAATCACCTTCATCGGG
ATGCCGGCAGAAACGGTACCCTCCTCCATAAACCGACGGATGCGTTCGGCCGCAGTGGCC
TTGTTGCAGAAGGATCTGTAGTCCCTCGCGTTTGACAATGGAGACGGACCGAACAAGTCC
TTGAACCAAACATCTTCGTTCGCCATCGCCTCCAAAAAGTGCTGCGATCTCGTCTCTGAG
ATTGTGGACGCAGCGCACCAGCCCTGCTCATCGCATGAAAGTTGAGCGCGAAGTGACGAC
CCTGTAAGAAGCATGGTGGCCCCGCCGCCTGGTTCCACCCTCGTGCC
>at03247
CCCAAAAGAGGTTTTTTTTTCTTTTGATACAGCGCGGTCCCCACTCATAAGTCAACCTTC
GGTCTAAAGCGGGCCGTCACAACCGCGGGGCAGGTCACGGCAGGACCTTGACGCTGATGA
TGTTGCCGTTGAGCTCGCCTCGGTCGAAGGTGTCCATGGCCTTGACAGCGTGCCGAGGCT
CCAGGAACGTGATGTAGGCGGTGCCCCGGTCCATCTCGAAGCTCGAGATCCTGCCCGTGG
CCGCCTCGAAGGCGTCCCGGATGTCCCGCTGCGGCAGGTCCGCCGGGATGTTCGTCACCT
TCACGCGCTTCGCCGGCTGGCCCTGCGGCGGCGGCGCCGGCGCCTCCTCCAGCCGGTGCT
TCCGCGCGCCGGATGCACCGGGGGTCGCGTCCGCTGGGTGTTGGGAGCTGGCGTTCAGGA
ACGCGCCTGCGGAGCCCCTGGGTATGGCGACGGTCCTGCCATCATTGGGGACGCCGACCG
CCCTTGCGGATCCTCTCACTGCAGGCTCAGGGTCGGGCTTCGATGTGCTGTAGCCGCCAC
CTTCTCCGCCCCAGGCGCCGTCGCCCTTCCCTCGCGCGTACGAGCTGCTCGA
>at03248

TTTCTTGCAAACAGTCACGGGCTCTGCTCCACCCAACAGTGGACAAGGATGGGTATGTGC
CTACACATCGCACAAGTATACACATCTTACAAGATGCCTGTCATGCTGATGCATCAGCCA
ATTTTCACGATGAAGCATGAACGTGCAATGTGGCTAACCTCACTTGCCGCATCCACCCCA
GTCCTTGCCCTTCTTGCCCTCGCCTTTGGCCCCCCCTTTCTTGTGTTCTTCGTCTTCAAA
AAGAGACTGGAGCCGTCTGCAAGTGTGAATCCATTGAATGTGTTGATGACGGTTGAAGCG
ACCTGCGGGTCGAGAAAGTCTACAAAGCCAACGCTCGTGCAAGTACCGTCAGGGTGAGTC
ATCGCCGTCACACCTCTTTGAGCTATGGCGCCAAAAGGTGACATGAGCTTGTAGAGCTCA
AGGTCTGTGAAATCTGACGGCAGGCCTTTGATGTAAACACAAGCCTCATCTGTCTTCGGA
CCTCCCCCTGGCAGGATGCCTTGCTTCGAGGCAGCGATAATGATTGACTTGATGGTGCTG
CCACTTCCACCTTTGCCAACACTCTTGCCACCATCCTTGCCACCCTCCTTGCCGCCCTCC
TTGCCGCCCTGCTCATAGGGTGCAGATCGGTCGCTCTCAGACCAGCCACTGCCTGCCTTG
CCATATGTGCCGTTCTTGCCGCCACCCTTGCCATTGTTGGCATACTTGACCATAATGGGT
TCACTAAGGCCTTCGGGCATGTTTCCATTGAGATTTTCTACAATCCATGATGCCTCCTGT
GTGGCCGCGAATCGGACCAACGCAGCACCCTTCTGGCCCGGCTGTTTGGGTGGCAGCACC
TTGAGTGACGTGATAGTGCCATAGGCACCAAATATCTGCTGCACGGTGGCCTCGTCGATC
CCAATGGGGAGGTCTGAGATGTAAAGATTATCGCCCGCCATCCCCAGGGGGGGGGCTTCC
GCGTGGGTGCCCCCTGGCAATGATGTGCTTGAAGACGATCCAAAACCTGGGCCAAAAGGA
CTTGAGCCAAAATGGCTACGG
>at03249
CAGCTCAGACCAGGCGCAGCGAAGAAGATAGACGCCAACGGCGGCGAGTGCAGATCGAGC
AAGAGAAGGAGGGTAGGCGGTTGCAGACGTCGGCAGCACTTGCGATTCAGCGCCACTATC
GCGGCACGTTGGGGCGGAGAGCTGCTTTCGTCCAGCGTCGCGCGGTGATGTCACACCGAA
GGAGAGCACAGAGGCTCCCTGCAAAGCTGCAGACACCACTGCCAGAGGCGCCGCGGCGGC
TGGTCTCAAGCAGAGGTCGCGACACTGGAGTGCCATCTCGTTCTGGTCGCGCTGGCTGAT
GCCAGGAAACAAAGACCTGGCTGGAGCGGTGCACCACGATCGCCGACAGTGTGCAACAGG
CCTAGGACCCCACGTGTACTTGTACAAGCTCTGAACNGTTCGACTGGCAGCG
>at03250
TTTTTTTTTTTTTTTTTGCTCTTGCTAACGCCGTTGCAGCTGATGCTGCCGCTCCTTGT
GATTTGGTTGATTCAAACACAGCGGCAATCAGGTCTCGTCACACCCAACCCACATGCACA
CACGCCTCAAACTAAAGGCATATTCTCAAGGCCGCGCCCAACGCCCTCGATGTCCTCTTG
CAGGGTGTGCGGCCCGGTCACTGCAGATTGCTTGCAAACGCCCCAGGTGCTGCATTGCCG
TGGCAAGAGACGAATTAAGGATCTCATTCAGCTCATCGGCCTCAGCCTCAGCACTGCTGA
CAGCAGGAAGGGCCCTGCCAGCCGGAGCACTGTCGCCGTGACCCAGGATCGGCGCAAGGC
ACTGCGTGGTGCTAGTGTGCGGGAGCATGGGTGAACTCGATGGCTGAGCCCGGCACAATG
AATCAGCAGCCTGGGGGGCCGCTGGCGGACCGCAAGTCTGTGCAGCACACCCCTCGAGCT
CCATCCCTCCAGGGCTGCTGGCGACACACGATCTGCTGGCTGCATACCCCCCCCAAGCGC
CGCTGCCTGCCCGAGCTACATTGCCAACACCTACACTGGCTGCATCGAGTGCTGGTCCAT
GATCATCCATGTGCTTACTTGCCTCATGGCTACGGTTGCGCATGGGCCCTCGATTGCGAG
CCGGTGTGGGCTGGGACTCAATGGGCCTTCGATTGCGAGCCGGTGTGGGCTGGGACTCGA
CGCCCAGATGCTCCACAGGAGGTTNGGCTAATCGCCATGGGTCTTGGCCTTGAGCCGAAA
GCTTGACTCTCCAGGGACCCCTTGGCAATCCTACTTTGTGAAGAGAGACCACGCATCTCT
TGACGGACAGCTTGCCTCTGTACCCTGGGCAGCTCAGGCGAAGGTTGCTTTTGCCATGTC
CCCGCAAGCAATCCATCGGAATTCCGGCGTGTGTCCATGAAACTCTGGATGTTCACACTC
CCTGTGGTGTCAGCATGTGAATTCCATTGGGTTGGTCCACGTAGACTTCCCGATCCTCTT
GACCGCACCACCGGAATGAGCTGCTGGTCTCCCTCTTGGACTTTCGCAAACTACCAAAGT
CTGGAGGACTCCGAGGTCTTTGCGGCCGCTCGATGCGGTCTTTTGGTTCTGGCAGCGCCT
GTGCAAGTGTCCTTAGGCATTGCAGCCGTGCGCCCCCAGAGGAGAGCGAAAGCTCATAGT
GCTTTCAGCCGAGCGTGATCGCTGAAGAACTCTTCCAGGAGCAAGTGGATTGGTTGGAGG
ACCATGTGCTGTGTTCCCGGACATAAATGTCACGGCACCTGCAGGCAGAGTGCTCTTTGA
AACATCCTCTTCTCCATCTACTCTCTGCTCATCTTGCCAACTGAAGAAATGCTGCTTCTG
GTTTGGCCGAGGCTGTTGTCGAGCATCTGGTGCACGCACTGATTGAAACAGCTCATCCCC
TGAAGTAAGCCCTTGCGAGGTCCCCAGAGTCTGTGCGGGAGGCAAACGCTCTGGAGATCT
GGAGGTCTGCCGAGTTGGAGGTGCCGACGATCTCAGCTGCATCGATTCCAGTGATCTTGA
CCGCTGTCTAACAC
>at03251

TTGTCTAGTCTGTCTNTNTTNTTNTTGTTNTTNTTTNTTTNTTTTNTTTTNTTTTNTTTT
TTNTTTTTTTTTTTTTTTTTNTTTTTTTTTTNTTTTTTTTTTTTNTTTTTTTTTTTTTTN
TTTTTTTTTTTTTTTTNTTTTTTTTTTTTTTTTTNTNTTTcNTAGGGtCtAGGCACtTCC
AATCGTCGCCTCTGTGGCCTCTGCACTTGCCTCTGCACTCAAGCACCCAACGCGGCTCCC
GCTCAGGCGTCGTCTCCGCCGATCTTGCGTGTGTAGATCATCTGCGCATGGTGCTTGTCA
CCAACATGCACAATCAGGCCCTTCTCTTCCAAGTGCCTGATGGCCTCTCGGGCAACGGAG
CCGTTCACCTTCAGCCGCTCACTGACGACAGAGGGCGTAATGAGTTTTGCCTTGGGAATC
TCCTTCATGAGCTTGTCGTATGTGGGCTTATCGAACTTGACCATGTTAGCGAGCTTCTCC
TTGACCTTGCCCTTGCTCCACTTCTTCTTCCTCGACTTTCCGCCAGCCATGGCAGCCTTC
ATCTTAGCCTCTTTGGACTTTTGCACCACCTTATTTGCACCCATGATGACAAATTCTCAC
AAGGACGCCACTGCAACTGATACCAAATAACCCCGTGGCTCGAGCCGAAATGGCTCTTGA
GCCAAAAATGGCTACGGA
>at03252
GCCCGGACCTTTGCCCTACAAATTGAGTTTCCATAGAGTCTCTTGTCTTTGGCCGACAAG
GTGGAAGACTTCTTAGTACCGCTCTGAGGACATATGTATATCAGATTTCCAGGATTTTGC
TTCCAGTGCTGCTGTGGCAAAGACTGGACCAAATGGTAAGCGCATGAATCAGGATGAGGA
TAAACTGTGAAGGCGACGCCTGAGGCTACGCACATCTTAGCAAAAGCTGGCACCCAAGAC
TGGCCTTCATGTTGGAGATAGCCGAGCTCCACCAGCAAGGGCATGACAAGCGGACGGCAA
ATGTGCACAATCTCGCTTTGAGAGGCCACTTTAGTGAACAGGTCTTTCGAACACGGCCTC
CGAATGATGCTCGAACCGTTCGGATGGAGCTCGATGCAAAGATTAGCAGGCTTCACACCA
GCGCTGTCAACCTTGTAGGGGACGCACTCCGCTCCACAGGCATCTAAAAATGGCGACAAT
GGTCTGTGTGCCGGATGAATCCACAAGGCACGGCAGCCGCAGGAGATGA
>at03253
TTTTTTTTTTTTTTTTTGCAACTTCGAAGCCTTCACATGTCGAGTCCTGACTCGCGACA
AAAGCCGAAGCCCACTCACTCCGAGCAAGGTCTCCAATGCCTTCCTCTTTTATCAAAGAG
CAGCGGCAGGGAGTGGTACGACGTGCGCTTCCACTCGATCTTCCAGACTGCCCGCTCATC
ATCACCTGTGTTCTCCTTGAGGGAAAACCTTGCCTTCACGCGCCAGTCGGGCGGCAACCA
TGAGTCGGTGGTCACCTTGAAGAGTGCCCAGATAAAGACCGATGTGCTGCTTTGGAAGTC
CACTTCGTACTCCACCATCTCGAGGCCTTTGAGCGCTGTCCACAGCTCTTTGAGGGCACT
CCTCTTGAGCACCCGGGCAAAATCCGAGTCCGAGATGGGTCCTCCAAGTTCTTCCTCAAA
CCTCTTCAAGCGTCTGAGCTGTCCACGCAAGTACACCCAGTCCAGTTCCTTCATATTCGA
CCTCAACGTCTCACTTCGTATGTTGATGAAGGTAGCGTGCGGGGCCATCAACTGCCTCAG
AAAAAGCTCTGAAGTTGTCGTCCCGGTCACGCACGTTCACGATAAATTCCTTTTTCAGGC
ACTGTCCCATTTCTGGCACATCGCTATCACGATCAGGGGTGAGGACTCCATCATAGCGAG
ACCAGACCTCATTGCGAGACCCGGCCACAATCACCGAAAGGATGCCATAAATGCCATACA
TGATGGAGAGCACGTTCATGGAAGGATGCAGCAAGAGACGGGGCAACACTGACACTCAGC
CCTGAACCTGGCGCCTGGAGCCTCGAGCCAAGG
>at03254
GGTGGCTTCGGGCCGCTGGTCCCCATACGAGACCCGCCGAAAGCCTACCTCACCCGTTGG
CGTCTGCTCTCTGCAGGGACCTCAAACGCCTCATCCAGGTGCCTGTCCACACCTTGACGG
CAAGGTGTGGAGGCCTAGCCTCATCCACCACCACGCTGGGGCCCTTCCAAAGCGTTGCCC
TCCTCCGCCGCCCTCTTCGTAGGCGGGGGCTTTCGGACGTACTTCATTCGCTCCGCCTCC
TTCTTGCTCTCCTCTGCCGCCTTGGCATGCCGCTCCTTGCGGATCTGCTGATACTTGGCC
TCCGTGGCATACCCCTGCAGCATGTCGTGAGCCATCTTCGCCTGAGCACTTCGGTCCAGA
GGCAAGGCCAGGGCACCTGCATTCTCGCGGAAGCCCACGGCCTCCAGGTATCCACGTGCC
CCGTTGAACTTCAAGATCTTCTTCTGGATGGCTGCATTGTCGATCTTGAGGGAAAAGTAT
TTCGCCTCACCAGGGTTGGCGACAATGTTGCCCAACACCTTCAGCATGGTCATGTAGCAG
AGCTCTTGGTCTTCCCAAGGTTCTACCTCGCTGAGCTTCACCAGCTCCGCAAGCGCAACT
GGAGCATTGATGTCCCCATCGGATGTGGTTGATTGAAATTCCACTACAGCCCCACCGTGC
CAATTTTGCAAAGGTGGCACCAGAGAATGGCCCGAGGACAGCGCAAATGAGAATCCACCT
TCTTCCACGGCGTCACCAATGTACCACCTCTCTGACTTCTCGGAAAAGTAAATGATCCTC
TCCCCCTTGTCCTTGGACTTTGGCGCGGCACCCTCGGCAGCCTCTTCCTCCTTCATGTCT
TCATCTTCGGAGGGAGCCTTGTGCCACACAGCTCTGCCATTGTATGGCACCGCAGGGAGA
TACACCCCGTTGGTTTTCTCATTGGTGCACTTGGAGACAGTGATCTGAACCGCCTGAAAG
CCAGAGGTTGGCACTGGGCCATGCCGGCCTTTGACCACTCCATTCCCTTCTGCAACTGCC

ATCGGGGCGTCAGTCGATGCCTCACCGGCTCCGGGCTCGCCCAAGAATATGACGTCTCCC
TCGCGGTACTGCACGTCACCAAAGGAGGAGTCATCATCTGGAAGCACCTTCCCATTCAGA
GTGAATTTGTAAGTGTCTGCGGGTCCAAGGTCAGGGTTCACGGACTCAATTAGACACTTC
AGAGTTGCCACAGAGTCAGAAGTGTCCAGGTCAAAGTCCATGGCCTCCCCCTTCGCGGTC
TTGACCACCACCTTCATTTGAACAAAGATCGACGCTGCCCTGTCTATGCAGGTGGCAGCT
TGTGCCCTCGAATGCTGGAGTGTGTGCTGCAATGCCTAAGCTTGTCCCATCTAAATCTCA
TGCTAAGGCCTGCTGAGCCAACGTGGCTTGAGCCAAAATGGCTACGG
>at03255
CCGTAGCCATTTTGGCTCAAGGTATCCAATGGCTCGAATGCACTCAACAGTGGCATCGCT
GGGCAGAAGGGGCTCTCGTCACCAGCATTCTCAGACACATCTTGGAGGTGTTTCGCAGCC
GTGGCGCTTCTTCGATCACGCACAGCTTACCCTTACAGTGCTTACACAGCGGCATTGGCA
GATGTCCAACTTCCTCAAGCATTGGGCTTGCTCACGCCACAAGCACATTTGAAATCGAAG
TGTGCTTCTGTTTGGATTGAAATGTATTCGGCATGCAAAAAAAAGAAAAAAAAACAA
>at03256
TTTGTTTTTTTTTCTTTTTTTTTCAGAAACTAAGAGGCTGCCCACAACGTGCAACGTATG
ACGCTGCCTCGAGGACGTGGATGCCCGAAAAGCGCAATGAGCCGCTGCCTGCCAACTGCT
CAGATGCTTTGGATGCGAAGGCATCCTCAGCGCGAGGCTCCTCTTACCCGACTCAGGCCT
TCAGGGCCGCGAGCATGGAGGGGGCGAAGGAGTCGTCGTCCATGCCGAGCTTGGTGATCT
TGCCGTCGTCGATGATCATGGCGTAGCGCTTCGAGCGGATGCTGCCCAGCTTCCCAGTGG
CGTCCAGCTCCATGTCCAAGGCCTTGGTCAGCTCGCACTTGGTGTCCGCCAGCATGCGCA
CCTTGCCCTCCGCCTCCTGCTGCTTGCCCCAGGCGGCCATCACGAAAGGGGTCGTTCACG
GAGACGCACGCAATGACCTCAGCTCCCGCCGCCTTGAGCTCCTCCGCACCCTCCAAGAAG
CCGGGCAGGTGCTTCTCGGAGCAGGTGGGCGTGAAGGCGCCCGGCACCGCGAAGAGGATG
CCCTTCTTGCCCTTGAAGAGCTCGAGGACGTTCACGGTCTCGCCGGGCTTGCCCTCGTCC
ACGTCCACTGACGGCAGCTCGTCGCCCTCCGCCGCCCTCAGCGACACCACCTCCACGCTG
CAGCGGCCTTGGGGCGCAGCCGAGCCGCGGAGACCAGACACGAAGGCAGGCGCCTCC
>at03257
CCGTAGCCGTTTTGGCTCAAGCGGGACCCAGCTCAGCTGCCTCACATTCCGGTCCGCAAG
TCTCCTCTGCCGCAGCTTGCCGGTCTCGATGGCCAGCTCTTCTCCCGCCTTTGCGCTGTG
GCTGGCCCTGGCCTCGTCATGCGGCACCTGGGCTGGGAGCATCCCAGCCGCGGGAAGCGA
AGGCGAGAGGGGCGGCTTCCAGCAGCAATACACACACCAGTACGCCAAACAGTTTGAGCA
GTACATGAGTGCAGGAGACAAGGGGGAGCACGCCTCAGGCTTCCAGAAGTACCTGGACAG
GCCCGGCTCCAGCGCCACAGCAGGAAAGAGCGGTGACTTTGAGGAGTACATGGTCCGGTC
AGCTGGACAAGCGGGTGCTTCGGCACAGGGCGGCAGCTACC
>at03258
TTTGTTTTTTTTTCTTTTTTGTTTGTTTCAAACTCCGCACTCTGTCCGTCCGACGACACA
ATGGAAGCCCTACCTGCTGCATACCTCATAAATCGGCACCCTGACCTGAGAGCAAACCCG
CCACCGTCCAAGTTGCGAGCAGAACTGCAGTGGCAAGCGGCATCACAGCGATGCAAATCA
AGAAGAGCTGAGAGAAGGGCCGCTTGCTCCATGGGTCAGTAAGCACAAAGGCCATTCCGA
CTAGGACCTGCATCCACATACCTCCTAAGCTGGTGAACTTGTCTCCGATCAACACCAGGT
GAATGAAGCCACCCCATCCACTCAAGTTTCCGATGATAATGCTGCCGCCCAGCAAGCTGT
AATCCACAAGTGTGGCAAGAGCTCCCACGATCTCAAAGTAAGTCTCCACGCCGGCCCTCG
CTGCATCTTGGAAATCTCCTTTGGCGAGCCCAGCCTTCGTTTGCTCAATGTGGTGCGGGA
TCAGATCACAATAGCTGAGGAGCACCCAAAGGCACATCACCTTATGGGAGCAGTACGCAA
TCATCTTGTGCTTTTTGATGAGGGCTTCATTCTGCGTTGCTGCTTTCCCTGCACTTTCCT
TTTTGGCCATCCGTGCTGCCTAACTGAGCAAGACGAGGTACGGGCACAATTGGAGCTTTA
TGCTGCACAGGAGCCGGAACCAAAGCTTCAATAGAAGCGGCTTGAGCCAAAATGGCTACG
G
>at03259
GATGTGGGTGATCGTGCTCACCATTGCGTGCTCAAGGCTCTCGAGCGCGCTGTGGCACAC
GTCAACGAAGACGTCCCAGACGCCAGCGCAGCACTCCTGAAGACTACCAACAACCTGGGG
GATTTCCTGCACGCATGCACAGGCAGCCTCCTTGTTTGCACAGGAGCTGATGCAGTCAAG
AGGCTCCGTGAAGCAGTGGGCACACTCATACGGGCAGGAGGAATTGCTCAGCTCTGCCTT
GAAGGCGTCCATGCGAGTTTTCACACAATGCTTTATCTCTG
>at03260

CCGTAGCCATTTTGGCTCAAGCCAGTGCCAAACTCGGGAACGGACCCCTCCATCGGCACA
GGATGCTCCTGCCTTTGCCGTCACCTCCCATGCAGATAGGGCCACCAACGGAGTATGCCA
GAAGCAGGTCACCCTGGATGTTGGCCCTCCTCGTCATCCAGAGCGTCCTGTGCGTCCTCC
GAATTGCCTTCATCCTCGACATCATGGGCGGATTCATAATGTTGATCATGGTGGGCATCG
GCTGGTACGCTTGGAAGGAGCACCTGCACATTACCTTCGTCTGCTCCTGGGGCCTCCTGT
GCCTCGTCAACGGCTCCTTCGATGCTGTGAGGCTCATCGACGCTGCCGTGAAGTCACACA
TGCCCTTGTTCTCTAGTAAGTTCAACTTCTGGTACAACCTGAGCTCTGCTGTCCAGATCT
TGATTCCCGTGTTCACTCTGGCTGGCG
>at03261
CCGTAGCCATTTTGGCTCAAGCCACTGTTGAGCTGAGCAGCTTGCCGCCCCCCCTGGCCA
ACGTAGCTTTCTGACCAGCGAGTACAGATCCCCGAAGCAATGGCGGCCGTCGCAATGAAG
CTTGCAGTTGTCGTGCTGGCGCTCTCCAACGCAGTGGCGGCGGCGGGCCGCTCCGGGGCC
AAGCACACCGGGCCGGAGGACCAGCTGGCGCAGGAGATGACCCACGACCTGGAGATGAAC
TTCAACAAGATCGCGCCCTTTGGCAAGGAGGACACCGCGAAGGAA
>at03262
TTTTTTTTTTTTTTTTTCACATTGTGAGCTTCAGACTGTTTGCCACGCTAATGCAAAAT
TGGATGGATATGAGCGGCCGCTGTACCAGCCGCCACATCACCAGCCCGCCCGCAAAACCA
GCAAAATCGCAACTGAACACAGGAAAACAGACTCAGCACATGCAGGAGCGGCACTGCCAC
GATGCCGCTTTTAATTTCGAATGACCCTGCTCACTTACGGGTGGAGAGAGTGCGGGACAG
CCAGTCCAGGCCCTCGTACAGCCCATCACCCGTAGTCGCGCACGCCGACTGAATGAACCA
TTGGCGGTGCCGCAAATTGTGCAACCCAAGCTTCTCCGTGATCTCAGCGGCCGTCATGGC
GTTAGGCAAGTCCTGCTTGTTCGCGAAAACGAGAAGCACCGCATCACGCATCTCGTCCTC
GTTCAGCATCTTTGTGAGCTCCTCGCGAGCGTCCTCCACGCGGTCGCGGTCGTTGCTGTC
AACCACGTAGATGAGACCGTTGGTGCCCTGGTAGTAGTGACGCCAGAGAGGGCGGATCTT
GTCCTGGCCACCCACGTCCCACACGGTGAATGACAGGTTCTTATACTCTACCGTCTCCAC
GTTGAAGCCAATCGTGGGGATGGTGGTTACCACTTCTCCCAACTTGAGCTTGTAAAGAAT
TGTTGTTTTGCCTGCCGCATCCAGGCCGACCATGAGGATGCGCATGTCCAACTTGCCGAT
GAGCCGCTGCCAGATCTTGGTGAAGGCGAGACCCATGGCTACCTCACAAGCCTGCAGACA
CAGGGGCAGAGGAGAAAGCTCGTGCC
>at03263
TTTTTTTTTTTTTTTTTCCCTGGCATGGCTCAGAGGTCTCCTGCACCGCCACAGTCTGC
GCCCAAGTTGCCAAGCGTAAGCTCAGCCTTCATCTTCTCGAGCCAATCAGGCGAATCCTT
CAACCTGCAGAGCGCTTGGATGCCGTTGGCCTGCTTGATGGGGCCATGCGATGATGGGCT
CAAGGCCAGGTTCTTGAGAGCCTGGCCAGCACCCCAGGCCTCTATGTTCTGCGAGAAACG
CACGTCTTTGTGAGCCCTGGAGGGCCACTGGTCCTCCGCGACCCCTGGGTACGCCGGGCC
CTTCGCGATCGTCCGCACCAGCGCCTCTACAAGCCCAGGGTAGTCCACCAGCAACCTGCG
CACACCCTCACTGGTCTCGAAAGGGACGTTGCCGTAGGCTGCTGCCAGGTTGCCCAGCGC
GGCCCCAGCCCACATCCGGCCCTTGGCCGCCTCGTGCC
>at03264
CCCGTCTTTGTTTTCTTGGGTCTCATCTTCGTGGCAGCCTTGGAGCTACGGGGACTTCTG
CATTGCCAATTCACGCTCCTACTCGGTTGTCTTCTGGTGTTGAATGCAGTCCTCACTGGC
CTCATTGTGCTGTCCAGCTTCTGGTTTGTGAAGCTTGTCGGTGCCTTGACGTTGAAGGTG
GTCACGCAGGCCCGNACTTTGGCGTTGATTCTTGTCTCAGTCCTGTTCTTCCACGAAGAT
TGCTCCTCCATGCAATACTTGGGTTACGTGCTAACCCTGATCGGCATGGGGTTCTATGAC
CAAGCAAAATGCCAGCTTGCAGAGGCAAAAC
>at03265
AGATTTTGGGTTTCGAGTGTGGCGGCTCGCAGTGGGTGTTGGAGAACTGCTTTCCTTGTG
GCACCATCCAGAAGCCATCCTTGGCTGACATTGACTACCTTGTGGAGATCAAGAAGAACA
TTGAGCGTGAGCGGATTGCTGCAGCATTGCCCATAGAGCAGCGCTGGACATCCAGGAGCA
CCAGCCCCCTGAGCCCTGCCTACTCACCGAACGGCGACGACATCTTCTCGTGGGTCGGTG
TCATCATGTACATCACGGACGAGGGAAGCGCGCCAGCCATCAAAGCAAAGTTCAGGGAGT
ACGCCATGCGCCACGCAGACCTGACCTTCGCGTACAATGGCGTTTTCCACTGGGGAAAGA
TAGATCTTAACTTCCATGCTGGACAGGAGCGCTTGACAGCCATGCAGCAGGGCATTCGTC
GGCGTTTTGACGTTGACGGCTTCACCAAGCTTCGCAAGCGTCTGGATCCCAACAATGTCC
TCGGCAACACTCTCACTGACGAAGTTTTCAAGGCTTGAAGGCTGAGTCTGAGGTGCTTAG

ACCAGAGGCATGGCTGATTGCGAAACAAAAAAAAAGAAAAAAAAACAAA
>at03266
TTTTTTTTTTTTTTTTTAGCCAGGACTGCCCCAGCCAGAGTGCCCACGTGCGGGCGCTG
CCACGTGCGCGTCCCTGGCCGTCGGGGGCGCCAAGGCCCCGCCCGCCTCACCAGCTGCCG
ACATTCGAGGCGTACCCGGAGCTCTCCAGGTCGCCCCTAAAATGCGGCGCCTGCCAGCTC
GCCTGCCAGTTGCTCGGCAGCAGCGAGCTCCGCCTCGAGCTCGCCCTTTAGCGGGGGTCC
CTTGCTGGTCGCCCGGAAGAGCGGGCGCTGCAACTTGCGCATCTTCCAAGTCTGCACGAT
CACATGCGAGCCCTTGATCTCATGGACCTCGTTGAGCATCAGCTTCCTCAGCTCCATGTT
TGGGTTCAAGAACTTCACGGAACCGCTCATCTCCCCAGAGTCGCCGTGCGCTCTCAGGGT
CACGTCTTCAATAGCCCCGAAGGTGCTGAAGTAGTTGCGCAATCCTCCTCTGTCGCGTCC
TGCGG
>at03267
TTGTTTTTTTTTTCTTTTTTTTTTGTACTTCAGGCCAGGATGCTGACACCCGCAGATCCC
CTCGCACTACGTTGTCAGAGTGGCAATCTATGCCACGTCGTGAGCTGCCCCATTACTGGC
TGCTCCCTCGACCCCATTTGAAGGTGCAGGATCAGCTTCCAGAATGGTGAGGTCCCCAGA
GACCTCATCCTCAGCTGCCCGCACAAAGGCGGAGGTCATGACCAGGCACTGGCCGAACTT
CTCGTTGCAAACAGCTCGGCAGCAACCGAGCTCCAGGATTTCGAAGCCCAAGCTCCGGTA
TCGCTCATCGCACTCCAGCAGCGTTGTGGCGGAATCGCTATGGAACGGAGCTCCTGTGGA
CGGGTCGATGAAGTCACACCAGCGGTCAGTGGCATCGTAGTGGGCCTTTAGAGCCTGCAT
GCGCTGAACTAGGACCTTGAAGCACTGCTGCCTCTCCTCCATCATCTCCGTTGACATCGC
GGTCTGCCGATGCTTAGTCTGCAATGCCAGCGTGATGATCACGTGCGGCTCCCTCAGCAG
GTCGCGCTCAGGGAAGAGGCCGTCGAAGTTCCGCGCCAGTCGCCTGTTCACCGCCTGGAC
GCGCACCTCGCACATCGCCGGTACTTCTGCACCTTCAGCCACAGCAAGACGTGGGTGGCA
CGCCTGAAGGGCCTCAAAGCCTGCATGCGCACTTTGAGCCCTGCACAGCTTGAGCCAAAA
TGGCTACGG
>at03268
TTTTTTTTTTTTTTTCTGCACACTATGTCCGCAAAACAGCATGAGGGTCGGGAGCCAGG
TGCGCTGCGCGGGGGACCGCCATTCCCGCCGCCCAGCCTCACAGGAATGACACATCCACT
GCCTCCAGCTTTCCACCTGATCCCTGAGCCACCCTGAACTGGATCTGGTCGCCAGCCGCC
GGCTCCCGCGGCAGCTGCGCGTTGCTCTTGACGCCGATGAACAGGTCGCCGTCGAAGGCG
TCGGCCTGCACGAAGCCCCAGGTGCCCCGGAAGCTCCTGACCCAGCCATTGAGAATGGAG
CCAACCGCGTTGGCGGGGCTGCGCCCCCCCAGCGCGCCGGGAGCGTTGGGCGGCGCGCCC
AACGCCGGCTGGCCCAGCGTGCCGAGCATGACGGGCCGGGGTGAGCGCGAGCGGGCTTCC
GTCAGGCCCCGCGCGCCGCTGGGAGCCGACACAGGGGCTCCTTTTGGAGTCGCTGTGAGA
GTGGTGCTACCAGACCCCTCCACCTTCACATTGACAGCCTCGAAACCAGTTTGCGACTTG
TTTCCAGCGCTGCGCTGCACCATGAACTCAACGTTGTCGCCAAGCTTGAGAGGCACAGTC
AAGTGGGGGTTGCTTCGCATGCCGACAAAGAGGTCACCGGGATACTTGTGCGACGTCACG
AAACCCCATGTGTCGTGGAAGCTGCGCACACGGCCCGAGCACTGG
>at03269
TTTTTTTTTTTTTTTTTAGTTGGCCAATGCATAACCAGGGGTCGACGCTCAGCCCTGCA
GCTGCGTTCCCTCCTGCCGCGGCTCACTTGCGGACTGCGGTGCCTGCCCAGCAAGAGACA
TCAATGACGGACCACTGGCGCCCGACAGTTGCAGCGTCTCCGGCATGGGAGGCAGCCTGT
CCTCCTGCTGCGCTGCGATCTCGGCAAGATGCTTCTCCCCTCTCCCCCCTCTGCGGTGCC
AGCCCAAGCAGCTGATTGAGCATCGTCCGGTTCCTCTGAAACTGCCTGTTGCTATTGAAG
CGAGCGCTTGCCTTCAGCATCTCGAGAACGCCCTTTTGGCCCTCGGCAAATCCATAATTC
GGCAGGAGCTTGTTCTGCACCGTCAGGAAGAGCTCCGTGTGCGCCTGGCCGTAGCTCTGC
CGCGCGGTGCCGTGCACCGCCTCCACCTCGGCCAGTCTCCTCTGGAAGCCCTCGTCCGCG
AAGGCGACGTAGAGCTCCCGCTGCAGCCGCAGGACCCTCTCCAGCGTCAAGCCACCTTCG
GCGGGATCGGCCTCCTGAGGTTGGCCCTCAGGCGCCTCGAGCGCCGGAGCCGGCCTGGCG
GAAGCAACCGGCTCTGGCACGTCCGCCTCTGTGTCGCGGAGGGCCCTGAGGGCGCGGGTG
AAGGCGCTGCGGTGGCCAGGGTGCTCCACGCCCAGGTCTGCCAAGAAGTCCTGTGGCCGG
TCCGCGTAGCGGTCCCTGACGTCCTTGACGCCACTCACCATCCCGCACAGCTTCTGCCAG
TACTGCGCCAGCTTGGGGCCGTCCACGGATCGGAGCCACGCGCCGAGTTCCTCCGCAGCT
GGCCCCTCCAGCGTCTGCTTGCCGGAGTTGTTGGTGGCTTGCCCTGATGCCTCCTCTTGG
CGGCGGGTCCATGAAGACCCATTGCTCCAGGAGATCTTGCTCCCATCAGCAGCCAGCGTG

CCCTGGGAGACCTTGGAGGCGACCTTGAGTGCGCATTTCTTCCTGCTCACGACTGCGAAA
TCCAAGGCCTCACCGCTCGGGCCAGTAGCCTTGGCGCCGTTGACTTTCACTATCCCGCCG
GAATCATCCTCCCAGAGACCCGCGAACGGCGCTGTGTCAGCGGCTGCTGGCATGCCAGGC
CTTCGGGGGGAAGTGACCTGAGAATTCTTGAGCCAAAATGGCTACGG
>at03270
TTTTTTTTTTTTTTTTTCCCCACGCTTCGTGTAGTCGTAACCTTCCTCCTTGCGCCCTG
CCTCCTTGAAGGCGGCCTCCGTCTCCTCCTGGAGGACCTTGGCGCCCTCGCCCATGGACC
GCCAATCGTCCCACTCGCGCGTGATGGCGCCGCTGGCGGAGAGCTGGCGCAGCCACTCGC
TGACCAGGTCACTGAAGTCCCGATCCTCGAACAGCTTGCTGGAGGTCACACCCTTGTCGA
AGACCATGCCGTCAACCTCGTCCACAATGAGGATGGTCTCCCGGAGGCTGTCCTCGTACT
CCTCGGCCTGCCCCTCCGTCAGCGAGTCCAAGCAAGATCCTTCGACGTCGTCTCCAGTGC
AATACACGATCAGGCAGTCCTGGTCCTTCAGATCAGCAGGGTTGTTGCTTGCGCGGATGC
CCAAGGCCTTGAACATCTTCTCAATGACAGCAAAGTCGCGCTGGGTGAGCAACCTGTCAT
CGTTGATGACGTGAGCCCGCATGCCCTTCTTTGCGACGAATGCTGCCAGCATCCCAATGA
TCATGGACTTGCCCTCACCGGTGCCGACGCGAGCTAACAACGTCTTAGGCAGCTCCCGGA
AGCCTGCACCGGAGGAGCCGGCGCCGCAGGCCCGGAAGCCGAACATCAGCAGGGCGATGA
TCTGCGTGTGATGAGGAAGCATCAGGATGTTGTATCGCTGCTTCATGGCATCCGCAGCTG
CCGCGGCAATGTCCAGGACTTTATCCCAATCGGATGGCAAGAAGGAGATCCCGCGCCACA
TGACGTTCTTCGTCAGCTTCGGGGCCTTGCTCGTGATGAGCTGCTTGACATCCATATCTG
GAGTGAT
>at03271
TTTTTTTTTTTTTTTTTGACGACGGCAATGCCTCAAGGAAAATCGCAGCCCGGAGCCTC
TTGACCATTGATCCGCTCAGTGCCATCAAATGCTTGGCGCTTTCAGCGTCATCTGTGTTT
TAGGCCTGTGCAAGGCGACCAGCTAAGGCAGGCCTCTGCCACATGCTCGCAGGTCAGGAC
CGTGCATTGGCTCACGTCTTGACCACAGCGATTATCGTCGAGGAGGGAACGGCCTTCTTG
TACTTCCTGAGCAAGGCCGCGGTGTGGCACAGATCACGATTGCATACTGCCGCGACTCCG
TTCACTTCCTTGTTGATGCTGCGAAGAAAGGGTGCCATTGTCACGTCCTTGTCATCCAGC
GTTGCAGTGTCAACGATCAGTTTCAAGCACTCAGGAGGCACCTCGTATTGCTTCGATATA
ATAACTCGCATGTCGAAGACACACGTCGTCGGGTCCAGCCCAATAGTCCATGCCTCCCCA
GACAATCCGTTGACAGTAACATCGAACATTGGACCAGTTCCTAACACAAGTTAGCCGCAC
AGTAGCCCGGCCATTCCATACGGCCTTCAGCCGAAATGGCAACGGTTCTTGAGCCAAAAT
GGCTACGG
>at03272
CCGCTGACGGAATCGCCCAAAGCCGCTGCCACGTTCAACCGCAAAGATTGCACAGATGTT
GTTGGTGCCACAGCTTGTGGTCCCCAGGCTAAATTCCCAGACATACCCAATACGCTGACA
ATGACTTGCTCAGAATATGCAGCTTGGATCTCTGCTTTATCTACGGTGAACACCCGAGCC
AGAAAGTCCTCGTGGCAGGGTTCCCCGCCAGGGGATCGCAAGCGTCCCAAGATCAGGTCA
GTCTCGCTGGGCTGGCATCCAATCTTCTGCAACATGCTCACAAAGTCCGATTGCGCTATC
TCACCTGTGATCCCGGCATCAAGCTTCTCCAAGACAAGCGCAGCACGCTTCGGCTGCGTC
CATGATTGCTGCGACCGCGGCTGCATGAGTCTAGGATCGCGGGATCGCCGGAGCCGAGCT
TGAACCTTGAGCCAAAATGGCTACGG
>at03273
CCGTTTCGGCTTCAGCCAGCAGGCGCGGCGCAGCGGGGATGCCGAGCGATGAAGGAGATG
AGCTGGAGTCCGAAATCGACGAGGAGGACATCCCTGAGGACGACTTTGAGTCCGAGTCTG
CTGATGGTGAGGAGAGCGAGGAGGCCGAGGTTGAGCGTTTTGAGCCAGGCCGGACTTGAA
ACGGCCAGCACC
>at03274
GCCGACGTTCTCTTTACGTGCCTCTTATGTCGCGCGGTATGGTCAACACCTCTTGTTGCA
GAGATTCAAGACCAGGTGCATGTTGCCGAATATTGGGACATGGTTCAGTCCCGTGCCTCT
TTCAAGTCAGCTGACATGTGGCCAGCGATGCGACCAGGGAAACTGGTTGGGATGTTGGGT
CCAAAGCTTGCTATGCCATGTTGCTGCTGTTGCTTCGTCCTTCCAGCCTTGGTCCTCTTG
GTGTTGTATTTCTGTGGCGTCGTGTCGTTTTGAGGAGCTCGGGCACTCAGTGGCTGCAGC
CTTAGGGGCGTTTGGTGCTCTTGCCGCCGCCGCCGGATGTGGAACTCACTGCTACACTCT
GCACTCAAGCCAGCCCACGCTG
>at03275

CCATAAGCCCACAGCCCGGTCGTACCACTGCGCCTTGGCGCACGCGTGGAGCGCGGAGTC
GTAGAGCAGCGCGTCGGGCCGGTCGACGCCCGCGAAGACCTCCAGGGCGCCCCGGTGGTC
GCGGTCCCGCACGCACTGCTGCATCTCCCGGGAGGCGCGGACGTGCTCAGCGTCTCGGCT
GGCCGGCTGCGTCCGCGTCGGCACCTTGTCAGGCGCGTCTTCCGCGAGCTTGCTGTTCGG
TCCGTCAAAGTTGGCATCTCTCTGCTTCCGTTGTGAAGCCCGAACGGTGATGCTCCTCAA
AATACTCCTCTGGGCACATGGGACTGTCGGCAGTCTTCTCCAGCTCCGCAGAAGGCTTTG
AGCAGCAGCGAAGCGGGCAGAGCCAAGCATTCCGTGATGGTGGGG
>at03276
TTTTTTTTTTTTTTTTTGATCAGTGCGGACAGAACCGTGCAACTGCGCGGGGGCTCTAG
CAACGGATGCCAGCATCCCTATTAAAACAAGAGGATCCTGGCCATGTAAGCCAGAGGTCT
TACAAAAAATCCGGAACGCCGCCACTTTCAAAGTTGGAGGCTCCCTTCAGAAGAGGCTCC
CCTCCAGGAAAGAGGCTCCCTTCAGGAAGAGGCTTCTGACATTGAGATGTCAGAGACGCA
AGGCTTCCTGCAAGGCCTCGTAGGCTTCCTGAACCTTCAGGAACATCTGCTTTGCAAACT
CGACATCCCCCACATACTTGTCTGGGTGGTATTGCAAGACCATCTGATGGTACTTTCGCC
TGATCTCTTCCTTTGAAGCTGTCGGCGCCAGCTCCAGGACCTGAAAGTAACGATCAAGCT
TGCCAGTGTTGAATGGTGAGCTGGGATCATCCGTGTGCTCACGGAACTCGTCTTTGTCAT
CGCCTGGCTCTTTTGCCCAGTCTGGCATTCTGTCGAGACCAAGCCACTTCAAGACGTCAG
GCCTCAAGCGCTTGACGACGAACGGCAGAGCCCGCTTGTCTTGCTTGACAGCCGTAATCG
CCATTTCCAGATCTTGCACCTTTGGGTTCGCGAGCCCAAGTGCCG
>at03277
GGTGCCGGCGCGAAATATGGCAGGAGACTCTAGGGATAGGGCAAAGCGCGGCGCCGTGCG
GCGGCGCAGTGGCACTGGCGGGGTCGCCGACAAGGAGGAGAAGAGTACTCCGGTGTTGGC
AGCGAGTGAGGACTGGGAGGTTAGGGACACCAAGACCAAGGGTCGGGGCCTTTTCGCGGC
ACGGGACTTCCAAATTGGCGAGTGCATCATCAGTGAGGCTCCCCTCATCGAGGGCGTCCG
GAACTGGCGCGGAGCTTGGGTAGGGCTGCTGTCTCTGGCGGCACTTGGCTGGCTCCTCAC
AGGCCCCGCAGAGAGCCTGTTTGGCTGGTCCTACCGATGCACCATGGTCATCGTGCTGAA
GCCCGCGGTCTTCGCGTGCTATGAGGCCCTCGTCCTTTGGTCCC
>at03278
CCGTAGCCATTTTGGCTCAAGCCGCTTTGGCTTCAGCTCCTTGGCGAGAGCCGTATTGAA
GTGCGCATTGTTGGCTGTTTCAATGACATCCCTGGCGGCCCACCTTCTTGCTGAGGCGCA
GTCTGGCACGGGTGATGTTACAGTGGCTACTTGGGGAGGGGAAGGCAAAGAGAAAACGGT
GTGGCATGGCTGTGTGTTGTCGCGCTCGGAATACTTCAGAGCTGGGTTAGCACACAAACT
TCCAGGAAAATGGCAGTAAGGAATTCAGTTTTGATGATATCGCCACAAGGCACA
>at03279
ATCTCCTTCAGCTCCGTCGTGCGGAGTTTGGTTACCCTTCCCCTTTCAGTCTCGGGACCG
AAGGCCAGCAGCCGCCGCGCGCGCATCTCCGAGACCAACAGCCTGCTGTCAGGCATCCAG
CCCAGTCCGCTGGGCCCGCGCATGTTGTCCTCGGCGTCCGGCACCCTCGTGACAGTGCGC
CGCTGCTTGGTCTCCGGATCCACGGCGATGACCTCCTGGGAGCCAAAGTCGCTGAAGTAG
AGAGCGCCTCGGCGCCACCGGGCCCC
>at03280
ATCGTCTTGCCCAAGCTCCTGGAGAAGGGCCTGCTCTGCGCCGGCACCATCGACGTGCTC
TTCATCGAGTTCCACGAGACGAAGCCGACCGGCTACGAGCAGTACAGCCCCGAGGCCCTG
AAGGCGCAGATCGCCGAGGGCGAGCACTGCGCCGGCCGCTCGCCCACGTAGATCATCGAC
CTCGACGACGAGTCGTACGCTGCTGACGGGGCNCCACTGCCTGGGGATCCTNATGAAGAG
GCATTGCGTTGATTCCACGGAATTCAAGCCGAGCCTAGAAGCTCATACCCGAACGTTTTC
GCCGCCAGCATCGGACTCGGAGCGAGACAGCATCATGATA
>at03281
TTTTTTTTTTTTTTTTTGGGCGCGAGGTCCTTCAGCGTCCGCCGCAACGAGAAGATCGC
ATGCCACGTCACCGTGCGCGGCGAGAAGGCGCGGCCGCGCGGAGGCCATTGTTCAGCCCT
CTCCAATTCAACTGCCATCTTTCAGCCTATTCTGACGTCTTAAAAAAAAGATTGAAAGAA
TCCAAATCGAAAAAAGCAAAGAATTGGGATGGCAGAGGCCAGTCTGCATACTCTGAGCAA
GCGCTGGAGCAAGTAGTTTGGCCGGCAGAAGCCGCTGCCGTTGCCTTCCCCTGCGCAGAG
GAGCCGCCCCAGGCCTCAGCCGCCTCACAGCTTGGACTGCCCCGCCGCTATCCTGGAGTA
GACCTCCGCGCCGTACGGCTCGTAGGCGCCGGACTCGACGTTGAACCACCAGACCTCGTC
CGCGATCTTGGCCGGATCGCACCCCTCGTTGCGGTAGTCCACCTTCTGATGCTTGAAGGT

GCCGGTGAGGTTGATCTCCTCCGCCAAGAAGCGCACGAAGAGCGGCACGGCGTAGGGAGG
CAGGTTT
>at03282
TTGTTTTTTTTCTTTTTTTTTGTTGATTCTTCTTCACAGACTGCCACAATGCTCACAAAG
CATGCCTGCTGCACATGCAGATGCGCATGCCTTTCACAAGGGGCGTCAAATTATGTGAAA
AAAGCCAGAGGCGCTTGTTGCGGCAGAGCCGGCTGTTGCGGCAGAGAAAGCTAGCCCAAA
G
>at03283
TAGTCCTATTTTTTGGTCTTCAAGGAGAGCACGACGTGATATCGACGCTTTGATAGGGCC
AGCCATGGGTAAGCGCAAGGCTGCTTGGCGCAAGGCCAAAATTGAAGATGTCGAGGAGGC
GCTCGAGGACGACAAGCTGGTGAACAAGCTTCGGAAGAAGGGCACGAGCAGGAAGGAGGC
GGCCGACGTCCACGATCTTTTCACCGTCGACACCGTCGGCGCCGGCGAAGGCCTGTCGGC
GCGCACCCGCCGCGAGATCGCGCGGGCCAAGCTCTTCCCGCCCAAGGGCCCCAACATCGG
CCTCTCGGCCTACGAGGAGGCCAAGATCGAGCGCGCCGGCCGCGCGCTCGAGGCGAGCAA
GGCGCCCAAGGCGCAGGGGCCGGAGGTCTTCGACATCCTGGTCCGGAGAAGACCAAGATG
GAGAAGGTCAAGGAGAGCAGGGACGGCTCCACGGGTCGAAGC
>at03284
TTTTTTTTTTTTTTTTGTCCGTATGAATGAGTTAATAATCCTCCAATTGCCAGCGTTGGA
GCAAATGGTTATACATGGTCGAGGCCTAGCATAGGTGCATGCGAAAGGCAAAGGCAGGGA
GCCAGCTACCTTGCAACATTGCACCGCTAAAGAGTAGACAATCCAGGCCAAATCGCAGCT
GCAAGCAGCACCTGTGCAGAAGTACCACTCCGGGTGAAACTGGTCCCAAACGGTCCGTCT
CCTGAGCTGGCCTCATTTCTCGCAGCTCTCGCCTCTTGCCCACTCGAAGTCCTCTGCAGA
GTCAAGCGGCTGATAGCCGAGCTTGCTGATGCTCTCTTCCAAATCGAAGACGCGCTCGGC
GTTGTTGCTCGAGGGCGTAAGCGCAGAGATAGCCGCCATCCTCGTCGGCGGGATCAGCGC
CGTCAGCGACGCCAGGCAGAAGCCTATGCAATCACGGCGCGAGAGGAACATGGCTCGCAG
GTAGCGCTCGAACTTGGTGCCCTTGAGCTCCGTGGGGTCGTCGTACAAGACCCAGCCAAT
GCGAAGCGAGACCAGCTCGAAGCTCTTCCAGACCTTGGAGTACAGCCTCGTGCC
>at03285
TGGCAATCGTACTACAATAGACGGGCATCTTCACCATCACCTGAAGAGATTGGCACTTTG
AATCTGCTGCGCACGCCGCAACACATCGGTCGAGTAGTTGTTCCCAGTCGTGCCACGGTC
GATGTTCCCCTGCGTCTGCACGTTCCGTACACCCATGTTGTATGCTGCCACGCCACCCTG
AGTCTTCTTGCTTTCTGGCCAGGATGGATGCTTGGCTGCAACCGCATGGATACTTTCTGA
CAAGATCTTCCACCCCCTGCTCGATATGGTCGCCTGACTGCGGCCCCTTGCTGGTATCCA
CCAAATGA
>at03286
TTTTTTTTTTTTTTTTTTACTCCAAAGAATTGGCCGCGAGTTGCTGCCGTTCTCTGTGCA
TCGGAGGATGAAAACTAGGAAACCGGGCATACATCGTCGTGTGCGTGGATGCCCAAAGAA
GCACACCCATGTGCAGGTCGAGAAGGAGCTGCACTCGCATGCTTTGAAGTTGGCGTCAAA
TTGTTGATAACCTTCAGCCAACGCCTACAGCGTCTCGAGCAGCCGATCGTTGCATCCTCA
CTCCTTCTTGCCCCACCAATGGCCGATCACGTTGAAGGGGAAGACGGGAAACACGCTGGG
AGGCCGCTTGGGTGGATTGGCAATCTCCTCGTTGTCCCAGTTCAGCGTCTTCAGGTAATG
GACGATGTCCACACGTGTCTGGAAGTCGTCGATGCCGCGGAAGTTCATCTGGACGTTCTG
CTGCGTCATCTGCCGTGGATTCTTCATGTAGTTCATGAGTGGGCCCTCGAACCACACGAG
CCCATCCGCCCGCCCTCCCATGACCTGCTTCTGCTGGATCTCTGCCTCCCCACTGGCCCG
GCCATAGATGTTGAAGAGCGTGGGGCCGAGCTGGAAGGCGCCGCTCTGCACGATGCGGTT
GTCAGGATGGATGGAGTGGCACTGGCCGCAGTGCTTCTTGAACAGCTTGCGCCCTCTCAA
CACGTCGCCCTCGGGCAGCACGAAGTCCAGACTCACGTCACTGAAGTCGCTCCGTACCTT
GCCCCCGCCGACGTTTTGACCTACAGAGGCCGGGATGAAGGAGCGCCTCGAGGGCAGCCG
CTCGAGTGTCTCG
>at03287
ATACATCCTTGGCATCATGCCCCATGATCTGGCCATCTTGGCTTTTCCGAGCACAGTGGT
GGATACCATGACCACTGCCACTATGGCCGTCAAGCTAAGCTCGATTGTGACAGGGGGAAG
ACCATCTGCAAGCTTGCAACACACAAAGGCCAAGGACAGCATCAGCGTACTCCCAATTGC
ACCCAAGAGGCAAATGCGCACCATATGAACCTGACGTGCTCGGCCATTGTCACCAGCCTC
AACGAGGAGTTGCGACACAATATGAGCCAGGGCAGCTAATGTACCAAGAGTGCCTAAGCT

GAGGGCTGCCTTCACACACCAGATCAATGTGGTCTGGCGCTGTGCTATGAATACTGCTT
>at03288
AAGACACTCTGTGGTCGTCCGATTGTTTGTGATGCCTTCTACGGCATGGCCAATATGCCA
GCTAACTATCTTCTCATCTTTGCCACTGCCCTTGGGTTCAGTATCATTCGGGTCGACCGT
CTTGTCCTTGAGCTCTTTTGCGTCTTCTGGCAACTTCGTCACGTTCTCACGAAGCTCTAA
CCGGTCGGTCCCCAACACTGCTCCCAAGCGAGAGACTGCCGTCAATCTTGATACCACCCA
GGACAAACTCCTCAGCCATGATGTCCTCGGAGTCCGCCTGCATCACATCCCGCCTCTCAT
GG
>at03289
TTTTTTTTTCTTTTTTTTTTTTTTTGTTCACAGCATTGCTTGCGTTGAGTGCGCTATGAAC
GACGAGGAGTCCGCCCAGTGGACCTGGCAGGATGCGCCCTACGTGCGTCCACCAACCTCG
GCACCAGCCCTTTGGCTTCTCCCGCCGGCGAGCGCAAAAGCTTCCAGGGGCGCCCTAAGT
TGAGGCTTGCACCGCCCGCGGCAATTGCCTGCCCCTGCTTGGCGACTCCCTCGACCTGGG
GCTCCGAGGGTACGAGGCCACCGCCTCCACCGGAGCGTAGAGATGCTGCACAGCCCTCCG
CTTTTTCAGCCGGTGGCAGCTGCGAAGCAACTCCTCCGCGAGGAGCTCCGACTGCACCGC
GATGAAGATGAGGACCTGGCCACCCTCTAGCAGCGTGCGCCGCACGTTGAGCTGCACCAT
GGAGGCGTAGGGCGGCCTCACACGCTCCTGTGTCCTCGCGCTGCCCTTCGTGGACGGTGG
GC
>at03290
TTTTTTTTTTTTTNTTCCGAGAGCCGACAGCGCACACGAATCGAGGGAGTCATCTTGCA
AGCGATCGAGAAAAACTGGCAGGCTCAGCTCATGGAGACGAGGCCCTGCGGCTCAAGAGC
TGCCCAAGCACACTCACTCCTCCTCCAGCGCGCCCATGAACTCGCCAAACTT
>at03291
GAGAACTGCGAGCGGCTGGCGCCGCTCTGGCCAGGCGATGTCTCGTCGCTGTCGATGAAG
GTCAGCACCGAGTGCTGGTTGGCCCCCCGCGGGCTCGGCGCCACGAAGGTCACCACGCCC
CGCCCGGATGCGCGAGCCCACGGTGCAGCAAGCTTCGGCCGGGAGAGGGCTTCTGCCGTC
GCACGACGAGTCGGAGTCAGAGAAGCCCTCGCTTTCTTCCAGGCACAGGTAATACATCTC
CAAGCATACGCAGTGATGCTCGCGGAAGTACGTGGCCTGAGTGATGCAGGTTGTCCGCGA
GGAGAGAAGCTCCTCGATCATCAGCGGATGGAAGTACATCTTGATGCCCATGCGGATCAC
TTCCGAGGGCCGGGCGTCGTCAATGCGGAGCCAAATCACTGAGTGCTGGTTGTCGTGGTC
GAAGCCGGTCTTTGCGAAGTATCCGCCG
>at03292
GCTGCTTAAAGGTGCTGGATTTGAGTTTGTGTGGCGCTCAAGGAATCGATGGCGAGTGAG
ATACGGTAGCCATGAAACAACGGTATGGTTCTTCACCAAGGATACCATGCTGACAGATAC
GACCCTGGTGCTTTCAGTTACGGTAACACTCGCCATGTGCATGACTTGCCAACTCTTGGT
GTGGTTGATGCAGTGCGTGAGCTCTCTGAAGACCAGCACATCGCCGATCTCGGGAGAACG
GTCATCGTGATGGGCACTGCGCGCAAGGCCAAGCTCTGCCACAGTCAGTGCTTGCTTGTG
CATGACTTCGACCGGGCAGCGGTGACAGAGAACAACTGGCAGGGTTTGGAGCGCGTAGTG
CG
>at03293
NNNNNNNNNNNNNNNNNNNNNGCGCACTATGACCTGATGCAACATCCTTGGACTTCACGG
GTGGGAAGCTTGGCATGCACCCGTTCAACGCCGGAAGTGGAGAGTCCTCAACTTCCGTGC
GTTCCATGGACAAGGCCCGCCGTGAAGCCTTGGATGTCTGTCTCTGCATGCAGCCAGGGC
CAGCGAGCGGAGGCGCGGATCGCCGCCGACTTCTGCAGTGGCGCTTTTTTTGGCTGGTGG
GCGTCAACATGCTGGCCAACAGAAGCTGAGCCTCCTGCATCCTGGCTTCCTCTGGAGTGG
CTGTGGCTGTAGCTGTCTCCGTCCGCTCGCATGACTCCGGGGGTGTAACAACGATGGTTG
GAACTTCAGGTCCGTATGCACCACTGGCGCTCATAGCAGCCACCACATCGTCAACGCATG
TCAATGGCAAAGATACGACATGCCCATCTGTATAAGTTGA
>at03294
CCGTAGCCATTTTGGCTCAAGCCAACGCTTGGGCCTCGTGGGCACGGGGTATGAGGGAAG
GCACTTGCAATATCCGGGGCCGGCATGAGTCTTGGTATGGCAGGCACTTCTGAAGTCATC
AACAGCTTTGCCGTGAAGGTTTCTGTGTGAGCTTGGGCTGTGGAGTGATGGACGGATCCG
TGACAGCCTCACCAGCGACAAGCTCAGACGCGGATGATGCCCCAAACTTGGAAATCTTTG
TGAACGTGCTGATTGTCCTTGTGGCAGGCTTCTTCCTCTTCCTTGTGTGGGGTTTGAAGG
GGCTCTGGTGGCCACTGCTCTGGGAGAATACGGTCAGCAAGTTGAGGCTTCAGTCGCCGA

GGAATGCATATTGCATTGCATGGATCACCTCCTGCTTGTGTCCTTGTTGCTTCAACAGGT
TCCATCCGCCGTTTCGGCTACGACTCGTGGTGCACGAGGCATGGAACTTGCGGAGGATTG
ACGTGATGAACCACATGGAGTGCTTTGTTGAAGCGAGATGTGGTCTGAACCCGAGCAAGA
CGACGGCGATCCAGGCAGTACCCTTGAACAACAGGAGTGAACCTGTGATCTGGAATGACG
CCGTCGACTTGGAGGTCCAGATCACAGACGAGATTCTCGTACTCCAAGTGCACAACTCCA
CCCCTGTGTCGCCAGACCAGCTGATCGGTAGCGCCCACTTGTCCGTGTCGGATGAGTTCT
CGCGCATGCAGGGCAGTCTGGAGAAGGTCAAGAGCATCGAGCGCGAGACGGCAAAGCTGA
TGTGGGTCAGAGAGGATGGCAACTTGGAGGACGCTGGGAAGATTGTCTTCTCACTCTATG
CCACTAAGCCTCAGACGCCTTTGCCCCCAGTGCTGCCCGGCATGGACGTGAGCAAGCATA
TGGAGTTCCTACAAGATGATTCCACCGATGCACTTCTTCCGTGAGGATGGCGAGGTCTCT
ATGCCACTTGGCCTCAGTGGCCTTTGCCCCCGGTGCTGCCCGACATGGACGTGAGCAAGC
GTGTGGAGTTCCTGGTGCATGGTTCCGAGAATGCACTTCCCTTCGTGAGGATGGCGAGGT
GTGAGCGGCCAGAGATCAGCACTGACAGCTTCCAAAAAGGAAAAAAAAAACAA
>at03295
TTTTTTTTTTTTTTTTTCCATGACAAGACCGCAATCCGATCACAGGACAGCCAAGCCTA
CCCCCACCCAGCAGGTGGTCGTCACAATTGTTGATGCGTACATGACCAAGTAGAGCATAG
CCACAGACTGGATAATTGCCTTGTCCAATGCAGCCCTGTGGCAAATCACGACCAAGTTGA
GGAAGGATGGTGCAGACCACTGCATCACCAGCATAGTCCGGAACAGGCGGTCGTCGGGCC
AGAGGTGCGCCAGGAGGCTGCGCACCACCGCTGAGGCTGCAAGGCAATCATCCTGAGCGC
TGCACTGGGAAGTGGGATACTCAGCCTGCAACTTGCTGAGCTTGGCAAAGGTCAGGAAGC
CTACTGCAGGCAGAAGCACCAACTTCCCAAAGACCACCGCTGCCAGCCACGGTCGCAGCC
TGCCCAGCGTGACTCGCTGAACTTTAGCAGTGCTCTCCTCAGCAGCCTGGAATTCTCGCA
AGGCACAACCGAGCGTTCCACTCAATATCTGGAGGCCCATCGCGGGCACTGGTGTGCTGA
TCCCCTTCATTGCGTCACCCAGCGGCTTTAGAGGAGCTGCGGGACCAAACACCAGACCAC
GCAGGGCTGGATTGAGCCCAATTGGAACGCCCACAAGCATTGCCATCAAGATGGGGTTGC
AAGAAAGCACCGCCTGACAAATCGACGAGACCATATGCTGCACCCAGCCTGGCAACCACT
GACGGCCAAGAGCACAACCGTC
>at03296
AGACACTTGAATGCCTATCACCGATTCATCCAATGGTTGGAACTCTCCTTCCCATGGATT
CTTCATTCTTTGCGTGTGTGCTATTTTTTCATTAAATCACTGCTGCATGGAATGGGCACA
CACGATTTTTCATTCTGTAATTTCATCATATCATTCTAAGAATCAAAAGATTAAGATCAA
GAAAACAAGAATTACATGAAAATACCTAAGAAAAAGAGAAAGAATGTGAATCCAGAGATA
TTTTTCCAATGATTCACCATATTTTTCTAAGAGATAAGATAATTCCCAAACCAAAAGAGA
AAAATTGCTTGAATGTTCCCATATTTTCTATCAAATGAATCAAAAAATGAAATCAGGAAA
AGAAAAAAAGCCATGAAATCACTAAAGACATCCATTGACCACGGCAGCAGCATTCTTGAA
AACCTTTCCGTGAGTCATTACACCATTTCATTCACCAGCTTTTATAAACACGCGTGGTGT
CTTCATGGGACTCACCTTCATATTTTTCATCTTTTTCTCACAGTAATTCTTGAAGATTTC
TAAAATAATTGGTTGAATTTGTTAGGCTCTTTCCCTTATTCTCTCTAGTGTATTGGGAAT
GAATAAAAGTAAAGCCATATTATAATCTCTTATCACTTATTATTAGTAAAATTTAATAGA
GATAGAGTATTGGAACAGGATTAAGGAAAGACATTCTAGAGATCACTTCTTTATTCAAGT
AATTTCTCTTTCAGATGGTTAAGATCTTCAAACAAATAATATAATTTCTCTAAGTTTATA
ACTATCCTAATATATTACAGATGATTATAGAAGAGAAGAATCCAATGAAAATTCAGTTAA
TTCTATCATTAATTTCCTAAAGTTAAAGATCTGTGAAACAAAGAAAGCAAGAGATGTAAG
TTATGAATGCCTGAAATTTTGTTCTTTTCCAACTGTAATTCAAGAGAAAAGAGAGAGATA
ATAAACAAGAAAAGAAATGAAAAACTACAACCCCAAAAGAAAAGACCTAGAAGAAAAAGA
CCAAGGAGAAGATGAAAGAAATGTAATCCTGTAAGAAAAAAGAAGACACAACTATAAACT
GAATCATTAATTGATAAACCCATGATCTGAAATTCTTTGATTTGCAAACTAATAAATGTC
CAAGCTAATATGAAAGAGAATAATGAAAAGAAAAACAAAAATTGTGATGAAATTTCTAAA
CTTACAAAAGCACCTCCTAAAGATACAGCAGCATTAGATAAAAGAAGTGTATTAGCAAAA
GTTAATTCACAAGGATCTGGAAGATAGAAACCTTCTCCAAGACAAATCCCAAAAGTTGGT
GAAGATAATGAATGAAAAGATCCCCAAAAGAAGGATACAAATAACAGACCTTCAGAAACA
AGAAATAGAAGAAAAAGAAGAAACAAATAAATTAATAAGACTTCATATTTTTTATCTAAT
TCTCTTAATAAATCTCTGAACCAAAAATGAAAAGCAAAGATAAGCCCAACAATAATAAAG
ATAAAGAAAAGAAAATTACTCCATGAGAAGTGAAGTCCTTTCCAGCAGTAGAGATTAATA
AAAGAGAAGAAGAGTTCAAAGACGTATAGAACAAAGACTAATAACCAAGAACTATAAAGA

AGAAAACAAAATGAACCTGATGCAACTTCCAGATGAATAGAATTCCAAAAGACAAGTCTC
CAGGCAGAGATAAAGACAATAAAACTCGAGTGTGATGAAAAGAATGAAAGAAAAAANACT
GACCAATGACCATACGATTAGCGCTTGATCGCGTGTCATTCACAACTCTTCAAGGAATAG
AAGACAGAAGACAAGAGAATCCCACACGCGTTTACAAGCGATTCTTCTTGGCATAACATT
AAATCCTAAGAAATGCATTGGGGAAAAGGTTAGAAGAATACCAACAAATGTTAAAACCAA
A
>at03297
TTGTTTTTTTTTCTTTTTTTTTATTGTGAGATCGGCCTTATTGTCGTGACCCCATTGATT
ACTTGGGTGTGAGCCACCAAACGCCATCAGCGCTGACTTCAACACCTGACAGTGACAGCT
TGTGGGCAAAACCAAGAGCTGCAACCACGGTCTCAAACCAATAAGTAGTGAAGTCATGCC
CTGATATGATACCCCCTGGCGCCAACACCTTGAGTGCATCCTGTAAGTCTTGCAGTACAC
CATCGTGGTGATGGTCTGCATCCACAAAGATGAGATGCATAGAGTTTGGCGGCAAATGCA
ACATGGCCTCGGAGGACGAGACTCTCATCAGGTGCGCCTTGAAATGACGTAGCCGATCCG
TGGCGGTCTGGCGTATTCCCTCCACGTGTTCAAAGGTGCACTTCTGACAGTCATCCATAA
GAGTGTGATCCCACGTGTCCACGCCAATGTATTGCAACCATGGGCGGCTTTCCAGAACAC
TGTGCGCATTCCTTCCATCATCCACGCCCAGCTCCAGGAATGTCAGCACTTCCCGCACGT
CTTCCGCTTGACCGCGCGCAACACGGTCCACGAGTGTCTGAAGCACGGTGCTGCGCAGAC
CTTGAACTTCGTTGAAGAATACTTCGGGCATGGACAGGTGTGTGTTGCGGACGTAGTGCA
CAAAGACAGGCCCGGAGGATCCGGCGAGCATGGGGATCTTACACCTTCCTA
>at03298
GACCGACGTGCGTCCGTCAACGCAGCGGACAAAGACGGCTTCACCGCACTGGGCCATGCC
GTCATGGTGCATGATGCTGAGACTTGCAGGTTGCTGATGGAGTACAGAGCAAATCCATGC
ATGCCGATGGGTCCACAAGGGAGAACGCCCTCAGAGCGCGCGAGCGCAATGCCAGACATC
ATGGACGCCCTGCAGGGGAGTCCGCCACAGGATCGAGTCGTGCCCCAGCTGAAAGCATGG
CTCTTAGGGGGGAGGGCACAGGAAGCATGAAGGCGCGCGGCGAGCGGCAGCATGTCGTGT
TTAGTTGTGCCGTCGCGGACTAGGACAGATGG
>at03299
TTTTTTTTTTTTTTTTTTCCACTGTGTCACTAGCCGCCCTCTTGGGGGGAACAGTGTGTC
CGTAGTCCGAAGTCCAGGGTCGACAATGAATGGTGCACAATCAGCAATACAATTGTTCAT
ACGTTGGATGATGAAATTGTGTGCCTCAGGCTCCCTCTCAAGATCCGGAGCATCCACTTG
TAAATGTACACTTCTCGACCCCAGTGCCATGTATTCAAAGCAATTTTCGGCCTGGGACCT
GTTTCAAGAGACTCCAGGGTATCGCCAACTGCTCGGACGAGATCCCCAACTGCCGGATCA
GTTGCAAAGCCAGCCAATTGCGGTCGCCAGCATTCCATCTCCATGTCCAGCGCGGCGGCA
CAAAAACGCGCAGCCTCCGGCACGGACCCCGTCATTGCTGCTCGGCATTGCGATGCCGAA
AGCAACACCTCCATCGCCACTGGATCATCGCCCTCCGCAATCGGCGCGGCTTGAAAACCT
GCATGCTCAGTACCACCCTCGTGCC
>at03300
TGGGGTGACGGGAGGCGGTCTCTCACTTGTGGTAGGTTCCTTCGTAGGCAAGCCTTTGAG
GCTCTTGGAGCAACCAGCAACGTGGACTACGCACGAGCGCTGAAGCACCTTGGCACGAGC
TGCCGAGACGACGACGGTGACTGTGAGGCCGCACTCGTTATGTACGAATGCGCGCGGGAA
GCGTATGCAGCAAGTGGGCAATGCATTCTCCTGGATTTGCAGACCTACTCTCAAACATGG
CGAGCTTCGTTGCATGCAGAATGATGGCAAAGCCGGACTCCATGCCTTTGAGGAGGCCCA
GGCAATCTATGAGGCCATTGGCGCAATAAGCGGTGTGGCTACGCGCGATG
>at03301
TTGCTTTTTTTTCTTTTTTTTTCGTGATACTAGTGGTATTAACGACATTCGTCCACCAGGT
CTTCGAATGTCACGCCGCATCGGCCCACTCATTTCGCCAAAGATTGCAAGCGCGGCCAAC
CGCGAACAATGCCTTCCCCCTTGTGTTTCACCCCGGTAGTGTCTGATATGACCTTCCAAA
GCACGTGCTCCTTGGTCAAGTCATGGTGGAAGTCCTGCGTGCAGAAGCTGCTGGAGGCTT
TCTCCCAGTAGTGCTGCCTGCAATCGGAGGCCTCAAGAAAGCCGTAGTGGAGAACCTCCT
TGCACAGGTTCATGAGGAAGAGCCCTCCCATCATGCCACTGGTGCTCCAGGTTGTCGAGT
TCAGGCGTCTGAGCGAGAGGGGCCCCTGCTCGA
>at03302
CCGTAGCCATTTTGGCTCAAGTCAGTTGGGCTCAGGTTTGTCTGCGGCTCTGAGGTGATC
TGCTCTTGCACCCTTGTTGCATCCGTGATGTCCGCGTCCAGCGCTGCAGTTGCAGAGCAA
CAGGAGGGTCAGACTTTCGACATTCAGCTCATGGGCGGGGAAGTTGTGGCGGTAAGTGTA

GGAGGAGACCGAGTGGGCGAGCTGAAGAGGGCTTTTCTCGCTGTCAAGCCTTGTCCGACG
GACAAGGTCGTGAAGCTGATGCAAGGGCCCACCATCCTGAAGGACGTCGACACGTTGGCC
ACTCTTGATCCTAAGGGCATCATGGCTGTGTTCACTACCCCGGAGATGTCTTACTACATC
GGCGTGGATGGTGAGGTCCGCTTGGTCAGCGAGGGCGACGCGGACTTTGAGGAAGAGCAA
GCAAAGCGCTCCCAAGTGAGCCCAGACCGATGGGTGCCGTCTCCTCCCAACCACCCTGAG
TGCCACAACCTTGCCACGCTCGGTGAAGGTGCCAGCGTGACTGCTTCTTCTAACATCTGG
GGGGCCGGCGCGGTCTCTGAGCAGAAGCTGCTGAACGTCTTGAGGTTAGGTAGCGTGCAC
GGCAGTCCCCGCTTCGTGGGAGGAGACAATTCATTTATCTTTCGGGAAAACCAGCCAAAT
CAGATCCTGACGATTGACCTCGGCAAAGAGCGCACGCTCGTCCGCATTGGAAGCATGTGC
TATGGCGACCGCTGGCTCAGTCGCTTTTGCGTGCGTACGCAGGCTGATGGCGAAGATGAC
CAGATAGAATGGGGCGACGACACAACGAGGCGCTCAGAGGGTGGGGTTGCCTTCTTTGAC
CGCGAGCCAACACCTGTCCGGATCATCCACATGACGTGCAAGAGCGGGCACTATTATGGC
GCAGGTGCAAGGATAGGCCCTGTGTTTGCGTATGGGTGGGAAGCAGGAGATGGCACCCTT
GCTGCACAAGTCTAAGTGCCACGTGCTGCTCCCAACAAGTATGGAAGGCCAGAGCAAAGG
AGTGTTCTCAGCAGGTGCCTACCATTGCGGCAATGTCCTACTGATGCGTC
>at03303
TACCCATGGAACCACCCATGGATCCACCTAAGGGATTGCCTATGCCACTGCTCATGGGCC
GCCCGAGTCGCCGCACATAGGACTGCACATAGGAGCACCCATGTTATCGCCCATGGGACA
ACCTGCGCCACTACTCATCGGAGAGCCCATGGGACCAGCTATGGGACTGCCCATCGCACC
AGTCATGGGTCCACCCATAGCACTGCTCAAGTGACAGCCCGAAGGCTGGCCCATGGCACC
ACCCATGGAG
>at03304
TTTTTTTTTTTTTTTTTGGGATGGCCAGTGTCTCAGCGGGAACTGAAGCCAGGGTTCGT
GTACTGCTCAGGCCGAGGCGGGGACTCGCTCAGTCTAACGAAGGCCGCCCGCCGCCGCCG
TCCAGCCACGACGAGCAGCTCTTCACGCAGGAGTTCCATCCCATTACGCAGGCCCTTCAG
CACTCTCCAGTTGCAGACGTCTTCGCCCTGCACAATCAGCCGCAGCTGCTGCAGGCGGCC
TGCGGTCACGGGGCAGCCAGACTCGCCCGTGTTCGGGATGCACTGCCGTAAGCGGACGCA
CCGTACAGCTCCCGGCCGGCACACGCCGACGACGCCTAGGCTGACGGCACCAGCCGCGAA
GGTGGCGGAGCTCGACACCCAGGCGGAAGTCTCGTTTCCGTCGAAAGCGTTGCTGGACGT
GTGGGCTCCGAAGTGGCCTGTGGAGCCAGACTTCGCCCAGCGGTAGGCATCGCCCGAGCA
GTCCGAAGCTTCGAAGAATGCGAGCTCGATGACCCCCCACTGCGCTGTAGACGCTTCGGC
ATTCACCAGGAAGATGCTGTCCTGGGTGCCAACGTCGCAGAGTTCGACCCCAACCCCCTG
GGGGCTCGTTCCAAGGGGGGACTTGGTGAAGACGAAGATGTGCGACCGGTTGCCCGCCGC
CGAGATCAAGGACGTGTTCGGGGGCACTTCCAAGGAGTTCTCCCCGACCGGGACGAAGCC
CAAAAGTGCTTGCACCGATGCGGTCCCTATGCTGTCCGTCAAATACACATCATACCCTAT
GACTTCGGAGACGTCTGCAGGCGGCACCCATGACACGGTTCCCTTGATCGCGCTAACGTT
AAGGTCAAAGTCCTGGAAGTCGACATTGGAAACCAGCGGCAGAGCTACCTGCAGAGTCCT
GGGACTGGACTGTCCTTCTACCTCCTCAGAGCCACGGACTCTGCCAAGGAGAAGCTCTAA
GAGGGCCAAGGCAGAAGGCAGAAGGTGCC
>at03305
TTTTTTTTTTTTTTTTTGATTTGGATCTTCGCATTGGGTCCTTAAGCTCTAGAAATTGC
CTGCAAGCCAGTCCTTACTTGAAGTTGGGCGCCAGCAACCGCCTGTTTGCCCTGCGTATT
GGATCGCTGCACCTGCGCGCACAAGCCCATGTGTACATGCTCTGGCAAGTCACTGCGACT
TCAGCTTGATGACTCCAGGACCTGCCACTCCTCTGAGCTCGGGAGCTTCAGGAACTCGTG
GGGTTGCTGTCGAGAGCTCGACGGCAAAGTGCTCCGGGAGGAAGTGACCACGGCCCCAGC
ACCAGATCTAGAGGGAGGCCTTTGCGCATAGGTGCAGGGCCCATGCCGCAGCGTTGATGT
CACCGCGGAACCAGGCTGAGAGTGGTGAGATAAAGAAAGGAGAGGGCGCCAAGTCGCATC
AGCGACGCAGCGGTGGCCTCGCTGCCAACGAAGTGCCCGGCTCCGGGGGCCGCTCGCTGG
TGGCCAGCCTGCAGAGCCGCCGGACGGGCTCCGTCCAGGCCTCGCTGGCTCGAACCCTCT
CACCCACCGCCTCCTCGTGCC
>at03306
AGCAGTGGAAGCCCGGCTGGACCGAGCTTGGCGAGGTCACTGACGAGAAGGTGGCGGCCT
TCTTCGCGCCGCTAGAGCCCGGACATCGGCGAGGCGAGCTGCGCTTTGATGACCCCAAGG
TTTGGAGCGAGGCTGGCAGGAAGCCCTTCCCAGAGGTCTTGGTTCGCTCCAAGCTCTGAG
CCTTACCTGCCCGGCTGCCCGCGCGCACGCGCGGCGGGGGAAGGCGCTTTGAACGGCCTC

TCTGAGCCGAGGGCGAGATCCGCGATGACTCCAGGATTCCTCTTTT
>at03307
TTTTTTTTTTTTTTTCTTGCTCTGTATCGTTGCAGACACATAGATGGTGGCGGAACTCAG
TCCACCCTGGCCCGTCCCGTTGTGTGGCTCGCCTACGGTCCGCCGTCACCATGTCCACAC
GGCTGTGGCCGCAGTGACTGCCTCCAATCTCAGATGCTGCAGGATGGATCCCCAGCAGTC
CAACTCGATCGTCTCGATGCGCACCACGTGGTTTTGGTCGACGCACCACACGCGAGCATT
CGAGTCACAACCCACGGCCACAGCCCTTCATTCTATGTCCGGTGGAATAGGTGGGAAGAA
ACACAAGCAGATTAAAACAGTGACCGCGACTAGGCCGAGTGAAGAAACAATGCCGGCAAT
GGAGTTGGCTATCGGGTTGTCCAAGACTTCAGGTAGGAGGTCTCTGAAGAACAAACTCGC
CAGGCGGTTGCTTTTCTTCAGCTTCTCGATTTCAGCTTTCACCGAATCTTTGTCCAGGCC
ACCGCCACCGTTCTTCATAAGTTCGGTGATGGCCGTGACGCTCTTCAGGTCCTTGGCCTT
CTTCACTGCCTTGCGGAACACCTTCATGTAGCCGTCCTGCTGCTTCAGCGTCATGCCCAG
GACCTCGTACTTGCGCTTGTAGATGGGGTTCCCCTTGCTGGCGTCGCCCGCCGAGAAGAC
GTACTGCTTGTCCGGCATCATGCCGAGGCGCGCCTCGTGCC
>at03308
TTTTTTTTTTTTTTTGTGAACGCGGCAGGATGTATATCAGTGCCTTCCATAGCTGCAGA
CTCCACTGCCAGGAGCTCTACACAGCAGTCTACCCAATGGCCAACTGATGATGGCGGCAC
GCGGCAAAACCTGCCTCAAATGCGCTGTCCCACCAGGGACAGCCAGAGGGGTGGCATTGG
TCAAAGAGCACGAGGCAAGGCCACGTGAGCCTGCCAGTGACGACGTGCATACCTTGCTGG
GACAGTGTCTGCCGTGCCTGTACGTCCTACCACTGCACAGAACCCTCCCACGCACGAGGT
TTCACGGAGGCTCCACCCAGCTTCATGCTTGGGAGGAGGCTGTCCATCCAGCTTCATGCC
CGGGAGGAGGCTGCTCCACCCAGCTTCATGCCTGGAAGGAGGCTGCTCCGCCCAGCTTCA
TGCCTGGGTGGAGGTTCCACTGCCCATGACCCATCAATGCGGATAGGCTGCGGCGTGGAC
ATGCTCAAGCCTCTGGTTTGGCTTGTTGGGCCTTGGAAACAGGCATGGCCTCAGCGACGA
AAGGGTGCTGCAGCAAGTCGTCCGGCGTCGCTCTCTTCTCCGGGTCCAGCGTCGTCATCT
GCGTGAGCAAGTCGGCAAACTGCTTTGCCTCCTCTTGACGAATACAGCTGATCTTCCCTG
CTGT
>at03309
AGCATCTTTTAGTGCCCATGCTGGTGGTCATCCCGTCTCAACATGCCACTTCTGCAGCTC
TCTTCGGAGCGATGCACATCCTTGCGAGCGGCAAGTAGCGAGTGTGGCAATGGAGAATCC
GACGAGGAGGCCTTGCCAGTGATCGATGCCACAACGCCATCCTCTTGCTCAGGGGCTTTG
ACTCCGCTTCATGTGCAAAAAGGGCGTTGCAACGAACCTGCATCTCCCGCAAGCTGTCCA
TGCCTCCCTCCTTGTGGACCTAGTGGTGATTCGAGTTCGTCACGTGTGCGACAG
>at03310
GATTTTCGGCTCCTTCGTCCAATTGCCATCCTGCCCCTCCCTCCCCCTTGAGGATGATCA
CGGCCTTCCTGGCTCGCTCACGCTATCAACCCAAAGCAAACGACCATGAACACCGTCTGG
ACTACATTGATGGGCTCCAGAGGGTGAAGTCTACTGCCCTGTTGTTTGTCGGCTCCTTGC
TGGGCCTCCTGGGGTTCATCTTCGCGGCCTTCCTGGGCTACTGGAACGTGATCAACGAGG
CCTCCCACATAAACTTCCGCGGGAAGCCAATGGACATCTATTTCCCGGCGACCGTGAGTG
AGATGGTATACAACAGCACTACTGGCTCTGGCAAGTGCTTCTTCGCCTTTGTGCTCATCG
GCGGCATTTGCATGCTGATGTCGTGGTATCCTTGGGCGCTGCGGAATGTCTACATCGGAG
ACGACAAGATGCTGTTCTCAGGCTGGATAGGCAATGACGTCAGTGGCAATCCCCGAGGGA
TCAAGGTGCTGATGTTGCGCCAATTCCTGCCGCCCATTGGGATCATGATGGTCGCGTGCA
TCCCGGCGCCGCCGGCGGCGAATCGCCTGTTCAGCGACACCGTGAGTTCGCTCATTCACA
CCTTGGGAGCAACCATGTCGATTGGAGGATATGCGTTTATCGAATTAGCCACGCTTACTG
TATGGTCAGACAAAGTCGTGTTTGATACGCCAATCAATGGACAA
>at03311
ACCAGTGAGGAGGATGTGCATGTGCCTGAGATCATGCAGCGCGAAGGCATCACCCGGAAG
GCCGTTGAGCAGCTTGTTGGGAGGTGCCCGCGCTGATCGGCTTGGAGGACGGGCATGCGC
CAGAGATCACGCAGCAGGAGTGCATCACCCAGCAGATGCCAGAGACCATGCAGTGTGAGT
GCATCATGAAGTGTGATCAGTGTGGTCAGCAGTACACGCTTGGTGAAACCTTGGGATTGC
CAGAGTGGCCTCTTAGATCCATTCCACACCATTGTGACTGTTTCCATCCAGCGCTGAAGA
AGAAGAAGAAGAAAAATGTGATGAAGAGCTAGCGGTGATAGCTTAGCATATGTGGT
>at03312
TTGTTTTTTTTTCTTTTTTTTTCTTTGTAGAGAGCCTATTTCTTTTCACTCTCACACTGC

CGGTCTTAGGGCACGTGGTGGTAGGTGACGTAGAGGTGTCCAGTCCATCAGCTTTAGTCA
TCTACGATGAGGGGGCTGCCATTTCGACAAATATCATGTTCGAAAGCCCTTCAGGGTTGT
CGAAATACAGATGATGCCCACTGTCTGACAGAATATGGAATCTGCAATTAGGGATCTCCT
CGCTGGTAGCTTGGTCCATCCAATCCCTGTCTCCATAGATCACAAACATTGGCGTCTTCA
ATCCTGCAAGCCTGGCTTTAATCGTGCCGCCTGCCATTTCAACGGGCTTGCAGAAAGGCT
CGAAG
>at03313
TTTTTTTTTTTTTTTTTCCTTCAAACCTCGGAACTGCAAAGTAAGTCCTCTGTCTGGCT
CGTCTCATGCAGGTGCAGAATCGCCCGGAAGCAAGCATTGGCTCCAGGCACACACATTGG
CGACAAGCTTCAGCAGAGGGAAAAAAACGCGCGAGGCAAGCTCTAGCTACTGGGGGGGCC
CGCTGCACTGAGTGCCAAGACGGCAGCATGGCGCGTAGGCGCACCACTGGTGCTGCAATA
CATGGCAAGCGCAGCTTGGCCTCCACGGCAGAAATTCCAGTCGTGCACTTCATACCCGGA
TTCGTGGCTCAGGTGTCAAGCCATCTCGTTCTCTTTCCTACCTCCCACTCGCCCTTTCTA
GATTGCCCCTTTGCGTCAACATAGACGCCGATGCCGTGCTGCTTGCCGCCCTTCCACTGC
CCCACGTAAGATCTGCCATCAGGCCAGCAGAACTTGCCTTCTCCAGACTTCCTATCTCCC
ACATACCCGCCCTCGTACCATCGGCCATCTGGCCACTTCATGTGTCCAGTGCCAGTCATC
TGACTGTCGCGCCATTGGCCTTGGTAAACGCGGCCGTCGGAGAATGTGTAAGTTCCCTCG
CCGTCCATGGCATCCTCAGTGAATTGCCCTGCAAAGGAAGAGCCGTCTGACGATGTGAAG
ACTCCATAGCCGTGCTTCTTNCCANTCNNGTATTCCTTGTTATTTCTTCCTTTTGTAATC
TGGAGCCGTCATGCCACATCTCGAAGCCATGGCCTGACTTCATATCCTGCAACCAGTCGC
CCTCATATGAAGATCCATCCGAGTGGAGAAACTTACCCTTGCCGTGTGCCTTGTCGTTCA
GCCATTGGCCATCATAGAGGTCGCCATTCGAATGTGCAAACTTCCCGGAACCACAAGCCC
GATCGTTTTCGAACTGGCCCTCGTACTTGCCGCCATCTGGCCTGACATGATGCCGTGCCC
GTGTCGATGTCCATCACGCCACTGTCCGGTGACGTACNCCATTATCCAGCTCCACTGGAC
CTTTC
>at03314
CTTGAAGTCCCCGCGACCAGAACGGCTGCATTGCGATCGGCCCCTTCTTCATGGTTTGTC
GTGCGCAAAGTGAACATCAGGCAGACGCGCGTGCAATTGGTTCACTATGACGTGCTGGGC
TGCGGTTATCTGAGGGAGAAGGACATGGAGAATTTCATCTTTGAGCTGATTCCCACATTA
CCGCAGCTGGACTCTTTGCAAGAGGAGTTCTATCCANTCTACGTCTTCACAGCGGTGAGG
AAGtNTTTCTNCTTTCTCGACCCGAAGAGGACGGGAAGGCTTCGCATACGTGACTTGTTG
TCATCCTCCATCCTACCTGAGCTCTACGAGCTCCGGCAAGAGCAGCCATTGGGGGCACAA
GAGGCGGAAAACAATTGGTTCAGCATGCAGTCTGCATTAAGAGTGTACGGGCATACTTGG
AGCTCGACCAGGATCAGAACGGATGCTGAGCAAGAGTGA
>at03315
CTTCAGCGTTAACATGAAAGTGGAGGTCTTCAGCCTGAGCTTCGAAGAGTCCACCTCGTG
CCTCGTTTGCGATGACGTCTGCGTCTTCAAGCCCTTCCTTCCCACGGTCCCCACCATCTA
CATGCGCACTGCCAAGTGCCCATCGGATGAGTTCGGGGAGATGCTGAACATCATGCCCAA
GATCAACCTGACCGACTTCCCGAAGTTCGTTCGCGTTCATGTAAAGACCGCGGCAATCTT
GCACCGGCAATCAGGGGAGAAGGTCTTCGAGGCTACCATGGAAGAATTTATCTGAGGCGG
GCCTAGCCACTGGAGGCAAAACGAACTGAGGGGCGGCGCTTCAGCAGAACG
>at03316
CCGACCATCACGCCAGATTGGGAAGGTGCTGCACTCCGGGCCATCAGAGTGCCCGTAGCT
CACATGTGATTTTGTCATGCACACCCCACATGCGCGCTTGCGCATGTATGCATGCAGTAC
TGTCATCGCCCACCGCACGAGTCGCTACGTCGCGTTCGCATCACACGAGTGCCGACGCAG
CCCCATCTCAG
>at03317
TTTTCGGCTCAAGGCTTGAGACGTCCGCGCCAGTTCTGCGCAGAGCTTACCTAAACATCA
GATCCGAGGATCCTAGCNCCCATCGGCAAGAAATGGCCAAGCTGTGGGGTGGTGCAGGGC
TTCACCCACTTGTTGAGGCCTACACGGTGGGGGAGGACTTCCGTATCGATAGCGAGCACA
TGTTGCCGTATGACATCCGCGCGAGCAAGGCGCACGCTGAGATGCTGCATGGCATCGGCG
TGCTCACAGCCGAGGAGGTCGCCACTTTGGCGAAGGCGCTCCAGGAGATCCAGGAGCTTT
GGCAGAAGGGTGAGTTCAAGGTCGAGCGCAGCCAGGAGGACGGGCAC
>at03318
GGGAGAAACCAGTGGTTTTGAGCTGTGTAGACGTGGACCATTCCATACATTGTCGGAGCC

CCCATATCTTGATAGCCAATGATAACACCGGCAAACGAGTAAAATACGGTAAGCCCGGTC
ATGGCTGTGATCGGCTTCCAAGAGAAGTATGCGGTAGACCGATTGGGCGAGGCAGCGGAC
AACCTTGGCGTGGAATCTGCCGCATCGCTGTTTGCAATGGAGGAAGCCTTGTCCATCCTT
CTTCCAAGCATGTTCAGCATTGCTGGCAAATAGATGATGAGCAGGAACGTGTAAGCAGTA
GAAGCaAAAATCCTCAACGTGATGTCCGTAAGTTCCTGCCACCATGACGAGCACCAGCCT
GCATACCAAATCAACtAGCGTTCGCCCGGGTCCAATTGGTAAATGATCGCAGCGAtCTGA
GGCACTCTTCGGGAAAGAAGAGCACGTATTTGGTGGCAGCAATCACAGAAAAGCCGCCGG
CGCCGCAATAAGCCCATGGAAGCCGAGGCCAAGCAGCAGGGCTGTGAATAACAGCAATGG
GTTGCTGCGTCCCGTGGTGCCGCTCCTCCCAAGCACACCACTACCTTTGTACTGGTGAAA
GCCGATCAGAAAGAGAGGCCCGATCAGGAGCTCCAGAATTGATACAAGCGCCGGGGTGAG
GTACATCACAGCATGTGCAAGAAACGGGGATGTCACAACCAGCTCCTGAACGCCGGTACT
GCGCACAGTTCCAGCGGACAACACACTCACTACCCAATCAAATA
>at03319
CCGTAGCCATTTTGGCTCAAGCCATTCGGGCTCAGAGAGTGCCTGCTCATCCCGTGTGCA
ACCCCCGATGGTAGCCGCATCCAGGCTTTACGCTCAGGTGCTGGCTCGATGCCCACTGGC
CACGCACTCCGTGACAGCAGGTGCCTTGTACGGCGGAGGTGACTTCTTGGCGCAGCGGCT
GGAGGAAGCCTTCCAGATACAGTCGCCCGGGAAGGCGGAGTACAACCTCGACCGAACTCT
GCGCATGGCCACGTTCGGCCTGCTCTTCGCGGGACCCCTCGTGAGGGCTTGGTACAGCTG
CTTGCATAGGCTCCTGCCTGCTCGGTGCCAGGCTGCTTCGACAGCGTCGGCCTCGGAAGT
GCGGGGAATGTACTGGCCCGCGGATCCGCGCGAGCAGAGCAAGACGCTTCATGTGATGGC
GAAGGTCGTGCTGGACCACCTCGTCTTTCAGCTGCCGTTCCTGAATCTTTACATGTTTGC
AACATGCATGCTGGAAGGTCGGCCATTCGACAAGTGCTTGGATCGGTGCAGGAATGACTT
CTATGGGACGTGGGCGTACTCGATCGTCTTCTGGTGTCCCCTGCAGACGCTCAACTTCTA
CATGGTCCCGCCCACGTACACAGCCTTCACCGCGAATGTGTTCGGTGGCGTGTGGACGAC
GTTCCTCTCCTTGCTGTGGCACCGCCGCGACTACGGTGCCGCCCGAAGCCCTGCCAGCGG
CCGCGAAGAGGAATGACGTGCTGGCGGCCCTGCCTGAGCAAACGGGCCGGAGATGTGGGA
GTCCAGCACGACACTGCAAGCTGCAGCGGAGATCGCC
>at03320
AAGGTGCAGAAGCTCCTGAAGACTTCGACATAGTAGTGGTTGGCATGGCCGCCTTGGAGG
ACGAGATCTTGCTGCACCATAGGCCATCAAAGAGCCTCTTGGTGGCGGACTTCGTTTCTT
CGCCAGCGCCGACAAGGAGGATCCTGGGGGCGTGCC
>at03321
TTTTTTTTTTTTTTTTTGACACTTTGCCGCACATTCGTCGGTAGTTGGATTTTCTCGTC
GTGTACATTTGATGCCTGCACTCAGTTTACTCCTGGATTGCTTCCAGCTGCCCACGAATG
TGCAGGACCAGGTTGTCATCCTCCAATTGAAGTGGCAACGCCTCATCCAAAGGCCTCCTG
GTAGAGGCGCGACGCAGCAGCCTCATCGCCAGCCAGCATTGCCCCTTCGGCTTCAGTGGC
CATTGTCTCCGCAGCTTCCCGAGACAAGGTCGCTTCCCCGAGTGTGCTGCGGATGAAGGC
GCGGCGCGATCTGGCTGCAGCTATGGCGGCCTCAGCCTCACCGCGCCAGAGGTGCGCCTC
AGCAGCCAGGTCAGAGAGCACAGCGGCCAGCCAGTGAGCGCCAGGCACCGGAGCCGGCGA
CCCCTCAACCATGCCGCTGCCCTCAAGCAGCGTTGCATGGAGCGTCGTCAAGGCTTCAAG
CCCTGAGCCGGATGGGGCCGTCTCGCGTCGCCCGGCCGAGCCTGCTGCATAGCGCCTATT
GCGGACGCCGTACAGCACCTGGCTCTCCATGAATGGCAACGATTCATGCCGCATCAGCCA
GTCCGCCTCGCACTTCTCCAAAAGAGCCACTGTGCCACTGCTGCCACACGCACGGCATGG
CCCCCATGACCCTCCATCTCCTGGGCTCACCCGTCCTGCGGCGCATGTGGGGCAGCCCAA
GGAGCGCGTGTCATCATCAGAGGCACATCGCTCGCAACCGCAGTGGAAACCCCAAGGCTC
AAGCCGTCTTCGGCGCTCCGCTGTTGGCTGCAGAAGGCTGTCCTCAGAGAGGTAGGACAC
GGTCACTTCCTCACCGGCAAGCACTGGCCGAATAGCAATGATCCGGAGCCGATCTGAAGA
AGGGCGCAGCAAGGCAATTT
>at03322
CATTCGAACATGCCACGTGGAAACATGATCGATAGACACTCCAGAGTGAGGACGTTCATT
ACTCCGCGGGGCCGACCTACCTTGCTTGAATCAGCCGCCAGTCATCCACGGCTGCTCATG
TGCTGCGTTTGGAAGACGAATCCTTGTTGTGCGCCCCGATGCTCTTTTGATTCATCCGAC
CTTTCGGAGAGCGTGCTGCCTGGGAAGCTTTGCCCGAGTGGAACTCTGCAGGTAATGAGC
CGATTTCAGCCAGTCCAACTTCAGCCCCATCATCGGTGCAAACACAATGCATCGGTCCTG
ACCGTCACACGTCTTTTTCTCGGCCTGTAGTCCCGCTACGGTCACATCGCAGCTCGTGAT

ATCACCATTTTCTGGCTTGCACCGGCAAGCTTCGCCCGGGTGGCATCCTGTGTCATCCCT
GCACACCATTCCCGGTTGTATGAACGCCCCTTTTGTTGTTTTCAAGCGTTCGCAAATGTC
ATTCATTGAGCGGATTTCCGCCAGTCCAACTCCAGTCCCATCATCAGTTCCTTCCGCTTC
CTCCGGACCGTCATCCTCGACCCGCTCTGCCCACGATTGCAGCACTGCATACAAGGTCAG
CAGGCGAAGATGATCATCGACATCGTTGATGCGTGG
>at03323
TTTTTTTTTTTTTTTTTTGCACCCATCATCTGCTGCAGGCATCGTGCGCCATGCCTCGGG
TGAGTCGGTCTCTTTAAGGCCCCCTAGCTTGAGCTTTTGATGGGGAAAAGTTGCAGCGCG
GCCACAGCCACCGCCTCGTCCGCACTCGCGTGAGGCCGACCTGCCGCACTTATCTAGGGC
TGCCGATGAGGCAGCCAGGCGCAGCAAGCGGAGTGGGGACCGCCGGGCCGCCTGCCTAGC
CGCTTATCGTCAGCGCAGCCTGGAGGCGAGCGCAGAGGCGGCCTGCCAGCGACAAGAGTC
TCAGGCCCCGGCTCGGCGGCCGCAGTCGCTTGGCCGCGGCCTGCTTCCCGTGCTCCGAGC
GGAATCCTGAGGACCGTGTCGTCACAAGCCTTGCGGCAACGGCCGCCAGCCGCGATGGAG
GGCACCCACGTCAGAGGCCACCTTGCGCTTCCTCAGCTCGTCCATGCTTGGCGCCCCCGC
GGCGGAGCGGCCACTTCTGCAGTCCTCCAAGCGAGAGACTCTGTGCACCGCTTGGGCTTC
TGCTGCCCACTTCCGTCCGGTGTCGTCAGGCACTCATCGCCTGACCCGTAGCGGCGCGAC
CTCGTGCC
>at03324
TCGTACTTATAGTGTGTCGAAGCAGTCATTAACTGTTGACCGCATTATCCCTTCAAAGGG
ATACGTTCCAGGGAACGTCCGATTCCTTCCCTTCTGGCTCAACAGGTCCTTGCACATCTT
TTCAGACAAGAAAGCCTTGCGCATCGCAGAGCGCTTAGCTGGGCTTCGGCCGAGGCTCTC
TGTACACAGCATTAGTGAAGATCAGCACGCAAGGGCAAGAGCAATCTTAGGGTTCAGCAG
AAGCAGGGCTAGAAGGATCGGAACTTTCGTCGACGACTGCTTTGATTTTGCATCGTTGCT
TCGCGCGGGTCGATTCACTGAGCGTTGTCCATTTAGTGACATACCCTTGGCGTACTACAA
GAAGCTGGATGGAAAGCTGCTACAAAGCAGCCCTTCTTTTGACAGAATTGACCCTAGGGT
GGCTACTCATTGGCAACGTTCAGATTGTTGCTATGTCTGTCAATACG
>at03325
TTTTTTTTTTTTTTTTTTGACCGTCTTGGGAATACTGAGAAAGCTGCTCTCCTGACGACA
AGCGCTTCATCGGGCGGCATGTGCACCCTTCCTGCGCTCGCTTAAACCAAAACGTGCAGG
ACCTGTGCCCGCGCAAGTGGCCGATGGCATGTGCACTCCAGGGTTCGCCAAGAGCTGCAG
GTCACAGGCGTGGTGCTTCTGGCTGATGCCTGCGCAGGTGGCAGGCACTAGCTTGAGCAC
ATGAAGCGCGAGCGCCCCCTTTACAATGGAGCTGTGCATGCACTGGCCTGCCCCGGAAAT
CAGGAGCTGAAAAGATCCGTCCTCGCAAGGAGGTGCGAGGCATTGAAGTGTCCGATGTTG
GGAAAGGCCTCGGCGCGCTGACCCGGCTCCAGCCCCATCCACTCAGCAATGGGCACCATC
ATGCTCTCCCAGGGGTANTCCGGGATGAGCCGCCCCCGCCCCAGGTCGTGGTCGTTGCCG
GCCCGAAAGGACGCCGCGAACTGGTTCAGCACCCGCCGGCCGCGCAGGCTCCCCTCGTGC
C
>at03326
GGTACCCCAGCCTCTGCCAAAAGGACATTCATCTGACCGGGCATGCGGCCAGCAACCGGA
TGGATGCCGAAGCGCACCTCGATATCCTTGTCCCGGAGGGTCTTTGCTATGGCAGCCACT
GAGTTCTGGCAACGTGCAACTGCCGTACCGTAGCCAGGAACAATCATCACCGACTTGGAC
TCAGCTAGGGCGGCAGCAGCGCCTTCTGCGTTAATCTCAGTATGCTCCTTCTGTTCGACT
TCACCCTCTTTCTTGGGCGCAACATTGATACCTCCGAAGATGACGTTCATGATGTCGCGG
TTCATCGCATCACACATAATCTTTGTGAGGATCGCGC
>at03327
CTAGtCtTACGGCTCGATGAGGTGTACAGTGAGCAATAAAAATACAAAATAGAGAGCGAG
GACTGGCACGTCCGTACGCCATGGTTTAGCGTATGCGCCCTCgGCTCGTCCNGCAGCTGG
GACGAAAAGCAGCTGCACGCTGGCAAATCAGCGGCATATTTCGCCGGATGTGCTCTAAAT
CGTGATGCCGCCGAGTGATGCTGCAAGGCAGCGTGCNACTCCTTACCTGGGCTAAGATGG
ACACATGGTACGAAGAAAACAATTGATACCACTCGCATGGCGAGTGATACGAGGCGTTGC
GAGGGAACCGGAGAAGCCTTCGCGACAAATGCAAGCCAACGCCGACATTCAAGGGTGCAA
ATAATCCTGCCAACCCCACCATAGTACAAAAGAACC
>at03328
GCATTTTGGGCTCCGGAACGATGCTCGGATCCGCACAAGCGGGAGTCTGTTTGTCAGAAA
GGGTGGTGGCCCCTTGCAAGAGCAGTCCCAAGTTACTCGTCAAGGCGGCGGCACTGTGGG

CGCTCTGGCTCACTTGCAGGCTCCCTTTGCTTCTCGTTGGC
>at03329
CTACTTTCTCATGGGAGCCGTCGACGCGCGCCCGGGGGGCGACATGGAGCTGCTGCAGAC
CTACCACGCTGAGTTCGTCCGTGCTGGCGGCACGCTCAGTCTCAGCGAGCTCTTGGCCGA
CACGGCTGCCGCAGTGCTCCTGCTGGCCTCGGCGGCTCTCTTCCACTATCCCATCTACTC
GTGGCCTGGGGGGTCCGCATCATCAGCTGCTTCGTGGATCCGAACAACATGCCCGACATT
CCGTCCAAGCTCCGGGAGGCGGAGAAGCTCTACAGGATGGTCTACCGAATGATCCAGCGC
GTGCCGCTGATCTTGGGCGATGAGA
>at03330
TTTTTTTTTTTTTTTCTTGTCCTTCGACCTCTACCACAGGCAAAGCAACTCTGAAGTGGT
ATTGCTGTGGCTTCCACAGCAGGGCCACAAGTGCTCTCGTGCCAGCCTGCAGTGACACCG
ACAACTGCAGGCGATCAGTCGAGACTGTTGAAGCTGGAGCTCTCAGGCCTCTCGCCACTC
ACGTCTGGGCAGCCAACCAGCTCGGATGCGGCTCTCTCCTCCTGCCCTGTGGGCTGGGCC
TCTTGCTCTGTGGACAACGCCTCCGCCTTGGCCGCGTGCGGGACAACCTCCTCCTGGATG
GCCTCCTGCACTGCAGGCTGGGGCTGCTGCTCTGCAGGCAACGCCTCTACGGGCTTGACC
CCGCGTCTAGCAAACTCGTCCAGGATGGCCTTGCTGTGGTGTGGGTTGGTCTCTGCAAAC
ACGACCGGGGTCTTCCCCTTCTTGGCCTGCACGGCGGGATCTGCCCTGCAGTCCAGGAGC
AGCTTGACAGCCTTAACGTCCCCGCGGCCCGCAGCCCAGTGCAGAGGCGTGAAGCCCAGG
CTGTCCTGCGCGTTCACGTCCGCCCTGTGCTTGCAGAGGCACTCCATGGCGCCGATGTTG
AACTGTTCCGCGGCGACGTGCAAGGGGCTGCGGCAGTTCTTGGGGAGCTCCGCCGATATG
TCGCACCTGGCTGCCAGGAGCTCCTTCATCGAGGGGGGGTGGTGCAGGGCGTGGAAGAGG
ACAGGTTTACCGTGACAGTCCCTGTGATCGAGATCTGCTCGGGCCTCGACGAGAACCCGG
ATCAGGTTGACAAAGGGCGTTGGCCGTTTCAAGGCGGCGACGAGTGGCCACTGCTCGTTG
AGCTCATCGCGTTCGTTCACAAGCGCTCCTCGCCGCACCAGGTCTATGGCCTCCTCTTCC
GGAATGCTTCGCAGCGGCTGTAGCCAGTCGTCTTTCCGTAGGGGGGCGATCCGGCTTCGG
GCGCGGCCACTTCAGGGTGGCGAGTG
>at03331
GTCATCATTATCTCTTCCGTCGTGATACATTTTCCCAGCATCGCCTTTTCAAGGTATTTA
ACCAAGTGAGCCGGCATCCGGATGAGCTTCTCAGTCGGATCGTACGTGGCACGATTGTGC
CAAACCGACGCGGCCTCAAACGGCCCAATCCGAATCTCACTCTCCTCGAAGGCTTGCTCA
AACGTTTCCAGCGCAAACGACACATCCATGTCCAGGGCTTCGCGGCCTATGGCATTCATG
TCCACGATTTGAGGCGCCTCGTGGTTAGATTCCATAAGACTGGAACTCTGCACAGAACTT
GACGTCTGCCTTGTCAAAGTCATACTTGGCCCATGTGCGGCAACCACATCTTGGCATCCT
GCAGGGGCTGCGG
>at03332
TGGCTGTTTGGGGCGAAAGAAGGAAGGTGCTGTTTGCACTCTTCATGCCTCAAGCCAATG
GACATCCTGCCAGGAGAGCCGTCCTTCAGCAGCCACTTCGAGGAGTACCGCTTCCGGGTG
CCCAGCCCTGGAAGCGACAGCGAGGATGGCACTGGCGAGGCTCCGCGTGGAGGCATGGTT
GAGGGAGGAGGGCGCCGGGAGGGGGGGAGGGGAGGCGGGAGGGCTT
>at03333
TCCCTGATCTCGAAGGGCTTTCGCATGAAGACCGCATGCGGCATCTCGATGGTGTCCAAC
TCGACTGAGCACTCAACTGTGAGCATGCTCCGAGAACTGCTGCCCATGAGCTTCTTGAAG
GAGGAGAAGCTGTAGAAACTGGACCCAGCTGCGGCCTTCTTCGACTGTGCCACTCCCGCG
CTCTCTGCACTTGCTCTCATGGAAATGCCCTCCTTGGTCAGCAAGGTTTCTAGTAGCTGA
GACTCACCCAGGAGCCGCAATTTCTGCTTGCTCATCTGCCTGAAACTAGAAATTGAAGCC
CAAGCACTGCCCAAGTGATCACCAATGACTGTGCAGTCGCCTCCATCATCGTCATCGTAA
ACATCAAAGATCAAGAGCATCGGTTCATC
>at03334
GCCGCAGAAGCTCAGAGTGTGCTGGATGCCTGTCCCTCCATCAGCCGAGATGCACCAAAC
GCGGTACCGACCTGCTCTGCCCCGACCGCCTGGAATGTATAGTGTGCATGACATGCAAAT
GTGACTTACTGTGATTTTCCTGCGTGTGCCATGGACGATTGCTGTACCGTGGATGCTGGA
TCTAACTTGTTCCTGCTTCATTCTTGCCATGCTTTTCCAGGCTGTGCCATCGGATGCCAT
GTCTTGGAGTGGGAGGGTCTTCAAGGAGGGCTTGAGACAAACAAAAAAAAAAGAAAAAAA
AAAAAAAAAAAAAAANAAAAAAAAAAAAAAAAAAANAAAAAAAAAAAAAAAAAANAAAA
>at03335

TCTTTCtACAGCCGAAGATGGCGCTGTCAGTCCACACGAGCCGCGAAGGATGCAATCCCG
ACTGGCCAAGAGGCCCTGGCGCATGCGTTGGTCTTCACGCACCATCCTACTTTGGGCACT
GGCCCCAGTGGCAGCAGGCTGTGCACTCTGCAGCCGCGCTAGAGCGAGGCGTCATCGTAG
TCCAACTCCTCTTGCCGCATGCTCGGGTTGACCTCCCTGACGGAGGATTGCATCAGGACG
GCCTCCTCGAAGTCGTTCTCGCACTCCTCCTCTTCCTCTACGACCGGGGCGGGCCGGCCG
GTGCGGCGCTTGACCTTCGTCGGCTCGGGGGTTCACGCCCACCTGGATGAGGCTCAGGCC
ATCGTGCTGAT
>at03336
TTTTTTTTTTTTTTTTGTGCGCTGCTTCTTTCAAACAGCAGGCGGAATCGCAGCACGGTC
AGGGCGCTTGAGTTGAGGATCTCAGCGTGATCTCTTCCTTCCGGTACAAAACCGTGGACA
GGTCATCGATCTCCGTGGACGGGGTGCCTTCCTTCAGGTGGGTCAGGATAAGATACATGT
TGGTGCCGTGACCCGGCAGCGCCTGCATGTTGACGTCCCCCCCGAAGTACTGCGCGTGCA
GACGTGTCAGCGGCAGGCCGAAGCCGTAGCCCGCCAGTTCGCTCTGCTTCCTCCTTCTGT
CCCTCGCGGCGGCGCTCGCGGCGGCCCCGCCCCAGGTACAGGACTCGTCCGCGTCGGAGC
GCACCGAGGTCCAGCCGTACTGCCAGACCTCCTGCTGCACCTGCTTGGGCATGCCGCCCC
CCTGGTCCGCGATCTTGATGATCACGTGGATGTCGCCCTGCTGCAACTCCACCTGCACCG
CCGGGATGGTTGGCCTCCTGCCGATGCCGCCGTTCCTGGAGGCGTGACGCTCGACGGTCG
CACGGAAGGCGTTTTTGAGGAGCTCCCGGACCATGTAGTGCACGTGTCGCGGGATGTAGT
CCAGCACACAGTCCATGTTCCCGCAGTACTCCACCTCCGGCGCGATGCCGTACAGCGACT
GCGACAG
>at03337
GATGAAGGTTGAGCACTTCAATGACTGGGGCGCCTCTTTCACGCCCCTCATGGCCCCAGC
AGCGGAGGAGGTTGGCGAGGCGCTTGACGAGATGCTGCCAAGGATGAACCCACCGAGGCA
CACTgGGTTTACAGTGACCGCCACTGCAGAGCCCATCAAGCCGGGAACCAACCCCAAAGA
GGTGGTGGAGTTGCTCAAGCGCCAACTCACAGCCCAATCAAGTGGGAGTCCTCTGTTCGG
TCAATGATCAAGTGTGGCATCACCGAGTTCCATCTTGTGGGTCCCAGCTGGCACATGCGT
GGGTGGATGAAGCATATTGATCCG
>at03338
TTTTTTTTTTTTTTTTTGAAGAAAAAAACGCTGCAACTAAATCAAAGTGTGGTGTGCCTC
CAAACTGTACATCCATCAACAAGCTGCGGCACAGCAATGCGTGGATGTCCTAAGGAGTCC
TAAGTGTGACCAAATGCTCTGGGTACGCCTGGGTGCCTCTAGGAGCCACGGTCTCCACGT
GTGGCCGAAGTCCTGCAGGGTTGCACAGCAAAGAATGCAGGTCTAGGGAGTCCTGAAGAT
GACCAAATATTCTGGGTACGCCTGGGTGCCTCTGGGAGCCATAATCTCCATGTGTGACTG
GTGCTCTGCAGGGTGGCCTTCCATCGGCCCCTCTGGAGCAACGACAGAATCGTACCTCTC
CCCAGCATGGTGTGGTAGCTCTGGGGGCAACCGTTCTGCCGAGCGACAGCGTGGTGTCAG
ATACGGCTCACCCACCAAGACGCGAGAGATGAGGAGCGCCTGCATGCCGCTGGAGTCTGG
CTGGCAATACTGAGCAGCCTTGCAAGCTCGCGGAGAGAAGTACGTTCCGATCCCATACAT
GGTGTTAGCGTCCCGTGCAAGCCTATCGTCAAAGCCTTGCTGGCAAATCCTTGGAACTGC
TTCCCACGGTGACCCATGAAACAGGAAGACCTCGTTCATGGCGGCATGCAACGGGCAAGG
GGGCCCAGCTGACGAATCCTCAAGCTCAGTGAAGAAGTCGTTGGAATTTGGCACTTGGAG
CTGCATCTGTGCTCTCTGTAGCCCAGGCTTCCATGCATGCGCTGCCTCGTGCCGA
>at03339
CAGGTCGTCATCGTCGTAGCGCACTATTGAAACCTAGAAGTCCTCCGTGCACGACTCGGC
CAGGGACTCCCTGTACCCGCCGGACCTGTGGAATGTCTTGGGCCCCCATCGCTGTCTACA
ACAGAATTAAGGGGCTGGAATTGCGGATGGACCGTGGACCCATCCGACGCGTCAATCGCC
ATGGTCGCGGTGTATGCATCTTCGCCTTCGTACAGCAGCTTACCAGCGTTAGCCTTTTCG
GCTTGGGACTTGCCTCCTTCCAGATGTCAAGGGGCTGAGGCTGGGGGATCTGTTCGTCGA
AGCGCTCCTCGCTGATCACTACGTTCTGTAGGTGCACGGACGAGGCCCGGCAGGAGCGAT
>at03340
CTGGAGGTCAGAGTCGGCAGTTGCGCGCGGTTCAACATAAAGTACTGGAGGCGGCTGCGG
GAGGTGCTTGAGGTTCACGGTCGCAAGGTGGTCGGACTGAAGATCCGGCAGAGGAAGGGC
CACTTGCATCCAGACGCGCTGGAGGATCTCTGCCTCCTGCTGCCCGCCTCTCTTCAGTCT
CTGGACCTGTCCTGGGCTCTGCCAGGTGCGCATGGACTGAGCCTCTTGGTTGAGGGTCTG
CCTCCAGGCCTGCAGCGGCTGGAGCTGAGAGGTTGCGTGGACGAGGGGAGACAGTTCGCC
ATGGTCCTTGGCCATTTGTTGCGCAGACTGGTAGACCTCAAGTACGTGCATGCAACAGGG

GATTGGGGCGGTGCAGACCCGCCATCAACGAGCTTCGGGCTGCTGCAGGAAGGTCCTGCG
AGTTGGCTTTCGTCTGACCAGTTGTGGGGGAGAGCAGAATGGTCGGCTCTGCTTCTCATG
GCGGCCGCACCACAGAGGGTGATACTTGCCGTCACTGTTAGCCTGTGAATGTCTGGCGCT
GGAGCTTG
>at03341
TTTTTTTTTTTTTTTTTGCGCAGACTAAGTTAACAAACCATTACCAAGCTCCGTGCAGG
GCCTAGCACCCACTCAAAAGCTGGCGTGGGGTTGAGATCGTGCGCACTCAAGGACGTGCA
CAAGGCTGATAGCTGATGTGGTCCATGCGAAAATGGTCAGGCCCAAGAAAATGGCGGCCG
TCAAACCTGCCGCAAGCACCAGGCTCTGCCACGTGTACGCCGCGCTGACAAAACCCACGA
TGACCCCCTCAAATACCGTAAAGAACAGCAGGAAACAGTAGTTCTGGGGGAACTTGCGGC
AGATGTCGTGGCAGCAGCAGAGTACGCACATCGAGACCAGATTCATGGCCACGGAGACGT
ACAGCAGCCAAGCGTTGGCCTTCACAAAGGCCTGTGCCTGGGAAAGTGGTGCCGCAATGG
CGACGGTCAACAGAAGCTGCATGGAGAGGATACCGTAAACCTTGCGAACAAAGCCGATGC
GGACCTCAGTGGCTGCGTCCTTGATGTCATTCCGGCCAAGGAGACTTGAAGCCTCGGAGG
GCTGCATGGACAGCCTCGCAGACTGCCTTGCGGCCATATCCTGGCCCTGAGGAGCTGCAG
GGCAACCAAGGAAGTGCGGTTGAAGGAAGGCAGAGGCGTAACT
>at03342
TTTTTTTTTTTTTTTTGTAAGGCTTCCGAATCCAGACTCGGCCGCAAGTCTTGGCGCAGT
ACTCTCAAGACTTGTCGATGGGCATCGTGCGGTTGCAAGCGCTCTGGCCGTTGTACACCA
GCATCAGAGGATCCTTTTCCGTGTGCTCGTTCTCGTATCCCAGAGGGTGGCTGATGTACA
TCACGCCATCGTCATTCCTTGCGTGCTTCTTCCTTGCGTGACCAAAGATGTGGAGCCTGC
TCCGCAGCGACGGCCACCGGATCTGCTGGTCCAGCTCCTCGCAACCCATGACCTTGGCGA
TCTTTGGCACGGCTCGGTCGAAGGGCAGGCCGTGCCTCGGCAGGAAGTGCGAGAAGGTGA
TCACAGTACCCTCATAGGGCAGCTCCAAGTGGAGGTTGTTCAGCTTCAGGAAGAACCTCC
AGACCTCCTGCTCGGCGCTCATTGGCCATCTGCAGTGCGCGTCGAAGGACAGCTTGGGGT
CAGGGAACGGATCGCCCTCGTCGTACTCCGCGTTGTACCACGAGAAGAGCGGCACGATGT
AGCAGTCCTCAGCGATGGGCGCCGGGAAGACGTCGACGCCCAGCTCGTCGCAGGCGTCCA
TGATGGCGTTGAACTTCGCAATGGAGTCGGGGTATTTCTTCACCTCCGACGGGTGGATCC
ACATCTCGTGGGTGCCAGGAGTGA
>at03343
TTTTTTTTTTTTTTTTTGATTTTCGAATGATTCGATCAGGTAGCCATCCCGCAGGCCAT
TGACAAGCTTAGCACAGAGGAATTGGCACTGTCTTCCACGGGCCGTTTCCAATTAGCCAG
ACCTCCTCGCCATTGCCGGAGCACACTCGCAGGTGGTCCTCATAAACTCACTGGAGTCAA
GACTTTCTCTCTCTATGCCTGGCTGCACTTACGACATGCCTGGCTACACTTGACGCTCAC
GGTGATGGGGGCCAGGCCGACGTCCACGGGGACACCCACCAGTCATTAACTGGGGGGTGG
TTCTCCGGCAGTGAGCAGTCAAGGGTTCCCTGCATGTCCGCGTAGAGAGAGCTGCTGGCC
ATGCAGTGGCAGCCCGGGACGCTTGTCAGAACGTCTACGTTCACGCCGATGCCGCGCGAT
GAGAAGTAAGCCTCGAAGCCGCTGCCGAAGAGGCTCGCCTCCACGACCTGAGACTGCTGG
ACGCCGGCATCGATCGATAACTCCGTCCGGGTCCAGATGCGCATGGGCGGGAAGCAGTGG
TCTTCCGGGGGGAACATCACTGGAAAGCCCAGGGAGGCGCAGCTCGTGGCCGAGACTGTG
TCGTGGATGTGCATCGGCAGCTGCGCTGAGCCCTTGAGGACCCCCATCGCCAGGGTCGCC
AGAACGAACGGCCCTCATCGCACATCAGCTCCTTGCCAGTGTCCGGGTCGTGGTGGACC
CAGGCGCCGGGCTGGCTGCTCAGCGCGTCACACTGGCTCCCCCGGGCGGCAACGAGCGCG
CTGCTGGTGTGGCAGGTGCAGT
>at03344
GGACGTGGACCACATCGGCCGGGAAGGCTCCTCAGCTCTGAGCATCGCTTGCCGCTGTGC
GCACAAAGAAGTGGTCGAGCTGCTCTTCTCCGCCGGAGCCTCGCCATTCTGTGTGCCAAT
GGGGGAACGGCAATTTCGACAGCGCTGCAGGAGATGAGATCCTGGAAATGATCCGTGGAC
ACAGGCCGGGTCGCAACACAGCGAGCGTGGGCACGCCTCAGTCTACCACCTTGCGGATGT
AGTCGGCAGGTCCCCGCCTCTTCCGCCGTCTTTGCACTGCTCGAGAGTGCCGTGGAAGGG
AGCGGGCGGATTTCAGCGCAGCAAGATGGCTTAGTTT
>at03345
TTTTTTTTTTTTTTTTTTGCGCGCCGACAGCAGCACCAGCACCGTCAACACGCGTGCCCC
CCAAAGCACCTGTATCAAAGGATCGTGCACTGCATCAGATGACAGGGGCTTGAATTTCCT
GCAAAAGAGCACTTCAAGCAGAATTGCCCTGCCTGGAATCGCAGGCCTGCCAGCCATTCA

CCAACAGGGGCGTCTCTGCGATCAAAAGGTGGTTAAACAGTCTCAGAGATTCCAGCGCGG
ACTGCCACATCCGCCTGTGCGTGACCGCCTTTACGGGTCGGGGCTTACCAAACCTCCCAT
GCGCCCTCTGCTTCGCCATCGCGCGGGATTGCTCGCGCGAGACTCGGGCACCCGGGCGGG
TGGCTCAGCCCAGCGCCGCACAACAGCGGCCGGCCGCGAGGATGAGATGGATCCTCCCTC
AGAGGGCGAAGTCCGAGGCCTTGAGCGCCTTGTCGCCGCTCGCGAGGTGCTCCTCCAGCT
GCGTCAGCGTGAACATGGGCGCGAAGCAGGCGCAGATGAAGTCCGTGC
>at03346
TTTTTTTTTTTTTTTTGGCTCACAAAGTGGGCATCGGCATGAGTTCGCTGCCCATTGCT
AAACACAATATGCAAAACAAAAGAAAGCAAGCAGGACGGCGTATGGTCCGCAGACCTCTC
TCTCTCATGGAACTACTGTAACCAGCACATCAGCCAATACAAGCACCAGCACGTCAAGTG
CTGCCAAGTTGGAGCCACAGCATGCCTGCCCAAAGCATCGGCATCGATTCAACAACAGGG
CTTGGAACGGTGCTTTTGACGGTAGAGGCAAACGTAGAGCCGACTTGCTGTTAAAAGTAC
AAGCAGACCAAGCCCTTGAAAATGCGGGGTCCCTAACACGGCGTGTCAATGTTTGCAATT
CGCATGAGCGCTCCTGCGCATGACCTGGTAATCATTCCCAGGAGCTCTTGATTGGTGTTC
TCAAGCACATCCTGGCTCGAGTGGTAGGCTGGATAGCGCATGTCGTCTCCATGAATAGCG
AGCATCGCATGGAGGTTCTCGTTGAGGAAACTCATGTGATCCGAGCCAAAAGGAGCAGTG
TTGACTTGAAGGTTCAACCCATCCCCGTTGTATTCCTTGGAGACCTGAGCCACCTCTTCC
GTCACGTCTGTAGACCAGGGTTTGGTCTCCAGCGTCAGTGTGGGAGTTGGNAACTCTGCG
TTCGTGCT
>at03347
TCTTCTTGAAGTCCAAAACTTACTGGCTCTCCTTGAACGCCTCCGTGTCCTTCATCCACT
GAATCTTGGCCTCCTGCTCCTTCTCCCTGAGCTCAGCCTTCTCCTTCTCATCAGCCTTCA
TGTACAGCTTCATCAGTGCGTCACGGCCCTTGCCCTCACCCTTACCCGGCTCCACCTCGC
TCTTGTGCGCGAGCGCATACATTGAGAAGGCACTCGCGGGGCGCTTGGGCAGCTCGCGCA
GGCCAAGGAGTTTTATTCTCCTTGGCCTTCACCATCTGGCGCTCCAGCAAGTACTCAGTG
TACTTCTGCTTCTTCTTGAACGCAAGCAAGTCCTCATCGTACTTGACCTTCAGCTTTGCC
GCCTCTTCCTGGTAGTTCTGCTTCTTGTCCTGCGGGACGTCCACCCACATCTGGGCATCT
TCTCC
>at03348
CCGTAGCCATTTTGGCTCAAGGCGTTTGTCAGTCATGCCCACACTAGTTGCGTTGTTGTC
CATCCAAACTCCCTCTGTACAGCCATGGCGACATCGCTTTGGGCTTCAGTCCTGATGTCC
TTTCTGGCTGCATACCTTGGCACATACAAGCCCCATCGCGACTTCTTCTTGTTCTTCCTC
ACGTTTTGGGCAGACTGCATCCAAACAGAACTTCCTCTGGAGCCCGCAAGCGATCCAAAT
GGCACGTTGGACACCATCCATGTGGTCTATGCGAGCGAGTCCCAGAACTTTGCCGGACTC
CTCAACAGCATGCTGTCCCTGGCCGAGAACTTGCAGGTG
>at03349
TTTTTTTTTTTTTGCGCCCGCCGCCGGCACATTCACCCACGCGAGTGTTCTCCGCTCCA
CTTTCCGGGCCCGGCGCTCTCCGCAAGGCCCGACTAGCCAAAGGAGGTGTTCCACTTCCC
GGGCCCAGCGCTCTCCGCAAGGCCCAGCTAGCAAAAGGCGACGCTTCACTTTCCAGGCCT
GGCGCTCTCCGCAAGGCCCGATTAGCCAAAGGCGGCACAGCCTCAGAGCCGCGCTTCAGG
GGGCGCAGCCGCCGATCCGGGAACCGTGAGGGCCCGGTAGGCGTCCGTGTACAACTCCGT
CTTCATCTGCGTGAACATCTTCGGGTTGAAGCGCGCCAGCTTCAGGCTCGAGGGCAGGTT
GGCCCTTGCCATCCTCTGCGCCTCCTCTAGCAGCACCTCCCCGGCGCACTCGGCGTCGAT
GATGCCAGCAGCTAGGGCCTCAGCGCTGCTGAAGCGCTTGCCTGCCAGCACACCCTGTCG
CATGGCTGAGGGCGAGAGCTTCGCCTTCGCAAGCTCCGCGAAGGCCTTCGAAAGCCGCAT
GCCTAGATTCAGCTCCGGGAAGTTCAAGAAGCCCCTGGTTGGTCCGCATGATTCGCCAGT
CGCAGGCCAGGGCGACGAAGAGCCCCGCGCCGAAGGCGTGGCCGTTCACGGCGCAGACGG
CGTGGCAGTCCATGACCAGCAGCCGCGCCAACACGCGCCAGAACGACTCCAGCATGGCTG
CGGTCTCTGCTCCGTGCTTCTGCATCCACTCCACGTCCAGGCCGTTGCAGAAGAACTTGC
CGGTGCCAGTGATGACCAGGGCCTTCGGGTGCGGCGCCTTCTCAACCGCAGACAGGGCGT
CCGAGAGAAGCCGATGCTGGTGGGTTGATCCGGCACTCGCCTCGGTCGAGGACCAAGGTC
GA
>at03350
TTTTTTTTTTTTTTTTTCAAAGTCAAATGCGCCCAAAAGAAACACACACAGCCACCACG
TTAAGTCCTATGCTCGCGAGCAGTTCCCGTCGTCATACACTGTCCGGAGTACTCCAACCC

GCAGAGAGTAAAACGATGGAGGATGAATTTGTGTGGAATCAGTAGACCCCCGGTGTTCCA
CCACCCTGTACACATAAGGGAAATCCACTTGTGGGCAGCTGCCCCACAGTATAATTCAAA
TACGTGTCCAAGGTCTCACATCAGAATCCAGCTTCCTAAGGCACCCCCGTCCTGGCCCTG
CAAGCCATAAGCAACCGTGGACACGTGTGCAAAGCAGATCAAGGATTCGGATCACCACAG
GCAGCGGGGAGGACCAATCAGCATCACGACTGCGATGGACTCAGCAGGCGTCTTGTTGAG
ATGTCTCCGACATCTTCAGCCAATGCTGTGGCATTCACAATCCCCTGGACATCTCTCCTC
AGCCAGTCTGTCACCTCGACATTCAGAAAGGCGTCCCTTAAGGATTTATCTGTTTGTGCT
CGTCTCGTCTTTAGGAAAGCAAGCAGCCCTTTGAGTCGGTCCTTTGCCTTGGGAATCTTG
TCACAGGCCTGGTATATGGCAGCACCAGCTTCAGCCACCCCAGTGAAACCTGTGATGAGA
CCATTAATGCCAATGCCACAAAGTGCTTTCACGTTCATTCTCTTGGTGCAT
>at03351
CAGGGTCTCTGTCTGCAGCCCCACCGTGGAGTCGCGGCAACTCTTCCACGAGCAGCTCTG
GAACGTGTTCAATGCGAGCGACTGGCCGGACAAGGAGTTGATCGTCATAGAGACGTATCA
GGGGTCGCCCTCGGCCTTCTTCAAAAGCAAGGCCGAGGAGGACCAGAGAGTCATCTACGT
GGCCTTCCAGAGGCCGCGCTACGACGACTGGTCCATCGGCCTGAAGCGCAACATGTGCAC
ACACCTGGCCACGGGAGAGTTCATCGCGAACTTCGACGACGACGACCTGTACGCGCCGAA
CTACATCACCACGATGATCGAGGCGATGCGAAAGAGCAGGAGCTTTGGTGTGACCCTCAG
CAGCTGGTACGTCTACGACTGGGCTGCTGCAAGATGGGCTTCGTGGACCCGCAGGCTATG
TGC
>at03352
CTGAGTTTCGGCCATCGAGCTCGCGAGCAGCCGGCCCCTTAGGCCTGGCGCCCCATGGAC
CCGTGGACCCACTGATCAGGGCACTCGCGGCCGGGCATGGCGGCCACCTATTCCAATGGA
GACTCGCAGATGGTGGTGGACCCCCCGGTGGTGCAGACCAACGGCTCGCTGGGGGGACCG
CCTACGGCGGCGCTGCGCATCCCGCTGGGCCACCCGCCGGTCGTCTTCCCCCNGCCCGGC
GGCCAGG
>at03353
TTTTTTTTTTTTTTTTTCCAAAGACAAATGATGCAGTGCAGAGCCACTCCCTGTGACAA
CCCATCTCAACACCCTCAAGCGATGACAACTCAAGTCCACCAGCTCAGACTCCTCGCATC
CAGCATCCGAAAGGGTGTAGAGCCCTTCGGCACTTGGAAGTACCAGCCCCACGGCAGCAG
CCTACATTTCCGACGCAAACTTCCAGCCGCCGCCCTTTTGCTTCAGGTACATCTTGCCAG
CGCTGGCCGCCGGCATCTCGCGGATCGGGTCACCAGTGTCGTAGGGGGAGTAGATGATGC
CTTCAGTGTAGTGGTCGTACCGAATGGTCTTGGCCTTGGCCTGGTACTCCGGCGAGCCGA
TTCCGGGGATGAGCTCCCACCGGTACATGACGGGGACGATCTTGGACGCCGCGTAGAGGC
CGACCATGCCTCCAACAACAGGCATGATCTTGTCCATGGCGACTGCGAGGCAGCGCCAAG
CCAAGAGCAAGTAGGCGACGCGCCGCCCGTGCAGGTGGCAATGCTGCTGTTCAAGCCGAA
ATGGCTTTTTTTTTCTTTTTTTTTTTCCCACATGATCCGGCTTCAGGGGAGTGCATCGAG
TCATGTATCCAGTGCCAAGTGGTGATGATGCGGATGGTGATCAGACGCACCTACCGCCTG
TAGTTCAGTCACTCTGAGAGGCCATGACGGGCAAATGCCAGTCAAGGTTGCGCTGACGCG
TGCAAGGCTGACGCACTGCCAACACACCGTGGATACACGCTTCTCAGCTCAGAGCATCTG
ATCCCGGCGATAGCAGAGGGT
>at03354
GTCGTGATGCAACCAAGATCCTTTGTACTCGTCCAGCTGGTCGCACTTGCCCCGATTGCT
TGCCAGATCAACGCTGTCCGAATGGCAAGTGCATGCTGCCATCAAAGAGACGACACCCTC
TGCCAGCCCCCACTTGGAGGCATATCCCTTCATGACTTTTTCCTCTGCAGCCAAATACGC
CTTTGGCCCGGAACTGTCCTGGAAGGATGACATGACGCCAGGATGGCAGGTGTCCTCTCC
TCCCGGCACATTGAAGCCCAAATCCGAGCATGTCTGGTTGACGTGTGCGTAGGTCAGGTT
GTAAAGTCCTCCTTTCACCCCAGACTTTGCCAAAGCCAGCGCCGACTCCACACTTCGTGA
GGGGCCATCTTTACACACCACACCATCGTCAGAGATGAAGGCGCTCCTGCCTGCCGACGA
CCCTCTCTCGATCCCAACAGCCGAGCTTACATGCATGCCTGAAGCTTGGAGTATCAGCGT
ACACAGCGTGGCCAACGCTGCTGCAAGATGTGAGCAGGCCATGTCAAGGGTGAGTTCAGA
GGCTTGCGGTTGACGGAAGAGGCGGTTGGCGGCAGGGGAGAATAAGTACCCGGAGCGGAA
ATGGCTTGAGCCAAAATGGCTACGG
>at03355
TTTTTTTTTTTTTTCATTGACCTTGGCGCAACACTGTCTGCCAAACCATCCTAACTTGTA
AAGTACCTACGGGCTTGCCCAAATGTGTCAGAGGCAACTCACCATCAAATCCTTGGTCGG

ATACAGATGACAAGGCCTGAACTGACGATGCGATGAAGCGATCACCAACGTGTGGGTGTC
GTGACATCACAGTGAATTCGATTTTGTGGGCTTGCCCACTGACTGGCAAAATAACAGAGC
AATTCCAAAGAGGATCCGTACCTCGTGCC
>at03356
TAGTTCCTATTTTTGGTCTCAAGAATCATTTCGGCTCTAGGGCAGGTGAGTGATATGCAC
AGGCAGAACTGGCATCACCACatGTCGACGNGTGTGANCGCGCTTGCGTGCGGCTACTGG
CGCATGCGGGTCGAGGCGTCCACAGCACGTNCGTGATATGAGGGGATCGTCTATCGAAAT
ACCATGTGCGGCCCTGCTGTCATTGGCCGCTGGATGCATTAGCCTCGTCGTNGTCCTATG
CCTGAGGCCGCTCAGCACGCTGGCTGTCCGGGAGATTGTGCCATGCCAGTCCTCCGCAAA
CCTCAGCACGGCCA
>at03357
TTTTTTTTTTTTTTTTTGACGATGACACAACCTCAAGATCAGTCCATTCGTGCTAGCAG
CTCCATCCAAGCCACTCAGACTATTCCAGAGATGCCCCCTTCGCCCTTCTCTTCTTCTTC
TTCTTGACCTGGGACTTCTGCGGCTCAGTTGGTGACTCCGAGGGGCCCTGAGCAACTGAT
GCTTCAGGGGCTGGTGCAGCTGCTGCAACCTTCCTCTTTTTCACCTTCTTCTTCTTCGCC
TCCTGGGGAGCTGACGCCTCCCCCTCGGGTTGCGTCGCTGACTCCCCCACGGGTTGCGCT
GCCGCCTCGTGCCTCGCCGCCTTCGCGAGCGATGCCTCAGCCTCCTCCTTGCCAGCCTTG
TCTGCATCATCCGCTACCGCCTCCTCAGCCTTGGTCTCTGCCTCGTGCC
>at03358
TTTTTTTTTTTTTTTTTACGCTTGCGCAGAGCCTTCGGCGCTTTCGCCGGAAGTAGCTA
GGGATCCGGTGTCGGCCGTCTCAGAGTCGGTGTGAGCGGCGGCCTGCAGCCTGGCGGCCT
GCAGCTTGCCTCTCCTGGAGAGAATCTCCTCCATGCGCCGCCCGATCTCCGCGCTCGTCT
CCAGCAAGTGGCTCCGGGCCTGCTGCACCACCAGCGCCTCTGCGGGCCCTCTGCCTGCAG
TGGTGCCGGCCGGCTCTGCAGGTGGACCCTTGGCCTCGCCGCCTGCAGCTGCTGATGAAG
CTGCTGCGCGTCAGGATTAATACCAGCGCCATCGACACCTGCATCAAGCAGGTTGAATTT
CGAAATGTGAGGCGAAGCTGCAGTGGCAAAGGCGTGCAAAGGGTCTGCGAGTGCCAGGGT
CGCCATGGAGGGTGGTACGGCACGGTCCATGTGGTCCCTAAGGTGGTCGCAAGCACGAAT
TGAAGGGGCACTGCTGGGCCTTGAGCTCATCGACGTGCCAGCTTCACCGCCAGCGCCCTC
CTGTTCCGGGTGATCGGGGCAGTGTTGATCATTGCTGCCGCGACGGCCGCGGATGGGGAT
GGGCGCACTGTTGAGCAACACCACTGGTGCCTGGCCTCGCACTCGCGAGCTCAGGGCCGC
CTCCCTGTAACCGTTGGCCGCAGAGCTGTGAGACCTGGCATGGGCAAGGGAGCCTGCCGA
GGAGGACTGCTTCAGGCCTGCGGTGACCGGCGACTGGGCTCTCGAAGTCTGGCCGGAAGG
CTG
>at03359
TGTGGCCACCTTGTATGTCACACAGCTTCTATCATCGGCAGCCAGAAAAGGCTTCAGTGA
TCTTGCCGCGCATTGTGGCTTGCGGTGCGgCTCCTTGGCACTGCTCTGCTGACCTTGCTG
GCACGACGAGCATCGGGGCACTCTCTGGGATCATCACGCTATACCTCTACTACTTTGTTT
TGCCTTGGACAAACATCGAGTTTCTGAACCTCTTGGACTCCCTGACAGGAACGGAGAGCA
ATGACAACTTGCTGCCTACGCGTTTCTGGTCAAAGACGTGCTGAGCAGTCTACTGGGCAA
CCTCCGGAACATGATCTTGATGTGGCTGCTCGCGGCCACCGGCTTCTACGAGCCCGACTG
CAACGCTCTGTGCGCATCCGACGCGGACCGACAGCGCTGCGTTGACAAGTGCCATGCGGA
CTCTCATGCCAGCATCACCGGAGCAACGGTGGCGTTGGTACGTCTGCTGTACGTGGTGGT
GACACCAGGAGCTTACCTTCTTGCCTGCCTGATCATGCGGTGGTATTCCAGGGCCGGTGC
CCAGGAGTACTCAGAGATTGCACCTCACGTTCTAGGTGTGGGCCAGGATGTGAGCGGGAT
GTCCGGAAGAGTCAAGCGAAGAGGAGGAGATTCAGAGTGCTGAAGTGGGCCGCTTTGATA
CAGGCAGTC
>at03360
TTTTGGAGGTATTGTGAGCATGCAGATCTACGATTGTTGCTTCCGAGCCCTTCCAACTAC
TCCAGCGTCCGGGGTTCATTCGTGCAGCACTGTGCCCAAAAACACCGGCATCGCCCCACA
TCCATCTGTACATTGCGGGCATGGTCTTGCGAGACTTGGACGCGAACTGAATGTGCTCAT
TCAAGGCGGCGGCCCAGTCCTCCGGTGAATGTTCGGAAGAACTTGCATCAAACACAAAGA
TTCGATCAGTGCCTGCACCGGTGAAACTGCCGTTCTGCCATTTTTCCTCCAATTGCCTTC
AGGGCAAAACCTTCCATCGGCACTGGACCTCACCTCGCATGGGCTGGAAAGGTCAACCTT
TCCTGAAGGCGGCTCTCCAACACCCGATGACCAGTACAGGCGCCTTTCTCAAGGNTGACA
TACCTTCGGTCCATCAGGCCTCCGCACATGCTCTGAC

>at03361
TTTTTTTTTTTTTTTTTTGCACTCGCAATGGACCTCATTTCTGAAATGTACAGTCCGCCA
AACGTATTTGAAATGGCCTGCACACGCTGAATCTGTGAGTCAGTCAGGCTCAAGATCCCT
TGCTGTGGCTAGTATGGATGTCCTCCAAACTGCACAATGGATCTTTCAAGAGTGTAAAAT
TTCCGCGCAACTGGACTTCAATGCTTGGCTCCAAACTGTGTCTCTGTGACCCCGGCACTC
ATGATGCCTGTGGAAGAGGTCCCTCCCGAGATGATGTTGCCAGCCTCGTCCACAATCTCG
GAGCCGCTCCATTTCTGGTGCGTCAAGGTCTCGACACCGTTCTCCCGCTCCTGCCGCTGG
ATGAGCTGCTACGTAGGCCGCTGCTCCACGCTTCGCGTAGTCCCTCGCGAACAAGTCGAT
GCTGAGCGAATCGCAGTGGAAGCCAGCAAGTGTGATGAACTGCCAGCAGAAGCCCATCTT
TGCCAAATCCCAGATGAAGGACTCCATCTGGGCGTCCGTCATGCCGGAGGCGTCCCAGTT
GAAGGACGGGCTGAGGTTGTAGGCCAGCATCTGATGGGGGACCGCGGCGCGAACCTCTGT
TGCGAATTGAGTGGCCTGGCTCAAGATGGGCTTGCCGGTCTCCATCCAGATGCAGTCGGC
ATACGGCGCAAAAGCGAATGGCTCGCTGGATGCAGTAGTCAGTCGAGCCCTTGATTCGGT
AGTAGCCTTCGACGGAGCGCGCAATGTCCCAATCGAAGAAGACGTCCCCAGCACCGAGGC
CCGCGGCTGCCTTCCTCATCTCAGGCAGCGACATCTTCAGGGAGTCCTTGAGCCACTTGC
TGGCATCGACGCCTTTGGACTTCAGGACCTTGGCCACGGCATCCGGGTATGTCATGCAGC
CGGCGCGCTCCTCCCAATCTTTGTCTGTGCCCTTCCGAATTGCCTCAAACAGTGGCTCAA
CGCCTGCAACTGTTGCGCCCTTGATGTGCGGATGGTCGATGGGGTCAATGTTGGTGTCGA
TCATGGTGGCTGCCTCGGCATCAGTCCGCGCAACCAAGACCAAGGGAGAGTTCAGGATGT
CAGCCTGCAGGCGGCAAGCAATGAGCCGCTGGATGTGCTCCCAAGTGGAAACCAAGACCT
TACCCCCCATGTGGCCGCACTTCTTGGTCCCAGGCTTCTGGTCCTCGATGTGGATGCCTG
CCGCGCCATTCTCAATGAACATCTTGGCAAGCTTCATCGTTGCCGTCAGCCCACCGTGGC
CAGTATCGGCATCCGCGATGATCGGGTTGAAGTAGTCCACCACAGGTGTCTTTGCTCGCT
CCTCTGGCGACATCCGGCGGCGCTCCTCGTACTGCTTCCTGTCGTGGAAGAGCTGGGCCT
TGAAGAGCTGATCGACCTTGTTGGGCACGGTGTCGTACGGGTAGTCCGCGACGTCGGGGC
CTGGCTCGTTGGACGTGGAGGCGGTGGAGCTCGACTGCCAGCCACTCACGTAGACGGTGC
TGAGGTACTTCGCCATCTGCACGACCTGGACGGGGTCCAGGGCGCCGAAGGTGTGGGAGC
AAGTGCCCTTGGCCTGGTGGTCGCGCATCATCTTGTAGAGCTTGTCGGAGACCTTCGCTC
CCGTGAAGTGCAGCGGCATGGTGCCCTGCAGCTTCACCACGTCCTC
>at03362
TTTTTTTTTTTTTTTTATGCTGATGGTTTGACGCTCATGAGTATGATCACACTGCGAGCC
TTAAGCCCGACTTGAGTAGCTCATGGCACCAGCATTAGCAGCGTCCTGACAGCATAGAGC
ACCCGAGCAATGCCCAAGGTGCTTGCAGGAATGAAGCTCGGATACATGGCAATGAGGCCA
CCTGCTTCCAACTCCGAGCCATAGCCGTCGAACGCGCCAACGAAGCCGCCACCGACCGCC
GGCACGATGCTCGTCACGTGCCCTCCAAACCTGTCGAACCCCCGGCCCGAAGCGCCGCAG
AGCCTCCGCGAGGCCGCGTACGCCGCGTCCTCGGCGTACGCGCTGGCGCTGACGTCCAGC
ACCCTCCACGCGCGCGCCATGAGGCCGTACGCTATCCTGATATCCTGCCCCGGACGGTAA
AGCCGGCCGTCCTCGTCGCGGAAGAAGCCGCCGGCCCCAGCCACGCGGTGGGCGCCCTGC
GCCACCGGACTCAGAGGGTGCGGCCGCCACGGCAGCTGCCGAGCGCTGCCCACGAAGACC
TCCAGCTCGTCGGCGAAGTTCGCCGCGAAGAGCAGCGCATTGCCGTCGGCCGCCTCCAGG
AGCGTGGGGTNNNNNNNNNNNNNNNNNNNNNAGGCCACCGCGCCCACCTCCCGCCAGAGC
CCCGGGAAGTCCACGGCGCGCCAGAGCAGCAGGGAGCCCTGCCACTCGCTGGCCAT
>at03363
AGGCGGGGATGCTTTGCAACGAGCTTAACTACCTCCTTAGCCTTTTCGACATCAAAGACA
CCATACTGTCCTGCAATGCCTTTGGCTTTTAGATCAACACCGCGACGCAAACCCCCCTGC
ATGTCAATGAAGTACTGCCCGTCGTCGGGTATCAGCCACTTGAGCCTCTCAAGCTGAGGA
ATCACGTTCTCAGGCCCGAATCCGCCTGCATATCCACATCTCAGATGCACGTCGGGGCGT
TTGAAGACTGTGACTTCTCTGCCGCAGCCTTGCGACTCGTCAAAGAGGAGATACATGTTC
TTTGGTTGCCGGGGATGCGTCGTCAAACCCTTGTCAAGCTCGCTGACATTTTCCCCTCTG
CATGAGGAAAAA
>at03364
CCATTTGGCACTGAGCTGCTACGTGTGGGGCGGCATGGCGCTGTCTCTGCGTGAGGTCCT
CACGGAAATCTGGGCGGCTGTGATGCCCAGGGATGAAAAGCGGCGTCTTTCTCGAGGCTT
GGCACTTGAGGGCGTGCGTGGGTTTGCTGCTGCGGTGGCGGCAGCA
>at03365

TTTTTTTTTTTTTTTTGTGCTCGAACGGGCAGAGGGAGGGTCTAATTCACGACCTGTTCT
AACGGCTGACCCTGTAATGGCAGTTCTCAGCAGTTCGAGAGCAAACTCAAGAGTCCACCC
CCCTCACCGTACCTGCAACCCCAACTCAACCAAGTGCCGAGCAACTCTGGCCTGATACTC
AGGACAGTACTCTCTGGCCCTCGCAGCACCCACTTGCCAGGCGACATACTGTAACAATGG
TGCACTTTCAGCCTCGCCATAGCCCTCGGCTGCAGTAACCGTCACGGCCTGCAACTGCTG
CTCTGTATCTGTCCTCACAACCATCAGCGGCACGAGCGTGTACCGTCGGGATCGTCTGCC
TCCGGTGAGATCGATACGCCTGCAAGTCTTCCTTCGAGATGCGCCAGGCTGTGCCGTGCA
TGCATGACTCCGGCTCTCTCTGCACAGGATTCATGTGAACACGCGAGTCATCATTGATCA
GGTGCCACTCCATGCACCAGCCATGAGCAATTGCGGTGACCTCTTCGAAAACAGCCACGG
CATTCGACGCGA
>at03366
AGGGGGGCGCAGCCCAATGGCAAGCAGAGCATAGGCAGGATTGATAGTCCGGGTGGCGAC
ACCTACACCCCTAATTCTGGAATTTGGCAGACGGTGTGGCTGGAGCGTGTGCCCACGAAC
TACATTTCCAAGCTCAAGATCGACCAGGCTTCGGAGACAACAGTCTCCGTCTGGGCAACT
CTGGTGGGTGGCAACTCGTTTACAGTTGACGTACTTGACGGCTCCAGAGTCATTGCTCGT
GGCAGTGGTACCGGTGCTGCCCCAGCTCGGATCTCATTGCCATCTCCACAGCTCTGGTCG
CCGGCATCTCCCCACCTTTAT
>at03367
CTTCTTAAGCACAGTTCGCGAACTCGAAACCATGGCACAGCGCTTCGGCGCTGGCCACAT
TGCATGCAGGTCGGCTTCTGTGGACATGTTTATGAGCAGCAGGTTGCTCCGGAAGACGAT
CGGCACCTGCCGCAGCTCCGACATGATTCTACCATAATCCTGTCCCACCATTCGTCTGCT
GGTGGCGACTTGGAGCATGGATGGGCAACGTGCATGGCTTGCGGCGTCTCTGCTGTAGTA
GTCCGCATCGCACTCCAAAACGCCTGCCGCAAGCACAACGGAGGCACAAAGGAAGGCGAT
CTTGGCGCACATCAGGCAAAAGGTCCTCCGGGCAGTCTGCGGAGGAAGATATGGAGTGGC
TGGTATGCAGAGCCAAAGGGGCACGAGGGTGTGCCGAAATGGC
>at03368
TTTTTTTTTTTTTTTTTCATACTCCTATTAAGCTGAGTGAATGCGGGGGGACAGTGGCA
GACGGAAGGAATGGCAAGGGACAGCACGGCACATCTAGTACTCGACAGGCGTGAGACCTT
ACAGCCTCATCCTTCAGGAGTTTCTCAACGGAAGACGCCTCACGCCTCCAGAACGGCAAC
GCCACCGGTGAAGGTGTACCTTGGAGGAGTGTCCCACTTCCATGTCACCACCTTCCTTCT
GTCGATCGCATTGAAGTCGAGCAAGATCTCAAACCCGGCACCGACCTGCCAGCTCTTCAC
AGTGAACGGCCTCCCCTCACTACCTCGGTCGGGCCCCCTGACGATGCACTCTCCAGTGCT
GGCAAAGCCAACCTCTGGGTAGAAGGAAAATTCTGGGTCCTCGTCAACTATCACGTTGAA
GAACTCGCAGAAGCCGCGGAACTTCTCGTCGTAATTCTCGCGCACGACGCCCCGGCATCT
GAAGAGGCCCGGCACGTCCGGGTCCATCTCCATCTGCTTGCAGGGCCCGCCGTGGAAGGC
CACCCAGTAGTCGTGCCGGTTCCAGCCACTTAGGCTCTGCCAGACCTTCTCTCCCTTCGT
CTCCGCCTCGTGCC
>at03369
CACCCAAAATGGATTGCCGCAACGTTTCACTGCGGCCGACCGGGCCCTCACTCCACAGAG
GGCGTGCGTGCGAACCTCAATGATCTCCTCCAGCCCGACCCGGCCATGGGACGCGCAGGC
AGGCACCTCACCCCCGCCGGTGCCGCCCCTTCGGCCTGGGGCTGCCGCTGCCGGAGGCGG
CCTCGCCCTCGCGCGCCTGCCGCTTGCCGCCCTCGGCCTTGCCGCCTCCT
>at03370
TTTTTTTTTTTTTTTTTTGCCAAAGCCACTGACGGCCGCCTGTGCCCGCACCCCGCTCAC
TGGCCGTCCACGTCGATGACGTCGTCCGGCTTCTTCTGCTCGCCGCCGGGCGGTGGCGCG
GCGCCGCCAGGCGGAGGGGCCGCGCCGCCCTCGGGCGCAGCGCCCGTGTTGGCGTAGGCG
GCCTGGCCGATCTTCATGAGCTCCTCCTGGAGGTCCTTGGTCATCGTCTTCAGCAGCTCC
GGGTCCGGCTCCGCCTCCGCGACCTTCTCCTTCAGGGCCGCGAGCTTGGGGTCGATGGCG
GCCTTGAGGTCGGCGGGGACCTTGTCAGCGAGCTCCTGAAGCTGCTTCTCCGTCTGATAC
ACGAGGGACTCTGCGTTGTTCTTGGCCTCGACACCGTCCTTGCGCTTCTCGTCCTCCGCG
GCGTTCGCCTCGGCATCCTTCCCTCGTGCC
>at03371
CGCTTGTATGACCGCCGGGACGTTGCGGACGGCGCAAGCCCAAAGCCTGATTTCCTGCTG
CTCCTGTACCCCGGAGTCCCTTTGGCAGAATTCTCGGCGAATCCCGACGCATTTGACCTG
AGTCACGGGCCACCCATCTTCGTCGCCGCTGGCGCACAGGACGCATGCTGCAGTGTGGAC

AACCTCTACAAGTTCCACTTGCTCCTCAAGTCTCAAGGCGATCCAGTGGTCGAGTTGAAC
ATATATACTGACGTGGGCCACGCCTTTACCATGTGCTACCGGATTGAAGGGGTGTTGGGA
GGCCATGCAGCACGAGGCTTGCGTCTGGATGCTGCGTGCTCGGCGGTTCATCATGTGGAA
TGTGGAGAGAAAGGCGCCAAGAGACGTTGATCAGTGGTACG
>at03372
TTTTTTTTTTTTTTTTATACATCAATATGCACAGAAACACATGTCATCCGATACCTTTA
GAGCCCAACACAGAAGTGTTGATGCGCGTGGGCATCTTTCCTCAGACCAGGCCAGGATGC
GCACGGGCCGCTGCAGCCGCCCTCAAAGCTGCGGCTGCACCCTCTTCCGCCTGCTGGTCT
TGGGTAGTCTTGGCGCAGCGTGGTCCTTTTCTGGAGTCCCCAAGTCAGGGTGCCAGAAGG
TGACCATCAGCACATAGCGCGGTTCAGACCCATCATGCCAGGCCTCGTGCTCGAAGGAGT
CGTCGAAGCAGAGTGTGCGGCCCTCGGCCCAGCGCCGGGGCGGCCCGGCGCCCACGCGCA
GCCGTGGGCCGTCGGGCACCAGGAGCCCAAGGTGGCAGTAGAGGTGGAAGTTGACCGGCC
CAGTGTGTGGCTTTAGCCTGCTGCCAGGCAGCAACTGGAAGTAGGCAGCCTCCTTTGGCA
AGTTGACATTGTGGGTGCGCGCGTGGGGAACGTGCCCACGAGCCTCGTGCC
>at03373
CTGCAGATGCTCAAAAACTTCATTGCGGAGTTTGACGGTGAGGGATGCTATTGCGAGCAG
CACATTGAGGACCTTGCCAAGGAGGTTGAGGAGATAGAAGGCATTCCAATCAAGCGCTAC
CACATCAAGAAGCAGCAGGATTGGGAGTGGCAGCATTTCTGCTTGGAGCATGACATCAAT
CCTGATGGCAATATGGCCTGAGAACGTGTCAGCACGTGTGGCTGGCGCGCTGTTGCCAGC
AGCCGGTGCCGATCTTCGGCGTGCATTTANGCGCACCGGTAATAAAA
>at03374
TTGTTTTTTTTTCTTTTTTTTTTGTAATCTCACATGCGGACCTTGGCACGACAATCACGG
TCTGTGCATCTCGGTGCTCCCATGACCAGCAGATGCGAGCAGCGCGGAGCACCGAGTTGC
ATGCATGCTGAGCCTGCCGCGGTCGCAGTCGTCAGTCTATACACATGCACGGAGAGGTGA
TCACAGGATGCAGGCTACTGCGGCAACACCGCGAAGTACGGCCACCTGCCGTCGGGCAAG
GCTTAAAAAGGTACTGGACTAAGGCGGCGCGGGCTCTCATGGCCCAGCAGCAAGTTCAGG
CGGACCCGGTTTAAGATTGAAGACCTGGACTGCCAAGCCTGCAAACCTCACTGTTTGTGG
CCCTGACACAGAGTTGAAAGTCAGCAGGATCTCGTCACTGGCTGCGTCCAGAACAGTCTT
TGCATCGACACAGACGTAGTGCCACTTGTCCGGCCCCAGACCATCGACCCAGTCGTTGTG
CACGACACTCTGTACTTTGATCCCGAAATCTGCAGATCTGGTTGGTACCCGCTCTACGAA
TTTTATCCAGACAGACACACGGATCCATCGCCCTTCTGGCACGCGCCCTTGGTGCCAGCC
ACACCATTGCCAAGGTTCTCCATCGCCCGACCTCTTGGTCCACTCGATGGAATATGAGCT
GCAGGCGTGCACATCGAGGCCCGTGGGGAAGTCGTCGGTGCAGAACGTCATCCTACCTCT
TCCTGCAACAGCTGCCAGGCCTCTCATACGGAAGCCCATCATGTACACGCTCGAGTGCCC
CAAGTCCCAAATGCGAACAGGCCAAGGCGGATGGCCTATGACAAGCTTGAGCAGGTCATA
CCAGTCGCGGCACACAGCCGCCACTGCTGCCGGAGATGTTGGCGGGAGAGCCCTGAGTGC
CAATACAAACACTTCGAGTTGAAACGACCTGCCACCATGCTCCAGGAGCATCTGCCCCTT
TCTCGCCGGAGCGCAAACGCTTACCATCTTCAAGAGCATCGTAGACCAAGTGCAGCCTGT
GACCCACAGCAGAGGTCCATGTTTGTGGTGGAACCCATTCAGAGAAGCCCATGCAGCGCT
CAGGTCATGCTGAGTCATGACTGATGCAGCTTCGTAACATTGGTTTGCAACGATACCAAG
GCACAACGTCCTATACCAAACGGGGCGCGTGCCCTTGAGCCAAAAATGGCTACGG
>at03375
GTAACGACTTCATGAATGGCAAAGCAGGTGCGCAGTCGCTGAAGGAGTGGCTCACGCCGT
TCCTCAAGGACCTCCACTCGGTCGTGGAAGAGGCAGTGAGGAGCGCACCCGCCATCCGCA
TCAACGCCGTCTCGACGGATTTTCTGGGCATCAGCAAGCCTCTGGATCTTGCGCTGAGGT
TGAATGGCCTGGGCTCCGCTCATGCGAAGCAAGCCAAGAGCTCCTCGTTTCTAAGCTGGC
TTACCCTTTGCTGCGGCCGTTGAGCCATCCATCCAACCATACAGCCAATTCAATCCACTT
TTGTGTGCAGACTTGTGTGTAGACCTCTCGCCTTTCCACAAATCGCCTGCGCCGCTGCTC
GAGTTTTTTCCCTGGGATCATCACTCGTTCAATCGCGTTCTTTTGATCTGCCTGGCCCAG
TGCTGACTGCGCTGAGTGACA
>at03376
TTTTTTTTTTTTTTTTTTGGTCTGCGTCACACCCTTACAATGTCAGTGCAACACTGTCTC
ACACACCGTGCTTGGATTTCTGCATCTATGCACAAAACGTGCCATCTTGAACGAACCACC
AAATCCCTACACAAAAGCAGCTGGGCCAAGCTAGAATATTGGTATTGGTCTGGACAGCGC
AACATGCCTAAGTCTACTGCGCATCAGCTTCCACCAAGTGGTCTTCAGTTGATCTTCAAC

AGCTCAACCTCAAACACGAGGTCGGCATTGGGTGGGATCACACCACCTGCGCCCCTCTCG
CCATAGCCAAGCTCCGAAGGTATGTGGAGAGTTGCCTTCTCACCCAACGACATCTTCATA
ACTCCCTCATCCCATCCTTGGATCACCTGCCCAACACCGATCTGGAATGAGAACGGCTGG
CCGCGGTCGCGAGAGCTGTCAAATTTCGCACCTGTCGAGGCCAAGGTGCCAGTATAATGC
ATGGTGAGCTGGTCGCCCGCACTTGGGTATGTCTTCCCATCGCCGGGGGAGATTGTCTTC
ACTACGACCTGTGGGGCAAGCTCGCATGACACGAGCGTGACCGTGAAGTTGAGAACCTTC
CCTGCCAGCTCGTGGTTCAAGTCGACTGTCGCCGTGTCGCCTTCAACTTTGGTGACCAAG
GCCTTCCCACCAGCCGAGGTTCGAAGGGCATCGCCAACCTTGACATTGGCAGGGAGGCGG
TCCAAGGGTACCTCCTGCACGCCACGAGGGTCATGATCACCATACGCATCAGCTGGCTCG
AGGCGCAGCTGCTTCGTCTCGCCCACCTTCATGCCCACAACGCCCTTGTCAAAGCCAGGT
ATCATCTGCCCCCACCCCACCACGTGAAAGCAAGTGGCTGCTTACCCTCTGAGGAGTCGA
AGACGCTTCCATCTTCCAGCTTGCCAACATAGTCGATCTTCACTCTGAAACCGCTGGCTG
CCACCGGTGCGCTCTCGGCTGAGCAGAACCGCTGGGAAAGGACGGCGGTAGGTGCCGCGG
CTCCGACGGTCGCCGACAAGACTGCAATGCGGCGATTCGAGGTCGCAAGTGATCTCATGG
CAGCAGAAGAGGTAGCAGCCAGGGGCACGCCTTTGATCGCGCGGCTTGCTGCGCGCACCC
CTGGACGTGTAAGCATAGACATCATGGGCAGTCGGCTCCAAAACCGCCGCAATGTCGTGA
GGCAAAATTGCTTGAGCCAAAATGGCTACGG
>at03377
GCGCTGGCAGCTTCAGGGGGTATGGCTTCGTACTCTTTGAGGACAGGAGCGTGGCTGAGA
GAGTCTTCCAGACCAAAGGCCACGAGTCAATGGCAAGGCCATTGATTGCCGTCGTCCGTT
GGAGTCTGGCGGACCCAGCGACAGCAACGACAACAAAGTGTTCGTCGGTGGCCTGCCGAA
GACGTGTACGAAGCAGAGTGTTGAGGCGTACTTTGAGGCCAGGTTCGGCAAGGTGGCGAG
CCTGCAGCTCAAGTACGACTCGGATGA
>at03378
GCCAAACTTCAGCACCTGCGACGCCAGAGCGAGCTCAATGAACTGCCTCTGGTAGGGCTG
CAAACTCTGCCTCAAGGAAGCCTCGCCAGCCGTGCTCTCCTCCCCATGCAGCTTCACCAC
CTCTGCGCGCACGGCGTTCATGGCGTCCCGGAACTCCTTGGCAGCCCTCCCAGCGTCCAC
CGCCGCGGAGATGCCGCGCGAGATCGGCAGCAGCAGGCCGTAGCCGTCGCCCGTGATGGC
GGCTCGGCAGCAGGCCTCCAAGTCGCCGCCCTGTGCGCCCACGCCAGGAGCGAGTATCCA
GCCCGATGGGTTCACCCGGCGCACGGCCTTGATCGCAGCAATGTCGGTGGCTCCGACGAC
GAGGCCAACATTGCTGTGCTGGTTCCAGTCCTGACCGGGCACCGTGAGCATTGCTACTCT
CTCAAAGACACACCGGTGTTCGCCCACAATCTTGAGCATTTGTATCTGCGATGAAGATGG
GTTCGAAGTCTTGCAAAGGACAAAAAGCCCCCTTTGTTGCGCCATACTTGCCGGCAGGAT
CTGCAAATGGCGCAAGCGAATCCCAGCCCATGTACGGAGAGAGCGTGATTGCGTCAGCTT
CAATCTCATCGAAGGCCGACGCAGCGTATGCATCAGAGGTCGTCCCGATGTCTCCACGCT
TGGCATCTAACAGGACTAAGGCACCTGTCGCCTTGGCAGCCTTCACGACTCGCTTCAGCA
CAGCAATGCCCTCCT
>at03379
TTTTTTTTTTTTTTTTTTCCTCACAATAGGTACGCCAGTTTGCTGCGTGCCGGTGCCTTC
GGGGGAACAACGACATCGCCACCAGAATCCTCAACAGGCGGCGTGCCTAGGGCCTCCGGA
GTCGCACTCTCGCTAGCAGCTGGCATTGTGCTCTCAACTTGTCCCACTGGAGGAGCAGCC
TCGCTTGCCCCAGCAACCTGTTGCGCAGGGTCACTTGGCTGCGCTATGGGCTGTCCACTG
GTGGAGGCCGTTGCAGGAGCATCATTCGTCGCATTTGTCGCATCAATGTTTTGAAGAGCT
GCTGACAAAGCACCATCACAAGACGGCTTTTCATCAGTGGCAGGGCTTTCAACCGGCTGC
CCAACAGGTTGTTGCAGTTGTGCTGCATGCAGTGGTGAAGCCGCTGGACTGCCAGCAACC
TGGTCTGCTTCTTCCTTTGATGGCGTAGCGGTCACAGCAGCAGGTGGCCCTGAATGTCGT
GGGGGTCCCAACAGCCAACAACGCACCTTCCTCTCCATGGAGCTCCTGGAGAAACCAACG
GCTTGACAACCTCTCTGTACAGTTCTTCAAGCTCCATCTGCTCCCCTCCTCGGCGGAGCT
GCATCACGTGCCTCACCTCGTTGTGATCACCCCTGCAGAGATGAATGCTGAGCCTTGCAC
GCGACTCCTCACCCCANGCCTGCCACGCGCCCACGCCCTCGTCCTTCGTCCAAACACACC
ACAGGTCGAACTCG
>at03380
GTTTGTGGGCGCCTCCGTGCGGACGCCCGTTACCTTTGCCATGATCTTGATTGCCATCTT
CATTGCCTCGAGCTCCCTGGCGCGAGTCTCGGACCTCCTTGCCCACTCGGACTTCTTGAC
GGTGCACGCATTCTCAGTCTGCGTGAGGGTGGCCGAGTCCGCCTGTAGTTCGTCCCGCGT

GCTGTTCAAGGAGCCTTCCGCTTCATTCAGTGCAGTCCTCGCGTCGCCCCGAGCGGTGTC
CTTGGCAGTCTTAGAGTTGGTGGCCTTGTTAGTGAGGTCCTGGCGAGCCGCCCTCGCGAG
CTCGTAGGCGTTCAGAGAAGCCGTCTCGCCCTTTGTCGCCGCATTCTTCTCGTCCTCAAA
CTTCAGTTTCAGCTCCTTCAGCATCTCGATCACGCTGTTGGACTTGAAGGCATACTTCTT
GACGTGTGCA
>at03381
GCAAACGCATCCTCAGCGTTGTGCTTGATGACCATACCCGTGATGATTGGCAGAGAAGTG
GTGTGTGCGCATTGTGTATTGATGACAACAGTGCGCGTCGCCATTGTTCTGTGAAGGCGT
TGGGCCTTCCTTGTGTTGTGGTTTTTGATTGCATGCATGCAGACCTCTGAGAAATGGTGT
GTGCAATCGGCAAGTCTTCAGTCATATGCGGTCATGGAGCTGAAGATCTGGCAAGCTCTT
TGAGCCGGTGATCCAGCCTTTTGAAGATCTGCCCATGAGTTGTAATGAAGTCATTGCCCC
AGCTCCTTTGCAGAATGGGCCTCAGAGCTTGCCATCACATTTCCTTCCTGCAGCCTGTGA
GAAGCCGGCCACTCCAGTGAAGAAGAAGACTGGGGATGTGGAAGATTAGCGCACTGGCAA
GTAGGACTAAGACGAAGTCTTGCAGTCGTGCTCGTGGAAACACTGGGGGCTGCTGCATGG
GGTTGCAGACAGCAGCTATCTGACTTATTGTAAGCGCTTCTCGGGCCAACGCGGATTAAG
CACTTTTATCTGTGCGGCGAAGGCATTGCGCTGGAGTAGACAAAAAAAAAAAAGAAAAAA
AAA
>at03382
GAGTGGAAGAACTTAATCGCCATCCTGTAGTTCTTAATTAAGTAAACATTCGCGATATGT
GCGGAAATAGTGGTCAAAGCAGCGGCTCAACTCAGCCCAGCACTGCCATGCGGACAGGAG
CACCCATGGCAGCGCAACACCCTCAACCACTGCACACACCATCACCATCACCAGTAGCCC
GATACGACAGCACCTCGCGACGTGCATGACACACGAGGAATCACCGAGCAGCACACCAGA
GCCAACCTAGCACACATCAGAGGAGTCGCCACC
>at03383
TTGTTTTTTTTTCTTTTTTTTTGGGCAGCGATTCCATCCACTCATAGGTGCAATCAGGTA
TCCTTGGAGTTCAGCATATTCAAACATGTCAACACCCTCGTGGACACTGGGAGGGAGGAA
TGAATGCATGTCCGTTGCTTCGGGCAAGCTCCTCGCATCCTTGCACGCGGCATCATTCCA
TAGTTGAGCATCTAATAAATAAAGCAAGCCTGTCACGACCATGCATGAACCAGGTGGCCA
ATGGCACTCCATGCCATCAGTCATCTGAGCTTCTACACGCCAAGACAGCCAAGTTTCATT
CTGTGACGGCCGAAGCACGTCACATCCCTGCAGCGGCTCAGCAGATGTACGCACTGAGAT
GCGGAGTGCACTGACTGTCTCGCCCTTCGCTTTGGAGCAAATGAACTCGCACCTGTAATC
GTCTAGAGCTCCTCTAATCCAGCGGCCTCGTCAAAGCCCAACGCCAAGTTGAGGTTCTGC
ACGCAGGCACCGCTTGCGCCCTTCCCCAGATTGTCAAGCCGTGCGAGAAGCCAGAGAGTG
CCCTTGGCTTCGTTGCCGAAGCAGAAGATCTCCAGGTGATTCGTGTTGTTCAGCAGGTCG
GGGCGCAGGAAGGCCCCACGTTCAAGCGAATCTGTTGGGTTCAGCGGTGCCACCGAGACG
AAGCGCTTGCCCTCGTAGTACTTGGCAAGGGCGTCGTGCACGGCGGCAAGCGTGGTGCCT
TCCGCGAGCTGCGACATCCTCAGAGGCACGGACACGACCATGCCCTGCTTGAAGTCACCG
ACAGAGGGGCAGAAGATCGGTTCCTGCTCAAGGCCTGTCCACCGGGCCATCTCCGGCAGG
TGCTTGTGGTTCAGGTTGAAGCCGTAAGCACCCCAGGGCTCGTGCCCAGGGCTTTCGTAG
AGCTCGATGAGCCCCTTGCCGCCGCCTGAGTAGCCGCTCACTGCGTGCACCATGAGGTCG
GCGCTACTGGGCAGCAAGCCAGCGTCCACCAATGGGCCGGACCATGCTGATGAAACCTGT
GGGGTAGCAGCCCGGGTTGGCGATGCGCTTGGAGGCAGCAATCTCATCAGCGTGGCCCGG
GGCTAGCTCCGGGAAGCCGTAGATCCAACCTTCGCTGACACGATGTGCTGTGCTCGCATC
AACGACAACAGTGTCAGCTCCCTCGAGGAGAGCCACCGCAGCTCGAGCGGCGTCATCTGG
TAGGCAGAGGACGGCTGCATCTGCAGACCGTAGCGCATCGCGCCTTGCCGCCTCGTCTTT
TCGCAGGCTGGGCTCCAGGCTCAGAAGCTCGATCTTTGGGTGCTTTTCAAGACGCTCCCG
CACTTGCAGTCCTGTGGTCCCAGCCTCGCCGTCGATGAAGACGACGGCCTGCCGCACGAC
GCAGCGAGCGCCCTTCTTCTGCCGGGTGCTTCCACGCAAGAATGCAGCCGCAGTGGCCGC
AGCAGCGGAGCTCAGAGCCACTGTTGCGTGCGCATAACCATACGTGGATCTGCTGCGCCC
GAGAGGTGCTGTGGCCTCCTGTTGCTTGGGAACGGATGGGGCCCTTGCTTGAGTTCCCAG
ACCTGTGAGTGGGATTGAAAATGTTGACAGCATGGCTATTCCCTGAAGGCCTCTGCAGGA
GTTGGAGCCGGAAAAACGTACTTGAGCCAAAATGGCTACGG
>at03384
CGAAGCAGCTGGCCAAGCCAACAAGGAAGCTTGGACAAGATGGAGGACCTCTCCGAGCAG
GTCGCCGAGGACCGGAAGCTGCTGCAGGTCCAGCAGGCCCTGCAGAGGATGCTGGAGAGC

GACTTCGAGGTCTGTAATTGGTCGCTGACGGTTGAGATTCCCGTGGAACAGCCAGAGATC
TGAGCACCGGTTGCACGGAATTTGTCGCAGTTGTTTAGAGTTAGGATTTTCGTCCACTGA
TAGACATGAAGGAAGCGACCAATGACTGTGCCAGCCCCAGCAATGCTGTACATTGTTACG
TGATACGTTTCGTAGCGCTGGCATCGTATTACGTTTGAGGAAG
>at03385
TTTTTTTTTTTTTTTTTAAGCATCGTTTGGGCCCAACAATCTCCGGTCAGCTCGCAACC
CTGCCTCCAGCCTTGATTTGCCGCCTGCCTCGGACCGCGAAGCAGATGCCAGCGCTGGAG
CGCCATACTCCTGCAGCACTTGCTCCACCGCTCGCCTCCAAGCCGCTGCCTTCGGATCGC
GCAGCCGGGCAAAGCTGAAGGACACGAGTACCCGGAGGCCTGCAAGTGGCGATCCGTCGG
TGCCACCTGCTGCAAGGCCAAGCGAGAATGCCGCGGCTGAGAAAGGCTGCGTGCGGTGTG
CAAGCTTCCCATCGAAGACGGCAGCCTCGTTCACCCGGTAGCGGTGCAGGAACGTCTCGC
CGTCGGCGTCCCAGGGCTGGTACTCCAAGTTGCCCTCGTGCTCGGGGAACGACAGCGGAT
GCAAGGGCGCCAGAGCCGTGGCCGTGGCGAAGCGTGGGATGCGAGCCCGCCTCGTGCC
>at03386
CCATTTGCGGACAATCATCAGAGATGGCAACCTCCAGCACCATAGCCCCTCACAGTGTGA
GTGCCGTCACAAAGGGGCGGGGTCTACGTGTTGCAATCATAGCAAAAGGGAGTCGTGGTG
ACGTGCAGCCAGTTGTTGCGCTGGCTTTGAGGCTGCAGGAGAGAGGAGTCCATGTGCGTG
TCTTCACCAACAGTGGGACTGAGGGTTTTGTTCGCAGCTTCTCGGTTGAAGCAGAGGACT
ATCCGTGGGAGGCAGATCCTGCGCGGGATGTCAGAGCAAGGACGCAAGAAGAAGTCCTAG
AGGTTGTTTGTCCAAGCTGGGCCACTGGTGAGGAGCCTCGCGCGATATACAGAAAGGTCA
AGCAGTTCATGCCGTGTCTAGTGCTCTACAAGAGATTCAATGTGTTGGAGG
>at03387
TTTTTTTTTTTTTTTTTAACAATCACAACAAAGGATGCACTTCACTCCGACAAGAATGC
CGCACGTCAGCGCGGCGCAAGTCCTGTGCAAGGACCCGAGACTCTGCGCAAAAGCTGCCA
AAGCGACCCTCTGCGCATGCCGCCCTCCGCACGCCGCGTCTCCTCATGCGGGCTACTCCC
TCGCCTGCTGCACTCCTCTGCCTGCGGAGCGCCGCAGCGGTGGCCCTGCGCAGAGCCGGA
CGCAGCCCCCCCCACCGCACTCAGCTCCAGACGGCCCCCGCCTGCCCACTTCACGCGCTC
AGTCCTCCATGTCATCGGAGCGCTTGGGCGGCTTGCCGCAGGGCTGGATCATGGCCTGGA
GGCAGTTGAATCGCACACGTGCTACAGGCTCGTTCTCACACAGAGCGAAGTACGCGAGGC
GTGTGTAGTGGCCGACGTGGGACGCCATGGTGTCTCTGTGGATGTTGGTGGCCCATTCCC
ACTTGGAGGTGTCAGCTCCCCCCGGTGCCGTGGTACTTTGTCTGCAGGTGCTCCATCTGG
GCGTGGACGTGGAAGCGGTCGTACACGCCGAGGTCAACAGTCTGGCCGGGAGCTGCCGCC
ATGGCTGCCGGAGGGTAGCAGGCCCAGAGGCGGTGCCGCACGGACCCGGCAGGAGTTCCC
CAAAATGGGAGGGCGGAGATGCAGGGGGAGGGC
>at03388
CCGTGGCCATTTTGGCTCAAGTTTCAAATTTCTAGGGCCGGCGTTCTGCGCGGGCCGGGC
CCCTCCCACAGATAGCCGCCCGGCTCTTCCCCCCCTCGGGGAGCGCCCCCACCGGCTGCC
ATGATCAAGCGGGCGCTCGGCCTCTGGCTGGACGGCAAGCAGGACAGCGACGACGACAGC
AGCAATGCCGAGGAGGTCACCGAGGTCGAACGCCAGAAGCTGTACAGCCGGATGTTGCCG
GGTGCATCCGAGGGATCCAAAAAGAGGTGGACGCGGCCGCGGGCGCGGCCGAGGCCGCGT
CGCCGCGCGAGCTGACGGCTGTCCGGTGGACAGCGACGACAGCAGCGACGCCGAGG
>at03389
TCGACTTTTTCAGCGGTCACCTAACGGCAGCGATCTCGCATGTCGCACTAATGAGCTCCT
CCGCAGGGAAGGGGACCACCGTCCTCCTCTTCCTCCTTTGCCGTGTCGGGTAATGCCGCC
AAGACGTCTCGGGCTGCATGACGGACATCTTCGTCAAAGTGCCCAAGCAAGGGCCCGATC
AGGATGCTGGCATGATGGGTCACCACCTGGGCAAGGGCAGCACCGGCAGCATCACGCACC
AGCCAGTTGCTGTCCTCTAGCCTACCTTGCCAACATGGAGATCACTGTCTCGTCACCTCG
CGCTGTGATATGCCCCAGAGCCTCGCAAGCCCCTTGTCTGGAAATCTCGTCGCAGTCCGA
CAGGTG
>at03390
TTTTTTTTTTTTTTTTTGCTGGAGGCAATGATGACCGCAATACTCGTGATGAGGAGCAT
CACATCACACCAGCCGGTCTGAAGCACCTCTCGAAATAGGTTACTTTGGTGGGGCTGCAG
TAGTGGAAAATGCTGAATTCCCATCACACGTCTGAGAACGCGAGTGAATGGCAACTTGGA
GAGAACGCCTGTGGCATGGACAAGTCACGGTGCACCTGTCTGCAAAATCGCCTACAAACA
AGCAAGGCATGCACGCTTTCCATGCGGGCATCTTCCGTTGCGATCGGGGCACACCAGGTG

TGTAGTCCGGAGACCTATGGAAAACAGAAAAAAACCTAGTCAACAGGTTCAATGAGGTCT
CGGATGTCCTTCCCGCAACCCAGGAAGTAGCCTCCATCATAGATGGTGTTCTCCCCAACT
GGCAACTCAGCAACATTGGAGAAGAAATCGCTCTCGGCCCTGTTGACGCGCATCATGATG
ATGTTGAAGACGGTCTCAAGATCAATCTGCTTAGCTTCACACTCCTCCTTACTTGCTCTC
TCCCCTCTCGTGTCCATCCATTCCTTGATCATTTTGATCCTCGTGACATCCACGTTGTCC
TTCTCGACCTGCGAGACCTTGCCTTGAACGTCATCCCAGCTCACCCTCACGCGGTTGATG
TAGTTTCTCAG
>at03391
GAAACTCCTCCACTCGATGAACCATCATTGGGACAAGCTTCTCCATGAGCTCACTGTCGA
ATGCCCGGACACGTCCAAAGGCTGTTGCCAATCGCTTCATGTCCACAGCGGCCATTCGGG
AGTCTCTGGAATCACCATCTTGAATGCATTCCAGAATGTGAGACGAGAATCGCGAAAAGA
GTGCAAAGTCTTGCACTCTTGCAGTGGAGAATGCGGCAGCAAGCCGCACGGAATCTGTAG
CCGACAACCCTGTCAGGAGCTCTGAAGCTCGAGCTGCGTAAGTCTGCCACAAGTCTCGCT
CCAGGTCACCTTCAGCAAGGGAGCGACGTGTTGCACGGCTGAGAGCGGCACAGTCCATCG
ACGCAACTTGCAATGCGGAACGAAGTGCGCGCCCAATTTGCATCATGGAGT
>at03392
CCCATCTTCTGCTCATCCTGGGCTTTTGGttAGtAAGGCTTCCTTGGCAGCGTTGAGCGC
ACCAACACTCCCCTCCAGCGCTTTCTCGGCGGCATCCTCTGCCCGGGTCTTCTCCTCAAC
CACTGCCTTCATGCTGGCCTCCTCACCCTCACGCCCTGCCTTATCCGCTTGCACCTTGCT
AACCCCCTCATCTGCAACTTTCAGCTGCTCCTGGATGTTGGCCTCGGAGCTCTTCAAGAC
CTCAGCCATCATCTCGACCATCCGTGCCTGGAAAGCATGCCGCTCATCTTTGGCAATGCT
AAGGCATTGATGCAGGGTGTCAGTCAGCATCGCCAGCACCTC
>at03393
TACGCAGGACAGCCGTACCAAGGGAGTCATGGTGTGCCTCATGGTCAATGCTGCCCATGG
CCTGAGCACACCGTGGATCGTTCCGCCGGGGCTCAAGATGCATGCCGGCACCTACCAGCA
GGTCCTAGAGGAGGTGTTGCTGCCAGAGGTTCTGGCAAAGTCGGGGCAGGACTCCTACAT
TGTCCACGACAACGCTCCCTCGCACGCGGCTGCGAGCACGCAGCGCTACCTCGCGGACGT
TGCGCAGGCGCACAGGATCTTCTGCGTGGCGCAGCCGCCGGCGAGCCCCGACCTGCAGCC
ACTGGACTACGCGTTCTGGGCAGCCTTGCAGCGGCGCGTCAAGGACGTCGAGAGCATCCC
TGCACTGATTGCGGAAATCAAGAAGGCCGTCAATGAGATAAATTCTGAGTGGG
>at03394
CCGTAGCCATTTTGGCTCAAGTGGCCACGTTGCCTCGCCTTTTGCGACTCCTTTTGCAGG
CTTGGCATGCATATGTGGCAGCTGGCCTCCTTGCTTCTGAGTGGGCTGTGTGCCGGCAGC
ACAGGCAATGCTGCTGCGGAGTTGAACTGTAGTCGAGCAGTGGTCGGATTGGAATCGACC
GAGCCTGGACGTCCTGCACCAGAAATGCTTTCCACGTGCGGTGTGCTGCTGCTACGAGCC
ACGCTGGATTTGCGAGTGGTGGAACAGCTGCTGGAAAACTTTCTGCAGCTGCCTGCAGAG
ACGCTAGAGGCTCTGCATCATGGAAAAGTGCGCGAGGGACGGCACCAGACGCACCTCCCA
TTCTTG
>at03395
TCGAAGCAAGCGCACCTGTACAGGAAAGGACTCTGGTGGAAGAGCGACGTCCTTTGCAGA
AAGCAGCTCAATCGGCTTCCCGGAGCAGTCATTGGCCCAGATACACACTACCTCGTCGGA
CTCTGGCAATCGGCTTGCCACTGACTGGAGCAACTCGTCGAGCGAGTTGCAACCATCCAC
GGTGAGTGAAGTGCTGGGTTGCTCTCTGGATTCGCAGGCCTGCTGCAAGTCCTCAGACGC
CGCTGCCCGACTGCCTTCATTTCCAAGCATCGCGCCTGCCCAGCAAGCAGCAATACCAGC
CCCCATTGGAGCCCAAGCGGGAATGCGGAGCGGGCCACCTGCGACGCCACCCTTGCCGCT
ATCATTCTCGCAAGACGTGGGGCTTGCAGACCGAGGGGATGTGCCTTTGAGCTTCAACTG
CTTCTTGAATGACGAGCGCATGAACAGAGCCTGTAGATCCCTGTCGCACCTCTCTGCGAT
GTCGAGGCACGAGCTGCCGCCCGCGGTGCGCAAGGCCAGGTCAGCGCCACGGTCCAACAA
GAAGCTGATGGCCTCTGTGCCTCGTGCC
>at03396
GGAGCGGCATTCTGCGGCAGAAGGCAGTAGGCCAAGTTCAAGGATTCTTCCTTGACCAAC
TTGGATTCCTGCTCAAAACGATCGTGGAGGAGCTCAACAATCCAGCGTGCTTTGCCGGTG
TCAAAGCCCAGTATCTTGACTGGTTCGTTTGCATATCTTGTGTCCTGAACGTTGAGGTAT
GCCACGGAGGACCGGTGGAAAGATGCTTTCAAGCCCTGCAACATGCATTGACTCCTCGAG
CAGACCAGAATCGGTCTCCTGAGAGAACCTTAGAGTTGTCAACAAGGTATGGCCCCGGTC

AAAGTCAGTGTCAGAACATGGCCAAGAGGATCGTCAGAGCGGGTTCAATGCACACAGGTC
TGGTGGAATGAGCAACGCTGCAGTTGGAAATGCCCGCGACCAATCACAAAA
>at03397
TTTTTTTTTTTTTTTTCTCGAGCACCGTGACTTGCTTCCTGCAGCACGCATGCTCTCAGC
GCCATCATTACCTGGCATTGCTGCCTCAGACTTCCTCTACTTCCTCCATGCATCCGCATC
CGCACATGTATCCAGCACACCTCGCCGTTTCCTCCATCTTGGCCTCGACACTTCACCGGT
CCATGTAAGTCTGAGCATCCAGCCTTTGCACGCCAGGTGCATCCAGTGGTGCCGCTCGCG
GACACCAGCACACCCGATACCACGCGCGCCTGAAGCAAGCAGCTCGAGCGGCTCAATCAT
ACGGGGCCCACGCAGGGGCTCCCTTCAAGCGCCCTCTGGCCAGCCAGGTCTCAGCCGCTT
CTGCGCTGGCCCGCTCATGAAGGTGCCGTCTTGGGCGACTCGCTTCAGGTCGTGCTCGAC
CCGTATCTGCTGGGCCCGTTCCTTGCCACTCTTCATGTCAACCCCAGGAGTGAAGCTCAG
GTGCGTTCCCAGCGGAGGCTCTGCGCCGTCAGGGAACTGCTCAGCAAACTCTGACTTGTG
CGCAAAATACCTCTTTCCCGGTGGATTGCTGCATTCTATGAAGCCACAAGCACTCTTGGC
CTTCCAGTCTGTCAATGTGCCAAACAATCGCGGAGGCCCAAGTGAATCCTGGACGGCAGA
GTTGTCTTCAGCTCGAATATCCTGCGCGCGCTCTTTGCCGCTCCTGGCATCCACTCCCAG
AGTGAAGCTAAACAGAGTCCCAGGAGGCGGTTCGTTTGCATCGGCAAACTGATCTGAAAA
CTCGGACTTGTGAG
>at03398
TTTTTTTTTTTTTTTTTCGCGCGCGCCCGCTCGGAAGCCTCCAGCCGCGCCACTCCGGC
CCTCTCCCAATCCGCGGCCGCGGCGGCCCCGCCGCCCCGCCGCGGCCGCCCGCCCCGCCC
CGCGGGAGCCGCCGCGCGGCTCGAGGGGGGGAGGGGCCGGAAGATCCGCCCGGGGGCCGG
AGCGGCAGCGGCCTGCTTCCGCGCGGTGCCGCCTATGATCCTGATAAGCGAGGAGGCCGC
ACGACGCTGCACCCCTCCCCAAGGCCCCTGCTCCGGCTAGTGGCCCATGGCCGCGCCCGT
CTGGAGGCAGCCGTCCAGGAGCGAGGGGCTGTAGCCCAGTTCCCGCTCCCAGTCGTCGCT
GCAGTCCTGGAGCTCGACGACGCCCATGGCCTGGTCCGTGTGCAGGGCCTTGTTGGAGCA
GCAGACGCGGTACCGCGGCCCGTAGCCCAAGCCACCGATCCGGAAGCCCATGTAGAAGTT
GGGCACGGGGACGGGCCTGAACTTGTGTCCTGTGATCGGCCCTTCCTTCTTGACTCCCTC
ATCTGCCACCAGACCGTTGTCCTTCAGGAAAGTCATGCACGATTCCGGGTCCTTGGAGAT
CACCTTATAGATGGTTGCCTCCATGTCAGTTTTCGTGAGCGCGCCCCAGTGCTTGTTCAG
CTTCTTGTTTGTTTCCTGGAACTTCTCCACGTCCCTGGCCGTAAGGTCCCGCGCGAGCTC
ATTCAAGAGAGCCAGTGCATACTTCCTCGTGGGAGCCTTGTCGAGCTTGCCGCGGGCGAC
CCAGAGATCGGTGAGCG
>at03399
TTTTTTTTTTTTTTTTTCATAGGCAAACTGGACATGGCCAGCTCCCCCCCCTGCCAGGC
AGCAGGCGGCGCTCACCCGAGGGGCACCACGCCGCCGCCCCCGCACGGCTCCAGCAGCGA
GTCCACGTCGCCGGGGCCCAGGCGCTGGAAGCTCTCCAGGTTCACGCTGGAGCCCAGGTG
GATCCGCAGGATGGCCCCCGCGAGCAGCCTGGAGATGTCCACGACCTTCAGGCGATCTCC
CATGCCCAGGGAGGCCAGCCTCTGCAGGGCCCCCGCCTGCGAGATGCTGTTGGTCACCGC
GATCCACTGGAGGCGGCAGGTCTCCACCATCTCCTTCACACGCAGGTGCGCATCTGCAGA
GAAGTAGCCATGCGTCGCGACGCCGTAGATCTTTGAGTCAGGACACAGCTTGTGCACGGC
TCTTCCGACTTCGGCTATCCGGCTGCAGGTGTCGCAGATGTCGTCCACAATGATGATGTC
CTTGACGTGGTCGGCCTTGCCGCAGATGATCTTGATCTCCCCGACGCCTCCGTCCTTGGG
ACGCATGTGGTCCGCCATCATGAAACCCGCCCCCAGCCTCGTGCCGAATTC
>at03400
TTTTTTTTTTTTTTTTTGCGTCACACACGGATGAATGTATGCATCTGGCAAGTTCCCTC
AAGCTTACATGGTGCTGATCATGAGAAGCTCACTAAACATGAAGGATGCCACTTGCCGCC
GAATGGGGCGGGGCAAAAATCGGCCCATCCCTACAATAAGTCTGGCTCGCATCCCGTAAG
CGTCGGCCTCTTCAAGGACAGACTTCCCATCGTGATTGACCATCTTAGTAGCGGCGCCCA
TGTCAGGGACCTCAAGAAGCAGTATCCACAACCCGCCCAGGTCGTTCGCACGGGCACAGA
GGTGCATGGGCGTGTCACCGTGTCGGTCTTGAAGTGTAGGGTCAGCGCCAAAGTCGAGCA
GCAGCTCCAGCATCTCGTAGCGCTTGGCCTTGAAGCCAGGGTTCTTCAAGGGCCTGCTCA
GGCGGCGGAGGGCCACGTGCAGAGGCGACTCCAAGTTAGACTGTGCCGCCCCCATCTCGG
CAGGGGCTGTGGCCTCAATGGTAAGCAGATTCTCACGTCCCTCGCCGCCTTTCATGCTGA
GGAGGTAGTGCACAATGTCTAAGTGTCCACAGGCAACTGCTCTGTGCACCAGTGCATAAC
CGTAGGCATCGTAGCAGCCCAGGTCGGTCCGGACATCTTGCACCAATCTGCGGACGACCT

CGAGGTGTCCTTCAGAACAGGCCAGCGCCACACAGGACGCACCTTGCTCGTCCCTCATTG
AAGGGTTTGCACCAAATCGCAACAAAGCTGACATCACCTGTAAGCTTCCAAATCTGCAAG
CAACCGCCAGCGCTGTTTCCTTTTGTTCGGTAATGGGTGCGTCAATTCCAGTACGCAAAG
CACGTAGCAAATTGAAAGCTGTCCGTACCTTATCCATGCGGATAGCATAGACAAGATCTG
CATGCGTTTGGTCTGTGCTCTGATTGGGGCCAACTTCAACGTTTCGCGCTGCATCGGCTG
AATACCACGTTCTCAGTGGTGCGTCTGCCTTTGCAGCCCCAGAGACGGTGCTCTCACCGG
TGCTCGAGTCACAGCGGGTAAGCGGAGGTACAATCATGGCCGCAGCTGCAACCAGAGCGG
CTGAAGCCAACTGCCATTGGCCTGGCGAACGCCTCTTCAGATCATCGCAGCTCCTCCTTA
CAGAGGCGAGGTACTCAGACAGTGATCTTTGATACCAGCGATGACGAGAGTACTTGGGAC
CAGTGCCAACATGTTTCCAACATTGAGGCAGTTGCCCAAAGCCAAATAGTAAACGCAGAT
CGCCTTCTACTCTGGATCGCGCCAGTATACGGGACGCCATGGAACAGGCCAGCAACCGCC
CACACCTGTCCTTTCCAACCACTACCTTGAGCCAAAATGGCTACGG
>at03401
TTTTTTTTTTTTTTTTTTCAAGCGATCGTCCGAGATGTTGCAGACACGCCAGCCTGAATT
GGAATGCTTGCTCTCAGCTTTGCTGAAACCTCAAGCTGCGTTGGCCTGAACCCATCGTTT
CAGCACACCAACCCATCGCTCTCCCACAGCCTGGATCTGGACAGGCACATTCTGCTCCAC
CATTTTCTTGAGCCAGATCGAGGAGACCCAATCGAGAGCACAGGTGCGGACCATGCGTAT
CTGGTCTGATCCTTCGCTGCAGGTGAACGTGTCGCAGACCACAACGTTGAAGCTTGTACC
ATACGGCACATCGATGGACATGGTGACCGACTCCAAGACAACCTTGGATTTGCTCACAGA
TACGTGCCAAGTGCTCTGGCACCGAGTCTCCTTCGGGCACATGGGTGCTGGTGGCACCGG
TATCCTGAAATCAATGGACCGCGCCTTGCCATGCACCCCTGCCAAGCTCGTCCACTCATG
CACATCCAGGTCTTCAGCCTGCACCTCTTCAAAGTACATCGCCATGAGCTGCCCTGGGCT
TCTCCAATCCTCGGACTCCACGCTCGCGAGGTGGTGCT
>at03402
TGCTGCGCTTTCTCCATGATGCCAAGGATGTTGGCCGCCTTCTTCTCGTCGCCCCAGGTG
GAGAGGAAGGTGTAGGCGTCGCCCTTGTCCCCCGCGCGGCCGGTGCGGCCGATTCTGTGG
ACGTAGCTCTCTGCGTCGTTGGGCGCGTCGTAGTTCACTACGATGGNGATGTTCCGGATG
TCGAGGCCGCGCGCCGCCACGTCGGTGGCCACGAGCACGCGGACCTCGCCGCTGCGGAAG
CTGCTCAGGGCGGTGTCGCGCGCCCGCTGGTCCAGGTCGCCGTGGAGCTCCAGTGCGGAG
AGGCCCAGGcCNCCTGTCGTTCCCGAGCTCCCAGGCCAGGTCTCTGCACTTCCTCTTGGT
GTTGCAGAAGACGAGGCAGTTGCCGCTGTGGCCGAGGTAcCCCCAGGATCCTCTTCAGCG
CCTCATCCTTCTGCTTCTGGTCGGAGCAGACCTCGACGTGCTGCCGGATGtTTTGGTTCG
TCGTGATATCGTCCGAGCCAATcCTGGATCTGCACCGGGTCCCTGATGTACCTCTCCGCC
AGCTTCTTGCACTCCGGGCCACGTCGCCGTGAC
>at03403
TTTTTTTTTTTTTTTTTTCGAGCAAGCAATGAGAGGGTGTGCATGTTTGCAAGAACAGAT
CTCGCTGCCCACAGGATGTCGGTGATGGTCAAACCAGCGCAGTGCCCGCCGAACGCGGGC
GTTGTCAAGAGGTGGGACCGCAAAGAGAAGCCAGCGTGGCCTGCTCGGGCCGTCCCAGGG
CACCACATGCCAGTGCAGCTACATGACCTCCATGTAAACGGTGCCCTGGTTGCCAACGGT
GGTGACCCAACCAGTCATGCCATCGGACTTGACCTTGCACTTCATCCGCATGAGACCGGA
CTTGTCTTCCTTCCGAGCCCATTCTCTAACCTCCACAATCTCGCCCTCTTTCAGCTTCCT
GGTGGTGTCCTTCAACTTCCGCGTTGCATCCTTGGAGCCGCCACCATCCAGCTCAAAGGA
CTCCGTGAGGATGGTGTCCTTGACGACTTTGAACAAGCTACCACCGTCCTCAAGGAAGAC
CGTGCCCTGGTTGCCTGCCAGTGTGATCCAGCCCTCCAGCTCGTCTTTCATGACCTTCGC
CTTCACTCTCAACACATCCACAGTGTCCTCCTTTGTGGGGCCCTCCAGAATCTCTACAAC
CTCGCCCACTTCCAGCCTCCTCAAAGTCTTGCTCTCCTTGATGCTGATGCCAGTCGTGAT
GACTGTGTCCTTAGACACCTTCATGAAGGTCCGAATGACGTTCACGAACTTGTCCTTGTT
GATGGACTCCTCNACCTCATCATCCAAGCCAGCAAAACCTTG
>at03404
CCGTAGCCATTTTGGCTCAAGCACTTTCGGCCCCATTTTTGAGCTTCTCGCCCATTGTAC
ACAGCTCGACCCCCTGCCCTCCCACCCATGCACTCGCCCACAGCTCCCGCATCGGCAGCG
GGTGAGCCAGAGCCGGCGCCGGAGTCTCCCGCCTCCCAGGAGCGAGCCAAGCGCGACGCC
GCGGCAGCTGCGGCCCCCGCGCCGCCATCGAAGAGGCCGCGCTACGACGGCAAGGCCTTC
AGCGAGCTGACGATTGCGGACCTCGAGCGCATCGTGAGGGAGAACACGGAGCTGGAGGAA
>at03405

TTTTTTTTTTTTTTTTTGAGCAGGGGGGGGCCCTCCTGCCCGGGGGCGGCCGCGCCTCC
GCGGCGCGGTCCGAGCCTCGGGCGAGGCGCCGGGGCTCCTGCCCGAGGGCCCGGGCGCGG
GAGCGGGGAACCGCCGCCGGTCAAATGTTCACGCGGCGCTCCCCGCGCGGCGGCAGGGCC
TCGAGCCGCGCCAGCCGGGCGCTCGAGGCCTCCGTGCTCGAGGCGAGCTCCCGCACGTAC
CCCTGGATGTCCTGGACCAACGTCTGGTTGCTGTACGAAATCGTCCCGCCCTCTCCGTCC
CCGCCGCGGCCCAACGCGCGCAGCTTCTCCTGCGTGAGCCACTGAACCAAGAAGCTCGCC
TGCCGCTGCTGCATGCCCGGGAAGGCGCTCAACAACGACTGCTCCGAAACCTCGTTGTCG
GGGTGCGCGGGGTCGTCGTCCCTCTCCAGCATCAGCCAGATCTTGTGCAGGCGGATGAAG
CCGCGCGTGTCCAGCCTCCACCGCGTCCACTCCTTGGACTGCTGCCAAAGGGTCATGGTG
CCCGCGTTGGCCTGCGTGACTGCCGTGTANTGGGCCAGGATGATGGCCAGCAGCATGTTC
AGCCTCGTGCC
>at03406
CCAGTGCCAGCCTACACGGCTCCTCGCTACGATGGGTTTCGGCTTTGGAGACACCACTGC
GCGCATGAACAAGTTCGTGGCTGCTTGTAGCGAGAGCCTGGGCTTGGGGCACACTGGGAG
TGACACTGACACTCCCACACCAATGCCAGAATCAGAACCAGTCCGGAAGTGCATCACGTG
CCAGGAGAGCTACGTACAGCAGGTCTCCCAGATGGCCGACCCCAATGGATGCATGATGCA
CTGTACCCCAGCCATCTCCTGCCTCCAGGGAGTGAGTGACGCCTGCCTCCGCAAGCTCCA
CCCATGCTTGCGGTGCCACTCGAGAAAGCTGGCAGCATATGACAAATGTGCCGGCACTGA
GGAGAGCGCAAAGATGATCAGCATCCTGGGGGATCGTCTCCAAGGTAGTGAAAACAGGCA
CTGTTGAGAAGGACGGTGTGAGCG
>at03407
CCTACTTCCATGTGATCGTTTGGGCAAGCTTCCTCGTGCGTGTGTAGGTGCACATGCATG
CACAAGCCCAACGCAGCCAGGAACGCTCAGTACGGCCTGGCTGTGAGGTCAGCTCGGCGT
GTGCCTCTGTGAAGTTCTCGCTGTTCGGCCATGGGCCTGGCCGCCGTGCCCCGCGAGCAT
GTGATCAGGGCCCG
>at03408
GGGTCGTAAACCCAGGCAACACCAGGCCATAATAAAATGTCGATCGGTGGCAATGCTCAT
CTGCATAGGGCGAGTCTTGAGCTGTGTGCCACGAAGGGTGGTTTGAGGGTCAGGGACCTG
TCAAGATGAGGCTCCACAGCAGATGCCTGTTGCAGTTGGTAGAACGACTGCACAGAGAGT
GGTGGATGGGATGGGCTGGGCGGCAGGGGCCAGAACATGAACCTAGAATGGTAAAGGCTT
GGGGGGCTTGGGTAGCGCAGGCCTTCCTCGTAACAAAATAGC
>at03409
TTTTTTTTTTTTTTTTCTCTTCTCTTCTCATTCTGGCAGGATCCCTAGCTCCACCAATCC
CCCCTCGCGGCCACTCAGCCCAAAGCCAACAGCCCCTTCGCGAACAGCAGGCGGTGGCCC
ACAGGCTTGACGTTGAGGACCTCGAAGAAGGACGGCTCGACGCAGCCCAGCGTGCCGGGC
GCGATGGGCTGGGAGAGCCGGGCGGCGCCGATCTGGGCGAAGTCCGCGTCGAACTCGTTC
TTGATGGCCTCGAAGTACTGGAGCATGGAGCCGCGGCCTCCGTCCAGGCTCTCGAGCCAG
TCCCACACGCGCTGGTCGGAGTCGCCCTCGGGGCGCGAGACCAGCGTGTCGGTGCCGGAC
GCCGCGGGTGGGGCCATCTGCTCTGCGTCTGCAGCTCCAGCCACCTCCTTGTGTCCCCTC
TCTGGNCGGTGGCCCCACAGCCGAGCTGGCCTTGCTGCGGGCGGCCACCGCGACCTGCGG
TACGGGCTCCACCGGCTCCGGCCTCCGGCCGGCGCCACTGCCGTCTGTCGCGACACTCGT
GGTACTCATCATCGCCTTGTCCCTCGTGCC
>at03410
ACCTCATTTTTCTTCGTCGTCCTGTGGGATGTCTATGCCCAGTCGATCCAGATGACGAC
GCGAGATGCGTCCAAGCCATGCTTCGACATGATACGCTTGATGCCTTTGCGCAATCTAGT
GTACTTCTCGCCCTCGACGTTGTCGGGGCATATGCGGTCCCACCAGCGGTGGCTGACGAA
GATGACGATGTCGCTCTCTaGTCCAGAGCCGGGTGAGGTTATCGGAGCTCCGCGGGAAGC
GGCCTATGCCGAGGAAACCAGCCCAGGGAATCACTTGCATTGGCCACCGTTCCAGGCTGA
GCATCTGGCTGTACATGAGCTGTGCCAGCTGGTCTAGACTGGTCTGCGTTGCCATCAAGG
ATGCCCTCTGGTTGGTTGTGCTCTTCGAGTCCTCAGAGACAAAGTTGTCCACGCTCAGGC
TTTCGCTTGAGTGCTGCGCTGGAAG
>at03411
CTAATTTTGGCCCAAGCTGACTGGCATTCGTTCCACCACAGGCTCGAAAGATCCAGGCTC
GCGTCTCCTCACCAGCCCTAGCCATTCGTGAGAAGAGGTGCCATAACTCGTCTGATGGAT
AGAATGGAGGGATGCACACATTCCCGCTGACGTTTTGTTGTACTTTCTCTTTCAACCCTG

GAGACTTCTGCCACATGTTTCACCTTGAA
>at03412
GCTCGTGACTTTCTTGGGGCAATGCGCATGGGACTAGTCTTACCCATGCTTGTGAACGGC
ACCGGCTTTGTGACCAAGGCCATCTGCTTCAACTATTCAGCGGTGTTCAACAAATACTCT
ATGTTGATAGATGCAGCAGCTATGACACTTAGTGTAACCACGGAAGCTATATGCCTATTT
GTCATTATTGGTGCGTTCAAGAGACAGCGTCCAGCATTCAAAGATGAGGGCGAACCGCCG
GAGCACTTCATCCTGGCAGAGCACCGAGCCACCTCCGACTCGTGGAACGCCAAGGTCGCC
GACTTGGCCAGCCGCGGCATGAGCATCGCCGCTCTGTTGGACTTCTTCGAGCTGCTGGGC
CGGAGGCAGCGTTATGCCAGACTTCGACCTGCAGCGGTCCACGACCAACGACGTAGTCCG
GCAGGCC
>at03413
GACATGCACATGCGCGTACACAGCCGCACGGCACACAAGCAATCAGTGGCGGAGCAAGAC
AAGGCAACTCAAAAAGGCAAAGTAGGAGGCTTGAAAATCATTGCCTGCCAACTTCGCGCC
CGCTGGCAAGGGGCGTTCCGCGCTCAAAGAGCCATGGTGTCAGATGACGTTGCCCCCGAA
CTGTTGCTGGACCGCCTTGTGGGGAGACTCGGTTGCCCCTGGGGCCCAGTCGCTTGTTGG
CGCGCCACCTGCTCCGCGGTCGAGGCCGCAAGCTTCCTGAAGCGGTCGCGCTTGTTCTTG
CCTATTCTGGCGATCTCCCGCTTGCTGTGCCCGGCGTGGCAGAAGTTGCACCGCTCGCCC
GAGGTGCAGCCGATGGCCTCGAACAGGAAGAAGCATGGCCTGCACCGCCCGCTCGCGTGC
GCCGCGCTCCCTCGAGAAGGCAGGCTCGGAGGCTGCGCCAAGTGCGCGGGCTCCGCTA
>at03414
TTGTTTTTTTTTCTTTTTTTTTGGTTTTTCCTATCAAACGTCCAGCGGAATGGTTGCTC
TCAAAACGGTGCGCGCTGGCACACCTTGGACATGTCCGCCGTAACCTCCGCGTGCATGTA
TTTACACATATGATGGCAATGGTGTTTGCCTGTCTGGCATGGATGCTTGGACCCTCACAG
AGCATCCTTCACACGAGCACGGCCACTGTCGATCTCAAGCAACTTCACCACGTAGTCCAT
CGGATCGGACTCCCTGTCGCTGCTC
>at03415
TTGTTTTTTTCTTTTTTTGTTGCTCTTCAAGACTTGAATTGCGCAGTCAGATACGGAAT
GCAGAGTAACTCCAGCTTGGTGTGGGACGAAGAAGCCCTCCAAACCAATTACTGATGCCG
AGCATGAAACCACCCCTCAAGTTGTGCACGGTGCAAATCGCATCAATCATTGCGCAATCA
TGGAAAATCTGTCGCTGTCAGCAACAGGCTCAAACCCGGAGCAAGACTGCACATAAAATG
ATGCTCACAGGTTATCCAAGATGCCGAAATTCTGCACGATGAAAACGATCATTGCCCTCA
CCAAATTTCTTCGTTGAGCACTTCCGTTGTGAGCACTTCCCAAGCAGTGTCCATTTCCTT
AGAAGGTGTGTCTGCAAACCATGAAGGGAAGCTATCTCCAGCATGGTCTATGGACACAGT
CGCCACTGCTTTCAAAGTGTGGCCCCAAAAGTGCAAGCATGCAAAGTACTCGTAAACCCA
CTTCCGGGAAACTTTCAAGTGGCAAAAGTCCCGCATCTTCCGGTTCAGAGCCCTCCACAT
GCCTCAAGAAACGAACGGCACCACTCTCAGTGACCTGAATCCAAACACGAAGAGACTCCG
GCACACTCGTGCC
>at03416
TTGTTTTTTTCTTTTTTTGGAAAGGAAGACGGTTGACTCTCACCGCTGGTGCGTGAGAA
GCACGCGCCGATCCAAGTTCCCCCAAGGCAAGCGAGCGACGAGAGACCAAGGATCCGCAG
GACTTGGCTGACGGACCAACTACAACTGTACAGAATCAAGCTCGAGGAAGGCCTAGGAAT
TCACGCAGGCAGCCTACCTCCGCTCCTCCTCGAGCTTCTCCAGGACCTTCGAGCGGACGT
CGAAGGGGAGCTCCGCCGTCTCCCCCCGGAGCTTGGCGTTCTTGGCCATGATGACGCCCA
CCTTCACGCGGAACTTCGTGCTGGCCTGCTCGAGGCCCGCCAGCATCGTCGCCACGCCCT
TCTTGGTGGGGTCGGACGCGGCGCTCCGGTTGCTGGCCACGACCAGGTCCTCAACCTTCT
TCAGGTACGCCTGGGCCCTGAGGTCTCCCATGGCTGCTTCGGATCTGCAAGGGGGGGCAA
GGCACCGCTGTGGCCTACCGGAGCTCGAGCTTGAGCCAAAATGGCTTGAGCCAAAATGGC
TACGG
>at03417
AGCTCTCAGGTTCTCTGGCAACTGGACGCAGATGGCTGGCTTTTCCGCACAGCTTCGGCG
CGCAGGCGCGGCCTGCTTTCTGCTGCCGCCTCGCTTGGCACTTGGGTGGTATCCGCATGC
CTCCAGAAGCACCTTCGAGTGTGACGTGAGCTGCTGAAAGGCCTCGGGGCTGCCACCCTC
TTTGTCCGGATGCTTGGTGAGCGACAACTTCCGGTAGGCTGACACCACCTCTGCCGGACT
CCACTTGGCCAGCTCGGCCTTGACCGAACTCACGTCAGGCAAAGGCAAAGACCCGTCTGC
AGGCGCGGCGCCTGCGGCAGCAAGCATGTTCAGG

>at03418
TTTTTTTTTTTTTTTTTTGCTTTTTGCAAGCCTCTTGTTGGGTCTTTGGCCCGAGCACTG
CGAGGGGAGACCATATGAAGGGCAAACACCCCAGTTTGCTGTGTGGGGGTTGTACTCGAG
CCACAGCGAGTACAGGAACACTAAGTCGGCCCACGGGTCCTCGCCGGCGCCCCCGCCGCC
GCTCGCCGAGGCCTCCTCGCGGCCCAGCAGCGCCCGGACCTCCGCGAGGTGGCCCTCGCG
CGCGCGGCTGCGCCAGGGCGAGCCGTGGTAGACCTGGGGCATGCCCTCTTTCTGCGGGGC
CAGATACCACTGGAGGACCTCCATGAGCTTTCGCGCCGCTCCTTTCCAGTGACTCTCCAG
CCGTCTGTGCCGCGGACGCTCCCTGATCGTGCATTGGAGGCGTTCCTCCTGTGCCTCCGA
GTGCTGCGAGGCAACCACCACTGCCACTCCACCAGCCAGTGCCACGGCACCCGCACCCGC
GCCCACGAGGGCTGCAGGCGAGGCCTCCGCGAAGACGGCCGCGGCGGAGACCAAAGCTGC
GCGGGCACCCGCGGCAGTGGCGGCCAGGGCCGTCTGGACAGCGGGCGTCTCGATGACCTC
GCTGGCGGCGCACCAGGCGCCGAACGCCAGCACGGGGTTTGGGAAAGAGGGACGCAGCCA
GACGTGGGTGCCCTCACTGTCCACCTCGCCGTCCTCCCCATGCCACCCTGTACGGCAGAA
GGGGCAGCGGCGAAGGGCTTCACTGCATACT
>at03419
TTTTTTTTTTTTTTTTTTGCATGTGTTCGAGCTTGCGCACTCCCCCAAATCACAAGATTA
CAGAAACTCAAAACCTGACAAGTGAGGAGGGGAGAGCTGTAGGAGAATGTTCATGCCAAC
GAATTAGTGCCAGGATTCGGATGGTCTGGACCCGGTGCAAATCACAGCCGGTCCAATCAT
TATATTATTGTGTAGACTGCATTTCGGCAATCGCAGCCTGTACCAGTCTGGAAAGAAAGC
CCATGCTGCGTGCCTACATGCCACACGCACATACGTGCATGACGGCATCACGCACACAAT
GCCTAGATCTCAGATCAAGTCGGCAATGTCGTTTGGCATTTCTTCAATCTGGGTGTGATA
GTACCTCTCCAGGTCCTTCATGTTGCGGACGTCGTTGTTTGTGACAAAATTGATTGCAAC
ACCTTTCCGGCCGAACCGCCCACTGCGTCCGATGCGGTGCAGGTAGTTCTCCATGTTTGA
TGGAAGGTCATAGTTGATCACGAGGGACACCTGCTGCACGTCGATGCCTCGAGCAAGCAA
GTCAGTCGAGATAAGCACGCGTGAGGAGCCGGAGCGAAATTCTCGCATGACCAAGTCGCG
CTCCTTCTGGTCCAGCTCCGCGTGCATGGTTGACACAGTGAAGTCGCGCTTGGTCAGCTG
GTCCTGGAGGAAGTCGACCTTACGGCGCGTGTTGCAATAGATGATGCACTGGGTGATGGT
CAATGTCTCGTACAAGTCGACCAGGGTGTCGAGCTTCCACTCCTCCTTCTCAATTGCCAC
GTAGAACTGTCGGATACCCTCCAGAGTCAACTCGTCCTTCTTCACAAGAATCCTGACAGC
ATCACGCATGAACTTGGTGGTCATCTCCAAGATTTCTGGGGCCATGGTGGCAGAGAAGAG
GCACACCTGGACGTTAGGTGGCAAGCACTTGAAGATGTCGTAGATCTGGTCCTTGAACCC
ACGTGACATCAGCTCGTCTGCCTCGTCCAAAACAAACACCTGAAGATCATCTACCCGGAG
GTTGCGCTTGCTCACCATGTCATAAACACGGCCAGGTGTGCCTACAACAAGATGTTGTCC
ATCCCGGAGCTTCTCAATGTCATCGCGCACGGAGGTGCCACCAATGCACACATGTGCTCT
GATATTGCAGTAGTCACCCAGTGCCAAGGCGACCTTGTGAATCTGGTTAGCAAGCTCACG
GGTCGGTGCAAGAATTAGCGCCTGCGTTGCCTTCAATGAAAAGTCAATACGTTGCAGAGC
ACCAATCGTGAACGTAGCCGTCTTCCCGGTACCAGACTGTGCTTGGCCAATTGTGTCCCG
GCCATCGAGAATGGGCTTGATGCCTCGCTGTTGGATGGCACTTGGCTTTTCAAAGCCATA
AGAGTAGATGCCGCGCAATAAGTTCTCGTTAAGGTCGTAATCGTCAAAGGACTCAAAGAC
TGGATAGTCCTCAGCGCCAGTAACAGTCACACCCTCTGATGACATGGCGCTTTCCTGGAT
GGAGAGCAGCAATCACACCACAACTAGATCTCGCCTTGATCCCAACTTGAGCCAAAATGG
CTACGG
>at03420
GTGACCTTGTCAAGCACCTTTGTTGCCGGCCTTCACAGTGAGATCACAAATCAAACACCA
CAATGCGGTCATTGTAGCTAAGATTGTGAGCGCTCCATTGTTAGTGTGGATGTCATTCCG
GAGATGTCGAGTGCACGGACGTGTCGCGCGACCATGCCACCGAGTACCACAGGCTGAGAG
CTCTGATCAGTGTGAGGTGGGCAGGTCATCCAGCGAACTTCTTGCTTGGGAGCATTGGGA
CTGGACAATGTGGCAGGACTACCATCGCATCCGCATCAAATGCCCAGTCCCTCGCTCTTT
CAAGCTTGACGGCAAGACTGAAGCGCTAGTGGAGCAGCTGACGAGAGAGAGTCACATGGT
TGCCATGTTGCAGTCTAGAATTGGTCTTGGCATGGTGTCGCAGCTTGAGCAGGTATTGCA
GCAAAGGACACGATCTGTGCGTTTCGTGTTTGAAGCACCTGGTAACCTGAGCAACCTCTG
GGCTTGTCTCAGGACAATGGACTCGTTCGGCGTTCAGTACGGGGAGATCATTGACCACCA
GGATGCAGGATCTGAGTGGAATGACTACATGTTTCGGGCTGCTGCTTCTCAGTCTTGGAT
CACCGTGCGCCGCTGGTCCTGTGCGACGACATGTGTAGAGGCCCTCCACTCCGAGGGCTA
CCAAGTTCTGCTTCTCGACTTGGATCCTACTAGTAGAAACATTGACGAGTTGCTTCCCAT

ATGGGCAGGAGTAGATTGTGACAGCCAGGGAGGAAGGCTCCCGCTTGCCTTGGTCATTGG
AAATGAGTTGGATGGGGTCTCGGATGCAATGCGTCAGGTGGCCGATGAACGGGCCTTCTT
CGAGACTCATGGCATGTGCCAGTCCTTCAATATGAGCGTGGCCTGCACGCTGATGCTGGC
ATCCCTTAGCGCACATGGGCTGTTGATTCCTGGTAACCTTTCGGCTGAGGAGGAGGCACG
CTTACGCCTCTGCTGGCTTGTGGATGCGTACGGCAGCCAGGACGCGGAAGCCTTGCTTCT
TTCCAGTTCGCAAGACTCCTCTTTGAGCTCTCTTCAACGTACAGGCCCAGCCTAGCCCCA
GCCCAATGCAAAGTCAGATTCTGATGTTGTACACTCCGTAGGTGTTTC
>at03421
CACCCGGACGCAGCATGGAGAAGATGTCCGCCAAGCGGGGTCCGATCCCAGCGAGGCGGT
GGGCCCAAAATGCGCCGGCGTGTGGACTCAGAGGATCGGAGGCCCGGCTCAGCGCCGGCT
TGCTCCAGGCACGGACAAGGTGAAGGTGATCTCGCGGCTGTGACGAAGGACCTTCTCGGC
GGCCCCTCACCGGTGCCAGCCCCAAGCCGGATGCCCCCAGGGCGAACTCCGGCGGCAGCG
TCCAGGAGGGCTGCGCTGGTGCGTCCCAGGGCTGCGGGGCGGCGGAGGGCGCCTCGCCGC
CGGCTGCGGGGGTCTCCTTTGCTTGGGACATCCCTGCCTTGAGCATACACAGGTGCCATG
TGGGACTGGGGCGGCGGAAACCAAGCCTCGTGCAACACCGCGGGGAGAAGAACCCGCGGG
CGCGGAGGCAGGCGGTATCCAAGGTGCA
>at03422
CTCAGGAGAGGCTGGGTTGGTTCACCTCCATTGCCTCTCCTTCCGTAGGCCTCCTTGCAT
GAGCTAGATGTTTGAATTCCTTCGCGAGAACCTTCTCTGCAGCTCGGATGACTCGCCAAA
GCGTGAATTGAATTCAAGGAGAAGGCCTGGCGAGGTCATCTCGTCGCAGGGCAACCCCGT
GCACCCGCTGCAGCAGCG
>at03423
TTTTTTTTTTTTTTTTCTCTCCAACGAACTCAAGCGCGTGTTCCACGGCAAGCTCCTCAT
GCGTCCTTTGCCGCACAGTCGGTGCCAGCCGTCAAAGCATTGCCCTCAGAGACCCTCCAT
CATTGGCTCAGTCTCCATCTCCGTCTCCGTCTCCGCCGGAGAGCGGCGGGTGGCCTCAGG
GCCTGAAGGTCTTGCCGGCGGCCATGGAGCTGCAGCTGAGCTCGAAGCCGAGCTGCGAGG
CCACGTGCGTCACGCCCGTGCTCAGCGCGTTCTTGGTCACGCCCTCTGCCACACTGGCCA
CGGTGTGGACGTGCCGTGCGAAGATGCCAAGGAGGCCACCGCCAGAAGGCGTTGGAGCGC
TGGGCGCGGGTGGGGGCTTCGGCTTGAGGCTCGCGCAGCGCGTCTCCGTCTGGCGGCCCT
CGCGCACGGGGAACTCCGCAAGCCAATTCACCAAGGGCGTCTCATCACCGGAGATCTTGT
TGCCGGTAAAGCTCAGGAACTTACCCATGGCGCCTCCCTTCTTGGTGACCTGGTTGCCCA
CGGGGGTGGTCTGGTACATCACGTCGTTGTTGAGGTACATGTGCTGGCCGCTGTTTACCG
AGAAGGACACCCAGTTTGGCTTCATGTTGTAGCGGTCGTACTCGGACATGATCAGACTGG
CAAATTGTTCCGGGCCCACGTGATCCATGTAGCCGAAGAGAGACTTGTGCACGACCTTGT
AGAACGCAATCTGCACAAAGTCAATCTTGGAGTCCAGATTTGCAAAGGCAACCTTTGGGT
ACTCCCACATGGCCTGCTCGAAGATCCGCTGCACGTCGACCGTCCCGGTGTTGCACACAA
AGCTCAAGGTGTTGGTAAGCAAACCATTGTTGCACATGCCGAAGCCCTTCATCATGCCCC
CCTGCACGCCGAACGGGAAGATCGCCGCATAGGAGTCTGCAATGACCGCGGCGGCGCGGT
AGGAGAACTCCCCCAGCAGCGCCCGCGACCAGATCTGCGTGCCCAGGGCGCCGGCGGAAG
AGCCGGTGATGATCAGCGAGTCCAGGGTCTG
>at03424
TTTTTTTTTTTTTTTTTCGCTGAGCTTGTCAAAGTAAAGGATGGGGCGGGAACCCATGA
GGCAGTTTCCCGCCAGCCTGACCTCTCCGGTGGTGTGGATGTTCAGGACCTGGAACTTGA
GGCTCGGCCCCGAGGGCACCCTGGCCATCCACATGTAGAGGTCCGTGTGCTTGCGCCCTT
CGAAGTACATCACGTTGTTGCAGCTCTTCAGCTCACAGATCTCGTTGATCTCGTGCAGCT
TTTCCCGCTTCTCGAACTTGGGCTCCGCCTTGTGGTGGGGGAGGAGCTTCTTAAAGTCCT
CTACGAGATGCCTGGACTGCGCGTTGATGCCGCGGCTCCCGAAGATCAGCGTCCGCTGCT
TGTTGCGCCAGCGGGTCTCCTTCTGGATGTACACCGCGTCGGCCATTAGGTAGGGGTCCG
GCTTCTCGTCGGCATCGGCCCCCTGCTTGTTGGCCTCCGCCTCCTCCTCGGCCTTGGCCG
CCTCCGCCGCCTCGCGCTCGGCCGGCGTGGGCATCTCGGGGGGCAGCATGTCCTCCTCGG
CCTCGGCGGCTTCCTGAGCGTCCCGCATCGCTTTGTAGTCTTT
>at03425
TTGTTTTTTTTTTCTTTTTTTTTTGGACATGACAATGAACATTCATGGAGGAGCTTACTT
GTCACTCGGTCATTGAGGCCTCACATCACTTACTCAGCTTGGTCCACTCAGCCCGGCAAC
TGACCAACGCCGCAATGCAACCAGCAATGCCAATGTATGCATCGTGCCACCCGTGTAGAT

GGCTTATGCGCAGCCAAATGGCAAGGGTTGTTTGTGGGAAGTACGTTGCCACATCCGCGG
TGAGCTTTATGACAGCAAACATGGTTTCTCTCCGCTCCTTCTCTGCCGCATTCTTGTCCT
GGGATATTGCAAGACGACCTCGTTCATCGGCATTTGTCTCACCAATGGAGCCTAAAGAGG
CCTTCTGAGCCAACGCCAGAACGTGCTGCGACAAACGGCGATGCCGCACGCAAGAAGCAA
CGAGTCCAACGGAGTCGGACATGGCTCGCCAATTCTTTTGGCGCTGGTTTGCTGAGCTGT
TGCTCCACCATGGCGAGTCCGGTCTTACGATGAGTCCAACAGCGGAGTGCGTGAAAAAGA
ACATGTTGTCCCAGAAGATGTGCATGGCCATCAGCAACCACTGCAGCGCACTCAACCCAT
TGACCACATCGGAAGAACCACCCAAAAATGCTAGGAAGTGGTCAATGGCAAGTTTCAGAT
CAGCCGACCAACGGAAAGCACGGAAGGCCTTCCGTGAAAGTGACAGCTGGGCGTACAATT
GGAAAAGCATTCGCCCGATGTCGTGCTCCTTATTGCTCCTGAGTATCAAGAAGGCCAGCC
CACGGCTGCCATACTGCACAAACCGTAACTGCTTGTCTTGGAAATCACGAATATTGAGCG
CTCGGGCCCACATTGCACCAGACATGATATCCTCTCGAGTGGTGCAGCTGAAGCATCACT
CTGTTTGTAAGAGCAAGCATACGGCAACGGATTGCAAACCCATT
>at03426
CCGTAGCCATTTTGGCTCAAGGGGCCCTCGCGCCGCGGCGGCCGCAGCGGAGGCCGCCCC
CGCCGCCCTCCCGAGCCCATGGGCGTGGAGGTGGTGACCGTGGAGCCGGGGGATGGAAGG
ACGTACCCGAAGCCAGGGGACAAGCTGCGGATGCACTACGTCGGCAAGG
>at03427
CATTTGCCCCCACCAGAGGAGGAGGAGTTCCCATTCTGTCAATTCCCTCCTCCCTGGCCT
GGTATTGCGAACACGCTGCCCCCGCCATCCATGGAATGTCAGCGTTCTGGGCTGGGACTT
CTGACATGCCGCCGCCAACGGCCATGGAAGATGGTTGGTATTCGTGTCTGTATCAGGTTC
TTGCGAGGGCCTCCTGGTTGGTTTCGTGCGTCAGATCCTTCTGGGAACTATGCAGTTTCC
ACCGGTGGAGAGCCTCTGGACTCGCTGCGACAATTTACAAATGCCAGCGGCAGCGGCGGC
ACCAACAACGGATTGACTTCGAAGTTGTTTGGGCAGTCGAGAAACG
>at03428
ATTCTGGTTTCAGCCGACGTCACCTCCCAGTCGCGAGAGAGGGCTGAGTGGAGCCATGTC
AGACCGCAAGGCCGTGATCAAGAATGCGGACATGGCGGAGGACATGCAACAGGACGCGAT
CGACTGCGCCACGCAGGCGCTGGAGAAGTACAACATCGAGAAGGACATCGCTGCCTTCAT
CAAGAAGGAGTTCGACAAGAAGTACAACCCCACCTGGCACGCCATCGTGGGCCGGAACTT
CGGCTCCTACGTGACGCACGAGACGAAGCACTTCATCTACTTCTACCTCGGCCAGGTGGC
CATCCTGCTCTTCAAGTCCGGCTGAGCGGGCTTCTGCCGGGCGCACTTGGAGCGGGCGGA
TTGCCGGAACCGCCGCGA
>at03429
GTTGAGATACCCAGATCCCGCGGCTTCGAAGGACTGGGAAACCACGGTGGTGGATCAGAT
CTCGGAGACGGAGTTCCATGTCCTGCGGACACTTGACAGAGATCACCCCAGGGTTCAGCA
GCTCAAGCTGTTGGATACATGGGACAAGGTCACGCTTGTCAAACAGGACGGGGCCATTGT
TGCGGAAACCATCAACAGCAAGGGCGAACTACAGGTGGTTACTTGGTACGTGATTCACAG
GAGTCCAGTTTGTGTTGAGTGCTGGTGCGACGGCCCCCCACAGCGGAAGGCTGGGACTTT
CGTGAAGGCGAGACTGAGCAACTTGCTGGGCGTCATCCTGGAGCGGGCAACCTGCGCCCA
GAGCCTCATGCAAACCCTCGGCGACATGGATGACTTGGACGCGTTCGAGGATGCGGCAGA
CGAGGAGAGTCAGGTGGAGGACAGTACAGAG
>at03430
GCCTAAATTGGCAAAGTTCGCTGAGTGGGATGAAGGCATGAGATGGTTGCTGGTTCGCTT
CTACGCCATGCTGAGAGAGAAAGGCGATCTGGGGCTGATCCAGTGCTGCGCGCTGGTCAA
GCGCCACGACATCTTCCCCGGAGTCAGCTGGGGCACTGCTTCGGAGGAGGAGCAGCAGTG
GTGGAAGGACACCCAGTGCGACATGCTGGTGGGCGCCGACGACCCAGAGGGGGGCGGCCGG
CGCCGTCTACTGTCCCCAAGATGGCAAGCTTGCAAAGATGCTGCCGCCAGAGGAGCCTTC
CGCCAGCGTGGTGCTCGCGTCCGAGGACGGCCTCGGCCACGGCCGTGACGGCGCCGGGTG
CGATGAAGAAGACCGTATGGAGCTCAACAACTCCTACATAGACAGGGACAAGGCCAACGC
CATGAGTGACCAGTGCAGGGAGTTCTACCTGATCCGTGACGGAGTG
>at03431
TCCGATCCATTCACTATCTTTCGAATGTGTGACAGCAACTCTGGCTTATCTCGCTGGAAA
CATGGGTGTTTAAATTCCAAAACTTTCTCAAAGCCGCTTTGTTGCATTTCAATCAATTGA
TCAGACCAAACTTTGCGGAAGCCATAAAAAAATTGAGTTGTCTAACAAATGAAGAGAAATT
GCTGTGTTTGAAATATTGCGGGATGATTGAATCTACCAAGCGGACTTGATCAATAATGTA

AAAATGAAAGTCCATCTTCTGCCCAGACAGCAATATCTGGTGGACAAGACTTGAGCATCG
CGTAAGTTTTGTTCAGAAACAAAGGGTACTTCTTGTCGAAATCCTCTTGTTTTTGTGGCG
ATGGCGCCCTCACTGTTCCGGTTGCTTTCATGATTGATGCTGTACAAATTTCAAAGGTAT
CAATGATAATAGAATTACTCTGTTGTTGCAACTGATTTTGTCTC
>at03432
ACAGCTTGTGCTTCTGGCGACACGCGACACTCTTAGGGAGGCCCTGATAGCAGACCCAGA
CATGTGCTACTGTCTGCGGCGCCGGATCTCAGACCTCCTGGAGCATCTCTGGTGCGACAT
CATGACCGAGGTGGAGATCATGGTGGAGGCGGCCCGAGTAGGGGTTGCAGGCCACAGCAT
GCACGATGTGGAGAGTCTTGCCCTGATTGGGTGGGAGCCCCCCCACATCCTCCACCCGTT
GGATATCCGCGGCTTCCTGCTGTACCATTGGCTGCCCTACGACAAATCGGTCTTTGGGCA
GCTCAAGGACCCTTTCTACCTCCCTTTCTTGGTAATCTCCTTCGTGCCAGTACTGCGAAA
CTTCCTGT
>at03433
TAGATGCACCCTCGAATACCAATGTGCATGTCCACGCCCCTGTAAACGTCCACCATTGTC
GCCCAGCATCTCAGAAGGAGGACCTGACCCTGTCAGCAAAGCTGCGCGGTGGGGATGCAC
GCTCAACCTCATGCTCAGTCTTCTGCCGCTTGGCATGGTTGCGCTGCGGTGCAAAGGCCT
GCTTTGTGAAAATCTTCCCGCCAGAGTGCTTGTTGCTGGCTCCCACAGCAGCCCAAACCT
GATTGAGCGGCATGCCACAGAGAGAAAAGGAGACCTGAACCCGCCGTAACTCCTCTGGTG
TGAACTCAGGTCGTCTTTGAAGCTACTTCTTCG
>at03434
GTCTCCTTACAGAGGTCGGCCTTGGATTCGGCATTGGTGTTAGGAGTTTGAATGCATTGA
GGAGCGACAGATGGTCGCCAAACCCGGAGGCGCAGGACTTGTGCGCGCGGTCAGCCGCCT
TCTGTGCGTGCCGTGGGCGCTGGAAGGGCGGGGGCACGGAGATCATAGCAACGATCGCGA
TGGCCTCGTCGCAGCAGCCCCTGTTTGCAGCAGCGATCACGACGGCGTCCCAGGTGTGGG
TCGATTGGTAGCTCCGCAATATGCTCGCCGATCTTGGTGAGATGGCCCTNATCATCGATG
GCACCGAGAAAGTGGAGCATCTCCAGCGCGCGCATCATTGACTCAGGCGATGGTCGCCTC
CATAAAGTCGAAGTGGACAAGGTCGTTGATG
>at03435
CATCCTCACCGTGCAAGCGCCCATCCAACCACCTTGCACCATGCTGGCGCTGCGCCCCTC
CGCCATCCGTGTTGGCAGCCGCCGCTGCCTCGCATGCCTTGCGGTGGCCTTAGTTATCTG
CCTCCTGTCAGGAGGCAGTAGTGGATCCTCATTTAGCGTTGGCCCACTCAGGGCAGGTAC
TCAGGTCGATGCGAGCCCAGCCGTCCCTGGCGAGGCAGTGCAGCCTGCACTGCCCTCGAG
GTGGGGAGGCAGCGCGGCGGCTGCCGGCTTGTCAGCGCATGGCCTTTGCGCCGCACTGGC
CATGGCTGCTTGTGCAGTGCGGGGCCTGGCCGTCTTGGCAAGGCGGTCTCGCAAGGACGG
GCGACGAGAGCCGGACATGTCTCGTCGCTCGGTTGTCCTGCCTGCTGCAGTCCTCGCCAG
>at03436
CCGTTAGCCATTTTTGGCTCAAGTTGCGACCAGTGCTCCTCCAAGCGATCCAAGGATAAC
CCCAGCGGATGGTACCTCGGGATGACGCCGCGCGTTGGCTTCAACTACCCCCCAGACTAC
AACTCCATCTCGCTTGGAACCCAATCGAATTTTATCCCATGGGAGGGCGGCTTGGTCAAG
AAACCAAACATCGGCATCAGCAATGACGTTGGAGAGGGCGATAAATTCTTCGGCAGCGAT
CGCTTCTTCGGCCTCGGCGCCCTCTTTGATAGGTGACATCCTCGGCAGCGACCGCTGCAG
GTCTTGAACAGCAGCATGTCATACAAAAAGCAAAAAAAAAAAAGAAAAAAAACA
>at03437
TCCGTAGCCATTTTGGCTCAAGCAGCAATTTTGCGTCAAGCGCATTTTTCGAGGCCATCT
GGACACACTGCGCTGCCAGCAGCCCCATCATGGCGTCCAACACGAGGGTGCTCCCAGTTC
TCCTTGCCGCGGCAGTTGGCACGATGCTCTTGCGCAGCTTGCTCGTGCCAAGCCACGGCG
AGGATGCAGCCTTCGCCACGCCAAGGCTTGTGGCGACCTCGGTTCACCACAACTGCCGGC
CAGTGCGAAGCTCCGGAGCACTCCTTGCCCAGGGGGGTTTGCCCGCTGTGGTCAATGGGG
TGCCAGCCCGTCAAGGTGTTGCAGCGCACTTCAAGGTGACATTGGAGACCCCTGATGGCA
CACAGGAGTTTGAGTGCCCCGGAGGACGTTTACATCCTGGACCAGGCCGAGGAGGAGGGC
CTGGAGCTGCCCTACTCCTGCCGCGCGGGCTCCTGCTCCAGCTGCGCTGGGAAGGTCCTC
TCGGGCTCCATCGACCAGTCTGACCAGGCATTCTTGGACGATGATCAGATGGGCGATGGC
TACTGCCTCACGTGCGTCACGTATGCCACCTCTGACGTTACCATCAAGACTCACTGTGAG
GATGAGCTCTGAGCGCCCAGTACCATGGTGATTTTCTCCCGTTCCTGAACACTCGAGGTT
TTCATAGAAGCGGCTGAGTGTTGCAAAATCCTAAGAAAAAAAAAAAAGAAAAAAAAAAACAA

A
>at03438
TCCACTTCCGCAAGCGTTGCAATGCGGtCCttTCCtTCGtCCACCCTCAAAGGGCATCAC
CGCGCGAGGATCGTCCTCAAGCGCCGCCGCCGCCTCCTCAGCAGTATAGCCGAGCTGCTC
CAACGTCGGCAAGAAGTGCAGGGCTTCCTGCAGCTCCTTTCCAGGCTCTGCTTCGGACAG
CTCCGGCAGcCGGCGGGAGCCGCGCGGGCACGTTGAGCGGCTCGCGGATGGTGCCCCGGN
TCTCGGCCTTGTTCTTGAAGGACGTGAAGAGGAGCGGGATGTTGTTCGCCGTGCAGCCGC
ACTCGTGCGGCAAGTACAGGGTGCCGCCCCAGGACCC
>at03439
TGAAACAGAAGGCAGTGAAGGAGCGCCTGAAGGCGAAGGCGAAGGCAAAGGCCGAGAAGG
AGAAGCTCAAGGTGAAGGCTTTGATGGAGAAGGAAAAGGCTGAGCGCAAGGCTCAGAAAG
AAAGGGAGAAGGCCAAGGCGCTGAAGACGGTGAAGAAAACCGCCGTCAAGAAAGCGAAGG
AGAGGGTTATGGGTTAGAGATTGCCTCTGTCTCCCAGTATTGCTCTGCATTGTACATAAG
AAGTTCATCAGGACGGCTGCTGCTAGTTTCAGTTCGGGGCCTGCCCCCGAATTACGAGGT
GAGTGTGAGCCCGCTCACACCATGCAAGACATGACTCACTTATCAAATTCAGATGTCGCT
TGTGCGTGTGGGGCAGGCAGGCGTACAGTCTGAAGCGCTCGGTTAGATCGGCAGCTACAG
GGCGTCGAGTCATCCACACAGAACCATGTCAGTGAAGCTT
>at03440
GATGGGGATAATGAAACGATAGACGCAAATCTGCTGCGCTCTATCAAAAGACCTGAGAAA
GGGCGATGCAGGATCCTCCAGGGAGCCAATGCACTTCCCGAAACCGATCTCACCTATGGT
ATCCAGCGTGAAGCGATTCATCAAGTTGAAGACGTCTATGGCTTGTCAGGCTCCACTCTC
TCCAAAATGTTGCGAAGCTTGCGGGCGTTTCGCTGCACCACAACCCAAATGTGATCCTTG
AAGAGCTTTGCAGTGAACATCTGGCTCGTCGTTTGTCGTTGGTGATACCAGTCATGACCG
TTTTTTTCGACATTGAAGATGCCTTGG
>at03441
GGAGGAGGGTGCCCAGGCTGACGACGGTGATTCCCAAGTTGTCTAAGCGACAGAAAGTGG
CAGCGCAGGGTGAGCCAGCCGTGCCGATTGATTTCCAGTGGACCGGACTGGGGTTTGGCA
TGAGTGATGTGGCAATGCACTTGAGCCACTCCGTGGCCGTTGCGGCGTTGGATGGGGCAG
GGGAGCGTGCATTGGTTGAGCTGTACCACCGCGCTCTGGTGGAAGCAGCTGGGAGGTCCA
GGATGGCCTTCTACACCATGGATGTGGCCTGGCGCCACTATTGCTTGGGCGTGGTGGATT
ATGCACGTGTGGTTCTAGGGTGCTTCTTCAAGGACGCCTCGCAGTCAGCGTTTGCTGCTC
GCAGCAAACAGCCGAATGTTGGCTTGGCATACCGTGATGTGAATGCATCCCTGCTATTCG
TGGAGCGCGTTGACCGATGCC
>at03442
CCTGGGCAAAGGCCTTCAGCGAGAACAGGTCCTTGTACGTGCTCCCGCCGCTGTTCGGGG
ACTTCACGGAGCGAACGCAGTACACGCACACGTGCCTGCCCAGGATGTCGAAGCACGGCT
GGGTGCTGTCGTGCTTTGCGAGCTCGTAGTAGACGCCCCAGAAGGCATCGAGGCTGAAAT
TGCTCTTCACGAAGTCGGACTGAAACTCCT
>at03443
TTAGTTTTCCTATTTTGGCTCAAGTCAATTGCCATTCTgGATCAAGGTaCGTAgGCGACT
TGTTATCGCCCTGTTTCAGAAGTAAGAGCAATGGTTAcCTCTTGATGGAATTGCTGCTAC
TTTGGCTAAGAGGACTGAAGAGGCCAATGAAAGGCACCATGCCACTGATGCCAAAAACAC
CTCACCAGAAGCGAGTGCAACGAGGATGGCTGAACAG
>at03444
CCGTAGCCATTTTGGCTCAAGGCCCGAACACTTCCGTTGCAGCCCCCAACGTAGCATCAT
ACGGCTTGCCATGGCGCGCTCAGTGGTCGTTGCCTTTGTCGCGCTGGCTTGCTTTGCGGT
CCCTTGCATTGCGACTCGGAACTTTCGTGTCCCGAACACGACACTTGAGAAGGGGGGCAA
CTTGACGAACTCGTCGATCCCCTCTCCAACGCACCAGTTCATGGAGGTCAAGTTGGGACC
GTTCGCGTCAGCAGTGGAGGCCTGCGACTACTGCTTCGGCTCATTCACCAAGTCCGGCAC
GCCGCCCGCGGGGCCCGTGGCCCCGGCCTGCGTCTGCATGGCCTACCCGGAAGGCAGCGA
GTTCAACATGTTTTGTGCAACGCCGACGTCAGCTGCTGGCTACATCTCGGAGAAGAGCGG
TTGCCGTTGCAAAGGCCGCGACATGGAGCAGATGGCTGCCACCACGTGCGAGCCGATCAG
CTGAGCGGGCTGAAGTGGGGCTGCGGCCGCTTCGGATGAGGAGCAGTGGGAGGGTTGGCG
CCTCGAGCGCGAAGGGTGATAGCCAACGACGAAAAAAAAAAGAAAAAAAAAACAA
>at03445

GCTCAAGCCTACTTTGGAAAAAGTCGGGCCCGCTTAACGGACTTTGATCAGCTATCGAAC
CGCCGTTGTTCTCTATCAGACATGCTTGGCGCCATTCTCTTGCTGACCAGTGCGCGCGCA
TGCGCTTCACTCCGGTCAATCCATCAGATGCCACACCAATCAGGCAAGACCAGCGCGGCG
CGGGAAAGCACAGCATCAGTGGTCAGCCCTCTCAATCCATGGACTTACCAGCCTTTTGCA
AGATTGTCAAATTTGTCTCGCTTCCCCAACGACGATTACAACGATCCATCATGCGCTCAA
CACT
>at03446
TCACCTCCCGGACTCGGCGCCGTCCTCGCCGGCCCCAAGGGTCTCCGGCTCGGGCGCGTT
GATCCTCGCCAGCTCGAGGTCGCCGAACTGCTTCGCCTCGCCCTGGGGCTCTGGCGCCGG
CGTCCGCGGCTCCTTCGCCGCGGGCGTCCTGTTGGAGGAGCTCTTGTAGAAGAGGGAGAA
GAAGACCAGGGCCAGCCCCAACAGCTGCAGGATGCTGATGGGGTGGGCGTACACGGCGTA
GGAGATGAGGATGGAAATAACCTGTCGCAAGTTCATTGTTGCGGCAAGGACAAGTGCCCC
AAACTCCTTAACCTCTGAGTAGATGAAGAACTGGCCGGCAACAGCCGCAGCGCTGAGGGC
AGAGGTGTGCACTACGAAGTCGGGGTGCCGGGAACAGAAGGACAAGGCTGTCAAGGTCCG
TCCCGTAACGACGAGGGTGATGAGAGAGACGAGGCCAGAGCCACTATTCACGTAGAGCAT
CTGGTTGTACTTAGACGTCTCGTGGTCATGGAACAGCTTCTCCTGGAAGGTGGACGTGAA
CCCGTCGCAGGCGAGAAAGGCGAGGAGCAGAAGCAAGCCTTTGAAGCTGCTCTCCTTGGC
GGAGTGCTTCGCGCTGATGTCACCCTC
>at03447
TTTTTTTTTTTTTTTGTCGTGGCCATGCCCATGACAGTGTCCATGCAGATGCCATGCCC
ATGCCCATACCCAGCTCACTCTCCGTACTCATGCCCACGGCCACGTCAACATAGCGGTGT
GCATCGCTGCAGACTCTTCAAGGCTACATTGTCAAGAAGCGTCTGCAAGGGGACAACACG
CTGGGTCCTGCTCACGCAGAGGCACACGCATGATGACGCAGCGGCCCATCTTCGGTTCAG
CGGCAGTACGGTGTCCCCTCCCAGTAACATCCTCCACGAGAAGGCAATTCCCGCGATGAA
GCAAGACTGGTGCCCCAACTCCCGGCCACAGCCACATCCCTCCACTGATTACGATGACCC
CACTGCCGCCTTGAACAATTGTGGAATACGTCCAGCCGTTGACCAGGTGACCATTTGCCC
TCACACGTGTTGGCCACCGGATGCGCCGTGTTTTGGGGTGGTGGTACCTCCAATAGACCA
GGCATTTCACCGAGAAGCGGAACGGACCGTGCGAGCGGTGACCCAGCGGAGAAGTGGAGT
GGGCGAATGCGTGACTCGCCATCAGGGCCTGTGAACAAGCGTAGTAGCACCAGGCTGGAA
GAGTCTTGACAAGTCGGCTTGCTGGCACAAGGCCTCGTGGCCTTCACAGCCCTCGCTGCA
GCTTCCAGATTGCTCTTAGATAGGCGGGCGCGAGAGACAAGGCCATAGTGGTGACAGCGA
NATGTCGTCGACACCGCAGTGTCATGTCAAGTCAGATGTANGAGCGCGGCAGGATATGCA
NGGACGCCATGGGATGC
>at03448
TCCGAGGGTCAAGATCATCTTGCAGGGGAGCTCGCATGCCAATGCCCTCTGACCTCGTTT
GGTCCTGAATCTTGGCTTGGGTCGTGTCATCTTGATTAGGTATGCAATCAGACCGGCCAT
TGCCTGGTCTTCTTGGACTTGTAGCAGTTGTGCTCCTTGCAGTCGCACTGTGGGATGATC
ACTTTTTTGCTCCATGTCTTGTCTACTCCGGTTGCCTATGCACAATTTCGTGGGACTGCC
ACCCAGCGGCAGCGGTGTCAGCAGCTGCAGCAGGGACGGGAGCAGCAGCGGGGCGAGCGT
AGGAAACCACCAAAGCGGATTGTGACAACTGGCGATCCAGTGAGTACAGATGCGGACCAA
CGGAAGGCTCCCCTTTGTGCTCAGTGGGTTGTCAGTGTGGTCGTCAGAGCTCCAGGTACC
CCTACGGCTCAAGCGTGG
>at03449
CCGTAGCCATTTTGGCTCAAGCTTTCCATTGGCCCTAAGCACTGGGCACCGCCTCTTCTT
ATCCCGCCCCGTCGAGAGGCCCCATCCCTGGCCTCTTGGTGGGGATGGCCAAGAACCCAC
GTGTCTATCTGGACATTAGCATCGGGTCCCGAGCCGGTGGTCGTGTAGTCTTCGAGCTTT
TCGCTGATGTCACACCGCGCACTGCTGAGAACTTCCGGGGACTATGCACAGGGGAGTATG
GCCAGGGTCGCACGACGAGGAAGCCATTGAGCTACATGGGTTGCAACTTCTTCCGGTCCG
TCAAGGGGGTGATGGTGCAATCCGGGGACTTCCAGTTTAACAACGGAGACGGTGGTGAGA
GTGTTTACGGCGGCACGTTCAATGATGAAGACTTCTCGCGGCGCCACACGCAGGCTGGCA
TCCTCAGCATGGCAAACAAGGGCCGCAACAGCAATGGCTCACAGTTCTTCATCACTCTCA
AACGCCATCCTCTCCTCGATGGAAAGCACATAGTATTCGGCCAAGTGATAGCTGGTATGG
ACGTGCTTCGTGCCATTGGTCAGGTACCCACTGACAGGGACGAGCGGCCGCGGGTCCCAG
TGACAATTGTAGGATGTGGGGAGGTGGGCAAGACAAACAAGAGCCCAGCCAATTTGCATG
TGCAAGTAGAGAAGCACATCAATGACTTGAATGAGGAAATAGCACCCACTGGCGCGAAGG

TGCCTGAGGCAATAAAGGCCAAAGCTATCCTCACTGGCAAGCAGGGTGGTGCTGCTGTCG
AAGATGCCACACCAGTGAGCAGACGTCCTGATGATAAGAACGAAGAGGCTGACGAAGGCG
AGCAGGGCACCACCACCGTCCCTGCACGCAATGAACGTGAGCGTCGCCTTTTCGAGCTGA
GGCTGAAGATGAACCAATGCCGGGCATCGAACAACAAAGAGGTAGTGGAGGAGCAGAAGC
GTGCTGCAGATCCTGAATACGCCATGCGACAGGCACGGGCGCAACAGAAGAGGGAAAGGG
AGGAGAAGGCAGATGCGCAACAGGAGGGTGATGAGGCGCGAGCCAAAACAGTGCCCAAAG
GAAAAGAGTACATGCTTGAGACTGTCGAGACAGTTGAGATGAAGGATGCAAAAGAAGAAG
AAGGGGAACCCCGAGGCCTTTGGCTGGGATGTCTTCAACCAGGACTCGCTTCTGCGCGCG
CACGAGAAGCGTCTGAAGCACATCAAGTTCGAGGAGAAGGCTTACAAGGAACAGAAGGAG
CAGCTGCAGGGCGACACCTCATTATTTGCAGGCTTTGACCACAAGCCCACTGAAGAGGCA
AAGGAGCGCCTGCAAGGGGCGGTGGAGAAGATCTTGGACAAGAAGAAGGACTTCTCCAGG
CGCCGCGAGTATTGCCCCGAGGAGGATGTCACGTACATCAACGAGCGCAACCGCCACTTC
AACAAGAAGATGCAGCGCTACTTCGGGGCGTACACCGAGGAGATCCGACAGAACCTCGAG
CGCGGCACGGCACTCTGATGGGTCTCCGCAGGAGCTCCGGTGTCCTGTCCGTAACTCTGC
TGACGTATGTTCTGATGTCGTGCCTTGAC
>at03450
TTTTTTTTTTTTTTTTTCCGGCTAAACCTCCTCCAGGTCGTCGCAAAGGGTGCATGTGA
CCATGGAACAGTTGTCCTCCGCGCCCAAACTCAGCCTGACCTCCATGGCAAGGCCAGCAG
GCCCGACCACCCGCCACTTCAGTCCATCGCTTCTGCCATCGGGGCCGCAAACGAGGCCTT
CTCCAGGCGGGGCGCACGCACTGGAAGGATACAGCACCATGTCCCAGTCGTGGTTGACCA
GGATCTGGAACTCCTCCCAGGAGTGCTCCATGACAAAGCGCGCCGCGTAGATGTCAGCGG
TGCCCTCCAGACGGTGCATCTCCACAAGGCCGCTTCCACTCCAAGAGCCCGCGAGCGAGT
ACACGTCCTCGGAGACCTCCGGGCCGCTCTGCCAGGTGACGCTGCCCATCTCGGCCGTCG
TGGCCGTGATCTCGAAGTCCCTGCCGCGCGCGCGGAGCTTGACTGTCAGCGGNTCCCCTG
GCGCGCCCCAGGCGACCAGTTCGCCGGGGGGCCCGCGGACCGGNCCCATGATGGCGCCGC
AAGGTCCGG
>at03451
TTGTTTTTTTTTCTTTTTTTTTTGTCGATGCGAACACCGTTGACGGCCAGAGTCTGTTGT
ACACTGTGCTACTACATGGCCGCCTCACGGCCACAGCCCCCGCCTCACTATGCCCCTCAC
TCCTCGGGCCCCTCGGGGCAGGTCCGTACTTGGCCCACCAGTTACACATGCACTGGCCTT
CCGCTGGGCCTCACCCCTATGCATTCTCACAAGTGCGGCGGCACACCAGGCCCCCAAGCC
CCAATACGCTCCTGTAAGAAATAAAATGCTTCTATGCATTGCATGGCAGCCTGAGGCCAC
TCAGCACATCTCCTGAAAGACTCTTCAATCGAGGCCCAGCCATTCTCACATTGCCCGCAG
CGTCCACGGCCTAAGCCTGCGCTTCTACCATTCACCTCGCCACGAGCTGCTGGAATTCAC
TGACATCTCAAGCCATCACGAGGGGGGGGCAAGAACATGTGCCCTAGCCTTCACCACGCC
ATCATCTCTACCTTGATCCTTGATGGCTGTTCTTGAGCCAAAATGGCTACGG
>at03452
TCTTCGCCCTTGGCTAGCCCACTGTTGTGGTCAGGAGGATTTCAGATACAAATCTTCGGT
CTACGATCTACGATCTAGGCACTTCCCCATGCAATGGGGAAGGACAATTCTCAATCCATC
ATGCTCAAGCTCTTCATGAGCTTGGTAAGCGACTCAAACTCTGCCTTGAGCTTCTCGGAG
TGCTCATTCTTCTTCTTCTTCTCATCCTCATCCTCAATGCCAGACCTCACTCGGCTGGCC
TGCAAAAGNTGCATCAGGCAG
>at03453
CCGTAGCCATTTTGGCTCAAGGCTGTTCAATTTGGACGTGCGCAAGCTGCCCAGAAGTTC
TCTTGTGGTGGTGCCCTCGATGCATGCGCCACGCCGCTTTGCGCATGCCTTGCACGTATC
ATTCCTGCTATGTATGACAGTCGGCACTTCTGCGGTCGAGCCGACTGTCCTGGTGACAGG
AGCTACTGGCCGGACTGGAGCTTTGCTGTACAACCAGCTGAAGGACGCCGGCGTGAGAGT
GCGCGGTTTGGTGAAGAACGTGACGAAAGCCCGTCAACTGCTGAATTGTGACAAGTGCGA
CAGTTCTGAAGGCATTTTTGTGGCAGATATCACAAAGAAAGATACCCTGGGGCCAGCGAT
GCAGGGTGCAACAGCCTTGGCCATTGTCACAAGCTCCGTGCCGATTTGCGACCCATTCCC
CCATTGCAGCTTTCCAAAAGGGGGAGAGCCCATCAACGTAGACTGGCTCGGCCAGAAGAA
TCAGATCGAGGCCTTTGCAGAGTCACGTGGCAAGGGGCAAGCTCTTGGCCCTGTGGTCCT
TTGCTCCACTATGGGCACAACAGAGCCAGAGGATGGCAAAGGCGGCATTCCTGGTTTCAT
CGGCTTCTACAAGCTCAATGCCGAGGCGTTTTTGATGTCCTCGGGCGTGCCTTTCACCAT
TGTGAAACCATGTGGACTTGGCAACGGACCAGCAGGAGAGCAGGAGTTGGTTGTCGGGCA

TGATGACTCACTTCAGCTAAATCCGCCTATGATTGCTCGTGCAGATGTCGCGCGGGTCAT
GGCAGGGGCCTTACAGTCTGGGGAGCTCGCTGCCGGCCTTCGTTTTGACCTTTGTGCCAA
GAGCTCCGGAACGCCAACTGCAGATGTGCGGGAGGGCTGCGTGCCGCGCACTACCCTTGG
GAGAAGGGAGCTGCTGCAACCTCAG
>at03454
TTTTTTTTTTTTTTTTTGCGGGCAGGCTTGCTTTTGGGCTACACGCTGGCAAGACCAGC
GCGTGATCAGAGCGTTGTGCGCACCCTTTGGTGTCACTCAAGTTGCACTACTTGCCAGCT
CATCTGCCATGTTCTTCGTGTGAGTACATGACCCTCGTTGTGAAAGGTATCCACAGTCCC
AGCACACCCCAACTTCATACGTAGCTGCCCACGTCACTTACAGTTTGGTGGCCCAGAACA
AAAAGCGCTTTCGCCTCACTGTCTTCATGCAGGTTGCCAGTGTCCTAGTGCAACCAGTGC
AAGGCCGTTGAATTCAGGCGAGCACCGAGGCCCGAGGCTTCAGCACCTTGCAGGTAGTCA
TTCGAATCAGAGGGCCAGGCAGCCAGTGGTCTCAGGCCACTCTCGTCCGGCGAAGGCAGT
CATCGCCTGTCTTCCAGCGCCTTCACGCCGTCATCATCCCCAACTGACTGAATTAGGGAC
TTTGCGGCTTGAACAATCTCGTCAAAGCGCTCCTGCTTCTTTGTCCTGGCCTGTTCAAGC
TTTTTAAGTTCTGCTTCACGTTTCATTATGTACCACGCCAAGGCTTCCTGGTCGTGATGA
ATCTCCTTCAGCTTCTTCAGCTCGCCCTTCTGAAGCTCCTTCTTCATGGCCAAAGCGCTC
TTCCACTTTTGCTGCGCCTCGTGCCGAATTCT
>at03455
GAGGCAGCGAAGCAAGCCGGCGTCGGGCCACTTTCTGCCGTGGTGTCCCGCGGGGACTCC
ACACTGCGAGGCCCTACCCTTTGGAGACCGACGCGCTGGCGCGCGGCCTGCAATGGGGGA
CCGGAGAGGGCTCCGGCGCCAGTGCTCCCACCTTGATCCTGGCGCCATTCTTCAAGGAAG
GCGGCCGCCTCACGGCGGGGGACTACCACTATGTTGCAGGTCCACCTGGCTCTGGGGAGG
ATGGGAGTGCAAAGTTGACGCTGGCAGGGGGACACGGAGTTCGCCAAGGATAAGGCATTT
GGATACAAGGCTAGCTTCCTGCCGGCCTGGGTCCAGGAGAAGACTCCCAGTGGGGCAACT
CCACCAGATGTTCGATGCCTGAGCTTGGAGACCATTCGACGTGGC
>at03456
TTTATTTTGTTTCTTACTTGGCTCACCTAAGGTTGTGTGCGCGCTTGCCTGGATCTGAGA
TGTCACTCTTACAAGCGAATAAATCTGCGCAGGCCCCCGCCGCCCTCCTCCTGTGCAACA
CCCACAGCAGGCTCGCCTTCTTGAGACATACCGATGTAGAACAAAAGACCACCTCCAGCT
CTCGGTACAAAGTGCGGAGCGCTGACAGTCTTTTTCATCCCAAGCACCTGCCTCCGACGA
AGGCTGGAAGACAGGCTCGGTACCTT
>at03457
TTTTTTTTTTTTTTTTTGATGCCTGTTACCTTGCCTGCCGGCAGATCGCGGTCTGGTCT
GTGGCTCGAAGGAAGCACACCCTGTCACGCTAGGTCCACTCTTTGGCGGACACGACAAAT
TGTCTGAGAAGAAATTATTCTATGCCATCAATCGGCCCAACGTACCGGTTGGCAAGAAGC
TGGCCGCCTTGTACGGCCAACACCCAAAACATGCGGATTCGCCTCCGAACCCACAGAAGG
CAAGATTCCGACGAAGCCGGCTCATCGTCCTGGTGCTTCGCTAGGTTTTCTGCTGTGTCG
CATTGATGGCTCCTCCGTGCTGAAGAAGGCTGCACATGCTGCTCAAGACATTTTCTTCTG
CGCGATGACCTGGTATGCCGTCGTGAACATGCGCTCCTTGTCCATCGCGATGAACGAGGT
ACCATGGTCTGTGAATCGCTTCAGAAGCCTGTGGAAGTGACCCGGGAGGATGCCGAACTT
GCAGAGGTATCCGATCAAAGCCTCCAGGCCCTCGAAGAGCCACCTCTCCTCCTCGATCAG
CGGAGCTTGCAAGTCGCCCACGACCTCCTCGAAGATGACCTCCATACCTGCCTTCTCGAT
GGTGTCNCTCCAGTAGGCCGTGTGCCATAAGCCACCGAGCCTCGTCAC
>at03458
TTTTTTTTTTTTTCTTTCTTCAGGGACCTGGCGCGACAACATCAAAGGTATGAGACGGA
ATTCATGAGCGTCCAAAAGCACCTTTCACTACACAGACTATACAGTGCACCCGCACATGA
AACCACCTCAAATCCTTTTGTCAGTCTACCTAATCCAAGCCACTTCTCATCACTTCTTCT
TGCCACCTCCAGCTGCGTCCTCCTTCCTTTCACAAGAGATAACCTTCCCCAGCATGACAA
CACCGTTCCCGTCCATGAACGCAACGCGAGAGAGACCCTCACAATTCTTGAAAGTGTCGC
AAACAAGCGGCTGCTGAGGCTGGAAGCTGCACTGTGCCATCTCATTTGACTTGAGAGAGT
GCGGATCTTCCATTTTTTTCCCGCCGGTCTCTTTTCCCATCTTCCACTTGAGCTTGGAAA
TGCGGCACGCAGAGCGACCACACCTGACAAAACCAATTGGTGAATAACCAACCTTGATCT
CATTGGGGATATCCAGGACCTGCATCTGGGCGTCAAACTCGCGCGTCTGACCCAATGTGG
TGTCCTTCTTGTAAACCATGACGTCACCAGAGCGCGGCATGTTGTTCTTGTCCAGTCCCT
TGATGTTCAAGCCAACGTTATCACCAGGATTTGCAAAGTCCACCCGCTGGTGGTGCATCT

CCACTGTGAACACCTTGCCAGTGCATGGATTGGAGGCTGTGTGCGTTGGCAAGAACACCA
CCTCTTTCGCAGGCTTTCACACCCCCTGCTCCCACCGGCNCGGCCAGGACATCGCCGACG
CCTTTGATCTTGTAGATGCCAGAGATCGGCATCCGCATGGGCGCACTGACAGGCCTCTCC
GGCACGCGGCAAAGCTTGT
>at03459
CTTGAGCGGCCGCCGCCCAGAGAGCCCGGACCCGTCCGGCCCGGGCATGGCACCCATGGA
GTCCACGAAGGCAGAGGATGGCCTCAGCCGCGAGGAACTCGAGAAGGCCCTTGGCAACTG
CGGCCTGGAAGAGCTGCAGGAGATGAGCAAGCGCTTGGGCAAGGGCATGGAGCAGTTCAG
AGAGATGCTGGACGACCTGCAGGCTGAGCAGACTGCCTTGAGGGAGGAGAACGGGCAGCT
GAACGACACCATCACGATGATGA
>at03460
CATCCTTGTGCGTGTCAACTACAAAGTTCCCGTCCCGCTGCTGGCGCCTCTAGAGTTCAC
CTCCAACCGTTCGCTGTGTGACCTACTGCATTGTGAGGGTAGCTTCAATGACAAGAAGGG
ATCGTTTCCGGCTGGCTTTGCTGCCATCGAGTGGGACACCACCACCGGAGATGGCAAGAC
CTGGCGAGGGATGGAGATGCCTCCCACCACCAAGGGCCACAGCTCATTTATGGGAGGAAC
ATTGCATTGTTTGCCGAGATCGTAGAAGGCGGCGTGCGGCT
>at03461
GGCGGGGACAAGCCTTCATGTGGCAAACAGACTCCATCCTCCACACTTGCAAACCAAGTG
GGGAATACAGGTCCCGCTATACGCTCGTTTGAAGCATGCATGCACAAACTTCATGGATCC
TGGAACGGACTTACAGCGCTGAGCAGAGCGAGAGCAGCAAAACCCGTGGGAACAAAAAAT
GTGCGGCAGCAGACCGTACAAGCAAACCAGCACGCACATCCAATGCTCAGTAGCCCAAGT
CTGTACGATAGGGTATGGAGCAAGATCGCGCACAGCGACACTgGGCAGCAGNCACTGGCA
GTACCCATAACCGGTAAGGGAAGCGTAAGAGGAATATCTCCACACTAGCCCTCACTGAAT
ATGGAGAGCCCATTGAACCACTCCACCTTGTAGGCGCCGCGCACGTAGCCGCCACTGAGC
AACATTCCACCAGAAGCTCCAGGCAATGGTCACAGCCGCAGTTATGCGGTAGGCGTCCCG
ATAGCCAACCGCCGTCATCACCGGAGCCCAAACCACTGCGCCGCAGAAAGCGGACAGCTG
GAAGTACAGGCCCGACGTGAGCCCCTGAAAGCGCTGCTTGTGGGCGTCCGGGGCGTAGCG
CCGCCCGAAGTCCACGGCGGCGCACCACGTCGCCGCGAAGGTGAAGCCGTGGAGCGGCTC
GACGGCGAGCACCAGCCACGGGCGCTCCCTCGGCAGCGCCGAGTACAGCGCACACTTCAA
TGCCAGGATGAAGTGGCTCAAAGTGAACAGCGGCACGAAG
>at03462
TTTTTTTTTTTTTTTTCTCCGTCTCCGACTCTGCTTCTAGCTTTGTCTCTTGGCCGACCA
AGGCCGCTGGAGGGGCCCGCGGGTCACTTGTACTCGGTGGCCTTCCTCTTGAGGATGTCC
TCCAGCGGACCCAGCAGCATCTTGAAGGTCCTCCCGGACAAGGTTAGGAGCCTGCAGTCG
GTGGAGGCTACGACGCTGGCTGCCCGTGGCTCATTCATGAGCAACGCGAGCTCGCCGAAG
TAGTCTCCGCTTTTGTACTGCATGACCTCCTGCGGCGGCGTGTCCCGCACGTACACCTTC
TGGGCGATGCACTCGCCCTCCTCTAGGATGAAGAAGATGTCGCCACTGGCCCCCTGCTGC
ACTATGGTCTCACCCTTCTTGAAGGTCACCGTCTGCAGCGCATCGCACATGGAGGTGAGC
TCATAGGCCTCCATGCTCTCCAAGATCGGCACGCTCTTGAGGAACTCCTCGTAGCGCTCG
CGCCGCTTGCAAGCGGCGCCGCGGACGATGTGGTTGAAGGTCTCCCGGTCGAGCTGCCAC
AGAACGCACCTATCGTGAGCCTTCACAGATGCCGCCCTTGGGCAGTTGTAGAGCAGAGCA
AGCTCGCCGAAGGCGTCGCCGGCCTTGCACTCCTTAACCAGCTTGTCCTCGCCATCGGCC
TGCCTCTTGTAGC
>at03463
CCCACGTGGGGTGCAGCCAGCCTAGATGGCTCCAGGCACCTCCAGGAAGCGTCTCCTGAG
CACCGGCAGCGGAGGAGCTGTGCGCGAAGCATCTGGAGGAACAGTCCTGCACGCCAGTGC
CCAGCGGCAAAGCAGCCTGCTGCGAAGGCTTCCGTTGCGCAGATGGCGGCCCGCTCAGCC
CAGCCAAGCTCCTCCAGATAGGACTCGGCAAGGGAGAGGGCACCAATCTTGAGGCCCTGC
CACCAAGGA
>at03464
TTTTTTTTCTTTTTTTTTGGTCCAGTGTGGTTCTTGTACAGAATCACGGTACCACTTGCG
CGCTTGCCAGCAGAGGGCCGTCACTCCTTGCCCTGCCACAAGTCAAGCACCAAGGGACTG
CCCTCCAACTCAGTGCCATTGAGCAACAGCAGCGCGTTGTTGGCTTCCTCCGA
>at03465
TTGTTTTTTTTTCTTTTTTTTTAGTACGCTCACACAGCTGGCACAGCAAACAAAACCAAG

CTGCCTCTCACAGCTTGGCCCAAGAACTCACACAACGCGCAAAACCAAACTGCCCCTCAC
AGCCTGGCCATGCTTGTATTTCGGACCCGGCGCAATGCCGCCCAGGCTCGAGCCACAGCG
CCCCCCACAGCCACGTTGTTCTTCACTAGCGCTATGTTGGCGCGCAGAGATTCTCCGCCA
GTCAGCTCGTTTATCCGCTTCAGCAGAAAGGGCGTCACCTCATTGCCCTTGATGCCTTGG
TCCTGGGACTCCCTCAAAGCCTTCAAAGTGGCCTCCTCCACACTGGCAGCCTCAGCTGCG
AGGTCGCCCGGCACGGGGACGGCCATCACGACACCGGATGATATCCCGAGGGAGTGATGG
CTCTGCAATAACTCAGCCATGGCCTCAGGGCTGTCCACGCGGCAGGCCAGGGGCAAGCCG
CTGTGCGGCGTGAAGAAGGCCGGGAAGTCGTCAGTACCGTAGCCTGCCACGCACACGCCC
TGCGTCTCCAGGTACTCCAATGTCTTGGGCAGGTCCAAAATGCTCTTTGCACCGGCGCAG
ACCACGCAAACTGGCGTTCTTCCAAGCTCGGTTAGGTCGGCTGAAATGTCCCATGTCTCC
TCTGCACCGCGGTGGACACCGCCTATGCCACCCGGAACGAAGACTGGAATGCCAGCACTA
TGCGCCAAGAGCATGGTGGCCGAGACTGTTGTGGCCCCGTTCTGCTTTGCAGCGACGGTT
GCAGCAAGGTCGCGTCGAGACACCTTGCGACACTCAAGACCAAGCCGGCCGAGCTCCTCG
AGCTGCTCAGAAGTCATTCCGATGTGGA
>at03466
TTTTTTTTTTTTTTTTTGGCCTTGGCAGCTGCCATTGCTGCCTGGTCCGCGGTCGGTCC
CCCCTTCCGCCGAGGCGCGCTCAGCCCAGCGGGACGAAGGGGGAGCCCTTCATGCCCGCG
CGGCCGTGCTTGATGGGCTTGTAGGTGATGGAGAACTCGGCCAGGTAGTGGCCGATCATC
TCGGGCTTCACCTCCACGTTGATGTACTGCTTCCCGTTGTACACACCCACAACACTGCCG
ATCATCTCTGGCACAATGACCATGTTCCTGAGGTGTGTCTTCACTGCCTCGGGCTTCTCT
CCGTAGGCCGTGTCACGCTTTGCCTTCCGCAACTTCTTGAGGAGAGTGATCGGCCCCCTC
TTGATGCCACGGCTGAACTTCCGGCGCTGGCGGGCCCGGAAGAGCTCCATGAGGTCCTGG
TTGCTCATGTCCAAGAGCTTGTCAAGGTCAATGCCGCGGTAGGAGTACTTGCGGAAGGTG
CGCTTCTTCGGCTGCTCGTCACCCATCCTGCCGAATCCGAGCGATCTGTTGGCTGTAGGA
GGCAGCGGAGATTGCTCCAAATCCCTTGAGCCAAAATGGCTACGGACTCTG
>at03467
TTTTTTTTTTTTTTCTCCGATCACCGCGGCAGTGCCATGCGCTCCTGGTTTGCCTGGTC
ACGCATCCGGTCATGGGCGGCCGACAGGAGTTCTCTGAACTCCCTCACATTGCACCCACG
TGACCTGAGCAGCGCCTCGCACTGCTCCTTGTACTCCGGCTTCAGCGCGTCCAGCAGCGC
GAACCTTCCGAAGAAGACGTCCCAGCGCTGCTCCGCCATCTCGATCTGGTCCAGGAAGGC
GAGCTTCCCCGCGGCGTCGAAGCCGCCGTAGAACTCTCCCAGCTTGATGAGATTCCCATG
GTCGCGATAGAGTGCGTCGTACTCCTCCCGCACCAGGGGGCTCTCGAAGACCTCTTGGTC
GAAGACGGAGGAGAGCTCGATCAGCTTGCCGGAGCTGAAGAGCGCCTGGGGATCGCCCAG
GCCCTGCGGCCTCCCCCCCGCCGACTCTGGTTCCATTCCCAGGCCGGGGTCCGGCGGCGG
GGGCCTGCCCTCGCTGAAGGCGAGCAGCGTGTCGATCTCGTGGCGCATCCACCGGCCGAT
CCGCTCCAAGGACTGGCCGCGGCGCGCCAGCAAGGCGTTGGTGAACTCGAAGCGTTCACG
CAGCTGGAAGTCCTGGCAGTCNCGCTGCCGCTCCACCAGTGCCTCCCAGCCCCCCTCGTG
CCGAATTCT
>at03468
CGCTGGCCCTCCGCAGCACCTCTCGAAGAGGCTCTTGTCGCCCTCGGGCTCCAGGCCGCA
CAGGCTGCGTACTCGCACCTGGCAACACGCCGCCCTCACCGCGCTGCTCCTCCAGCAGCT
CCCCGGCCCCTCGGTCACCGCTTTTCTCTGCCCTCGGATGAGAGTGGCTCCGACCGCCGG
GTGCCGCTGGGCACAGCAGCGCTTGAAGGTGTACTGCCCACGCCAGCAAGCGCTGTTGTG
TCCAGCCGAACAGCACAGACTCTTCATGAGCCATGGGAAGCCGAGCATGGCGTCATCCTC
ACCGCCCCTTGGCGGCCAATGCTCACGCAGCGCCGTCGGCTGCCAGCAGCGCGGTCACCC
CAAATTACGGTCCCGCACGGTCGCCTGCGCTGC
>at03469
GTGGCCTGCTGCCGCGCGAGGCTTTGAAGCTCGTCGGCTTGGTGGCCAAGGAGGGGTTGT
GCGGCATGGAGGTCGTCGAGGTCTCGCCGCCGTATGACCACGCTGACATCACGTCGCTGA
TGGCGCTCAGGATCATTGTTGACACATTGGGTACCCTGGTCGCTTACGGGAAGCTGGGCG
CGCACAAGTCCATTATTGACAGGCCGTGGAAGCCATTGTGACCGGCCGCATACGCAGTAA
AGCCGCAGCACGGCTAGGTTTCGACACACGCGAACGCCGGCAAGTGATCCGGCTGACTTT
CAAACTTGCCACAATTCTATGATGACGCTCATGTG
>at03470
CCGTAGCCATTTTGGCTCAAGCCATTTTGGCACAGGGTAGCAAGAGCAAGTCTGTCGATC

CCTTGCGACCTTCAGCGCATCGAGGAAGACTCCAGGCAGCAAACCCCAGCACAGATGTCT
GCCGAGCTTGTGGAGGCCATGAGCGCTATCAGCCTCGAGGCACAGACAGAGGAGCAGCAG
AAGCCGAGCAGCGACTGCCTGACCATGAAGGTCAAGGTCACAGGTGGGCCCTCCTTCGAA
CTGGAGTTGGAGCCGACGATGGCGGTCAGGGAGGTGAAGAAGCTCGCCAGGGAGCACTGC
AACATCGAGCCCGAGCACATGCGCATCATCTACAAGGACCAGGTCCTGAAAGACGCGGAC
ACCATGGCCAGCTGCAAGCACCTCGACGCCGCCGAGCCCTTGCGCGTGTGCTTCACGGCC
GGCCATGCCTCGCTGATGGGCGGCTCGAAGCCGGCCCTGCAGCAGCGCAACCCCTTCGTC
ACGCCCGTGCGCGGCCTCCCGGGCTCCAAGGGCAACCGCCCTTCTCGCATGTCTGGCCGC
CTGGGAGGCATGGGCCTCATCCGCTGCTACGGCATCATGATGAAGCGACAGGAGTTCCGC
GAGAAGGCTGTGGAGATCGGCTTCGTCAAGTACCGCTGAGCGCTTGAGAGGGCCCTTCGG
GAGGCTTGCGGAGGGATGCGGACAGATGTCGTCATGAAAAAA
>at03471
TTTTTTTTTTTTTTTTTCCTGCGTCAGCTCAAAACTGCCGAATTCCCGCATGCTGCCGA
CCTCAGCCCAAGGTCAGCAAGCAAAAGCCACCGAAGACCTAGCTGAACCAGGAGCCACTC
TTCTCAGCGCCCTTGATGCACGAGTCAACTGCACCTTGGATGATCCCATGCAACTGGGCC
GGAGTCTTGCGTCCTGGCTGCACCTCCTTGTAGAACTCGACGCGAAGGGGGTCGCGGAGC
ACCTTGGAGGTGTACGAGTCGATGCACGAGGCTTCCGACAAGGCCTCGTCGTTCTCGCGC
GTGGAGGAGACAAGGAGGTCCTTCTCCGTGTCGAAGGCAAACTTCTCATGCGTGACGAGG
TTCTCGAAGCTGTCGATGACCTCGAAGTCACCACCGTCGAGCTCCTTGACCTTGTGCTCC
TTGATCCCTTCCTTGCCGAGGCCGTCCTTGATGACGCTCACATAGAGGGCCCAGAACTGA
TCTGGGGTGATGCTGGCGTCCAAGGCTTCCGACACCGCACATGGCTTGCCGTCCGAGGAG
GTCACGCCTGCCTTGCAGACCGGGGAGCCGTTGGTCACCCCGGCACGCTTCGCCACCTGC
CCCAAGAGAGCCGATAGGATCCCCGCCATGGCAGAGCCAGCCACGCGCTGCTCAACGACT
TCGGTCCAAGCTCAATCCGCATGGGGAAGA
>at03472
TTTTTTTTTTTTTTTTTTTTTTTTTAGCGCCGTAATGCCGTCGGTAAGCGAAAGCTTC
TCAGGAAACCGTTCCCATGGCTAGTCACAGCACATGATAGCCAGTACTCTGGCAGCTCAT
CACTCTGCCACTCACTCGGCGGGAACCCCGGCATCAGTTCACCTTGCCAGCGTGCCGCCT
CATCGATAAATCCCAGGTTCCTGTAGAATTGGAGCCCGGTGGGGTTGTTGACCTTGCACG
TGAGCGCCAAGCGGGGCAGCTTCAGCTCCAGGCAACGCTGCCGAGCGTGTTGCAGCAGCC
TCCGGCCATGACCGCATCGGCGCTGCGCGGGGTCAACGGCCAGATGCTTCACCGTCAGCT
GGGCGCTCCCGTCGCGGCGACCGAGCACGATGAAGCCCAAGCAGCGGCCCCCTTGCGCCA
GCACCACGCCCTCCCAATGCTCGCTCATGTCGCGAAGTCTGGAACTCTTGGTGAGGCACT
GCTGCAGGGTATCCCTCTCAAACTCTACCTCTGATAATGCGCGAGCCGCTTTTGCCAGGT
CAGCAGACAAGCGACCGTCCACGNGTGATCAGGCTCGGGCCTGCCAGATCGGCAATGGCG
CCAACATCTGCAACTGGCGAGGGTGCAGGCTTGGAAGCAGCCACTTCCCCTCCCTCGCCG
GCGTCTTGAGCAGGCTCCTCGCACATCTCGGCGAGCTCGGCCCAAGGCCCGGCAGCTGCG
TCGGGGGCCACCTCTTGGCTCATGGCACGCGGACGGCGTCCTTGCTGGGTCTCAGTATTT
GGACTTCATCTTGAGCCAAAATGGCTACGG
>at03473
TACAAGCATAAGTTGACTTTGCAATGCGACATCCTCCCCTCGGAGCAATGAGGCAAACCT
AGCCCAGTCTTCTCAGCTGTAAAGCTTCAGGTCGTACTTCACCTTCTCAATGCCCATCGG
GTTCAGCGTGCGGATGAGGGTCTCGACCTCTTCTGATGTCCTGTAGTTGACGGTGTACAT
CCAAGCGAAGGGCACGCACGTGAGGCCTATGCACGCGAGGTTCCAGTTGTACCACTTGAT
GCGGTCCTCCTCCATCATGTTGTAGCGCCTCGCGAAGCGGCTCTTCGTGAACTCTGGGAG
CTCGATGCGCATGAGTGAGCGCCTGGACAGAGCAAGCGCGCCACGCATCGCCATGGCGGC
AGTGCTGCTCGAGGAGAGCTGGGTGGGTCTAGTGGGCGCTGCACCGTACTTGAGCCAAAA
TGGCTACGG
>at03474
TTTTTTTTTTTTTTTTTGCCTTGCGCTGGCCCTGACACCGACCGCGTGCCACTCCCAAG
CTCGGCCCTTGGGGAATGGCTGCGAGAAAGCCTCCAAGCTCAGAAGTCAGCTGCAGCCTC
TCAGCAGCCAGAAAGTCCGATCATCGGTTCCGCTGGATGAGGGTGGCAGAGCGATGCCCG
AAATCAACATCCCTGTCGTCGGCATAGTTGACCCAGGTATTTCCTTCAAGGAGGTCGTCG
AGTTCGGACATCAGCTCCTGCTCATCGTAGCCATTGTACAGAACCTGCTCCCAGAAGCGC
ATGAACTCCTCCAGTGTAATGAGGCCGTCTCTGTCCTCATCAACCTCATTGAACATGGCA

TTGGCGCTGATCTCCGGAAACTTCTTGAAGAACTTCTTAGCCTCTTCCTTCGTAAGCCGT
TTGTTCCCATCCAAATCCATCAGGTTGTACAAGGCCTCCACCTTTGCACTGAGCTCCGTG
GACAGTCCGGTACGTGTGACCGTGTCCAACATGACCGTCGGCAGCGGAGAGAATACTCTG
CCCTTGTTCGGTGGCATGAAGCCGCAGCGAACGCTCTCCGCAGGCCTGACCGCCGGCGTG
GAGGTGCTCCTCCGCATTGGCTTCTGCGACACGGAGGAGGAGACCTTCCTCGGCTCGACG
GTGGGAGTGCCGAGGCGGAGGGAACCCTGGACGTCGCCCTTCACGTTGTATATCCGGACA
GAAGAGGCTTCGCTGGATGCTGCAGTGGCTCACCCAAGTGCCTTTTGGGCTGCGTCCTGN
ACCCGACAATCTGCATCGCGGNACGCGACATTGTCGACACTCGCTGCAGAATGGCGGCAC
ATCGCTCTTCCTCTTGCTGACTATGT
>at03475
CCGTTAGTCCATTTTGGCTCAAGTGAGAGCGCCCAGTATTGTATGGGTATGCTGCACAAT
GGTGCCACGAGGTCCATGCAGGCAGGTGAAGTCCTTCAAAACAAAGGACGTATGCTTTTC
ACACGACCCTCGTGTTGGCGGTTCCTGCCCTCTAGGCGTTCTCTGCACAAAGGAGCATCT
TGACACGTTTGAGGAAGAGTGCGCGACGCGCTTCGACGACGCAATTTCCGCTTGGAACGC
CGCCTTAGCGCGGCGAAGGGGAAACGGCGACAGTTGATGCAGCCTCTGAAGGCCTGCAGG
AAGATGTGCTAGAGCCGCGCGCCATCACCAAGGCACTGTTCGCTTCCACGGACTACGTAC
TGCCCAAAGGCGTGTTTGATGTGCCTT
>at03476
GTGAAGGTCGTCAAGGCTGACAAGTCCACCTTCTTCGTCGGCAAGGGGCCGCTGGGAGGG
TGGATCTGGATGACGCACCAGATTATCGGAATCCAAGCTGTGCACTTTGCTTTTTCCCTC
CTGGCTCCGCTGCTCAACCGACGGCTTGCGCTGGGAACCTACCGGCTGTCAGTGCTGCTT
GGGGGCCTTGGCACATTCGTGACGATTCAATTCTTCTGCCTTGTCTACCCGACGACTGCC
TTCACCGAGGAATGCCGCATATGGGCTCCACGGGTCCCGCAATACAGGCTCATCCAGTCC
TACTTGCACCTCCCGCAACTGATTCTGGCAGTCACTGACGTATGCTTGCTGAAGCAGAGG
AGCACGCTGCTCCTCGCCTCGCCGCC
>at03477
TTTTTTTTTTTTTTTTTGCCACTCCACAGCCTTGTTGATCTTGTTCAATTCATGGATTG
CTTCCTTGTCACTAGTCCACTAGTTCTGGTCTGCTGCACGACAAACAGGGAACTGGTTAC
GGGCCCCCGCCGTGAAGGATACACAAACATCTATACATGATTGTGTTGCCGCTCATTTGC
CCCGGCCTCGAAAATAACTGGATCGTATCAGGGCCATCCACTTCTTTGAAACCATGATAC
AGCCCATCGCCTGAAAATACGCAACTCTGGACTTTGTGGAGCGTCATCCTCTGATTCGCA
CATATTGCTCCGTTCGAGAGTCAAGTCAAGAGTTGAGTTGTTCTTTGCGATGATAAAGAT
CCACACATTGAGCATCCCGCCTCCAGTCGCACAACCGCAGAGGCAAACGACACCTGCCAT
GTCATTTGCACAATACGTAGACAAATGAGGATCGGCAGTGATGGCTGCAAGTAGACGTTA
CCCAGGTCGGCATACCTTCTGCAAAGAGGTGATGCTTCCCGGAGGCAGCTTGGGGTGCAG
TGTCTTCCAGATGATATACAGTTGTTATCAGTCAATCGCAGTCCAACTTGCTGCTACTCC
AGTCGATACACGCGTTTGGCTGTGCCCCCAAACAATTCCGTCATCTCCTCCTGAGTGCAC
CCACGACCCTTTGCAATCCGCTTGAATGCGTTCACAACACGGTGTATGAGCAGCTGCCCT
TATCCATGGGGAAGTTTGACTCAAACATGCAACGCTCTGCACCAAAGGCATCGATCACAA
ATTGGTAAAACGGCCAANCGATGGCAGCCACCTCCTCCGAGCTCGGTGGCAATGGTCG
>at03478
TTTTTTTTTTTTTCGTTCAGGTCTTGCATGCTATGATGACTCGTTCCACCTGGCAAAAA
GTCAGAAGCTCATCAGCTTCTGACGCTGCATCTGAATTAGCTACCGGAGGCATGCTCCCT
ACCCACCCCAGCCACTTCAGCAGGAAGGATCACGTAGCCAAACATACATGAGAGAGTCTC
CTAGCATCAAAGAGCTAGATGCCACGTGCCCTGCTGATCGAGCAAAGGCTAAGAGCAAAG
ACGGAACTCCATCATGACCGCATGGGGCAGGCGGCCCCCTGCAGAGGTGGTGTGCTTGTT
GCGACTTGCCCACTAGGCACTATCAAGCAAAACACACTGTACAACTCCCAGGAAAGATCT
GTACAAGAATGCCCACATCTGGAATGACTTAAGTCAGTGCACACCATATAAGACCCATCA
GCATGATCAAAAGGGCAATGCTGTTCATTGAATTGGGACATCCAACTCCTCCTCCAGCTT
TAGAACCAAGTCCAGCGCCTCCTTCGATGAAGGCTGCGAGACGACAAGCTGGCACAGCCA
CGTTGCAAGACCGTCCTCGCGGGCAACCACCGTTGTACCAGCATGGACGAGCGGATCTGA
AGAGATGACGCCAGGGCGAGCCCGCAGAGCCTCGAAGCACTGTGCACTGAGGAGCTTCCC
CGAACGCCTTGCGTTGTANAAGCAGTAGCCCACGCAAGTGAGCGGATGCGCTGGCACAGG
AGGTCGCGCGGGGAATGCCAAGGCAATGAAGATCGCCTCCTCGACAAGGTCTACCCCCCA
GACAAGAC

>at03479
TTTTTTTTTTTTTTTTNTTTTTTTTTTTTTTTTTTTTTGTTGATTCCGTGCGCACATC
AGGCATGTCCTGCCTCACTCATCACAACTCGCATCCCTTGTCCTTTGCCCGGGGTGCCGA
ATCACTGGAAAAAGGCTCGTTGGCCAAATGGCCGGTGCCCAGCCGGGCACTTGCTTCAGA
GTCCGACTTGCAGGAGAATGGATGCCTGGATTGAAGCGCTGTCTGGTCAAAGCTTCGTTC
TGATTTGTTTCGTAGAATGTTTGAAGTGTGAAGCAAAACTGTGTCTGGACAACCCATGAG
CCTGAATTGCTGGTCGCATTGCTCCGCACCACAGGGCCATGAAAACGCCGGGTCGTCCTC
CGGGGGCACAGGATCCTGAAGAAGCAGGATCCGGCAGCTGTGGTCCGTGAAGTCTGGGTG
CAGACGGAGGGGCATACCAAGCTCGTTGGCCATGAATTCTTCGGTTCCCGCCTTGCAGAG
ATTGAGGCAGAGGTTCTTGCAACCGCCTGACTCCTCCATGAAGCCTCGTGCC
>at03480
TTTTTTTTTTTTTTTTTGATTCAAAAACACTCATGGACGGCCAAACGGCCGGAGCCTTG
GCGCAGAGAAGAATGAAGACAGGTCAGAGAAGACACCAGTGCACACCACGTNGAAGGAGA
TACAACACACATCACTCGACATGAGTCGACGTGCATGTCAGCCAGCTTGCGTCAGCCCTT
CTCCGATCCTGCCCGGAGGCGGCAGGCACCACGCCTGACCAGGGGTCTTCGAAAATGGCG
CTCAGGCCCCAAGCACCTCGGCCTTGGGTAGTGCCTCCCGCAGCTGCCTCTGACACCGCA
GTCCCAGCACCACTCCGCAGTCCCAGCACCACTCCGCGGAGCACCAGTCTGCAGAAGCAG
CCAGCTCCCCAGCGCCGCTTCGAAGCGGGTCCCCCACTGAAGGACAGAGTCCTCCGGCCC
TGGCGTCATAGCAACCTCACAATCCTGCTGGTCAGCGCCTCCCGCCTCCACAGGATCTCT
CGGATCTCCGGCGCCGTGGTTTCTGGCGAGCGCCAGGTGCAGAGGGCCTCCGCCGACGCC
CTTGGCTCTTGGAGCACCGGCTCTGCTGCCAGAACCGCTGCCAGCAGGCAGCGGTGTGCA
AGCACAGCTGGTTGCCACCCCCGAATCACAAACTCATGCCCCANAGTCTCGAGACTGCTG
CANAGATGATTTGCTCGATATGCCTCAGATCTGCACATCGAGGAACATGCGAAGGTCCTT
CACGCCAAGCAGCGACTTGCACACGTCATAGAGGAAGACTGGGCGCTGATGATGCATCCA
GCCCAGACGCATCGTG
>at03481
TTTTTTTTTTTTTTTTTCAAAGGCTGGGAATGGCGCTCATGCCCCCCCGGGCCCCGTCA
CGCGTCGGCACTCCACCAAGGCCCCTCTATCAGGCGTTGAAGGTCTGCACGGTCTTCGTG
ATCTGCGCGCGGCAGAGCGGGCAGGTGGTGACCTGGTCAAGGCATCGCGCGCACACCGTG
CGGTGGTTGCAGTTGAGGATGACCGTGTCGATCTCGCGCTCGTAGCAGATCTTGCAGAGG
TGCCTCTCCAATTCCGTCTCCAAGCGCTTCTGCAGCTCGTGCACCTGCAGCTGCAGCCTA
TAGGCCTCAGAGTTCTGATCCGCCGCCTGCGGCTGGCTGTCCGCACCGCTGTCCGGCTCC
GCCGACGCGTCCTCGCCCAAGGCTCCGAGCGTGGACACCGGCCTCCGCCTCCGCTTCTCT
TCCCGCGGGAGCTGTTCCGGCAGCGCTATCCTGTACATGTCGTTCCTGCGCTGGCCGTCA
AAGCCGCCCAGGATGTACATCGCCCCCCCGTACATCGCGGCGCAGTGGAAGGTCCGCGCT
GATGGCGGGTCGCCAAAGGTGTTCAGGCGCGACCACGACCTCGTATCGAAGTTGAACTCG
CAGAGGTCGGCGAAGCGCGCCTGGCGCTTGTCCACGCCCCCGAAGACGAACATGCAGCAG
CCGTAAACGGCGGCGCTGTGGCGGTACCGCGAGGGCGGCACGTCCCCTCGACCAGGTATG
CTATACCAGTGCTTTGTGGTCATGGAGAACTCGTACAGGTCATCAAGCGTGTCGTGGCCG
TTCCAGCCACCGAAGACAAACATTCCTGTCTTGCAGACTACCGCGGAGTGGCCGAAGCGG
CCCATGGGCGCGGCACCCTCTGGCCGGATCTCCACCCACTCGCGCTCGTCGATGCTGTAC
TGGTGGAGGTCCTGGAAACGCGACCTCGCATCGAATCCGCCGAAGATGTAGAGGCTTGCC
TCGTACATGATGCAGGAGTGGTCCGTCCGAACGCTTGGCTGGTTCGCAGCGTCGATGCGG
GACCAGGTCGCGCGAGGGATGTTGAACTCGAAGAGGTCGTTGAAGTAGTCCCCATCCTTC
TTTGTGTACCCTCCAAAGAAGAAGATGCTGTCGCGACAGATGACCGCCTTCGAGCCTGAC
CTCGCAGAGGGCGCGTGGCCGCCGGCGGGCTCCACCAGGGCCCAAGTTCTCTTCTGCACA
TCGTACTTGTAGATGTCGTTCTGGCGCGCATTCTCGTCCGTCNNCGCCATGAGATAGAAA
CAGTGGTTCCCAGACACACGGCGTGGCCCGTGCGCGGCGTGTACTTGTCGCCCATCTGGC
TGTTTGACGGGGCTGCACGTCACATGTCCTCCGGCATCAGCGCGCAAGGCGCGTCCTGCG
CGTCGCCC
>at03482
CTTTGTTATTGTCTAGCTCCGCATCTTGCATAGGGCACGGATTGGAGGCCATGCCTCAAA
GTCCAACAAAGGCTGCCGCAAAGCTTGTGCTGCAGCGCACTGCTTCTGCCGCGAATGTCG
GAAGCGCGGGACAGGCACGGAGAAAGAGCAACCCTGCGGTACGCTCAGTCAAACCTGTTG
GGGGGCAGCCTCTACGGCCTATGTAACTTCGATAAGTCTCAGGTGGCGGCACGGAAGAAG

GATAAGGGCTCCATCGCTGTGCCAGAAGACGCAGTTCCTTCCACCGATGCCTCACCGATG
CCACCTCCG
>at03483
TTTTTTTTTTTTTTTTTTAGGCTTGACGCTCGAGGCCTCATTCTCAACGACTGACCCTGT
ATATCGCCTCTACACTGCCTGCTTCCCAGCAACCTCCGCCCGCCGAACACGGCCATCCGC
CCCTGCTCAGTCGATGGGCGGCAGGTCGTCCTCCTCGTCCCCGCCCGCAGGCACGGCGGG
CTCGGCGGCGGGCGCAGCGGCGGCCTCCTGGAGGCCCTTTGTCTTCCAGTCTGGGTCGTT
CTTGTGCTGGAAGCAGGGATGCCAGAAATGGACGGTGAACACTCACGCAGTCTCCATCCG
CAAACTCGTCCAGTTCCTCGTAGTGCTCAAAGGAGTCGTCAAAGACTATGCACTGCCCGT
CGATCCACGGCCGCGGATCTTGCCCTCCGATAACCACCCCAGGCTCTGCACCGAGTGGTA
CACTGATCCCCAGCTGGCAAGTGAGTCGCGCATTGGTTGGGCCACAATGCGGCTTCAGCT
TCGACCCGGGGCTGAGAATTCTAAAAGCGACCTCACCACCACCGCAGAATGCCAGTCCTG
TGGCGTCGGCACACCGTTCTGTGAGCGTCCGTACTGTCCCT
>at03484
TGCACAGGAAGCAGCTGCAGAGAATGGCCTCGCCTCTTTGCAGCAGCAGCGCGACCACGG
CGAGGCGCTCAAGAATGCACTGTTCTACAAGAATCAGGGCAACAGGCGGCTCTCCGAGGG
CGATGGCAAAGCTGCGGCAGACGAGTACACCAGCGGCTTAGAGTGGCTGGAGGACTTGCC
GCGCGGTGACCAAAACGTGCGCGAGATCCGCATTGCACTGCTGTCAAACCGGGCTCAAGC
GCACCTGAAGCGCAAGCTCTGGCAGGACGCTCTCGATGACGTCGAGGCGGTGCTCCGCGA
AGACGCGCGGCATCCCAAGGCGAAATTTCGAAAGGCCAAGGCGCTCGTCGAACTCGCGCG
GTACAGCGAGGCTGCCTCGGAGCTGCGGCAGATCGCTGAGGCAGGAGCCGGGGCAAGTGA
GGATGTGAAGGA
>at03485
GAACCATGCAACCAATATGACGCCTGCTGCTCGCGGATACCATACACAAGGTCATGAACT
CCATAACCCAGCGTTATCTGGGGCAGCACCTCTGCAAGCGGCGTGGCAGGCCCCAGGCAC
AGGTATGATCTCAGTGGTTGCTGTACGTCAGCCGCAGCCTCAGATGCCCACACCAAGAGA
GCGCCGCACCCAATGCAAACATGGTGGACTAGATTTGAGAGGAAGAGTTCGCTGCTTCGA
CGAACTCCGAGCTTTCGGAACCGCGCTGCCGGAATCAGCGTGGCAACATAGTCCAGCGCA
AACCAAAACAGGCAACTCACGAGGCCCACAAGCAGCCATCTCGGAGCCGTCACTATCATC
CCCGGTGTGGCAACGTAGCAGTCAGTAGGGTCTGGAGACAAAGAGGCGTACTGTCTATGA
TCTGGCGCACAAATTGACCGCCGAGCAGATATGACCGTCAATTTGCTTGAAAGCTTGAGC
TTGAGCCAAAATGGCTACGG
>at03486
TTGGCATTGGCAGAGGAATGGGGCCACACAGAGGTGGCAAGGGTCCTTCGCGGAGACATT
GGCGTCTGAGTCGGACGGCATGGGTGCGGCACGTCTTGGTAAGGGTAGGAAGGGCAGATC
TGCCGTTGCCCACTGTCTCGACTTTGTTGTTGGCTGCCAGTTTGATTTCCAAGAGGTTAT
GGGCCACAGATGGGCTGATCCTCACTGCTTTAGGCGGAAGGTGCATTGCGTTGTTTGGCT
TTGCCCGTGGCATATACAGAAGCGTAGACTATTTCCTCCTTGCTGTCTGCCCTGCAGAGT
GCCGCCCACATTGCCACGGTGCAGTGGCTTCTGCACCTAGGTTCACAAGTGTTGAGCATG
GCACCGGCACAGCCAGGAA
>at03487
TTTTTTTTTTTTTTTTTTGACCCACATGTCCCTCCGCTCACGTCCATCATACTGGCAAGG
CCGCCACGGTTCAAGCTCGCTAGGGGGGGCTGGACGGTCTGCTGGCGTTCCCAGGCCCCT
GTCGCCACCTCGGCACGCTGCACCTTCAGAGCAGCGAGAGCTGCACAGCCTAGTCCTCGC
GGGGAGACTTCTTCTTCTCGCGGTCCCGGTCGCGGCTCCGGGACCGGTCGCGCTTCTTGT
CGCGGTCCCGATCCTTCCCCTCCTTGGGGCGCGGGGCGTCCTGATCCAAATCCTTATCCT
TATCCTTCTCCTTCTCCTTCTCTTTATCCTTAGTCTTCTCCTTGTCTTTGACGGCTTGCC
CTNNNNNNNNNNNNNNNNNNNNNNNNNNAGCCTCCTTGGCATCCAACCCTCTACGGTTCG
CCCCCTTTCGACGTCCACCATCACGCGCCGGCCATCGATCTTCTTACCATCGCCCTGCTT
GTACGCGTTCTTCAAGTCTCGCTCGTGCTCGTACTCGATGAACGCGTAACCGCGCGGTTT
[truncated: 467,278 more chars]
