# Supplementary material for: Transcriptome Profiling of a Toxic Dinoflagellate Reveals a Gene-Rich Protist and a Potential Impact on Gene Expression Due to Bacterial Presence
Source: PLoS One. 2010 Mar 12;5(3):e9688. doi: 10.1371/journal.pone.0009688 (PMC2837391; doi:10.1371/journal.pone.0009688)
Supplement: Figure S2 — The unique set of Alexandrium MPSS signatures derived from this work and their expression levels under the different culture conditions that were studied. (71.88 MB PDF) [file pone.0009688.s002.pdf]

Tag	F	G	N	O	P
GATCAAAAAAAAAAAAAAA	7907.07	3996.97	5290.77	4902.74	6733.[illegible]
GATCAAAAAAAAAAAAAAC	122.24	52.05	60.14	64.15	90.92
GATCAAAAAAAAAAAAAAG	42.35	7.81	11.45	8.83	9.99
GATCAAAAAAAAAAAAAAN	29.99	0.00	0.00	3.05	0.69
GATCAAAAAAAAAAAAAAT	1244.10	408.29	671.51	699.91	859.2[illegible]
GATCAAAAAAAAAAAAACA	9.16	6.83	7.73	10.52	13.09
GATCAAAAAAAAAAAAACC	2.29	1.95	2.58	3.39	6.20
GATCAAAAAAAAAAAAACG	5.95	2.60	3.15	5.09	5.17
GATCAAAAAAAAAAAAACN	11.22	0.00	0.00	0.00	0.00
GATCAAAAAAAAAAAAACT	4.81	0.65	2.00	1.02	1.38
GATCAAAAAAAAAAAAAGA	9.16	6.18	6.87	4.07	2.41
GATCAAAAAAAAAAAAAGC	2.29	0.98	0.00	0.68	2.07
GATCAAAAAAAAAAAAAGG	1.37	0.00	0.57	0.68	0.34
GATCAAAAAAAAAAAAAGN	0.46	0.00	0.00	0.00	0.00
GATCAAAAAAAAAAAAAGT	3.43	0.00	0.57	0.34	1.03
GATCAAAAAAAAAAAAANA	0.00	0.65	0.57	4.07	1.38
GATCAAAAAAAAAAAAANC	0.00	0.00	0.00	0.00	0.00
GATCAAAAAAAAAAAAANG	0.00	0.00	0.00	0.00	0.00
GATCAAAAAAAAAAAAANN	31.36	0.00	0.00	1.36	1.72
GATCAAAAAAAAAAAAANT	0.00	0.00	0.00	0.00	0.00
GATCAAAAAAAAAAAAATA	5.04	1.95	5.15	2.04	4.82
GATCAAAAAAAAAAAAATC	149.02	40.67	73.59	78.07	89.54
GATCAAAAAAAAAAAAATG	3.66	0.65	2.00	0.68	1.38
GATCAAAAAAAAAAAAATN	0.92	0.00	0.00	0.00	0.00
GATCAAAAAAAAAAAAATT	2.75	0.65	2.29	0.68	1.03
GATCAAAAAAAAAAAACAA	5.72	2.93	4.30	6.11	12.40
GATCAAAAAAAAAAAACAC	0.69	0.65	0.00	0.68	1.72
GATCAAAAAAAAAAAACAG	0.00	0.33	0.29	0.00	0.00
GATCAAAAAAAAAAAACAN	0.00	0.00	0.00	0.00	0.00
GATCAAAAAAAAAAAACAT	1.60	1.30	0.86	0.34	1.03
GATCAAAAAAAAAAAACCA	0.46	0.33	0.29	0.00	0.69
GATCAAAAAAAAAAAACCC	0.00	0.00	0.29	0.00	0.34
GATCAAAAAAAAAAAACCG	0.23	0.00	0.29	0.00	0.34
GATCAAAAAAAAAAAACCT	0.00	0.00	0.00	0.34	0.00
GATCAAAAAAAAAAAACGA	0.00	0.00	0.00	0.34	0.34
GATCAAAAAAAAAAAACGG	0.00	0.00	0.00	0.00	0.00

GATCAAAAAAAAAAAAACGT	0.00	0.65	0.00	0.00	1.03
GATCAAAAAAAAAAAAACNN	5.49	0.00	0.00	0.00	0.00
GATCAAAAAAAAAAAAACTA	0.23	0.00	0.00	0.00	0.00
GATCAAAAAAAAAAAAACTC	0.23	0.00	0.00	0.68	0.34
GATCAAAAAAAAAAAAAGAA	8.70	4.88	6.30	5.09	3.79
GATCAAAAAAAAAAAAAGAC	0.00	0.00	0.57	0.00	0.00
GATCAAAAAAAAAAAAAGAG	0.23	0.00	0.86	0.00	0.00
GATCAAAAAAAAAAAAAGAT	0.69	0.00	0.00	0.00	0.69
GATCAAAAAAAAAAAAAGCA	0.00	0.00	0.00	0.34	0.00
GATCAAAAAAAAAAAAAGCG	0.23	0.00	0.29	0.00	0.00
GATCAAAAAAAAAAAAAGGA	0.46	0.33	0.00	0.00	0.00
GATCAAAAAAAAAAAAAGGG	0.00	0.00	0.29	0.34	0.00
GATCAAAAAAAAAAAAAGNN	0.23	0.00	0.00	0.00	0.00
GATCAAAAAAAAAAAAAGTA	0.00	0.33	0.00	0.00	0.00
GATCAAAAAAAAAAAAAGTC	0.00	0.33	0.00	0.00	0.00
GATCAAAAAAAAAAAAANAA	0.00	0.00	0.00	2.04	0.69
GATCAAAAAAAAAAAAANAC	0.00	0.00	0.00	0.34	0.00
GATCAAAAAAAAAAAAANAN	0.00	0.00	0.00	0.34	0.69
GATCAAAAAAAAAAAAANAT	0.00	0.00	0.00	0.00	0.34
GATCAAAAAAAAAAAAANNN	2.75	0.00	0.29	0.00	0.00
GATCAAAAAAAAAAAAATAA	1.60	0.65	1.43	0.34	0.69
GATCAAAAAAAAAAAAATAC	0.00	0.00	0.29	0.00	0.00
GATCAAAAAAAAAAAAATAG	1.14	0.65	0.00	0.34	0.00
GATCAAAAAAAAAAAAATAN	0.23	0.00	0.00	0.00	0.00
GATCAAAAAAAAAAAAATAT	0.23	0.00	0.00	0.00	0.34
GATCAAAAAAAAAAAAATCA	1.37	0.00	0.00	0.34	0.00
GATCAAAAAAAAAAAAATCC	0.00	0.00	0.29	0.00	0.00
GATCAAAAAAAAAAAAATCG	10.53	3.25	5.15	6.45	9.30
GATCAAAAAAAAAAAAATCT	0.00	0.00	0.57	0.00	0.34
GATCAAAAAAAAAAAAATGA	0.00	0.33	0.00	0.00	0.00
GATCAAAAAAAAAAAAATGG	0.46	0.00	0.00	0.00	0.00
GATCAAAAAAAAAAAAATGT	0.00	0.00	0.29	0.00	0.00
GATCAAAAAAAAAAAAATNA	0.00	0.00	0.00	0.34	0.00
GATCAAAAAAAAAAAAATTC	0.00	0.00	0.00	0.00	0.00
GATCAAAAAAAAAAAACAAA	5.49	7.81	6.30	5.43	13.43
GATCAAAAAAAAAAAACAAC	0.46	0.33	0.57	0.00	0.00
GATCAAAAAAAAAAAACAAG	0.00	0.00	0.00	0.34	0.00

GATCAAAAAAAAAAAACAAN	0.00	0.00	0.00	0.34	0.00
GATCAAAAAAAAAAAACAAT	0.69	0.00	0.00	1.02	1.03
GATCAAAAAAAAAAAACACA	0.00	0.00	0.29	0.68	1.03
GATCAAAAAAAAAAAACACT	0.00	0.00	0.00	0.00	0.34
GATCAAAAAAAAAAAACAGA	0.23	0.33	0.00	0.00	0.00
GATCAAAAAAAAAAAACANN	3.20	0.00	0.00	0.00	0.00
GATCAAAAAAAAAAAACATA	0.00	0.00	0.00	0.00	0.69
GATCAAAAAAAAAAAACATC	0.00	0.00	0.00	0.00	0.00
GATCAAAAAAAAAAAACCAA	0.23	0.33	0.29	0.34	0.69
GATCAAAAAAAAAAAACCCA	0.00	0.00	0.00	0.00	0.34
GATCAAAAAAAAAAAACCNN	2.75	0.00	0.00	0.00	0.00
GATCAAAAAAAAAAAACCTC	0.00	0.00	0.00	0.00	0.34
GATCAAAAAAAAAAAACGAG	0.00	0.00	0.00	0.00	0.34
GATCAAAAAAAAAAAACGTC	0.00	0.33	0.00	0.00	0.00
GATCAAAAAAAAAAAACNNN	5.72	0.00	0.00	0.00	0.00
GATCAAAAAAAAAAAACTAA	0.23	0.00	0.00	0.00	0.00
GATCAAAAAAAAAAAACTCG	0.23	0.00	0.00	0.00	0.00
GATCAAAAAAAAAAAAGAAA	7.10	5.21	5.15	3.39	2.76
GATCAAAAAAAAAAAAGAAC	0.00	0.00	0.00	0.34	0.00
GATCAAAAAAAAAAAAGAAG	0.23	0.65	0.00	0.00	0.00
GATCAAAAAAAAAAAAGAAT	0.69	0.65	0.86	0.34	0.69
GATCAAAAAAAAAAAAGACA	0.00	0.00	0.00	0.00	0.00
GATCAAAAAAAAAAAAGACG	0.23	0.00	0.00	0.00	0.00
GATCAAAAAAAAAAAAGAGA	0.00	0.00	0.00	0.34	0.00
GATCAAAAAAAAAAAAGAGC	0.00	0.00	0.00	0.34	0.00
GATCAAAAAAAAAAAAGAGT	0.00	0.00	0.29	0.00	0.00
GATCAAAAAAAAAAAAGANN	0.23	0.00	0.00	0.00	0.00
GATCAAAAAAAAAAAAGATC	0.23	0.33	0.00	0.00	0.34
GATCAAAAAAAAAAAAGATG	0.00	0.00	0.29	0.00	0.00
GATCAAAAAAAAAAAAGCAA	0.00	0.00	0.29	0.00	0.00
GATCAAAAAAAAAAAAGCCA	0.00	0.00	0.00	0.00	0.34
GATCAAAAAAAAAAAAGCNN	0.23	0.00	0.00	0.00	0.00
GATCAAAAAAAAAAAAGGAA	0.23	0.33	0.00	0.00	0.00
GATCAAAAAAAAAAAAGGGA	0.00	0.00	0.57	0.00	0.00
GATCAAAAAAAAAAAAGTAT	0.00	0.00	0.00	0.00	0.00
GATCAAAAAAAAAAAANAAA	0.00	0.00	0.86	2.04	22.04
GATCAAAAAAAAAAAANAAC	0.00	0.00	0.00	0.00	0.34

GATCAAAAAAAAAAAANAAN	0.00	0.00	0.00	0.00	0.69
GATCAAAAAAAAAAAANAAT	0.00	0.00	0.00	0.34	2.76
GATCAAAAAAAAAAAANACA	0.00	0.00	0.00	0.00	0.34
GATCAAAAAAAAAAAANANA	0.00	0.00	0.29	0.00	0.00
GATCAAAAAAAAAAAANANN	1.37	0.00	0.00	0.00	0.34
GATCAAAAAAAAAAAANANT	0.00	0.00	0.29	0.00	0.00
GATCAAAAAAAAAAAANATC	0.00	0.00	0.00	0.00	0.69
GATCAAAAAAAAAAAANCNN	0.92	0.00	0.00	0.00	0.00
GATCAAAAAAAAAAAANNNN	8.70	0.00	0.00	0.00	0.00
GATCAAAAAAAAAAAANNNT	0.00	0.00	0.00	0.00	0.00
GATCAAAAAAAAAAAATAAA	1.83	0.98	0.57	0.68	0.34
GATCAAAAAAAAAAAATAAG	0.00	0.00	0.00	0.00	0.00
GATCAAAAAAAAAAAATAAT	0.23	0.00	0.00	0.34	0.00
GATCAAAAAAAAAAAATAGA	0.00	0.00	0.29	0.00	0.00
GATCAAAAAAAAAAAATAGC	0.00	0.00	0.00	0.00	0.34
GATCAAAAAAAAAAAATCAA	0.69	0.00	0.00	0.00	0.00
GATCAAAAAAAAAAAATCAT	0.00	0.00	0.00	0.34	0.00
GATCAAAAAAAAAAAATCGA	0.00	0.00	0.00	0.68	0.00
GATCAAAAAAAAAAAATCGN	0.00	0.00	0.00	0.00	0.00
GATCAAAAAAAAAAAATCGT	0.92	0.00	0.57	1.02	2.41
GATCAAAAAAAAAAAATCTA	0.00	0.00	0.29	0.00	0.69
GATCAAAAAAAAAAACAAAA	5.72	1.95	1.43	5.09	5.51
GATCAAAAAAAAAAACAAAC	0.00	0.00	0.57	0.68	0.00
GATCAAAAAAAAAAACAAAG	0.00	0.00	0.00	0.00	0.34
GATCAAAAAAAAAAACAAAT	0.00	0.00	0.00	1.02	0.69
GATCAAAAAAAAAAACAACA	0.00	0.00	0.57	0.34	0.00
GATCAAAAAAAAAAACAACC	0.46	0.00	0.00	0.00	0.00
GATCAAAAAAAAAAACAACT	0.00	0.00	0.00	0.34	0.00
GATCAAAAAAAAAAACAANN	0.00	0.00	0.00	0.00	0.00
GATCAAAAAAAAAAACAATC	0.00	0.00	0.00	0.00	0.34
GATCAAAAAAAAAAACACAA	0.00	0.00	0.00	0.00	0.34
GATCAAAAAAAAAAACACAC	0.00	0.00	0.00	0.00	0.69
GATCAAAAAAAAAAACACCC	0.23	0.00	0.29	0.00	0.00
GATCAAAAAAAAAAACAGAA	0.00	0.33	0.00	0.00	0.00
GATCAAAAAAAAAAACAGCA	0.00	0.00	0.00	0.00	0.34
GATCAAAAAAAAAAACANNN	0.00	0.00	0.00	0.00	0.00
GATCAAAAAAAAAAACCAAA	0.00	0.00	0.00	0.00	0.69

GATCAAAAAAAAAAACCANN	0.23	0.00	0.00	0.00	0.00
GATCAAAAAAAAAAACGAAA	0.00	0.00	0.00	0.00	0.34
GATCAAAAAAAAAAACNANN	0.23	0.00	0.00	0.00	0.00
GATCAAAAAAAAAAACNCNN	0.69	0.00	0.00	0.00	0.00
GATCAAAAAAAAAAACNNNN	4.58	0.00	0.00	0.00	0.00
GATCAAAAAAAAAAAGAAAA	1.37	6.51	6.01	6.45	6.20
GATCAAAAAAAAAAAGAAAG	0.00	0.00	0.29	0.00	0.00
GATCAAAAAAAAAAAGAAAN	0.23	0.00	0.00	0.00	0.00
GATCAAAAAAAAAAAGAAAT	0.69	0.33	0.29	0.00	0.00
GATCAAAAAAAAAAAGAACA	0.00	0.00	0.00	0.00	0.34
GATCAAAAAAAAAAAGAGAA	0.00	0.00	0.00	0.00	0.00
GATCAAAAAAAAAAAGATGT	0.00	0.33	0.00	0.00	0.00
GATCAAAAAAAAAAAGCGAG	0.00	0.00	0.00	0.00	0.00
GATCAAAAAAAAAAAGGAAA	0.00	0.33	0.00	0.00	0.00
GATCAAAAAAAAAAATAAAA	2.29	0.00	1.15	1.02	0.34
GATCAAAAAAAAAAATAAAT	0.46	0.00	0.00	0.00	0.00
GATCAAAAAAAAAAATCGTA	0.23	0.33	0.00	0.00	0.00
GATCAAAAAAAAAAATCTAT	0.00	0.00	0.00	0.00	0.34
GATCAAAAAAAAAAATGAAA	0.00	0.00	0.00	0.00	0.00
GATCAAAAAAAAAAATTAAA	0.00	0.00	0.29	0.00	0.00
GATCAAAAAAAAAACAAAAA	2.06	1.63	1.43	3.39	3.44
GATCAAAAAAAAAACAAAAC	0.00	0.00	0.00	0.00	0.69
GATCAAAAAAAAAACAAAAT	0.00	0.33	0.00	0.00	0.34
GATCAAAAAAAAAACAAACA	0.23	0.00	0.00	0.34	0.00
GATCAAAAAAAAAACAAACC	0.00	0.00	0.00	0.00	0.00
GATCAAAAAAAAAACAAATC	0.00	0.00	0.00	0.00	0.00
GATCAAAAAAAAAACAACAA	0.46	0.00	0.00	0.00	0.69
GATCAAAAAAAAAACAACAC	0.00	0.00	0.00	0.34	0.00
GATCAAAAAAAAAACAATAG	0.00	0.00	0.29	0.00	0.00
GATCAAAAAAAAAACACAAA	0.00	0.00	0.29	0.00	0.34
GATCAAAAAAAAAACACAAC	0.00	0.00	0.00	0.34	0.34
GATCAAAAAAAAAACACCAA	0.00	0.00	0.00	0.00	0.00
GATCAAAAAAAAAACACCAC	0.00	0.00	0.29	0.00	0.00
GATCAAAAAAAAAACACCCA	0.00	0.33	0.00	0.00	0.00
GATCAAAAAAAAAACAGACA	0.00	0.00	0.29	0.00	0.00
GATCAAAAAAAAAACANACA	0.00	0.00	0.00	0.00	0.34
GATCAAAAAAAAAACATACA	0.00	0.00	0.00	0.34	0.00

GATCAAAAAAAAAACATATA	0.23	0.00	0.00	0.00	0.00
GATCAAAAAAAAAACCAAAC	0.00	0.00	0.00	0.00	0.34
GATCAAAAAAAAAACCAAAT	0.00	0.00	0.00	0.00	0.00
GATCAAAAAAAAAACCAACC	0.00	0.00	0.29	0.00	0.00
GATCAAAAAAAAAACCCCAA	0.00	0.00	0.00	0.00	0.34
GATCAAAAAAAAAACCCCCT	0.23	0.00	0.00	0.00	0.00
GATCAAAAAAAAAACCNNNN	0.23	0.00	0.00	0.00	0.00
GATCAAAAAAAAAAGAAAAA	4.12	3.25	4.58	2.04	5.17
GATCAAAAAAAAAAGAAAAC	0.00	0.00	0.00	0.34	0.00
GATCAAAAAAAAAAGAAAAT	0.23	0.00	0.00	0.68	0.00
GATCAAAAAAAAAAGAAAGA	0.23	0.00	0.00	0.00	0.00
GATCAAAAAAAAAAGAAGAA	0.23	0.00	0.00	0.00	0.34
GATCAAAAAAAAAAGANNNN	0.23	0.00	0.00	0.00	0.00
GATCAAAAAAAAAAGATGCA	0.00	0.00	0.29	0.00	0.00
GATCAAAAAAAAAAGCAAAA	0.00	0.00	0.00	0.34	0.00
GATCAAAAAAAAAAGGAAAA	0.00	0.00	0.00	0.00	1.38
GATCAAAAAAAAAAGTAGAA	0.00	0.00	0.00	0.00	0.34
GATCAAAAAAAAAATAAAAA	0.23	0.33	0.29	0.68	0.00
GATCAAAAAAAAAATATTTC	0.00	0.00	0.00	0.00	0.00
GATCAAAAAAAAAATCAAAA	0.46	0.00	0.00	0.00	0.00
GATCAAAAAAAAAATCGTAN	0.23	0.00	0.00	0.00	0.00
GATCAAAAAAAAAATCGTAT	1.14	0.00	0.00	0.00	0.00
GATCAAAAAAAAAATCTAAT	0.00	0.00	0.00	0.34	0.00
GATCAAAAAAAAACAAAAAA	8.93	0.98	1.43	5.43	2.41
GATCAAAAAAAAACAAAAAT	0.23	0.00	0.00	0.00	1.38
GATCAAAAAAAAACAAAACA	0.46	0.33	0.00	0.00	0.34
GATCAAAAAAAAACAAAACT	0.23	0.00	0.00	0.00	0.00
GATCAAAAAAAAACAAAAGA	0.00	0.00	0.00	0.00	0.34
GATCAAAAAAAAACAAAANN	0.23	0.00	0.00	0.00	0.00
GATCAAAAAAAAACAAAATA	0.00	0.00	0.29	0.00	0.00
GATCAAAAAAAAACAAACAA	0.23	0.00	0.00	0.00	0.34
GATCAAAAAAAAACAAATAA	0.00	0.00	0.00	0.00	0.34
GATCAAAAAAAAACAACAAC	0.00	0.00	0.00	0.00	0.34
GATCAAAAAAAAACAACNNN	0.23	0.00	0.00	0.00	0.00
GATCAAAAAAAAACAACTAA	0.00	0.00	0.00	0.00	0.34
GATCAAAAAAAAACAAGAAA	0.23	0.00	0.00	0.00	0.00
GATCAAAAAAAAACAAGAAC	0.00	0.00	0.00	0.00	0.00

GATCAAAAAAAAACACAAAA	0.00	0.00	0.00	0.34	0.34
GATCAAAAAAAAACACACCA	0.00	0.00	0.00	0.00	0.34
GATCAAAAAAAAACACCACA	0.00	0.00	0.00	0.00	0.00
GATCAAAAAAAAACAGAGGA	0.00	0.00	0.00	0.34	0.00
GATCAAAAAAAAACCAAAAA	0.00	0.00	0.00	0.00	0.34
GATCAAAAAAAAACCAAAAT	0.00	0.00	0.00	0.00	0.34
GATCAAAAAAAAACCAAACC	0.00	0.00	0.00	0.00	0.00
GATCAAAAAAAAACCAGANA	0.00	0.00	0.00	0.34	0.00
GATCAAAAAAAAACCCAAAA	0.23	0.00	0.00	0.00	0.00
GATCAAAAAAAAACCCAACC	0.23	0.00	0.00	0.00	0.00
GATCAAAAAAAAAGAAAAAA	2.52	5.53	8.30	5.43	2.76
GATCAAAAAAAAAGAAAAAC	0.00	0.00	0.00	0.00	0.69
GATCAAAAAAAAAGAAAAAG	0.23	0.00	0.00	0.00	0.00
GATCAAAAAAAAAGAAAAAN	0.00	0.00	0.00	0.00	0.34
GATCAAAAAAAAAGAAAAAT	0.00	0.00	0.29	0.00	0.00
GATCAAAAAAAAAGAAAACA	0.00	0.00	0.57	0.00	0.00
GATCAAAAAAAAAGAAAAGA	0.00	0.33	0.00	0.00	0.00
GATCAAAAAAAAAGAAAATC	0.23	0.00	0.00	0.00	0.00
GATCAAAAAAAAAGAAAGAA	0.00	0.00	0.29	0.00	0.00
GATCAAAAAAAAAGAAATAA	0.00	0.33	0.00	0.00	0.00
GATCAAAAAAAAAGAANAAA	0.00	0.00	0.00	0.00	0.34
GATCAAAAAAAAAGAATAAA	0.00	0.00	0.29	0.00	0.00
GATCAAAAAAAAAGAGAAAA	0.00	0.00	0.00	0.34	0.00
GATCAAAAAAAAAGCCAAAA	0.00	0.00	0.29	0.00	0.00
GATCAAAAAAAAAGGAAAAA	0.00	0.00	0.29	0.00	0.00
GATCAAAAAAAAATAAAAAA	1.14	2.28	1.72	0.68	0.34
GATCAAAAAAAAATAAAAAT	0.00	0.33	0.00	0.68	0.00
GATCAAAAAAAAATAAAGAA	0.00	0.33	0.00	0.00	0.00
GATCAAAAAAAAATAATAAA	0.00	0.00	0.00	0.00	1.03
GATCAAAAAAAAATCAAAAA	0.46	0.33	0.29	0.00	0.00
GATCAAAAAAAAATCGTATG	0.00	0.00	0.00	0.34	0.00
GATCAAAAAAAAATCTAAAA	0.00	0.00	0.57	0.34	0.00
GATCAAAAAAAACAAAAAAA	4.81	1.63	1.72	2.72	5.85
GATCAAAAAAAACAAAAAAC	0.23	0.33	0.00	0.00	0.34
GATCAAAAAAAACAAAAAAG	0.23	0.00	0.00	0.00	0.00
GATCAAAAAAAACAAAAAAT	0.23	0.00	0.00	0.00	0.69
GATCAAAAAAAACAAAAACA	0.00	0.00	0.00	0.34	0.34

GATCAAAAAAAACAAAAACC	0.00	0.00	0.00	0.00	0.34
GATCAAAAAAAACAAAAACT	0.00	0.00	0.00	0.00	0.34
GATCAAAAAAAACAAAAAGA	0.00	0.00	0.29	0.00	0.00
GATCAAAAAAAACAAAAANA	0.00	0.00	0.00	1.02	0.00
GATCAAAAAAAACAAAAATA	0.00	0.00	0.29	0.00	0.00
GATCAAAAAAAACAAAACAA	0.00	0.00	0.00	0.34	0.69
GATCAAAAAAAACAAAACCA	0.00	0.00	0.00	0.00	0.34
GATCAAAAAAAACAAAATAT	0.00	0.00	0.00	0.34	0.00
GATCAAAAAAAACAAAATCA	0.00	0.00	0.00	0.34	0.00
GATCAAAAAAAACAAACAAA	0.23	0.00	0.29	0.00	0.00
GATCAAAAAAAACAAACAAC	0.00	0.33	0.00	0.00	0.00
GATCAAAAAAAACAAACACA	0.00	0.00	0.00	0.34	0.00
GATCAAAAAAAACAAACCAA	0.00	0.00	0.00	0.00	0.34
GATCAAAAAAAACAAACCAC	0.00	0.33	0.00	0.00	0.00
GATCAAAAAAAACAAAGGAA	0.00	0.00	0.00	0.34	0.00
GATCAAAAAAAACAAANAAA	0.00	0.00	0.00	0.00	0.69
GATCAAAAAAAACAAATGTG	0.00	0.00	0.00	0.00	0.00
GATCAAAAAAAACAACAAAA	0.00	0.00	0.00	0.00	0.69
GATCAAAAAAAACAACAAAC	0.00	0.00	0.00	0.34	0.00
GATCAAAAAAAACAACACCA	0.23	0.00	0.00	0.00	0.00
GATCAAAAAAAACAACCCAA	0.23	0.00	0.00	0.00	0.00
GATCAAAAAAAACAATAAAA	0.23	0.00	0.00	0.00	0.00
GATCAAAAAAAACACAAAAA	0.00	0.00	0.29	0.34	0.00
GATCAAAAAAAACACAACAA	0.00	0.00	0.00	0.34	0.00
GATCAAAAAAAACAGCGCAA	0.00	0.00	0.00	0.00	0.00
GATCAAAAAAAACCAAAAAA	0.00	0.00	0.00	0.00	0.34
GATCAAAAAAAACCAAAAAT	0.00	0.00	0.00	0.34	0.00
GATCAAAAAAAACCAACACA	0.00	0.00	0.00	0.00	0.00
GATCAAAAAAAACCACAACC	0.00	0.00	0.00	0.00	0.34
GATCAAAAAAAACGCAATCG	0.00	0.00	0.00	0.00	0.34
GATCAAAAAAAACTGCTGCT	0.00	0.33	0.00	0.00	0.00
GATCAAAAAAAACTGTTTCT	0.00	0.00	0.29	0.00	0.00
GATCAAAAAAAAGAAAAAAA	1.37	2.93	6.59	6.79	4.82
GATCAAAAAAAAGAAAAAAC	0.00	0.00	0.57	0.00	0.00
GATCAAAAAAAAGAAAAAAT	0.92	0.00	0.00	0.00	1.38
GATCAAAAAAAAGAAAAGAG	0.00	0.00	0.00	0.34	0.00
GATCAAAAAAAAGAAANAAA	0.00	0.00	0.00	0.00	0.34

GATCAAAAAAAAGAACAAAA	0.23	0.00	0.29	0.00	0.00
GATCAAAAAAAAGAAGCAAC	0.00	0.00	0.00	0.00	0.34
GATCAAAAAAAAGAAGCTAG	0.00	0.00	0.00	0.00	0.34
GATCAAAAAAAAGAGAAAAA	0.00	0.33	0.00	0.00	0.00
GATCAAAAAAAAGATAAAAA	0.92	0.00	0.00	0.00	0.00
GATCAAAAAAAAGGAAAAAA	0.00	0.00	0.00	0.68	0.00
GATCAAAAAAAAGTAAAAAA	0.00	0.00	0.00	0.00	0.00
GATCAAAAAAAAGTAAAAAG	0.00	0.00	0.29	0.00	0.00
GATCAAAAAAAAGTCGAGTG	0.00	0.00	0.29	0.00	0.00
GATCAAAAAAAATAAAAAAA	1.14	0.33	0.29	0.34	1.72
GATCAAAAAAAATAAAAAAT	0.00	0.00	0.00	0.34	0.00
GATCAAAAAAAATCAAAAAA	0.69	0.33	0.29	0.00	0.34
GATCAAAAAAAATCGTATGC	0.00	0.00	0.00	0.00	0.34
GATCAAAAAAAATCTAAAAA	0.00	0.65	0.57	0.00	0.34
GATCAAAAAAAATGAAAAAA	0.00	0.00	0.00	0.00	0.00
GATCAAAAAAAATTAAAAAA	0.00	0.00	0.29	0.00	0.00
GATCAAAAAAAATTTTTAAA	0.23	0.00	0.00	0.00	0.00
GATCAAAAAAACAAAAAAAA	5.26	0.98	1.72	3.05	1.38
GATCAAAAAAACAAAAAAAC	0.23	0.00	0.29	0.00	0.34
GATCAAAAAAACAAAAAAAG	0.23	0.00	0.00	0.00	0.00
GATCAAAAAAACAAAAAAAT	0.46	0.00	0.00	0.00	0.00
GATCAAAAAAACAAAAAACA	0.00	0.00	0.00	0.34	0.69
GATCAAAAAAACAAAAACAA	0.00	0.00	0.29	0.00	0.34
GATCAAAAAAACAAAAAGAA	0.00	0.33	0.00	0.00	0.00
GATCAAAAAAACAAAACAAC	0.00	0.00	0.00	0.00	0.34
GATCAAAAAAACAAAACAAG	0.00	0.00	0.00	0.00	0.00
GATCAAAAAAACAAAACCAA	0.00	0.00	0.00	0.00	0.34
GATCAAAAAAACAAACAAAA	0.23	0.00	0.00	0.00	0.00
GATCAAAAAAACAAATTCAA	0.00	0.00	0.00	0.00	0.00
GATCAAAAAAACAACAAAAA	0.23	0.00	0.00	0.00	0.34
GATCAAAAAAACAACACAAA	0.00	0.00	0.00	0.00	0.00
GATCAAAAAAACAACACACA	0.23	0.00	0.00	0.00	0.00
GATCAAAAAAACAACCAAAC	0.23	0.00	0.00	0.00	0.00
GATCAAAAAAACAACGACAT	0.23	0.00	0.00	0.00	0.00
GATCAAAAAAACACAAAAAA	0.23	0.00	0.29	0.34	0.34
GATCAAAAAAACACAAAACA	0.23	0.00	0.00	0.00	0.00
GATCAAAAAAACACAGCACA	0.23	0.00	0.00	0.00	0.00

GATCAAAAAAACATCTAACC	0.00	0.65	0.00	0.00	0.34
GATCAAAAAAACATCTAACT	0.00	0.00	0.57	0.00	0.00
GATCAAAAAAACATTGTAAA	0.00	0.00	0.00	0.00	0.00
GATCAAAAAAACCAAAAAAA	0.23	0.00	0.00	0.00	0.34
GATCAAAAAAACCAAAAAAT	0.00	0.00	0.00	0.00	0.34
GATCAAAAAAACCAACAACA	0.00	0.00	0.00	0.00	0.34
GATCAAAAAAACCACAACCT	0.23	0.00	0.00	0.00	0.00
GATCAAAAAAACCCAAAACA	0.00	0.00	0.29	0.00	0.00
GATCAAAAAAACCCCCCCAC	0.23	0.00	0.00	0.00	0.00
GATCAAAAAAACGATTCTTT	0.00	0.00	0.00	0.34	0.00
GATCAAAAAAACGTGTTCTC	0.00	0.00	0.29	0.00	0.34
GATCAAAAAAACTAAAAAAA	0.00	0.65	0.00	0.00	0.00
GATCAAAAAAACTGCTGCTC	0.46	1.95	0.00	0.68	0.00
GATCAAAAAAAGAAAAAAAA	3.66	0.98	2.58	2.72	3.10
GATCAAAAAAAGAAAAAAAT	0.23	0.00	0.29	0.00	0.34
GATCAAAAAAAGAAAAAACA	0.00	0.00	0.00	0.00	0.69
GATCAAAAAAAGAAAAACAA	0.00	0.00	0.29	0.00	0.00
GATCAAAAAAAGAAAACAAA	0.00	0.00	0.29	0.00	0.00
GATCAAAAAAAGAAAACAAG	0.00	0.00	0.00	0.00	0.34
GATCAAAAAAAGAAAAGAAG	0.00	0.00	0.57	0.00	0.00
GATCAAAAAAAGAAAAGGAA	0.23	0.00	0.00	0.00	0.00
GATCAAAAAAAGAAACAANN	0.00	0.00	0.00	0.34	0.00
GATCAAAAAAAGAAAGAAAA	0.00	0.00	0.00	0.00	0.34
GATCAAAAAAAGAAATAGAA	0.00	0.65	0.00	0.00	0.00
GATCAAAAAAAGAAGAAAAA	0.00	0.00	0.29	0.00	0.00
GATCAAAAAAAGAATAAAAA	0.00	0.33	0.00	0.00	0.00
GATCAAAAAAAGACTGAGAG	0.00	0.00	0.00	0.00	0.00
GATCAAAAAAAGAGAAAAAA	0.00	0.00	0.57	0.00	0.00
GATCAAAAAAAGAGACATAT	0.00	0.00	0.29	0.00	0.00
GATCAAAAAAAGCAAAAAAA	0.00	0.00	0.00	0.34	0.00
GATCAAAAAAAGCTACGTTT	0.00	0.00	0.00	0.00	0.00
GATCAAAAAAAGGAAAAAAA	0.00	0.00	0.29	0.00	0.69
GATCAAAAAAAGGAAANNNN	0.23	0.00	0.00	0.00	0.00
GATCAAAAAAAGGAAGGCAG	0.00	0.00	0.00	0.34	0.00
GATCAAAAAAAGGACAAGAA	0.00	0.00	0.29	0.00	0.00
GATCAAAAAAAGGACCAAAA	0.00	0.00	0.00	0.00	0.00
GATCAAAAAAAGGAGGTTGA	0.00	0.00	0.00	0.00	0.00

GATCAAAAAAAGGCAAAAAA	0.00	0.00	0.00	0.00	0.00
GATCAAAAAAAGGGAAAAGA	0.00	0.00	0.00	0.00	1.03
GATCAAAAAAAGTGTACCTG	0.46	0.00	0.29	0.00	0.34
GATCAAAAAAATAAAAAAAA	2.06	0.33	0.00	1.02	0.00
GATCAAAAAAATAAAAAAAT	0.00	0.00	0.00	0.34	0.00
GATCAAAAAAATAAAAAACC	0.00	0.00	0.00	0.00	0.34
GATCAAAAAAATAAAACAAA	0.00	0.00	0.00	0.34	0.00
GATCAAAAAAATAAACAAAA	0.00	0.00	0.00	0.00	0.00
GATCAAAAAAATAAAGAAAA	0.00	0.00	0.00	0.00	0.00
GATCAAAAAAATAAGAAATG	0.00	0.00	0.00	0.00	0.69
GATCAAAAAAATACCTCCCC	0.00	0.00	0.00	1.02	0.00
GATCAAAAAAATACGGGGTC	0.23	0.00	0.00	0.00	0.00
GATCAAAAAAATAGTCTCAA	0.23	0.00	0.00	0.00	0.00
GATCAAAAAAATCGGAGAAG	0.00	0.00	0.29	0.00	0.00
GATCAAAAAAATCTAAAAAA	0.00	0.00	0.86	0.68	0.00
GATCAAAAAAATGAAAAAAN	0.00	0.00	0.00	0.00	0.00
GATCAAAAAAATGAAATTAA	0.00	0.65	0.00	0.00	0.00
GATCAAAAAAATGCTCTGTC	0.00	0.00	0.00	0.00	1.03
GATCAAAAAAATGTACAAAA	0.00	0.00	0.29	0.00	0.00
GATCAAAAAAATGTACTGAT	0.00	0.00	0.00	0.00	0.00
GATCAAAAAAATGTTGAGGA	0.00	0.33	0.00	0.00	0.00
GATCAAAAAAATTGAGAGAG	0.00	0.00	0.00	0.34	0.00
GATCAAAAAAATTGCACGCA	0.00	0.33	0.00	0.34	0.00
GATCAAAAAAATTGCACTCT	0.23	0.00	0.00	0.00	0.00
GATCAAAAAAATTGCCGAAT	0.92	0.00	0.00	0.00	0.00
GATCAAAAAAATTGTAAAAA	0.00	0.00	0.00	0.00	0.00
GATCAAAAAACAAAAAAAAA	5.72	2.28	2.58	3.05	4.82
GATCAAAAAACAAAAAAAAC	0.00	0.00	0.29	0.00	0.69
GATCAAAAAACAAAAAAAAG	0.23	0.00	0.00	0.34	0.00
GATCAAAAAACAAAAAAAAT	0.00	0.00	0.29	0.34	0.34
GATCAAAAAACAAAAAAACA	0.00	0.00	0.00	0.34	0.00
GATCAAAAAACAAAAAAACC	0.23	0.00	0.00	0.00	0.00
GATCAAAAAACAAAAAAACT	0.00	0.33	0.00	0.00	0.00
GATCAAAAAACAAAAAACAC	0.00	0.00	0.00	0.34	0.00
GATCAAAAAACAAAAACAAA	0.00	0.33	0.00	0.00	0.00
GATCAAAAAACAAAAACAAC	0.00	0.00	0.00	0.34	0.00
GATCAAAAAACAAAAACAAG	0.00	0.00	0.00	0.00	0.34

GATCAAAAAACAAAACAAAA	0.00	0.00	0.00	0.00	0.69
GATCAAAAAACAAAACAAAC	0.00	0.00	0.29	0.00	0.00
GATCAAAAAACAAAACCAAC	0.00	0.00	0.00	0.00	0.00
GATCAAAAAACAAAAGCAAC	0.00	0.00	0.00	0.00	0.34
GATCAAAAAACAAACAAAAA	0.00	0.00	0.00	0.00	0.34
GATCAAAAAACAACAAAAAA	0.00	0.00	0.00	0.00	0.34
GATCAAAAAACAACAAAAAC	0.00	0.00	0.00	0.00	0.00
GATCAAAAAACAACAACAAA	0.23	0.00	0.00	0.00	0.34
GATCAAAAAACAACAACACG	0.00	0.00	0.00	0.00	0.34
GATCAAAAAACAACACCACA	0.00	0.33	0.00	0.00	0.00
GATCAAAAAACAACCACAAC	0.00	0.00	0.00	0.34	0.00
GATCAAAAAACAACCCCACC	0.00	0.33	0.00	0.00	0.00
GATCAAAAAACAACTGCACC	0.00	0.00	0.57	0.00	0.00
GATCAAAAAACAAGTTCCAG	0.00	0.00	0.00	0.34	0.00
GATCAAAAAACACAAAAAAA	0.00	0.00	0.00	0.00	0.34
GATCAAAAAACACAAAAACA	0.00	0.00	0.00	0.00	0.34
GATCAAAAAACACAAACAAA	0.00	0.00	0.00	0.00	0.34
GATCAAAAAACACAAACCAC	0.23	0.00	0.00	0.00	0.00
GATCAAAAAACACACAAAGA	0.00	0.00	0.00	0.00	0.34
GATCAAAAAACACACGACAT	0.23	0.00	0.00	0.00	0.00
GATCAAAAAACAGCCTGCTC	0.00	0.00	0.29	0.00	0.00
GATCAAAAAACAGCGAGAAC	0.00	0.00	0.29	0.00	0.00
GATCAAAAAACAGGCCCCAG	0.00	0.00	0.00	0.00	0.00
GATCAAAAAACAGTCCGACA	0.23	0.00	0.29	0.00	0.00
GATCAAAAAACAGTTTCCAG	0.23	0.00	0.00	0.00	0.00
GATCAAAAAACATGAGCGGT	0.00	0.33	0.00	0.00	0.00
GATCAAAAAACATGTGGCAA	0.23	0.00	0.00	0.00	0.00
GATCAAAAAACATTATTCTG	0.00	0.00	0.00	0.00	0.00
GATCAAAAAACATTGTAAAA	0.46	0.65	0.00	0.00	0.00
GATCAAAAAACATTGTATTT	0.00	0.00	0.57	0.00	0.00
GATCAAAAAACCAAACCACA	0.00	0.00	0.00	0.00	0.34
GATCAAAAAACCAGGCCATG	0.00	0.00	0.29	0.00	0.00
GATCAAAAAACCCCCACCCC	2.75	0.00	0.00	0.00	0.00
GATCAAAAAACCCCCACCCG	0.23	0.00	0.00	0.00	0.00
GATCAAAAAACCCCCACCCT	0.23	0.00	0.00	0.00	0.00
GATCAAAAAACCCCCCCCCC	0.46	0.00	0.00	0.00	0.00
GATCAAAAAACCCCCCGCCC	0.23	0.00	0.00	0.00	0.00

GATCAAAAAACCCCGCCCC	1.14	0.00	0.00	0.00	0.00
GATCAAAAAACCCCTCCCC	0.46	0.00	0.00	0.00	0.00
GATCAAAAAACCCCTCCCT	0.46	0.00	0.00	0.00	0.00
GATCAAAAAACCTAGGCTTG	0.00	0.00	0.00	0.00	0.00
GATCAAAAAACGGCATTCTC	0.00	0.00	0.29	0.00	0.00
GATCAAAAAACGGCCCGGGC	0.00	0.00	0.29	0.00	0.00
GATCAAAAAACGTATAGATT	0.00	0.00	0.57	0.00	0.00
GATCAAAAAACGTGTGCCTG	0.23	0.00	0.00	0.00	0.00
GATCAAAAAACTCACCACAC	0.00	0.33	0.00	0.00	0.00
GATCAAAAAACTGAGTTCCT	0.00	0.00	0.29	0.00	0.00
GATCAAAAAAGAAAAAAAAA	2.98	4.55	6.01	4.07	4.82
GATCAAAAAAGAAAAAAAAN	0.00	0.00	0.00	0.34	0.00
GATCAAAAAAGAAAAAAAAT	0.00	0.00	0.57	0.00	0.34
GATCAAAAAAGAAAAAAACA	0.00	0.33	0.00	0.00	0.00
GATCAAAAAAGAAAAAAAGA	0.23	0.00	0.00	0.34	0.00
GATCAAAAAAGAAAAAAATC	0.23	0.00	0.00	0.00	0.00
GATCAAAAAAGAAAAAACAA	0.23	0.00	0.00	0.00	0.00
GATCAAAAAAGAAAAGAAAA	0.00	0.00	0.00	0.00	0.00
GATCAAAAAAGAAAAGACAA	0.00	0.00	0.00	0.00	0.34
GATCAAAAAAGAAACAAGAG	0.00	0.00	0.00	0.00	0.34
GATCAAAAAAGAAGAAAAAA	0.00	0.00	0.00	0.00	0.00
GATCAAAAAAGAAGAGAAAA	0.00	0.33	0.00	0.00	0.00
GATCAAAAAAGACAAAATGA	0.00	0.00	0.00	0.00	0.34
GATCAAAAAAGACCAACTTG	0.00	0.00	0.29	0.00	0.00
GATCAAAAAAGAGAAAAAAA	0.00	0.00	0.00	0.68	0.00
GATCAAAAAAGATAAGAAAA	0.00	0.00	0.00	0.00	0.00
GATCAAAAAAGATACTACAA	0.00	0.00	0.00	0.00	0.34
GATCAAAAAAGATTCCGTGA	0.23	0.00	0.00	0.00	0.00
GATCAAAAAAGCAAAAAAAA	0.00	0.33	0.57	0.34	0.00
GATCAAAAAAGCCCCAGACA	0.00	0.00	0.00	0.00	0.34
GATCAAAAAAGCGACGCGAG	0.00	0.00	0.00	0.00	0.00
GATCAAAAAAGCGATGGGGG	0.00	0.00	0.00	0.00	0.34
GATCAAAAAAGCGTCATTCT	0.00	0.00	0.00	0.34	0.00
GATCAAAAAAGCGTCTAAAA	0.00	0.00	0.29	0.00	0.00
GATCAAAAAAGCTTGCTCAG	0.00	0.00	0.00	0.00	0.00
GATCAAAAAAGGAAAAAAGG	0.00	0.00	0.00	0.00	0.34
GATCAAAAAAGGCAAAAAAA	0.00	0.00	0.00	0.00	0.00

GATCAAAAAAGGCAATTCCA	0.00	0.00	0.29	0.00	0.00
GATCAAAAAAGGCGCTTTTC	0.00	0.00	0.00	0.00	0.00
GATCAAAAAAGGCTTGAGAG	0.00	0.33	0.00	0.00	0.00
GATCAAAAAAGGGCTGAGGC	0.00	0.00	0.00	0.00	0.00
GATCAAAAAAGGTCCTGCTT	0.00	0.00	0.00	0.68	0.00
GATCAAAAAAGTAATATGGA	0.23	0.00	0.00	0.00	0.00
GATCAAAAAAGTCACTTCTT	0.69	0.00	0.00	0.00	0.00
GATCAAAAAAGTCCGTTGTG	0.00	0.33	0.00	0.00	0.00
GATCAAAAAAGTGATGCTTG	0.00	0.00	0.00	0.00	0.00
GATCAAAAAATAAAAAAAAA	0.46	0.00	0.57	0.00	1.03
GATCAAAAAATAAAAAAAAT	0.00	0.33	0.00	0.00	0.34
GATCAAAAAATAAAAAAACA	0.00	0.00	0.00	0.34	0.00
GATCAAAAAATAAAACAATA	0.00	0.00	0.00	0.00	0.00
GATCAAAAAATAACAAAACA	0.00	0.00	0.00	0.00	0.34
GATCAAAAAATACATATCAA	0.00	0.00	0.00	0.00	0.34
GATCAAAAAATACCTCCCCA	0.00	0.00	0.00	0.00	0.00
GATCAAAAAATACCTCCCCG	2.98	1.95	1.15	1.02	0.34
GATCAAAAAATAGCACACAC	0.46	0.00	0.00	0.00	0.00
GATCAAAAAATAGGCATGGG	0.00	0.00	0.00	0.68	0.00
GATCAAAAAATAGTTTCAAA	0.00	0.33	0.00	0.00	0.00
GATCAAAAAATATAGCTTCT	0.00	0.00	0.00	0.00	0.00
GATCAAAAAATATGAAAAAA	0.00	0.65	0.29	0.00	0.00
GATCAAAAAATATGAATAAA	0.00	0.33	0.00	0.00	0.00
GATCAAAAAATCAAAAAAAA	0.00	0.00	0.00	0.68	0.00
GATCAAAAAATCATCTGCTC	0.00	0.00	0.00	0.34	0.00
GATCAAAAAATCCCAACACT	0.00	0.00	0.00	0.00	0.00
GATCAAAAAATCGAACCGTT	0.23	0.00	0.00	0.00	0.00
GATCAAAAAATCGTATGCCG	0.00	0.00	0.57	0.00	0.00
GATCAAAAAATCTAAAAAAA	0.00	0.00	0.29	0.00	0.00
GATCAAAAAATCTTGGGGGG	0.00	0.33	0.00	0.00	0.69
GATCAAAAAATGAATAAAGC	0.23	0.00	0.00	0.00	0.00
GATCAAAAAATGACCCAGAA	0.23	0.00	0.00	0.00	0.00
GATCAAAAAATGCAAAAAAA	0.00	0.00	0.29	0.00	0.00
GATCAAAAAATGCCTCAAGA	0.46	0.00	0.00	0.00	0.00
GATCAAAAAATGGTATGAAA	0.00	0.33	0.00	0.00	0.00
GATCAAAAAATGGTATGAAC	0.00	0.00	0.00	0.00	0.00
GATCAAAAAATGGTATGAAG	2.29	3.58	2.00	3.73	1.38

GATCAAAAAATTGGGTTTTT	0.00	0.00	0.00	0.00	1.03
GATCAAAAACAAAAAAAAAA	103.47	26.68	35.22	35.98	53.73
GATCAAAAACAAAAAAAAAC	0.46	0.33	0.00	0.00	1.03
GATCAAAAACAAAAAAAAAG	0.23	0.00	0.00	0.00	0.00
GATCAAAAACAAAAAAAAAN	0.23	0.00	0.00	0.00	0.00
GATCAAAAACAAAAAAAAAT	2.75	0.33	2.58	1.02	1.38
GATCAAAAACAAAAAAAACA	0.00	0.00	0.00	0.34	0.00
GATCAAAAACAAAAAAAACN	0.23	0.00	0.00	0.00	0.00
GATCAAAAACAAAAAAAAGA	0.00	0.00	0.00	0.00	0.00
GATCAAAAACAAAAAAAANN	0.46	0.00	0.00	0.00	0.00
GATCAAAAACAAAAAAAATA	0.00	0.00	0.00	0.00	0.34
GATCAAAAACAAAAAAACAA	0.00	0.00	0.00	0.00	0.34
GATCAAAAACAAAAAAACNN	0.23	0.00	0.00	0.00	0.00
GATCAAAAACAAAAAAAGAA	0.00	0.00	0.00	0.34	0.00
GATCAAAAACAAAAAACAAA	0.23	0.00	0.29	0.34	0.00
GATCAAAAACAAAAAACAAC	0.00	0.00	0.00	0.00	0.00
GATCAAAAACAAAAAACCCA	0.00	0.33	0.00	0.00	0.00
GATCAAAAACAAAAAATAAA	0.23	0.00	0.00	0.00	0.00
GATCAAAAACAAAAACAAAA	1.14	0.00	0.29	0.34	0.00
GATCAAAAACAAAAACGAAA	0.00	0.00	0.00	0.00	0.34
GATCAAAAACAAAAAGAAAA	0.69	0.00	0.00	0.00	0.00
GATCAAAAACAAAAAGGAAA	0.00	0.00	0.29	0.00	0.00
GATCAAAAACAAAAATCTAA	0.00	0.00	0.00	0.68	0.00
GATCAAAAACAAAACAAAAA	0.00	0.00	0.00	0.00	0.34
GATCAAAAACAAAACAAAAC	0.00	0.00	0.00	0.00	0.34
GATCAAAAACAAAAGAAAAA	0.23	0.00	0.00	0.00	0.00
GATCAAAAACAAAATGTATT	0.00	0.00	0.00	0.00	0.34
GATCAAAAACAAACAAAAAA	0.23	0.00	0.00	0.00	0.69
GATCAAAAACAAACAAAACA	0.00	0.00	0.00	0.34	0.00
GATCAAAAACAAACAATGAA	0.00	0.00	0.00	0.00	0.00
GATCAAAAACAAACCAATCG	0.00	0.00	0.00	0.00	0.34
GATCAAAAACAAAGAAAAAA	18.54	7.16	4.30	9.84	9.64
GATCAAAAACAAAGAAAAAC	0.23	0.00	0.00	0.00	0.00
GATCAAAAACAAAGAAAAAG	0.00	0.00	0.00	0.34	0.00
GATCAAAAACAAAGAAAACN	0.23	0.00	0.00	0.00	0.00
GATCAAAAACAAAGAAAAGA	0.46	0.00	0.00	0.00	0.00
GATCAAAAACAAAGAAAAGC	0.00	0.33	0.00	0.00	0.00

GATCAAAAACAAAGAAAAGG	0.92	0.65	2.00	1.36	2.76
GATCAAAAACAAAGAACACA	0.00	0.00	0.00	0.00	0.34
GATCAAAAACAAAGAANNNN	0.23	0.00	0.00	0.00	0.00
GATCAAAAACAAAGCAAAAA	0.00	0.33	0.00	0.00	0.00
GATCAAAAACAAATAAAAAA	0.00	0.00	0.00	0.00	0.34
GATCAAAAACAAATCAAAAA	1.37	0.33	0.57	0.68	0.34
GATCAAAAACAAATCAAACN	0.23	0.00	0.00	0.00	0.00
GATCAAAAACAAATCAACAA	0.00	0.00	0.00	0.00	0.34
GATCAAAAACAAATCAACAC	0.23	0.00	0.29	0.68	0.00
GATCAAAAACAAATGAAAAA	0.00	0.00	0.29	0.00	0.00
GATCAAAAACAAATGAAATA	0.23	0.00	0.00	0.00	0.00
GATCAAAAACAAATTCCTGC	0.23	1.30	1.72	2.04	1.72
GATCAAAAACAACAAAAAAA	0.00	0.00	0.00	0.00	0.34
GATCAAAAACAACACAAAAA	0.00	0.00	0.00	0.34	0.00
GATCAAAAACAACGAAAANA	0.00	0.00	0.00	0.34	0.00
GATCAAAAACAAGAAAAAAA	0.00	0.33	0.00	0.00	0.00
GATCAAAAACAAGAAAAAAT	0.00	0.00	0.00	0.00	0.34
GATCAAAAACAAGAACACAA	0.00	0.33	0.00	0.00	0.00
GATCAAAAACAAGGAAAAAA	0.00	0.00	0.00	0.00	0.00
GATCAAAAACAAGGAAAGGC	0.00	0.33	0.00	0.00	0.00
GATCAAAAACAATATAAAAA	0.00	0.00	0.00	0.00	1.72
GATCAAAAACAATCCGGGCA	0.69	0.65	0.00	0.00	0.00
GATCAAAAACAATGCTTTCG	0.00	0.00	0.57	0.00	0.00
GATCAAAAACAATGTTCACA	0.00	0.00	0.29	0.00	0.00
GATCAAAAACACAAAAAAAA	0.00	0.33	0.00	0.00	0.00
GATCAAAAACACAAAAAACA	0.23	0.00	0.00	0.00	0.00
GATCAAAAACACAAAAACGC	0.00	0.00	0.00	0.00	0.00
GATCAAAAACACAACTCCAG	0.00	0.00	0.00	0.34	0.00
GATCAAAAACACACACACCA	0.00	0.33	0.00	0.00	0.00
GATCAAAAACACACATAAAA	0.00	0.00	0.00	0.00	0.00
GATCAAAAACACACCAAACA	0.00	0.00	0.00	0.00	0.34
GATCAAAAACACACCAAACC	0.00	0.33	0.00	0.00	0.00
GATCAAAAACACAGACACAG	0.00	0.00	0.29	0.00	0.00
GATCAAAAACACAGCTTTGG	0.00	0.00	0.29	0.00	0.00
GATCAAAAACACCAAACAAA	0.00	0.00	0.00	0.00	0.00
GATCAAAAACACCACGGTCG	0.00	0.00	0.00	0.00	0.00
GATCAAAAACACCCAGTTTT	0.00	0.00	0.00	0.34	0.00

GATCAAAAACACCTTGACTC	0.00	0.00	0.00	0.00	0.34
GATCAAAAACACGGAATTGG	0.00	0.00	0.00	0.00	0.00
GATCAAAAACACGGATGTGG	0.00	0.33	0.00	0.00	0.00
GATCAAAAACACGGCAACAC	0.00	0.00	0.00	0.00	0.00
GATCAAAAACACGGCCTTGG	0.00	0.00	0.00	0.34	0.00
GATCAAAAACACGGCGTTGG	0.23	0.00	0.00	0.00	0.00
GATCAAAAACACGGCTCTGG	0.00	0.00	0.00	0.00	0.00
GATCAAAAACACGGCTGTGG	0.23	0.00	0.00	0.00	0.00
GATCAAAAACACGGCTNTGG	0.00	0.00	0.00	0.00	0.69
GATCAAAAACACGGCTTAGG	0.23	0.00	0.00	0.34	0.00
GATCAAAAACACGGCTTNNN	0.23	0.00	0.00	0.00	0.00
GATCAAAAACACGGCTTTAG	0.00	0.00	0.00	0.00	0.00
GATCAAAAACACGGCTTTCA	0.00	0.00	0.00	0.00	0.34
GATCAAAAACACGGCTTTCG	0.00	0.00	0.00	0.34	0.00
GATCAAAAACACGGCTTTGG	27.47	49.12	39.52	37.34	32.03
GATCAAAAACACGGCTTTTG	0.00	0.33	0.29	0.34	0.00
GATCAAAAACACGTAGACAT	0.00	0.00	0.00	0.00	0.69
GATCAAAAACACGTTCATTG	0.00	0.33	0.00	0.00	0.00
GATCAAAAACACTCTGGAGG	0.00	0.33	0.00	0.00	0.00
GATCAAAAACAGAAAAAAAA	0.46	0.00	0.00	0.34	0.34
GATCAAAAACAGAGAAAAAA	0.23	0.00	0.00	0.00	0.00
GATCAAAAACAGAGGAGGGA	0.00	0.00	0.57	0.00	0.00
GATCAAAAACAGAGTTAGGA	0.00	0.00	0.00	0.00	0.34
GATCAAAAACAGCAAAAAAA	3.20	0.65	1.43	0.34	0.69
GATCAAAAACAGCAAGAAAA	0.00	0.65	0.00	0.00	0.00
GATCAAAAACAGCAGCCAAC	0.00	0.00	0.29	0.00	0.00
GATCAAAAACAGCATCAAAA	0.46	0.33	0.29	0.00	0.69
GATCAAAAACAGCATCAAGA	0.23	0.00	0.00	0.34	0.00
GATCAAAAACAGCGTACAGT	0.00	0.33	0.00	0.00	0.00
GATCAAAAACAGGACATGAG	0.00	0.00	0.00	0.34	0.00
GATCAAAAACAGGCGACAGA	3.20	0.33	0.00	0.68	0.69
GATCAAAAACAGGTGTTGAC	0.00	0.33	0.00	0.00	0.00
GATCAAAAACAGTTCAAAAA	0.00	0.33	0.00	0.00	0.00
GATCAAAAACAGTTCGAAAA	0.23	0.00	0.00	0.34	0.00
GATCAAAAACAGTTCGAAAC	0.00	0.00	0.29	0.00	0.00
GATCAAAAACATAAAAAAAA	0.00	0.00	0.00	1.02	0.00
GATCAAAAACATAAACAAGA	0.00	0.00	0.00	0.68	0.00

GATCAAAAACATCAAAAAAA	0.23	0.33	0.29	0.00	0.00
GATCAAAAACATCATGGCGG	0.00	0.00	0.00	0.68	0.00
GATCAAAAACATCCGTGACT	0.00	0.00	0.00	0.00	0.00
GATCAAAAACATGCCACAAT	0.00	0.00	0.00	0.00	0.34
GATCAAAAACATGTACTAAA	0.00	0.33	0.00	0.00	0.00
GATCAAAAACATGTACTGAA	0.00	0.00	0.00	0.00	0.00
GATCAAAAACATGTACTGAC	0.00	0.00	0.29	0.00	0.00
GATCAAAAACATGTACTGAT	0.23	1.30	2.29	1.02	0.34
GATCAAAAACATTAAAAAAA	0.00	0.00	0.00	0.00	0.00
GATCAAAAACATTGAGGCTG	0.00	0.00	0.00	0.34	0.00
GATCAAAAACCAAAAAAAAA	0.46	0.00	0.00	0.34	0.00
GATCAAAAACCAAAAAACAC	0.00	0.00	0.29	0.00	0.00
GATCAAAAACCAAAAACACA	0.00	0.33	0.00	0.00	0.00
GATCAAAAACCAAACAAACC	0.00	0.00	0.00	0.00	0.34
GATCAAAAACCAAACACAAC	0.00	0.00	0.29	0.00	0.00
GATCAAAAACCAACACACCA	0.00	0.00	0.00	0.00	0.34
GATCAAAAACCAACACGAAA	0.00	0.00	0.00	0.00	0.00
GATCAAAAACCACAAAAAAG	0.00	0.00	0.00	0.00	0.00
GATCAAAAACCACCACAATC	0.00	0.00	0.29	0.00	0.00
GATCAAAAACCACCTCCAGC	0.23	0.00	0.00	0.00	0.00
GATCAAAAACCAGACGCCTT	0.00	0.00	0.00	0.34	0.00
GATCAAAAACCATCCTCAGT	0.00	0.00	0.00	0.00	0.34
GATCAAAAACCCAACAAAAC	0.23	0.00	0.00	0.00	0.00
GATCAAAAACCCAACACCAG	0.00	0.33	0.00	0.00	0.00
GATCAAAAACCCAACTCCAG	0.00	0.00	0.29	0.00	0.00
GATCAAAAACCCACAGTAGA	0.00	0.00	0.00	0.00	0.00
GATCAAAAACCCCACAAAAC	0.00	0.00	0.29	0.00	0.00
GATCAAAAACCCCACAACCA	0.23	0.00	0.00	0.00	0.00
GATCAAAAACCCCCCACCCC	0.23	0.00	0.00	0.00	0.00
GATCAAAAACCCTTCCTCTG	0.00	0.00	0.29	0.00	0.00
GATCAAAAACCGCACGCAAA	0.00	0.00	0.00	0.00	0.34
GATCAAAAACCGCAGCATCC	0.00	0.00	0.00	0.00	0.34
GATCAAAAACCGCATCAAAA	0.46	0.00	0.00	0.00	0.00
GATCAAAAACCGCATGCTCG	0.00	0.00	0.29	0.34	0.00
GATCAAAAACCGGCCCAAAT	0.00	0.00	0.29	0.00	0.00
GATCAAAAACCGGGTGGTGT	0.00	0.00	0.29	0.00	0.00
GATCAAAAACCGTCAACAGT	0.23	0.00	0.00	0.00	0.00

GATCAAAAACCGTTAAAAGA	0.46	0.00	0.00	0.00	0.00
GATCAAAAACCTGGATGCTG	0.00	0.00	0.00	0.00	0.34
GATCAAAAACGAAAAAAAAA	0.00	0.65	0.00	0.34	0.00
GATCAAAAACGAAAAACAAA	0.00	0.00	0.00	0.00	0.00
GATCAAAAACGAACACTAAG	0.00	0.00	0.57	0.00	0.00
GATCAAAAACGAAGAAAAAA	0.00	0.00	0.00	0.34	0.00
GATCAAAAACGAAGAAAAGA	0.00	0.33	0.00	0.00	0.00
GATCAAAAACGAAGCAACAG	0.00	0.33	0.00	0.00	0.00
GATCAAAAACGAATTCTTTG	0.00	0.00	0.00	0.00	0.00
GATCAAAAACGACCACAAAA	0.00	0.00	0.00	0.34	0.00
GATCAAAAACGACCACACAA	0.00	0.33	0.00	0.00	0.00
GATCAAAAACGACCACACGC	0.46	0.00	0.00	0.00	0.00
GATCAAAAACGACCACTAAG	0.92	0.33	0.00	0.68	0.34
GATCAAAAACGACCGGGCAT	0.23	0.00	0.00	0.00	0.00
GATCAAAAACGACCTGGCAC	0.00	0.00	0.00	0.34	0.00
GATCAAAAACGACCTGGCAT	2.29	3.25	0.86	4.07	2.07
GATCAAAAACGACCTGNCAT	0.00	0.00	0.00	0.00	0.34
GATCAAAAACGAGAAAAAAA	0.00	0.33	0.00	0.00	0.00
GATCAAAAACGATTTCTGGA	0.00	0.00	0.00	0.34	0.00
GATCAAAAACGCAAAATGCC	0.00	0.00	0.29	0.00	0.00
GATCAAAAACGCCGCAGACT	0.92	0.65	0.57	1.36	0.00
GATCAAAAACGCCGTCTCAA	0.00	0.00	0.57	0.00	0.00
GATCAAAAACGGACGCTCCC	0.00	0.00	0.00	0.00	0.00
GATCAAAAACGGCAAGAAGA	0.00	0.00	0.29	0.00	0.00
GATCAAAAACGGCCGCTGGC	0.00	0.33	0.00	0.00	0.00
GATCAAAAACGGCCGTGTTG	0.00	0.33	0.00	0.00	0.69
GATCAAAAACGGCCGTTTGG	0.00	0.00	0.29	0.00	0.00
GATCAAAAACGGGAAGAAGA	0.00	0.00	0.29	0.00	0.00
GATCAAAAACGGGCAGCTGT	0.23	0.00	0.57	0.00	0.00
GATCAAAAACGGGCTTNTNT	0.00	0.33	0.00	0.00	0.00
GATCAAAAACGGGTTTGCAC	0.00	0.65	0.00	0.00	0.00
GATCAAAAACGGTAAGAAGA	8.93	29.93	53.55	25.80	39.26
GATCAAAAACGGTAAGAAGC	0.00	0.33	0.57	0.00	0.00
GATCAAAAACGGTAAGAAGG	0.23	0.00	0.00	0.00	0.00
GATCAAAAACGGTAAGAAGT	0.00	0.00	0.29	0.00	0.00
GATCAAAAACGGTAAGAATC	0.00	0.00	0.57	0.68	0.00
GATCAAAAACGGTAAGCAGA	0.00	0.33	0.00	0.00	0.00

GATCAAAAACGGTAAGCATA	0.00	0.00	0.00	0.34	0.00
GATCAAAAACGGTATGGCAG	0.00	0.00	0.00	0.00	0.00
GATCAAAAACGGTCTTGTGT	0.00	0.33	0.00	0.00	0.00
GATCAAAAACGGTGCGGTCT	0.92	0.00	0.00	0.34	0.69
GATCAAAAACGGTGTGGTCT	0.69	0.00	0.00	0.00	0.00
GATCAAAAACGTAAAAAAAA	0.00	0.00	0.57	0.00	0.00
GATCAAAAACGTCCCTTTTT	0.00	0.00	0.00	0.00	0.00
GATCAAAAACTAAAAAAAAA	0.00	0.00	0.00	0.00	0.00
GATCAAAAACTAAAATAAAT	0.23	0.00	0.00	0.00	0.00
GATCAAAAACTAACGAAGAA	0.00	0.00	0.29	0.00	0.00
GATCAAAAACTACATACAAT	0.00	0.00	0.00	0.00	0.34
GATCAAAAACTCCAGCAAAA	0.69	0.00	0.00	0.00	0.69
GATCAAAAACTCCAGCAAAT	0.46	0.65	2.00	4.41	3.10
GATCAAAAACTCCAGCACNN	0.23	0.00	0.00	0.00	0.00
GATCAAAAACTCCAGCANNN	0.23	0.00	0.00	0.00	0.00
GATCAAAAACTCCCAAAGTC	0.00	0.33	0.86	0.00	0.00
GATCAAAAACTCCGTCATCG	0.00	0.00	0.29	1.02	0.34
GATCAAAAACTCGAGCGTTC	0.00	0.00	0.00	0.00	0.00
GATCAAAAACTGAGAAGGCT	0.00	0.00	0.00	0.00	0.34
GATCAAAAACTGCATGTCCG	0.00	0.00	0.00	0.00	0.00
GATCAAAAACTGGAGTGCTC	0.00	0.33	0.00	0.00	0.00
GATCAAAAACTGGATGCGGA	0.00	0.00	0.00	0.34	0.00
GATCAAAAACTGGCCTGAAG	0.00	0.00	0.00	0.00	0.00
GATCAAAAACTGGGAAAAAA	0.00	0.00	0.00	0.00	1.03
GATCAAAAACTGGGAAAAAC	0.00	0.00	0.00	0.00	0.34
GATCAAAAACTGGTATCCCT	0.00	0.00	0.00	0.00	0.00
GATCAAAAACTGGTATGAAA	0.00	0.00	0.57	0.00	0.00
GATCAAAAACTGGTATGAAG	9.39	5.21	8.02	16.97	5.85
GATCAAAAACTGGTATGAAT	0.46	0.00	0.00	0.00	0.00
GATCAAAAACTGGTATGACG	0.00	0.00	0.29	0.00	0.00
GATCAAAAACTGGTATGATC	0.00	0.00	0.00	0.00	0.34
GATCAAAAACTGGTGCTGCA	0.00	0.00	0.57	0.00	0.00
GATCAAAAACTGGTGTGAAG	0.00	0.00	0.29	0.00	0.00
GATCAAAAACTGGTTTGAGG	0.00	0.00	0.00	0.34	0.00
GATCAAAAACTTCGACCTCA	0.00	0.00	0.29	0.68	0.00
GATCAAAAACTTCGGAGTGT	0.00	0.00	0.00	0.00	0.34
GATCAAAAACTTGCAGGTCC	0.00	0.00	0.00	1.02	0.00

GATCAAAAACTTGGGCATGT	0.00	0.33	0.00	0.00	0.00
GATCAAAAACTTTGTCTGCA	2.75	0.33	0.00	1.02	0.69
GATCAAAAACTTTGTCTGNN	0.00	0.00	0.00	0.00	0.00
GATCAAAAAGAAAAAAAAAA	47.84	18.54	22.34	21.04	24.80
GATCAAAAAGAAAAAAAAAG	0.00	0.00	0.00	0.00	0.34
GATCAAAAAGAAAAAAAAAT	1.60	0.33	0.00	0.34	0.00
GATCAAAAAGAAAAAAAACA	0.23	0.00	0.00	0.34	0.00
GATCAAAAAGAAAAAAAAGA	0.23	0.00	0.00	0.00	0.00
GATCAAAAAGAAAAAAAATA	0.23	0.00	0.00	0.00	0.00
GATCAAAAAGAAAAAAATAA	0.23	0.00	0.00	0.00	0.00
GATCAAAAAGAAAAAAATCT	0.00	0.00	0.57	0.00	0.00
GATCAAAAAGAAAAAAGAAA	0.23	0.00	0.00	0.00	0.00
GATCAAAAAGAAAAAANAAA	0.00	0.00	0.00	0.00	0.34
GATCAAAAAGAAAAAATAAA	0.23	0.00	0.00	0.00	0.00
GATCAAAAAGAAAAACAAAA	0.23	0.00	0.00	0.00	0.00
GATCAAAAAGAAAAACAACA	0.00	0.00	0.00	0.00	0.34
GATCAAAAAGAAAAAGAAAA	0.00	0.00	0.29	0.00	0.00
GATCAAAAAGAAAAGAAAAA	0.00	0.00	0.00	0.68	0.00
GATCAAAAAGAAAAGAAAAT	0.23	0.00	0.00	0.00	0.00
GATCAAAAAGAAACTTTGTT	0.23	0.00	0.00	0.00	0.00
GATCAAAAAGAAAGGCGAGC	0.00	0.00	0.00	0.00	0.34
GATCAAAAAGAAAGGCGAGG	0.00	0.65	0.00	0.34	0.00
GATCAAAAAGAAAGGCGGGG	0.23	0.00	0.00	0.00	0.00
GATCAAAAAGAAAGGTTGCT	0.00	0.00	0.00	0.00	0.00
GATCAAAAAGAAATCATTTC	0.92	0.00	0.00	0.00	0.00
GATCAAAAAGAACAAAAAAA	18.77	3.58	0.86	6.79	6.54
GATCAAAAAGAACAAAAAAC	0.23	0.00	0.00	0.00	0.00
GATCAAAAAGAACAAAAAAG	0.23	0.00	0.00	0.00	0.00
GATCAAAAAGAACAAAAAAN	0.23	0.00	0.00	0.00	0.00
GATCAAAAAGAACAATAATA	0.23	0.00	0.00	0.00	0.00
GATCAAAAAGAACAGAAAAA	0.00	0.33	0.00	0.00	0.00
GATCAAAAAGAACCCAAACA	0.46	0.00	0.00	0.00	0.00
GATCAAAAAGAAGAAGAAGC	0.00	0.00	0.29	0.00	0.69
GATCAAAAAGAAGATTGCTG	0.00	0.00	0.00	0.00	0.00
GATCAAAAAGAAGCCCTTCA	0.46	1.95	0.29	0.68	0.00
GATCAAAAAGAAGCCGGTCC	0.00	0.00	0.00	0.00	0.00
GATCAAAAAGAAGGAGGAGG	0.00	0.33	0.00	0.00	0.00

GATCAAAAAGAAGTGCAAAA	0.00	0.00	0.00	0.00	0.00
GATCAAAAAGAAGTGCAACT	0.00	0.00	0.29	0.00	0.00
GATCAAAAAGAATAAAAAAA	0.00	0.00	0.00	0.68	0.00
GATCAAAAAGAATCTAAAAA	0.46	0.00	0.00	0.00	0.00
GATCAAAAAGACAAAAAAAA	0.00	0.00	0.29	0.00	0.00
GATCAAAAAGACAAGCCCTT	0.00	0.33	0.00	0.00	0.00
GATCAAAAAGACACACCCTA	0.00	0.65	0.00	0.00	0.00
GATCAAAAAGACACCAACCT	0.00	0.00	0.00	0.00	0.34
GATCAAAAAGACGAAGCAAG	0.00	0.00	0.00	0.00	0.00
GATCAAAAAGACGAAGGAGG	1.83	5.53	3.72	3.73	2.41
GATCAAAAAGACGAAGGAGT	0.00	0.00	0.00	0.34	0.00
GATCAAAAAGACGAAGGCGG	0.00	0.33	0.00	0.00	0.00
GATCAAAAAGACGTAGCGCC	0.00	0.00	0.00	0.34	0.00
GATCAAAAAGACTAAAAAAA	0.23	0.00	0.00	0.00	0.00
GATCAAAAAGACTAAGGCTC	0.00	0.00	0.29	0.00	0.00
GATCAAAAAGACTGCCAAAC	0.00	0.00	0.00	0.00	0.34
GATCAAAAAGACTGTCAAGT	0.69	0.00	0.00	0.00	0.00
GATCAAAAAGACTTACATAT	0.00	0.33	0.00	0.00	0.00
GATCAAAAAGAGAAAAAAAA	1.14	0.00	0.00	0.68	0.00
GATCAAAAAGAGAAGAAAAA	0.00	0.00	0.00	0.34	0.00
GATCAAAAAGAGAAGTGCGC	0.00	0.00	0.00	0.34	0.00
GATCAAAAAGAGAGAATAAG	0.23	0.00	0.00	0.00	0.00
GATCAAAAAGAGCTTCAAGA	0.46	0.00	0.00	0.00	0.00
GATCAAAAAGAGGAAAAAAA	0.00	0.00	0.00	0.00	0.00
GATCAAAAAGAGGGCCTGGC	0.00	0.00	0.57	0.00	0.00
GATCAAAAAGAGGTGCGACT	0.00	0.00	0.00	0.00	0.00
GATCAAAAAGAGTAAAAAAA	0.00	0.65	0.00	0.00	0.00
GATCAAAAAGAGTGAATAAG	4.81	3.25	4.01	6.11	3.10
GATCAAAAAGAGTGAATAAT	0.23	0.00	0.00	0.00	0.00
GATCAAAAAGAGTGAATCAG	0.00	0.00	0.00	0.00	0.00
GATCAAAAAGAGTGACATGC	0.00	0.33	0.00	0.00	0.00
GATCAAAAAGAGTGAGTAAG	0.00	0.00	0.29	0.00	0.00
GATCAAAAAGATAAAAAAAA	1.37	0.00	0.00	0.00	0.00
GATCAAAAAGATAATTAGCT	0.46	0.00	0.00	0.00	0.00
GATCAAAAAGATACACACTA	0.00	0.00	0.00	0.00	0.00
GATCAAAAAGATACACCCGA	0.00	0.33	0.00	0.34	0.00
GATCAAAAAGATACACCCTA	13.73	14.97	21.48	9.84	15.15

GATCAAAAAGATACACCCTC	0.00	0.00	0.00	0.34	0.00
GATCAAAAAGATACACGCTA	0.23	0.00	0.00	0.00	0.00
GATCAAAAAGATACACTCGT	0.00	0.33	0.00	0.00	0.00
GATCAAAAAGATACGCCCTA	0.00	0.00	0.29	0.00	0.00
GATCAAAAAGATGAAAAAAA	0.92	0.33	0.00	0.00	0.00
GATCAAAAAGATGATGCATT	0.00	0.00	0.00	0.00	0.00
GATCAAAAAGATGTTCACAA	0.00	0.65	0.00	0.00	0.00
GATCAAAAAGATTGAGAACG	0.00	0.33	0.00	0.00	0.00
GATCAAAAAGCAAAGTCGCT	0.00	0.00	0.00	0.00	0.34
GATCAAAAAGCAACGCAGCT	0.00	0.00	0.29	0.00	0.00
GATCAAAAAGCAACGTCGCC	0.00	0.00	0.00	0.00	0.69
GATCAAAAAGCAACGTCGCT	0.00	0.00	16.61	0.68	24.11
GATCAAAAAGCAACGTNGCT	0.00	0.00	0.00	0.00	0.34
GATCAAAAAGCAAGGCGCGG	0.23	0.00	0.00	0.00	0.00
GATCAAAAAGCAATGGCCTC	0.00	0.00	0.00	0.00	0.00
GATCAAAAAGCAATGTCGCT	0.00	0.00	0.29	0.00	0.69
GATCAAAAAGCACACGCAAA	0.00	0.00	0.00	0.34	0.00
GATCAAAAAGCACGGTCAAT	0.00	0.00	0.00	0.34	0.34
GATCAAAAAGCACGGTCGCA	0.00	0.00	0.00	0.34	0.00
GATCAAAAAGCACGGTCGCT	0.00	0.33	0.00	0.00	0.00
GATCAAAAAGCACTGCGAAG	0.00	0.00	0.00	0.34	0.00
GATCAAAAAGCACTGTATTT	0.00	0.00	0.57	0.34	0.00
GATCAAAAAGCAGATGGGGG	0.00	0.33	0.00	0.00	0.00
GATCAAAAAGCAGCAGAAGC	0.00	0.00	0.00	0.00	0.00
GATCAAAAAGCAGCCCAAGG	0.00	0.00	0.00	0.68	0.34
GATCAAAAAGCAGGCCAAGC	0.00	0.00	0.00	0.00	0.34
GATCAAAAAGCAGGCCAAGG	0.00	0.00	0.00	0.00	0.34
GATCAAAAAGCAGTACAAGG	0.00	0.00	0.00	0.00	0.34
GATCAAAAAGCAGTCAAAGA	0.00	0.00	0.00	0.34	0.00
GATCAAAAAGCAGTCCAAGC	0.00	0.00	0.29	0.00	0.34
GATCAAAAAGCAGTCCAAGG	8.93	74.50	154.35	44.47	97.81
GATCAAAAAGCAGTCCACGG	0.00	0.00	0.29	0.00	0.00
GATCAAAAAGCAGTCCANGG	0.00	0.00	0.00	0.34	0.00
GATCAAAAAGCAGTCCATGG	0.00	0.00	0.29	0.00	0.00
GATCAAAAAGCAGTCCCAGG	0.00	0.00	0.00	0.00	0.34
GATCAAAAAGCAGTCCGAGG	0.00	0.00	0.29	0.00	0.00
GATCAAAAAGCAGTGCAAGG	0.00	0.00	0.29	0.00	0.00

GATCAAAAAGCATAGAGCCA	0.00	0.00	0.00	0.00	0.00
GATCAAAAAGCATGGTGGCG	0.00	0.00	0.29	0.00	0.00
GATCAAAAAGCATGGTGGTC	0.69	0.65	0.29	0.00	0.00
GATCAAAAAGCATTGGATAG	0.00	0.00	0.00	0.34	0.00
GATCAAAAAGCCAAAAAAAA	0.46	3.25	2.86	1.70	4.13
GATCAAAAAGCCAAAAAAAT	0.00	0.33	0.00	0.00	0.00
GATCAAAAAGCCAACAAACA	0.00	0.00	0.00	0.00	0.00
GATCAAAAAGCCAACACAAA	0.00	0.00	0.00	0.00	0.00
GATCAAAAAGCCATTCAAAA	0.00	0.00	0.29	0.00	0.00
GATCAAAAAGCCCAAGCGCG	0.00	0.00	0.00	0.00	0.00
GATCAAAAAGCCCCTTTCCA	0.00	0.00	0.29	0.00	0.00
GATCAAAAAGCCGAGAAAAA	0.00	0.00	0.00	0.34	0.00
GATCAAAAAGCCGAGCCATC	0.00	0.33	0.86	0.00	0.34
GATCAAAAAGCCGTCCAAGG	0.00	0.33	0.00	0.00	0.00
GATCAAAAAGCCTGGAGAAG	0.46	0.00	0.29	0.00	0.00
GATCAAAAAGCGAAAAAAAA	0.00	0.00	0.00	0.34	0.00
GATCAAAAAGCGCAAAAAAA	0.23	0.00	0.00	0.00	0.00
GATCAAAAAGCGCACGCAAA	2.06	6.83	5.73	7.13	4.13
GATCAAAAAGCGCACGCACA	0.00	0.00	0.00	0.00	0.00
GATCAAAAAGCGCAGCGTAG	0.00	0.00	0.57	0.00	0.00
GATCAAAAAGCGCAGGAGGA	0.00	0.00	0.00	0.34	0.00
GATCAAAAAGCGGTCCAAGG	0.00	0.00	0.00	0.00	0.34
GATCAAAAAGCGTACCTAGT	0.00	0.33	0.29	0.00	0.00
GATCAAAAAGCTAAAAAAAA	0.92	0.33	0.00	0.34	0.00
GATCAAAAAGCTACACCCTA	0.00	0.00	0.00	0.00	0.34
GATCAAAAAGCTCAGGATTG	0.00	0.00	0.00	0.00	0.00
GATCAAAAAGCTCAGGCTTG	0.00	0.33	0.29	0.00	0.00
GATCAAAAAGCTGATGAGCC	0.00	0.00	0.00	0.00	0.00
GATCAAAAAGCTTGACCGGG	0.00	0.33	0.00	0.00	0.00
GATCAAAAAGCTTGGCTTTT	0.00	0.00	0.00	0.00	0.00
GATCAAAAAGCTTTCGCAGG	0.00	0.00	0.00	0.00	0.00
GATCAAAAAGGAAAAAAAAA	6.41	3.58	3.15	6.11	3.10
GATCAAAAAGGAAAAAAAAG	0.23	0.00	0.00	0.00	0.00
GATCAAAAAGGAAAAAAGAA	0.46	0.00	0.00	0.00	0.00
GATCAAAAAGGAACTACGAA	0.00	0.00	0.00	0.00	0.00
GATCAAAAAGGACATAGGAA	0.00	0.00	0.00	0.00	1.03
GATCAAAAAGGACGCACATG	0.00	0.00	0.00	0.00	0.00

GATCAAAAAGGACGGCCAAA	0.00	0.65	0.00	0.00	0.00
GATCAAAAAGGACGGTTGTT	0.00	0.00	0.00	0.00	0.34
GATCAAAAAGGACTGGAGGT	0.00	0.00	0.29	0.00	0.00
GATCAAAAAGGAGAAAAAAA	0.69	0.00	0.00	0.00	0.69
GATCAAAAAGGAGAAAGTTT	0.00	0.33	0.00	0.00	0.00
GATCAAAAAGGAGAACNNNN	0.23	0.00	0.00	0.00	0.00
GATCAAAAAGGAGAGCTGAG	0.00	0.00	0.00	0.00	0.00
GATCAAAAAGGAGCGTGTGG	0.00	0.00	0.57	0.00	0.00
GATCAAAAAGGAGGCAAAAA	0.00	0.00	0.00	0.34	0.00
GATCAAAAAGGAGGCTAAAA	0.92	0.00	0.00	0.00	0.00
GATCAAAAAGGAGGCTAAGA	0.00	0.00	0.00	0.00	0.00
GATCAAAAAGGAGGCTAAGC	0.23	0.00	0.00	0.00	0.00
GATCAAAAAGGAGGCTGAAA	0.00	0.00	0.29	0.00	0.00
GATCAAAAAGGAGGCTGAGC	16.71	9.11	8.59	13.58	8.61
GATCAAAAAGGAGGCTGAGG	0.23	0.00	0.00	0.00	0.00
GATCAAAAAGGAGGCTGTGC	0.23	0.00	0.00	0.00	0.00
GATCAAAAAGGAGGGCGCCA	0.00	0.33	0.00	0.00	0.00
GATCAAAAAGGAGGGGAGCA	0.00	0.00	0.00	0.00	0.34
GATCAAAAAGGATGCCAAGA	1.14	0.00	0.00	0.68	1.03
GATCAAAAAGGCAAAAAAAA	1.14	0.33	0.00	0.00	0.00
GATCAAAAAGGCAATCTCAA	0.00	0.00	0.29	0.00	0.00
GATCAAAAAGGCAATTGAGA	0.00	0.00	0.00	0.00	0.34
GATCAAAAAGGCATCTCGAT	0.00	0.33	0.00	0.00	0.00
GATCAAAAAGGCCAAGCAAA	0.00	0.00	0.00	0.00	0.34
GATCAAAAAGGCCGCAACAG	0.00	0.00	0.00	0.00	0.00
GATCAAAAAGGCCGCAACCG	0.00	0.00	0.29	0.34	0.00
GATCAAAAAGGCCGCGCCGA	0.00	0.33	0.00	0.00	0.00
GATCAAAAAGGCGAAGCCAT	0.00	0.00	0.00	0.00	0.00
GATCAAAAAGGCGCAGCAGA	1.37	0.65	0.00	0.34	0.00
GATCAAAAAGGCGCAGGTCG	0.00	0.33	0.00	0.00	0.00
GATCAAAAAGGCGCGGGATC	0.00	0.00	0.00	0.00	0.00
GATCAAAAAGGCGCTGATAA	0.00	0.00	0.00	0.00	0.00
GATCAAAAAGGCGGAGGCCC	0.23	0.00	0.57	0.34	0.00
GATCAAAAAGGCGGAGGCCT	0.00	0.33	0.00	0.00	0.00
GATCAAAAAGGCGGTCACTT	0.00	0.65	0.29	0.00	0.00
GATCAAAAAGGCGTAAATGA	0.69	0.00	0.00	0.00	0.00
GATCAAAAAGGCGTCCATAC	0.00	0.00	0.00	0.00	0.34

GATCAAAAAGGCTAGGAATG	0.00	0.00	0.29	0.00	0.00
GATCAAAAAGGCTGGCGCTC	0.00	0.00	0.00	0.00	0.34
GATCAAAAAGGCTGGGAGTG	0.00	0.00	0.29	0.00	0.00
GATCAAAAAGGCTTTAGTGT	0.00	0.00	0.00	0.00	0.00
GATCAAAAAGGGAAGAATAA	0.00	0.00	0.00	0.34	0.00
GATCAAAAAGGGAAGACGTA	0.23	0.00	0.00	0.00	0.00
GATCAAAAAGGGAAGCACTG	0.00	0.65	0.86	0.00	0.00
GATCAAAAAGGGAGTGTGGC	0.00	0.00	0.00	0.00	0.34
GATCAAAAAGGGATGAGGCA	0.00	0.00	0.00	0.34	0.00
GATCAAAAAGGGCAAACACC	0.00	0.00	0.00	0.00	0.34
GATCAAAAAGGGCATGCCTT	0.00	0.00	0.00	0.00	0.00
GATCAAAAAGGGCCAGTGGC	0.00	0.00	0.00	0.00	0.00
GATCAAAAAGGGCCATCTAG	0.00	0.00	0.00	0.00	0.34
GATCAAAAAGGGCCCATGCT	0.00	0.00	0.00	0.00	0.00
GATCAAAAAGGGCCCTGCTG	0.00	0.00	0.00	0.00	0.00
GATCAAAAAGGGCGAGACAG	0.00	0.00	0.00	0.34	0.00
GATCAAAAAGGGCGAGACTG	0.00	0.00	0.00	0.00	0.00
GATCAAAAAGGGCGGAGGCA	0.00	0.00	0.29	0.00	0.00
GATCAAAAAGGGCTAAGGCA	0.23	0.33	0.29	0.00	0.00
GATCAAAAAGGGCTGAAGCA	0.00	0.00	0.00	0.00	0.00
GATCAAAAAGGGCTGAGGAA	0.23	0.33	0.00	0.00	0.00
GATCAAAAAGGGCTGAGGCA	39.83	44.90	28.64	48.54	39.95
GATCAAAAAGGGCTGAGGCC	0.46	0.00	0.00	0.68	0.00
GATCAAAAAGGGCTGAGGCG	0.23	0.00	0.00	0.00	0.00
GATCAAAAAGGGCTGAGGCN	0.00	0.00	0.00	0.00	0.00
GATCAAAAAGGGCTGAGGCT	0.23	0.33	0.00	0.00	0.00
GATCAAAAAGGGCTGAGGGA	0.23	0.00	0.00	0.00	0.00
GATCAAAAAGGGCTGANGCA	0.00	0.00	0.00	0.00	0.34
GATCAAAAAGGGCTGANNNA	0.00	0.00	0.29	0.00	0.00
GATCAAAAAGGGCTGANNNN	0.23	0.00	0.00	0.00	0.00
GATCAAAAAGGGCTGCGGAA	0.00	0.00	0.29	0.00	0.00
GATCAAAAAGGGCTGCGGAG	0.00	0.00	0.00	0.00	0.34
GATCAAAAAGGGCTGTGAGA	0.00	0.33	0.00	0.00	0.00
GATCAAAAAGGGCTTCAAGC	0.00	0.00	0.29	0.00	0.00
GATCAAAAAGGGGAGGCTGT	0.00	0.00	0.00	1.02	0.00
GATCAAAAAGGGGAGGGATA	0.00	0.00	0.29	0.00	0.00
GATCAAAAAGGGGGCTGAGC	0.00	0.00	0.00	0.34	0.00

GATCAAAAAGGGGTAAAAAA	0.00	0.00	0.29	0.00	0.00
GATCAAAAAGGGGTCGGAGG	0.00	0.00	0.29	0.00	0.00
GATCAAAAAGGGTAAAAAAA	0.46	0.00	0.00	0.00	0.34
GATCAAAAAGGGTGGACTTT	0.00	0.00	0.00	0.00	0.00
GATCAAAAAGGGTTGAGGCA	0.00	0.00	0.29	0.00	0.00
GATCAAAAAGGTATTCTTGG	0.00	0.00	0.00	0.00	0.00
GATCAAAAAGGTCAACCACG	0.00	0.00	0.00	0.00	0.00
GATCAAAAAGGTCATGCCTT	0.00	1.63	0.29	0.34	0.34
GATCAAAAAGGTCATGGTCC	0.00	0.00	0.00	0.00	0.00
GATCAAAAAGGTCCGGCAGG	0.00	0.00	0.29	0.34	0.00
GATCAAAAAGGTGAGAAGCA	0.00	0.00	0.00	0.34	0.00
GATCAAAAAGGTGAGCAGCC	0.00	0.33	0.00	0.00	0.00
GATCAAAAAGGTGAGCTTTG	0.00	0.00	0.29	0.00	0.00
GATCAAAAAGGTTAAAAAAA	0.00	0.00	0.00	0.00	0.00
GATCAAAAAGGTTCCAAAAA	0.00	0.00	0.57	0.00	0.00
GATCAAAAAGGTTCCAAACA	0.00	0.00	0.00	0.34	0.00
GATCAAAAAGGTTCCAAATG	1.37	0.00	0.00	0.00	0.00
GATCAAAAAGGTTCCAATTG	0.00	0.00	0.00	0.34	0.00
GATCAAAAAGGTTGAAATGA	0.00	0.33	0.00	0.00	0.00
GATCAAAAAGGTTGAATTCN	0.00	0.00	0.00	0.00	0.00
GATCAAAAAGGTTGAATTCT	6.41	5.21	2.86	4.41	3.79
GATCAAAAAGGTTGAATTNN	0.23	0.00	0.00	0.00	0.00
GATCAAAAAGTAAAAAAAAA	6.41	1.30	0.57	1.02	3.10
GATCAAAAAGTAAAAAAAAC	0.23	0.00	0.00	0.00	0.00
GATCAAAAAGTAAAAAATAA	0.00	0.00	0.29	0.00	0.00
GATCAAAAAGTACCCAGGCT	0.00	0.00	0.00	0.00	0.34
GATCAAAAAGTAGCCATTCC	0.00	0.00	0.29	0.00	0.00
GATCAAAAAGTAGCTAGAAC	0.00	0.00	0.29	0.00	0.00
GATCAAAAAGTAGTCCAAGG	0.00	0.33	0.00	0.00	0.00
GATCAAAAAGTCAAAAAAAA	42.58	9.11	11.17	21.38	8.61
GATCAAAAAGTCAAAAAAAN	0.23	0.00	0.00	0.00	0.00
GATCAAAAAGTCAAAAAAAT	0.23	0.33	0.29	0.00	0.00
GATCAAAAAGTCAAAAAACN	0.23	0.00	0.00	0.00	0.00
GATCAAAAAGTCAAAAAANN	0.23	0.00	0.00	0.00	0.00
GATCAAAAAGTCAAAAAGTC	0.00	0.00	0.00	0.68	0.00
GATCAAAAAGTCAAACAAAA	0.23	0.00	0.00	0.00	0.00
GATCAAAAAGTCAAAGGACA	0.00	0.00	0.29	0.00	0.00

GATCAAAAAGTCAAGAAAAA	0.00	0.00	0.00	0.00	0.34
GATCAAAAAGTCAAGTGCTG	0.00	0.33	0.00	0.00	0.00
GATCAAAAAGTCAGAAAAAA	0.46	0.33	0.00	0.00	0.34
GATCAAAAAGTCAGCAGCCA	0.00	0.00	0.00	0.00	0.00
GATCAAAAAGTCAGTTCCAC	0.00	0.00	0.00	0.00	0.34
GATCAAAAAGTCATGAAAAA	0.00	0.00	0.57	0.00	0.00
GATCAAAAAGTCCACACCGC	0.00	0.00	0.29	0.00	0.00
GATCAAAAAGTCCCAGAAAA	0.00	0.00	0.00	0.00	0.34
GATCAAAAAGTCCGCGGGAG	0.00	0.33	0.00	0.00	0.00
GATCAAAAAGTCCGCGGGCA	0.00	0.00	0.29	0.00	0.00
GATCAAAAAGTCGAAAAAAA	0.00	0.00	0.29	0.00	0.69
GATCAAAAAGTCGGCAAAAA	0.00	0.00	0.29	0.00	0.00
GATCAAAAAGTCTAAAAAAA	6.18	0.65	1.43	2.72	2.07
GATCAAAAAGTCTAAAAAAN	0.23	0.00	0.00	0.00	0.00
GATCAAAAAGTCTAGTGGAA	0.00	0.00	0.00	0.00	0.34
GATCAAAAAGTCTCGTATGC	0.23	0.00	0.00	0.00	0.00
GATCAAAAAGTCTGAAAAAA	0.00	0.98	0.57	0.34	0.00
GATCAAAAAGTCTGACAAAA	0.00	0.00	0.00	0.00	0.34
GATCAAAAAGTCTGAGAAAA	0.00	0.00	0.57	0.00	0.00
GATCAAAAAGTCTGCAAAAA	14.88	5.21	4.87	10.18	7.23
GATCAAAAAGTCTGCAAACA	0.00	0.00	0.00	0.34	0.00
GATCAAAAAGTCTGCAAAGA	0.00	0.00	0.29	0.00	0.00
GATCAAAAAGTCTGCAACAA	0.00	0.00	0.00	0.34	0.00
GATCAAAAAGTCTGCAACGA	0.00	0.00	0.00	0.00	0.34
GATCAAAAAGTCTGCATAAA	0.23	0.00	0.00	0.00	0.00
GATCAAAAAGTCTGCCAAAA	0.00	0.00	0.00	0.34	0.00
GATCAAAAAGTCTGCCACCA	0.00	0.00	0.29	0.00	0.00
GATCAAAAAGTCTTGCATGA	0.00	0.65	0.00	0.00	0.00
GATCAAAAAGTCTTTGTATG	0.00	0.00	0.00	0.00	0.34
GATCAAAAAGTGAAAGCGAA	0.00	0.00	0.29	0.00	0.00
GATCAAAAAGTGCATGGGTC	0.00	0.33	0.00	0.00	0.00
GATCAAAAAGTGCCCGTTTT	0.00	0.00	0.00	1.02	0.00
GATCAAAAAGTGGGCCAGCT	0.00	0.00	0.00	0.34	0.00
GATCAAAAAGTGTGTTGACG	0.00	0.65	0.00	0.00	0.00
GATCAAAAAGTTACTTGAGA	0.00	0.00	0.00	0.34	0.00
GATCAAAAAGTTGACGCCAG	0.00	0.00	0.00	0.34	0.00
GATCAAAAAGTTGACTTGAC	0.00	0.33	0.00	0.00	0.00

GATCAAAAAGTTGAGCAGCT	0.00	0.00	0.57	0.00	0.34
GATCAAAAAGTTGAGCTTTG	0.00	0.98	0.00	2.38	2.07
GATCAAAAAGTTGAGGAAAC	0.00	0.33	0.00	0.00	0.69
GATCAAAAAGTTGAGGACAC	0.00	0.33	0.00	0.00	0.00
GATCAAAAAGTTGAGGAGAT	1.14	0.00	1.72	1.02	1.03
GATCAAAAAGTTGAGGCAGG	0.00	0.00	0.00	0.34	0.00
GATCAAAAAGTTGAGGCCAA	0.00	0.00	0.00	0.68	0.00
GATCAAAAAGTTGAGGCCAC	2.06	1.95	3.72	4.75	3.79
GATCAAAAAGTTGAGGCCAN	0.23	0.00	0.00	0.00	0.00
GATCAAAAAGTTGAGGCCAT	0.00	0.00	0.00	0.00	0.00
GATCAAAAAGTTGAGGCCNC	0.00	0.00	0.00	0.34	0.00
GATCAAAAAGTTGAGTTTTT	0.00	0.00	0.29	0.00	1.03
GATCAAAAAGTTGCCAAAAA	0.23	0.00	0.00	0.00	0.00
GATCAAAAAGTTGCCAGGTG	0.00	0.00	0.00	0.00	0.00
GATCAAAAAGTTGTTACACT	0.00	0.00	0.00	0.00	0.34
GATCAAAAAGTTTAAAGCAA	2.06	0.33	0.00	0.00	0.00
GATCAAAAAGTTTGTTATTG	0.00	0.00	0.00	0.00	0.00
GATCAAAAAGTTTTGCCAAA	0.00	0.00	0.00	0.00	0.00
GATCAAAAAGTTTTGCCACG	0.23	0.33	0.86	0.00	0.00
GATCAAAAATAAAAAAAAAA	3.20	0.33	0.29	0.34	2.07
GATCAAAAATAAAAAAAAAT	0.23	0.00	0.00	0.00	0.34
GATCAAAAATAAAAAAGAAA	0.00	0.00	0.29	0.00	0.00
GATCAAAAATAAAAAATAAA	0.00	0.00	0.29	0.00	0.00
GATCAAAAATAAAGAGGGCC	0.92	0.00	0.29	0.00	0.00
GATCAAAAATAAATTAGTGA	0.23	0.00	0.00	0.00	0.00
GATCAAAAATAACAAAAAAC	0.00	0.00	0.00	0.00	0.34
GATCAAAAATAAGAAAAAAA	0.00	0.33	0.00	0.00	0.00
GATCAAAAATAAGGTTAGGT	0.00	0.00	0.00	0.00	0.69
GATCAAAAATAATCTCGTAT	0.00	0.00	0.00	0.00	0.00
GATCAAAAATAATTTGCGCA	0.46	0.00	1.15	0.00	0.00
GATCAAAAATACATATAANA	0.00	0.00	0.00	0.00	0.34
GATCAAAAATACATCTAACC	0.69	2.60	1.43	1.02	0.34
GATCAAAAATACATTCATCG	0.00	0.00	0.29	0.00	0.00
GATCAAAAATACCACGAAAA	0.00	0.00	0.00	0.00	0.00
GATCAAAAATAGAAAAAAAA	0.23	0.00	0.00	0.00	0.00
GATCAAAAATAGCACGCTAA	0.00	0.00	0.29	0.00	0.00
GATCAAAAATAGGCCTCAGG	0.00	0.00	0.00	0.00	0.00

GATCAAAAATAGGGGTTTTG	0.00	0.00	0.00	0.00	0.00
GATCAAAAATAGTGGCACAG	0.00	0.00	0.00	0.00	0.34
GATCAAAAATATAAGAAAAA	0.00	0.00	0.00	0.00	0.69
GATCAAAAATATATAACTGT	0.00	0.00	0.00	0.00	0.69
GATCAAAAATATATCACTGT	0.00	0.00	0.00	0.00	0.34
GATCAAAAATATCACGAAAA	0.00	0.00	0.00	0.00	0.00
GATCAAAAATATCGAGACTG	0.00	0.00	0.00	1.02	0.00
GATCAAAAATATGTCAGGGG	0.00	0.00	0.00	0.00	0.34
GATCAAAAATATGTCAGGTG	0.23	1.63	0.00	1.36	0.34
GATCAAAAATATTGTTTTGG	0.00	0.00	0.29	0.00	0.00
GATCAAAAATCAAAAAAAAA	0.92	0.65	0.57	0.00	0.34
GATCAAAAATCAATACAGGG	0.00	0.00	0.00	0.00	0.34
GATCAAAAATCAATGATGAG	0.00	0.33	0.00	0.00	0.00
GATCAAAAATCACACAAAAT	0.00	0.00	0.29	0.00	0.00
GATCAAAAATCAGCTACTTG	0.23	0.00	0.00	0.00	0.00
GATCAAAAATCATGACAATT	0.00	0.00	0.29	0.00	0.00
GATCAAAAATCATTGTAAAA	0.00	0.00	0.00	0.00	0.00
GATCAAAAATCATTGTATTT	0.00	0.33	0.57	0.00	0.34
GATCAAAAATCATTGTGTCG	0.00	0.00	0.29	0.00	0.00
GATCAAAAATCCCCACAGAC	0.00	0.33	0.00	0.34	0.34
GATCAAAAATCCGCCCCCCG	0.00	0.00	0.29	0.00	0.00
GATCAAAAATCCTGAAGCTT	0.00	0.00	0.00	0.00	0.00
GATCAAAAATCCTGCAACAA	0.23	0.00	0.00	0.00	0.00
GATCAAAAATCGAAAAAACA	0.00	0.00	0.00	0.34	0.00
GATCAAAAATCGAAAACCAA	0.00	0.00	0.00	0.00	0.34
GATCAAAAATCGGGCACCCA	0.00	0.00	0.29	0.00	0.00
GATCAAAAATCGGGGATGGA	0.00	0.00	0.29	0.00	0.00
GATCAAAAATCGTATGCCGT	0.23	0.00	0.00	0.00	0.34
GATCAAAAATCGTGACAGAA	0.00	0.00	0.29	0.00	0.00
GATCAAAAATCGTGACCGAA	5.49	11.06	13.46	11.54	9.64
GATCAAAAATCGTGACCGAG	0.23	0.00	0.00	0.00	0.00
GATCAAAAATCGTGACCGCA	0.00	0.00	0.00	0.00	0.00
GATCAAAAATCGTGACCGGA	0.00	0.33	0.00	0.00	0.00
GATCAAAAATCGTGACCGNA	0.00	0.33	0.00	0.00	0.00
GATCAAAAATCGTGACGAAT	0.00	0.00	0.00	0.00	0.34
GATCAAAAATCGTGACGGCC	0.00	0.00	0.00	0.00	0.00
GATCAAAAATCGTGCCCGAA	0.00	0.00	0.29	0.00	0.00

GATCAAAAATCGTGTCTGCT	0.00	0.00	0.00	0.68	0.00
GATCAAAAATCTAAAAAAAA	0.23	3.25	6.01	9.84	10.33
GATCAAAAATCTAAAAAAAC	0.00	0.00	0.00	0.34	0.00
GATCAAAAATCTCGACCACT	0.00	0.00	0.00	0.00	0.00
GATCAAAAATCTCGCCCACT	1.37	0.33	0.86	1.70	1.72
GATCAAAAATCTCGCCCAGA	0.00	0.33	0.00	0.00	0.00
GATCAAAAATCTCGCCCATA	0.00	0.33	0.00	0.00	0.00
GATCAAAAATCTGTCTCGGT	0.00	0.00	0.29	0.00	0.00
GATCAAAAATCTTAAAAAAA	0.00	0.00	0.00	0.00	0.34
GATCAAAAATGAAAAAAAAA	0.00	0.33	0.00	0.00	0.34
GATCAAAAATGAAATGTCTG	0.00	0.00	0.00	0.00	0.00
GATCAAAAATGAAGAAAAAA	0.00	0.00	0.00	0.00	0.00
GATCAAAAATGAAGTTCCAG	0.00	0.00	0.00	0.00	0.00
GATCAAAAATGAATGCCCGA	0.00	0.00	0.00	0.34	0.00
GATCAAAAATGAATGCCTGG	1.60	0.98	0.86	2.04	0.34
GATCAAAAATGAATGCGTGG	0.00	0.00	0.00	0.34	0.00
GATCAAAAATGACCAGTTTT	0.23	0.00	0.00	0.00	0.00
GATCAAAAATGAGCCCCTCA	0.69	0.33	0.00	0.34	0.00
GATCAAAAATGATACCCCCT	0.00	0.00	0.00	0.00	0.00
GATCAAAAATGATACCTCAT	0.00	0.33	0.00	0.00	0.00
GATCAAAAATGATACCTCCG	0.00	0.00	0.00	0.00	0.00
GATCAAAAATGATACCTCCT	27.24	68.32	42.95	81.80	59.93
GATCAAAAATGATACCTCNN	0.23	0.00	0.00	0.00	0.00
GATCAAAAATGATATCTCCT	0.00	0.00	0.00	0.00	0.34
GATCAAAAATGATGCCTCCT	0.00	0.00	0.00	0.00	0.34
GATCAAAAATGATTCGTATG	0.00	0.00	0.00	0.00	0.69
GATCAAAAATGCATTGGCTC	0.00	0.00	0.29	0.00	0.00
GATCAAAAATGCATTGGCTT	0.00	0.00	0.29	0.00	0.00
GATCAAAAATGCCACCGCCC	1.37	0.00	0.00	0.00	0.00
GATCAAAAATGCCAGGAATG	0.00	0.33	0.00	0.00	0.00
GATCAAAAATGGACGCCTTG	0.23	0.00	0.00	0.00	0.00
GATCAAAAATGGCCGAATGG	0.00	0.00	0.29	0.00	0.00
GATCAAAAATGGCCGTCTGT	0.00	0.33	0.00	0.34	0.00
GATCAAAAATGGCTTCAAGG	3.66	0.98	0.86	1.36	0.00
GATCAAAAATGGCTTCCAGG	0.23	0.00	0.00	0.00	0.00
GATCAAAAATGTAAAAAAAA	0.00	0.00	0.29	0.00	0.00
GATCAAAAATGTAATCAAAA	0.00	0.00	0.00	0.00	0.34

GATCAAAAATGTCAAAAAAA	0.00	0.33	0.00	0.00	0.00
GATCAAAAATGTCCATGCAA	0.00	0.00	0.00	0.34	0.00
GATCAAAAATGTGTAAAAAA	0.00	0.00	0.00	0.00	0.00
GATCAAAAATTAAAAAAAAA	0.23	0.00	0.29	0.34	0.34
GATCAAAAATTAAGAAAAAA	0.00	0.33	0.00	0.00	0.00
GATCAAAAATTCGGTTGCTA	0.23	0.00	0.00	0.00	0.69
GATCAAAAATTCTGAGCCGT	0.00	0.00	0.00	0.68	0.00
GATCAAAAATTGAAAATAAA	0.00	0.00	0.00	0.00	1.38
GATCAAAAATTGAAAATAAT	0.46	0.00	0.00	0.00	0.00
GATCAAAAATTGATACTACT	0.00	0.00	0.00	0.34	0.00
GATCAAAAATTGCAAAAAAA	0.00	0.33	0.00	0.00	0.00
GATCAAAAATTGCCGATTTC	0.00	0.00	0.00	0.34	0.00
GATCAAAAATTGGCCGGCCA	0.00	0.00	0.29	0.00	0.00
GATCAAAAATTTAAAAAAAA	0.00	0.00	0.00	0.00	0.00
GATCAAAAATTTGAAATTCC	0.00	0.00	0.29	0.00	0.00
GATCAAAAATTTGACGGGCA	0.92	1.95	2.86	2.38	1.72
GATCAAAAATTTGGGCCGAC	0.00	0.33	0.00	0.00	0.00
GATCAAAAATTTTGCTCCCG	0.00	0.33	0.00	0.00	0.00
GATCAAAACAAAAAAAAAAA	49.44	14.64	30.07	25.12	35.47
GATCAAAACAAAAAAAAAAC	0.23	0.00	0.29	0.34	0.00
GATCAAAACAAAAAAAAAAG	0.23	0.00	0.00	0.00	0.00
GATCAAAACAAAAAAAAAAN	0.00	0.00	0.00	0.34	0.00
GATCAAAACAAAAAAAAAAT	0.92	0.65	1.43	2.04	2.07
GATCAAAACAAAAAAAAACA	0.23	0.00	0.00	0.00	0.00
GATCAAAACAAAAAAAAACC	0.00	0.00	0.00	0.34	0.00
GATCAAAACAAAAAAAAAGA	0.00	0.00	0.00	0.00	0.34
GATCAAAACAAAAAAAAANA	0.00	0.00	0.00	0.34	0.00
GATCAAAACAAAAAAAACAA	0.23	0.00	0.00	0.00	0.00
GATCAAAACAAAAAAAACAC	0.00	0.00	0.00	0.00	0.34
GATCAAAACAAAAAAAACCC	0.00	0.00	0.29	0.00	0.00
GATCAAAACAAAAAAAAGAA	0.00	0.00	0.00	0.00	0.00
GATCAAAACAAAAAAAATAA	0.00	0.00	0.29	0.00	0.00
GATCAAAACAAAAAAACAAA	0.00	0.00	0.00	0.34	0.34
GATCAAAACAAAAAAACAAC	0.23	0.00	0.00	0.00	0.00
GATCAAAACAAAAAAACCAC	0.00	0.33	0.00	0.00	0.00
GATCAAAACAAAAAAACGGA	0.23	0.00	0.00	0.00	0.00
GATCAAAACAAAAAAAGAAA	0.00	0.00	0.29	0.34	0.00

GATCAAAACAAAAAAANAAC	0.00	0.00	0.00	0.00	0.34
GATCAAAACAAAAAACAAAA	0.00	0.00	0.00	0.00	0.34
GATCAAAACAAAAAACAACA	0.00	0.00	0.00	0.00	0.00
GATCAAAACAAAAAACACCA	0.00	0.00	0.00	0.00	0.00
GATCAAAACAAAAAAGAAAA	0.00	0.00	0.00	0.00	0.34
GATCAAAACAAAAAATAAAA	0.00	0.00	0.00	0.00	0.34
GATCAAAACAAAAACACAAA	0.23	0.00	0.00	0.00	0.00
GATCAAAACAAAAACACATA	0.00	0.00	0.29	0.00	0.00
GATCAAAACAAAAACATCAA	0.46	0.00	0.00	0.00	0.00
GATCAAAACAAAAACCCAAA	0.00	0.00	0.29	0.00	0.00
GATCAAAACAAAAACTGACT	0.00	0.00	0.00	0.00	0.00
GATCAAAACAAAAAGAAAAA	0.00	0.00	0.00	0.34	0.34
GATCAAAACAAAAAGAAGCA	0.00	0.00	0.00	0.00	0.69
GATCAAAACAAAAAGATAAA	0.00	0.00	0.00	0.00	0.00
GATCAAAACAAAAATAATTA	0.00	0.00	0.00	0.00	1.38
GATCAAAACAAAACAAAAAA	0.23	0.00	0.00	0.68	0.00
GATCAAAACAAAACAAAAAG	0.00	0.00	0.00	0.00	0.34
GATCAAAACAAAACAAAACA	0.00	0.00	0.29	0.00	4.48
GATCAAAACAAAACAAAATC	0.00	0.00	0.00	0.00	0.34
GATCAAAACAAAACAAAGCA	0.00	0.00	0.00	0.00	0.69
GATCAAAACAAAACAAATAA	0.00	0.00	0.00	0.34	0.00
GATCAAAACAAAACAACAAC	0.00	0.33	0.00	0.00	0.00
GATCAAAACAAAACACAACA	0.00	0.00	0.29	0.00	0.00
GATCAAAACAAAACAGAACA	0.00	0.00	0.00	0.00	0.34
GATCAAAACAAAACATACAC	0.00	0.00	0.00	0.00	0.69
GATCAAAACAAAAGAAAAAA	0.00	0.00	0.00	0.34	0.00
GATCAAAACAAAATTTGGGA	0.46	0.33	0.29	0.00	0.00
GATCAAAACAAACAAAAAAA	0.23	0.00	0.00	0.00	0.00
GATCAAAACAAACAAAAACA	0.23	0.00	0.00	0.00	0.00
GATCAAAACAAACAAAACCA	0.23	0.00	0.00	0.00	0.00
GATCAAAACAAACAAAAGCC	0.00	0.00	0.00	0.00	0.34
GATCAAAACAAACAAACAAA	0.00	0.00	0.00	0.00	0.34
GATCAAAACAAACAAACATG	0.00	0.00	0.00	0.00	0.69
GATCAAAACAAACAAACCCA	0.00	0.00	0.00	0.00	0.00
GATCAAAACAAACAACAAAA	0.23	0.00	0.00	0.00	0.69
GATCAAAACAAACAACACAC	0.00	0.00	0.00	0.00	0.69
GATCAAAACAAACAAGTCGA	0.00	0.00	0.00	0.00	1.03

GATCAAAACAAACAATCCAG	0.23	0.00	0.00	0.00	0.00
GATCAAAACAAACACACCAC	0.00	0.00	0.00	0.00	0.34
GATCAAAACAAACAGACANN	0.00	0.00	0.00	0.00	0.00
GATCAAAACAAAGAAAAAAA	7.10	0.33	2.86	2.04	5.17
GATCAAAACAAAGAAAAGAA	0.00	0.00	0.00	0.00	0.00
GATCAAAACAAAGAAAAGGG	0.00	0.33	0.00	0.00	0.00
GATCAAAACAAAGAAACAAA	0.00	0.00	0.86	0.00	0.69
GATCAAAACAAAGAAACATC	0.00	0.00	0.00	0.00	0.34
GATCAAAACAAAGAAGAAAA	0.00	0.00	0.29	0.00	0.00
GATCAAAACAAAGCAACACA	0.00	0.00	0.00	0.00	0.34
GATCAAAACAAAGTAGACGC	0.00	0.33	0.00	0.00	0.00
GATCAAAACAAATAATGAAT	0.00	0.00	0.00	0.34	0.00
GATCAAAACAACAAAAAAAA	0.00	0.00	0.00	0.34	0.00
GATCAAAACAACAAAAACAC	0.00	0.00	0.29	0.00	0.00
GATCAAAACAACAAAAGAAA	0.00	0.00	0.00	0.00	0.34
GATCAAAACAACAACAACAA	0.00	0.00	0.00	0.00	0.34
GATCAAAACAACAACAAGAA	0.00	0.00	0.00	0.00	0.34
GATCAAAACAACAACACCCG	0.23	0.00	0.00	0.00	0.00
GATCAAAACAACACAAAACA	0.00	0.00	0.00	0.00	0.34
GATCAAAACAACAGAATTCA	0.00	0.00	0.29	0.00	0.00
GATCAAAACAACAGGCCCCG	0.00	0.00	0.29	0.00	0.00
GATCAAAACAACCAACCAAA	0.23	0.00	0.00	0.00	0.00
GATCAAAACAACCCACACCA	0.00	0.33	0.00	0.00	0.00
GATCAAAACAACCTCCATCA	1.83	0.65	0.29	2.04	0.34
GATCAAAACAACGGCAGACT	0.23	0.00	0.00	0.00	0.00
GATCAAAACAACGGCAGTCT	0.23	0.00	0.00	0.00	0.00
GATCAAAACAACTCGGGCCA	0.00	0.00	0.00	0.34	0.00
GATCAAAACAACTTGACGGC	0.00	0.00	0.00	0.00	0.00
GATCAAAACAAGAAAAAAAA	0.00	0.00	0.86	0.00	0.00
GATCAAAACAAGAAAAGAAA	0.00	0.00	0.00	0.34	0.00
GATCAAAACAAGACCTCGTG	0.00	0.33	0.00	0.00	0.00
GATCAAAACAAGAGTGCATG	0.00	0.00	0.00	0.00	0.34
GATCAAAACAAGCAAAAAAA	1.37	0.00	0.29	1.70	1.72
GATCAAAACAAGCACACACA	0.23	0.00	0.00	0.00	0.00
GATCAAAACAAGCATTGGAC	0.00	1.95	2.29	0.34	1.03
GATCAAAACAAGCGTGCCCC	0.00	0.00	0.29	0.00	0.00
GATCAAAACAAGCTCGTTCT	0.92	0.00	0.00	0.00	0.00

GATCAAAACAAGGAGTCCCT	0.00	0.65	0.00	0.34	0.00
GATCAAAACAAGGAGTTGAA	0.00	0.00	0.86	0.00	0.00
GATCAAAACAAGGATATCTC	0.00	0.00	0.29	0.00	0.00
GATCAAAACAAGGCCAATGC	0.00	0.00	0.00	0.00	0.69
GATCAAAACAATAGAATCAA	0.00	0.00	0.29	0.00	0.69
GATCAAAACAATATGAGTAC	0.00	0.00	0.00	0.00	0.00
GATCAAAACAATCAAAAAAA	0.00	0.00	0.00	0.00	0.34
GATCAAAACAATCATANGAA	0.00	0.00	0.00	0.00	0.34
GATCAAAACAATCATATGAA	0.00	0.00	0.00	0.00	0.34
GATCAAAACAATCGTATGCC	0.00	0.00	0.29	0.00	0.00
GATCAAAACAATGCTGACCA	0.00	0.00	0.00	0.00	0.00
GATCAAAACAATGTAGACGC	0.46	0.00	0.00	0.00	0.00
GATCAAAACAATTCCAGGGG	0.00	0.33	0.00	0.00	0.00
GATCAAAACACAAAAAAAAA	1.14	0.00	0.29	0.00	0.34
GATCAAAACACAAAAAAAAT	0.00	0.00	0.29	0.00	0.00
GATCAAAACACAAAATTGGC	0.00	0.00	0.57	0.00	0.00
GATCAAAACACAAACAAAAT	0.00	0.00	0.00	0.34	0.00
GATCAAAACACAAACCCAAA	0.00	0.00	0.29	0.00	0.00
GATCAAAACACAACAAGCTA	0.00	0.00	0.00	0.00	0.34
GATCAAAACACAACACAACG	0.00	0.00	0.00	0.00	0.34
GATCAAAACACAACATCACA	0.00	0.00	0.00	0.00	0.69
GATCAAAACACAACGCGCCA	0.00	0.00	0.00	0.00	0.69
GATCAAAACACAATTCTCCG	0.00	0.00	0.00	0.00	0.00
GATCAAAACACACAAAGCAC	0.00	0.33	0.00	0.00	0.00
GATCAAAACACACAACACAC	0.00	0.00	0.00	0.00	0.69
GATCAAAACACACAACCAAC	0.00	0.00	0.00	0.00	0.00
GATCAAAACACACACACAAC	0.00	0.00	0.29	0.00	0.00
GATCAAAACACACACACACA	0.00	0.00	0.29	0.00	0.34
GATCAAAACACACACACATA	0.00	0.00	0.00	0.00	0.34
GATCAAAACACACAGGGGGG	0.00	0.00	0.57	0.00	0.00
GATCAAAACACACCAACCCA	0.23	0.00	0.00	0.00	0.00
GATCAAAACACACCAAGGAA	0.00	0.00	0.00	0.00	0.34
GATCAAAACACAGAGCAGAC	0.00	0.00	0.00	0.00	0.34
GATCAAAACACAGAGGGGCA	0.00	0.00	0.00	0.00	0.69
GATCAAAACACAGCCCGTAA	0.00	0.00	0.29	0.00	0.00
GATCAAAACACAGGCATAGC	0.00	0.00	0.29	0.00	0.00
GATCAAAACACAGGCGGGTA	0.00	0.00	0.29	0.00	0.00

GATCAAAACACATCATGGCC	0.00	0.00	0.29	0.00	0.00
GATCAAAACACATCGTATTT	0.00	0.00	0.00	0.00	0.00
GATCAAAACACATCGTCGCC	0.00	0.00	0.00	0.00	0.69
GATCAAAACACATCGTGTTT	0.00	0.33	0.00	0.00	0.00
GATCAAAACACATGTTTTTT	0.23	0.00	0.00	0.00	0.00
GATCAAAACACATTGCAACC	0.23	0.00	0.29	0.00	0.00
GATCAAAACACCAAAAAATA	0.00	0.00	0.00	0.34	0.00
GATCAAAACACCAAAACAAA	0.00	0.00	0.00	0.00	0.00
GATCAAAACACCAACCGCAC	0.00	0.00	0.00	0.00	0.69
GATCAAAACACCAGTTCTGN	0.00	0.00	0.00	0.00	0.00
GATCAAAACACCCACCCACC	0.00	0.00	0.29	0.00	0.00
GATCAAAACACCGTGGGGCC	0.00	0.00	0.00	0.00	0.00
GATCAAAACACCTCGCTCTT	0.00	0.00	0.00	0.00	0.00
GATCAAAACACCTTGCAACG	0.00	0.33	1.15	1.02	0.34
GATCAAAACACCTTGCTGTC	0.00	0.33	0.00	0.00	0.00
GATCAAAACACGAATCCATA	0.00	0.33	0.00	0.00	0.00
GATCAAAACACGAATGCATG	0.00	0.00	0.00	0.00	0.00
GATCAAAACACGACACACCA	0.00	0.00	0.00	0.00	0.69
GATCAAAACACGCAGAACAA	0.00	0.00	0.00	0.00	0.34
GATCAAAACACGCCAAACAA	0.23	0.00	0.00	0.00	0.00
GATCAAAACACGGACTTCCA	0.23	0.00	0.00	0.00	0.00
GATCAAAACACGTACATGCC	0.00	0.00	0.00	0.00	0.00
GATCAAAACACTAAAAAAAA	0.92	0.00	0.00	0.00	0.00
GATCAAAACACTCACTGAGC	0.00	0.00	0.00	0.00	0.00
GATCAAAACACTCAGCAAGC	0.00	0.00	0.00	0.34	0.00
GATCAAAACACTCAGCGAGC	1.14	2.93	3.72	7.13	3.10
GATCAAAACACTCAGCGAGG	0.00	0.00	0.00	0.34	0.00
GATCAAAACACTCAGTGAAC	0.00	0.00	0.00	0.00	0.00
GATCAAAACACTCAGTGAGC	0.00	0.00	0.00	0.00	0.00
GATCAAAACACTCAGTGANC	0.00	0.00	0.00	0.00	0.00
GATCAAAACACTCCAAAACA	0.23	0.00	0.00	0.00	0.00
GATCAAAACACTCGACAGAA	1.37	0.00	0.00	0.00	0.00
GATCAAAACACTGCTTAATC	0.00	0.00	0.00	0.00	0.34
GATCAAAACACTTCTTCAGA	0.00	0.65	0.00	0.00	0.00
GATCAAAACACTTGGAGTAA	0.00	0.00	0.00	0.00	0.34
GATCAAAACAGAAAAAAAAA	0.00	0.00	0.57	0.00	0.00
GATCAAAACAGAAAGAATAA	0.00	0.00	0.57	0.00	0.00

GATCAAAACAGAACAGAAAT	0.00	0.00	0.29	0.00	0.00
GATCAAAACAGAACAGAACA	0.00	0.00	0.00	0.00	0.34
GATCAAAACAGAACAGAGCA	0.00	0.00	0.00	0.00	0.34
GATCAAAACAGAACATGACT	0.00	0.00	0.29	0.00	0.00
GATCAAAACAGAACATGNCT	0.00	0.00	0.00	0.00	0.34
GATCAAAACAGACAAAAAAA	0.00	0.00	0.00	0.00	0.00
GATCAAAACAGACGAGGAAG	0.00	0.00	0.00	0.34	0.00
GATCAAAACAGACTGGTCCG	0.00	0.00	0.29	0.00	0.00
GATCAAAACAGAGAACAAAA	0.00	0.00	0.00	0.00	0.34
GATCAAAACAGAGCAGAGCA	0.00	0.00	0.29	0.00	0.34
GATCAAAACAGAGGACATCC	0.00	0.00	0.00	0.00	0.00
GATCAAAACAGATGTTCGTC	0.00	0.00	0.29	0.34	0.34
GATCAAAACAGCCGAGGGCG	0.00	0.00	0.00	0.34	0.00
GATCAAAACAGCCGCTATGC	0.00	0.00	0.29	0.00	0.00
GATCAAAACAGCCGTGCACA	0.00	0.00	0.29	0.68	0.69
GATCAAAACAGCCTGCTCGC	0.00	0.00	0.00	0.00	0.34
GATCAAAACAGCTCTGGAGC	0.23	0.00	0.00	0.00	0.00
GATCAAAACAGCTGCTGGGT	0.00	0.00	0.00	0.34	0.00
GATCAAAACAGGACATGCCA	0.00	0.00	0.00	0.00	0.34
GATCAAAACAGGAGCGTTGC	0.00	0.00	0.00	0.00	0.00
GATCAAAACAGGAGCTTTGC	0.23	0.00	0.29	0.00	0.00
GATCAAAACAGGATGATTGG	0.00	0.00	0.00	0.00	0.34
GATCAAAACAGGCAAAAAAA	0.00	0.00	0.00	0.00	0.34
GATCAAAACAGGCACCAGGA	0.00	0.00	0.00	0.00	0.00
GATCAAAACAGGCACTTTGC	0.00	0.00	0.29	0.00	0.00
GATCAAAACAGGCCTCTTGG	0.00	0.33	0.00	0.00	0.00
GATCAAAACAGGCGATGTTC	0.00	0.33	0.00	0.00	0.00
GATCAAAACAGGCGCACAGC	0.00	0.00	0.00	0.00	0.00
GATCAAAACAGGCGCTCTGC	0.00	0.00	0.29	0.34	0.00
GATCAAAACAGGCGCTTAGC	0.00	0.00	0.00	0.00	0.00
GATCAAAACAGGCGCTTCGG	0.00	0.00	0.00	0.34	0.00
GATCAAAACAGGCGCTTGCT	0.00	0.00	0.00	0.34	0.00
GATCAAAACAGGCGCTTNNN	0.23	0.00	0.00	0.00	0.00
GATCAAAACAGGCGCTTTCC	0.00	0.00	0.00	0.00	0.00
GATCAAAACAGGCGCTTTGA	0.00	0.33	0.29	0.00	0.34
GATCAAAACAGGCGCTTTGC	21.97	50.10	39.80	45.82	37.88
GATCAAAACAGGCGCTTTNN	0.00	0.00	0.00	0.00	0.00

GATCAAAACAGGCGCTTTTC	0.00	0.00	0.00	0.34	0.34
GATCAAAACAGGCGGTTTGC	0.00	0.00	0.29	0.00	0.00
GATCAAAACAGGCTCTTTGC	0.00	0.00	0.00	0.00	0.34
GATCAAAACAGGGAGCCGTG	0.23	0.00	0.00	0.00	0.00
GATCAAAACAGGGCTCGAGC	0.69	0.00	0.00	0.00	0.00
GATCAAAACAGGGGTCGAGC	0.00	0.33	0.00	0.00	0.00
GATCAAAACAGGGTCAGGGC	0.00	0.33	0.00	0.34	0.34
GATCAAAACAGGTATGAACT	0.00	0.00	0.29	0.00	0.00
GATCAAAACAGTGAATCCCG	0.00	0.00	0.00	0.00	0.00
GATCAAAACAGTGACTCCGC	0.00	0.00	0.00	0.34	0.00
GATCAAAACAGTGACTGGCT	0.00	0.00	0.00	1.02	0.00
GATCAAAACAGTGGGTAAGT	0.00	0.65	0.00	0.34	0.00
GATCAAAACAGTTTCAAAGT	0.00	0.00	0.00	0.68	0.00
GATCAAAACATAAAAATACA	0.00	0.33	0.00	0.00	0.00
GATCAAAACATAACAGAGCA	0.00	0.00	0.00	0.00	0.69
GATCAAAACATACGATAAGA	0.00	0.00	0.00	0.00	0.34
GATCAAAACATAGCAGAACA	0.00	0.00	0.00	0.00	0.69
GATCAAAACATAGCGCACTG	0.00	0.00	0.00	0.00	0.34
GATCAAAACATAGGCAATTT	0.00	0.00	0.29	0.00	0.00
GATCAAAACATATCCAGAGG	0.00	0.00	0.29	0.00	0.00
GATCAAAACATCAAACTTTC	0.00	0.65	0.00	0.00	0.00
GATCAAAACATCAAGACACC	0.00	0.00	0.00	0.00	0.34
GATCAAAACATCACGCGTGA	0.00	0.00	0.00	0.34	0.00
GATCAAAACATCCCAATCCC	0.00	0.00	0.00	0.00	0.00
GATCAAAACATCCTGCTATG	0.00	0.33	0.00	0.00	0.00
GATCAAAACATGATTGCCAC	0.00	0.00	0.00	0.00	1.03
GATCAAAACATGCAATCTTG	0.00	0.33	0.00	0.00	0.00
GATCAAAACATGCATGAAGA	0.00	0.00	0.29	0.00	0.00
GATCAAAACATGCTAAGTCA	0.00	0.00	0.57	0.00	0.00
GATCAAAACATGGTAAAAAA	0.92	0.00	0.00	0.00	0.00
GATCAAAACATGGTCAAAAA	0.23	0.00	0.00	0.00	0.34
GATCAAAACATGGTCAAAGC	0.00	0.00	0.00	0.00	1.03
GATCAAAACATGGTCGATGG	0.00	0.00	0.29	0.00	0.00
GATCAAAACATGGTTGATGG	0.00	0.00	0.00	0.68	0.00
GATCAAAACATGTGTAAACA	0.00	0.00	0.29	0.00	0.00
GATCAAAACATTCCCTTTCC	0.00	0.00	0.29	0.00	0.00
GATCAAAACATTGAACATTG	0.00	0.00	0.00	0.00	0.34

GATCAAAACATTGCTGACGG	0.92	0.00	0.00	0.00	0.00
GATCAAAACATTGCTTAATC	0.00	0.00	0.00	0.00	0.69
GATCAAAACCAAAAAAAAAA	5.72	0.00	0.86	1.70	5.17
GATCAAAACCAAAAAAAAGA	0.92	0.00	0.00	0.00	0.00
GATCAAAACCAAAAAACCCA	0.00	0.00	0.00	0.34	0.00
GATCAAAACCAAAACAAAAA	0.00	0.00	0.29	0.00	0.34
GATCAAAACCAAACAACCAC	0.23	0.00	0.00	0.00	0.00
GATCAAAACCAAACACAAAC	0.00	0.00	0.29	0.00	0.00
GATCAAAACCAACAAAAAAA	0.00	0.00	0.00	0.00	0.34
GATCAAAACCAACCTCTTCC	0.69	0.00	0.00	0.68	0.00
GATCAAAACCAACTTTCCAA	0.00	0.00	0.57	0.00	0.00
GATCAAAACCAAGAACACGA	0.00	0.00	0.00	0.00	0.34
GATCAAAACCAAGAACAGGA	0.00	0.00	0.00	0.00	0.34
GATCAAAACCAAGGCAGCCC	0.00	0.33	0.00	0.00	0.00
GATCAAAACCAAGGTGCTGT	0.00	0.00	0.00	0.00	0.00
GATCAAAACCAAGTTCGCCT	0.00	0.00	0.29	0.00	0.00
GATCAAAACCAATCGCCTCT	0.00	0.00	0.29	0.00	0.00
GATCAAAACCAATTAACACT	0.00	0.33	0.00	0.00	0.00
GATCAAAACCACAGAAATGG	0.00	0.00	0.29	0.00	0.00
GATCAAAACCACCGAGACAT	0.00	0.00	0.29	0.00	0.00
GATCAAAACCACCGTTGGGC	0.00	0.00	0.29	0.00	0.00
GATCAAAACCACCTGCATGC	0.00	0.33	0.00	0.00	0.34
GATCAAAACCACCTGTCGCC	0.00	0.00	0.57	0.00	0.00
GATCAAAACCACGGCGTTGG	0.00	0.33	0.00	0.00	0.00
GATCAAAACCACGGCTTTGG	0.00	0.33	0.00	0.00	0.00
GATCAAAACCACTGGAGCTC	0.00	0.00	0.57	0.00	0.00
GATCAAAACCAGACCGTTGA	0.23	0.00	0.00	0.00	0.00
GATCAAAACCAGACGCCAGC	0.00	0.00	0.00	1.02	0.00
GATCAAAACCAGACGCCATC	0.00	0.00	0.00	0.00	0.34
GATCAAAACCAGACGCCTTC	0.23	0.00	0.00	0.34	0.00
GATCAAAACCAGCGGTCGGA	0.00	0.00	0.00	0.00	0.00
GATCAAAACCAGTCTTGACC	0.00	0.00	0.29	0.00	0.00
GATCAAAACCATACGAAGAA	0.00	0.00	1.43	0.00	0.34
GATCAAAACCATCCAGGAAG	0.00	0.00	0.00	0.00	0.00
GATCAAAACCATCGTGGCCT	0.23	0.00	0.00	0.00	0.00
GATCAAAACCATGAGTGATA	0.23	0.00	0.00	0.00	0.00
GATCAAAACCATGCGAAAGC	0.00	0.00	0.00	0.00	0.00

GATCAAAACCATGTTTTCAC	0.00	0.00	0.29	0.00	0.00
GATCAAAACCATTCTGCGAA	0.00	0.00	0.00	0.00	0.00
GATCAAAACCCAATGCAAGC	0.00	0.00	0.29	0.00	0.00
GATCAAAACCCACAAACCAA	0.00	0.00	0.00	0.00	0.00
GATCAAAACCCACACCACAC	0.00	0.00	0.00	0.00	0.34
GATCAAAACCCACACTCGAC	0.00	0.00	0.29	0.00	0.00
GATCAAAACCCACAGCGAAG	0.00	0.00	0.29	0.34	0.00
GATCAAAACCCACCATTTAC	0.00	0.00	0.00	0.00	0.00
GATCAAAACCCACTGCGAAG	11.45	42.62	65.00	38.70	52.00
GATCAAAACCCACTGCGAAT	0.00	0.00	0.29	0.00	0.00
GATCAAAACCCACTGCGACG	0.00	0.33	0.57	0.34	0.69
GATCAAAACCCACTGCGCAG	0.46	0.65	0.00	0.34	0.34
GATCAAAACCCACTGCGCCG	0.00	0.33	0.00	0.00	0.00
GATCAAAACCCACTGCGGAG	0.00	0.00	0.29	0.00	0.34
GATCAAAACCCACTGCGTAN	0.23	0.00	0.00	0.00	0.00
GATCAAAACCCACTGGCCTG	0.00	0.00	0.29	0.00	0.00
GATCAAAACCCACTGTGAAG	0.00	0.00	0.00	0.00	0.34
GATCAAAACCCAGAATGGCC	0.92	0.00	0.29	0.00	0.00
GATCAAAACCCATGCTCTGA	0.00	0.00	0.00	0.00	0.69
GATCAAAACCCCACAGTGGC	0.00	0.00	0.00	0.34	0.00
GATCAAAACCCCACATTGGT	0.00	0.00	0.00	0.34	0.00
GATCAAAACCCCCCGCAGAA	0.00	0.00	0.00	0.68	0.00
GATCAAAACCCCCTGCGACN	0.00	0.00	0.00	0.00	0.00
GATCAAAACCCGATGCTTGA	0.00	0.33	0.00	0.00	0.00
GATCAAAACCCGCAGTGGGC	0.46	0.98	1.43	2.04	0.69
GATCAAAACCCGCTGTGGGC	0.00	0.00	0.00	0.00	0.00
GATCAAAACCCTACAAAGGC	0.69	0.65	0.29	0.00	0.34
GATCAAAACCCTCAAGTCTG	0.00	0.00	0.29	0.34	0.00
GATCAAAACCCTCCAGATGA	0.23	0.00	0.00	0.00	0.00
GATCAAAACCCTCGTATGCC	0.00	0.00	0.00	0.00	0.69
GATCAAAACCCTCTTCAGGG	0.00	0.00	0.00	0.00	0.00
GATCAAAACCGACCCTGACT	0.00	0.00	0.00	0.34	0.00
GATCAAAACCGACTGTATTG	0.00	0.00	0.29	0.00	0.69
GATCAAAACCGCAAGTACCT	0.23	0.00	0.00	0.00	0.00
GATCAAAACCGCAGGCCTGC	0.00	0.00	0.00	0.00	0.00
GATCAAAACCGCCACCAAGG	0.00	0.00	0.00	0.00	0.00
GATCAAAACCGCCGCGGCAC	0.46	0.00	0.00	0.00	0.00

GATCAAAACCGCCGGTAGGT	0.00	0.33	0.00	0.00	0.00
GATCAAAACCGCCTTGACCA	0.00	0.00	0.00	0.00	0.34
GATCAAAACCGCGAAAAATG	0.00	0.00	0.00	0.34	0.00
GATCAAAACCGGAACCTCCG	0.00	0.00	0.00	0.00	0.34
GATCAAAACCGGACTCTTCG	0.00	0.00	0.29	0.00	0.00
GATCAAAACCGGAGCTGGTT	0.00	0.00	0.29	0.00	0.00
GATCAAAACCGGAGCTGTTT	0.00	0.00	0.00	0.34	0.34
GATCAAAACCGGATGCTTAC	0.00	0.00	0.29	0.00	0.00
GATCAAAACCGGCGGGTTTG	0.00	0.00	0.29	0.00	0.00
GATCAAAACCGGCTTAAGCC	0.00	0.00	0.00	0.00	0.34
GATCAAAACCGTAAAGGACG	0.00	0.00	0.00	0.00	0.00
GATCAAAACCGTCAAGGACG	0.00	0.65	0.86	0.00	0.34
GATCAAAACCGTCAAGGACT	0.00	0.00	0.00	0.00	0.00
GATCAAAACCGTCAAGGATG	0.00	0.00	0.00	0.00	0.00
GATCAAAACCGTGACCTTCG	0.00	0.00	0.00	0.68	0.00
GATCAAAACCGTGCCAGTCA	0.00	0.00	0.00	0.00	0.00
GATCAAAACCGTGCTGTTGA	0.00	0.00	0.00	0.34	0.00
GATCAAAACCTAAAAAAAAA	3.89	0.65	0.00	0.00	0.00
GATCAAAACCTAAAAAACAA	0.00	0.33	0.00	0.00	0.00
GATCAAAACCTACTATGACC	0.00	0.00	1.43	1.02	0.00
GATCAAAACCTAGACCAAGA	0.00	0.00	0.29	0.00	0.00
GATCAAAACCTAGGAAAAAA	0.23	0.00	0.00	0.00	0.00
GATCAAAACCTCCAAGCCCT	0.00	0.33	0.29	1.02	0.00
GATCAAAACCTCGATGTGGC	0.23	0.00	0.29	0.00	0.00
GATCAAAACCTCGCAAAAGG	0.00	0.00	0.29	0.00	0.34
GATCAAAACCTCGCCGGGAA	0.23	0.65	0.29	0.68	0.00
GATCAAAACCTCGCCGGGAC	0.00	0.00	0.00	0.00	0.00
GATCAAAACCTCGTCGCGGA	0.00	0.00	0.00	0.34	0.00
GATCAAAACCTCGTCGGGAA	0.23	0.00	0.00	0.00	0.00
GATCAAAACCTCTGCTTGTT	0.23	0.00	0.00	0.00	0.00
GATCAAAACCTGCAACCTGC	0.00	0.00	0.00	0.00	0.34
GATCAAAACCTGCGCAGAAA	0.00	0.00	0.00	0.00	0.00
GATCAAAACCTGGAGTGATC	0.00	0.33	0.29	0.00	0.00
GATCAAAACCTGGAGTGCNC	0.00	0.00	0.29	0.00	0.00
GATCAAAACCTGGAGTGCTC	20.83	19.84	33.50	11.88	23.07
GATCAAAACCTGGAGTGCTG	0.00	0.00	0.29	0.00	0.00
GATCAAAACCTGGAGTGCTT	0.00	0.00	0.29	0.00	0.00

GATCAAAACCTGGAGTGTTC	0.00	0.33	0.00	0.00	0.00
GATCAAAACCTGGTTGATTT	0.00	0.00	0.00	0.00	0.34
GATCAAAACCTGTGTTTCCA	0.00	0.00	0.00	0.00	0.00
GATCAAAACCTGTTTTGTCA	0.00	0.00	0.00	0.00	1.03
GATCAAAACCTTAAAAAAAA	0.69	0.33	0.00	0.00	0.00
GATCAAAACCTTCGTATGCC	0.00	0.00	0.00	0.00	0.00
GATCAAAACCTTGGAGAGGT	0.23	0.00	0.00	0.00	0.00
GATCAAAACCTTGTCACAAG	0.69	0.00	0.00	0.00	0.00
GATCAAAACCTTGTCGAGAT	0.00	0.00	0.00	0.34	0.00
GATCAAAACGAAAAAAAAAA	6.64	0.65	1.15	1.70	1.72
GATCAAAACGAAAAAAAAAT	0.00	0.00	0.00	0.00	0.00
GATCAAAACGAAAAAAACAA	0.23	0.00	0.00	0.00	0.00
GATCAAAACGAAACCAAACA	0.00	0.00	0.00	0.00	0.69
GATCAAAACGAAAGAAAAAA	0.00	0.00	0.00	0.00	0.34
GATCAAAACGAACAGCGAAG	0.23	0.00	0.00	0.00	0.00
GATCAAAACGAACCCAACGA	0.00	0.00	0.29	0.00	0.00
GATCAAAACGAACGACCTGT	0.00	0.00	0.00	0.00	0.00
GATCAAAACGAACTGCGAAG	0.00	0.33	0.00	0.00	0.00
GATCAAAACGAAGGAGGAAC	0.00	0.00	0.00	0.00	0.34
GATCAAAACGAAGGCGACAA	0.00	0.00	0.00	1.02	1.03
GATCAAAACGAAGGTCTCGT	0.00	0.00	0.57	0.00	0.00
GATCAAAACGACAGTATGCG	0.23	0.33	0.00	0.00	0.00
GATCAAAACGACGCCAGGTG	0.00	0.00	0.00	0.34	0.00
GATCAAAACGACGGCTTGCG	0.00	0.00	0.00	0.00	0.00
GATCAAAACGACGTCTGGAA	1.60	0.33	0.86	1.02	0.69
GATCAAAACGACGTCTGGGA	0.23	0.00	0.00	0.00	0.00
GATCAAAACGACTCTCGGCA	0.46	0.00	0.00	0.00	0.00
GATCAAAACGAGAAAAAAAA	0.00	0.33	0.00	0.00	0.34
GATCAAAACGAGAATCCTCC	0.00	0.00	0.00	0.00	0.00
GATCAAAACGAGACACCGTG	0.23	0.00	0.00	0.00	0.00
GATCAAAACGAGACATAGTG	0.23	0.00	0.00	0.00	0.00
GATCAAAACGAGACATCATG	0.00	0.00	0.29	0.00	0.00
GATCAAAACGAGACATCGTG	13.28	9.11	2.00	4.75	0.69
GATCAAAACGAGACATCGTT	0.92	0.00	0.00	0.00	0.00
GATCAAAACGAGACATCTTG	0.00	0.00	0.29	0.00	0.00
GATCAAAACGAGACATTGTG	0.23	0.00	0.00	0.00	0.00
GATCAAAACGAGATGACCAG	0.00	0.00	0.00	0.34	0.00

GATCAAAACGAGCCCCAGCG	0.00	0.00	0.29	0.00	0.00
GATCAAAACGAGCCCGTCCA	0.00	0.00	0.00	0.00	0.00
GATCAAAACGAGCGGGTGTG	0.00	0.00	0.00	0.00	0.34
GATCAAAACGAGGAAAAAAA	0.23	0.00	0.00	0.00	0.00
GATCAAAACGAGGACCTGCC	0.23	0.00	0.00	0.00	0.00
GATCAAAACGAGGCATCGTG	0.23	0.00	0.00	0.00	0.00
GATCAAAACGAGGGCGGCTG	0.00	0.00	0.00	0.00	0.00
GATCAAAACGAGTTCTTTCT	0.00	0.00	0.29	0.00	0.00
GATCAAAACGATAAAAAAAA	0.00	0.98	0.29	0.00	0.00
GATCAAAACGATATAAAAAA	0.46	0.00	0.00	0.00	0.00
GATCAAAACGATATGAAAAA	1.37	0.33	0.00	0.34	0.00
GATCAAAACGATATGAAAAT	0.00	0.00	0.00	0.34	0.00
GATCAAAACGATATGAAGCA	0.23	0.00	0.00	0.00	0.00
GATCAAAACGATATGACTAA	0.00	0.00	0.00	0.00	0.00
GATCAAAACGATATGAGAAA	0.23	0.00	0.00	0.00	0.00
GATCAAAACGATGTCGCTGA	0.46	0.00	0.00	0.00	0.69
GATCAAAACGCAAAAAAAAA	4.35	0.65	3.72	2.72	1.72
GATCAAAACGCAAAAAAAAT	0.00	0.00	0.00	0.00	0.00
GATCAAAACGCAAAAAAAGA	0.23	0.00	0.00	0.00	0.00
GATCAAAACGCAAAGCAGTT	0.00	0.00	0.29	0.00	0.00
GATCAAAACGCAAGAGTTGT	0.23	0.00	0.00	0.00	0.00
GATCAAAACGCAAGGAGTCG	1.37	0.33	0.86	0.68	0.69
GATCAAAACGCAATCATACT	0.00	0.00	0.57	0.00	0.00
GATCAAAACGCAATGCGAAG	0.00	0.00	0.00	0.34	0.00
GATCAAAACGCACAGCGAAG	0.00	0.00	0.57	0.00	0.00
GATCAAAACGCACCGCGAAG	0.23	0.00	0.00	0.00	0.00
GATCAAAACGCACGGCGATG	0.00	0.33	0.00	0.00	0.00
GATCAAAACGCACGTGCTGG	0.00	0.33	0.29	0.00	0.34
GATCAAAACGCACTCCATTG	0.00	0.33	0.00	0.00	0.00
GATCAAAACGCACTCGTCTG	0.00	0.00	0.29	0.00	0.00
GATCAAAACGCACTGAGACG	0.00	0.33	0.00	0.00	0.00
GATCAAAACGCACTGCGAAG	47.15	60.84	116.26	89.27	82.66
GATCAAAACGCACTGCGAAN	0.23	0.00	0.00	0.00	0.00
GATCAAAACGCACTGCGAAT	0.00	0.00	0.57	0.00	0.00
GATCAAAACGCACTGCGACC	0.00	0.00	0.00	0.00	0.00
GATCAAAACGCACTGCGACG	0.23	0.00	1.15	0.00	0.00
GATCAAAACGCACTGCGAGG	0.23	0.00	0.00	0.34	0.00

GATCAAAACGCACTGCGANN	0.23	0.00	0.00	0.00	0.00
GATCAAAACGCACTGCGCAC	0.00	0.00	0.00	0.00	0.34
GATCAAAACGCACTGCGCAG	0.00	0.00	0.29	0.68	0.69
GATCAAAACGCACTGCGCCG	0.00	0.00	0.00	0.34	0.00
GATCAAAACGCACTGCGCNN	0.23	0.00	0.00	0.00	0.00
GATCAAAACGCACTGCGGAG	0.23	0.00	0.00	0.00	0.00
GATCAAAACGCACTGCGTAG	0.23	0.00	0.00	0.00	0.00
GATCAAAACGCACTGCNAAG	0.00	0.00	0.00	0.00	1.72
GATCAAAACGCACTGCNGAG	0.00	0.00	0.00	0.00	0.34
GATCAAAACGCACTGCNNNN	0.23	0.00	0.00	0.00	0.00
GATCAAAACGCACTGCTAAT	0.00	0.00	0.29	0.00	0.00
GATCAAAACGCACTGCTCGT	0.00	0.00	0.57	0.00	0.34
GATCAAAACGCACTGTGTGG	0.00	0.00	0.29	0.00	0.00
GATCAAAACGCAGAAGGGAG	0.23	0.00	0.00	0.00	0.00
GATCAAAACGCAGAAGGNNN	0.23	0.00	0.00	0.00	0.00
GATCAAAACGCAGAAGGTAG	8.01	9.11	6.01	9.84	5.51
GATCAAAACGCAGAAGGTAN	0.23	0.00	0.00	0.00	0.00
GATCAAAACGCAGAAGGTTC	0.00	0.00	0.00	0.00	0.00
GATCAAAACGCAGCAGGTAG	0.23	0.00	0.00	0.00	0.00
GATCAAAACGCAGCCTGTCT	0.00	0.00	0.29	0.00	0.00
GATCAAAACGCAGGAGGTAG	0.00	0.00	0.00	0.34	0.00
GATCAAAACGCATACAAGTC	0.00	0.00	0.00	0.00	0.00
GATCAAAACGCCAATTTGAA	0.00	0.00	0.00	0.00	0.00
GATCAAAACGCCAGAGCTGT	0.23	0.00	0.00	0.00	0.00
GATCAAAACGCCATGCGAAG	0.00	0.33	0.00	0.00	0.00
GATCAAAACGCCCATCTATG	0.00	0.00	0.00	0.00	0.00
GATCAAAACGCCCGCACCCC	0.23	0.00	0.00	0.00	0.00
GATCAAAACGCCCTGCGAAG	0.23	0.00	0.00	0.00	0.00
GATCAAAACGCCCTGCGCCG	0.23	0.00	0.00	0.00	0.00
GATCAAAACGCCGAGCGAGA	0.00	0.00	0.00	0.00	0.69
GATCAAAACGCCTGTGCGGG	0.00	0.00	0.00	0.00	0.69
GATCAAAACGCGAAAAAAAA	0.92	0.33	1.72	0.00	3.79
GATCAAAACGCGAAAAAACA	0.00	0.00	0.29	0.00	0.00
GATCAAAACGCGAAAAAAGA	0.00	0.00	0.00	0.00	0.34
GATCAAAACGCGAAAAGAAA	0.00	0.00	0.00	0.00	0.34
GATCAAAACGCGACGAGACC	0.00	0.00	0.00	0.00	0.00
GATCAAAACGCGAGAAAAAA	1.37	0.33	2.29	1.36	0.69

GATCAAAACGCGAGAAAAAC	0.23	0.00	0.00	0.00	0.00
GATCAAAACGCGAGAAACAA	0.00	0.00	0.00	0.00	0.34
GATCAAAACGCGAGAAACTG	0.00	0.00	0.29	0.00	0.00
GATCAAAACGCGAGAACAAA	0.00	0.00	0.00	0.00	0.00
GATCAAAACGCGAGAACCAA	0.23	0.00	0.29	0.00	0.69
GATCAAAACGCGAGAACCTA	0.92	1.30	1.15	0.34	1.03
GATCAAAACGCGAGAACCTG	9.39	20.82	15.75	11.54	13.43
GATCAAAACGCGAGAACCTT	0.00	0.00	0.00	0.00	1.03
GATCAAAACGCGAGAACGTG	0.00	0.00	0.00	0.00	0.00
GATCAAAACGCGAGAACNNN	0.23	0.00	0.00	0.00	0.00
GATCAAAACGCGAGACCCTA	0.00	0.00	0.00	0.00	0.34
GATCAAAACGCGAGCACCTG	0.00	0.00	0.00	0.00	0.34
GATCAAAACGCGCTGCGAAG	0.00	0.33	0.00	0.00	0.00
GATCAAAACGCGGTGGCGGA	0.00	0.00	0.29	0.00	0.00
GATCAAAACGCGTGAACCTG	0.23	0.00	0.00	0.00	0.00
GATCAAAACGCGTTATGTTC	0.00	0.00	0.00	0.00	0.00
GATCAAAACGCGTTGCCGAG	0.46	1.30	0.57	2.04	0.00
GATCAAAACGCGTTTAACCT	0.00	0.00	0.00	0.00	0.00
GATCAAAACGCTGCCTGACN	0.23	0.00	0.00	0.00	0.00
GATCAAAACGCTGCCTGACT	0.92	0.65	0.57	2.38	0.69
GATCAAAACGCTGGTCCCAG	0.00	0.33	0.00	0.00	0.00
GATCAAAACGGAAAAAAAAA	0.00	0.00	0.00	0.00	0.00
GATCAAAACGGAAAAGAGGA	0.00	0.00	0.00	0.00	0.00
GATCAAAACGGAAAGAGGCG	0.00	0.00	0.29	0.00	0.00
GATCAAAACGGAACAGGAGC	0.00	0.33	0.00	0.00	0.00
GATCAAAACGGAACGGAGCA	0.00	0.00	0.29	0.00	0.00
GATCAAAACGGAATGCTTGC	0.00	0.00	0.00	0.00	0.00
GATCAAAACGGAATGCTTGT	2.29	0.98	1.15	0.00	1.03
GATCAAAACGGACAAGAGCC	0.23	0.00	0.00	0.34	0.00
GATCAAAACGGACCAGGAGC	0.00	0.00	0.29	0.00	0.00
GATCAAAACGGACTGACGAG	0.69	1.30	1.43	0.34	0.69
GATCAAAACGGACTTACGAG	0.00	0.00	0.00	0.00	0.00
GATCAAAACGGAGCAACCCT	1.37	0.65	1.43	0.34	0.34
GATCAAAACGGATTGACGAG	0.00	0.33	0.00	0.00	0.00
GATCAAAACGGCACAGGAGC	0.46	0.00	0.00	0.00	0.00
GATCAAAACGGCATGGAAGG	0.00	0.00	0.00	0.00	0.00
GATCAAAACGGCATGGCAGG	0.00	0.00	0.00	0.00	0.00

GATCAAAACGGCCAGGAGCT	0.00	0.00	0.00	0.00	0.00
GATCAAAACGGCCATGTCCC	0.00	0.00	0.00	0.00	0.00
GATCAAAACGGCCCAGGAGA	0.23	0.00	0.00	0.00	0.00
GATCAAAACGGCCCAGGAGC	36.40	16.27	8.59	13.58	12.74
GATCAAAACGGCCCAGGATC	0.46	0.00	0.00	0.00	0.00
GATCAAAACGGCCCAGGCGC	0.23	0.00	0.00	0.00	0.34
GATCAAAACGGCCCAGGCGG	0.23	0.00	0.00	0.00	0.00
GATCAAAACGGCCCAGNCNN	0.23	0.00	0.00	0.00	0.00
GATCAAAACGGCCCCGGGGC	0.00	0.00	0.00	0.00	0.00
GATCAAAACGGCGCGGGCTC	0.00	0.00	0.00	1.70	0.00
GATCAAAACGGCGGAGTCTT	0.69	0.00	0.00	0.00	0.00
GATCAAAACGGCTCAGACTC	0.00	0.00	0.00	0.00	0.00
GATCAAAACGGCTCGGACTC	0.00	0.33	0.00	0.68	0.00
GATCAAAACGGGAAAAAAAA	0.00	0.00	0.00	0.00	0.00
GATCAAAACGGGCATGTCCC	2.06	2.93	3.15	5.09	2.76
GATCAAAACGGGCGCTTTGC	0.00	0.00	0.00	0.34	0.34
GATCAAAACGGGCTGCGGCA	0.00	0.00	0.00	0.00	0.00
GATCAAAACGGGGAACTTGC	0.00	0.00	0.29	0.00	0.00
GATCAAAACGGTAAGAAGAT	0.00	0.00	0.00	0.00	0.34
GATCAAAACGGTACGGGGGT	0.23	0.00	0.29	0.34	0.69
GATCAAAACGGTCGGCAGCG	0.00	0.00	0.00	0.34	0.69
GATCAAAACGGTCGGCCGCC	0.00	0.00	0.00	0.00	0.00
GATCAAAACGGTCGGCGGCG	0.00	0.00	0.00	0.00	0.34
GATCAAAACGGTGGGCATGA	0.00	0.00	0.00	0.00	0.00
GATCAAAACGGTTGGCAGCG	0.00	0.00	0.29	0.00	0.00
GATCAAAACGTACTGCGAAG	0.00	0.00	0.00	0.00	0.34
GATCAAAACGTAGACGAAGA	0.23	0.00	0.57	0.00	0.00
GATCAAAACGTAGACGAAGC	0.23	0.00	0.00	0.00	0.00
GATCAAAACGTAGCGCGGAG	0.23	0.00	0.00	0.00	0.00
GATCAAAACGTCAAAAAAAA	0.00	0.33	0.00	0.00	0.00
GATCAAAACGTCACAAAAAA	0.23	0.33	0.00	0.00	0.00
GATCAAAACGTCAGCAAATG	0.00	0.00	0.29	0.00	0.00
GATCAAAACGTCCAAGCAAG	0.00	0.33	0.00	0.00	0.00
GATCAAAACGTCCGATCCAA	0.00	0.00	0.29	0.00	0.00
GATCAAAACGTCCGTGCCAA	0.00	0.00	0.00	0.00	0.00
GATCAAAACGTCCGTTCCAA	6.41	8.13	9.74	7.13	6.20
GATCAAAACGTCCGTTCCAC	0.00	0.65	0.29	0.00	0.00

GATCAAAACGTCCGTTCCAN	0.23	0.00	0.00	0.00	0.00
GATCAAAACGTCCGTTCCCA	0.00	0.00	0.57	0.00	0.34
GATCAAAACGTCCGTTCCCG	0.23	0.00	0.00	0.00	0.00
GATCAAAACGTCCGTTCCTA	0.23	0.00	0.00	0.00	0.00
GATCAAAACGTCGACAGCTT	0.00	0.00	0.00	0.00	0.00
GATCAAAACGTGGAAAAAAA	0.00	0.00	0.00	0.00	0.00
GATCAAAACGTGGAACATGC	0.00	0.33	0.00	0.00	0.00
GATCAAAACGTGGAAGCAGA	0.00	0.33	0.00	0.00	0.00
GATCAAAACGTGGAGCTGCC	0.00	0.00	0.29	0.00	0.00
GATCAAAACGTTCAACTACA	0.00	0.00	0.29	0.00	0.00
GATCAAAACGTTGTACAGCT	0.00	0.00	0.00	0.00	0.34
GATCAAAACTAAAAAAAAAA	38.46	9.43	11.17	14.26	13.78
GATCAAAACTAAAAAAAAAC	0.23	0.00	0.00	0.34	0.00
GATCAAAACTAAAAAAAAAT	0.46	0.98	0.00	1.36	0.00
GATCAAAACTAAAAAAAATC	0.00	0.00	0.00	0.00	0.00
GATCAAAACTAAAAAAAGAA	0.23	0.00	0.00	0.00	0.00
GATCAAAACTAAAAAACACA	0.00	0.00	0.00	0.00	0.34
GATCAAAACTAAAAAAGAAA	0.00	0.00	0.00	0.34	0.00
GATCAAAACTAAAAAANNNN	0.00	0.00	0.00	0.00	0.00
GATCAAAACTAAAAACAAAA	0.00	0.00	0.00	0.00	0.34
GATCAAAACTAAAAGAAAAA	0.23	0.00	0.00	0.00	0.00
GATCAAAACTAACAACAACA	0.00	0.00	0.00	0.00	0.00
GATCAAAACTAACAACAACC	0.23	0.00	0.00	0.00	0.00
GATCAAAACTAATGGCATGC	0.23	0.00	0.00	0.00	0.00
GATCAAAACTACTGGTACTG	0.00	0.00	0.00	0.34	0.00
GATCAAAACTAGCGCAACAC	0.00	0.00	0.29	0.00	0.00
GATCAAAACTAGGCGTTTGC	0.23	0.33	0.29	0.68	0.00
GATCAAAACTATGATTTTGA	0.00	0.00	0.00	0.00	0.00
GATCAAAACTCAAAAAAAAA	0.92	0.33	0.57	0.34	0.00
GATCAAAACTCAAACCCCAA	0.00	0.00	0.00	0.00	0.00
GATCAAAACTCAAAGAGGTC	0.00	0.00	0.00	0.34	0.00
GATCAAAACTCAATCCATCA	0.00	0.00	0.86	0.00	0.00
GATCAAAACTCACTGCGAAG	0.00	0.00	0.00	0.00	0.00
GATCAAAACTCATGAACGCT	0.46	0.00	0.00	0.00	0.00
GATCAAAACTCATGGCCTTA	0.69	0.33	0.00	0.34	0.00
GATCAAAACTCATTAAGTTG	0.00	0.00	0.29	0.00	0.00
GATCAAAACTCCAACGTCCG	0.00	0.65	1.43	1.36	1.72

GATCAAAACTCCCATCTTCC	0.00	0.00	0.00	1.70	0.00
GATCAAAACTCCCTTGTAGG	0.00	0.00	0.86	0.00	0.00
GATCAAAACTCCGAAGCCCG	0.00	0.00	0.29	0.00	0.00
GATCAAAACTCCGTCCTCAA	0.92	0.98	1.72	0.68	1.38
GATCAAAACTCCTCCGCATT	0.00	0.00	0.00	0.68	0.00
GATCAAAACTCGAAAAAAAA	0.23	0.00	0.57	0.34	0.00
GATCAAAACTCGAGAAAAAA	0.00	0.00	0.00	0.00	0.34
GATCAAAACTCGATTTTCAA	0.00	0.00	0.57	0.00	0.00
GATCAAAACTCGCCAGGAAG	0.00	0.00	0.29	0.00	0.00
GATCAAAACTCGGCAAAAAA	0.00	0.00	0.57	0.68	0.34
GATCAAAACTCGGCCAAAAA	0.23	0.00	0.00	0.00	0.00
GATCAAAACTCGGCCTCGTA	0.46	0.00	0.00	0.00	0.00
GATCAAAACTCGGCCTTCTA	0.00	0.00	0.00	0.00	0.00
GATCAAAACTCGGCCTTTGA	0.00	0.00	0.00	0.00	0.34
GATCAAAACTCGGCCTTTTA	11.22	8.78	4.01	8.15	4.13
GATCAAAACTCGGCCTTTTT	0.00	0.00	0.57	0.00	0.34
GATCAAAACTCGTGACGGCC	0.00	0.00	0.00	0.00	0.00
GATCAAAACTCTAATCGAGA	0.00	0.00	0.00	0.34	0.00
GATCAAAACTCTCAGGGCTG	9.61	7.81	4.87	3.39	3.10
GATCAAAACTCTCAGGGCTN	0.23	0.00	0.00	0.00	0.00
GATCAAAACTCTCAGGGCTT	0.00	0.00	0.00	0.34	0.00
GATCAAAACTCTCAGGGNNN	0.23	0.00	0.00	0.00	0.00
GATCAAAACTCTCCGGGCTG	0.00	0.00	0.00	0.00	0.34
GATCAAAACTCTCGGGGCTG	0.23	0.00	0.00	0.00	0.00
GATCAAAACTCTGAAGCCCG	0.23	0.33	0.00	0.00	0.34
GATCAAAACTCTTGCCGAAC	0.00	0.00	0.00	0.00	0.00
GATCAAAACTGAAAAAAAAA	12.36	2.93	2.58	1.36	2.41
GATCAAAACTGAAAAACAAA	0.00	0.00	0.00	0.00	0.34
GATCAAAACTGAAAGAGGTC	0.23	0.98	0.57	0.68	0.34
GATCAAAACTGAATGATTGG	0.00	0.00	0.00	0.34	0.00
GATCAAAACTGACAATTTGT	0.00	0.00	0.86	0.00	0.00
GATCAAAACTGAGATGGAGC	0.00	0.00	0.00	0.00	0.00
GATCAAAACTGAGGACGTGC	0.00	0.00	0.00	0.34	0.00
GATCAAAACTGATGGCAAAA	0.00	0.00	0.29	0.00	0.00
GATCAAAACTGATGGCATGA	0.00	0.00	0.00	0.00	0.00
GATCAAAACTGATGGCATGC	1.83	1.95	2.29	4.41	2.41
GATCAAAACTGATGGCATGT	0.00	0.00	0.29	0.00	0.00

GATCAAAACTGCAAAAAAAA	9.16	2.93	1.72	4.41	3.79
GATCAAAACTGCAAAAAAAT	0.23	0.00	0.00	0.00	0.00
GATCAAAACTGCAAAGACGC	2.52	3.25	2.29	2.38	2.76
GATCAAAACTGCAAATTCGC	0.00	0.00	0.00	0.34	0.00
GATCAAAACTGCAGAAAGAA	0.00	0.00	0.00	0.34	0.00
GATCAAAACTGCAGAGAAAA	0.00	0.00	0.00	0.34	0.00
GATCAAAACTGCAGGATGAC	0.23	0.00	0.00	0.00	0.00
GATCAAAACTGCAGGCNNNN	0.23	0.00	0.00	0.00	0.00
GATCAAAACTGCATGCCAAA	0.00	0.00	0.00	0.00	0.00
GATCAAAACTGCATGTAAAA	0.00	0.00	0.29	0.00	0.00
GATCAAAACTGCATGTTAAA	0.00	0.00	0.00	0.00	0.69
GATCAAAACTGCATGTTATT	7.55	3.58	1.72	2.72	3.10
GATCAAAACTGCATGTTTTT	0.00	0.00	0.00	0.34	0.00
GATCAAAACTGCATTGTTGT	0.23	0.00	0.00	0.00	0.00
GATCAAAACTGCCGGAAGCC	0.00	0.00	0.00	0.00	0.00
GATCAAAACTGCCGGGGAGA	0.00	0.00	0.00	0.00	0.34
GATCAAAACTGCCGGTAGGG	0.00	0.00	0.00	0.34	0.00
GATCAAAACTGCCGGTAGGT	2.29	2.60	1.15	1.02	0.69
GATCAAAACTGCGGACGCCT	0.00	0.33	0.00	0.00	0.00
GATCAAAACTGCTCGTAGCT	0.00	0.00	0.00	0.00	0.00
GATCAAAACTGCTCTTTGTT	0.23	0.00	0.00	0.00	0.00
GATCAAAACTGCTGGTAGGT	0.00	0.00	0.00	0.00	0.00
GATCAAAACTGCTTGGCCGG	0.00	0.00	0.29	0.00	0.00
GATCAAAACTGCTTGGTGCG	0.23	0.00	0.00	0.00	0.00
GATCAAAACTGCTTGGTGTG	0.23	0.00	0.00	0.00	0.00
GATCAAAACTGCTTTAGAAG	0.00	0.33	0.00	0.00	0.00
GATCAAAACTGGCGCTTTGC	0.00	0.00	0.00	0.00	0.00
GATCAAAACTGGCGTCCACA	0.00	0.00	0.00	0.00	0.00
GATCAAAACTGGGAGCCGAA	0.00	0.00	0.00	0.00	0.00
GATCAAAACTGGGAGCCGAT	0.00	0.00	0.00	0.68	0.00
GATCAAAACTGGGAGTTCTC	0.23	0.00	0.00	0.00	0.00
GATCAAAACTGGGCAACCTA	0.23	0.00	0.00	0.00	0.00
GATCAAAACTGGGTGTCAGT	0.00	0.00	0.00	0.00	0.00
GATCAAAACTGGTACTCACG	0.00	0.65	0.00	0.00	0.00
GATCAAAACTGGTTCTGTCG	0.00	0.00	0.00	0.00	0.00
GATCAAAACTGGTTTCAATA	0.00	0.33	0.00	0.68	0.69
GATCAAAACTGGTTTGTGAT	0.46	0.00	0.00	0.00	0.00

GATCAAAACTGTAGGCACCT	0.00	0.00	0.00	0.34	0.00
GATCAAAACTGTCGCATTTC	0.00	0.00	0.29	0.00	0.00
GATCAAAACTGTGGCTGACC	0.00	0.00	0.29	0.00	0.00
GATCAAAACTGTGGGCATGA	0.00	0.33	0.00	0.00	0.00
GATCAAAACTTATACCTCCT	0.00	0.00	0.29	0.00	0.00
GATCAAAACTTCGGTCACCT	0.00	0.00	0.29	0.00	0.00
GATCAAAACTTCTACGTTTG	0.00	0.00	0.00	0.00	0.00
GATCAAAACTTGAGTTTGAT	0.00	0.65	0.00	0.00	0.00
GATCAAAACTTGATGGACAA	0.00	0.00	0.00	0.00	0.34
GATCAAAACTTGCATCAAGC	0.00	0.65	0.00	0.00	0.00
GATCAAAACTTGGACACATT	0.00	0.00	0.00	0.00	0.00
GATCAAAACTTGGCCTTTTA	0.00	0.00	0.00	0.34	0.00
GATCAAAACTTGGTGAGGCG	0.00	0.00	0.00	0.68	0.00
GATCAAAACTTGTCAATACC	0.00	0.00	0.00	0.00	0.00
GATCAAAACTTTATGTTGCT	0.00	0.00	0.29	0.00	0.00
GATCAAAACTTTTAGCAGGC	0.00	0.00	0.00	0.00	0.00
GATCAAAAGAAAAAAAAAAA	43.03	12.36	21.76	19.35	16.19
GATCAAAAGAAAAAAAAAAC	0.23	0.00	0.00	0.00	0.00
GATCAAAAGAAAAAAAAAAG	0.00	0.00	0.00	0.00	0.34
GATCAAAAGAAAAAAAAAAT	2.75	0.33	1.15	1.02	1.72
GATCAAAAGAAAAAAAAACA	0.23	0.00	0.00	0.00	0.00
GATCAAAAGAAAAAAAAAGA	0.46	0.00	0.00	0.00	0.69
GATCAAAAGAAAAAAAAANN	0.23	0.00	0.00	0.00	0.00
GATCAAAAGAAAAAAAACNN	0.23	0.00	0.00	0.00	0.00
GATCAAAAGAAAAAAAAGAA	0.00	0.00	0.29	0.00	0.00
GATCAAAAGAAAAAAAGAAA	0.46	0.00	0.00	0.00	0.00
GATCAAAAGAAAAAAGAAAA	0.00	0.33	0.00	0.00	0.00
GATCAAAAGAAAAACAACAA	0.00	0.00	0.00	0.00	0.34
GATCAAAAGAAAAACAAGCA	0.23	0.00	0.00	0.00	0.00
GATCAAAAGAAAAAGAAAAA	0.23	0.00	0.00	0.00	0.00
GATCAAAAGAAAAAGAAGAA	0.00	0.00	0.57	0.00	0.00
GATCAAAAGAAAAAGTAAAA	0.00	0.00	0.00	0.00	0.69
GATCAAAAGAAAAAGTAGAA	0.00	0.00	0.00	0.00	0.34
GATCAAAAGAAAACCACACT	0.00	0.00	0.57	0.00	0.00
GATCAAAAGAAAAGAAAAAA	0.00	0.00	0.00	0.00	0.00
GATCAAAAGAAAAGAAAGAA	0.00	0.33	0.00	0.00	0.00
GATCAAAAGAAAATCTGAAC	0.00	0.00	0.86	0.00	0.00

GATCAAAAGAAAATCTGATG	0.69	0.65	0.57	1.36	1.72
GATCAAAAGAAACAACACTG	0.00	0.00	0.57	0.00	0.00
GATCAAAAGAAACACAAAAA	0.00	0.00	0.00	0.00	0.34
GATCAAAAGAAACACACGGA	0.00	0.00	0.00	0.00	1.72
GATCAAAAGAAAGAAAAAAA	1.37	0.00	0.00	0.00	2.76
GATCAAAAGAAAGAAAGAAA	0.00	0.33	0.00	0.00	0.00
GATCAAAAGAAAGAATTGAA	0.46	0.00	0.00	0.00	0.00
GATCAAAAGAAAGAATTGGA	0.69	0.00	0.00	0.00	0.00
GATCAAAAGAAAGACAAANN	0.23	0.00	0.00	0.00	0.00
GATCAAAAGAAAGACAGAGA	0.00	0.33	0.00	1.02	0.00
GATCAAAAGAAAGACCCCAA	0.00	0.00	0.29	0.00	0.00
GATCAAAAGAAAGACGTATG	0.00	0.00	0.00	0.00	0.00
GATCAAAAGAAAGATAAAAA	2.29	0.00	0.00	0.34	0.69
GATCAAAAGAAAGATAAAAN	0.23	0.00	0.00	0.00	0.00
GATCAAAAGAAAGATAGACT	0.00	0.00	0.57	0.00	0.34
GATCAAAAGAAAGATCCCAA	0.00	0.33	0.00	0.00	0.00
GATCAAAAGAAAGATTCCAA	0.92	0.00	0.00	0.00	0.00
GATCAAAAGAAAGCAGGCCA	0.00	0.00	0.00	0.00	0.34
GATCAAAAGAAAGGGATANN	0.23	0.00	0.00	0.00	0.00
GATCAAAAGAAAGGTTTTCA	0.00	0.00	0.57	0.00	0.00
GATCAAAAGAAAGTGTTCGA	0.00	0.00	0.00	0.00	0.00
GATCAAAAGAAATCAAAAAA	0.00	0.33	0.00	0.00	0.00
GATCAAAAGAAATTATATCT	0.23	0.00	0.00	0.00	0.00
GATCAAAAGAAATTGTAAAA	0.00	0.00	0.00	0.00	0.00
GATCAAAAGAACAAAACACA	0.00	0.33	0.00	0.00	0.00
GATCAAAAGAACAGCGAGAA	0.23	0.00	0.00	0.00	0.00
GATCAAAAGAACATAAGGCA	0.00	0.00	0.00	0.00	0.00
GATCAAAAGAACATAGGGCA	0.00	0.00	0.00	0.34	0.00
GATCAAAAGAACGCGATTGA	0.00	0.00	0.00	0.34	0.00
GATCAAAAGAACGGAACCGA	0.23	0.00	0.00	0.00	0.00
GATCAAAAGAACTTTATGAA	0.23	0.00	0.00	0.00	0.00
GATCAAAAGAAGACGAAAAA	1.83	0.33	0.00	1.70	0.00
GATCAAAAGAAGACTGAGCA	0.23	0.00	0.00	0.00	0.00
GATCAAAAGAAGAGAAAGGG	0.00	0.33	0.00	0.00	1.38
GATCAAAAGAAGCAAAAAAA	0.69	0.33	0.29	0.00	0.00
GATCAAAAGAAGCAGAAGGA	0.00	0.00	0.29	0.00	0.00
GATCAAAAGAAGCATCCAAG	0.00	0.33	0.00	0.00	0.00

GATCAAAAGAAGCATCCATG	0.00	0.00	0.00	0.00	0.00
GATCAAAAGAAGCCAAAAAA	0.00	0.00	0.29	0.00	0.00
GATCAAAAGAAGCCAGCGGG	0.00	0.00	0.00	0.00	0.00
GATCAAAAGAAGCGAAGCAC	0.00	0.00	0.00	0.34	0.00
GATCAAAAGAAGCGCCCAAG	0.00	0.00	0.00	0.00	0.34
GATCAAAAGAAGCGTACAAA	0.00	0.00	0.29	0.00	0.00
GATCAAAAGAAGCGTCAAAA	0.00	0.00	0.29	3.05	1.03
GATCAAAAGAAGCGTCAAAT	0.23	0.00	0.00	0.00	0.00
GATCAAAAGAAGCGTCCAAA	6.18	6.51	10.02	4.41	6.89
GATCAAAAGAAGCGTCCAAT	11.90	12.69	17.18	10.18	21.35
GATCAAAAGAAGCGTCCACA	0.00	0.00	0.00	0.34	0.00
GATCAAAAGAAGCGTCCACN	0.23	0.00	0.00	0.00	0.00
GATCAAAAGAAGCGTCCCNN	0.23	0.00	0.00	0.00	0.00
GATCAAAAGAAGCGTCCGAA	0.00	0.00	0.00	0.68	0.00
GATCAAAAGAAGCTCGACGA	0.00	0.00	0.00	0.68	0.00
GATCAAAAGAAGCTTCCTGA	0.00	0.00	0.00	0.00	0.34
GATCAAAAGAAGGAGAAGAA	0.00	0.00	0.00	0.00	2.76
GATCAAAAGAAGGCCTGACG	0.00	0.00	0.29	0.00	0.00
GATCAAAAGAAGGTTCGAGA	0.00	0.00	0.00	0.00	0.34
GATCAAAAGAAGTAAAAAAA	0.00	0.00	0.86	0.68	0.00
GATCAAAAGAAGTACGCAGG	0.00	0.65	0.00	0.34	1.38
GATCAAAAGAAGTCACGAGA	0.00	0.00	0.00	0.34	0.00
GATCAAAAGAAGTGACAGGC	0.23	0.33	0.00	0.34	0.34
GATCAAAAGAAGTGGACTGC	0.00	0.00	0.00	0.00	0.34
GATCAAAAGAATAAAAAAAA	1.37	0.00	0.29	0.34	0.69
GATCAAAAGAATAGTGATGC	2.06	0.33	0.29	0.00	1.72
GATCAAAAGAATCAAGAGAA	0.00	0.00	0.00	0.00	0.34
GATCAAAAGAATCAATGGCG	0.00	0.00	0.00	0.00	0.00
GATCAAAAGAATCATTTAGA	0.00	0.00	0.00	0.00	0.00
GATCAAAAGAATCGCGTAAG	0.00	0.00	0.00	0.00	0.69
GATCAAAAGAATGAAAAAAA	0.00	0.00	0.00	0.00	0.34
GATCAAAAGAATGTGTGGTA	0.92	0.00	0.00	0.34	0.00
GATCAAAAGAATTACATTAC	0.00	0.00	0.00	0.00	0.34
GATCAAAAGAATTGACAGAA	0.00	0.00	0.00	0.34	0.00
GATCAAAAGAATTGACAGCC	0.23	0.00	0.00	0.00	0.00
GATCAAAAGAATTGCAAAAA	0.23	0.65	0.29	0.68	0.00
GATCAAAAGAATTTGGTCAA	0.46	0.00	0.00	0.00	0.34

GATCAAAAGACAAAAAAAAA	3.20	1.95	2.29	3.05	2.07
GATCAAAAGACAAAGGATGA	0.00	0.33	0.00	0.00	0.00
GATCAAAAGACACAAAGTGT	0.00	0.00	0.00	0.68	0.00
GATCAAAAGACAGCAACGAC	0.00	0.33	0.00	0.00	0.00
GATCAAAAGACAGCACTGTC	0.69	0.33	0.00	0.00	0.00
GATCAAAAGACAGCTTGGCC	0.46	0.00	0.00	0.00	0.00
GATCAAAAGACAGCTTTGGA	0.00	0.00	0.00	0.00	0.00
GATCAAAAGACATATCAAGT	0.00	0.00	0.00	0.00	0.69
GATCAAAAGACATGAATGGT	0.00	0.00	0.29	0.00	0.00
GATCAAAAGACATTGTACAA	0.00	0.00	0.00	0.00	0.00
GATCAAAAGACCCAACGACG	0.00	0.00	0.00	0.34	0.34
GATCAAAAGACCCAACGACT	0.00	0.00	0.29	0.34	0.34
GATCAAAAGACCCCTGCTCA	0.00	0.00	0.00	0.00	0.00
GATCAAAAGACCCTCCACCT	1.60	4.55	1.15	3.73	1.72
GATCAAAAGACCCTCCACGT	0.00	0.00	0.29	0.00	0.00
GATCAAAAGACCCTCCCCCT	0.00	0.98	0.00	0.00	0.00
GATCAAAAGACCCTCCGCCT	0.00	0.00	0.00	0.00	0.34
GATCAAAAGACCGAGTGTTG	0.00	0.00	0.29	0.00	0.00
GATCAAAAGACCTGCGAATG	0.00	0.33	0.00	0.00	0.00
GATCAAAAGACGAAATACAG	0.00	0.00	0.00	0.00	0.34
GATCAAAAGACGAAGATGGA	0.23	0.00	0.00	0.00	0.00
GATCAAAAGACGAAGGAGGC	0.46	0.00	0.00	0.00	0.00
GATCAAAAGACGACCTACGC	0.00	0.33	0.00	0.00	0.00
GATCAAAAGACGCACATGTC	0.00	0.33	0.00	0.00	0.00
GATCAAAAGACGCTGGCTCG	0.00	0.00	0.29	0.00	0.00
GATCAAAAGACGGACTGAGC	0.00	0.33	0.00	0.00	0.00
GATCAAAAGACGGGCCGGGG	0.00	0.00	0.00	0.00	0.34
GATCAAAAGACGGGCGCTGG	0.46	0.00	0.00	0.34	0.34
GATCAAAAGACGTCGGAATG	0.00	0.00	0.00	0.00	0.00
GATCAAAAGACGTGACCAAC	0.00	0.00	0.00	0.00	0.34
GATCAAAAGACGTTTAAAGG	0.00	0.00	0.00	0.00	0.69
GATCAAAAGACTCGATTCCA	0.23	0.00	0.00	0.00	0.00
GATCAAAAGACTGGAGGCTG	0.00	0.33	0.00	0.00	0.00
GATCAAAAGACTTACACCTC	0.00	0.00	0.00	0.00	0.00
GATCAAAAGACTTACGCCAT	0.00	0.00	0.00	0.00	0.00
GATCAAAAGACTTTTGGCTA	0.00	0.00	0.29	0.00	0.00
GATCAAAAGAGAAAAAAAAA	0.46	0.00	0.00	0.68	1.03

GATCAAAAGAGAACAACAAA	0.00	0.00	0.00	0.00	0.00
GATCAAAAGAGAAGCGGCGC	0.00	0.00	0.29	0.00	0.00
GATCAAAAGAGAAGGGACGG	0.00	0.65	0.00	0.00	0.00
GATCAAAAGAGAAGGGCCGC	0.23	0.00	0.29	0.00	0.00
GATCAAAAGAGAATCTGATG	0.00	0.00	0.00	0.34	0.00
GATCAAAAGAGACAGAATGG	0.00	0.00	0.00	0.00	0.34
GATCAAAAGAGACATGGGAC	0.00	0.00	0.00	0.34	0.00
GATCAAAAGAGACCGACCAT	0.00	0.00	0.00	0.00	0.34
GATCAAAAGAGAGAAATATC	0.23	0.00	0.00	0.00	0.00
GATCAAAAGAGAGGGGCAGA	0.00	0.00	0.29	0.00	0.00
GATCAAAAGAGAGGTAACAT	0.00	0.00	0.00	0.00	0.69
GATCAAAAGAGATGGCAGGC	0.23	0.00	0.00	0.00	0.00
GATCAAAAGAGCAAAAAAAA	0.00	0.00	0.00	0.00	0.00
GATCAAAAGAGCATGCTCAG	0.00	0.00	0.00	0.00	0.34
GATCAAAAGAGCATGGCGAT	0.00	0.00	0.00	0.00	0.00
GATCAAAAGAGCCCAAGAAG	0.00	0.00	0.00	0.34	0.00
GATCAAAAGAGCCGGCGTCA	0.00	0.33	0.00	0.00	0.00
GATCAAAAGAGCCTGCATGC	0.00	0.33	0.00	0.00	0.00
GATCAAAAGAGCCTGCCCTG	0.00	0.33	0.00	0.00	0.00
GATCAAAAGAGCCTGCGAAA	5.72	4.23	7.16	6.11	3.79
GATCAAAAGAGCCTGCGAAG	0.00	0.00	0.00	0.34	0.00
GATCAAAAGAGCCTGCGACA	0.23	0.00	0.00	0.00	0.00
GATCAAAAGAGCCTGCGCAA	0.00	0.33	0.00	0.00	0.00
GATCAAAAGAGCCTGCGNNN	0.23	0.00	0.00	0.00	0.00
GATCAAAAGAGCTCGCTCAG	0.00	0.00	0.29	0.00	0.00
GATCAAAAGAGCTTACTCAG	0.23	0.00	0.00	0.00	0.00
GATCAAAAGAGCTTGCNCAG	0.00	0.00	0.00	0.00	0.34
GATCAAAAGAGCTTGCTCAC	0.00	0.00	0.00	0.00	0.00
GATCAAAAGAGCTTGCTCAG	17.63	22.77	38.37	16.29	23.07
GATCAAAAGAGCTTGCTCAN	0.00	0.00	0.00	0.00	0.00
GATCAAAAGAGCTTGCTCAT	0.00	0.00	0.00	0.00	0.00
GATCAAAAGAGCTTGCTCCG	0.00	0.33	0.00	0.00	0.00
GATCAAAAGAGCTTGCTCGC	0.00	0.00	0.00	0.34	0.00
GATCAAAAGAGCTTGCTCTC	0.00	0.00	0.00	0.00	0.00
GATCAAAAGAGCTTGCTCTG	0.00	0.00	0.00	0.00	0.00
GATCAAAAGAGCTTGCTGAG	0.23	0.00	0.00	0.00	0.00
GATCAAAAGAGCTTGCTGGA	0.00	0.00	0.00	0.00	0.00

GATCAAAAGAGCTTGGCTGG	0.00	0.00	0.00	0.00	0.00
GATCAAAAGAGGAGGAGAGA	0.00	0.33	0.00	0.00	0.00
GATCAAAAGAGGGGATGCCA	0.23	0.00	0.00	0.00	0.00
GATCAAAAGAGGGGGACAAG	0.00	0.00	0.00	0.00	0.00
GATCAAAAGAGGGGGATGAT	0.00	0.00	0.00	0.34	0.00
GATCAAAAGAGGTCATCCAA	1.14	0.65	0.57	0.34	0.00
GATCAAAAGAGGTCGAGGCC	0.00	0.00	0.00	0.00	0.00
GATCAAAAGAGGTCTAAAAA	0.00	0.00	0.00	0.68	0.00
GATCAAAAGAGGTCTAAGAA	0.00	0.00	0.00	0.00	0.34
GATCAAAAGAGGTCTGGCAA	0.92	0.33	0.00	0.34	0.00
GATCAAAAGAGGTCTGGCAG	1.37	0.65	1.43	3.73	0.00
GATCAAAAGAGGTCTGGCTG	0.00	0.00	0.00	0.00	0.69
GATCAAAAGAGGTGGCAGGT	0.00	0.00	0.00	0.00	0.34
GATCAAAAGAGGTTGAGGCC	1.83	0.98	2.29	0.68	1.03
GATCAAAAGAGTGTTCCAAG	0.69	0.33	0.57	0.34	1.03
GATCAAAAGAGTGTTCCATT	0.00	0.00	0.00	0.00	0.69
GATCAAAAGAGTTGTAGGCA	1.37	0.00	0.29	0.00	0.00
GATCAAAAGAGTTTGCCTCC	0.00	0.00	0.00	0.00	0.00
GATCAAAAGATAAAGGATGA	0.00	0.65	0.00	0.00	0.34
GATCAAAAGATAAAGGTTGA	0.00	0.00	0.00	0.00	0.00
GATCAAAAGATAAATAACGA	0.23	0.00	0.00	0.00	0.00
GATCAAAAGATAATAAAGGA	0.23	0.00	0.00	0.00	0.00
GATCAAAAGATACACCCTAC	0.69	0.00	0.00	0.00	0.00
GATCAAAAGATACTCGTTGT	0.00	0.00	0.86	0.00	0.00
GATCAAAAGATACTTAAGGA	0.00	0.00	0.00	0.00	0.34
GATCAAAAGATACTTACGCA	0.00	0.00	0.29	0.00	0.00
GATCAAAAGATAGAAAAAAA	0.46	1.30	0.00	0.00	0.00
GATCAAAAGATAGAAACATC	0.00	0.00	0.00	0.00	0.34
GATCAAAAGATAGAAATATC	0.69	0.00	0.00	0.00	0.00
GATCAAAAGATAGCAGGAAA	0.00	0.00	0.00	0.00	0.00
GATCAAAAGATATACTTAGT	0.00	0.00	0.00	0.00	0.34
GATCAAAAGATCGACTATTC	0.00	0.00	0.00	0.00	0.00
GATCAAAAGATGAAAAAAAA	0.00	0.00	0.00	0.00	0.34
GATCAAAAGATGAGATGTTC	0.00	0.00	0.29	0.00	0.00
GATCAAAAGATGGAAAAAAA	0.23	0.00	0.00	0.00	0.34
GATCAAAAGATGGACACCCA	0.00	0.00	0.00	0.34	0.00
GATCAAAAGATGGACTAAAA	0.00	0.00	0.00	0.00	0.00

GATCAAAAGATGGACTGTCA	0.00	0.00	0.29	0.00	0.00
GATCAAAAGATGGACTGTNN	0.23	0.00	0.00	0.00	0.00
GATCAAAAGATGGACTGTTA	1.37	0.00	0.00	0.00	1.03
GATCAAAAGATGGACTGTTG	4.35	2.60	0.57	2.38	1.38
GATCAAAAGATGGAGTGTTG	0.23	0.00	0.00	0.00	0.00
GATCAAAAGATGGCCCAAAC	0.00	0.00	0.00	0.00	0.34
GATCAAAAGATGGCCCACTT	0.00	0.33	0.29	0.00	0.00
GATCAAAAGATGGCCTGTTG	0.00	0.00	0.00	0.00	0.00
GATCAAAAGATGTAGAATTT	0.23	0.00	0.00	0.00	0.00
GATCAAAAGATTAGAATGGG	1.14	0.00	0.00	0.34	0.00
GATCAAAAGATTAGAATGNN	0.23	0.00	0.00	0.00	0.00
GATCAAAAGATTATTAGGTA	0.00	0.00	0.00	0.00	0.34
GATCAAAAGATTGGTATGAA	0.00	0.00	0.00	0.34	0.00
GATCAAAAGATTTCTGACGT	0.00	0.00	0.00	0.00	0.00
GATCAAAAGATTTCTTTGGT	0.00	0.00	0.00	0.00	0.00
GATCAAAAGCAAAAAAAAAA	48.53	17.24	39.23	26.82	39.61
GATCAAAAGCAAAAAAAAAC	0.46	0.00	0.00	0.00	0.00
GATCAAAAGCAAAAAAAAAT	2.06	0.33	0.57	0.34	2.07
GATCAAAAGCAAAAAAAANN	0.23	0.00	0.00	0.00	0.00
GATCAAAAGCAAAAAAAATC	0.00	0.00	0.29	0.00	0.00
GATCAAAAGCAAAAAAACAA	0.00	0.00	0.00	0.00	0.69
GATCAAAAGCAAAAAAAGAA	0.00	0.00	0.00	0.00	0.00
GATCAAAAGCAAAAAAANAA	0.00	0.00	0.00	0.00	0.34
GATCAAAAGCAAAAAACAAA	0.23	0.00	0.00	0.00	0.00
GATCAAAAGCAAAAAACCAA	0.00	0.00	0.00	0.00	0.34
GATCAAAAGCAAAAAANAAA	0.00	0.00	0.00	0.00	0.34
GATCAAAAGCAAAAATCTAA	0.00	0.00	0.00	0.68	0.00
GATCAAAAGCAAAACAACAA	0.00	0.00	0.00	0.34	0.00
GATCAAAAGCAAAAGAAAAA	0.00	0.00	0.00	0.00	0.00
GATCAAAAGCAAACAAAAAA	0.00	0.33	0.00	0.00	0.00
GATCAAAAGCAAACGCCCCG	0.00	0.00	0.29	0.00	0.00
GATCAAAAGCAAAGAAAAAA	0.00	0.00	0.00	0.00	0.00
GATCAAAAGCAAATAAAAAA	0.00	0.00	0.00	0.00	0.00
GATCAAAAGCAACAAAAAAA	0.00	0.00	0.00	0.00	0.00
GATCAAAAGCAACATCAAAA	0.00	0.00	0.00	0.34	0.00
GATCAAAAGCAACATCAGAA	0.00	0.00	0.00	0.00	0.34
GATCAAAAGCAACCGCGCCT	0.00	0.00	0.00	0.00	0.00

GATCAAAAGCAACGAAAAAA	0.00	0.00	0.57	0.00	0.00
GATCAAAAGCAACGCAATGA	0.00	0.00	0.29	0.00	0.00
GATCAAAAGCAACTCAGGAA	0.00	0.33	0.29	0.00	0.00
GATCAAAAGCAACTGCACAT	0.23	0.00	0.00	0.00	0.00
GATCAAAAGCAAGAAAAAAA	0.00	0.33	0.00	0.00	0.00
GATCAAAAGCAAGACTGGAA	0.00	0.00	0.57	0.00	0.00
GATCAAAAGCAAGAGGAGGA	0.00	0.00	0.29	0.00	0.00
GATCAAAAGCAAGATGATGA	0.00	0.00	0.29	0.00	0.00
GATCAAAAGCAAGGAAAAAA	0.00	0.65	0.00	0.00	0.00
GATCAAAAGCAAGGAGGTAA	0.00	0.00	0.29	1.02	0.00
GATCAAAAGCAATCTTTAAT	0.00	0.00	0.00	0.00	0.34
GATCAAAAGCAATGCGGGCA	0.00	0.00	0.00	0.00	0.00
GATCAAAAGCACAAAAAAAA	0.00	0.00	0.29	0.00	0.00
GATCAAAAGCACAAAGCAGA	0.23	0.00	0.00	0.00	0.00
GATCAAAAGCACATCCATGA	0.00	0.00	0.00	0.00	0.34
GATCAAAAGCACATCCGACA	0.00	0.00	0.00	0.00	0.00
GATCAAAAGCACCGCGGGCA	0.00	0.33	0.00	0.00	0.00
GATCAAAAGCACCGGCAACG	0.00	0.33	0.00	0.00	0.00
GATCAAAAGCACGAGCTCTC	0.46	0.00	0.00	0.00	0.00
GATCAAAAGCACGCGGCTAT	0.00	0.00	0.29	0.00	0.00
GATCAAAAGCACGCTTCCCT	1.37	0.65	0.00	1.02	0.34
GATCAAAAGCACGCTTCTCG	0.00	0.00	0.00	1.36	0.00
GATCAAAAGCACGCTTCTTG	0.00	0.00	0.00	1.36	0.34
GATCAAAAGCACGGCGGGCA	0.00	0.00	0.00	0.34	0.00
GATCAAAAGCACGGGAGAAA	0.00	0.00	0.00	0.00	0.00
GATCAAAAGCACTCGGGCTT	0.46	0.00	0.00	0.00	0.34
GATCAAAAGCACTCTTTCAG	0.00	0.00	0.00	0.00	0.00
GATCAAAAGCACTGAGCCGC	0.00	0.00	0.00	0.00	0.00
GATCAAAAGCACTGAGGGCA	0.00	0.00	0.00	0.34	0.00
GATCAAAAGCACTGCCTTGG	0.00	0.00	0.00	0.00	0.00
GATCAAAAGCACTGCGCACA	0.00	0.00	0.00	0.00	0.00
GATCAAAAGCACTGCGCCTC	0.23	0.00	0.00	0.00	0.00
GATCAAAAGCACTGCGGAGG	1.14	0.00	0.00	0.68	0.00
GATCAAAAGCACTGCGGCTT	0.23	0.33	0.00	0.00	0.00
GATCAAAAGCACTGCGGGAA	0.23	0.00	0.00	0.34	0.00
GATCAAAAGCACTGCGGGAC	0.23	0.00	0.00	0.34	0.00
GATCAAAAGCACTGCGGGAG	0.00	0.00	0.00	0.34	0.00

GATCAAAAGCACTGCGGGCA	35.02	43.59	14.60	29.19	7.23
GATCAAAAGCACTGCGGGCC	1.37	0.65	0.29	0.68	0.69
GATCAAAAGCACTGCGGGCG	0.23	0.00	0.00	0.00	0.00
GATCAAAAGCACTGCGGGCT	9.61	5.21	1.72	3.73	1.38
GATCAAAAGCACTGCGGTCA	0.23	0.33	0.00	0.00	0.00
GATCAAAAGCACTGCGGTGA	0.23	0.00	0.00	0.00	0.00
GATCAAAAGCACTGCGNGNN	0.23	0.00	0.00	0.00	0.00
GATCAAAAGCACTGCTGGAG	0.23	0.00	0.00	0.00	0.00
GATCAAAAGCACTGCTGTTT	1.14	0.33	0.86	0.00	0.00
GATCAAAAGCACTGGAGAAA	0.00	0.65	0.00	0.34	0.00
GATCAAAAGCACTGGAGGCC	0.23	0.33	0.00	0.34	0.00
GATCAAAAGCACTGGATGTC	0.00	0.00	0.00	0.00	0.00
GATCAAAAGCACTGGCTTCA	0.23	0.00	0.00	0.00	0.00
GATCAAAAGCACTGGGACTG	0.00	0.00	0.00	0.00	0.00
GATCAAAAGCAGAAAAAAAA	0.00	0.00	0.00	0.00	1.03
GATCAAAAGCAGAAGAGCAC	0.23	0.00	0.00	0.00	0.00
GATCAAAAGCAGACGAAAAA	0.00	0.33	0.00	0.00	0.00
GATCAAAAGCAGACGAGAGA	0.00	0.33	0.00	0.00	0.00
GATCAAAAGCAGACGAGAGC	1.14	1.63	2.29	2.38	1.03
GATCAAAAGCAGACGAGGGC	0.23	0.00	0.00	0.00	0.00
GATCAAAAGCAGACTCGTAT	0.00	0.00	0.29	0.00	0.00
GATCAAAAGCAGCACCGGAT	0.23	0.00	0.00	0.00	0.00
GATCAAAAGCAGCCAGGGAC	0.00	0.00	0.00	0.34	0.00
GATCAAAAGCAGCGAAAGGG	0.00	0.00	0.00	0.00	0.00
GATCAAAAGCAGCGTCCTCT	0.00	0.00	0.00	0.00	0.69
GATCAAAAGCAGGGCAACGA	0.69	0.33	0.29	0.00	0.00
GATCAAAAGCAGGGCTGCCC	0.00	0.00	0.00	0.00	0.00
GATCAAAAGCAGGGGACTGC	0.00	0.00	0.00	0.00	0.00
GATCAAAAGCAGTATCTCTC	0.00	0.00	0.00	0.34	0.00
GATCAAAAGCAGTGAAAATG	0.00	0.00	0.29	0.00	0.00
GATCAAAAGCAGTGGAATGC	0.00	0.33	0.00	0.00	0.00
GATCAAAAGCAGTGGACTAC	0.00	0.00	0.29	0.00	0.00
GATCAAAAGCAGTGGACTCC	0.00	0.00	0.00	0.00	0.00
GATCAAAAGCAGTGGACTGC	15.79	10.09	12.60	8.15	14.46
GATCAAAAGCAGTGGCTTCG	0.00	0.00	0.00	0.34	0.00
GATCAAAAGCATAAAAAAAA	0.23	0.00	0.00	0.00	0.00
GATCAAAAGCATAAGCTCTC	0.00	0.00	0.00	0.00	0.00

GATCAAAAGCATCAAAAAAA	0.00	0.33	0.00	0.00	0.00
GATCAAAAGCATCATCAGTA	0.00	0.00	0.00	0.00	0.00
GATCAAAAGCATCGAACGAA	0.00	0.00	0.29	0.00	0.34
GATCAAAAGCATCGAACGCA	0.00	0.00	0.00	0.00	0.34
GATCAAAAGCATCGAACGTA	8.47	12.36	4.30	9.16	8.61
GATCAAAAGCATCGAACGTT	0.00	0.33	0.00	0.00	0.00
GATCAAAAGCATCGAACTTA	0.00	0.00	0.00	0.00	0.34
GATCAAAAGCATCGAGCACG	0.00	0.65	0.00	0.00	0.00
GATCAAAAGCATCGCGATAT	0.00	0.00	0.00	0.34	0.00
GATCAAAAGCATCGCGCTAT	0.00	0.33	0.57	1.02	0.34
GATCAAAAGCATCGCGGTCT	0.00	0.00	0.00	0.00	0.34
GATCAAAAGCATCGTACGTA	0.00	0.00	0.00	0.00	0.00
GATCAAAAGCATCTCGTGGA	0.00	0.00	0.57	0.00	0.00
GATCAAAAGCATGAAAAAAA	2.75	0.33	0.29	0.68	0.34
GATCAAAAGCATGACCTCTC	0.00	0.00	0.00	0.68	0.00
GATCAAAAGCATGAGCGCTC	0.69	0.00	0.00	0.00	0.00
GATCAAAAGCATGAGCNCTC	0.00	0.00	0.00	0.00	0.34
GATCAAAAGCATGAGCTCCC	0.23	0.00	0.00	0.00	0.00
GATCAAAAGCATGAGCTCCT	0.00	0.00	0.00	0.34	0.00
GATCAAAAGCATGAGCTCNC	0.00	0.00	0.00	0.00	0.00
GATCAAAAGCATGAGCTCNN	0.46	0.00	0.00	0.00	0.00
GATCAAAAGCATGAGCTCTA	0.69	0.65	0.00	0.68	0.00
GATCAAAAGCATGAGCTCTC	116.74	62.46	14.32	69.24	39.26
GATCAAAAGCATGAGCTCTG	0.00	0.33	0.00	0.00	0.00
GATCAAAAGCATGAGCTCTN	0.46	0.00	0.00	0.00	0.00
GATCAAAAGCATGAGCTNNN	0.23	0.00	0.00	0.00	0.00
GATCAAAAGCATGAGGTTTG	0.23	0.00	0.00	0.00	0.00
GATCAAAAGCATGCCTTTGA	0.00	0.00	0.00	0.00	0.00
GATCAAAAGCATGCGCTCNC	0.00	0.00	0.00	0.00	0.34
GATCAAAAGCATGCGCTCTC	0.23	0.00	0.00	0.00	0.00
GATCAAAAGCATGCGGAGCA	0.00	0.00	0.00	0.68	0.00
GATCAAAAGCATGCGTTCTC	0.23	0.00	0.00	0.00	0.00
GATCAAAAGCATGGCGCAAA	0.00	0.33	0.00	0.00	0.00
GATCAAAAGCATGTGCTCTC	0.23	0.00	0.00	0.00	0.00
GATCAAAAGCATGTTGGAAA	0.00	0.00	0.00	0.00	0.34
GATCAAAAGCATTGCTGTGT	0.23	0.00	0.00	0.00	0.00
GATCAAAAGCCAAAAAAAAA	0.46	0.98	0.00	0.00	0.00

GATCAAAAGCCAACTCTTGC	0.00	0.00	0.00	0.00	0.00
GATCAAAAGCCAAGGACCAT	0.00	0.00	0.00	0.00	0.34
GATCAAAAGCCACTGGTCAA	0.00	0.33	0.00	0.00	0.00
GATCAAAAGCCAGAGGAGCC	0.69	0.00	0.00	0.00	0.00
GATCAAAAGCCAGATTTGAA	0.00	0.00	0.00	0.00	0.34
GATCAAAAGCCAGCAACGGA	0.00	0.00	0.00	0.34	0.00
GATCAAAAGCCAGCACAGGA	0.00	0.33	0.00	0.00	0.00
GATCAAAAGCCAGCACCGAA	0.69	0.00	0.00	0.00	0.00
GATCAAAAGCCAGCACCGCC	0.00	0.00	0.00	0.34	0.00
GATCAAAAGCCAGCACCGGA	29.99	26.68	10.88	16.97	11.02
GATCAAAAGCCAGCACCGGC	0.23	0.00	0.00	0.00	0.00
GATCAAAAGCCAGCACCGGN	0.23	0.00	0.00	0.00	0.00
GATCAAAAGCCAGCATCGTA	0.23	0.00	0.00	0.00	0.00
GATCAAAAGCCAGCCTTGGA	0.23	0.00	0.00	0.00	0.00
GATCAAAAGCCAGCGAGATG	0.00	0.00	0.00	0.00	0.00
GATCAAAAGCCAGCGTTGGA	0.00	0.00	0.00	0.34	0.00
GATCAAAAGCCAGCTTTGGA	46.47	69.95	20.62	80.11	37.54
GATCAAAAGCCAGCTTTGGC	0.00	0.65	0.00	0.34	0.00
GATCAAAAGCCAGCTTTGGG	0.23	0.00	0.00	0.00	0.00
GATCAAAAGCCAGCTTTGGN	0.23	0.00	0.00	0.00	0.34
GATCAAAAGCCAGCTTTGGT	0.23	0.00	0.00	0.00	0.00
GATCAAAAGCCAGCTTTGTA	0.00	0.33	0.00	0.34	0.00
GATCAAAAGCCAGGACTTGC	0.00	0.00	0.00	0.00	1.03
GATCAAAAGCCAGGTTTGGA	0.23	0.00	0.00	0.00	0.00
GATCAAAAGCCAGTCCCGAG	0.00	0.33	0.00	0.00	0.00
GATCAAAAGCCAGTGCTTGT	0.00	0.33	0.86	0.00	0.00
GATCAAAAGCCATATCGCTC	0.00	0.00	0.00	0.00	0.00
GATCAAAAGCCATTGCTTGT	0.00	0.00	0.00	0.68	0.00
GATCAAAAGCCCAGTGTCTG	0.00	0.00	0.00	0.34	0.00
GATCAAAAGCCCATAGTGTG	0.00	0.33	0.00	0.00	0.00
GATCAAAAGCCCGCACGAGG	0.00	0.33	0.00	0.00	0.00
GATCAAAAGCCCGCACTGGA	0.00	0.00	0.00	0.34	0.00
GATCAAAAGCCCGCTCTGGA	0.23	0.00	0.00	0.00	0.00
GATCAAAAGCCCGCTTTGGA	0.23	0.00	0.00	0.00	0.00
GATCAAAAGCCGCATGGTGT	0.00	0.00	0.00	0.00	0.00
GATCAAAAGCCGGCCCGAAA	0.00	0.00	0.29	0.00	0.00
GATCAAAAGCCGGCTTGCTG	0.00	0.00	0.29	0.00	0.00

GATCAAAAGCCGGTCCAAGA	0.00	0.00	0.00	0.34	0.00
GATCAAAAGCCGTGGACTGC	0.00	0.00	0.29	0.00	0.00
GATCAAAAGCCGTGGTGACA	0.00	0.00	0.00	0.00	0.00
GATCAAAAGCCGTTGTTCAC	0.00	0.00	0.00	0.34	0.00
GATCAAAAGCCGTTGTTCCC	3.66	1.30	0.86	3.39	2.41
GATCAAAAGCCGTTGTTCNN	0.23	0.00	0.00	0.00	0.00
GATCAAAAGCCGTTGTTGCC	0.23	0.00	0.00	0.00	0.00
GATCAAAAGCCTAAAAAAAA	0.00	0.00	0.29	0.00	0.00
GATCAAAAGCCTATTTTGCC	0.23	0.00	0.00	0.00	0.00
GATCAAAAGCCTCAAAAAAA	0.46	0.00	0.00	0.00	0.00
GATCAAAAGCCTCACCAGTC	0.00	0.00	0.00	0.00	0.00
GATCAAAAGCCTCATCAGTG	0.00	0.33	0.86	0.00	0.34
GATCAAAAGCCTCATCGAAA	0.00	0.00	0.00	0.34	0.00
GATCAAAAGCCTCCAAAAAA	0.00	0.33	0.00	0.00	0.00
GATCAAAAGCCTCCACATGC	3.66	2.93	1.43	1.36	1.72
GATCAAAAGCCTCCACATGT	0.00	0.00	0.29	0.00	0.00
GATCAAAAGCCTCCGCAAGG	0.00	0.33	0.00	0.34	0.00
GATCAAAAGCCTCCTTATTT	0.23	0.00	0.00	0.00	0.00
GATCAAAAGCCTCGAGTCCT	0.00	0.00	0.00	0.34	0.00
GATCAAAAGCCTCGATTCCA	4.35	1.30	1.15	2.04	1.72
GATCAAAAGCCTCGATTTTC	0.00	0.00	0.00	0.00	0.00
GATCAAAAGCCTCGCAGAAC	0.23	0.00	0.00	0.00	0.00
GATCAAAAGCCTCGTTTCCG	0.00	0.00	0.00	0.00	0.00
GATCAAAAGCCTGTTTAAGG	0.00	0.00	0.00	0.00	0.00
GATCAAAAGCCTTGGCGCCG	0.00	0.00	0.00	0.68	0.00
GATCAAAAGCGAAAAAAAAA	5.26	0.33	1.15	2.04	2.07
GATCAAAAGCGAAGGACATG	0.23	0.00	0.00	0.00	0.00
GATCAAAAGCGAGCGGTCGC	1.60	0.65	0.29	0.34	0.69
GATCAAAAGCGATCCAGCAA	0.00	0.00	0.29	0.00	0.00
GATCAAAAGCGCAAACCCCT	0.00	0.00	0.00	0.00	0.34
GATCAAAAGCGCAGAGTTGC	0.00	0.65	0.00	0.00	0.00
GATCAAAAGCGCAGTTCTGC	0.00	0.33	0.00	0.00	0.00
GATCAAAAGCGCATAGTGTG	0.00	0.33	0.00	0.00	0.00
GATCAAAAGCGCGTACGAAG	0.46	0.00	0.86	0.68	0.34
GATCAAAAGCGCTTGCTCAG	0.00	0.00	0.29	0.00	0.00
GATCAAAAGCGGCAAAAAAA	0.46	0.33	0.00	0.00	0.00
GATCAAAAGCGGCATCAAGT	0.00	0.00	0.00	0.00	0.34

GATCAAAAGCGGCATGGACT	0.00	0.00	0.00	0.00	0.00
GATCAAAAGCGGCATGGTCT	0.00	0.00	0.00	0.00	0.00
GATCAAAAGCGGCCCGGCGG	0.00	0.00	0.00	0.00	0.34
GATCAAAAGCGGCCTGCCGC	0.00	0.00	0.00	0.34	0.00
GATCAAAAGCGGCGATGGCT	0.23	0.00	0.00	0.00	0.00
GATCAAAAGCGGCGCGCCGT	0.00	0.33	0.00	0.00	0.00
GATCAAAAGCGGCGTGATTC	0.00	0.00	0.00	0.34	0.00
GATCAAAAGCGGCGTGATTN	0.23	0.00	0.00	0.00	0.00
GATCAAAAGCGGCGTTCGTA	0.00	0.00	0.00	0.00	0.34
GATCAAAAGCGGCGTTTTTT	0.00	0.00	0.00	0.34	0.34
GATCAAAAGCGGGATCAAGG	0.00	0.00	0.00	0.34	0.00
GATCAAAAGCGGTTGCCTCT	0.00	0.00	0.00	0.00	0.69
GATCAAAAGCGGTTTTGGGA	0.23	0.00	0.00	0.00	0.00
GATCAAAAGCGGTTTTGGTA	3.20	0.33	0.00	0.34	0.00
GATCAAAAGCGTAAAAAAAA	1.83	0.00	0.00	0.00	0.00
GATCAAAAGCGTCACTAGTC	0.00	0.33	0.00	0.00	0.00
GATCAAAAGCGTCATGAAGA	0.00	0.00	0.29	0.00	0.00
GATCAAAAGCGTCATGGTAA	1.37	0.00	0.29	1.02	0.00
GATCAAAAGCGTCGGAAGGA	0.23	0.00	0.00	0.34	0.00
GATCAAAAGCGTCGGAAGGG	0.00	0.00	0.00	0.00	0.34
GATCAAAAGCGTCGGACGGA	0.23	0.00	0.00	0.00	0.00
GATCAAAAGCGTCGGCATGG	0.00	0.00	0.29	0.00	0.00
GATCAAAAGCGTCTCAAAAA	0.00	0.00	0.57	0.00	0.00
GATCAAAAGCGTGAAAAAAA	0.00	0.00	0.29	0.00	0.00
GATCAAAAGCGTGAACACGT	0.00	0.33	0.00	0.00	0.00
GATCAAAAGCGTGAGGCACA	0.23	0.00	0.00	0.00	0.00
GATCAAAAGCGTGCAAAAAA	0.69	0.00	0.00	0.34	0.00
GATCAAAAGCGTGCAGGGAA	0.23	0.00	0.00	0.34	0.00
GATCAAAAGCGTGCAGGGAT	0.69	0.00	0.00	0.00	0.00
GATCAAAAGCGTGCAGGGCT	0.23	0.00	0.00	0.00	0.00
GATCAAAAGCGTTGCATTGG	0.00	0.00	0.00	0.00	0.00
GATCAAAAGCTAACCCAACC	0.00	0.00	0.00	0.00	0.00
GATCAAAAGCTACTAGCGTG	0.23	0.00	0.00	0.00	0.00
GATCAAAAGCTATCTGCGCG	0.00	0.00	0.00	0.00	0.00
GATCAAAAGCTCACTGTGTG	0.23	0.00	0.00	0.00	0.00
GATCAAAAGCTCAGCACAGA	0.00	0.00	0.00	0.00	0.00
GATCAAAAGCTCAGGCCTGC	0.00	0.00	0.00	0.34	0.00

GATCAAAAGCTCAGTGTGTG	0.00	0.00	0.00	0.34	0.00
GATCAAAAGCTCATAGGGTG	0.23	0.00	0.00	0.00	0.00
GATCAAAAGCTCATAGTGAG	0.00	0.00	0.00	0.00	0.00
GATCAAAAGCTCATAGTGGG	0.23	0.00	0.00	0.00	0.00
GATCAAAAGCTCATAGTGTG	22.20	9.76	6.87	11.88	7.23
GATCAAAAGCTCATAGTGTN	0.23	0.00	0.00	0.00	0.00
GATCAAAAGCTCATAGTGTT	0.00	0.00	0.00	0.00	0.00
GATCAAAAGCTCATATTGTG	0.00	0.00	0.00	0.68	0.00
GATCAAAAGCTCATCGTACG	0.23	0.00	0.00	0.00	0.00
GATCAAAAGCTCATCGTATG	0.69	0.33	0.00	0.34	0.69
GATCAAAAGCTCATCGTGTG	0.23	0.00	0.00	0.00	0.00
GATCAAAAGCTCATGTGTGC	0.00	0.00	0.00	0.00	0.00
GATCAAAAGCTCATTGCGTG	0.23	0.00	0.00	0.00	0.00
GATCAAAAGCTCATTGGGTG	0.00	0.65	0.00	0.00	0.00
GATCAAAAGCTCATTGTGAG	0.00	0.00	0.00	0.00	0.34
GATCAAAAGCTCATTGTGCG	0.23	0.00	0.00	0.00	0.00
GATCAAAAGCTCATTGTGNN	0.23	0.00	0.00	0.00	0.00
GATCAAAAGCTCATTGTGTG	35.71	21.15	7.16	12.22	5.51
GATCAAAAGCTCATTGTGTT	0.00	0.33	0.00	0.00	0.00
GATCAAAAGCTCATTGTTCG	0.23	0.00	0.00	0.00	0.00
GATCAAAAGCTCCCTCGTCT	0.00	0.00	0.29	0.00	0.00
GATCAAAAGCTCCCTTTACA	0.23	0.00	0.00	0.00	0.34
GATCAAAAGCTCCGGCATGC	0.00	0.00	0.00	0.00	0.34
GATCAAAAGCTCCGGCCTGC	1.14	2.28	1.15	1.36	1.38
GATCAAAAGCTCGACTATTC	0.00	0.65	0.00	0.00	0.69
GATCAAAAGCTCGCTAAGGC	0.00	0.00	0.00	0.00	0.00
GATCAAAAGCTCGTTGTGTG	0.00	0.00	0.00	0.34	0.00
GATCAAAAGCTCTTACACTC	0.00	0.00	0.00	0.00	0.00
GATCAAAAGCTGAAGAAGCA	0.00	0.98	0.00	0.00	0.00
GATCAAAAGCTGAGCCGCGG	0.00	0.00	0.00	0.00	0.00
GATCAAAAGCTGAGCTCTCG	0.00	0.00	0.00	0.34	0.00
GATCAAAAGCTGAGCTTGAA	0.00	0.65	0.00	0.00	0.00
GATCAAAAGCTGATTGATTG	0.00	0.00	0.29	0.00	0.00
GATCAAAAGCTGCAGCAAGC	0.69	0.00	0.29	0.00	0.00
GATCAAAAGCTGCCTTCAAA	0.46	0.00	0.29	0.00	0.00
GATCAAAAGCTGCTCGCACT	0.00	0.00	0.00	0.00	0.00
GATCAAAAGCTGCTTGGTCA	0.00	0.33	0.00	0.00	0.00

GATCAAAAGCTGGAATCTAA	0.00	0.33	0.00	0.00	0.00
GATCAAAAGCTGGATGGCTG	0.00	0.00	0.00	0.00	0.34
GATCAAAAGCTGGGTTGAGA	0.00	0.00	0.00	0.00	0.00
GATCAAAAGCTGTGAGCAAA	0.00	0.00	0.00	0.00	0.34
GATCAAAAGCTGTGCCGGCC	0.23	0.33	0.00	0.00	0.00
GATCAAAAGCTGTGCGACCA	0.23	0.00	0.00	0.00	0.00
GATCAAAAGCTTATTCATCT	0.23	0.00	0.00	0.00	0.00
GATCAAAAGCTTCCACGGTG	0.23	0.00	0.00	0.00	0.00
GATCAAAAGCTTCCACGNNN	0.23	0.00	0.00	0.00	0.00
GATCAAAAGCTTCCGTATTC	0.00	0.00	0.29	0.00	0.00
GATCAAAAGCTTCTGGCCGG	0.00	0.00	0.00	0.00	0.34
GATCAAAAGCTTGACAGCAC	0.23	0.00	0.57	0.00	0.00
GATCAAAAGCTTGGGAATGG	0.00	0.33	0.00	0.00	0.00
GATCAAAAGCTTGGGGAAAA	0.00	0.00	0.29	0.00	0.00
GATCAAAAGCTTGGTGCCAT	0.00	0.00	0.00	0.00	0.00
GATCAAAAGCTTGTTCTTGG	0.00	0.00	0.00	0.00	0.34
GATCAAAAGCTTTGCATTGG	0.00	0.00	0.57	0.00	0.00
GATCAAAAGGAAAAAAAAAA	10.07	6.18	2.00	5.09	3.10
GATCAAAAGGAAAAAAAAAG	0.23	0.00	0.00	0.00	0.00
GATCAAAAGGAAAAAAAAAT	0.00	0.00	0.29	0.00	0.34
GATCAAAAGGAAAAAAAGAA	0.00	0.00	0.00	0.34	0.00
GATCAAAAGGAAAACAAAAA	0.00	0.00	0.29	0.00	0.00
GATCAAAAGGAAAAGAAAAA	0.23	0.00	0.00	0.00	0.00
GATCAAAAGGAAAGAAAAAA	0.00	0.00	0.57	0.00	0.00
GATCAAAAGGAAATCTGATG	0.00	0.00	0.00	0.34	0.00
GATCAAAAGGAACAAGGGAG	0.00	0.00	0.86	0.00	0.00
GATCAAAAGGAACCTATAAC	0.00	0.65	0.00	1.02	0.69
GATCAAAAGGAACCTATGAC	0.00	0.00	0.00	0.00	0.34
GATCAAAAGGAACGCGATAG	0.00	0.00	0.29	0.00	0.00
GATCAAAAGGAACGGACAGC	0.46	0.33	0.00	1.36	0.00
GATCAAAAGGAAGAAAAAAA	0.46	0.00	0.29	0.00	0.00
GATCAAAAGGAAGACAAAAA	0.00	0.33	0.00	0.00	0.00
GATCAAAAGGAAGACAAGTG	0.23	0.98	0.00	0.34	0.34
GATCAAAAGGAAGAGAAGGC	0.23	0.00	0.00	0.00	0.00
GATCAAAAGGAAGCAAAAAA	0.23	0.00	0.00	0.00	0.00
GATCAAAAGGAAGCAAAGAC	0.46	0.00	0.00	0.00	0.00
GATCAAAAGGAAGCCAAAAA	0.46	0.00	0.29	1.36	0.00

GATCAAAAGGAAGCCACAAA	0.00	0.00	0.00	0.00	0.00
GATCAAAAGGAAGCCACATT	0.00	0.33	0.00	0.00	0.00
GATCAAAAGGAAGCCACCAA	0.00	0.00	0.00	1.02	0.00
GATCAAAAGGAAGCCACCTA	0.00	0.00	0.00	0.00	0.00
GATCAAAAGGAAGCCACCTT	2.06	2.28	0.57	2.38	0.00
GATCAAAAGGAAGCCTCCGT	0.00	0.00	0.29	0.00	0.00
GATCAAAAGGAAGCCTGCGA	0.46	0.33	0.00	0.34	0.34
GATCAAAAGGAAGCCTGCGC	0.00	0.00	0.00	0.34	0.00
GATCAAAAGGAAGGAGGCCG	0.23	0.00	0.00	0.00	0.00
GATCAAAAGGAAGGTCGACA	0.23	0.00	0.00	0.68	0.00
GATCAAAAGGAATCTCATCA	0.00	0.00	0.00	0.00	0.00
GATCAAAAGGACAAAAAAAA	0.00	0.00	0.00	0.00	0.69
GATCAAAAGGACAAGGATGC	0.00	0.00	0.00	0.00	0.00
GATCAAAAGGACACGTGAGA	0.00	0.00	0.29	0.00	0.00
GATCAAAAGGACACGTTCAG	0.00	0.00	0.00	0.00	0.34
GATCAAAAGGACACGTTGCG	0.00	0.33	0.00	0.00	0.00
GATCAAAAGGACAGCTGTGC	0.00	0.00	0.00	0.34	0.00
GATCAAAAGGACAGTGGGGT	0.00	0.33	0.00	0.00	0.00
GATCAAAAGGACCATATATC	0.00	0.33	0.00	0.00	0.00
GATCAAAAGGACGAAAAAAA	0.00	0.33	0.00	0.00	0.00
GATCAAAAGGACGCAAAAAA	0.00	0.00	0.00	0.00	0.34
GATCAAAAGGACGCCACCTT	0.23	0.00	0.00	0.00	0.00
GATCAAAAGGACGCCTAGAT	0.00	0.65	0.29	0.34	0.34
GATCAAAAGGACGGAGGCCG	0.23	0.00	0.00	0.00	0.00
GATCAAAAGGACGGGCCAGA	0.00	0.00	0.00	0.00	0.00
GATCAAAAGGACGGGCGGCG	0.00	0.00	0.00	0.00	0.34
GATCAAAAGGACGTTTGCCT	0.00	0.00	0.00	0.00	0.69
GATCAAAAGGACTTCAAAAA	0.00	0.33	0.00	0.00	0.00
GATCAAAAGGACTTCCACAA	1.14	0.00	0.00	0.00	0.00
GATCAAAAGGACTTCGGAGT	0.00	0.00	0.00	0.34	0.00
GATCAAAAGGACTTCGGCAT	5.49	2.93	2.58	5.09	4.82
GATCAAAAGGAGAAAAAAAA	0.00	0.00	0.00	0.00	1.38
GATCAAAAGGAGAAGAAGAA	0.00	0.00	0.29	0.00	0.00
GATCAAAAGGAGAAGAAGCC	0.00	0.00	0.57	0.00	0.00
GATCAAAAGGAGAGGCCTTC	0.92	0.00	0.57	0.00	0.00
GATCAAAAGGAGCAAAAAAA	0.46	0.00	0.29	0.00	0.00
GATCAAAAGGAGCGCCCATG	0.00	0.00	0.00	0.00	0.34

GATCAAAAGGAGCGGCCGTC	0.23	0.00	0.00	0.00	0.00
GATCAAAAGGAGCGGTCCGG	0.00	0.00	0.00	0.00	1.03
GATCAAAAGGAGGAAAAAAA	0.00	0.00	0.00	0.00	0.00
GATCAAAAGGAGGAGAAGGA	0.00	0.00	0.00	0.00	0.34
GATCAAAAGGAGGAGGAGGA	0.00	0.00	0.00	0.00	0.69
GATCAAAAGGAGGGAACAAG	0.00	0.33	0.00	0.00	0.00
GATCAAAAGGAGGGAGATGG	0.23	0.00	0.00	0.00	0.00
GATCAAAAGGAGGGCCAAGC	0.00	0.00	0.00	0.68	0.00
GATCAAAAGGAGGGCTCCTC	0.00	0.33	0.00	0.00	0.00
GATCAAAAGGAGGGGGATGA	0.00	0.00	0.29	0.00	0.34
GATCAAAAGGAGGTTCTGTC	0.00	0.00	0.00	0.00	0.34
GATCAAAAGGAGTGAACAGT	0.00	0.00	0.00	0.00	0.00
GATCAAAAGGAGTGGCAAAA	0.00	0.00	0.29	0.00	0.00
GATCAAAAGGATAAAAAAAA	2.06	0.00	0.00	0.00	1.03
GATCAAAAGGATAAAAAATA	0.00	0.00	0.57	0.00	0.00
GATCAAAAGGATAAATGGCA	1.37	0.65	0.86	0.68	1.03
GATCAAAAGGATGAAAAAAA	43.95	17.89	25.20	19.01	24.45
GATCAAAAGGATGAAAAAAN	0.00	0.00	0.00	0.00	0.00
GATCAAAAGGATGAAAAAAT	0.00	0.00	0.57	0.00	0.00
GATCAAAAGGATGAAAAACN	0.23	0.00	0.00	0.00	0.00
GATCAAAAGGATGAAAAANN	0.23	0.00	0.00	0.00	0.00
GATCAAAAGGATGAAAAATA	0.69	0.00	0.00	0.00	0.00
GATCAAAAGGATGAAAAGCA	0.00	0.00	0.00	0.00	0.00
GATCAAAAGGATGAAAAGTG	4.35	1.95	0.57	0.68	0.00
GATCAAAAGGATGAAAAGTT	0.00	0.00	0.00	0.00	0.00
GATCAAAAGGATGAAACANN	0.23	0.00	0.00	0.00	0.00
GATCAAAAGGATGAAAGAAA	0.00	0.33	0.00	0.00	1.38
GATCAAAAGGATGAAAGGTG	0.00	0.00	0.00	0.00	0.00
GATCAAAAGGATGAATTTCT	0.46	0.00	0.00	0.00	0.00
GATCAAAAGGATGAGAAAAA	0.23	0.00	0.29	0.00	0.00
GATCAAAAGGATGAGCTCCG	0.00	0.00	0.00	0.00	0.34
GATCAAAAGGATGAGGCTGA	0.00	0.00	0.00	0.00	0.00
GATCAAAAGGATGATCGTAT	0.00	0.00	0.00	0.34	0.00
GATCAAAAGGATGCAAAAAA	0.00	0.98	0.00	0.00	0.00
GATCAAAAGGATGCAAAAAC	0.23	0.00	0.00	0.00	0.00
GATCAAAAGGATGCAAAAAT	0.23	0.00	0.00	0.00	0.00
GATCAAAAGGATGGAAAAAA	0.00	0.00	0.57	0.00	0.00

GATCAAAAGGATGGATTCTT	0.00	0.00	0.57	0.00	0.00
GATCAAAAGGATGTAAAAAA	0.00	0.00	0.00	0.00	0.00
GATCAAAAGGATTAAAAAAA	0.23	0.00	0.00	0.00	0.00
GATCAAAAGGATTCGTATGC	0.00	0.00	0.00	0.00	0.00
GATCAAAAGGATTCTCTCCT	0.00	0.00	0.29	0.00	0.00
GATCAAAAGGATTGCCGGCA	0.00	0.33	0.00	0.00	0.00
GATCAAAAGGCAAAAAAAAA	8.47	3.58	4.01	5.77	5.85
GATCAAAAGGCAAAGAAAAA	0.23	0.00	0.00	0.00	0.00
GATCAAAAGGCAACGCATGA	0.00	0.00	0.29	0.00	0.00
GATCAAAAGGCAACGTCGCT	0.00	0.00	0.29	0.00	0.00
GATCAAAAGGCAAGCCTGTG	0.00	0.00	0.00	0.00	0.34
GATCAAAAGGCAATTGTTCA	0.00	0.00	0.00	0.00	0.00
GATCAAAAGGCACCCGCTTG	0.00	0.00	0.29	0.00	0.00
GATCAAAAGGCACGCGCTCG	0.00	0.00	0.00	0.00	0.69
GATCAAAAGGCAGAAAAAAA	0.46	0.00	0.00	0.00	0.00
GATCAAAAGGCAGAAACAAA	0.00	0.00	0.00	0.34	0.00
GATCAAAAGGCAGAGAAAAA	0.00	0.00	0.00	0.00	0.34
GATCAAAAGGCAGAGCAAAA	1.14	0.00	0.29	0.00	1.03
GATCAAAAGGCAGAGCGAAA	0.69	0.33	0.86	0.00	0.34
GATCAAAAGGCAGAGCGACA	3.89	1.63	2.29	2.38	2.07
GATCAAAAGGCAGAGCGACG	0.00	0.00	0.00	0.34	0.00
GATCAAAAGGCAGAGCGCTT	0.00	0.00	0.00	0.00	0.34
GATCAAAAGGCAGAGCTTAA	0.00	0.33	0.00	0.00	0.00
GATCAAAAGGCAGCCCACGT	0.00	0.98	0.29	0.68	0.34
GATCAAAAGGCAGCCCCATC	0.23	0.00	0.00	0.00	0.00
GATCAAAAGGCAGCCCGGAA	0.23	0.00	0.29	0.00	0.00
GATCAAAAGGCAGTCCAAGG	0.00	0.33	0.00	0.00	0.00
GATCAAAAGGCAGTTGGCAT	0.00	0.00	0.00	0.00	0.00
GATCAAAAGGCATCAATCTG	0.00	0.65	0.00	0.00	0.00
GATCAAAAGGCATCGTTGCA	0.00	0.00	0.00	0.34	0.00
GATCAAAAGGCATCTGAAGC	0.00	0.00	0.00	0.00	0.00
GATCAAAAGGCCAAAAAAAA	0.00	0.33	0.00	0.00	0.00
GATCAAAAGGCCAGCCGCGG	0.23	0.00	0.00	0.00	0.00
GATCAAAAGGCCAGCCGCGT	0.00	0.33	0.86	0.00	0.34
GATCAAAAGGCCCTGCTCGA	0.00	0.00	0.00	0.00	0.34
GATCAAAAGGCCGCCACGTT	0.00	0.00	0.00	0.00	0.69
GATCAAAAGGCCGGAAAANN	0.00	0.00	0.00	0.00	0.00

GATCAAAAGGCCGGGGTGGA	0.00	0.33	0.00	0.00	0.00
GATCAAAAGGCCGTTTAAAA	0.00	0.33	0.00	0.00	0.00
GATCAAAAGGCCTGGCAGAC	0.00	0.00	0.00	0.00	0.34
GATCAAAAGGCCTGGTCGCT	0.00	0.33	0.00	0.68	0.34
GATCAAAAGGCGAAAAAAAA	0.69	0.00	0.57	0.34	0.00
GATCAAAAGGCGAAGCGGGA	4.35	1.95	0.57	1.36	1.38
GATCAAAAGGCGAAGCGGGG	0.00	0.00	0.00	0.34	0.00
GATCAAAAGGCGAAGCGGNN	0.23	0.00	0.00	0.00	0.00
GATCAAAAGGCGACGAAAAG	0.23	0.00	0.00	0.00	0.00
GATCAAAAGGCGAGGCATTG	0.00	0.00	0.00	0.00	0.00
GATCAAAAGGCGATGAAAAA	6.64	3.25	4.30	7.13	4.13
GATCAAAAGGCGATGAAAAG	3.89	1.63	2.58	3.39	0.34
GATCAAAAGGCGATGAAANN	0.23	0.00	0.00	0.00	0.00
GATCAAAAGGCGATGAACNN	0.23	0.00	0.00	0.00	0.00
GATCAAAAGGCGATGCAAAA	0.00	0.00	0.29	0.00	0.00
GATCAAAAGGCGATGGAAAA	0.00	0.00	0.29	0.00	0.00
GATCAAAAGGCGCCTGAAGC	0.00	0.00	0.00	0.00	0.34
GATCAAAAGGCGCTGAAAAG	0.23	0.00	0.00	0.00	0.00
GATCAAAAGGCGGAAAAAAA	0.00	0.33	0.57	0.00	0.34
GATCAAAAGGCGGAAATTAA	0.00	0.00	0.00	0.34	0.00
GATCAAAAGGCGGAAGAAGA	0.00	0.00	0.00	0.00	0.00
GATCAAAAGGCGGAAGGAAT	0.00	0.00	0.00	0.00	0.00
GATCAAAAGGCGGAAGGACT	1.60	0.65	0.29	1.70	0.69
GATCAAAAGGCGGATATGTT	0.00	0.00	0.00	0.00	0.69
GATCAAAAGGCGGCAACAAT	0.00	0.00	0.00	0.00	0.34
GATCAAAAGGCGGGAAAAAA	0.23	0.33	0.29	0.00	1.03
GATCAAAAGGCGGGAACAAA	1.37	1.63	0.29	1.70	1.03
GATCAAAAGGCGGGAACAAG	5.95	4.23	2.29	7.81	2.76
GATCAAAAGGCGGGAACANG	0.00	0.00	0.00	0.00	0.00
GATCAAAAGGCGGGAACCTG	0.00	0.00	0.00	0.00	0.00
GATCAAAAGGCGGGATATCA	0.00	0.33	0.00	0.00	0.00
GATCAAAAGGCGTCAGGCTA	0.00	0.00	0.00	0.34	0.00
GATCAAAAGGCGTGTGGACT	0.23	0.00	0.00	0.00	0.00
GATCAAAAGGCTACCGGGCA	0.00	0.00	0.00	0.34	0.00
GATCAAAAGGCTAGGAATGG	0.00	0.98	0.00	0.00	0.00
GATCAAAAGGCTCAAAAAAA	0.46	0.33	0.00	0.68	0.00
GATCAAAAGGCTCACCTATC	0.46	0.00	0.00	0.68	0.00

GATCAAAAGGCTCACTTATC	0.23	0.00	0.00	0.00	0.00
GATCAAAAGGCTCCAAAAAA	0.92	0.65	0.00	0.34	0.69
GATCAAAAGGCTGACGACGT	0.00	0.00	0.00	0.00	1.03
GATCAAAAGGCTGCAGAAGA	0.00	0.00	0.00	0.00	0.00
GATCAAAAGGCTTGAGAGGA	0.00	0.00	0.29	0.00	0.00
GATCAAAAGGCTTGAGCCGG	5.72	1.95	1.72	2.38	1.72
GATCAAAAGGGAAAAAAAAA	6.64	2.28	0.57	4.75	1.72
GATCAAAAGGGAAAAAAAAT	0.00	0.00	0.00	0.34	0.00
GATCAAAAGGGAAAAAGAAA	0.00	0.00	0.00	0.00	0.00
GATCAAAAGGGAAACAATTC	0.00	0.00	0.00	0.00	0.34
GATCAAAAGGGAAAGAAAAA	0.00	0.00	0.00	0.00	0.34
GATCAAAAGGGAAAGGGTAG	0.00	0.33	0.00	0.00	0.00
GATCAAAAGGGAACAAAAAA	0.00	0.00	0.29	0.00	0.00
GATCAAAAGGGAACAGAACA	0.00	0.00	0.00	0.00	0.69
GATCAAAAGGGAAGAGCAGC	0.00	0.00	0.00	0.00	0.00
GATCAAAAGGGAATGCTGAC	0.00	0.33	1.15	0.00	0.00
GATCAAAAGGGACAAAAAAA	1.83	0.00	0.29	1.02	0.69
GATCAAAAGGGACAAGCATA	0.00	0.00	0.00	0.00	0.00
GATCAAAAGGGACAAGCATC	0.00	0.00	0.00	0.00	0.00
GATCAAAAGGGACACAAAAA	0.00	0.00	0.00	0.68	0.00
GATCAAAAGGGACACAAAGA	0.00	0.33	0.00	0.00	0.00
GATCAAAAGGGACACAATTC	15.34	12.36	4.30	9.50	13.09
GATCAAAAGGGACACACTTC	0.00	0.00	0.00	0.00	0.00
GATCAAAAGGGACATAATTC	0.00	0.00	0.00	0.00	0.00
GATCAAAAGGGACCAAAAAA	3.43	0.65	1.72	2.38	1.72
GATCAAAAGGGACCAAGGCT	0.46	0.33	0.00	0.68	0.00
GATCAAAAGGGACCCAATTC	0.23	0.00	0.00	0.00	0.00
GATCAAAAGGGACCGAAAAA	0.00	0.00	0.00	0.00	0.34
GATCAAAAGGGACCGCAAAA	0.00	0.33	0.00	0.00	0.00
GATCAAAAGGGATAAAAAAA	1.60	0.00	0.57	1.02	0.69
GATCAAAAGGGATACAAAAA	0.00	0.98	0.00	0.00	0.00
GATCAAAAGGGATACAAGTC	0.23	0.00	0.00	0.00	0.00
GATCAAAAGGGATACAATTC	21.97	10.41	3.44	11.54	6.54
GATCAAAAGGGATACAGAAA	0.00	0.00	0.00	1.02	0.00
GATCAAAAGGGATACAGTTC	0.23	0.00	0.00	0.00	0.00
GATCAAAAGGGATACCATTC	0.00	0.00	0.00	0.00	0.00
GATCAAAAGGGATACTCGTA	0.00	0.00	0.00	0.00	0.00

GATCAAAAGGGATCCCATTC	0.00	0.00	0.00	0.00	0.00
GATCAAAAGGGCAATGCTGT	0.46	0.00	0.00	0.00	0.00
GATCAAAAGGGCACAGCCGC	0.00	0.00	0.00	0.00	0.00
GATCAAAAGGGCACGTTTGC	0.00	0.33	0.00	0.00	0.00
GATCAAAAGGGCCAAAAAAA	0.00	0.00	0.00	0.00	0.34
GATCAAAAGGGCCCAGGAGC	0.00	0.00	0.00	0.34	0.00
GATCAAAAGGGCTCGCATTG	0.00	0.00	0.00	0.00	0.69
GATCAAAAGGGCTGAGGCAC	0.00	0.33	0.00	0.00	0.00
GATCAAAAGGGGAAAAAAAA	0.23	0.00	0.00	0.00	0.00
GATCAAAAGGGGAAAAAAAC	0.23	0.00	0.00	0.00	0.00
GATCAAAAGGGGAAACAAAA	0.00	0.33	0.00	0.34	0.34
GATCAAAAGGGGAAAGGGGG	0.00	0.33	0.00	0.00	0.00
GATCAAAAGGGGAATGACCA	0.00	0.00	0.00	0.00	0.34
GATCAAAAGGGGAGGAGGCG	0.00	0.00	0.57	0.00	0.69
GATCAAAAGGGGAGGTAAAC	0.00	0.00	0.29	0.00	0.00
GATCAAAAGGGGCAAAAATA	0.23	0.00	0.00	0.00	0.00
GATCAAAAGGGGCACAATTC	0.23	0.00	0.00	0.34	0.00
GATCAAAAGGGGCAGAGGGC	0.23	0.00	0.00	0.00	0.00
GATCAAAAGGGGCCAAAAAA	0.00	0.00	0.00	0.00	0.00
GATCAAAAGGGGCCTCCTCT	0.00	0.00	0.00	0.34	0.00
GATCAAAAGGGGTAAGAGGA	0.00	0.00	0.00	0.00	0.34
GATCAAAAGGGGTACAATTC	0.46	0.00	0.00	0.00	0.00
GATCAAAAGGGGTACAGTTC	0.23	0.00	0.00	0.00	0.00
GATCAAAAGGGGTGGCCAAA	0.00	0.00	0.29	0.00	0.00
GATCAAAAGGGTAAAAAAAA	1.83	0.65	0.00	0.00	0.34
GATCAAAAGGGTAAAAAANN	0.23	0.00	0.00	0.00	0.00
GATCAAAAGGGTAAGCAGCC	0.23	0.00	0.00	0.00	0.00
GATCAAAAGGGTATCTTGGA	0.23	0.00	0.00	0.00	0.00
GATCAAAAGGGTATCTTGGG	0.69	0.00	0.00	0.00	0.00
GATCAAAAGGGTCAAAAAAA	13.05	4.23	2.86	6.79	4.82
GATCAAAAGGGTCAAAAATC	0.00	0.00	0.00	0.00	0.34
GATCAAAAGGGTCAAACAAA	0.46	0.00	0.00	0.00	0.00
GATCAAAAGGGTCAAAGAAA	0.00	0.00	0.00	0.34	0.34
GATCAAAAGGGTCAACTGTA	0.00	0.00	0.00	0.00	0.34
GATCAAAAGGGTCAATGGTC	0.00	0.33	0.00	0.00	0.00
GATCAAAAGGGTCACAAAAA	0.92	0.00	0.00	0.00	0.00
GATCAAAAGGGTCAGACACG	0.00	0.00	0.57	0.00	0.00

GATCAAAAGGGTCAGATGGA	16.48	11.39	4.58	3.39	5.85
GATCAAAAGGGTCAGATGGC	0.23	0.33	0.00	0.00	0.00
GATCAAAAGGGTCAGATGNN	0.23	0.00	0.00	0.00	0.00
GATCAAAAGGGTCATAAAAA	0.23	0.00	0.00	0.00	0.00
GATCAAAAGGGTCGGATGGA	0.00	0.00	0.00	0.00	0.34
GATCAAAAGGGTGAGCAGCN	0.23	0.00	0.00	0.00	0.00
GATCAAAAGGGTGAGGTGTT	0.46	0.00	0.00	0.00	0.00
GATCAAAAGGGTGCCACGGA	0.23	0.65	0.29	0.00	0.00
GATCAAAAGGGTGTGCACGC	0.00	0.00	0.00	0.00	0.69
GATCAAAAGGGTTGAATTCT	0.00	0.00	0.00	0.00	0.00
GATCAAAAGGGTTGGGCATC	0.92	0.65	0.29	0.00	0.34
GATCAAAAGGTAAAAAAAAA	0.00	0.00	0.00	0.00	0.00
GATCAAAAGGTAGTTAAAAG	0.23	0.00	0.00	0.00	0.00
GATCAAAAGGTATACAAATC	0.00	0.33	0.00	0.00	0.00
GATCAAAAGGTCAAAAAAAA	0.00	0.33	0.29	0.34	0.34
GATCAAAAGGTCACCTCCAA	0.00	0.00	0.29	0.00	0.00
GATCAAAAGGTCAGATAGGC	0.00	0.00	0.29	0.00	0.00
GATCAAAAGGTCAGATTGGA	0.00	0.33	0.29	0.68	0.00
GATCAAAAGGTCAGATTGGC	3.20	3.90	4.87	6.79	4.48
GATCAAAAGGTCAGCAGTCT	0.00	0.00	0.00	0.00	0.34
GATCAAAAGGTCCGCTGGCT	0.00	0.00	0.00	0.00	0.34
GATCAAAAGGTCGGACATGA	0.00	0.00	0.00	0.68	0.00
GATCAAAAGGTCGGACCATG	2.52	1.95	0.00	2.38	0.00
GATCAAAAGGTCTACGAGTT	0.23	0.00	0.00	0.00	0.00
GATCAAAAGGTCTGCAAAAA	0.00	0.00	0.00	0.00	0.00
GATCAAAAGGTCTTGGTCTT	0.00	0.00	0.00	0.00	0.00
GATCAAAAGGTGAAGTCCAC	0.00	0.00	0.00	0.00	0.00
GATCAAAAGGTGAAGTCCGA	0.00	0.65	0.57	0.00	0.34
GATCAAAAGGTGCAATATGT	0.00	0.00	0.00	0.00	0.00
GATCAAAAGGTGCAGAAGGA	0.92	0.65	0.57	0.00	1.03
GATCAAAAGGTGCAGGTATA	0.00	0.00	0.57	0.00	0.00
GATCAAAAGGTGCAGTGGGA	0.23	0.00	0.00	0.00	0.00
GATCAAAAGGTGCGCCAAAA	0.00	0.33	0.00	0.00	0.00
GATCAAAAGGTGGCTGAGCT	0.00	0.33	0.00	0.00	0.00
GATCAAAAGGTGTAGAATCT	0.00	0.00	0.00	0.00	0.00
GATCAAAAGGTGTGGGAGAG	0.23	0.00	0.00	0.00	0.00
GATCAAAAGGTTAAGATGGA	0.00	0.33	0.00	0.00	0.00

GATCAAAAGGTTGAGGAAGT	0.00	0.00	0.00	0.00	0.00
GATCAAAAGGTTGGCTGTGA	0.69	0.00	0.00	0.00	0.00
GATCAAAAGGTTTGAGGTTC	0.00	0.00	0.00	0.00	0.34
GATCAAAAGGTTTTATAACG	0.23	0.00	0.00	0.00	0.00
GATCAAAAGTAAAAAAAAAA	1.14	0.00	0.00	1.02	1.03
GATCAAAAGTAAGAAAAAAA	0.46	0.00	0.00	0.00	0.00
GATCAAAAGTAAGACGTATG	0.00	0.00	0.00	0.00	0.00
GATCAAAAGTAAGATAAAAA	0.00	0.00	0.29	0.00	0.00
GATCAAAAGTAATCTGTAAT	0.23	0.00	0.00	0.34	0.00
GATCAAAAGTAATTCTATAT	0.00	0.00	0.00	0.34	0.00
GATCAAAAGTACACCGACTG	0.69	0.00	0.29	0.00	0.00
GATCAAAAGTACACCTGCAA	0.00	0.00	0.00	0.00	0.69
GATCAAAAGTACCCAACAAC	0.00	0.00	0.00	0.00	0.00
GATCAAAAGTACCCCAAAAA	0.00	0.33	0.00	0.00	0.00
GATCAAAAGTACCCCAAAGT	0.00	0.33	0.00	0.00	0.00
GATCAAAAGTACGAGAACTC	0.00	0.00	0.29	0.00	0.00
GATCAAAAGTACGCAAAAAA	0.23	0.00	0.00	0.34	0.00
GATCAAAAGTACGCAAGCAA	0.00	0.00	0.29	0.00	0.69
GATCAAAAGTACGCAAGCCA	1.60	1.30	0.57	1.02	1.03
GATCAAAAGTACGTAAGCAA	0.92	0.00	0.29	0.00	0.00
GATCAAAAGTACGTAAGCCA	0.00	0.33	0.00	0.00	0.00
GATCAAAAGTACGTAAGCTC	0.00	0.33	0.00	0.00	0.00
GATCAAAAGTACGTAAGNNN	0.23	0.00	0.00	0.00	0.00
GATCAAAAGTACGTGAGCCA	0.00	0.00	0.29	0.00	0.00
GATCAAAAGTACGTGCCTGC	0.00	0.00	0.00	0.00	0.00
GATCAAAAGTAGCAGCGCTC	0.00	0.00	0.29	0.00	0.00
GATCAAAAGTAGGAGGAGTA	0.00	0.00	0.29	0.00	0.00
GATCAAAAGTAGGAGGATGA	0.00	0.00	0.57	0.00	0.34
GATCAAAAGTAGTCAGTCAA	0.00	0.00	0.29	0.00	0.00
GATCAAAAGTAGTGTCCGCA	0.00	0.00	0.29	0.00	0.00
GATCAAAAGTATATGGCCAC	0.00	0.00	0.29	0.00	0.00
GATCAAAAGTATCGAACGTA	0.00	0.00	0.00	0.00	0.34
GATCAAAAGTATGAAAAAAA	0.00	0.00	0.00	0.00	0.00
GATCAAAAGTATGAAGACCA	0.00	0.00	0.00	0.00	0.00
GATCAAAAGTATGAAGAGAA	0.00	0.00	0.29	0.00	0.00
GATCAAAAGTATGCCGTCCC	0.00	0.00	0.29	0.00	0.00
GATCAAAAGTATGGGCAAAA	0.46	0.00	0.00	0.68	0.00

GATCAAAAGTATGGGCACAA	0.00	0.00	0.29	0.00	0.00
GATCAAAAGTATGGGCATTA	0.23	0.00	0.00	0.00	0.00
GATCAAAAGTATTGCTGGCC	0.23	0.00	0.00	1.02	0.00
GATCAAAAGTATTTGATGGG	0.46	0.00	0.00	0.00	0.00
GATCAAAAGTCAAAAAAAAA	0.00	0.65	0.00	0.34	0.00
GATCAAAAGTCAAGAAGCAA	0.00	0.33	0.00	0.00	0.34
GATCAAAAGTCAATTCAACG	0.00	0.00	0.00	0.00	0.00
GATCAAAAGTCAATTCATCG	10.07	12.36	14.60	13.58	17.22
GATCAAAAGTCATGTACACG	0.00	0.00	0.00	0.00	0.34
GATCAAAAGTCCAAAAAAAA	0.00	0.00	0.00	0.00	0.00
GATCAAAAGTCCGGAGCTGN	0.00	0.00	0.00	0.00	0.00
GATCAAAAGTCCGGAGCTGT	0.92	0.65	0.00	1.02	0.00
GATCAAAAGTCCGTTCTGAA	0.00	0.00	0.00	0.00	0.00
GATCAAAAGTCCTCCTGCTA	0.00	0.00	0.00	0.34	0.00
GATCAAAAGTCGATCCATCG	0.23	0.00	0.00	0.00	0.00
GATCAAAAGTCGGAGGCCCT	0.00	0.00	0.00	0.00	0.00
GATCAAAAGTCGTGCGATTC	0.00	0.33	0.00	0.00	0.00
GATCAAAAGTCTCTGTGTCA	0.00	0.00	0.00	0.00	0.00
GATCAAAAGTCTTAAAAAAA	0.00	0.00	0.29	0.00	0.00
GATCAAAAGTCTTGCTGGCC	0.00	0.65	0.00	0.34	3.10
GATCAAAAGTGAAAAAAAAA	0.69	0.00	0.00	0.68	0.00
GATCAAAAGTGAAGGTTTCG	0.46	0.33	0.29	0.68	0.00
GATCAAAAGTGAATTCATCG	0.00	0.00	0.29	0.00	0.00
GATCAAAAGTGACAAAAAAA	0.00	0.00	0.00	0.00	0.00
GATCAAAAGTGATAAAAAAA	0.00	0.00	0.00	0.00	0.34
GATCAAAAGTGATAGCCAAA	0.23	0.00	0.00	0.00	0.00
GATCAAAAGTGATAGCCGAA	0.46	0.00	0.00	0.00	0.00
GATCAAAAGTGATAGCCGAC	0.23	0.00	0.00	0.00	0.00
GATCAAAAGTGATAGCCGAG	0.46	1.30	0.57	1.36	0.34
GATCAAAAGTGATAGCCGTG	0.23	0.00	0.00	0.00	0.00
GATCAAAAGTGCAGAGAGAG	0.00	0.00	0.00	0.00	0.00
GATCAAAAGTGCAGCCATGG	0.00	0.00	0.00	0.00	0.34
GATCAAAAGTGCGAGTGATG	0.00	0.00	0.00	0.34	0.00
GATCAAAAGTGCTGGGCTCA	0.00	0.00	0.00	0.00	0.34
GATCAAAAGTGGCAAAAAAA	0.00	0.00	0.00	0.34	0.00
GATCAAAAGTGGCATCAAGG	0.00	0.00	0.00	0.00	0.34
GATCAAAAGTGGCATCAAGT	0.00	0.00	0.00	0.34	0.00

GATCAAAAGTGGCCACAAGC	0.00	0.00	0.29	0.00	0.00
GATCAAAAGTGGCCACATAA	0.00	0.00	0.00	0.00	0.34
GATCAAAAGTGGCCACATAC	1.37	1.30	3.72	4.75	4.48
GATCAAAAGTGGCCACATGC	0.92	4.23	10.60	10.52	10.33
GATCAAAAGTGGCCACGTGC	0.00	0.00	0.29	0.00	0.34
GATCAAAAGTGGCCACTTGC	0.00	0.00	0.00	1.02	0.00
GATCAAAAGTGGCCATACCA	0.00	0.00	0.00	0.00	0.34
GATCAAAAGTGGCCGCATGC	0.00	0.00	0.29	0.00	0.00
GATCAAAAGTGGGGGCAAAC	0.00	0.00	0.00	0.00	0.34
GATCAAAAGTGGTCACATAC	0.69	0.00	0.00	0.00	0.00
GATCAAAAGTGGTGCAAAAA	0.00	0.33	0.00	0.00	0.00
GATCAAAAGTGGTGCAAAAT	0.00	0.00	0.00	0.00	0.34
GATCAAAAGTGGTGCAAATG	5.04	1.30	0.29	1.36	1.72
GATCAAAAGTGTCTTGGGTT	0.46	0.00	0.00	0.00	0.00
GATCAAAAGTGTGACCTTGC	0.00	0.00	0.00	0.34	0.00
GATCAAAAGTGTGCCAAGGC	0.23	0.00	0.00	0.00	0.00
GATCAAAAGTTAACTGGACC	0.00	0.00	0.00	0.00	0.00
GATCAAAAGTTAACTGGACT	0.00	0.00	0.00	0.00	0.00
GATCAAAAGTTGAAAAAAAA	0.23	0.00	0.29	0.00	0.00
GATCAAAAGTTGAAGAATTT	0.00	0.00	0.00	0.00	0.00
GATCAAAAGTTGCCAAAATC	0.00	0.00	0.00	0.00	1.03
GATCAAAAGTTGGAGAATTT	0.00	0.00	0.00	0.00	0.34
GATCAAAAGTTGGTGCAGAC	0.00	0.00	0.00	0.00	0.00
GATCAAAAGTTGTAAAAAAA	3.43	0.00	0.00	0.68	0.00
GATCAAAAGTTGTAAAAAGA	0.00	0.00	0.00	0.00	0.34
GATCAAAAGTTGTAGAAAAA	1.37	0.00	0.00	0.00	1.03
GATCAAAAGTTGTAGAAAAN	0.23	0.00	0.00	0.00	0.00
GATCAAAAGTTGTAGAAATC	0.00	0.00	0.00	0.00	0.34
GATCAAAAGTTGTAGAATAA	0.69	0.00	0.00	0.00	0.00
GATCAAAAGTTGTAGAATTC	0.23	0.00	0.29	0.00	0.00
GATCAAAAGTTGTAGAATTG	0.23	0.00	0.00	0.00	0.00
GATCAAAAGTTGTAGAATTN	0.00	0.00	0.00	0.00	0.00
GATCAAAAGTTGTAGAATTT	49.90	12.36	28.64	17.65	42.02
GATCAAAAGTTGTAGACTTG	0.00	0.00	0.29	0.00	0.00
GATCAAAAGTTGTAGANNNN	0.23	0.00	0.00	0.00	0.00
GATCAAAAGTTGTATCGTAT	0.00	0.00	0.29	0.00	0.00
GATCAAAAGTTGTCTCGGAT	0.00	0.00	0.00	0.34	0.00

GATCAAAAGTTTAACACAGA	0.00	0.00	0.00	0.68	0.00
GATCAAAAGTTTGAAAAAAA	0.00	0.00	0.00	1.02	0.00
GATCAAAAGTTTGAGGACGG	0.23	0.00	0.00	0.00	0.00
GATCAAAATAAAAAAAAAAA	0.69	0.65	0.86	0.68	2.41
GATCAAAATAAAAAAAAAAT	0.00	0.00	0.29	0.34	0.34
GATCAAAATAAAAAAAAGAA	0.00	0.00	0.00	0.00	1.03
GATCAAAATAAAAAATAAAA	0.46	0.00	0.00	0.00	0.00
GATCAAAATAAAAAATGAAT	0.00	0.33	0.00	0.00	0.00
GATCAAAATAAAAACACTTG	0.23	0.00	0.00	0.00	0.00
GATCAAAATAAAACTAGCAA	0.00	0.33	0.00	0.00	0.00
GATCAAAATAAAACTGGCAA	0.00	0.00	0.00	0.00	0.34
GATCAAAATAAAATTTTGAA	0.23	0.00	0.00	0.00	0.00
GATCAAAATAAACAAAATAA	0.00	0.00	0.29	0.00	0.00
GATCAAAATAAACAGCTCTG	0.00	0.00	0.00	0.00	0.00
GATCAAAATAAACTTTTGAA	1.14	0.00	0.00	0.00	0.00
GATCAAAATAAACTTTTGNN	0.23	0.00	0.00	0.00	0.00
GATCAAAATAACAATGTCTC	1.14	0.00	0.00	0.34	0.00
GATCAAAATAACAGCCAAAT	0.23	0.00	0.00	0.00	0.00
GATCAAAATAACATCCCACG	0.00	0.00	0.00	0.00	0.00
GATCAAAATAACTGTGTCGT	0.00	0.00	0.29	0.00	0.00
GATCAAAATAATCCGCGCCA	0.46	0.98	0.57	0.00	0.34
GATCAAAATAATGACTGTAT	0.00	0.00	0.29	0.00	0.00
GATCAAAATAATGTAGAGGG	0.00	0.00	0.00	0.00	0.00
GATCAAAATACAACTATCCA	0.23	0.00	0.00	0.00	0.00
GATCAAAATACAAGAAAAAA	0.00	0.65	0.00	0.00	0.00
GATCAAAATACACAACAGAA	0.00	0.00	0.00	0.00	0.34
GATCAAAATACACTGCGAGC	0.00	0.00	0.00	0.00	0.00
GATCAAAATACAGAATTTAG	0.00	0.00	0.00	0.00	0.00
GATCAAAATACAGCGAGCCG	0.00	0.65	0.00	0.00	0.00
GATCAAAATACAGGTGTTCG	0.00	0.00	0.00	0.00	0.00
GATCAAAATACATTCCCTTA	0.00	0.00	0.00	0.00	0.34
GATCAAAATACGTGAGCACG	0.00	0.00	0.00	0.34	0.00
GATCAAAATACTGCCTATAC	0.00	0.00	0.57	0.00	0.00
GATCAAAATACTTGCTTCCC	0.00	0.33	0.00	0.00	0.00
GATCAAAATAGAACGAAGGC	0.00	0.00	0.00	0.00	0.34
GATCAAAATAGAACGCAGAA	0.00	0.00	0.00	0.68	0.00
GATCAAAATAGAACGCAGGA	0.23	0.00	0.00	0.00	0.34

GATCAAAATAGAACGGAGGA	0.23	0.00	0.00	0.00	0.00
GATCAAAATAGAACGGCCAG	0.00	0.00	0.00	0.00	0.00
GATCAAAATAGAACGTAAAA	0.00	0.00	0.00	0.34	0.00
GATCAAAATAGAACGTACAA	0.00	0.00	0.00	0.34	0.00
GATCAAAATAGAACGTAGAA	0.23	0.00	0.00	0.00	0.00
GATCAAAATAGAACGTAGCA	0.00	0.00	0.00	0.00	0.00
GATCAAAATAGAACGTAGGA	13.28	9.43	8.88	16.63	11.02
GATCAAAATAGAACGTAGGC	0.00	0.00	0.00	0.00	0.34
GATCAAAATAGAACGTAGGG	0.00	0.00	0.00	0.00	0.00
GATCAAAATAGAACGTAGGN	0.00	0.00	0.00	0.00	0.00
GATCAAAATAGAACGTAGNN	0.23	0.00	0.00	0.34	0.00
GATCAAAATAGAACGTAGTA	0.00	0.00	0.86	0.00	0.00
GATCAAAATAGAACGTCCAA	0.00	0.00	0.29	0.00	0.00
GATCAAAATAGAACGTCGGA	0.00	0.00	0.00	0.34	0.00
GATCAAAATAGAACGTGGGA	0.00	0.00	0.00	0.00	0.00
GATCAAAATAGAACGTGGGG	0.23	0.00	0.00	0.00	0.00
GATCAAAATAGAACTGCGGA	0.00	0.00	0.00	0.00	0.00
GATCAAAATAGAGCGTAGGA	0.00	0.33	0.00	0.00	0.00
GATCAAAATAGCCAAACGCA	0.00	0.00	0.00	0.00	1.72
GATCAAAATAGCGAAGCGTG	0.23	0.00	0.00	0.00	0.00
GATCAAAATAGCGTGGTCTC	0.00	0.00	0.29	0.34	0.34
GATCAAAATAGCTATTGCTA	0.00	0.00	0.00	0.00	0.00
GATCAAAATAGTTTGAAGGA	0.00	0.00	0.00	0.00	0.00
GATCAAAATATACAGTGGTT	0.23	0.00	0.00	0.00	0.00
GATCAAAATATGACCACAAG	0.00	0.33	0.00	0.00	0.00
GATCAAAATATGTCTTCATT	0.00	0.00	0.00	0.00	0.34
GATCAAAATATTCTTGTAAC	0.00	0.00	0.00	0.00	0.00
GATCAAAATATTGACAAACT	0.00	0.00	0.00	0.00	0.34
GATCAAAATATTGAGGAGAG	0.46	0.00	0.00	0.00	0.00
GATCAAAATATTGAGGATAA	0.00	0.33	0.29	0.00	0.00
GATCAAAATATTGAGGCTGA	0.00	0.00	0.00	0.00	0.00
GATCAAAATATTGAGTGCAG	0.00	0.00	0.57	0.00	0.00
GATCAAAATATTGGATAAGA	0.00	0.00	0.29	0.00	0.00
GATCAAAATCAAAAAAAAAA	23.35	3.58	6.30	5.43	4.48
GATCAAAATCAAAAAAAAAT	0.23	0.00	0.00	0.34	0.00
GATCAAAATCAAAAAAAANN	0.23	0.00	0.00	0.00	0.00
GATCAAAATCAAAAAGTCCA	0.23	0.00	0.00	0.00	0.00

GATCAAAATCAAAACAAAAA	0.00	0.00	0.00	0.00	0.00
GATCAAAATCAAAACAAACC	0.00	0.00	0.00	0.00	0.34
GATCAAAATCAAAAGCAAAA	0.69	0.00	0.00	0.00	0.00
GATCAAAATCAAACAAAAAA	0.23	0.00	0.00	0.00	0.00
GATCAAAATCAAACTCAAAA	0.00	0.00	0.00	0.00	0.34
GATCAAAATCAAACTCTGCG	0.00	0.00	0.00	0.00	0.00
GATCAAAATCAACGCAGGAA	0.23	0.00	0.00	0.00	0.00
GATCAAAATCAAGAGGAAGG	0.00	0.00	0.00	0.00	0.00
GATCAAAATCAAGAGTACTT	0.00	0.00	0.29	0.00	0.00
GATCAAAATCAAGCAGACAA	0.00	0.00	0.00	0.68	0.00
GATCAAAATCAAGCGGTGAG	0.23	0.00	0.00	0.00	0.00
GATCAAAATCAAGCGGTGGG	0.23	0.00	0.00	0.00	0.00
GATCAAAATCAAGGGGGCTT	0.23	0.00	0.00	0.00	0.00
GATCAAAATCAATCAAATTA	0.00	0.00	0.29	0.00	0.00
GATCAAAATCAATCGTATGC	0.46	0.00	0.00	0.00	0.00
GATCAAAATCACAAAAAACA	0.00	0.33	0.00	0.00	0.00
GATCAAAATCACCGCATTCA	0.00	0.00	0.29	0.00	0.00
GATCAAAATCACGACGATGA	0.00	0.00	0.00	0.34	0.00
GATCAAAATCACGACGATGG	1.37	4.88	1.72	3.39	1.72
GATCAAAATCACTCCCAAAA	0.00	0.00	0.00	0.00	0.00
GATCAAAATCACTCCCAAGT	0.23	0.00	0.00	0.00	0.00
GATCAAAATCACTTTTAAAA	0.00	0.00	0.00	0.00	0.69
GATCAAAATCAGACAGCATT	0.00	0.00	0.57	0.00	0.00
GATCAAAATCAGAGGAAAAA	0.00	0.00	0.00	0.00	0.34
GATCAAAATCAGCTAAAAAA	0.00	0.00	0.00	0.00	0.69
GATCAAAATCAGCTACGTGA	0.00	0.00	0.00	0.34	0.00
GATCAAAATCAGCTACTTGA	3.43	1.95	2.58	1.02	3.10
GATCAAAATCAGCTACTTGT	0.00	0.00	0.29	0.00	0.00
GATCAAAATCAGCTCCTTGA	0.00	0.00	0.00	0.00	0.34
GATCAAAATCAGGCCTTGCC	0.00	0.00	0.00	0.34	0.00
GATCAAAATCAGGCTGAGCA	0.00	0.00	0.00	0.00	0.34
GATCAAAATCATAAAAAAAA	0.00	0.00	0.00	0.00	0.00
GATCAAAATCATAGCGGATG	0.00	0.00	0.00	0.34	0.00
GATCAAAATCATCATCAAAA	0.00	0.00	0.00	0.00	0.00
GATCAAAATCATCATCAAAG	0.00	0.00	0.57	0.00	0.00
GATCAAAATCATCATCATAA	0.46	0.00	0.00	0.00	0.00
GATCAAAATCATCTCCAGCG	0.00	0.00	0.86	0.00	0.34

GATCAAAATCATCTTCATCC	0.00	0.00	0.29	0.00	0.00
GATCAAAATCATGACGACGA	0.00	0.00	0.00	0.34	0.00
GATCAAAATCATGATGGTGT	0.00	0.00	0.00	0.34	0.00
GATCAAAATCATGGTTTAGA	0.46	0.33	0.29	0.00	0.00
GATCAAAATCATTGTATTCT	0.00	0.00	0.00	0.00	0.00
GATCAAAATCATTTAAACTC	0.00	0.00	0.00	0.00	0.00
GATCAAAATCATTTGGCGCA	0.00	0.00	0.29	0.00	0.00
GATCAAAATCCAATGCAGTG	0.00	0.33	0.00	0.00	0.00
GATCAAAATCCAGAACAAGC	0.00	0.00	0.00	0.68	0.00
GATCAAAATCCAGCACCCCA	0.00	0.00	0.57	0.00	0.00
GATCAAAATCCAGTGCCGCG	0.00	0.00	0.00	0.00	0.00
GATCAAAATCCAGTGCCGTG	0.00	0.00	0.00	0.00	0.00
GATCAAAATCCCAAGCGCTC	0.00	0.00	0.00	1.02	0.00
GATCAAAATCCCAGAAACAA	0.00	0.00	0.00	0.00	0.34
GATCAAAATCCCCAGCCGCG	0.00	0.33	0.29	0.34	0.00
GATCAAAATCCCCAGCTGCT	0.23	0.00	0.00	0.00	0.00
GATCAAAATCCCCATAGGGA	0.00	0.33	0.00	0.00	0.00
GATCAAAATCCCGACATGTG	0.69	0.00	0.00	0.00	0.34
GATCAAAATCCCGAGCTAAG	0.00	0.00	0.57	0.00	0.00
GATCAAAATCCCGAGCTACG	0.00	0.00	0.29	0.00	0.00
GATCAAAATCCGCCCTTGGT	0.00	0.00	0.29	0.00	0.00
GATCAAAATCCGGGGCCGCA	0.00	0.00	0.00	0.00	0.00
GATCAAAATCCTATTTAGAA	0.23	0.00	0.00	0.00	0.00
GATCAAAATCCTCCCGGACG	0.00	0.00	0.00	0.34	0.00
GATCAAAATCCTCCTTTAAA	0.00	0.00	0.00	0.00	0.00
GATCAAAATCCTCTCAATCC	0.00	0.00	0.00	0.00	0.34
GATCAAAATCCTCTCCAGCC	0.00	0.00	0.29	0.00	0.34
GATCAAAATCCTCTCCTTGC	0.00	0.00	0.00	0.00	0.34
GATCAAAATCCTGCTGCCTA	0.00	0.00	0.00	0.34	0.00
GATCAAAATCCTGCTGCCTG	0.00	0.00	0.00	0.34	0.34
GATCAAAATCCTGGCATCTT	0.00	0.00	0.00	0.34	0.00
GATCAAAATCCTGGCTATTT	0.00	0.00	0.29	0.00	0.00
GATCAAAATCCTGGTCAGGA	0.23	0.00	0.00	0.00	0.00
GATCAAAATCCTTGAGGACT	0.00	0.33	0.00	0.00	0.00
GATCAAAATCGAAAAAAAAA	0.00	0.00	0.00	0.00	0.00
GATCAAAATCGACGTGGGCG	0.00	0.00	0.00	0.00	0.34
GATCAAAATCGAGCTTGCTG	0.23	0.00	0.57	0.34	0.00

GATCAAAATCGCCTTCAACG	0.00	0.00	0.00	0.00	0.34
GATCAAAATCGCCTTCAGTG	0.00	0.65	0.29	0.00	0.69
GATCAAAATCGCTCTTGACA	0.00	0.00	0.00	0.00	0.00
GATCAAAATCGCTGGGTCAT	0.00	0.00	0.00	0.00	0.34
GATCAAAATCGCTGGTTGCG	0.00	0.00	0.00	0.00	0.00
GATCAAAATCGCTTCCGCGG	0.00	0.00	0.00	0.00	0.34
GATCAAAATCGCTTCTTGAG	0.00	0.00	0.29	0.00	0.00
GATCAAAATCGGCGTATGCC	0.00	0.00	0.00	0.00	1.03
GATCAAAATCGGCTAAAAAA	0.00	0.00	0.29	0.00	0.00
GATCAAAATCGGTACGTGAC	0.46	0.00	0.00	0.00	0.00
GATCAAAATCGTATGCCGTC	0.00	0.00	0.00	0.00	0.00
GATCAAAATCGTCGAGGTCA	0.00	0.00	0.00	0.34	0.00
GATCAAAATCGTGGTGTTAC	0.23	0.98	0.29	0.34	0.00
GATCAAAATCTAAAAAAAAA	1.37	0.00	0.00	0.00	0.00
GATCAAAATCTACCAGACAC	0.23	0.00	0.00	0.00	0.00
GATCAAAATCTCAAAAAAAA	0.00	0.00	0.00	0.68	0.00
GATCAAAATCTCAACCTTTT	0.00	1.95	0.00	0.00	0.00
GATCAAAATCTGACTGTCAC	0.00	0.00	0.00	0.00	0.34
GATCAAAATCTGCACAAGCG	0.00	0.00	0.00	0.00	0.34
GATCAAAATCTGCCAGACAC	10.99	6.83	6.01	6.45	3.44
GATCAAAATCTGCCAGACAT	0.00	0.00	0.00	0.00	0.34
GATCAAAATCTGCCAGACTC	0.00	0.33	0.00	0.00	0.00
GATCAAAATCTGCCAGCCAC	0.23	0.00	0.00	0.00	0.00
GATCAAAATCTGCCAGGCAC	0.23	0.00	0.00	0.00	0.00
GATCAAAATCTGCCAGNNNN	0.46	0.00	0.00	0.00	0.00
GATCAAAATCTGCCAGTCAC	0.00	0.00	0.00	0.00	0.69
GATCAAAATCTGCCCGACAC	0.00	0.33	0.00	0.00	0.00
GATCAAAATCTGCCTGTTGG	0.00	0.00	0.00	0.68	0.00
GATCAAAATCTGGCAAGGCG	0.00	0.00	0.00	0.00	0.00
GATCAAAATCTGGGCAAGTG	0.00	0.00	0.00	0.00	0.34
GATCAAAATCTTACATTGGA	0.00	0.00	0.00	0.00	0.00
GATCAAAATCTTCACGGAGG	0.00	0.33	0.00	0.00	0.00
GATCAAAATCTTCAGGGCCG	0.00	0.00	0.29	0.00	0.00
GATCAAAATCTTCCCGGAAG	0.00	0.00	0.86	0.00	0.00
GATCAAAATCTTCCCGGACA	0.00	0.00	0.00	0.34	0.00
GATCAAAATCTTCCCGGACG	11.67	18.87	12.03	19.69	12.05
GATCAAAATCTTCCCGGATG	0.00	0.00	0.29	0.00	0.00

GATCAAAATCTTCCCGGCCG	0.00	0.33	0.00	0.00	0.00
GATCAAAATCTTCCCGGCGT	0.00	0.00	0.00	0.00	0.00
GATCAAAATCTTCCCGGTCG	0.23	0.00	0.00	0.00	0.00
GATCAAAATCTTCCTTCCTG	0.00	0.33	0.29	0.00	0.00
GATCAAAATCTTGAGAAGCC	0.00	0.00	0.57	0.00	0.00
GATCAAAATCTTGATTACAA	0.00	0.00	0.57	0.00	0.00
GATCAAAATGAAAAAAAAAA	20.83	0.65	5.73	6.79	5.85
GATCAAAATGAAAAAAAAAT	0.92	0.00	0.29	0.00	0.00
GATCAAAATGAAAAAAAANN	0.00	0.00	0.00	0.00	0.00
GATCAAAATGAAAAAAACAA	0.00	0.00	0.00	0.00	0.00
GATCAAAATGAAAAAACNNN	0.23	0.00	0.00	0.00	0.00
GATCAAAATGAAAAAAGAAA	0.23	0.00	0.00	0.00	0.00
GATCAAAATGAAAAGAAAAA	0.46	0.98	0.29	0.68	0.00
GATCAAAATGAAAAGAAATA	0.46	0.00	0.29	0.00	0.00
GATCAAAATGAAAAGGAGAA	0.23	0.00	0.00	0.00	0.00
GATCAAAATGAAACAAAAAA	0.00	0.00	0.29	0.00	0.00
GATCAAAATGAAAGAAAAAA	0.46	0.33	0.00	0.00	0.00
GATCAAAATGAAAGAAAACN	0.23	0.00	0.00	0.00	0.00
GATCAAAATGAAAGACCCAG	0.00	0.00	0.00	0.34	0.00
GATCAAAATGAAAGCACCAA	0.00	0.00	0.29	0.34	0.00
GATCAAAATGAAAGCACCAT	0.00	0.33	0.00	0.00	1.38
GATCAAAATGAAAGCACCCT	0.00	0.00	0.00	0.00	0.34
GATCAAAATGAAAGCACCGT	3.89	2.28	1.72	3.39	3.10
GATCAAAATGAAAGCACCNN	0.23	0.00	0.00	0.00	0.00
GATCAAAATGAAAGGAAATA	0.23	0.00	0.00	0.00	0.00
GATCAAAATGAAAGGGAAGA	0.23	0.00	0.00	0.00	0.00
GATCAAAATGAAATTAAGGC	0.46	0.00	0.00	0.00	0.00
GATCAAAATGAACATAGAAA	0.00	0.00	0.29	0.00	0.00
GATCAAAATGAACATCACAA	0.00	0.00	0.00	0.00	0.00
GATCAAAATGAACATCGACA	19.46	16.59	21.19	13.92	12.05
GATCAAAATGAACATCGACC	0.00	0.00	0.29	0.68	0.00
GATCAAAATGAACATCGAGA	0.23	0.00	0.00	0.00	0.00
GATCAAAATGAACATCGATA	0.00	0.00	0.00	0.00	0.00
GATCAAAATGAACATCNNNN	0.23	0.00	0.00	0.00	0.00
GATCAAAATGAACGACATGT	0.00	0.00	0.29	0.34	0.00
GATCAAAATGAAGGCACCGC	0.00	0.00	0.00	0.00	0.00
GATCAAAATGAATAAAAAAA	0.46	0.00	0.00	0.00	0.00

GATCAAAATGAATGTCAAAA	0.00	0.00	0.29	0.00	0.00
GATCAAAATGAATGTCGATG	0.00	0.00	0.29	0.00	0.00
GATCAAAATGACAAAAAAAA	1.14	0.00	0.57	0.34	1.72
GATCAAAATGACCCTCTAAC	0.00	0.00	0.29	0.00	0.00
GATCAAAATGACGCTAAACA	0.00	0.00	0.29	0.00	0.00
GATCAAAATGAGAAAAAAAA	0.00	0.00	0.00	0.00	0.69
GATCAAAATGAGAAAGAGAA	0.00	0.00	0.00	0.00	0.00
GATCAAAATGAGAAGTCAAA	0.00	0.00	0.00	0.00	0.00
GATCAAAATGAGAAGTCGTG	0.00	0.33	0.00	0.00	0.00
GATCAAAATGAGAAGTCTAT	0.00	0.00	0.29	0.00	0.00
GATCAAAATGAGAGCAACCA	0.23	0.00	0.00	0.00	0.00
GATCAAAATGAGCAAGGAGG	0.00	0.00	0.29	0.00	0.00
GATCAAAATGAGCATGCCTC	0.00	0.00	0.29	0.00	0.00
GATCAAAATGAGCCTCTTGA	0.00	0.00	0.29	0.00	0.00
GATCAAAATGAGGCAATGAA	0.00	0.00	0.00	0.00	0.00
GATCAAAATGAGTCAACCAC	0.00	0.33	0.00	0.00	0.34
GATCAAAATGAGTTCCCTTG	0.00	0.00	0.00	0.00	0.00
GATCAAAATGATAAAAAAAA	1.83	0.00	0.00	0.00	2.07
GATCAAAATGATAAAAAAAN	0.23	0.00	0.00	0.00	0.00
GATCAAAATGATAAAATAAA	0.00	0.00	0.00	0.00	0.00
GATCAAAATGATACCTCCTG	0.23	0.00	0.00	0.00	0.00
GATCAAAATGATATAAAAAA	0.23	0.00	0.00	0.00	0.00
GATCAAAATGATATGAAGTG	0.46	0.65	0.00	0.00	0.69
GATCAAAATGATGAAAAAAA	0.00	0.00	0.00	0.00	1.72
GATCAAAATGATGAAATCAA	0.23	0.00	0.00	0.00	0.00
GATCAAAATGATGACGAGTT	0.23	0.00	0.00	0.00	0.00
GATCAAAATGATGAGGAAAA	0.23	0.00	0.00	0.00	0.00
GATCAAAATGATGAGGAGGC	0.00	0.00	0.29	0.00	0.00
GATCAAAATGATTTGTCAAT	0.00	0.65	0.00	0.00	0.00
GATCAAAATGCAAAAAAAAA	1.60	2.28	0.00	2.04	1.72
GATCAAAATGCAAATCAAGC	0.00	0.00	0.00	0.34	0.00
GATCAAAATGCAAGCACATA	0.23	0.00	0.00	0.00	0.00
GATCAAAATGCAAGCACCAG	0.46	0.00	0.00	0.00	0.00
GATCAAAATGCAAGCACCAT	0.00	0.00	0.57	0.00	0.34
GATCAAAATGCAAGGACATA	0.00	0.00	0.00	0.68	0.00
GATCAAAATGCACCGTCTGC	0.00	0.00	0.00	0.34	0.00
GATCAAAATGCACTGCGAAG	0.00	0.00	0.00	0.34	0.69

GATCAAAATGCAGACTCTTG	0.23	0.98	0.00	0.00	0.34
GATCAAAATGCAGAGCCTTC	0.00	0.00	0.00	0.68	0.00
GATCAAAATGCAGCATCGCA	0.00	0.00	0.29	0.00	0.00
GATCAAAATGCAGCGTCCCT	0.23	0.00	0.00	0.00	0.00
GATCAAAATGCAGGCAAAAA	0.00	0.00	0.00	0.00	0.00
GATCAAAATGCAGGCAAATT	0.00	0.00	0.00	0.00	0.00
GATCAAAATGCAGGGATATC	0.46	0.33	0.57	0.00	0.34
GATCAAAATGCATGAGAAGA	0.00	0.00	0.00	0.00	0.00
GATCAAAATGCATGATGATG	0.00	0.00	0.29	0.00	0.00
GATCAAAATGCATGGCGATG	0.23	0.00	0.00	0.00	0.00
GATCAAAATGCATGTAAAAA	0.00	0.00	0.00	1.36	0.00
GATCAAAATGCATGTCAAAA	1.37	0.33	2.86	3.73	1.38
GATCAAAATGCATGTCAGAA	0.00	0.00	0.00	0.34	0.00
GATCAAAATGCATGTCGAAA	0.00	0.00	0.00	0.34	0.00
GATCAAAATGCATGTCGATG	7.32	1.30	3.15	5.09	2.41
GATCAAAATGCATGTCGCTG	0.23	0.00	0.00	0.00	0.00
GATCAAAATGCATTTAAGGA	0.00	0.00	0.00	0.00	0.00
GATCAAAATGCCAAAAAAAA	0.69	1.30	1.15	0.68	1.03
GATCAAAATGCCACCACAAA	0.00	0.00	0.57	0.00	0.00
GATCAAAATGCCAGCGTTTC	0.00	0.00	0.00	0.00	0.00
GATCAAAATGCCATGTAAAA	0.00	0.00	0.29	0.00	0.00
GATCAAAATGCCCAGGATGG	0.00	0.00	0.00	0.34	0.00
GATCAAAATGCCCGGAATAA	0.00	0.00	0.29	0.00	0.00
GATCAAAATGCCCTACCCCT	0.00	0.00	0.00	0.34	0.00
GATCAAAATGCCCTTGATGG	0.00	0.00	0.00	0.68	0.00
GATCAAAATGCCGGCTGCCG	0.23	0.33	0.00	0.00	0.34
GATCAAAATGCCTAAAAAAA	1.60	0.00	0.00	0.00	1.38
GATCAAAATGCCTGAGGAAA	0.00	0.00	0.00	0.00	0.00
GATCAAAATGCCTGAGGCAA	0.23	0.98	0.57	0.68	0.34
GATCAAAATGCGAAAAAAAA	0.00	0.33	0.57	0.68	0.34
GATCAAAATGCGAGAACCTG	0.23	0.00	0.00	0.00	0.00
GATCAAAATGCGAGTCTGTG	0.00	0.00	0.29	0.00	0.00
GATCAAAATGCGCATGCCTA	0.00	0.33	0.00	0.00	0.00
GATCAAAATGCGCATGCCTC	0.00	0.00	0.00	0.00	0.34
GATCAAAATGCGCTGCTGCT	0.00	0.33	0.00	0.00	0.00
GATCAAAATGCGGGAAAAAA	0.00	0.33	0.29	0.34	0.00
GATCAAAATGCGGGCAAAAA	0.69	1.63	3.15	5.09	0.34

GATCAAAATGCGGGCAACCA	7.10	10.74	6.30	16.29	6.54
GATCAAAATGCGGGCAACCC	0.00	0.00	0.00	0.00	0.00
GATCAAAATGCGGGCAACTC	0.46	0.00	0.00	0.00	0.00
GATCAAAATGCGGGCAAGCA	0.00	0.33	0.00	0.00	0.00
GATCAAAATGCGGGCAATCA	0.00	0.00	0.00	0.34	0.00
GATCAAAATGCGGGGAACCA	0.00	0.00	0.00	0.34	0.00
GATCAAAATGCGTACAGGAG	0.23	0.00	0.00	0.00	0.00
GATCAAAATGCTAAAACATG	0.00	0.00	0.00	0.00	0.00
GATCAAAATGCTACAAAAAA	0.00	0.00	0.00	0.68	0.00
GATCAAAATGCTCAAAAAAA	0.00	0.00	0.00	0.00	0.34
GATCAAAATGCTCAATGCGG	0.00	0.00	0.00	0.00	0.00
GATCAAAATGCTCCAGCTAG	0.23	0.65	0.00	0.00	0.34
GATCAAAATGCTGCAAAAAA	1.14	0.00	0.00	0.34	0.34
GATCAAAATGCTGCCCCCTC	0.00	0.00	0.57	0.00	0.00
GATCAAAATGCTGCCTATGC	0.00	0.00	0.00	0.00	0.34
GATCAAAATGCTGGAGTCTT	0.23	0.00	0.00	0.00	0.00
GATCAAAATGCTGGAGTGTT	0.00	0.33	0.00	0.00	0.00
GATCAAAATGCTGGGGGAAA	0.23	0.00	0.00	0.00	0.00
GATCAAAATGCTGGGGGAGA	0.23	0.00	0.00	0.00	0.00
GATCAAAATGCTGGGGGGAA	0.23	0.00	0.00	0.00	0.00
GATCAAAATGCTGTAGAGCC	0.23	0.33	0.00	0.34	0.00
GATCAAAATGCTGTAGAGGG	0.23	0.00	0.00	0.00	0.00
GATCAAAATGGAAAAAAACAA	0.23	0.00	0.00	0.00	0.00
GATCAAAATGGAAAGATGGA	0.23	0.00	0.00	0.00	0.00
GATCAAAATGGAACGTAGGA	0.00	0.00	0.00	0.00	0.69
GATCAAAATGGAATCTCTCG	0.00	0.00	0.00	0.00	0.34
GATCAAAATGGACGAGCAAG	0.00	0.98	0.00	0.00	0.00
GATCAAAATGGAGCCGCGCA	0.23	0.65	0.00	0.00	0.34
GATCAAAATGGAGGTGAATG	0.00	0.00	0.00	0.00	0.34
GATCAAAATGGAGGTGTCGT	0.00	0.33	0.00	0.00	0.00
GATCAAAATGGAGTCATAAG	0.23	0.00	0.00	0.68	0.00
GATCAAAATGGAGTCCACTA	0.00	0.00	0.00	0.00	0.00
GATCAAAATGGAGTGACTTC	0.00	0.00	0.00	0.00	0.34
GATCAAAATGGAGTGGAAGG	0.00	0.00	0.29	0.00	0.00
GATCAAAATGGCAAAAAAAA	0.00	0.33	0.00	0.34	0.00
GATCAAAATGGCCACATTGG	0.00	4.23	4.58	2.72	7.58
GATCAAAATGGCCACATTGN	0.23	0.00	0.00	0.00	0.00

GATCAAAATGGCCCCAAAGA	0.23	0.00	0.00	0.00	0.00
GATCAAAATGGCCCCGGAGG	0.00	0.00	0.00	0.00	0.34
GATCAAAATGGCCTATGGGG	0.00	0.00	0.00	0.00	0.34
GATCAAAATGGCCTTCCGGG	0.00	0.00	0.00	0.00	0.34
GATCAAAATGGCGTGGAAGG	2.29	2.93	3.15	3.05	3.44
GATCAAAATGGCGTGGACGG	0.23	0.00	0.00	0.00	0.00
GATCAAAATGGCTGCTTCAC	0.23	0.00	0.00	0.00	0.00
GATCAAAATGGCTTGAGCCA	0.00	0.00	0.29	0.00	0.00
GATCAAAATGGCTTGAGCCC	0.00	0.00	0.57	0.00	0.00
GATCAAAATGGGCATGGGGG	0.00	0.00	0.00	0.34	0.00
GATCAAAATGGGCCGCCGCC	0.00	0.00	0.29	0.00	0.00
GATCAAAATGGGCTTGGCTA	0.00	0.00	0.29	0.00	0.00
GATCAAAATGGTCAAATGAC	0.00	0.33	0.00	0.00	0.00
GATCAAAATGGTCGACGAGA	0.92	1.95	0.00	0.68	1.38
GATCAAAATGGTCGGCTTGG	0.00	0.00	0.29	0.00	0.00
GATCAAAATGGTCGGGTTCA	0.00	0.00	0.00	0.00	0.69
GATCAAAATGGTGATAGCTT	0.00	0.00	0.00	0.34	0.00
GATCAAAATGTAAAAAAAAA	9.84	1.63	4.01	3.05	2.07
GATCAAAATGTAAAAAAAAC	0.00	0.00	0.00	0.00	0.34
GATCAAAATGTAAAAAACAA	0.00	0.33	0.00	0.00	0.00
GATCAAAATGTAAACAAAAA	0.00	0.00	0.00	0.00	0.34
GATCAAAATGTACAAAAAAA	0.00	0.00	0.00	0.68	0.00
GATCAAAATGTAGATATGGC	0.00	0.00	0.00	0.00	0.00
GATCAAAATGTCAAAAAAAA	24.72	6.51	14.03	16.63	18.94
GATCAAAATGTCAAAAAAAC	0.00	0.00	0.00	0.34	0.00
GATCAAAATGTCAAAAAAAT	0.00	0.00	0.00	0.34	0.00
GATCAAAATGTCAAAAATTT	0.00	0.00	0.29	0.00	0.00
GATCAAAATGTCAAAAGAAA	0.23	0.00	0.00	0.00	0.00
GATCAAAATGTCAAACACAC	0.00	0.00	0.00	0.00	0.00
GATCAAAATGTCAAGAAAAA	0.23	0.00	0.00	0.00	0.00
GATCAAAATGTCACAAAAAA	0.23	0.65	0.00	0.00	0.00
GATCAAAATGTCACAAGAAA	0.00	0.00	0.00	0.00	0.34
GATCAAAATGTCACCAAAAA	0.46	0.33	0.29	0.00	0.00
GATCAAAATGTCACCTCCAA	0.92	0.00	0.00	0.00	0.00
GATCAAAATGTCACCTCCTA	0.00	0.00	0.29	0.00	0.00
GATCAAAATGTCCAAAAAAA	0.00	0.00	0.00	0.00	0.34
GATCAAAATGTCCAAAATCA	0.00	0.00	0.29	0.00	0.00

GATCAAAATGTCCTGAGCTC	0.00	0.33	0.00	0.00	0.00
GATCAAAATGTCGAAAAAAA	0.00	0.00	0.57	0.00	0.00
GATCAAAATGTCGGCTCATC	0.00	0.00	0.00	0.00	0.00
GATCAAAATGTCTAAAAAAA	0.23	0.00	0.00	0.00	0.00
GATCAAAATGTCTCAAATGT	0.00	0.00	0.00	0.00	0.00
GATCAAAATGTCTGCTNCAA	0.00	0.00	0.00	0.00	0.34
GATCAAAATGTCTGCTTAAA	0.00	0.33	0.00	0.00	0.00
GATCAAAATGTCTGCTTCAA	3.89	5.21	4.30	5.09	2.07
GATCAAAATGTCTGTGCGCG	0.00	0.00	0.00	0.00	1.03
GATCAAAATGTGAAAAAAAA	1.83	0.33	0.29	1.02	2.07
GATCAAAATGTGAGGAACGC	0.23	0.00	0.57	0.68	0.69
GATCAAAATGTGCATCTGAG	0.00	0.98	0.29	0.34	1.03
GATCAAAATGTGGGCAACCA	0.00	0.00	0.00	0.34	0.00
GATCAAAATGTTAAAAAAAA	0.92	0.00	0.00	0.00	0.00
GATCAAAATGTTAAAAAAAC	0.00	0.00	0.00	0.00	0.34
GATCAAAATGTTAGTCTCGA	0.00	0.33	0.00	0.00	0.00
GATCAAAATGTTATTGTTTG	0.23	0.00	0.00	0.00	0.00
GATCAAAATGTTCCTGAAGC	0.69	0.00	0.00	0.00	0.00
GATCAAAATGTTGAAAAAAA	0.00	0.33	0.00	0.00	0.00
GATCAAAATGTTGAGGATTA	0.00	0.00	0.29	0.00	0.00
GATCAAAATGTTGAGGATTC	0.69	2.93	0.29	1.70	0.34
GATCAAAATGTTGAGGCTGA	0.00	0.33	0.00	0.34	0.00
GATCAAAATGTTGATTACAA	0.00	0.00	0.29	0.00	0.00
GATCAAAATGTTGCGAACCA	0.00	0.00	0.00	0.00	0.00
GATCAAAATGTTGGGGACAG	0.00	0.65	0.00	0.00	0.00
GATCAAAATGTTTAAAAAAA	3.89	0.33	0.57	0.00	0.34
GATCAAAATGTTTGAAAAAA	0.00	0.00	0.00	0.34	0.00
GATCAAAATGTTTGATTGCA	0.23	0.00	0.00	0.34	0.00
GATCAAAATGTTTGATTGCC	0.23	0.00	0.00	0.00	0.69
GATCAAAATGTTTGCGTGTG	0.00	0.00	0.29	0.00	0.00
GATCAAAATGTTTGTGGATG	0.00	0.00	0.00	0.00	0.00
GATCAAAATTAAAAAAAAAA	4.58	0.98	1.72	0.34	1.03
GATCAAAATTAAAAAAAAAN	0.23	0.00	0.00	0.00	0.00
GATCAAAATTAAAAAAAAAT	0.23	0.00	0.00	0.00	0.00
GATCAAAATTAAATGCCTTT	0.00	0.00	0.00	0.00	0.00
GATCAAAATTAACATCAAAA	0.00	0.00	0.00	0.00	0.00
GATCAAAATTAATTTCATGA	0.46	0.00	0.00	0.00	0.00

GATCAAAATTACACCGTGGC	0.00	0.00	0.00	0.00	0.34
GATCAAAATTAGAGACTCAA	0.00	0.00	0.00	0.00	0.00
GATCAAAATTAGTTTTTTTT	0.00	0.65	0.00	0.00	0.00
GATCAAAATTATCGCCAAAG	0.00	0.33	0.00	0.00	0.00
GATCAAAATTATGCCTCAGT	0.00	0.00	0.57	0.00	0.00
GATCAAAATTATTATATCTA	1.60	0.00	0.00	0.00	0.00
GATCAAAATTCAAGTACATC	0.00	0.00	0.00	0.00	0.34
GATCAAAATTCACAGTGCAG	0.00	0.00	0.29	0.00	0.00
GATCAAAATTCAGAACTGGA	0.00	0.00	0.00	0.00	0.00
GATCAAAATTCAGCAAGTGC	0.00	0.00	0.29	0.00	0.00
GATCAAAATTCAGGGGCGCG	0.00	0.00	0.00	0.34	0.00
GATCAAAATTCCAATGCAGC	0.23	0.00	0.00	0.00	0.00
GATCAAAATTCCAGCGCCGC	0.00	0.00	0.00	0.00	0.00
GATCAAAATTCCAGTGAAGC	0.00	0.00	0.00	0.00	0.00
GATCAAAATTCCAGTGCAGA	0.23	0.00	0.00	0.00	0.34
GATCAAAATTCCAGTGCAGC	36.62	43.27	48.39	36.66	34.10
GATCAAAATTCCAGTGCATC	0.00	0.33	0.00	0.34	0.00
GATCAAAATTCCAGTGCCGC	0.23	0.00	0.00	0.00	0.34
GATCAAAATTCCAGTGCCNN	0.23	0.00	0.00	0.00	0.00
GATCAAAATTCCAGTGCNNN	0.23	0.00	0.00	0.00	0.00
GATCAAAATTCCAGTGNNNN	0.92	0.00	0.00	0.00	0.00
GATCAAAATTCCCGTGCCGC	0.23	0.00	0.00	0.00	0.00
GATCAAAATTCCTGAGGTGA	0.00	0.00	0.00	0.00	0.00
GATCAAAATTCCTGTGCAGC	0.00	0.00	0.00	0.00	0.00
GATCAAAATTCGAAAATTCG	0.00	0.00	0.29	0.00	0.00
GATCAAAATTCGCTCTCGCA	0.23	0.00	0.00	0.68	0.00
GATCAAAATTCTAGTGCAGC	0.23	0.00	0.00	0.00	0.00
GATCAAAATTCTGCCTATTC	0.00	0.65	0.00	0.00	0.00
GATCAAAATTGAACTACGGT	0.00	0.00	0.00	0.00	0.00
GATCAAAATTGACACCAAGA	2.52	1.95	1.15	1.02	2.07
GATCAAAATTGACACCAAGC	0.00	0.00	0.29	0.00	0.00
GATCAAAATTGAGAACATCT	0.23	1.63	1.15	2.38	3.44
GATCAAAATTGAGAACATGC	0.23	0.00	0.00	0.00	0.00
GATCAAAATTGAGCCTTGTC	1.37	0.00	0.00	0.68	0.00
GATCAAAATTGATGCGGCTC	0.00	0.00	0.00	0.00	0.00
GATCAAAATTGATTTGTGCG	0.00	0.33	0.00	0.00	0.00
GATCAAAATTGCAAAAAAAA	0.23	0.00	0.57	0.68	0.00

GATCAAAATTGCCAGATTTA	0.00	0.00	0.00	0.00	0.34
GATCAAAATTGCTCTAATGT	0.69	0.00	1.72	0.68	0.34
GATCAAAATTGCTGAATGTC	0.00	0.00	0.00	0.00	0.34
GATCAAAATTGCTGCAATGC	0.00	0.00	0.29	0.00	0.00
GATCAAAATTGCTGCGCCTC	0.00	0.00	0.00	0.00	0.00
GATCAAAATTGCTGCGCTCA	0.00	0.00	0.00	0.00	0.00
GATCAAAATTGCTGCGGAAA	0.46	0.65	0.29	0.34	0.00
GATCAAAATTGCTGCGGATC	0.23	0.00	0.00	0.00	0.00
GATCAAAATTGCTGCGGCAA	0.00	0.00	0.00	0.00	0.00
GATCAAAATTGCTGCGGCAC	0.00	0.00	0.57	0.00	0.00
GATCAAAATTGCTGCGGCCT	0.00	0.00	0.00	0.00	0.00
GATCAAAATTGCTGCGGCTA	0.00	0.00	0.00	0.34	0.34
GATCAAAATTGCTGCGGCTC	8.24	6.18	6.01	4.75	3.79
GATCAAAATTGCTGCGGTTG	0.00	0.00	0.00	0.00	0.00
GATCAAAATTGCTGCTCGCC	0.00	0.00	0.57	0.00	0.00
GATCAAAATTGCTGGGCCAC	0.00	0.00	0.00	0.00	0.34
GATCAAAATTGCTGGGCTCT	0.00	0.33	0.00	0.00	0.00
GATCAAAATTGCTGGGGCAT	0.23	0.00	0.00	0.00	0.00
GATCAAAATTGCTGGGNNNN	0.23	0.00	0.00	0.00	0.00
GATCAAAATTGCTGGGTAAT	0.00	0.00	0.00	0.00	0.00
GATCAAAATTGCTGGGTCAA	0.00	0.33	0.00	0.00	0.00
GATCAAAATTGCTGGGTCAT	7.10	3.90	7.73	3.05	8.61
GATCAAAATTGCTGGGTCCT	0.00	0.00	0.00	0.00	0.00
GATCAAAATTGCTGGGTCTT	0.00	0.00	0.00	0.00	0.00
GATCAAAATTGCTGGGTTAT	0.23	0.00	0.00	0.00	0.00
GATCAAAATTGCTGGGTTCG	0.00	0.00	0.00	0.00	0.34
GATCAAAATTGCTTAATTTG	0.23	0.00	0.00	0.00	0.00
GATCAAAATTGGAACGATTG	0.69	0.00	0.00	0.00	0.00
GATCAAAATTGGATGCAAGC	0.23	0.00	0.00	0.00	0.00
GATCAAAATTGGCTCTGCTA	0.00	0.00	0.00	0.00	0.69
GATCAAAATTGGTTTGCTTT	0.00	0.00	0.00	0.00	0.69
GATCAAAATTGTAAAAAAAA	0.00	0.00	0.00	0.00	0.00
GATCAAAATTGTCGCGCCAA	0.00	0.33	0.00	0.00	0.00
GATCAAAATTGTGAAAAAAA	0.23	0.00	0.00	0.00	0.00
GATCAAAATTGTTTGAATGT	0.00	0.00	0.00	0.00	0.69
GATCAAAATTTAGACGTTGC	0.46	0.65	0.00	0.00	0.00
GATCAAAATTTCAACCAACA	0.00	0.00	0.29	0.00	0.00

GATCAAAATTTCGAGGCCTC	0.00	0.00	0.29	0.00	0.00
GATCAAAATTTGAATGATGA	0.00	0.00	0.86	0.00	0.00
GATCAAAATTTGGGAGCTAT	0.23	0.65	0.00	1.36	0.00
GATCAAAATTTTACATNATA	0.00	0.00	0.00	0.00	0.34
GATCAAAATTTTACCGCACA	0.00	0.00	0.00	0.00	0.00
GATCAAAATTTTATTGGAGG	0.00	0.00	0.29	0.00	0.00
GATCAAAATTTTGATTTCAG	0.00	0.00	0.57	0.00	0.00
GATCAAAATTTTGGGTTGCT	0.00	0.33	0.00	0.00	0.00
GATCAAACAAAAAAAAAAAA	102.09	23.10	31.50	41.75	45.46
GATCAAACAAAAAAAAAAAC	0.46	0.00	0.29	0.34	0.00
GATCAAACAAAAAAAAAAAG	0.00	0.00	0.00	0.00	0.69
GATCAAACAAAAAAAAAAAN	0.46	0.00	0.00	0.00	0.00
GATCAAACAAAAAAAAAAAT	6.18	1.30	2.29	2.38	4.82
GATCAAACAAAAAAAAAACA	0.00	0.00	0.29	0.00	0.69
GATCAAACAAAAAAAAAAGA	0.00	0.00	0.00	0.00	0.00
GATCAAACAAAAAAAAAANN	0.46	0.00	0.00	0.00	0.00
GATCAAACAAAAAAAAAATC	0.23	0.00	0.00	0.34	0.00
GATCAAACAAAAAAAAACAA	0.00	0.33	0.00	0.00	0.00
GATCAAACAAAAAAAAACAC	0.23	0.00	0.00	0.00	0.00
GATCAAACAAAAAAAAACAT	0.23	0.00	0.00	0.00	0.00
GATCAAACAAAAAAAAAGAA	0.00	0.33	0.00	0.00	0.00
GATCAAACAAAAAAAAANNN	0.23	0.00	0.00	0.00	0.00
GATCAAACAAAAAAAACAAA	0.23	0.00	0.00	0.00	0.34
GATCAAACAAAAAAAACACA	0.00	0.00	0.00	0.00	0.34
GATCAAACAAAAAAAACCCA	0.00	0.33	0.00	0.00	0.00
GATCAAACAAAAAAAACCNN	0.23	0.00	0.00	0.00	0.00
GATCAAACAAAAAAAACTCT	0.00	0.00	0.29	0.00	0.00
GATCAAACAAAAAAAAGAAA	0.00	0.00	0.00	0.00	0.00
GATCAAACAAAAAAACAAAA	0.00	0.00	0.29	0.00	0.34
GATCAAACAAAAAACAAAAA	0.23	0.00	0.00	0.00	0.00
GATCAAACAAAAAACAAGAA	0.23	0.00	0.00	0.00	0.00
GATCAAACAAAAAACAATCC	0.00	0.00	0.00	0.68	0.00
GATCAAACAAAAAACACACA	0.00	0.00	0.00	0.00	0.34
GATCAAACAAAAAACCAACA	0.00	0.00	0.00	0.00	0.34
GATCAAACAAAAAACCACAC	0.00	0.00	0.00	0.34	0.00
GATCAAACAAAAAAGAAAAA	0.00	0.33	0.00	0.68	0.00
GATCAAACAAAAACAAAAAC	0.00	0.00	0.00	0.00	0.00

GATCAAACAAAAACAAACCA	0.00	0.33	0.00	0.00	0.00
GATCAAACAAAAATCAAGAT	0.00	0.00	0.29	0.00	0.00
GATCAAACAAAAATCCAAAA	0.00	0.00	0.29	0.00	0.00
GATCAAACAAAAATGGGAAA	1.83	0.00	0.29	0.68	0.34
GATCAAACAAAAATGGGAAG	0.23	0.00	0.00	0.00	0.00
GATCAAACAAAAATGGGCAA	0.00	0.00	0.00	0.34	1.03
GATCAAACAAAACAAAAAAA	27.01	3.90	5.44	5.09	5.85
GATCAAACAAAACAAAAAAT	0.00	0.00	0.00	0.00	0.34
GATCAAACAAAACAAAAACN	0.23	0.00	0.00	0.00	0.00
GATCAAACAAAACAAAAANN	0.23	0.00	0.00	0.00	0.00
GATCAAACAAAACAAAACAA	0.00	0.00	0.00	0.00	0.00
GATCAAACAAAACAAATGGG	0.00	0.00	0.29	0.00	0.34
GATCAAACAAAACAAATGNN	0.69	0.00	0.00	0.00	0.00
GATCAAACAAAACAAATGTG	9.61	4.23	6.01	7.81	8.61
GATCAAACAAAACAAATGTN	0.00	0.00	0.00	0.00	0.00
GATCAAACAAAACAAGATGT	0.00	0.00	0.00	0.00	0.34
GATCAAACAAAACAGAAAAA	0.00	0.00	0.00	0.00	0.00
GATCAAACAAAACAGACCCA	0.00	0.00	0.29	0.00	0.00
GATCAAACAAAACGAAAAAA	0.00	0.00	0.29	0.00	0.69
GATCAAACAAAACGAAACGA	0.00	0.00	0.29	0.00	0.00
GATCAAACAAAAGAAAAAAA	0.00	0.00	0.00	0.00	0.34
GATCAAACAAAAGAAATAGG	0.00	0.00	0.00	0.00	0.69
GATCAAACAAAAGACCCAAG	0.00	0.00	0.00	0.00	0.00
GATCAAACAAAAGACCCGCG	0.00	0.00	0.00	0.00	0.00
GATCAAACAAAAGACTGACA	0.23	0.00	0.00	0.00	0.00
GATCAAACAAAAGATGAAGG	0.00	0.00	0.00	0.00	0.00
GATCAAACAAAAGCATTCTA	0.23	0.00	0.00	0.34	0.00
GATCAAACAAAAGGTTTGAC	0.46	0.00	0.00	0.00	0.00
GATCAAACAAAAGTTATGGT	0.23	0.00	0.00	0.00	0.00
GATCAAACAAAATAAAAAAA	0.00	0.00	0.00	0.00	0.34
GATCAAACAAAATATGTTGC	0.00	0.00	0.29	0.00	0.00
GATCAAACAAACAAAAAAAA	0.46	0.00	0.00	0.00	0.69
GATCAAACAAACAAAAATAA	0.00	0.00	0.29	0.00	0.00
GATCAAACAAACAAAACAAA	0.00	0.00	0.00	0.00	0.34
GATCAAACAAACAAACAAAA	0.00	0.33	0.00	0.00	0.00
GATCAAACAAACAAACAAAC	0.00	0.00	0.00	0.00	0.69
GATCAAACAAACAAACACAC	0.00	0.00	0.29	0.00	0.00

GATCAAACAAACAAAGTCGT	0.00	0.00	0.29	0.00	0.00
GATCAAACAAACAACACACA	0.00	0.00	0.00	0.00	0.34
GATCAAACAAACAACCGACC	0.00	0.00	0.00	0.00	0.69
GATCAAACAAACAATAGCGG	0.00	0.00	0.29	0.00	0.00
GATCAAACAAACACAAAAAA	0.00	0.33	0.00	0.00	0.00
GATCAAACAAACACACAACA	0.46	0.33	0.29	0.34	0.00
GATCAAACAAACACACAACC	0.23	0.00	0.00	0.00	0.00
GATCAAACAAACACAGAATT	0.00	0.00	0.00	0.00	0.00
GATCAAACAAACACATTGTA	0.00	0.33	0.00	0.00	0.00
GATCAAACAAACACCGGCCG	0.23	0.00	0.00	0.00	0.00
GATCAAACAAACAGACACAC	0.00	0.00	0.29	0.00	0.00
GATCAAACAAACATGCATAC	0.00	0.00	0.00	0.00	0.00
GATCAAACAAACCAAAAACA	0.00	0.00	0.00	0.34	0.00
GATCAAACAAACCAAACAAC	0.00	0.00	0.00	0.00	0.34
GATCAAACAAACCACAAAAC	0.23	0.00	0.00	0.00	0.00
GATCAAACAAACGCGTTGCG	0.00	0.00	0.00	0.00	0.00
GATCAAACAAACTCCGGCCG	0.92	0.98	0.29	1.70	0.34
GATCAAACAAAGAAAACAAC	0.00	0.00	0.00	0.00	1.03
GATCAAACAAAGAAGTGGGG	0.00	0.00	0.29	0.00	0.00
GATCAAACAAAGACGCCGAG	0.00	0.00	0.00	0.00	0.34
GATCAAACAAAGAGGGAACC	0.00	0.33	0.00	0.00	0.00
GATCAAACAAAGCAAAGCAA	0.23	0.00	0.00	0.00	0.00
GATCAAACAAAGCCAGACAT	0.00	0.00	0.29	0.00	0.00
GATCAAACAAAGGAAACGCT	0.00	0.00	0.00	0.00	0.34
GATCAAACAAAGGCGTGCCG	0.00	0.00	0.86	0.00	0.00
GATCAAACAAAGGGGTTCTG	0.00	0.00	0.29	0.00	0.00
GATCAAACAAATAAGCGCGG	0.00	0.00	0.29	0.00	0.00
GATCAAACAAATAATCGCCG	0.00	0.33	0.00	0.00	0.00
GATCAAACAAATAATCGCGC	0.00	0.00	0.00	0.34	0.00
GATCAAACAAATAATCGCGG	7.10	15.29	14.32	9.16	20.32
GATCAAACAAATAATCGCTC	0.00	0.00	0.00	1.02	0.00
GATCAAACAAATAATCGTGG	0.00	0.00	0.29	0.00	0.00
GATCAAACAAATACTCGCGG	0.00	0.33	0.00	0.00	0.00
GATCAAACAAATGATATCAT	0.00	0.00	0.00	0.00	0.00
GATCAAACAAATGTGAACAA	0.00	0.00	0.57	0.00	0.00
GATCAAACAAATTGGTTTTA	0.46	0.00	0.00	0.00	0.00
GATCAAACAAATTTAAAAAT	0.00	0.33	0.00	0.00	0.00

GATCAAACAACAAAAAAACA	0.00	0.00	0.00	0.00	0.34
GATCAAACAACAAAAAAACT	0.00	0.00	0.29	0.00	0.00
GATCAAACAACAAAACAAAA	0.00	0.00	0.00	0.34	0.00
GATCAAACAACAAAACAAAC	0.00	0.00	0.00	0.00	0.34
GATCAAACAACAAAACAACA	0.00	0.00	0.00	0.00	0.34
GATCAAACAACAAATATTCA	0.00	0.00	0.29	0.00	0.00
GATCAAACAACAACAAAACA	0.23	0.00	0.00	0.00	0.00
GATCAAACAACAACACACAA	0.00	0.00	0.00	0.00	0.00
GATCAAACAACAAGGCTTGC	0.00	0.00	0.00	0.00	0.00
GATCAAACAACAATGCACAA	0.00	0.33	0.00	0.00	0.00
GATCAAACAACACAAAAAAC	0.00	0.00	0.00	0.00	0.00
GATCAAACAACACAAACATG	0.00	0.00	0.29	0.00	0.00
GATCAAACAACACAAACCAC	0.00	0.00	0.00	0.00	0.34
GATCAAACAACACAACACAA	0.00	0.00	0.00	0.00	0.34
GATCAAACAACACAACACGG	0.00	0.00	0.29	0.00	0.00
GATCAAACAACACACACACA	0.23	0.00	0.00	0.00	0.00
GATCAAACAACACACAGCAC	0.00	0.00	0.86	0.00	0.00
GATCAAACAACACACCACGC	0.00	0.00	0.00	0.00	0.34
GATCAAACAACACACGAAAA	0.00	0.00	0.00	0.00	0.34
GATCAAACAACACCATGAAC	0.00	0.33	0.00	0.00	0.00
GATCAAACAACACGCACCAC	0.00	0.00	0.29	0.00	0.00
GATCAAACAACACGTACAAA	0.00	0.00	0.57	0.00	0.00
GATCAAACAACAGAACAGAA	0.00	0.00	0.00	0.00	0.69
GATCAAACAACAGAGCCCCA	0.00	0.00	0.00	0.00	0.34
GATCAAACAACATAAAGGAT	0.23	0.00	0.00	0.00	0.00
GATCAAACAACATTGTATTT	0.00	0.00	0.00	0.00	0.34
GATCAAACAACCAAACTACA	0.23	0.00	0.00	0.00	0.00
GATCAAACAACCAGTGTGTT	0.00	0.00	0.57	0.00	0.00
GATCAAACAACCATAAGCCG	0.00	0.00	0.57	0.00	0.00
GATCAAACAACCCCAACAAC	0.00	0.00	0.00	0.00	0.69
GATCAAACAACCCCCACCCC	0.23	0.00	0.00	0.00	0.00
GATCAAACAACCCCCCCCCC	0.23	0.00	0.00	0.00	0.00
GATCAAACAACCCCCTAGGA	0.00	0.00	0.00	0.00	0.00
GATCAAACAACCGAGACACC	0.92	0.00	0.00	0.00	0.00
GATCAAACAACCGAGCTGGG	0.00	0.00	0.00	0.34	0.00
GATCAAACAACCGCAGCATC	0.00	0.00	0.29	0.00	0.00
GATCAAACAACCTGAAGCTC	0.00	0.00	0.00	0.00	0.34

GATCAAACAACGAAGAGAGC	0.00	0.00	0.29	0.00	0.00
GATCAAACAACGCACACCCA	0.00	0.00	0.00	0.00	0.34
GATCAAACAACGCCAGACTC	0.00	0.00	0.00	0.00	1.03
GATCAAACAACGCCATCTAC	0.00	0.00	0.00	0.00	0.00
GATCAAACAACGCGCATTGC	0.00	0.00	0.00	0.00	0.34
GATCAAACAACGCGCGCGGA	0.00	0.33	0.00	0.00	0.00
GATCAAACAACGGCACCAAC	0.00	0.00	0.29	0.00	0.00
GATCAAACAACGTAAGGTAA	0.23	0.00	0.00	0.00	0.00
GATCAAACAACGTGGAATGA	0.00	0.00	0.57	0.00	0.00
GATCAAACAACGTGGAATGC	0.00	0.33	0.86	0.00	0.00
GATCAAACAACGTGGACTGC	0.00	0.00	0.29	0.00	0.00
GATCAAACAACGTGGANTGC	0.00	0.00	0.29	0.00	0.00
GATCAAACAACTAAAAAAAA	0.00	0.00	0.29	1.02	0.00
GATCAAACAACTAATCGAGG	0.00	0.00	0.29	0.00	0.00
GATCAAACAACTACATGTGC	0.00	0.00	0.00	0.00	0.34
GATCAAACAACTCAGAACTC	0.00	0.00	0.29	0.00	0.00
GATCAAACAACTCCCACACC	0.00	0.00	0.00	0.00	0.34
GATCAAACAACTCTACGGCA	0.46	0.33	0.00	0.00	0.00
GATCAAACAACTCTGTGCCC	0.00	0.00	0.00	0.00	0.00
GATCAAACAACTGAATGTGT	0.00	0.00	0.00	0.00	0.34
GATCAAACAACTGTTCCCAG	0.00	0.00	0.00	0.00	0.00
GATCAAACAACTGTTTCCAT	0.00	0.00	0.00	0.00	0.34
GATCAAACAACTTTTGATGT	0.00	0.00	0.00	0.00	0.34
GATCAAACAAGAAAAAAAAA	0.00	0.00	0.29	0.00	0.00
GATCAAACAAGACAACGCAA	0.00	0.00	0.00	0.00	0.34
GATCAAACAAGACAAGACAA	0.00	0.00	0.00	0.00	0.34
GATCAAACAAGACAAGACCA	0.00	0.00	0.00	0.00	1.03
GATCAAACAAGACTGATTGT	0.00	0.00	0.00	0.00	0.00
GATCAAACAAGAGAATAATG	0.00	0.00	0.29	0.00	0.00
GATCAAACAAGAGGAGGGAG	0.00	0.00	0.29	0.00	0.00
GATCAAACAAGAGTAAAGCC	0.69	0.00	0.00	0.00	0.00
GATCAAACAAGCAAAAAAAA	0.00	0.00	0.29	0.00	0.00
GATCAAACAAGCAAGACAAA	0.00	0.00	0.00	0.00	0.34
GATCAAACAAGCACACCGAG	0.00	0.00	0.00	0.34	0.00
GATCAAACAAGCACAGGCCA	0.00	0.00	0.00	0.34	0.00
GATCAAACAAGCACTGCCAC	0.00	0.00	0.29	0.00	0.00
GATCAAACAAGCACTGGGCC	0.69	1.95	1.15	0.34	0.00

GATCAAACAAGCCAAAAAAA	0.00	0.00	0.29	0.00	0.00
GATCAAACAAGCCGGATTCG	0.00	0.00	0.00	0.00	0.00
GATCAAACAAGCGCATTGTG	0.23	0.00	0.00	0.00	0.00
GATCAAACAAGCGCCAAGAG	0.00	0.33	0.00	0.00	0.00
GATCAAACAAGCGCCTTGTG	0.00	0.33	0.29	0.00	0.00
GATCAAACAAGCGGCACTCT	0.00	0.00	0.29	0.00	0.00
GATCAAACAAGGACGGGACG	0.00	0.33	0.29	0.00	0.00
GATCAAACAAGGACGGGCAG	0.00	0.33	0.00	0.00	0.00
GATCAAACAAGGACGGGCCG	0.23	0.65	3.15	0.00	0.00
GATCAAACAAGGACGGGCGG	0.00	0.00	0.29	0.00	0.00
GATCAAACAAGGACGGGCTG	0.00	0.00	0.29	0.00	0.00
GATCAAACAAGGCCTATTGG	0.00	0.00	0.00	0.00	0.34
GATCAAACAAGGCTGGCGTG	0.00	0.00	0.00	0.00	0.69
GATCAAACAAGGGCCGCCGC	0.23	0.00	0.00	0.34	0.00
GATCAAACAAGGGCCTGCTT	0.00	0.00	0.00	0.00	0.00
GATCAAACAAGGGCGTCGGG	0.00	0.00	0.29	0.00	0.00
GATCAAACAAGGGTGATTGT	0.00	0.00	0.00	0.00	0.34
GATCAAACAAGGGTTTTTGG	0.00	0.00	0.29	0.00	0.00
GATCAAACAAGGTCGCGGCA	0.00	0.00	0.29	0.00	0.00
GATCAAACAAGGTGCGTCTC	0.00	0.00	0.29	0.00	0.00
GATCAAACAAGGTGTAAGAC	0.00	0.33	0.00	0.00	0.34
GATCAAACAAGGTTGAAAAA	0.46	0.33	0.00	0.00	0.34
GATCAAACAAGGTTGCAAAA	1.14	0.33	0.29	0.68	0.69
GATCAAACAAGGTTGCATTT	5.26	0.65	2.29	2.38	3.79
GATCAAACAAGTAAAAAAAA	0.23	0.00	0.00	0.00	0.00
GATCAAACAAGTAATCGAGA	0.00	0.00	0.57	0.00	0.00
GATCAAACAAGTACCACCCC	1.14	0.00	0.00	0.00	0.00
GATCAAACAAGTAGAGCGGG	0.00	0.00	0.00	0.00	0.00
GATCAAACAAGTATCACCCC	0.00	0.00	0.00	0.00	0.00
GATCAAACAAGTCAAACGCC	0.46	0.00	0.00	0.00	0.00
GATCAAACAAGTCCACTGCT	0.69	0.00	0.00	0.00	0.00
GATCAAACAAGTCGTGCAGA	0.00	0.00	0.00	0.68	0.00
GATCAAACAAGTCTGGGGCA	0.46	0.00	0.29	0.00	0.00
GATCAAACAAGTGATTGCTC	0.69	1.30	0.29	2.38	1.38
GATCAAACAAGTGCAAGAAG	0.00	0.00	0.00	0.00	0.00
GATCAAACAAGTGCAGAGCT	0.92	0.00	0.00	0.00	0.00
GATCAAACAAGTGCCAACAA	0.00	0.33	0.29	0.00	0.00

GATCAAACAAGTGTGTGTGT	0.00	0.00	0.00	0.00	0.34
GATCAAACAAGTTCATGGTG	0.00	0.00	0.00	0.00	0.00
GATCAAACAAGTTCCAAAAA	0.23	0.33	0.00	0.00	0.00
GATCAAACAAGTTCTTTTCT	0.00	0.00	0.57	0.00	0.00
GATCAAACAAGTTGCACTCG	0.00	0.00	0.29	0.00	0.00
GATCAAACAAGTTGCCAAGA	0.00	0.33	0.00	0.00	0.00
GATCAAACAAGTTGGGGAAG	0.00	0.00	0.57	0.00	0.00
GATCAAACAAGTTGTCTTTC	0.00	0.00	0.00	0.00	0.00
GATCAAACAATAAACACGTT	0.00	0.00	0.00	0.00	0.00
GATCAAACAATAATAAAATA	0.00	0.00	0.00	0.00	0.00
GATCAAACAATACAAATGTG	0.00	0.00	0.00	0.00	0.00
GATCAAACAATAGAATCAAT	0.00	0.00	0.00	0.00	1.03
GATCAAACAATATGGTTTGA	0.00	0.00	0.29	0.00	0.00
GATCAAACAATCAAAAAAAA	0.46	1.30	0.00	0.00	0.00
GATCAAACAATCAAAATAAA	0.00	0.33	0.00	0.00	0.00
GATCAAACAATCAAAATACA	0.00	0.33	0.00	0.00	0.00
GATCAAACAATCAAACATAT	0.00	0.65	2.00	2.38	0.34
GATCAAACAATCCAAAAAAA	0.00	0.00	0.00	0.00	0.69
GATCAAACAATCCAAATACA	0.00	0.00	0.00	0.00	0.34
GATCAAACAATCCACAACAC	0.00	0.00	0.00	0.00	0.34
GATCAAACAATCCAGAGAAA	0.69	0.00	0.00	0.00	0.69
GATCAAACAATCCCGAGAAA	0.00	0.00	0.00	0.00	0.34
GATCAAACAATCCCTCTCCT	0.23	1.30	0.29	0.34	0.69
GATCAAACAATCGACCCTGT	0.46	0.00	0.00	0.34	0.00
GATCAAACAATCTACTGAAG	0.00	0.00	0.29	0.00	0.00
GATCAAACAATCTCCAAGAA	0.00	0.00	0.29	0.00	0.00
GATCAAACAATGACCTGCTT	4.12	4.88	2.29	3.05	2.41
GATCAAACAATGACCTTCGT	0.23	0.00	0.00	0.00	0.00
GATCAAACAATGCGCCACGG	0.00	0.00	0.29	0.00	0.00
GATCAAACAATGTCGAGGTG	0.00	0.00	0.29	0.00	0.00
GATCAAACAATGTTGGGGCA	0.00	0.00	0.00	0.00	0.69
GATCAAACAATTAAAAAAAA	0.00	0.00	0.00	0.34	0.00
GATCAAACAATTCCGACCTA	0.23	0.00	0.29	0.34	0.00
GATCAAACAATTCCGTGACG	0.00	0.33	0.57	0.68	0.00
GATCAAACAATTGCAAGAAC	0.00	0.00	0.00	0.68	0.00
GATCAAACACAAAAAAAAAA	0.00	0.00	0.00	0.00	0.00
GATCAAACACAAAAACAACA	0.00	0.00	0.00	0.00	0.00

GATCAAACACAAAACAAAAA	0.00	0.00	0.29	0.00	0.00
GATCAAACACAAAACTGGCG	0.00	0.00	0.00	0.00	0.69
GATCAAACACAAACAAAGCG	0.00	0.00	0.29	0.00	0.00
GATCAAACACAAACACAAAC	0.00	0.00	0.29	0.00	0.00
GATCAAACACAAACCACAAA	0.00	0.00	0.00	0.00	0.34
GATCAAACACAACAAATAAA	0.00	0.00	0.00	0.00	0.34
GATCAAACACAACACAAACA	0.23	0.00	0.00	0.00	0.00
GATCAAACACAACACAACAA	0.00	0.00	0.00	0.00	0.34
GATCAAACACAACACAACAC	0.00	0.00	0.29	0.00	2.76
GATCAAACACAACACCAAAA	0.00	0.00	0.00	0.00	0.34
GATCAAACACAACATGGCGT	0.00	0.00	0.29	0.00	0.00
GATCAAACACAACCGCAACC	0.00	0.00	0.57	0.00	0.00
GATCAAACACAACGAAACAA	0.00	0.00	0.00	0.00	0.34
GATCAAACACAACTGCCGAA	0.00	0.00	0.00	0.00	0.34
GATCAAACACAAGAGATTGT	2.06	2.60	0.57	2.38	0.69
GATCAAACACAAGCGAGGGA	0.00	0.00	0.00	0.00	0.34
GATCAAACACAAGGACTTCT	0.00	0.00	0.00	1.02	0.34
GATCAAACACAAGTATAGAC	0.00	0.00	0.00	0.00	0.34
GATCAAACACAATGAGAAGA	0.00	0.00	0.00	0.00	0.00
GATCAAACACAATGCTAGCT	0.00	0.00	0.00	0.00	0.00
GATCAAACACAATTCANAAG	0.00	0.00	0.00	0.00	0.34
GATCAAACACAATTTATTTG	0.00	0.00	0.00	0.00	0.34
GATCAAACACACAACACAAC	0.00	0.00	0.29	0.00	0.00
GATCAAACACACAAGGACAT	0.00	0.00	0.00	0.00	0.69
GATCAAACACACAATGTGGG	0.00	0.00	0.00	0.00	0.34
GATCAAACACACAATTCAAA	0.00	0.00	0.00	0.00	0.69
GATCAAACACACACACACCC	0.00	0.00	0.00	0.00	0.34
GATCAAACACACACACACTC	0.00	0.00	0.29	0.00	0.00
GATCAAACACACACACCTAT	0.00	0.00	0.00	0.00	0.00
GATCAAACACACACCACAAC	0.00	0.00	0.00	0.00	0.34
GATCAAACACACACGCGCTC	0.00	0.00	0.00	0.00	0.34
GATCAAACACACACTCCTTT	0.00	0.00	0.00	0.00	0.34
GATCAAACACACAGACGCAC	0.00	0.00	0.00	0.00	0.00
GATCAAACACACATATCACT	0.00	0.00	0.00	0.00	1.38
GATCAAACACACCAACAAAC	0.00	0.00	0.00	0.00	0.69
GATCAAACACACCACACAAC	0.00	0.00	0.00	0.00	0.69
GATCAAACACACCACCACAC	0.00	0.00	0.00	0.00	0.69

GATCAAACACACCCCAAGGT	0.00	0.00	0.00	0.00	0.00
GATCAAACACACCCTGAGCC	0.92	0.33	0.00	0.00	0.69
GATCAAACACACGCCGGGGT	0.00	0.00	0.29	0.00	0.00
GATCAAACACACGCGAAGGT	0.00	0.00	0.00	0.00	0.69
GATCAAACACACGCGAGGGT	0.00	0.00	0.29	0.00	0.69
GATCAAACACACGCGATGGT	0.00	0.00	0.00	0.00	0.69
GATCAAACACACGCGGGGGG	0.00	0.00	0.29	0.00	0.00
GATCAAACACACGCGGGGGT	0.00	0.00	0.57	0.00	0.34
GATCAAACACACGCGGTGGT	0.00	0.00	0.00	0.00	0.34
GATCAAACACACGCTCTGGG	0.00	0.00	0.29	0.00	0.00
GATCAAACACACGGAACCAG	0.00	0.00	0.00	0.00	0.34
GATCAAACACACGGACAAGC	0.00	0.00	0.00	0.00	0.34
GATCAAACACACGGACACAG	0.00	0.00	0.00	0.00	0.34
GATCAAACACACGGCTTTGT	0.00	0.00	0.00	0.34	0.00
GATCAAACACACTTGTCCGT	0.00	0.00	0.29	0.00	0.00
GATCAAACACACTTGTGCCA	0.00	0.00	0.00	0.00	0.00
GATCAAACACAGAAGGACNN	0.23	0.00	0.00	0.00	0.00
GATCAAACACAGAAGGACTG	1.60	1.63	3.15	0.34	1.03
GATCAAACACAGACGAGGGC	0.00	0.00	0.00	0.00	0.34
GATCAAACACAGATGTGGCT	0.00	0.00	0.29	0.00	0.00
GATCAAACACAGGCGAGGGT	0.00	0.00	0.29	0.00	0.00
GATCAAACACAGGGGTTATG	0.00	0.00	0.29	0.00	0.00
GATCAAACACAGGGGTTCTG	0.23	0.33	0.29	0.34	0.00
GATCAAACACAGTGTTGACT	0.00	0.00	0.57	0.00	0.00
GATCAAACACATACGGAAAT	0.00	0.00	0.00	0.00	0.34
GATCAAACACATCAATCACC	0.00	0.00	0.00	0.00	1.38
GATCAAACACATGCTGGTCA	0.00	0.00	0.00	0.00	0.00
GATCAAACACATGGAGAGCA	0.00	0.00	0.00	0.34	0.00
GATCAAACACATGGCAAAGG	0.00	0.00	0.00	0.34	0.00
GATCAAACACATGGCCAAGG	0.23	0.00	0.00	0.00	0.00
GATCAAACACATTGGGTCTC	0.23	0.00	0.00	0.00	0.00
GATCAAACACATTTTCGGCC	1.37	0.00	0.00	0.34	0.00
GATCAAACACATTTTTCTCA	0.46	0.00	0.00	0.00	0.00
GATCAAACACCAAAAAAAAA	0.23	0.00	0.57	0.00	1.03
GATCAAACACCAAAACAATA	0.00	0.00	0.57	0.00	0.00
GATCAAACACCAACGAAACA	0.00	0.00	0.29	0.00	0.00
GATCAAACACCACACCACAA	0.00	0.00	0.00	0.00	0.34

GATCAAACACCAGAACAGAA	0.00	0.00	0.29	0.00	0.00
GATCAAACACCAGTGACTTC	0.00	0.00	0.00	1.36	0.00
GATCAAACACCATCAGTGCT	0.00	0.00	0.00	0.00	0.00
GATCAAACACCATGGAATCC	0.00	0.00	0.00	0.00	0.00
GATCAAACACCATGGAGCAA	0.00	0.65	0.29	0.00	0.00
GATCAAACACCATGGAGCAC	2.29	1.63	4.01	1.70	4.48
GATCAAACACCATGGAGCAG	0.00	0.00	0.00	0.00	0.34
GATCAAACACCATGGAGCCC	0.00	0.00	0.00	0.00	0.34
GATCAAACACCATGGAGCGA	0.00	1.30	0.57	1.70	1.03
GATCAAACACCATGGAGCGC	0.23	0.00	0.00	0.00	0.00
GATCAAACACCATGGAGNAC	0.00	0.00	0.00	0.34	0.00
GATCAAACACCCACCCCAAA	0.23	0.00	0.00	0.00	0.00
GATCAAACACCCACTTCAAA	0.00	0.00	0.00	0.00	0.34
GATCAAACACCCAGCGCTGC	0.23	0.00	0.29	0.34	0.00
GATCAAACACCCCACGGATA	0.00	0.00	0.00	0.00	0.34
GATCAAACACCCCACTCAAG	0.46	0.33	0.00	0.34	0.00
GATCAAACACCCCCACCCGA	0.00	0.00	0.00	0.00	0.34
GATCAAACACCCGAATGACT	0.00	0.00	0.29	0.00	0.00
GATCAAACACCCGGCATGCA	0.00	0.33	0.00	0.00	0.00
GATCAAACACCCGGCCAGCA	0.00	0.33	0.00	0.00	0.00
GATCAAACACCCGGCCTGCA	0.23	0.00	0.00	0.00	0.00
GATCAAACACCCTGGCTGCG	0.00	0.00	0.00	0.00	0.34
GATCAAACACCGAAGCTCAA	0.00	0.00	0.00	0.00	1.03
GATCAAACACCGAAGCTGGT	0.00	0.00	0.00	0.00	0.00
GATCAAACACCGCCCCACAC	0.00	0.00	0.00	0.00	0.34
GATCAAACACCGCCCGCCGG	0.00	0.00	0.29	0.00	0.00
GATCAAACACCGCCGCATCC	0.00	0.00	0.00	0.00	0.00
GATCAAACACCGCTGCTCAG	0.23	0.33	0.00	0.68	1.03
GATCAAACACCGGACCACAG	0.00	0.33	0.00	0.00	0.00
GATCAAACACCGGAGGAGCC	0.00	0.00	0.00	0.00	0.00
GATCAAACACCGGCAGGTGC	0.00	0.00	0.29	0.00	0.00
GATCAAACACCGGCCTGCTT	0.00	0.00	0.00	0.00	0.34
GATCAAACACCGGGCACCAA	0.00	0.33	0.00	0.00	0.00
GATCAAACACCGGGGTTCTG	0.00	0.00	0.29	0.00	0.00
GATCAAACACCGGTCCCGTT	0.00	0.00	0.29	0.00	0.00
GATCAAACACCGTGAGCAAT	0.00	0.33	0.29	0.68	0.69
GATCAAACACCTAGTGACGA	0.00	0.00	0.00	0.00	0.00

GATCAAACACCTCACTGCGC	0.00	0.00	0.29	0.00	0.00
GATCAAACACCTCTGCTACA	0.00	0.00	0.00	0.00	0.34
GATCAAACACCTTAGCACCA	0.00	0.00	0.29	0.00	0.00
GATCAAACACCTTGAAGGCC	0.00	0.00	0.00	0.00	0.00
GATCAAACACCTTTACCCTT	0.00	0.00	0.00	0.00	0.00
GATCAAACACGACACAACAC	0.00	0.00	0.00	0.00	0.34
GATCAAACACGACACGGGCC	0.00	0.00	0.29	0.00	0.00
GATCAAACACGACAGAACAC	0.00	0.00	0.29	0.00	0.00
GATCAAACACGACCGCACGT	0.00	0.00	0.00	0.00	0.00
GATCAAACACGAGTTTGACC	0.00	0.00	0.57	0.34	0.00
GATCAAACACGATAAACCAA	0.00	0.00	0.29	0.00	0.00
GATCAAACACGATGAGCATG	0.00	0.00	0.00	0.34	0.00
GATCAAACACGATGATAACA	0.00	0.00	0.29	0.00	0.00
GATCAAACACGATTTGGGCC	0.00	0.00	0.00	0.00	0.69
GATCAAACACGCACAACACA	0.00	0.00	0.00	0.00	0.34
GATCAAACACGCACGCACAA	0.00	0.00	0.00	0.00	0.34
GATCAAACACGCACTTGTGA	0.00	0.00	0.00	0.00	0.00
GATCAAACACGCAGAAGCCT	0.23	0.00	0.00	0.00	0.00
GATCAAACACGCAGGTCTCA	0.00	0.33	0.00	0.00	0.00
GATCAAACACGCCAATGTCC	0.00	0.33	0.29	0.00	0.00
GATCAAACACGCCCTGGCCA	0.00	0.00	0.00	0.34	0.34
GATCAAACACGCCTGCATTG	0.23	0.00	0.00	0.00	0.00
GATCAAACACGCGAGACCGC	0.00	0.00	0.29	0.00	0.00
GATCAAACACGCGCACACGC	0.00	0.00	0.00	0.00	0.69
GATCAAACACGCGGGGCATA	0.00	0.00	0.29	0.00	0.00
GATCAAACACGCTGCCAGGA	0.00	0.00	0.00	0.00	0.34
GATCAAACACGCTTGTCCTT	0.00	0.00	0.00	0.00	0.69
GATCAAACACGGAATCTCCC	0.00	0.00	0.00	0.00	0.00
GATCAAACACGGCACATCAC	0.00	0.00	0.00	0.00	0.69
GATCAAACACGGCATGGGGA	0.00	0.00	0.29	0.00	0.00
GATCAAACACGGCCGCGTCA	2.75	2.60	2.58	4.41	1.72
GATCAAACACGGCCGCGTCC	0.23	0.00	0.29	0.00	0.00
GATCAAACACGGCCGCGTCN	0.00	0.00	0.00	0.00	0.00
GATCAAACACGGCCGCGTCT	0.00	0.00	0.00	0.34	0.00
GATCAAACACGGCCGCGTGG	0.00	0.33	0.00	0.00	0.00
GATCAAACACGGCCGCTGGC	0.00	0.00	0.00	0.00	0.00
GATCAAACACGGCCGCTTCT	0.00	0.00	0.00	0.00	0.00

GATCAAACACGGCGATAAAG	0.00	0.33	0.00	0.00	0.00
GATCAAACACGGCGATTGCA	0.00	0.00	0.00	0.00	0.00
GATCAAACACGGCGTCCCCA	0.23	0.00	0.00	0.00	0.00
GATCAAACACGGCTGACTGC	0.00	0.00	0.00	0.34	0.00
GATCAAACACGGGCATAGAG	0.00	0.33	0.00	0.00	0.00
GATCAAACACGGGCCAAATG	0.00	0.00	0.00	0.00	0.34
GATCAAACACGGGCGCGTCA	0.00	0.00	0.00	0.34	0.00
GATCAAACACGGGCGGGTCA	0.23	0.00	0.00	0.00	0.00
GATCAAACACGGGGTAAGAC	0.00	0.00	0.00	0.00	0.34
GATCAAACACGGTCGACGAA	0.00	0.33	0.00	0.00	0.00
GATCAAACACGGTCGCGCCC	0.00	0.00	0.00	0.34	0.00
GATCAAACACGGTCGCGGGA	0.23	0.00	0.00	0.00	0.00
GATCAAACACGGTCGCGNNN	0.23	0.00	0.00	0.00	0.00
GATCAAACACGGTCGCGTCA	23.58	16.92	4.58	14.26	7.58
GATCAAACACGGTCGCGTCC	0.23	0.33	0.00	0.00	0.00
GATCAAACACGGTCGCGTGA	0.46	4.55	0.29	2.38	1.03
GATCAAACACGGTCGCGTNC	0.00	0.00	0.00	0.00	0.00
GATCAAACACGGTCGCGTTA	0.23	0.00	0.00	0.00	0.00
GATCAAACACGGTCGCGTTC	0.00	0.33	0.00	0.00	0.00
GATCAAACACGGTCGGGTCC	0.23	0.00	0.00	0.00	0.00
GATCAAACACGGTTAATGTG	0.00	0.00	0.00	0.00	0.34
GATCAAACACGTACAACACA	0.00	0.00	0.00	0.00	0.34
GATCAAACACGTACACATTA	0.00	0.00	0.00	0.00	0.34
GATCAAACACGTCGTCGTCC	0.00	0.00	0.00	0.00	0.34
GATCAAACACGTCTGCATGG	0.00	0.33	0.57	0.34	1.03
GATCAAACACGTGGGTAGGT	0.00	0.33	0.00	0.00	0.00
GATCAAACACGTGTGGTTAC	0.00	0.00	0.29	0.00	0.00
GATCAAACACGTTTTTTGCA	0.00	0.00	0.00	0.00	0.34
GATCAAACACTAGAAACCGG	0.23	0.65	0.00	0.00	0.00
GATCAAACACTATCAACGCG	0.00	0.00	0.00	0.00	0.69
GATCAAACACTCAAAATACA	0.00	0.00	0.00	0.00	0.00
GATCAAACACTCCGCACAGC	0.46	0.33	0.57	0.34	0.00
GATCAAACACTCCGCACCGC	0.23	0.00	0.00	0.00	0.00
GATCAAACACTCCGGCATGA	0.46	0.00	0.00	0.00	0.00
GATCAAACACTCGAGAAAAA	0.00	0.00	0.00	0.00	0.00
GATCAAACACTCTTACAACT	0.00	0.00	0.00	0.00	0.69
GATCAAACACTGAACATGAT	0.00	0.00	0.00	0.00	0.00

GATCAAACACTGCAGAGCCA	0.00	0.00	0.00	0.00	0.34
GATCAAACACTGCCGCCAAA	0.00	0.00	0.29	0.00	0.00
GATCAAACACTGCTGGAATC	0.00	0.00	0.00	0.00	0.34
GATCAAACACTGCTGGCAAG	0.00	0.00	0.00	0.00	0.00
GATCAAACACTGGAAACACA	0.00	0.00	0.00	0.00	0.69
GATCAAACACTGGCCTGCTT	3.66	6.18	3.15	5.77	4.13
GATCAAACACTGGGAGAGGC	0.00	0.00	0.00	0.00	0.00
GATCAAACACTGGGGAAAAA	0.00	0.00	0.57	0.00	0.00
GATCAAACACTGGGGAAAAC	0.00	0.00	0.00	0.00	0.00
GATCAAACACTGGTATGAAG	1.37	3.58	1.72	4.41	1.38
GATCAAACACTGGTATGAAT	0.00	0.98	0.00	0.00	0.00
GATCAAACACTGGTATGCCG	0.00	0.00	0.29	0.00	0.00
GATCAAACACTGGTGCGGAA	0.00	0.33	0.00	0.00	0.00
GATCAAACACTGTCCCGCTC	0.00	0.33	0.00	0.34	0.34
GATCAAACACTGTCCCTTGT	0.00	0.00	0.00	0.34	0.00
GATCAAACACTGTGATGACA	0.00	0.00	0.00	0.00	0.00
GATCAAACACTGTGGTTTCA	0.23	0.00	0.00	0.00	0.00
GATCAAACACTTACAGGCCA	0.00	0.33	0.00	1.02	0.00
GATCAAACACTTATACAAGA	0.00	0.00	0.29	0.00	0.00
GATCAAACACTTCCTTTTTT	0.23	0.00	0.00	0.00	0.00
GATCAAACACTTCTTGCTGC	0.00	0.00	0.00	0.00	0.00
GATCAAACACTTGGAGACAA	0.00	0.00	0.29	0.00	0.00
GATCAAACACTTGGAGGTCC	0.00	0.00	0.00	0.34	0.00
GATCAAACACTTTTGGGGCA	0.00	0.00	0.29	0.00	0.00
GATCAAACAGAAAAAAAAAA	1.60	0.33	1.15	1.02	0.00
GATCAAACAGAAAAAACAAA	0.00	0.00	0.00	0.00	0.00
GATCAAACAGAAAAGGCAGG	0.23	0.00	0.00	0.00	0.00
GATCAAACAGAACATGACAT	0.00	0.00	0.00	0.00	0.34
GATCAAACAGAACTTGCTGT	0.00	0.00	0.00	0.00	0.00
GATCAAACAGAAGAACGAGG	0.00	0.33	0.00	0.00	0.00
GATCAAACAGAAGAAGCAGA	0.00	0.00	0.00	0.00	0.34
GATCAAACAGAAGGTCAAAC	0.00	0.00	0.00	0.00	0.69
GATCAAACAGAAGTGCACTG	0.23	0.00	0.00	0.00	0.00
GATCAAACAGAATGACCGGG	0.00	0.33	0.00	0.00	0.00
GATCAAACAGAATTGATGTT	0.00	0.00	0.00	0.00	0.34
GATCAAACAGACAAGACGTC	0.00	0.00	0.00	0.00	0.34
GATCAAACAGACACAGGCAC	0.00	0.00	0.29	0.00	1.03

GATCAAACAGACAGACAGAC	0.00	0.00	0.29	0.00	0.00
GATCAAACAGACAGGAAAAA	0.00	0.00	0.00	0.00	0.00
GATCAAACAGACAGGACCCC	3.20	1.95	2.00	0.68	1.38
GATCAAACAGACAGGACCCG	0.00	0.00	0.29	0.00	0.00
GATCAAACAGACAGGACCGC	0.00	0.00	0.00	0.00	0.00
GATCAAACAGACAGGACCGT	0.23	0.00	0.00	0.00	0.34
GATCAAACAGACAGGACTCA	0.00	0.65	0.86	0.34	0.00
GATCAAACAGACAGGCACGT	0.00	0.00	0.29	0.00	0.00
GATCAAACAGACAGTATCAA	0.00	0.00	0.29	0.00	0.00
GATCAAACAGACAGTGAGAA	0.00	0.00	0.00	0.68	0.00
GATCAAACAGACCCGTGCTG	0.00	0.00	0.00	0.00	0.00
GATCAAACAGACCTGCGTGC	0.00	0.00	0.00	0.00	0.34
GATCAAACAGACGGCTGGCG	0.23	0.98	0.57	1.02	0.00
GATCAAACAGACGGGCGCCA	0.00	0.33	0.00	0.00	0.00
GATCAAACAGACTTACACGT	0.23	0.00	0.00	0.00	0.00
GATCAAACAGAGCAACAGGA	0.00	0.33	0.00	0.00	0.00
GATCAAACAGAGCCACGAGA	0.00	0.00	0.00	0.34	0.00
GATCAAACAGAGCGCCAGCG	0.00	0.00	0.29	0.00	0.00
GATCAAACAGAGCGTTGTTG	0.00	0.00	0.00	0.00	0.00
GATCAAACAGAGGAGACTCA	0.00	0.00	0.00	0.00	0.34
GATCAAACAGAGGAGCCAGG	0.00	0.00	0.00	0.34	0.00
GATCAAACAGAGGAGCCGCA	0.23	0.00	0.00	0.34	0.00
GATCAAACAGAGGAGCCTCA	15.57	8.13	7.73	14.60	15.50
GATCAAACAGAGGAGCCTCG	0.23	0.00	0.00	0.00	0.00
GATCAAACAGAGGAGCNTNN	0.23	0.00	0.00	0.00	0.00
GATCAAACAGAGGAGGATAA	0.23	0.00	0.00	0.00	0.00
GATCAAACAGAGGTAACCAG	0.00	0.00	0.29	0.00	0.00
GATCAAACAGATACACACTA	0.00	0.00	0.00	0.34	0.00
GATCAAACAGATGAGGAAGA	0.00	0.00	0.57	0.00	0.00
GATCAAACAGATGATTGATG	0.00	0.00	0.29	0.00	0.00
GATCAAACAGATGCAATGGT	0.00	0.00	0.29	0.00	0.00
GATCAAACAGATGGGAAGGA	0.00	0.00	0.00	0.00	0.34
GATCAAACAGATTAGGGTGC	0.23	0.00	0.00	0.00	0.00
GATCAAACAGATTTCGGCCA	0.00	0.00	0.00	0.00	0.00
GATCAAACAGCAAAAAAAAA	4.58	3.25	0.57	3.05	2.41
GATCAAACAGCAAAAGAAAA	0.00	0.00	0.00	0.00	0.34
GATCAAACAGCAAACTTGCA	0.00	0.00	0.57	0.00	0.00

GATCAAACAGCAAACTTGCT	0.46	0.33	0.00	0.00	0.34
GATCAAACAGCAAAGAAAAA	0.00	0.00	0.00	0.00	0.00
GATCAAACAGCAAAGGCAGA	0.23	0.00	0.00	0.00	0.00
GATCAAACAGCAAATAAAAA	0.23	0.00	0.00	0.00	0.00
GATCAAACAGCAACATCACC	0.00	0.00	0.29	0.00	0.00
GATCAAACAGCAACGCATGA	0.00	0.00	0.57	0.00	0.00
GATCAAACAGCAATGCCAGG	0.00	0.65	0.00	0.00	0.00
GATCAAACAGCAATGGAGCC	0.00	0.00	0.00	0.00	0.34
GATCAAACAGCACGACNTCT	0.00	0.00	0.00	0.00	0.34
GATCAAACAGCACGACTTCT	0.23	0.65	0.29	0.34	0.00
GATCAAACAGCACTTGGGGA	0.00	0.00	0.00	0.00	0.00
GATCAAACAGCACTTGGTGA	1.37	2.28	0.29	1.70	0.00
GATCAAACAGCACTTTGAGG	0.00	0.00	0.00	0.00	0.00
GATCAAACAGCAGCGTGCTC	0.00	0.65	0.00	0.00	0.00
GATCAAACAGCAGCTCGCGA	0.00	0.33	0.00	0.00	0.00
GATCAAACAGCAGGTGGCCA	0.46	0.00	0.00	0.34	0.00
GATCAAACAGCAGGTGGCCG	0.00	0.33	0.00	0.00	0.00
GATCAAACAGCAGTCCAAGG	0.00	0.00	0.29	0.00	0.00
GATCAAACAGCAGTGCGAAG	0.00	0.00	0.29	0.00	0.00
GATCAAACAGCAGTGTGAAG	0.46	0.33	0.86	0.34	0.69
GATCAAACAGCATACACATG	0.46	0.33	0.57	0.00	0.00
GATCAAACAGCATATTGCCT	0.00	0.33	0.29	0.00	0.00
GATCAAACAGCATATTGCTG	0.46	1.63	0.29	0.68	1.38
GATCAAACAGCATATTGCTT	0.00	0.00	0.00	0.00	0.69
GATCAAACAGCATTCCAGAA	0.00	0.00	0.00	0.00	0.00
GATCAAACAGCATTTCGATG	0.00	0.00	0.00	0.00	0.00
GATCAAACAGCCACGACATC	0.00	0.00	0.00	0.00	0.00
GATCAAACAGCCAGAGAGCN	0.23	0.00	0.00	0.00	0.00
GATCAAACAGCCAGGCTCAA	0.69	1.30	0.29	0.34	0.00
GATCAAACAGCCAGGCTCAG	0.00	0.00	0.00	0.34	0.00
GATCAAACAGCCATGCGCAC	0.00	0.00	0.29	0.34	0.00
GATCAAACAGCCATTGCACT	0.00	0.33	0.00	0.00	0.00
GATCAAACAGCCATTGCTCA	0.00	0.33	0.00	0.00	0.00
GATCAAACAGCCATTGCTCC	0.00	0.33	0.57	0.00	0.00
GATCAAACAGCCGACTACCA	0.00	0.33	0.00	0.00	0.00
GATCAAACAGCCGAGGCAGG	0.69	0.65	0.86	0.34	0.00
GATCAAACAGCCGAGGCGGA	0.00	0.33	0.00	0.00	0.00

GATCAAACAGCCGGGCGTGG	0.00	0.33	0.00	0.00	0.00
GATCAAACAGCCGTATCCGC	0.23	0.33	0.00	0.00	0.00
GATCAAACAGCCGTGCCTGG	0.92	0.00	0.57	0.00	0.34
GATCAAACAGCCGTGCCTGN	0.23	0.00	0.00	0.00	0.00
GATCAAACAGCCTAGAATGC	0.00	0.00	0.00	0.00	0.34
GATCAAACAGCCTGCATGGC	0.00	0.00	0.00	0.34	0.00
GATCAAACAGCCTGCTCGCC	0.00	0.00	0.00	0.00	0.00
GATCAAACAGCCTGGACGTC	0.00	0.00	0.57	0.00	0.00
GATCAAACAGCGAAAAAAAA	0.69	0.33	0.00	0.34	0.69
GATCAAACAGCGAGGAAACG	0.00	0.00	0.00	0.00	0.34
GATCAAACAGCGATGACGAC	0.00	0.00	0.29	0.34	0.00
GATCAAACAGCGCAAAAAAA	20.37	4.88	5.44	5.43	3.79
GATCAAACAGCGCAAAAACN	0.23	0.00	0.00	0.00	0.00
GATCAAACAGCGCAAAACAA	0.23	0.00	0.00	0.00	0.00
GATCAAACAGCGCAAACANN	0.23	0.00	0.00	0.00	0.00
GATCAAACAGCGCAAAGAAA	0.23	0.00	0.00	0.00	0.00
GATCAAACAGCGCAAANNNN	0.23	0.00	0.00	0.00	0.00
GATCAAACAGCGCAAATAAA	0.00	0.00	0.00	0.00	0.34
GATCAAACAGCGCAACCCTG	0.00	0.33	0.00	0.00	0.00
GATCAAACAGCGCAACNNNN	0.23	0.00	0.00	0.00	0.00
GATCAAACAGCGCACCAAAA	1.60	0.33	0.29	0.34	0.00
GATCAAACAGCGCACCAGAA	0.00	0.00	0.29	0.00	0.00
GATCAAACAGCGCACCCAAA	0.92	0.00	0.00	0.00	1.03
GATCAAACAGCGCACCCANN	0.23	0.00	0.00	0.00	0.00
GATCAAACAGCGCACCCCAA	0.00	0.00	0.00	0.34	1.03
GATCAAACAGCGCACCCCCC	0.00	0.00	0.00	0.34	0.00
GATCAAACAGCGCACCCCTC	9.61	5.21	4.87	4.75	4.13
GATCAAACAGCGCACCCCTT	0.00	0.00	0.00	0.00	0.69
GATCAAACAGCGCGAACATG	0.00	0.00	0.29	0.00	0.00
GATCAAACAGCGCGAACATT	0.00	0.00	0.57	0.00	0.00
GATCAAACAGCGCGGGCTAC	0.23	0.00	0.57	0.34	0.00
GATCAAACAGCGGCTTGGTA	0.00	0.00	0.00	0.00	0.34
GATCAAACAGCGTGAAAAAA	0.00	0.00	0.29	0.00	0.00
GATCAAACAGCGTTGGCCAC	0.00	0.00	0.00	0.34	0.00
GATCAAACAGCTAAAAAAAA	0.00	0.33	0.00	0.00	0.00
GATCAAACAGCTCAAAAAAA	0.00	0.00	0.00	0.00	0.34
GATCAAACAGCTCACGGGCA	3.66	4.23	1.15	3.05	0.69

GATCAAACAGCTCATCCCCA	0.00	0.00	0.00	0.34	0.00
GATCAAACAGCTCCGCCCAC	0.00	0.00	0.00	0.00	0.34
GATCAAACAGCTCGGTGTGC	0.00	0.00	0.00	0.00	0.34
GATCAAACAGCTGAGGGAGC	0.23	0.98	0.29	0.00	0.00
GATCAAACAGCTGAGGGGGC	0.00	0.00	0.29	0.00	0.00
GATCAAACAGCTGCAGCGCC	33.65	4.23	2.29	3.73	0.34
GATCAAACAGCTGCAGCGGC	0.46	0.00	0.00	0.00	0.00
GATCAAACAGCTGCAGCGNN	0.23	0.00	0.00	0.00	0.00
GATCAAACAGCTGCAGGGCC	0.23	0.00	0.00	0.00	0.00
GATCAAACAGCTGGAGCGGG	0.00	0.00	0.00	0.00	0.00
GATCAAACAGCTGGAGGAGG	0.00	0.00	0.00	0.00	0.00
GATCAAACAGCTGGCCTTTG	0.23	0.00	0.00	0.00	0.00
GATCAAACAGCTTAAGTGTA	0.00	0.00	0.00	0.68	0.00
GATCAAACAGCTTAAGTGTC	0.46	0.00	0.00	0.00	0.00
GATCAAACAGCTTACACGTG	0.00	0.00	0.00	0.00	0.00
GATCAAACAGCTTCAAATTG	0.00	0.33	0.29	0.34	0.00
GATCAAACAGCTTCAGCAGC	1.14	0.33	0.29	0.34	0.34
GATCAAACAGCTTGGTACAA	0.00	0.00	0.00	0.00	0.00
GATCAAACAGCTTGTAGAAG	0.00	0.00	0.29	0.00	0.00
GATCAAACAGGAAAAAAAAA	1.14	0.00	1.15	1.02	1.03
GATCAAACAGGAAAAGAAAA	0.00	0.00	0.00	0.34	0.00
GATCAAACAGGAAAAGGGAA	0.23	1.63	0.29	0.00	0.00
GATCAAACAGGACATGAGGC	0.00	0.00	0.00	0.00	0.00
GATCAAACAGGACCGCGGGA	0.00	0.00	0.00	0.00	0.00
GATCAAACAGGACCGTCAGG	0.00	0.33	0.00	0.00	0.00
GATCAAACAGGACTTTGGTC	0.00	0.00	0.29	0.00	0.00
GATCAAACAGGAGCTGAAAA	1.37	0.00	0.00	1.02	0.69
GATCAAACAGGAGCTGAAAG	0.92	0.00	0.00	0.68	0.00
GATCAAACAGGATGCAAGAC	0.00	0.00	0.00	0.34	0.00
GATCAAACAGGCAATCGCAG	0.00	0.00	0.00	0.00	0.34
GATCAAACAGGCAATGCTTG	0.00	0.00	0.00	0.00	0.00
GATCAAACAGGCACTCATCA	0.00	0.00	0.29	0.00	0.00
GATCAAACAGGCCATCCACG	0.00	0.33	0.57	0.00	0.00
GATCAAACAGGCCTTGACGA	0.00	0.00	0.00	0.00	0.34
GATCAAACAGGCGAACACGC	0.00	0.00	0.00	0.00	0.34
GATCAAACAGGCGCTTTGCT	0.00	0.00	0.00	0.00	0.00
GATCAAACAGGCGGAGTCTG	0.69	0.00	0.00	0.00	0.00

GATCAAACAGGCTGGAGGAG	0.00	1.30	0.00	1.02	1.03
GATCAAACAGGGAAACAGGT	0.00	0.00	0.00	0.34	0.00
GATCAAACAGGGACACAGGA	0.23	0.00	0.00	0.00	0.00
GATCAAACAGGGCAACAGGA	0.00	0.65	0.00	0.68	0.00
GATCAAACAGGGCAACAGGC	0.00	0.00	0.29	0.00	0.00
GATCAAACAGGGCAATTCCA	0.00	0.00	0.00	0.00	0.00
GATCAAACAGGGCACAGACT	0.00	0.00	0.00	0.34	0.00
GATCAAACAGGGGAGCCTGA	0.23	0.00	0.00	0.00	0.00
GATCAAACAGGGGAGGGTGG	0.23	0.00	0.00	0.00	0.00
GATCAAACAGGTAGGGAAAA	0.00	0.00	0.29	0.00	0.00
GATCAAACAGGTATTCACAA	0.00	0.00	0.29	0.00	0.00
GATCAAACAGGTCTGGAGCC	1.14	0.33	0.86	0.00	0.00
GATCAAACAGGTCTGGAGGA	0.00	0.00	0.29	0.00	0.00
GATCAAACAGGTCTGGAGGG	0.46	0.00	0.29	0.68	0.00
GATCAAACAGGTGAGCGATT	0.23	0.00	0.00	0.00	0.00
GATCAAACAGGTGGAGTGCA	0.00	0.00	0.00	0.34	0.00
GATCAAACAGGTTGAAGCCC	0.00	0.00	0.00	0.00	0.34
GATCAAACAGTAAAAAAAAA	0.46	0.00	0.00	0.00	0.00
GATCAAACAGTAAACCAACC	0.23	0.00	0.00	0.00	0.00
GATCAAACAGTAAAGGCAGA	0.23	0.00	0.00	0.00	0.34
GATCAAACAGTAATTCACAA	0.00	0.00	0.00	0.00	0.00
GATCAAACAGTAATTCACAG	0.00	0.00	0.00	0.00	0.34
GATCAAACAGTACGCGGAGG	0.00	0.65	2.00	0.00	0.00
GATCAAACAGTAGTCCATGC	0.00	0.00	0.00	0.00	0.00
GATCAAACAGTATTCAAGGA	0.69	1.30	0.57	0.68	0.69
GATCAAACAGTATTCACGGA	0.00	0.00	0.00	0.00	0.00
GATCAAACAGTCAACAGAGG	0.00	0.00	0.29	0.00	0.34
GATCAAACAGTCCGAGCGGT	0.00	0.00	0.00	0.00	1.03
GATCAAACAGTCGAATTGCT	0.00	0.00	0.00	1.36	0.00
GATCAAACAGTCGGGAGCAG	0.00	0.00	0.29	0.00	0.00
GATCAAACAGTCGTATCATT	0.00	0.00	0.00	0.00	0.00
GATCAAACAGTCTGAGCCGT	0.23	0.00	0.00	0.00	0.00
GATCAAACAGTGAAAAAAAA	0.00	0.33	0.00	0.34	0.00
GATCAAACAGTGAAAGCGAA	0.00	0.33	0.86	0.34	0.00
GATCAAACAGTGAACCACAG	0.00	0.00	0.00	0.34	0.00
GATCAAACAGTGAATCGAGG	0.00	0.65	0.00	0.34	0.00
GATCAAACAGTGACGGGGAC	0.23	0.00	0.00	0.00	0.00

GATCAAACAGTGCAAAGTCA	0.00	0.00	0.00	0.00	0.00
GATCAAACAGTGCAGAGTAT	0.00	0.00	0.00	0.00	0.34
GATCAAACAGTGCCAACTCA	0.23	0.00	0.00	0.00	0.00
GATCAAACAGTGCCATGCTG	0.00	0.00	0.00	0.00	0.00
GATCAAACAGTGCCCGTTTT	0.00	0.00	0.00	0.34	0.00
GATCAAACAGTGCGGGCTAC	0.00	0.33	0.00	0.00	0.00
GATCAAACAGTGCTGCAGGC	1.37	0.65	0.29	0.34	0.00
GATCAAACAGTGGCAAAAAA	0.00	0.33	0.00	0.00	0.00
GATCAAACAGTGGCCGCGTC	3.20	2.28	3.72	1.36	0.34
GATCAAACAGTGGCCGCGTG	0.23	0.00	0.00	0.00	0.00
GATCAAACAGTGGCTGCGGC	0.00	0.00	0.00	0.00	0.00
GATCAAACAGTGGCTGCGTC	0.00	0.00	0.00	0.00	0.00
GATCAAACAGTGGTGAGTGT	0.00	0.00	0.00	0.00	0.00
GATCAAACAGTGGTTAAACA	0.00	0.00	0.29	0.00	0.00
GATCAAACAGTGTCACCCTG	0.00	0.33	0.00	0.00	0.00
GATCAAACAGTTAGACAGGA	0.00	0.00	0.29	0.00	0.00
GATCAAACAGTTCAACTTTG	0.00	0.98	0.29	0.00	0.00
GATCAAACAGTTGAGTTTGT	0.00	0.00	0.57	0.00	0.00
GATCAAACAGTTGAGTTTTT	0.00	0.00	0.29	0.00	0.00
GATCAAACAGTTGCATGCAG	0.00	0.00	0.00	0.00	0.00
GATCAAACAGTTGCGAAAAA	0.00	0.00	0.00	0.00	0.00
GATCAAACAGTTGGCTGTGA	0.00	0.98	0.86	1.70	0.00
GATCAAACAGTTGTATCAGG	0.23	0.00	0.00	0.00	0.00
GATCAAACAGTTTGCTCTCC	0.69	0.33	0.00	0.00	0.00
GATCAAACAGTTTGGACATC	0.00	0.00	0.57	0.00	0.00
GATCAAACATAAAAAAAAAA	2.06	0.65	0.00	2.04	0.00
GATCAAACATAAAAACTGAA	0.23	0.00	0.00	0.00	0.00
GATCAAACATAACACAACAA	0.00	0.00	0.00	0.00	0.34
GATCAAACATAACACAACAC	0.00	0.00	0.00	0.00	0.34
GATCAAACATAACAGGATGC	0.00	0.00	0.00	0.00	0.00
GATCAAACATACAAAAAAAA	0.23	0.00	0.00	0.00	0.00
GATCAAACATACCAACAATG	0.00	0.00	0.29	0.00	0.00
GATCAAACATACCGAGCTGA	0.00	0.00	0.00	0.00	0.00
GATCAAACATACCGGTTGCC	0.00	0.00	0.00	0.00	0.34
GATCAAACATACGTCGGCAG	0.00	0.00	0.29	0.00	0.00
GATCAAACATACGTCGGCAT	4.58	0.65	1.15	1.70	2.41
GATCAAACATAGAATCGTAG	0.00	0.00	0.00	0.00	0.00

GATCAAACATAGCACGTGTT	0.00	0.00	0.29	0.00	0.00
GATCAAACATAGGCAGATGC	0.00	0.00	0.00	0.00	0.34
GATCAAACATAGTAGGGTGC	0.00	0.00	0.00	0.00	0.34
GATCAAACATATAAGGTAAT	0.00	0.00	0.57	0.00	0.34
GATCAAACATATCTAAAACA	0.23	0.00	0.00	0.00	0.00
GATCAAACATATCTGGGCGC	0.00	0.00	0.00	0.00	0.34
GATCAAACATATGCTGCCGG	0.00	0.00	0.29	0.00	0.00
GATCAAACATATGTCAGTCA	0.00	0.00	0.29	0.00	0.00
GATCAAACATATTTGGAGGA	0.46	0.00	0.00	0.00	0.00
GATCAAACATCAAAAAAAAA	0.69	0.00	0.00	0.00	0.00
GATCAAACATCAAAATACAT	0.00	0.00	0.00	0.00	0.34
GATCAAACATCAACACCATA	0.00	0.00	0.29	0.00	0.00
GATCAAACATCAACTGGATT	0.00	0.33	0.00	0.00	0.00
GATCAAACATCAAGAGAACT	0.00	0.00	0.00	0.68	0.00
GATCAAACATCACCCATGCC	0.00	0.00	0.00	0.00	1.38
GATCAAACATCACCCGATCA	0.00	0.00	0.29	0.00	0.00
GATCAAACATCAGCACGTAC	0.69	0.00	0.00	0.00	0.34
GATCAAACATCAGCATCATG	0.46	0.98	0.00	0.00	0.69
GATCAAACATCAGGGATTTG	0.23	0.00	0.00	0.00	0.00
GATCAAACATCATTGCGAGT	0.00	0.00	0.00	0.34	0.00
GATCAAACATCCAGAGAAAT	0.00	0.65	0.00	0.00	0.00
GATCAAACATCCAGAGAACT	41.89	18.54	12.03	10.18	12.40
GATCAAACATCCAGAGCACT	0.23	0.00	0.29	0.00	0.00
GATCAAACATCCAGAGGACT	0.23	0.00	0.00	0.00	0.00
GATCAAACATCCAGATAACT	0.46	0.00	0.00	0.00	0.00
GATCAAACATCCATGCGGCT	0.00	0.00	0.00	0.00	0.00
GATCAAACATCCTCCGTCAC	0.00	0.65	0.00	0.34	0.00
GATCAAACATCCTGCTCCCG	0.00	0.33	0.00	0.00	0.00
GATCAAACATCCTGCTGCCG	0.00	0.33	0.00	0.00	0.34
GATCAAACATCCTGCTTCCG	0.00	0.33	0.00	0.00	0.00
GATCAAACATCGAAGGCGTT	0.00	0.00	0.00	0.00	0.34
GATCAAACATCGACTGATTT	0.00	0.33	0.00	0.00	0.00
GATCAAACATCGACTGGAGT	0.00	0.00	0.00	0.34	0.34
GATCAAACATCGACTGGANN	0.23	0.00	0.00	0.00	0.00
GATCAAACATCGACTGGATT	14.42	18.87	14.03	11.88	14.12
GATCAAACATCGACTGGCTT	0.00	0.00	0.00	0.00	0.00
GATCAAACATCGCAAATGGG	0.00	0.00	0.00	0.00	0.34

GATCAAACATCGCCTGGATT	0.00	0.00	0.29	0.00	0.00
GATCAAACATCGCCTGGCTT	0.23	0.00	0.00	0.00	0.00
GATCAAACATCGCTCGCGTC	0.00	0.00	0.00	0.00	0.69
GATCAAACATCGGTGGTGGC	0.00	0.65	0.00	0.00	0.00
GATCAAACATCGGTGGTGGT	0.00	0.00	0.00	0.34	0.00
GATCAAACATCGTAGCTCGG	0.23	0.00	0.00	0.00	0.00
GATCAAACATCTACTGGATT	0.00	0.00	0.00	0.00	0.34
GATCAAACATCTCGCCCAGA	0.00	0.00	0.00	0.34	0.00
GATCAAACATCTGCTGTGCG	0.00	0.00	0.29	0.00	0.00
GATCAAACATCTGGAAGCTG	0.23	0.00	0.00	0.00	0.34
GATCAAACATCTGTAAAGCC	0.00	0.65	0.00	0.00	0.00
GATCAAACATCTTGCATTGG	0.00	0.00	0.86	0.00	0.00
GATCAAACATGAAAAAAAAA	0.69	0.00	0.29	1.36	1.03
GATCAAACATGAACGAAGTA	0.00	0.00	0.00	0.00	0.34
GATCAAACATGAACGGGAAC	0.00	0.00	0.29	0.00	0.00
GATCAAACATGAATGCCTCG	0.23	0.00	0.00	0.00	0.00
GATCAAACATGACAGACCTA	0.00	0.00	0.00	0.00	1.72
GATCAAACATGACAGGGATC	0.00	0.00	0.00	0.00	0.00
GATCAAACATGACTCTGGGC	0.00	0.00	0.00	0.00	0.34
GATCAAACATGATAGCTTCG	0.23	0.00	0.00	0.00	0.00
GATCAAACATGCAAAAAAAA	0.00	0.00	0.00	0.00	0.00
GATCAAACATGCAAGCAAAC	0.00	0.00	0.29	0.00	0.00
GATCAAACATGCAAGCACCT	0.00	0.00	2.00	0.00	2.76
GATCAAACATGCACGCACCT	0.00	0.00	0.57	0.00	0.00
GATCAAACATGCAGGACCCT	0.00	0.00	0.00	0.00	0.00
GATCAAACATGCATGACCAT	0.00	0.00	0.00	0.00	0.00
GATCAAACATGCATGCCGCC	0.00	0.00	0.29	0.00	0.00
GATCAAACATGCATGTCCGG	0.00	0.00	0.57	0.00	0.00
GATCAAACATGCCAACTTGC	0.00	0.00	0.29	0.00	0.00
GATCAAACATGCCACGATGC	0.00	0.00	0.29	0.00	0.00
GATCAAACATGCCACTTTCA	0.00	0.33	0.00	0.00	0.00
GATCAAACATGCCAGGCTCC	0.46	0.00	0.00	0.00	0.34
GATCAAACATGCCCCGTTCA	0.00	0.00	0.00	0.00	0.00
GATCAAACATGCCCTGCTTG	0.00	0.00	0.29	0.00	0.00
GATCAAACATGCCCTGGCCA	0.00	0.00	0.29	0.00	0.00
GATCAAACATGCCGTCCTGT	0.00	0.00	0.00	0.00	0.00
GATCAAACATGCCTGAGGAT	0.00	0.00	0.29	0.00	0.00

GATCAAACATGCGACACCGG	0.00	0.00	0.00	0.00	0.00
GATCAAACATGCGCGATTGG	0.23	0.00	0.00	0.00	0.00
GATCAAACATGCGGAGCAAA	0.00	0.00	0.00	0.34	0.00
GATCAAACATGCGGCACCGG	0.00	0.00	0.29	0.34	0.34
GATCAAACATGCGGTCGGAG	0.00	0.00	0.00	0.00	0.00
GATCAAACATGCTCAACTCA	0.00	0.00	0.29	0.00	0.00
GATCAAACATGCTGAGCCGT	0.23	0.00	0.00	0.00	0.00
GATCAAACATGCTTCCAGGC	0.00	0.00	0.29	0.00	0.69
GATCAAACATGCTTGTTATT	0.46	0.00	0.00	0.00	0.00
GATCAAACATGGAAAAAAAA	0.00	0.00	0.00	0.00	0.00
GATCAAACATGGAAAAGCNN	0.23	0.00	0.00	0.00	0.00
GATCAAACATGGAAAAGCTG	1.37	0.98	0.57	1.02	1.72
GATCAAACATGGAAAGAAAA	0.00	0.00	0.00	0.00	0.00
GATCAAACATGGAAAGCTGC	0.00	0.00	0.00	0.00	0.00
GATCAAACATGGACAACAAG	0.00	0.00	0.29	0.00	0.00
GATCAAACATGGACACCGCG	0.23	0.00	0.00	0.00	0.00
GATCAAACATGGACACCTCG	0.23	0.33	0.00	0.00	0.00
GATCAAACATGGACCCTGCC	0.00	0.00	0.29	0.00	0.00
GATCAAACATGGAGGATAAA	0.00	0.00	0.00	0.00	0.00
GATCAAACATGGAGGCAACC	0.00	0.00	0.00	0.34	0.00
GATCAAACATGGAGGGAAAG	0.00	0.00	0.00	0.00	0.00
GATCAAACATGGAGGGAACG	0.46	1.30	0.29	1.36	1.03
GATCAAACATGGAGGGACCG	0.00	0.00	0.00	0.00	0.00
GATCAAACATGGATATATCT	0.00	0.00	0.29	0.00	0.00
GATCAAACATGGCAAAAAAA	0.00	0.00	0.00	0.00	0.00
GATCAAACATGGCCTTTGCA	0.23	0.00	0.29	0.34	0.00
GATCAAACATGGCCTTTGCC	0.23	0.00	0.00	0.00	0.00
GATCAAACATGGCTTCAAGG	0.00	0.98	0.00	0.00	0.00
GATCAAACATGGGCGTTGGT	0.69	0.65	1.43	0.68	0.34
GATCAAACATGGTAGGCGGT	0.00	0.00	0.29	0.00	0.00
GATCAAACATGGTCAGACTC	0.00	0.00	0.00	0.34	0.00
GATCAAACATGGTCGCATGG	0.00	0.00	0.00	0.00	0.34
GATCAAACATGGTCGCGCCA	0.00	0.00	0.00	0.68	0.00
GATCAAACATGGTCGCGTCA	6.18	4.23	0.86	5.09	0.34
GATCAAACATGGTCGCGTCC	0.00	0.33	0.00	0.00	0.00
GATCAAACATGGTCGCGTNN	0.00	0.00	0.00	0.00	0.00
GATCAAACATGGTCGCTCGT	0.00	0.00	0.00	0.00	0.34

GATCAAACATGGTCTGCATG	0.00	0.00	0.00	0.00	0.00
GATCAAACATGGTGTTCCTT	0.00	0.33	0.29	0.34	0.00
GATCAAACATGGTTTCGTAT	0.00	0.00	0.00	0.00	0.00
GATCAAACATGGTTTGGTAT	0.23	0.33	0.00	0.68	0.00
GATCAAACATGTAAAAAAAA	0.00	0.00	0.00	0.34	0.00
GATCAAACATGTACAGCGGT	0.00	0.00	0.00	0.34	0.34
GATCAAACATGTCAAAAAAA	0.69	0.00	0.00	0.00	0.00
GATCAAACATGTCATCAACC	0.00	0.00	0.00	0.00	0.34
GATCAAACATGTCCATCAAC	0.69	0.00	0.00	0.00	0.00
GATCAAACATGTCGAGACCC	0.00	0.00	0.00	0.34	0.00
GATCAAACATGTCGAGACCG	0.00	0.33	0.00	0.00	0.00
GATCAAACATGTCGAGCACG	0.00	0.00	0.00	0.00	0.00
GATCAAACATGTCGAGGAAA	0.00	0.33	0.00	0.00	0.00
GATCAAACATGTCGAGGAAG	0.00	0.00	0.29	0.00	0.00
GATCAAACATGTCGAGGCAA	0.23	0.00	0.00	0.00	0.34
GATCAAACATGTCGAGGCAG	0.00	0.00	0.00	0.34	0.00
GATCAAACATGTCGAGGCCA	4.81	5.21	2.58	8.49	7.92
GATCAAACATGTCGAGGCCG	7.55	8.78	2.86	9.50	6.89
GATCAAACATGTCGAGGCCN	0.23	0.00	0.00	0.00	0.00
GATCAAACATGTCGAGGCCT	0.00	0.00	0.00	0.00	0.00
GATCAAACATGTCGAGGCNN	0.00	0.00	0.00	0.00	0.34
GATCAAACATGTCGAGGCTA	0.00	0.00	0.00	0.00	0.00
GATCAAACATGTCGAGGCTG	0.00	0.33	0.00	0.00	0.34
GATCAAACATGTCGAGGGCG	0.23	0.00	0.00	0.00	0.00
GATCAAACATGTCGAGGTAA	0.00	0.00	0.00	0.68	0.00
GATCAAACATGTCGAGTCCT	0.00	0.33	0.00	0.00	0.00
GATCAAACATGTCGTGGTTC	0.00	0.00	0.00	0.00	0.00
GATCAAACATGTGTGCCTGG	0.69	0.00	0.00	0.34	0.00
GATCAAACATGTTCGTATGC	0.00	0.00	0.00	0.00	0.69
GATCAAACATGTTGACGTCC	0.46	0.00	0.00	0.00	0.00
GATCAAACATGTTGCTGTCG	0.00	0.00	0.29	0.00	0.00
GATCAAACATGTTGGGGGGG	0.00	0.00	0.29	0.00	0.00
GATCAAACATGTTGTCCAAC	0.00	0.00	0.29	0.00	0.00
GATCAAACATGTTGTTGCGC	0.00	0.00	0.00	0.00	0.00
GATCAAACATTAAAGTTTAA	0.23	0.00	0.00	0.00	0.00
GATCAAACATTACACCACCA	0.00	0.00	0.00	0.00	0.00
GATCAAACATTACATCGGCC	0.00	0.00	0.29	0.00	0.00

GATCAAACATTATGAGCCGG	0.00	0.00	0.00	0.00	0.34
GATCAAACATTATGAGCCGT	0.23	0.00	0.29	0.00	0.00
GATCAAACATTATGAGTCGT	0.00	0.33	0.00	0.00	0.00
GATCAAACATTCAGAGCCGT	0.00	0.00	0.29	0.00	0.00
GATCAAACATTCCGAGCCGT	0.00	0.00	0.00	0.00	0.34
GATCAAACATTCCGCGCAGC	0.00	0.00	0.00	0.00	0.00
GATCAAACATTCGCAGCCCA	0.00	0.00	0.00	0.00	0.34
GATCAAACATTCGGCTCCGT	0.23	0.00	0.00	0.00	0.00
GATCAAACATTCTAAGCCGT	0.00	0.33	0.00	0.34	0.00
GATCAAACATTCTCAAGAAA	0.46	0.00	0.00	0.34	0.00
GATCAAACATTCTGAACAGC	0.00	0.00	0.00	0.00	0.34
GATCAAACATTCTGAACTCG	0.00	0.33	0.00	0.00	0.00
GATCAAACATTCTGACCCGT	0.00	0.00	0.00	0.00	0.34
GATCAAACATTCTGAGACGC	0.00	0.00	0.29	0.00	0.00
GATCAAACATTCTGAGACGT	0.46	0.00	0.57	0.34	0.69
GATCAAACATTCTGAGCAAC	0.00	0.00	0.00	0.00	0.34
GATCAAACATTCTGAGCAGA	0.00	0.33	0.00	0.00	0.00
GATCAAACATTCTGAGCAGC	0.00	0.00	0.29	0.00	0.00
GATCAAACATTCTGAGCAGT	0.23	0.00	0.57	0.34	0.00
GATCAAACATTCTGAGCCAA	2.52	1.95	0.86	1.36	3.10
GATCAAACATTCTGAGCCAG	0.00	0.00	0.29	0.00	0.00
GATCAAACATTCTGAGCCAT	0.00	0.00	0.00	0.34	0.00
GATCAAACATTCTGAGCCCC	0.00	0.00	0.29	0.00	0.00
GATCAAACATTCTGAGCCCT	0.00	0.00	0.00	0.00	0.00
GATCAAACATTCTGAGCCGA	0.00	0.00	0.00	0.00	0.34
GATCAAACATTCTGAGCCGC	0.46	0.33	0.29	0.34	0.69
GATCAAACATTCTGAGCCGG	0.69	0.33	0.57	0.00	0.00
GATCAAACATTCTGAGCCGN	1.37	0.00	0.00	0.68	0.00
GATCAAACATTCTGAGCCGT	229.82	140.22	162.37	160.89	149.47
GATCAAACATTCTGAGCCNN	0.69	0.00	0.00	0.00	0.00
GATCAAACATTCTGAGCCNT	0.00	0.00	0.00	0.68	0.00
GATCAAACATTCTGAGCCTC	0.00	0.33	0.00	0.00	0.00
GATCAAACATTCTGAGCCTG	0.23	0.33	0.29	0.00	0.34
GATCAAACATTCTGAGCCTT	0.00	0.00	0.00	0.00	0.34
GATCAAACATTCTGAGCGGC	0.00	0.33	0.00	0.00	0.00
GATCAAACATTCTGAGCGTA	0.00	0.00	0.00	0.34	0.00
GATCAAACATTCTGAGCTGT	0.00	0.00	0.29	0.00	0.00

GATCAAACATTCTGAGCTTC	0.00	0.00	0.29	0.00	0.00
GATCAAACATTCTGAGGCGA	0.00	0.33	0.00	0.00	0.00
GATCAAACATTCTGAGNCGT	0.00	0.00	0.00	0.00	0.69
GATCAAACATTCTGAGNNNN	0.23	0.00	0.00	0.00	0.00
GATCAAACATTCTGAGTAGT	0.23	0.00	0.00	0.00	0.00
GATCAAACATTCTGAGTCGT	4.12	1.30	2.00	0.34	1.03
GATCAAACATTCTGAGTCTA	0.00	0.65	0.00	0.00	0.00
GATCAAACATTCTGATACGT	0.23	0.00	0.00	0.00	0.00
GATCAAACATTCTGCGACGT	0.23	0.00	0.00	0.00	0.00
GATCAAACATTCTGCGCCGT	0.23	0.00	0.00	0.00	0.34
GATCAAACATTCTGCTCCGT	0.23	0.00	0.00	0.00	0.00
GATCAAACATTCTGCTTGTG	0.00	0.00	0.00	0.34	0.00
GATCAAACATTCTGGGCCGT	0.23	0.00	0.00	0.00	0.00
GATCAAACATTCTGTGCCAA	0.00	0.00	0.00	0.00	0.00
GATCAAACATTCTTAGCAGT	0.00	0.00	0.00	0.00	0.34
GATCAAACATTGAACAGCTT	0.23	0.00	0.00	0.00	0.00
GATCAAACATTGAAGGTGGC	0.00	0.00	0.57	0.00	0.00
GATCAAACATTGACTAACAG	0.00	0.00	0.00	0.34	0.00
GATCAAACATTGATGACATG	0.23	0.00	1.15	0.00	0.00
GATCAAACATTGATGTGGCA	0.00	0.00	0.00	0.00	0.00
GATCAAACATTGCAAATGGG	0.00	0.00	0.00	0.00	0.00
GATCAAACATTGCCTCGCTA	0.00	0.00	0.00	0.00	0.00
GATCAAACATTGCGCGCAGT	0.00	0.33	0.00	0.00	0.00
GATCAAACATTGCGCTCAAG	0.00	0.00	0.29	0.00	0.00
GATCAAACATTGGAAGTAGG	0.00	0.00	0.29	0.00	0.00
GATCAAACATTGGTTGACGT	0.00	0.00	0.29	0.00	0.34
GATCAAACATTGTGAGCCGT	0.23	0.00	0.00	0.00	0.00
GATCAAACATTTAAGGATTT	0.00	0.00	0.00	0.00	0.34
GATCAAACATTTCTCTGGCA	0.00	0.00	0.00	0.34	0.00
GATCAAACATTTGAGAGCCA	0.00	0.33	0.00	0.00	0.00
GATCAAACATTTGGTATGCC	0.46	0.65	0.00	0.00	0.00
GATCAAACATTTGTGGGAGG	0.00	0.33	0.00	0.34	0.00
GATCAAACATTTTCAGCAAA	0.00	0.00	0.00	0.00	0.34
GATCAAACATTTTCAGCAAG	0.00	0.00	0.00	0.00	0.00
GATCAAACATTTTCAGCCAA	0.00	0.98	0.00	0.00	0.69
GATCAAACATTTTCAGCCAG	0.46	0.65	0.86	0.00	1.38
GATCAAACATTTTGCCAACA	0.00	0.00	0.00	0.00	0.00

GATCAAACCAAAAAAAAAAA	8.47	0.33	0.86	0.68	2.76
GATCAAACCAAAAAAAAAAC	0.00	0.00	0.00	0.00	0.34
GATCAAACCAAAAAAAAATC	0.00	0.00	0.00	0.00	0.34
GATCAAACCAAAAAAAACAA	0.00	0.00	0.00	0.00	0.34
GATCAAACCAAAAAAACACA	0.23	0.00	0.00	0.00	0.00
GATCAAACCAAAAAAAGCCA	0.23	0.00	0.00	0.00	0.00
GATCAAACCAAAAAACAAAA	0.23	0.00	0.00	0.00	0.00
GATCAAACCAAAAAACCATA	0.00	0.00	0.00	0.00	0.00
GATCAAACCAAAATTGGCAG	0.00	0.00	0.00	0.00	0.34
GATCAAACCAAACAAAACAA	0.00	0.00	0.00	0.00	0.34
GATCAAACCAAACCAAACCA	0.00	0.00	0.00	0.00	0.34
GATCAAACCAAACGTGTGTT	0.00	0.00	0.00	0.00	0.69
GATCAAACCAAAGCTCTATC	0.23	0.00	0.86	0.68	0.00
GATCAAACCAAAGCTGCCCT	0.92	2.28	0.57	0.34	0.34
GATCAAACCAAAGCTGCCTT	0.00	0.33	0.00	0.00	0.00
GATCAAACCAAATCAAACCA	0.00	0.00	0.86	0.00	0.00
GATCAAACCAAATCGAATCA	0.00	0.00	0.29	0.00	0.00
GATCAAACCAAATGCGGCTG	0.00	0.00	0.29	0.00	0.00
GATCAAACCAAATGGCGCAT	0.00	0.00	0.00	0.00	0.69
GATCAAACCAAATTCAAGAA	0.00	0.00	0.29	0.00	0.00
GATCAAACCAACAAAAAAAA	0.23	0.00	0.00	0.00	0.00
GATCAAACCAACAACAGAGG	0.00	0.00	0.00	0.00	0.00
GATCAAACCAACAACATACC	0.00	0.00	0.00	0.34	0.00
GATCAAACCAACACAACACA	0.00	0.00	0.00	0.00	0.34
GATCAAACCAACAGAAGCAC	0.00	0.00	0.29	0.00	0.00
GATCAAACCAACAGAGCTGA	0.00	0.00	0.00	0.34	0.00
GATCAAACCAACAGCGCTGA	0.00	0.00	0.00	0.00	0.00
GATCAAACCAACAGTGAAGA	0.00	0.33	0.00	0.00	0.00
GATCAAACCAACAGTGCTGA	4.12	14.31	18.61	11.20	6.20
GATCAAACCAACAGTGCTNN	0.23	0.00	0.00	0.00	0.00
GATCAAACCAACATCTGACT	0.00	0.65	0.00	0.00	0.00
GATCAAACCAACCAAACCAA	0.00	0.00	0.00	0.00	0.34
GATCAAACCAACGCACATCA	0.23	0.00	0.00	0.00	0.00
GATCAAACCAACGCCACTGG	0.00	0.00	0.29	0.00	0.00
GATCAAACCAACGGTGCTGA	0.00	0.00	0.00	0.00	0.00
GATCAAACCAACTTGGACCT	0.00	0.00	0.57	0.00	1.03
GATCAAACCAACTTTCATCG	0.00	0.00	0.00	0.34	0.00

GATCAAACCAACTTTTATAG	0.00	0.00	0.00	0.00	0.34
GATCAAACCAACTTTTATCG	11.45	17.57	6.59	19.35	9.64
GATCAAACCAACTTTTATCT	0.23	0.00	0.00	0.00	0.00
GATCAAACCAACTTTTATNN	0.00	0.00	0.00	0.00	0.00
GATCAAACCAACTTTTCTCG	0.00	0.33	0.00	0.00	0.00
GATCAAACCAACTTTTTCGG	0.00	0.33	0.00	0.00	0.00
GATCAAACCAAGAACATGTG	0.00	0.00	0.00	0.00	0.34
GATCAAACCAAGACAAGACA	0.00	0.00	0.29	0.00	0.00
GATCAAACCAAGCAAAAAAA	0.23	0.00	0.00	0.00	0.00
GATCAAACCAAGCACGACTC	0.00	0.00	0.00	0.00	0.34
GATCAAACCAAGCACGACTG	0.00	0.65	0.29	0.00	0.34
GATCAAACCAAGGCCCAGGC	0.23	0.00	0.00	0.00	0.00
GATCAAACCAAGGGACCTTA	0.00	0.00	0.29	0.00	0.00
GATCAAACCAAGGGCAAGAA	0.00	0.00	0.00	0.00	0.00
GATCAAACCAAGGTCGTGCT	0.00	0.00	0.00	0.34	0.00
GATCAAACCAAGTGTTGTTC	0.00	0.00	0.00	0.00	1.03
GATCAAACCAATCAAATACA	0.23	0.00	0.00	0.00	0.00
GATCAAACCAATCAACAGAT	0.23	0.00	0.00	0.00	0.00
GATCAAACCAATCCAGAGGG	0.00	0.00	0.00	0.00	0.34
GATCAAACCAATCCAGTGGG	2.29	3.25	1.43	3.73	1.03
GATCAAACCAATCGCATGGA	0.92	1.95	0.29	0.34	1.38
GATCAAACCAATCGCATGGC	0.00	0.00	0.00	0.00	0.34
GATCAAACCAATCGCTTGGA	0.00	0.33	0.00	0.00	0.00
GATCAAACCAATCGTATGGA	0.00	0.33	0.00	0.00	0.00
GATCAAACCAATGCCGGCCT	0.00	0.00	0.00	0.00	0.34
GATCAAACCAATTCAAGAAT	0.00	0.00	0.29	0.00	0.00
GATCAAACCACAAAAAAAAA	0.00	0.00	0.00	0.00	0.00
GATCAAACCACAAAACCGAC	0.00	0.00	0.00	0.34	0.00
GATCAAACCACAAAAGAAAA	0.00	0.00	0.00	0.00	0.34
GATCAAACCACAAAAGGCTT	0.00	0.00	0.00	0.34	0.00
GATCAAACCACAAACACACC	0.00	0.00	0.00	0.00	0.00
GATCAAACCACAACCCCACA	0.00	0.00	0.00	0.00	0.69
GATCAAACCACACACAACAT	0.00	0.00	0.00	0.00	0.69
GATCAAACCACACACACAAA	0.00	0.00	0.00	0.00	0.69
GATCAAACCACACCAAACTG	0.00	0.00	0.29	0.00	0.00
GATCAAACCACACCACACGA	0.00	0.00	0.00	0.00	0.00
GATCAAACCACACCAGACCA	0.00	0.00	0.00	0.00	0.34

GATCAAACCACACCATACCA	0.00	0.00	0.00	0.00	0.34
GATCAAACCACACCCAACCA	0.00	0.00	0.00	0.00	0.34
GATCAAACCACACCCCCCAC	0.23	0.00	0.00	0.00	0.00
GATCAAACCACACCGCGAAA	0.00	0.00	0.00	0.00	0.69
GATCAAACCACACTCTCGGC	0.23	0.00	0.00	0.00	0.00
GATCAAACCACAGCACAACA	0.00	0.00	0.29	0.00	0.00
GATCAAACCACAGCGCCAGT	0.00	0.00	0.00	0.00	0.00
GATCAAACCACAGGAATAGC	0.00	0.00	0.00	0.00	0.00
GATCAAACCACAGGCANNGC	0.00	0.00	0.00	0.34	0.00
GATCAAACCACAGGCATAGC	6.41	6.51	3.72	5.43	5.85
GATCAAACCACAGGCATAGT	0.00	0.33	0.00	0.00	0.00
GATCAAACCACAGGCATANN	0.23	0.00	0.00	0.00	0.00
GATCAAACCACAGGCATGGC	0.23	0.00	0.00	0.00	0.00
GATCAAACCACAGGCCTAGC	0.23	0.00	0.00	0.00	0.00
GATCAAACCACAGGCCTCAG	0.92	0.00	0.00	0.00	0.00
GATCAAACCACATAGACAAA	0.00	0.00	0.57	0.00	0.00
GATCAAACCACATAGCAAAG	0.00	0.00	0.29	0.00	0.00
GATCAAACCACATGGCGCAA	0.00	0.00	0.00	0.00	0.34
GATCAAACCACATGGCGCAT	0.00	0.00	0.00	0.00	0.69
GATCAAACCACATTGCTCGA	0.00	0.00	0.00	0.00	0.69
GATCAAACCACCAAAATACA	0.00	0.00	0.00	0.34	0.00
GATCAAACCACCAAACACAC	0.23	0.00	0.00	0.00	0.00
GATCAAACCACCAAAGTCGG	0.00	0.65	0.00	0.00	0.00
GATCAAACCACCAAGCCAGC	0.00	0.00	0.29	0.00	0.00
GATCAAACCACCAAGGCATG	0.00	0.00	0.29	0.00	0.00
GATCAAACCACCAAGGCCCA	0.00	0.00	0.00	0.34	0.00
GATCAAACCACCAAGGCGNN	0.00	0.00	0.00	0.00	0.00
GATCAAACCACCAAGGCGTT	0.00	0.98	3.15	2.38	3.10
GATCAAACCACCAAGGTGTA	0.00	0.00	0.00	0.34	0.00
GATCAAACCACCATAGCGGA	0.00	0.33	0.29	0.00	0.00
GATCAAACCACCCAAATACA	0.00	0.65	0.00	0.00	0.00
GATCAAACCACCCTGTAAGG	0.00	0.00	0.29	0.00	0.00
GATCAAACCACCGACCTGTG	0.00	0.00	0.29	0.00	0.00
GATCAAACCACCGGCGGCCT	0.00	0.00	0.00	0.34	0.00
GATCAAACCACCGGCTTCGC	0.23	0.00	0.00	0.00	0.00
GATCAAACCACCGTTTTTCG	0.23	0.00	0.00	0.00	0.00
GATCAAACCACGACGTGCCA	0.23	0.00	0.00	0.00	0.00

GATCAAACCACGAGTGGTGA	0.00	0.00	0.29	0.00	0.00
GATCAAACCACGCAGCAGAG	0.00	0.00	0.29	0.00	0.00
GATCAAACCACGCAGCAGAT	0.00	0.00	0.29	0.00	0.00
GATCAAACCACGCCACCACC	0.00	0.00	0.00	0.00	0.34
GATCAAACCACGGCTGGATT	0.23	0.00	0.00	0.00	0.00
GATCAAACCACGGGGGCGTC	0.00	0.00	0.00	0.00	0.00
GATCAAACCACTCACTGGAC	0.92	0.00	0.00	0.34	0.00
GATCAAACCACTCAGCAGAT	0.00	0.00	0.29	0.00	0.00
GATCAAACCACTGGGGCGTC	0.00	0.33	0.00	0.00	0.00
GATCAAACCACTGTGCATGA	0.00	0.00	0.29	0.00	0.00
GATCAAACCACTTCGCGGCC	0.00	0.65	0.00	0.00	0.34
GATCAAACCAGAAAACAGAC	0.00	0.00	0.29	0.00	0.34
GATCAAACCAGAACATACCA	0.00	0.00	0.00	0.00	0.69
GATCAAACCAGACAACAAAA	0.00	0.33	0.00	0.00	0.00
GATCAAACCAGACCCGGACC	0.00	0.00	0.29	0.00	0.00
GATCAAACCAGACGCAGGAG	0.23	0.00	0.00	0.00	0.00
GATCAAACCAGACTCGTATG	0.23	0.00	0.00	0.00	0.00
GATCAAACCAGACTGGTCCA	0.00	0.00	0.00	0.00	0.34
GATCAAACCAGACTTCGAAA	0.00	0.00	0.00	0.00	0.34
GATCAAACCAGAGCGAGAGG	0.00	0.00	0.29	0.00	0.00
GATCAAACCAGAGTGCCCCT	0.46	0.00	0.00	0.00	0.00
GATCAAACCAGCAGCAGAGG	0.00	0.00	0.00	0.00	0.34
GATCAAACCAGCCAAATACA	0.00	0.00	0.00	0.00	0.34
GATCAAACCAGCCAATGACC	0.00	0.00	0.00	0.00	0.00
GATCAAACCAGCTTTTATCG	0.23	0.00	0.00	0.68	0.00
GATCAAACCAGGACTGTTGC	0.23	0.00	0.00	0.00	0.00
GATCAAACCAGGAGCACCAG	0.00	0.33	0.00	0.00	0.00
GATCAAACCAGGCAATCTCT	0.00	0.33	0.00	0.00	0.00
GATCAAACCAGGCAGTCTGT	0.23	2.93	3.15	0.68	1.72
GATCAAACCAGGCATCCAAC	9.61	6.83	4.30	5.77	2.41
GATCAAACCAGGCATCCAAN	0.23	0.00	0.00	0.00	0.00
GATCAAACCAGGCATCCACN	0.23	0.00	0.00	0.00	0.00
GATCAAACCAGGCCCCTTGG	0.00	0.33	0.57	0.00	0.00
GATCAAACCAGGCCGTCTGT	0.23	0.00	0.00	0.00	0.00
GATCAAACCAGGCCTCTGGG	0.00	0.00	0.00	0.34	0.00
GATCAAACCAGGCCTCTTCG	0.00	0.00	0.00	0.34	0.00
GATCAAACCAGGCCTCTTGG	28.16	49.45	28.35	44.47	34.10

GATCAAACCAGGCCTCTTNN	0.23	0.00	0.00	0.00	0.00
GATCAAACCAGGCCTCTTTC	0.23	0.00	0.00	0.00	0.00
GATCAAACCAGGCGCTCTTC	0.23	0.00	0.00	0.00	0.00
GATCAAACCAGGCGCTTTGC	0.00	0.00	0.00	0.34	0.00
GATCAAACCAGGCGGCAATT	0.23	0.00	0.57	0.34	1.03
GATCAAACCAGGCGGCCANT	0.00	0.00	0.00	0.34	0.00
GATCAAACCAGGCGGCCATT	0.00	0.33	0.00	0.68	0.69
GATCAAACCAGGCGGCCTGA	0.00	0.00	0.00	0.00	0.34
GATCAAACCAGGCGGCCTTC	0.00	0.33	0.29	0.00	0.00
GATCAAACCAGGCGGCCTTT	0.00	0.00	0.29	0.00	0.00
GATCAAACCAGGCGGGCAGA	0.00	0.00	0.00	0.00	0.34
GATCAAACCAGGCGGGCCGA	0.00	0.00	0.00	0.00	0.34
GATCAAACCAGGCTGATGCA	0.23	0.00	0.00	0.00	0.00
GATCAAACCAGGTATTCACA	0.00	0.00	0.29	0.00	0.00
GATCAAACCAGGTCCAGCGC	0.00	0.00	0.00	0.00	0.34
GATCAAACCAGTCGAGACCG	0.23	0.00	0.00	0.00	0.00
GATCAAACCAGTCTCTCCCG	0.00	0.33	0.00	0.00	0.00
GATCAAACCAGTGAAGCTGC	0.00	0.33	0.00	0.00	0.00
GATCAAACCAGTTCCAGCGC	0.00	0.00	0.00	0.00	0.00
GATCAAACCATAAAAAAAAA	6.18	0.00	0.29	0.00	0.34
GATCAAACCATAAAAATACA	0.00	0.00	0.29	0.00	0.00
GATCAAACCATAATCATCAA	1.83	0.00	0.57	0.00	0.00
GATCAAACCATACAAATACA	0.23	0.00	0.00	0.00	0.00
GATCAAACCATACAATGCAC	0.00	0.00	0.00	0.00	0.00
GATCAAACCATACAATGCAG	0.00	0.00	0.00	0.00	0.00
GATCAAACCATAGCAAACCC	0.00	0.00	0.00	0.00	0.34
GATCAAACCATCAAAAAAAA	12.59	1.30	5.15	2.38	4.13
GATCAAACCATCAAAAAAAG	0.23	0.00	0.00	0.00	0.00
GATCAAACCATCAAAAATCT	0.00	0.00	0.00	0.34	0.00
GATCAAACCATCAAAAGAAA	0.00	0.33	0.00	0.00	0.00
GATCAAACCATCAAAAGACA	0.23	0.00	0.00	0.00	0.00
GATCAAACCATCAAAATAAA	1.83	0.98	0.00	0.00	0.34
GATCAAACCATCAAAATACA	108.96	48.80	46.68	29.19	42.36
GATCAAACCATCAAAATACG	0.00	0.00	0.29	0.00	0.00
GATCAAACCATCAAAATACN	1.37	0.00	0.00	0.00	0.00
GATCAAACCATCAAAATACT	0.00	0.33	1.15	0.34	1.03
GATCAAACCATCAAAATANN	0.69	0.00	0.00	0.00	0.00

GATCAAACCATCAAAATGCA	0.00	0.00	0.00	0.00	0.00
GATCAAACCATCAAACNNNN	0.46	0.00	0.00	0.00	0.00
GATCAAACCATCAAACTACA	0.00	0.00	0.00	0.00	0.00
GATCAAACCATCAAATACAA	0.00	0.00	0.00	0.00	0.00
GATCAAACCATCAACAGACA	0.23	0.00	0.00	0.00	0.00
GATCAAACCATCAAGACTGA	0.00	0.00	0.00	0.68	0.00
GATCAAACCATCAAGATACA	0.23	0.00	0.00	0.00	0.00
GATCAAACCATCAATATACA	0.46	0.00	0.00	0.00	0.00
GATCAAACCATCAGAAAAAA	0.23	0.33	0.00	0.00	0.00
GATCAAACCATCAGAATACA	0.00	0.00	0.29	0.00	0.00
GATCAAACCATCAGTGCTGA	0.00	0.00	0.00	0.00	0.00
GATCAAACCATCATCAGTGC	0.00	0.00	0.00	0.00	0.00
GATCAAACCATCCAAAAAAA	0.23	0.00	0.00	0.00	0.00
GATCAAACCATCCAAAAACA	0.00	0.00	0.00	0.34	0.00
GATCAAACCATCCAAACACA	0.00	0.00	0.00	0.34	0.34
GATCAAACCATCCAAAGACA	0.46	0.00	0.00	0.00	0.00
GATCAAACCATCCAAATAAA	0.46	0.33	0.00	0.00	0.00
GATCAAACCATCCAAATACA	130.48	77.10	90.49	75.35	83.69
GATCAAACCATCCAAATACC	0.23	0.00	0.00	0.00	0.34
GATCAAACCATCCAAATACG	0.00	0.00	0.00	0.00	0.00
GATCAAACCATCCAAATACN	0.92	0.00	0.00	0.00	0.00
GATCAAACCATCCAAATACT	0.23	0.00	0.00	0.00	0.00
GATCAAACCATCCAAATAGA	0.00	0.33	0.00	0.00	0.00
GATCAAACCATCCAAATANA	0.00	0.00	0.00	0.00	0.00
GATCAAACCATCCAAATANN	0.46	0.00	0.00	0.00	0.00
GATCAAACCATCCAAATATA	0.00	0.00	0.29	0.00	0.00
GATCAAACCATCCAAATCCA	0.00	0.00	0.00	0.00	0.00
GATCAAACCATCCAAATNCA	0.00	0.00	0.00	0.34	0.00
GATCAAACCATCCAAATNNN	0.23	0.00	0.00	0.00	0.00
GATCAAACCATCCAAATTCA	0.00	0.33	0.00	0.00	0.00
GATCAAACCATCCAACTACA	0.00	0.00	0.00	0.00	0.00
GATCAAACCATCCAAGTACA	0.23	0.00	0.00	0.00	0.00
GATCAAACCATCCATGATGG	0.00	0.00	0.00	0.00	0.69
GATCAAACCATCCCAATACA	0.46	0.00	0.00	0.00	0.00
GATCAAACCATCCCACTCAA	0.23	0.00	0.00	0.00	0.00
GATCAAACCATCCCCATCCC	0.46	0.00	0.00	0.00	0.00
GATCAAACCATCCTTGCTCT	0.00	0.00	0.29	0.00	0.00

GATCAAACCATCGAAAAAAA	0.00	0.33	0.00	0.00	0.00
GATCAAACCATCGAAATACA	0.23	0.00	0.00	0.00	0.00
GATCAAACCATCGCAACTCG	0.00	0.33	0.00	0.00	0.00
GATCAAACCATCGCTGCCCC	0.00	0.00	0.00	0.00	0.34
GATCAAACCATCGGAGGTGC	0.00	0.00	0.00	0.00	0.34
GATCAAACCATCTCGTATGC	0.23	0.00	0.00	0.00	0.00
GATCAAACCATGAAAATACA	0.00	0.00	0.00	0.34	0.00
GATCAAACCATGAAATCATG	0.00	0.00	0.00	0.00	0.34
GATCAAACCATGAAGTCTTT	0.00	0.00	0.00	0.00	0.34
GATCAAACCATGACTAAACA	0.00	0.00	0.29	0.00	0.00
GATCAAACCATGACTAAATC	0.00	0.00	0.00	0.00	0.34
GATCAAACCATGCAACCAGG	0.00	0.65	0.00	0.00	0.00
GATCAAACCATGCAAGTAGA	0.00	0.00	0.00	0.34	0.00
GATCAAACCATGCCAAATCT	0.00	0.00	0.00	0.00	0.34
GATCAAACCATGCCAAATGC	0.92	0.00	0.00	1.02	0.00
GATCAAACCATGCCTGAACC	0.00	0.00	0.29	0.00	0.00
GATCAAACCATGCGGTGGGT	0.00	0.00	0.29	0.00	0.00
GATCAAACCATGCTCATATC	0.00	0.00	0.00	0.00	0.34
GATCAAACCATGGTCAAGCC	0.00	0.00	0.29	0.00	0.00
GATCAAACCATGGTGACTTG	0.00	0.00	0.29	0.00	0.00
GATCAAACCATGTCTGCAGA	0.00	0.33	0.00	0.00	0.00
GATCAAACCATTCAAAAAAA	0.00	0.00	0.00	0.00	0.00
GATCAAACCATTGCACTCCA	0.00	0.00	0.29	0.00	0.00
GATCAAACCATTGCACTNCA	0.00	0.00	0.29	0.00	0.00
GATCAAACCATTGCAGGGTG	0.00	0.00	0.29	0.00	0.00
GATCAAACCATTGCAGGGTT	0.00	0.00	0.29	0.00	0.00
GATCAAACCATTTCACAATA	0.00	0.00	0.29	0.00	0.00
GATCAAACCATTTGAAGCAA	0.00	0.33	0.00	0.00	0.00
GATCAAACCCAAAAAAAAAA	1.83	0.00	0.57	1.36	0.00
GATCAAACCCAAAAACAAAC	0.23	0.00	0.00	0.00	0.00
GATCAAACCCAAAACACAAA	0.23	0.00	0.00	0.00	0.00
GATCAAACCCAAACAAACCA	0.00	0.00	0.29	0.00	0.34
GATCAAACCCAAACAACACC	0.00	0.00	0.00	0.00	0.00
GATCAAACCCAAACACAAAC	0.00	0.00	0.00	0.00	0.34
GATCAAACCCAAACATGTGT	0.00	0.00	0.29	0.00	0.00
GATCAAACCCAACATTTGAA	0.00	0.33	0.00	0.00	0.00
GATCAAACCCAACCAACATA	0.00	0.00	0.00	0.00	0.34

GATCAAACCCAACCACCCAG	0.23	0.00	0.00	0.00	0.00
GATCAAACCCAACCCTGAAC	0.00	0.00	0.00	0.00	0.00
GATCAAACCCAACCGGAGCC	0.00	0.00	0.00	0.00	0.00
GATCAAACCCAACGCAACAT	0.00	0.33	0.00	0.00	0.00
GATCAAACCCAACGCGTCAG	0.46	0.33	0.00	0.00	0.00
GATCAAACCCAAGACACAGC	0.00	0.33	0.57	0.00	0.00
GATCAAACCCAAGCCCAGAC	0.00	0.00	0.00	0.00	0.34
GATCAAACCCAAGCTGCTTG	0.00	0.33	0.57	0.34	0.00
GATCAAACCCAAGTCAAAAG	0.00	0.00	0.00	0.34	0.00
GATCAAACCCAATAACAAAT	0.00	0.00	0.00	0.00	0.69
GATCAAACCCAATCCAACAT	0.00	0.00	0.00	0.00	0.00
GATCAAACCCAATTCAAAAA	0.00	0.00	0.00	0.00	0.69
GATCAAACCCAATTCAAAAG	2.29	0.65	2.00	1.70	2.76
GATCAAACCCAATTCAAAAN	0.23	0.00	0.00	0.00	0.00
GATCAAACCCAATTTATGAT	0.00	0.00	0.00	0.34	0.00
GATCAAACCCAATTTATGCT	0.92	0.65	0.00	0.00	0.00
GATCAAACCCACAGAAATGG	0.00	0.00	0.00	0.00	1.72
GATCAAACCCACAGCCGGAA	0.00	0.00	0.57	0.00	0.00
GATCAAACCCACAGCCTCAT	0.00	0.00	0.00	0.34	0.00
GATCAAACCCACAGCTATTA	8.24	5.53	2.86	4.75	2.76
GATCAAACCCACAGCTATTG	0.23	0.00	0.00	0.00	0.00
GATCAAACCCACAGTTGATA	0.00	0.00	0.29	0.00	0.00
GATCAAACCCACCATTGAAC	0.00	0.00	0.00	0.00	0.00
GATCAAACCCACCGCTAAAA	0.00	0.33	0.00	0.00	0.00
GATCAAACCCACGAGCCATG	1.14	0.98	0.00	0.00	0.34
GATCAAACCCACGCTGCGTT	0.00	0.00	0.29	0.00	0.00
GATCAAACCCACGGCGAACG	0.00	0.33	0.00	0.00	0.00
GATCAAACCCACTCACGCCG	0.23	0.00	0.00	0.00	0.00
GATCAAACCCACTGCGAAGA	0.00	0.00	0.29	0.00	0.00
GATCAAACCCACTGCGAATG	0.00	0.00	0.29	0.00	0.00
GATCAAACCCAGAACGGACC	0.00	0.00	0.29	0.00	0.00
GATCAAACCCAGAAGTGCCG	0.00	0.00	0.00	0.00	0.34
GATCAAACCCAGCAGAAGAA	0.23	0.00	0.29	0.00	0.00
GATCAAACCCAGCCAAGAGN	0.00	0.00	0.00	0.00	0.00
GATCAAACCCAGCGCGGCCA	0.00	0.00	0.00	0.00	0.34
GATCAAACCCAGCGGCCTGC	0.00	0.00	0.00	0.00	0.00
GATCAAACCCAGGCAGCACA	0.46	0.00	0.00	0.00	0.34

GATCAAACCCAGGCTTCTTC	0.00	0.00	0.00	0.00	0.34
GATCAAACCCAGGTTGTCGG	0.23	0.00	0.00	0.00	0.00
GATCAAACCCAGGTTGTGTC	0.00	0.00	0.29	0.00	0.00
GATCAAACCCAGTCGCCAAT	0.00	0.33	0.29	0.00	0.69
GATCAAACCCATATCATAAA	0.00	0.00	0.00	0.00	0.00
GATCAAACCCATATCATACA	0.00	0.00	0.00	0.00	1.03
GATCAAACCCATCTGACAAC	0.23	0.00	0.00	0.00	0.00
GATCAAACCCATGCGCGAGC	0.23	0.00	0.00	0.00	0.00
GATCAAACCCATGCGCGCGC	0.23	0.00	0.00	0.00	0.00
GATCAAACCCATGCGCGTGC	0.46	0.33	0.29	0.68	0.34
GATCAAACCCATGTTCACGA	0.00	0.33	0.00	0.34	0.69
GATCAAACCCATTCTTGATG	0.00	0.33	0.00	0.00	0.00
GATCAAACCCATTTGGCGCA	0.00	0.00	0.29	0.00	0.00
GATCAAACCCCAAAAGCTTG	0.00	0.00	0.29	0.34	0.00
GATCAAACCCCAAACCCTGG	0.00	0.33	0.00	0.00	0.69
GATCAAACCCCAACCCTGGG	1.37	1.63	2.00	2.72	1.38
GATCAAACCCCACAAACCCC	0.00	0.00	0.00	0.00	0.00
GATCAAACCCCACAACGGAC	0.00	0.00	0.29	0.00	0.00
GATCAAACCCCACCACAACC	0.00	0.00	0.00	0.00	0.00
GATCAAACCCCATTTGCCCG	0.00	0.00	0.29	0.00	0.00
GATCAAACCCCCACCACCCA	0.00	0.33	0.00	0.00	0.00
GATCAAACCCCCCCCCACCC	0.00	0.00	0.00	0.00	0.34
GATCAAACCCCCCTCTTCCC	0.00	0.00	0.00	0.00	0.34
GATCAAACCCCCCTGAGGCC	0.00	0.00	0.00	0.00	0.00
GATCAAACCCCCGCGTACTG	0.00	0.00	0.00	0.00	0.69
GATCAAACCCCCTGCGAATG	0.23	1.63	0.57	0.34	0.69
GATCAAACCCCCTTACATGG	0.00	0.00	0.00	0.00	0.00
GATCAAACCCCGCAAATCAT	0.00	0.00	0.00	0.00	0.34
GATCAAACCCCGCCCGCCGG	0.00	0.00	0.29	0.34	0.00
GATCAAACCCCGGCTTGAAA	0.00	0.00	0.00	0.68	0.00
GATCAAACCCCTAAACCACC	0.00	0.00	0.00	0.00	0.34
GATCAAACCCCTAGGCGTTT	0.00	0.33	0.00	0.00	0.00
GATCAAACCCCTCACCACGG	0.00	0.00	0.00	0.00	0.00
GATCAAACCCCTCAGATGGG	0.00	0.00	0.00	0.00	0.00
GATCAAACCCCTGGAGCGTG	0.00	0.00	0.00	0.00	0.00
GATCAAACCCCTGGTGAAGC	0.00	0.00	0.00	0.34	0.00
GATCAAACCCCTTTCTCCCT	0.00	0.00	0.00	0.00	0.00

GATCAAACCCGAACAGAGCT	0.00	0.00	0.00	0.00	0.69
GATCAAACCCGAAGTTCAAG	0.23	0.00	0.00	0.00	0.00
GATCAAACCCGACGACCGCC	0.00	0.00	0.29	0.00	0.00
GATCAAACCCGCACACCTAC	0.00	0.00	0.00	0.00	0.00
GATCAAACCCGCACTATGCG	0.23	0.00	0.00	0.00	0.00
GATCAAACCCGCCCGCTCAC	0.00	0.00	0.00	0.34	0.00
GATCAAACCCGCCGCCTGCC	0.00	0.00	0.00	0.34	0.00
GATCAAACCCGCGTACGGAG	0.00	0.00	0.00	0.00	0.34
GATCAAACCCGGACTATTTG	0.00	0.00	0.00	0.34	0.00
GATCAAACCCGGAGATGCGT	0.00	0.33	0.00	0.00	0.00
GATCAAACCCGGCAGCCAAG	0.00	0.00	0.29	0.00	0.00
GATCAAACCCGGCTGCCTCT	0.23	0.00	0.00	0.00	0.00
GATCAAACCCGGCTGCCTGT	0.23	0.00	0.00	0.00	0.00
GATCAAACCCGGCTTGCTCA	0.00	0.00	0.00	0.00	0.00
GATCAAACCCGGCTTGCTCC	0.69	0.00	0.00	0.00	0.00
GATCAAACCCGTGTTGCAAG	0.00	0.00	0.00	0.00	0.00
GATCAAACCCTAGGTCTACC	0.23	0.00	0.00	0.00	0.00
GATCAAACCCTCAAAAAAAC	0.23	0.00	0.00	0.00	0.00
GATCAAACCCTCCAAATACA	0.00	0.00	0.00	0.34	0.00
GATCAAACCCTGACTCTACC	0.00	0.00	0.29	0.00	0.00
GATCAAACCCTGCCATTGCT	0.00	0.00	0.00	0.00	0.00
GATCAAACCCTGGAGAAGAG	0.00	0.00	0.29	0.00	0.00
GATCAAACCCTTCTGGCAAG	0.46	0.98	0.57	0.34	0.00
GATCAAACCCTTGCTTGGCC	0.00	0.00	0.00	0.34	0.00
GATCAAACCGAAAGAGGAAA	0.00	0.00	0.00	0.00	1.03
GATCAAACCGAACACGCTCG	0.00	0.00	0.00	0.00	0.34
GATCAAACCGAACACTCACA	0.23	0.65	0.29	0.00	0.69
GATCAAACCGAACGCAACCT	0.00	0.00	0.00	0.34	0.00
GATCAAACCGACAACGGTAC	0.00	0.00	0.00	0.68	0.00
GATCAAACCGACGTCGGACT	0.00	0.00	0.00	0.00	0.00
GATCAAACCGACTTTCAGTG	2.98	6.18	4.01	7.81	3.44
GATCAAACCGACTTTCNGTG	0.00	0.00	0.00	0.34	0.00
GATCAAACCGACTTTTGTGA	0.00	0.00	0.29	0.00	0.00
GATCAAACCGAGCCTCTTCG	0.00	0.00	0.00	0.00	0.34
GATCAAACCGATGACCCTCG	0.00	0.00	0.29	0.00	0.00
GATCAAACCGATTCTTAATG	0.00	0.00	0.00	0.00	0.00
GATCAAACCGCAAAAAAAAA	0.23	0.00	0.00	0.34	0.00

GATCAAACCGCAACACGGCG	0.00	0.00	0.00	0.00	0.34
GATCAAACCGCAAGTCAAAA	0.00	0.00	0.00	0.00	0.00
GATCAAACCGCAGAGGGACC	0.92	0.00	0.57	0.00	0.00
GATCAAACCGCATAGCACAT	0.23	0.00	0.00	0.00	0.00
GATCAAACCGCCAAAGCGGC	0.00	0.00	0.00	0.34	0.00
GATCAAACCGCCATGGAGCG	0.23	0.00	0.29	0.00	0.00
GATCAAACCGCCCGGGCACA	0.00	0.00	0.00	0.00	0.00
GATCAAACCGCCCTAAAAAA	0.00	0.00	0.00	0.34	0.00
GATCAAACCGCCGCGGCCGC	0.00	0.00	0.00	0.34	0.00
GATCAAACCGCCGTGCACCC	0.00	0.00	0.00	0.00	0.00
GATCAAACCGCGACTCCAAG	0.00	0.00	0.00	0.00	0.00
GATCAAACCGCGCAAAAAAA	0.23	0.00	0.00	0.00	0.00
GATCAAACCGCGCCGCGGCG	6.64	1.95	0.29	4.75	1.72
GATCAAACCGCGTACGCCCA	0.00	0.00	0.00	0.00	0.00
GATCAAACCGCTCTCCCCTC	0.00	0.00	0.00	0.00	0.34
GATCAAACCGCTGCCTGACT	0.46	0.00	0.00	0.00	0.00
GATCAAACCGCTGGACATGA	0.00	0.00	0.00	0.00	0.34
GATCAAACCGCTGGAGACGT	0.00	0.65	0.29	0.34	0.00
GATCAAACCGCTGGGCCTGC	0.00	0.00	0.00	0.00	0.00
GATCAAACCGGAAAGCGTGG	0.00	0.00	0.00	0.34	0.00
GATCAAACCGGACGCAAACT	0.00	0.00	0.00	0.34	0.00
GATCAAACCGGACGCAACAT	0.00	0.00	0.00	0.00	0.34
GATCAAACCGGACGCAACCA	0.00	0.00	0.00	0.34	0.00
GATCAAACCGGACGCAACCN	0.23	0.00	0.00	0.00	0.00
GATCAAACCGGACGCAACCT	15.11	11.39	12.31	19.01	17.22
GATCAAACCGGACGCAACNN	0.00	0.00	0.00	0.00	0.34
GATCAAACCGGACGCAANNN	0.00	0.00	0.00	0.00	0.34
GATCAAACCGGACGCACCCT	0.00	0.00	0.29	0.00	0.00
GATCAAACCGGACGCAGTGG	0.00	0.33	0.00	0.00	0.00
GATCAAACCGGACGCCACCT	0.00	0.00	0.00	0.00	0.34
GATCAAACCGGACGCCTTTA	0.00	0.00	0.00	0.34	0.00
GATCAAACCGGAGCAAAGAA	0.00	0.00	0.00	0.00	0.00
GATCAAACCGGAGGCAACCT	0.00	0.00	0.00	0.34	0.00
GATCAAACCGGCACGAATGG	0.00	0.00	0.00	0.00	0.00
GATCAAACCGGCAGCAACCT	0.00	0.33	0.00	0.00	0.00
GATCAAACCGGCATGCAGCC	0.00	0.00	0.00	0.00	0.34
GATCAAACCGGCCAATGGAA	0.00	0.00	0.00	0.00	0.00

GATCAAACCGGCCCGGGTGT	0.00	0.00	0.00	0.00	0.00
GATCAAACCGGCGCTGAGAA	0.00	0.00	0.00	0.68	0.34
GATCAAACCGGCTTAGAGAC	1.14	0.33	1.15	2.04	2.76
GATCAAACCGGCTTAGAGCC	0.00	0.00	0.00	0.00	0.00
GATCAAACCGGGACAATAGG	0.00	0.00	0.57	0.00	0.00
GATCAAACCGGGAGCTCTGC	0.00	0.98	0.00	0.00	0.34
GATCAAACCGGGCAAAAGCC	0.00	0.33	0.00	0.00	0.00
GATCAAACCGGGCACCAACC	0.23	0.00	0.00	0.00	0.00
GATCAAACCGGGCCGAGCCG	0.00	0.00	0.00	0.00	0.69
GATCAAACCGGGCCTCTTGG	0.00	0.00	0.00	0.00	0.00
GATCAAACCGGGCGTCCCAG	0.00	0.00	0.00	0.00	0.34
GATCAAACCGGGGCTCGTCT	0.00	0.98	0.00	0.68	0.34
GATCAAACCGGTGATGGNNN	0.23	0.00	0.00	0.00	0.00
GATCAAACCGGTGTACGTCT	0.00	1.63	0.29	1.02	2.07
GATCAAACCGTAACGCTGAG	0.00	0.00	0.29	0.00	0.00
GATCAAACCGTAATCACACA	0.00	0.00	0.00	0.00	0.00
GATCAAACCGTACCAACTGT	0.00	0.00	0.00	0.00	0.00
GATCAAACCGTACCGAGGCG	0.00	0.00	0.00	0.00	0.00
GATCAAACCGTACCGCCGAG	0.00	0.00	0.00	0.34	0.00
GATCAAACCGTACCGCTCTG	0.00	0.00	0.00	0.00	0.34
GATCAAACCGTACCGCTGAA	0.00	0.65	0.00	0.00	0.00
GATCAAACCGTACCGCTGAG	0.69	0.98	2.86	3.05	0.69
GATCAAACCGTACCGCTGTT	0.00	0.33	0.00	0.00	0.00
GATCAAACCGTAGACAGATT	0.00	0.00	0.29	0.00	0.00
GATCAAACCGTCAAAATACA	0.23	0.00	0.00	0.00	0.34
GATCAAACCGTCCACCCGGC	2.98	2.60	1.15	2.72	1.38
GATCAAACCGTCCACTTGGT	0.00	0.00	0.00	0.00	0.00
GATCAAACCGTCCCTGGCCA	0.00	0.33	0.00	0.00	0.00
GATCAAACCGTCGACAGCCT	0.00	0.00	0.00	0.00	0.69
GATCAAACCGTCGACAGCTC	0.00	0.00	0.00	0.00	0.34
GATCAAACCGTCGACAGCTT	13.96	7.48	6.87	4.75	11.37
GATCAAACCGTCGACAGNTN	0.00	0.00	0.29	0.00	0.00
GATCAAACCGTGAAGTGGTC	0.23	0.00	0.00	0.34	0.69
GATCAAACCGTGCCGCTTTC	0.23	0.00	0.29	0.00	0.00
GATCAAACCGTGCTAGCGAG	0.23	0.00	0.00	0.00	0.00
GATCAAACCGTGGGCACAGC	0.00	0.00	0.00	1.02	0.00
GATCAAACCGTTCCCTCCTA	0.23	0.33	0.00	0.00	0.00

GATCAAACCGTTCGTATGCC	0.00	0.00	0.00	0.34	0.00
GATCAAACCGTTGACAGCTT	0.23	0.00	0.00	0.00	0.00
GATCAAACCTAATCATCGCG	0.92	0.00	0.00	0.34	0.00
GATCAAACCTACACCTCTTG	0.00	0.00	0.29	0.00	0.00
GATCAAACCTACAGCTATTA	0.23	0.00	0.00	0.00	0.00
GATCAAACCTACGACCCAGT	0.00	0.00	0.29	0.00	0.00
GATCAAACCTAGAGAAAAAT	0.00	0.33	0.00	0.00	0.00
GATCAAACCTAGGTTGTCGG	0.00	0.33	0.00	0.00	0.00
GATCAAACCTAGTGTGTCAG	0.00	0.00	0.00	0.34	0.00
GATCAAACCTAGTTTGTGTC	0.00	0.00	0.29	0.00	0.00
GATCAAACCTATCTGTACGC	0.00	0.00	0.00	1.02	0.00
GATCAAACCTATGTACGCTA	0.00	0.00	0.00	0.00	0.00
GATCAAACCTCAACAACAAC	0.00	0.00	0.00	0.00	0.00
GATCAAACCTCAAGAAAAAA	0.69	0.00	0.00	0.00	1.38
GATCAAACCTCAAGAAAAAT	0.00	0.00	0.00	1.02	0.00
GATCAAACCTCAGACTGGGT	0.00	0.00	0.57	0.00	0.00
GATCAAACCTCAGCCACTGT	0.00	0.00	0.00	0.00	0.00
GATCAAACCTCAGCGTGCGG	0.00	0.33	0.00	0.00	0.00
GATCAAACCTCCACCCAGGA	0.00	1.30	0.86	0.34	0.00
GATCAAACCTCCCAAAAAAG	0.00	0.33	0.00	0.00	0.00
GATCAAACCTCCTCGTGTGT	0.23	0.00	0.00	0.00	0.00
GATCAAACCTCGAGTCAAAC	0.00	0.00	0.00	0.00	0.00
GATCAAACCTCGCACTCAAG	0.00	0.00	0.00	0.34	0.00
GATCAAACCTCGCGGGATGG	0.00	0.00	0.29	0.00	0.00
GATCAAACCTCTCACGCTGA	0.00	0.00	0.29	0.00	0.00
GATCAAACCTCTGCAATACG	0.00	0.33	0.00	0.00	0.00
GATCAAACCTCTGCACAGGA	0.46	0.33	0.00	0.00	0.00
GATCAAACCTCTTGACATGC	0.00	0.00	0.29	1.02	0.00
GATCAAACCTCTTGGTTGCT	0.00	0.00	0.00	0.00	0.34
GATCAAACCTGACAAACAAA	0.00	0.00	0.29	0.00	0.00
GATCAAACCTGACAAACAAG	0.23	0.00	0.00	0.00	0.00
GATCAAACCTGACAACTGGT	0.00	0.00	0.00	0.00	0.00
GATCAAACCTGAGACGTGAC	0.00	0.00	0.00	0.00	0.00
GATCAAACCTGAGGACATGA	0.46	0.00	0.00	0.00	0.00
GATCAAACCTGATTGGTCAA	0.00	0.00	0.00	0.00	0.00
GATCAAACCTGCCACATTCG	0.00	0.00	0.00	0.00	0.34
GATCAAACCTGCCATATGCT	0.00	0.33	0.00	0.00	0.00

GATCAAACCTGCCCCACGGA	0.00	0.33	0.86	0.00	0.00
GATCAAACCTGCCCCCCCGC	0.23	0.00	0.00	0.00	0.00
GATCAAACCTGCCGCTTCTA	0.00	0.00	0.00	0.00	0.00
GATCAAACCTGCCGGTGAAG	0.00	0.98	0.29	0.34	0.34
GATCAAACCTGCCGGTGACG	0.46	0.00	0.00	0.00	0.00
GATCAAACCTGCGATTCTGA	0.23	0.00	0.00	0.00	0.00
GATCAAACCTGCTGAACTGG	0.00	0.00	0.00	0.00	0.34
GATCAAACCTGGAAAGAGCA	0.00	0.98	0.00	0.00	0.00
GATCAAACCTGGAAAGAGCC	0.00	0.33	0.00	1.02	0.69
GATCAAACCTGGAAAGAGCN	0.00	0.00	0.00	0.00	0.00
GATCAAACCTGGACTATGGT	0.00	0.33	0.00	0.00	0.00
GATCAAACCTGGAGACCGAC	0.00	0.00	0.00	0.00	0.00
GATCAAACCTGGAGAGCTCA	0.00	0.00	0.00	0.00	0.34
GATCAAACCTGGCCAAATGT	0.00	0.00	0.29	0.00	0.00
GATCAAACCTGGCCTTTGCA	0.46	0.00	0.00	0.00	0.00
GATCAAACCTGGCGACGCCT	0.00	0.00	0.00	0.00	0.34
GATCAAACCTGGCGACGTCC	0.00	0.65	0.00	0.00	0.00
GATCAAACCTGGCTTGGACT	0.46	0.00	0.00	0.00	0.00
GATCAAACCTGGTGCCCCCT	0.23	0.00	0.00	0.00	0.00
GATCAAACCTGGTTGACTCA	0.00	0.00	0.00	0.00	0.34
GATCAAACCTGTGACTCAAA	0.00	0.00	0.29	0.00	0.00
GATCAAACCTGTGACTCCAA	0.00	0.00	0.29	0.00	0.00
GATCAAACCTGTTTGAACAA	0.00	0.00	0.29	0.00	0.00
GATCAAACCTTAGGACTCAC	0.00	0.00	0.00	0.00	0.00
GATCAAACCTTCGCGTGGTG	0.00	0.00	0.00	0.00	0.00
GATCAAACCTTCTACCTCCA	0.00	0.00	0.29	0.34	0.00
GATCAAACCTTCTACTTTGC	0.23	0.00	0.00	0.00	0.34
GATCAAACCTTCTGAGCCGT	0.23	0.00	0.00	0.00	0.00
GATCAAACCTTGAGCCAAAA	0.00	0.33	0.00	0.00	0.00
GATCAAACCTTGCAACACAG	0.00	0.00	0.29	0.00	0.00
GATCAAACCTTTAATTAATA	1.14	0.00	0.00	0.00	0.00
GATCAAACCTTTCCCCAAGC	0.00	0.33	0.00	0.00	0.00
GATCAAACCTTTGATTCCTT	0.00	0.00	0.00	0.34	0.00
GATCAAACCTTTGCTGATTT	1.14	0.33	1.15	1.02	0.69
GATCAAACCTTTGGACTAAC	0.00	0.00	0.00	0.00	0.00
GATCAAACCTTTGGACTCAC	0.00	0.00	0.00	0.00	0.00
GATCAAACCTTTTGAAAAAA	0.69	0.00	0.00	0.34	0.00

GATCAAACGAAAAAAAAAAA	2.06	1.63	1.72	0.68	1.03
GATCAAACGAAAAAAAAAAT	0.00	0.00	0.29	0.34	0.34
GATCAAACGAAAAAAAAAGA	0.00	0.33	0.00	0.00	0.00
GATCAAACGAAAAAACNNNN	0.23	0.00	0.00	0.00	0.00
GATCAAACGAAAAGAAACAC	0.00	0.00	0.00	0.00	0.34
GATCAAACGAAATGAACAAC	0.23	0.00	0.00	0.00	0.00
GATCAAACGAACACCATGCA	0.00	0.33	0.57	0.00	0.00
GATCAAACGAACCAACACAC	0.00	0.00	0.00	0.00	0.34
GATCAAACGAACGAAGAACA	0.00	0.00	0.00	0.00	0.34
GATCAAACGAACGAGCGAGG	0.00	0.00	0.29	0.00	0.00
GATCAAACGAACGGTCATCA	0.00	0.00	0.57	0.00	0.00
GATCAAACGAACTGCCGCCA	0.00	0.00	0.00	0.00	0.00
GATCAAACGAACTTTTATCG	0.00	0.33	0.00	0.00	0.00
GATCAAACGAAGAAGAAGAA	0.00	0.00	0.00	0.00	1.03
GATCAAACGAAGACGAAGAA	0.00	0.00	0.29	0.00	0.00
GATCAAACGAAGCGAAGCAC	0.00	0.00	0.29	0.00	0.00
GATCAAACGAAGCGTCCAAT	0.00	0.00	0.00	0.34	0.00
GATCAAACGAAGCTGGAGTC	0.23	0.00	0.00	0.00	0.00
GATCAAACGAAGGAGAAGGG	0.00	0.00	0.29	0.00	0.34
GATCAAACGAAGGAGCAAGA	0.00	0.00	0.29	0.00	0.34
GATCAAACGAATCAGAAGAA	0.00	0.00	0.29	0.00	0.00
GATCAAACGAATCAGCGCAG	0.00	0.00	0.00	0.00	0.00
GATCAAACGAATCGCCGGCG	0.23	0.00	0.00	0.00	0.00
GATCAAACGAATGGATACCT	0.00	0.00	0.29	0.00	0.00
GATCAAACGAATGTTGATTC	0.23	0.00	0.00	0.00	0.00
GATCAAACGACAAAAAAAAA	6.18	2.93	0.86	1.36	1.72
GATCAAACGACAAAAAAAAN	0.23	0.00	0.00	0.00	0.00
GATCAAACGACAAAAAAAAT	0.00	0.00	0.00	0.00	0.69
GATCAAACGACAAACGGGCC	0.23	0.00	0.00	0.00	0.00
GATCAAACGACAAGCAACAG	0.00	0.33	0.00	0.00	0.00
GATCAAACGACAAGCAACGC	0.92	1.30	1.15	1.36	0.00
GATCAAACGACAATATGCTT	0.00	0.00	0.29	0.00	0.00
GATCAAACGACACACATAAC	0.00	0.00	0.00	0.00	0.69
GATCAAACGACAGAAAAAAA	0.00	0.00	0.29	0.00	0.34
GATCAAACGACAGAACTTCG	0.46	0.00	0.00	0.00	0.00
GATCAAACGACAGAAGTTCT	0.00	0.00	0.29	0.00	0.00
GATCAAACGACAGAATAGCG	0.00	0.00	0.00	0.34	0.00

GATCAAACGACAGAATCTCG	0.00	0.00	0.00	0.34	0.00
GATCAAACGACAGAATNTCG	0.00	0.00	0.00	0.00	0.34
GATCAAACGACAGAATTGCG	0.00	0.00	0.00	0.68	0.00
GATCAAACGACAGAATTTAG	0.00	0.00	0.00	0.34	0.00
GATCAAACGACAGAATTTCG	35.71	23.10	7.16	22.06	23.42
GATCAAACGACAGAATTTTG	0.00	0.33	0.00	0.00	0.00
GATCAAACGACAGCACAACA	0.00	0.00	0.00	0.00	0.69
GATCAAACGACAGCATTACG	0.00	0.33	0.00	0.00	0.00
GATCAAACGACCAAAAAGGA	0.00	0.00	0.00	0.00	0.00
GATCAAACGACCAACACGGA	3.89	2.28	6.30	5.43	4.13
GATCAAACGACCAGTGCCAT	0.00	0.00	0.00	0.00	0.00
GATCAAACGACCCCCATGTC	0.00	0.00	0.00	0.00	0.34
GATCAAACGACCGAACCATG	0.00	0.00	0.00	0.00	0.34
GATCAAACGACCTGCTGCTG	0.00	0.00	0.00	0.00	0.34
GATCAAACGACCTTGTCACT	0.00	0.00	0.29	0.00	0.00
GATCAAACGACGCATCAAAA	0.00	0.00	0.00	0.00	0.00
GATCAAACGACGCCGCGATG	0.00	0.33	0.00	0.00	0.00
GATCAAACGACGGAGGAAGG	0.00	0.00	0.00	0.00	0.34
GATCAAACGACGTACCATCA	0.00	0.00	0.29	0.00	0.00
GATCAAACGACTTCATCCTC	0.46	0.00	0.00	0.00	0.00
GATCAAACGACTTCTTGTTT	0.00	0.00	0.29	0.00	0.00
GATCAAACGAGAACGGAAGC	0.00	0.00	0.00	0.00	0.69
GATCAAACGAGACCGTTCCA	0.00	0.00	0.00	0.68	0.00
GATCAAACGAGACCTTGTGC	0.00	0.00	0.00	0.34	0.00
GATCAAACGAGACCTTGTTC	0.23	0.65	0.57	1.36	1.72
GATCAAACGAGACGGGGGTA	0.00	0.00	0.00	0.00	0.34
GATCAAACGAGAGAAGAGCA	0.00	0.00	0.57	0.00	0.00
GATCAAACGAGAGCTTGCTT	0.23	0.33	0.00	0.34	0.00
GATCAAACGAGAGGCGATGA	0.00	0.00	0.00	0.00	0.69
GATCAAACGAGCAAAAAAAA	0.00	0.00	0.00	0.34	0.00
GATCAAACGAGCAAATGCGA	0.00	0.00	0.00	0.00	0.00
GATCAAACGAGCACTGCAGC	0.23	0.00	0.00	0.00	0.00
GATCAAACGAGCAGCTTCAG	0.69	3.90	9.74	4.07	4.48
GATCAAACGAGCGAGCTCGG	0.00	0.33	0.00	0.00	0.00
GATCAAACGAGCGCGAGATG	0.00	0.00	0.00	0.00	0.69
GATCAAACGAGCTCCGACCT	0.00	0.33	0.00	0.00	0.00
GATCAAACGAGCTTCTGAAT	0.00	0.00	0.00	0.00	0.34

GATCAAACGAGGAGAGCACA	0.00	0.00	0.00	0.68	0.00
GATCAAACGAGGCACAAAAA	0.23	0.00	0.00	0.00	0.00
GATCAAACGAGGCACACCAG	0.00	0.00	0.29	0.00	0.00
GATCAAACGAGGCATCAGAG	0.00	0.00	0.00	0.00	0.00
GATCAAACGAGGCCAATACA	0.00	0.00	0.29	0.00	0.00
GATCAAACGAGGCCTTGGTC	0.00	0.00	0.00	0.00	0.00
GATCAAACGAGGCGTCGGAT	0.00	0.33	0.00	0.00	0.00
GATCAAACGAGGCGTTCATT	0.23	0.98	0.00	0.00	0.34
GATCAAACGAGGGCTACGGA	0.00	0.00	0.00	0.00	0.00
GATCAAACGAGGGGGGGGGC	0.00	0.00	0.00	0.00	0.34
GATCAAACGAGTCATGCAAG	0.00	0.33	0.29	0.00	0.00
GATCAAACGAGTGACCAACC	0.00	0.00	0.00	0.00	0.34
GATCAAACGAGTTTGCCTCC	0.00	0.00	0.29	0.00	0.34
GATCAAACGATAGGCTCCAC	0.00	0.00	0.00	0.00	0.34
GATCAAACGATCTAAAAAAA	0.00	0.00	0.00	0.00	0.34
GATCAAACGATGAAGGGATG	0.00	0.33	0.00	0.00	0.00
GATCAAACGATGAGAGCCCA	0.00	0.33	0.00	0.00	0.00
GATCAAACGATGGAGGAGTT	0.23	0.00	0.00	0.00	0.00
GATCAAACGATGGTGTTATT	0.00	0.00	0.29	0.00	0.00
GATCAAACGATTTTCTCTTC	0.00	0.00	0.00	0.00	0.00
GATCAAACGCAAAAAAAAAA	1.37	0.00	0.00	0.34	0.34
GATCAAACGCAAACGAGGGC	0.00	0.00	0.00	0.00	0.00
GATCAAACGCAAACGCCCCA	0.00	0.00	0.29	0.00	0.00
GATCAAACGCAAACGCGGGC	0.00	0.00	0.00	0.00	0.34
GATCAAACGCAACCACAAAC	0.00	0.00	0.00	0.00	0.69
GATCAAACGCAACGACGCGA	0.00	0.33	0.00	0.00	0.00
GATCAAACGCAACGCACCGC	0.00	0.00	0.29	0.00	0.00
GATCAAACGCAACGCGCTCA	0.00	0.00	0.29	0.00	0.00
GATCAAACGCAAGACGGGGT	0.00	0.00	0.29	0.00	0.00
GATCAAACGCAAGAGCTAGC	0.00	0.00	0.57	0.00	0.00
GATCAAACGCAAGCAGGGGT	0.00	0.00	0.29	0.00	0.00
GATCAAACGCAAGCCTGTTT	0.00	0.00	0.00	0.00	0.34
GATCAAACGCAAGCGAAGAT	0.00	0.00	0.29	0.00	0.00
GATCAAACGCAAGCGAGGGT	0.00	0.00	0.00	0.00	0.34
GATCAAACGCAAGCTGGTCT	0.00	0.00	0.00	0.00	0.34
GATCAAACGCAAGGAAGGGG	0.00	0.00	0.29	0.00	0.00
GATCAAACGCAAGGGATGGT	0.00	0.00	0.00	0.00	0.34

GATCAAACGCAATCGAGGGT	0.00	0.00	0.00	0.00	0.34
GATCAAACGCAATGGCCTTG	0.23	0.00	0.00	0.00	0.00
GATCAAACGCAATTTGAAAA	0.00	0.00	0.29	0.00	0.00
GATCAAACGCACAAAAAAAA	0.46	0.00	0.00	0.68	0.00
GATCAAACGCACAAACACAT	0.00	0.00	0.00	0.00	0.34
GATCAAACGCACAACCTTTA	0.00	0.00	0.00	0.00	0.00
GATCAAACGCACAAGCCCGG	0.00	0.00	0.00	0.00	0.34
GATCAAACGCACACACAGAG	0.00	0.00	0.00	0.00	0.34
GATCAAACGCACAGTTGAAG	0.23	0.00	0.00	0.00	0.00
GATCAAACGCACCAAAAAAA	0.46	1.95	0.29	0.34	0.34
GATCAAACGCACCAAAAAAT	0.46	0.00	0.00	0.00	0.00
GATCAAACGCACCAAACGTC	0.46	0.65	0.00	2.04	0.34
GATCAAACGCACCACACGTA	0.23	0.00	0.00	0.00	0.00
GATCAAACGCACGCGAGGGT	0.00	0.00	0.00	0.00	1.38
GATCAAACGCACGGGTGCTC	0.00	0.00	0.29	0.00	0.00
GATCAAACGCACGTGAGGGT	0.00	0.00	0.00	0.00	0.00
GATCAAACGCACTCGCTGGT	0.23	0.00	0.00	0.34	0.00
GATCAAACGCACTGCGCATG	0.00	0.00	0.29	0.00	0.00
GATCAAACGCACTGCGGGCA	0.00	0.00	0.29	0.00	0.00
GATCAAACGCACTGGGCGCA	0.00	0.00	0.00	0.00	0.34
GATCAAACGCAGAAAAAAAA	0.00	0.00	0.29	0.00	0.00
GATCAAACGCAGAAGAATGA	0.00	0.00	1.72	0.00	3.44
GATCAAACGCAGAAGAATGC	0.00	0.00	0.00	0.00	0.34
GATCAAACGCAGACAGAGCA	0.00	0.00	0.00	0.00	0.34
GATCAAACGCAGACGAATGA	0.00	0.00	0.57	0.00	0.00
GATCAAACGCAGACGTCCAA	1.37	0.00	0.00	0.00	0.00
GATCAAACGCAGACGTGCCA	0.23	0.00	0.00	0.00	0.00
GATCAAACGCAGAGCAGAGA	0.00	0.00	0.00	0.00	0.69
GATCAAACGCAGATGACGGT	0.00	0.00	0.29	0.00	0.00
GATCAAACGCAGCCTTGCTC	0.23	0.33	0.00	0.00	0.00
GATCAAACGCAGCGCAATCG	0.00	0.00	0.29	0.00	0.00
GATCAAACGCAGGAACAAAA	0.00	0.00	0.00	0.34	0.00
GATCAAACGCAGGAACAGGC	0.23	1.30	2.86	0.68	1.03
GATCAAACGCAGGCCGGGGT	0.00	0.00	0.29	0.00	0.00
GATCAAACGCAGGCGAGGGT	0.00	0.00	0.00	0.00	0.69
GATCAAACGCAGGCGGCCAG	0.23	0.00	0.00	0.00	0.00
GATCAAACGCAGGGCGGGGT	0.00	0.00	0.29	0.00	0.00

GATCAAACGCAGGGGCGGGG	0.00	0.00	0.29	0.00	0.00
GATCAAACGCAGGTTTGGTC	0.00	0.33	0.00	0.00	0.00
GATCAAACGCATATCAGAAC	0.00	0.00	0.00	0.00	0.69
GATCAAACGCATCCTTGCCT	0.00	0.00	0.00	0.34	0.00
GATCAAACGCATCTGGCCGT	0.00	0.00	0.00	0.68	0.00
GATCAAACGCATCTGGTCGC	0.46	0.65	0.00	0.00	0.69
GATCAAACGCATGATAGGTT	0.00	0.00	0.29	0.00	0.00
GATCAAACGCATGATCTCTC	0.00	0.33	0.00	0.00	0.00
GATCAAACGCATGCATGCGC	0.00	0.00	0.29	0.00	0.00
GATCAAACGCATGGAATATT	0.00	0.00	0.00	0.00	0.34
GATCAAACGCATGGAGAGCA	0.00	0.00	0.00	0.00	0.00
GATCAAACGCATGTATCCTG	0.00	0.33	0.00	0.00	0.00
GATCAAACGCATGTGCTCTC	0.23	0.00	0.00	0.00	0.00
GATCAAACGCATGTGGGGAA	0.00	0.00	0.29	0.00	0.00
GATCAAACGCATTGAAAAAC	0.00	0.00	0.00	0.00	0.34
GATCAAACGCATTGCATATC	0.00	0.00	0.00	0.00	0.34
GATCAAACGCATTTGCCGCA	0.00	0.00	0.00	0.34	0.00
GATCAAACGCATTTGGCGCA	0.23	0.00	0.00	0.00	0.00
GATCAAACGCATTTTTGTGC	0.23	0.00	0.29	0.00	0.00
GATCAAACGCCAAAGTGGGA	0.00	0.33	0.00	0.00	0.00
GATCAAACGCCAAAGTGTGA	0.00	0.00	0.86	0.00	0.00
GATCAAACGCCAAGCATGTA	0.00	0.00	0.29	0.00	0.00
GATCAAACGCCACCCCGATT	0.00	0.33	0.57	0.68	0.00
GATCAAACGCCACTTGCAGA	0.00	0.33	0.00	0.00	0.00
GATCAAACGCCAGAAAGGGA	0.00	0.00	0.00	0.00	0.34
GATCAAACGCCAGAACATAC	0.00	0.00	0.29	0.00	0.00
GATCAAACGCCCAACGATTC	0.00	0.00	0.29	0.00	0.00
GATCAAACGCCCCCAAACCC	0.00	0.00	0.29	0.00	0.00
GATCAAACGCCCGAGGCGCA	0.00	0.00	0.29	0.00	0.00
GATCAAACGCCCGTCCCGGC	0.00	0.33	0.00	0.00	0.00
GATCAAACGCCCTTGGTCGC	0.23	0.00	0.00	0.00	0.00
GATCAAACGCCGAAGAATGT	0.00	0.00	0.29	0.00	0.00
GATCAAACGCCGAGTGTGAC	0.00	0.00	0.29	0.00	0.00
GATCAAACGCCGATCTATTC	0.00	0.00	0.29	0.00	0.00
GATCAAACGCCGCGACTCGC	0.00	0.00	0.00	0.00	0.34
GATCAAACGCCGCGGGGTCC	0.00	0.00	0.00	0.34	0.00
GATCAAACGCCGCTTCCTCT	0.00	0.65	0.29	0.68	0.00

GATCAAACGCCTCGAGGTGC	0.00	0.33	0.00	0.00	0.00
GATCAAACGCCTCGTGCTCA	0.00	0.33	0.00	0.00	0.00
GATCAAACGCCTGAATGATG	0.23	0.00	0.00	0.00	0.00
GATCAAACGCCTGACCTTCG	0.00	0.00	0.00	0.00	0.34
GATCAAACGCCTGCATAGAC	0.00	0.00	0.00	0.34	0.00
GATCAAACGCCTGGCGGATG	0.00	0.33	0.29	0.00	0.00
GATCAAACGCCTGGCGGGTG	0.00	0.00	0.29	0.00	0.00
GATCAAACGCCTGGGGAAGG	0.00	0.00	0.00	0.34	0.00
GATCAAACGCCTGGTGAAGG	0.46	0.00	0.00	1.70	0.69
GATCAAACGCCTTGAGTCAG	0.00	0.00	0.00	0.34	0.00
GATCAAACGCCTTTGGCGCA	0.00	0.33	0.00	0.00	0.00
GATCAAACGCGAAACGCGTG	0.00	0.00	0.00	0.00	0.69
GATCAAACGCGAAAGAAAAA	0.00	0.00	0.29	0.00	0.00
GATCAAACGCGAAAGAGATG	0.00	0.00	0.29	0.00	0.00
GATCAAACGCGACACCCCCT	0.00	0.00	0.00	0.00	0.34
GATCAAACGCGACGTTATTT	0.46	0.00	0.00	0.00	0.00
GATCAAACGCGAGCAAAAAA	0.00	0.00	0.00	0.34	0.00
GATCAAACGCGAGGAGGAGT	0.00	0.00	0.00	0.34	0.00
GATCAAACGCGCACACACGC	0.00	0.00	0.57	0.00	0.00
GATCAAACGCGCAGCAAGGG	0.00	0.00	0.29	0.00	0.00
GATCAAACGCGCCGAGATCC	0.00	0.00	0.29	0.00	0.00
GATCAAACGCGCCTACGACG	0.00	0.00	0.00	0.00	0.34
GATCAAACGCGCGCGATGGT	0.00	0.00	0.29	0.00	0.00
GATCAAACGCGCGCGTTGAG	0.00	0.00	0.29	0.00	0.00
GATCAAACGCGCGCTGCGCG	2.75	0.33	0.29	0.34	0.00
GATCAAACGCGGCAAAAAAA	0.00	0.00	0.00	0.00	0.34
GATCAAACGCGGCACGGTCC	0.00	0.00	0.00	0.00	0.00
GATCAAACGCGGCCAACTGG	0.00	0.00	0.00	0.34	0.00
GATCAAACGCGGCCCTCAGC	0.00	0.00	0.00	0.00	0.34
GATCAAACGCGGCGTCAAAA	0.00	0.33	0.00	0.00	0.00
GATCAAACGCGGCGTCCCCA	2.98	1.95	0.29	3.05	1.03
GATCAAACGCGGCGTCCCCG	0.00	0.00	0.00	0.00	0.00
GATCAAACGCGGGAAATGTT	0.00	0.33	0.00	0.00	0.00
GATCAAACGCGGGCAGACCT	0.00	0.00	0.00	0.00	0.34
GATCAAACGCGGGCAGTCCC	0.00	0.33	0.00	0.00	0.00
GATCAAACGCGGGCAGTTCC	0.23	0.00	0.29	0.00	1.03
GATCAAACGCGGTGGCGGTC	0.00	0.00	0.29	0.00	0.00

GATCAAACGCGTCCTGCAAG	0.00	0.00	0.29	0.00	0.00
GATCAAACGCGTGGAAGTTT	0.00	0.00	0.00	0.00	0.00
GATCAAACGCGTGGACCATG	0.00	0.00	0.00	0.68	0.00
GATCAAACGCGTGGACTCCT	0.00	0.00	0.29	0.00	0.00
GATCAAACGCGTGGGGCATA	0.00	0.00	0.29	0.00	0.00
GATCAAACGCTACCCCAAAT	0.23	0.00	0.00	0.00	0.00
GATCAAACGCTACCCCAGAG	0.00	0.00	0.29	0.00	0.00
GATCAAACGCTACCTGGATG	0.00	0.00	0.29	0.00	0.00
GATCAAACGCTACTGTCACA	0.00	0.00	0.00	0.34	0.00
GATCAAACGCTATGGTGACC	0.00	0.00	0.00	0.00	0.00
GATCAAACGCTATTGTGCCA	0.46	0.00	0.57	0.34	0.00
GATCAAACGCTCAACCACTG	0.69	0.65	0.29	0.00	0.00
GATCAAACGCTCATTGTGTG	0.23	0.00	0.00	0.00	0.00
GATCAAACGCTCCACTCTCG	0.46	0.65	0.00	0.34	0.34
GATCAAACGCTCCTCGGGCG	0.00	0.00	0.29	0.00	0.00
GATCAAACGCTCGCTAGTCG	0.00	0.00	0.29	0.00	0.00
GATCAAACGCTCGTATGCCG	0.00	0.00	0.00	0.00	0.34
GATCAAACGCTCTCTCCAGC	0.00	0.33	0.00	0.00	0.00
GATCAAACGCTGAAAGGCAA	0.00	0.00	0.00	0.00	0.00
GATCAAACGCTGACAACGAC	0.00	0.00	0.00	0.00	0.34
GATCAAACGCTGACCAGACA	0.00	0.00	0.29	0.00	0.00
GATCAAACGCTGCCCGCCGG	0.00	0.00	0.00	0.00	0.34
GATCAAACGCTGCCGCAAAC	0.00	0.00	0.29	0.00	0.00
GATCAAACGCTGCGGAGGAG	0.00	0.00	0.00	0.00	0.34
GATCAAACGCTGTTCGGATG	0.00	0.00	0.29	0.00	0.00
GATCAAACGCTTCCGTATTC	5.04	6.18	0.29	2.72	1.72
GATCAAACGCTTCCTATCTT	0.23	0.00	0.00	0.00	0.00
GATCAAACGCTTGAATTTGG	0.23	0.00	0.00	0.00	0.00
GATCAAACGCTTGCTGCTTA	0.00	0.00	0.29	0.00	0.00
GATCAAACGCTTGGAGTGTG	2.52	0.98	0.57	0.68	1.38
GATCAAACGCTTGGATCGTA	0.00	0.00	0.00	0.00	0.69
GATCAAACGCTTGGCCTCGG	0.00	0.65	1.15	0.00	0.34
GATCAAACGCTTGGCGTGTG	0.23	0.33	0.00	0.00	0.00
GATCAAACGCTTGTCCGACA	0.00	0.00	0.00	0.00	0.00
GATCAAACGCTTTCGTATTC	0.23	0.00	0.00	0.00	0.00
GATCAAACGGAAAAAAAAAA	0.00	0.00	0.00	0.68	0.00
GATCAAACGGAACATGACAT	0.00	0.00	0.00	0.00	0.34

GATCAAACGGAACGCGCAAA	0.00	0.00	0.57	0.00	0.00
GATCAAACGGAACGTACATA	0.00	0.00	0.00	0.00	0.34
GATCAAACGGAAGAGGAAGA	0.00	0.00	0.29	0.00	0.69
GATCAAACGGACACGGCCAA	0.00	0.00	0.29	0.00	0.00
GATCAAACGGACATTGTGAA	0.00	0.00	0.00	0.34	0.00
GATCAAACGGACGACGACAA	0.00	0.00	0.00	0.00	0.34
GATCAAACGGACGTTGTCCA	0.23	0.33	0.00	0.00	0.00
GATCAAACGGACTCGTGTCA	0.00	0.00	0.00	0.00	0.00
GATCAAACGGACTTGTGTAA	0.00	0.33	0.00	0.00	0.00
GATCAAACGGACTTGTGTCA	10.76	7.48	4.30	17.31	4.82
GATCAAACGGACTTGTGTCN	0.00	0.00	0.00	0.00	0.00
GATCAAACGGACTTGTGTNA	0.00	0.00	0.00	0.00	0.34
GATCAAACGGACTTGTNNNN	0.23	0.00	0.00	0.00	0.00
GATCAAACGGAGAAAAAAAA	0.00	0.00	0.29	0.00	0.00
GATCAAACGGAGAAATCGGG	0.00	0.00	0.00	0.00	0.34
GATCAAACGGAGAGAGAACA	0.00	0.00	0.00	0.00	0.00
GATCAAACGGAGAGAGAGCA	0.00	0.00	0.57	0.00	0.00
GATCAAACGGAGAGGCCTTC	0.23	0.00	0.00	0.00	0.00
GATCAAACGGAGATTGTGAG	0.00	0.00	0.00	0.00	0.34
GATCAAACGGAGCAAAAAAA	2.98	0.00	0.29	0.00	0.34
GATCAAACGGAGCAAGAAAA	0.46	0.00	0.00	0.00	0.00
GATCAAACGGAGCAAGCCAA	0.00	0.33	0.00	0.00	0.00
GATCAAACGGAGCAAGCCGT	0.23	0.00	0.00	0.00	0.00
GATCAAACGGAGCAAGCCTT	0.46	0.33	0.57	0.68	0.00
GATCAAACGGAGCTTCTGGT	0.00	0.00	0.00	0.00	0.00
GATCAAACGGAGGCGACGAC	0.00	0.00	0.29	0.00	0.00
GATCAAACGGAGGTGACCAC	0.00	0.33	0.00	0.00	0.34
GATCAAACGGATAATCAGAC	0.00	0.00	0.00	0.00	0.34
GATCAAACGGATGACCCTGT	0.00	0.00	0.00	0.00	0.34
GATCAAACGGATGAGAATGA	0.00	0.00	0.57	0.00	0.34
GATCAAACGGATTCCTCACA	7.55	2.28	1.15	4.07	0.69
GATCAAACGGATTCGCCCGG	0.00	0.00	0.57	0.00	0.00
GATCAAACGGCAAAAAAAAA	0.00	0.00	0.00	0.34	0.00
GATCAAACGGCAACCCACTT	0.00	0.00	0.00	0.00	0.34
GATCAAACGGCAAGCAAGTG	0.00	0.00	0.00	0.68	0.00
GATCAAACGGCAATCGAAAA	0.00	0.00	0.00	0.00	0.34
GATCAAACGGCACTGAGCAT	0.00	0.00	0.00	0.00	0.00

GATCAAACGGCACTGCTAAA	0.00	0.00	0.00	0.34	0.00
GATCAAACGGCAGAAAAAAA	0.00	0.00	0.00	0.00	0.34
GATCAAACGGCAGAACTCTT	0.00	0.00	0.00	0.00	0.00
GATCAAACGGCAGTCCATGG	0.00	0.33	0.00	0.00	0.00
GATCAAACGGCAGTGCTTGG	0.00	0.65	0.57	0.68	0.69
GATCAAACGGCAGTGCTTTG	0.00	0.00	0.00	0.34	0.00
GATCAAACGGCAGTGTTTGG	0.69	0.00	0.29	0.00	0.00
GATCAAACGGCAGTGTTTTG	0.00	0.00	0.00	0.34	0.00
GATCAAACGGCATAAATCTT	0.00	0.00	0.57	0.00	0.00
GATCAAACGGCATCAACGCT	0.00	0.00	0.00	0.00	0.00
GATCAAACGGCCCAAGCTTG	0.00	0.00	0.00	0.34	0.00
GATCAAACGGCCGATGTGGG	0.00	0.33	0.00	0.00	0.00
GATCAAACGGCCTCGTGCAA	0.00	0.65	0.00	0.00	0.00
GATCAAACGGCCTTAACAGA	0.00	0.00	0.29	0.00	0.00
GATCAAACGGCGAAAAAAAA	0.00	0.98	0.57	0.00	0.69
GATCAAACGGCGAAAAATGT	0.00	0.00	0.00	0.00	0.69
GATCAAACGGCGAAGATTTA	0.00	0.00	0.00	0.00	0.34
GATCAAACGGCGAAGGCCTG	0.00	0.00	0.00	0.34	0.00
GATCAAACGGCGACAGTGGC	0.00	0.00	0.29	0.00	0.00
GATCAAACGGCGAGACAAGG	0.00	0.00	0.00	0.34	0.00
GATCAAACGGCGAGGCAAGG	0.00	0.33	0.00	0.00	0.00
GATCAAACGGCGAGGCTGAA	0.00	0.00	0.00	0.68	0.34
GATCAAACGGCGAGGCTGGA	0.23	0.00	0.00	0.00	0.00
GATCAAACGGCGATGATTTC	0.00	0.00	0.00	0.00	0.69
GATCAAACGGCGATTCACAA	0.00	0.00	0.29	0.00	0.00
GATCAAACGGCGCACGACTT	0.00	0.00	0.00	0.00	0.00
GATCAAACGGCGCCCCGTCG	0.23	0.00	0.00	0.00	0.00
GATCAAACGGCGCCGGCAAC	0.00	0.00	0.00	0.00	0.34
GATCAAACGGCGGAAAAAAA	1.83	3.90	7.45	1.70	5.51
GATCAAACGGCGGAAAAAAT	0.23	0.00	0.00	0.00	0.34
GATCAAACGGCGGAAAACAA	0.00	0.65	0.29	0.00	0.00
GATCAAACGGCGGAAACTAA	0.00	0.00	0.57	0.00	0.00
GATCAAACGGCGGAAATAAA	0.00	0.00	0.00	0.00	1.03
GATCAAACGGCGGAAATCAA	0.00	0.00	0.86	0.00	0.00
GATCAAACGGCGGAAATCGT	0.00	0.00	0.00	0.00	0.34
GATCAAACGGCGGAAATNAA	0.00	0.00	0.29	0.00	0.00
GATCAAACGGCGGAAATTAA	10.53	24.72	46.39	40.73	48.91

GATCAAACGGCGGAAATTAC	0.00	0.00	0.00	0.00	0.34
GATCAAACGGCGGAAATTAT	0.23	0.00	0.29	0.00	0.34
GATCAAACGGCGGAAATTCA	0.23	0.00	0.00	0.00	0.00
GATCAAACGGCGGAAATTGA	0.23	0.00	0.00	0.00	0.34
GATCAAACGGCGGAAGTTAA	0.23	0.00	0.00	0.34	0.00
GATCAAACGGCGGACATTAA	0.00	0.00	0.29	0.00	0.00
GATCAAACGGCGGAGAAAAA	0.00	0.33	0.00	0.00	0.00
GATCAAACGGCGGAGTAAAA	0.00	0.00	0.00	0.00	0.34
GATCAAACGGCGGCGCGCCC	0.00	0.00	0.29	0.00	0.00
GATCAAACGGCGGCTTGGTA	0.00	0.33	0.00	0.00	0.00
GATCAAACGGCGGGCGCCGG	0.00	0.00	0.00	0.34	0.00
GATCAAACGGCGGGCGCGGG	0.00	0.00	0.00	0.34	0.00
GATCAAACGGCGGGGAAAAA	0.00	0.00	0.00	0.34	0.00
GATCAAACGGCGTACCTTTG	0.00	0.33	0.00	0.00	0.00
GATCAAACGGCGTCCGGGAG	0.00	0.00	0.29	0.00	0.00
GATCAAACGGCGTCTTCTTC	0.00	0.00	0.00	0.00	0.00
GATCAAACGGCGTTGTTTCA	0.00	0.00	0.00	0.00	0.00
GATCAAACGGCTCAAAAAAA	0.00	0.00	0.00	0.00	0.34
GATCAAACGGCTCAGTCAAG	0.00	0.00	0.00	0.00	0.00
GATCAAACGGCTCCAGAAGG	0.00	0.33	0.00	0.00	0.00
GATCAAACGGCTGCGTTTGC	0.23	0.00	0.00	0.00	0.00
GATCAAACGGCTGCGTTTGG	0.00	0.00	0.00	0.00	0.00
GATCAAACGGCTGGCTCTGG	0.00	0.00	0.00	0.00	0.34
GATCAAACGGCTGTCTCTGG	0.23	0.00	0.29	0.00	0.00
GATCAAACGGCTGTCTCTTG	0.00	0.00	0.00	0.00	0.34
GATCAAACGGCTTCGCTTTG	0.46	0.00	0.00	0.00	0.00
GATCAAACGGCTTGAATTAG	0.00	0.00	0.00	1.02	0.00
GATCAAACGGCTTGTTGGGC	0.00	0.00	0.00	0.00	0.00
GATCAAACGGGAAAGACCGT	0.23	0.00	0.00	0.00	0.00
GATCAAACGGGAAAGGAGGC	0.00	0.00	0.00	0.34	0.00
GATCAAACGGGAACCCCAAA	0.00	0.00	0.29	0.00	0.00
GATCAAACGGGACACAATTC	0.00	0.00	0.00	0.34	0.00
GATCAAACGGGAGAGGCAGC	0.00	0.00	0.29	0.00	0.00
GATCAAACGGGAGAGGCCGC	0.00	0.98	0.29	0.68	1.03
GATCAAACGGGAGCAGAAAA	0.00	0.00	0.00	0.34	0.00
GATCAAACGGGATGCCATGG	0.00	0.33	0.00	0.00	0.00
GATCAAACGGGCAAGAGGGG	1.83	0.65	3.15	4.41	0.34

GATCAAACGGGCAGCGTGTC	0.00	0.00	0.00	0.34	0.00
GATCAAACGGGCCTATGCGA	0.00	0.00	0.00	0.00	0.34
GATCAAACGGGCCTGGCGGG	0.00	0.33	0.00	0.00	0.00
GATCAAACGGGCGGAGTGCT	0.00	0.33	0.00	0.00	0.00
GATCAAACGGGCGGATGGGA	0.00	0.00	0.00	0.00	0.00
GATCAAACGGGCTCAGTAAA	0.00	0.00	0.86	0.00	0.00
GATCAAACGGGGAAAAAAAA	0.00	0.00	0.00	0.68	0.00
GATCAAACGGGGCAGTCAAA	0.00	0.33	0.00	0.00	0.00
GATCAAACGGGGCGCTGGGC	0.00	0.00	0.00	0.34	0.00
GATCAAACGGGGGCGCCAAC	0.00	0.33	0.00	0.00	0.00
GATCAAACGGGGGGCGTACC	0.00	0.00	0.00	0.00	0.00
GATCAAACGGGGTAACAGGG	0.00	0.00	0.29	0.00	0.00
GATCAAACGGGTAGATTTGC	0.00	0.00	0.00	0.00	0.69
GATCAAACGGGTCGATGAGC	0.46	0.98	0.00	1.02	0.00
GATCAAACGGGTGGCAACAC	0.00	0.00	0.00	0.68	0.00
GATCAAACGGTATGCCCGCC	0.00	0.00	0.00	0.00	0.34
GATCAAACGGTCGGAGGTCC	0.00	0.00	0.29	0.00	0.00
GATCAAACGGTGAAAAAAAA	0.00	0.00	0.29	0.00	0.00
GATCAAACGGTGAAGGTGAA	0.00	0.00	0.29	0.00	0.00
GATCAAACGGTGCAGAAAGC	0.00	0.00	0.00	0.00	0.34
GATCAAACGGTGCCGCGCTC	0.92	0.00	0.29	0.00	0.00
GATCAAACGGTGCCTACAAG	0.00	0.33	0.00	0.00	0.00
GATCAAACGGTGCGCCACTG	0.00	0.00	0.00	0.00	0.34
GATCAAACGGTGGAACAAAA	0.00	0.00	0.00	1.36	0.00
GATCAAACGGTGGAGGAAGG	0.46	0.00	0.00	0.34	0.00
GATCAAACGGTTGGCTGTGA	0.92	0.98	0.29	1.02	0.34
GATCAAACGGTTGGCTGTGC	0.23	0.00	0.00	0.00	0.00
GATCAAACGGTTGGGCACGG	0.00	0.00	0.00	0.00	0.34
GATCAAACGGTTGGTTGTGA	0.00	0.00	0.00	0.00	0.00
GATCAAACGTAAAAAAAAAA	0.23	0.65	0.00	0.00	0.00
GATCAAACGTAAAAATCTAA	0.00	0.00	0.00	0.00	0.34
GATCAAACGTAAGAAGAAAA	0.23	0.00	0.00	0.00	0.34
GATCAAACGTAGAATCTGTT	0.00	0.00	0.00	0.34	0.00
GATCAAACGTAGATTCAGGC	0.00	0.00	0.00	0.00	0.00
GATCAAACGTAGGAGGATGA	0.00	0.00	0.57	0.00	0.34
GATCAAACGTAGGGGAACGA	0.00	0.00	0.57	0.00	0.69
GATCAAACGTATACATTTGG	0.00	0.00	0.00	0.00	0.34

GATCAAACGTATACGGCGAT	0.00	0.00	0.29	0.00	0.00
GATCAAACGTATCAAAAAAA	2.06	0.33	0.29	0.00	0.34
GATCAAACGTATCAATGTAG	0.00	0.00	0.00	0.68	0.00
GATCAAACGTATCACGTGCC	0.00	0.00	0.00	0.00	0.34
GATCAAACGTATCCCGTAGA	0.00	0.00	0.00	0.00	0.00
GATCAAACGTATGCCGTTTC	0.00	0.00	0.00	0.00	1.03
GATCAAACGTATGTATCCCA	0.00	0.00	0.00	0.00	0.00
GATCAAACGTATGTTCAACC	0.00	0.00	0.00	0.00	0.34
GATCAAACGTCAAAAAAAAA	0.23	0.00	0.00	0.00	0.00
GATCAAACGTCAACCGCCGG	0.00	0.00	0.00	0.00	0.34
GATCAAACGTCAGAAAAAAA	0.00	0.00	0.29	0.00	0.00
GATCAAACGTCCACGGGTCC	0.00	0.00	0.00	0.00	0.00
GATCAAACGTCCAGACCCGG	0.00	0.00	0.00	0.00	0.00
GATCAAACGTCCAGACCGCT	0.69	0.00	0.00	0.00	0.00
GATCAAACGTCCCGGTACCC	0.00	0.00	0.29	0.00	0.00
GATCAAACGTCGACTGGATT	0.00	0.00	0.29	0.00	0.00
GATCAAACGTCGCACCGACG	0.46	1.95	0.86	1.02	1.03
GATCAAACGTCGGGAGGTGG	0.00	0.00	0.00	0.00	0.00
GATCAAACGTCGGGGCCAAA	0.00	0.00	0.00	0.00	0.00
GATCAAACGTCTGTCTTGGT	0.00	0.00	0.00	0.00	0.34
GATCAAACGTCTGTGCTGGA	0.00	0.65	0.29	0.34	0.69
GATCAAACGTGAAAGGACCA	0.00	0.00	0.00	0.00	0.00
GATCAAACGTGAAAGGACTG	0.00	0.00	0.00	0.68	0.00
GATCAAACGTGAAGACCGTA	0.00	0.00	0.29	0.00	0.00
GATCAAACGTGACATGCAGG	0.00	0.00	0.00	0.00	0.00
GATCAAACGTGACTCCCGAG	0.00	0.00	0.00	0.68	0.00
GATCAAACGTGAGTCGGCGT	0.00	0.00	0.00	0.00	0.34
GATCAAACGTGCAGCAGAGG	0.00	0.00	0.29	0.00	0.00
GATCAAACGTGCATTCTTCC	0.00	0.00	0.00	0.00	0.00
GATCAAACGTGCCACCACCA	0.00	0.00	0.00	0.34	0.00
GATCAAACGTGCCCGGCCCG	0.00	0.00	0.00	0.00	0.00
GATCAAACGTGCCCGGCTCG	0.00	0.00	0.00	0.00	0.34
GATCAAACGTGCCCTGCAAA	0.46	0.65	0.00	0.00	0.00
GATCAAACGTGCCCTGCCCG	0.00	0.33	0.57	0.00	0.69
GATCAAACGTGCCCTGCCGT	0.00	0.00	0.00	0.00	0.69
GATCAAACGTGCGACTGGTC	0.00	0.00	0.00	0.00	0.34
GATCAAACGTGCGCGGGAGG	0.00	0.00	0.86	0.00	0.00

GATCAAACGTGGACTTGCCT	0.23	0.00	0.00	0.00	0.00
GATCAAACGTGGATGCAAAT	0.00	0.00	0.29	0.00	0.00
GATCAAACGTGGCCCGGCAT	0.00	0.00	0.29	0.00	0.00
GATCAAACGTGGGTGCATGT	0.92	0.00	0.00	0.68	0.00
GATCAAACGTGGTGACTGGT	0.00	0.00	0.29	0.00	0.00
GATCAAACGTGGTGGAATGG	0.00	0.00	0.29	0.34	0.00
GATCAAACGTGGTTGGCTCA	0.00	0.00	0.29	0.00	0.00
GATCAAACGTGTCACAGCGC	0.00	0.00	0.57	0.00	0.00
GATCAAACGTGTCTTGTCTA	0.92	0.33	0.00	0.00	0.00
GATCAAACGTGTGCCGGGAT	0.00	0.65	0.00	0.00	0.00
GATCAAACGTGTGCGCGCAG	0.00	0.00	0.29	0.00	0.00
GATCAAACGTGTGCTGGATG	0.00	0.00	0.00	0.34	0.00
GATCAAACGTGTGTACAGAA	0.23	0.00	0.00	0.00	0.00
GATCAAACGTTAGATGGTGC	0.00	0.00	0.00	0.34	0.00
GATCAAACGTTATTGTAGGG	0.00	0.00	0.00	0.34	0.00
GATCAAACGTTCAAACGACC	0.00	0.00	0.57	0.00	0.00
GATCAAACGTTCAAGCTCGA	0.92	0.33	0.57	0.34	0.00
GATCAAACGTTCGCGGTTCC	0.23	0.00	0.00	0.00	0.00
GATCAAACGTTCTGAGCCGT	0.69	0.00	0.00	0.00	0.00
GATCAAACGTTGCCTAATCT	0.00	0.00	0.00	0.00	0.00
GATCAAACGTTGGGAAAGTG	0.00	0.00	0.00	0.00	0.00
GATCAAACGTTGGTTTAGGT	0.00	0.00	0.00	0.00	0.34
GATCAAACGTTTGAAAAAAA	0.00	0.00	0.29	0.00	0.00
GATCAAACGTTTGACTTGAT	0.00	0.33	0.00	0.00	0.00
GATCAAACTAAAAAAAAAAA	0.00	0.00	0.00	0.00	0.00
GATCAAACTAAAACCACCTG	0.00	0.00	0.00	0.00	0.00
GATCAAACTAAAGTTGAAAA	0.92	0.00	0.00	0.00	0.00
GATCAAACTAACAATGAGAA	0.00	0.00	0.57	0.00	0.00
GATCAAACTAAGCCGACTTT	0.00	0.00	0.00	0.00	0.00
GATCAAACTAATAAGAAATA	0.00	0.00	0.00	0.00	1.03
GATCAAACTAATACAGCAAA	0.00	0.00	0.00	0.00	0.00
GATCAAACTAATCTGACGGG	0.00	0.33	0.00	0.00	0.00
GATCAAACTAATGCGGTGCG	0.00	0.00	0.57	0.00	0.00
GATCAAACTAATGCTGTACA	2.75	0.00	0.00	1.02	0.00
GATCAAACTAATGCTGTCCA	0.23	0.00	0.00	0.00	0.00
GATCAAACTAATTTGGCGCA	0.00	0.33	0.00	0.00	0.00
GATCAAACTAATTTGGCTCG	0.00	0.00	0.00	0.34	0.00

GATCAAACTACAAAAAAAAA	0.00	0.00	0.00	0.00	0.00
GATCAAACTACAAAAAAAGA	0.00	0.00	0.00	0.00	0.00
GATCAAACTACACTGGAGAC	0.23	0.00	0.00	0.00	0.00
GATCAAACTACACTTGGGCA	0.00	0.00	0.00	0.00	0.34
GATCAAACTACAGAAAAAAA	0.00	0.00	0.00	0.00	0.00
GATCAAACTACAGAAGAACA	0.00	0.00	0.00	0.00	0.00
GATCAAACTACAGAATTCCC	0.00	0.00	0.00	0.00	0.00
GATCAAACTACAGAATTTCA	0.00	0.00	0.00	0.00	0.00
GATCAAACTACAGAATTTCG	0.00	0.00	0.00	0.00	0.00
GATCAAACTACAGAATTTCN	0.00	0.00	0.00	0.00	0.00
GATCAAACTACAGAATTTCT	0.00	0.00	0.00	0.00	0.00
GATCAAACTACAGATTTTCG	0.00	0.00	0.00	0.00	0.00
GATCAAACTACAGCGCAACC	0.00	0.00	0.29	0.00	0.00
GATCAAACTACATACATTAC	0.00	0.00	0.57	0.00	0.00
GATCAAACTACATATTGTAA	0.23	0.00	0.00	0.00	0.00
GATCAAACTACATGCTTCAA	1.37	0.33	0.00	1.36	1.38
GATCAAACTACATGTGTGTA	0.00	0.00	0.00	0.00	0.00
GATCAAACTACATTGTAATA	0.00	0.00	0.00	0.00	0.34
GATCAAACTACCAATGGGGC	0.00	0.00	0.00	0.00	0.00
GATCAAACTACCAGCCACCT	0.23	0.00	0.00	0.00	0.00
GATCAAACTACCATATGCGG	0.23	0.00	0.57	0.00	1.38
GATCAAACTACCCTGACCTA	0.00	0.00	0.00	0.00	0.00
GATCAAACTACCCTGACCTT	0.00	0.00	0.29	0.00	0.00
GATCAAACTACCTATTTGAG	0.00	0.00	0.00	0.00	0.00
GATCAAACTACGAAAAAAAA	0.00	0.00	0.00	0.00	0.00
GATCAAACTACGATGAATGC	0.46	0.00	0.00	0.00	0.00
GATCAAACTACTACTAAACA	0.00	0.00	0.29	0.00	0.00
GATCAAACTAGAACTCATAT	0.23	0.00	0.00	0.00	0.00
GATCAAACTAGACCGGAATC	0.00	0.00	0.00	0.00	0.34
GATCAAACTAGAGCAATAGC	0.00	0.00	0.29	0.00	0.00
GATCAAACTAGATTTTTTTT	0.00	0.33	0.00	0.00	0.00
GATCAAACTAGCAAAGTGCT	0.00	0.00	0.00	0.00	0.00
GATCAAACTAGCACCGTACC	0.00	0.00	0.29	0.00	0.00
GATCAAACTAGCACTGNCNN	0.23	0.00	0.00	0.00	0.00
GATCAAACTAGCACTGTAGC	9.84	5.86	6.01	3.73	7.58
GATCAAACTAGCACTGTATC	0.00	0.00	0.00	0.00	0.34
GATCAAACTAGCATGAGAGC	0.69	0.00	0.00	0.00	0.00

GATCAAACTAGCCCTTGAGA	0.00	0.00	0.29	0.00	0.00
GATCAAACTAGCGCGGCTGC	0.23	0.00	0.00	0.00	0.00
GATCAAACTAGCGCGGTTGT	0.00	0.00	0.57	0.00	0.00
GATCAAACTAGCGGAACATG	0.46	0.00	0.00	0.00	0.00
GATCAAACTAGGAGATTAAC	0.00	0.00	0.00	0.00	0.00
GATCAAACTAGGATTTAACT	3.89	0.00	0.00	0.00	0.00
GATCAAACTAGGCCTCTTGG	0.00	0.00	0.29	0.00	0.00
GATCAAACTAGGGAGAGAGC	0.00	0.00	0.57	0.00	0.00
GATCAAACTAGTTGTATATC	0.00	0.00	0.00	0.00	0.00
GATCAAACTAGTTTTTTTTT	0.00	0.00	0.00	0.00	0.34
GATCAAACTATAAACACAAC	0.00	0.00	0.00	0.00	0.34
GATCAAACTATAGACTTCAG	0.00	0.00	0.29	0.00	0.00
GATCAAACTATAGTGCCGTG	0.00	0.00	0.00	0.00	0.00
GATCAAACTATCTGACCGTC	0.00	0.00	0.00	0.00	0.00
GATCAAACTATGAAAGACCT	0.00	0.33	0.00	0.00	0.00
GATCAAACTATGGGAATGGA	0.00	0.33	0.00	0.00	0.00
GATCAAACTATTACAGGCAC	0.00	0.33	0.00	0.00	0.00
GATCAAACTATTGAACAGCT	0.46	0.00	0.00	0.00	0.00
GATCAAACTATTGTGGTCCG	0.00	0.00	0.57	0.00	0.00
GATCAAACTATTTCTGAAGT	0.00	0.00	0.00	0.00	0.00
GATCAAACTCAAAATTGCCG	0.00	0.00	0.00	0.34	0.00
GATCAAACTCAAACACAAAA	0.00	0.00	0.00	0.00	0.34
GATCAAACTCAAACCAAGTG	0.00	0.00	0.00	0.00	0.00
GATCAAACTCAAACTGCCGC	1.60	6.83	9.74	15.27	10.68
GATCAAACTCAAACTGCCGN	0.23	0.00	0.00	0.00	0.00
GATCAAACTCAAACTGCGGC	0.23	0.00	0.00	0.00	0.00
GATCAAACTCAAACTTGCAG	0.00	0.00	0.00	0.00	0.34
GATCAAACTCAACATCAATT	0.69	0.00	0.00	0.00	0.00
GATCAAACTCAAGAGACGGA	0.00	0.33	0.00	0.00	0.00
GATCAAACTCAAGAGCCGGG	0.69	0.00	0.00	0.00	0.00
GATCAAACTCAAGCGCGGGG	0.00	0.00	0.00	0.34	0.00
GATCAAACTCAAGGCCGCGG	0.00	0.00	0.00	0.00	1.03
GATCAAACTCAAGGGAGGCG	0.23	0.00	0.00	0.00	0.00
GATCAAACTCAAGTGCGCCC	0.00	0.00	0.00	0.00	0.34
GATCAAACTCAATGGGTTGA	0.46	0.33	0.29	0.00	1.72
GATCAAACTCAATTGGCGCA	0.00	0.00	0.00	0.00	0.00
GATCAAACTCACAACCAAGC	0.00	0.00	0.00	0.00	0.34

GATCAAACTCACAATCACGC	0.00	0.00	0.00	0.00	0.34
GATCAAACTCACAGAATCGG	0.00	0.00	0.00	0.00	0.00
GATCAAACTCACGCGGTATT	0.00	0.00	0.29	0.00	0.00
GATCAAACTCACGCGGTCTT	0.69	0.65	0.57	0.68	0.00
GATCAAACTCACTCACTATG	0.00	0.00	0.57	0.34	0.34
GATCAAACTCACTGCAATTG	0.00	0.00	0.00	0.00	0.34
GATCAAACTCACTTGGCACA	0.00	0.00	0.29	0.00	0.00
GATCAAACTCACTTGGCGCA	0.00	0.00	0.00	0.34	0.00
GATCAAACTCACTTGGCTGC	0.00	0.00	0.00	0.00	0.34
GATCAAACTCACTTTGGAGG	0.46	0.00	0.00	0.00	0.69
GATCAAACTCAGATTCACCT	0.00	0.33	0.00	0.00	0.00
GATCAAACTCAGGACCTCGG	0.00	0.00	0.00	0.34	0.00
GATCAAACTCAGGAGTCATC	0.00	0.00	0.00	0.00	0.00
GATCAAACTCAGGGCCGCGC	0.00	0.00	0.00	0.00	0.00
GATCAAACTCAGTTGGCGCA	0.00	0.33	0.29	0.00	0.34
GATCAAACTCATATGGCGTC	0.00	0.00	0.00	0.00	0.00
GATCAAACTCATCCTAGCTT	0.00	0.00	0.00	0.00	0.00
GATCAAACTCATCGACTTTG	0.00	0.00	0.00	0.00	0.00
GATCAAACTCATCTGAAGGG	0.00	0.33	0.00	0.00	0.00
GATCAAACTCATCTGACCAC	0.00	0.00	0.00	0.00	0.00
GATCAAACTCATGAGGGCAG	0.00	0.00	0.00	0.00	0.00
GATCAAACTCATGTGGATGC	0.00	0.00	0.29	0.00	0.00
GATCAAACTCATGTGGCTGC	0.00	0.00	0.29	0.00	0.00
GATCAAACTCATTAGGCGCA	0.00	0.00	0.00	0.34	0.00
GATCAAACTCATTATGCCCC	0.00	0.00	0.00	0.00	0.00
GATCAAACTCATTCAGCGCC	0.00	0.00	0.00	0.00	0.00
GATCAAACTCATTCCAGACA	0.00	0.00	0.00	0.00	0.34
GATCAAACTCATTCGGCACG	0.00	0.00	0.00	0.00	0.00
GATCAAACTCATTCGGCGCA	0.00	0.00	0.29	0.00	0.00
GATCAAACTCATTGGCGCAA	0.00	0.00	0.00	0.00	0.00
GATCAAACTCATTTAGCGCA	0.00	0.00	0.29	0.00	0.34
GATCAAACTCATTTAGCGCC	0.00	0.00	0.57	0.00	0.00
GATCAAACTCATTTAGGCAA	0.00	0.00	0.00	0.00	0.00
GATCAAACTCATTTCGCGTC	0.00	0.00	0.00	0.00	0.69
GATCAAACTCATTTCGGGGT	0.23	0.00	0.00	0.00	0.34
GATCAAACTCATTTGAAGAA	0.00	0.00	0.00	0.00	0.69
GATCAAACTCATTTGAAGCA	0.00	0.00	0.00	0.00	0.00

GATCAAACTCATTTGAATGC	0.00	0.00	0.00	0.00	0.00
GATCAAACTCATTTGACAGA	0.00	0.00	0.00	0.00	0.00
GATCAAACTCATTTGACGCA	0.00	0.00	0.00	0.00	0.00
GATCAAACTCATTTGACGGA	0.00	0.00	0.29	0.00	0.00
GATCAAACTCATTTGGAGCA	0.00	0.98	1.15	0.68	1.72
GATCAAACTCATTTGGAGCC	0.00	0.00	0.29	0.00	0.00
GATCAAACTCATTTGGCACA	0.23	1.30	0.00	0.34	0.00
GATCAAACTCATTTGGCACC	0.00	0.00	0.00	0.00	0.69
GATCAAACTCATTTGGCACT	0.00	0.00	0.00	0.00	0.00
GATCAAACTCATTTGGCAGC	0.00	0.00	0.00	0.00	0.34
GATCAAACTCATTTGGCATT	0.00	0.00	0.00	0.00	0.00
GATCAAACTCATTTGGCGAA	0.00	0.33	0.57	0.34	0.34
GATCAAACTCATTTGGCGAC	0.23	0.00	0.00	0.34	0.00
GATCAAACTCATTTGGCGCA	111.48	187.71	345.64	229.79	255.89
GATCAAACTCATTTGGCGCC	0.46	0.33	2.29	0.68	2.07
GATCAAACTCATTTGGCGCG	0.00	0.00	0.29	0.34	0.00
GATCAAACTCATTTGGCGCN	0.00	0.00	0.00	0.68	0.00
GATCAAACTCATTTGGCGCT	0.00	0.00	0.29	0.00	0.00
GATCAAACTCATTTGGCGGA	0.23	0.00	1.15	1.36	0.34
GATCAAACTCATTTGGCGNA	0.00	0.00	0.00	0.34	0.00
GATCAAACTCATTTGGCGNN	0.46	0.00	0.00	0.00	0.34
GATCAAACTCATTTGGCGTA	0.00	0.33	0.29	0.00	0.00
GATCAAACTCATTTGGCGTC	0.00	0.00	0.00	0.00	0.00
GATCAAACTCATTTGGCGTG	0.00	0.00	0.00	0.00	0.34
GATCAAACTCATTTGGCGTT	0.00	0.00	0.57	0.00	0.00
GATCAAACTCATTTGGCNNN	0.00	0.00	0.00	0.00	0.34
GATCAAACTCATTTGGCTCA	0.00	0.33	0.29	0.34	0.00
GATCAAACTCATTTGGCTCG	0.00	0.00	0.00	1.02	0.00
GATCAAACTCATTTGGCTGC	0.00	0.00	0.00	0.00	0.00
GATCAAACTCATTTGGCTTA	0.00	0.00	0.29	0.00	0.00
GATCAAACTCATTTGGGAAA	0.00	0.33	0.00	0.00	0.00
GATCAAACTCATTTGGGTCA	0.00	0.00	0.00	0.00	0.34
GATCAAACTCATTTGGNGCA	0.00	0.00	0.00	0.00	1.03
GATCAAACTCATTTGGNGNA	0.00	0.33	0.00	0.00	0.00
GATCAAACTCATTTGGNNNN	0.23	0.00	0.00	0.00	0.00
GATCAAACTCATTTGGTCGT	0.00	0.00	0.57	0.00	0.00
GATCAAACTCATTTGGTCTT	0.00	0.00	0.29	0.00	0.00

GATCAAACTCATTTGGTGAA	0.00	0.00	0.00	0.00	0.34
GATCAAACTCATTTGGTGCA	1.14	0.00	1.43	0.00	0.00
GATCAAACTCATTTGGTGCG	0.00	0.00	0.29	0.00	0.00
GATCAAACTCATTTGGTGCN	0.00	0.00	0.00	0.00	0.00
GATCAAACTCATTTGTCGCA	0.00	0.00	0.29	0.00	0.00
GATCAAACTCATTTTTCTCA	0.00	0.00	0.29	0.00	0.00
GATCAAACTCCAAACCCACA	0.00	0.00	0.29	0.00	0.00
GATCAAACTCCACAAGCACC	0.00	0.00	0.00	0.00	0.34
GATCAAACTCCACACCACCG	0.00	0.00	0.29	0.00	0.00
GATCAAACTCCACAGCAAAA	0.00	0.00	0.57	0.00	0.00
GATCAAACTCCACAGGAGCT	0.00	0.00	0.29	0.00	0.00
GATCAAACTCCACGTCGGAT	3.66	8.13	3.72	2.72	2.07
GATCAAACTCCACGTCGGCT	0.00	0.33	0.00	0.00	0.34
GATCAAACTCCACGTCNGAT	0.00	0.00	0.00	0.00	0.34
GATCAAACTCCACTTTCACG	0.00	0.33	0.86	0.00	0.00
GATCAAACTCCAGAGGACGA	0.00	0.00	0.00	0.00	0.69
GATCAAACTCCAGAGGACGG	13.05	21.47	7.45	24.78	15.84
GATCAAACTCCAGAGGACGN	0.00	0.00	0.00	0.00	0.00
GATCAAACTCCAGAGGACGT	0.00	0.00	0.00	0.34	0.00
GATCAAACTCCAGAGGACTC	0.00	0.00	0.29	0.00	0.00
GATCAAACTCCAGAGGCCGG	0.00	0.00	0.29	0.00	0.00
GATCAAACTCCAGAGGNNNN	0.23	0.00	0.00	0.00	0.00
GATCAAACTCCAGAGTACGG	0.00	0.33	0.00	0.00	0.00
GATCAAACTCCAGGGGACGG	0.00	0.00	0.00	0.00	0.34
GATCAAACTCCAGTGGACGG	0.23	0.00	0.00	0.00	0.00
GATCAAACTCCCACACGAGC	0.00	0.33	0.29	0.34	0.00
GATCAAACTCCCCAAGACAC	0.00	0.00	0.00	0.00	0.69
GATCAAACTCCCGAATGCCG	0.00	0.00	0.29	0.00	0.00
GATCAAACTCCGCTATGAAG	0.46	0.00	0.29	0.00	0.34
GATCAAACTCCGGTCTACCA	0.00	0.00	0.00	0.34	0.00
GATCAAACTCCGGTCTCAGT	0.23	0.00	0.00	0.00	0.00
GATCAAACTCCTCACTCCTC	0.00	0.00	0.29	0.00	0.00
GATCAAACTCCTCCCCAGCC	0.00	0.00	0.00	0.00	0.00
GATCAAACTCCTCCGTCAGT	0.00	0.00	0.00	0.00	0.00
GATCAAACTCCTGAACCACA	0.00	0.00	0.00	0.34	0.00
GATCAAACTCCTGCTGACCC	0.00	0.00	0.00	0.68	0.00
GATCAAACTCCTTGGTGTGA	0.00	0.00	0.00	0.68	0.00

GATCAAACTCCTTTCAGGTC	0.00	0.00	0.00	0.00	0.34
GATCAAACTCCTTTGACGCC	0.23	0.00	0.00	0.00	0.00
GATCAAACTCCTTTGACTGC	0.00	0.00	0.29	0.00	0.00
GATCAAACTCGAATTACAGG	0.00	0.00	0.00	0.00	0.34
GATCAAACTCGATGAGTTGG	0.00	0.00	0.29	0.00	0.00
GATCAAACTCGATGTTGCCT	0.00	0.00	0.29	0.00	0.00
GATCAAACTCGCAAAGAAAA	0.00	0.00	0.00	0.00	0.69
GATCAAACTCGCACACAACC	0.00	0.00	0.29	0.00	0.00
GATCAAACTCGCCGACGGGC	0.00	0.65	0.00	0.00	0.00
GATCAAACTCGCGCGCCATG	0.00	0.00	0.00	0.00	0.34
GATCAAACTCGCGCGGGCTT	0.23	0.33	0.00	0.00	0.00
GATCAAACTCGCGCGGNNNN	0.23	0.00	0.00	0.00	0.00
GATCAAACTCGCGCGGTCTA	0.00	0.33	0.00	0.00	0.00
GATCAAACTCGCGCGGTCTG	0.23	0.00	0.00	0.00	0.00
GATCAAACTCGCGCGGTCTT	5.72	3.90	1.15	3.05	0.69
GATCAAACTCGCGCGGTGTT	0.23	0.00	0.00	0.00	0.00
GATCAAACTCGCTCCATGCG	0.00	0.33	0.00	0.34	0.34
GATCAAACTCGCTGCTTCTA	0.00	0.00	0.00	1.02	0.34
GATCAAACTCGGCTTATGCC	0.00	0.00	0.29	0.00	0.00
GATCAAACTCGGGAATACAC	0.00	0.00	0.29	0.00	1.03
GATCAAACTCGGGGGGCTCC	0.00	0.00	0.00	0.00	0.34
GATCAAACTCGTCAGCTCAA	0.92	0.00	0.00	0.00	0.00
GATCAAACTCGTCATCGCAG	0.00	0.00	0.00	0.34	0.00
GATCAAACTCGTGTTTGATG	0.23	0.00	0.00	0.00	0.00
GATCAAACTCGTTTGGCGTT	0.00	0.00	0.00	0.00	0.34
GATCAAACTCTAACAGCTGT	0.00	0.00	0.00	0.00	0.34
GATCAAACTCTAGAGGACGG	0.00	0.33	0.00	0.00	0.00
GATCAAACTCTAGTGATGCC	0.46	0.65	0.00	0.34	0.00
GATCAAACTCTCTCTCTCTC	0.23	0.00	0.00	0.00	0.00
GATCAAACTCTGAAAGGAAG	0.00	0.00	0.00	0.68	0.00
GATCAAACTCTGACTTGAGC	0.00	0.33	0.00	0.00	0.00
GATCAAACTCTGCAAGGCGA	0.00	0.00	0.29	0.00	0.34
GATCAAACTCTGCAGGCCGA	0.23	0.00	0.00	0.00	0.00
GATCAAACTCTGCCTGACCT	0.00	0.00	0.00	0.00	0.69
GATCAAACTCTGGGGCCGTG	0.23	0.00	0.86	0.00	0.00
GATCAAACTCTGTTCACAAC	0.00	0.00	0.29	0.00	0.00
GATCAAACTCTTACTAGCTT	0.00	0.00	0.29	0.00	0.00

GATCAAACTCTTCAAGAACC	7.10	0.33	2.58	1.02	0.00
GATCAAACTCTTCAGGCTTA	0.00	0.00	0.00	0.00	0.00
GATCAAACTCTTCCTAGCTT	14.65	11.71	12.03	14.26	10.68
GATCAAACTCTTCTTGCTGC	0.00	0.00	0.29	0.00	0.00
GATCAAACTCTTTAAGAACC	0.23	0.00	0.00	0.00	0.00
GATCAAACTCTTTCCATTCG	0.00	0.33	0.00	0.34	0.00
GATCAAACTCTTTTGGCGCA	0.00	0.00	0.57	0.00	0.34
GATCAAACTGAAAAAAAAAA	0.00	0.00	0.29	0.68	0.00
GATCAAACTGAAATCCCCAG	0.00	0.00	0.00	0.00	0.34
GATCAAACTGACATCTTCCT	0.00	0.00	0.00	0.00	0.69
GATCAAACTGAGAACTGGGG	0.23	0.00	0.00	0.00	0.00
GATCAAACTGAGACTCCCTC	0.00	0.00	0.29	0.00	0.00
GATCAAACTGAGCCTCTGGA	0.00	0.00	0.29	0.00	0.00
GATCAAACTGATGCACCCGT	0.00	0.33	0.00	0.00	0.00
GATCAAACTGATGGTAACGG	0.00	0.00	0.00	0.00	0.34
GATCAAACTGATTGGCCTCC	0.00	0.33	0.00	0.00	0.00
GATCAAACTGCAAAAAAAAA	0.00	0.00	0.00	0.00	0.00
GATCAAACTGCAACACACAC	0.00	0.00	0.00	0.00	0.34
GATCAAACTGCAAGATGCGC	0.00	0.00	0.00	0.00	0.00
GATCAAACTGCAAGCACCAT	0.00	0.65	0.29	0.34	0.00
GATCAAACTGCAGAAGAGCC	0.00	0.00	0.00	0.00	0.00
GATCAAACTGCAGGATGTCC	0.00	0.00	0.00	0.34	0.00
GATCAAACTGCAGTGGAAAT	0.00	0.33	0.00	0.00	0.00
GATCAAACTGCATACGTCAT	0.00	0.00	0.00	0.00	0.69
GATCAAACTGCATCCGTCCT	0.00	0.00	0.00	0.00	0.34
GATCAAACTGCATGGACGAG	0.00	0.00	0.29	0.00	0.00
GATCAAACTGCATGTTGATG	0.69	0.00	0.00	0.00	0.00
GATCAAACTGCATTGTCCTC	0.00	0.00	0.00	0.34	0.00
GATCAAACTGCATTGTCCTG	0.00	0.00	0.57	0.00	0.00
GATCAAACTGCCACGACATC	0.00	0.00	0.00	0.34	0.00
GATCAAACTGCCGTCGAGGG	0.00	0.00	0.29	0.00	0.00
GATCAAACTGCGAGCAATGG	0.00	0.00	0.00	0.00	0.00
GATCAAACTGCGCGAAGAAT	0.00	0.00	0.00	0.00	0.00
GATCAAACTGCGCGCAGAGA	0.00	0.00	0.00	0.00	0.00
GATCAAACTGCGGCGCGGTA	0.00	0.00	0.00	0.00	0.00
GATCAAACTGCGTTTGATGG	0.00	0.00	0.00	0.00	0.00
GATCAAACTGCTCACGCACA	0.00	0.00	0.29	0.00	0.00

GATCAAACTGCTCCAGCAAG	0.00	0.00	0.29	0.00	0.00
GATCAAACTGCTCGACTTTT	0.69	0.65	0.86	0.68	1.03
GATCAAACTGCTCGAGCCCA	0.00	0.65	0.00	0.34	0.00
GATCAAACTGCTCGCGCCCA	0.00	0.00	0.29	0.00	0.00
GATCAAACTGCTCTTCCTGG	0.00	0.33	0.00	0.34	0.00
GATCAAACTGCTCTTCCTTG	0.00	0.00	0.29	0.00	0.00
GATCAAACTGCTCTTCTTGG	0.00	0.00	0.29	0.00	0.00
GATCAAACTGCTGAAGTACA	0.00	0.33	0.00	0.00	0.00
GATCAAACTGCTGAAGTCCT	0.00	0.00	0.57	0.00	0.00
GATCAAACTGCTGACAAACA	0.23	0.00	0.00	0.00	0.00
GATCAAACTGCTGGCAGACT	0.00	0.00	0.00	0.34	0.00
GATCAAACTGCTGGCTCGGA	0.00	0.00	0.00	0.00	0.34
GATCAAACTGCTGTGAGTGG	0.00	0.00	0.29	0.00	0.00
GATCAAACTGCTTCGATTGA	0.00	0.00	0.00	0.00	0.00
GATCAAACTGCTTTGAGAAG	0.00	0.00	0.00	0.00	0.34
GATCAAACTGGAACTGGCAC	0.00	0.00	0.00	0.00	0.34
GATCAAACTGGACCGCCTGG	0.23	0.00	0.00	0.00	0.00
GATCAAACTGGACGGAATGA	0.00	0.00	0.29	0.00	0.00
GATCAAACTGGAGGACTCTT	0.00	0.00	0.00	0.00	0.00
GATCAAACTGGAGGAGCTGA	0.23	0.65	0.00	0.68	0.00
GATCAAACTGGAGTTCAATC	0.00	0.00	0.00	0.00	0.00
GATCAAACTGGCAACAAAGG	0.46	0.00	0.00	0.00	0.00
GATCAAACTGGCAACGCTGA	0.00	0.00	0.29	0.00	0.00
GATCAAACTGGCAGAGGGGC	0.00	0.00	0.00	0.34	0.00
GATCAAACTGGCAGAGGTGC	0.00	0.33	0.00	0.00	0.00
GATCAAACTGGCAGCATTGG	0.00	0.98	1.72	2.04	5.17
GATCAAACTGGCAGCCTTGG	0.00	0.00	0.00	0.00	0.00
GATCAAACTGGCATGTCGCC	0.00	0.00	0.00	0.00	0.69
GATCAAACTGGCCGCTTGGA	0.00	0.00	0.00	0.34	0.00
GATCAAACTGGCCGGAATAT	0.00	0.00	0.00	0.00	0.69
GATCAAACTGGCCTCTGCAG	0.00	0.00	0.00	0.00	0.34
GATCAAACTGGCTGTGTGCA	0.00	0.00	0.29	0.00	0.00
GATCAAACTGGGAACTGTGG	0.00	0.33	0.00	0.00	0.00
GATCAAACTGGGCAGACCAA	0.92	0.65	1.15	1.70	2.41
GATCAAACTGGGCATCTGTA	0.23	0.00	0.00	0.00	0.00
GATCAAACTGGGCCAGAGCT	0.00	0.00	0.29	0.00	0.00
GATCAAACTGGGCCGCCCGC	0.00	0.00	0.29	0.00	0.00

GATCAAACTGGGCGCGCTGC	0.46	0.33	0.29	0.00	0.34
GATCAAACTGGGTGCGCTCG	0.00	0.00	0.29	0.00	0.00
GATCAAACTGGTACGCAACC	0.00	0.00	0.57	0.00	0.00
GATCAAACTGGTACTCGGAA	0.00	0.00	0.57	0.00	0.00
GATCAAACTGGTATTGTCCA	0.00	0.00	0.00	0.00	0.69
GATCAAACTGGTCGGAGCTG	0.00	0.33	0.00	0.00	0.00
GATCAAACTGGTCGTATGCC	0.00	0.00	0.86	0.00	0.00
GATCAAACTGGTTCATGCTC	0.46	0.00	0.00	0.00	0.00
GATCAAACTGGTTCCGATAA	0.46	0.00	0.00	0.00	0.00
GATCAAACTGGTTCCGATGA	0.23	0.00	0.00	0.00	0.00
GATCAAACTGGTTGACTGCC	0.00	0.00	0.00	0.00	0.34
GATCAAACTGTAATTAAGAA	0.23	0.00	0.00	0.00	0.00
GATCAAACTGTATCCTTGAA	0.92	0.00	0.00	0.68	0.00
GATCAAACTGTATCTCAACA	0.00	0.00	0.00	0.00	0.00
GATCAAACTGTATCTCTCCA	5.72	3.90	7.45	6.79	6.54
GATCAAACTGTATCTCTCTA	0.00	0.00	0.00	0.00	0.34
GATCAAACTGTATGCGCGCG	0.00	0.00	0.29	0.00	0.00
GATCAAACTGTATGCTGTCT	0.00	0.00	0.00	0.00	0.00
GATCAAACTGTCCCATCTAC	0.00	0.00	0.00	0.00	0.00
GATCAAACTGTCCCTGGCCA	2.06	0.33	0.57	0.68	0.00
GATCAAACTGTCGACTAACA	0.00	0.00	0.00	0.34	0.00
GATCAAACTGTCTGGTCATT	2.29	0.00	0.00	0.00	0.00
GATCAAACTGTGAGGCCTGG	0.00	0.00	0.29	0.00	0.34
GATCAAACTGTGCAACAAAC	0.00	0.00	0.86	0.00	0.00
GATCAAACTGTGCATCTGAA	0.23	0.00	0.29	0.00	0.00
GATCAAACTGTGCCAGATGT	0.00	0.00	0.00	0.00	0.00
GATCAAACTGTGCTCCAGCC	0.00	0.33	0.00	0.00	0.00
GATCAAACTGTGGACCCCGG	1.14	1.63	0.86	2.04	2.07
GATCAAACTGTGGCTATTGT	0.00	0.33	0.00	0.00	0.00
GATCAAACTGTGGGTGCACT	0.00	0.00	0.00	0.00	0.34
GATCAAACTGTGTGGATACA	0.00	0.98	0.00	0.00	0.00
GATCAAACTGTGTGGATACN	0.23	0.00	0.00	0.00	0.00
GATCAAACTGTTCATGAAGA	0.23	0.00	0.00	0.00	0.00
GATCAAACTGTTGCCTTTGC	0.46	0.00	0.00	0.00	0.00
GATCAAACTGTTGGCTGTGA	0.00	0.00	0.00	0.34	0.00
GATCAAACTGTTTGGCACCG	0.23	0.98	0.00	0.00	0.34
GATCAAACTTAAAACAGAGT	0.00	0.00	0.00	0.00	0.34

GATCAAACTTAAACGAGATC	0.00	0.00	0.29	0.00	0.00
GATCAAACTTAAAGAGAACT	0.46	0.00	0.00	0.00	0.00
GATCAAACTTAAGCTATGTT	0.00	0.00	0.29	0.00	0.00
GATCAAACTTACACATCCCT	0.00	0.00	0.29	0.00	0.00
GATCAAACTTACAGCAGCGT	0.00	0.00	0.00	0.00	0.00
GATCAAACTTACCTCCCTTT	0.00	0.00	0.00	0.00	0.00
GATCAAACTTACCTGAGGCT	0.00	0.33	0.00	0.00	0.00
GATCAAACTTACCTTAGTTT	0.00	0.00	0.00	0.00	0.00
GATCAAACTTAGGACGGTGA	0.00	0.00	0.29	0.00	0.00
GATCAAACTTAGTAGGCTTT	0.00	0.00	0.00	0.00	0.34
GATCAAACTTATCAAAAAAA	0.23	0.00	0.00	0.00	0.00
GATCAAACTTATCATCAGTG	0.00	0.00	0.00	0.00	0.00
GATCAAACTTCAAACTGCCG	0.00	0.00	0.57	0.00	0.00
GATCAAACTTCAATGTGCCT	0.00	0.00	0.00	0.34	0.00
GATCAAACTTCACGCAGGTC	0.00	0.00	0.00	0.68	0.00
GATCAAACTTCAGCCAATTT	0.00	0.00	0.00	0.00	0.69
GATCAAACTTCAGCCACTTG	0.00	0.00	0.00	0.00	0.00
GATCAAACTTCAGCCAGGAC	0.00	0.98	0.00	1.02	0.34
GATCAAACTTCAGTAAGAAA	0.00	0.00	0.00	0.00	0.69
GATCAAACTTCAGTGTGTCA	0.00	0.00	0.00	0.00	0.00
GATCAAACTTCATTTCAGAA	0.00	0.00	0.00	0.00	0.34
GATCAAACTTCCAGCCTGAA	0.23	0.00	0.00	0.00	0.00
GATCAAACTTCGCGGTCGCT	0.00	0.00	0.57	0.00	0.00
GATCAAACTTCGGCGTTCGG	0.00	0.00	0.00	0.00	0.34
GATCAAACTTCGTGAGGTTC	0.00	0.00	0.00	0.34	0.00
GATCAAACTTCGTGAGTTGA	0.00	0.00	0.29	0.00	0.00
GATCAAACTTCTTCACAACC	0.00	0.00	0.00	0.00	0.00
GATCAAACTTCTTTGAGAAA	0.00	0.00	0.29	0.00	0.00
GATCAAACTTGAAAAAAAAA	0.46	0.00	0.00	0.00	0.00
GATCAAACTTGAATATGAAC	0.23	0.00	0.00	0.00	0.00
GATCAAACTTGAATATGACC	4.35	0.65	0.86	0.34	0.00
GATCAAACTTGAATATGACT	0.23	0.00	0.00	0.00	0.00
GATCAAACTTGAGAACGGCA	0.00	0.00	0.00	0.00	0.00
GATCAAACTTGAGAACGGCC	0.69	0.33	0.00	0.00	0.00
GATCAAACTTGAGCGCGGGC	0.00	0.00	0.00	0.00	0.34
GATCAAACTTGAGGGCCCAA	0.00	0.00	0.29	0.00	0.00
GATCAAACTTGAGTTTGATN	0.46	0.00	0.00	0.00	0.00

GATCAAACTTGAGTTTGATT	19.00	11.71	17.18	10.18	16.88
GATCAAACTTGAGTTTGCTT	0.00	0.00	0.00	0.00	0.34
GATCAAACTTGAGTTTGGTT	0.23	0.00	0.00	0.00	0.00
GATCAAACTTGAGTTTGTTT	0.00	0.00	0.29	0.00	0.00
GATCAAACTTGAGTTTTCGT	0.23	0.00	0.00	0.00	0.00
GATCAAACTTGATAAAATGA	0.69	0.00	0.00	0.00	0.00
GATCAAACTTGATGTTGCTG	0.00	0.00	0.00	0.00	0.34
GATCAAACTTGATTGAGCCA	0.00	0.65	0.00	0.00	0.00
GATCAAACTTGCACTTTGTT	0.00	0.00	0.00	0.00	0.34
GATCAAACTTGCATGCCACC	2.75	7.48	2.29	6.45	5.51
GATCAAACTTGCATTCATCC	0.00	0.00	0.00	0.00	0.34
GATCAAACTTGCCCACTCCA	0.00	0.00	0.29	0.00	0.00
GATCAAACTTGCCCTGCTGG	0.00	0.33	0.00	0.00	0.00
GATCAAACTTGCCCTGCTNN	0.23	0.00	0.00	0.00	0.00
GATCAAACTTGCCCTGCTTG	2.98	4.88	5.44	8.15	4.13
GATCAAACTTGCCTGAACTT	0.00	0.00	0.29	0.00	0.00
GATCAAACTTGCCTGACCTT	1.14	1.30	0.86	2.04	1.72
GATCAAACTTGCCTGGCTCT	0.00	0.33	0.00	0.00	0.00
GATCAAACTTGCCTTTGCCA	0.00	0.33	0.00	0.00	0.00
GATCAAACTTGCTGACGGGA	0.00	0.33	0.00	0.00	0.00
GATCAAACTTGCTGCCCGCG	0.23	0.00	0.00	0.00	0.00
GATCAAACTTGCTGGTGAAG	0.46	0.00	0.29	0.00	0.00
GATCAAACTTGCTTCCAGAG	0.00	0.00	0.29	0.00	0.00
GATCAAACTTGCTTGACAAT	0.00	0.33	0.00	0.00	0.00
GATCAAACTTGCTTGACCAC	0.23	0.00	0.00	0.00	0.00
GATCAAACTTGCTTGACCAT	5.26	2.28	0.86	3.05	2.76
GATCAAACTTGCTTGGACTC	0.46	0.98	0.29	0.00	0.00
GATCAAACTTGGAATAGGCA	0.00	0.33	0.00	0.00	0.00
GATCAAACTTGGAATCGGCG	2.52	0.65	1.72	3.05	1.72
GATCAAACTTGGAATCGGCN	0.23	0.00	0.00	0.00	0.00
GATCAAACTTGGAATCGTTG	0.00	0.00	0.00	0.00	0.00
GATCAAACTTGGAGCGAGGG	0.00	0.00	0.00	0.00	0.00
GATCAAACTTGGAGTCGAAA	0.00	0.00	0.00	0.00	0.69
GATCAAACTTGGATTATCCA	1.83	1.30	0.00	0.68	0.34
GATCAAACTTGGCACGAACC	0.00	0.00	0.00	0.00	0.69
GATCAAACTTGGCCGAAAGA	0.00	0.00	0.00	0.00	0.00
GATCAAACTTGGCCGACCTT	0.00	0.00	0.29	0.00	0.00

GATCAAACTTGGCCGGCTGG	0.00	0.00	0.29	0.00	0.00
GATCAAACTTGGCGAAAAAC	0.00	0.00	0.00	0.34	0.00
GATCAAACTTGGGCGTTGGT	0.00	0.00	0.57	0.00	0.00
GATCAAACTTGGTACCTATA	0.69	0.65	0.00	0.00	0.00
GATCAAACTTGGTCAGCATC	0.00	0.00	0.29	0.00	0.00
GATCAAACTTGGTCGAAGCA	0.00	0.33	0.00	0.00	0.00
GATCAAACTTGGTCTCAGCA	0.69	0.98	1.15	2.04	2.07
GATCAAACTTGGTCTCAGTA	0.00	0.00	0.29	0.00	0.00
GATCAAACTTGGTTTACTCA	0.00	0.00	0.00	0.00	2.07
GATCAAACTTGTTGGTAACA	0.00	0.00	0.00	0.00	0.34
GATCAAACTTGTTTTAATAT	0.00	0.00	0.00	0.00	0.69
GATCAAACTTGTTTTTTTTT	0.23	0.00	0.00	0.00	0.00
GATCAAACTTTAAATGGGTA	0.00	0.00	0.00	0.00	0.00
GATCAAACTTTACTTCTTTA	0.00	0.00	0.00	0.00	0.34
GATCAAACTTTATTGGAGGA	0.00	0.00	0.29	0.00	0.00
GATCAAACTTTCACGACGCA	0.00	0.00	0.00	0.00	0.00
GATCAAACTTTCTACCCCAT	0.00	0.00	0.00	0.00	0.34
GATCAAACTTTCTGAGCCGT	0.00	0.00	0.29	0.00	0.00
GATCAAACTTTGAAGTCAAC	0.00	0.00	0.00	0.34	0.00
GATCAAACTTTGCGGACGGA	0.00	0.00	0.00	0.34	0.00
GATCAAACTTTGCGGACGGC	1.37	0.33	0.29	0.68	0.34
GATCAAACTTTGGCGGCGAC	0.00	0.00	0.00	0.00	0.34
GATCAAACTTTGGTGTCTCT	0.00	0.00	0.29	0.00	0.00
GATCAAACTTTTATTCATAC	0.00	0.00	0.00	0.00	0.34
GATCAAACTTTTTAACATTC	0.23	0.00	0.00	0.00	0.00
GATCAAAGAAAAAAAAAAAA	14.88	7.81	12.03	11.20	17.56
GATCAAAGAAAAAAAAAAAC	0.00	0.00	0.00	0.00	0.00
GATCAAAGAAAAAAAAAAAT	0.69	0.33	2.00	0.00	1.03
GATCAAAGAAAAAAAAAAGA	0.00	0.00	0.29	0.00	0.69
GATCAAAGAAAAAAAAAANN	0.00	0.00	0.00	0.00	0.34
GATCAAAGAAAAAAAAAATA	0.00	0.00	0.00	0.34	0.00
GATCAAAGAAAAAAAAAATC	0.23	0.00	0.00	0.00	0.00
GATCAAAGAAAAAAAAAGAA	0.00	0.33	0.86	0.00	0.00
GATCAAAGAAAAAAAGAAAA	0.00	0.33	0.00	0.68	0.00
GATCAAAGAAAAAAGAAAAA	0.00	0.33	0.00	0.00	0.69
GATCAAAGAAAAAAGAAAAG	0.00	0.00	1.43	0.00	0.00
GATCAAAGAAAAACATGGCC	0.00	0.00	0.29	0.00	0.00

GATCAAAGAAAAACGAAGAG	0.00	0.00	0.00	0.00	0.00
GATCAAAGAAAAACTAGAAA	0.00	0.33	0.00	0.00	0.00
GATCAAAGAAAAAGAAAAAA	0.00	0.00	0.00	0.00	0.00
GATCAAAGAAAAAGAAAGAG	0.23	0.00	0.00	0.00	0.00
GATCAAAGAAAAAGCAGCGG	0.00	0.33	0.29	0.00	0.34
GATCAAAGAAAACAAAAAAA	0.00	0.00	0.29	0.00	0.00
GATCAAAGAAAACAAACAAG	0.00	0.00	0.29	0.00	0.00
GATCAAAGAAAACAATGCCA	0.00	0.00	0.00	0.00	0.69
GATCAAAGAAAACCACAGCG	0.00	0.00	0.29	0.00	0.00
GATCAAAGAAAAGAAAAAAA	0.46	0.33	0.29	0.00	0.34
GATCAAAGAAAAGAAAGAAA	0.00	0.00	0.29	0.00	0.00
GATCAAAGAAAAGAAATGGA	0.00	0.00	0.00	0.00	0.69
GATCAAAGAAAAGAAGAAAA	0.00	0.33	0.00	0.00	0.00
GATCAAAGAAAAGAGAAAAA	0.23	0.00	0.00	0.00	0.00
GATCAAAGAAAAGAGAATTA	0.00	0.00	0.00	0.00	0.69
GATCAAAGAAAAGAGGCAGA	0.00	0.00	0.00	0.00	0.00
GATCAAAGAAAAGCAAAAAA	0.00	0.00	0.00	0.34	0.00
GATCAAAGAAAAGCATAGTA	0.00	0.00	0.00	0.00	0.34
GATCAAAGAAAAGGAAAGGT	0.00	0.00	0.00	0.00	0.00
GATCAAAGAAAAGGACATCA	0.00	0.00	0.00	0.00	0.00
GATCAAAGAAAAGTAGAGAA	0.00	0.00	0.00	0.00	0.34
GATCAAAGAAAAGTGAAAAA	0.00	0.00	0.57	0.00	0.00
GATCAAAGAAAAGTGAGGGC	0.69	0.33	0.57	0.68	0.69
GATCAAAGAAAAGTGGCAAA	0.00	0.00	0.00	0.00	0.00
GATCAAAGAAAAGTGGCAGA	11.90	5.86	2.00	5.43	5.17
GATCAAAGAAAAGTGGCAGC	0.23	0.00	0.00	0.00	0.00
GATCAAAGAAAAGTGGCAGG	0.23	0.00	0.00	0.00	0.00
GATCAAAGAAAAGTGGCCGA	0.00	0.00	0.00	0.34	0.00
GATCAAAGAAAAGTGTAAAA	0.00	0.00	0.00	0.00	0.34
GATCAAAGAAAAGTGTAAAG	0.23	0.00	0.00	0.00	0.00
GATCAAAGAAAAGTGTATTA	0.69	0.98	0.57	0.00	1.38
GATCAAAGAAAAGTTGTGTC	0.00	0.00	0.57	0.00	0.00
GATCAAAGAAAATGACAAAA	0.00	0.00	0.86	0.00	0.00
GATCAAAGAAAATGATGAAC	0.00	0.00	0.00	0.00	0.34
GATCAAAGAAAATGCGGCGC	0.92	0.00	0.00	0.68	0.00
GATCAAAGAAAATTAAGATT	0.00	0.00	0.00	0.34	0.00
GATCAAAGAAACAAAAAAAA	0.00	0.00	0.00	0.00	0.34

GATCAAAGAAACAGCAGAAG	0.00	0.00	0.00	1.02	0.00
GATCAAAGAAACGGTTTCTG	0.46	0.65	0.29	0.00	0.69
GATCAAAGAAACGTGAAAGA	0.23	0.00	0.00	0.00	0.00
GATCAAAGAAAGAAAAAAAA	0.00	0.00	0.00	0.00	0.00
GATCAAAGAAAGAAAAGAAA	0.00	0.00	0.29	0.00	0.00
GATCAAAGAAAGACAAAAAA	0.23	0.00	0.00	0.00	0.00
GATCAAAGAAAGACGGGGAA	0.00	0.00	0.00	0.00	0.00
GATCAAAGAAAGCAATGAAA	0.00	0.00	0.29	0.00	1.38
GATCAAAGAAAGCAATGAAT	0.00	0.00	0.00	0.00	0.69
GATCAAAGAAAGCATCGTAT	0.00	0.00	0.57	0.00	0.00
GATCAAAGAAAGCGCAGTCC	0.00	0.00	0.00	0.34	0.00
GATCAAAGAAAGCGCAGTCT	0.46	0.33	0.00	0.00	0.34
GATCAAAGAAAGGAGCTAGG	0.00	0.33	0.00	0.00	0.00
GATCAAAGAAAGGCATAAGG	0.00	0.00	0.29	0.00	0.00
GATCAAAGAAAGGGATGTAA	0.00	0.00	0.00	0.00	0.00
GATCAAAGAAAGGGCAACCG	0.00	0.00	0.00	0.00	0.00
GATCAAAGAAAGGGCGANNN	0.00	0.00	0.00	0.00	0.00
GATCAAAGAAAGGGCGATTG	2.06	3.25	0.86	2.04	1.72
GATCAAAGAAAGGGGGCGGG	0.00	0.33	0.00	0.00	0.00
GATCAAAGAAAGTATGGAGA	0.23	0.00	0.00	0.00	0.00
GATCAAAGAAATAAATAGAT	0.00	0.00	0.57	0.00	0.00
GATCAAAGAAATAATTTCCT	0.23	0.00	0.00	0.00	0.00
GATCAAAGAAATCTGAGGAA	0.23	0.00	0.00	0.00	0.00
GATCAAAGAAATCTTAGCCT	0.92	0.33	0.00	0.34	0.69
GATCAAAGAAATCTTGCAGG	0.00	0.33	0.00	0.00	0.34
GATCAAAGAAATGCGTACTT	0.00	0.00	0.00	0.00	0.00
GATCAAAGAAATGGACACAG	0.00	0.33	0.00	0.00	0.00
GATCAAAGAAATGGACATCA	0.00	0.00	0.00	0.34	0.34
GATCAAAGAAATGGACATCC	0.00	0.00	0.00	0.00	0.00
GATCAAAGAAATTCGGCAGA	0.00	0.00	0.00	0.00	0.00
GATCAAAGAAATTGCCAACT	0.69	0.33	0.29	0.34	0.00
GATCAAAGAAATTGGAACTG	0.00	0.00	0.29	0.34	0.34
GATCAAAGAAATTGGAACTT	0.00	0.00	0.00	0.00	0.34
GATCAAAGAACAAAAAAGCA	0.23	0.00	0.00	0.00	0.00
GATCAAAGAACAAGAAACAC	0.00	0.00	0.00	0.00	0.34
GATCAAAGAACACACAAAGG	0.00	0.00	0.29	0.00	0.00
GATCAAAGAACACGTAACGC	0.00	0.00	0.57	0.00	0.00

GATCAAAGAACAGAGCACGG	0.00	0.00	0.00	0.00	0.34
GATCAAAGAACAGAGGAAGG	0.23	0.00	0.00	0.00	0.00
GATCAAAGAACAGCACAGAT	0.00	0.00	0.29	0.00	0.00
GATCAAAGAACAGCACAGGA	0.00	0.00	0.00	0.00	0.34
GATCAAAGAACAGTGAAGCC	0.00	0.00	0.00	0.00	0.00
GATCAAAGAACATCATGTGT	0.00	0.00	0.57	0.00	0.00
GATCAAAGAACATCATTAAT	0.00	0.00	0.29	0.00	0.00
GATCAAAGAACATGTCCCCC	0.23	0.33	0.00	0.00	0.00
GATCAAAGAACCCCATCTTC	0.00	0.00	0.29	0.00	0.00
GATCAAAGAACCCCCACCCT	0.00	0.00	0.00	0.00	0.34
GATCAAAGAACCCGAAGAAA	0.00	0.00	0.00	0.00	0.00
GATCAAAGAACCGCCATGGT	0.00	0.00	0.00	0.34	0.00
GATCAAAGAACCGCCTTGGT	0.00	0.65	0.57	1.36	1.38
GATCAAAGAACGAAAACATA	0.23	0.00	0.00	0.00	0.00
GATCAAAGAACGAAGAACGG	0.00	0.00	0.29	0.00	0.00
GATCAAAGAACGAGGACAAC	0.00	0.33	0.00	0.34	0.00
GATCAAAGAACGCCCTCTGC	0.23	0.00	0.00	0.00	0.00
GATCAAAGAACGCGTGTGAA	0.00	0.00	0.00	0.00	0.00
GATCAAAGAACGGAGGGGGG	0.00	0.33	0.00	0.00	0.00
GATCAAAGAACGGATGAGAC	0.92	0.65	0.57	1.02	0.69
GATCAAAGAACGGCGGAGAC	0.23	0.00	0.00	0.00	0.00
GATCAAAGAACGGCGGCGCC	0.23	0.00	0.00	0.00	0.00
GATCAAAGAACGGCGGCGGC	0.23	0.00	0.00	0.00	0.00
GATCAAAGAACGGCGGGGCC	0.00	0.00	0.00	0.00	0.00
GATCAAAGAACGGCGGGGCG	0.23	0.00	0.00	0.00	0.00
GATCAAAGAACGGCGGNNNC	0.00	0.00	0.29	0.00	0.00
GATCAAAGAACGTCAGAGAG	0.00	0.65	0.00	0.00	0.00
GATCAAAGAACGTCAGAGCG	0.23	0.00	0.00	0.00	0.00
GATCAAAGAACGTCAGAGGC	0.00	0.00	0.00	0.00	0.00
GATCAAAGAACGTCAGAGGG	0.00	0.33	0.00	0.00	0.00
GATCAAAGAACGTCAGAGTG	14.42	13.01	4.58	13.24	5.17
GATCAAAGAACTCATGGAAG	0.00	0.00	0.86	0.34	0.34
GATCAAAGAACTCGAGCTGC	0.23	0.65	0.00	0.00	0.00
GATCAAAGAACTCGCTTGCA	0.00	0.00	0.00	0.34	0.00
GATCAAAGAAGAAAAAAAAA	0.23	0.00	0.29	0.00	0.00
GATCAAAGAAGAAGAAGAAG	0.00	0.00	0.29	0.00	0.34
GATCAAAGAAGAAGAAGACA	0.00	0.00	0.00	0.00	0.34

GATCAAAGAAGAAGAAGCAG	0.00	0.00	0.29	0.00	0.34
GATCAAAGAAGAAGACNNNN	0.23	0.00	0.00	0.00	0.00
GATCAAAGAAGAAGGAGGAG	0.00	0.00	0.00	0.00	0.34
GATCAAAGAAGAATTTGTGG	0.00	0.00	0.29	0.00	0.00
GATCAAAGAAGACAGAAGAC	0.00	0.00	4.87	0.00	1.38
GATCAAAGAAGACCAGCTTG	0.00	0.00	0.00	0.00	0.00
GATCAAAGAAGACGAAGAAG	0.00	0.00	0.57	0.00	0.00
GATCAAAGAAGACGAGCTGT	0.00	0.33	0.00	0.00	0.00
GATCAAAGAAGAGAAAGAGA	0.00	0.00	0.57	0.00	0.69
GATCAAAGAAGAGAAGAGAA	0.00	0.00	0.00	0.00	0.69
GATCAAAGAAGAGACGAGGA	0.23	0.00	0.00	0.00	0.00
GATCAAAGAAGAGAGCCCAG	1.14	0.65	0.29	1.02	0.34
GATCAAAGAAGAGAGGGTCA	0.00	1.30	0.29	0.68	0.00
GATCAAAGAAGAGCGACAGG	0.00	0.00	0.00	0.00	0.00
GATCAAAGAAGAGCTTGCAC	0.00	0.00	0.29	0.68	0.00
GATCAAAGAAGAGGACAGGC	0.00	0.00	0.00	0.00	0.00
GATCAAAGAAGAGGATACAC	0.00	0.00	0.00	0.00	0.34
GATCAAAGAAGAGGATAGAC	0.00	0.00	0.29	0.00	0.00
GATCAAAGAAGAGGTGCAGG	0.00	0.33	0.00	0.00	0.00
GATCAAAGAAGAGTTGGAGG	0.00	0.00	0.00	0.00	0.00
GATCAAAGAAGCAAAAAAAA	0.00	0.00	0.00	0.34	0.00
GATCAAAGAAGCAGCATGCT	0.00	0.00	0.00	0.00	0.00
GATCAAAGAAGCAGTTGCTG	0.00	0.00	0.00	0.34	0.00
GATCAAAGAAGCATGCATAC	0.00	0.00	0.29	0.00	0.00
GATCAAAGAAGCATTTGTGG	0.00	0.00	0.29	0.00	0.00
GATCAAAGAAGCCAACTGGT	1.37	0.00	0.00	0.00	0.00
GATCAAAGAAGCGACAGAGG	0.00	0.33	0.00	0.00	0.00
GATCAAAGAAGCGACGAAGA	0.00	0.00	0.00	0.00	0.00
GATCAAAGAAGCGCCTGACC	0.92	0.00	0.00	0.00	0.00
GATCAAAGAAGCGCTTGGGG	0.00	0.00	0.00	1.02	0.00
GATCAAAGAAGCGGCGAAGA	0.00	0.00	0.00	0.34	0.34
GATCAAAGAAGCGGCGAAGC	21.06	34.48	35.22	26.48	18.94
GATCAAAGAAGCGGCGAAGG	0.00	0.00	0.00	0.00	0.00
GATCAAAGAAGCGGCGACGC	0.23	0.33	0.00	0.00	0.00
GATCAAAGAAGCGGCGAGGC	0.23	0.00	0.00	0.00	0.00
GATCAAAGAAGCGGCGCAGC	0.23	0.00	0.00	0.00	0.00
GATCAAAGAAGCGGCGCNNN	0.23	0.00	0.00	0.00	0.00

GATCAAAGAAGCGGCGGAGC	0.46	0.00	0.00	0.00	0.00
GATCAAAGAAGCGGCGTAGC	0.00	0.00	0.00	0.00	0.00
GATCAAAGAAGCGGCTCGTA	0.23	0.00	0.00	0.00	0.00
GATCAAAGAAGCGGTCTGGG	0.00	0.00	0.00	0.00	0.34
GATCAAAGAAGCGGTGGAAG	0.00	0.33	0.29	0.34	0.00
GATCAAAGAAGCTCGTATGC	0.69	0.00	0.00	0.00	0.00
GATCAAAGAAGCTGCGAAGC	0.00	0.00	0.00	0.34	0.00
GATCAAAGAAGCTGGCGCTC	0.00	0.00	0.00	0.00	0.00
GATCAAAGAAGCTGGTTGCC	0.00	0.00	0.00	0.68	0.00
GATCAAAGAAGCTTCCAAAA	0.00	0.00	0.00	0.34	0.00
GATCAAAGAAGCTTCCTGAT	0.00	0.33	0.00	0.34	0.34
GATCAAAGAAGCTTGGAACA	0.23	0.00	0.00	0.00	0.00
GATCAAAGAAGCTTTCGCTG	0.00	0.00	0.00	0.68	0.00
GATCAAAGAAGGAAACGGAC	0.00	0.00	0.00	0.00	0.00
GATCAAAGAAGGAAGAATAG	0.00	0.00	0.00	0.34	0.00
GATCAAAGAAGGACAGCTGC	0.00	0.00	0.29	0.00	0.00
GATCAAAGAAGGAGAGGAGG	0.23	0.00	0.00	0.00	0.00
GATCAAAGAAGGATTATGCT	0.00	1.30	1.15	0.00	0.00
GATCAAAGAAGGCTGGCTGG	0.00	0.00	0.00	0.34	0.00
GATCAAAGAAGGGCTCCAGA	0.00	0.33	0.00	0.00	0.00
GATCAAAGAAGTACCGCGCC	1.60	0.00	0.00	0.00	0.00
GATCAAAGAAGTATCACAGG	1.14	0.98	0.29	1.02	1.38
GATCAAAGAAGTATCACAGN	0.00	0.00	0.00	0.00	0.00
GATCAAAGAAGTATTTTAAT	0.23	0.00	0.00	0.00	0.00
GATCAAAGAAGTATTTTCAT	0.23	0.00	0.00	0.00	0.00
GATCAAAGAAGTCCCGAAGG	0.00	0.00	0.00	0.34	0.00
GATCAAAGAAGTGAAGGTGA	0.00	0.00	0.00	0.34	0.00
GATCAAAGAAGTGACGAGGC	0.46	0.00	0.29	0.00	0.69
GATCAAAGAAGTGCAGCGCA	0.00	0.33	0.00	0.00	0.00
GATCAAAGAAGTGCAGCGCG	0.00	0.00	0.00	0.34	0.00
GATCAAAGAAGTGGAATGAG	0.00	0.00	0.00	0.34	0.00
GATCAAAGAAGTGGACAGCT	0.00	0.65	0.00	0.00	0.00
GATCAAAGAAGTGGCTCTGG	0.00	0.00	0.00	0.00	0.00
GATCAAAGAAGTGGCTGGCG	0.00	0.00	0.00	0.00	0.00
GATCAAAGAAGTTGTACGAC	0.00	0.00	0.00	0.00	0.00
GATCAAAGAAGTTTCAAAGG	0.00	0.00	0.00	0.00	0.00
GATCAAAGAAGTTTGAAGAG	0.00	0.33	0.00	0.00	0.00

GATCAAAGAAGTTTTTTTAA	0.00	0.00	0.29	0.00	0.00
GATCAAAGAATAAGAAGAAG	0.23	0.00	0.00	0.00	0.00
GATCAAAGAATACAACAGAT	0.00	0.00	0.00	0.00	0.00
GATCAAAGAATAGGCTGGGA	0.00	0.00	0.00	0.00	0.00
GATCAAAGAATATCCCGTAC	0.00	0.00	0.00	0.00	0.00
GATCAAAGAATATCCCGTGC	0.00	0.00	0.00	0.00	0.00
GATCAAAGAATCAAGGACGT	0.00	0.00	0.00	0.00	0.00
GATCAAAGAATCATGAACTG	0.00	0.00	0.00	0.00	0.34
GATCAAAGAATCATGGCTGC	0.00	0.00	0.00	0.00	0.00
GATCAAAGAATCCCAACAGA	0.00	0.00	0.00	0.00	0.00
GATCAAAGAATCCGCATGCT	0.00	0.65	0.29	0.00	0.34
GATCAAAGAATCGAAGCATC	0.00	0.00	0.00	0.00	0.00
GATCAAAGAATCGTGAGGAC	0.00	0.00	0.00	0.00	0.00
GATCAAAGAATCTGGAAAGA	0.00	0.00	0.00	0.00	0.34
GATCAAAGAATGCTGGAAGT	0.00	0.00	0.00	0.00	0.00
GATCAAAGAATGCTGGATTC	0.23	0.33	0.57	0.00	0.34
GATCAAAGAATGCTTGAAAC	0.00	0.00	0.86	0.00	0.00
GATCAAAGAATGGATGGGGN	0.23	0.00	0.00	0.00	0.00
GATCAAAGAATGGCCATCGC	0.00	0.00	0.00	0.34	0.00
GATCAAAGAATGGGCCCCCG	0.00	0.00	0.00	0.34	0.00
GATCAAAGAATGGTCTTGCT	0.00	0.33	0.00	0.00	0.00
GATCAAAGAATGTATGGAGG	24.95	10.09	6.59	6.79	7.92
GATCAAAGAATGTATGGAGN	0.23	0.00	0.00	0.00	0.00
GATCAAAGAATGTGCTCCCT	0.00	0.00	0.00	0.00	0.00
GATCAAAGAATGTGCTGCTT	0.00	0.00	0.00	0.00	0.00
GATCAAAGAATTCACAGTTN	0.23	0.00	0.00	0.00	0.00
GATCAAAGAATTCGCCCCCG	0.00	0.00	0.00	0.00	0.34
GATCAAAGAATTCGGGTCGG	0.00	0.00	0.00	0.34	0.00
GATCAAAGAATTGGCACCCG	0.00	0.33	0.29	0.34	0.00
GATCAAAGAATTGGCCACCG	0.00	0.65	0.00	0.00	0.00
GATCAAAGAATTGGCCCCAG	0.00	0.00	0.00	0.00	0.00
GATCAAAGAATTGGCCCCCA	0.00	0.00	0.00	0.00	0.00
GATCAAAGAATTGGCCCCCG	17.17	53.68	26.06	32.92	25.83
GATCAAAGAATTGGCCCCCN	0.00	0.00	0.00	0.00	0.00
GATCAAAGAATTGGCCCCNN	0.46	0.00	0.00	0.00	0.00
GATCAAAGAATTGGCCCTCG	0.00	0.00	0.29	0.00	0.00
GATCAAAGAATTGGCGCCCG	0.23	0.00	0.00	0.00	0.00

GATCAAAGAATTTCTTCCAA	0.00	0.00	0.00	0.68	0.00
GATCAAAGAATTTCTTCCCA	0.69	0.00	0.00	0.00	0.00
GATCAAAGAATTTCTTCCCC	0.00	0.33	0.00	0.00	0.00
GATCAAAGAATTTCTTCCCT	12.13	13.34	6.87	10.86	4.48
GATCAAAGAATTTCTTCCNN	0.23	0.00	0.00	0.00	0.00
GATCAAAGAATTTCTTNCNN	0.23	0.00	0.00	0.00	0.00
GATCAAAGAATTTGCGCCCA	0.00	0.33	0.00	0.00	0.00
GATCAAAGAATTTGCTCACT	0.23	0.00	0.00	0.00	0.00
GATCAAAGAATTTGCTCCCT	7.55	6.51	9.16	13.92	15.15
GATCAAAGAATTTGCTNNNN	0.23	0.00	0.00	0.00	0.00
GATCAAAGACAAAAAAAAAA	0.00	0.33	0.57	1.02	0.34
GATCAAAGACAAAAAACATC	0.00	0.00	0.00	0.00	0.00
GATCAAAGACAAACTTCGGA	0.00	0.65	0.00	0.00	0.00
GATCAAAGACAACAGAGGCA	0.00	0.00	0.00	0.00	0.34
GATCAAAGACAACGAGCAGA	0.00	0.00	0.00	0.00	0.00
GATCAAAGACAACGAGCAGT	0.00	0.00	0.00	0.34	0.00
GATCAAAGACAAGAATCAAC	0.69	0.33	0.00	1.36	1.03
GATCAAAGACAAGACCTCCA	0.00	0.00	0.00	0.34	0.00
GATCAAAGACAAGACCTTCA	0.23	1.63	0.57	3.39	1.03
GATCAAAGACAAGCATGGCT	0.00	0.33	0.00	0.00	0.00
GATCAAAGACAAGGACCGCC	0.69	0.00	0.00	0.00	0.00
GATCAAAGACAAGGCAGGGG	0.00	0.00	0.00	0.00	0.00
GATCAAAGACAAGTGGCAGA	0.00	0.00	0.00	0.00	0.00
GATCAAAGACAAGTGTGGTG	0.00	0.33	0.00	0.00	0.00
GATCAAAGACAATGTCTCTA	0.00	0.33	0.00	0.00	0.00
GATCAAAGACACAGAAGAAC	0.00	0.00	0.00	0.00	0.00
GATCAAAGACACCCTCCCAA	0.00	0.00	0.29	0.00	0.00
GATCAAAGACACCCTCGCCC	0.69	0.00	0.00	0.00	0.00
GATCAAAGACACCCTTGGCG	0.00	0.65	0.29	0.00	0.00
GATCAAAGACACCCTTGGGG	0.00	0.00	0.00	0.00	0.34
GATCAAAGACACCGTGGCGG	0.00	0.00	0.00	0.00	0.00
GATCAAAGACACCGTTGACA	0.00	0.00	0.00	0.00	0.00
GATCAAAGACACCTCCGCCG	0.00	0.33	0.00	0.00	0.00
GATCAAAGACACGATGGTCG	0.00	0.00	0.00	0.00	0.00
GATCAAAGACACGCTCAGCA	0.00	0.00	0.00	0.00	0.00
GATCAAAGACACGGCAATAA	0.23	0.00	0.29	0.00	0.00
GATCAAAGACACGGCAATAG	0.92	0.33	2.00	0.34	0.34

GATCAAAGACACGGTCCCCA	0.00	0.33	0.00	0.00	0.00
GATCAAAGACACGTGCCCCG	0.00	0.33	0.00	0.34	0.00
GATCAAAGACACTCCCAAGT	0.00	0.00	0.00	0.34	0.00
GATCAAAGACACTGCCCTCG	0.92	0.65	0.29	0.34	0.00
GATCAAAGACACTGCGCCAG	0.00	0.33	0.00	0.00	0.00
GATCAAAGACACTGCGTCTC	0.46	0.00	0.00	0.00	0.00
GATCAAAGACAGAAACAAGG	0.00	0.00	0.00	0.34	0.00
GATCAAAGACAGAACGCCAG	0.00	0.00	0.00	0.00	0.00
GATCAAAGACAGAAGCAAGG	0.00	0.00	0.29	0.00	0.00
GATCAAAGACAGAAGCATGG	2.29	10.09	17.75	11.88	10.33
GATCAAAGACAGACGGGGAA	0.00	0.00	0.00	0.00	0.34
GATCAAAGACAGAGCAGCCT	0.00	0.00	0.00	0.34	0.00
GATCAAAGACAGAGGGAAAA	0.00	0.00	0.29	0.00	0.00
GATCAAAGACAGCAAGTTCG	0.00	0.33	0.00	0.00	0.00
GATCAAAGACAGCGGCGACA	0.23	0.00	0.00	0.00	0.00
GATCAAAGACAGCGGTGTCT	0.00	0.00	0.00	0.00	0.00
GATCAAAGACAGCTCATCGA	0.00	0.00	0.00	0.34	0.00
GATCAAAGACAGGAAGCGGA	0.23	0.00	0.00	0.00	0.00
GATCAAAGACAGGGAGGAAC	0.23	0.33	0.00	0.00	0.00
GATCAAAGACAGTCAGCGTC	0.00	0.00	0.00	0.00	0.00
GATCAAAGACAGTGGCCAGA	0.00	0.33	0.00	0.00	0.00
GATCAAAGACAGTGGCGAGA	6.64	4.88	2.58	4.07	2.41
GATCAAAGACAGTGGCGAGC	0.23	0.00	0.00	0.00	0.00
GATCAAAGACAGTGGCGAGG	2.29	0.00	0.00	0.00	0.00
GATCAAAGACAGTGGCGAGN	0.23	0.00	0.00	0.00	0.00
GATCAAAGACAGTGGCGGGG	0.23	0.00	0.00	0.00	0.00
GATCAAAGACATACCCAAGA	0.23	0.00	0.29	0.34	0.34
GATCAAAGACATCAGGAGCT	0.00	0.00	0.57	0.00	0.00
GATCAAAGACATCCTCATCC	0.23	0.00	0.00	0.00	0.00
GATCAAAGACATCCTCATGG	0.00	0.00	0.29	0.00	0.00
GATCAAAGACATCGAATTCA	0.00	0.00	3.44	0.00	2.41
GATCAAAGACATCGACTTCA	0.00	0.00	0.00	0.00	0.34
GATCAAAGACATCGTCCGCA	0.00	0.65	0.00	0.00	0.00
GATCAAAGACATCTGACACT	0.00	0.33	0.00	0.00	0.00
GATCAAAGACATCTTCCAAG	0.00	0.00	0.00	0.00	0.00
GATCAAAGACATCTTCCAGA	0.00	0.33	0.00	1.36	0.00
GATCAAAGACATGCCTGAGG	0.00	0.00	0.00	0.00	0.00

GATCAAAGACATGCCTGGTC	0.00	0.00	0.00	0.00	0.00
GATCAAAGACATGGACATCA	0.00	0.00	0.00	0.00	0.00
GATCAAAGACATGGACATCC	0.00	0.65	0.00	0.00	0.00
GATCAAAGACATGGCCAAAA	0.00	0.00	0.00	0.00	0.00
GATCAAAGACATGGCCATCA	0.00	0.33	0.00	0.00	0.00
GATCAAAGACATGGCCGGCT	0.00	0.33	0.00	0.00	0.00
GATCAAAGACATGTTCTCGT	0.00	0.33	0.00	0.00	0.00
GATCAAAGACATGTTCTGGT	6.41	4.55	2.86	1.36	1.38
GATCAAAGACCAAAAAAAAA	0.46	0.00	0.00	0.00	0.34
GATCAAAGACCAAGACTGAC	0.00	0.00	0.00	0.00	0.00
GATCAAAGACCAAGAGACGG	0.00	0.00	0.00	0.00	0.00
GATCAAAGACCACACCACAC	0.00	0.00	0.00	0.00	0.34
GATCAAAGACCACACTGCTC	0.00	1.30	0.57	0.00	0.00
GATCAAAGACCACATACAGG	0.00	0.00	0.00	0.34	0.00
GATCAAAGACCACGCACACA	0.00	0.00	0.29	0.00	0.00
GATCAAAGACCACGGCTGAC	0.00	0.00	0.29	0.00	0.00
GATCAAAGACCACTATCTTG	0.00	0.00	0.00	0.68	0.00
GATCAAAGACCAGATGTGGC	0.00	0.00	0.00	0.00	0.00
GATCAAAGACCAGCACTGTC	0.23	0.00	0.00	0.00	0.00
GATCAAAGACCAGCAGAACA	0.00	0.00	0.00	0.00	0.34
GATCAAAGACCAGCCGCATC	0.00	0.00	0.00	0.00	0.34
GATCAAAGACCAGGAAAAGG	0.00	0.00	0.00	0.00	0.00
GATCAAAGACCAGGAGCAGT	0.00	0.00	0.00	0.00	0.00
GATCAAAGACCAGGAGTTTG	0.00	0.00	0.00	0.00	0.00
GATCAAAGACCAGTGAAGCA	0.00	0.00	0.00	0.00	0.00
GATCAAAGACCAGTGAAGCC	9.39	8.13	0.57	9.50	2.41
GATCAAAGACCAGTGAAGNN	0.23	0.00	0.00	0.00	0.00
GATCAAAGACCAGTGAGGCC	0.23	0.00	0.00	0.00	0.00
GATCAAAGACCAGTGANGCC	0.00	0.00	0.00	0.00	0.34
GATCAAAGACCATACACTTG	0.46	0.00	0.00	0.00	0.00
GATCAAAGACCATACCATTC	0.23	0.00	0.00	0.00	0.00
GATCAAAGACCATCGCTTCA	0.00	0.00	0.00	0.00	0.00
GATCAAAGACCCACGGCCCG	0.00	0.00	0.00	0.00	0.00
GATCAAAGACCCAGAANNNG	0.00	0.00	0.00	0.00	0.00
GATCAAAGACCCAGCATTGG	0.00	0.00	0.29	0.00	0.00
GATCAAAGACCCCCATGCAG	0.00	0.00	0.00	0.00	0.34
GATCAAAGACCCCGGCCAAG	0.00	0.00	0.29	0.00	0.00

GATCAAAGACCCGATTCATA	0.00	0.00	0.00	0.34	0.00
GATCAAAGACCCGGCCTGAA	0.00	0.00	0.29	0.00	0.00
GATCAAAGACCCGTGAGGAG	0.00	0.00	0.00	0.00	0.00
GATCAAAGACCCTGAGCACC	0.00	0.00	0.00	0.00	0.69
GATCAAAGACCCTTAACTGA	0.00	0.00	0.00	0.34	0.00
GATCAAAGACCGAATACGGA	0.00	0.00	0.00	0.34	0.00
GATCAAAGACCGAATACGGG	0.00	0.00	0.00	0.34	0.00
GATCAAAGACCGACGTCCAT	0.00	0.00	0.00	0.00	0.34
GATCAAAGACCGCAAAAAAA	0.00	0.00	0.29	0.00	0.00
GATCAAAGACCGCAAACAAA	0.00	0.00	0.29	0.00	0.00
GATCAAAGACCGCAGCATCC	0.00	0.00	0.00	0.00	0.69
GATCAAAGACCGCAGCCAAA	0.00	0.00	0.00	0.00	0.00
GATCAAAGACCGCAGTGAAA	0.00	0.00	0.00	0.00	0.34
GATCAAAGACCGCAGTGCTT	0.00	0.00	0.00	0.00	0.00
GATCAAAGACCGCAGTGTTT	5.72	5.53	10.88	8.83	8.27
GATCAAAGACCGCATCAAAA	0.00	0.00	0.00	0.00	0.34
GATCAAAGACCGCATCATCC	0.00	0.00	0.29	0.00	0.69
GATCAAAGACCGCATCGCTT	0.00	0.33	0.00	0.00	0.00
GATCAAAGACCGCATCTGTG	0.00	0.00	0.29	0.00	0.00
GATCAAAGACCGCATGAGAT	0.00	0.00	0.00	0.00	0.00
GATCAAAGACCGCATGATTT	0.00	0.00	0.00	0.00	0.34
GATCAAAGACCGCATGCAAA	0.00	0.33	0.00	0.00	0.00
GATCAAAGACCGCATGCAGC	0.00	0.33	0.29	1.02	0.00
GATCAAAGACCGCATGCAGT	0.00	0.33	0.00	0.00	0.00
GATCAAAGACCGCATGCCAA	0.00	0.00	0.00	0.00	0.00
GATCAAAGACCGCATGCCGT	0.00	0.65	1.43	0.68	0.00
GATCAAAGACCGCATGCGGC	1.14	0.98	1.15	2.04	1.72
GATCAAAGACCGCATGCTAC	0.00	0.00	0.29	0.00	0.00
GATCAAAGACCGCATGCTCG	0.46	0.65	0.29	0.68	0.34
GATCAAAGACCGCATGCTGA	0.00	0.00	0.00	0.00	0.34
GATCAAAGACCGCATGGAAA	0.69	0.00	1.43	0.34	0.34
GATCAAAGACCGCATGGAAC	0.00	0.00	0.00	0.00	0.00
GATCAAAGACCGCATGGACC	0.00	0.00	0.29	0.00	0.00
GATCAAAGACCGCATGGACG	0.00	0.00	0.29	0.00	0.00
GATCAAAGACCGCATGGAGA	0.00	0.00	0.00	0.00	0.00
GATCAAAGACCGCATGGAGC	0.00	0.00	0.00	0.00	0.69
GATCAAAGACCGCATGGAGG	0.00	0.00	0.00	0.68	0.00

GATCAAAGACCGCATGGATA	0.00	0.00	0.00	0.00	0.00
GATCAAAGACCGCATGGATC	0.69	1.30	3.15	0.68	3.79
GATCAAAGACCGCATGGATG	0.00	0.33	0.57	0.00	0.34
GATCAAAGACCGCATGGATT	2.29	0.65	1.15	0.68	0.34
GATCAAAGACCGCATGGCAA	0.00	0.00	0.00	0.00	0.00
GATCAAAGACCGCATGGCCG	0.00	0.00	0.00	0.34	0.00
GATCAAAGACCGCATGGCGT	0.00	0.00	0.00	0.00	0.00
GATCAAAGACCGCATGGTCT	0.00	0.00	0.00	0.00	0.00
GATCAAAGACCGCATGTTGG	0.00	0.00	0.29	0.00	0.00
GATCAAAGACCGCCAAAAAA	0.00	0.00	0.00	0.00	1.03
GATCAAAGACCGCCGTGAAA	0.00	0.33	0.29	0.00	1.03
GATCAAAGACCGCCGTGANA	0.00	0.00	0.00	0.00	0.34
GATCAAAGACCGCCTCCAGT	0.00	0.00	0.00	0.34	0.00
GATCAAAGACCGCCTGAAAA	0.46	0.00	0.00	0.00	0.00
GATCAAAGACCGCCTGCTCA	0.00	0.33	0.00	0.00	0.00
GATCAAAGACCGCCTGCTCG	0.00	0.00	0.29	0.00	0.34
GATCAAAGACCGCCTTGACA	0.00	0.33	0.00	0.00	0.00
GATCAAAGACCGCGCGGGGA	0.00	0.00	0.00	0.00	0.34
GATCAAAGACCGCGCGTGAT	0.00	0.00	0.00	0.00	0.00
GATCAAAGACCGCGGCATCC	0.00	0.00	0.00	0.00	0.00
GATCAAAGACCGCGGTGTGA	0.00	0.33	0.00	0.00	0.00
GATCAAAGACCGCGGTGTTT	0.00	0.65	0.00	1.02	0.34
GATCAAAGACCGCGTGAAAA	0.23	0.33	0.29	1.02	0.00
GATCAAAGACCGCGTGAACA	0.00	0.00	0.00	0.00	0.00
GATCAAAGACCGCGTGACAA	0.00	0.00	0.00	0.34	0.00
GATCAAAGACCGCGTGATAA	0.00	0.00	0.29	0.00	0.00
GATCAAAGACCGCGTGCAAA	0.23	0.00	0.00	0.00	0.34
GATCAAAGACCGCGTGCAAC	0.00	0.00	0.00	0.34	0.00
GATCAAAGACCGCGTGCCGT	0.46	0.00	0.00	0.00	0.00
GATCAAAGACCGCGTGCGGC	0.00	0.33	0.00	0.00	0.00
GATCAAAGACCGCGTGCTCA	0.00	0.00	0.00	0.34	0.00
GATCAAAGACCGCGTGCTCG	0.00	0.00	0.57	0.00	0.34
GATCAAAGACCGCGTGGAAA	1.60	0.33	0.57	0.00	0.34
GATCAAAGACCGCGTGGAAG	0.00	0.00	0.00	0.00	0.34
GATCAAAGACCGCGTGGACG	0.00	0.00	0.00	0.00	0.00
GATCAAAGACCGCGTGGAGA	0.00	0.33	0.00	0.34	0.34
GATCAAAGACCGCGTGGAGG	0.00	0.00	0.57	0.00	0.00

GATCAAAGACCGCGTGGATA	0.00	0.65	0.29	0.34	0.34
GATCAAAGACCGCGTGGATC	1.14	0.65	0.57	1.02	1.72
GATCAAAGACCGCGTGGATG	0.23	0.00	1.15	0.68	0.00
GATCAAAGACCGCGTGGATT	0.23	0.33	0.57	0.00	0.00
GATCAAAGACCGCGTGGCAA	0.00	0.65	0.00	0.00	0.00
GATCAAAGACCGCGTGGCGT	0.00	0.33	0.00	0.34	0.00
GATCAAAGACCGCGTGGGAA	0.00	0.00	0.00	0.34	0.00
GATCAAAGACCGCGTGGGGA	0.00	0.00	0.00	0.00	0.34
GATCAAAGACCGCGTGGTGT	0.00	0.00	0.00	0.00	0.00
GATCAAAGACCGCGTGTAAA	7.55	5.53	6.87	8.15	3.10
GATCAAAGACCGCGTGTAAC	0.00	0.33	0.00	0.00	0.00
GATCAAAGACCGCGTGTAAT	0.00	0.00	0.00	0.00	0.34
GATCAAAGACCGCGTGTAGC	0.00	0.33	0.00	0.00	0.00
GATCAAAGACCGCGTGTATC	1.14	0.65	1.72	0.68	2.07
GATCAAAGACCGCGTGTCAC	0.00	0.00	0.57	0.00	0.00
GATCAAAGACCGCGTGTCTT	0.23	0.00	0.00	0.00	0.00
GATCAAAGACCGCGTGTGAA	8.47	3.58	12.31	7.81	6.89
GATCAAAGACCGCGTGTGAG	0.23	0.00	0.00	0.00	0.00
GATCAAAGACCGCGTGTGAT	1.37	0.65	1.15	0.34	1.38
GATCAAAGACCGCGTGTGCA	0.23	0.00	0.00	0.00	0.00
GATCAAAGACCGCGTGTTTC	0.46	0.98	2.00	1.02	1.38
GATCAAAGACCGCTCCAAGC	0.46	0.00	0.00	0.00	0.00
GATCAAAGACCGCTCCATTG	0.92	0.98	2.29	1.70	0.34
GATCAAAGACCGCTCCCCAG	0.69	0.65	0.00	0.34	1.03
GATCAAAGACCGCTCGCCGT	0.00	0.33	0.29	0.00	0.34
GATCAAAGACCGCTCGTATG	0.00	0.00	0.00	0.68	0.00
GATCAAAGACCGCTGATGTT	0.00	0.00	0.00	0.00	0.00
GATCAAAGACCGGAAGGACG	1.60	0.65	0.00	0.68	0.34
GATCAAAGACCGGATTGCTC	0.00	0.00	0.00	0.00	0.34
GATCAAAGACCGGCATTAAG	0.23	0.00	0.00	0.00	0.00
GATCAAAGACCGGCGCGCAA	0.00	0.00	0.00	0.00	0.00
GATCAAAGACCGGGCTGCGA	0.00	0.00	0.00	0.34	0.00
GATCAAAGACCGTACGCCAG	3.20	2.93	1.43	3.73	1.72
GATCAAAGACCGTACGGCAG	0.23	0.00	0.00	0.00	0.00
GATCAAAGACCGTGGGTTGA	0.00	0.00	0.00	0.00	0.34
GATCAAAGACCGTGTAGTAG	0.00	0.00	0.00	0.00	0.00
GATCAAAGACCGTTCCATTG	0.00	0.33	4.30	0.00	2.07

GATCAAAGACCGTTCCCTTG	0.00	0.00	0.29	0.00	0.00
GATCAAAGACCTACCGCCCC	0.46	1.95	0.29	0.34	0.00
GATCAAAGACCTAGGGGAAG	0.00	0.33	0.00	0.00	0.00
GATCAAAGACCTCACCAACA	0.00	0.33	0.00	0.00	0.00
GATCAAAGACCTCCAGAGCG	0.00	0.33	0.00	0.00	0.00
GATCAAAGACCTCCCAAGGA	0.46	0.00	0.00	0.00	0.00
GATCAAAGACCTCCCAGCCT	0.00	0.00	0.29	0.00	0.00
GATCAAAGACCTCCTGCGTG	0.00	0.00	0.29	0.00	0.00
GATCAAAGACCTCGTCTGGC	0.00	0.00	0.00	0.34	0.00
GATCAAAGACCTCTCGGAGA	0.00	0.00	0.29	0.00	0.00
GATCAAAGACCTCTTCGGGA	0.00	0.33	0.00	0.00	0.00
GATCAAAGACCTGATTTCAA	0.00	0.33	0.00	0.00	0.00
GATCAAAGACCTGCAAAAAA	0.00	0.00	0.00	0.00	0.34
GATCAAAGACCTGCTTGTCC	0.00	0.33	0.29	0.00	0.00
GATCAAAGACCTGTGGAGGA	0.00	0.00	0.00	0.00	0.00
GATCAAAGACCTGTGGTGTG	0.00	0.00	0.00	0.00	0.34
GATCAAAGACCTTCAAGATG	0.00	0.00	0.00	0.00	0.00
GATCAAAGACCTTCAGCGCC	0.00	0.00	0.00	0.34	0.00
GATCAAAGACCTTCTCCAGG	0.00	0.33	0.00	0.00	0.00
GATCAAAGACCTTGATAGGT	0.00	0.00	0.00	0.00	0.00
GATCAAAGACCTTGGGGAAG	0.00	0.33	0.00	0.00	0.00
GATCAAAGACCTTTCGTATG	0.00	0.65	0.00	0.00	0.00
GATCAAAGACCTTTGCATGA	0.00	0.00	0.00	0.34	0.00
GATCAAAGACCTTTGGCGTG	0.00	0.00	0.00	0.00	0.00
GATCAAAGACCTTTGGGGTG	0.23	0.00	0.00	0.00	0.00
GATCAAAGACCTTTGGTCTG	0.00	0.00	0.00	0.00	0.34
GATCAAAGACCTTTGGTGAG	0.00	0.33	0.00	0.00	0.00
GATCAAAGACCTTTGGTGTC	0.00	0.00	0.00	0.00	0.34
GATCAAAGACCTTTGGTGTG	7.78	11.39	14.89	9.84	9.30
GATCAAAGACCTTTGGTGTN	0.23	0.00	0.00	0.00	0.00
GATCAAAGACCTTTGGTGTT	0.23	0.00	0.00	0.00	0.00
GATCAAAGACCTTTGTGGGA	0.00	0.00	0.00	0.68	0.00
GATCAAAGACCTTTTGAGCC	0.00	0.00	0.00	0.00	0.00
GATCAAAGACCTTTTGTGTG	0.00	0.33	0.00	0.00	0.00
GATCAAAGACGAAAAAAAAA	0.00	0.00	0.00	0.34	0.00
GATCAAAGACGAAGTTCAAC	0.00	0.00	0.00	0.00	0.00
GATCAAAGACGACAGCTCTG	0.00	0.00	0.29	0.00	0.00

GATCAAAGACGACCCGGTGA	0.00	0.00	0.29	0.00	0.00
GATCAAAGACGACCGGCCGA	0.46	0.98	0.00	0.68	0.00
GATCAAAGACGACCGGCCGG	0.23	0.00	0.00	0.00	0.00
GATCAAAGACGAGCTGTGCC	0.00	0.00	0.00	0.00	0.34
GATCAAAGACGAGGAGCGCC	0.00	0.33	0.00	0.00	0.00
GATCAAAGACGAGGAGGGCA	0.00	0.33	0.00	0.00	0.00
GATCAAAGACGAGGAGGGCT	0.23	0.00	0.00	0.00	0.00
GATCAAAGACGAGTCAGGCG	0.00	0.00	0.00	0.00	0.00
GATCAAAGACGAGTTGGAAG	0.00	0.00	0.00	0.00	0.69
GATCAAAGACGATGAGGCAA	0.00	0.00	0.00	0.00	0.00
GATCAAAGACGATGCCCCAA	0.00	0.00	0.00	0.34	0.00
GATCAAAGACGCAAATGGCC	0.00	0.00	0.57	0.00	0.00
GATCAAAGACGCAGACCTCT	0.00	0.33	0.29	0.00	0.00
GATCAAAGACGCAGACCTGC	0.00	0.00	0.29	0.00	0.00
GATCAAAGACGCAGACCTTC	0.00	0.00	0.29	0.00	0.00
GATCAAAGACGCAGCCCTCT	0.23	0.00	0.00	0.00	0.00
GATCAAAGACGCATTCTGCT	0.00	0.00	0.00	0.00	0.34
GATCAAAGACGCCATTTTCC	0.23	0.00	0.00	0.00	0.00
GATCAAAGACGCCGACGAGG	0.00	0.00	0.00	0.34	0.00
GATCAAAGACGCCGACGTCG	0.69	0.00	0.00	0.00	0.00
GATCAAAGACGCCGCAGAGG	0.00	0.00	0.29	0.00	0.00
GATCAAAGACGCCGGCATGG	0.00	0.00	0.29	0.00	0.00
GATCAAAGACGCCGTCTTCC	0.00	0.00	0.00	0.00	0.00
GATCAAAGACGCCGTGAAAA	0.00	0.00	0.00	0.00	0.34
GATCAAAGACGCCGTGCCAT	0.46	0.33	0.00	0.00	0.69
GATCAAAGACGCCGTGCTCA	0.00	0.00	0.29	0.00	0.00
GATCAAAGACGCCGTGGAAA	0.00	0.00	0.29	0.00	0.00
GATCAAAGACGCCGTGGAGC	0.00	0.00	0.29	0.00	0.00
GATCAAAGACGCCGTGGAGG	0.00	0.00	0.29	1.02	0.00
GATCAAAGACGCCTTTGTTG	0.00	0.33	0.29	0.34	0.00
GATCAAAGACGCTATGGAAG	0.00	0.00	0.00	0.00	0.00
GATCAAAGACGCTATGGCTG	0.00	0.00	0.00	0.00	0.00
GATCAAAGACGCTGCCAAAG	0.00	0.00	0.00	0.34	0.00
GATCAAAGACGCTGCCAAAT	0.00	0.00	0.00	0.00	0.00
GATCAAAGACGCTTGCTCGC	0.00	0.00	0.00	0.00	0.34
GATCAAAGACGCTTGCTTCG	0.00	0.00	0.00	0.00	0.00
GATCAAAGACGCTTGTAAAA	0.00	0.33	0.00	0.00	0.00

GATCAAAGACGGAACATTGC	0.00	0.00	0.00	0.00	0.34
GATCAAAGACGGAAGCATGG	0.00	0.00	0.29	0.00	0.00
GATCAAAGACGGAATGCCTA	0.00	0.00	0.29	0.00	0.00
GATCAAAGACGGAATGGGTC	0.00	0.65	0.00	0.00	0.69
GATCAAAGACGGAATGGGTN	0.00	0.00	0.00	0.00	0.00
GATCAAAGACGGAGAGGGAA	0.00	0.00	0.29	0.00	0.00
GATCAAAGACGGAGAGTGCA	0.23	0.00	0.00	0.34	0.00
GATCAAAGACGGATGTGCAG	0.00	0.00	0.00	0.00	0.34
GATCAAAGACGGCAGCNTGA	0.00	0.00	0.00	0.00	0.34
GATCAAAGACGGCAGCTTGA	0.00	0.65	0.57	0.34	0.69
GATCAAAGACGGCTTCAGAC	0.23	0.00	0.00	0.00	0.00
GATCAAAGACGGGGAANAGC	0.00	0.00	0.29	0.00	0.00
GATCAAAGACGGGTGTGTCA	0.00	0.00	0.00	0.00	0.34
GATCAAAGACGGTCAAGCGA	0.00	0.00	0.00	0.00	0.34
GATCAAAGACGGTCTCGTCG	0.00	0.00	0.29	0.00	0.00
GATCAAAGACGGTGCAGGTG	0.69	2.28	0.57	1.36	0.00
GATCAAAGACGGTGCTCTGG	0.00	0.33	0.00	0.00	0.00
GATCAAAGACGTCAAGGAAG	0.69	0.98	0.00	0.34	0.00
GATCAAAGACGTGCATTTTG	0.00	0.00	0.00	0.34	0.00
GATCAAAGACGTGGAGACAA	0.00	0.00	0.00	0.34	0.00
GATCAAAGACGTGGGCAAAA	0.00	0.33	0.00	0.00	0.00
GATCAAAGACGTGGGGCTGT	0.00	0.00	0.00	0.00	0.00
GATCAAAGACGTGGTCATTG	0.46	0.65	0.00	1.02	0.69
GATCAAAGACGTGTACTGAA	0.00	0.00	0.00	0.00	0.00
GATCAAAGACGTGTACTGAC	7.78	3.25	3.15	2.04	2.76
GATCAAAGACGTGTTCTGGT	0.00	0.00	0.29	0.00	0.00
GATCAAAGACGTTCAACAGT	2.52	3.90	1.15	1.36	2.07
GATCAAAGACGTTCGACAGT	0.00	0.00	0.00	0.00	0.34
GATCAAAGACGTTGATGGAA	0.00	0.00	0.00	0.34	0.00
GATCAAAGACTAAAAAAAAA	0.00	0.00	0.00	0.34	0.00
GATCAAAGACTAAGAGACAG	0.00	0.00	0.00	0.00	0.34
GATCAAAGACTAAGAGACGG	0.00	0.00	0.29	0.34	0.69
GATCAAAGACTACAAGCCGT	0.00	0.00	0.00	0.34	0.00
GATCAAAGACTACAGCCGTG	0.00	0.00	0.29	0.00	0.00
GATCAAAGACTACATGCTGT	0.23	0.00	0.00	0.00	0.00
GATCAAAGACTACTTGGAGC	0.00	0.00	0.00	0.68	0.34
GATCAAAGACTATTCAACCT	0.69	0.33	0.57	0.00	1.38

GATCAAAGACTATTCTGAGC	0.00	0.00	0.00	0.00	0.00
GATCAAAGACTCAAAAAAAA	0.46	0.00	0.29	0.00	0.69
GATCAAAGACTCAATGATTA	0.00	0.00	0.00	0.34	0.00
GATCAAAGACTCAATGATTG	0.00	0.00	0.29	0.00	0.00
GATCAAAGACTCAATGATTT	0.00	0.00	0.29	0.00	0.00
GATCAAAGACTCACGACTGA	0.00	0.00	0.00	0.00	0.00
GATCAAAGACTCATGGCAAA	0.00	0.00	0.00	0.00	0.00
GATCAAAGACTCATGGGCTT	0.00	0.00	0.00	0.00	0.00
GATCAAAGACTCCAAGGGCT	0.46	0.00	0.00	0.00	0.00
GATCAAAGACTCCAGCTGCA	0.23	0.00	0.00	0.00	0.00
GATCAAAGACTCCATTCCCG	0.00	0.33	0.00	0.00	0.00
GATCAAAGACTCCATTCCNN	0.23	0.00	0.00	0.00	0.00
GATCAAAGACTCCATTCCTG	8.47	15.29	10.88	15.95	19.29
GATCAAAGACTCCATTCCTN	0.23	0.00	0.00	0.00	0.00
GATCAAAGACTCCATTNCTG	0.00	0.00	0.00	0.00	0.34
GATCAAAGACTCCCTACCTG	0.23	0.00	0.00	0.00	0.00
GATCAAAGACTCCGTTCCTG	0.00	0.00	0.00	0.34	0.00
GATCAAAGACTCGCTCATGC	0.00	0.00	0.00	0.68	0.00
GATCAAAGACTCGGCACGGC	0.00	0.00	0.29	0.00	0.00
GATCAAAGACTCTGAGCACC	0.00	0.00	0.00	0.00	0.34
GATCAAAGACTCTGAGCCGT	0.00	0.00	0.29	0.00	0.00
GATCAAAGACTCTGGTTTCA	0.23	0.00	0.00	0.00	0.00
GATCAAAGACTCTTGCAGGC	0.00	0.33	0.00	0.00	0.00
GATCAAAGACTGAAATCAAC	0.00	0.00	0.00	0.00	0.34
GATCAAAGACTGAAGGAGGG	0.00	0.00	0.29	0.00	0.00
GATCAAAGACTGACATCTAT	0.23	0.33	0.86	0.68	0.34
GATCAAAGACTGACGGATGA	0.00	0.65	0.00	0.00	0.00
GATCAAAGACTGATGTAAAA	0.00	0.00	0.00	1.36	0.00
GATCAAAGACTGCACCATGG	0.23	0.33	0.00	0.00	0.00
GATCAAAGACTGCACTCCGC	0.00	0.33	0.00	0.00	0.00
GATCAAAGACTGCACTCGCA	0.00	0.00	0.00	0.00	0.00
GATCAAAGACTGCATGGGCG	0.00	0.00	0.00	0.00	0.00
GATCAAAGACTGCCGGCGAT	0.00	0.00	0.00	0.34	0.00
GATCAAAGACTGCCGGCTGC	0.00	0.33	0.00	0.34	0.00
GATCAAAGACTGCCGGCTGG	0.00	0.33	0.00	0.00	0.00
GATCAAAGACTGCCTCAATG	0.00	0.33	0.00	0.00	0.00
GATCAAAGACTGCGCCCTCG	4.35	2.28	3.15	1.70	2.76

GATCAAAGACTGCGCCCTNN	0.00	0.00	0.00	0.00	0.34
GATCAAAGACTGCGGCGACC	0.00	0.00	0.00	0.00	0.34
GATCAAAGACTGCGGCTATG	0.00	0.00	0.00	0.00	0.00
GATCAAAGACTGCGGGCACT	0.00	0.00	0.29	0.00	0.00
GATCAAAGACTGCGTGTGAA	0.00	0.00	0.00	0.00	0.69
GATCAAAGACTGCTGGACGG	0.00	0.00	0.00	0.00	0.00
GATCAAAGACTGCTTGCCGC	0.00	0.33	0.00	0.00	0.00
GATCAAAGACTGGCGCGCGG	0.69	1.30	0.29	1.36	1.38
GATCAAAGACTGGCTGGGGG	0.23	0.00	0.00	0.00	0.00
GATCAAAGACTGGGTTAAGG	0.00	0.00	0.00	0.00	0.00
GATCAAAGACTGGTTCGCGA	0.00	0.00	0.00	0.00	1.03
GATCAAAGACTGTACATGCG	0.00	0.00	0.57	1.02	0.34
GATCAAAGACTGTAGTGTGG	0.00	0.00	0.29	0.00	0.00
GATCAAAGACTGTATGGAGG	0.23	0.00	0.00	0.00	0.00
GATCAAAGACTGTCCATCAG	0.00	0.00	0.00	0.34	0.00
GATCAAAGACTGTCTAAAAA	0.00	0.00	0.29	0.00	0.00
GATCAAAGACTGTCTAACAG	0.00	0.98	2.58	0.00	0.00
GATCAAAGACTGTCTACCAC	0.00	0.00	0.00	0.34	0.00
GATCAAAGACTGTCTACCAG	8.24	24.72	19.19	24.78	31.68
GATCAAAGACTGTCTACCAN	0.00	0.00	0.00	0.00	0.00
GATCAAAGACTGTCTACCCG	0.00	0.65	0.29	0.00	0.00
GATCAAAGACTGTCTGCCAG	0.00	0.00	0.00	0.00	0.34
GATCAAAGACTGTGGCTGGA	0.00	0.65	0.86	0.00	0.00
GATCAAAGACTTAATGCCGG	0.46	0.33	0.00	0.34	0.34
GATCAAAGACTTATCTACCG	0.00	0.00	0.00	0.00	0.00
GATCAAAGACTTCAAAGAAC	0.00	0.00	0.00	0.00	0.00
GATCAAAGACTTCCGGAGGA	0.00	0.00	0.00	0.00	0.00
GATCAAAGACTTCTTCGTCG	1.14	0.65	0.29	1.02	0.34
GATCAAAGACTTGAGTTCAT	0.00	0.00	0.00	0.34	0.00
GATCAAAGACTTGCGCCAGG	0.00	0.00	0.00	0.00	0.00
GATCAAAGACTTGCTCCAGG	0.92	0.98	1.72	1.02	1.38
GATCAAAGACTTGTTGAGCC	0.00	0.00	0.00	0.00	0.00
GATCAAAGACTTTGCATTGG	0.00	0.33	0.00	0.00	0.00
GATCAAAGACTTTTGAGCCC	0.00	0.00	0.57	0.00	0.00
GATCAAAGACTTTTTGAGAC	0.00	0.00	0.29	0.00	0.00
GATCAAAGACTTTTTGAGCC	53.56	31.23	12.31	27.15	16.53
GATCAAAGACTTTTTGAGNC	0.00	0.00	0.00	0.00	0.34

GATCAAAGACTTTTTGAGNN	0.46	0.00	0.00	0.00	0.00
GATCAAAGACTTTTTGATCC	0.23	0.00	0.00	0.00	0.00
GATCAAAGACTTTTTGCGCC	0.23	0.00	0.00	0.34	0.00
GATCAAAGACTTTTTGCNNN	0.23	0.00	0.00	0.00	0.00
GATCAAAGAGAAAAAAAAAA	0.23	0.00	0.00	0.00	0.34
GATCAAAGAGAAAATAGAAG	0.00	0.00	0.00	0.00	0.00
GATCAAAGAGAAAATGATGA	0.00	0.00	0.00	0.00	0.00
GATCAAAGAGAAAGAAAAAA	0.00	0.00	0.00	0.00	0.00
GATCAAAGAGAACGAGGAGG	0.00	0.00	0.00	0.00	0.00
GATCAAAGAGAACGATGGCA	0.00	0.00	0.57	0.00	0.00
GATCAAAGAGAACGGGACCG	0.00	0.00	0.00	0.00	0.00
GATCAAAGAGAAGAAAGAGG	0.23	0.00	0.29	0.00	0.00
GATCAAAGAGAAGAACAAAG	0.00	0.33	0.00	0.00	0.00
GATCAAAGAGAAGAACAAGG	0.00	0.00	0.00	0.00	0.69
GATCAAAGAGAAGAACGAGG	18.77	51.08	40.66	31.57	40.98
GATCAAAGAGAAGAACGAGN	0.23	0.00	0.00	0.00	0.00
GATCAAAGAGAAGAACGAGT	0.00	0.33	0.00	0.00	0.00
GATCAAAGAGAAGAACGANN	0.23	0.00	0.00	0.00	0.00
GATCAAAGAGAAGAACGCGG	0.00	0.00	0.00	0.00	0.00
GATCAAAGAGAAGAACGGGG	0.00	0.00	0.29	0.00	0.00
GATCAAAGAGAAGAACNAGG	0.00	0.00	0.00	0.34	0.00
GATCAAAGAGAAGAAGGAAT	0.00	0.33	1.15	0.68	0.69
GATCAAAGAGAAGACACGCA	0.00	0.33	0.00	0.00	0.00
GATCAAAGAGAAGAGTATGA	0.00	0.00	0.00	0.00	0.34
GATCAAAGAGAAGCACAAAG	27.01	40.02	32.07	23.76	20.32
GATCAAAGAGAAGCACAACG	0.00	0.00	0.29	0.00	0.00
GATCAAAGAGAAGCACAAGG	0.23	0.00	0.00	0.00	0.00
GATCAAAGAGAAGCACACAG	0.23	0.33	0.00	0.34	0.00
GATCAAAGAGAAGCACAGAG	0.00	0.33	0.29	0.00	0.34
GATCAAAGAGAAGCACCAAG	0.23	0.33	0.00	0.00	0.34
GATCAAAGAGAAGCACCCCG	0.23	0.00	0.00	0.00	0.00
GATCAAAGAGAAGCACNANN	0.23	0.00	0.00	0.00	0.00
GATCAAAGAGAAGCACNNAG	0.00	0.00	0.00	0.00	0.00
GATCAAAGAGAAGCACTCGT	0.00	0.00	0.00	0.00	0.34
GATCAAAGAGAAGCAGAAAG	0.00	0.00	0.29	0.00	0.00
GATCAAAGAGAAGCAGCAGC	0.00	0.33	0.00	0.00	0.00
GATCAAAGAGAAGCAGCCGG	0.00	0.33	0.00	0.00	0.00

GATCAAAGAGAAGCAGCTGG	0.00	0.33	0.00	0.34	0.34
GATCAAAGAGAAGCATGAAA	0.00	0.65	0.00	0.00	0.00
GATCAAAGAGAAGCCCAAAG	0.00	0.00	0.00	0.00	0.34
GATCAAAGAGAAGCGAAGCT	0.00	0.00	0.57	0.00	0.00
GATCAAAGAGAAGCGCAGCA	0.00	0.00	0.00	0.00	0.00
GATCAAAGAGAAGCTTCGCC	0.00	0.33	0.00	0.00	0.00
GATCAAAGAGAAGGACAAGC	2.98	1.30	0.86	1.02	0.00
GATCAAAGAGAAGGAGGGAG	0.00	0.00	0.00	0.00	0.00
GATCAAAGAGAAGGGAGAGG	0.00	0.00	0.00	0.00	0.00
GATCAAAGAGAAGGGTGAGG	0.23	1.30	2.86	1.02	0.34
GATCAAAGAGAAGTGAGGAC	0.00	0.33	0.00	0.00	0.00
GATCAAAGAGAAGTTCGACG	0.00	0.33	0.29	0.34	0.00
GATCAAAGAGAAGTTTCTAT	0.00	0.00	0.00	0.00	0.69
GATCAAAGAGAATAAGAGGC	0.23	0.98	0.57	0.00	0.00
GATCAAAGAGAATAAGAGNN	0.00	0.00	0.00	0.00	0.00
GATCAAAGAGAATGACGGAT	0.00	0.00	0.29	0.00	0.00
GATCAAAGAGAATTCCAAAA	0.00	0.00	0.00	0.00	1.03
GATCAAAGAGAATTCCTCTA	2.52	0.33	0.00	0.68	0.00
GATCAAAGAGAATTTCTTTG	0.00	0.00	0.00	0.00	0.00
GATCAAAGAGACAAGCGCCC	0.00	0.33	0.00	0.00	0.34
GATCAAAGAGACACCCTGAG	0.00	0.00	0.00	0.00	0.00
GATCAAAGAGACGCACAAAG	0.00	0.00	0.00	0.34	0.00
GATCAAAGAGACGGCGACAA	0.00	0.00	0.00	0.00	0.00
GATCAAAGAGACTGAGCGAA	0.00	0.65	0.00	0.00	0.00
GATCAAAGAGAGACATTTGA	0.00	0.00	0.29	0.00	0.00
GATCAAAGAGAGACCCCCTG	0.00	0.00	0.00	0.00	0.00
GATCAAAGAGAGACCCTCTA	0.00	0.00	0.00	0.00	0.69
GATCAAAGAGAGCAGAAAAG	0.23	0.65	0.00	0.00	0.00
GATCAAAGAGAGCAGGCAGA	0.00	0.00	0.00	0.34	0.00
GATCAAAGAGAGCATTTAGG	0.23	0.98	0.00	0.34	0.34
GATCAAAGAGAGCCACCCAA	0.00	0.00	0.29	0.00	0.00
GATCAAAGAGAGCGCCTCAT	0.00	0.33	0.00	0.00	0.00
GATCAAAGAGAGCGTTTAGG	0.00	0.00	0.00	0.00	0.34
GATCAAAGAGAGCTTTTAGG	0.00	0.33	0.00	0.00	0.00
GATCAAAGAGAGGGGGGGGA	0.00	0.00	0.00	0.00	0.34
GATCAAAGAGAGGGGTTCGT	0.00	0.00	0.00	0.34	0.00
GATCAAAGAGATAAAAAAAA	0.00	0.00	0.00	0.00	0.00

GATCAAAGAGATGCACCTTG	3.66	2.93	0.57	1.36	0.69
GATCAAAGAGATGGACATCA	0.00	0.65	0.00	0.34	0.00
GATCAAAGAGATGGAGACGT	0.00	0.00	0.00	0.00	0.00
GATCAAAGAGATGGAGACTT	0.00	0.00	0.29	0.00	0.00
GATCAAAGAGATGGCACACG	0.00	0.00	0.00	0.34	0.00
GATCAAAGAGATTATGTGCA	0.00	0.00	0.00	0.68	0.00
GATCAAAGAGATTCGGAAGA	0.00	0.33	0.29	0.00	0.34
GATCAAAGAGATTCGGCAGA	8.70	35.14	43.81	25.46	37.54
GATCAAAGAGATTCGGCAGC	0.23	0.00	0.00	0.00	0.00
GATCAAAGAGATTCGGCAGG	0.23	0.00	0.00	0.00	0.00
GATCAAAGAGATTCGGCCGA	0.00	0.00	0.00	0.34	0.00
GATCAAAGAGATTCGGCCGC	0.00	0.00	0.29	0.00	0.00
GATCAAAGAGATTCGGCGGA	0.00	0.00	0.00	0.00	0.00
GATCAAAGAGATTCTCGTAT	0.23	0.00	0.00	0.00	0.00
GATCAAAGAGATTGGATGCT	0.00	0.33	0.00	0.00	0.00
GATCAAAGAGATTGGCAGAG	0.00	0.00	0.00	0.00	0.00
GATCAAAGAGCAAAAAAAAA	0.69	0.00	0.00	0.68	1.03
GATCAAAGAGCAAACTCATT	0.00	0.00	0.00	0.00	0.34
GATCAAAGAGCAAGAAAAAG	0.00	0.00	0.00	0.68	0.34
GATCAAAGAGCAAGGTTGAA	0.00	0.33	0.00	0.00	0.00
GATCAAAGAGCAAGTTGATG	0.00	0.00	0.00	0.00	0.00
GATCAAAGAGCACAAACGAA	0.00	0.00	0.00	0.00	0.00
GATCAAAGAGCACAATCAAA	0.23	0.00	0.00	0.00	0.00
GATCAAAGAGCACACACAAG	0.00	0.00	0.00	0.00	0.34
GATCAAAGAGCACACCACAT	0.00	0.33	0.00	0.68	0.00
GATCAAAGAGCACACCACTG	0.00	0.00	0.00	0.00	0.34
GATCAAAGAGCACCACATGC	0.00	0.00	0.00	0.00	0.34
GATCAAAGAGCACCACCTGC	0.00	0.00	0.00	0.00	0.34
GATCAAAGAGCAGAACGAGG	0.00	0.00	0.00	0.00	0.34
GATCAAAGAGCAGAGTGCTG	0.00	0.65	0.00	0.00	0.00
GATCAAAGAGCAGCACAAAG	0.23	0.00	0.00	0.00	0.00
GATCAAAGAGCAGCACAGTA	0.00	0.00	0.00	0.00	0.00
GATCAAAGAGCAGCACCAAG	0.00	0.00	0.00	0.00	0.00
GATCAAAGAGCAGCATAGGC	0.00	0.33	0.00	0.00	0.00
GATCAAAGAGCAGCGCGAGC	0.00	0.33	0.00	0.00	0.00
GATCAAAGAGCAGGCTTGGT	0.00	0.00	0.00	0.34	0.34
GATCAAAGAGCAGGGCCAGT	0.00	0.00	0.00	0.00	0.00

GATCAAAGAGCAGGTGCCCT	0.00	0.00	0.00	0.00	0.34
GATCAAAGAGCAGTCCGAGG	0.23	0.00	0.00	0.00	0.00
GATCAAAGAGCAGTCCGTGG	0.00	0.00	0.00	0.00	0.00
GATCAAAGAGCAGTTTACGG	0.00	0.00	0.00	0.00	0.00
GATCAAAGAGCATCATGAGA	0.00	0.33	0.00	0.00	0.34
GATCAAAGAGCATCTGCTCT	0.46	0.00	0.00	0.00	0.00
GATCAAAGAGCATGGCGCTA	2.75	4.88	2.29	1.70	0.34
GATCAAAGAGCATGGCGTTA	0.00	0.33	0.00	0.00	0.00
GATCAAAGAGCCAATTGTTG	0.00	0.33	0.00	0.00	0.00
GATCAAAGAGCCATGATATC	0.00	0.00	0.57	0.00	0.00
GATCAAAGAGCCGGGCCCAA	0.00	0.00	0.00	0.34	0.00
GATCAAAGAGCCTTGCGGTA	0.00	0.33	0.00	0.00	0.00
GATCAAAGAGCGACAAAAGG	0.00	0.00	0.00	0.00	0.34
GATCAAAGAGCGACGCCGTG	0.23	0.00	0.00	0.00	0.00
GATCAAAGAGCGAGAACAGG	0.69	0.00	0.86	0.00	0.00
GATCAAAGAGCGAGGGCCTT	0.00	0.33	0.00	0.00	0.00
GATCAAAGAGCGCAAAGAGG	0.00	0.00	0.00	0.00	0.00
GATCAAAGAGCGCAAAGGTG	0.00	0.00	0.00	0.00	0.00
GATCAAAGAGCGCAATCAGC	3.43	4.88	3.72	3.73	2.41
GATCAAAGAGCGCAATCAGG	0.00	0.00	0.29	0.00	0.00
GATCAAAGAGCGCATCGTAT	0.00	0.00	0.00	0.00	0.00
GATCAAAGAGCGCATGAATA	0.00	0.00	0.00	0.00	0.34
GATCAAAGAGCGCATGCACC	0.00	0.00	0.00	0.00	0.00
GATCAAAGAGCGCATGCANN	0.46	0.00	0.00	0.00	0.00
GATCAAAGAGCGCATGCATC	40.29	20.50	24.34	15.61	16.53
GATCAAAGAGCGCATGCATG	0.23	0.00	0.00	0.00	0.00
GATCAAAGAGCGCATGCATN	0.23	0.00	0.00	0.00	0.00
GATCAAAGAGCGCATGCATT	0.00	0.00	0.00	0.00	0.00
GATCAAAGAGCGCATGCCTC	0.23	0.00	0.00	0.00	0.00
GATCAAAGAGCGCATGNATC	0.00	0.00	0.00	0.00	0.34
GATCAAAGAGCGCATGNNNN	0.23	0.00	0.00	0.00	0.00
GATCAAAGAGCGCCTACGTC	0.23	0.00	0.00	0.00	0.00
GATCAAAGAGCGCCTGCATC	0.00	0.33	0.00	0.00	0.00
GATCAAAGAGCGCCTGCCTC	0.00	0.00	0.00	0.00	0.34
GATCAAAGAGCGCGACCCTG	0.00	0.33	0.00	0.00	0.00
GATCAAAGAGCGCGTGCGCC	0.00	0.00	0.00	0.34	0.00
GATCAAAGAGCGCGTGCTCC	1.37	2.28	1.43	2.38	0.00

GATCAAAGAGCGCTGCATTG	0.00	0.00	0.29	0.00	0.00
GATCAAAGAGCGCTGCGTGG	0.00	0.00	0.00	0.34	0.34
GATCAAAGAGCGGAAAAAAA	0.00	0.33	0.00	0.00	0.00
GATCAAAGAGCGGAATCAGC	0.23	0.00	0.00	0.00	0.00
GATCAAAGAGCGGCCTTTGT	0.00	0.00	0.00	0.68	0.00
GATCAAAGAGCGGCGAGAAA	0.00	0.00	0.00	0.68	0.00
GATCAAAGAGCGGCTCCACA	0.00	0.00	0.00	0.68	0.00
GATCAAAGAGCGGCTCGACA	0.92	1.95	0.57	2.72	1.38
GATCAAAGAGCGGCTCGACC	0.00	0.00	0.00	0.00	0.00
GATCAAAGAGCGGCTCGCCC	0.00	0.00	0.00	0.34	0.00
GATCAAAGAGCGGCTTGGAC	0.00	0.33	0.00	0.00	0.00
GATCAAAGAGCGGGACTGCC	0.00	0.00	0.29	0.00	0.00
GATCAAAGAGCGGGGAAGAA	0.00	0.00	0.29	0.00	0.00
GATCAAAGAGCGTTCCGGAC	0.46	0.65	0.29	0.68	0.00
GATCAAAGAGCTAGAGTATC	0.00	0.00	0.29	0.00	0.00
GATCAAAGAGCTCAACGAGT	0.23	0.00	0.00	0.00	0.00
GATCAAAGAGCTCCGACACG	1.14	0.65	0.57	1.70	0.34
GATCAAAGAGCTCCGCCACG	0.00	0.00	0.00	0.00	0.34
GATCAAAGAGCTCCTTCCAG	0.00	0.00	0.00	0.00	0.00
GATCAAAGAGCTCCTTCCCG	0.00	0.00	0.00	0.34	0.00
GATCAAAGAGCTCGTATGCC	0.23	0.00	0.00	0.00	0.00
GATCAAAGAGCTCTCCGAGC	0.00	0.00	0.00	0.34	0.00
GATCAAAGAGCTGAACTGAG	0.23	0.00	0.00	0.00	0.00
GATCAAAGAGCTGACAGACA	0.00	0.00	0.00	0.00	0.34
GATCAAAGAGCTGAGCCTCC	0.00	0.00	0.00	0.00	0.00
GATCAAAGAGCTGATGCAAA	0.00	0.33	0.00	0.00	0.69
GATCAAAGAGCTGATTCGCC	0.00	0.00	0.00	0.00	0.00
GATCAAAGAGCTGCACGGCC	0.00	0.00	0.00	0.00	0.34
GATCAAAGAGCTGGACCCGG	0.00	0.33	0.00	0.00	0.00
GATCAAAGAGCTGGAGAGGG	0.00	0.00	0.00	0.00	0.00
GATCAAAGAGCTGGATTAGC	0.00	0.00	0.00	0.00	0.00
GATCAAAGAGCTGGCCCGGA	0.00	0.00	0.00	0.34	0.00
GATCAAAGAGCTGGCTGTTC	0.00	0.00	0.00	0.00	0.34
GATCAAAGAGCTGGGGCTGT	0.00	0.33	0.00	0.00	0.00
GATCAAAGAGCTGGGTATTG	0.00	0.33	0.00	0.00	0.34
GATCAAAGAGCTGGTGCTTC	0.00	0.65	0.00	0.00	0.00
GATCAAAGAGCTGTACTTTG	1.37	0.98	0.86	2.04	0.34

GATCAAAGAGCTGTCAGTTC	0.23	0.00	0.00	0.00	0.00
GATCAAAGAGCTGTGTGCTT	0.00	0.00	0.00	0.00	0.00
GATCAAAGAGCTGTTTGTCC	0.00	0.00	0.00	0.00	0.00
GATCAAAGAGCTTCTTGCAC	0.00	0.00	0.00	0.00	0.34
GATCAAAGAGCTTGAGAGGG	0.00	0.00	0.00	0.00	0.00
GATCAAAGAGCTTGAGCATG	0.00	0.00	0.00	0.34	0.00
GATCAAAGAGCTTGCTCATG	0.00	0.00	0.00	0.00	0.00
GATCAAAGAGCTTGGCGCAG	0.00	0.00	0.00	0.00	0.34
GATCAAAGAGGAAAAAAAAA	2.98	0.98	1.15	1.36	2.41
GATCAAAGAGGAAAAAAAAN	0.23	0.00	0.00	0.00	0.00
GATCAAAGAGGAAAAAAAGA	0.00	0.33	0.00	0.00	0.00
GATCAAAGAGGAAAAANNNN	0.23	0.00	0.00	0.00	0.00
GATCAAAGAGGAAAACAAAA	0.00	0.00	0.00	0.00	0.34
GATCAAAGAGGAAAAGAAGG	0.00	0.00	0.00	0.00	0.00
GATCAAAGAGGAAAGAGGAA	0.00	0.00	0.29	0.00	0.00
GATCAAAGAGGAAATAGGAG	0.00	0.00	0.00	0.00	0.00
GATCAAAGAGGAACAGACTG	0.23	0.00	0.00	0.00	0.00
GATCAAAGAGGAACAGCATG	0.00	0.00	0.29	0.00	0.00
GATCAAAGAGGAACAGCCTG	9.61	4.23	5.15	4.41	3.44
GATCAAAGAGGAAGAGCCTG	0.00	0.00	0.00	0.00	0.00
GATCAAAGAGGAAGTAAGCT	0.00	0.00	0.29	0.00	0.00
GATCAAAGAGGACACTGTGA	0.00	0.00	0.57	0.00	0.00
GATCAAAGAGGACAGACATG	0.00	0.33	0.00	0.00	0.00
GATCAAAGAGGACATCCTTC	0.00	0.00	0.00	0.00	0.00
GATCAAAGAGGACGAGCCAA	0.00	0.98	1.43	0.00	3.44
GATCAAAGAGGACGAGCCAG	0.00	0.00	0.00	0.00	0.69
GATCAAAGAGGACGAGCCTC	0.00	0.00	0.00	0.00	0.34
GATCAAAGAGGACGGACTGA	0.00	0.00	0.00	0.34	0.00
GATCAAAGAGGACGGTGTCG	0.00	0.33	0.00	0.00	0.00
GATCAAAGAGGACGTGCTGG	0.23	0.00	0.00	0.00	0.00
GATCAAAGAGGACTACTACA	0.00	0.00	0.00	0.00	0.00
GATCAAAGAGGACTGTTATT	0.00	0.33	0.00	0.00	0.00
GATCAAAGAGGACTTGCCTG	0.00	0.00	0.57	0.00	0.00
GATCAAAGAGGAGAAAAAAA	0.23	0.00	0.00	0.00	0.00
GATCAAAGAGGAGAACGAGG	0.00	0.33	0.00	0.00	0.00
GATCAAAGAGGAGAAGAGCC	0.23	0.65	0.00	0.34	0.00
GATCAAAGAGGAGAAGATGC	0.00	0.65	0.00	0.00	0.00

GATCAAAGAGGAGAGAAAAA	0.46	0.00	0.00	0.00	2.76
GATCAAAGAGGAGAGAAATA	0.00	0.33	0.00	0.00	0.00
GATCAAAGAGGAGAGAGAAA	0.00	0.33	0.00	0.00	0.00
GATCAAAGAGGAGAGAGATA	23.35	17.89	10.31	18.67	13.43
GATCAAAGAGGAGAGAGATG	0.46	0.00	0.00	0.00	0.00
GATCAAAGAGGAGAGAGCTA	0.23	0.00	0.00	0.00	0.00
GATCAAAGAGGAGAGAGCTG	0.23	0.00	0.00	0.00	0.00
GATCAAAGAGGAGAGAGTAT	0.00	0.33	0.00	0.00	0.00
GATCAAAGAGGAGAGAGTGA	0.00	0.33	0.00	0.00	0.00
GATCAAAGAGGAGAGGCCTG	0.23	0.33	0.29	0.34	0.00
GATCAAAGAGGAGCACAAAG	0.00	0.00	0.00	0.34	0.00
GATCAAAGAGGAGCCATGTG	0.23	0.00	0.00	0.00	0.00
GATCAAAGAGGAGCGTGCAT	0.00	0.00	0.00	0.34	0.00
GATCAAAGAGGAGCTGGCAC	0.46	0.33	0.00	0.00	0.34
GATCAAAGAGGAGCTGGCCA	0.23	0.00	0.00	0.00	0.34
GATCAAAGAGGAGCTGGCCT	0.00	0.33	0.00	0.00	0.00
GATCAAAGAGGAGGAAGAAG	0.00	0.00	0.00	0.00	0.69
GATCAAAGAGGAGGAGATGC	0.00	0.33	0.00	0.00	0.00
GATCAAAGAGGAGGAGGAGG	0.00	0.00	0.00	0.00	0.34
GATCAAAGAGGAGGCGCCAG	0.00	0.00	0.00	0.00	0.00
GATCAAAGAGGAGGCGTGTT	0.00	0.00	0.00	0.34	0.00
GATCAAAGAGGAGGCTGTGC	0.00	0.00	0.00	0.00	0.00
GATCAAAGAGGAGGTCTAGT	0.00	0.00	0.00	0.00	0.00
GATCAAAGAGGAGGTGGAGA	0.46	0.33	0.00	0.34	0.00
GATCAAAGAGGAGGTGGAGG	0.23	0.00	0.00	0.00	0.00
GATCAAAGAGGAGTGAGGGC	0.00	0.00	0.00	0.00	0.00
GATCAAAGAGGATAACAATT	0.00	0.33	0.00	0.00	0.00
GATCAAAGAGGATGACAAGA	0.23	0.00	0.00	0.34	0.34
GATCAAAGAGGCAAAAAAAA	0.23	0.33	0.00	0.34	0.00
GATCAAAGAGGCAATCTGCT	0.23	0.33	0.00	0.00	0.00
GATCAAAGAGGCACAGCCTG	0.23	0.00	0.00	0.00	0.34
GATCAAAGAGGCACAGGAGC	0.00	0.00	0.29	0.00	0.00
GATCAAAGAGGCAGAGCATA	0.00	0.00	0.00	0.00	0.00
GATCAAAGAGGCAGCCAACG	0.00	0.00	0.00	0.00	0.34
GATCAAAGAGGCAGCGAAGG	0.00	0.00	0.00	0.00	0.34
GATCAAAGAGGCAGCGCAGA	0.00	0.00	0.00	0.34	0.00
GATCAAAGAGGCAGTCAAGT	0.00	0.00	0.00	0.00	0.34

GATCAAAGAGGCAGTGAAGC	0.00	0.00	0.00	0.00	0.00
GATCAAAGAGGCAGTGAAGG	0.00	0.65	0.00	0.00	1.38
GATCAAAGAGGCATCTAGCT	0.00	0.00	0.00	0.00	0.34
GATCAAAGAGGCATCTGCGC	0.00	0.00	0.00	0.00	0.00
GATCAAAGAGGCATGTGTCA	0.00	0.00	0.00	0.00	0.00
GATCAAAGAGGCATTCAAGC	0.69	0.65	0.57	0.34	0.00
GATCAAAGAGGCCCTGTGTC	0.00	0.00	0.00	0.00	0.00
GATCAAAGAGGCCGCGTCCT	0.00	0.00	0.00	0.34	0.00
GATCAAAGAGGCCGTCGGGC	0.00	0.00	0.00	0.00	0.00
GATCAAAGAGGCCGTCTACC	0.23	0.00	0.00	0.00	0.00
GATCAAAGAGGCCGTGAACA	0.00	0.00	0.29	0.00	0.00
GATCAAAGAGGCCGTGAAGC	1.37	1.95	3.44	1.36	1.72
GATCAAAGAGGCCTGCACGA	0.00	0.00	0.00	0.00	0.00
GATCAAAGAGGCGAAGCGGC	0.46	0.00	0.00	0.00	0.00
GATCAAAGAGGCGCCGGCGC	0.23	0.00	0.00	0.00	0.00
GATCAAAGAGGCGGAATGTT	0.00	0.00	0.00	0.00	0.00
GATCAAAGAGGCGGAGCAAT	0.46	0.00	0.00	1.02	0.00
GATCAAAGAGGCGGCGAAGC	0.00	0.00	0.00	0.00	0.00
GATCAAAGAGGCGGCGCAGC	0.23	0.00	0.00	0.00	0.00
GATCAAAGAGGCGGCGGAAG	0.00	0.00	0.00	0.00	0.00
GATCAAAGAGGCGGGCGCGG	0.00	0.00	0.29	0.00	0.00
GATCAAAGAGGCGGGCGGCG	0.00	0.33	0.00	0.00	0.00
GATCAAAGAGGCGGGCGGGG	0.00	0.00	0.29	0.00	0.00
GATCAAAGAGGCGGGGGGGC	0.23	0.00	0.00	0.00	0.00
GATCAAAGAGGCGGTGGTGG	0.00	0.00	0.00	0.00	0.00
GATCAAAGAGGCGTGAAGCA	0.00	0.33	0.00	0.00	0.00
GATCAAAGAGGCTGGCAACG	0.00	0.00	0.00	0.00	0.00
GATCAAAGAGGCTGGGAACG	0.00	0.00	0.29	0.00	0.00
GATCAAAGAGGCTGGGAAGA	0.00	0.00	0.29	0.00	0.00
GATCAAAGAGGCTGGGAAGC	0.00	0.00	0.00	1.02	2.07
GATCAAAGAGGCTGGGAAGG	0.00	0.00	0.29	0.34	0.69
GATCAAAGAGGCTGGGAGGA	0.00	0.00	0.00	0.00	0.00
GATCAAAGAGGCTGGGCAGG	0.23	0.00	0.00	0.00	0.00
GATCAAAGAGGCTGGGGAAC	0.00	0.00	0.00	0.00	0.34
GATCAAAGAGGCTGGGGAGC	0.00	0.00	0.00	1.02	0.00
GATCAAAGAGGCTGGGGAGG	0.00	0.00	0.00	0.68	0.00
GATCAAAGAGGCTGGGTCCG	0.00	0.33	0.00	0.00	0.00

GATCAAAGAGGCTGTGAAGC	0.23	2.93	5.73	0.68	0.34
GATCAAAGAGGCTGTGAAGG	0.23	0.33	0.00	0.00	0.00
GATCAAAGAGGCTGTGCAGA	0.00	0.00	0.29	0.00	0.00
GATCAAAGAGGCTGTGGAGC	0.23	0.00	0.57	0.00	0.00
GATCAAAGAGGCTTGGTGAC	0.00	0.00	0.00	0.00	0.34
GATCAAAGAGGGAAACTCTG	0.00	0.33	0.00	0.00	0.00
GATCAAAGAGGGAACCTCAT	0.23	0.00	0.29	0.00	0.00
GATCAAAGAGGGACAGCCTG	0.00	0.00	0.29	0.00	0.00
GATCAAAGAGGGACGCATCA	0.00	0.65	0.00	0.34	0.00
GATCAAAGAGGGAGGAGGGA	0.00	0.00	0.00	0.00	0.34
GATCAAAGAGGGAGGTCGCA	0.23	0.00	0.00	0.00	0.00
GATCAAAGAGGGCAAAAAAA	0.23	0.00	0.00	0.00	0.00
GATCAAAGAGGGCAACCGAG	0.23	0.33	0.57	0.34	0.00
GATCAAAGAGGGCAGCACGA	0.00	0.00	0.00	0.00	0.34
GATCAAAGAGGGCGAGCTGT	0.00	0.00	0.29	0.00	0.00
GATCAAAGAGGGCGCCATCG	0.00	0.00	0.00	0.00	0.00
GATCAAAGAGGGCGCCTGCT	0.00	0.00	0.29	0.00	0.00
GATCAAAGAGGGCGCGAAGA	0.00	0.00	0.00	0.34	0.00
GATCAAAGAGGGCGGATGGG	0.00	0.00	0.00	0.00	0.69
GATCAAAGAGGGCGTCCTTA	0.23	0.00	0.00	0.00	0.00
GATCAAAGAGGGCGTTGGAG	0.00	0.00	0.00	0.34	0.00
GATCAAAGAGGGCTGGCTAG	0.00	0.00	0.00	0.00	0.00
GATCAAAGAGGGCTGGCTCA	0.46	0.65	0.00	0.68	0.00
GATCAAAGAGGGCTTTCTCT	0.00	0.33	0.00	0.00	0.00
GATCAAAGAGGGGACCTTCA	0.00	0.33	0.00	0.00	0.00
GATCAAAGAGGGGACGAAGG	0.00	0.00	0.57	0.00	0.00
GATCAAAGAGGGGCTTTGTG	0.23	0.65	0.00	2.04	0.00
GATCAAAGAGGGGGAAAAAA	0.00	0.00	0.29	0.00	0.00
GATCAAAGAGGGGGAAAGAG	0.00	0.00	0.00	0.00	0.34
GATCAAAGAGGGGGCAAGAC	0.00	0.00	0.00	0.00	0.00
GATCAAAGAGGGGGCAAGAG	3.66	2.28	2.58	3.05	1.72
GATCAAAGAGGGTGACCTGA	0.92	0.00	0.00	0.00	0.00
GATCAAAGAGGTAAAAAAAA	11.22	1.95	2.86	6.79	4.48
GATCAAAGAGGTAAAAAAAN	0.23	0.00	0.00	0.00	0.00
GATCAAAGAGGTAAAAAAAT	0.92	0.00	0.00	0.00	0.00
GATCAAAGAGGTAAAGCTTT	0.00	0.00	0.29	0.00	0.00
GATCAAAGAGGTAGGAAGGG	0.23	0.00	0.00	0.00	0.00

GATCAAAGAGGTCAGGCAGC	0.00	0.00	0.00	0.34	0.00
GATCAAAGAGGTCCTCCTAC	0.00	0.00	0.00	0.00	0.34
GATCAAAGAGGTCGACAAGA	0.00	0.00	0.00	0.00	0.34
GATCAAAGAGGTCGGTTTCA	0.00	0.00	0.00	0.34	0.00
GATCAAAGAGGTCTTCTCTG	0.00	0.00	0.29	0.00	0.00
GATCAAAGAGGTCTTGCGCC	0.00	0.00	0.00	0.00	0.00
GATCAAAGAGGTCTTGCGCT	0.23	0.00	0.00	0.00	0.00
GATCAAAGAGGTGAAGCCGC	0.00	0.00	0.00	0.00	0.00
GATCAAAGAGGTGAAGCTGC	0.69	0.00	0.00	0.00	0.34
GATCAAAGAGGTGGAAAAAA	0.00	0.00	0.00	0.00	0.00
GATCAAAGAGGTGGAGAAGG	0.00	0.00	0.29	0.00	0.00
GATCAAAGAGGTGGAGGGGA	0.00	0.00	0.00	0.00	0.00
GATCAAAGAGGTGGCACCCC	0.23	0.00	0.00	0.00	0.00
GATCAAAGAGGTGGGACTGC	0.00	0.00	0.00	0.00	0.34
GATCAAAGAGGTGTCTGTTT	0.46	0.00	0.00	0.00	0.00
GATCAAAGAGGTGTGCTCCG	0.00	0.00	0.00	0.00	0.00
GATCAAAGAGGTGTGTCAAA	0.00	0.65	0.00	0.00	0.00
GATCAAAGAGGTGTGTCATA	0.23	0.00	0.00	0.34	0.00
GATCAAAGAGGTGTGTCGAA	0.00	0.33	0.00	0.00	0.00
GATCAAAGAGGTGTGTGTTT	3.43	0.33	1.15	0.00	0.00
GATCAAAGAGTAACAGCCTG	0.23	0.00	0.00	0.00	0.00
GATCAAAGAGTACATGCAGG	0.00	0.33	0.00	0.00	0.00
GATCAAAGAGTACATGTAGG	0.00	0.00	0.00	0.00	0.00
GATCAAAGAGTACGAACGGG	0.69	0.00	0.00	0.00	0.00
GATCAAAGAGTACGAGCGGG	0.00	0.98	0.29	0.34	0.00
GATCAAAGAGTACGAGGGCC	0.00	0.33	0.00	1.36	0.00
GATCAAAGAGTACGAGGGCN	0.00	0.00	0.00	0.00	0.00
GATCAAAGAGTATGAGGGCC	0.00	0.33	0.00	0.00	0.00
GATCAAAGAGTATGTGCTGT	0.00	0.00	0.00	0.00	0.00
GATCAAAGAGTCACCTGCGT	0.00	0.00	0.00	0.34	0.00
GATCAAAGAGTCAGGAAGAA	0.00	0.00	0.00	0.34	0.00
GATCAAAGAGTCCATGCCTT	0.00	0.00	0.00	0.00	0.34
GATCAAAGAGTCCGAGCCCA	0.00	0.00	0.00	0.34	0.00
GATCAAAGAGTCCGAGTTGA	0.00	0.00	0.00	0.00	0.00
GATCAAAGAGTCGCCTTCGA	0.00	0.33	0.00	0.00	0.00
GATCAAAGAGTCGGCCTGCT	0.00	0.00	0.00	0.34	0.00
GATCAAAGAGTGACAGGAAC	0.23	0.00	0.00	0.00	0.00

GATCAAAGAGTGACTGGAGA	0.00	0.00	0.00	0.34	0.00
GATCAAAGAGTGCAAAAAAA	0.46	0.65	0.00	0.00	0.00
GATCAAAGAGTGCAAATGCA	0.00	0.00	0.00	0.00	0.00
GATCAAAGAGTGCACCTGCT	0.00	0.00	0.00	0.00	0.34
GATCAAAGAGTGCACTTGGC	0.00	0.00	0.00	0.68	0.00
GATCAAAGAGTGCACTTGTT	0.00	0.00	0.00	0.00	0.34
GATCAAAGAGTGCATGGAAC	0.00	0.00	0.00	0.00	0.34
GATCAAAGAGTGCCAAAAAA	0.00	0.00	0.00	0.00	0.00
GATCAAAGAGTGCCTGCACC	0.00	0.00	0.29	0.00	0.00
GATCAAAGAGTGCCTGGACA	10.07	5.21	4.30	7.13	4.48
GATCAAAGAGTGCCTGGACC	0.23	0.00	0.00	0.00	0.00
GATCAAAGAGTGCCTGGACG	0.00	0.33	0.00	0.00	0.00
GATCAAAGAGTGCCTGGATA	0.46	0.00	0.00	0.00	0.00
GATCAAAGAGTGCCTGGGCA	0.23	0.00	0.00	0.00	0.00
GATCAAAGAGTGCCTTCGTA	0.00	0.00	0.00	0.00	0.00
GATCAAAGAGTGCGCCGGCT	0.23	0.00	0.00	0.00	0.00
GATCAAAGAGTGCGCCNGCT	0.00	0.00	0.00	0.00	0.34
GATCAAAGAGTGCGCCTGCT	6.18	4.23	4.01	6.45	7.23
GATCAAAGAGTGCGCCTGGT	0.00	0.33	0.00	0.00	0.00
GATCAAAGAGTGCGCTTTAC	0.00	0.00	0.29	0.00	0.00
GATCAAAGAGTGGAGAGACC	0.00	0.00	0.00	0.00	0.00
GATCAAAGAGTGGGCAAGAG	0.00	0.00	0.29	0.00	0.00
GATCAAAGAGTGGGGCTGCC	0.00	0.00	0.00	0.00	0.34
GATCAAAGAGTGTCAGCGAC	0.00	0.33	0.00	0.00	0.00
GATCAAAGAGTGTCAGCGAT	0.23	0.98	0.57	0.34	0.00
GATCAAAGAGTGTCAGCGCT	0.23	0.00	0.00	0.34	0.00
GATCAAAGAGTGTTCACGAT	0.00	0.33	0.00	0.00	0.00
GATCAAAGAGTTCGAAGCAG	0.00	0.00	0.00	0.34	0.00
GATCAAAGAGTTCGACGCAG	0.00	0.00	0.00	0.00	0.00
GATCAAAGAGTTCGATAGGC	0.00	0.00	0.00	0.00	0.00
GATCAAAGAGTTCGCCTCCC	0.00	0.00	0.00	0.00	0.00
GATCAAAGAGTTCGCGTGTA	0.00	0.00	0.57	0.00	0.00
GATCAAAGAGTTCGCGTGTG	0.00	0.00	0.00	0.00	0.00
GATCAAAGAGTTCGCTCCAA	0.00	0.65	1.15	0.00	0.00
GATCAAAGAGTTCGGAAGTG	0.00	0.00	0.29	0.00	0.00
GATCAAAGAGTTCGGCCTAA	0.00	0.00	0.57	0.00	0.00
GATCAAAGAGTTCTAACATT	0.00	0.33	0.00	0.00	0.00

GATCAAAGAGTTGCGCCTGC	0.00	0.00	0.00	0.00	0.00
GATCAAAGAGTTTGACACCA	0.00	0.00	0.00	0.34	0.00
GATCAAAGAGTTTGCCCAGC	0.00	0.00	0.00	0.00	0.00
GATCAAAGAGTTTGGCAAGC	0.00	0.00	0.00	0.34	0.00
GATCAAAGAGTTTGGCCTCA	0.00	0.00	0.00	0.00	0.00
GATCAAAGAGTTTGTTTGTA	1.14	0.00	0.29	0.34	0.00
GATCAAAGAGTTTTAAGGTA	0.00	0.33	0.00	0.00	0.00
GATCAAAGATAAAAAAAAAA	2.98	0.33	0.57	0.00	0.00
GATCAAAGATAAACTCGAAC	0.00	0.00	0.29	0.00	0.00
GATCAAAGATAAATAGACGA	0.00	0.00	0.00	0.00	0.34
GATCAAAGATAACCAGAAAC	0.23	0.00	0.00	0.00	0.00
GATCAAAGATAAGCCCGTGG	0.92	0.33	0.00	0.00	1.03
GATCAAAGATAAGCCGTGCT	0.00	0.00	0.00	0.00	0.00
GATCAAAGATAAGGTCAGCA	0.00	0.00	0.00	0.34	0.00
GATCAAAGATAATGTCCCGT	0.00	0.00	0.00	0.00	0.34
GATCAAAGATAATGTTCCAA	0.92	0.00	0.00	1.02	1.03
GATCAAAGATACAGTGATGG	15.34	6.83	3.72	4.07	8.61
GATCAAAGATACAGTGATGT	0.00	0.33	0.00	0.00	0.00
GATCAAAGATACAGTGCTGG	0.00	0.00	0.00	0.00	0.00
GATCAAAGATACAGTGGTGG	0.23	0.00	0.00	0.00	0.00
GATCAAAGATACCAAAAGAA	0.00	0.00	0.00	0.00	0.00
GATCAAAGATACTAGCCCTG	0.00	0.00	0.00	0.00	0.00
GATCAAAGATAGAGCCCCCT	0.00	0.33	0.00	0.00	0.00
GATCAAAGATAGCGAGGCAG	0.92	0.00	0.00	0.34	0.00
GATCAAAGATAGCGGGCCCG	0.00	0.00	0.00	0.00	0.00
GATCAAAGATAGTCTGGTAC	0.00	0.00	0.00	0.00	0.00
GATCAAAGATATAAAAAAAA	0.46	0.00	0.00	0.00	0.00
GATCAAAGATATAGTTACGT	0.00	0.98	0.29	0.00	2.07
GATCAAAGATATGAACAAGC	0.00	0.33	0.00	0.00	0.00
GATCAAAGATATGATATACA	0.00	0.00	0.00	0.00	0.34
GATCAAAGATATGCGAGATG	0.00	0.00	0.00	0.00	0.00
GATCAAAGATATGCTGCCCC	0.00	0.33	0.00	0.00	0.00
GATCAAAGATATGGCTTTTT	0.00	0.00	0.00	0.00	0.00
GATCAAAGATATTGTTGAGC	0.00	0.33	0.29	0.00	0.00
GATCAAAGATCATCCAAGTG	0.00	0.00	0.00	0.34	0.00
GATCAAAGATCGATGTCTGG	0.00	0.00	0.00	0.00	0.00
GATCAAAGATCGTATGCCGT	0.23	0.00	0.86	0.00	0.34

GATCAAAGATGAAGTGGACG	0.00	0.33	0.00	0.00	0.00
GATCAAAGATGAAGTTGACC	0.69	0.00	0.00	0.00	0.00
GATCAAAGATGACAATTTGT	0.00	0.00	0.00	0.00	0.00
GATCAAAGATGACATTTGAG	1.14	1.30	0.00	0.00	0.00
GATCAAAGATGACTTGGTAG	0.00	0.00	0.29	0.00	0.34
GATCAAAGATGAGATTGAGA	0.00	0.00	0.00	0.34	0.34
GATCAAAGATGAGCACCGAT	0.00	0.00	0.00	0.00	0.00
GATCAAAGATGAGCACTCAC	0.00	0.00	0.00	0.00	0.00
GATCAAAGATGAGCTCCGTG	0.23	0.00	0.00	0.00	0.34
GATCAAAGATGAGGTGAAGA	2.52	0.33	0.00	0.68	0.00
GATCAAAGATGAGGTGAAGN	0.23	0.00	0.00	0.00	0.00
GATCAAAGATGAGGTGATGG	0.46	0.00	0.00	0.00	0.00
GATCAAAGATGAGGTGCAGT	0.00	0.00	0.00	0.00	0.00
GATCAAAGATGAGTCCGAAG	0.00	0.00	0.57	0.00	0.00
GATCAAAGATGCAAAGGAGC	0.00	0.00	0.00	0.00	0.00
GATCAAAGATGCAAAGGAGN	0.23	0.00	0.00	0.00	0.00
GATCAAAGATGCAAAGGAGT	2.29	0.33	0.00	0.68	0.00
GATCAAAGATGCAAAGGCGT	0.00	0.00	0.00	0.00	0.00
GATCAAAGATGCAGGAGTGA	0.00	0.00	0.00	0.00	0.00
GATCAAAGATGCAGGATCCG	0.00	0.00	0.29	0.00	0.00
GATCAAAGATGCAGGCTCCG	0.00	0.98	0.57	0.00	0.69
GATCAAAGATGCAGGTCTCG	0.00	0.33	0.00	0.00	0.00
GATCAAAGATGCATATGAGA	0.23	0.00	0.00	0.00	0.00
GATCAAAGATGCCAAAAAAA	0.23	0.00	0.29	0.00	0.00
GATCAAAGATGCCAAAAGAA	1.14	0.33	0.00	0.68	1.72
GATCAAAGATGCCAGTCTGC	0.00	0.00	0.00	0.34	0.00
GATCAAAGATGCCCAGCCTG	0.00	0.00	0.29	0.00	0.00
GATCAAAGATGCCCAGGCTG	0.46	1.63	0.00	0.34	0.69
GATCAAAGATGCCGCCCGTC	0.00	0.00	0.29	0.00	0.00
GATCAAAGATGCCGCCCTTC	0.00	0.33	0.00	0.00	0.00
GATCAAAGATGCCGTGAAGC	0.92	1.63	2.58	0.68	1.03
GATCAAAGATGCCGTTCTGC	0.00	0.00	0.00	0.00	0.34
GATCAAAGATGCGATGAGCG	0.00	0.65	0.29	0.68	0.00
GATCAAAGATGCGATGAGGC	0.00	0.00	0.00	0.00	0.00
GATCAAAGATGCGCACTCTG	0.00	0.00	0.00	0.00	0.00
GATCAAAGATGCGCATTTGT	0.00	0.33	0.00	0.00	0.00
GATCAAAGATGCGCGATGGG	0.00	0.00	0.00	0.00	0.34

GATCAAAGATGCTCATGAAA	0.00	0.00	0.00	0.00	0.00
GATCAAAGATGCTGGCTAGC	0.00	0.00	0.00	0.00	0.00
GATCAAAGATGCTGTGCAAG	0.00	0.00	0.29	0.00	0.00
GATCAAAGATGGAAAAAAAA	0.69	0.00	0.29	0.00	0.00
GATCAAAGATGGAGGAAACG	0.00	0.00	0.00	0.00	0.00
GATCAAAGATGGAGGAAGGC	0.23	0.00	0.00	0.00	0.00
GATCAAAGATGGAGTCCAGT	0.00	0.00	0.29	0.00	0.00
GATCAAAGATGGATTCAAGG	0.00	0.00	0.00	0.00	0.00
GATCAAAGATGGCAAAAAAA	6.41	0.65	0.86	1.70	1.72
GATCAAAGATGGCAAAAAAN	0.00	0.00	0.00	0.00	0.00
GATCAAAGATGGCAAGGGAA	0.00	0.00	0.29	0.00	1.03
GATCAAAGATGGCAAGGGCA	0.23	0.00	0.00	0.00	0.00
GATCAAAGATGGCAAGGGCC	0.00	0.00	0.57	0.00	0.00
GATCAAAGATGGCAAGGGGG	0.00	0.00	0.00	0.00	0.00
GATCAAAGATGGCAAGGGTA	6.64	3.90	5.15	6.79	5.51
GATCAAAGATGGCAAGGGTT	0.46	0.00	0.29	0.34	0.00
GATCAAAGATGGCAGGGAAG	0.00	0.00	0.00	0.34	0.69
GATCAAAGATGGCATCTCCA	0.00	0.33	0.00	0.00	0.00
GATCAAAGATGGCATGCCGC	0.00	0.00	0.00	0.34	0.00
GATCAAAGATGGCATGTTGG	0.69	0.33	0.00	0.00	0.00
GATCAAAGATGGCCGAAAAA	0.00	0.00	0.29	0.00	0.00
GATCAAAGATGGCGAACTGA	0.00	0.00	0.00	0.34	0.00
GATCAAAGATGGCGACAGGG	0.00	0.33	0.00	0.00	0.00
GATCAAAGATGGCGAGGCAG	0.69	0.65	1.15	0.00	0.34
GATCAAAGATGGCGATGCAG	0.00	0.00	0.00	0.00	0.34
GATCAAAGATGGCGCGCAAA	0.00	0.00	0.00	0.00	0.00
GATCAAAGATGGCGCGCCCA	0.23	0.00	0.00	0.00	0.00
GATCAAAGATGGCGCGCCCC	0.23	0.00	0.00	0.00	0.00
GATCAAAGATGGCGCGCGAC	0.92	0.00	0.00	0.00	0.00
GATCAAAGATGGCGTTACTG	0.00	0.00	0.00	0.00	0.00
GATCAAAGATGGCTTCAAGG	0.00	0.00	0.00	0.34	0.00
GATCAAAGATGGCTTGCGGC	0.00	0.00	0.00	0.68	0.00
GATCAAAGATGGCTTTGAGT	0.00	0.00	0.00	0.00	1.38
GATCAAAGATGGGAAAAAAA	0.00	0.00	0.00	0.00	0.00
GATCAAAGATGGGAGAACCA	0.00	0.00	0.29	0.00	0.00
GATCAAAGATGGGAGAAGGA	0.00	0.33	0.00	0.00	0.00
GATCAAAGATGGGAGTAGGC	0.46	0.00	0.00	1.02	0.00

GATCAAAGATGGGCGATGGG	0.23	0.00	0.57	0.00	0.00
GATCAAAGATGGGCGCGAAA	0.23	0.00	0.00	0.00	0.00
GATCAAAGATGGGCGGGGGG	0.23	0.00	0.00	0.00	0.00
GATCAAAGATGGGCGTGAAA	0.69	0.00	0.00	0.68	0.00
GATCAAAGATGGGGCGTGAG	0.00	0.00	0.29	0.00	0.00
GATCAAAGATGGGGTTACTG	0.23	0.00	0.00	0.00	0.00
GATCAAAGATGGTAAGGGTA	0.00	0.00	0.00	0.00	0.00
GATCAAAGATGGTATGTCCA	0.00	0.00	0.00	0.68	0.00
GATCAAAGATGGTGTTACTG	12.82	6.18	5.44	5.77	7.92
GATCAAAGATGGTGTTACTN	0.23	0.00	0.00	0.00	0.00
GATCAAAGATGGTGTTACTT	0.00	0.00	0.00	0.00	0.00
GATCAAAGATGTAAGGAAGC	0.00	0.00	0.29	0.00	0.00
GATCAAAGATGTAATTAAAA	0.00	0.00	0.00	0.00	0.00
GATCAAAGATGTCACCTGAA	0.00	0.00	0.00	0.00	0.00
GATCAAAGATGTCGTTGAGA	0.00	0.00	0.00	0.00	0.00
GATCAAAGATGTCTTTGATT	0.00	0.00	0.00	0.00	0.00
GATCAAAGATGTGCGTCGTG	0.00	0.00	0.00	0.00	0.00
GATCAAAGATGTGTCCGGAC	0.00	0.00	0.57	0.00	1.03
GATCAAAGATGTTCATCCCA	0.00	0.00	0.00	0.68	0.00
GATCAAAGATGTTCCCAAAA	0.00	0.33	0.00	0.00	0.00
GATCAAAGATGTTGAAAAAA	0.00	0.00	0.29	0.00	0.34
GATCAAAGATGTTGATGCGG	0.00	0.00	0.29	0.00	0.00
GATCAAAGATGTTGCCATAA	0.23	0.00	0.00	0.00	0.00
GATCAAAGATGTTGCCATAT	0.23	0.00	0.00	0.00	0.34
GATCAAAGATGTTGCTGTGT	1.37	1.63	1.72	0.68	1.38
GATCAAAGATGTTTGAAAAA	1.14	0.00	0.00	0.34	0.34
GATCAAAGATGTTTGCAAAA	27.93	13.01	13.17	16.97	7.92
GATCAAAGATGTTTGCAAAC	0.46	0.00	0.00	0.00	0.00
GATCAAAGATGTTTGCAAAT	0.00	0.00	0.00	0.00	0.69
GATCAAAGATGTTTGCAACT	0.00	0.00	0.00	0.34	0.00
GATCAAAGATGTTTGCAAGA	0.00	0.00	0.29	0.00	0.00
GATCAAAGATGTTTGCAANN	0.23	0.00	0.00	0.00	0.00
GATCAAAGATGTTTGCACAA	0.23	0.00	0.29	0.00	0.00
GATCAAAGATGTTTGCAGAA	0.00	0.00	0.00	0.00	0.69
GATCAAAGATGTTTGCGAAA	0.23	0.00	0.29	0.00	0.00
GATCAAAGATGTTTGCTAAA	0.00	0.00	0.00	0.00	0.00
GATCAAAGATGTTTGCTCGT	0.00	0.00	0.00	0.34	0.00

GATCAAAGATTAAGCCATGC	0.00	0.00	0.00	0.00	0.00
GATCAAAGATTCAACGAGTA	0.00	0.00	0.00	1.02	0.00
GATCAAAGATTCATGTCCAA	0.00	0.00	0.29	0.00	0.00
GATCAAAGATTCGGCCCACG	0.00	0.33	0.00	0.00	0.34
GATCAAAGATTCGGCCCCCA	0.00	0.00	0.00	0.00	0.00
GATCAAAGATTCGTATGCCG	0.23	0.00	0.00	0.00	0.00
GATCAAAGATTCTATGAAAA	0.00	0.00	0.00	0.00	0.00
GATCAAAGATTCTGAGCCGG	0.00	0.00	0.00	0.34	0.00
GATCAAAGATTCTGAGCCGT	2.06	0.00	0.86	0.68	0.34
GATCAAAGATTCTGAGCCNN	0.23	0.00	0.00	0.00	0.00
GATCAAAGATTCTGCGCCGT	0.00	0.00	0.29	0.00	0.00
GATCAAAGATTGCAAGGGTA	0.00	0.00	0.00	1.02	0.00
GATCAAAGATTGCGACGGGC	0.00	0.00	0.00	0.00	0.00
GATCAAAGATTGCGATTGCG	0.00	0.00	0.00	0.00	0.69
GATCAAAGATTGCGGCGCAA	0.00	0.00	0.00	0.00	0.00
GATCAAAGATTGCGTGCTGC	0.00	0.00	0.00	0.00	0.00
GATCAAAGATTGCGTGGACA	0.00	0.33	0.00	0.34	0.00
GATCAAAGATTGCTGCCTTC	0.00	0.33	0.00	0.00	0.00
GATCAAAGATTGGATGCCGC	0.00	0.00	0.00	0.00	0.00
GATCAAAGATTGTGTGAAAA	0.00	0.00	0.00	0.00	0.00
GATCAAAGATTTAGAATGTC	0.00	0.00	0.00	0.00	0.00
GATCAAAGATTTTCTTCCCT	0.00	0.33	0.00	0.00	0.00
GATCAAAGCAAAAAAAAAAA	32.96	6.18	10.60	8.49	11.71
GATCAAAGCAAAAAAAAAAC	0.00	0.00	0.29	0.00	0.00
GATCAAAGCAAAAAAAAAAG	0.23	0.00	0.00	0.00	0.00
GATCAAAGCAAAAAAAAAAT	0.46	0.33	0.57	0.00	0.69
GATCAAAGCAAAAAAACGAA	0.23	0.00	0.00	0.00	0.00
GATCAAAGCAAAAAACNNNN	0.23	0.00	0.00	0.00	0.00
GATCAAAGCAAAACCTGTGA	0.00	0.33	0.00	0.00	0.00
GATCAAAGCAAAACGTGCAA	0.00	0.65	0.00	0.00	0.00
GATCAAAGCAAAAGCGAAAA	0.00	0.00	0.29	0.00	0.00
GATCAAAGCAAACAAAAAAA	0.46	0.00	0.00	0.00	0.00
GATCAAAGCAAACAACACAA	0.00	0.00	0.00	0.00	0.34
GATCAAAGCAAACACATCCC	0.00	0.00	0.00	0.00	0.00
GATCAAAGCAAACTCACTGG	0.00	0.00	0.29	0.00	0.00
GATCAAAGCAAAGAAAAAAA	3.66	1.63	2.00	2.04	0.69
GATCAAAGCAAAGAAAACAA	0.23	0.00	0.00	0.00	0.00

GATCAAAGCAAAGAAACGAG	0.00	0.00	0.00	0.00	0.00
GATCAAAGCAAAGAAGGAGG	0.00	0.33	0.29	0.34	0.00
GATCAAAGCAAAGACCTGTG	0.00	0.00	0.00	0.00	0.34
GATCAAAGCAAAGAGAAAAA	0.00	0.33	0.00	0.00	0.00
GATCAAAGCAAAGATATGCA	0.00	0.00	0.00	0.00	0.00
GATCAAAGCAAAGCAAAGCG	0.00	0.00	0.00	0.00	0.69
GATCAAAGCAAAGGAAAAAA	0.23	0.00	0.00	0.00	0.00
GATCAAAGCAAATCCAATCA	0.00	0.00	0.00	0.00	0.00
GATCAAAGCAAATGTCACCA	0.92	0.98	0.00	0.68	0.34
GATCAAAGCAAATGTCACCG	0.00	0.00	0.00	0.00	0.00
GATCAAAGCAAATTTGCCGA	0.23	0.00	1.15	1.02	0.00
GATCAAAGCAAATTTGTCGA	0.23	0.00	0.00	0.00	0.00
GATCAAAGCAACAGCGCCGA	0.00	0.00	0.00	0.00	0.00
GATCAAAGCAACATCCTTCA	0.00	0.00	0.29	0.00	0.00
GATCAAAGCAACCAAGGTGC	0.00	0.00	0.00	0.68	0.00
GATCAAAGCAACCGGTTCAC	0.00	0.00	0.29	0.00	0.00
GATCAAAGCAACGAAGAGAA	0.00	0.00	0.00	0.00	1.03
GATCAAAGCAACGAGTTAGC	0.00	0.00	0.29	0.00	0.00
GATCAAAGCAACGAGTTCGA	0.00	0.00	0.00	0.00	0.34
GATCAAAGCAACGAGTTCGC	0.92	0.33	2.29	0.68	0.69
GATCAAAGCAACGAGTTCGT	0.00	0.00	0.00	0.00	0.00
GATCAAAGCAACGCGGCGCA	0.00	0.00	0.29	0.00	0.00
GATCAAAGCAACGTCCTTTC	0.00	0.00	0.00	0.00	0.00
GATCAAAGCAACTCAACACA	0.00	0.00	0.00	0.00	0.34
GATCAAAGCAACTGAACGTC	0.00	0.00	0.00	0.00	0.00
GATCAAAGCAACTGGGGGGC	0.00	0.33	0.00	0.00	0.00
GATCAAAGCAACTGGGGTGC	9.61	15.94	18.33	12.22	12.05
GATCAAAGCAACTGGGGTGN	0.23	0.00	0.00	0.00	0.00
GATCAAAGCAACTGGGGTGT	0.00	0.33	0.00	0.34	0.00
GATCAAAGCAACTTAGGGGG	0.23	0.00	0.00	0.00	0.00
GATCAAAGCAAGAAAAAAAA	0.00	0.00	0.29	0.00	0.00
GATCAAAGCAAGAAGGCGAC	0.23	0.00	0.00	0.00	0.00
GATCAAAGCAAGAATTTCGA	0.00	0.00	0.00	0.00	0.00
GATCAAAGCAAGACACGAGG	0.00	1.30	0.29	1.02	0.34
GATCAAAGCAAGACAGCCTT	0.00	0.00	0.00	0.00	0.00
GATCAAAGCAAGAGAAAGAG	0.92	0.00	0.00	0.00	0.00
GATCAAAGCAAGCCCGTCCC	0.00	0.33	0.00	0.00	0.00

GATCAAAGCAAGGAAAAAAA	0.23	0.00	0.00	0.00	0.00
GATCAAAGCAAGGACATCTC	0.00	0.00	0.29	0.00	0.00
GATCAAAGCAAGGCAGAAAA	0.00	0.00	0.00	0.00	0.34
GATCAAAGCAAGGCAGACGG	0.92	0.65	0.57	0.34	0.69
GATCAAAGCAAGGCGGCCCC	0.00	0.00	0.29	0.00	0.00
GATCAAAGCAAGGGGCTCCA	0.23	0.00	0.00	0.00	0.00
GATCAAAGCAAGGGGGCAAC	0.00	0.00	0.29	0.00	0.00
GATCAAAGCAAGTGCACCGC	0.00	0.33	0.00	0.00	0.00
GATCAAAGCAAGTGCTCAGC	0.23	0.00	0.00	0.00	0.00
GATCAAAGCAATACCTCATC	0.00	0.00	0.57	0.00	0.00
GATCAAAGCAATATCCCTGA	2.75	0.65	2.58	0.68	2.41
GATCAAAGCAATATCCTTGA	0.00	0.00	0.29	0.00	0.00
GATCAAAGCAATCAGAACTT	0.00	0.00	0.00	0.00	0.00
GATCAAAGCAATCCGGGCAA	0.00	0.00	0.29	0.34	0.00
GATCAAAGCAATGCTTAACG	0.00	0.00	0.29	0.00	0.00
GATCAAAGCAATGTCCTCTT	0.00	0.00	0.00	0.34	0.00
GATCAAAGCAATTCAGGCCA	2.06	3.90	0.29	0.34	0.00
GATCAAAGCAATTGGTAGCA	0.00	0.00	0.00	0.34	0.00
GATCAAAGCAATTTAAGGCA	0.00	0.33	0.00	0.00	0.00
GATCAAAGCACAAAAAAAAA	0.00	0.00	0.00	0.00	0.34
GATCAAAGCACAAACAAAAA	0.00	0.00	0.00	0.00	0.00
GATCAAAGCACAACACAACA	0.00	0.00	0.29	0.00	0.69
GATCAAAGCACAAGAACAAC	0.00	0.00	0.29	0.00	0.00
GATCAAAGCACAAGCAAGTG	0.00	0.00	0.00	0.00	0.00
GATCAAAGCACAAGCATGTC	0.00	0.00	0.29	0.00	0.00
GATCAAAGCACACGCACTTT	0.00	0.00	0.00	0.00	0.34
GATCAAAGCACACTCTGTGC	0.00	0.33	0.00	0.00	0.00
GATCAAAGCACACTGCTGTG	0.00	0.33	0.00	0.00	0.00
GATCAAAGCACACTGTGTTT	0.00	0.00	0.29	0.00	0.00
GATCAAAGCACAGAGCAACG	0.00	0.00	0.29	0.00	0.00
GATCAAAGCACAGGCACGTA	0.00	0.33	0.29	0.68	0.00
GATCAAAGCACAGGCAGTAT	0.00	0.33	0.00	0.00	0.00
GATCAAAGCACAGGTCGACC	0.00	0.65	0.00	0.00	0.00
GATCAAAGCACAGTCCGCAT	0.00	0.00	0.00	0.00	0.00
GATCAAAGCACATGAGCTCG	0.00	0.00	0.29	0.00	0.00
GATCAAAGCACATGGGTGCC	0.23	0.00	0.00	0.00	0.00
GATCAAAGCACATTCCACAT	0.00	0.00	0.00	0.00	0.00

GATCAAAGCACCAAACAAAA	0.46	0.00	0.00	0.00	0.00
GATCAAAGCACCATTCAAGG	0.69	0.00	0.00	0.34	0.00
GATCAAAGCACCATTCAAGT	1.37	0.98	1.15	1.02	0.34
GATCAAAGCACCCAACTTCT	0.00	0.00	0.00	0.00	0.69
GATCAAAGCACCCCCGCCCT	0.23	0.00	0.00	0.00	0.00
GATCAAAGCACCGAAGCATC	0.23	0.00	0.00	0.00	0.00
GATCAAAGCACCGAAGTATC	0.00	0.00	0.00	0.00	0.00
GATCAAAGCACCGGCTGCGG	0.00	0.00	0.00	0.00	0.00
GATCAAAGCACCGGCTGGGC	0.00	0.00	0.29	0.00	0.34
GATCAAAGCACCGGCTGGGG	0.23	0.00	0.29	0.34	0.00
GATCAAAGCACCGGCTGTGC	0.00	0.33	0.00	0.00	0.00
GATCAAAGCACCTGGGGAGC	0.00	0.00	0.00	0.00	0.00
GATCAAAGCACCTTCAAGCT	0.00	0.00	0.29	0.00	0.00
GATCAAAGCACGAAGACAAA	0.00	0.00	0.29	0.00	0.00
GATCAAAGCACGCAAGATGA	0.00	0.00	0.00	0.00	0.34
GATCAAAGCACGCACCCCTT	0.00	0.00	0.29	0.00	0.00
GATCAAAGCACGCCGTTTGC	0.00	0.65	0.00	0.00	0.00
GATCAAAGCACGCGCGTGTC	0.00	0.00	0.00	0.00	0.34
GATCAAAGCACGGCGGCCGC	0.00	0.33	0.00	0.00	0.00
GATCAAAGCACGGCGGCGCC	0.46	0.98	0.00	0.34	0.34
GATCAAAGCACGGCGGCGGA	0.23	0.33	0.00	0.00	0.00
GATCAAAGCACGGCGGCGGC	0.23	0.00	0.00	0.00	0.00
GATCAAAGCACGGCGGGCAC	0.23	0.00	0.00	0.00	0.00
GATCAAAGCACGGCGGGGGC	0.23	0.00	0.00	0.00	0.00
GATCAAAGCACGGCGGTGAC	0.23	0.00	0.00	0.00	0.00
GATCAAAGCACGTGTCATCA	0.00	0.00	0.00	0.34	0.00
GATCAAAGCACTAAAAAAAA	0.00	0.00	0.29	0.00	0.00
GATCAAAGCACTCCGGCAAC	0.00	0.00	0.00	0.00	0.00
GATCAAAGCACTCCGTGCAT	0.23	0.00	0.57	0.34	0.00
GATCAAAGCACTCGCACTTT	0.00	0.33	0.86	0.34	0.69
GATCAAAGCACTCGCCTTTT	0.00	0.00	0.00	0.00	0.00
GATCAAAGCACTGCGGGCAT	0.23	0.00	0.00	0.00	0.00
GATCAAAGCACTGGTAAAAA	0.00	0.00	0.00	0.34	0.34
GATCAAAGCACTGGTAGAGC	4.35	2.60	1.15	3.73	0.00
GATCAAAGCACTGGTAGCGC	0.00	0.00	0.00	0.34	0.00
GATCAAAGCACTTCGCAAAA	0.00	0.00	0.29	0.00	0.00
GATCAAAGCACTTGCGTTTG	0.00	0.65	0.00	0.00	0.69

GATCAAAGCAGAAACAATGA	0.00	0.00	0.00	0.00	0.00
GATCAAAGCAGAACAGAACA	0.46	0.00	0.00	0.00	0.00
GATCAAAGCAGAACAGAGCA	0.00	0.00	0.00	0.00	0.34
GATCAAAGCAGAACAGGACA	0.00	0.00	0.00	0.00	0.34
GATCAAAGCAGAACATAACA	0.00	0.00	0.00	0.00	0.34
GATCAAAGCAGAACCACACC	0.23	0.00	0.00	0.00	0.00
GATCAAAGCAGAACCAGTCA	0.00	0.00	0.00	0.00	1.03
GATCAAAGCAGAACCATCCC	2.75	1.95	0.29	2.04	0.69
GATCAAAGCAGAACCATCTC	0.00	0.33	0.00	0.00	0.00
GATCAAAGCAGAACCGGTAA	0.00	0.00	0.00	0.00	0.34
GATCAAAGCAGAACCGGTAG	0.00	0.00	0.29	0.00	0.00
GATCAAAGCAGAACCGGTCA	0.00	0.00	0.57	0.34	0.34
GATCAAAGCAGAACCGGTCC	0.00	0.00	0.00	0.00	0.00
GATCAAAGCAGAACGGAGCA	0.00	0.00	0.00	0.00	0.34
GATCAAAGCAGAACGGGACC	0.23	0.00	0.00	0.00	0.00
GATCAAAGCAGAATCCACTG	0.00	0.00	0.00	0.00	0.34
GATCAAAGCAGACGAGCGTA	0.00	0.00	0.00	0.00	0.00
GATCAAAGCAGACGGTGTGG	0.00	0.00	0.00	0.00	0.00
GATCAAAGCAGACTATGCTT	0.00	0.00	0.29	0.00	0.00
GATCAAAGCAGAGCAGAACA	0.00	0.00	0.29	0.00	0.00
GATCAAAGCAGAGCAGAGCA	0.00	0.00	0.00	0.00	0.69
GATCAAAGCAGAGCTACACA	0.00	0.00	0.00	0.00	0.00
GATCAAAGCAGAGTTACAAA	0.23	0.00	0.00	0.68	0.00
GATCAAAGCAGAGTTACAGA	2.75	0.98	0.00	1.36	1.03
GATCAAAGCAGAGTTACANA	0.00	0.00	0.00	0.00	0.00
GATCAAAGCAGATTCAAAGT	0.00	0.00	0.00	0.00	0.34
GATCAAAGCAGCAACCCTAC	0.46	0.33	0.00	0.34	0.34
GATCAAAGCAGCAAGGAAAA	0.00	0.00	0.57	0.00	0.00
GATCAAAGCAGCAAGGCCTT	0.00	0.98	0.00	0.00	0.00
GATCAAAGCAGCACAAGTTC	0.23	0.00	0.00	0.00	0.00
GATCAAAGCAGCACATGGAG	0.00	0.00	0.00	0.00	0.00
GATCAAAGCAGCACCGAACA	0.00	0.00	0.00	0.00	0.00
GATCAAAGCAGCACGCGCCT	0.46	0.00	0.00	0.00	0.00
GATCAAAGCAGCAGAAATCT	0.00	0.00	0.00	0.00	0.34
GATCAAAGCAGCAGAAGTTC	0.00	0.33	0.00	0.00	0.00
GATCAAAGCAGCAGACATCT	0.00	0.00	0.00	0.00	0.34
GATCAAAGCAGCAGACCTCT	0.00	0.00	0.00	0.34	0.34

GATCAAAGCAGCAGGAGTCG	0.00	0.00	0.00	0.00	0.00
GATCAAAGCAGCATAAGCTC	0.23	0.00	0.00	0.34	0.00
GATCAAAGCAGCATAAGGTC	0.00	0.00	0.00	0.00	0.00
GATCAAAGCAGCATAAGTNN	0.23	0.00	0.00	0.00	0.00
GATCAAAGCAGCATAAGTTC	10.76	10.74	7.16	5.43	9.30
GATCAAAGCAGCATAATCGT	0.00	0.00	0.29	0.00	0.00
GATCAAAGCAGCATACCACT	0.00	0.33	0.00	0.00	0.00
GATCAAAGCAGCATCAGTTC	0.23	0.00	0.00	0.00	0.00
GATCAAAGCAGCATCCTTCA	0.00	0.00	0.00	0.00	0.00
GATCAAAGCAGCATGCAAAA	0.00	0.00	0.00	0.34	0.00
GATCAAAGCAGCATTTTGGC	0.00	0.00	0.00	0.00	0.00
GATCAAAGCAGCCAAGGAAA	0.23	0.00	0.00	0.00	0.00
GATCAAAGCAGCCACGCAGC	0.00	0.00	0.00	0.00	0.00
GATCAAAGCAGCCACTGTCG	0.00	0.00	0.29	0.00	0.00
GATCAAAGCAGCCAGCTCCG	0.00	0.00	0.00	0.00	0.34
GATCAAAGCAGCCCGTCGCC	0.23	0.00	0.00	0.00	0.00
GATCAAAGCAGCCGAAACGG	0.00	0.00	0.00	0.34	0.00
GATCAAAGCAGCCGGCGTCC	0.00	0.00	0.00	0.00	0.00
GATCAAAGCAGCGATCATCG	0.00	0.00	0.00	0.34	0.00
GATCAAAGCAGCGCAGAGCA	0.00	0.00	0.00	0.00	0.34
GATCAAAGCAGCGCCGAACA	2.52	0.98	4.01	3.05	3.10
GATCAAAGCAGCGCCGAACC	0.00	0.00	0.57	0.00	0.00
GATCAAAGCAGCGCCGACCA	0.00	0.00	0.29	0.00	0.00
GATCAAAGCAGCGCCGCACA	0.00	0.00	0.29	0.00	0.00
GATCAAAGCAGCGCTCGGCA	0.00	0.00	0.00	0.34	0.34
GATCAAAGCAGCGGAGGAGT	0.00	0.00	0.00	0.00	0.00
GATCAAAGCAGCGGCGAAGA	0.00	0.00	0.00	0.34	0.00
GATCAAAGCAGCGGTGGCCC	0.00	0.33	0.00	0.00	0.00
GATCAAAGCAGCTCCCCGGA	0.00	0.00	0.29	0.00	0.00
GATCAAAGCAGCTCGACCTG	0.23	0.00	0.00	0.34	0.00
GATCAAAGCAGCTCGCGTTG	0.00	0.00	0.00	0.00	0.00
GATCAAAGCAGCTCTGAGGG	0.00	0.00	0.00	0.00	0.00
GATCAAAGCAGCTGCTCACG	0.00	0.00	0.00	0.00	0.00
GATCAAAGCAGCTGTGCAAC	0.23	0.00	0.00	0.00	0.00
GATCAAAGCAGCTTCAAAGT	0.00	0.00	0.00	0.00	0.00
GATCAAAGCAGCTTCTTTTC	0.00	0.00	0.00	0.00	0.00
GATCAAAGCAGGAAAGTTGG	0.00	0.00	0.29	0.00	0.00

GATCAAAGCAGGAACCTCCA	0.00	0.00	0.00	0.34	0.00
GATCAAAGCAGGAACCTCCG	0.23	0.00	0.00	0.00	0.00
GATCAAAGCAGGAGCTCAGC	0.00	0.00	0.00	0.34	0.00
GATCAAAGCAGGATTCTTGC	0.69	0.00	0.00	0.34	0.00
GATCAAAGCAGGCAAACGCG	0.00	0.33	0.00	0.00	0.00
GATCAAAGCAGGCAACGCTG	0.00	0.65	0.00	0.34	0.00
GATCAAAGCAGGCGCTCAGC	0.23	0.00	0.00	0.00	0.00
GATCAAAGCAGGCTTCCCGG	0.00	0.00	0.00	0.00	0.00
GATCAAAGCAGGCTTGTGGG	0.00	0.00	0.57	0.00	0.34
GATCAAAGCAGGGAGAAGAT	0.23	0.00	0.00	0.00	0.00
GATCAAAGCAGGGCAGTGGC	0.23	0.00	0.00	0.00	0.00
GATCAAAGCAGGGCGATGAG	0.00	0.00	0.00	0.00	0.00
GATCAAAGCAGGGGCTCAGC	0.00	0.00	0.00	0.34	0.00
GATCAAAGCAGGGGGCTCTC	0.23	0.00	0.00	0.00	0.00
GATCAAAGCAGGGTTCCAGA	0.00	0.33	0.00	0.00	0.00
GATCAAAGCAGGTCCTCTTG	0.00	0.00	0.29	0.00	0.00
GATCAAAGCAGGTCGCAAGG	0.00	0.33	0.00	0.00	0.00
GATCAAAGCAGGTGCTCAGA	0.23	0.00	0.00	0.34	0.00
GATCAAAGCAGGTGCTCAGC	33.65	30.58	8.30	30.89	19.98
GATCAAAGCAGGTGCTCAGT	0.00	0.00	0.00	0.68	0.00
GATCAAAGCAGGTGCTCANN	0.23	0.00	0.00	0.00	0.00
GATCAAAGCAGGTGCTCNNN	0.00	0.00	0.00	0.00	0.00
GATCAAAGCAGGTGCTTAGC	0.00	0.00	0.00	0.00	0.00
GATCAAAGCAGGTTCGGCGC	0.00	0.00	0.00	0.34	0.00
GATCAAAGCAGTAAAAAAAA	0.00	0.00	0.00	0.00	0.34
GATCAAAGCAGTACTTGCGC	0.00	0.00	0.00	0.34	0.00
GATCAAAGCAGTAGACCTGT	0.00	0.00	0.00	0.00	0.00
GATCAAAGCAGTATTCTTGC	0.00	0.00	0.00	0.34	0.00
GATCAAAGCAGTATTTACAT	0.00	0.00	0.00	0.34	0.00
GATCAAAGCAGTATTTTCAT	0.00	0.00	0.29	0.68	0.34
GATCAAAGCAGTATTTTCTT	0.00	0.00	0.00	0.34	0.00
GATCAAAGCAGTCCGAAGTT	0.00	0.00	0.00	0.00	0.00
GATCAAAGCAGTCCTTGCGC	0.00	0.00	0.00	0.00	0.00
GATCAAAGCAGTCCTTGGGC	0.00	0.00	0.00	0.00	0.00
GATCAAAGCAGTCGACGCCA	0.00	0.00	0.29	0.00	0.00
GATCAAAGCAGTCTGCCGCC	0.00	0.00	0.00	0.00	0.00
GATCAAAGCAGTCTGCTTGC	0.23	0.00	0.00	0.00	0.69

GATCAAAGCAGTGAACTTGG	0.00	0.00	0.00	0.00	0.34
GATCAAAGCAGTGGACCCCA	0.23	0.33	0.00	1.02	0.00
GATCAAAGCAGTTACCCCCT	0.00	0.00	0.57	0.00	0.00
GATCAAAGCAGTTCCAGAGC	0.00	0.00	0.00	0.00	0.00
GATCAAAGCAGTTGAAAGGC	0.00	0.00	0.00	0.00	0.69
GATCAAAGCAGTTGAAGAGA	0.00	0.00	0.00	0.00	0.34
GATCAAAGCAGTTGAAGCCT	0.00	0.33	0.00	0.00	0.00
GATCAAAGCAGTTTCAAAAA	0.23	0.00	0.00	0.34	0.00
GATCAAAGCAGTTTCAAACT	0.00	0.00	0.00	0.00	0.00
GATCAAAGCAGTTTCAAAGA	0.00	0.00	0.57	0.00	0.69
GATCAAAGCAGTTTCAAAGG	0.23	0.00	0.57	0.00	0.00
GATCAAAGCAGTTTCAAAGN	0.46	0.00	0.00	0.00	0.00
GATCAAAGCAGTTTCAAAGT	59.29	32.86	32.64	33.94	31.68
GATCAAAGCAGTTTCAAANT	0.00	0.33	0.00	0.00	0.00
GATCAAAGCAGTTTCAACGG	0.00	0.33	0.00	0.00	0.00
GATCAAAGCAGTTTCAACGT	0.00	0.65	0.00	0.00	0.34
GATCAAAGCAGTTTCAACTT	0.00	0.00	0.00	0.00	0.00
GATCAAAGCAGTTTCAATGT	0.23	0.00	0.00	0.00	0.00
GATCAAAGCAGTTTCACAGT	0.23	0.00	0.00	0.00	0.00
GATCAAAGCAGTTTCACANN	0.23	0.00	0.00	0.00	0.00
GATCAAAGCAGTTTCANNNN	0.23	0.00	0.00	0.00	0.00
GATCAAAGCAGTTTCATAGT	0.00	0.00	0.00	0.00	0.34
GATCAAAGCAGTTTCCAAGT	0.00	0.00	0.00	0.00	0.34
GATCAAAGCAGTTTCGCAGT	0.23	0.00	0.00	0.00	0.00
GATCAAAGCAGTTTCGCCGA	0.23	0.00	0.00	0.00	0.00
GATCAAAGCAGTTTCTAAGT	0.23	0.00	0.00	0.00	0.00
GATCAAAGCAGTTTCTGAGC	0.00	0.00	0.86	0.00	0.00
GATCAAAGCATAAAAAAAAA	0.00	0.00	0.00	0.34	0.00
GATCAAAGCATAAAAAGCCA	0.46	0.00	0.00	0.00	0.00
GATCAAAGCATACATCTGCA	0.00	0.00	0.00	0.00	0.00
GATCAAAGCATACTTTGGCT	0.00	0.00	0.00	0.00	0.00
GATCAAAGCATAGACGCATC	0.00	0.00	0.00	0.00	0.34
GATCAAAGCATAGGGAGCCT	0.00	0.00	1.15	0.34	0.00
GATCAAAGCATAGTCCAGCG	0.23	0.00	0.00	0.00	0.00
GATCAAAGCATAGTGTTGCC	0.00	0.00	0.00	0.34	0.00
GATCAAAGCATATAGAAAAT	0.92	0.00	0.00	0.00	0.00
GATCAAAGCATATGAGAGCA	0.00	0.00	0.29	0.00	0.00

GATCAAAGCATCAAAAAAAA	0.00	0.65	1.15	0.00	0.34
GATCAAAGCATCAAAATACA	0.23	0.00	0.00	0.00	0.00
GATCAAAGCATCAAACAAAA	0.23	0.00	0.00	0.00	0.00
GATCAAAGCATCAAAGCATC	0.00	0.33	0.00	0.00	0.00
GATCAAAGCATCACAATCAA	0.23	0.00	0.00	0.00	0.00
GATCAAAGCATCACCCCCGC	0.00	0.00	0.29	0.00	0.00
GATCAAAGCATCACGCTGGA	0.46	0.00	0.00	0.00	0.00
GATCAAAGCATCATGGCATG	0.00	0.33	0.00	1.02	0.69
GATCAAAGCATCCAAAAAAA	0.00	0.00	0.00	0.34	0.00
GATCAAAGCATCCATCCGCG	0.46	0.00	0.00	0.00	0.00
GATCAAAGCATCCTCGCATG	0.23	0.98	0.57	1.36	1.03
GATCAAAGCATCGAAAAAAA	2.52	1.30	0.57	1.36	0.34
GATCAAAGCATCGAAAAGAA	0.23	0.00	0.00	0.00	0.00
GATCAAAGCATCGAAGAAAA	0.46	0.00	0.00	0.00	0.00
GATCAAAGCATCGAAGAAAG	0.00	0.00	0.00	0.00	0.34
GATCAAAGCATCGAAGAATA	0.00	0.00	0.00	0.00	0.00
GATCAAAGCATCGAAGAATC	0.00	0.33	0.57	0.00	0.00
GATCAAAGCATCGAAGCAAA	2.06	3.90	1.15	1.36	0.69
GATCAAAGCATCGAAGCAAG	0.23	0.00	0.00	0.00	0.00
GATCAAAGCATCGAAGCACA	0.00	0.33	0.29	0.00	0.00
GATCAAAGCATCGAAGCACC	0.00	0.00	0.00	0.00	0.00
GATCAAAGCATCGAAGCACG	0.00	0.00	0.00	0.00	0.00
GATCAAAGCATCGAAGCAGA	0.00	0.00	0.29	0.00	0.00
GATCAAAGCATCGAAGCAGC	0.00	0.00	0.00	0.34	0.34
GATCAAAGCATCGAAGCANN	1.14	0.00	0.00	0.00	0.00
GATCAAAGCATCGAAGCATA	0.23	0.00	0.29	0.00	0.34
GATCAAAGCATCGAAGCATC	97.06	55.63	50.97	50.91	48.91
GATCAAAGCATCGAAGCATG	0.00	0.33	0.00	0.00	0.00
GATCAAAGCATCGAAGCATN	0.69	0.00	0.00	0.00	0.00
GATCAAAGCATCGAAGCCNN	0.23	0.00	0.00	0.00	0.00
GATCAAAGCATCGAAGCCTC	0.00	0.33	0.00	0.00	0.34
GATCAAAGCATCGAAGCCTT	0.00	0.33	0.00	0.00	0.00
GATCAAAGCATCGAAGCGGA	0.00	0.00	0.57	0.00	0.00
GATCAAAGCATCGAAGCGGC	0.00	0.00	0.00	0.34	0.00
GATCAAAGCATCGAAGCGTC	0.69	0.00	0.00	0.00	0.00
GATCAAAGCATCGAAGCNNN	0.23	0.00	0.00	0.00	0.00
GATCAAAGCATCGAAGCNTC	0.00	0.00	0.00	0.00	0.34

GATCAAAGCATCGAAGGATA	0.00	0.33	0.00	0.00	0.00
GATCAAAGCATCGAAGNANN	0.23	0.00	0.00	0.00	0.00
GATCAAAGCATCGAAGNCTC	0.00	0.00	0.00	0.00	0.34
GATCAAAGCATCGAAGNNNN	0.46	0.00	0.00	0.00	0.00
GATCAAAGCATCGAAGTAAA	0.00	0.33	0.00	0.00	0.00
GATCAAAGCATCGAGATGCA	0.23	0.33	0.00	0.00	0.00
GATCAAAGCATCGAGCTTGC	0.00	0.00	0.00	0.00	0.00
GATCAAAGCATCGAGGCATC	0.23	0.00	0.00	0.34	0.00
GATCAAAGCATCGAGGTCGC	0.00	0.33	0.00	0.00	0.00
GATCAAAGCATCGCAGCATC	0.23	0.00	0.00	0.00	0.00
GATCAAAGCATCGCCACCTC	0.23	0.00	0.00	0.00	0.00
GATCAAAGCATCGGAGCAAA	0.00	0.00	0.00	0.00	0.00
GATCAAAGCATCGGTGAAGG	0.00	0.65	0.00	0.68	0.69
GATCAAAGCATCGTATGCCG	0.23	0.00	0.00	0.00	0.00
GATCAAAGCATCTGAGAGTG	0.00	0.00	0.00	0.34	0.00
GATCAAAGCATCTGCATGGG	0.00	0.00	0.00	0.68	0.00
GATCAAAGCATCTGCGAGTG	0.23	0.65	0.57	0.34	0.00
GATCAAAGCATCTGGAACGA	0.23	0.00	0.00	0.00	0.34
GATCAAAGCATCTGGTTCAG	0.00	0.65	0.00	0.00	0.00
GATCAAAGCATCTGTGAGTG	0.00	0.00	0.00	0.00	0.00
GATCAAAGCATCTGTGTGAT	0.00	0.00	0.00	0.00	0.34
GATCAAAGCATCTTGGTGAT	0.00	0.00	0.00	0.00	0.00
GATCAAAGCATGAGAGGAGT	0.46	0.00	0.00	0.68	0.00
GATCAAAGCATGAGCCTTGG	0.00	0.33	0.00	0.00	0.00
GATCAAAGCATGAGCTCTCG	0.00	0.00	0.00	0.00	0.00
GATCAAAGCATGAGTCCGAG	0.00	0.00	0.00	0.00	0.34
GATCAAAGCATGATTGTTCA	0.00	0.00	0.00	0.00	0.00
GATCAAAGCATGCAAACAGT	0.00	0.00	0.00	0.68	0.00
GATCAAAGCATGCACCTGCC	0.00	0.00	0.29	0.00	0.00
GATCAAAGCATGCACGGACA	0.00	0.00	0.00	0.00	0.00
GATCAAAGCATGCAGCACGG	0.00	0.00	0.57	0.00	0.00
GATCAAAGCATGCAGCACTC	0.46	0.00	0.57	0.00	0.00
GATCAAAGCATGCCCATATC	0.00	0.00	0.29	0.00	0.00
GATCAAAGCATGCCCCATGC	0.23	0.00	0.29	0.00	0.00
GATCAAAGCATGCGGAAAAG	0.00	0.00	0.00	0.34	0.00
GATCAAAGCATGCGTGTGAT	0.23	0.00	0.00	0.00	0.00
GATCAAAGCATGGACGGCCG	0.23	0.00	0.00	0.00	0.00

GATCAAAGCATGGACGTCCG	0.23	0.00	0.00	0.00	0.00
GATCAAAGCATGGAGCGCTG	0.00	0.00	0.29	0.00	0.00
GATCAAAGCATGGAGGATCA	0.00	0.00	0.00	0.00	0.34
GATCAAAGCATGGGCCATTC	0.00	0.00	0.29	0.00	0.00
GATCAAAGCATGGGGAGAGG	0.00	0.00	0.00	0.00	0.34
GATCAAAGCATGGTGCCCAA	0.00	0.00	0.00	0.34	0.00
GATCAAAGCATGTACGTACA	0.00	0.33	0.00	0.00	0.34
GATCAAAGCATTAAACAAAA	0.00	0.00	0.00	0.00	0.00
GATCAAAGCATTAAACAACT	0.00	0.00	0.00	0.00	0.00
GATCAAAGCATTAAGAGGCA	0.00	0.00	0.00	0.00	0.34
GATCAAAGCATTAAGATGCA	0.00	0.00	0.29	0.00	0.00
GATCAAAGCATTACGTTGGC	0.00	0.00	0.00	0.00	0.00
GATCAAAGCATTAGTGGCCG	0.69	0.00	0.00	0.00	0.00
GATCAAAGCATTAGTGGGAG	0.23	0.00	0.00	0.00	0.00
GATCAAAGCATTCAGCCTTC	0.00	0.00	0.00	0.00	0.34
GATCAAAGCATTCATGTGAA	0.00	0.00	0.57	0.00	0.00
GATCAAAGCATTCATGTTGC	0.00	0.98	0.00	0.00	0.00
GATCAAAGCATTCCCTCCAG	0.23	0.00	0.00	0.00	0.00
GATCAAAGCATTCCGCGGAA	0.00	0.00	0.00	0.00	0.00
GATCAAAGCATTCGACGGTA	0.00	0.00	0.29	0.00	0.00
GATCAAAGCATTCGTGTGAT	0.23	0.00	0.00	0.00	0.00
GATCAAAGCATTCTATCTGG	0.00	0.00	0.00	0.00	0.00
GATCAAAGCATTGAATTGCT	0.00	0.00	0.00	0.00	0.00
GATCAAAGCATTGAGATGCA	0.46	0.00	0.29	0.00	0.00
GATCAAAGCATTGCACGCTA	2.75	4.23	0.86	4.75	0.69
GATCAAAGCATTGGAGGACC	0.00	0.00	0.00	0.34	0.00
GATCAAAGCATTGGAGGCAA	0.00	0.33	0.00	0.00	0.00
GATCAAAGCATTGGCTGTTA	0.00	0.00	0.29	0.00	0.00
GATCAAAGCATTGGGCCAGG	0.00	0.00	0.00	0.00	0.00
GATCAAAGCATTGTGCCCAG	0.00	0.00	0.00	0.00	0.00
GATCAAAGCATTGTGGGGGC	0.23	0.00	0.00	0.00	0.00
GATCAAAGCATTTCAGCTGT	0.00	0.33	0.00	0.00	0.00
GATCAAAGCATTTCTGTTCT	0.00	0.33	0.00	0.00	0.00
GATCAAAGCATTTGAGAGAT	0.00	0.33	0.00	0.00	0.00
GATCAAAGCATTTGCGTGAA	0.00	0.00	0.00	0.34	0.00
GATCAAAGCATTTGCGTGAT	0.00	0.00	0.00	0.00	0.34
GATCAAAGCATTTGGGCGAG	0.00	0.00	0.00	0.00	0.34

GATCAAAGCATTTGTGCAGC	0.00	0.00	0.00	0.34	0.00
GATCAAAGCATTTGTGCGAT	0.00	0.33	0.00	0.00	0.00
GATCAAAGCATTTGTGGGAA	0.00	0.33	0.00	0.00	0.00
GATCAAAGCATTTGTGNGAT	0.00	0.00	0.00	0.00	0.34
GATCAAAGCATTTGTGNNNN	0.23	0.00	0.00	0.00	0.00
GATCAAAGCATTTGTGTAAT	0.00	0.00	0.00	0.34	0.00
GATCAAAGCATTTGTGTGAA	0.00	0.33	0.00	0.00	1.03
GATCAAAGCATTTGTGTGAC	0.00	0.00	0.00	0.00	0.00
GATCAAAGCATTTGTGTGAN	0.23	0.00	0.00	0.00	0.34
GATCAAAGCATTTGTGTGAT	55.17	42.62	20.33	38.02	28.59
GATCAAAGCATTTGTGTGCT	0.23	0.33	0.00	0.00	0.00
GATCAAAGCATTTGTGTGGG	0.23	0.00	0.00	0.00	0.00
GATCAAAGCATTTGTGTGNN	0.46	0.00	0.00	0.00	0.00
GATCAAAGCATTTGTGTGNT	0.00	0.33	0.00	0.00	0.00
GATCAAAGCATTTGTGTGTG	0.00	0.33	0.00	0.00	0.00
GATCAAAGCATTTGTGTNAT	0.00	0.00	0.00	0.00	0.34
GATCAAAGCATTTGTGTTCT	0.00	0.00	0.00	0.00	0.00
GATCAAAGCATTTGTTTTGC	0.00	0.00	0.00	0.00	0.00
GATCAAAGCATTTTGTCCTG	0.00	0.33	0.00	0.00	0.00
GATCAAAGCATTTTTTGGAT	0.00	0.00	0.00	0.00	0.34
GATCAAAGCCAAAAAAAAAA	0.00	0.33	0.29	0.00	0.00
GATCAAAGCCAAAGCATTGG	0.69	0.33	0.00	0.68	0.00
GATCAAAGCCAAATCTCACT	0.00	0.00	0.00	0.00	0.69
GATCAAAGCCAAATCTGAGG	0.00	0.00	0.00	0.00	0.34
GATCAAAGCCAAATCTGATT	0.23	0.00	0.00	0.34	0.00
GATCAAAGCCAAATCTGTTT	0.00	0.00	0.00	0.34	0.00
GATCAAAGCCAACAAGCGTG	0.00	0.00	0.00	0.00	0.34
GATCAAAGCCAACTACCTCT	0.00	0.00	0.00	0.00	0.00
GATCAAAGCCAAGAAGTCAA	0.00	0.00	0.00	0.34	0.00
GATCAAAGCCAAGACGGCAA	0.00	0.00	0.00	0.00	0.00
GATCAAAGCCAAGACGTCAA	0.23	0.33	0.29	1.02	0.69
GATCAAAGCCAAGCACGTCT	0.00	0.00	0.00	0.00	0.34
GATCAAAGCCAAGGACCGCG	0.00	0.00	0.29	0.00	0.00
GATCAAAGCCAAGTAAAAGG	0.00	0.00	0.00	0.00	1.03
GATCAAAGCCAATCACAATG	0.00	0.00	0.00	0.34	0.00
GATCAAAGCCAATGACACCT	0.00	0.00	0.00	0.00	0.34
GATCAAAGCCAATGGCGTGC	0.00	0.00	0.29	0.68	0.00

GATCAAAGCCAATTGAGGGG	0.00	0.00	0.00	0.00	0.00
GATCAAAGCCACACCCAGNN	0.00	0.00	0.00	0.00	0.00
GATCAAAGCCACACCCAGTC	0.23	0.65	0.00	0.34	0.00
GATCAAAGCCACACCGCCAC	1.60	0.33	0.29	0.34	0.00
GATCAAAGCCACACGAAGCA	0.00	0.00	0.86	0.00	0.00
GATCAAAGCCACAGACCTCT	0.23	0.00	0.00	0.68	0.34
GATCAAAGCCACAGCAACCA	0.92	0.65	0.00	0.34	0.34
GATCAAAGCCACAGCCCTCT	0.00	0.00	0.29	0.00	0.00
GATCAAAGCCACAGCTATGA	0.23	0.00	0.00	0.00	0.00
GATCAAAGCCACCAATGCGA	0.00	0.33	0.00	0.00	0.00
GATCAAAGCCACCAATGCGC	1.37	1.30	0.00	0.00	0.34
GATCAAAGCCACCCATACCA	0.00	0.00	0.00	0.00	0.69
GATCAAAGCCACCGAAGACG	0.92	0.00	0.00	0.00	0.00
GATCAAAGCCACCGCCGTCA	0.00	0.00	0.29	0.00	0.00
GATCAAAGCCACCTATCGGA	0.00	0.33	0.00	0.00	0.00
GATCAAAGCCACCTATCGGG	0.00	0.00	0.00	0.00	0.00
GATCAAAGCCACGCTCCCGA	0.00	0.00	0.00	0.00	0.00
GATCAAAGCCACTGGACACC	0.00	0.00	0.29	0.00	0.00
GATCAAAGCCACTGGTACTG	0.00	0.00	0.00	0.34	0.00
GATCAAAGCCACTGGTGTGC	0.00	0.00	0.00	0.00	0.00
GATCAAAGCCACTTGACACA	0.00	0.00	0.00	0.34	0.00
GATCAAAGCCACTTGCCCTC	0.00	0.00	0.00	0.00	0.69
GATCAAAGCCAGAACCTCCG	0.00	0.00	0.29	0.00	0.00
GATCAAAGCCAGAAGTTCAA	0.00	0.00	0.00	0.34	0.00
GATCAAAGCCAGAATATGCT	0.00	0.00	0.00	0.00	0.00
GATCAAAGCCAGACACTTAC	0.46	0.00	0.57	0.00	0.00
GATCAAAGCCAGACCTCTGG	0.00	0.00	0.00	0.00	0.00
GATCAAAGCCAGACGCCTTC	0.23	0.00	0.00	0.34	0.00
GATCAAAGCCAGCACCTCCA	0.00	0.00	0.29	0.00	0.00
GATCAAAGCCAGCACGAGGG	0.00	0.00	0.00	0.00	0.00
GATCAAAGCCAGCATCCCGG	0.46	0.00	0.00	0.00	0.00
GATCAAAGCCAGCCTCGTGT	0.00	0.00	0.00	0.00	0.00
GATCAAAGCCAGCGTCTTAG	0.46	0.00	0.00	0.00	0.00
GATCAAAGCCAGGCGTGTAG	0.00	0.00	0.00	0.00	0.00
GATCAAAGCCAGGGCTCTTA	0.00	0.00	0.57	0.00	0.00
GATCAAAGCCAGGGCTCTTC	0.23	0.98	0.00	1.02	0.00
GATCAAAGCCAGGTCATCCG	0.00	0.33	0.00	0.00	0.00

GATCAAAGCCAGTCCGCGCA	0.00	0.00	0.00	0.00	0.34
GATCAAAGCCAGTCTTCACA	0.23	0.00	0.00	0.00	0.00
GATCAAAGCCAGTCTTCAGC	12.36	4.23	1.43	4.07	4.82
GATCAAAGCCAGTCTTCATC	0.00	0.00	0.00	0.00	0.00
GATCAAAGCCAGTGCTATCA	0.23	0.00	0.00	0.00	0.00
GATCAAAGCCAGTGTCTGGT	0.00	0.00	0.00	0.68	0.00
GATCAAAGCCAGTTGCTGGC	0.00	0.33	0.00	0.00	0.00
GATCAAAGCCATCTCCGCCC	0.00	0.00	0.00	0.00	0.00
GATCAAAGCCATCTCCTCCG	0.00	0.00	0.57	0.00	0.00
GATCAAAGCCATGAAGCAAG	0.46	0.33	0.00	0.00	1.03
GATCAAAGCCATGAAGNAAG	0.00	0.00	0.00	0.00	0.34
GATCAAAGCCATGACAGGGT	0.00	0.00	0.00	0.00	0.69
GATCAAAGCCATGGCGTTGC	0.00	0.00	0.00	0.00	0.34
GATCAAAGCCATGGTGGCTG	0.00	0.00	0.00	0.00	0.00
GATCAAAGCCCAAGACCTCC	0.00	0.33	0.00	0.00	0.00
GATCAAAGCCCAAGCGTGGG	0.00	0.00	0.00	0.00	0.34
GATCAAAGCCCAAGGCAAAA	0.00	0.00	0.00	0.00	0.00
GATCAAAGCCCAAGGCCCTG	0.00	0.65	0.29	0.34	0.69
GATCAAAGCCCACACCCCGC	0.00	0.00	0.00	0.34	0.00
GATCAAAGCCCACCGGGCCC	0.00	0.00	0.00	0.00	0.34
GATCAAAGCCCACGCGAACC	0.00	0.00	0.00	0.00	0.00
GATCAAAGCCCACTCCAAGG	0.00	0.00	0.00	0.68	0.00
GATCAAAGCCCACTCGCTCC	0.00	0.00	0.00	0.00	0.00
GATCAAAGCCCACTGCGAAG	0.00	0.33	0.00	0.34	0.00
GATCAAAGCCCACTTTGCAC	0.69	1.63	0.29	2.04	0.69
GATCAAAGCCCAGAAAAGCT	0.00	0.33	0.00	0.00	0.00
GATCAAAGCCCAGCTGCGGA	0.00	0.00	0.00	0.34	0.00
GATCAAAGCCCAGCTGCGGC	0.00	0.00	0.00	0.34	0.00
GATCAAAGCCCAGGGCAGGG	0.00	0.00	0.00	0.00	0.34
GATCAAAGCCCATAGCTCGA	0.69	0.33	0.00	0.00	1.03
GATCAAAGCCCCAGGCACGG	0.00	0.00	0.00	0.34	0.00
GATCAAAGCCCCAGTGCTGC	0.00	0.00	0.00	0.00	0.34
GATCAAAGCCCCCCCCACCC	0.00	0.00	0.29	0.00	0.00
GATCAAAGCCCCGTTGTCCA	0.00	0.00	0.00	0.00	0.34
GATCAAAGCCCCTGAGGGTT	0.00	0.00	0.00	0.34	0.00
GATCAAAGCCCCTGCCAATG	0.00	0.00	0.00	0.34	0.00
GATCAAAGCCCCTTCCGCAG	0.00	0.33	0.57	0.00	0.69

GATCAAAGCCCCTTCCGCCG	0.00	0.00	0.00	0.00	0.00
GATCAAAGCCCCTTCGCGTG	0.00	0.00	0.00	0.00	0.00
GATCAAAGCCCCTTGCCTCT	0.92	0.65	0.00	1.36	0.00
GATCAAAGCCCGACGGCCAT	0.00	0.00	0.00	0.00	0.34
GATCAAAGCCCGCAGCGGGC	0.23	0.00	0.00	0.00	0.00
GATCAAAGCCCGCAGTGGGC	1.14	1.95	2.00	1.36	1.38
GATCAAAGCCCGCAGTGGTC	0.00	0.33	0.00	0.00	0.00
GATCAAAGCCCGCGGACCCA	0.00	0.00	0.57	0.00	0.00
GATCAAAGCCCGCGTGTAAC	0.00	0.00	0.29	0.00	0.00
GATCAAAGCCCGCGTTTGTT	0.00	0.00	0.00	0.00	0.00
GATCAAAGCCCGCTGTTCCC	0.00	0.00	0.29	0.00	0.00
GATCAAAGCCCGGCCGGTCC	0.23	0.00	0.00	0.00	0.00
GATCAAAGCCCGGCCTTCGA	0.00	0.00	0.00	0.00	0.00
GATCAAAGCCCTCATGCGTC	0.00	0.00	0.00	0.00	0.34
GATCAAAGCCCTCCACTCTG	0.23	0.00	0.00	0.00	0.00
GATCAAAGCCCTCGACTCGG	0.00	0.00	0.00	0.00	0.34
GATCAAAGCCCTCTCCTCGC	0.23	0.00	0.00	0.00	0.00
GATCAAAGCCCTCTGCCTTG	0.00	0.00	0.00	0.00	0.34
GATCAAAGCCCTTGCTGGCG	0.00	0.00	0.00	0.00	0.00
GATCAAAGCCCTTTGGTGGG	0.23	0.00	0.00	0.00	0.00
GATCAAAGCCGAAAGCGGGG	0.00	0.33	0.00	0.00	0.00
GATCAAAGCCGAAAGTCCAG	0.23	0.00	0.00	0.00	0.00
GATCAAAGCCGAACCTCCGC	0.00	0.00	0.00	0.34	0.00
GATCAAAGCCGAAGACATAT	0.00	0.33	0.00	0.00	0.00
GATCAAAGCCGAAGACCTCT	0.00	0.33	0.29	0.00	0.00
GATCAAAGCCGAAGACGTCG	0.00	0.33	0.00	0.00	0.00
GATCAAAGCCGAAGAGTGCC	0.00	0.00	0.00	0.00	0.00
GATCAAAGCCGAAGCCCTGT	0.00	0.00	0.00	0.00	0.00
GATCAAAGCCGACAGCAACG	0.00	0.00	0.00	0.00	0.34
GATCAAAGCCGACAGCGCCG	0.23	0.00	0.00	0.00	0.00
GATCAAAGCCGACAGCTACG	0.00	0.00	0.29	0.34	0.00
GATCAAAGCCGACAGCTCAG	0.00	0.00	0.00	0.00	0.34
GATCAAAGCCGACAGCTCCG	0.23	0.65	0.86	0.00	1.03
GATCAAAGCCGACAGCTCTG	0.00	0.00	0.00	0.00	0.00
GATCAAAGCCGACAGCTGCG	0.00	0.00	0.00	0.00	0.00
GATCAAAGCCGACCTTTCGT	0.00	0.00	0.00	0.34	0.00
GATCAAAGCCGAGCTCAAGA	0.46	0.65	0.29	0.34	0.69

GATCAAAGCCGAGCTCATGA	0.00	0.00	0.00	0.00	0.00
GATCAAAGCCGATAGATCCG	0.00	0.00	0.29	0.00	0.00
GATCAAAGCCGATCCGGAGA	0.00	0.00	0.00	0.00	0.34
GATCAAAGCCGATTCGGAGA	0.23	0.00	0.00	0.00	0.00
GATCAAAGCCGCAAACCTCT	0.00	0.00	0.00	0.00	0.00
GATCAAAGCCGCAAATAGAG	0.00	0.00	0.00	0.00	0.69
GATCAAAGCCGCAGAAATCT	0.00	0.33	0.00	0.00	0.00
GATCAAAGCCGCAGAACTCT	0.23	1.63	1.15	0.00	0.34
GATCAAAGCCGCAGACACCT	0.00	0.00	0.00	0.68	0.00
GATCAAAGCCGCAGACATAT	0.00	0.00	0.00	0.00	0.00
GATCAAAGCCGCAGACATCG	0.00	0.33	0.00	0.00	0.00
GATCAAAGCCGCAGACATCT	0.00	0.33	1.15	0.34	0.00
GATCAAAGCCGCAGACATGT	0.00	0.00	0.29	0.00	0.00
GATCAAAGCCGCAGACCAAC	0.00	0.00	0.29	0.00	0.00
GATCAAAGCCGCAGACCAAT	0.00	0.00	0.00	0.34	0.00
GATCAAAGCCGCAGACCACT	0.00	0.00	0.29	0.34	0.00
GATCAAAGCCGCAGACCCCT	0.23	0.65	0.29	0.34	0.00
GATCAAAGCCGCAGACCGCT	0.46	0.00	1.15	0.00	0.00
GATCAAAGCCGCAGACCNCN	0.00	0.00	0.00	0.34	0.00
GATCAAAGCCGCAGACCTAA	0.00	0.00	0.29	0.00	0.00
GATCAAAGCCGCAGACCTAC	0.00	0.00	0.00	0.00	0.34
GATCAAAGCCGCAGACCTAT	0.00	0.00	0.86	0.34	1.03
GATCAAAGCCGCAGACCTCA	0.00	0.00	0.29	0.68	0.69
GATCAAAGCCGCAGACCTCC	0.23	0.00	0.29	0.34	0.69
GATCAAAGCCGCAGACCTCG	0.23	0.65	0.86	0.68	0.00
GATCAAAGCCGCAGACCTCN	0.46	0.00	0.00	0.00	0.00
GATCAAAGCCGCAGACCTCT	176.49	274.90	383.72	290.21	271.7[illegible]
GATCAAAGCCGCAGACCTGT	0.46	0.00	0.00	0.00	0.00
GATCAAAGCCGCAGACCTNN	1.14	0.00	0.00	0.00	0.00
GATCAAAGCCGCAGACCTNT	0.00	0.00	0.00	0.68	0.34
GATCAAAGCCGCAGACCTTC	0.00	0.00	0.29	0.00	0.00
GATCAAAGCCGCAGACCTTT	0.00	0.00	0.29	0.34	0.34
GATCAAAGCCGCAGACGTCT	0.00	0.00	0.29	0.00	0.00
GATCAAAGCCGCAGACNNNN	0.46	0.00	0.00	0.00	0.00
GATCAAAGCCGCAGACNTCT	0.00	0.00	0.00	0.00	0.34
GATCAAAGCCGCAGACNTNN	0.23	0.00	0.00	0.00	0.00
GATCAAAGCCGCAGACTCGT	0.23	0.00	0.57	0.00	0.00

GATCAAAGCCGCAGAGCTCA	0.00	0.00	0.00	0.00	0.00
GATCAAAGCCGCAGAGCTCT	0.23	0.00	0.29	0.00	0.00
GATCAAAGCCGCAGATCTAT	0.00	0.00	0.29	0.00	0.00
GATCAAAGCCGCAGATCTCT	0.00	0.00	0.29	0.00	0.00
GATCAAAGCCGCAGCCCACT	0.00	0.00	0.00	0.00	0.34
GATCAAAGCCGCAGCCCCCT	0.23	0.00	0.00	0.00	0.00
GATCAAAGCCGCAGCCCTCT	0.23	0.00	0.57	0.34	0.34
GATCAAAGCCGCAGCGCAGA	0.23	0.33	0.00	0.34	0.00
GATCAAAGCCGCAGGACTCT	0.00	0.00	0.00	0.34	0.00
GATCAAAGCCGCAGGCCGCT	0.23	0.00	0.00	0.00	0.00
GATCAAAGCCGCAGGCCTCT	0.46	0.00	0.29	0.00	0.00
GATCAAAGCCGCAGTCCTCT	0.00	0.00	0.00	0.00	0.00
GATCAAAGCCGCAGTTCTGT	0.00	0.00	0.29	0.00	0.00
GATCAAAGCCGCAGTTTTGC	0.00	0.00	0.00	0.00	0.00
GATCAAAGCCGCATACCTCT	0.69	0.00	0.00	0.00	0.00
GATCAAAGCCGCATGGAAAG	0.00	0.00	0.00	0.34	0.00
GATCAAAGCCGCATTGCTCT	0.00	0.00	0.00	0.34	0.00
GATCAAAGCCGCCATCATGC	0.00	0.00	0.00	0.00	0.00
GATCAAAGCCGCCCAAGCAA	0.23	0.00	0.00	0.00	0.00
GATCAAAGCCGCCGAACTGC	0.00	0.00	0.29	0.00	0.00
GATCAAAGCCGCCGACCTCT	0.00	0.33	0.00	0.34	0.00
GATCAAAGCCGCCGCGCTCT	0.23	0.00	0.00	0.00	0.00
GATCAAAGCCGCCTACCGCC	0.00	0.00	0.86	0.00	0.00
GATCAAAGCCGCCTCCTTCA	0.23	0.00	0.00	0.00	0.00
GATCAAAGCCGCCTTCCTGG	0.23	0.33	0.00	0.00	0.00
GATCAAAGCCGCCTTGCTCG	0.00	0.00	0.00	0.00	0.00
GATCAAAGCCGCGAAAAATG	0.23	0.00	0.00	0.00	0.00
GATCAAAGCCGCGAAAACTG	0.23	0.00	0.00	0.00	0.00
GATCAAAGCCGCGAGCTCCT	0.69	0.00	0.29	0.68	0.34
GATCAAAGCCGCGCATAGAA	0.00	0.33	0.00	0.68	0.00
GATCAAAGCCGCGGACCTCT	0.00	0.00	0.00	0.00	0.69
GATCAAAGCCGCGGCAATTA	0.00	0.33	0.00	0.00	0.00
GATCAAAGCCGCGGGCAACC	0.46	0.00	0.00	0.68	0.69
GATCAAAGCCGCGTCATGAT	0.00	0.33	0.00	0.00	0.00
GATCAAAGCCGCTAGCTACA	0.00	0.00	0.29	0.00	0.00
GATCAAAGCCGCTCGTATGC	0.00	0.00	0.29	0.00	0.00
GATCAAAGCCGCTGGCTTCA	0.00	0.00	0.00	2.38	0.00

GATCAAAGCCGGAAACTCCG	0.00	0.00	0.29	0.00	0.00
GATCAAAGCCGGAACACCCG	0.00	0.00	0.00	0.00	0.00
GATCAAAGCCGGAACATACG	0.23	0.00	0.00	0.00	0.00
GATCAAAGCCGGAACCCCCG	0.00	0.00	0.00	0.34	0.00
GATCAAAGCCGGAACCGCCG	0.69	0.00	0.00	0.34	0.00
GATCAAAGCCGGAACCNCCG	0.00	0.00	0.00	0.00	0.69
GATCAAAGCCGGAACCNNNN	0.23	0.00	0.00	0.00	0.00
GATCAAAGCCGGAACCTACG	0.00	0.00	0.29	0.00	0.00
GATCAAAGCCGGAACCTCAG	0.00	0.00	0.29	0.68	0.69
GATCAAAGCCGGAACCTCCA	0.00	0.33	0.00	0.34	0.00
GATCAAAGCCGGAACCTCCC	0.00	0.00	0.00	0.00	0.00
GATCAAAGCCGGAACCTCCG	94.31	80.68	67.87	95.04	77.83
GATCAAAGCCGGAACCTCCN	0.92	0.00	0.00	0.00	0.00
GATCAAAGCCGGAACCTCCT	0.00	0.00	0.57	0.00	0.34
GATCAAAGCCGGAACCTCNN	0.23	0.00	0.00	0.00	0.00
GATCAAAGCCGGAACCTCTC	0.23	0.00	0.00	0.00	0.00
GATCAAAGCCGGAACCTCTG	0.23	0.00	0.00	0.00	0.00
GATCAAAGCCGGAACCTGCG	0.23	0.00	0.00	0.00	0.00
GATCAAAGCCGGAACCTNNN	0.23	0.00	0.00	0.00	0.00
GATCAAAGCCGGAACCTTCG	0.23	0.00	0.00	0.00	0.00
GATCAAAGCCGGAACTTCCG	0.00	0.00	0.29	0.00	0.00
GATCAAAGCCGGAAGAAAAG	0.00	0.00	0.29	0.00	0.00
GATCAAAGCCGGAAGAAAGA	0.00	0.33	0.00	0.00	0.00
GATCAAAGCCGGAAGAAGAA	0.23	0.00	0.00	0.00	0.00
GATCAAAGCCGGAAGATCAA	0.00	0.00	0.00	0.34	0.00
GATCAAAGCCGGAAGCTCCG	0.23	0.00	0.00	0.00	0.00
GATCAAAGCCGGAAGGTCAA	0.23	0.00	0.00	0.00	0.00
GATCAAAGCCGGAAGGTCAC	0.00	0.00	0.00	0.34	0.00
GATCAAAGCCGGAAGTCCAA	0.00	0.00	0.29	0.00	0.00
GATCAAAGCCGGAAGTGCAA	0.23	0.00	0.00	0.00	0.00
GATCAAAGCCGGAAGTNCAA	0.00	0.00	0.00	0.00	0.34
GATCAAAGCCGGAAGTNCNN	0.00	0.00	0.00	0.34	0.00
GATCAAAGCCGGAAGTNNNN	0.23	0.00	0.00	0.00	0.00
GATCAAAGCCGGAAGTTAAA	0.00	0.33	0.29	0.00	0.00
GATCAAAGCCGGAAGTTCAA	27.47	30.58	27.49	45.48	34.10
GATCAAAGCCGGAAGTTCAC	0.00	0.00	0.29	0.00	0.00
GATCAAAGCCGGAAGTTCAN	0.23	0.00	0.00	0.00	0.00

GATCAAAGCCGGAAGTTCAT	0.00	0.33	0.00	0.00	0.34
GATCAAAGCCGGAAGTTCGA	0.00	0.00	0.00	0.00	0.34
GATCAAAGCCGGAAGTTCTA	0.00	0.00	0.00	0.00	0.34
GATCAAAGCCGGAAGTTGAA	0.00	0.00	0.00	0.34	0.00
GATCAAAGCCGGAAGTTTAA	0.00	0.00	0.00	0.00	0.34
GATCAAAGCCGGAAGTTTTG	0.00	0.33	0.00	0.00	0.00
GATCAAAGCCGGAATCTCCG	0.00	0.00	0.00	0.34	0.00
GATCAAAGCCGGACATGTTT	0.00	0.33	0.29	0.00	0.00
GATCAAAGCCGGACCTCTGC	0.00	0.00	0.00	0.00	0.00
GATCAAAGCCGGACGGGCAC	0.23	0.00	0.00	0.00	0.00
GATCAAAGCCGGACGTTCAA	0.00	0.00	0.00	0.34	0.00
GATCAAAGCCGGAGCCTCCG	0.00	0.00	0.00	0.34	0.00
GATCAAAGCCGGAGGTGGGA	0.00	0.00	0.29	0.00	0.00
GATCAAAGCCGGAGGTTCAA	0.23	0.00	0.00	0.34	0.00
GATCAAAGCCGGAGTCATCA	0.00	0.00	0.00	0.00	0.34
GATCAAAGCCGGCACCTCAG	0.00	0.00	0.00	0.00	0.00
GATCAAAGCCGGCACCTCCG	0.00	0.00	0.29	0.00	0.00
GATCAAAGCCGGCAGATTAG	0.00	0.98	0.29	0.34	0.34
GATCAAAGCCGGCCAGCGCA	0.00	0.00	0.00	0.00	0.34
GATCAAAGCCGGCGCGCAAA	0.23	0.00	0.00	0.00	0.00
GATCAAAGCCGGCTGGTCGG	0.23	0.00	0.00	0.00	0.00
GATCAAAGCCGGCTTCTGCA	0.00	0.33	0.00	0.00	0.69
GATCAAAGCCGGCTTCTGCG	1.60	0.98	2.29	2.04	0.69
GATCAAAGCCGGCTTCTGGA	0.00	0.33	0.00	0.00	0.00
GATCAAAGCCGGGACCTCCG	0.46	0.33	0.00	0.00	0.00
GATCAAAGCCGGGACCTTGG	0.23	0.00	0.00	0.00	0.00
GATCAAAGCCGGGGCAATTA	0.23	0.00	0.00	0.00	0.00
GATCAAAGCCGGGGGCTGGG	0.00	0.00	0.00	0.00	0.34
GATCAAAGCCGGTGTCTGGT	0.00	0.00	0.00	0.34	0.00
GATCAAAGCCGTAGTCAAGA	0.00	0.00	0.29	0.00	0.00
GATCAAAGCCGTCCTTGCTT	0.00	0.00	0.00	0.34	0.00
GATCAAAGCCGTCCTTTCGG	0.23	0.00	0.00	0.00	0.00
GATCAAAGCCGTCCTTTCGT	5.49	5.21	7.45	14.93	8.27
GATCAAAGCCGTGAGGGAGA	0.23	0.00	0.00	0.00	0.00
GATCAAAGCCGTGCAAGCCT	0.00	0.00	0.00	0.00	1.38
GATCAAAGCCGTGCCTCTGC	0.00	0.33	0.00	0.00	0.00
GATCAAAGCCGTGCGGCTTA	0.00	0.00	0.29	0.00	0.00

GATCAAAGCCGTGGAACTTA	0.00	0.00	0.00	0.00	0.34
GATCAAAGCCGTGGCAAATA	0.23	0.00	0.00	0.00	0.00
GATCAAAGCCGTGGCAATGA	0.00	0.00	0.29	0.00	0.00
GATCAAAGCCGTGGCAATNA	0.00	0.00	0.00	0.34	0.00
GATCAAAGCCGTGGCAATTA	11.22	7.16	20.90	11.20	15.84
GATCAAAGCCGTGGCAATTG	0.00	0.33	0.00	0.00	0.00
GATCAAAGCCGTGGCAATTN	0.00	0.00	0.00	0.00	0.00
GATCAAAGCCGTGGCACTTA	0.00	0.00	0.29	0.00	0.00
GATCAAAGCCGTTGAAAAAA	0.00	0.33	0.86	0.34	0.69
GATCAAAGCCGTTGAAAAGA	2.75	3.58	2.00	2.04	3.10
GATCAAAGCCGTTGAAAAGT	0.00	0.00	0.86	0.00	0.00
GATCAAAGCCGTTGCCCGCA	0.00	0.00	0.00	0.00	0.34
GATCAAAGCCTAATGCGTTG	0.00	0.00	0.00	0.34	0.34
GATCAAAGCCTACAGCATCG	0.00	0.00	0.00	0.00	0.00
GATCAAAGCCTATGCCAGGG	0.23	0.00	0.00	0.00	0.00
GATCAAAGCCTATGCCAGGT	1.83	1.63	1.43	0.34	0.34
GATCAAAGCCTCACAGTGCT	0.92	0.00	1.15	0.00	0.34
GATCAAAGCCTCACCACGGC	0.00	0.00	0.00	0.00	0.00
GATCAAAGCCTCAGACCTCT	0.00	0.00	0.00	0.34	0.00
GATCAAAGCCTCATGCATTT	0.00	1.30	0.29	0.34	0.00
GATCAAAGCCTCCAAGAATG	0.00	0.65	0.00	0.00	0.00
GATCAAAGCCTCCAAGCCCT	0.00	0.00	0.29	0.00	0.00
GATCAAAGCCTCGACGACGA	0.00	0.00	0.00	0.00	0.00
GATCAAAGCCTCGCCGCCTC	0.00	0.33	0.00	0.00	0.00
GATCAAAGCCTCGCGTGAAG	0.00	0.00	0.29	0.00	0.00
GATCAAAGCCTCGTTTCACA	0.00	0.00	0.00	0.00	0.00
GATCAAAGCCTCTGCATTGC	0.00	0.00	0.00	0.00	0.34
GATCAAAGCCTCTGGTTTCA	0.23	0.00	0.29	0.00	0.00
GATCAAAGCCTGAAACTATG	0.00	0.00	0.00	0.34	0.00
GATCAAAGCCTGAAGTGCAC	0.00	0.33	0.00	0.00	0.00
GATCAAAGCCTGAGGCCATC	0.00	0.00	0.00	0.00	0.00
GATCAAAGCCTGCACGGAGG	0.00	0.00	0.00	0.00	0.00
GATCAAAGCCTGCAGGCGGC	0.00	0.00	0.00	0.00	0.00
GATCAAAGCCTGCTCGCTGT	0.00	0.00	0.00	0.00	0.00
GATCAAAGCCTGCTGGACGG	18.54	11.39	10.02	8.49	11.37
GATCAAAGCCTGCTGGCCGG	0.23	0.00	0.00	0.00	0.00
GATCAAAGCCTGCTGGCNNN	0.23	0.00	0.00	0.00	0.00

GATCAAAGCCTGCTGGGCGG	0.00	0.00	0.00	0.34	0.00
GATCAAAGCCTGCTGGNNNN	0.46	0.00	0.00	0.00	0.00
GATCAAAGCCTGCTGTGTCG	0.46	0.00	0.00	0.00	0.00
GATCAAAGCCTGGCAGAGAC	0.00	0.00	0.00	0.00	0.34
GATCAAAGCCTGGCAGAGCC	0.00	0.00	0.00	0.00	0.34
GATCAAAGCCTGGCATTTCA	0.00	0.00	0.00	0.00	0.00
GATCAAAGCCTGGTATGACT	0.00	0.00	0.00	0.00	0.00
GATCAAAGCCTGGTTGGCTG	0.00	0.00	0.00	0.00	0.00
GATCAAAGCCTGTATCGGTA	0.46	0.33	0.00	0.00	0.34
GATCAAAGCCTGTGCCAGGT	1.60	3.25	1.72	3.39	2.41
GATCAAAGCCTTATGCGTTG	0.00	0.00	0.00	0.34	0.00
GATCAAAGCCTTATTCCTTC	0.00	0.33	0.00	0.34	0.00
GATCAAAGCCTTCAGCCTNN	0.00	0.00	0.00	0.00	0.34
GATCAAAGCCTTCAGCCTTC	1.37	4.23	1.15	1.02	2.07
GATCAAAGCCTTCGTATGCC	0.00	0.00	0.29	0.00	0.00
GATCAAAGCCTTCGTGGGCC	0.00	0.00	0.00	0.00	0.00
GATCAAAGCCTTCTGGCAAG	0.23	0.00	0.00	0.00	0.00
GATCAAAGCCTTGCACCATT	0.00	0.33	0.00	0.00	0.00
GATCAAAGCCTTGGAGAGAG	0.00	0.33	0.86	0.34	0.00
GATCAAAGCCTTGGTTCAGC	0.00	0.00	0.00	0.34	0.00
GATCAAAGCCTTTGGCGAGA	0.00	0.00	0.00	0.00	0.34
GATCAAAGCCTTTGTGTGAT	0.00	0.00	0.00	0.34	0.00
GATCAAAGCCTTTTTGATCC	0.00	0.00	0.00	0.00	0.00
GATCAAAGCGAAGCACAAAG	0.23	0.00	0.00	0.00	0.00
GATCAAAGCGAAGCAGAGCA	0.00	0.00	0.00	0.34	0.00
GATCAAAGCGAAGGGCCTTT	0.00	0.00	0.00	0.00	0.00
GATCAAAGCGAATCGCGTTT	0.00	0.00	0.00	0.00	0.00
GATCAAAGCGAATTGGCTGC	0.23	0.33	0.00	0.00	0.00
GATCAAAGCGACAACTGTTC	0.23	0.00	0.00	0.00	0.00
GATCAAAGCGACATTTTGAG	0.00	0.00	0.00	0.00	0.00
GATCAAAGCGACCACCTCCG	0.00	0.00	0.00	0.00	0.34
GATCAAAGCGACCAGCGAAT	0.00	0.00	0.29	0.00	0.00
GATCAAAGCGACCTTGTGAG	0.00	0.00	0.00	0.00	0.00
GATCAAAGCGACGTCGACAT	0.00	0.00	0.00	0.00	0.00
GATCAAAGCGACTCCAGCGA	0.00	0.00	0.00	0.34	0.00
GATCAAAGCGACTTGACACA	0.92	0.33	1.15	1.70	0.00
GATCAAAGCGACTTGACACC	0.23	0.00	0.00	0.00	0.00

GATCAAAGCGACTTGACCCA	0.00	0.00	0.00	0.34	0.00
GATCAAAGCGACTTGACCGA	0.00	0.00	0.00	0.34	0.00
GATCAAAGCGAGAAGGGGGG	0.00	0.33	0.00	0.00	0.00
GATCAAAGCGAGAGAGGAGG	0.23	0.33	0.00	0.68	0.00
GATCAAAGCGAGCAGTTTTG	0.00	0.00	0.00	0.68	0.00
GATCAAAGCGAGGATGAAGT	0.00	0.33	0.00	0.00	0.00
GATCAAAGCGAGGCTCGAGC	0.00	0.33	0.00	0.34	0.00
GATCAAAGCGAGTGAGAGAA	0.23	0.00	0.00	0.00	0.69
GATCAAAGCGAGTGAGAGAT	0.00	0.00	0.29	0.00	0.00
GATCAAAGCGAGTGAGTCCA	0.00	0.33	0.00	0.00	0.00
GATCAAAGCGAGTGCCAGCC	0.00	0.33	0.00	0.00	0.00
GATCAAAGCGATGGAGACGT	0.00	0.00	0.29	0.00	0.34
GATCAAAGCGATTCTGTCTT	0.00	0.00	0.00	0.34	0.00
GATCAAAGCGCAAAAAAAAA	0.00	0.00	0.29	0.00	0.00
GATCAAAGCGCAACGAGCGG	0.00	0.00	0.29	0.00	0.00
GATCAAAGCGCAAGGAATTC	0.46	0.00	0.00	0.34	0.00
GATCAAAGCGCAAGGCCGCT	0.23	0.00	0.00	0.00	0.00
GATCAAAGCGCAAGGCCGTG	0.00	0.00	0.00	0.34	0.00
GATCAAAGCGCAAGGCTGCT	4.12	3.25	1.15	1.70	1.38
GATCAAAGCGCAAGGCTGGT	0.00	0.00	0.29	0.00	0.00
GATCAAAGCGCAAGGGTAGA	0.00	0.00	0.29	0.00	0.00
GATCAAAGCGCACAGAGCAT	0.00	0.00	0.00	0.34	0.00
GATCAAAGCGCAGACAGCCG	0.00	0.33	0.00	0.00	0.00
GATCAAAGCGCAGCACAGCG	0.00	0.00	0.00	0.00	0.69
GATCAAAGCGCAGCCGGCCA	0.00	0.00	0.00	0.00	0.00
GATCAAAGCGCAGCGGCTCC	0.00	0.33	0.00	0.00	0.00
GATCAAAGCGCAGCTCTTGA	0.00	0.33	0.00	0.00	0.00
GATCAAAGCGCAGGACCGGC	0.00	1.63	0.00	0.34	0.00
GATCAAAGCGCAGGCGGCAG	0.00	0.33	0.00	0.00	0.00
GATCAAAGCGCAGGCTCACA	0.00	0.00	0.00	0.00	0.34
GATCAAAGCGCAGGCTTGGC	0.00	0.33	0.00	0.00	0.00
GATCAAAGCGCAGGCTTGGT	0.23	0.00	0.00	0.00	0.34
GATCAAAGCGCATTGAGCGA	0.69	0.00	0.29	0.00	0.34
GATCAAAGCGCATTGAGCGG	1.37	0.33	0.57	0.00	1.03
GATCAAAGCGCCAAAAAAAA	0.00	0.33	0.00	0.00	0.00
GATCAAAGCGCCAAAAAAAC	0.00	0.00	0.29	0.00	0.00
GATCAAAGCGCCAGAAAAAA	0.00	0.00	0.00	0.34	0.00

GATCAAAGCGCCAGAGCTGC	0.00	0.33	0.00	0.00	0.00
GATCAAAGCGCCAGCATTTC	0.00	0.00	0.29	0.00	0.00
GATCAAAGCGCCAGCCGCTC	0.00	0.00	0.00	0.00	0.34
GATCAAAGCGCCAGGGCTGC	0.00	0.00	0.29	0.00	0.00
GATCAAAGCGCCAGTCCTCC	0.00	0.65	0.00	0.00	0.00
GATCAAAGCGCCAGTCCTGT	0.00	0.00	0.29	0.00	0.00
GATCAAAGCGCCAGTGATGC	0.00	0.00	0.00	0.00	0.34
GATCAAAGCGCCAGTGCCAT	0.00	0.00	0.29	0.00	0.00
GATCAAAGCGCCAGTGCCGC	0.00	0.00	0.00	0.34	0.34
GATCAAAGCGCCAGTGCCTC	0.00	0.00	0.00	0.34	0.00
GATCAAAGCGCCAGTGCTCC	0.00	0.00	0.00	0.34	0.00
GATCAAAGCGCCAGTGCTGA	0.00	0.00	0.29	0.00	0.34
GATCAAAGCGCCAGTGCTGC	9.39	16.92	14.60	24.10	22.04
GATCAAAGCGCCAGTGCTGG	0.00	0.33	0.00	0.00	0.00
GATCAAAGCGCCAGTGGCAC	0.00	0.00	0.00	0.34	0.00
GATCAAAGCGCCAGTGNNNN	0.23	0.00	0.00	0.00	0.00
GATCAAAGCGCCATTTTGAG	0.46	0.00	0.29	1.02	0.69
GATCAAAGCGCCATTTTGAT	0.00	0.00	0.00	0.00	0.00
GATCAAAGCGCCATTTTGTG	0.00	0.00	0.00	0.00	0.34
GATCAAAGCGCCCAGCGGCG	0.23	0.00	0.00	0.00	0.00
GATCAAAGCGCCCGTGCTGC	0.23	0.00	0.00	0.00	0.00
GATCAAAGCGCCGAAGTCGA	0.00	0.00	0.00	0.00	0.00
GATCAAAGCGCCGGATTAGC	0.00	0.00	0.29	0.00	0.00
GATCAAAGCGCCGGTGCTGC	0.00	0.00	0.29	0.00	0.00
GATCAAAGCGCCGTCTGCTG	0.00	0.00	0.29	0.00	0.00
GATCAAAGCGCCGTCTGTCG	0.00	0.00	0.29	0.00	0.00
GATCAAAGCGCCTCATGGCG	0.00	0.00	0.00	0.00	0.00
GATCAAAGCGCCTTCGCCGG	0.00	0.00	0.00	0.00	0.00
GATCAAAGCGCGAACACCAA	0.00	0.00	0.00	0.00	0.34
GATCAAAGCGCGAAGCGCCC	0.00	0.33	0.00	0.00	0.00
GATCAAAGCGCGAGGGCATT	0.00	0.00	0.00	0.34	0.00
GATCAAAGCGCGAGGGCCTT	2.98	5.86	2.29	5.09	0.34
GATCAAAGCGCGATGTCTGC	0.00	0.00	0.00	0.34	0.00
GATCAAAGCGCGCATGCAAC	0.00	0.00	0.00	0.00	0.00
GATCAAAGCGCGCGAGCATG	0.00	0.00	0.00	0.00	0.34
GATCAAAGCGCGCGAGCGTG	3.43	4.23	2.86	1.02	2.76
GATCAAAGCGCGCTGGGTGG	0.00	0.00	0.29	0.00	0.00

GATCAAAGCGCGGAAGAAGC	0.00	0.00	0.29	0.00	0.00
GATCAAAGCGCGGAAGCAGT	0.46	0.00	0.00	0.00	0.00
GATCAAAGCGCGGACTGCCA	0.00	0.33	0.00	0.00	0.00
GATCAAAGCGCGGAGAACCG	0.00	0.33	0.00	0.68	0.00
GATCAAAGCGCGGGCATTCT	0.00	0.00	0.29	0.00	0.00
GATCAAAGCGCGGGGTATTT	0.00	0.00	0.29	0.00	0.00
GATCAAAGCGCGTGCTTGTT	0.23	0.00	0.00	0.00	0.00
GATCAAAGCGCTACCTTGCT	0.00	0.00	0.00	0.34	0.00
GATCAAAGCGCTCCACAACC	0.00	0.00	0.00	0.00	0.00
GATCAAAGCGCTCCGTGTTC	0.00	0.00	0.00	0.00	0.00
GATCAAAGCGCTCGATTAGC	0.00	0.00	0.29	0.00	0.00
GATCAAAGCGCTCGCAGGGC	2.75	0.33	0.57	0.00	0.00
GATCAAAGCGCTCGGCGGGC	0.00	0.00	0.00	0.00	0.00
GATCAAAGCGCTGCAGTCAG	0.00	0.00	0.00	0.34	0.00
GATCAAAGCGCTGCGCTCCT	0.00	0.00	0.29	0.00	0.00
GATCAAAGCGCTGCTACTGT	0.00	0.00	0.00	0.00	0.00
GATCAAAGCGCTGCTGAACG	0.00	0.65	0.00	0.34	1.03
GATCAAAGCGCTGCTGCACG	0.00	0.00	0.00	0.00	0.00
GATCAAAGCGCTGGATCAGC	0.00	0.00	0.00	0.34	0.00
GATCAAAGCGCTGGATNNNN	0.23	0.00	0.00	0.00	0.00
GATCAAAGCGCTGGATTAGA	0.23	0.33	0.29	0.00	0.00
GATCAAAGCGCTGGATTAGC	30.44	49.78	25.77	48.20	29.62
GATCAAAGCGCTGGATTAGN	0.23	0.00	0.00	0.00	0.00
GATCAAAGCGCTGGATTAGT	0.00	0.33	0.00	0.00	0.00
GATCAAAGCGCTGGATTANN	0.23	0.00	0.00	0.00	0.00
GATCAAAGCGCTGGATTCGC	0.00	0.00	0.00	0.00	0.34
GATCAAAGCGCTGGCGTAGC	0.23	0.00	0.00	0.00	0.00
GATCAAAGCGCTGGGTTAGC	0.23	0.00	0.29	0.00	0.00
GATCAAAGCGCTTCCCAACA	0.00	0.00	0.29	0.00	0.00
GATCAAAGCGCTTCTGCTGG	0.00	0.33	0.29	0.00	0.00
GATCAAAGCGGAAGCGACCA	0.23	0.00	0.00	0.00	0.00
GATCAAAGCGGAAGTACTAG	0.00	0.00	0.29	0.00	0.00
GATCAAAGCGGAATGCTGCA	0.00	0.00	0.29	0.00	0.00
GATCAAAGCGGAATTACNNN	0.00	0.00	0.29	0.00	0.00
GATCAAAGCGGAATTACTCA	0.00	0.00	0.29	0.00	0.00
GATCAAAGCGGAATTACTCC	13.05	21.15	12.03	12.56	16.88
GATCAAAGCGGAATTACTNN	0.00	0.00	0.00	0.00	0.00

GATCAAAGCGGAATTACTTC	0.00	0.00	0.00	0.00	0.00
GATCAAAGCGGAATTATTCC	0.00	0.00	0.00	0.00	0.00
GATCAAAGCGGACAGATCAG	0.00	0.33	0.00	0.00	0.00
GATCAAAGCGGACAGCTCAG	0.00	0.33	0.00	0.34	0.00
GATCAAAGCGGACTCTCCCG	0.46	0.00	0.00	0.00	0.00
GATCAAAGCGGAGAAATGGC	0.00	0.33	0.00	0.00	0.00
GATCAAAGCGGAGCGGAGTG	0.23	0.00	0.00	0.00	0.00
GATCAAAGCGGAGGAGGAGT	0.00	0.00	0.00	0.00	0.00
GATCAAAGCGGAGGCAGCAG	0.00	0.00	0.00	0.00	0.34
GATCAAAGCGGAGGGGATGT	0.23	0.00	0.00	0.00	0.00
GATCAAAGCGGATGTGAATA	0.23	1.63	0.00	0.68	0.00
GATCAAAGCGGATGTTGGGC	0.00	0.00	0.00	0.34	0.00
GATCAAAGCGGCACCGCCGC	0.00	0.00	0.29	0.00	0.00
GATCAAAGCGGCAGAGGGAT	0.00	0.00	0.00	0.00	0.00
GATCAAAGCGGCCATTGGAC	0.00	0.33	0.00	0.00	0.00
GATCAAAGCGGCCCGTCGGG	0.00	0.00	0.29	0.00	0.00
GATCAAAGCGGCGGAAGAGT	0.00	0.00	0.00	0.34	0.00
GATCAAAGCGGCGGACCTCT	0.00	0.33	0.00	0.00	0.00
GATCAAAGCGGCGGAGGAGA	0.23	0.33	0.00	0.00	0.00
GATCAAAGCGGCGGAGGAGC	0.23	0.00	0.00	0.00	0.00
GATCAAAGCGGCGGAGGAGG	1.37	0.00	0.00	0.00	0.00
GATCAAAGCGGCGGAGGAGN	0.00	0.00	0.00	0.00	0.00
GATCAAAGCGGCGGAGGAGT	15.11	10.41	2.00	10.18	4.13
GATCAAAGCGGCGGAGGCGT	0.69	0.00	0.00	0.00	0.00
GATCAAAGCGGCGGAGGGGG	1.14	0.00	0.00	0.34	0.00
GATCAAAGCGGCGGAGGGGT	5.04	0.33	0.00	0.34	0.00
GATCAAAGCGGCGGAGGTGT	0.23	0.00	0.00	0.00	0.00
GATCAAAGCGGCGGCGGAGG	0.00	0.33	0.00	0.00	0.00
GATCAAAGCGGCGGCGGCGT	0.23	0.00	0.00	0.00	0.00
GATCAAAGCGGCGGGGGAGC	0.23	0.00	0.00	0.00	0.00
GATCAAAGCGGCGGGGGAGG	0.00	0.00	0.00	0.00	0.34
GATCAAAGCGGCGGGGGAGT	0.23	0.00	0.00	0.00	0.00
GATCAAAGCGGCGGGGGCGG	0.23	0.00	0.00	0.00	0.00
GATCAAAGCGGCGGGGGGGC	0.23	0.00	0.00	0.00	0.00
GATCAAAGCGGCGGGGGGGG	0.23	0.00	0.00	0.00	0.00
GATCAAAGCGGCGGGGGGGT	0.92	0.00	0.00	0.00	0.00
GATCAAAGCGGCTAGGCGGA	0.00	0.00	0.00	0.00	0.00

GATCAAAGCGGCTATGTCTC	0.00	0.00	0.00	0.00	0.00
GATCAAAGCGGCTCGTAGGG	0.00	0.00	0.00	0.00	0.00
GATCAAAGCGGCTGGGGCAC	0.00	0.00	0.00	0.00	0.00
GATCAAAGCGGCTGGGGCAT	0.23	0.00	0.29	0.00	0.69
GATCAAAGCGGCTGGGGCCT	0.00	0.00	0.29	0.34	0.00
GATCAAAGCGGCTGGGGGAT	0.00	0.65	0.57	1.02	0.34
GATCAAAGCGGCTGGGGGGT	0.00	0.00	0.00	0.34	0.69
GATCAAAGCGGCTGTGGCAT	0.00	0.33	0.00	0.00	0.00
GATCAAAGCGGCTGTGGCGT	0.00	0.00	0.29	0.00	0.00
GATCAAAGCGGCTGTGGGAT	0.00	0.00	0.00	0.00	0.34
GATCAAAGCGGCTGTGGGCT	0.00	0.33	0.00	0.00	0.00
GATCAAAGCGGCTTTTGCGC	0.00	0.00	0.00	0.00	0.69
GATCAAAGCGGGAAAGCGCT	0.00	0.00	0.00	0.00	0.00
GATCAAAGCGGGAACGTCCG	0.23	0.00	0.00	0.00	0.00
GATCAAAGCGGGACACCACC	0.00	0.00	0.57	0.00	0.00
GATCAAAGCGGGCGCCTAGT	0.00	0.00	0.00	0.34	0.00
GATCAAAGCGGGCGCCTCGT	0.23	0.65	0.00	0.34	0.00
GATCAAAGCGGGCGCCTGGT	0.69	0.00	0.00	0.00	0.34
GATCAAAGCGGGGACAACAA	0.23	0.00	0.00	0.00	0.00
GATCAAAGCGGGGCGATGGG	0.00	0.00	0.00	0.00	0.34
GATCAAAGCGGGGCGGGGGG	0.00	0.00	0.29	0.00	0.00
GATCAAAGCGGGGCGTTGGG	0.23	0.00	0.00	0.00	0.00
GATCAAAGCGGTAGTATTCG	0.00	0.65	0.57	0.00	0.00
GATCAAAGCGGTATTACTCC	0.00	0.00	0.29	0.00	0.00
GATCAAAGCGGTCTTGCTGC	0.69	0.00	0.00	0.00	0.00
GATCAAAGCGGTGGATTAGC	0.00	0.00	0.00	0.00	0.00
GATCAAAGCGGTGGCAGCAC	0.00	0.00	0.29	0.00	0.00
GATCAAAGCGGTGTTTGCCG	0.23	0.33	0.00	1.02	0.34
GATCAAAGCGTAGATGATGC	0.69	0.98	0.86	1.70	1.03
GATCAAAGCGTCAGGATATC	0.00	0.00	0.29	0.00	0.00
GATCAAAGCGTCCGCAGTTG	0.00	0.00	0.00	0.00	0.34
GATCAAAGCGTCCGTCAGCC	0.00	0.33	0.00	0.00	0.00
GATCAAAGCGTCCGTCAGGA	0.00	0.00	0.00	0.00	0.00
GATCAAAGCGTCGAAAAAAA	0.23	0.00	0.00	0.00	0.00
GATCAAAGCGTCGTGGAAGG	0.00	0.00	0.00	0.00	0.34
GATCAAAGCGTCGTGGTCCN	0.23	0.00	0.00	0.00	0.00
GATCAAAGCGTCTGAGTAAA	0.23	0.00	0.00	0.00	0.00

GATCAAAGCGTGCCCAGGTG	0.00	0.33	0.00	0.00	0.00
GATCAAAGCGTGCCGCCGGC	0.00	0.00	0.00	0.00	0.34
GATCAAAGCGTGCCGGAAAA	0.00	0.00	0.00	0.34	0.00
GATCAAAGCGTGCCGGAAAC	0.46	0.00	0.00	0.00	0.00
GATCAAAGCGTGGCTGAAGA	0.46	0.65	0.00	0.68	1.03
GATCAAAGCGTGGCTTCGAA	0.00	0.00	0.00	0.00	0.00
GATCAAAGCGTGGCTTCGAC	4.12	0.98	0.00	0.00	0.00
GATCAAAGCGTGTGGAATGC	0.00	0.00	0.00	0.00	0.00
GATCAAAGCGTTAAAAAAAA	0.23	0.00	0.00	0.00	0.00
GATCAAAGCGTTCGTGGACT	0.00	1.63	0.00	1.02	0.00
GATCAAAGCGTTGAAAAAAA	0.00	0.00	0.00	0.34	0.00
GATCAAAGCGTTGGTGCATA	0.23	0.00	0.00	0.00	0.00
GATCAAAGCGTTGTTTTGAG	0.00	0.00	0.00	0.00	0.00
GATCAAAGCGTTTGAGAGGG	0.00	0.00	0.29	0.00	0.00
GATCAAAGCGTTTTGATCGG	0.00	0.00	0.00	0.00	0.34
GATCAAAGCTAAAAAAAAAA	1.37	0.00	0.29	0.00	0.00
GATCAAAGCTAACGGTTTTG	0.00	0.00	0.00	0.34	0.00
GATCAAAGCTAATGCCAGAC	0.46	4.55	1.43	3.73	2.76
GATCAAAGCTAATGCCAGCC	0.00	0.33	0.00	0.00	0.00
GATCAAAGCTACAGTGATGG	0.23	0.00	0.00	0.00	0.00
GATCAAAGCTACAGTTGGCC	0.46	0.33	0.00	0.00	0.00
GATCAAAGCTACGCCCAGTA	0.23	0.00	0.00	1.70	0.00
GATCAAAGCTACTCAAGGCA	0.00	0.00	0.00	0.00	0.00
GATCAAAGCTACTTGGCTGC	0.00	0.00	0.00	0.00	0.00
GATCAAAGCTAGAACAAGGC	0.00	0.00	0.00	0.34	0.00
GATCAAAGCTAGAACCGAGA	0.23	0.33	0.00	0.68	0.00
GATCAAAGCTAGATCTCTGG	0.00	0.00	0.00	0.34	0.00
GATCAAAGCTAGGCGATGGG	0.00	0.00	0.29	0.00	0.00
GATCAAAGCTAGTCTTCGAA	0.00	0.33	0.00	0.00	0.00
GATCAAAGCTATCGGCCCTG	0.00	0.00	0.00	0.34	0.00
GATCAAAGCTATCTTCAACT	0.46	1.30	0.00	1.02	0.00
GATCAAAGCTATCTTGCTCC	0.00	0.00	0.00	0.00	0.00
GATCAAAGCTATGCATGAGA	0.00	0.00	0.29	0.00	0.00
GATCAAAGCTATGCCGGTCA	0.00	0.98	0.29	0.00	0.00
GATCAAAGCTATTAGCCCCA	0.00	0.33	0.00	0.00	0.00
GATCAAAGCTCAAGACTGAC	0.00	0.00	0.00	0.00	1.03
GATCAAAGCTCAAGAGCTCG	0.00	0.00	0.00	0.00	0.00

GATCAAAGCTCAATGTCTGG	0.00	0.00	0.00	0.00	0.00
GATCAAAGCTCAGCGTGGGC	0.69	0.00	0.00	0.00	0.00
GATCAAAGCTCAGCTGCGGC	0.00	0.00	0.00	0.00	0.00
GATCAAAGCTCAGGTCTGGG	0.23	0.33	0.00	0.00	1.72
GATCAAAGCTCAGTTAAGCG	0.92	0.33	0.00	0.68	0.34
GATCAAAGCTCATCAGTGAA	0.00	0.00	0.00	0.68	0.00
GATCAAAGCTCATCGGGGCA	0.00	0.00	0.29	0.00	0.00
GATCAAAGCTCCACGTCTCC	0.00	0.00	0.00	0.00	0.00
GATCAAAGCTCCAGCGAAGG	0.00	0.00	0.00	0.00	0.00
GATCAAAGCTCCATCTTGTC	0.23	0.00	0.00	0.00	0.00
GATCAAAGCTCCATCTTGTG	0.00	0.00	0.00	0.34	0.00
GATCAAAGCTCCATGCACGT	0.00	0.00	0.00	0.00	0.34
GATCAAAGCTCCATTCTGTA	0.00	0.33	0.00	0.00	0.00
GATCAAAGCTCCGCTCAGGA	0.00	0.00	0.00	0.34	0.00
GATCAAAGCTCCGCTCCCAG	0.23	0.00	0.00	0.34	0.00
GATCAAAGCTCCGGGGCAGT	0.00	0.00	0.00	0.00	0.34
GATCAAAGCTCGACGTCTGG	0.00	0.00	0.00	0.00	0.00
GATCAAAGCTCGAGGTCGGA	0.00	0.00	0.00	0.34	0.00
GATCAAAGCTCGAGGTCTGG	0.23	0.00	0.00	0.00	0.00
GATCAAAGCTCGATATCTGG	0.00	0.00	0.00	0.00	0.34
GATCAAAGCTCGATCGTATG	0.23	0.00	0.00	0.00	0.00
GATCAAAGCTCGATGACGGA	0.00	0.00	0.00	0.00	0.34
GATCAAAGCTCGATGATTGA	0.00	0.00	0.00	0.68	0.00
GATCAAAGCTCGATGCATGG	0.00	0.00	0.00	0.34	0.00
GATCAAAGCTCGATGCCTGA	0.00	0.00	0.29	0.00	0.00
GATCAAAGCTCGATGCCTGG	0.23	0.33	0.00	0.00	0.00
GATCAAAGCTCGATGGCTGG	0.23	0.00	0.00	0.00	0.00
GATCAAAGCTCGATGTCAAA	0.00	0.00	0.00	0.00	0.00
GATCAAAGCTCGATGTCAAG	0.00	0.33	0.00	0.00	0.00
GATCAAAGCTCGATGTCCAA	0.00	0.00	0.00	0.68	0.00
GATCAAAGCTCGATGTCCAG	0.00	0.33	0.00	0.00	0.00
GATCAAAGCTCGATGTCCGA	0.00	0.00	0.00	0.00	0.00
GATCAAAGCTCGATGTCCGC	0.23	0.00	0.00	0.00	0.00
GATCAAAGCTCGATGTCCGG	0.00	0.00	0.00	0.34	0.00
GATCAAAGCTCGATGTCCTC	0.00	0.00	0.00	0.00	1.38
GATCAAAGCTCGATGTCGCG	0.00	0.00	0.00	0.34	0.00
GATCAAAGCTCGATGTCGGG	0.23	0.00	0.00	0.00	0.00

GATCAAAGCTCGATGTCGTG	0.00	0.00	0.00	0.34	0.00
GATCAAAGCTCGATGTCNNN	0.23	0.00	0.00	0.00	0.00
GATCAAAGCTCGATGTCTAA	0.00	0.33	0.00	0.00	0.34
GATCAAAGCTCGATGTCTAC	0.00	0.33	0.00	0.00	0.00
GATCAAAGCTCGATGTCTAG	0.00	0.00	0.29	0.00	0.69
GATCAAAGCTCGATGTCTCA	0.00	0.65	0.00	0.00	0.00
GATCAAAGCTCGATGTCTCC	0.00	0.00	0.00	0.34	0.00
GATCAAAGCTCGATGTCTCG	0.00	0.00	0.29	0.00	0.00
GATCAAAGCTCGATGTCTCN	0.00	0.00	0.00	0.34	0.00
GATCAAAGCTCGATGTCTGA	2.52	2.28	1.15	2.72	1.38
GATCAAAGCTCGATGTCTGC	0.00	1.30	0.86	0.00	0.34
GATCAAAGCTCGATGTCTGG	154.74	88.81	54.69	97.42	81.62
GATCAAAGCTCGATGTCTGN	0.23	0.00	0.00	0.00	0.00
GATCAAAGCTCGATGTCTGT	0.00	0.65	0.57	0.34	0.00
GATCAAAGCTCGATGTCTNN	0.23	0.00	0.00	0.34	0.00
GATCAAAGCTCGATGTCTTG	0.00	0.00	0.00	0.00	0.69
GATCAAAGCTCGATGTCTTT	0.00	0.00	0.00	0.00	0.00
GATCAAAGCTCGATGTGAGG	0.00	0.00	0.29	0.00	0.00
GATCAAAGCTCGATGTGGAC	0.00	0.33	0.00	0.00	0.00
GATCAAAGCTCGATGTNNNN	0.46	0.00	0.00	0.00	0.00
GATCAAAGCTCGATGTNTGG	0.00	0.00	0.00	0.00	0.34
GATCAAAGCTCGATGTTTGC	0.00	0.00	0.00	0.00	0.00
GATCAAAGCTCGATGTTTGG	0.23	0.00	0.57	0.00	0.00
GATCAAAGCTCGATTTCTGG	0.23	0.00	0.00	0.34	0.00
GATCAAAGCTCGCTGACTGG	0.23	0.00	0.00	0.00	0.00
GATCAAAGCTCGCTGCCTGG	0.23	0.00	0.00	0.00	0.00
GATCAAAGCTCGCTGCTAGC	0.00	0.00	0.00	0.34	0.00
GATCAAAGCTCGCTGTCTGG	0.23	0.00	0.00	0.34	0.00
GATCAAAGCTCGGCCTGAAA	0.00	0.00	0.00	0.00	0.69
GATCAAAGCTCGGGGAGCGC	0.00	0.00	0.00	0.00	0.00
GATCAAAGCTCGGTGCCTGG	0.23	0.00	0.00	0.00	0.00
GATCAAAGCTCGTATGCCGN	0.23	0.00	0.00	0.00	0.00
GATCAAAGCTCGTATGCCGT	0.23	0.00	0.00	0.00	0.00
GATCAAAGCTCGTTGCTGAG	0.00	0.00	0.00	0.00	0.34
GATCAAAGCTCGTTGTCTGG	0.00	0.00	0.29	0.00	0.00
GATCAAAGCTCTGAAGCAGC	0.00	0.33	0.00	0.00	0.00
GATCAAAGCTCTGAGGTTCG	0.00	0.00	0.00	0.00	0.34

GATCAAAGCTCTGGACTTCC	0.00	0.00	0.29	0.00	0.00
GATCAAAGCTCTGGCATGCG	0.00	0.00	0.00	0.00	0.00
GATCAAAGCTCTGTCACCTG	0.69	0.65	1.43	0.00	0.34
GATCAAAGCTCTTCACTGCT	0.00	0.00	0.00	0.00	0.00
GATCAAAGCTCTTCCAGGGC	0.00	0.00	0.00	0.00	0.00
GATCAAAGCTCTTCTCAGCT	0.00	0.00	0.00	0.00	0.00
GATCAAAGCTCTTCTCTGCT	7.78	11.71	12.31	9.50	9.99
GATCAAAGCTCTTCTCTGNN	0.00	0.00	0.00	0.00	0.00
GATCAAAGCTCTTTCAGGGC	0.00	0.00	0.00	0.00	0.34
GATCAAAGCTCTTTCGTATG	0.00	0.00	0.00	0.00	0.00
GATCAAAGCTGAAAAAAAAA	0.00	0.65	0.00	0.00	0.00
GATCAAAGCTGAAATGTTTT	0.23	0.00	0.00	0.00	0.00
GATCAAAGCTGAACGTCTTT	0.00	0.00	0.00	0.34	0.00
GATCAAAGCTGAAGACGTCT	0.23	0.00	0.00	0.00	0.00
GATCAAAGCTGAAGTCATCA	0.00	0.00	0.00	0.00	0.00
GATCAAAGCTGACAGCTCCG	0.00	0.33	0.00	0.00	0.00
GATCAAAGCTGACCAAAGAA	0.69	0.00	0.00	0.00	0.00
GATCAAAGCTGACGGCAAGG	0.00	0.00	0.00	0.00	0.34
GATCAAAGCTGAGAAACAGA	0.00	0.00	0.29	0.00	0.00
GATCAAAGCTGAGCGATGGG	0.00	0.00	0.00	0.00	0.34
GATCAAAGCTGAGGCTGGAG	0.00	0.00	0.29	0.00	0.00
GATCAAAGCTGAGGGCACAA	0.00	0.00	0.00	0.00	0.00
GATCAAAGCTGAGGGTCACA	0.00	0.00	0.00	0.34	0.00
GATCAAAGCTGAGGGTCACT	0.23	0.65	0.29	0.00	0.00
GATCAAAGCTGAGTGTAGCG	0.00	0.00	0.00	0.00	0.34
GATCAAAGCTGATGGCATGC	0.23	0.00	0.00	0.00	0.00
GATCAAAGCTGATGTGTTTC	0.00	0.00	0.86	0.00	0.00
GATCAAAGCTGCACAATGAA	0.00	0.00	0.00	0.68	0.34
GATCAAAGCTGCAGAAGTTG	0.23	0.33	0.00	0.00	0.00
GATCAAAGCTGCAGACCTCT	0.00	0.33	0.29	0.34	0.34
GATCAAAGCTGCAGCCCTCT	0.23	0.00	0.00	0.00	0.00
GATCAAAGCTGCATCAGAGC	0.23	0.00	0.00	0.00	0.00
GATCAAAGCTGCCAAGCTCG	0.00	0.33	0.29	0.00	0.00
GATCAAAGCTGCCCACGGAC	0.00	0.00	0.00	0.00	0.00
GATCAAAGCTGCCGTCATGA	0.00	0.00	0.00	0.00	0.34
GATCAAAGCTGCCGTGACGA	0.69	1.63	0.86	1.70	3.44
GATCAAAGCTGCCGTGCGCA	0.00	0.00	0.00	0.34	0.00

GATCAAAGCTGCCTACGAGG	2.06	1.63	2.29	0.00	2.41
GATCAAAGCTGCCTCAACAG	0.00	0.00	0.29	0.00	0.00
GATCAAAGCTGCCTTGATAA	0.00	0.33	0.00	0.00	0.34
GATCAAAGCTGCGAGCTCCT	0.00	0.00	0.29	0.00	0.00
GATCAAAGCTGCGCAAGAGA	0.00	0.00	0.00	0.00	0.00
GATCAAAGCTGCGCATGAGA	0.00	0.00	0.29	0.00	0.00
GATCAAAGCTGCGGTGACGT	0.00	0.33	0.00	0.00	0.34
GATCAAAGCTGCTACAGGCA	0.00	0.00	0.29	0.00	0.00
GATCAAAGCTGCTCAAGGAA	0.23	0.00	0.00	0.00	0.00
GATCAAAGCTGCTCAAGGCA	18.77	26.68	23.77	33.94	29.96
GATCAAAGCTGCTCAAGGCC	0.00	0.33	0.00	0.00	0.00
GATCAAAGCTGCTCAAGGCG	0.00	0.00	0.00	0.34	0.00
GATCAAAGCTGCTCAAGGCN	0.23	0.00	0.00	0.00	0.00
GATCAAAGCTGCTCAAGGCT	0.23	0.00	0.00	0.00	0.00
GATCAAAGCTGCTCACATGC	0.00	0.00	0.00	0.00	0.34
GATCAAAGCTGCTCAGGGCA	0.00	0.00	0.00	0.00	0.00
GATCAAAGCTGCTCCAGGCA	0.23	0.00	0.00	0.00	0.00
GATCAAAGCTGCTCCATTTC	0.00	0.00	0.00	0.34	0.00
GATCAAAGCTGCTCGCAAAA	0.00	0.33	0.00	0.00	0.00
GATCAAAGCTGCTCGCATGA	0.00	0.00	0.00	0.34	0.00
GATCAAAGCTGCTCGCATGC	17.17	10.09	8.59	8.49	11.37
GATCAAAGCTGCTCGCATNN	0.23	0.00	0.00	0.00	0.00
GATCAAAGCTGCTCGCCTGC	0.23	0.00	0.00	0.00	0.00
GATCAAAGCTGCTCGTATGC	0.00	0.00	0.29	0.00	0.34
GATCAAAGCTGCTCTGGGAC	0.00	0.00	0.00	0.00	0.00
GATCAAAGCTGCTGGAGGTC	0.00	0.00	0.00	0.00	0.00
GATCAAAGCTGCTTGGTCTG	0.00	0.00	0.29	0.00	0.00
GATCAAAGCTGGACACCTCT	0.00	0.00	0.00	0.34	0.00
GATCAAAGCTGGACGATGGG	0.00	0.33	0.00	0.00	0.00
GATCAAAGCTGGACTGCGTC	0.23	0.00	0.00	0.00	0.00
GATCAAAGCTGGAGCAGCTG	0.00	0.33	0.00	0.00	0.00
GATCAAAGCTGGAGGCCGTG	0.00	0.00	0.57	0.00	0.00
GATCAAAGCTGGAGGGGCGG	0.23	0.33	0.00	0.00	0.00
GATCAAAGCTGGCAAGGGGA	0.00	0.00	0.29	0.00	0.00
GATCAAAGCTGGCGACTTCC	0.23	0.33	0.86	0.00	0.00
GATCAAAGCTGGCGCTGGCC	0.00	0.00	0.00	0.00	0.34
GATCAAAGCTGGCGGCAAGG	0.00	0.00	0.00	0.34	0.00

GATCAAAGCTGGCGGGAGTC	0.00	0.00	0.29	0.00	0.00
GATCAAAGCTGGCGGGCGTC	0.23	0.33	0.00	0.00	0.00
GATCAAAGCTGGCGTGACGC	0.46	0.33	0.00	0.68	0.00
GATCAAAGCTGGCTCGCCGA	0.00	0.65	0.57	0.68	0.34
GATCAAAGCTGGCTTCGAAG	0.00	0.00	0.00	0.68	0.00
GATCAAAGCTGGGAGATGGA	0.00	0.00	0.00	0.00	0.34
GATCAAAGCTGGGAGATGGG	0.23	0.98	0.00	0.00	0.34
GATCAAAGCTGGGAGATGGT	0.00	0.00	0.29	0.00	0.00
GATCAAAGCTGGGAGGTGGG	0.23	0.00	0.00	0.00	0.00
GATCAAAGCTGGGCAATGGG	0.00	0.00	0.29	0.00	0.34
GATCAAAGCTGGGCATAATC	0.00	0.00	0.29	0.00	0.00
GATCAAAGCTGGGCGAAGCG	0.00	0.00	0.00	0.00	0.34
GATCAAAGCTGGGCGAAGGG	0.23	0.00	1.15	0.34	0.00
GATCAAAGCTGGGCGACCGT	0.00	0.00	0.00	0.00	0.00
GATCAAAGCTGGGCGACGGG	0.46	0.33	0.29	0.00	0.69
GATCAAAGCTGGGCGAGGGG	0.46	0.33	0.86	0.34	0.34
GATCAAAGCTGGGCGANGGG	0.00	0.33	0.00	0.00	1.03
GATCAAAGCTGGGCGATCGA	0.00	0.00	0.00	0.34	0.00
GATCAAAGCTGGGCGATGAG	0.00	0.00	0.00	0.00	0.34
GATCAAAGCTGGGCGATGCC	0.00	0.00	0.29	0.00	0.00
GATCAAAGCTGGGCGATGGC	0.23	0.00	0.29	0.00	0.34
GATCAAAGCTGGGCGATGGG	122.92	149.00	184.99	113.03	147.0[illegible]
GATCAAAGCTGGGCGATGGN	0.00	0.00	0.00	0.34	0.00
GATCAAAGCTGGGCGATGGT	0.00	0.33	0.57	0.00	0.34
GATCAAAGCTGGGCGATGNG	0.00	0.00	0.00	0.34	0.00
GATCAAAGCTGGGCGATGNN	0.46	0.00	0.00	0.00	0.00
GATCAAAGCTGGGCGATNNN	0.23	0.00	0.00	0.00	0.00
GATCAAAGCTGGGCGATTGG	0.00	0.33	0.00	0.00	0.00
GATCAAAGCTGGGCGATTGT	0.00	0.00	0.00	0.00	0.34
GATCAAAGCTGGGCGCTGAG	0.00	0.00	0.00	0.34	0.00
GATCAAAGCTGGGCGCTGGG	0.00	0.00	0.00	1.02	0.34
GATCAAAGCTGGGCGGGGGG	0.23	0.00	0.00	0.00	0.00
GATCAAAGCTGGGCGGTGGG	0.23	0.00	0.00	0.00	0.00
GATCAAAGCTGGGCGTCGGG	0.23	0.00	0.00	0.00	0.00
GATCAAAGCTGGGCGTTGGG	0.23	0.00	0.29	0.00	0.00
GATCAAAGCTGGGCTGCAGT	0.00	0.00	0.00	0.00	0.00
GATCAAAGCTGGGCTGCTGG	0.23	0.00	0.00	0.00	0.00

GATCAAAGCTGGGCTTTCAG	0.00	0.00	0.29	0.00	0.00
GATCAAAGCTGGGGCCCTAG	0.00	0.00	0.00	0.00	0.34
GATCAAAGCTGGGGGTTGGG	0.23	0.00	0.00	0.00	0.00
GATCAAAGCTGGGTGATGGG	0.00	0.00	0.00	0.00	0.34
GATCAAAGCTGGTACCGCGC	0.00	0.00	0.00	0.00	0.34
GATCAAAGCTGGTACCGCGN	0.00	0.00	0.00	0.00	0.00
GATCAAAGCTGGTATGCCAC	4.35	2.28	1.15	2.72	2.07
GATCAAAGCTGGTATGCCCC	0.00	0.33	0.00	0.00	0.00
GATCAAAGCTGGTGGGAGTG	0.00	0.00	0.29	0.00	0.00
GATCAAAGCTGTATGCAGAT	0.00	0.00	0.00	0.00	0.00
GATCAAAGCTGTGACTTTGC	0.00	0.00	0.00	0.00	0.69
GATCAAAGCTGTGGCTGGCA	0.69	0.33	0.86	0.34	0.00
GATCAAAGCTGTGGCTGGCC	0.23	0.33	0.00	0.00	0.00
GATCAAAGCTGTGGCTGGCG	0.46	0.00	0.00	0.00	0.00
GATCAAAGCTGTGGCTGGGA	0.00	0.00	0.57	0.68	0.00
GATCAAAGCTGTGGCTGGGC	0.00	0.00	0.00	0.00	0.00
GATCAAAGCTGTGGCTGGGG	0.46	0.00	0.00	0.34	0.69
GATCAAAGCTGTGGCTGGTA	0.00	0.33	0.00	0.34	0.00
GATCAAAGCTGTGGCTGTCA	0.92	0.98	0.29	0.34	0.00
GATCAAAGCTGTGGCTGTCC	0.23	0.65	0.00	0.00	0.00
GATCAAAGCTGTGGCTGTGA	0.46	0.00	0.00	0.00	0.00
GATCAAAGCTGTGGCTGTTA	0.00	0.33	0.29	0.00	0.00
GATCAAAGCTGTGGCTGTTC	0.00	0.00	0.29	0.00	0.00
GATCAAAGCTGTTGCACTGG	0.00	0.65	0.00	0.00	0.00
GATCAAAGCTGTTTAAAAGT	0.23	0.00	0.00	0.00	0.00
GATCAAAGCTTAAGGCTCTT	0.23	0.00	0.00	0.00	0.00
GATCAAAGCTTATGCTGACA	0.00	0.00	0.00	0.34	0.00
GATCAAAGCTTATGGAGGAA	0.00	0.00	0.00	0.00	0.00
GATCAAAGCTTCAAGAAGTA	0.23	0.00	0.00	0.00	0.00
GATCAAAGCTTCACGACAAC	0.00	0.00	0.29	0.00	0.00
GATCAAAGCTTCACGACAAG	0.00	0.00	0.57	0.34	1.03
GATCAAAGCTTCATCGAGTG	0.00	0.00	0.00	0.00	0.69
GATCAAAGCTTCCGCGACGA	0.00	0.33	0.00	0.00	0.00
GATCAAAGCTTCCGTGTCTC	0.00	0.65	0.00	1.02	1.03
GATCAAAGCTTCTCCACCTA	1.37	0.65	1.15	0.00	0.69
GATCAAAGCTTCTGCACCCT	0.00	0.00	0.00	1.02	0.00
GATCAAAGCTTCTGCACCTC	0.00	0.33	0.00	0.00	0.00

GATCAAAGCTTCTGCACCTT	3.43	2.28	1.72	4.07	2.41
GATCAAAGCTTGAAAACAGG	0.00	0.00	0.00	0.00	0.34
GATCAAAGCTTGAAGTCACG	0.00	0.33	0.00	0.00	0.00
GATCAAAGCTTGAGATTGTG	0.23	0.00	0.00	0.00	0.00
GATCAAAGCTTGAGGCTCTT	0.23	0.00	0.00	0.00	0.00
GATCAAAGCTTGAGGGGAAG	0.00	0.00	0.00	0.00	0.00
GATCAAAGCTTGATGTCTGG	0.00	0.00	0.29	0.00	0.00
GATCAAAGCTTGCATGTGAG	0.00	0.00	0.00	0.34	0.00
GATCAAAGCTTGCATTTGTC	0.00	0.65	0.29	0.00	0.34
GATCAAAGCTTGCGCAGGTA	0.00	0.00	0.00	0.00	0.00
GATCAAAGCTTGCGGCGCAA	0.00	0.00	0.00	0.34	0.00
GATCAAAGCTTGCGGCGCCA	0.00	0.00	0.00	0.34	0.00
GATCAAAGCTTGCTCGGGCG	0.00	0.00	0.00	0.00	0.34
GATCAAAGCTTGCTTCGTGT	0.46	0.33	0.57	0.34	0.69
GATCAAAGCTTGCTTCGTTC	0.23	0.33	0.00	0.00	0.00
GATCAAAGCTTGGCAGCAGC	0.23	0.65	0.29	1.36	0.00
GATCAAAGCTTGGCCGTTGT	0.23	0.00	0.00	0.00	0.00
GATCAAAGCTTGGCTGTTGG	0.00	0.33	0.00	0.00	0.00
GATCAAAGCTTGGGCCACAA	0.00	0.33	0.00	0.00	0.00
GATCAAAGCTTGGGCTTGAA	1.60	2.28	1.72	2.04	0.34
GATCAAAGCTTGGGCTTGGA	0.46	0.00	0.00	0.00	0.00
GATCAAAGCTTGTCTGCAAC	0.00	0.33	0.00	0.00	0.00
GATCAAAGCTTGTGCCGTTC	0.00	0.00	0.00	0.34	0.00
GATCAAAGCTTTGCCGCTGT	0.46	0.00	0.00	0.00	0.00
GATCAAAGCTTTGCCGGTGT	0.46	0.00	0.00	0.00	0.00
GATCAAAGCTTTGCCGGTTT	0.00	0.33	0.00	0.00	0.00
GATCAAAGCTTTGCCGTTAT	0.23	0.00	0.00	0.00	0.00
GATCAAAGCTTTGCCGTTGA	0.00	0.00	0.29	0.00	0.34
GATCAAAGCTTTGCCGTTGG	0.46	0.00	0.00	0.00	0.00
GATCAAAGCTTTGCCGTTGN	0.46	0.00	0.00	0.00	0.00
GATCAAAGCTTTGCCGTTGT	83.32	35.14	23.77	28.85	20.66
GATCAAAGCTTTGCCGTTNT	0.00	0.00	0.00	0.34	0.00
GATCAAAGCTTTGCCGTTTT	0.00	0.00	0.29	0.00	0.00
GATCAAAGCTTTGCTCTTGT	0.00	0.33	0.00	0.00	0.00
GATCAAAGCTTTGGGGTCTT	0.00	0.00	0.00	0.00	0.34
GATCAAAGCTTTTGCACCTT	0.00	0.00	0.00	0.34	0.00
GATCAAAGGAAAAAAAAAAA	3.66	0.33	0.86	1.02	1.38

GATCAAAGGAAAAAAAAAAT	0.23	0.00	0.00	0.00	0.00
GATCAAAGGAAAAAAAAGAA	0.00	0.00	0.00	0.68	0.00
GATCAAAGGAAAAAAACNNN	0.23	0.00	0.00	0.00	0.00
GATCAAAGGAAAAAAAGAAA	0.00	0.00	0.00	0.00	0.00
GATCAAAGGAAAAAAANCNN	0.23	0.00	0.00	0.00	0.00
GATCAAAGGAAAAAGAATAA	0.23	0.00	0.00	0.00	0.00
GATCAAAGGAAAAAGGAAAG	0.23	0.00	0.00	0.00	0.00
GATCAAAGGAAAATGGTGAC	0.00	0.00	0.00	0.34	0.34
GATCAAAGGAAACAGGTCCG	0.00	0.00	0.00	0.00	0.34
GATCAAAGGAAAGAGAAGAG	0.00	0.00	0.00	0.00	0.34
GATCAAAGGAAAGGCTAAAG	0.46	0.00	0.00	0.00	0.00
GATCAAAGGAAATCAGAGAT	0.00	0.00	0.29	0.00	0.00
GATCAAAGGAAATCAGAGGT	0.00	0.00	0.29	0.00	0.00
GATCAAAGGAAATCAGAGTT	0.00	0.00	0.57	0.00	0.00
GATCAAAGGAAATCAGCGTT	0.00	0.00	0.00	0.00	0.00
GATCAAAGGAAATCAGNGNN	0.23	0.00	0.00	0.00	0.00
GATCAAAGGAAATCAGTCGT	0.00	0.33	0.00	0.00	0.00
GATCAAAGGAAATCAGTGNN	0.23	0.00	0.00	0.00	0.00
GATCAAAGGAAATCAGTGTC	0.00	0.00	0.00	0.00	0.34
GATCAAAGGAAATCAGTGTN	0.23	0.00	0.00	0.00	0.00
GATCAAAGGAAATCAGTGTT	21.75	31.88	29.21	26.82	28.24
GATCAAAGGAAATGTGACTC	0.23	0.00	0.00	0.00	0.00
GATCAAAGGAACAAAACAAA	0.69	0.00	0.00	0.00	0.00
GATCAAAGGAACCCGGCCTG	0.00	0.00	0.29	0.00	0.00
GATCAAAGGAACGATTACAG	0.00	0.00	0.00	0.00	0.00
GATCAAAGGAACGCCCATGC	0.00	0.00	0.29	0.00	0.00
GATCAAAGGAACGTCAAGAG	0.46	0.65	0.00	0.68	0.34
GATCAAAGGAACTCAGTGCT	0.00	0.00	0.00	0.00	0.34
GATCAAAGGAACTGGCCATG	0.23	0.98	0.29	1.02	0.00
GATCAAAGGAAGCACGAGGA	0.23	0.00	0.00	0.00	0.00
GATCAAAGGAAGCATCCCAA	0.00	0.00	0.00	0.34	0.00
GATCAAAGGAAGCGAAAAAA	0.46	0.00	0.00	0.68	0.34
GATCAAAGGAAGCGAAGCAA	0.23	0.00	0.00	0.68	0.34
GATCAAAGGAAGCGAAGCAC	4.58	4.88	3.44	3.73	4.48
GATCAAAGGAAGCGAAGNNN	0.23	0.00	0.00	0.00	0.00
GATCAAAGGAAGCGACATTC	0.00	0.33	0.00	0.00	0.00
GATCAAAGGAAGCGGAAAAA	0.00	0.00	0.00	0.34	0.00

GATCAAAGGAAGCGGCGTCT	0.00	0.00	0.00	0.00	0.00
GATCAAAGGAAGGAGAAAAC	0.00	0.00	0.29	0.00	0.34
GATCAAAGGAAGGAGAAGAA	0.00	0.00	0.00	0.00	0.69
GATCAAAGGAAGGAGAAGGG	0.00	0.00	1.43	0.00	1.72
GATCAAAGGAAGGAGTAAGG	0.00	0.00	0.29	0.00	0.00
GATCAAAGGAAGGCGGCGTC	0.00	0.00	0.29	0.00	0.00
GATCAAAGGAAGTGGAATCT	0.00	0.00	0.29	0.00	0.69
GATCAAAGGAATGCTTAGTT	0.46	0.00	0.00	0.34	0.00
GATCAAAGGACAAAAGACCA	0.00	0.00	0.00	0.00	0.00
GATCAAAGGACAAAGCTGAT	0.00	0.00	0.00	0.68	0.69
GATCAAAGGACAATGACCCT	0.00	0.00	0.29	0.00	0.00
GATCAAAGGACACGAATGGA	0.00	0.00	0.00	0.00	0.00
GATCAAAGGACACTGCTGTT	0.00	0.00	0.00	0.00	0.34
GATCAAAGGACAGAAAAGCT	0.00	0.00	0.00	0.34	0.00
GATCAAAGGACAGACGCCGC	0.00	0.00	0.29	0.00	0.00
GATCAAAGGACAGACGCCGG	0.00	0.00	1.15	0.00	1.03
GATCAAAGGACAGACTCCAT	0.00	0.33	0.00	0.34	0.00
GATCAAAGGACAGCGGCCGC	0.00	0.00	0.57	0.00	0.00
GATCAAAGGACATCCGTGTT	0.23	0.00	0.00	0.00	0.00
GATCAAAGGACATCGTACAA	0.00	0.00	0.57	0.00	0.00
GATCAAAGGACATCTGNNNN	0.23	0.00	0.00	0.00	0.00
GATCAAAGGACATGAATGGG	0.23	0.00	0.00	0.00	0.00
GATCAAAGGACATGAATGGT	3.43	4.23	2.58	0.68	0.69
GATCAAAGGACATGAATGNN	0.23	0.00	0.00	0.00	0.00
GATCAAAGGACATGCATGGT	0.00	0.00	0.29	0.00	0.00
GATCAAAGGACATGGCGCGA	0.00	0.00	0.29	0.00	0.00
GATCAAAGGACATGGTACAA	0.00	0.00	0.00	0.00	0.00
GATCAAAGGACATGTACAGG	0.00	0.00	0.29	0.00	0.00
GATCAAAGGACATTCGTATG	0.00	0.00	0.00	0.00	0.34
GATCAAAGGACATTGAACAA	0.00	0.00	0.00	0.34	0.00
GATCAAAGGACATTGCAAAA	0.00	0.00	0.00	0.00	0.00
GATCAAAGGACATTGCACAA	0.00	0.00	0.00	0.34	0.00
GATCAAAGGACATTGCACAG	0.00	0.00	0.29	0.00	0.00
GATCAAAGGACATTGGACAA	0.00	0.00	0.29	0.34	0.00
GATCAAAGGACATTGTAAAA	0.00	0.00	0.00	0.00	0.69
GATCAAAGGACATTGTACAA	20.60	44.24	44.39	59.74	57.17
GATCAAAGGACATTGTACAC	0.23	0.33	0.00	0.00	0.00

GATCAAAGGACATTGTACAG	0.00	0.00	0.00	0.00	0.34
GATCAAAGGACATTGTACNA	0.00	0.00	0.00	0.00	0.00
GATCAAAGGACATTGTAGAA	0.00	0.33	0.00	0.00	0.00
GATCAAAGGACATTGTCCNN	0.23	0.00	0.00	0.00	0.00
GATCAAAGGACATTGTNCAA	0.00	0.00	0.00	0.00	0.00
GATCAAAGGACATTGTTAAA	0.00	0.00	0.00	0.00	0.00
GATCAAAGGACCGCAGGATG	5.26	3.25	6.59	6.45	6.20
GATCAAAGGACCGCAGGGTG	0.00	0.00	0.29	0.00	0.00
GATCAAAGGACCGTGTCATC	0.00	0.00	0.00	0.00	0.00
GATCAAAGGACCTGGGGACA	0.46	0.00	0.00	0.00	0.00
GATCAAAGGACCTGGGGCCA	0.23	0.00	0.00	0.00	0.00
GATCAAAGGACCTTACTAGC	0.00	0.33	0.00	0.00	0.00
GATCAAAGGACCTTGTACAC	0.00	0.33	0.00	0.00	0.00
GATCAAAGGACGATAAAAAA	0.00	0.00	0.00	0.00	0.00
GATCAAAGGACGATAAAAGC	0.00	0.00	0.00	0.00	0.00
GATCAAAGGACGCACAGATG	0.00	0.00	0.00	0.00	0.00
GATCAAAGGACGCACGAGCT	0.00	0.33	0.29	0.00	0.00
GATCAAAGGACGCAGCGGCC	0.23	0.65	0.00	0.34	0.00
GATCAAAGGACGGAGCGATG	0.00	0.33	0.00	0.00	0.00
GATCAAAGGACGGAGCTAGG	0.23	0.00	0.00	0.00	0.00
GATCAAAGGACGGAGGCGCC	0.00	0.00	0.00	0.34	0.00
GATCAAAGGACGGAGGGGAA	0.00	0.00	0.29	0.00	0.00
GATCAAAGGACGGATGGATT	0.00	0.00	0.00	0.00	0.00
GATCAAAGGACGGCGGAGCC	0.23	0.00	0.00	0.00	0.00
GATCAAAGGACGGCGGCCCC	0.00	0.00	0.00	0.00	0.34
GATCAAAGGACGGCGGCGAC	0.23	0.00	0.00	0.00	0.00
GATCAAAGGACGGCGGCGCC	1.83	0.33	0.00	0.00	0.00
GATCAAAGGACGGCGGCGCG	0.23	0.00	0.00	0.00	0.00
GATCAAAGGACGGCGGCGGC	0.69	0.00	0.00	0.00	0.00
GATCAAAGGACGGCGGCGGG	0.23	0.00	0.00	0.00	0.00
GATCAAAGGACGGCGGGGCC	0.23	0.00	0.29	0.00	0.00
GATCAAAGGACGGCGGGGCG	0.23	0.00	0.00	0.00	0.00
GATCAAAGGACGGCGGGGGC	0.00	0.00	0.00	0.34	0.00
GATCAAAGGACGGCGGGGGT	0.00	0.00	0.00	0.34	0.00
GATCAAAGGACGGTTGCAGA	0.00	0.00	0.00	0.34	0.00
GATCAAAGGACGTGGATATT	0.00	0.00	0.00	0.00	0.00
GATCAAAGGACGTGGCCAGG	0.00	0.00	0.57	0.68	1.38

GATCAAAGGACGTGTCTGAA	0.23	0.00	0.00	0.00	0.00
GATCAAAGGACTCCGGCCCC	0.00	0.00	0.00	0.34	0.00
GATCAAAGGACTTTGTACAA	0.00	0.00	0.29	0.00	0.00
GATCAAAGGACTTTTCTGGG	0.00	0.00	0.00	0.68	0.00
GATCAAAGGAGAAGAATAGG	0.23	0.00	0.00	0.00	0.00
GATCAAAGGAGAAGCTCCCT	0.00	0.00	0.00	0.00	0.00
GATCAAAGGAGAAGTCTTCC	0.00	0.00	0.00	0.00	0.34
GATCAAAGGAGACGCCGGAA	0.00	0.00	0.00	0.34	0.00
GATCAAAGGAGACGCCGGAG	0.00	0.33	0.29	0.34	0.00
GATCAAAGGAGAGAAGTTAA	0.23	0.00	0.00	0.00	0.00
GATCAAAGGAGAGACCTCTC	0.00	0.33	0.00	0.00	0.34
GATCAAAGGAGAGAGTCTGA	0.00	0.33	0.00	0.00	0.00
GATCAAAGGAGAGATGCGAG	0.69	0.00	0.00	0.00	0.00
GATCAAAGGAGAGGCCTTCC	0.00	0.00	0.00	0.00	0.00
GATCAAAGGAGAGTGGCCAT	0.00	0.33	0.29	0.00	0.34
GATCAAAGGAGATGACTTCC	0.00	0.00	0.00	0.34	0.00
GATCAAAGGAGATGCCACGC	0.00	0.00	0.57	0.00	0.00
GATCAAAGGAGATGGACGTG	0.00	0.33	0.00	0.00	0.00
GATCAAAGGAGATGGCTTCC	0.23	0.00	0.00	0.00	0.00
GATCAAAGGAGATGTATTAC	0.00	0.00	0.00	0.00	0.00
GATCAAAGGAGATGTCCTCC	0.00	0.00	0.00	0.00	0.00
GATCAAAGGAGATGTCTCCC	0.00	0.00	0.00	0.00	0.00
GATCAAAGGAGATGTCTTAA	0.00	0.33	0.00	0.00	0.00
GATCAAAGGAGATGTCTTAC	0.00	0.33	0.29	0.34	0.00
GATCAAAGGAGATGTCTTCA	0.00	0.00	0.00	0.00	0.34
GATCAAAGGAGATGTCTTCC	6.87	24.72	49.54	32.92	41.67
GATCAAAGGAGATGTCTTCG	0.00	0.00	0.00	0.00	0.00
GATCAAAGGAGATGTCTTCN	0.00	0.00	0.00	0.00	0.00
GATCAAAGGAGATGTCTTCT	0.00	0.00	0.29	0.00	0.00
GATCAAAGGAGATGTCTTGA	0.00	0.00	0.57	0.00	0.00
GATCAAAGGAGATGTCTTGC	0.00	0.00	0.00	0.00	0.34
GATCAAAGGAGATGTCTTTC	0.00	0.00	0.29	0.00	0.00
GATCAAAGGAGATGTGAAAA	0.00	0.00	0.00	0.00	0.00
GATCAAAGGAGCAACCATCT	0.00	0.00	0.00	0.00	0.00
GATCAAAGGAGCACGCAGAA	0.00	0.00	0.00	0.00	0.34
GATCAAAGGAGCAGAGTGCG	0.00	0.00	0.00	0.00	0.00
GATCAAAGGAGCAGGCTTCC	0.00	0.00	0.00	0.00	0.00

GATCAAAGGAGCCACATGGG	0.00	0.00	0.29	0.00	0.00
GATCAAAGGAGCCCGAGGAG	0.00	0.00	0.00	0.00	0.00
GATCAAAGGAGCCGTCTTCC	0.00	0.00	0.29	0.00	0.00
GATCAAAGGAGCGACAGATA	0.00	0.00	0.00	0.00	0.00
GATCAAAGGAGCGGCCTCAG	0.69	2.93	1.72	2.72	4.82
GATCAAAGGAGCGGCCTGAG	0.00	0.00	0.00	0.34	0.00
GATCAAAGGAGCGGCGAAGC	0.23	0.00	0.00	0.00	0.00
GATCAAAGGAGCGGCGAGTC	0.23	0.00	0.00	0.00	0.00
GATCAAAGGAGCGGCTTGGA	0.46	0.65	0.00	0.00	0.34
GATCAAAGGAGCGGCTTTGA	0.00	0.00	0.00	0.00	0.34
GATCAAAGGAGCTGCTGGGC	0.00	0.00	0.00	0.00	0.00
GATCAAAGGAGGAAGAAGAA	0.00	0.00	0.57	0.00	0.00
GATCAAAGGAGGACGTGGTC	0.00	0.00	0.00	0.00	0.00
GATCAAAGGAGGAGAAAAAG	0.46	0.65	0.00	0.68	0.34
GATCAAAGGAGGAGTCATGG	0.46	0.00	0.00	0.00	0.00
GATCAAAGGAGGCAACCTGA	0.00	0.00	0.00	0.34	0.00
GATCAAAGGAGGCACCCAGG	3.20	0.65	0.86	0.34	0.34
GATCAAAGGAGGCACCCCCT	0.00	0.00	0.00	0.34	0.00
GATCAAAGGAGGCACCCTGG	0.23	0.00	0.00	0.00	0.00
GATCAAAGGAGGCAGACGTT	0.00	0.00	0.00	0.68	0.69
GATCAAAGGAGGCCTGATGT	0.00	0.00	0.00	0.00	0.34
GATCAAAGGAGGCGCGTCGA	0.00	0.00	0.29	0.34	0.00
GATCAAAGGAGGCGCTTAGC	0.23	0.00	0.00	0.00	0.00
GATCAAAGGAGGGAACGAAA	0.00	0.33	0.00	0.00	0.00
GATCAAAGGAGGGAACGACA	0.00	0.98	1.43	0.00	1.03
GATCAAAGGAGGGGCCAGGT	0.00	0.00	0.00	0.00	0.00
GATCAAAGGAGGGGGGCGTG	0.23	0.00	0.00	0.00	0.00
GATCAAAGGAGGGGGGGGGN	0.23	0.00	0.00	0.00	0.00
GATCAAAGGAGGTGACTGTC	0.00	0.00	0.00	0.00	0.00
GATCAAAGGAGGTGGATGGG	0.00	0.00	0.00	0.00	0.00
GATCAAAGGAGGTGGGGCGC	0.00	0.00	0.29	0.00	0.00
GATCAAAGGAGTGAGGGCAG	0.00	0.00	0.00	0.00	0.34
GATCAAAGGAGTGCAGACGT	0.69	0.00	0.00	0.00	0.00
GATCAAAGGAGTGCGCATCT	0.00	0.00	0.29	0.00	0.00
GATCAAAGGAGTGCTAGACG	0.00	0.33	0.29	0.00	0.00
GATCAAAGGAGTGGCAAGGC	0.00	0.00	0.00	0.00	0.34
GATCAAAGGAGTTGGAGATG	0.00	0.00	0.00	0.00	0.00

GATCAAAGGAGTTGTCTTCC	0.00	0.00	0.00	0.34	0.00
GATCAAAGGAGTTTGAAGGA	0.23	0.00	0.00	0.00	0.00
GATCAAAGGATAAAAAAAAA	0.00	0.00	0.00	0.00	0.34
GATCAAAGGATAATACCAAC	0.23	0.00	0.00	0.00	0.00
GATCAAAGGATAGGCTGGGA	0.00	0.33	0.00	0.00	0.00
GATCAAAGGATATAATGAGC	0.00	0.00	0.00	0.00	0.00
GATCAAAGGATCATGTGTAG	0.23	0.00	0.00	0.00	0.00
GATCAAAGGATCTAGCCAAA	0.23	0.00	0.00	0.00	0.00
GATCAAAGGATCTGGAACGA	0.46	0.00	0.00	0.00	0.00
GATCAAAGGATCTGGATCGA	0.23	0.00	0.00	0.00	0.00
GATCAAAGGATGAAAAAAAA	0.92	0.00	0.86	0.34	0.34
GATCAAAGGATGAAAAAGAA	0.00	0.33	0.00	0.00	0.00
GATCAAAGGATGAATGTTTC	0.00	0.00	0.00	0.00	0.00
GATCAAAGGATGAATGTTTT	0.00	0.00	0.00	0.00	0.00
GATCAAAGGATGCAAAAAAA	0.00	0.33	0.00	0.00	0.00
GATCAAAGGATGCCCTGGCC	0.00	0.00	0.00	0.34	0.00
GATCAAAGGATGGACCGGCC	0.00	0.00	0.57	0.00	0.00
GATCAAAGGATGGAGCTATG	0.00	0.00	0.29	0.00	0.00
GATCAAAGGATGGCAACGGG	0.00	0.00	0.29	0.00	0.00
GATCAAAGGATGGTCAAAAA	0.00	0.00	0.00	0.00	0.00
GATCAAAGGATGTGATATAG	0.00	0.00	0.00	0.34	0.00
GATCAAAGGATGTGCCCCTG	0.00	0.33	0.00	0.00	0.00
GATCAAAGGATTATGTTTTT	0.92	0.65	0.29	0.00	1.72
GATCAAAGGATTCAGCGCCT	0.00	0.00	0.29	0.34	0.00
GATCAAAGGATTCAGCGCGA	0.00	0.00	0.29	0.68	0.00
GATCAAAGGATTCGGATTGG	0.23	0.00	0.00	0.00	0.00
GATCAAAGGATTGAATGGCG	0.00	0.65	0.00	0.00	0.00
GATCAAAGGATTGATGGGGC	0.00	0.00	0.00	0.00	1.03
GATCAAAGGATTGCAGCAGT	0.69	0.00	0.00	0.00	0.00
GATCAAAGGATTGCCCCCCG	0.00	0.00	0.00	0.34	0.00
GATCAAAGGATTGGCCCCCG	0.00	0.33	0.00	0.00	0.00
GATCAAAGGATTTGCAGCGC	0.00	0.00	0.00	0.00	0.00
GATCAAAGGATTTGCATTGG	0.23	0.33	0.29	0.00	0.00
GATCAAAGGATTTGCGTTGG	0.00	0.00	0.29	0.00	0.00
GATCAAAGGCAAAAAAAAAA	10.30	3.90	2.00	6.79	5.17
GATCAAAGGCAAAAAAAAAN	0.00	0.00	0.00	0.00	0.00
GATCAAAGGCAAAAAAAAAT	0.00	0.00	0.00	0.68	0.00

GATCAAAGGCAAAAGAAAAA	0.00	0.00	0.00	0.34	0.34
GATCAAAGGCAAAAGAGATG	0.46	0.98	0.57	1.02	1.72
GATCAAAGGCAAACTGTCAT	0.00	0.00	0.00	0.00	0.34
GATCAAAGGCAAATTAAGCA	0.00	0.00	0.86	0.00	0.00
GATCAAAGGCAACAAACTAC	0.00	0.00	0.00	0.34	0.00
GATCAAAGGCAACACGACAC	0.00	0.33	0.57	0.00	0.00
GATCAAAGGCAACACGACCC	0.00	0.00	0.00	0.00	0.00
GATCAAAGGCAACACGCCAC	0.00	0.00	0.00	0.00	0.00
GATCAAAGGCAACCGCTGCA	0.46	0.00	0.00	0.00	0.00
GATCAAAGGCAACGAGCGTA	0.00	0.00	0.00	0.00	0.69
GATCAAAGGCAACTGAACGG	0.00	0.00	0.00	0.00	0.34
GATCAAAGGCAAGAAGCGCA	0.00	0.00	0.00	0.00	0.00
GATCAAAGGCAAGACCTTCG	0.00	0.00	0.00	0.00	0.00
GATCAAAGGCAAGAGGAAGA	0.00	0.00	0.29	0.00	0.00
GATCAAAGGCAAGAGGATGA	0.00	0.00	1.72	0.00	0.00
GATCAAAGGCAAGAGTTCGG	0.00	0.00	0.00	0.00	0.00
GATCAAAGGCAAGCCGAACA	0.00	0.00	0.00	0.00	0.34
GATCAAAGGCAAGGGCCAAG	0.00	0.00	0.29	0.00	0.00
GATCAAAGGCAAGGGGGATT	0.23	0.00	0.00	0.00	0.00
GATCAAAGGCAAGGGGGCTT	0.23	0.00	0.00	0.00	0.00
GATCAAAGGCAAGTGCACGT	0.00	0.00	0.00	0.00	0.00
GATCAAAGGCAAGTTCAAGT	0.00	0.00	0.00	0.00	0.00
GATCAAAGGCAATCGGCGAC	0.00	0.33	0.00	0.00	0.00
GATCAAAGGCAATCTTGATG	0.00	0.33	0.00	0.00	0.00
GATCAAAGGCAATGCAGGGA	0.46	0.00	0.00	0.00	1.03
GATCAAAGGCACAGAGGCAC	0.00	0.00	0.29	0.00	0.00
GATCAAAGGCACAGTCAGGG	0.00	0.00	0.00	0.00	0.00
GATCAAAGGCACCGACCTCG	0.23	0.00	0.29	0.00	0.34
GATCAAAGGCACCGCTCGGT	0.00	0.00	0.00	0.00	0.34
GATCAAAGGCACGAGCAAGG	0.00	0.00	0.00	0.00	0.00
GATCAAAGGCACGAGCAGCT	0.23	0.00	0.00	0.00	0.00
GATCAAAGGCACGCGCAGCT	0.23	0.00	0.00	0.00	0.00
GATCAAAGGCACGCTGCTGG	0.00	0.00	0.00	0.34	0.00
GATCAAAGGCACGGGCACGG	0.00	0.33	0.00	0.00	0.00
GATCAAAGGCACGTCATTTG	1.14	1.63	2.00	2.38	0.00
GATCAAAGGCACGTGAGACG	0.00	0.33	0.29	0.34	0.34
GATCAAAGGCACGTGAGAGC	0.00	0.00	0.00	0.00	0.00

GATCAAAGGCACGTGCAATG	0.00	0.00	0.00	0.00	0.34
GATCAAAGGCACTCGGAGAC	0.00	0.00	0.00	0.34	0.00
GATCAAAGGCACTGCATTCG	0.00	0.00	0.00	0.00	0.69
GATCAAAGGCACTGCGCCCT	0.23	0.00	0.29	0.00	0.00
GATCAAAGGCACTGCTTGGA	0.00	0.00	0.00	0.34	0.00
GATCAAAGGCACTGTCGTGC	0.00	0.00	0.00	0.34	0.34
GATCAAAGGCAGAAACGAGG	0.00	0.33	0.00	0.00	0.00
GATCAAAGGCAGAACAGAGT	0.23	0.00	0.00	0.00	0.00
GATCAAAGGCAGAAGATTCG	0.00	0.33	0.29	0.00	0.69
GATCAAAGGCAGAAGCATGG	0.00	0.00	0.00	0.00	0.00
GATCAAAGGCAGAATCAGAA	0.00	0.00	0.00	0.00	0.34
GATCAAAGGCAGAATCGAGC	0.00	0.00	0.00	0.34	0.00
GATCAAAGGCAGACACGTGG	0.69	1.95	0.57	1.02	0.34
GATCAAAGGCAGACTCGTTT	0.00	0.00	0.00	0.00	0.00
GATCAAAGGCAGAGCTGCTC	0.00	0.00	0.00	0.00	0.00
GATCAAAGGCAGATGTGAAC	0.00	0.00	0.29	0.00	0.00
GATCAAAGGCAGCAAAAAAA	0.00	0.00	0.29	0.00	0.00
GATCAAAGGCAGCAAGAATG	0.00	0.00	0.00	0.00	0.00
GATCAAAGGCAGCACCTTCA	0.00	0.00	0.00	0.00	0.00
GATCAAAGGCAGCAGGTTGG	0.23	0.00	0.00	0.00	0.00
GATCAAAGGCAGCATCCTTC	0.00	0.00	0.29	0.00	0.00
GATCAAAGGCAGCCAGGACA	0.00	0.33	0.57	0.34	0.00
GATCAAAGGCAGCGACAGGA	0.00	0.33	0.00	0.00	0.00
GATCAAAGGCAGCTAGAATA	0.23	0.00	0.00	0.00	0.00
GATCAAAGGCAGCTGATGGC	0.00	0.33	0.00	0.00	0.34
GATCAAAGGCAGGCCAAAGG	0.23	0.00	0.00	0.00	0.00
GATCAAAGGCAGTCTAAAAA	0.46	0.00	0.00	0.68	0.00
GATCAAAGGCAGTGAAGGAC	0.00	0.00	0.29	0.00	0.00
GATCAAAGGCAGTGCAAAAA	0.00	0.00	0.29	0.34	0.00
GATCAAAGGCAGTGCACGTA	0.00	0.33	0.29	0.00	0.00
GATCAAAGGCAGTGCTGGAT	0.00	0.00	0.29	0.00	0.00
GATCAAAGGCAGTGTTTCGA	0.00	0.00	0.00	0.00	0.34
GATCAAAGGCAGTTTGCGTC	0.00	0.00	0.00	0.34	0.34
GATCAAAGGCATAACAGGAT	0.00	0.33	0.00	0.00	0.00
GATCAAAGGCATCAATCCCN	0.00	0.00	0.00	0.00	0.00
GATCAAAGGCATCCATCCCC	1.60	0.33	0.57	0.00	1.72
GATCAAAGGCATCCGACACG	0.00	0.00	0.00	0.00	0.34

GATCAAAGGCATCGTATGCC	0.23	0.00	0.00	0.00	0.00
GATCAAAGGCATGAAAAAAA	0.23	0.00	0.00	0.00	0.00
GATCAAAGGCATGAACAAAA	0.46	0.00	0.00	0.00	0.34
GATCAAAGGCATGAACAGAA	1.37	0.33	0.29	0.68	0.00
GATCAAAGGCATGACAGTGC	0.00	0.00	0.00	0.00	0.00
GATCAAAGGCATGAGCTCTC	0.00	0.00	0.29	0.00	0.00
GATCAAAGGCATGGGATGAG	0.23	0.00	0.00	0.00	0.00
GATCAAAGGCATGGTCCGTT	0.69	0.00	0.00	0.00	0.00
GATCAAAGGCCAACGACCAC	0.00	0.00	0.00	0.00	1.03
GATCAAAGGCCAAGCGGCCC	0.00	0.00	0.29	0.00	0.00
GATCAAAGGCCACGAACGTG	0.00	0.00	0.00	0.00	0.34
GATCAAAGGCCACTCAGCCA	0.00	0.00	0.86	0.00	0.00
GATCAAAGGCCAGAAAGGTC	0.23	0.00	0.00	0.00	0.00
GATCAAAGGCCAGAATCTGG	0.00	0.00	0.00	0.00	0.34
GATCAAAGGCCAGAGGCCCT	1.37	1.95	0.57	2.04	1.38
GATCAAAGGCCAGAGTCGGC	0.00	0.33	0.00	0.00	0.00
GATCAAAGGCCAGCACCGGA	0.00	0.00	0.29	0.00	0.00
GATCAAAGGCCAGCGCCGGC	0.00	0.00	0.00	0.00	0.34
GATCAAAGGCCAGCGGCGGC	0.23	0.00	0.00	0.00	0.00
GATCAAAGGCCAGCGGTGCC	0.00	0.00	0.29	0.00	0.00
GATCAAAGGCCAGCGTCGGC	18.54	7.48	5.44	4.41	8.27
GATCAAAGGCCAGCGTCGGG	0.23	0.00	0.00	0.00	0.34
GATCAAAGGCCAGGCTGGTC	0.00	0.00	0.29	0.00	0.00
GATCAAAGGCCATCAACACG	0.00	0.00	1.15	0.00	0.00
GATCAAAGGCCATCTCAAGC	0.00	0.00	0.29	0.34	0.00
GATCAAAGGCCATGATAGCA	0.00	0.00	0.00	0.00	0.69
GATCAAAGGCCATTCCGGCC	0.69	0.00	0.57	0.00	0.00
GATCAAAGGCCCACTTGTCT	0.46	0.00	0.00	0.68	0.00
GATCAAAGGCCCAGAGATGC	0.00	0.00	0.00	0.34	0.00
GATCAAAGGCCCATAGCAAT	0.00	0.00	0.00	0.34	0.00
GATCAAAGGCCCCCCTCCCC	0.23	0.00	0.00	0.00	0.00
GATCAAAGGCCCTCCGTGCG	1.37	3.90	1.43	0.68	2.07
GATCAAAGGCCGAGAGACGG	0.00	0.00	0.00	0.00	0.00
GATCAAAGGCCGAGGACGGG	0.00	0.33	0.00	0.00	0.00
GATCAAAGGCCGCAACAACT	0.00	0.00	0.00	0.00	0.00
GATCAAAGGCCGCCACCACG	0.00	0.00	0.29	0.00	0.00
GATCAAAGGCCGCCTGGCGG	0.00	0.65	0.29	0.00	0.34

GATCAAAGGCCGCGCTTGCT	0.00	0.00	0.29	0.00	0.00
GATCAAAGGCCGCGTGTTGT	0.00	0.33	0.00	0.00	0.00
GATCAAAGGCCGCTCCTCGG	0.00	0.00	0.00	0.00	0.00
GATCAAAGGCCGGAAAAAAA	0.00	0.00	0.29	0.00	0.00
GATCAAAGGCCGGCAAAGCC	0.23	0.00	0.00	0.00	0.00
GATCAAAGGCCGGCCCCGCA	0.00	0.33	0.00	0.00	0.00
GATCAAAGGCCGGCGGCAGG	0.00	0.00	0.00	0.34	0.00
GATCAAAGGCCGGTTTCCCG	0.00	0.00	0.00	0.00	0.34
GATCAAAGGCCGTACGGTTG	0.00	0.00	0.00	0.34	0.34
GATCAAAGGCCGTGGTGGCA	0.00	0.00	0.00	0.00	0.34
GATCAAAGGCCGTGTGGGCC	0.00	0.00	0.00	0.00	0.00
GATCAAAGGCCTAGTCTTGG	0.23	0.00	0.00	0.00	0.00
GATCAAAGGCCTCCGCCGTT	0.00	0.00	0.00	0.00	0.00
GATCAAAGGCCTCCTGCGTC	2.29	0.33	0.86	1.02	0.00
GATCAAAGGCCTCCTGCTGC	0.00	0.00	0.29	0.00	0.00
GATCAAAGGCCTCCTGNGTC	0.00	0.00	0.00	0.34	0.00
GATCAAAGGCCTCGCCTGGT	0.23	0.00	0.00	0.00	0.00
GATCAAAGGCCTCGTGGACT	0.00	0.00	0.00	0.34	0.00
GATCAAAGGCCTGACTCCGG	0.69	1.30	0.00	0.34	0.34
GATCAAAGGCCTGATGAAGT	0.23	0.65	0.00	0.00	0.69
GATCAAAGGCCTGATGGAGT	0.00	0.00	0.29	0.00	0.00
GATCAAAGGCCTGCAACATG	0.00	0.00	0.00	0.00	0.00
GATCAAAGGCCTGCAATGCC	0.00	0.00	0.00	0.00	0.00
GATCAAAGGCCTGCCCTGCT	0.00	0.00	0.00	0.00	0.00
GATCAAAGGCCTGCGCCTCC	0.00	0.33	0.00	0.00	0.00
GATCAAAGGCCTGGACGCCT	0.00	0.00	0.00	0.34	0.00
GATCAAAGGCCTGGGGAAAC	0.00	0.00	0.00	0.68	0.00
GATCAAAGGCCTGGGTCGGG	1.14	0.00	0.00	0.34	0.00
GATCAAAGGCCTGTGCTGGC	0.00	0.00	0.00	0.00	0.00
GATCAAAGGCCTTAGTGGCA	0.46	0.00	0.00	0.00	0.00
GATCAAAGGCCTTGCCTTCG	0.00	0.00	0.00	0.00	0.00
GATCAAAGGCCTTTCAGAGC	0.00	0.00	0.00	0.00	0.00
GATCAAAGGCGACACCAGTG	0.23	0.00	0.00	0.00	0.00
GATCAAAGGCGACTCGTATG	0.00	0.00	0.29	0.00	0.00
GATCAAAGGCGAGAAATATA	0.00	0.33	0.00	0.00	0.00
GATCAAAGGCGAGAACTCTA	0.00	0.00	0.00	0.00	0.00
GATCAAAGGCGAGAACTCTC	0.00	0.00	0.00	0.00	0.00

GATCAAAGGCGAGACATCTA	0.00	0.00	0.00	0.34	0.00
GATCAAAGGCGAGACATCTC	0.00	0.00	0.00	0.00	0.00
GATCAAAGGCGAGACCACTC	0.00	0.33	0.00	0.00	0.69
GATCAAAGGCGAGACCGCTC	0.46	0.00	0.00	0.00	0.00
GATCAAAGGCGAGACCTATC	0.00	0.00	0.00	0.00	0.00
GATCAAAGGCGAGACCTCGC	0.23	0.00	0.00	0.00	0.00
GATCAAAGGCGAGACCTCNN	1.14	0.00	0.00	0.00	0.00
GATCAAAGGCGAGACCTCTA	0.00	0.00	0.00	0.00	0.34
GATCAAAGGCGAGACCTCTC	61.12	88.49	56.70	76.37	54.76
GATCAAAGGCGAGACCTCTN	0.23	0.00	0.00	0.00	0.00
GATCAAAGGCGAGACCTTTC	0.00	0.00	0.29	0.00	0.00
GATCAAAGGCGAGACTTCTC	0.00	0.00	0.29	0.00	0.00
GATCAAAGGCGAGACTTCTT	0.00	0.33	0.00	0.00	0.00
GATCAAAGGCGAGCCCTCGC	0.23	0.00	0.00	0.00	0.00
GATCAAAGGCGAGCCCTCTC	0.00	0.00	0.00	0.34	0.00
GATCAAAGGCGAGCTCACGG	0.00	0.33	0.57	0.00	1.38
GATCAAAGGCGAGCTCACGN	0.00	0.00	0.00	0.00	0.00
GATCAAAGGCGAGGCCTCTC	0.46	0.00	0.00	0.00	0.00
GATCAAAGGCGAGGGCTGCG	0.00	0.65	0.00	0.00	0.00
GATCAAAGGCGATACCTCTC	0.00	0.00	0.00	0.00	0.34
GATCAAAGGCGATGCCTTGT	0.23	0.33	0.57	0.00	0.00
GATCAAAGGCGCAAAAAAAA	0.69	0.65	0.57	0.68	0.00
GATCAAAGGCGCATGCTGGC	0.00	0.00	0.00	0.00	0.00
GATCAAAGGCGCATTGCAAA	0.00	0.00	0.00	0.00	0.34
GATCAAAGGCGCATTGCACT	0.00	0.00	0.29	0.00	0.00
GATCAAAGGCGCCAAGGACC	0.23	0.00	0.00	0.68	0.00
GATCAAAGGCGCCACCCCGC	0.00	0.33	0.00	0.00	0.00
GATCAAAGGCGCCAGCGGTC	0.00	0.00	0.00	0.00	0.00
GATCAAAGGCGCCCAGAGGG	0.00	0.00	0.00	0.00	0.00
GATCAAAGGCGCCCGGCAGG	0.00	0.00	0.29	0.00	0.00
GATCAAAGGCGCCCTGGACA	0.46	0.00	0.00	0.00	0.00
GATCAAAGGCGCCCTGGACG	0.23	0.00	0.00	0.00	0.00
GATCAAAGGCGCGACCTCTC	0.00	0.33	0.00	0.00	0.00
GATCAAAGGCGCGAGGAGGC	0.00	0.00	0.00	0.00	0.00
GATCAAAGGCGCGCGAGGCC	0.23	0.33	0.29	0.34	0.00
GATCAAAGGCGCGCTTCATC	0.00	0.00	0.00	0.00	0.00
GATCAAAGGCGCGGAATAGG	0.00	0.33	0.00	0.00	0.00

GATCAAAGGCGCTGGCTTCA	0.23	0.00	0.00	0.00	0.00
GATCAAAGGCGCTGGTTTCA	0.23	0.00	0.00	0.00	0.00
GATCAAAGGCGCTTGCCCAC	0.00	0.00	0.00	0.34	0.00
GATCAAAGGCGCTTGCCCAG	0.69	2.28	1.72	1.36	3.44
GATCAAAGGCGCTTGCCCCG	0.23	0.00	0.00	0.34	0.00
GATCAAAGGCGCTTGCCCTG	0.23	0.00	0.00	0.00	0.00
GATCAAAGGCGGAGCGAAGG	0.00	0.33	0.00	0.00	0.00
GATCAAAGGCGGCGCCCGAC	0.46	0.00	0.00	0.00	0.00
GATCAAAGGCGGCGTCGCCA	0.23	0.00	0.00	0.00	0.00
GATCAAAGGCGGCTTGGAGA	0.00	0.00	0.00	0.34	0.00
GATCAAAGGCGGGCCGGAGT	0.00	0.33	0.00	0.00	0.00
GATCAAAGGCGGGCCGGGGT	0.00	0.00	0.29	0.00	0.00
GATCAAAGGCGGGCCGGTGT	2.98	0.98	0.86	1.36	0.69
GATCAAAGGCGGGCCGGTTC	0.23	0.00	0.00	0.00	0.00
GATCAAAGGCGGGGAGGAGG	0.00	0.00	0.29	0.00	0.00
GATCAAAGGCGGGTGAAGTC	0.00	0.00	0.00	0.34	0.00
GATCAAAGGCGGGTGTTATG	0.00	0.33	0.00	0.00	0.00
GATCAAAGGCGTCAAGAATT	0.00	0.00	0.00	0.34	0.34
GATCAAAGGCGTCACCTTGA	0.00	0.00	0.00	0.00	0.00
GATCAAAGGCGTCATGTGTA	0.00	0.00	0.00	0.00	0.00
GATCAAAGGCGTCCAGTACG	0.23	0.00	0.00	0.00	0.00
GATCAAAGGCGTCCAGTCCG	0.69	0.65	0.29	0.00	0.34
GATCAAAGGCGTCCTCGCGA	0.00	0.00	0.00	0.00	0.00
GATCAAAGGCGTCGAGGAGG	0.00	0.00	0.00	0.00	0.00
GATCAAAGGCGTCGGCGACG	0.23	0.65	0.57	0.00	0.69
GATCAAAGGCGTCGGCGATG	1.14	2.28	2.86	3.05	3.79
GATCAAAGGCGTCGGCNNNN	0.23	0.00	0.00	0.00	0.00
GATCAAAGGCGTCTTTGATG	0.23	0.00	0.00	0.00	0.00
GATCAAAGGCGTGGAAGTGC	0.00	0.00	0.00	0.00	0.00
GATCAAAGGCGTTGGCGATG	0.00	0.00	0.29	0.00	1.38
GATCAAAGGCGTTGTAATCG	0.23	0.00	0.00	0.00	0.00
GATCAAAGGCTACAAGAAGG	0.00	0.00	1.15	0.34	0.00
GATCAAAGGCTACAGCCCTG	0.00	0.33	0.00	0.00	0.00
GATCAAAGGCTACAGCCGGG	0.23	0.00	0.00	0.00	0.00
GATCAAAGGCTACAGCCGTG	10.76	5.21	2.86	4.75	5.85
GATCAAAGGCTACAGGCGAG	0.23	0.00	0.00	0.00	0.00
GATCAAAGGCTACCCGGGCC	0.00	0.00	0.29	0.00	0.00

GATCAAAGGCTAGAAGGGCC	0.00	0.00	0.00	0.00	0.34
GATCAAAGGCTAGGACTGCC	0.00	0.00	0.29	0.00	0.00
GATCAAAGGCTAGGCAGAGG	0.00	0.00	0.00	0.00	0.00
GATCAAAGGCTAGTCGGAGG	0.00	0.00	0.00	0.00	0.00
GATCAAAGGCTATTCAAAAG	0.69	0.00	0.29	0.00	0.00
GATCAAAGGCTATTGTTAAA	0.00	0.00	0.00	0.00	0.34
GATCAAAGGCTCACAAGAGG	0.00	0.00	0.00	0.00	0.00
GATCAAAGGCTCACGATGCC	0.23	0.33	0.00	0.68	0.00
GATCAAAGGCTCAGCGACTG	0.00	0.00	0.00	0.68	0.00
GATCAAAGGCTCATGGATTT	0.00	0.00	0.29	0.00	0.00
GATCAAAGGCTCATGGCTGC	0.23	0.00	0.00	0.00	0.00
GATCAAAGGCTCCAACTCGT	0.00	0.00	0.00	0.00	0.00
GATCAAAGGCTCCACCGACT	0.00	0.00	0.00	0.00	1.03
GATCAAAGGCTCCGAAGACT	0.23	0.00	0.00	0.00	0.00
GATCAAAGGCTCCGGTGGAG	0.00	0.00	0.00	0.68	0.34
GATCAAAGGCTCGCAAGAGG	0.00	0.00	0.29	0.00	0.00
GATCAAAGGCTCGCCCGCAG	0.23	0.33	0.00	0.00	1.03
GATCAAAGGCTCGGCGCTGC	0.00	0.00	0.00	0.00	0.00
GATCAAAGGCTCGTTGTGTG	0.23	0.00	0.00	0.00	0.00
GATCAAAGGCTCTAAGTTAG	0.00	0.00	0.00	1.36	0.00
GATCAAAGGCTCTCCTCCTG	0.46	1.30	0.00	0.00	0.34
GATCAAAGGCTCTCCTCCTN	0.23	0.00	0.00	0.00	0.00
GATCAAAGGCTCTGCATTGG	0.00	0.33	0.00	0.00	0.00
GATCAAAGGCTCTGGTTTCA	0.92	0.33	0.29	0.00	0.00
GATCAAAGGCTCTTTCCTCT	0.00	0.00	0.00	0.00	0.00
GATCAAAGGCTGACACTTCA	0.00	0.00	0.00	0.00	0.00
GATCAAAGGCTGATTTTGCA	0.00	0.00	0.00	0.34	0.00
GATCAAAGGCTGCGCCAGGT	0.00	0.33	0.00	0.00	0.00
GATCAAAGGCTGCTCGCTGA	0.00	0.00	0.57	0.00	0.00
GATCAAAGGCTGGACATTCG	0.00	0.33	0.00	0.00	0.00
GATCAAAGGCTGGGATGTGG	0.00	0.00	0.00	0.34	0.00
GATCAAAGGCTGGGATTTGG	0.00	0.00	0.00	0.34	0.00
GATCAAAGGCTGGGGCAAGA	0.00	0.33	0.00	0.00	0.00
GATCAAAGGCTGGTTGGAGA	0.00	0.00	0.00	0.00	0.00
GATCAAAGGCTGGTTTTTGT	0.00	0.00	0.00	0.00	0.00
GATCAAAGGCTGTGCATTGG	0.00	0.00	0.00	0.34	0.00
GATCAAAGGCTGTGCGGTGG	0.00	0.00	0.00	0.34	0.00

GATCAAAGGCTGTGCGTTGG	0.23	0.33	0.00	0.00	0.00
GATCAAAGGCTGTGGTTTCA	0.00	0.33	0.00	0.00	0.00
GATCAAAGGCTGTGTCGATC	0.00	0.00	0.00	0.34	0.00
GATCAAAGGCTGTGTTAGGT	0.00	0.00	0.00	0.00	0.00
GATCAAAGGCTGTTGGCTGG	0.00	0.00	0.00	0.00	0.00
GATCAAAGGCTTAGCATTGG	0.00	0.33	0.00	0.34	0.00
GATCAAAGGCTTCAATTATT	0.00	0.00	0.00	0.00	0.00
GATCAAAGGCTTCACCTCCC	0.23	0.00	0.00	0.00	0.00
GATCAAAGGCTTCGCATTGG	0.00	0.00	0.29	0.00	0.00
GATCAAAGGCTTCGCGTTGG	0.00	0.00	0.29	0.00	0.00
GATCAAAGGCTTCGTCGAGT	0.00	0.33	0.00	0.00	0.34
GATCAAAGGCTTGATTTCAG	0.00	0.00	0.00	0.68	0.00
GATCAAAGGCTTGCGTTGGA	0.00	0.00	0.00	0.00	0.34
GATCAAAGGCTTGGGCTCCA	0.00	0.00	0.00	0.00	0.00
GATCAAAGGCTTTGAATTGG	0.00	0.00	0.00	0.00	0.00
GATCAAAGGCTTTGACTACG	0.00	0.00	0.00	0.00	0.00
GATCAAAGGCTTTGAGTCGG	0.00	0.00	0.00	0.34	0.00
GATCAAAGGCTTTGCACGGG	0.00	0.00	0.00	0.00	0.00
GATCAAAGGCTTTGCAGCAA	0.00	0.00	0.00	0.34	0.00
GATCAAAGGCTTTGCANTGG	0.00	0.33	0.00	0.00	0.34
GATCAAAGGCTTTGCATAGG	0.00	0.00	0.00	0.00	0.34
GATCAAAGGCTTTGCATGCT	0.00	0.00	0.00	0.00	0.00
GATCAAAGGCTTTGCATNGG	0.00	0.00	0.00	0.34	0.00
GATCAAAGGCTTTGCATNNN	0.23	0.00	0.00	0.00	0.00
GATCAAAGGCTTTGCATTGA	0.00	0.00	0.00	0.00	0.00
GATCAAAGGCTTTGCATTGG	121.09	121.35	93.35	89.27	83.00
GATCAAAGGCTTTGCATTGN	0.92	0.00	0.00	0.00	0.00
GATCAAAGGCTTTGCATTGT	0.00	0.33	0.29	0.68	0.00
GATCAAAGGCTTTGCATTNG	0.00	0.00	0.29	0.34	0.00
GATCAAAGGCTTTGCATTNN	0.69	0.00	0.00	0.34	0.00
GATCAAAGGCTTTGCATTTG	0.00	0.00	0.00	0.34	0.00
GATCAAAGGCTTTGCCCCAA	0.00	0.00	0.00	0.00	0.00
GATCAAAGGCTTTGCCCCCC	0.00	0.00	0.29	0.34	0.00
GATCAAAGGCTTTGCCCGAA	0.46	0.65	1.15	0.68	0.00
GATCAAAGGCTTTGCCCGAC	0.46	1.63	0.29	1.02	1.03
GATCAAAGGCTTTGCCTGAA	0.00	0.00	0.29	0.00	0.00
GATCAAAGGCTTTGCCTTNG	0.00	0.00	0.00	0.34	0.00

GATCAAAGGCTTTGCGCTCG	0.23	0.00	0.00	0.00	0.00
GATCAAAGGCTTTGCGCTGG	0.00	0.00	0.00	0.00	0.00
GATCAAAGGCTTTGCGGTGG	0.23	0.00	0.29	0.34	0.00
GATCAAAGGCTTTGCGNNNN	0.23	0.00	0.00	0.00	0.00
GATCAAAGGCTTTGCGTAGG	0.00	0.00	0.00	0.00	0.00
GATCAAAGGCTTTGCGTCGG	0.00	0.00	0.00	0.34	0.00
GATCAAAGGCTTTGCGTGAA	0.00	0.00	0.00	0.34	0.00
GATCAAAGGCTTTGCGTTAG	0.00	0.00	0.00	0.00	0.34
GATCAAAGGCTTTGCGTTGC	0.00	0.00	0.00	0.00	0.34
GATCAAAGGCTTTGCGTTGG	65.24	67.34	63.57	46.16	55.79
GATCAAAGGCTTTGCGTTGN	0.23	0.00	0.00	0.00	0.00
GATCAAAGGCTTTGCGTTGT	0.00	0.00	0.00	0.34	0.00
GATCAAAGGCTTTGCGTTTG	0.00	0.00	0.00	0.00	0.34
GATCAAAGGCTTTGCTCGTA	0.00	0.33	0.29	0.00	0.00
GATCAAAGGCTTTGCTTTGG	0.00	0.00	0.00	0.00	0.00
GATCAAAGGCTTTGGATTGG	0.00	0.33	0.00	0.00	0.00
GATCAAAGGCTTTGGGTTGG	0.00	0.00	0.29	0.00	0.00
GATCAAAGGCTTTGTGCTCA	0.00	0.00	0.00	0.00	0.00
GATCAAAGGGAAAAAAAAAA	0.00	0.00	0.29	0.00	0.00
GATCAAAGGGAAAAAAAACA	0.00	0.00	0.29	0.00	0.00
GATCAAAGGGAAAAGAGTCA	0.00	0.00	0.00	0.00	0.34
GATCAAAGGGAAACAGGAAC	0.00	0.33	0.00	0.00	0.00
GATCAAAGGGAAATCTGGGG	0.00	0.00	0.00	0.34	0.00
GATCAAAGGGAACATCAGAA	0.00	0.00	0.00	0.00	0.69
GATCAAAGGGAAGAATAGGC	0.00	0.00	0.00	0.00	0.00
GATCAAAGGGAAGAGTGGGT	0.00	0.33	0.57	0.00	0.00
GATCAAAGGGAAGAGTGTGT	0.00	0.00	0.00	0.00	0.00
GATCAAAGGGAAGGCTTGGT	0.00	0.00	0.29	0.00	0.00
GATCAAAGGGAAGGTCCAAA	0.00	0.33	0.00	0.00	0.00
GATCAAAGGGAAGGTCCGCT	0.46	0.65	0.29	0.00	0.34
GATCAAAGGGACAAAAAAAA	0.69	0.00	0.00	0.00	0.00
GATCAAAGGGACAAACAAAA	0.00	0.00	0.00	0.34	0.00
GATCAAAGGGACAAACAGTT	0.00	0.00	0.57	0.00	0.00
GATCAAAGGGACAAGAGCCG	0.00	0.00	0.29	0.00	0.00
GATCAAAGGGACAAGAGTCA	1.60	0.98	2.29	2.38	4.48
GATCAAAGGGACAAGAGTCG	0.00	0.00	0.00	1.02	0.00
GATCAAAGGGACAAGGACAT	0.00	0.65	0.29	1.70	0.00

GATCAAAGGGACACAATTCT	0.00	0.00	0.00	0.34	0.00
GATCAAAGGGACCGTGCTAT	0.00	0.00	0.29	0.00	0.00
GATCAAAGGGACTGTCACGG	0.00	0.00	0.00	0.00	0.00
GATCAAAGGGAGACCTGTCC	0.00	0.00	0.29	0.00	0.00
GATCAAAGGGAGATTGAGCC	0.00	0.00	0.00	0.00	0.00
GATCAAAGGGAGCAAAAAAA	0.23	0.00	0.29	0.00	0.00
GATCAAAGGGAGCAAATGAA	0.00	0.00	0.00	0.34	0.00
GATCAAAGGGAGCAAGAAAA	0.46	0.00	0.00	0.00	0.34
GATCAAAGGGAGCAAGCCTT	0.69	0.33	1.43	0.00	0.34
GATCAAAGGGAGCAGTGGAA	0.00	0.00	0.29	0.00	0.00
GATCAAAGGGAGCGACCTTG	0.00	0.00	0.00	0.00	0.00
GATCAAAGGGAGCTTCTGCG	0.00	0.00	0.00	0.34	0.00
GATCAAAGGGAGGAGGAGGA	0.00	0.00	0.86	0.00	0.69
GATCAAAGGGAGGCCGCAAA	0.00	0.00	0.00	0.00	0.34
GATCAAAGGGAGGGCCTGAG	0.46	1.95	1.43	1.70	1.03
GATCAAAGGGAGGGCCTGCG	0.00	0.00	0.00	0.00	0.34
GATCAAAGGGAGGGGACAGG	0.00	0.33	0.00	0.00	0.00
GATCAAAGGGAGTGAGAAAA	0.00	0.00	0.29	0.00	0.00
GATCAAAGGGAGTGAGAGAG	0.00	0.00	0.00	0.00	0.00
GATCAAAGGGATACTCTGAC	0.46	0.00	0.00	0.00	0.00
GATCAAAGGGATCACGTGCC	0.46	0.33	0.00	0.00	0.34
GATCAAAGGGATCCGGCACA	0.00	0.33	0.00	0.00	0.00
GATCAAAGGGATCCGGCCCA	0.00	0.00	0.00	0.00	0.00
GATCAAAGGGATGAGCAGGG	0.00	0.33	0.00	0.00	0.00
GATCAAAGGGATGGAGACGT	0.46	0.33	0.29	0.00	0.34
GATCAAAGGGATGGAGACTC	0.00	0.00	0.29	0.00	0.00
GATCAAAGGGATGGAGTCGT	0.23	0.00	0.00	0.00	0.00
GATCAAAGGGATGGCGCCGT	0.00	0.00	0.00	0.00	0.00
GATCAAAGGGATTCGGAAGA	0.23	0.33	0.00	1.02	0.00
GATCAAAGGGATTCGGCGGT	0.23	0.00	0.00	0.00	0.00
GATCAAAGGGATTGAAGGGA	0.00	0.00	0.00	0.00	1.03
GATCAAAGGGCAAAAAAAAA	0.00	0.00	0.00	0.34	0.00
GATCAAAGGGCAAAGGCAAA	0.00	1.63	1.15	1.70	1.38
GATCAAAGGGCAAAGGCAAG	0.46	0.00	0.00	0.34	0.00
GATCAAAGGGCAAAGGCTTG	0.00	0.00	0.00	0.00	0.00
GATCAAAGGGCAACAGGAAC	0.00	0.33	0.00	0.34	0.00
GATCAAAGGGCAACTCTCCT	0.00	0.00	0.00	0.00	0.69

GATCAAAGGGCAAGACAAGC	0.00	0.00	0.00	0.68	0.00
GATCAAAGGGCAAGACATAG	0.00	0.00	0.00	0.00	0.34
GATCAAAGGGCACAAAGTCT	0.23	0.00	0.00	0.00	0.34
GATCAAAGGGCACACCACAT	0.00	0.33	0.00	0.00	0.00
GATCAAAGGGCACCCGATCC	0.00	0.33	0.00	0.34	0.00
GATCAAAGGGCACCCGCCCC	0.00	0.33	0.00	0.00	0.00
GATCAAAGGGCACCCGCTCA	0.23	0.00	0.00	0.00	0.00
GATCAAAGGGCACCCGCTCC	12.13	23.42	6.30	19.01	3.79
GATCAAAGGGCACCCGCTCN	0.23	0.00	0.00	0.00	0.00
GATCAAAGGGCACCTGCTCC	0.00	0.00	0.00	0.00	0.00
GATCAAAGGGCACGTGGCAA	0.00	0.00	0.00	0.00	0.69
GATCAAAGGGCACTTCTAGG	0.00	0.00	0.00	0.00	0.34
GATCAAAGGGCAGATGGGCA	0.00	0.00	0.00	0.00	0.00
GATCAAAGGGCAGCGGAAAT	0.00	0.33	0.00	0.00	0.34
GATCAAAGGGCAGGCGTGGT	0.23	0.00	0.00	0.00	0.00
GATCAAAGGGCAGGCTTGGG	0.23	0.00	0.00	0.00	0.00
GATCAAAGGGCAGGCTTGGT	1.60	0.00	0.57	0.34	0.34
GATCAAAGGGCAGGGGCTGG	0.00	0.00	0.00	0.00	0.00
GATCAAAGGGCAGGGTGAGG	0.23	0.00	0.00	0.00	0.00
GATCAAAGGGCAGGTTGAGG	0.23	0.00	0.29	0.00	0.00
GATCAAAGGGCAGTGTATAA	0.00	0.00	0.29	0.00	0.00
GATCAAAGGGCATGCCCTTG	0.00	0.33	0.00	0.00	0.00
GATCAAAGGGCATGTGGATG	0.00	0.00	0.00	0.00	0.34
GATCAAAGGGCATTCCCAGG	0.23	0.00	0.00	0.00	0.00
GATCAAAGGGCCACGCCCAA	0.00	0.00	0.00	0.34	0.00
GATCAAAGGGCCACGCCCCA	0.00	0.00	0.00	0.34	0.00
GATCAAAGGGCCCCCGCTCC	0.00	0.00	0.00	0.34	0.00
GATCAAAGGGCCGAGTTAGT	0.00	0.00	0.00	0.00	0.34
GATCAAAGGGCCGAGTTCGT	1.14	1.30	0.00	0.34	0.69
GATCAAAGGGCCGAGTTTGT	0.00	0.33	0.00	0.00	0.00
GATCAAAGGGCCGCGTCCGG	0.46	1.30	0.29	0.34	0.34
GATCAAAGGGCCGGGACTTT	0.23	0.00	0.00	0.00	0.00
GATCAAAGGGCCTTCGTATT	0.00	0.00	0.29	0.00	0.00
GATCAAAGGGCGAAAGGCCT	0.00	0.00	0.00	0.00	0.00
GATCAAAGGGCGAAGCAAGC	0.92	0.33	0.29	0.00	0.00
GATCAAAGGGCGAATGCTGG	0.00	0.00	0.00	0.68	0.00
GATCAAAGGGCGCCCGTGGC	0.00	0.00	0.00	0.00	0.00

GATCAAAGGGCGCCGCGTTG	0.00	0.00	0.00	0.34	0.00
GATCAAAGGGCGGAAGTCCT	0.00	0.98	0.29	0.00	0.69
GATCAAAGGGCGGAATGGCT	0.00	0.00	0.57	0.00	0.00
GATCAAAGGGCGGCGCGCTA	0.00	0.33	0.00	0.00	0.00
GATCAAAGGGCGGGCCTGAG	0.00	0.00	0.00	0.34	0.00
GATCAAAGGGCTAAAAAAAA	0.46	0.00	0.00	0.00	0.00
GATCAAAGGGCTACGGCACA	0.00	0.00	0.29	0.00	0.00
GATCAAAGGGCTACGGCCCA	0.00	0.00	0.00	0.34	0.00
GATCAAAGGGCTCACTGAGC	0.46	0.00	0.29	0.00	0.69
GATCAAAGGGCTCAGGCACA	0.00	0.00	0.00	0.00	0.00
GATCAAAGGGCTCAGGCCCA	0.00	0.00	0.00	0.34	0.00
GATCAAAGGGCTCCAGCCCA	0.00	0.33	0.00	0.00	0.00
GATCAAAGGGCTCCAGCCTG	0.00	0.00	0.57	0.00	0.00
GATCAAAGGGCTCCGACCCA	0.00	0.33	0.00	0.00	0.00
GATCAAAGGGCTCCGAGAGA	0.00	0.00	0.00	0.00	0.00
GATCAAAGGGCTCCGGACCA	0.00	0.33	0.29	0.00	0.00
GATCAAAGGGCTCCGGACGA	0.23	0.00	0.00	0.00	0.00
GATCAAAGGGCTCCGGCACA	0.46	0.65	1.15	0.00	0.34
GATCAAAGGGCTCCGGCCAA	0.23	0.65	0.00	1.02	0.00
GATCAAAGGGCTCCGGCCCA	20.14	38.06	28.35	58.72	39.61
GATCAAAGGGCTCCGGCCCC	0.23	0.33	0.00	0.34	0.00
GATCAAAGGGCTCCGGCCCG	0.00	0.33	0.00	0.00	0.00
GATCAAAGGGCTCCGGCCCN	0.00	0.00	0.00	0.34	0.00
GATCAAAGGGCTCCGGCCCT	0.00	0.00	0.00	0.34	0.00
GATCAAAGGGCTCCGGCCGA	0.00	0.00	0.29	0.00	0.00
GATCAAAGGGCTCCGGCCNA	0.00	0.00	0.00	0.00	0.00
GATCAAAGGGCTCCGGCGCA	0.46	0.00	0.00	0.00	0.00
GATCAAAGGGCTCCGGCTCA	0.00	0.33	0.00	0.00	0.00
GATCAAAGGGCTCCGGTCCA	0.00	0.33	0.29	0.00	0.00
GATCAAAGGGCTCCGGTCCC	0.00	0.00	0.29	0.00	0.00
GATCAAAGGGCTCCGTCCCA	0.00	0.00	0.00	0.34	0.00
GATCAAAGGGCTCGGGCTCC	0.23	0.00	0.00	0.00	0.00
GATCAAAGGGCTCGTGAAGA	0.00	1.30	0.29	0.00	1.72
GATCAAAGGGCTCGTGACGA	0.00	0.00	0.00	0.00	0.00
GATCAAAGGGCTCGTGGTGT	0.00	0.00	0.00	0.00	0.00
GATCAAAGGGCTGCAGTTCA	0.00	0.00	0.00	0.00	0.34
GATCAAAGGGCTGGAGAAGT	0.00	0.00	0.00	0.00	0.00

GATCAAAGGGCTGGAGTTGT	0.00	0.00	0.00	0.00	0.00
GATCAAAGGGCTGGCACCCT	0.00	0.00	0.00	0.00	0.00
GATCAAAGGGCTGGGAATGG	0.00	0.00	0.00	0.00	1.03
GATCAAAGGGCTGGTGCTGT	0.00	0.33	0.00	0.00	0.00
GATCAAAGGGCTTGAGCCGG	0.23	0.00	0.00	0.00	0.00
GATCAAAGGGCTTGCAGCTG	0.00	0.33	0.00	0.00	0.00
GATCAAAGGGCTTGGAATGG	0.00	0.00	0.00	0.00	0.00
GATCAAAGGGGAAAAAAAAA	0.00	0.00	0.00	0.68	0.00
GATCAAAGGGGACATTCAAG	1.83	3.90	2.58	1.70	3.44
GATCAAAGGGGACCGGAGCC	0.00	0.00	0.00	0.34	0.00
GATCAAAGGGGAGAAGAACA	0.69	0.65	0.57	0.68	1.03
GATCAAAGGGGAGAATCGTA	0.00	0.00	0.00	0.34	0.00
GATCAAAGGGGAGACCTCTC	0.00	0.00	0.00	0.34	0.00
GATCAAAGGGGAGACGAGTA	0.00	0.00	0.00	0.00	0.00
GATCAAAGGGGAGCACGCGA	0.00	0.00	0.00	0.00	0.34
GATCAAAGGGGAGCACGTTT	0.00	0.65	0.00	0.00	0.00
GATCAAAGGGGAGCCAAAAA	0.00	0.00	0.00	0.34	0.00
GATCAAAGGGGAGGAGTGCT	0.23	0.00	0.00	0.00	0.00
GATCAAAGGGGAGGTGTGTG	0.00	0.33	0.00	0.00	0.00
GATCAAAGGGGCAGTGTTTG	0.00	0.00	0.29	0.00	0.00
GATCAAAGGGGCATCTTCGG	0.23	0.00	0.00	0.34	0.00
GATCAAAGGGGCCACGCTCG	0.00	0.00	0.00	0.00	0.00
GATCAAAGGGGCCACGCTTG	0.00	0.00	0.00	0.00	0.00
GATCAAAGGGGCCATGCTTG	0.00	0.00	0.29	0.00	0.00
GATCAAAGGGGCCATGTTTG	0.00	0.00	0.29	0.00	0.00
GATCAAAGGGGCGGCCTGGG	0.23	0.00	0.00	0.00	0.00
GATCAAAGGGGCGGCTACT	0.00	0.00	0.00	0.00	0.34
GATCAAAGGGGCGGGGGGGC	0.23	0.00	0.00	0.00	0.00
GATCAAAGGGGCGGGGGTCG	0.00	0.00	0.00	0.00	0.34
GATCAAAGGGGGAAGGGAAG	0.00	0.00	0.00	0.00	0.34
GATCAAAGGGGGAGAAAAGG	0.00	0.00	0.00	0.00	0.34
GATCAAAGGGGGAGACGAGA	0.00	0.00	0.00	0.00	0.69
GATCAAAGGGGGAGACGAGG	0.00	0.00	0.00	0.34	0.00
GATCAAAGGGGGAGACGAGN	0.23	0.00	0.00	0.00	0.00
GATCAAAGGGGGAGACGAGT	7.10	31.23	26.63	24.10	13.09
GATCAAAGGGGGAGACGCAT	0.00	0.00	0.00	0.00	0.34
GATCAAAGGGGGAGACGCGC	0.00	0.00	0.00	0.00	0.00

GATCAAAGGGGGAGACGCGG	0.00	0.00	0.00	0.00	0.00
GATCAAAGGGGGAGACGCGT	0.00	0.33	0.00	0.00	0.00
GATCAAAGGGGGAGACGGGT	0.23	0.00	0.00	0.00	0.00
GATCAAAGGGGGAGAGTAGT	0.23	0.00	0.00	0.00	0.00
GATCAAAGGGGGAGCCGAGT	0.23	0.00	0.00	0.00	0.00
GATCAAAGGGGGAGGCGAGA	0.23	0.00	0.00	0.00	0.00
GATCAAAGGGGGAGGCGAGT	0.00	0.33	0.00	0.00	0.00
GATCAAAGGGGGCAGCTCTT	0.00	0.00	0.29	0.00	0.00
GATCAAAGGGGGCGACGAGT	0.00	0.00	0.29	0.00	0.00
GATCAAAGGGGGCGTCGCCA	0.46	0.00	0.00	0.00	0.00
GATCAAAGGGGGCTTGGATG	0.23	0.00	0.00	0.00	0.00
GATCAAAGGGGGGAAGGGGA	0.00	0.00	0.00	0.00	0.34
GATCAAAGGGGGGCCCGAGT	0.23	0.00	0.00	0.00	0.00
GATCAAAGGGGGGCTGGTGG	0.00	0.00	0.29	0.00	0.00
GATCAAAGGGGGGGAACGGC	0.00	0.00	0.00	0.00	0.00
GATCAAAGGGGGGGGCGAGG	0.23	0.00	0.00	0.00	0.00
GATCAAAGGGGGGGGGGGGG	0.23	0.00	0.00	0.00	0.34
GATCAAAGGGGGGTCACTCT	0.00	0.00	0.29	0.00	0.00
GATCAAAGGGGGTGTTAAGT	0.00	0.00	0.00	0.00	0.34
GATCAAAGGGGTCAACCCGC	0.23	0.00	0.00	0.34	0.00
GATCAAAGGGGTCAACCCTG	0.00	0.00	0.29	0.00	0.00
GATCAAAGGGGTTGGCGACG	0.00	0.00	0.00	0.34	0.00
GATCAAAGGGGTTTCGCCTG	1.83	4.55	4.30	4.75	3.44
GATCAAAGGGGTTTCGCCTN	0.00	0.00	0.00	0.34	0.00
GATCAAAGGGGTTTCGTATG	0.00	0.00	0.00	0.34	0.00
GATCAAAGGGGTTTCGTCTG	0.00	0.33	0.00	0.00	0.00
GATCAAAGGGTAAAAAAAAA	0.46	0.00	0.00	0.00	0.00
GATCAAAGGGTAAGGGGAAG	0.00	0.33	0.00	0.00	0.00
GATCAAAGGGTAAGGGTAAG	0.46	0.00	0.00	0.34	0.00
GATCAAAGGGTATTCGTTGG	0.00	0.00	0.00	0.00	0.00
GATCAAAGGGTCAAAAAAAA	0.23	0.00	0.00	0.00	0.00
GATCAAAGGGTCACCAGAGC	0.92	0.33	0.00	0.00	0.00
GATCAAAGGGTCACCAGAGG	0.23	0.00	0.00	0.00	0.00
GATCAAAGGGTCCAATGTGC	0.00	0.00	0.00	0.00	0.00
GATCAAAGGGTCGAAAAAAA	0.00	0.33	0.00	0.00	0.00
GATCAAAGGGTCGCAAAAAA	0.00	0.65	0.00	0.00	0.00
GATCAAAGGGTCGTAAAAAA	0.23	0.00	0.29	0.68	0.34

GATCAAAGGGTCGTACTTCA	0.23	0.33	0.00	0.34	0.00
GATCAAAGGGTCGTACTTCG	1.60	0.33	0.00	1.36	0.69
GATCAAAGGGTCTGTATTGG	0.23	0.00	0.00	0.00	0.00
GATCAAAGGGTTCCAGTGCG	0.00	0.00	0.00	0.00	0.00
GATCAAAGGGTTCTGCGTCA	0.00	0.00	0.00	1.02	0.00
GATCAAAGGGTTTGAAAAAA	0.00	0.00	0.00	0.00	0.34
GATCAAAGGTAAAAAAAAAA	0.69	0.33	0.00	0.00	2.41
GATCAAAGGTAAAAAAAAAC	0.23	0.00	0.00	0.00	0.00
GATCAAAGGTAAGGGCTGGA	0.00	0.00	0.00	0.00	0.69
GATCAAAGGTAAGGTTTGAA	0.00	0.33	0.57	0.34	0.00
GATCAAAGGTACCACGTGCC	0.23	0.00	0.00	0.00	0.00
GATCAAAGGTACCCCTATTG	0.23	0.00	0.00	0.00	0.34
GATCAAAGGTACGTAGGGGA	0.23	0.00	0.00	0.00	0.00
GATCAAAGGTACTGCCCTCA	0.00	0.00	0.00	0.00	0.00
GATCAAAGGTACTGCTATGC	0.46	0.00	0.00	0.00	0.34
GATCAAAGGTACTGCTGCCC	0.00	0.00	0.00	0.00	0.00
GATCAAAGGTAGCACGAGCC	0.00	0.00	0.00	0.34	0.00
GATCAAAGGTAGCATGTGCT	0.23	0.00	0.00	0.00	0.00
GATCAAAGGTAGCTAGAAAA	0.23	0.00	0.00	0.00	0.00
GATCAAAGGTAGCTAGAATA	2.75	0.98	4.30	1.36	6.89
GATCAAAGGTAGCTAGCATA	0.00	0.00	0.00	0.00	0.34
GATCAAAGGTAGCTTCCAAC	0.00	0.33	0.00	0.00	0.00
GATCAAAGGTAGGAAGGAAC	0.00	0.00	0.00	0.00	0.00
GATCAAAGGTAGGAAGGACC	1.14	0.65	0.00	0.68	1.03
GATCAAAGGTAGTATTCGCA	0.46	0.98	0.86	0.34	0.00
GATCAAAGGTAGTATTCGCT	0.69	0.33	0.00	0.34	0.00
GATCAAAGGTAGTATTTGCA	0.23	0.00	0.00	0.00	0.00
GATCAAAGGTAGTGAGAGAG	0.00	0.00	0.29	0.00	0.00
GATCAAAGGTATAACCTGCC	0.00	0.00	0.29	0.00	0.00
GATCAAAGGTATCAAAAAAA	0.92	0.00	0.00	0.00	0.00
GATCAAAGGTATCAAGTGAC	0.00	0.00	0.29	0.00	0.00
GATCAAAGGTATCAAGTGCC	0.00	0.00	0.00	0.00	0.00
GATCAAAGGTATCACGAAAA	0.00	0.33	0.00	0.00	0.00
GATCAAAGGTATCACGAGCA	0.00	0.00	0.00	0.00	0.34
GATCAAAGGTATCACGAGCC	0.23	0.33	0.00	0.00	0.00
GATCAAAGGTATCACGCGCA	0.00	0.00	0.00	0.00	0.34
GATCAAAGGTATCACGCGCC	0.00	0.65	0.00	0.00	0.00

GATCAAAGGTATCACGCGCT	0.00	0.00	0.00	0.34	0.00
GATCAAAGGTATCACGCGTG	0.00	0.00	0.29	0.00	0.00
GATCAAAGGTATCACGGGCC	0.23	0.00	0.00	0.34	0.00
GATCAAAGGTATCACGTAGA	0.00	0.00	0.00	0.34	0.00
GATCAAAGGTATCACGTGAC	0.00	0.33	0.29	0.00	0.69
GATCAAAGGTATCACGTGCA	2.06	1.63	1.43	0.34	0.69
GATCAAAGGTATCACGTGCC	32.28	33.83	39.80	37.68	42.02
GATCAAAGGTATCACGTGCN	0.23	0.00	0.00	0.00	0.00
GATCAAAGGTATCACGTGCT	0.00	0.33	0.00	0.34	0.69
GATCAAAGGTATCACGTGGC	0.23	0.00	0.00	0.00	0.00
GATCAAAGGTATCACGTGTA	0.00	0.00	0.00	0.00	0.34
GATCAAAGGTATCACGTGTC	0.00	0.00	0.29	0.00	0.34
GATCAAAGGTATCACGTNCC	0.00	0.00	0.00	0.34	0.00
GATCAAAGGTATCACGTTCC	0.00	0.33	0.00	0.00	0.00
GATCAAAGGTATCACGTTGC	0.00	0.00	0.00	0.00	0.00
GATCAAAGGTATCACTCGTA	0.23	0.00	0.00	0.00	0.00
GATCAAAGGTATCAGGAGCG	0.00	0.00	0.57	0.00	0.00
GATCAAAGGTATCAGGTGGA	0.00	0.00	0.29	0.00	0.00
GATCAAAGGTATCATGTGCC	0.00	0.00	0.29	0.00	0.00
GATCAAAGGTATCCCGTGCC	0.00	0.00	0.00	0.00	0.34
GATCAAAGGTATCGCGTGAC	0.00	0.00	0.00	0.00	0.00
GATCAAAGGTATGAACAGCC	0.00	0.00	0.29	0.00	0.00
GATCAAAGGTATGACGAATC	5.26	6.83	0.29	3.73	0.69
GATCAAAGGTCAAGGGCTCA	0.00	0.00	0.00	0.34	0.00
GATCAAAGGTCACAAAGACG	0.23	0.00	0.00	0.00	0.00
GATCAAAGGTCACAAAGGCT	0.00	0.00	0.00	0.00	0.00
GATCAAAGGTCACAAAGTCT	19.00	20.82	6.30	11.88	12.05
GATCAAAGGTCACGAAGTCT	0.00	0.00	0.00	0.00	0.34
GATCAAAGGTCAGCGACGAT	0.00	0.00	0.00	0.00	0.00
GATCAAAGGTCAGCGACGGA	0.00	2.60	2.00	2.38	0.69
GATCAAAGGTCAGCGACGGC	0.00	0.00	0.29	0.00	0.00
GATCAAAGGTCATCCAAGTG	0.00	0.00	0.00	0.00	0.00
GATCAAAGGTCATCTGCGGA	0.00	0.00	0.29	0.00	0.00
GATCAAAGGTCATGAGGGCA	0.00	0.00	0.00	0.00	0.34
GATCAAAGGTCCAGACTACA	0.00	0.33	0.00	0.00	0.00
GATCAAAGGTCCCTGTGTAC	0.00	0.65	0.00	0.00	0.00
GATCAAAGGTCCCTGTGTGC	0.00	0.33	0.00	0.00	0.00

GATCAAAGGTCCTGGCCCCA	0.00	0.65	0.00	0.00	0.00
GATCAAAGGTCGCCAATGGC	0.00	0.33	0.00	0.00	0.00
GATCAAAGGTCGGCAATCGA	0.00	0.00	0.29	0.00	0.00
GATCAAAGGTCGGCTGCAGT	0.00	0.00	0.00	0.34	0.00
GATCAAAGGTCGGTAAAAAA	0.00	0.00	0.00	0.34	0.00
GATCAAAGGTCGGTTTACTG	0.00	0.00	0.00	0.00	0.00
GATCAAAGGTCGTGCGCACA	0.00	0.00	0.00	0.00	0.00
GATCAAAGGTCTCAGAGGAG	0.00	0.33	0.00	0.00	0.00
GATCAAAGGTCTCCCGTGCC	0.23	0.00	0.00	0.00	0.00
GATCAAAGGTCTGCATCTGC	0.00	0.00	0.00	0.34	0.00
GATCAAAGGTCTGCCATGCA	0.00	0.00	0.00	0.00	0.69
GATCAAAGGTCTGGATGCAA	0.00	0.00	0.00	0.00	0.00
GATCAAAGGTCTGGTGCTGT	0.00	0.33	0.00	0.34	0.00
GATCAAAGGTCTTCTTCCAG	0.00	0.00	0.00	0.34	0.00
GATCAAAGGTCTTGATGGAA	0.46	0.00	0.00	0.00	0.00
GATCAAAGGTCTTGCACCTG	0.00	0.33	0.00	0.00	0.00
GATCAAAGGTCTTGCCTCTT	0.23	0.33	0.00	0.00	0.00
GATCAAAGGTCTTTCTTGTC	0.00	0.00	0.00	0.00	0.00
GATCAAAGGTGAAAAAAAAA	0.00	0.00	0.29	0.00	0.00
GATCAAAGGTGAACCGGCCT	0.00	0.33	0.00	0.00	0.69
GATCAAAGGTGAACGGGCAC	0.00	0.00	0.00	0.34	0.00
GATCAAAGGTGACTTGTGCA	0.00	0.00	0.00	0.00	0.00
GATCAAAGGTGAGACCTCTC	0.00	0.00	0.00	0.34	0.00
GATCAAAGGTGAGTCGCTCA	0.00	0.00	0.29	0.00	0.00
GATCAAAGGTGCAAAAAAAA	0.23	0.00	0.00	0.00	0.00
GATCAAAGGTGCAAAGAAAA	0.00	0.00	0.00	0.34	0.00
GATCAAAGGTGCAAGTTCAT	0.00	0.00	0.00	0.00	0.34
GATCAAAGGTGCAGATGTAA	0.23	0.33	0.00	0.00	0.34
GATCAAAGGTGCAGATGTAC	0.00	0.00	0.00	0.00	0.00
GATCAAAGGTGCCAGAACTC	0.46	0.00	0.00	0.34	0.00
GATCAAAGGTGCCAGAATTC	0.00	0.00	0.00	0.00	0.00
GATCAAAGGTGCTCCATTTC	0.00	0.00	0.00	0.34	0.00
GATCAAAGGTGCTGTCAAGA	0.23	0.00	0.00	0.00	0.69
GATCAAAGGTGCTTGCATTG	0.00	0.00	0.00	0.00	0.00
GATCAAAGGTGGAGAGAAGT	2.06	2.60	0.86	0.34	0.34
GATCAAAGGTGGCAGCTTCC	0.23	0.00	0.00	0.00	0.00
GATCAAAGGTGGCGTCGCCA	11.90	1.95	0.29	1.36	1.03

GATCAAAGGTGGCGTCGCCN	0.23	0.00	0.00	0.00	0.00
GATCAAAGGTGGCGTCGGCC	0.23	0.00	0.00	0.00	0.00
GATCAAAGGTGGCGTCNNNN	0.23	0.00	0.00	0.00	0.00
GATCAAAGGTGGCGTCTTGC	0.00	0.00	0.00	0.34	0.00
GATCAAAGGTGGCGTGAGGA	0.00	0.00	0.29	0.00	0.00
GATCAAAGGTGGCTGATTGA	0.00	0.00	0.00	0.00	0.00
GATCAAAGGTGGCTGATTGG	2.06	0.00	0.29	2.38	1.03
GATCAAAGGTGGCTTAGCTC	0.00	0.00	0.00	0.00	0.00
GATCAAAGGTGGGCGATGNN	0.23	0.00	0.00	0.00	0.00
GATCAAAGGTGGGCGGAAGG	0.46	0.00	0.00	0.00	0.00
GATCAAAGGTGGGGATGTCG	0.00	0.00	0.00	0.34	0.00
GATCAAAGGTGGGGCCAGGT	0.00	0.00	0.00	1.02	0.00
GATCAAAGGTGGTCCCCTCC	0.23	0.00	0.00	0.00	0.00
GATCAAAGGTGGTCCTCTCC	1.37	0.00	0.00	0.00	0.00
GATCAAAGGTGGTCGACATG	0.00	0.00	0.29	0.00	0.00
GATCAAAGGTGGTGCACTGA	0.00	0.00	0.00	0.00	0.69
GATCAAAGGTGGTGCGTCAG	0.00	0.33	0.00	0.00	0.00
GATCAAAGGTGGTGCTCTGG	0.00	0.00	0.00	0.34	0.00
GATCAAAGGTGGTTGTGGTG	0.46	0.65	0.00	0.00	0.69
GATCAAAGGTGTAATCTTTT	0.00	0.00	0.00	0.00	0.00
GATCAAAGGTGTAGCACCCC	0.00	0.00	0.57	0.34	0.00
GATCAAAGGTGTATGAGCCA	0.00	0.00	0.00	0.00	0.00
GATCAAAGGTGTCGGCGACG	0.00	0.98	0.29	0.34	0.00
GATCAAAGGTGTCGGCGATG	0.23	0.00	0.86	0.00	1.03
GATCAAAGGTGTCGGCNATG	0.00	0.00	0.00	0.00	0.34
GATCAAAGGTGTCGGCTTAT	0.00	0.33	0.00	0.00	0.00
GATCAAAGGTGTGTGTGGTG	0.00	0.33	0.00	0.00	0.34
GATCAAAGGTGTTGGCGACG	0.00	0.00	0.29	0.00	0.00
GATCAAAGGTGTTGGCGATG	2.06	3.58	4.30	2.04	2.76
GATCAAAGGTGTTGGCGATN	0.00	0.00	0.00	0.00	0.00
GATCAAAGGTGTTGGTGACG	0.00	0.00	0.29	0.00	0.34
GATCAAAGGTTAACCGCTGC	0.69	0.00	0.00	0.00	0.00
GATCAAAGGTTCCTGGAAGA	0.00	0.00	0.00	0.00	0.00
GATCAAAGGTTCGTATGCCG	0.00	0.00	0.00	0.00	0.34
GATCAAAGGTTCTATGCTGA	0.23	0.00	0.00	0.00	0.00
GATCAAAGGTTCTGCCTCGG	0.00	0.00	0.00	0.00	0.34
GATCAAAGGTTCTGCCTTGG	0.00	0.00	0.00	0.00	0.00

GATCAAAGGTTCTGGGGACC	0.00	0.00	0.00	0.34	0.00
GATCAAAGGTTGAAATCTTC	0.46	0.00	0.00	0.00	0.00
GATCAAAGGTTGCAGTCCTG	0.00	0.00	0.00	0.00	0.00
GATCAAAGGTTGGAAAAAAA	0.00	0.00	0.00	0.34	0.34
GATCAAAGGTTGGAAAAGGC	0.23	0.00	0.29	1.02	0.00
GATCAAAGGTTGGCTCTCAG	0.23	0.00	0.00	0.00	0.00
GATCAAAGGTTGGGCGGAGG	0.00	0.33	0.00	0.34	0.00
GATCAAAGGTTGGGCGGCGG	8.24	9.43	2.86	2.04	1.72
GATCAAAGGTTGGGCGGCNN	0.23	0.00	0.00	0.00	0.00
GATCAAAGGTTGGGTCTCGT	0.23	0.00	0.00	0.00	0.00
GATCAAAGGTTGTAGAATTT	0.00	0.00	0.00	0.00	0.00
GATCAAAGGTTTCATCATCT	0.23	0.00	0.00	0.00	0.00
GATCAAAGGTTTCTATCCGG	0.00	0.00	0.29	0.00	0.00
GATCAAAGGTTTGCCACTGG	0.00	0.00	0.00	0.00	0.34
GATCAAAGGTTTGGCGGCGG	0.00	0.00	0.00	0.34	0.00
GATCAAAGTAAAAAAAAAAA	7.55	0.65	1.43	1.70	2.76
GATCAAAGTAAAAAAAAAAT	0.46	0.00	0.00	0.00	0.00
GATCAAAGTAAAAGCCCAGT	0.00	0.00	0.00	0.00	0.69
GATCAAAGTAAAATCAAAAA	0.69	0.33	0.29	1.02	0.00
GATCAAAGTAAAATCAAATT	2.06	4.23	2.86	1.02	2.76
GATCAAAGTAAAGACGAGGG	0.23	0.00	0.00	0.00	0.00
GATCAAAGTAAATACAATCA	0.00	0.00	0.00	0.00	0.34
GATCAAAGTAAATCGTGTGC	0.00	0.00	0.00	0.00	0.69
GATCAAAGTAAATGTGCAGT	2.29	0.00	0.00	1.36	1.38
GATCAAAGTAAATTCAAAAA	0.69	0.33	0.00	0.34	0.00
GATCAAAGTAAATTGAGTAA	0.23	0.00	0.00	0.00	0.00
GATCAAAGTAAGGCGGAGAC	0.00	0.33	0.00	0.00	0.00
GATCAAAGTAAGGCGGCCCC	0.00	0.00	0.00	0.34	0.00
GATCAAAGTAAGGCGGCGCC	0.00	0.00	0.00	0.00	0.34
GATCAAAGTAAGGCGGCGGC	0.23	0.00	0.00	0.00	0.00
GATCAAAGTAATGGAGACGT	0.00	0.00	0.00	0.00	0.34
GATCAAAGTACAACGGCCAG	0.00	0.00	0.00	0.68	0.00
GATCAAAGTACAATCAACAA	0.00	0.00	0.00	0.00	0.34
GATCAAAGTACAGAAAAGCG	0.23	0.00	0.00	0.00	0.00
GATCAAAGTACAGAAAAGCT	17.40	19.19	7.73	11.88	9.30
GATCAAAGTACAGAAAAGNN	0.00	0.00	0.00	0.00	0.00
GATCAAAGTACAGAGTCCTG	0.00	0.00	0.00	0.00	0.00

GATCAAAGTACAGAGTGTCG	0.00	0.00	0.29	0.00	0.00
GATCAAAGTACAGCGGCGCC	0.00	0.33	0.00	0.34	0.00
GATCAAAGTACAGCTGACTC	0.00	0.00	0.29	0.00	0.00
GATCAAAGTACAGGCTTGGT	0.00	0.33	0.00	0.00	0.00
GATCAAAGTACAGTGCAGGA	0.00	0.00	0.00	0.00	0.34
GATCAAAGTACATCGGCGCC	0.00	0.00	0.00	0.00	0.00
GATCAAAGTACCAAGAGGCT	0.00	0.00	0.00	0.00	0.34
GATCAAAGTACCAGAGGGGC	0.23	0.00	1.43	0.00	0.69
GATCAAAGTACCGAGCAGAA	0.00	0.65	0.00	0.00	0.00
GATCAAAGTACCTGGACAAC	0.00	0.00	0.29	0.00	0.00
GATCAAAGTACCTTCGGAGG	0.23	0.00	0.00	0.00	0.00
GATCAAAGTACGACGGCGCC	0.00	0.00	0.00	0.00	0.00
GATCAAAGTACGACTTTGCG	0.00	0.00	0.00	0.00	0.00
GATCAAAGTACGCGGCGCGC	0.00	0.00	0.00	0.00	0.00
GATCAAAGTACGCTCGTGAC	0.00	0.33	0.00	0.00	0.00
GATCAAAGTACGCTTCTGCC	0.00	0.00	0.29	0.00	0.00
GATCAAAGTACGGAGGACCA	0.00	0.33	0.00	0.00	0.00
GATCAAAGTACGGAGGAGAC	0.00	0.00	0.00	0.00	0.34
GATCAAAGTACGGAGGAGCC	0.00	0.00	0.29	0.00	0.00
GATCAAAGTACGGAGGCGCC	0.46	0.00	0.00	0.34	0.00
GATCAAAGTACGGAGGCGCG	0.00	0.33	0.00	0.00	0.00
GATCAAAGTACGGAGGCGGC	0.00	0.00	0.29	0.00	0.00
GATCAAAGTACGGAGGGGCA	0.00	0.00	0.00	0.34	0.00
GATCAAAGTACGGAGGGGGC	0.23	0.00	0.00	0.00	0.00
GATCAAAGTACGGAGGTCCC	0.23	0.00	0.00	0.00	0.00
GATCAAAGTACGGAGGTGCC	0.23	0.00	0.00	0.00	0.00
GATCAAAGTACGGCAACTGC	0.00	0.00	0.29	0.00	0.00
GATCAAAGTACGGCAGCATC	0.00	0.00	0.29	0.00	0.00
GATCAAAGTACGGCAGCGCC	0.00	0.33	0.29	0.00	0.00
GATCAAAGTACGGCGACGCC	0.23	0.33	0.00	0.34	0.34
GATCAAAGTACGGCGCCACC	0.00	0.00	0.00	0.00	0.34
GATCAAAGTACGGCGCGCCA	0.00	0.33	0.00	0.00	0.00
GATCAAAGTACGGCGGACCA	0.00	0.00	0.00	0.00	0.34
GATCAAAGTACGGCGGACCC	0.00	0.00	0.29	0.00	0.00
GATCAAAGTACGGCGGAGAC	0.00	0.00	0.29	0.00	0.34
GATCAAAGTACGGCGGAGCA	0.00	0.00	0.29	0.68	0.00
GATCAAAGTACGGCGGAGCC	0.46	4.23	1.15	1.02	1.72

GATCAAAGTACGGCGGAGCG	0.00	0.65	0.00	0.00	0.00
GATCAAAGTACGGCGGAGGA	0.00	0.00	0.00	0.34	0.00
GATCAAAGTACGGCGGAGGC	0.46	1.30	0.00	0.00	0.00
GATCAAAGTACGGCGGAGTG	0.00	0.00	0.00	0.00	0.34
GATCAAAGTACGGCGGCACC	0.23	0.33	0.57	0.00	0.00
GATCAAAGTACGGCGGCAGC	0.00	0.00	0.29	0.00	0.00
GATCAAAGTACGGCGGCCAC	0.00	0.00	0.00	0.00	0.34
GATCAAAGTACGGCGGCCCC	0.92	0.33	0.29	1.36	0.34
GATCAAAGTACGGCGGCCGC	0.00	0.00	0.00	0.34	0.00
GATCAAAGTACGGCGGCGAC	0.46	1.30	2.29	1.70	1.03
GATCAAAGTACGGCGGCGCA	0.00	1.63	0.00	2.04	1.03
GATCAAAGTACGGCGGCGCC	406.08	383.89	190.14	196.19	161.87
GATCAAAGTACGGCGGCGCG	2.06	0.33	1.15	1.36	0.34
GATCAAAGTACGGCGGCGCN	1.83	0.00	0.00	0.00	0.00
GATCAAAGTACGGCGGCGCT	0.92	0.65	0.00	0.34	0.00
GATCAAAGTACGGCGGCGGA	0.00	0.33	0.00	0.00	0.34
GATCAAAGTACGGCGGCGGC	38.00	26.03	20.05	25.12	11.71
GATCAAAGTACGGCGGCGGG	0.46	0.33	0.57	0.34	0.69
GATCAAAGTACGGCGGCGGN	0.69	0.00	0.00	0.00	0.00
GATCAAAGTACGGCGGCGGT	0.00	0.33	0.00	0.00	0.34
GATCAAAGTACGGCGGCGNC	0.00	0.00	0.29	0.00	0.00
GATCAAAGTACGGCGGCGNN	1.83	0.00	0.00	0.00	0.00
GATCAAAGTACGGCGGCGTC	0.23	0.33	0.29	0.00	0.00
GATCAAAGTACGGCGGCTCC	0.23	0.33	0.00	0.00	0.00
GATCAAAGTACGGCGGCTCG	0.23	0.33	0.00	0.00	0.00
GATCAAAGTACGGCGGGCCC	0.00	0.00	0.00	0.34	0.34
GATCAAAGTACGGCGGGCGC	0.00	0.00	0.00	0.00	0.00
GATCAAAGTACGGCGGGGAA	0.00	0.33	0.00	0.00	0.00
GATCAAAGTACGGCGGGGAC	0.00	0.00	0.29	0.34	0.00
GATCAAAGTACGGCGGGGAN	0.00	0.00	0.00	0.00	0.00
GATCAAAGTACGGCGGGGCA	0.00	0.65	0.29	1.02	0.00
GATCAAAGTACGGCGGGGCC	12.36	19.19	20.90	64.83	18.94
GATCAAAGTACGGCGGGGCG	0.00	0.98	0.00	1.70	0.69
GATCAAAGTACGGCGGGGCN	0.46	0.00	0.00	0.00	0.00
GATCAAAGTACGGCGGGGCT	0.00	0.00	0.29	0.00	0.00
GATCAAAGTACGGCGGGGGA	0.00	0.65	0.00	0.34	0.34
GATCAAAGTACGGCGGGGGC	6.64	6.83	4.58	30.89	8.61

GATCAAAGTACGGCGGGGGG	0.23	0.33	0.00	1.36	0.00
GATCAAAGTACGGCGGGGGN	0.00	0.00	0.00	0.34	0.00
GATCAAAGTACGGCGGGGGT	0.00	0.00	0.00	0.00	0.00
GATCAAAGTACGGCGGGGNG	0.00	0.00	0.00	0.34	0.00
GATCAAAGTACGGCGGGGNN	0.23	0.00	0.00	0.00	0.00
GATCAAAGTACGGCGGGGTC	0.00	0.00	0.29	0.00	0.00
GATCAAAGTACGGCGGGTCC	0.23	0.00	0.00	0.00	0.00
GATCAAAGTACGGCGGGTTG	0.00	0.00	0.00	0.00	0.00
GATCAAAGTACGGCGGNGCA	0.00	0.00	0.00	0.00	0.34
GATCAAAGTACGGCGGNGCC	0.00	0.00	0.00	0.00	0.69
GATCAAAGTACGGCGGNGNN	0.23	0.00	0.00	0.00	0.00
GATCAAAGTACGGCGGNNNN	0.69	0.00	0.00	0.00	0.00
GATCAAAGTACGGCGGTGAC	0.00	0.00	0.29	0.00	0.00
GATCAAAGTACGGCGGTGCC	0.00	0.65	0.00	0.00	0.34
GATCAAAGTACGGCGGTGCG	0.23	0.00	0.00	0.00	0.00
GATCAAAGTACGGCGGTGGC	0.00	0.33	0.29	0.00	0.00
GATCAAAGTACGGCGTAGCC	0.00	0.00	0.00	0.00	0.34
GATCAAAGTACGGCGTCTGC	0.00	0.33	0.00	0.00	0.00
GATCAAAGTACGGCTGAGCC	0.00	0.33	0.00	0.00	0.00
GATCAAAGTACGGGGGCGCC	0.23	0.00	0.00	0.00	0.00
GATCAAAGTACGGTCGTATG	0.23	0.00	0.00	0.00	0.00
GATCAAAGTACGGTGGCGCC	0.23	0.00	0.00	0.34	0.00
GATCAAAGTACGGTGGCGTG	0.00	0.00	0.00	0.34	0.00
GATCAAAGTACGGTTGCCGC	0.00	0.00	0.00	0.00	0.34
GATCAAAGTACGGTTGCGCC	0.00	0.00	0.00	0.00	0.00
GATCAAAGTACGTAGGCGCC	0.00	0.00	0.00	0.00	0.34
GATCAAAGTACGTTCAGCAA	0.00	0.00	0.00	0.00	0.69
GATCAAAGTACTAGAGAGAC	0.00	0.00	0.00	0.00	0.00
GATCAAAGTACTGAAAAGCT	0.00	0.00	0.00	0.00	0.34
GATCAAAGTACTGATGACAA	0.00	0.00	0.00	0.34	0.00
GATCAAAGTACTGCGGCGCC	0.00	0.33	0.00	0.00	0.34
GATCAAAGTACTTAGCCTTC	0.00	0.00	0.00	0.00	0.34
GATCAAAGTACTTGAAGGTT	0.00	0.00	0.29	0.00	0.00
GATCAAAGTACTTTGATGTT	0.23	0.00	0.00	0.00	0.00
GATCAAAGTAGAAAAAAAAA	1.14	0.00	0.00	0.68	1.38
GATCAAAGTAGAATCAAAAA	1.60	0.00	0.00	0.68	1.03
GATCAAAGTAGAATCAACAA	46.47	17.24	21.48	18.67	23.76

GATCAAAGTAGAATCAACAG	0.00	0.00	0.29	0.00	0.00
GATCAAAGTAGAATCAACAT	0.00	0.33	0.00	0.00	0.00
GATCAAAGTAGAATCAACCC	0.00	0.00	0.29	0.00	0.00
GATCAAAGTAGAATCAACCT	0.00	0.00	0.00	0.00	0.34
GATCAAAGTAGAATCAACGA	0.00	0.00	0.29	0.00	0.00
GATCAAAGTAGAATCAACTA	0.00	0.00	0.00	0.34	0.00
GATCAAAGTAGAATCAACTC	0.00	0.00	0.00	0.00	0.00
GATCAAAGTAGAATCAACTG	0.23	0.00	0.00	0.00	0.00
GATCAAAGTAGAATCAATTA	0.00	0.00	0.00	0.00	0.00
GATCAAAGTAGAATCATCAA	0.23	0.00	0.00	0.00	0.00
GATCAAAGTAGAATCATTCA	0.00	0.00	0.00	0.00	0.00
GATCAAAGTAGAATCGAAAA	0.00	0.00	0.00	0.00	0.00
GATCAAAGTAGACTCAACAA	0.23	0.00	0.00	0.00	0.00
GATCAAAGTAGAGGCGAAGG	0.23	0.00	0.00	0.00	0.00
GATCAAAGTAGAGTTCTCAA	0.23	0.00	0.00	0.34	0.00
GATCAAAGTAGCCTCCGACG	0.23	0.00	0.00	0.34	0.00
GATCAAAGTAGCTACCTCGA	0.00	0.65	0.00	0.00	0.00
GATCAAAGTAGGACAGGAGG	0.00	0.00	0.00	0.00	0.00
GATCAAAGTAGGAGGGTTCG	0.00	0.00	0.29	0.00	0.00
GATCAAAGTAGGCGGCGCCT	0.00	0.00	0.00	0.00	0.34
GATCAAAGTAGGGATGTAAA	0.23	0.65	0.00	0.00	0.00
GATCAAAGTAGGGCGGCGGC	0.23	0.00	0.00	0.00	0.00
GATCAAAGTAGGGTTGACCA	0.00	0.00	0.29	0.00	0.00
GATCAAAGTAGGTTTGACAA	0.00	0.00	0.00	0.34	0.00
GATCAAAGTAGGTTTGACCA	0.00	0.00	0.00	0.00	0.00
GATCAAAGTAGTAAAAAAAA	0.69	0.00	0.86	2.04	0.69
GATCAAAGTAGTAACGATGT	0.00	0.00	0.00	0.00	0.34
GATCAAAGTAGTAAGGAGTA	0.00	0.00	0.00	0.00	0.00
GATCAAAGTAGTAAGGATGT	2.75	4.23	2.58	1.70	2.76
GATCAAAGTAGTAGTGGAAA	0.00	0.00	0.00	0.34	0.00
GATCAAAGTAGTCCTAATCA	0.00	0.00	0.57	0.00	0.00
GATCAAAGTAGTCGCCTCCG	0.23	0.00	0.00	0.00	0.00
GATCAAAGTAGTTGCAAATC	0.00	0.00	0.00	0.00	0.00
GATCAAAGTAGTTTGAAAAA	0.00	0.00	0.00	0.00	0.00
GATCAAAGTAGTTTGAAACC	0.00	0.00	0.00	0.00	0.00
GATCAAAGTAGTTTGAACAA	0.00	0.00	0.00	0.00	0.00
GATCAAAGTAGTTTGAACAC	0.46	0.00	0.00	0.00	0.00

GATCAAAGTAGTTTGAACAG	0.00	0.65	0.00	0.00	0.00
GATCAAAGTAGTTTGAAGAC	0.00	0.00	0.00	0.34	0.00
GATCAAAGTAGTTTGAAGAG	6.64	3.25	4.58	7.13	3.79
GATCAAAGTAGTTTGAAGGA	3.20	4.88	3.72	5.77	7.23
GATCAAAGTAGTTTGAAGNN	0.23	0.00	0.00	0.00	0.00
GATCAAAGTAGTTTGAAGTC	0.00	0.00	0.00	0.00	0.00
GATCAAAGTAGTTTGAAGTG	0.00	0.00	0.00	0.00	0.00
GATCAAAGTAGTTTGATCAC	0.00	0.00	0.00	0.00	0.69
GATCAAAGTAGTTTGCTCAC	0.23	0.00	0.00	0.00	0.00
GATCAAAGTAGTTTGGATGA	0.00	0.00	0.00	0.00	0.00
GATCAAAGTAGTTTGGATGT	1.14	1.30	2.29	2.72	0.34
GATCAAAGTAGTTTGGCGGC	0.23	0.00	0.00	0.00	0.00
GATCAAAGTAGTTTGGTCAC	0.23	0.00	0.00	0.00	0.00
GATCAAAGTAGTTTTTTTAA	0.00	0.00	0.29	0.00	0.00
GATCAAAGTATAGAAAAGCG	0.00	0.00	0.00	0.00	0.00
GATCAAAGTATATGGAACGA	0.00	0.00	0.29	0.00	0.00
GATCAAAGTATCAAGACTAT	1.60	0.00	0.86	1.70	0.00
GATCAAAGTATCACAGTCCG	0.00	0.00	0.00	0.00	0.00
GATCAAAGTATCAGCGGTTG	0.23	0.00	0.00	0.00	0.00
GATCAAAGTATCAGCGTTTG	2.98	2.93	0.00	2.04	1.72
GATCAAAGTATCAGGAACGA	0.00	0.00	0.29	0.00	0.00
GATCAAAGTATCTCTCTCAA	0.00	0.33	0.00	0.00	0.00
GATCAAAGTATCTGAAACAA	0.00	0.00	0.00	0.34	0.00
GATCAAAGTATCTGGAAAAA	0.00	0.33	0.00	0.00	0.00
GATCAAAGTATCTGGAAAGA	0.23	0.00	0.29	0.00	0.00
GATCAAAGTATCTGGAACAA	0.92	0.33	0.29	0.34	0.34
GATCAAAGTATCTGGAACAG	0.00	0.00	0.29	0.00	0.00
GATCAAAGTATCTGGAACGA	61.80	21.80	20.62	20.03	21.01
GATCAAAGTATCTGGAACGG	0.23	0.00	0.86	0.00	0.00
GATCAAAGTATCTGGAACGN	0.23	0.00	0.00	0.00	0.00
GATCAAAGTATCTGGAACGT	0.23	0.65	0.00	0.00	0.00
GATCAAAGTATCTGGAACNA	0.00	0.00	0.00	0.00	0.34
GATCAAAGTATCTGGAACTG	0.00	0.00	0.29	0.00	0.00
GATCAAAGTATCTGGAATTA	0.23	0.00	0.00	0.00	0.00
GATCAAAGTATCTGGAGGTA	0.00	0.00	0.00	0.00	0.34
GATCAAAGTATCTGGANCGA	0.00	0.33	0.00	0.00	0.00
GATCAAAGTATCTGGANCNA	0.00	0.33	0.00	0.00	0.00

GATCAAAGTATCTGGANCNN	0.23	0.00	0.00	0.00	0.00
GATCAAAGTATGAAAGGCAC	0.46	0.00	0.29	0.34	1.03
GATCAAAGTATGCCTGCGGG	0.00	0.00	0.29	0.00	0.00
GATCAAAGTATGGAGATATG	0.23	0.00	0.00	0.00	0.00
GATCAAAGTATGGAGCTATG	2.29	2.60	0.86	2.38	2.41
GATCAAAGTATGGCATGTGC	0.00	0.00	0.00	0.00	0.00
GATCAAAGTATGGCGGCGCC	0.69	0.00	0.29	0.00	0.00
GATCAAAGTATGGCGGCGGC	0.23	0.00	0.00	0.00	0.00
GATCAAAGTATGGCGGGGCC	0.23	0.00	0.00	0.00	0.00
GATCAAAGTATGTTGCGGGT	0.00	0.00	0.00	0.00	0.00
GATCAAAGTATTCTCGTTGG	0.23	0.00	0.00	0.68	0.00
GATCAAAGTATTTTTCCAGC	0.00	0.00	0.00	0.00	0.00
GATCAAAGTATTTTTGAATG	0.00	0.00	0.00	0.00	0.34
GATCAAAGTCAAAAAAAAAA	1.37	0.33	1.43	0.34	0.69
GATCAAAGTCAAAAAAAGAA	0.00	0.00	0.29	0.00	0.00
GATCAAAGTCAAAACAACCA	0.00	0.33	0.00	0.00	0.00
GATCAAAGTCAAAGATTTGA	0.23	0.00	0.29	0.00	0.00
GATCAAAGTCAACGGGACTG	0.00	0.33	0.00	0.68	0.00
GATCAAAGTCAAGACCCTCA	0.00	0.33	0.00	0.00	0.00
GATCAAAGTCAAGACTTGTC	0.00	0.00	0.00	0.00	0.00
GATCAAAGTCAAGAGTTTCG	1.37	0.00	0.00	0.34	0.00
GATCAAAGTCAAGGGGGATG	0.00	0.00	0.29	0.00	0.00
GATCAAAGTCAAGGGGGATN	0.23	0.00	0.00	0.00	0.00
GATCAAAGTCAAGGGGGATT	15.11	9.76	2.00	7.13	3.79
GATCAAAGTCAAGGGGGCTT	0.69	0.00	0.00	0.34	0.00
GATCAAAGTCAAGGGGGGTT	1.37	0.00	0.29	0.00	0.00
GATCAAAGTCAAGGGGTTAT	0.00	0.33	0.00	0.00	0.00
GATCAAAGTCAAGGTGGATT	0.23	0.00	0.00	0.00	0.00
GATCAAAGTCAAGTGATGCG	0.00	0.00	0.00	0.34	0.00
GATCAAAGTCAAGTTCCGGG	0.00	0.00	0.00	0.34	0.00
GATCAAAGTCAATCGATTTC	0.46	0.33	0.00	0.34	0.00
GATCAAAGTCAATGATGGGG	0.00	0.33	0.00	0.00	0.00
GATCAAAGTCAATGCCTACG	1.37	0.00	0.29	0.00	0.00
GATCAAAGTCAATGGGACTG	0.00	0.00	0.00	0.00	0.00
GATCAAAGTCAATTCATTCC	0.00	0.00	0.29	0.00	0.00
GATCAAAGTCACAAACACAA	1.37	0.98	0.00	0.00	0.00
GATCAAAGTCACAGTCTTTG	0.00	0.00	0.00	0.34	0.00

GATCAAAGTCACCACCAAAA	0.00	0.00	0.00	0.00	0.34
GATCAAAGTCACGGTCTTGC	0.00	0.00	0.00	0.34	0.00
GATCAAAGTCACGGTGAATG	0.00	0.00	0.00	0.00	0.69
GATCAAAGTCACTCACCAGT	0.23	0.00	0.00	0.00	0.00
GATCAAAGTCACTCACTATG	0.00	0.00	0.29	0.00	0.00
GATCAAAGTCACTCCCAAAA	0.92	0.00	0.57	0.68	0.00
GATCAAAGTCACTCCCAAGC	0.00	0.00	0.00	0.68	0.00
GATCAAAGTCACTCCCAAGN	0.23	0.00	0.00	0.00	0.00
GATCAAAGTCACTCCCAAGT	17.85	10.74	8.88	14.93	8.27
GATCAAAGTCACTCCCAANN	0.23	0.00	0.00	0.00	0.00
GATCAAAGTCACTCCCCAGT	0.00	0.00	0.00	0.34	0.00
GATCAAAGTCACTCCCTAGT	0.00	0.33	0.00	0.00	0.00
GATCAAAGTCACTCCGCCGT	0.23	0.00	0.00	0.00	0.00
GATCAAAGTCACTGGAGTTC	0.00	0.00	0.00	0.34	0.00
GATCAAAGTCAGACAAAAGT	0.23	0.00	0.00	0.00	0.00
GATCAAAGTCAGACACCATC	0.00	0.00	0.00	0.00	0.00
GATCAAAGTCAGACGTGCGG	0.00	0.00	0.00	0.34	0.00
GATCAAAGTCAGATGTGACC	0.00	0.00	0.00	1.36	0.34
GATCAAAGTCAGCACCGTGC	0.00	0.00	0.00	0.34	0.00
GATCAAAGTCAGCCCTCAAG	0.00	0.00	0.00	0.34	0.00
GATCAAAGTCAGTCAACTAC	0.00	0.00	0.29	0.00	0.00
GATCAAAGTCATACGTTAGG	0.00	0.33	0.00	0.00	0.00
GATCAAAGTCATCAAGGGCA	0.00	0.00	0.29	0.00	0.00
GATCAAAGTCATCAGACGGT	0.23	0.00	0.00	0.00	0.00
GATCAAAGTCATGACACTCA	0.00	0.33	0.00	0.00	0.00
GATCAAAGTCATGACATTCA	1.37	0.65	0.29	0.34	1.03
GATCAAAGTCATGACTTTCT	0.00	0.00	0.00	0.34	0.00
GATCAAAGTCATGATTGTGT	0.00	0.00	0.29	0.00	0.00
GATCAAAGTCATGCGGTGTG	0.00	0.00	0.00	0.00	0.34
GATCAAAGTCATGGAGACGT	0.00	0.00	0.00	0.00	0.34
GATCAAAGTCATGGATGGTA	0.00	0.00	0.00	0.00	0.00
GATCAAAGTCATGTCGGCAA	0.00	0.33	0.00	0.00	0.00
GATCAAAGTCATTGCGGTGG	0.00	0.00	0.00	0.00	0.00
GATCAAAGTCATTTGGCGCA	0.23	0.00	0.00	0.00	0.00
GATCAAAGTCCAACATGTCT	0.00	0.00	0.00	0.00	1.03
GATCAAAGTCCAGACAGGTT	0.00	0.00	0.00	0.00	0.34
GATCAAAGTCCAGCAGGAGA	0.00	0.00	0.00	0.00	0.00

GATCAAAGTCCAGGGGCTCG	0.00	0.00	0.00	0.00	0.00
GATCAAAGTCCATATCCTAC	0.00	0.00	0.29	0.00	0.00
GATCAAAGTCCATATCCTCA	0.00	0.00	0.00	0.00	0.00
GATCAAAGTCCATATCCTCC	0.46	1.95	1.43	2.38	2.07
GATCAAAGTCCATATCTTCC	9.16	5.21	2.00	9.16	6.89
GATCAAAGTCCATATCTTCN	0.46	0.00	0.00	0.00	0.00
GATCAAAGTCCATCTGCTCT	0.00	0.00	0.00	0.34	0.00
GATCAAAGTCCATGAAGTCC	0.46	0.33	0.00	0.34	0.00
GATCAAAGTCCATGACGCAA	0.00	0.00	0.00	0.00	0.34
GATCAAAGTCCCACGGGATT	0.69	0.00	0.00	0.34	0.34
GATCAAAGTCCCCAGGAAGC	0.23	0.00	0.29	1.02	0.34
GATCAAAGTCCCCATGCCAG	0.00	0.00	0.00	0.00	0.00
GATCAAAGTCCCCCGGGCAG	0.00	0.00	0.00	0.00	0.34
GATCAAAGTCCCGGGCAGAT	0.00	0.00	0.00	0.00	0.69
GATCAAAGTCCCGTGGCCTT	0.00	0.00	0.00	0.00	0.00
GATCAAAGTCCCTACGAGAA	0.00	0.00	0.00	0.34	0.00
GATCAAAGTCCCTACGCGCA	0.46	0.33	0.00	0.00	0.69
GATCAAAGTCCCTCCTCGAG	0.23	0.00	0.00	0.00	0.00
GATCAAAGTCCCTGCCCCAA	0.00	0.33	0.00	0.68	0.00
GATCAAAGTCCCTTTCCTGC	0.00	0.33	0.29	0.00	0.34
GATCAAAGTCCGAAGCCGGA	0.23	0.00	0.00	0.00	0.00
GATCAAAGTCCGACCACACC	0.00	0.33	0.00	0.00	0.00
GATCAAAGTCCGACCTGCCT	0.00	0.00	0.00	0.34	0.00
GATCAAAGTCCGAGGGGACA	0.00	0.33	0.00	0.00	0.34
GATCAAAGTCCGGAGGAGCG	0.23	0.00	0.00	0.34	0.00
GATCAAAGTCCGGCGCCTGA	0.00	0.00	0.00	0.00	0.34
GATCAAAGTCCGGGCCATAC	0.00	0.00	0.00	0.00	0.34
GATCAAAGTCCGGGTATTAG	0.00	0.33	0.00	0.00	0.00
GATCAAAGTCCTCAGCAGAC	0.00	0.00	0.29	0.00	0.00
GATCAAAGTCCTCGCGAAGG	0.00	1.30	0.00	0.00	0.00
GATCAAAGTCCTGAAGCACA	0.00	0.00	0.00	0.00	0.00
GATCAAAGTCCTGAAGCTAA	0.23	0.00	2.58	0.00	0.34
GATCAAAGTCCTGAATCGAT	0.00	0.00	0.29	0.00	0.00
GATCAAAGTCCTGCACGAAG	0.00	0.33	0.00	1.36	0.00
GATCAAAGTCCTGTGAGCTT	0.00	0.00	0.00	0.00	0.34
GATCAAAGTCCTGTGCAGTT	0.00	0.00	0.00	0.00	0.34
GATCAAAGTCGAAAAAAAAA	0.00	0.00	0.00	0.00	0.69

GATCAAAGTCGAAGGAAAAA	0.00	0.00	0.29	0.00	0.00
GATCAAAGTCGAATTCTCTC	0.00	0.00	0.00	0.00	0.34
GATCAAAGTCGAATTTGGGC	0.00	0.00	0.00	0.68	0.00
GATCAAAGTCGAATTTGGGT	0.00	0.33	0.57	0.68	1.03
GATCAAAGTCGACTCGGGGG	0.23	0.00	0.00	0.00	0.00
GATCAAAGTCGACTCGGTGG	1.83	2.60	0.00	2.38	0.34
GATCAAAGTCGAGAACACGG	0.00	1.30	0.00	0.34	0.00
GATCAAAGTCGAGAACACGN	0.23	0.00	0.00	0.00	0.00
GATCAAAGTCGAGGATGAGG	0.23	0.00	0.00	0.34	0.00
GATCAAAGTCGAGTCCAGGC	0.00	0.00	0.00	0.00	0.34
GATCAAAGTCGAGTCGAGCA	0.00	0.00	0.00	0.00	0.00
GATCAAAGTCGATGACGTTG	0.00	1.30	0.57	1.02	0.34
GATCAAAGTCGCAGCCGGGC	0.00	0.00	0.57	0.00	0.00
GATCAAAGTCGCAGCGTCGA	0.00	0.00	0.00	0.00	0.00
GATCAAAGTCGCAGGCCTCT	0.00	0.00	0.00	0.00	0.00
GATCAAAGTCGCAGTCGAGG	0.00	0.65	0.00	0.00	0.34
GATCAAAGTCGCAGTCGAGT	0.00	0.00	0.00	0.00	0.00
GATCAAAGTCGCCGCGGGGC	0.00	0.00	0.00	0.00	0.00
GATCAAAGTCGCCGCGTGGC	0.69	0.33	0.29	0.00	0.00
GATCAAAGTCGCCTCCTAGC	0.00	0.00	0.00	0.00	0.34
GATCAAAGTCGCGCTCGAAA	0.00	0.00	0.00	0.34	0.00
GATCAAAGTCGCGGGCGCTC	0.00	0.33	0.00	0.00	0.00
GATCAAAGTCGCGTGGCCTT	1.83	2.60	2.86	3.39	2.07
GATCAAAGTCGCTCCCGTTA	0.23	0.00	0.00	0.00	0.00
GATCAAAGTCGCTCCGCCCC	0.23	0.00	0.00	0.00	0.00
GATCAAAGTCGCTGGTTTCA	0.23	0.00	0.00	0.00	0.00
GATCAAAGTCGGAACCTCCG	0.00	0.00	0.00	0.34	0.00
GATCAAAGTCGGCCCCACGA	0.00	0.00	0.00	0.34	0.00
GATCAAAGTCGGCGAAGCGT	0.00	0.00	0.00	1.02	0.00
GATCAAAGTCGGCGCTAAGG	0.00	0.33	0.00	0.00	0.00
GATCAAAGTCGGCGCTCACG	0.00	0.00	0.00	0.00	0.00
GATCAAAGTCGGCGCTCTTG	0.00	0.00	0.00	0.34	0.00
GATCAAAGTCGGCGTACCTG	0.00	0.00	0.00	0.00	0.34
GATCAAAGTCGGTCCTCAAG	0.00	0.00	0.00	0.00	0.00
GATCAAAGTCGGTGCCATCC	0.00	0.00	0.29	0.00	0.69
GATCAAAGTCGGTGGCGCTC	0.46	0.65	0.00	0.68	0.34
GATCAAAGTCGGTGGTAGAC	0.23	1.30	0.86	0.00	1.03

GATCAAAGTCGTAGAATCAG	0.00	0.00	0.00	0.00	0.34
GATCAAAGTCGTCATGAAAA	0.00	1.30	0.57	0.34	1.03
GATCAAAGTCGTCATGATAA	0.00	0.00	0.00	0.00	0.34
GATCAAAGTCGTCATGATGC	0.00	0.00	0.00	0.00	0.34
GATCAAAGTCGTCATGATGT	1.60	1.30	2.58	2.38	3.79
GATCAAAGTCGTCGCTGCTG	0.00	0.00	0.00	0.00	0.00
GATCAAAGTCGTCTGAGGGG	0.00	0.00	0.29	0.00	0.00
GATCAAAGTCGTTACCATCC	0.00	0.00	0.00	0.00	0.34
GATCAAAGTCGTTGAGCCGA	0.00	0.33	0.00	0.00	0.34
GATCAAAGTCGTTGCCGGTG	0.00	0.00	0.29	0.00	0.00
GATCAAAGTCTACCACACCT	0.00	0.00	0.00	0.00	0.00
GATCAAAGTCTAGTGAGATT	0.23	0.00	0.00	0.00	0.00
GATCAAAGTCTATGGTTTCA	0.00	0.33	0.00	0.00	0.00
GATCAAAGTCTCAAAAAAAA	0.46	0.00	0.29	0.00	0.00
GATCAAAGTCTCAAAAAGCC	0.00	0.00	0.00	0.00	0.00
GATCAAAGTCTCACTGGCTG	0.00	0.00	0.29	0.00	0.00
GATCAAAGTCTCCAGCAGCA	0.23	0.00	0.00	0.00	0.00
GATCAAAGTCTCCGGTTTCA	0.46	0.00	0.00	0.00	0.00
GATCAAAGTCTCGGTTGTGA	0.00	0.33	0.00	0.00	0.00
GATCAAAGTCTCTAAAAAAA	0.92	0.00	0.29	0.00	0.00
GATCAAAGTCTCTCATTATG	0.69	0.00	0.00	0.34	0.00
GATCAAAGTCTCTCCTCCCC	0.23	0.00	0.00	0.00	0.00
GATCAAAGTCTCTGATTAAA	0.00	0.00	0.29	0.00	0.00
GATCAAAGTCTCTGATTTTC	0.00	0.00	0.00	0.00	0.00
GATCAAAGTCTCTGGATTCA	0.00	0.00	0.29	0.00	0.00
GATCAAAGTCTCTGGATTCC	0.23	0.00	0.00	0.00	0.00
GATCAAAGTCTCTGGCCATG	0.00	0.00	0.00	0.00	0.00
GATCAAAGTCTCTGGCTTCA	0.23	0.00	0.00	0.00	0.00
GATCAAAGTCTCTGGCTTCC	0.46	0.00	0.00	0.00	0.00
GATCAAAGTCTCTGGGTTCA	0.69	0.00	0.00	0.00	0.00
GATCAAAGTCTCTGGGTTCG	0.23	0.00	0.00	0.00	0.00
GATCAAAGTCTCTGGTAAAA	1.37	0.00	0.00	0.00	0.00
GATCAAAGTCTCTGGTCTCA	0.23	0.33	0.00	0.00	0.34
GATCAAAGTCTCTGGTGAAA	0.00	0.00	0.29	0.00	0.00
GATCAAAGTCTCTGGTGTCA	0.00	0.33	0.00	0.00	0.00
GATCAAAGTCTCTGGTNTCA	0.00	0.00	0.00	0.00	0.34
GATCAAAGTCTCTGGTTAAA	1.14	0.00	0.00	0.00	0.00

GATCAAAGTCTCTGGTTCCA	0.23	0.00	0.00	0.00	0.34
GATCAAAGTCTCTGGTTGCA	0.23	0.00	0.00	0.00	0.00
GATCAAAGTCTCTGGTTTAA	0.23	0.00	0.29	0.34	0.00
GATCAAAGTCTCTGGTTTAN	0.00	0.00	0.00	0.00	0.34
GATCAAAGTCTCTGGTTTCA	249.05	67.67	72.16	51.59	68.54
GATCAAAGTCTCTGGTTTCC	0.69	0.65	0.57	0.00	0.34
GATCAAAGTCTCTGGTTTCG	0.92	0.00	0.29	0.00	0.00
GATCAAAGTCTCTGGTTTCN	0.92	0.00	0.00	0.00	0.00
GATCAAAGTCTCTGGTTTCT	0.23	0.00	0.00	0.00	0.00
GATCAAAGTCTCTGGTTTNA	0.00	0.00	0.00	0.00	0.00
GATCAAAGTCTCTGGTTTNN	1.37	0.00	0.00	0.00	0.00
GATCAAAGTCTCTGGTTTTA	0.23	0.00	0.00	0.00	0.00
GATCAAAGTCTCTGTTTTCA	0.23	0.00	0.00	0.00	0.00
GATCAAAGTCTCTTGTTTCA	0.23	0.00	0.00	0.00	0.00
GATCAAAGTCTGAAACATGG	0.00	0.33	0.57	0.34	1.38
GATCAAAGTCTGACGTTTTC	0.00	0.00	0.00	0.34	0.00
GATCAAAGTCTGACTGCAAT	0.00	0.00	0.29	0.00	0.00
GATCAAAGTCTGACTGNCAC	0.00	0.00	0.00	0.00	0.34
GATCAAAGTCTGACTGTAAA	0.46	0.00	0.29	0.00	0.34
GATCAAAGTCTGACTGTAAC	0.00	0.00	0.00	0.00	0.00
GATCAAAGTCTGACTGTCAA	2.75	1.63	0.86	2.38	1.72
GATCAAAGTCTGACTGTCAC	32.28	26.03	16.04	28.51	28.93
GATCAAAGTCTGACTGTCAG	0.00	0.00	0.00	0.00	0.00
GATCAAAGTCTGACTGTCAT	0.00	0.00	0.00	0.00	0.34
GATCAAAGTCTGACTGTCCC	0.00	0.00	0.00	0.00	0.34
GATCAAAGTCTGACTGTCTC	0.00	0.00	0.29	0.34	0.00
GATCAAAGTCTGATCAACAT	0.46	0.00	0.00	0.00	0.00
GATCAAAGTCTGATTAAAAA	0.00	0.00	0.00	0.00	0.00
GATCAAAGTCTGATTGTCAC	0.00	0.00	0.00	0.00	0.69
GATCAAAGTCTGCAAAAAAA	0.00	0.33	0.00	0.00	0.00
GATCAAAGTCTGCCAGGGAT	0.00	0.00	0.00	0.34	0.00
GATCAAAGTCTGCCGTGGAT	0.00	0.00	0.29	0.34	0.00
GATCAAAGTCTGCCGTTCCA	0.00	0.00	0.29	0.00	0.00
GATCAAAGTCTGCCTTGCCT	0.00	0.00	0.00	0.00	1.03
GATCAAAGTCTGCGACTTTG	0.00	0.00	0.00	0.34	0.00
GATCAAAGTCTGCGTTGTCA	0.00	0.33	0.00	0.00	0.00
GATCAAAGTCTGCTCCGCCC	0.00	0.00	0.57	0.00	0.34

GATCAAAGTCTGCTCCGCCT	0.00	0.00	0.00	0.00	0.34
GATCAAAGTCTGGACATGGT	0.00	0.00	0.29	0.00	0.00
GATCAAAGTCTGGGACATTC	0.00	0.00	0.00	0.00	0.00
GATCAAAGTCTGTCCAATCG	0.00	0.00	0.00	0.34	0.00
GATCAAAGTCTTAAGAAGAA	0.23	0.00	0.00	0.00	0.00
GATCAAAGTCTTAAGCAGCA	0.00	0.00	0.00	0.00	0.00
GATCAAAGTCTTCAGCAAAA	0.69	0.00	0.00	0.00	0.00
GATCAAAGTCTTCAGCAGCA	11.67	3.58	3.72	8.49	6.89
GATCAAAGTCTTCAGCAGCC	0.00	0.00	0.29	0.34	0.00
GATCAAAGTCTTCAGCAGTC	0.00	0.00	0.00	0.00	0.00
GATCAAAGTCTTCCCAGTGC	0.00	0.98	0.00	0.00	0.00
GATCAAAGTCTTCCGCAGAA	0.00	0.00	0.00	0.00	0.00
GATCAAAGTCTTCGCGCAGC	0.00	0.00	0.00	0.00	0.00
GATCAAAGTCTTCTCTGAAA	0.00	0.00	0.00	0.34	0.00
GATCAAAGTCTTGAGAGCCG	0.00	0.00	0.29	0.00	0.00
GATCAAAGTCTTGAGGTCAA	0.00	0.00	0.29	0.00	0.00
GATCAAAGTCTTGCGAGGTC	0.00	0.00	0.00	0.00	0.34
GATCAAAGTCTTGGAAAGGT	0.00	0.33	0.00	0.00	0.00
GATCAAAGTCTTTAGCAGCA	0.00	0.00	0.00	0.34	0.00
GATCAAAGTCTTTGGGTTGG	0.00	0.00	0.00	0.00	0.34
GATCAAAGTCTTTGGTTTCA	0.23	0.00	0.00	0.00	0.00
GATCAAAGTCTTTGTCTTCC	0.00	0.00	0.00	0.00	0.34
GATCAAAGTCTTTTCATTGG	0.00	0.00	0.00	0.34	0.00
GATCAAAGTCTTTTGCGCTC	0.00	0.00	0.00	0.00	0.00
GATCAAAGTGAAAAAAAAAA	6.18	2.60	3.15	5.43	5.85
GATCAAAGTGAAAAAGTGTA	0.23	0.00	0.00	0.00	0.00
GATCAAAGTGAAAGAGTTCA	0.00	0.00	0.00	0.00	0.00
GATCAAAGTGAACGAGTTCG	0.00	0.00	0.00	0.00	0.00
GATCAAAGTGAACGGAAAAG	0.23	0.00	0.00	0.00	0.00
GATCAAAGTGAAGGAGACGT	0.00	0.00	0.29	0.00	0.00
GATCAAAGTGAAGGCCCCGC	0.00	0.00	0.29	0.00	0.00
GATCAAAGTGAAGGGCAAAA	0.23	0.00	0.00	0.00	0.00
GATCAAAGTGAAGGGCAAGT	0.23	0.33	0.00	0.00	0.00
GATCAAAGTGAAGGGCAATG	0.23	0.00	0.00	0.00	0.00
GATCAAAGTGAAGGGGGGTT	0.23	0.00	0.00	0.00	0.00
GATCAAAGTGAAGGTCCCTG	0.00	0.33	0.00	0.34	0.00
GATCAAAGTGAATGGAGCCG	0.00	0.00	0.57	0.00	0.00

GATCAAAGTGAATTTGAAGG	0.23	0.00	0.00	0.00	0.34
GATCAAAGTGACAAAGTTGC	0.00	0.00	0.00	0.00	0.34
GATCAAAGTGACACCTAATG	0.00	0.00	0.00	0.00	0.34
GATCAAAGTGACATACGGAA	0.23	0.00	0.00	0.00	0.00
GATCAAAGTGACATCCGGAA	1.14	0.98	0.29	0.00	0.34
GATCAAAGTGACATCCGGAG	0.00	0.33	0.00	0.00	0.00
GATCAAAGTGACATTCAACA	0.00	0.33	0.00	0.00	0.00
GATCAAAGTGACCAAAAAAA	0.46	0.00	0.00	0.00	0.00
GATCAAAGTGACGAGAAGTA	0.23	0.00	0.00	0.00	0.00
GATCAAAGTGACGCGCCGTC	0.00	0.33	0.00	0.00	0.00
GATCAAAGTGACGGAGACGT	0.00	0.00	0.00	0.00	0.34
GATCAAAGTGACGTGTAAGG	0.00	0.00	0.00	0.00	0.00
GATCAAAGTGACGTTTGAGG	0.00	0.00	0.00	0.00	0.00
GATCAAAGTGAGAAAAAAAA	1.83	1.63	1.15	2.04	0.00
GATCAAAGTGAGAAAAAAAN	0.23	0.00	0.00	0.00	0.00
GATCAAAGTGAGAAAACAAA	0.00	0.00	0.29	0.00	0.00
GATCAAAGTGAGAAGGGGGG	6.64	7.48	6.30	10.86	9.64
GATCAAAGTGAGACCAAGCC	0.00	0.33	0.00	0.00	0.00
GATCAAAGTGAGAGGGGGGG	0.00	0.00	0.00	0.34	0.00
GATCAAAGTGAGCACGGTGC	0.00	0.33	0.00	0.68	0.00
GATCAAAGTGAGCCAGGGAC	0.23	0.00	0.00	0.00	0.00
GATCAAAGTGAGCCAGGTCC	0.00	0.00	0.00	0.00	0.34
GATCAAAGTGAGCCAGTGAC	1.37	2.60	4.01	3.73	0.69
GATCAAAGTGAGCGAAACGG	0.00	0.33	0.00	0.00	0.00
GATCAAAGTGAGCGAACCGG	0.00	0.33	0.00	0.00	0.34
GATCAAAGTGAGGAAAAAAA	0.23	0.00	0.00	0.34	0.00
GATCAAAGTGAGGCACAGTG	0.00	0.00	0.00	0.00	0.34
GATCAAAGTGAGGGAGACGT	0.00	0.00	0.29	0.00	0.00
GATCAAAGTGAGGGGAAAAA	0.00	0.00	0.00	0.00	0.00
GATCAAAGTGATAAAAAAAA	0.00	0.00	0.57	0.00	0.00
GATCAAAGTGATGAGACGTT	0.00	0.33	0.29	0.00	0.00
GATCAAAGTGATGCGACGTG	0.00	0.00	0.00	0.00	0.34
GATCAAAGTGATGGAAAAAA	0.00	0.00	0.29	0.00	0.00
GATCAAAGTGATGGAGAAGT	0.23	0.00	0.00	0.00	0.00
GATCAAAGTGATGGAGAATG	0.00	0.00	0.00	0.00	0.00
GATCAAAGTGATGGAGACGA	0.00	0.00	0.29	0.34	0.34
GATCAAAGTGATGGAGACGC	0.00	0.00	0.57	0.00	0.00

GATCAAAGTGATGGAGACGG	0.69	0.00	0.00	0.00	0.69
GATCAAAGTGATGGAGACGN	0.00	0.00	0.00	0.34	0.00
GATCAAAGTGATGGAGACGT	49.67	121.02	163.80	109.30	163.59
GATCAAAGTGATGGAGACNT	0.00	0.00	0.00	0.34	0.00
GATCAAAGTGATGGAGACTC	0.00	0.00	0.00	0.00	1.03
GATCAAAGTGATGGAGACTT	0.00	0.00	0.00	0.34	0.00
GATCAAAGTGATGGAGANNN	0.00	0.00	0.00	0.00	0.00
GATCAAAGTGATGGAGATGG	0.00	0.00	0.29	0.00	0.00
GATCAAAGTGATGGAGATGT	0.00	0.00	0.00	0.00	0.34
GATCAAAGTGATGGAGCAGT	0.00	0.33	0.00	0.00	0.00
GATCAAAGTGATGGAGCCGC	0.23	0.00	0.00	0.00	0.00
GATCAAAGTGATGGAGCCGT	0.23	0.65	0.29	0.00	0.34
GATCAAAGTGATGGAGCNNN	0.46	0.00	0.00	0.00	0.00
GATCAAAGTGATGGAGGCGT	0.46	0.00	0.00	0.00	0.00
GATCAAAGTGATGGAGGTGC	0.00	0.00	0.00	1.02	0.00
GATCAAAGTGATGGAGNCGT	0.00	0.00	0.00	0.00	0.34
GATCAAAGTGATGGAGNCNT	0.00	0.00	0.00	0.34	0.00
GATCAAAGTGATGGAGNNNN	0.69	0.00	0.00	0.00	0.00
GATCAAAGTGATGGAGTCGT	0.00	0.00	0.29	0.00	1.03
GATCAAAGTGATGGAGTTTG	0.00	0.33	0.00	0.00	0.00
GATCAAAGTGATGGCGAACA	0.00	0.00	0.29	0.00	0.00
GATCAAAGTGATGGCGACGT	0.00	0.00	0.29	0.00	0.00
GATCAAAGTGATGGCGGCGT	0.23	0.00	0.00	0.00	0.00
GATCAAAGTGATGGGCCAGG	0.00	0.00	0.00	0.00	0.00
GATCAAAGTGATGGGGACGT	0.00	0.00	0.00	0.34	0.34
GATCAAAGTGATGGGGTGGT	0.00	0.00	0.00	0.00	0.34
GATCAAAGTGATGTAGACGT	0.00	0.00	0.00	0.00	0.00
GATCAAAGTGATGTTCCATG	0.00	0.00	0.57	0.00	0.00
GATCAAAGTGATTCTGCATT	0.00	0.00	0.29	0.00	0.00
GATCAAAGTGATTGAGACGN	0.00	0.00	0.00	0.00	0.00
GATCAAAGTGCAAAAAAAAA	6.18	0.33	1.43	1.36	2.07
GATCAAAGTGCAAAAAAAAT	0.00	0.00	0.00	0.00	0.00
GATCAAAGTGCAAAAATCAA	0.00	0.00	0.00	0.00	0.00
GATCAAAGTGCAAAAGGCAG	0.00	0.00	0.00	0.00	0.00
GATCAAAGTGCAAAGAAAAT	0.23	0.00	0.00	0.00	0.00
GATCAAAGTGCAAAGCATCT	0.00	0.00	0.00	0.00	0.34
GATCAAAGTGCAACTCACAT	0.00	0.00	0.00	0.00	0.34

GATCAAAGTGCAAGCTTGGT	0.00	0.00	0.29	0.00	0.00
GATCAAAGTGCAAGGATTGA	0.00	0.65	0.00	0.00	0.00
GATCAAAGTGCACAAGGCCG	0.00	0.00	0.00	0.00	0.34
GATCAAAGTGCACCCGCTCC	0.00	0.33	0.00	0.00	0.00
GATCAAAGTGCACTCAATCC	0.00	0.33	0.29	0.00	0.00
GATCAAAGTGCACTCAATCG	0.00	0.00	0.00	0.34	0.00
GATCAAAGTGCAGGATGGGA	0.00	0.33	0.00	0.00	0.00
GATCAAAGTGCAGGATTGGG	0.00	0.00	0.00	0.00	0.34
GATCAAAGTGCAGGCCTGGT	0.46	0.00	0.00	0.00	0.00
GATCAAAGTGCAGGCGGCAG	1.37	1.30	2.86	0.68	1.03
GATCAAAGTGCAGGCGTGAT	0.00	0.33	0.00	0.00	0.00
GATCAAAGTGCAGGCTAAAA	0.23	0.00	0.57	0.00	0.34
GATCAAAGTGCAGGCTAAGA	0.00	0.00	0.57	0.00	0.00
GATCAAAGTGCAGGCTCGGT	0.23	0.00	0.00	0.34	0.00
GATCAAAGTGCAGGCTGGGT	0.23	0.00	0.29	0.00	0.00
GATCAAAGTGCAGGCTNGGT	0.00	0.00	0.00	0.00	0.69
GATCAAAGTGCAGGCTTAAA	0.46	0.33	0.00	0.00	0.00
GATCAAAGTGCAGGCTTCGT	0.23	0.33	0.00	0.00	0.00
GATCAAAGTGCAGGCTTGAA	0.00	0.33	0.29	0.00	0.00
GATCAAAGTGCAGGCTTGAG	0.00	0.00	0.29	0.00	0.00
GATCAAAGTGCAGGCTTGGA	0.00	0.00	0.29	0.00	0.69
GATCAAAGTGCAGGCTTGGC	0.00	0.65	0.57	0.00	0.34
GATCAAAGTGCAGGCTTGGG	1.83	0.00	0.86	2.04	3.44
GATCAAAGTGCAGGCTTGGT	126.36	68.32	125.43	47.86	104.01
GATCAAAGTGCAGGCTTGNN	0.92	0.00	0.00	0.00	0.00
GATCAAAGTGCAGGCTTNGT	0.00	0.00	0.00	0.00	0.34
GATCAAAGTGCAGGCTTTGG	0.00	0.00	0.00	0.00	0.00
GATCAAAGTGCAGGCTTTGT	0.00	0.00	0.57	1.70	0.34
GATCAAAGTGCAGGTAAAAA	0.00	0.00	0.00	0.00	0.34
GATCAAAGTGCAGTCTTTGT	0.00	0.00	0.29	0.00	0.00
GATCAAAGTGCAGTGCAGCC	0.00	0.00	0.29	0.00	0.00
GATCAAAGTGCAGTGGCTTG	0.00	0.00	0.00	0.00	0.34
GATCAAAGTGCAGTTTTAGT	0.00	0.00	0.00	0.34	0.00
GATCAAAGTGCATGACAGGG	0.00	0.00	0.00	0.00	0.00
GATCAAAGTGCATGCAGAAA	0.00	0.00	0.29	0.00	0.00
GATCAAAGTGCATGCTTTCA	0.00	0.00	0.29	0.00	0.00
GATCAAAGTGCATTTTTAAG	0.00	0.33	0.00	0.00	0.00

GATCAAAGTGCCAAGCATCT	2.29	0.98	1.43	0.68	0.69
GATCAAAGTGCCAGTGTGGT	0.23	0.00	0.00	0.00	0.00
GATCAAAGTGCCCGTGGGTG	0.00	0.65	0.57	0.68	0.00
GATCAAAGTGCCGCAGCGCC	0.46	0.00	0.00	0.00	0.00
GATCAAAGTGCCGGCGTGGT	0.46	0.00	0.00	0.00	0.00
GATCAAAGTGCCGGCTTGGT	0.23	0.00	0.00	0.00	0.00
GATCAAAGTGCCGTTCAATG	0.00	0.33	0.00	0.00	0.00
GATCAAAGTGCCTGTCTTGC	0.69	0.00	0.29	0.34	0.34
GATCAAAGTGCGAGAAGGAC	0.00	0.00	0.00	0.34	0.00
GATCAAAGTGCGAGGCAGAA	1.14	0.33	0.29	1.36	0.00
GATCAAAGTGCGAGGCATTG	0.00	0.00	0.00	0.34	0.00
GATCAAAGTGCGCCAAAGTT	0.00	0.33	0.00	0.00	0.00
GATCAAAGTGCGCCTGTACC	0.23	0.00	0.00	0.00	1.38
GATCAAAGTGCGCCTGTGCT	0.00	0.00	0.00	0.34	0.00
GATCAAAGTGCGCCTGTTGC	0.00	0.00	0.29	0.00	0.00
GATCAAAGTGCGGCGGCGCC	0.00	0.00	0.00	0.34	0.00
GATCAAAGTGCGGGAACGTC	0.00	0.00	0.29	0.00	0.00
GATCAAAGTGCGGGGAAGAA	1.37	2.60	3.44	2.38	4.13
GATCAAAGTGCGGGGAAGAN	0.00	0.00	0.00	0.00	0.00
GATCAAAGTGCGGGGAAGCA	0.00	0.00	0.00	0.34	0.00
GATCAAAGTGCGGGGATGAA	0.00	0.00	0.29	0.00	0.00
GATCAAAGTGCGGGGGAGGA	0.23	0.00	0.00	0.00	0.00
GATCAAAGTGCGGGGGGCAG	0.00	0.00	0.00	0.34	0.00
GATCAAAGTGCTAAAAAAAA	11.67	0.98	2.86	4.07	4.13
GATCAAAGTGCTAAAACAAA	0.23	0.00	0.00	0.00	0.00
GATCAAAGTGCTAGAAAAAA	0.23	0.00	0.00	0.00	0.00
GATCAAAGTGCTCCGGCCCA	0.00	0.00	0.00	0.34	0.00
GATCAAAGTGCTCCTCCCAG	0.00	0.00	0.00	0.00	0.00
GATCAAAGTGCTCCTGGCAG	0.00	0.00	0.00	0.00	0.00
GATCAAAGTGCTCTGTGGCT	0.00	0.00	0.00	0.00	0.00
GATCAAAGTGCTGAAAAACA	0.00	0.00	0.00	0.00	0.00
GATCAAAGTGCTGAAGCCAA	0.00	0.00	0.29	0.00	0.00
GATCAAAGTGCTGCAAAAAA	0.00	0.00	0.00	0.68	0.00
GATCAAAGTGCTGCAAGAAA	0.00	0.00	0.00	0.00	0.34
GATCAAAGTGCTGCATCGTA	0.00	0.00	0.00	0.00	0.00
GATCAAAGTGCTGCTAAAAA	0.69	0.00	0.00	0.00	0.00
GATCAAAGTGCTGCTACAAC	0.23	0.00	0.00	0.00	0.00

GATCAAAGTGCTGCTACGTA	0.23	0.00	0.00	0.00	0.00
GATCAAAGTGCTGCTACGTG	1.37	0.00	0.00	0.34	0.00
GATCAAAGTGCTGCTTGCCA	0.00	0.00	0.29	0.00	0.00
GATCAAAGTGCTGGAGAAGT	0.00	0.00	0.00	0.00	0.34
GATCAAAGTGCTGGCGCATG	0.00	0.33	0.29	0.00	0.00
GATCAAAGTGCTGGCGCCTG	0.00	0.00	0.29	0.00	0.00
GATCAAAGTGCTGGGAAAAA	0.00	0.00	0.29	0.00	0.00
GATCAAAGTGCTGGGGAAGT	0.00	0.33	0.00	0.00	0.00
GATCAAAGTGCTGGGGCAGT	0.00	0.33	0.00	0.00	0.00
GATCAAAGTGCTGTGGTACT	0.00	0.00	0.00	0.00	0.00
GATCAAAGTGCTTAAAAAAA	0.00	0.33	0.00	0.00	0.00
GATCAAAGTGCTTGTGACTC	0.00	0.00	0.00	0.00	0.00
GATCAAAGTGCTTGTGCCTC	0.00	0.00	0.00	0.00	0.00
GATCAAAGTGCTTTCTCTTG	0.00	0.00	0.29	0.00	0.00
GATCAAAGTGGAAAAAAAAA	0.46	0.00	0.00	0.34	0.00
GATCAAAGTGGAAATCGTAA	0.00	0.00	0.29	0.00	0.00
GATCAAAGTGGAAGAGTTGA	0.00	0.00	0.00	0.00	0.00
GATCAAAGTGGAATCAACAA	0.23	0.00	0.00	0.00	0.00
GATCAAAGTGGACAAACCAC	0.00	0.00	0.00	0.00	0.00
GATCAAAGTGGACGAGGGCG	0.00	0.00	0.00	0.34	0.00
GATCAAAGTGGACGAGTGCG	5.26	2.60	4.30	4.07	3.10
GATCAAAGTGGACGAGTTCG	0.00	0.00	0.00	0.00	0.34
GATCAAAGTGGACGGGTGCG	0.00	0.00	0.29	0.00	0.00
GATCAAAGTGGACTACCAGT	0.00	0.33	0.00	0.00	0.00
GATCAAAGTGGACTATGTGA	0.00	0.33	0.29	0.00	0.69
GATCAAAGTGGAGAGAGTGG	0.00	0.00	0.00	0.00	0.34
GATCAAAGTGGAGATGCGGA	0.23	0.33	0.00	1.70	0.00
GATCAAAGTGGAGATTGACA	0.00	0.33	0.00	0.00	0.00
GATCAAAGTGGAGGCGTGGT	0.00	0.33	0.00	0.00	0.00
GATCAAAGTGGAGGCTTGGT	0.00	0.00	0.29	0.00	0.00
GATCAAAGTGGAGGTGTCGT	0.00	0.00	0.00	0.00	0.00
GATCAAAGTGGATATAGCCG	0.00	0.00	0.00	0.00	0.00
GATCAAAGTGGATGCACACC	0.00	0.00	0.00	0.00	0.00
GATCAAAGTGGATTCTCGCT	0.00	0.00	0.00	0.00	0.00
GATCAAAGTGGCAAAAAAAA	0.00	0.98	0.57	0.00	0.00
GATCAAAGTGGCACCAGACC	0.46	0.00	0.29	0.00	0.00
GATCAAAGTGGCAGAGAGGC	0.23	0.00	0.00	0.00	0.00

GATCAAAGTGGCAGCCATAT	0.00	0.00	0.00	0.00	0.00
GATCAAAGTGGCAGGCGCCT	0.00	0.00	0.29	0.00	0.00
GATCAAAGTGGCCGGTGCTG	1.37	2.28	1.15	1.36	0.34
GATCAAAGTGGCGAAAAAAA	0.46	0.00	0.00	0.00	0.00
GATCAAAGTGGCGATTTGAA	0.00	0.33	0.00	0.00	0.00
GATCAAAGTGGCGCCCAAGG	0.00	0.00	0.00	0.00	0.00
GATCAAAGTGGCGGACGACA	0.00	0.00	0.00	0.00	0.00
GATCAAAGTGGCGGACTGCT	0.69	0.00	0.29	0.34	0.00
GATCAAAGTGGCGGCAGGGG	0.46	0.00	0.29	0.00	0.00
GATCAAAGTGGCGGCCTTAA	0.00	0.00	0.00	0.00	0.00
GATCAAAGTGGCGGGAGGAC	0.00	0.00	0.00	0.00	0.34
GATCAAAGTGGCGGGAGGGG	0.23	0.00	0.00	0.00	0.00
GATCAAAGTGGCGGGGAAAA	0.00	0.00	0.29	0.00	0.00
GATCAAAGTGGCGGGGGAGA	0.00	0.00	0.00	0.34	0.00
GATCAAAGTGGCGGGGGCGG	1.14	0.00	0.00	0.00	0.34
GATCAAAGTGGCGGGGGGGC	0.92	0.33	0.00	0.68	0.00
GATCAAAGTGGCGGGGGGGG	8.01	2.93	2.86	33.60	12.74
GATCAAAGTGGCGGGGGGGT	0.00	0.00	0.00	0.68	0.00
GATCAAAGTGGCGGGGGGTG	0.23	0.00	0.00	0.34	0.34
GATCAAAGTGGCGGGGGTAG	0.00	0.00	0.00	0.34	0.00
GATCAAAGTGGCGGGGGTGC	0.00	0.00	0.00	0.00	0.34
GATCAAAGTGGCGGGGGTGG	0.00	0.33	0.57	0.68	0.34
GATCAAAGTGGCGGGGGTGT	0.00	0.00	0.00	0.00	0.34
GATCAAAGTGGCGGGGTGGC	0.00	0.00	0.00	0.34	0.00
GATCAAAGTGGCGGGGTGGG	0.00	0.33	0.57	0.34	0.00
GATCAAAGTGGCGGGGTGGT	0.00	0.33	0.29	0.00	0.00
GATCAAAGTGGCGGGTGCGG	0.00	0.00	0.29	0.00	0.00
GATCAAAGTGGCGGGTGGGA	0.00	0.33	0.00	0.00	0.00
GATCAAAGTGGCGGGTGGGC	0.69	0.33	0.00	0.00	0.34
GATCAAAGTGGCGGGTGGGG	4.58	1.30	0.29	0.34	0.34
GATCAAAGTGGCGGGTGGGT	0.00	0.00	0.29	0.00	0.00
GATCAAAGTGGCGGGTGGTG	0.00	0.33	0.00	0.00	0.00
GATCAAAGTGGCGGGTGTGC	0.00	0.00	0.29	0.00	0.00
GATCAAAGTGGCGGGTGTGG	0.23	0.33	0.00	0.00	0.00
GATCAAAGTGGCGGGTGTTG	0.00	0.00	0.29	0.00	0.00
GATCAAAGTGGCGGTGGGGC	0.00	0.00	0.00	0.34	0.00
GATCAAAGTGGCGGTTGCGC	0.23	0.00	0.00	0.00	0.00

GATCAAAGTGGCGGTTGTGG	0.00	0.33	0.00	0.00	0.00
GATCAAAGTGGCTAGCTGAT	0.00	0.33	0.00	0.00	0.00
GATCAAAGTGGCTCATGGCC	0.00	0.33	0.00	0.00	0.00
GATCAAAGTGGCTGAAATGA	0.00	0.00	0.00	0.00	0.00
GATCAAAGTGGGAAAAGAGC	0.00	0.33	0.57	0.00	0.00
GATCAAAGTGGGAGGGGGGG	0.23	0.00	0.00	0.00	0.00
GATCAAAGTGGGCACGGTAA	0.00	0.00	0.86	0.34	0.00
GATCAAAGTGGGCCAATCGA	0.00	0.33	0.00	0.00	0.00
GATCAAAGTGGGCCCAAAAA	0.00	0.00	0.00	0.00	0.00
GATCAAAGTGGGCCGAAGGC	0.00	0.00	0.00	0.68	0.00
GATCAAAGTGGGCCGGAGAA	0.00	0.00	0.29	0.00	0.00
GATCAAAGTGGGCGCACGAG	0.00	1.30	1.43	0.68	0.69
GATCAAAGTGGGCGCGCGAG	0.00	0.00	0.00	0.34	0.34
GATCAAAGTGGGCGCGTGTT	0.00	0.00	0.29	0.00	0.00
GATCAAAGTGGGCGGCGAGC	0.00	0.33	0.00	1.70	0.00
GATCAAAGTGGGCTGGACTG	0.00	0.00	0.29	0.00	0.00
GATCAAAGTGGGCTTTCTCT	0.00	0.00	0.29	0.00	0.00
GATCAAAGTGGGGAAGAAGG	0.00	0.00	0.29	0.00	0.00
GATCAAAGTGGGGACGAAGG	16.02	21.80	23.77	22.74	12.40
GATCAAAGTGGGGACGAANN	0.00	0.00	0.00	0.00	0.00
GATCAAAGTGGGGACGAGGG	0.23	0.00	0.29	0.00	0.00
GATCAAAGTGGGGACGANGN	0.00	0.00	0.00	0.34	0.00
GATCAAAGTGGGGACGCAGG	0.00	0.00	0.00	0.34	0.00
GATCAAAGTGGGGACGCNNN	0.23	0.00	0.00	0.00	0.00
GATCAAAGTGGGGACGGAGG	0.23	0.00	0.00	0.00	0.00
GATCAAAGTGGGGACGNNNN	0.23	0.00	0.00	0.00	0.00
GATCAAAGTGGGGACGTGCC	0.00	0.00	0.00	0.00	0.00
GATCAAAGTGGGGAGGAAGG	0.00	0.33	0.29	0.00	0.00
GATCAAAGTGGGGCCGAAGG	0.23	0.00	0.00	0.00	0.00
GATCAAAGTGGGTACGAAGG	0.00	0.00	0.00	0.00	0.00
GATCAAAGTGGTAAGCCTTC	0.00	0.00	0.29	0.00	0.00
GATCAAAGTGGTCATACGCC	0.00	0.00	0.00	0.00	0.00
GATCAAAGTGGTCCCCGATT	0.00	0.00	0.00	0.00	0.00
GATCAAAGTGGTGAAGACCG	0.00	0.00	0.00	0.34	0.00
GATCAAAGTGGTGCAGCGTA	0.00	0.00	0.86	0.00	0.00
GATCAAAGTGGTGGAGGCGT	0.23	0.00	0.00	0.00	0.00
GATCAAAGTGGTGGGTGCGC	0.00	0.00	0.00	0.00	0.34

GATCAAAGTGTACCAGGCAG	0.00	0.00	0.00	0.00	0.34
GATCAAAGTGTACCAGGCGG	0.00	0.00	0.00	0.00	0.34
GATCAAAGTGTATGGCACGG	0.00	0.00	0.00	0.34	0.00
GATCAAAGTGTATTCAGTGT	0.00	0.33	0.00	0.00	0.00
GATCAAAGTGTCACAGACCG	0.00	0.33	0.00	0.00	0.00
GATCAAAGTGTCACAGTCCG	1.83	4.88	2.58	7.47	2.41
GATCAAAGTGTCACATTCCG	0.00	0.00	0.29	0.00	0.00
GATCAAAGTGTCAGCACAAA	0.00	0.00	0.57	0.00	0.00
GATCAAAGTGTCAGCACAAT	0.00	0.00	0.29	0.00	0.00
GATCAAAGTGTCAGCGAAAA	0.00	0.33	0.00	0.00	0.00
GATCAAAGTGTCCCGGATAT	0.23	0.00	0.00	0.00	0.00
GATCAAAGTGTCGGATTGTT	0.00	0.00	0.00	0.00	0.34
GATCAAAGTGTCTCCAAGCG	0.23	0.00	0.00	0.00	0.00
GATCAAAGTGTGACCGCCTA	0.00	0.33	0.00	0.00	0.00
GATCAAAGTGTGAGGAAAGC	0.23	0.00	0.00	0.00	0.00
GATCAAAGTGTGCAGAGTGT	0.00	0.33	0.00	0.00	0.00
GATCAAAGTGTGCAGTCTGG	0.00	0.00	0.00	0.00	0.34
GATCAAAGTGTGGCAGGTAT	0.00	0.33	0.00	0.68	0.00
GATCAAAGTGTGGCTTCTCT	0.00	0.00	0.00	0.00	0.34
GATCAAAGTGTGGGGAAGAA	0.00	0.00	0.00	0.00	0.00
GATCAAAGTGTTCAGGTTAT	0.00	0.00	0.00	0.00	0.34
GATCAAAGTGTTCGCTGTCA	0.00	0.33	0.00	0.00	0.00
GATCAAAGTGTTGGAGACGT	0.00	0.00	0.00	0.00	0.34
GATCAAAGTGTTGGGCGCGG	0.00	0.00	0.29	0.00	0.00
GATCAAAGTGTTTACATGCC	0.00	0.00	0.00	0.00	0.34
GATCAAAGTGTTTGAATTAG	2.98	7.16	2.86	10.18	5.17
GATCAAAGTGTTTGATGCAA	0.00	0.00	0.00	0.00	0.69
GATCAAAGTGTTTGATNCNN	0.00	0.00	0.00	0.00	0.34
GATCAAAGTGTTTGCATTAG	0.00	0.00	0.00	0.00	0.34
GATCAAAGTGTTTGGGTTCC	0.00	0.00	0.00	0.00	0.00
GATCAAAGTGTTTGTGTCGT	0.00	0.00	0.00	0.00	0.00
GATCAAAGTTAAAAAAAAAA	0.92	0.00	0.00	0.00	0.00
GATCAAAGTTAAGATTCAGT	0.00	0.00	0.00	0.00	0.00
GATCAAAGTTAAGCCCAAGA	0.00	0.00	0.00	0.00	0.00
GATCAAAGTTACAAAAAAAA	0.00	0.00	0.00	0.00	0.34
GATCAAAGTTACATGTCAAA	0.23	0.00	0.00	0.00	0.00
GATCAAAGTTACATGTCACT	0.00	0.00	0.57	0.00	0.34

GATCAAAGTTACCCACATGT	0.00	0.00	0.00	0.00	0.00
GATCAAAGTTACTCAGCTTG	0.46	0.33	0.00	0.00	0.00
GATCAAAGTTAGAAGGTTGG	0.00	0.00	0.00	0.34	0.00
GATCAAAGTTAGCCATATGG	0.00	0.00	0.00	0.00	0.34
GATCAAAGTTAGCTCCTTGC	0.23	0.00	0.00	0.00	0.00
GATCAAAGTTAGCTCTCGCC	0.00	0.00	0.00	0.34	0.00
GATCAAAGTTAGCTGCATGC	0.00	0.00	0.00	0.00	0.34
GATCAAAGTTAGGAGCCATG	0.00	0.00	0.00	0.00	0.00
GATCAAAGTTAGGTGTGTTG	0.00	0.00	0.29	0.00	0.00
GATCAAAGTTATGAGTAAGG	1.60	0.33	0.29	0.00	0.69
GATCAAAGTTATGCGTCATG	0.00	0.00	0.29	0.00	0.00
GATCAAAGTTATGCGTCGGG	0.23	0.00	0.00	0.00	0.00
GATCAAAGTTCAAAAAAAAA	0.00	0.00	0.00	0.00	0.34
GATCAAAGTTCAACGACTTC	0.00	0.00	0.00	0.00	0.00
GATCAAAGTTCAAGCCCTGC	0.23	0.00	0.57	0.00	0.00
GATCAAAGTTCAAGCTGATA	0.00	0.00	0.00	0.00	0.00
GATCAAAGTTCACACTGCAG	0.00	0.00	0.00	0.00	1.03
GATCAAAGTTCACATGCACA	0.00	0.33	0.00	0.34	0.00
GATCAAAGTTCACCGTCAGC	0.00	0.00	0.00	1.02	0.00
GATCAAAGTTCACCTTAACC	0.00	0.00	0.00	0.00	0.34
GATCAAAGTTCAGCGGAGCA	0.00	0.00	0.00	0.34	0.00
GATCAAAGTTCATACTCTGA	0.00	0.65	0.00	0.68	0.00
GATCAAAGTTCCAGGGCCCG	0.00	0.00	0.00	0.00	0.34
GATCAAAGTTCCTGCAGTGC	0.00	0.33	0.29	0.34	0.34
GATCAAAGTTCCTTGTTAAA	0.00	0.00	0.00	0.68	0.00
GATCAAAGTTCCTTGTTACA	0.00	0.33	1.15	0.00	0.00
GATCAAAGTTCGAAAGTCCT	0.00	0.00	0.29	0.34	0.00
GATCAAAGTTCGGCGGGGGC	0.00	0.00	0.00	0.34	0.00
GATCAAAGTTCGTATGCCGT	0.00	0.00	0.00	0.00	0.00
GATCAAAGTTCTACTTTGGG	0.00	0.00	0.00	0.00	0.00
GATCAAAGTTCTGAGTAAGG	0.00	0.00	0.00	0.00	0.34
GATCAAAGTTCTGCCATGCA	0.00	0.00	0.00	0.00	0.69
GATCAAAGTTCTGGATCCGT	0.00	0.33	0.00	0.00	0.00
GATCAAAGTTCTTCATGGAG	0.00	0.00	0.29	0.00	0.00
GATCAAAGTTGAAAACAAAG	0.23	0.00	0.00	0.00	0.00
GATCAAAGTTGAAAAGAAAA	0.00	0.00	0.00	0.00	0.00
GATCAAAGTTGAACAGAACA	0.00	0.00	0.00	0.00	0.34

GATCAAAGTTGAACTGTTTA	0.69	0.33	0.57	0.68	1.38
GATCAAAGTTGAAGGGCAGG	0.23	0.00	0.00	0.00	0.00
GATCAAAGTTGAAGGTCGCA	0.69	1.30	0.00	1.02	0.00
GATCAAAGTTGAAGGTCGCG	0.00	0.33	0.00	0.34	0.00
GATCAAAGTTGACAAAAAAA	0.00	0.33	0.00	0.00	0.00
GATCAAAGTTGACCAGGGCC	0.00	0.00	0.00	0.34	0.00
GATCAAAGTTGACGAGGTAC	0.23	0.33	0.00	0.00	0.00
GATCAAAGTTGAGACCACGA	0.00	0.00	0.00	0.34	0.00
GATCAAAGTTGAGAGTTTTC	0.00	0.00	0.00	0.00	0.00
GATCAAAGTTGAGTGATACA	0.00	0.00	0.00	0.00	0.34
GATCAAAGTTGAGTGTAATG	0.00	0.00	0.00	0.00	0.34
GATCAAAGTTGAGTGTAGCG	0.46	0.00	0.00	0.00	0.00
GATCAAAGTTGAGTGTTACA	0.00	0.00	0.00	0.00	0.69
GATCAAAGTTGCACATGCAT	0.46	0.00	0.00	0.00	0.00
GATCAAAGTTGCACTGACCA	0.00	0.33	0.00	0.00	0.00
GATCAAAGTTGCATTCCGCT	0.00	0.65	0.00	0.00	0.00
GATCAAAGTTGCCAGCAAAA	0.00	0.00	0.29	0.00	0.00
GATCAAAGTTGCCCAAATTG	0.23	0.00	0.00	0.00	0.00
GATCAAAGTTGCCCCGCAAA	0.00	0.00	0.29	0.00	0.00
GATCAAAGTTGCCTCCGATG	0.00	0.00	0.00	0.00	0.00
GATCAAAGTTGCGACCATGG	0.00	0.00	0.00	0.00	0.00
GATCAAAGTTGCGCAGCCTG	0.00	0.33	0.00	0.00	0.34
GATCAAAGTTGCGCATACCG	0.00	0.00	0.29	0.00	0.00
GATCAAAGTTGCGCGAGGCG	0.00	0.00	0.00	0.00	0.34
GATCAAAGTTGCGGGGGGGG	0.23	0.00	0.00	0.00	0.00
GATCAAAGTTGGATGCTGGC	0.00	0.00	0.00	0.00	0.69
GATCAAAGTTGGGAAATCAT	0.00	0.00	0.00	0.00	0.69
GATCAAAGTTGGGCGATGGG	0.23	0.33	0.00	0.00	0.00
GATCAAAGTTGGGGCCTTAC	0.23	0.00	0.00	0.00	0.00
GATCAAAGTTGGGTAGACGA	0.23	0.00	0.00	0.00	0.00
GATCAAAGTTGGGTGTTGTG	0.23	0.00	0.00	0.00	0.00
GATCAAAGTTGGGTTATGGG	0.00	0.00	0.29	0.00	0.00
GATCAAAGTTGGTCCCGTGC	0.23	0.00	0.00	0.00	0.00
GATCAAAGTTGGTCGCAAAA	0.00	0.00	0.00	0.34	0.00
GATCAAAGTTGGTCGCACAA	0.23	0.00	0.00	0.00	0.00
GATCAAAGTTGGTCGCAGAA	0.46	0.00	0.00	0.00	0.00
GATCAAAGTTGGTCGCCCCT	0.92	0.65	0.57	0.34	0.00

GATCAAAGTTGGTCGCCCGT	0.00	0.00	0.29	0.00	0.00
GATCAAAGTTGGTCGGAAGT	1.60	0.00	0.00	0.00	0.34
GATCAAAGTTGGTGGTGGTG	0.23	0.00	0.00	0.00	0.00
GATCAAAGTTGGTTACTCGC	0.00	0.00	0.00	0.00	0.34
GATCAAAGTTGTCCGAGTCA	0.00	0.00	0.00	0.00	0.00
GATCAAAGTTGTCCTCAGAT	0.00	0.00	0.00	0.00	0.00
GATCAAAGTTGTCGCGCCTC	0.00	0.00	0.00	0.68	0.34
GATCAAAGTTGTCGCGTGCT	0.00	0.00	0.00	0.00	0.69
GATCAAAGTTGTGTTTGGCG	0.00	0.00	0.00	0.00	0.34
GATCAAAGTTGTTCCTAGCC	0.00	0.00	0.00	0.34	0.00
GATCAAAGTTGTTGCGCCTC	0.23	0.33	0.00	0.00	0.69
GATCAAAGTTGTTTTTCGGG	0.00	0.00	0.29	0.00	0.00
GATCAAAGTTTAGGCGTGAA	0.00	0.00	0.00	0.00	0.34
GATCAAAGTTTATGGTGCGC	0.00	0.00	0.00	0.00	0.34
GATCAAAGTTTATTGGAGGT	0.00	0.33	0.00	0.00	0.00
GATCAAAGTTTCCATCCACC	0.00	0.00	0.00	0.00	0.00
GATCAAAGTTTCCGACTGCC	0.00	0.33	0.00	0.00	0.00
GATCAAAGTTTCTATGATGA	0.00	0.00	0.00	0.34	0.00
GATCAAAGTTTCTATGCTGA	0.23	3.90	3.72	4.07	2.07
GATCAAAGTTTCTCGTGCGC	0.00	0.00	0.00	0.34	0.34
GATCAAAGTTTCTGGTCTTC	0.00	0.00	0.00	0.00	0.34
GATCAAAGTTTGAACCTGTG	0.00	0.00	0.00	0.00	0.00
GATCAAAGTTTGCCATTACA	0.00	0.00	0.00	0.00	0.00
GATCAAAGTTTGGAAAAAAA	0.00	0.00	0.00	0.00	0.00
GATCAAAGTTTGGCATGTGC	4.35	3.58	2.58	2.72	3.44
GATCAAAGTTTGGGTCTAAT	0.00	0.00	0.00	0.00	0.00
GATCAAAGTTTGGGTCTCAA	2.06	0.65	0.29	1.02	0.00
GATCAAAGTTTGGGTCTCAC	0.00	0.00	0.00	0.34	0.00
GATCAAAGTTTGGGTCTCAT	15.34	19.52	7.73	19.35	13.09
GATCAAAGTTTGGGTCTCCT	0.23	0.00	0.00	0.00	0.00
GATCAAAGTTTGGGTCTCGT	0.23	0.65	0.29	0.00	0.00
GATCAAAGTTTGGGTCTCNN	0.23	0.00	0.00	0.00	0.00
GATCAAAGTTTGGGTCTTAA	0.00	0.33	0.00	0.00	0.00
GATCAAAGTTTGGTAGACGA	0.23	0.65	0.29	1.02	0.00
GATCAAAGTTTGTCGAGAAG	0.00	0.00	0.00	0.34	0.00
GATCAAAGTTTGTCGCTGAG	0.00	0.00	0.29	0.00	0.00
GATCAAAGTTTTCCGAGGAA	0.00	0.00	0.00	0.00	0.00

GATCAAAGTTTTCCGAGGAC	2.06	1.63	0.57	2.72	0.34
GATCAAAGTTTTCCGAGGAN	0.00	0.00	0.00	0.00	0.00
GATCAAAGTTTTCCGAGGCC	0.00	0.00	0.00	0.00	0.00
GATCAAAGTTTTCTCACAGT	0.23	0.00	0.00	0.34	0.00
GATCAAATAAAAAAAAAAAA	6.64	4.88	1.43	4.41	2.76
GATCAAATAAAAAAAAAAGA	0.23	0.00	0.00	0.00	0.00
GATCAAATAAAAAAAAAATC	0.00	0.00	0.00	0.00	0.00
GATCAAATAAAAAAAAGAAA	0.23	0.00	0.00	0.00	0.00
GATCAAATAAAAATTTTAGA	0.23	0.00	0.00	0.00	0.00
GATCAAATAAAACGAGCACA	0.00	0.00	0.00	0.00	0.34
GATCAAATAAAACTCAAGTA	0.00	0.00	0.29	0.00	0.00
GATCAAATAAAAGAAAGAAA	0.00	0.00	0.00	0.00	1.38
GATCAAATAAAATAAGAGAG	0.00	0.00	0.00	0.00	0.69
GATCAAATAAACAAACACTA	0.00	0.00	0.29	0.00	0.00
GATCAAATAAACTAAAAAAA	0.69	0.00	0.00	0.00	0.00
GATCAAATAAACTAGTAAAA	0.00	0.33	0.00	0.34	0.00
GATCAAATAAACTAGTAACC	0.23	0.00	0.00	0.00	0.00
GATCAAATAAACTATCGCGC	0.00	0.00	0.00	0.00	0.00
GATCAAATAAACTATCGCGG	0.46	0.00	0.00	0.34	0.34
GATCAAATAAACTCAGGTGT	0.00	0.00	0.00	0.00	0.34
GATCAAATAAACTGCCCGTC	0.00	0.00	0.00	0.00	0.00
GATCAAATAAAGACTANTAA	0.00	0.00	0.00	0.00	0.34
GATCAAATAAAGGCCGAAGG	0.00	0.00	0.00	0.00	0.00
GATCAAATAAAGGGTCGCCC	0.00	0.00	0.00	0.34	0.00
GATCAAATAAAGTTATAAAA	0.69	0.00	0.00	0.00	0.00
GATCAAATAAATAAAAAAAA	0.46	0.00	0.00	0.00	0.00
GATCAAATAAATAACAAATA	0.00	0.33	0.00	0.00	0.00
GATCAAATAAATAATGCTGG	0.00	0.00	0.00	0.00	0.00
GATCAAATAAATATATATTC	0.00	0.00	0.29	0.00	0.00
GATCAAATAAATCCCAAGAA	0.00	0.00	0.29	0.00	0.00
GATCAAATAAATCTGATGAC	0.00	0.00	0.29	0.00	0.00
GATCAAATAAATGAAGAACT	0.00	0.00	0.29	0.00	0.00
GATCAAATAAATTCAGGCCA	0.00	0.00	0.00	0.34	0.69
GATCAAATAACAAGGGCTCT	0.00	0.00	0.00	0.00	0.00
GATCAAATAACAATAAAACA	0.00	0.00	0.00	0.00	0.34
GATCAAATAACAATACGTTG	0.00	0.00	0.00	0.68	0.00
GATCAAATAACAATATGTTG	0.00	0.33	0.00	0.00	0.34

GATCAAATAACAATATTTTG	0.00	0.00	0.00	0.00	0.00
GATCAAATAACCTACCCGTC	0.00	0.00	0.00	0.34	0.00
GATCAAATAACCTGCACGAC	0.00	0.00	0.00	0.34	0.00
GATCAAATAACCTGCCCAAA	0.00	0.00	0.00	0.00	0.34
GATCAAATAACCTGCCCGTA	0.00	0.00	0.00	0.00	0.00
GATCAAATAACCTGCCCGTC	8.24	8.46	4.58	7.13	5.17
GATCAAATAACCTGCCCGTN	0.00	0.00	0.00	0.00	0.00
GATCAAATAACCTGCCNGTC	0.00	0.00	0.00	0.00	0.00
GATCAAATAACCTGCCTGTC	0.23	0.00	0.00	0.00	0.00
GATCAAATAACGACGCATCT	0.00	0.00	0.00	0.34	0.00
GATCAAATAACGAGGGATCT	0.00	0.00	0.57	0.34	0.34
GATCAAATAACGAGGGCGCT	0.00	0.00	0.57	0.00	0.00
GATCAAATAACGAGGGCTCC	0.00	0.00	0.00	0.00	0.00
GATCAAATAACGAGGGCTCN	0.23	0.00	0.00	0.00	0.00
GATCAAATAACGAGGGCTCT	29.07	44.90	37.51	46.50	37.54
GATCAAATAACGAGGGCTNN	0.23	0.00	0.00	0.00	0.00
GATCAAATAACGAGGGCTTT	0.00	0.00	0.00	0.00	0.00
GATCAAATAACGAGGGGTCT	0.00	0.33	0.00	0.00	0.00
GATCAAATAACGTATGATCC	0.00	0.00	0.00	0.34	0.00
GATCAAATAACTCGCTCCTT	0.00	0.00	0.00	0.34	0.00
GATCAAATAACTGCTACACA	0.00	0.00	0.00	0.00	0.34
GATCAAATAACTTGGATAAA	0.00	0.00	0.00	0.00	0.00
GATCAAATAACTTTGCATTA	0.00	0.00	0.00	0.00	0.69
GATCAAATAAGAAAAAAAAA	0.00	0.65	0.00	0.00	0.00
GATCAAATAAGAAGAATATG	0.00	0.00	0.29	0.00	0.00
GATCAAATAAGAGCGCCTTC	0.00	0.33	0.00	0.00	0.00
GATCAAATAAGCACCTGGTC	0.23	0.00	0.00	0.00	0.00
GATCAAATAAGCAGCGCAGA	0.00	0.33	0.00	0.00	0.00
GATCAAATAAGCGGCGCTTT	0.00	0.00	0.00	0.34	0.00
GATCAAATAAGGAGGACTGG	0.00	0.00	0.00	0.00	0.00
GATCAAATAAGTCCATACCG	0.23	0.00	0.00	0.00	0.00
GATCAAATAAGTGATTGTTT	0.69	0.00	0.00	0.00	0.00
GATCAAATAAGTGGCAAAAA	0.00	0.00	0.00	0.00	0.34
GATCAAATAAGTGGCGCTTT	2.06	1.63	0.29	2.04	2.41
GATCAAATAAGTGTACAGTC	0.00	0.00	0.00	0.34	0.00
GATCAAATAATAAGAAGGTA	0.00	0.00	0.00	0.00	0.00
GATCAAATAATCAAACATAT	0.00	0.00	0.00	0.00	0.69

GATCAAATAATCCAAATACA	0.00	0.00	0.00	0.00	0.00
GATCAAATAATCCGAACCTC	0.00	0.00	0.29	0.00	0.00
GATCAAATAATCGGGAGAGG	0.00	0.00	0.00	0.00	0.00
GATCAAATAATCTTACAAAT	0.00	0.33	0.00	0.00	0.00
GATCAAATAATGAAAAAAAA	0.00	0.00	0.00	0.00	0.00
GATCAAATAATGAGGGCTCT	0.00	0.00	0.00	0.34	0.00
GATCAAATAATGAGGTGTTT	0.00	0.00	0.00	0.00	0.00
GATCAAATAATGATTGTGCT	0.69	0.00	0.00	0.00	0.00
GATCAAATAATGCCACAAGA	0.23	0.00	0.00	0.34	0.00
GATCAAATAATGCCACAAGT	0.00	0.33	0.00	0.00	0.00
GATCAAATAATGCCATGGAA	0.00	0.00	0.29	0.00	0.00
GATCAAATAATGGAAGAAGT	0.23	0.00	0.00	0.00	0.00
GATCAAATAATGGAAGCAGT	2.52	1.30	0.57	0.68	0.34
GATCAAATAATGTGAATAGG	0.00	0.00	0.00	0.00	1.38
GATCAAATAATTCACGCTCG	0.23	0.65	0.00	0.00	0.00
GATCAAATAATTCACGTCCT	0.23	0.33	0.86	0.68	1.72
GATCAAATAATTTATGCCTG	0.00	0.00	0.29	0.00	0.00
GATCAAATACAAAAAAAAAA	0.00	0.00	0.00	0.00	0.00
GATCAAATACAAAACAAAAA	0.00	0.00	0.00	0.00	0.34
GATCAAATACAAACAAAACA	0.23	0.00	0.00	0.00	0.00
GATCAAATACAACATAACGA	0.46	1.30	0.00	0.00	0.00
GATCAAATACAAGGACGAGA	0.00	0.00	0.00	0.00	0.00
GATCAAATACAAGGATACAA	0.23	0.33	0.00	0.00	0.00
GATCAAATACACGCCACAAT	0.00	0.00	0.29	0.00	0.00
GATCAAATACAGCAACAAGC	0.00	0.00	0.00	0.34	0.00
GATCAAATACAGCCCTGGCC	0.00	0.00	0.00	0.00	0.00
GATCAAATACAGCCTTTCTC	0.00	0.00	0.00	0.00	0.00
GATCAAATACAGCTAACCAA	0.00	0.00	0.00	0.34	0.00
GATCAAATACAGGCCACAGG	0.00	0.00	0.00	0.00	0.00
GATCAAATACAGGCCGAAGG	0.00	0.00	0.00	0.34	0.00
GATCAAATACAGGCCGCAGG	27.01	29.93	16.32	19.35	22.04
GATCAAATACAGGCCGCCGG	0.00	0.98	0.57	0.00	0.34
GATCAAATACAGGCCGGGGG	0.23	0.00	0.00	0.00	0.00
GATCAAATACAGGCTCCTAT	0.00	0.00	0.00	0.00	0.34
GATCAAATACAGGCTCGGAT	0.00	0.00	0.00	0.00	0.34
GATCAAATACAGGCTCGTAT	0.00	0.00	0.00	0.00	0.34
GATCAAATACAGGGCGCAGG	0.00	0.00	0.00	0.34	0.00

GATCAAATACATAGTCACAC	0.00	0.00	0.00	0.00	0.34
GATCAAATACATATGTGTGG	0.00	0.00	0.00	0.00	0.00
GATCAAATACATCGGATTCA	0.00	0.00	0.00	0.00	0.00
GATCAAATACATGACAATGC	0.00	0.00	0.29	0.00	0.00
GATCAAATACATGACACTAC	0.00	0.00	0.29	0.00	0.00
GATCAAATACATGCCAAAGC	0.00	0.33	0.00	0.00	0.00
GATCAAATACATGCCAANGC	0.00	0.00	0.00	0.00	0.34
GATCAAATACATGCCAATCC	0.00	0.00	0.00	0.00	0.00
GATCAAATACATGCCAATGA	0.00	0.00	0.29	0.00	0.00
GATCAAATACATGCCAATGC	11.22	8.78	16.32	15.95	16.88
GATCAAATACATGCCAATGT	0.00	0.00	0.00	0.00	0.00
GATCAAATACATGCCAGAGC	0.00	0.33	0.00	0.00	0.00
GATCAAATACATGGATGGCG	0.00	0.00	0.29	0.00	0.00
GATCAAATACATGTTCTACG	0.00	0.00	0.00	0.34	0.00
GATCAAATACCACAGACCAA	0.00	0.00	0.00	0.00	1.03
GATCAAATACCAGCCGCAGC	0.23	0.00	0.00	0.00	0.00
GATCAAATACCATTACTCTT	0.00	0.00	0.00	0.00	0.00
GATCAAATACCCGCGCCGTC	0.00	0.33	0.00	0.00	0.00
GATCAAATACCGAGGGCTCT	0.00	0.00	0.29	0.00	0.00
GATCAAATACCGCATGCTAC	0.00	0.00	0.29	0.00	0.00
GATCAAATACCGCGGGCTCT	0.00	0.00	0.29	0.00	0.00
GATCAAATACCGGCCGCAGG	0.00	0.00	0.00	0.00	0.00
GATCAAATACCGGCCGGTCA	0.00	0.00	0.00	0.00	0.34
GATCAAATACCGTCCCACGC	0.00	0.00	0.00	0.00	0.00
GATCAAATACCGTCCGGACG	0.00	0.00	0.29	0.00	0.00
GATCAAATACCGTCCGGTAA	0.00	0.33	0.00	0.00	0.00
GATCAAATACCGTCCGGTCA	0.00	0.33	0.00	0.00	0.00
GATCAAATACCGTCCTAGTC	0.00	0.00	0.00	0.68	0.00
GATCAAATACCTAGAGTATC	0.00	0.00	0.29	0.00	0.00
GATCAAATACCTCTTTCANN	0.00	0.00	0.00	0.00	0.00
GATCAAATACCTCTTTCATC	5.95	4.55	2.58	2.04	4.13
GATCAAATACCTGATAATGT	0.00	0.33	0.00	0.00	0.00
GATCAAATACCTGCCATGCG	0.00	0.00	0.00	0.00	0.34
GATCAAATACCTGGTCTACC	0.46	0.00	0.00	0.00	0.00
GATCAAATACCTGTGGGAAG	0.00	0.00	0.29	0.00	0.00
GATCAAATACCTTGCAGTAA	0.23	0.33	0.00	0.00	0.34
GATCAAATACGAGGCAGCGG	0.46	0.00	0.00	0.00	0.00

GATCAAATACGCACTGTCAC	0.00	0.00	0.29	0.00	0.00
GATCAAATACGCGACACATG	0.00	0.00	0.00	0.00	0.00
GATCAAATACGGCAACTACT	0.00	0.00	0.29	0.00	0.00
GATCAAATACGGCCAGCTCC	0.00	0.65	0.00	0.34	0.00
GATCAAATACGGGCCGCAGG	0.23	0.00	0.00	0.00	0.00
GATCAAATACGGTGTGGGTG	0.23	0.33	0.00	0.00	0.00
GATCAAATACGTCGTGGAGG	0.00	0.00	0.29	0.00	0.00
GATCAAATACGTCTGCTGTA	0.00	0.00	0.00	0.00	0.00
GATCAAATACGTGCAACGAA	0.00	0.00	0.00	0.00	0.00
GATCAAATACGTGCAATTCC	0.00	0.00	0.00	0.00	0.34
GATCAAATACGTGCCATAGA	1.14	0.65	0.86	1.02	1.72
GATCAAATACGTGCCATCGA	0.00	0.00	0.00	0.00	0.34
GATCAAATACGTGCCATGGA	0.46	0.00	0.00	0.00	0.00
GATCAAATACTAAAAAAAAA	0.00	0.00	0.00	0.34	0.00
GATCAAATACTCCTTGTGGG	0.46	0.00	0.00	0.00	0.00
GATCAAATACTGTCCGGTCA	0.00	0.00	0.29	0.00	0.00
GATCAAATACTGTGCAAACG	0.00	0.00	0.00	0.34	0.00
GATCAAATACTTCAGGCCTT	0.46	0.65	0.00	0.00	0.00
GATCAAATACTTGACGCCAT	0.00	0.00	0.00	0.00	0.34
GATCAAATACTTGGGCCTGC	0.23	0.33	0.29	0.00	0.00
GATCAAATACTTGTGTGTTG	0.69	0.65	0.00	0.00	0.34
GATCAAATACTTGTGTTCTG	0.00	0.00	0.00	0.68	0.00
GATCAAATAGAAAAAAAAAA	1.14	0.65	0.57	0.68	1.72
GATCAAATAGAACAGATAAT	0.00	0.00	0.57	0.00	0.00
GATCAAATAGAACAGCACTG	0.00	0.00	0.00	0.68	0.00
GATCAAATAGAAGACAGAAA	0.23	0.00	0.00	0.34	0.00
GATCAAATAGACAAAAAAAA	0.00	0.98	0.29	3.05	1.38
GATCAAATAGACTGAATCTT	0.00	0.33	0.00	0.34	0.34
GATCAAATAGAGGTCAAATA	0.00	0.00	0.00	0.00	0.00
GATCAAATAGATAGAGTATC	0.00	0.00	0.00	0.00	0.00
GATCAAATAGATAGAGTATN	0.00	0.00	0.00	0.00	0.00
GATCAAATAGATGCGCAGGT	0.00	0.33	0.29	0.00	0.00
GATCAAATAGATTCTTCAGA	0.00	0.00	0.00	0.34	0.00
GATCAAATAGCAATCCCATC	0.00	0.00	0.00	0.34	0.00
GATCAAATAGCACGAGACTC	0.00	0.00	0.00	0.00	0.34
GATCAAATAGCAGAATGAAG	0.00	0.00	0.29	0.00	0.00
GATCAAATAGCAGGTTGAGG	0.00	0.00	0.00	0.00	0.34

GATCAAATAGCCAGAGTATC	0.23	0.00	0.00	0.00	0.00
GATCAAATAGCCATGCTTGG	0.00	0.00	0.29	0.00	0.00
GATCAAATAGCCTCTCTGCC	0.00	0.00	0.00	0.00	0.00
GATCAAATAGCGAGTATCTG	0.00	0.33	0.00	0.00	0.00
GATCAAATAGCTAGAGAATC	0.00	0.00	0.00	0.00	0.00
GATCAAATAGCTAGAGCATC	0.00	0.00	0.29	0.00	0.00
GATCAAATAGCTAGAGGATC	0.23	0.33	0.00	0.00	0.00
GATCAAATAGCTAGAGTAAC	0.00	0.33	0.00	0.00	0.00
GATCAAATAGCTAGAGTACC	0.00	0.33	0.00	0.00	0.00
GATCAAATAGCTAGAGTAGC	0.00	0.00	0.00	0.00	0.00
GATCAAATAGCTAGAGTAGG	0.00	0.65	0.00	0.00	0.00
GATCAAATAGCTAGAGTANC	0.00	0.00	0.00	0.00	0.00
GATCAAATAGCTAGAGTATA	0.00	0.33	0.00	0.00	0.34
GATCAAATAGCTAGAGTATC	17.40	38.71	20.62	52.61	31.34
GATCAAATAGCTAGAGTATN	0.46	0.00	0.00	0.00	0.00
GATCAAATAGCTAGAGTATT	0.00	0.00	0.00	0.00	0.34
GATCAAATAGCTAGAGTCTC	0.00	0.00	0.00	0.34	0.00
GATCAAATAGCTAGAGTCTG	0.23	0.00	0.00	0.00	0.00
GATCAAATAGCTAGAGTTTC	0.46	0.33	0.29	0.00	0.00
GATCAAATAGCTAGCGTATC	0.00	0.00	0.00	0.00	0.00
GATCAAATAGCTAGTGTCTG	0.00	0.00	0.00	0.00	0.00
GATCAAATAGCTCTCCAAGC	0.00	0.33	0.00	0.00	0.00
GATCAAATAGCTGGGGGATC	0.23	0.00	0.00	0.00	0.00
GATCAAATAGGAAAAAAAAA	0.00	0.00	0.29	0.00	0.00
GATCAAATAGGATGCTTGCC	0.00	0.33	0.00	0.00	0.00
GATCAAATAGGCTAATTACC	0.00	0.00	0.00	0.00	0.00
GATCAAATAGGGCAAATTTT	0.00	0.33	0.00	0.00	0.00
GATCAAATAGGGCGTGTCTT	0.23	0.00	0.00	0.00	0.00
GATCAAATAGGGCTGCAGCT	0.00	0.00	0.00	0.34	0.00
GATCAAATAGGTATAGGGCA	0.00	0.33	0.00	0.00	0.00
GATCAAATAGGTGAGGTGGC	0.00	0.00	0.00	0.00	0.00
GATCAAATAGTAAAAAAAAA	0.00	0.00	0.00	0.00	0.34
GATCAAATAGTCAAAACATG	0.00	0.33	0.00	0.00	0.00
GATCAAATAGTCGTGCTTGC	0.00	0.00	0.29	0.00	0.00
GATCAAATAGTGAAAAGAAA	0.00	0.00	0.29	0.00	0.00
GATCAAATAGTGCCCAGAGA	0.00	0.00	0.00	0.34	0.00
GATCAAATAGTGGCACAGTG	0.00	0.33	0.00	0.00	0.00

GATCAAATAGTGGGCGCTTT	0.00	0.00	0.00	0.34	0.00
GATCAAATAGTTAAAAAAAA	2.29	0.33	0.57	0.00	1.38
GATCAAATAGTTGAAAAAAA	1.14	0.00	0.00	0.00	0.34
GATCAAATAGTTGATAAAAA	0.92	0.00	0.00	0.00	0.00
GATCAAATATAAAAACTTAA	0.92	0.00	0.00	0.00	0.00
GATCAAATATAATTTCAAGT	0.00	0.00	0.29	0.00	0.00
GATCAAATATAGCATTTGTG	0.00	0.33	0.00	0.00	0.00
GATCAAATATAGCATTTGTT	0.00	0.00	0.00	0.34	0.00
GATCAAATATAGTATTGCTA	0.00	0.00	1.72	0.68	0.00
GATCAAATATAGTTTTGCTA	0.00	0.00	0.00	0.68	0.00
GATCAAATATCACAAAAAAA	0.00	0.00	0.57	0.00	0.00
GATCAAATATCACTAAATAC	0.00	0.00	0.00	0.00	0.00
GATCAAATATCACTCTCCTA	1.14	0.00	0.00	0.00	0.00
GATCAAATATCCACGAGGTG	0.00	0.00	0.00	0.00	0.00
GATCAAATATCGTGTTCAGC	1.60	0.00	0.86	0.00	0.00
GATCAAATATCTGTCTGGTC	0.00	0.00	0.00	0.00	0.00
GATCAAATATGAAAAAAAAA	0.00	0.00	0.00	0.00	0.69
GATCAAATATGACAGACCTC	0.00	0.00	0.00	0.00	0.00
GATCAAATATGATGATGAGA	0.00	0.00	0.00	0.00	0.00
GATCAAATATGATGTTGTGC	0.00	0.00	0.00	0.00	0.00
GATCAAATATGCAAAAAAAA	0.00	0.00	0.00	0.68	0.00
GATCAAATATGCCCAAGCCG	0.23	0.00	0.00	1.02	0.00
GATCAAATATGCTACATGGC	0.00	0.00	0.29	0.00	0.00
GATCAAATATGCTTTTGGGA	0.23	0.00	0.00	0.00	0.00
GATCAAATATGGATAGGTAC	0.00	0.00	0.00	0.00	0.00
GATCAAATATGGCATCAACA	0.00	0.00	0.00	0.00	0.00
GATCAAATATGGGCTTTCAG	0.00	0.00	0.00	0.34	0.00
GATCAAATATGGGCTTTCNG	0.00	0.00	0.00	0.00	0.34
GATCAAATATGGGCTTTTAT	0.00	0.00	0.29	0.00	0.00
GATCAAATATGGTGTTGATG	0.00	0.00	0.00	0.00	0.00
GATCAAATATGGTTCCCACA	0.00	0.33	0.00	0.00	0.00
GATCAAATATGGTTCCCACG	0.23	0.33	0.57	0.34	0.34
GATCAAATATGTCTGCGTGG	0.23	0.65	0.00	0.00	0.00
GATCAAATATTAAGCATTGC	0.00	0.00	0.00	0.00	1.03
GATCAAATATTCAACCACAC	0.00	0.00	0.00	0.00	0.00
GATCAAATATTCAACTCTTT	0.00	0.00	0.00	0.00	0.34
GATCAAATATTCAGAAAGGC	0.00	0.00	0.00	0.00	0.00

GATCAAATATTCAGCCCACA	3.66	9.76	3.15	6.11	2.41
GATCAAATATTCGTGCAGCC	0.00	0.00	0.00	0.00	0.00
GATCAAATATTCGTGCAGTC	0.23	0.00	0.00	0.34	0.00
GATCAAATATTCTAAAGATG	0.00	0.00	0.00	0.00	0.00
GATCAAATATTCTACAGTCC	0.00	0.00	0.00	0.00	0.00
GATCAAATATTGAACAGCTT	0.00	0.00	0.00	0.34	0.00
GATCAAATATTGGAGATTGC	0.23	0.33	0.00	0.00	0.00
GATCAAATATTGTGCCAATA	0.00	0.00	0.00	0.34	0.00
GATCAAATATTTGATTTTAA	0.46	0.00	0.00	0.00	0.00
GATCAAATATTTGTGCAGTC	0.00	0.00	0.00	0.00	0.00
GATCAAATATTTTTACAAAA	0.00	0.00	0.00	0.00	0.34
GATCAAATCAAAAAAAAAAA	7.32	1.63	2.29	1.70	0.69
GATCAAATCAAAAAAAAAAC	0.23	0.00	0.00	0.00	0.00
GATCAAATCAAAAAAAAAAT	0.23	0.00	0.00	0.00	0.00
GATCAAATCAAAAAAAAANN	0.23	0.00	0.00	0.00	0.00
GATCAAATCAAAACAAAAAA	0.00	0.00	0.00	0.34	0.00
GATCAAATCAAAACACAGCA	0.00	0.00	0.00	0.00	0.69
GATCAAATCAAAACACCATC	0.00	0.00	0.00	0.00	0.34
GATCAAATCAAAAGTCTCAA	0.00	0.00	0.00	0.00	0.00
GATCAAATCAAAATGACGGT	0.23	0.00	0.00	0.00	0.00
GATCAAATCAAAATGACGTT	0.69	0.00	0.00	0.00	0.00
GATCAAATCAAAATTCTTTT	0.23	0.00	0.00	0.00	0.00
GATCAAATCAAAATTTAAAG	1.37	0.65	0.00	0.00	0.34
GATCAAATCAAAATTTAGGG	0.00	0.00	0.29	0.00	0.00
GATCAAATCAAAATTTTGGA	0.23	0.00	0.00	0.00	0.00
GATCAAATCAAAATTTTTTG	0.00	0.00	0.29	0.00	0.00
GATCAAATCAAAATTTTTTT	0.46	0.00	0.00	0.00	0.00
GATCAAATCAAACACAACAA	0.00	0.00	0.00	0.00	0.69
GATCAAATCAAACTTAACAC	0.00	0.00	0.29	0.00	0.00
GATCAAATCAAAGAGCCCAC	0.00	0.00	0.00	0.00	0.00
GATCAAATCAAAGGCATCAA	0.00	0.00	0.29	0.00	0.00
GATCAAATCAAAGGTCTCAA	0.00	0.00	0.29	0.00	0.00
GATCAAATCAAAGGTCTCCG	0.00	0.00	0.00	0.00	0.34
GATCAAATCAAATCAAAAAA	0.23	0.33	0.00	0.00	0.00
GATCAAATCAAATCAAATCG	0.00	0.00	0.86	0.00	0.00
GATCAAATCAAATCATTGTC	0.23	0.00	0.00	0.00	0.00
GATCAAATCAAATCTGGCAA	0.69	0.00	0.00	0.00	0.00

GATCAAATCAAATTAGGTGG	0.00	0.00	0.00	0.00	0.34
GATCAAATCAACAGGCGACA	0.00	0.00	0.29	0.00	0.00
GATCAAATCAACATAGGTGG	0.00	0.00	0.29	0.00	0.00
GATCAAATCAACCCTTGCTG	0.46	0.00	0.00	0.00	0.00
GATCAAATCAACGAACACAC	0.00	0.00	0.29	0.00	0.00
GATCAAATCAACGAGCTGGC	0.00	0.00	0.00	0.00	0.00
GATCAAATCAACTCAGGTGG	0.00	0.00	0.00	0.34	0.00
GATCAAATCAACTGAGGCGG	0.23	0.00	0.00	0.00	0.00
GATCAAATCAACTGAGGTTA	0.00	0.00	0.29	0.00	0.00
GATCAAATCAACTTACGTGG	0.00	0.00	0.29	0.00	0.00
GATCAAATCAACTTAGGAGG	0.00	0.00	0.57	0.00	0.34
GATCAAATCAACTTAGGCAA	0.00	0.33	0.00	0.00	0.00
GATCAAATCAACTTAGGCCG	0.00	0.00	0.29	0.00	0.00
GATCAAATCAACTTAGGCGG	0.00	0.33	0.00	0.00	0.00
GATCAAATCAACTTAGGGCG	0.00	0.00	0.00	0.00	0.34
GATCAAATCAACTTAGGGGG	0.46	0.00	0.29	0.34	0.00
GATCAAATCAACTTAGGGGN	0.23	0.00	0.00	0.00	0.00
GATCAAATCAACTTAGGTAG	0.46	0.00	0.00	0.00	0.00
GATCAAATCAACTTAGGTCG	0.00	0.33	0.00	0.00	0.69
GATCAAATCAACTTAGGTGA	0.00	0.00	0.00	0.00	0.34
GATCAAATCAACTTAGGTGC	0.00	0.00	0.57	0.00	0.34
GATCAAATCAACTTAGGTGG	105.07	66.37	59.56	54.65	61.99
GATCAAATCAACTTAGGTGN	0.46	0.00	0.00	0.00	0.00
GATCAAATCAACTTAGGTGT	0.00	0.98	0.57	0.00	0.00
GATCAAATCAACTTAGGTNN	0.46	0.00	0.00	0.00	0.00
GATCAAATCAACTTAGGTTG	0.23	0.00	0.00	0.00	0.00
GATCAAATCAACTTAGNNNN	0.23	0.00	0.00	0.00	0.00
GATCAAATCAACTTAGTGTG	0.00	0.65	0.00	0.00	0.00
GATCAAATCAACTTAGTTGG	0.23	0.65	0.00	0.00	0.00
GATCAAATCAAGACTTTTTC	0.00	0.00	0.00	0.34	0.00
GATCAAATCAAGCCAATTAA	0.00	0.33	0.00	0.00	0.00
GATCAAATCAAGCGCAAAGC	0.00	0.33	0.00	0.00	0.00
GATCAAATCAAGCGCGACCA	0.00	0.33	0.29	0.00	0.00
GATCAAATCAAGGATTGCCT	0.00	0.00	0.29	0.00	0.00
GATCAAATCAAGGGTGCGCT	0.23	0.00	0.00	0.00	0.00
GATCAAATCAAGGTTGCGCT	0.00	0.65	0.86	0.68	0.34
GATCAAATCAAGTCAGCGTC	0.69	0.98	2.00	0.68	0.00

GATCAAATCAAGTCTTTTTT	0.00	0.00	0.29	0.00	0.00
GATCAAATCAAGTGGACAAG	0.00	0.00	0.00	0.00	0.00
GATCAAATCAATACCACACA	0.00	0.00	0.00	0.00	0.69
GATCAAATCAATCAAAGAGA	0.00	0.00	0.00	0.00	0.00
GATCAAATCAATCTGAACTT	0.00	0.00	0.00	0.34	0.00
GATCAAATCAATGAGAACAG	0.00	0.33	0.00	0.00	0.00
GATCAAATCAATTAAAATTA	0.00	0.00	0.00	0.00	0.69
GATCAAATCAATTCCAGGAG	0.00	0.00	0.00	0.00	0.69
GATCAAATCAATTCTGATGT	0.00	0.00	0.00	0.34	0.00
GATCAAATCAATTTTTTGGA	0.00	0.00	0.00	0.00	0.69
GATCAAATCACAGAGAAAAT	0.00	0.00	0.00	0.34	0.00
GATCAAATCACATCACAACA	0.00	0.00	0.29	0.00	0.69
GATCAAATCACATCCCAACA	0.00	0.00	0.00	0.00	0.34
GATCAAATCACATGGCGTCA	0.00	0.00	0.29	0.00	0.00
GATCAAATCACATGGTGTCA	0.23	0.00	0.00	0.00	0.00
GATCAAATCACCAAAAAAAA	0.00	0.00	0.00	0.34	0.34
GATCAAATCACCACAAACCC	0.00	0.00	0.00	0.34	0.00
GATCAAATCACCGCACAGGA	0.00	0.00	0.00	0.00	0.00
GATCAAATCACCGCGGAAGG	0.00	0.33	0.00	0.00	0.00
GATCAAATCACCTTAGGTGG	0.23	0.00	0.00	0.00	0.00
GATCAAATCACGCACGTAGA	0.00	0.00	0.00	0.00	0.00
GATCAAATCACGCATTGCAA	0.00	0.00	0.29	0.00	0.00
GATCAAATCACGCGGGCTCT	0.00	0.00	0.00	0.00	0.00
GATCAAATCACGCTACAACA	0.00	0.00	0.00	0.00	0.34
GATCAAATCACGGCCATGCT	0.00	0.00	0.29	0.00	0.00
GATCAAATCACGGCTGCGCT	0.23	0.33	0.00	0.00	0.00
GATCAAATCACGGGGGCCGT	0.00	0.00	0.00	0.00	0.34
GATCAAATCACGTCAATGCT	0.00	0.33	0.00	0.00	0.00
GATCAAATCACGTGCCATGC	0.00	0.00	0.00	0.34	0.00
GATCAAATCACTCACACTTC	0.00	0.33	0.00	0.00	0.00
GATCAAATCACTCTCTCGAG	0.00	0.00	0.00	0.00	0.00
GATCAAATCACTGGAGCTCA	0.00	0.00	0.00	0.34	0.00
GATCAAATCACTGGCGCTGC	0.00	0.00	0.00	0.00	0.00
GATCAAATCACTGTGAGAGA	0.00	0.00	0.00	0.00	0.00
GATCAAATCACTGTGAGCAG	1.60	0.65	0.86	0.00	0.00
GATCAAATCACTGTGTTGCT	0.00	0.33	0.00	0.34	0.00
GATCAAATCAGAAAAAAAAA	0.00	0.33	0.00	0.00	0.00

GATCAAATCAGAAAGCTCAC	0.46	0.33	0.00	0.34	0.00
GATCAAATCAGAACAAACCA	0.00	0.00	0.00	0.00	0.34
GATCAAATCAGAACAGAACA	0.00	0.00	0.00	0.00	0.69
GATCAAATCAGAATTGAGCC	0.00	0.33	0.00	0.00	0.00
GATCAAATCAGAATTGCCGC	0.00	0.00	0.29	0.00	0.00
GATCAAATCAGAATTGCGGA	0.23	0.00	0.00	0.00	0.00
GATCAAATCAGAATTGCGGC	12.59	7.48	3.44	8.83	4.13
GATCAAATCAGAATTGCGGN	0.23	0.00	0.00	0.00	0.00
GATCAAATCAGAATTGCTGC	0.00	0.33	0.00	0.00	0.00
GATCAAATCAGAATTGNNNN	0.23	0.00	0.00	0.00	0.00
GATCAAATCAGACGTTTTTT	0.00	0.33	0.00	0.00	0.00
GATCAAATCAGAGAAGTTAA	0.23	0.00	0.00	0.00	0.00
GATCAAATCAGAGACCAGAA	0.00	0.00	0.00	0.00	0.34
GATCAAATCAGAGCCGAACA	0.00	0.00	0.00	0.00	0.34
GATCAAATCAGAGCGAGGTC	0.00	0.33	0.00	1.02	0.00
GATCAAATCAGATTGGCACT	0.00	0.00	0.00	0.00	0.34
GATCAAATCAGCATCAGTCT	0.00	0.00	0.00	0.34	0.00
GATCAAATCAGCCAGGTCCT	0.46	0.00	0.00	0.00	0.00
GATCAAATCAGCGAAATGAA	0.00	0.00	0.29	0.00	0.00
GATCAAATCAGCGTAAAATG	0.23	0.00	0.00	0.00	0.00
GATCAAATCAGCGTTGCTGG	0.00	0.00	0.00	0.00	0.00
GATCAAATCAGCGTTGCTGT	0.23	0.00	0.00	0.00	0.00
GATCAAATCAGCTTCTGAAT	0.00	0.00	0.00	0.00	0.00
GATCAAATCAGCTTCTGGGC	0.00	0.33	0.00	0.00	0.00
GATCAAATCAGCTTGCACGC	0.00	0.00	0.00	0.68	0.34
GATCAAATCAGGAATAGTCA	0.00	0.00	0.00	0.00	0.34
GATCAAATCAGGAGGACGAA	1.37	0.33	0.00	0.00	0.00
GATCAAATCAGGAGGACGCA	0.00	0.00	0.00	0.00	0.00
GATCAAATCAGGCAGCCAAC	0.00	0.00	0.00	0.34	0.00
GATCAAATCAGGGCTTTCAG	0.00	0.00	0.29	0.00	0.00
GATCAAATCAGGGGGTACAA	0.23	0.00	0.00	0.00	0.00
GATCAAATCAGGGTGTACAA	1.37	0.00	0.00	0.00	0.00
GATCAAATCAGGTCCTGAAA	0.23	0.00	0.00	0.00	0.00
GATCAAATCAGGTGATTCAT	0.23	0.65	0.86	0.34	1.03
GATCAAATCAGTCGTGTTCG	2.06	0.00	0.00	0.00	0.34
GATCAAATCAGTGTTGACCT	0.00	0.00	0.29	0.00	0.00
GATCAAATCAGTTCAGGCCT	0.69	0.00	0.00	0.00	0.00

GATCAAATCATACCAGCCAG	0.00	0.00	0.00	0.00	0.00
GATCAAATCATACTTGAAAT	0.00	0.00	0.00	0.00	0.34
GATCAAATCATATCACAACA	0.00	0.00	0.29	0.00	0.00
GATCAAATCATATCTCAACA	0.00	0.00	0.00	0.00	0.69
GATCAAATCATATGGAGAGA	0.00	0.33	0.00	0.00	0.00
GATCAAATCATCAAAGAAGC	0.00	0.00	0.29	0.00	0.00
GATCAAATCATCATCGCTCC	0.00	0.33	0.00	0.00	0.00
GATCAAATCATCCAAATACA	0.00	0.00	0.00	0.34	0.00
GATCAAATCATCGGGAGAGA	0.92	0.65	0.00	0.34	0.00
GATCAAATCATCGGGAGAGG	0.23	0.00	0.00	0.00	0.00
GATCAAATCATCGGGAGAGT	0.23	0.00	0.00	0.00	0.00
GATCAAATCATCGTCCTTGG	3.20	1.95	2.29	0.68	6.20
GATCAAATCATCGTCCTTGT	0.00	0.00	0.00	0.00	0.34
GATCAAATCATCGTCCTTTG	0.00	0.33	0.00	0.00	0.00
GATCAAATCATGAATTGCAG	0.00	0.33	0.29	0.34	0.00
GATCAAATCATGACGTTTTT	0.23	0.00	0.00	0.00	0.00
GATCAAATCATGAGTTTGGC	0.00	0.00	0.00	0.00	0.69
GATCAAATCATGATTTCCGC	0.00	0.00	0.00	0.34	0.00
GATCAAATCATGCGTAAATC	0.00	0.00	0.00	0.68	0.00
GATCAAATCATGCTCAAAAA	0.00	0.00	0.00	0.34	0.00
GATCAAATCATGGCAGGCAA	0.00	0.00	0.29	0.00	0.00
GATCAAATCATGGCGTCATG	0.00	0.00	0.00	0.00	0.00
GATCAAATCATGTCCCAAAA	0.00	0.00	0.29	0.00	0.00
GATCAAATCATGTCGGAAAA	0.23	0.33	0.29	0.68	0.00
GATCAAATCATGTCTCAAAA	0.00	0.33	0.00	0.34	0.00
GATCAAATCATGTCTCAAAG	1.37	1.63	0.29	0.34	0.69
GATCAAATCATTATGCTGAC	0.00	0.00	0.00	0.00	0.69
GATCAAATCATTGTCACTCC	0.00	0.65	0.00	0.00	0.00
GATCAAATCATTTCATAACA	0.00	0.00	0.86	0.00	0.00
GATCAAATCATTTGGAGACT	0.00	0.00	0.29	0.00	0.00
GATCAAATCATTTGGCGCAA	0.00	0.00	0.00	0.00	0.34
GATCAAATCATTTTTGGAGA	0.23	0.00	0.00	0.00	0.00
GATCAAATCATTTTTGGAGT	0.23	0.00	0.00	0.00	0.00
GATCAAATCCAAAAAAAAAA	0.46	0.00	0.29	0.00	0.00
GATCAAATCCAAAGAAGTCC	0.46	0.00	0.00	0.00	0.00
GATCAAATCCAAAGACACAG	0.00	0.00	0.00	0.00	0.34
GATCAAATCCAACATGACGG	0.00	0.00	0.00	0.00	0.00

GATCAAATCCAACCAGCGGT	0.00	0.00	0.00	0.00	0.00
GATCAAATCCAATCGGGGCG	0.00	0.00	0.00	0.00	0.00
GATCAAATCCAATTTAGGCG	0.00	0.33	0.29	0.00	0.00
GATCAAATCCACAGCACTCC	0.00	0.00	0.29	0.00	0.00
GATCAAATCCACCTGTCTTC	0.23	0.00	0.00	0.00	0.00
GATCAAATCCACGAAAAATG	0.00	0.00	0.00	0.00	0.34
GATCAAATCCACGCGAGCCA	0.23	0.00	0.00	0.68	0.34
GATCAAATCCACGGCATAGC	0.00	0.00	0.00	0.00	0.00
GATCAAATCCACTTAGGTGG	0.00	0.00	0.57	0.34	0.00
GATCAAATCCAGAAGTGCCG	0.00	1.30	0.29	0.68	1.38
GATCAAATCCAGACAACTCT	0.00	0.00	0.00	0.68	0.00
GATCAAATCCAGAGTGTTGA	8.70	4.55	4.58	5.09	3.79
GATCAAATCCAGAGTGTTGC	0.00	0.00	0.00	0.00	0.00
GATCAAATCCAGAGTGTTNA	0.00	0.00	0.00	0.34	0.00
GATCAAATCCAGAGTGTTNN	0.23	0.00	0.00	0.00	0.00
GATCAAATCCAGATTTAATG	0.00	0.00	0.57	0.00	0.00
GATCAAATCCAGCCGGCAGA	0.00	0.00	0.00	0.34	0.00
GATCAAATCCAGGAGTGGGC	1.60	3.25	0.57	0.68	0.69
GATCAAATCCAGGCCGCAGT	0.00	0.00	0.00	0.00	0.00
GATCAAATCCAGGCCGTCGC	0.00	0.00	0.00	0.00	0.00
GATCAAATCCAGGTCTGAAT	0.00	0.33	0.57	0.00	0.00
GATCAAATCCAGTATTCCTG	0.00	0.00	0.00	0.00	0.00
GATCAAATCCATCAAATTCA	0.00	0.00	0.29	0.00	0.00
GATCAAATCCATCAACTCCA	0.00	0.00	0.00	0.00	0.34
GATCAAATCCATCCGAGCGC	0.00	0.00	0.00	0.34	0.34
GATCAAATCCATCCGAGCTC	1.60	0.00	0.29	1.36	0.00
GATCAAATCCCACAGCNGNN	0.23	0.00	0.00	0.00	0.00
GATCAAATCCCACAGCTGTC	0.69	0.00	0.00	0.00	0.00
GATCAAATCCCAGTGTGTCC	0.00	0.00	0.00	0.00	0.00
GATCAAATCCCATTCCAGTC	0.46	0.00	0.29	0.00	0.34
GATCAAATCCCCTTACATGG	0.00	0.65	0.00	0.68	0.69
GATCAAATCCCCTTACCATG	0.00	0.33	0.00	0.00	0.00
GATCAAATCCCCTTACGTGG	0.00	0.00	0.00	0.34	0.00
GATCAAATCCCCTTAGGTGG	0.23	0.00	0.00	0.00	0.00
GATCAAATCCCCTTTCATGG	0.00	0.00	0.00	0.00	0.00
GATCAAATCCCGAGGCAAAA	0.00	0.00	0.00	0.34	0.00
GATCAAATCCCGATGAGGCA	0.00	0.00	0.00	0.00	0.00

GATCAAATCCCGGGGCCGCC	0.00	0.00	0.29	0.00	0.00
GATCAAATCCCGGTTGTCCT	0.23	1.63	0.00	0.68	0.34
GATCAAATCCCTATGGAAGA	0.00	0.00	0.00	0.00	0.34
GATCAAATCCCTTTTCTGTT	0.23	0.00	0.00	0.00	0.00
GATCAAATCCGAAAAAAAAA	0.00	0.00	0.00	0.00	0.00
GATCAAATCCGAAAACAAAA	0.00	0.00	0.29	0.00	0.00
GATCAAATCCGAGAAAAAAA	0.00	0.65	0.00	0.00	0.00
GATCAAATCCGAGAAAAATA	0.00	0.00	0.29	0.00	0.00
GATCAAATCCGAGAAGTCGT	0.00	0.00	0.57	0.00	0.00
GATCAAATCCGAGCAGGATA	0.00	0.00	0.29	0.34	0.00
GATCAAATCCGAGCTGAGGC	0.00	0.00	0.00	0.00	0.00
GATCAAATCCGAGGAGCGCA	0.00	0.00	0.00	0.00	0.34
GATCAAATCCGAGGTGCAGA	0.00	0.00	0.57	0.34	0.34
GATCAAATCCGAGGTGGCAC	0.00	0.00	0.00	0.00	0.00
GATCAAATCCGCAATTAAAA	0.00	0.00	0.57	0.00	0.00
GATCAAATCCGCACAAAAAA	0.00	0.00	0.00	0.00	0.00
GATCAAATCCGCAGACCTCT	0.00	0.00	0.29	0.00	0.00
GATCAAATCCGCAGCGACGG	0.00	0.00	0.00	0.00	0.00
GATCAAATCCGCATGAGTGC	0.00	0.33	0.29	0.00	0.34
GATCAAATCCGCCATGGTCG	0.00	0.00	0.00	0.00	0.00
GATCAAATCCGCCATGGTGG	5.72	5.21	6.30	6.79	7.92
GATCAAATCCGCCCACAGGT	0.00	0.00	0.00	0.00	0.00
GATCAAATCCGCGAAAAAAA	1.37	0.98	1.15	3.05	0.34
GATCAAATCCGCGAAAAACG	0.00	0.00	0.29	1.36	0.00
GATCAAATCCGCGAAAAATA	0.00	0.33	0.00	0.00	0.00
GATCAAATCCGCGAAAAATC	0.00	0.00	0.29	0.00	0.00
GATCAAATCCGCGAAAAATG	62.95	33.18	39.80	46.16	27.21
GATCAAATCCGCGAAAAATN	0.46	0.00	0.00	0.00	0.00
GATCAAATCCGCGAAAAATT	0.69	0.00	0.00	0.00	0.00
GATCAAATCCGCGAAAACAA	0.46	0.00	0.00	0.34	0.34
GATCAAATCCGCGAAAACCT	0.00	0.00	0.00	0.00	0.00
GATCAAATCCGCGAAAACTG	15.11	3.58	6.30	8.83	5.17
GATCAAATCCGCGAAAACTN	0.23	0.00	0.00	0.00	0.00
GATCAAATCCGCGAAAAGTG	0.46	0.00	0.00	0.00	0.00
GATCAAATCCGCGAAAATCT	0.46	0.00	0.00	0.00	0.00
GATCAAATCCGCGAAACCNN	0.23	0.00	0.00	0.00	0.00
GATCAAATCCGCGAAAGATG	0.23	0.00	0.00	0.00	0.34

GATCAAATCCGCGAAANNNN	0.46	0.00	0.00	0.00	0.00
GATCAAATCCGCGAACAAGC	0.00	0.33	0.00	0.00	0.00
GATCAAATCCGCGACCACTG	0.23	0.00	0.00	0.00	0.00
GATCAAATCCGCGAGAACTG	0.23	0.00	0.00	0.00	0.00
GATCAAATCCGCGCAAAAAA	0.00	0.00	0.57	0.34	0.00
GATCAAATCCGCGCAAAATG	0.00	0.00	0.29	0.00	0.00
GATCAAATCCGCGCAAATTG	0.23	0.00	0.00	0.00	0.00
GATCAAATCCGCGCCACCTG	0.23	0.00	0.00	0.00	0.00
GATCAAATCCGCGGAAAAAA	0.00	0.00	0.29	0.00	0.00
GATCAAATCCGCGGAAACAA	0.00	0.00	0.00	0.00	0.34
GATCAAATCCGCGGAAACTG	6.41	4.88	4.58	2.38	3.44
GATCAAATCCGCGGAAACTT	0.00	0.00	0.00	0.34	0.00
GATCAAATCCGCGGCAACTG	0.23	0.00	0.00	0.00	0.00
GATCAAATCCGCGGCATCCT	0.00	0.00	0.00	0.00	0.00
GATCAAATCCGCTCGTATGC	0.00	0.00	0.57	0.00	0.00
GATCAAATCCGCTCGTCGCG	0.00	0.33	0.00	0.34	0.00
GATCAAATCCGCTCTTGAGG	0.00	0.00	0.00	0.00	0.00
GATCAAATCCGCTGGGAAAA	0.00	0.33	0.00	0.00	0.00
GATCAAATCCGGAACCTCCG	0.00	0.00	0.00	0.34	0.00
GATCAAATCCGGAAGTTCAA	0.00	0.00	0.00	0.00	0.69
GATCAAATCCGGCATCAAGA	0.00	0.00	0.00	0.68	0.00
GATCAAATCCGGCCTGTGCA	0.23	0.00	0.00	0.00	0.00
GATCAAATCCGGGCGATGGC	0.00	0.00	0.00	0.00	0.34
GATCAAATCCGGTAGGACTC	0.00	0.00	0.00	0.00	0.00
GATCAAATCCGGTGCAGAGG	0.00	0.00	0.00	0.68	0.00
GATCAAATCCGGTGGTACAC	0.00	0.00	0.00	0.00	0.34
GATCAAATCCGTAAATGTGC	0.46	0.00	0.00	0.00	0.00
GATCAAATCCGTCATTGGCA	0.00	0.00	0.00	0.00	0.00
GATCAAATCCGTCGGCAAAG	0.00	0.33	0.00	0.00	0.00
GATCAAATCCGTGCCTTTGC	0.00	0.00	0.00	0.00	0.00
GATCAAATCCGTTACAGTGC	0.23	0.33	0.00	0.00	0.00
GATCAAATCCTAAAAAAAAA	0.00	0.00	0.57	0.00	0.00
GATCAAATCCTAAGCGCAAC	0.00	0.00	0.00	0.34	0.00
GATCAAATCCTACCGCTTGA	0.00	0.00	0.00	0.00	0.69
GATCAAATCCTACTCAGGTA	0.00	0.00	0.29	0.00	0.00
GATCAAATCCTATAGAAAAT	0.00	0.00	0.86	0.00	0.00
GATCAAATCCTCCATGGTGG	0.00	0.00	0.00	0.00	0.00

GATCAAATCCTCCGCTTTTC	0.00	0.33	0.00	0.00	0.00
GATCAAATCCTCGCCATGTG	0.00	0.00	0.00	0.00	0.00
GATCAAATCCTCGCCTGGCC	0.00	0.00	0.00	0.00	0.00
GATCAAATCCTGCAACGAGT	0.00	0.00	0.00	0.00	0.00
GATCAAATCCTGCGCATCCT	0.00	0.00	0.00	0.00	0.34
GATCAAATCCTGCGGAAGTT	0.00	0.00	0.00	0.00	0.00
GATCAAATCCTGCGTGCAGC	0.00	0.00	0.00	1.36	0.00
GATCAAATCCTGGTCCCACA	0.69	0.00	0.00	0.00	0.00
GATCAAATCCTGGTCCGGCA	0.23	0.00	0.29	0.00	0.00
GATCAAATCCTGGTGCCCAC	0.00	0.00	0.86	0.34	0.00
GATCAAATCCTGGTGCCCCC	0.00	0.33	0.00	0.00	0.00
GATCAAATCCTTCGAGGAGG	0.23	0.00	0.00	0.00	0.00
GATCAAATCCTTCTTCAGCG	0.00	0.00	0.00	0.00	0.00
GATCAAATCCTTCTTCAGTG	0.69	0.98	0.00	0.34	0.00
GATCAAATCCTTGCCCCGTT	0.00	0.00	0.00	0.00	0.00
GATCAAATCCTTGCCGTGCT	0.23	0.00	0.29	2.04	0.34
GATCAAATCCTTGGCGTACG	0.00	0.00	0.00	0.00	0.00
GATCAAATCCTTTGCACCCG	0.00	0.00	0.00	0.00	0.00
GATCAAATCCTTTTCCGGGG	0.23	0.00	0.00	0.00	0.00
GATCAAATCCTTTTTAAAAA	0.00	0.00	0.00	0.00	0.00
GATCAAATCCTTTTTCAAAA	0.00	0.33	0.29	0.00	0.00
GATCAAATCCTTTTTCCGGG	0.00	0.00	0.00	0.34	0.00
GATCAAATCCTTTTTCGCGG	0.00	0.00	0.00	0.00	0.00
GATCAAATCCTTTTTCGGGC	0.23	0.00	0.00	0.00	0.00
GATCAAATCCTTTTTCGGGG	18.77	29.28	23.77	25.80	25.83
GATCAAATCCTTTTTCGGGT	0.00	0.00	0.57	0.00	0.00
GATCAAATCCTTTTTCGGNG	0.00	0.33	0.00	0.00	0.00
GATCAAATCCTTTTTCGGTG	0.00	0.00	0.00	0.00	0.34
GATCAAATCCTTTTTCGTAT	0.00	0.00	0.00	0.34	0.00
GATCAAATCCTTTTTCGTGG	0.23	0.65	0.29	0.00	0.00
GATCAAATCCTTTTTCTTGG	0.00	0.00	0.00	0.00	0.00
GATCAAATCCTTTTTTGGGG	0.00	0.00	0.00	0.00	0.34
GATCAAATCGAAAAAAAAAA	0.69	0.33	0.29	0.34	0.00
GATCAAATCGACCCTGTCCT	0.00	0.00	0.29	0.00	0.00
GATCAAATCGACTCGATTAG	0.00	0.00	0.29	0.00	0.00
GATCAAATCGACTTAGGTGG	0.00	0.00	0.00	0.34	0.00
GATCAAATCGACTTCAACCA	0.00	0.33	0.00	0.00	0.34

GATCAAATCGACTTCCACCA	0.00	0.00	0.29	0.00	0.00
GATCAAATCGACTTCCCGTG	0.00	0.00	0.57	0.00	0.00
GATCAAATCGAGCTCTCTCT	9.16	1.95	1.72	3.39	2.41
GATCAAATCGAGCTCTCTNN	0.23	0.00	0.00	0.00	0.00
GATCAAATCGATAAAAAAAA	0.69	0.00	0.00	0.00	0.00
GATCAAATCGATACAAAAAA	0.00	0.00	0.00	0.00	1.38
GATCAAATCGATGAGAAAAA	0.00	0.00	0.29	0.00	0.00
GATCAAATCGATGAGAACAA	0.00	0.33	0.29	0.68	0.34
GATCAAATCGATGAGAACAG	0.00	0.98	0.57	0.34	1.38
GATCAAATCGATGAGGACAG	0.00	0.00	0.00	0.00	0.00
GATCAAATCGATGCTTAAAA	0.00	0.00	0.00	0.00	0.34
GATCAAATCGATGGCGCCGT	0.00	0.00	0.29	0.00	0.00
GATCAAATCGATTAAAAAAA	0.00	0.00	0.00	0.00	0.34
GATCAAATCGATTCAAAAAA	3.20	0.00	0.00	0.00	0.34
GATCAAATCGATTCAAAANN	0.23	0.00	0.00	0.00	0.00
GATCAAATCGATTCACTCAC	1.60	0.00	0.00	0.00	0.00
GATCAAATCGATTCTAATGA	0.46	0.00	0.00	0.00	0.00
GATCAAATCGATTCTATCTA	0.00	0.00	0.00	0.00	1.03
GATCAAATCGATTCTCAAAA	0.00	0.00	0.00	0.00	0.69
GATCAAATCGATTCTTAAAA	29.53	2.28	2.86	3.39	8.27
GATCAAATCGATTCTTAAAN	0.69	0.00	0.00	0.00	0.00
GATCAAATCGATTCTTAACA	0.23	0.00	0.00	0.00	0.00
GATCAAATCGATTCTTAACC	0.46	0.00	0.00	0.00	0.00
GATCAAATCGATTCTTAACN	0.23	0.00	0.00	0.00	0.00
GATCAAATCGATTCTTAAGA	0.23	0.00	0.00	0.00	0.00
GATCAAATCGATTCTTAAGC	0.23	0.00	0.00	0.00	0.00
GATCAAATCGATTCTTAANN	0.23	0.00	0.00	0.00	0.00
GATCAAATCGATTCTTAATA	5.26	0.65	0.00	0.34	0.34
GATCAAATCGATTCTTAATC	0.00	0.00	0.29	0.00	0.00
GATCAAATCGATTCTTAATG	30.44	9.76	15.46	14.26	20.66
GATCAAATCGATTCTTAATT	0.23	0.00	0.00	0.00	0.00
GATCAAATCGATTCTTAGAA	0.23	0.00	0.00	0.00	0.00
GATCAAATCGATTCTTANTG	0.00	0.00	0.00	0.34	0.00
GATCAAATCGATTCTTATTG	0.00	0.00	0.00	0.00	0.34
GATCAAATCGATTCTTCATA	0.00	0.33	0.00	0.00	0.00
GATCAAATCGATTCTTCGTA	0.46	0.00	0.00	0.00	0.00
GATCAAATCGATTCTTGTCT	0.00	1.63	0.00	0.00	0.69

GATCAAATCGATTCTTTCAT	0.23	0.00	0.29	0.00	0.69
GATCAAATCGATTCTTTCTT	0.00	0.00	0.00	0.68	0.34
GATCAAATCGCAAGAAAAAA	0.00	0.00	0.29	0.00	0.00
GATCAAATCGCAATGTTACG	0.23	0.00	0.00	0.00	0.00
GATCAAATCGCAGTCCTACC	0.00	0.65	0.29	0.00	0.34
GATCAAATCGCCGCGCTGGG	0.00	0.00	0.29	0.00	0.00
GATCAAATCGCGAAGGCCAG	0.00	0.00	0.57	0.68	0.00
GATCAAATCGCGAAGGCCTG	0.00	0.00	0.00	0.00	0.00
GATCAAATCGCGAGAACCTA	0.00	0.00	0.00	0.34	0.00
GATCAAATCGCGCAGATGTG	0.00	0.00	0.00	0.00	0.00
GATCAAATCGCGCAGCAAAA	0.00	0.00	0.29	0.00	0.00
GATCAAATCGCGCATCTTGG	0.00	0.00	0.00	0.00	0.00
GATCAAATCGCGCCCTTGGG	0.00	0.33	0.00	0.00	0.00
GATCAAATCGCTCAGGAGTT	0.46	0.00	0.00	0.00	0.00
GATCAAATCGCTCCGTGACG	0.00	0.00	0.00	0.34	0.00
GATCAAATCGCTTCATGCCA	0.00	0.00	0.00	0.00	0.69
GATCAAATCGCTTCTACCTG	0.23	0.00	0.00	0.00	0.00
GATCAAATCGCTTCTTCGCT	0.00	0.00	0.29	0.34	1.03
GATCAAATCGCTTGATGTCC	0.46	0.00	0.00	0.00	0.00
GATCAAATCGCTTGCTAAAA	0.00	0.00	0.00	0.34	0.00
GATCAAATCGGAATCTAATC	0.00	0.00	0.29	0.00	0.00
GATCAAATCGGCAACAAACT	0.00	0.65	0.29	0.34	0.34
GATCAAATCGGCAACAGATG	0.00	0.33	0.00	0.00	0.00
GATCAAATCGGCAACAGCTG	0.00	0.98	0.29	0.68	0.69
GATCAAATCGGCCACAAACT	0.00	0.00	0.00	0.00	0.00
GATCAAATCGGCCAGTCTGC	0.00	0.33	0.29	0.00	0.00
GATCAAATCGGCTAATCACC	0.00	0.00	0.00	0.00	0.34
GATCAAATCGGGCATTGCGG	0.00	0.00	0.00	0.00	0.00
GATCAAATCGGGCTTGGAGT	0.23	0.00	0.00	0.00	0.00
GATCAAATCGGGCTTGTTGG	10.30	5.53	4.01	3.05	3.10
GATCAAATCGGGCTTGTTGN	0.00	0.00	0.00	0.00	0.00
GATCAAATCGGGCTTGTTNN	0.23	0.00	0.00	0.00	0.00
GATCAAATCGGGCTTGTTTG	0.00	0.65	0.00	0.00	0.00
GATCAAATCGGGGCAGTGAA	0.00	0.00	0.00	0.00	0.34
GATCAAATCGGGGTTGTCAG	0.46	0.00	0.00	0.00	0.00
GATCAAATCGGTCCCAGAGC	0.00	0.00	0.00	0.00	0.69
GATCAAATCGGTGCGTGACA	0.00	0.00	0.00	0.00	0.34

GATCAAATCGGTGGTCTCCT	0.00	0.33	0.00	0.00	0.00
GATCAAATCGGTTCTTAATG	0.00	0.33	0.00	0.00	0.00
GATCAAATCGTACTCAGGTA	0.00	0.00	0.29	0.00	0.00
GATCAAATCGTATGAAATCT	0.46	0.00	0.00	0.00	0.00
GATCAAATCGTATGCCGTCT	0.23	0.00	0.00	0.00	0.00
GATCAAATCGTCCAAAGTCA	0.00	0.00	0.29	0.00	0.00
GATCAAATCGTGAATTGACG	0.00	0.00	0.00	0.34	0.00
GATCAAATCGTGCTCCCAAA	0.00	0.00	0.57	0.00	0.00
GATCAAATCGTGGACATTGG	0.00	0.33	0.00	0.00	0.00
GATCAAATCGTGGAGCAGCA	0.00	0.00	0.00	0.00	0.34
GATCAAATCGTGTGAGGCTC	0.00	0.33	0.00	0.00	0.00
GATCAAATCGTGTGCATCTG	0.00	0.00	0.00	0.00	0.34
GATCAAATCGTGTGCATTTG	0.00	0.00	1.15	0.68	0.00
GATCAAATCGTGTGTGCGCG	0.00	0.00	0.29	0.00	0.00
GATCAAATCGTTCCGAATCC	0.00	0.00	0.00	0.00	0.34
GATCAAATCGTTGTGGCAAG	0.00	0.00	0.29	0.00	0.00
GATCAAATCGTTTCGATGTC	0.00	0.00	0.00	0.00	0.00
GATCAAATCGTTTGTAACCG	0.00	0.00	0.00	0.34	0.00
GATCAAATCTAAGCACGTGG	0.00	0.33	0.00	0.00	0.00
GATCAAATCTAAGGACCTCC	0.00	0.65	0.00	0.00	0.34
GATCAAATCTAAGGACCTCG	0.46	0.98	0.29	1.02	0.69
GATCAAATCTAAGGACCTGT	0.69	0.00	0.00	0.00	0.00
GATCAAATCTAATTGTGAGC	0.00	0.00	0.00	0.00	0.00
GATCAAATCTAATTTAGGCG	0.00	0.00	0.00	0.00	0.00
GATCAAATCTACCTGTATGG	0.00	0.00	0.00	0.00	0.00
GATCAAATCTACGTAGGGGT	0.00	0.00	0.00	0.00	0.00
GATCAAATCTACTTCTGAAC	0.00	0.00	0.00	0.00	0.00
GATCAAATCTAGTAAGAAAC	2.06	3.25	1.43	1.02	1.03
GATCAAATCTCACGATTATC	0.00	0.00	0.00	1.36	0.00
GATCAAATCTCAGGCCGACT	0.23	0.00	0.00	0.00	0.00
GATCAAATCTCCACGCACAT	0.00	0.00	0.00	0.00	0.34
GATCAAATCTCCGCATTCGC	0.23	0.00	0.29	0.00	0.00
GATCAAATCTCGATGTCTGG	0.00	0.00	0.00	0.00	0.00
GATCAAATCTCGCGCAAGCT	0.00	0.00	0.00	0.34	0.00
GATCAAATCTCGCGGGGAAA	0.00	0.00	0.00	0.00	0.00
GATCAAATCTCGGAAGACGT	0.00	0.33	0.00	0.00	0.00
GATCAAATCTCTCGCCTCGG	0.00	0.00	0.29	0.00	0.00

GATCAAATCTCTCGCCTTGG	0.69	4.23	2.58	4.07	3.10
GATCAAATCTCTGCGGGTGG	0.23	0.33	0.29	0.00	0.00
GATCAAATCTCTTTTTTGCC	0.00	0.00	0.00	0.00	0.00
GATCAAATCTGAAAGAAAAA	0.00	0.00	0.00	0.00	0.00
GATCAAATCTGACGACGGGA	0.46	0.00	0.86	1.70	2.07
GATCAAATCTGAGCACCTCG	0.00	0.00	0.00	0.00	0.00
GATCAAATCTGAGCGTGTAG	0.00	0.00	0.29	0.00	0.00
GATCAAATCTGAGCTGAAGG	0.00	0.00	0.00	0.34	0.00
GATCAAATCTGAGCTTGGAG	0.23	0.00	0.00	0.00	0.00
GATCAAATCTGAGCTTGTAG	4.12	7.81	3.72	5.43	1.03
GATCAAATCTGAGCTTGTNN	0.23	0.00	0.00	0.00	0.00
GATCAAATCTGAGCTTTCAG	0.00	0.00	0.00	0.00	0.00
GATCAAATCTGAGGCTTAAG	0.00	0.00	0.00	0.00	0.00
GATCAAATCTGAGTCTATTC	0.00	0.00	0.29	0.00	0.00
GATCAAATCTGATGACGACC	0.00	0.33	0.00	0.00	0.00
GATCAAATCTGCAACGGGGC	0.00	0.00	0.29	0.00	0.00
GATCAAATCTGCAACGGGGT	0.00	0.00	0.29	0.00	0.00
GATCAAATCTGCAACTGAGA	0.00	0.00	0.00	0.00	0.00
GATCAAATCTGCAAGCACGG	0.00	0.33	0.00	0.00	0.00
GATCAAATCTGCGAAAAAAA	0.00	0.00	0.00	0.00	0.00
GATCAAATCTGCGCCGACCG	0.00	0.00	0.00	0.00	0.00
GATCAAATCTGCGGAGCGAG	0.00	0.33	0.00	0.00	0.00
GATCAAATCTGCGGTGACGT	0.00	0.00	0.00	0.00	0.34
GATCAAATCTGCTCAAGGCA	0.00	0.33	0.00	0.00	0.00
GATCAAATCTGCTGGGCCGT	0.00	0.00	0.29	0.00	0.00
GATCAAATCTGCTTTGCCGG	0.00	0.00	0.00	0.00	0.00
GATCAAATCTGGACTTTCAT	0.00	0.00	0.00	0.34	0.00
GATCAAATCTGGAGGGTATT	0.46	0.00	0.00	0.00	0.00
GATCAAATCTGGCGCAAGCT	0.00	0.00	0.00	0.34	0.00
GATCAAATCTGGGAGCACAA	0.00	0.33	0.00	0.00	0.00
GATCAAATCTGGGATTTCAT	0.00	0.33	0.00	0.00	0.00
GATCAAATCTGGGCAATCCT	0.00	0.00	0.00	0.00	0.00
GATCAAATCTGGGCGAAGGG	0.00	0.33	0.00	0.00	0.00
GATCAAATCTGGGCTCCCAT	0.00	0.00	0.00	0.00	0.00
GATCAAATCTGGGCTCTCAG	0.00	0.00	0.00	0.34	0.00
GATCAAATCTGGGCTGTCAG	0.00	0.00	0.00	0.34	0.00
GATCAAATCTGGGCTTCCTG	0.00	0.00	0.00	0.00	0.00

GATCAAATCTGGGCTTGTAG	0.00	0.33	0.00	0.00	0.00
GATCAAATCTGGGCTTNCAC	0.00	0.00	0.00	0.00	0.34
GATCAAATCTGGGCTTTAAG	0.00	0.00	0.29	0.34	0.00
GATCAAATCTGGGCTTTCAA	0.00	0.65	0.57	0.00	0.34
GATCAAATCTGGGCTTTCAC	0.00	0.00	0.00	0.34	0.00
GATCAAATCTGGGCTTTCAG	39.60	67.67	76.74	78.41	82.66
GATCAAATCTGGGCTTTCAT	0.00	0.00	0.29	0.34	0.34
GATCAAATCTGGGCTTTCCG	0.00	0.33	0.00	0.34	0.00
GATCAAATCTGGGCTTTCCN	0.46	0.00	0.00	0.00	0.00
GATCAAATCTGGGCTTTCGG	0.23	0.00	0.29	0.00	0.00
GATCAAATCTGGGCTTTCGT	0.23	0.00	0.00	0.00	0.00
GATCAAATCTGGGCTTTCNN	0.46	0.00	0.00	0.00	0.00
GATCAAATCTGGGCTTTTGA	0.00	0.00	0.00	0.68	0.00
GATCAAATCTGGGGTTTCAG	0.00	0.00	0.29	0.00	0.00
GATCAAATCTGGTCTGATTT	0.00	0.00	0.00	0.00	0.00
GATCAAATCTGGTCTTTCAG	0.00	0.00	0.00	0.00	0.34
GATCAAATCTGTGCAGTTCG	0.00	0.33	0.00	0.00	0.00
GATCAAATCTGTGCTTTCCG	0.00	0.33	0.00	0.00	0.00
GATCAAATCTGTGGTGGGGC	0.00	0.00	0.00	0.68	0.00
GATCAAATCTGTGGTGGTGC	1.37	0.33	0.00	0.00	0.34
GATCAAATCTGTGGTGGTGG	0.46	0.00	0.00	0.00	0.00
GATCAAATCTGTTCCCATCC	0.00	0.00	0.00	0.00	0.00
GATCAAATCTGTTCTGTTCT	0.00	0.00	0.00	0.00	0.34
GATCAAATCTGTTTTGGCTG	0.00	0.00	0.00	0.00	0.00
GATCAAATCTTAAATTAGAC	0.00	0.00	0.29	0.00	0.00
GATCAAATCTTAGACCAGGC	0.00	0.00	0.00	0.00	0.00
GATCAAATCTTAGCCTCAGG	8.47	7.16	2.29	4.07	2.76
GATCAAATCTTAGCCTCAGN	0.23	0.00	0.00	0.00	0.00
GATCAAATCTTAGCCTCCGG	0.00	0.00	0.29	0.00	0.00
GATCAAATCTTATTGGAGGA	0.00	0.00	0.00	0.00	0.34
GATCAAATCTTCAGACTACC	0.00	0.33	0.00	0.00	0.00
GATCAAATCTTCAGACTCCC	0.00	0.00	0.00	0.00	0.34
GATCAAATCTTCAGACTCTG	0.23	0.33	0.29	0.00	0.00
GATCAAATCTTCGAGCGCGC	0.00	0.00	0.00	0.00	0.00
GATCAAATCTTCGGCACTCA	0.00	0.00	0.00	0.00	0.00
GATCAAATCTTCGTCGGGCC	0.00	0.00	0.00	0.34	0.00
GATCAAATCTTCGTCGTAGT	0.00	0.00	0.00	0.00	0.00

GATCAAATCTTCGTCGTCGT	0.00	0.33	0.00	0.00	0.00
GATCAAATCTTCGTCGTGGA	0.00	0.00	0.29	0.00	0.00
GATCAAATCTTCGTCGTGGG	0.23	0.00	0.00	0.00	0.00
GATCAAATCTTCGTCGTGGT	5.72	16.59	10.31	14.26	5.17
GATCAAATCTTCGTCGTGNT	0.00	0.00	0.00	0.00	0.34
GATCAAATCTTCTGATACGC	0.00	0.00	0.29	0.00	0.34
GATCAAATCTTGAAAAAAAA	0.00	0.00	0.00	0.00	0.34
GATCAAATCTTGAATGGAAG	4.58	7.16	7.73	8.83	9.30
GATCAAATCTTGAATGGATG	0.00	0.00	0.00	0.68	0.00
GATCAAATCTTGACATAACT	0.00	0.00	0.00	0.34	0.00
GATCAAATCTTGACCCGCTC	0.00	0.00	0.00	0.00	0.00
GATCAAATCTTGCCGGAGTT	0.00	0.00	0.00	0.00	0.00
GATCAAATCTTGCGCAACCT	0.00	0.00	0.00	0.00	0.00
GATCAAATCTTGCGCAAGCA	0.00	0.33	0.00	0.00	0.00
GATCAAATCTTGCGCAAGCT	9.39	12.36	18.33	19.35	11.02
GATCAAATCTTGCGGGGGCA	0.00	0.00	0.00	0.00	0.00
GATCAAATCTTGCGGGGGCT	0.46	0.00	0.00	0.00	0.00
GATCAAATCTTGCGGGGGGC	0.23	0.00	0.00	0.00	0.00
GATCAAATCTTGCGTGGAGC	0.00	0.00	0.00	0.68	0.00
GATCAAATCTTGGCGAGGCG	0.00	0.00	0.00	0.00	0.00
GATCAAATCTTGGCGTGGGC	0.00	0.33	0.00	0.00	0.00
GATCAAATCTTGGCTTTACG	0.00	0.00	0.00	0.00	0.34
GATCAAATCTTGGCTTTCAG	0.23	0.00	0.00	0.00	0.00
GATCAAATCTTGGTTTTAGG	0.00	0.33	0.00	0.00	0.00
GATCAAATCTTGTCAGCAGC	0.00	0.00	0.00	0.00	0.00
GATCAAATCTTTATGTCGCT	0.00	0.00	1.15	0.00	0.00
GATCAAATCTTTCGCCTTGG	0.23	0.00	0.86	0.00	1.72
GATCAAATCTTTGGATATTG	0.00	0.33	0.00	0.00	0.00
GATCAAATCTTTTCCACACT	0.00	0.00	0.00	0.00	0.34
GATCAAATCTTTTTTTTGCT	0.00	0.00	0.29	0.00	0.00
GATCAAATGAAAAAAAAAAA	1.60	0.65	1.72	1.36	4.82
GATCAAATGAAAAAAAAAAT	0.23	0.00	0.00	0.00	0.00
GATCAAATGAAACCGTTTTT	0.00	0.00	0.29	0.00	0.00
GATCAAATGAAAGAAACAAA	0.00	0.00	0.29	0.00	0.00
GATCAAATGAAAGAAACACA	0.00	0.00	0.29	0.00	0.00
GATCAAATGAAATCAGTGTT	0.00	0.00	0.00	0.00	0.34
GATCAAATGAAATTAAAAAA	0.23	0.00	0.00	0.00	0.00

GATCAAATGAACACAAAATC	0.00	0.33	0.00	0.34	0.00
GATCAAATGAACATGAGCGA	0.00	0.00	0.00	0.00	0.00
GATCAAATGAACCACAAAAA	0.00	0.33	0.00	0.00	0.00
GATCAAATGAACGCAAAATC	0.00	0.00	0.00	0.00	0.00
GATCAAATGAACTGTCATGC	0.92	0.00	0.00	0.00	0.00
GATCAAATGAAGAAAAAGAG	0.23	0.00	0.00	0.00	0.00
GATCAAATGAAGAAGAGGGA	0.00	0.00	0.00	0.00	0.34
GATCAAATGAAGACCACTTG	0.00	0.00	0.00	0.00	0.00
GATCAAATGAAGATGACAAG	0.00	0.00	0.00	0.00	0.34
GATCAAATGAAGCTCAGGAT	0.23	0.00	0.00	0.00	0.00
GATCAAATGAAGGAGCTAAA	0.00	0.33	0.00	0.00	0.00
GATCAAATGAAGTAAAAGTA	0.00	0.00	0.29	0.00	0.00
GATCAAATGAAGTAACTGTC	0.00	0.00	0.00	0.00	0.34
GATCAAATGAAGTACGCAGG	0.00	0.00	0.00	0.00	0.00
GATCAAATGAAGTCCAGACA	0.00	0.00	0.29	0.00	0.00
GATCAAATGAAGTGGCGGCA	0.00	0.00	0.00	0.00	0.00
GATCAAATGAAGTTCCTCGT	0.00	0.65	0.00	0.00	0.00
GATCAAATGAATAAAAAAAA	0.00	0.00	0.00	0.00	1.03
GATCAAATGAATCCTCAATC	0.00	0.00	0.00	0.00	0.00
GATCAAATGAATGGCATATC	0.23	0.00	0.00	0.00	0.00
GATCAAATGAATGGGAGAAG	0.00	0.00	0.00	0.00	0.34
GATCAAATGACAAAAAAAAA	0.23	0.00	0.29	0.00	0.00
GATCAAATGACACACAATGG	0.23	0.00	0.00	0.68	0.00
GATCAAATGACACCAGAGAT	0.00	0.00	0.00	0.34	0.00
GATCAAATGACACTGAGGGA	0.23	0.00	0.29	0.34	0.00
GATCAAATGACACTGAGGGC	0.00	0.00	0.29	0.00	0.00
GATCAAATGACATCGCGCTC	0.00	0.00	0.00	0.34	0.00
GATCAAATGACATCGCGGAA	0.00	0.00	0.00	0.34	0.00
GATCAAATGACGCTCCGCGG	0.00	0.00	0.00	0.00	0.34
GATCAAATGACGGAGGATGA	0.00	0.00	0.00	0.00	0.34
GATCAAATGACGTAGTACGT	0.00	0.33	0.00	0.00	0.00
GATCAAATGACGTTTTTTGG	0.23	0.00	0.29	0.34	0.00
GATCAAATGACTCATGTGTG	0.00	0.00	0.29	0.00	0.00
GATCAAATGACTGGCCTCTG	0.00	0.00	0.00	0.00	0.00
GATCAAATGACTGTTGAGAC	0.00	0.00	0.00	1.02	0.00
GATCAAATGACTTTGTAAAA	0.00	0.00	0.86	0.00	0.00
GATCAAATGAGAAAAAAAAA	3.20	0.65	0.00	0.00	1.03

GATCAAATGAGAAAAAAAAT	0.00	0.33	0.00	0.00	0.00
GATCAAATGAGAAAAAAGAN	0.23	0.00	0.00	0.00	0.00
GATCAAATGAGAAAAGGACA	0.00	0.00	0.00	0.00	0.00
GATCAAATGAGAAAAGTTAA	0.23	0.00	0.00	0.00	0.00
GATCAAATGAGAACTCCAAG	0.00	0.33	0.00	0.00	0.00
GATCAAATGAGAATGGTTTT	0.00	0.00	0.00	0.00	0.34
GATCAAATGAGAATTATGAA	0.00	0.00	0.00	0.00	0.00
GATCAAATGAGACATCCAAG	0.00	0.00	0.00	0.00	0.34
GATCAAATGAGACCTCCAAG	1.60	1.63	1.43	4.75	3.79
GATCAAATGAGACCTCCAAN	0.23	0.00	0.00	0.00	0.00
GATCAAATGAGAGAAAAAAA	15.57	10.09	3.72	10.86	5.51
GATCAAATGAGAGAAAAGAA	0.00	0.00	0.00	0.68	0.00
GATCAAATGAGAGAAAGAAA	0.00	0.00	0.29	0.00	0.00
GATCAAATGAGAGAACCGAC	0.00	0.00	0.00	0.00	0.00
GATCAAATGAGAGAAGAAAA	0.23	0.33	0.29	0.68	1.03
GATCAAATGAGAGAAGAAAC	0.00	0.33	0.00	0.00	0.00
GATCAAATGAGAGAAGAAAG	0.00	0.00	0.57	0.00	0.00
GATCAAATGAGAGAAGAATC	0.00	0.33	0.00	0.00	0.00
GATCAAATGAGAGAAGATAA	0.23	0.00	0.00	0.34	0.00
GATCAAATGAGAGAAGATGA	0.00	0.00	0.00	0.34	0.00
GATCAAATGAGAGAAGCAAA	0.00	0.00	0.00	0.00	0.00
GATCAAATGAGAGAAGCCAA	0.00	0.00	0.00	0.00	0.00
GATCAAATGAGAGAAGCTAA	0.23	0.33	0.00	0.00	0.00
GATCAAATGAGAGAAGCTGA	0.00	0.00	0.00	0.00	0.69
GATCAAATGAGAGAAGGAAA	0.00	0.65	0.00	0.00	0.00
GATCAAATGAGAGAAGGTAA	0.46	0.33	0.00	0.00	0.00
GATCAAATGAGAGAAGGTAG	0.23	0.00	0.00	0.00	0.00
GATCAAATGAGAGAAGGTAT	0.00	0.00	0.00	0.34	0.00
GATCAAATGAGAGAAGNNNN	0.92	0.00	0.00	0.00	0.00
GATCAAATGAGAGAAGNTAA	0.00	0.00	0.00	0.00	0.34
GATCAAATGAGAGAAGNTAC	0.00	0.00	0.00	0.34	0.00
GATCAAATGAGAGAAGNTNN	0.46	0.00	0.00	0.00	0.00
GATCAAATGAGAGAAGTAAA	9.16	2.60	0.86	9.50	4.82
GATCAAATGAGAGAAGTCAA	0.00	0.33	0.57	0.34	0.34
GATCAAATGAGAGAAGTCCA	0.00	0.00	0.29	0.00	0.00
GATCAAATGAGAGAAGTGAA	0.23	0.00	0.00	0.00	0.00
GATCAAATGAGAGAAGTNAA	0.00	0.00	0.00	0.34	0.00

GATCAAATGAGAGAAGTTAA	222.50	94.67	38.94	113.37	84.38
GATCAAATGAGAGAAGTTAC	1.14	0.00	0.00	1.02	0.00
GATCAAATGAGAGAAGTTAG	0.00	0.65	0.00	0.68	0.00
GATCAAATGAGAGAAGTTAN	1.14	0.00	0.00	0.00	0.00
GATCAAATGAGAGAAGTTAT	0.00	0.00	0.00	0.00	0.34
GATCAAATGAGAGAAGTTCA	0.23	0.00	0.00	0.34	0.00
GATCAAATGAGAGAAGTTCT	0.00	0.00	0.00	0.00	0.00
GATCAAATGAGAGAAGTTGA	0.23	0.00	0.00	0.00	0.69
GATCAAATGAGAGAAGTTNN	0.92	0.00	0.00	0.00	0.00
GATCAAATGAGAGAAGTTTA	0.23	0.00	0.00	0.34	0.00
GATCAAATGAGAGAATTTAC	0.23	0.00	0.00	0.00	0.00
GATCAAATGAGAGACCTTCC	0.00	0.00	0.00	0.00	0.00
GATCAAATGAGAGACGTTGA	0.23	0.00	0.00	0.00	0.00
GATCAAATGAGAGAGGAGCT	0.00	0.00	0.00	0.68	0.00
GATCAAATGAGAGAGGATAT	0.00	0.00	0.00	0.00	0.00
GATCAAATGAGAGAGGTGCC	0.00	0.33	0.00	0.00	0.00
GATCAAATGAGAGAGGTTAA	0.23	0.00	0.00	0.00	0.00
GATCAAATGAGAGAGGTTCA	0.00	0.00	0.00	0.00	0.00
GATCAAATGAGAGATGCTCC	0.46	0.00	0.00	0.00	0.00
GATCAAATGAGAGATGTTAA	0.23	0.00	0.00	0.00	0.00
GATCAAATGAGAGCAGATGA	0.23	0.00	0.00	0.00	0.00
GATCAAATGAGAGCAGCTAG	0.23	0.00	0.00	0.00	0.00
GATCAAATGAGAGCAGTGTG	0.00	0.33	0.00	0.00	0.00
GATCAAATGAGAGCAGTTAA	0.46	0.00	0.00	0.00	0.34
GATCAAATGAGAGGAGTTAA	0.69	0.00	0.00	0.00	0.00
GATCAAATGAGAGGAGTTAG	0.23	0.00	0.00	0.00	0.00
GATCAAATGAGAGTGGAGAT	0.00	0.33	0.00	0.00	0.00
GATCAAATGAGAGTTGATTA	0.00	0.00	0.29	0.00	0.00
GATCAAATGAGATAAGTTAA	0.00	0.00	0.00	0.00	0.00
GATCAAATGAGCACGAGTCA	0.00	0.00	0.00	0.00	0.00
GATCAAATGAGCACGTCCTG	0.23	0.00	0.29	0.00	0.69
GATCAAATGAGCAGTCCTAC	0.00	0.33	0.00	0.00	0.00
GATCAAATGAGCATGGTTTT	0.00	0.00	0.00	0.00	0.00
GATCAAATGAGCATTGAAAA	0.00	0.00	0.00	0.00	0.00
GATCAAATGAGCCGGACTGG	0.00	0.00	0.00	0.00	0.00
GATCAAATGAGCCTCGACAC	4.12	1.63	1.15	0.68	0.69
GATCAAATGAGCCTGGTACT	0.00	0.00	0.00	0.34	0.00

GATCAAATGAGCCTTCCCCT	0.00	0.00	0.00	0.00	0.00
GATCAAATGAGCGAAGTTCA	0.23	0.00	0.00	0.00	0.00
GATCAAATGAGCGACGGTAA	0.23	0.00	0.00	0.00	0.00
GATCAAATGAGCGCCGTTCC	0.23	0.00	0.00	0.00	0.00
GATCAAATGAGCGCGAATCG	0.00	0.00	0.57	0.00	0.00
GATCAAATGAGCGCGAGAGG	0.00	0.33	0.00	0.00	0.00
GATCAAATGAGCGGAAAGGA	0.00	0.00	0.00	0.00	0.34
GATCAAATGAGCGGCGTTAC	0.23	0.00	0.00	0.00	0.00
GATCAAATGAGCGTGAATAG	0.00	0.33	0.00	0.00	0.00
GATCAAATGAGCGTGAGAGG	0.00	0.00	0.00	0.00	0.34
GATCAAATGAGGAAGGGCTA	0.00	0.00	0.00	0.00	0.00
GATCAAATGAGGCAGACTGG	0.00	0.00	0.00	0.00	0.69
GATCAAATGAGGCCTCCTTG	0.00	0.00	0.00	0.00	0.00
GATCAAATGAGGCGGAATGG	0.00	0.00	0.00	0.00	0.00
GATCAAATGAGGCGGACNNN	0.00	0.00	0.00	0.00	0.00
GATCAAATGAGGCGGACTGC	0.00	0.00	0.00	0.00	0.00
GATCAAATGAGGCGGACTGG	0.00	0.00	0.00	0.00	0.00
GATCAAATGAGGCGGCCCGG	0.00	0.00	0.00	0.00	0.00
GATCAAATGAGGGAAGCTCT	0.00	0.00	0.00	0.34	0.00
GATCAAATGAGGGAAGGTAA	0.23	0.00	0.00	0.00	0.00
GATCAAATGAGGGAAGTTAA	0.46	0.00	0.29	0.34	0.00
GATCAAATGAGGGGGGCACC	0.00	0.00	0.57	0.00	0.00
GATCAAATGAGGGGGGGGGG	0.00	0.00	0.29	0.00	0.00
GATCAAATGAGGGTTGCGGG	0.00	0.65	0.00	1.02	0.00
GATCAAATGAGGTGCTCACC	0.00	0.00	0.00	0.00	0.00
GATCAAATGAGTGACGAGGA	0.00	0.00	0.00	0.00	0.00
GATCAAATGAGTGACGATGC	0.00	0.65	0.29	1.70	0.69
GATCAAATGAGTGCGAGGAA	0.00	0.00	0.00	0.00	0.00
GATCAAATGAGTGCGCAAAA	0.00	0.00	0.29	0.00	0.00
GATCAAATGAGTGCTGTGTT	0.23	0.00	0.00	0.00	0.00
GATCAAATGAGTGTATTGCC	0.00	0.33	0.00	0.00	0.00
GATCAAATGAGTGTGAGAAA	0.00	0.00	0.00	0.00	0.34
GATCAAATGATAAAAAAAAA	0.00	0.00	0.29	0.00	1.03
GATCAAATGATACAGACACA	0.00	0.00	0.00	0.68	0.00
GATCAAATGATACAGACACC	0.00	0.00	0.29	0.00	0.00
GATCAAATGATAGAAGTTAC	0.00	0.00	0.00	0.00	0.34
GATCAAATGATAGCCAGCAA	0.00	0.33	0.00	0.00	0.00

GATCAAATGATAGCCGCTGC	1.37	0.65	1.15	0.00	1.72
GATCAAATGATAGCCGCTGG	0.00	0.33	0.00	0.00	0.00
GATCAAATGATAGCCGCTTG	0.23	0.00	0.00	0.00	0.00
GATCAAATGATAGGCTGCTT	0.00	0.33	0.00	0.00	0.00
GATCAAATGATAGTTGTGTG	3.89	3.58	1.72	3.73	2.07
GATCAAATGATCGCAACGCG	0.23	0.00	0.00	0.00	0.00
GATCAAATGATCGCAAGAGG	0.00	0.00	0.00	0.00	0.34
GATCAAATGATCGCCAGAGG	0.00	0.00	0.29	0.00	0.00
GATCAAATGATGACTGCAGC	0.00	0.00	0.57	0.00	0.00
GATCAAATGATGCAAGCTTG	0.92	0.65	0.00	1.02	0.00
GATCAAATGATGCAGGACTT	0.00	0.00	0.00	0.34	0.00
GATCAAATGATGCCAAAACG	0.00	0.00	0.29	0.00	0.00
GATCAAATGATGCGTTTTTG	0.23	0.00	0.00	0.00	0.00
GATCAAATGATGCTTTTTTG	0.46	0.00	0.00	0.00	0.00
GATCAAATGATGGGGGAGCA	0.46	0.00	0.00	1.02	0.00
GATCAAATGATGTAGTACGT	3.20	5.21	3.15	3.05	3.44
GATCAAATGATTAAACTTTA	0.23	0.00	0.00	0.00	0.00
GATCAAATGATTGACCTAGG	0.00	0.00	0.00	0.00	0.69
GATCAAATGATTGTTGTGTG	0.00	0.33	0.00	0.00	0.00
GATCAAATGATTTAAAAAAA	0.00	0.65	0.00	0.00	0.00
GATCAAATGATTTAGTACGT	0.00	0.00	0.00	0.00	0.34
GATCAAATGATTTCAAAAAA	0.00	1.30	1.15	0.00	1.03
GATCAAATGATTTCATTTAA	0.00	0.00	0.29	0.00	0.00
GATCAAATGATTTGTCTTGC	0.00	0.33	0.00	0.00	0.00
GATCAAATGCAAAAAAAAAA	1.37	0.00	0.00	0.00	1.03
GATCAAATGCAAAAAAAAAT	0.00	0.00	0.00	0.00	0.34
GATCAAATGCAAAGTGGACT	0.00	0.33	0.00	0.00	0.00
GATCAAATGCAACACAGAAA	0.00	0.00	0.29	0.00	0.00
GATCAAATGCAACCTCACAA	0.23	0.00	0.00	0.00	0.00
GATCAAATGCAATTTAAGAG	0.00	0.33	0.00	0.00	0.00
GATCAAATGCACACACAAAA	0.23	0.00	0.29	0.00	0.00
GATCAAATGCACACACAGAA	8.70	8.13	8.88	2.38	4.13
GATCAAATGCACACACAGAC	0.23	0.00	0.00	0.00	0.00
GATCAAATGCACACACAGAN	0.23	0.00	0.00	0.00	0.00
GATCAAATGCACACACAGNN	0.00	0.00	0.00	0.00	0.34
GATCAAATGCACACACCNNN	0.23	0.00	0.00	0.00	0.00
GATCAAATGCACACGCAGAA	0.00	0.00	0.29	0.00	0.00

GATCAAATGCACACTCCGCG	0.00	0.00	0.00	0.34	0.00
GATCAAATGCACATGCGCAA	0.00	0.00	0.00	0.00	0.34
GATCAAATGCACATGCTGAG	0.00	0.00	0.00	0.00	0.00
GATCAAATGCACCGGCCTGA	0.00	0.00	0.29	0.00	0.00
GATCAAATGCACCGGGCTTT	0.00	0.00	0.29	0.00	0.00
GATCAAATGCACCTGGCCCC	0.00	0.00	0.00	0.00	0.00
GATCAAATGCACGCAAGAGG	0.00	0.00	0.29	0.00	0.00
GATCAAATGCACGCGCTCGG	0.69	0.00	0.00	0.00	0.00
GATCAAATGCACGTAACAAA	0.00	0.33	0.00	0.00	0.00
GATCAAATGCACTTTGGCCC	0.46	0.00	0.00	0.00	0.00
GATCAAATGCAGAGGCCTTG	0.23	0.00	0.00	0.00	0.00
GATCAAATGCAGAGTCCTTG	0.92	0.00	0.00	0.00	0.00
GATCAAATGCAGAGTGTTGA	0.23	0.00	0.00	0.00	0.00
GATCAAATGCAGCAAAAAAA	0.00	0.33	0.29	0.00	1.03
GATCAAATGCAGCAAAAATC	0.00	0.00	0.29	0.00	0.00
GATCAAATGCAGCAACAAAA	1.60	2.93	1.72	2.38	2.41
GATCAAATGCAGCAACAAGA	0.00	0.00	0.29	0.00	0.00
GATCAAATGCAGCAACAAGC	13.05	16.92	4.30	12.56	12.05
GATCAAATGCAGCAACAAGG	0.23	0.00	0.29	0.00	0.00
GATCAAATGCAGCAACAANC	0.00	0.00	0.00	0.34	0.00
GATCAAATGCAGCAACAANN	0.23	0.00	0.00	0.00	0.00
GATCAAATGCAGCAACACGC	0.00	0.33	0.00	0.00	0.00
GATCAAATGCAGCAACATGC	0.00	0.33	0.00	0.00	0.00
GATCAAATGCAGCATCAGTT	0.00	0.33	0.00	0.00	0.00
GATCAAATGCAGCCAAAGGG	0.00	0.33	0.00	0.00	0.00
GATCAAATGCAGCCAAAGTG	0.23	0.00	0.00	0.00	0.00
GATCAAATGCAGCTGTCTTG	0.00	0.33	0.00	0.00	0.00
GATCAAATGCAGGGGCTTCA	0.00	0.00	0.00	0.00	0.34
GATCAAATGCAGGTGCTTAA	0.00	0.00	0.29	0.00	0.00
GATCAAATGCAGGTGCTTCA	2.29	1.95	0.57	0.68	0.69
GATCAAATGCAGTATTCCCA	0.00	0.00	0.00	0.34	0.00
GATCAAATGCAGTATTCCTG	0.00	0.00	0.57	0.68	0.00
GATCAAATGCAGTGAAAAAA	0.00	0.33	0.00	0.00	0.00
GATCAAATGCAGTGTAGGCA	0.00	0.00	0.00	0.00	0.00
GATCAAATGCATACCAAAGT	0.00	0.00	0.00	0.34	0.00
GATCAAATGCATCAGACCTT	0.46	0.00	0.00	0.00	0.00
GATCAAATGCATCGAGAGTT	0.23	0.00	0.00	0.00	0.00

GATCAAATGCATGTACAAGG	0.00	0.00	0.57	0.00	0.00
GATCAAATGCATGTGCCATT	0.00	0.00	0.00	0.00	0.34
GATCAAATGCATTGCGAGTG	0.00	0.00	0.00	0.00	0.00
GATCAAATGCATTGGGGATG	0.00	0.00	0.29	0.00	0.00
GATCAAATGCCAATGTGCAA	1.37	0.33	0.29	0.00	1.38
GATCAAATGCCAATGTGCAC	0.23	0.00	0.00	0.00	0.00
GATCAAATGCCAATGTGCGT	0.00	0.00	0.00	0.34	0.00
GATCAAATGCCAATGTGGCT	0.00	0.00	0.00	0.00	0.00
GATCAAATGCCACAGAACTC	0.00	0.00	0.00	0.00	0.69
GATCAAATGCCACTTGGGCG	0.00	0.33	0.00	0.68	0.00
GATCAAATGCCAGCGGACCT	0.00	0.00	0.00	0.00	0.00
GATCAAATGCCAGTCTTGAG	0.46	0.00	0.00	0.68	0.00
GATCAAATGCCAGTCTTGAT	0.23	0.00	0.00	0.00	0.00
GATCAAATGCCATGGTGGTG	0.00	0.00	0.29	0.00	0.00
GATCAAATGCCATTGCATGC	0.00	0.00	0.00	0.00	0.69
GATCAAATGCCCGCAAGAGG	0.00	0.00	0.00	0.00	0.00
GATCAAATGCCCGCCGCGCG	0.00	0.00	0.29	0.00	0.00
GATCAAATGCCGACTTCCTG	0.00	0.00	0.00	0.00	0.34
GATCAAATGCCGATTAAAGG	0.00	0.00	0.57	0.00	0.00
GATCAAATGCCGCAGCCGCT	0.00	0.33	0.00	0.00	0.00
GATCAAATGCCGCCAAAGTG	0.00	0.33	0.00	0.00	0.00
GATCAAATGCCGCCGCCTTG	0.00	0.00	0.00	0.34	0.00
GATCAAATGCCGCTCTGCAA	0.00	0.00	0.29	0.00	0.00
GATCAAATGCCGCTCTGTAA	0.00	0.00	0.00	0.00	0.00
GATCAAATGCCGGCCTCCCA	0.00	0.00	0.00	0.00	1.38
GATCAAATGCCGGCTGCCGA	0.46	0.33	0.00	0.00	0.00
GATCAAATGCCGGGCTTCAG	0.00	0.00	0.00	0.00	0.00
GATCAAATGCCGGTCTTCAG	2.29	2.60	2.00	0.34	0.00
GATCAAATGCCGGTCTTCTG	0.00	0.00	0.29	0.00	0.00
GATCAAATGCCTAAAAAAAA	0.92	0.00	0.00	0.00	0.00
GATCAAATGCCTGCAGAGAC	0.00	0.00	0.00	0.00	0.34
GATCAAATGCCTGTGGCAAA	0.00	0.00	0.29	0.00	0.00
GATCAAATGCCTTGAAGTGA	0.00	0.00	0.00	0.00	0.00
GATCAAATGCGAAAATTGTT	0.00	0.00	0.00	0.00	0.00
GATCAAATGCGAAGACTGGA	0.00	0.00	0.00	0.00	0.34
GATCAAATGCGACGCAGGTG	0.00	0.00	0.00	0.00	0.00
GATCAAATGCGAGAAAAAAG	0.23	0.00	0.00	0.00	0.00

GATCAAATGCGAGAAGTTAA	0.23	0.00	0.00	0.00	0.00
GATCAAATGCGAGACCTTTC	0.00	0.00	0.00	0.00	0.00
GATCAAATGCGAGAGCAGAG	0.46	0.00	0.00	0.00	0.00
GATCAAATGCGAGCTCCCGC	0.23	0.00	0.00	0.00	0.00
GATCAAATGCGAGGTGCAAT	0.00	0.00	0.00	0.68	0.00
GATCAAATGCGAGTTACATG	0.00	0.00	0.00	0.00	0.00
GATCAAATGCGATGACGGGG	0.00	0.00	0.29	0.34	0.00
GATCAAATGCGATGTTGTCG	0.00	0.00	0.00	0.00	0.00
GATCAAATGCGCACGCCTGC	0.00	0.00	0.00	0.34	0.00
GATCAAATGCGCAGGCGCAG	0.23	0.00	0.00	0.00	0.00
GATCAAATGCGCATGCTGTC	0.00	0.00	0.00	0.00	0.34
GATCAAATGCGCCAAAGCTG	0.00	0.00	0.00	0.00	0.34
GATCAAATGCGCCGCACTTG	0.00	0.00	0.29	0.00	0.00
GATCAAATGCGCCTAAAGGT	0.00	0.00	0.57	0.34	0.00
GATCAAATGCGCGCAAGAGG	0.69	0.00	0.00	0.00	0.00
GATCAAATGCGCGCAAGCGG	0.00	0.00	0.29	0.00	0.00
GATCAAATGCGCGCACGGGG	0.23	0.00	0.00	0.00	0.00
GATCAAATGCGCGCCGCTGT	0.00	0.00	0.00	0.00	0.34
GATCAAATGCGCGCGAAGCT	0.46	0.00	0.29	0.00	0.00
GATCAAATGCGCGGCATTCT	0.00	0.65	0.00	0.00	0.34
GATCAAATGCGCTCTAAAAA	0.00	0.00	0.00	0.00	0.00
GATCAAATGCGCTTTCGCCC	0.23	0.00	0.00	0.00	0.00
GATCAAATGCGGAGTTTTTT	0.00	0.00	0.29	0.00	0.00
GATCAAATGCGGCAGCCTCT	0.00	0.00	0.00	0.00	0.00
GATCAAATGCGGCAGCGGCG	0.00	0.00	0.00	0.34	0.00
GATCAAATGCGGCGAATTTT	0.23	0.00	0.00	0.00	0.00
GATCAAATGCGGCGGTGTTT	0.00	0.00	0.00	0.00	0.00
GATCAAATGCGGCGTGTTTC	0.00	0.00	0.00	0.00	0.34
GATCAAATGCGGCGTGTTTT	0.00	0.00	0.00	0.00	0.00
GATCAAATGCGGCGTTGTTC	0.00	0.00	0.29	0.00	0.00
GATCAAATGCGGCGTTTGCA	0.00	0.00	0.00	0.00	0.00
GATCAAATGCGGCGTTTTTN	0.23	0.00	0.00	0.00	0.00
GATCAAATGCGGCGTTTTTT	0.69	0.33	2.00	0.68	3.79
GATCAAATGCGGGAAGTTTG	0.00	0.00	0.29	0.00	0.00
GATCAAATGCGGGCGGTGGG	0.23	0.00	0.00	0.00	0.00
GATCAAATGCGGGGACACGA	0.00	0.33	0.00	0.00	0.00
GATCAAATGCGGTGAGAGTA	0.00	0.00	0.29	0.00	0.00

GATCAAATGCGGTGCTTTCT	0.00	0.00	0.00	0.34	0.00
GATCAAATGCGTGCAAAAAA	0.00	0.33	0.00	0.00	0.00
GATCAAATGCGTGCGAAAAA	1.60	2.93	1.72	3.39	2.41
GATCAAATGCGTGCGAGAAA	0.00	0.00	0.00	0.00	0.00
GATCAAATGCGTGGTGGTTG	0.92	0.00	0.00	0.68	0.00
GATCAAATGCGTTGCAATTG	0.00	0.00	0.29	0.00	0.00
GATCAAATGCGTTGTAAGAG	0.00	0.00	0.00	0.00	0.34
GATCAAATGCGTTGTAAGTC	0.00	0.00	0.00	0.00	0.00
GATCAAATGCGTTGTAATAG	0.00	0.33	0.00	0.00	0.00
GATCAAATGCGTTGTAATTA	0.00	0.00	0.00	0.00	0.00
GATCAAATGCGTTGTAATTG	42.35	8.13	6.30	14.93	4.48
GATCAAATGCGTTGTACNNN	0.23	0.00	0.00	0.00	0.00
GATCAAATGCGTTGTAGTTG	0.00	0.00	0.00	0.00	0.00
GATCAAATGCTACAGAGGTG	0.00	0.00	0.00	0.00	0.34
GATCAAATGCTAGCAAGAGG	0.00	0.33	0.00	0.34	0.00
GATCAAATGCTCACAACCCC	0.00	0.00	0.29	0.00	0.00
GATCAAATGCTCACAAGAGG	0.00	0.00	0.00	0.00	0.00
GATCAAATGCTCACTTGGGC	2.52	0.33	0.00	0.34	0.00
GATCAAATGCTCATGGCTGA	0.00	0.00	0.00	0.34	0.00
GATCAAATGCTCATGGCTGC	1.60	0.33	0.86	1.02	1.03
GATCAAATGCTCATGGCTGG	0.00	0.00	0.00	0.00	0.34
GATCAAATGCTCCGGAGAGT	0.00	0.98	0.29	1.02	0.00
GATCAAATGCTCGAAAGAGG	0.00	0.00	0.00	0.00	0.00
GATCAAATGCTCGCAAAAGC	0.00	0.00	0.00	0.34	0.00
GATCAAATGCTCGCAAAGTT	0.00	0.00	0.57	0.00	0.00
GATCAAATGCTCGCAACAGG	0.00	0.00	0.00	0.34	0.34
GATCAAATGCTCGCAACATG	0.23	0.00	0.00	0.00	0.00
GATCAAATGCTCGCAAGAAA	0.00	0.00	0.00	0.00	0.00
GATCAAATGCTCGCAAGAAG	0.00	0.00	0.29	0.00	0.00
GATCAAATGCTCGCAAGACG	0.00	0.00	0.00	0.00	0.00
GATCAAATGCTCGCAAGAGA	0.00	0.33	0.29	0.00	0.00
GATCAAATGCTCGCAAGAGC	0.00	0.00	0.00	0.34	0.00
GATCAAATGCTCGCAAGAGG	165.50	195.20	303.83	333.66	242.8[illegible]
GATCAAATGCTCGCAAGAGN	0.46	0.00	0.29	0.00	0.00
GATCAAATGCTCGCAAGAGT	0.46	0.00	0.57	0.34	0.34
GATCAAATGCTCGCAAGANG	0.00	0.00	0.00	0.34	0.00
GATCAAATGCTCGCAAGANN	0.69	0.00	0.00	0.00	0.00

GATCAAATGCTCGCAAGCGG	0.69	1.30	0.57	0.34	0.34
GATCAAATGCTCGCAAGCNN	0.23	0.00	0.00	0.00	0.00
GATCAAATGCTCGCAAGGGG	0.00	0.00	0.86	0.00	0.34
GATCAAATGCTCGCAAGGGT	0.00	0.00	0.29	0.00	0.00
GATCAAATGCTCGCAAGNGG	0.00	0.33	0.00	0.00	0.00
GATCAAATGCTCGCAAGNGN	0.00	0.00	0.00	0.34	0.00
GATCAAATGCTCGCAAGTGG	0.23	0.00	0.29	0.00	0.00
GATCAAATGCTCGCAANAGG	0.00	0.00	0.00	0.00	0.34
GATCAAATGCTCGCAANNNN	0.23	0.00	0.00	0.00	0.00
GATCAAATGCTCGCACAGAA	0.00	0.00	0.29	0.00	0.00
GATCAAATGCTCGCACGACG	0.00	0.00	0.00	0.34	0.00
GATCAAATGCTCGCACGAGG	0.23	0.00	0.29	0.00	0.00
GATCAAATGCTCGCACGCGG	0.00	0.00	0.29	0.34	0.00
GATCAAATGCTCGCACNANN	0.23	0.00	0.00	0.00	0.00
GATCAAATGCTCGCAGAGTT	0.00	0.00	0.00	0.68	0.00
GATCAAATGCTCGCAGGAGG	0.00	0.33	0.29	0.34	0.00
GATCAAATGCTCGCCAAACG	0.00	0.00	0.29	0.00	0.00
GATCAAATGCTCGCCAGAGG	0.23	0.00	0.86	0.34	0.00
GATCAAATGCTCGCCAGAGT	0.00	0.00	0.29	0.00	0.00
GATCAAATGCTCGCCAGCGG	0.23	0.00	0.29	0.00	0.00
GATCAAATGCTCGCCCCCGG	0.00	0.00	0.00	0.00	0.00
GATCAAATGCTCGCGAGAGG	0.23	0.00	0.00	0.00	0.00
GATCAAATGCTCGCGAGGAG	0.00	0.00	0.00	0.00	0.00
GATCAAATGCTCGCGAGGCC	0.00	0.00	0.00	0.00	0.34
GATCAAATGCTCGCGGGAGG	0.23	0.00	0.00	0.00	0.00
GATCAAATGCTCGCTAGAGG	0.00	0.00	0.00	0.00	0.34
GATCAAATGCTCGGAAGAAG	0.00	0.00	0.00	0.00	0.00
GATCAAATGCTCGGTTGCAG	0.00	0.00	0.00	0.00	0.00
GATCAAATGCTCGTAAGAGG	0.00	0.33	0.29	0.00	0.00
GATCAAATGCTCGTATGCCG	0.00	0.00	0.00	0.34	0.34
GATCAAATGCTCTCACGGTG	0.00	0.00	0.00	0.68	0.00
GATCAAATGCTCTTACCCTG	0.23	0.00	0.00	0.00	0.00
GATCAAATGCTGACTGATTG	0.00	0.00	0.00	0.00	0.00
GATCAAATGCTGATCTTCTG	0.00	0.00	0.00	0.34	0.00
GATCAAATGCTGCACAGTGA	0.00	0.00	0.00	0.68	0.00
GATCAAATGCTGCACAGTGG	0.00	0.65	0.00	1.02	0.69
GATCAAATGCTGGCAAGAGG	0.00	0.00	0.00	0.00	0.00

GATCAAATGCTGGCAGTGAA	0.00	0.00	0.00	0.34	0.00
GATCAAATGCTGGCCCCATC	0.00	0.33	0.00	0.00	0.00
GATCAAATGCTGGGCGCTTG	0.00	0.00	0.57	0.00	0.00
GATCAAATGCTGGGCGGCAA	0.00	0.33	0.00	0.00	0.00
GATCAAATGCTGGGCGGGGA	0.00	0.00	0.00	0.00	0.00
GATCAAATGCTGGGCGGGGT	0.00	0.00	0.29	0.00	0.00
GATCAAATGCTGGGCTCTGG	0.00	0.00	0.00	0.00	0.00
GATCAAATGCTGGTCTTATG	0.00	0.00	0.00	0.00	0.00
GATCAAATGCTGGTCTTCCG	0.00	0.00	0.00	0.34	0.00
GATCAAATGCTGGTCTTCTG	6.87	6.51	11.45	8.49	14.81
GATCAAATGCTGGTCTTCTT	0.00	0.00	0.00	0.34	0.00
GATCAAATGCTGTGGGACCT	0.00	0.33	0.00	0.00	0.00
GATCAAATGCTTAAAAAAAA	0.69	0.00	0.00	0.00	0.00
GATCAAATGCTTCTTGACCC	0.00	0.33	0.00	0.00	0.00
GATCAAATGCTTCTTGGAGG	0.00	0.33	0.00	0.00	0.00
GATCAAATGCTTGCAAGAGG	0.00	0.33	0.29	0.00	0.00
GATCAAATGCTTGCAATAGC	0.00	0.33	0.00	0.00	0.00
GATCAAATGCTTGTTGAGGT	0.46	0.00	0.57	0.00	0.34
GATCAAATGCTTTCCGGTTT	0.00	0.00	0.29	0.00	0.00
GATCAAATGCTTTGATGATG	0.00	0.33	0.00	0.00	0.00
GATCAAATGCTTTGCATTGA	0.46	0.00	0.29	0.00	0.00
GATCAAATGCTTTGCATTGG	0.46	0.00	0.00	0.00	0.00
GATCAAATGCTTTGCGTAGC	0.23	0.00	0.00	0.00	0.00
GATCAAATGGAAAAAAAAAA	0.00	0.33	0.00	0.00	0.00
GATCAAATGGAAATGACTCG	0.00	0.00	0.00	0.34	0.00
GATCAAATGGAACGAATCGA	0.23	0.33	0.29	0.00	0.00
GATCAAATGGAACGTTTGCG	0.00	0.33	0.00	0.00	0.00
GATCAAATGGAAGATAGCAC	0.00	0.00	0.00	0.00	0.34
GATCAAATGGAAGGTTGAGG	0.00	0.33	0.00	0.00	0.00
GATCAAATGGAATCCATGTC	1.83	2.60	0.86	1.36	2.76
GATCAAATGGAATCTCGCTC	0.00	0.00	0.29	0.00	0.00
GATCAAATGGAATTCTATAT	0.00	0.00	0.00	0.34	0.00
GATCAAATGGACCGAGCCAG	0.00	0.00	0.00	0.00	0.34
GATCAAATGGACGCTGGCGG	0.00	0.00	0.00	0.00	0.00
GATCAAATGGACTGTAGTTG	0.23	0.00	0.00	0.00	0.00
GATCAAATGGAGAAAAAAAA	0.00	0.00	0.00	0.00	0.69
GATCAAATGGAGAGGCACTC	0.46	0.33	0.00	0.34	0.00

GATCAAATGGAGCAAAGAAA	0.23	0.00	0.00	0.00	0.00
GATCAAATGGAGCGCCACTT	0.00	0.00	0.00	0.00	0.34
GATCAAATGGAGCTGATTGC	0.00	0.33	0.00	0.00	0.00
GATCAAATGGAGGGCGCGAA	0.00	0.00	0.00	0.00	0.00
GATCAAATGGAGTGAAAAAA	0.46	0.00	0.00	0.00	0.00
GATCAAATGGAGTGAAATCT	0.00	0.00	0.00	0.34	0.00
GATCAAATGGAGTGATTAAA	0.00	0.00	0.29	0.00	0.00
GATCAAATGGAGTGGATGAC	0.00	0.33	0.57	0.34	0.34
GATCAAATGGATACGAAGAA	0.00	0.00	0.29	0.00	0.00
GATCAAATGGATACGCAGAA	0.00	0.00	0.29	0.00	0.00
GATCAAATGGATACTTTTAA	0.23	0.00	0.00	0.00	0.00
GATCAAATGGATAGATAAAA	0.00	0.00	0.29	0.00	0.00
GATCAAATGGATAGATAAAC	0.00	0.00	0.00	0.00	0.00
GATCAAATGGATAGATAAAT	0.69	0.00	0.00	0.00	0.34
GATCAAATGGATAGATTAAA	0.00	0.00	0.00	0.00	0.00
GATCAAATGGATGAATGTCC	0.00	0.00	0.00	0.00	0.00
GATCAAATGGATTAGAGCTC	0.46	0.00	0.00	0.00	0.00
GATCAAATGGCAAAAAAAAA	0.69	0.98	0.29	0.00	0.34
GATCAAATGGCAAAAAAAAT	0.00	0.00	0.00	0.00	0.00
GATCAAATGGCAACACAGCT	0.00	0.00	0.29	0.00	0.00
GATCAAATGGCAAGACGACA	0.23	0.00	0.00	0.00	0.00
GATCAAATGGCAAGTAGTCG	0.00	0.00	0.00	0.00	0.00
GATCAAATGGCACAACATCC	1.83	0.00	0.00	0.34	0.00
GATCAAATGGCACGCCCTCG	0.00	0.00	0.57	0.00	0.00
GATCAAATGGCACGTTGAGG	0.23	0.00	0.00	0.00	0.00
GATCAAATGGCACTGGCGTC	0.00	0.00	0.00	0.34	0.00
GATCAAATGGCAGACGACGC	0.00	0.00	0.00	0.00	0.00
GATCAAATGGCAGAGTCAAG	0.00	0.33	0.00	0.00	0.00
GATCAAATGGCAGATTGAGG	0.00	0.00	0.29	0.00	0.00
GATCAAATGGCAGGATGAGG	0.00	0.00	0.29	0.34	0.00
GATCAAATGGCAGGCTGAGG	0.23	0.00	0.29	0.00	0.34
GATCAAATGGCAGGCTTAAG	0.00	0.00	0.00	0.34	0.00
GATCAAATGGCAGGGGGAGG	0.00	0.00	0.00	0.00	0.00
GATCAAATGGCAGGGTGACG	0.00	0.00	0.00	0.34	0.00
GATCAAATGGCAGGGTGAGG	0.92	1.95	3.15	6.45	4.48
GATCAAATGGCAGGGTGCGG	0.00	0.00	0.00	0.34	0.00
GATCAAATGGCAGGGTGGGG	0.00	0.33	0.57	0.34	0.34

GATCAAATGGCAGGGTTAGG	0.00	0.00	0.00	0.68	0.00
GATCAAATGGCAGGTCGACG	0.00	0.00	0.29	0.00	0.00
GATCAAATGGCAGGTCGAGG	0.00	0.00	0.00	0.00	0.00
GATCAAATGGCAGGTGGAGC	0.23	0.00	0.00	0.00	0.00
GATCAAATGGCAGGTGGAGG	0.00	0.00	0.57	0.00	0.00
GATCAAATGGCAGGTTAAGG	0.00	0.00	0.00	0.00	0.00
GATCAAATGGCAGGTTGACC	0.00	0.00	0.00	0.00	0.00
GATCAAATGGCAGGTTGACG	0.00	0.33	0.00	0.00	0.00
GATCAAATGGCAGGTTGAGA	0.00	0.00	0.29	0.00	0.00
GATCAAATGGCAGGTTGAGC	0.00	0.00	0.29	0.00	0.00
GATCAAATGGCAGGTTGAGG	51.05	130.78	235.67	66.19	116.7[illegible]
GATCAAATGGCAGGTTGAGT	0.00	0.33	0.00	0.00	0.00
GATCAAATGGCAGGTTGATG	0.00	0.33	0.00	0.00	0.69
GATCAAATGGCAGGTTGCGG	0.69	0.33	0.57	0.00	1.03
GATCAAATGGCAGGTTGGGG	0.69	0.00	2.29	0.00	0.00
GATCAAATGGCAGGTTGGTG	0.00	0.00	0.29	0.00	0.00
GATCAAATGGCAGGTTGTGG	0.00	0.00	0.57	0.00	0.00
GATCAAATGGCAGGTTNAGG	0.00	0.00	0.00	0.00	0.00
GATCAAATGGCAGGTTTACG	0.00	0.00	0.00	0.68	0.00
GATCAAATGGCAGGTTTAGG	0.00	0.33	0.86	1.02	2.07
GATCAAATGGCAGGTTTGGG	0.00	0.00	0.29	0.00	0.00
GATCAAATGGCAGTCTGTCC	0.23	0.00	0.00	0.00	0.34
GATCAAATGGCATCATGAGT	0.00	0.00	0.29	0.34	0.00
GATCAAATGGCATGCCCTTG	0.00	0.65	0.29	0.00	0.00
GATCAAATGGCATGCCGGAT	0.00	0.00	0.29	0.00	0.00
GATCAAATGGCATGGCAGCC	0.00	0.00	0.00	0.00	0.34
GATCAAATGGCATGGCCACC	0.23	0.00	0.00	0.00	0.00
GATCAAATGGCATGGCGGAT	2.52	0.33	2.00	2.04	1.03
GATCAAATGGCATGGCGGCC	0.00	0.00	0.00	0.00	0.34
GATCAAATGGCATGGCNNNN	0.23	0.00	0.00	0.00	0.00
GATCAAATGGCATTGCCAAC	0.00	0.00	0.29	0.00	0.00
GATCAAATGGCATTGCCACC	13.73	15.62	12.89	15.95	8.27
GATCAAATGGCATTGCCACT	0.00	0.00	0.00	0.00	0.00
GATCAAATGGCATTGCCCCC	0.00	0.00	0.00	0.00	0.00
GATCAAATGGCATTGCTCGT	0.00	0.00	0.00	0.00	0.34
GATCAAATGGCATTGCTGAT	0.00	0.00	0.00	0.34	0.00
GATCAAATGGCATTGTCACA	0.00	0.00	0.00	0.00	0.00

GATCAAATGGCCACACTTGT	0.00	0.33	0.00	0.00	0.34
GATCAAATGGCCACATGCAC	2.06	0.98	0.86	1.70	1.03
GATCAAATGGCCACATGCAN	0.23	0.00	0.00	0.00	0.00
GATCAAATGGCCACGCCTAA	0.69	0.00	0.00	0.00	0.00
GATCAAATGGCCACGTAGCA	0.00	0.33	0.00	0.00	0.00
GATCAAATGGCCAGGCGGAC	1.60	0.33	0.29	1.70	0.34
GATCAAATGGCCCATAAGAA	0.00	0.00	0.29	0.00	0.00
GATCAAATGGCCGGTTGAGG	0.00	0.00	0.29	0.00	0.00
GATCAAATGGCCGGTTGAGN	0.00	0.00	0.00	0.00	0.00
GATCAAATGGCCGGTTGAGT	0.00	0.00	0.29	0.00	0.00
GATCAAATGGCCTTGGGGGC	0.00	0.00	0.29	0.00	0.00
GATCAAATGGCGAGAGTATC	0.00	0.33	0.00	0.00	0.00
GATCAAATGGCGAGCGGGAC	0.00	0.00	0.29	0.00	0.00
GATCAAATGGCGCAGGGCGG	0.00	0.00	0.00	0.00	0.00
GATCAAATGGCGCGAGCCGG	0.00	0.00	0.00	0.00	0.00
GATCAAATGGCGGAAAAAAA	0.00	0.00	0.29	0.00	0.00
GATCAAATGGCGGGTTCAGG	0.23	0.00	0.00	0.00	0.00
GATCAAATGGCGGGTTGAGG	0.23	0.00	0.00	0.00	0.00
GATCAAATGGCGTTGCTCAA	0.46	0.00	0.00	0.00	0.00
GATCAAATGGCGTTGCTCAT	7.10	0.98	2.29	3.05	1.38
GATCAAATGGCGTTGCTCCN	0.23	0.00	0.00	0.00	0.00
GATCAAATGGCGTTGCTCCT	0.23	0.00	0.00	0.00	0.00
GATCAAATGGCTAGAGTATC	0.00	0.33	0.57	0.00	0.00
GATCAAATGGCTATCATCAT	0.00	0.00	0.00	0.00	0.34
GATCAAATGGCTCGGCGAGC	0.00	0.00	0.86	0.00	0.00
GATCAAATGGCTCTCATGCA	0.00	0.00	0.00	0.34	0.00
GATCAAATGGCTGGGGAACG	0.00	0.33	0.00	0.00	0.00
GATCAAATGGCTGGTTGAGG	0.00	0.00	0.00	0.00	0.00
GATCAAATGGCTGTGGCGGG	0.00	0.00	0.00	0.00	0.00
GATCAAATGGCTTAGAGATA	0.00	0.00	0.00	0.00	0.00
GATCAAATGGCTTCAAAAGC	0.23	0.00	0.86	0.00	0.00
GATCAAATGGGAAAAAAAAA	0.00	0.00	0.00	0.00	0.00
GATCAAATGGGAAACAAAAA	0.46	0.00	0.00	0.00	0.00
GATCAAATGGGAAACAAAAG	0.46	0.65	0.00	0.68	0.00
GATCAAATGGGACGACCTTA	0.46	0.00	0.00	0.00	0.00
GATCAAATGGGACTCCCAAA	0.00	0.65	0.00	0.00	0.00
GATCAAATGGGAGCAGAAGC	0.00	0.33	0.29	0.34	0.34

GATCAAATGGGAGCTTCAAA	0.23	0.00	0.00	0.00	0.00
GATCAAATGGGAGGAAAGGG	0.00	0.00	0.29	0.00	0.00
GATCAAATGGGAGGACTCCG	0.00	0.00	0.00	0.00	0.34
GATCAAATGGGAGTTTCAAA	0.00	0.33	0.00	0.00	0.00
GATCAAATGGGCAGCGTGTC	2.75	5.21	7.73	7.13	5.17
GATCAAATGGGCCATGGTGG	0.00	0.00	0.00	0.00	0.34
GATCAAATGGGCGATGGCTA	0.23	0.00	0.00	0.00	0.00
GATCAAATGGGCGCGTCAAG	0.00	0.33	0.00	0.00	0.00
GATCAAATGGGCGCTGGGAG	0.00	0.00	0.00	0.00	0.00
GATCAAATGGGGAGAAGAGA	0.00	0.00	0.29	0.00	0.00
GATCAAATGGGGCAAATTTT	0.00	0.00	0.00	0.00	0.00
GATCAAATGGGGCGGCGACT	0.00	0.00	0.29	0.00	0.00
GATCAAATGGGGCTTTGGCA	0.00	0.00	0.00	0.00	0.34
GATCAAATGGGGGAAGTTTT	0.23	0.00	0.00	0.00	0.00
GATCAAATGGGGGGCGGGCT	0.23	0.00	0.00	0.00	0.00
GATCAAATGGGGGTTGACCA	0.00	0.65	0.29	0.34	0.00
GATCAAATGGGGTCTTGGCG	0.00	0.00	0.57	0.00	0.00
GATCAAATGGGTGCAGGCAT	0.00	0.00	0.00	0.00	0.00
GATCAAATGGGTGCGGGCTT	0.00	0.33	0.00	0.00	0.00
GATCAAATGGTAATAGCAGG	0.00	0.00	0.00	0.68	0.00
GATCAAATGGTACAGCGGCA	0.00	0.00	0.00	0.00	0.00
GATCAAATGGTACAGTGGCC	0.00	0.33	0.00	0.00	0.00
GATCAAATGGTAGAGCGCTC	0.00	0.00	0.00	0.00	0.34
GATCAAATGGTAGGTTGAGG	0.00	0.00	0.00	0.00	0.34
GATCAAATGGTCAAAAAAAA	0.00	0.00	0.00	0.34	0.00
GATCAAATGGTCCAGTGGCA	0.00	0.00	0.00	0.00	0.34
GATCAAATGGTCCCGTGGAA	0.00	0.33	0.00	0.00	0.00
GATCAAATGGTCCGCTCTGG	0.00	0.00	0.00	0.34	0.00
GATCAAATGGTCCTCACCAC	0.23	0.00	0.29	0.00	0.00
GATCAAATGGTGAAGGAGGA	0.00	0.00	0.00	0.00	1.03
GATCAAATGGTGACGGCACG	0.00	0.00	0.00	0.00	0.34
GATCAAATGGTGCTCACAGA	0.00	0.00	0.00	0.34	0.00
GATCAAATGGTGCTCACAGC	0.69	1.30	0.86	1.02	2.76
GATCAAATGGTGGCCAATGA	0.23	0.33	0.57	0.00	0.00
GATCAAATGGTGGGGCATCA	0.00	0.00	0.00	0.34	0.00
GATCAAATGGTGGGGCATCG	0.00	0.00	0.29	0.00	0.69
GATCAAATGGTGGGTAGAAC	0.00	0.00	0.00	0.34	0.00

GATCAAATGGTGTTGCCCTG	0.00	0.00	0.00	0.00	0.34
GATCAAATGGTTCAGGACAT	0.00	0.00	0.00	0.00	0.00
GATCAAATGGTTGACTAAAA	0.00	0.65	0.00	0.00	0.00
GATCAAATGGTTGACTGTTA	0.23	0.00	0.00	0.00	0.00
GATCAAATGGTTGCTGAGTC	0.23	0.00	0.00	0.00	0.00
GATCAAATGGTTGGCTGTGA	0.23	0.00	0.00	0.00	0.34
GATCAAATGGTTTGGCATGC	0.00	0.00	0.00	0.00	0.34
GATCAAATGGTTTTGCCAGT	0.23	0.00	0.00	0.00	0.00
GATCAAATGTAAAAAAAAAA	0.46	0.00	0.00	0.00	0.00
GATCAAATGTAAACCGAGAG	0.23	0.00	0.00	0.68	0.69
GATCAAATGTAAATCTCTAT	0.00	0.00	0.00	0.00	0.34
GATCAAATGTAAGTTATATC	0.00	0.00	0.00	0.00	0.34
GATCAAATGTAATAAAAAAA	1.14	0.00	0.00	0.00	0.00
GATCAAATGTAATGGAATGG	0.00	0.00	0.00	0.34	0.00
GATCAAATGTAATTAAAAAA	5.26	0.00	0.00	0.68	1.03
GATCAAATGTAATTCAAAAA	1.60	0.33	1.15	0.34	0.69
GATCAAATGTAATTCAAAGA	0.00	0.00	0.29	0.00	0.00
GATCAAATGTAATTCCAAAG	0.23	0.00	1.15	0.00	0.00
GATCAAATGTAATTCTAAAA	1.37	0.00	0.00	0.00	0.34
GATCAAATGTAATTCTAAGG	0.00	0.00	0.00	0.68	0.00
GATCAAATGTAATTCTAGAT	1.14	0.33	0.86	3.05	2.41
GATCAAATGTAATTCTANNN	0.23	0.00	0.00	0.00	0.00
GATCAAATGTAATTCTATAA	0.46	0.33	0.00	0.00	0.00
GATCAAATGTAATTCTATAN	0.23	0.00	0.00	0.00	0.00
GATCAAATGTAATTCTATAT	38.46	17.57	25.20	15.27	64.75
GATCAAATGTAATTCTATCT	0.00	0.00	0.00	0.00	0.00
GATCAAATGTAATTCTATGT	0.23	0.00	0.00	0.00	0.00
GATCAAATGTAATTCTATNN	0.46	0.00	0.00	0.00	0.00
GATCAAATGTAATTCTCTGA	0.00	0.00	0.00	0.00	0.00
GATCAAATGTAATTCTCTNN	0.23	0.00	0.00	0.00	0.00
GATCAAATGTAATTCTCTTT	0.23	0.00	0.00	0.00	0.00
GATCAAATGTAATTCTGTAT	0.00	0.00	0.00	0.00	0.00
GATCAAATGTAATTCTNTAT	0.00	0.00	0.00	0.00	0.34
GATCAAATGTAATTCTTAAA	0.46	0.00	0.00	0.00	0.00
GATCAAATGTAATTCTTGTG	0.46	0.00	0.00	0.00	0.00
GATCAAATGTACCTGGACCC	0.00	0.33	0.00	0.00	0.00
GATCAAATGTACCTGGCACC	0.23	0.00	0.57	0.00	0.00

GATCAAATGTACCTGGCCCC	7.32	12.36	10.60	6.11	8.61
GATCAAATGTACCTGGCCCT	0.00	0.00	0.00	0.34	0.00
GATCAAATGTACCTGGCCGA	0.00	0.00	0.00	0.00	0.00
GATCAAATGTACTCAGTTGC	0.00	0.00	0.29	0.00	0.00
GATCAAATGTAGCAGGGTGC	0.23	0.00	0.00	0.00	0.00
GATCAAATGTAGCTGAGCAC	0.00	0.00	0.00	0.00	0.00
GATCAAATGTATCGTTTGTG	0.23	0.00	0.00	0.00	0.00
GATCAAATGTATGGAGTCCG	0.00	0.00	0.00	0.00	0.34
GATCAAATGTCATAAAAAAA	0.46	0.00	0.00	0.34	0.69
GATCAAATGTCATCCATTGT	0.00	0.00	0.00	0.00	0.00
GATCAAATGTCCAAAGCGAC	2.29	0.00	0.00	1.36	1.38
GATCAAATGTCCAAAGCGTC	0.23	0.00	0.00	0.00	0.00
GATCAAATGTCCAGAGCGAC	0.23	0.00	0.00	0.00	0.00
GATCAAATGTCCGCATNGAG	0.00	0.00	0.00	0.00	0.34
GATCAAATGTCGCGAGAGTG	0.00	0.98	0.57	0.00	0.34
GATCAAATGTCGGGCTGATT	0.00	0.00	0.00	0.34	0.00
GATCAAATGTCTATGTGTCC	0.00	0.00	0.00	0.00	0.34
GATCAAATGTCTCCAAGCCC	0.92	0.00	0.00	0.34	0.00
GATCAAATGTCTCTGTGTCA	0.23	0.00	0.29	0.00	0.34
GATCAAATGTCTCTTTGTCA	0.00	0.00	0.29	0.00	0.00
GATCAAATGTCTGCTTGGCA	0.00	0.00	0.57	0.00	0.00
GATCAAATGTCTGGTTGATG	0.23	0.00	0.00	0.00	0.00
GATCAAATGTGAAAAAAAAA	0.00	0.00	0.00	0.00	0.00
GATCAAATGTGACATCCCTA	0.23	0.33	0.29	1.36	0.00
GATCAAATGTGACATCCCTC	0.00	0.00	0.00	0.00	0.00
GATCAAATGTGACGCAGAGG	0.00	0.00	0.00	0.34	0.00
GATCAAATGTGACGCTGAGG	3.20	7.81	3.15	8.83	2.41
GATCAAATGTGAGGAAANNN	0.23	0.00	0.00	0.00	0.00
GATCAAATGTGATTCTATAT	0.46	0.00	0.00	0.00	0.00
GATCAAATGTGCAAGAAAGA	0.00	0.33	0.00	0.00	0.00
GATCAAATGTGCAGGTTCCC	0.00	0.65	0.00	0.00	0.00
GATCAAATGTGCATGTCTTC	0.00	0.00	0.00	0.34	0.00
GATCAAATGTGCCACCGTAA	0.00	0.33	0.29	0.00	0.00
GATCAAATGTGCCGCATTCG	0.46	1.30	0.29	0.00	0.69
GATCAAATGTGCCTGGCCCC	0.00	0.00	0.00	0.34	0.00
GATCAAATGTGCGCTTCGCG	0.00	0.00	0.00	0.00	0.00
GATCAAATGTGGAAAAAAAA	1.37	0.00	0.29	0.00	0.00

GATCAAATGTGGCAAAAAAA	0.69	0.00	0.00	0.68	0.00
GATCAAATGTGGCACAAGGA	0.00	0.00	0.00	0.34	0.00
GATCAAATGTGGCATCTCCC	0.00	0.00	0.00	0.00	0.34
GATCAAATGTGGCATGATGG	0.69	0.65	1.15	1.02	0.00
GATCAAATGTGGCGGAAGCC	0.00	0.00	0.00	0.00	0.34
GATCAAATGTGGCGTATTGC	0.00	0.00	0.00	0.00	0.00
GATCAAATGTGGCTCTCCTA	0.00	0.00	0.29	0.00	0.00
GATCAAATGTGGCTCTCCTC	0.00	0.00	0.29	0.00	0.00
GATCAAATGTGGTGCCAATG	0.46	0.33	0.00	0.00	0.00
GATCAAATGTGTCAATGCAA	0.00	0.00	0.00	0.00	0.34
GATCAAATGTGTCTTGGGTT	0.00	0.00	0.00	0.34	0.00
GATCAAATGTGTGATGACCT	0.00	0.00	0.00	0.00	0.34
GATCAAATGTGTGGCCTTTG	0.00	0.00	0.00	0.00	0.00
GATCAAATGTGTGGTGGGAA	0.00	0.00	0.29	0.00	0.00
GATCAAATGTGTGGTGGGCA	0.46	0.00	2.58	2.38	0.34
GATCAAATGTGTGGTGNGNN	0.23	0.00	0.00	0.00	0.00
GATCAAATGTGTTCTTTGTG	0.00	0.00	0.29	0.00	0.00
GATCAAATGTTAAAAAAAAA	0.00	0.65	0.29	0.68	0.00
GATCAAATGTTACTTTCACT	0.00	0.00	0.29	0.00	0.00
GATCAAATGTTAGATGAAGC	0.00	0.65	0.00	1.02	0.34
GATCAAATGTTAGATGATGC	0.00	0.00	0.00	0.00	0.00
GATCAAATGTTAGATGCAGC	0.00	0.00	0.00	0.00	0.00
GATCAAATGTTAGCGCGATG	0.00	0.00	0.29	0.00	0.00
GATCAAATGTTATAAGCCCA	0.00	0.00	0.00	0.00	0.00
GATCAAATGTTATTGGAGGA	0.00	0.00	0.00	0.34	0.34
GATCAAATGTTCAAGTCAAT	0.00	0.33	0.00	0.00	0.00
GATCAAATGTTCCAGAGACT	0.00	0.33	0.00	0.68	0.00
GATCAAATGTTCCCCACCCC	0.23	0.00	0.00	0.00	0.00
GATCAAATGTTCGCAAGAGG	0.23	0.00	0.00	0.00	0.00
GATCAAATGTTCGCCCACGC	0.00	0.00	0.00	0.00	0.00
GATCAAATGTTGAAAAAAAA	0.69	0.33	0.86	1.02	3.79
GATCAAATGTTGAAAAAAAT	0.00	0.00	0.00	0.34	0.00
GATCAAATGTTGAGAAAAAA	0.00	0.00	0.00	0.00	0.00
GATCAAATGTTGATTATTTT	0.00	0.00	0.29	0.00	0.00
GATCAAATGTTGCAAAAAAA	3.43	0.98	2.58	4.41	4.48
GATCAAATGTTGCACAAAAA	0.00	0.00	0.29	0.34	0.69
GATCAAATGTTGCACAAAGA	0.00	0.00	0.00	0.00	0.00

GATCAAATGTTGCACAATTG	0.00	0.00	0.00	0.00	0.00
GATCAAATGTTGCACACTTG	1.37	2.60	1.43	1.36	0.34
GATCAAATGTTGCCAACAAG	0.00	0.00	0.29	0.00	0.00
GATCAAATGTTGCCAACACA	0.00	0.00	0.29	0.00	0.00
GATCAAATGTTGCCAACACC	0.00	0.00	1.15	0.00	0.00
GATCAAATGTTGCCAACATC	0.00	0.00	0.29	0.00	0.00
GATCAAATGTTGCCTCAAGC	0.00	0.00	0.29	0.00	0.00
GATCAAATGTTGCGAGACAA	0.00	0.00	0.00	0.00	0.00
GATCAAATGTTGCGCAAGCT	0.00	0.00	0.29	0.00	0.00
GATCAAATGTTGCGCGAGAA	0.00	0.33	0.00	0.00	0.00
GATCAAATGTTGGAAATAAA	0.00	0.00	0.00	0.34	0.00
GATCAAATGTTGGGCTACTC	0.00	0.00	0.00	0.00	0.00
GATCAAATGTTGGTTTGACC	1.37	0.00	0.29	0.68	0.34
GATCAAATGTTGTTAGACAG	0.92	0.00	0.00	0.00	0.00
GATCAAATGTTGTTGTTGTT	0.00	0.00	0.00	0.00	0.34
GATCAAATGTTTAAAAAAAA	0.23	0.00	0.00	0.00	0.00
GATCAAATGTTTCGAAGAGG	0.00	0.00	0.00	0.34	0.00
GATCAAATGTTTCGGGACGG	0.46	0.00	0.00	0.00	0.34
GATCAAATGTTTGAACTTGT	0.00	0.00	0.00	0.00	0.00
GATCAAATGTTTGGGGCACG	0.00	0.00	0.29	0.68	0.00
GATCAAATGTTTGTCATCCC	0.00	0.00	0.00	0.34	0.34
GATCAAATTAAAAAAAAAAA	0.00	0.00	0.00	0.34	0.34
GATCAAATTAAAACCCCCCC	0.00	0.00	0.29	0.00	0.00
GATCAAATTAAAGCTTGGCC	0.00	0.00	0.00	0.00	0.00
GATCAAATTAAATAAACCAC	0.00	0.00	0.00	0.00	0.69
GATCAAATTAAATTAAGTTG	0.00	0.00	0.00	0.00	0.69
GATCAAATTAAATTGAATCA	0.00	0.00	0.29	0.00	0.00
GATCAAATTAAATTGCGTTA	0.00	0.00	0.57	0.00	0.00
GATCAAATTAACGTGATGCG	0.00	0.00	0.29	0.00	0.00
GATCAAATTAACTCAGCCGG	0.00	0.33	0.00	0.00	0.34
GATCAAATTAAGAAGGACAG	0.00	0.00	0.00	0.00	0.00
GATCAAATTAAGATTAACAC	0.00	0.00	0.29	0.00	0.00
GATCAAATTAAGCATAATTT	1.37	0.00	0.00	0.00	0.00
GATCAAATTAAGCATACTNN	0.23	0.00	0.00	0.00	0.00
GATCAAATTAAGTGCCTCTG	0.69	0.33	0.00	0.00	0.00
GATCAAATTAATAAATTATT	0.00	0.00	0.00	0.00	0.00
GATCAAATTAATCCCCCTTG	0.00	0.00	0.29	0.00	0.00

GATCAAATTAATGAGCTCTC	0.00	0.00	0.00	0.34	0.00
GATCAAATTAATGATTATTA	0.23	0.00	0.00	0.00	0.00
GATCAAATTAATGCGCATGG	0.00	0.00	0.29	0.00	0.00
GATCAAATTAATGGTTATTA	0.23	0.00	0.00	0.00	0.00
GATCAAATTAATTGTAGCCT	0.23	0.00	0.29	0.00	0.00
GATCAAATTAATTTGCATTA	0.00	0.00	0.29	0.00	0.00
GATCAAATTACAAAAATCAC	0.00	0.00	0.00	0.00	0.34
GATCAAATTACATGTGATTT	0.00	0.00	0.00	0.00	0.69
GATCAAATTACATTAATTCA	0.00	0.00	0.29	0.00	0.00
GATCAAATTACATTGTTTTT	0.00	0.00	0.00	0.00	0.34
GATCAAATTACATTTCATTA	0.00	0.00	0.00	0.00	1.03
GATCAAATTACCCCGCTTCG	0.69	0.33	0.00	0.68	0.00
GATCAAATTACGCCCGGTGT	0.00	0.00	0.29	0.00	0.00
GATCAAATTACGGTACAGTG	0.00	0.33	0.00	0.00	0.00
GATCAAATTAGAGAAGTTAA	0.46	0.00	0.00	0.00	0.00
GATCAAATTAGAGGATGTAA	0.23	0.00	0.00	0.00	0.00
GATCAAATTAGCACTTTTGA	0.23	0.00	0.00	0.00	0.00
GATCAAATTAGCCCCCCCCC	0.00	0.00	0.00	0.34	0.00
GATCAAATTAGTATTCCATC	0.00	0.00	0.00	0.00	0.34
GATCAAATTAGTGCAAGGCC	0.00	0.33	0.00	0.68	0.00
GATCAAATTAGTGCACTGCC	0.00	0.00	0.00	0.00	0.00
GATCAAATTAGTGGCAAAAA	0.00	0.00	0.00	0.00	0.00
GATCAAATTAGTGGCACCTG	0.00	0.00	0.00	0.00	0.00
GATCAAATTAGTGGCACTAA	0.46	0.00	0.00	0.00	0.00
GATCAAATTAGTGGCACTTA	0.00	0.00	0.00	0.00	0.00
GATCAAATTAGTGGTGCTGG	0.00	0.00	0.29	0.00	0.00
GATCAAATTAGTTTTGTTTT	0.00	0.00	0.00	0.00	0.00
GATCAAATTATAGGAAAAAA	0.23	0.00	0.00	0.00	0.00
GATCAAATTATAGGACAAAA	0.00	0.00	0.00	0.00	0.00
GATCAAATTATAGGAGAAAA	0.46	0.33	0.00	0.00	0.00
GATCAAATTATAGGAGAGAA	4.12	1.63	2.29	1.02	1.38
GATCAAATTATAGGAGAGGA	0.23	0.00	0.00	0.00	0.00
GATCAAATTATAGGAGAGNN	0.23	0.00	0.00	0.00	0.00
GATCAAATTATATTAATTCA	0.00	0.00	0.86	0.00	0.00
GATCAAATTATATTGGAGGA	0.00	0.00	0.29	0.00	0.00
GATCAAATTATCGAGGTGAA	0.46	0.65	0.86	0.00	0.00
GATCAAATTATGCCCCGACT	0.00	0.00	0.00	0.34	0.00

GATCAAATTATGGCACGCAA	0.00	0.00	0.00	0.00	0.00
GATCAAATTATGGCACGCCA	0.00	0.33	0.86	0.00	0.00
GATCAAATTATGGCAGCCCT	0.69	0.00	0.00	0.00	0.00
GATCAAATTATGGCAGGCCC	0.00	0.00	0.29	0.00	0.00
GATCAAATTATGGCAGGCCT	0.23	0.00	0.00	0.00	0.00
GATCAAATTATGGCGCCAAT	0.00	0.00	0.29	0.00	0.00
GATCAAATTATTACAGNNNN	0.23	0.00	0.00	0.00	0.00
GATCAAATTATTATAGCTGG	0.00	0.00	0.00	0.00	0.00
GATCAAATTATTCTTTCTGC	0.00	0.00	0.00	0.00	0.00
GATCAAATTATTTTAAGAGG	0.23	0.00	0.00	0.00	0.00
GATCAAATTATTTTTTTGTG	0.23	0.00	0.00	0.00	0.00
GATCAAATTCAAAAAAAAAA	0.69	0.00	1.72	1.02	0.00
GATCAAATTCAAAAAAACAA	0.00	0.00	0.29	0.00	0.00
GATCAAATTCAAAGTAGTCC	0.00	0.00	0.29	0.00	0.00
GATCAAATTCAACAAAAAAA	1.14	0.00	0.00	0.00	0.00
GATCAAATTCAACAAAACTT	0.92	0.00	0.29	0.68	0.00
GATCAAATTCAACAAATTCA	0.00	0.00	0.57	0.00	0.00
GATCAAATTCAACACAAAAA	0.00	0.00	0.29	0.00	0.00
GATCAAATTCAACCCCGGAG	1.14	0.98	0.29	1.02	0.34
GATCAAATTCAACCTAGGGA	0.00	0.00	0.00	0.00	0.34
GATCAAATTCAACTTCAACC	0.00	0.33	0.00	0.00	0.00
GATCAAATTCAACTTCAACT	0.00	0.00	0.00	0.34	0.00
GATCAAATTCAAGAAGATGA	0.69	0.65	0.29	1.36	0.69
GATCAAATTCAAGAAGATGG	0.00	0.00	0.00	0.34	0.00
GATCAAATTCAAGATGATGA	0.00	0.00	0.00	0.00	0.00
GATCAAATTCAAGATGATGN	0.00	0.00	0.00	0.00	0.00
GATCAAATTCAATAAATTTA	0.00	0.00	0.00	0.00	0.34
GATCAAATTCACACTCGAAC	0.00	0.33	0.29	0.68	0.00
GATCAAATTCACAGACATGT	0.00	0.00	0.00	0.00	0.00
GATCAAATTCACCTCCCCCC	0.00	0.00	0.00	0.00	0.00
GATCAAATTCACGGACAAGC	0.23	0.00	0.00	0.00	0.00
GATCAAATTCACGGACCTGC	0.23	0.33	0.00	2.38	0.34
GATCAAATTCACGGCAGAAA	0.23	0.00	0.00	0.00	0.00
GATCAAATTCACGGCCCTGC	0.00	0.00	0.00	0.00	0.00
GATCAAATTCACGGTGGGCA	0.00	0.00	0.29	0.00	0.00
GATCAAATTCACTCCAAAAT	0.00	0.00	0.00	0.00	0.00
GATCAAATTCACTCTGTGCC	0.00	0.33	0.00	0.00	0.00

GATCAAATTCAGATTGGTCA	0.00	0.00	0.00	0.68	0.00
GATCAAATTCAGCAAATGCT	0.00	0.00	0.00	0.68	0.00
GATCAAATTCAGCTTCTATT	0.00	0.00	0.00	0.00	0.00
GATCAAATTCAGGGCTTTTG	0.00	0.00	0.00	0.00	0.00
GATCAAATTCAGGTCCAAGC	0.00	0.65	0.00	0.34	0.00
GATCAAATTCAGTAATCAAC	0.69	0.00	0.00	0.34	0.00
GATCAAATTCAGTCACGACT	0.00	0.33	0.29	0.00	0.00
GATCAAATTCAGTGCAGTCA	0.00	0.00	0.00	0.00	0.69
GATCAAATTCATAGCAGTCA	0.00	0.00	0.29	0.00	0.00
GATCAAATTCATCAAAATGA	0.00	0.00	0.29	0.00	0.00
GATCAAATTCATCAAATTCA	0.00	0.00	0.00	0.00	0.34
GATCAAATTCATCAATTCTC	0.23	0.00	0.00	0.00	0.00
GATCAAATTCATCGAATTCA	0.00	0.00	2.86	0.00	3.10
GATCAAATTCATCGAGTTCA	0.00	0.00	0.29	0.00	0.00
GATCAAATTCATCTGACGGG	0.23	0.00	0.00	0.00	0.00
GATCAAATTCATCTTCAAGA	0.00	0.00	0.00	0.00	0.00
GATCAAATTCATGCATAAAA	0.00	0.00	0.29	0.00	0.00
GATCAAATTCATGCCCTTTT	0.00	0.00	0.29	0.00	0.00
GATCAAATTCATGGAATTCA	0.00	0.00	0.00	0.00	1.03
GATCAAATTCATGGAGAATT	0.00	0.00	0.00	0.68	0.00
GATCAAATTCATGGATGACA	0.00	0.33	0.00	0.00	0.00
GATCAAATTCATGGATGAGG	0.00	0.00	0.00	0.34	0.00
GATCAAATTCATGGATGATA	0.00	0.00	0.29	0.00	0.00
GATCAAATTCATGGATGCTA	29.07	25.05	10.02	12.90	13.78
GATCAAATTCATGGATGCTC	0.00	0.00	0.00	0.00	0.34
GATCAAATTCATGGATGCTT	0.69	0.00	0.00	0.00	0.00
GATCAAATTCATGGATGGTA	0.23	0.00	0.00	0.00	0.00
GATCAAATTCATGGATNCNN	0.23	0.00	0.00	0.00	0.00
GATCAAATTCATGGATNCTA	0.00	0.00	0.00	0.00	0.34
GATCAAATTCATGGATNNNN	0.23	0.00	0.00	0.00	0.00
GATCAAATTCATGGATTCTA	0.00	0.33	0.00	0.00	0.00
GATCAAATTCATGGCTGCTC	0.23	0.00	0.00	0.00	0.00
GATCAAATTCATGGGTGCTA	0.00	0.00	0.00	0.34	0.00
GATCAAATTCATGGTTGCTA	0.00	0.00	0.29	0.00	0.00
GATCAAATTCATGTTAATCT	0.00	0.00	0.86	0.00	0.00
GATCAAATTCATTCAAATTC	0.00	0.00	0.57	0.00	0.00
GATCAAATTCATTCAGGCCG	0.00	0.00	0.00	0.00	0.34

GATCAAATTCATTCTAGCCG	0.00	0.00	0.00	0.00	1.03
GATCAAATTCATTCTATCCG	0.00	0.00	0.00	0.34	0.00
GATCAAATTCATTGATGCTT	0.00	0.00	0.00	0.00	0.34
GATCAAATTCCAAAAAAAAA	0.00	0.00	0.00	0.00	1.38
GATCAAATTCCAAAGAAAAA	0.00	0.00	0.00	0.00	0.00
GATCAAATTCCAATACATCA	1.14	0.98	1.43	1.36	0.00
GATCAAATTCCACAGACCGA	0.00	0.00	0.29	0.00	0.00
GATCAAATTCCACCAAACTT	0.00	0.00	0.29	0.00	0.00
GATCAAATTCCAGTGGATGC	0.46	0.00	0.00	0.34	0.00
GATCAAATTCCCCCGCTTCG	0.46	0.00	0.00	0.00	0.00
GATCAAATTCCCGGTGAACC	0.00	0.00	0.00	0.34	0.00
GATCAAATTCCGAAACGAAT	0.00	0.00	0.00	0.00	0.00
GATCAAATTCCGCACCTGGG	0.00	0.00	0.00	0.34	0.00
GATCAAATTCCGCGCTGCTT	0.69	0.00	0.00	0.00	0.00
GATCAAATTCCGCGCTGGCA	0.00	0.00	0.00	0.34	0.00
GATCAAATTCCGGAAAAAAA	0.46	0.00	0.00	0.00	0.00
GATCAAATTCCGGAAACGAA	4.35	4.55	0.86	2.38	0.34
GATCAAATTCCGGAAACGAG	0.00	0.33	0.00	0.00	0.00
GATCAAATTCCGGAAACGAN	0.23	0.00	0.00	0.00	0.00
GATCAAATTCCGGAAGCGAA	1.37	0.00	0.00	0.00	0.69
GATCAAATTCCGGAGACGAC	0.00	0.00	0.29	0.00	0.00
GATCAAATTCCGGCCATGGG	0.00	0.00	0.00	0.00	0.00
GATCAAATTCCGTATTCCAT	0.23	0.00	0.00	0.00	0.00
GATCAAATTCCGTATTCCNN	0.23	0.00	0.00	0.00	0.00
GATCAAATTCCTACGGAGCA	0.00	0.00	0.00	0.34	0.00
GATCAAATTCCTCGAGATTG	0.00	0.33	0.00	0.00	0.00
GATCAAATTCCTCGCATGAG	1.14	0.33	0.00	0.34	0.69
GATCAAATTCCTCGCCGCCA	0.00	0.00	0.00	0.00	0.00
GATCAAATTCCTCGCCGCCG	0.69	0.33	0.57	1.70	0.00
GATCAAATTCCTCGCTGCCC	0.00	0.00	0.00	0.00	0.00
GATCAAATTCCTCGGCGTGA	0.00	0.00	0.00	0.00	0.00
GATCAAATTCCTCGGGAACT	0.00	0.33	0.00	0.00	0.00
GATCAAATTCCTCTGCCCCT	0.00	0.00	0.00	0.00	0.00
GATCAAATTCCTGGGCGCGC	0.23	0.00	0.00	0.00	0.00
GATCAAATTCCTGGGCTCGC	2.75	2.60	1.72	1.36	1.38
GATCAAATTCCTTGGAACGC	0.00	0.00	0.00	0.68	0.00
GATCAAATTCCTTGTTGGAC	0.00	0.00	0.00	0.00	0.00

GATCAAATTCCTTTCCGCCT	0.00	0.00	0.00	0.00	0.00
GATCAAATTCGAAAAAAAAA	0.00	0.00	0.00	0.00	0.34
GATCAAATTCGAAGAAGAAG	0.00	0.00	0.00	0.00	0.34
GATCAAATTCGAAGAAGAGG	0.00	0.00	0.57	0.34	0.00
GATCAAATTCGAAGGGGCTC	0.00	0.33	0.00	0.00	0.00
GATCAAATTCGACACCCGGA	0.00	0.33	0.00	0.00	0.00
GATCAAATTCGACCTTGTTG	0.00	0.00	0.29	0.00	0.00
GATCAAATTCGAGAAAAAAA	0.00	0.33	0.00	0.00	0.00
GATCAAATTCGAGGCAAAAA	0.00	0.33	0.86	0.00	0.34
GATCAAATTCGAGGCAAATC	0.46	0.33	0.00	0.00	0.00
GATCAAATTCGAGGCAGCGC	0.00	0.33	0.00	0.00	0.00
GATCAAATTCGCAGCAGCAG	0.00	0.00	0.00	0.00	0.00
GATCAAATTCGCCTCCGGTG	0.92	0.98	0.57	0.00	0.00
GATCAAATTCGGAGTTGTTT	0.00	0.00	0.00	0.34	0.00
GATCAAATTCGGCGCCGACT	0.23	0.00	0.57	1.36	0.34
GATCAAATTCGGGAAAAGAG	0.92	0.65	0.57	0.00	1.03
GATCAAATTCGGGAAAAGGA	0.00	0.00	0.00	0.00	0.00
GATCAAATTCGGGAGAAGAG	0.00	0.00	0.29	0.00	0.00
GATCAAATTCGTCGTTTCAC	0.00	0.33	0.00	0.00	0.00
GATCAAATTCGTGATTGCGG	0.00	0.65	0.00	0.00	0.00
GATCAAATTCGTGCGAATGT	0.00	0.00	0.00	0.00	0.00
GATCAAATTCGTGTGAAAAA	0.00	0.00	0.00	0.68	0.00
GATCAAATTCGTGTGAGAAA	0.00	0.33	0.00	0.00	0.00
GATCAAATTCGTGTGAGCAC	2.75	0.65	0.86	1.02	1.72
GATCAAATTCGTGTGAGCGG	0.00	0.00	0.00	0.00	0.69
GATCAAATTCGTGTGTCACG	0.00	0.33	0.00	0.00	0.00
GATCAAATTCGTTGATTTCA	0.00	0.00	0.00	0.00	0.34
GATCAAATTCTAAAAAAAAA	29.76	5.86	9.45	9.16	13.09
GATCAAATTCTAAAAAAAAN	0.23	0.00	0.00	0.00	0.00
GATCAAATTCTAAAAAAAAT	0.23	0.00	0.29	0.00	0.00
GATCAAATTCTAAAAAAAGG	0.00	0.00	0.00	0.00	0.00
GATCAAATTCTAAAAAAANN	0.23	0.00	0.00	0.00	0.00
GATCAAATTCTAAAAAAATA	0.23	0.00	0.00	0.00	0.00
GATCAAATTCTAAAAAACAA	0.00	0.00	0.00	0.00	0.34
GATCAAATTCTAAAAAGAAA	0.00	0.00	0.00	0.68	0.00
GATCAAATTCTAAAAAGGAA	0.00	0.00	0.00	0.00	0.00
GATCAAATTCTAAACAACAC	0.00	0.00	0.00	0.00	0.34

GATCAAATTCTAAAGAAAAA	0.00	0.00	0.29	0.00	0.00
GATCAAATTCTAAATAAAAA	0.00	0.00	0.29	0.00	0.00
GATCAAATTCTAAGAAAAAA	0.00	0.00	0.29	0.34	0.00
GATCAAATTCTAAGGAAAAA	0.00	0.00	0.57	0.00	0.00
GATCAAATTCTACAAAAAAA	0.00	0.00	0.00	0.68	0.69
GATCAAATTCTAGAAAAAAA	0.00	0.00	0.00	0.00	0.00
GATCAAATTCTATAAGCCAA	0.00	0.00	0.00	0.00	0.00
GATCAAATTCTATTGATGGG	0.00	0.00	0.00	0.00	0.34
GATCAAATTCTCCTGCGCTG	0.00	0.00	0.00	0.00	0.00
GATCAAATTCTCGCAAGAGG	0.00	0.00	0.00	0.00	0.00
GATCAAATTCTCTGGTTTCA	0.00	0.00	0.29	0.00	0.00
GATCAAATTCTGAAAAAAAA	0.46	0.00	0.00	0.34	0.00
GATCAAATTCTGAAAAAANA	0.00	0.00	0.00	0.00	0.00
GATCAAATTCTGATCANGAT	0.00	0.00	0.00	0.00	0.34
GATCAAATTCTGATTACGCT	0.00	0.00	0.00	0.34	0.00
GATCAAATTCTGATTCAAAA	0.23	0.00	0.57	0.00	0.00
GATCAAATTCTGATTCAAGA	0.00	0.33	0.00	0.00	0.00
GATCAAATTCTGATTCAAGG	0.00	0.00	0.00	0.00	0.00
GATCAAATTCTGATTCAGCT	0.00	0.00	0.00	0.00	0.00
GATCAAATTCTGATTCAGGA	13.73	12.04	10.31	15.27	11.02
GATCAAATTCTGATTCAGGC	0.00	0.00	0.00	0.00	1.03
GATCAAATTCTGATTCAGGG	0.23	0.00	0.57	0.00	0.00
GATCAAATTCTGATTCAGTA	0.00	0.33	0.00	0.00	0.00
GATCAAATTCTGATTTGCCA	0.69	1.30	2.00	2.04	1.03
GATCAAATTCTGATTTGCCT	0.69	0.00	0.00	0.00	0.00
GATCAAATTCTGATTTGCTC	0.00	0.00	0.00	0.00	0.34
GATCAAATTCTGATTTNCCA	0.00	0.00	0.00	0.34	0.00
GATCAAATTCTGCCATTCGT	0.69	0.00	0.00	0.00	0.00
GATCAAATTCTGGAAAAAAA	0.00	0.00	0.00	0.00	0.00
GATCAAATTCTGGGCAGGCC	0.00	0.00	0.00	0.00	0.34
GATCAAATTCTGGTGACTCC	0.00	0.00	0.00	0.00	0.00
GATCAAATTCTTCAACAAGT	0.00	0.33	0.00	0.00	0.00
GATCAAATTCTTCGTCTTTT	0.00	0.33	0.00	0.00	0.00
GATCAAATTCTTGAAAAAAA	0.00	0.33	0.00	0.00	0.34
GATCAAATTCTTGGATGCTA	0.00	0.00	0.00	0.00	0.00
GATCAAATTGAAAAAAAAAA	0.92	0.00	0.00	0.00	0.00
GATCAAATTGAAAGACTTCG	0.23	0.00	0.00	0.00	0.00

GATCAAATTGAACGTCAAAA	0.00	0.65	0.00	0.00	0.00
GATCAAATTGAACTCATAAC	0.00	0.33	0.00	0.34	0.00
GATCAAATTGAAGAGCAGCG	0.23	0.00	0.00	0.00	0.00
GATCAAATTGAAGCTGAAAG	0.00	0.00	0.00	0.00	0.34
GATCAAATTGACAGCCGCTG	0.69	0.00	0.00	0.34	0.34
GATCAAATTGACATTGTCAA	0.23	0.00	0.00	0.00	0.00
GATCAAATTGACGGGTCACG	0.00	0.33	0.29	0.00	0.34
GATCAAATTGACTGGGTGGC	0.00	0.00	0.00	0.00	0.00
GATCAAATTGAGCTGGTTTG	0.00	0.00	0.00	0.00	0.34
GATCAAATTGAGGTTGTGAC	0.00	0.33	0.29	0.34	0.00
GATCAAATTGAGTAAATCCT	0.00	0.00	0.57	0.00	0.00
GATCAAATTGAGTTTGCTGG	0.00	0.00	0.00	0.68	0.00
GATCAAATTGATATTATTTG	0.46	0.00	0.00	0.00	0.00
GATCAAATTGATGGCAAGAG	0.00	0.00	0.57	0.00	0.00
GATCAAATTGATTCATAATC	0.23	0.00	0.00	0.00	0.00
GATCAAATTGATTCTTAATG	0.00	0.00	0.00	0.00	0.34
GATCAAATTGATTGAGTGAT	0.00	0.00	0.00	0.00	0.00
GATCAAATTGATTTATTTCN	0.23	0.00	0.00	0.00	0.00
GATCAAATTGCAAAAAAAAA	0.00	0.00	0.00	0.34	0.00
GATCAAATTGCAACTGCAAA	0.23	0.00	0.00	0.00	0.00
GATCAAATTGCACAAGACAC	0.00	0.00	0.86	0.00	0.00
GATCAAATTGCACCTGGTTG	0.00	0.00	0.00	0.00	0.34
GATCAAATTGCAGACGACGA	0.00	0.00	0.00	0.00	0.00
GATCAAATTGCAGGCTTGGT	0.00	0.00	0.00	0.34	0.00
GATCAAATTGCAGGTGCGGC	0.00	0.00	0.29	0.00	0.00
GATCAAATTGCAGGTGCTGC	1.60	0.33	0.00	0.34	0.34
GATCAAATTGCATACGTCAT	0.00	0.00	0.29	0.00	0.00
GATCAAATTGCCACGACCTG	0.00	0.00	0.29	0.00	0.00
GATCAAATTGCCCAACATGC	0.00	0.00	0.00	0.00	0.00
GATCAAATTGCCGAGGTATT	0.00	0.00	0.00	0.34	0.00
GATCAAATTGCCGCCTTTTT	0.00	0.00	0.86	0.00	0.00
GATCAAATTGCCGGGAACAG	0.00	0.00	0.00	0.34	0.00
GATCAAATTGCCGGGAACTA	0.00	0.00	0.00	0.00	0.34
GATCAAATTGCCGGGAACTC	1.83	2.28	0.57	2.38	2.76
GATCAAATTGCCGGGAACTT	0.46	0.00	0.00	0.00	0.00
GATCAAATTGCCGGGCAGTC	0.23	0.00	0.00	0.00	0.00
GATCAAATTGCGAAAACTTT	0.23	0.00	0.00	0.00	0.00

GATCAAATTGCGCAAACGTT	0.00	0.00	0.00	0.00	0.34
GATCAAATTGCGCAAATTTT	0.23	0.00	0.00	0.00	0.00
GATCAAATTGCGGTCCACNN	0.23	0.00	0.00	0.00	0.00
GATCAAATTGCGGTCCACTG	6.18	9.76	6.30	5.77	4.48
GATCAAATTGCGGTCCCCTG	0.00	0.00	0.00	0.00	0.00
GATCAAATTGCTAAAAAAAA	0.00	0.33	0.00	0.00	0.00
GATCAAATTGCTAGAGTATC	0.00	0.00	0.00	0.00	0.00
GATCAAATTGCTGCTGTCCA	0.00	0.33	0.29	0.00	0.00
GATCAAATTGCTGCTGTTCA	0.00	0.00	0.29	0.00	0.00
GATCAAATTGCTGTCCACTG	0.00	0.00	0.00	0.00	0.34
GATCAAATTGCTTCCTTTTT	0.00	0.00	0.00	0.00	0.00
GATCAAATTGCTTCGATGCC	0.00	0.00	0.29	0.00	0.00
GATCAAATTGCTTGGTGATG	0.00	0.00	0.00	0.00	0.00
GATCAAATTGCTTTCCACGG	0.00	0.00	0.00	0.34	0.00
GATCAAATTGGAAAAATTTT	0.00	0.33	0.00	0.00	0.00
GATCAAATTGGAAACCCTCC	0.00	0.33	0.00	0.00	0.00
GATCAAATTGGACAGGTTTT	0.23	0.00	0.00	0.00	0.00
GATCAAATTGGACCCCTGGT	0.46	0.00	0.00	0.00	0.00
GATCAAATTGGATAGGTTTN	0.23	0.00	0.00	0.00	0.00
GATCAAATTGGATAGGTTTT	2.52	3.58	2.58	5.77	4.13
GATCAAATTGGATTGCGCTT	0.00	0.00	0.00	0.00	0.00
GATCAAATTGGCAAATTGGG	0.92	1.30	2.86	1.70	1.03
GATCAAATTGGCAAATTGGT	0.00	0.00	0.00	0.00	0.34
GATCAAATTGGCAACGCCCA	0.00	0.00	0.00	0.00	0.34
GATCAAATTGGCAATCTCTG	0.00	0.33	0.00	0.00	0.00
GATCAAATTGGCACCCCCCA	0.00	0.33	0.00	0.00	0.00
GATCAAATTGGCACCGCCCT	0.23	0.00	0.00	0.00	0.00
GATCAAATTGGCACGCACAA	0.00	0.00	0.00	0.00	0.34
GATCAAATTGGCACGCCACC	0.00	0.00	0.00	0.00	0.34
GATCAAATTGGCACGCCCAA	0.00	0.00	0.29	0.00	0.00
GATCAAATTGGCACGCCCCA	28.38	24.40	10.02	19.35	12.05
GATCAAATTGGCACGCCCCC	0.00	0.65	0.00	0.00	0.00
GATCAAATTGGCACGCCCCG	0.23	0.00	0.00	0.00	0.00
GATCAAATTGGCACGCCCNN	0.46	0.00	0.00	0.00	0.00
GATCAAATTGGCACGCCTCA	0.00	0.00	0.00	0.00	0.00
GATCAAATTGGCACGCNNNN	0.23	0.00	0.00	0.00	0.00
GATCAAATTGGCACGCTCCA	0.23	0.00	0.00	0.00	0.00

GATCAAATTGGCATAGAGTC	0.00	0.00	0.00	0.00	0.00
GATCAAATTGGCATGCCCCC	0.00	0.33	0.00	0.00	0.00
GATCAAATTGGCCGGCAGAG	0.00	0.00	0.00	0.00	0.00
GATCAAATTGGCCGGCAGGG	0.00	0.00	0.29	0.00	0.00
GATCAAATTGGCCGGGGGAG	0.23	0.00	0.00	0.00	0.00
GATCAAATTGGCCGTGCACA	0.69	0.00	0.00	0.00	0.00
GATCAAATTGGCGCTCAAGA	0.00	0.00	0.00	0.00	0.00
GATCAAATTGGCGGCCACTG	0.00	0.00	0.00	0.00	0.34
GATCAAATTGGCGGGCAGAG	0.00	0.00	0.00	0.00	0.00
GATCAAATTGGCTCCAGGTT	0.00	0.00	0.00	0.00	0.34
GATCAAATTGGGAAAATGAC	0.23	0.00	0.00	0.00	0.00
GATCAAATTGGGAAAATTTT	0.00	0.00	0.00	0.00	0.00
GATCAAATTGGGAAATCATG	0.00	0.00	0.29	0.00	0.00
GATCAAATTGGGAAGCTCTG	0.00	0.00	0.00	0.34	0.00
GATCAAATTGGGACGCCCCA	0.00	0.00	0.00	0.34	0.00
GATCAAATTGGGATGATGTT	0.00	0.00	0.29	0.00	0.00
GATCAAATTGGGCAAATATT	0.00	0.00	0.00	0.00	0.34
GATCAAATTGGGCAAATGTT	0.00	0.00	0.29	0.00	0.00
GATCAAATTGGGCAAATTNN	0.23	0.00	0.00	0.00	0.00
GATCAAATTGGGCAAATTTN	0.23	0.00	0.00	0.00	0.00
GATCAAATTGGGCAAATTTT	14.19	3.90	10.31	7.47	7.58
GATCAAATTGGGCAACTTTT	0.00	0.33	0.00	0.00	0.00
GATCAAATTGGGCCACATTT	0.00	0.33	0.00	0.00	0.00
GATCAAATTGGGTAGCTGGG	0.00	0.00	0.00	0.34	0.00
GATCAAATTGGGTTAGATGG	0.23	0.00	0.00	0.00	0.00
GATCAAATTGGTCAAATTTT	0.00	0.00	0.29	0.00	0.00
GATCAAATTGGTGCGCCGAA	0.00	0.00	0.29	0.00	0.00
GATCAAATTGGTGTGGTGTT	0.23	0.00	0.00	0.00	0.00
GATCAAATTGGTTCCACTTG	0.00	0.00	0.29	0.00	0.00
GATCAAATTGGTTGCCAGGC	0.00	0.33	0.00	0.00	0.00
GATCAAATTGGTTTCGAGAT	0.00	0.00	0.00	0.00	0.00
GATCAAATTGTACACGCCAA	0.00	0.00	0.29	0.00	0.00
GATCAAATTGTATCTTTCCC	0.69	0.00	0.00	0.00	0.34
GATCAAATTGTATTGGAGGA	0.23	0.00	0.00	0.34	0.00
GATCAAATTGTATTTAAAGA	0.69	0.00	0.00	0.34	0.00
GATCAAATTGTATTTAAATA	0.00	0.33	0.00	0.00	0.00
GATCAAATTGTATTTGTGAC	0.00	0.00	0.00	0.00	0.00

GATCAAATTGTCAAGTAATT	0.00	0.00	0.00	0.34	0.00
GATCAAATTGTCAAGTCATT	0.00	0.33	0.00	0.00	0.00
GATCAAATTGTCTGTCTTCT	0.23	0.00	0.00	0.00	0.00
GATCAAATTGTGAGTGTGCA	0.23	0.00	0.00	0.00	0.00
GATCAAATTGTGCACCAAGG	0.00	0.00	0.29	0.00	0.00
GATCAAATTGTGCAGAGGGA	0.23	0.00	0.00	0.00	0.00
GATCAAATTGTGCAGCCGCC	0.00	0.00	0.00	0.00	0.00
GATCAAATTGTGGACTATGT	0.00	0.00	0.00	0.00	0.00
GATCAAATTGTGGAGTGATG	0.00	0.00	0.29	0.00	0.00
GATCAAATTGTGGCGAGGCG	0.23	0.00	0.00	0.00	0.00
GATCAAATTGTGGGCTTCTT	0.00	0.00	0.00	0.00	0.34
GATCAAATTGTGTCTCCCCC	0.00	0.00	0.00	0.00	0.00
GATCAAATTGTGTGAGAGCA	0.00	0.00	0.29	0.00	0.00
GATCAAATTGTGTTTTCGCA	0.00	0.00	0.00	0.00	0.00
GATCAAATTGTTGAAGGACA	0.00	0.00	0.00	0.68	0.00
GATCAAATTGTTGTTGCAAA	0.23	0.00	0.00	0.00	0.00
GATCAAATTGTTGTTGCAGA	0.00	0.00	0.00	0.34	0.00
GATCAAATTGTTGTTGCAGC	3.20	1.95	0.86	2.72	0.34
GATCAAATTGTTTGCAACCC	0.00	0.00	0.00	0.00	0.34
GATCAAATTGTTTGCATTAT	0.00	0.33	0.00	0.00	0.00
GATCAAATTTAAAGGCTTTG	0.23	0.00	0.00	0.68	0.00
GATCAAATTTAAGTTCAAAA	0.00	0.00	0.00	0.00	0.00
GATCAAATTTACCACGGAAA	0.00	0.00	0.00	0.00	0.00
GATCAAATTTAGAGCTGAAT	0.00	0.00	0.00	0.00	0.00
GATCAAATTTATAAGTTATT	0.23	0.00	0.00	0.00	0.00
GATCAAATTTATCACTCATT	0.46	0.00	0.00	0.00	0.00
GATCAAATTTATCGAAATCA	0.00	0.00	0.29	0.00	0.00
GATCAAATTTATCGAATTCA	0.00	0.00	0.00	0.00	1.72
GATCAAATTTATGGATGGTA	0.00	0.00	0.00	0.34	0.00
GATCAAATTTATGTGTTAAA	0.00	0.00	0.86	0.00	0.00
GATCAAATTTATTTTGTTTG	0.00	0.00	0.00	0.00	0.00
GATCAAATTTCAAAAAAAAA	0.00	0.00	0.00	0.34	0.00
GATCAAATTTCAAAAACTGC	0.00	0.00	0.00	0.00	0.34
GATCAAATTTCAAGAAGGAC	0.69	0.00	0.00	0.00	0.34
GATCAAATTTCACGGCTTCC	0.00	0.00	0.29	0.00	0.00
GATCAAATTTCAGTTGTGCA	0.00	0.00	0.00	0.00	0.00
GATCAAATTTCATCCAGACT	0.00	0.00	0.00	0.00	0.00

GATCAAATTTCATTGGAGGA	0.00	0.00	0.00	0.00	0.34
GATCAAATTTCCCGAGAACT	0.00	0.00	0.29	0.00	0.00
GATCAAATTTCGGCGAGGCG	0.00	0.00	0.00	0.00	0.34
GATCAAATTTCTTGATGTGT	0.00	0.00	0.29	0.00	0.00
GATCAAATTTGAAGATGTGC	0.00	0.00	0.00	0.00	0.34
GATCAAATTTGACAACACCA	1.37	0.65	1.15	1.02	1.38
GATCAAATTTGACAACTCCG	0.00	0.00	0.00	0.34	0.00
GATCAAATTTGATGGCGACG	0.00	0.00	0.00	0.34	0.00
GATCAAATTTGATGGGAGTT	0.00	0.00	0.00	0.00	0.34
GATCAAATTTGCGTGCGTGG	0.00	0.65	0.00	0.00	0.00
GATCAAATTTGCTTCGTGGT	0.00	0.00	0.29	0.00	0.00
GATCAAATTTGGACCTAGAG	0.00	0.00	0.00	0.00	0.00
GATCAAATTTGGAGCCGGTG	0.23	0.33	0.00	0.34	0.00
GATCAAATTTGGCAAATTTT	0.00	0.33	0.00	0.00	0.00
GATCAAATTTGGCCAATTTT	0.00	0.00	0.00	0.00	0.00
GATCAAATTTGGGCTTTCAG	0.00	0.00	0.00	0.00	0.00
GATCAAATTTGGTGCGTTGA	0.00	0.00	0.29	0.00	0.00
GATCAAATTTGTAAAATTTT	0.23	0.00	0.00	0.00	0.00
GATCAAATTTGTATGTAACT	0.00	0.00	0.29	0.00	0.00
GATCAAATTTGTGACCTGCC	0.00	0.00	0.00	0.00	0.34
GATCAAATTTGTTCAAGCCC	0.46	0.00	0.00	0.00	0.00
GATCAAATTTGTTGCAAATT	0.00	0.00	0.00	0.00	1.38
GATCAAATTTTAAAAAAAAA	12.82	3.25	2.86	6.79	2.41
GATCAAATTTTAAAAAAAAT	0.00	0.00	0.00	0.00	0.34
GATCAAATTTTAAAAACACA	0.23	0.00	0.00	0.00	0.00
GATCAAATTTTAAAAGGCTN	0.23	0.00	0.00	0.00	0.00
GATCAAATTTTAAAAGGCTT	1.83	0.65	0.57	0.34	0.34
GATCAAATTTTAACTCCCTT	2.06	0.65	0.29	0.34	1.38
GATCAAATTTTAACTCGCTT	0.00	0.00	0.00	0.00	0.34
GATCAAATTTTAAGAAAAAA	0.00	0.00	0.29	0.00	0.00
GATCAAATTTTAATGGAGGA	0.00	0.00	0.00	0.00	0.00
GATCAAATTTTAATGTTAAA	0.00	0.00	0.29	0.00	0.00
GATCAAATTTTACAACACCA	0.00	0.00	0.00	0.00	0.34
GATCAAATTTTACTATTTAA	0.23	0.00	0.00	0.00	0.00
GATCAAATTTTACTGGAGGA	0.23	0.00	0.00	0.00	0.00
GATCAAATTTTAGCAGGCCA	0.00	0.00	0.00	0.00	0.34
GATCAAATTTTAGTGGAGGA	0.23	0.00	0.00	0.00	0.00

GATCAAATTTTATAAAAAAA	0.00	0.00	0.00	0.00	0.00
GATCAAATTTTATAAGCACA	0.69	0.00	0.00	0.00	0.34
GATCAAATTTTATAAGCACC	0.23	0.00	0.00	0.00	0.00
GATCAAATTTTATAAGCCCA	57.91	35.46	21.48	27.83	22.73
GATCAAATTTTATAAGCCCG	0.23	0.00	0.00	0.00	0.00
GATCAAATTTTATAAGCCCT	0.00	0.33	0.00	0.00	0.00
GATCAAATTTTATAAGCCNN	0.23	0.00	0.00	0.00	0.00
GATCAAATTTTATAAGCCTC	0.00	0.00	0.00	0.00	0.34
GATCAAATTTTATAAGCNNN	0.23	0.00	0.00	0.00	0.00
GATCAAATTTTATAAGTCCA	0.00	0.00	0.00	0.00	0.00
GATCAAATTTTATAATCCCA	0.00	0.33	0.00	0.00	0.00
GATCAAATTTTATACGACAC	0.23	0.00	0.00	0.00	0.00
GATCAAATTTTATCGGAGTA	0.00	0.00	0.00	0.00	0.34
GATCAAATTTTATTAGGGGA	0.00	0.00	0.00	0.00	0.00
GATCAAATTTTATTGAAGGT	0.00	0.00	0.57	0.00	0.00
GATCAAATTTTATTGCCTTG	0.00	0.00	0.57	0.00	0.00
GATCAAATTTTATTGGAGCA	0.00	0.00	0.00	0.34	0.00
GATCAAATTTTATTGGAGGA	186.33	135.66	134.30	144.60	145.68
GATCAAATTTTATTGGAGGC	2.75	0.33	0.86	0.00	0.00
GATCAAATTTTATTGGAGGG	2.52	0.00	0.57	0.00	0.00
GATCAAATTTTATTGGAGGN	0.69	0.00	0.00	0.00	0.00
GATCAAATTTTATTGGAGGT	0.69	0.00	0.00	0.00	0.69
GATCAAATTTTATTGGAGNA	0.00	0.00	0.00	0.68	0.00
GATCAAATTTTATTGGAGNN	0.23	0.00	0.00	0.00	0.00
GATCAAATTTTATTGGAGTA	0.23	0.33	0.00	0.00	0.69
GATCAAATTTTATTGGAGTG	0.00	0.00	0.29	0.00	0.00
GATCAAATTTTATTGGANNN	0.00	0.00	0.00	0.00	0.00
GATCAAATTTTATTGGATGA	0.00	0.00	0.00	0.34	0.00
GATCAAATTTTATTGGATGC	0.00	0.33	0.00	0.00	0.00
GATCAAATTTTATTGGCGGA	0.23	0.33	0.00	0.00	0.00
GATCAAATTTTATTGGCGGC	0.00	0.00	0.00	0.00	0.00
GATCAAATTTTATTGGCNNN	0.23	0.00	0.00	0.00	0.00
GATCAAATTTTATTGGGCAG	0.00	0.00	0.00	0.00	0.34
GATCAAATTTTATTGGGGGA	0.00	0.00	0.00	0.00	0.34
GATCAAATTTTATTGGGGGC	0.00	0.00	0.00	0.00	0.34
GATCAAATTTTATTGGNGGA	0.00	0.00	0.00	0.00	0.34
GATCAAATTTTATTGGNGNN	0.23	0.00	0.00	0.00	0.00

GATCAAATTTTATTGGNNNN	0.69	0.00	0.00	0.00	0.00
GATCAAATTTTATTGGTGGA	0.23	0.00	0.00	0.00	0.34
GATCAAATTTTATTGTAGGA	0.00	0.33	0.00	0.00	0.00
GATCAAATTTTCTAAGCCCA	0.00	0.33	0.00	0.00	0.00
GATCAAATTTTCTCCGCCCC	0.23	0.00	0.00	0.00	0.00
GATCAAATTTTCTGATACAC	0.00	0.00	0.00	0.68	0.00
GATCAAATTTTCTGATACGA	0.00	0.98	0.00	0.00	0.00
GATCAAATTTTCTGATACGC	5.04	11.06	20.05	19.69	11.02
GATCAAATTTTCTGATATGC	0.00	0.00	0.00	0.34	0.00
GATCAAATTTTCTGATNCGC	0.00	0.00	0.00	0.00	0.34
GATCAAATTTTCTGGCGCAC	0.00	0.00	0.00	0.00	0.69
GATCAAATTTTCTGGTACGC	0.00	0.00	0.00	0.00	0.34
GATCAAATTTTCTGGTGAAA	0.00	0.00	0.00	0.00	0.34
GATCAAATTTTCTGGTGAGA	0.00	0.33	0.29	0.00	0.00
GATCAAATTTTCTGGTGAGC	0.00	0.00	0.29	0.00	0.00
GATCAAATTTTCTGGTGCAA	0.69	0.33	0.57	1.02	0.34
GATCAAATTTTCTGGTGCAC	5.72	12.04	12.89	23.42	15.50
GATCAAATTTTCTGGTGCAN	0.23	0.00	0.00	0.00	0.00
GATCAAATTTTCTGGTGCCC	0.00	0.00	0.29	0.00	0.00
GATCAAATTTTCTGGTGCGA	0.00	0.00	0.29	0.00	0.00
GATCAAATTTTCTGGTGCGC	2.75	9.76	13.75	18.67	11.02
GATCAAATTTTCTGGTGCGG	0.00	0.00	0.00	0.00	0.34
GATCAAATTTTCTGGTGCNN	0.23	0.00	0.00	0.00	0.00
GATCAAATTTTCTGGTTAGC	0.00	0.00	0.29	0.00	0.00
GATCAAATTTTCTGGTTCAC	0.00	0.00	0.00	0.00	0.34
GATCAAATTTTCTGGTTCGC	0.00	0.00	0.00	0.34	0.00
GATCAAATTTTCTTGGAGTA	0.00	0.33	0.00	0.00	0.00
GATCAAATTTTGAACTCAAA	0.00	0.00	0.29	0.00	0.00
GATCAAATTTTGACGAGGCG	0.23	0.00	0.00	0.00	0.00
GATCAAATTTTGCAAATTCT	0.00	0.00	0.00	0.00	0.34
GATCAAATTTTGCCCATTTT	0.00	0.00	0.00	0.00	0.00
GATCAAATTTTGGAGACTAT	0.00	0.00	0.00	0.00	0.34
GATCAAATTTTGGCACGCCA	0.00	0.00	0.29	0.00	0.00
GATCAAATTTTGGCGAGCAC	0.00	0.00	0.00	0.00	0.00
GATCAAATTTTGGCGAGGAG	0.00	0.65	0.00	0.34	0.00
GATCAAATTTTGGCGAGGCG	20.83	28.95	13.75	28.17	18.25
GATCAAATTTTGGCGAGGCT	0.00	0.00	0.00	0.34	0.00

GATCAAATTTTGGCGAGGNN	0.23	0.00	0.00	0.00	0.00
GATCAAATTTTGGCGCGGTG	0.00	0.00	0.29	0.00	0.00
GATCAAATTTTGGCGGGGCG	0.00	0.00	0.00	0.00	0.00
GATCAAATTTTGTAATCCGA	0.00	0.00	0.00	0.34	0.00
GATCAAATTTTGTAATCCTC	0.00	0.00	0.00	0.34	0.00
GATCAAATTTTGTATGCATG	0.00	0.00	0.00	0.00	0.00
GATCAAATTTTGTATGCTTT	0.00	0.00	0.00	0.00	0.00
GATCAAATTTTGTCAGCGGA	0.00	0.00	0.00	0.00	0.00
GATCAAATTTTGTCGAGGCG	0.00	0.00	0.29	0.00	0.00
GATCAAATTTTGTCTTGCCC	0.00	0.00	0.29	0.00	0.00
GATCAAATTTTGTTGCGAAA	0.00	0.00	0.57	0.00	0.00
GATCAAATTTTGTTGCGGAA	0.46	0.00	0.00	0.00	0.00
GATCAAATTTTGTTGCGGAC	2.06	1.63	0.57	0.00	1.38
GATCAAATTTTGTTGCGGGC	0.00	0.00	0.00	0.34	0.00
GATCAAATTTTGTTGGAGGA	0.00	0.00	0.00	0.00	0.34
GATCAAATTTTTAAAAAAAA	0.00	0.00	0.00	0.00	0.34
GATCAAATTTTTAAAGCCCA	0.00	0.00	0.00	0.00	0.00
GATCAAATTTTTGGAGACTA	0.00	0.33	0.00	0.00	0.00
GATCAAATTTTTTGGTGCAC	0.23	0.00	0.00	0.00	0.00
GATCAAATTTTTTTGGAGGA	0.69	0.00	0.86	0.68	0.69
GATCAAATTTTTTTTTTTTT	0.00	0.00	0.29	0.00	0.00
GATCAACAAAAAAAAAAAAA	89.96	31.56	49.25	48.20	78.87
GATCAACAAAAAAAAAAAAC	0.46	0.33	0.00	0.34	1.03
GATCAACAAAAAAAAAAAAG	0.00	0.00	0.00	0.34	0.00
GATCAACAAAAAAAAAAAAT	6.41	1.63	5.15	3.05	4.82
GATCAACAAAAAAAAAAACA	0.23	0.00	0.00	0.68	1.38
GATCAACAAAAAAAAAAACG	0.00	0.00	0.00	0.00	0.34
GATCAACAAAAAAAAAAACT	0.00	0.33	0.00	0.00	0.00
GATCAACAAAAAAAAAAAGA	0.23	0.00	0.00	0.00	0.00
GATCAACAAAAAAAAAAAGG	0.23	0.00	0.00	0.00	0.00
GATCAACAAAAAAAAAAANN	0.69	0.00	0.00	0.00	0.00
GATCAACAAAAAAAAAAATA	0.00	0.00	0.00	0.34	0.00
GATCAACAAAAAAAAAAATC	0.46	0.33	0.57	0.34	0.69
GATCAACAAAAAAAAAACAA	0.00	0.33	0.00	0.00	0.34
GATCAACAAAAAAAAAACAC	0.00	0.00	0.00	0.00	0.34
GATCAACAAAAAAAAAACAT	0.00	0.00	0.00	0.00	0.34
GATCAACAAAAAAAAAACNN	0.23	0.00	0.00	0.00	0.00

GATCAACAAAAAAAAAACTC	0.00	0.00	0.00	0.34	0.00
GATCAACAAAAAAAAAANNN	0.00	0.00	0.00	0.00	0.00
GATCAACAAAAAAAAACAAA	0.23	0.00	0.00	0.00	1.03
GATCAACAAAAAAAAACNNN	0.46	0.00	0.00	0.00	0.00
GATCAACAAAAAAAAAGAAA	0.23	0.33	0.00	0.00	0.00
GATCAACAAAAAAAAAGCAA	0.23	0.00	0.00	0.00	0.00
GATCAACAAAAAAAACACAA	0.00	0.00	0.00	0.00	0.34
GATCAACAAAAAAAACNNNN	0.23	0.00	0.00	0.00	0.00
GATCAACAAAAAAACAAAAA	0.23	0.00	0.29	0.00	0.00
GATCAACAAAAAAACAAAAT	0.00	0.00	0.00	0.00	0.34
GATCAACAAAAAAACAATAT	0.69	0.33	0.29	0.00	0.00
GATCAACAAAAAAACACAAN	0.00	0.00	0.00	0.00	0.00
GATCAACAAAAAAACACACA	0.00	0.33	0.00	0.00	0.00
GATCAACAAAAAAACACAGA	0.23	0.00	0.00	0.00	0.00
GATCAACAAAAAAACACCAG	0.00	0.00	0.00	0.00	0.00
GATCAACAAAAAAAGTAAAA	0.00	0.00	0.00	0.00	0.00
GATCAACAAAAAACAAAAAA	0.00	0.00	0.29	0.00	0.00
GATCAACAAAAAACAAAACA	0.00	0.00	0.00	0.00	0.00
GATCAACAAAAAATAAAAAA	0.23	0.00	0.00	0.00	0.00
GATCAACAAAAAATAAACAA	0.00	0.00	0.00	0.00	0.34
GATCAACAAAAACAAAAACC	0.00	0.00	0.00	0.00	0.34
GATCAACAAAAACAAAACAA	0.00	0.00	0.00	0.00	0.34
GATCAACAAAAACACAAATT	0.00	0.00	0.00	0.00	0.34
GATCAACAAAAACCAAAAAA	0.00	0.33	0.00	0.00	0.00
GATCAACAAAAACCAAAACA	0.23	0.00	0.00	0.00	0.00
GATCAACAAAAACCAACCCA	0.00	0.33	0.00	0.00	0.00
GATCAACAAAAACGAAAATC	0.69	0.00	0.00	0.00	0.00
GATCAACAAAAAGAAAAAAA	0.00	0.00	0.00	0.00	0.00
GATCAACAAAAAGAATAAGA	0.00	0.00	0.57	0.00	0.00
GATCAACAAAAAGAATAAGC	0.00	0.00	0.29	0.00	0.00
GATCAACAAAAAGCAAGAAT	0.46	0.00	0.00	0.00	0.00
GATCAACAAAAAGGACCAGC	0.00	0.98	0.00	0.34	1.03
GATCAACAAAAAGTGTCCAG	0.23	0.00	0.00	0.00	0.00
GATCAACAAAAATAAAAAAA	0.00	0.00	0.00	0.00	0.00
GATCAACAAAAATCTAAAAA	0.00	0.00	0.29	0.00	0.00
GATCAACAAAACAAAAAAAA	0.00	0.00	0.00	0.34	0.00
GATCAACAAAACAAACACAC	0.00	0.00	0.29	0.00	0.00

GATCAACAAAACAAAGAAAC	0.00	0.00	0.00	0.34	0.00
GATCAACAAAACAACACCCA	0.00	0.00	0.00	0.00	0.00
GATCAACAAAACAAGGACAT	0.00	0.00	0.00	0.00	0.69
GATCAACAAAACACACCCCA	0.00	0.00	0.00	0.00	0.34
GATCAACAAAACAGAACAAA	0.23	0.00	0.00	0.00	0.00
GATCAACAAAACAGTCTTGA	0.00	0.33	0.00	0.00	0.00
GATCAACAAAACATAAGCAA	0.00	0.00	0.00	0.00	0.69
GATCAACAAAACCACGATCC	0.00	0.33	0.00	0.00	0.00
GATCAACAAAACCAGCTCCT	0.00	0.33	0.00	0.00	0.00
GATCAACAAAACCCCGGAGC	0.00	0.00	0.29	0.00	0.00
GATCAACAAAACGAAACACA	0.00	0.00	0.00	0.00	0.34
GATCAACAAAACGAATGGTC	0.00	0.33	0.00	0.00	0.00
GATCAACAAAACTATACAGT	0.00	0.00	0.00	0.00	0.00
GATCAACAAAAGAAAAAAAA	0.23	0.00	0.00	0.34	0.34
GATCAACAAAAGCAAAAAAA	0.00	0.00	0.29	0.00	0.00
GATCAACAAAAGCAAGACGG	0.00	0.00	0.00	0.34	0.00
GATCAACAAAAGCATTACAC	0.23	0.00	0.29	0.00	0.00
GATCAACAAAAGCCTCCTGT	0.23	0.00	0.00	0.00	0.00
GATCAACAAAAGCGAGCAGG	0.00	0.00	0.00	0.00	0.00
GATCAACAAAAGGAACAGAG	0.00	0.00	0.00	0.00	0.34
GATCAACAAAAGGAGGCGCG	0.00	0.33	0.00	0.34	0.34
GATCAACAAAAGGCGAGCCG	0.00	0.00	0.00	0.00	0.34
GATCAACAAAAGGCGTGCCG	0.00	0.00	0.00	0.00	0.00
GATCAACAAAAGGCTTCAGG	0.00	0.33	0.00	0.00	0.00
GATCAACAAAAGGTGCATGC	4.12	5.21	2.58	3.05	5.51
GATCAACAAAAGGTTCATGC	0.23	0.00	0.00	0.00	0.00
GATCAACAAAAGTCAGGTCA	0.00	0.33	0.00	0.00	0.00
GATCAACAAAATAGCAAAAA	0.00	0.00	0.00	0.00	0.34
GATCAACAAAATATGTTGGG	0.00	0.00	0.29	0.00	0.00
GATCAACAAAATATTTCAAA	0.00	0.00	0.00	0.00	0.00
GATCAACAAAATCAACACGG	0.00	0.33	0.00	0.00	0.34
GATCAACAAAATCAACAGGC	0.00	0.00	0.00	0.00	0.00
GATCAACAAAATCAAGCAGC	0.00	0.00	0.00	0.00	0.00
GATCAACAAAATCCACCTCA	0.00	0.00	0.00	0.00	1.03
GATCAACAAAATCCCAATGA	0.00	0.00	0.29	0.00	0.00
GATCAACAAAATGAAAAAAA	0.00	0.00	0.00	0.00	0.34
GATCAACAAAATGAGGCGTG	0.00	0.00	0.29	0.00	0.00

GATCAACAAAATGATGAGCT	0.00	0.33	0.57	0.00	0.00
GATCAACAAAATTAAAAAAA	0.92	0.00	0.00	0.00	0.00
GATCAACAAAATTAAAAGAA	0.92	0.33	0.00	0.00	0.00
GATCAACAAACAAAAAAAAA	0.46	0.33	0.29	2.04	0.34
GATCAACAAACAAAAAACAA	0.00	0.00	0.00	0.00	0.00
GATCAACAAACAAAAAGTTC	0.00	0.33	0.29	0.00	0.00
GATCAACAAACAAAAAGTTT	0.00	0.00	0.29	0.00	0.00
GATCAACAAACAAAACCAAA	0.00	0.00	0.29	0.00	0.00
GATCAACAAACAAACAAAAA	0.00	0.00	0.00	0.00	0.34
GATCAACAAACAAACAAACA	0.00	0.00	0.57	0.00	0.00
GATCAACAAACAACAAACAA	0.00	0.00	0.00	0.00	0.34
GATCAACAAACAACATATAA	0.00	0.65	0.00	0.00	0.00
GATCAACAAACAACCACATG	0.00	0.00	0.00	0.00	0.34
GATCAACAAACAACGACTGT	0.00	0.00	0.00	0.00	0.00
GATCAACAAACAACGCCACA	0.00	0.00	0.29	0.00	0.00
GATCAACAAACAAGAACAAG	0.00	0.00	0.00	0.00	0.69
GATCAACAAACACAAACAAA	0.00	0.00	0.00	0.00	0.34
GATCAACAAACACAACAAAT	0.23	0.00	0.00	0.00	0.00
GATCAACAAACACAACAATA	0.00	0.00	0.00	0.00	1.72
GATCAACAAACACAACACGT	0.00	0.00	0.29	0.00	0.00
GATCAACAAACACACAAACA	0.00	0.00	0.29	0.00	0.69
GATCAACAAACACACACATA	0.00	0.00	0.00	0.00	0.69
GATCAACAAACACAGACACA	0.00	0.00	0.00	0.00	0.34
GATCAACAAACACATTGATG	0.00	0.00	0.00	0.34	0.00
GATCAACAAACACCAACAAT	0.00	0.00	0.29	0.00	0.00
GATCAACAAACACCACTACC	0.23	0.00	0.00	0.00	0.00
GATCAACAAACACTGGAAGT	0.00	0.00	0.00	0.68	0.00
GATCAACAAACAGAGGTTGA	0.00	0.33	0.00	0.00	0.00
GATCAACAAACATTGTAAAA	0.00	0.00	0.00	0.00	0.69
GATCAACAAACATTGTATTT	0.00	0.00	0.29	0.00	0.00
GATCAACAAACCAAAAAACA	0.00	0.00	0.29	0.00	0.00
GATCAACAAACCAGGCTTGA	0.00	0.00	0.00	0.34	0.00
GATCAACAAACCAGTCTTGA	0.00	0.33	0.00	0.00	0.00
GATCAACAAACCCCCCTTAA	0.00	0.00	0.00	0.68	0.34
GATCAACAAACCCGCCTTGA	0.00	0.00	0.57	0.00	0.00
GATCAACAAACCCTCCCTAA	0.46	0.00	0.00	0.00	0.00
GATCAACAAACCCTCCTTAA	0.69	0.33	0.00	0.68	0.00

GATCAACAAACCCTGTTCTC	0.00	0.00	0.00	0.00	0.34
GATCAACAAACCCTTAGCCC	0.00	0.00	0.29	0.00	0.00
GATCAACAAACCCTTCGACA	0.00	0.65	0.00	0.00	0.00
GATCAACAAACCGATGCGTG	0.00	0.00	0.00	0.00	0.00
GATCAACAAACCGGTTGGAC	0.00	0.00	0.00	0.00	0.34
GATCAACAAACCTACTAGGC	0.00	0.00	0.00	0.34	0.00
GATCAACAAACGAAAGAAAA	0.00	0.00	0.00	0.00	0.00
GATCAACAAACGAACCAACA	0.00	0.00	0.00	0.00	0.00
GATCAACAAACGAAGGAGGT	0.00	0.00	0.00	0.00	0.00
GATCAACAAACGAGTCCTTG	0.00	0.00	0.00	0.00	0.00
GATCAACAAACGATAACACG	0.00	0.00	0.29	0.00	0.00
GATCAACAAACGCTCCGCGG	0.23	0.00	0.00	0.00	0.00
GATCAACAAACGCTGATGCG	0.00	0.00	0.57	0.00	0.00
GATCAACAAACGGCTGGATT	0.00	0.00	0.00	0.00	0.00
GATCAACAAACTAAAAAAAA	0.00	0.00	0.00	0.34	0.00
GATCAACAAACTAATCCTGC	0.00	0.00	0.00	0.00	0.34
GATCAACAAACTCACACGAC	0.00	0.00	0.00	0.00	0.34
GATCAACAAACTCACGCCGT	0.00	0.00	0.29	0.00	0.00
GATCAACAAACTCCTCGCGG	0.00	0.00	0.00	0.00	0.34
GATCAACAAACTCGGGCTGT	0.00	0.00	0.29	0.00	0.00
GATCAACAAACTGCTGCAGA	0.00	0.00	0.00	0.00	0.00
GATCAACAAACTGCTGCTGC	0.00	0.00	0.00	0.00	0.00
GATCAACAAACTGCTGTAGA	0.00	0.00	0.29	0.00	0.69
GATCAACAAACTGCTGTGCT	0.23	0.00	0.00	0.00	0.00
GATCAACAAACTGGTGGCCA	0.00	0.00	0.00	0.00	0.34
GATCAACAAACTGTGTCTCC	0.00	0.00	0.00	0.00	0.00
GATCAACAAACTTGTTCACA	0.00	0.00	0.00	0.00	0.00
GATCAACAAAGAAAAAAAAA	0.69	0.00	0.00	0.00	0.00
GATCAACAAAGAAAGGCCAA	0.00	0.00	0.00	0.00	0.00
GATCAACAAAGAAGGCCTTG	0.00	0.00	0.29	0.00	0.69
GATCAACAAAGAAGTGAAAA	0.00	0.00	0.29	0.00	0.00
GATCAACAAAGACACAGGCA	0.46	0.65	0.00	0.00	0.00
GATCAACAAAGACATCACCA	0.23	1.30	0.57	0.00	1.38
GATCAACAAAGACATCCAAC	0.23	0.00	0.00	0.00	0.00
GATCAACAAAGACCATAACA	0.00	0.00	0.00	0.00	0.69
GATCAACAAAGACCGGTACC	0.00	0.00	0.00	0.00	0.34
GATCAACAAAGACCGGTTTG	0.00	0.65	0.29	0.34	0.00

GATCAACAAAGACGAAGATG	0.00	0.00	0.00	0.00	0.00
GATCAACAAAGACTAAGAGA	0.00	0.00	0.00	0.00	0.34
GATCAACAAAGACTGGTGCG	0.00	0.33	0.00	0.00	0.00
GATCAACAAAGAGACGCGTG	0.23	0.00	0.00	0.00	0.00
GATCAACAAAGAGCGCGCGA	0.23	0.00	0.00	0.00	0.00
GATCAACAAAGATGGTTTGG	0.00	0.00	0.00	0.00	0.69
GATCAACAAAGCAACGCGAG	0.00	0.00	0.00	0.00	0.00
GATCAACAAAGCAACGTGTG	0.00	0.00	0.00	0.00	0.00
GATCAACAAAGCAACTACTC	0.00	0.00	0.00	0.00	0.00
GATCAACAAAGCATCCNNNN	0.23	0.00	0.00	0.00	0.00
GATCAACAAAGCATGCACGC	0.00	0.00	0.00	0.00	0.34
GATCAACAAAGCCACATTTT	0.00	0.00	0.00	0.00	0.34
GATCAACAAAGCCCGCATGG	0.00	0.00	0.29	0.00	0.00
GATCAACAAAGCCCTCCGGA	0.69	0.00	0.00	0.68	0.00
GATCAACAAAGCCGGAGGAG	0.00	0.00	0.00	0.34	0.00
GATCAACAAAGCGAAGGCGC	0.00	0.00	0.00	0.00	0.00
GATCAACAAAGCGACATGTG	0.00	0.33	0.00	0.00	0.00
GATCAACAAAGCGACGAGTG	0.00	0.00	0.00	0.34	0.00
GATCAACAAAGCGACGCGCG	0.00	0.00	0.00	0.00	0.00
GATCAACAAAGCGACGCGTG	0.69	0.00	0.00	0.34	0.34
GATCAACAAAGCGACGGGGG	0.23	0.00	0.00	0.00	0.00
GATCAACAAAGCGACGGGTG	0.69	0.00	0.29	0.34	0.69
GATCAACAAAGCGACGNGTG	0.00	0.00	0.00	0.00	0.69
GATCAACAAAGCGACGTGAG	0.00	0.00	0.00	0.00	0.00
GATCAACAAAGCGACGTGCG	0.23	0.00	0.00	0.00	0.69
GATCAACAAAGCGACGTGGC	0.00	0.00	0.00	0.00	0.34
GATCAACAAAGCGACGTGGG	0.69	0.00	0.00	0.00	0.34
GATCAACAAAGCGACGTGNG	0.00	0.00	0.00	0.68	0.00
GATCAACAAAGCGACGTGNN	0.69	0.00	0.00	0.00	0.00
GATCAACAAAGCGACGTGTA	0.00	0.00	0.29	0.00	0.00
GATCAACAAAGCGACGTGTG	83.09	74.17	99.94	83.84	98.15
GATCAACAAAGCGACGTGTN	0.23	0.00	0.00	0.00	0.00
GATCAACAAAGCGACGTGTT	0.00	0.00	0.29	1.02	0.00
GATCAACAAAGCGACGTNNN	0.23	0.00	0.00	0.00	0.34
GATCAACAAAGCGACGTTCG	0.00	0.00	0.00	0.00	0.00
GATCAACAAAGCGACGTTTG	0.23	0.00	0.00	0.00	0.00
GATCAACAAAGCGAGGTGTG	0.00	0.00	0.00	0.00	0.00

GATCAACAAAGCGCACATGG	0.00	0.00	0.00	0.34	0.00
GATCAACAAAGCGCCGTGTG	0.46	0.00	0.00	0.00	0.00
GATCAACAAAGCGCCTTGTG	0.00	0.00	0.00	0.00	0.00
GATCAACAAAGCGCTATTTG	0.00	0.33	0.00	0.00	0.00
GATCAACAAAGCGGAGGCGG	0.00	0.00	0.29	0.34	0.34
GATCAACAAAGCGGAGGGCA	0.00	0.00	0.57	0.00	0.00
GATCAACAAAGCGGCGTGTG	0.00	0.00	0.57	0.00	0.34
GATCAACAAAGGACCCTTGC	0.00	0.00	0.00	0.00	0.00
GATCAACAAAGGACGGCCGG	0.00	0.00	0.00	0.00	0.34
GATCAACAAAGGACGGGCCG	0.00	0.00	0.00	0.00	0.00
GATCAACAAAGGAGAGTCGC	0.00	0.00	0.00	0.00	0.00
GATCAACAAAGGATGAAGTT	0.00	0.00	0.00	0.00	0.00
GATCAACAAAGGCATCAAGN	0.00	0.00	0.00	0.00	0.00
GATCAACAAAGGCCGCCTTG	0.23	0.00	0.00	0.00	0.00
GATCAACAAAGGCGGGATAA	0.00	0.33	0.00	0.00	0.00
GATCAACAAAGGGAAAAAAA	0.00	0.00	0.00	0.00	0.69
GATCAACAAAGGGCTTGACG	0.00	0.00	0.00	0.00	0.34
GATCAACAAAGGGGAGCGGG	0.00	0.00	0.00	0.00	0.00
GATCAACAAAGGTGCTGCCT	0.00	0.33	0.00	0.00	0.00
GATCAACAAAGTCCTGGCCG	0.23	0.33	0.00	0.34	0.00
GATCAACAAAGTCCTGGCGG	0.00	0.00	0.29	0.00	0.00
GATCAACAAAGTCTTTTTCG	0.00	0.00	0.00	0.00	0.34
GATCAACAAAGTGAACTTCC	0.00	0.33	0.00	0.00	0.00
GATCAACAAAGTGGATGCGC	0.00	0.00	0.29	0.00	0.00
GATCAACAAAGTTGCACTCG	0.00	0.00	0.00	0.34	0.00
GATCAACAAAGTTGGAAAAG	0.00	0.00	0.29	0.00	0.00
GATCAACAAAGTTGGGATGG	0.00	0.00	0.00	0.00	0.00
GATCAACAAAGTTGTGCAAG	0.00	0.33	0.00	0.00	0.00
GATCAACAAATAAAAAAAAA	1.37	0.65	0.29	0.00	0.00
GATCAACAAATAACATCAAC	0.00	0.00	0.00	0.00	0.34
GATCAACAAATACAAAAAAA	1.60	0.00	0.00	0.00	0.00
GATCAACAAATACAAAGGCC	0.00	0.00	0.00	0.00	1.03
GATCAACAAATATATTTGTG	0.00	0.33	0.00	0.00	0.00
GATCAACAAATATGGTAATA	0.00	0.00	0.00	0.00	0.69
GATCAACAAATATTGCATTT	0.00	0.98	0.00	0.68	0.34
GATCAACAAATATTGTCAGA	0.00	0.00	0.86	0.00	0.00
GATCAACAAATATTTGCCTC	0.00	0.00	0.00	0.00	0.69

GATCAACAAATCAAACGAGA	0.00	0.00	0.29	0.00	0.00
GATCAACAAATCAGATGGGC	0.00	0.00	0.00	0.68	0.00
GATCAACAAATCCAACTGAA	1.60	0.00	0.00	1.36	0.00
GATCAACAAATCCAAGCTGA	0.00	0.00	0.29	0.34	0.34
GATCAACAAATCCCGTGTTC	0.23	0.00	0.00	0.34	0.00
GATCAACAAATCGACGTGAG	0.00	0.00	0.00	0.00	0.34
GATCAACAAATGAACCCTGC	0.00	0.00	0.29	0.00	0.00
GATCAACAAATGAACTGAAC	0.00	0.00	0.00	0.00	0.00
GATCAACAAATGAGAAGAAG	0.00	0.00	0.29	0.00	0.00
GATCAACAAATGAGGGTGTG	0.00	0.00	0.29	0.00	0.00
GATCAACAAATGCATCCCGG	0.00	0.00	0.00	0.00	0.00
GATCAACAAATGGAGACCGC	0.00	0.00	0.00	0.00	0.00
GATCAACAAATGGCCGATGG	0.00	0.00	0.00	0.00	0.34
GATCAACAAATGGCTTGAGC	0.23	0.00	0.00	0.00	0.00
GATCAACAAATGGTGCCTGA	0.00	0.00	0.00	0.00	0.00
GATCAACAAATGTTAAAGGG	0.00	0.00	0.29	0.00	0.00
GATCAACAAATTCACGCGCC	0.46	0.00	0.00	0.00	0.00
GATCAACAAATTCATGCGCC	0.46	0.00	0.00	0.00	0.00
GATCAACAAATTCATTGTTT	0.00	0.65	0.00	0.00	0.00
GATCAACAAATTGAAGTGTG	0.00	0.00	0.29	0.00	0.00
GATCAACAAATTGGCCAACA	0.23	0.00	0.00	0.68	0.00
GATCAACAAATTGGGTGTCT	0.00	0.00	0.29	0.00	0.00
GATCAACAAATTTTCTGGTG	0.00	0.00	0.00	0.00	0.00
GATCAACAACAAAAAAAAAA	1.14	0.00	0.57	1.70	2.07
GATCAACAACAAAAAAAACA	0.00	0.00	0.00	0.34	0.34
GATCAACAACAAAAACAACC	0.00	0.00	0.00	0.00	0.00
GATCAACAACAAAACAAAAC	0.00	0.00	0.00	0.00	1.72
GATCAACAACAAAACAAANA	0.00	0.00	0.00	0.00	0.34
GATCAACAACAAAACCACAA	0.00	0.00	0.00	0.00	1.03
GATCAACAACAAAATCAACA	0.00	0.33	0.00	0.00	0.00
GATCAACAACAAAATCTTGC	0.23	0.00	0.00	0.00	0.00
GATCAACAACAAACAAAACA	0.00	0.00	0.00	0.00	0.34
GATCAACAACAAACACAAAA	0.00	0.00	0.00	0.00	0.00
GATCAACAACAAACACTGCA	0.00	0.33	0.00	0.00	0.00
GATCAACAACAAACAGAACA	0.00	0.00	0.00	0.00	0.34
GATCAACAACAAACCAAACA	0.00	0.00	0.00	0.34	1.03
GATCAACAACAAACCAACAG	0.00	0.00	0.00	0.00	0.34

GATCAACAACAAACCACACA	0.00	0.00	0.29	0.00	0.69
GATCAACAACAAACCGAACA	0.00	0.00	0.00	0.00	0.34
GATCAACAACAAAGAAAGGG	0.00	0.00	0.00	0.00	0.34
GATCAACAACAAATTTGTGT	0.23	0.00	0.00	0.00	0.00
GATCAACAACAACAAAACCA	0.00	0.00	0.29	0.00	0.00
GATCAACAACAACAAAATAT	0.00	0.00	0.29	0.00	0.00
GATCAACAACAACAACAAAA	0.00	0.00	0.00	0.00	0.34
GATCAACAACAACAACAACA	0.00	0.00	0.57	0.00	1.03
GATCAACAACAACAACAATC	0.00	0.00	0.29	0.00	0.00
GATCAACAACAACAACACGA	0.00	0.00	0.00	0.00	0.34
GATCAACAACAACAACACTA	0.00	0.00	0.00	0.00	1.03
GATCAACAACAACAACAGAA	0.00	0.00	0.00	0.00	1.38
GATCAACAACAACAACAGAC	0.00	0.00	0.29	0.00	0.00
GATCAACAACAACAACCAAC	0.00	0.00	0.00	0.00	0.34
GATCAACAACAACAACCGCA	0.00	0.00	0.00	0.00	0.34
GATCAACAACAACAACGAGA	0.00	0.00	0.00	0.00	0.34
GATCAACAACAACAACGGTC	0.00	0.00	0.00	0.00	0.34
GATCAACAACAACAACNAAC	0.00	0.00	0.00	0.00	0.34
GATCAACAACAACAAGAGGA	0.00	0.00	0.29	0.00	0.00
GATCAACAACAACACACAAC	0.00	0.00	0.00	0.00	0.69
GATCAACAACAACACCACCA	0.00	0.00	0.57	0.00	0.00
GATCAACAACAACACCCGCA	0.00	0.00	0.29	0.00	0.00
GATCAACAACAACAGAAGAT	0.00	0.00	0.57	0.00	0.00
GATCAACAACAACAGATGAT	0.00	0.00	0.00	0.00	0.69
GATCAACAACAACAGCAACA	0.00	0.00	0.00	0.00	0.34
GATCAACAACAACATACGCA	0.00	0.00	0.29	0.00	0.00
GATCAACAACAACCACTTCG	0.00	0.33	0.00	0.34	0.00
GATCAACAACAACCATCATA	0.00	0.00	0.00	0.00	0.34
GATCAACAACAACCGGCCCA	0.00	0.00	0.29	0.00	0.00
GATCAACAACAACGACCGCA	0.00	0.00	0.57	0.00	0.00
GATCAACAACAACGAGGATC	0.00	0.00	0.00	0.00	0.34
GATCAACAACAACGCACACA	0.00	0.00	0.29	0.00	0.00
GATCAACAACAACGGCAGCG	0.00	0.00	0.00	0.00	0.00
GATCAACAACAACGGCATCG	0.23	0.33	0.29	0.00	0.34
GATCAACAACAACTACTCGG	0.00	0.65	2.58	0.34	1.38
GATCAACAACAACTCAATGG	0.00	0.00	0.29	0.00	0.00
GATCAACAACAACTGCTCGG	0.00	0.00	0.00	0.00	0.00

GATCAACAACAAGAACAAAA	0.00	0.00	0.00	0.00	0.34
GATCAACAACAAGAACTCCT	0.00	0.00	0.00	0.34	0.00
GATCAACAACAAGAATAAGA	0.00	0.00	0.57	0.00	1.38
GATCAACAACAAGAATTGGC	0.00	0.00	0.00	0.00	0.00
GATCAACAACAAGAGGAGGA	0.00	0.00	0.57	0.00	0.00
GATCAACAACAAGATGCAGT	0.00	0.00	0.00	0.00	0.00
GATCAACAACAAGCAAGGCC	0.00	0.00	0.00	0.00	0.00
GATCAACAACAAGCCCTACA	0.00	0.00	0.00	0.34	0.00
GATCAACAACAAGCCGCCCT	0.00	0.00	0.00	0.00	0.00
GATCAACAACAAGCGCAGAA	0.00	0.00	0.00	0.00	0.34
GATCAACAACAAGGCCAAAC	0.00	0.00	0.29	0.00	0.00
GATCAACAACAAGTCAACAC	0.00	0.00	0.00	0.00	0.34
GATCAACAACAATACCAACA	0.00	0.00	0.29	0.00	0.00
GATCAACAACAATATTATCA	0.00	0.00	0.86	0.00	0.00
GATCAACAACAATCAAGCGC	0.23	0.00	0.00	0.68	0.00
GATCAACAACAATCAATCGC	0.00	0.00	0.00	0.00	0.00
GATCAACAACAATCCTCGTC	0.00	0.00	0.00	0.00	0.00
GATCAACAACAATGCTGTTT	0.00	0.00	0.00	0.00	0.34
GATCAACAACAATGTCCTTC	0.00	0.65	0.00	0.00	0.00
GATCAACAACAATTGGGACG	0.23	0.00	0.29	0.34	0.69
GATCAACAACACAAAAAAAC	0.23	0.00	0.00	0.00	0.00
GATCAACAACACAAAACAAA	0.00	0.00	0.00	0.00	1.38
GATCAACAACACAAAACAAC	0.00	0.00	0.29	0.34	0.00
GATCAACAACACAAACAACA	0.00	0.00	0.00	0.00	1.03
GATCAACAACACAAACAACC	0.00	0.00	0.29	0.00	0.00
GATCAACAACACAAACGGCG	0.00	0.00	0.00	0.00	1.03
GATCAACAACACAACACAAC	0.00	0.00	0.00	0.00	2.07
GATCAACAACACAACACAAG	0.00	0.00	0.00	0.00	0.69
GATCAACAACACAACACACA	0.00	0.00	0.29	0.00	0.00
GATCAACAACACAACACACC	0.00	0.00	0.00	0.00	0.34
GATCAACAACACAAGACTAA	0.00	0.00	0.00	0.00	0.34
GATCAACAACACAAGGAACA	0.00	0.00	0.00	0.00	0.34
GATCAACAACACAAGTCGAA	0.00	0.00	0.00	0.00	0.34
GATCAACAACACACAACACA	0.00	0.00	0.00	0.00	0.69
GATCAACAACACACACCACC	0.00	0.00	0.00	0.00	0.34
GATCAACAACACACACTGAC	0.00	0.00	0.00	0.00	0.34
GATCAACAACACACCACAAC	0.00	0.00	0.00	0.00	2.41

GATCAACAACACACCACAAN	0.00	0.00	0.00	0.00	0.00
GATCAACAACACACCGAACA	0.00	0.00	0.00	0.00	0.69
GATCAACAACACACTCTTGG	0.00	0.00	0.00	0.00	0.00
GATCAACAACACATCAAAGA	0.00	0.00	0.00	0.00	0.34
GATCAACAACACATCCACGC	0.00	0.00	0.00	0.00	0.34
GATCAACAACACCAAAAAAT	0.00	0.33	0.00	0.00	0.00
GATCAACAACACCAACAACA	0.00	0.00	0.57	0.00	0.00
GATCAACAACACCACAACAC	0.00	0.00	0.00	0.00	0.34
GATCAACAACACCACCAACA	0.00	0.00	0.00	0.00	0.34
GATCAACAACACCACGCTGA	0.00	0.33	0.00	0.00	0.00
GATCAACAACACCACTCAAC	0.00	0.00	0.00	0.00	0.34
GATCAACAACACCAGAAGAA	0.00	0.00	0.57	0.00	0.34
GATCAACAACACCAGCAACC	0.23	0.00	0.00	0.00	0.00
GATCAACAACACCATCAAGA	0.00	0.33	0.00	0.68	0.00
GATCAACAACACCATGCCAC	0.00	0.00	0.00	0.00	0.00
GATCAACAACACCCAACACA	0.00	0.00	0.00	0.00	0.00
GATCAACAACACCCACACAC	0.00	0.00	0.00	0.00	0.34
GATCAACAACACCGAATACA	0.00	0.33	0.00	0.00	0.00
GATCAACAACACCGCCCGCG	0.00	0.00	0.00	0.00	0.34
GATCAACAACACCTGCCCAC	0.00	0.33	0.00	0.00	0.00
GATCAACAACACCTTCGGTC	0.00	0.33	0.00	0.00	0.00
GATCAACAACACGACACAAA	0.00	0.00	0.29	0.00	0.00
GATCAACAACACGACCAGAC	0.00	0.00	0.00	0.00	0.34
GATCAACAACACGAGCAACA	0.00	0.00	0.29	0.00	0.00
GATCAACAACACGCTCGAGA	0.00	0.33	0.00	0.00	0.00
GATCAACAACACGGAAGCGG	0.00	0.00	0.00	0.00	0.00
GATCAACAACACGGACGCGG	12.36	13.01	10.88	10.18	6.54
GATCAACAACACGGACGCTG	0.00	0.00	0.00	0.00	0.00
GATCAACAACACGGACTCGT	0.00	0.00	0.00	0.00	0.34
GATCAACAACACGGAGGCGG	0.23	0.00	0.00	0.00	0.00
GATCAACAACACGTTGGACC	0.00	0.00	0.00	0.34	0.00
GATCAACAACACTAGCACGC	0.00	0.00	0.29	0.00	0.00
GATCAACAACACTGAATACA	0.00	0.33	0.00	0.00	0.00
GATCAACAACACTGGCGGGC	0.23	0.00	0.00	0.00	0.00
GATCAACAACACTGGCTGGC	0.92	0.00	0.00	0.00	0.00
GATCAACAACACTGGCTTCC	0.00	0.00	0.00	0.00	0.00
GATCAACAACACTGGGCACA	0.00	0.00	0.00	0.00	0.69

GATCAACAACACTGGGTGCT	0.00	0.00	0.00	0.00	0.34
GATCAACAACACTGTGGGGG	0.00	0.00	0.00	0.00	0.00
GATCAACAACAGAAAAAGAC	0.00	0.00	0.57	0.00	0.00
GATCAACAACAGAAACATAA	0.00	0.00	0.57	0.00	0.00
GATCAACAACAGAACTTGTT	0.00	0.00	0.00	0.00	0.00
GATCAACAACAGAAGAACGC	0.00	0.00	0.00	0.00	0.69
GATCAACAACAGAAGGTGTA	0.00	0.00	0.00	0.00	0.69
GATCAACAACAGACATCACA	0.00	0.00	0.00	0.00	1.03
GATCAACAACAGCAGGCGGG	0.00	0.00	0.29	0.00	0.00
GATCAACAACAGCCAACAGA	0.00	0.33	0.00	0.00	0.00
GATCAACAACAGCCGTATCC	0.00	0.00	0.00	0.00	0.00
GATCAACAACAGCCTCGGTC	0.00	0.33	0.00	0.00	0.00
GATCAACAACAGCGGCCTCA	0.00	0.00	0.29	0.00	0.00
GATCAACAACAGCGGCCTTA	0.00	0.00	0.29	0.00	0.00
GATCAACAACAGCGGGCGGG	0.00	0.00	0.29	0.00	0.00
GATCAACAACAGCTAACATG	0.00	0.00	0.00	0.00	0.34
GATCAACAACAGCTCCGAGG	0.00	0.33	0.00	0.00	0.00
GATCAACAACAGCTGATGGC	1.14	0.00	0.00	1.02	0.34
GATCAACAACAGGAAATAAC	0.00	0.00	0.29	0.00	0.00
GATCAACAACAGGCCTAATT	0.00	0.00	0.00	0.00	0.00
GATCAACAACAGGGACATGG	0.00	0.00	0.00	0.00	0.34
GATCAACAACAGGGGCCAAT	0.00	0.00	0.29	0.00	0.00
GATCAACAACAGGGGTTCTG	0.00	0.33	0.00	0.00	0.00
GATCAACAACAGTAGCTATC	0.00	0.00	0.00	0.00	0.34
GATCAACAACAGTGCGCTGA	0.23	0.00	0.00	0.00	0.00
GATCAACAACAGTTGCCTAC	0.69	0.00	0.00	0.00	0.00
GATCAACAACAGTTGTCGTC	0.23	0.00	0.00	0.00	0.00
GATCAACAACATACAACAAC	0.00	0.00	0.00	0.00	0.69
GATCAACAACATACATCTGG	0.00	0.00	0.00	0.00	0.34
GATCAACAACATATCAAATG	0.00	0.00	0.00	0.00	0.34
GATCAACAACATCAACAACA	0.00	0.00	0.00	0.00	0.34
GATCAACAACATCAACCAGA	0.00	0.00	0.29	0.00	0.00
GATCAACAACATCAAGAACC	0.23	0.00	0.00	0.00	0.00
GATCAACAACATCAAGAAGA	0.00	0.00	0.00	0.00	0.00
GATCAACAACATCAAGAAGC	20.37	24.07	18.61	20.03	18.60
GATCAACAACATCAAGAAGN	0.00	0.00	0.00	0.34	0.00
GATCAACAACATCAAGAANN	0.23	0.00	0.00	0.00	0.00

GATCAACAACATCAAGCNNN	0.23	0.00	0.00	0.00	0.00
GATCAACAACATCAAGGCTA	0.00	0.33	0.00	0.00	0.00
GATCAACAACATCAAGTCTG	0.00	0.00	0.00	0.00	0.00
GATCAACAACATCAATAAGC	0.00	0.00	0.29	0.00	0.00
GATCAACAACATCACCGTGG	0.00	0.00	0.00	0.34	0.00
GATCAACAACATCAGCAACA	0.00	0.00	0.00	0.00	1.38
GATCAACAACATCAGGAAGC	0.23	0.00	0.00	0.00	0.00
GATCAACAACATCATATTCC	0.00	0.00	0.00	0.00	0.00
GATCAACAACATCATGGAGA	0.00	0.00	0.29	0.00	0.00
GATCAACAACATCCAGAAGC	0.00	0.00	0.00	0.00	0.34
GATCAACAACATCCTCATTA	3.20	0.65	0.57	2.72	0.69
GATCAACAACATCCTCCTTA	0.23	0.00	0.00	0.00	0.00
GATCAACAACATCGAATGCA	0.00	0.00	0.29	0.00	0.00
GATCAACAACATCGACCTTG	0.00	0.00	0.00	0.00	0.00
GATCAACAACATCGCAAGTC	0.00	0.33	0.00	0.00	0.00
GATCAACAACATCGCGCTCC	0.00	0.33	0.00	0.00	0.00
GATCAACAACATCTAGAAGC	0.00	0.00	0.00	0.00	0.34
GATCAACAACATCTTTGGCC	0.23	0.33	0.00	0.00	0.00
GATCAACAACATGAAAGGAG	0.23	0.00	0.00	0.00	0.00
GATCAACAACATGAGAAGAC	0.00	0.00	0.00	0.00	0.34
GATCAACAACATGATGCCAG	0.23	0.00	0.00	0.00	0.00
GATCAACAACATGCAACTTA	0.00	0.00	0.00	0.00	0.34
GATCAACAACATGCACATGA	0.00	0.00	0.00	0.00	1.03
GATCAACAACATGCAGCAAA	0.00	0.00	0.00	0.00	0.00
GATCAACAACATGCAGTGTG	0.23	0.00	0.00	0.00	0.00
GATCAACAACATGCATCCCT	0.00	0.00	0.29	0.00	0.00
GATCAACAACATGCATTCTT	0.23	0.00	0.00	0.00	0.00
GATCAACAACATGCGCACGA	0.00	0.00	0.00	0.00	0.34
GATCAACAACATGCGGCGCA	0.00	0.00	0.00	0.00	0.00
GATCAACAACATGCGGGGGA	0.00	0.00	0.00	0.00	0.34
GATCAACAACATGCGGGGGG	11.90	7.48	3.72	5.43	3.79
GATCAACAACATGCTCCTCC	0.00	0.00	0.29	0.00	0.00
GATCAACAACATGCTCGCTG	0.00	0.00	0.57	0.00	0.00
GATCAACAACATGCTTTTGT	5.26	3.90	2.00	0.68	3.10
GATCAACAACATGGTGGAGG	0.23	0.00	0.00	0.00	0.00
GATCAACAACATGTCAACAC	0.00	0.00	0.00	0.00	0.34
GATCAACAACATTGCGCAAA	0.00	0.00	0.00	0.00	0.00

GATCAACAACATTGCGCCAA	0.00	0.00	0.00	0.00	0.00
GATCAACAACATTGCGCCGA	0.00	0.33	0.00	0.00	0.00
GATCAACAACATTGCGCTAA	0.00	0.00	0.29	0.00	0.69
GATCAACAACATTGCGCTCA	0.00	0.00	0.57	0.00	0.00
GATCAACAACATTGCGCTGA	14.65	10.09	8.59	8.15	9.30
GATCAACAACATTGCGCTGC	0.23	0.00	0.00	0.00	0.00
GATCAACAACATTGCGCTGN	0.00	0.00	0.00	0.00	0.00
GATCAACAACATTGCGCTNA	0.00	0.00	0.00	0.34	0.00
GATCAACAACATTGCGGTGA	0.00	0.00	0.00	0.00	0.00
GATCAACAACATTGGAAGGC	0.23	0.00	0.00	0.00	0.34
GATCAACAACATTGTCACAT	0.00	0.00	0.00	0.00	0.00
GATCAACAACCAAAAAAAAA	2.06	0.00	0.29	0.34	0.34
GATCAACAACCAAAAACACA	0.00	0.00	0.00	0.00	0.00
GATCAACAACCAACAAACAC	0.23	0.00	0.00	0.00	0.00
GATCAACAACCAACAAAGAC	0.23	0.00	0.00	0.00	0.00
GATCAACAACCAACCTGCTC	0.00	0.33	0.00	0.34	0.00
GATCAACAACCACAAACATG	0.00	0.00	0.29	0.00	0.00
GATCAACAACCACAGGAAGA	1.37	0.33	1.15	0.68	1.03
GATCAACAACCACCCCCATG	0.00	0.00	0.00	0.00	0.00
GATCAACAACCACCTCCAGA	0.00	0.00	0.00	0.34	0.00
GATCAACAACCACCTCCAGC	34.34	18.22	5.73	15.95	10.33
GATCAACAACCACCTCCAGN	0.23	0.00	0.00	0.00	0.00
GATCAACAACCACCTCCANN	0.23	0.00	0.00	0.00	0.00
GATCAACAACCACCTCCCGC	0.00	0.33	0.00	0.00	0.00
GATCAACAACCACCTCCGGC	0.00	0.00	0.00	0.00	0.00
GATCAACAACCACCTCGAGC	0.00	0.00	0.00	0.00	0.00
GATCAACAACCACCTGGCGG	0.00	0.00	0.00	0.00	0.00
GATCAACAACCACGACAACA	0.00	0.00	0.00	0.00	0.00
GATCAACAACCACTGCTGCA	0.00	0.00	0.00	0.00	0.00
GATCAACAACCAGAACAAGA	0.00	0.00	0.00	0.00	0.34
GATCAACAACCAGACACGTG	0.00	0.00	0.00	0.00	0.34
GATCAACAACCAGCAAGACC	0.46	0.00	0.00	0.00	0.00
GATCAACAACCAGCAGGACA	0.00	0.65	0.00	0.00	0.00
GATCAACAACCAGCATGACA	0.23	0.00	0.00	0.00	0.00
GATCAACAACCAGCCGGCTC	0.46	0.98	0.00	0.34	0.00
GATCAACAACCAGCCTACTC	0.46	0.00	0.00	0.00	0.00
GATCAACAACCAGCCTGCCA	0.00	0.33	0.00	0.00	0.00

GATCAACAACCAGCCTGCTC	1.14	0.65	0.86	0.00	0.00
GATCAACAACCAGCGCATGT	1.14	2.28	0.00	2.04	1.03
GATCAACAACCAGTTTGAAT	0.00	0.00	0.29	0.00	0.00
GATCAACAACCATAACATAA	0.00	0.00	0.00	0.00	0.69
GATCAACAACCATAGGCATA	0.00	0.00	0.29	0.00	0.00
GATCAACAACCATCTCACGT	0.00	0.00	0.00	0.00	0.00
GATCAACAACCATGAGCACA	0.23	0.00	0.00	0.68	0.00
GATCAACAACCATGAGCTGC	0.00	0.00	0.00	0.00	0.00
GATCAACAACCATGGTGGCC	0.00	0.00	0.00	0.34	0.00
GATCAACAACCATGTAATGG	0.00	0.00	0.00	0.68	0.00
GATCAACAACCCACAGTGGC	0.00	0.00	0.29	0.00	0.00
GATCAACAACCCAGCCTTTG	0.00	0.33	0.29	0.68	0.34
GATCAACAACCCAGGAATGG	0.23	0.00	0.00	0.00	0.00
GATCAACAACCCATACATCC	0.69	0.00	0.00	0.34	0.00
GATCAACAACCCATGATTAC	0.00	0.00	0.00	0.00	0.34
GATCAACAACCCCAATTGGA	0.00	0.33	0.00	0.00	0.00
GATCAACAACCCCCAGGTGC	0.23	0.00	0.00	0.00	0.00
GATCAACAACCCCCGGAACC	0.00	0.00	0.00	0.34	0.00
GATCAACAACCCCTACGAGG	0.00	0.00	0.00	0.00	0.00
GATCAACAACCCGCAAGTGC	0.00	0.00	0.00	0.00	0.00
GATCAACAACCCGCGCCTCT	0.00	0.00	0.00	0.00	0.00
GATCAACAACCCGCGCTACG	0.00	0.33	0.00	0.00	0.00
GATCAACAACCCGGCCTGAA	0.00	0.00	0.00	0.00	0.00
GATCAACAACCCGGCCTGCA	0.00	0.00	0.29	0.00	0.00
GATCAACAACCCTACTCTGC	0.00	0.00	0.00	0.00	0.00
GATCAACAACCCTTGACACT	0.00	0.00	0.00	0.00	0.00
GATCAACAACCGAACACAAC	0.00	0.00	0.00	0.00	0.69
GATCAACAACCGAAGAACGA	0.00	0.00	1.15	0.00	0.00
GATCAACAACCGACAGTGAT	0.00	0.00	0.00	0.00	0.00
GATCAACAACCGCAGCATCC	0.46	0.00	0.57	0.34	0.69
GATCAACAACCGCATCTCCG	0.00	0.00	0.00	0.00	0.00
GATCAACAACCGCTCCGAGG	28.16	15.94	7.73	14.26	4.48
GATCAACAACCGCTCCGAGN	0.46	0.00	0.00	0.00	0.00
GATCAACAACCGCTCCGCGG	0.23	0.00	0.00	0.00	0.00
GATCAACAACCGCTCCGNNN	0.00	0.00	0.00	0.00	0.00
GATCAACAACCGCTCCGTGG	0.23	0.00	0.00	0.00	0.00
GATCAACAACCGCTCCNCNN	0.23	0.00	0.00	0.00	0.00

GATCAACAACCGCTGGCAAC	0.00	0.00	0.00	0.00	0.34
GATCAACAACCGGAAAAAAA	0.00	0.00	0.00	0.00	0.00
GATCAACAACCGGACTTGCA	0.00	0.00	0.29	0.00	0.00
GATCAACAACCGGCCGTACC	0.00	0.33	0.00	0.00	0.00
GATCAACAACCGTGCGGTAA	0.00	0.00	0.00	0.34	0.00
GATCAACAACCGTGCTCCGG	0.00	0.00	0.00	0.00	0.00
GATCAACAACCTAAAAAAAA	0.69	0.00	0.00	0.00	0.00
GATCAACAACCTACACAAAA	0.00	0.65	0.00	0.00	0.00
GATCAACAACCTACAGAGAT	0.00	0.00	0.00	0.00	0.00
GATCAACAACCTACAGTGAA	0.69	0.00	0.00	0.00	0.34
GATCAACAACCTACAGTGAC	0.23	0.00	0.00	0.00	0.00
GATCAACAACCTACAGTGAT	6.41	3.58	4.87	1.36	2.41
GATCAACAACCTACAGTGGT	0.69	0.00	0.00	0.00	0.00
GATCAACAACCTACAGTGNT	0.00	0.00	0.00	0.00	0.00
GATCAACAACCTACAGTGTT	0.69	0.00	0.00	0.00	0.00
GATCAACAACCTACTGTGAT	0.00	0.33	0.00	0.00	0.00
GATCAACAACCTATGGCATG	0.00	0.00	0.00	0.00	0.00
GATCAACAACCTCAAGCCTG	0.00	0.65	0.00	0.34	0.00
GATCAACAACCTCAAGGAAC	0.69	1.63	2.58	1.02	1.03
GATCAACAACCTCATGGAGG	0.23	0.00	0.00	0.00	0.00
GATCAACAACCTCCACCAGA	0.00	0.00	0.00	0.00	0.00
GATCAACAACCTCCACGAGG	0.00	0.33	0.29	0.34	0.69
GATCAACAACCTCCTCGAGA	0.00	0.00	0.00	0.00	0.00
GATCAACAACCTCGGCGGTT	0.00	0.00	0.29	0.00	0.00
GATCAACAACCTCGTGAAGA	0.00	0.00	0.00	0.00	0.34
GATCAACAACCTCGTGGTAC	0.00	0.65	0.00	0.00	0.00
GATCAACAACCTCGTGGTCC	0.00	1.30	0.57	0.68	0.00
GATCAACAACCTCTACAACC	0.00	0.00	0.00	0.34	0.00
GATCAACAACCTCTACAGGA	0.00	0.00	0.00	0.00	0.00
GATCAACAACCTCTCGGCGC	0.00	0.00	0.00	0.00	0.00
GATCAACAACCTCTGAAGTT	0.00	0.00	0.00	0.00	0.34
GATCAACAACCTGAAGACCT	1.37	0.33	0.29	0.00	0.34
GATCAACAACCTGAGGGGCA	0.00	0.00	0.29	0.00	0.00
GATCAACAACCTGCAGGTGA	4.35	4.55	0.57	2.38	2.07
GATCAACAACCTGCAGTGAT	0.00	0.00	0.00	0.34	0.00
GATCAACAACCTGCCTAGCG	0.00	0.00	0.00	0.34	0.34
GATCAACAACCTGCTCTCGA	0.00	0.00	0.00	0.68	0.00

GATCAACAACCTGCTTGAAA	0.00	0.00	0.00	0.68	0.34
GATCAACAACCTTAGAGCTG	0.00	0.00	0.00	0.00	0.00
GATCAACAACCTTGACAGCA	0.00	0.00	0.57	0.00	0.34
GATCAACAACCTTGCGCTGA	0.00	0.00	0.29	0.00	0.00
GATCAACAACCTTGGTTTCA	0.00	0.00	0.00	0.00	0.00
GATCAACAACCTTGTAAGAA	0.00	0.00	0.00	0.00	0.00
GATCAACAACCTTTGAGCTG	0.00	0.00	0.00	0.00	0.00
GATCAACAACGAAAAAAAAA	0.46	0.00	0.00	0.00	0.00
GATCAACAACGAAAACTGCG	0.00	0.00	0.00	0.00	0.00
GATCAACAACGAAACTAATA	0.00	0.00	0.00	0.34	0.00
GATCAACAACGAAGCCCGCG	0.00	0.65	0.00	0.00	0.00
GATCAACAACGAAGTTTTGC	0.00	0.00	0.57	0.00	0.00
GATCAACAACGAATGCACGC	0.00	0.00	0.00	0.00	0.00
GATCAACAACGACATGTGGA	0.00	0.00	0.00	0.34	0.00
GATCAACAACGACCACGGCC	0.00	0.00	0.29	0.00	0.00
GATCAACAACGACCAGGACT	0.00	0.00	0.00	0.68	0.00
GATCAACAACGACCCCGACT	0.00	0.00	0.00	0.00	0.00
GATCAACAACGACCTCTCCA	0.00	0.00	0.00	0.00	0.34
GATCAACAACGACCTCTTCA	0.00	0.98	0.00	1.02	0.00
GATCAACAACGACCTGCGAG	0.46	0.65	0.00	0.00	0.00
GATCAACAACGACGACAACA	0.00	0.00	0.57	0.00	0.00
GATCAACAACGACGGTGGCA	0.00	0.33	0.00	0.00	0.00
GATCAACAACGACTCCCCGC	0.00	0.00	0.00	0.00	0.00
GATCAACAACGACTCCGGCG	0.23	0.00	0.00	0.34	0.00
GATCAACAACGACTCTGCGG	0.00	0.00	0.00	0.00	0.00
GATCAACAACGAGAACAGCA	0.00	0.00	0.00	0.00	0.00
GATCAACAACGAGACCACGA	0.00	0.00	0.00	0.00	0.69
GATCAACAACGAGACGAAGG	0.23	0.33	0.00	0.00	0.00
GATCAACAACGAGACGTTGG	0.00	0.00	0.00	0.00	0.00
GATCAACAACGAGAGAAGGG	0.00	0.00	0.29	0.00	0.00
GATCAACAACGAGAGTCCTG	0.00	0.00	0.00	0.00	0.34
GATCAACAACGAGATGCAGT	0.00	0.00	0.00	0.00	0.00
GATCAACAACGAGCGAGAGA	0.00	0.00	0.00	0.00	0.69
GATCAACAACGAGGAGGCAA	0.00	0.33	0.00	0.00	0.00
GATCAACAACGAGGCTCCCT	0.00	0.00	0.29	0.00	0.00
GATCAACAACGAGTCAGACT	0.00	0.00	0.00	0.00	0.34
GATCAACAACGATGCAGATG	0.23	0.00	0.00	0.68	0.00

GATCAACAACGATGGTCACA	0.00	0.00	0.29	0.00	0.00
GATCAACAACGATTAATTTT	2.52	0.00	0.00	0.00	0.00
GATCAACAACGATTCCAGTG	0.00	0.00	0.00	0.00	0.00
GATCAACAACGATTCCGACG	0.00	0.00	0.00	0.00	0.00
GATCAACAACGATTCCGGCC	0.00	0.00	0.00	0.00	0.00
GATCAACAACGATTCCGGCG	0.23	0.33	0.57	1.02	0.34
GATCAACAACGATTCCGGCT	0.00	0.00	0.00	0.00	0.00
GATCAACAACGATTCCGGGT	0.00	0.00	0.00	0.34	0.00
GATCAACAACGATTCCGGTG	0.00	0.00	0.00	0.34	0.00
GATCAACAACGATTCCGTGC	0.00	0.00	0.00	0.00	0.00
GATCAACAACGATTCCTGCG	0.00	0.00	0.00	0.00	0.00
GATCAACAACGATTGCACGC	0.00	0.00	0.00	0.00	0.34
GATCAACAACGATTGCACGT	0.00	0.33	0.00	0.00	0.00
GATCAACAACGATTGCCAGC	0.00	0.00	0.00	0.00	0.00
GATCAACAACGATTTTTGGT	0.00	0.00	0.00	0.00	0.34
GATCAACAACGCACAACACA	2.06	0.98	0.29	1.36	0.69
GATCAACAACGCACGGGTAA	0.00	0.00	0.00	0.00	0.00
GATCAACAACGCACTCTCGC	0.23	0.00	0.00	0.00	0.00
GATCAACAACGCAGAGGCCG	0.00	0.00	0.00	0.00	0.00
GATCAACAACGCAGAGGCTG	0.00	0.00	0.57	0.34	0.00
GATCAACAACGCAGGTGGGA	0.00	0.00	0.00	0.00	0.00
GATCAACAACGCCAAAAAAA	0.00	0.00	0.00	0.34	0.69
GATCAACAACGCCAAAAGCA	0.00	0.00	0.00	0.34	0.00
GATCAACAACGCCAAAAGCC	0.00	0.00	0.00	0.00	0.34
GATCAACAACGCCAACACAC	0.00	0.00	0.00	0.00	0.34
GATCAACAACGCCAATGCTC	0.00	0.00	0.00	0.00	0.69
GATCAACAACGCCAGGAGAC	0.00	0.33	0.00	0.00	0.00
GATCAACAACGCCAGTGCCC	0.00	0.00	0.00	0.00	1.38
GATCAACAACGCCATTTGGA	0.00	0.00	0.29	0.00	0.00
GATCAACAACGCCCCACCCC	0.00	0.00	0.00	0.00	0.00
GATCAACAACGCCCCTAAGA	0.00	0.00	0.00	0.00	0.00
GATCAACAACGCCCCTAAGC	0.23	0.00	0.29	0.34	0.69
GATCAACAACGCCCGCGCCA	0.00	0.00	0.29	0.00	0.00
GATCAACAACGCCGCACTCC	0.00	0.00	0.00	0.00	0.34
GATCAACAACGCCGCACTTC	0.00	0.00	0.00	0.00	0.00
GATCAACAACGCCGGCATCA	0.69	0.00	0.00	0.00	0.00
GATCAACAACGCCTACGAGG	0.00	0.00	0.00	0.34	0.00

GATCAACAACGCCTTAATAT	0.00	0.00	0.29	0.00	0.00
GATCAACAACGCGACGTGTC	0.00	0.00	0.00	0.00	0.34
GATCAACAACGCGACGTGTG	0.00	0.00	0.29	0.00	0.00
GATCAACAACGCGCAACAAC	0.00	0.00	0.00	0.00	0.69
GATCAACAACGCGCACGGAG	0.00	0.00	0.29	0.00	0.00
GATCAACAACGCGCCAAAAA	0.00	0.33	0.29	0.00	0.00
GATCAACAACGCGCCCAACA	0.00	0.00	0.00	0.00	0.34
GATCAACAACGCTACTCCTT	0.00	0.00	0.00	0.00	0.00
GATCAACAACGCTGAAAAAG	0.00	0.00	0.00	0.34	0.00
GATCAACAACGCTGGCATCG	0.00	0.00	0.00	0.00	0.34
GATCAACAACGCTTACCTGG	0.00	0.00	0.00	0.00	0.00
GATCAACAACGCTTCTGCGC	0.00	0.00	0.00	0.00	0.00
GATCAACAACGGAACGTCGG	0.00	0.00	0.00	0.00	0.00
GATCAACAACGGACGCCTCG	0.00	0.00	0.00	0.00	0.00
GATCAACAACGGACTGTCCA	0.00	0.00	0.00	0.34	0.00
GATCAACAACGGAGCGCCCA	0.00	0.33	0.29	0.68	0.00
GATCAACAACGGATATCACA	0.00	0.00	0.00	0.34	0.00
GATCAACAACGGCAATGTCG	0.00	0.00	0.00	0.00	0.00
GATCAACAACGGCCAAGGGC	0.00	0.33	0.00	0.00	0.00
GATCAACAACGGCCATCATC	0.69	0.33	0.00	0.00	0.00
GATCAACAACGGCCCACAGG	0.00	0.00	0.00	0.34	0.00
GATCAACAACGGCCCAGTCT	0.00	0.00	0.00	0.00	0.34
GATCAACAACGGCCCCACAC	0.00	0.00	0.00	0.00	0.34
GATCAACAACGGCCCTAATT	0.00	0.00	0.00	0.00	0.69
GATCAACAACGGCCGCCTCG	1.83	0.98	1.43	3.39	0.69
GATCAACAACGGCCGCCTCT	0.00	0.33	0.00	0.00	0.00
GATCAACAACGGCCGCCTGG	0.00	0.65	0.00	1.02	0.00
GATCAACAACGGCCGCCTTG	0.00	0.00	0.00	0.34	0.00
GATCAACAACGGCCGCGTTG	0.00	0.00	0.00	0.34	0.00
GATCAACAACGGCCGGTTGG	0.00	0.00	0.00	0.00	0.00
GATCAACAACGGCCGGTTTG	0.00	0.33	0.00	0.00	0.00
GATCAACAACGGCCGTTTGG	1.14	3.25	0.57	0.68	0.34
GATCAACAACGGCCGTTTTG	0.00	0.00	0.00	0.00	0.34
GATCAACAACGGCCTCAAGA	0.00	0.00	0.00	0.00	0.00
GATCAACAACGGCCTGAGTG	0.00	0.00	0.00	0.00	0.34
GATCAACAACGGCGTGGAGA	0.00	0.00	0.00	0.00	0.34
GATCAACAACGGCGTGTCTG	0.00	0.00	0.00	0.00	0.00

GATCAACAACGGCTCATGCC	0.00	0.00	0.00	0.00	0.34
GATCAACAACGGCTCTGGCT	0.00	0.00	0.00	0.00	0.00
GATCAACAACGGCTGAATTG	0.00	0.00	0.00	0.34	0.00
GATCAACAACGGCTGGAAAA	0.46	0.00	0.00	0.00	0.00
GATCAACAACGGCTGGAAGC	0.00	0.33	0.00	0.00	0.00
GATCAACAACGGCTGGTGAG	0.00	0.00	0.00	0.00	0.00
GATCAACAACGGGCAGGTGG	0.23	0.00	0.00	0.34	0.00
GATCAACAACGGGCGCACGC	0.00	0.00	0.00	0.34	0.00
GATCAACAACGGGCGCGTTG	0.00	0.00	0.00	0.00	0.00
GATCAACAACGGGCGCTTTG	0.92	0.98	0.86	1.02	1.38
GATCAACAACGGGGAGATGG	0.00	0.00	0.00	0.00	0.00
GATCAACAACGGGGAGATGT	0.00	0.00	0.00	0.68	0.00
GATCAACAACGGGGATTATT	0.00	0.33	0.00	1.36	0.00
GATCAACAACGGGGCAGTGG	0.00	0.33	0.00	0.00	0.00
GATCAACAACGGTACCCTGG	0.00	0.00	0.29	0.00	0.00
GATCAACAACGGTCTACTGC	0.00	0.00	0.00	0.00	0.00
GATCAACAACGGTGAGAAAG	0.00	0.33	0.00	0.00	0.00
GATCAACAACGTAAACACCA	0.00	0.00	0.00	0.00	0.69
GATCAACAACGTACACTTAA	0.00	0.00	0.00	0.00	0.34
GATCAACAACGTCAAGAAGC	0.00	0.00	0.00	0.34	0.00
GATCAACAACGTCCACGTCC	0.23	0.00	0.57	0.34	0.00
GATCAACAACGTCGGCATGG	0.00	0.00	0.00	0.34	0.00
GATCAACAACGTGAGCATGA	0.92	0.00	0.00	0.00	0.00
GATCAACAACGTGCTTTTGT	0.00	0.00	0.29	0.00	0.00
GATCAACAACGTGGACTACA	0.00	0.00	0.00	0.34	0.69
GATCAACAACGTGGAGACGG	0.00	0.00	0.00	0.00	0.00
GATCAACAACGTGGCGCCGA	0.00	0.00	0.00	0.00	0.34
GATCAACAACGTGGGCGCAA	0.00	0.33	0.00	0.00	0.00
GATCAACAACGTGGGGGCAA	0.00	0.00	0.00	0.00	0.00
GATCAACAACGTGGTATTGC	0.00	0.00	0.29	0.00	0.00
GATCAACAACGTGGTTGTTC	0.00	0.00	0.29	0.00	0.00
GATCAACAACGTGTACGTTG	0.00	0.00	0.29	0.68	0.00
GATCAACAACGTTAAAAAAA	2.29	0.00	0.29	0.34	0.34
GATCAACAACGTTAAACATT	0.00	0.00	0.00	0.00	0.00
GATCAACAACGTTCAGGACT	0.00	0.00	0.00	0.00	0.00
GATCAACAACGTTCGAAGTA	0.00	0.00	0.00	0.00	0.00
GATCAACAACGTTCTTGGGT	0.23	1.95	0.29	0.68	0.34

GATCAACAACGTTGAGCCAA	0.00	0.00	0.29	0.00	0.00
GATCAACAACTAAAAAAAAA	0.46	0.00	0.57	0.00	0.00
GATCAACAACTAGATGTGTT	0.00	0.00	0.00	0.00	0.00
GATCAACAACTAGGCATATC	0.00	0.00	0.29	0.00	0.00
GATCAACAACTAGGCATCTC	0.46	0.65	0.86	0.00	1.03
GATCAACAACTAGGCATGTC	0.23	0.00	0.00	0.00	0.00
GATCAACAACTATGAGAGGA	0.00	0.00	0.57	0.00	0.00
GATCAACAACTCACTCACAC	0.00	0.00	0.00	0.00	0.34
GATCAACAACTCATACCATA	0.00	0.00	0.00	0.00	0.34
GATCAACAACTCCAAGCCCA	0.00	0.00	0.29	0.00	0.00
GATCAACAACTCCAAGTGGG	0.00	0.00	0.00	0.00	0.00
GATCAACAACTCCCTGGAGC	0.00	0.00	0.00	0.00	0.00
GATCAACAACTCCCTGGGCC	0.00	0.00	0.57	0.00	1.03
GATCAACAACTCCTTCCCCT	0.00	0.00	0.00	0.34	0.00
GATCAACAACTCGATTTTGG	0.00	0.00	0.29	0.00	0.00
GATCAACAACTCGCTTGCGC	0.00	0.00	0.00	0.00	0.00
GATCAACAACTCGTAATGCG	0.69	0.00	0.00	0.00	0.00
GATCAACAACTGCAACATCA	0.00	0.00	0.29	0.00	0.00
GATCAACAACTGCAACCAGC	0.00	0.00	0.00	0.00	0.34
GATCAACAACTGCCAGCGCG	0.00	0.00	0.00	0.34	0.00
GATCAACAACTGCGGCCTGG	0.00	0.00	0.00	0.00	0.00
GATCAACAACTGCGTCGGAT	0.92	0.33	0.00	0.68	0.00
GATCAACAACTGCGTCGGTG	0.46	0.00	0.00	0.00	0.00
GATCAACAACTGCGTGGGCT	0.00	0.00	0.00	0.34	0.00
GATCAACAACTGCTTGCCCA	0.00	0.00	0.00	0.00	0.69
GATCAACAACTGGAACTTGG	0.00	0.00	0.00	0.34	0.00
GATCAACAACTGGCCATTCT	0.00	0.00	0.00	0.00	0.69
GATCAACAACTGGGACGTGG	0.00	0.00	0.00	0.00	0.00
GATCAACAACTGGGTGACGC	0.00	0.00	0.00	0.00	0.00
GATCAACAACTGTCAGCAGC	0.00	0.00	0.00	0.00	0.00
GATCAACAACTGTCTAGTCG	0.00	0.00	0.00	0.00	0.34
GATCAACAACTGTGTGGGAT	0.00	0.00	0.29	0.34	0.00
GATCAACAACTGTTTGCCGC	0.00	0.33	0.00	0.00	0.00
GATCAACAACTTCAAGAGCT	0.23	0.00	0.00	0.00	0.00
GATCAACAACTTCACGCCCC	0.00	0.00	0.29	0.00	0.00
GATCAACAACTTCAGGAGGG	0.00	0.00	0.00	0.00	0.00
GATCAACAACTTCAGTGGAG	0.00	0.00	0.00	0.00	0.34

GATCAACAACTTCCTCTTTT	0.23	0.00	0.00	0.00	0.00
GATCAACAACTTCCTGCATC	0.00	0.00	0.00	0.00	0.00
GATCAACAACTTCCTGGTGC	0.00	0.33	0.00	0.00	0.00
GATCAACAACTTCGAATCGG	0.00	0.00	0.29	0.00	0.00
GATCAACAACTTCGCCGGGG	0.00	0.33	0.00	0.00	0.00
GATCAACAACTTCTACGCGC	0.00	0.00	0.00	0.00	0.00
GATCAACAACTTCTCCGGGG	0.00	0.33	0.00	0.00	0.00
GATCAACAACTTGCCCGTAT	0.00	0.00	0.00	0.00	0.34
GATCAACAACTTGCGGACTC	0.00	0.00	0.00	0.00	0.00
GATCAACAACTTGCGGCCTG	0.00	0.00	0.00	0.00	0.00
GATCAACAACTTGCGGCGGA	0.00	0.00	0.00	0.00	0.00
GATCAACAACTTGCGGCTGA	0.00	0.65	0.57	0.00	0.69
GATCAACAACTTGGCCACGG	0.46	0.33	0.00	0.00	0.00
GATCAACAACTTGGGCACNN	0.00	0.00	0.00	0.00	0.00
GATCAACAACTTGGGGACAG	0.00	0.00	0.29	0.00	0.00
GATCAACAACTTGGGGGCAG	0.00	0.00	0.29	0.00	0.00
GATCAACAACTTGTCCACAG	0.00	0.65	0.00	0.34	0.00
GATCAACAACTTGTTCCCGC	0.00	0.00	0.57	0.00	1.03
GATCAACAACTTTGCCACGG	0.00	0.00	0.00	0.34	0.00
GATCAACAACTTTGCCTTGC	0.00	0.00	0.00	0.34	0.00
GATCAACAACTTTGCGCTGA	0.00	0.00	0.00	0.34	0.00
GATCAACAAGAAAAAAAAAA	27.01	7.16	14.03	11.88	14.81
GATCAACAAGAAAAAAAAAG	0.00	0.00	0.29	0.00	0.00
GATCAACAAGAAAAAAAAAN	0.23	0.00	0.00	0.00	0.00
GATCAACAAGAAAAAAAAAT	0.23	0.33	0.29	0.00	0.34
GATCAACAAGAAAAAAAAGA	0.23	0.00	0.00	0.00	0.00
GATCAACAAGAAAAAAACAA	0.00	0.00	0.00	0.00	0.34
GATCAACAAGAAAAAGAAAA	0.00	0.00	0.29	0.00	0.00
GATCAACAAGAAACTCGTGT	0.00	0.33	0.00	0.00	0.00
GATCAACAAGAAACTCTACC	1.60	0.00	0.29	1.02	0.34
GATCAACAAGAAAGACTAAG	0.00	0.00	0.00	0.00	0.00
GATCAACAAGAAATGCATTT	0.00	0.00	0.29	0.00	0.00
GATCAACAAGAACAAAAAAA	0.00	0.00	0.00	0.00	0.00
GATCAACAAGAACAAGAGCA	0.00	0.00	0.00	0.00	1.03
GATCAACAAGAACACACACA	0.00	0.00	0.29	0.00	0.00
GATCAACAAGAACAGCCTGC	0.00	0.00	0.29	0.00	0.00
GATCAACAAGAACATCAACT	0.00	0.00	0.00	0.00	0.69

GATCAACAAGAACCCGCTCG	0.00	0.00	0.29	0.00	0.00
GATCAACAAGAACCCGGTCG	0.00	0.00	0.00	0.00	0.00
GATCAACAAGAACCCGTTCG	0.92	0.65	0.00	0.00	1.03
GATCAACAAGAACCGCTCCG	0.00	0.00	0.00	0.00	0.34
GATCAACAAGAACTAAAAAG	0.23	0.00	0.00	0.00	0.00
GATCAACAAGAACTCTACCT	0.00	0.00	0.29	0.00	0.00
GATCAACAAGAAGAAAAAAA	0.00	0.00	0.00	0.00	0.00
GATCAACAAGAAGAAGAAGA	0.00	0.00	0.57	0.00	0.69
GATCAACAAGAAGAAGCGCG	0.00	0.33	0.00	0.00	0.00
GATCAACAAGAAGACCATCG	0.00	0.98	0.29	0.34	0.34
GATCAACAAGAAGACTCTCT	0.00	0.00	0.29	0.00	0.00
GATCAACAAGAAGAGCATTT	0.23	0.00	0.00	0.00	0.00
GATCAACAAGAAGAGGAGCG	0.00	0.00	0.00	0.00	0.00
GATCAACAAGAAGAGGTGGA	0.00	0.00	1.15	0.00	0.00
GATCAACAAGAAGATTGATG	0.00	0.00	0.00	0.00	0.34
GATCAACAAGAAGATTGCGG	0.00	0.98	0.29	0.34	1.03
GATCAACAAGAAGATTGCTC	0.00	0.33	0.00	0.00	0.00
GATCAACAAGAAGATTGCTG	5.04	16.27	14.60	8.49	13.43
GATCAACAAGAAGATTGTAG	0.00	0.00	0.29	0.00	0.00
GATCAACAAGAAGCAAAAAA	0.23	0.00	0.00	0.00	0.00
GATCAACAAGAAGCAAGTGG	0.00	0.33	0.00	0.00	0.00
GATCAACAAGAAGCACGAGC	0.00	0.00	0.00	0.00	0.00
GATCAACAAGAAGCCAAAGG	0.00	0.33	0.00	0.00	1.03
GATCAACAAGAAGCCGCGCG	0.00	0.00	0.57	0.00	0.00
GATCAACAAGAAGCGTCTGG	0.69	0.00	0.29	0.68	0.00
GATCAACAAGAAGCTCTACC	0.00	0.65	0.00	0.00	0.00
GATCAACAAGAAGGAAGGAA	0.00	0.00	0.86	0.00	0.00
GATCAACAAGAAGGAAGTTC	0.00	0.00	0.29	0.00	0.00
GATCAACAAGAAGGACAACC	0.00	0.00	0.00	0.00	0.00
GATCAACAAGAAGGACCGCT	0.00	0.33	0.00	0.00	0.00
GATCAACAAGAAGGAGAAGA	0.00	0.00	0.29	0.00	0.00
GATCAACAAGAAGGCGAGGA	0.00	0.00	0.86	0.00	0.00
GATCAACAAGAAGGCGCGCG	0.23	0.00	0.00	0.00	0.00
GATCAACAAGAAGGGAGAGT	0.00	0.00	0.00	0.34	0.00
GATCAACAAGAAGGGAGGGA	0.00	0.00	0.00	0.34	0.00
GATCAACAAGAAGGGAGTTG	0.00	0.00	0.00	0.00	0.00
GATCAACAAGAAGGGCATTT	0.00	0.33	0.00	0.00	0.00

GATCAACAAGAAGGGTCTAT	0.00	0.00	0.00	0.68	0.00
GATCAACAAGAAGTAGCGCG	0.00	0.33	0.00	0.00	0.00
GATCAACAAGAAGTCGCGCC	0.00	0.33	0.00	0.00	0.00
GATCAACAAGAAGTCGCGCG	8.93	14.97	11.17	8.49	7.92
GATCAACAAGAAGTCGCGCN	0.00	0.00	0.29	0.00	0.00
GATCAACAAGAAGTCGCGCT	0.00	0.00	0.29	0.00	0.00
GATCAACAAGAAGTCGCGNN	0.46	0.00	0.00	0.00	0.00
GATCAACAAGAAGTCGNGCG	0.00	0.00	0.00	0.00	0.34
GATCAACAAGAAGTGCAAAA	0.00	0.00	0.00	0.00	0.00
GATCAACAAGAAGTGCAATT	0.00	0.00	0.00	0.34	0.00
GATCAACAAGAAGTGCACAA	0.00	0.33	0.00	0.00	0.00
GATCAACAAGAAGTGCACGC	0.00	0.00	0.00	0.00	0.69
GATCAACAAGAAGTGCATCT	0.00	0.00	0.00	0.00	0.00
GATCAACAAGAAGTGCATGT	0.00	0.00	0.00	0.34	0.00
GATCAACAAGAAGTGCATNN	0.00	0.00	0.00	0.00	0.00
GATCAACAAGAAGTGCATTC	0.00	0.00	0.29	0.00	0.00
GATCAACAAGAAGTGCATTG	0.00	0.33	0.00	0.00	0.00
GATCAACAAGAAGTGCATTN	0.23	0.00	0.00	0.00	0.00
GATCAACAAGAAGTGCATTT	16.48	11.39	13.75	13.24	9.99
GATCAACAAGAAGTGCGTTT	0.00	0.00	0.00	0.00	0.00
GATCAACAAGAAGTGCTCGT	0.23	0.33	0.00	0.00	0.00
GATCAACAAGAAGTGTCAGA	0.00	0.00	0.00	0.00	0.34
GATCAACAAGAAGTTCATTT	0.00	0.33	0.00	0.00	0.00
GATCAACAAGAATCACCGCA	0.00	0.00	0.00	0.00	0.69
GATCAACAAGAATGCCCGCC	0.00	0.00	0.00	0.34	0.00
GATCAACAAGAATGGAGTAA	0.69	0.33	0.00	0.00	0.00
GATCAACAAGAATGTTTCGA	0.00	0.00	0.00	0.34	0.00
GATCAACAAGACAAAAAAAA	1.14	1.95	2.00	1.02	2.41
GATCAACAAGACAAAACAAA	0.69	0.33	0.86	0.68	0.69
GATCAACAAGACAAAACAAG	0.23	0.00	0.00	0.00	0.00
GATCAACAAGACAAAACACA	0.00	0.98	0.86	0.68	1.72
GATCAACAAGACAAAACGAA	0.00	0.00	0.29	0.00	0.00
GATCAACAAGACAAAATGGA	0.00	0.33	0.00	0.00	0.00
GATCAACAAGACACTAGTGT	0.00	0.00	0.00	0.00	0.00
GATCAACAAGACACTCGTGT	0.00	0.65	0.00	0.00	0.00
GATCAACAAGACAGGGAAGA	0.00	0.00	0.00	0.00	0.00
GATCAACAAGACCAACAAGA	0.00	0.00	0.57	0.00	1.72

GATCAACAAGACCAGCGCCA	0.00	0.00	0.29	0.00	0.00
GATCAACAAGACCAGGAAGA	0.00	0.00	0.00	0.34	0.34
GATCAACAAGACCATCGTAT	0.00	0.00	0.29	0.34	0.00
GATCAACAAGACCCACGTGT	0.00	0.33	0.00	0.00	0.00
GATCAACAAGACCCGCGTCA	0.00	0.00	0.00	0.34	0.00
GATCAACAAGACCCGCGTGT	0.00	0.00	0.00	0.00	0.34
GATCAACAAGACCCGCTTCG	0.23	0.00	0.00	0.00	0.00
GATCAACAAGACCCTCGAGA	0.00	0.00	0.00	0.00	0.34
GATCAACAAGACCCTCGAGC	0.23	0.00	0.00	0.00	0.00
GATCAACAAGACCCTCGCGT	0.00	0.00	0.00	0.00	0.00
GATCAACAAGACCCTCGGGT	0.23	0.00	0.00	0.00	0.00
GATCAACAAGACCCTCGNNN	0.23	0.00	0.00	0.00	0.00
GATCAACAAGACCCTCGTAT	0.69	0.65	0.00	0.68	0.00
GATCAACAAGACCCTCGTGA	0.23	0.00	0.29	0.00	0.00
GATCAACAAGACCCTCGTGG	0.00	0.00	0.00	1.02	0.00
GATCAACAAGACCCTCGTGT	49.44	55.63	29.21	37.34	28.93
GATCAACAAGACCCTCGTNN	0.00	0.00	0.00	0.00	0.00
GATCAACAAGACCCTCGTTC	0.00	0.00	0.00	0.00	0.69
GATCAACAAGACCCTCTTCC	0.00	0.00	0.00	0.34	0.00
GATCAACAAGACCGACAAGA	0.00	0.00	0.00	0.00	0.34
GATCAACAAGACCGACCTGC	0.00	0.33	0.00	0.34	0.00
GATCAACAAGACCGACGTCT	0.00	0.00	0.00	0.00	0.00
GATCAACAAGACCGCCACGG	0.00	0.00	0.29	0.00	0.00
GATCAACAAGACCGGGAAGG	0.23	0.00	0.00	0.00	0.00
GATCAACAAGACCGTGAAGG	8.93	2.60	1.15	3.39	2.07
GATCAACAAGACCGTGGAGG	0.46	0.00	0.00	0.00	0.00
GATCAACAAGACGATCATTG	0.00	0.00	0.00	0.00	0.00
GATCAACAAGACGATTGCTG	0.23	0.00	0.00	0.00	0.00
GATCAACAAGACGCTGATAA	0.00	0.33	0.00	0.00	0.00
GATCAACAAGACGTGCATNN	0.00	0.00	0.00	0.00	0.00
GATCAACAAGACTCTCGTAT	0.00	0.00	0.29	0.00	0.00
GATCAACAAGACTGGAGCTG	0.00	0.33	0.00	0.00	0.00
GATCAACAAGAGAAAGAGGA	0.00	0.00	0.00	0.00	0.34
GATCAACAAGAGAAGAAGAA	0.00	0.00	0.00	0.00	0.69
GATCAACAAGAGAAGAAGAC	0.00	0.00	0.00	0.00	0.34
GATCAACAAGAGACCGTCAA	0.00	0.33	0.86	0.34	1.38
GATCAACAAGAGAGAGACTT	0.00	0.00	0.00	0.00	0.34

GATCAACAAGAGAGCAACAA	0.00	0.00	0.00	0.00	0.34
GATCAACAAGAGATGAAGAA	0.00	0.00	0.29	0.00	1.72
GATCAACAAGAGCAACAAGA	0.00	0.00	0.00	0.00	0.00
GATCAACAAGAGCAACTCGG	0.00	0.00	0.00	1.02	0.34
GATCAACAAGAGCACCTGTC	0.00	0.00	0.00	0.68	0.00
GATCAACAAGAGCATGCGCA	0.00	0.00	0.00	0.00	0.00
GATCAACAAGAGCCGCAAGA	0.00	0.00	0.00	0.00	0.34
GATCAACAAGAGCCTCCTCG	0.00	0.00	0.57	0.34	0.00
GATCAACAAGAGCCTTTTAC	0.00	0.00	0.00	0.68	0.00
GATCAACAAGAGCGCGAGCT	0.23	0.00	0.00	0.00	0.00
GATCAACAAGAGCTCCGGCC	0.00	0.00	0.57	0.00	0.00
GATCAACAAGAGGAATGACC	0.23	0.00	0.00	0.00	0.00
GATCAACAAGAGGAGGAGGA	0.00	0.00	0.57	0.00	1.03
GATCAACAAGAGGGCCATCT	0.00	0.00	0.00	0.34	0.00
GATCAACAAGAGGTCCAATG	0.00	0.00	0.00	0.00	0.00
GATCAACAAGAGGTCCACGC	0.00	0.00	0.00	0.34	0.00
GATCAACAAGAGGTGCAGCA	0.00	0.33	0.00	0.00	0.00
GATCAACAAGAGTGTTGGCC	0.00	0.33	0.29	0.00	0.00
GATCAACAAGAGTGTTGGCN	0.00	0.00	0.00	0.00	0.00
GATCAACAAGATAAAAAAAA	0.00	0.00	0.00	0.00	0.69
GATCAACAAGATAGTTTGCC	0.00	0.00	0.00	0.34	0.00
GATCAACAAGATAGTTTGCG	0.00	0.00	0.29	0.00	0.00
GATCAACAAGATCCTCGCAG	0.00	0.33	0.00	0.00	0.00
GATCAACAAGATCCTCGCGG	0.00	0.00	0.29	0.00	0.00
GATCAACAAGATGAAGGAGA	0.00	0.00	0.00	0.00	1.38
GATCAACAAGATGCACTTGT	0.00	0.00	0.29	0.00	0.00
GATCAACAAGATGCGGCCCG	0.00	0.00	0.29	0.00	0.00
GATCAACAAGATGGAGATAT	0.00	0.00	0.00	0.00	0.00
GATCAACAAGATGGAGATGC	0.00	0.00	0.00	0.68	0.69
GATCAACAAGATGGGGCACG	0.00	0.00	0.00	0.00	0.34
GATCAACAAGATGGTCGTCG	1.14	2.60	2.86	2.04	2.07
GATCAACAAGATGGTGGTCG	0.23	0.00	0.00	0.00	0.00
GATCAACAAGATTATCAAGG	0.00	0.00	0.00	0.34	0.00
GATCAACAAGATTGAGACAG	0.00	0.00	0.00	0.00	0.34
GATCAACAAGATTGCGGAGA	0.00	0.00	0.00	0.00	1.03
GATCAACAAGATTGCGGGCA	0.00	0.00	0.00	0.00	0.00
GATCAACAAGATTGGCACGT	0.23	0.33	0.00	0.00	0.69

GATCAACAAGATTGTTCAGC	0.00	0.00	0.00	0.00	0.00
GATCAACAAGCAAAAAAAAA	30.44	3.25	7.45	8.15	9.99
GATCAACAAGCAAAAAAAAT	0.23	0.00	0.00	0.00	0.00
GATCAACAAGCAAAAAAACN	0.23	0.00	0.00	0.00	0.00
GATCAACAAGCAAAAAAANN	0.23	0.00	0.00	0.00	0.00
GATCAACAAGCAAAAATAAA	0.00	0.00	0.00	0.34	0.00
GATCAACAAGCAACAACGGC	0.00	0.00	0.00	0.00	0.34
GATCAACAAGCAACCGCGAG	0.00	0.00	0.00	0.34	0.00
GATCAACAAGCAATGTGTTC	0.46	0.00	0.00	0.00	0.00
GATCAACAAGCAATTAAAAA	3.20	0.00	0.29	0.34	0.34
GATCAACAAGCAATTACATG	0.92	0.33	0.29	0.68	1.03
GATCAACAAGCAATTACCTG	0.00	0.33	0.00	0.00	0.00
GATCAACAAGCACAACGAGC	0.00	0.00	0.00	0.00	0.00
GATCAACAAGCACACCGCAC	0.00	0.00	0.00	0.00	1.03
GATCAACAAGCACACGCTGA	0.00	0.00	0.00	0.00	0.00
GATCAACAAGCACCTGGACA	0.00	0.33	0.00	0.00	0.00
GATCAACAAGCACGGCATGG	0.00	0.00	0.00	0.00	0.00
GATCAACAAGCACGTGCTCA	0.00	0.33	0.00	0.00	0.00
GATCAACAAGCACTCCAGCG	0.00	0.00	0.00	0.00	0.00
GATCAACAAGCACTTCCGCA	0.00	0.00	0.00	0.00	0.00
GATCAACAAGCAGAGAAGAA	0.23	0.00	0.00	0.00	0.00
GATCAACAAGCAGAGCATCC	0.00	0.33	0.00	0.00	0.00
GATCAACAAGCAGAGCCCAG	0.00	0.00	0.29	0.00	0.00
GATCAACAAGCAGATGTTCG	0.23	0.65	0.00	0.00	0.00
GATCAACAAGCAGCACCTTT	0.00	0.00	0.00	0.00	0.00
GATCAACAAGCAGCCGCGCG	0.00	0.00	0.29	0.00	0.00
GATCAACAAGCAGCGCATCC	0.00	2.28	2.86	3.05	2.76
GATCAACAAGCAGCGCTTTC	0.00	0.00	0.29	0.00	0.00
GATCAACAAGCAGCGGAAAA	0.00	0.33	0.00	0.00	0.00
GATCAACAAGCAGGACCTGA	0.23	0.98	0.29	1.02	0.00
GATCAACAAGCAGGCGATGC	0.00	0.00	0.00	0.34	0.00
GATCAACAAGCAGGGCCGGA	0.00	0.00	0.29	0.00	0.00
GATCAACAAGCAGGGCCTGA	0.23	0.00	0.00	0.34	0.00
GATCAACAAGCAGGTAAGCC	0.00	0.33	0.00	0.00	0.00
GATCAACAAGCAGTGCGCTG	0.00	0.00	0.00	0.00	0.69
GATCAACAAGCAGTGCTTGG	0.00	0.00	0.00	0.34	0.00
GATCAACAAGCATGGCGACC	0.00	0.00	0.00	0.00	0.00

GATCAACAAGCATGTTGACC	0.00	0.00	0.00	0.00	0.00
GATCAACAAGCCAAGCGCTG	0.00	0.00	0.86	0.00	0.00
GATCAACAAGCCACACCTGG	0.23	0.00	0.00	0.00	0.00
GATCAACAAGCCACGACCCA	0.00	0.00	0.00	0.00	0.34
GATCAACAAGCCACGCTCTC	0.69	0.33	0.00	0.68	0.00
GATCAACAAGCCAGCGCAAA	0.00	0.00	0.00	1.02	0.00
GATCAACAAGCCATGCGCCG	0.00	0.00	0.29	0.00	0.00
GATCAACAAGCCATTACATG	0.23	0.00	0.00	0.00	0.00
GATCAACAAGCCCAAGCTCA	0.00	0.00	0.29	0.00	0.00
GATCAACAAGCCCACCCACA	0.00	0.00	0.00	0.00	0.69
GATCAACAAGCCCAGCGTCC	0.00	0.33	0.00	0.00	0.00
GATCAACAAGCCCAGGATGT	0.00	0.00	0.00	0.00	0.00
GATCAACAAGCCCATCCTCA	0.00	0.00	0.00	0.34	0.00
GATCAACAAGCCCATGGCTA	0.00	0.00	0.00	0.00	1.03
GATCAACAAGCCCCATGGCT	0.00	0.00	0.00	0.00	0.34
GATCAACAAGCCCGACCATG	0.00	0.00	0.00	0.34	0.00
GATCAACAAGCCCGTGGAGA	0.00	0.33	0.00	0.68	0.00
GATCAACAAGCCCTATGGCA	0.23	0.00	0.00	0.00	0.00
GATCAACAAGCCCTCACAGG	0.00	0.00	0.00	0.00	0.00
GATCAACAAGCCCTTCCTTC	0.00	0.33	0.00	0.00	0.00
GATCAACAAGCCGAGGTGGC	0.00	0.00	0.00	0.00	0.34
GATCAACAAGCCGATATCAT	0.46	0.00	0.00	0.00	0.00
GATCAACAAGCCGCGCTTGC	0.00	0.00	0.00	0.00	0.00
GATCAACAAGCCGGAACTCC	0.23	0.00	0.00	0.00	0.00
GATCAACAAGCCGGAGCTCT	0.00	0.00	0.00	0.34	0.34
GATCAACAAGCCGGAGGACA	0.00	0.33	0.00	0.00	0.00
GATCAACAAGCCGGCAACTT	0.00	0.00	0.00	0.00	0.69
GATCAACAAGCCGGGTGGGG	0.00	0.00	0.00	0.34	0.00
GATCAACAAGCCGTGCAGCC	0.92	0.98	0.29	0.34	0.00
GATCAACAAGCCGTGCAGGG	0.00	0.00	0.00	0.00	0.00
GATCAACAAGCCGTTCGTGG	0.00	0.00	0.00	0.34	0.00
GATCAACAAGCCGTTTTTTT	0.00	0.00	0.00	0.00	0.00
GATCAACAAGCCTGAGCTCC	0.00	0.33	0.00	0.00	0.00
GATCAACAAGCCTGAGCTGC	0.00	0.00	0.00	0.00	0.34
GATCAACAAGCCTTCTCTGC	0.00	1.30	0.00	0.00	0.00
GATCAACAAGCCTTTCCTGG	0.00	0.00	0.00	0.34	0.00
GATCAACAAGCGAAACATCG	0.00	0.33	0.00	0.00	0.00

GATCAACAAGCGAAAGGAGA	0.00	0.33	0.00	0.00	0.00
GATCAACAAGCGACCTTTTC	0.00	0.00	0.00	0.00	0.00
GATCAACAAGCGACGTGTGA	0.00	0.00	0.00	0.00	0.34
GATCAACAAGCGACTCCTGC	0.00	0.00	0.00	0.00	0.00
GATCAACAAGCGACTCTCCC	0.00	0.00	0.00	0.68	0.00
GATCAACAAGCGACTCTTGC	0.23	0.00	0.00	0.34	0.34
GATCAACAAGCGAGAAAAAA	0.00	0.00	0.00	0.00	0.00
GATCAACAAGCGAGCTTCGA	0.00	0.00	0.00	0.00	0.00
GATCAACAAGCGCAAGCTCC	0.23	0.00	0.00	0.00	0.00
GATCAACAAGCGCACGAAAA	0.00	0.00	0.29	0.00	0.00
GATCAACAAGCGCACGAGCG	0.00	0.33	0.00	0.00	0.00
GATCAACAAGCGCACGCGCG	0.00	0.33	0.29	0.00	0.00
GATCAACAAGCGCACTCGGG	0.00	0.00	0.29	0.00	0.00
GATCAACAAGCGCATGCGCA	0.46	0.65	0.57	0.68	1.38
GATCAACAAGCGCGGCATGG	0.23	0.00	0.00	0.00	0.00
GATCAACAAGCGGAACAACG	0.23	0.00	0.00	0.00	0.00
GATCAACAAGCGGAACACCG	0.00	0.00	0.00	0.00	0.00
GATCAACAAGCGGAACATCG	3.66	5.53	1.43	5.09	1.72
GATCAACAAGCGGCGGGAGT	0.00	0.00	0.29	0.00	0.00
GATCAACAAGCGGCTCTTCG	1.14	0.00	0.00	0.00	0.00
GATCAACAAGCGGGACTTGA	0.00	0.33	0.00	0.34	0.00
GATCAACAAGCGGGCTCGCT	0.00	0.33	0.00	0.00	0.00
GATCAACAAGCGGGGCATGG	0.23	0.00	0.29	0.00	0.00
GATCAACAAGCGTTCGTATG	0.00	0.33	0.00	0.00	0.00
GATCAACAAGCGTTTCAACA	0.00	0.00	0.29	0.00	0.00
GATCAACAAGCTAAAAAAAA	0.46	0.00	0.00	0.00	0.00
GATCAACAAGCTAAGGCTGA	0.00	0.00	0.00	0.00	0.00
GATCAACAAGCTAATCACAC	0.00	0.00	0.29	0.00	0.00
GATCAACAAGCTACCCCACG	0.46	0.33	0.57	1.02	0.69
GATCAACAAGCTCAACGACC	0.23	0.65	0.00	0.34	0.00
GATCAACAAGCTCATCCTCC	0.00	0.00	0.29	0.00	0.00
GATCAACAAGCTCATCGCCG	0.00	0.00	0.00	0.00	0.00
GATCAACAAGCTCATTGGGG	0.00	0.33	0.00	0.00	0.00
GATCAACAAGCTCCGGGAGC	0.00	0.00	0.29	0.68	0.69
GATCAACAAGCTCCTCGAGG	0.00	0.00	0.29	0.00	0.00
GATCAACAAGCTCCTCGCGG	8.24	10.09	11.45	4.75	6.20
GATCAACAAGCTCCTCGCGT	0.00	0.00	0.00	0.00	0.00

GATCAACAAGCTCCTCGCTC	0.46	0.00	0.00	0.00	0.00
GATCAACAAGCTCGAACAAT	0.00	0.00	0.00	0.00	0.34
GATCAACAAGCTCGCACGCT	0.00	0.00	0.00	0.68	0.00
GATCAACAAGCTCGCATGGC	0.00	0.00	0.00	0.00	0.34
GATCAACAAGCTCGCCGTGA	0.00	0.00	0.00	0.00	0.00
GATCAACAAGCTCGCGGGCT	0.00	0.00	0.29	0.00	0.00
GATCAACAAGCTCGGCATGG	0.00	0.00	0.29	0.00	0.00
GATCAACAAGCTCTGCCGGA	0.23	0.00	0.00	0.00	0.00
GATCAACAAGCTGAGGGTGC	0.00	0.00	0.29	0.00	0.00
GATCAACAAGCTGATGAGGG	0.00	0.33	0.00	0.00	0.00
GATCAACAAGCTGATGGCCG	0.00	0.00	0.29	0.00	0.00
GATCAACAAGCTGCACCCGA	0.00	0.33	0.00	0.00	0.00
GATCAACAAGCTGCATTTGC	0.00	0.00	0.29	0.00	0.00
GATCAACAAGCTGCCGGGCA	2.06	2.60	0.57	1.70	3.10
GATCAACAAGCTGCCGGGTC	0.23	0.00	0.00	0.00	0.00
GATCAACAAGCTGCGGACCG	0.23	0.00	0.00	0.00	0.34
GATCAACAAGCTGCGGCACG	0.00	0.00	0.00	0.00	0.00
GATCAACAAGCTGCGGCCCC	0.00	0.00	0.00	0.34	0.00
GATCAACAAGCTGCGGCCCG	5.49	7.81	4.30	5.09	6.54
GATCAACAAGCTGCGGCCGA	0.00	0.00	0.00	0.34	0.00
GATCAACAAGCTGCGGCGCG	0.23	0.00	0.00	0.00	0.00
GATCAACAAGCTGCGGTACG	0.00	0.33	1.72	0.68	0.34
GATCAACAAGCTGCTAGAGC	0.00	0.00	0.29	0.00	0.00
GATCAACAAGCTGGGCTTCA	0.00	0.65	0.00	0.00	0.00
GATCAACAAGCTGGGTCACA	0.00	0.00	0.29	0.00	0.00
GATCAACAAGCTGGGTCACG	0.00	0.33	0.00	0.00	0.00
GATCAACAAGCTGTCGGGCG	0.23	0.00	0.00	0.00	0.00
GATCAACAAGCTTCAGTGTG	0.46	0.00	0.00	0.00	0.00
GATCAACAAGCTTCCCCTCT	0.00	0.00	0.00	0.00	0.00
GATCAACAAGCTTCTGAACA	0.00	0.33	0.00	0.00	0.00
GATCAACAAGCTTGGCACGG	0.00	0.00	0.00	0.00	0.34
GATCAACAAGCTTGGCTGCA	0.00	0.00	0.00	0.00	0.34
GATCAACAAGCTTGGCTTCA	0.00	0.33	0.00	0.00	0.00
GATCAACAAGCTTGGGACTG	0.00	0.00	0.00	0.00	0.34
GATCAACAAGCTTTTCAAGG	0.00	0.00	0.00	0.68	0.00
GATCAACAAGGAAAGCGCGG	0.00	0.00	0.00	0.00	0.00
GATCAACAAGGACAACTCGT	0.00	0.00	0.00	0.34	0.00

GATCAACAAGGACAACTNNC	0.00	0.00	0.00	0.00	0.34
GATCAACAAGGACAACTTAA	0.00	0.33	0.00	0.00	0.00
GATCAACAAGGACAACTTAC	17.85	25.70	20.90	24.44	19.63
GATCAACAAGGACAAGGAGG	0.00	0.00	0.00	0.00	0.34
GATCAACAAGGACAGAGTGG	0.00	0.00	0.00	0.00	0.69
GATCAACAAGGACAGCTGGC	0.00	0.00	0.00	0.00	0.00
GATCAACAAGGACATCATTG	0.00	0.00	0.29	0.68	0.00
GATCAACAAGGACATGCGCG	0.00	0.00	0.00	0.00	0.00
GATCAACAAGGACCCCGATG	0.00	0.00	0.00	0.00	0.00
GATCAACAAGGACCGCAAGG	0.00	0.00	0.29	0.00	0.34
GATCAACAAGGACGGACTGC	0.00	0.00	0.00	0.34	0.34
GATCAACAAGGACGGGCTGC	0.00	0.00	0.00	0.00	0.34
GATCAACAAGGACGGTCTGC	0.46	0.00	0.00	0.00	0.00
GATCAACAAGGACGTCGAGC	0.00	0.33	0.00	0.00	0.00
GATCAACAAGGACGTTCTCC	0.00	0.00	0.00	0.00	0.34
GATCAACAAGGACTCCTTTG	0.00	0.00	0.00	0.00	0.34
GATCAACAAGGACTTCTGCA	0.00	0.00	0.00	0.00	0.00
GATCAACAAGGACTTGAGAG	0.00	0.00	0.00	0.68	0.00
GATCAACAAGGAGAACGACC	0.00	0.33	0.57	1.02	0.69
GATCAACAAGGAGATGCTGG	0.00	0.00	0.29	0.00	0.00
GATCAACAAGGAGCAAGAAG	0.00	0.00	0.00	0.34	0.00
GATCAACAAGGAGCAGAGCG	0.00	0.00	0.00	0.68	0.00
GATCAACAAGGAGCTCCTGC	0.00	0.00	0.00	0.34	0.69
GATCAACAAGGAGCTGATAA	0.00	0.00	0.00	0.00	0.00
GATCAACAAGGAGGACCGTG	0.00	0.00	0.29	0.00	0.00
GATCAACAAGGAGGACCTCG	0.00	0.00	0.00	0.34	0.00
GATCAACAAGGAGGACGCAA	0.00	0.00	0.00	0.00	0.00
GATCAACAAGGAGGCAAAGT	0.00	0.00	0.00	0.00	0.00
GATCAACAAGGAGGCCACAG	0.00	0.00	0.00	0.34	0.00
GATCAACAAGGAGGCTTTGC	0.00	0.00	0.00	0.00	0.00
GATCAACAAGGAGGGAGCCA	0.00	0.65	0.00	0.34	0.00
GATCAACAAGGAGGGCGACA	0.00	0.00	0.29	0.00	0.00
GATCAACAAGGAGGGCGCAA	0.00	0.00	0.29	0.00	0.00
GATCAACAAGGAGGGCGCAC	0.00	0.00	0.29	0.00	0.00
GATCAACAAGGAGGGCGCCA	7.32	27.98	32.07	19.35	33.41
GATCAACAAGGAGGGCGCCC	0.00	0.33	0.29	0.00	0.00
GATCAACAAGGAGGGCGCCN	0.00	0.00	0.00	0.00	0.00

GATCAACAAGGAGGGCGCNN	0.23	0.00	0.00	0.00	0.00
GATCAACAAGGAGGGCGCTA	0.00	0.00	0.00	0.34	0.00
GATCAACAAGGAGGGCGGCA	0.23	0.00	0.00	0.00	0.00
GATCAACAAGGAGGGGGCAA	0.00	0.00	0.00	0.00	0.34
GATCAACAAGGAGGGTGATA	0.46	0.00	0.29	0.00	1.03
GATCAACAAGGAGGGTGATT	0.23	0.00	0.00	0.00	0.00
GATCAACAAGGAGGTCAGAT	0.00	0.00	0.00	0.00	0.00
GATCAACAAGGAGGTTGAGA	0.00	0.00	0.00	0.00	0.00
GATCAACAAGGAGTGCATTA	0.23	0.00	0.00	0.00	0.00
GATCAACAAGGAGTGCTCTT	0.00	0.00	0.00	0.34	0.00
GATCAACAAGGATAAAAAAA	0.00	0.00	0.00	0.00	0.00
GATCAACAAGGATATTGCGG	0.23	0.65	0.00	0.00	0.00
GATCAACAAGGCAAAAAAAA	0.69	0.33	0.29	0.34	0.34
GATCAACAAGGCAACGCCGC	0.00	0.00	0.29	0.00	0.00
GATCAACAAGGCACAACACA	0.00	0.00	0.57	0.00	0.00
GATCAACAAGGCACAGCGTG	0.00	0.00	0.00	0.34	0.00
GATCAACAAGGCAGCATGCT	0.00	0.00	0.29	0.00	0.00
GATCAACAAGGCAGTGCATC	0.00	0.00	0.00	0.00	0.00
GATCAACAAGGCAGTTATCA	0.00	0.33	0.00	0.34	0.00
GATCAACAAGGCATCACGGT	0.00	0.00	0.29	0.00	0.00
GATCAACAAGGCATCACGTC	0.00	0.00	0.00	0.00	0.34
GATCAACAAGGCATCACGTT	5.04	0.65	2.00	1.02	0.69
GATCAACAAGGCCAAGCCCG	0.00	0.00	0.00	0.00	0.00
GATCAACAAGGCCAGAGATG	0.00	0.00	0.00	0.00	0.00
GATCAACAAGGCCATGCAGC	0.00	0.33	0.00	0.00	0.00
GATCAACAAGGCCCACCAGG	0.23	0.00	0.00	0.00	0.00
GATCAACAAGGCCCACGAGG	0.00	0.65	0.00	0.00	0.00
GATCAACAAGGCCCCGATGC	0.23	0.00	0.00	0.00	0.00
GATCAACAAGGCCCTCGTGT	0.23	0.00	0.29	0.00	0.00
GATCAACAAGGCCGAGGCGC	0.00	0.00	0.00	0.34	0.00
GATCAACAAGGCCGAGGTGA	0.00	0.00	0.29	0.00	0.00
GATCAACAAGGCCGAGGTGC	0.23	0.33	0.57	2.72	2.41
GATCAACAAGGCCGCGGTGC	0.00	0.33	0.00	0.00	0.00
GATCAACAAGGCCGTGCTCG	0.00	0.33	0.00	0.00	0.00
GATCAACAAGGCCGTTATCA	0.00	0.33	0.00	0.00	0.00
GATCAACAAGGCCTCCAAGA	0.00	0.00	0.29	0.00	0.00
GATCAACAAGGCCTTGCTAG	0.00	0.00	0.00	0.00	0.00

GATCAACAAGGCCTTGGCTG	0.00	0.00	0.00	0.00	0.00
GATCAACAAGGCCTTGTCTG	0.00	0.00	0.00	0.34	0.34
GATCAACAAGGCGAAGGCCA	0.00	0.00	0.29	0.00	0.34
GATCAACAAGGCGAGAGCCA	0.00	0.33	0.00	0.00	0.00
GATCAACAAGGCGCTACTAA	0.23	0.00	0.00	0.00	0.00
GATCAACAAGGCGCTGAAAA	0.00	0.00	0.00	0.34	0.00
GATCAACAAGGCGCTGACAA	0.00	0.00	0.00	0.00	0.69
GATCAACAAGGCGCTGAGAA	0.23	0.00	0.29	0.00	0.00
GATCAACAAGGCGCTGATAA	22.43	21.15	33.22	31.57	32.37
GATCAACAAGGCGCTGATAC	0.00	0.00	0.29	0.00	0.00
GATCAACAAGGCGCTGATAN	0.23	0.00	0.00	0.00	0.00
GATCAACAAGGCGCTGATGA	0.23	0.00	0.00	0.00	0.00
GATCAACAAGGCGCTGATTT	0.00	0.00	0.00	0.00	0.34
GATCAACAAGGCGCTGCTAA	0.00	0.00	0.00	0.00	0.00
GATCAACAAGGCGCTGGTAA	0.00	0.00	0.29	0.00	0.00
GATCAACAAGGCGGACCTCG	0.00	0.00	0.00	0.00	0.34
GATCAACAAGGCGGAGAGCA	0.00	0.33	0.00	0.00	0.00
GATCAACAAGGCGGAGCGCA	0.00	0.00	0.57	0.34	0.00
GATCAACAAGGCGGAGCGCC	0.00	0.00	0.00	0.34	0.00
GATCAACAAGGCGGAGTCCG	0.00	0.33	0.00	0.68	0.00
GATCAACAAGGCGGATTCCT	0.00	0.00	0.00	0.00	0.00
GATCAACAAGGCGGGCCAAG	0.00	0.00	0.00	0.34	0.00
GATCAACAAGGCGGGCCGCC	0.00	0.00	0.00	0.34	0.34
GATCAACAAGGCGGGGCGCC	0.23	0.00	0.00	0.00	0.00
GATCAACAAGGCGGGTGATA	0.23	0.00	0.00	0.00	0.00
GATCAACAAGGCGTCGCGTG	0.23	0.00	0.00	0.00	0.00
GATCAACAAGGCGTTGATAA	0.00	0.00	0.29	0.00	0.00
GATCAACAAGGCGTTTGAGA	0.00	0.98	0.86	1.02	0.00
GATCAACAAGGCGTTTGATC	0.00	0.00	0.00	0.00	0.34
GATCAACAAGGCTCCGGCAA	0.00	0.00	0.29	0.00	0.00
GATCAACAAGGCTCCTATGT	0.00	0.00	0.00	0.00	0.34
GATCAACAAGGCTCCTCTGT	1.14	0.65	1.15	1.36	0.69
GATCAACAAGGCTCGGCGCA	0.00	0.00	0.00	0.00	0.00
GATCAACAAGGCTCTCTGGT	0.00	0.00	0.00	0.00	0.34
GATCAACAAGGCTGGGGAGT	0.00	0.00	0.00	0.00	0.00
GATCAACAAGGCTGGGGCAT	0.00	0.00	0.29	0.00	0.00
GATCAACAAGGCTGTCACTC	0.00	0.00	0.00	0.00	0.69

GATCAACAAGGCTGTGAATG	0.00	0.33	0.00	0.00	0.00
GATCAACAAGGCTGTGCCGT	0.00	0.00	0.29	0.00	0.00
GATCAACAAGGCTGTGGAAT	0.00	0.00	0.00	0.00	0.00
GATCAACAAGGCTGTGGAGC	0.00	0.00	0.00	0.00	0.34
GATCAACAAGGCTGTGGAGG	0.00	0.00	0.00	0.34	0.00
GATCAACAAGGCTGTGGAGT	17.63	10.74	10.31	6.79	8.61
GATCAACAAGGCTGTGGCGT	0.23	0.00	0.00	0.68	0.00
GATCAACAAGGCTGTGGGGT	0.23	0.00	0.00	0.00	0.00
GATCAACAAGGCTGTGGTTT	0.00	0.00	0.00	0.00	0.34
GATCAACAAGGCTGTGNGNN	0.23	0.00	0.00	0.00	0.00
GATCAACAAGGCTGTGTAGT	0.00	0.00	0.00	0.68	0.00
GATCAACAAGGCTGTTGCCG	0.00	0.33	0.00	0.00	0.00
GATCAACAAGGGAATACCGC	0.00	0.33	0.57	0.34	0.00
GATCAACAAGGGAGCGGAGA	0.00	0.33	0.00	0.00	0.00
GATCAACAAGGGAGCGGTGG	0.00	0.33	0.00	0.34	0.00
GATCAACAAGGGCAAGGAGC	0.00	0.00	0.00	0.34	0.00
GATCAACAAGGGCAAGGCCA	0.00	0.00	0.00	0.34	0.00
GATCAACAAGGGCACAAACG	1.37	0.98	1.72	0.00	0.69
GATCAACAAGGGCACACACG	0.00	0.00	0.00	0.00	0.00
GATCAACAAGGGCACAGGGG	0.00	0.00	0.00	0.00	0.00
GATCAACAAGGGCATACTCG	0.00	0.00	1.15	0.00	1.03
GATCAACAAGGGCATGGTGG	0.00	0.00	0.00	0.00	0.00
GATCAACAAGGGCCGCCTTG	0.00	0.00	0.29	0.00	0.00
GATCAACAAGGGCCGCGATA	0.00	0.00	0.57	0.00	0.00
GATCAACAAGGGCCGCGTGC	0.00	0.00	0.00	0.00	0.34
GATCAACAAGGGCCGGCTGG	0.23	0.00	0.00	0.00	0.00
GATCAACAAGGGCGCCACGG	0.00	0.00	0.00	0.00	0.34
GATCAACAAGGGCTGAGGCC	0.23	0.00	0.00	0.00	0.00
GATCAACAAGGGCTGAGGGA	0.23	0.00	0.00	0.00	0.00
GATCAACAAGGGGAATGAAG	0.00	0.33	0.00	0.00	0.00
GATCAACAAGGGGAATGACC	2.06	1.30	0.29	2.38	0.34
GATCAACAAGGGGAATGCCC	0.00	0.33	0.00	0.00	0.00
GATCAACAAGGGGCGTATGG	0.00	0.00	0.00	0.00	0.69
GATCAACAAGGGGGACATCG	0.00	0.00	0.00	0.00	0.34
GATCAACAAGGGGGACCTCG	0.23	0.00	0.00	0.00	0.00
GATCAACAAGGGGGCCATCT	0.46	0.33	0.00	0.00	0.00
GATCAACAAGGGGGCCATGG	0.00	0.00	0.00	0.00	0.00

GATCAACAAGGGGGGAGGGA	0.00	0.00	0.00	0.00	0.00
GATCAACAAGGGTGTGGTCG	0.00	0.00	0.00	0.00	0.00
GATCAACAAGGTAATCCGCC	0.00	0.00	0.29	0.00	0.00
GATCAACAAGGTAGGCATAA	0.00	0.00	0.00	0.00	0.34
GATCAACAAGGTAGTCCACC	0.00	0.00	0.29	0.00	0.00
GATCAACAAGGTCAATTTCA	0.00	0.00	0.00	0.68	0.00
GATCAACAAGGTCAGAAGAA	0.23	0.00	0.00	0.00	0.00
GATCAACAAGGTCAGGAGTG	0.00	0.00	0.00	0.34	0.00
GATCAACAAGGTCATCCTAC	0.00	0.33	0.00	0.00	0.00
GATCAACAAGGTCATCCTCA	0.00	0.33	0.00	0.00	0.00
GATCAACAAGGTCATCCTCC	17.85	9.11	14.32	5.77	6.89
GATCAACAAGGTCATCCTCT	0.92	0.00	0.00	0.00	0.00
GATCAACAAGGTCATCCTTC	0.23	0.00	0.00	0.00	0.00
GATCAACAAGGTCATCTTCC	0.00	0.00	0.29	0.00	0.00
GATCAACAAGGTCATGGGTG	0.00	0.33	0.57	0.00	0.69
GATCAACAAGGTCATTGGTG	0.00	0.00	0.00	0.00	0.00
GATCAACAAGGTCCAGCTCC	0.00	0.00	0.00	0.00	0.34
GATCAACAAGGTCCAGGTGA	0.23	0.00	0.00	0.00	0.00
GATCAACAAGGTCCTGAAGT	0.00	0.00	0.00	0.00	0.00
GATCAACAAGGTCCTTCGTG	0.00	0.00	0.00	0.00	0.34
GATCAACAAGGTCGAGGTCT	0.00	0.00	0.00	0.00	0.00
GATCAACAAGGTCTCTTACG	0.00	0.33	0.00	0.00	0.00
GATCAACAAGGTCTTGAGCC	0.00	0.00	0.00	0.34	0.00
GATCAACAAGGTGAAGAACG	0.00	0.00	0.00	0.00	0.00
GATCAACAAGGTGAAGGGCT	0.23	0.00	0.00	0.00	0.00
GATCAACAAGGTGAGCACGG	0.23	0.00	0.00	0.00	0.00
GATCAACAAGGTGCGGCCCG	0.23	0.00	0.00	0.00	0.00
GATCAACAAGGTGCTGACCG	0.00	0.33	0.00	0.00	0.00
GATCAACAAGGTGCTGATAC	0.23	0.00	0.00	0.00	0.00
GATCAACAAGGTGGACATCG	0.23	0.00	0.00	0.00	0.00
GATCAACAAGGTGGACCCCA	0.00	0.00	0.00	0.34	0.00
GATCAACAAGGTGGACCGCG	0.23	0.00	0.00	0.00	0.00
GATCAACAAGGTGGACCTCG	11.45	22.77	17.75	12.90	17.91
GATCAACAAGGTGGACCTGG	0.00	0.33	0.00	0.00	0.00
GATCAACAAGGTGGAGCGGC	0.23	0.00	0.00	0.00	0.00
GATCAACAAGGTGGAGCTCG	0.23	0.00	0.00	0.00	0.00
GATCAACAAGGTGGTGGTGG	0.69	0.00	0.29	0.34	0.00

GATCAACAAGGTTCTCAAGG	0.00	0.33	0.00	0.00	0.00
GATCAACAAGGTTGCTGGTG	0.00	0.00	0.29	0.00	0.00
GATCAACAAGGTTGGCAAGG	0.00	0.00	0.29	0.00	0.00
GATCAACAAGGTTGGCCCGG	0.00	0.00	0.00	0.00	0.69
GATCAACAAGGTTGGGAACC	0.00	0.00	0.00	0.00	0.00
GATCAACAAGGTTGTAAAGG	0.00	0.00	0.29	0.00	0.00
GATCAACAAGGTTGTACCGG	0.23	0.00	0.00	0.00	0.00
GATCAACAAGGTTGTCAAGG	12.59	6.83	4.87	9.50	7.92
GATCAACAAGTAAAAAAAAA	0.00	0.00	0.00	0.34	0.00
GATCAACAAGTAAAAACCAT	0.23	0.00	0.00	0.00	0.00
GATCAACAAGTAAGAGGAGA	0.00	0.00	0.00	0.00	0.34
GATCAACAAGTACAAAAAGC	0.00	0.00	0.00	0.34	0.00
GATCAACAAGTACCAACCGC	0.00	0.00	0.00	0.68	0.00
GATCAACAAGTACGCGGCTG	0.00	0.00	0.00	0.00	0.34
GATCAACAAGTACGGGGAAA	0.00	0.33	0.00	0.00	0.00
GATCAACAAGTATAAGCTGG	0.00	0.00	0.00	0.00	0.00
GATCAACAAGTATAAGCTGT	0.00	0.00	0.29	0.00	0.00
GATCAACAAGTATCCTTCCG	0.00	0.00	0.00	0.00	0.00
GATCAACAAGTATCTTGACC	0.00	0.33	0.00	0.34	0.00
GATCAACAAGTATGGCGCAG	0.00	0.00	0.00	0.34	0.34
GATCAACAAGTCAGTGGCCA	0.00	0.00	0.00	0.00	0.00
GATCAACAAGTCATGGCTTG	0.00	0.00	0.00	0.00	0.34
GATCAACAAGTCCACGCCTC	0.00	0.00	0.00	0.00	0.34
GATCAACAAGTCCCCGATGC	0.46	0.33	0.00	0.00	0.00
GATCAACAAGTCCCTGACGG	0.00	0.33	0.57	0.00	0.00
GATCAACAAGTCCCTGTTGG	0.00	1.30	0.57	0.34	0.00
GATCAACAAGTCCGGGGTGC	0.00	0.00	0.29	0.00	0.00
GATCAACAAGTCCTCACAAA	0.92	0.00	0.00	0.00	0.00
GATCAACAAGTCCTGCAAGA	0.92	0.00	0.57	0.00	0.34
GATCAACAAGTCGCTCACGG	0.00	0.00	0.00	0.00	0.00
GATCAACAAGTCGCTCACTG	0.23	0.00	0.00	0.00	1.03
GATCAACAAGTCGCTCTCAG	0.00	0.00	0.00	0.00	0.00
GATCAACAAGTCTCCAAGTG	0.00	0.00	0.00	0.00	0.00
GATCAACAAGTCTCTTGACC	0.00	0.00	0.29	0.00	0.00
GATCAACAAGTGAGCCATCA	0.00	0.00	0.00	0.00	0.34
GATCAACAAGTGCACTGCAA	0.00	0.33	0.00	0.00	0.00
GATCAACAAGTGCACTGCAC	0.00	0.00	0.57	0.68	0.00

GATCAACAAGTGCAGAAATC	1.37	0.00	0.00	0.00	0.00
GATCAACAAGTGCTGCATGA	0.00	0.65	0.00	1.02	0.00
GATCAACAAGTGGACGGGGA	0.00	0.00	0.00	0.00	0.00
GATCAACAAGTGGATGGTGG	0.23	0.00	0.00	1.02	0.00
GATCAACAAGTGGCATGCAC	0.00	0.33	0.00	0.00	0.00
GATCAACAAGTGGCTCAGTA	0.00	0.00	0.00	0.00	0.34
GATCAACAAGTGGGACATCT	0.00	0.33	0.00	0.34	0.00
GATCAACAAGTGGGATCGAT	0.00	0.00	0.29	0.00	0.00
GATCAACAAGTGGGCATCTC	0.00	0.00	0.00	0.34	0.00
GATCAACAAGTGGGCCAACT	0.00	0.00	0.00	0.00	0.00
GATCAACAAGTGGGCCATCN	0.46	0.00	0.00	0.00	0.00
GATCAACAAGTGGGCCATCT	31.36	21.47	10.31	21.72	19.98
GATCAACAAGTGGGCCATNN	0.23	0.00	0.00	0.00	0.00
GATCAACAAGTGGGCCCTCT	0.23	0.00	0.00	0.00	0.00
GATCAACAAGTGGGCCNNNN	0.23	0.00	0.00	0.00	0.00
GATCAACAAGTGGGCGCGCT	0.23	0.00	0.00	0.00	0.00
GATCAACAAGTGGGCTATCT	0.23	0.00	0.00	0.00	0.00
GATCAACAAGTGGGCTCGAT	0.00	0.33	0.00	0.00	0.00
GATCAACAAGTGGGCTCGCC	0.00	0.00	0.00	0.00	0.34
GATCAACAAGTGGGCTCGCN	0.00	0.00	0.00	0.00	0.00
GATCAACAAGTGGGCTCGCT	15.11	26.35	9.16	27.49	19.29
GATCAACAAGTGGGCTCGGT	0.23	0.00	0.00	0.00	0.00
GATCAACAAGTGGGCTCGTA	0.00	0.00	0.00	0.34	0.00
GATCAACAAGTGGGCTTGCT	0.00	0.00	0.00	0.00	0.00
GATCAACAAGTGGGGCATCT	0.00	0.33	0.00	0.00	0.00
GATCAACAAGTGGTCCATCT	0.00	0.00	0.00	0.00	0.00
GATCAACAAGTGGTGGACTG	0.23	0.00	0.00	0.68	0.00
GATCAACAAGTGGTTTGAGC	0.00	0.00	0.00	0.00	0.00
GATCAACAAGTGTACTTCGC	0.00	0.00	0.29	0.00	0.00
GATCAACAAGTGTCGTCTTA	0.00	0.00	0.00	0.68	0.00
GATCAACAAGTGTGCCATCT	0.00	0.33	0.00	0.00	0.00
GATCAACAAGTTAAACTTTG	0.00	0.00	0.00	0.00	0.00
GATCAACAAGTTAGAAAAAA	0.00	0.00	0.00	0.00	0.69
GATCAACAAGTTCACGCCCG	0.00	0.00	0.00	0.34	0.00
GATCAACAAGTTCCAGCTCC	0.23	0.00	0.00	0.00	0.00
GATCAACAAGTTCCAGGGCC	0.00	0.00	0.00	0.00	0.00
GATCAACAAGTTCCAGGTGA	0.23	0.00	0.00	0.68	0.00

GATCAACAAGTTCCAGGTGG	0.46	0.00	0.00	0.00	0.00
GATCAACAAGTTCCTCTTCC	0.00	0.33	0.00	0.34	0.00
GATCAACAAGTTCGCTGCGA	0.00	0.65	0.29	3.73	1.03
GATCAACAAGTTCTACGACT	0.00	0.33	0.00	0.00	0.00
GATCAACAAGTTGAGGCTCG	0.00	0.00	0.00	0.00	0.00
GATCAACAAGTTGATGTAAA	0.00	0.00	0.29	0.00	0.00
GATCAACAAGTTGATGTCAA	2.75	1.30	0.57	2.04	0.00
GATCAACAAGTTGATGTTAA	0.23	0.00	0.00	0.00	0.00
GATCAACAAGTTGCTTAGCG	0.00	0.00	0.00	0.00	0.00
GATCAACAAGTTGGCCATCT	0.00	0.00	0.29	0.00	0.00
GATCAACAAGTTGGTGTCAA	0.00	0.00	0.00	0.00	0.00
GATCAACAAGTTGTTGGTGT	0.00	0.00	0.00	0.00	0.69
GATCAACAAGTTTGCGAGGA	0.00	0.00	0.00	0.00	0.69
GATCAACAAGTTTGCGCACC	0.00	0.00	0.00	0.34	0.00
GATCAACAAGTTTGCTGCGA	0.23	0.00	0.00	0.68	0.00
GATCAACAATAAAAAAAAAA	0.00	0.33	0.00	0.00	0.00
GATCAACAATAAAGAAGAAA	0.00	0.00	0.57	0.00	0.00
GATCAACAATAACGTTTAGT	0.00	0.00	0.00	0.00	0.69
GATCAACAATAATGACAATA	0.00	0.00	0.00	0.00	0.34
GATCAACAATAATGAGGCCG	0.00	0.00	0.00	0.00	0.00
GATCAACAATACCAGAAGGC	0.00	0.00	0.00	0.00	0.00
GATCAACAATACGCTGAAAA	0.00	0.00	0.00	0.34	0.00
GATCAACAATACGCTGCTTA	0.00	0.00	0.00	0.00	0.34
GATCAACAATACTAGGAATG	0.23	0.00	0.00	0.00	0.00
GATCAACAATAGAAAAAAAA	0.00	0.00	0.00	0.00	0.00
GATCAACAATAGCGAGTCCT	0.00	0.00	0.00	0.00	0.00
GATCAACAATAGCTTGTTTT	0.00	0.00	0.00	0.34	0.00
GATCAACAATAGGGGCACAG	0.00	0.00	0.00	0.00	0.00
GATCAACAATAGTGGCACAG	5.04	14.31	8.02	6.79	7.23
GATCAACAATAGTGGCACCG	0.23	0.00	0.00	0.00	0.00
GATCAACAATAGTGGCACCN	0.23	0.00	0.00	0.00	0.00
GATCAACAATATCAACACCA	0.00	0.00	0.00	0.00	1.03
GATCAACAATATCTGTGTGC	0.00	0.00	0.00	0.00	0.34
GATCAACAATATCTTTGTGT	0.23	0.00	0.29	0.34	0.00
GATCAACAATATTGCGCTGA	0.00	0.00	0.00	0.00	0.00
GATCAACAATCAAAAAAAAA	0.00	0.00	0.00	0.00	0.34
GATCAACAATCAAACGCAAC	0.00	0.00	0.00	0.00	0.34

GATCAACAATCAAGCAAAGG	0.00	0.00	0.00	0.00	0.34
GATCAACAATCACGAGAACA	0.00	0.00	0.00	0.00	0.00
GATCAACAATCAGAGAAGGC	0.00	0.00	0.29	0.00	0.00
GATCAACAATCAGGAGTCCG	0.23	0.00	0.00	0.00	0.00
GATCAACAATCAGGAGTCTG	10.99	5.86	2.00	3.39	1.38
GATCAACAATCAGGAGTGTG	0.00	0.00	0.00	0.00	0.00
GATCAACAATCATTGTATTT	0.00	0.00	0.29	0.00	0.00
GATCAACAATCATTTGAACG	0.00	0.00	0.00	0.00	0.00
GATCAACAATCCCGGTCGCA	0.23	0.00	0.00	0.00	0.34
GATCAACAATCCGACGCGAG	0.00	0.33	0.00	0.00	0.00
GATCAACAATCCGCACGTCT	0.00	0.00	0.00	0.00	0.00
GATCAACAATCGAACAGTCG	0.00	0.00	0.00	0.00	0.69
GATCAACAATCGAGCAGCTG	0.00	0.33	0.00	0.00	0.00
GATCAACAATCGCCGCCTTG	0.00	0.33	0.00	0.00	0.00
GATCAACAATCGCTGTCATG	0.00	0.00	0.00	0.00	0.00
GATCAACAATCGGGCCGTCA	0.00	0.00	0.00	0.00	0.00
GATCAACAATCTCATCTCAT	0.00	0.00	0.00	0.00	0.34
GATCAACAATCTCATGAATC	0.23	0.00	0.86	0.00	0.69
GATCAACAATCTCCGACCCC	0.00	0.33	0.00	0.00	0.00
GATCAACAATCTCCTCCAGG	0.00	0.00	0.29	0.00	0.00
GATCAACAATCTCCTTTCCA	0.00	0.00	0.00	0.34	0.00
GATCAACAATCTGCATGCCT	0.00	0.00	0.29	0.00	0.00
GATCAACAATGAAACGGGAA	0.00	0.00	0.00	0.34	0.00
GATCAACAATGAAGACCAGG	0.00	0.00	0.00	0.00	0.00
GATCAACAATGAATGGACAA	0.00	0.33	0.00	0.00	0.00
GATCAACAATGACCAATGCG	0.00	0.00	0.00	0.34	0.00
GATCAACAATGACCGCACGG	0.00	0.00	0.57	0.00	0.00
GATCAACAATGACCGCGTCT	0.00	0.00	0.00	0.00	0.00
GATCAACAATGACCTCTTCA	0.46	0.33	0.57	0.68	1.38
GATCAACAATGACGCCACAT	0.00	0.00	0.29	0.00	0.00
GATCAACAATGACGCCACGA	0.00	0.00	0.00	0.00	0.00
GATCAACAATGACTATATTT	0.00	0.00	0.29	0.00	0.00
GATCAACAATGACTTGGGAA	0.00	0.00	0.29	0.00	0.00
GATCAACAATGAGAAGCCGC	0.00	0.00	0.00	0.00	0.34
GATCAACAATGAGCGTCTCA	0.23	0.00	0.00	0.00	0.00
GATCAACAATGAGCTCAAGG	0.00	0.00	0.00	0.00	0.00
GATCAACAATGAGCTCTGTG	0.00	0.00	0.00	0.00	0.00

GATCAACAATGAGTGGGACA	3.43	0.98	1.72	1.02	1.38
GATCAACAATGATGTAATGG	0.00	0.00	0.00	0.00	0.69
GATCAACAATGCAAAAAAAA	0.23	0.00	0.00	0.34	0.00
GATCAACAATGCAAACGCAC	0.00	0.00	0.00	0.00	0.00
GATCAACAATGCAACTGCTT	0.00	0.65	0.29	0.68	0.00
GATCAACAATGCAGGCAACC	0.23	0.00	0.00	0.00	0.00
GATCAACAATGCAGGGAAAA	0.00	0.00	0.00	0.00	0.00
GATCAACAATGCAGGGAACC	0.00	0.00	0.86	0.00	0.00
GATCAACAATGCAGGGACAT	0.00	0.00	0.00	0.00	0.00
GATCAACAATGCAGGGACTT	0.46	0.65	0.29	1.36	0.34
GATCAACAATGCAGGGAGAG	0.00	0.00	0.00	0.00	0.34
GATCAACAATGCAGGGCAAC	0.23	0.00	0.00	0.00	0.00
GATCAACAATGCAGGGGGCA	0.00	0.00	0.00	0.34	0.00
GATCAACAATGCAGTGATAG	0.00	0.00	0.00	0.68	0.00
GATCAACAATGCCAGAAGGC	0.00	0.00	0.00	0.00	0.00
GATCAACAATGCCAGTGTCC	0.00	0.00	0.00	0.00	0.00
GATCAACAATGCCCCTCAGT	0.00	0.33	0.57	0.34	0.00
GATCAACAATGCCCGCCTTG	0.00	0.00	0.00	0.34	0.00
GATCAACAATGCCGAAAAGG	0.00	0.00	0.00	0.00	0.34
GATCAACAATGCCTACCAGG	0.00	0.00	0.00	0.68	0.34
GATCAACAATGCCTCATTCG	0.00	0.00	0.00	0.00	1.03
GATCAACAATGCGAAAAAAA	0.69	0.00	0.00	0.00	0.00
GATCAACAATGCGAACATGA	0.00	0.00	0.00	0.00	0.00
GATCAACAATGCGAAGGCAA	0.00	0.00	0.29	0.00	0.00
GATCAACAATGCGATGTCGA	0.00	0.00	0.00	0.34	0.00
GATCAACAATGCGTTGATGT	0.23	0.00	0.00	0.00	0.00
GATCAACAATGCTAAAGATT	0.00	0.00	0.00	0.00	0.00
GATCAACAATGCTCTTGACT	0.00	0.00	0.00	0.00	0.69
GATCAACAATGCTTAACGAA	0.00	0.00	0.00	0.00	0.34
GATCAACAATGGAAGAGGCA	0.00	0.00	0.00	0.34	0.00
GATCAACAATGGACAGGAAA	0.00	0.00	0.00	0.34	0.00
GATCAACAATGGACAGTGAG	0.00	0.33	0.00	0.00	0.00
GATCAACAATGGACGCCTTG	0.00	0.00	0.00	0.00	0.00
GATCAACAATGGAGACTACT	0.00	0.33	0.00	0.00	0.00
GATCAACAATGGAGGCACAT	0.00	0.00	0.00	0.00	0.69
GATCAACAATGGCAAAGAAC	0.00	0.00	0.00	0.00	0.00
GATCAACAATGGCAAGGAGG	0.00	2.60	1.15	2.04	2.41

GATCAACAATGGCAGCCTCG	0.00	0.00	0.00	0.00	0.34
GATCAACAATGGCAGCCTTG	0.46	0.00	0.00	0.00	0.00
GATCAACAATGGCAGCGTTT	0.00	0.33	0.00	0.00	0.00
GATCAACAATGGCAGGGAGG	0.00	0.33	0.00	0.00	0.00
GATCAACAATGGCATCAAGT	0.00	0.00	0.00	0.00	0.00
GATCAACAATGGCATGTTTG	0.00	0.00	0.29	0.00	0.00
GATCAACAATGGCCACCTTG	0.00	0.00	0.00	0.34	0.00
GATCAACAATGGCCATCATC	0.92	1.30	0.00	2.72	0.00
GATCAACAATGGCCGAAAGG	0.00	0.00	0.29	0.00	0.00
GATCAACAATGGCCGACTTG	0.00	0.00	0.00	0.34	0.00
GATCAACAATGGCCGCATTG	0.00	0.00	0.00	0.34	0.69
GATCAACAATGGCCGCCCTG	0.23	0.98	0.00	0.34	0.00
GATCAACAATGGCCGCCGTG	0.69	0.65	0.00	0.00	0.34
GATCAACAATGGCCGCCNNN	0.23	0.00	0.00	0.00	0.00
GATCAACAATGGCCGCCTAG	0.00	0.00	0.00	0.00	0.34
GATCAACAATGGCCGCCTCC	0.00	0.00	0.00	0.00	0.34
GATCAACAATGGCCGCCTCG	1.60	2.28	0.00	2.04	0.00
GATCAACAATGGCCGCCTGG	0.23	0.98	0.00	0.34	0.00
GATCAACAATGGCCGCCTNN	1.14	0.00	0.00	0.00	0.00
GATCAACAATGGCCGCCTTA	0.00	0.00	0.00	0.00	0.69
GATCAACAATGGCCGCCTTC	0.00	0.00	0.00	0.00	0.34
GATCAACAATGGCCGCCTTG	157.94	163.31	66.72	144.26	58.89
GATCAACAATGGCCGCCTTN	0.23	0.00	0.00	0.00	0.00
GATCAACAATGGCCGCCTTT	0.00	0.33	0.29	0.68	0.69
GATCAACAATGGCCGCGCGG	0.00	0.00	0.00	0.34	0.00
GATCAACAATGGCCGCGTTG	0.23	0.00	0.00	0.00	0.00
GATCAACAATGGCCGCNNNN	0.23	0.00	0.00	0.00	0.00
GATCAACAATGGCCGGCTGG	0.23	0.00	0.00	0.00	0.00
GATCAACAATGGCCGTCTGG	0.23	0.33	0.00	0.00	0.00
GATCAACAATGGCCGTCTTC	0.23	0.00	0.00	0.00	0.00
GATCAACAATGGCCGTCTTG	0.23	0.00	0.00	0.00	0.00
GATCAACAATGGCCTGGGCA	0.00	0.00	0.29	0.00	0.34
GATCAACAATGGCGATTATT	0.00	0.00	0.00	0.34	0.00
GATCAACAATGGCTGCCTTG	0.23	0.00	0.00	0.00	0.00
GATCAACAATGGCTTCATCC	0.00	0.00	0.00	0.00	0.00
GATCAACAATGGCTTCATCG	0.00	0.00	0.00	0.00	0.00
GATCAACAATGGGCAATCGC	0.00	0.00	0.57	0.00	0.00

GATCAACAATGGGCGGCCCG	0.00	0.00	0.29	0.00	0.00
GATCAACAATGGGCTGCGAT	0.00	0.33	0.00	0.00	0.00
GATCAACAATGGGGATTATT	0.00	0.00	0.00	0.68	0.69
GATCAACAATGGGGGCTGAT	0.23	0.00	0.00	0.00	0.00
GATCAACAATGGTCGATTGG	1.37	0.33	0.57	4.07	0.69
GATCAACAATGGTCGATTGT	0.00	0.00	0.00	0.00	0.34
GATCAACAATGGTGGAAAGG	0.00	0.00	0.00	0.34	0.00
GATCAACAATGGTGGTCACG	0.00	0.00	0.57	0.00	0.00
GATCAACAATGTACAAATAC	0.23	0.00	0.00	0.00	0.00
GATCAACAATGTACTAATTG	0.23	0.33	0.29	1.02	0.00
GATCAACAATGTCAGCTTGG	0.00	0.00	0.00	0.00	0.34
GATCAACAATGTCCACCTGG	0.00	0.00	0.57	0.00	0.00
GATCAACAATGTCTGAAATC	0.00	0.00	0.00	0.00	0.00
GATCAACAATGTCTGTGGCG	0.00	0.00	0.29	0.00	0.00
GATCAACAATGTGCCTGTCT	0.00	0.00	0.00	0.00	0.00
GATCAACAATGTGCTCATTG	0.00	0.00	0.00	0.00	0.00
GATCAACAATGTGCTGCCAA	0.00	0.00	0.00	0.00	0.00
GATCAACAATGTGCTGCGAT	0.00	0.33	0.00	0.00	0.00
GATCAACAATGTGCTGCTCT	0.00	0.65	0.00	0.00	0.00
GATCAACAATGTGCTGTGTG	0.00	0.00	0.29	0.00	0.00
GATCAACAATGTGGACGTAG	0.00	0.33	0.00	0.00	0.00
GATCAACAATGTGTTTGTCA	0.00	0.00	0.00	0.00	0.34
GATCAACAATGTTGCCCATG	0.00	0.00	0.00	0.00	0.69
GATCAACAATGTTGTGGCGG	0.00	0.00	0.00	0.68	0.00
GATCAACAATTACGAGTTTG	0.00	0.00	0.00	0.00	0.00
GATCAACAATTACTTGCGGT	0.00	0.00	0.00	0.34	0.00
GATCAACAATTAGCACTGGG	0.00	0.00	0.29	0.00	0.00
GATCAACAATTCCAAGAGCT	0.00	0.00	0.00	0.34	0.00
GATCAACAATTCGAAGGAGT	0.23	0.00	0.00	0.00	0.00
GATCAACAATTCTACGTTGG	0.00	0.00	0.29	0.00	0.00
GATCAACAATTGAAAAGGGC	0.00	0.00	0.00	0.00	0.34
GATCAACAATTGAAAAGTGC	0.00	0.00	0.00	0.00	0.34
GATCAACAATTGAGGTCTTT	0.00	0.33	0.00	0.00	0.00
GATCAACAATTGCAAGAGCT	0.00	0.33	0.00	0.00	0.00
GATCAACAATTGCACCCAAG	0.00	0.00	0.00	0.00	0.69
GATCAACAATTGCGTTGACC	0.00	0.00	0.00	0.00	0.00
GATCAACAATTGCGTTGGAC	0.00	0.00	0.00	0.00	0.00

GATCAACAATTGCTCCAACC	0.00	0.00	0.00	0.00	0.34
GATCAACAATTGGAAGTAGC	0.46	0.00	0.00	0.00	0.00
GATCAACAATTGGAAGTGTT	0.00	0.00	0.00	0.00	0.34
GATCAACAATTGTGGCACAG	0.00	0.33	0.00	0.00	0.00
GATCAACAATTTAATTGCTA	0.46	0.00	0.00	0.00	0.00
GATCAACAATTTATACCATT	0.00	0.00	0.00	0.00	0.00
GATCAACAATTTCAAGAGAC	0.00	0.00	0.00	0.00	0.34
GATCAACAATTTCAAGAGCN	0.23	0.00	0.00	0.00	0.00
GATCAACAATTTCAAGAGCT	56.54	42.29	42.09	27.83	31.34
GATCAACAATTTCAAGAGNN	0.23	0.00	0.00	0.00	0.00
GATCAACAATTTCAAGAGNT	0.00	0.00	0.00	0.34	0.00
GATCAACAATTTCAAGANCT	0.00	0.00	0.29	0.00	0.00
GATCAACAATTTCAAGANNN	0.00	0.00	0.00	0.00	0.00
GATCAACAATTTCAAGCGCT	0.69	0.00	0.00	0.00	0.00
GATCAACAATTTCAAGGGCT	0.23	0.00	0.00	0.00	0.00
GATCAACAATTTCAAGNGCT	0.00	0.00	0.00	0.00	0.69
GATCAACAATTTCAAGNNNN	0.46	0.00	0.00	0.00	0.00
GATCAACAATTTCAAGTTCT	0.00	0.00	0.00	0.00	0.34
GATCAACAATTTCACGAGCT	0.00	0.00	0.00	0.00	0.00
GATCAACAATTTCAGGAGCT	0.00	0.00	0.00	0.00	0.34
GATCAACAATTTCCCAATCT	0.00	0.00	0.00	0.00	0.00
GATCAACAATTTCCCCCGCT	0.23	0.00	0.00	0.00	0.00
GATCAACAATTTCTCGGGAC	0.00	0.00	0.00	0.00	0.34
GATCAACAATTTTAAGAGGT	0.00	0.00	0.00	0.00	0.00
GATCAACAATTTTTCAAGGC	0.00	0.00	0.00	0.00	0.00
GATCAACAATTTTTTTACCG	0.00	0.00	0.00	0.34	0.00
GATCAACACAAAAAAAAAAA	8.01	1.30	6.01	2.38	4.48
GATCAACACAAAAAAAAAAT	0.00	0.33	0.00	0.00	0.00
GATCAACACAAAAAAAAANA	0.00	0.00	0.00	0.00	0.00
GATCAACACAAAAAAAAGAA	0.00	0.00	0.57	0.00	0.00
GATCAACACAAAAAAAANNN	0.23	0.00	0.00	0.00	0.00
GATCAACACAAAAAATGTCC	0.00	0.00	0.00	0.00	0.34
GATCAACACAAAAACAAAAA	0.00	0.00	0.29	0.00	0.00
GATCAACACAAAAACAAGAG	0.00	0.00	0.57	0.00	0.34
GATCAACACAAAAACCCCAC	0.00	0.00	0.29	0.00	0.00
GATCAACACAAAAAGAAAAA	2.98	1.30	2.00	4.75	5.51
GATCAACACAAAAAGAAGAA	0.00	0.00	0.29	0.00	0.00

GATCAACACAAAAAGACAAA	0.00	0.00	0.00	0.00	0.00
GATCAACACAAAAAGAGAAA	0.46	0.65	1.43	1.02	4.48
GATCAACACAAAAAGAGAAG	0.46	0.33	0.57	0.68	0.34
GATCAACACAAAAAGCANAG	0.00	0.00	0.00	0.00	0.34
GATCAACACAAAAATCTAAA	0.00	0.00	0.00	0.34	0.00
GATCAACACAAAACAAAAAC	0.00	0.00	0.00	0.00	0.69
GATCAACACAAAACAAAACA	0.00	0.00	0.00	0.00	0.69
GATCAACACAAAACAACACA	0.23	0.00	0.00	0.00	0.34
GATCAACACAAAACACACAC	0.00	0.00	0.00	0.00	1.72
GATCAACACAAAACATGTTG	0.00	0.00	0.00	0.00	0.00
GATCAACACAAAACCGCACC	0.00	0.00	0.00	0.00	1.03
GATCAACACAAAAGAAAAAA	0.00	0.00	0.00	0.00	0.00
GATCAACACAAAAGGAACGA	0.00	0.00	0.29	0.00	0.00
GATCAACACAAAATAATAAA	0.00	0.00	0.29	0.00	0.00
GATCAACACAAAATCAAATC	0.00	0.00	0.29	0.00	0.00
GATCAACACAAACAAGTTTT	0.00	0.00	0.00	0.34	0.00
GATCAACACAAACACAACAA	0.00	0.00	0.29	0.00	0.00
GATCAACACAAACACAAGCC	0.00	0.00	0.00	0.00	0.34
GATCAACACAAACACACAGG	0.00	0.00	0.29	0.00	0.00
GATCAACACAAACACAGAAA	0.00	0.00	0.00	0.00	0.34
GATCAACACAAACACGGGCA	0.00	0.00	0.00	0.00	0.00
GATCAACACAAACGGCTCCG	0.00	0.33	0.00	0.00	0.00
GATCAACACAAACTTATCAA	0.00	0.00	0.29	0.00	0.00
GATCAACACAAAGAGAGAAA	0.23	0.00	0.57	0.00	0.00
GATCAACACAAAGCATATTC	0.00	0.00	0.00	0.34	0.00
GATCAACACAAATAAAAGAA	0.00	0.00	0.00	0.00	0.69
GATCAACACAAATAGTATGG	0.00	0.00	0.29	0.00	0.00
GATCAACACAACAAACACGG	0.00	0.00	0.00	0.00	0.34
GATCAACACAACAAATCAAA	0.00	0.00	0.00	0.00	0.34
GATCAACACAACAACACCAA	0.00	0.00	0.29	0.00	0.00
GATCAACACAACAACANACA	0.00	0.00	0.00	0.00	0.34
GATCAACACAACAACGTCGA	0.00	0.00	0.57	0.00	0.00
GATCAACACAACAACTCAAG	0.00	0.00	0.29	0.00	0.00
GATCAACACAACAAGACAAA	0.00	0.00	0.00	0.00	0.00
GATCAACACAACAAGAGGAA	0.00	0.00	0.00	0.00	0.69
GATCAACACAACAAGCACGC	0.00	0.00	0.00	0.00	0.34
GATCAACACAACACAAACAC	0.00	0.00	0.29	0.00	0.00

GATCAACACAACACAAACGA	0.00	0.00	0.00	0.00	0.34
GATCAACACAACACAACAAC	0.00	0.00	0.29	0.00	0.00
GATCAACACAACACAACACA	0.00	0.00	2.86	0.00	18.94
GATCAACACAACACAACACG	0.00	0.00	0.00	0.00	1.38
GATCAACACAACACAACACT	0.00	0.00	0.29	0.00	0.00
GATCAACACAACACAACCCA	0.00	0.00	0.29	0.00	0.00
GATCAACACAACACAACGCA	0.00	0.00	0.57	0.00	0.00
GATCAACACAACACACAAAC	0.00	0.00	0.00	0.00	0.34
GATCAACACAACACACAACA	0.00	0.00	0.57	0.00	1.38
GATCAACACAACACACACCA	0.00	0.00	0.00	0.00	0.69
GATCAACACAACACACATCA	0.00	0.00	0.00	0.00	0.34
GATCAACACAACACACCACA	0.00	0.00	0.57	0.00	2.07
GATCAACACAACACACCACC	0.00	0.00	0.00	0.00	0.34
GATCAACACAACACACCACG	0.00	0.00	0.00	0.00	0.34
GATCAACACAACACACCAGA	0.23	0.00	0.00	0.00	0.00
GATCAACACAACACACTACA	0.00	0.00	0.29	0.00	0.00
GATCAACACAACACAGACCA	0.00	0.00	0.00	0.00	0.34
GATCAACACAACACAGACGC	0.00	0.00	0.00	0.00	1.03
GATCAACACAACACAGCACA	0.00	0.00	0.00	0.00	1.72
GATCAACACAACACAGCATA	0.00	0.00	0.57	0.00	0.00
GATCAACACAACACATCACA	0.00	0.00	0.29	0.00	2.76
GATCAACACAACACATGCGG	0.00	0.00	0.29	0.00	0.00
GATCAACACAACACCAAACC	0.00	0.00	0.00	0.00	0.34
GATCAACACAACACCACACA	0.00	0.00	0.00	0.00	0.34
GATCAACACAACACCCCCAT	0.00	0.00	0.00	0.00	0.34
GATCAACACAACACCGCACA	0.00	0.00	0.00	0.00	0.34
GATCAACACAACACGACACA	0.00	0.00	0.00	0.00	0.34
GATCAACACAACACGACACG	0.00	0.00	0.29	0.00	0.00
GATCAACACAACACGTCACT	0.00	0.00	0.57	0.00	0.00
GATCAACACAACACTCAACA	0.00	0.00	0.86	0.00	0.00
GATCAACACAACAGTCAGCA	0.00	0.33	0.00	0.00	0.00
GATCAACACAACATAACACA	0.00	0.00	0.00	0.00	0.34
GATCAACACAACATAACCCT	0.00	0.00	0.57	0.00	0.00
GATCAACACAACATACTTGC	0.00	0.00	0.00	0.00	0.34
GATCAACACAACATCTGTAT	0.00	0.00	0.00	0.00	0.00
GATCAACACAACCAAGCTGA	0.00	0.00	0.00	0.00	0.00
GATCAACACAACCACATAAC	0.00	0.00	0.00	0.00	0.69

GATCAACACAACCACCAACG	0.00	0.00	0.57	0.00	0.00
GATCAACACAACCAGACACG	0.00	0.00	0.00	0.00	0.34
GATCAACACAACCATCACAC	0.00	0.00	0.57	0.00	0.00
GATCAACACAACCCAACACA	0.00	0.00	0.00	0.00	0.34
GATCAACACAACCGAACCAA	0.00	0.00	0.29	0.00	0.00
GATCAACACAACCGAGCTCA	0.00	0.00	0.00	0.34	0.00
GATCAACACAACCGCACACA	0.00	0.00	0.57	0.00	0.00
GATCAACACAACCGCAGCAT	0.00	0.00	0.00	0.00	0.00
GATCAACACAACGAAAAAAG	0.00	0.00	0.00	0.00	0.34
GATCAACACAACGAACACAC	0.00	0.00	0.00	0.00	1.38
GATCAACACAACGCAACACA	0.00	0.00	0.00	0.00	1.38
GATCAACACAACGCAACACG	0.00	0.00	0.29	0.00	0.00
GATCAACACAACGCAACGAC	0.00	0.00	0.00	0.00	0.34
GATCAACACAACGCAACGCC	0.00	0.00	0.29	0.00	0.00
GATCAACACAACGCACATCA	0.00	0.00	0.29	0.00	0.00
GATCAACACAACGCCACACT	0.00	0.00	0.29	0.00	0.00
GATCAACACAACGCCCAAGA	0.00	0.00	0.00	0.00	0.34
GATCAACACAACGCTGTCGT	0.00	0.33	0.29	0.68	0.00
GATCAACACAACGGGTGGCC	0.00	0.00	0.00	0.00	0.69
GATCAACACAACGGTCAACA	0.00	0.00	0.29	0.00	0.34
GATCAACACAACTGCACCAC	0.00	0.00	0.00	0.00	0.00
GATCAACACAACTGGCTCAG	0.69	0.00	0.00	0.00	0.00
GATCAACACAACTGGCTCCG	0.23	0.00	0.00	0.00	0.00
GATCAACACAAGAAGAAGCA	0.00	0.00	0.29	0.00	0.00
GATCAACACAAGACACACAA	0.00	0.00	0.00	0.00	1.03
GATCAACACAAGACGGAACA	0.00	0.00	0.57	0.00	0.00
GATCAACACAAGAGACGAAT	0.23	0.33	0.57	0.00	0.00
GATCAACACAAGCAACGAAG	0.00	0.00	0.00	0.34	0.00
GATCAACACAAGCAAGCAAG	0.00	0.00	0.29	0.00	0.00
GATCAACACAAGCAAGTAAG	0.00	0.00	0.57	0.00	0.00
GATCAACACAAGCCACGCGA	0.00	0.00	0.29	0.00	0.00
GATCAACACAAGCCCACAGG	0.23	0.00	0.00	0.00	0.00
GATCAACACAAGCCCATAGG	0.23	0.00	0.00	0.00	0.00
GATCAACACAAGCCGACAGC	0.00	0.00	0.00	0.00	0.00
GATCAACACAAGCCGCACGC	0.00	0.00	0.00	0.00	0.34
GATCAACACAAGCCTTGAAC	0.00	0.00	0.00	0.34	0.00
GATCAACACAAGGCAGCACG	0.00	0.00	0.00	0.00	0.00

GATCAACACAAGGGGAAGAA	0.00	0.00	0.00	0.00	0.34
GATCAACACAAGTAGAACGC	0.00	0.00	0.57	0.00	0.00
GATCAACACAAGTCAGCACA	0.00	0.00	0.00	0.00	0.00
GATCAACACAAGTCAGCACG	2.52	3.90	2.58	6.45	2.76
GATCAACACAAGTCAGNNNN	0.23	0.00	0.00	0.00	0.00
GATCAACACAAGTTCAGCTC	0.23	0.00	0.00	0.00	0.00
GATCAACACAATAACAACTG	0.00	0.00	0.00	0.00	1.03
GATCAACACAATAATCATGA	0.00	0.33	0.00	0.00	0.34
GATCAACACAATACACCACA	0.00	0.00	0.29	0.00	0.00
GATCAACACAATACCAGACA	0.00	0.00	0.29	0.00	0.00
GATCAACACAATCACCGCAA	0.00	0.00	0.00	0.00	0.34
GATCAACACAATCCGGCTCA	0.00	0.00	0.00	0.00	0.34
GATCAACACAATCGACTGAA	0.00	0.00	0.00	0.00	0.00
GATCAACACAATGAACACGA	0.00	0.00	0.29	0.00	0.34
GATCAACACAATGAAGGAGA	0.00	0.00	0.00	0.00	0.00
GATCAACACAATGAGGGACG	0.00	0.33	0.00	0.00	0.00
GATCAACACAATGATATGGC	0.00	0.00	0.00	0.00	0.00
GATCAACACAATGATGACCA	0.00	0.00	0.00	0.00	1.03
GATCAACACAATGATGGAGG	0.23	0.33	0.00	0.34	0.00
GATCAACACAATGCAGCAAA	0.00	0.98	0.00	0.68	0.00
GATCAACACAATGCATGTGT	0.00	0.00	0.00	0.00	0.34
GATCAACACAATGTTCACCA	0.00	0.00	0.29	0.00	0.00
GATCAACACAATGTTTCTTC	0.00	0.00	0.00	0.00	0.34
GATCAACACAATTCAAAACA	0.00	0.00	0.00	0.00	0.34
GATCAACACAATTTGATAGA	0.00	0.00	0.00	0.00	0.34
GATCAACACACAAAAATTGG	0.00	0.00	0.29	0.00	0.00
GATCAACACACAAAACACCG	0.00	0.00	0.29	0.00	0.00
GATCAACACACAAACAAGAG	0.00	0.00	0.00	0.00	0.34
GATCAACACACAAACACAAA	0.00	0.00	0.00	0.00	0.69
GATCAACACACAAACCATGG	0.00	0.00	0.00	0.00	0.00
GATCAACACACAAAGAAAGA	0.00	0.00	0.29	0.00	0.00
GATCAACACACAACAAACAA	0.00	0.00	0.00	0.00	0.34
GATCAACACACAACAACACA	0.00	0.00	0.29	0.00	1.03
GATCAACACACAACAACACG	0.00	0.00	0.00	0.00	1.03
GATCAACACACAACAAGAAC	0.00	0.00	0.29	0.00	0.00
GATCAACACACAACAATCAA	0.00	0.00	0.00	0.00	0.34
GATCAACACACAACACAACA	0.00	0.00	0.29	0.00	0.69

GATCAACACACAACACAAGC	0.00	0.00	0.00	0.00	0.34
GATCAACACACAACACACAA	0.00	0.00	0.29	0.00	0.00
GATCAACACACAACACACCA	0.00	0.00	1.15	0.00	0.69
GATCAACACACAACACACTC	0.00	0.00	0.00	0.00	0.34
GATCAACACACAACACAGCA	0.00	0.00	0.29	0.00	0.00
GATCAACACACAACACCACA	0.00	0.00	0.00	0.00	0.34
GATCAACACACAACACGACA	0.00	0.00	0.00	0.00	0.34
GATCAACACACAACAGAACG	0.00	0.00	0.00	0.00	0.34
GATCAACACACAACATCAAA	0.00	0.00	0.00	0.00	0.34
GATCAACACACAACCCACAA	0.00	0.00	0.00	0.00	0.00
GATCAACACACAACCGACAT	0.00	0.00	0.00	0.00	0.34
GATCAACACACAACGCAACA	0.00	0.00	0.00	0.00	0.34
GATCAACACACAACGCACAC	0.00	0.00	0.00	0.00	0.34
GATCAACACACAATGGTATA	0.00	0.00	0.00	0.00	0.34
GATCAACACACACAAAAAAA	0.00	0.00	0.29	0.00	0.00
GATCAACACACACAACACAA	0.00	0.00	0.00	0.00	1.72
GATCAACACACACAACACAC	0.00	0.00	0.00	0.00	0.34
GATCAACACACACAACACGC	0.00	0.00	0.29	0.00	0.00
GATCAACACACACAACATAC	0.00	0.00	0.29	0.00	0.00
GATCAACACACACAACGCAC	0.00	0.00	0.29	0.00	0.00
GATCAACACACACAATAAAG	0.00	0.33	0.00	0.00	0.00
GATCAACACACACACACAAT	0.00	0.00	0.00	0.00	0.34
GATCAACACACACACACACA	0.00	0.00	0.00	0.00	1.38
GATCAACACACACACACACC	0.00	0.00	0.00	0.00	0.34
GATCAACACACACACACACG	0.00	0.00	0.00	0.00	0.34
GATCAACACACACACACCAA	0.00	0.00	0.57	0.00	0.00
GATCAACACACACACACGCT	0.00	0.00	0.00	0.00	0.34
GATCAACACACACACCAAGC	0.00	0.00	0.00	0.00	0.34
GATCAACACACACACCACCA	0.00	0.00	0.00	0.00	0.34
GATCAACACACACAGACACA	0.00	0.00	0.00	0.00	0.34
GATCAACACACACATACACC	0.00	0.00	0.00	0.00	0.34
GATCAACACACACCAAAACA	0.00	0.00	0.00	0.00	0.34
GATCAACACACACCAACACA	0.00	0.00	0.00	0.00	0.34
GATCAACACACACCACACAA	0.00	0.00	0.00	0.00	0.34
GATCAACACACACCACAGAA	0.00	0.00	0.00	0.00	0.69
GATCAACACACACCACAGCG	0.00	0.00	0.00	0.00	0.34
GATCAACACACACCCCACGC	0.00	0.00	0.00	0.00	0.34

GATCAACACACACGCAGACA	0.00	0.00	0.00	0.00	1.03
GATCAACACACACTCACACA	0.00	0.00	0.00	0.00	0.34
GATCAACACACACTGAGATG	0.00	0.00	0.00	0.00	0.34
GATCAACACACACTTACACT	0.00	0.00	0.00	0.00	0.34
GATCAACACACAGAAACCGA	0.00	0.00	0.00	0.00	0.34
GATCAACACACAGACGAGAA	0.23	0.00	0.00	0.00	0.00
GATCAACACACAGCACAGCA	0.00	0.00	0.29	0.00	0.00
GATCAACACACAGCAGATGG	0.00	0.33	0.00	0.00	0.00
GATCAACACACAGGAGGAGG	0.00	0.00	0.57	0.00	0.00
GATCAACACACAGGTGCTTT	0.00	0.00	0.29	0.00	0.00
GATCAACACACAGTGGCAGA	0.00	0.00	0.29	0.00	0.00
GATCAACACACAGTGGCCGC	0.00	0.00	0.00	0.00	0.34
GATCAACACACAGTGTGTGT	0.00	0.00	0.29	0.00	0.00
GATCAACACACATAAAACAA	0.00	0.00	0.00	0.00	0.34
GATCAACACACATAACAGAA	0.00	0.00	0.29	0.00	0.34
GATCAACACACATAACGCAC	0.00	0.00	0.00	0.00	0.34
GATCAACACACATATCGGGA	0.00	0.00	0.00	0.00	0.34
GATCAACACACATCGCTCCT	0.00	0.00	0.29	0.00	0.00
GATCAACACACATGGGCGAT	0.00	0.00	0.00	0.34	0.00
GATCAACACACCAAACCTGG	0.00	0.00	0.00	0.00	0.00
GATCAACACACCACAACACA	0.00	0.00	0.86	0.00	0.69
GATCAACACACCACAACACG	0.00	0.00	0.00	0.00	0.34
GATCAACACACCACAACACT	0.00	0.00	0.00	0.00	0.34
GATCAACACACCACAAGGAG	0.00	0.00	0.00	0.00	0.69
GATCAACACACCACACAACA	0.00	0.00	0.29	0.00	1.03
GATCAACACACCACACACCA	0.00	0.00	0.29	0.00	0.00
GATCAACACACCACAGTGGC	0.00	0.00	0.00	0.00	0.00
GATCAACACACCACGAAGAA	0.00	0.00	0.00	0.00	0.69
GATCAACACACCACGCCACG	0.00	0.00	0.29	0.00	0.00
GATCAACACACCACGCGCTG	0.00	0.00	0.29	0.00	0.00
GATCAACACACCACTCCAAG	0.00	0.00	0.00	0.00	0.69
GATCAACACACCACTGAGAA	0.00	0.33	0.00	0.00	0.00
GATCAACACACCACTGCTGG	0.00	0.00	0.00	0.00	0.34
GATCAACACACCACTGGAGG	0.00	0.00	0.00	0.00	0.34
GATCAACACACCACTGGTGG	0.23	0.00	0.00	0.00	0.00
GATCAACACACCAGCCAGAG	0.00	0.00	0.29	0.00	0.00
GATCAACACACCAGGGAGTT	0.00	0.33	0.00	0.00	0.00

GATCAACACACCAGGTGCGA	0.00	0.00	0.00	0.00	0.00
GATCAACACACCAGTAATCC	0.00	0.00	0.00	0.00	0.00
GATCAACACACCATTGGTGG	0.00	0.00	0.00	0.68	0.00
GATCAACACACCCAAGCTGA	0.00	0.33	0.00	0.00	0.00
GATCAACACACCCAATCCAC	0.00	0.00	0.00	0.00	0.00
GATCAACACACCCACACGCC	0.00	0.33	0.00	0.00	0.00
GATCAACACACCCCTCAACA	0.00	0.00	0.00	0.00	0.69
GATCAACACACCCGTCTTCC	0.00	0.00	0.29	0.00	0.00
GATCAACACACCGAACAGAT	0.00	0.00	0.00	0.00	0.34
GATCAACACACCGAGTCCCG	0.00	0.00	0.00	0.00	0.34
GATCAACACACCGATGAGGC	0.00	0.00	0.00	0.00	0.00
GATCAACACACCGCAACACA	0.00	0.00	0.29	0.00	0.34
GATCAACACACCGCACCACA	0.00	0.00	0.86	0.00	0.00
GATCAACACACCGGTAATCC	0.23	0.00	0.00	0.00	0.00
GATCAACACACCGTCAATGT	0.00	0.00	0.00	0.34	0.00
GATCAACACACGACAACACA	0.00	0.00	0.00	0.00	0.69
GATCAACACACGCAAACTGC	0.00	0.33	0.00	0.00	0.00
GATCAACACACGCAACACAA	0.00	0.00	0.00	0.00	0.34
GATCAACACACGCACGCGCG	0.00	0.00	0.00	0.00	0.34
GATCAACACACGCCAAACAC	0.00	0.00	0.00	0.00	0.34
GATCAACACACGCCGGATGC	0.00	0.00	0.00	0.34	0.00
GATCAACACACGCGCGCACA	0.00	0.00	0.00	0.00	0.69
GATCAACACACGGCACACCG	0.00	0.00	0.29	0.00	0.00
GATCAACACACGTACGCACG	0.00	0.33	0.00	0.00	0.00
GATCAACACACTAAAACAAC	0.00	0.00	0.29	0.00	0.00
GATCAACACACTAGAATCAA	0.00	0.00	0.00	0.00	0.34
GATCAACACACTCCAAAATC	0.00	0.33	0.29	0.00	0.34
GATCAACACACTCCAACATC	0.00	0.00	0.00	0.00	0.00
GATCAACACACTCCACACAC	0.00	0.00	0.29	0.00	0.00
GATCAACACACTCGCACACG	0.00	0.00	0.00	0.00	0.34
GATCAACACACTCGTCTTCA	0.00	0.00	0.00	0.00	0.00
GATCAACACACTCGTCTTCC	0.00	1.63	0.86	0.00	0.69
GATCAACACACTGAACAACA	0.00	0.00	0.00	0.00	0.34
GATCAACACACTGATTCATG	0.00	0.00	0.00	0.00	0.00
GATCAACACACTGCAAAGAT	0.00	0.00	0.00	0.00	0.00
GATCAACACACTGCACAAGG	0.00	0.00	0.00	0.00	0.00
GATCAACACACTGCGCACAC	0.00	0.00	0.00	0.00	0.34

GATCAACACACTGCGCCATA	0.00	0.00	0.29	0.34	0.00
GATCAACACACTGCGCCGAA	0.00	0.00	0.00	0.00	0.34
GATCAACACACTGCTCCAGT	0.23	0.33	0.00	0.68	0.00
GATCAACACACTGCTCCCGT	0.00	0.33	0.00	0.00	0.00
GATCAACACACTGGACTATA	0.00	0.00	0.29	0.00	0.00
GATCAACACACTGTTCAGTG	0.00	0.00	0.00	0.68	0.00
GATCAACACACTTCCTTCCC	0.00	0.33	0.00	0.34	0.00
GATCAACACACTTGTGTTCA	0.00	0.00	0.00	0.00	0.00
GATCAACACACTTGTGTTCC	0.00	0.00	0.29	0.00	0.34
GATCAACACACTTGTGTTCT	0.00	0.00	0.00	0.00	0.00
GATCAACACAGAAAAAAAAA	0.00	0.33	0.57	0.00	0.00
GATCAACACAGAACAAAACA	0.00	0.00	0.00	0.00	0.34
GATCAACACAGAACAAAACC	0.00	0.65	0.00	0.00	0.00
GATCAACACAGAACAACACA	0.00	0.00	0.00	0.00	0.69
GATCAACACAGAACAACACC	0.00	0.00	0.00	0.00	0.69
GATCAACACAGAACAGAACA	0.00	0.00	0.00	0.00	4.13
GATCAACACAGAACAGAACC	0.00	0.00	0.00	0.00	0.69
GATCAACACAGAACAGAGCA	0.00	0.00	0.00	0.00	0.34
GATCAACACAGAACAGCACA	0.00	0.00	0.00	0.00	0.69
GATCAACACAGAACATAAAC	0.00	0.00	0.00	0.00	0.34
GATCAACACAGAACATATGG	0.00	0.00	0.00	0.00	0.34
GATCAACACAGAAGAATTGG	0.00	0.00	0.29	0.00	0.00
GATCAACACAGAAGATAACA	0.00	0.00	0.00	0.00	1.38
GATCAACACAGAAGCGCAGA	0.00	0.00	0.00	0.34	0.00
GATCAACACAGACAACACAC	0.00	0.00	0.00	0.00	0.34
GATCAACACAGACATACAAC	0.00	0.00	0.00	0.34	0.00
GATCAACACAGACATCAAAC	0.00	0.00	0.00	0.34	0.00
GATCAACACAGACATCCAAA	1.60	1.30	2.58	3.05	1.38
GATCAACACAGACATCCAAC	48.99	63.44	44.10	58.38	38.57
GATCAACACAGACATCCAAG	0.23	0.00	0.00	0.00	0.00
GATCAACACAGACATCCACT	0.00	0.00	0.00	0.00	0.69
GATCAACACAGACATCCAGC	0.23	0.00	0.00	0.00	0.00
GATCAACACAGACATCCANN	0.23	0.00	0.00	0.00	0.00
GATCAACACAGACATCCCAC	0.00	0.00	0.57	0.34	0.00
GATCAACACAGACATCGCCC	0.00	0.00	0.29	0.00	0.00
GATCAACACAGACATCNAAC	0.00	0.00	0.00	0.00	0.34
GATCAACACAGACATCTAAA	0.00	0.00	0.00	0.00	0.69

GATCAACACAGACATCTAAC	0.00	0.33	0.00	0.34	0.00
GATCAACACAGACATCTGAC	0.00	0.00	0.00	0.00	0.34
GATCAACACAGACCAGAACA	0.00	0.00	0.00	0.00	0.34
GATCAACACAGACGAATACG	0.00	0.00	0.00	0.00	0.34
GATCAACACAGACGTCCAAC	0.23	0.00	0.00	0.00	0.00
GATCAACACAGAGAACAAGA	0.00	0.00	0.00	0.00	0.34
GATCAACACAGAGAACAGAG	0.00	0.00	0.00	0.00	0.69
GATCAACACAGAGATAGAGA	0.00	0.00	0.29	0.00	0.00
GATCAACACAGAGCACAACA	0.00	0.00	0.00	0.00	0.34
GATCAACACAGAGCAGAGCA	0.00	0.00	0.29	0.00	1.03
GATCAACACAGCAAGTGGAG	0.00	0.00	0.00	0.00	0.34
GATCAACACAGCACAACACA	0.00	0.00	0.57	0.00	2.07
GATCAACACAGCACACAACG	0.00	0.00	0.29	0.00	0.00
GATCAACACAGCACAGCACA	0.00	0.00	0.00	0.00	0.34
GATCAACACAGCACATCAAA	0.00	0.00	0.29	0.00	0.00
GATCAACACAGCACATCACA	0.00	0.00	0.29	0.00	0.34
GATCAACACAGCACATGGCC	0.00	0.33	0.00	0.00	0.00
GATCAACACAGCACGGCGCG	0.00	0.00	0.00	0.00	0.34
GATCAACACAGCACTTGATG	0.00	0.00	0.00	0.00	0.00
GATCAACACAGCATCACAGG	0.00	0.33	0.00	1.02	0.34
GATCAACACAGCCAAGCTGA	0.00	0.00	0.00	0.00	0.00
GATCAACACAGCCAGCCCTG	0.00	0.33	0.00	0.00	0.00
GATCAACACAGCCAGGCACT	0.92	0.00	0.00	0.34	0.00
GATCAACACAGCCCAACACA	0.00	0.00	0.00	0.00	1.03
GATCAACACAGCCCTCCCCC	0.23	0.00	0.00	0.00	0.00
GATCAACACAGCGACGTGTG	0.23	0.00	0.00	0.00	0.00
GATCAACACAGCGCAGAGCC	0.00	0.00	0.00	0.00	0.34
GATCAACACAGCGGCCGTGC	0.00	0.00	0.00	0.00	0.00
GATCAACACAGCGTCTGGCG	0.00	0.00	0.00	0.00	0.00
GATCAACACAGCTCAACACA	0.00	0.00	0.00	0.00	0.34
GATCAACACAGCTGCATCCA	0.00	0.00	0.00	0.00	0.00
GATCAACACAGCTGCCTTCG	0.00	0.00	0.00	0.34	0.00
GATCAACACAGCTGTCGTGA	0.00	0.33	0.00	0.00	0.00
GATCAACACAGGATGTCAAC	0.23	0.33	0.00	1.36	0.00
GATCAACACAGGCAACCTGC	0.00	0.00	0.00	0.00	0.34
GATCAACACAGGCCCTTCGT	0.23	0.00	0.00	0.00	0.00
GATCAACACAGGCCTGGTCA	0.00	0.00	0.00	0.00	0.34

GATCAACACAGGCGGCACCG	0.00	0.00	0.00	0.00	0.00
GATCAACACAGGCGGGGGCA	0.23	0.00	0.00	0.00	0.00
GATCAACACAGGCTGGGGAA	0.00	0.00	0.57	0.34	0.00
GATCAACACAGGCTGGGGCA	2.75	5.86	5.15	4.07	3.10
GATCAACACAGGCTGGGGCC	0.00	0.65	0.29	1.02	0.00
GATCAACACAGGCTGGGGCT	0.00	0.00	0.00	0.34	0.00
GATCAACACAGGCTGGGGGA	0.00	0.00	0.00	0.00	0.00
GATCAACACAGGCTGGGGGG	0.23	0.00	0.00	0.00	0.00
GATCAACACAGGGGCAACGA	0.00	0.00	0.29	0.00	0.00
GATCAACACAGGGTCCTTCT	0.00	0.00	0.00	0.34	0.00
GATCAACACAGGGTGCACGG	0.00	0.33	0.29	0.00	1.38
GATCAACACAGGGTGTGTTT	0.00	0.00	0.29	0.00	0.00
GATCAACACAGGTGTCATGG	0.00	0.00	0.00	0.34	0.00
GATCAACACAGGTTGGGGTC	0.00	0.00	0.00	0.00	0.00
GATCAACACAGTAGCATATG	0.00	0.33	0.00	0.00	0.00
GATCAACACAGTCAAAGCGG	0.00	0.00	0.00	0.00	0.00
GATCAACACAGTCAACAAGC	0.00	0.00	0.00	0.00	1.03
GATCAACACAGTCCTGCACA	0.23	0.33	0.00	0.00	0.00
GATCAACACAGTCCTGCCTA	0.00	0.98	0.00	0.34	0.00
GATCAACACAGTCCTTGCGC	0.00	0.00	0.29	0.00	0.00
GATCAACACAGTCGGAACAT	0.00	0.33	0.00	0.00	0.00
GATCAACACAGTCGGACCAT	2.06	2.60	0.57	1.02	0.34
GATCAACACAGTCGGGGTGT	0.00	0.00	0.00	0.34	0.00
GATCAACACAGTGACCTTCT	0.00	0.00	0.00	0.00	0.34
GATCAACACAGTGACGAGAA	0.00	0.00	0.29	0.00	0.34
GATCAACACAGTTCCGGGCA	0.00	0.00	0.57	0.00	0.00
GATCAACACAGTTTTGAGTG	1.14	0.33	0.00	0.34	0.34
GATCAACACATAACAACACA	0.00	0.00	0.29	0.00	0.00
GATCAACACATAACAAGAAA	0.00	0.00	0.00	0.00	0.34
GATCAACACATAACACAACA	0.69	0.00	0.00	0.00	0.00
GATCAACACATAACACATCA	0.00	0.00	0.00	0.00	0.34
GATCAACACATAACAGAACA	0.00	0.00	0.00	0.00	0.34
GATCAACACATAACAGCTGA	0.00	0.00	0.00	0.00	0.34
GATCAACACATAACATCCGC	0.00	0.00	0.29	0.00	0.00
GATCAACACATAATTGAACA	0.00	0.00	0.00	0.00	1.03
GATCAACACATACCATGACC	0.00	0.00	0.00	0.00	0.69
GATCAACACATACGAAGACA	0.00	0.00	0.00	0.00	0.69

GATCAACACATACTAGCTGA	0.00	0.00	0.29	0.00	0.00
GATCAACACATAGCAACAAC	0.00	0.00	0.00	0.00	0.69
GATCAACACATAGCATAGCG	0.00	0.00	0.00	0.00	0.34
GATCAACACATAGCATCACC	0.00	0.00	0.29	0.00	0.00
GATCAACACATATAGCAGCA	0.00	0.00	0.00	0.00	0.34
GATCAACACATATGAGCACA	0.00	0.00	0.29	0.00	0.00
GATCAACACATCAAAACACT	0.00	0.00	0.86	0.00	1.38
GATCAACACATCAAACAGTC	0.00	0.00	0.00	0.00	0.00
GATCAACACATCAAAGCACA	0.00	0.00	0.00	0.00	0.00
GATCAACACATCAAAGCCGA	0.00	0.00	0.00	0.00	0.00
GATCAACACATCAAAGCTGA	0.00	0.33	0.00	0.00	0.00
GATCAACACATCAAGATTTA	0.00	0.00	0.00	0.00	0.34
GATCAACACATCAAGCACAG	0.00	0.00	0.00	0.00	0.34
GATCAACACATCAAGGACAC	0.23	0.00	0.00	0.00	0.00
GATCAACACATCACAACACA	0.00	0.00	0.29	0.00	0.00
GATCAACACATCACAACGCA	0.00	0.00	0.00	0.00	0.34
GATCAACACATCACAAGAAC	0.00	0.00	0.00	0.00	0.34
GATCAACACATCACACCACA	0.00	0.00	0.57	0.00	0.00
GATCAACACATCACACCACG	0.00	0.00	0.00	0.00	0.69
GATCAACACATCACACCGCA	0.00	0.00	0.29	0.00	0.00
GATCAACACATCACAGAACA	0.00	0.00	0.00	0.00	0.69
GATCAACACATCACATAACA	0.00	0.00	0.00	0.00	0.00
GATCAACACATCACATCACA	0.00	0.00	0.29	0.00	0.00
GATCAACACATCACCAACAA	0.00	0.00	0.29	0.00	0.00
GATCAACACATCACCGCACA	0.00	0.00	0.00	0.00	0.34
GATCAACACATCACGCCACA	0.00	0.00	0.00	0.00	0.34
GATCAACACATCACTCAACA	0.00	0.00	0.00	0.00	1.03
GATCAACACATCAGCAGACG	0.00	0.00	0.00	0.00	0.00
GATCAACACATCAGGGAGTT	0.00	0.00	0.29	0.00	0.00
GATCAACACATCAGTAACCC	0.00	0.00	0.29	0.00	0.00
GATCAACACATCATCGTCGT	0.00	0.00	0.29	0.00	0.00
GATCAACACATCATTGTGGT	0.00	0.00	0.29	0.00	0.00
GATCAACACATCCAAGATGA	0.00	0.00	0.00	0.00	0.69
GATCAACACATCCAAGCAGA	0.00	0.00	0.00	0.00	0.34
GATCAACACATCCAAGCCGA	0.00	0.00	0.00	0.00	0.34
GATCAACACATCCAAGCGGA	0.00	0.00	0.29	0.34	0.00
GATCAACACATCCAAGCGTC	0.00	0.00	0.00	0.00	0.00

GATCAACACATCCAAGCNGN	0.00	0.00	0.00	0.00	0.00
GATCAACACATCCAAGCTCA	0.00	0.00	0.00	0.00	0.34
GATCAACACATCCAAGCTGA	58.60	95.65	58.42	94.02	57.86
GATCAACACATCCAAGCTGC	0.23	0.00	0.00	0.00	0.69
GATCAACACATCCAAGCTGG	0.00	0.98	0.00	0.00	0.00
GATCAACACATCCAAGCTGN	0.23	0.00	0.00	0.00	0.00
GATCAACACATCCAAGCTGT	0.00	0.00	0.00	0.00	0.00
GATCAACACATCCAAGCTNA	0.00	0.00	0.00	0.34	0.00
GATCAACACATCCAAGCTNC	0.00	0.00	0.00	0.00	0.00
GATCAACACATCCAAGCTNN	0.23	0.00	0.00	0.00	0.00
GATCAACACATCCAAGCTTA	0.00	0.00	0.00	0.00	0.00
GATCAACACATCCAAGNTGA	0.00	0.00	0.00	0.00	0.34
GATCAACACATCCAAGTTGA	0.00	0.33	0.00	0.00	0.69
GATCAACACATCCACGCTGA	0.23	0.00	0.00	0.00	0.00
GATCAACACATCCAGGCTGA	0.92	0.33	0.00	0.00	0.00
GATCAACACATCCCAGCTGA	0.23	0.00	0.00	0.00	0.00
GATCAACACATCCCCGCTGC	0.23	0.00	0.00	0.00	0.00
GATCAACACATCCGAGCACG	0.00	0.00	0.29	0.00	0.00
GATCAACACATCCGAGCTGA	0.00	0.00	0.00	0.68	0.69
GATCAACACATCCTAGCTGA	0.23	0.00	0.00	0.68	0.00
GATCAACACATCCTAGCTNN	0.23	0.00	0.00	0.00	0.00
GATCAACACATCGAAGCTGA	0.00	0.00	0.00	0.34	0.00
GATCAACACATCGCACCACC	0.00	0.00	0.29	0.00	0.00
GATCAACACATCGTGGCGAA	0.00	0.00	0.00	0.00	1.03
GATCAACACATCTAAGCTGA	0.23	0.00	0.00	0.00	0.69
GATCAACACATCTCGCGCCA	0.00	0.33	0.00	0.00	0.00
GATCAACACATGAACCAAAT	0.00	0.00	0.29	0.00	0.00
GATCAACACATGAAGACATG	0.00	0.00	0.29	0.00	0.00
GATCAACACATGACAATGCG	0.00	0.00	0.00	0.00	0.34
GATCAACACATGAGCGGTTA	0.00	0.00	0.00	0.00	1.03
GATCAACACATGAGCTCGAA	0.00	0.00	0.00	0.34	0.00
GATCAACACATGCAACTCAG	0.00	0.00	0.00	0.00	0.34
GATCAACACATGCACGGTGC	0.00	0.33	0.00	0.34	0.34
GATCAACACATGCATTTGTT	0.00	0.33	0.00	0.00	0.00
GATCAACACATGCCAGCGTG	0.00	0.00	0.29	0.00	0.00
GATCAACACATGCCTCGTAT	0.00	0.00	0.00	0.00	0.34
GATCAACACATGCCTCGTTA	1.83	2.60	3.44	1.36	1.38

GATCAACACATGCCTTCGGA	0.00	0.00	0.00	0.00	0.69
GATCAACACATGCGAGGCCA	0.00	0.00	0.00	0.00	1.03
GATCAACACATGCGCATGAA	0.00	0.00	0.00	0.00	0.69
GATCAACACATGCTCCGATG	0.00	0.00	0.29	0.00	0.00
GATCAACACATGCTTCACCT	0.00	0.00	0.00	0.34	0.00
GATCAACACATGGAGCATCC	1.14	0.65	0.00	0.00	2.07
GATCAACACATGGCGCATGG	0.00	0.00	0.00	0.00	0.34
GATCAACACATGGCGTTCAC	0.00	0.00	0.00	0.34	0.00
GATCAACACATGGGGCAACA	0.00	0.00	0.29	0.00	0.00
GATCAACACATGGTCAATGG	0.00	0.00	0.57	0.00	0.00
GATCAACACATGGTGCATAG	0.00	0.00	0.00	0.00	0.34
GATCAACACATGGTGCATGG	14.19	8.13	8.88	6.79	5.17
GATCAACACATGTCGATGTG	0.00	0.00	0.29	0.00	0.00
GATCAACACATGTGGATGAG	0.00	0.33	0.00	0.00	0.00
GATCAACACATTCAGGGCAT	0.00	0.00	0.29	0.00	0.00
GATCAACACATTGCAGGGAG	0.00	0.33	0.00	0.00	0.00
GATCAACACCAAAAAAAAAA	0.92	1.30	0.00	0.34	1.38
GATCAACACCAAAAAAAACA	0.69	0.00	0.00	0.00	0.00
GATCAACACCAAAAACAAAC	0.23	0.00	0.00	0.00	0.00
GATCAACACCAAAATCATCG	0.00	0.00	0.29	0.00	0.00
GATCAACACCAAACTCTTTA	0.00	0.00	0.00	0.00	0.00
GATCAACACCAAAGCAGCGT	0.00	0.00	0.00	0.00	0.00
GATCAACACCAACAACACAA	0.00	0.00	0.00	0.00	0.34
GATCAACACCAACAACAGAA	0.00	0.00	0.29	0.00	0.34
GATCAACACCAACAACAGAC	0.00	0.00	0.00	0.00	0.00
GATCAACACCAACACCAATG	0.00	0.00	0.00	0.00	0.00
GATCAACACCAACACCACAC	0.69	0.00	0.00	0.00	0.00
GATCAACACCAACACTCCAC	0.00	0.00	0.00	0.00	0.34
GATCAACACCAACATCATGG	0.00	0.00	0.00	0.00	0.34
GATCAACACCAACCACACAA	0.00	0.00	0.57	0.00	0.00
GATCAACACCAACTCCAACA	0.00	0.00	0.00	0.00	0.34
GATCAACACCAACTCCGTGA	0.23	0.98	0.00	0.00	0.00
GATCAACACCAAGAACACCG	0.00	0.00	0.00	0.00	0.00
GATCAACACCAAGAACTCCG	0.00	0.00	0.00	0.00	0.00
GATCAACACCAAGACCAAGA	0.00	0.00	0.00	0.00	0.34
GATCAACACCAAGAGCAAAC	0.00	0.00	0.00	0.00	0.34
GATCAACACCAAGCGCTGCT	0.00	0.00	0.00	0.00	0.00

GATCAACACCAAGGAGAAGG	0.00	0.00	0.29	0.00	0.00
GATCAACACCAAGGAGCGGC	0.46	0.00	0.00	0.68	0.00
GATCAACACCAAGGAGGAGC	0.00	0.00	0.00	0.00	0.00
GATCAACACCAAGGCCACTG	0.00	0.33	0.00	0.00	0.34
GATCAACACCAAGGGCTTTG	0.00	0.33	0.00	0.00	0.00
GATCAACACCAAGTGCCACT	0.00	0.00	0.00	0.00	0.00
GATCAACACCAATCTCTTCG	0.69	0.98	0.00	0.00	0.00
GATCAACACCACAACACACC	0.00	0.00	0.00	0.00	0.34
GATCAACACCACAACACCAA	0.00	0.33	0.00	0.00	0.00
GATCAACACCACAACCAAAC	0.00	0.00	0.29	0.00	0.00
GATCAACACCACACAACACA	0.00	0.00	0.29	0.00	0.34
GATCAACACCACAGCCCCCA	0.00	0.00	0.00	0.00	0.69
GATCAACACCACCAACAAAA	0.00	0.00	0.00	0.00	0.00
GATCAACACCACCAACAANN	0.00	0.00	0.00	0.00	0.00
GATCAACACCACCAACACCA	0.00	0.00	0.00	0.00	0.34
GATCAACACCACCAAGCGGA	0.00	0.00	0.00	0.68	0.00
GATCAACACCACCACAACAA	0.00	0.00	0.29	0.00	0.00
GATCAACACCACCACCGGGG	0.00	0.33	0.00	0.00	0.00
GATCAACACCACCACGAAGC	0.46	0.00	0.00	0.00	0.00
GATCAACACCACCATCACCA	0.00	0.00	0.57	0.00	0.00
GATCAACACCACCCACACCA	0.00	0.00	0.29	0.00	0.34
GATCAACACCACGCCACACC	0.00	0.00	0.00	0.00	0.34
GATCAACACCACGGAGGACT	0.00	0.00	0.00	0.00	0.00
GATCAACACCACGGCCAAAA	0.00	0.00	0.00	0.00	0.69
GATCAACACCACGTGCTTTG	0.00	0.00	0.29	0.00	0.00
GATCAACACCACTGGCACGC	0.00	0.00	0.00	0.00	0.69
GATCAACACCAGACAATACA	0.00	0.00	0.00	0.34	0.00
GATCAACACCAGACCAACAC	0.00	0.00	0.00	0.00	0.69
GATCAACACCAGATGCGTTG	0.00	0.00	0.57	0.00	0.00
GATCAACACCAGCATCATCA	0.00	0.00	0.29	0.00	0.00
GATCAACACCAGCCGGTCTG	0.00	0.00	0.00	0.00	0.00
GATCAACACCAGCGTGTGGG	0.00	0.33	0.00	0.00	0.00
GATCAACACCAGGCCATGGA	0.00	0.00	0.00	0.34	0.00
GATCAACACCAGGGCACAGC	0.00	0.00	0.00	0.00	0.34
GATCAACACCAGGGGGACTG	0.00	0.00	0.00	0.00	0.34
GATCAACACCAGGGTGGGGG	0.00	0.00	0.00	0.34	0.00
GATCAACACCAGTATGTACT	0.00	0.00	0.00	0.00	0.69

GATCAACACCAGTCAGCGCG	0.23	0.00	0.00	0.00	0.00
GATCAACACCAGTGAATTGC	0.00	0.33	0.00	0.00	0.00
GATCAACACCAGTGAATTGT	0.92	0.98	0.86	0.34	1.38
GATCAACACCATACCAAGGC	0.00	0.00	0.29	0.00	0.00
GATCAACACCATACCACACC	0.00	0.00	0.00	0.00	0.69
GATCAACACCATACTGTGCG	0.00	0.00	0.00	0.00	1.03
GATCAACACCATATGTTCAC	0.00	0.00	0.00	0.00	0.34
GATCAACACCATCACGGACT	0.00	0.00	0.00	0.00	0.00
GATCAACACCATCATCGAGG	0.00	0.33	0.29	0.00	0.00
GATCAACACCATCATCGTGC	0.00	0.65	0.00	0.00	0.00
GATCAACACCATCATGTAGG	0.92	0.00	0.00	0.00	0.00
GATCAACACCATCCAAGGCA	0.69	0.00	0.00	0.00	0.00
GATCAACACCATCCAAGGCT	0.00	0.00	0.00	0.00	0.00
GATCAACACCATCCAGGGCA	0.00	0.00	0.00	0.00	0.00
GATCAACACCATCCGCTCGC	0.00	0.00	0.00	0.68	0.00
GATCAACACCATCCTCGTTG	0.00	0.33	0.00	0.00	0.00
GATCAACACCATCCTCTTAG	0.00	0.00	0.00	0.00	0.34
GATCAACACCATCTGACAGA	0.00	0.00	0.29	0.00	0.00
GATCAACACCATCTGCGGCC	0.00	0.00	0.00	0.00	0.34
GATCAACACCATCTGTTCAC	13.73	9.11	5.73	8.15	9.64
GATCAACACCATCTGTTCAT	0.00	0.00	0.00	0.00	0.00
GATCAACACCATCTGTTCCC	0.23	0.00	0.00	0.00	0.00
GATCAACACCATCTGTTCTC	0.00	0.00	0.00	0.68	0.00
GATCAACACCATGATGGAGG	0.00	2.28	0.57	2.72	1.03
GATCAACACCATGATGGCGG	0.00	0.00	0.00	0.34	0.00
GATCAACACCATGATGGGGG	0.00	0.00	0.00	0.00	0.34
GATCAACACCATGCAATATG	0.00	0.00	0.29	0.00	0.00
GATCAACACCATGCACGCAC	0.00	0.00	0.57	0.34	0.00
GATCAACACCATGCACTTCT	0.23	0.00	0.00	0.00	0.00
GATCAACACCATGCAGCTGC	0.00	0.00	0.00	0.00	0.00
GATCAACACCATGCATCCAT	0.00	0.00	0.57	0.00	0.00
GATCAACACCATGCGCCCCT	0.23	0.00	0.00	0.00	0.34
GATCAACACCATGGACTCAA	0.00	0.00	0.29	0.00	0.00
GATCAACACCATGGAGGCGG	0.00	0.00	0.00	0.00	0.00
GATCAACACCATGGAGGTGG	0.00	0.00	0.00	0.34	0.00
GATCAACACCATGGCAGCCG	0.00	0.00	0.00	0.00	0.34
GATCAACACCATGGCGGTGG	0.00	0.00	0.29	0.00	0.00

GATCAACACCATGGCTGCGG	0.00	0.00	0.29	0.00	0.00
GATCAACACCATGGGGGCGG	0.00	0.00	0.00	0.00	0.00
GATCAACACCATGGTGGAGG	0.00	0.33	0.00	0.68	0.00
GATCAACACCATGGTGGCGG	0.00	0.00	0.00	0.00	0.34
GATCAACACCATGGTGGGGG	1.14	0.00	0.29	0.34	1.03
GATCAACACCATGGTGGGNN	0.23	0.00	0.00	0.00	0.00
GATCAACACCATGGTGGNNN	0.00	0.00	0.00	0.00	0.00
GATCAACACCATGGTGGTGA	0.23	0.00	0.00	0.00	0.00
GATCAACACCATGGTGGTGG	29.99	40.02	46.39	27.83	35.47
GATCAACACCATGGTGGTTG	0.00	0.33	0.00	0.00	0.00
GATCAACACCATGGTGTGGA	0.00	0.00	0.00	0.00	0.34
GATCAACACCATGGTGTGTC	0.00	0.00	0.29	0.00	0.00
GATCAACACCATGGTGTTGG	0.00	0.00	0.00	0.34	0.00
GATCAACACCATGTATGCCT	0.00	0.00	0.29	0.00	0.00
GATCAACACCATGTGAACGT	0.00	0.00	0.29	0.00	0.00
GATCAACACCATTTTGGTGC	0.46	0.00	0.57	0.34	0.00
GATCAACACCCAAAAAAAAA	0.23	0.00	0.00	0.00	0.00
GATCAACACCCAAACCGGAA	0.00	0.00	0.00	0.00	0.34
GATCAACACCCAACTCGAAG	0.00	0.00	0.00	0.00	0.34
GATCAACACCCAACTCTCGA	0.00	0.00	0.00	0.00	0.69
GATCAACACCCACAAGTGCC	0.00	0.33	0.00	0.00	0.00
GATCAACACCCACAAGTGTC	0.00	0.33	0.00	0.34	0.69
GATCAACACCCACACACTGC	0.00	0.00	0.00	0.00	0.34
GATCAACACCCACAGCCACA	0.46	0.33	0.00	0.34	0.34
GATCAACACCCACATTGTCA	0.00	0.00	0.00	0.34	0.00
GATCAACACCCACCATACGA	0.00	0.00	0.29	0.00	0.00
GATCAACACCCACCTCGGAG	0.00	0.33	0.00	0.00	0.00
GATCAACACCCACTGCGAAG	0.23	0.00	0.00	0.00	0.00
GATCAACACCCAGAATCTGT	1.37	0.00	0.00	0.00	0.00
GATCAACACCCAGACCGAGA	0.00	0.00	0.29	0.00	0.00
GATCAACACCCAGCTCCGGG	0.00	0.33	0.00	0.00	0.00
GATCAACACCCAGTGGGTAC	0.00	0.00	0.00	0.00	0.34
GATCAACACCCATTGCAGCT	0.00	0.00	0.29	0.00	0.00
GATCAACACCCCAAAGTGGC	0.00	0.33	0.29	0.00	0.00
GATCAACACCCCACACTGGC	0.00	0.33	0.00	0.00	0.00
GATCAACACCCCACAGAGGC	0.00	0.00	0.29	0.00	0.00
GATCAACACCCCACAGCGGC	0.00	0.00	0.86	0.00	0.34

GATCAACACCCCACAGGCGC	0.00	0.00	0.00	0.00	0.00
GATCAACACCCCACAGGGGC	0.00	0.65	0.29	0.00	0.00
GATCAACACCCCACAGNGGC	0.00	0.00	0.00	0.00	1.03
GATCAACACCCCACAGTAGC	0.23	0.00	0.00	0.00	0.00
GATCAACACCCCACAGTCGC	0.00	0.00	0.00	0.34	0.00
GATCAACACCCCACAGTGGA	0.00	0.00	0.57	0.34	0.34
GATCAACACCCCACAGTGGC	18.77	48.15	65.29	82.14	81.28
GATCAACACCCCACAGTGGN	0.00	0.00	0.00	0.00	0.00
GATCAACACCCCACAGTGNN	0.00	0.00	0.00	0.00	0.00
GATCAACACCCCACAGTTGC	0.00	0.00	0.00	0.00	0.34
GATCAACACCCCACATTGGC	0.00	0.00	0.00	0.34	0.00
GATCAACACCCCACCGCGGA	0.00	0.00	0.29	0.00	0.00
GATCAACACCCCATACGAAT	0.00	0.00	0.00	0.00	0.34
GATCAACACCCCCAGGGCAG	0.00	0.00	0.00	0.00	0.00
GATCAACACCCCCAGTATAG	0.23	0.00	0.00	0.00	0.00
GATCAACACCCCGAAGTCCT	0.00	0.00	0.00	0.00	0.00
GATCAACACCCGACAGTGGC	0.00	0.00	0.00	0.00	0.00
GATCAACACCCGCACAGAAC	0.00	0.00	0.57	0.00	0.00
GATCAACACCCGCATCATGG	0.00	0.33	0.00	0.00	0.00
GATCAACACCCGCGAGGCGC	0.00	0.00	0.00	0.00	0.34
GATCAACACCCGGCTCGGAG	0.00	0.00	0.00	0.34	0.00
GATCAACACCCGGCTCGGCG	1.83	1.63	1.15	2.72	0.34
GATCAACACCCGGGGACCGA	0.00	0.00	0.00	0.00	0.00
GATCAACACCCGTGGCACAA	0.00	0.00	0.00	1.02	0.00
GATCAACACCCGTGGCATGC	0.00	0.00	0.00	0.00	0.34
GATCAACACCCGTGGTGTCT	0.00	0.00	0.00	0.00	0.34
GATCAACACCCTAAGTGGGA	0.00	0.00	0.00	0.34	0.00
GATCAACACCCTATTTTTGT	0.00	0.00	0.00	0.00	0.34
GATCAACACCCTCGCCTGCA	0.00	0.00	0.29	0.00	0.00
GATCAACACCCTCTATGATG	0.00	0.00	0.29	0.00	0.00
GATCAACACCCTCTCGGAGC	0.23	0.00	0.00	0.34	0.00
GATCAACACCCTCTCGGAGG	0.00	0.00	0.00	0.00	0.34
GATCAACACCCTGAAGTGCA	0.00	0.00	0.00	0.00	0.34
GATCAACACCCTGCAGTACA	0.00	0.00	0.00	0.00	0.00
GATCAACACCCTGCCTTGCC	0.23	0.00	0.00	0.00	0.00
GATCAACACCCTGGGCAAGA	0.00	0.00	0.29	0.00	0.00
GATCAACACCCTTGCCAGCA	0.00	0.00	0.00	0.00	0.34

GATCAACACCCTTGGGCTCT	0.00	0.33	0.00	0.00	0.00
GATCAACACCGAACAGAAGA	0.00	0.00	0.29	0.00	0.00
GATCAACACCGAACCGAACC	0.00	0.00	0.57	0.00	0.00
GATCAACACCGACAACGACA	0.00	0.00	0.29	0.00	0.00
GATCAACACCGACAGCTCCG	0.00	0.65	0.29	2.04	0.69
GATCAACACCGACAGCTCTG	0.23	0.00	0.29	0.00	0.00
GATCAACACCGACCAACACC	0.00	0.00	0.29	0.00	0.00
GATCAACACCGACCCAAAGG	0.00	0.33	0.00	0.00	0.00
GATCAACACCGACCTTGCCG	0.00	0.00	0.00	0.00	0.69
GATCAACACCGACTGCTACG	0.00	0.00	0.00	0.00	0.00
GATCAACACCGAGACCGTCT	0.00	0.00	0.00	0.00	1.03
GATCAACACCGAGAGGGAGT	0.00	0.00	0.00	0.34	0.00
GATCAACACCGAGCAGCACA	0.00	0.00	0.00	0.00	0.69
GATCAACACCGAGCCGCTCG	0.00	0.00	0.29	0.00	0.00
GATCAACACCGAGTAGAACA	0.00	0.00	0.00	0.00	0.34
GATCAACACCGATAGCTCCG	0.00	0.00	0.00	0.00	0.00
GATCAACACCGATGCTCCGC	0.00	0.00	0.00	0.34	0.00
GATCAACACCGATTGTTTCT	1.83	0.65	0.29	1.02	0.00
GATCAACACCGCAAGAGTGA	0.00	0.33	0.00	0.00	0.00
GATCAACACCGCACAACACA	0.00	0.00	0.00	0.00	1.03
GATCAACACCGCACAGTGGC	0.00	0.00	0.00	0.00	0.00
GATCAACACCGCACCCCGGC	0.00	0.00	0.00	0.00	0.00
GATCAACACCGCAGCGCCGG	0.00	0.00	0.29	0.00	0.00
GATCAACACCGCCAAAACGC	0.00	0.00	0.00	0.00	0.34
GATCAACACCGCCACAATGA	0.00	0.00	0.29	0.00	0.00
GATCAACACCGCCGCGCTGC	0.00	0.00	0.00	0.00	0.00
GATCAACACCGCCGGCCCGA	0.00	0.33	0.00	0.00	0.00
GATCAACACCGCCGTCCAAC	0.00	0.00	0.29	0.00	0.34
GATCAACACCGCCTGCGTCT	0.00	0.00	0.29	0.00	0.00
GATCAACACCGCCTTGACCA	0.00	0.00	0.29	0.00	0.00
GATCAACACCGCCTTGGCAA	0.00	0.00	0.00	0.00	0.00
GATCAACACCGCGAGAATGA	0.23	0.00	0.29	0.34	0.00
GATCAACACCGCGCAGTTTT	0.00	0.00	0.00	0.34	0.00
GATCAACACCGCGGCTATTC	0.00	0.00	0.29	0.00	0.00
GATCAACACCGCGTGCGCTC	0.00	0.33	0.00	0.00	0.00
GATCAACACCGCTCGATCCG	0.00	0.33	0.00	0.00	0.00
GATCAACACCGCTGCGCGCA	0.00	0.00	0.00	0.00	0.00

GATCAACACCGCTGGGGTGC	0.00	0.00	0.57	0.00	0.00
GATCAACACCGCTGTGCGCG	0.00	0.33	0.29	0.00	0.34
GATCAACACCGGAAGTAGCT	0.69	0.00	0.00	0.00	0.00
GATCAACACCGGAGGCCAAG	0.00	0.00	0.00	1.02	0.00
GATCAACACCGGAGGTCAAG	0.00	0.33	0.00	0.00	0.00
GATCAACACCGGAGTGGGGA	0.00	0.00	0.00	0.34	0.00
GATCAACACCGGCAAGATGC	0.00	0.00	0.00	0.34	0.00
GATCAACACCGGCCAATTCA	0.00	0.00	0.29	0.00	0.00
GATCAACACCGGCCGCTTCT	0.00	0.00	0.00	0.00	0.00
GATCAACACCGGCGCACTGC	0.00	0.00	0.00	0.00	0.34
GATCAACACCGGCGGCCTCN	0.23	0.00	0.00	0.00	0.00
GATCAACACCGGCGGCCTCT	0.69	0.00	0.00	0.00	0.00
GATCAACACCGGCGGCCTTT	0.00	0.00	0.00	0.00	0.00
GATCAACACCGGGCTGGCTG	0.00	0.33	0.00	0.00	0.00
GATCAACACCGGGGACCGAG	0.00	0.00	0.00	0.34	0.00
GATCAACACCGGGGACCGCG	0.00	0.00	0.00	0.00	0.00
GATCAACACCGGGGGCTTGA	0.00	0.00	0.00	0.00	0.00
GATCAACACCGGTGACATCG	0.00	0.00	0.00	1.36	0.00
GATCAACACCGGTGACGCTT	0.00	0.00	0.29	0.68	0.00
GATCAACACCGTAAATGCCN	0.23	0.00	0.00	0.00	0.00
GATCAACACCGTAAATGCCT	1.37	0.65	0.29	0.34	0.69
GATCAACACCGTACTGGCCG	0.00	0.00	0.00	0.00	0.00
GATCAACACCGTACTGGCTG	0.00	0.00	0.00	0.34	0.00
GATCAACACCGTATCGTCGG	0.00	0.00	0.29	0.00	0.00
GATCAACACCGTATTCACGC	0.00	0.00	0.00	0.00	0.00
GATCAACACCGTCAGGAGGC	0.00	0.33	0.00	0.00	0.00
GATCAACACCGTCGATGCAA	0.00	0.65	0.00	0.00	0.00
GATCAACACCGTCTACAGCC	0.00	0.33	0.00	0.00	0.00
GATCAACACCGTCTTCGCTG	0.00	0.33	0.29	0.00	0.00
GATCAACACCGTGCGAACCA	0.00	0.33	0.00	0.00	0.00
GATCAACACCGTGCGAGCCA	0.23	1.63	3.15	3.39	1.03
GATCAACACCGTGGTGGAGC	0.00	0.00	0.00	0.00	0.00
GATCAACACCGTGTAGTACC	0.00	0.00	0.00	0.34	0.00
GATCAACACCGTTGCCGTAA	0.00	0.00	0.00	0.00	0.34
GATCAACACCGTTGGGAATG	0.00	0.00	0.00	0.34	0.00
GATCAACACCGTTGGGCCGC	0.00	0.00	0.29	0.00	0.00
GATCAACACCGTTGTGCAGA	0.00	0.00	0.00	0.00	0.00

GATCAACACCTAACCGAAAT	0.00	0.00	0.57	0.00	0.00
GATCAACACCTAAGAGTTGC	0.23	0.00	0.00	0.00	0.00
GATCAACACCTACCGAGTAC	0.00	0.00	0.29	0.00	0.00
GATCAACACCTACCGCATAT	0.00	0.00	0.00	0.34	0.00
GATCAACACCTACGGCATCC	0.00	0.00	0.29	0.34	0.34
GATCAACACCTAGGAGCAGG	0.00	0.33	0.00	0.00	0.00
GATCAACACCTATATCGGGC	0.00	0.00	0.00	0.00	0.69
GATCAACACCTCACACACCT	0.00	0.00	0.29	0.00	0.00
GATCAACACCTCACAGTGGC	0.00	0.00	0.00	0.00	0.34
GATCAACACCTCACCCTGCA	0.46	0.65	0.00	1.02	0.00
GATCAACACCTCACGTTGGA	0.00	0.00	0.00	0.00	0.34
GATCAACACCTCCAACAGTG	0.00	0.00	0.00	0.34	0.00
GATCAACACCTCCATTGGCG	0.00	0.00	0.00	0.00	0.34
GATCAACACCTCCATTGGTG	0.00	0.00	0.00	0.00	0.34
GATCAACACCTCCCGATGTG	0.00	0.00	0.00	0.34	0.00
GATCAACACCTCCGGGGTGC	0.00	0.00	0.00	0.00	0.34
GATCAACACCTCCTAGGATT	0.00	0.00	0.29	0.00	0.00
GATCAACACCTCCTCATCCT	0.00	0.00	0.29	0.00	0.00
GATCAACACCTCCTCGATCG	0.00	0.00	0.00	0.34	0.00
GATCAACACCTCCTTTTTCG	0.00	0.00	0.00	0.00	0.69
GATCAACACCTCGCAGGCTG	0.00	0.33	0.00	0.00	0.00
GATCAACACCTCGCCGAAGG	0.00	0.00	0.00	0.00	0.00
GATCAACACCTCGGAGAAGA	0.00	0.00	0.00	0.68	0.34
GATCAACACCTCGGAGAAGN	0.23	0.00	0.00	0.00	0.00
GATCAACACCTCGGGGGTGC	0.00	0.00	0.57	0.00	0.00
GATCAACACCTCGTCTCTTT	0.00	0.33	0.00	0.00	0.00
GATCAACACCTCTACGGTTG	0.23	0.00	0.00	0.00	0.00
GATCAACACCTCTACGTTTG	0.46	0.00	0.00	0.00	0.00
GATCAACACCTCTGCATGTC	0.00	0.00	0.29	0.68	0.00
GATCAACACCTCTGGGGCGC	0.00	0.00	0.29	0.00	0.00
GATCAACACCTCTGGGGTCC	0.00	0.00	0.00	0.34	0.00
GATCAACACCTCTGGGGTGC	7.78	9.76	17.47	15.27	14.81
GATCAACACCTCTGGGGTGG	0.00	0.00	0.00	0.00	0.00
GATCAACACCTGAACAAGAC	0.00	0.33	0.00	0.34	0.00
GATCAACACCTGAGAAAAAA	0.00	0.00	0.00	0.00	0.00
GATCAACACCTGAGAAAGAC	0.00	0.00	0.29	0.00	0.00
GATCAACACCTGAGCCAGTA	0.00	0.00	0.29	0.00	0.00

GATCAACACCTGCAAAAACC	0.00	0.00	0.00	0.00	0.34
GATCAACACCTGCAAAACGC	0.00	0.00	0.29	0.00	0.00
GATCAACACCTGCAACATGC	0.00	0.00	0.29	0.00	0.00
GATCAACACCTGCAACCCCC	0.00	0.00	0.29	0.00	0.00
GATCAACACCTGCAACCCGA	0.00	0.33	0.00	0.00	0.00
GATCAACACCTGCAACCCGC	0.00	0.00	0.29	0.68	0.00
GATCAACACCTGCAACCGGC	0.00	0.33	0.00	0.00	0.34
GATCAACACCTGCAACCGTG	0.00	0.00	0.00	0.00	0.00
GATCAACACCTGCAACCTCC	0.00	0.00	0.29	0.00	0.00
GATCAACACCTGCAACCTCG	0.00	0.00	0.00	0.00	0.34
GATCAACACCTGCAACCTGA	0.00	0.33	0.29	0.00	0.00
GATCAACACCTGCAACCTGC	2.52	22.12	57.56	19.35	57.52
GATCAACACCTGCAACCTGG	0.00	0.00	0.29	0.00	0.00
GATCAACACCTGCAACCTGT	0.00	0.33	0.57	0.00	0.00
GATCAACACCTGCAACGTCC	0.00	0.00	0.00	0.00	0.00
GATCAACACCTGCAACTGCC	0.00	0.00	0.00	0.00	0.34
GATCAACACCTGCAATCGTA	0.00	0.00	0.00	0.34	0.00
GATCAACACCTGCACCCTGA	0.00	0.00	0.29	0.00	0.00
GATCAACACCTGCAGCATTG	0.00	0.00	0.00	0.00	0.00
GATCAACACCTGCAGCCTTG	0.00	0.00	0.29	0.00	0.00
GATCAACACCTGCAGTGATG	0.00	0.00	0.00	0.00	0.00
GATCAACACCTGCATCTACC	0.00	0.00	0.00	0.34	0.00
GATCAACACCTGCCACATGC	0.00	0.33	0.00	0.00	0.00
GATCAACACCTGCCCGATGC	0.00	0.00	0.29	0.00	0.34
GATCAACACCTGCCCGTCCT	0.00	0.00	0.29	0.00	0.00
GATCAACACCTGCCGTTGAA	0.00	0.00	0.00	0.34	0.00
GATCAACACCTGCCTCTTTT	0.00	0.00	0.29	0.00	0.00
GATCAACACCTGCGAGCTGC	0.00	0.00	0.00	0.00	0.34
GATCAACACCTGCGAGNTGC	0.00	0.00	0.00	0.00	0.34
GATCAACACCTGCGTCCTGC	0.00	0.00	0.00	0.00	0.34
GATCAACACCTGCTGCTGCG	0.00	0.00	0.00	0.00	0.00
GATCAACACCTGCTGGAAGA	0.00	0.00	0.00	0.00	0.00
GATCAACACCTGGCTGGATT	0.00	0.00	0.29	0.00	0.00
GATCAACACCTGGGAGCCTG	0.00	0.00	0.29	0.00	0.00
GATCAACACCTGGGATACGA	1.14	0.33	0.00	1.36	0.34
GATCAACACCTGGGCACGCG	0.00	0.00	0.00	0.00	0.34
GATCAACACCTGGGCGAACA	0.00	0.00	0.57	0.00	0.00

GATCAACACCTTAAGCAGGA	0.00	0.00	0.29	0.00	0.00
GATCAACACCTTCACCCTGA	0.00	0.33	0.00	0.00	0.00
GATCAACACCTTCATCCTGA	0.00	0.00	0.00	0.34	0.00
GATCAACACCTTCCCCGAGG	0.00	0.00	0.86	0.00	0.00
GATCAACACCTTGAAACCAA	0.00	0.00	0.00	0.00	0.34
GATCAACACCTTGAACAATG	0.00	0.33	0.00	0.00	0.00
GATCAACACCTTGAAGCTGG	0.00	0.00	0.00	0.68	0.00
GATCAACACCTTGCCAGCTA	0.00	0.33	0.00	0.00	0.00
GATCAACACCTTGGCCAAGC	0.23	0.00	0.00	0.00	0.00
GATCAACACCTTGTAAAAAA	0.46	0.00	0.00	0.00	0.00
GATCAACACCTTGTCAAAAA	0.00	0.00	0.57	0.00	0.00
GATCAACACCTTGTCTCGTA	0.00	0.00	0.29	0.00	0.00
GATCAACACCTTGTCTCTTT	5.49	5.21	7.16	10.86	8.27
GATCAACACCTTTGGGGTGC	0.00	0.00	0.00	0.00	0.34
GATCAACACCTTTGGTCTGC	0.00	0.00	0.57	0.00	0.00
GATCAACACCTTTGTCGCGC	0.00	0.00	0.00	0.00	0.00
GATCAACACGAAAAAAAAAA	0.23	0.33	0.00	0.00	0.00
GATCAACACGAAAATCAAGA	0.00	0.00	0.57	0.00	0.00
GATCAACACGAAACCCAGCC	0.00	0.00	0.00	0.00	0.00
GATCAACACGAAAGTTTGCT	0.00	0.00	0.57	0.00	0.00
GATCAACACGAACAAAACAA	0.00	0.00	0.00	0.00	0.34
GATCAACACGAACATGCAGG	0.00	0.00	0.00	0.00	0.00
GATCAACACGAACCGATTGC	0.00	0.00	0.00	0.00	0.00
GATCAACACGAACGGCCGTC	0.00	0.33	0.00	0.00	0.00
GATCAACACGAACTTCAATC	0.00	0.00	0.00	0.00	0.00
GATCAACACGAACTTCCCAT	0.00	0.00	0.00	0.00	0.00
GATCAACACGAAGAAGCTGA	0.00	0.00	0.29	0.00	0.00
GATCAACACGAAGGAGGAAC	0.00	0.00	0.00	0.00	0.00
GATCAACACGAAGGAGGACA	0.00	0.00	0.57	0.00	0.00
GATCAACACGAAGGAGGAGA	0.23	0.33	0.57	0.00	0.34
GATCAACACGAAGGAGGAGC	27.70	46.20	69.87	43.45	65.44
GATCAACACGAAGGAGGAGN	0.00	0.00	0.00	0.68	0.00
GATCAACACGAAGGAGGAGT	0.00	0.00	0.00	0.00	0.34
GATCAACACGAAGGAGGANN	0.00	0.00	0.00	0.00	0.00
GATCAACACGAAGGAGGCGA	0.00	0.00	0.00	0.00	0.00
GATCAACACGAAGGAGGCGC	0.23	0.33	0.00	0.00	0.34
GATCAACACGAAGGAGGGGC	0.92	0.33	0.00	0.00	0.00

GATCAACACGAAGGAGGGGT	0.00	0.00	0.29	0.00	0.00
GATCAACACGAAGGAGGNGC	0.00	0.00	0.00	0.34	0.00
GATCAACACGAAGGAGGTCT	0.00	0.00	0.00	0.00	0.00
GATCAACACGAAGGAGGTGC	0.23	0.00	0.00	0.00	0.00
GATCAACACGAAGGAGGTGG	0.00	0.00	0.00	0.00	0.00
GATCAACACGAAGGAGNAGC	0.00	0.00	0.00	0.34	0.34
GATCAACACGAAGGCGGAGC	0.00	0.33	0.00	0.00	0.00
GATCAACACGAATAATCCGC	0.00	0.00	0.57	0.00	0.69
GATCAACACGAATCAGCATG	0.00	0.00	0.00	0.34	0.00
GATCAACACGAATCCCAAAA	0.00	0.00	0.29	0.34	0.00
GATCAACACGACAAAGTTTG	0.46	0.00	0.00	0.00	0.00
GATCAACACGACAAATAACA	0.00	0.00	0.00	0.00	0.34
GATCAACACGACACAACACA	0.00	0.00	0.00	0.00	0.34
GATCAACACGACACCGCANC	0.00	0.00	0.29	0.00	0.00
GATCAACACGACACGACACG	0.00	0.00	0.00	0.00	0.34
GATCAACACGACACGGCACG	0.00	0.00	0.00	0.00	1.03
GATCAACACGACATCTCTTC	0.00	0.00	0.00	0.34	0.00
GATCAACACGACCCGAGCCC	0.23	0.00	0.00	0.00	0.00
GATCAACACGACCCGGCAAG	0.00	0.00	0.00	0.00	0.34
GATCAACACGACCCGGCCCG	0.00	0.00	0.29	0.00	0.00
GATCAACACGACCCTCGTGG	0.00	0.00	0.00	0.00	0.34
GATCAACACGACCCTGTCTT	0.00	0.00	0.00	0.00	0.34
GATCAACACGACGAATCAAC	0.00	0.00	0.00	0.00	0.69
GATCAACACGACTCTCGGCA	0.00	0.00	0.00	0.00	0.34
GATCAACACGACTGCATGCG	0.00	0.00	0.57	0.00	0.00
GATCAACACGACTGTGTGGC	0.00	0.00	0.00	0.00	0.34
GATCAACACGACTTCCTACC	0.00	0.00	0.00	0.00	0.34
GATCAACACGAGAAAGAATT	0.00	0.00	0.00	0.00	0.00
GATCAACACGAGAAAGACTT	0.69	4.88	1.15	4.07	2.41
GATCAACACGAGAAAGGCGA	0.23	0.00	0.00	0.00	0.00
GATCAACACGAGAAGAAGGA	0.00	0.00	0.29	0.00	0.00
GATCAACACGAGAAGGATAG	0.46	0.33	0.00	0.00	0.00
GATCAACACGAGACAAGACG	0.00	0.00	0.00	0.00	0.00
GATCAACACGAGCAACAAGA	0.00	0.00	0.29	0.00	0.00
GATCAACACGAGCAACCATA	0.00	0.00	0.00	0.00	0.34
GATCAACACGAGCAAGGGCA	0.00	0.00	0.29	0.00	0.00
GATCAACACGAGCACATGTG	0.00	0.00	0.00	0.00	0.34

GATCAACACGAGCACGCGGC	0.00	0.33	0.00	0.34	0.00
GATCAACACGAGCCACATGG	0.00	0.00	0.00	0.00	0.00
GATCAACACGAGCGAACTCA	0.00	0.00	0.00	0.34	0.00
GATCAACACGAGCGCGCTGC	0.00	0.00	0.00	0.00	0.34
GATCAACACGAGCGCGGCGC	0.00	0.00	0.29	0.00	0.00
GATCAACACGAGCGTCCCAC	0.00	0.00	0.00	0.00	0.69
GATCAACACGAGCTCATGGC	4.81	2.93	2.58	5.77	3.10
GATCAACACGAGCTGGCCAA	0.00	0.00	0.00	0.34	0.00
GATCAACACGAGCTGGCCAC	0.46	0.00	1.15	0.34	0.00
GATCAACACGAGCTGGCCNN	0.00	0.00	0.00	0.00	0.00
GATCAACACGAGGAAGTCTC	0.00	0.33	0.00	0.00	0.00
GATCAACACGAGGACTGAAG	0.00	0.00	0.57	0.00	0.00
GATCAACACGAGGGAGGAGC	0.00	0.00	0.29	0.00	0.00
GATCAACACGAGGGGGCGCG	0.23	0.65	0.00	0.00	0.00
GATCAACACGAGTGAAAGCT	0.00	0.33	0.00	0.00	0.00
GATCAACACGAGTTGAGAGC	0.00	0.00	0.29	0.00	0.00
GATCAACACGATAAATGTGG	0.69	0.33	0.00	0.34	0.34
GATCAACACGATGAACTTCT	0.00	0.00	0.00	0.00	0.00
GATCAACACGATGAAGTGCG	0.00	0.00	0.00	0.00	0.00
GATCAACACGATGCCGCAAT	0.00	0.33	0.86	0.00	0.00
GATCAACACGATGCGTCATC	0.23	0.33	0.00	0.00	0.00
GATCAACACGATGGAGGGCG	0.00	0.65	0.00	0.00	0.00
GATCAACACGATGGTCTGTC	0.00	0.33	0.00	0.00	0.00
GATCAACACGATGTAGGTGG	0.23	1.63	1.72	1.02	0.00
GATCAACACGATTTCGTTGA	0.00	0.00	0.00	0.34	0.00
GATCAACACGATTTGCTGCA	0.00	0.00	0.00	0.34	0.00
GATCAACACGCAAAAGTGTG	0.00	0.00	0.00	0.68	0.00
GATCAACACGCAACACACAA	0.00	0.00	0.00	0.00	0.34
GATCAACACGCAACATCACG	0.00	0.00	0.00	0.00	0.34
GATCAACACGCAAGGTGTGA	0.23	0.33	0.00	0.34	0.00
GATCAACACGCACACTAAGC	0.00	0.00	0.29	0.00	0.00
GATCAACACGCACAGCATGG	0.00	0.00	0.29	0.00	0.00
GATCAACACGCACATCCTAA	0.00	0.00	0.00	0.00	0.34
GATCAACACGCACCAGGGAC	0.00	0.00	0.29	0.00	0.00
GATCAACACGCACCAGGGCC	2.06	3.58	2.86	4.75	2.07
GATCAACACGCACCAGGGCT	0.23	0.00	0.00	0.00	0.34
GATCAACACGCACCCGGCAA	0.00	0.00	0.00	0.00	0.34

GATCAACACGCACGGGTGGA	0.00	0.00	0.00	0.00	0.00
GATCAACACGCACGTCCATG	0.00	0.00	0.29	0.00	0.00
GATCAACACGCACTAGAACT	0.00	0.00	0.00	0.00	1.03
GATCAACACGCACTGTACGC	0.00	0.00	0.00	0.00	0.00
GATCAACACGCAGGACCTTA	0.23	0.00	0.00	0.00	0.00
GATCAACACGCAGGAGGCGC	0.00	0.33	0.00	0.00	0.00
GATCAACACGCCACAACACA	0.00	0.00	0.29	0.00	0.00
GATCAACACGCCACACACCA	0.00	0.00	0.00	0.00	0.34
GATCAACACGCCACGCCACG	0.00	0.00	0.00	0.00	0.34
GATCAACACGCCACTGTCCA	0.00	0.00	0.00	0.00	0.00
GATCAACACGCCCAAAAAAC	0.00	0.00	0.29	0.00	0.00
GATCAACACGCCCACCCTGA	0.00	0.65	0.00	0.68	0.00
GATCAACACGCCCAGGCCCC	0.00	0.00	0.00	0.34	0.00
GATCAACACGCCCCACCCAC	0.00	0.00	0.29	0.00	0.00
GATCAACACGCCCCCCAGCC	0.00	0.00	0.00	0.00	0.00
GATCAACACGCCCCGGCCCG	0.00	0.00	0.00	0.00	0.34
GATCAACACGCCCTACCCCT	0.00	0.00	0.00	0.00	0.00
GATCAACACGCCCTGCCTTG	0.00	0.00	0.00	0.00	0.00
GATCAACACGCCGAGCAACA	0.00	0.00	0.00	0.00	0.34
GATCAACACGCCTCTCAAAG	0.00	0.00	0.00	0.00	0.00
GATCAACACGCCTGATTGAC	0.00	0.00	0.00	0.34	1.03
GATCAACACGCGAATGAGAC	0.23	0.00	0.00	0.00	0.00
GATCAACACGCGAATGAGTC	0.00	0.00	0.57	0.00	0.00
GATCAACACGCGAGTGCACA	0.00	0.00	0.29	0.00	0.00
GATCAACACGCGCAAGATAG	0.00	0.00	0.00	0.00	0.00
GATCAACACGCGCAAGATTG	0.00	0.00	0.00	0.00	0.34
GATCAACACGCGCCATCGCG	0.00	0.00	0.00	0.00	0.00
GATCAACACGCGCCTCGCCA	0.23	2.28	0.00	0.34	0.69
GATCAACACGCGCTGGGAGC	0.23	0.00	0.00	0.00	0.00
GATCAACACGCGGAATCCCT	0.00	0.00	0.00	0.68	0.00
GATCAACACGCGGAGAAAGC	0.00	0.00	0.29	0.00	0.00
GATCAACACGCGTTTGTACA	0.00	0.00	0.00	0.00	0.00
GATCAACACGCTCAACGGCC	0.00	0.00	0.00	0.00	0.00
GATCAACACGCTCAAGCGAC	0.00	0.00	0.00	0.00	0.00
GATCAACACGCTCATTGAGA	0.00	0.33	0.00	0.00	0.00
GATCAACACGCTCCTCCCGC	0.00	0.00	0.29	0.00	0.00
GATCAACACGCTCGAGGGCA	0.00	0.65	0.00	0.00	0.00

GATCAACACGCTCGGACTGG	0.00	0.00	0.00	0.34	0.00
GATCAACACGCTCGTCTTCA	0.00	0.65	2.58	3.73	2.41
GATCAACACGCTCGTCTTCC	0.00	0.33	0.57	0.00	1.72
GATCAACACGCTCTATGCCC	0.00	0.65	0.00	0.00	0.00
GATCAACACGCTCTTGGAAC	0.00	0.33	0.00	0.34	0.00
GATCAACACGCTGACCAATA	0.00	0.00	0.00	0.00	0.00
GATCAACACGCTGCAGTTCG	0.46	0.00	0.00	0.00	0.00
GATCAACACGCTGCGCCTGG	0.00	0.65	0.29	0.34	0.69
GATCAACACGCTGCGCCTNN	0.00	0.00	0.00	0.00	0.00
GATCAACACGCTGCGGAACT	0.92	0.00	0.57	0.68	0.00
GATCAACACGCTGGCCGGCA	0.00	0.00	0.29	0.00	0.00
GATCAACACGCTGGGCATGG	0.00	0.00	0.00	0.00	0.69
GATCAACACGCTGGTCTTCA	0.00	0.00	0.86	0.00	0.00
GATCAACACGCTGGTCTTCC	0.00	0.65	0.29	1.02	0.34
GATCAACACGCTGGTTTGAA	0.00	0.00	0.00	0.00	0.00
GATCAACACGCTGTTGGAAG	0.00	0.00	0.00	0.00	0.00
GATCAACACGCTTGCATTGT	0.00	0.00	0.00	0.00	0.34
GATCAACACGCTTGTCTTCC	0.00	0.33	0.86	0.34	0.69
GATCAACACGGAACAACACA	0.00	0.00	0.00	0.00	0.69
GATCAACACGGAACGTGCTC	0.00	0.00	0.29	0.00	0.00
GATCAACACGGAATAGAGGC	0.00	0.00	0.29	0.00	0.00
GATCAACACGGACAAGAGAA	0.00	0.00	0.00	0.00	0.34
GATCAACACGGACAGCTACG	0.00	0.33	0.29	0.00	0.34
GATCAACACGGACAGCTCAG	0.00	0.00	0.29	0.00	0.00
GATCAACACGGACAGCTTTC	0.00	0.00	0.00	0.00	0.00
GATCAACACGGACGCATGGC	0.69	0.65	0.57	3.05	0.34
GATCAACACGGACGCATTGA	0.23	0.00	0.00	0.00	0.00
GATCAACACGGACGCATTGG	0.00	0.33	0.00	0.00	0.00
GATCAACACGGACGGGCTCC	0.00	0.00	0.00	0.00	0.00
GATCAACACGGACTGCAGCA	0.00	0.00	0.00	0.00	0.00
GATCAACACGGACTTCGACG	1.37	1.30	0.57	0.68	0.34
GATCAACACGGAGCACGCGT	0.00	0.00	0.00	0.00	0.34
GATCAACACGGAGCGAGTGC	0.00	0.00	0.00	0.00	0.69
GATCAACACGGAGCGCGGAG	0.00	0.00	0.00	0.00	0.00
GATCAACACGGAGGACACCT	0.00	0.33	0.00	0.00	0.00
GATCAACACGGAGGAGGAGC	0.23	0.00	0.00	0.00	0.00
GATCAACACGGAGGCACGCT	0.00	0.00	0.00	0.00	0.00

GATCAACACGGAGGTCTCGG	0.00	0.00	0.00	0.68	0.00
GATCAACACGGATGCCGAGT	0.00	0.00	0.00	0.00	0.00
GATCAACACGGATGGTGATG	0.00	0.33	0.00	0.00	0.00
GATCAACACGGCAACACATT	0.00	0.00	0.00	0.00	0.00
GATCAACACGGCAAGAACGC	0.00	0.00	0.00	0.00	0.00
GATCAACACGGCAAGGGAGA	0.00	0.00	0.00	0.00	0.00
GATCAACACGGCAATGCACC	0.00	0.00	0.29	0.00	0.00
GATCAACACGGCAATTGGCC	0.00	0.00	0.00	0.00	0.00
GATCAACACGGCACACATTG	0.00	0.00	0.29	0.00	0.00
GATCAACACGGCAGCTGCAC	0.00	0.33	0.00	0.00	0.00
GATCAACACGGCCACCCTGC	0.00	0.00	0.29	0.00	0.00
GATCAACACGGCCCAGGAGC	0.00	0.00	0.00	0.34	0.00
GATCAACACGGCCCGGAGTC	0.00	0.00	0.00	0.34	0.00
GATCAACACGGCCCGGCATG	0.00	0.65	0.86	0.34	0.00
GATCAACACGGCCCTTGGCC	0.00	0.00	0.00	0.00	0.00
GATCAACACGGCCGGGACCG	0.00	0.00	0.00	0.00	0.34
GATCAACACGGCCGTGAACG	0.00	0.00	0.00	0.34	0.00
GATCAACACGGCCGTGACCG	0.46	0.00	0.29	0.68	0.69
GATCAACACGGCCGTGCAGC	0.00	0.65	0.00	0.68	0.00
GATCAACACGGCCGTGCATA	0.00	0.00	0.00	0.00	0.00
GATCAACACGGCGATTAGGA	0.23	0.00	0.00	0.00	0.00
GATCAACACGGCGCGGTGCG	0.00	0.00	0.00	0.00	0.00
GATCAACACGGCGTACAAGC	0.46	0.00	0.00	0.34	0.34
GATCAACACGGCGTACTAGC	0.00	0.33	0.00	0.00	0.00
GATCAACACGGCGTCCCTTG	0.00	0.00	0.00	0.00	0.00
GATCAACACGGCGTTCGACT	0.00	0.33	0.00	0.00	0.00
GATCAACACGGGAAGCTCCT	0.00	0.00	0.00	0.34	0.00
GATCAACACGGGACAGGCAG	0.00	0.33	0.00	0.00	0.00
GATCAACACGGGACGCTTTT	0.00	0.33	0.00	0.00	0.00
GATCAACACGGGACTACATC	0.00	0.00	0.00	0.00	0.00
GATCAACACGGGACTGATGG	0.23	0.00	0.00	0.00	0.00
GATCAACACGGGCAACGGAG	2.98	1.30	1.43	2.72	0.69
GATCAACACGGGCAAGGATG	0.00	0.33	0.00	0.00	0.00
GATCAACACGGGCACACGCT	0.00	0.00	0.29	0.00	0.00
GATCAACACGGGCACGAACA	0.00	0.00	0.00	0.00	0.00
GATCAACACGGGCAGATGGC	0.00	0.00	0.00	0.34	0.00
GATCAACACGGGCATCAAGT	0.23	0.00	0.00	0.00	0.00

GATCAACACGGGCATGATGC	0.00	0.00	0.00	0.34	0.00
GATCAACACGGGCATGCGTG	0.00	0.00	0.00	0.00	0.00
GATCAACACGGGCATTTGTT	0.00	0.00	0.29	0.00	0.34
GATCAACACGGGCCACTTTA	0.00	0.00	0.57	0.00	0.00
GATCAACACGGGCCTGAGGG	0.23	0.00	0.00	0.00	0.00
GATCAACACGGGCCTGATGG	9.84	2.28	0.57	0.34	0.69
GATCAACACGGGCCTGATGN	0.23	0.00	0.00	0.00	0.00
GATCAACACGGGCCTGCTGG	0.23	0.00	0.00	0.00	0.00
GATCAACACGGGCCTGGTGG	0.23	0.00	0.00	0.00	0.00
GATCAACACGGGCGAGAATA	0.00	0.33	0.00	0.00	0.00
GATCAACACGGGGAAACTTA	0.00	0.00	0.00	0.00	0.00
GATCAACACGGGGCTCTGCG	0.00	0.33	0.00	0.00	0.34
GATCAACACGGGGGGCCTGA	0.00	0.00	0.00	0.00	0.34
GATCAACACGGGGTCGACAC	0.00	0.00	0.29	0.00	0.00
GATCAACACGGGGTTGAATT	0.23	0.00	0.00	0.00	0.00
GATCAACACGGTCAACCACG	0.00	0.00	0.00	0.00	0.00
GATCAACACGGTCACTTTGG	0.00	0.33	0.86	0.34	0.69
GATCAACACGGTCGTTGTCG	0.00	0.00	0.00	0.00	0.00
GATCAACACGGTCTACAGCC	0.00	0.33	0.00	0.00	0.00
GATCAACACGGTGACGAGAA	0.00	0.00	0.29	0.00	0.00
GATCAACACGGTGATAATCA	0.00	0.00	0.00	0.34	0.00
GATCAACACGGTGGCCGACC	0.00	0.33	0.29	0.00	0.00
GATCAACACGGTGTACAGGC	0.00	0.00	0.00	0.00	0.00
GATCAACACGGTGTTGCGCA	0.00	0.00	0.29	0.00	0.00
GATCAACACGGTTGCGGCAG	0.00	0.00	0.00	0.00	0.34
GATCAACACGGTTGTAGACA	0.00	0.00	0.00	0.00	0.34
GATCAACACGTAAGGACGAC	0.00	0.00	0.57	0.00	0.00
GATCAACACGTACCAAGGAG	0.00	0.00	0.00	0.34	0.00
GATCAACACGTACCGAAGAG	1.37	0.33	0.29	0.68	0.00
GATCAACACGTACCTGGACT	0.00	0.00	0.00	1.36	0.34
GATCAACACGTACGCAACGC	1.60	5.53	3.72	5.43	4.48
GATCAACACGTACGCGACGC	0.00	0.00	0.29	0.00	0.00
GATCAACACGTATGAGGAGG	0.00	0.00	0.00	0.00	0.00
GATCAACACGTATGTCCTTG	0.00	0.00	0.00	0.00	0.00
GATCAACACGTCAAACTTTG	0.00	0.00	0.00	0.00	0.69
GATCAACACGTCAAGAAGGA	0.23	0.33	0.00	0.00	0.00
GATCAACACGTCAGGAATGC	0.00	0.00	0.00	0.00	0.69

GATCAACACGTCAGGGGGCT	0.46	0.00	0.00	0.00	0.00
GATCAACACGTCCAAGCTGA	0.00	0.00	0.00	0.34	0.00
GATCAACACGTCCCGCCGCG	0.23	0.00	0.00	0.00	0.00
GATCAACACGTCGGAATTGC	0.00	0.00	0.29	0.00	0.00
GATCAACACGTCTACAAGGT	0.00	0.00	0.29	0.00	0.00
GATCAACACGTGAATTTGTT	0.00	0.00	0.00	0.00	0.34
GATCAACACGTGAGCTACCT	0.00	0.33	0.00	0.00	0.00
GATCAACACGTGATTTTCTC	0.00	0.00	0.00	0.34	0.00
GATCAACACGTGCAAAAAAA	0.00	0.00	0.00	0.00	0.34
GATCAACACGTGCAAAGAAA	0.23	0.00	0.00	0.00	0.00
GATCAACACGTGCACTTGTT	0.00	0.00	0.29	0.00	0.00
GATCAACACGTGCAGAAAAA	0.00	0.00	0.00	0.00	0.00
GATCAACACGTGCATGATAC	0.00	0.33	0.00	0.00	0.00
GATCAACACGTGCATGATGA	0.00	0.00	0.29	0.00	0.00
GATCAACACGTGCATTNNNN	0.23	0.00	0.00	0.00	0.00
GATCAACACGTGCATTTGCT	0.00	0.00	0.29	0.00	0.00
GATCAACACGTGCATTTGGT	0.00	0.65	0.00	0.00	0.00
GATCAACACGTGCATTTGNN	0.69	0.00	0.00	0.00	0.00
GATCAACACGTGCATTTGTC	0.00	0.33	0.00	1.02	0.00
GATCAACACGTGCATTTGTN	0.46	0.00	0.00	0.00	0.00
GATCAACACGTGCATTTGTT	42.81	16.92	27.20	30.89	25.14
GATCAACACGTGCATTTTGT	0.00	0.00	0.00	0.00	0.00
GATCAACACGTGCCCGGTGG	0.00	0.00	0.00	0.00	0.00
GATCAACACGTGCCTTATGG	0.46	0.00	0.00	0.00	0.00
GATCAACACGTGCCTTTGTT	0.23	0.00	0.00	0.00	0.00
GATCAACACGTGCGCCCAGG	0.92	0.00	1.72	0.00	0.69
GATCAACACGTGCGCGTCGA	0.00	0.00	0.29	0.00	0.00
GATCAACACGTGCGCGTTGA	0.00	0.00	0.57	0.00	0.00
GATCAACACGTGCGGCGGGG	0.00	0.00	0.29	0.00	0.00
GATCAACACGTGCGTTTGAA	0.00	0.00	0.29	0.00	0.00
GATCAACACGTGGAGAAAGT	0.00	0.00	0.29	0.00	0.00
GATCAACACGTGGTAAGTTC	0.00	0.00	0.00	0.00	0.34
GATCAACACGTGTGTCTACT	0.00	0.00	0.00	0.00	0.00
GATCAACACGTTAGCGGGGT	0.00	0.00	0.00	0.00	0.34
GATCAACACGTTCAGTAGCG	0.00	0.00	0.00	0.34	0.34
GATCAACACGTTCGTATGCC	0.00	0.00	0.29	0.00	0.00
GATCAACACGTTGGCATGCG	0.00	0.00	0.00	0.00	0.34

GATCAACACGTTGTTGCCGT	0.00	0.33	0.00	0.00	0.00
GATCAACACTAAAAAAAAAA	13.05	0.98	4.01	6.11	6.54
GATCAACACTAAAAAAAAAT	0.00	0.33	0.00	0.68	0.00
GATCAACACTAAAAAGAAAA	0.00	0.33	0.00	0.00	0.00
GATCAACACTAATTAACGTA	0.00	0.00	0.00	0.00	0.00
GATCAACACTAATTAATGTA	1.83	1.63	0.29	0.68	0.34
GATCAACACTAATTGGATGT	0.00	0.00	0.00	0.00	0.34
GATCAACACTACAGCGGGAG	0.00	0.33	0.00	0.00	0.00
GATCAACACTACGTCCTTAG	0.00	0.00	0.29	0.00	0.00
GATCAACACTACTCCTTGCA	0.00	0.33	0.00	0.00	0.34
GATCAACACTACTTAATGAA	0.00	0.00	0.00	0.34	0.00
GATCAACACTAGCCACTGCT	0.00	0.00	0.00	0.00	0.00
GATCAACACTAGCCTACTCC	0.00	0.00	0.29	0.00	0.00
GATCAACACTAGCTTGTGTT	0.00	0.00	0.00	0.00	0.00
GATCAACACTAGGGGGGCCC	0.00	0.00	0.29	0.00	0.00
GATCAACACTAGGGGGGCTC	0.00	0.00	0.29	0.00	0.00
GATCAACACTATCAACAACA	0.00	0.00	0.57	0.00	0.00
GATCAACACTATGGTGGTGG	0.00	0.00	0.00	0.34	0.00
GATCAACACTATTCGACATG	0.46	0.00	0.00	0.00	0.00
GATCAACACTATTGCATGCC	0.00	0.00	1.15	0.00	0.00
GATCAACACTATTTTCCCGA	0.00	0.00	0.00	0.00	0.34
GATCAACACTCAAAAAAAAA	0.00	0.00	0.00	0.00	0.69
GATCAACACTCAAAAAGCCA	0.00	0.00	0.29	0.00	0.00
GATCAACACTCAACACACAC	0.00	0.00	0.57	0.00	0.00
GATCAACACTCAACAGGTCG	0.00	0.00	0.00	0.00	0.00
GATCAACACTCACGTGGGTG	0.00	0.00	0.29	0.34	0.00
GATCAACACTCAGGGCTAGG	0.00	0.00	0.00	0.34	0.00
GATCAACACTCAGGTGGGTG	0.00	0.65	0.00	0.34	0.34
GATCAACACTCAGTGCTCTC	0.46	0.33	0.00	0.00	0.00
GATCAACACTCAGTTCCACA	0.00	0.00	0.00	0.00	0.69
GATCAACACTCATATGAACT	0.46	0.00	0.29	0.68	0.34
GATCAACACTCATGGCCTAC	0.92	0.00	0.29	0.00	0.00
GATCAACACTCATGGCCTCC	0.00	0.00	0.29	0.00	0.00
GATCAACACTCCAGAAGCCA	0.00	0.00	0.29	0.00	0.00
GATCAACACTCCGTGCATGA	0.23	1.30	0.57	1.70	0.34
GATCAACACTCCTCAACGCG	0.00	0.00	0.00	0.00	0.34
GATCAACACTCCTTTCCAGA	0.23	0.00	0.00	0.00	0.00

GATCAACACTCCTTTCTCTC	0.00	0.00	0.00	0.00	0.00
GATCAACACTCGCCAGGCAT	0.00	0.00	0.00	0.00	0.00
GATCAACACTCGGACAAGCC	0.00	0.00	0.00	0.00	0.00
GATCAACACTCGGCCTTTTA	0.00	0.00	0.00	0.34	0.00
GATCAACACTCGGGCGCCAG	0.00	0.00	0.00	0.00	0.34
GATCAACACTCGGTGCCAAG	0.00	0.00	0.00	0.00	0.00
GATCAACACTCGTATGCCGT	0.00	0.00	0.29	0.00	0.00
GATCAACACTCGTGGCACAC	0.00	0.00	0.00	0.00	0.34
GATCAACACTCGTTTCCAGA	0.00	0.00	0.00	0.00	0.00
GATCAACACTCTACTCCCTG	0.00	0.00	0.00	0.00	0.34
GATCAACACTCTCAGTCATG	0.46	0.00	0.29	0.00	0.34
GATCAACACTCTCATCCACG	0.00	0.00	0.00	0.00	0.34
GATCAACACTCTCCTCAATG	0.00	0.00	0.00	0.34	0.00
GATCAACACTCTCCTCACTG	3.43	6.83	4.58	6.79	7.92
GATCAACACTCTCCTCACTN	0.00	0.00	0.00	0.00	0.00
GATCAACACTCTCCTCACTT	0.00	0.33	0.00	0.00	0.00
GATCAACACTCTCGAGCGAG	0.00	0.00	0.00	0.34	0.00
GATCAACACTCTCGCGAAGG	0.92	0.00	0.00	0.00	0.00
GATCAACACTCTCTGAATGC	0.46	0.00	0.00	0.00	0.00
GATCAACACTCTCTGGTTCA	0.69	1.63	2.86	1.36	0.69
GATCAACACTCTGACCGACA	0.00	0.33	0.29	0.00	0.00
GATCAACACTCTGGCGCTGA	0.00	0.00	0.00	0.00	0.00
GATCAACACTCTGTGCAATC	0.00	0.00	0.00	0.00	0.00
GATCAACACTCTGTGCCCGG	0.00	0.00	0.29	0.00	0.00
GATCAACACTCTTAACTGGG	0.00	0.00	0.00	0.00	0.34
GATCAACACTCTTCTTTGCA	0.00	0.00	0.29	0.00	0.00
GATCAACACTCTTTTCAAGA	0.00	0.00	0.29	0.00	0.00
GATCAACACTCTTTTCCAGA	44.18	43.92	14.03	29.87	18.94
GATCAACACTCTTTTCCAGC	0.23	0.00	0.00	0.00	0.00
GATCAACACTCTTTTCCAGG	0.46	0.00	0.00	0.00	0.00
GATCAACACTCTTTTCCAGT	0.00	0.00	0.00	0.34	0.00
GATCAACACTCTTTTCCATC	0.00	0.00	0.00	0.34	0.00
GATCAACACTCTTTTCCCGA	0.00	0.33	0.00	0.00	0.00
GATCAACACTCTTTTCCCGC	0.00	0.33	0.00	0.00	0.00
GATCAACACTCTTTTGCCGC	0.23	0.00	0.00	0.00	0.00
GATCAACACTGAAAAAAAAA	0.23	0.00	0.00	0.34	0.34
GATCAACACTGAACACATCA	0.00	0.00	0.29	0.00	0.00

GATCAACACTGAACGCATAC	0.00	0.00	0.57	0.00	0.00
GATCAACACTGACAGCTCCG	0.00	0.00	0.00	0.34	0.34
GATCAACACTGACATCCAAC	0.23	0.00	0.00	0.00	0.00
GATCAACACTGACCATAACA	0.00	0.00	0.29	0.00	0.00
GATCAACACTGACCATGGGC	0.00	0.00	0.00	0.00	0.00
GATCAACACTGACCCCTTCT	0.00	0.00	0.29	0.00	0.00
GATCAACACTGACGTCTTTC	0.00	1.30	0.00	0.00	0.34
GATCAACACTGACGTCTTTT	0.00	0.33	0.00	1.36	0.00
GATCAACACTGAGACCTTNN	0.00	0.00	0.00	0.00	0.00
GATCAACACTGAGAGGCACG	0.00	0.33	0.00	0.00	0.00
GATCAACACTGAGGGGCATC	1.37	0.33	0.00	1.02	0.69
GATCAACACTGAGTACAGGA	0.00	0.33	0.00	0.00	0.00
GATCAACACTGAGTTTGGTG	0.00	0.00	0.00	0.34	0.34
GATCAACACTGATCCCTCCT	0.00	0.00	0.00	0.00	0.34
GATCAACACTGATCGTATGC	0.00	0.00	0.29	0.00	0.00
GATCAACACTGATGACAAAA	0.00	0.00	0.00	0.68	0.00
GATCAACACTGATGGAAAAA	0.00	0.00	0.00	0.34	0.00
GATCAACACTGATGGCAAAA	0.23	0.65	0.86	0.00	0.69
GATCAACACTGATGGCAAGC	0.00	0.33	0.00	0.00	0.00
GATCAACACTGATGGCACAC	0.00	0.00	0.00	0.00	0.34
GATCAACACTGATGGCAGCG	0.00	0.00	0.00	0.34	0.00
GATCAACACTGATGGCAGGC	0.23	0.00	0.00	0.00	0.00
GATCAACACTGATGGCATAA	0.00	0.00	0.00	0.00	0.00
GATCAACACTGATGGCATAT	0.00	0.00	0.00	0.00	0.00
GATCAACACTGATGGCATCA	0.00	0.00	0.00	0.34	0.00
GATCAACACTGATGGCATCC	0.46	0.00	0.00	0.00	0.00
GATCAACACTGATGGCATGA	0.00	0.33	1.43	0.34	0.34
GATCAACACTGATGGCATGC	26.78	11.39	18.04	17.31	19.29
GATCAACACTGATGGCATGG	0.00	0.00	0.00	0.00	0.00
GATCAACACTGATGGCATGN	0.46	0.00	0.00	0.00	0.00
GATCAACACTGATGGCATNN	0.23	0.00	0.00	0.00	0.00
GATCAACACTGATGGCATTC	0.00	0.00	0.00	0.34	0.34
GATCAACACTGATGGCCAAA	0.00	0.00	0.00	0.00	0.00
GATCAACACTGATGGCCATC	0.00	0.00	0.00	0.00	0.34
GATCAACACTGATGGCGTGG	0.00	0.00	0.00	0.34	0.00
GATCAACACTGATGGCNNNN	0.23	0.00	0.00	0.00	0.00
GATCAACACTGATGGCTTGG	0.00	0.00	0.00	0.00	0.34

GATCAACACTGATGGTATGC	0.00	0.00	0.00	0.00	0.34
GATCAACACTGCAAAAGCAG	0.00	0.00	0.00	0.00	0.00
GATCAACACTGCAAGAAAAA	0.23	0.00	0.00	0.00	0.00
GATCAACACTGCACACGACT	0.23	0.00	0.00	0.00	0.00
GATCAACACTGCACACTGCG	0.00	0.33	0.00	0.00	0.00
GATCAACACTGCACAGCGCG	0.00	0.33	0.00	0.00	0.00
GATCAACACTGCACGTTTTT	0.00	0.00	0.00	0.00	0.00
GATCAACACTGCACTGCGGA	0.46	0.00	0.00	0.34	0.00
GATCAACACTGCACTTTTGG	0.00	0.00	0.29	0.00	0.00
GATCAACACTGCCAATGCTC	0.00	0.00	0.29	0.00	0.00
GATCAACACTGCCACTGCCG	0.00	0.00	0.29	0.00	0.00
GATCAACACTGCCCCGATCA	0.00	0.00	0.00	0.34	0.00
GATCAACACTGCCGAATGCA	0.00	0.00	0.29	0.00	0.00
GATCAACACTGCCGAGATTC	0.00	0.00	0.29	0.00	0.00
GATCAACACTGCCTTTGGTC	0.00	0.33	0.00	0.00	0.00
GATCAACACTGCGACCTGCT	0.23	0.00	0.00	0.00	0.00
GATCAACACTGCGACCTTCT	0.69	0.65	0.29	1.02	1.38
GATCAACACTGCGAGTGTGG	0.00	0.33	0.00	0.00	0.00
GATCAACACTGCGATTGGCA	0.00	0.00	0.29	0.00	0.00
GATCAACACTGCGCTGCCCG	0.00	0.00	0.29	0.00	0.00
GATCAACACTGCGCTGCCTG	0.00	0.00	0.86	0.34	0.34
GATCAACACTGCGGGGCATC	0.00	0.33	0.00	0.00	0.00
GATCAACACTGCGGTCTTCG	0.00	0.00	0.00	0.00	0.00
GATCAACACTGCTAAAAAAA	0.00	0.00	0.00	1.02	0.00
GATCAACACTGCTACGTTTG	0.23	0.00	0.00	0.00	0.00
GATCAACACTGCTCAAAAAA	0.69	0.00	0.00	0.34	0.00
GATCAACACTGCTCAACANN	0.23	0.00	0.00	0.00	0.00
GATCAACACTGCTCAAGAAG	0.00	0.33	0.00	0.00	0.00
GATCAACACTGCTCAAGAGC	0.00	0.00	0.00	0.34	0.00
GATCAACACTGCTCAAGAGG	0.00	0.00	0.00	0.00	0.00
GATCAACACTGCTCAAGAGT	3.43	12.69	8.02	10.52	17.56
GATCAACACTGCTCAAGGGT	0.00	0.00	0.29	0.00	0.00
GATCAACACTGCTCAGGAGT	0.46	0.00	0.00	0.00	0.00
GATCAACACTGCTGACAACG	0.00	0.33	0.00	0.00	0.00
GATCAACACTGCTGGTGGTA	0.00	0.00	0.00	0.00	0.69
GATCAACACTGCTGGTGGTC	0.00	0.00	0.00	0.00	0.00
GATCAACACTGCTTGGTGTG	0.23	0.00	0.00	0.00	0.00

GATCAACACTGGAAAAAAAA	0.00	0.00	0.29	0.00	0.00
GATCAACACTGGAATTAGTA	0.00	0.00	0.00	0.00	1.03
GATCAACACTGGAATTGGTA	0.00	0.00	0.00	0.00	0.34
GATCAACACTGGCCAGAATC	0.00	0.00	0.29	0.00	0.00
GATCAACACTGGCGAGGCGT	0.00	0.33	0.00	0.00	0.00
GATCAACACTGGCGCTAAGC	0.00	0.00	0.00	0.34	0.00
GATCAACACTGGCGGGGCCG	0.00	0.00	0.00	0.00	0.34
GATCAACACTGGCTTGTTGA	0.00	0.00	0.00	0.00	0.00
GATCAACACTGGGCTACAGG	0.00	0.00	0.86	0.34	0.00
GATCAACACTGGGTCCGAGG	0.00	0.00	0.00	0.00	0.00
GATCAACACTGTAATGTTTT	0.00	0.00	0.00	0.00	0.34
GATCAACACTGTCACGCTGT	0.00	0.00	0.00	0.00	0.00
GATCAACACTGTCAGCGCAT	0.00	0.00	0.00	0.68	0.00
GATCAACACTGTCCACAATA	0.00	0.00	0.00	0.00	0.00
GATCAACACTGTCCCACTTG	0.00	0.00	0.00	0.00	0.00
GATCAACACTGTCCCCCGGC	1.37	0.00	0.00	0.00	0.00
GATCAACACTGTCCCCCGNN	0.23	0.00	0.00	0.00	0.00
GATCAACACTGTCGTATGCC	0.00	0.00	0.29	0.00	0.00
GATCAACACTGTCTGCGCAA	0.00	0.00	0.00	0.68	0.00
GATCAACACTGTCTTTGGCA	1.14	0.65	0.57	1.36	0.69
GATCAACACTGTGATGTGCA	0.00	0.00	0.00	0.00	0.00
GATCAACACTGTGCCTCTGA	0.00	0.00	0.00	0.00	0.34
GATCAACACTGTGCTCCTGC	0.00	0.00	0.00	0.00	0.00
GATCAACACTGTGCTGATGA	0.00	0.00	0.00	0.00	0.00
GATCAACACTGTGCTGATTA	0.00	0.00	0.29	0.00	0.00
GATCAACACTGTGCTGCCGA	0.00	0.00	0.00	0.00	0.00
GATCAACACTGTGCTGCNNN	0.00	0.00	0.00	0.00	0.00
GATCAACACTGTGCTGCTCT	0.46	0.00	0.00	0.00	0.00
GATCAACACTGTGCTGCTGA	25.87	31.56	24.91	23.76	16.19
GATCAACACTGTGCTGCTGC	0.23	0.00	0.29	0.68	0.00
GATCAACACTGTGCTGCTGG	0.00	0.00	0.00	0.00	0.34
GATCAACACTGTGCTGCTGT	0.23	0.00	0.00	0.00	0.00
GATCAACACTGTGCTGCTNN	0.23	0.00	0.00	0.00	0.00
GATCAACACTGTGCTGCTTA	0.00	0.33	0.00	0.00	0.00
GATCAACACTGTGCTGGTGA	0.00	0.00	0.00	0.00	0.00
GATCAACACTGTGCTGTTGA	0.00	0.00	0.29	0.00	0.00
GATCAACACTGTGTACAGAC	0.00	0.00	0.00	0.00	0.00

GATCAACACTGTTCAAGAGT	0.00	0.00	0.00	0.00	0.34
GATCAACACTGTTCTGTTCT	0.00	0.00	0.57	0.00	0.00
GATCAACACTGTTGCCGGAA	0.00	0.00	0.00	0.00	0.00
GATCAACACTGTTGCGCGAG	0.00	0.00	0.29	0.00	0.00
GATCAACACTGTTGGTCGGA	0.00	0.00	0.29	0.00	0.00
GATCAACACTTAAACTCAAA	0.00	0.00	0.00	0.00	0.34
GATCAACACTTACCGATTGC	0.00	0.00	0.00	0.68	0.34
GATCAACACTTATCCTGAAG	0.00	0.00	0.00	0.34	0.00
GATCAACACTTCAAGCACTA	0.00	0.00	0.29	0.00	0.00
GATCAACACTTCAAGCATTA	0.23	1.63	2.00	1.70	0.34
GATCAACACTTCACAGGTGC	0.00	0.00	0.00	0.00	0.00
GATCAACACTTCACGTTTGC	0.00	0.00	0.00	0.00	0.34
GATCAACACTTCCACGTTTG	0.00	0.00	0.00	0.00	0.34
GATCAACACTTCCCAGTGGC	0.00	0.00	0.00	0.34	0.00
GATCAACACTTCCCGGCTCC	0.23	0.00	0.29	0.00	0.00
GATCAACACTTCCTCGTATG	0.00	0.00	0.29	0.00	0.00
GATCAACACTTCCTTGCGCG	0.00	0.00	0.00	0.00	0.00
GATCAACACTTCGACGTTTG	0.00	0.00	0.00	0.34	0.00
GATCAACACTTCTAAGTTTG	0.00	0.00	0.00	0.00	0.00
GATCAACACTTCTACAATTC	0.00	0.00	0.29	0.00	0.00
GATCAACACTTCTACATTTG	0.00	0.00	0.00	0.00	0.00
GATCAACACTTCTACGATGG	0.00	0.00	0.00	0.00	0.34
GATCAACACTTCTACGATTG	0.00	0.00	0.00	0.00	0.00
GATCAACACTTCTACGGTTG	0.69	0.00	0.29	0.34	0.00
GATCAACACTTCTACGNNNN	0.23	0.00	0.00	0.00	0.00
GATCAACACTTCTACGNTNN	0.00	0.00	0.00	0.00	0.00
GATCAACACTTCTACGNTTG	0.00	0.00	0.00	0.00	1.03
GATCAACACTTCTACGTCGC	0.00	0.33	0.00	0.00	0.00
GATCAACACTTCTACGTCGT	0.00	0.00	0.29	0.00	0.00
GATCAACACTTCTACGTGTG	0.23	0.00	0.29	0.00	0.00
GATCAACACTTCTACGTTAC	0.00	0.00	0.00	0.00	0.34
GATCAACACTTCTACGTTGG	0.23	0.00	0.00	0.00	0.00
GATCAACACTTCTACGTTNG	0.00	0.00	0.00	0.34	0.00
GATCAACACTTCTACGTTNN	0.69	0.00	0.00	0.00	0.00
GATCAACACTTCTACGTTTC	0.00	0.00	0.00	0.34	0.00
GATCAACACTTCTACGTTTG	171.91	62.46	49.54	81.80	43.05
GATCAACACTTCTACGTTTN	0.69	0.00	0.00	0.00	0.00

GATCAACACTTCTACGTTTT	0.69	0.00	0.00	0.00	0.00
GATCAACACTTCTACTCGTA	0.23	0.00	0.00	0.00	0.00
GATCAACACTTCTCAGGGCA	0.46	0.33	0.29	0.00	0.34
GATCAACACTTCTCCGTTCG	0.00	0.00	0.00	0.00	0.00
GATCAACACTTCTCCGTTTG	0.23	0.00	0.00	0.00	0.00
GATCAACACTTCTGCGCACA	0.00	0.00	0.29	0.00	0.00
GATCAACACTTCTGTACAGT	0.00	0.00	0.00	0.00	1.03
GATCAACACTTCTGTGTCTG	0.00	0.00	0.00	0.00	0.34
GATCAACACTTCTTCGTATG	0.00	1.63	0.00	0.68	0.34
GATCAACACTTGACCTGCCT	0.00	0.00	0.00	0.00	0.00
GATCAACACTTGCAAAGGGG	0.00	0.00	0.00	0.34	0.00
GATCAACACTTGCAACGCCG	0.23	0.00	0.00	0.00	0.00
GATCAACACTTGCAGGGCAC	0.00	0.00	0.00	0.34	0.00
GATCAACACTTGCCCATTAG	0.00	0.00	0.57	0.00	0.00
GATCAACACTTGCCCCCGTG	0.00	0.00	0.00	0.00	0.00
GATCAACACTTGCCGACAAG	0.00	0.00	0.29	0.00	0.00
GATCAACACTTGCGCAGTCC	0.00	0.00	0.00	0.00	0.00
GATCAACACTTGGAACAAGG	0.00	0.00	0.00	0.00	0.00
GATCAACACTTGGAACTGGT	0.00	0.33	0.00	0.00	0.00
GATCAACACTTGGAGCCAGG	0.00	0.00	0.29	0.00	0.00
GATCAACACTTGGCGAAAAG	0.92	0.00	0.00	0.00	0.00
GATCAACACTTGGGACCGGC	0.00	0.00	0.00	0.00	0.00
GATCAACACTTGGTGGGGTC	0.00	0.33	0.00	0.00	0.00
GATCAACACTTGTAAATATC	0.00	0.00	0.29	0.00	0.00
GATCAACACTTGTAGATATC	0.00	0.00	0.00	0.00	0.34
GATCAACACTTGTATTATTT	0.00	0.00	0.00	0.34	0.00
GATCAACACTTTACCAGCTA	0.00	0.00	0.00	0.00	0.00
GATCAACACTTTCCAGCGCT	0.23	0.00	0.00	0.00	0.00
GATCAACACTTTGCCAGATA	0.00	0.33	0.00	0.00	0.00
GATCAACACTTTGCCAGCTA	11.67	24.72	13.75	15.95	22.73
GATCAACACTTTTAAAATTC	0.00	0.00	0.29	0.00	0.00
GATCAACACTTTTACGTTTG	0.00	0.00	0.29	0.00	0.00
GATCAACACTTTTTTCCAGA	0.00	0.00	0.00	0.00	0.00
GATCAACACTTTTTTCCGGT	0.23	0.00	0.00	0.00	0.00
GATCAACAGAAAAAAAAAAA	1.37	0.33	0.00	0.68	0.69
GATCAACAGAAAAAAAAAGA	0.00	0.00	0.29	0.00	0.00
GATCAACAGAAACATGCAAA	0.00	0.00	0.00	0.34	0.00

GATCAACAGAAACCATTGAT	0.69	0.00	0.00	0.00	0.00
GATCAACAGAAACCCACATC	0.00	0.00	0.00	0.00	0.34
GATCAACAGAAACCGGCTGC	0.00	0.00	0.00	0.00	0.34
GATCAACAGAAACTCAAGAG	0.00	0.00	0.00	0.00	0.34
GATCAACAGAAAGGAGAACA	0.00	0.00	0.00	0.34	0.00
GATCAACAGAAAGGGTTGGT	0.00	0.00	0.00	0.00	0.69
GATCAACAGAACACTACCCG	0.00	0.00	0.00	0.00	0.34
GATCAACAGAACAGAAAAGA	0.00	0.00	0.00	0.00	0.34
GATCAACAGAACAGAACAGA	0.00	0.00	0.00	0.00	0.34
GATCAACAGAACAGCAGAGC	0.00	0.33	0.00	0.00	0.00
GATCAACAGAACATGAACAC	0.00	0.00	0.57	0.00	0.34
GATCAACAGAACCGTTAACA	0.00	0.00	0.00	0.00	0.69
GATCAACAGAACGCAAATTA	0.00	0.33	0.00	0.00	0.00
GATCAACAGAACGCAGAACG	0.00	0.00	0.86	0.00	0.69
GATCAACAGAACGCAGAAGG	0.00	0.00	0.00	0.00	0.34
GATCAACAGAACTATCATCG	0.00	0.00	0.00	0.00	0.34
GATCAACAGAACTTGGAAGA	3.89	2.28	1.15	3.05	1.03
GATCAACAGAAGAAGAAGAA	0.00	0.00	4.87	0.00	5.51
GATCAACAGAAGAAGAAGAC	0.00	0.00	0.29	0.00	0.34
GATCAACAGAAGAAGAAGGA	0.00	0.00	0.57	0.00	0.00
GATCAACAGAAGAAGAAGGC	0.00	0.00	0.29	0.00	0.00
GATCAACAGAAGAAGAAGTA	0.00	0.00	0.00	0.00	0.34
GATCAACAGAAGAAGCAACA	0.00	0.00	0.29	0.00	0.00
GATCAACAGAAGAAGCAGAC	0.00	0.00	0.00	0.00	0.34
GATCAACAGAAGAAGCAGCA	0.00	0.00	0.29	0.00	0.00
GATCAACAGAAGAAGCCGAA	0.00	0.00	0.00	0.00	0.69
GATCAACAGAAGAAGCGAGT	0.00	0.00	0.00	0.00	0.00
GATCAACAGAAGAAGGAGGA	0.00	0.00	1.43	0.00	1.38
GATCAACAGAAGAAGGTCTA	0.00	0.00	0.29	0.00	0.00
GATCAACAGAAGAATACGCA	0.00	0.00	0.57	0.00	0.00
GATCAACAGAAGAATCATCA	0.00	0.00	0.29	0.00	0.00
GATCAACAGAAGAGAAGAAA	0.00	0.00	0.00	0.00	0.00
GATCAACAGAAGATGTTGGA	0.00	0.33	0.00	0.00	0.00
GATCAACAGAAGCAACAGAA	0.00	0.00	0.29	0.00	0.00
GATCAACAGAAGCCATGGTA	0.00	0.33	0.00	0.00	0.00
GATCAACAGAAGCCGAAAGC	0.00	0.00	0.29	0.00	0.00
GATCAACAGAAGCGGATGAA	0.00	0.00	0.00	0.00	0.34

GATCAACAGAAGCGGGAGGG	0.00	0.00	0.00	0.00	0.00
GATCAACAGAAGCTTGGTCG	0.00	0.00	0.00	0.00	0.34
GATCAACAGAAGGAGACGGA	0.00	0.00	0.29	0.00	0.00
GATCAACAGAAGGAGGCTGA	0.00	0.00	0.86	0.00	0.69
GATCAACAGAAGGATAATAA	0.00	0.00	0.00	0.00	1.03
GATCAACAGAAGGATACGAT	0.00	0.00	0.29	0.00	0.00
GATCAACAGAAGGATGAGGA	0.00	0.00	0.00	0.00	0.69
GATCAACAGAAGGGGAGGAG	0.00	0.00	2.00	0.00	0.69
GATCAACAGAAGTACCGCCC	0.00	0.33	0.00	0.00	0.00
GATCAACAGAAGTATTTGAG	0.00	0.00	0.00	0.34	0.00
GATCAACAGAATAAACAAGG	0.00	0.00	0.86	0.00	0.00
GATCAACAGAATAAGAAGAA	0.00	0.00	0.29	0.00	0.00
GATCAACAGAATAAGAAGAC	0.00	0.00	0.00	0.00	1.72
GATCAACAGAATCTTGGCGG	0.00	0.00	0.00	0.00	0.00
GATCAACAGAATGAACTGCG	0.00	0.00	0.29	0.00	0.00
GATCAACAGAATGTCACCGC	0.00	0.00	0.00	0.00	0.34
GATCAACAGAATGTCGCCGC	0.23	2.60	2.29	3.05	3.44
GATCAACAGAATGTTCGTAT	0.00	0.00	0.00	0.34	0.00
GATCAACAGAATTTTGTGGC	0.00	0.00	0.00	0.00	0.00
GATCAACAGACAAAAAAAAA	0.00	0.00	0.00	0.00	1.03
GATCAACAGACAAGAGCACA	0.00	0.33	0.00	0.00	0.00
GATCAACAGACACACATACA	0.00	0.00	0.00	0.00	0.00
GATCAACAGACACAGACAGT	0.46	0.33	0.29	0.00	0.00
GATCAACAGACACCACAACA	0.00	0.00	0.86	0.00	0.00
GATCAACAGACACCATGGTA	0.00	0.00	0.00	0.00	0.00
GATCAACAGACACTACTCGG	0.23	0.00	0.00	0.00	0.00
GATCAACAGACACTGGGGGT	0.46	0.00	0.00	0.00	0.00
GATCAACAGACAGAGGTTGA	0.00	0.00	0.00	0.00	0.00
GATCAACAGACAGTATCGGG	0.00	0.00	0.00	1.02	0.00
GATCAACAGACAGTATGGTA	0.00	0.00	0.00	0.00	0.00
GATCAACAGACCAAAAAAAA	0.46	0.00	0.00	0.00	0.00
GATCAACAGACCACGCCACA	0.00	0.00	0.00	0.00	0.34
GATCAACAGACCACTTGCTC	0.00	0.65	0.29	0.34	0.34
GATCAACAGACCCAACAAAC	0.00	0.00	0.29	0.00	0.00
GATCAACAGACCCGAGCTGG	0.00	0.00	0.00	0.00	0.00
GATCAACAGACCCGAGTTTC	0.00	0.00	0.00	0.00	0.00
GATCAACAGACCCTTCTCAG	4.12	2.60	0.29	2.04	0.69

GATCAACAGACCCTTGACGA	0.00	0.00	0.00	0.34	0.00
GATCAACAGACCCTTGACTC	0.46	0.33	0.00	0.00	0.00
GATCAACAGACCCTTGGAAA	0.46	0.00	0.00	0.00	0.00
GATCAACAGACCGAACTTTG	0.00	0.33	0.00	0.00	0.00
GATCAACAGACCGCCTCCCC	0.00	0.00	0.00	0.34	0.00
GATCAACAGACCTCAAAAAA	0.00	0.00	0.29	0.00	0.00
GATCAACAGACCTGTTACTG	0.92	0.00	0.57	0.00	1.72
GATCAACAGACCTTCACAAG	0.92	4.23	2.86	3.73	3.44
GATCAACAGACGAAGAAGAA	0.00	0.00	0.57	0.00	0.00
GATCAACAGACGAAGGATAA	0.00	0.00	2.00	0.00	0.00
GATCAACAGACGACAAAAAA	0.23	0.00	0.57	0.00	0.00
GATCAACAGACGACCTTGTA	0.00	0.00	0.29	0.00	0.00
GATCAACAGACGACGCACCA	0.00	0.00	0.29	0.00	0.00
GATCAACAGACGAGCGCCCA	0.00	0.65	0.00	0.00	0.00
GATCAACAGACGAGCGCCGC	0.46	0.00	0.29	0.34	0.00
GATCAACAGACGAGGCTCTG	0.00	0.00	0.00	0.00	0.34
GATCAACAGACGAGGGCAGC	0.69	0.00	1.43	0.68	1.03
GATCAACAGACGAGGGCTCT	0.00	0.00	0.29	0.00	0.00
GATCAACAGACGCCAAGGTA	0.00	0.00	0.00	0.00	0.00
GATCAACAGACGCCAGGGTA	0.23	0.00	0.00	0.34	0.00
GATCAACAGACGCCATAGTA	0.00	0.00	0.29	0.00	0.00
GATCAACAGACGCCATGATA	0.23	0.00	0.00	0.00	0.00
GATCAACAGACGCCATGGAA	0.00	0.00	0.00	0.34	0.34
GATCAACAGACGCCATGGAG	0.00	0.00	0.00	0.00	0.34
GATCAACAGACGCCATGGCA	0.00	0.00	0.00	0.34	0.34
GATCAACAGACGCCATGGGT	0.00	0.33	0.00	0.00	0.00
GATCAACAGACGCCATGGTA	20.83	31.88	40.95	41.41	48.22
GATCAACAGACGCCATGGTC	0.23	0.00	0.57	0.34	0.34
GATCAACAGACGCCATGGTN	0.00	0.00	0.00	0.34	0.00
GATCAACAGACGCCATGGTT	0.46	0.00	0.00	0.00	0.34
GATCAACAGACGCGAGGCCT	0.00	0.33	0.00	0.00	0.00
GATCAACAGACGCGATGGTA	0.00	0.00	0.00	0.00	0.00
GATCAACAGACGGGAGGCCT	0.69	0.65	0.00	0.68	0.34
GATCAACAGACTCGGCCTTG	0.00	0.00	0.00	0.00	0.00
GATCAACAGACTCTTTCTCC	0.00	0.33	0.00	0.00	0.00
GATCAACAGACTGAAATGCG	0.00	0.00	0.29	0.00	0.00
GATCAACAGACTGAACCGCG	0.23	0.00	0.00	0.00	0.00

GATCAACAGACTGAACCTGG	0.00	0.33	0.00	0.00	0.00
GATCAACAGACTGAACTGAG	0.00	0.33	0.00	0.00	0.34
GATCAACAGACTGAACTGCG	9.61	21.15	29.21	19.35	32.72
GATCAACAGACTGAACTGCT	1.37	0.00	0.00	0.34	1.03
GATCAACAGACTGAACTGTG	0.00	0.00	0.29	0.00	0.34
GATCAACAGACTGAGGCGGG	0.00	0.00	0.00	0.00	0.34
GATCAACAGACTGAGGCGGT	0.23	0.00	0.00	0.00	0.00
GATCAACAGACTGCAGCGGG	0.00	0.00	0.00	0.34	0.00
GATCAACAGACTGCCAGAGC	0.00	0.00	0.29	0.00	0.00
GATCAACAGACTGGACCTCA	0.00	0.00	0.00	0.00	0.00
GATCAACAGACTGGAGCAAA	0.69	0.33	0.86	1.70	1.38
GATCAACAGACTGTACTGCG	0.00	0.00	0.00	0.00	0.00
GATCAACAGAGAAACACGAT	0.00	0.00	0.00	0.00	0.00
GATCAACAGAGAAGGAGAAG	0.00	0.00	0.86	0.00	0.00
GATCAACAGAGACAGAGACA	0.00	0.00	0.00	0.00	0.69
GATCAACAGAGACAGTGTAT	0.00	0.00	0.00	0.00	0.34
GATCAACAGAGACATCGTCT	0.00	0.00	0.00	0.00	0.00
GATCAACAGAGACATCGTGC	0.00	0.00	0.29	0.00	0.00
GATCAACAGAGACCAGAGGC	0.00	0.00	0.00	0.34	0.00
GATCAACAGAGACCTCATGC	0.00	0.33	0.29	0.00	0.00
GATCAACAGAGAGAACCAGA	0.00	0.00	0.00	0.00	0.34
GATCAACAGAGAGAGACAGA	0.00	0.00	0.00	0.00	0.34
GATCAACAGAGAGAGAGAAA	0.00	0.00	0.29	0.00	0.00
GATCAACAGAGAGCAGCACA	0.00	0.00	0.00	0.00	0.00
GATCAACAGAGAGCGCGCGA	0.92	0.00	0.29	0.68	0.34
GATCAACAGAGAGCGCGCGN	0.23	0.00	0.00	0.00	0.00
GATCAACAGAGAGTGCGCGA	1.83	0.00	0.86	0.00	0.34
GATCAACAGAGATGCATGCG	0.00	0.33	0.00	0.00	0.00
GATCAACAGAGATGCGAAGC	0.00	0.00	0.00	0.00	0.34
GATCAACAGAGCACACACGC	0.00	0.00	0.29	0.00	0.00
GATCAACAGAGCACAGAAGT	0.00	0.00	0.00	0.00	0.34
GATCAACAGAGCAGACCGCC	0.00	0.33	0.00	0.00	0.00
GATCAACAGAGCAGCCGCAG	0.00	0.00	0.29	0.00	0.00
GATCAACAGAGCATATACGG	0.00	0.00	0.00	0.00	0.34
GATCAACAGAGCATGCCAAC	0.00	0.00	0.29	0.00	0.00
GATCAACAGAGCCAACAGCG	0.00	0.00	0.00	0.00	0.34
GATCAACAGAGCCAACCGCG	0.00	0.00	0.00	0.00	0.34

GATCAACAGAGCCAACGGCG	0.00	0.00	0.00	0.00	0.00
GATCAACAGAGCCAACTCCC	0.00	0.00	0.00	0.00	0.34
GATCAACAGAGCCAACTGAG	0.23	0.33	0.00	0.00	0.00
GATCAACAGAGCCAACTGCG	9.84	27.33	37.23	27.15	26.17
GATCAACAGAGCCAACTGCN	0.23	0.00	0.00	0.00	0.00
GATCAACAGAGCCAACTGCT	0.00	1.30	1.43	1.02	1.03
GATCAACAGAGCCAACTGGG	0.00	0.00	0.29	0.00	0.00
GATCAACAGAGCCAACTGTG	0.00	0.33	0.00	0.00	0.00
GATCAACAGAGCCAACTTCG	0.00	0.00	0.00	0.00	0.00
GATCAACAGAGCCCAGCAAT	0.00	0.00	0.00	0.00	0.34
GATCAACAGAGCCTCGACCG	0.00	0.00	0.00	0.00	0.00
GATCAACAGAGCCTGGAGGT	0.00	0.33	0.00	0.00	0.00
GATCAACAGAGCGACGTACG	0.00	0.00	0.00	0.00	0.34
GATCAACAGAGCGCAGATTG	0.00	0.33	0.00	0.00	0.00
GATCAACAGAGCGCAGTAGT	0.00	0.00	0.00	0.00	0.00
GATCAACAGAGCTAACTGCG	0.00	0.00	0.29	0.00	0.00
GATCAACAGAGCTCATGACA	0.00	0.00	0.00	0.00	0.00
GATCAACAGAGCTGAACGTG	0.00	0.00	0.00	0.00	0.00
GATCAACAGAGCTTGCCTTT	0.23	0.00	0.00	0.00	0.00
GATCAACAGAGCTTTTAGGT	0.00	0.00	0.00	0.00	0.00
GATCAACAGAGGAAATGATT	0.00	0.00	0.00	0.00	0.34
GATCAACAGAGGAACAGATT	0.00	0.00	0.00	0.34	0.00
GATCAACAGAGGAACAGCCT	0.00	0.00	0.29	0.00	0.00
GATCAACAGAGGAACGGTTT	0.23	0.00	0.00	0.00	0.00
GATCAACAGAGGAACTGANN	0.23	0.00	0.00	0.00	0.00
GATCAACAGAGGAACTGATA	0.00	0.00	0.00	0.00	0.00
GATCAACAGAGGAACTGATT	19.91	20.50	5.44	15.61	9.30
GATCAACAGAGGAACTGCTT	0.00	0.33	0.29	0.00	0.00
GATCAACAGAGGAACTGGTT	0.00	0.33	0.00	0.00	0.00
GATCAACAGAGGACCTGATT	0.23	0.00	0.00	0.00	0.00
GATCAACAGAGGACGGCGGG	0.23	0.00	0.00	0.00	0.00
GATCAACAGAGGCAACCGGA	0.00	0.00	0.00	0.00	0.34
GATCAACAGAGGCATCAAGT	0.00	0.65	0.00	0.00	0.34
GATCAACAGAGGCCATTGAG	0.00	0.00	0.00	0.00	0.00
GATCAACAGAGGCCTCCAGT	0.00	0.00	0.00	0.00	0.00
GATCAACAGAGGCGCACGTG	0.00	0.00	0.00	0.00	0.34
GATCAACAGAGGCGCAGGTG	0.00	0.00	0.00	0.00	0.00

GATCAACAGAGGCTCAGTTG	0.23	0.65	0.00	0.00	0.00
GATCAACAGAGGGAAGCTGT	0.00	0.98	0.29	0.68	0.00
GATCAACAGAGGGGAATGAA	0.00	0.00	0.00	0.34	0.00
GATCAACAGAGGGGAATGAT	9.61	11.71	11.74	21.04	9.99
GATCAACAGAGGGGAATGCT	0.23	0.00	0.00	0.00	0.00
GATCAACAGAGGGGCTTGCT	0.23	0.00	0.00	0.00	0.00
GATCAACAGAGGGGGATGAT	0.69	0.00	0.00	0.00	0.00
GATCAACAGAGGGGGCTGGT	0.23	0.00	0.00	0.00	0.00
GATCAACAGAGGGTGAGCGT	0.00	0.00	0.29	0.00	0.00
GATCAACAGAGGGTTGGTTC	0.00	0.00	0.29	0.00	0.00
GATCAACAGAGGTATGCAAA	0.46	0.00	0.00	0.00	0.00
GATCAACAGAGGTCGCTGCC	0.00	0.00	0.00	1.02	0.00
GATCAACAGAGGTGCGCAAA	0.00	0.00	0.00	0.34	0.00
GATCAACAGAGGTGCGCGAA	1.14	0.33	1.15	2.04	1.03
GATCAACAGAGGTGCGCGAC	0.23	0.00	0.57	0.00	0.00
GATCAACAGAGTACGCTGAG	0.23	0.00	0.00	0.00	0.00
GATCAACAGAGTAGCTCGAG	0.00	0.00	0.00	0.34	0.00
GATCAACAGAGTCCTCAATG	0.46	0.00	0.29	0.00	0.00
GATCAACAGAGTCGGCACTG	0.00	0.33	0.00	0.00	0.00
GATCAACAGAGTCGGCATGG	0.00	0.65	0.00	0.00	0.00
GATCAACAGAGTGGGCTTAG	0.23	0.00	0.00	0.00	0.00
GATCAACAGAGTTTACATCA	0.00	0.00	0.57	0.00	0.00
GATCAACAGATAAACGTGGG	0.23	0.00	0.00	0.00	0.00
GATCAACAGATAACAGAACA	0.00	0.00	0.00	0.00	0.34
GATCAACAGATACCACATGC	0.00	0.00	0.00	0.00	0.00
GATCAACAGATACCTCACGC	0.00	0.00	0.00	0.00	0.00
GATCAACAGATACCTCATGA	0.00	0.00	0.00	0.00	0.00
GATCAACAGATACCTCATGC	26.10	17.89	8.02	16.97	11.02
GATCAACAGATACCTCCTGC	0.00	0.00	0.00	0.00	0.00
GATCAACAGATACCTCGTGC	0.23	0.00	0.00	0.00	0.00
GATCAACAGATACCTCTCGT	0.23	0.00	0.00	0.00	0.00
GATCAACAGATACTGCCGCG	0.00	0.00	0.00	0.00	0.00
GATCAACAGATCCCTACTGC	0.23	0.00	0.00	0.00	0.00
GATCAACAGATGCAAATTGC	0.00	0.98	0.00	0.34	0.00
GATCAACAGATGCATGCCCA	0.00	0.00	0.29	0.00	0.00
GATCAACAGATGCCTCATGC	0.23	0.00	0.00	0.00	0.00
GATCAACAGATGGAAAAGGC	0.46	0.65	0.00	0.00	0.00

GATCAACAGATGGGCATAAG	0.00	0.33	0.00	0.00	0.00
GATCAACAGATGTCTGAGGC	0.23	0.00	0.00	0.00	0.00
GATCAACAGATGTCTGAGGG	0.23	0.00	0.00	0.00	0.00
GATCAACAGATGTCTGAGGT	0.23	0.33	0.29	1.70	0.00
GATCAACAGATTAGAAACCG	0.00	0.00	0.29	0.00	0.00
GATCAACAGATTAGAAATCG	0.00	0.00	0.29	0.00	0.00
GATCAACAGATTGTCACCGC	0.00	0.00	0.00	0.00	0.00
GATCAACAGATTGTCGCCGC	0.00	0.00	0.00	0.00	0.00
GATCAACAGATTTAGATGTC	0.00	0.00	0.00	0.00	0.00
GATCAACAGATTTCGGACAG	3.66	6.18	4.30	5.77	3.79
GATCAACAGATTTCGGGCAG	0.00	0.33	0.00	0.00	0.00
GATCAACAGATTTCTAAAGG	0.00	0.00	0.00	0.00	1.03
GATCAACAGATTTCTAAAGT	0.00	0.00	0.00	0.34	0.00
GATCAACAGATTTGCTACTT	0.00	0.00	0.00	0.00	0.00
GATCAACAGATTTTATAACG	0.00	0.00	0.00	0.34	0.00
GATCAACAGCAAAAAAAAAA	0.23	0.00	0.57	0.00	0.00
GATCAACAGCAAAAATCTAA	0.00	0.00	0.00	0.00	0.00
GATCAACAGCAAAACAAAAA	0.00	0.00	0.00	0.00	0.00
GATCAACAGCAAACATACGC	0.00	0.00	0.00	0.00	0.34
GATCAACAGCAAAGAAAGGA	0.00	0.00	0.00	0.00	0.00
GATCAACAGCAAAGAAAGGC	0.00	0.33	0.00	0.00	0.00
GATCAACAGCAACAGCGATG	0.00	0.00	0.00	0.00	0.00
GATCAACAGCAACAGGCACG	0.00	0.33	0.00	0.00	0.34
GATCAACAGCAACAGGCAGC	0.00	0.00	0.00	0.00	0.00
GATCAACAGCAACATTAAAA	0.00	0.00	0.00	0.34	0.00
GATCAACAGCAACATTAATT	3.20	3.25	0.57	3.05	0.69
GATCAACAGCAACCAATATC	0.00	0.00	0.00	0.68	0.00
GATCAACAGCAACGCAACAC	0.00	0.00	0.29	0.00	0.00
GATCAACAGCAACGCCACCT	0.00	0.00	0.00	0.00	1.03
GATCAACAGCAACGGGCAGC	0.00	0.00	0.00	0.00	0.34
GATCAACAGCAACTCCACCA	0.00	0.33	0.00	1.02	0.00
GATCAACAGCAACTCCACCC	0.00	0.00	0.29	0.00	0.00
GATCAACAGCAACTCCACCG	0.00	0.00	0.00	0.00	0.00
GATCAACAGCAACTCCGGCC	0.00	0.00	0.00	0.00	0.00
GATCAACAGCAAGAAAAAAA	0.00	0.00	0.00	0.34	0.00
GATCAACAGCAAGAACGCAC	0.00	0.00	0.00	0.00	0.34
GATCAACAGCAAGAGCACCT	0.00	0.00	0.00	0.00	0.00

GATCAACAGCAAGAGCACTT	0.23	0.00	0.00	0.00	0.00
GATCAACAGCAAGAGCTACA	0.00	0.00	0.29	0.00	0.00
GATCAACAGCAAGAGGACTT	0.00	0.65	0.00	0.00	0.00
GATCAACAGCAAGAGTAAAG	0.00	0.00	0.29	0.00	0.00
GATCAACAGCAAGAGTACCT	0.00	0.00	0.29	0.00	0.00
GATCAACAGCAAGAGTACGT	0.23	0.00	0.00	0.00	0.00
GATCAACAGCAAGAGTACNT	0.00	0.33	0.00	0.34	0.00
GATCAACAGCAAGAGTACTN	0.23	0.00	0.00	0.00	0.00
GATCAACAGCAAGAGTACTT	47.61	27.00	26.35	31.23	25.83
GATCAACAGCAAGAGTNNNN	0.23	0.00	0.00	0.00	0.00
GATCAACAGCAAGATGGACG	0.00	0.33	0.00	0.00	0.00
GATCAACAGCAAGCACCACA	0.23	0.00	0.00	0.00	0.00
GATCAACAGCAAGCACGGAA	0.00	0.00	0.00	0.00	0.00
GATCAACAGCAAGCCTTGTC	0.00	0.00	0.00	0.00	0.34
GATCAACAGCAAGCGTACTT	0.00	0.00	0.00	0.00	0.34
GATCAACAGCAAGCGTCACC	0.00	0.00	0.00	0.34	0.00
GATCAACAGCAAGGAGTACA	0.00	0.00	0.00	0.34	0.00
GATCAACAGCAAGGCGCTGC	0.00	0.33	0.00	0.00	0.34
GATCAACAGCAAGGGAAAAA	0.00	0.00	0.29	0.00	0.00
GATCAACAGCAAGGGCAGAA	0.00	0.00	0.29	0.00	0.00
GATCAACAGCAAGGTGGAGC	0.00	0.00	0.29	0.00	0.00
GATCAACAGCAAGTACGTGG	0.46	0.33	0.00	0.34	0.00
GATCAACAGCAAGTGCCACG	0.00	0.00	0.00	0.00	0.00
GATCAACAGCAAGTTTGTCG	0.00	0.00	0.00	0.00	0.69
GATCAACAGCAATAATTGAC	0.00	0.33	0.57	0.00	0.00
GATCAACAGCAATCCTGCAG	0.92	3.58	0.29	2.04	0.34
GATCAACAGCAATTTTGCAT	0.00	0.00	0.00	0.00	0.00
GATCAACAGCACACAACACA	0.23	0.00	0.00	0.00	0.00
GATCAACAGCACAGAATCGG	0.00	0.00	0.00	0.34	0.00
GATCAACAGCACCAAAAAAC	0.00	0.00	0.29	0.00	0.00
GATCAACAGCACCAAAAGCT	0.00	0.00	0.00	0.00	0.00
GATCAACAGCACCAAGCAGT	0.23	0.00	0.00	0.00	0.00
GATCAACAGCACCAAGTGCC	0.00	0.65	0.00	0.68	0.00
GATCAACAGCACCACACCAC	0.00	0.00	0.00	0.34	0.00
GATCAACAGCACCACATGCT	0.69	0.33	0.29	0.34	0.00
GATCAACAGCACCATCTCCA	0.00	0.33	0.00	0.00	1.03
GATCAACAGCACCATTTCGA	0.00	0.00	0.00	0.00	0.00

GATCAACAGCACCATTTGAC	0.00	0.00	0.00	0.34	0.00
GATCAACAGCACCTCCGACA	0.00	0.00	0.00	0.34	0.00
GATCAACAGCACGCCACGGC	0.23	0.00	0.00	0.00	0.00
GATCAACAGCACGCCCCCCA	0.00	0.00	0.00	0.00	0.00
GATCAACAGCACGCGGGCTT	0.23	0.00	0.00	0.00	0.00
GATCAACAGCACGGACGACT	6.64	0.65	2.86	0.68	1.38
GATCAACAGCACGGACGCCT	0.00	0.00	0.00	0.00	0.00
GATCAACAGCACTAGCCGGT	0.00	0.00	0.29	0.00	0.00
GATCAACAGCACTATAGCGT	0.00	0.33	0.00	0.00	0.00
GATCAACAGCACTCGGGCTT	0.46	0.00	0.00	0.34	0.00
GATCAACAGCACTGCACCAT	0.00	0.00	0.00	0.00	0.69
GATCAACAGCACTGCGGGCA	0.23	0.00	0.00	0.00	0.00
GATCAACAGCACTTGCCTGG	0.00	0.00	0.00	0.00	0.34
GATCAACAGCACTTGGGGCC	0.23	0.00	0.00	0.00	0.00
GATCAACAGCACTTGGGTCC	0.23	0.65	0.00	0.68	0.34
GATCAACAGCACTTTCTGGA	0.00	0.00	0.00	0.00	0.00
GATCAACAGCAGAAGGAAAA	0.00	0.00	0.29	0.00	0.00
GATCAACAGCAGAAGGATAA	0.00	0.00	1.72	0.00	0.00
GATCAACAGCAGAAGGGTCA	0.00	0.00	0.86	0.00	0.69
GATCAACAGCAGAAGTTCCG	0.00	0.00	0.00	0.68	0.00
GATCAACAGCAGACAATTGC	0.00	0.00	0.00	0.00	0.00
GATCAACAGCAGACACGGGG	0.00	0.00	0.29	0.00	0.00
GATCAACAGCAGAGAGAAAA	0.00	0.00	0.57	0.00	0.00
GATCAACAGCAGAGGAGGAC	0.00	0.00	0.29	0.00	0.00
GATCAACAGCAGAGTCGAGG	0.00	0.00	0.00	0.34	0.00
GATCAACAGCAGATGCCGCA	0.00	0.33	0.00	0.00	0.00
GATCAACAGCAGATGCGGCA	0.00	0.00	0.00	0.34	0.00
GATCAACAGCAGCAAAAAAA	2.06	1.30	1.43	0.68	1.38
GATCAACAGCAGCAAAGAAA	1.14	0.33	0.86	0.68	1.38
GATCAACAGCAGCAAAGAGA	0.92	0.33	0.00	0.00	0.69
GATCAACAGCAGCAACCAGG	0.00	0.00	0.00	0.00	0.34
GATCAACAGCAGCAAGGTCG	0.00	0.00	0.00	0.00	0.69
GATCAACAGCAGCACGAGCC	0.00	0.00	0.00	0.34	0.00
GATCAACAGCAGCAGGCATG	0.00	0.65	0.00	0.00	0.00
GATCAACAGCAGCAGGTCCT	0.00	0.00	0.00	0.34	0.00
GATCAACAGCAGCATCAAGT	0.00	0.65	0.00	0.00	0.00
GATCAACAGCAGCATCAATA	0.00	0.33	0.00	0.00	0.00

GATCAACAGCAGCGGACCGC	0.23	0.00	0.00	0.00	0.00
GATCAACAGCAGCGGGAGGG	0.00	0.00	0.00	0.34	0.00
GATCAACAGCAGCGGGCCGG	0.00	0.00	0.00	0.00	0.00
GATCAACAGCAGCGGGCGGG	8.93	11.06	10.88	9.50	7.92
GATCAACAGCAGCGGGCTCG	0.00	0.00	0.00	0.34	0.00
GATCAACAGCAGCGGGGGGG	0.23	0.00	0.00	0.34	0.00
GATCAACAGCAGCGGGTGGG	0.00	0.33	0.00	0.00	0.00
GATCAACAGCAGCGTCATGA	0.00	0.00	0.00	0.34	0.00
GATCAACAGCAGCGTCCCAA	0.00	0.00	0.00	0.00	0.00
GATCAACAGCAGCTCGTATG	0.00	0.00	0.00	0.00	0.34
GATCAACAGCAGCTTCGTCA	0.00	0.00	0.00	0.00	0.00
GATCAACAGCAGGAACTCCA	0.00	0.98	0.00	0.00	0.00
GATCAACAGCAGGAGGACTG	0.46	0.00	0.00	0.00	0.00
GATCAACAGCAGGAGGAGGG	0.00	0.00	0.00	0.00	0.34
GATCAACAGCAGGATTTGCC	0.00	0.00	0.29	0.00	0.00
GATCAACAGCAGGCGTTGAC	0.00	0.00	0.00	0.34	0.00
GATCAACAGCAGGGCTGTCT	0.00	0.00	0.29	0.00	0.00
GATCAACAGCAGGGCTTGAC	0.00	0.33	0.00	0.00	0.00
GATCAACAGCAGGGTTGCTC	0.00	0.00	0.00	0.00	0.34
GATCAACAGCAGGTCACCCA	0.23	0.00	0.00	0.00	0.00
GATCAACAGCAGTATCAAGA	0.23	0.00	0.00	0.00	0.00
GATCAACAGCAGTCCAAGAA	0.00	0.98	0.29	0.68	0.00
GATCAACAGCAGTCCCCGGA	0.00	0.33	0.00	0.00	0.00
GATCAACAGCAGTGCAGTTC	0.00	0.00	0.57	0.00	0.00
GATCAACAGCAGTGGGGGGG	0.23	0.00	0.00	0.00	0.00
GATCAACAGCAGTTCTCCTA	0.00	0.00	0.00	0.00	0.00
GATCAACAGCATAGCCTGAT	0.00	0.00	0.00	0.00	0.34
GATCAACAGCATAGCTGTGT	0.00	0.00	0.29	0.00	0.00
GATCAACAGCATCAGAAGAA	0.00	0.00	0.29	0.00	0.34
GATCAACAGCATCATCAAGG	0.00	0.00	0.57	0.00	0.00
GATCAACAGCATCCGCTCGG	0.46	0.00	0.57	0.00	0.00
GATCAACAGCATCCTAAGAT	0.23	0.00	0.00	0.00	0.00
GATCAACAGCATCCTGGACG	0.23	0.00	0.00	0.68	0.00
GATCAACAGCATCCTTCGGA	0.00	0.00	0.00	0.00	0.00
GATCAACAGCATCGAACTTA	0.00	0.00	0.00	0.34	0.00
GATCAACAGCATCGTCCTCG	0.00	0.00	0.00	0.34	0.00
GATCAACAGCATCTCGGCGG	0.00	0.00	0.00	0.34	0.00

GATCAACAGCATCTCGGTTG	0.00	0.00	0.00	0.00	0.00
GATCAACAGCATCTGGCGTG	0.00	0.00	0.00	0.34	0.00
GATCAACAGCATCTGGGCGG	0.23	0.00	0.00	0.00	0.00
GATCAACAGCATCTTAAGCA	1.60	0.00	0.00	0.00	0.00
GATCAACAGCATCTTAAGCC	0.23	0.00	0.00	0.00	0.00
GATCAACAGCATCTTANNNN	0.23	0.00	0.00	0.00	0.00
GATCAACAGCATCTTGCCGG	0.00	0.00	0.00	0.68	0.00
GATCAACAGCATCTTGGAGG	0.00	0.00	0.00	0.34	0.00
GATCAACAGCATCTTGGCGG	9.84	17.57	10.02	18.33	6.54
GATCAACAGCATCTTGGCTG	0.00	0.00	0.00	0.34	0.00
GATCAACAGCATGAAAAAAA	0.23	0.00	0.00	0.00	0.00
GATCAACAGCATGAAGAAGA	0.00	0.33	0.29	0.00	0.00
GATCAACAGCATGAAGGAAA	0.00	0.00	0.00	0.34	0.00
GATCAACAGCATGAAGGCAA	0.00	0.00	0.00	0.34	0.00
GATCAACAGCATGACCATGC	0.00	0.00	0.00	0.00	0.00
GATCAACAGCATGAGGCGGT	0.23	0.00	0.00	0.00	0.00
GATCAACAGCATGCAACCCA	0.23	0.00	0.00	0.00	0.00
GATCAACAGCATGCCCAAGT	0.00	0.00	0.29	0.00	0.00
GATCAACAGCATGCCGGTGT	0.00	0.00	0.29	0.00	0.00
GATCAACAGCATGGAGGGCA	0.00	0.00	0.00	0.68	0.00
GATCAACAGCATGGGTCTGC	0.23	0.00	0.00	0.00	0.00
GATCAACAGCATGGTCTCAA	0.00	0.00	0.29	0.00	0.00
GATCAACAGCATGGTTCGTC	0.00	0.00	0.57	0.00	0.00
GATCAACAGCATGTCAAGTC	0.00	0.00	0.00	0.00	0.34
GATCAACAGCATTGCCGTGT	0.00	0.00	0.00	0.00	0.34
GATCAACAGCATTGCTGGGT	0.23	0.00	0.29	0.00	0.00
GATCAACAGCATTGCTGTGC	0.00	0.00	0.00	0.00	0.00
GATCAACAGCATTGCTGTGT	10.30	24.72	28.06	22.06	23.76
GATCAACAGCATTGCTGTNN	0.23	0.00	0.00	0.00	0.00
GATCAACAGCATTGTCTTCG	0.23	0.33	0.00	0.34	0.34
GATCAACAGCCAATACTTGC	0.00	0.00	0.00	0.00	0.00
GATCAACAGCCAATTGTCCA	0.00	0.00	0.00	0.00	0.00
GATCAACAGCCAATTTTTTC	0.00	0.00	0.29	0.00	0.00
GATCAACAGCCACACACACA	0.00	0.00	0.29	0.00	0.00
GATCAACAGCCACATAGGCC	0.23	0.00	0.29	0.00	0.00
GATCAACAGCCACTCCCAGG	0.00	0.00	0.00	0.00	0.00
GATCAACAGCCAGAGCATGC	0.00	0.33	0.00	0.00	0.00

GATCAACAGCCAGATCGTGG	0.00	0.00	0.00	0.00	0.00
GATCAACAGCCAGATGCTCG	0.00	0.00	0.00	0.00	0.00
GATCAACAGCCAGCACTTGG	0.00	0.00	0.00	0.00	0.00
GATCAACAGCCAGCATCCGC	0.00	0.65	0.00	0.00	0.00
GATCAACAGCCAGCGGGCCC	0.23	0.98	0.29	0.34	0.00
GATCAACAGCCAGCTTTGGA	0.00	0.00	0.29	0.00	0.00
GATCAACAGCCAGGAGCAGC	0.69	0.00	0.00	1.02	0.00
GATCAACAGCCAGGAGGCTC	0.00	0.00	0.00	0.00	0.00
GATCAACAGCCAGGCGCCGC	0.00	0.00	0.29	0.00	0.00
GATCAACAGCCAGGCTGAGT	0.00	0.00	0.29	0.00	0.00
GATCAACAGCCATGCGTGAG	0.00	0.00	0.00	0.00	0.00
GATCAACAGCCCAAAGATTT	0.23	0.00	0.00	0.00	0.00
GATCAACAGCCCCATTCTCG	0.00	0.00	0.00	0.00	0.34
GATCAACAGCCCCGAAGTCC	2.06	1.30	0.00	0.34	0.69
GATCAACAGCCCGAACAACC	0.00	0.00	0.00	0.00	0.34
GATCAACAGCCCGAGCCAAA	0.00	0.00	0.29	0.00	0.00
GATCAACAGCCCGAGCCCTA	0.00	0.00	0.00	0.00	0.34
GATCAACAGCCCGAGGCGGT	0.00	0.00	0.00	0.00	0.00
GATCAACAGCCCGGTGGGCT	0.00	0.00	0.29	0.00	0.00
GATCAACAGCCCGTCGAAAA	0.00	0.00	0.00	0.34	0.34
GATCAACAGCCCGTCGNAAA	0.00	0.00	0.00	0.00	0.34
GATCAACAGCCCGTTGCTTG	0.00	0.00	0.00	0.34	0.00
GATCAACAGCCCTCGTGGCG	0.00	0.33	0.29	0.34	0.69
GATCAACAGCCCTTGTGCAA	0.00	0.33	0.00	0.00	0.00
GATCAACAGCCCTTTCACGG	0.46	0.00	0.00	0.00	0.00
GATCAACAGCCGCACATGGG	0.46	0.33	0.00	0.00	0.00
GATCAACAGCCGCATCACCC	0.00	0.00	0.00	0.34	0.00
GATCAACAGCCGCCAGCTGC	0.00	0.00	0.00	0.00	0.34
GATCAACAGCCGCCATGGTA	0.00	0.00	0.00	0.34	0.00
GATCAACAGCCGCCGAGAAG	0.00	0.00	0.00	0.00	0.00
GATCAACAGCCGCCTCACCC	0.00	0.00	0.00	0.34	0.00
GATCAACAGCCGCGCCACAC	0.00	0.00	0.00	0.68	0.00
GATCAACAGCCGGAATATTT	0.00	0.33	0.00	0.00	0.00
GATCAACAGCCGGAGGGACC	0.00	0.00	0.00	0.00	0.00
GATCAACAGCCGGGCAGCGG	0.00	0.00	0.00	0.00	0.00
GATCAACAGCCGGGGTGTAG	0.00	0.33	0.00	0.00	0.00
GATCAACAGCCGGTGCCACA	0.46	0.00	0.00	0.00	0.00

GATCAACAGCCGTATCCAGG	0.00	0.00	0.00	0.34	0.00
GATCAACAGCCGTCGATGAT	0.00	0.00	0.00	0.00	0.34
GATCAACAGCCGTGTGTCGG	0.69	0.00	0.00	0.00	0.00
GATCAACAGCCGTGTGTTAG	0.00	0.00	0.29	0.00	0.00
GATCAACAGCCTAACTGCTG	2.06	3.25	0.29	2.72	1.72
GATCAACAGCCTAACTGCTN	0.23	0.00	0.00	0.00	0.00
GATCAACAGCCTAGATGGCT	0.00	0.00	0.00	0.00	0.00
GATCAACAGCCTCAGGGACC	0.23	0.00	0.00	0.00	0.00
GATCAACAGCCTCCCAGCCT	0.00	0.00	0.29	0.00	0.00
GATCAACAGCCTCCTGACGC	0.00	0.00	0.00	0.00	0.69
GATCAACAGCCTGAACTGCG	0.23	0.00	0.00	0.00	0.00
GATCAACAGCCTGAACTGCT	0.00	0.00	0.29	0.00	0.00
GATCAACAGCCTGAAGCTGG	0.00	0.00	0.29	0.00	0.00
GATCAACAGCCTGAGCTCAA	0.23	0.00	0.00	0.00	0.00
GATCAACAGCCTGAGCTCTC	0.23	0.00	0.00	0.00	0.00
GATCAACAGCCTGAGGAGGT	0.00	0.33	0.00	0.00	0.00
GATCAACAGCCTGAGGCGGA	0.23	0.00	0.00	0.00	0.00
GATCAACAGCCTGAGGCGGC	0.00	0.00	0.29	0.34	0.00
GATCAACAGCCTGAGGCGGG	2.29	0.98	1.15	5.09	2.41
GATCAACAGCCTGAGGCGGN	0.23	0.00	0.00	0.00	0.00
GATCAACAGCCTGAGGCGGT	15.34	16.59	10.88	10.52	6.20
GATCAACAGCCTGAGGCGNT	0.00	0.00	0.00	0.00	0.00
GATCAACAGCCTGATTGCGG	0.23	0.00	0.00	0.00	0.00
GATCAACAGCCTGCCTCCTA	0.46	0.00	0.00	0.00	0.00
GATCAACAGCCTGCGGCGGT	0.00	0.33	0.00	0.00	0.34
GATCAACAGCCTGCGGGGGA	0.46	0.00	0.00	0.00	0.00
GATCAACAGCCTGCTCGCAA	0.00	0.00	0.57	0.00	0.00
GATCAACAGCCTGCTCGCCG	0.00	2.93	0.57	1.02	1.38
GATCAACAGCCTGCTCGCCT	0.00	0.00	0.00	0.00	0.34
GATCAACAGCCTGCTCGTCG	0.00	0.00	0.29	0.00	0.00
GATCAACAGCCTGGGGGACT	0.23	0.00	0.00	0.00	0.00
GATCAACAGCCTGTACATCG	0.00	0.00	0.00	0.34	0.00
GATCAACAGCCTGTCGAAAA	0.00	0.00	0.00	0.34	0.00
GATCAACAGCCTTCAGGCCC	0.00	0.00	0.29	0.00	0.69
GATCAACAGCCTTCCGGCCC	0.00	0.00	0.00	0.34	0.00
GATCAACAGCCTTCTCCGCG	0.00	0.33	0.00	0.00	0.00
GATCAACAGCCTTGGCTCAC	0.00	0.00	0.57	0.00	0.00

GATCAACAGCCTTGGGTGTT	0.00	0.00	0.00	0.00	0.00
GATCAACAGCCTTGTCCTGA	0.46	0.00	0.00	0.34	0.00
GATCAACAGCCTTTACCAAG	0.46	0.33	0.29	1.02	0.00
GATCAACAGCCTTTACCCAG	0.00	0.00	0.00	0.00	0.00
GATCAACAGCGAATTGAAAG	0.00	0.00	0.00	0.00	0.69
GATCAACAGCGACAGGTGGG	0.00	0.00	0.00	0.00	0.00
GATCAACAGCGACAGTGACT	0.00	0.00	0.29	0.00	0.00
GATCAACAGCGACATGCCCA	0.00	0.00	0.00	0.00	0.00
GATCAACAGCGACCATGGGC	0.00	0.00	0.29	0.00	0.00
GATCAACAGCGACCCCGGGC	0.00	0.00	0.00	0.00	0.00
GATCAACAGCGACCCGGGCC	0.00	0.33	0.00	0.00	0.34
GATCAACAGCGACCCTGGGC	0.46	1.95	1.72	1.70	0.69
GATCAACAGCGACCGCAAAA	0.23	0.00	0.29	0.00	0.00
GATCAACAGCGACCGCCACG	0.00	0.00	0.00	0.34	0.00
GATCAACAGCGACGACGTCG	0.00	0.00	0.00	0.34	0.00
GATCAACAGCGACGGCATGA	0.00	0.00	0.00	0.00	0.34
GATCAACAGCGACGGCATGG	0.00	0.33	0.00	0.34	0.00
GATCAACAGCGACGGCCTGA	0.00	1.95	0.57	1.36	0.34
GATCAACAGCGACTACAACT	0.00	0.33	0.00	0.00	0.00
GATCAACAGCGACTACTACG	0.00	0.00	0.00	0.00	0.34
GATCAACAGCGACTGCAGCA	0.00	0.00	0.00	0.00	0.00
GATCAACAGCGACTGGACGT	0.00	0.33	0.00	0.00	0.34
GATCAACAGCGAGAACTTCC	0.00	0.00	0.00	0.00	0.00
GATCAACAGCGAGAAGCGCG	0.00	0.33	0.57	0.34	1.03
GATCAACAGCGAGCTCATCG	0.00	0.00	0.00	0.00	0.34
GATCAACAGCGAGTTGACGT	0.23	0.00	0.00	0.00	0.00
GATCAACAGCGATGGCCTGG	0.00	0.00	0.00	0.00	0.34
GATCAACAGCGATTTACGGG	0.00	0.00	0.00	0.00	0.00
GATCAACAGCGCAAACCCCG	0.00	0.00	0.00	0.00	0.34
GATCAACAGCGCAAACCCNN	0.00	0.00	0.00	0.00	0.00
GATCAACAGCGCAAACTAAA	0.00	0.00	0.00	0.00	0.00
GATCAACAGCGCAACACACA	0.00	0.00	0.00	0.00	0.34
GATCAACAGCGCACACACCA	0.00	0.00	0.00	0.34	0.00
GATCAACAGCGCACACACGA	0.00	0.00	0.00	0.34	0.00
GATCAACAGCGCACACACTA	0.00	0.00	0.00	0.00	0.00
GATCAACAGCGCACATGTTC	0.00	0.00	0.00	0.34	0.00
GATCAACAGCGCACCATTGT	0.00	0.00	0.00	0.00	0.69

GATCAACAGCGCACCTCANT	0.00	0.00	0.00	0.00	0.00
GATCAACAGCGCAGCCTCGT	0.00	0.00	0.00	0.00	0.00
GATCAACAGCGCATGTCGGT	0.00	0.00	0.00	0.34	0.00
GATCAACAGCGCCAAGTCGC	0.00	0.00	0.00	0.34	0.00
GATCAACAGCGCCAGATTTG	0.46	0.00	1.43	0.68	0.69
GATCAACAGCGCCAGCGTGG	1.14	0.98	0.29	2.38	1.38
GATCAACAGCGCCCACTGCG	0.23	0.00	0.00	0.00	0.00
GATCAACAGCGCCGACGACA	0.00	0.00	0.00	0.00	0.69
GATCAACAGCGCCGGCGGCG	0.00	0.00	0.00	0.34	0.00
GATCAACAGCGCCGTGCGCG	0.00	0.33	0.00	0.00	0.00
GATCAACAGCGCCTAGCAAA	0.00	0.00	0.00	0.00	0.00
GATCAACAGCGCCTCGATGC	0.46	0.00	0.00	0.00	0.00
GATCAACAGCGCCTTCAACA	0.23	0.00	0.00	0.00	0.00
GATCAACAGCGCGCACCAGC	0.00	0.00	0.57	0.34	0.00
GATCAACAGCGCGCACCCCG	0.23	0.00	0.00	0.34	0.00
GATCAACAGCGCGCACCTGC	0.00	0.00	0.00	0.00	0.00
GATCAACAGCGCGCCCCCCG	0.00	0.00	0.00	0.00	0.34
GATCAACAGCGCGCCTCAAA	0.00	0.33	0.00	0.00	0.00
GATCAACAGCGCGCTCAACT	0.00	0.00	0.00	0.00	0.00
GATCAACAGCGCGCTGGCGG	0.46	0.00	0.00	0.34	0.34
GATCAACAGCGCGGAGGGCT	0.69	0.33	0.00	0.00	0.00
GATCAACAGCGCGGCCGGGG	0.00	0.00	0.00	0.00	0.00
GATCAACAGCGCGGCTTAGG	0.00	0.00	0.00	0.00	0.34
GATCAACAGCGCGGGCTTAG	0.00	0.00	0.00	0.00	0.00
GATCAACAGCGCGTAGTGCC	0.23	0.00	0.00	0.00	0.00
GATCAACAGCGCGTCCTGCA	0.00	0.00	0.00	0.00	0.00
GATCAACAGCGCTTCTAATG	0.00	0.00	0.00	0.34	0.00
GATCAACAGCGCTTGCAACT	0.46	0.00	0.00	0.68	0.69
GATCAACAGCGGAAAGGAGC	0.00	0.00	0.00	0.34	0.00
GATCAACAGCGGAATCAAGT	0.00	0.00	0.57	0.00	0.34
GATCAACAGCGGACTGCTGG	0.00	0.00	0.29	0.34	0.00
GATCAACAGCGGAGGTCTCT	0.00	0.00	0.00	0.34	0.00
GATCAACAGCGGATGCGGTG	0.00	0.33	0.00	0.00	0.00
GATCAACAGCGGATGTAGAT	0.23	0.00	0.00	0.00	0.00
GATCAACAGCGGCAAGCAGC	0.00	0.33	0.00	0.00	0.00
GATCAACAGCGGCAAGGAGA	0.00	0.33	0.00	0.34	0.00
GATCAACAGCGGCAAGGAGC	8.93	14.64	12.89	14.93	8.61

GATCAACAGCGGCAAGGANN	0.00	0.00	0.00	0.34	0.00
GATCAACAGCGGCAAGGCGC	0.23	0.00	0.00	0.34	0.00
GATCAACAGCGGCACCAAGT	0.23	0.00	0.29	0.00	0.00
GATCAACAGCGGCACCACGT	0.00	0.33	0.00	0.00	0.00
GATCAACAGCGGCACCCTGC	0.23	0.00	0.00	0.00	0.00
GATCAACAGCGGCAGAAAGA	0.00	0.33	0.00	0.00	0.00
GATCAACAGCGGCAGCAAGT	0.46	0.33	0.00	0.00	0.00
GATCAACAGCGGCATAAAGT	0.00	0.00	0.00	0.00	0.00
GATCAACAGCGGCATACAGC	0.23	0.00	0.00	0.00	0.00
GATCAACAGCGGCATACTTG	0.00	0.00	0.29	0.00	0.00
GATCAACAGCGGCATATAAC	0.00	0.00	0.00	0.00	0.00
GATCAACAGCGGCATATCAC	4.35	14.64	7.16	8.83	3.44
GATCAACAGCGGCATATTAC	0.00	0.00	0.29	0.00	0.00
GATCAACAGCGGCATCAAAC	0.00	0.33	0.00	0.00	0.00
GATCAACAGCGGCATCAAAT	0.00	0.00	0.29	0.34	0.34
GATCAACAGCGGCATCAACT	0.46	0.00	0.00	0.00	0.00
GATCAACAGCGGCATCAAGA	0.92	0.33	0.29	1.02	2.07
GATCAACAGCGGCATCAAGC	1.14	0.00	0.00	1.02	0.69
GATCAACAGCGGCATCAAGG	2.06	0.65	0.29	0.34	0.34
GATCAACAGCGGCATCAAGN	1.37	0.00	0.00	0.00	0.00
GATCAACAGCGGCATCAAGT	235.31	224.15	144.33	157.84	149.47
GATCAACAGCGGCATCAANN	0.23	0.00	0.00	0.00	0.00
GATCAACAGCGGCATCAANT	0.00	0.00	0.00	0.00	0.34
GATCAACAGCGGCATCAATC	0.00	0.65	0.00	0.00	0.69
GATCAACAGCGGCATCAATT	0.00	0.33	0.00	0.00	0.00
GATCAACAGCGGCATCACGC	0.23	0.00	0.00	0.00	0.00
GATCAACAGCGGCATCACGG	0.00	0.00	0.00	0.00	0.34
GATCAACAGCGGCATCACGT	0.23	0.00	0.57	0.34	0.69
GATCAACAGCGGCATCAGCA	0.46	0.00	0.00	0.00	0.00
GATCAACAGCGGCATCAGGT	0.69	0.98	0.57	0.00	0.34
GATCAACAGCGGCATCCAAT	0.23	0.00	0.00	0.00	0.00
GATCAACAGCGGCATCCAGA	0.00	0.00	0.00	0.00	0.00
GATCAACAGCGGCATCCAGT	1.14	1.30	0.00	0.34	0.00
GATCAACAGCGGCATCCTGT	0.23	0.00	0.00	0.00	0.00
GATCAACAGCGGCATCGAGG	0.46	0.00	0.00	0.00	0.00
GATCAACAGCGGCATCGAGT	1.14	0.00	0.57	0.34	0.00
GATCAACAGCGGCATCGTAT	0.23	0.00	0.00	0.00	0.00

GATCAACAGCGGCATCNAGT	0.00	0.00	0.00	0.00	0.69
GATCAACAGCGGCATCNANT	0.00	0.33	0.00	0.00	0.00
GATCAACAGCGGCATCNNNN	0.23	0.00	0.00	0.00	0.00
GATCAACAGCGGCATCTAGT	0.00	0.00	0.29	0.00	0.00
GATCAACAGCGGCATGAAGT	0.00	0.00	0.00	0.00	0.00
GATCAACAGCGGCATGCCCG	0.00	0.00	0.00	0.34	0.00
GATCAACAGCGGCATGTCAC	0.23	0.00	0.00	0.00	0.00
GATCAACAGCGGCATTAAGT	0.00	0.00	0.00	0.34	0.00
GATCAACAGCGGCCACACCT	0.00	0.00	0.00	0.00	0.69
GATCAACAGCGGCCACACTT	0.00	0.00	0.00	0.00	0.00
GATCAACAGCGGCCAGAGGA	0.00	0.00	0.00	0.00	0.34
GATCAACAGCGGCCTACCGT	0.23	0.00	0.00	0.00	0.00
GATCAACAGCGGCCTCAAGT	0.00	0.00	0.00	0.34	0.34
GATCAACAGCGGCCTGAAAA	0.00	0.00	0.00	0.00	0.34
GATCAACAGCGGCGACGGCC	0.23	0.00	0.00	0.00	0.00
GATCAACAGCGGCGCAGGGC	0.23	0.00	0.00	0.00	0.00
GATCAACAGCGGCGCGAGAG	0.00	0.00	0.29	0.00	0.00
GATCAACAGCGGCGCGAGCG	0.92	0.65	1.72	0.68	0.00
GATCAACAGCGGCGCTCTCC	0.46	0.00	0.00	0.00	0.00
GATCAACAGCGGCGGCATGG	0.23	0.00	0.00	0.34	0.00
GATCAACAGCGGCGGCATTG	0.00	0.00	0.29	0.00	0.00
GATCAACAGCGGCGTCAAGT	0.23	0.00	0.00	0.00	0.00
GATCAACAGCGGCGTGATTC	0.00	0.00	0.29	0.00	0.00
GATCAACAGCGGCGTGTACT	0.00	0.00	0.00	0.00	0.34
GATCAACAGCGGCGTGTGCA	0.00	0.00	0.00	0.34	0.00
GATCAACAGCGGCTGAGCAT	0.00	0.33	0.00	0.00	0.00
GATCAACAGCGGCTGCCTCG	0.23	0.00	0.00	0.00	0.00
GATCAACAGCGGCTGGCAGC	0.23	0.00	0.00	0.00	0.00
GATCAACAGCGGCTTCAAGT	0.23	0.00	0.00	0.00	0.00
GATCAACAGCGGCTTCCAGT	0.00	0.00	0.29	0.00	0.00
GATCAACAGCGGCTTGGCGG	0.00	0.00	0.00	0.00	0.34
GATCAACAGCGGGAAATGTG	0.23	0.00	0.00	0.00	0.00
GATCAACAGCGGGATCAAGT	0.23	0.00	0.00	0.00	0.00
GATCAACAGCGGGCGCGTTG	0.00	0.00	0.00	0.00	0.00
GATCAACAGCGGGGGAAAGG	0.00	0.33	0.00	0.00	0.00
GATCAACAGCGGGGGCTTAG	0.00	0.00	0.00	0.00	0.34
GATCAACAGCGGGTGCAGAG	0.00	0.00	0.00	0.34	0.00

GATCAACAGCGGTATGAAGC	0.23	0.00	0.00	0.00	0.00
GATCAACAGCGGTCACACTT	0.00	0.00	0.00	0.68	0.00
GATCAACAGCGGTGGAAAGG	0.00	0.00	0.29	0.00	0.00
GATCAACAGCGTAGGCATGG	0.00	0.00	0.29	0.00	0.00
GATCAACAGCGTCAAGTCAG	0.69	0.00	0.00	0.00	0.00
GATCAACAGCGTCACCTCAT	0.00	0.00	0.00	0.68	0.00
GATCAACAGCGTCAGCATGG	0.00	0.65	0.29	0.00	0.00
GATCAACAGCGTCATCCAGC	0.00	0.00	0.00	0.00	0.00
GATCAACAGCGTCGACATGG	0.00	0.33	0.00	0.00	0.00
GATCAACAGCGTCGCAAACA	0.00	0.00	0.00	0.00	0.00
GATCAACAGCGTCGCTTGAC	0.23	0.00	0.00	0.00	0.00
GATCAACAGCGTCGGAAAGG	0.00	0.00	0.00	0.00	0.34
GATCAACAGCGTCGGAATGG	0.00	0.00	0.86	0.00	0.00
GATCAACAGCGTCGGCAAGG	0.00	0.00	0.00	0.00	0.00
GATCAACAGCGTCGGCACGG	0.00	0.00	0.29	0.68	0.34
GATCAACAGCGTCGGCAGGG	0.23	0.65	0.00	0.00	0.00
GATCAACAGCGTCGGCANGG	0.00	0.00	0.00	0.34	0.00
GATCAACAGCGTCGGCATAG	0.23	0.00	0.00	0.34	0.00
GATCAACAGCGTCGGCATGA	0.00	0.33	0.00	0.00	0.00
GATCAACAGCGTCGGCATGG	69.13	185.76	231.09	146.63	180.47
GATCAACAGCGTCGGCATGN	0.46	0.00	0.00	0.34	0.00
GATCAACAGCGTCGGCATGT	0.00	0.00	0.00	0.00	0.34
GATCAACAGCGTCGGCATNG	0.00	0.00	0.00	0.34	0.00
GATCAACAGCGTCGGCATNN	0.46	0.00	0.00	0.00	0.00
GATCAACAGCGTCGGCATTG	0.00	0.00	0.00	0.34	0.00
GATCAACAGCGTCGGCCNNN	0.23	0.00	0.00	0.00	0.00
GATCAACAGCGTCGGCCTCG	0.00	0.00	0.00	0.00	0.00
GATCAACAGCGTCGGCCTGG	0.23	0.65	0.86	0.34	0.00
GATCAACAGCGTCGGCGGGG	0.00	0.00	0.57	0.00	0.00
GATCAACAGCGTCGGCGTGG	0.46	0.00	0.00	0.00	0.34
GATCAACAGCGTCGGCNTGG	0.00	0.00	0.00	0.00	0.34
GATCAACAGCGTCGGCTGGG	0.23	0.00	0.00	0.00	0.00
GATCAACAGCGTCGGCTTGG	0.23	0.00	0.00	0.00	0.00
GATCAACAGCGTCGGGATGG	0.00	0.00	0.29	0.00	0.00
GATCAACAGCGTCGGTAGGA	0.00	0.00	0.00	0.00	0.00
GATCAACAGCGTCGGTATGG	0.00	0.33	0.00	0.00	0.00
GATCAACAGCGTCGTCATGG	0.00	0.00	0.29	0.00	0.00

GATCAACAGCGTCTGCATGG	0.00	0.33	0.00	0.00	0.00
GATCAACAGCGTCTTTAGAC	0.00	0.00	0.00	0.00	0.00
GATCAACAGCGTGAAATGGG	0.23	0.00	0.00	0.00	0.00
GATCAACAGCGTGAAATGTG	10.53	13.34	11.74	12.22	16.53
GATCAACAGCGTGAAATGTT	0.46	0.00	1.15	0.00	0.00
GATCAACAGCGTGAAATTCG	0.00	0.00	0.00	0.00	0.69
GATCAACAGCGTGAACTGTT	0.00	0.00	0.00	0.00	0.00
GATCAACAGCGTGAAGAAGA	0.46	0.00	1.15	0.00	0.00
GATCAACAGCGTGAAGTGTG	0.00	0.33	0.00	0.00	0.00
GATCAACAGCGTGAATCGTA	0.00	0.00	0.00	0.00	0.34
GATCAACAGCGTGACATGTG	0.00	0.00	0.00	0.00	0.00
GATCAACAGCGTGACCTTGT	0.00	0.00	0.00	0.00	0.00
GATCAACAGCGTGCAATGCG	0.23	0.00	0.00	0.00	0.00
GATCAACAGCGTGCACACGT	0.00	0.00	0.00	0.00	0.34
GATCAACAGCGTGCCGCTCC	0.00	0.00	0.00	0.00	0.00
GATCAACAGCGTGGAAAGGG	0.00	0.00	0.29	0.00	0.00
GATCAACAGCGTGGCCCTGC	0.00	0.33	0.00	0.34	0.00
GATCAACAGCGTGGGATTAG	0.00	0.00	0.00	0.34	0.00
GATCAACAGCGTGGGCTGAG	0.00	0.00	0.29	0.00	0.00
GATCAACAGCGTGGGCTTAC	0.00	0.33	0.00	0.00	0.00
GATCAACAGCGTGGGCTTAG	8.93	7.16	3.72	7.81	3.44
GATCAACAGCGTGGTGCCAC	0.00	0.00	0.29	0.34	0.00
GATCAACAGCGTGGTGGGCT	0.00	0.00	0.00	0.34	0.00
GATCAACAGCGTGTGGGAGT	0.00	0.00	0.00	0.00	0.34
GATCAACAGCGTTCCGCTGA	0.00	0.00	0.00	0.00	0.00
GATCAACAGCGTTGTCATCA	0.00	0.00	0.00	0.34	0.00
GATCAACAGCTAAGAGTTGC	0.23	0.00	0.00	0.00	0.00
GATCAACAGCTAAGCGGGGC	0.00	0.00	0.29	0.00	0.00
GATCAACAGCTACCAAGTGC	0.00	0.00	0.29	0.00	0.00
GATCAACAGCTACCATTGCG	0.00	0.00	0.00	0.00	0.00
GATCAACAGCTACCTCATGC	0.00	0.00	0.00	0.00	0.00
GATCAACAGCTACTCGAATG	0.00	0.00	0.00	0.00	0.00
GATCAACAGCTATGCGCGGC	0.00	0.00	0.86	0.00	0.00
GATCAACAGCTCAATGTTAA	0.00	0.00	0.29	0.00	0.00
GATCAACAGCTCAGGATGGC	0.00	0.00	0.00	0.00	0.00
GATCAACAGCTCATTGAGCT	0.00	0.00	0.86	0.00	0.00
GATCAACAGCTCCATTCTCG	0.69	0.33	1.15	1.02	1.72

GATCAACAGCTCCCCGATAC	0.00	0.00	0.00	0.00	1.03
GATCAACAGCTCCGCCGTAG	0.00	0.00	0.00	0.00	0.69
GATCAACAGCTCCGTCGAGG	1.14	0.65	0.00	1.02	0.00
GATCAACAGCTCCTTCTCCT	0.00	0.00	0.00	0.00	0.00
GATCAACAGCTCGAAACATC	0.00	0.00	0.00	0.00	0.00
GATCAACAGCTCGCCGGGCT	0.00	0.00	0.00	0.00	0.00
GATCAACAGCTCGCGCCACG	0.00	0.00	0.00	0.34	0.00
GATCAACAGCTCGCTGCGGC	0.00	0.33	0.00	0.00	0.00
GATCAACAGCTCGGTGCTGG	0.00	0.00	0.00	0.34	0.00
GATCAACAGCTCGTGCAGGC	0.00	0.00	0.00	0.00	0.00
GATCAACAGCTCTGGTCGGC	0.00	0.00	0.00	0.00	0.00
GATCAACAGCTGAGCTCGAG	0.23	0.00	0.00	0.00	0.00
GATCAACAGCTGAGCTGTTG	0.69	0.33	0.00	0.00	0.00
GATCAACAGCTGCAAAAGAG	0.23	0.00	0.29	0.00	0.34
GATCAACAGCTGCCCTTTCG	0.00	0.00	0.00	0.00	0.34
GATCAACAGCTGCCTCCATC	0.23	0.00	0.00	0.00	0.00
GATCAACAGCTGCCTGCCTC	0.00	0.00	0.57	0.00	0.00
GATCAACAGCTGCGAGTTCA	0.23	0.00	0.00	0.00	0.00
GATCAACAGCTGGCATTGGG	0.00	0.00	0.00	0.00	0.00
GATCAACAGCTGGGTGGTGA	0.23	0.00	0.00	0.00	0.00
GATCAACAGCTGTGATGCCC	0.00	0.65	0.00	2.38	0.00
GATCAACAGCTGTGTGGAGA	0.00	0.33	0.29	0.34	0.00
GATCAACAGCTGTGTGGGGA	0.00	0.33	0.00	0.00	0.00
GATCAACAGCTGTTGGCCAG	0.00	0.00	0.00	0.00	0.00
GATCAACAGCTGTTGTCACG	0.00	0.00	0.00	0.00	0.00
GATCAACAGCTTCAGCATGG	0.00	0.00	0.00	0.00	0.34
GATCAACAGCTTCCCATGGT	0.00	0.33	0.29	0.00	0.69
GATCAACAGCTTCGCAGGCG	0.00	0.00	0.00	0.00	1.03
GATCAACAGCTTCGCGCAGG	0.00	0.00	0.29	0.00	0.00
GATCAACAGCTTCTACCCAA	0.00	0.33	0.00	0.00	0.00
GATCAACAGCTTCTCCTTGG	0.00	0.00	0.00	0.00	0.00
GATCAACAGCTTGACAAAAA	0.00	0.00	0.00	0.00	0.00
GATCAACAGCTTGAGCTGAA	0.00	0.00	0.00	0.00	0.34
GATCAACAGCTTGAGCTGAC	0.00	0.00	0.29	0.00	0.00
GATCAACAGCTTGCCATCTG	0.00	0.00	0.29	0.00	0.00
GATCAACAGCTTGGCCTTGC	0.00	0.00	0.00	0.34	0.00
GATCAACAGCTTGGGCATGG	0.00	0.00	0.29	0.00	0.00

GATCAACAGCTTGGGCGAGG	0.00	0.00	0.00	0.00	0.00
GATCAACAGCTTGGGGGAAG	0.00	0.00	0.00	0.34	0.00
GATCAACAGCTTGGGGGAGG	2.98	4.88	2.58	0.68	1.03
GATCAACAGCTTGGGGGCGG	0.46	0.00	0.00	0.00	0.00
GATCAACAGCTTGGGGGGGG	1.60	0.00	0.00	0.00	0.00
GATCAACAGCTTTGCCGTGG	0.00	0.00	0.00	0.00	0.69
GATCAACAGGAAAAAAAAAT	0.00	0.33	0.00	0.00	0.00
GATCAACAGGAAAGTGACAG	0.00	0.00	0.57	0.00	0.00
GATCAACAGGAACAAGAGGA	0.00	0.00	0.86	0.00	0.34
GATCAACAGGAACATCTCTG	0.00	0.00	0.00	0.00	0.00
GATCAACAGGAACATCTGCC	0.00	0.00	0.00	0.00	0.00
GATCAACAGGAACATCTGNN	0.00	0.00	0.00	0.00	0.00
GATCAACAGGAACCCCCCCC	0.00	0.00	0.00	0.34	0.00
GATCAACAGGAACCCTGACC	0.00	0.00	0.57	0.00	0.00
GATCAACAGGAACCCTGTAC	0.00	0.00	0.00	0.00	0.00
GATCAACAGGAACCTGAAGA	0.00	0.00	0.00	0.00	0.00
GATCAACAGGAACTTGGAAC	0.00	0.00	0.00	0.00	0.00
GATCAACAGGAAGACGAAGA	0.00	0.00	0.00	0.00	0.00
GATCAACAGGAAGAGGAGAG	0.23	0.00	0.00	0.00	0.00
GATCAACAGGAAGCAAATCC	0.00	0.00	0.00	0.34	0.00
GATCAACAGGAAGCACGTAG	0.00	0.00	0.00	0.34	0.00
GATCAACAGGAAGCCCGCAG	0.23	0.00	0.00	0.00	0.00
GATCAACAGGAAGGCCGCAT	0.00	0.00	0.00	0.00	0.34
GATCAACAGGAATCTACGCT	0.00	0.00	0.00	0.34	0.00
GATCAACAGGAATGCCCTTG	0.00	0.33	0.00	0.00	0.00
GATCAACAGGACCCTCGAGG	0.00	0.00	0.00	0.00	0.00
GATCAACAGGACCGCCGCCN	0.00	0.00	0.00	0.00	0.34
GATCAACAGGACCGCCGCCT	2.98	3.90	2.58	4.75	3.10
GATCAACAGGACCGCCGCNN	0.00	0.00	0.00	0.00	0.00
GATCAACAGGACGCCGAAGA	0.00	0.00	0.00	0.00	0.34
GATCAACAGGACGCGCGCGG	0.00	0.00	0.00	0.34	0.34
GATCAACAGGACTTGGCTGC	0.00	0.00	0.00	0.00	0.34
GATCAACAGGAGAAGAAGGA	0.00	0.00	0.00	0.00	0.34
GATCAACAGGAGAAGGTGGG	0.00	0.33	0.00	0.00	0.00
GATCAACAGGAGAGGGCTCA	0.46	0.00	0.00	0.00	0.00
GATCAACAGGAGAGGTCTGA	0.23	0.33	0.00	0.34	0.00
GATCAACAGGAGAGTGATGG	0.46	0.00	0.00	0.34	0.00

GATCAACAGGAGCAACTCGC	0.00	0.00	0.00	0.00	0.34
GATCAACAGGAGCAATGAGG	0.00	0.33	0.00	0.00	0.00
GATCAACAGGAGCAATGTGC	0.00	0.00	0.00	0.34	0.00
GATCAACAGGAGCAATGTGN	0.23	0.00	0.00	0.00	0.00
GATCAACAGGAGCAATGTGT	2.75	1.95	1.72	2.04	4.82
GATCAACAGGAGCAATGTNN	0.23	0.00	0.00	0.00	0.00
GATCAACAGGAGCACGGGTA	0.00	0.33	0.00	0.68	0.00
GATCAACAGGAGCACGGGTG	0.00	0.00	0.00	0.00	0.00
GATCAACAGGAGCAGAAGAA	0.00	0.00	1.15	0.00	1.38
GATCAACAGGAGCAGATGAA	0.92	0.00	0.00	0.00	0.00
GATCAACAGGAGCAGCGGCT	0.00	0.00	0.00	0.34	0.00
GATCAACAGGAGCATGGGCA	0.00	0.00	0.00	0.00	0.00
GATCAACAGGAGCGGCCAAG	0.23	0.00	0.00	0.00	0.00
GATCAACAGGAGGACCGGTC	0.00	0.33	0.00	0.34	0.00
GATCAACAGGAGGACCTTGA	0.00	0.00	0.00	0.34	0.00
GATCAACAGGAGGAGATGGA	0.00	0.00	0.00	0.34	0.00
GATCAACAGGAGGAGATTGG	0.00	0.00	0.00	0.34	0.00
GATCAACAGGAGGAGGAGAA	0.00	0.00	0.00	0.00	0.34
GATCAACAGGAGGAGGAGGG	0.00	0.33	0.57	0.00	0.34
GATCAACAGGAGGAGGCGCG	0.00	0.00	0.00	0.00	0.34
GATCAACAGGAGGAGGCGCT	0.00	0.00	0.29	0.00	0.00
GATCAACAGGAGGAGGCGGA	0.00	0.00	0.29	0.00	0.00
GATCAACAGGAGGAGGCGGG	0.23	0.00	0.00	0.00	0.69
GATCAACAGGAGGAGGGGGG	2.29	0.00	0.00	1.02	0.34
GATCAACAGGAGGAGGNGGG	0.00	0.00	0.00	0.00	0.34
GATCAACAGGAGGAGGTGCG	0.00	0.00	0.00	0.34	0.00
GATCAACAGGAGGAGGTGGA	0.00	0.00	0.00	0.00	0.00
GATCAACAGGAGGAGGTGGG	12.82	29.28	29.21	28.85	31.34
GATCAACAGGAGGAGGTGGT	0.00	0.00	0.00	0.34	0.00
GATCAACAGGAGGAGGTTGT	0.00	0.33	0.00	0.00	0.00
GATCAACAGGAGGATATGGG	0.00	0.00	0.00	0.00	0.00
GATCAACAGGAGGCAGCCTG	0.00	0.00	0.29	0.00	0.00
GATCAACAGGAGGCCGAGCA	0.00	0.00	0.00	0.00	0.34
GATCAACAGGAGGCCTTAAG	0.46	0.00	0.00	0.34	0.00
GATCAACAGGAGGCGGGGGG	0.23	0.00	0.00	0.00	0.00
GATCAACAGGAGGCGGTGGG	0.00	0.00	0.00	0.00	0.00
GATCAACAGGAGGGAGTCTA	0.00	0.00	0.00	0.00	0.34

GATCAACAGGAGGGGGAGGG	0.23	0.00	0.00	0.00	0.00
GATCAACAGGAGGGGGCGCC	0.23	0.00	0.00	0.00	0.00
GATCAACAGGAGGGGGTGGG	0.92	0.00	0.00	0.00	0.00
GATCAACAGGAGTAGGCGAC	0.00	0.00	0.29	0.00	0.00
GATCAACAGGAGTCCATCTA	0.00	0.00	0.00	0.34	0.00
GATCAACAGGAGTGAAGAAC	0.00	0.33	0.00	0.00	0.00
GATCAACAGGAGTTGTGGGC	0.00	0.00	0.00	0.00	0.00
GATCAACAGGATAAGAAGAA	0.00	0.00	1.43	0.00	0.00
GATCAACAGGATGAAAAAAA	0.00	0.00	0.29	0.00	0.00
GATCAACAGGATGAACACCA	0.00	0.00	0.00	0.00	0.00
GATCAACAGGATGCAGACTC	0.00	0.00	0.00	0.00	0.34
GATCAACAGGATGCAGGCGG	0.00	0.00	0.29	0.00	0.00
GATCAACAGGATGCCAGACG	0.00	0.00	0.29	0.00	0.00
GATCAACAGGATGGCCGCTG	0.00	0.33	0.29	0.00	0.00
GATCAACAGGATGGCTTGTG	0.00	0.33	0.00	0.00	0.00
GATCAACAGGATTGAAGAAC	2.29	5.21	3.15	4.41	5.51
GATCAACAGGATTGAAGAAN	0.00	0.00	0.00	0.00	0.00
GATCAACAGGATTGTTCTTG	0.00	0.00	0.00	0.00	0.00
GATCAACAGGATTGTTTACA	0.23	0.00	0.00	0.00	0.00
GATCAACAGGATTGTTTGTG	0.00	0.00	0.00	0.00	0.00
GATCAACAGGCAAAGCTGGT	0.00	0.00	0.00	0.34	0.00
GATCAACAGGCAAGCGATTG	0.00	0.33	0.00	0.00	0.00
GATCAACAGGCAAGCGCGCA	0.00	0.00	0.29	0.00	0.00
GATCAACAGGCAATTTTTTG	0.00	0.33	0.00	0.00	0.00
GATCAACAGGCACCACGATG	0.00	0.00	0.00	0.00	0.69
GATCAACAGGCACCAGGAGG	0.00	0.00	0.29	0.00	0.00
GATCAACAGGCACCCCGTCA	0.00	0.33	0.57	1.36	0.00
GATCAACAGGCACCCCTTAG	0.00	0.00	0.00	0.00	0.34
GATCAACAGGCACCCTGTGA	2.06	0.98	0.57	1.02	0.69
GATCAACAGGCACGCCCTCG	0.23	0.00	0.00	0.00	0.00
GATCAACAGGCACGCGCTTG	0.00	0.00	0.00	0.34	0.00
GATCAACAGGCACGGCGTCG	0.00	0.00	0.00	0.00	0.00
GATCAACAGGCACGTGAGCA	0.00	0.33	0.00	0.00	0.00
GATCAACAGGCAGAATTTCC	0.00	0.00	0.00	0.00	0.34
GATCAACAGGCAGAGCGCCA	0.23	0.00	0.00	0.00	0.00
GATCAACAGGCAGAGGCGGA	0.00	0.00	0.29	0.00	0.00
GATCAACAGGCAGCAGAGTG	0.00	0.00	0.00	0.00	0.00

GATCAACAGGCAGCGCGAGA	0.00	0.33	0.00	0.00	0.00
GATCAACAGGCAGCGCGAGG	0.69	0.00	0.00	0.00	0.00
GATCAACAGGCAGCGGCGCC	0.23	0.00	0.00	0.00	0.00
GATCAACAGGCAGCTTGAGA	0.00	0.00	0.00	0.00	0.00
GATCAACAGGCAGGAGATGG	0.00	0.33	0.00	0.00	0.69
GATCAACAGGCAGGCCTGCC	0.69	0.65	0.57	0.00	0.69
GATCAACAGGCAGGGAATGC	0.00	0.00	0.00	0.00	0.00
GATCAACAGGCAGTGCCACA	0.00	0.00	0.00	0.00	0.00
GATCAACAGGCATCGCATGA	0.00	0.00	0.00	0.00	0.34
GATCAACAGGCATCGCCTGA	0.00	0.00	0.00	0.00	0.00
GATCAACAGGCATGACAGGG	0.00	0.00	0.29	0.00	0.00
GATCAACAGGCATGCCCTGG	0.00	0.00	0.00	0.34	0.00
GATCAACAGGCATGCCCTTG	0.23	0.33	0.00	0.00	0.00
GATCAACAGGCATGGGCTGC	0.23	0.00	0.00	0.00	0.00
GATCAACAGGCATTGACATG	0.00	0.00	0.00	0.00	0.00
GATCAACAGGCATTGCCGCT	0.00	0.00	0.00	0.00	0.69
GATCAACAGGCATTGCCGGG	0.00	0.00	0.29	0.34	0.00
GATCAACAGGCCCCACCCCT	0.00	0.33	0.00	0.00	0.00
GATCAACAGGCCCCTTCTTA	0.00	0.00	0.00	0.00	0.00
GATCAACAGGCCCGAGCAGG	0.00	0.00	0.00	0.00	0.34
GATCAACAGGCCCTACCCCT	0.23	0.33	0.29	0.00	0.00
GATCAACAGGCCCTGGCTGC	0.00	0.00	0.29	0.00	0.00
GATCAACAGGCCCTTCTCAG	0.23	0.00	0.00	0.00	0.00
GATCAACAGGCCGGAACCGA	0.00	0.33	0.00	0.00	0.00
GATCAACAGGCCGTGCCTGT	0.00	0.00	0.00	0.00	0.00
GATCAACAGGCCGTGGTGGG	0.00	0.65	0.00	0.00	0.00
GATCAACAGGCCTCGGCAGG	0.23	0.00	0.00	0.00	0.00
GATCAACAGGCCTCGGCATG	0.23	0.00	0.00	0.00	0.00
GATCAACAGGCCTCGTATGC	0.00	0.00	0.00	0.34	0.00
GATCAACAGGCCTGACCCAC	0.00	0.00	0.00	0.34	0.00
GATCAACAGGCCTGCAGAGG	0.00	0.00	0.29	0.00	0.00
GATCAACAGGCCTGTCCCAC	0.92	0.98	0.29	1.02	0.00
GATCAACAGGCCTTAGCTGC	0.00	0.00	0.00	0.00	0.34
GATCAACAGGCCTTCAATGC	0.00	0.00	0.00	0.00	0.34
GATCAACAGGCCTTGGACGC	0.00	0.00	0.00	0.00	0.00
GATCAACAGGCCTTGGCCGC	0.00	0.00	0.00	0.00	0.00
GATCAACAGGCCTTGGCCGG	0.00	0.00	0.00	0.34	0.00

GATCAACAGGCCTTGGCTGA	0.00	0.00	0.00	0.00	0.00
GATCAACAGGCCTTGGCTGC	22.20	18.22	5.44	18.33	12.40
GATCAACAGGCCTTGGCTGG	0.00	0.00	0.00	0.34	0.00
GATCAACAGGCCTTGGCTNN	0.23	0.00	0.00	0.00	0.00
GATCAACAGGCCTTGGTCGT	0.00	0.00	0.00	0.34	0.00
GATCAACAGGCCTTGGTTGC	0.23	0.00	0.29	0.00	0.00
GATCAACAGGCCTTGTCGGG	0.23	0.00	0.00	0.00	0.00
GATCAACAGGCGAAGAAGAA	0.00	0.00	0.29	0.00	0.00
GATCAACAGGCGAATGGCTT	0.00	0.00	0.00	0.00	0.00
GATCAACAGGCGACTCTTGC	0.00	0.00	0.00	0.00	0.00
GATCAACAGGCGCGGCTAAT	0.00	0.00	0.00	0.00	0.34
GATCAACAGGCGCGGCTACT	0.00	1.30	0.00	0.34	0.34
GATCAACAGGCGGAGGTGGG	0.23	0.33	0.00	0.00	0.00
GATCAACAGGCGGCCATCAA	0.00	0.00	0.00	0.00	0.34
GATCAACAGGCGGGCCAGAG	0.00	0.00	0.00	0.00	0.34
GATCAACAGGCGTCGGGACA	0.00	0.00	0.00	0.00	0.34
GATCAACAGGCGTTGTCTCC	0.00	0.00	0.29	0.00	0.00
GATCAACAGGCTAGCCAGGG	0.00	0.33	0.29	0.00	0.00
GATCAACAGGCTCATGTGAC	0.00	0.00	0.00	0.68	0.00
GATCAACAGGCTCATGTGGC	1.14	0.98	0.00	0.34	0.00
GATCAACAGGCTCCAACACG	0.00	0.00	0.00	0.00	0.34
GATCAACAGGCTCCCGAGCA	0.00	0.00	0.00	0.00	0.00
GATCAACAGGCTCCTGACAA	0.00	0.98	0.00	0.68	0.34
GATCAACAGGCTCCTGATGC	0.00	0.65	0.00	1.02	0.34
GATCAACAGGCTCGAGCAGG	10.30	12.36	2.58	6.79	5.85
GATCAACAGGCTCGAGCCGG	0.00	0.00	0.29	0.00	0.00
GATCAACAGGCTCGAGNAGG	0.00	0.00	0.29	0.00	0.00
GATCAACAGGCTCTGCCGGA	1.14	0.33	0.00	0.00	0.00
GATCAACAGGCTCTGCCGGC	0.23	0.00	0.00	0.00	0.00
GATCAACAGGCTGAACTGCG	0.00	0.00	0.29	0.00	0.34
GATCAACAGGCTGACAGCAG	0.00	1.30	1.15	1.02	0.69
GATCAACAGGCTGACCACGA	0.00	0.00	0.00	0.34	0.00
GATCAACAGGCTGACTGAGG	0.23	0.00	0.00	0.00	0.00
GATCAACAGGCTGCAGTCCA	0.00	0.33	0.00	0.00	0.00
GATCAACAGGCTGCTCGACA	0.00	0.00	0.29	0.00	0.00
GATCAACAGGCTGCTGCAGG	0.46	0.00	0.00	0.34	0.00
GATCAACAGGCTGGTTGTGG	0.00	0.33	0.00	0.00	0.00

GATCAACAGGCTTATTAGAA	0.23	0.00	0.00	0.00	0.00
GATCAACAGGCTTGCAAATC	0.00	0.00	0.00	0.00	0.00
GATCAACAGGCTTTGGCTGC	0.23	0.00	0.00	0.00	0.00
GATCAACAGGGACACAATTC	0.00	0.00	0.00	0.00	0.00
GATCAACAGGGACAGCCGAA	0.23	0.00	0.00	0.00	0.00
GATCAACAGGGACATGCTGA	0.00	0.00	0.00	0.34	0.00
GATCAACAGGGACCAGTTCC	0.00	0.33	0.00	0.34	0.69
GATCAACAGGGACCGATGCT	0.00	0.00	0.00	0.00	0.34
GATCAACAGGGACGACTACG	0.23	0.65	0.29	0.34	0.00
GATCAACAGGGAGAAGAGGG	0.00	0.00	0.00	0.00	0.00
GATCAACAGGGAGCTCCCTT	0.00	0.00	0.00	0.00	0.69
GATCAACAGGGAGCTTGGGG	0.23	0.00	0.00	0.00	0.00
GATCAACAGGGAGGCATTTG	0.00	0.00	0.00	0.34	0.00
GATCAACAGGGAGGCCGCGA	0.00	0.00	0.00	0.00	0.34
GATCAACAGGGAGTTCCTCG	0.00	0.98	0.29	0.34	0.00
GATCAACAGGGATGACTACG	0.00	1.30	1.15	1.02	1.38
GATCAACAGGGCAAAGGGCC	0.00	0.33	0.29	1.02	0.00
GATCAACAGGGCCAGCCACG	0.00	0.00	0.00	0.00	0.00
GATCAACAGGGCCATCGAGG	0.00	0.65	0.29	0.00	1.38
GATCAACAGGGCCGAAGCGC	0.46	0.00	0.00	0.00	0.00
GATCAACAGGGCCGAAGTGC	2.75	1.30	0.00	0.34	1.72
GATCAACAGGGCCGTCAACG	0.23	0.00	0.00	0.00	0.00
GATCAACAGGGCCGTGGCAA	0.00	0.00	0.00	0.00	0.00
GATCAACAGGGCCTAAAAAA	0.00	0.00	0.00	0.34	0.00
GATCAACAGGGCCTCACAAC	0.00	0.00	0.00	0.34	0.00
GATCAACAGGGCGCTGACCG	0.00	0.33	0.00	0.00	0.00
GATCAACAGGGCGCTGCCGG	0.00	0.00	0.29	0.00	0.00
GATCAACAGGGCGCTGCTGG	0.00	0.33	0.00	0.00	0.00
GATCAACAGGGCGCTGTTGG	0.00	0.00	0.00	0.00	0.00
GATCAACAGGGCGTCGCTGG	0.00	0.00	0.00	0.00	0.00
GATCAACAGGGCGTGGCTGG	0.00	0.00	0.00	0.00	0.34
GATCAACAGGGCGTTCCTGG	0.00	0.00	0.00	0.00	0.34
GATCAACAGGGCGTTGATGG	0.23	0.00	0.00	0.00	0.00
GATCAACAGGGCGTTGCNNN	0.00	0.00	0.00	0.00	0.00
GATCAACAGGGCGTTGCTGG	14.42	26.35	21.48	12.90	18.25
GATCAACAGGGCGTTGGTGG	0.00	0.00	0.00	0.00	0.00
GATCAACAGGGCGTTGTTGG	0.00	0.00	0.00	0.34	0.00

GATCAACAGGGCTACAGTTC	0.23	0.00	0.00	0.00	0.00
GATCAACAGGGCTACCATTG	0.00	0.00	0.29	0.00	0.00
GATCAACAGGGCTGATACGA	0.00	0.00	0.00	0.34	0.00
GATCAACAGGGCTGGGCGTA	0.00	0.00	0.00	0.34	0.00
GATCAACAGGGCTTGCAATC	0.00	0.33	0.00	0.00	0.00
GATCAACAGGGCTTGCTTGA	0.00	0.00	0.00	0.00	0.69
GATCAACAGGGGAATCAAGT	0.00	0.00	0.29	0.00	0.00
GATCAACAGGGGACGGAGAG	0.00	0.00	0.00	0.00	0.34
GATCAACAGGGGAGAGTGAT	0.23	0.00	0.00	0.00	0.00
GATCAACAGGGGAGTGATAC	0.00	0.33	0.00	0.00	0.00
GATCAACAGGGGATGAGGAC	0.00	0.00	1.15	0.00	2.76
GATCAACAGGGGCAAGGCCG	0.00	0.00	0.00	0.34	0.00
GATCAACAGGGGCAATTATT	0.00	0.33	0.00	0.00	0.00
GATCAACAGGGGCACCAAGT	0.23	0.00	0.00	0.00	0.00
GATCAACAGGGGCATCAAGG	0.23	0.00	0.00	0.00	0.00
GATCAACAGGGGCATCAAGT	1.37	0.00	0.86	0.00	0.34
GATCAACAGGGGCATGTGGT	0.00	0.00	0.00	0.00	0.00
GATCAACAGGGGCTCAGTTG	0.00	0.00	0.29	0.00	0.00
GATCAACAGGGGCTCATTCA	0.00	0.00	0.00	0.00	0.00
GATCAACAGGGGGAGGTGGG	0.00	0.00	0.29	0.00	0.00
GATCAACAGGGGGGCCCCGG	0.00	0.33	0.00	0.00	0.00
GATCAACAGGGGGGGAAAGG	0.46	0.00	0.00	0.00	0.00
GATCAACAGGGGGGGGCTAC	0.00	0.33	0.00	0.00	0.00
GATCAACAGGGGTGGAAAGG	0.23	0.00	0.29	0.00	0.00
GATCAACAGGGGTTGCCCGC	0.00	0.00	0.00	0.00	0.00
GATCAACAGGGTCGTATGCC	0.00	0.00	0.00	0.00	0.34
GATCAACAGGGTGCAGGAGA	0.00	0.00	0.00	0.00	0.00
GATCAACAGGGTGGCCCCGA	0.00	0.00	0.00	0.34	0.00
GATCAACAGGGTGGCCCCGG	0.23	0.00	0.29	1.02	0.00
GATCAACAGGGTGTAAGAAA	0.00	0.00	0.29	0.00	0.00
GATCAACAGGGTGTTGCTGG	0.00	0.00	0.29	0.00	0.00
GATCAACAGGGTTAGCGAAT	0.00	0.00	0.29	0.00	0.00
GATCAACAGGTAAATTCACG	0.00	0.33	0.00	0.00	0.00
GATCAACAGGTAGGTTGGTG	0.00	0.33	0.00	0.00	0.00
GATCAACAGGTATTCCAGCG	0.00	0.33	0.00	0.00	0.00
GATCAACAGGTCAATGAGCA	0.23	0.00	0.00	0.00	0.00
GATCAACAGGTCAGGCGCCG	0.23	0.00	1.15	0.00	0.00

GATCAACAGGTCAGGCGNNN	0.00	0.00	0.00	0.00	0.00
GATCAACAGGTCATCCTCTG	0.00	0.00	0.00	0.00	0.00
GATCAACAGGTCCAACAAGA	0.00	0.00	0.29	0.00	0.00
GATCAACAGGTCCCTCACAG	0.69	0.00	0.29	0.00	0.00
GATCAACAGGTCCGGCGGCA	0.23	0.00	0.00	0.00	0.00
GATCAACAGGTCCTCTTCGC	0.00	0.33	0.00	0.00	0.00
GATCAACAGGTCGCCCGCCA	0.00	0.00	0.00	0.00	0.00
GATCAACAGGTCTGTGGGCC	0.00	0.65	0.00	0.00	0.00
GATCAACAGGTCTGTGGGTG	0.23	0.33	0.00	0.34	0.00
GATCAACAGGTCTGTGGGTT	0.00	0.33	0.00	0.00	0.00
GATCAACAGGTCTTGGCTGC	0.00	0.00	0.00	0.34	0.00
GATCAACAGGTGAACAGGCA	0.00	0.65	0.00	0.00	0.00
GATCAACAGGTGAACTCTGC	1.14	0.00	0.00	0.00	0.34
GATCAACAGGTGAATGACTT	0.46	0.00	0.00	0.00	0.00
GATCAACAGGTGCGGCAGCT	0.00	0.33	0.00	0.68	0.00
GATCAACAGGTGCGGCAGGG	0.00	0.00	0.00	0.34	0.00
GATCAACAGGTGCTAAAGGA	0.00	0.00	0.00	0.00	0.00
GATCAACAGGTGGAACTATG	0.00	0.00	0.00	0.00	0.00
GATCAACAGGTGGTAGTCCC	0.92	0.00	0.00	0.00	0.00
GATCAACAGGTGGTCGCGGG	0.00	0.00	0.29	0.00	0.00
GATCAACAGGTGTGAAAAGG	0.00	0.33	0.00	0.00	0.34
GATCAACAGGTGTGAAGAAA	0.00	0.00	0.29	0.00	0.00
GATCAACAGGTGTGGTTTGG	0.00	0.00	0.00	0.00	0.69
GATCAACAGGTGTTCACCCA	0.00	0.00	0.29	0.00	0.00
GATCAACAGGTTCACATATT	0.00	0.00	0.00	0.00	0.34
GATCAACAGGTTCACGAGTG	0.00	0.00	0.00	0.00	0.34
GATCAACAGGTTCATGTGGC	0.23	0.00	0.00	0.00	0.00
GATCAACAGGTTGAGGGTGC	0.23	0.33	0.00	0.34	0.00
GATCAACAGGTTGCCTCGTC	0.00	0.00	0.00	0.00	0.00
GATCAACAGGTTGTCAGCGG	0.00	0.00	0.29	0.00	0.00
GATCAACAGGTTGTCATGAG	0.23	0.00	0.00	0.00	0.00
GATCAACAGGTTGTCTTGGT	0.00	0.00	0.00	0.00	0.00
GATCAACAGGTTTCAGTCAT	0.00	0.00	0.00	0.00	0.00
GATCAACAGGTTTGGGGAGT	0.00	0.00	0.00	0.00	0.69
GATCAACAGGTTTTATAACG	5.95	5.86	3.44	6.11	7.58
GATCAACAGTAAAAAAAAAA	3.20	0.00	0.00	0.00	0.00
GATCAACAGTAAAAAAAAAC	0.23	0.00	0.00	0.00	0.00

GATCAACAGTAAACGATATT	0.00	0.00	0.00	0.00	0.69
GATCAACAGTACATCGAGCC	0.00	0.00	0.29	0.00	0.00
GATCAACAGTACGCATGTTT	0.00	0.33	0.00	0.00	0.00
GATCAACAGTACGCGGAGGT	0.00	0.33	0.00	0.00	0.00
GATCAACAGTACGGAGCTGA	1.14	1.30	0.29	0.00	0.00
GATCAACAGTAGACGGACGA	0.00	0.00	0.00	0.00	0.00
GATCAACAGTAGATGGACGT	0.69	0.65	0.29	0.34	1.72
GATCAACAGTAGCATCAAGT	0.00	0.00	0.00	0.00	0.00
GATCAACAGTAGGAGTGGTT	0.00	0.00	0.00	0.00	0.34
GATCAACAGTAGGGTTTGTT	0.00	0.00	0.29	0.00	0.00
GATCAACAGTAGGTAAGAGT	0.00	0.00	0.00	0.00	0.00
GATCAACAGTAGGTAATAGT	0.00	0.65	2.00	1.36	3.10
GATCAACAGTAGGTTCGTAT	0.00	0.00	0.29	0.00	0.00
GATCAACAGTAGTGGCACAG	0.00	0.33	0.00	0.00	0.00
GATCAACAGTAGTGGTGTTG	0.00	0.00	0.00	0.00	0.00
GATCAACAGTATATCTACAG	0.23	0.65	0.29	0.34	0.69
GATCAACAGTATATTTACAG	0.00	0.00	0.00	0.00	0.00
GATCAACAGTATCGTCATCT	0.00	0.33	0.00	0.00	0.00
GATCAACAGTATCTTGGCGG	0.00	0.33	0.00	0.00	0.00
GATCAACAGTATGGCACTTT	0.00	0.00	0.86	0.00	0.00
GATCAACAGTCAAAAAAAAA	0.00	0.00	0.00	0.00	0.00
GATCAACAGTCAAAACTTGG	0.00	0.00	0.00	0.00	0.00
GATCAACAGTCAATTCATCG	0.00	0.33	0.00	0.00	0.00
GATCAACAGTCACCAGATGC	0.00	0.00	0.29	0.00	0.00
GATCAACAGTCACTTGCGTG	2.29	0.00	0.29	0.34	0.00
GATCAACAGTCATATCCCGT	0.69	0.33	0.86	0.68	0.69
GATCAACAGTCATATGTCAA	0.00	0.00	0.00	0.68	0.00
GATCAACAGTCATCATTGCG	0.00	0.33	0.00	0.00	0.00
GATCAACAGTCATCATTGCT	4.12	4.55	2.00	6.45	3.79
GATCAACAGTCATCGAAAAA	0.00	0.00	0.29	0.00	0.00
GATCAACAGTCATCGCTAAA	0.23	0.00	0.00	0.00	0.00
GATCAACAGTCATCGCTAAT	0.23	0.00	0.00	0.00	0.00
GATCAACAGTCATCGGAATG	0.00	0.00	0.00	0.00	0.00
GATCAACAGTCATCTCATGG	0.00	0.00	0.00	0.00	0.00
GATCAACAGTCCAAAAAAAA	0.23	0.00	0.29	0.00	0.00
GATCAACAGTCCAAAGCAAA	0.46	0.00	0.29	0.68	0.00
GATCAACAGTCCAAAGCAAG	0.69	0.00	0.00	0.00	0.00

GATCAACAGTCCCCGCTTCT	0.00	0.00	0.00	0.00	0.00
GATCAACAGTCCCGCATGTG	0.00	0.00	0.00	0.00	0.69
GATCAACAGTCCCTTTGCAT	0.00	0.00	0.86	0.00	0.00
GATCAACAGTCCGAGTGCAT	0.00	0.33	0.00	0.00	0.00
GATCAACAGTCCGCGCCAAA	0.69	0.33	0.57	0.68	0.00
GATCAACAGTCCGCGCCCNN	0.23	0.00	0.00	0.00	0.00
GATCAACAGTCCGCGGAAAG	0.23	0.00	0.00	0.00	0.00
GATCAACAGTCCGGCAGCTG	0.92	1.63	1.15	1.36	0.00
GATCAACAGTCCTAGGAATG	0.23	0.00	0.00	0.00	0.00
GATCAACAGTCCTCGGAAAA	0.00	0.00	0.00	0.00	1.03
GATCAACAGTCCTCGGAAAG	0.00	0.00	0.00	0.34	0.00
GATCAACAGTCCTCGGAACA	0.00	0.00	0.29	0.00	0.00
GATCAACAGTCCTCGGAANN	0.23	0.00	0.00	0.00	0.00
GATCAACAGTCCTCGGAATC	0.00	0.65	0.00	0.00	0.00
GATCAACAGTCCTCGGAATG	40.97	39.69	23.77	36.66	21.70
GATCAACAGTCCTCGGAATN	0.00	0.00	0.00	0.34	0.00
GATCAACAGTCCTCGGACAA	0.00	0.00	0.29	0.00	0.00
GATCAACAGTCCTCGGATGA	0.46	0.00	0.00	0.00	0.00
GATCAACAGTCCTCGGCATG	0.23	0.00	0.00	0.00	0.00
GATCAACAGTCCTCGGCCTG	0.46	0.00	0.00	0.00	0.00
GATCAACAGTCCTCGGCGTG	0.23	0.00	0.00	0.00	0.00
GATCAACAGTCCTCGGGATG	0.46	0.33	0.00	0.00	0.00
GATCAACAGTCCTGCACAAC	0.00	0.00	0.57	0.00	0.00
GATCAACAGTCCTTGTCGTT	0.00	0.00	0.00	0.00	0.34
GATCAACAGTCGAATGTTGG	0.00	0.00	0.00	0.00	0.00
GATCAACAGTCGCAGAAGTC	0.00	0.00	0.29	0.00	0.00
GATCAACAGTCGCTTCGACC	0.00	0.98	0.00	0.00	0.00
GATCAACAGTCGGAGAATGT	0.00	0.00	0.00	0.00	0.34
GATCAACAGTCGGCAGTCCA	0.00	0.00	0.00	0.00	0.00
GATCAACAGTCGTATGCCGT	0.00	0.00	0.29	0.00	0.00
GATCAACAGTCGTCTGCCTT	0.23	0.00	0.00	0.00	0.00
GATCAACAGTCTGAACGCCA	0.00	0.00	0.00	0.00	0.34
GATCAACAGTCTGCAGAACA	0.00	0.00	0.00	0.34	0.00
GATCAACAGTCTGCAGGCCA	0.23	0.00	0.00	0.00	0.00
GATCAACAGTCTGCAGTCCA	10.99	15.29	17.75	19.01	14.46
GATCAACAGTCTGCAGTCCC	0.00	0.00	0.29	0.68	0.00
GATCAACAGTCTGCAGTCCN	0.00	0.00	0.00	0.00	0.00

GATCAACAGTCTGCAGTCNN	0.00	0.00	0.00	0.00	0.00
GATCAACAGTCTGCAGTTCA	0.00	0.00	0.00	0.00	0.00
GATCAACAGTCTGCCGTCCC	0.00	0.00	0.00	0.00	0.00
GATCAACAGTCTGCCGTTCA	0.00	0.00	0.00	0.00	0.00
GATCAACAGTCTGCGGTCCA	0.00	0.00	0.00	0.00	0.00
GATCAACAGTCTGCTCGTAT	0.00	0.00	0.00	0.34	0.00
GATCAACAGTCTGGAATGGA	0.46	0.00	0.00	0.00	0.00
GATCAACAGTCTTCTACCCA	0.00	0.33	0.00	0.00	0.00
GATCAACAGTCTTGATTTGT	0.00	0.00	0.29	0.00	0.00
GATCAACAGTCTTGTCGTTG	0.00	0.00	0.29	0.00	0.00
GATCAACAGTGAAAAAAAAA	0.46	0.00	0.29	0.00	0.00
GATCAACAGTGAAGACCTAC	0.00	0.00	0.00	0.34	0.00
GATCAACAGTGAAGGCAGGG	0.00	0.33	0.00	0.00	0.00
GATCAACAGTGACCAGCTGG	0.00	0.33	0.00	0.00	0.00
GATCAACAGTGACGAGGATG	0.00	0.00	0.00	0.34	0.69
GATCAACAGTGACGCTTCGG	0.23	0.00	0.00	0.00	0.00
GATCAACAGTGACTGAGCGC	0.00	0.33	0.00	0.00	0.00
GATCAACAGTGAGCAGCGTT	0.00	0.00	0.00	0.00	0.34
GATCAACAGTGAGCATAAGA	0.46	0.00	0.00	0.68	0.00
GATCAACAGTGAGCGATGGG	0.00	0.00	0.00	0.34	0.00
GATCAACAGTGATGGCCTCT	0.00	0.00	0.00	0.34	0.00
GATCAACAGTGATGGCTTCT	0.00	0.00	0.00	0.00	0.00
GATCAACAGTGATGTTCTGG	0.00	0.33	0.00	0.00	0.00
GATCAACAGTGATTTTTTCT	1.60	0.00	0.00	0.34	0.69
GATCAACAGTGCAACCATGG	0.00	0.00	0.29	0.00	0.00
GATCAACAGTGCAATCGCCC	0.46	0.00	0.00	0.00	0.00
GATCAACAGTGCACAAAGCG	0.00	0.00	0.00	0.00	0.00
GATCAACAGTGCACCTCTGC	0.23	0.00	0.57	0.68	0.69
GATCAACAGTGCACTCACGA	0.00	0.00	0.29	0.00	0.00
GATCAACAGTGCATAGACAG	0.00	0.00	0.00	0.00	0.00
GATCAACAGTGCATCATCTC	0.00	0.00	0.00	0.00	1.38
GATCAACAGTGCATGAGGTT	0.00	0.00	0.57	0.00	0.00
GATCAACAGTGCCAACCTGC	0.00	0.00	0.00	0.00	0.00
GATCAACAGTGCCAAGAAGG	0.00	0.00	0.00	0.00	0.00
GATCAACAGTGCCACCTGTT	0.00	0.00	0.00	0.34	0.00
GATCAACAGTGCCAGAATCA	0.00	0.00	0.00	0.00	0.00
GATCAACAGTGCCATCAAGT	0.00	0.00	0.00	0.68	0.00

GATCAACAGTGCCTGTTTGC	0.23	0.00	0.00	0.00	0.00
GATCAACAGTGCGAACCCTG	0.46	0.33	0.29	0.34	1.38
GATCAACAGTGCGCAAAGCG	0.23	0.00	0.29	0.00	0.00
GATCAACAGTGCGCGCCCCC	0.23	0.00	0.00	0.00	0.00
GATCAACAGTGCGTGCCATC	0.23	0.00	0.00	0.00	0.00
GATCAACAGTGCTGAGTCCT	0.00	0.00	0.00	0.00	0.34
GATCAACAGTGCTGATAAAA	0.23	0.00	0.00	0.34	0.00
GATCAACAGTGCTTCAGACC	0.00	0.00	0.00	0.34	0.00
GATCAACAGTGCTTGCAACT	0.23	0.00	0.00	0.00	0.00
GATCAACAGTGCTTGCACCA	0.00	0.00	0.00	0.34	0.00
GATCAACAGTGCTTGCCAAA	0.00	0.00	0.00	0.00	0.69
GATCAACAGTGCTTGCCATC	24.26	10.74	5.44	14.60	8.27
GATCAACAGTGCTTGCCATG	0.00	0.00	0.00	0.00	0.34
GATCAACAGTGCTTGCCATN	0.23	0.00	0.00	0.00	0.00
GATCAACAGTGCTTGCCCAG	0.00	0.00	0.00	0.00	0.69
GATCAACAGTGCTTGCCCTC	0.23	0.00	0.29	0.00	0.00
GATCAACAGTGCTTGCCTTC	0.00	0.00	0.00	0.00	0.00
GATCAACAGTGCTTGCGTGA	0.00	0.33	0.00	0.00	0.00
GATCAACAGTGCTTGCNNNN	0.23	0.00	0.00	0.00	0.00
GATCAACAGTGCTTGGGCCT	0.00	0.33	0.00	0.00	0.00
GATCAACAGTGCTTGTGTGT	0.00	0.00	0.29	0.00	0.00
GATCAACAGTGGAACATGAA	0.69	0.65	0.86	3.39	0.69
GATCAACAGTGGAAGGAGAG	0.23	0.00	0.00	0.00	0.00
GATCAACAGTGGACGGAGAG	6.87	15.62	6.30	9.50	7.23
GATCAACAGTGGACGGAGAN	0.23	0.00	0.00	0.00	0.00
GATCAACAGTGGACGGAGCG	0.46	0.00	0.29	0.00	0.00
GATCAACAGTGGACGGAGGG	0.92	0.00	0.00	0.34	0.00
GATCAACAGTGGACGGAGNG	0.00	0.00	0.00	0.00	0.00
GATCAACAGTGGACGGCGAG	0.00	0.33	0.00	0.00	0.34
GATCAACAGTGGACGGGGAG	0.23	0.00	0.00	0.00	0.00
GATCAACAGTGGACGGNGGG	0.00	0.00	0.00	0.00	0.34
GATCAACAGTGGACGGNNNN	0.23	0.00	0.00	0.00	0.00
GATCAACAGTGGAGAAATGC	0.00	0.00	0.00	0.34	0.00
GATCAACAGTGGAGGAAAGG	0.00	0.00	0.00	0.00	0.00
GATCAACAGTGGCAACAGCG	0.00	0.33	0.00	0.00	0.00
GATCAACAGTGGCAGCAAGC	0.23	0.00	0.00	0.00	0.00
GATCAACAGTGGCAGCAAGT	0.69	0.00	0.00	0.00	0.00

GATCAACAGTGGCATAAAGT	0.23	0.00	0.29	0.34	0.00
GATCAACAGTGGCATAACGA	0.00	0.00	0.00	0.34	0.00
GATCAACAGTGGCATCAACA	0.00	0.00	0.00	0.00	0.00
GATCAACAGTGGCATCAAGA	0.00	0.00	0.29	0.68	0.00
GATCAACAGTGGCATCAAGC	0.69	0.65	0.57	0.68	0.69
GATCAACAGTGGCATCAAGG	1.14	0.65	0.57	0.68	0.34
GATCAACAGTGGCATCAAGN	0.69	0.00	0.00	0.00	0.00
GATCAACAGTGGCATCAAGT	177.63	190.32	123.71	155.46	130.8[illegible]
GATCAACAGTGGCATCAANT	0.00	0.00	0.29	0.34	0.00
GATCAACAGTGGCATCAATA	0.00	0.33	0.00	0.00	0.00
GATCAACAGTGGCATCAATT	0.00	0.33	0.00	0.00	0.00
GATCAACAGTGGCATCACGT	0.46	0.65	0.86	0.34	0.00
GATCAACAGTGGCATCACNN	0.46	0.00	0.00	0.00	0.00
GATCAACAGTGGCATCAGGT	0.00	0.65	0.00	0.00	0.34
GATCAACAGTGGCATCANGT	0.00	0.00	0.00	0.34	0.00
GATCAACAGTGGCATCATGT	0.23	0.00	0.00	0.34	0.00
GATCAACAGTGGCATCCAGT	0.23	1.63	0.57	0.00	0.34
GATCAACAGTGGCATCGAGT	0.69	0.00	0.00	0.00	0.00
GATCAACAGTGGCATCGTAT	0.00	0.00	0.00	0.00	0.69
GATCAACAGTGGCATCNAGT	0.00	0.00	0.29	0.00	0.69
GATCAACAGTGGCATCNNNN	0.69	0.00	0.00	0.00	0.00
GATCAACAGTGGCCACATGC	0.00	0.00	0.00	0.00	0.00
GATCAACAGTGGCCCCATAC	0.00	0.33	0.00	0.00	0.00
GATCAACAGTGGCCTCAAGA	0.00	0.33	0.00	0.00	0.00
GATCAACAGTGGCCTCNNGT	0.00	0.00	0.00	0.34	0.00
GATCAACAGTGGCCTGCCGG	0.00	0.00	0.00	0.68	0.00
GATCAACAGTGGCGAATGGT	0.23	0.00	0.29	0.00	0.00
GATCAACAGTGGCGAGCATG	0.00	0.33	0.00	0.00	0.00
GATCAACAGTGGCGAGGACG	0.00	0.00	0.00	0.00	0.00
GATCAACAGTGGCGCCATCA	0.00	0.00	0.29	0.00	0.00
GATCAACAGTGGCGTCAAGT	0.23	0.00	0.00	0.00	0.00
GATCAACAGTGGCGTCGAGT	0.23	0.00	0.00	0.00	0.00
GATCAACAGTGGCTTTGTCG	0.00	0.00	0.00	0.00	0.00
GATCAACAGTGGGATCAAGG	0.23	0.00	0.00	0.00	0.00
GATCAACAGTGGGGGAAAGG	0.46	0.33	0.29	0.00	0.00
GATCAACAGTGGTAGAAAAA	0.00	0.00	0.00	0.68	0.00
GATCAACAGTGGTAGTAAGG	0.23	0.00	0.00	0.00	0.00

GATCAACAGTGGTATCAAGG	0.00	0.00	0.29	0.00	0.00
GATCAACAGTGGTATCAAGT	10.30	7.16	6.01	5.09	4.13
GATCAACAGTGGTATCTCGT	0.00	0.33	0.00	0.00	0.00
GATCAACAGTGGTCGTATGC	0.00	0.00	0.00	0.68	0.00
GATCAACAGTGGTGCATGGA	0.00	0.00	0.29	0.00	0.00
GATCAACAGTGGTGGAAAAA	0.69	0.65	0.29	1.70	0.69
GATCAACAGTGGTGGAAAAG	0.00	0.00	0.29	0.00	0.00
GATCAACAGTGGTGGAAAGA	0.00	0.00	0.00	0.00	0.34
GATCAACAGTGGTGGAAAGG	56.54	74.50	47.25	63.81	58.20
GATCAACAGTGGTGGAAANG	0.00	0.00	0.29	0.00	0.00
GATCAACAGTGGTGGAAATG	0.00	0.00	0.29	0.00	0.00
GATCAACAGTGGTGGAACGG	0.23	0.00	0.00	0.34	0.34
GATCAACAGTGGTGGAAGGG	0.23	0.65	0.29	0.00	0.00
GATCAACAGTGGTGGACAGG	0.69	0.00	0.00	0.34	0.00
GATCAACAGTGGTGGACNNN	0.23	0.00	0.00	0.00	0.00
GATCAACAGTGGTGGAGAGG	0.46	0.00	0.29	0.00	0.00
GATCAACAGTGGTGGANNNN	0.23	0.00	0.00	0.00	0.00
GATCAACAGTGGTGGATAGG	0.23	0.00	0.00	0.00	0.00
GATCAACAGTGGTGGGAAGG	0.69	0.00	0.00	0.00	0.69
GATCAACAGTGGTGGGGAGG	0.23	0.00	0.00	0.00	0.00
GATCAACAGTGGTGGTAAGG	0.00	0.00	0.00	0.00	0.00
GATCAACAGTGGTGGTGGGG	0.00	0.00	0.00	0.00	0.34
GATCAACAGTGGTTAAAAGG	0.00	0.00	0.00	0.00	0.00
GATCAACAGTGGTTGAAAAG	0.00	0.00	0.00	0.00	0.00
GATCAACAGTGTACAGGCTG	0.00	0.00	0.00	0.00	0.00
GATCAACAGTGTCAGTGCCG	0.00	1.30	0.29	0.00	0.00
GATCAACAGTGTCATGTCAT	0.00	0.00	0.00	0.00	0.00
GATCAACAGTGTCATTGTCA	0.23	0.00	0.00	0.00	0.00
GATCAACAGTGTCGACTCGT	0.23	0.00	0.00	0.00	0.00
GATCAACAGTGTCGGCATGG	0.00	0.00	0.00	0.00	0.00
GATCAACAGTGTGCACGTGC	0.00	0.00	0.00	0.00	0.34
GATCAACAGTGTGGCCCGGA	0.00	0.00	0.00	0.00	0.00
GATCAACAGTGTTGACGTCG	0.00	0.00	0.86	0.00	0.00
GATCAACAGTTACAACTACA	0.00	0.33	0.00	0.00	0.00
GATCAACAGTTCAGTCATCA	0.46	0.00	0.00	0.00	0.69
GATCAACAGTTCCGATCCAG	0.00	0.65	0.00	0.34	0.00
GATCAACAGTTCCTCGCTGA	0.00	0.65	0.00	0.00	1.03

GATCAACAGTTCGGACTGAC	0.00	0.00	0.00	0.00	0.00
GATCAACAGTTCGTATGCCG	0.23	0.00	0.00	0.00	0.00
GATCAACAGTTCTCCGTCTT	0.00	0.98	0.29	0.00	0.00
GATCAACAGTTGAAAACGAC	0.00	0.00	0.00	0.00	0.00
GATCAACAGTTGAACGTTTA	0.00	0.00	0.29	0.00	0.00
GATCAACAGTTGAGCCTCAA	0.00	0.00	0.29	0.00	0.00
GATCAACAGTTGATTATGAG	0.00	0.33	0.00	0.00	0.00
GATCAACAGTTGCAGAATTT	0.00	0.00	0.00	0.00	0.00
GATCAACAGTTGCATGCGTT	0.23	0.00	0.29	0.34	0.00
GATCAACAGTTGGATATCAA	0.00	0.00	0.00	0.00	0.34
GATCAACAGTTGGCGAGTCC	0.46	0.00	0.00	0.00	0.00
GATCAACAGTTGTAGAATTT	0.23	0.00	0.00	0.00	0.00
GATCAACAGTTGTGCATCGT	0.00	0.00	0.29	0.00	0.00
GATCAACAGTTGTTCTGTTC	0.46	0.00	0.00	0.00	0.00
GATCAACAGTTTAGGAGCGC	0.92	0.00	0.00	0.00	0.00
GATCAACAGTTTCTTCCCAG	0.00	0.00	0.00	0.00	0.34
GATCAACAGTTTGACACTAT	0.92	0.00	0.00	0.68	0.00
GATCAACAGTTTTAGGTTGT	0.00	0.00	0.00	0.00	0.34
GATCAACAGTTTTCAGGATG	0.92	0.98	0.86	0.34	1.38
GATCAACATAAAAAAAAAAA	2.75	0.98	0.00	0.00	1.38
GATCAACATAAAAAAGTAAA	0.23	0.00	0.00	0.00	0.00
GATCAACATAAAACAATTGC	0.00	0.00	0.29	0.00	0.00
GATCAACATAAAAGGCCAGG	0.00	0.00	0.00	0.00	0.34
GATCAACATAACAATAATCG	0.00	0.00	0.00	0.00	0.69
GATCAACATAACACATAACA	0.00	0.00	0.00	0.00	0.34
GATCAACATAACACCTGCGG	0.00	0.00	0.29	0.00	0.00
GATCAACATAACAGCGAACA	0.00	0.00	0.00	0.00	0.34
GATCAACATAACAGGAGTGG	0.00	0.00	0.00	0.00	0.00
GATCAACATAACATATGGTG	0.00	0.00	0.00	1.02	0.00
GATCAACATAACGATAATAG	0.00	0.00	0.00	0.00	0.34
GATCAACATAAGAAATCAAA	0.00	0.00	0.29	0.00	0.00
GATCAACATAAGAACACGGT	0.00	0.00	0.29	0.00	0.00
GATCAACATAAGAAGAAGAA	0.00	0.00	0.57	0.00	0.00
GATCAACATAAGAAGAAGAG	0.00	0.00	0.57	0.00	1.38
GATCAACATAAGACATAACA	0.00	0.00	0.00	0.00	0.00
GATCAACATAAGACATCACA	0.69	2.60	6.01	4.07	6.54
GATCAACATAAGACCCTGAG	0.00	0.00	0.00	0.00	0.69

GATCAACATAAGATGCAGAC	0.00	0.00	0.29	0.00	0.34
GATCAACATAAGCCGGGGCG	0.00	0.00	0.00	0.00	0.00
GATCAACATAAGCCGTTACT	0.00	0.00	0.00	0.00	0.69
GATCAACATAAGCTTCTGCT	0.00	0.00	0.00	0.00	0.00
GATCAACATAAGGAAAGGTG	0.00	0.00	0.00	0.00	0.34
GATCAACATAAGGAGACAGA	0.00	0.00	0.00	0.00	0.69
GATCAACATAAGGTATCGGG	0.46	3.25	2.29	2.72	1.38
GATCAACATAAGTGACATTG	0.00	0.00	0.00	0.00	0.00
GATCAACATAATATTCAAAA	0.00	0.00	0.00	0.00	0.34
GATCAACATAATGCCCCTAG	0.00	0.00	0.29	0.00	0.00
GATCAACATAATGTGTGTTT	0.00	0.00	0.29	0.00	0.00
GATCAACATAATTTTTGCTG	0.00	0.00	0.29	0.00	0.34
GATCAACATACAACGGACAA	0.00	0.00	0.00	0.00	1.03
GATCAACATACACATTCTGC	0.00	0.00	0.86	0.00	0.00
GATCAACATACACCACGCGG	0.00	0.00	0.00	0.00	0.34
GATCAACATACACCCACTTG	0.00	0.00	0.29	0.00	0.00
GATCAACATACACGCAGACA	0.00	0.00	0.29	0.00	0.00
GATCAACATACAGCGAACGC	0.23	0.00	0.29	0.00	0.34
GATCAACATACCATACCCCT	0.00	0.00	0.00	0.34	0.00
GATCAACATACCCGACCGCA	0.00	0.00	0.00	0.00	0.69
GATCAACATACCCTACCCCT	0.00	0.00	0.00	0.00	0.00
GATCAACATACCGATTGAAA	0.00	0.00	0.00	0.00	0.00
GATCAACATACCGTGCAGCA	0.00	0.00	0.00	0.00	0.00
GATCAACATACGAAGAAGAA	0.00	0.00	0.00	0.00	0.69
GATCAACATACGCGCCTTCA	0.00	0.00	0.00	0.00	0.00
GATCAACATACGCTGGCAGA	0.23	0.00	0.00	0.00	0.00
GATCAACATACGGAAATCCG	0.00	0.00	0.29	0.00	0.00
GATCAACATACGGAGCTAAC	0.00	0.00	0.00	0.00	0.00
GATCAACATACTAACAGAGA	0.00	0.00	0.00	0.00	0.69
GATCAACATACTTCCGCCTC	0.00	0.00	0.29	0.00	0.00
GATCAACATAGAAACGATGC	0.00	0.00	0.00	0.00	0.00
GATCAACATAGAACGTAGGA	0.00	0.00	0.00	0.34	0.00
GATCAACATAGAAGAAGAGG	0.00	0.00	0.00	0.34	0.00
GATCAACATAGAAGAGACCG	0.00	0.00	0.00	0.68	0.00
GATCAACATAGACAAAAAAA	0.00	0.00	0.00	0.00	0.00
GATCAACATAGACGAGCGTC	0.00	0.33	0.00	0.00	0.00
GATCAACATAGACGCCATGC	0.00	0.00	0.00	0.00	0.00

GATCAACATAGAGTGCTTCA	8.24	3.90	1.43	3.05	1.38
GATCAACATAGATGAGACTG	0.00	0.33	0.00	0.00	0.00
GATCAACATAGATGCCGGCC	0.00	0.00	0.29	0.00	0.00
GATCAACATAGCACGGGCAA	0.00	0.00	0.00	0.00	0.34
GATCAACATAGCATCACACC	0.00	0.00	0.00	0.00	0.34
GATCAACATAGCCAACTGCG	0.00	0.00	0.00	0.00	0.00
GATCAACATAGCCCGTCTGA	0.00	0.00	0.00	0.00	0.00
GATCAACATAGGAACTTATT	0.00	0.00	0.29	0.00	0.00
GATCAACATAGGCTTCAACA	0.00	0.00	0.00	0.00	0.00
GATCAACATAGTAACCGTCG	0.00	0.00	0.00	0.00	0.34
GATCAACATAGTCTTCAACA	9.16	1.95	1.15	1.36	1.03
GATCAACATAGTGAACATGT	0.00	0.00	0.00	0.00	0.69
GATCAACATATAAGAGTTGC	0.23	0.00	0.00	0.00	0.00
GATCAACATATAAGGTTTTA	0.00	0.00	0.00	0.00	0.34
GATCAACATATACATCAGGC	0.00	0.33	0.00	0.00	0.00
GATCAACATATACCATCTGG	0.00	0.00	0.00	0.00	0.00
GATCAACATATATGCTGGTA	0.00	0.00	0.00	0.00	0.00
GATCAACATATATTATGGGA	0.00	0.00	0.29	0.00	0.00
GATCAACATATCACTGCTGA	0.46	0.00	0.00	0.00	0.00
GATCAACATATCACTGCTGN	0.23	0.00	0.00	0.00	0.00
GATCAACATATCAGCCGCCA	0.00	0.00	0.00	0.00	0.00
GATCAACATATCAGTAAGAA	0.46	0.00	0.00	0.00	0.00
GATCAACATATCGACCGCGC	0.00	0.65	0.00	0.00	0.00
GATCAACATATGAGCCTCAA	0.23	0.00	0.00	0.00	0.00
GATCAACATATGCAATGGCC	0.00	0.00	0.00	0.00	0.34
GATCAACATATGCGCTCGTT	2.29	1.95	1.15	2.38	0.34
GATCAACATATGCGGCGAGG	0.00	0.00	0.29	0.00	0.00
GATCAACATATGCGGGGGGT	0.23	0.00	0.00	0.00	0.00
GATCAACATATGCGGTCGGT	0.00	0.33	0.00	0.00	0.00
GATCAACATATGCGGTGGGG	1.14	0.00	0.29	0.00	0.00
GATCAACATATGCGGTGGGT	12.13	12.69	14.32	12.90	9.30
GATCAACATATGCGGTGGTG	0.00	0.00	0.57	0.00	0.00
GATCAACATATGCGGTTGCT	0.00	0.00	0.29	0.00	0.00
GATCAACATATGCGGTTGGT	0.23	0.00	0.00	0.00	0.00
GATCAACATATGCTATCTTA	0.00	0.00	0.29	0.00	0.00
GATCAACATATGCTCGAAGG	0.00	0.33	0.00	0.00	0.00
GATCAACATATGTCTGAGGT	0.00	0.00	0.00	0.00	0.00

GATCAACATATGTTAAGAAC	0.23	0.00	0.00	0.00	0.00
GATCAACATATTCATATTAT	0.23	0.00	0.00	0.00	0.00
GATCAACATATTCGCATCCC	0.00	0.00	0.00	0.00	0.00
GATCAACATATTTGCCAGAC	0.00	0.00	0.00	0.00	0.34
GATCAACATATTTGCTCGGG	0.00	0.00	0.00	0.00	0.34
GATCAACATCAAAAAAAAAA	2.75	0.33	0.57	1.70	0.00
GATCAACATCAAAAAAAAAT	0.00	0.33	0.00	0.00	0.00
GATCAACATCAAAAAAGTCA	0.00	0.00	0.00	0.00	0.00
GATCAACATCAAAAAATCAC	0.00	0.00	0.86	0.00	0.00
GATCAACATCAAAAAGAAAC	0.00	0.00	0.57	0.00	0.00
GATCAACATCAAAAAGCTAA	0.46	0.00	0.00	0.00	0.00
GATCAACATCAAAAAGCTGC	0.00	0.00	0.00	0.00	0.00
GATCAACATCAAAAAGTCAA	0.00	0.00	0.00	0.34	0.00
GATCAACATCAAAAAGTCAC	2.98	5.53	5.44	8.49	3.79
GATCAACATCAAAAAGTCAG	0.00	0.00	0.00	0.34	0.00
GATCAACATCAAAAAGTTGC	0.00	0.00	0.29	0.00	0.00
GATCAACATCAAAAGGTCAC	0.00	0.00	0.00	0.00	0.00
GATCAACATCAAAGAAAGCA	0.23	0.00	0.00	0.00	0.34
GATCAACATCAAATCCATGG	0.00	0.00	0.57	0.00	0.00
GATCAACATCAACAAACAAC	0.00	0.00	0.29	0.00	0.00
GATCAACATCAACAACAACA	0.00	0.00	0.29	0.00	1.38
GATCAACATCAACAACAGGA	0.00	0.00	0.29	0.00	0.34
GATCAACATCAACAATCGAA	0.00	0.00	0.86	0.00	0.69
GATCAACATCAACACCCAGA	0.00	0.00	0.29	0.00	0.00
GATCAACATCAACACGCCCT	0.00	0.00	0.29	0.00	0.00
GATCAACATCAACACGTCCA	0.00	0.00	0.29	0.00	0.00
GATCAACATCAACAGAAGAA	0.00	0.00	3.15	0.00	3.10
GATCAACATCAACAGAAGAT	0.00	0.00	0.29	0.00	0.34
GATCAACATCAACAGCAACA	0.00	0.00	0.00	0.00	0.34
GATCAACATCAACAGCACAG	0.00	0.00	0.00	0.00	0.34
GATCAACATCAACAGCATCA	0.00	0.00	0.00	0.00	0.00
GATCAACATCAACAGGAGAA	0.00	0.00	0.29	0.00	0.00
GATCAACATCAACATCAACA	0.00	0.00	0.00	0.00	0.34
GATCAACATCAACATCAACG	0.00	0.00	0.29	0.00	0.00
GATCAACATCAACATCAATC	0.92	0.00	0.57	0.00	0.00
GATCAACATCAACATCAGCT	0.23	0.00	0.00	0.00	0.00
GATCAACATCAACCACAACT	0.00	0.00	0.00	0.00	0.00

GATCAACATCAACCCTCTGC	0.23	0.00	0.00	0.00	0.00
GATCAACATCAACCGTAGCC	0.00	0.00	0.00	0.00	0.69
GATCAACATCAACGAAAAAA	0.23	0.00	0.00	0.00	0.00
GATCAACATCAACGAAAGAA	0.00	0.00	0.00	0.00	0.34
GATCAACATCAACGGTCGCA	0.00	0.00	0.00	0.00	0.34
GATCAACATCAACGTTGTTG	0.00	0.00	0.00	0.34	0.00
GATCAACATCAACTAAAACA	0.00	0.00	0.29	0.00	0.00
GATCAACATCAACTGAAGGC	0.00	0.00	0.00	0.00	0.00
GATCAACATCAAGAACCACA	0.00	0.00	0.00	0.00	0.00
GATCAACATCAAGAAGTTGA	1.14	0.98	0.00	0.34	0.00
GATCAACATCAAGAGCTTGC	0.00	0.00	0.00	0.00	0.00
GATCAACATCAAGATGCCCA	0.00	0.00	0.29	0.00	0.00
GATCAACATCAAGCAATGAG	0.00	0.00	0.00	0.34	0.00
GATCAACATCAAGCACACAT	0.00	0.00	0.00	0.34	0.00
GATCAACATCAAGCACCACA	24.26	20.17	4.30	7.13	5.85
GATCAACATCAAGCACCACG	0.00	0.00	0.29	0.00	0.00
GATCAACATCAAGCACCACN	0.23	0.00	0.00	0.00	0.00
GATCAACATCAAGCACCCNN	0.23	0.00	0.00	0.00	0.00
GATCAACATCAAGCACNNNN	0.23	0.00	0.00	0.00	0.00
GATCAACATCAAGCAGCACA	0.00	0.00	0.00	0.34	0.00
GATCAACATCAAGCATCAAG	0.46	0.98	0.29	0.34	0.34
GATCAACATCAAGGCTCTGC	0.00	0.33	0.00	0.00	0.00
GATCAACATCAAGGTCCAAC	0.00	0.00	0.57	0.00	0.00
GATCAACATCAATGACCCTA	0.00	0.00	0.00	0.34	0.00
GATCAACATCAATGAGGACT	0.00	0.00	0.00	0.00	0.00
GATCAACATCACAAACGGCG	0.00	0.00	0.00	0.00	0.69
GATCAACATCACAAAGGAAA	0.69	0.00	0.00	0.00	3.44
GATCAACATCACAACACCAC	0.00	0.00	0.00	0.00	0.34
GATCAACATCACAATCATCA	0.00	0.00	0.57	0.00	0.00
GATCAACATCACACACACAC	0.00	0.00	0.00	0.00	0.34
GATCAACATCACACACCAAC	0.00	0.00	0.00	0.00	0.34
GATCAACATCACACATATGA	0.00	0.00	0.29	0.00	0.00
GATCAACATCACACCGGTTT	0.00	0.00	0.00	0.00	0.34
GATCAACATCACACCTAGGA	0.23	0.00	0.00	0.00	0.00
GATCAACATCACACCTATGA	18.31	11.06	12.03	8.49	13.78
GATCAACATCACACCTATGG	0.00	0.00	0.00	0.34	0.00
GATCAACATCACACCTATGT	0.00	0.00	0.00	0.00	0.69

GATCAACATCACACCTCNNN	0.23	0.00	0.00	0.00	0.00
GATCAACATCACACCTCTNN	0.23	0.00	0.00	0.00	0.00
GATCAACATCACACGGCACA	0.00	0.00	0.29	0.00	0.00
GATCAACATCACAGAAAGAG	0.00	0.33	0.00	0.00	0.00
GATCAACATCACAGGTGATG	8.24	5.21	3.15	5.43	4.13
GATCAACATCACAGGTGATT	0.46	0.00	0.00	0.00	0.00
GATCAACATCACAGGTGCTG	0.00	0.00	0.00	0.00	0.00
GATCAACATCACAGGTTCGT	0.00	0.00	0.00	0.00	0.69
GATCAACATCACAGTCTTCG	0.00	0.00	0.00	0.00	0.34
GATCAACATCACATCACATC	0.00	0.00	0.00	0.00	0.34
GATCAACATCACATCTAAGC	0.00	0.00	0.00	0.00	1.03
GATCAACATCACCAACAACA	0.00	0.00	0.00	0.00	0.34
GATCAACATCACCAACTCGC	0.00	0.00	0.00	0.00	0.34
GATCAACATCACCAGCAAGG	0.46	0.00	0.57	0.00	0.69
GATCAACATCACCATCACCA	0.00	0.00	0.00	0.00	0.00
GATCAACATCACCATGATTC	0.00	0.00	0.29	0.00	0.00
GATCAACATCACCCATCTGA	0.00	0.00	0.00	0.00	0.34
GATCAACATCACCTACAAGT	0.00	0.00	0.29	0.00	0.00
GATCAACATCACCTTCAACA	0.23	0.00	0.00	0.00	0.69
GATCAACATCACCTTCAACC	0.00	0.00	0.00	0.00	1.03
GATCAACATCACCTTGAGAG	0.00	0.33	0.00	0.00	0.00
GATCAACATCACCTTGAGCG	9.39	2.93	2.00	6.45	2.41
GATCAACATCACCTTTGGCT	0.00	0.00	0.00	0.34	0.00
GATCAACATCACGAAGGAGA	3.43	3.58	3.44	5.43	3.44
GATCAACATCACGAAGGAGG	0.23	0.00	0.00	0.00	0.00
GATCAACATCACGAAGGCGA	0.00	0.33	0.00	0.00	0.00
GATCAACATCACGAGCAATA	0.00	0.00	0.00	0.00	0.00
GATCAACATCACGGACATCG	0.00	0.00	0.00	1.36	0.00
GATCAACATCACGGAGGAGT	0.00	0.00	0.00	0.00	0.00
GATCAACATCACGGGCTTCT	0.00	0.00	0.29	0.00	0.00
GATCAACATCACGGTGCCCG	0.00	0.00	0.00	0.34	0.00
GATCAACATCACGGTGTTGA	0.00	0.00	0.00	0.34	0.00
GATCAACATCACGTGCCTCG	0.00	0.00	0.29	0.00	0.00
GATCAACATCACTCTCCTCG	0.00	0.00	0.00	0.00	0.34
GATCAACATCACTGGATGCG	0.00	0.33	0.00	0.00	0.00
GATCAACATCACTGGCNGNG	0.00	0.33	0.00	0.00	0.00
GATCAACATCACTGGCTGGG	0.00	0.00	0.29	0.00	0.00

GATCAACATCAGAACCGTTG	0.00	0.00	0.00	0.00	0.00
GATCAACATCAGACAAGGGC	0.00	0.00	0.00	0.00	0.34
GATCAACATCAGACATCACA	0.23	0.00	0.00	0.00	0.00
GATCAACATCAGAGTGAGGG	0.00	0.00	0.00	0.34	0.00
GATCAACATCAGAGTGCGAA	0.00	0.33	0.00	0.00	0.00
GATCAACATCAGAGTGCGGA	5.26	5.21	2.00	2.04	3.44
GATCAACATCAGAGTGCGGC	0.00	0.33	0.00	0.00	0.00
GATCAACATCAGAGTGCGGN	0.23	0.00	0.00	0.00	0.00
GATCAACATCAGCAATGGCC	0.00	0.00	0.29	0.00	0.00
GATCAACATCAGCACGCAAA	0.00	0.00	0.00	0.68	0.00
GATCAACATCAGCACGCGAA	2.29	2.93	0.86	0.00	1.03
GATCAACATCAGCACGCGGA	0.23	0.00	0.00	0.00	0.00
GATCAACATCAGCAGTAAAA	0.00	0.00	0.00	0.00	0.00
GATCAACATCAGCATGCAGG	0.00	0.00	0.00	0.00	0.34
GATCAACATCAGCGCATCAA	0.00	0.00	0.00	0.00	0.34
GATCAACATCAGCGCCACCC	0.00	0.33	0.00	0.34	0.00
GATCAACATCAGCGGAGCCA	0.00	0.00	0.29	0.00	0.00
GATCAACATCAGGTACGCGG	1.14	1.63	1.15	1.36	0.34
GATCAACATCAGGTACGCTC	0.00	0.00	0.29	0.00	0.00
GATCAACATCAGTGTGTCGG	3.89	3.58	2.58	3.05	2.07
GATCAACATCAGTGTGTGGG	0.00	0.00	0.29	0.00	0.00
GATCAACATCAGTTGAAGCA	0.00	0.33	0.00	0.00	0.00
GATCAACATCATACTTGTGC	0.00	0.00	0.57	0.00	0.00
GATCAACATCATAGAATACA	0.00	0.00	0.00	0.00	0.00
GATCAACATCATAGTGCGGA	0.23	0.00	0.00	0.00	0.00
GATCAACATCATATGTTTGT	0.00	0.00	0.00	0.00	0.69
GATCAACATCATCAACCTTG	0.00	0.00	0.00	0.00	0.69
GATCAACATCATCACCAAGC	0.00	0.00	0.00	0.00	0.69
GATCAACATCATCAGAAGAA	0.00	0.00	0.29	0.00	0.34
GATCAACATCATCAGGGAGA	0.00	0.00	0.29	0.34	0.00
GATCAACATCATCATCATCA	0.00	0.00	0.00	0.00	0.00
GATCAACATCATCATCGCAA	0.00	0.00	0.00	0.00	1.03
GATCAACATCATCCGACGAC	0.00	0.00	0.00	0.00	0.00
GATCAACATCATCCTCACCG	0.00	0.00	0.29	0.00	0.00
GATCAACATCATCCTCTTCT	0.00	0.00	0.29	0.00	0.00
GATCAACATCATCCTGCGCG	0.00	0.00	0.00	0.00	0.00
GATCAACATCATCGTCATCA	0.00	0.00	0.00	0.00	0.34

GATCAACATCATCTGAAGGG	0.00	0.00	0.29	0.00	0.00
GATCAACATCATCTGACGGG	0.46	0.00	0.00	0.00	0.34
GATCAACATCATGAACCACA	0.00	0.00	0.00	0.00	0.00
GATCAACATCATGACACCTC	0.00	0.00	0.00	0.00	0.00
GATCAACATCATGAGGTAGA	0.00	0.00	0.29	0.00	0.00
GATCAACATCATGCAGAGGC	0.00	0.00	0.00	0.34	0.00
GATCAACATCATGGCGCCTG	0.00	0.00	0.00	0.00	0.00
GATCAACATCATGTAGGTCT	0.00	0.33	0.00	0.00	0.00
GATCAACATCATTGCATCCA	0.00	0.00	0.00	0.00	0.34
GATCAACATCATTGCGATGA	0.00	0.33	0.00	0.00	0.00
GATCAACATCATTTCCATCA	0.00	0.00	0.86	0.00	0.00
GATCAACATCATTTGCAAGA	0.00	0.33	0.00	0.00	0.00
GATCAACATCCAAAAAAAAA	0.23	0.00	0.29	0.34	0.00
GATCAACATCCAAGCTGACT	0.00	0.00	0.00	0.00	0.00
GATCAACATCCAAGTCAGGA	0.00	0.00	0.86	1.02	0.00
GATCAACATCCACATAGGAC	0.00	0.33	0.00	0.34	0.00
GATCAACATCCACATAGGCC	7.78	3.58	3.72	3.73	5.17
GATCAACATCCACATTCACA	4.35	1.95	2.58	3.73	1.38
GATCAACATCCACTGGAGTC	0.00	0.00	0.00	0.34	0.00
GATCAACATCCACTTGTTCA	0.23	0.00	0.00	0.00	0.00
GATCAACATCCAGAACAGCC	0.00	0.00	0.00	0.00	0.34
GATCAACATCCAGACGTCCG	0.46	0.00	0.00	0.00	0.00
GATCAACATCCAGATTGTTG	0.00	0.33	0.00	0.00	0.00
GATCAACATCCAGCACAAAA	0.00	0.00	0.00	0.00	0.00
GATCAACATCCAGCACAGCC	15.11	17.57	12.03	17.31	13.78
GATCAACATCCAGCACAGGC	0.23	0.00	0.00	0.00	0.00
GATCAACATCCAGCACCGCC	0.23	0.65	0.00	0.00	0.00
GATCAACATCCAGCACNGCC	0.00	0.00	0.00	0.34	0.00
GATCAACATCCAGCACNNNN	0.23	0.00	0.00	0.00	0.00
GATCAACATCCAGCAGCGGG	0.92	0.00	0.00	0.00	0.00
GATCAACATCCAGCGAACAT	0.00	0.00	0.00	0.00	0.00
GATCAACATCCAGGTGCCAT	0.00	0.00	0.00	0.00	0.69
GATCAACATCCCAAATTCAT	0.23	0.00	0.00	0.00	0.00
GATCAACATCCCACATCAAA	0.46	0.33	0.00	0.00	0.00
GATCAACATCCCACATCCGT	0.00	0.65	0.29	0.00	0.34
GATCAACATCCCACATCCNN	0.00	0.00	0.00	0.00	0.00
GATCAACATCCCACCCCCAC	0.00	0.00	0.00	0.00	0.00

GATCAACATCCCCACATTGC	0.00	0.00	0.00	0.34	0.00
GATCAACATCCCCAGGCCCC	0.00	0.00	0.00	0.00	0.00
GATCAACATCCCCGAGACGG	0.00	0.00	0.29	0.00	0.00
GATCAACATCCCCTACCCAT	0.00	0.00	0.00	0.00	0.00
GATCAACATCCCGTGGCCAC	0.23	0.00	0.00	0.00	0.00
GATCAACATCCCTGAGGACT	0.00	0.00	0.00	0.00	0.34
GATCAACATCCCTGAGGGAC	0.46	0.65	0.29	0.34	0.00
GATCAACATCCCTTCGGCCC	0.00	0.00	0.00	0.00	0.00
GATCAACATCCCTTTGGCCA	0.00	0.00	0.00	0.34	0.00
GATCAACATCCGAAGATTGC	0.00	0.00	0.29	0.00	0.00
GATCAACATCCGCACATGGG	0.00	0.00	0.29	0.00	0.00
GATCAACATCCGCGCCACAC	0.00	0.00	0.00	0.68	0.00
GATCAACATCCGCGCCCGCT	0.00	0.00	0.00	0.00	0.34
GATCAACATCCGCTCACGCA	0.00	0.00	0.00	0.00	0.00
GATCAACATCCGGACAATGC	0.00	0.00	0.00	0.34	0.00
GATCAACATCCGGACGTCGC	0.00	0.00	0.29	0.00	0.00
GATCAACATCCGGCCGCGGC	0.00	0.00	0.29	0.00	0.00
GATCAACATCCGGGCACTGC	0.00	0.00	0.29	0.34	0.00
GATCAACATCCGGTTGCAAG	0.00	0.33	0.00	0.00	0.00
GATCAACATCCGTGGTGCAG	0.46	0.00	0.57	0.34	0.00
GATCAACATCCGTGGTGGAA	0.00	0.00	0.00	0.34	0.00
GATCAACATCCTATGATGCA	0.46	0.00	0.00	0.00	0.00
GATCAACATCCTATTGCCCG	0.00	0.00	0.00	0.34	0.00
GATCAACATCCTCAACAGCC	1.60	0.33	0.00	0.00	0.00
GATCAACATCCTCAACATCC	0.00	0.33	0.00	0.00	0.00
GATCAACATCCTCATAAGTC	0.00	0.00	0.00	0.00	0.00
GATCAACATCCTCATGGATG	0.00	0.00	0.00	0.68	0.00
GATCAACATCCTCGGTGCTG	0.00	0.00	0.00	0.00	0.34
GATCAACATCCTCGTATGCC	0.00	0.00	0.00	0.00	0.00
GATCAACATCCTCTGGATTG	0.00	0.00	0.29	0.00	0.00
GATCAACATCCTGAAGTCTG	0.00	0.00	0.00	0.00	0.00
GATCAACATCCTGAGGGACA	0.00	0.00	0.00	0.34	0.00
GATCAACATCCTGCACCAAA	0.00	0.00	0.00	0.34	0.00
GATCAACATCCTGGAGAAAA	0.00	0.00	0.00	0.00	0.00
GATCAACATCCTGGAGCACC	0.00	0.00	0.00	0.00	0.00
GATCAACATCCTGGAGGACT	2.98	1.63	2.58	3.05	1.72
GATCAACATCCTGGAGGAGT	0.00	0.00	0.00	0.68	0.00

GATCAACATCCTGGAGGANN	0.23	0.00	0.00	0.00	0.00
GATCAACATCCTGGAGGTCG	0.00	0.33	0.00	0.00	0.00
GATCAACATCCTGGCAGGAA	0.00	0.00	0.29	0.00	0.00
GATCAACATCCTGGCCATCA	0.00	0.00	0.00	0.00	0.00
GATCAACATCCTGGGGAGGA	0.00	0.33	0.00	0.00	0.00
GATCAACATCCTGGGGGACT	0.23	0.00	0.29	0.00	0.34
GATCAACATCCTGGTGCGCG	0.00	0.33	0.00	0.00	0.00
GATCAACATCCTGGTGGAGG	0.92	0.33	0.00	0.34	0.34
GATCAACATCCTGTTACCCG	0.23	0.00	0.00	0.00	0.00
GATCAACATCCTGTTGCACG	0.00	0.00	0.29	0.00	0.00
GATCAACATCCTGTTGCCAG	0.23	0.00	0.00	0.00	0.00
GATCAACATCCTGTTGCCCG	8.01	16.92	11.17	16.63	14.12
GATCAACATCCTGTTGCCCN	0.00	0.00	0.00	0.00	0.00
GATCAACATCCTGTTGCTCG	0.00	0.33	0.00	0.00	0.00
GATCAACATCCTGTTTCCCT	0.00	0.00	0.00	0.00	0.34
GATCAACATCCTTCTCCGCG	0.00	0.00	0.00	0.00	0.00
GATCAACATCCTTGAGAACC	0.00	0.00	0.00	0.00	0.00
GATCAACATCCTTGAGACCT	0.00	0.00	0.00	0.00	0.34
GATCAACATCCTTGAGGACA	0.00	0.00	0.29	0.00	0.00
GATCAACATCCTTGAGGACC	0.00	0.00	0.29	0.00	0.00
GATCAACATCCTTGAGGACN	0.46	0.00	0.00	0.00	0.00
GATCAACATCCTTGAGGACT	33.88	47.82	39.52	47.86	33.75
GATCAACATCCTTGAGGANN	0.23	0.00	0.00	0.00	0.00
GATCAACATCCTTGAGGCCT	0.00	0.65	0.57	0.00	0.00
GATCAACATCCTTGAGGCNN	0.00	0.00	0.00	0.00	0.00
GATCAACATCCTTGAGGCTT	0.00	0.00	0.00	0.34	0.00
GATCAACATCCTTGAGNNNN	0.23	0.00	0.00	0.00	0.00
GATCAACATCCTTGAGTACT	0.00	0.00	0.00	0.00	0.00
GATCAACATCCTTGCGGACT	0.00	0.00	0.29	0.00	0.00
GATCAACATCCTTGTAATCA	0.00	0.00	0.00	0.00	0.00
GATCAACATCGAAAAAAAAA	0.23	0.00	0.00	0.00	0.00
GATCAACATCGAAACTCACA	0.00	0.00	0.00	0.00	0.00
GATCAACATCGAAGAGTACG	0.00	0.00	0.00	0.00	0.00
GATCAACATCGACAAGGCCA	0.00	0.00	0.00	0.00	0.00
GATCAACATCGACAAGGTAA	0.00	0.00	0.00	0.34	0.00
GATCAACATCGACACGAACG	0.00	0.00	0.00	0.68	0.00
GATCAACATCGACAGGACCA	0.00	0.00	0.00	0.34	0.00

GATCAACATCGACAGTTGTG	0.00	0.00	0.00	0.00	0.00
GATCAACATCGACCAGGTGC	0.69	0.00	0.00	0.34	0.00
GATCAACATCGACCGGATAG	0.00	0.65	0.00	0.00	0.00
GATCAACATCGACCGTGAGG	0.00	0.00	0.29	0.00	0.00
GATCAACATCGACGAGCAAC	0.00	0.00	0.29	0.00	0.00
GATCAACATCGACGGAACAA	0.23	0.00	0.00	0.00	0.00
GATCAACATCGACGGCCGGG	0.00	0.00	0.29	0.34	0.00
GATCAACATCGACGTGCAAC	0.23	0.00	0.00	0.00	0.00
GATCAACATCGAGATGCAGC	0.92	0.33	0.00	0.00	0.00
GATCAACATCGAGATTGTCC	0.00	0.00	0.00	0.00	0.34
GATCAACATCGAGCAGGCTG	0.00	0.65	0.57	1.02	2.76
GATCAACATCGAGCAGGCTT	0.00	0.00	0.00	0.00	0.00
GATCAACATCGAGGACGCCG	0.00	0.33	0.00	1.02	0.00
GATCAACATCGAGGCCCTCA	0.00	0.00	0.00	0.00	0.69
GATCAACATCGATGAGTTGG	0.00	0.00	0.00	0.00	0.00
GATCAACATCGCAAACAGCA	0.69	0.33	0.00	0.68	0.00
GATCAACATCGCAAAGGCCA	0.00	0.00	0.29	0.00	0.00
GATCAACATCGCAAAGTAAG	0.00	0.00	0.00	0.00	0.00
GATCAACATCGCAACCATAA	0.00	0.00	0.57	0.00	0.00
GATCAACATCGCAATGCCCG	0.00	0.00	0.00	0.00	0.34
GATCAACATCGCACCTATGA	0.00	0.00	0.00	0.00	0.00
GATCAACATCGCAGCCGAGG	0.00	0.00	0.00	0.00	0.00
GATCAACATCGCAGGGCTGG	0.00	0.33	0.00	0.00	0.00
GATCAACATCGCAGTATCCC	0.46	0.00	0.00	0.00	0.00
GATCAACATCGCCAAAGCCG	0.00	0.00	0.00	0.00	0.00
GATCAACATCGCCAACAGGG	0.00	0.00	0.86	0.00	0.00
GATCAACATCGCCAAGGCCA	11.90	5.21	3.44	9.84	3.10
GATCAACATCGCCAAGGCCC	0.00	0.65	0.00	0.00	0.00
GATCAACATCGCCAAGGCCG	0.23	0.00	0.00	0.00	0.00
GATCAACATCGCCAAGGCCN	0.23	0.00	0.00	0.00	0.00
GATCAACATCGCCAAGGCTC	0.69	0.00	0.00	0.00	0.00
GATCAACATCGCCAAGGTCA	0.00	0.00	0.00	0.00	0.00
GATCAACATCGCCACATCCT	0.00	0.00	0.00	0.00	0.34
GATCAACATCGCCACTCCAA	0.00	0.00	0.00	0.00	0.00
GATCAACATCGCCAGACACG	0.00	0.00	0.00	0.00	0.00
GATCAACATCGCCATCCCCA	0.00	0.00	0.29	0.00	0.00
GATCAACATCGCCCGGCGGC	0.00	0.33	0.00	0.00	0.00

GATCAACATCGCCCTCATTC	0.00	0.00	0.00	0.00	0.00
GATCAACATCGCCGCGGCCT	0.00	0.00	0.00	0.00	0.34
GATCAACATCGCCGCGTTCC	0.00	0.00	0.00	0.00	0.00
GATCAACATCGCCGCTGCCC	0.00	0.00	0.00	0.00	0.00
GATCAACATCGCCGCTTTTT	0.00	0.00	0.00	0.00	0.00
GATCAACATCGCCGGGAAAA	0.00	0.00	0.00	0.00	0.00
GATCAACATCGCCGGGACTT	0.92	0.33	0.00	0.68	0.34
GATCAACATCGCCGGGTGCG	0.23	0.33	0.00	0.00	0.00
GATCAACATCGCCTATGTTG	0.00	0.00	0.00	0.00	0.34
GATCAACATCGCCTCATGCC	0.00	0.00	0.00	0.00	1.38
GATCAACATCGCGAGCACGC	0.00	0.00	0.57	0.00	0.00
GATCAACATCGCGGACCGCG	0.00	0.00	0.00	0.34	0.00
GATCAACATCGCGGAGGACG	0.46	0.00	0.00	0.00	0.00
GATCAACATCGCGGTTGTTC	0.00	0.00	0.00	0.00	0.00
GATCAACATCGCTCGCTTGG	0.00	0.00	0.29	0.00	0.34
GATCAACATCGCTTCGGCAG	0.00	0.00	0.00	0.00	0.00
GATCAACATCGGAGGCATGG	0.23	0.00	0.29	0.00	0.00
GATCAACATCGGCAGTGCTC	0.23	0.00	0.00	0.00	0.00
GATCAACATCGGCATCAAGT	0.00	0.00	0.00	0.34	0.00
GATCAACATCGGCATCATGC	0.23	0.00	0.29	0.00	0.00
GATCAACATCGGCATGTCCC	0.00	0.00	0.00	0.00	0.00
GATCAACATCGGCCGGTAGG	0.00	0.00	0.00	0.00	0.00
GATCAACATCGGCGAGAACA	0.00	0.00	0.29	0.00	0.00
GATCAACATCGGCGCATGCA	0.00	0.00	0.00	0.00	0.00
GATCAACATCGGCGCGCCGT	0.23	0.00	0.00	0.00	0.00
GATCAACATCGGCGCGGCGC	0.23	0.00	0.00	0.00	0.00
GATCAACATCGGCGTCAACA	0.00	0.00	0.00	0.00	0.00
GATCAACATCGGCTAAGCGG	0.00	0.00	0.00	0.34	0.00
GATCAACATCGGCTACCCGG	0.23	0.00	0.00	0.00	0.00
GATCAACATCGGCTACTCGG	0.46	0.33	0.00	0.34	0.00
GATCAACATCGGGAGTCACA	0.00	0.00	0.29	0.00	0.00
GATCAACATCGGGCGACGTC	0.00	0.33	0.00	0.68	0.69
GATCAACATCGGTAAAACAT	0.00	0.00	0.00	0.00	0.00
GATCAACATCGGTAGGTGGA	0.23	0.00	0.00	0.00	0.00
GATCAACATCGGTAGGTGGG	0.23	0.00	0.00	0.00	0.00
GATCAACATCGTATCGGGTG	0.00	0.00	0.00	0.34	0.00
GATCAACATCGTCACCGAGG	0.00	0.00	0.00	0.00	0.34

GATCAACATCGTCAGGGCTC	0.00	0.00	0.00	0.00	0.00
GATCAACATCGTCAGTGATC	0.00	0.00	0.00	0.00	0.34
GATCAACATCGTCAGTGCTC	4.12	12.04	18.04	9.50	12.40
GATCAACATCGTCCAGGGTT	0.00	0.00	0.00	0.00	0.34
GATCAACATCGTCCGTTGTG	0.46	0.00	0.00	0.00	0.00
GATCAACATCGTCGAGACGG	0.92	0.00	0.00	0.68	0.00
GATCAACATCGTCGATTTCG	0.00	0.00	0.00	0.00	0.00
GATCAACATCGTCGGCAATC	0.00	0.00	0.57	0.68	0.00
GATCAACATCGTCGTCGACG	0.23	0.65	0.29	0.68	0.00
GATCAACATCGTCTTCAACA	1.83	0.33	0.29	0.00	0.69
GATCAACATCGTCTTCACCA	0.23	0.00	0.00	0.00	0.00
GATCAACATCGTCTTCATAA	0.00	0.00	0.00	0.00	0.00
GATCAACATCGTGACGGAGA	0.46	0.00	0.00	0.68	0.00
GATCAACATCGTGACTGAGA	0.00	0.00	0.00	0.68	0.00
GATCAACATCGTGCAAAAAA	0.00	0.33	0.00	0.68	0.00
GATCAACATCGTGCCGTCAA	0.00	0.00	0.00	0.00	0.34
GATCAACATCGTGCCGTCAT	0.23	0.98	0.00	0.34	0.34
GATCAACATCGTGCCGTCGA	0.00	0.00	0.00	0.00	0.00
GATCAACATCGTGCTCATAC	0.00	0.33	0.00	0.00	0.00
GATCAACATCGTGGACACGC	0.23	0.33	0.00	0.00	0.00
GATCAACATCGTGGCAAAAA	0.23	0.00	0.00	0.00	0.00
GATCAACATCGTGTTGAAGG	0.00	0.00	0.00	0.00	0.00
GATCAACATCTAAATTCACG	0.00	0.00	0.00	0.00	0.34
GATCAACATCTAAGAGCTGC	0.00	0.00	0.00	0.00	0.00
GATCAACATCTAAGAGGTGC	0.23	0.00	0.00	0.00	0.00
GATCAACATCTAAGAGNTGC	0.00	0.00	0.00	0.00	0.34
GATCAACATCTAAGAGTAGC	0.00	0.33	0.00	0.00	0.00
GATCAACATCTAAGAGTNNN	0.00	0.00	0.00	0.00	0.34
GATCAACATCTAAGAGTTGC	57.23	36.44	24.05	27.49	32.72
GATCAACATCTAAGAGTTGN	0.00	0.00	0.00	0.34	0.00
GATCAACATCTAAGCGTCGC	0.00	0.00	0.00	0.00	0.34
GATCAACATCTAAGCGTTGC	0.23	0.00	0.00	0.00	0.00
GATCAACATCTAAGGGGTGC	0.23	0.00	0.00	0.00	0.00
GATCAACATCTACAAGGTCC	0.00	0.00	0.00	0.00	0.34
GATCAACATCTACGCCCCGG	0.00	0.00	0.00	0.00	0.00
GATCAACATCTACGGGCACC	0.23	0.00	0.00	0.00	0.00
GATCAACATCTACGTGCAAC	0.00	0.00	0.29	0.00	0.00

GATCAACATCTACGTGCACC	34.79	21.15	10.31	12.56	7.58
GATCAACATCTACGTGCACT	0.00	0.00	0.29	0.34	0.00
GATCAACATCTACGTGCANC	0.00	0.00	0.00	0.34	0.00
GATCAACATCTACGTGCGCC	0.23	0.00	0.00	0.00	0.00
GATCAACATCTACTGCCTGC	0.00	0.00	0.00	0.00	0.00
GATCAACATCTAGGAGTTGC	0.23	0.00	0.00	0.00	0.00
GATCAACATCTATCAACGTC	0.00	0.00	0.00	0.00	0.34
GATCAACATCTCAAAAAAAA	0.00	0.00	0.00	0.00	0.00
GATCAACATCTCAAAAACAA	0.00	0.00	0.00	0.00	0.00
GATCAACATCTCACACGTGT	0.00	0.00	0.00	0.00	0.00
GATCAACATCTCACCACAGG	0.00	0.00	0.86	0.00	0.00
GATCAACATCTCAGAGTTGC	0.23	0.00	0.00	0.00	0.00
GATCAACATCTCATATACGC	0.00	0.00	0.00	0.00	0.00
GATCAACATCTCCCTCCTAG	0.00	0.00	0.29	0.00	0.00
GATCAACATCTCGAAGCACA	0.00	0.00	0.00	0.00	0.00
GATCAACATCTCGCCATGGT	0.00	0.00	0.00	0.00	0.34
GATCAACATCTCGCGAGCGT	0.00	0.00	0.00	0.00	0.00
GATCAACATCTCGCGAGTGT	0.23	3.58	2.86	2.72	2.76
GATCAACATCTCGCTCGTAT	0.00	0.65	0.00	0.00	0.00
GATCAACATCTCGGCGTTGC	0.23	0.00	0.00	0.00	0.00
GATCAACATCTCGTGCAGGT	0.00	0.00	0.00	0.00	0.00
GATCAACATCTCTGACCATG	0.00	0.33	0.00	0.34	0.00
GATCAACATCTGAGAGTTGC	0.00	0.00	0.00	0.34	0.00
GATCAACATCTGAGCCTCAA	0.00	0.33	0.00	0.00	0.00
GATCAACATCTGAGTGCGGA	0.23	0.00	0.00	0.00	0.00
GATCAACATCTGATGGTTTG	0.00	0.33	0.00	0.00	0.00
GATCAACATCTGCACGCAGC	0.00	0.00	0.00	1.02	0.00
GATCAACATCTGCACTCCTC	0.00	0.00	0.00	0.00	0.00
GATCAACATCTGCATATGTG	0.69	0.65	0.00	0.68	0.00
GATCAACATCTGCATGTGCT	0.23	0.33	0.00	0.00	0.34
GATCAACATCTGCGGGACCT	0.00	0.00	0.00	0.00	0.00
GATCAACATCTGCGGGGAGG	0.00	0.33	0.00	0.00	0.00
GATCAACATCTGCGTGCACC	0.23	0.00	0.00	0.00	0.00
GATCAACATCTGGAAGAATG	0.00	0.33	0.86	1.36	0.00
GATCAACATCTGGAAGGATG	0.00	0.65	0.00	0.00	0.00
GATCAACATCTGGAGATGAT	0.23	0.00	0.00	0.00	0.00
GATCAACATCTGGCTCAACG	0.00	0.33	0.00	0.00	0.00

GATCAACATCTGGCTGATAA	0.00	0.33	0.00	0.00	0.00
GATCAACATCTGGGCGAGGT	0.00	0.00	0.00	0.00	0.34
GATCAACATCTGGGGCCGTG	0.00	0.00	0.00	0.34	0.34
GATCAACATCTGGGGCTGGT	0.00	0.00	0.00	0.34	0.00
GATCAACATCTGGGGCTGTA	0.00	0.00	0.00	0.34	0.00
GATCAACATCTGGGGCTGTT	0.00	0.98	0.00	0.68	0.00
GATCAACATCTTCATGTTCG	0.00	0.00	0.00	0.00	0.00
GATCAACATCTTCCATGCTT	0.00	0.33	0.00	0.00	0.00
GATCAACATCTTCGAGGAGG	0.46	0.33	0.00	0.00	0.00
GATCAACATCTTCTACGCGC	0.00	0.00	0.00	0.00	0.00
GATCAACATCTTCTTGCTGC	0.00	0.00	0.29	0.00	0.00
GATCAACATCTTGAAGCAGA	0.46	0.00	0.00	0.00	0.00
GATCAACATCTTGCAGACTC	0.00	0.00	0.00	0.00	0.00
GATCAACATCTTGCAGTTCT	0.00	0.00	0.00	0.00	0.00
GATCAACATCTTGCAGTTNT	0.00	0.00	0.00	0.00	0.00
GATCAACATCTTTGGAAGCC	0.00	0.00	0.86	0.00	0.00
GATCAACATCTTTGTCAGCA	0.00	0.00	0.00	0.00	0.34
GATCAACATCTTTTCTTGCC	0.00	0.00	0.00	0.00	0.00
GATCAACATGAAAAAAAAAA	0.00	0.33	0.00	0.00	0.34
GATCAACATGAAACCGTTTC	0.00	0.33	0.00	0.00	0.00
GATCAACATGAAAGAGAAGG	0.00	0.33	0.00	0.00	0.00
GATCAACATGAAATTCAGCT	0.69	0.00	0.00	0.34	0.00
GATCAACATGAACAACATAC	0.00	0.00	0.29	0.00	0.00
GATCAACATGAACAAGAAGG	0.00	0.33	0.00	0.34	0.00
GATCAACATGAACACAAGGA	0.00	0.00	0.00	0.00	0.69
GATCAACATGAACACGACCT	0.23	0.33	0.00	0.00	0.00
GATCAACATGAACAGGTGTC	0.00	0.00	0.00	0.00	0.34
GATCAACATGAACATTCTCA	1.14	0.33	0.00	0.00	0.34
GATCAACATGAACGATGGGC	0.23	0.00	0.29	0.34	0.00
GATCAACATGAACTGTGTCC	0.00	0.00	0.00	0.00	0.00
GATCAACATGAACTGTGTCG	1.14	3.58	5.15	2.72	10.68
GATCAACATGAACTGTGTTG	0.00	0.00	0.00	0.34	0.00
GATCAACATGAAGAACATCT	0.00	0.33	0.00	0.00	1.03
GATCAACATGAAGACGAAGC	0.00	0.00	0.29	0.00	0.00
GATCAACATGAAGAGCACAC	0.00	0.65	0.00	0.68	0.00
GATCAACATGAAGCCGCACA	0.00	0.00	0.00	0.34	0.00
GATCAACATGAAGCCGCAGG	0.00	0.33	0.00	0.00	0.00

GATCAACATGAAGCTGGAGA	0.00	0.00	0.00	0.00	0.00
GATCAACATGAAGGTTTTTG	0.46	0.00	0.00	0.00	0.00
GATCAACATGAAGTCACAGG	0.00	0.00	0.00	0.00	0.00
GATCAACATGAATATCTTGC	0.00	0.65	0.00	0.00	0.00
GATCAACATGAATCGCCAAT	0.00	0.00	0.00	0.00	0.00
GATCAACATGAATCGTGTTC	0.00	0.00	0.00	0.00	0.34
GATCAACATGACAAAGACAA	0.00	0.00	0.29	0.00	0.00
GATCAACATGACAACTACGA	0.00	0.33	0.00	0.00	0.00
GATCAACATGACAATCCGCT	0.00	0.00	0.00	0.34	0.00
GATCAACATGACAGTGCCGT	0.00	0.00	0.00	0.00	0.00
GATCAACATGACAGTGGGGA	0.00	0.00	0.29	0.00	0.00
GATCAACATGACATACCCCT	0.00	0.00	0.00	0.34	0.34
GATCAACATGACCACGATTG	0.00	0.33	0.00	0.34	0.00
GATCAACATGACCCTTCTGG	0.00	0.00	0.00	0.34	0.00
GATCAACATGACCGCAGGCC	0.00	0.00	0.00	0.00	0.00
GATCAACATGACCTACCCCT	0.00	0.00	0.00	0.34	0.34
GATCAACATGACCTTCAACA	0.00	0.00	0.00	0.00	0.00
GATCAACATGACGAAAATAC	0.00	0.00	0.57	0.00	0.00
GATCAACATGACGAGGACGA	0.00	0.00	0.00	0.00	0.00
GATCAACATGACGGAGGTGG	0.00	0.33	0.00	0.00	0.00
GATCAACATGACGTTCGTGG	0.00	0.00	0.00	0.00	0.00
GATCAACATGACTGACCACG	0.00	0.00	0.00	0.00	0.00
GATCAACATGACTGATGTGC	0.00	0.00	0.00	0.34	0.00
GATCAACATGACTTTTTAAC	0.00	0.00	0.00	0.00	0.00
GATCAACATGAGAAGAAAGG	0.00	0.00	0.29	0.00	0.00
GATCAACATGAGAAGGATAG	0.23	0.00	0.00	0.00	0.00
GATCAACATGAGCATTGGCA	0.00	0.00	0.00	0.00	0.00
GATCAACATGAGCATTGGCT	0.69	0.00	0.00	0.00	0.00
GATCAACATGAGCCCGAACA	0.00	0.00	0.29	0.34	0.69
GATCAACATGAGCCCGTGCG	0.00	0.00	0.00	0.34	0.00
GATCAACATGAGCCTAAATG	0.00	0.00	0.29	0.00	0.00
GATCAACATGAGCCTGAAAG	0.00	0.00	0.00	0.00	0.00
GATCAACATGAGCGTGAAGG	0.23	0.00	0.00	0.00	0.00
GATCAACATGAGCTCGAAAG	0.00	0.00	0.00	0.34	0.00
GATCAACATGAGCTTGAAAG	12.36	11.06	4.01	10.86	3.79
GATCAACATGAGCTTGAAAT	0.00	0.00	0.00	0.34	0.00
GATCAACATGAGCTTGAANG	0.00	0.00	0.00	0.34	0.00

GATCAACATGAGCTTGAATG	0.23	0.00	0.00	0.00	0.00
GATCAACATGAGCTTGAGAG	0.23	0.00	0.00	0.00	0.00
GATCAACATGAGCTTGAGCT	0.00	0.00	0.00	0.00	0.00
GATCAACATGAGCTTGNNNN	0.00	0.00	0.00	0.00	0.00
GATCAACATGAGCTTTAAAG	0.00	0.00	0.00	0.00	0.34
GATCAACATGAGGAGCATGA	0.00	0.00	0.29	0.00	0.00
GATCAACATGAGGCAGGATC	0.00	0.33	0.00	0.00	0.00
GATCAACATGAGGCGGTAGC	0.00	0.00	0.00	0.00	0.00
GATCAACATGAGGGCAGGGC	0.00	0.00	0.00	0.00	0.00
GATCAACATGAGGGTGCGCG	0.00	0.65	0.00	0.00	0.00
GATCAACATGAGGTTGGTGN	0.00	0.00	0.00	0.00	0.00
GATCAACATGAGTGAATTCA	0.00	0.00	0.00	0.00	0.69
GATCAACATGAGTGAGAGCA	0.00	0.00	0.57	0.00	0.34
GATCAACATGAGTGAGGCCG	0.00	0.00	0.00	0.00	0.00
GATCAACATGAGTGCAGCTG	0.23	0.33	0.00	0.34	0.00
GATCAACATGAGTTTGGTGC	0.00	0.33	0.00	0.00	0.00
GATCAACATGATAATCATGG	0.00	0.00	0.00	0.00	0.00
GATCAACATGATAATCCTGT	0.00	0.00	0.00	0.00	0.00
GATCAACATGATAGTAAAAA	0.92	0.00	0.86	0.00	0.00
GATCAACATGATGAACATCT	0.00	0.00	0.00	0.00	0.34
GATCAACATGATGAACTTAT	0.23	0.00	0.00	0.00	0.00
GATCAACATGATGAACTTCT	10.30	19.52	14.60	9.16	12.40
GATCAACATGATGAACTTNN	0.23	0.00	0.00	0.00	0.00
GATCAACATGATGCTAAGGC	0.23	0.00	0.00	0.00	0.00
GATCAACATGATGCTAATGT	0.00	0.00	0.00	0.00	0.00
GATCAACATGATGGACCTCG	0.00	0.33	0.00	0.00	0.00
GATCAACATGATGGAGGAGG	0.00	0.00	0.00	0.00	0.00
GATCAACATGATGGAGGGCA	0.00	0.00	0.00	0.00	0.00
GATCAACATGATGGAGGGGG	0.00	0.33	0.29	0.00	0.00
GATCAACATGATGGTAAAAA	0.69	0.98	0.00	1.70	0.00
GATCAACATGATGGTATGGT	0.00	0.33	0.00	0.00	0.00
GATCAACATGATGTACTTCT	0.00	0.00	0.00	0.00	0.69
GATCAACATGATGTGAACAT	0.00	0.00	0.00	0.00	0.34
GATCAACATGATGTTAAAAA	0.00	0.00	0.00	0.00	0.00
GATCAACATGATTGGCATCC	0.23	0.00	0.00	0.00	0.00
GATCAACATGCAAAAAAAAA	0.23	0.33	0.00	0.68	0.00
GATCAACATGCAAAACGAAG	0.00	0.00	0.00	0.00	0.00

GATCAACATGCAAAGCAGCC	0.00	0.00	0.00	0.00	0.34
GATCAACATGCAAATTCACG	0.00	0.33	0.00	0.00	0.00
GATCAACATGCAACAAAAAA	0.00	0.00	0.00	0.00	0.34
GATCAACATGCAACACAATG	0.00	0.00	0.00	0.00	0.34
GATCAACATGCAACTAGAAC	0.00	0.33	0.86	0.00	0.00
GATCAACATGCAAGGTTTGA	0.00	0.00	0.29	0.00	0.00
GATCAACATGCAATACCCCA	0.00	0.00	0.00	0.00	0.34
GATCAACATGCACAGGCCCC	0.00	0.00	0.00	0.34	0.00
GATCAACATGCACATCAGGN	0.00	0.00	0.00	0.00	0.00
GATCAACATGCACCACTTAC	0.00	0.00	0.00	0.00	0.00
GATCAACATGCACCGCCGGC	0.00	0.00	0.00	0.00	0.34
GATCAACATGCACGAGGCAG	0.00	0.00	0.00	0.00	0.34
GATCAACATGCACGCAGCTG	0.00	0.00	0.00	0.00	0.00
GATCAACATGCACGGCCTGG	0.23	0.00	0.00	0.34	0.00
GATCAACATGCACTACCCCT	0.23	0.33	0.86	0.00	0.00
GATCAACATGCACTGAGCCT	0.00	0.00	0.00	0.34	0.00
GATCAACATGCACTTGTTGT	0.46	0.00	0.00	0.00	0.00
GATCAACATGCAGAACTCCG	0.00	0.00	0.00	0.68	0.00
GATCAACATGCAGACACGCC	0.00	0.65	0.00	0.00	0.00
GATCAACATGCAGCAAAAGA	0.00	0.33	0.00	0.00	0.00
GATCAACATGCAGCCCTACT	0.00	0.33	0.00	0.00	0.00
GATCAACATGCAGCCGTGCC	0.00	0.00	0.29	0.00	0.00
GATCAACATGCAGCGCTATG	0.00	0.33	0.00	0.68	0.00
GATCAACATGCAGGGCGGGG	0.00	0.33	0.00	0.00	0.00
GATCAACATGCAGGGGACAA	0.00	0.00	0.29	0.00	0.00
GATCAACATGCAGGTCATCG	0.00	0.00	0.00	0.00	0.00
GATCAACATGCAGTCGGACA	0.00	0.00	0.00	0.00	0.00
GATCAACATGCATCATGGGC	0.00	0.33	0.00	0.00	0.00
GATCAACATGCCAAAAAAAA	0.00	0.00	0.57	0.00	0.00
GATCAACATGCCAAACACAA	0.00	0.00	0.57	0.00	0.00
GATCAACATGCCAACATCGT	0.00	0.00	0.00	0.00	0.69
GATCAACATGCCAAGAGCCA	0.00	0.00	0.00	0.00	0.69
GATCAACATGCCAAGAGCCG	1.83	0.65	1.15	2.04	1.38
GATCAACATGCCAAGAGCCT	0.00	0.00	0.29	0.00	0.00
GATCAACATGCCAAGAGCGG	0.00	0.00	0.00	0.34	0.00
GATCAACATGCCAGCCTACT	0.00	0.33	0.00	0.34	0.00
GATCAACATGCCATAACCAT	0.00	0.00	0.00	0.00	0.00

GATCAACATGCCATACACCT	0.00	0.00	0.00	0.34	0.00
GATCAACATGCCATACCACT	0.00	0.00	0.00	0.00	0.69
GATCAACATGCCATACCCAT	0.23	0.00	0.29	0.00	0.00
GATCAACATGCCATACCCCA	0.00	0.00	0.00	0.00	0.34
GATCAACATGCCATACCCCG	0.23	0.33	0.00	0.34	0.00
GATCAACATGCCATACCCCT	20.37	41.32	63.29	51.25	47.87
GATCAACATGCCATACGCCT	0.00	0.00	0.00	0.34	0.00
GATCAACATGCCATACTCGT	0.00	0.00	0.57	0.00	0.00
GATCAACATGCCATGCCCCT	0.00	0.00	0.00	0.00	0.00
GATCAACATGCCATGTACAC	0.00	0.00	0.00	0.00	0.00
GATCAACATGCCCAACACGG	0.00	0.00	0.00	0.00	0.00
GATCAACATGCCCAACCCCT	0.00	0.00	0.29	0.34	0.00
GATCAACATGCCCAAGCCCC	0.00	0.00	0.00	0.00	0.00
GATCAACATGCCCAGGCCAC	0.00	0.00	0.29	0.00	0.00
GATCAACATGCCCAGGCCCA	0.00	0.00	0.29	0.00	0.00
GATCAACATGCCCAGGCCCC	8.47	7.81	5.44	11.88	5.17
GATCAACATGCCCCACCCCT	0.00	0.00	0.00	0.00	0.00
GATCAACATGCCCGACGGCG	0.00	0.00	0.00	0.00	0.00
GATCAACATGCCCGACGGNN	0.23	0.00	0.00	0.00	0.00
GATCAACATGCCCGACGGTG	0.00	0.98	0.29	0.34	0.00
GATCAACATGCCCGAGTATG	0.23	0.33	0.29	0.00	0.00
GATCAACATGCCCGGCAGAG	0.23	0.00	0.00	0.00	0.00
GATCAACATGCCCGGCCGCG	0.00	0.00	0.00	0.00	0.00
GATCAACATGCCCGGCCTTC	0.00	0.00	0.00	0.00	0.00
GATCAACATGCCCGGGACAA	0.23	0.00	0.00	0.00	0.00
GATCAACATGCCCGGGCCAC	0.23	0.00	0.00	0.00	0.00
GATCAACATGCCCGGGTATG	0.00	0.00	0.00	0.34	0.00
GATCAACATGCCCGTTGTTG	0.23	0.00	0.00	0.68	0.00
GATCAACATGCCCTAACCCT	0.00	0.00	0.57	0.00	0.34
GATCAACATGCCCTACACCT	0.00	0.33	0.29	0.00	0.34
GATCAACATGCCCTACCAAC	0.00	0.00	0.00	0.00	0.34
GATCAACATGCCCTACCACT	0.23	0.98	0.00	0.34	0.34
GATCAACATGCCCTACCCAA	0.00	0.33	0.00	0.00	0.00
GATCAACATGCCCTACCCAC	0.00	0.33	0.00	0.00	0.00
GATCAACATGCCCTACCCAT	0.23	0.00	0.00	0.34	0.34
GATCAACATGCCCTACCCCA	1.37	0.00	0.29	0.34	0.00
GATCAACATGCCCTACCCCC	0.23	0.33	0.57	0.00	0.34

GATCAACATGCCCTACCCCG	0.00	0.33	0.00	0.34	0.34
GATCAACATGCCCTACCCCN	0.00	0.00	0.00	0.00	0.00
GATCAACATGCCCTACCCCT	132.76	187.71	224.51	194.83	183.91
GATCAACATGCCCTACCCGA	0.23	0.00	0.00	0.00	0.00
GATCAACATGCCCTACCCNN	0.23	0.00	0.00	0.00	0.00
GATCAACATGCCCTACCCTC	0.23	0.00	0.00	0.00	0.34
GATCAACATGCCCTACCCTT	0.46	0.00	0.00	0.00	0.34
GATCAACATGCCCTACCGCA	0.00	0.00	0.29	0.00	0.00
GATCAACATGCCCTACCNNN	0.00	0.00	0.00	0.00	0.00
GATCAACATGCCCTACCTCT	0.00	0.33	0.00	0.00	0.00
GATCAACATGCCCTACGAAT	0.23	0.00	0.00	0.00	0.00
GATCAACATGCCCTACNCCT	0.00	0.00	0.29	0.00	0.34
GATCAACATGCCCTACNCNN	0.00	0.00	0.29	0.00	0.00
GATCAACATGCCCTACTCCT	0.00	0.00	0.00	0.00	0.34
GATCAACATGCCCTATCCCT	0.00	0.00	0.00	0.00	0.00
GATCAACATGCCCTCGTATG	0.00	0.00	0.00	0.68	0.00
GATCAACATGCCCTGCCCCG	0.23	0.00	0.00	0.00	0.00
GATCAACATGCCCTGCCCCT	0.00	0.33	0.00	0.68	0.34
GATCAACATGCCCTGCCGCT	0.23	0.00	0.00	0.00	0.00
GATCAACATGCCCTGGAACT	0.00	0.65	0.00	0.00	0.00
GATCAACATGCCGCCCATGG	0.00	0.00	0.29	0.00	0.00
GATCAACATGCCGCGGTGAA	0.23	0.33	0.00	0.34	0.00
GATCAACATGCCGCGGTGGC	0.23	0.00	0.00	0.00	0.00
GATCAACATGCCGCGGTGGG	0.23	0.00	0.00	0.00	0.00
GATCAACATGCCGCTGGGNN	0.23	0.00	0.00	0.00	0.00
GATCAACATGCCGCTGGGTG	1.14	0.98	0.29	1.02	0.00
GATCAACATGCCGCTGTGAG	0.00	0.33	0.00	0.00	0.00
GATCAACATGCCGGAGAACA	0.00	0.00	0.57	0.00	0.00
GATCAACATGCCGGAGAACC	0.00	0.00	0.29	0.00	0.00
GATCAACATGCCGGCGGCAA	0.00	0.33	0.00	0.00	0.00
GATCAACATGCCGGCGGCGT	0.00	0.33	0.00	0.00	0.00
GATCAACATGCCGTCAGTGT	0.00	0.00	0.29	0.00	0.00
GATCAACATGCCGTCCAGCG	0.00	0.00	0.00	0.00	0.69
GATCAACATGCCTCTGGCCC	0.46	0.33	0.29	1.70	0.00
GATCAACATGCCTCTGGCTG	0.00	0.00	0.00	0.34	0.00
GATCAACATGCCTGCACTGC	0.23	0.33	0.00	0.00	0.00
GATCAACATGCCTGGCACTC	0.00	0.00	0.00	0.00	0.00

GATCAACATGCCTGTGTTTG	0.00	0.00	0.00	0.34	0.00
GATCAACATGCCTTACCCCT	0.23	0.00	0.00	0.00	0.00
GATCAACATGCCTTACGGCA	0.00	0.33	0.00	0.00	0.00
GATCAACATGCGACCGCGCC	0.00	0.00	0.00	0.34	0.00
GATCAACATGCGAGTGAAGC	0.00	0.00	0.29	0.00	0.00
GATCAACATGCGCGCATGGG	0.00	0.00	0.00	0.00	0.34
GATCAACATGCGCGTCTACA	0.00	0.00	0.00	0.00	0.00
GATCAACATGCGCGTGCGTG	0.00	0.33	0.00	0.00	0.00
GATCAACATGCGCTTGCCCG	0.23	0.00	0.00	0.00	0.00
GATCAACATGCGGAAGAAGC	0.00	0.00	0.00	0.00	0.34
GATCAACATGCGGCAGGTTC	0.23	0.00	0.00	0.68	0.00
GATCAACATGCGGCTCCTGG	0.00	0.00	0.00	0.00	0.00
GATCAACATGCGGGCATGAC	0.00	0.00	0.57	0.00	0.00
GATCAACATGCGGTGTGCCG	0.00	0.00	0.29	0.00	0.00
GATCAACATGCGTTGGCTCT	0.00	0.00	0.00	0.00	0.00
GATCAACATGCGTTGTCGCT	0.00	0.00	0.29	0.00	0.34
GATCAACATGCTAAACGAGT	0.23	0.00	0.00	0.00	0.00
GATCAACATGCTAACAGAAG	0.23	0.00	0.00	0.00	0.00
GATCAACATGCTATTTTTAG	0.00	0.00	0.00	0.00	0.34
GATCAACATGCTCAAAGAGC	0.23	0.00	0.00	0.00	0.00
GATCAACATGCTCAAAGAGG	0.00	0.00	0.00	0.00	0.00
GATCAACATGCTCAAAGAGT	1.37	0.98	0.57	0.68	1.72
GATCAACATGCTCAACATGA	0.00	0.00	0.29	0.00	0.00
GATCAACATGCTCAAGACTC	0.00	0.00	0.00	0.34	0.00
GATCAACATGCTCACAGAGG	0.00	0.00	0.00	0.00	0.00
GATCAACATGCTCACAGCAG	0.23	0.00	0.00	0.34	0.00
GATCAACATGCTCACAGCGG	0.00	0.00	0.00	0.00	0.00
GATCAACATGCTCACAGTAG	21.52	10.41	4.58	10.18	4.48
GATCAACATGCTCACAGTCN	0.23	0.00	0.00	0.00	0.00
GATCAACATGCTCACAGTNN	0.23	0.00	0.00	0.00	0.00
GATCAACATGCTCACTCGTA	0.00	0.00	0.00	0.00	0.34
GATCAACATGCTCATCATAA	0.00	0.00	0.00	0.34	0.00
GATCAACATGCTCATCATGA	0.00	0.98	1.43	1.70	1.38
GATCAACATGCTCCATGGGA	0.00	0.00	0.00	0.68	0.69
GATCAACATGCTCGCAAACC	0.00	0.00	0.00	0.00	0.34
GATCAACATGCTCGGCCCCC	0.00	0.00	0.29	0.00	0.00
GATCAACATGCTCGGTCAAA	0.00	0.33	0.00	0.00	0.00

GATCAACATGCTCGGTCCAA	0.00	0.00	0.29	0.00	0.00
GATCAACATGCTCGGTCCCC	1.60	0.00	0.29	0.00	0.69
GATCAACATGCTCGTATGCC	0.00	0.00	0.29	0.00	0.00
GATCAACATGCTCTACCCCT	0.00	0.00	0.00	0.00	0.00
GATCAACATGCTCTTCCTGG	0.00	0.00	0.00	0.34	0.00
GATCAACATGCTGAAGAAGA	0.00	0.00	0.00	0.00	0.00
GATCAACATGCTGAGCGCTG	0.00	0.00	0.29	0.00	0.00
GATCAACATGCTGAGCTGTC	0.00	0.00	0.00	0.00	0.69
GATCAACATGCTGAGTGGCA	0.00	0.00	0.00	0.00	0.00
GATCAACATGCTGAGTGGGC	0.00	0.00	0.00	0.34	0.00
GATCAACATGCTGCATTGGT	0.46	0.00	0.00	0.00	0.00
GATCAACATGCTGCCAGTGT	0.00	0.65	0.00	0.00	0.00
GATCAACATGCTGCCGCAAA	0.00	0.00	0.29	0.00	0.00
GATCAACATGCTGCCGCGCT	0.69	0.00	0.00	0.00	0.00
GATCAACATGCTGCCTTTGC	0.00	0.00	0.00	0.00	0.34
GATCAACATGCTGGAGAAAA	0.00	0.00	0.00	0.34	0.00
GATCAACATGCTGGCGGGCG	0.00	0.00	0.00	0.00	0.34
GATCAACATGCTGTGAAAAC	0.00	0.00	0.00	0.00	0.00
GATCAACATGCTTACTTCCT	0.00	0.00	0.00	0.00	0.00
GATCAACATGCTTCTGCAGC	2.06	0.00	0.00	1.02	0.00
GATCAACATGCTTCTGCCGG	0.00	0.00	0.29	0.00	0.00
GATCAACATGCTTGACATGT	0.00	0.00	0.00	0.00	0.00
GATCAACATGCTTGGTCCCC	0.00	0.00	0.00	0.34	0.00
GATCAACATGCTTTAGGTCC	0.00	0.00	0.57	0.00	0.00
GATCAACATGGAAAAAAAAA	0.46	0.00	0.00	0.00	0.00
GATCAACATGGAAACTCTCG	0.00	0.00	0.00	0.00	0.34
GATCAACATGGAACCGCTCG	0.00	0.00	0.00	0.34	0.00
GATCAACATGGAAGACACCA	0.00	0.33	0.00	0.68	0.00
GATCAACATGGAATCTCTAG	0.00	0.33	0.00	0.00	0.00
GATCAACATGGAATCTCTCG	12.82	54.98	44.39	27.15	41.33
GATCAACATGGAATCTCTCN	0.00	0.00	0.00	0.00	0.00
GATCAACATGGAATCTCTCT	0.00	0.33	0.00	0.00	0.00
GATCAACATGGAATCTCTNN	0.23	0.00	0.00	0.00	0.00
GATCAACATGGAATTTCTCG	0.23	0.00	0.00	0.00	0.00
GATCAACATGGACAGGAATG	0.00	0.00	0.00	0.00	0.00
GATCAACATGGACATCTGCA	0.00	0.00	0.00	0.00	0.34
GATCAACATGGACCAGGTCA	0.23	0.65	0.00	0.68	0.00

GATCAACATGGACCTTCAAG	0.00	0.00	0.00	0.34	0.00
GATCAACATGGACGCCTCCA	0.23	0.00	0.00	0.00	0.00
GATCAACATGGACGGATCTG	0.00	0.00	0.00	0.00	0.00
GATCAACATGGACGGCTCCG	0.92	0.65	0.57	0.34	0.34
GATCAACATGGACGGCTCTG	5.72	4.23	4.01	3.39	1.72
GATCAACATGGACTGTGGGT	0.00	0.00	0.00	0.00	0.69
GATCAACATGGACTTGCCCA	0.00	0.33	0.00	0.34	0.00
GATCAACATGGAGAACGAGC	0.46	0.33	0.00	0.00	0.00
GATCAACATGGAGACGGAAA	0.00	0.00	0.29	0.00	0.00
GATCAACATGGAGACGGCAG	0.23	0.00	0.00	0.00	0.00
GATCAACATGGAGACGGGAG	0.69	0.33	0.00	0.00	0.00
GATCAACATGGAGACGGTAA	0.92	0.33	0.57	0.00	0.00
GATCAACATGGAGACGGTAG	12.59	4.88	4.58	5.43	2.07
GATCAACATGGAGACGGTAN	0.23	0.00	0.00	0.00	0.00
GATCAACATGGAGACGGTGG	0.00	0.00	0.29	0.00	0.00
GATCAACATGGAGACGGTNN	0.23	0.00	0.00	0.00	0.00
GATCAACATGGAGCTGCAGC	0.23	0.00	0.57	0.68	0.34
GATCAACATGGAGCTGCNNN	0.00	0.00	0.00	0.00	0.00
GATCAACATGGAGGAAGCCG	0.00	0.00	0.00	0.68	0.00
GATCAACATGGAGGCAGCGA	0.00	0.33	0.00	0.00	0.00
GATCAACATGGAGTGCGTCA	0.23	0.00	0.00	0.00	0.00
GATCAACATGGAGTGGCCTG	0.00	0.00	0.00	0.34	0.00
GATCAACATGGATGAGGCCA	0.00	0.33	0.00	0.00	0.00
GATCAACATGGATGCTCTGC	0.00	0.00	0.00	0.00	0.00
GATCAACATGGATGTGAGAG	0.00	0.00	0.00	0.00	0.00
GATCAACATGGCAATCTTTG	0.46	0.00	0.00	0.00	0.00
GATCAACATGGCACACAACG	0.00	0.00	0.00	0.00	0.00
GATCAACATGGCAGACCAGG	0.00	0.00	0.00	0.34	0.00
GATCAACATGGCAGAGAGAT	0.00	0.00	0.00	0.00	0.00
GATCAACATGGCATGCATTA	0.00	0.33	0.00	0.00	0.00
GATCAACATGGCCAACAAGC	0.23	0.00	0.00	0.00	0.00
GATCAACATGGCCACCGCGC	0.00	0.65	0.00	0.00	0.34
GATCAACATGGCCAGCTCCA	0.46	0.00	0.00	0.00	0.00
GATCAACATGGCCGCAGACA	0.00	0.00	0.00	0.00	0.34
GATCAACATGGCCGGCTCCG	0.23	0.00	0.00	0.00	0.00
GATCAACATGGCCTCTTACT	0.00	0.00	0.00	0.00	0.00
GATCAACATGGCCTCTTCAG	0.23	0.00	0.00	0.00	0.00

GATCAACATGGCCTGGGCGT	0.00	0.00	0.29	0.00	0.00
GATCAACATGGCCTTAAGAT	0.46	0.00	0.00	0.00	0.00
GATCAACATGGCCTTCAACA	0.00	0.00	0.00	0.68	0.00
GATCAACATGGCGGCAAGAG	0.00	0.00	0.00	0.68	0.34
GATCAACATGGCGGCGCTCA	1.14	0.00	0.57	1.02	0.00
GATCAACATGGCGGCGCTCC	0.00	0.00	0.00	0.34	0.00
GATCAACATGGCTAGAGGTA	0.00	0.33	0.00	0.00	0.00
GATCAACATGGCTATGGTCC	0.00	0.00	0.00	0.00	0.34
GATCAACATGGCTCTGGATT	0.23	0.00	0.00	0.00	0.00
GATCAACATGGCTGAAGAGT	1.14	0.33	1.15	1.70	0.69
GATCAACATGGCTGAAGANT	0.00	0.00	0.00	0.34	0.00
GATCAACATGGCTGAAGGGT	0.23	0.00	0.00	0.00	0.00
GATCAACATGGCTGGGCAAA	0.00	0.00	0.00	0.00	0.00
GATCAACATGGCTGGGGAAA	0.00	0.65	0.00	0.00	0.00
GATCAACATGGCTGGGGGAA	0.00	0.00	0.00	0.34	0.00
GATCAACATGGGATCTCTCG	0.00	0.00	0.29	0.00	0.34
GATCAACATGGGCAAATTTT	0.00	0.00	0.29	0.00	0.00
GATCAACATGGGCCTCCAGG	0.00	0.33	0.00	0.00	0.00
GATCAACATGGGCGACTGGG	0.00	0.00	0.00	0.00	0.34
GATCAACATGGGCGTTTGAT	0.00	0.00	0.29	0.00	0.00
GATCAACATGGGGAGCCCCC	0.00	0.00	0.00	0.00	0.00
GATCAACATGGGGCACGCCC	0.00	0.00	0.29	0.00	0.00
GATCAACATGGGGGAACCAG	0.00	0.00	0.29	0.34	0.00
GATCAACATGGGGGGATGAG	0.00	0.00	0.57	0.00	0.00
GATCAACATGGGGGGGAGGG	0.00	0.00	0.57	0.00	0.00
GATCAACATGGGTTGTGTCG	0.46	0.65	0.29	0.00	0.34
GATCAACATGGTAATCGCAC	0.46	0.33	0.57	0.00	0.00
GATCAACATGGTCAAAAAAA	0.00	0.65	0.00	0.00	0.00
GATCAACATGGTCAAAGGCA	0.00	0.00	0.00	0.00	0.00
GATCAACATGGTCCAGGCGG	0.00	0.00	0.00	0.34	0.00
GATCAACATGGTCCGCAGCA	0.00	0.00	0.00	0.00	0.00
GATCAACATGGTCGTTAAAA	0.00	0.00	0.00	0.00	0.00
GATCAACATGGTGAACTTCT	0.00	0.00	0.29	0.00	0.00
GATCAACATGGTGACGGTAG	0.23	0.00	0.00	0.00	0.00
GATCAACATGGTGAGCACCT	0.00	0.00	0.00	0.68	0.34
GATCAACATGGTGAGCGCCG	0.00	0.00	0.00	0.00	0.00
GATCAACATGGTGATGGGGG	0.00	0.00	0.00	0.00	0.00

GATCAACATGGTGGAGCCCT	0.00	0.00	0.29	0.00	0.00
GATCAACATGGTGGTGCTCC	0.00	0.00	0.00	0.00	0.00
GATCAACATGGTGGTGTGTG	0.00	0.00	0.00	0.00	0.00
GATCAACATGGTGTTGGGGG	0.00	0.33	0.00	0.00	0.00
GATCAACATGGTTCGGAATC	0.00	0.00	0.00	0.00	0.00
GATCAACATGGTTGGATTAC	0.00	0.00	0.00	0.00	0.34
GATCAACATGGTTGGCGCAT	0.00	0.00	0.00	0.00	0.69
GATCAACATGGTTGTGCGAG	0.00	0.00	0.00	0.34	0.00
GATCAACATGGTTGTGCGCG	0.00	0.00	0.00	0.00	0.00
GATCAACATGGTTGTGTAAG	0.00	0.33	0.00	0.00	0.00
GATCAACATGGTTGTGTGAG	10.76	14.97	14.89	11.54	16.19
GATCAACATGGTTGTGTGTG	0.23	0.00	0.00	0.00	0.00
GATCAACATGGTTGTGTTAG	0.00	0.00	0.00	0.34	0.00
GATCAACATGTAAAAAAAAA	0.00	0.00	0.00	0.68	0.00
GATCAACATGTAAACACTAC	0.92	0.00	0.00	0.00	0.00
GATCAACATGTAAACTCACG	0.00	0.00	0.00	0.00	0.34
GATCAACATGTAAATTAAAA	0.00	0.00	0.00	0.00	0.00
GATCAACATGTAAATTCAAA	0.69	0.33	0.00	0.00	0.00
GATCAACATGTAAATTCACG	30.90	27.33	15.18	14.93	15.50
GATCAACATGTAAATTCACN	0.46	0.00	0.00	0.00	0.00
GATCAACATGTAAATTCACT	0.92	0.65	0.29	0.00	0.00
GATCAACATGTAAATTCATG	0.00	0.00	0.00	0.00	0.00
GATCAACATGTAAATTCCCG	0.23	0.00	0.00	0.00	0.00
GATCAACATGTAAATTCGCC	0.00	0.00	0.00	0.00	0.00
GATCAACATGTAAATTCGCG	0.23	0.00	0.00	0.00	0.00
GATCAACATGTAACACTCAA	0.00	0.00	0.00	0.00	0.34
GATCAACATGTACAAGTTGC	0.00	0.33	0.29	0.00	0.00
GATCAACATGTACACCAGGC	0.00	0.00	0.00	0.00	0.00
GATCAACATGTACAGAGAGG	0.00	0.00	0.00	0.34	0.00
GATCAACATGTACATCACGC	0.00	0.00	0.00	0.00	0.00
GATCAACATGTACATCAGGC	33.19	23.42	30.93	26.82	24.80
GATCAACATGTACATCAGNN	0.23	0.00	0.00	0.00	0.00
GATCAACATGTACATCAGTC	0.00	0.33	0.00	0.00	0.00
GATCAACATGTACCGAGTTC	0.00	0.00	0.00	0.00	0.00
GATCAACATGTACGCCTCGC	0.00	0.00	0.00	0.00	0.34
GATCAACATGTAGGTTCACC	0.00	0.00	0.00	0.00	0.69
GATCAACATGTATATCAGGC	0.00	0.00	0.00	0.00	0.00

GATCAACATGTATCTTCGAA	0.00	0.00	0.00	0.00	0.34
GATCAACATGTATGCATCAA	0.00	0.00	0.00	0.00	0.34
GATCAACATGTCAAAAATCG	0.23	2.60	0.00	1.36	0.00
GATCAACATGTCAACACTAA	0.00	0.00	0.29	0.00	0.00
GATCAACATGTCAGGGGAGG	0.00	0.00	0.29	0.68	0.00
GATCAACATGTCAGGGNNNN	0.23	0.00	0.00	0.00	0.00
GATCAACATGTCCAGACAGG	0.00	0.00	0.00	0.00	0.00
GATCAACATGTCCAGCCAGT	0.00	0.65	0.00	0.00	0.00
GATCAACATGTCCAGTCAGG	6.18	4.55	1.72	2.38	3.10
GATCAACATGTCCAGTNNNN	0.00	0.00	0.00	0.00	0.00
GATCAACATGTCCATCAGGC	0.00	0.00	0.00	0.00	0.00
GATCAACATGTCCATGCCCT	0.00	0.33	0.00	0.00	0.00
GATCAACATGTCCATTCCGT	0.00	0.00	0.29	0.00	0.00
GATCAACATGTCCGCCGGCA	0.46	0.00	0.00	0.00	0.00
GATCAACATGTCCGGGTGGG	0.00	0.00	0.00	0.00	0.00
GATCAACATGTCCGGTCAGG	0.00	0.33	0.00	0.00	0.00
GATCAACATGTCCTACCCCT	0.00	0.33	0.29	0.00	0.00
GATCAACATGTCGGAAGAAA	0.23	0.33	0.00	0.00	0.00
GATCAACATGTCGGAAGAAC	5.49	1.30	0.00	1.70	0.00
GATCAACATGTCGGGCGTGG	0.00	0.33	0.00	0.00	0.00
GATCAACATGTCGGGCNTGG	0.00	0.00	0.00	0.00	0.34
GATCAACATGTCGTGGCTGG	0.00	0.00	0.00	0.00	0.34
GATCAACATGTCGTGTTAGT	0.00	0.00	0.00	0.00	0.00
GATCAACATGTCTAGTCAGG	0.00	0.00	0.00	0.00	0.00
GATCAACATGTCTGAGTTCA	0.00	0.33	0.00	0.34	0.00
GATCAACATGTGACTTTTGC	0.00	0.00	0.00	0.34	0.00
GATCAACATGTGCAAACACA	0.00	0.00	0.57	0.00	0.00
GATCAACATGTGCAAAGACA	0.00	0.00	0.00	0.00	0.34
GATCAACATGTGCAATGTTC	0.00	0.00	0.00	0.00	0.34
GATCAACATGTGCACACTGC	0.00	0.00	0.29	0.00	0.00
GATCAACATGTGCACGCGTC	0.00	0.00	0.29	0.00	0.00
GATCAACATGTGCATTCCGC	0.00	0.33	0.00	0.00	0.00
GATCAACATGTGCGCCCTGC	0.00	0.00	0.29	0.00	0.00
GATCAACATGTGCGCTCGTT	0.00	0.00	0.00	0.00	0.00
GATCAACATGTGCTTCAAGT	1.37	0.00	0.86	0.34	0.00
GATCAACATGTGCTTCAGGT	0.23	0.00	0.00	0.00	0.00
GATCAACATGTGCTTGCGTT	0.00	0.00	0.00	0.00	0.00

GATCAACATGTGGAGCGAAT	0.00	0.00	0.57	0.00	0.00
GATCAACATGTGGCAAAAAA	1.83	0.33	0.86	0.00	0.34
GATCAACATGTGGGCTGATT	0.92	0.00	0.29	1.02	0.00
GATCAACATGTGTCTTTCAA	0.00	0.00	0.00	0.00	0.34
GATCAACATGTTCACTGTGG	0.23	0.00	0.00	0.00	0.00
GATCAACATGTTCAGCAGCA	0.00	0.00	0.00	0.00	0.00
GATCAACATGTTCCGGACTT	0.00	0.00	0.29	0.00	0.00
GATCAACATGTTCCTGAAGC	0.23	0.00	0.00	0.00	0.00
GATCAACATGTTCCTGAGGC	0.00	0.00	0.00	0.68	0.00
GATCAACATGTTCCTGCTTG	0.00	0.00	0.00	0.00	0.00
GATCAACATGTTCGGCAGGG	0.00	0.00	0.00	0.00	0.34
GATCAACATGTTCTAATCTG	0.00	0.00	0.00	0.00	0.00
GATCAACATGTTGGAAGACC	0.00	0.00	0.00	0.00	0.00
GATCAACATGTTGGAAGATG	0.00	0.00	0.00	0.00	0.00
GATCAACATGTTGGCAGACG	0.00	0.00	0.00	0.00	0.69
GATCAACATGTTGGTCTTCC	0.00	0.00	0.00	0.00	0.00
GATCAACATGTTTAGTGTTG	0.00	0.00	0.29	0.00	0.00
GATCAACATGTTTGACGAGG	0.00	0.00	0.00	0.00	0.69
GATCAACATGTTTGAGGGCG	0.00	0.00	0.29	0.00	0.00
GATCAACATGTTTGAGTGCG	0.00	0.00	0.29	0.00	0.00
GATCAACATGTTTGAGTGGG	0.00	0.00	0.00	0.34	0.34
GATCAACATGTTTGGGGCAG	0.00	0.00	0.00	0.00	0.69
GATCAACATGTTTTAAGTTA	0.00	0.00	0.00	0.00	0.34
GATCAACATGTTTTAAGTTG	2.06	4.23	9.74	7.13	12.74
GATCAACATGTTTTAATTTG	0.00	0.00	0.00	0.00	0.34
GATCAACATTAAAAAAAAAA	0.92	0.65	0.29	0.34	3.79
GATCAACATTAAAAAAAAAT	0.00	0.00	0.29	0.00	0.00
GATCAACATTAAGGCCCTGC	0.00	0.00	0.00	0.00	0.00
GATCAACATTAATAAAAAAA	0.00	0.33	0.00	0.00	0.00
GATCAACATTAATCCATCCG	0.00	0.33	0.00	0.00	0.00
GATCAACATTACATTCATTG	0.46	0.00	0.57	0.00	0.34
GATCAACATTACTCCAAGCC	0.00	0.00	0.00	0.00	0.00
GATCAACATTAGCAAGGAGG	0.00	0.33	0.00	0.00	0.00
GATCAACATTAGCTCTGGTG	0.00	0.00	0.00	0.00	0.00
GATCAACATTAGCTCTTGCA	0.00	0.00	0.00	0.34	0.00
GATCAACATTATCAGGAGGG	0.00	0.98	0.00	0.00	0.00
GATCAACATTCAAAAAAAAA	0.00	0.00	0.86	0.00	0.00

GATCAACATTCAAAGCAGCC	0.00	0.65	0.57	0.00	0.00
GATCAACATTCAAAGCAGTC	0.00	0.00	0.00	0.00	0.34
GATCAACATTCAACGCTGTC	0.00	0.00	0.00	0.00	0.34
GATCAACATTCAAGCAGCAT	0.23	0.00	0.00	0.00	0.69
GATCAACATTCACCAGATTG	0.23	0.00	0.29	0.00	0.00
GATCAACATTCAGACTGCGA	0.00	0.00	0.00	0.00	0.00
GATCAACATTCAGGAGGACA	0.00	0.33	0.00	0.00	0.00
GATCAACATTCATGTGTGTG	0.00	0.00	0.29	0.00	0.00
GATCAACATTCATTGGAATA	0.00	0.00	0.00	0.00	0.34
GATCAACATTCATTGGNATA	0.00	0.00	0.00	0.00	0.34
GATCAACATTCCATACCCCT	0.00	0.00	0.00	0.00	0.34
GATCAACATTCCCAAGCACA	1.83	1.30	1.43	1.02	1.03
GATCAACATTCCCATGTGTC	0.69	0.00	0.00	1.02	0.00
GATCAACATTCCGCCCTCGA	0.00	0.00	0.00	0.00	0.00
GATCAACATTCCGGAGGACC	0.00	0.00	0.00	0.00	0.00
GATCAACATTCCTTGCTCCT	1.83	1.30	1.43	2.04	2.07
GATCAACATTCGAGGGTTTC	0.00	0.00	0.29	0.00	0.00
GATCAACATTCGTATGCCGT	0.00	0.00	0.00	0.34	0.00
GATCAACATTCGTGGAAGCA	0.23	0.00	0.00	0.00	0.00
GATCAACATTCGTGGCAGCG	0.23	0.00	0.00	0.00	0.00
GATCAACATTCGTTCCAAAA	0.00	0.00	0.29	0.00	0.00
GATCAACATTCGTTCCCACA	0.00	0.00	0.29	0.00	0.00
GATCAACATTCTCATGGATG	0.00	0.00	0.00	0.34	0.00
GATCAACATTCTCCTGCGAA	0.00	0.33	0.00	0.00	0.00
GATCAACATTCTCGAGGACT	0.00	0.00	0.00	0.34	0.00
GATCAACATTCTCGGCAGAA	0.00	0.00	0.00	0.00	0.00
GATCAACATTCTCTAGTTCA	0.00	0.33	0.00	0.00	0.00
GATCAACATTCTCTGGATCA	0.00	0.00	0.00	0.00	0.34
GATCAACATTCTCTGGTTCA	0.00	5.21	3.72	2.04	2.76
GATCAACATTCTGAGCACAC	0.00	0.00	0.00	0.00	0.34
GATCAACATTCTGGACGTAC	0.00	0.00	0.29	0.00	0.00
GATCAACATTCTGGAGGACA	0.00	0.00	0.00	0.00	0.69
GATCAACATTCTGGAGGACT	2.52	5.86	3.72	11.54	2.41
GATCAACATTCTGGAGGATA	0.00	0.00	0.00	0.00	0.34
GATCAACATTCTGGAGTTTG	0.00	0.00	0.00	0.00	0.00
GATCAACATTCTGTTGCCCG	0.00	0.00	0.00	0.00	0.00
GATCAACATTCTTACCAGGG	0.00	0.00	0.00	0.00	0.00

GATCAACATTCTTAGCAAAA	0.00	0.00	0.00	0.34	0.00
GATCAACATTCTTGGCAAAA	0.00	0.00	0.00	0.68	0.00
GATCAACATTCTTGGCAAGA	6.18	3.90	0.29	2.04	0.34
GATCAACATTCTTGGCAAGC	0.23	0.00	0.00	0.00	0.00
GATCAACATTCTTGGCAAGG	0.00	0.00	0.29	0.00	0.00
GATCAACATTCTTGGGCCGC	0.23	0.00	0.00	0.00	0.00
GATCAACATTGAAAAAAAAA	0.00	0.00	0.29	0.00	0.00
GATCAACATTGAAACANCAT	0.00	0.00	0.00	0.00	0.34
GATCAACATTGAAACATCAT	0.00	0.00	0.00	0.00	0.34
GATCAACATTGAATCTCTCG	0.00	0.33	0.00	0.00	0.00
GATCAACATTGACACTTGGA	0.00	0.00	0.00	0.00	0.00
GATCAACATTGACGACAGGG	0.00	0.00	0.00	0.00	0.00
GATCAACATTGACGACGGGG	0.23	0.00	0.00	0.00	0.00
GATCAACATTGACGACTGAG	0.00	0.00	0.00	0.00	0.00
GATCAACATTGACGACTGGA	0.00	0.65	0.00	0.00	0.00
GATCAACATTGACGACTGGG	40.52	17.57	2.58	10.52	5.51
GATCAACATTGACGACTNNN	0.23	0.00	0.00	0.00	0.00
GATCAACATTGACGCCTGGT	0.23	0.00	0.00	0.00	0.00
GATCAACATTGACGTCGCAA	0.00	0.00	0.00	0.00	0.34
GATCAACATTGACTACTGGG	0.46	0.00	0.00	0.00	0.00
GATCAACATTGACTCCTGGA	0.00	0.00	0.00	0.00	0.00
GATCAACATTGACTCGTTGC	0.23	0.33	0.00	0.34	0.00
GATCAACATTGAGATGGCGT	0.00	0.00	0.00	0.00	0.00
GATCAACATTGAGCTGCCTA	0.00	0.65	0.00	0.00	0.00
GATCAACATTGAGGAGGACG	0.00	0.00	0.00	0.00	0.00
GATCAACATTGAGGAGTTCG	0.00	0.00	0.00	0.68	0.00
GATCAACATTGAGTATGCCA	0.23	0.00	0.00	0.00	0.00
GATCAACATTGATAGACATG	0.00	0.00	0.29	0.00	0.00
GATCAACATTGATGAGGAGG	0.00	0.00	0.00	0.00	0.00
GATCAACATTGATGCTCCTG	0.00	0.00	0.29	0.00	0.00
GATCAACATTGATGGCATGC	0.00	0.00	0.29	0.00	0.00
GATCAACATTGCAAATGGCA	0.00	0.00	0.00	0.00	0.00
GATCAACATTGCAACTTGGG	0.00	0.00	0.00	0.00	0.00
GATCAACATTGCAGAAGCCA	0.46	0.65	0.29	0.34	0.00
GATCAACATTGCAGACGCCA	0.00	0.00	0.00	0.34	0.00
GATCAACATTGCAGCACAGT	0.00	0.00	0.00	0.34	0.00
GATCAACATTGCAGTCATTA	0.23	0.33	0.00	0.00	0.00

GATCAACATTGCAGTGGAGC	0.00	0.00	0.00	0.00	0.00
GATCAACATTGCATCTGTCT	0.00	0.00	0.29	0.00	0.00
GATCAACATTGCCAAAAAAA	0.00	0.00	0.29	0.00	0.00
GATCAACATTGCCAAGGCCA	0.00	0.00	0.29	0.00	0.00
GATCAACATTGCCACCATGT	0.00	0.00	0.00	0.00	0.00
GATCAACATTGCCACGCATT	0.00	0.00	0.00	0.00	0.34
GATCAACATTGCCAGCAGGT	0.00	0.00	0.00	0.34	0.00
GATCAACATTGCCAGCAGTT	3.43	7.81	4.30	12.56	6.54
GATCAACATTGCCATATAAA	0.00	0.00	0.29	0.00	0.00
GATCAACATTGCCATATACA	0.00	0.00	0.57	0.00	0.00
GATCAACATTGCCGCCTGGG	0.23	0.00	0.00	0.00	0.00
GATCAACATTGCCTTCTTCT	0.92	0.00	0.00	0.00	0.34
GATCAACATTGCGAAGGACG	0.00	0.00	0.00	0.00	0.00
GATCAACATTGCGTGAGCGC	0.23	0.00	0.00	0.00	0.00
GATCAACATTGCGTGAGCTC	4.81	1.95	2.29	2.38	1.72
GATCAACATTGCGTGGGCTC	0.00	0.00	0.00	0.00	0.34
GATCAACATTGCTCCGTTGG	0.00	0.33	0.00	0.00	0.00
GATCAACATTGCTCCGTTGT	0.46	0.00	0.00	0.00	0.00
GATCAACATTGCTCTAATGT	0.00	0.00	0.00	0.00	0.00
GATCAACATTGCTGCCCTAG	0.00	0.00	0.29	0.00	0.00
GATCAACATTGCTGGGCGTT	0.92	0.33	0.00	0.68	0.34
GATCAACATTGCTTTTCATG	0.00	0.65	0.00	0.00	0.00
GATCAACATTGGAAGGACAA	0.46	0.33	0.57	0.00	1.03
GATCAACATTGGAGGGAAGT	0.00	0.00	0.00	0.34	0.00
GATCAACATTGGCATTGTCA	0.00	0.00	0.00	0.00	0.34
GATCAACATTGGCCAGCCAC	0.00	0.00	0.29	0.00	0.00
GATCAACATTGGCCGTAGGG	0.00	0.00	0.00	0.34	0.00
GATCAACATTGGCTGATTGG	0.00	0.00	0.29	0.00	0.00
GATCAACATTGGCTTCAGCC	0.00	0.00	0.57	0.00	0.00
GATCAACATTGGCTTGCATC	0.92	0.00	0.29	0.00	0.00
GATCAACATTGGGCGGTGGC	0.00	0.33	0.00	0.00	0.00
GATCAACATTGGTCTGCACA	0.23	0.00	0.00	0.00	0.00
GATCAACATTGGTCTGTACA	0.00	0.00	0.00	0.00	0.00
GATCAACATTGTAAAAAAAA	2.52	2.60	4.58	2.38	3.79
GATCAACATTGTAAAAAACA	0.23	0.00	0.00	0.00	0.00
GATCAACATTGTAAAAAACN	0.23	0.00	0.00	0.00	0.00
GATCAACATTGTAACAAAAA	0.00	0.00	0.00	0.00	0.00

GATCAACATTGTACGGAAGT	0.00	0.00	0.29	0.00	0.00
GATCAACATTGTAGAAAAAA	0.00	0.00	0.57	0.00	0.69
GATCAACATTGTCAAAAAAA	3.20	3.25	13.75	5.43	11.37
GATCAACATTGTCAAAAAAT	0.00	0.00	0.00	0.00	0.00
GATCAACATTGTCAAAAGAA	0.23	0.00	0.00	0.00	0.00
GATCAACATTGTCAAANAAA	0.00	0.00	0.00	0.00	0.34
GATCAACATTGTCAAGAAAA	0.00	0.00	0.00	0.00	0.00
GATCAACATTGTCATAGAAA	0.00	0.00	0.00	0.00	0.00
GATCAACATTGTCATCGTAT	0.69	0.00	0.00	0.00	0.00
GATCAACATTGTCATGGTCA	0.00	0.00	0.29	0.00	0.00
GATCAACATTGTCATTACCG	0.00	0.00	0.00	0.00	0.00
GATCAACATTGTCATTGTAA	0.69	0.00	0.00	0.68	0.00
GATCAACATTGTCATTGTAG	0.00	0.00	0.00	0.00	0.34
GATCAACATTGTCATTGTCA	33.65	33.18	44.10	38.02	45.12
GATCAACATTGTCATTGTCC	0.23	0.65	0.29	0.00	0.00
GATCAACATTGTCATTGTCG	0.00	0.00	0.00	0.68	0.00
GATCAACATTGTCATTGTCT	0.00	0.00	0.00	0.00	0.34
GATCAACATTGTCATTGTGA	0.00	0.00	0.00	0.00	0.00
GATCAACATTGTCATTGTTC	0.00	0.00	0.00	0.34	0.00
GATCAACATTGTCATTNTCA	0.00	0.00	0.00	0.00	0.69
GATCAACATTGTCATTTCGA	0.00	0.33	0.00	0.00	0.00
GATCAACATTGTCATTTTCA	0.00	0.00	0.00	0.00	0.00
GATCAACATTGTCCAAAAAA	0.00	0.00	0.00	0.00	1.03
GATCAACATTGTCCAGCACT	0.00	0.00	0.00	0.00	0.00
GATCAACATTGTCCTCTCTA	0.00	0.00	0.00	0.00	0.34
GATCAACATTGTCCTGGTCC	0.23	0.00	0.00	0.00	0.00
GATCAACATTGTCGAAAAAA	0.00	0.00	0.29	0.00	0.00
GATCAACATTGTCGCTNTTG	0.00	0.00	0.00	0.00	0.34
GATCAACATTGTCTCGTATG	0.00	0.33	0.00	0.00	0.00
GATCAACATTGTCTGTTCGT	0.00	0.00	0.57	0.00	0.00
GATCAACATTGTCTTTATGA	0.00	0.33	0.00	0.00	0.00
GATCAACATTGTCTTTGTCA	0.00	0.00	0.00	0.00	1.03
GATCAACATTGTGCTGCTGA	0.00	0.33	0.00	0.00	0.00
GATCAACATTGTGGAGGTTC	0.00	0.00	0.00	0.00	0.34
GATCAACATTGTGGAGGTTT	0.00	0.00	0.29	0.00	0.00
GATCAACATTGTGGGCTCCA	0.00	0.33	0.57	0.00	0.00
GATCAACATTGTTAAAAAAA	0.23	0.00	0.00	0.00	0.00

GATCAACATTGTTGAAAAAA	0.23	0.00	0.57	0.68	0.00
GATCAACATTGTTGTGTGAG	0.00	0.00	0.00	0.34	0.00
GATCAACATTTAAAAAACAA	0.00	0.00	0.29	0.00	0.00
GATCAACATTTACCGGTGGC	0.00	0.00	0.29	0.00	0.00
GATCAACATTTAGAAGGGCT	0.00	0.33	0.00	0.00	0.00
GATCAACATTTATCCGAGGG	0.00	0.00	0.00	0.34	0.00
GATCAACATTTCACACGTGT	0.00	0.33	0.00	0.00	0.00
GATCAACATTTCAGCCTCAA	0.00	0.33	0.00	0.00	0.00
GATCAACATTTCAGCTGTGT	0.23	0.00	0.00	0.00	0.00
GATCAACATTTCCAAACCAA	0.00	0.00	0.00	0.00	0.34
GATCAACATTTCTACTCAGA	0.00	0.00	0.00	0.68	0.00
GATCAACATTTCTACTCCCT	0.00	0.00	0.00	0.00	0.34
GATCAACATTTCTAGATTGG	0.00	0.33	0.00	0.00	0.00
GATCAACATTTCTGCATGCC	0.00	0.00	0.00	0.00	0.00
GATCAACATTTCTTCATGGT	0.46	0.00	0.57	0.34	0.00
GATCAACATTTCTTGCTTGT	0.00	0.00	0.00	0.00	0.69
GATCAACATTTGAAGTAAAA	0.00	0.00	0.00	0.00	0.00
GATCAACATTTGAGACTCAA	0.00	0.65	0.29	0.00	0.00
GATCAACATTTGAGCATCAA	0.00	0.00	0.00	0.00	0.69
GATCAACATTTGAGCATCAC	0.23	0.00	0.00	0.00	0.00
GATCAACATTTGAGCCAATC	0.00	0.00	0.00	0.00	0.34
GATCAACATTTGAGCCCCAA	0.00	0.00	0.00	0.00	0.00
GATCAACATTTGAGCCCCCA	0.00	0.00	0.00	0.00	0.00
GATCAACATTTGAGCCCCTT	0.00	0.00	0.00	0.00	0.00
GATCAACATTTGAGCCNCAA	0.00	0.00	0.00	0.00	0.34
GATCAACATTTGAGCCNNNN	0.46	0.00	0.00	0.00	0.00
GATCAACATTTGAGCCTAAA	0.23	0.33	0.00	0.68	0.69
GATCAACATTTGAGCCTAAC	0.00	0.00	0.00	0.34	0.00
GATCAACATTTGAGCCTACA	0.00	0.33	0.00	0.00	0.00
GATCAACATTTGAGCCTCAA	145.13	217.32	116.55	135.77	92.99
GATCAACATTTGAGCCTCAC	0.23	0.00	0.29	0.34	0.34
GATCAACATTTGAGCCTCAN	0.23	0.00	0.00	0.00	0.00
GATCAACATTTGAGCCTCAT	0.00	0.33	0.00	0.00	0.00
GATCAACATTTGAGCCTCCA	0.46	0.00	0.57	0.34	0.00
GATCAACATTTGAGCCTCCC	0.23	0.00	0.00	0.00	0.00
GATCAACATTTGAGCCTCCN	0.00	0.00	0.00	0.00	0.00
GATCAACATTTGAGCCTCGA	0.46	0.00	0.00	0.00	0.00

GATCAACATTTGAGCCTCNA	0.00	0.00	0.00	0.00	0.34
GATCAACATTTGAGCCTCNN	0.92	0.00	0.00	0.00	0.00
GATCAACATTTGAGCCTCTA	0.00	0.00	0.00	0.34	0.00
GATCAACATTTGAGCCTCTC	0.00	0.00	0.29	0.00	0.00
GATCAACATTTGAGCCTNAA	0.00	0.00	0.00	0.00	0.34
GATCAACATTTGAGCTTCAA	0.00	0.00	0.00	0.00	0.34
GATCAACATTTGAGTCTCAA	0.23	0.00	0.57	0.00	0.00
GATCAACATTTGCAACGCCG	0.00	0.00	0.00	0.00	0.00
GATCAACATTTGCAACGTCG	0.69	0.33	0.00	0.34	0.00
GATCAACATTTGCACTGACT	0.00	0.00	0.29	0.00	0.00
GATCAACATTTGCAGAGGCA	0.00	0.00	0.00	0.00	0.00
GATCAACATTTGCAGCCAGC	0.00	0.00	0.00	0.34	0.00
GATCAACATTTGCATGATTC	0.00	0.00	0.00	0.34	0.00
GATCAACATTTGCGCCTCAC	0.00	0.33	0.00	0.00	0.00
GATCAACATTTGCGCTTCCC	0.23	0.00	0.00	0.00	0.00
GATCAACATTTGGCTCAATG	0.00	0.33	0.00	0.00	0.00
GATCAACATTTGGGCCTCAA	0.23	0.00	0.00	0.00	0.00
GATCAACATTTGGGTTCAAA	0.92	0.00	0.00	0.34	0.00
GATCAACATTTGGGTTCAAG	1.83	0.00	1.43	1.36	1.03
GATCAACATTTGTCGTATGC	0.00	0.00	0.00	0.00	0.00
GATCAACATTTGTGCCTCAA	0.00	0.00	0.00	0.34	0.00
GATCAACATTTTCAAGAGCT	0.00	0.00	0.00	0.68	0.00
GATCAACATTTTCCACTGTG	0.00	0.65	0.00	0.00	0.00
GATCAACATTTTCCTGGCCG	0.00	0.00	0.00	0.00	0.00
GATCAACATTTTCTACCTTA	0.00	0.33	0.00	0.00	0.00
GATCAACATTTTCTTCAACG	0.00	0.00	0.00	0.00	0.00
GATCAACATTTTGCGCTGCT	0.00	0.00	0.00	0.00	0.00
GATCAACATTTTTGGTGGCA	1.14	0.98	0.00	2.04	0.00
GATCAACCAAAAAAAAAAAA	30.22	7.16	4.58	9.84	12.05
GATCAACCAAAAAAAAAAAC	0.23	0.00	0.00	0.00	0.00
GATCAACCAAAAAAAAAAAT	1.60	0.33	0.57	0.34	0.69
GATCAACCAAAAAAAAAACN	0.23	0.00	0.00	0.00	0.00
GATCAACCAAAAAAAAAATC	0.23	0.00	0.00	0.00	0.34
GATCAACCAAAAAAAATAAA	0.00	0.00	0.00	0.00	1.03
GATCAACCAAAAAACAAAAC	0.00	0.00	0.00	0.00	0.00
GATCAACCAAAAAACAAAAT	0.23	0.00	0.00	0.00	0.00
GATCAACCAAAAAACCCAAC	0.00	0.00	0.00	0.00	0.34

GATCAACCAAAAACAAAAAA	0.00	0.00	0.00	0.34	0.00
GATCAACCAAAAACAGATGA	0.00	0.33	0.00	0.00	0.34
GATCAACCAAAAACAGTTGA	0.00	0.00	0.29	0.00	0.00
GATCAACCAAAAACCCAAAC	0.00	0.00	0.00	0.00	0.00
GATCAACCAAAAATCTAAAA	0.00	0.00	0.57	0.00	0.00
GATCAACCAAAACACATATC	0.00	0.00	0.00	0.00	0.69
GATCAACCAAAAGAAAAAAA	0.00	0.00	0.00	0.00	0.34
GATCAACCAAAAGCGACAGA	0.00	0.00	0.00	0.00	0.00
GATCAACCAAAATCCAGATG	0.00	0.00	0.00	0.00	0.69
GATCAACCAAACACAAACAA	0.00	0.00	0.29	0.00	0.00
GATCAACCAAACACATACAA	0.00	0.00	0.29	0.00	0.00
GATCAACCAAACACCACAAA	0.00	0.00	0.00	0.00	0.00
GATCAACCAAACACTCTCCG	0.00	0.00	0.29	0.00	0.00
GATCAACCAAACAGGTGCCA	0.46	0.00	0.00	0.00	0.00
GATCAACCAAACATCTCTCT	0.00	0.33	0.00	1.02	0.34
GATCAACCAAACATGTTCCA	0.00	0.00	0.00	0.00	0.34
GATCAACCAAACCTATCCCA	0.00	0.00	0.57	0.00	0.00
GATCAACCAAACCTATCCCG	0.00	0.65	1.15	2.04	0.69
GATCAACCAAACCTTCGACT	0.00	0.00	0.00	0.00	0.00
GATCAACCAAACTACAAAGC	0.00	0.00	0.00	0.00	0.00
GATCAACCAAAGAACAACAG	0.00	0.00	0.00	0.00	0.00
GATCAACCAAAGACCAGACA	0.00	0.00	0.00	0.00	0.34
GATCAACCAAAGACGGGCCG	0.00	0.00	0.00	0.00	0.00
GATCAACCAAAGCCCGAAGC	0.00	0.00	0.00	0.00	0.00
GATCAACCAAAGCCCTCCTC	0.00	0.00	0.00	0.68	0.00
GATCAACCAAAGCGTCATCT	0.00	0.00	0.00	0.00	0.00
GATCAACCAAAGGAGTGCCG	0.00	0.00	0.00	0.00	0.00
GATCAACCAAAGGCCACAGG	0.00	0.00	0.00	0.34	0.00
GATCAACCAAAGGCGTCCGA	0.00	0.00	0.00	0.00	0.69
GATCAACCAAAGGCGTGAAG	0.00	0.00	0.29	0.00	0.00
GATCAACCAAAGGCGTGACG	0.00	0.33	0.00	0.00	0.34
GATCAACCAAAGGCGTGCCG	14.88	24.72	32.36	20.71	34.78
GATCAACCAAAGGCGTGTCA	0.00	0.00	0.29	0.00	0.00
GATCAACCAAAGGCGTGTCG	0.00	0.00	0.00	0.00	0.00
GATCAACCAAAGGCGTNANN	0.00	0.00	0.00	0.00	0.34
GATCAACCAAAGGCTCGTAT	0.00	0.33	0.00	0.00	0.00
GATCAACCAAAGGGGTGATT	0.00	0.00	0.00	0.00	0.34

GATCAACCAAAGTCCAAAAA	0.23	0.00	0.00	0.00	0.00
GATCAACCAAAGTCGTGCCG	0.00	0.00	0.00	0.00	0.34
GATCAACCAAATAAAAAAAA	0.46	0.00	0.00	0.00	0.00
GATCAACCAAATAAGTTTTC	0.00	0.00	0.00	0.00	0.69
GATCAACCAAATCAAAGGTC	0.00	0.00	0.00	0.00	0.34
GATCAACCAAATCAAGCACA	0.00	0.00	0.00	0.00	0.00
GATCAACCAAATCGGCTGTC	0.00	0.00	0.29	0.00	0.00
GATCAACCAAATGCCTGGGC	0.00	0.00	0.00	0.00	0.00
GATCAACCAAATTGGTTTTA	4.58	1.63	0.00	1.36	0.69
GATCAACCAACAAAAATAGC	0.00	0.00	0.00	0.34	0.00
GATCAACCAACAAATGGCTA	0.00	0.00	0.29	0.00	0.00
GATCAACCAACAACAACAAC	0.23	0.00	0.00	0.00	0.00
GATCAACCAACAACACATAC	0.00	0.00	0.29	0.00	0.69
GATCAACCAACAAGGCAAGG	0.00	0.33	0.00	0.00	0.00
GATCAACCAACAAGGCAATG	2.29	1.95	0.00	1.36	0.34
GATCAACCAACAAGGCAGTT	0.00	0.00	0.00	0.00	0.00
GATCAACCAACAAGGCTCGT	0.00	0.65	0.00	0.00	0.00
GATCAACCAACAATCAATCC	0.00	0.00	0.00	0.00	0.34
GATCAACCAACACAAAACAA	0.23	0.00	0.00	0.00	0.00
GATCAACCAACACAACAAGA	0.23	0.00	0.00	0.00	0.00
GATCAACCAACACAACACAA	0.00	0.00	0.00	0.00	1.38
GATCAACCAACACAACTAAC	0.00	0.00	0.29	0.00	0.00
GATCAACCAACACAAGAATG	0.00	0.00	0.29	0.00	0.00
GATCAACCAACACACAACAG	0.00	0.00	0.00	0.00	0.34
GATCAACCAACACATGCGGA	0.00	0.00	0.29	0.00	0.00
GATCAACCAACACTTGCATG	0.00	0.00	0.00	0.34	0.00
GATCAACCAACAGCCTGCTC	0.00	0.00	0.00	0.00	0.00
GATCAACCAACAGCTGGGCT	0.23	0.00	0.00	0.00	0.00
GATCAACCAACAGTATTTCT	0.00	0.00	0.00	0.00	0.00
GATCAACCAACAGTGCTGAG	0.00	0.33	0.00	0.00	0.00
GATCAACCAACATAACACAC	0.00	0.00	0.57	0.00	0.00
GATCAACCAACATCACATGC	0.00	0.00	0.00	0.00	0.34
GATCAACCAACCAACCAACC	0.00	0.00	0.00	0.00	0.34
GATCAACCAACCAACCACAC	0.00	0.00	0.00	0.00	0.34
GATCAACCAACCACCACATA	0.00	0.00	0.00	0.00	0.00
GATCAACCAACCACGCAGTC	0.00	0.00	0.00	0.00	0.00
GATCAACCAACCACTTCCTC	0.00	0.00	0.00	0.68	0.00

GATCAACCAACCACTTCCTT	0.00	0.00	0.57	0.00	0.00
GATCAACCAACCAGCCAATG	0.00	0.33	0.00	0.00	0.00
GATCAACCAACCATCGTCGG	0.00	0.00	0.29	0.00	0.00
GATCAACCAACCATTGCAGC	0.23	0.33	0.00	0.00	0.00
GATCAACCAACCCAGGTTGC	0.00	0.00	0.00	0.00	0.69
GATCAACCAACCCGAGTGGG	0.00	0.00	0.00	0.00	0.34
GATCAACCAACCCGATTGTG	0.00	0.00	0.29	0.00	0.00
GATCAACCAACCGGTCGTCC	0.00	0.00	0.29	0.00	0.00
GATCAACCAACCGTATGTGA	0.00	0.00	0.29	0.00	0.00
GATCAACCAACCTACGGGCA	0.00	0.00	0.00	0.00	1.03
GATCAACCAACGAAAGACCG	0.00	0.00	0.29	0.00	0.00
GATCAACCAACGAATGGTAT	0.00	0.00	0.86	0.00	0.34
GATCAACCAACGAATGGTGT	0.00	0.00	0.29	0.00	0.00
GATCAACCAACGCCCTGACC	0.00	0.00	0.00	0.00	0.34
GATCAACCAACGCCGCCGCG	0.00	0.00	0.00	0.00	0.00
GATCAACCAACGCGGAATGC	0.23	0.00	0.00	0.00	0.00
GATCAACCAACGGAAGCCTC	0.00	0.00	0.00	0.34	0.00
GATCAACCAACGGACGTCGA	0.00	0.00	0.00	0.68	0.00
GATCAACCAACGGCATACAC	0.00	0.00	0.29	0.00	0.00
GATCAACCAACGGGGAATGC	0.23	0.00	0.00	0.00	0.00
GATCAACCAACGGGGTTCTG	0.00	0.00	0.00	0.00	0.34
GATCAACCAACGGTGAATGC	0.00	0.00	0.29	0.00	0.00
GATCAACCAACGTCCGTTGG	0.00	0.00	0.00	0.00	0.00
GATCAACCAACGTCGCGTCC	0.00	0.33	0.00	0.00	0.00
GATCAACCAACGTCGTATGC	0.46	0.98	2.00	0.34	0.34
GATCAACCAACGTGGAAAAG	0.00	0.00	0.00	0.00	0.00
GATCAACCAACGTGGAAAGA	0.00	0.00	0.00	0.34	0.00
GATCAACCAACGTGGAACGC	0.00	0.00	0.00	0.68	0.00
GATCAACCAACGTGGAAGCG	0.00	0.00	0.29	0.00	0.00
GATCAACCAACGTGGAAGGC	0.00	0.00	0.29	0.00	0.00
GATCAACCAACGTGGAATAC	0.00	0.00	0.00	0.00	0.34
GATCAACCAACGTGGAATCC	0.00	0.00	0.00	0.00	0.34
GATCAACCAACGTGGAATGA	0.00	0.00	0.57	0.00	0.00
GATCAACCAACGTGGAATGC	62.03	49.12	58.70	61.10	87.48
GATCAACCAACGTGGAATGG	0.00	0.00	0.00	0.34	0.00
GATCAACCAACGTGGAATGN	0.00	0.00	0.00	0.34	0.00
GATCAACCAACGTGGAATGT	0.00	0.00	0.00	0.00	0.34

GATCAACCAACGTGGAATNC	0.00	0.00	0.00	0.68	0.00
GATCAACCAACGTGGACNNN	0.23	0.00	0.00	0.00	0.00
GATCAACCAACGTGGACTGA	0.00	0.00	0.00	0.34	0.00
GATCAACCAACGTGGACTGC	0.23	0.00	0.00	0.00	0.69
GATCAACCAACGTGGAGTGC	0.69	0.00	0.00	0.34	0.00
GATCAACCAACGTGGTCTGC	0.23	0.00	0.00	0.00	0.00
GATCAACCAACGTGGTGCGT	0.00	0.00	0.00	0.00	0.00
GATCAACCAACTAAACAACC	0.00	0.00	0.00	0.00	0.69
GATCAACCAACTCAACCAAC	0.00	0.00	0.00	0.00	0.34
GATCAACCAACTCCCCAAGA	0.00	0.00	0.00	0.00	0.34
GATCAACCAACTGAGACCCC	0.00	0.00	0.00	0.00	0.00
GATCAACCAACTGCCCGTCG	0.00	0.00	0.00	0.00	0.34
GATCAACCAACTGTTTTCGG	0.00	0.00	0.00	0.00	0.00
GATCAACCAACTTGCGCGGC	0.00	0.00	0.00	0.00	0.00
GATCAACCAACTTTGCGAGA	0.00	0.00	0.00	0.34	0.00
GATCAACCAAGAAAAAAAAA	9.39	1.95	1.15	1.36	2.76
GATCAACCAAGAAAAAAAAG	0.23	0.00	0.00	0.00	0.00
GATCAACCAAGAAAAAAAAN	0.23	0.00	0.00	0.00	0.00
GATCAACCAAGAACAGCCTG	0.00	0.00	0.00	0.00	0.00
GATCAACCAAGAAGAAAAAA	0.23	1.30	1.15	0.68	1.72
GATCAACCAAGAAGATAAAA	0.00	0.00	0.00	0.00	0.00
GATCAACCAAGAAGATGTCA	0.00	0.00	0.00	0.68	0.00
GATCAACCAAGAAGATGTTG	1.14	0.00	0.57	1.02	0.34
GATCAACCAAGAATCCTCCG	0.00	0.00	0.00	0.00	0.34
GATCAACCAAGACAAGACAA	0.00	0.00	0.00	0.00	0.34
GATCAACCAAGACAGACGAT	0.00	0.00	0.00	0.00	0.69
GATCAACCAAGACGAGCAAG	0.00	0.00	0.29	0.00	0.00
GATCAACCAAGAGATAGCCG	0.00	0.33	0.00	0.00	0.00
GATCAACCAAGAGATGAGAG	0.00	0.00	0.00	0.00	0.34
GATCAACCAAGAGGCAGGGC	0.00	0.00	0.00	0.00	0.34
GATCAACCAAGAGGCTTGGC	0.00	0.00	0.00	0.68	1.03
GATCAACCAAGATGGCGTAC	0.00	0.00	0.00	0.00	0.00
GATCAACCAAGCAAAAAAAA	0.46	0.00	0.00	0.00	0.00
GATCAACCAAGCAAACACGA	0.00	0.00	0.29	0.00	0.00
GATCAACCAAGCAAGCAACC	0.00	0.00	0.29	0.00	0.00
GATCAACCAAGCACGGGCCG	0.23	0.00	0.00	0.00	0.00
GATCAACCAAGCACTCGTGT	0.00	0.00	0.00	0.00	0.00

GATCAACCAAGCATGAAGGC	0.00	0.00	0.29	0.00	0.00
GATCAACCAAGCCATGCCTC	0.92	0.00	0.00	0.34	0.00
GATCAACCAAGCCGTCCACG	0.23	0.00	0.00	0.00	0.00
GATCAACCAAGCGAAAAAAA	0.00	0.33	0.00	0.00	0.00
GATCAACCAAGCGAAGCAAC	0.00	0.00	0.00	0.00	0.00
GATCAACCAAGCGAAGCGCA	0.00	0.33	0.29	0.00	0.00
GATCAACCAAGCGACGTGTG	0.00	0.00	0.00	0.00	0.00
GATCAACCAAGCGACTTGTG	0.00	0.98	0.00	0.00	0.00
GATCAACCAAGCGCCATGTG	0.46	0.00	0.00	0.00	0.00
GATCAACCAAGCGCCCAAAA	0.00	0.00	0.00	0.00	0.00
GATCAACCAAGCGCCCTGGT	0.00	0.00	0.00	0.00	0.00
GATCAACCAAGCGCCCTGTG	0.23	0.00	0.00	0.00	0.00
GATCAACCAAGCGCCTAAAA	0.46	0.00	0.00	0.00	0.00
GATCAACCAAGCGCCTATCG	0.00	0.00	0.00	0.00	0.34
GATCAACCAAGCGCCTGGTG	0.23	0.00	0.00	0.00	0.00
GATCAACCAAGCGCCTTGCG	0.23	0.00	0.00	0.34	0.00
GATCAACCAAGCGCCTTGGG	0.00	0.00	0.00	0.00	0.00
GATCAACCAAGCGCCTTGNN	0.23	0.00	0.00	0.00	0.00
GATCAACCAAGCGCCTTGTA	0.23	0.00	0.00	0.68	0.00
GATCAACCAAGCGCCTTGTG	45.09	33.18	11.45	30.89	19.29
GATCAACCAAGCGCCTTGTN	0.23	0.00	0.00	0.00	0.00
GATCAACCAAGCGCCTTGTT	0.00	0.33	0.00	0.00	0.00
GATCAACCAAGCGCCTTTGT	0.00	0.00	0.00	0.00	0.34
GATCAACCAAGCGCGACATA	0.00	0.00	0.00	0.00	0.34
GATCAACCAAGCGTATTGTG	0.00	0.00	0.00	0.00	0.34
GATCAACCAAGCGTCTTGTG	0.00	0.00	0.00	0.34	0.00
GATCAACCAAGCTTGGACCG	0.00	0.33	0.00	0.00	0.00
GATCAACCAAGGAACGGCTG	0.00	0.00	0.00	0.00	0.00
GATCAACCAAGGAAGGGCCG	0.00	0.00	0.29	0.34	0.00
GATCAACCAAGGAATGCCAG	0.00	0.00	0.00	0.68	0.00
GATCAACCAAGGACAGATGC	0.00	0.00	0.00	0.00	0.00
GATCAACCAAGGACAGGCCG	0.00	0.65	0.00	0.00	0.00
GATCAACCAAGGACCGGTCC	0.00	0.00	0.00	0.00	0.34
GATCAACCAAGGACGAACCT	0.00	0.00	0.00	0.00	0.00
GATCAACCAAGGACGAGCCG	0.00	0.00	0.00	0.34	0.00
GATCAACCAAGGACGAGCGA	0.00	0.33	0.00	0.00	0.00
GATCAACCAAGGACGAGCGC	0.00	0.33	0.00	0.00	0.00

GATCAACCAAGGACGGCCCG	0.00	0.00	0.00	0.00	0.34
GATCAACCAAGGACGGCCGG	0.00	0.00	0.00	0.00	0.00
GATCAACCAAGGACGGGAAG	0.00	0.00	0.00	0.00	0.34
GATCAACCAAGGACGGGACG	0.00	0.65	0.86	0.00	1.03
GATCAACCAAGGACGGGAGC	0.00	0.00	0.00	0.34	0.00
GATCAACCAAGGACGGGCAG	0.00	0.33	0.57	1.02	0.34
GATCAACCAAGGACGGGCCA	0.00	0.00	0.00	0.34	0.00
GATCAACCAAGGACGGGCCC	0.00	0.33	0.00	0.00	0.00
GATCAACCAAGGACGGGCCG	33.65	103.13	71.30	143.92	92.99
GATCAACCAAGGACGGGCCN	0.23	0.00	0.00	0.00	0.00
GATCAACCAAGGACGGGCGC	0.00	0.00	0.00	0.00	0.00
GATCAACCAAGGACGGGCGG	0.00	0.00	0.00	0.34	0.00
GATCAACCAAGGACGGGCNG	0.00	0.33	0.00	0.34	0.00
GATCAACCAAGGACGGGCTC	0.23	0.00	0.00	0.00	0.00
GATCAACCAAGGACGGGCTG	0.00	0.33	0.00	1.02	0.00
GATCAACCAAGGACGGGGCG	0.69	0.00	0.00	0.00	0.00
GATCAACCAAGGACGGGTCG	0.23	0.00	0.00	0.00	0.00
GATCAACCAAGGACGGNCCG	0.00	0.00	0.00	0.00	1.03
GATCAACCAAGGACGTGCGG	0.00	0.00	0.29	0.00	0.00
GATCAACCAAGGACTGGCCT	0.00	0.33	0.00	0.00	0.00
GATCAACCAAGGACTGGGGC	0.00	0.33	0.00	0.00	0.00
GATCAACCAAGGACTTGATG	0.00	0.00	0.29	0.00	0.00
GATCAACCAAGGAGATGTCT	0.00	0.33	0.00	0.00	0.00
GATCAACCAAGGAGGGGCCG	0.00	0.00	0.29	0.00	0.00
GATCAACCAAGGATGGGCCG	0.00	0.00	0.29	0.34	0.00
GATCAACCAAGGATTGAACT	1.14	0.00	0.00	0.00	0.00
GATCAACCAAGGCAAGGAGG	0.00	0.33	0.00	0.68	0.00
GATCAACCAAGGCAGCAAAG	0.00	1.30	0.00	0.34	0.00
GATCAACCAAGGCAGGCGCG	0.00	0.00	0.00	0.00	0.00
GATCAACCAAGGCCGGGACG	0.00	0.00	0.29	0.00	0.00
GATCAACCAAGGGCGGGCCG	0.00	0.33	0.00	0.00	0.34
GATCAACCAAGGGCTGGTTA	0.00	0.00	0.00	0.00	0.00
GATCAACCAAGGGGAAGGGC	0.23	0.00	0.00	0.00	0.00
GATCAACCAAGGGTTTTTGG	0.69	0.00	0.00	0.00	0.00
GATCAACCAAGGTATCGATG	0.00	0.00	0.57	0.00	0.00
GATCAACCAAGGTCGGGCCG	0.00	0.33	0.00	0.00	0.00
GATCAACCAAGTGGAACGCG	0.00	0.00	0.00	0.00	0.00

GATCAACCAAGTGGCACTCG	0.23	0.00	0.00	0.00	0.00
GATCAACCAAGTGGCCTTCC	0.00	0.00	0.00	0.34	0.00
GATCAACCAAGTTGCACTAG	0.00	0.00	0.00	0.68	0.00
GATCAACCAAGTTGCACTCG	15.34	27.00	13.75	18.33	19.63
GATCAACCAAGTTGCCAAAA	0.69	0.00	0.00	0.00	0.00
GATCAACCAAGTTGCCAAAT	0.23	0.00	0.00	0.00	0.00
GATCAACCAAGTTGCCAAGA	0.23	0.00	0.57	1.02	0.34
GATCAACCAAGTTGCCAAGC	0.00	0.00	0.29	0.00	0.00
GATCAACCAAGTTGGTTTTA	0.23	0.00	0.00	0.00	0.00
GATCAACCAAGTTGTGTCAA	0.00	0.00	0.00	0.00	0.00
GATCAACCAATAAAAATCTC	0.23	0.00	0.00	0.00	0.00
GATCAACCAATAAACATCAG	0.00	0.00	0.00	0.00	0.00
GATCAACCAATACCAACGTA	0.00	0.00	0.00	0.00	0.00
GATCAACCAATACGTCATTG	0.23	0.00	0.00	0.00	0.00
GATCAACCAATCAAGCAGGC	0.00	0.00	0.00	0.00	0.00
GATCAACCAATCAATCCTAT	0.46	0.33	0.29	0.34	0.00
GATCAACCAATCAATGATGA	0.00	0.00	0.57	0.00	0.00
GATCAACCAATCCAGAAAGA	0.00	0.00	0.29	0.00	0.00
GATCAACCAATCGTATGCCG	0.00	0.00	0.00	0.00	0.34
GATCAACCAATGAAAAAGAC	0.00	0.00	0.29	0.00	0.00
GATCAACCAATGACACAGCC	0.00	0.00	0.00	0.00	0.34
GATCAACCAATGACGCAAAA	0.46	0.00	0.00	0.00	0.00
GATCAACCAATGACGCCAAA	0.69	1.63	0.86	0.00	3.79
GATCAACCAATGACGCCAAC	0.00	0.33	0.00	0.00	0.00
GATCAACCAATGACGCTCAA	0.00	0.65	0.00	0.00	0.00
GATCAACCAATGACTCGAAA	0.00	0.00	0.00	0.00	0.00
GATCAACCAATGCGGCGAAA	0.00	0.00	0.00	0.00	0.34
GATCAACCAATGCGGCTTCA	0.00	0.00	0.00	0.00	0.00
GATCAACCAATGCGTCCAGT	0.00	0.00	0.00	0.34	0.00
GATCAACCAATGCTTCTTCG	1.14	0.98	0.29	0.34	0.34
GATCAACCAATGGCTTCCCC	0.23	0.00	0.00	0.00	0.00
GATCAACCAATGGGAATCGT	0.00	0.00	0.00	0.00	0.00
GATCAACCAATGGGCCTGCG	0.00	0.00	0.00	0.00	0.34
GATCAACCAATGGTCATCCA	0.46	0.65	0.00	0.00	0.00
GATCAACCAATGTACTGCTC	0.00	0.00	0.00	0.34	0.00
GATCAACCAATTAAAAAAAA	0.00	0.00	0.00	0.00	0.00
GATCAACCAATTAGCCCACA	0.23	0.65	0.00	0.34	0.00

GATCAACCAATTAGCTTCTT	1.14	0.33	0.57	1.02	1.38
GATCAACCAATTCGCTGTGA	0.00	0.00	0.00	0.00	0.00
GATCAACCAATTCTCGACAG	0.00	0.00	0.29	0.00	0.00
GATCAACCAATTCTCTTGGA	0.00	0.00	0.00	0.00	0.00
GATCAACCAATTGCAGTTAC	0.00	0.00	0.00	0.00	0.34
GATCAACCAATTGGCTCAAG	0.00	0.00	0.00	0.34	0.00
GATCAACCAATTGGCTGCGA	0.23	0.00	0.00	0.00	0.00
GATCAACCAATTGGCTGGGA	0.00	0.00	0.00	0.34	1.03
GATCAACCAATTGGCTGGGG	0.00	0.00	0.00	0.68	0.00
GATCAACCAATTGGCTGTGA	0.46	1.95	0.29	0.68	1.03
GATCAACCAATTGGCTGTGG	2.06	0.00	0.29	0.34	0.00
GATCAACCAATTGGCTGTGT	0.00	0.00	0.00	0.34	0.00
GATCAACCAATTGGCTGTNG	0.00	0.00	0.00	0.00	0.00
GATCAACCACAAAAAACACC	0.00	0.00	0.29	0.00	0.00
GATCAACCACAAAACCAACA	0.00	0.00	0.29	0.00	0.00
GATCAACCACAAACCAAACA	0.00	0.00	0.00	0.00	2.41
GATCAACCACAAATGTCGGA	0.00	0.00	0.00	0.00	0.00
GATCAACCACAACAACAAAC	0.00	0.00	0.00	0.00	0.34
GATCAACCACAACAACAACA	0.00	0.00	0.00	0.00	0.34
GATCAACCACAACAACACAC	0.00	0.00	0.00	0.00	0.34
GATCAACCACAACAACCCGA	0.00	0.00	0.00	0.00	0.34
GATCAACCACAACACAAACT	0.23	0.00	0.00	0.00	0.00
GATCAACCACAACACAACAC	0.00	0.00	0.00	0.00	0.34
GATCAACCACAACAGACACA	0.00	0.00	0.29	0.00	0.00
GATCAACCACAACATCGAGG	0.46	0.00	0.00	0.34	0.00
GATCAACCACAACCACACAC	0.00	0.00	0.29	0.00	0.00
GATCAACCACAACCGAGCAT	0.00	0.00	0.00	0.00	0.69
GATCAACCACAACGGTAGCT	2.29	1.30	1.15	2.04	2.41
GATCAACCACAACTGCCTGC	0.00	0.00	0.29	0.00	0.00
GATCAACCACAACTTAACAG	0.00	0.00	0.00	0.00	0.34
GATCAACCACAAGAAGGTGC	0.00	0.00	0.00	0.00	0.00
GATCAACCACAAGAATGACA	0.23	0.00	0.00	0.00	0.69
GATCAACCACAAGAATGCCC	0.46	0.65	0.29	1.02	1.03
GATCAACCACAAGAATGCCG	0.00	0.00	0.00	0.34	0.00
GATCAACCACAAGCAGCGGT	0.00	0.00	0.29	0.00	0.00
GATCAACCACAAGGCATGGA	0.00	0.33	0.00	0.00	0.00
GATCAACCACAAGGGCAGTG	0.00	0.00	0.57	0.34	0.00

GATCAACCACAATAACTATG	0.00	0.00	0.29	0.00	0.00
GATCAACCACAATACAGCAC	0.00	0.00	0.29	0.00	0.00
GATCAACCACACAACACACA	0.00	0.00	0.00	0.00	0.34
GATCAACCACACACAACCAC	0.00	0.00	0.00	0.00	0.34
GATCAACCACACACACAAAT	0.00	0.00	0.29	0.00	0.00
GATCAACCACACACACACAG	0.00	0.00	0.86	0.00	0.34
GATCAACCACACACAGGGAA	0.00	0.00	0.00	0.00	0.34
GATCAACCACACACTCGTGT	0.00	0.33	0.00	0.00	0.00
GATCAACCACACAGCCTCGT	0.00	0.33	0.00	0.00	0.00
GATCAACCACACCAACACAA	0.00	0.00	0.00	0.00	0.34
GATCAACCACACCAACATCA	0.00	0.00	0.00	0.00	0.69
GATCAACCACACCACAACAC	0.00	0.00	0.00	0.00	0.34
GATCAACCACACCACCCCAA	0.00	0.00	0.00	0.00	0.00
GATCAACCACACCAGCATTA	0.00	0.00	0.00	0.00	0.34
GATCAACCACACCCAACAAC	0.00	0.00	0.00	0.00	0.34
GATCAACCACACCCCAAGAA	0.00	0.00	0.29	0.68	0.00
GATCAACCACACCCCATAGT	0.00	0.00	0.29	0.00	0.00
GATCAACCACACCCTCCCCC	0.00	0.00	0.00	0.00	0.00
GATCAACCACACGCTCCGGG	0.00	0.00	0.00	0.34	0.00
GATCAACCACACGCTCGGGA	0.00	0.00	0.00	0.00	0.00
GATCAACCACACGCTCTGGG	0.69	1.30	0.57	1.02	1.03
GATCAACCACACGCTCTGGT	0.00	0.00	0.00	0.00	0.34
GATCAACCACACGGACGGTC	1.37	0.00	0.00	0.34	0.00
GATCAACCACACGGCCTCGT	0.00	0.00	0.00	0.00	0.00
GATCAACCACACGGGGAGAA	0.00	0.00	0.00	0.00	0.34
GATCAACCACACGGGGTCTG	0.23	0.00	0.00	0.00	0.00
GATCAACCACACTAGCACGC	0.00	0.00	0.57	0.00	0.00
GATCAACCACACTCAAGTTG	0.00	0.00	0.29	0.00	0.00
GATCAACCACAGAACAGAAC	0.00	0.00	0.00	0.00	0.34
GATCAACCACAGAACTCCGC	0.00	0.33	0.00	0.00	0.00
GATCAACCACAGAGCGATGA	0.00	0.00	0.00	0.00	0.34
GATCAACCACAGAGCGATGT	0.00	0.65	0.29	0.68	1.03
GATCAACCACAGAGCGCCTG	0.00	0.00	0.00	0.00	0.00
GATCAACCACAGAGGATGTC	0.00	0.00	0.29	0.00	0.00
GATCAACCACAGAGGGAAAG	0.00	0.00	0.29	0.00	0.00
GATCAACCACAGAGGTTCTG	0.23	0.00	0.00	0.00	0.00
GATCAACCACAGCAGGCTCT	0.00	0.00	0.00	0.00	0.00

GATCAACCACAGCGACGCGG	0.00	0.00	0.00	0.00	0.34
GATCAACCACAGCTTCGGAG	0.69	0.00	0.00	0.34	0.00
GATCAACCACAGGACCGCGC	0.00	0.00	0.00	0.00	0.00
GATCAACCACAGGACCGTGC	2.29	0.65	0.00	0.34	0.34
GATCAACCACAGGACGCGCT	0.00	0.00	0.29	0.00	0.00
GATCAACCACAGGACGCGTT	0.00	0.00	0.00	0.00	0.34
GATCAACCACAGGACGTGAA	0.00	0.00	0.29	0.00	0.00
GATCAACCACAGGAGCGCGC	0.00	0.00	0.00	0.00	0.00
GATCAACCACAGGGACTCTG	0.00	0.00	0.00	0.00	0.34
GATCAACCACAGGGATTCTG	0.00	0.00	0.00	0.34	0.00
GATCAACCACAGGGCTCCTT	0.00	0.98	0.00	0.00	0.00
GATCAACCACAGGGGACATG	0.00	0.00	0.00	0.34	0.00
GATCAACCACAGGGGATCAG	0.00	0.33	0.00	0.00	0.00
GATCAACCACAGGGGCTCTG	0.69	0.00	0.00	0.34	0.00
GATCAACCACAGGGGGACTG	0.23	0.00	0.00	0.00	0.00
GATCAACCACAGGGGGGCTG	0.23	0.00	0.00	0.00	0.00
GATCAACCACAGGGGGTCTG	0.92	0.00	0.29	0.34	0.00
GATCAACCACAGGGGGTCTT	0.00	0.00	0.00	0.34	0.00
GATCAACCACAGGGGTACTG	0.00	0.00	0.00	0.34	0.00
GATCAACCACAGGGGTAGTG	0.00	0.33	0.00	0.00	0.00
GATCAACCACAGGGGTCCTG	0.00	0.33	0.00	0.00	0.00
GATCAACCACAGGGGTGCGG	0.00	0.00	0.00	0.34	0.00
GATCAACCACAGGGGTGCTG	0.92	0.00	0.00	0.00	0.00
GATCAACCACAGGGGTNCNN	0.23	0.00	0.00	0.00	0.00
GATCAACCACAGGGGTNNNN	0.92	0.00	0.00	0.00	0.00
GATCAACCACAGGGGTTATG	0.00	0.00	0.29	0.68	0.00
GATCAACCACAGGGGTTCAG	0.00	0.00	0.00	0.34	0.00
GATCAACCACAGGGGTTCCG	0.23	0.00	0.00	0.00	0.00
GATCAACCACAGGGGTTCGG	0.46	0.65	0.00	0.00	0.00
GATCAACCACAGGGGTTCGT	0.00	0.00	0.00	0.00	0.34
GATCAACCACAGGGGTTCNG	0.00	0.00	0.00	0.34	0.00
GATCAACCACAGGGGTTCNN	1.60	0.00	0.00	0.00	0.00
GATCAACCACAGGGGTTCTA	0.00	0.00	0.00	0.00	0.34
GATCAACCACAGGGGTTCTC	0.00	0.33	0.00	0.00	0.00
GATCAACCACAGGGGTTCTG	179.00	156.81	58.99	84.18	65.78
GATCAACCACAGGGGTTCTN	0.69	0.00	0.00	0.00	0.00
GATCAACCACAGGGGTTCTT	0.00	0.00	0.29	0.00	0.34

GATCAACCACAGGGGTTGTG	0.23	0.00	0.00	0.00	0.00
GATCAACCACAGGGGTTNNN	0.69	0.00	0.00	0.00	0.00
GATCAACCACAGTGGTTCTG	0.69	0.00	0.00	0.00	0.00
GATCAACCACAGTTGGTTGG	0.00	0.00	0.00	0.00	0.00
GATCAACCACATAATCAGGA	0.00	0.00	0.00	0.00	0.34
GATCAACCACATCACAATCA	0.00	0.00	0.57	0.00	0.00
GATCAACCACATCATGGAGT	0.00	0.00	0.00	0.68	0.00
GATCAACCACATCTCGGCGC	0.00	0.00	0.29	0.00	0.00
GATCAACCACATCTCGGTGC	2.06	0.98	2.00	1.70	1.72
GATCAACCACATCTGACGGG	0.00	0.00	0.00	0.00	0.00
GATCAACCACATGAAAATTT	0.00	0.00	0.29	1.36	0.00
GATCAACCACATGACTGAAA	0.00	0.00	0.00	0.00	0.00
GATCAACCACATGAGAAGGA	0.00	0.00	0.00	0.00	0.34
GATCAACCACATGCACAAGC	0.00	0.00	0.29	0.00	0.00
GATCAACCACATGCCAGTTA	0.00	0.33	0.00	0.00	0.00
GATCAACCACATGGAGAACA	0.00	0.00	0.00	0.00	0.69
GATCAACCACATGGCCGGGC	0.00	0.33	0.00	0.34	0.34
GATCAACCACATTGAAACCA	0.00	0.00	0.00	0.00	0.34
GATCAACCACATTGGCTGCG	0.00	0.33	0.00	0.00	0.00
GATCAACCACCAAAAAAAAA	0.00	0.00	0.00	0.00	0.00
GATCAACCACCAAAACACTC	0.23	0.00	0.00	0.00	0.00
GATCAACCACCAAAAGTAAA	0.00	0.00	0.00	0.00	0.34
GATCAACCACCAAAGTCTCG	0.00	0.00	0.29	0.00	0.00
GATCAACCACCACATACTTC	0.00	0.98	0.00	0.00	1.38
GATCAACCACCACATGGCTG	0.00	0.00	0.00	0.00	0.34
GATCAACCACCACCACAATC	0.23	0.00	0.00	0.00	0.00
GATCAACCACCACCGCGGAT	0.00	0.00	0.00	0.34	0.00
GATCAACCACCACCGCGGGT	0.00	0.65	0.00	0.00	0.00
GATCAACCACCACCGCTGAT	0.00	0.33	0.00	0.00	0.00
GATCAACCACCACCGGTACC	0.00	0.00	0.29	0.00	0.00
GATCAACCACCACGCCCCGC	0.00	0.00	0.00	0.00	0.69
GATCAACCACCACGTGCGNA	0.00	0.00	0.00	0.00	0.00
GATCAACCACCACTTTGGAT	0.00	0.00	0.00	0.34	0.00
GATCAACCACCAGCAACCAC	0.00	0.00	0.00	0.00	0.69
GATCAACCACCAGCACCTAT	0.00	0.00	0.00	1.02	0.00
GATCAACCACCAGCGAAGTG	0.00	0.00	0.29	0.00	0.00
GATCAACCACCAGCGCCTCN	0.23	0.00	0.00	0.00	0.00

GATCAACCACCAGCGCCTCT	4.81	9.11	3.44	6.11	4.48
GATCAACCACCAGCGTCTCT	0.00	0.00	0.00	0.34	0.00
GATCAACCACCAGGAATTGG	0.00	0.00	0.00	0.00	0.00
GATCAACCACCAGGCCTGCA	0.00	0.33	0.00	0.00	0.00
GATCAACCACCATTGAAGCC	0.00	0.00	0.00	0.00	0.69
GATCAACCACCCAACAACAA	0.23	0.00	0.00	0.00	0.00
GATCAACCACCCAACATCAC	0.00	0.00	0.29	0.00	0.00
GATCAACCACCCAATATGGG	0.00	0.00	0.00	0.34	0.00
GATCAACCACCCCGCTGATG	0.23	0.00	0.00	0.00	0.00
GATCAACCACCCGAAGGTGA	0.00	0.00	0.29	0.00	0.34
GATCAACCACCCGAAGGTGC	0.00	0.00	0.00	0.00	0.00
GATCAACCACCCGACCGCCC	0.00	0.00	0.29	0.00	0.00
GATCAACCACCCGCCACCCG	0.00	0.00	0.29	0.00	0.00
GATCAACCACCCGGCACAGA	0.00	0.00	0.00	0.34	0.00
GATCAACCACCCGGCACTCA	0.00	0.00	0.00	0.00	0.00
GATCAACCACCCGGCAGACA	0.00	0.00	0.00	0.00	0.34
GATCAACCACCCGGCATGCA	0.00	0.00	0.00	0.00	0.00
GATCAACCACCCGGCCCGCA	0.00	0.00	0.29	0.00	0.00
GATCAACCACCCGGCCCGCC	0.00	0.00	0.00	0.00	0.00
GATCAACCACCCGGCCGGCA	0.00	0.33	0.00	0.00	0.00
GATCAACCACCCGGCCTGAA	0.00	0.00	0.00	0.34	0.00
GATCAACCACCCGGCCTGAC	0.00	0.00	0.00	0.00	0.00
GATCAACCACCCGGCCTGCA	14.88	49.45	51.83	45.14	50.63
GATCAACCACCCGGCCTGCC	0.00	0.65	0.86	0.00	0.00
GATCAACCACCCGGCCTGCG	0.23	0.00	0.00	0.00	0.00
GATCAACCACCCGGCCTGCN	0.00	0.00	0.00	0.68	0.00
GATCAACCACCCGGCCTGCT	0.00	0.00	0.00	0.00	0.34
GATCAACCACCCGGCCTGNA	0.00	0.00	0.00	0.00	0.00
GATCAACCACCCTGGCAAGT	0.00	0.00	0.00	0.00	0.69
GATCAACCACCGACGCGCAC	0.00	0.00	0.00	0.00	0.34
GATCAACCACCGATGCAGGA	0.00	0.00	0.00	0.00	0.00
GATCAACCACCGCAATCAGG	0.00	0.00	0.00	0.00	0.34
GATCAACCACCGCACGCCCA	0.00	0.00	0.29	0.00	0.00
GATCAACCACCGCATCTCGC	0.00	0.00	0.29	0.00	0.00
GATCAACCACCGCCAAAGCG	0.00	0.33	0.00	0.00	0.00
GATCAACCACCGCGCGGAGG	0.00	0.00	0.00	0.34	0.00
GATCAACCACCGCGCGGATG	0.00	0.00	0.00	0.00	0.34

GATCAACCACCGCTCCGANN	0.00	0.00	0.00	0.00	0.00
GATCAACCACCGGGGGGGGG	0.00	0.00	0.00	0.00	0.00
GATCAACCACCGGGGGTCTG	0.23	0.00	0.00	0.00	0.00
GATCAACCACCGGGGTTCTG	0.00	0.65	0.00	0.00	0.00
GATCAACCACCGGGTGCACA	0.00	0.00	0.00	0.00	0.34
GATCAACCACCGGTCAACTG	0.00	0.00	0.00	0.00	0.00
GATCAACCACCGTCCGCCGC	0.00	0.00	0.00	0.00	0.69
GATCAACCACCTCATCGAGG	0.00	0.33	0.00	1.02	0.00
GATCAACCACCTCATCTCAG	0.00	0.00	0.29	0.00	0.00
GATCAACCACCTCATCTCGA	0.00	0.00	0.00	0.00	0.00
GATCAACCACCTCGGGCAAC	0.00	0.00	0.00	0.00	0.69
GATCAACCACCTGAGAGAAC	0.92	0.00	0.29	0.68	0.69
GATCAACCACCTGAGGGAGC	0.00	0.00	0.00	0.34	0.00
GATCAACCACCTGCGCGCAA	0.00	0.00	0.00	0.00	0.34
GATCAACCACCTGTAGTGAG	0.00	0.00	0.29	0.00	0.00
GATCAACCACCTTGGATTTT	0.00	0.00	0.00	0.00	0.00
GATCAACCACCTTGGGGCCC	0.23	0.00	0.00	0.00	0.00
GATCAACCACCTTGTGGCCA	0.00	0.00	0.00	0.00	0.34
GATCAACCACCTTGTGTTGG	0.00	0.00	0.57	0.00	0.00
GATCAACCACGAAAAAAAAA	0.00	0.00	0.00	0.34	0.00
GATCAACCACGACACCAACC	0.00	0.00	0.00	0.00	0.34
GATCAACCACGACACGCTTG	0.00	0.00	0.00	0.00	0.00
GATCAACCACGACATTGGAA	0.00	0.00	0.00	0.00	1.38
GATCAACCACGACCAGATGC	0.00	0.00	0.00	0.00	0.00
GATCAACCACGACCGTGGCG	0.00	0.00	0.00	0.00	0.00
GATCAACCACGACCTGAACC	0.23	0.00	0.00	0.68	0.00
GATCAACCACGACCTTTGCT	0.00	0.00	0.00	0.00	0.00
GATCAACCACGACGAGAGCT	0.23	0.00	0.00	0.00	0.00
GATCAACCACGACGAGGGCT	0.23	0.00	0.00	0.00	0.00
GATCAACCACGACGAGGGNN	0.23	0.00	0.00	0.00	0.00
GATCAACCACGACGAGTGCN	0.23	0.00	0.00	0.00	0.00
GATCAACCACGACGAGTGCT	19.00	5.86	4.01	4.07	2.76
GATCAACCACGACGATCGTA	0.23	0.00	0.00	0.00	0.00
GATCAACCACGAGCGATCAG	0.00	0.00	0.00	0.00	0.00
GATCAACCACGAGCTCAAGG	0.00	0.00	0.00	0.34	0.00
GATCAACCACGAGGAACAGC	0.00	0.00	0.29	0.00	0.00
GATCAACCACGAGGAGACGA	0.00	0.00	0.00	0.00	0.00

GATCAACCACGAGGTGGCCG	0.00	0.00	0.00	0.34	0.00
GATCAACCACGAGTGTTGGA	0.46	0.00	0.00	0.00	0.00
GATCAACCACGATTTTTTCG	0.23	0.00	0.00	0.00	0.00
GATCAACCACGCAAACAAGA	0.00	0.00	0.29	0.00	0.00
GATCAACCACGCAACAACCA	0.00	0.00	0.00	0.00	0.34
GATCAACCACGCAATGTTCG	0.00	0.00	0.00	0.00	0.34
GATCAACCACGCACGGCAGC	0.00	0.00	0.00	0.00	0.34
GATCAACCACGCAGCTGCTG	0.00	0.00	0.00	0.00	0.34
GATCAACCACGCAGGTCTGC	0.46	0.33	0.29	1.02	0.00
GATCAACCACGCATTATTCC	0.00	0.00	0.00	0.00	0.34
GATCAACCACGCCAAGAAGA	0.00	0.00	0.57	0.00	0.00
GATCAACCACGCCAAGGAGG	0.69	1.30	0.00	0.34	0.00
GATCAACCACGCCACACCGC	0.00	0.00	0.29	0.00	0.00
GATCAACCACGCCACCCCCA	0.00	0.00	0.00	0.00	0.34
GATCAACCACGCCACTATGC	0.00	0.33	0.00	0.00	0.00
GATCAACCACGCCATCACCG	0.00	0.00	0.00	0.00	0.34
GATCAACCACGCCGTCCTCA	0.00	0.00	0.00	0.00	0.34
GATCAACCACGCCGTCCTCT	7.10	6.51	5.73	6.79	5.17
GATCAACCACGCCGTCCTNN	0.00	0.00	0.00	0.00	0.00
GATCAACCACGCCTGTTCCA	0.23	0.00	0.00	0.00	0.00
GATCAACCACGCGACATCGC	0.23	0.00	0.00	0.00	0.00
GATCAACCACGCGCAATCGA	0.00	0.00	0.00	0.00	0.00
GATCAACCACGCGCCTTGTG	0.00	0.00	0.29	0.00	0.00
GATCAACCACGCGGGCATGC	0.23	0.00	0.00	0.00	0.00
GATCAACCACGCTAACACAC	0.00	0.00	0.00	0.00	0.00
GATCAACCACGCTCGCCGTC	0.46	0.00	0.00	0.00	0.00
GATCAACCACGCTGCAAGGA	6.18	16.59	6.30	7.81	10.33
GATCAACCACGCTGCAAGGC	0.00	0.00	0.00	0.00	0.00
GATCAACCACGCTGCAAGGN	0.00	0.00	0.00	0.00	0.00
GATCAACCACGCTGCAAGNN	0.23	0.00	0.00	0.00	0.00
GATCAACCACGCTGCACGGA	0.00	0.00	0.00	0.00	0.00
GATCAACCACGCTGCACGGT	0.23	0.00	0.00	0.00	0.00
GATCAACCACGCTGCACTGA	0.00	0.00	0.00	0.34	0.00
GATCAACCACGGACACAACC	0.00	0.00	0.29	0.00	0.00
GATCAACCACGGACAGTTGA	0.00	0.00	0.00	0.00	0.00
GATCAACCACGGACGGGCAG	0.23	0.00	0.00	0.00	0.00
GATCAACCACGGACGGGCCG	0.23	0.00	0.00	0.34	0.00

GATCAACCACGGACGGGCGA	0.23	0.00	0.00	0.00	0.00
GATCAACCACGGCCAGCAGC	0.00	0.00	0.00	0.00	0.00
GATCAACCACGGCCCGCCAC	0.00	0.00	0.00	0.00	0.34
GATCAACCACGGCCGTGGGA	0.23	0.00	0.00	0.00	0.00
GATCAACCACGGCGGGAAGG	0.00	0.00	0.00	0.34	0.00
GATCAACCACGGGGAGGCCA	0.00	0.33	0.29	0.00	0.00
GATCAACCACGGGGGCTCTC	0.23	0.00	0.00	0.00	0.00
GATCAACCACGGGTGGGTGA	0.00	0.00	0.00	1.36	0.00
GATCAACCACGGTGGGAAGG	0.00	0.00	0.00	0.34	0.00
GATCAACCACGTAAGTCTTT	0.00	0.00	0.00	0.00	0.00
GATCAACCACGTACACACAC	0.00	0.00	0.00	0.00	0.34
GATCAACCACGTAGTGGTCT	0.23	0.33	0.29	0.34	0.00
GATCAACCACGTCCGCGGGG	0.00	0.00	0.29	0.00	0.00
GATCAACCACGTCCTAGCTT	0.00	0.00	0.00	0.00	0.00
GATCAACCACGTCGTAGTCT	0.00	0.00	0.00	0.34	0.00
GATCAACCACGTCTCCTTCG	0.00	0.00	0.00	0.00	0.00
GATCAACCACGTGAAGCGCG	0.00	0.00	0.00	0.00	0.00
GATCAACCACGTGACCTCCC	0.00	0.98	0.29	0.00	0.00
GATCAACCACGTGCCGGCGG	0.00	0.00	0.00	0.00	0.00
GATCAACCACGTGGCGACGC	0.00	0.33	0.00	0.00	0.00
GATCAACCACGTGGCGGCGG	0.23	0.00	0.00	0.34	0.00
GATCAACCACGTGGCGGCGT	3.43	5.86	3.15	5.77	4.13
GATCAACCACGTGGTGGTCT	0.00	0.00	0.29	0.00	0.00
GATCAACCACGTTGCACTCG	0.00	0.33	0.00	0.00	0.00
GATCAACCACGTTTTGTTGT	0.46	0.00	0.00	0.00	0.00
GATCAACCACTAATGGCAAA	0.46	0.00	0.00	0.00	0.00
GATCAACCACTACAAGGTAA	0.00	0.00	0.00	0.00	0.34
GATCAACCACTACAGCTTCG	0.00	0.00	0.00	0.00	0.69
GATCAACCACTACGATGCGG	0.00	0.00	0.00	0.00	0.00
GATCAACCACTACGGCATGT	0.23	0.00	0.00	0.00	0.00
GATCAACCACTACGGCCGGA	0.00	0.00	0.00	0.68	0.00
GATCAACCACTATGATGATT	0.00	0.00	0.00	0.68	0.00
GATCAACCACTATTTGTTGA	0.00	0.00	0.00	0.34	0.00
GATCAACCACTCAATGTTAA	0.00	0.00	0.00	0.00	0.69
GATCAACCACTCACAACACA	0.00	0.00	0.00	0.00	0.34
GATCAACCACTCCTGCCTGC	0.23	0.00	0.29	0.00	0.00
GATCAACCACTCCTTCCGTA	0.00	0.00	0.00	0.00	0.00

GATCAACCACTCGAAAGAGC	0.00	0.00	0.00	0.00	0.00
GATCAACCACTCGCAGAATC	0.00	0.33	0.00	0.00	0.00
GATCAACCACTCGTGCCGGC	1.60	0.65	0.00	0.34	0.00
GATCAACCACTCTCACACTC	0.00	0.00	0.29	0.00	0.00
GATCAACCACTCTTCAGCTG	0.00	0.00	0.86	0.00	0.00
GATCAACCACTGACATGTGT	0.00	0.00	0.00	0.00	0.69
GATCAACCACTGATGACATG	0.00	0.00	0.00	0.00	0.00
GATCAACCACTGCCAGCAAA	0.00	0.00	0.00	0.34	0.00
GATCAACCACTGCCCGCAAG	0.00	0.00	0.00	0.00	0.00
GATCAACCACTGCCTGCAGA	0.00	0.00	0.00	0.00	0.00
GATCAACCACTGCCTGGAGG	3.66	0.33	0.29	3.73	1.38
GATCAACCACTGCCTGGAGT	0.00	0.00	0.00	0.00	0.00
GATCAACCACTGCCTGGCGG	0.00	0.00	0.00	0.00	0.00
GATCAACCACTGCCTGTAGC	0.00	0.33	0.00	0.00	0.00
GATCAACCACTGCGTCCGGC	0.00	0.00	0.29	0.00	0.00
GATCAACCACTGCTTCTTCT	0.00	0.33	0.00	0.00	0.00
GATCAACCACTGGATCGATC	0.00	0.00	0.29	0.00	0.00
GATCAACCACTGGCCACTTC	0.00	0.00	0.00	0.00	0.00
GATCAACCACTGGGAATCGT	0.00	0.00	0.00	0.34	0.00
GATCAACCACTGGGATGAAA	0.00	0.33	0.00	0.00	0.00
GATCAACCACTGGGATGAAC	5.04	4.88	7.45	5.77	5.85
GATCAACCACTGGGATGACG	0.00	0.00	0.29	0.00	0.00
GATCAACCACTGGGATGACN	0.23	0.00	0.00	0.00	0.00
GATCAACCACTGGGATGTAC	0.00	0.00	0.00	0.00	0.00
GATCAACCACTGGGGCACGG	0.00	0.00	0.00	0.00	0.34
GATCAACCACTGGGGGGGGG	0.00	0.00	0.00	0.00	0.34
GATCAACCACTGGGGTTCTG	0.00	0.00	0.57	0.00	0.00
GATCAACCACTGTAGCATAC	0.00	0.33	0.00	0.34	0.00
GATCAACCACTGTTTAAGTG	0.00	0.33	0.00	0.00	0.00
GATCAACCACTTAGCTTCTT	0.00	0.00	0.29	0.00	0.00
GATCAACCACTTCATCGCCT	0.00	0.00	0.00	0.00	0.69
GATCAACCACTTCGCACACG	0.00	0.00	0.00	0.00	0.00
GATCAACCACTTCGCCCACG	0.46	0.65	1.15	2.72	0.00
GATCAACCACTTGCAGCGCT	0.00	0.33	0.00	0.00	0.00
GATCAACCACTTGCGCAGCC	0.00	0.00	0.00	0.00	0.00
GATCAACCACTTGCGCTACC	0.00	0.00	0.00	0.00	0.34
GATCAACCACTTGGAGCAGA	0.00	0.00	0.00	0.00	0.69

GATCAACCACTTGTACCAAA	0.00	0.00	0.00	0.34	0.00
GATCAACCACTTGTGGGTGC	0.00	0.33	0.00	0.00	0.00
GATCAACCACTTTCTTGAAA	0.00	0.00	0.29	0.00	0.34
GATCAACCACTTTGAGCTGG	0.00	0.33	0.00	0.00	0.00
GATCAACCACTTTGCGGCCC	0.00	0.33	0.00	0.00	0.00
GATCAACCAGAAAAAAAAAA	1.83	0.33	0.57	1.36	0.34
GATCAACCAGAAAAACTTGG	0.00	0.65	0.00	0.68	0.00
GATCAACCAGAACAACAATG	0.00	0.00	1.15	0.00	0.00
GATCAACCAGAACAAGCGAC	0.00	0.33	0.00	0.00	0.00
GATCAACCAGAACCAAGGGG	0.23	0.00	0.00	0.00	0.00
GATCAACCAGAACCAGAAGC	0.00	0.00	0.00	0.34	0.00
GATCAACCAGAACCTCAAGA	0.69	0.65	0.00	0.68	0.00
GATCAACCAGAACCTCAGGA	0.00	0.33	0.00	0.00	0.00
GATCAACCAGAACCTGGATG	0.00	0.00	0.00	0.00	0.00
GATCAACCAGAACGGAAGGG	0.23	0.00	0.00	0.00	0.00
GATCAACCAGAAGACACTAT	0.00	0.00	0.00	0.00	0.00
GATCAACCAGAAGAGCAGCG	0.00	0.00	0.00	0.00	0.34
GATCAACCAGAAGGAACCAC	0.00	0.00	0.00	0.00	0.00
GATCAACCAGAAGGGCGGCG	0.00	0.00	0.00	0.00	0.00
GATCAACCAGAAGGTCTTGG	0.69	0.65	0.00	0.34	0.34
GATCAACCAGAAGTGGGCGG	0.00	0.33	0.00	0.68	0.00
GATCAACCAGAAGTTCGATC	0.00	0.00	0.00	0.00	0.34
GATCAACCAGAATCGCCAGG	0.92	0.98	0.00	3.39	1.03
GATCAACCAGAATCGCCTGG	0.23	0.00	0.00	0.00	0.00
GATCAACCAGAATCGCTTCA	0.00	0.00	0.00	0.00	0.34
GATCAACCAGACACAACAAC	0.00	0.00	0.00	0.00	0.69
GATCAACCAGACAGAAAGAC	0.00	0.00	0.29	0.00	0.00
GATCAACCAGACAGAAGGGT	0.00	0.00	0.29	0.00	0.00
GATCAACCAGACATGCAAGA	0.00	0.00	0.29	0.00	0.00
GATCAACCAGACATGCCAGA	1.14	0.65	0.57	0.34	0.00
GATCAACCAGACATGTCCTA	0.00	0.00	0.00	0.00	0.34
GATCAACCAGACCAGGGACG	0.23	0.00	0.00	0.00	0.00
GATCAACCAGACCATCTACG	0.00	0.00	0.00	0.00	0.00
GATCAACCAGACCATGCCTG	0.00	0.33	0.00	0.00	0.00
GATCAACCAGACCATGTCAA	0.00	0.00	0.00	0.00	0.34
GATCAACCAGACCCTCGTGT	0.00	0.33	0.00	0.00	0.00
GATCAACCAGACCGACACAC	0.00	0.00	0.00	0.00	1.38

GATCAACCAGACGCCGGGCT	0.00	0.00	0.00	0.00	0.00
GATCAACCAGACGCGGCTTG	0.00	0.00	0.00	0.00	0.34
GATCAACCAGACGGCGCAGA	0.00	0.00	0.00	0.00	0.00
GATCAACCAGAGAAAAAAAA	0.46	0.98	0.29	1.36	1.03
GATCAACCAGAGAACAAAAA	8.24	1.63	2.86	2.04	3.10
GATCAACCAGAGAACAACAA	0.23	0.33	0.29	1.36	2.07
GATCAACCAGAGAACAACAG	11.45	4.23	3.72	7.81	6.54
GATCAACCAGAGAACAACAN	0.23	0.00	0.00	0.00	0.00
GATCAACCAGAGAACAACGG	0.00	0.00	0.00	0.00	0.00
GATCAACCAGAGAACAACNN	0.23	0.00	0.00	0.00	0.00
GATCAACCAGAGAACAAGAG	0.00	0.00	0.00	0.34	0.00
GATCAACCAGAGAACAGCCT	0.00	0.00	0.00	0.00	0.34
GATCAACCAGAGAACGAAAA	0.00	0.00	0.00	0.34	0.00
GATCAACCAGAGAAGAAAAA	0.00	0.00	0.00	0.34	0.00
GATCAACCAGAGAAGAATGG	0.00	0.00	0.00	0.00	0.69
GATCAACCAGAGAAGAGCGA	0.00	0.00	0.29	0.00	0.00
GATCAACCAGAGAGATGGGG	0.00	0.00	0.29	0.00	0.00
GATCAACCAGAGCAGCGGCA	0.00	0.00	0.57	0.68	0.00
GATCAACCAGAGCAGCGGCC	0.00	0.00	0.00	0.00	0.69
GATCAACCAGAGCCTCACTG	0.00	0.00	0.00	0.68	1.03
GATCAACCAGAGCTGGTCTT	0.69	0.00	0.00	0.00	0.00
GATCAACCAGAGGATGGCGG	0.00	0.33	0.00	0.00	0.00
GATCAACCAGAGGCACCGTG	0.00	0.00	0.00	0.00	0.00
GATCAACCAGAGGCCAGGAA	0.00	0.00	0.00	0.00	0.00
GATCAACCAGAGGGGAGACA	0.00	0.00	0.00	0.00	0.00
GATCAACCAGAGGTACCCGG	0.00	0.00	0.29	0.00	0.00
GATCAACCAGAGTCTGTCTG	0.00	0.00	0.00	0.34	0.00
GATCAACCAGATAGTTAATT	0.00	0.00	0.00	0.00	0.00
GATCAACCAGATAGTTATTC	0.00	0.33	0.00	0.00	0.00
GATCAACCAGATAGTTNNNN	0.23	0.00	0.00	0.00	0.00
GATCAACCAGATATTGTCCG	0.69	0.33	0.00	0.34	0.69
GATCAACCAGATCGTATGCC	0.00	0.00	0.00	0.00	0.00
GATCAACCAGATGCAAAGGG	0.23	0.00	0.00	0.00	0.00
GATCAACCAGATGCAAGGGA	0.00	0.00	0.00	0.00	0.34
GATCAACCAGATGCAATGTC	0.23	0.33	1.15	3.39	2.76
GATCAACCAGATGCCACACA	0.23	0.33	0.00	0.00	0.00
GATCAACCAGATGCGCTCGA	0.00	0.00	0.29	0.00	0.00

GATCAACCAGATGGCGGGGC	0.00	0.00	0.00	0.34	0.00
GATCAACCAGATGGGCGATG	0.00	0.00	0.00	0.00	0.00
GATCAACCAGATTAAATTCA	0.00	0.33	0.29	0.34	0.00
GATCAACCAGATTCATATGG	0.00	0.00	0.00	0.00	0.00
GATCAACCAGATTGATTTAA	0.00	0.00	0.00	0.00	0.00
GATCAACCAGATTGGAAATC	0.00	0.33	0.00	0.00	0.00
GATCAACCAGCAAAAACATG	0.00	0.00	0.00	0.00	0.34
GATCAACCAGCAAACCGGAC	0.00	0.33	0.00	0.00	0.00
GATCAACCAGCAAACGAAAC	0.00	0.00	0.00	0.00	0.00
GATCAACCAGCACCTCCAGA	0.00	0.00	0.00	0.00	0.00
GATCAACCAGCACCTGCAGA	0.00	0.00	0.00	0.00	0.34
GATCAACCAGCACCTTCAAA	0.00	0.00	0.00	0.00	0.69
GATCAACCAGCACGCCGGGG	0.00	0.00	0.00	0.00	0.00
GATCAACCAGCACGTTAGAA	0.00	0.00	0.00	0.00	0.00
GATCAACCAGCAGACAAACA	0.00	0.00	0.00	0.00	0.34
GATCAACCAGCAGACGAGTG	0.00	0.33	0.00	0.00	0.00
GATCAACCAGCAGAGGACTG	0.23	0.00	0.00	0.00	0.00
GATCAACCAGCAGCAGCTGG	0.00	0.65	0.00	0.00	0.00
GATCAACCAGCAGCCGGTGT	0.46	0.00	0.00	0.00	0.00
GATCAACCAGCAGCGGACTG	0.00	0.00	0.00	0.34	0.00
GATCAACCAGCAGCGGCCTG	0.00	0.00	0.00	0.00	0.00
GATCAACCAGCAGCTGGGCT	5.49	2.93	4.58	5.77	5.17
GATCAACCAGCAGCTGGGNN	0.00	0.00	0.00	0.00	0.00
GATCAACCAGCAGCTGGTCT	0.00	0.00	0.00	0.34	0.00
GATCAACCAGCAGGTCGTCA	0.00	0.33	0.00	0.00	0.00
GATCAACCAGCATCGGCACT	0.00	0.33	0.00	0.00	0.00
GATCAACCAGCATGCACCAA	0.00	0.33	0.00	0.00	0.00
GATCAACCAGCATGGGGGCG	0.00	0.00	0.00	0.00	0.00
GATCAACCAGCATTGGCACT	1.83	1.95	2.58	0.34	1.72
GATCAACCAGCATTGTTAGT	0.00	0.00	0.00	0.00	0.00
GATCAACCAGCCACAGTAGC	0.00	0.00	0.00	0.00	0.00
GATCAACCAGCCATGTTGGG	0.00	0.00	0.29	0.00	1.03
GATCAACCAGCCCGCATAGG	0.00	0.00	0.00	0.00	0.00
GATCAACCAGCCCTCCAGGG	0.00	0.00	0.00	0.00	0.34
GATCAACCAGCCGAGCCTCT	0.00	0.33	0.29	0.00	0.69
GATCAACCAGCCGCCCGATG	0.00	0.00	0.00	0.00	0.34
GATCAACCAGCCGCTTCGTA	0.00	0.00	0.00	0.34	0.00

GATCAACCAGCCGGCCGGCA	0.00	0.00	0.00	0.00	0.00
GATCAACCAGCCGGGAGTCA	0.00	0.00	0.00	0.00	0.34
GATCAACCAGCCGGGCGACA	0.00	0.00	0.00	0.34	0.00
GATCAACCAGCCGGTAGTCA	0.00	0.00	0.00	0.00	0.00
GATCAACCAGCCGGTCGCCA	0.00	0.00	0.00	0.00	0.34
GATCAACCAGCCGGTCGGCA	0.23	0.00	0.29	0.00	0.00
GATCAACCAGCCGGTCGTAA	0.00	0.00	0.00	0.34	0.00
GATCAACCAGCCGGTCGTCA	11.90	23.10	19.76	20.71	17.91
GATCAACCAGCCGGTCGTCC	0.00	0.65	0.00	0.00	0.34
GATCAACCAGCCGGTCGTCG	0.00	0.00	0.29	0.00	0.00
GATCAACCAGCCGGTCNTCA	0.00	0.00	0.00	0.00	0.34
GATCAACCAGCCGGTCNTNA	0.00	0.00	0.29	0.00	0.00
GATCAACCAGCCGTACGATG	0.00	0.00	0.00	0.00	0.34
GATCAACCAGCCGTACGCTG	0.00	0.00	0.00	0.00	0.34
GATCAACCAGCCTGATCACC	0.00	0.00	0.00	0.00	0.00
GATCAACCAGCCTTTACGCG	0.00	0.00	0.00	0.00	0.34
GATCAACCAGCGAGCCATGG	0.00	0.00	0.00	0.00	0.00
GATCAACCAGCGAGTCATGG	0.23	0.00	0.00	0.00	0.00
GATCAACCAGCGCAGAGGAG	0.00	0.00	0.00	0.68	0.00
GATCAACCAGCGCGACGTCG	0.00	0.00	0.00	0.34	0.00
GATCAACCAGCGCGTGAGCC	0.46	0.65	0.00	0.34	0.69
GATCAACCAGCGCTCGGGCG	0.00	0.33	0.00	0.00	0.00
GATCAACCAGCGGCGAGCAG	0.00	0.00	0.29	0.00	0.00
GATCAACCAGCGGCTGGAGG	0.00	0.00	0.29	0.00	0.00
GATCAACCAGCGGCTTACCT	0.00	0.00	0.00	0.00	0.34
GATCAACCAGCGTGGAATGC	0.00	0.00	0.00	0.00	0.00
GATCAACCAGCGTGGAGACA	4.81	26.03	4.58	19.35	6.54
GATCAACCAGCGTGGAGACC	0.00	0.00	0.00	0.34	0.00
GATCAACCAGCGTGGAGACG	0.00	0.00	0.00	0.34	0.00
GATCAACCAGCGTGGAGATA	0.23	0.33	0.57	0.34	0.69
GATCAACCAGCGTGGAGCAA	0.00	0.00	0.00	0.00	0.00
GATCAACCAGCGTGGAGCCA	0.46	0.00	0.00	0.00	0.00
GATCAACCAGCGTGGATGTT	0.46	0.00	0.00	0.00	0.00
GATCAACCAGCGTGGCGCGA	0.23	0.00	0.00	0.00	0.00
GATCAACCAGCGTGGCTTGA	0.00	0.00	0.00	0.68	0.00
GATCAACCAGCGTGGTGTCG	0.00	0.00	0.00	0.00	0.00
GATCAACCAGCTAGCAACTC	0.69	0.33	0.00	0.34	0.00

GATCAACCAGCTCAACTATG	0.00	0.00	0.00	0.00	0.00
GATCAACCAGCTCACCATGG	0.00	0.00	0.00	0.68	0.34
GATCAACCAGCTCACCGTGC	0.00	0.00	0.00	0.34	0.00
GATCAACCAGCTCCACATCT	0.46	0.65	0.00	0.00	0.00
GATCAACCAGCTCCCTCTGA	0.00	0.00	0.00	0.00	0.00
GATCAACCAGCTCCGCGCAG	0.23	0.00	0.00	0.00	0.00
GATCAACCAGCTCCGCGGCC	0.00	0.33	0.00	0.00	0.00
GATCAACCAGCTCCTGGCCA	0.00	0.00	0.00	0.00	0.00
GATCAACCAGCTCTACGCGG	0.00	0.33	0.00	0.00	0.00
GATCAACCAGCTCTGGCATT	0.00	0.00	0.29	0.00	0.00
GATCAACCAGCTCTTGAACA	0.00	0.00	0.29	0.00	0.00
GATCAACCAGCTGAACGCGT	0.00	0.00	0.00	0.00	0.00
GATCAACCAGCTGAACGTGA	0.00	0.00	0.29	0.00	0.00
GATCAACCAGCTGACCTCCA	0.00	0.00	0.00	0.34	0.34
GATCAACCAGCTGAGAGAGT	0.00	0.00	0.00	0.00	0.00
GATCAACCAGCTGAGGGAAC	0.00	0.00	0.29	0.00	0.00
GATCAACCAGCTGAGGGACC	0.00	0.00	0.00	0.00	0.00
GATCAACCAGCTGAGGGACG	0.00	0.00	0.00	0.00	0.00
GATCAACCAGCTGAGGGAGA	0.00	0.33	0.00	0.34	0.00
GATCAACCAGCTGAGGGAGC	26.78	22.45	12.60	26.82	13.43
GATCAACCAGCTGAGGGAGG	0.00	0.00	0.00	0.34	0.00
GATCAACCAGCTGAGGGAGN	0.23	0.00	0.00	0.00	0.00
GATCAACCAGCTGAGGGAGT	0.23	0.00	0.00	0.34	0.00
GATCAACCAGCTGAGGGCGC	0.23	0.00	0.29	0.00	0.00
GATCAACCAGCTGAGGGGGC	0.00	0.00	0.00	0.00	0.00
GATCAACCAGCTGAGGGTGC	0.23	0.00	0.00	0.00	0.00
GATCAACCAGCTGAGGNNNN	0.23	0.00	0.00	0.00	0.00
GATCAACCAGCTGCGCAATG	0.23	0.00	0.00	0.00	0.00
GATCAACCAGCTGCGGGGGC	0.23	0.00	0.00	0.00	0.00
GATCAACCAGCTGCTGAAGA	0.46	0.00	0.00	0.00	0.00
GATCAACCAGCTGCTGAAGN	0.23	0.00	0.00	0.00	0.00
GATCAACCAGCTGCTGGGCA	0.00	0.00	0.29	0.34	0.00
GATCAACCAGCTGCTTCAGG	0.00	0.00	0.00	0.00	0.00
GATCAACCAGCTGGCCGCGG	0.23	0.00	0.00	0.00	0.00
GATCAACCAGCTGGCCGTGG	0.23	0.00	0.29	0.34	0.00
GATCAACCAGCTGGCGAGGC	0.00	0.00	0.29	0.68	0.00
GATCAACCAGCTGGCGGATG	0.00	0.00	0.00	0.00	0.34

GATCAACCAGCTGGGCCTCA	0.00	0.00	0.00	0.00	0.00
GATCAACCAGCTGGTCTGTC	0.00	0.33	0.00	0.00	0.00
GATCAACCAGCTGTGACTCT	0.23	0.00	0.00	0.00	0.00
GATCAACCAGCTGTGGCCTA	0.00	0.00	0.00	0.00	0.00
GATCAACCAGCTGTTCTTGG	0.00	0.00	0.00	0.00	0.00
GATCAACCAGCTGTTGAATC	0.69	0.00	0.00	0.00	0.00
GATCAACCAGCTTATCAAGC	0.00	0.00	0.00	0.00	0.00
GATCAACCAGCTTCACATCT	1.37	0.33	0.00	0.34	0.00
GATCAACCAGCTTGACGCCG	0.00	0.00	0.00	0.34	0.00
GATCAACCAGCTTGGAAACC	0.00	0.33	0.00	0.00	0.00
GATCAACCAGCTTGTCAGCG	0.00	0.00	0.00	0.00	0.00
GATCAACCAGCTTGTTAGCG	0.23	0.33	0.00	0.34	0.00
GATCAACCAGCTTTGTAGAG	1.60	1.95	0.29	5.09	0.69
GATCAACCAGCTTTGTAGAN	0.23	0.00	0.00	0.00	0.00
GATCAACCAGGAAATCTGGA	0.00	0.00	0.00	0.00	0.69
GATCAACCAGGAACTCCGTG	0.00	0.00	0.00	0.34	0.00
GATCAACCAGGAACTTGTCA	0.00	0.00	0.00	0.00	0.00
GATCAACCAGGAATTCACAA	0.00	0.00	0.00	0.00	0.34
GATCAACCAGGACCACCACG	0.00	0.33	0.00	0.00	0.00
GATCAACCAGGACGAGCTTG	0.00	0.33	0.00	0.34	0.00
GATCAACCAGGACGCGGAGG	0.46	0.00	0.00	0.00	0.00
GATCAACCAGGACGCGTGGG	1.14	0.00	0.00	0.34	0.34
GATCAACCAGGACGGCGGTA	0.00	0.33	0.00	0.00	0.00
GATCAACCAGGACGGGCGCC	0.00	0.00	0.00	0.00	0.34
GATCAACCAGGACGGGGTGA	0.00	0.00	0.29	0.00	0.00
GATCAACCAGGACGTCACTC	0.00	2.60	3.15	0.68	2.41
GATCAACCAGGACGTCCCNN	0.23	0.00	0.00	0.00	0.00
GATCAACCAGGACTACACTT	0.00	0.00	0.29	0.00	0.00
GATCAACCAGGACTACATCC	0.00	0.33	0.00	0.00	0.00
GATCAACCAGGACTACTCTT	1.60	0.65	0.29	1.36	0.00
GATCAACCAGGAGAGCCAGC	0.46	0.00	0.00	0.34	0.00
GATCAACCAGGAGAGCCGCA	0.23	0.00	0.00	0.00	0.00
GATCAACCAGGAGATGTCTC	0.00	0.00	0.00	0.00	0.00
GATCAACCAGGAGCACACGC	0.00	0.00	0.00	0.00	0.00
GATCAACCAGGAGCCAGGGG	0.00	0.00	0.00	0.34	0.00
GATCAACCAGGAGGAGGGTT	0.00	0.33	0.00	0.00	0.00
GATCAACCAGGAGGCAAGCG	0.00	0.00	0.00	0.00	0.34

GATCAACCAGGAGGTACGGA	0.23	0.00	0.00	0.00	0.00
GATCAACCAGGAGGTGCCAT	0.00	0.00	0.00	0.68	0.00
GATCAACCAGGATATGAAGG	0.23	0.00	0.00	0.00	0.00
GATCAACCAGGATTGCTTTG	0.00	0.00	0.57	0.00	0.00
GATCAACCAGGCAAAAATCT	0.00	0.00	0.00	0.68	0.00
GATCAACCAGGCAAGGAGAA	0.00	0.00	0.00	0.68	0.00
GATCAACCAGGCAATAAGAA	0.00	0.00	0.29	0.00	0.00
GATCAACCAGGCAATACACG	0.00	0.00	0.00	0.00	0.34
GATCAACCAGGCAATCCCCG	0.00	0.00	0.00	0.00	0.34
GATCAACCAGGCAATGGAGC	0.00	0.00	0.00	0.00	0.00
GATCAACCAGGCACGGGAGA	0.23	0.00	0.00	0.00	0.00
GATCAACCAGGCAGAGAGAC	0.00	0.00	0.00	0.00	0.69
GATCAACCAGGCATGTCGGA	0.00	0.00	0.00	0.00	0.69
GATCAACCAGGCCAAGGATT	0.00	0.00	0.00	0.00	0.00
GATCAACCAGGCCACGGATG	0.00	0.00	0.00	0.68	0.00
GATCAACCAGGCCAGACACC	0.00	0.00	0.00	0.34	0.00
GATCAACCAGGCCATCAACG	0.00	0.00	0.00	0.34	0.00
GATCAACCAGGCCATCACAG	0.00	0.00	0.29	0.00	0.00
GATCAACCAGGCCATCATCT	0.00	0.00	0.00	0.34	0.00
GATCAACCAGGCCATCCAAG	0.00	0.33	0.00	0.34	0.00
GATCAACCAGGCCATCCACA	0.00	0.00	0.00	0.34	0.00
GATCAACCAGGCCATCCACG	30.67	54.33	34.94	50.91	34.44
GATCAACCAGGCCATCCACN	0.23	0.00	0.00	0.00	0.00
GATCAACCAGGCCATCCACT	0.46	0.00	0.00	0.00	0.00
GATCAACCAGGCCATCCAGG	0.23	0.00	0.00	0.00	0.00
GATCAACCAGGCCATCCANG	0.00	0.00	0.00	0.34	0.00
GATCAACCAGGCCATCCCCG	0.00	0.65	0.00	0.00	0.00
GATCAACCAGGCCATCCCNN	0.23	0.00	0.00	0.00	0.00
GATCAACCAGGCCATCTACG	0.00	0.00	0.29	0.00	0.00
GATCAACCAGGCCCCGTCAC	0.00	0.00	0.00	0.34	0.00
GATCAACCAGGCCGGCATGG	0.00	0.00	0.29	0.00	0.00
GATCAACCAGGCCTCAATGC	0.00	0.00	0.29	0.00	0.00
GATCAACCAGGCCTTTGAGC	0.00	0.33	0.00	0.00	0.00
GATCAACCAGGCGAATCAGT	0.00	0.00	0.00	0.34	0.00
GATCAACCAGGCGACAAAGT	0.00	0.00	0.00	0.00	0.00
GATCAACCAGGCGTGACAGG	0.00	0.65	0.00	0.00	0.00
GATCAACCAGGCGTGTCGGA	0.00	0.00	0.00	0.34	0.00

GATCAACCAGGCGTGTCGGG	0.00	0.00	0.57	0.00	0.00
GATCAACCAGGCGTGTTGGA	0.00	0.00	0.00	0.00	0.69
GATCAACCAGGCTGACATGC	0.00	0.00	0.00	0.68	0.00
GATCAACCAGGCTGACGTGG	0.00	0.00	0.00	0.00	0.00
GATCAACCAGGGACGGGCCG	0.00	0.00	0.00	0.34	0.00
GATCAACCAGGGAGCACAAA	0.00	0.00	0.00	0.00	0.34
GATCAACCAGGGCAGGAGGA	0.00	0.00	0.00	0.00	0.00
GATCAACCAGGGCTTGCAGC	0.00	0.65	0.00	0.34	0.34
GATCAACCAGGGGAGCGAGA	0.00	0.33	0.00	0.00	0.00
GATCAACCAGGGGAGGGGGG	0.23	0.00	0.00	0.00	0.00
GATCAACCAGGGGAGGGTGG	0.23	0.98	0.29	0.00	0.69
GATCAACCAGGGGTGTCGGA	0.00	0.00	0.00	0.34	0.00
GATCAACCAGGTAACAAAAA	0.00	0.00	0.00	0.00	0.00
GATCAACCAGGTAATAATTC	0.00	0.00	0.00	0.00	0.00
GATCAACCAGGTAGCGCTGG	0.00	0.00	0.57	0.00	0.69
GATCAACCAGGTAGCGTTGG	0.00	0.00	0.00	0.00	0.00
GATCAACCAGGTAGGCATAG	0.00	0.00	0.29	0.00	0.00
GATCAACCAGGTAGGCATGG	0.00	0.00	0.00	0.34	0.00
GATCAACCAGGTAGTCTAGG	0.00	0.33	0.00	0.00	0.00
GATCAACCAGGTAGTCTGTG	0.00	0.00	0.00	0.00	0.00
GATCAACCAGGTATTCACAA	0.00	0.33	15.18	0.00	108.83
GATCAACCAGGTATTCACAC	0.00	0.00	0.29	0.00	0.34
GATCAACCAGGTATTCATAT	0.00	0.00	0.29	0.00	0.00
GATCAACCAGGTATTCNCAA	0.00	0.00	0.00	0.00	1.03
GATCAACCAGGTCAACTTTG	0.23	0.00	0.00	0.00	0.00
GATCAACCAGGTCATGCGTT	0.00	0.00	0.00	0.00	0.34
GATCAACCAGGTCCAACGGT	0.00	0.00	0.00	0.34	0.00
GATCAACCAGGTCCCGCACC	0.00	0.00	0.00	0.68	0.00
GATCAACCAGGTCCGGGGGG	0.00	0.00	0.00	0.00	0.00
GATCAACCAGGTCCTCTTCG	0.00	0.00	0.00	0.34	0.00
GATCAACCAGGTCGCCAAGA	0.00	0.00	0.00	0.00	0.34
GATCAACCAGGTCGGCANGG	0.00	0.00	0.00	0.34	0.00
GATCAACCAGGTCGGCATGG	5.72	16.27	25.20	15.95	11.71
GATCAACCAGGTCTTGAGCA	0.00	0.00	0.00	0.00	0.00
GATCAACCAGGTGAAGACAT	0.00	0.33	0.00	0.68	1.03
GATCAACCAGGTGACCATTG	0.00	0.33	0.00	0.00	0.00
GATCAACCAGGTGAGCAACG	0.23	0.65	0.29	0.34	0.00

GATCAACCAGGTGAGCCTGA	0.00	0.00	0.00	0.34	0.00
GATCAACCAGGTGGAGTTCG	0.00	0.00	0.29	0.00	0.00
GATCAACCAGGTGTCAAAAA	0.00	0.00	0.00	0.00	0.00
GATCAACCAGGTTATCCGCC	0.00	0.00	0.00	0.00	0.34
GATCAACCAGTAAAGCTTCT	1.60	0.33	0.00	0.34	0.00
GATCAACCAGTAAGAGGAGG	0.00	0.00	0.00	0.00	0.34
GATCAACCAGTAAGCGGAGG	0.00	0.33	0.00	0.00	0.00
GATCAACCAGTACACGGAGG	0.00	0.00	0.00	0.00	0.34
GATCAACCAGTACGAGCAGG	0.00	1.30	0.57	1.02	0.69
GATCAACCAGTACGAGGAGG	0.00	0.00	0.00	0.34	0.00
GATCAACCAGTACGCAGAGG	0.00	0.00	0.00	0.00	0.00
GATCAACCAGTACGCGGAAG	0.23	0.00	0.00	0.00	0.00
GATCAACCAGTACGCGGACG	0.00	0.33	0.00	0.00	0.00
GATCAACCAGTACGCGGAGC	0.00	0.00	0.00	0.00	0.00
GATCAACCAGTACGCGGAGG	33.42	62.46	28.92	58.04	47.18
GATCAACCAGTACGCGGAGN	0.23	0.00	0.00	0.00	0.00
GATCAACCAGTACGCGGANG	0.00	0.00	0.00	0.34	0.00
GATCAACCAGTACGCGGCGG	0.00	1.30	0.00	0.34	0.00
GATCAACCAGTACGCGGGGG	0.00	0.00	0.29	0.34	0.00
GATCAACCAGTACGCGGTGG	0.00	0.00	0.00	0.00	0.00
GATCAACCAGTACGCGNAGG	0.00	0.00	0.00	0.00	0.34
GATCAACCAGTACGCGNGNN	0.23	0.00	0.00	0.00	0.00
GATCAACCAGTACGGCTTTA	0.00	0.00	0.00	0.00	0.00
GATCAACCAGTACGTGCCGC	0.00	0.33	0.00	0.00	0.34
GATCAACCAGTAGAAGAGGA	0.00	0.00	0.57	0.00	0.00
GATCAACCAGTAGCACCTGG	0.46	0.00	0.00	0.00	0.00
GATCAACCAGTAGTCATATG	0.00	0.00	0.00	0.00	0.00
GATCAACCAGTATGGACATC	0.23	0.00	0.00	0.00	0.00
GATCAACCAGTCAAGCAGTC	0.00	0.00	0.00	0.00	0.34
GATCAACCAGTCAATCAGTC	0.00	0.00	0.29	0.00	0.00
GATCAACCAGTCAATGCCCT	0.00	0.33	0.00	0.34	0.69
GATCAACCAGTCACGTTCTA	0.23	0.00	0.00	0.00	0.00
GATCAACCAGTCACTCAGCA	0.00	0.00	0.00	0.00	0.34
GATCAACCAGTCAGGCAGTC	0.00	0.00	0.57	0.00	0.00
GATCAACCAGTCATCCATGC	0.00	0.00	0.00	0.00	0.00
GATCAACCAGTCATTCATCC	0.00	0.00	0.00	0.00	0.00
GATCAACCAGTCCAGAGCCG	0.00	0.33	0.00	0.00	0.00

GATCAACCAGTCCATCCGAG	0.23	0.33	0.29	0.00	0.00
GATCAACCAGTCCCAGTTTG	0.00	0.98	0.00	0.00	0.00
GATCAACCAGTCCCGAGTCT	0.00	0.00	0.00	0.68	0.00
GATCAACCAGTCCCTGCTGG	0.00	0.00	0.00	0.00	0.00
GATCAACCAGTCCCTGGACA	0.00	0.00	0.00	0.34	0.00
GATCAACCAGTCCCTGGGCA	0.46	0.00	0.00	0.00	0.00
GATCAACCAGTCCGAAGTCC	0.00	0.00	0.00	0.00	0.00
GATCAACCAGTCCTTCTGGG	0.46	0.00	0.00	0.00	0.00
GATCAACCAGTCGAGCACTG	0.00	0.00	0.00	0.00	0.00
GATCAACCAGTCGAGCGGGC	0.23	0.00	0.00	0.00	0.00
GATCAACCAGTCGCTCCTGG	0.00	0.33	0.00	0.68	0.00
GATCAACCAGTCGGTCGGTC	0.23	0.00	0.00	0.00	0.00
GATCAACCAGTCTGAACACA	0.00	0.00	0.29	0.00	0.69
GATCAACCAGTCTGGCAAGT	0.00	0.65	0.00	0.00	0.00
GATCAACCAGTGAACGCCCT	0.00	0.00	0.00	0.00	0.00
GATCAACCAGTGAAGGTGCT	0.00	0.00	0.00	0.00	0.00
GATCAACCAGTGACGGGGCT	0.23	0.00	0.00	0.00	0.34
GATCAACCAGTGACGGTGCC	0.00	0.33	0.29	0.00	0.00
GATCAACCAGTGACGGTGCG	0.00	0.33	0.00	0.00	0.00
GATCAACCAGTGACGGTGCT	7.78	11.39	1.72	9.84	7.23
GATCAACCAGTGACGGTGGC	0.23	0.00	0.00	0.00	0.00
GATCAACCAGTGACGGTGGT	0.23	0.00	0.00	0.00	0.00
GATCAACCAGTGACTCGTAT	0.00	0.00	0.00	0.34	0.00
GATCAACCAGTGATGACATG	0.00	0.00	0.00	0.00	0.00
GATCAACCAGTGCACGCCCA	0.46	0.33	0.00	0.00	0.00
GATCAACCAGTGCATGGAGG	0.46	0.33	0.29	0.34	0.00
GATCAACCAGTGCCCCCAGC	0.00	0.00	0.00	0.00	0.00
GATCAACCAGTGCCGGTGCT	0.23	0.00	0.00	0.00	0.00
GATCAACCAGTGCCTTGAGT	0.00	0.33	0.29	0.68	0.00
GATCAACCAGTGCGGATGAT	0.00	0.00	0.00	0.00	0.34
GATCAACCAGTGCTCTCGCG	0.00	0.00	0.00	0.34	0.00
GATCAACCAGTGCTTTCTGG	0.00	0.00	0.00	0.00	0.00
GATCAACCAGTGGAAATTTC	0.69	0.00	0.00	1.02	0.00
GATCAACCAGTGGCGGCGCT	0.00	0.00	0.00	0.00	0.00
GATCAACCAGTGGGTAGATT	0.00	0.00	0.00	0.00	0.34
GATCAACCAGTGGTACTCCC	0.00	0.00	0.00	0.34	0.00
GATCAACCAGTGGTCCCTAG	0.00	0.33	0.00	0.00	0.00

GATCAACCAGTGGTTGCCCG	1.83	1.63	0.57	0.34	0.34
GATCAACCAGTGTGGCACGG	0.00	0.00	0.00	0.00	0.00
GATCAACCAGTGTGGGGAAC	0.00	0.00	0.29	0.00	0.00
GATCAACCAGTGTTGAAGTC	0.00	0.00	0.00	0.00	0.34
GATCAACCAGTGTTGAATTC	0.23	0.00	0.29	0.00	0.00
GATCAACCAGTTAGGACATC	0.00	0.33	0.00	0.00	0.00
GATCAACCAGTTAGGCAGTC	0.00	0.00	0.00	0.00	0.34
GATCAACCAGTTCAACGACA	0.00	0.00	0.00	0.00	0.00
GATCAACCAGTTCAACNTTG	0.00	0.00	0.00	0.00	0.69
GATCAACCAGTTCAACTACG	0.00	0.00	0.00	0.00	0.00
GATCAACCAGTTCAACTATG	1.14	2.93	4.01	3.05	3.44
GATCAACCAGTTCAACTTNN	0.23	0.00	0.00	0.00	0.00
GATCAACCAGTTCAACTTTA	0.00	0.33	0.00	0.00	0.00
GATCAACCAGTTCAACTTTG	36.40	47.50	67.29	35.98	46.49
GATCAACCAGTTCAACTTTT	0.00	2.28	1.72	1.70	2.41
GATCAACCAGTTCATCGTAT	0.00	0.00	0.00	0.34	0.00
GATCAACCAGTTCATGAAGG	0.00	0.00	0.00	0.00	0.00
GATCAACCAGTTCCAGGACC	1.83	1.30	0.29	0.00	0.00
GATCAACCAGTTCCATCATC	0.00	0.00	0.00	0.34	0.00
GATCAACCAGTTCCCGGACC	0.00	0.33	0.00	0.00	0.00
GATCAACCAGTTCCGTATGC	0.00	0.00	0.00	0.34	0.00
GATCAACCAGTTCGACCCGT	0.00	0.00	0.00	0.00	0.00
GATCAACCAGTTCGACTTTG	0.00	0.00	0.29	0.00	0.00
GATCAACCAGTTCGTATGCC	0.00	0.33	0.00	0.00	0.00
GATCAACCAGTTCTCCATTT	0.00	0.00	0.00	0.34	0.00
GATCAACCAGTTGAGTGGAG	0.00	0.33	0.00	0.00	0.00
GATCAACCAGTTGGGCGAAC	0.00	0.00	0.00	0.00	0.00
GATCAACCAGTTGGGCGACC	0.00	0.00	0.00	0.34	0.00
GATCAACCAGTTGTTGTTGC	0.00	0.00	0.00	0.00	0.00
GATCAACCAGTTTAAGGGAG	0.00	0.00	0.00	0.00	0.00
GATCAACCAGTTTCCAATAG	0.00	0.00	0.00	0.00	0.34
GATCAACCAGTTTGGAAAAA	0.00	0.33	0.00	0.00	0.00
GATCAACCAGTTTGGAAACA	0.00	0.33	0.00	0.00	0.00
GATCAACCAGTTTGGAAACC	28.16	31.56	9.74	32.25	15.50
GATCAACCAGTTTGGAAANN	0.23	0.00	0.00	0.00	0.00
GATCAACCAGTTTGGAANCN	0.00	0.00	0.00	0.00	0.34
GATCAACCAGTTTGGACACC	0.23	0.33	0.00	0.34	0.00

GATCAACCAGTTTGGACANN	0.23	0.00	0.00	0.00	0.00
GATCAACCAGTTTGGACATC	36.85	9.43	4.87	8.15	4.13
GATCAACCAGTTTGGACTCG	0.00	0.00	0.00	0.34	0.00
GATCAACCAGTTTGGAGACA	0.00	0.00	0.00	0.00	0.00
GATCAACCAGTTTGGAGACC	0.00	0.00	0.00	0.68	0.00
GATCAACCAGTTTGGAGACT	0.00	0.00	0.00	0.68	0.00
GATCAACCAGTTTGGAGAGT	0.00	0.33	0.00	0.00	0.00
GATCAACCAGTTTGGAGATC	0.00	0.00	0.29	0.68	0.00
GATCAACCAGTTTGGAGATG	0.00	0.00	0.00	0.00	0.00
GATCAACCAGTTTGGAGATT	0.00	0.00	0.00	0.34	0.00
GATCAACCAGTTTGGAGGTC	0.23	0.00	0.00	0.00	0.00
GATCAACCAGTTTGGAGTCT	0.00	0.33	0.00	0.34	0.00
GATCAACCAGTTTGGAGTTC	0.00	0.00	0.00	0.34	0.00
GATCAACCAGTTTGGANATC	0.00	0.00	0.00	0.00	0.34
GATCAACCAGTTTGGATATC	0.23	0.00	0.29	0.00	0.00
GATCAACCAGTTTGGCAATG	0.00	0.00	0.00	0.00	0.34
GATCAACCAGTTTGGCCCCC	0.00	0.00	0.00	0.00	0.00
GATCAACCAGTTTGGCGTGT	0.00	0.00	0.00	0.00	0.34
GATCAACCAGTTTGGCTTCT	0.00	0.00	0.00	0.00	0.34
GATCAACCAGTTTGGGAACC	0.46	0.00	0.00	0.00	0.00
GATCAACCAGTTTGGGAACT	0.00	0.00	0.00	0.00	0.34
GATCAACCAGTTTGGGACCA	0.00	0.00	0.29	0.00	0.00
GATCAACCAGTTTGGGTGCC	0.00	0.33	0.00	0.00	0.00
GATCAACCATAAAGGCAAGC	0.00	0.00	0.00	0.00	0.34
GATCAACCATAACCCCAGAG	0.00	0.00	0.29	0.00	0.00
GATCAACCATAAGCATCAGT	0.00	0.65	0.00	0.00	0.00
GATCAACCATAATCGTATGC	0.00	0.00	0.29	0.00	0.00
GATCAACCATAATCGTATTT	0.00	0.65	0.86	0.34	0.00
GATCAACCATAATGATAATG	0.00	0.00	0.29	0.00	0.00
GATCAACCATACAAAAAAAA	0.00	0.00	0.29	0.00	0.00
GATCAACCATACATTACCCA	0.00	0.00	0.00	0.00	0.34
GATCAACCATACCAAAGTAA	0.00	0.00	0.00	0.00	0.69
GATCAACCATACCAAAGTAT	0.23	0.00	0.00	0.00	0.00
GATCAACCATACCAAAGTCA	0.00	0.00	0.00	0.00	0.34
GATCAACCATAGAAAAAAAA	0.69	0.00	0.00	0.00	0.00
GATCAACCATAGACAAAAAA	0.23	0.00	0.00	0.00	0.00
GATCAACCATAGCACCATAA	0.00	0.00	0.29	0.00	0.00

GATCAACCATAGGGGTTCTG	0.23	0.65	0.00	0.00	0.00
GATCAACCATATCACACCAC	0.00	0.00	0.00	0.00	0.34
GATCAACCATATCGTTGTAC	0.46	2.28	0.57	0.00	0.00
GATCAACCATCACCACCATC	0.00	0.00	0.00	0.00	0.00
GATCAACCATCACCATCACC	0.00	0.00	0.00	0.00	0.34
GATCAACCATCACCATCATC	0.00	0.00	0.57	0.00	0.00
GATCAACCATCACGCACCTC	0.00	0.00	0.29	0.00	0.00
GATCAACCATCACGCACCTG	0.00	0.00	0.00	0.00	0.00
GATCAACCATCACGCCCCCC	0.00	0.33	0.00	0.00	0.34
GATCAACCATCACGCCTCCC	0.00	0.00	0.00	0.00	0.00
GATCAACCATCAGAGTCTTC	0.00	0.33	0.00	0.00	0.00
GATCAACCATCAGATCATCA	0.00	0.33	0.00	0.00	0.00
GATCAACCATCAGGCAAAGA	0.00	0.00	0.00	0.00	0.00
GATCAACCATCAGTCTGGCC	0.46	0.33	0.00	0.00	0.34
GATCAACCATCAGTGCCAGA	0.00	0.00	0.29	0.00	0.00
GATCAACCATCATAGGCAGA	0.00	0.00	0.29	0.00	0.00
GATCAACCATCATCTATGGC	0.00	0.33	0.00	0.00	0.00
GATCAACCATCATCTGTCCC	0.23	0.00	0.00	0.00	0.00
GATCAACCATCCACCGCTTA	0.00	0.00	0.29	0.00	0.00
GATCAACCATCCAGCGTGGA	0.00	0.00	0.00	0.00	0.00
GATCAACCATCCCGAGGTGC	0.00	0.33	0.00	0.34	0.69
GATCAACCATCCCGCCTTCG	0.46	0.00	0.00	0.00	0.00
GATCAACCATCCGGAGGACA	0.23	0.00	0.00	0.00	0.00
GATCAACCATCCGGCCGAAA	0.00	0.00	0.29	0.00	0.00
GATCAACCATCCGGCCTGAA	0.00	0.00	0.00	0.34	0.00
GATCAACCATCCGGCCTGCA	0.00	0.00	0.00	0.34	0.00
GATCAACCATCCGGCGCAGA	0.23	0.00	0.00	0.00	0.00
GATCAACCATCCTGGGAAAC	0.00	0.00	0.00	0.00	0.00
GATCAACCATCGCGTGTTTG	0.00	0.00	0.00	0.00	0.34
GATCAACCATCGGTGGAGAG	0.00	0.00	0.00	0.00	0.00
GATCAACCATCGTATGCCGT	0.00	0.00	0.00	0.34	0.00
GATCAACCATCTAAGCTAAG	0.00	0.33	0.00	0.00	0.00
GATCAACCATCTCACCTCAG	0.00	0.00	0.00	0.34	0.00
GATCAACCATCTCAGCTCAG	0.23	0.00	0.00	0.00	0.00
GATCAACCATCTCATATCAG	0.23	0.00	0.00	0.00	0.00
GATCAACCATCTCATCCTGG	0.00	0.00	0.29	0.00	0.00
GATCAACCATCTCATCGCAG	0.00	0.00	0.00	0.00	0.00

GATCAACCATCTCATCNCAG	0.00	0.00	0.00	0.00	0.34
GATCAACCATCTCATCTAAG	0.00	0.33	0.00	0.00	0.00
GATCAACCATCTCATCTCAG	17.85	24.72	28.35	28.51	32.72
GATCAACCATCTCATCTCAT	0.00	0.33	0.00	0.00	0.34
GATCAACCATCTCATCTCCG	0.00	0.00	0.00	0.00	0.00
GATCAACCATCTCATCTCGG	0.00	0.00	0.00	0.00	0.00
GATCAACCATCTCATCTCNN	0.00	0.00	0.00	0.00	0.00
GATCAACCATCTCATCTTAG	0.23	0.00	0.00	0.00	0.00
GATCAACCATCTCCGGCCAC	0.00	0.00	0.00	0.00	0.00
GATCAACCATCTCCTCTCCG	0.00	0.00	0.00	0.00	0.00
GATCAACCATCTCTCGTATG	0.00	0.00	0.00	0.00	0.00
GATCAACCATCTGACCGACC	0.00	0.00	0.00	0.34	0.00
GATCAACCATCTGACTTGAA	0.00	0.33	0.00	0.00	0.00
GATCAACCATCTGAGAACCA	0.00	0.00	0.00	0.00	0.34
GATCAACCATCTGAGACACC	0.00	0.00	0.00	0.00	0.34
GATCAACCATCTGAGACCCC	3.43	7.48	1.72	7.47	5.17
GATCAACCATCTGAGACCNC	0.00	0.00	0.00	0.00	0.00
GATCAACCATCTGGCACATC	0.00	0.00	0.00	0.00	0.00
GATCAACCATGAAGATGAAA	0.00	0.00	0.29	0.00	0.00
GATCAACCATGAATTCACCC	0.00	0.00	0.00	0.00	0.34
GATCAACCATGACCAACATC	0.00	0.00	0.00	0.34	0.00
GATCAACCATGACCCATTCG	0.00	0.00	0.00	0.00	0.00
GATCAACCATGACGAGTGCT	0.00	0.00	0.29	0.00	0.00
GATCAACCATGACTGAGGAG	0.00	0.00	0.29	0.00	0.00
GATCAACCATGAGAGCACAG	0.00	0.00	0.00	0.00	0.69
GATCAACCATGAGCTTGTAC	0.00	0.00	0.00	0.00	0.00
GATCAACCATGAGTGAAGAG	0.00	0.00	0.00	0.34	0.00
GATCAACCATGATCGTCCGA	0.00	0.33	0.00	0.00	0.00
GATCAACCATGCAAAAAAAA	7.78	1.30	3.72	1.70	3.44
GATCAACCATGCAAAAAACA	0.46	0.33	0.29	0.00	0.00
GATCAACCATGCAAAAATCA	27.24	4.23	5.73	5.43	3.10
GATCAACCATGCAAAAATCG	0.00	0.00	0.29	0.00	0.00
GATCAACCATGCAAAAATCN	0.46	0.00	0.00	0.00	0.00
GATCAACCATGCAAAAATCT	0.23	0.00	0.00	0.00	0.00
GATCAACCATGCAAAAGAAA	0.23	0.00	0.00	0.00	0.00
GATCAACCATGCAAAAGACA	0.00	0.00	0.29	0.00	0.00
GATCAACCATGCAAAANAAA	0.00	0.00	0.00	0.00	0.34

GATCAACCATGCAAACACCT	0.00	0.00	0.29	0.00	0.69
GATCAACCATGCAAATTTTG	0.00	0.00	0.00	0.34	0.00
GATCAACCATGCAACAGATG	0.00	0.00	0.00	0.00	0.69
GATCAACCATGCACACACAT	0.00	0.00	0.00	0.00	0.34
GATCAACCATGCACAGACAA	0.00	0.00	0.00	0.00	0.34
GATCAACCATGCACAGGCAC	0.00	0.00	0.00	0.00	0.00
GATCAACCATGCACCACAAA	0.23	0.00	0.00	0.00	0.00
GATCAACCATGCAGCCAATG	0.00	0.00	0.00	0.00	0.00
GATCAACCATGCAGGCGCTG	0.23	0.33	0.00	0.00	0.00
GATCAACCATGCATTCACCT	0.00	0.33	0.00	0.00	0.00
GATCAACCATGCCACTTGGC	0.00	0.00	0.29	0.00	0.00
GATCAACCATGCCCACGGAG	0.23	0.00	0.00	0.00	0.00
GATCAACCATGCCCACGGCG	1.14	0.65	0.57	0.00	0.34
GATCAACCATGCCCACGGCT	0.23	0.00	0.00	0.00	0.00
GATCAACCATGCCCAGGTGA	0.00	0.00	0.00	0.00	0.00
GATCAACCATGCCGCCACCG	0.00	0.00	0.29	0.00	0.00
GATCAACCATGCGCAACGAC	0.00	0.00	0.00	0.00	0.00
GATCAACCATGCGGTCTCAA	0.00	0.00	0.00	0.34	0.00
GATCAACCATGCGGTTGTGC	0.00	0.00	0.00	0.68	0.00
GATCAACCATGCGTCTAGCG	0.00	0.00	0.29	0.00	0.00
GATCAACCATGCTAGGCAGA	0.00	0.00	0.00	0.00	0.34
GATCAACCATGCTGCGTGGA	0.00	0.00	0.00	0.00	0.69
GATCAACCATGCTGTGCCCA	0.00	0.00	0.00	0.00	0.34
GATCAACCATGGACGCATGA	0.00	0.00	0.00	0.00	0.34
GATCAACCATGGACGCATTG	0.00	0.33	0.00	0.00	0.00
GATCAACCATGGATAAGGTG	0.00	0.00	0.00	0.00	0.00
GATCAACCATGGATGCATGG	0.00	0.00	0.29	0.34	0.34
GATCAACCATGGATGCGTGT	0.00	0.00	0.29	0.00	0.00
GATCAACCATGGCAATGTGA	0.00	0.00	0.00	0.00	0.00
GATCAACCATGGCCGACTTG	0.23	0.00	0.00	0.00	0.00
GATCAACCATGGCCTCGCGA	0.00	0.00	0.00	0.00	0.00
GATCAACCATGGCCTCGGTC	0.23	0.00	0.00	0.00	0.00
GATCAACCATGGCCTCGTNN	0.23	0.00	0.00	0.00	0.00
GATCAACCATGGCCTCGTTC	1.14	0.65	0.00	2.04	1.03
GATCAACCATGGCCTCGTTT	0.23	0.00	0.00	0.00	0.00
GATCAACCATGGCGCAGTTA	0.00	0.00	0.29	0.00	0.00
GATCAACCATGGCGCGAAAC	0.00	0.00	0.00	0.00	0.69

GATCAACCATGGCGGCACCT	0.00	0.00	0.00	0.34	0.00
GATCAACCATGGGAAAAAAA	0.00	0.00	0.00	0.34	0.00
GATCAACCATGGGATTCTCA	0.00	0.65	0.57	1.36	0.34
GATCAACCATGGGCGTTGGC	0.00	0.00	0.00	0.00	0.00
GATCAACCATGGGCTTTCCG	0.00	0.00	0.00	0.00	0.00
GATCAACCATGGGTGGCCCG	0.23	0.00	0.00	0.00	0.00
GATCAACCATGGTACAAACC	0.00	0.00	0.29	0.00	0.00
GATCAACCATGGTGCAGACC	0.00	0.00	0.29	0.00	0.00
GATCAACCATGGTGGTTAGG	0.00	0.00	0.00	0.00	0.00
GATCAACCATGTACCATTCG	0.00	0.00	0.00	0.00	0.00
GATCAACCATGTACTCATCC	0.00	0.00	0.00	0.00	0.34
GATCAACCATGTAGCGGTTG	0.00	0.00	0.00	0.00	0.34
GATCAACCATGTAGGTTGCC	0.00	0.00	0.00	0.00	0.69
GATCAACCATGTCACAATCG	0.00	0.00	0.00	0.00	0.34
GATCAACCATGTCAGGAAGC	0.00	0.00	0.00	1.36	0.00
GATCAACCATGTCAGTCATG	2.98	2.93	2.00	2.04	2.41
GATCAACCATGTCCATTCGG	0.00	0.00	0.00	0.00	0.00
GATCAACCATGTCCCATNNN	0.23	0.00	0.00	0.00	0.00
GATCAACCATGTCCCATTAG	0.00	0.00	0.00	0.00	0.34
GATCAACCATGTCCCATTCA	0.00	0.00	0.00	0.00	0.00
GATCAACCATGTCCCATTCC	0.00	0.00	0.00	0.00	0.34
GATCAACCATGTCCCATTCG	3.43	11.71	19.19	21.04	21.70
GATCAACCATGTCCGATTCG	0.00	0.00	0.00	0.34	0.00
GATCAACCATGTCGCATCGG	0.00	0.00	0.00	0.00	0.00
GATCAACCATGTCGCTGCGG	0.00	0.00	0.00	0.34	0.00
GATCAACCATGTGGCACAAA	0.00	0.00	0.00	0.00	0.34
GATCAACCATGTGTCTGGGT	0.00	0.00	0.00	0.00	0.00
GATCAACCATGTTGCATGTG	0.00	0.33	0.00	0.00	0.00
GATCAACCATGTTTATTGCG	0.00	0.00	0.00	0.00	0.00
GATCAACCATTAATGACATG	0.00	0.00	0.00	0.00	0.00
GATCAACCATTCGTCCACTC	0.00	0.00	0.00	0.00	0.00
GATCAACCATTCTGAGCCGT	0.00	0.00	0.00	0.68	0.00
GATCAACCATTGAAAGCGCG	0.00	0.33	0.00	0.00	0.00
GATCAACCATTGAACAGCTT	0.23	0.00	0.00	0.00	0.00
GATCAACCATTGACCAGCTT	0.00	0.00	0.00	0.34	0.00
GATCAACCATTGATAATCAA	0.00	0.00	0.00	0.00	0.34
GATCAACCATTGATATCATG	0.00	0.00	0.00	0.00	0.00

GATCAACCATTGATGACAGG	0.00	0.00	0.00	0.00	0.00
GATCAACCATTGATGACATG	21.75	20.50	24.63	18.33	29.27
GATCAACCATTGATGACGTG	0.23	0.00	0.00	0.00	0.00
GATCAACCATTGATGATATG	0.00	0.00	0.00	0.00	0.34
GATCAACCATTGCAAAATTG	0.00	0.00	0.00	0.00	0.34
GATCAACCATTGCAACATGG	0.00	0.00	0.00	0.34	0.00
GATCAACCATTGCAACATTG	2.98	1.95	2.58	0.00	1.03
GATCAACCATTGCAACCTNN	0.23	0.00	0.00	0.00	0.00
GATCAACCATTGCAACCTTT	0.00	0.00	0.00	0.00	0.34
GATCAACCATTGCACTTGAT	0.00	0.00	0.00	0.00	0.34
GATCAACCATTGCAGGGCCC	0.00	0.00	0.00	0.34	0.00
GATCAACCATTGCTACACCT	0.00	0.00	0.29	0.00	0.00
GATCAACCATTGCTGACCTG	0.46	0.00	0.00	0.00	0.00
GATCAACCATTGGAACTGCT	0.00	0.00	0.00	0.00	0.00
GATCAACCATTGGAAGCAGC	0.46	0.00	0.00	0.00	0.00
GATCAACCATTGGAAGGAGC	0.92	0.00	0.00	0.00	0.00
GATCAACCATTGGAAGNNNN	0.23	0.00	0.00	0.00	0.00
GATCAACCATTGGAAGTAAA	0.23	0.00	0.00	0.34	0.00
GATCAACCATTGGAAGTAGA	0.00	0.00	0.00	0.34	0.00
GATCAACCATTGGAAGTAGC	26.32	11.71	7.16	14.26	9.64
GATCAACCATTGGAAGTAGN	0.23	0.00	0.00	0.00	0.00
GATCAACCATTGGAAGTANN	0.46	0.00	0.00	0.00	0.00
GATCAACCATTGGAAGTCGC	0.23	0.00	0.00	0.00	0.00
GATCAACCATTGGAAGTGAC	0.23	0.00	0.00	0.00	0.00
GATCAACCATTGGAAGTGGC	0.23	0.00	0.00	0.00	0.00
GATCAACCATTGGCAGACGC	0.00	0.00	0.00	0.00	0.00
GATCAACCATTGGCAGGAGC	0.23	0.00	0.00	0.00	0.00
GATCAACCATTGGCAGTAGC	0.23	0.00	0.00	0.00	0.00
GATCAACCATTGGGAGTAGC	0.23	0.00	0.00	0.00	0.00
GATCAACCATTGGTCACTTG	0.00	0.00	0.29	0.00	0.00
GATCAACCATTGGTGACATG	0.23	0.00	0.00	0.00	0.00
GATCAACCATTGGTGGCAAC	0.00	0.00	0.00	0.00	0.34
GATCAACCATTGTAAATGAG	0.00	0.00	0.29	0.00	0.00
GATCAACCATTGTTCGCCCC	0.00	0.00	0.29	0.00	0.00
GATCAACCATTTCAAAAAAA	0.00	0.00	0.29	0.00	0.00
GATCAACCATTTCACGAGCT	0.00	0.00	0.29	0.00	0.00
GATCAACCATTTCACGCTGC	0.00	0.65	0.86	1.02	0.00

GATCAACCATTTCTTCCCAG	0.00	0.00	0.00	0.00	0.00
GATCAACCATTTGCCAAGTT	0.00	0.33	0.00	0.34	0.00
GATCAACCATTTGGATGCCG	0.69	1.63	0.00	2.04	1.03
GATCAACCATTTGGTGCAGA	0.00	0.00	0.00	0.00	0.34
GATCAACCATTTGTCCCCAC	0.00	0.00	0.00	0.00	0.34
GATCAACCCAAAAAAAAAAA	6.18	0.33	0.57	2.38	1.38
GATCAACCCAAAAAAAAAAT	0.23	0.00	0.00	0.00	0.34
GATCAACCCAAAAAACCAAC	0.23	0.00	0.00	0.00	0.00
GATCAACCCAAAACAACACA	1.37	0.00	0.00	0.00	0.00
GATCAACCCAAAACACCACC	0.69	0.00	0.00	0.00	0.00
GATCAACCCAAAAGAAAAAA	0.00	0.33	0.00	0.00	0.00
GATCAACCCAAAAGGATTCG	0.00	0.00	0.00	0.00	0.34
GATCAACCCAAAAGGCTCAA	0.00	0.33	0.00	1.02	0.00
GATCAACCCAAAAGGCTCCA	1.37	0.65	1.43	0.34	2.07
GATCAACCCAAAAGGCTCCG	3.20	2.28	0.29	1.36	0.69
GATCAACCCAAAAGTTGGTA	0.00	0.00	0.29	0.00	0.00
GATCAACCCAAACAAAAAAA	0.00	0.00	0.00	0.34	0.00
GATCAACCCAAACACACACA	0.00	0.00	0.29	0.00	0.00
GATCAACCCAAAGACGCACT	0.00	0.00	0.00	0.00	0.34
GATCAACCCAAAGCGATAAA	0.00	0.00	0.29	0.00	0.00
GATCAACCCAAAGTGGTATG	0.23	0.00	0.00	0.00	0.00
GATCAACCCAAAGTGGTCTG	2.06	2.60	2.00	1.02	1.72
GATCAACCCAAAGTTGTCTG	0.23	0.00	0.00	0.00	0.00
GATCAACCCAAATCGGACTT	0.00	0.00	0.00	0.00	0.00
GATCAACCCAACAAAANAAA	0.00	0.00	0.00	0.00	0.34
GATCAACCCAACACAACACA	0.00	0.00	0.00	0.00	0.34
GATCAACCCAACACACAGCA	0.00	0.00	0.00	0.00	0.34
GATCAACCCAACACACCACA	0.00	0.00	0.29	0.00	0.34
GATCAACCCAACACCGCATC	0.00	0.00	0.00	0.00	0.34
GATCAACCCAACACGCACCA	0.00	0.00	0.00	0.00	0.34
GATCAACCCAACATGGGACT	0.00	0.33	0.00	0.00	0.00
GATCAACCCAACATGTAGAC	0.00	0.00	0.00	0.00	0.34
GATCAACCCAACCACCTGCC	0.00	0.00	0.00	0.34	0.00
GATCAACCCAACCACGGCAA	0.00	0.00	0.00	0.34	0.00
GATCAACCCAACCATACGGT	0.00	0.00	0.57	0.00	0.00
GATCAACCCAACCATCCGCA	0.00	0.33	0.00	0.00	0.00
GATCAACCCAACCATCCGCT	0.46	0.00	0.29	0.68	0.00

GATCAACCCAACCATCTGCT	0.00	0.00	0.00	0.00	0.34
GATCAACCCAACCATGGCAC	0.00	0.00	0.29	0.00	0.00
GATCAACCCAACCCAACCCA	0.00	0.00	0.29	0.00	0.00
GATCAACCCAACCCATGCCA	0.00	0.00	0.29	0.00	0.00
GATCAACCCAACGCGTTGAG	0.00	0.00	0.29	0.00	0.00
GATCAACCCAACGGGAAGGG	0.00	1.63	0.00	0.68	0.00
GATCAACCCAACTCACAAGC	0.23	0.00	1.43	1.36	0.69
GATCAACCCAACTCCCAAAC	0.00	0.00	0.00	0.68	0.00
GATCAACCCAAGAAGCGGGG	0.00	0.00	0.29	0.00	0.00
GATCAACCCAAGACAAGAAA	0.00	0.00	0.00	0.00	0.00
GATCAACCCAAGATGACTAC	0.00	0.00	0.00	0.00	0.00
GATCAACCCAAGCAACCCGA	0.00	0.00	0.00	0.00	0.34
GATCAACCCAAGCACAGCCT	0.00	0.00	0.00	0.00	0.00
GATCAACCCAAGCAGCGGGG	0.00	0.00	0.29	0.00	0.00
GATCAACCCAAGCCTTCCAA	0.00	0.00	0.00	0.34	0.34
GATCAACCCAAGCGCTGCAA	0.23	0.00	0.00	0.00	0.00
GATCAACCCAAGCTTGTGTT	0.00	0.00	0.29	0.00	0.00
GATCAACCCAAGTCCATGAG	0.00	0.00	0.00	0.00	0.00
GATCAACCCAATACGTTTTC	0.00	0.00	0.00	0.00	0.34
GATCAACCCAATGCCATGCA	0.00	0.00	0.29	0.00	0.00
GATCAACCCAATGCTGATTC	0.00	0.00	0.29	0.00	0.00
GATCAACCCAATGCTGCTTC	0.00	0.00	0.00	0.34	0.00
GATCAACCCAATGCTGTTTT	0.00	0.33	0.00	0.00	0.00
GATCAACCCACAAAAAAAAA	0.00	0.00	0.00	0.34	0.00
GATCAACCCACAATAGCGGA	0.00	0.00	0.00	0.00	0.34
GATCAACCCACACCATGGAG	0.00	0.00	0.00	0.00	0.69
GATCAACCCACACCCAAAGA	0.00	0.00	0.00	0.00	0.00
GATCAACCCACACTGCATTA	0.00	0.00	0.00	0.00	0.34
GATCAACCCACAGAGGAGAG	0.00	0.00	0.00	0.00	0.00
GATCAACCCACATAAAGACT	0.00	0.00	0.29	0.00	0.00
GATCAACCCACATGCAAGGT	0.00	0.00	0.29	0.00	0.00
GATCAACCCACCAAAACAAA	0.00	0.00	0.00	0.00	0.00
GATCAACCCACCAACCCAGG	0.00	0.33	0.00	0.00	0.00
GATCAACCCACCACAACCAA	0.00	0.00	0.00	0.00	0.34
GATCAACCCACCACAACGCA	0.00	0.00	0.00	0.00	0.34
GATCAACCCACCACAGCGGA	0.00	0.00	0.00	0.68	0.00
GATCAACCCACCACCCTTCT	0.00	0.00	0.86	0.00	0.00

GATCAACCCACCACCGGTTT	0.23	0.00	0.00	0.00	0.00
GATCAACCCACCAGAACAGA	0.00	0.00	0.00	0.00	0.34
GATCAACCCACCAGTCAACA	0.00	0.00	0.00	0.00	1.03
GATCAACCCACCATAGCACA	0.00	0.00	0.29	0.00	0.00
GATCAACCCACCATAGCGAA	0.00	0.00	0.00	0.34	0.00
GATCAACCCACCATAGCGGA	5.95	6.83	9.45	4.41	11.37
GATCAACCCACCATAGCGGC	0.23	0.00	0.00	0.00	0.00
GATCAACCCACCATAGCGGT	0.00	0.00	0.00	0.00	0.34
GATCAACCCACCATAGCNNN	0.23	0.00	0.00	0.00	0.00
GATCAACCCACCGGAGAAGT	0.23	0.00	0.00	0.00	0.00
GATCAACCCACCTACAATGC	0.00	0.00	0.00	0.00	0.00
GATCAACCCACGCCGCTGTT	0.69	0.33	0.29	0.00	1.03
GATCAACCCACGCCGCTTGA	0.00	0.33	0.00	0.00	0.00
GATCAACCCACGCGGAATGC	0.23	0.00	0.00	0.00	0.00
GATCAACCCACGGGACTACG	0.46	0.00	0.29	0.34	0.00
GATCAACCCACGGGAGCACA	0.00	0.00	0.00	0.34	0.00
GATCAACCCACTCAAGGCAT	0.00	0.00	0.86	0.00	0.00
GATCAACCCACTCAAGGCTT	0.23	0.98	0.00	1.36	0.00
GATCAACCCACTCCTGTGCA	0.00	0.00	0.00	0.00	0.00
GATCAACCCACTCTGGCCTT	0.00	0.00	0.00	0.00	0.00
GATCAACCCACTGAGGGCAG	0.00	0.00	0.00	0.00	0.69
GATCAACCCACTGATGCTGA	0.00	0.00	0.00	0.00	0.34
GATCAACCCACTGGGCTGGC	0.00	0.00	0.29	0.00	0.00
GATCAACCCACTGTTGGTGA	0.23	0.00	0.00	0.00	0.00
GATCAACCCAGAAACAGCAC	0.00	0.00	0.29	0.00	0.00
GATCAACCCAGAATTCGAGG	0.23	0.00	0.00	0.00	0.69
GATCAACCCAGAATTCGAGN	0.23	0.00	0.00	0.00	0.00
GATCAACCCAGACACGNNNN	0.00	0.00	0.00	0.00	0.00
GATCAACCCAGACACGTGGG	0.00	0.65	0.00	0.00	0.00
GATCAACCCAGACCACCCAC	0.00	0.00	0.00	0.00	0.34
GATCAACCCAGACCCACAAA	0.23	0.00	0.00	0.00	0.00
GATCAACCCAGACCGCCTAG	0.00	0.00	0.00	0.00	0.34
GATCAACCCAGACGACCCGA	1.37	0.00	0.00	0.00	0.00
GATCAACCCAGACGTGTTGT	0.00	0.33	0.00	0.34	0.00
GATCAACCCAGACTGGACCG	0.00	0.00	0.29	0.34	0.00
GATCAACCCAGACTGGCATG	0.00	0.00	0.00	0.00	0.69
GATCAACCCAGACTGGCCCA	0.23	0.00	0.00	0.00	0.00

GATCAACCCAGACTGGGCCG	0.46	0.00	0.00	0.00	0.34
GATCAACCCAGACTGGTAAA	0.00	0.00	0.00	0.34	0.00
GATCAACCCAGACTGGTACG	0.00	0.33	0.86	0.00	0.00
GATCAACCCAGACTGGTCAA	0.46	0.00	0.29	0.00	0.00
GATCAACCCAGACTGGTCAG	0.00	0.33	0.00	0.00	0.00
GATCAACCCAGACTGGTCCA	16.94	8.78	7.16	12.90	5.17
GATCAACCCAGACTGGTCCC	0.00	0.00	0.00	0.34	0.00
GATCAACCCAGACTGGTCCG	22.20	20.17	13.17	29.53	18.25
GATCAACCCAGACTGGTCCN	0.23	0.00	0.00	0.00	0.00
GATCAACCCAGACTGGTCNN	0.69	0.00	0.00	0.00	0.00
GATCAACCCAGACTGGTTCG	0.00	0.00	0.29	0.00	0.00
GATCAACCCAGACTGTTGCG	0.00	0.00	0.00	0.00	0.00
GATCAACCCAGACTTGGTGA	0.00	0.00	0.00	0.34	0.00
GATCAACCCAGAGAACAGGA	0.00	0.00	0.00	0.00	0.69
GATCAACCCAGAGAGAGCGC	0.00	0.00	0.29	0.00	0.00
GATCAACCCAGAGGCATGCA	1.83	0.00	0.00	0.34	0.34
GATCAACCCAGAGGTGTACG	0.00	0.00	0.00	0.68	0.34
GATCAACCCAGAGTGTAGAC	1.14	2.28	0.29	0.68	0.34
GATCAACCCAGATGAAAAAA	0.00	0.00	0.00	1.02	0.00
GATCAACCCAGATGCAATGA	0.00	0.00	0.00	0.00	0.00
GATCAACCCAGATGTAAAAA	0.69	0.00	0.00	0.00	0.00
GATCAACCCAGATGTAAAAN	0.46	0.00	0.00	0.00	0.00
GATCAACCCAGATGTGNTNN	0.23	0.00	0.00	0.00	0.00
GATCAACCCAGATGTGTNGT	0.00	0.00	0.00	0.00	0.34
GATCAACCCAGATGTGTTAA	0.23	1.63	0.29	0.00	0.00
GATCAACCCAGATGTGTTGG	0.00	0.00	0.29	0.00	0.00
GATCAACCCAGATGTGTTGT	15.34	11.06	3.15	13.92	6.54
GATCAACCCAGATGTGTTNN	0.23	0.00	0.00	0.00	0.00
GATCAACCCAGATTGCCCGA	0.00	0.00	0.29	0.00	0.00
GATCAACCCAGCAGTGATGG	3.89	0.98	0.29	2.72	1.03
GATCAACCCAGCATGAGCAC	0.00	0.00	0.29	0.00	0.00
GATCAACCCAGCATGCACCA	0.00	0.33	0.00	0.00	0.00
GATCAACCCAGCCATCCTTA	0.23	0.98	0.00	0.00	0.69
GATCAACCCAGCCATCTGCT	0.00	0.00	0.00	0.00	0.34
GATCAACCCAGCCCTCCCGT	0.00	0.33	0.00	0.00	0.00
GATCAACCCAGCCTGGACGT	0.00	0.00	0.00	0.00	0.34
GATCAACCCAGCCTGGTCCA	0.23	0.00	0.00	0.00	0.00

GATCAACCCAGCGATGAGCT	0.69	0.00	0.00	0.00	0.00
GATCAACCCAGCGCCTNGTG	0.00	0.00	0.00	0.00	0.34
GATCAACCCAGCGCCTTGTG	0.00	0.00	0.00	0.00	0.34
GATCAACCCAGCGTTAGATG	1.37	0.98	0.86	0.68	1.03
GATCAACCCAGCGTTCCTGA	0.00	0.33	0.00	0.00	0.00
GATCAACCCAGCTCTTCTGA	0.00	0.00	0.00	0.00	0.00
GATCAACCCAGCTGCATCCA	0.46	3.90	3.15	2.04	3.44
GATCAACCCAGCTGCATCCC	0.23	0.00	0.00	0.00	0.00
GATCAACCCAGCTGCATCCT	0.00	0.00	0.00	0.00	1.03
GATCAACCCAGCTGCATTCA	0.00	0.00	0.29	0.00	0.00
GATCAACCCAGCTGCTCGCG	0.00	0.00	0.29	0.00	0.00
GATCAACCCAGCTGTGTTGG	0.23	0.00	0.00	0.00	0.00
GATCAACCCAGCTGTTCTTC	0.00	0.00	0.00	0.00	0.34
GATCAACCCAGGACGGGCCG	0.23	0.00	0.00	0.34	0.00
GATCAACCCAGGACGGGCGG	0.00	0.00	0.00	0.34	0.00
GATCAACCCAGGAGAGCTAG	0.00	0.00	0.00	0.34	0.00
GATCAACCCAGGCAAAAAAA	0.00	0.00	0.57	0.00	0.00
GATCAACCCAGGCATCCAAC	0.00	0.00	0.00	0.34	0.00
GATCAACCCAGGCGGCCCAG	0.00	0.00	0.00	0.00	0.34
GATCAACCCAGGGATGAGAG	0.00	0.00	0.00	0.34	0.00
GATCAACCCAGGGGGTAGAG	0.23	0.00	0.00	0.00	0.00
GATCAACCCAGGGGTGCCCC	0.23	0.00	0.00	0.00	0.00
GATCAACCCAGGTAAGTATC	1.37	0.65	0.00	0.34	0.00
GATCAACCCAGTAGAGACAA	0.00	0.00	0.29	0.00	0.00
GATCAACCCAGTAGAGGAGC	0.46	0.00	0.00	0.00	0.00
GATCAACCCAGTAGTAGCAG	0.00	0.00	0.29	0.00	0.00
GATCAACCCAGTATTGCCGC	0.00	0.65	0.57	0.68	0.69
GATCAACCCAGTCGACGAGA	0.00	0.00	0.00	0.00	0.00
GATCAACCCAGTCTACTGGG	0.00	0.33	0.00	0.00	0.00
GATCAACCCAGTCTGGACCG	0.00	0.00	0.29	0.00	0.00
GATCAACCCAGTGAAGCGAA	0.00	0.00	0.00	0.00	0.34
GATCAACCCAGTGAGCGCTT	0.00	0.00	0.29	0.00	0.00
GATCAACCCAGTGAGGTATG	0.00	0.00	0.29	0.00	0.00
GATCAACCCAGTGCGGTGGT	0.00	0.00	0.00	0.00	0.00
GATCAACCCAGTGTAGACCC	0.00	0.00	0.00	0.34	0.00
GATCAACCCAGTTGTGTTGT	0.00	0.00	0.00	0.34	0.00
GATCAACCCATAACAGAACA	0.00	0.00	0.00	0.00	0.69

GATCAACCCATAACGAACAG	0.00	0.00	0.00	0.00	0.69
GATCAACCCATATCATACAT	0.46	0.00	0.00	0.00	0.00
GATCAACCCATCAAAAAAAA	0.00	0.00	0.00	0.34	0.00
GATCAACCCATCAAAATACA	0.23	0.00	0.00	0.00	0.00
GATCAACCCATCAAGTGGTT	0.00	0.00	0.00	0.00	0.00
GATCAACCCATCAATGGCCC	0.00	0.00	0.29	0.00	0.00
GATCAACCCATCACAATACA	0.23	0.00	0.00	0.00	0.00
GATCAACCCATCATTCAGTC	0.00	0.00	0.00	0.34	0.00
GATCAACCCATCATTGAGCC	0.00	0.00	0.00	0.00	0.34
GATCAACCCATCCAACCACC	0.23	0.00	0.00	0.00	0.00
GATCAACCCATCCCACTCCA	0.00	0.00	0.29	0.00	0.00
GATCAACCCATCGATGAGTC	0.00	0.00	0.29	0.00	0.00
GATCAACCCATCGCGTGAGT	0.00	0.33	0.00	0.00	0.00
GATCAACCCATCGGGACAGG	0.23	0.00	0.00	0.00	0.00
GATCAACCCATCTCGCCCGC	0.00	0.00	0.00	0.00	0.00
GATCAACCCATCTTCCGCAA	0.00	0.00	0.00	0.00	0.00
GATCAACCCATCTTGTGACC	0.00	0.00	0.00	0.00	0.00
GATCAACCCATCTTGTGCCC	0.00	0.00	0.00	0.00	0.00
GATCAACCCATGCAACAATA	0.00	0.00	0.00	0.00	0.69
GATCAACCCATGCGTTGCTC	0.00	0.33	0.00	0.34	0.00
GATCAACCCATGGACGGAAA	0.00	0.00	0.00	0.00	0.00
GATCAACCCATGGCTCCCAC	0.00	0.00	0.00	0.00	0.69
GATCAACCCATGGCTCCCCC	0.00	0.00	0.29	0.00	0.00
GATCAACCCATGGGACGACA	0.00	0.33	0.00	0.00	0.00
GATCAACCCATTGCAACTTG	0.00	0.00	0.00	0.00	0.00
GATCAACCCATTGCAGCGTG	0.00	0.00	0.29	0.00	0.00
GATCAACCCATTGCAGGCTG	0.00	0.00	0.29	0.00	0.00
GATCAACCCATTGCAGGGCG	0.00	0.00	0.29	0.00	0.00
GATCAACCCATTGCAGGGGG	0.00	0.00	0.00	0.34	0.00
GATCAACCCATTGCAGGGTG	5.95	16.27	28.35	14.93	19.98
GATCAACCCATTGCAGGTTG	0.23	0.00	0.00	0.00	0.00
GATCAACCCATTGCAGNNNN	0.00	0.00	0.00	0.00	0.00
GATCAACCCATTGCCGGGTG	0.00	0.00	0.00	0.00	0.34
GATCAACCCATTGGCTTGCC	0.00	0.33	0.00	0.00	0.00
GATCAACCCCAAAGACAACG	0.00	0.33	0.00	0.00	0.00
GATCAACCCCAACAAGAGTA	0.00	0.00	0.00	0.34	0.00
GATCAACCCCAACACCATGG	0.00	0.00	0.00	0.00	0.00

GATCAACCCCAACACGCAAC	0.00	0.00	0.29	0.00	0.34
GATCAACCCCAACCAACAAA	0.00	0.00	0.00	0.00	0.00
GATCAACCCCAACCACCAGT	0.00	0.00	0.00	0.00	0.34
GATCAACCCCAACCACCCAA	0.00	0.00	0.00	0.00	0.34
GATCAACCCCAACCACCCAG	0.92	0.33	0.57	2.04	1.72
GATCAACCCCAACCACCCCG	0.69	0.98	0.86	0.34	0.69
GATCAACCCCAACCACCCTG	0.00	0.00	0.29	1.02	0.00
GATCAACCCCAACCGAGCGA	0.00	0.00	0.00	0.00	0.00
GATCAACCCCAACCTGGACG	0.00	0.00	0.00	0.34	0.00
GATCAACCCCAACCTGGACT	0.00	0.00	0.00	0.00	0.00
GATCAACCCCAACGACACGG	0.00	0.00	0.00	0.00	0.34
GATCAACCCCAACTTCCGGG	0.00	0.00	0.29	0.00	0.00
GATCAACCCCAAGAACGCCG	0.00	0.00	0.00	0.34	0.00
GATCAACCCCAAGAACTCAG	0.00	0.00	0.00	0.00	0.34
GATCAACCCCAAGAACTCCG	14.88	5.86	3.72	2.72	2.76
GATCAACCCCAAGACAATGA	0.00	0.00	0.00	0.00	0.00
GATCAACCCCAAGATGGAGG	0.00	0.00	0.00	0.00	0.00
GATCAACCCCAAGCACACCA	0.00	0.00	0.57	0.34	0.69
GATCAACCCCAAGCACACCC	0.00	0.00	0.00	0.00	0.34
GATCAACCCCAAGCACGCGG	0.00	0.65	0.29	0.34	0.00
GATCAACCCCAAGCACNCCA	0.00	0.00	0.00	0.00	0.34
GATCAACCCCAAGCACTACA	0.00	0.00	0.00	0.34	0.00
GATCAACCCCAAGCACTCCA	1.60	2.28	6.30	2.72	5.51
GATCAACCCCAAGCACTGTG	0.00	0.00	0.00	0.00	0.00
GATCAACCCCAAGCAGCCCC	0.00	0.00	0.29	0.00	0.00
GATCAACCCCAAGCATCGAT	0.00	0.00	0.29	0.00	0.00
GATCAACCCCAAGCTTGGTC	0.00	0.00	0.00	0.00	0.69
GATCAACCCCAAGGACCTGC	0.00	0.00	0.00	0.00	0.00
GATCAACCCCAAGGGCTCCT	0.00	0.33	0.00	0.00	0.00
GATCAACCCCAAGTGCAAGG	0.00	0.00	0.00	0.34	0.00
GATCAACCCCAAGTGCTCTC	0.00	0.00	0.29	0.00	0.00
GATCAACCCCAAGTTCGTCT	0.00	0.00	0.00	0.00	0.34
GATCAACCCCAATCAACCCG	0.00	0.00	0.00	0.00	0.00
GATCAACCCCAATCACACCG	0.00	0.00	0.29	0.00	0.00
GATCAACCCCAATCACCCCG	21.75	15.94	15.75	9.84	10.33
GATCAACCCCAATCATCCTG	0.23	0.33	1.43	0.34	0.69
GATCAACCCCAATCCCCCCG	0.23	0.00	0.00	0.00	0.00

GATCAACCCCACAAAACACA	0.23	0.00	0.00	0.00	0.00
GATCAACCCCACACACCACG	0.00	0.00	0.29	0.00	0.00
GATCAACCCCACCAGCATCG	0.46	0.65	0.86	1.02	0.69
GATCAACCCCACCCACAACC	0.00	0.00	0.00	0.00	0.34
GATCAACCCCACCTGCCGGT	0.00	0.00	0.00	0.34	0.00
GATCAACCCCACGCCAGCTT	0.46	0.00	0.00	0.00	0.00
GATCAACCCCACTACCCACA	0.23	0.00	0.00	0.00	0.00
GATCAACCCCACTTCTCGGT	0.00	0.33	0.00	0.00	0.00
GATCAACCCCAGAACCCCTC	0.00	0.00	0.29	0.00	0.00
GATCAACCCCAGAGACAATG	0.00	0.00	0.00	0.00	0.00
GATCAACCCCAGAGACACGT	0.00	0.00	0.00	0.00	0.34
GATCAACCCCAGCACCGCAA	0.00	0.00	0.00	0.00	0.00
GATCAACCCCAGCGAAGTCG	0.00	0.00	0.29	0.00	0.00
GATCAACCCCAGCGGCACGA	0.00	0.00	0.00	0.00	0.00
GATCAACCCCAGCGTGAGAC	0.00	0.33	0.00	0.00	0.00
GATCAACCCCAGCTTGGGTT	0.23	0.00	0.00	0.00	0.00
GATCAACCCCAGCTTGTGTT	0.00	0.00	0.00	0.00	0.00
GATCAACCCCAGGATGACAG	0.00	0.00	0.29	0.00	0.00
GATCAACCCCAGGCACTCCA	0.00	0.00	0.29	0.00	0.00
GATCAACCCCAGGCAGGGTA	0.00	0.33	0.00	0.00	0.00
GATCAACCCCAGGCAGGTTA	1.37	1.63	0.57	3.05	2.41
GATCAACCCCAGGCATGCGC	0.00	0.00	0.00	0.00	0.00
GATCAACCCCAGGCCCAGCA	0.00	0.00	0.00	0.34	0.00
GATCAACCCCAGGCTCATCT	0.00	0.00	0.00	1.36	0.00
GATCAACCCCAGGGACAACC	0.00	0.00	0.00	0.68	0.34
GATCAACCCCAGGGCGTATC	0.00	0.33	0.00	0.34	0.00
GATCAACCCCAGGGGGCCAG	0.00	0.33	0.00	0.00	0.00
GATCAACCCCAGGGTGACAG	0.00	0.33	0.29	0.00	0.00
GATCAACCCCAGGTGCTCTT	0.00	0.33	0.00	0.00	0.00
GATCAACCCCAGGTTAAACC	0.00	0.00	0.00	0.00	0.69
GATCAACCCCAGTACGTCCC	0.00	0.00	0.00	0.00	0.00
GATCAACCCCAGTATGTCCC	0.00	0.00	0.29	0.00	0.00
GATCAACCCCAGTCGTCGCC	0.00	0.33	0.00	0.00	0.00
GATCAACCCCAGTGAGGCTC	0.00	0.65	0.00	0.00	0.00
GATCAACCCCATAACAACAA	0.00	0.00	0.00	0.00	0.34
GATCAACCCCATACAGAACT	0.00	0.00	0.00	0.00	0.00
GATCAACCCCATCACATTCC	0.00	0.33	0.00	0.34	0.00

GATCAACCCCATCACCAGAG	0.00	0.00	0.00	0.34	0.00
GATCAACCCCATCGAACCGG	0.00	0.33	0.00	0.00	0.00
GATCAACCCCATCGACCCAG	1.14	3.25	0.29	1.02	1.03
GATCAACCCCATCGACCCGG	0.00	0.65	0.57	0.00	0.34
GATCAACCCCATCTGACGGG	0.23	0.00	0.00	0.00	0.34
GATCAACCCCATCTGGACTT	0.00	0.00	0.00	0.00	0.00
GATCAACCCCATCTGTTCAC	0.00	0.00	0.00	0.68	0.00
GATCAACCCCATGATGTGGC	0.00	0.00	0.00	0.00	0.00
GATCAACCCCATGGGCTAAA	0.00	0.00	0.29	0.00	0.00
GATCAACCCCATGTGATAGT	0.00	0.00	0.00	0.00	0.69
GATCAACCCCATTAGCGCAA	0.00	0.00	0.00	0.34	0.00
GATCAACCCCATTGCAACGG	0.00	0.00	0.00	0.00	0.00
GATCAACCCCCAAAAAAAAA	0.23	0.00	0.00	0.00	0.00
GATCAACCCCCAAAAAAACA	0.23	0.00	0.00	0.00	0.00
GATCAACCCCCAAAAACCCC	0.23	0.00	0.00	0.00	0.00
GATCAACCCCCACAAGCCGG	0.00	0.00	0.00	0.68	0.00
GATCAACCCCCACACTGATG	0.00	0.00	0.57	0.00	0.00
GATCAACCCCCACATCGAGG	0.46	0.33	0.00	1.02	0.00
GATCAACCCCCACCTGGGGG	0.23	0.00	0.00	0.00	0.00
GATCAACCCCCACCTGGGTG	0.23	0.00	0.00	0.00	0.00
GATCAACCCCCACGGATGTC	0.00	0.00	0.29	0.00	0.00
GATCAACCCCCACGGCTCGG	0.00	0.00	0.29	0.00	0.00
GATCAACCCCCAGACGAACC	0.00	0.00	0.00	0.00	0.34
GATCAACCCCCAGCATGCAC	0.00	0.00	0.00	0.34	0.00
GATCAACCCCCAGCTCAAGG	0.00	0.00	0.00	0.00	0.34
GATCAACCCCCAGCTCCTGG	1.83	2.60	1.15	3.39	2.76
GATCAACCCCCCAAAAAAAA	0.00	0.00	0.29	0.00	0.00
GATCAACCCCCCAACAAAAA	0.00	0.33	0.00	0.00	0.00
GATCAACCCCCCAACGAAAA	0.46	0.00	0.00	0.00	0.00
GATCAACCCCCCAACGAACC	0.00	0.00	0.00	0.00	0.00
GATCAACCCCCCACCGCCAC	0.00	0.00	0.29	0.00	0.00
GATCAACCCCCCAGCTGAGT	0.00	0.00	0.29	0.00	0.00
GATCAACCCCCCCAAGCGTC	0.00	0.33	0.00	0.00	0.00
GATCAACCCCCCCACCACAC	0.00	0.00	0.29	0.00	0.00
GATCAACCCCCCCCCCACCC	0.00	0.00	0.00	0.00	0.34
GATCAACCCCCCCCCCCCAC	0.00	0.00	0.00	0.00	0.34
GATCAACCCCCCCCCCCCCC	0.00	0.00	0.00	0.00	0.69

GATCAACCCCCCCTCCTCCC	0.00	0.00	0.00	0.00	0.34
GATCAACCCCCCCTGGGCAT	0.00	0.00	0.00	0.00	0.00
GATCAACCCCCCCTTTTGCC	0.00	0.00	0.00	0.00	0.34
GATCAACCCCCCGACCAAGG	0.00	0.00	0.00	0.00	0.34
GATCAACCCCCCGGACGTCG	0.92	0.00	0.00	0.00	0.00
GATCAACCCCCCGTGGCTNN	0.00	0.00	0.00	0.00	0.00
GATCAACCCCCCGTGGCTTC	0.69	0.98	0.00	1.02	0.34
GATCAACCCCCCGTGGCTTN	0.00	0.00	0.00	0.00	0.00
GATCAACCCCCCTGTGCCAG	0.00	0.00	0.29	0.68	0.00
GATCAACCCCCGAGTGACTG	0.23	0.65	0.00	2.04	0.69
GATCAACCCCCGCTGGCAAG	0.00	0.00	0.00	0.00	0.00
GATCAACCCCCGCTGGCAAT	2.29	2.60	0.29	2.38	1.72
GATCAACCCCCGCTGGCANN	0.00	0.00	0.00	0.00	0.00
GATCAACCCCCGCTGGCCAT	0.00	0.00	0.29	0.00	0.00
GATCAACCCCCGCTTTGTTC	0.00	0.33	0.00	0.00	0.00
GATCAACCCCCGGGTCACAG	0.00	0.98	0.00	0.68	0.00
GATCAACCCCCGGGTGACAG	0.00	0.00	0.00	0.34	0.00
GATCAACCCCCGGTACCAGG	0.00	0.33	0.00	0.00	0.00
GATCAACCCCCGTCAGCACT	0.00	0.33	0.00	0.68	0.00
GATCAACCCCCGTGGAGTGC	0.23	0.00	0.00	0.00	0.00
GATCAACCCCCGTTTGAAAA	0.00	0.00	0.00	0.68	0.00
GATCAACCCCCTCAACACAA	0.00	0.00	0.00	0.00	0.34
GATCAACCCCCTCTGGCCTT	1.37	1.30	2.29	1.02	1.72
GATCAACCCCCTCTTCCCTC	0.00	0.33	0.29	0.00	0.00
GATCAACCCCCTCTTCTCCG	0.00	0.00	0.00	0.00	0.00
GATCAACCCCCTGACGCTGC	0.00	0.00	0.00	0.00	0.69
GATCAACCCCCTGCCCTCCC	0.00	0.00	0.00	0.34	0.00
GATCAACCCCCTGCCTGCGT	0.00	0.00	0.00	0.00	0.34
GATCAACCCCCTGGCCAAGG	0.23	0.33	0.00	0.34	0.00
GATCAACCCCCTGGCTGTGC	0.00	0.00	0.00	0.34	0.00
GATCAACCCCCTGTCAACAC	0.00	0.00	0.00	0.00	0.00
GATCAACCCCCTTACCATGG	0.00	0.33	0.00	0.00	0.00
GATCAACCCCCTTTCTTTGG	0.00	0.00	0.00	0.00	0.00
GATCAACCCCGACAAGATGG	0.00	0.65	0.00	0.00	0.00
GATCAACCCCGACAAGCTGG	0.00	0.00	0.00	0.00	0.00
GATCAACCCCGACACCAAAC	0.00	0.00	0.00	0.00	0.34
GATCAACCCCGACAGCCCCG	0.00	0.00	0.00	0.00	0.34

GATCAACCCCGACAGCTCCG	0.00	0.00	0.29	0.00	0.00
GATCAACCCCGACGTCGGCT	0.00	0.00	0.00	0.00	0.69
GATCAACCCCGACTCGGGGA	0.46	0.65	0.00	1.02	0.00
GATCAACCCCGACTCGGGGN	0.23	0.00	0.00	0.00	0.00
GATCAACCCCGAGAACGCCG	0.00	0.00	0.00	0.34	0.00
GATCAACCCCGAGAACTCCG	0.00	0.00	0.00	0.00	0.34
GATCAACCCCGAGAGCCCGA	0.00	0.33	0.00	0.00	0.00
GATCAACCCCGAGAGGCTCA	0.23	0.00	0.00	0.00	0.00
GATCAACCCCGAGCAGCACT	0.00	0.00	0.00	0.00	0.00
GATCAACCCCGAGGGTGGCG	0.23	0.00	0.00	0.00	0.00
GATCAACCCCGCACCTTTGC	0.00	0.00	0.00	0.00	0.00
GATCAACCCCGCAGCCATCA	0.00	0.00	0.00	0.00	0.00
GATCAACCCCGCAGCCCATG	0.00	0.00	0.00	0.00	0.34
GATCAACCCCGCCCAAAAAA	0.00	0.00	0.00	0.34	0.00
GATCAACCCCGCCCGCTCAC	0.00	0.00	0.00	0.00	0.34
GATCAACCCCGCCTGGGCCA	0.46	0.33	0.00	0.00	0.00
GATCAACCCCGCGGTGCCCT	0.00	0.00	0.00	0.00	0.69
GATCAACCCCGCGTCCGCGG	0.00	0.00	0.00	0.34	0.00
GATCAACCCCGCTCCCCTGA	0.00	0.00	0.00	0.00	0.00
GATCAACCCCGGCAACTCGG	0.46	0.00	0.00	0.34	0.00
GATCAACCCCGGCAGGCTCG	0.23	0.00	0.00	0.00	0.00
GATCAACCCCGGCGTCAGGG	0.00	0.00	0.00	0.34	0.00
GATCAACCCCGGCGTCATGG	0.00	0.33	1.15	0.34	1.03
GATCAACCCCGGCGTGCGTG	0.00	0.00	0.00	0.00	0.34
GATCAACCCCGGCTGGAAGG	0.00	0.00	0.00	0.34	0.00
GATCAACCCCGGGCCCGCTA	0.23	0.00	0.00	0.00	0.00
GATCAACCCCGGGGATGTGT	0.00	0.33	0.00	0.00	0.00
GATCAACCCCGGTGGTACAC	0.00	0.00	0.00	0.00	0.00
GATCAACCCCGGTTGCGAGT	0.00	0.00	0.00	0.00	0.34
GATCAACCCCGTAGACGTGG	0.00	0.33	0.00	0.68	0.00
GATCAACCCCGTCCTCTGGA	0.00	0.33	0.00	0.34	0.34
GATCAACCCCGTCGCCAAGC	0.00	0.00	0.00	0.68	0.00
GATCAACCCCGTGAAGCCGG	0.00	0.00	0.00	0.34	0.00
GATCAACCCCGTGACGGCGC	0.00	0.00	0.00	0.00	0.00
GATCAACCCCGTGCTCTCGC	0.00	0.00	0.29	0.34	0.00
GATCAACCCCGTGCTGACGA	0.00	0.33	0.00	0.00	0.00
GATCAACCCCGTGCTGCCGA	0.00	0.33	0.00	0.00	0.00

GATCAACCCCGTGGCCAGTC	0.00	0.00	0.29	0.34	0.00
GATCAACCCCGTGGCGTAGC	0.00	0.00	0.00	0.00	0.00
GATCAACCCCGTGGGCATCG	0.00	0.33	0.00	0.00	0.00
GATCAACCCCTACACATCCG	0.00	0.00	0.00	0.00	0.00
GATCAACCCCTACGATGTGC	0.00	0.00	0.00	0.34	0.00
GATCAACCCCTACGCCTCCG	0.00	0.00	0.00	0.00	0.00
GATCAACCCCTACGCGCAGT	1.14	0.00	0.29	0.34	0.34
GATCAACCCCTACGCGTCCG	0.00	0.00	0.00	0.00	0.34
GATCAACCCCTAGGGCCGTG	0.00	0.00	0.00	0.00	0.34
GATCAACCCCTATAACGCCC	0.00	0.00	0.00	0.00	0.34
GATCAACCCCTATGCCTCCG	0.00	0.00	0.00	0.00	0.34
GATCAACCCCTCAAAATACA	0.23	0.00	0.00	0.00	0.00
GATCAACCCCTCACACGTGG	0.00	0.00	0.00	0.00	0.00
GATCAACCCCTCAGAGACTT	0.00	0.00	0.00	0.00	0.00
GATCAACCCCTCATCGATTG	0.23	0.00	0.00	0.34	0.34
GATCAACCCCTCATTTTCGG	0.00	0.00	0.29	0.00	0.00
GATCAACCCCTCCCAATGAA	0.00	0.00	0.57	0.00	0.00
GATCAACCCCTCGGTAGCGA	0.00	0.00	0.00	0.00	0.00
GATCAACCCCTCGGTGCTGA	0.00	0.00	0.00	0.00	0.00
GATCAACCCCTCGTGGCTTC	0.00	0.33	0.00	0.00	0.00
GATCAACCCCTCGTGGTACA	0.00	0.00	0.00	0.00	0.00
GATCAACCCCTCTAACGCGA	0.00	0.00	0.00	0.00	0.34
GATCAACCCCTCTAGAGTTG	0.00	0.33	0.00	0.00	0.34
GATCAACCCCTGCGGGCAGG	0.00	0.33	0.00	0.00	0.00
GATCAACCCCTGCTACACTT	0.00	0.00	0.00	0.00	0.34
GATCAACCCCTGGAAGGACA	0.23	0.00	0.00	0.00	0.00
GATCAACCCCTGGAAGTACA	1.14	1.30	1.43	0.68	1.72
GATCAACCCCTGGACAGAGA	0.00	0.00	0.00	0.00	0.00
GATCAACCCCTGGATAGAGC	0.00	0.65	0.00	0.00	0.00
GATCAACCCCTGGCCAGGTG	0.46	0.33	0.57	0.00	0.34
GATCAACCCCTGGCCAGGTT	0.00	0.33	0.00	0.00	0.00
GATCAACCCCTGGGGCCGTG	0.00	0.00	0.00	0.00	0.34
GATCAACCCCTGGTCCAAGA	0.00	0.00	0.00	0.68	0.00
GATCAACCCCTGTGCGCGTG	0.00	0.00	0.00	0.00	0.34
GATCAACCCCTTAAAAGATG	0.00	0.00	0.00	0.00	0.00
GATCAACCCCTTCAACCTGC	0.00	0.00	0.00	0.00	0.34
GATCAACCCCTTCAACGGGC	0.00	0.00	0.00	0.34	0.00

GATCAACCCCTTCACCGGCT	0.00	0.33	0.00	0.00	0.00
GATCAACCCCTTCACCTGCT	2.06	1.30	3.15	0.34	2.41
GATCAACCCCTTCCCCATCT	0.00	0.00	0.00	0.00	0.00
GATCAACCCCTTCGCCGAGC	0.23	0.00	0.29	1.02	0.00
GATCAACCCCTTCTCCAAGT	0.00	0.33	0.00	0.00	0.00
GATCAACCCCTTCTTGCTGC	0.00	0.00	0.00	0.00	0.34
GATCAACCCCTTGGAGGGGG	0.46	0.00	1.43	0.00	0.00
GATCAACCCCTTTGAGTGGG	0.00	0.00	0.00	0.00	0.69
GATCAACCCCTTTTCCAAGT	0.00	0.00	0.00	0.00	0.00
GATCAACCCCTTTTCTTTGC	0.00	0.00	0.00	0.00	0.00
GATCAACCCGAAACAATGGC	0.00	0.00	0.00	0.00	0.34
GATCAACCCGAACACGGTCT	0.00	0.00	0.57	0.68	0.00
GATCAACCCGAACGAGAACA	0.23	0.33	0.00	0.00	0.00
GATCAACCCGAAGATGGGAA	10.07	5.53	8.59	9.16	8.27
GATCAACCCGAAGATGGGCA	0.00	0.00	0.00	0.00	0.34
GATCAACCCGAAGATGGGNN	0.00	0.00	0.00	0.00	0.00
GATCAACCCGAAGGAGGAGC	0.00	0.33	0.00	0.00	0.00
GATCAACCCGAAGTACCTGG	0.23	0.00	0.00	0.00	0.00
GATCAACCCGAAGTACTTGG	1.14	0.00	0.00	0.68	0.34
GATCAACCCGACCAATAGCA	0.00	0.00	0.00	0.00	0.00
GATCAACCCGACCACCGCCC	0.23	2.28	0.00	1.36	1.03
GATCAACCCGACCCCTAGGC	0.00	0.00	0.29	0.00	0.00
GATCAACCCGACCGTGATGC	0.00	0.00	0.00	0.00	0.00
GATCAACCCGACGCCGCTGG	0.23	0.00	0.00	0.00	0.00
GATCAACCCGACGCCTGCTC	0.00	0.33	0.00	0.00	0.00
GATCAACCCGACGGTCATCA	0.00	0.00	0.00	0.00	0.00
GATCAACCCGACGTCGGACT	0.00	0.00	0.00	0.34	0.00
GATCAACCCGACTTTCGTGA	0.00	0.00	0.00	0.00	0.00
GATCAACCCGACTTTTGTGA	0.46	0.98	0.57	0.34	0.69
GATCAACCCGAGACCACCCG	0.23	0.00	0.00	0.00	0.00
GATCAACCCGAGCAAGATGG	0.46	0.00	0.00	0.00	0.00
GATCAACCCGAGCTAGGCCC	0.00	0.00	0.00	0.00	0.34
GATCAACCCGAGGAGGAGCC	0.00	0.00	0.00	0.68	0.00
GATCAACCCGAGGCGCCTCA	0.23	0.00	0.00	0.00	0.00
GATCAACCCGAGGCGGCACA	0.23	0.00	0.00	0.00	0.00
GATCAACCCGAGGCGGGCAG	0.00	0.00	0.00	0.34	0.00
GATCAACCCGAGGTCCGAGG	0.46	0.33	0.00	0.00	0.34

GATCAACCCGAGGTGCAATG	0.00	0.00	0.29	0.00	0.00
GATCAACCCGAGTCCGCGAT	0.00	0.00	0.00	0.68	0.00
GATCAACCCGATGAAGGCTT	0.00	0.00	0.29	0.00	0.00
GATCAACCCGATGCAATGTC	0.00	0.00	0.00	0.00	0.00
GATCAACCCGCAAGCCGTCG	0.00	0.33	0.00	0.00	0.00
GATCAACCCGCACAACCCGC	0.46	0.00	0.00	0.68	0.69
GATCAACCCGCACCAGTTGA	0.00	0.00	0.00	0.00	0.34
GATCAACCCGCACCGTTGGA	0.00	0.00	0.00	0.00	0.00
GATCAACCCGCAGATGGGAA	0.00	0.00	0.29	0.00	0.00
GATCAACCCGCAGCAAGCGT	0.00	0.00	0.29	0.00	0.00
GATCAACCCGCAGCACTTCG	0.00	0.00	0.00	0.34	0.00
GATCAACCCGCAGCATCCTT	0.00	0.33	0.00	0.00	0.00
GATCAACCCGCAGGTCTACA	0.00	0.65	0.00	0.00	0.00
GATCAACCCGCAGGTGCTCT	0.00	0.33	1.15	0.00	1.03
GATCAACCCGCATCCGGAGC	0.00	0.00	0.00	0.00	0.00
GATCAACCCGCCACAGCCGC	0.00	0.33	0.00	0.00	0.00
GATCAACCCGCCCAAATGCA	0.00	0.00	0.00	0.00	0.34
GATCAACCCGCCCCTTGAGA	0.00	0.00	0.00	0.68	0.34
GATCAACCCGCCGAACATAT	0.00	0.00	0.00	0.00	0.34
GATCAACCCGCCGACACATC	0.00	0.00	0.00	0.00	0.69
GATCAACCCGCCGACAGCGG	0.00	0.00	0.29	0.34	0.00
GATCAACCCGCCGAGAGGGG	0.00	0.00	0.29	0.00	0.00
GATCAACCCGCCGCAAGCCC	0.23	0.00	0.00	0.00	0.00
GATCAACCCGCCGGTCTTGT	0.00	0.00	0.00	0.00	0.34
GATCAACCCGCCGGTTTTGT	0.69	0.65	0.57	0.34	1.03
GATCAACCCGCGAGTAGTTG	0.00	0.00	0.00	0.00	0.00
GATCAACCCGCGCGCGCGCG	0.00	0.00	0.00	0.00	0.69
GATCAACCCGCGCGCGTGCA	0.00	0.00	0.00	0.00	0.34
GATCAACCCGCGGAAAGGCT	0.00	0.00	0.00	0.00	0.00
GATCAACCCGCGGTACCAGG	0.23	0.00	0.00	0.00	0.00
GATCAACCCGCGTACGCTCA	3.66	3.25	2.29	7.81	3.44
GATCAACCCGCGTACGCTCC	0.00	0.00	0.00	0.00	0.00
GATCAACCCGCGTACGCTNA	0.00	0.00	0.00	0.34	0.00
GATCAACCCGCGTACGNTCA	0.00	0.00	0.00	0.34	0.00
GATCAACCCGCTAGCTTTCT	0.23	0.65	0.00	0.00	0.00
GATCAACCCGCTCGCCACGG	0.00	0.00	0.00	0.00	0.00
GATCAACCCGCTCTGGCCCT	0.00	0.00	0.00	0.00	0.00

GATCAACCCGCTCTTCTCGG	0.23	0.00	0.00	0.00	0.00
GATCAACCCGCTGAGCATGC	0.00	0.33	0.00	0.00	0.00
GATCAACCCGCTGCGGTAGA	0.23	0.00	0.00	0.00	0.00
GATCAACCCGCTGCTGCACA	0.00	0.00	0.00	0.00	0.34
GATCAACCCGCTGCTGCCCT	0.00	0.33	0.00	0.00	1.38
GATCAACCCGCTGGCTGGCC	0.00	0.00	0.00	0.34	0.00
GATCAACCCGCTGGGTGAGG	0.00	0.65	0.86	0.00	1.72
GATCAACCCGGAACGACGTT	0.00	0.98	0.29	0.34	0.34
GATCAACCCGGAATACTTGG	0.00	0.33	0.00	0.00	0.00
GATCAACCCGGACACGATTG	0.00	0.65	0.00	0.00	0.00
GATCAACCCGGACAGCTCAG	0.00	0.33	0.00	0.00	0.00
GATCAACCCGGACAGGTGAT	0.00	0.00	0.00	0.00	0.00
GATCAACCCGGACCGAGCAC	0.00	0.00	0.29	0.00	0.00
GATCAACCCGGACTACGTCC	0.00	0.00	0.00	0.00	0.00
GATCAACCCGGACTCAGGCC	0.00	0.00	0.00	0.34	0.00
GATCAACCCGGACTTCCAGA	0.00	0.00	0.86	0.00	0.00
GATCAACCCGGACTTCCANN	0.00	0.00	0.00	0.00	0.00
GATCAACCCGGACTTTTTGA	0.00	0.33	0.00	0.00	0.00
GATCAACCCGGAGAAGCTTG	0.00	0.00	0.00	0.68	0.00
GATCAACCCGGAGAAGGAGA	0.46	0.00	0.00	0.00	0.34
GATCAACCCGGAGGAGGTGC	0.23	0.00	0.00	0.00	0.00
GATCAACCCGGAGGTCGCTG	0.00	0.00	0.00	0.00	0.00
GATCAACCCGGAGGTTCGGT	0.00	0.00	0.00	0.34	0.00
GATCAACCCGGAGTGGCTTG	0.00	0.33	0.00	0.00	0.00
GATCAACCCGGAGTTCGAAG	0.00	0.00	0.29	0.00	0.00
GATCAACCCGGCCGCGGACA	0.00	0.00	0.00	0.00	0.00
GATCAACCCGGCGAATTGGA	0.46	0.98	0.00	0.34	0.34
GATCAACCCGGCGACGAACC	0.00	0.00	0.29	0.00	0.00
GATCAACCCGGCGATTCTGA	0.00	0.00	0.29	0.00	0.00
GATCAACCCGGCGTCACGCT	3.20	0.33	0.29	0.00	0.00
GATCAACCCGGGACCAACCA	0.00	0.00	0.29	0.00	0.00
GATCAACCCGGGAGCTGTTG	0.00	0.00	0.29	0.00	0.00
GATCAACCCGGGATTATTGC	0.00	0.00	0.00	0.00	0.34
GATCAACCCGGGCCTGACGG	0.23	0.00	0.00	0.00	0.00
GATCAACCCGGGCTCCGATG	0.00	0.00	0.00	0.00	0.00
GATCAACCCGGGGAACCCCA	0.00	0.00	0.00	0.00	0.00
GATCAACCCGGGGAACCGGC	0.00	0.65	0.00	0.00	0.00

GATCAACCCGGGGACTCGCG	0.00	0.00	0.00	0.68	0.00
GATCAACCCGGGGCACCGGC	0.00	0.00	0.00	0.00	0.69
GATCAACCCGGGTATCCCTC	0.00	0.00	0.00	0.00	0.00
GATCAACCCGGTATTCCCAA	0.00	0.00	0.00	0.00	0.34
GATCAACCCGGTCGGAGCTG	0.00	0.00	0.00	0.00	0.00
GATCAACCCGGTCGTGCAGG	0.46	0.00	0.29	0.00	0.00
GATCAACCCGGTGAGGGCGG	0.23	0.00	0.00	0.00	0.00
GATCAACCCGGTGATGGCGG	2.75	1.95	0.29	2.04	1.03
GATCAACCCGGTGCGGTCTG	0.00	0.00	0.29	0.00	0.00
GATCAACCCGGTTGAAAACC	0.23	0.00	0.00	0.00	0.00
GATCAACCCGTACACCTTCG	0.00	0.33	0.00	0.00	0.00
GATCAACCCGTACGCGGAGN	0.00	0.00	0.00	0.00	0.34
GATCAACCCGTACTACGGCA	0.00	0.00	0.00	0.00	0.34
GATCAACCCGTAGATCCGAT	0.00	0.00	0.00	0.00	0.00
GATCAACCCGTAGTTGACTC	0.00	0.00	0.00	0.34	0.00
GATCAACCCGTCCATCAACC	0.00	0.00	0.00	0.00	0.00
GATCAACCCGTCCATTTGCG	0.23	0.00	0.00	0.00	0.00
GATCAACCCGTCTTGCATAC	0.00	0.00	0.00	0.00	0.00
GATCAACCCGTCTTGCATGC	0.00	0.33	0.00	0.00	0.00
GATCAACCCGTGCAGCCCTT	0.00	0.00	0.00	0.34	0.00
GATCAACCCGTGCCAGGGTG	0.00	0.33	0.00	0.00	0.00
GATCAACCCGTGCTGTGGTG	0.23	0.00	0.00	0.00	0.00
GATCAACCCGTGCTTCAACC	0.00	0.00	0.00	0.68	0.00
GATCAACCCGTGGCGCGGCA	0.00	0.00	0.29	0.00	0.00
GATCAACCCGTGGCGTTGCG	0.00	0.00	0.29	0.00	0.00
GATCAACCCGTGGCTCCTGC	0.23	0.00	0.00	0.00	0.00
GATCAACCCGTGGGACTGGG	0.00	0.00	0.00	0.00	0.00
GATCAACCCGTTCATGCGTT	0.00	0.00	0.29	0.00	0.00
GATCAACCCGTTCCTTATTG	0.00	0.00	0.29	0.00	0.00
GATCAACCCGTTCGTCGTCG	0.00	0.00	0.00	0.00	0.00
GATCAACCCGTTCGTCTTCG	0.00	0.33	0.00	0.34	0.69
GATCAACCCGTTGATGCTGG	0.00	0.00	0.57	0.34	0.00
GATCAACCCGTTGGTGCCTT	0.00	0.00	0.00	1.02	0.00
GATCAACCCGTTTATGAAAG	0.00	0.00	0.00	0.00	0.00
GATCAACCCGTTTGGAAACC	0.00	0.00	0.00	0.00	0.00
GATCAACCCTAAAAAAAAAA	2.06	0.00	1.72	0.00	1.72
GATCAACCCTAACCACCCCG	0.00	0.00	0.00	0.34	0.00

GATCAACCCTAACTCGATTT	0.00	0.00	0.00	0.00	0.00
GATCAACCCTAACTTTCCCT	0.00	0.00	0.29	0.34	0.00
GATCAACCCTAGATTGTGTT	0.23	0.00	0.00	0.68	0.34
GATCAACCCTAGCAAAAAAA	0.23	0.00	0.00	0.00	0.69
GATCAACCCTAGCACGTGTG	0.00	0.00	0.00	0.34	0.00
GATCAACCCTAGCATGTGTT	0.00	0.00	0.00	0.34	0.34
GATCAACCCTAGCCTGTGGT	0.00	0.00	0.00	0.34	0.00
GATCAACCCTAGCCTGTGTT	0.00	0.00	0.29	0.00	0.00
GATCAACCCTAGCGTGTGGT	0.23	0.00	0.00	0.00	0.00
GATCAACCCTAGCGTGTGTT	0.23	0.00	0.00	0.00	0.00
GATCAACCCTAGCTAAAAAA	0.23	0.00	0.29	0.00	0.69
GATCAACCCTAGCTAGTTTT	0.00	0.00	0.00	0.00	0.34
GATCAACCCTAGCTCATGCA	0.00	0.00	0.00	0.00	0.34
GATCAACCCTAGCTCGTATG	0.00	0.00	0.00	0.34	0.00
GATCAACCCTAGCTCTGTTC	0.00	0.00	0.00	0.68	0.00
GATCAACCCTAGCTGGTGTT	0.00	0.00	0.00	0.34	0.00
GATCAACCCTAGCTTATGAA	0.00	0.00	0.29	0.00	0.00
GATCAACCCTAGCTTATGTT	0.23	0.00	0.00	0.00	0.00
GATCAACCCTAGCTTCTTTG	0.00	0.00	0.00	0.00	0.34
GATCAACCCTAGCTTGAGTT	0.23	0.33	0.29	0.00	0.00
GATCAACCCTAGCTTGCGTT	0.00	0.00	0.29	0.34	0.00
GATCAACCCTAGCTTGGGTT	0.69	0.00	0.29	0.34	0.00
GATCAACCCTAGCTTGNGNN	0.23	0.00	0.00	0.00	0.00
GATCAACCCTAGCTTGNNNN	0.23	0.00	0.00	0.00	0.00
GATCAACCCTAGCTTGTAAA	0.23	0.00	0.29	0.00	0.34
GATCAACCCTAGCTTGTATT	0.00	0.00	0.00	0.00	1.38
GATCAACCCTAGCTTGTCTT	0.00	0.00	0.00	0.34	0.00
GATCAACCCTAGCTTGTGAA	0.92	0.33	1.43	0.68	0.00
GATCAACCCTAGCTTGTGAC	0.00	0.00	0.00	0.34	0.00
GATCAACCCTAGCTTGTGAT	0.00	0.00	0.00	0.00	0.34
GATCAACCCTAGCTTGTGCA	0.00	0.00	0.29	0.00	0.00
GATCAACCCTAGCTTGTGCC	0.00	0.33	0.29	0.00	0.00
GATCAACCCTAGCTTGTGCT	0.00	0.00	0.00	0.34	0.00
GATCAACCCTAGCTTGTGGT	0.23	0.00	0.00	0.00	0.00
GATCAACCCTAGCTTGTGNN	0.23	0.00	0.00	0.00	0.00
GATCAACCCTAGCTTGTGTA	0.00	0.00	0.57	0.34	0.34
GATCAACCCTAGCTTGTGTC	0.00	0.65	0.00	0.00	0.34

GATCAACCCTAGCTTGTGTG	0.23	0.00	0.00	0.00	0.00
GATCAACCCTAGCTTGTGTN	0.23	0.00	0.00	0.00	0.00
GATCAACCCTAGCTTGTGTT	134.83	108.66	138.88	139.51	142.58
GATCAACCCTAGCTTGTNNN	0.00	0.00	0.00	0.00	0.00
GATCAACCCTAGCTTGTTTT	0.00	0.00	0.29	0.00	0.00
GATCAACCCTAGCTTTAAAA	0.00	0.00	0.00	0.34	0.00
GATCAACCCTAGGCTGTGCG	0.00	0.00	0.00	0.00	0.00
GATCAACCCTAGGGTGACAG	0.23	0.65	1.15	1.02	1.03
GATCAACCCTAGGGTGACAT	0.00	0.00	0.00	0.00	0.00
GATCAACCCTAGTTGACCCT	0.46	0.33	0.00	0.34	0.00
GATCAACCCTATATCCTTCG	0.00	0.00	0.57	0.00	0.00
GATCAACCCTATCTTGTGTT	0.00	0.00	0.00	0.34	0.00
GATCAACCCTCAAAATCACG	0.00	0.00	0.00	0.00	0.34
GATCAACCCTCACATGAAGC	0.00	0.00	0.00	0.00	0.00
GATCAACCCTCAGGACTTCA	0.23	0.00	0.00	0.34	0.00
GATCAACCCTCAGTACCAGG	0.23	0.00	0.00	0.00	0.00
GATCAACCCTCCACCAGAGG	0.00	0.00	0.00	0.00	0.34
GATCAACCCTCCCATGTGAA	0.00	0.65	0.00	0.00	0.00
GATCAACCCTCCCCAGAGCG	0.00	0.00	0.00	0.68	0.00
GATCAACCCTCCCCAGATGC	0.00	0.00	0.00	1.02	0.00
GATCAACCCTCCCTTCTCGC	0.00	0.00	0.00	0.00	0.00
GATCAACCCTCCGCTTGCGG	0.00	0.00	0.00	0.00	0.00
GATCAACCCTCCTCCACCTG	0.00	0.00	0.57	0.00	0.00
GATCAACCCTCCTCTTCTTG	0.00	0.00	0.00	0.34	0.00
GATCAACCCTCGACATAGGA	0.00	0.00	0.00	0.00	0.34
GATCAACCCTCGACTGGCTT	0.23	0.00	0.00	0.00	0.00
GATCAACCCTCGATTTTCGA	0.00	0.33	0.00	0.00	0.00
GATCAACCCTCGCAGGATGC	0.23	0.00	0.00	0.00	0.00
GATCAACCCTCGCTTGTGTT	0.23	0.00	0.00	0.34	0.00
GATCAACCCTCGCTTTTGTT	0.00	0.00	0.29	0.00	0.00
GATCAACCCTCGGCACCAGG	0.23	0.00	0.00	0.00	0.00
GATCAACCCTCGGGACCAGG	0.23	0.00	0.00	0.00	0.00
GATCAACCCTCGGTACCAGA	0.23	0.00	0.00	0.00	0.00
GATCAACCCTCGGTACCAGG	60.43	26.03	12.03	17.65	13.09
GATCAACCCTCGGTACCAGN	0.92	0.00	0.00	0.00	0.00
GATCAACCCTCGGTACCANN	0.23	0.00	0.00	0.00	0.00
GATCAACCCTCGGTACCCGG	0.46	0.65	0.00	0.00	0.00

GATCAACCCTCGGTACCNNN	0.46	0.00	0.00	0.00	0.00
GATCAACCCTCGGTACNAGG	0.00	0.00	0.00	0.00	0.34
GATCAACCCTCGGTACNNNN	0.46	0.00	0.00	0.00	0.00
GATCAACCCTCGGTCACAGG	0.23	0.00	0.00	0.00	0.00
GATCAACCCTCGGTGCCTGG	0.23	0.00	0.00	0.00	0.00
GATCAACCCTCGGTTGCGTT	0.00	0.00	0.29	0.00	0.00
GATCAACCCTCGTATGCCGT	0.23	0.00	0.00	0.00	0.00
GATCAACCCTCGTGGCGCCA	0.00	0.00	0.00	0.00	0.00
GATCAACCCTCGTGGTAAGC	0.00	0.00	0.00	0.00	0.00
GATCAACCCTCTACAGGCAC	0.00	0.33	0.00	0.68	0.00
GATCAACCCTCTCAGCGGTT	0.00	0.00	0.00	0.34	0.00
GATCAACCCTCTCCTCCCAC	0.00	0.33	0.00	0.00	0.00
GATCAACCCTCTCGACGCGA	0.23	0.65	0.29	1.70	0.34
GATCAACCCTCTCGTATGCC	0.00	0.33	0.00	0.00	0.34
GATCAACCCTCTCGTCATGC	0.92	0.33	0.86	0.68	0.00
GATCAACCCTCTCGTCATGN	0.00	0.00	0.00	0.00	0.00
GATCAACCCTCTCGTCATGT	0.00	0.00	0.00	0.00	0.00
GATCAACCCTCTCGTTATGC	0.00	0.33	0.00	0.00	0.69
GATCAACCCTCTGATACCCC	0.00	0.00	0.00	0.34	0.00
GATCAACCCTCTGCTGTACA	0.00	0.00	0.57	0.00	0.34
GATCAACCCTCTGGGAGCTC	0.00	0.00	0.29	0.00	0.00
GATCAACCCTCTGGGCGGCT	0.23	0.00	0.00	0.00	0.00
GATCAACCCTCTGGTCATGC	0.00	0.00	0.00	0.34	0.00
GATCAACCCTCTGGTCATGT	0.46	1.63	0.57	2.72	2.41
GATCAACCCTCTGGTCCTGT	0.00	0.00	0.00	0.00	0.00
GATCAACCCTCTGTGATGGA	0.00	0.33	0.00	0.00	0.00
GATCAACCCTCTTGAGGGAG	0.00	0.00	0.00	0.00	0.00
GATCAACCCTGAACGTGTGG	0.00	0.33	0.00	0.00	0.00
GATCAACCCTGACAAGCTGG	0.00	0.00	0.00	0.00	0.00
GATCAACCCTGACGAATCTC	0.00	0.00	0.00	0.34	0.00
GATCAACCCTGACGTTGGCA	0.00	0.00	0.00	0.00	0.34
GATCAACCCTGACTCAGGTA	0.00	0.00	0.00	0.00	0.00
GATCAACCCTGAGGCATGGC	0.00	0.00	0.29	0.00	0.00
GATCAACCCTGATAAGGAGA	0.00	0.33	0.29	0.00	0.69
GATCAACCCTGATGAGGCTG	0.00	0.00	0.00	0.34	0.00
GATCAACCCTGATTCGGGGA	0.00	0.00	0.00	0.00	0.00
GATCAACCCTGCAAAAAAAA	0.23	0.00	0.00	0.00	0.00

GATCAACCCTGCAAGCGCAC	0.00	0.00	0.00	0.34	0.00
GATCAACCCTGCAGCACACG	0.00	0.00	0.00	0.00	0.34
GATCAACCCTGCATGCATGG	0.00	0.33	0.00	0.00	0.00
GATCAACCCTGCATGTGCTG	0.00	0.00	0.00	0.00	0.00
GATCAACCCTGCCAAACACG	0.00	0.00	0.29	0.00	0.00
GATCAACCCTGCCAACGAGG	0.00	0.00	0.00	0.00	0.69
GATCAACCCTGCCACCCAGT	0.00	0.00	0.00	0.68	0.00
GATCAACCCTGCCACCGAGG	0.00	0.00	0.29	0.00	0.00
GATCAACCCTGCCCGACTTC	0.00	0.00	0.00	0.00	0.00
GATCAACCCTGCCCGCCTGG	0.00	0.00	0.29	0.00	0.00
GATCAACCCTGCCCGGCTNN	0.00	0.00	0.00	0.00	0.00
GATCAACCCTGCCCGGCTTC	0.00	0.65	1.72	2.04	0.69
GATCAACCCTGCCTGGAGGC	0.00	0.00	0.29	0.00	0.00
GATCAACCCTGCGATTCTGA	2.06	8.78	3.72	7.47	3.10
GATCAACCCTGCGATTCTGN	0.00	0.00	0.00	0.00	0.00
GATCAACCCTGCGCAGAAGG	0.23	0.00	0.00	0.00	0.00
GATCAACCCTGCGCCGCGCA	0.00	0.00	0.57	0.00	0.69
GATCAACCCTGCGCCGCTCA	0.00	0.00	0.00	0.34	0.00
GATCAACCCTGCGGCCAGTG	0.00	0.00	0.00	0.00	0.00
GATCAACCCTGCGGGCAGGG	0.00	0.00	0.00	0.00	0.00
GATCAACCCTGCGTTAGTAG	0.00	0.00	0.29	0.00	0.00
GATCAACCCTGCGTTGGAGC	0.00	0.00	0.00	0.00	0.00
GATCAACCCTGCTCAAGAGT	0.00	0.00	0.00	0.00	0.00
GATCAACCCTGCTGTTATGC	0.46	0.00	0.00	0.00	0.00
GATCAACCCTGCTTCTTCAG	0.92	0.00	0.00	0.00	0.00
GATCAACCCTGCTTCTTCNN	0.23	0.00	0.00	0.00	0.00
GATCAACCCTGCTTTAACGC	0.00	0.00	0.00	0.00	0.34
GATCAACCCTGGAAACAGTG	0.23	0.33	0.00	0.00	0.00
GATCAACCCTGGAAATAGCG	0.00	0.33	0.00	0.00	0.00
GATCAACCCTGGAACATTGG	0.00	0.00	0.00	0.00	0.69
GATCAACCCTGGACACGTAC	0.00	0.65	1.15	1.70	1.03
GATCAACCCTGGAGCAAAAA	0.00	0.00	0.00	0.00	0.34
GATCAACCCTGGCAACTCCG	0.00	0.33	0.00	0.00	0.34
GATCAACCCTGGCAGAGAGT	0.92	1.95	2.00	0.68	0.34
GATCAACCCTGGCAGGCTGA	0.00	0.00	0.00	0.00	0.00
GATCAACCCTGGCGAGCTGG	0.00	0.00	0.00	0.00	0.34
GATCAACCCTGGCTACGAAG	0.00	0.00	0.00	0.00	0.00

GATCAACCCTGGCTGATCGC	0.00	0.00	0.00	0.00	0.34
GATCAACCCTGGCTGATTGC	0.23	0.65	0.57	0.34	0.00
GATCAACCCTGGCTTGTGTT	0.00	0.00	0.00	0.00	0.00
GATCAACCCTGGGAACAGTG	0.00	0.33	0.00	0.00	0.00
GATCAACCCTGGGAGCGTTG	0.00	0.00	0.00	0.34	0.00
GATCAACCCTGGGCTTATAG	0.00	0.33	0.29	0.00	1.38
GATCAACCCTGGGGACCGGG	0.00	0.00	0.00	0.00	0.34
GATCAACCCTGGGTCCGAGG	0.46	0.65	2.00	1.02	0.34
GATCAACCCTGGGTCCGGCG	0.00	0.00	0.29	0.00	0.00
GATCAACCCTGGTCACGCCT	0.00	0.00	0.00	0.00	0.00
GATCAACCCTGGTCTTTGTA	0.00	0.00	0.29	0.00	0.00
GATCAACCCTGGTGCCCTGC	0.00	0.00	0.00	0.00	0.00
GATCAACCCTGGTGTTGCGC	0.00	0.00	0.00	0.34	0.00
GATCAACCCTGTACATCTAT	0.23	0.33	0.29	0.00	0.00
GATCAACCCTGTCATGTGCG	0.00	0.00	0.00	0.00	0.34
GATCAACCCTGTCCCATTCG	0.00	0.00	0.00	0.00	0.34
GATCAACCCTGTCCTCTGGA	0.69	0.00	0.00	0.34	0.00
GATCAACCCTGTCCTCTGGC	0.00	0.00	0.00	0.34	0.00
GATCAACCCTGTGAAAAAAA	0.23	0.00	0.00	0.00	0.00
GATCAACCCTGTGAACAAAA	0.23	0.00	0.00	0.00	0.00
GATCAACCCTGTGCGGCGCA	0.00	0.00	0.00	0.00	0.00
GATCAACCCTGTGCTGCTGC	0.23	0.00	0.00	0.00	0.00
GATCAACCCTGTGCTGGACA	0.92	0.00	0.86	0.34	0.00
GATCAACCCTGTGGACTCCC	0.23	0.00	0.00	0.00	0.00
GATCAACCCTGTGGCCGGGC	0.00	0.00	0.00	0.00	0.00
GATCAACCCTGTGGTTGGCC	0.00	0.33	0.00	0.00	0.00
GATCAACCCTGTTAAGTTGT	0.00	0.00	0.29	0.00	0.00
GATCAACCCTTACAGTAGCT	0.00	0.98	0.00	0.00	0.00
GATCAACCCTTCCACATCGC	0.00	0.33	0.00	0.00	0.00
GATCAACCCTTCCCCATCGC	4.81	7.81	3.72	2.72	5.17
GATCAACCCTTCGTCATTGG	0.92	0.65	0.57	1.70	1.72
GATCAACCCTTCGTCCTTGG	7.78	3.25	1.72	4.41	2.41
GATCAACCCTTCGTCCTTGN	0.23	0.00	0.00	0.00	0.00
GATCAACCCTTCGTCCTTNN	0.23	0.00	0.00	0.00	0.00
GATCAACCCTTCGTCCTTTG	0.00	0.00	0.00	0.34	0.00
GATCAACCCTTGATATGAAG	0.00	0.33	0.29	0.00	0.34
GATCAACCCTTGCAGATACT	0.00	0.00	0.00	0.00	0.00

GATCAACCCTTGCCCGTGGA	0.00	0.00	0.29	0.00	0.00
GATCAACCCTTGCGGATAGC	0.00	0.00	0.57	0.34	0.00
GATCAACCCTTGCGTACAGC	0.23	0.00	0.00	0.00	0.00
GATCAACCCTTGCGTCCAGG	0.00	0.00	0.00	0.34	0.00
GATCAACCCTTGCTTATTTC	0.00	0.00	0.00	0.34	0.00
GATCAACCCTTGCTTGTGTT	0.00	0.00	0.00	1.36	0.69
GATCAACCCTTGCTTTCTCT	0.00	0.00	0.00	0.00	0.00
GATCAACCCTTGGAGCAGCT	0.00	0.00	0.00	0.00	0.34
GATCAACCCTTGTGACGTGC	0.00	0.00	0.00	0.00	0.34
GATCAACCCTTTATTCCAAG	0.23	0.00	0.00	0.00	0.00
GATCAACCCTTTCAAGAATA	0.00	0.33	0.00	0.00	0.00
GATCAACCCTTTCAAGAATT	15.11	10.41	10.31	12.22	2.76
GATCAACCCTTTCAAGACTT	0.23	0.00	0.00	0.00	0.00
GATCAACCCTTTCAAGCATT	0.23	0.00	0.00	0.00	0.00
GATCAACCCTTTCAAGTTGC	0.00	0.00	0.00	0.00	0.00
GATCAACCCTTTCACGAATT	0.23	0.00	0.00	0.00	0.00
GATCAACCCTTTCAGGAATT	0.00	0.00	0.00	0.00	0.34
GATCAACCCTTTCTCGTATG	0.00	0.00	0.00	0.00	0.00
GATCAACCCTTTTTTCCGGT	0.23	0.00	0.00	0.00	0.00
GATCAACCGAAAAAAAAAAA	6.64	1.30	1.43	2.38	1.72
GATCAACCGAAAAAAAAAAN	0.23	0.00	0.00	0.00	0.00
GATCAACCGAAAAAAAAAAT	0.46	0.00	0.00	0.00	0.00
GATCAACCGAAAAAAANAAT	0.00	0.00	0.00	0.00	0.34
GATCAACCGAAAAAGAAAAA	15.57	3.58	3.15	7.13	4.48
GATCAACCGAAAAAGAAATT	0.00	0.00	0.00	0.00	1.03
GATCAACCGAAAAAGAAGAA	0.92	0.65	0.57	0.68	0.00
GATCAACCGAAAAAGAAGAC	0.00	0.00	0.00	0.00	0.34
GATCAACCGAAAAAGCAGCA	0.23	0.00	0.00	0.00	0.00
GATCAACCGAAAAAGGAAAA	0.00	0.00	0.00	0.00	0.34
GATCAACCGAAAACGTGTGT	0.00	0.00	0.29	0.00	0.00
GATCAACCGAAAAGGAGAAC	0.00	0.00	0.00	0.00	2.07
GATCAACCGAAAGACTCTGG	0.00	0.00	0.00	0.00	0.00
GATCAACCGAAATGATTCCT	0.00	0.00	0.00	0.00	0.34
GATCAACCGAAATGCTTCCT	5.95	3.25	4.01	4.41	6.89
GATCAACCGAAATGCTTCGT	0.00	0.00	0.00	0.00	0.00
GATCAACCGAACAATAAATG	0.00	0.00	0.00	0.00	0.69
GATCAACCGAACACTGGTTN	0.00	0.00	0.00	0.34	0.00

GATCAACCGAACAGCCCACT	0.00	0.00	0.00	0.00	0.00
GATCAACCGAACGAAACTCA	0.00	0.00	0.00	0.00	0.34
GATCAACCGAACGTTCCAGG	0.00	0.33	0.00	0.00	0.00
GATCAACCGAACTCCGCCCG	0.23	0.00	0.00	1.02	0.00
GATCAACCGAACTCCGCCGC	0.00	0.00	0.00	0.34	0.00
GATCAACCGAACTGTACAGA	0.00	0.00	0.00	0.00	0.00
GATCAACCGAAGAAGAAGAA	0.00	0.00	0.86	0.00	0.00
GATCAACCGAAGACCCCACC	0.00	0.00	0.29	0.00	0.00
GATCAACCGAAGAGATGCCG	0.00	0.33	0.00	0.00	0.00
GATCAACCGAAGAGGGTTGC	0.00	0.00	0.29	0.00	0.00
GATCAACCGAAGAGGTCTGC	0.23	0.00	0.00	0.00	0.00
GATCAACCGAAGAGGTTTCC	0.00	0.00	0.00	0.00	0.00
GATCAACCGAAGAGGTTTGA	0.00	0.00	0.00	0.00	0.00
GATCAACCGAAGAGGTTTGC	17.17	9.43	9.45	15.61	14.12
GATCAACCGAAGAGGTTTGG	0.23	0.00	0.00	0.00	0.00
GATCAACCGAAGAGGTTTGN	0.00	0.00	0.00	0.00	0.00
GATCAACCGAAGAGTTTTGC	0.00	0.65	0.00	0.00	0.00
GATCAACCGAAGATACGAGA	1.14	0.65	0.29	0.68	1.03
GATCAACCGAAGATACNNNN	0.23	0.00	0.00	0.00	0.00
GATCAACCGAAGCAAAAAAA	0.92	0.65	0.00	1.70	1.72
GATCAACCGAAGCAACAGAA	0.00	0.00	0.29	0.00	0.00
GATCAACCGAAGCAGAAAAA	0.00	0.00	0.57	1.02	0.34
GATCAACCGAAGCAGAAAGG	0.46	0.33	0.29	0.34	0.69
GATCAACCGAAGCGGAAAAA	0.00	0.00	0.00	0.00	0.00
GATCAACCGAAGCTCTGAAG	0.00	0.00	0.00	0.00	0.00
GATCAACCGAAGCTGCAGTT	0.00	0.00	0.00	0.00	0.34
GATCAACCGAAGGCTGGCGG	0.00	0.00	0.29	0.00	0.00
GATCAACCGAAGGTCCTTGG	0.00	0.00	0.00	0.34	0.00
GATCAACCGAAGTGGATGTA	0.00	0.00	0.00	0.00	0.00
GATCAACCGAAGTTCCTTGG	0.00	0.00	0.00	0.00	0.00
GATCAACCGAATCCCCATCC	0.00	0.00	0.29	0.00	0.00
GATCAACCGAATCGAGAGGG	0.00	0.00	0.00	0.00	0.00
GATCAACCGAATCTGCCGGG	0.00	0.00	0.00	0.00	0.00
GATCAACCGAATGAGCAGCG	0.00	0.00	0.00	0.68	0.00
GATCAACCGAATGAGGCCAA	0.23	0.33	0.00	0.00	0.34
GATCAACCGAATGCTGCTAG	0.00	0.00	0.00	0.00	0.00
GATCAACCGACAAAAAAAAA	0.00	0.00	0.00	0.00	0.34

GATCAACCGACAAAGTCTGA	0.00	0.00	0.00	0.00	0.34
GATCAACCGACAACCAGGCA	0.23	0.00	0.00	0.00	0.00
GATCAACCGACAACCGAGCA	0.00	0.00	0.00	0.00	0.00
GATCAACCGACAACCGGCCA	0.23	0.00	0.00	0.00	0.00
GATCAACCGACAACCGGGAA	0.23	0.00	0.00	0.00	0.00
GATCAACCGACAACCGGGCA	9.39	10.74	4.30	6.11	4.48
GATCAACCGACAACCGGGGA	0.00	0.00	0.29	0.00	0.00
GATCAACCGACAACTGGTCG	0.00	0.00	0.29	0.00	0.00
GATCAACCGACAAGGAAAAA	0.00	0.00	0.00	0.00	0.00
GATCAACCGACAAGGCCATG	0.00	0.00	0.00	0.00	0.69
GATCAACCGACAAGTCCATG	0.23	0.00	0.00	0.00	0.00
GATCAACCGACACAGCCGGA	0.00	0.00	0.00	0.00	0.69
GATCAACCGACATCCGAGAC	0.00	0.00	0.29	0.00	0.00
GATCAACCGACATCGATGCG	0.00	0.00	0.00	0.00	0.34
GATCAACCGACATGCAACCA	0.00	0.00	0.00	0.00	0.34
GATCAACCGACCAAACTGGC	0.23	0.00	0.00	0.00	0.00
GATCAACCGACCAAGAGGGG	0.00	0.00	0.00	0.00	0.00
GATCAACCGACCCGTGCAGA	0.23	0.00	0.00	0.00	0.00
GATCAACCGACCGAGAGGGG	0.00	0.00	0.00	0.00	0.00
GATCAACCGACCGCATCCTG	0.00	0.00	0.00	0.34	0.00
GATCAACCGACCGTCCGACT	0.00	0.00	0.00	0.00	0.69
GATCAACCGACCTCTCGACC	0.46	0.00	0.00	0.00	0.00
GATCAACCGACGCAAAACCC	0.00	0.00	0.00	0.00	0.00
GATCAACCGACGCAAGTAGT	1.14	0.00	0.00	0.34	0.00
GATCAACCGACGCAGAACCC	0.00	0.33	0.00	0.00	0.00
GATCAACCGACGCGGTTTGC	0.00	0.00	0.00	0.00	0.34
GATCAACCGACGGACGCTGG	0.00	0.33	0.00	0.00	0.00
GATCAACCGACGGCACCTTC	0.00	0.33	0.00	0.00	0.00
GATCAACCGACGGTGGGCTT	0.00	0.65	0.00	0.00	0.00
GATCAACCGACGTGGGGCAG	0.00	0.00	0.00	0.00	0.34
GATCAACCGACGTGGTCCCA	0.00	0.00	0.00	0.00	0.00
GATCAACCGACGTGTTTAAT	0.00	0.00	0.00	0.34	0.00
GATCAACCGACTTGACCCAC	0.00	0.00	0.00	0.34	0.00
GATCAACCGAGAAGAGTGTG	0.69	0.00	0.00	0.00	0.00
GATCAACCGAGACAACATGC	0.00	0.00	0.29	0.00	0.00
GATCAACCGAGACTGGTCCA	0.23	0.00	0.00	0.00	0.00
GATCAACCGAGAGACAGCAG	0.00	0.00	0.57	0.00	0.00

GATCAACCGAGAGGGAGAGG	0.00	0.00	0.29	0.00	0.00
GATCAACCGAGAGTTGAAGC	0.00	0.00	0.00	0.00	0.00
GATCAACCGAGATCATTAGA	0.00	0.00	0.00	0.00	0.00
GATCAACCGAGCAAGATCAA	0.00	0.00	0.00	0.00	0.00
GATCAACCGAGCCATGCAAG	0.00	0.00	0.00	0.00	0.69
GATCAACCGAGCCGACGCTT	0.46	0.00	0.00	0.34	0.00
GATCAACCGAGCCTCTTATG	0.00	0.65	0.29	0.00	0.00
GATCAACCGAGCGATGTCCA	0.00	0.00	0.00	0.00	0.00
GATCAACCGAGCGCCTCGAC	0.00	0.00	0.29	0.00	0.00
GATCAACCGAGCGCCTTGTG	0.00	0.00	0.00	0.34	0.00
GATCAACCGAGCGGGCCAAC	0.00	0.00	0.00	0.00	0.34
GATCAACCGAGCGTTGGGAT	0.00	0.00	0.00	0.00	0.00
GATCAACCGAGCTGTCGAAC	0.00	0.00	0.00	0.34	0.00
GATCAACCGAGCTGTCGAAG	0.00	0.00	0.00	0.00	0.00
GATCAACCGAGGACGGGCCG	0.00	0.33	0.00	0.00	0.00
GATCAACCGAGGAGGCGGCT	0.00	0.00	0.00	0.00	0.34
GATCAACCGAGGCAAAGGAA	0.23	0.00	0.00	0.00	0.00
GATCAACCGAGGCACAGGTC	0.23	0.00	0.00	0.00	0.00
GATCAACCGAGGCAGACACC	0.46	0.00	0.00	0.00	0.00
GATCAACCGAGGCATAGCTG	0.00	0.00	0.29	0.68	0.00
GATCAACCGAGGCCGCCTCC	0.23	0.00	0.00	0.00	0.00
GATCAACCGAGGGTGTTCCA	0.00	0.00	0.00	0.00	0.00
GATCAACCGAGGTCTATCCC	2.06	0.65	0.29	0.00	1.03
GATCAACCGAGTAACAACGG	0.00	0.00	0.29	0.00	0.00
GATCAACCGAGTCAATGACA	0.00	0.33	0.00	0.00	0.00
GATCAACCGAGTCATGCAAG	2.29	2.93	6.30	3.39	9.64
GATCAACCGAGTCATGCACG	0.00	0.00	0.00	0.00	0.00
GATCAACCGAGTCGTGGGAG	0.00	0.33	0.29	0.34	0.00
GATCAACCGAGTCTGTCTGA	0.00	0.00	0.00	0.00	0.00
GATCAACCGAGTCTGTCTTC	0.00	0.00	0.00	0.00	0.00
GATCAACCGAGTCTGTTAAA	0.00	0.00	0.00	0.00	0.00
GATCAACCGAGTGACCAACC	0.00	0.00	0.00	0.00	0.00
GATCAACCGAGTGACCACCC	0.00	0.00	0.29	0.00	0.00
GATCAACCGAGTTGGCCATG	0.00	0.00	0.00	0.34	0.00
GATCAACCGATGAAAAAAAA	0.69	0.00	0.00	0.00	0.00
GATCAACCGATGACAGCAAA	0.00	0.00	0.00	0.00	0.69
GATCAACCGATGACGCTGAG	0.00	0.00	0.00	0.00	0.00

GATCAACCGATGAGAGTCCA	2.75	1.30	2.29	1.02	0.34
GATCAACCGATGAGAGTCCN	0.23	0.00	0.00	0.00	0.00
GATCAACCGATGCCACAGGA	0.00	0.00	0.00	0.00	0.00
GATCAACCGATGCCCAGTTG	0.00	0.00	0.00	0.00	0.00
GATCAACCGATGGAAGGAGC	0.46	0.00	0.00	0.00	0.00
GATCAACCGATGGATGGAGC	0.23	1.30	0.00	0.68	0.69
GATCAACCGATGGGTCCTCC	0.00	0.00	0.29	0.00	0.00
GATCAACCGATGGTCTTTGA	5.95	3.25	0.86	6.11	1.38
GATCAACCGATTGAGTACGT	0.00	0.00	0.29	0.00	0.00
GATCAACCGATTTTCTCGGT	0.00	0.00	0.00	0.00	0.00
GATCAACCGCAAAAAAAAAA	0.00	0.00	0.00	0.34	0.34
GATCAACCGCAACACAACAC	0.00	0.00	0.00	0.00	0.34
GATCAACCGCAACAGCACAT	0.00	0.00	0.00	0.00	0.34
GATCAACCGCAACCACATCG	0.00	0.00	0.29	0.00	0.34
GATCAACCGCAACCACTGGC	0.00	0.00	0.29	0.00	0.00
GATCAACCGCAACTCCAACG	0.00	0.00	0.29	0.00	0.00
GATCAACCGCAACTTCCGCT	0.00	0.33	0.00	0.34	0.00
GATCAACCGCAAGAAATTTC	0.00	0.00	0.00	0.00	0.00
GATCAACCGCAAGAAGAGGT	0.00	0.00	0.00	0.00	0.00
GATCAACCGCAAGAAGCGGT	0.00	0.00	0.29	0.00	0.00
GATCAACCGCAAGCAGAGGT	0.00	0.00	0.29	0.34	1.03
GATCAACCGCAAGCAGCGGA	0.00	0.00	0.29	0.00	0.34
GATCAACCGCAAGCAGCGGC	0.23	0.00	0.29	0.00	0.34
GATCAACCGCAAGCAGCGGG	0.92	0.33	1.43	1.36	1.38
GATCAACCGCAAGCAGCGGT	11.45	35.79	52.12	25.80	44.77
GATCAACCGCAAGCAGCGNN	0.00	0.00	0.00	0.00	0.00
GATCAACCGCAAGCAGCGTC	0.00	0.00	0.00	0.00	0.00
GATCAACCGCAAGCAGCNNN	0.23	0.00	0.00	0.00	0.00
GATCAACCGCAAGCAGCTCG	0.00	0.00	0.29	0.00	0.00
GATCAACCGCAAGCAGGGGT	0.00	0.00	0.00	0.00	0.00
GATCAACCGCAAGCAGNGGT	0.00	0.00	0.00	0.00	0.34
GATCAACCGCAAGCAGNGNN	0.23	0.00	0.00	0.00	0.00
GATCAACCGCAAGCAGTGGT	0.00	0.00	0.00	0.00	0.00
GATCAACCGCAAGCGAGGGT	0.00	0.00	0.86	0.00	0.00
GATCAACCGCAAGGACGGTG	0.00	0.33	0.00	0.00	0.00
GATCAACCGCAAGTACGACT	4.58	3.58	2.58	2.38	2.76
GATCAACCGCAAGTTCACAA	0.00	0.33	0.00	0.00	0.00

GATCAACCGCAAGTTCACAG	0.00	0.00	0.00	0.00	0.00
GATCAACCGCAATGCAAAAT	0.00	0.00	0.00	0.00	0.34
GATCAACCGCAATGCTTGCA	0.00	0.00	0.00	0.00	0.69
GATCAACCGCAATGGGCTTG	0.00	0.00	0.00	0.00	0.34
GATCAACCGCAATTGCACAT	0.46	0.00	0.00	0.00	0.00
GATCAACCGCACACACACAA	0.00	0.00	0.00	0.34	0.00
GATCAACCGCACAGACCAGT	0.00	0.00	0.00	0.00	0.34
GATCAACCGCACAGGCAGTG	0.00	0.33	0.00	0.00	0.00
GATCAACCGCACAGGTACCT	0.00	0.00	0.00	0.34	0.00
GATCAACCGCACATCCGTGC	0.00	0.00	0.00	0.00	0.34
GATCAACCGCACCAACACAA	0.00	0.00	0.00	0.00	0.34
GATCAACCGCACCACAACAC	0.00	0.00	0.00	0.00	0.34
GATCAACCGCACCCAACAAC	0.00	0.00	0.00	0.00	0.00
GATCAACCGCACCCACTCGG	0.00	0.00	0.00	0.00	0.00
GATCAACCGCACCCGGGGAC	0.00	0.00	0.00	0.00	0.00
GATCAACCGCACCCTGCAAG	0.00	0.00	0.00	0.34	0.00
GATCAACCGCACCCTGCTCC	0.92	0.33	0.00	0.00	0.34
GATCAACCGCACCCTGTGTT	0.00	0.00	0.00	0.00	0.00
GATCAACCGCACCGCTTCCT	0.00	0.00	0.29	0.00	0.00
GATCAACCGCACCGTATACG	0.69	0.00	0.00	0.00	0.00
GATCAACCGCACCTCTTAGA	0.00	0.33	0.00	0.00	0.00
GATCAACCGCACCTTCCGAG	0.00	0.33	0.00	0.00	0.00
GATCAACCGCACCTTCTTTG	0.00	0.33	0.00	0.00	0.00
GATCAACCGCACCTTGATAG	0.00	0.33	0.00	0.00	0.00
GATCAACCGCACGAACATCA	0.00	0.00	0.00	0.00	0.00
GATCAACCGCACGACCGTCG	0.00	0.33	0.00	0.34	0.34
GATCAACCGCACGAGCATCA	0.23	0.98	0.86	0.34	0.34
GATCAACCGCACGATGTCGG	2.29	3.58	2.00	2.04	1.38
GATCAACCGCACGCTGAGAA	0.00	0.00	0.00	0.00	0.69
GATCAACCGCACGCTGCTAA	0.00	0.00	0.00	0.34	0.00
GATCAACCGCACGGAGTTCA	0.00	0.00	0.00	0.00	0.00
GATCAACCGCACGGCCAAAA	0.00	0.00	0.29	0.00	0.00
GATCAACCGCACGGCGATGA	0.00	0.33	0.00	0.00	0.00
GATCAACCGCACGGCGCTGA	0.69	0.33	0.57	0.00	0.00
GATCAACCGCACTCACAGAC	0.00	0.00	0.00	0.00	0.00
GATCAACCGCACTCTCACTG	0.00	0.33	0.00	0.00	0.00
GATCAACCGCAGACCGCGCT	0.00	0.33	0.00	0.00	0.00

GATCAACCGCAGACGAGGAA	0.00	0.00	0.00	0.34	0.00
GATCAACCGCAGACTGGCGG	0.00	0.00	0.00	0.00	0.00
GATCAACCGCAGAGACATGT	0.00	0.00	0.29	0.00	0.00
GATCAACCGCAGAGCGAGTG	0.00	0.00	0.00	0.00	0.00
GATCAACCGCAGATGAAGAA	0.23	0.00	0.00	0.00	0.00
GATCAACCGCAGATGAGGAA	3.89	5.21	1.43	5.09	3.10
GATCAACCGCAGATGAGGAT	0.23	0.00	0.00	0.68	0.00
GATCAACCGCAGATGGGGAA	0.23	0.65	0.00	0.00	0.00
GATCAACCGCAGATGGGGCA	0.23	0.00	0.00	0.00	0.00
GATCAACCGCAGATTTGAAC	0.00	0.00	0.00	0.00	0.34
GATCAACCGCAGCAAAAAAA	0.00	0.00	0.00	0.68	0.00
GATCAACCGCAGCAGGCCAA	0.00	0.00	0.00	0.00	0.00
GATCAACCGCAGCATCCNNN	0.23	0.00	0.00	0.00	0.00
GATCAACCGCAGCATCCTTC	1.37	0.33	1.72	0.34	6.20
GATCAACCGCAGCCACCTCC	0.00	0.00	0.00	0.00	0.00
GATCAACCGCAGCGCCNTCG	0.00	0.00	0.00	0.34	0.00
GATCAACCGCAGCGCCTTCG	1.83	1.30	0.29	1.02	0.34
GATCAACCGCAGCGGTTGAG	0.00	0.00	0.00	0.00	0.00
GATCAACCGCAGCGGTTTGC	0.00	0.33	0.00	0.00	1.03
GATCAACCGCAGCTACACCG	0.00	0.00	0.29	0.00	0.00
GATCAACCGCAGCTGGGAGA	1.37	0.33	0.57	0.68	1.72
GATCAACCGCAGCTGGGAGG	0.46	0.00	0.00	0.00	0.00
GATCAACCGCAGCTGGGCGC	0.23	0.00	0.00	0.00	0.00
GATCAACCGCAGGAACAAGG	0.00	0.00	0.29	0.00	0.00
GATCAACCGCAGGAACTATG	0.00	0.00	0.57	1.70	0.00
GATCAACCGCAGGACCTACG	0.00	0.00	0.00	0.00	0.00
GATCAACCGCAGGATGGCGG	0.00	0.00	0.00	0.00	0.00
GATCAACCGCAGGCCTGCCA	0.00	0.00	0.29	0.00	0.00
GATCAACCGCAGGCTAGCGG	0.00	0.00	0.00	0.00	0.00
GATCAACCGCAGGCTGACGA	0.23	0.98	0.86	1.02	0.34
GATCAACCGCAGGCTGACGC	0.00	0.33	0.00	0.00	0.00
GATCAACCGCAGGCTGACGG	0.00	0.00	0.00	0.00	0.00
GATCAACCGCAGGCTGACTG	0.00	0.00	0.00	0.00	0.00
GATCAACCGCAGGCTGGAGG	0.00	0.33	0.00	0.68	0.00
GATCAACCGCAGGCTGGCAG	0.00	0.00	0.00	0.00	0.69
GATCAACCGCAGGCTGGCCG	0.00	0.65	0.00	0.00	0.00
GATCAACCGCAGGCTGGCGG	9.84	31.56	48.11	31.57	40.29

GATCAACCGCAGGCTGGCGN	0.23	0.00	0.00	0.00	0.00
GATCAACCGCAGGCTGGCGT	0.00	0.00	0.00	0.34	0.00
GATCAACCGCAGGCTGGCTA	0.00	0.00	0.00	0.34	0.00
GATCAACCGCAGGCTGGGGG	0.23	0.00	2.00	0.34	0.69
GATCAACCGCAGGCTGGTGG	0.00	0.33	0.00	0.00	0.00
GATCAACCGCAGGCTGNCGG	0.00	0.00	0.00	0.00	0.34
GATCAACCGCAGGCTTACGT	0.00	0.00	0.00	0.00	0.00
GATCAACCGCAGGGGTTCTG	0.46	0.33	0.00	0.34	0.00
GATCAACCGCAGGGTGGAGC	0.00	0.00	0.00	0.34	0.00
GATCAACCGCAGGTCGTATG	0.00	0.33	0.00	0.00	0.00
GATCAACCGCAGTCTAAGGT	0.00	0.00	0.29	0.00	0.00
GATCAACCGCAGTCTTCCTG	0.00	0.00	0.00	0.00	0.34
GATCAACCGCAGTGTTTATG	0.00	0.00	0.00	0.00	0.00
GATCAACCGCAGTGTTTCAG	0.23	0.65	0.00	2.04	1.72
GATCAACCGCAGTGTTTCNG	0.00	0.00	0.00	0.00	0.00
GATCAACCGCATCAAGGCGA	0.00	0.00	0.29	0.00	0.00
GATCAACCGCATCACTATAC	0.46	0.33	0.29	1.02	0.34
GATCAACCGCATCATGGACT	0.00	0.00	0.00	0.00	0.00
GATCAACCGCATCCTGAGCA	0.00	0.00	0.00	0.00	0.00
GATCAACCGCATCTCCTACA	0.00	0.00	0.00	0.34	0.00
GATCAACCGCATCTCCTGCA	0.00	0.00	0.00	0.00	0.00
GATCAACCGCATCTCTCGAT	0.00	0.00	0.00	0.00	0.00
GATCAACCGCATCTGACGGG	0.46	0.00	0.00	0.00	0.00
GATCAACCGCATCTTTGCGC	0.00	0.00	0.00	0.00	0.34
GATCAACCGCATCTTTGCGG	0.00	0.00	0.00	0.00	0.34
GATCAACCGCATGAAGCAGC	0.00	0.98	0.00	0.68	0.34
GATCAACCGCATGCACCTAC	0.00	0.00	0.29	0.00	0.00
GATCAACCGCATGCTGCGTG	0.00	0.00	0.00	0.00	0.69
GATCAACCGCATTCAAGGCA	0.00	0.00	0.00	0.00	0.34
GATCAACCGCATTGACCTAG	0.00	0.00	0.00	0.68	0.00
GATCAACCGCATTGACCTTG	0.00	0.33	0.00	0.68	0.00
GATCAACCGCATTGATAACA	0.00	0.00	0.00	0.00	0.34
GATCAACCGCATTGTAGAGT	0.00	0.00	0.00	0.34	0.00
GATCAACCGCCAAAAGGTTG	0.00	0.00	0.00	0.34	0.00
GATCAACCGCCAAAGCGTGA	0.00	0.00	0.00	0.00	0.00
GATCAACCGCCAAAGTGAGA	0.00	0.00	0.00	0.00	0.34
GATCAACCGCCAAAGTGAGC	0.00	0.00	0.29	0.00	0.00

GATCAACCGCCAAAGTGCGA	0.23	0.00	0.00	0.00	0.00
GATCAACCGCCAAAGTGGGA	0.69	0.00	0.29	0.00	0.00
GATCAACCGCCAAAGTGGGC	0.23	0.00	0.00	0.00	0.00
GATCAACCGCCAAAGTGGGG	0.23	0.00	0.00	0.00	0.00
GATCAACCGCCAAAGTGTAA	0.00	0.00	0.00	0.00	0.34
GATCAACCGCCAAAGTGTGA	28.38	11.71	9.74	23.42	24.80
GATCAACCGCCAAAGTGTGC	0.00	0.00	0.29	0.00	0.00
GATCAACCGCCAAAGTGTGG	0.00	0.00	0.00	0.00	0.00
GATCAACCGCCAAAGTGTNN	0.23	0.00	0.00	0.00	0.00
GATCAACCGCCAACCGGGCA	0.23	0.00	0.00	0.00	0.00
GATCAACCGCCAACTGTGAA	0.00	0.33	0.00	0.00	0.00
GATCAACCGCCAATTCGACC	0.23	0.00	0.00	0.00	0.00
GATCAACCGCCAATTCGACT	0.00	0.00	0.00	0.34	0.00
GATCAACCGCCACAAGAAGG	0.00	0.00	0.00	0.00	0.34
GATCAACCGCCACCCACTTG	0.00	0.00	0.29	0.00	0.00
GATCAACCGCCACCGCAACA	0.00	0.00	0.29	0.00	0.00
GATCAACCGCCACCTAGGCC	0.00	0.00	0.29	0.00	0.00
GATCAACCGCCACGACGGCC	0.00	0.00	0.00	0.00	0.34
GATCAACCGCCACTTCAACG	0.00	0.00	0.29	0.00	0.00
GATCAACCGCCAGAATCTCT	0.00	0.33	0.00	0.34	0.00
GATCAACCGCCAGAATCTTT	0.00	0.00	0.29	0.00	0.00
GATCAACCGCCAGAGTGCGC	0.00	0.00	0.29	0.00	0.00
GATCAACCGCCAGCAACGTC	0.00	0.00	0.00	0.00	0.00
GATCAACCGCCAGCACTTGG	0.23	0.00	0.00	0.00	0.00
GATCAACCGCCAGGAGCTCG	0.23	0.00	0.00	0.00	0.00
GATCAACCGCCAGGAGTTCA	0.00	0.00	0.00	0.68	0.00
GATCAACCGCCAGGCTTGTG	0.23	0.00	0.00	0.00	0.00
GATCAACCGCCATATACACA	0.00	0.00	0.29	0.00	0.00
GATCAACCGCCATGGCAGCC	0.00	0.00	0.00	0.00	0.69
GATCAACCGCCCCCCCCAAN	0.00	0.00	0.00	0.00	0.00
GATCAACCGCCCCCCCCCAC	0.00	0.00	0.29	0.00	0.00
GATCAACCGCCCCCTCTCTG	0.00	0.00	0.00	0.00	0.69
GATCAACCGCCCCCTCTCTT	0.00	0.00	0.00	0.34	0.00
GATCAACCGCCCGACACGCC	0.00	0.00	0.00	0.00	0.34
GATCAACCGCCCGAGCCTCA	0.00	0.00	0.00	0.00	0.00
GATCAACCGCCCGAGCTTGG	0.00	0.00	0.29	0.00	0.00
GATCAACCGCCCGGCGGACG	0.23	0.00	0.00	0.00	0.00

GATCAACCGCCCGTACCGGC	0.00	0.00	0.29	0.00	0.00
GATCAACCGCCCGTCCCGGC	0.69	2.93	0.86	1.70	1.72
GATCAACCGCCCTCCTTATC	0.00	0.00	0.00	0.00	0.34
GATCAACCGCCCTCTCATTG	0.69	0.65	0.57	2.04	0.69
GATCAACCGCCCTGGGTGTG	0.00	0.33	0.00	0.00	0.00
GATCAACCGCCGACGACAAG	0.00	0.00	0.00	0.00	0.34
GATCAACCGCCGCGCGGGTG	0.00	0.00	0.29	0.00	0.00
GATCAACCGCCGCTGGGTCC	0.00	0.33	1.15	0.68	0.34
GATCAACCGCCGCTGTGGCC	0.00	0.33	0.00	0.00	0.00
GATCAACCGCCGCTTCCAGC	0.00	0.00	0.00	0.00	0.34
GATCAACCGCCGGCCTGGCC	0.00	0.00	0.00	0.00	0.00
GATCAACCGCCGGCTGGCGG	0.00	0.00	0.00	0.00	0.34
GATCAACCGCCGGCTGGCGT	0.00	0.00	0.29	0.00	0.00
GATCAACCGCCGGGTGCTCG	0.23	0.00	0.00	0.00	0.00
GATCAACCGCCGGTGGGTCC	0.00	0.00	0.00	0.34	0.00
GATCAACCGCCGTGGCACCT	0.00	0.00	0.29	0.00	0.00
GATCAACCGCCGTTCGCGTT	0.00	0.00	0.00	0.00	0.00
GATCAACCGCCTAAGTCTCT	0.00	0.00	0.00	0.00	0.00
GATCAACCGCCTCACACCGG	0.00	0.00	0.57	0.00	0.00
GATCAACCGCCTCACGGCGG	0.00	0.00	0.00	0.00	0.00
GATCAACCGCCTCATAGACT	0.00	0.00	0.00	0.00	0.00
GATCAACCGCCTCCACAAAA	0.23	0.00	0.00	0.00	0.00
GATCAACCGCCTCCCCGGCC	0.00	0.00	0.00	0.00	0.34
GATCAACCGCCTCCTAGAGG	0.23	0.33	0.00	0.34	0.00
GATCAACCGCCTCCTGAAGG	0.92	0.65	0.57	0.68	1.03
GATCAACCGCCTCGAACGTA	0.00	0.33	0.00	0.00	0.00
GATCAACCGCCTCGGCTTCG	0.00	0.00	0.00	0.00	0.00
GATCAACCGCCTCTGGGCCC	0.00	0.00	0.00	0.34	0.00
GATCAACCGCCTGAAGAAGG	0.00	0.33	0.00	0.00	0.00
GATCAACCGCCTGACCTTCG	0.00	0.00	0.00	0.00	0.34
GATCAACCGCCTGACGGATG	0.00	0.00	0.29	0.00	0.00
GATCAACCGCCTGGAGCTCA	0.23	0.00	0.00	0.00	0.00
GATCAACCGCCTGGAGGATG	0.00	0.00	0.00	0.00	0.00
GATCAACCGCCTGGAGGCAG	1.60	0.00	0.00	0.00	0.00
GATCAACCGCCTGGATGATG	0.00	0.33	0.00	0.00	0.00
GATCAACCGCCTGGCCGCCA	0.00	0.00	0.29	0.00	0.00
GATCAACCGCCTGGCGGAAG	0.00	0.00	0.00	0.00	0.34

GATCAACCGCCTGGCGGAGG	0.00	0.33	0.00	0.34	0.00
GATCAACCGCCTGGCGGATG	7.78	21.47	16.61	27.49	21.70
GATCAACCGCCTGGCGGATN	0.00	0.00	0.00	0.34	0.00
GATCAACCGCCTGGCGGATT	0.00	0.00	0.00	0.34	0.00
GATCAACCGCCTGGCGGCTG	0.00	0.65	0.57	0.68	0.00
GATCAACCGCCTGGCGGGTG	0.46	0.00	0.00	0.00	0.00
GATCAACCGCCTGGGAACCT	2.52	2.93	0.86	5.43	1.03
GATCAACCGCCTGGGCTTCT	0.00	0.33	0.00	0.34	0.00
GATCAACCGCCTGGGGCATC	0.00	0.00	0.00	0.34	0.00
GATCAACCGCCTGGGGGATG	0.23	0.00	0.00	0.00	0.00
GATCAACCGCCTGGTCGTAT	0.00	0.00	0.00	0.34	0.00
GATCAACCGCCTGGTGCTCA	12.36	12.36	9.74	13.24	10.68
GATCAACCGCCTGGTGCTCC	0.23	0.00	0.00	0.00	0.00
GATCAACCGCCTGGTGCTCG	0.00	0.00	0.29	0.00	0.00
GATCAACCGCCTGGTGCTTA	0.00	0.00	0.29	0.00	0.00
GATCAACCGCCTTGACTTCC	0.00	0.00	0.29	0.00	0.00
GATCAACCGCCTTGTGCACT	0.23	0.00	0.00	0.00	0.00
GATCAACCGCGAACGATTTT	0.00	0.00	0.00	0.00	0.00
GATCAACCGCGAACGTGGTC	0.00	0.00	0.57	0.00	0.00
GATCAACCGCGAATGACACC	0.23	0.00	0.00	0.00	0.00
GATCAACCGCGACAGAGGGT	0.00	0.00	0.00	0.34	0.34
GATCAACCGCGACAGCTTCG	0.00	1.30	0.29	0.68	1.03
GATCAACCGCGACGATCACA	0.00	0.00	0.00	0.00	0.34
GATCAACCGCGACGGCCAAC	0.23	0.00	0.00	0.00	0.00
GATCAACCGCGACGGGGCTC	0.00	0.00	0.00	0.00	0.34
GATCAACCGCGAGATCGTAG	0.00	0.33	0.00	0.00	0.00
GATCAACCGCGAGCACCACC	0.46	0.00	0.00	0.00	0.00
GATCAACCGCGAGCTGGATT	0.00	0.00	0.00	1.02	0.00
GATCAACCGCGAGGCGGGCG	0.00	0.33	0.00	0.00	0.00
GATCAACCGCGAGGTGGATG	0.46	0.33	0.29	0.68	0.34
GATCAACCGCGAGGTGGCGC	0.00	0.00	0.00	0.34	0.00
GATCAACCGCGAGGTTCCCA	0.69	0.00	0.00	0.00	0.00
GATCAACCGCGAGTGCAGCT	0.23	0.00	0.00	0.00	0.00
GATCAACCGCGATGCGAGCG	0.00	0.33	0.00	0.00	0.34
GATCAACCGCGCAACGCGCA	0.00	0.00	0.29	0.00	0.00
GATCAACCGCGCACGTCCGG	0.00	0.00	0.00	0.00	0.00
GATCAACCGCGCACTGCAGC	0.00	0.00	0.00	0.00	0.00

GATCAACCGCGCAGGAATGC	0.00	0.00	0.00	0.34	0.00
GATCAACCGCGCAGGGATGC	0.00	0.00	0.00	0.00	0.00
GATCAACCGCGCAGTGAAAA	0.00	0.00	0.00	0.00	0.34
GATCAACCGCGCATAGAAGT	0.00	0.65	0.00	0.00	0.00
GATCAACCGCGCCAAAAAAA	0.00	0.00	0.00	0.68	0.00
GATCAACCGCGCCAACTGCG	0.23	0.00	0.00	0.00	0.00
GATCAACCGCGCCAAGAAAA	0.00	0.00	0.00	0.00	0.00
GATCAACCGCGCCCGCGGGA	0.00	0.33	0.00	0.00	0.00
GATCAACCGCGCCCGCGGTC	0.23	0.00	0.00	0.00	0.00
GATCAACCGCGCCGCGGCGC	0.00	0.65	0.00	0.34	0.00
GATCAACCGCGCCGTGCGTT	0.00	0.33	0.57	0.00	0.00
GATCAACCGCGCCGTGGCCG	0.00	0.00	0.00	0.00	0.00
GATCAACCGCGCCTCGCAGG	0.00	0.65	0.00	0.00	0.34
GATCAACCGCGCCTGCTCCA	0.00	0.00	0.29	0.00	0.00
GATCAACCGCGCCTTGGCTG	3.89	1.95	0.86	4.07	2.41
GATCAACCGCGCCTTGTGTC	0.46	0.00	0.00	0.00	0.00
GATCAACCGCGCGAGCATCA	0.00	0.00	0.00	0.34	0.00
GATCAACCGCGCGCAGCCCG	0.00	0.00	0.00	0.00	0.00
GATCAACCGCGCGCGTGGTA	0.00	0.00	0.00	0.34	0.00
GATCAACCGCGCGGCCCATT	0.00	0.00	0.00	0.00	0.00
GATCAACCGCGCGGCCTTCC	0.00	0.00	0.00	0.34	0.00
GATCAACCGCGCTAAAAAAA	0.23	0.00	0.00	1.02	0.00
GATCAACCGCGCTAGTGGAA	0.00	0.00	0.00	0.34	0.00
GATCAACCGCGCTCATCACA	0.00	0.00	0.29	0.00	0.00
GATCAACCGCGCTCGAAAAA	0.00	0.33	0.00	0.00	0.00
GATCAACCGCGCTCGCGGAA	0.00	0.00	0.29	0.00	0.00
GATCAACCGCGCTCGTGAAA	0.00	0.00	0.00	0.34	0.69
GATCAACCGCGCTCGTGAGA	0.00	0.33	0.00	0.00	0.00
GATCAACCGCGCTCGTGCAA	0.00	0.00	0.00	0.34	0.34
GATCAACCGCGCTCGTGGAA	4.58	10.09	14.60	20.71	20.32
GATCAACCGCGCTCGTGGAC	0.00	0.00	0.00	0.00	0.00
GATCAACCGCGCTCGTGGCA	0.00	0.00	0.57	0.68	0.00
GATCAACCGCGCTCGTGGCC	0.00	0.00	0.00	0.00	0.00
GATCAACCGCGCTCGTGGGA	0.00	0.00	0.00	0.00	0.00
GATCAACCGCGCTCGTGGTA	0.00	0.00	0.00	0.00	0.34
GATCAACCGCGCTCGTGTAA	0.00	0.00	0.00	0.00	0.00
GATCAACCGCGCTGAGATGC	0.23	0.65	0.00	0.00	0.34

GATCAACCGCGCTGAGTTCT	0.23	0.00	0.00	0.00	0.00
GATCAACCGCGCTGGCTCAG	0.00	0.00	0.00	0.34	0.00
GATCAACCGCGGAAAGCGTA	0.00	0.00	0.00	0.00	0.00
GATCAACCGCGGAAAGCGTT	0.46	0.00	0.29	0.00	0.00
GATCAACCGCGGAGGTGTTA	6.18	2.28	0.86	1.36	1.03
GATCAACCGCGGAGTGGAAG	1.37	3.58	3.72	3.73	3.44
GATCAACCGCGGAGTGGACG	0.23	0.33	0.29	0.00	0.00
GATCAACCGCGGATGGGCCT	0.00	0.00	0.00	0.00	0.00
GATCAACCGCGGCAAGACGC	0.00	0.00	0.00	0.34	0.00
GATCAACCGCGGCAAGCGCC	0.00	0.00	0.00	0.34	0.00
GATCAACCGCGGCATAAATA	0.00	0.33	0.00	0.00	0.00
GATCAACCGCGGCATCAAGT	0.23	0.00	0.29	0.00	0.34
GATCAACCGCGGCATCAATT	0.00	0.00	0.00	0.00	0.00
GATCAACCGCGGCATCACGT	0.00	0.00	0.29	0.00	0.00
GATCAACCGCGGCGCGCGGT	0.00	0.00	0.29	0.00	0.69
GATCAACCGCGGCGCTGTTC	0.00	0.00	0.00	0.34	0.00
GATCAACCGCGGCGGCGGAG	0.00	0.00	0.00	0.00	0.00
GATCAACCGCGGCTACGTCA	0.00	0.65	0.29	0.34	0.00
GATCAACCGCGGCTCCAAGC	0.00	0.00	0.00	0.00	0.00
GATCAACCGCGGGAAGCTGT	0.00	0.00	0.00	0.00	0.00
GATCAACCGCGGGCTGGCGG	0.00	0.00	0.29	0.00	0.00
GATCAACCGCGGGGGGGTTT	0.23	0.00	0.00	0.00	0.00
GATCAACCGCGGTGAGAAAG	0.00	0.00	0.00	0.68	0.00
GATCAACCGCGGTGGTAAAA	0.23	0.00	0.00	0.00	0.00
GATCAACCGCGGTGGTGGAA	0.23	0.00	0.00	0.00	0.00
GATCAACCGCGTAAAGGCCA	0.69	0.00	0.00	0.00	0.00
GATCAACCGCGTAAAGGGTG	0.23	0.00	0.00	0.00	0.00
GATCAACCGCGTCAGGCTGA	0.00	0.00	0.00	0.00	0.00
GATCAACCGCGTCGGCACTT	0.00	0.00	0.00	0.00	0.34
GATCAACCGCGTCGGCATGG	0.00	0.33	0.00	0.00	0.34
GATCAACCGCGTCGGCATTG	0.00	0.00	0.00	0.00	0.00
GATCAACCGCGTCGGCGATG	0.00	0.00	0.00	0.34	0.00
GATCAACCGCGTGAAATCCG	0.00	0.00	0.29	0.00	0.00
GATCAACCGCGTGATGACAC	0.00	0.00	0.00	0.00	0.00
GATCAACCGCGTGGAGTACG	0.00	0.00	0.29	1.36	0.34
GATCAACCGCGTGGTGTGTG	0.00	0.00	0.00	0.00	0.34
GATCAACCGCGTGTTCCTGC	0.00	0.00	0.00	0.00	0.00

GATCAACCGCTAGTGCATTC	0.00	0.00	0.00	0.00	0.00
GATCAACCGCTCACTGCCGC	0.00	0.00	0.00	0.00	0.69
GATCAACCGCTCAGAGTTAA	0.00	0.00	0.00	0.34	0.00
GATCAACCGCTCCCGCGAGC	0.23	0.00	0.00	0.00	0.00
GATCAACCGCTCCGCGTGCG	0.69	0.33	0.00	0.00	0.00
GATCAACCGCTCCGTGCGGC	0.46	0.00	0.00	0.00	0.00
GATCAACCGCTCCTGCACGC	0.00	0.00	0.00	0.00	0.34
GATCAACCGCTCCTTCGCAC	0.00	0.00	0.57	0.00	0.34
GATCAACCGCTCCTTGACGA	0.46	0.33	0.57	1.36	0.00
GATCAACCGCTCGAGCGGTG	0.00	0.00	0.00	0.00	0.00
GATCAACCGCTCGCAGTGGA	0.00	0.00	0.00	0.00	0.00
GATCAACCGCTCGGCCCCCG	0.00	0.00	0.00	0.00	0.00
GATCAACCGCTCGTATGCCG	0.00	0.00	0.00	0.00	0.34
GATCAACCGCTCTTTGCTGG	0.00	0.00	0.00	0.00	0.00
GATCAACCGCTGCAGAAAAA	0.00	0.00	0.00	0.00	0.69
GATCAACCGCTGCAGGAAGC	0.00	0.00	0.00	0.00	0.00
GATCAACCGCTGCATCCTTC	0.00	0.00	0.00	0.00	0.00
GATCAACCGCTGCCGAACGC	0.00	0.00	0.29	0.00	0.00
GATCAACCGCTGCCGAATGC	0.00	0.00	0.00	0.00	0.00
GATCAACCGCTGCCGCACGC	0.23	0.00	0.00	0.00	0.34
GATCAACCGCTGCCGTACAC	0.23	0.33	0.86	0.34	1.38
GATCAACCGCTGCCGTACCG	0.00	0.33	0.00	0.00	0.00
GATCAACCGCTGCCGTACGC	0.00	2.60	0.86	2.38	0.69
GATCAACCGCTGCCGTACTC	0.00	0.33	0.00	0.34	0.00
GATCAACCGCTGCCGTCCCC	0.00	0.00	0.00	0.00	0.00
GATCAACCGCTGCCGTCCGC	0.00	0.00	0.00	0.00	0.00
GATCAACCGCTGCGCTTGGA	0.00	0.00	0.00	0.00	0.34
GATCAACCGCTGCGGCAAGC	0.00	0.00	0.29	0.00	0.00
GATCAACCGCTGCTTTCGGG	0.00	0.00	0.00	0.00	0.69
GATCAACCGCTGGAGTTGCC	0.23	0.65	0.29	0.00	0.00
GATCAACCGCTGGAGTTGCT	0.00	0.00	0.00	0.00	0.34
GATCAACCGCTGGCTGACGA	0.00	0.33	0.00	0.00	0.00
GATCAACCGCTGGGACAGTC	0.23	0.00	0.00	0.34	0.69
GATCAACCGCTGGGACCATG	0.00	0.00	0.00	0.00	0.00
GATCAACCGCTGGGGCCGTG	0.23	0.00	0.00	0.00	0.00
GATCAACCGCTGGTGTGGGT	0.00	0.00	0.00	0.00	0.34
GATCAACCGCTGTCCCTACC	0.00	0.00	0.00	0.00	0.34

GATCAACCGCTGTCGTATGC	0.00	0.00	0.00	0.00	0.00
GATCAACCGCTGTGAGCAAG	0.00	0.00	0.00	0.00	0.00
GATCAACCGCTGTGAGCAAN	0.00	0.00	0.00	0.00	0.00
GATCAACCGCTGTTCAATCC	0.00	0.00	0.00	0.00	0.69
GATCAACCGCTTCAGCGACG	0.00	0.00	0.00	0.00	0.00
GATCAACCGCTTCGACATCG	0.00	0.98	0.00	0.34	1.38
GATCAACCGCTTCGCGGACG	0.23	0.33	0.00	0.34	0.00
GATCAACCGCTTCGCGGAGG	0.00	0.00	0.00	0.00	0.69
GATCAACCGCTTCGCTAGCA	0.00	0.33	0.00	0.00	0.00
GATCAACCGCTTCGGCTTCG	0.00	0.00	0.00	0.00	0.69
GATCAACCGCTTCTACATCG	0.00	0.33	0.00	0.00	0.00
GATCAACCGCTTCTTCCAGC	0.00	0.00	0.00	0.00	0.00
GATCAACCGCTTGCAGACCT	0.00	0.00	0.00	0.68	0.00
GATCAACCGCTTGCTGATTA	0.00	0.00	0.29	0.00	0.00
GATCAACCGCTTGCTGCTTA	1.14	3.25	4.01	1.36	3.79
GATCAACCGCTTGGCCCCCG	0.00	0.33	0.00	0.00	0.00
GATCAACCGCTTGGGAACCT	0.00	0.00	0.00	0.00	0.00
GATCAACCGCTTGGGTGCTC	0.00	0.33	0.00	0.00	0.00
GATCAACCGCTTTCAGCTGC	0.00	0.00	0.29	0.00	0.34
GATCAACCGCTTTGCCNNNN	0.23	0.00	0.00	0.00	0.00
GATCAACCGCTTTGCCTGCC	0.46	0.00	0.00	0.00	0.00
GATCAACCGCTTTGGTGGCC	0.00	0.00	0.00	0.00	0.69
GATCAACCGGAAAAAAAAAA	0.00	0.00	0.00	3.05	0.00
GATCAACCGGAAAGAGCAGG	0.00	0.00	0.00	0.00	0.69
GATCAACCGGAAATCGTATG	0.00	0.33	0.00	0.00	0.00
GATCAACCGGAACAAGAGAC	0.00	0.00	0.00	0.00	0.00
GATCAACCGGAACTCCGTCA	0.00	0.00	0.00	0.00	0.00
GATCAACCGGAATGGATTGA	0.00	0.00	0.29	0.00	0.00
GATCAACCGGACAACTCAAA	0.00	0.00	0.00	0.00	0.69
GATCAACCGGACAAGGAGTT	0.00	0.33	0.00	0.00	0.34
GATCAACCGGACAATGAGAA	0.00	0.00	0.00	0.00	0.34
GATCAACCGGACAGAGCGGG	0.00	0.00	0.00	0.00	0.00
GATCAACCGGACAGAGGGGG	0.23	0.00	0.00	0.00	0.00
GATCAACCGGACAGAGTGGG	0.46	1.30	0.29	1.36	0.69
GATCAACCGGACCATTCGCG	0.00	0.33	0.00	0.00	0.00
GATCAACCGGACGAGCCCTG	0.00	0.00	0.29	0.00	0.00
GATCAACCGGACGATGCGCT	0.46	0.98	0.00	0.00	0.00

GATCAACCGGACGCAGGCGC	0.00	0.00	0.00	0.00	0.00
GATCAACCGGACTCCCCAGC	0.00	0.00	0.29	0.00	0.00
GATCAACCGGACTCCTTGTA	0.00	0.00	0.00	0.00	0.00
GATCAACCGGACTGCGTCTA	0.23	0.65	0.00	0.34	0.00
GATCAACCGGACTTGTGAAC	0.00	0.00	0.29	0.00	0.00
GATCAACCGGAGAGGAGACA	0.00	0.00	0.00	0.00	0.34
GATCAACCGGAGCATCAGGA	0.23	0.00	0.00	0.00	0.00
GATCAACCGGAGCATTTCGT	0.00	0.00	0.00	0.34	0.00
GATCAACCGGAGCGAGTTTG	0.00	0.33	0.00	0.00	0.00
GATCAACCGGAGCGGGCGGA	0.00	0.00	0.00	0.00	0.34
GATCAACCGGAGGAGAAGGA	0.00	0.00	0.00	0.00	0.69
GATCAACCGGAGGAGGTAGA	0.00	0.00	0.57	0.00	0.00
GATCAACCGGAGGGGCCGGG	0.00	0.00	0.00	0.00	0.34
GATCAACCGGAGTCGCATTA	0.00	0.00	0.00	0.00	0.34
GATCAACCGGATAAAAAAAA	0.00	0.00	0.57	0.34	0.00
GATCAACCGGATGTCTCGGA	0.00	0.65	0.00	0.00	0.00
GATCAACCGGATTAAATTCA	0.00	0.00	0.00	0.00	0.00
GATCAACCGGATTCACCACC	0.23	0.00	0.00	0.00	0.00
GATCAACCGGATTCGCATGA	0.00	0.00	0.00	0.00	0.34
GATCAACCGGCAAACCAAAA	0.00	0.00	0.29	0.00	0.00
GATCAACCGGCACAAGACTT	0.00	0.00	0.00	0.00	0.34
GATCAACCGGCACATCGCCA	0.00	0.00	0.00	0.00	0.69
GATCAACCGGCACGCCCTCG	0.00	0.33	0.00	0.00	0.00
GATCAACCGGCACGCGCTTG	0.00	0.33	0.00	0.00	0.00
GATCAACCGGCAGACATCCA	0.00	0.00	0.29	0.00	0.00
GATCAACCGGCAGACTTTCC	0.00	3.58	5.44	2.04	4.48
GATCAACCGGCAGACTTTCT	0.00	0.00	0.00	0.00	0.69
GATCAACCGGCAGCTGGAGG	0.00	0.33	0.00	0.00	0.00
GATCAACCGGCAGGAGCAGC	0.00	0.00	0.00	0.34	0.00
GATCAACCGGCAGGGCAGAG	0.00	0.00	0.00	0.34	0.00
GATCAACCGGCAGGTCACGC	0.00	0.00	0.00	0.00	0.00
GATCAACCGGCAGTTTGCCG	0.69	0.00	0.00	0.00	0.69
GATCAACCGGCATCATGGCG	0.00	0.00	0.00	0.00	0.00
GATCAACCGGCATCGATACA	0.00	0.00	0.00	0.00	0.34
GATCAACCGGCATCGGCGCA	0.00	0.00	0.00	0.34	0.00
GATCAACCGGCATGCCCTTG	0.23	0.00	0.00	0.68	0.00
GATCAACCGGCATTCCATGC	0.00	0.00	0.29	0.00	0.00

GATCAACCGGCATTGCGAAA	0.00	0.00	0.00	0.00	0.00
GATCAACCGGCCACCGAAGA	0.00	0.00	0.00	0.00	0.34
GATCAACCGGCCAGAGCCAC	0.00	0.00	0.00	0.00	0.00
GATCAACCGGCCATACCCCG	0.00	0.00	0.00	0.00	0.00
GATCAACCGGCCATGCTTTT	0.00	0.00	0.00	0.34	0.00
GATCAACCGGCCCATCACGA	0.00	0.00	0.00	0.68	0.00
GATCAACCGGCCCATCACGC	0.00	0.65	0.00	0.00	0.34
GATCAACCGGCCCATTGACG	0.00	0.33	0.00	0.00	0.00
GATCAACCGGCCCCAAGAAT	0.00	0.00	0.00	0.34	0.00
GATCAACCGGCCCTGCTTTT	0.69	0.00	0.00	0.00	0.00
GATCAACCGGCCGCGGTGAT	0.00	0.98	0.00	0.34	0.34
GATCAACCGGCCGCGGTGCT	0.00	0.00	0.29	0.00	0.00
GATCAACCGGCCGCTGGACG	0.23	0.33	0.00	0.00	0.00
GATCAACCGGCCGGAGCCGC	0.00	0.00	0.00	0.00	0.00
GATCAACCGGCCGGCCCCGA	0.00	0.00	0.00	0.00	1.03
GATCAACCGGCCGGCCCTGA	0.00	0.00	0.00	0.00	0.34
GATCAACCGGCCGTGCTGAA	1.83	0.33	0.00	0.68	0.00
GATCAACCGGCCTTCCACGC	0.23	0.00	0.29	0.00	0.00
GATCAACCGGCCTTCCATGC	15.79	11.39	13.46	7.81	7.58
GATCAACCGGCCTTCCATGN	0.00	0.00	0.00	0.00	0.00
GATCAACCGGCCTTCCCTGC	0.00	0.00	0.29	0.00	0.00
GATCAACCGGCCTTCCGTGC	0.00	0.00	0.29	0.00	0.00
GATCAACCGGCCTTGAATCT	0.00	0.33	0.00	0.34	0.69
GATCAACCGGCGAAGAAGAC	0.00	0.00	0.00	0.00	0.34
GATCAACCGGCGATGCCTGA	0.46	0.33	0.00	0.00	0.00
GATCAACCGGCGCAAGTCGA	0.00	0.00	0.00	1.02	0.00
GATCAACCGGCGCAGCGGCG	0.00	0.00	0.00	0.00	0.34
GATCAACCGGCGCCTTCGCC	0.46	0.00	0.29	0.00	0.69
GATCAACCGGCGGAAGGTCT	0.00	0.33	0.57	0.34	1.72
GATCAACCGGCGGAAGGTGC	0.00	0.33	0.00	1.02	0.34
GATCAACCGGCGGACTTTCC	0.00	0.00	0.00	0.00	0.00
GATCAACCGGCGGCGGCTCA	0.00	0.00	0.00	0.00	0.34
GATCAACCGGCGGCGGCTCT	0.00	0.98	0.00	0.68	0.34
GATCAACCGGCGGCGGGTCT	0.00	0.00	0.29	0.00	0.00
GATCAACCGGCGGGCCATGT	0.23	0.00	0.00	0.00	0.00
GATCAACCGGCGTCATGCAT	0.00	0.00	0.00	0.00	0.00
GATCAACCGGCGTGCCCTTG	0.23	0.00	0.00	0.00	0.00

GATCAACCGGCGTGCTCGGC	0.00	0.00	0.00	0.34	0.00
GATCAACCGGCTCATGGCGA	0.00	0.00	0.00	0.00	0.00
GATCAACCGGCTCCTGCTGC	0.00	0.00	0.29	0.68	0.00
GATCAACCGGCTCTTCCTGG	0.00	0.00	0.00	0.00	0.00
GATCAACCGGCTCTTCGACG	0.00	0.00	0.00	0.00	0.00
GATCAACCGGCTGCGCAGGG	0.00	0.00	0.29	0.34	0.00
GATCAACCGGCTGGGCGCAG	0.00	0.00	0.00	0.00	0.00
GATCAACCGGCTGGGGGATG	0.23	0.00	0.00	0.00	0.00
GATCAACCGGCTGTTCTTGG	0.00	0.00	0.29	0.00	0.00
GATCAACCGGCTTGAGCAAT	0.46	0.00	0.00	0.00	0.00
GATCAACCGGCTTGCCCCAC	0.00	0.00	0.00	0.34	0.00
GATCAACCGGCTTGCCCGAA	0.69	0.33	0.29	0.00	0.00
GATCAACCGGCTTGCCCGAC	8.70	5.21	3.44	2.72	2.07
GATCAACCGGCTTGCCCGCC	0.23	0.00	0.00	0.34	0.34
GATCAACCGGCTTGCCCGGC	0.23	0.00	0.00	0.00	0.00
GATCAACCGGCTTGCCCTAC	0.23	0.00	0.00	0.00	0.00
GATCAACCGGCTTTGCTAGT	0.00	0.00	0.00	0.00	0.69
GATCAACCGGGAAGCACANG	0.00	0.33	0.00	0.00	0.00
GATCAACCGGGAATCCTTGG	0.23	0.00	0.00	0.34	0.00
GATCAACCGGGACCCACTTC	0.23	0.00	0.00	0.00	0.00
GATCAACCGGGACCGCGTGG	0.00	0.33	0.00	0.00	0.00
GATCAACCGGGACCGTGAGC	0.00	0.00	0.00	0.00	0.34
GATCAACCGGGACTGCGGGG	0.00	0.00	0.00	0.00	0.00
GATCAACCGGGAGATTGCCN	0.00	0.00	0.00	0.00	0.00
GATCAACCGGGAGCTGGCGA	0.00	1.30	0.29	3.05	1.03
GATCAACCGGGAGCTGGCGC	0.23	0.00	0.00	0.00	0.00
GATCAACCGGGAGCTGGCTG	0.00	0.33	0.00	0.00	0.00
GATCAACCGGGAGTTTGGGG	0.23	0.65	0.00	0.00	0.00
GATCAACCGGGATTGGTACC	0.46	0.00	0.00	0.00	0.00
GATCAACCGGGATTGGTCTG	0.00	0.00	0.00	0.00	0.00
GATCAACCGGGCAACCACAT	0.00	0.00	0.00	0.00	0.34
GATCAACCGGGCACATGCGC	0.00	0.00	0.29	0.00	0.00
GATCAACCGGGCAGTCATGG	0.00	0.00	0.00	0.34	0.00
GATCAACCGGGCCACCCGCG	0.23	0.00	0.00	0.00	0.00
GATCAACCGGGCCGAAGGGC	0.00	0.00	0.00	0.00	0.00
GATCAACCGGGCCTGGCCCC	0.00	0.33	0.00	0.00	0.00
GATCAACCGGGCGCCCTTCT	0.23	0.00	0.00	0.00	0.00

GATCAACCGGGCGCCGCAGC	0.46	0.00	0.00	0.00	0.00
GATCAACCGGGCGCGCACGT	0.00	0.00	0.29	0.00	0.00
GATCAACCGGGCGGCCGGCC	0.23	0.00	0.00	0.00	0.00
GATCAACCGGGCTGCGCCCA	0.00	0.00	0.00	0.00	0.34
GATCAACCGGGCTTCCATGC	0.00	0.00	0.00	0.00	0.00
GATCAACCGGGGACGCCTCG	0.23	0.00	0.00	0.00	0.00
GATCAACCGGGGCACAGTCG	0.23	0.00	0.00	0.00	0.00
GATCAACCGGGGCTGCATTA	0.23	0.00	0.00	0.34	0.00
GATCAACCGGGGGAGCGCAC	0.00	0.00	0.00	0.34	0.00
GATCAACCGGGTCACGGGCC	0.23	0.00	0.00	0.00	0.00
GATCAACCGGGTCACGGGCG	0.23	0.00	0.00	0.00	0.00
GATCAACCGGGTCGGCATGG	0.00	0.00	0.00	0.00	0.00
GATCAACCGGGTCTCCTTCA	0.00	0.00	0.00	0.00	0.34
GATCAACCGGGTCTCCTTTA	0.00	0.65	0.00	0.34	0.00
GATCAACCGGGTGATGAAGA	0.46	0.00	0.00	0.68	0.00
GATCAACCGGGTGGAGCACG	0.00	0.33	0.00	0.00	0.00
GATCAACCGGTAAAGCTTCT	0.23	0.00	0.00	0.00	0.00
GATCAACCGGTAAGTGGTCA	0.00	0.00	0.00	0.00	0.34
GATCAACCGGTAATAGATGG	0.00	0.00	0.00	0.00	0.00
GATCAACCGGTACGCGGAGG	0.23	0.00	0.00	0.00	0.00
GATCAACCGGTACTACTCCG	0.00	0.00	0.00	0.00	0.00
GATCAACCGGTATGAGGCGG	0.00	0.00	0.29	0.00	0.00
GATCAACCGGTCCTCCGCGA	0.00	0.00	0.00	0.00	0.00
GATCAACCGGTCGATGAGTA	0.00	0.00	0.29	0.00	0.00
GATCAACCGGTCGGCACTGC	0.00	0.00	0.00	0.00	0.00
GATCAACCGGTCTGTCAGCC	0.00	0.33	0.00	0.00	0.00
GATCAACCGGTGCCTCATCC	0.00	0.00	0.00	0.00	0.34
GATCAACCGGTGGATGGAGC	0.69	2.28	0.57	1.36	0.34
GATCAACCGGTGGGACTCCG	0.00	0.00	0.00	0.34	0.00
GATCAACCGGTGGTCTTGTC	0.00	0.00	0.00	0.34	0.00
GATCAACCGGTGGTCTTTTC	0.00	0.00	0.00	0.34	0.00
GATCAACCGGTTGCAAGAGG	0.69	0.00	0.00	0.00	0.00
GATCAACCGGTTGCCACATG	0.00	0.00	0.00	0.00	0.00
GATCAACCGTAAAAAAGAAC	0.00	0.00	0.29	0.00	0.00
GATCAACCGTAAGAGTGCTT	0.23	0.00	0.00	0.00	0.00
GATCAACCGTAAGTGCGAGA	0.00	0.00	0.00	0.00	0.00
GATCAACCGTACGTCGCCAA	0.00	0.00	0.00	0.00	0.00

GATCAACCGTAGCAGAAAAA	0.00	0.33	0.00	0.00	0.00
GATCAACCGTAGCATCCTTC	0.00	0.00	0.00	0.00	0.34
GATCAACCGTAGCTGTTCAG	0.00	0.00	0.00	0.00	0.00
GATCAACCGTAGCTGTTCGT	0.00	0.00	0.00	0.00	0.34
GATCAACCGTAGCTGTTCTG	3.20	7.81	3.44	7.47	7.23
GATCAACCGTAGCTGTTCTT	0.00	0.33	0.00	0.00	0.00
GATCAACCGTAGGATGGCGT	0.00	0.00	0.00	0.00	0.34
GATCAACCGTATCCTTGAGT	0.00	0.00	0.00	0.00	0.00
GATCAACCGTATGAAAAGAC	0.00	0.00	0.29	0.00	0.00
GATCAACCGTATGCAAGGAA	0.00	0.00	0.00	0.00	0.00
GATCAACCGTATTACCGAGG	0.00	0.00	0.00	0.00	0.34
GATCAACCGTCAAAAAAAAA	0.00	0.33	0.00	0.00	0.00
GATCAACCGTCACTGCCTGA	0.00	0.00	0.29	0.00	0.00
GATCAACCGTCACTGGNNNN	0.00	0.00	0.00	0.00	0.00
GATCAACCGTCAGTGCGAGA	0.00	0.33	0.29	0.00	0.00
GATCAACCGTCATGGTTTCC	0.00	0.00	0.00	0.34	0.34
GATCAACCGTCCCCATATGC	0.00	0.00	0.00	0.00	0.00
GATCAACCGTCCCCCAGAGC	0.00	0.00	0.00	0.00	0.00
GATCAACCGTCCCCGGAGGT	0.00	0.00	0.00	0.00	0.00
GATCAACCGTCCCGACCGTG	0.00	0.00	0.00	0.34	0.00
GATCAACCGTCCCGGCCGTC	0.00	0.65	0.57	0.34	0.00
GATCAACCGTCCTATCAAAG	0.00	0.33	0.57	0.34	0.00
GATCAACCGTCCTATCGCTG	0.00	0.00	0.57	0.00	0.00
GATCAACCGTCCTGCCTCGC	0.00	0.00	0.00	0.00	0.34
GATCAACCGTCGCCCCGGCT	0.00	0.33	0.00	0.00	0.00
GATCAACCGTCGGCACTTAC	0.23	0.00	0.00	0.00	0.00
GATCAACCGTCGGTGAGGAC	0.00	0.00	0.00	0.00	0.34
GATCAACCGTCGTATGGGGT	0.23	0.00	0.00	0.00	0.00
GATCAACCGTCGTATGTGAA	0.00	0.00	0.00	0.34	0.00
GATCAACCGTCGTGGACGGA	0.00	0.00	0.00	0.00	0.34
GATCAACCGTCTCATCTCAG	0.00	0.00	0.00	0.00	0.00
GATCAACCGTCTCGTAGAAT	0.00	0.00	0.00	0.00	0.34
GATCAACCGTCTGATGCCAG	0.00	0.00	0.00	0.00	0.34
GATCAACCGTCTGCAGACTG	0.00	0.33	0.00	0.00	0.00
GATCAACCGTCTGGAAGCGG	0.00	0.00	0.00	0.34	0.00
GATCAACCGTCTGGAGCTTG	0.46	0.00	0.00	0.00	0.00
GATCAACCGTCTGGCATCCA	0.00	0.00	0.00	0.34	0.00

GATCAACCGTCTGGCATTTA	0.00	0.33	0.00	0.34	0.00
GATCAACCGTCTGGCGCAAA	0.00	0.00	0.00	0.00	0.34
GATCAACCGTCTGGCGGAGG	0.00	0.00	0.00	0.34	0.00
GATCAACCGTCTGTGGAGGA	0.00	0.00	0.29	0.00	0.00
GATCAACCGTCTGTGGCGGA	0.00	0.00	0.00	0.34	0.34
GATCAACCGTCTTTCGTCAT	0.00	0.00	0.29	0.00	0.00
GATCAACCGTGAACTCACAG	0.23	0.00	0.00	0.00	0.00
GATCAACCGTGAGGCGAAGC	0.00	0.00	0.00	0.00	0.34
GATCAACCGTGATGGTAAGA	1.83	1.63	1.15	2.04	1.72
GATCAACCGTGATGGTAAGC	0.23	0.00	0.00	0.00	0.00
GATCAACCGTGATGGTCAGA	0.00	0.00	0.00	0.34	0.00
GATCAACCGTGCACCGAGGA	0.00	0.00	0.00	0.00	0.00
GATCAACCGTGCATCCGGTG	0.46	0.00	0.00	0.00	0.00
GATCAACCGTGCATGTGTGT	0.23	0.00	0.00	0.00	0.00
GATCAACCGTGCCAGCGCTC	0.00	0.00	0.57	0.34	0.00
GATCAACCGTGCCAGGGCAG	0.00	0.65	0.00	1.02	0.00
GATCAACCGTGCCATGGGCC	0.00	0.00	0.00	0.00	1.03
GATCAACCGTGCCGACGCTC	0.00	0.33	0.00	0.00	0.00
GATCAACCGTGCCGACGCTT	0.00	0.00	0.29	0.34	0.00
GATCAACCGTGCCGTCGCCA	0.00	0.00	0.00	0.00	0.69
GATCAACCGTGCCTTGGACA	0.00	0.00	0.00	0.00	0.00
GATCAACCGTGCGCTCAACA	0.00	0.00	0.00	0.00	0.00
GATCAACCGTGCGGACTGTC	1.60	1.95	0.86	1.02	0.69
GATCAACCGTGCGGCATAAC	0.00	0.00	0.00	0.00	0.00
GATCAACCGTGCTCGTTGTA	0.00	0.00	0.00	0.00	0.00
GATCAACCGTGCTGCTTGGG	0.23	0.33	0.00	0.68	0.00
GATCAACCGTGCTTCCCCGG	0.00	0.00	0.29	0.00	0.00
GATCAACCGTGGCAGCACGC	0.00	0.00	0.00	0.00	0.34
GATCAACCGTGGCATCCAGT	0.00	0.00	0.00	0.00	0.00
GATCAACCGTGGCCTCACGT	0.00	0.33	0.00	0.00	0.00
GATCAACCGTGGCCTCGTTC	0.23	0.00	0.00	0.00	0.00
GATCAACCGTGGCTGGGTGT	0.00	0.00	0.00	0.00	0.00
GATCAACCGTGGGTACGGGA	0.23	0.00	0.00	0.00	0.00
GATCAACCGTGGGTTGCGCC	0.00	0.00	0.29	0.00	0.00
GATCAACCGTGGTCTGAAGC	0.00	0.00	0.00	0.34	0.00
GATCAACCGTGGTGGAACGG	0.00	0.00	0.00	0.00	0.00
GATCAACCGTGGTTGGAAGG	0.23	0.00	0.00	0.00	0.00

GATCAACCGTGTAACGGGCC	0.00	0.33	0.00	0.00	0.00
GATCAACCGTGTACGCATGG	0.00	0.00	0.00	0.00	0.00
GATCAACCGTGTCAAGAAGG	0.00	0.33	0.00	0.00	0.00
GATCAACCGTGTCACAGGCC	0.00	0.00	0.00	0.34	0.00
GATCAACCGTGTCACGGGAC	0.00	0.00	0.00	0.00	0.34
GATCAACCGTGTCACGGGCC	9.84	17.89	7.16	11.88	5.51
GATCAACCGTGTCACGGGCN	0.00	0.00	0.00	0.00	0.00
GATCAACCGTGTCACGGGCT	1.14	0.00	0.00	1.02	0.00
GATCAACCGTGTCACGGGNC	0.00	0.00	0.00	0.34	0.00
GATCAACCGTGTCACGGGNN	0.23	0.00	0.00	0.00	0.00
GATCAACCGTGTCACGGGNT	0.00	0.00	0.00	0.34	0.00
GATCAACCGTGTCACGGGTC	0.00	0.00	0.00	0.00	0.34
GATCAACCGTGTCCTCAAGC	0.00	0.00	0.00	0.00	1.03
GATCAACCGTGTCGCGGGCC	0.00	0.00	0.00	0.00	0.00
GATCAACCGTGTCGGGGCAC	0.00	0.00	0.00	0.00	0.34
GATCAACCGTGTGCACAGGT	0.00	0.00	0.00	0.34	0.00
GATCAACCGTGTGCTTGGCC	2.52	5.21	2.86	6.11	3.10
GATCAACCGTGTGCTTGNCC	0.00	0.00	0.00	0.34	0.00
GATCAACCGTGTGCTTGNNN	0.23	0.00	0.00	0.00	0.00
GATCAACCGTGTGGGATTCG	0.00	0.00	0.00	0.00	0.34
GATCAACCGTGTTGCTTGGT	0.00	0.00	0.29	0.00	0.00
GATCAACCGTTACAACTCCA	0.00	0.33	0.00	0.00	0.00
GATCAACCGTTACTGCGGGC	0.00	0.00	0.00	0.00	0.69
GATCAACCGTTCAAACCGAT	0.00	0.00	0.29	0.00	0.00
GATCAACCGTTCCTCGCACG	0.00	0.00	0.00	0.00	0.00
GATCAACCGTTCGCGACCCA	0.00	0.00	0.00	0.00	0.00
GATCAACCGTTCGCGCTCTC	0.00	0.00	0.00	0.00	0.00
GATCAACCGTTCGTATGCCG	0.46	0.00	0.00	0.00	0.00
GATCAACCGTTCGTGTATTT	0.23	0.00	0.00	0.00	0.00
GATCAACCGTTGATGACATG	0.00	0.00	0.00	0.00	0.34
GATCAACCGTTGCCACCTTG	0.00	0.00	0.00	0.00	0.34
GATCAACCGTTGCCTCCAGT	0.23	0.00	0.00	0.00	0.00
GATCAACCGTTGCCTCTGGA	0.00	0.33	0.00	0.00	0.00
GATCAACCGTTGGGCTATTC	0.00	0.00	0.00	0.00	0.00
GATCAACCGTTTCGAAGCGG	0.00	0.00	0.00	0.00	0.00
GATCAACCGTTTCTTCCCAG	15.11	26.68	10.60	20.37	16.53
GATCAACCGTTTCTTCCCAT	0.00	0.00	0.00	0.34	0.00

GATCAACCGTTTCTTCCCCG	0.00	0.00	0.00	0.00	0.00
GATCAACCGTTTCTTCCCTG	0.23	0.00	0.00	0.00	0.34
GATCAACCGTTTCTTCNCAG	0.00	0.00	0.00	0.00	0.34
GATCAACCGTTTCTTCNNNN	0.23	0.00	0.00	0.00	0.00
GATCAACCGTTTGAGGCAGG	0.23	0.00	0.00	0.00	0.00
GATCAACCGTTTGAGGCTCA	2.06	3.25	0.57	1.02	0.00
GATCAACCGTTTTAAAGATT	0.00	0.00	0.29	0.00	0.00
GATCAACCTAAAAAAAAAAA	11.45	0.98	1.72	4.07	4.13
GATCAACCTAAAAAAACNNN	0.23	0.00	0.00	0.00	0.00
GATCAACCTAAAAAATAAAT	0.00	0.00	0.00	0.00	0.00
GATCAACCTAAAAATAAAAA	0.00	0.00	0.00	0.00	0.00
GATCAACCTAAACTAAAAAA	0.00	0.33	0.00	0.00	0.00
GATCAACCTAAAGCAAAAAA	0.00	0.00	0.00	0.00	0.34
GATCAACCTAACAAAGGAAG	0.00	0.00	0.00	0.34	0.00
GATCAACCTAAGAGGTTTGC	0.00	0.00	0.00	0.00	0.00
GATCAACCTAATCACAATTC	0.00	0.00	0.00	0.00	0.00
GATCAACCTAATCATGAAGN	0.00	0.00	0.00	0.00	0.00
GATCAACCTAATCCACCTGA	0.46	0.00	0.00	0.00	0.00
GATCAACCTAATCTGACGGG	0.00	0.00	0.00	0.00	0.00
GATCAACCTAATTCTTTAGT	0.23	0.00	0.00	0.00	0.00
GATCAACCTACAAAAAAAAA	0.23	0.00	0.00	0.00	0.00
GATCAACCTACAAAACTCTT	0.23	0.00	0.00	0.00	0.00
GATCAACCTACAAGGCTACA	0.00	0.00	0.00	0.34	0.00
GATCAACCTACACATCCGTG	0.00	0.00	0.00	0.00	0.34
GATCAACCTACAGCATCCTT	0.00	0.00	0.00	0.00	0.00
GATCAACCTACAGCGAACCC	1.37	0.00	0.29	0.00	1.38
GATCAACCTACAGCGANNNN	0.23	0.00	0.00	0.00	0.00
GATCAACCTACAGCGCTACA	0.23	0.00	0.00	0.00	0.00
GATCAACCTACAGCGCTACC	9.84	8.46	2.86	6.45	2.07
GATCAACCTACAGCGCTACG	0.23	0.00	0.00	0.00	0.00
GATCAACCTACAGCGCTACN	0.23	0.00	0.00	0.00	0.00
GATCAACCTACAGGATGCCA	0.00	0.00	0.29	0.00	0.00
GATCAACCTACATGTTGGCC	0.00	0.00	0.29	0.00	0.00
GATCAACCTACATTGCGGGT	0.00	0.00	0.00	0.00	0.69
GATCAACCTACATTGTAATA	0.00	0.00	0.00	0.00	0.69
GATCAACCTACCACCCTTTA	0.00	0.00	0.29	0.00	0.00
GATCAACCTACCATTGACAA	0.00	0.33	0.00	0.00	0.00

GATCAACCTACCCGGGCAGA	0.23	0.00	0.00	0.00	0.00
GATCAACCTACCCGTCATTG	0.46	0.00	0.00	0.00	0.00
GATCAACCTACCCGTCGCAC	0.00	0.00	0.00	0.34	0.00
GATCAACCTACCCGTGCAGA	19.46	6.18	8.02	6.45	3.44
GATCAACCTACCCGTGCAGC	0.00	0.33	0.29	0.00	0.00
GATCAACCTACCCGTGCAGN	0.00	0.00	0.00	0.00	0.00
GATCAACCTACCCGTGCAGT	0.46	0.00	0.00	0.00	0.00
GATCAACCTACCCGTGCATA	0.00	0.98	0.00	1.36	0.34
GATCAACCTACCCGTGCCGC	0.23	0.00	0.00	0.00	0.00
GATCAACCTACCGTGCAGCA	0.00	0.33	0.00	0.00	0.00
GATCAACCTACGCATTGCAG	0.00	0.00	0.00	0.34	0.00
GATCAACCTACGCGGCAAAC	0.00	0.00	0.00	0.00	0.34
GATCAACCTACTATTCCTGG	0.00	0.00	0.29	0.00	0.00
GATCAACCTACTCCACCTCG	0.23	0.00	0.00	0.00	0.00
GATCAACCTACTCCGGCTCC	0.00	0.00	0.00	0.00	0.00
GATCAACCTACTCCTCCTGG	0.00	0.00	0.00	0.00	0.34
GATCAACCTACTCGAATGCC	0.00	0.00	0.00	0.34	0.00
GATCAACCTACTCTGAGCGA	0.00	0.00	0.29	0.00	0.00
GATCAACCTACTCTTAATGG	0.00	0.00	0.29	0.00	0.00
GATCAACCTACTCTTACTGG	0.23	0.00	0.00	0.00	0.34
GATCAACCTACTCTTCATGG	0.00	0.00	0.29	0.00	0.00
GATCAACCTACTCTTCCNNN	0.00	0.00	0.00	0.00	0.34
GATCAACCTACTCTTCCTAG	0.00	0.00	0.00	0.00	0.34
GATCAACCTACTCTTCCTGG	50.13	83.61	93.64	56.35	76.80
GATCAACCTACTCTTCCTGN	0.23	0.00	0.00	0.00	0.00
GATCAACCTACTCTTCCTGT	0.00	0.00	0.57	0.00	0.00
GATCAACCTACTCTTCCTNG	0.00	0.00	0.00	0.00	0.00
GATCAACCTACTCTTCGTGG	0.00	0.00	0.29	0.00	0.00
GATCAACCTACTCTTCNNNN	0.23	0.00	0.00	0.00	0.00
GATCAACCTACTCTTCNTGG	0.00	0.00	0.00	0.00	0.34
GATCAACCTACTCTTCTGGN	0.23	0.00	0.00	0.00	0.00
GATCAACCTACTGATAGCGC	0.23	0.00	0.00	0.00	0.00
GATCAACCTAGAAAAAAAAA	0.46	0.00	0.00	0.00	0.69
GATCAACCTAGAAAAAAAAT	0.00	0.00	0.00	0.00	0.34
GATCAACCTAGAGCAAAAAA	4.58	3.25	2.86	4.07	6.20
GATCAACCTAGAGCAAAAAT	0.00	0.00	0.00	0.00	0.34
GATCAACCTAGAGCAAAACN	0.23	0.00	0.00	0.00	0.00

GATCAACCTAGAGCAACAAA	0.00	0.33	0.29	0.00	0.00
GATCAACCTAGAGCAATAAA	0.23	0.00	0.57	0.00	2.07
GATCAACCTAGAGCAATAGA	0.23	0.00	0.00	0.00	0.00
GATCAACCTAGAGCAATAGC	12.82	9.11	9.74	11.20	11.71
GATCAACCTAGAGCAATAGT	0.00	0.00	0.57	0.00	0.00
GATCAACCTAGAGCAATCNN	0.23	0.00	0.00	0.00	0.00
GATCAACCTAGAGCACTAGC	0.00	0.00	0.00	0.00	0.34
GATCAACCTAGAGCCAAAAA	0.00	0.00	0.29	0.00	0.00
GATCAACCTAGAGCGGTTGT	0.23	0.00	0.00	0.00	0.00
GATCAACCTAGAGCTCTCTG	0.00	0.00	0.29	0.00	0.00
GATCAACCTAGAGGAAAAAA	0.00	0.00	0.00	0.00	0.69
GATCAACCTAGCCACGTGCT	0.23	0.00	0.00	0.00	0.00
GATCAACCTAGCCGTGCAGA	0.00	0.33	0.00	0.00	0.00
GATCAACCTAGCGCGGCAGC	0.00	0.00	0.00	0.34	0.00
GATCAACCTAGCGCGGCTGT	0.00	0.00	0.00	0.00	0.34
GATCAACCTAGCGCGGGTGT	0.00	0.33	0.00	0.00	0.00
GATCAACCTAGCGCGGNTGT	0.00	0.00	0.00	0.00	0.34
GATCAACCTAGCGCGGTNNN	0.23	0.00	0.00	0.00	0.00
GATCAACCTAGCGCGGTTCA	0.00	0.00	0.00	0.00	0.00
GATCAACCTAGCGCGGTTGC	0.23	0.00	0.00	0.00	0.00
GATCAACCTAGCGCGGTTGG	0.23	0.00	0.00	0.34	0.00
GATCAACCTAGCGCGGTTGN	0.00	0.00	0.00	0.34	0.00
GATCAACCTAGCGCGGTTGT	27.70	17.24	4.87	22.40	11.37
GATCAACCTAGCGCGGTTNN	0.23	0.00	0.00	0.00	0.34
GATCAACCTAGCTCTCGCTT	0.46	0.98	0.00	0.34	0.34
GATCAACCTAGGACGGGCCG	0.00	0.00	0.00	0.34	0.00
GATCAACCTAGGCATTGCAG	0.00	0.00	0.00	0.00	0.00
GATCAACCTAGGCTTGTTTG	0.00	0.33	0.00	0.00	0.00
GATCAACCTAGGGCTGGGCG	0.00	0.00	0.29	0.00	0.00
GATCAACCTAGGTTCCCGAG	0.00	0.00	0.29	0.00	0.00
GATCAACCTAGTCAGGACTT	0.00	0.00	0.00	0.34	0.00
GATCAACCTAGTCTGAGAGA	0.23	0.00	0.00	0.00	0.00
GATCAACCTAGTCTGAGCGA	2.52	1.30	2.00	0.68	2.07
GATCAACCTAGTCTGAGCTA	0.46	0.00	0.00	0.00	0.00
GATCAACCTAGTGATGGAAA	0.00	0.00	0.00	0.00	0.00
GATCAACCTAGTGCGGTTGT	0.23	0.00	0.00	0.00	0.00
GATCAACCTAGTGGGCGTCA	0.00	0.00	0.57	0.00	0.00

GATCAACCTATGGCGCCTGG	0.00	0.00	0.00	0.00	0.00
GATCAACCTATGGGCGTTCC	0.23	0.00	0.00	0.00	0.00
GATCAACCTATGGGGCCGTG	0.00	0.33	0.00	0.34	0.00
GATCAACCTATGTGGCCCGC	0.00	0.00	0.00	0.00	0.00
GATCAACCTATTCCCACACC	0.00	0.00	0.00	0.00	0.34
GATCAACCTCAAAGAGGCTT	0.00	0.00	0.00	0.00	0.69
GATCAACCTCAAATTCGGGG	1.14	0.00	0.29	1.02	0.00
GATCAACCTCAACATGGTTT	0.00	0.00	0.57	0.00	0.00
GATCAACCTCAACCTCCCAG	0.00	0.00	0.00	0.00	0.00
GATCAACCTCAACGAGGGCG	0.00	0.00	0.00	0.00	0.34
GATCAACCTCAACTCCGTGG	0.23	0.00	0.00	0.34	0.00
GATCAACCTCAACTGTCGCT	0.00	0.00	0.00	0.34	0.00
GATCAACCTCAAGACCATGA	0.00	0.00	0.00	0.00	0.00
GATCAACCTCAAGCAGCTGG	0.00	0.00	0.00	0.34	0.00
GATCAACCTCAAGCGCGGGG	11.90	18.54	6.59	6.79	8.61
GATCAACCTCAAGCGCGGNN	0.00	0.00	0.00	0.00	0.00
GATCAACCTCAAGCGGAACA	0.00	0.00	0.29	0.00	0.00
GATCAACCTCAAGCGGACCA	0.69	0.65	2.29	0.00	0.34
GATCAACCTCAAGCGGCCCC	0.23	0.00	0.00	0.00	0.00
GATCAACCTCAAGCTTGGTC	0.00	0.33	0.00	0.00	0.00
GATCAACCTCAAGTTCGGGG	0.00	0.00	0.29	0.00	0.34
GATCAACCTCAATGATGGGA	0.00	0.33	0.00	0.00	0.00
GATCAACCTCACAAAGGAAG	1.37	2.93	1.43	4.75	3.79
GATCAACCTCACAAAGGACC	0.00	0.00	0.00	0.00	0.00
GATCAACCTCACAAAGGACG	0.00	0.33	0.00	0.00	0.00
GATCAACCTCACAAGCTCTT	0.00	0.65	0.00	0.00	0.00
GATCAACCTCACAAGTCCGA	0.00	0.00	0.00	0.00	0.34
GATCAACCTCACAATCAACA	0.00	0.00	0.29	0.00	0.00
GATCAACCTCACAGCATGTC	0.00	0.00	0.29	0.00	0.00
GATCAACCTCACAGCGAAGG	0.00	0.00	0.29	0.00	0.00
GATCAACCTCACATGGCGGT	0.00	0.33	0.00	0.00	0.00
GATCAACCTCACCACCGGGA	0.00	0.00	0.00	0.00	0.00
GATCAACCTCACCACCTCTT	0.23	0.00	0.00	0.00	0.00
GATCAACCTCACCCGACCTG	0.00	0.33	0.00	0.00	0.00
GATCAACCTCACCCGTCCGG	0.00	0.33	0.00	0.00	0.00
GATCAACCTCACCGACACCC	0.00	0.00	0.00	0.00	0.34
GATCAACCTCACCGCGTAGG	0.00	0.00	0.29	0.00	0.00

GATCAACCTCACCGGCCCTC	2.06	0.98	0.00	1.02	0.34
GATCAACCTCACCGGCGGAC	0.23	0.00	0.00	0.00	0.00
GATCAACCTCACCGGCNNNN	0.23	0.00	0.00	0.00	0.00
GATCAACCTCACCGTGGCCG	0.23	0.33	0.00	0.34	0.00
GATCAACCTCACCTGACGGG	0.00	0.00	0.00	0.34	0.00
GATCAACCTCACCTGGCGCT	0.23	0.00	0.00	0.00	0.00
GATCAACCTCACCTGGCGTN	0.23	0.00	0.00	0.00	0.00
GATCAACCTCACCTGGCGTT	11.45	4.88	2.29	2.72	2.07
GATCAACCTCACCTGTCGGT	0.00	0.00	0.00	0.00	0.00
GATCAACCTCACCTTCAACA	0.00	0.33	0.29	0.00	0.00
GATCAACCTCACCTTCAACT	0.23	0.00	0.00	0.68	0.00
GATCAACCTCACGATAGACT	0.00	0.98	0.00	0.00	0.00
GATCAACCTCACGCTTCTCT	0.46	0.33	0.29	0.34	2.41
GATCAACCTCACGGACCCCC	0.00	0.00	0.00	0.00	0.00
GATCAACCTCACGGACGCTG	0.23	0.00	0.00	0.34	0.00
GATCAACCTCACGGACGCTT	0.00	0.00	0.00	0.34	0.00
GATCAACCTCACGGGGCGGA	0.23	0.00	0.00	0.00	0.00
GATCAACCTCACGTGGCGAA	0.00	0.00	0.00	0.34	0.00
GATCAACCTCACGTGGCGTA	5.49	4.55	11.74	5.77	6.20
GATCAACCTCACGTGGGGGA	0.00	0.00	0.29	0.00	0.00
GATCAACCTCACTCGCGGCC	0.00	0.00	0.00	0.00	0.00
GATCAACCTCACTCGTATGC	0.69	0.00	0.00	0.00	0.00
GATCAACCTCACTGATGCAG	0.23	0.33	0.57	0.00	0.00
GATCAACCTCACTGGTGACA	0.23	0.00	0.00	0.00	0.00
GATCAACCTCACTGGTGCAA	0.00	0.00	0.00	0.34	0.00
GATCAACCTCACTGGTGCCA	21.97	5.21	2.00	1.70	0.00
GATCAACCTCACTGGTGCCC	0.23	0.00	0.00	0.00	0.00
GATCAACCTCACTGGTGCCT	0.46	0.00	0.00	0.00	0.00
GATCAACCTCACTGGTGCNN	0.23	0.00	0.00	0.00	0.00
GATCAACCTCACTGGTGCTA	0.23	0.00	0.00	0.00	0.00
GATCAACCTCAGAACCAGAC	0.00	0.33	0.00	0.00	0.00
GATCAACCTCAGACCCTGAC	0.00	0.00	0.00	0.00	0.34
GATCAACCTCAGAGAAGTCA	0.00	0.33	0.00	0.00	0.00
GATCAACCTCAGCAACGAGA	0.00	0.00	0.00	0.00	0.00
GATCAACCTCAGCACAGAGG	16.25	3.25	1.43	7.13	2.76
GATCAACCTCAGCACGGAGG	0.00	0.00	0.00	0.34	0.00
GATCAACCTCAGCAGGGAAA	0.00	0.00	0.00	0.68	0.00

GATCAACCTCAGCAGGGACG	0.46	0.00	0.00	0.00	0.00
GATCAACCTCAGCAGGGCAT	0.00	0.00	0.00	0.00	0.34
GATCAACCTCAGCATCCTTC	0.00	0.00	0.00	0.34	0.00
GATCAACCTCAGCGCCTCTG	0.00	0.00	0.00	0.00	0.34
GATCAACCTCAGCGCTGAAT	0.00	0.00	0.29	0.00	0.00
GATCAACCTCAGCGGAGAAG	0.00	0.00	0.29	0.00	0.00
GATCAACCTCAGCGTCTTGG	0.00	0.00	0.29	0.00	0.00
GATCAACCTCAGCGTGACCT	0.00	0.00	0.00	0.68	0.00
GATCAACCTCAGCGTGGACA	0.00	0.65	0.00	0.00	0.00
GATCAACCTCAGCTGACGGG	0.46	0.00	0.00	0.00	0.00
GATCAACCTCAGGATTCATC	0.46	0.00	0.86	0.68	0.00
GATCAACCTCAGGGGCAAGC	0.00	0.00	0.00	0.00	0.34
GATCAACCTCAGTACTGAAA	0.00	0.00	0.00	0.00	0.34
GATCAACCTCATAAAAAAAA	0.00	0.00	0.00	0.00	0.34
GATCAACCTCATACCCAGAT	0.00	0.33	0.00	0.00	0.00
GATCAACCTCATACCCAGCT	0.23	0.33	0.00	0.00	0.34
GATCAACCTCATAGACCTTA	0.00	0.00	0.00	0.00	0.00
GATCAACCTCATAGACCTTG	1.37	0.00	0.00	0.00	0.00
GATCAACCTCATAGACTCCC	0.00	0.33	0.00	0.00	0.00
GATCAACCTCATATATGCCC	0.00	0.33	0.00	0.00	0.00
GATCAACCTCATATGACGGG	0.00	0.00	0.00	0.00	0.34
GATCAACCTCATCAAAAAAA	0.00	0.33	0.00	0.00	0.00
GATCAACCTCATCAAACGGA	0.00	0.00	0.00	0.00	0.69
GATCAACCTCATCACAAAAG	0.00	0.00	0.57	0.00	0.00
GATCAACCTCATCAGACGGG	0.00	0.33	0.00	0.00	0.00
GATCAACCTCATCAGGGAGG	0.00	0.00	0.00	0.34	0.00
GATCAACCTCATCATAAAGC	0.00	0.00	0.00	0.34	0.00
GATCAACCTCATCATCCAGA	0.00	0.00	0.00	0.00	0.69
GATCAACCTCATCATGAAGA	0.00	0.00	0.00	0.00	0.00
GATCAACCTCATCATGAAGC	19.69	24.07	16.61	30.21	17.91
GATCAACCTCATCATGAAGN	0.00	0.00	0.00	0.00	0.00
GATCAACCTCATCATGAANC	0.00	0.00	0.00	0.34	0.00
GATCAACCTCATCATGAANN	0.23	0.00	0.00	0.00	0.00
GATCAACCTCATCATGCAGC	0.00	0.00	0.00	0.00	0.00
GATCAACCTCATCATGNAGC	0.00	0.00	0.00	0.00	0.34
GATCAACCTCATCATGTAGC	0.00	0.00	0.00	0.00	0.00
GATCAACCTCATCATTCGTA	0.00	0.33	0.00	0.00	0.00

GATCAACCTCATCCACCAGG	0.46	0.00	0.00	0.00	0.00
GATCAACCTCATCCGACGGG	0.00	0.33	0.00	0.34	0.00
GATCAACCTCATCCGCCGCC	0.00	0.00	0.00	0.00	0.69
GATCAACCTCATCCTGAAGC	0.00	0.00	0.00	0.68	0.00
GATCAACCTCATCCTGGCGC	0.23	0.00	0.00	0.00	0.00
GATCAACCTCATCGCAGGTC	0.00	0.33	0.00	0.00	0.00
GATCAACCTCATCGCCAGCA	0.00	0.00	0.57	0.00	0.00
GATCAACCTCATCGCCTGGA	0.00	0.33	0.00	0.00	0.00
GATCAACCTCATCGCGAGGA	0.00	0.00	0.00	0.34	0.00
GATCAACCTCATCGGACGCG	0.00	0.00	0.29	0.00	0.00
GATCAACCTCATCGGACGGG	0.23	0.00	0.00	0.00	0.00
GATCAACCTCATCGGGCGGG	0.00	0.00	0.00	0.00	0.00
GATCAACCTCATCGGTGCGA	0.00	0.00	0.29	0.00	0.00
GATCAACCTCATCTAACAGG	0.00	0.00	0.00	0.00	0.34
GATCAACCTCATCTAACGGG	0.00	0.00	0.00	0.00	0.00
GATCAACCTCATCTCACGGG	0.00	0.00	0.29	0.00	0.00
GATCAACCTCATCTGAAAAA	0.00	0.00	0.29	0.00	0.00
GATCAACCTCATCTGAAGGA	0.00	0.00	0.29	0.00	0.00
GATCAACCTCATCTGAAGGG	0.00	0.00	0.00	0.00	0.69
GATCAACCTCATCTGACAAG	0.00	0.00	0.00	0.34	0.00
GATCAACCTCATCTGACAAT	0.00	0.00	0.29	0.00	0.00
GATCAACCTCATCTGACAGG	0.00	0.00	0.29	0.00	0.00
GATCAACCTCATCTGACATT	0.00	0.00	0.00	0.00	0.00
GATCAACCTCATCTGACCAC	0.00	0.00	0.00	0.00	0.34
GATCAACCTCATCTGACCCC	0.00	0.00	0.00	0.00	0.00
GATCAACCTCATCTGACCGG	0.00	0.00	0.00	0.34	0.00
GATCAACCTCATCTGACGAA	0.00	0.00	0.00	0.68	0.00
GATCAACCTCATCTGACGAG	0.00	0.33	0.00	0.00	0.34
GATCAACCTCATCTGACGAT	0.00	0.00	0.00	0.34	0.00
GATCAACCTCATCTGACGCG	0.00	0.33	0.00	0.00	0.00
GATCAACCTCATCTGACGGA	0.46	0.65	0.29	0.34	1.03
GATCAACCTCATCTGACGGC	0.00	0.00	0.29	0.00	0.00
GATCAACCTCATCTGACGGG	301.01	192.59	191.57	183.29	194.59
GATCAACCTCATCTGACGGN	0.69	0.00	0.00	0.00	0.34
GATCAACCTCATCTGACGGT	0.00	0.33	0.57	1.36	1.03
GATCAACCTCATCTGACGNN	0.69	0.00	0.00	0.00	0.00
GATCAACCTCATCTGACGTG	0.00	0.00	0.29	0.34	0.69

GATCAACCTCATCTGACNGG	0.00	0.00	0.00	0.00	0.34
GATCAACCTCATCTGACTCG	0.23	0.33	0.00	0.00	0.00
GATCAACCTCATCTGACTGG	0.23	0.33	0.00	0.34	0.00
GATCAACCTCATCTGACTNG	0.00	0.00	0.00	0.00	0.34
GATCAACCTCATCTGAGGGG	0.00	0.00	0.00	0.00	0.34
GATCAACCTCATCTGAGGNN	0.23	0.00	0.00	0.00	0.00
GATCAACCTCATCTGANGGG	0.00	0.00	0.00	0.34	0.34
GATCAACCTCATCTGATGAG	0.00	0.00	0.29	0.00	0.00
GATCAACCTCATCTGATGGG	0.00	0.00	0.29	0.00	0.00
GATCAACCTCATCTGCCGGG	0.23	0.33	0.00	0.00	0.00
GATCAACCTCATCTGCNGNN	0.23	0.00	0.00	0.00	0.00
GATCAACCTCATCTGCNNNN	0.46	0.00	0.00	0.00	0.00
GATCAACCTCATCTGGCAGT	0.00	0.00	0.00	0.00	0.00
GATCAACCTCATCTGGCGGA	0.00	0.00	0.00	0.34	0.00
GATCAACCTCATCTGGCGGG	0.46	0.33	0.00	0.34	0.00
GATCAACCTCATCTGGCTGA	0.00	0.00	0.00	0.00	0.69
GATCAACCTCATCTGGCTGG	0.00	0.00	0.00	0.00	0.34
GATCAACCTCATCTGTCGGG	0.00	0.00	0.00	0.00	0.00
GATCAACCTCATCTGTTCGG	0.00	0.00	0.00	0.00	0.00
GATCAACCTCATCTGTTTGC	0.00	0.00	0.00	0.00	0.34
GATCAACCTCATCTTACGCG	0.00	0.00	0.00	0.34	0.00
GATCAACCTCATCTTGAAGC	0.00	0.00	0.00	0.00	0.34
GATCAACCTCATGATGCGTC	0.00	0.00	0.00	0.34	0.00
GATCAACCTCATGCCCGCGC	0.00	0.65	0.29	1.36	0.00
GATCAACCTCATGCCCGCGT	0.00	0.00	0.00	0.00	0.00
GATCAACCTCATGCTGCAGG	0.00	0.33	0.00	0.00	0.00
GATCAACCTCATGGTCTATG	0.00	0.00	0.00	0.00	0.34
GATCAACCTCATGTGGCGTA	0.00	0.00	0.29	0.00	0.00
GATCAACCTCATTCGACGGG	0.00	0.33	0.57	0.34	0.00
GATCAACCTCATTGACTCCC	0.23	1.30	0.00	1.02	1.38
GATCAACCTCATTGGCTTCT	0.46	0.00	0.29	0.34	0.34
GATCAACCTCATTGGCTTGT	0.00	0.00	0.00	0.00	0.00
GATCAACCTCATTGGGTCAG	0.00	0.00	0.00	0.68	0.00
GATCAACCTCATTTGACGGG	0.00	0.33	0.00	0.00	0.00
GATCAACCTCATTTGCGAAG	0.00	0.00	0.00	0.00	0.00
GATCAACCTCATTTGGCGCA	0.00	0.00	0.29	0.00	0.00
GATCAACCTCCAACGGCTGC	0.00	0.00	0.29	0.00	0.00

GATCAACCTCCAAGTGTGCA	0.00	0.00	0.00	0.00	0.00
GATCAACCTCCACAAATTCG	0.00	0.00	0.00	0.34	0.00
GATCAACCTCCACACGCAAA	0.00	0.00	0.57	0.00	0.00
GATCAACCTCCACAGGTCGT	0.00	0.00	0.00	0.34	0.00
GATCAACCTCCACCGTGTGC	0.00	0.00	0.00	0.68	0.00
GATCAACCTCCACGACACAC	0.00	0.00	0.00	0.34	0.00
GATCAACCTCCACGACGCAC	2.29	0.98	1.15	2.04	0.34
GATCAACCTCCACGAGATGA	0.00	0.00	0.00	0.00	0.00
GATCAACCTCCACGGCGAAC	0.00	0.00	0.00	0.00	0.00
GATCAACCTCCACGGCGCAC	3.66	1.30	2.58	1.36	3.79
GATCAACCTCCACGGCGCCC	0.00	0.00	0.00	0.00	0.00
GATCAACCTCCACGGCGCTC	0.23	0.00	0.00	0.00	0.00
GATCAACCTCCACGGGGGGC	0.00	0.00	0.00	0.00	0.00
GATCAACCTCCACTCCTCCT	0.00	0.00	0.29	0.68	0.00
GATCAACCTCCACTTCTGAT	0.00	0.33	0.00	0.00	0.00
GATCAACCTCCAGCACGGCC	0.23	0.00	0.00	0.00	0.00
GATCAACCTCCAGCGCGGGG	0.00	0.33	0.00	0.00	0.00
GATCAACCTCCAGCGCTACC	0.23	0.00	0.00	0.00	0.00
GATCAACCTCCAGCTCGCCT	0.46	0.00	0.00	0.00	0.00
GATCAACCTCCAGGTGAGGG	0.00	0.00	0.00	0.00	0.00
GATCAACCTCCCACTGCGAT	0.00	0.00	0.29	0.00	0.00
GATCAACCTCCCCAGGCGCT	0.00	0.00	0.00	0.00	0.00
GATCAACCTCCCCCTCCCCT	0.23	0.33	0.00	0.00	0.00
GATCAACCTCCCCGCTCAGC	0.00	0.00	0.00	0.34	0.00
GATCAACCTCCCCTCAATAA	0.00	0.00	0.00	0.00	0.34
GATCAACCTCCCGAAGGAGA	0.00	0.00	0.00	0.00	0.00
GATCAACCTCCCGACATTGA	0.23	0.00	0.00	0.00	0.00
GATCAACCTCCCGGCAGGCG	0.00	0.00	0.00	0.00	0.00
GATCAACCTCCCTGCAACGT	0.00	0.33	0.29	0.00	0.00
GATCAACCTCCGAGCACAGA	0.00	0.00	0.00	0.00	0.34
GATCAACCTCCGAGTCCTGG	0.00	0.33	0.29	0.00	0.00
GATCAACCTCCGCCAACTGA	0.00	0.00	0.00	0.00	0.34
GATCAACCTCCGCTGGGCCA	0.00	0.00	0.00	0.00	0.00
GATCAACCTCCGGCGCTGGA	0.00	0.00	0.00	0.00	0.00
GATCAACCTCCGGGGCCGTG	0.00	0.00	0.00	0.34	0.00
GATCAACCTCCGTGCAGACT	0.00	0.33	0.00	0.00	0.00
GATCAACCTCCTCAAGATGG	0.46	0.00	0.00	0.00	0.34

GATCAACCTCCTCATCATTG	0.00	0.00	0.29	0.00	0.00
GATCAACCTCCTCATGAAGC	0.00	0.00	0.00	0.00	0.00
GATCAACCTCCTCCACAGTG	0.00	0.00	0.00	0.00	0.34
GATCAACCTCCTCCAGGAGA	0.00	0.00	0.00	0.00	0.69
GATCAACCTCCTCCCAATCC	0.00	0.00	0.29	0.00	0.00
GATCAACCTCCTCCCGTACC	0.00	0.00	0.57	1.02	0.00
GATCAACCTCCTCCGACCAA	0.00	0.00	0.00	0.00	0.34
GATCAACCTCCTCCGCCGTC	0.00	0.00	0.57	0.00	0.00
GATCAACCTCCTCCGCGATG	0.00	0.00	0.00	0.00	0.34
GATCAACCTCCTCCTCAGCG	0.00	0.33	0.00	0.00	0.00
GATCAACCTCCTCCTCCGCT	0.00	0.00	0.29	0.00	0.00
GATCAACCTCCTCGGAAGGA	0.00	0.00	0.29	0.00	0.00
GATCAACCTCCTCTATGTCC	0.00	0.00	0.00	0.00	0.00
GATCAACCTCCTCTCCGCCA	0.00	0.33	0.00	0.00	0.00
GATCAACCTCCTCTGACGGG	0.46	0.33	0.00	0.00	0.00
GATCAACCTCCTCTGCCGGG	0.23	0.00	0.00	0.00	0.00
GATCAACCTCCTCTGGCGGG	0.23	0.00	0.00	0.00	0.00
GATCAACCTCCTCTTCCTGG	0.00	0.00	0.00	0.34	0.00
GATCAACCTCCTCTTGCTGC	0.00	0.00	0.29	0.00	0.00
GATCAACCTCCTGAGGCACA	0.00	1.63	0.00	0.34	0.69
GATCAACCTCCTGCACCCTC	0.00	0.00	0.00	0.00	0.00
GATCAACCTCCTGGCCAAGG	0.00	0.00	0.00	0.00	0.00
GATCAACCTCCTGGCCTGCC	0.00	0.65	0.00	1.70	0.34
GATCAACCTCCTGTTGCCCG	0.00	0.00	0.00	0.00	0.00
GATCAACCTCCTTCACAGCG	0.00	0.33	0.00	0.00	0.00
GATCAACCTCCTTCACAGTG	1.14	3.58	3.44	3.05	3.44
GATCAACCTCCTTCACAGTT	0.00	0.00	0.00	0.00	0.69
GATCAACCTCCTTCACCGTG	0.00	0.00	0.00	0.00	0.00
GATCAACCTCCTTCATAGTG	0.23	0.00	0.00	0.00	0.00
GATCAACCTCCTTGGCCTTC	0.00	0.33	0.00	0.34	0.00
GATCAACCTCCTTTGCCCTA	0.00	0.00	0.00	0.00	0.00
GATCAACCTCGAATCCCAGA	0.00	0.00	0.00	0.00	0.00
GATCAACCTCGACAGCTGCC	0.00	0.00	0.00	1.70	0.00
GATCAACCTCGACAGGTTCC	0.00	0.00	0.00	0.00	0.34
GATCAACCTCGACCTCGAGC	0.46	0.00	0.00	0.00	0.00
GATCAACCTCGACGTCCTGC	0.23	0.00	0.00	0.00	0.00
GATCAACCTCGACGTCCTGN	0.00	0.00	0.00	0.00	0.00

GATCAACCTCGACGTCNNNN	0.23	0.00	0.00	0.00	0.00
GATCAACCTCGACTGCAACT	0.00	0.33	0.00	0.34	0.34
GATCAACCTCGACTGGAGAG	0.00	0.33	0.00	0.00	0.00
GATCAACCTCGAGAACCCGG	0.00	0.00	0.00	0.00	0.34
GATCAACCTCGAGCAGAGGA	1.83	1.30	0.00	0.68	0.00
GATCAACCTCGAGCCCTTCT	0.00	0.33	0.00	0.34	0.34
GATCAACCTCGAGGACCTCC	0.00	0.33	0.57	1.02	0.34
GATCAACCTCGAGGAGGTGC	0.23	0.00	0.00	0.34	0.00
GATCAACCTCGAGGCCATCC	0.00	0.00	0.00	0.00	0.00
GATCAACCTCGATGAGCAAC	0.00	0.00	0.00	0.00	0.34
GATCAACCTCGATGAGTTGG	4.12	3.90	3.44	3.73	6.20
GATCAACCTCGATGTGTTGG	0.00	0.33	0.00	0.00	0.00
GATCAACCTCGCAATGCCCG	2.29	7.81	2.29	9.50	4.13
GATCAACCTCGCCAGGCTGT	0.46	0.00	0.00	0.00	0.00
GATCAACCTCGCCGCCTGGA	0.00	0.33	0.00	0.00	0.00
GATCAACCTCGCCGGGAACG	2.52	1.63	0.86	0.68	0.69
GATCAACCTCGCCGGGGACG	0.23	0.00	0.00	0.00	0.00
GATCAACCTCGCCTCCGCCG	0.00	0.00	0.00	0.00	0.34
GATCAACCTCGCGAAGCCGG	0.00	0.00	0.00	0.00	0.34
GATCAACCTCGCGAAGCTGG	0.23	0.00	0.00	0.00	0.34
GATCAACCTCGCGCACCATT	0.00	0.00	0.00	0.00	0.34
GATCAACCTCGCGCAGCACC	0.00	0.00	0.00	0.34	0.00
GATCAACCTCGCGCCGCAGG	0.69	0.00	0.00	0.00	0.00
GATCAACCTCGCGCTTGAGT	0.00	0.00	0.00	0.00	0.00
GATCAACCTCGCGGTATGAC	0.00	0.00	0.00	0.00	0.00
GATCAACCTCGCGGTCATGG	0.00	0.00	0.00	0.34	0.00
GATCAACCTCGCTGATAACT	0.00	0.00	0.57	0.00	0.00
GATCAACCTCGGCAACGAAT	2.06	0.33	0.57	0.00	0.34
GATCAACCTCGGCAACGGAT	0.23	0.00	0.00	0.00	0.00
GATCAACCTCGGCGACGCGG	0.00	0.00	0.00	0.00	0.00
GATCAACCTCGGCGAGGAGC	0.00	0.00	0.00	1.02	0.00
GATCAACCTCGGCTACCGCT	0.69	0.00	0.00	0.00	0.00
GATCAACCTCGGGAGCAACG	0.00	0.00	0.00	0.34	0.00
GATCAACCTCGGGGGCCGTG	0.00	0.00	0.29	0.00	0.00
GATCAACCTCGGTCTAAAAA	0.23	0.00	0.00	0.00	0.00
GATCAACCTCGGTCTACCCT	0.92	1.95	1.15	0.68	0.00
GATCAACCTCGTATGCCGTC	0.00	0.00	0.00	0.00	0.69

GATCAACCTCGTCACGCGCA	0.00	0.00	0.00	0.00	0.00
GATCAACCTCGTCGCACGCG	1.37	0.00	0.86	1.02	0.00
GATCAACCTCGTCGCCCCCG	0.00	0.00	0.00	0.00	0.00
GATCAACCTCGTCTCCTGGG	0.92	3.58	1.15	2.72	1.03
GATCAACCTCGTCTGACGGG	0.23	0.00	0.00	0.00	0.00
GATCAACCTCGTCTTCAACA	0.69	0.00	0.00	0.00	0.00
GATCAACCTCGTCTTCTGGG	0.00	0.00	0.00	0.00	0.34
GATCAACCTCGTGAGGTATA	0.00	0.00	0.00	0.00	0.00
GATCAACCTCGTGCCCGTGC	0.00	0.00	0.00	0.34	0.00
GATCAACCTCGTGCGAACGC	0.00	0.00	0.29	0.00	0.00
GATCAACCTCGTGCTGGGCA	0.23	0.00	0.00	0.00	0.00
GATCAACCTCGTGGACATGC	0.00	0.00	0.00	0.00	0.34
GATCAACCTCGTGGAGCATG	0.00	0.33	0.00	0.00	0.00
GATCAACCTCGTGGCAGGCC	0.00	0.00	0.00	0.34	0.00
GATCAACCTCGTGGCAGGGC	0.00	0.00	0.00	0.34	0.00
GATCAACCTCGTGGCGCATC	0.69	0.98	0.57	0.68	0.34
GATCAACCTCGTGGCGCCAC	0.00	0.00	0.00	0.00	0.00
GATCAACCTCGTGGCGGGCC	1.14	2.28	0.29	2.38	0.69
GATCAACCTCGTGGCGGGGC	0.46	0.00	0.00	0.68	0.34
GATCAACCTCGTGGGAGCAG	0.00	0.00	0.29	0.00	0.00
GATCAACCTCGTGGGCACCG	0.00	0.00	0.00	1.02	0.00
GATCAACCTCGTGGGGGGAG	0.00	0.00	0.29	0.00	0.00
GATCAACCTCTACGAATGGG	0.00	0.00	0.00	0.34	0.00
GATCAACCTCTACGGCTTCC	0.00	0.00	0.29	0.00	0.00
GATCAACCTCTACGGGACCA	0.00	0.00	0.00	0.00	0.00
GATCAACCTCTACGGGACCT	0.46	0.33	0.00	0.00	0.34
GATCAACCTCTACGGGACGG	0.00	0.33	0.00	0.00	0.00
GATCAACCTCTACGGGACGT	0.23	0.33	0.86	0.00	0.34
GATCAACCTCTACGTCAACC	0.46	0.00	0.00	0.00	0.00
GATCAACCTCTAGTGAGGCC	0.23	0.00	0.00	0.00	0.00
GATCAACCTCTATGCAGGCC	0.23	0.00	0.00	0.00	0.34
GATCAACCTCTCACACGGGC	0.00	0.33	0.00	0.00	0.00
GATCAACCTCTCAGCGGTGA	0.00	0.00	0.29	0.00	0.00
GATCAACCTCTCAGTGACAG	0.00	0.00	0.00	0.00	0.00
GATCAACCTCTCCAGGCAGT	0.00	0.00	0.29	0.00	0.00
GATCAACCTCTCCCACCCCT	0.00	0.00	0.29	0.00	0.00
GATCAACCTCTCCCCCTTCA	0.23	0.00	0.00	0.00	0.00

GATCAACCTCTCCGACGGGC	0.00	0.00	0.00	0.00	0.00
GATCAACCTCTCCGAGCCCA	0.46	0.00	0.00	0.00	0.00
GATCAACCTCTCCGCCTGGA	4.12	4.55	1.43	3.73	2.07
GATCAACCTCTCCGCCTGGC	0.23	0.00	0.00	0.00	0.00
GATCAACCTCTCCGCCTGGG	0.23	0.00	0.00	0.00	0.00
GATCAACCTCTCCGGGACCA	0.00	0.00	0.00	0.00	0.34
GATCAACCTCTCCGTGAATG	1.14	0.00	0.00	0.00	0.00
GATCAACCTCTCCTCGGCGG	0.00	0.00	0.00	0.34	0.00
GATCAACCTCTCCTGGAATC	0.00	0.00	0.00	0.00	0.00
GATCAACCTCTCGCCCTTCA	0.23	0.00	0.00	0.68	0.00
GATCAACCTCTCGCTCTCGG	0.00	0.00	0.00	0.68	0.00
GATCAACCTCTCTGCTGCAA	0.00	0.00	0.00	0.00	0.00
GATCAACCTCTCTGGATGGA	0.00	0.00	0.00	0.00	1.03
GATCAACCTCTCTGTAAAGC	0.00	0.00	0.29	0.00	0.00
GATCAACCTCTGAGCCGAGA	0.00	0.33	0.00	0.00	0.00
GATCAACCTCTGAGGCGCTG	0.23	0.00	0.00	0.00	0.00
GATCAACCTCTGCATTTGTT	0.00	0.00	0.00	0.00	0.00
GATCAACCTCTGCCAGATGA	0.00	0.33	0.29	0.00	0.00
GATCAACCTCTGCCTTGCGG	0.00	0.33	0.29	0.00	0.00
GATCAACCTCTGCCTTTGAG	0.00	0.00	0.00	1.36	0.34
GATCAACCTCTGCGCAGCGC	0.00	0.00	0.00	0.34	0.00
GATCAACCTCTGCGGACGCC	0.00	0.33	0.00	0.00	0.00
GATCAACCTCTGCGGCCGCC	2.52	1.30	0.29	1.70	0.69
GATCAACCTCTGCGTCCGCG	0.23	0.00	0.00	0.00	0.00
GATCAACCTCTGCTTCAGGC	0.00	0.00	0.00	0.00	0.00
GATCAACCTCTGGAAATTAG	0.00	0.00	0.29	0.34	0.00
GATCAACCTCTGGCATCGCA	0.00	0.00	0.00	0.00	0.34
GATCAACCTCTGGCGCCGTG	0.23	0.33	0.29	0.00	0.00
GATCAACCTCTGGGACCGTG	0.00	0.00	0.00	0.34	0.00
GATCAACCTCTGGGACTGTG	0.00	0.00	0.57	0.00	0.00
GATCAACCTCTGGGCCCACG	0.00	0.00	0.00	0.00	0.00
GATCAACCTCTGGGCCCGTG	0.00	0.00	0.00	0.34	0.00
GATCAACCTCTGGGCCGTGG	0.00	0.00	0.00	0.00	0.00
GATCAACCTCTGGGGCAGTG	0.46	0.65	0.29	0.68	0.69
GATCAACCTCTGGGGCCGAG	0.00	0.00	0.00	0.00	0.00
GATCAACCTCTGGGGCCGCG	0.69	0.00	0.29	0.34	1.38
GATCAACCTCTGGGGCCGGG	0.92	0.33	0.29	0.00	0.34

GATCAACCTCTGGGGCCGGT	0.00	0.65	0.00	0.00	0.00
GATCAACCTCTGGGGCCGNG	0.00	0.00	0.00	0.00	0.00
GATCAACCTCTGGGGCCGNN	0.23	0.00	0.00	0.00	0.00
GATCAACCTCTGGGGCCGTC	0.46	1.30	0.00	0.00	0.00
GATCAACCTCTGGGGCCGTG	104.15	120.70	91.92	95.38	108.1[illegible]
GATCAACCTCTGGGGCCGTN	0.69	0.00	0.00	0.00	0.00
GATCAACCTCTGGGGCCGTT	0.69	0.65	0.29	0.00	0.00
GATCAACCTCTGGGGCGGGG	0.23	0.00	0.00	0.00	0.00
GATCAACCTCTGGGGCGGTG	0.46	0.00	0.00	0.00	0.00
GATCAACCTCTGGGGCNNNN	0.46	0.00	0.00	0.00	0.00
GATCAACCTCTGGGGCTGTG	0.00	0.33	0.29	0.34	0.34
GATCAACCTCTGGGGGCGCG	0.00	0.00	0.29	0.00	0.00
GATCAACCTCTGGGGGCGTG	0.00	0.33	0.00	0.00	0.00
GATCAACCTCTGGGGTCGGG	0.23	0.00	0.00	0.00	0.00
GATCAACCTCTGGGGTCGTG	0.00	0.33	0.00	0.00	0.00
GATCAACCTCTGGTGCCTCA	0.00	0.00	0.29	0.00	0.00
GATCAACCTCTGGTTCCGTG	0.00	0.00	0.00	0.00	0.34
GATCAACCTCTGTGCACTGC	0.00	0.00	0.00	0.34	0.00
GATCAACCTCTGTGCAGCAC	0.00	0.33	0.00	0.00	0.00
GATCAACCTCTGTGCAGCCC	6.41	8.78	2.58	2.04	5.17
GATCAACCTCTGTGGCCGTG	0.00	0.33	0.00	0.00	0.00
GATCAACCTCTGTGGGCGCG	0.00	0.00	0.00	0.00	0.00
GATCAACCTCTTCAACGACC	0.46	0.65	0.29	0.68	0.00
GATCAACCTCTTCAAGGACC	0.00	0.00	0.00	0.00	0.00
GATCAACCTCTTCATCCAGA	0.69	0.00	0.00	0.00	0.00
GATCAACCTCTTCATCCTTC	0.00	0.00	0.00	0.00	0.00
GATCAACCTCTTCATGAAGC	0.23	0.00	0.00	0.00	0.00
GATCAACCTCTTCCTGCAGG	0.00	0.00	0.00	0.00	0.00
GATCAACCTCTTCGAATCAT	0.46	0.33	0.00	0.00	0.00
GATCAACCTCTTCGAATCNT	0.00	0.00	0.00	0.00	0.00
GATCAACCTCTTCGAGGCCG	1.14	2.93	0.57	2.04	0.69
GATCAACCTCTTCGATGTGG	0.00	0.33	0.00	0.00	0.00
GATCAACCTCTTCGCCGTTC	0.00	0.00	0.00	0.00	0.34
GATCAACCTCTTCTGATATT	1.37	0.33	0.57	0.00	0.00
GATCAACCTCTTCTTGCACC	0.23	0.00	0.00	0.00	0.00
GATCAACCTCTTCTTGCCGC	0.23	0.00	0.00	0.00	0.00
GATCAACCTCTTCTTGCTGA	0.00	0.00	0.00	0.34	0.00

GATCAACCTCTTCTTGCTGC	27.93	35.79	28.06	28.51	31.34
GATCAACCTCTTCTTGCTGN	0.69	0.00	0.00	0.00	0.00
GATCAACCTCTTCTTGCTGT	0.00	0.00	0.00	0.34	0.00
GATCAACCTCTTCTTGCTTC	0.00	0.00	0.00	0.34	0.00
GATCAACCTCTTCTTGTTGA	0.00	0.33	0.00	0.00	0.00
GATCAACCTCTTCTTGTTGC	0.00	0.33	0.00	0.00	0.00
GATCAACCTCTTCTTTCTGC	0.00	0.00	0.00	0.34	0.00
GATCAACCTCTTGCGCGCAG	0.00	0.00	0.29	0.00	0.00
GATCAACCTCTTGCTCCACA	0.00	0.00	0.00	0.00	0.00
GATCAACCTCTTGTGGAAGG	0.46	0.00	0.00	0.00	0.00
GATCAACCTCTTTAATGACC	0.69	0.33	0.00	0.00	0.00
GATCAACCTCTTTCCCACAC	0.00	0.00	0.00	0.34	0.00
GATCAACCTCTTTGCAAAGG	0.00	0.00	0.00	0.00	0.00
GATCAACCTCTTTGTGAAGG	0.00	0.00	0.00	0.00	0.69
GATCAACCTGAAAAAGACGA	0.00	0.00	0.00	0.00	0.00
GATCAACCTGAAAGACAAAA	0.00	0.00	0.00	0.00	0.00
GATCAACCTGAAAGCACGCC	0.00	0.33	0.00	0.00	0.00
GATCAACCTGAAATGCAGAA	0.00	0.65	0.00	0.00	0.00
GATCAACCTGAACACGAAAC	0.00	0.00	0.29	0.00	0.00
GATCAACCTGAACCTCTGGA	0.00	0.33	0.00	0.00	0.00
GATCAACCTGAACGACTTCA	0.00	0.00	0.29	0.00	0.00
GATCAACCTGAACGTGAACA	0.23	0.00	0.00	0.00	0.00
GATCAACCTGAACTGCCGGG	0.00	0.00	0.00	0.34	0.00
GATCAACCTGAACTGGCGTT	0.00	0.33	0.00	0.00	0.00
GATCAACCTGAACTGGGTGC	0.00	0.00	0.00	0.00	0.00
GATCAACCTGAAGACCTTCC	0.00	0.00	0.00	0.00	0.00
GATCAACCTGAAGCTCCAGG	0.00	0.00	0.00	0.00	0.00
GATCAACCTGAAGTTTGGGG	0.00	0.00	0.00	0.00	0.00
GATCAACCTGAATCTCCGCA	0.00	0.33	0.00	0.00	0.00
GATCAACCTGACACAGACGA	0.69	0.00	0.00	0.68	0.34
GATCAACCTGACACTCCTCT	0.23	0.00	0.00	0.00	0.00
GATCAACCTGACCAAAGTCA	0.00	0.33	0.57	0.00	1.72
GATCAACCTGACCAGCATCA	0.00	0.00	0.00	0.00	0.00
GATCAACCTGACCATGTCCT	0.00	0.00	0.00	0.00	0.00
GATCAACCTGACCGACNTCC	0.00	0.00	0.00	0.00	0.00
GATCAACCTGACCGACTTCC	4.58	11.06	6.01	7.47	5.17
GATCAACCTGACCGAGTTCC	0.00	0.00	0.00	0.34	0.00

GATCAACCTGACCGCCCAGA	0.00	0.00	0.00	0.00	0.34
GATCAACCTGACGCACAAGG	0.00	0.00	0.00	0.34	0.00
GATCAACCTGACGCAGTACC	0.00	0.33	0.00	0.34	0.00
GATCAACCTGACGTACGAGC	0.00	0.00	0.00	1.02	0.00
GATCAACCTGACTGGGTGGC	0.00	0.33	0.00	0.00	0.00
GATCAACCTGACTTGAATGC	0.00	0.00	0.00	0.00	0.00
GATCAACCTGAGAACTGCGG	0.00	0.33	0.00	0.00	0.00
GATCAACCTGAGAACTGGGG	0.23	0.00	0.00	0.00	0.00
GATCAACCTGAGAACTGTGG	13.96	10.41	5.15	10.86	5.17
GATCAACCTGAGATCGCACC	0.00	0.33	0.00	0.00	0.00
GATCAACCTGAGCATCATTG	0.00	0.00	0.00	0.00	0.00
GATCAACCTGAGCCTCTGGA	5.95	22.77	12.31	20.71	16.53
GATCAACCTGAGCCTCTGGT	0.23	0.00	0.00	0.00	0.00
GATCAACCTGAGCGGCTACA	0.00	0.00	0.00	0.00	0.34
GATCAACCTGAGGACCTGCT	0.00	0.00	0.00	0.00	0.00
GATCAACCTGAGGACGAACA	0.00	0.00	0.00	0.00	0.00
GATCAACCTGAGGACGCACC	0.00	0.00	0.00	0.00	0.00
GATCAACCTGAGGGAGGGGG	0.00	0.00	0.00	0.34	0.00
GATCAACCTGAGGGAGGGTG	1.14	0.33	0.00	0.34	0.00
GATCAACCTGAGGGCGCACC	0.00	0.00	0.00	0.00	0.00
GATCAACCTGAGGTCGCAAC	0.00	0.00	0.29	0.00	0.00
GATCAACCTGAGGTCGCACC	5.26	9.11	14.03	12.56	11.37
GATCAACCTGAGGTCGCACN	0.23	0.00	0.00	0.00	0.00
GATCAACCTGAGGTCGCCAC	0.00	0.00	0.00	0.00	0.34
GATCAACCTGAGGTCGCTCG	0.00	0.00	0.00	0.34	0.00
GATCAACCTGAGGTGGCCGC	0.00	0.00	0.29	0.00	0.00
GATCAACCTGAGGTGGNACC	0.00	0.00	0.00	0.00	0.00
GATCAACCTGAGGTTGCACC	0.00	0.00	0.00	0.00	0.69
GATCAACCTGAGGTTGTGAG	1.37	2.60	0.00	2.04	1.38
GATCAACCTGAGGTTGTGCG	0.00	0.33	0.00	0.00	0.00
GATCAACCTGAGTCGTATGC	0.00	0.33	0.00	0.00	0.00
GATCAACCTGAGTGCCGCCT	0.00	0.33	0.00	0.00	0.00
GATCAACCTGAGTTAAAAAA	0.00	0.33	0.00	0.00	0.00
GATCAACCTGAGTTATCGAG	0.00	0.00	0.29	0.34	0.69
GATCAACCTGAGTTATCGAT	0.00	0.00	0.00	0.00	0.00
GATCAACCTGATACAATTTA	0.00	0.00	0.00	0.00	0.34
GATCAACCTGATACGACTCG	0.23	0.00	0.00	0.00	0.00

GATCAACCTGATATCCCACT	2.75	0.00	0.00	0.68	0.69
GATCAACCTGATCTTCTTGC	0.00	0.00	0.00	0.00	0.00
GATCAACCTGATGCCTGGCA	0.00	0.33	0.00	0.00	0.00
GATCAACCTGATGTTCGGGG	0.00	0.33	0.00	0.00	0.00
GATCAACCTGCAAAAAAAAA	0.00	0.00	0.00	0.00	0.00
GATCAACCTGCAAAGAGGGT	0.23	0.00	0.00	0.00	0.00
GATCAACCTGCAAAGAGGTT	0.46	0.00	0.00	0.00	0.00
GATCAACCTGCAAGGACATG	0.23	0.00	0.00	0.00	0.00
GATCAACCTGCACACTCTGT	0.00	0.00	0.29	0.00	0.00
GATCAACCTGCACCAGCCCC	0.00	0.00	0.00	0.00	0.00
GATCAACCTGCACGAAGAAG	0.00	0.00	0.00	0.00	0.00
GATCAACCTGCACGGCATCG	0.00	0.00	0.00	0.34	0.00
GATCAACCTGCACTTCTGCA	0.00	0.33	0.00	0.00	0.00
GATCAACCTGCACTTGCGCG	0.00	0.00	0.29	0.00	0.34
GATCAACCTGCACTTGTCAG	0.23	0.33	0.29	1.36	0.00
GATCAACCTGCAGACATAGC	0.00	0.00	0.00	0.00	0.00
GATCAACCTGCAGCAGAAGA	0.23	0.00	0.00	0.00	0.00
GATCAACCTGCAGCAGGCCA	0.00	0.00	0.00	0.00	0.00
GATCAACCTGCAGCCACTGA	0.00	0.00	0.00	0.68	0.00
GATCAACCTGCAGCCACTGG	1.37	1.63	0.29	1.02	0.00
GATCAACCTGCAGGAGCACA	0.46	0.00	0.00	0.00	0.34
GATCAACCTGCAGGAGCACC	0.00	0.33	0.00	0.00	0.00
GATCAACCTGCAGGGCTACA	0.00	0.00	0.00	0.00	0.00
GATCAACCTGCAGGGTCGAT	0.00	0.00	0.00	0.34	0.00
GATCAACCTGCAGGTTTACG	0.46	0.00	0.00	0.00	0.00
GATCAACCTGCATCGATTCA	0.00	0.00	0.00	0.00	0.00
GATCAACCTGCCACTTCCAC	0.00	0.00	0.57	0.00	0.00
GATCAACCTGCCATGCGTTG	0.00	0.33	0.00	0.00	0.00
GATCAACCTGCCCACTGCCT	0.00	0.00	0.29	0.00	0.00
GATCAACCTGCCCACTTTCC	0.00	0.00	0.00	0.00	0.34
GATCAACCTGCCCTTCCTGG	0.00	0.33	0.00	0.00	0.00
GATCAACCTGCCGAGCGTGT	0.00	0.00	0.00	0.00	0.00
GATCAACCTGCCGAGGCCTA	0.00	0.00	0.00	0.00	0.00
GATCAACCTGCCGATGAGTC	0.23	0.00	0.00	0.00	0.00
GATCAACCTGCCGCCGAAGC	0.00	0.00	0.00	0.00	0.00
GATCAACCTGCCGCGCATGT	0.00	0.65	0.00	0.34	0.00
GATCAACCTGCCGCGCGTGT	0.00	0.33	0.00	0.34	0.34

GATCAACCTGCCGGATGGAG	0.46	0.33	0.29	0.00	0.69
GATCAACCTGCCGGATGGGG	0.23	0.00	0.00	0.00	0.00
GATCAACCTGCCGTCCATGC	0.23	0.00	0.00	0.00	0.00
GATCAACCTGCCGTCTGAGT	0.46	0.00	0.00	0.00	0.00
GATCAACCTGCCGTTGAAAA	0.00	0.00	0.57	0.00	0.00
GATCAACCTGCCTCGTGGCT	0.00	0.00	0.00	0.00	0.34
GATCAACCTGCCTGGCTGTG	0.46	0.00	0.00	0.00	0.00
GATCAACCTGCCTTGAATTC	0.00	0.33	0.00	0.00	0.00
GATCAACCTGCCTTGGCACC	0.00	0.00	0.00	0.00	0.00
GATCAACCTGCCTTGGCAGG	0.23	0.00	0.00	0.00	0.00
GATCAACCTGCGACCCCATT	0.00	0.33	0.00	0.00	0.00
GATCAACCTGCGAGTAGGTA	0.00	0.00	0.00	0.00	0.34
GATCAACCTGCGCAACTAGC	0.00	0.00	0.29	0.00	0.00
GATCAACCTGCGCATCTGGG	0.00	0.33	0.00	0.00	0.00
GATCAACCTGCGCCTCTGGA	0.00	0.00	0.29	0.00	0.00
GATCAACCTGCGCGCAGAGG	0.00	0.00	0.29	0.34	0.00
GATCAACCTGCGGCAATTGG	0.00	0.33	0.00	0.00	0.00
GATCAACCTGCGGCGGGACC	0.00	0.00	0.00	0.00	0.00
GATCAACCTGCGGCGGTACC	0.23	0.00	0.00	0.00	0.00
GATCAACCTGCGTACGCAGC	0.00	0.00	0.29	0.34	0.00
GATCAACCTGCGTACGCTCA	0.00	0.00	0.29	0.00	0.00
GATCAACCTGCGTGAAGCTT	0.00	0.00	0.00	0.00	0.00
GATCAACCTGCGTTCATGCA	0.23	0.00	0.29	0.34	0.00
GATCAACCTGCTAAAAAAAA	0.23	0.00	0.29	0.00	0.34
GATCAACCTGCTAGCAGCAT	0.00	0.00	0.00	0.00	0.00
GATCAACCTGCTATTTCAAA	0.00	0.00	0.00	0.00	0.69
GATCAACCTGCTATTTCTCA	5.95	3.90	1.43	5.77	5.51
GATCAACCTGCTATTTCTNA	0.00	0.00	0.00	0.00	0.00
GATCAACCTGCTCAAGCTGT	0.00	0.33	0.00	0.00	0.00
GATCAACCTGCTCCACTTGT	0.00	0.00	0.57	0.34	0.34
GATCAACCTGCTCCGGGAGC	0.00	0.00	0.00	0.00	0.00
GATCAACCTGCTCGAAGAGT	0.00	0.00	0.00	0.00	0.34
GATCAACCTGCTCGAGACCG	0.00	0.65	0.00	0.00	0.00
GATCAACCTGCTCGCCGTGA	0.00	0.00	0.29	0.00	0.34
GATCAACCTGCTCGCGTGAG	0.00	0.00	0.00	0.34	0.00
GATCAACCTGCTCGGCCCGA	0.00	0.00	0.00	0.00	0.69
GATCAACCTGCTCGTACCNN	0.23	0.00	0.00	0.00	0.00

GATCAACCTGCTCGTATGCC	0.00	0.00	0.00	0.34	0.00
GATCAACCTGCTCTACTTGG	0.00	0.00	0.00	0.34	0.00
GATCAACCTGCTCTCCCTGG	0.00	0.00	0.00	0.34	0.00
GATCAACCTGCTCTTCATGG	0.00	0.00	0.29	0.00	0.34
GATCAACCTGCTCTTCATGT	0.00	0.33	0.00	0.00	0.00
GATCAACCTGCTCTTCCAAA	0.00	0.00	0.57	0.00	0.00
GATCAACCTGCTCTTCCAGG	0.00	0.33	0.00	0.00	0.00
GATCAACCTGCTCTTCCCGG	0.23	0.33	0.00	0.00	0.00
GATCAACCTGCTCTTCCGGG	0.00	0.33	0.29	0.00	0.34
GATCAACCTGCTCTTCCTCG	0.00	0.33	0.00	0.00	0.34
GATCAACCTGCTCTTCCTGA	0.00	0.00	0.00	0.34	0.00
GATCAACCTGCTCTTCCTGG	49.21	127.20	93.64	125.25	88.51
GATCAACCTGCTCTTCCTGN	0.23	0.00	0.00	0.00	0.00
GATCAACCTGCTCTTCCTGT	0.00	0.98	0.29	0.34	0.00
GATCAACCTGCTCTTCCTNN	0.23	0.00	0.00	0.00	0.00
GATCAACCTGCTCTTCCTTG	0.00	0.00	0.00	0.34	0.00
GATCAACCTGCTCTTCGTAT	0.00	0.00	0.00	0.00	0.34
GATCAACCTGCTCTTCGTGG	0.23	0.00	0.00	0.00	0.00
GATCAACCTGCTCTTCNNNN	0.23	0.00	0.00	0.00	0.00
GATCAACCTGCTCTTCNTGG	0.00	0.00	0.00	0.34	0.69
GATCAACCTGCTCTTCTGGG	0.00	0.00	0.00	0.34	0.00
GATCAACCTGCTCTTCTTCA	0.00	0.00	0.00	0.34	0.00
GATCAACCTGCTCTTCTTGC	0.00	0.33	0.00	0.00	0.00
GATCAACCTGCTCTTCTTGG	44.64	89.14	57.84	81.80	55.10
GATCAACCTGCTCTTCTTGT	0.00	0.65	0.57	0.00	0.00
GATCAACCTGCTCTTCTTNG	0.00	0.00	0.00	0.00	0.00
GATCAACCTGCTCTTCTTNN	0.00	0.00	0.00	0.00	0.00
GATCAACCTGCTCTTCTTTG	0.00	0.98	0.29	0.00	0.00
GATCAACCTGCTCTTCTTTT	0.00	0.00	0.00	0.34	0.00
GATCAACCTGCTGAATTTAA	0.00	0.00	0.00	0.00	0.00
GATCAACCTGCTGAATTTAT	0.00	0.00	0.00	0.00	0.00
GATCAACCTGCTGACCAGCC	0.00	0.33	0.00	0.00	0.00
GATCAACCTGCTGATACCTC	0.23	0.00	0.00	0.00	0.00
GATCAACCTGCTGATAGCGC	32.05	8.78	1.72	9.50	2.07
GATCAACCTGCTGATAGCGN	0.23	0.00	0.00	0.00	0.00
GATCAACCTGCTGATAGCTC	0.23	0.00	0.00	0.00	0.00
GATCAACCTGCTGCAGCGCT	0.00	0.33	0.00	0.34	0.00

GATCAACCTGCTGCATCAGC	0.00	0.00	0.00	0.34	0.00
GATCAACCTGCTGCATTGAA	0.00	0.00	0.00	0.34	0.00
GATCAACCTGCTGCGAAGTC	0.00	0.00	0.00	0.00	0.00
GATCAACCTGCTGCGCAAAC	0.00	0.00	0.00	0.00	0.34
GATCAACCTGCTGCTAGCGC	0.00	0.00	0.00	0.00	0.00
GATCAACCTGCTGCTCAAAA	0.00	0.00	0.00	0.00	0.00
GATCAACCTGCTGCTGTTGC	0.46	0.98	0.00	0.00	0.34
GATCAACCTGCTGCTGTTTC	0.00	0.00	0.00	0.34	0.00
GATCAACCTGCTGGACTCCG	0.00	0.00	0.00	0.00	0.00
GATCAACCTGCTGGGAAAAA	0.00	0.00	0.00	0.00	0.00
GATCAACCTGCTGGTCGTGC	0.00	0.00	0.00	0.34	0.00
GATCAACCTGCTGTTCCTGG	0.00	0.00	0.57	0.00	0.69
GATCAACCTGCTGTTCGTAT	0.00	0.00	0.00	0.68	0.00
GATCAACCTGCTGTTCTTGG	0.00	1.95	3.15	0.68	2.41
GATCAACCTGCTTCGTGAGC	0.00	0.00	0.00	0.00	0.34
GATCAACCTGCTTTTTCTCA	0.00	0.00	0.00	0.00	0.00
GATCAACCTGGAAAAAAAAA	0.23	0.00	0.00	0.00	0.00
GATCAACCTGGAAGCATGCC	0.23	0.00	0.00	0.00	0.00
GATCAACCTGGAATCCCCTG	0.23	0.00	0.00	0.00	0.00
GATCAACCTGGAATCTCTCG	0.00	0.00	0.00	0.00	0.34
GATCAACCTGGACAAAAAAA	0.00	0.00	0.29	0.00	0.00
GATCAACCTGGACATCACCC	0.00	0.65	0.00	0.00	0.00
GATCAACCTGGACATCTTCA	0.00	0.00	0.00	0.00	0.00
GATCAACCTGGACCGGTACT	0.00	0.33	0.00	0.00	0.00
GATCAACCTGGACGAGAGGA	0.00	0.00	0.00	0.00	0.00
GATCAACCTGGACGAGTTCA	0.00	0.00	0.00	0.00	0.00
GATCAACCTGGACTACAAGC	0.23	0.33	0.29	0.00	0.34
GATCAACCTGGAGAGCATAT	0.46	0.00	0.00	0.00	0.34
GATCAACCTGGAGCGGCATC	0.00	0.33	0.00	0.00	0.00
GATCAACCTGGAGCGTGTCA	0.00	0.00	0.00	0.00	0.34
GATCAACCTGGAGCTCTACG	1.60	0.00	0.00	0.00	0.00
GATCAACCTGGAGGTGTGTT	0.00	0.00	0.57	0.00	0.34
GATCAACCTGGAGTACCTGT	0.00	0.33	0.00	0.00	0.00
GATCAACCTGGAGTCCCGAA	0.00	0.00	0.29	0.34	0.00
GATCAACCTGGATGATGACC	0.00	0.00	0.00	0.00	0.00
GATCAACCTGGATGGCGTGG	0.00	0.33	0.00	0.00	0.00
GATCAACCTGGCAATCAACT	0.69	0.33	0.29	0.68	0.00

GATCAACCTGGCAGCACGGA	0.00	0.00	0.00	0.68	0.34
GATCAACCTGGCAGCACGNN	0.00	0.00	0.00	0.00	0.00
GATCAACCTGGCAGCCGGCC	0.00	0.00	0.29	0.00	0.00
GATCAACCTGGCAGTAGTTG	0.00	0.00	0.00	0.34	0.00
GATCAACCTGGCCACCACTG	0.00	0.00	0.00	0.00	0.00
GATCAACCTGGCCACCGCTT	0.92	0.98	0.00	0.00	0.00
GATCAACCTGGCCAGCATGT	0.00	0.33	0.00	0.00	0.00
GATCAACCTGGCCCAGAACG	0.00	0.00	0.00	0.34	0.00
GATCAACCTGGCCGAGCCAA	0.23	0.00	0.00	0.00	0.00
GATCAACCTGGCGACCGGCC	0.00	0.00	0.29	0.00	0.00
GATCAACCTGGCGATGAGTC	0.23	0.00	0.00	0.00	0.00
GATCAACCTGGCGATGGGGC	0.23	0.00	0.00	0.00	0.00
GATCAACCTGGCGCCGGTAG	0.23	0.00	0.00	0.00	0.00
GATCAACCTGGCGCGGGCGG	0.00	0.00	0.00	0.00	0.00
GATCAACCTGGCGGAAGTAA	0.00	0.00	0.00	0.34	0.00
GATCAACCTGGCGGCAGGCA	0.00	0.00	0.00	0.00	0.00
GATCAACCTGGCGGCCGGAC	0.23	0.33	0.00	0.00	0.69
GATCAACCTGGCGGCCGGCC	16.02	21.47	14.03	20.37	12.74
GATCAACCTGGCGGCCGGGC	0.46	0.00	0.29	0.34	0.00
GATCAACCTGGCGGCCGGNC	0.00	0.00	0.00	0.34	0.00
GATCAACCTGGCTACGCTAC	0.00	0.00	0.00	0.00	0.34
GATCAACCTGGCTCAAGGTC	0.00	0.00	0.00	0.00	0.00
GATCAACCTGGCTGCGCTGG	0.00	0.33	0.00	0.00	0.34
GATCAACCTGGCTTCTGCCA	0.00	0.00	0.29	0.00	0.00
GATCAACCTGGGAAAAAAAA	0.00	0.00	0.29	0.34	0.34
GATCAACCTGGGAGACGTCC	0.00	0.00	0.00	0.00	0.00
GATCAACCTGGGCAAAAAAA	0.00	0.00	0.00	0.00	1.03
GATCAACCTGGGCAAAGCAG	0.00	0.33	0.29	0.00	0.34
GATCAACCTGGGCAGACGAT	0.00	0.33	0.00	0.00	0.00
GATCAACCTGGGCATCTCAT	0.69	0.98	1.15	0.00	0.00
GATCAACCTGGGCCACTGTG	0.00	0.00	0.29	0.00	0.00
GATCAACCTGGGCCAGCTCG	0.00	0.00	0.00	0.00	0.34
GATCAACCTGGGCCATCGGC	0.00	0.00	0.29	0.00	0.00
GATCAACCTGGGCCGGGCCC	0.23	0.00	0.00	0.00	0.00
GATCAACCTGGGCCTCTGGA	0.00	0.00	0.00	0.68	0.00
GATCAACCTGGGCGGAAGAG	0.00	0.33	0.00	0.00	0.00
GATCAACCTGGGCGGAGCTG	0.00	0.00	0.29	0.00	0.00

GATCAACCTGGGCTCACATA	0.00	0.00	0.00	0.34	0.00
GATCAACCTGGGGAGGAAGA	0.00	0.33	0.00	0.00	0.00
GATCAACCTGGGGATAGGAC	1.83	0.00	0.29	0.00	0.00
GATCAACCTGGGGATATAGA	0.00	0.33	0.00	0.00	0.00
GATCAACCTGGGGATTGGGA	0.00	0.00	0.00	0.00	0.69
GATCAACCTGGGGCAAGCGC	0.00	0.00	0.00	0.00	0.00
GATCAACCTGGGGCCCTGCC	0.00	0.00	0.00	0.00	0.69
GATCAACCTGGGGCCGTGGT	0.00	0.00	0.00	0.34	0.00
GATCAACCTGGGGCGCATGA	0.23	0.00	0.00	0.00	0.00
GATCAACCTGGGGCGGAAGA	12.82	16.92	9.45	10.86	12.74
GATCAACCTGGGGCGGAAGC	0.23	0.00	0.00	0.00	0.00
GATCAACCTGGGGCGGAAGG	0.00	0.00	0.00	0.00	0.00
GATCAACCTGGGGCGGACGA	0.00	0.00	0.00	0.00	0.69
GATCAACCTGGGGCGGACGT	0.00	0.00	0.00	0.34	0.00
GATCAACCTGGGGCGGCAAG	0.00	0.98	0.00	0.00	0.00
GATCAACCTGGGGCGGCAGA	0.00	0.00	0.00	0.00	0.00
GATCAACCTGGGGCGGCGGA	0.23	0.00	0.00	0.00	0.00
GATCAACCTGGGGCGGGAGA	0.23	0.00	0.00	0.00	0.00
GATCAACCTGGGGCGGGAGG	0.23	0.00	0.00	0.00	0.00
GATCAACCTGGGGCGGTAGA	0.23	0.00	0.00	0.00	0.00
GATCAACCTGGGGGCCGGCC	0.00	0.33	0.00	0.00	0.00
GATCAACCTGGGGGCGTCCT	0.00	0.00	0.00	0.00	0.00
GATCAACCTGGGGGTAGGGC	0.23	0.00	0.00	0.00	0.00
GATCAACCTGGGGGTCAAAC	0.00	0.00	0.29	0.00	0.00
GATCAACCTGGGTAATCTCA	0.00	0.00	0.29	0.00	0.00
GATCAACCTGGGTCACTACG	0.00	0.33	0.00	0.00	0.00
GATCAACCTGGGTCTCTCTC	0.23	0.00	0.00	0.00	0.00
GATCAACCTGGTAGGAGCTG	0.00	0.00	0.00	0.34	0.00
GATCAACCTGGTAGGTACAC	0.00	0.00	0.00	0.00	0.69
GATCAACCTGGTATGGGTGT	0.00	0.00	0.00	0.00	0.34
GATCAACCTGGTCGGAGATG	0.00	0.00	0.00	0.34	0.00
GATCAACCTGGTCGGAGCCG	0.00	0.00	0.00	0.00	0.00
GATCAACCTGGTCGGAGCTA	0.00	0.00	0.00	0.00	0.00
GATCAACCTGGTCGGAGCTC	0.00	0.00	0.00	0.00	0.00
GATCAACCTGGTCGGAGCTG	8.01	25.38	25.77	29.87	22.04
GATCAACCTGGTCGGANCTG	0.00	0.00	0.00	0.00	0.34
GATCAACCTGGTCGGCATCC	0.00	0.00	0.00	0.00	0.00

GATCAACCTGGTCGGCGCTG	0.00	0.00	0.00	0.00	0.00
GATCAACCTGGTCTTCTTGG	0.00	0.00	0.00	0.00	0.00
GATCAACCTGGTGACGAGGC	0.00	0.00	0.00	0.00	0.00
GATCAACCTGGTGCGGGAAC	0.00	0.33	0.00	0.00	0.00
GATCAACCTGGTGGTCAACT	0.00	0.33	0.00	0.00	0.00
GATCAACCTGGTGTCGGGAA	0.00	0.33	0.00	0.00	0.00
GATCAACCTGGTGTCTGCTG	0.00	0.00	0.00	0.34	0.00
GATCAACCTGGTTACCAGCA	0.46	0.00	0.29	0.00	0.00
GATCAACCTGGTTGCCATTG	0.00	0.00	0.29	0.00	0.00
GATCAACCTGTACAAAAAAA	0.00	0.33	0.00	0.00	0.00
GATCAACCTGTACAAGAGCT	0.00	0.00	0.00	0.00	0.34
GATCAACCTGTACAAGTATT	0.00	0.98	0.00	0.00	0.34
GATCAACCTGTACAAGTGAT	0.23	0.00	0.00	0.00	0.00
GATCAACCTGTACAAGTGCG	0.00	0.00	0.57	0.00	0.00
GATCAACCTGTACAAGTGCT	4.81	3.58	8.02	10.18	6.54
GATCAACCTGTACACAGCAC	0.00	0.00	0.00	0.00	0.00
GATCAACCTGTACACATTCA	0.23	0.00	0.00	0.00	0.00
GATCAACCTGTACACGTGCT	0.00	0.00	0.00	0.00	0.00
GATCAACCTGTACTTCCACC	0.00	0.65	0.00	0.68	0.00
GATCAACCTGTAGAGGTTTG	0.00	0.00	0.00	0.00	0.00
GATCAACCTGTAGCATGTCA	0.00	0.00	0.29	0.00	0.00
GATCAACCTGTATGACAAGC	0.00	0.00	0.00	0.00	0.00
GATCAACCTGTCCAGGGTTT	0.00	0.00	0.29	0.00	0.00
GATCAACCTGTCCGTCGAAA	2.52	3.25	0.29	1.36	1.72
GATCAACCTGTCCGTCGACN	0.46	0.00	0.00	0.00	0.00
GATCAACCTGTCCGTCGCAA	0.00	0.33	0.00	0.00	0.00
GATCAACCTGTCGATGAGAA	0.00	0.00	0.00	0.00	0.00
GATCAACCTGTCGATGAGCC	0.23	0.00	0.00	0.34	0.00
GATCAACCTGTCGATGAGGC	0.46	0.00	0.00	0.00	0.00
GATCAACCTGTCGATGAGNC	0.00	0.00	0.00	0.00	0.00
GATCAACCTGTCGATGAGTA	0.00	0.00	0.00	0.00	0.00
GATCAACCTGTCGATGAGTC	32.73	26.03	12.03	14.26	11.02
GATCAACCTGTCGATGAGTG	0.00	0.00	0.29	0.00	0.00
GATCAACCTGTCGATGAGTN	0.46	0.00	0.00	0.00	0.00
GATCAACCTGTCGATGGGTC	0.23	0.00	0.00	0.00	0.00
GATCAACCTGTCGATGNNNN	0.23	0.00	0.00	0.00	0.00
GATCAACCTGTCGGGCAGGG	0.00	0.00	0.29	0.00	0.00

GATCAACCTGTCTGAGGACA	0.00	0.00	0.00	0.34	0.00
GATCAACCTGTCTTACCTCG	0.00	0.65	0.00	0.34	0.00
GATCAACCTGTGCAGACCCC	0.00	0.00	0.00	0.34	0.00
GATCAACCTGTGCGTGCAGG	0.00	0.00	0.00	0.00	0.00
GATCAACCTGTGGCTCCATG	0.00	0.33	0.00	0.00	0.00
GATCAACCTGTGGGCGTGTC	0.00	0.00	0.29	0.00	0.00
GATCAACCTGTGGGCTGCGA	0.00	0.33	0.00	0.00	0.00
GATCAACCTGTTCATTCCAC	0.00	0.00	0.00	0.00	0.00
GATCAACCTGTTCCGGGTGC	0.00	0.00	0.00	0.34	0.00
GATCAACCTGTTCCTGCCCG	0.00	0.00	0.00	0.00	0.34
GATCAACCTGTTCGAACAAG	0.00	0.00	0.29	0.00	0.00
GATCAACCTGTTCTTATGCC	0.00	0.00	0.00	0.00	0.34
GATCAACCTGTTCTTCTTGG	0.00	0.00	0.00	0.34	0.00
GATCAACCTGTTCTTTGAAA	0.00	0.00	0.29	0.00	0.00
GATCAACCTGTTGAAGATGA	0.00	0.00	0.29	0.00	0.00
GATCAACCTGTTGCCATTTG	0.00	0.00	0.00	0.00	0.00
GATCAACCTGTTGGACGCTG	0.00	0.00	0.29	0.00	0.00
GATCAACCTTAAAAAAAAAA	0.00	0.65	0.86	0.00	0.00
GATCAACCTTAATGGAGGCG	0.00	0.00	0.00	0.34	0.00
GATCAACCTTACATGGCCAT	0.00	0.00	0.00	0.00	0.00
GATCAACCTTACATGGCGCT	0.00	0.33	0.00	0.00	0.00
GATCAACCTTACCTGGCGAT	0.00	0.00	0.00	0.00	0.00
GATCAACCTTACCTGGCGCT	12.82	16.27	7.16	11.54	12.05
GATCAACCTTACCTGGCGNN	0.23	0.00	0.00	0.00	0.00
GATCAACCTTACCTGGCGTT	0.00	0.00	0.00	0.00	0.00
GATCAACCTTACGTGGCGTA	0.00	0.00	0.29	0.00	0.00
GATCAACCTTAGAATCGATT	0.00	0.33	0.00	0.00	0.00
GATCAACCTTAGTTTTCACA	0.00	0.00	0.29	0.00	0.00
GATCAACCTTATCACCTCGC	0.00	0.98	0.00	0.34	0.00
GATCAACCTTATCATGAAGC	0.00	0.00	0.00	0.00	0.00
GATCAACCTTATCTGACGGG	0.00	0.33	0.29	0.00	0.34
GATCAACCTTCAAAAAAAAA	0.00	0.00	0.00	0.00	0.34
GATCAACCTTCAAGCGTCAT	0.00	0.00	0.00	0.00	0.34
GATCAACCTTCACATACAAG	0.00	0.00	0.00	0.00	0.00
GATCAACCTTCCATGGTTGC	0.00	0.00	0.00	0.00	0.00
GATCAACCTTCCATTGAATC	0.23	0.00	0.00	0.00	0.00
GATCAACCTTCCATTGCTTC	0.46	0.00	0.29	0.00	0.34

GATCAACCTTCCCAAGCGTT	0.00	0.00	0.29	0.00	0.00
GATCAACCTTCCCAGGAGTT	0.00	0.33	0.00	0.00	0.00
GATCAACCTTCCCAGGCGCT	0.00	0.00	0.29	0.00	0.00
GATCAACCTTCCCAGGCGGT	0.23	0.00	0.00	0.00	0.00
GATCAACCTTCCCAGGCGTN	0.23	0.00	0.00	0.00	0.00
GATCAACCTTCCCAGGCGTT	13.05	7.81	7.16	9.16	2.07
GATCAACCTTCCTCATGGGA	0.00	0.33	0.00	0.00	0.00
GATCAACCTTCGAAAGGGTG	0.00	0.00	0.29	0.00	0.00
GATCAACCTTCGACGACAGC	0.00	0.00	0.00	0.00	0.34
GATCAACCTTCGAGAATGCT	0.46	0.00	0.00	0.00	0.00
GATCAACCTTCGAGCGCATG	0.00	0.33	0.00	0.00	0.00
GATCAACCTTCGAGCGCTTT	0.92	0.98	0.57	1.02	0.69
GATCAACCTTCGAGCGGAGT	0.46	0.33	0.29	0.34	0.34
GATCAACCTTCGAGCGGGGT	0.23	0.00	0.00	0.00	0.00
GATCAACCTTCGCATGAGCT	0.00	0.33	0.00	0.00	0.00
GATCAACCTTCGTAAGGGGA	0.00	0.00	0.29	0.00	0.00
GATCAACCTTCGTATGAAAA	0.00	0.00	0.00	0.00	0.34
GATCAACCTTCGTATGAAGA	0.00	0.00	0.00	0.00	0.00
GATCAACCTTCGTATGCCGT	0.00	0.00	0.00	0.00	1.72
GATCAACCTTCGTATGCGGA	0.00	0.00	0.00	0.34	0.00
GATCAACCTTCGTATGGAGA	0.00	0.00	0.00	0.00	0.34
GATCAACCTTCGTATGGGAA	0.46	0.33	1.15	0.34	0.00
GATCAACCTTCGTATGGGCA	0.00	0.00	0.00	0.00	0.00
GATCAACCTTCGTATGGGGA	11.67	11.71	21.76	14.60	17.22
GATCAACCTTCGTATGGGGC	0.23	0.00	0.00	0.00	0.34
GATCAACCTTCGTATGGGGG	0.92	0.00	0.00	0.00	0.00
GATCAACCTTCGTATGGGGT	0.23	0.00	0.00	0.00	0.00
GATCAACCTTCGTATGGGNN	0.23	0.00	0.00	0.00	0.00
GATCAACCTTCGTATGGGTA	0.00	0.65	0.00	0.00	0.00
GATCAACCTTCGTATTCAGC	0.00	0.00	0.00	0.00	0.34
GATCAACCTTCGTCAAGGTC	0.00	0.00	0.29	0.00	0.00
GATCAACCTTCGTGTGGGGA	0.00	0.33	0.00	0.00	0.00
GATCAACCTTCGTTAAGAAA	0.00	0.00	0.00	0.68	0.00
GATCAACCTTCGTTGGGAAA	0.00	0.00	0.00	0.00	0.34
GATCAACCTTCTAAAAAAAA	0.23	0.00	0.00	0.00	0.00
GATCAACCTTCTAACAAAAA	0.00	0.00	0.00	0.00	0.34
GATCAACCTTCTAACCATAC	0.46	0.00	0.29	0.00	0.00

GATCAACCTTCTCAGCGCCC	0.00	0.00	0.00	0.00	0.00
GATCAACCTTCTCTCTCCAG	0.23	0.00	0.00	0.00	0.00
GATCAACCTTCTGAACGAGA	0.00	0.00	0.00	0.00	0.00
GATCAACCTTCTGACACAAA	0.00	0.00	0.00	0.68	0.00
GATCAACCTTCTGGCGGACT	0.00	0.33	0.00	0.00	0.00
GATCAACCTTCTGGTCATGT	0.00	0.33	0.00	0.00	0.00
GATCAACCTTCTGTTCTTGA	0.00	0.00	0.00	0.00	0.00
GATCAACCTTCTGTTCTTGG	0.69	0.98	0.00	2.04	1.72
GATCAACCTTGAAAAAAAAA	0.00	0.65	0.00	0.68	0.00
GATCAACCTTGACAGCGAGC	0.00	0.00	0.00	0.34	0.34
GATCAACCTTGACCCGGGGG	0.00	0.00	0.00	0.00	0.00
GATCAACCTTGACCGTCCAG	0.00	0.00	0.00	0.34	0.00
GATCAACCTTGACGACCTCG	0.00	0.33	0.00	0.00	0.00
GATCAACCTTGAGAATCCCT	0.00	0.00	0.29	0.00	0.00
GATCAACCTTGAGGCCAAGC	0.23	0.00	0.00	0.00	0.00
GATCAACCTTGAGGCCATGC	0.69	0.00	0.00	0.00	0.00
GATCAACCTTGAGGCCTGGC	0.00	0.00	0.29	0.00	0.00
GATCAACCTTGAGGTATGAC	0.00	0.33	0.29	0.68	0.00
GATCAACCTTGAGGTATGCC	0.23	0.00	0.00	0.00	0.00
GATCAACCTTGATTCCCGAG	0.00	0.00	0.00	0.34	0.00
GATCAACCTTGCAACAGAAG	0.00	0.00	0.00	0.00	0.34
GATCAACCTTGCAATGCCNG	0.00	0.00	0.29	0.00	0.00
GATCAACCTTGCAGTCTACT	0.00	0.98	0.86	0.00	0.34
GATCAACCTTGCATGCACGC	0.00	0.00	0.29	0.00	0.00
GATCAACCTTGCATGCTCCT	0.46	0.33	0.29	0.00	2.07
GATCAACCTTGCATGGTTTA	0.00	0.00	0.00	0.00	0.34
GATCAACCTTGCCAAGCGGC	0.00	0.00	0.00	0.00	0.00
GATCAACCTTGCCACAGCCT	0.00	0.33	0.00	0.34	0.00
GATCAACCTTGCCAGCCAGT	0.00	0.00	0.00	0.00	0.00
GATCAACCTTGCCCGACTTT	0.00	0.00	0.00	0.00	0.00
GATCAACCTTGCCGACACGC	0.00	0.00	0.00	0.00	0.34
GATCAACCTTGCCGGACTGG	0.00	0.00	0.00	0.00	0.34
GATCAACCTTGCCTGGCGCT	0.00	0.00	0.00	0.00	0.00
GATCAACCTTGCGAGAGCTG	0.00	0.00	0.00	0.00	0.34
GATCAACCTTGCGCGAAGAG	0.00	0.00	0.00	0.00	0.00
GATCAACCTTGCGCGAAGGC	0.00	0.00	0.00	0.00	0.34
GATCAACCTTGCGGCGCTGG	0.00	0.00	0.29	0.00	0.69

GATCAACCTTGCGTGCAAAA	0.00	0.00	0.00	0.34	0.00
GATCAACCTTGCTCACCTCA	1.14	0.65	0.00	0.34	0.00
GATCAACCTTGCTCACCTNN	0.23	0.00	0.00	0.00	0.00
GATCAACCTTGCTCCCCGAG	0.00	0.00	0.29	0.00	0.00
GATCAACCTTGCTGATGTTT	0.00	0.00	0.00	0.00	0.34
GATCAACCTTGCTGGACTGG	0.46	1.63	0.57	1.70	1.38
GATCAACCTTGCTTCACGAG	0.00	0.33	0.00	0.00	0.00
GATCAACCTTGCTTCCAGAG	0.00	0.00	0.29	0.00	0.00
GATCAACCTTGCTTCCAGCG	0.00	0.00	0.29	0.00	0.00
GATCAACCTTGCTTCCCGAC	0.00	0.33	0.00	0.00	0.00
GATCAACCTTGCTTCCCGAG	24.04	31.88	47.25	34.28	48.22
GATCAACCTTGCTTCCCGAN	0.23	0.00	0.00	0.00	0.00
GATCAACCTTGCTTCCCGAT	0.00	0.33	0.00	0.34	0.00
GATCAACCTTGCTTCCCGCG	0.00	0.00	0.00	0.00	0.00
GATCAACCTTGCTTCCCGCN	0.23	0.00	0.00	0.00	0.00
GATCAACCTTGCTTCCCGNG	0.00	0.00	0.00	0.00	0.00
GATCAACCTTGCTTCCGGAG	0.00	0.33	0.00	0.00	0.00
GATCAACCTTGCTTCCNGNN	0.23	0.00	0.00	0.00	0.00
GATCAACCTTGCTTGACCTT	1.37	0.33	3.44	3.39	2.41
GATCAACCTTGCTTGCCCTC	0.00	0.00	0.00	0.00	0.00
GATCAACCTTGGAACTTTTG	0.00	0.00	0.00	0.00	0.34
GATCAACCTTGGACCTAGAG	0.00	0.00	0.00	0.00	0.00
GATCAACCTTGGACGTGCGT	0.00	0.00	0.00	0.00	0.00
GATCAACCTTGGACTGCAAC	0.23	0.33	0.00	0.00	0.00
GATCAACCTTGGCAACGAAT	0.23	0.00	0.00	0.00	0.00
GATCAACCTTGGCAAGAACG	0.00	0.98	0.57	0.68	1.72
GATCAACCTTGGCAAGTTTT	0.00	0.00	0.00	0.34	0.00
GATCAACCTTGGCACGAACG	0.00	0.33	0.57	0.00	0.00
GATCAACCTTGGCCCGTCTG	0.23	0.00	0.00	0.00	0.00
GATCAACCTTGGCCTTAGGG	0.00	0.00	0.00	0.00	0.00
GATCAACCTTGGCTCTTCTG	0.23	0.00	0.00	0.00	0.00
GATCAACCTTGGCTTATCCA	0.23	0.00	0.00	0.00	0.00
GATCAACCTTGGCTTCAGCC	0.00	0.00	0.00	0.00	0.34
GATCAACCTTGGGGAAAAAA	0.00	0.00	0.00	0.00	0.34
GATCAACCTTGGGGAAAAGA	0.00	0.00	0.57	0.00	0.00
GATCAACCTTGGGGCATGAG	0.23	0.00	0.00	0.00	0.00
GATCAACCTTGGGGGAAAAA	0.00	3.90	2.29	4.07	2.76

GATCAACCTTGGGGGAAACA	0.46	0.33	0.00	0.00	0.00
GATCAACCTTGGGGGAAANN	0.23	0.00	0.00	0.00	0.00
GATCAACCTTGGGGGGGGCT	0.00	0.00	0.00	0.34	0.00
GATCAACCTTGGGGTGAAAA	0.00	0.00	0.00	0.34	0.00
GATCAACCTTGGGTGAAAAA	0.00	0.33	0.00	0.00	0.00
GATCAACCTTGGTGCCCCAA	0.00	0.00	0.00	0.00	0.00
GATCAACCTTGGTGCCCCCT	0.00	0.98	0.00	0.68	0.00
GATCAACCTTGTCATGGCTT	0.00	0.00	0.00	1.02	0.00
GATCAACCTTGTCGCGCGCG	0.00	0.00	0.00	1.02	0.00
GATCAACCTTGTGTCTGGTG	0.00	0.00	0.00	0.00	0.00
GATCAACCTTGTGTGCTGAG	0.00	0.98	0.00	0.00	0.34
GATCAACCTTTAAAAAAAAA	0.00	0.00	0.00	0.00	0.34
GATCAACCTTTACTGTTCGC	0.00	0.65	0.00	0.00	0.00
GATCAACCTTTAGGACAACT	0.00	0.00	0.00	0.00	0.00
GATCAACCTTTATCCCACCG	0.00	0.00	0.00	1.02	0.00
GATCAACCTTTCACCAGCAC	0.00	0.00	0.29	0.00	0.00
GATCAACCTTTCATGCTCCT	0.00	0.00	0.00	0.34	0.00
GATCAACCTTTCCGTCTGGG	0.00	0.00	0.00	0.00	0.00
GATCAACCTTTCCTTGTTGG	0.00	0.00	0.00	0.34	0.00
GATCAACCTTTCGGAAAACG	0.00	0.00	0.29	0.00	0.00
GATCAACCTTTCGGACAACC	0.00	0.00	0.00	0.00	0.00
GATCAACCTTTCGGACAACT	0.92	0.33	1.15	1.36	1.03
GATCAACCTTTCGGAGCGGA	0.00	0.00	0.00	0.34	0.00
GATCAACCTTTCTTGCTTGT	0.00	0.00	0.29	0.00	0.00
GATCAACCTTTGAGGATTGC	0.00	0.00	0.00	0.00	0.34
GATCAACCTTTGCAACATTG	0.00	0.00	0.00	0.00	0.00
GATCAACCTTTGCATCCAGC	0.00	0.00	0.29	0.00	0.34
GATCAACCTTTGCCACCCAC	0.00	0.00	0.00	0.00	0.34
GATCAACCTTTGCCCAAAAA	0.00	0.00	0.00	0.00	0.34
GATCAACCTTTGCCCAGAAA	0.00	0.00	0.00	0.68	0.00
GATCAACCTTTGCCCAGGAA	0.23	0.00	0.29	0.34	0.34
GATCAACCTTTGCCCAGGAT	0.00	0.33	0.00	0.00	0.00
GATCAACCTTTGCGCTTCGA	0.00	0.00	0.29	0.00	0.00
GATCAACCTTTGCTGCTGTG	0.00	0.00	0.00	0.00	0.69
GATCAACCTTTGGAACAGCC	0.00	0.00	0.00	0.00	0.00
GATCAACCTTTGGAACAGCN	0.00	0.00	0.00	0.00	0.00
GATCAACCTTTGGGACAGCC	0.00	0.65	0.29	0.00	0.00

GATCAACCTTTGGGGACGTG	0.00	0.00	0.29	0.00	0.00
GATCAACCTTTGGGGCCGTG	0.46	0.33	0.29	0.34	0.69
GATCAACCTTTGTTTAGCTG	0.00	0.00	0.00	0.00	0.34
GATCAACCTTTTACAGCAAA	0.00	0.00	0.29	0.00	0.00
GATCAACCTTTTACCTACCA	0.00	0.00	0.00	0.00	0.34
GATCAACCTTTTCCAAAGTC	0.00	0.00	0.00	0.00	0.00
GATCAACCTTTTCCAGAGTC	0.00	0.00	0.00	0.00	0.00
GATCAACCTTTTGAATGAGA	0.00	0.00	0.00	0.00	0.00
GATCAACCTTTTTCTGGCGG	0.00	0.65	0.00	0.00	0.00
GATCAACCTTTTTTACTATG	0.23	0.00	0.00	0.00	0.00
GATCAACGAAAAAAAAAAAA	0.69	0.33	2.00	0.68	0.34
GATCAACGAAAAAAAAAAAT	0.00	0.00	0.00	0.00	0.34
GATCAACGAAAACAAACGGG	0.00	0.00	0.29	0.00	0.00
GATCAACGAAAACCGGCTCT	0.00	0.00	0.00	0.00	0.00
GATCAACGAAAAGAGATGTG	0.00	0.00	0.29	0.00	0.00
GATCAACGAAAAGCGAGCAG	0.46	0.00	0.00	1.02	0.00
GATCAACGAAAAGCTTCAGG	0.00	0.33	0.00	0.00	0.00
GATCAACGAAAAGGAAAGGT	0.00	0.00	0.57	0.00	0.00
GATCAACGAAAAGGAAATAG	0.00	0.00	0.00	0.34	0.00
GATCAACGAAAAGGTGAGGC	0.00	0.00	0.00	0.34	0.00
GATCAACGAAAATCCATCGC	0.46	0.00	0.00	0.00	0.00
GATCAACGAAACACCGCACA	0.00	0.00	0.00	0.00	0.34
GATCAACGAAACCCACTTGA	0.00	0.00	0.00	0.00	0.34
GATCAACGAAACCCACTTGC	3.89	0.98	0.86	3.39	1.72
GATCAACGAAACCCACTTGN	0.23	0.00	0.00	0.00	0.00
GATCAACGAAACCCTGCTCA	0.00	0.00	0.00	0.00	0.00
GATCAACGAAACGACACACT	0.00	0.00	0.00	0.00	0.34
GATCAACGAAACGAGGAGCT	0.00	0.00	0.00	0.34	0.00
GATCAACGAAAGAAAAAAAA	0.23	0.00	0.00	0.00	0.00
GATCAACGAAAGACTTCAGG	0.00	0.00	0.00	0.00	0.34
GATCAACGAAAGCCCCTCCA	0.00	0.00	0.57	0.00	0.00
GATCAACGAAAGCCTTCGAA	0.00	0.00	0.00	0.00	0.34
GATCAACGAAAGCGTTGGCG	0.00	0.00	0.00	0.00	0.00
GATCAACGAAAGCTGAAAGG	0.00	0.00	0.00	0.00	0.00
GATCAACGAAAGCTTTGCCC	0.00	0.00	0.00	0.34	0.34
GATCAACGAAAGGAGTGAAG	0.00	0.33	0.00	0.00	0.00
GATCAACGAAAGGATTCAGG	0.00	0.00	0.29	0.34	0.00

GATCAACGAAAGGATTCAGT	0.00	0.00	0.00	0.34	0.00
GATCAACGAAAGGATTCCGG	0.00	0.00	0.29	0.00	0.00
GATCAACGAAAGGCAGCTTT	0.00	0.00	0.00	0.34	0.00
GATCAACGAAAGGCCTCAGG	0.00	0.00	0.29	0.00	0.00
GATCAACGAAAGGCGTAAGG	0.00	0.00	0.00	0.00	0.00
GATCAACGAAAGGCGTCAGG	0.00	0.00	0.00	0.68	0.00
GATCAACGAAAGGCTCCAGG	0.00	0.33	0.00	0.00	0.00
GATCAACGAAAGGCTCGTAT	0.00	0.00	0.00	0.00	0.34
GATCAACGAAAGGCTGCAGG	0.00	0.00	0.00	0.00	0.34
GATCAACGAAAGGCTGTCGG	0.00	0.33	0.00	0.00	0.00
GATCAACGAAAGGCTTAAGG	0.00	0.00	0.86	0.00	1.03
GATCAACGAAAGGCTTAATG	0.00	0.00	0.29	0.00	0.00
GATCAACGAAAGGCTTACTA	0.00	0.00	0.00	0.00	0.34
GATCAACGAAAGGCTTCAAG	0.00	0.00	0.00	0.00	0.34
GATCAACGAAAGGCTTCACG	0.00	0.00	0.29	0.00	0.00
GATCAACGAAAGGCTTCAGG	43.72	180.23	361.10	186.69	248.31
GATCAACGAAAGGCTTCAGN	0.23	0.00	0.00	0.34	0.00
GATCAACGAAAGGCTTCAGT	0.00	0.00	0.00	0.68	0.69
GATCAACGAAAGGCTTCANG	0.00	0.00	0.00	0.34	0.34
GATCAACGAAAGGCTTCATC	0.00	0.00	0.00	0.00	0.34
GATCAACGAAAGGCTTCATG	0.00	0.00	0.29	0.00	0.00
GATCAACGAAAGGCTTCCGG	0.00	1.30	2.29	0.34	0.34
GATCAACGAAAGGCTTCCGT	0.00	0.00	0.29	0.00	0.00
GATCAACGAAAGGCTTCCNN	0.23	0.00	0.00	0.00	0.00
GATCAACGAAAGGCTTCGGG	0.00	0.00	0.57	0.00	0.34
GATCAACGAAAGGCTTCGNN	0.23	0.00	0.00	0.00	0.00
GATCAACGAAAGGCTTCTGG	0.00	0.00	0.00	0.00	0.00
GATCAACGAAAGGCTTGAGG	0.23	0.00	0.00	0.00	0.00
GATCAACGAAAGGCTTNAGG	0.00	0.00	0.29	0.00	0.34
GATCAACGAAAGGCTTTAGG	0.00	0.65	0.00	0.00	0.00
GATCAACGAAAGGGTTCAGG	0.00	0.33	0.00	0.00	0.00
GATCAACGAAAGGTTGTTTA	0.00	0.00	0.29	0.00	0.00
GATCAACGAAAGGTTTCAGG	0.00	0.00	0.29	0.00	0.00
GATCAACGAAAGTCTTCAGG	0.00	0.00	0.29	0.00	0.00
GATCAACGAAAGTCTTCAGT	0.00	0.00	0.00	0.00	0.34
GATCAACGAAAGTGAAAAAA	0.00	0.00	0.00	0.00	0.00
GATCAACGAAATCAACGGCT	0.00	0.00	0.00	0.00	0.69

GATCAACGAAATCATCGCGA	0.00	0.00	0.29	0.00	0.34
GATCAACGAACACAATGAAA	0.00	0.00	0.29	0.00	0.00
GATCAACGAACACACCACCA	0.00	0.00	0.00	0.00	0.00
GATCAACGAACACCAAACCA	0.23	0.00	0.00	0.00	0.00
GATCAACGAACACGATGCAC	0.00	0.00	0.00	0.00	0.00
GATCAACGAACAGCTGGCGG	0.00	0.00	0.00	0.00	0.00
GATCAACGAACAGGGGACAG	0.00	0.00	0.00	0.00	0.34
GATCAACGAACAGTCGTATG	0.00	0.00	0.00	0.34	0.00
GATCAACGAACATCGAACAA	0.00	0.00	0.00	0.00	1.38
GATCAACGAACCAAAGTTGT	0.00	0.00	0.00	0.34	0.00
GATCAACGAACCACGAGCGC	0.00	0.00	0.00	0.00	0.00
GATCAACGAACCACGCGAGC	3.43	3.25	1.43	7.13	5.17
GATCAACGAACCACGCGAGT	0.23	0.00	0.00	0.00	0.00
GATCAACGAACCACGCGCGC	0.00	0.00	0.29	0.00	0.00
GATCAACGAACCACGCGGGC	0.00	0.00	0.00	0.34	0.34
GATCAACGAACCGCCCAACC	0.00	0.00	0.29	0.00	0.00
GATCAACGAACGAAAAAAAA	0.00	0.00	0.00	0.34	0.00
GATCAACGAACGAGGGTTGC	0.00	0.00	0.29	0.00	0.00
GATCAACGAACGGCTGCCGG	0.23	0.00	0.00	0.00	0.00
GATCAACGAACGGCTGGCGC	0.23	0.00	0.00	0.00	0.00
GATCAACGAACGGCTTCAGG	0.00	0.00	0.29	0.34	0.00
GATCAACGAACGGGTTCAGT	0.00	0.33	0.00	0.00	0.00
GATCAACGAACGTCAGAGTG	0.23	0.00	0.00	0.00	0.00
GATCAACGAACGTGTCTGAA	0.23	0.00	0.00	0.00	0.00
GATCAACGAACGTGTGAGCA	0.00	0.33	0.00	0.00	0.00
GATCAACGAACTCATCTCCG	0.00	0.00	0.00	0.00	0.00
GATCAACGAACTCGGCCAGT	0.00	0.00	0.00	0.00	0.00
GATCAACGAACTCTCCGTAG	0.00	0.00	0.29	0.00	0.00
GATCAACGAACTGAACGGCT	0.00	0.00	0.29	0.00	0.00
GATCAACGAACTGCTGCCCC	0.23	0.00	0.29	0.34	0.00
GATCAACGAACTGCTTCCGC	0.00	0.00	0.00	0.00	0.00
GATCAACGAACTTTGACACG	0.00	0.00	0.00	0.00	0.34
GATCAACGAAGAACGCAGCC	0.00	0.00	0.29	0.00	0.00
GATCAACGAAGAAGAAAAAA	0.69	0.00	0.00	0.00	0.00
GATCAACGAAGAATCGATGA	0.00	0.00	0.29	0.00	0.00
GATCAACGAAGACAACATGC	0.00	0.00	0.00	0.00	0.34
GATCAACGAAGACAGAAGAC	0.00	0.00	1.43	0.00	0.69

GATCAACGAAGACAGAAGGC	0.00	0.00	0.29	0.00	0.00
GATCAACGAAGACCGAAGAC	0.00	0.00	1.15	0.00	0.34
GATCAACGAAGAGACGCACA	0.00	0.00	0.00	0.00	0.34
GATCAACGAAGAGCAGAGGA	0.00	0.00	0.29	0.00	0.34
GATCAACGAAGAGGCAACCT	0.00	0.00	0.00	0.00	0.34
GATCAACGAAGAGGCAATCC	0.00	0.00	0.29	0.00	0.00
GATCAACGAAGAGGCACTCC	1.60	0.33	0.00	0.00	0.00
GATCAACGAAGAGGCGGAAG	0.23	0.00	0.00	0.00	0.00
GATCAACGAAGAGGTGCGGA	0.00	0.00	0.29	0.00	0.00
GATCAACGAAGATGGTGAGG	0.23	0.33	0.00	1.36	0.00
GATCAACGAAGCAAACTGAC	0.00	0.00	0.00	0.34	0.00
GATCAACGAAGCACTCCACA	0.00	0.33	0.29	0.00	0.00
GATCAACGAAGCAGCCATCA	0.00	0.33	0.00	0.00	0.00
GATCAACGAAGCATGGTGAT	0.23	0.00	0.00	0.00	0.00
GATCAACGAAGCCCATCCTC	0.00	0.00	0.00	0.00	0.34
GATCAACGAAGCGAAGTGAC	0.00	0.00	0.00	0.00	0.00
GATCAACGAAGCGACGTGTG	0.00	0.00	0.00	0.00	0.00
GATCAACGAAGCGAGGCCAG	0.00	0.00	0.00	0.00	0.34
GATCAACGAAGCGCACAAGA	0.46	0.98	0.57	1.70	0.00
GATCAACGAAGCGCATCCTT	0.00	0.00	0.00	0.00	0.00
GATCAACGAAGCGCCCCGCA	0.00	0.00	0.00	0.34	0.00
GATCAACGAAGCGCCCTTCC	0.00	0.00	0.00	0.00	0.00
GATCAACGAAGCGCCGCCGC	0.00	0.00	0.29	0.00	0.00
GATCAACGAAGCGCGGCCAG	0.46	0.33	0.00	0.34	0.34
GATCAACGAAGCGCGTGACA	0.00	0.00	0.57	0.00	1.38
GATCAACGAAGCGGACGTGA	0.00	0.00	0.00	0.00	0.34
GATCAACGAAGCTGAGATGG	0.69	0.00	0.00	0.00	0.00
GATCAACGAAGGAAGAAAGA	0.00	0.00	0.57	0.00	0.00
GATCAACGAAGGACAACACT	0.23	0.00	0.00	0.00	0.00
GATCAACGAAGGACAGATCG	0.00	0.00	0.00	0.34	0.00
GATCAACGAAGGCATCTGCG	0.00	0.00	0.00	0.00	0.00
GATCAACGAAGGCATGATGG	0.00	0.00	0.00	0.00	0.00
GATCAACGAAGGCTTCAGGG	0.46	0.00	0.29	0.00	0.00
GATCAACGAAGGGAGGTACT	0.46	0.00	0.00	0.00	0.00
GATCAACGAAGGGATGTCTG	0.00	0.00	0.00	0.00	0.00
GATCAACGAAGGGCTTCAGG	0.23	0.00	0.00	0.00	0.00
GATCAACGAAGGGGGAATCA	0.00	0.00	0.00	0.00	0.00

GATCAACGAAGGGGGAATTC	0.00	0.33	0.00	0.00	0.00
GATCAACGAAGGGTTCCAGA	0.00	0.00	0.29	0.00	0.00
GATCAACGAAGTCCTTGCGA	0.00	0.33	0.00	0.00	0.00
GATCAACGAAGTCGAGAAGG	0.00	0.00	0.29	0.00	0.00
GATCAACGAAGTCGAGCAGG	0.00	0.00	0.29	0.00	0.00
GATCAACGAAGTCGCAGACC	0.00	0.00	0.00	0.00	0.34
GATCAACGAAGTCGCANACC	0.00	0.00	0.00	0.00	0.34
GATCAACGAAGTCTGGACTG	0.23	0.00	0.00	0.00	0.00
GATCAACGAAGTGCGCTTGA	0.23	0.00	0.00	0.00	0.00
GATCAACGAAGTGCGTCCTG	0.00	0.00	0.00	0.00	0.00
GATCAACGAAGTGGAAGCCT	0.00	0.00	0.00	0.00	0.00
GATCAACGAAGTGGACGCCG	0.00	0.00	0.00	0.00	0.00
GATCAACGAAGTGGATGCAA	0.00	0.00	0.00	0.68	0.00
GATCAACGAAGTGGATGCAC	0.00	0.00	0.00	0.00	0.00
GATCAACGAAGTGGATGCCG	0.00	0.65	0.86	0.00	0.00
GATCAACGAAGTGGATGCTG	0.00	0.33	0.57	1.02	2.07
GATCAACGAAGTGGATGGGG	0.00	0.00	0.00	0.34	0.00
GATCAACGAATAACAGAACA	0.00	0.00	0.00	0.00	0.34
GATCAACGAATAAGAATAAG	0.00	0.00	0.00	0.00	1.03
GATCAACGAATACCACCTGA	1.37	0.33	0.00	0.34	0.00
GATCAACGAATAGAAGAAGA	0.00	0.00	0.00	0.00	0.34
GATCAACGAATAGGCAGGCG	0.00	0.33	0.00	0.00	0.00
GATCAACGAATCAACCTCAN	0.00	0.00	0.00	0.00	0.34
GATCAACGAATCAGAGATCC	0.00	0.00	0.00	0.00	0.00
GATCAACGAATCAGAGGGCC	0.23	0.00	0.00	0.00	0.00
GATCAACGAATCAGAGGTAG	0.46	0.00	0.29	0.00	0.00
GATCAACGAATCAGAGGTCC	0.00	0.33	2.58	1.70	1.38
GATCAACGAATCATTCCCAC	0.92	0.00	0.00	0.00	0.00
GATCAACGAATCCTGCCAAG	0.00	0.00	0.29	0.00	0.00
GATCAACGAATCGCAGATGC	0.00	0.00	0.00	0.34	0.00
GATCAACGAATCTTTCCCAC	0.00	0.65	0.00	0.00	0.00
GATCAACGAATGGCCGAACG	0.00	0.00	0.29	0.00	0.00
GATCAACGAATGGCCGAGCG	0.00	0.00	0.29	0.34	0.00
GATCAACGAATGGCCTAGCG	0.00	0.33	0.00	0.34	0.69
GATCAACGAATGTTCCTGGA	0.23	0.00	0.00	0.00	0.00
GATCAACGAATTCAAACTGC	0.00	0.00	0.29	0.34	0.34
GATCAACGAATTTGTGAACG	0.00	0.00	0.57	0.00	0.00

GATCAACGACAACAAGCAGT	0.46	0.00	0.00	0.00	0.00
GATCAACGACAACAAGGACC	0.46	0.00	0.00	0.00	0.00
GATCAACGACAACAGAAGTG	0.00	0.00	0.00	0.00	0.00
GATCAACGACAACATCAGCA	0.00	0.00	0.00	0.00	0.00
GATCAACGACAACCATCAGC	0.00	0.00	0.29	0.00	0.00
GATCAACGACAACCCGTACA	0.00	0.00	0.00	0.00	0.00
GATCAACGACAACCGGCGCG	0.00	0.98	0.00	0.00	0.00
GATCAACGACAACGAGTGGG	0.00	0.00	0.00	0.00	0.00
GATCAACGACAACGATTTCA	0.00	0.00	0.00	0.00	0.00
GATCAACGACAACTACGTGA	0.46	0.98	0.00	0.34	0.00
GATCAACGACAACTACGTGT	0.23	0.00	0.00	0.00	0.00
GATCAACGACAACTACTGGT	0.00	0.00	0.29	0.34	0.00
GATCAACGACAACTGCACCA	0.00	0.00	0.29	0.00	0.00
GATCAACGACAACTGGAGCG	0.00	0.00	0.00	0.00	0.00
GATCAACGACAAGAAGGAGA	0.00	0.00	0.00	0.00	0.00
GATCAACGACAAGACAAAAC	0.00	0.00	0.00	0.00	0.69
GATCAACGACAAGACCTTCA	0.00	0.00	0.00	0.34	0.00
GATCAACGACAAGCACTGGC	0.00	0.00	0.00	0.00	0.00
GATCAACGACAAGCAGGTCG	0.00	0.00	0.00	0.00	0.34
GATCAACGACAAGCTTGCCA	0.00	0.65	0.00	1.02	0.34
GATCAACGACAAGCTTTCGA	0.00	0.00	0.00	0.00	0.00
GATCAACGACAAGGCCAGGA	0.00	0.00	0.29	0.00	0.00
GATCAACGACAAGGCGAGTA	0.00	0.33	0.29	0.00	0.00
GATCAACGACAAGGTCATGG	0.00	0.00	0.86	0.00	0.00
GATCAACGACAAGGTCCTGG	0.00	0.00	0.29	0.00	0.00
GATCAACGACAAGGTCGACA	0.00	0.00	0.00	0.00	0.34
GATCAACGACAAGTGGTTTG	0.00	0.00	0.00	0.00	0.34
GATCAACGACAAGTTTGCTG	0.00	0.33	0.00	0.00	0.00
GATCAACGACAAGTTTGGCA	0.00	0.00	0.00	0.00	0.00
GATCAACGACAATGACGTGA	0.00	0.00	0.00	0.34	0.00
GATCAACGACACAAAGTAAT	0.00	0.33	0.00	0.68	0.00
GATCAACGACACAACACCAA	0.00	0.00	0.57	0.00	0.00
GATCAACGACACAGACCTCA	0.23	0.00	0.00	0.34	0.00
GATCAACGACACCAACACAC	0.00	0.00	0.00	0.00	0.34
GATCAACGACACCAATGAGG	0.00	0.00	0.00	0.00	0.69
GATCAACGACACCATCGGTC	0.00	0.00	0.00	0.00	0.34
GATCAACGACACCATGATGG	0.00	0.33	0.00	0.00	0.00

GATCAACGACACCCAGTGCT	0.00	0.00	0.00	0.00	0.00
GATCAACGACACCCCCTTGA	0.00	0.33	0.00	0.00	0.00
GATCAACGACACCCGGGAGG	0.00	0.33	0.00	0.00	0.00
GATCAACGACACCGCGCTGC	0.69	0.00	0.00	0.00	0.00
GATCAACGACACCGGCTAAC	0.00	0.00	0.00	0.00	0.34
GATCAACGACACCGGTTGCG	0.00	0.00	0.00	0.00	0.34
GATCAACGACACCTCCGGCC	0.00	0.00	0.00	0.00	0.00
GATCAACGACACCTGCGGTC	0.23	0.00	0.00	0.00	0.00
GATCAACGACACCTTCGGCC	0.00	0.33	0.00	0.00	0.34
GATCAACGACACCTTCGGGC	0.00	0.33	0.00	0.00	0.00
GATCAACGACACCTTCGGTC	23.12	32.53	24.34	31.91	35.82
GATCAACGACACCTTCGGTG	0.00	0.33	0.00	0.00	0.00
GATCAACGACACCTTCGTGA	0.46	0.00	0.00	0.00	0.00
GATCAACGACACCTTCNNNN	0.23	0.00	0.00	0.00	0.00
GATCAACGACACCTTCTCCT	0.00	0.00	0.00	0.00	0.00
GATCAACGACACCTTTGTGA	0.00	0.00	0.00	0.00	0.00
GATCAACGACACGCTCATGA	0.00	0.00	0.00	0.00	0.00
GATCAACGACACGCTGCGTG	0.23	0.00	0.00	0.00	0.00
GATCAACGACACGGCGTACC	0.00	0.00	0.00	0.34	0.00
GATCAACGACACGTGCATTG	0.00	0.00	0.29	0.00	0.00
GATCAACGACACTGCACCGG	0.00	0.00	0.00	0.34	0.00
GATCAACGACACTGCGTTGT	0.00	0.00	0.29	0.00	0.00
GATCAACGACACTGTGAAGC	0.69	0.98	0.86	0.34	1.03
GATCAACGACACTGTGAAGN	0.00	0.00	0.00	0.00	0.00
GATCAACGACAGACGGCCGC	0.23	0.00	0.00	0.00	0.00
GATCAACGACAGCACGCCCA	0.00	0.33	0.29	0.00	0.00
GATCAACGACAGCATCAGGA	0.69	0.00	0.29	0.00	0.34
GATCAACGACAGCATCTCAA	0.00	0.00	0.29	0.00	0.00
GATCAACGACAGCCGAGGGG	0.00	0.00	0.00	0.00	0.34
GATCAACGACAGCTACTCTG	0.00	0.33	0.00	0.00	0.34
GATCAACGACAGCTCGAGTT	0.00	0.00	0.00	0.00	0.00
GATCAACGACAGCTGGAGCG	0.00	0.00	0.00	0.00	0.34
GATCAACGACAGCTTCACTA	0.00	0.33	0.00	0.00	0.00
GATCAACGACAGGAGCAACA	0.00	0.00	0.00	0.00	0.00
GATCAACGACAGGCTTCAGG	0.00	0.00	0.00	0.34	0.00
GATCAACGACAGGCTTCCGT	0.00	0.00	0.29	0.00	0.00
GATCAACGACAGGGACAACC	0.00	0.00	0.00	0.00	0.00

GATCAACGACAGGGACGACC	1.37	3.25	3.72	7.81	3.79
GATCAACGACAGGGACGGCC	0.00	0.33	0.00	0.00	0.00
GATCAACGACAGGGGAAAGT	0.00	0.00	0.00	0.00	0.34
GATCAACGACAGTACTAAGG	0.46	0.00	0.00	0.00	0.00
GATCAACGACAGTCCGGCGA	0.00	0.00	0.00	0.00	0.00
GATCAACGACAGTCCGGTCA	0.00	0.00	0.00	0.00	0.34
GATCAACGACAGTGCGAGGG	0.00	0.00	0.00	0.68	0.00
GATCAACGACAGTGCTGTCG	0.00	0.00	0.00	0.00	0.34
GATCAACGACAGTTGTACTC	0.46	0.33	0.29	1.70	0.00
GATCAACGACATAAGAAGCA	0.00	0.00	0.29	0.00	0.00
GATCAACGACATAGGCCACT	0.00	0.33	0.00	0.00	0.00
GATCAACGACATATCGTTCT	0.00	0.00	0.00	0.00	0.00
GATCAACGACATCAACGTCG	0.00	0.00	0.00	0.00	0.00
GATCAACGACATCAACGTGG	1.37	0.00	0.00	0.00	0.00
GATCAACGACATCAACGTGN	0.23	0.00	0.00	0.00	0.00
GATCAACGACATCAACGTGT	0.00	0.33	0.00	0.00	0.00
GATCAACGACATCAAGAAGC	0.23	0.00	0.00	0.00	0.00
GATCAACGACATCAGCCTAC	0.00	0.00	0.29	0.00	0.00
GATCAACGACATCATCGCGG	0.00	0.00	0.00	0.34	0.00
GATCAACGACATCCAGTCTG	0.00	0.33	0.00	0.00	0.00
GATCAACGACATCCTCGCGA	0.46	0.00	0.29	0.34	0.00
GATCAACGACATCCTGGGTC	0.00	0.33	0.00	0.00	0.00
GATCAACGACATCGGGGATT	0.00	0.00	0.00	0.00	0.34
GATCAACGACATCGTATTGT	0.00	0.00	0.00	0.34	0.00
GATCAACGACATCTCTGAAG	0.00	0.00	0.00	0.34	0.00
GATCAACGACATCTCTGACG	0.23	0.65	0.29	1.02	0.69
GATCAACGACATCTGGTTGC	0.00	0.00	0.00	0.34	0.00
GATCAACGACATGAAGGAGG	0.00	0.00	0.00	0.34	0.00
GATCAACGACATGAGGAGCC	0.46	0.33	0.00	1.02	0.00
GATCAACGACATGGACATCA	0.92	0.00	0.00	0.00	0.00
GATCAACGACATGGAGGAGG	0.00	0.33	0.00	0.00	0.00
GATCAACGACATGGCAGATG	0.00	0.00	0.00	0.00	0.00
GATCAACGACATGGCGGAGG	0.00	0.00	0.00	0.00	0.00
GATCAACGACATGGGGATGT	0.00	0.00	0.29	0.00	0.00
GATCAACGACATGGGGGAGG	0.23	0.00	0.00	0.00	0.00
GATCAACGACATGGTCTATC	0.00	0.00	0.00	0.00	0.00
GATCAACGACATGGTGGAGG	8.93	6.18	2.86	4.75	2.41

GATCAACGACATGGTGGCGG	0.23	0.00	0.00	0.00	0.00
GATCAACGACATGGTGGGGG	0.00	0.00	0.00	0.00	0.00
GATCAACGACATGGTGGTGG	0.23	0.00	0.00	0.00	0.00
GATCAACGACATGTACCAGT	0.00	0.00	0.00	1.02	0.00
GATCAACGACATGTCCATGT	0.00	0.00	0.29	0.00	0.00
GATCAACGACATGTGCATGG	0.00	0.00	0.00	0.00	0.00
GATCAACGACATTAACAAAG	0.23	0.33	0.00	0.00	0.00
GATCAACGACATTCGAAGGG	0.46	0.00	0.00	0.00	0.00
GATCAACGACATTGAGCTGT	0.23	0.00	0.00	0.00	0.00
GATCAACGACATTGCAGAGG	1.37	0.00	0.00	0.68	0.00
GATCAACGACATTGCAGATG	0.00	0.00	0.00	0.00	0.00
GATCAACGACATTGTCCAGG	0.00	0.00	0.00	0.00	0.00
GATCAACGACATTGTCGACA	0.00	0.33	0.00	0.00	0.00
GATCAACGACCAACACTCAT	0.00	0.00	0.00	0.00	0.34
GATCAACGACCAAGCAACAC	0.00	0.00	0.00	0.00	0.34
GATCAACGACCAAGCACTGG	0.00	0.00	0.29	0.00	0.00
GATCAACGACCAAGCCCTGG	0.00	0.00	0.00	0.00	0.00
GATCAACGACCAAGGGCCTT	0.00	0.98	0.00	1.36	0.34
GATCAACGACCAATCTGAGC	0.00	0.00	0.00	0.00	0.34
GATCAACGACCACACCGACA	0.00	0.00	0.00	0.00	0.69
GATCAACGACCACACGTGGT	0.23	0.00	0.00	0.00	0.00
GATCAACGACCACAGGAAAA	0.00	0.00	0.29	0.00	0.00
GATCAACGACCACAGGAAGG	8.01	1.95	7.45	3.39	12.40
GATCAACGACCACATAGGAT	0.92	0.33	0.29	0.00	0.00
GATCAACGACCACGTCTGCG	0.00	0.00	0.29	0.00	0.00
GATCAACGACCACTGTGGAT	0.46	0.00	0.00	0.00	0.00
GATCAACGACCACTGTGGNN	0.23	0.00	0.00	0.00	0.00
GATCAACGACCACTTCAAGT	0.00	0.00	0.00	0.34	0.34
GATCAACGACCAGAAACTTG	0.00	0.00	0.29	0.00	0.00
GATCAACGACCAGGCAGAGT	0.23	0.00	0.00	0.00	0.34
GATCAACGACCAGGGCCCTT	1.60	0.98	0.86	0.34	0.00
GATCAACGACCAGTTCATGG	0.00	0.00	0.00	0.00	0.00
GATCAACGACCATAACCATA	0.00	0.00	0.00	0.00	0.34
GATCAACGACCATGTGGTGC	0.00	0.00	0.00	0.34	0.00
GATCAACGACCATTGAAGGC	0.00	0.33	0.00	0.00	0.00
GATCAACGACCCAAACATGG	0.00	0.00	0.57	0.00	0.00
GATCAACGACCCAGAGCTCA	0.46	0.00	0.00	0.34	0.34

GATCAACGACCCATGCTCAC	0.00	0.00	0.00	0.00	0.00
GATCAACGACCCATTGGTGT	0.00	0.00	0.00	0.00	0.69
GATCAACGACCCCATGCTGT	0.00	0.00	0.29	0.00	0.00
GATCAACGACCCCATTTCTC	0.00	0.33	0.00	0.00	0.00
GATCAACGACCCCCGCCACA	0.00	0.00	0.00	0.00	0.34
GATCAACGACCCCGCAGAAC	0.00	0.00	0.00	0.34	0.00
GATCAACGACCCCTTAGTCG	0.00	0.00	0.00	0.00	0.34
GATCAACGACCCGCGCGAGG	0.00	0.00	0.00	0.00	0.00
GATCAACGACCCGGCCTGCA	0.00	0.00	0.00	0.34	0.00
GATCAACGACCCGTACAAGA	0.00	0.00	0.00	0.00	0.00
GATCAACGACCCGTCAAAGC	0.00	0.65	0.00	0.34	0.00
GATCAACGACCCGTCCAAGC	0.00	0.00	0.00	0.00	0.00
GATCAACGACCCGTCGAGCC	0.00	0.00	0.00	0.00	0.00
GATCAACGACCCGTCTGTGG	0.00	0.00	0.00	1.02	0.34
GATCAACGACCCGTTCATGG	0.23	3.90	2.29	2.72	5.51
GATCAACGACCCGTTCATTC	0.00	0.00	0.00	0.00	0.00
GATCAACGACCCGTTCNTGG	0.00	0.00	0.00	0.00	0.34
GATCAACGACCCGTTCTTGG	0.00	0.33	0.00	0.00	0.00
GATCAACGACCCTGACGTGT	0.00	0.98	0.00	0.00	0.00
GATCAACGACCCTGCAAAGG	0.00	0.00	0.00	0.00	0.00
GATCAACGACCCTGCCGAAG	0.00	0.65	0.00	0.34	0.00
GATCAACGACCCTTACTCAA	0.00	0.00	0.29	0.00	0.00
GATCAACGACCGAGTGGACG	0.23	0.00	0.00	0.00	0.00
GATCAACGACCGCAGTTTCG	0.00	0.00	0.00	0.00	0.00
GATCAACGACCGCCGCTTCG	0.00	0.00	0.29	0.00	0.00
GATCAACGACCGCGTCGACG	0.00	0.00	0.00	0.34	0.00
GATCAACGACCGCGTTTTAT	0.00	0.00	0.29	0.00	0.00
GATCAACGACCGCTCGAGGT	0.00	0.65	0.00	0.00	0.00
GATCAACGACCGCTGACTGG	0.00	0.00	0.00	0.00	0.00
GATCAACGACCGCTTCCTTC	0.00	0.33	0.00	0.00	0.69
GATCAACGACCGGAACATGT	0.00	0.00	0.00	0.00	0.34
GATCAACGACCGGAAGCGGC	0.00	0.00	0.00	0.00	0.34
GATCAACGACCGGAAGGCGA	0.92	0.33	0.00	0.34	0.34
GATCAACGACCGGCCAGTGA	0.23	0.00	0.00	0.00	0.00
GATCAACGACCGGCCGGTCA	0.23	0.00	0.00	0.00	0.00
GATCAACGACCGGCTGGCGG	0.00	0.00	0.29	0.34	0.00
GATCAACGACCGGGAAGAGG	0.00	0.00	0.00	0.00	0.00

GATCAACGACCGTCCGGCCA	0.00	0.33	0.00	0.00	0.00
GATCAACGACCGTCTAGGAG	0.00	0.00	0.29	0.00	0.00
GATCAACGACCGTTGTGAGC	0.00	0.00	0.00	0.00	0.00
GATCAACGACCGTTTCCCGC	0.00	0.00	0.29	0.00	0.00
GATCAACGACCTCAACGGTC	0.00	0.33	0.00	0.00	0.00
GATCAACGACCTCATCATAG	0.00	0.00	0.00	0.34	0.00
GATCAACGACCTCCTCCCTC	0.00	0.00	0.00	0.00	0.34
GATCAACGACCTCGAGGCCC	0.23	0.65	0.00	1.36	0.00
GATCAACGACCTCGCCACGC	0.23	0.00	0.00	0.00	0.00
GATCAACGACCTCGTCTGGA	0.00	0.33	0.00	0.34	0.00
GATCAACGACCTCGTCTGGT	0.00	0.33	0.00	0.00	0.00
GATCAACGACCTCGTGAAAG	0.00	0.00	0.57	0.00	0.34
GATCAACGACCTCGTGGAGG	0.92	0.00	0.00	0.34	0.00
GATCAACGACCTCGTGNCGC	0.00	0.33	0.00	0.00	0.00
GATCAACGACCTCTACGCAT	0.00	0.00	0.00	0.00	0.00
GATCAACGACCTCTCCGCGG	0.00	0.00	0.00	0.00	0.69
GATCAACGACCTCTCGCGCC	0.00	0.00	0.00	0.34	0.00
GATCAACGACCTCTCTGACG	0.00	0.00	0.00	0.00	0.00
GATCAACGACCTGCGCTGCC	0.00	0.33	0.00	1.02	0.00
GATCAACGACCTGCGTATCT	0.00	0.00	0.29	0.00	0.00
GATCAACGACCTGGCCTACA	0.23	0.33	0.00	0.00	0.34
GATCAACGACCTGGCGAGCT	0.00	0.00	0.57	0.00	0.00
GATCAACGACCTGGTACCAC	0.00	0.00	0.00	0.00	0.00
GATCAACGACCTTCTCCTCC	0.00	0.00	0.29	0.00	0.00
GATCAACGACCTTCTTGAAT	0.00	0.00	0.29	0.00	0.00
GATCAACGACGAAAGACTGG	0.00	0.00	0.00	0.00	0.34
GATCAACGACGACAAGCCAG	0.00	0.00	0.00	0.00	0.34
GATCAACGACGACAGAAGAC	0.00	0.00	0.29	0.00	0.34
GATCAACGACGACAGCGCCG	0.00	0.00	0.00	0.00	0.00
GATCAACGACGACAGCTAAG	0.00	0.00	0.00	0.00	0.00
GATCAACGACGACAGCTCCG	0.00	0.33	0.29	0.00	0.69
GATCAACGACGACAGCTCTG	0.23	0.00	0.00	0.00	0.00
GATCAACGACGACAGCTGCG	0.00	0.00	0.00	0.34	0.00
GATCAACGACGACATGTACA	0.23	0.00	0.00	0.00	0.00
GATCAACGACGACCGCACCG	0.00	0.00	0.00	0.00	0.00
GATCAACGACGACCGCTCCG	0.00	0.00	0.00	0.00	0.00
GATCAACGACGACGACAACT	0.69	0.00	0.86	0.34	0.00

GATCAACGACGACGAGAAGT	0.23	0.33	0.00	0.00	0.00
GATCAACGACGACGCCACAA	0.00	0.00	0.00	0.34	0.00
GATCAACGACGACGGGCGGC	0.00	0.00	0.00	0.00	0.00
GATCAACGACGACGGTGAGT	0.23	0.00	0.29	0.00	0.00
GATCAACGACGACTTCGTGG	0.00	0.33	0.00	0.34	0.00
GATCAACGACGAGAACCCAC	0.00	0.00	0.00	0.00	0.00
GATCAACGACGAGCACGTGT	0.00	0.00	0.00	0.00	0.34
GATCAACGACGAGCCGCAAC	0.00	0.00	0.29	0.00	0.00
GATCAACGACGAGCTTGAGC	4.12	2.60	0.86	3.73	0.34
GATCAACGACGAGCTTGAGG	0.23	0.00	0.00	0.00	0.00
GATCAACGACGAGCTTGTGC	0.23	0.00	0.00	0.00	0.00
GATCAACGACGAGCTTNAGC	0.00	0.00	0.00	0.34	0.00
GATCAACGACGAGCTTTAGC	0.00	0.00	0.00	0.00	0.00
GATCAACGACGAGGAGAAGC	0.00	0.00	0.29	0.00	0.00
GATCAACGACGAGGTGCGGA	0.00	0.33	0.00	0.00	0.00
GATCAACGACGAGTGACGAA	1.83	1.30	0.29	0.68	1.38
GATCAACGACGAGTGACGCG	0.00	0.00	0.00	0.00	0.00
GATCAACGACGAGTTCAGGA	0.00	0.00	0.00	0.34	0.00
GATCAACGACGATAAGGAGG	0.00	0.33	0.00	0.00	0.00
GATCAACGACGATAGCTCCG	0.23	0.33	0.29	0.00	0.00
GATCAACGACGCAATCATCT	0.00	0.33	0.00	0.00	0.00
GATCAACGACGCACTCGCTT	0.00	0.33	0.00	0.00	0.00
GATCAACGACGCACTGGGGG	0.00	0.00	0.29	0.00	0.00
GATCAACGACGCAGGCCTGG	0.00	0.00	0.00	0.34	0.00
GATCAACGACGCCAAGCATG	0.00	0.00	0.00	0.00	0.00
GATCAACGACGCCACTGACC	0.00	0.33	0.29	0.34	0.34
GATCAACGACGCCAGGAGGA	0.00	0.00	0.00	0.34	0.00
GATCAACGACGCCATGACTG	0.00	0.00	0.00	0.00	0.00
GATCAACGACGCCATGAGCG	0.00	0.33	0.29	0.34	0.34
GATCAACGACGCCATGCATG	0.00	0.00	0.00	0.34	0.00
GATCAACGACGCCATGCCCC	0.00	0.00	0.00	0.34	0.00
GATCAACGACGCCATGCCTG	11.22	10.41	3.72	11.54	3.44
GATCAACGACGCCATGCCTN	0.00	0.00	0.00	0.00	0.00
GATCAACGACGCCATGCTGT	0.23	0.00	0.00	0.00	0.00
GATCAACGACGCCCGGCCCA	0.00	0.00	0.00	0.00	0.00
GATCAACGACGCCGAGGAGG	0.00	0.00	0.00	0.00	0.00
GATCAACGACGCCGATATGT	0.00	0.00	0.00	0.00	0.34

GATCAACGACGCCTCCATCT	0.00	0.00	0.29	0.00	0.00
GATCAACGACGCCTCGCCTA	0.23	0.00	0.00	0.00	0.00
GATCAACGACGCCTTCCGAC	0.00	0.00	0.00	0.00	0.00
GATCAACGACGCGAAGCTGT	0.46	0.00	0.00	0.00	0.00
GATCAACGACGCGCACGCGT	0.00	0.65	0.00	0.00	0.00
GATCAACGACGCGCGAGGCG	0.69	0.00	0.00	0.00	0.00
GATCAACGACGCGCTTGAGC	0.23	0.00	0.00	0.00	0.00
GATCAACGACGCGGACAACG	0.00	0.00	0.29	0.00	0.00
GATCAACGACGCGGGGAGGG	0.69	0.00	0.00	0.00	0.00
GATCAACGACGCGGGGGGGG	0.23	0.00	0.00	0.00	0.00
GATCAACGACGCGGTGCCTC	0.00	0.00	0.00	0.00	0.00
GATCAACGACGCGTAAGGGA	0.00	0.00	0.00	0.34	0.00
GATCAACGACGCGTGCAAGA	0.00	0.33	0.57	0.00	0.69
GATCAACGACGCTGAGTAAA	0.00	0.33	0.00	0.00	0.00
GATCAACGACGCTGGAGGGC	0.00	0.00	0.00	0.00	0.00
GATCAACGACGCTGTTGAAG	0.46	0.00	0.00	0.00	0.00
GATCAACGACGGACAGGACG	0.00	0.00	0.00	0.00	0.34
GATCAACGACGGCAACAAGA	0.00	0.00	0.29	0.00	0.00
GATCAACGACGGCACCATGA	0.00	0.00	0.00	0.00	0.00
GATCAACGACGGCACCATGC	0.00	0.33	0.00	0.34	0.00
GATCAACGACGGCCAGCCCC	0.46	0.00	0.00	0.00	0.00
GATCAACGACGGCGAAGATG	0.23	0.33	0.00	0.00	0.00
GATCAACGACGGCGAGAAGG	0.00	0.00	0.29	0.00	0.00
GATCAACGACGGCGAGCCCT	0.00	0.00	0.00	0.00	0.00
GATCAACGACGGCTGCACCT	0.46	0.00	0.29	0.00	0.34
GATCAACGACGGCTTCATGA	0.00	0.33	0.00	0.00	0.00
GATCAACGACGGGAGGGCTG	0.00	0.00	0.00	0.00	1.03
GATCAACGACGGGCAGCAGC	0.00	0.00	0.00	0.34	1.03
GATCAACGACGGGCGGCGGC	0.00	0.00	0.29	0.00	0.00
GATCAACGACGGGCTGCACA	0.00	0.33	0.00	0.00	0.00
GATCAACGACGGTCCAGGGC	0.00	0.00	0.00	0.34	0.00
GATCAACGACGGTGAAGGAC	0.00	0.33	0.00	0.00	0.00
GATCAACGACGTAGAGAAGG	0.00	0.33	0.00	0.00	0.34
GATCAACGACGTCAGCCCGA	0.69	1.95	0.29	1.36	1.03
GATCAACGACGTCAGCGTGG	0.00	0.00	0.29	0.34	0.00
GATCAACGACGTCGAGAAGG	21.52	36.76	48.39	39.03	39.95
GATCAACGACGTCGAGAANG	0.00	0.00	0.00	0.34	0.00

GATCAACGACGTCGAGACGG	0.00	0.00	0.00	0.34	0.00
GATCAACGACGTCGAGAGGG	0.00	0.33	0.00	0.00	0.00
GATCAACGACGTCGAGCAGG	0.46	0.00	0.00	0.34	0.00
GATCAACGACGTCGAGGAGG	0.23	0.00	0.00	0.34	0.00
GATCAACGACGTCGCGAAGG	0.00	0.00	0.29	0.00	0.00
GATCAACGACGTCGGCGAAG	0.46	0.00	0.00	0.00	0.00
GATCAACGACGTCGGCGCNN	0.23	0.00	0.00	0.00	0.00
GATCAACGACGTCGTGAAGG	0.00	0.00	0.00	0.00	0.00
GATCAACGACGTCTATCAGA	0.46	0.00	0.29	0.00	0.00
GATCAACGACGTGAAAGCAG	0.00	0.33	0.00	0.00	0.00
GATCAACGACGTGAAGGAGG	0.23	0.00	0.00	0.00	0.00
GATCAACGACGTGAAGGGAG	0.00	0.00	0.00	0.68	0.00
GATCAACGACGTGAAGGGTG	0.00	0.00	0.00	0.00	0.00
GATCAACGACGTGAAGGTGA	3.66	1.30	0.00	0.68	0.34
GATCAACGACGTGACCTTCA	0.00	0.00	0.00	0.00	0.00
GATCAACGACGTGCCCAAAT	0.00	0.00	0.00	0.34	0.00
GATCAACGACGTGGACCTCC	0.00	0.33	0.00	0.00	0.34
GATCAACGACGTGGAGCGGG	0.00	0.00	0.00	0.00	0.00
GATCAACGACGTGGCCCTGG	0.00	0.00	0.00	0.00	0.34
GATCAACGACGTGGCCTGGC	0.23	0.00	0.00	0.34	0.00
GATCAACGACGTGGCGTGGC	0.00	0.00	0.00	0.34	0.00
GATCAACGACGTGGCTCTGC	0.00	0.33	0.29	0.68	0.34
GATCAACGACGTGGCTGGCC	0.46	0.00	0.00	0.00	0.00
GATCAACGACGTGGGGCCAG	0.00	0.33	0.00	0.00	0.00
GATCAACGACGTTGAGAAGG	0.00	0.00	0.00	0.68	0.00
GATCAACGACGTTGTCGGCG	0.00	0.00	0.29	0.00	0.69
GATCAACGACTACCACCGGC	0.69	0.65	0.29	0.00	0.00
GATCAACGACTACCACCGGT	0.00	0.00	0.00	0.34	0.00
GATCAACGACTACCTGGACC	0.00	0.00	0.00	0.00	0.00
GATCAACGACTACTTCCGCT	0.00	0.00	0.57	0.00	0.00
GATCAACGACTACTTGGCCC	0.69	0.33	0.00	0.00	0.00
GATCAACGACTATCCCACGA	0.00	0.00	0.00	0.00	0.34
GATCAACGACTATGCTCAAG	0.00	0.00	0.00	0.00	0.34
GATCAACGACTATGCTCCAG	7.10	4.55	2.58	3.39	2.07
GATCAACGACTATGCTCCCG	0.00	0.00	0.29	0.00	0.00
GATCAACGACTCAAGTTCTG	0.00	0.00	0.57	0.00	0.00
GATCAACGACTCACGGCCTA	0.00	0.00	0.00	0.34	0.00

GATCAACGACTCAGAAGTAC	0.00	0.00	0.00	0.00	0.00
GATCAACGACTCCAAGCTTT	0.23	0.00	0.00	0.00	0.00
GATCAACGACTCCACGCTGT	0.00	0.33	0.00	0.00	0.00
GATCAACGACTCCATCTTGG	0.00	0.00	0.00	0.34	0.00
GATCAACGACTCCATGATGT	0.00	0.00	0.29	0.00	0.00
GATCAACGACTCCATGCTGA	0.00	0.33	0.29	0.00	0.00
GATCAACGACTCCATGCTGG	0.00	0.00	0.29	0.00	0.00
GATCAACGACTCCATGCTGT	13.28	16.92	21.19	27.83	30.31
GATCAACGACTCCATGCTNN	0.00	0.00	0.00	0.00	0.00
GATCAACGACTCCATGCTTC	0.00	0.00	0.29	0.00	0.00
GATCAACGACTCCATGNTGT	0.00	0.00	0.00	0.34	0.00
GATCAACGACTCCCAGCTGC	0.00	0.00	0.00	0.00	0.00
GATCAACGACTCCCTCTTCG	0.46	0.00	0.00	0.00	0.00
GATCAACGACTCCGTGCTGT	0.46	0.00	0.00	0.00	0.00
GATCAACGACTCCTACAAGG	0.23	0.00	0.00	0.00	0.00
GATCAACGACTCCTGACCGG	0.23	0.00	0.00	0.00	0.00
GATCAACGACTCCTGCAAGA	0.00	0.00	0.00	0.00	0.00
GATCAACGACTCCTGCAAGG	50.13	23.75	8.59	13.24	12.40
GATCAACGACTCCTGCACGG	0.46	0.00	0.00	0.00	0.34
GATCAACGACTCCTGCCAGG	0.00	0.33	0.00	0.00	0.00
GATCAACGACTCCTGCCAGT	0.00	0.00	0.00	0.00	0.34
GATCAACGACTCCTGCNANN	0.23	0.00	0.00	0.00	0.00
GATCAACGACTCCTGCNNNN	0.23	0.00	0.00	0.00	0.00
GATCAACGACTCCTTCATAA	0.00	0.00	0.00	0.00	0.00
GATCAACGACTCGAACCCAA	0.23	0.00	0.00	0.00	0.69
GATCAACGACTCGAAGCAGC	0.00	0.00	0.00	0.00	0.00
GATCAACGACTCGAAGCGTG	0.00	0.00	0.00	0.00	0.00
GATCAACGACTCGAGCGTAA	0.00	0.33	0.00	0.00	0.00
GATCAACGACTCGAGTGTTT	0.46	0.00	0.00	0.00	0.00
GATCAACGACTCGCGGCAGC	0.69	0.98	0.00	1.02	0.00
GATCAACGACTCGCGGCCGC	0.00	0.00	0.00	0.00	0.00
GATCAACGACTCGCTTGCTT	0.00	0.00	0.00	0.00	0.00
GATCAACGACTCGGAAGTAC	0.00	0.00	0.00	0.00	0.00
GATCAACGACTCGGAGGCGC	0.69	0.00	0.00	0.00	0.00
GATCAACGACTCGTGCAAGG	0.00	0.33	0.00	0.00	0.00
GATCAACGACTCTATGCTGT	0.00	0.00	0.00	0.00	0.34
GATCAACGACTCTATTCTGA	0.00	0.00	0.29	0.00	0.00

GATCAACGACTCTCGGCAAC	0.00	0.00	0.00	0.34	0.00
GATCAACGACTCTTGTTGCA	0.00	0.00	0.29	0.00	0.00
GATCAACGACTCTTTAGGGA	0.23	0.33	0.00	0.00	0.34
GATCAACGACTGACCAGCCG	0.00	0.33	0.29	0.34	0.00
GATCAACGACTGCAAAGGTG	0.00	0.00	0.00	0.00	0.00
GATCAACGACTGCAAGGTGG	0.23	0.00	0.00	0.00	0.00
GATCAACGACTGCACGGAGG	0.00	0.00	0.00	0.00	0.34
GATCAACGACTGCACGGCGG	0.00	0.65	0.29	0.34	0.00
GATCAACGACTGCACGGGGG	0.00	0.33	0.00	0.00	0.69
GATCAACGACTGCACGGTCG	0.00	0.00	0.00	0.00	0.00
GATCAACGACTGCACGGTGG	17.85	13.66	20.90	13.92	8.27
GATCAACGACTGCATCGACA	0.23	0.00	0.57	0.34	0.69
GATCAACGACTGCCTGGACT	0.00	0.00	0.00	0.00	0.00
GATCAACGACTGCGAATTAT	0.00	0.00	0.00	0.00	0.00
GATCAACGACTGCGCGGTGG	0.23	0.00	0.00	0.00	0.00
GATCAACGACTGCTGCAAGG	0.00	0.33	0.00	0.00	0.00
GATCAACGACTGCTTGGGAC	0.23	0.98	1.43	1.70	0.00
GATCAACGACTGGCCAAACA	0.23	0.00	0.00	0.00	0.00
GATCAACGACTGGCCAGCCG	0.23	0.00	0.00	0.00	0.00
GATCAACGACTGGGCTGCAA	0.00	0.00	0.00	0.00	0.00
GATCAACGACTGGGTTGCAA	0.23	0.00	0.29	0.34	0.00
GATCAACGACTGGTCCTGAG	0.00	0.33	0.00	0.00	0.00
GATCAACGACTTCAAAGTC	0.00	0.00	0.00	0.00	0.00
GATCAACGACTTCACGCCCG	0.00	0.00	0.29	0.00	0.00
GATCAACGACTTCCTCCTCG	1.37	0.33	0.57	0.00	1.03
GATCAACGACTTCCTCCTGG	0.00	0.00	0.00	0.00	0.00
GATCAACGACTTCCTGGAGG	0.00	0.00	0.29	1.02	0.00
GATCAACGACTTCGTCTACA	0.00	0.33	0.00	0.00	0.00
GATCAACGACTTCGTGTACG	0.00	0.00	0.00	0.00	0.00
GATCAACGACTTCTCGTGCC	0.00	0.00	0.29	0.00	0.00
GATCAACGACTTCTTCCTCC	0.00	0.00	0.00	0.34	0.00
GATCAACGACTTCTTCGTCC	0.00	0.00	0.00	0.34	0.00
GATCAACGACTTGCGCTACC	0.23	0.00	0.00	0.00	0.00
GATCAACGACTTGCGTGAGG	0.00	0.33	0.00	0.00	0.00
GATCAACGACTTGCTTGCTT	0.00	0.00	0.00	0.00	0.00
GATCAACGACTTGGACGAGA	0.00	0.00	0.00	0.00	0.00
GATCAACGACTTGTGGACAC	0.00	0.00	0.00	0.00	0.00

GATCAACGACTTGTGGCAAT	0.00	0.00	0.29	0.00	0.00
GATCAACGACTTGTGGCCAT	2.06	0.98	4.58	3.39	4.82
GATCAACGACTTGTTGAAGG	0.00	0.00	0.00	0.34	0.69
GATCAACGACTTTCGGAAGG	0.00	0.00	0.00	0.00	0.00
GATCAACGACTTTGTGTACG	0.00	0.00	0.00	0.68	0.00
GATCAACGACTTTTTGAGTC	0.23	0.00	0.00	0.00	0.00
GATCAACGACTTTTTGCGCC	0.23	0.00	0.00	0.00	0.00
GATCAACGAGAAAATTTGAG	0.23	0.00	0.00	0.00	0.00
GATCAACGAGAAACAGTTGC	0.00	0.00	0.00	0.00	0.00
GATCAACGAGAAAGGCTTTT	0.23	0.00	0.00	0.00	0.00
GATCAACGAGAAATGATAGG	0.00	0.00	0.29	0.00	1.38
GATCAACGAGAAATGTGCGC	0.00	0.33	0.00	0.00	0.00
GATCAACGAGAAATGTGCTG	0.23	0.00	0.00	0.00	0.00
GATCAACGAGAACCCCAGGC	0.00	0.00	0.00	0.00	0.00
GATCAACGAGAACGCCTATT	0.23	0.65	0.00	0.00	0.00
GATCAACGAGAACGCCTTCC	0.00	0.00	0.00	0.34	0.00
GATCAACGAGAACGGCGGGC	0.00	0.65	0.00	0.00	0.34
GATCAACGAGAACGGCGGGG	0.00	0.00	0.00	0.34	0.00
GATCAACGAGAACTCCTATT	0.00	0.00	0.00	0.34	0.00
GATCAACGAGAACTTCTTGA	0.00	0.00	0.00	0.34	0.00
GATCAACGAGAACTTGGTCT	0.00	0.33	0.00	0.00	0.00
GATCAACGAGAAGAACGTGG	0.00	0.00	0.00	0.00	0.00
GATCAACGAGAAGAGAATGA	0.00	0.00	0.00	0.00	0.00
GATCAACGAGAAGAGGCGGA	0.00	0.00	0.00	0.00	0.00
GATCAACGAGAAGATTGAGC	0.00	0.00	0.00	0.34	0.00
GATCAACGAGAAGCACAAGG	0.00	0.33	0.00	0.00	0.00
GATCAACGAGAAGCAGAGCC	0.00	0.00	0.00	1.02	0.00
GATCAACGAGAAGCAGGCCG	0.00	0.00	0.00	0.00	0.00
GATCAACGAGAAGCAGGGCT	0.00	0.00	0.00	0.00	0.00
GATCAACGAGAAGCCTCGGC	0.00	0.00	0.00	0.34	0.00
GATCAACGAGAAGCGCAACG	0.00	0.00	0.29	0.00	0.00
GATCAACGAGAAGCGGGAAG	0.00	0.00	0.00	0.00	0.00
GATCAACGAGAAGCTGCGCA	0.00	0.00	0.00	0.34	0.00
GATCAACGAGAAGCTGGGCG	0.00	0.65	0.00	0.00	0.34
GATCAACGAGAAGCTGGTCN	0.00	0.00	0.00	0.34	0.00
GATCAACGAGAAGCTGGTCT	2.06	0.00	0.57	0.00	0.00
GATCAACGAGAAGGAGGGAC	0.00	0.00	0.29	0.00	0.00

GATCAACGAGAAGGAGGTGG	0.00	0.00	0.00	0.34	1.03
GATCAACGAGAAGGCCCGCG	0.23	0.33	0.00	0.34	0.00
GATCAACGAGAAGGTGGTGG	0.00	0.33	0.00	0.00	0.00
GATCAACGAGAAGTGCATTT	0.00	0.33	0.00	0.00	0.00
GATCAACGAGAAGTTCAAGC	0.00	0.65	0.29	0.34	0.00
GATCAACGAGAAGTTCAAGN	0.00	0.00	0.00	0.00	0.00
GATCAACGAGAATGCGATTG	0.00	0.00	0.00	0.00	0.00
GATCAACGAGAATGCGCGGA	0.69	0.00	0.00	0.00	0.00
GATCAACGAGACAGAGATTG	0.00	0.65	0.86	0.68	0.00
GATCAACGAGACATATTGAC	0.00	0.00	0.00	0.00	0.34
GATCAACGAGACATTGTCGC	0.23	0.00	0.00	0.00	0.00
GATCAACGAGACCAAGGACG	0.23	0.65	0.00	0.68	0.34
GATCAACGAGACCCTCTGGG	0.23	0.33	0.00	0.00	0.00
GATCAACGAGACCCTGGGTG	0.46	0.00	0.00	0.00	0.00
GATCAACGAGACCGTGAAGG	0.23	0.00	0.00	0.00	0.00
GATCAACGAGACCTCCGAGT	0.23	0.00	0.00	0.00	0.00
GATCAACGAGACCTTCGGTC	0.00	0.00	0.00	0.00	0.34
GATCAACGAGACCTTTGGTA	0.00	0.33	0.00	0.00	0.00
GATCAACGAGACGACCACAC	0.00	0.00	0.29	0.00	0.00
GATCAACGAGACGCTGGGGG	0.23	0.00	0.00	0.00	0.00
GATCAACGAGACGGAGAGCT	0.00	0.00	0.00	0.00	0.00
GATCAACGAGACGGAGATCG	0.00	0.00	0.00	0.00	0.69
GATCAACGAGACGGAGGGAC	0.00	0.00	0.00	0.34	0.00
GATCAACGAGACGGAGGGCC	0.00	0.00	0.29	0.68	0.34
GATCAACGAGACTATCCCGA	0.00	0.00	0.00	0.00	0.00
GATCAACGAGACTCGGGAAT	0.23	0.00	0.00	0.00	0.00
GATCAACGAGACTGGCATTG	0.00	0.00	0.00	0.00	0.00
GATCAACGAGAGAGGGAGAA	0.00	0.00	0.29	0.00	0.00
GATCAACGAGAGCATCCAGA	0.00	0.33	0.00	0.00	0.00
GATCAACGAGAGCATGGTGG	0.23	0.00	0.00	0.00	0.00
GATCAACGAGAGCGCCCGCG	0.00	0.00	0.00	0.00	0.34
GATCAACGAGAGCTACGCGC	0.00	0.98	0.00	0.00	0.00
GATCAACGAGAGCTAGCCGA	0.00	0.00	0.00	0.00	0.00
GATCAACGAGAGCTTGTGAT	0.00	0.33	0.00	0.00	0.00
GATCAACGAGAGGACTACTC	0.23	0.00	0.00	0.00	0.00
GATCAACGAGAGGACTTCCC	0.00	0.00	0.00	0.00	0.00
GATCAACGAGAGGCAGCTCA	0.00	0.00	0.00	0.00	0.00

GATCAACGAGAGGCTTCAGG	0.00	0.65	0.29	0.00	0.34
GATCAACGAGAGGGTTCAGG	0.00	0.33	0.00	0.00	0.00
GATCAACGAGAGTTGAGACC	0.00	0.33	0.00	0.00	0.00
GATCAACGAGAGTTTTCAGG	0.46	0.00	0.00	0.00	0.00
GATCAACGAGATACAGCCCA	0.00	0.00	0.00	0.00	0.00
GATCAACGAGATATGGGCGA	0.00	0.00	0.00	0.00	0.34
GATCAACGAGATCATGATGT	0.00	0.00	0.00	0.68	0.34
GATCAACGAGATGAAGCAGG	0.23	0.00	0.00	0.00	0.00
GATCAACGAGATGAAGGCGG	0.46	0.65	0.29	0.68	0.34
GATCAACGAGATGAAGTGAG	0.00	0.00	0.00	0.00	0.00
GATCAACGAGATGACTGATC	0.00	0.00	0.00	0.00	0.00
GATCAACGAGATGACTTCTC	0.00	0.33	0.00	0.00	0.00
GATCAACGAGATGATGGTGG	0.00	0.00	0.00	0.68	0.00
GATCAACGAGATGCAACCCA	0.00	0.00	0.00	0.00	0.34
GATCAACGAGATGCAGCAGA	0.00	0.00	0.00	0.34	0.00
GATCAACGAGATGCAGCAGG	15.79	6.83	6.30	7.81	6.20
GATCAACGAGATGCAGCAGN	0.23	0.00	0.00	0.00	0.00
GATCAACGAGATGCAGCGGG	0.00	0.00	0.00	0.00	0.00
GATCAACGAGATGCAGGACG	0.00	0.00	0.00	0.34	0.00
GATCAACGAGATGCCAGAGC	0.00	0.00	0.00	0.00	0.00
GATCAACGAGATGCGCCTGG	0.69	0.33	0.00	0.00	0.34
GATCAACGAGATGCGGAAGC	0.00	0.33	0.00	1.02	1.03
GATCAACGAGATGGAGGAGA	0.00	0.00	0.00	0.34	0.00
GATCAACGAGATGGCCCTGG	0.00	0.33	0.00	0.00	0.00
GATCAACGAGATGGCCTTGG	12.82	9.11	3.44	4.07	2.41
GATCAACGAGATGGCCTTTG	0.00	0.33	0.00	0.00	0.00
GATCAACGAGATGGGTCACA	0.00	0.00	0.00	0.00	0.00
GATCAACGAGATGGTCCAGG	0.00	0.33	0.00	0.00	0.69
GATCAACGAGATGGTGCAGG	1.83	0.98	0.57	0.00	1.03
GATCAACGAGATGGTGCTGA	0.00	0.33	0.00	0.00	0.00
GATCAACGAGATGGTGNANN	0.23	0.00	0.00	0.00	0.00
GATCAACGAGATGTGCTACA	0.00	0.00	0.00	0.00	0.00
GATCAACGAGATTTGGAGAA	0.00	0.00	0.00	0.34	0.00
GATCAACGAGCAAAAAAAAT	0.00	0.00	0.29	0.00	0.00
GATCAACGAGCAACAACCAA	0.00	0.00	0.00	0.00	0.34
GATCAACGAGCAACAAGAAC	0.00	0.00	0.57	0.00	0.00
GATCAACGAGCAACGCGCAT	0.00	0.00	0.00	0.00	0.69

GATCAACGAGCAATGGTTGA	0.00	0.00	0.00	0.00	0.00
GATCAACGAGCACAACAGTG	0.00	0.00	0.00	0.34	0.00
GATCAACGAGCACAACGAGT	0.00	0.00	0.00	0.00	0.34
GATCAACGAGCACACATCAC	0.00	0.00	0.29	0.00	0.00
GATCAACGAGCACACCNNNN	0.23	0.00	0.00	0.00	0.00
GATCAACGAGCACACCTACG	21.29	7.16	4.58	6.11	3.10
GATCAACGAGCACACCTACN	0.23	0.00	0.00	0.00	0.00
GATCAACGAGCACACCTCCG	0.23	0.00	0.00	0.00	0.00
GATCAACGAGCACCGCCCGC	0.00	0.00	0.00	0.00	0.00
GATCAACGAGCACCGGCGAA	0.00	0.00	0.29	0.00	0.00
GATCAACGAGCACGAGCTTT	0.69	0.33	0.29	0.34	0.69
GATCAACGAGCACGGCCACC	0.46	0.00	0.00	0.00	0.00
GATCAACGAGCACGGCCTGC	0.00	0.33	0.00	0.34	0.00
GATCAACGAGCACTACTCGG	1.83	1.30	0.00	3.73	3.44
GATCAACGAGCACTCAGCCT	0.00	0.00	0.00	0.34	0.00
GATCAACGAGCACTTGAGGC	0.00	0.00	0.00	0.34	0.00
GATCAACGAGCAGAGAGCAG	0.00	0.00	0.00	0.00	0.34
GATCAACGAGCAGCCGAAGA	0.46	0.00	0.00	0.00	0.00
GATCAACGAGCAGCGTGTGG	0.00	0.00	0.00	0.68	0.00
GATCAACGAGCAGCTCGCCG	0.00	0.65	0.00	0.34	0.34
GATCAACGAGCAGGAGCGCG	0.23	0.00	0.29	1.02	0.00
GATCAACGAGCAGGCGCGAG	0.23	0.00	0.00	0.00	0.00
GATCAACGAGCAGGTCCGGC	0.00	0.33	0.00	0.34	0.00
GATCAACGAGCAGTGCCGTC	0.46	0.00	0.00	0.00	0.00
GATCAACGAGCAGTGCGGGA	0.00	0.00	0.57	0.00	0.00
GATCAACGAGCAGTGCGGGG	1.60	1.30	1.72	0.34	0.00
GATCAACGAGCAGTGTGGGC	0.00	0.00	0.00	0.00	0.00
GATCAACGAGCATGAGGAGC	0.00	0.00	0.00	0.00	0.00
GATCAACGAGCATGAGGAGG	0.00	0.00	0.00	0.00	0.00
GATCAACGAGCATGAGGAGT	0.00	0.00	0.00	0.34	0.00
GATCAACGAGCATGGCATTC	0.00	0.00	0.29	0.00	0.00
GATCAACGAGCATTGGCATA	0.00	0.33	0.00	0.00	0.00
GATCAACGAGCCAACTGTTC	0.00	0.00	0.00	0.00	0.34
GATCAACGAGCCAAGCTCCA	0.00	0.00	0.00	0.00	0.34
GATCAACGAGCCAATGGACT	0.00	0.00	0.00	0.00	0.34
GATCAACGAGCCAATGTAAT	0.00	0.00	0.00	0.34	0.00
GATCAACGAGCCAATGTACT	0.69	0.00	0.00	0.68	0.34

GATCAACGAGCCACACACGC	0.00	0.00	0.00	0.00	0.34
GATCAACGAGCCACATGTTC	0.23	0.00	0.00	0.00	0.00
GATCAACGAGCCAGCTCCTG	0.00	0.33	0.57	0.00	0.34
GATCAACGAGCCATCCGNNN	0.00	0.00	0.00	0.00	0.00
GATCAACGAGCCCGAGGACC	0.23	0.65	0.29	0.00	0.00
GATCAACGAGCCCGAGGACG	0.00	0.00	0.00	0.34	0.00
GATCAACGAGCCCGCTCCCC	0.00	0.00	0.29	0.00	0.00
GATCAACGAGCCCGCTCTGG	0.00	0.00	0.29	0.00	0.00
GATCAACGAGCCCGGCCCCG	0.00	0.00	0.29	0.00	0.00
GATCAACGAGCCCTGCCTGT	0.00	0.33	0.00	0.00	0.00
GATCAACGAGCCGCATCGCA	0.00	0.00	0.00	0.00	0.00
GATCAACGAGCCGCCGACGA	0.00	0.00	0.00	0.00	0.00
GATCAACGAGCCGCTGCCTG	0.00	0.00	0.00	0.00	0.00
GATCAACGAGCCGGGCGGGG	1.60	2.28	0.00	0.68	1.72
GATCAACGAGCCGTGCAAGG	0.00	0.33	0.00	0.00	0.00
GATCAACGAGCCGTGCCAGG	0.00	0.00	0.00	0.00	0.00
GATCAACGAGCCTCGCGCAT	0.46	0.00	0.00	0.00	0.00
GATCAACGAGCCTGCCAAGA	0.00	0.00	0.00	0.00	0.00
GATCAACGAGCCTGCGTGGA	0.00	0.33	0.00	0.00	0.00
GATCAACGAGCGAAAAAAAA	0.00	0.00	0.00	0.00	0.00
GATCAACGAGCGAACCCGCG	0.46	0.00	0.00	0.00	0.00
GATCAACGAGCGAAGTAAGA	0.00	0.00	0.00	0.34	0.00
GATCAACGAGCGACAAACGG	0.00	0.98	0.86	0.68	0.34
GATCAACGAGCGACCTACCG	0.00	0.00	0.00	0.00	0.00
GATCAACGAGCGACCTACCT	0.00	0.00	0.29	0.00	0.00
GATCAACGAGCGAGCAACGA	0.00	0.00	0.00	0.00	0.34
GATCAACGAGCGCAAAAAAA	0.00	0.33	0.00	0.00	0.00
GATCAACGAGCGCAAGCACC	0.23	0.00	0.00	0.00	0.00
GATCAACGAGCGCAATCAGC	0.00	0.00	0.29	0.00	0.00
GATCAACGAGCGCAGGCCAG	0.00	0.00	0.00	0.34	0.00
GATCAACGAGCGCATCAAGA	0.00	0.00	0.29	0.00	0.00
GATCAACGAGCGCATCACTC	0.00	0.00	0.57	0.34	0.34
GATCAACGAGCGCCACGTGG	0.00	0.00	0.00	0.00	0.00
GATCAACGAGCGCCTGCATC	0.00	0.00	0.29	0.00	0.00
GATCAACGAGCGCCTGGGCG	0.00	0.00	0.00	0.00	0.34
GATCAACGAGCGCGACGAGT	0.23	0.00	0.00	0.00	0.00
GATCAACGAGCGCGCCCCAT	0.00	0.00	0.00	0.00	0.69

GATCAACGAGCGCGGTGGCG	0.00	0.00	0.29	0.68	0.00
GATCAACGAGCGCTGGCTGG	0.00	0.00	0.00	0.00	0.00
GATCAACGAGCGGAATGGCA	0.00	0.00	0.29	0.00	0.00
GATCAACGAGCGGACGGAGC	0.23	0.00	0.00	0.00	0.00
GATCAACGAGCGGCAAGGGC	0.00	0.00	0.00	0.00	0.00
GATCAACGAGCGGCAGCTGA	0.00	0.00	0.29	0.00	0.00
GATCAACGAGCGGCGGGGCT	0.00	0.00	0.00	0.00	0.00
GATCAACGAGCGGCTGGGAG	0.00	0.00	0.00	0.00	0.34
GATCAACGAGCGGGAGTACG	0.23	0.33	0.00	0.00	0.00
GATCAACGAGCGGGCTTCAA	0.00	0.33	0.00	0.34	0.00
GATCAACGAGCGGTCCTTCG	8.70	2.93	0.86	3.05	0.34
GATCAACGAGCGTCAAAAGC	0.23	0.33	0.00	0.00	0.00
GATCAACGAGCTCAAAGAGT	0.00	0.00	0.29	0.00	0.00
GATCAACGAGCTCAACGAGC	0.00	0.00	0.00	0.00	0.00
GATCAACGAGCTCAACGAGN	0.46	0.00	0.00	0.00	0.00
GATCAACGAGCTCAACGAGT	37.54	29.60	15.46	16.97	16.19
GATCAACGAGCTCAACGANN	0.23	0.00	0.00	0.00	0.00
GATCAACGAGCTCAACGCGT	0.00	0.00	0.00	0.00	0.00
GATCAACGAGCTCAACNCNN	0.23	0.00	0.00	0.00	0.00
GATCAACGAGCTCAACNNNN	0.46	0.00	0.00	0.00	0.00
GATCAACGAGCTCAAGGAGC	0.00	0.00	0.00	0.00	0.34
GATCAACGAGCTCAAGGCCC	0.00	0.00	0.00	0.34	0.00
GATCAACGAGCTCACCTCCG	0.00	0.00	0.00	0.34	0.00
GATCAACGAGCTCAGCGAGT	0.00	0.33	0.00	0.00	0.00
GATCAACGAGCTCATGAGGA	0.00	0.00	0.00	0.00	1.03
GATCAACGAGCTCATGCGGA	0.00	0.00	0.00	0.00	0.00
GATCAACGAGCTCCAGCAGC	0.00	1.63	0.86	2.38	0.69
GATCAACGAGCTCCGGCGGG	0.00	0.00	0.00	0.00	0.00
GATCAACGAGCTCCGGGTGC	0.23	0.00	0.29	0.34	0.00
GATCAACGAGCTCGGCCTNN	0.00	0.00	0.00	0.00	0.00
GATCAACGAGCTCGGGGGCT	0.00	0.00	0.00	0.34	0.00
GATCAACGAGCTCTCGGAGC	0.00	0.00	0.00	0.00	0.34
GATCAACGAGCTCTCGGGGC	0.00	0.00	0.00	0.00	0.34
GATCAACGAGCTCTGCCACG	0.00	0.00	0.00	0.68	0.00
GATCAACGAGCTCTGCTACG	0.00	0.00	0.00	0.00	0.00
GATCAACGAGCTCTGCTACT	0.46	0.00	0.00	0.00	0.00
GATCAACGAGCTCTGGGCCG	0.00	0.00	0.29	0.00	0.00

GATCAACGAGCTCTTACACG	0.00	0.00	0.00	0.00	0.34
GATCAACGAGCTCTTCGTGG	0.00	0.00	0.00	0.68	0.69
GATCAACGAGCTCTTGTCNG	0.00	0.00	0.00	0.00	0.00
GATCAACGAGCTCTTGTCTG	0.00	1.63	0.86	1.36	0.69
GATCAACGAGCTGAAGTGCA	0.00	0.00	0.00	0.00	0.34
GATCAACGAGCTGACGTCTG	0.00	0.33	0.00	0.68	0.00
GATCAACGAGCTGACTCGCC	0.00	0.33	0.00	0.00	0.00
GATCAACGAGCTGATGCGCC	0.00	0.00	0.29	0.00	0.00
GATCAACGAGCTGATTAGCC	0.00	0.00	0.00	0.00	0.00
GATCAACGAGCTGATTCGAA	0.00	0.00	0.00	0.00	0.34
GATCAACGAGCTGATTCGAC	0.00	0.33	0.00	0.00	0.00
GATCAACGAGCTGATTCGCC	19.23	20.50	27.20	30.55	27.90
GATCAACGAGCTGATTCGCG	0.00	0.00	0.00	0.00	0.00
GATCAACGAGCTGATTCGCT	0.23	0.00	0.00	0.00	0.00
GATCAACGAGCTGATTCGNN	0.23	0.00	0.00	0.00	0.00
GATCAACGAGCTGCACACCG	0.00	0.00	0.00	0.00	0.00
GATCAACGAGCTGCACGTGG	0.00	0.00	0.00	1.36	0.00
GATCAACGAGCTGCAGCAGG	0.00	0.00	0.00	0.00	0.00
GATCAACGAGCTGCGGGCGG	0.00	0.00	0.00	0.00	0.00
GATCAACGAGCTGCGTGGTG	0.23	0.00	0.00	1.02	0.00
GATCAACGAGCTGCTACGGC	0.00	0.00	0.00	0.00	0.69
GATCAACGAGCTGCTGGGTA	1.14	0.33	0.29	0.68	1.03
GATCAACGAGCTGGAGGGGT	0.00	0.00	0.00	0.00	0.00
GATCAACGAGCTGGATGCTG	0.00	0.00	0.00	0.00	0.34
GATCAACGAGCTGGCGAGTG	0.00	0.00	0.00	0.00	0.00
GATCAACGAGCTGGCGGAGG	0.00	0.00	0.00	0.00	0.34
GATCAACGAGCTGGCTGCGA	0.00	1.63	0.29	0.00	0.00
GATCAACGAGCTGGGCGAGG	0.00	0.33	0.00	0.00	0.00
GATCAACGAGCTGGTTCGGG	0.00	0.00	0.00	0.00	0.00
GATCAACGAGCTGTGAGCGC	0.00	0.00	0.00	0.00	0.00
GATCAACGAGCTGTGGTGCT	0.00	0.00	0.29	0.00	0.00
GATCAACGAGCTTATCCGTG	0.46	0.65	0.86	0.00	0.00
GATCAACGAGCTTATTCGCC	0.92	0.00	0.00	0.00	0.00
GATCAACGAGCTTCAGTCTC	0.00	0.00	0.00	0.00	0.00
GATCAACGAGCTTCGGTCGG	0.00	0.00	0.00	0.00	0.00
GATCAACGAGCTTCTGTCTT	0.23	0.00	0.00	0.00	0.00
GATCAACGAGCTTTGCTACA	0.23	0.00	0.29	0.68	1.38

GATCAACGAGGAAAAAAACG	0.23	0.00	0.00	0.00	0.00
GATCAACGAGGAACTCGGAT	0.00	0.00	0.00	0.00	0.00
GATCAACGAGGAATGGGTCA	0.00	0.00	0.00	0.00	0.00
GATCAACGAGGACAACATTG	0.46	0.00	0.00	0.00	0.34
GATCAACGAGGACAAGGCTT	0.00	0.00	0.00	0.00	0.00
GATCAACGAGGACAAGTCGG	0.00	0.00	0.29	0.00	0.00
GATCAACGAGGACAGCTCAG	0.00	0.33	0.00	0.34	0.34
GATCAACGAGGACAGCTCCG	0.00	0.00	0.00	0.00	0.34
GATCAACGAGGACCACGCGA	0.00	0.00	0.00	0.00	0.00
GATCAACGAGGACCAGCGCC	0.00	0.00	0.00	0.00	0.00
GATCAACGAGGACCGCCTCT	0.00	0.00	0.29	0.34	0.00
GATCAACGAGGACCGGTTCT	0.00	0.33	0.00	0.00	0.00
GATCAACGAGGACCTCAAGG	0.00	0.00	0.00	0.68	0.00
GATCAACGAGGACCTGCTGC	0.00	0.00	0.29	0.00	0.00
GATCAACGAGGACCTGGCTG	0.00	0.00	0.00	0.00	0.00
GATCAACGAGGACCTTGTAG	0.00	0.00	0.00	0.00	0.69
GATCAACGAGGACGAACAGC	0.00	0.00	0.29	0.00	0.00
GATCAACGAGGACGAACTGC	0.69	0.00	0.29	0.00	0.69
GATCAACGAGGACGACCTGC	0.23	0.33	0.86	4.41	1.38
GATCAACGAGGACGAGAAGG	0.00	0.33	0.00	0.00	0.00
GATCAACGAGGACGAGACCA	0.23	0.00	0.00	0.00	0.00
GATCAACGAGGACGAGGATG	0.00	0.00	0.00	0.00	0.34
GATCAACGAGGACGGAGGAC	0.00	0.00	0.00	0.00	0.69
GATCAACGAGGACTACGAGG	0.00	0.00	0.00	0.00	0.00
GATCAACGAGGACTGACAGG	0.00	0.00	0.00	0.00	0.00
GATCAACGAGGACTGAGAGC	0.00	0.00	0.00	0.00	0.00
GATCAACGAGGACTGGGGCA	0.00	0.33	0.00	0.00	0.00
GATCAACGAGGACTGGGTCA	1.60	3.90	2.58	1.36	3.44
GATCAACGAGGACTGGGTCG	0.00	0.00	0.00	0.00	0.34
GATCAACGAGGACTGGNTCA	0.00	0.00	0.00	0.00	0.34
GATCAACGAGGACTTTGAGG	0.00	0.00	0.00	0.34	0.00
GATCAACGAGGACTTTGCCA	0.00	0.00	0.00	0.00	0.00
GATCAACGAGGAGAAATTGA	0.00	0.00	0.00	0.00	0.00
GATCAACGAGGAGAAGTTGA	0.00	0.00	0.00	0.00	0.00
GATCAACGAGGAGACCGAGC	0.00	0.00	0.00	0.00	0.00
GATCAACGAGGAGAGGAAAG	0.00	0.00	0.29	0.00	0.00
GATCAACGAGGAGAGGGCGG	0.00	0.00	0.00	0.00	0.34

GATCAACGAGGAGATGGCAA	0.00	0.00	0.29	0.00	0.00
GATCAACGAGGAGATGGCCA	0.46	0.98	0.00	0.00	0.00
GATCAACGAGGAGCACAACG	0.00	0.00	0.00	0.68	0.00
GATCAACGAGGAGCACGCGT	0.00	0.33	0.00	0.00	0.00
GATCAACGAGGAGCCCATTG	0.00	0.00	0.00	0.00	0.34
GATCAACGAGGAGCCCTTCA	0.00	0.00	0.00	0.00	0.34
GATCAACGAGGAGGAGCTGC	0.00	0.33	0.00	0.00	0.00
GATCAACGAGGAGGAGGAGC	0.00	0.00	0.29	0.00	0.00
GATCAACGAGGAGGAGTTCT	0.00	0.00	0.00	0.00	0.00
GATCAACGAGGAGGATGACA	0.23	0.00	0.00	0.00	0.69
GATCAACGAGGAGGCCAAGG	0.00	0.33	0.00	0.00	0.00
GATCAACGAGGAGGCTACAG	0.00	0.65	0.29	0.00	0.00
GATCAACGAGGAGGTGGAGG	0.00	0.33	0.00	0.34	0.00
GATCAACGAGGAGGTTCGCT	0.00	0.33	0.00	0.34	0.00
GATCAACGAGGAGTCCATTG	0.46	0.33	0.29	0.34	0.00
GATCAACGAGGAGTGGGAGG	1.14	0.33	0.57	0.00	1.38
GATCAACGAGGAGTGGGGGG	0.23	0.00	0.00	0.00	0.00
GATCAACGAGGAGTTGGCGG	0.00	0.33	0.00	0.34	0.69
GATCAACGAGGATAAGTAAT	0.00	0.00	0.29	0.00	0.00
GATCAACGAGGATGAGATCA	0.00	0.00	0.29	0.00	0.00
GATCAACGAGGATGAGCACC	0.00	0.33	0.00	0.00	0.00
GATCAACGAGGATGAGCGCA	0.23	0.00	0.00	0.00	0.00
GATCAACGAGGATGAGCTCA	0.00	0.00	0.00	0.00	0.34
GATCAACGAGGATGAGCTCC	12.82	14.31	6.87	11.88	14.46
GATCAACGAGGATGAGCTCN	0.23	0.00	0.00	0.00	0.00
GATCAACGAGGATGAGCTGC	0.46	0.00	0.00	0.00	0.00
GATCAACGAGGATGAGCTNC	0.00	0.00	0.00	0.34	0.00
GATCAACGAGGATGGCGTCA	0.00	0.33	0.00	0.34	0.00
GATCAACGAGGATGGGCTCC	0.00	0.00	0.29	0.00	0.00
GATCAACGAGGATGGGGCTC	0.00	0.00	0.29	0.00	0.00
GATCAACGAGGCAAACCCGC	0.00	0.00	0.00	0.00	0.00
GATCAACGAGGCAACCCCGC	6.64	5.21	2.29	6.45	2.07
GATCAACGAGGCAACTACGC	0.00	0.00	0.00	0.34	0.00
GATCAACGAGGCAAGGGCCC	0.23	0.00	0.00	0.00	0.00
GATCAACGAGGCAATGCGGG	0.92	1.30	0.57	1.36	0.00
GATCAACGAGGCAATTGGCC	0.00	0.00	0.00	0.34	0.00
GATCAACGAGGCACCTCTCC	0.00	0.00	0.00	0.00	0.34

GATCAACGAGGCAGACAAGA	0.23	0.00	0.00	0.00	0.00
GATCAACGAGGCAGCTCTCA	0.00	0.33	0.00	0.00	0.00
GATCAACGAGGCAGTTAAGA	0.00	0.00	0.00	0.00	0.34
GATCAACGAGGCAGTTGTTG	0.00	0.33	0.00	0.00	0.00
GATCAACGAGGCATTGCCGC	0.00	0.00	0.00	0.00	0.34
GATCAACGAGGCCAAATCAA	0.00	0.00	0.00	0.00	0.34
GATCAACGAGGCCAAATNAN	0.00	0.00	0.00	0.00	0.34
GATCAACGAGGCCAAGGAGA	0.69	0.65	0.29	1.02	0.34
GATCAACGAGGCCAAGGAGC	0.23	0.00	0.29	0.00	0.00
GATCAACGAGGCCACCCCGC	0.00	0.00	0.29	0.00	0.00
GATCAACGAGGCCAGAAAGC	0.00	0.00	0.29	0.00	0.00
GATCAACGAGGCCATGCGAG	0.23	0.00	0.00	0.00	0.00
GATCAACGAGGCCATGCGGG	1.37	0.33	0.57	1.02	0.00
GATCAACGAGGCCATGGCGC	0.00	0.00	0.00	0.00	0.34
GATCAACGAGGCCATTGGCC	0.00	0.00	0.00	0.00	0.00
GATCAACGAGGCCCACGCGG	0.46	0.33	0.00	0.00	0.00
GATCAACGAGGCCCATGCGG	0.00	0.00	0.00	0.00	0.00
GATCAACGAGGCCCTCTTCA	0.00	0.33	0.00	0.00	0.34
GATCAACGAGGCCCTGGACC	0.00	0.33	0.29	0.00	0.00
GATCAACGAGGCCGAGCAGT	0.00	0.00	0.00	0.00	0.00
GATCAACGAGGCCGAGGGTC	0.00	0.00	0.00	0.00	0.69
GATCAACGAGGCCGCGGACG	0.00	0.00	0.00	0.00	0.34
GATCAACGAGGCCGCTCTGG	0.00	0.00	0.00	1.02	0.00
GATCAACGAGGCCGTCCGCT	0.00	0.00	0.00	0.00	0.00
GATCAACGAGGCCTCCCACG	0.00	0.33	0.00	0.00	0.00
GATCAACGAGGCCTCGCACG	0.00	0.00	0.00	0.34	0.00
GATCAACGAGGCCTTCGCAT	0.00	0.33	0.00	0.00	0.00
GATCAACGAGGCCTTCTTCC	0.00	0.00	0.29	0.00	0.00
GATCAACGAGGCCTTTCCTT	0.00	0.00	0.00	0.34	0.00
GATCAACGAGGCGACCGTTC	0.00	0.00	0.00	0.00	0.00
GATCAACGAGGCGACCGTTG	0.00	0.00	0.00	0.00	0.00
GATCAACGAGGCGCGCACCT	0.00	0.33	0.00	0.34	0.34
GATCAACGAGGCGCTCGACA	0.00	0.33	0.00	0.00	0.00
GATCAACGAGGCGCTGATAA	0.00	0.00	0.29	0.00	0.00
GATCAACGAGGCGGTCGACC	0.00	0.00	0.00	0.00	0.00
GATCAACGAGGCGGTCGAGC	0.23	0.00	0.00	0.00	0.00
GATCAACGAGGCGGTGCCTG	0.23	0.00	0.00	0.00	0.00

GATCAACGAGGCGTCCTATC	0.00	0.33	0.00	0.00	0.00
GATCAACGAGGCGTCCTTCG	0.46	0.00	0.29	0.00	0.34
GATCAACGAGGCGTCGTGCA	0.00	0.00	0.00	0.34	0.00
GATCAACGAGGCGTTAGCCC	0.23	0.00	0.00	0.00	0.00
GATCAACGAGGCGTTCGAAC	0.00	0.33	0.57	0.00	0.00
GATCAACGAGGCGTTCGACA	0.00	0.33	0.00	1.02	0.00
GATCAACGAGGCGTTCGACC	58.60	55.31	30.64	50.58	27.55
GATCAACGAGGCGTTCGACT	0.00	0.00	0.29	0.00	0.00
GATCAACGAGGCGTTCGAGC	0.23	0.00	0.00	0.00	0.00
GATCAACGAGGCGTTCGANC	0.00	0.00	0.00	0.00	0.00
GATCAACGAGGCGTTCGANN	0.46	0.00	0.00	0.00	0.00
GATCAACGAGGCGTTCGATC	0.00	0.00	0.00	0.34	0.00
GATCAACGAGGCGTTCGCCC	0.23	0.33	0.00	0.34	0.34
GATCAACGAGGCGTTCGCCT	0.00	0.65	1.72	0.00	0.00
GATCAACGAGGCGTTCGGCC	0.23	0.00	0.00	0.00	0.00
GATCAACGAGGCGTTCGTCA	0.00	0.00	0.00	0.00	0.00
GATCAACGAGGCGTTCGTCC	0.00	0.33	0.00	0.00	0.00
GATCAACGAGGCGTTCNCNN	0.23	0.00	0.00	0.00	0.00
GATCAACGAGGCTCGGCGCA	0.00	0.00	0.00	0.68	0.00
GATCAACGAGGCTCGGCGCN	0.00	0.00	0.00	0.34	0.00
GATCAACGAGGCTGCACAGC	0.46	1.30	0.57	1.02	1.72
GATCAACGAGGCTGCTCAGT	0.00	0.00	0.00	0.00	0.00
GATCAACGAGGCTGGACAGC	0.00	0.00	0.00	0.00	0.00
GATCAACGAGGCTGTCATCA	0.00	0.00	0.00	0.34	0.00
GATCAACGAGGCTTCTTTCG	0.00	0.00	0.00	0.00	0.00
GATCAACGAGGCTTGATACG	0.00	0.00	0.00	0.00	0.34
GATCAACGAGGCTTGCCGGC	0.00	0.00	0.00	0.00	0.34
GATCAACGAGGCTTTCGACC	0.23	0.00	0.00	0.00	0.00
GATCAACGAGGGAATGGTGG	0.00	0.33	0.00	0.00	0.00
GATCAACGAGGGCACGCACA	0.00	0.00	0.00	0.00	1.38
GATCAACGAGGGCAGGGTGG	0.00	0.00	0.29	0.00	0.00
GATCAACGAGGGCATAGTGG	0.00	0.00	0.00	0.00	0.00
GATCAACGAGGGCATGGAGG	0.23	0.65	0.00	1.02	0.00
GATCAACGAGGGCATGGCAG	0.00	0.00	0.00	0.00	0.00
GATCAACGAGGGCATGGCGG	0.92	0.65	0.57	0.34	0.69
GATCAACGAGGGCATGGGGG	0.92	0.98	0.00	0.00	0.34
GATCAACGAGGGCATGGTCG	0.00	0.00	0.00	0.00	0.00

GATCAACGAGGGCATGGTGG	46.47	86.21	71.02	83.84	75.42
GATCAACGAGGGCATGGTNG	0.00	0.00	0.00	0.00	0.00
GATCAACGAGGGCATGGTNN	0.00	0.00	0.00	0.00	0.00
GATCAACGAGGGCATGNNNN	0.23	0.00	0.00	0.00	0.00
GATCAACGAGGGCATGNTGG	0.00	0.00	0.00	0.34	0.00
GATCAACGAGGGCCGCGCAC	0.00	0.00	0.00	0.00	0.34
GATCAACGAGGGCCTGTGAG	0.00	0.33	0.00	0.34	0.00
GATCAACGAGGGCGCGAAGC	0.23	0.00	0.00	0.00	0.00
GATCAACGAGGGCGTGGTGG	0.00	0.00	0.00	0.68	0.00
GATCAACGAGGGCTTCAAGT	0.00	0.00	0.57	1.02	0.34
GATCAACGAGGGGAAAAGGA	0.23	0.00	0.00	0.00	0.00
GATCAACGAGGGGAACATGC	0.00	0.00	0.00	0.00	0.00
GATCAACGAGGGGAATTGCC	0.23	0.00	0.00	0.00	0.00
GATCAACGAGGGGATGGTGG	0.23	0.00	0.00	0.00	0.00
GATCAACGAGGGGGGGAACA	0.00	0.33	0.00	0.68	0.00
GATCAACGAGGGGGGGGACA	0.69	0.00	0.00	0.00	0.00
GATCAACGAGGGGTACAGAC	0.00	0.00	0.29	0.00	0.69
GATCAACGAGGGGTACATAC	0.00	0.00	0.57	0.00	1.72
GATCAACGAGGGGTCTTGCC	0.23	0.33	0.00	1.02	0.34
GATCAACGAGGGTATGGTGG	0.00	0.00	0.00	0.00	0.34
GATCAACGAGGGTGACGTGG	0.00	0.00	0.00	0.00	0.00
GATCAACGAGGGTGCGCCCG	0.00	0.00	0.00	0.00	0.00
GATCAACGAGGTAGCGAAGC	0.00	0.00	0.29	0.00	0.00
GATCAACGAGGTCATGCCCA	0.00	0.00	0.00	0.68	0.34
GATCAACGAGGTCCAGGCGA	0.00	0.00	0.00	0.00	0.69
GATCAACGAGGTCCCAGATT	0.00	0.00	0.00	0.68	0.34
GATCAACGAGGTCCCGCTGC	0.00	0.00	0.00	0.00	0.00
GATCAACGAGGTCGCGAAGA	0.00	0.00	0.29	0.00	0.00
GATCAACGAGGTCGCGAAGC	0.23	1.95	0.86	0.34	0.00
GATCAACGAGGTCGCGACGC	0.00	0.33	0.00	0.00	0.00
GATCAACGAGGTCGCGCAGC	0.00	0.00	0.00	0.00	0.00
GATCAACGAGGTCGCGGAGC	0.23	0.00	0.00	0.00	0.00
GATCAACGAGGTCGTGGAAA	0.00	0.00	0.29	0.00	0.00
GATCAACGAGGTCGTGGACA	0.00	0.00	0.00	0.68	0.34
GATCAACGAGGTCTCGCTGC	0.69	0.98	0.00	0.34	0.34
GATCAACGAGGTCTCGTGAA	0.00	0.00	0.00	0.00	0.34
GATCAACGAGGTCTTCCAGG	0.46	0.00	0.00	0.00	0.00

GATCAACGAGGTGAAGTACT	0.00	0.00	0.29	0.00	0.00
GATCAACGAGGTGAATAGTC	0.00	0.00	0.00	0.34	0.00
GATCAACGAGGTGATGCTGA	0.00	0.00	0.00	0.00	0.00
GATCAACGAGGTGCCATCCG	0.00	0.00	0.00	0.00	0.00
GATCAACGAGGTGCCATCCT	0.00	0.00	0.00	0.68	0.00
GATCAACGAGGTGCCGGCGG	0.46	0.00	0.00	0.34	0.00
GATCAACGAGGTGCGCATGC	0.00	0.00	0.29	0.00	0.00
GATCAACGAGGTGCTGACCA	0.00	0.33	0.00	0.68	0.00
GATCAACGAGGTGCTGCCCA	0.46	0.00	0.00	0.00	0.00
GATCAACGAGGTGCTGGCCG	0.00	0.00	0.00	0.34	0.00
GATCAACGAGGTGCTGGGTT	0.00	0.00	0.29	0.00	0.34
GATCAACGAGGTGCTGGTCA	0.46	0.00	0.29	0.34	0.34
GATCAACGAGGTGCTGGTCG	0.00	0.00	0.00	0.00	0.00
GATCAACGAGGTGGATGCAG	0.00	0.00	0.00	0.00	0.00
GATCAACGAGGTGGATGCCG	0.46	0.33	1.43	0.68	1.38
GATCAACGAGGTGGATGCGC	0.00	0.00	0.00	0.00	0.69
GATCAACGAGGTGGATGCTG	0.69	3.90	7.16	4.41	5.17
GATCAACGAGGTGGATGCTT	0.00	0.00	0.29	0.00	0.00
GATCAACGAGGTGGCCGGGC	0.00	0.00	0.29	0.00	0.00
GATCAACGAGGTGGGTGCTG	0.00	0.00	0.00	0.00	0.00
GATCAACGAGGTTCTTCGGG	0.23	0.00	0.00	1.02	0.69
GATCAACGAGGTTCTTCGGN	0.00	0.00	0.00	0.00	0.00
GATCAACGAGGTTGATGCTG	0.46	0.00	0.00	0.00	1.38
GATCAACGAGTAACAAAAAA	0.00	0.33	0.57	0.00	0.00
GATCAACGAGTACAAGAACT	0.00	0.00	0.00	0.34	0.00
GATCAACGAGTACCACCTCA	0.23	1.63	0.29	1.36	0.34
GATCAACGAGTACGACACCG	0.69	0.00	0.00	0.00	0.00
GATCAACGAGTACGACTTGA	0.00	0.00	0.00	0.00	0.00
GATCAACGAGTACGAGTCCG	0.00	0.00	0.00	0.34	0.00
GATCAACGAGTACGAGTCTG	0.69	0.00	0.00	1.36	0.69
GATCAACGAGTACGCGTTGA	0.00	0.00	0.00	0.00	0.34
GATCAACGAGTACTACCTGA	0.00	0.33	0.00	0.68	0.00
GATCAACGAGTATCCCTCGG	0.00	0.00	0.29	0.00	0.00
GATCAACGAGTATCCTTCCG	1.37	0.65	0.86	3.05	0.00
GATCAACGAGTATCCTTCGT	0.00	0.33	0.00	0.00	0.00
GATCAACGAGTATCCTTTAG	0.00	0.00	0.29	0.00	0.00
GATCAACGAGTATCGGTTTG	0.00	0.00	0.00	0.00	0.00

GATCAACGAGTATGAGTCAG	0.00	0.65	0.00	0.34	0.34
GATCAACGAGTATGCAGCAG	0.00	0.00	0.00	0.00	0.00
GATCAACGAGTCCACGGGCG	0.23	0.00	0.00	0.00	0.00
GATCAACGAGTCCAGCCAGC	0.23	0.00	0.00	0.34	0.00
GATCAACGAGTCCAGCTGTA	0.00	0.00	0.00	0.34	0.69
GATCAACGAGTCCGCCACTT	0.00	0.00	0.00	0.00	0.00
GATCAACGAGTCGTATGCCA	0.00	0.00	0.00	0.00	0.00
GATCAACGAGTCTCATTCCG	0.23	0.00	0.00	0.00	0.00
GATCAACGAGTGCAAAAGAC	0.00	0.33	0.00	0.00	0.00
GATCAACGAGTGCAAAGACT	0.00	0.00	0.00	0.00	1.38
GATCAACGAGTGCAAAGCCT	0.00	0.00	0.00	0.00	0.34
GATCAACGAGTGCAAGGAAC	0.00	0.00	0.00	0.00	0.00
GATCAACGAGTGCAAGGAAG	0.00	0.00	0.00	0.00	0.00
GATCAACGAGTGCAAGGACA	0.00	0.65	0.29	0.68	0.34
GATCAACGAGTGCACGCGCG	0.69	0.00	0.00	0.34	0.34
GATCAACGAGTGCACGGCCT	0.00	0.00	0.57	0.00	0.00
GATCAACGAGTGCATGCGGA	0.00	0.00	0.57	0.00	0.00
GATCAACGAGTGCATGGAGG	0.00	0.00	0.00	0.34	0.00
GATCAACGAGTGCCAGATGG	0.00	0.00	0.29	0.00	0.00
GATCAACGAGTGCGCGCAGG	0.00	0.00	0.57	0.34	0.34
GATCAACGAGTGCGTTGGTG	0.00	0.00	0.00	0.00	0.00
GATCAACGAGTGCTACACCG	0.00	0.00	0.29	0.00	0.00
GATCAACGAGTGCTTAACGC	0.00	0.33	0.00	0.00	0.00
GATCAACGAGTGCTTCCTAG	0.00	0.00	0.86	0.00	0.00
GATCAACGAGTGCTTGGAGG	1.60	1.63	0.86	2.72	0.69
GATCAACGAGTGCTTGGGGC	0.00	0.00	0.00	0.00	0.00
GATCAACGAGTGCTTGGTAG	0.00	0.00	0.00	0.00	0.00
GATCAACGAGTGCTTGTATG	0.00	0.33	0.00	0.00	0.00
GATCAACGAGTGGAAAGAGA	0.00	0.00	0.00	0.00	0.00
GATCAACGAGTGGACCTGGA	0.00	0.00	0.00	0.34	0.00
GATCAACGAGTGGCCATCGC	0.23	0.00	0.00	0.00	0.00
GATCAACGAGTGGCTTAAGG	0.00	0.00	0.29	0.00	0.00
GATCAACGAGTGGGCGCCCC	0.00	0.33	0.29	0.00	0.34
GATCAACGAGTGGGGCTACT	0.00	0.00	0.29	0.00	0.00
GATCAACGAGTGGTACCTCT	0.00	0.00	0.00	0.34	0.00
GATCAACGAGTGGTACCTNN	0.00	0.00	0.00	0.00	0.00
GATCAACGAGTGGTCCTCCT	0.00	0.00	0.00	0.00	0.00

GATCAACGAGTGGTTGGCAG	0.00	0.00	0.00	0.34	0.00
GATCAACGAGTTAGAGGCTG	0.00	0.00	0.29	0.00	0.00
GATCAACGAGTTATCGGAGG	0.00	0.00	0.00	0.00	0.00
GATCAACGAGTTATGTGCTG	0.23	0.00	0.00	0.00	0.00
GATCAACGAGTTATGTGCTT	0.00	0.33	0.00	0.00	0.00
GATCAACGAGTTCAGCCAGA	0.00	0.00	0.00	0.34	0.00
GATCAACGAGTTCAGCCAGC	25.64	23.75	9.74	17.31	11.02
GATCAACGAGTTCAGCCAGN	0.23	0.00	0.00	0.00	0.00
GATCAACGAGTTCAGCCANN	0.23	0.00	0.00	0.00	0.00
GATCAACGAGTTCAGCCATC	0.00	0.00	0.00	0.34	0.34
GATCAACGAGTTCAGCCCGC	0.46	0.00	0.00	0.00	0.00
GATCAACGAGTTCAGCCGGC	0.23	0.33	0.00	0.00	0.00
GATCAACGAGTTCCACCACA	0.00	0.00	0.29	0.00	0.00
GATCAACGAGTTCCAGTTTA	0.00	0.00	0.29	0.00	0.00
GATCAACGAGTTCGTATGCC	0.00	0.00	0.29	0.00	0.00
GATCAACGAGTTCTCGAGGC	0.00	0.00	0.00	0.00	0.00
GATCAACGAGTTCTCGGAGG	16.25	16.59	5.15	8.15	7.58
GATCAACGAGTTCTCGGCGG	0.23	0.33	0.00	0.00	0.00
GATCAACGAGTTCTCTGAGG	0.00	0.00	0.00	0.00	0.00
GATCAACGAGTTGAAGTGCA	2.75	1.63	2.29	0.68	1.03
GATCAACGAGTTGGGCGAGG	0.00	0.00	0.00	0.00	0.00
GATCAACGAGTTGGGCGCCC	0.23	0.00	0.00	0.00	0.00
GATCAACGAGTTTAGCCAGC	0.23	0.00	0.00	0.00	0.00
GATCAACGAGTTTTCAGCAG	0.00	0.33	0.00	0.00	0.00
GATCAACGATAAAAAAAAAA	0.92	0.00	0.29	0.00	0.00
GATCAACGATAAAAAAATAT	1.14	0.00	0.00	0.00	0.00
GATCAACGATAAATCAAGCT	0.00	0.00	0.00	0.00	0.34
GATCAACGATAAGATGAGGC	0.00	0.00	0.86	0.00	0.00
GATCAACGATAAGCATAGGC	0.00	0.00	0.00	0.34	0.00
GATCAACGATAATGAACGGA	0.00	0.00	0.86	0.00	1.03
GATCAACGATACAGTGATGT	0.23	0.00	0.00	0.00	0.00
GATCAACGATACCACCGAAA	0.00	0.33	0.00	0.00	0.00
GATCAACGATACCAGGCTGT	0.00	0.00	0.00	0.00	0.34
GATCAACGATACGCCCGGTT	0.00	0.00	0.00	0.00	0.00
GATCAACGATACTGGGCTCT	0.23	0.65	0.29	0.34	0.00
GATCAACGATAGAACCTGCC	0.00	0.33	0.00	0.00	0.00
GATCAACGATAGCAAAAAAA	0.00	0.33	0.00	0.00	0.00

GATCAACGATAGCAACGACA	0.00	0.33	0.00	0.00	0.34
GATCAACGATAGCACAGAGA	0.00	0.00	0.29	0.00	0.00
GATCAACGATAGCACCAAAA	0.00	0.00	0.00	0.00	0.34
GATCAACGATAGCACCAGAC	0.00	0.33	0.00	0.00	0.00
GATCAACGATAGCACCCACA	0.00	0.00	0.29	0.34	0.00
GATCAACGATAGCACCGAAA	1.37	1.30	1.72	1.70	0.69
GATCAACGATAGCACCGAAG	0.00	0.00	0.00	0.00	0.34
GATCAACGATAGCACCGACA	33.42	27.00	28.64	47.52	26.52
GATCAACGATAGCACCGACC	0.00	0.00	0.00	0.34	0.00
GATCAACGATAGCACCGACG	0.23	0.65	0.00	1.36	0.00
GATCAACGATAGCACCGACT	0.23	0.00	0.00	0.00	0.34
GATCAACGATAGCACCGAGA	0.23	0.00	0.29	0.00	0.00
GATCAACGATAGCACCGAGC	0.00	0.00	0.00	0.00	0.00
GATCAACGATAGCACCGANN	0.23	0.00	0.00	0.00	0.00
GATCAACGATAGCACCGATA	0.00	0.33	0.00	0.00	0.69
GATCAACGATAGCACCGCCA	0.00	0.00	0.00	0.34	0.00
GATCAACGATAGCACCGGCA	0.92	0.00	0.29	0.00	0.00
GATCAACGATAGCACCGGCC	0.00	0.00	0.00	0.00	0.34
GATCAACGATAGCACCGGGG	0.00	0.00	0.00	0.00	0.00
GATCAACGATAGCACCGTAG	0.00	0.00	0.29	0.00	0.00
GATCAACGATAGCACCGTCA	0.00	0.00	0.00	0.00	0.00
GATCAACGATAGCACCGTGG	0.00	0.33	0.00	0.00	0.00
GATCAACGATAGCACCTCCA	0.00	0.00	0.29	0.00	0.00
GATCAACGATAGCACCTTGG	0.00	0.00	0.00	0.00	0.34
GATCAACGATAGCACTCAAA	0.00	0.65	0.00	0.00	0.00
GATCAACGATAGCATGCAGG	0.00	0.00	0.00	0.00	0.34
GATCAACGATAGCCCCATCA	0.00	0.00	0.00	0.00	0.00
GATCAACGATAGCGCCGACG	0.00	0.00	0.00	0.00	0.34
GATCAACGATAGCTGGACAC	0.00	0.00	0.00	0.00	0.00
GATCAACGATAGCTTTCACA	0.00	0.00	0.00	0.00	0.00
GATCAACGATAGGGCAGCTG	0.00	0.00	0.00	0.34	0.00
GATCAACGATAGTACCGACA	0.23	0.00	0.00	0.00	0.00
GATCAACGATATACGGAGTG	0.00	0.00	0.00	0.00	0.00
GATCAACGATATCATGATGT	0.23	0.33	0.00	0.00	0.00
GATCAACGATATTGGGCTCT	0.00	0.33	0.86	0.68	0.34
GATCAACGATCAAGGAAGCT	0.00	0.00	0.00	0.00	0.00
GATCAACGATCGGTCGTTGA	0.00	0.00	0.00	0.00	0.34

GATCAACGATGAAGAGCCCT	0.00	0.00	0.00	0.00	0.00
GATCAACGATGAAGCGCTCC	0.00	0.00	0.00	0.34	0.00
GATCAACGATGAAGGAAGGA	0.92	0.00	0.29	0.00	0.34
GATCAACGATGAAGGACCGA	0.23	0.00	0.00	0.00	0.00
GATCAACGATGAAGTCAGAA	0.92	0.00	0.29	0.34	0.34
GATCAACGATGACAACCAGA	0.00	0.00	0.00	0.00	0.34
GATCAACGATGACAGCTCCG	0.00	0.65	0.29	0.00	0.00
GATCAACGATGACAGGTGTT	0.00	0.00	0.00	0.00	1.38
GATCAACGATGACCCGTTGC	0.00	0.00	0.00	0.00	0.00
GATCAACGATGACCGCAAAA	0.46	0.33	0.00	0.68	0.34
GATCAACGATGACCGCACAA	0.00	0.00	0.00	0.00	0.34
GATCAACGATGACCGCGAAA	0.23	0.00	0.00	0.68	0.00
GATCAACGATGACCGCGAAG	0.00	0.00	0.00	0.34	0.00
GATCAACGATGACCGCGACG	0.23	0.00	0.00	0.00	0.00
GATCAACGATGACCGCGATG	4.81	2.93	0.57	4.07	2.41
GATCAACGATGACCGTGATG	0.00	0.00	0.29	0.00	0.00
GATCAACGATGACTATGGTG	0.00	0.00	0.00	0.00	0.34
GATCAACGATGAGAAGGGTG	0.00	0.00	0.00	0.68	0.00
GATCAACGATGAGAATGAGG	0.00	0.00	0.29	0.00	0.00
GATCAACGATGAGAGAGAGA	0.00	0.00	0.29	0.00	0.00
GATCAACGATGAGGCCTCAT	0.00	0.00	0.00	0.68	0.00
GATCAACGATGAGGTGAATC	0.00	0.33	0.00	0.00	0.00
GATCAACGATGAGGTGACTC	0.00	0.00	0.00	0.00	0.00
GATCAACGATGAGGTGCCTC	0.00	0.00	0.29	0.00	0.00
GATCAACGATGATAGCTCCG	0.00	0.00	0.00	0.00	0.00
GATCAACGATGCAAGAGACC	0.00	0.00	0.00	0.00	0.00
GATCAACGATGCAGCAGACC	0.00	0.00	0.00	0.00	0.00
GATCAACGATGCAGCAGAGC	0.00	0.00	0.00	0.00	0.00
GATCAACGATGCAGCCAAGT	0.00	0.00	0.29	0.00	0.00
GATCAACGATGCAGGGCCAC	0.00	0.33	0.00	0.00	0.00
GATCAACGATGCAGTGCCTC	0.00	0.33	0.00	0.00	0.00
GATCAACGATGCAGTTGCCA	0.00	0.33	0.00	0.00	0.00
GATCAACGATGCCAAGACCG	0.00	0.98	0.57	0.68	0.34
GATCAACGATGCCACTGACC	0.46	0.65	0.00	0.00	0.00
GATCAACGATGCCATCTCTT	0.00	0.00	0.00	0.00	0.00
GATCAACGATGCCGAGACGA	0.00	0.00	0.00	0.00	0.00
GATCAACGATGCCGCGAAGT	0.00	0.00	0.29	0.00	0.00

GATCAACGATGCCGCGCAAG	0.00	0.00	0.00	0.34	0.00
GATCAACGATGCCGGTCGCC	0.00	0.00	0.00	0.00	0.00
GATCAACGATGCCTTGGAGG	0.00	0.00	0.00	0.34	0.00
GATCAACGATGCGCGGGGCT	0.00	0.00	0.00	0.00	0.00
GATCAACGATGCGGAGCCTC	0.00	0.00	0.00	0.00	0.00
GATCAACGATGCGGAGGTGT	0.00	0.00	0.00	0.00	0.00
GATCAACGATGCGGCCGAGT	0.69	0.98	0.29	0.68	0.34
GATCAACGATGCGGGGCCCC	0.23	0.00	0.00	0.00	0.00
GATCAACGATGCGGGGCTGC	0.00	0.33	0.00	0.00	0.00
GATCAACGATGCGGTGACCC	0.00	0.00	0.29	0.00	0.00
GATCAACGATGCGGTGACTC	0.00	0.00	0.00	0.34	0.00
GATCAACGATGCGGTGAGTC	0.00	0.33	0.00	0.00	0.00
GATCAACGATGCGGTGCATC	0.00	0.00	0.57	0.00	0.00
GATCAACGATGCGGTGCATG	0.00	0.00	0.29	0.00	0.00
GATCAACGATGCGGTGCCAC	0.00	0.00	0.00	0.00	0.34
GATCAACGATGCGGTGCCCC	0.46	0.00	0.00	0.00	0.00
GATCAACGATGCGGTGCCGC	0.23	0.00	0.00	0.00	0.00
GATCAACGATGCGGTGCCNN	0.00	0.00	0.00	0.00	0.00
GATCAACGATGCGGTGCCTC	35.71	37.74	26.63	23.08	19.63
GATCAACGATGCGGTGCCTG	0.46	0.00	0.00	0.00	0.00
GATCAACGATGCGGTGCCTT	0.92	0.00	0.00	0.00	0.00
GATCAACGATGCGGTGCTTC	0.00	0.00	0.29	0.00	0.00
GATCAACGATGCGGTGGCTC	0.00	0.00	0.00	0.00	0.00
GATCAACGATGCGGTTCCTC	0.23	0.00	0.00	0.00	0.00
GATCAACGATGCTATCGTTG	0.00	0.00	0.00	0.00	0.00
GATCAACGATGCTCGTATGC	0.46	0.00	0.00	0.00	0.00
GATCAACGATGCTGAGCTCT	0.00	0.00	0.00	0.00	0.00
GATCAACGATGCTGCCAAGT	0.00	0.33	0.29	0.00	0.00
GATCAACGATGCTGTGCCTC	0.23	0.00	0.00	0.00	0.00
GATCAACGATGGAAAAAACG	0.00	0.33	0.00	0.00	0.00
GATCAACGATGGAAAACAAA	0.46	0.00	0.00	0.00	0.00
GATCAACGATGGAAAACACG	0.00	1.63	1.72	1.02	0.69
GATCAACGATGGACATGTGC	0.00	0.00	0.29	0.00	0.00
GATCAACGATGGAGCTGCAG	0.00	0.00	0.00	0.34	0.00
GATCAACGATGGCAGGAAGC	0.23	0.00	0.00	0.00	0.00
GATCAACGATGGCATGATTC	0.00	0.00	0.00	0.00	0.34
GATCAACGATGGCCGCAAAA	0.00	0.00	0.00	0.00	0.00

GATCAACGATGGCGCAGTTT	0.00	0.00	0.29	0.68	0.34
GATCAACGATGGCGCGAGTA	0.23	0.00	0.00	0.00	0.00
GATCAACGATGGCTACAGCA	0.92	0.65	0.29	0.00	0.00
GATCAACGATGGCTACAGCC	0.23	0.00	0.00	0.00	0.00
GATCAACGATGGCTTGAACT	0.00	0.65	0.00	0.34	0.00
GATCAACGATGGGTCATTGC	0.23	0.00	0.00	0.00	0.00
GATCAACGATGGTAAAAAAA	0.23	0.00	0.00	0.00	0.00
GATCAACGATGTATTCTACC	0.00	0.33	0.00	0.00	0.00
GATCAACGATGTCAATGCGT	2.06	0.00	0.29	0.00	0.34
GATCAACGATGTCAGCAGGC	0.92	0.00	0.00	0.00	0.00
GATCAACGATGTCAGGAAAC	0.00	0.00	0.29	0.00	0.00
GATCAACGATGTCAGGAACA	0.00	0.00	0.00	0.00	0.00
GATCAACGATGTCAGGAAGA	0.00	0.33	0.00	0.34	0.00
GATCAACGATGTCAGGAAGC	36.40	23.10	24.05	20.37	18.60
GATCAACGATGTCAGGAAGN	0.46	0.00	0.00	0.00	0.00
GATCAACGATGTCAGGAANN	0.23	0.00	0.00	0.00	0.00
GATCAACGATGTCAGGAGGC	0.46	0.00	0.00	0.00	0.00
GATCAACGATGTCAGGCAGC	0.23	0.33	0.00	0.00	0.34
GATCAACGATGTCAGGNCNN	0.23	0.00	0.00	0.00	0.00
GATCAACGATGTCAGGNNNN	0.46	0.00	0.00	0.00	0.00
GATCAACGATGTCAGGTAGC	0.23	0.00	0.00	0.00	0.00
GATCAACGATGTCCACATGC	0.23	0.00	0.00	1.02	0.34
GATCAACGATGTCCACATNN	0.00	0.00	0.00	0.00	0.00
GATCAACGATGTCCTGATTG	0.23	0.00	0.00	0.00	0.00
GATCAACGATGTCGAGAAGG	0.00	0.00	0.29	0.34	0.00
GATCAACGATGTCGGGAAGC	0.00	0.00	0.00	0.00	0.34
GATCAACGATGTCTCGTATG	0.00	0.00	0.29	0.00	0.00
GATCAACGATGTGCGATGGC	0.00	0.00	0.57	0.00	0.00
GATCAACGATGTGCGCTCAA	0.00	0.00	0.00	0.34	0.00
GATCAACGATTACTCTGGTT	0.00	0.00	0.00	0.00	0.00
GATCAACGATTCAAGGCACC	0.23	0.00	0.00	0.00	0.00
GATCAACGATTCCATGCTGT	0.00	0.00	0.00	0.00	0.34
GATCAACGATTCCGCGGCCG	0.23	0.00	0.00	0.00	0.00
GATCAACGATTCTTTGATGA	0.23	0.00	0.00	0.00	0.00
GATCAACGATTGATTACAAC	0.00	0.00	0.00	0.00	0.34
GATCAACGATTGCGAGAAGA	0.23	0.00	0.00	0.00	0.00
GATCAACGATTGCGTGAAGA	35.71	27.00	13.17	19.69	11.71

GATCAACGATTGCGTGAAGC	0.46	0.00	0.00	0.00	0.00
GATCAACGATTGCGTGAAGN	0.00	0.00	0.00	0.00	0.00
GATCAACGATTGCGTGAANN	0.23	0.00	0.00	0.00	0.00
GATCAACGATTGCGTGAATA	0.00	0.33	0.00	0.00	0.00
GATCAACGATTGCGTGACGA	0.00	0.00	0.00	0.00	0.00
GATCAACGATTGCGTGAGGA	0.00	0.33	0.00	0.00	0.00
GATCAACGATTGCGTGATGA	0.23	0.00	0.00	0.00	0.00
GATCAACGATTGCGTGCAGA	0.23	0.00	0.00	0.00	0.00
GATCAACGATTGCGTGGAGA	0.23	0.00	0.00	0.00	0.00
GATCAACGATTGCTACAAGC	0.00	0.00	0.29	0.00	0.00
GATCAACGATTGGAGTCTCG	0.00	0.33	0.00	0.00	0.00
GATCAACGATTGGCATGTCA	0.00	0.33	0.00	0.00	0.00
GATCAACGATTGTGTCAAGA	0.00	0.00	0.00	0.34	0.00
GATCAACGATTGTTTCGGGT	0.00	0.00	0.00	0.34	0.00
GATCAACGATTTCATCCAGC	0.23	0.00	0.00	0.00	0.00
GATCAACGATTTTGATGAGG	0.00	0.00	0.29	0.00	0.00
GATCAACGATTTTTTCAACA	0.00	0.00	0.00	0.00	0.00
GATCAACGATTTTTTCTTCA	0.00	0.00	0.29	0.00	0.00
GATCAACGCAAAAAAAAAAA	0.00	0.00	0.29	0.68	1.38
GATCAACGCAAAAAAAAAAT	0.00	0.00	0.00	0.00	0.00
GATCAACGCAAAAAAAAATA	0.00	0.00	0.00	0.00	0.00
GATCAACGCAAAAGGAAAAA	0.00	0.00	0.00	0.00	0.34
GATCAACGCAAACAAGAAAG	0.00	0.00	0.00	0.00	1.03
GATCAACGCAAACAAGNAAG	0.00	0.00	0.00	0.00	0.34
GATCAACGCAAACACAGCAC	0.00	0.00	0.00	0.00	0.69
GATCAACGCAAACACATCGA	0.00	0.00	0.00	0.00	0.34
GATCAACGCAAACACCAACA	0.00	0.00	0.86	0.00	0.00
GATCAACGCAAACACCGTGC	0.00	0.00	0.00	0.00	0.00
GATCAACGCAAACACTGTTG	0.00	0.00	0.00	0.00	0.34
GATCAACGCAAACAGTACCG	0.00	0.00	0.00	0.00	0.34
GATCAACGCAAACATCGCCA	0.00	0.00	0.00	0.00	0.00
GATCAACGCAAACATGAAGG	0.46	1.30	0.29	0.00	0.69
GATCAACGCAAACATGTGCA	0.00	0.00	0.00	0.00	0.34
GATCAACGCAAACATTGTGA	0.00	0.00	0.00	0.00	0.34
GATCAACGCAAACCTCTGGC	0.00	0.00	0.00	0.00	0.34
GATCAACGCAAACTTGGACG	0.00	0.00	0.00	0.00	0.00
GATCAACGCAAAGACCTGGC	0.00	0.33	0.00	0.00	0.00

GATCAACGCAAAGCAACGCA	0.00	0.00	0.00	0.00	0.69
GATCAACGCAAAGCAAGGCG	0.00	0.00	0.00	0.00	0.69
GATCAACGCAAAGCATGTCC	0.00	0.00	0.00	0.34	0.00
GATCAACGCAAAGCGGCTGG	0.00	0.00	0.29	0.00	0.00
GATCAACGCAAAGCGTTGGG	0.00	0.00	0.00	0.34	0.00
GATCAACGCAAAGCGTTTGG	1.14	2.28	3.15	1.70	0.69
GATCAACGCAAAGCGTTTNN	0.23	0.00	0.00	0.00	0.00
GATCAACGCAAAGGCACATG	0.00	0.00	0.00	0.00	0.34
GATCAACGCAAAGGCATGCT	0.00	0.00	0.00	0.00	0.00
GATCAACGCAAATCAATGCA	0.00	0.00	0.00	0.00	0.69
GATCAACGCAAATCAGTGAA	0.23	0.00	0.00	0.00	0.00
GATCAACGCAAATCAGTGGA	1.14	0.00	0.86	0.00	1.38
GATCAACGCAACAACACAAA	0.00	0.00	0.00	0.00	0.34
GATCAACGCAACAACTACGA	0.00	0.00	0.00	0.00	0.34
GATCAACGCAACAATCAACA	0.00	0.00	0.00	0.00	1.03
GATCAACGCAACACAAAACA	0.00	0.00	0.00	0.00	0.34
GATCAACGCAACACAACACA	0.00	0.00	0.00	0.00	2.41
GATCAACGCAACACAACACG	0.00	0.00	0.00	0.00	0.34
GATCAACGCAACACAACGCA	0.00	0.00	0.00	0.00	0.69
GATCAACGCAACACACAACA	0.00	0.00	0.86	0.00	0.34
GATCAACGCAACACACACTG	0.00	0.00	0.29	0.00	0.00
GATCAACGCAACACAGAACA	0.00	0.00	0.00	0.00	0.34
GATCAACGCAACACAGNACA	0.00	0.00	0.00	0.00	0.34
GATCAACGCAACACATTGCT	0.00	0.00	0.29	0.00	0.00
GATCAACGCAACACCCACAC	0.00	0.00	0.29	0.00	0.00
GATCAACGCAACACCGACTC	0.23	1.30	0.00	0.00	0.00
GATCAACGCAACACCTCTCA	0.00	0.00	0.29	0.00	0.00
GATCAACGCAACATGAAGTG	0.00	0.00	0.29	0.00	0.00
GATCAACGCAACCATGAAAT	0.00	0.00	0.00	0.00	0.34
GATCAACGCAACCCACCTGG	0.00	0.00	0.00	0.34	0.34
GATCAACGCAACGACGAAGA	0.00	0.00	0.29	0.00	0.00
GATCAACGCAACGCAAAGCA	0.00	0.00	0.00	0.00	0.34
GATCAACGCAACGCAACGCA	0.00	0.00	0.00	0.00	1.38
GATCAACGCAACGGCATGGC	0.00	0.33	0.00	0.68	0.00
GATCAACGCAACGTTCAGGT	0.00	0.00	0.57	0.00	0.34
GATCAACGCAAGACCTTGCA	0.00	0.00	0.00	0.00	0.34
GATCAACGCAAGAGCAGACA	0.00	0.33	0.00	0.00	0.00

GATCAACGCAAGCACAGCAC	0.00	0.00	0.00	0.00	0.69
GATCAACGCAAGCACATGTG	0.00	0.00	0.00	0.00	0.34
GATCAACGCAAGCAGCCTGT	0.00	0.00	0.00	0.00	0.00
GATCAACGCAAGCGAGGCGT	0.23	0.00	0.00	0.00	0.00
GATCAACGCAAGGCGGAAAC	0.00	0.00	0.29	0.00	0.00
GATCAACGCAAGGCGGCAAC	0.46	0.00	0.00	0.00	0.00
GATCAACGCAAGGCTGTGGG	0.46	0.00	0.00	0.00	0.00
GATCAACGCAAGGCTTCAGG	0.00	0.33	0.00	0.00	0.00
GATCAACGCAAGGCTTGGTC	0.00	0.00	0.00	1.02	0.00
GATCAACGCAAGGGGGGTCG	0.23	0.00	0.00	0.00	0.00
GATCAACGCAAGGTGACAAC	0.46	0.00	0.00	0.00	0.00
GATCAACGCAAGGTGGAAAC	0.00	0.00	0.00	0.68	0.00
GATCAACGCAAGGTGGCAAC	21.06	21.47	26.35	18.67	23.76
GATCAACGCAAGGTGGCAAG	0.23	0.00	0.00	0.00	0.00
GATCAACGCAAGGTGGCAGC	0.00	0.00	0.29	0.00	0.00
GATCAACGCAAGGTGGCANN	0.23	0.00	0.00	0.00	0.00
GATCAACGCAAGGTGGCCAC	0.00	0.00	0.29	0.00	0.00
GATCAACGCAAGGTGGCTCG	0.00	0.00	0.00	0.34	0.00
GATCAACGCAAGGTGGTAAC	0.23	0.00	0.00	0.00	0.00
GATCAACGCAAGGTTGCAAC	0.00	0.00	0.29	0.00	0.00
GATCAACGCAAGTGCGAACA	0.00	0.00	0.00	0.00	0.00
GATCAACGCAAGTTGAAAGC	0.00	0.00	0.29	0.00	0.00
GATCAACGCAATAGCAACTC	0.00	0.00	0.29	0.00	0.00
GATCAACGCAATCCTAAGTA	0.00	0.00	0.29	0.00	0.00
GATCAACGCAATCCTTGCGC	0.23	0.00	0.00	0.00	0.00
GATCAACGCAATGACGCAAT	0.00	0.00	0.00	0.00	0.34
GATCAACGCAATGAGAGAAA	0.00	0.00	0.00	0.00	0.00
GATCAACGCAATGCAAAAGA	0.00	0.00	0.00	0.00	0.00
GATCAACGCAATGCCAAAGA	0.00	0.98	0.00	0.68	0.69
GATCAACGCAATGGGCGTGC	0.00	0.00	0.00	0.00	0.00
GATCAACGCAATTGCACGGG	0.00	0.33	0.00	0.00	0.00
GATCAACGCACAAAAAAAAA	0.00	0.00	0.29	0.00	0.00
GATCAACGCACAAAACACAC	0.00	0.00	0.29	0.00	0.00
GATCAACGCACAACAAAAGA	0.00	0.00	0.29	0.00	0.00
GATCAACGCACAACAACACA	0.00	0.00	0.00	0.00	0.34
GATCAACGCACAACAATAGC	0.23	0.33	0.00	0.00	0.00
GATCAACGCACAACACACAA	0.00	0.00	0.00	0.00	0.34

GATCAACGCACAACACACCG	0.00	0.00	0.00	0.00	0.34
GATCAACGCACAACACAGCA	0.00	0.00	0.00	0.00	0.34
GATCAACGCACAACATCACG	0.00	0.00	0.29	0.00	0.00
GATCAACGCACAACATGCCA	0.00	0.00	0.00	0.00	0.34
GATCAACGCACAACGACACA	0.00	0.00	0.00	0.00	0.34
GATCAACGCACAACGCAAGG	0.00	0.00	0.00	0.00	1.03
GATCAACGCACAACGCACAA	0.00	0.00	0.00	0.00	1.03
GATCAACGCACAAGAGATAA	0.00	0.00	0.00	0.00	0.34
GATCAACGCACAAGTTCTGT	0.00	0.00	0.00	0.34	0.00
GATCAACGCACACAAAGACG	0.00	0.00	0.00	0.00	0.34
GATCAACGCACACAACCGTA	0.00	0.00	0.00	0.00	0.34
GATCAACGCACACAGACCAA	0.00	0.00	0.57	0.00	0.00
GATCAACGCACACATACCGA	0.00	0.00	0.00	0.00	0.34
GATCAACGCACACCACAACA	0.00	0.00	0.00	0.00	1.38
GATCAACGCACACCACAGGG	0.00	0.00	0.00	0.00	0.34
GATCAACGCACACGACAGGG	0.00	0.00	0.00	0.34	0.00
GATCAACGCACAGAACATAT	0.00	0.00	0.00	0.00	0.34
GATCAACGCACAGGCGAGTG	0.46	0.00	0.00	0.34	0.69
GATCAACGCACAGGCGGGTG	0.46	0.00	0.00	0.00	0.00
GATCAACGCACAGTGGCAGA	0.23	0.00	0.00	0.00	0.00
GATCAACGCACAGTGGTAGG	0.00	0.33	0.00	0.00	0.00
GATCAACGCACATCACAGCG	0.00	0.00	0.00	0.00	0.69
GATCAACGCACATGTGTGCC	0.00	0.00	0.29	0.00	0.00
GATCAACGCACCAAAAAAAA	0.00	0.00	0.00	0.34	0.00
GATCAACGCACCAACTGTTC	0.23	0.00	0.00	0.00	0.00
GATCAACGCACCACAACGCA	0.00	0.00	0.00	0.00	0.34
GATCAACGCACCACACACAA	0.00	0.00	0.29	0.00	0.00
GATCAACGCACCACCGAGCG	0.00	0.00	0.00	0.00	0.69
GATCAACGCACCACGCACCA	0.00	0.00	0.00	0.00	0.34
GATCAACGCACCACGCCACG	0.00	0.00	0.00	0.00	0.69
GATCAACGCACCATACAAGA	0.00	0.00	0.00	0.00	1.03
GATCAACGCACCATACAGGT	0.00	0.00	0.00	0.00	0.34
GATCAACGCACCATGACGGA	0.00	0.33	0.00	0.00	0.00
GATCAACGCACCCATACTGA	0.00	0.00	0.29	0.00	0.00
GATCAACGCACCCCCCCCCC	0.00	0.00	0.00	0.00	0.34
GATCAACGCACCCCGATATG	0.00	0.00	0.00	0.00	1.03
GATCAACGCACCCGTGAAAA	0.23	0.00	0.00	0.00	0.00

GATCAACGCACCCTGTGACT	0.00	0.00	0.29	0.00	0.00
GATCAACGCACCGCAAAGCA	0.00	0.00	0.00	0.00	0.34
GATCAACGCACCGCAACACA	0.00	0.00	0.00	0.00	0.34
GATCAACGCACCGCAACGCA	0.00	0.00	0.29	0.00	0.69
GATCAACGCACCGCGGGCTA	0.00	0.00	0.00	0.00	0.34
GATCAACGCACCTCAATAGC	4.12	0.00	0.86	1.70	0.69
GATCAACGCACCTCCAAGNG	0.00	0.00	0.00	0.34	0.00
GATCAACGCACCTCCAAGTG	1.60	0.98	0.86	2.72	0.34
GATCAACGCACCTCCAGTTC	0.00	0.00	0.00	0.34	0.00
GATCAACGCACCTGGGAAGG	0.00	0.98	0.00	0.00	0.00
GATCAACGCACCTTAATAGC	0.23	0.00	0.00	0.00	0.00
GATCAACGCACCTTCTGAAC	0.00	0.00	0.29	0.00	0.00
GATCAACGCACGCCGCACGC	0.00	0.00	0.00	0.00	0.34
GATCAACGCACGCGATACAA	0.46	0.00	0.00	0.00	0.34
GATCAACGCACGGCGGAGTA	0.23	0.00	0.00	0.00	0.00
GATCAACGCACGGTGAGCGG	0.00	0.00	0.00	0.00	0.00
GATCAACGCACGGTTATGGG	0.00	0.00	0.00	0.34	0.00
GATCAACGCACTAAAAAAAA	1.37	0.33	0.00	0.68	0.34
GATCAACGCACTACAATGTA	0.00	0.00	0.00	0.00	1.03
GATCAACGCACTCCAATCGC	0.00	0.00	0.00	0.00	0.00
GATCAACGCACTCCGGAAAG	0.00	0.00	0.00	0.00	0.00
GATCAACGCACTCCTCCCTA	0.00	0.00	0.00	0.00	0.00
GATCAACGCACTCCTCCCTC	0.00	0.00	0.00	0.34	0.00
GATCAACGCACTGATGGTGA	0.00	0.00	0.00	0.00	0.00
GATCAACGCACTGCGGTCGA	0.00	0.33	0.00	0.00	0.00
GATCAACGCACTGCGGTCTC	0.00	0.00	0.00	0.00	0.00
GATCAACGCACTGCTAAAAA	4.58	0.00	0.29	1.36	0.00
GATCAACGCACTGCTGACGA	0.00	0.00	0.00	0.68	0.00
GATCAACGCACTGCTGGCGA	0.00	0.00	0.00	0.34	0.00
GATCAACGCACTGCTGNNNN	0.23	0.00	0.00	0.00	0.00
GATCAACGCACTGCTGTCAA	0.00	0.98	0.00	0.00	0.34
GATCAACGCACTGCTGTCCA	0.00	0.00	0.00	0.34	0.00
GATCAACGCACTGCTGTCGA	10.99	8.13	5.73	15.95	4.48
GATCAACGCACTGCTGTCGC	0.00	0.00	0.00	0.34	0.00
GATCAACGCACTGCTGTCGT	0.00	0.00	0.29	0.00	0.00
GATCAACGCACTGGAGAACC	0.00	0.00	0.29	0.00	0.00
GATCAACGCACTGGATGTGA	0.00	0.00	0.00	0.00	0.00

GATCAACGCACTGGTCATGA	0.00	0.33	0.00	0.34	0.00
GATCAACGCACTGGTCGTGA	0.00	0.00	0.00	0.00	0.00
GATCAACGCACTGTTGGGCA	0.00	0.00	0.29	0.00	0.00
GATCAACGCACTGTTGGTGA	0.00	0.33	0.00	0.00	0.00
GATCAACGCACTTGCACCAC	0.00	0.00	0.00	0.00	0.00
GATCAACGCACTTGGCTTGG	0.00	0.00	0.00	0.34	0.00
GATCAACGCAGAAAGCGAGC	0.00	0.00	0.00	0.34	0.00
GATCAACGCAGAACAAGACA	0.00	0.00	0.00	0.00	0.34
GATCAACGCAGAACAGAACA	0.00	0.00	0.00	0.00	0.34
GATCAACGCAGAACAGAAGA	0.00	0.33	0.00	0.00	0.00
GATCAACGCAGAACAGAGCA	0.00	0.00	0.00	0.00	0.34
GATCAACGCAGAACGCACTG	0.23	0.00	0.00	0.00	0.00
GATCAACGCAGAACGCCCAA	0.00	0.00	0.29	0.00	0.00
GATCAACGCAGAAGGCTATG	0.00	0.00	0.29	0.00	0.00
GATCAACGCAGACACAGAGG	0.00	0.33	0.00	0.00	0.00
GATCAACGCAGACAGATCAG	0.00	0.00	0.00	0.34	0.00
GATCAACGCAGACAGATCCG	0.00	0.00	0.29	0.00	0.00
GATCAACGCAGACAGCACCG	0.00	0.00	0.57	0.00	0.00
GATCAACGCAGACAGCCCCG	0.23	0.00	0.00	0.00	0.34
GATCAACGCAGACAGCTCAG	0.00	0.00	0.29	0.00	0.00
GATCAACGCAGACAGCTCCA	0.00	0.00	0.29	0.00	0.00
GATCAACGCAGACAGCTCCG	0.23	0.33	0.86	0.00	0.69
GATCAACGCAGACAGGCAGG	0.00	0.00	0.00	0.34	0.00
GATCAACGCAGACAGTTACG	0.00	0.00	0.00	0.00	0.34
GATCAACGCAGACATCCAAC	0.00	0.00	0.00	0.34	0.00
GATCAACGCAGACCAAGCTA	0.23	1.63	3.44	1.02	2.07
GATCAACGCAGACCAANCTA	0.00	0.00	0.00	0.00	0.00
GATCAACGCAGACCGATCTG	0.00	0.00	0.29	0.00	0.00
GATCAACGCAGACCGCTCAG	0.00	0.33	0.00	0.00	0.00
GATCAACGCAGACCGCTCCT	0.00	0.00	0.00	0.34	0.00
GATCAACGCAGACGAACCAT	0.00	0.00	0.00	0.34	0.00
GATCAACGCAGACGGACCCG	0.00	0.00	0.00	0.00	0.34
GATCAACGCAGACTAAGCGA	0.00	0.00	0.00	0.00	0.34
GATCAACGCAGACTACGCGA	2.29	3.25	2.86	2.38	6.54
GATCAACGCAGACTACGTGA	0.46	0.00	0.00	0.00	0.00
GATCAACGCAGACTGCCCCA	0.00	0.00	0.00	0.00	0.00
GATCAACGCAGAGCAACACA	0.00	0.00	0.00	0.00	0.34

GATCAACGCAGAGCAACGCA	0.00	0.00	0.00	0.00	0.34
GATCAACGCAGAGCAGAGCA	0.00	0.00	0.57	0.00	0.69
GATCAACGCAGAGCAGCCTC	0.00	0.33	0.00	0.00	0.00
GATCAACGCAGAGGACATAA	0.00	0.00	0.29	0.00	0.00
GATCAACGCAGAGGAGAGAA	0.00	0.00	0.00	0.00	0.34
GATCAACGCAGAGGAGTTGT	0.00	0.00	0.00	0.00	0.00
GATCAACGCAGAGTCTAGGT	0.00	0.00	0.29	0.00	0.00
GATCAACGCAGATAGCTCCG	0.00	0.00	0.00	0.34	0.34
GATCAACGCAGATGCCGAGG	0.00	0.00	0.86	1.70	0.69
GATCAACGCAGATGGCTGCA	0.23	0.00	0.00	0.00	0.00
GATCAACGCAGATTGAAAGA	0.00	0.00	0.00	0.00	0.34
GATCAACGCAGATTGAGACG	0.00	0.98	0.00	1.36	0.00
GATCAACGCAGATTTGCATG	0.00	0.65	0.00	0.00	0.00
GATCAACGCAGCAACGGCCC	0.00	0.00	0.00	0.68	0.00
GATCAACGCAGCAAGAGACA	0.00	0.00	0.00	0.00	0.00
GATCAACGCAGCAAGGACAC	0.00	0.00	0.00	0.00	0.00
GATCAACGCAGCAAGGTCAC	0.23	1.30	0.57	2.04	0.34
GATCAACGCAGCACAGAAGA	0.00	0.33	0.00	0.00	0.00
GATCAACGCAGCACGCTGCG	0.00	0.00	0.00	0.00	0.00
GATCAACGCAGCAGACAGAA	0.00	0.00	0.00	0.00	0.34
GATCAACGCAGCAGAGGAGC	0.00	0.00	0.00	0.00	0.34
GATCAACGCAGCAGCGACCG	0.00	0.00	0.00	0.34	0.00
GATCAACGCAGCAGGGGGCG	0.00	0.33	0.00	0.00	0.00
GATCAACGCAGCAGTGTGGT	0.00	0.00	0.00	0.00	0.00
GATCAACGCAGCATCCTTCA	0.00	0.00	0.00	0.34	0.00
GATCAACGCAGCATGGCCTG	0.00	0.00	0.29	0.00	0.34
GATCAACGCAGCATGTCAGG	0.00	0.00	0.00	0.00	0.00
GATCAACGCAGCCAAGCGGG	0.00	0.00	0.29	0.00	0.00
GATCAACGCAGCCAAGTCGT	0.46	0.00	0.29	0.00	0.34
GATCAACGCAGCCACATTGG	0.00	0.00	0.00	0.00	0.34
GATCAACGCAGCCCTACTTT	0.00	0.00	0.29	0.00	0.00
GATCAACGCAGCCGCGGAGG	1.14	0.33	0.29	1.36	1.03
GATCAACGCAGCCGCTGTTG	0.00	0.00	0.00	0.00	0.34
GATCAACGCAGCCTTGCCTC	0.00	0.00	0.29	0.00	0.34
GATCAACGCAGCCTTGTTGT	0.00	0.00	0.00	0.00	0.00
GATCAACGCAGCGCAGAACA	0.00	0.00	0.00	0.00	0.34
GATCAACGCAGCGCCATTGG	0.00	0.00	0.00	0.00	0.00

GATCAACGCAGCGCGGGGCT	0.00	0.00	0.00	0.00	0.34
GATCAACGCAGCTCAGGCAG	0.00	0.00	0.29	0.00	0.00
GATCAACGCAGCTCGCGCTG	0.00	0.00	0.00	0.34	0.00
GATCAACGCAGCTGCCAGCA	0.00	0.00	0.00	0.68	0.00
GATCAACGCAGCTGTTCCAT	0.00	0.00	0.00	0.00	0.34
GATCAACGCAGCTTTGTAGG	0.00	0.00	0.00	0.00	0.34
GATCAACGCAGGAGCCTTGA	0.00	0.00	0.00	0.34	0.00
GATCAACGCAGGCAAGCGGG	0.00	0.00	0.00	0.00	0.00
GATCAACGCAGGCACAGATA	0.00	0.00	0.00	0.00	0.34
GATCAACGCAGGCAGATTGA	0.00	0.00	0.00	0.00	0.00
GATCAACGCAGGCAGCAGGT	0.00	0.33	0.00	0.00	0.00
GATCAACGCAGGCATCCACA	0.00	0.00	0.57	1.70	0.69
GATCAACGCAGGCATGCATG	0.00	0.00	0.00	0.00	0.69
GATCAACGCAGGCCTTGCGC	0.46	0.00	0.00	0.00	0.00
GATCAACGCAGGCGGCCTAA	0.23	0.00	0.00	0.00	0.00
GATCAACGCAGGCGGGTCCT	0.00	0.00	0.00	0.00	0.34
GATCAACGCAGGCGTCAAGG	0.00	0.33	0.00	0.00	0.00
GATCAACGCAGGCTGGGGCA	0.23	0.00	0.29	0.34	0.34
GATCAACGCAGGGAAGTTGT	0.00	0.00	0.00	0.00	0.69
GATCAACGCAGGGAGGTCCG	0.00	0.00	0.00	0.00	0.69
GATCAACGCAGGGATCCAGA	0.00	0.00	0.00	0.00	0.00
GATCAACGCAGGGCGTTCAC	0.00	0.00	0.29	0.34	0.00
GATCAACGCAGGGCTCCAGA	0.23	0.00	0.00	0.00	0.00
GATCAACGCAGGGGTCCAGA	0.69	0.65	0.29	0.00	0.00
GATCAACGCAGGGGTCCCGA	0.23	0.00	0.00	0.00	0.00
GATCAACGCAGGGTAGTGGA	0.00	0.00	0.00	0.00	0.00
GATCAACGCAGGGTCATGAG	0.00	0.00	0.29	0.00	0.00
GATCAACGCAGGGTTACTGA	0.23	0.00	0.00	0.00	0.00
GATCAACGCAGGGTTCAAGA	0.00	0.33	0.00	0.34	0.00
GATCAACGCAGGGTTCCAGA	38.91	33.18	23.20	51.59	21.70
GATCAACGCAGGGTTCCAGC	0.23	0.00	0.29	0.00	0.00
GATCAACGCAGGGTTCCAGG	0.23	0.00	0.00	0.00	0.00
GATCAACGCAGGGTTCCAGN	0.23	0.00	0.00	0.00	0.00
GATCAACGCAGGGTTCCANA	0.00	0.00	0.00	0.34	0.00
GATCAACGCAGGGTTCCATA	0.00	0.00	0.00	0.00	0.34
GATCAACGCAGGGTTCCCGA	0.00	0.00	0.00	0.34	0.34
GATCAACGCAGGGTTCCGGA	0.00	0.00	0.29	0.00	0.00

GATCAACGCAGGGTTCCGGC	0.00	0.00	0.00	0.34	0.00
GATCAACGCAGGGTTCCNNN	0.00	0.00	0.00	0.00	0.00
GATCAACGCAGGGTTCGAGA	0.00	0.00	0.00	0.34	0.00
GATCAACGCAGGGTTGCAGA	0.23	0.00	0.00	0.00	0.00
GATCAACGCAGGTCGTGACT	0.00	0.00	0.00	0.00	0.34
GATCAACGCAGTACAGCAGG	0.00	0.00	0.00	0.00	0.00
GATCAACGCAGTACAGCTTG	0.00	0.33	0.00	0.00	0.00
GATCAACGCAGTACTTGCGC	0.00	0.00	0.29	0.00	0.00
GATCAACGCAGTCAGCAGGA	0.00	0.00	0.00	0.34	0.00
GATCAACGCAGTCAGCGGCG	0.23	0.00	0.00	0.00	0.00
GATCAACGCAGTCATTGCGA	0.00	0.33	0.00	0.00	0.00
GATCAACGCAGTCATTGCGC	0.00	0.00	0.00	0.00	0.34
GATCAACGCAGTCATTGCGG	0.00	0.00	0.29	0.00	0.00
GATCAACGCAGTCCCTGAGT	0.00	0.00	0.00	0.34	0.00
GATCAACGCAGTCCTCGCGC	0.00	0.00	0.29	0.00	0.00
GATCAACGCAGTCCTTGCCA	0.00	0.00	0.00	0.34	0.34
GATCAACGCAGTCCTTGCGA	0.00	0.33	0.29	0.34	0.69
GATCAACGCAGTCCTTGCGC	65.92	60.19	42.95	61.78	49.94
GATCAACGCAGTCCTTGCGN	0.23	0.00	0.00	0.00	0.00
GATCAACGCAGTCCTTGCGT	0.00	0.65	0.29	0.00	0.00
GATCAACGCAGTCCTTGCNN	0.23	0.00	0.00	0.00	0.00
GATCAACGCAGTCCTTGCTC	0.00	0.33	0.00	0.00	0.00
GATCAACGCAGTCCTTGTGC	0.00	0.33	0.00	0.00	0.00
GATCAACGCAGTCCTTNCGC	0.00	0.00	0.00	0.00	0.69
GATCAACGCAGTCCTTTCGC	0.00	0.00	0.00	0.34	0.00
GATCAACGCAGTCTCCGTCA	0.00	0.00	0.29	0.00	0.00
GATCAACGCAGTGGTATGCA	0.00	0.00	0.00	0.00	0.34
GATCAACGCAGTGTTTTGTT	0.00	0.98	0.00	0.00	0.00
GATCAACGCAGTTCCCTGTG	0.00	0.00	0.00	0.34	0.00
GATCAACGCAGTTGAAGCTG	0.46	0.00	0.00	0.00	1.03
GATCAACGCAGTTGTTCCAT	0.00	0.00	0.00	0.00	0.34
GATCAACGCATAAAAAAAAA	0.00	0.00	0.00	0.34	0.00
GATCAACGCATAACACATCA	0.00	0.00	0.00	0.00	0.69
GATCAACGCATAAGCACACA	0.00	0.00	0.57	0.00	0.00
GATCAACGCATAGCAACGCA	0.00	0.00	0.00	0.00	0.69
GATCAACGCATAGCACAACA	0.00	0.00	0.00	0.00	0.34
GATCAACGCATAGCACAACC	0.00	0.00	0.00	0.00	0.34

GATCAACGCATATATGATGC	0.00	0.33	0.00	0.00	0.00
GATCAACGCATATGATACAA	0.00	0.00	0.00	0.00	0.00
GATCAACGCATATTCCCATC	0.00	0.33	0.00	0.00	0.00
GATCAACGCATCAAAAAAAA	12.36	2.28	5.15	6.45	3.10
GATCAACGCATCAAAAAGCC	0.00	0.00	0.29	0.00	0.00
GATCAACGCATCAAAAATCA	0.00	0.00	0.00	0.68	0.00
GATCAACGCATCAAAAATCT	0.00	0.00	0.00	0.00	0.00
GATCAACGCATCAAAAGCAA	0.00	0.00	0.00	0.00	0.00
GATCAACGCATCAAAANCAA	0.00	0.00	0.00	0.34	0.00
GATCAACGCATCAAAATCAA	47.38	26.03	24.34	25.12	16.53
GATCAACGCATCAAAATCAC	0.23	0.00	0.00	0.34	0.00
GATCAACGCATCAAAATCAG	0.00	0.00	0.00	0.68	0.00
GATCAACGCATCAAAATCAN	0.23	0.00	0.00	0.00	0.00
GATCAACGCATCAAAATCNN	0.00	0.00	0.00	0.00	0.00
GATCAACGCATCAAACAACC	0.00	0.00	0.00	0.00	0.00
GATCAACGCATCAAACTCAA	0.92	0.00	0.00	0.00	0.00
GATCAACGCATCAAATCAAT	0.00	0.33	0.00	0.00	0.00
GATCAACGCATCAATCTTCT	0.00	0.33	0.00	0.00	0.00
GATCAACGCATCACAATCAA	0.00	0.00	0.00	0.34	0.00
GATCAACGCATCACACTGTG	0.00	0.00	0.00	0.00	0.00
GATCAACGCATCACAGCACA	0.00	0.00	0.00	0.00	0.34
GATCAACGCATCAGAAAAAA	0.23	0.00	0.00	0.00	0.00
GATCAACGCATCAGAATCAA	0.00	0.00	0.00	0.00	0.00
GATCAACGCATCAGAATGTC	0.00	0.00	0.00	0.68	0.00
GATCAACGCATCCAAAAAAA	0.00	0.33	0.00	0.00	0.00
GATCAACGCATCCAAATCAA	0.23	0.00	0.00	0.00	0.00
GATCAACGCATCCAAGCTGA	0.23	0.00	0.00	0.00	0.00
GATCAACGCATCCAAGTGCA	0.00	0.65	0.00	0.68	0.00
GATCAACGCATCCACTGCTC	0.23	0.00	0.00	0.00	0.00
GATCAACGCATCCTCAAGAA	0.00	0.00	0.00	0.00	0.34
GATCAACGCATCCTTCATCA	0.00	0.00	0.00	0.00	0.00
GATCAACGCATCGAAAAAAA	0.00	0.00	0.00	0.00	0.00
GATCAACGCATCGAAAAACA	0.23	0.00	0.00	0.00	0.00
GATCAACGCATCGACATCAA	0.00	0.65	0.00	0.00	0.00
GATCAACGCATCGAGCCGCC	0.92	0.00	0.00	0.00	0.00
GATCAACGCATCGCAACACA	0.00	0.00	0.00	0.00	1.38
GATCAACGCATCGCGATGCC	0.00	0.00	0.00	0.00	0.00

GATCAACGCATCGGCAATGG	0.00	0.00	0.00	0.00	0.34
GATCAACGCATCGGGAGATA	0.00	0.00	0.00	0.00	0.00
GATCAACGCATCGGGCACCA	0.00	0.00	0.00	0.00	0.00
GATCAACGCATCGGTGTAGT	0.23	0.00	0.00	0.00	0.00
GATCAACGCATCTCGTATGC	0.23	0.00	0.00	0.00	0.00
GATCAACGCATCTCTGATGT	0.00	0.00	0.00	0.00	0.69
GATCAACGCATCTTCTGAAC	0.00	0.00	0.29	0.00	0.00
GATCAACGCATCTTGGGCGG	0.46	0.00	0.00	0.00	0.00
GATCAACGCATGAACACGCG	0.00	0.00	0.00	0.00	0.34
GATCAACGCATGAAGGTCAT	0.00	0.00	0.29	0.00	0.00
GATCAACGCATGAAGTTCAT	0.00	0.00	0.29	0.00	0.00
GATCAACGCATGACACATCA	0.00	0.00	0.00	0.00	0.34
GATCAACGCATGATGAGCTA	0.00	0.00	0.29	0.00	0.00
GATCAACGCATGCAACATGG	0.00	0.00	0.29	0.00	0.00
GATCAACGCATGCAACTTGG	0.00	0.65	0.00	0.00	0.00
GATCAACGCATGCAGAAGTG	0.00	0.00	0.00	0.34	0.00
GATCAACGCATGCATGTTGC	0.00	0.00	0.00	0.00	0.34
GATCAACGCATGCCTAACTC	0.00	0.00	0.29	0.00	0.00
GATCAACGCATGCGAGCAGG	0.23	0.00	0.00	0.00	0.00
GATCAACGCATGCGATTTGT	0.00	0.00	0.00	0.00	0.00
GATCAACGCATGCGCGCGGT	0.00	0.00	0.29	0.00	0.00
GATCAACGCATGCGTCAAGG	4.81	0.65	0.57	0.68	0.69
GATCAACGCATGCGTCAANN	0.23	0.00	0.00	0.00	0.00
GATCAACGCATGCTAGTGTC	0.00	0.00	0.29	0.00	0.00
GATCAACGCATGCTCGCAAA	0.00	0.00	0.00	0.00	0.34
GATCAACGCATGGAAATGGG	0.00	0.00	0.29	0.00	0.00
GATCAACGCATGGAGTTAAC	0.23	0.00	0.00	0.00	0.00
GATCAACGCATGGCATTCAT	0.00	0.00	0.00	0.00	0.34
GATCAACGCATGGCCTCTCA	0.00	0.00	0.00	0.00	0.00
GATCAACGCATGGCGCATTT	0.23	0.00	0.00	0.00	0.00
GATCAACGCATGGCGCGGAC	0.00	0.00	0.00	0.34	0.00
GATCAACGCATGGCGCTCAC	0.00	0.00	0.00	0.34	0.00
GATCAACGCATGGCGGTCAC	0.23	0.00	0.00	0.00	0.34
GATCAACGCATGGCGTGCAC	0.23	0.00	0.00	0.00	0.00
GATCAACGCATGGCGTTCAA	0.23	0.65	0.00	0.00	0.00
GATCAACGCATGGCGTTCAC	19.00	28.63	16.04	33.60	6.54
GATCAACGCATGGCGTTCAG	0.00	0.00	0.00	0.00	0.00

GATCAACGCATGGCGTTCAN	0.00	0.00	0.00	0.00	0.00
GATCAACGCATGGCGTTCCC	0.00	0.00	0.00	0.00	0.34
GATCAACGCATGGCGTTCNN	0.23	0.00	0.00	0.00	0.00
GATCAACGCATGGCGTTGAC	0.46	0.00	0.00	0.00	0.00
GATCAACGCATGGCTTCGCG	0.23	0.00	0.00	0.00	0.00
GATCAACGCATGGGAGGCCT	0.00	0.00	0.29	1.02	0.00
GATCAACGCATGTCCGCTGA	0.00	0.33	0.00	0.00	0.00
GATCAACGCATGTGAAGGTT	0.00	0.00	0.29	0.00	0.00
GATCAACGCATGTGGAAAGG	0.00	0.65	0.00	0.00	0.69
GATCAACGCATGTTCGAGTA	0.00	0.00	0.00	0.00	0.00
GATCAACGCATGTTCGTGCA	0.00	0.00	0.00	0.00	0.34
GATCAACGCATGTTGGGAGT	0.00	0.00	0.00	0.00	0.69
GATCAACGCATTACACCAAG	0.00	0.00	0.00	0.00	0.00
GATCAACGCATTCACGAGGC	0.00	0.00	0.00	0.00	0.34
GATCAACGCATTCAGCTTGA	0.92	0.33	0.00	0.34	0.69
GATCAACGCATTCATGAGGA	0.00	0.00	0.00	0.00	0.00
GATCAACGCATTCATGAGGC	3.43	8.13	8.59	9.50	7.92
GATCAACGCATTCATGAGGN	0.00	0.00	0.00	0.34	0.00
GATCAACGCATTCATGCGGA	0.00	0.00	0.00	0.00	0.34
GATCAACGCATTCATGGGGC	0.00	0.00	0.00	0.00	0.00
GATCAACGCATTCATTAAGC	0.00	0.00	0.00	0.00	0.34
GATCAACGCATTCATTCAGC	2.98	2.28	1.15	3.05	1.38
GATCAACGCATTCATTCGGC	0.00	0.00	0.00	0.00	0.34
GATCAACGCATTCCAAGGAT	0.00	0.00	0.00	0.00	0.34
GATCAACGCATTCCGCGGAA	0.00	0.00	0.00	0.00	1.38
GATCAACGCATTCCGCGGAT	0.00	0.00	0.57	0.00	0.00
GATCAACGCATTCCTTTCGC	0.00	0.33	0.00	0.00	0.00
GATCAACGCATTGAAGAAGA	0.00	0.65	0.00	1.02	0.00
GATCAACGCATTGATGATTG	0.00	0.00	0.00	0.34	0.00
GATCAACGCATTGATGCACA	0.00	0.00	0.00	0.00	0.34
GATCAACGCATTGATTAGCT	0.00	0.00	0.29	0.00	0.00
GATCAACGCATTGCGCGCAA	0.00	0.33	0.00	0.00	0.00
GATCAACGCATTGGCATTTT	0.46	0.00	0.00	0.00	0.00
GATCAACGCATTGGCTTGCC	0.00	0.00	0.00	0.68	0.00
GATCAACGCATTGGCTTGGT	0.00	0.00	0.00	0.34	0.00
GATCAACGCATTGGCTTTTN	0.23	0.00	0.00	0.00	0.00
GATCAACGCATTGGCTTTTT	5.72	2.93	1.43	2.04	0.34

GATCAACGCATTGGGGGCT	0.00	0.00	0.00	0.00	0.00
GATCAACGCATTGTAAACCA	0.00	0.00	0.00	0.00	0.34
GATCAACGCATTGTGTCTGT	0.00	0.00	0.00	0.00	0.00
GATCAACGCATTGTTGCAGT	0.00	0.00	0.00	0.00	0.34
GATCAACGCATTTAAAGGAG	0.00	0.33	0.00	0.00	0.00
GATCAACGCATTTACATGAG	0.00	0.00	0.00	0.00	0.34
GATCAACGCATTTGTGTGAT	0.23	0.00	0.00	0.00	0.00
GATCAACGCCAAAAAAAAAA	0.23	0.00	0.00	0.00	0.00
GATCAACGCCAAACATTTCC	0.00	0.00	0.00	0.00	0.34
GATCAACGCCAAACCATGGT	0.00	0.00	0.00	0.00	0.00
GATCAACGCCAAACGCACTA	0.00	0.00	0.29	0.00	0.00
GATCAACGCCAAACGCCACG	0.00	0.00	0.00	0.00	1.03
GATCAACGCCAAAGCCTTGG	0.23	0.00	0.00	0.00	0.00
GATCAACGCCAACAAAGACG	0.00	0.00	0.57	0.00	0.00
GATCAACGCCAACAACGACG	0.00	0.00	0.00	0.00	0.69
GATCAACGCCAACACCAGTA	0.00	0.00	0.00	0.00	0.34
GATCAACGCCAACAGACTGA	0.00	0.33	0.00	0.34	0.00
GATCAACGCCAACAGCAGGT	0.00	0.33	0.00	0.00	0.00
GATCAACGCCAACAGCATGG	0.00	0.00	0.00	0.68	0.00
GATCAACGCCAACAGCTCCG	0.00	0.33	0.29	0.00	0.69
GATCAACGCCAACAGTTCGC	0.00	0.00	0.00	0.00	0.34
GATCAACGCCAACATCGAGA	0.00	0.33	0.00	0.00	0.00
GATCAACGCCAACATTGCCA	2.52	2.60	0.86	2.72	1.38
GATCAACGCCAACATTGCCG	0.23	0.00	0.00	0.00	0.00
GATCAACGCCAACCAGGGAG	0.00	0.00	0.00	0.00	0.00
GATCAACGCCAACCGCGCGC	0.00	0.00	0.00	0.00	0.69
GATCAACGCCAACCTCGGCA	0.00	0.33	0.00	0.00	0.00
GATCAACGCCAACGAAGGAA	0.00	0.00	0.00	0.00	0.34
GATCAACGCCAACGCATGGC	0.00	0.00	0.29	0.00	0.00
GATCAACGCCAACGGAATAA	0.00	0.00	0.29	0.00	0.00
GATCAACGCCAACGTGGGCA	0.00	0.00	0.00	0.00	0.34
GATCAACGCCAAGAATGCAA	0.00	0.00	0.00	0.34	0.00
GATCAACGCCAAGACCAAGA	0.00	0.00	0.00	0.00	0.34
GATCAACGCCAAGAGATGAG	0.00	0.00	0.00	0.00	0.00
GATCAACGCCAAGAGCTTGG	0.00	0.00	0.00	0.34	0.00
GATCAACGCCAAGCACTGGG	0.00	0.00	0.29	0.00	0.00
GATCAACGCCAAGCGTTGAG	0.00	0.00	0.00	0.00	0.00

GATCAACGCCAAGGACTTGG	0.69	0.33	0.00	0.00	0.00
GATCAACGCCAAGGAGCGCT	0.00	0.00	0.00	0.00	0.69
GATCAACGCCAAGGCAGTGC	0.00	0.00	0.00	0.00	0.00
GATCAACGCCAAGGCATGCT	0.23	0.98	3.44	2.72	4.13
GATCAACGCCAAGGCATGTT	0.00	0.00	0.29	0.00	0.00
GATCAACGCCAAGGCGCTGC	0.00	1.30	0.86	0.68	0.00
GATCAACGCCAAGGTCGGTA	0.00	0.33	0.00	0.00	0.00
GATCAACGCCAAGGTTGTCC	0.00	0.65	0.00	0.00	0.00
GATCAACGCCAAGTACGGCA	0.23	0.00	0.00	0.00	0.00
GATCAACGCCAAGTACTGCA	0.46	0.33	0.00	0.00	0.00
GATCAACGCCAAGTACTGNN	0.23	0.00	0.00	0.00	0.00
GATCAACGCCAAGTCGGGCT	0.00	0.00	0.00	0.00	0.00
GATCAACGCCAAGTCGTGCG	0.00	0.00	0.00	0.34	0.00
GATCAACGCCAAGTCGTGCT	0.23	0.98	0.57	0.00	0.69
GATCAACGCCAAGTTCTGCG	0.69	0.00	0.00	0.68	0.00
GATCAACGCCAATAGCTCCG	0.00	0.00	0.29	0.00	0.34
GATCAACGCCAATCATGGCA	0.00	0.00	0.29	0.00	0.00
GATCAACGCCAATGCGGCGC	0.00	0.00	0.00	0.00	0.34
GATCAACGCCACAATACGTG	0.00	0.33	0.00	0.00	0.00
GATCAACGCCACACAAAACA	0.00	0.00	0.00	0.00	0.69
GATCAACGCCACACAACACA	0.00	0.33	0.00	0.00	0.00
GATCAACGCCACACACGCGG	0.00	0.33	0.00	0.00	0.00
GATCAACGCCACACCCGCGG	0.00	0.33	0.00	0.34	0.00
GATCAACGCCACAGCCAGCC	0.00	0.00	0.00	0.00	0.69
GATCAACGCCACAGCGCCCG	0.00	0.00	0.29	0.00	0.00
GATCAACGCCACAGGCGTGA	0.23	0.00	0.00	0.00	0.00
GATCAACGCCACATGCACAA	0.00	0.00	0.00	0.00	0.34
GATCAACGCCACATTCAAGG	0.00	0.33	0.00	0.00	0.69
GATCAACGCCACCAACACCC	0.00	0.00	0.00	0.00	0.34
GATCAACGCCACCAAGCACG	0.00	0.00	0.00	0.00	0.00
GATCAACGCCACCAAGCACT	0.69	0.00	0.00	0.00	0.00
GATCAACGCCACCATGAGGG	0.00	0.33	0.00	0.00	0.00
GATCAACGCCACCCTCCGAC	0.00	0.00	0.00	0.00	0.00
GATCAACGCCACCGCCCTGC	0.00	0.00	0.00	0.00	0.34
GATCAACGCCACCGGTCACC	0.00	0.00	0.00	0.00	0.69
GATCAACGCCACCTACCACG	0.00	0.00	0.00	0.00	0.00
GATCAACGCCACCTACTACG	0.00	0.00	0.00	0.68	0.00

GATCAACGCCACGAAGGCCG	0.23	0.00	0.00	0.00	0.00
GATCAACGCCACGCACACCA	0.00	0.00	0.29	0.00	0.00
GATCAACGCCACGCACGTGC	0.00	0.00	0.00	0.00	0.34
GATCAACGCCACGCGATTGC	0.00	0.00	0.00	0.00	0.34
GATCAACGCCACGGACGACA	0.00	0.00	0.00	0.00	0.00
GATCAACGCCACGGACGCCA	3.20	2.93	1.43	2.72	0.34
GATCAACGCCACGGACGCCC	0.00	0.00	0.00	0.00	0.00
GATCAACGCCACGGACTCCA	0.00	0.00	0.00	0.00	0.00
GATCAACGCCACGGATGCCA	0.00	0.00	0.00	0.00	0.00
GATCAACGCCACGGCCGTTG	0.23	0.00	0.00	0.00	0.00
GATCAACGCCACGGTCGACG	0.46	0.65	0.00	0.34	0.00
GATCAACGCCACGTCAGCAG	0.00	0.00	0.29	0.00	0.00
GATCAACGCCACGTTTCAAC	0.00	0.00	0.29	0.00	0.00
GATCAACGCCACTCGCACCC	0.00	0.00	0.00	0.00	0.34
GATCAACGCCACTGCCTTTG	7.55	3.58	0.86	1.70	0.34
GATCAACGCCACTTGCGAAG	0.00	0.00	0.29	0.00	0.00
GATCAACGCCAGACAACGAG	0.00	0.00	0.00	0.00	0.00
GATCAACGCCAGACACAACA	0.00	0.00	0.00	0.00	0.34
GATCAACGCCAGAGCAGATC	0.00	0.00	0.00	0.00	0.00
GATCAACGCCAGCAGGCCCC	0.46	0.00	0.00	0.34	0.00
GATCAACGCCAGCAGTTGGG	0.00	0.00	0.00	0.00	0.00
GATCAACGCCAGCATCGACT	0.00	0.00	0.00	0.34	0.00
GATCAACGCCAGCATCGAGA	2.06	0.00	1.15	0.68	0.00
GATCAACGCCAGCATGCTGG	0.00	0.00	0.29	0.00	0.00
GATCAACGCCAGCCGGAATG	0.69	0.33	0.00	0.00	0.00
GATCAACGCCAGCCTGCTGC	0.00	0.00	0.00	0.34	0.00
GATCAACGCCAGCCTGCTGG	19.69	6.51	3.44	5.43	1.38
GATCAACGCCAGCCTGCTGN	0.23	0.00	0.00	0.00	0.00
GATCAACGCCAGCCTGCTGT	0.23	0.00	0.00	0.00	0.00
GATCAACGCCAGCCTGCTTG	0.00	0.00	0.00	0.00	0.00
GATCAACGCCAGCGACTCGC	0.23	0.00	0.00	0.00	0.00
GATCAACGCCAGCGAGGCCG	0.00	0.00	0.29	0.00	0.00
GATCAACGCCAGCGTGGTTG	0.00	0.00	0.00	0.34	0.00
GATCAACGCCAGGAAAAGTC	0.00	0.00	0.00	0.34	0.00
GATCAACGCCAGGAGTGCCT	0.00	0.00	0.29	0.00	0.00
GATCAACGCCAGGGCACACT	0.23	0.00	0.00	0.00	0.00
GATCAACGCCAGGGGGGGGN	0.00	0.00	0.00	0.00	0.34

GATCAACGCCAGGTAAGCAG	0.00	0.00	0.29	0.00	0.00
GATCAACGCCAGGTCAGAAG	0.00	0.00	0.29	0.00	0.00
GATCAACGCCAGGTCAGCAG	0.00	4.23	5.15	0.00	0.00
GATCAACGCCAGGTCAGGAG	0.00	0.00	0.29	0.00	0.00
GATCAACGCCAGGTTCAATG	0.00	0.00	0.00	0.00	0.34
GATCAACGCCAGTCAGCCTA	0.23	0.00	0.00	0.00	0.00
GATCAACGCCAGTCAGTCTA	5.04	4.88	5.44	6.11	3.44
GATCAACGCCAGTCCATTTT	0.00	0.00	0.29	0.00	0.00
GATCAACGCCAGTCTGGCTC	0.00	0.33	0.29	0.00	0.00
GATCAACGCCAGTCTTCAGC	0.23	0.00	0.00	0.00	0.00
GATCAACGCCAGTGGACTGA	0.23	0.33	0.00	0.68	0.00
GATCAACGCCATCAGTGGAA	0.00	0.00	0.29	0.00	0.00
GATCAACGCCATCATTGGCA	0.00	0.33	0.00	0.34	0.00
GATCAACGCCATCCATTCCA	0.23	0.00	0.00	0.00	0.00
GATCAACGCCATCCCCTTAG	0.00	0.00	0.00	0.34	0.00
GATCAACGCCATCCTCCGAC	2.06	0.33	0.57	1.02	1.38
GATCAACGCCATCGACTACT	0.00	0.00	0.00	0.00	0.00
GATCAACGCCATCTATACCG	0.00	0.00	0.29	0.00	0.00
GATCAACGCCATCTGAGGGT	0.00	0.00	0.00	0.00	0.34
GATCAACGCCATCTGTTACT	0.00	0.00	0.00	0.68	0.34
GATCAACGCCATGATGCAGA	0.00	0.00	0.00	0.00	0.69
GATCAACGCCATGATGTGGC	0.00	0.00	0.00	0.00	0.00
GATCAACGCCATGCCAAAGA	0.69	0.33	0.00	1.70	0.00
GATCAACGCCATGCGCGCAA	0.00	0.00	0.00	0.34	0.00
GATCAACGCCATGCGGCAAA	0.00	0.00	0.29	0.00	0.00
GATCAACGCCATGGACCTGC	0.00	0.00	0.00	0.00	0.00
GATCAACGCCATGTAGTCGC	0.23	0.00	0.00	0.00	0.00
GATCAACGCCATTGCCAGGG	0.00	0.00	0.29	0.00	0.00
GATCAACGCCATTGTTTCTT	0.00	0.00	0.29	0.00	0.00
GATCAACGCCCAAAAAAAAA	0.00	0.00	0.00	0.00	0.00
GATCAACGCCCAACGTTTCC	0.00	0.00	0.00	0.00	0.34
GATCAACGCCCAAGCACCGA	0.00	0.00	0.29	0.00	0.00
GATCAACGCCCAAGCGGCCC	0.00	0.00	0.29	0.00	0.00
GATCAACGCCCAAGTCGTCT	1.37	1.30	0.57	0.68	1.03
GATCAACGCCCAAGTGGCTG	0.00	0.00	0.00	0.00	0.69
GATCAACGCCCAATCGAGCC	0.00	0.00	0.00	0.00	0.00
GATCAACGCCCACAGCTCCG	0.23	0.00	0.00	0.00	0.00

GATCAACGCCCACAGGCCAC	0.23	0.65	0.57	1.36	2.07
GATCAACGCCCACAGGCCCC	0.00	0.00	0.00	0.00	0.00
GATCAACGCCCACAGGCTCC	0.00	0.00	0.00	0.00	0.00
GATCAACGCCCACAGGNCAC	0.00	0.00	0.00	0.00	0.34
GATCAACGCCCACATCAGGA	0.00	0.00	0.29	0.00	0.00
GATCAACGCCCACATGCAAC	0.00	0.00	0.86	0.00	0.00
GATCAACGCCCACCCCACGT	0.23	0.33	0.00	0.68	0.34
GATCAACGCCCACGTACATC	0.00	0.00	0.00	0.00	0.00
GATCAACGCCCACTGGGCCA	0.00	0.00	0.29	0.00	0.00
GATCAACGCCCAGAAGTCAG	2.06	2.60	0.00	0.68	1.38
GATCAACGCCCAGCACCTCC	0.23	0.33	0.00	0.00	0.34
GATCAACGCCCAGGCGCGGA	0.00	0.00	0.00	0.68	0.69
GATCAACGCCCAGTGGAACA	0.00	0.00	0.00	0.00	0.00
GATCAACGCCCAGTTTGAAG	0.00	0.33	0.00	0.00	0.00
GATCAACGCCCCAGATGACG	0.00	0.00	0.29	0.00	0.00
GATCAACGCCCCAGCCCTGG	0.00	0.00	0.29	0.00	0.34
GATCAACGCCCCAGGGTGCG	0.00	0.00	0.29	0.00	0.00
GATCAACGCCCCATGGAGGA	0.23	0.00	0.00	0.00	0.00
GATCAACGCCCCCAGGCGAC	0.00	0.00	0.29	0.00	0.00
GATCAACGCCCCCTGGAGGA	0.00	0.33	0.00	0.00	0.00
GATCAACGCCCCGCAGCACG	0.00	0.33	0.00	0.00	0.00
GATCAACGCCCCGCTCGCCG	0.00	0.00	0.00	0.00	0.34
GATCAACGCCCCGGCCGATC	0.00	0.00	0.00	0.00	0.34
GATCAACGCCCCGTCCTGGC	0.00	0.00	0.00	0.00	0.00
GATCAACGCCCCTAGACACT	0.00	0.00	0.00	0.34	0.00
GATCAACGCCCCTAGGCCGC	0.00	1.30	0.29	0.34	1.03
GATCAACGCCCCTGCTTTTG	0.23	0.00	0.00	0.00	0.00
GATCAACGCCCCTGGCTGCG	0.00	0.00	0.00	0.34	0.00
GATCAACGCCCGACCACCGC	0.00	0.00	0.00	0.00	0.34
GATCAACGCCCGAGGAAAGA	0.00	0.65	0.00	0.00	0.00
GATCAACGCCCGCAAAAGTA	0.00	0.00	0.00	0.00	0.34
GATCAACGCCCGCATCTGGA	0.00	0.00	0.00	0.34	0.34
GATCAACGCCCGCGGCAGGC	0.00	0.00	0.00	0.00	0.00
GATCAACGCCCGCTCCTCTG	0.00	0.00	0.57	0.00	0.00
GATCAACGCCCGGCATGAGC	0.00	0.00	0.00	0.00	0.00
GATCAACGCCCGGCCCAAAA	0.00	0.00	0.00	0.00	0.34
GATCAACGCCCGGCTGCCGG	0.00	0.00	0.00	1.02	0.00

GATCAACGCCCGGGAGCTGC	0.46	0.00	0.00	0.00	0.00
GATCAACGCCCGGGAGGCTG	0.00	0.00	0.00	1.02	0.00
GATCAACGCCCGGGCGCCCT	0.00	0.00	0.57	0.00	0.00
GATCAACGCCCGGGGTCCAG	0.00	0.00	0.00	0.00	0.00
GATCAACGCCCGGTGAATGC	0.00	0.00	0.00	0.00	0.69
GATCAACGCCCGGTGGCTTC	0.69	0.00	0.29	0.00	0.00
GATCAACGCCCGTATCTTCT	0.00	0.00	0.57	0.00	0.00
GATCAACGCCCGTCCGGTCC	0.23	0.00	0.00	0.00	0.00
GATCAACGCCCGTCCTGTCA	0.00	0.00	0.00	0.34	0.00
GATCAACGCCCGTGCTCGCC	0.00	0.00	0.29	0.00	0.00
GATCAACGCCCTAGCTCGGC	0.00	0.33	0.00	0.00	0.34
GATCAACGCCCTCATCGACG	0.23	0.00	0.00	0.00	0.00
GATCAACGCCCTCATGGCCA	0.00	0.00	0.00	0.00	0.00
GATCAACGCCCTCCACATCG	0.00	0.00	0.00	0.00	0.00
GATCAACGCCCTCCACCATA	0.00	0.00	0.00	0.00	0.00
GATCAACGCCCTCCGCGTGT	0.00	0.00	0.00	0.00	0.34
GATCAACGCCCTCCTCACTC	0.00	0.33	0.00	0.00	0.00
GATCAACGCCCTCCTCCCTA	0.00	0.00	0.29	0.00	0.34
GATCAACGCCCTCCTCCCTC	14.19	40.02	25.77	24.44	25.49
GATCAACGCCCTCCTCNCTC	0.00	0.00	0.00	0.34	0.00
GATCAACGCCCTCCTTCCTC	0.00	0.00	0.00	0.00	0.00
GATCAACGCCCTCGCGGCGT	0.00	0.00	0.00	0.00	0.34
GATCAACGCCCTCGGCAAGA	0.00	0.00	0.00	1.70	0.00
GATCAACGCCCTCGGCAGAG	0.00	0.00	0.00	0.00	0.00
GATCAACGCCCTCGTCGACA	0.00	0.00	0.00	0.00	0.00
GATCAACGCCCTCTACGTCC	0.00	0.33	0.00	0.00	0.00
GATCAACGCCCTCTCCCCGC	3.43	3.90	0.29	1.70	1.38
GATCAACGCCCTGAAGCACG	1.14	0.33	0.29	0.68	0.34
GATCAACGCCCTGCCGCACC	0.00	0.00	0.00	0.00	0.69
GATCAACGCCCTGCGGCCTC	0.00	0.00	0.00	0.34	0.00
GATCAACGCCCTGGAAAAAA	0.00	0.00	0.00	0.34	0.00
GATCAACGCCCTGGCGCAGA	0.00	0.00	0.00	0.00	0.00
GATCAACGCCCTGGTCGCCG	0.00	0.65	0.00	0.00	0.00
GATCAACGCCCTGTGGTCCG	0.00	0.00	0.00	0.00	0.34
GATCAACGCCCTTGCGCCGG	0.00	0.00	0.29	0.00	0.00
GATCAACGCCCTTGCGTTGC	0.00	0.00	0.00	0.00	0.00
GATCAACGCCCTTTCCCCGC	0.23	0.00	0.00	0.00	0.00

GATCAACGCCGAAAGATCCA	0.00	0.33	0.00	0.00	0.00
GATCAACGCCGAAAGATCCG	0.00	0.00	0.00	0.00	0.34
GATCAACGCCGAAAGCACCG	0.00	0.00	0.00	0.00	0.34
GATCAACGCCGAAAGCTAAG	0.00	0.00	0.00	0.34	0.00
GATCAACGCCGAAAGCTACG	0.00	0.65	0.00	0.00	0.34
GATCAACGCCGAAAGCTATG	0.00	0.33	0.00	0.00	0.00
GATCAACGCCGAAAGCTCCG	0.00	0.98	1.15	0.34	0.69
GATCAACGCCGAAAGCTCTG	0.23	0.00	0.86	0.00	0.00
GATCAACGCCGAAAGCTTCG	0.00	0.00	0.00	0.00	0.00
GATCAACGCCGAACGCTCCG	0.00	0.00	0.29	0.00	0.00
GATCAACGCCGAACGCTCTG	0.00	0.00	0.29	0.00	0.00
GATCAACGCCGAACTGAAAC	0.00	0.00	0.29	0.00	0.00
GATCAACGCCGAAGAAGAAG	0.00	0.00	0.29	0.00	0.00
GATCAACGCCGAATTAAAGG	0.00	0.33	0.00	0.00	0.00
GATCAACGCCGACAAAAAAA	0.00	0.00	0.00	0.00	0.34
GATCAACGCCGACAACTCCG	0.00	0.00	0.00	0.34	0.34
GATCAACGCCGACACCATCG	0.00	0.00	0.29	0.00	0.00
GATCAACGCCGACACCTCCG	0.00	0.00	0.00	0.00	0.34
GATCAACGCCGACAGAACCG	0.00	0.00	0.00	0.00	0.34
GATCAACGCCGACAGACCGG	0.00	0.00	0.29	0.00	0.00
GATCAACGCCGACAGATACG	0.00	0.00	0.00	0.00	0.00
GATCAACGCCGACAGATCAG	0.00	0.00	0.00	0.34	0.34
GATCAACGCCGACAGATCCG	0.00	0.33	1.15	2.04	2.41
GATCAACGCCGACAGATCTG	0.00	0.33	0.00	0.34	0.69
GATCAACGCCGACAGATGTA	0.00	0.33	0.00	0.00	0.00
GATCAACGCCGACAGCAAAC	0.23	0.00	0.00	0.00	0.00
GATCAACGCCGACAGCACCG	0.00	0.98	1.72	0.68	0.69
GATCAACGCCGACAGCACTG	0.00	0.00	0.57	0.00	0.00
GATCAACGCCGACAGCCACG	0.00	0.33	0.00	0.00	0.00
GATCAACGCCGACAGCCCCA	0.00	0.00	0.00	0.00	0.00
GATCAACGCCGACAGCCCCC	0.00	0.00	0.00	0.00	0.00
GATCAACGCCGACAGCCCCG	0.46	1.63	0.86	0.68	0.34
GATCAACGCCGACAGCCCTG	0.00	0.00	0.00	0.00	0.34
GATCAACGCCGACAGCGACG	0.00	0.00	0.29	0.00	0.00
GATCAACGCCGACAGCGCAC	0.00	0.00	0.00	0.34	0.00
GATCAACGCCGACAGCGCCG	0.92	0.33	2.29	1.02	2.41
GATCAACGCCGACAGCGCTG	0.23	0.00	0.00	0.00	0.00

GATCAACGCCGACAGCGGCG	0.00	0.00	0.00	0.00	0.00
GATCAACGCCGACAGCGGGA	0.00	0.00	0.00	0.00	0.00
GATCAACGCCGACAGCNCCG	0.00	0.00	0.00	0.00	4.13
GATCAACGCCGACAGCNCNG	0.00	0.00	0.29	0.00	0.00
GATCAACGCCGACAGCNNNN	0.46	0.00	0.00	0.00	0.00
GATCAACGCCGACAGCTAAG	0.00	0.00	0.57	0.00	0.69
GATCAACGCCGACAGCTACA	0.00	0.33	0.00	0.00	0.34
GATCAACGCCGACAGCTACG	0.46	1.30	2.00	1.36	3.44
GATCAACGCCGACAGCTAGG	0.00	0.00	0.00	0.34	0.00
GATCAACGCCGACAGCTATG	0.00	0.00	0.00	0.00	0.34
GATCAACGCCGACAGCTCAA	0.00	0.33	0.00	0.00	0.00
GATCAACGCCGACAGCTCAC	0.00	0.00	0.00	0.00	0.69
GATCAACGCCGACAGCTCAG	0.46	2.28	3.44	2.38	3.44
GATCAACGCCGACAGCTCAN	0.00	0.00	0.00	0.00	0.00
GATCAACGCCGACAGCTCCA	0.00	0.33	0.57	0.34	2.07
GATCAACGCCGACAGCTCCC	0.00	0.00	0.57	0.34	1.72
GATCAACGCCGACAGCTCCG	196.40	733.94	1018.29	777.64	987.4[illegible]
GATCAACGCCGACAGCTCCN	0.92	0.00	0.00	1.36	0.69
GATCAACGCCGACAGCTCCT	0.00	0.65	0.57	0.00	0.69
GATCAACGCCGACAGCTCGA	0.00	0.00	0.00	0.00	1.03
GATCAACGCCGACAGCTCGG	0.23	0.33	0.29	1.36	0.69
GATCAACGCCGACAGCTCGT	0.23	0.00	0.00	0.00	0.34
GATCAACGCCGACAGCTCNG	0.00	0.00	0.00	0.68	0.69
GATCAACGCCGACAGCTCNN	1.14	0.00	0.00	0.00	0.00
GATCAACGCCGACAGCTCTC	0.00	0.00	0.00	0.34	0.34
GATCAACGCCGACAGCTCTG	28.61	97.92	148.91	77.39	113.6[illegible]
GATCAACGCCGACAGCTCTN	0.23	0.00	0.00	0.00	0.00
GATCAACGCCGACAGCTCTT	0.00	0.00	0.00	0.34	0.00
GATCAACGCCGACAGCTGCG	0.23	1.30	0.57	0.00	0.00
GATCAACGCCGACAGCTNCG	0.00	0.00	0.00	0.00	0.69
GATCAACGCCGACAGCTNNG	0.00	0.00	0.00	0.34	0.00
GATCAACGCCGACAGCTNNN	0.23	0.00	0.00	0.00	0.00
GATCAACGCCGACAGCTTCG	0.00	0.00	0.57	0.00	0.34
GATCAACGCCGACAGCTTTG	0.00	0.00	0.00	0.34	0.00
GATCAACGCCGACAGGTACG	0.00	0.00	0.29	0.00	0.00
GATCAACGCCGACAGGTCCG	0.23	0.00	0.00	0.00	0.00
GATCAACGCCGACAGTGCCG	0.00	0.00	0.29	0.00	0.00

GATCAACGCCGACAGTGGCA	0.00	0.00	0.00	0.00	0.00
GATCAACGCCGACAGTTCCC	0.00	0.00	0.00	0.68	0.00
GATCAACGCCGACAGTTCCG	0.00	0.00	0.29	0.00	2.07
GATCAACGCCGACATCGACG	0.00	0.00	0.00	0.34	0.00
GATCAACGCCGACATCGAGG	0.00	0.00	0.00	0.34	0.00
GATCAACGCCGACATCTCCG	0.00	0.00	0.29	0.00	0.00
GATCAACGCCGACATCTCTT	0.00	0.00	0.00	0.34	0.00
GATCAACGCCGACATGCCCC	0.00	0.33	0.00	0.00	0.00
GATCAACGCCGACATTTGTG	0.00	0.00	0.00	0.00	0.34
GATCAACGCCGACCCTTACG	0.00	0.00	0.29	0.34	1.03
GATCAACGCCGACCCTTTGG	0.00	0.00	0.00	0.68	0.00
GATCAACGCCGACCGATCCG	0.00	0.00	0.29	0.00	0.00
GATCAACGCCGACCGCCGCG	0.00	0.00	0.29	0.00	0.00
GATCAACGCCGACCGCTCAG	0.00	0.33	0.00	0.00	0.00
GATCAACGCCGACCGCTCCG	0.23	0.33	0.86	0.34	0.69
GATCAACGCCGACCGCTCCT	0.00	0.00	0.00	0.00	0.00
GATCAACGCCGACCGCTCTG	0.00	0.00	0.00	0.00	0.00
GATCAACGCCGACCGCTGCG	0.00	0.00	0.29	0.00	0.00
GATCAACGCCGACCGCTTCG	0.00	0.33	0.00	0.00	0.00
GATCAACGCCGACCTACGTC	0.00	0.33	0.00	0.00	0.00
GATCAACGCCGACCTAGAGA	0.00	0.00	0.00	0.00	0.34
GATCAACGCCGACCTGGAAA	0.00	0.33	0.00	0.00	0.00
GATCAACGCCGACCTTGCCT	0.23	0.00	0.00	0.00	0.00
GATCAACGCCGACGCCAAGA	0.00	0.33	0.00	0.00	0.00
GATCAACGCCGACGCTCCGC	0.00	0.00	0.00	0.00	0.34
GATCAACGCCGACGGCGACG	0.00	0.33	0.57	0.00	0.00
GATCAACGCCGACGGCGACT	0.00	0.00	0.00	0.00	0.00
GATCAACGCCGACGGCGCCA	0.00	0.00	0.00	0.68	0.34
GATCAACGCCGACGGCTCCA	0.00	0.00	0.00	0.00	0.00
GATCAACGCCGACGGCTCCG	0.23	0.00	0.57	0.34	0.34
GATCAACGCCGACGTGGCCG	0.23	0.00	0.00	0.00	0.00
GATCAACGCCGACTACCGCT	0.00	0.00	0.00	0.34	0.00
GATCAACGCCGACTCGTATG	0.00	0.00	0.00	0.34	0.00
GATCAACGCCGACTGCGGGA	0.00	0.00	0.00	0.34	0.00
GATCAACGCCGACTGCTCGC	0.00	0.33	0.29	0.00	0.00
GATCAACGCCGACTTGCTTG	0.00	0.00	0.29	0.00	0.00
GATCAACGCCGAGAGCTACG	0.23	0.00	0.00	0.00	0.00

GATCAACGCCGAGAGCTCCG	0.00	0.00	0.00	0.00	0.34
GATCAACGCCGAGAGCTCTN	0.23	0.00	0.00	0.00	0.00
GATCAACGCCGAGCCCAAAT	0.00	0.00	0.00	0.34	0.00
GATCAACGCCGAGGAGGTTC	0.00	0.00	0.29	0.00	0.00
GATCAACGCCGAGGTGAAGA	0.46	0.33	0.00	0.00	0.00
GATCAACGCCGAGTACCCAG	0.46	1.63	1.15	1.36	0.69
GATCAACGCCGAGTTGCGCC	0.00	0.00	0.29	0.00	0.00
GATCAACGCCGATAGATACG	0.00	0.00	0.00	0.00	0.34
GATCAACGCCGATAGATCAA	0.00	0.00	0.00	0.34	0.00
GATCAACGCCGATAGCCCCG	0.00	0.00	0.29	0.34	0.00
GATCAACGCCGATAGCCCGC	0.00	0.00	0.00	0.00	0.00
GATCAACGCCGATAGCGCCG	0.00	0.33	0.00	0.00	0.00
GATCAACGCCGATAGCNCCG	0.00	0.00	0.00	0.00	0.34
GATCAACGCCGATAGCNCNN	0.23	0.00	0.00	0.00	0.00
GATCAACGCCGATAGCNNNN	0.00	0.00	0.00	0.00	0.00
GATCAACGCCGATAGCTAAG	0.00	0.00	0.00	0.00	0.34
GATCAACGCCGATAGCTACG	0.00	0.00	0.29	0.34	0.00
GATCAACGCCGATAGCTCAG	0.00	0.00	0.29	0.00	0.69
GATCAACGCCGATAGCTCCA	0.00	0.00	0.00	0.00	0.34
GATCAACGCCGATAGCTCCC	0.00	0.00	0.00	0.00	0.34
GATCAACGCCGATAGCTCCG	15.57	72.55	113.97	70.94	94.02
GATCAACGCCGATAGCTCCN	0.00	0.00	0.00	0.00	0.00
GATCAACGCCGATAGCTCCT	0.00	0.33	0.00	0.00	0.00
GATCAACGCCGATAGCTCNG	0.00	0.00	0.00	0.00	0.34
GATCAACGCCGATAGCTCNN	0.00	0.00	0.00	0.34	0.00
GATCAACGCCGATAGCTCTG	0.00	0.00	0.29	0.00	0.00
GATCAACGCCGATAGCTTCG	0.00	0.33	0.00	0.00	0.00
GATCAACGCCGATAGTTCCG	0.00	0.00	0.00	0.00	0.00
GATCAACGCCGATAGTTTCG	0.00	0.00	0.00	0.00	0.00
GATCAACGCCGATATAGGGG	0.00	0.00	0.29	0.00	0.00
GATCAACGCCGATCGAGAAG	0.00	0.00	0.00	0.00	0.34
GATCAACGCCGATCGCTCCG	0.00	0.33	0.00	0.00	0.00
GATCAACGCCGATGCGGCGC	0.00	0.00	0.00	0.00	0.00
GATCAACGCCGATGCTCTGC	0.00	0.00	0.29	0.00	0.00
GATCAACGCCGATGGCTCCG	0.00	0.00	0.29	0.00	0.00
GATCAACGCCGCAAACTAAA	0.00	0.00	0.00	0.00	0.00
GATCAACGCCGCAAAGGAGG	0.46	0.65	0.29	0.00	0.00

GATCAACGCCGCAAAGGCAG	0.23	0.00	0.00	1.02	0.00
GATCAACGCCGCAAAGGGGG	0.23	0.00	0.00	0.00	0.00
GATCAACGCCGCAAATGCCA	0.00	0.00	0.29	0.00	0.00
GATCAACGCCGCAAGGTACG	0.00	0.00	0.00	0.00	0.34
GATCAACGCCGCACGGGACC	0.00	0.00	0.00	0.00	0.00
GATCAACGCCGCAGCACTCT	0.23	0.00	0.00	0.00	0.00
GATCAACGCCGCAGCGCCGG	0.00	0.00	0.29	0.00	0.00
GATCAACGCCGCAGCGGAGA	0.23	0.00	0.00	0.00	0.00
GATCAACGCCGCAGGGGGCG	0.00	0.00	0.00	0.00	0.00
GATCAACGCCGCCAAACGGA	0.00	0.00	0.00	0.00	0.00
GATCAACGCCGCCACGCATA	0.00	0.00	0.29	0.00	0.00
GATCAACGCCGCCAGATCCG	0.00	0.00	0.00	0.00	0.34
GATCAACGCCGCCAGCAACC	0.00	0.00	0.00	0.00	0.34
GATCAACGCCGCCAGCCCGG	0.00	0.33	0.00	0.00	0.00
GATCAACGCCGCCAGCGACA	0.00	0.00	0.00	0.00	0.34
GATCAACGCCGCCAGCGCCA	0.92	0.00	0.00	0.68	0.00
GATCAACGCCGCCAGCGCCG	0.23	0.00	0.00	0.00	0.00
GATCAACGCCGCCAGCGCCN	0.23	0.00	0.00	0.00	0.00
GATCAACGCCGCCAGCGTGG	0.00	0.00	0.00	0.34	0.00
GATCAACGCCGCCAGCTACG	0.00	0.00	0.29	0.34	0.00
GATCAACGCCGCCAGCTCCG	0.23	0.00	1.15	0.00	0.69
GATCAACGCCGCCAGGGCAA	0.00	0.00	0.00	0.68	0.00
GATCAACGCCGCCAGTGGTC	0.23	0.33	0.57	0.00	0.69
GATCAACGCCGCCATCGTGG	0.69	0.00	0.00	0.00	0.00
GATCAACGCCGCCATGTGCG	0.00	0.00	0.00	0.00	0.34
GATCAACGCCGCCCGATCCG	0.23	0.00	0.00	0.00	0.00
GATCAACGCCGCCGACATGC	0.00	0.33	0.00	0.00	0.00
GATCAACGCCGCCGAGTTGA	0.23	0.00	0.00	0.00	0.00
GATCAACGCCGCCGCCTAGA	0.00	0.00	0.00	0.00	0.34
GATCAACGCCGCCTACTTTT	1.60	0.00	0.00	0.34	1.03
GATCAACGCCGCCTCCGCGT	0.00	0.00	0.00	0.34	0.00
GATCAACGCCGCCTTGAAGA	0.00	0.00	0.00	0.34	0.00
GATCAACGCCGCGAAGGCAG	0.23	0.00	0.00	0.00	0.00
GATCAACGCCGCGAGCGGCT	0.23	0.00	0.00	0.00	0.00
GATCAACGCCGCGATGAAGA	0.00	0.00	0.00	0.00	0.00
GATCAACGCCGCGCTTATCA	0.00	0.33	0.00	0.00	0.00
GATCAACGCCGCGGCGCCCG	0.46	0.00	0.00	0.00	0.00

GATCAACGCCGCGTGCGCTC	0.00	0.00	0.00	0.00	0.00
GATCAACGCCGCTAGCTCCG	0.00	0.00	0.00	0.00	0.00
GATCAACGCCGCTAGCTCCT	0.00	0.00	0.00	0.00	0.34
GATCAACGCCGCTAGTACGG	0.00	0.00	0.29	0.00	0.00
GATCAACGCCGCTCGCAGCG	0.00	0.00	0.00	0.00	0.00
GATCAACGCCGCTCTTGTGC	0.00	0.00	0.00	0.00	0.00
GATCAACGCCGCTGCATATG	0.00	0.00	0.00	0.00	0.34
GATCAACGCCGCTTTTCGAC	1.14	0.65	0.86	0.00	0.69
GATCAACGCCGCTTTTCGGC	0.23	0.00	0.00	0.00	0.00
GATCAACGCCGCTTTTCNNN	0.23	0.00	0.00	0.00	0.00
GATCAACGCCGGAAACGCCC	0.23	0.00	0.00	0.68	0.34
GATCAACGCCGGAAACTTTT	0.00	0.00	0.00	0.34	0.00
GATCAACGCCGGAAAGAAAA	0.00	0.00	0.00	0.00	0.34
GATCAACGCCGGAGACTGGG	0.00	0.00	0.00	0.00	0.00
GATCAACGCCGGAGCCTGAG	0.00	0.00	0.29	0.00	0.00
GATCAACGCCGGAGGGAAGC	0.00	0.00	0.00	0.00	0.34
GATCAACGCCGGCAGCCCTG	0.00	0.00	0.00	0.00	0.00
GATCAACGCCGGCAGCTCCG	0.23	0.33	0.29	0.00	0.00
GATCAACGCCGGCCACAGGC	0.00	0.00	0.00	0.00	0.34
GATCAACGCCGGCCGTATGG	0.00	0.00	0.29	0.00	0.00
GATCAACGCCGGCCGTTCAC	0.23	0.00	0.00	0.00	0.00
GATCAACGCCGGCGACTCGC	2.06	2.93	0.86	1.36	1.38
GATCAACGCCGGCGCCACCT	0.00	0.00	0.00	0.00	0.00
GATCAACGCCGGCGCCCTTG	0.00	0.00	0.00	0.00	0.00
GATCAACGCCGGCGGCGACC	0.23	0.00	0.00	0.00	0.00
GATCAACGCCGGCTGCGTCG	0.00	0.00	0.00	0.34	0.00
GATCAACGCCGGCTTCGACC	0.00	0.00	0.00	0.68	0.00
GATCAACGCCGGGAGCAGCA	0.00	0.00	0.00	1.36	0.00
GATCAACGCCGGGATGAGTT	0.00	0.00	0.00	0.00	0.00
GATCAACGCCGGGTTGTTCG	0.00	0.00	0.00	0.00	0.34
GATCAACGCCGGTCAACTGC	0.00	0.33	0.00	0.00	0.00
GATCAACGCCGGTGCCCTGG	0.23	0.00	0.00	0.00	0.00
GATCAACGCCGGTTGATTGG	0.00	0.00	0.00	0.00	0.00
GATCAACGCCGTCAAAAAAA	0.00	0.00	0.00	0.00	0.34
GATCAACGCCGTCAGCACGG	0.00	0.00	0.00	0.00	0.34
GATCAACGCCGTCAGCACTG	0.00	0.00	0.00	0.34	0.00
GATCAACGCCGTCAGCTCCG	0.00	0.00	0.00	0.00	0.00

GATCAACGCCGTCATCGAGG	0.23	0.00	0.00	0.00	0.00
GATCAACGCCGTCCGGATGG	0.46	0.00	0.00	0.68	0.00
GATCAACGCCGTCCTTGCGC	0.00	0.00	0.29	0.00	0.00
GATCAACGCCGTCGAAAAAA	5.95	4.88	4.30	6.79	1.38
GATCAACGCCGTCGAAACTA	0.00	0.33	0.00	0.34	0.34
GATCAACGCCGTCGAAACTN	0.00	0.00	0.00	0.00	0.00
GATCAACGCCGTCGAAACTT	7.78	1.30	2.00	1.70	2.07
GATCAACGCCGTCGAAGAAA	0.00	0.00	0.00	0.34	0.00
GATCAACGCCGTCGAATTGC	0.00	0.33	0.00	0.00	0.00
GATCAACGCCGTCGATAAAA	0.00	0.00	0.00	0.00	0.00
GATCAACGCCGTCGCAAAAA	0.00	0.00	0.29	0.00	0.00
GATCAACGCCGTCGGAAAAA	0.00	0.00	0.00	0.00	0.69
GATCAACGCCGTCTCCAAAT	0.00	0.00	0.29	0.00	0.00
GATCAACGCCGTCTTCGCGA	0.00	0.00	0.00	0.34	0.00
GATCAACGCCGTCTTCTGCT	0.00	0.00	0.00	0.00	0.34
GATCAACGCCGTGACAGTGC	0.00	0.00	0.00	0.00	0.34
GATCAACGCCGTGACAGTGT	0.00	0.65	0.57	0.00	0.00
GATCAACGCCGTGATAGTGT	0.00	0.00	0.00	0.00	0.00
GATCAACGCCGTGATTGTGG	0.23	0.33	0.57	0.00	0.34
GATCAACGCCGTGCACAAAT	0.00	0.00	0.00	0.00	0.00
GATCAACGCCGTGCCCAAAT	9.39	10.41	5.44	10.52	8.61
GATCAACGCCGTGCCCAACT	0.00	0.00	0.00	0.00	0.34
GATCAACGCCGTGCCCACAT	0.00	0.00	0.00	0.34	0.00
GATCAACGCCGTGCCCCAAT	0.00	0.00	0.00	0.00	0.00
GATCAACGCCGTGCCCGAAT	0.23	0.00	0.00	0.00	0.00
GATCAACGCCGTGCGCTCGG	0.00	0.00	0.00	0.00	0.34
GATCAACGCCGTGCGCTCTG	0.00	0.00	0.00	0.34	0.69
GATCAACGCCGTGCGGCGGC	0.00	0.00	0.00	0.00	0.00
GATCAACGCCGTGCTCAAGG	0.00	0.00	0.00	0.00	0.34
GATCAACGCCGTGCTCCTCA	0.00	0.00	0.00	0.00	0.00
GATCAACGCCGTGGAAAAAA	0.23	0.00	0.00	0.00	0.00
GATCAACGCCGTGGAGGAGG	0.00	0.00	0.00	0.00	0.00
GATCAACGCCGTGGCGGGGC	0.23	0.00	0.00	0.00	0.00
GATCAACGCCGTGGCGGTGC	0.00	0.00	0.00	0.00	0.00
GATCAACGCCGTGGGGCACG	0.00	0.00	0.29	0.00	0.00
GATCAACGCCGTGTGATTAG	0.00	0.00	0.00	0.00	0.00
GATCAACGCCGTTGGGAGAG	0.00	0.00	0.00	0.34	0.00

GATCAACGCCGTTTAACAGC	0.00	0.00	0.00	0.00	0.34
GATCAACGCCTAAAAAAAAT	0.00	0.00	0.00	0.00	0.00
GATCAACGCCTACAGCGCCG	0.00	0.00	0.00	0.34	0.00
GATCAACGCCTACAGCTCCG	0.23	0.00	0.29	0.00	0.00
GATCAACGCCTACAGTTCCG	0.00	0.00	0.29	0.00	0.00
GATCAACGCCTACCGGGGGG	0.00	0.33	0.00	0.00	0.00
GATCAACGCCTACCTGCTCT	0.00	0.00	0.00	0.34	0.00
GATCAACGCCTACCTGGAGA	0.23	0.00	0.00	0.00	0.00
GATCAACGCCTACGTGAAGG	0.00	0.00	0.00	3.05	0.00
GATCAACGCCTAGCAGCCGG	0.00	0.00	0.00	0.00	0.00
GATCAACGCCTAGCAGCGGC	0.00	0.33	0.00	0.00	0.00
GATCAACGCCTATGCCGGGA	0.00	0.00	0.29	0.00	0.00
GATCAACGCCTCAAAATCAC	0.00	0.00	0.29	0.00	0.00
GATCAACGCCTCAAAGCTCG	0.00	0.00	0.00	0.68	0.00
GATCAACGCCTCAAGGCGCG	0.69	0.00	0.00	0.00	0.69
GATCAACGCCTCACGAGCGA	0.00	0.33	0.00	0.00	0.00
GATCAACGCCTCACTGCCGG	0.00	0.00	0.29	0.00	0.00
GATCAACGCCTCCAACTCAA	0.23	0.00	0.00	0.00	0.00
GATCAACGCCTCCAAGGCGG	0.00	0.33	0.00	0.00	0.00
GATCAACGCCTCCAAGGTGG	1.83	0.98	1.72	1.36	0.69
GATCAACGCCTCCACTGACG	0.23	0.00	0.00	0.00	0.00
GATCAACGCCTCCGGCAGAG	0.00	0.00	0.29	0.00	0.00
GATCAACGCCTCCTACTACG	0.00	0.00	0.00	0.00	0.00
GATCAACGCCTCCTGCACGG	0.23	0.00	0.00	0.00	0.00
GATCAACGCCTCGCGTTGCA	0.00	0.00	0.00	0.00	0.00
GATCAACGCCTCGCTCTTCC	0.00	0.33	0.00	0.00	0.00
GATCAACGCCTCGGAGCTGG	0.23	0.00	0.00	0.00	0.00
GATCAACGCCTCGGCCTACG	0.00	0.00	0.29	0.00	0.00
GATCAACGCCTCGGCCTCCT	0.46	0.00	0.29	0.00	0.00
GATCAACGCCTCGTTCGCTG	0.00	0.33	0.00	0.00	1.03
GATCAACGCCTCTCCCTGGT	2.75	1.63	0.00	1.70	1.38
GATCAACGCCTCTGCCTGCC	0.23	0.00	1.15	0.68	0.69
GATCAACGCCTCTGGCAGCT	2.29	3.25	2.00	1.36	0.69
GATCAACGCCTCTGGGGTGC	0.23	0.00	0.00	0.00	0.00
GATCAACGCCTGAAACTGTG	0.00	0.00	0.29	0.00	0.00
GATCAACGCCTGAAGGCCAC	0.46	0.65	0.00	0.00	0.00
GATCAACGCCTGACAACTGG	0.00	0.00	0.00	0.00	0.00

GATCAACGCCTGACCTCCCT	0.00	0.00	0.00	0.00	0.34
GATCAACGCCTGAGCCCAAG	0.23	0.00	0.00	0.00	0.00
GATCAACGCCTGAGGTGGGG	0.00	0.00	0.00	0.00	0.34
GATCAACGCCTGCAAGGAGG	0.23	0.33	0.00	0.00	0.00
GATCAACGCCTGCACGCAGT	0.00	0.00	0.00	0.00	0.00
GATCAACGCCTGCACGCATG	0.00	0.00	0.29	0.00	0.00
GATCAACGCCTGCAGCTCCC	0.23	0.00	0.00	0.00	0.00
GATCAACGCCTGCATCTAAC	0.00	0.33	0.00	0.00	0.00
GATCAACGCCTGCATCTACA	0.00	0.00	0.00	0.00	0.00
GATCAACGCCTGCATCTACC	7.78	8.13	1.72	5.77	5.17
GATCAACGCCTGCATCTGCA	0.23	0.33	0.00	0.00	0.00
GATCAACGCCTGCATGGAGC	0.00	0.00	0.00	0.00	0.00
GATCAACGCCTGCATGGAGG	0.00	0.33	0.29	0.00	0.00
GATCAACGCCTGCATTGGTT	0.00	0.00	0.00	0.00	0.69
GATCAACGCCTGCCGGCAGT	0.00	0.00	0.00	0.00	0.34
GATCAACGCCTGCCTCGGCG	0.00	0.65	0.00	0.00	0.00
GATCAACGCCTGCCTTTGGG	0.00	0.00	0.00	0.00	0.00
GATCAACGCCTGCGAGGCCA	0.00	0.00	0.00	0.68	0.00
GATCAACGCCTGCGCAAGGG	0.00	0.00	0.00	0.00	0.00
GATCAACGCCTGCGCCAAGG	0.46	0.00	0.57	0.34	0.00
GATCAACGCCTGCGCCCAGG	0.00	0.33	0.00	0.00	0.34
GATCAACGCCTGCGCGAAGT	0.23	0.00	0.00	0.00	0.00
GATCAACGCCTGCGCTTCCC	0.00	0.00	0.00	0.00	0.00
GATCAACGCCTGCGGCGCGG	0.00	0.00	0.29	0.00	0.00
GATCAACGCCTGCGTCAAGG	0.00	0.00	0.00	0.00	0.00
GATCAACGCCTGCGTCTACC	0.00	0.00	0.00	0.00	0.00
GATCAACGCCTGCTGCTGGG	0.00	0.00	0.00	0.00	0.34
GATCAACGCCTGCTGTGTCG	0.23	0.00	0.00	0.00	0.00
GATCAACGCCTGGCAGCAGG	0.00	0.00	0.00	0.00	0.00
GATCAACGCCTGGCAGCCAG	0.00	0.00	0.00	0.00	0.00
GATCAACGCCTGGCAGCCGC	0.00	0.00	0.00	0.00	0.00
GATCAACGCCTGGCAGCCGG	3.89	4.88	2.58	1.70	0.34
GATCAACGCCTGGCAGCGAC	0.00	0.00	0.00	0.00	1.03
GATCAACGCCTGGGAGGCTG	0.00	0.00	0.00	0.00	0.00
GATCAACGCCTGGGCGCATA	0.00	0.00	0.00	0.00	0.34
GATCAACGCCTGGGGGGCGC	0.00	0.00	0.00	1.36	0.34
GATCAACGCCTGGGTGTGCT	0.00	0.00	0.00	0.34	0.00

GATCAACGCCTGGTGGAGCA	0.00	0.00	0.00	0.34	0.00
GATCAACGCCTGTCTTTGGG	0.00	0.00	0.00	0.00	0.00
GATCAACGCCTGTGCACTGG	0.23	0.00	0.00	0.00	0.00
GATCAACGCCTGTGCCCCGG	0.69	0.00	0.00	0.00	0.00
GATCAACGCCTGTGCCCCGT	0.23	0.00	0.00	0.00	0.00
GATCAACGCCTGTGCCCTGG	24.26	4.55	0.57	6.11	2.76
GATCAACGCCTGTGCCNNNN	0.23	0.00	0.00	0.00	0.00
GATCAACGCCTTCAAGGGCA	0.00	0.33	0.86	0.00	1.03
GATCAACGCCTTCAAGGGCT	0.23	0.00	0.00	0.34	0.00
GATCAACGCCTTCACGATGT	0.00	0.00	0.00	0.00	0.34
GATCAACGCCTTCACGCAGA	0.92	0.00	0.00	0.00	0.00
GATCAACGCCTTCACGCCGC	0.23	0.33	0.00	0.00	0.00
GATCAACGCCTTCATCAAGC	0.00	0.00	0.00	0.34	0.00
GATCAACGCCTTCATCCAGA	0.00	0.33	0.29	0.00	0.00
GATCAACGCCTTCATCCAGC	17.17	21.80	22.05	20.03	18.25
GATCAACGCCTTCATCCAGN	0.00	0.00	0.00	0.00	0.00
GATCAACGCCTTCATCCATC	0.00	0.00	0.00	0.00	0.34
GATCAACGCCTTCATCCCGC	0.00	0.33	0.00	0.00	0.00
GATCAACGCCTTCATCCTGC	0.23	0.00	0.00	0.00	0.00
GATCAACGCCTTCATCGTAT	0.00	0.00	0.00	0.00	0.00
GATCAACGCCTTCATCNAGC	0.00	0.00	0.00	0.00	0.34
GATCAACGCCTTCATTCCGG	0.00	0.00	0.00	0.34	0.00
GATCAACGCCTTCCCTCGTG	0.00	0.00	0.00	0.00	0.00
GATCAACGCCTTCCGGGCGG	0.00	0.00	0.00	0.34	0.00
GATCAACGCCTTCCTCCAGC	0.00	0.00	0.00	0.00	0.00
GATCAACGCCTTCCTTGCGA	0.00	0.00	0.29	0.00	0.00
GATCAACGCCTTCGGATTCG	0.00	0.00	0.29	0.00	0.00
GATCAACGCCTTCGTCCAGC	0.00	0.00	0.00	0.00	0.00
GATCAACGCCTTCTCGCTCT	0.00	0.33	0.00	0.34	0.00
GATCAACGCCTTCTGCATGG	0.00	0.33	1.43	0.00	1.72
GATCAACGCCTTGAAGCTGC	0.00	0.00	0.00	0.00	0.34
GATCAACGCCTTGCAACTTC	0.00	0.00	0.00	0.00	0.00
GATCAACGCCTTGCACCGCA	0.00	0.00	0.00	0.00	0.00
GATCAACGCCTTGCGGAACG	0.23	0.33	0.00	0.00	0.00
GATCAACGCCTTGCGGAGAG	0.00	0.00	0.29	0.00	0.00
GATCAACGCCTTGCGGAGCG	2.06	4.23	2.58	2.72	1.72
GATCAACGCCTTGGCAATTC	0.00	0.00	0.00	0.00	0.34

GATCAACGCCTTGGCAGATG	0.00	0.00	0.00	1.02	0.00
GATCAACGCCTTGGTGCAGT	0.00	0.00	0.00	0.00	0.00
GATCAACGCCTTGTCGAGAG	0.00	0.00	0.00	0.00	0.00
GATCAACGCCTTGTGATGCG	0.00	0.00	0.00	0.00	0.00
GATCAACGCCTTTCCACAGA	0.00	0.00	0.29	0.00	0.00
GATCAACGCCTTTGCCGAAG	0.00	0.00	0.57	0.00	1.38
GATCAACGCCTTTGCTGAAA	0.00	0.33	0.00	0.00	0.00
GATCAACGCCTTTTGCACAC	0.00	0.00	0.00	0.00	0.34
GATCAACGCCTTTTGGAGGA	0.23	0.00	0.00	0.00	0.00
GATCAACGCGAAAAAAAAAA	0.00	0.33	0.00	0.00	0.00
GATCAACGCGAAACCGTATC	0.00	0.00	0.00	0.34	0.00
GATCAACGCGAAACCGTTTC	3.20	1.95	2.00	1.70	1.38
GATCAACGCGAAAGATATAT	0.00	0.00	0.29	0.00	0.00
GATCAACGCGAACAAAGCCA	0.00	0.00	0.00	0.34	0.00
GATCAACGCGAACAACACCA	0.23	0.00	0.00	0.00	0.00
GATCAACGCGAACAAGGCGT	0.00	0.00	0.00	0.00	0.00
GATCAACGCGAACAGCCTGC	0.00	0.00	0.00	0.34	0.00
GATCAACGCGAACCCAAATT	0.00	0.00	0.00	0.00	0.34
GATCAACGCGAACCCCAATT	0.00	0.00	0.00	0.00	0.34
GATCAACGCGAACTACGACT	0.00	0.00	0.00	0.00	0.00
GATCAACGCGAAGCCTATCT	0.00	0.33	0.00	0.00	0.00
GATCAACGCGAAGCGGCCAT	0.00	0.00	0.00	0.34	0.00
GATCAACGCGAAGGAAGCAT	0.00	0.00	0.00	0.00	0.00
GATCAACGCGAAGGCCATGA	0.00	0.00	0.00	0.00	0.69
GATCAACGCGAAGGGCCAGC	0.23	0.00	0.00	0.68	0.00
GATCAACGCGAAGGGCCCGC	0.23	0.00	0.00	0.00	0.00
GATCAACGCGAAGGGTTTCT	0.46	0.00	0.29	0.00	0.00
GATCAACGCGAATATTTGAA	0.00	0.00	0.00	0.00	0.00
GATCAACGCGACACAGCTTC	0.00	0.00	0.29	0.00	0.00
GATCAACGCGACACTACACC	0.00	0.00	0.00	0.00	0.34
GATCAACGCGACAGAACAGG	0.00	0.00	0.00	0.00	0.34
GATCAACGCGACAGCGGACA	1.14	1.63	0.57	1.02	0.00
GATCAACGCGACAGCGGGCA	0.23	0.00	0.00	0.00	0.34
GATCAACGCGACAGGCATTG	0.00	0.00	0.00	0.00	0.34
GATCAACGCGACCACCGTAC	0.23	0.00	0.00	0.00	0.00
GATCAACGCGACCATGCCCC	0.00	0.00	0.00	0.34	0.00
GATCAACGCGACCCAAGGGT	0.23	0.00	0.00	0.00	0.00

GATCAACGCGACCCCCTCGG	0.23	0.00	0.00	0.00	0.00
GATCAACGCGACCCCGATGG	0.00	0.98	0.00	0.00	0.00
GATCAACGCGACCGGGAACC	0.00	0.00	0.00	0.34	0.00
GATCAACGCGACCTCCAACC	0.00	0.00	0.00	1.02	0.00
GATCAACGCGACCTCCAGGG	0.00	0.00	0.00	0.00	0.00
GATCAACGCGACCTTGTGCT	0.00	0.00	0.00	0.34	0.00
GATCAACGCGACGACTTCTC	0.00	0.33	0.00	0.00	0.00
GATCAACGCGACGCACCTCG	0.23	0.00	0.00	0.00	0.00
GATCAACGCGACGCCCTGTA	0.23	0.00	0.00	0.68	0.00
GATCAACGCGACGGCGAGAC	0.00	0.33	0.00	0.00	0.00
GATCAACGCGAGATTGTGAT	0.23	0.00	0.00	0.00	0.00
GATCAACGCGAGATTGTGCT	0.00	0.00	0.00	0.34	0.00
GATCAACGCGAGCAGCCGAA	0.00	0.00	0.00	0.00	0.00
GATCAACGCGAGCATCGAGA	0.00	0.33	0.29	0.34	1.38
GATCAACGCGAGCATGGACA	0.00	0.00	0.00	0.00	0.00
GATCAACGCGAGCATTTGTT	0.23	0.00	0.00	0.00	0.00
GATCAACGCGAGCCGACACA	2.06	6.18	2.58	4.41	2.07
GATCAACGCGAGCCGACCCA	0.00	0.00	0.29	0.00	0.00
GATCAACGCGAGCGAGGAGC	0.00	0.00	0.29	0.00	0.00
GATCAACGCGAGCGAGGCAA	0.00	0.00	0.00	0.00	0.00
GATCAACGCGAGCGAGGCGA	0.23	0.00	0.00	0.00	0.00
GATCAACGCGAGCGAGGCGT	20.60	11.39	4.58	12.90	5.17
GATCAACGCGAGCGCAGGCT	0.00	0.00	0.00	0.00	0.00
GATCAACGCGAGCTACTCGA	0.69	0.00	0.00	0.34	0.00
GATCAACGCGAGCTACTCGN	0.00	0.00	0.00	0.00	0.00
GATCAACGCGAGCTCAATCA	0.00	0.00	0.00	0.00	0.34
GATCAACGCGAGCTCCATCA	0.46	0.00	0.00	0.00	0.00
GATCAACGCGAGCTCGTGCT	0.23	0.33	0.00	0.34	0.00
GATCAACGCGAGCTGCACGC	0.00	0.00	0.29	0.00	0.00
GATCAACGCGAGCTTGAGCT	0.00	0.00	0.29	0.00	0.00
GATCAACGCGAGCTTGCGCT	0.00	0.00	0.29	0.00	0.00
GATCAACGCGAGCTTGGGCT	0.23	0.33	0.00	0.00	0.34
GATCAACGCGAGCTTGNGCT	0.00	0.33	0.00	0.00	0.34
GATCAACGCGAGCTTGNGNT	0.00	0.00	0.29	0.00	0.00
GATCAACGCGAGCTTGTGAT	0.00	0.00	0.29	0.00	0.00
GATCAACGCGAGCTTGTGCG	0.23	0.33	0.00	0.00	0.00
GATCAACGCGAGCTTGTGCT	72.11	74.83	44.10	70.26	60.96

GATCAACGCGAGCTTGTGGT	0.00	0.33	0.00	0.00	0.00
GATCAACGCGAGCTTGTGNN	0.69	0.00	0.00	0.00	0.00
GATCAACGCGAGCTTGTTCG	0.00	0.00	0.29	0.00	0.00
GATCAACGCGAGCTTGTTCT	0.00	0.00	0.00	0.34	0.00
GATCAACGCGAGGACGAGAA	0.00	0.33	0.00	0.00	0.00
GATCAACGCGAGGACTTCTC	0.00	0.33	0.00	0.00	0.00
GATCAACGCGAGGCAAGAGG	0.23	0.00	0.00	0.00	0.00
GATCAACGCGAGGCAGCTGC	0.00	0.00	0.29	0.00	0.00
GATCAACGCGAGGCGGTATC	0.23	0.00	0.00	0.00	0.00
GATCAACGCGAGGGCAATTC	0.00	0.00	0.29	0.00	0.00
GATCAACGCGAGGTAGTGAT	0.00	0.00	0.00	0.00	0.00
GATCAACGCGAGTAGGGTTC	0.00	0.00	0.29	0.00	0.00
GATCAACGCGAGTCAAACTC	0.00	0.00	0.57	0.00	0.00
GATCAACGCGATAGCCTTTG	0.00	0.00	0.00	0.00	0.00
GATCAACGCGATGAATTCTC	0.00	0.00	0.00	0.00	0.00
GATCAACGCGATGACTTATC	0.00	0.00	0.00	0.00	0.34
GATCAACGCGATGACTTCCC	0.00	0.00	0.00	0.00	0.34
GATCAACGCGATGACTTCGC	0.00	0.00	0.00	0.00	0.00
GATCAACGCGATGACTTCNN	0.23	0.00	0.00	0.00	0.00
GATCAACGCGATGACTTCTA	0.00	0.00	0.00	0.00	0.00
GATCAACGCGATGACTTCTC	30.67	31.56	26.63	35.98	29.96
GATCAACGCGATGACTTCTG	0.00	0.00	0.00	0.00	0.00
GATCAACGCGATGACTTCTT	0.00	0.00	0.00	0.00	0.00
GATCAACGCGATGACTTNNN	0.23	0.00	0.00	0.00	0.00
GATCAACGCGATGCATTCTC	0.00	0.00	0.00	0.00	0.00
GATCAACGCGATGCCTGTGC	0.00	0.00	0.00	0.00	0.69
GATCAACGCGATGCGCGCAA	0.00	0.33	0.00	0.00	0.00
GATCAACGCGATGGCCCCGT	0.23	0.00	0.00	0.68	0.00
GATCAACGCGATGTACGACG	0.46	0.33	0.00	0.00	0.00
GATCAACGCGATTGCACAGC	0.00	0.00	0.00	0.00	0.34
GATCAACGCGATTTGACAAA	0.00	0.98	0.00	0.00	0.00
GATCAACGCGCAAAAGAGGA	0.00	0.00	0.00	0.00	0.34
GATCAACGCGCAAAGAAGCC	0.00	0.00	0.00	0.00	0.00
GATCAACGCGCAACCCTTCG	0.23	0.00	0.00	0.00	0.00
GATCAACGCGCAACGCACAA	0.00	0.00	0.00	0.00	0.34
GATCAACGCGCAACGCACAC	0.00	0.00	0.00	0.00	0.34
GATCAACGCGCAATGCAGCC	0.00	0.00	0.00	0.00	0.34

GATCAACGCGCACCAGCACC	0.00	0.00	0.00	0.00	0.00
GATCAACGCGCACCCTTCGG	0.00	0.00	0.29	0.00	0.00
GATCAACGCGCAGAACTGTG	0.00	0.00	0.00	0.00	0.34
GATCAACGCGCAGACGCAGT	0.23	0.00	0.00	0.00	0.00
GATCAACGCGCAGAGCCTCG	0.00	0.00	0.00	0.00	0.00
GATCAACGCGCAGAGCCTGC	0.00	0.00	0.00	0.34	0.00
GATCAACGCGCAGAGCCTGG	0.00	0.00	0.00	1.02	0.00
GATCAACGCGCAGAGGCCGA	0.23	0.00	0.00	0.00	0.00
GATCAACGCGCAGATGGAGC	0.00	0.00	0.29	0.00	0.00
GATCAACGCGCAGCCAGATG	0.00	0.00	0.29	0.00	0.00
GATCAACGCGCAGCCTGCGG	0.23	0.00	0.00	0.00	0.00
GATCAACGCGCAGCTCACGC	0.00	0.33	0.00	0.00	0.00
GATCAACGCGCAGCTCTTTG	0.00	0.33	0.00	0.00	0.00
GATCAACGCGCAGCTTACGC	0.00	0.00	0.00	0.34	0.00
GATCAACGCGCAGGCCCCTG	0.00	1.63	0.29	0.00	0.00
GATCAACGCGCAGGCCGCTG	0.00	0.33	0.00	0.00	0.00
GATCAACGCGCAGGCCGTGC	0.00	0.00	0.00	0.00	0.00
GATCAACGCGCAGGCGCCTA	0.00	0.33	0.00	0.00	0.00
GATCAACGCGCAGGGCTTTC	0.46	0.00	0.00	0.00	0.00
GATCAACGCGCAGTGCTACC	0.00	0.00	0.00	0.00	0.00
GATCAACGCGCAGTGGGAGA	0.00	0.00	0.29	0.68	0.00
GATCAACGCGCAGTTCGAAC	0.00	0.65	0.00	0.00	0.00
GATCAACGCGCATGCATTGA	0.46	0.33	0.00	0.00	0.00
GATCAACGCGCATGCATTGC	0.00	0.33	0.00	0.00	0.00
GATCAACGCGCATTGCCAAG	0.00	0.33	0.00	0.00	0.00
GATCAACGCGCCAACTGCNN	0.23	0.00	0.00	0.00	0.00
GATCAACGCGCCAACTGCTC	0.00	0.00	0.00	0.00	0.69
GATCAACGCGCCAACTGGTC	0.46	0.00	0.29	0.00	0.00
GATCAACGCGCCAACTGNNN	0.23	0.00	0.00	0.00	0.00
GATCAACGCGCCAACTGTCC	0.00	0.00	0.00	0.00	0.34
GATCAACGCGCCAACTGTGG	0.00	0.00	0.00	0.00	0.34
GATCAACGCGCCAACTGTNN	0.23	0.00	0.00	0.00	0.00
GATCAACGCGCCAACTGTTA	0.00	0.33	0.29	0.00	0.00
GATCAACGCGCCAACTGTTC	48.76	32.86	30.35	29.87	37.88
GATCAACGCGCCAACTGTTG	0.00	0.00	0.29	0.34	0.00
GATCAACGCGCCAACTGTTN	0.00	0.00	0.29	0.00	0.00
GATCAACGCGCCAACTNTNN	0.23	0.00	0.00	0.00	0.00

GATCAACGCGCCAATTGTTC	0.23	0.00	0.00	0.00	0.00
GATCAACGCGCCACAACTCA	0.00	0.00	0.00	0.00	0.34
GATCAACGCGCCACATCGCA	0.00	0.33	0.00	0.00	0.00
GATCAACGCGCCACATGCGC	0.00	0.00	0.00	0.00	0.00
GATCAACGCGCCACATGGAC	0.00	0.00	0.29	0.00	0.00
GATCAACGCGCCAGAGACGG	0.00	0.00	0.29	0.00	0.34
GATCAACGCGCCAGCCGGAG	0.00	0.00	0.00	0.00	0.00
GATCAACGCGCCAGCTGTGT	0.00	0.00	0.29	0.00	0.00
GATCAACGCGCCATACATAA	0.00	0.00	0.29	0.00	0.00
GATCAACGCGCCATCGCAAC	0.00	0.00	0.00	0.68	0.00
GATCAACGCGCCATCTGTTC	0.00	0.00	0.00	0.00	0.34
GATCAACGCGCCCACCGTTC	0.00	0.00	0.29	0.00	0.00
GATCAACGCGCCCACTGTTC	0.23	0.00	0.29	0.00	0.00
GATCAACGCGCCCAGCCTGG	0.00	0.00	0.29	0.00	0.00
GATCAACGCGCCCGAGCAGG	0.00	0.33	0.29	0.00	0.34
GATCAACGCGCCCGAGCCAG	0.00	0.00	0.00	0.34	0.00
GATCAACGCGCCCGAGCCGC	0.00	0.00	0.57	0.00	0.00
GATCAACGCGCCCGAGCCGG	16.94	18.54	17.75	19.35	18.25
GATCAACGCGCCCGAGCCNG	0.00	0.33	0.00	0.00	0.00
GATCAACGCGCCCGCGACGG	0.00	0.33	0.00	0.00	0.00
GATCAACGCGCCCGCGCCGG	0.00	0.00	0.29	0.00	0.00
GATCAACGCGCCCGGGCCGG	0.23	0.00	0.00	0.00	0.00
GATCAACGCGCCCGTGGACT	0.23	0.00	0.00	0.00	0.00
GATCAACGCGCCCGTGGTCT	1.14	1.30	0.57	1.02	0.69
GATCAACGCGCCCTGCCACG	0.00	0.00	0.86	0.00	0.69
GATCAACGCGCCCTGCCTGG	0.00	0.00	0.29	0.00	0.00
GATCAACGCGCCGCCGTAGG	0.23	0.00	0.00	0.00	0.00
GATCAACGCGCCGCTGCCGG	0.00	0.00	0.00	0.00	0.00
GATCAACGCGCCGTGTACCA	0.00	0.00	0.29	0.00	0.00
GATCAACGCGCCTGAGCTCT	0.00	0.00	0.00	0.00	0.00
GATCAACGCGCCTGTGCTAT	0.00	0.33	0.00	0.00	0.00
GATCAACGCGCCTGTGCTCT	0.23	0.33	0.00	0.34	0.34
GATCAACGCGCGACTCACCA	0.00	0.00	0.57	0.00	0.00
GATCAACGCGCGCAGACATG	0.00	0.00	0.29	0.00	0.00
GATCAACGCGCGCAGCTGTG	0.23	0.00	0.00	0.00	0.00
GATCAACGCGCGCATATGAG	0.00	0.00	0.00	0.00	0.00
GATCAACGCGCGCCCTCTTG	0.00	0.00	0.00	0.00	0.00

GATCAACGCGCGCCGCCCTC	0.00	0.33	0.00	0.00	0.00
GATCAACGCGCGCGCCGTCG	0.23	0.00	0.00	0.00	0.00
GATCAACGCGCGCGGCTGTG	0.23	0.00	0.00	0.00	0.00
GATCAACGCGCGCTTGTGCT	0.00	0.00	0.00	0.00	0.00
GATCAACGCGCGGACCGCAA	0.46	0.00	0.00	0.68	0.00
GATCAACGCGCGGACCGCGA	0.00	0.00	0.00	0.00	0.34
GATCAACGCGCGGAGCAGCC	0.00	0.00	0.00	0.00	0.00
GATCAACGCGCGGCACTTGC	0.92	0.33	0.29	1.02	0.69
GATCAACGCGCGGCCGATCC	0.00	0.65	0.00	0.00	0.34
GATCAACGCGCGGTGCCGGA	0.00	0.00	0.00	0.34	0.00
GATCAACGCGCGTCGGCGTG	0.00	0.33	0.00	0.00	0.00
GATCAACGCGCGTCGGTGTG	0.46	0.33	0.00	1.02	0.34
GATCAACGCGCGTCTCCTTG	0.92	0.98	0.29	1.02	0.34
GATCAACGCGCGTCTTCTGC	0.23	0.00	0.00	0.00	0.00
GATCAACGCGCGTGCTGAAC	0.00	0.33	0.00	0.00	0.00
GATCAACGCGCGTGCTGACC	0.00	0.00	0.00	0.00	0.00
GATCAACGCGCGTGCTTGGA	0.00	0.00	0.00	0.34	0.00
GATCAACGCGCTCACAATAC	0.92	0.00	0.00	0.00	0.00
GATCAACGCGCTCAGCACAA	0.00	0.00	0.00	0.00	0.00
GATCAACGCGCTCAGCTCGG	0.00	0.00	0.29	0.00	0.00
GATCAACGCGCTCATCGCGG	2.29	1.95	0.57	1.36	1.38
GATCAACGCGCTCATGCAGA	0.00	0.33	0.00	0.34	0.00
GATCAACGCGCTCCAACAAC	0.00	0.00	0.29	0.00	0.00
GATCAACGCGCTCCAGGTCA	0.23	0.33	0.00	0.00	0.00
GATCAACGCGCTCCCTAACC	0.00	0.00	0.29	0.00	0.00
GATCAACGCGCTCCTCGACG	0.00	0.00	0.29	0.00	0.00
GATCAACGCGCTCGTCATGA	0.46	1.30	0.57	1.02	0.34
GATCAACGCGCTCGTCATGG	0.23	0.00	0.00	0.00	0.00
GATCAACGCGCTCTGCCTGG	0.23	0.00	0.00	0.00	0.00
GATCAACGCGCTCTTCAGCA	0.00	0.00	0.00	0.34	0.00
GATCAACGCGCTGAATCTCG	0.00	0.33	0.00	0.34	0.00
GATCAACGCGCTGACGCGCT	0.00	0.00	0.00	0.00	0.00
GATCAACGCGCTGAGCCCGC	0.23	0.00	0.00	0.00	0.00
GATCAACGCGCTGAGCGTCC	0.00	0.00	0.00	0.34	0.00
GATCAACGCGCTGAGCTTCG	0.23	0.00	0.00	0.00	0.00
GATCAACGCGCTGATTAGCC	0.00	0.00	0.00	0.00	0.34
GATCAACGCGCTGCATGTAG	0.00	0.00	0.29	0.00	0.00

GATCAACGCGCTGCGCGCAA	0.23	0.00	0.00	0.00	0.00
GATCAACGCGCTGCGCGTCC	0.00	0.00	0.00	0.00	0.34
GATCAACGCGCTGCTGTGCA	0.00	0.00	0.00	0.00	0.69
GATCAACGCGCTGGCATGGC	0.00	0.00	0.29	0.00	0.00
GATCAACGCGCTGGCTGGTG	0.00	0.00	0.29	0.00	0.00
GATCAACGCGCTGTCCGAGG	0.23	0.00	0.00	0.00	0.00
GATCAACGCGCTGTCGCGAG	0.00	0.00	0.29	0.00	0.00
GATCAACGCGCTGTCTGTGG	0.00	0.00	0.29	0.00	0.00
GATCAACGCGCTGTGGAGGA	0.69	0.00	0.00	0.00	0.00
GATCAACGCGCTGTTCTGCT	0.00	0.00	0.00	0.00	0.34
GATCAACGCGCTTCGCAAGA	0.00	0.00	0.29	0.00	0.00
GATCAACGCGCTTGACCCCA	0.23	0.33	0.00	0.00	0.00
GATCAACGCGCTTGTGACCA	0.00	0.65	0.00	0.00	0.00
GATCAACGCGCTTTTTGGTG	0.46	0.00	0.00	0.34	0.00
GATCAACGCGGAAAGCTAAG	0.00	0.33	0.00	0.00	0.00
GATCAACGCGGAAAGCTCAG	0.00	0.00	0.57	0.00	0.69
GATCAACGCGGAAATTGGCC	0.00	0.00	0.00	0.00	0.34
GATCAACGCGGAACAAGGGA	0.00	0.00	0.00	0.00	1.03
GATCAACGCGGAAGTGTGGT	0.00	0.00	0.00	0.00	0.00
GATCAACGCGGAATTACTCC	0.00	0.33	0.00	0.00	0.00
GATCAACGCGGACAACTAAG	0.00	0.00	0.00	0.00	0.34
GATCAACGCGGACAAGTCCC	0.00	0.00	0.00	0.34	0.00
GATCAACGCGGACACATCAG	0.00	0.00	0.00	0.00	0.00
GATCAACGCGGACACCTCCG	0.00	0.00	0.00	0.00	0.00
GATCAACGCGGACAGACCAG	0.00	0.00	0.00	0.00	0.00
GATCAACGCGGACAGATCAG	0.00	0.98	0.29	0.00	0.34
GATCAACGCGGACAGATGAG	0.00	0.00	0.29	0.00	0.00
GATCAACGCGGACAGCACTG	0.00	0.00	0.00	0.34	0.00
GATCAACGCGGACAGCCCAA	0.00	0.00	0.29	0.00	0.00
GATCAACGCGGACAGCCCAG	0.00	0.00	0.00	0.00	0.34
GATCAACGCGGACAGCCCCG	0.00	0.00	0.00	0.34	0.00
GATCAACGCGGACAGCGCAG	0.23	0.00	0.29	0.00	0.00
GATCAACGCGGACAGCGCCG	0.00	0.33	0.00	0.34	0.00
GATCAACGCGGACAGCNCAG	0.00	0.00	0.00	0.00	0.34
GATCAACGCGGACAGCNNNN	0.23	0.00	0.00	0.00	0.00
GATCAACGCGGACAGCNTCG	0.00	0.00	0.00	0.00	0.34
GATCAACGCGGACAGCTAAG	0.00	0.33	1.43	0.00	0.00

GATCAACGCGGACAGCTCAA	0.00	0.33	0.00	0.34	0.00
GATCAACGCGGACAGCTCAC	0.00	0.00	0.00	0.34	0.34
GATCAACGCGGACAGCTCAG	44.87	122.97	205.61	124.57	173.23
GATCAACGCGGACAGCTCAN	0.46	0.00	0.00	0.00	0.00
GATCAACGCGGACAGCTCCG	0.00	1.63	2.86	1.36	1.03
GATCAACGCGGACAGCTCGC	0.00	0.00	0.00	0.34	0.00
GATCAACGCGGACAGCTCGG	0.46	0.33	0.00	0.00	0.00
GATCAACGCGGACAGCTCGT	0.46	0.00	0.00	0.00	0.00
GATCAACGCGGACAGCTCTG	0.00	0.00	0.57	0.00	0.00
GATCAACGCGGACAGCTGAG	0.00	0.00	0.29	0.00	0.00
GATCAACGCGGACAGCTTAG	0.00	0.00	0.29	0.00	0.00
GATCAACGCGGACAGCTTCG	0.00	0.00	0.00	0.00	0.34
GATCAACGCGGACAGGACAG	0.00	0.33	0.00	0.00	0.00
GATCAACGCGGACAGGTCAG	0.00	0.00	0.29	0.34	0.34
GATCAACGCGGACATCTCCT	0.00	0.00	0.00	0.00	0.34
GATCAACGCGGACATTCGAG	0.00	0.65	0.00	0.00	0.00
GATCAACGCGGACATTGCGA	0.00	0.00	0.00	0.00	0.34
GATCAACGCGGACCCCGGGA	0.00	0.00	0.00	0.00	0.00
GATCAACGCGGACCGCTCAG	0.00	0.00	0.57	0.00	0.34
GATCAACGCGGACCGGCACA	0.69	0.65	0.00	0.00	0.00
GATCAACGCGGACCTGAGGA	0.00	0.00	0.00	0.00	1.03
GATCAACGCGGACGACCTCG	0.23	0.00	0.00	0.34	0.00
GATCAACGCGGACGGCTCAG	0.00	0.33	0.00	0.00	0.00
GATCAACGCGGAGACGAGAA	0.00	0.00	0.00	0.00	0.34
GATCAACGCGGAGACGCGTG	0.00	0.00	0.00	0.68	0.00
GATCAACGCGGAGAGCAGAA	0.00	0.00	0.00	0.00	0.69
GATCAACGCGGAGAGCTCAG	0.23	0.00	0.00	0.00	0.00
GATCAACGCGGAGAGGGTGG	0.00	0.00	0.29	0.00	0.00
GATCAACGCGGAGCACGCGA	0.00	0.00	0.00	0.00	0.34
GATCAACGCGGAGCACGCGC	0.00	0.33	0.00	0.00	0.00
GATCAACGCGGAGCACGCGT	6.64	21.80	14.03	19.35	18.94
GATCAACGCGGAGCACGCTC	0.00	0.65	0.29	0.00	0.00
GATCAACGCGGAGCACNCGT	0.00	0.00	0.00	0.00	0.34
GATCAACGCGGAGCAGGTCG	0.00	0.00	0.29	0.00	0.00
GATCAACGCGGAGCATAATT	0.00	0.00	0.00	0.34	0.00
GATCAACGCGGAGCTCAAGG	0.00	0.33	0.00	0.00	0.00
GATCAACGCGGAGCTCACGA	0.00	0.33	0.00	0.00	0.00

GATCAACGCGGAGCTCGCGC	0.00	0.00	0.00	1.02	0.34
GATCAACGCGGAGCTGAGCA	0.00	0.33	0.00	0.00	0.00
GATCAACGCGGAGCTGCGCA	0.00	0.33	0.00	0.00	0.00
GATCAACGCGGAGCTGGACC	0.00	0.00	0.00	0.68	0.00
GATCAACGCGGAGCTTGCCA	0.00	0.00	0.00	0.00	0.34
GATCAACGCGGAGCTTGCCG	0.00	0.00	0.00	0.00	0.00
GATCAACGCGGAGCTTTGCG	0.00	0.00	0.00	0.00	0.34
GATCAACGCGGAGGCCCCGG	0.00	0.33	0.00	0.00	0.00
GATCAACGCGGAGGGTTTCT	0.23	0.00	0.00	0.00	0.00
GATCAACGCGGAGGTCAGGC	0.00	0.33	0.00	0.00	0.00
GATCAACGCGGAGTATGTTC	0.00	0.00	0.00	0.00	0.00
GATCAACGCGGAGTGATCCG	0.23	0.00	0.00	0.00	0.00
GATCAACGCGGAGTGCTCCA	0.00	0.33	0.00	0.00	0.00
GATCAACGCGGAGTGCTCCG	0.23	0.00	0.00	0.00	0.00
GATCAACGCGGAGTGGGACC	0.00	0.00	0.29	0.00	0.00
GATCAACGCGGATAGCTCAG	0.00	0.33	0.00	0.34	0.00
GATCAACGCGGATGACAGCG	0.00	0.00	0.29	0.00	0.00
GATCAACGCGGATGAGCTCC	0.00	0.33	0.00	0.00	0.00
GATCAACGCGGATGGCGCTG	1.37	0.33	1.43	0.00	0.34
GATCAACGCGGATTGGCCAT	0.00	0.00	0.00	0.34	0.00
GATCAACGCGGATTTGTTCA	0.00	0.33	0.00	0.00	0.00
GATCAACGCGGCAAACAGGG	0.00	0.00	0.29	0.00	0.00
GATCAACGCGGCAACTGGCC	0.00	0.00	0.00	0.00	0.00
GATCAACGCGGCAAGTCGGG	0.23	0.00	0.00	0.00	0.00
GATCAACGCGGCAAGTGGCC	0.00	0.00	0.00	0.00	0.00
GATCAACGCGGCAATTAGAC	0.00	0.00	0.00	0.00	0.00
GATCAACGCGGCAATTAGCC	0.00	0.00	0.00	0.34	0.00
GATCAACGCGGCAATTGACC	0.00	0.00	0.00	0.00	0.00
GATCAACGCGGCAATTGCCC	0.00	0.00	0.00	0.00	0.00
GATCAACGCGGCAATTGGAA	0.00	0.00	0.29	0.00	0.00
GATCAACGCGGCAATTGGAC	0.00	0.00	0.00	0.00	0.34
GATCAACGCGGCAATTGGCA	0.00	0.00	0.29	0.00	0.00
GATCAACGCGGCAATTGGCC	9.39	44.57	12.89	46.16	20.32
GATCAACGCGGCAATTGGCG	0.00	0.00	0.29	0.00	0.00
GATCAACGCGGCAATTGGCN	0.46	0.00	0.00	0.00	0.00
GATCAACGCGGCAATTGGCT	0.00	0.00	0.00	1.02	0.00
GATCAACGCGGCAATTGGGC	0.00	0.00	0.00	0.00	0.00

GATCAACGCGGCAATTGGTC	0.00	0.00	0.00	0.00	0.00
GATCAACGCGGCAATTGTCC	0.00	0.00	0.00	0.00	0.34
GATCAACGCGGCAGAAAAGG	0.23	0.00	0.00	0.00	0.00
GATCAACGCGGCAGAACAGG	7.32	13.34	5.15	6.11	6.89
GATCAACGCGGCAGAACGGG	0.00	0.00	0.00	0.00	0.00
GATCAACGCGGCAGAGGCGT	0.00	0.65	0.00	0.00	0.00
GATCAACGCGGCAGTTGGCC	0.23	0.00	0.00	0.00	0.00
GATCAACGCGGCATAGAGCA	0.00	0.33	0.00	0.00	0.00
GATCAACGCGGCATTGTCGN	0.00	0.00	0.00	0.00	0.00
GATCAACGCGGCATTGTCGT	0.69	0.33	0.57	0.68	0.69
GATCAACGCGGCCAACCCAC	0.00	0.00	0.00	0.00	0.34
GATCAACGCGGCCAAGCAGG	0.00	0.00	0.00	0.00	0.00
GATCAACGCGGCCAAGCGGG	1.14	5.21	3.15	2.04	3.79
GATCAACGCGGCCACGATTG	0.00	0.33	0.00	0.00	0.00
GATCAACGCGGCCACTTCCG	0.00	0.00	0.29	0.00	0.00
GATCAACGCGGCCAGCCCAT	0.00	0.00	0.00	0.00	0.00
GATCAACGCGGCCAGCTCAG	0.00	0.33	0.00	0.00	0.00
GATCAACGCGGCCATCGCAA	0.00	0.00	0.00	0.00	0.00
GATCAACGCGGCCATGGGCC	0.00	0.33	0.00	0.00	0.00
GATCAACGCGGCCATTAGCC	0.00	0.00	0.29	0.00	0.00
GATCAACGCGGCCATTCGCC	0.00	0.33	0.00	0.00	0.00
GATCAACGCGGCCATTGCCC	0.00	0.00	0.00	0.00	0.00
GATCAACGCGGCCATTGGAA	0.00	0.00	0.00	0.00	0.34
GATCAACGCGGCCATTGGAC	0.00	0.00	0.00	0.34	0.34
GATCAACGCGGCCATTGGCA	0.00	0.00	0.00	0.34	0.00
GATCAACGCGGCCATTGGCC	16.02	45.87	18.61	38.36	21.01
GATCAACGCGGCCATTGGCG	0.00	0.33	0.00	0.00	0.00
GATCAACGCGGCCATTGGCT	0.00	0.00	0.00	0.68	0.00
GATCAACGCGGCCATTGGNC	0.00	0.00	0.00	0.00	0.00
GATCAACGCGGCCATTGGNN	0.00	0.00	0.00	0.00	0.00
GATCAACGCGGCCATTGGTC	0.00	0.00	0.00	0.00	0.00
GATCAACGCGGCCATTGTGA	0.00	0.00	0.00	0.00	0.34
GATCAACGCGGCCCACAGAC	0.00	0.00	0.00	0.34	0.00
GATCAACGCGGCCCGATCAG	0.23	0.00	0.00	0.00	0.00
GATCAACGCGGCCCGCTCAG	0.00	0.00	0.29	0.00	0.00
GATCAACGCGGCCCTCGGCC	0.23	0.00	0.00	0.00	0.00
GATCAACGCGGCCCTTCCCT	0.00	0.00	0.00	0.00	0.00

GATCAACGCGGCCCTTGGCC	0.69	0.00	0.00	0.68	0.00
GATCAACGCGGCCGCGCCGG	0.00	0.33	0.00	0.00	0.00
GATCAACGCGGCCGGCCGCG	0.00	0.00	0.00	0.00	0.34
GATCAACGCGGCCGTCTTCC	0.00	0.00	0.00	0.68	0.00
GATCAACGCGGCCGTGTGCC	0.00	0.00	0.57	0.34	0.34
GATCAACGCGGCCGTGTTTG	0.00	0.00	0.29	0.00	0.00
GATCAACGCGGCCTACGCGG	0.00	0.00	0.00	0.00	0.34
GATCAACGCGGCCTGGGGCC	0.23	0.00	0.29	0.00	0.00
GATCAACGCGGCCTTGTGCC	0.00	0.00	0.00	0.00	0.00
GATCAACGCGGCGAAAATCA	0.00	0.00	0.00	0.00	0.00
GATCAACGCGGCGAAGCAGC	0.00	0.00	0.00	0.34	0.00
GATCAACGCGGCGAGCACCC	0.00	0.00	0.00	0.00	0.00
GATCAACGCGGCGCACTTCA	0.00	0.33	0.00	0.00	0.00
GATCAACGCGGCGCCGCGGG	0.23	0.00	0.00	0.00	0.00
GATCAACGCGGCGCCGGACG	0.00	0.00	0.00	0.34	0.00
GATCAACGCGGCGCCGGAGA	0.00	0.33	0.00	0.00	0.00
GATCAACGCGGCGGCAAGTT	0.23	0.00	0.00	0.00	0.00
GATCAACGCGGCGGCCAAGG	0.00	0.00	0.00	0.00	0.00
GATCAACGCGGCGGCGAGGC	0.00	0.00	0.00	0.34	0.00
GATCAACGCGGCGGCGGCGA	0.00	0.00	0.00	0.00	0.34
GATCAACGCGGCGGCTGGGG	0.00	0.33	0.00	0.00	0.00
GATCAACGCGGCGGGAACCG	0.00	0.00	0.00	0.34	0.00
GATCAACGCGGCGTACCGAG	0.00	0.00	0.00	0.00	0.00
GATCAACGCGGCGTCCTCTG	0.00	0.00	0.00	0.34	0.00
GATCAACGCGGCGTGGCGCC	0.00	0.00	0.00	0.00	0.34
GATCAACGCGGCGTGTGCAT	0.00	0.65	0.86	0.34	0.00
GATCAACGCGGCTCCTACGG	0.00	0.33	0.00	0.00	0.00
GATCAACGCGGCTCTGAATG	0.00	0.00	0.00	0.00	0.34
GATCAACGCGGCTGACACCC	0.00	0.33	0.00	0.00	0.00
GATCAACGCGGCTGACACTG	0.00	0.00	0.00	0.00	0.69
GATCAACGCGGCTGACCACC	0.00	0.00	0.00	0.34	0.00
GATCAACGCGGCTGAGAAGG	0.00	0.65	0.00	0.00	0.00
GATCAACGCGGCTGCAAAGA	0.23	0.65	0.57	0.68	0.00
GATCAACGCGGCTGTCTTCC	0.69	0.00	0.29	0.00	0.00
GATCAACGCGGGAATTGGCC	0.00	0.00	0.00	0.00	0.00
GATCAACGCGGGAGAAGAGG	0.23	0.00	0.00	0.00	0.00
GATCAACGCGGGAGAGGATA	0.23	0.00	0.00	0.00	0.00

GATCAACGCGGGAGAGGATG	0.23	0.00	0.00	0.00	0.00
GATCAACGCGGGAGCTGCTG	0.00	0.00	0.00	0.00	0.00
GATCAACGCGGGCATCAAGT	0.00	0.33	0.00	0.00	0.00
GATCAACGCGGGCATCGACT	0.00	0.00	0.00	0.00	0.00
GATCAACGCGGGCATCGCCA	0.23	0.00	0.00	0.00	1.72
GATCAACGCGGGCATGGTGG	0.00	0.00	0.00	0.34	0.00
GATCAACGCGGGCCCCGTGG	0.23	0.65	0.29	0.68	0.00
GATCAACGCGGGCGAGGCGT	0.23	0.00	0.00	0.00	0.00
GATCAACGCGGGCGTCGCTG	0.00	0.00	0.00	0.00	0.00
GATCAACGCGGGCTCCATCA	0.00	0.00	0.00	0.00	0.00
GATCAACGCGGGCTCCCTCA	0.00	0.00	0.00	0.00	0.00
GATCAACGCGGGCTTGTGCT	0.23	0.00	0.00	0.00	0.00
GATCAACGCGGGGGCCGTGA	0.00	0.00	0.00	0.00	0.00
GATCAACGCGGGGGGGGATG	0.23	0.00	0.00	0.00	0.00
GATCAACGCGGGGTATCAAC	0.00	0.00	0.00	0.00	0.34
GATCAACGCGGGGTGGAGCT	0.00	0.00	0.29	0.00	0.00
GATCAACGCGGGGTTCCAGA	0.23	0.00	0.00	0.00	0.00
GATCAACGCGGGGTTGGACT	0.00	0.00	0.00	0.34	0.00
GATCAACGCGGGTACGCCCT	0.23	0.00	0.00	0.00	0.00
GATCAACGCGGGTCAGCAAA	0.00	0.00	0.00	0.00	0.34
GATCAACGCGGGTCAGCACG	0.23	0.00	0.00	0.00	0.34
GATCAACGCGGTAGCCATGC	0.23	0.00	0.00	0.00	0.00
GATCAACGCGGTCCATCGAC	0.00	0.00	0.00	0.34	0.00
GATCAACGCGGTCGACTCCG	0.00	0.00	0.00	0.00	0.00
GATCAACGCGGTCTTCTCCG	0.00	0.00	0.00	0.00	0.00
GATCAACGCGGTGAAGGACG	0.23	0.00	0.00	0.00	0.00
GATCAACGCGGTGAAGGAGG	0.00	0.00	0.00	0.00	0.00
GATCAACGCGGTGACGAGAA	0.00	0.00	0.00	0.34	0.00
GATCAACGCGGTGCACTTTC	0.00	0.00	0.00	0.00	0.34
GATCAACGCGGTGCGCACCT	0.00	0.00	0.00	0.00	0.00
GATCAACGCGGTGCGCGCAA	0.69	0.00	0.00	0.00	0.00
GATCAACGCGGTGCGCGCGA	0.23	0.00	0.00	0.00	0.00
GATCAACGCGGTGGGGCTCG	0.00	0.65	0.00	0.00	1.03
GATCAACGCGGTGGGGCTNN	0.00	0.00	0.00	0.00	0.34
GATCAACGCGGTGTCCGGCT	0.00	0.00	0.00	0.00	0.34
GATCAACGCGGTTTGTGACA	0.00	0.00	0.00	0.00	0.34
GATCAACGCGTAAGTGGTTG	0.00	0.00	0.00	0.00	0.34

GATCAACGCGTAATGCCGCG	0.00	0.00	0.00	0.00	0.34
GATCAACGCGTACGCAGTGC	0.00	0.00	0.29	0.00	0.00
GATCAACGCGTAGCGCCGTG	0.00	0.00	0.29	0.00	0.00
GATCAACGCGTAGTTGCACA	0.46	0.00	0.00	0.00	0.00
GATCAACGCGTATCTGCCTG	0.00	0.33	0.00	0.00	0.00
GATCAACGCGTATTGACTCT	0.00	0.00	0.29	0.00	0.00
GATCAACGCGTCAGAGTCAA	0.23	0.00	0.00	0.00	0.00
GATCAACGCGTCCAGGTCTA	0.00	0.65	0.00	0.00	0.00
GATCAACGCGTCCATGTCAT	0.00	0.00	0.00	0.34	0.00
GATCAACGCGTCCGCGCTCA	0.00	0.00	0.00	0.00	0.00
GATCAACGCGTCCGGGCCAA	0.00	0.00	0.00	0.68	0.00
GATCAACGCGTCGACGCTTG	0.00	0.33	0.00	0.00	0.00
GATCAACGCGTCGCAGTTTA	0.00	0.00	0.00	0.00	0.00
GATCAACGCGTCGCGCGCAA	0.00	0.00	0.00	0.00	0.34
GATCAACGCGTCGTTGCATG	0.00	0.00	0.00	0.00	0.00
GATCAACGCGTCTCAATGCA	0.00	0.00	0.29	0.00	0.00
GATCAACGCGTCTCTGCAAA	0.00	0.00	0.00	0.00	0.00
GATCAACGCGTGACAGGGCG	0.00	0.00	0.29	0.00	0.00
GATCAACGCGTGACGGCTGT	0.46	0.65	0.29	0.00	0.00
GATCAACGCGTGACGGGCGG	0.23	0.00	0.00	0.00	0.00
GATCAACGCGTGATTCAACA	0.00	0.33	0.00	0.00	0.00
GATCAACGCGTGATTTACTC	0.00	0.00	0.00	0.00	0.00
GATCAACGCGTGATTTTATC	0.00	0.00	0.00	0.34	0.00
GATCAACGCGTGATTTTCTC	8.47	7.81	5.15	13.24	7.23
GATCAACGCGTGATTTTNNN	0.23	0.00	0.00	0.00	0.00
GATCAACGCGTGCATTTGTT	0.23	0.65	0.00	0.00	0.00
GATCAACGCGTGCCGAGTGG	0.00	0.00	0.00	0.00	0.34
GATCAACGCGTGCCGGGCGG	0.23	0.00	0.00	0.00	0.00
GATCAACGCGTGCCGGGGGG	0.46	0.00	0.00	0.34	0.00
GATCAACGCGTGCCGGGNNN	0.23	0.00	0.00	0.00	0.00
GATCAACGCGTGCCGGGTGG	5.95	7.81	5.73	4.07	3.10
GATCAACGCGTGCGAGGAGG	0.00	0.00	0.00	0.00	0.00
GATCAACGCGTGCGCCAAGG	0.00	0.00	0.00	0.34	0.00
GATCAACGCGTGCGCCAGGA	0.00	0.00	0.00	0.00	0.00
GATCAACGCGTGCGCGCGCG	0.00	0.00	0.00	0.34	0.00
GATCAACGCGTGCGGCGGTC	0.00	0.33	0.00	0.00	0.00
GATCAACGCGTGCGTCCAGC	0.00	0.00	0.29	0.00	0.00

GATCAACGCGTGCTAAAAGT	0.00	0.00	0.00	0.00	0.00
GATCAACGCGTGGAAGGCCC	0.00	0.33	0.00	0.00	0.00
GATCAACGCGTGGAATCTCG	0.00	0.00	0.00	0.00	0.34
GATCAACGCGTGGCCAATGT	0.00	0.00	0.00	0.00	0.34
GATCAACGCGTGGCGCGCAA	0.23	0.00	0.00	0.00	0.00
GATCAACGCGTGGCTCCTCC	0.23	0.00	0.00	0.00	0.00
GATCAACGCGTGGGCAGTGA	0.00	0.00	0.00	0.00	0.00
GATCAACGCGTGGGCCGTGA	2.06	4.55	8.59	11.20	7.23
GATCAACGCGTGGGCCGTGG	0.46	0.00	0.00	0.00	0.00
GATCAACGCGTGGGCCGTNA	0.00	0.00	0.00	0.00	0.00
GATCAACGCGTGGGCCTCTG	0.00	0.00	0.29	0.00	0.00
GATCAACGCGTGGTACATGA	0.23	0.00	0.00	0.00	0.00
GATCAACGCGTGGTGCAAGT	0.00	0.00	0.00	0.34	0.00
GATCAACGCGTGTGCTCTCT	0.00	0.00	0.29	0.00	0.00
GATCAACGCGTGTGTGGAAG	0.00	0.33	0.00	0.00	0.00
GATCAACGCGTGTGTGGCAG	0.69	0.33	0.86	0.34	1.03
GATCAACGCGTGTTATTGTT	0.23	0.00	0.00	0.00	0.00
GATCAACGCGTGTTTGGCAG	0.00	0.00	0.29	0.00	0.00
GATCAACGCGTGTTTTCAAG	0.00	0.33	0.00	0.00	0.00
GATCAACGCGTTCAGGAGAT	1.83	0.33	0.57	0.68	0.00
GATCAACGCGTTCAGGAGGT	0.23	0.00	0.00	0.00	0.00
GATCAACGCGTTCAGTAGCG	0.00	0.00	0.29	0.00	0.00
GATCAACGCGTTCATCCAGC	0.23	0.00	0.00	0.00	0.00
GATCAACGCGTTCGCGCAAA	0.00	0.00	0.00	0.34	0.00
GATCAACGCGTTCGCTCCAA	0.00	0.00	0.00	0.00	0.00
GATCAACGCGTTCTTGCCCA	0.23	0.00	0.00	0.00	0.00
GATCAACGCGTTCTTGCGCA	6.64	5.86	2.58	3.05	3.10
GATCAACGCGTTCTTGCGCC	0.23	0.00	0.00	0.00	0.00
GATCAACGCGTTCTTGCGGA	0.23	0.00	0.00	0.00	0.00
GATCAACGCGTTCTTGNNNN	0.23	0.00	0.00	0.00	0.00
GATCAACGCGTTGAACACAA	0.00	0.33	0.00	0.00	0.00
GATCAACGCGTTGAGCGCAA	0.00	0.00	0.29	0.34	0.00
GATCAACGCGTTGAGCGGCA	0.00	0.00	0.00	0.34	0.00
GATCAACGCGTTGATGCAAG	0.00	0.00	0.00	0.00	0.00
GATCAACGCGTTGCAAAAAA	0.00	0.00	0.00	0.00	0.00
GATCAACGCGTTGCAGTCGA	0.00	0.00	0.00	0.00	0.00
GATCAACGCGTTGCATGAAA	0.00	0.33	0.00	0.00	0.00

GATCAACGCGTTGCCGCACT	0.00	0.00	0.00	0.68	0.00
GATCAACGCGTTGCCGGATT	0.00	0.00	0.00	0.68	0.00
GATCAACGCGTTGCCTAAGT	0.00	0.33	0.00	0.00	0.00
GATCAACGCGTTGCGACAAA	0.00	0.33	0.00	0.34	0.00
GATCAACGCGTTGCGAGAAA	0.00	0.00	0.00	0.00	0.00
GATCAACGCGTTGCGAGCCG	0.00	0.00	0.29	0.00	0.00
GATCAACGCGTTGCGCACAA	0.23	0.33	0.00	0.00	0.00
GATCAACGCGTTGCGCAGAA	0.00	0.00	0.00	0.34	0.00
GATCAACGCGTTGCGCAGCA	0.00	0.00	0.00	0.00	0.00
GATCAACGCGTTGCGCATGC	0.00	0.00	0.00	0.34	0.00
GATCAACGCGTTGCGCATGG	0.00	0.00	0.29	0.00	0.00
GATCAACGCGTTGCGCCCAA	0.00	0.00	0.00	0.34	0.00
GATCAACGCGTTGCGCGAAA	0.00	1.63	0.00	1.36	0.00
GATCAACGCGTTGCGCGAGA	0.00	0.00	0.00	0.34	0.00
GATCAACGCGTTGCGCGCAA	87.67	51.40	24.34	85.20	43.74
GATCAACGCGTTGCGCGCAC	1.14	0.65	0.00	0.00	0.69
GATCAACGCGTTGCGCGCAG	0.23	0.00	0.00	0.00	0.34
GATCAACGCGTTGCGCGCAN	0.69	0.00	0.00	0.00	0.00
GATCAACGCGTTGCGCGCCA	0.92	0.33	0.00	1.02	0.00
GATCAACGCGTTGCGCGCCG	0.00	0.00	0.00	0.34	0.00
GATCAACGCGTTGCGCGCCN	0.69	0.00	0.00	0.00	0.00
GATCAACGCGTTGCGCGCCT	0.00	0.00	0.00	0.00	0.00
GATCAACGCGTTGCGCGCGA	0.23	0.00	0.29	0.68	0.00
GATCAACGCGTTGCGCGCNN	0.46	0.00	0.00	0.00	0.00
GATCAACGCGTTGCGCGCTG	0.00	0.00	0.29	0.00	0.00
GATCAACGCGTTGCGCGGAA	0.23	0.65	0.00	0.68	0.00
GATCAACGCGTTGCGCGGAG	0.00	0.00	0.29	0.00	0.00
GATCAACGCGTTGCGCGTAA	0.00	0.65	0.00	0.34	0.00
GATCAACGCGTTGCGCGTGA	0.00	0.00	0.29	0.00	0.00
GATCAACGCGTTGCGCNCNN	0.23	0.00	0.00	0.00	0.00
GATCAACGCGTTGCGCNTAA	0.00	0.00	0.00	0.00	0.34
GATCAACGCGTTGCGCTCAA	0.23	0.65	0.29	0.34	0.34
GATCAACGCGTTGCGCTCCC	0.00	0.00	0.29	0.00	0.00
GATCAACGCGTTGCGCTCGA	0.00	0.00	0.00	0.00	0.69
GATCAACGCGTTGCGCTGAA	0.00	0.00	0.00	0.34	0.00
GATCAACGCGTTGCGGACAG	0.00	0.00	0.00	0.00	0.69
GATCAACGCGTTGCGGCCTC	0.00	0.00	0.00	0.00	0.34

GATCAACGCGTTGCGGGCAA	0.23	0.00	0.29	0.00	0.00
GATCAACGCGTTGCGTCAAA	0.00	0.00	0.00	0.34	0.00
GATCAACGCGTTGCGTGAAA	0.00	0.00	0.00	0.00	0.00
GATCAACGCGTTGCGTGGAN	0.23	0.00	0.00	0.00	0.00
GATCAACGCGTTGCGTTCTC	0.00	0.00	0.00	0.68	0.00
GATCAACGCGTTGCTCAAAA	0.00	0.00	0.00	0.00	0.34
GATCAACGCGTTGCTCCAAA	0.00	0.00	0.00	0.00	0.00
GATCAACGCGTTGGCACAGC	0.00	0.00	0.00	0.00	0.34
GATCAACGCGTTGGGCGCAA	0.00	0.33	0.00	0.00	0.00
GATCAACGCGTTGTGTTGTA	0.00	0.33	0.00	0.00	0.34
GATCAACGCGTTTGCATGTG	0.46	0.65	0.00	0.00	0.00
GATCAACGCTAAAAAAAAAA	0.00	0.65	0.00	0.00	0.00
GATCAACGCTAACAGCCTGC	0.00	0.00	0.00	0.00	0.00
GATCAACGCTAACAGCTCCG	0.00	0.33	0.29	0.00	0.00
GATCAACGCTAACGCCGCGG	0.00	0.00	0.00	0.00	0.00
GATCAACGCTAAGATGCAGC	0.00	0.00	0.29	0.00	1.38
GATCAACGCTAAGGCCAACT	0.00	0.00	0.00	0.00	0.00
GATCAACGCTAAGTTTAAAA	0.00	0.00	0.00	0.00	0.34
GATCAACGCTAATTGAGGGA	0.46	1.95	2.00	0.00	0.34
GATCAACGCTAATTTGACAC	0.00	0.00	0.00	0.00	0.34
GATCAACGCTAATTTGAGGT	0.00	0.00	0.00	0.00	0.00
GATCAACGCTACAAAACGGC	0.00	0.00	0.00	0.00	0.34
GATCAACGCTACACGCCGNN	0.23	0.00	0.00	0.00	0.00
GATCAACGCTACACGCCGTG	0.23	0.00	0.00	0.00	0.00
GATCAACGCTACACGCCGTN	0.23	0.00	0.00	0.00	0.00
GATCAACGCTACACGCCGTT	18.31	13.34	14.89	10.18	13.78
GATCAACGCTACAGGGCAGC	0.46	0.00	0.00	0.00	0.00
GATCAACGCTACATGCCGTT	0.00	0.00	0.00	0.00	0.00
GATCAACGCTACCCGCCGCT	0.00	0.00	0.00	0.00	0.00
GATCAACGCTACCTTGTTCT	0.00	0.00	0.00	0.00	0.00
GATCAACGCTACGCTCTTGG	0.00	0.33	0.00	0.00	0.00
GATCAACGCTACGGAGGTGT	0.00	0.00	0.00	0.00	0.00
GATCAACGCTAGCGCCTCCA	0.23	0.00	0.00	0.00	0.00
GATCAACGCTAGCGCTGTTT	0.00	0.00	0.00	0.00	0.00
GATCAACGCTAGCTTGTGTT	0.00	0.33	0.00	0.00	0.00
GATCAACGCTATCATAAAGG	6.41	0.65	0.86	2.72	0.34
GATCAACGCTATCTGCTGCT	0.00	0.00	0.00	0.00	0.00

GATCAACGCTATGACCCCTG	0.00	0.00	0.29	0.00	0.00
GATCAACGCTATGACGTCGC	0.00	0.00	0.00	0.34	0.00
GATCAACGCTCAACACTTAA	0.00	0.00	0.00	0.00	1.03
GATCAACGCTCAACACTTAN	0.00	0.00	0.00	0.00	0.34
GATCAACGCTCAACGCACAA	0.00	0.00	0.00	0.00	1.03
GATCAACGCTCACAGCTCCG	0.00	0.33	0.00	0.00	0.34
GATCAACGCTCACTCCCGTG	0.00	0.00	0.00	0.00	0.00
GATCAACGCTCACTCCCTTC	0.00	0.00	0.00	0.34	0.00
GATCAACGCTCACTCCCTTG	0.00	0.65	0.29	0.68	0.00
GATCAACGCTCACTCCTTCG	0.00	0.00	0.00	0.00	0.00
GATCAACGCTCAGAGGAAGT	0.00	0.00	0.00	0.00	0.00
GATCAACGCTCAGATAGCGC	0.00	0.33	0.00	0.00	0.00
GATCAACGCTCAGCAATTGG	0.00	0.00	0.29	0.00	0.00
GATCAACGCTCAGCCAGGAC	0.00	0.00	0.00	0.34	0.00
GATCAACGCTCAGGCAGACT	0.00	0.00	0.00	0.34	0.00
GATCAACGCTCAGTACAACG	0.00	0.00	0.29	0.00	0.00
GATCAACGCTCAGTGGTTGA	0.00	0.33	0.00	0.00	0.00
GATCAACGCTCAGTTCCCGT	0.00	0.33	0.00	0.00	0.00
GATCAACGCTCATGACGGTA	0.00	0.00	0.00	0.00	0.34
GATCAACGCTCATTGCCACG	0.00	0.65	0.00	0.00	0.00
GATCAACGCTCATTTGTAAG	0.23	0.00	0.00	0.00	0.00
GATCAACGCTCCAACTGGAT	0.00	0.00	0.29	0.34	0.00
GATCAACGCTCCACCAAACC	0.00	0.00	0.00	0.34	0.00
GATCAACGCTCCAGCGCTAA	0.00	0.00	0.00	0.00	0.69
GATCAACGCTCCAGCGCTAG	0.00	0.00	0.00	0.00	0.34
GATCAACGCTCCAGCTCTGA	0.00	0.00	0.00	0.34	0.00
GATCAACGCTCCAGTGCTAG	0.23	0.00	0.00	0.00	0.00
GATCAACGCTCCATAGGCCG	0.00	0.33	0.00	0.00	0.00
GATCAACGCTCCGAACGACC	0.00	0.00	0.00	0.34	0.00
GATCAACGCTCCTCGGCAAA	0.00	0.00	0.29	0.00	0.00
GATCAACGCTCCTCGGCAGC	0.00	0.00	0.00	0.00	0.00
GATCAACGCTCCTCGGCAGG	0.00	0.00	0.00	0.34	0.00
GATCAACGCTCCTCGGCAGT	0.69	0.65	0.86	1.02	1.72
GATCAACGCTCCTCTAAGTG	0.00	0.00	0.00	1.02	0.00
GATCAACGCTCCTTTTGCCG	0.00	0.00	0.00	0.00	0.00
GATCAACGCTCGAGGCAGAC	0.00	0.00	0.00	0.34	0.00
GATCAACGCTCGAGGCAGGA	0.00	0.33	0.00	0.00	0.00

GATCAACGCTCGCAGGCTGC	0.23	0.00	0.00	0.00	0.00
GATCAACGCTCGCCCGAGAG	0.46	0.33	0.29	1.36	0.00
GATCAACGCTCGCGGGAAGT	0.00	0.00	0.00	0.00	0.00
GATCAACGCTCGCTGTCTGG	0.23	0.00	0.00	0.00	0.00
GATCAACGCTCGGACAAGCC	0.00	0.00	0.29	0.00	0.00
GATCAACGCTCGGCTCAAGC	0.00	0.00	0.00	0.00	0.69
GATCAACGCTCGTATGCCGT	0.46	0.00	0.86	0.34	1.03
GATCAACGCTCTCCAGGTGG	0.00	0.00	0.00	0.00	0.00
GATCAACGCTCTCCAGTGCT	0.00	0.33	0.00	0.00	0.00
GATCAACGCTCTCCCGGTGG	0.23	0.00	2.00	0.34	0.69
GATCAACGCTCTCCTCGACG	0.46	0.00	0.00	0.00	0.00
GATCAACGCTCTCGCGCACG	0.00	0.33	0.00	0.00	0.00
GATCAACGCTCTCTCTGCTA	0.00	0.00	0.00	0.00	0.34
GATCAACGCTCTGCAGTATG	0.00	0.00	0.00	0.00	0.00
GATCAACGCTCTGCTGTCGA	0.00	0.00	0.00	0.34	0.00
GATCAACGCTCTGGACCACA	0.00	0.00	0.00	0.34	0.34
GATCAACGCTCTGGAGGAAC	0.23	0.00	0.00	0.00	0.00
GATCAACGCTCTGGAGGACC	0.46	1.30	0.00	0.34	0.34
GATCAACGCTCTGGAGGCTC	0.00	2.93	1.72	1.36	0.69
GATCAACGCTCTGGGTTTTG	0.46	0.00	0.00	0.00	0.00
GATCAACGCTCTTCATCTGC	0.00	0.00	0.00	0.00	0.34
GATCAACGCTCTTGCGTTGC	8.70	8.46	6.30	10.18	7.58
GATCAACGCTCTTGCGTTGN	0.00	0.00	0.29	0.00	0.00
GATCAACGCTCTTGCGTTGT	0.00	0.33	0.00	0.00	0.00
GATCAACGCTGAAAGCTCCG	0.00	0.00	0.57	0.00	1.03
GATCAACGCTGAACACGCAA	0.00	0.33	0.00	0.00	0.00
GATCAACGCTGAACAGCCTG	0.00	0.00	0.00	0.00	0.69
GATCAACGCTGAAGAGTCAA	0.00	0.00	0.00	0.00	0.00
GATCAACGCTGACAACTCCG	0.00	0.00	0.00	0.00	0.34
GATCAACGCTGACAAGACCT	0.00	0.00	0.00	0.00	0.34
GATCAACGCTGACAAGGAGG	0.00	0.00	0.00	0.00	0.00
GATCAACGCTGACAAGGCGG	0.23	0.33	0.86	0.00	0.69
GATCAACGCTGACAGATCCG	0.00	0.00	0.00	0.00	0.34
GATCAACGCTGACAGCAACG	0.00	0.33	0.00	0.00	0.00
GATCAACGCTGACAGCATAG	0.00	0.33	0.00	0.00	0.00
GATCAACGCTGACAGCATCC	0.00	0.00	0.00	0.00	0.34
GATCAACGCTGACAGCCCAG	0.00	0.00	0.29	0.00	0.00

GATCAACGCTGACAGCCCCA	0.00	0.00	0.00	1.02	0.00
GATCAACGCTGACAGCCCCC	0.00	0.00	0.00	0.00	0.00
GATCAACGCTGACAGCCCCG	0.00	0.00	0.57	0.34	0.34
GATCAACGCTGACAGCCCGC	0.00	0.00	0.00	0.00	0.69
GATCAACGCTGACAGCCCTC	0.00	0.00	0.00	0.00	0.00
GATCAACGCTGACAGCGCCG	0.00	0.00	0.00	0.68	0.34
GATCAACGCTGACAGCGTCC	0.00	0.00	0.00	0.00	0.00
GATCAACGCTGACAGCNCCG	0.00	0.00	0.00	0.00	0.34
GATCAACGCTGACAGCNNNN	0.23	0.00	0.00	0.00	0.00
GATCAACGCTGACAGCTAAA	0.00	0.00	0.00	0.34	0.00
GATCAACGCTGACAGCTAAG	0.00	0.00	0.29	0.00	0.00
GATCAACGCTGACAGCTACA	0.00	0.00	0.29	0.00	0.00
GATCAACGCTGACAGCTACG	0.23	0.33	1.15	0.00	1.03
GATCAACGCTGACAGCTCAG	0.23	0.65	0.57	0.34	1.03
GATCAACGCTGACAGCTCCA	0.00	0.00	0.29	0.34	0.00
GATCAACGCTGACAGCTCCC	0.00	0.33	0.00	0.68	0.00
GATCAACGCTGACAGCTCCG	43.95	170.80	210.76	163.27	236.95
GATCAACGCTGACAGCTCCN	0.23	0.00	0.00	0.00	0.00
GATCAACGCTGACAGCTCCT	0.00	0.00	0.29	0.68	0.69
GATCAACGCTGACAGCTCGC	0.00	0.00	0.57	0.00	0.00
GATCAACGCTGACAGCTCNN	0.00	0.00	0.00	0.00	0.00
GATCAACGCTGACAGCTCTG	0.23	0.00	0.29	0.34	0.69
GATCAACGCTGACAGCTGCG	0.00	0.00	0.29	0.34	0.69
GATCAACGCTGACAGCTNNN	0.23	0.00	0.00	0.00	0.00
GATCAACGCTGACAGCTTCC	0.00	0.00	0.29	0.00	0.00
GATCAACGCTGACAGCTTCG	0.00	0.00	0.29	0.34	0.34
GATCAACGCTGACAGCTTGG	0.00	0.00	0.29	0.00	0.00
GATCAACGCTGACAGGACTG	0.00	0.33	0.00	0.00	0.00
GATCAACGCTGACAGGTCCG	0.00	0.00	0.00	0.00	0.34
GATCAACGCTGACAGGTCCT	0.00	0.00	0.00	0.00	0.34
GATCAACGCTGACAGGTGAG	0.00	0.00	0.00	0.00	0.00
GATCAACGCTGACAGTTCCG	0.00	0.00	0.00	0.00	0.00
GATCAACGCTGACATATTCG	0.00	0.00	0.00	0.00	0.00
GATCAACGCTGACATGCTGC	0.00	0.00	0.29	0.34	0.69
GATCAACGCTGACCGCTCCG	0.00	0.00	0.00	0.00	0.00
GATCAACGCTGACCGCTCCT	0.00	0.00	0.29	0.00	0.00
GATCAACGCTGACCTTCCTT	0.00	0.00	0.29	0.00	0.00

GATCAACGCTGACGGCTCCG	0.00	0.00	0.00	0.00	0.00
GATCAACGCTGACTCTGCTG	0.00	0.33	0.00	0.00	0.00
GATCAACGCTGACTGCTCGG	0.00	0.00	0.00	0.00	0.34
GATCAACGCTGAGAGCTCCG	0.00	0.00	0.00	0.00	0.00
GATCAACGCTGAGCCACTGG	2.06	0.33	0.29	0.34	0.00
GATCAACGCTGAGCCGCTGG	0.23	0.00	0.00	0.00	0.00
GATCAACGCTGAGGACTTTG	0.00	0.00	0.29	0.00	0.00
GATCAACGCTGAGGAGGTGN	0.23	0.00	0.00	0.00	0.00
GATCAACGCTGAGGAGGTGT	0.92	0.00	0.00	0.00	0.00
GATCAACGCTGAGGAGTGGT	0.23	0.00	0.00	0.00	0.00
GATCAACGCTGAGGAGTGTC	0.00	0.00	0.00	0.00	0.00
GATCAACGCTGAGGAGTTGC	0.00	0.00	0.29	0.00	0.34
GATCAACGCTGAGGAGTTGG	0.23	0.00	0.00	0.00	0.00
GATCAACGCTGAGGAGTTGT	13.73	15.62	14.32	14.93	17.22
GATCAACGCTGAGGAGTTNN	0.46	0.00	0.00	0.00	0.00
GATCAACGCTGAGGCGTTGT	0.23	0.00	0.00	0.00	0.00
GATCAACGCTGAGGCTGAGG	0.00	0.00	0.29	0.00	0.00
GATCAACGCTGAGGGCATCA	0.00	0.00	0.00	0.00	0.00
GATCAACGCTGAGGGCCCGC	0.00	0.00	0.00	0.00	0.34
GATCAACGCTGAGGGGGTGT	0.23	0.00	0.00	0.00	0.00
GATCAACGCTGAGGGTTGGG	0.00	0.33	0.00	0.00	0.00
GATCAACGCTGAGTTATCTG	0.00	0.00	0.00	0.00	0.00
GATCAACGCTGATAGCTACA	0.00	0.33	0.00	0.00	0.00
GATCAACGCTGATAGCTCCG	0.00	0.00	0.00	0.34	0.34
GATCAACGCTGATGAAGGCG	0.00	0.00	0.00	0.34	0.00
GATCAACGCTGATGCCTGCC	0.00	0.00	0.00	0.00	0.00
GATCAACGCTGATGCTGCTG	0.00	0.00	0.00	0.00	0.34
GATCAACGCTGATGTGCTTC	0.00	0.00	0.00	0.00	0.00
GATCAACGCTGCAAAAAAAA	0.23	0.00	0.00	0.00	0.00
GATCAACGCTGCAAGAAGCA	0.00	0.00	0.00	0.00	0.00
GATCAACGCTGCAAGCACCT	0.46	0.00	0.00	0.00	0.00
GATCAACGCTGCAGCGTAGT	0.00	0.00	0.00	0.34	0.00
GATCAACGCTGCAGCTCCTG	0.00	0.00	0.00	0.00	0.34
GATCAACGCTGCAGTCGACT	0.00	0.33	0.00	0.00	0.00
GATCAACGCTGCATTTAGCT	0.00	0.00	0.00	0.00	0.00
GATCAACGCTGCCAAAAAAA	0.00	0.00	0.00	0.00	0.69
GATCAACGCTGCCAAGGAAG	0.00	0.00	0.00	0.00	0.00

GATCAACGCTGCCAAGGAGG	0.00	0.00	0.00	0.00	0.00
GATCAACGCTGCCACACTCT	0.00	0.00	0.00	0.00	0.00
GATCAACGCTGCCCAACAGG	0.00	0.00	0.00	0.00	0.00
GATCAACGCTGCCCTGCTTC	0.00	0.00	0.00	0.00	0.00
GATCAACGCTGCCGCCAAGG	0.00	0.00	0.00	0.00	0.00
GATCAACGCTGCCGCCGAGC	0.00	0.00	0.86	0.00	0.00
GATCAACGCTGCCGCTACCC	0.00	0.33	0.00	0.00	0.00
GATCAACGCTGCCGGTCACT	0.00	0.00	0.29	0.00	0.00
GATCAACGCTGCCTGGGTCA	0.69	0.00	0.00	0.00	0.34
GATCAACGCTGCCTGTGACG	0.00	0.00	0.00	0.34	0.34
GATCAACGCTGCCTTCATGA	0.00	0.00	0.00	0.00	0.00
GATCAACGCTGCCTTGCTNN	0.00	0.00	0.00	0.00	0.00
GATCAACGCTGCCTTGCTTC	7.10	9.11	2.86	6.79	6.20
GATCAACGCTGCGAGCACCC	0.00	0.00	0.29	0.00	0.00
GATCAACGCTGCGCACGTAC	0.00	0.33	0.00	0.00	0.00
GATCAACGCTGCGCCCGCTT	0.00	0.00	0.29	0.00	0.00
GATCAACGCTGCGCGTCTTT	0.00	0.00	0.00	0.00	0.00
GATCAACGCTGCGGCCAAGG	0.00	0.00	0.00	0.00	0.00
GATCAACGCTGCGGCGTACG	0.23	0.33	0.00	0.00	0.00
GATCAACGCTGCGGGACCTC	0.23	0.33	0.86	0.68	2.41
GATCAACGCTGCGGGACTCC	1.14	0.33	1.72	1.70	2.41
GATCAACGCTGCGGGTGCCC	0.00	0.00	0.00	0.34	0.00
GATCAACGCTGCGTCCACGT	0.00	0.33	0.00	0.00	0.00
GATCAACGCTGCTCGTGAGA	0.00	0.00	0.00	0.00	0.00
GATCAACGCTGCTCTGCTCT	0.00	0.00	0.00	0.00	0.34
GATCAACGCTGCTGAGGCTC	0.00	0.00	0.00	0.00	0.00
GATCAACGCTGCTGATATGA	0.00	0.00	0.00	0.00	0.34
GATCAACGCTGCTGCGTTCG	0.00	0.00	0.00	0.00	0.00
GATCAACGCTGCTGCGTTTG	0.23	0.00	0.00	0.00	0.00
GATCAACGCTGCTGCTACGC	0.00	0.00	0.00	0.00	0.00
GATCAACGCTGCTGCTGCAA	0.69	1.30	0.29	0.68	0.34
GATCAACGCTGCTGCTGCAC	4.12	2.28	1.15	9.16	3.10
GATCAACGCTGCTGGACTTG	0.00	0.00	0.00	0.00	0.34
GATCAACGCTGCTGTCGCAG	0.00	0.65	0.00	0.00	0.00
GATCAACGCTGCTGTCGTCA	0.00	0.00	0.57	0.34	0.00
GATCAACGCTGCTTGCCATT	0.00	0.00	0.00	0.00	0.00
GATCAACGCTGCTTGTCTTG	0.00	0.65	0.86	2.38	0.34

GATCAACGCTGGAAAAAAAA	0.00	0.00	0.57	0.00	0.00
GATCAACGCTGGACCCCTCC	0.00	0.00	0.00	0.00	0.34
GATCAACGCTGGAGCTGCGC	0.00	0.00	0.29	0.00	0.00
GATCAACGCTGGATAAACGT	0.00	0.00	0.00	0.34	0.00
GATCAACGCTGGCAGCAACA	0.00	0.00	0.29	0.00	0.00
GATCAACGCTGGCAGCGCAG	0.00	0.00	0.29	0.00	0.00
GATCAACGCTGGCATAACCC	0.00	0.33	0.00	0.00	0.00
GATCAACGCTGGCATCGCTG	0.46	0.00	0.00	0.34	0.00
GATCAACGCTGGCATGAGCT	0.00	0.00	0.00	0.00	0.00
GATCAACGCTGGCCAAGCCC	0.00	0.00	0.00	0.00	0.00
GATCAACGCTGGCCGGGTAC	0.00	0.00	0.57	0.00	0.00
GATCAACGCTGGCGCCCAGG	0.00	0.00	0.29	0.00	0.00
GATCAACGCTGGCGGGGCAG	0.00	0.00	0.00	0.68	0.00
GATCAACGCTGGCGGGGCTC	0.00	0.00	0.00	0.34	0.00
GATCAACGCTGGCGTCCGCC	0.00	0.00	0.29	0.00	0.00
GATCAACGCTGGGAAAAAAA	0.00	0.00	0.29	0.34	0.00
GATCAACGCTGGGCATTCTT	0.00	0.00	0.00	0.00	0.34
GATCAACGCTGGGGAAAAAA	0.00	0.00	0.00	0.34	0.00
GATCAACGCTGGGGAGTTGT	0.00	0.00	0.00	0.00	0.00
GATCAACGCTGGGGGGGGGC	0.00	0.00	0.29	0.00	0.00
GATCAACGCTGGGTTGTTCG	0.00	0.65	0.29	0.34	0.69
GATCAACGCTGGGTTGTTGG	0.00	0.00	0.00	0.34	0.00
GATCAACGCTGGGTTGTTNG	0.00	0.00	0.00	0.34	0.00
GATCAACGCTGGTAAGGAGC	0.00	0.00	0.00	0.34	0.00
GATCAACGCTGGTAGATACT	0.00	0.33	0.00	0.00	0.00
GATCAACGCTGGTCCTCAGG	0.23	0.00	0.00	0.00	0.00
GATCAACGCTGGTCCTTTCT	0.00	0.00	0.00	0.34	0.00
GATCAACGCTGGTCGCTTGG	0.23	0.00	0.00	0.00	0.00
GATCAACGCTGGTGCCAGCA	0.00	0.00	0.00	0.00	0.00
GATCAACGCTGGTTCCAGCA	0.00	0.00	0.00	0.00	0.00
GATCAACGCTGTAGACCTTT	0.00	0.00	0.00	0.00	0.00
GATCAACGCTGTATACCGAA	0.00	0.00	0.00	0.00	0.00
GATCAACGCTGTCAAATGGG	0.00	0.00	0.00	0.00	0.00
GATCAACGCTGTCAGCTCCG	0.00	0.00	0.29	0.00	0.00
GATCAACGCTGTGAAGATAG	0.00	0.00	0.29	0.00	0.00
GATCAACGCTGTGATTCAGG	0.00	0.00	0.00	0.34	0.00
GATCAACGCTGTGCCGTTCC	0.00	0.33	0.00	0.00	0.00

GATCAACGCTGTGCTGCTGA	0.00	0.00	0.29	0.00	0.00
GATCAACGCTGTGGAACTTC	0.00	0.00	0.00	0.00	0.00
GATCAACGCTGTGGTAACAC	0.23	0.00	0.00	0.00	0.00
GATCAACGCTGTTATGTTCT	0.00	0.00	0.00	0.00	0.69
GATCAACGCTGTTCAACTCT	0.00	0.00	0.00	0.00	0.69
GATCAACGCTGTTCTGTTCT	0.00	0.00	0.00	0.00	1.03
GATCAACGCTGTTGAGTCGT	0.23	0.00	0.00	0.00	0.00
GATCAACGCTTAACACTTAA	0.00	0.00	0.00	0.00	0.34
GATCAACGCTTACAGCCCCT	0.00	0.00	0.00	0.00	0.34
GATCAACGCTTACAGCTGGA	0.00	0.00	0.00	0.00	0.00
GATCAACGCTTACTTCACTG	0.00	0.00	0.00	0.34	0.00
GATCAACGCTTCAAAAAAAA	0.00	0.00	0.00	0.00	0.00
GATCAACGCTTCCATCCAGC	0.23	0.00	0.00	0.00	0.00
GATCAACGCTTCCATTCATG	0.00	1.63	0.00	1.36	0.00
GATCAACGCTTCCGTCTGCG	0.00	0.00	0.00	0.34	0.00
GATCAACGCTTCGGGGGTCC	0.00	0.00	0.00	0.00	0.69
GATCAACGCTTCGTCTCTGG	0.00	0.00	0.29	0.00	0.00
GATCAACGCTTCTACGTTTG	0.23	0.00	0.00	0.00	0.00
GATCAACGCTTCTGGCAAGG	0.00	0.00	0.00	0.00	0.34
GATCAACGCTTCTGGCTCGG	0.00	0.00	0.29	0.00	0.00
GATCAACGCTTGAAGCCGCT	0.23	0.00	0.00	0.00	0.00
GATCAACGCTTGCAAAGAGG	0.00	0.00	0.00	0.00	0.00
GATCAACGCTTGCATCAGAG	0.00	0.00	0.00	0.00	0.00
GATCAACGCTTGCATGCCCA	0.00	0.00	0.00	0.68	0.00
GATCAACGCTTGCATGCCCG	0.00	0.00	0.57	0.00	0.34
GATCAACGCTTGCCAGGCGC	0.92	0.65	0.00	0.00	0.00
GATCAACGCTTGCCCATGAA	0.00	0.00	0.57	0.00	0.00
GATCAACGCTTGCCTCTGGA	0.23	0.00	0.00	0.00	0.00
GATCAACGCTTGCGAGAGGG	0.23	0.00	0.00	0.34	0.00
GATCAACGCTTGCGCCCGGG	0.00	0.00	0.00	0.00	0.00
GATCAACGCTTGCGTGAAGA	0.00	0.00	0.00	0.00	0.00
GATCAACGCTTGCTGAGAAA	0.00	0.00	0.00	0.34	0.00
GATCAACGCTTGGAAAAAAA	0.00	0.00	0.29	0.00	0.00
GATCAACGCTTGGAAGACCA	0.00	0.00	0.00	0.00	0.00
GATCAACGCTTGGAGACGCA	1.14	1.30	0.57	2.04	0.00
GATCAACGCTTGGAGACGGA	0.00	0.00	0.00	0.00	0.00
GATCAACGCTTGGCCTCTCA	0.00	0.00	0.29	0.00	0.00

GATCAACGCTTGGCGACGCA	0.00	0.00	0.00	0.00	0.00
GATCAACGCTTGGCGATGGG	0.00	0.00	0.00	0.34	0.00
GATCAACGCTTGGCTTGGCA	0.00	0.00	0.00	0.00	0.34
GATCAACGCTTGGTTAGACT	0.00	0.33	0.00	0.00	0.00
GATCAACGCTTGTGCCTATC	0.00	0.00	0.00	0.00	0.34
GATCAACGCTTGTTCCCGTG	0.00	0.00	0.00	0.00	0.34
GATCAACGCTTTAAGCATCC	0.00	0.00	0.00	0.00	0.00
GATCAACGCTTTAATACAGC	0.00	0.00	0.00	0.00	0.34
GATCAACGCTTTCATACAGA	0.00	0.00	0.00	0.00	0.00
GATCAACGCTTTCATACAGC	0.00	0.00	0.00	0.34	0.00
GATCAACGCTTTCATCCAAG	0.00	0.00	0.00	0.00	0.00
GATCAACGCTTTCATCCAGA	0.00	0.00	0.00	0.34	0.34
GATCAACGCTTTCATCCAGC	29.76	41.64	29.21	32.59	45.12
GATCAACGCTTTCATCCAGG	0.00	0.00	0.00	0.00	0.34
GATCAACGCTTTCATCCCGA	0.00	0.33	0.00	0.00	0.00
GATCAACGCTTTCATCCCGC	0.00	0.33	0.29	0.00	0.00
GATCAACGCTTTCATCCGGC	0.00	0.00	0.00	0.00	0.00
GATCAACGCTTTCATCNAGC	0.00	0.00	0.00	0.00	0.34
GATCAACGCTTTCATCTAGC	0.00	0.00	0.00	0.34	0.00
GATCAACGCTTTCATCTGGC	0.00	0.00	0.00	0.00	0.34
GATCAACGCTTTGACTGCCC	0.00	0.00	0.57	0.00	0.00
GATCAACGCTTTGGTGGCCA	0.00	0.00	0.29	0.00	0.00
GATCAACGCTTTTTATGCGC	0.00	0.00	0.00	0.00	0.34
GATCAACGCTTTTTTGATTC	0.00	0.00	0.00	0.00	0.00
GATCAACGCTTTTTTTTGAA	0.00	0.00	0.00	0.00	0.69
GATCAACGGAAAAAAAAAAA	0.46	0.33	0.00	0.34	0.00
GATCAACGGAAAAAAAAAAT	0.00	0.00	0.00	0.00	0.00
GATCAACGGAAAAAAAAATC	0.23	0.00	0.00	0.00	0.00
GATCAACGGAAAAAAAAGAA	0.00	0.00	0.00	0.00	0.00
GATCAACGGAAACTCGTGCC	0.23	0.00	0.00	0.00	0.00
GATCAACGGAAACTGGGACT	0.00	0.00	0.00	0.00	0.34
GATCAACGGAAAGTGAGCCT	0.00	0.00	0.57	0.00	0.00
GATCAACGGAAATGACGGTC	0.00	0.00	0.00	0.00	0.00
GATCAACGGAACAAAGAAGC	0.00	0.00	0.00	0.00	0.34
GATCAACGGAACATCGGCGA	0.23	0.00	0.00	0.00	0.00
GATCAACGGAACCAAGATCA	0.00	0.00	0.00	0.00	0.00
GATCAACGGAACCAAGGTCA	0.00	0.33	0.57	0.34	1.72

GATCAACGGAACCACGGTCA	0.00	0.33	0.00	0.00	0.00
GATCAACGGAACCATGGTGT	0.00	0.00	0.00	0.00	0.00
GATCAACGGAACGCAGGGCN	0.23	0.00	0.00	0.00	0.00
GATCAACGGAACTAGATACT	0.00	0.00	0.00	0.00	0.69
GATCAACGGAACTGAGCTGG	0.00	0.00	0.00	0.00	0.34
GATCAACGGAAGAAGGTTGA	0.23	0.00	0.00	0.00	0.00
GATCAACGGAAGCAATGGAC	2.06	3.25	2.29	2.38	1.03
GATCAACGGAAGCAATGGAG	0.00	0.33	0.00	0.00	0.00
GATCAACGGAAGCGGATGAA	0.23	0.00	0.00	0.00	0.00
GATCAACGGAAGCTGCACTT	0.00	0.33	0.00	0.00	0.34
GATCAACGGAAGCTTTTTCT	0.00	0.00	0.29	0.00	0.00
GATCAACGGAAGGAGAAGAA	0.00	0.00	0.57	0.00	1.03
GATCAACGGAAGGCACCGCT	0.00	0.00	0.00	0.00	0.34
GATCAACGGAAGGTTTTTCT	0.00	0.33	0.00	0.00	0.00
GATCAACGGAAGTCCTGCTG	0.00	0.00	0.29	0.00	0.00
GATCAACGGAAGTCTGCCCA	0.00	0.00	0.00	0.00	0.00
GATCAACGGAAGTTGTTGAC	0.00	0.00	0.00	0.00	0.69
GATCAACGGAATCAGAGGGA	0.00	0.00	0.29	0.00	0.00
GATCAACGGAATCCTATTCC	0.46	0.00	0.29	0.00	0.00
GATCAACGGAATTGGTTTGT	0.00	0.00	0.00	0.00	0.00
GATCAACGGACAAAACAAAA	0.23	0.00	0.00	0.00	0.00
GATCAACGGACAAAGAAGCA	0.00	0.00	0.00	0.00	1.03
GATCAACGGACAACACATAC	0.00	0.00	0.00	0.00	0.00
GATCAACGGACAACGCACAT	0.00	0.00	0.00	0.34	0.00
GATCAACGGACAAGGACAGA	4.12	1.95	4.01	2.38	4.13
GATCAACGGACACAGAGGAC	0.00	0.00	0.29	0.00	0.00
GATCAACGGACAGAATGAAG	0.23	0.33	0.00	0.00	0.00
GATCAACGGACAGACCGTTC	0.00	0.00	0.00	0.00	0.00
GATCAACGGACAGCTGGCAA	0.00	0.00	0.00	0.00	0.00
GATCAACGGACATAAAAACT	0.00	0.00	0.00	0.00	0.00
GATCAACGGACATCCATCTT	0.00	0.33	0.00	0.00	0.00
GATCAACGGACATTACATGC	0.00	0.00	0.00	0.00	0.00
GATCAACGGACCAAGATGAG	0.00	0.00	0.29	0.00	0.00
GATCAACGGACCACCCTTCA	0.00	0.00	0.00	0.00	0.34
GATCAACGGACCACGAGATT	0.00	0.00	0.00	0.68	0.00
GATCAACGGACCAGCGGGGC	0.00	0.00	0.00	0.00	0.34
GATCAACGGACCCGACAAAA	0.00	0.00	0.00	0.00	0.00

GATCAACGGACCCTCGTGCC	0.23	0.00	0.00	0.00	0.00
GATCAACGGACCCTGCAGAG	0.69	0.00	0.00	0.00	0.00
GATCAACGGACCCTGGAGCA	0.00	0.33	0.29	0.00	0.00
GATCAACGGACCGATTGTCT	0.00	0.00	0.00	0.00	0.34
GATCAACGGACCTCAACACA	0.00	0.00	0.00	0.00	0.34
GATCAACGGACCTTCACAAG	0.23	0.00	0.00	0.00	0.00
GATCAACGGACGAGACACTA	0.46	0.00	0.00	0.34	0.00
GATCAACGGACGAGGAGTGA	0.00	0.00	0.29	0.00	0.00
GATCAACGGACGCTTCTGGA	0.00	0.00	0.00	0.34	0.00
GATCAACGGACGGCAGCGCC	0.00	0.00	0.00	0.00	0.00
GATCAACGGACGGCAGCGCG	0.00	0.00	0.00	0.34	0.00
GATCAACGGACGGCGGTGGC	0.23	0.00	0.00	0.00	0.00
GATCAACGGACGGGTCTGAA	0.23	0.00	0.00	0.00	0.00
GATCAACGGACGGGTCTGGA	0.23	0.00	0.00	0.00	0.00
GATCAACGGACGTCCTGTGG	0.00	0.00	0.00	0.00	0.34
GATCAACGGACGTCTCTGGA	0.00	0.00	0.00	0.68	0.00
GATCAACGGACGTGCTGTTT	0.00	0.33	0.29	0.34	0.00
GATCAACGGACGTGGAAAAA	0.00	0.33	0.00	0.00	0.00
GATCAACGGACGTGGCTGAA	0.00	0.00	0.00	0.34	0.00
GATCAACGGACGTGTCAAAA	0.00	0.00	0.00	0.00	0.00
GATCAACGGACGTGTCCAAA	0.00	0.33	0.00	0.00	0.00
GATCAACGGACGTGTCCGAA	0.00	0.00	0.00	0.34	0.00
GATCAACGGACGTGTCGGAA	0.23	0.00	0.00	0.00	0.00
GATCAACGGACGTGTCNGAA	0.00	0.00	0.00	0.00	0.69
GATCAACGGACGTGTCTAAA	0.00	0.00	0.00	0.34	0.00
GATCAACGGACGTGTCTGAA	46.47	13.99	10.60	20.03	13.43
GATCAACGGACGTGTCTGAC	0.69	0.00	0.00	0.00	0.00
GATCAACGGACGTGTCTGAG	0.00	0.00	0.29	0.00	0.00
GATCAACGGACGTGTCTGCA	0.23	0.00	0.00	0.00	0.00
GATCAACGGACGTGTCTGGA	0.92	0.33	0.00	0.34	0.00
GATCAACGGACGTGTCTGNN	0.46	0.00	0.00	0.00	0.00
GATCAACGGACGTGTCTGTA	0.00	0.00	0.00	0.00	0.00
GATCAACGGACGTGTCTGTG	0.00	0.00	0.00	0.00	0.00
GATCAACGGACGTGTCTTAA	0.00	0.00	0.00	0.34	0.00
GATCAACGGACGTGTCTTCT	0.23	0.00	0.00	0.00	0.00
GATCAACGGACGTGTTTGAA	0.00	0.00	0.29	0.34	0.00
GATCAACGGACGTTGCTGAC	0.00	0.00	0.00	0.00	0.00

GATCAACGGACGTTGGGCAA	0.00	0.00	0.00	0.00	0.34
GATCAACGGACGTTTCAGTG	0.00	0.00	0.00	0.00	0.00
GATCAACGGACGTTTCCGAA	0.00	0.00	0.00	0.34	0.00
GATCAACGGACGTTTCCGCA	0.00	0.00	0.00	0.00	0.00
GATCAACGGACGTTTCTCGA	0.00	0.00	0.00	0.00	0.00
GATCAACGGACGTTTCTGAA	0.00	0.00	0.00	0.00	0.00
GATCAACGGACGTTTCTGCC	0.00	0.00	0.00	0.00	0.00
GATCAACGGACGTTTCTGGA	30.90	21.15	15.18	33.94	21.01
GATCAACGGACGTTTCTGGC	0.23	0.00	0.29	0.34	0.00
GATCAACGGACGTTTCTGGG	0.46	0.00	0.00	0.34	0.34
GATCAACGGACGTTTCTGGN	0.23	0.00	0.00	0.00	0.00
GATCAACGGACGTTTCTGGT	0.23	0.00	0.00	0.00	0.69
GATCAACGGACGTTTCTGTA	0.00	0.33	0.00	0.00	0.00
GATCAACGGACGTTTCTTGA	0.00	0.00	0.00	0.00	0.69
GATCAACGGACGTTTCTTTG	0.00	0.00	0.00	0.00	0.00
GATCAACGGACGTTTGTGGA	0.23	0.33	0.00	0.00	0.00
GATCAACGGACTACCTTGCA	0.00	0.00	0.00	0.00	0.00
GATCAACGGACTCCTGGGCG	0.00	0.00	0.29	0.34	0.00
GATCAACGGACTCGTATGCC	0.00	0.00	0.00	0.34	0.00
GATCAACGGACTCTGGCCGC	0.00	0.00	0.00	0.00	0.00
GATCAACGGACTGAGGTTCG	0.00	0.00	0.29	0.00	0.00
GATCAACGGACTGAGTATCT	0.00	0.00	0.00	0.00	0.00
GATCAACGGACTTAAGGCAG	0.69	0.00	0.00	0.00	0.69
GATCAACGGACTTGTGGTTG	0.00	0.00	0.00	0.00	0.00
GATCAACGGAGAAAAAAAAA	0.69	0.00	0.00	0.00	0.00
GATCAACGGAGAAAAAGTTC	0.00	0.98	0.29	0.00	0.00
GATCAACGGAGAACATGTGG	1.14	1.63	0.57	0.00	0.34
GATCAACGGAGAAGCTGTGC	0.00	0.00	0.00	0.34	0.00
GATCAACGGAGAATTGGACA	0.00	0.00	0.57	0.00	0.00
GATCAACGGAGACAGAGGCA	0.00	0.00	0.00	0.34	0.00
GATCAACGGAGAGCAGAACA	0.00	0.00	0.86	0.00	0.00
GATCAACGGAGAGCGACAGA	0.00	0.00	0.00	0.00	0.00
GATCAACGGAGAGGCCATCC	0.00	0.00	0.29	0.00	0.00
GATCAACGGAGAGGGCGTTG	0.00	0.33	0.00	0.34	0.00
GATCAACGGAGAGGGTCGTA	0.00	0.00	0.00	0.00	0.00
GATCAACGGAGAGGGTTCTG	7.10	6.51	1.43	4.75	1.38
GATCAACGGAGAGTGCAGAA	0.00	0.00	0.00	0.00	0.00

GATCAACGGAGATGAGGCAG	0.23	0.00	0.00	0.00	0.69
GATCAACGGAGATTAACCCA	0.00	0.33	0.00	0.34	0.00
GATCAACGGAGATTAACTCA	0.00	0.00	0.00	0.00	0.00
GATCAACGGAGATTAACTTA	0.69	0.00	0.00	0.00	0.00
GATCAACGGAGCATCTCCAT	0.00	0.33	0.00	0.00	0.00
GATCAACGGAGCATGGATTT	0.00	0.00	0.00	0.00	0.00
GATCAACGGAGCCAGGTCGT	0.00	0.00	0.00	0.00	0.00
GATCAACGGAGCCGAGGATG	0.00	0.65	0.00	0.34	0.00
GATCAACGGAGCCGTCCCCG	0.00	0.00	0.29	0.00	0.00
GATCAACGGAGCCGTTGGAA	0.00	0.00	0.00	0.00	0.34
GATCAACGGAGCCTTCGACT	0.00	0.00	0.00	0.34	0.00
GATCAACGGAGCGAGATGCG	0.00	0.00	0.00	0.00	0.34
GATCAACGGAGCGGCAAGTG	0.00	0.33	0.00	0.00	0.00
GATCAACGGAGCGGCGCTAG	0.23	0.33	0.57	0.00	0.00
GATCAACGGAGCGGCGCTCG	0.00	0.00	0.00	0.00	0.34
GATCAACGGAGCTCTTCCGA	0.00	0.33	0.00	0.00	0.00
GATCAACGGAGCTTCCGGGG	0.00	0.33	0.00	0.00	0.00
GATCAACGGAGGAAGGAAGG	0.00	0.00	0.29	0.00	0.00
GATCAACGGAGGAATTGGTT	0.00	0.00	0.29	0.00	0.00
GATCAACGGAGGAGCGCGGA	0.00	0.00	0.29	0.00	0.00
GATCAACGGAGGAGGAGCAG	0.00	0.00	0.29	0.00	0.00
GATCAACGGAGGATCTTGTT	0.00	0.00	0.00	0.00	0.34
GATCAACGGAGGATGCATGG	0.00	0.00	0.00	0.00	0.34
GATCAACGGAGGATTCAAAA	0.69	0.00	0.00	0.00	0.00
GATCAACGGAGGATTCAAAC	0.23	0.00	0.00	0.00	0.00
GATCAACGGAGGCAAAGACC	0.00	0.00	0.00	0.34	0.00
GATCAACGGAGGCGCGCGGA	0.00	0.00	0.00	0.00	0.00
GATCAACGGAGGCGGAAGAG	0.00	0.00	0.00	0.34	0.00
GATCAACGGAGGCGGCTCGG	0.23	0.00	0.00	0.00	0.00
GATCAACGGAGGCGGGGGTG	0.00	0.00	0.00	0.34	0.00
GATCAACGGAGGCTGATGGC	0.00	0.00	0.00	0.00	0.00
GATCAACGGAGGGGACAAGA	0.00	0.00	0.00	0.00	0.34
GATCAACGGAGGGTCCGTGA	0.00	0.00	0.00	0.00	0.34
GATCAACGGAGGTGACTCGA	0.00	0.00	0.00	0.34	0.00
GATCAACGGAGGTGACTCGG	0.00	0.00	0.00	0.34	0.00
GATCAACGGAGGTGCCTGTC	0.00	0.00	0.29	0.00	0.00
GATCAACGGAGGTGGCTGTA	0.00	0.00	0.00	0.34	0.00

GATCAACGGAGTCAGCTGCC	0.23	0.00	0.00	0.00	0.00
GATCAACGGAGTGGATGGGT	0.00	0.00	0.00	0.34	0.00
GATCAACGGAGTGGCAAACC	0.00	0.33	0.57	0.68	0.34
GATCAACGGAGTGGCCCTGA	0.00	0.00	0.00	0.34	0.00
GATCAACGGAGTGGGAAGTA	0.00	0.00	0.57	0.34	0.34
GATCAACGGAGTGGGAAGTG	0.00	0.33	0.00	0.00	0.00
GATCAACGGAGTGGGCGGCG	0.00	0.00	0.00	0.00	0.00
GATCAACGGAGTGGGGTCTG	0.23	0.00	0.00	0.00	0.00
GATCAACGGAGTGTTGCCCC	0.23	0.00	0.00	0.00	0.00
GATCAACGGAGTTGGTCAGT	0.00	0.00	0.00	0.00	0.00
GATCAACGGAGTTGGTTTGT	0.00	0.00	0.00	0.34	0.00
GATCAACGGATAAAAAAAAA	0.00	0.65	0.00	0.00	0.00
GATCAACGGATAAGGAAGGA	0.00	0.33	0.00	0.00	0.00
GATCAACGGATACACGTACG	0.00	0.00	0.00	0.00	0.69
GATCAACGGATAGAGAGACT	0.00	0.00	0.29	0.00	0.00
GATCAACGGATAGCATCTTG	0.23	0.00	0.00	0.00	0.00
GATCAACGGATAGGCTCAAG	0.00	0.00	0.00	0.00	0.34
GATCAACGGATAGTTTCATT	0.00	0.00	0.00	0.00	0.00
GATCAACGGATATGGATTGT	0.00	0.00	0.00	0.00	0.00
GATCAACGGATCGATGGATT	0.00	0.00	0.00	0.34	0.34
GATCAACGGATCGTATGCCG	0.00	0.00	0.00	0.00	0.34
GATCAACGGATGCACGCGCA	0.00	0.00	0.00	0.00	0.34
GATCAACGGATGCGAATAAT	0.00	0.00	0.00	0.00	0.00
GATCAACGGATGCGCCCGGG	0.23	0.00	0.00	0.00	0.00
GATCAACGGATGCTCCTGCA	0.00	0.00	0.00	0.00	0.00
GATCAACGGATGCTCCTGGA	0.00	0.65	0.00	0.00	0.00
GATCAACGGATGCTCCTGGG	0.23	0.00	0.00	0.00	0.00
GATCAACGGATGCTCCTTGC	0.23	0.00	0.00	0.00	0.00
GATCAACGGATGCTGCAGAA	0.23	0.00	0.00	0.00	0.00
GATCAACGGATGCTGGAGGA	0.00	0.00	0.29	0.00	0.00
GATCAACGGATGGATTGCAA	0.00	0.00	0.00	0.34	0.00
GATCAACGGATGGATTGCAC	0.00	0.00	0.29	0.00	0.34
GATCAACGGATGGCAATGAG	0.00	0.33	0.00	0.00	0.00
GATCAACGGATGGCCGCTGA	0.00	0.00	0.29	0.00	0.00
GATCAACGGATGGGCGCGGC	0.00	0.00	0.29	0.00	0.00
GATCAACGGATGGGGGCTCA	0.00	0.00	0.00	0.34	0.34
GATCAACGGATGGGGGCTCG	0.00	0.00	0.00	0.00	0.34

GATCAACGGATGGGTTGCCG	0.23	0.00	0.00	0.00	0.00
GATCAACGGATGGTACTGGA	0.23	0.00	0.00	0.00	0.00
GATCAACGGATGGTCATGGA	0.23	0.00	0.00	0.00	0.00
GATCAACGGATGGTCCNGNN	0.23	0.00	0.00	0.00	0.00
GATCAACGGATGGTCCNNNN	0.23	0.00	0.00	0.00	0.00
GATCAACGGATGGTCCTGGA	0.92	0.00	0.00	0.68	0.00
GATCAACGGATGGTCCTGGC	0.23	0.00	0.00	0.00	0.00
GATCAACGGATGGTCCTGGG	0.69	0.00	0.00	0.00	0.00
GATCAACGGATGGTCCTTGA	0.46	0.00	0.00	0.34	0.34
GATCAACGGATGGTTTGCCA	0.00	0.00	0.00	0.00	1.03
GATCAACGGATGGTTTGCCG	4.12	5.21	2.00	4.41	3.44
GATCAACGGATGGTTTGCCT	0.00	0.33	0.00	0.00	0.00
GATCAACGGATGGTTTGTCG	0.00	0.00	0.00	0.34	0.00
GATCAACGGATGGTTTTCCG	0.00	0.00	0.29	0.00	0.00
GATCAACGGATGTACCTTGA	0.00	0.00	0.00	0.34	0.00
GATCAACGGATGTCCACTCT	0.00	0.00	0.29	0.00	0.00
GATCAACGGATGTCCCAAAC	0.00	0.00	0.00	0.34	0.00
GATCAACGGATGTCCCTTGC	0.00	0.00	0.00	0.34	0.00
GATCAACGGATGTCCGCTGA	0.00	0.33	0.00	0.00	0.00
GATCAACGGATGTCTCTGGA	0.00	0.00	0.00	0.00	0.00
GATCAACGGATGTGAAGCAG	0.00	0.00	0.29	0.00	0.00
GATCAACGGATGTGGATGGC	0.00	0.00	0.00	0.00	0.34
GATCAACGGATGTTACTGGA	0.00	0.00	0.29	0.00	0.34
GATCAACGGATGTTACTTGA	0.00	0.00	0.29	0.00	0.00
GATCAACGGATGTTACTTGC	0.00	0.00	0.00	0.34	0.00
GATCAACGGATGTTCACGCT	0.00	0.00	0.29	0.00	0.00
GATCAACGGATGTTCATGGA	0.00	0.00	0.00	0.34	0.00
GATCAACGGATGTTCATGGT	0.00	0.00	0.29	0.00	0.00
GATCAACGGATGTTCCACAT	0.00	0.00	0.29	0.00	0.00
GATCAACGGATGTTCCCGAC	0.00	0.00	0.00	0.34	0.00
GATCAACGGATGTTCCCGGA	0.23	0.00	0.00	0.00	0.00
GATCAACGGATGTTCCCTGG	0.23	0.00	0.00	0.00	0.00
GATCAACGGATGTTCCGGGA	0.23	0.00	0.00	0.00	0.00
GATCAACGGATGTTCCGGGC	0.23	0.00	0.00	0.00	0.00
GATCAACGGATGTTCCNGNN	0.23	0.00	0.00	0.00	0.00
GATCAACGGATGTTCCNNNN	0.46	0.00	0.00	0.00	0.00
GATCAACGGATGTTCCTAAA	0.00	0.00	0.00	0.00	0.34

GATCAACGGATGTTCCTAGA	0.00	0.33	0.00	0.00	0.00
GATCAACGGATGTTCCTCAA	0.00	0.00	0.00	0.00	0.34
GATCAACGGATGTTCCTCCA	0.00	0.33	0.00	0.00	0.00
GATCAACGGATGTTCCTGAA	0.00	0.65	0.29	0.00	0.00
GATCAACGGATGTTCCTGCA	0.00	0.00	0.00	0.00	0.00
GATCAACGGATGTTCCTGGA	166.64	80.36	43.53	48.20	45.81
GATCAACGGATGTTCCTGGC	0.92	0.98	0.29	0.34	0.00
GATCAACGGATGTTCCTGGG	7.55	0.00	0.00	0.00	0.69
GATCAACGGATGTTCCTGGN	0.23	0.00	0.00	0.00	0.00
GATCAACGGATGTTCCTGGT	0.23	0.00	0.00	0.68	0.34
GATCAACGGATGTTCCTGNN	0.69	0.00	0.00	0.00	0.00
GATCAACGGATGTTCCTGTA	0.00	0.00	0.00	0.00	0.34
GATCAACGGATGTTCCTGTG	0.23	0.33	0.00	0.00	0.00
GATCAACGGATGTTCCTTAA	0.00	0.00	0.00	0.00	0.00
GATCAACGGATGTTCCTTCA	0.00	0.00	0.00	0.34	0.00
GATCAACGGATGTTCCTTCG	0.00	0.00	0.00	0.00	0.00
GATCAACGGATGTTCCTTCT	0.00	0.00	0.00	0.00	0.69
GATCAACGGATGTTCCTTGA	36.62	13.99	16.61	17.65	24.45
GATCAACGGATGTTCCTTGG	0.92	0.00	0.29	1.36	0.34
GATCAACGGATGTTCCTTGN	0.69	0.00	0.00	0.00	0.00
GATCAACGGATGTTCCTTGT	0.00	0.65	0.00	0.34	0.00
GATCAACGGATGTTCCTTNN	0.46	0.00	0.00	0.00	0.00
GATCAACGGATGTTCCTTTA	0.23	0.65	0.57	2.38	1.03
GATCAACGGATGTTCCTTTT	0.00	0.33	0.00	0.00	0.00
GATCAACGGATGTTCGTGGA	0.23	0.00	0.00	0.00	0.00
GATCAACGGATGTTCGTGGC	0.23	0.00	0.00	0.00	0.00
GATCAACGGATGTTCTTTGA	0.46	0.00	0.00	0.00	0.00
GATCAACGGATGTTCTTTGT	0.00	0.00	0.00	0.00	0.00
GATCAACGGATGTTTCTGGA	0.00	0.33	0.00	0.00	0.00
GATCAACGGATGTTTCTTGA	0.23	0.00	0.00	0.00	0.00
GATCAACGGATGTTTCTTGT	0.00	0.00	0.29	0.00	0.00
GATCAACGGATTATGTAAGA	0.00	0.00	0.00	0.00	0.00
GATCAACGGATTCGCTCTTG	0.00	0.00	0.00	0.34	0.00
GATCAACGGATTGGATGTGG	0.00	0.00	0.00	0.00	0.00
GATCAACGGATTTCTTCCTG	0.00	0.33	0.00	0.00	0.00
GATCAACGGATTTGCAGCAT	0.00	0.00	0.00	0.68	0.00
GATCAACGGATTTGCAGCGC	0.00	0.65	0.00	0.00	0.69

GATCAACGGCAAAAAAAAAA	0.23	0.00	0.00	0.00	0.00
GATCAACGGCAAATTCCCGC	0.23	0.00	0.00	0.00	0.00
GATCAACGGCAACAAAAAAA	0.69	0.00	0.00	0.00	0.00
GATCAACGGCAACATTAATT	0.00	0.00	0.00	0.00	0.00
GATCAACGGCAACATTCGAA	0.00	0.00	0.29	0.00	0.00
GATCAACGGCAACCACAAAT	0.00	0.00	0.00	0.00	0.34
GATCAACGGCAACGGCAACA	0.00	0.00	0.00	0.00	0.34
GATCAACGGCAACTCGGTCA	0.00	0.00	0.00	0.00	0.00
GATCAACGGCAACTGGCAAA	0.00	0.00	0.00	0.00	0.00
GATCAACGGCAAGAAGATGA	0.00	0.00	0.00	0.00	0.00
GATCAACGGCAAGCAGCACC	0.00	0.00	0.00	0.68	0.00
GATCAACGGCAAGCGCGTCG	0.23	0.00	0.00	0.00	0.00
GATCAACGGCAAGCTCTTCC	0.46	0.33	0.00	0.68	0.34
GATCAACGGCAAGCTGCCTG	0.00	2.60	0.86	0.00	0.00
GATCAACGGCAAGGGAGGCA	0.00	0.00	0.00	0.68	0.00
GATCAACGGCAAGGTCACAG	0.00	0.33	0.29	0.00	0.00
GATCAACGGCAAGTACGACC	0.00	0.00	0.00	0.00	0.00
GATCAACGGCAAGTGAGCGT	0.00	0.00	0.00	0.00	0.00
GATCAACGGCAAGTGCTGCT	0.00	0.33	0.00	0.00	0.00
GATCAACGGCAAGTTCTTCA	0.00	0.33	0.86	0.68	0.34
GATCAACGGCAATCCCAAGG	0.00	0.00	0.00	0.00	0.34
GATCAACGGCAATCGACTCA	0.00	0.00	0.00	0.00	0.34
GATCAACGGCAATCTGGAGT	0.00	0.00	0.00	0.00	0.34
GATCAACGGCAATGTGACAA	0.46	0.00	0.29	0.00	0.00
GATCAACGGCACAAAAGAAA	0.00	0.00	0.29	0.00	0.00
GATCAACGGCACAAAGGGAG	0.00	0.00	0.00	0.00	0.00
GATCAACGGCACACAGAGAG	0.00	0.00	0.00	0.00	0.34
GATCAACGGCACAGAGGCTC	0.00	0.00	0.00	0.34	0.00
GATCAACGGCACAGCGTCCA	0.00	0.00	0.00	0.34	0.00
GATCAACGGCACAGGTGGAC	0.23	0.00	0.29	0.00	0.00
GATCAACGGCACAGTTGAGG	0.46	0.00	0.29	0.00	0.00
GATCAACGGCACATTGACAC	0.00	0.00	0.00	0.00	0.34
GATCAACGGCACCACCAACT	0.00	0.00	0.00	0.00	0.34
GATCAACGGCACCACGGAGT	0.46	0.00	0.00	0.00	0.00
GATCAACGGCACCAGGCGTT	0.00	0.00	0.00	0.00	0.00
GATCAACGGCACCATTGTGG	0.00	0.00	0.00	0.00	0.00
GATCAACGGCACCCAGCAAT	0.00	0.00	0.00	0.00	0.00

GATCAACGGCACCCAGGAGC	0.23	0.00	0.00	0.00	0.00
GATCAACGGCACCCGAGCCG	0.23	1.30	0.00	0.00	0.00
GATCAACGGCACCTACGCCT	0.00	0.00	0.00	0.00	0.00
GATCAACGGCACCTCGACGG	0.00	0.33	0.00	0.00	0.00
GATCAACGGCACGAGCGGAG	0.00	0.00	0.00	0.00	0.00
GATCAACGGCACGCAGGAAC	0.00	0.33	0.29	0.00	0.00
GATCAACGGCACGCAGGACA	0.23	0.00	0.00	0.00	0.00
GATCAACGGCACGCAGGACC	7.55	9.43	4.87	7.81	3.79
GATCAACGGCACGCAGGACG	0.23	0.00	0.00	0.00	0.00
GATCAACGGCACGCAGGACN	0.23	0.00	0.00	0.00	0.00
GATCAACGGCACGCAGGAGC	0.23	0.00	0.00	0.00	0.00
GATCAACGGCACGCAGGANN	0.23	0.00	0.00	0.00	0.00
GATCAACGGCACGCAGNACC	0.00	0.00	0.00	0.00	0.34
GATCAACGGCACGCCGCGCA	0.00	0.00	0.00	0.00	0.00
GATCAACGGCACGCCGGACC	0.00	0.00	0.00	0.00	0.00
GATCAACGGCACGCGCCCCT	0.00	0.00	0.00	0.00	0.34
GATCAACGGCACGCGGAGCT	0.00	0.00	0.00	0.00	0.34
GATCAACGGCACGCGGCGCA	0.00	0.33	0.00	0.00	0.00
GATCAACGGCACGCTGGCAC	0.92	0.33	0.00	0.00	0.69
GATCAACGGCACGGCGCGCG	0.00	0.00	0.00	0.00	0.00
GATCAACGGCACGGCGGGCC	0.00	0.65	0.00	0.00	0.34
GATCAACGGCACGGCGGGCT	0.00	0.33	0.00	0.00	0.00
GATCAACGGCACGGCGGGGC	0.00	0.00	0.00	1.02	0.00
GATCAACGGCACGGCGGGGG	0.23	0.00	0.00	0.00	0.00
GATCAACGGCACGGGAACTG	0.00	0.00	0.00	0.34	0.00
GATCAACGGCACGGTCCAGC	0.00	0.00	0.00	0.00	0.00
GATCAACGGCACGTAGGACC	0.00	0.00	0.00	0.00	0.00
GATCAACGGCACGTCGCAGC	0.46	1.95	0.57	1.02	0.34
GATCAACGGCACGTCGCAGT	0.23	0.00	0.00	0.00	0.00
GATCAACGGCACGTGCTTTT	0.00	0.00	0.00	0.34	0.00
GATCAACGGCACTCGCATGG	1.83	1.30	0.29	0.68	0.00
GATCAACGGCACTCGCCTGG	0.00	0.00	0.29	0.00	0.00
GATCAACGGCACTGGATGCG	0.23	0.00	0.00	0.00	0.00
GATCAACGGCACTGGATGGG	0.23	0.00	0.00	0.00	0.00
GATCAACGGCACTTCTTCGC	0.00	0.33	0.00	0.00	0.00
GATCAACGGCAGAGCTTGGC	0.00	0.00	0.00	0.00	0.00
GATCAACGGCAGAGGAAGGA	0.00	0.33	0.00	0.00	0.00

GATCAACGGCAGAGGCCGGG	0.00	0.00	0.00	0.00	0.00
GATCAACGGCAGATACAACG	0.23	0.00	0.00	0.00	0.00
GATCAACGGCAGCAGTACGT	0.00	0.00	0.00	0.00	0.00
GATCAACGGCAGCGGCCCGG	0.00	0.00	0.00	0.00	0.00
GATCAACGGCAGCGTGCAAG	0.00	0.00	0.00	0.00	0.00
GATCAACGGCAGCGTGCACG	2.75	0.33	0.29	0.34	0.34
GATCAACGGCAGCTATGAAG	0.00	0.00	0.00	0.00	0.00
GATCAACGGCAGCTTGGACG	0.00	0.00	0.00	0.00	0.00
GATCAACGGCAGCTTTTTAT	0.00	0.00	0.00	0.00	0.00
GATCAACGGCAGCTTTTTCA	0.23	0.00	0.00	0.00	0.00
GATCAACGGCAGCTTTTTCT	16.02	27.33	14.03	5.77	12.40
GATCAACGGCAGCTTTTTNN	0.00	0.00	0.00	0.00	0.00
GATCAACGGCAGCTTTTTTT	0.00	0.33	0.57	0.68	0.34
GATCAACGGCAGGACGGAAC	0.00	0.00	0.29	0.00	0.00
GATCAACGGCAGGAGCGGGC	0.23	0.00	0.00	0.00	0.00
GATCAACGGCAGGCGCGTGG	0.00	0.00	0.00	0.34	0.00
GATCAACGGCAGGCTGGGGT	0.00	0.00	0.00	0.00	0.34
GATCAACGGCAGGGTCCCCA	0.00	0.00	0.00	0.00	1.03
GATCAACGGCAGGGTCCCCC	0.00	0.00	0.00	0.00	0.34
GATCAACGGCAGGTTCAAAA	0.00	0.33	0.00	0.00	0.00
GATCAACGGCAGGTTCCCCA	1.83	0.65	1.15	0.00	1.72
GATCAACGGCAGGTTCCCCC	0.23	0.33	0.00	0.00	0.34
GATCAACGGCAGGTTCCCTC	0.00	0.00	0.29	0.00	0.00
GATCAACGGCAGGTTCTCCT	0.00	0.00	0.29	0.00	0.00
GATCAACGGCAGGTTTTTCT	0.23	0.00	0.00	0.34	0.69
GATCAACGGCAGGTTTTTTT	0.00	0.00	0.00	0.68	0.34
GATCAACGGCAGTCTAGGAC	0.00	0.00	0.00	0.00	0.00
GATCAACGGCAGTGCCGCGA	0.00	0.00	0.00	0.00	0.00
GATCAACGGCATAACAGGAA	0.00	0.00	0.00	0.00	0.34
GATCAACGGCATAGACAAGT	0.00	0.00	0.00	0.00	0.00
GATCAACGGCATCAAGAAGG	0.00	0.00	0.00	0.00	0.00
GATCAACGGCATCAAGCAGC	0.00	0.33	0.00	0.34	0.34
GATCAACGGCATCACCCGTG	0.23	0.00	0.00	0.00	0.00
GATCAACGGCATCACCCGTT	0.23	0.00	0.00	0.00	0.00
GATCAACGGCATCATCCGCG	0.00	0.00	0.00	0.00	0.34
GATCAACGGCATCATCTGCC	0.00	0.33	0.00	0.00	0.00
GATCAACGGCATCATGTCCT	0.00	0.33	0.00	0.00	0.00

GATCAACGGCATCCCATGGT	0.00	0.00	0.00	0.00	0.00
GATCAACGGCATCCGCCACT	0.00	0.00	0.00	0.34	0.00
GATCAACGGCATCCTGTTCG	0.00	0.00	0.00	0.00	0.00
GATCAACGGCATCGCGTCCT	0.00	0.00	0.00	0.00	0.00
GATCAACGGCATCGGCTTCA	0.00	0.00	0.29	0.00	0.00
GATCAACGGCATCTCCAAGA	0.00	0.33	0.00	0.00	0.00
GATCAACGGCATCTCCAAGT	0.00	0.33	0.00	0.00	0.69
GATCAACGGCATCTCCGATC	0.23	0.00	0.00	0.00	0.00
GATCAACGGCATCTCCGGTA	0.00	0.00	0.00	0.00	0.34
GATCAACGGCATCTCCGGTC	0.23	0.33	0.00	0.34	0.00
GATCAACGGCATCTGCCAGG	0.00	0.00	0.00	0.00	0.34
GATCAACGGCATCTGCCTCG	0.00	0.00	0.00	0.00	0.00
GATCAACGGCATCTTGCCGA	0.23	0.00	0.00	0.00	0.69
GATCAACGGCATCTTTTTCT	0.00	0.00	0.57	0.00	0.00
GATCAACGGCATGAGGCTAT	0.00	0.00	0.00	0.00	0.00
GATCAACGGCATGATGCTGG	0.00	0.33	0.00	0.00	0.00
GATCAACGGCATGATTGTGT	0.23	0.00	0.00	0.00	0.00
GATCAACGGCATGCAGGGGA	0.23	0.00	0.00	0.00	0.00
GATCAACGGCATGCGCAACA	0.00	0.00	0.29	0.00	0.00
GATCAACGGCATGCGCAACG	0.00	0.33	0.00	0.34	0.34
GATCAACGGCATGGCCCAGC	0.00	0.00	0.00	0.00	0.00
GATCAACGGCATGGCCTGCG	0.00	0.33	0.00	0.00	0.00
GATCAACGGCATGGTCGCTT	0.00	0.00	0.00	0.00	0.34
GATCAACGGCATGGTTCGCG	0.00	0.00	0.00	0.00	0.34
GATCAACGGCATGTGCAAAA	0.00	0.00	0.29	0.00	0.00
GATCAACGGCATGTGCAACG	0.00	0.65	0.00	0.00	0.00
GATCAACGGCATTGGGGCCA	0.00	0.00	0.00	0.00	0.34
GATCAACGGCATTTATGAGC	0.00	0.00	0.00	0.00	0.00
GATCAACGGCCAAACGTGGT	0.00	0.00	0.00	0.00	0.34
GATCAACGGCCAACGGCCAA	0.00	0.00	0.29	0.00	0.00
GATCAACGGCCAAGCCTCCT	0.00	0.00	0.00	0.00	0.34
GATCAACGGCCAAGCCTTGG	0.00	0.00	0.00	0.34	0.00
GATCAACGGCCAAGTAAGTG	0.23	0.00	0.00	0.00	0.00
GATCAACGGCCAATGAGAGC	0.00	0.00	0.00	0.34	0.00
GATCAACGGCCACAACCACT	0.00	0.00	0.00	0.00	0.34
GATCAACGGCCACAACGTCA	0.00	0.00	0.00	0.00	0.00
GATCAACGGCCACACATGGT	0.23	0.00	0.00	0.00	0.00

GATCAACGGCCACACGAGGA	0.23	0.00	0.00	0.00	0.00
GATCAACGGCCACACGAGGT	0.23	0.33	0.00	0.34	0.34
GATCAACGGCCACACGCGGT	0.69	0.00	0.00	0.00	0.00
GATCAACGGCCACACGGGGA	0.00	0.00	0.00	0.00	0.00
GATCAACGGCCACACGGGGT	0.23	0.33	0.00	0.00	0.00
GATCAACGGCCACACGNNNN	0.23	0.00	0.00	0.00	0.00
GATCAACGGCCACACGTGGA	0.23	0.00	0.00	0.00	0.00
GATCAACGGCCACACGTGGC	0.00	0.00	0.00	0.00	0.34
GATCAACGGCCACACGTGGG	0.69	0.33	0.29	0.68	0.00
GATCAACGGCCACACGTGGT	38.23	22.12	24.34	26.48	22.04
GATCAACGGCCACACTCGTA	0.00	0.00	0.29	0.00	0.34
GATCAACGGCCACCAGAGGT	0.00	0.00	0.00	0.34	0.00
GATCAACGGCCACGATGTCC	0.00	0.00	0.00	0.00	0.00
GATCAACGGCCACGTGGGGG	0.23	0.00	0.00	0.00	0.00
GATCAACGGCCACGTGGTGG	1.14	0.00	0.00	0.34	0.69
GATCAACGGCCACTACAAGC	0.23	0.00	0.00	0.00	0.00
GATCAACGGCCACTCCTGAC	1.37	0.65	0.29	0.00	0.00
GATCAACGGCCAGACATAGT	0.00	0.00	0.00	0.00	0.34
GATCAACGGCCAGACGTGGT	0.23	0.00	0.00	0.00	0.00
GATCAACGGCCAGAGCGAAT	0.00	0.00	0.00	0.34	0.00
GATCAACGGCCAGAGCGCCC	0.46	0.00	0.00	0.00	0.34
GATCAACGGCCAGAGGTATT	0.00	0.00	0.00	0.00	0.00
GATCAACGGCCAGCGCGAGA	0.00	0.00	0.00	0.00	0.34
GATCAACGGCCAGCGGCGCA	0.00	0.33	0.00	0.00	0.00
GATCAACGGCCAGCGGGGCA	0.00	0.33	1.15	0.00	1.03
GATCAACGGCCAGCGGGGCC	0.00	0.00	0.29	0.00	0.00
GATCAACGGCCAGCTGGGAA	0.46	0.00	0.00	0.00	0.00
GATCAACGGCCAGCTGGGGA	0.23	0.00	0.00	0.00	0.00
GATCAACGGCCAGGGGCACT	0.00	0.00	0.00	0.00	0.00
GATCAACGGCCAGGGGCCCT	0.46	0.65	0.00	0.00	0.00
GATCAACGGCCAGTGACACT	0.23	0.00	0.00	0.00	0.00
GATCAACGGCCATACGTGGT	0.00	0.33	0.29	0.00	0.00
GATCAACGGCCATCGTGCCT	0.00	0.00	0.00	0.00	0.00
GATCAACGGCCATGGTGCAG	0.00	0.00	0.00	0.00	0.34
GATCAACGGCCATGTTGCAG	0.00	0.33	0.00	0.34	0.00
GATCAACGGCCCACGGTGAG	0.00	0.00	0.00	0.34	0.00
GATCAACGGCCCAGAGTGAG	0.00	0.00	0.00	0.34	0.00

GATCAACGGCCCAGCGGTGT	0.00	0.00	0.00	0.00	0.34
GATCAACGGCCCAGTCTGCT	0.00	0.00	0.00	0.34	0.00
GATCAACGGCCCAGTCTGGN	0.23	0.00	0.00	0.00	0.00
GATCAACGGCCCAGTCTGGT	0.92	0.00	0.00	1.02	0.00
GATCAACGGCCCATCCATGC	0.00	0.00	0.29	0.00	0.00
GATCAACGGCCCCGCTTAGA	0.00	0.33	0.00	0.00	0.00
GATCAACGGCCCCTACAAGC	0.46	0.00	0.00	0.00	0.00
GATCAACGGCCCCTCGACGG	0.00	0.00	0.00	0.00	0.00
GATCAACGGCCCGCAATTCA	0.23	0.00	0.00	0.00	0.00
GATCAACGGCCCGCGCTCAC	0.00	0.98	0.00	0.00	0.00
GATCAACGGCCCGCTGACCA	0.00	0.00	0.00	0.68	0.00
GATCAACGGCCCGCTGGGCC	0.00	0.00	0.00	0.00	0.00
GATCAACGGCCCGGAGGGCG	0.00	0.00	0.00	0.00	0.00
GATCAACGGCCCGGAGGTCA	0.00	0.33	0.00	0.00	0.00
GATCAACGGCCCGTCCCGGC	0.00	0.00	0.00	0.00	0.00
GATCAACGGCCCTGCCGGGT	0.00	0.00	0.00	0.00	0.34
GATCAACGGCCGACACTTGT	0.00	0.65	0.00	0.00	0.00
GATCAACGGCCGAGCCTGAA	0.00	0.00	0.00	0.00	0.00
GATCAACGGCCGAGGAAGGG	0.00	0.33	0.00	0.00	0.00
GATCAACGGCCGAGGACGGG	0.00	0.00	0.00	0.34	0.00
GATCAACGGCCGAGGCCGCG	0.00	0.00	0.00	0.34	0.00
GATCAACGGCCGAGGCCGGG	17.63	11.71	8.30	8.49	6.20
GATCAACGGCCGAGGCCGGN	0.23	0.00	0.00	0.00	0.00
GATCAACGGCCGAGGCCGNG	0.00	0.00	0.00	0.00	0.00
GATCAACGGCCGAGGCNGNN	0.23	0.00	0.00	0.00	0.00
GATCAACGGCCGAGTCACGG	0.23	0.00	0.00	0.00	0.69
GATCAACGGCCGAGTTTCCA	0.00	0.00	0.00	0.00	0.00
GATCAACGGCCGCAGCTCGC	2.98	0.33	0.00	0.00	0.00
GATCAACGGCCGCAGGGCAG	0.00	0.00	0.29	0.34	0.00
GATCAACGGCCGCATTGCAT	0.00	0.00	0.00	0.00	0.00
GATCAACGGCCGCCGCGGAC	0.00	0.00	0.00	0.34	0.00
GATCAACGGCCGCCTGCCGG	0.00	0.00	0.00	0.00	0.00
GATCAACGGCCGCCTGGCGG	0.00	0.00	0.29	0.00	0.00
GATCAACGGCCGCGCTCCGC	0.00	0.00	0.00	0.00	0.34
GATCAACGGCCGCGGGGGCG	0.23	0.00	0.00	0.00	0.00
GATCAACGGCCGCTGGGCGC	0.00	0.00	0.00	0.68	0.00
GATCAACGGCCGCTTTTTCT	0.00	0.00	0.00	0.00	0.00

GATCAACGGCCGGGGAGCCG	0.00	0.00	0.00	0.00	0.00
GATCAACGGCCGGTGCTTGG	0.00	0.00	0.00	0.00	0.00
GATCAACGGCCGTCTACCAG	0.00	0.00	0.00	0.00	0.69
GATCAACGGCCTCAACGCCC	0.46	0.33	0.29	0.00	0.00
GATCAACGGCCTCACGTTGT	0.00	0.00	0.00	0.34	0.00
GATCAACGGCCTCATCAGCC	0.00	0.00	0.00	0.00	0.00
GATCAACGGCCTCCTCCCTC	0.23	0.00	0.00	0.00	0.00
GATCAACGGCCTCCTCTACA	0.46	1.30	0.29	1.36	0.00
GATCAACGGCCTCCTCTACT	0.00	0.00	0.29	0.34	0.00
GATCAACGGCCTCGGTGTGT	0.00	0.00	0.00	0.00	0.00
GATCAACGGCCTCGTCTGCC	0.00	0.00	0.00	0.00	0.34
GATCAACGGCCTCGTGGAGC	1.83	0.33	1.72	1.02	1.03
GATCAACGGCCTCGTGGGGC	0.23	0.00	0.00	0.00	0.00
GATCAACGGCCTCTTCATGG	0.00	0.33	0.29	0.68	0.00
GATCAACGGCCTCTTCGTGG	0.00	0.00	0.00	0.34	0.00
GATCAACGGCCTGAAGAGCC	0.23	0.65	0.00	0.34	0.00
GATCAACGGCCTGAAGTTCG	0.00	0.00	0.00	0.00	0.00
GATCAACGGCCTGACAATAA	0.92	0.00	0.00	0.00	0.00
GATCAACGGCCTGAGGCGGT	0.23	0.00	0.00	0.00	0.00
GATCAACGGCCTGAGGCTCG	0.23	0.00	0.00	0.00	0.00
GATCAACGGCCTGAGGGTCG	0.00	0.00	0.00	0.34	0.00
GATCAACGGCCTGAGGTTCG	3.43	3.25	2.58	5.43	3.44
GATCAACGGCCTGCTGGTGG	0.00	0.00	0.29	0.00	0.00
GATCAACGGCCTGGAACGCC	0.00	0.00	0.00	0.00	0.00
GATCAACGGCCTGGACCGCC	0.00	0.00	0.00	0.34	0.00
GATCAACGGCCTGGGCCCGC	0.00	0.00	0.00	0.68	0.00
GATCAACGGCCTGGGCGGCG	0.00	0.33	0.00	0.00	0.00
GATCAACGGCCTGGTGTTGG	0.00	0.00	0.00	0.00	0.00
GATCAACGGCCTTCAAGGAG	0.00	0.00	0.57	0.00	0.00
GATCAACGGCCTTCAATGAG	0.00	0.00	0.29	0.00	0.00
GATCAACGGCGAAAACATGC	0.00	0.00	0.00	0.00	0.34
GATCAACGGCGAAAACATGN	0.23	0.00	0.00	0.00	0.00
GATCAACGGCGAAAACATGT	1.60	1.63	2.00	1.02	2.76
GATCAACGGCGAAAACNNNN	0.23	0.00	0.00	0.00	0.00
GATCAACGGCGAAAAGGGCC	0.00	0.33	0.00	0.00	0.00
GATCAACGGCGAACTCCGAT	0.00	0.00	0.00	0.34	0.00
GATCAACGGCGAAGGCACCT	0.00	0.00	0.00	0.00	0.00

GATCAACGGCGACAAGGGAC	0.00	0.00	0.00	0.00	0.00
GATCAACGGCGACAAGGGCC	0.00	0.33	0.00	0.68	0.00
GATCAACGGCGACAGCTCCG	0.00	0.33	0.00	0.00	0.00
GATCAACGGCGACAGGGGCC	0.00	0.00	0.00	0.34	0.00
GATCAACGGCGACCAAATGA	0.00	0.00	0.00	0.00	0.00
GATCAACGGCGACCCCCTGG	0.92	0.00	0.00	0.00	0.00
GATCAACGGCGACCCCGGGC	0.46	0.98	0.57	1.70	1.03
GATCAACGGCGACCCCTTGC	0.00	0.00	0.00	0.00	0.00
GATCAACGGCGACCCTGGGC	0.23	0.33	0.00	0.68	0.00
GATCAACGGCGACGCCACGC	0.00	0.00	0.29	0.00	0.00
GATCAACGGCGACGCGGACC	0.00	0.00	0.00	0.00	0.00
GATCAACGGCGACGGCAACA	0.00	0.00	0.00	0.00	0.34
GATCAACGGCGACGGCTCGG	0.00	0.00	0.29	0.00	0.00
GATCAACGGCGACTCTGTTG	0.00	0.00	0.00	0.00	0.34
GATCAACGGCGAGAAGCAAG	0.00	0.00	0.29	0.34	0.00
GATCAACGGCGAGAATGTGC	0.00	0.33	0.00	0.00	0.00
GATCAACGGCGAGATGCTGC	0.00	0.00	0.00	0.00	0.00
GATCAACGGCGAGATGTGGG	1.60	2.93	1.15	5.09	0.34
GATCAACGGCGAGCACTGCC	0.00	0.33	0.00	0.00	0.34
GATCAACGGCGAGCAGCACT	0.00	0.33	0.00	0.00	0.00
GATCAACGGCGAGCCAATGC	0.00	0.00	0.00	0.00	0.34
GATCAACGGCGAGCGACGAC	0.00	0.00	0.29	0.00	0.00
GATCAACGGCGAGCGCATGT	0.69	0.00	0.00	0.68	0.00
GATCAACGGCGAGCGCCTGT	0.00	0.00	0.00	0.00	0.00
GATCAACGGCGAGCTCATCG	0.00	0.33	0.00	0.00	0.00
GATCAACGGCGAGGACGCGC	0.00	0.00	0.00	0.00	0.00
GATCAACGGCGAGGAGGACG	0.00	0.65	0.86	0.00	0.00
GATCAACGGCGAGGAGTTCT	0.92	0.65	0.29	0.34	0.34
GATCAACGGCGAGGCTGCGA	0.00	0.00	0.00	0.00	0.00
GATCAACGGCGAGTCCATGC	0.00	0.00	0.00	0.00	0.00
GATCAACGGCGAGTCCGCGC	0.00	0.00	0.00	0.00	0.00
GATCAACGGCGAGTCCTACC	0.00	0.00	0.00	0.00	0.00
GATCAACGGCGATGAGGCCG	0.00	0.00	0.00	0.00	0.00
GATCAACGGCGATGCAATAG	0.00	0.00	0.00	0.00	0.00
GATCAACGGCGCAAACAAAA	0.00	0.00	0.00	0.00	0.34
GATCAACGGCGCACAGGTCC	0.00	0.00	0.00	0.00	0.00
GATCAACGGCGCACCCTCGG	0.00	0.00	0.00	0.00	0.34

GATCAACGGCGCAGACACGG	0.00	0.33	0.00	0.00	0.00
GATCAACGGCGCAGACGCCT	0.00	0.00	0.00	0.00	0.00
GATCAACGGCGCCAAGATGT	0.00	0.33	0.00	0.00	0.00
GATCAACGGCGCCATGCTTG	0.00	0.00	0.00	0.00	0.34
GATCAACGGCGCCCCGCTTC	0.00	0.00	0.00	0.34	0.00
GATCAACGGCGCCCGCGGGC	0.23	0.00	0.00	0.00	0.00
GATCAACGGCGCCGCGGCGG	0.23	0.00	0.00	0.00	0.00
GATCAACGGCGCCGCGNCNG	0.00	0.00	0.29	0.00	0.00
GATCAACGGCGCCGCGNNNN	0.23	0.00	0.00	0.00	0.00
GATCAACGGCGCCGCGTCGG	10.07	11.39	7.45	7.47	6.20
GATCAACGGCGCCGCGTTGG	0.00	0.00	0.29	0.00	0.00
GATCAACGGCGCCTACGACA	0.69	0.00	0.00	1.02	1.03
GATCAACGGCGCCTACGCCA	0.00	0.00	0.00	0.34	0.00
GATCAACGGCGCCTGCCCTC	0.00	0.00	0.00	0.34	0.00
GATCAACGGCGCGAGAAAGC	0.00	0.00	0.00	0.00	0.34
GATCAACGGCGCGATGCAAC	0.00	0.00	0.00	0.34	0.00
GATCAACGGCGCGCCGGCGG	0.23	0.00	0.00	0.00	0.00
GATCAACGGCGCGCGGAAAC	0.00	0.00	0.00	0.34	0.00
GATCAACGGCGCGCGGCACC	1.37	2.93	1.43	3.73	2.76
GATCAACGGCGCGCGGCAGC	0.00	0.00	0.00	0.00	0.00
GATCAACGGCGCGCGGCATC	0.00	0.00	0.00	0.00	0.00
GATCAACGGCGCGCGGCTCC	0.46	0.00	0.00	0.00	0.00
GATCAACGGCGCGCGGGCGG	0.23	0.00	0.00	0.00	0.00
GATCAACGGCGCGCTGACCC	0.00	0.33	0.00	0.00	0.00
GATCAACGGCGCGCTGCACA	0.00	0.00	0.00	0.00	0.34
GATCAACGGCGCGCTGCACC	8.70	9.76	6.30	8.83	5.51
GATCAACGGCGCGCTGCCAC	0.00	0.33	0.00	0.00	0.00
GATCAACGGCGCGCTGCTCT	0.00	0.00	0.00	0.00	0.00
GATCAACGGCGCGGACCCCT	0.00	0.00	0.00	0.34	0.00
GATCAACGGCGCGGACGCCT	0.00	0.00	0.00	0.34	0.00
GATCAACGGCGCGGAGGGGA	0.00	0.33	0.00	0.00	0.00
GATCAACGGCGCGGCGTCGG	0.00	0.00	0.00	0.00	0.00
GATCAACGGCGCGGGCGGCG	0.23	0.00	0.00	0.00	0.00
GATCAACGGCGCGGTCGCCT	0.00	0.00	0.00	0.34	0.00
GATCAACGGCGCGGTGGGCG	0.23	0.00	0.00	0.00	0.00
GATCAACGGCGCTCAACTGT	0.00	0.00	0.00	0.00	0.34
GATCAACGGCGGAACACGAC	0.00	0.00	0.29	0.00	0.00

GATCAACGGCGGACCTTGAA	0.00	0.00	0.00	0.34	0.00
GATCAACGGCGGAGAGGAGT	0.00	0.00	0.00	0.00	0.34
GATCAACGGCGGAGCTCTTC	0.00	0.00	0.00	0.34	0.00
GATCAACGGCGGAGCTTTTT	0.00	0.00	0.00	0.34	0.00
GATCAACGGCGGATGACGCA	0.23	0.00	0.00	0.00	0.00
GATCAACGGCGGATGCCCGC	0.00	0.00	0.00	0.00	0.34
GATCAACGGCGGCAAAAAAA	0.00	0.00	0.00	0.00	0.00
GATCAACGGCGGCAAGGAGC	0.00	0.33	0.00	0.00	0.00
GATCAACGGCGGCGACAACT	0.00	0.33	0.00	0.00	0.69
GATCAACGGCGGCGACTACC	0.00	0.00	0.00	0.00	0.00
GATCAACGGCGGCGACTACT	0.00	1.63	0.29	1.70	2.07
GATCAACGGCGGCTCTCGCA	0.46	0.00	0.86	0.00	0.34
GATCAACGGCGGCTCTCGCC	0.92	0.00	1.15	0.00	0.00
GATCAACGGCGGCTCTCGTA	0.23	0.00	0.00	0.00	0.00
GATCAACGGCGGGAACAACA	0.00	0.00	0.57	0.00	0.34
GATCAACGGCGGGAACAAGC	0.00	0.33	0.00	0.00	0.00
GATCAACGGCGGGAACTGTA	0.00	0.00	0.00	0.00	0.34
GATCAACGGCGGGAGCCACG	0.46	0.00	0.00	0.00	0.00
GATCAACGGCGGGAGCCACT	0.00	0.00	0.00	0.00	0.00
GATCAACGGCGGGAGCCCCG	0.00	0.33	0.00	0.00	0.00
GATCAACGGCGGGAGGGGGG	0.23	0.00	0.00	0.00	0.00
GATCAACGGCGGGATGCCAA	0.00	0.00	0.00	0.00	0.00
GATCAACGGCGGGCCACGCA	0.23	0.00	0.00	0.34	0.00
GATCAACGGCGGGCCAGGCC	0.00	0.00	0.00	0.00	0.00
GATCAACGGCGGGCCATGCA	6.41	3.90	2.29	4.41	4.13
GATCAACGGCGGGCCATGCC	0.00	0.00	0.57	0.00	0.00
GATCAACGGCGGGCCATGCG	0.23	0.00	0.00	0.00	0.00
GATCAACGGCGGGCCCTGCA	0.00	0.33	0.00	0.00	0.00
GATCAACGGCGGGGACAACA	0.00	0.00	0.00	0.34	0.00
GATCAACGGCGGGGACACGG	0.00	0.00	0.29	0.00	0.00
GATCAACGGCGGGGAGGAGT	0.00	0.00	0.00	0.34	0.00
GATCAACGGCGGGGAGGGGG	0.00	0.00	0.00	0.00	0.34
GATCAACGGCGGGGCAAGCA	0.00	0.00	0.00	0.34	0.00
GATCAACGGCGGGGCGGTCG	0.23	0.00	0.00	0.00	0.00
GATCAACGGCGGGGGAGGGC	0.00	0.33	0.00	0.00	0.00
GATCAACGGCGGGGGCGGCG	0.23	0.00	0.00	0.00	0.00
GATCAACGGCGGGGGGGAGG	0.00	0.00	0.00	0.34	0.00

GATCAACGGCGGGTCTCGGA	0.23	0.00	0.00	0.00	0.00
GATCAACGGCGGGTGGTTGG	0.23	0.00	0.00	0.00	0.00
GATCAACGGCGGTTTTGTCC	0.23	0.00	0.00	0.00	0.00
GATCAACGGCGTAGGTGGGC	0.00	0.00	0.00	0.00	0.00
GATCAACGGCGTCAAGGCGG	0.00	0.00	0.00	0.00	0.34
GATCAACGGCGTCACGGCGG	0.00	0.33	0.00	0.34	0.00
GATCAACGGCGTCATCACAG	0.00	0.00	0.00	0.00	0.00
GATCAACGGCGTCATCACTG	0.69	0.00	0.00	0.00	0.00
GATCAACGGCGTCCCGGCTA	0.23	0.00	0.00	0.00	0.00
GATCAACGGCGTCGACGCCC	0.00	0.00	0.00	0.00	0.34
GATCAACGGCGTCGAGGTGC	0.23	0.00	0.00	0.00	0.00
GATCAACGGCGTCGCCAAGC	0.00	0.33	0.00	0.00	0.00
GATCAACGGCGTCGTCAAGC	0.00	0.00	0.00	0.00	0.34
GATCAACGGCGTCGTGTTTG	0.00	0.00	0.00	0.34	0.00
GATCAACGGCGTCTACGTCC	0.46	0.00	0.00	0.00	0.69
GATCAACGGCGTCTACTGGC	0.23	0.00	0.00	0.00	0.00
GATCAACGGCGTCTGCCAGG	0.23	0.00	0.00	0.00	0.00
GATCAACGGCGTCTTTGATG	0.00	0.00	0.00	0.00	0.00
GATCAACGGCGTGAGGCGCT	0.00	0.00	0.00	0.00	0.00
GATCAACGGCGTGATGCCCA	0.00	0.00	0.00	0.34	0.00
GATCAACGGCGTGATGTGAT	0.23	0.00	0.00	0.00	0.00
GATCAACGGCGTGCCGCTGG	1.60	1.30	0.00	0.00	0.00
GATCAACGGCGTGCGAGTCT	0.00	0.00	0.00	0.00	0.00
GATCAACGGCGTGCGGTACG	0.00	0.00	0.00	0.00	0.00
GATCAACGGCGTGCGTGCCA	0.00	0.65	0.29	0.34	0.00
GATCAACGGCGTGGACGGCG	0.00	0.00	0.29	0.00	0.00
GATCAACGGCGTGGCTCTGC	0.23	0.00	0.00	0.34	0.00
GATCAACGGCGTGGGAGGCG	0.00	0.00	0.00	0.00	0.00
GATCAACGGCGTGGGCAAGC	0.00	0.00	0.00	0.34	0.00
GATCAACGGCGTGGGCGGAG	0.00	0.00	0.00	0.34	0.00
GATCAACGGCGTGGGCGGCG	5.49	11.71	6.01	8.49	5.51
GATCAACGGCGTGGGCGGCT	0.00	0.00	0.57	0.68	0.00
GATCAACGGCGTGGGCGGGG	0.23	0.00	0.00	0.00	0.00
GATCAACGGCGTGGGCGGTG	0.23	0.00	0.29	0.00	0.00
GATCAACGGCGTGGGGGGCG	0.23	0.00	0.00	0.00	0.00
GATCAACGGCGTGGTCCTGG	0.00	0.00	0.00	0.00	0.00
GATCAACGGCGTGGTGCATC	0.00	0.00	0.29	0.00	0.00

GATCAACGGCGTGTGGTCTC	0.23	0.00	0.57	0.00	0.69
GATCAACGGCGTTAGAATGC	0.00	0.00	0.29	0.00	0.00
GATCAACGGCGTTGATGAGG	0.00	0.33	0.00	0.00	0.00
GATCAACGGCGTTTACAAAC	0.00	0.00	0.29	1.02	0.34
GATCAACGGCTACGGGAAGG	0.00	0.00	0.00	0.34	0.00
GATCAACGGCTACGGTCTCT	0.00	0.00	0.00	0.00	0.00
GATCAACGGCTACTCGGAGA	0.00	0.65	0.57	0.34	0.00
GATCAACGGCTACTCGGAGG	0.00	0.00	0.29	0.00	0.00
GATCAACGGCTAGTTGAGCC	0.00	0.33	0.00	0.00	0.00
GATCAACGGCTATCGCATGA	0.00	0.00	0.29	0.00	0.00
GATCAACGGCTATCGCATTG	0.00	0.00	0.00	0.00	0.00
GATCAACGGCTATCGCCTGA	0.00	0.00	0.00	0.00	0.00
GATCAACGGCTATTCCCCCG	0.00	0.00	0.29	0.00	0.00
GATCAACGGCTCAGAGACGG	0.00	0.00	0.00	0.00	0.00
GATCAACGGCTCATCGATTT	0.23	0.00	0.00	0.00	0.00
GATCAACGGCTCATCGGTTT	0.23	0.00	0.00	0.00	0.00
GATCAACGGCTCATGTGTCG	0.00	0.98	0.00	0.00	0.00
GATCAACGGCTCCATGGGCC	0.00	0.00	0.00	0.00	0.00
GATCAACGGCTCCATGGTCA	0.00	0.00	0.29	0.00	0.00
GATCAACGGCTCCCTGCTGT	0.23	0.00	0.00	0.00	0.00
GATCAACGGCTCCTGCAAGG	0.23	0.00	0.00	0.00	0.00
GATCAACGGCTCGATCTTTG	0.00	0.00	0.00	0.00	0.69
GATCAACGGCTCGCAAGGCC	0.23	0.00	0.00	0.00	0.00
GATCAACGGCTCGCCGCCGC	0.00	0.00	0.00	0.00	0.00
GATCAACGGCTCGCCTGAGC	0.00	0.00	0.29	0.00	0.00
GATCAACGGCTCGGGGTTAA	0.00	0.00	0.29	0.00	0.00
GATCAACGGCTCGGTGGGTG	0.46	0.00	0.00	0.00	0.00
GATCAACGGCTCGGTGTGTG	0.00	0.00	0.29	0.00	0.34
GATCAACGGCTCGTCGTATG	0.69	0.00	0.00	0.00	0.00
GATCAACGGCTCGTTGCGTG	0.00	0.00	0.00	0.00	0.00
GATCAACGGCTCGTTGGGTG	0.23	0.00	0.00	0.00	0.00
GATCAACGGCTCGTTGTGGG	0.23	0.00	0.00	0.00	0.00
GATCAACGGCTCGTTGTGNN	0.23	0.00	0.00	0.00	0.00
GATCAACGGCTCGTTGTGTG	28.84	11.71	4.58	4.41	5.85
GATCAACGGCTCGTTGTGTN	0.23	0.00	0.00	0.00	0.00
GATCAACGGCTCGTTGTGTT	0.00	0.00	0.29	0.00	0.00
GATCAACGGCTCGTTGTNTN	0.00	0.00	0.00	0.34	0.00

GATCAACGGCTCGTTGTTTG	0.00	0.00	0.00	0.34	0.00
GATCAACGGCTCTCCGCCCC	0.00	0.00	0.29	0.00	0.00
GATCAACGGCTCTCCGCCGC	0.00	0.00	0.00	0.00	1.03
GATCAACGGCTCTCCGCCGT	0.00	0.33	0.00	0.00	0.00
GATCAACGGCTCTCCGGCGC	0.00	0.00	0.00	0.34	0.00
GATCAACGGCTCTGGTTTTT	0.00	0.33	0.00	0.00	0.00
GATCAACGGCTGAAAGACAG	0.00	0.00	0.00	0.00	0.00
GATCAACGGCTGAAAGCATG	2.29	1.30	1.72	0.34	1.03
GATCAACGGCTGAAAGCATN	0.23	0.00	0.00	0.00	0.00
GATCAACGGCTGAAGCGCCT	0.00	0.00	0.00	0.34	0.00
GATCAACGGCTGAAGGCATG	0.23	0.00	0.00	0.00	0.00
GATCAACGGCTGAGAGGAGA	0.00	0.00	0.29	0.00	0.00
GATCAACGGCTGATGAAGAA	0.00	0.00	0.00	0.34	0.00
GATCAACGGCTGATGAAGCA	13.28	7.81	4.58	10.18	7.58
GATCAACGGCTGATGAAGCC	0.23	0.98	0.29	0.00	0.34
GATCAACGGCTGATGAAGGA	0.23	0.00	0.00	0.00	0.00
GATCAACGGCTGCATCTACC	0.23	0.00	0.00	0.00	0.00
GATCAACGGCTGCCAGACTT	0.00	0.00	0.00	0.00	0.00
GATCAACGGCTGCCCGACGC	0.00	0.00	0.00	0.00	0.34
GATCAACGGCTGCGAATTAT	0.00	0.00	0.00	0.00	0.34
GATCAACGGCTGCGAGGTGC	0.00	0.00	0.00	0.00	0.00
GATCAACGGCTGCGCCAGGC	0.00	0.00	0.29	0.34	0.00
GATCAACGGCTGCGCCTGGA	0.00	0.00	0.00	0.00	0.00
GATCAACGGCTGGACCATCA	0.00	0.00	0.00	0.34	0.00
GATCAACGGCTGGCGCTACA	0.00	0.00	0.00	0.00	0.00
GATCAACGGCTGGCTCGGGG	0.00	0.33	0.00	0.00	0.00
GATCAACGGCTGGCTTTGCT	0.00	0.00	0.00	0.00	0.00
GATCAACGGCTGGGCCGACC	0.00	0.00	0.00	0.00	0.34
GATCAACGGCTGGTCTCACG	0.00	0.33	0.00	0.00	0.00
GATCAACGGCTGGTTTTGCT	0.00	0.33	0.86	0.00	0.00
GATCAACGGCTGTACCATCA	0.23	0.98	0.57	0.00	1.03
GATCAACGGCTGTACCCTCA	0.00	0.33	0.00	0.00	0.00
GATCAACGGCTGTTCCTGGC	0.23	0.00	0.00	0.00	0.00
GATCAACGGCTGTTCCTGTC	0.23	0.00	0.00	0.00	0.00
GATCAACGGCTGTTCCTTGA	0.00	0.00	0.00	0.34	0.00
GATCAACGGCTTCAAGGCCG	0.00	1.30	0.00	1.02	0.00
GATCAACGGCTTCGAGGAGG	0.23	0.00	0.29	0.34	0.00

GATCAACGGCTTCGCAAATA	0.00	0.33	0.00	0.00	0.00
GATCAACGGCTTCGGCCAGG	0.00	0.00	0.29	0.00	0.00
GATCAACGGCTTCTGCAAGG	0.00	0.33	0.00	0.00	0.00
GATCAACGGCTTCTGCACGG	0.00	0.00	0.00	0.00	0.00
GATCAACGGCTTCTGCCAGG	41.20	18.22	8.88	12.56	8.61
GATCAACGGCTTCTGCCCGG	0.23	1.63	0.00	0.00	0.00
GATCAACGGCTTCTGCNAGG	0.00	0.00	0.00	0.00	0.34
GATCAACGGCTTCTTCGGAG	0.00	0.00	0.57	0.00	0.00
GATCAACGGCTTGATGCTCA	0.00	0.00	0.00	0.00	0.00
GATCAACGGCTTGCATGCCG	0.00	0.00	0.00	0.00	0.00
GATCAACGGCTTGCTCGTCA	0.46	0.00	0.29	0.00	0.00
GATCAACGGCTTGCTGTACA	0.00	0.00	0.00	0.00	0.34
GATCAACGGCTTGGCGCTCT	0.46	0.00	0.00	0.00	0.00
GATCAACGGCTTGGGGGAGG	0.00	0.00	0.29	0.00	0.00
GATCAACGGCTTTCCAATCC	0.00	0.00	0.00	0.00	0.69
GATCAACGGCTTTGCATTGG	0.92	0.33	0.57	0.00	0.00
GATCAACGGCTTTGCCTTTT	0.23	0.00	0.00	0.00	0.00
GATCAACGGCTTTGCGTTGG	0.23	0.00	0.00	0.00	0.00
GATCAACGGCTTTGGTACGA	0.00	0.33	0.00	0.00	0.00
GATCAACGGCTTTGTCGACG	1.60	0.00	0.00	0.00	0.00
GATCAACGGCTTTGTTTAAA	0.46	0.00	0.00	0.68	0.00
GATCAACGGCTTTGTTTATC	16.71	4.55	7.45	7.13	10.68
GATCAACGGCTTTGTTTATT	0.00	0.33	0.29	0.68	0.00
GATCAACGGCTTTGTTTCTC	0.23	0.00	0.00	0.00	0.00
GATCAACGGCTTTGTTTGTC	0.23	0.00	0.00	0.00	0.00
GATCAACGGCTTTGTTTTTC	0.00	0.00	0.00	0.00	0.00
GATCAACGGCTTTTCAATCT	4.35	0.65	0.86	1.02	1.38
GATCAACGGCTTTTCGATCT	0.23	0.00	0.00	0.00	0.00
GATCAACGGGAAAGCGTCCT	0.69	0.33	0.00	0.00	0.00
GATCAACGGGAACGTCAGCG	0.00	0.33	0.00	0.00	0.00
GATCAACGGGAACTCCATGC	0.00	0.33	0.00	0.00	0.00
GATCAACGGGAAGACGCTGG	0.00	0.00	0.57	0.34	0.34
GATCAACGGGAAGAGAGAAG	0.00	0.00	0.00	0.00	0.34
GATCAACGGGAAGGTCCTGG	0.00	0.00	0.00	0.00	0.00
GATCAACGGGAAGGTTTTTG	0.23	0.00	0.00	0.00	0.00
GATCAACGGGAAGTCGCACG	0.00	0.33	0.00	0.34	0.00
GATCAACGGGAAGTTCCTCG	0.00	0.00	0.00	0.00	0.00

GATCAACGGGAATGCACAAA	0.00	0.33	0.00	0.00	0.00
GATCAACGGGACACAACGAG	0.00	0.00	0.00	0.00	0.34
GATCAACGGGACACAGATTT	0.00	0.00	0.29	0.00	0.00
GATCAACGGGACACAGCTTC	0.00	0.00	0.57	0.68	0.00
GATCAACGGGACACAGGTTC	0.23	0.00	0.00	0.00	0.00
GATCAACGGGACAGGCAACC	0.00	0.33	0.00	0.00	0.00
GATCAACGGGACATCAGCTG	0.00	0.00	0.00	0.00	0.00
GATCAACGGGACATCGGCGA	0.00	0.00	0.00	0.00	0.00
GATCAACGGGACCAATCCAC	0.00	0.00	0.00	0.00	0.00
GATCAACGGGACCCGCCGTG	0.00	0.33	0.00	0.00	0.00
GATCAACGGGACCCGCTGGG	0.23	0.00	0.00	0.00	0.00
GATCAACGGGACCCTCTGGG	0.23	0.00	0.29	0.00	0.00
GATCAACGGGACCCTGGATT	0.00	0.00	0.00	0.00	0.00
GATCAACGGGACCTCACGCG	0.00	0.33	0.00	0.00	0.00
GATCAACGGGACCTTCGGCT	0.00	0.33	0.00	0.68	0.00
GATCAACGGGACGGACCCCG	0.00	0.33	0.00	0.00	0.00
GATCAACGGGACGGACCCTG	0.00	0.00	0.00	0.34	0.00
GATCAACGGGACGGCCTTGG	0.23	0.00	0.00	0.00	0.00
GATCAACGGGACGGTGGGTG	0.00	0.00	0.00	0.00	0.34
GATCAACGGGACTACACGCC	0.00	0.00	0.00	0.00	0.34
GATCAACGGGACTGCCAAAT	0.23	0.00	0.00	0.00	0.00
GATCAACGGGACTGCCTGTC	0.00	0.00	0.00	0.00	0.00
GATCAACGGGAGAAGGTTGA	0.23	0.00	0.00	0.00	0.00
GATCAACGGGAGACAGAGGG	0.00	0.00	0.29	0.00	0.00
GATCAACGGGAGAGGTGCGC	0.00	0.00	0.00	0.34	0.00
GATCAACGGGAGCACAGTAC	0.00	0.00	0.29	0.00	0.00
GATCAACGGGAGCACAGTCG	0.00	0.33	0.86	0.68	0.00
GATCAACGGGAGCACTGACG	0.00	0.00	0.00	0.34	0.00
GATCAACGGGAGCACTGCAC	0.00	0.00	0.00	0.34	0.00
GATCAACGGGAGCACTGTAC	0.69	0.98	1.43	0.68	1.72
GATCAACGGGAGCACTGTCC	0.23	0.00	0.00	0.00	0.00
GATCAACGGGAGCACTGTCG	0.46	0.00	0.00	0.34	0.00
GATCAACGGGAGCAGCATCA	0.00	0.00	0.29	0.68	0.34
GATCAACGGGAGCCATAAGA	0.46	0.65	0.29	0.68	0.69
GATCAACGGGAGCGAAGCAG	0.00	0.00	0.00	0.00	0.00
GATCAACGGGAGCGACGAGG	0.23	0.00	0.00	0.00	0.00
GATCAACGGGAGCGCCGAGG	7.78	5.21	2.29	3.73	3.44

GATCAACGGGAGCGCCGAGN	0.23	0.00	0.00	0.00	0.00
GATCAACGGGAGCGCCGANN	0.23	0.00	0.00	0.00	0.00
GATCAACGGGAGCGCCGGGG	0.00	0.00	0.29	0.00	0.00
GATCAACGGGAGCTGTCAAG	0.00	0.00	0.00	0.00	0.00
GATCAACGGGAGCTTCCTTG	2.29	2.28	0.57	1.02	0.69
GATCAACGGGAGCTTGTGGT	0.00	0.00	0.00	0.00	0.00
GATCAACGGGAGGAGCGCTC	0.00	0.00	0.00	0.00	0.69
GATCAACGGGAGGAGGATGG	0.00	0.00	0.29	0.00	0.00
GATCAACGGGAGGGAGGAAG	0.23	0.00	0.00	0.00	0.00
GATCAACGGGAGGTTTCCAT	0.00	0.33	0.00	0.00	0.00
GATCAACGGGAGTCCACAAG	0.00	0.00	0.00	0.34	0.00
GATCAACGGGAGTGAGCAAG	0.00	0.33	0.29	0.00	0.00
GATCAACGGGAGTGCGATTC	0.00	0.00	0.00	0.00	0.00
GATCAACGGGAGTTAGACAG	0.00	0.33	0.00	0.00	0.00
GATCAACGGGATGCGGTCGG	0.00	0.00	0.29	0.00	0.00
GATCAACGGGATTTGCCATG	0.00	0.00	0.00	0.34	0.00
GATCAACGGGCAACAACCAA	0.00	0.00	0.00	0.00	0.34
GATCAACGGGCAACCACACC	0.00	0.00	0.00	0.00	0.00
GATCAACGGGCAACCTGAAA	0.00	0.00	0.00	0.68	0.00
GATCAACGGGCAAGGCATGG	0.00	0.00	0.29	0.00	0.00
GATCAACGGGCAAGGCCACA	0.92	0.65	0.86	0.68	0.34
GATCAACGGGCAATCATTCG	0.00	0.00	0.00	0.00	0.34
GATCAACGGGCACAGACCTA	0.00	0.00	0.29	0.00	0.00
GATCAACGGGCACAGCCGCT	0.00	0.00	0.00	0.34	0.00
GATCAACGGGCACAGCGTGG	0.00	0.00	0.00	0.00	0.00
GATCAACGGGCACATGGCCT	0.00	0.00	0.00	0.00	0.00
GATCAACGGGCACCCGCTCC	0.23	0.00	0.00	0.00	0.00
GATCAACGGGCACCTCGTGC	0.00	0.00	0.00	0.00	0.00
GATCAACGGGCACCTCGTGG	0.00	0.00	0.00	0.00	0.00
GATCAACGGGCACGACCGGG	0.23	0.65	0.29	0.68	0.69
GATCAACGGGCACGCCCTCG	0.23	0.00	0.00	0.00	0.00
GATCAACGGGCACGCGCTTG	0.23	0.00	0.00	0.00	0.00
GATCAACGGGCACTTGCATG	0.23	0.00	0.00	0.00	0.00
GATCAACGGGCAGAAGAAGA	0.00	0.00	0.00	0.00	0.34
GATCAACGGGCAGCAGGAGC	0.23	0.00	0.00	0.00	0.00
GATCAACGGGCAGCCCCAGC	0.00	0.00	0.00	0.00	0.00
GATCAACGGGCAGCCCCAGT	0.00	0.00	0.00	1.36	0.00

GATCAACGGGCAGCGCCACC	0.00	0.00	0.29	0.00	0.00
GATCAACGGGCAGGCTGGTT	0.00	0.00	0.00	0.00	0.34
GATCAACGGGCAGGTTGGTT	0.00	0.65	0.00	0.68	0.00
GATCAACGGGCATGAACGTG	0.00	0.00	0.00	0.34	0.00
GATCAACGGGCATGAGGGAG	0.00	0.00	0.00	0.00	0.00
GATCAACGGGCATGCCCTTG	1.37	0.65	0.29	1.02	0.34
GATCAACGGGCCAGACACGC	0.00	0.00	0.00	0.34	0.00
GATCAACGGGCCCGATGTGG	0.23	0.00	0.00	0.00	0.00
GATCAACGGGCCCTGAGCAG	0.00	0.00	0.00	0.00	0.00
GATCAACGGGCCGATGGGTC	0.00	0.00	0.29	0.00	0.00
GATCAACGGGCCGGACACGC	0.00	0.00	0.00	0.68	0.00
GATCAACGGGCCGGGCGCGC	0.23	0.00	0.00	0.00	0.00
GATCAACGGGCCTCCCCTTC	0.00	0.00	0.00	0.00	0.00
GATCAACGGGCGAGGCCGGG	0.23	0.00	0.00	0.00	0.00
GATCAACGGGCGCAGCAGCC	0.00	0.00	0.29	0.00	0.00
GATCAACGGGCGCAGGAACG	0.00	0.33	0.00	0.00	0.00
GATCAACGGGCGCAGGCACG	0.69	0.00	0.00	0.34	0.00
GATCAACGGGCGCAGGGACG	0.00	0.33	0.00	0.00	0.00
GATCAACGGGCGCGAGACAG	0.00	0.33	0.00	0.00	0.00
GATCAACGGGCGGGCGGACG	0.00	0.00	0.29	0.00	0.00
GATCAACGGGCGGGCTTGGT	0.23	0.00	0.00	0.00	0.00
GATCAACGGGCGTGACTCCT	0.00	0.00	0.29	0.00	0.00
GATCAACGGGCGTGATGCGT	0.00	0.00	0.00	0.00	0.34
GATCAACGGGCGTGCCATTG	0.23	0.00	0.00	0.00	0.00
GATCAACGGGCGTGTGGTTA	0.00	0.00	0.00	0.00	0.00
GATCAACGGGCTCTCGGAGG	0.00	0.00	0.00	0.34	0.00
GATCAACGGGCTGACATAAG	0.00	0.00	0.29	0.00	0.00
GATCAACGGGCTGCAGGAGC	0.46	0.00	2.29	0.00	1.38
GATCAACGGGCTGCTGCCAT	0.00	0.00	0.00	0.00	0.34
GATCAACGGGCTGCTGTGAG	0.00	1.30	0.29	0.00	0.00
GATCAACGGGCTGGAGGCAG	0.00	0.00	0.00	0.00	0.00
GATCAACGGGCTGGTCATGG	0.23	0.00	0.00	0.00	0.00
GATCAACGGGCTGGTGGTCG	0.00	0.00	0.29	0.00	0.00
GATCAACGGGGAACAGCTCT	0.00	0.00	0.86	0.00	0.00
GATCAACGGGGAACAGGTCC	0.23	0.33	0.00	0.00	0.00
GATCAACGGGGAAGTGCCTC	0.00	0.00	0.00	0.00	0.00
GATCAACGGGGAATACGAGG	0.00	0.00	0.00	0.34	0.00

GATCAACGGGGACAGCTCAG	0.00	0.00	0.00	0.00	0.00
GATCAACGGGGACAGCTTAG	0.00	0.00	0.29	0.00	0.00
GATCAACGGGGACGTGCGCG	0.00	0.00	0.00	0.00	0.00
GATCAACGGGGACTACGAGC	0.00	0.00	0.00	0.34	0.00
GATCAACGGGGACTACGAGG	50.82	19.84	4.87	14.60	9.30
GATCAACGGGGACTACGAGN	0.23	0.00	0.00	0.00	0.00
GATCAACGGGGACTACGANN	0.46	0.00	0.00	0.00	0.00
GATCAACGGGGACTACGCGG	0.23	0.00	0.00	0.00	0.00
GATCAACGGGGACTACNAGG	0.00	0.00	0.00	0.00	0.34
GATCAACGGGGACTCCGCGG	0.00	0.00	0.00	0.34	0.00
GATCAACGGGGACTGGACGC	0.23	0.00	1.72	0.00	0.00
GATCAACGGGGACTTTGCCG	0.00	0.33	0.00	0.34	0.00
GATCAACGGGGAGCACGGGT	0.23	0.00	0.00	0.00	0.00
GATCAACGGGGAGTTTGAGC	0.23	0.00	0.00	0.34	0.00
GATCAACGGGGCAACGGCCG	0.00	0.00	0.29	0.00	0.00
GATCAACGGGGCACATGATT	0.23	0.00	0.29	0.34	0.00
GATCAACGGGGCAGCCTGCT	0.00	0.00	0.00	0.00	0.34
GATCAACGGGGCAGCTCAGG	0.00	0.00	0.29	0.00	0.00
GATCAACGGGGCATGTTTCA	0.00	0.33	0.00	0.00	0.00
GATCAACGGGGCCGCGTCGG	0.23	0.00	0.00	0.00	0.00
GATCAACGGGGCCGGCCCGG	0.00	0.00	0.00	0.00	0.69
GATCAACGGGGCCTACGAGG	0.46	0.00	0.00	0.00	0.00
GATCAACGGGGCCTCAGCGG	0.23	0.00	0.00	0.00	0.00
GATCAACGGGGCCTGGGGCG	0.00	0.00	0.00	0.00	0.34
GATCAACGGGGCGCGAAAAA	0.00	0.00	0.00	0.00	0.34
GATCAACGGGGCGCTGCACC	0.00	0.00	0.00	0.00	0.00
GATCAACGGGGCGCTGTGGG	0.23	0.00	0.00	0.00	0.00
GATCAACGGGGCGGCGTGAG	0.23	0.00	0.00	0.00	0.00
GATCAACGGGGCTCATCTCA	0.00	0.00	0.29	0.00	0.00
GATCAACGGGGCTGAAGCTA	0.00	0.00	0.29	0.00	0.00
GATCAACGGGGCTGGCGACG	0.00	0.00	0.00	0.00	0.00
GATCAACGGGGCTTGGGCGA	0.00	0.00	0.29	0.00	0.00
GATCAACGGGGGACGTGACG	0.00	0.00	0.29	0.00	0.00
GATCAACGGGGGAGTGGCTC	0.23	0.00	0.00	0.00	0.00
GATCAACGGGGGCAACGAGT	0.23	0.00	0.00	0.00	0.00
GATCAACGGGGGCAACGTCG	0.00	0.00	0.00	0.68	0.34
GATCAACGGGGGCATGGTCT	0.00	0.00	0.29	0.00	0.00

GATCAACGGGGGCCAGAAGT	0.00	0.00	0.00	0.00	0.00
GATCAACGGGGGCCGCGCGA	0.00	0.33	0.00	0.00	0.00
GATCAACGGGGGCCTGCTCT	0.23	0.00	0.00	0.34	0.00
GATCAACGGGGGCGCCGCGG	0.23	0.00	0.00	0.00	0.00
GATCAACGGGGGCTACGAGG	0.69	0.00	0.00	0.00	0.00
GATCAACGGGGGCTGCGGCT	0.23	0.00	0.00	0.00	0.00
GATCAACGGGGGCTTGGTTG	0.00	0.00	0.00	0.00	0.00
GATCAACGGGGGGACCATTC	0.00	0.33	0.00	0.00	0.00
GATCAACGGGGGGACCTTGA	0.00	0.00	0.29	0.00	0.00
GATCAACGGGGGGAGCCGGA	0.00	0.00	0.29	0.68	0.00
GATCAACGGGGGGAGCCGGC	0.00	0.33	0.00	0.00	0.00
GATCAACGGGGGGCAGCAGG	0.23	0.00	0.00	0.00	0.00
GATCAACGGGGGGCCCAGAC	0.00	0.33	0.00	0.00	0.00
GATCAACGGGGGGCCCAGCA	0.00	0.00	0.00	0.00	0.00
GATCAACGGGGGGCCCAGCC	3.20	13.01	2.29	9.84	11.02
GATCAACGGGGGGCCCAGTC	0.00	0.00	0.00	0.00	0.34
GATCAACGGGGGGCCCCGCC	0.46	0.00	0.00	0.00	0.00
GATCAACGGGGGGCGCCGCA	0.00	0.00	0.00	0.34	0.00
GATCAACGGGGGGGACGTTT	0.00	0.00	0.29	0.00	0.00
GATCAACGGGGGGTACTTGT	0.23	0.00	0.00	0.00	0.00
GATCAACGGGGGGTCCTCTC	0.00	0.00	0.00	0.00	0.34
GATCAACGGGGGGTCCTGGA	0.23	0.00	0.00	0.00	0.00
GATCAACGGGGGGTTCTGGT	0.23	0.00	0.00	0.00	0.00
GATCAACGGGGGTCGTGCGG	0.00	0.33	0.00	0.00	0.00
GATCAACGGGGGTGGCAACC	0.00	0.00	0.29	0.00	0.00
GATCAACGGGGGTTCTTGTT	0.46	2.60	0.29	1.70	0.34
GATCAACGGGGTCAAGCTGC	0.00	0.33	0.00	0.00	0.00
GATCAACGGGGTCTTTTGTA	0.00	0.00	0.00	0.00	0.00
GATCAACGGGGTGGACATAG	0.00	0.00	0.00	0.00	0.00
GATCAACGGGGTGGCCGAGC	0.00	0.00	0.00	0.00	0.00
GATCAACGGGGTTGGCAACC	0.23	0.00	0.29	0.34	0.00
GATCAACGGGTAAAAAAAAA	0.00	0.00	0.29	0.00	0.00
GATCAACGGGTACCACACTC	0.23	0.00	0.00	0.00	0.00
GATCAACGGGTAGTGCTGAC	0.00	0.00	0.00	0.68	0.00
GATCAACGGGTAGTGCTGTA	0.00	0.33	0.00	0.00	0.00
GATCAACGGGTATCGCGAGG	0.00	0.00	0.00	0.34	0.00
GATCAACGGGTATTGGCATT	0.00	0.00	0.29	0.00	0.00

GATCAACGGGTCAGTGTCCG	0.00	0.00	0.29	0.00	0.00
GATCAACGGGTCATTACAGG	0.00	0.33	0.00	0.34	0.69
GATCAACGGGTCGCATGACC	0.00	0.00	0.00	0.00	0.00
GATCAACGGGTCGTGGTTGC	0.23	0.00	0.00	0.00	0.00
GATCAACGGGTCTGAGAGCG	0.00	0.00	0.00	0.34	0.00
GATCAACGGGTCTTTCTGAT	0.00	0.00	0.29	0.00	0.00
GATCAACGGGTGAGGGGCCT	0.46	0.00	0.00	0.34	0.00
GATCAACGGGTGATGGTTCC	0.46	0.65	0.57	1.36	0.69
GATCAACGGGTGCATACCTG	0.00	0.00	0.00	0.00	0.34
GATCAACGGGTGCATCCGTG	0.23	0.00	0.00	0.00	0.00
GATCAACGGGTGCATCTCGG	0.46	0.00	0.00	0.00	0.00
GATCAACGGGTGCGGAACGC	0.00	0.00	0.00	0.00	0.34
GATCAACGGGTGCTCCAGAT	0.00	0.00	0.00	0.34	0.00
GATCAACGGGTGGCCCGCTG	0.00	0.00	0.29	0.00	0.00
GATCAACGGGTGGCCCTGGG	0.69	0.00	0.00	0.00	0.00
GATCAACGGGTGGGGGTAGC	0.23	0.00	0.00	0.00	0.00
GATCAACGGGTGGTACTCTC	0.00	0.00	0.00	0.34	0.00
GATCAACGGGTGGTCATCTC	0.00	0.00	0.29	0.00	0.00
GATCAACGGGTGGTCATGAG	0.23	0.00	0.00	0.00	0.00
GATCAACGGGTGGTCCTCTC	4.12	8.78	5.73	7.47	11.71
GATCAACGGGTGGTCCTCTT	0.00	0.00	0.00	0.68	0.00
GATCAACGGGTGGTCCTGGA	0.00	0.33	0.00	0.00	0.00
GATCAACGGGTGGTCCTGTC	0.00	0.00	0.00	0.34	0.00
GATCAACGGGTGGTGCAAAA	0.00	0.00	0.00	0.34	0.00
GATCAACGGGTGGTGCATCG	0.00	0.33	0.29	0.00	0.00
GATCAACGGGTGGTGCCGAG	0.00	0.00	0.00	0.00	0.34
GATCAACGGGTGTAAGAGGG	0.23	0.00	0.00	0.00	0.00
GATCAACGGGTGTTCCTGGA	0.00	0.00	0.00	0.34	0.34
GATCAACGGGTTCGCGAACA	0.46	0.00	0.00	0.34	0.00
GATCAACGGGTTCTTCACTG	0.00	0.98	0.29	0.34	0.69
GATCAACGGGTTGAGCGCAA	0.00	0.33	0.00	0.00	0.00
GATCAACGGGTTGCCGGATT	0.00	0.00	0.57	0.00	0.00
GATCAACGGGTTGGCATGAG	0.23	0.00	0.00	0.00	0.00
GATCAACGGGTTGTCANGAG	0.00	0.00	0.00	0.00	0.34
GATCAACGGGTTGTCATGAG	23.35	22.12	47.82	40.05	49.94
GATCAACGGGTTGTCATGCG	0.23	0.00	0.29	0.00	0.00
GATCAACGGGTTGTCATGGG	0.00	0.00	0.29	0.00	0.00

GATCAACGGGTTGTCATGNG	0.00	0.00	0.00	0.34	0.00
GATCAACGGGTTGTCATGNN	0.00	0.00	0.00	0.34	0.00
GATCAACGGGTTGTCATNNN	0.00	0.00	0.00	0.00	0.34
GATCAACGGGTTGTCCTGAG	0.23	0.00	0.00	0.00	0.00
GATCAACGGGTTGTCCTGCG	0.00	0.33	0.00	0.00	0.00
GATCAACGGGTTGTGATGAG	0.00	0.00	0.29	0.00	0.00
GATCAACGGGTTGTGTNTGG	0.00	0.00	0.29	0.00	0.00
GATCAACGGGTTTGCGAACA	0.00	0.65	0.57	0.00	1.03
GATCAACGGTAATCTTTTGC	0.00	0.00	0.00	0.00	0.00
GATCAACGGTAATGGCGACA	0.00	0.00	0.00	0.00	0.34
GATCAACGGTACCCGAGCTG	0.00	0.33	0.00	0.00	0.00
GATCAACGGTAGCTGACAAC	0.23	0.00	0.00	0.00	0.00
GATCAACGGTAGCTGACTTT	0.69	0.00	0.00	0.00	0.00
GATCAACGGTAGGAAAAAAA	0.00	0.00	0.00	0.00	0.00
GATCAACGGTAGTACGAGAC	0.00	0.00	0.57	0.00	0.00
GATCAACGGTAGTATTCGCA	0.00	0.98	0.29	0.00	0.69
GATCAACGGTATCAACAGCG	0.00	0.00	0.00	0.34	0.00
GATCAACGGTATCACGTGCC	0.23	0.00	0.00	0.00	0.00
GATCAACGGTATCCCGGTGG	0.00	0.00	0.29	0.00	0.00
GATCAACGGTATCTACTCGC	0.00	0.33	0.00	0.00	0.00
GATCAACGGTATCTACTTGC	0.00	0.00	0.00	0.68	0.69
GATCAACGGTATGCCGTCCC	0.00	0.33	0.00	0.00	0.00
GATCAACGGTATGTCGGGTG	0.00	0.00	0.00	0.00	0.34
GATCAACGGTATGTGGGGGG	0.00	0.00	0.00	0.00	0.00
GATCAACGGTCAAAAAAAAT	0.23	0.00	0.00	0.00	0.00
GATCAACGGTCAATCATTCT	0.00	0.00	0.00	0.00	1.03
GATCAACGGTCACAACCTAC	0.00	0.00	0.00	0.00	1.03
GATCAACGGTCAGAAAGCCT	0.46	0.00	0.00	0.34	0.00
GATCAACGGTCATGGAAGAC	0.00	0.00	0.29	0.00	0.00
GATCAACGGTCCAATCTGTG	0.00	0.00	0.00	0.34	0.00
GATCAACGGTCCCGACGCTT	0.00	0.00	0.00	0.68	0.34
GATCAACGGTCCCGTGAATA	0.46	0.00	0.00	0.68	0.00
GATCAACGGTCCGCGTAAGA	0.00	0.00	0.00	0.00	0.34
GATCAACGGTCCTCGGAATG	0.23	0.00	0.00	0.34	0.00
GATCAACGGTCGAGGCGGCT	0.00	0.00	0.00	0.00	0.00
GATCAACGGTCGCGGAAGGC	0.23	0.00	0.00	0.00	0.00
GATCAACGGTCGCTAGCAAA	0.23	0.00	0.00	0.00	0.00

GATCAACGGTCGCTGGAGGA	0.00	0.00	0.00	0.00	0.00
GATCAACGGTCGGCACCCTC	0.00	0.00	0.00	0.00	0.00
GATCAACGGTCGGGCAAGTA	0.00	0.00	0.29	0.00	0.00
GATCAACGGTCGTGCTGTTT	0.00	0.00	0.00	0.34	0.00
GATCAACGGTCGTGGCGGCG	0.00	0.00	0.00	0.00	0.00
GATCAACGGTCTCGACGAAC	0.00	0.33	0.00	0.00	0.00
GATCAACGGTCTCTTCTTGT	0.00	0.33	0.00	0.00	0.00
GATCAACGGTCTGACTCTCG	0.00	0.00	0.00	0.00	0.00
GATCAACGGTCTGAGCTTCG	0.23	0.00	0.00	0.00	0.00
GATCAACGGTCTGCAAGGCA	0.46	0.33	0.00	0.34	0.00
GATCAACGGTCTGCAATGAG	0.46	0.00	0.00	0.00	0.00
GATCAACGGTCTGCAGACCT	0.00	0.00	0.00	0.00	0.00
GATCAACGGTGAATACGGTG	0.00	0.00	0.00	0.00	0.00
GATCAACGGTGAATGCAATG	0.00	0.00	0.00	0.00	0.34
GATCAACGGTGACAAGGGGC	0.00	0.33	0.00	3.39	0.34
GATCAACGGTGACAGAGTCT	0.00	0.00	0.00	0.00	0.00
GATCAACGGTGACAGCCGCC	0.00	0.65	0.00	0.34	0.34
GATCAACGGTGACAGCTCGG	0.00	0.00	0.00	0.00	0.00
GATCAACGGTGAGATGAATG	0.00	0.00	0.00	0.00	0.00
GATCAACGGTGAGATGACCT	0.00	0.33	0.00	0.00	0.00
GATCAACGGTGAGGCAGTGC	0.00	0.00	0.00	0.00	0.00
GATCAACGGTGAGTAGCGCG	0.00	0.00	0.00	0.34	0.00
GATCAACGGTGAGTGGTGGT	0.00	0.00	0.00	0.34	0.00
GATCAACGGTGCAAACAAAA	0.00	0.00	0.00	0.00	0.00
GATCAACGGTGCAGAAGAAA	0.46	0.33	0.00	1.02	0.00
GATCAACGGTGCAGCTGGCC	0.00	0.33	0.00	0.00	0.00
GATCAACGGTGCAGGACTTC	0.00	0.33	0.00	0.00	0.00
GATCAACGGTGCAGGCCACG	0.00	0.00	0.00	0.34	0.00
GATCAACGGTGCAGGGCCAC	0.23	0.65	0.00	0.00	0.00
GATCAACGGTGCCATGCGTT	0.00	0.33	0.00	0.00	0.00
GATCAACGGTGCCATTGCAG	0.00	0.00	0.00	0.00	0.00
GATCAACGGTGCCCTGGGGA	0.00	0.00	0.00	0.00	0.34
GATCAACGGTGCCGACGTTT	0.00	0.00	0.00	0.00	0.34
GATCAACGGTGCCGCCGAAA	0.00	0.65	0.00	0.00	0.00
GATCAACGGTGCCGCCGAGC	0.00	0.65	0.29	0.00	0.00
GATCAACGGTGCCTGGGCCT	0.00	0.00	0.00	0.00	0.00
GATCAACGGTGCTCACACAT	0.46	0.00	0.29	0.00	0.00

GATCAACGGTGCTGAAACAG	0.00	0.33	0.00	0.00	0.00
GATCAACGGTGCTGAACCAG	0.23	0.00	0.57	0.34	0.00
GATCAACGGTGCTGATAATG	0.00	0.00	0.00	0.00	0.69
GATCAACGGTGCTGCTGACC	0.23	0.00	0.00	0.00	0.00
GATCAACGGTGCTGTCATCC	0.00	0.00	0.00	0.34	0.00
GATCAACGGTGCTTCCTCAC	0.00	0.33	0.00	0.00	0.00
GATCAACGGTGCTTTCGACG	0.46	0.33	0.57	0.00	0.00
GATCAACGGTGCTTTGCGAA	1.60	0.98	0.00	0.34	0.34
GATCAACGGTGGAAGAAGAG	0.46	0.00	0.00	0.00	0.00
GATCAACGGTGGAAGCNNNN	0.23	0.00	0.00	0.00	0.00
GATCAACGGTGGACGAAGAG	0.23	0.00	0.00	0.00	0.00
GATCAACGGTGGAGCCAAGA	0.46	0.00	0.00	0.00	0.00
GATCAACGGTGGAGGAAAGG	0.23	0.00	0.00	0.00	0.00
GATCAACGGTGGCAATTTNN	0.23	0.00	0.00	0.00	0.00
GATCAACGGTGGCAGGAAGC	0.23	0.00	0.00	0.00	0.00
GATCAACGGTGGCAGGCGCT	0.00	0.00	0.00	0.00	0.00
GATCAACGGTGGCATCAAGT	0.00	0.00	0.00	0.00	0.00
GATCAACGGTGGCCTTCTTT	0.00	0.00	0.00	0.00	0.34
GATCAACGGTGGCGGCCTGG	0.00	0.00	0.00	0.00	0.00
GATCAACGGTGGCTCTACAC	0.00	0.00	0.00	0.00	0.00
GATCAACGGTGGGAGGGCGA	0.00	0.65	0.00	0.00	0.00
GATCAACGGTGGGCGCTTGT	0.23	0.00	0.00	0.00	0.00
GATCAACGGTGGGGGCCACC	0.00	0.33	0.00	0.00	0.00
GATCAACGGTGGTATCTCAA	0.00	0.00	0.00	0.00	0.00
GATCAACGGTGGTGGACGCG	0.23	0.00	0.00	0.00	0.00
GATCAACGGTGGTTTTGTCT	0.23	0.98	1.72	0.00	0.34
GATCAACGGTGGTTTTTTCT	0.00	0.00	0.00	0.00	0.34
GATCAACGGTGTAAAAAAAA	0.46	0.00	0.00	0.00	0.00
GATCAACGGTGTAATCTTAA	0.00	0.00	0.00	0.00	0.00
GATCAACGGTGTAATCTTTT	3.20	1.63	0.86	0.00	2.41
GATCAACGGTGTAGAAGCGT	0.00	0.00	0.29	0.00	0.00
GATCAACGGTGTATCCTCTT	0.23	0.00	0.00	0.00	0.00
GATCAACGGTGTCACGGCAA	0.23	0.00	0.00	0.00	0.00
GATCAACGGTGTCAGGACGC	0.23	0.00	0.00	0.00	0.00
GATCAACGGTGTCCATGCCT	0.00	0.00	0.00	0.00	0.00
GATCAACGGTGTCCCGGACA	0.00	0.00	0.29	0.00	0.00
GATCAACGGTGTCCCGGACN	0.23	0.00	0.00	0.00	0.00

GATCAACGGTGTCCCGGCAA	27.70	16.92	6.01	9.50	5.17
GATCAACGGTGTCCCGGCCA	0.23	0.00	0.00	0.00	0.00
GATCAACGGTGTCCCGGCGA	0.23	0.00	0.00	0.00	0.00
GATCAACGGTGTGACACCGC	0.23	0.00	0.00	0.00	0.00
GATCAACGGTGTGACTAGGG	0.00	0.33	0.00	0.00	0.00
GATCAACGGTGTGCCAGTCT	0.00	0.33	0.00	0.00	0.00
GATCAACGGTGTGGACTTGG	0.69	1.63	0.00	0.68	0.00
GATCAACGGTGTGGCTGGAC	0.00	0.33	0.00	0.00	0.00
GATCAACGGTGTGGCTGGGC	0.23	0.00	0.00	0.00	0.00
GATCAACGGTGTGGCTGGGG	0.00	0.33	0.00	0.00	0.00
GATCAACGGTGTGGGACCAG	0.00	0.33	0.00	0.34	0.34
GATCAACGGTGTGTATTCCT	0.23	0.00	0.00	0.00	0.00
GATCAACGGTGTGTGCCCGT	0.00	0.00	0.00	0.00	0.00
GATCAACGGTGTGTGGCCAG	0.00	0.00	0.00	0.34	0.34
GATCAACGGTTATGATAAAA	0.00	0.00	0.57	0.00	0.00
GATCAACGGTTCAAGACCTC	0.00	0.00	0.29	0.00	0.00
GATCAACGGTTCATGTCTGC	0.00	0.98	0.29	0.00	0.00
GATCAACGGTTCCAAGTACA	0.00	0.00	0.00	0.34	0.00
GATCAACGGTTCCAGTGACG	0.00	0.00	0.00	0.00	0.00
GATCAACGGTTCCGGGGTAA	0.00	0.00	0.29	0.00	0.00
GATCAACGGTTCGTATGCCG	0.00	0.00	0.00	0.00	0.34
GATCAACGGTTGATTACACG	0.23	0.00	0.00	0.00	0.00
GATCAACGGTTGCAAGTGCT	0.00	0.00	0.29	0.00	0.00
GATCAACGGTTGCGTATTTG	0.00	0.00	0.00	0.00	0.00
GATCAACGGTTGCTGGAGGA	4.58	15.62	11.74	14.93	8.61
GATCAACGGTTGCTGGAGGC	0.00	0.00	0.29	0.34	0.00
GATCAACGGTTGCTGGAGGG	0.46	0.00	0.29	0.00	0.00
GATCAACGGTTGCTGGAGGT	0.23	0.00	0.00	0.00	0.00
GATCAACGGTTGCTGGAGTA	0.00	0.00	0.29	0.00	0.00
GATCAACGGTTGCTGGATGA	0.00	0.00	0.00	0.00	0.34
GATCAACGGTTGCTGGCGGA	0.00	0.00	0.00	0.34	0.00
GATCAACGGTTGCTGGGGGA	0.00	0.33	0.00	0.00	0.00
GATCAACGGTTGGACATGGG	0.23	0.00	0.00	0.00	0.00
GATCAACGGTTGGGCGGCGG	0.23	0.00	0.00	0.00	0.00
GATCAACGGTTGGGGCAAGA	0.00	0.00	0.29	0.00	0.00
GATCAACGGTTGTATCAGAT	0.00	0.00	0.00	0.00	0.00
GATCAACGGTTGTGCTAAAA	0.00	0.00	0.00	0.00	0.69

GATCAACGGTTGTGCTTGCA	0.23	0.00	0.29	0.68	0.00
GATCAACGGTTGTGCTTGCG	0.69	0.00	0.00	0.00	0.69
GATCAACGGTTGTTCCTGGA	0.23	0.00	0.00	0.00	0.00
GATCAACGGTTTCCCCGTGC	0.00	0.00	0.00	0.00	0.00
GATCAACGGTTTCCTGAGAA	0.00	0.33	0.00	0.00	0.00
GATCAACGGTTTCGAAGCGG	0.23	0.00	0.00	0.00	0.00
GATCAACGGTTTCTCCATCA	0.46	0.65	0.29	0.68	0.00
GATCAACGGTTTCTCCATCG	1.37	2.28	0.29	0.34	0.69
GATCAACGGTTTTCCGACAG	0.00	0.00	0.29	0.00	0.00
GATCAACGGTTTTGCCGCCT	0.23	0.00	0.00	0.00	0.00
GATCAACGTAAAAAAAAAAA	0.00	0.00	0.00	0.34	0.00
GATCAACGTAAACAATGCAC	0.00	0.00	0.00	0.00	0.00
GATCAACGTAAAGGCATACA	0.23	0.65	0.00	0.00	0.00
GATCAACGTAAAGGCATTGC	0.00	0.00	0.00	0.34	0.00
GATCAACGTAACAATGCTAT	0.00	0.00	0.00	0.00	0.00
GATCAACGTAACACAGCTTC	0.00	0.00	0.00	0.34	0.00
GATCAACGTAACCGCCAGCG	0.00	0.00	0.29	0.34	0.00
GATCAACGTAACTGCTCTGA	0.00	0.00	0.00	0.00	0.00
GATCAACGTAAGAAAAAAAA	0.23	0.00	0.00	0.00	0.00
GATCAACGTAAGCGCAGCTA	0.00	0.00	0.00	0.00	0.00
GATCAACGTAAGCGCAGCTG	0.00	0.00	0.00	0.00	0.00
GATCAACGTAAGGCTTCAGG	0.00	0.00	0.57	0.00	0.00
GATCAACGTAAGTGTTCCGT	0.00	0.00	0.29	0.00	0.00
GATCAACGTAAGTTTTAGAT	0.00	0.00	0.00	0.00	0.00
GATCAACGTAATACTGTGTA	0.00	0.00	0.00	0.34	0.00
GATCAACGTAATATCACTGA	0.00	0.00	0.29	0.00	0.00
GATCAACGTACAGATGCGAG	0.00	0.00	0.00	0.00	0.00
GATCAACGTACAGCAAAGCT	0.23	0.00	0.00	0.00	0.00
GATCAACGTACCAAAGGACA	0.00	0.00	0.29	0.00	0.00
GATCAACGTACCCGCCCTCG	0.00	0.00	0.00	0.00	0.00
GATCAACGTACCGCAGAGCA	0.00	0.00	0.57	0.00	0.34
GATCAACGTACGCCCGCACC	0.23	0.00	0.00	0.00	0.00
GATCAACGTACGGCGGAGCC	0.23	0.00	0.00	0.00	0.00
GATCAACGTACGGCGGCGCC	0.23	0.00	0.00	0.34	0.00
GATCAACGTACTCGTCAACG	0.00	0.00	0.00	0.00	0.00
GATCAACGTACTTGCCACCG	0.46	0.00	0.00	0.00	0.00
GATCAACGTAGAATCAAAAC	0.23	0.00	0.00	0.00	0.00

GATCAACGTAGACTGCGACT	0.00	0.33	0.57	0.34	0.34
GATCAACGTAGAGCTCAAGG	0.00	0.00	0.29	1.02	1.38
GATCAACGTAGAGGAAGAGG	0.00	0.00	0.86	0.00	0.00
GATCAACGTAGATGGCCGTG	0.00	0.00	0.00	0.00	0.00
GATCAACGTAGCAGTGGCTC	0.00	0.33	0.00	0.00	0.00
GATCAACGTAGCCACAATGG	0.00	0.00	0.00	0.00	0.34
GATCAACGTAGGGCAGGGGG	0.00	0.00	0.00	0.00	0.34
GATCAACGTAGGGGACGAGC	0.00	0.00	0.00	0.00	0.00
GATCAACGTAGGGTTCCAGA	0.00	0.00	0.00	0.34	0.00
GATCAACGTAGGTTCCACTA	0.23	0.00	0.00	0.00	0.00
GATCAACGTAGTTGATGATA	0.00	0.33	0.00	0.00	0.00
GATCAACGTAGTTTTCCAGC	0.00	0.00	0.00	0.00	0.00
GATCAACGTATCAGCACTTT	0.00	0.00	0.00	0.00	0.34
GATCAACGTATCGATGGATT	0.00	0.00	0.00	0.34	0.00
GATCAACGTATCTGGTTTCA	0.23	0.00	0.00	0.00	0.00
GATCAACGTATGAAGTTCAT	0.69	0.00	0.00	0.00	0.00
GATCAACGTATGACCGCTGA	0.00	0.33	0.00	0.00	0.00
GATCAACGTATGCAAGAAAG	0.00	0.00	0.00	0.00	0.00
GATCAACGTATGCCCACCCT	0.00	0.00	0.00	0.00	0.34
GATCAACGTATGCTTCACTC	0.00	0.00	0.00	0.00	0.00
GATCAACGTATGGCCTCCTC	0.00	0.00	0.29	0.00	0.00
GATCAACGTATGGTGGATGC	0.00	0.33	0.00	0.00	0.00
GATCAACGTATGTACGCTGA	0.23	0.00	0.00	0.00	0.00
GATCAACGTATGTCCATGAA	0.00	0.00	0.29	0.00	0.00
GATCAACGTATGTCCGCAAA	0.00	0.33	0.00	0.00	0.00
GATCAACGTATGTCCGCCAA	0.00	0.00	0.29	0.00	0.00
GATCAACGTATGTCCGCTAA	0.00	0.00	0.29	0.00	0.00
GATCAACGTATGTCCGCTCA	0.00	0.33	0.00	0.00	0.00
GATCAACGTATGTCCGCTGA	18.31	28.30	28.92	38.36	31.00
GATCAACGTATGTCCGCTGC	0.46	0.33	0.00	0.34	0.00
GATCAACGTATGTCCGCTGG	0.46	0.33	0.29	0.00	0.00
GATCAACGTATGTCCGCTGT	0.00	0.33	0.00	0.00	0.00
GATCAACGTATGTCCGCTNA	0.00	0.00	0.00	0.00	0.00
GATCAACGTATGTCCGCTTA	0.00	0.00	0.00	0.00	0.00
GATCAACGTATGTCCGGTGG	0.23	0.00	0.00	0.00	0.00
GATCAACGTATGTTCCGTGA	0.00	0.00	0.00	0.00	0.34
GATCAACGTATTAAAAAAAA	0.00	0.33	0.00	0.00	0.00

GATCAACGTATTCGTGGTAT	0.00	0.00	0.00	1.02	0.00
GATCAACGTATTGCTCTGAC	0.00	0.00	0.00	0.34	0.00
GATCAACGTATTTCGGATGA	0.00	0.00	0.29	0.00	0.00
GATCAACGTATTTGGAGAGG	0.00	0.00	0.00	0.00	0.00
GATCAACGTATTTTTCAAGC	0.00	0.00	0.00	0.00	0.34
GATCAACGTATTTTTCCAGC	0.92	1.63	0.57	2.38	0.34
GATCAACGTCAAAAAAAAAA	0.00	0.00	0.29	0.00	0.34
GATCAACGTCAAGGAGAAGG	0.00	0.00	0.00	0.34	0.00
GATCAACGTCAAGGCAGAGG	0.00	0.33	0.00	0.34	0.00
GATCAACGTCAAGGTTTCCT	0.00	0.33	0.00	0.00	0.00
GATCAACGTCAAGTACAAGG	1.60	0.00	0.00	0.00	0.00
GATCAACGTCAAGTTCCTCG	0.23	0.00	0.00	0.00	0.00
GATCAACGTCACAAACTTCC	0.00	0.00	0.00	0.00	0.34
GATCAACGTCACAACTCTCA	0.00	0.00	0.29	0.00	0.00
GATCAACGTCACAACTCTGA	0.46	0.00	0.00	0.00	0.00
GATCAACGTCACAATCCAGC	0.00	0.33	0.00	0.00	0.00
GATCAACGTCACAGGATGCG	0.00	0.00	0.00	0.34	0.00
GATCAACGTCACCATGCTCT	0.00	0.00	0.00	0.34	0.00
GATCAACGTCACCCAGTGCA	0.46	0.00	0.00	0.00	0.00
GATCAACGTCACCGCGTGCC	0.00	0.00	0.57	0.00	0.00
GATCAACGTCACCGGCCACC	0.00	0.00	0.00	0.00	0.69
GATCAACGTCACCTCATACG	0.00	0.00	0.00	0.68	0.00
GATCAACGTCACCTCCTACG	0.00	0.00	0.00	0.00	0.00
GATCAACGTCACCTCGTACG	0.00	0.00	0.29	0.00	0.00
GATCAACGTCACCTTCAAGC	0.00	0.00	0.29	0.00	0.00
GATCAACGTCACGAAGAAGC	0.46	0.65	0.86	0.68	0.34
GATCAACGTCACGACCTACG	0.00	0.00	0.00	0.00	0.00
GATCAACGTCACGATTGTGT	0.00	0.00	0.29	0.00	0.00
GATCAACGTCACGCTGCCCG	0.00	0.00	0.00	0.34	0.00
GATCAACGTCACGGACACCA	0.00	0.00	0.00	0.00	0.00
GATCAACGTCACGGACGCAA	0.00	0.00	0.00	0.00	0.00
GATCAACGTCACGGACGCCA	0.46	2.93	2.00	2.38	1.72
GATCAACGTCACGGACGCCC	0.00	0.00	0.00	0.34	0.00
GATCAACGTCACGGACGCCG	0.00	0.00	0.00	0.00	0.00
GATCAACGTCACGGACGTCA	0.00	0.65	0.00	0.00	0.00
GATCAACGTCACGGGAGATG	0.00	0.00	0.00	0.00	0.00
GATCAACGTCACGGGATGCG	0.23	0.00	0.00	0.00	0.00

GATCAACGTCACGGGCGGCG	0.00	0.00	0.00	0.34	0.00
GATCAACGTCACGGTGCTGA	0.00	0.00	0.00	0.00	0.00
GATCAACGTCACGTCGTACG	0.00	0.00	0.00	0.00	0.00
GATCAACGTCACTCAAAATT	0.00	0.00	0.00	0.00	0.34
GATCAACGTCACTGGANGCG	0.00	0.00	0.00	0.00	0.34
GATCAACGTCACTGGATCTG	0.00	0.00	0.00	0.00	0.00
GATCAACGTCACTGGATGAG	0.00	0.65	0.00	0.00	0.00
GATCAACGTCACTGGATGCG	33.42	45.22	22.62	34.96	19.63
GATCAACGTCACTGGATGCN	0.00	0.00	0.00	0.00	0.00
GATCAACGTCACTGGATGGG	0.00	0.33	0.00	0.00	0.00
GATCAACGTCACTGGATGGT	0.00	0.00	0.00	0.00	0.00
GATCAACGTCACTGGATNNG	0.00	0.00	0.00	0.00	0.00
GATCAACGTCACTGGATTCG	0.23	0.00	0.00	0.00	0.00
GATCAACGTCACTGGCAGTG	0.00	0.00	0.29	0.00	0.00
GATCAACGTCACTGGGTGCG	0.00	0.00	0.00	0.34	0.00
GATCAACGTCACTGGTCGTA	0.23	0.00	0.00	0.00	0.00
GATCAACGTCACTTGAGACC	0.00	0.00	0.00	0.00	0.34
GATCAACGTCAGAGACATTA	0.00	0.00	0.00	0.00	0.00
GATCAACGTCAGAGAGCTGC	0.46	0.98	0.00	1.02	0.34
GATCAACGTCAGAGTCGTCA	0.00	0.00	0.00	0.00	0.34
GATCAACGTCAGAGTCGTTA	0.00	0.00	0.00	0.00	0.34
GATCAACGTCAGATACAAAA	0.00	0.00	0.00	0.00	0.00
GATCAACGTCAGCAAGAACA	0.23	0.00	0.00	0.00	0.00
GATCAACGTCAGCACCGCCT	0.46	0.00	0.00	0.00	0.34
GATCAACGTCAGCATGAAAG	0.00	0.00	0.00	0.00	0.00
GATCAACGTCAGCATGAACG	0.00	0.00	0.00	0.00	0.00
GATCAACGTCAGCATGAATG	0.23	0.33	0.00	0.68	0.00
GATCAACGTCAGCATGAATT	0.00	0.00	0.86	0.00	0.00
GATCAACGTCAGCATGNCNN	0.23	0.00	0.00	0.00	0.00
GATCAACGTCAGCCATCGGA	0.69	0.00	0.00	0.00	0.00
GATCAACGTCAGCCCCCTTG	0.23	0.00	0.00	0.00	0.00
GATCAACGTCAGCCTGCTGC	0.00	0.33	0.00	0.00	0.00
GATCAACGTCAGGCAGGTCG	0.00	0.00	0.00	0.00	0.00
GATCAACGTCAGGCTGCATG	0.00	0.00	0.00	0.34	0.00
GATCAACGTCAGGCTGGACA	0.23	0.00	0.29	0.00	0.00
GATCAACGTCAGGGCAGCCG	0.23	0.00	0.00	0.00	0.00
GATCAACGTCAGGTACAAGG	0.23	0.00	0.00	0.00	0.00

GATCAACGTCAGTGCAAGGG	0.00	0.00	0.00	0.00	0.00
GATCAACGTCATACCTGCTG	0.00	0.00	0.00	0.00	0.34
GATCAACGTCATCAAGGGCA	0.00	0.00	0.29	0.00	0.00
GATCAACGTCATCAATTTAG	0.00	0.00	0.00	0.00	0.00
GATCAACGTCATCACGGGCA	0.00	0.00	0.00	0.34	0.00
GATCAACGTCATCACGTGCA	0.00	0.00	0.00	0.34	0.69
GATCAACGTCATCATTGTTC	0.00	0.00	0.00	0.00	0.34
GATCAACGTCATCTGACGGG	0.00	0.00	0.00	0.00	0.00
GATCAACGTCATGAATGTGT	0.00	0.00	0.00	0.00	0.00
GATCAACGTCATGACGCACC	0.23	0.00	0.00	0.34	0.00
GATCAACGTCATGATTGGGT	0.00	0.33	0.00	0.00	0.00
GATCAACGTCATGATTGGTT	0.00	0.00	0.00	0.34	0.00
GATCAACGTCATGATTGTGN	0.46	0.00	0.00	0.00	0.00
GATCAACGTCATGATTGTGT	11.67	10.41	12.89	9.16	10.33
GATCAACGTCATGCTCATCA	0.00	0.00	0.29	0.00	0.00
GATCAACGTCATGGGCGTCA	0.92	0.00	0.00	0.00	0.00
GATCAACGTCATTGTCTTGA	0.00	0.98	0.00	0.00	0.00
GATCAACGTCCAAAAAAAAA	0.23	0.00	0.00	0.00	0.00
GATCAACGTCCAAAATGAAA	0.00	0.33	0.00	0.00	0.00
GATCAACGTCCAATGCGCCT	0.00	0.00	0.00	0.00	0.34
GATCAACGTCCACAACACCG	0.23	0.00	0.00	0.00	0.00
GATCAACGTCCACAAGACTG	0.00	0.00	0.00	0.00	0.00
GATCAACGTCCACTCCAACC	0.23	0.00	0.57	0.00	0.00
GATCAACGTCCACTGCCGCG	0.00	0.33	0.00	0.00	0.00
GATCAACGTCCAGATGGCAC	0.23	0.00	0.00	0.00	0.00
GATCAACGTCCAGCGACCAG	0.00	0.00	0.29	0.00	0.00
GATCAACGTCCAGCGGACGA	0.00	0.33	0.00	0.00	0.00
GATCAACGTCCAGGACATCC	0.00	0.00	0.00	0.00	0.34
GATCAACGTCCAGGACCATC	0.00	0.00	0.00	0.34	0.00
GATCAACGTCCAGGACTCTC	0.00	0.00	0.00	0.34	0.00
GATCAACGTCCAGGAGTTCC	0.00	0.00	0.00	0.00	0.00
GATCAACGTCCAGGTGCTCC	0.00	0.00	0.00	0.00	0.00
GATCAACGTCCATCTGCGAA	0.00	0.00	0.00	0.68	0.00
GATCAACGTCCATGGAGCTG	0.00	0.00	0.00	0.00	1.03
GATCAACGTCCATGGCGCTC	0.00	0.00	0.00	0.00	0.00
GATCAACGTCCCCAAGCCTG	0.00	0.00	0.29	0.00	0.00
GATCAACGTCCCCGCTTTGG	0.00	0.33	0.00	0.00	0.00

GATCAACGTCCCGGAGGTGG	0.46	0.00	0.00	0.00	0.00
GATCAACGTCCCGTCCAAAA	0.00	0.00	0.57	0.00	0.00
GATCAACGTCCCGTCCGCCA	0.00	0.00	0.29	0.00	0.00
GATCAACGTCCCTGGATGCG	0.00	0.33	0.00	0.00	0.00
GATCAACGTCCCTTTCCTGC	0.00	0.00	0.29	0.00	0.00
GATCAACGTCCGACCAAACC	0.00	0.00	0.00	0.00	0.00
GATCAACGTCCGCACAGGAC	0.00	0.00	0.00	0.00	0.34
GATCAACGTCCGCACAGGCA	0.00	0.00	0.00	0.34	0.00
GATCAACGTCCGCACAGGCC	7.55	3.25	1.15	2.38	0.69
GATCAACGTCCGCACGGAGA	0.00	0.00	0.29	0.00	0.00
GATCAACGTCCGCGATGCAC	0.00	0.00	0.00	0.00	0.34
GATCAACGTCCGCGATGTAC	0.00	0.33	0.00	1.36	0.34
GATCAACGTCCGCGTAGCTC	0.00	0.00	0.29	0.00	0.00
GATCAACGTCCGCTCAAGTC	0.23	0.00	0.00	0.00	0.00
GATCAACGTCCGGACTACAC	0.23	0.33	0.29	0.68	0.69
GATCAACGTCCGGATGCGCC	0.00	0.33	0.00	0.00	0.00
GATCAACGTCCGGCCAGTCT	0.00	0.00	0.00	0.00	0.00
GATCAACGTCCGGCGCAAAG	0.00	0.00	0.00	0.00	0.00
GATCAACGTCCGGGGTTGGG	0.00	0.00	0.00	0.00	0.34
GATCAACGTCCTCACCACGG	0.00	0.00	0.29	0.00	0.00
GATCAACGTCCTCAGGGAGA	0.23	0.00	1.15	1.02	0.34
GATCAACGTCCTCTGGAATG	0.00	0.00	0.00	0.00	0.00
GATCAACGTCCTCTTCATAC	0.00	0.33	0.00	0.00	0.00
GATCAACGTCCTGAACTGCA	0.00	0.00	0.00	0.00	0.00
GATCAACGTCCTGCCGTAGC	0.00	0.00	0.00	0.00	0.34
GATCAACGTCCTGCCGTCCC	0.46	0.98	0.00	1.36	0.34
GATCAACGTCCTGCCGTCGC	1.14	1.63	0.57	0.00	0.34
GATCAACGTCCTGCCGTCGT	0.00	0.00	0.00	0.34	0.00
GATCAACGTCCTGCGTGGGC	0.00	0.00	0.00	0.34	0.00
GATCAACGTCCTGGCTGGTG	0.00	0.00	0.00	0.00	0.34
GATCAACGTCCTGGTCAAGA	0.00	0.00	0.00	0.00	0.00
GATCAACGTCCTGGTGTGGG	0.00	0.00	0.00	0.00	0.69
GATCAACGTCCTTCGGGAAT	0.00	0.00	0.00	0.00	0.34
GATCAACGTCCTTGCAGGCC	0.23	0.00	0.00	0.00	0.00
GATCAACGTCCTTGGCACGA	0.00	0.00	0.00	0.00	0.00
GATCAACGTCGACAAAACAG	0.00	0.33	0.00	0.00	0.00
GATCAACGTCGACAACAAGA	0.92	0.33	0.00	0.34	0.34

GATCAACGTCGACAAGATGG	0.00	0.33	0.00	0.00	0.00
GATCAACGTCGACAGCTCCG	0.00	0.00	0.29	0.00	0.34
GATCAACGTCGACAGCTCTG	0.00	0.00	0.29	0.00	0.00
GATCAACGTCGACCGCGTGA	1.60	0.65	0.00	0.00	1.03
GATCAACGTCGACGCGAGCT	0.00	0.00	0.00	0.34	0.00
GATCAACGTCGAGACGCTGA	0.69	0.00	0.00	0.34	0.00
GATCAACGTCGAGACGGGCA	0.00	0.00	0.00	0.00	0.34
GATCAACGTCGAGCCCAACC	1.14	1.30	0.00	0.34	0.34
GATCAACGTCGAGTGGGGCT	0.46	0.00	0.00	0.00	0.00
GATCAACGTCGATGCGATTT	1.83	0.33	0.86	0.34	0.69
GATCAACGTCGATGCGCCTC	0.00	0.00	0.00	0.00	0.34
GATCAACGTCGCAAACAAAA	3.89	7.16	4.58	2.72	3.10
GATCAACGTCGCAAACAAAG	0.00	0.00	0.00	0.00	0.00
GATCAACGTCGCAAACAACA	0.00	0.00	0.00	0.00	0.00
GATCAACGTCGCAAACACAA	0.00	0.33	0.57	0.68	0.00
GATCAACGTCGCAAACTAAA	1.37	0.98	1.72	3.39	2.41
GATCAACGTCGCAAACTANA	0.00	0.00	0.00	0.00	0.00
GATCAACGTCGCAAACTGAG	0.00	0.00	0.00	0.00	0.00
GATCAACGTCGCAACACGGC	0.00	0.00	0.29	0.00	0.00
GATCAACGTCGCAGCTCGGC	0.00	0.33	0.00	0.00	0.00
GATCAACGTCGCATACAACG	0.00	0.00	0.00	0.00	0.00
GATCAACGTCGCCGGCTCGC	0.00	0.00	0.00	0.00	0.34
GATCAACGTCGCCTCGGTGT	0.00	0.00	0.00	0.00	0.00
GATCAACGTCGCCTTCCTTT	0.00	0.65	0.00	0.00	0.00
GATCAACGTCGCCTTTTTGG	0.00	0.00	0.29	0.00	0.00
GATCAACGTCGCGCAGCACG	0.00	0.00	0.00	0.00	0.00
GATCAACGTCGCGGAGGTGC	0.00	0.00	0.00	0.00	0.00
GATCAACGTCGCGGCCCACC	0.46	0.00	0.00	0.34	0.00
GATCAACGTCGCGGCTTGCC	0.00	0.00	0.00	0.00	0.00
GATCAACGTCGCGGGCCAAT	0.23	0.00	0.00	0.00	0.00
GATCAACGTCGCGTTCATCG	0.00	0.33	0.00	0.00	0.00
GATCAACGTCGCGTTCCTCG	0.00	0.33	0.00	0.68	0.00
GATCAACGTCGGACACAAAC	0.00	0.00	0.00	0.00	0.34
GATCAACGTCGGAGGACGGA	0.00	0.00	0.00	0.00	0.00
GATCAACGTCGGCGAAATAT	0.00	0.00	0.29	0.00	0.00
GATCAACGTCGGCGAGGGCT	0.46	0.00	0.00	0.68	0.00
GATCAACGTCGGCTACGAGT	0.00	0.00	0.29	0.34	0.00

GATCAACGTCGGCTTGCTGC	0.00	0.00	0.00	0.00	0.00
GATCAACGTCGGCTTGTTTG	0.00	0.00	0.29	0.00	0.34
GATCAACGTCGGGCAATCCC	0.00	0.00	0.00	0.00	0.00
GATCAACGTCGGGCAGATCC	0.00	0.00	0.00	0.00	0.00
GATCAACGTCGGGCAGGACG	0.00	0.00	0.00	0.00	0.69
GATCAACGTCGGGCAGGATG	0.00	0.00	0.00	0.00	0.34
GATCAACGTCGGGCAGGCGT	0.00	0.00	0.00	0.00	0.00
GATCAACGTCGGGCAGGCTG	0.00	0.00	0.29	0.00	0.00
GATCAACGTCGGGCAGGGCG	0.23	0.33	0.57	0.00	0.00
GATCAACGTCGGGCAGGGTG	0.00	0.00	0.29	0.34	0.34
GATCAACGTCGGGCAGGTAG	0.00	0.33	0.00	0.00	0.34
GATCAACGTCGGGCAGGTCG	9.84	26.03	24.05	20.71	25.83
GATCAACGTCGGGCAGGTCT	0.00	0.00	0.00	0.34	0.00
GATCAACGTCGGGCAGGTGG	0.00	0.00	0.29	0.00	0.00
GATCAACGTCGGGCAGGTTG	3.89	12.04	14.32	7.47	11.37
GATCAACGTCGGGCAGGTTT	0.00	0.33	0.00	0.00	0.00
GATCAACGTCGGGCAGNTTG	0.00	0.00	0.00	0.00	0.34
GATCAACGTCGGGCGGGTCG	0.00	0.00	0.29	0.00	0.00
GATCAACGTCGGTTTGTTGC	0.23	0.00	0.00	0.00	0.00
GATCAACGTCGTCAAGGCCA	0.69	0.98	0.29	1.70	1.72
GATCAACGTCGTCAAGGCCG	0.00	0.33	0.00	0.00	0.00
GATCAACGTCGTCATTAAAA	0.00	0.00	0.00	0.00	0.34
GATCAACGTCGTCCAGCGAA	0.00	0.33	0.00	0.00	0.00
GATCAACGTCGTCCTGCCGC	0.00	0.00	0.00	0.34	0.00
GATCAACGTCGTCCTGCGGC	0.00	0.00	0.00	0.00	0.34
GATCAACGTCGTCGGGCAAG	0.00	0.00	0.00	0.34	0.00
GATCAACGTCGTCTAGCCCC	0.00	0.00	0.00	0.00	0.00
GATCAACGTCGTCTCGAACA	0.00	0.00	0.00	0.00	0.00
GATCAACGTCGTCTTTTGGA	0.46	0.00	0.57	0.00	0.00
GATCAACGTCGTTCAGACTC	0.23	0.00	0.00	0.00	0.00
GATCAACGTCTAAAAAAAAA	0.46	0.00	0.00	0.00	0.00
GATCAACGTCTAAACGCATC	0.00	0.00	0.57	0.00	0.00
GATCAACGTCTACACCGATG	0.46	0.98	0.00	0.68	0.34
GATCAACGTCTAGAGCTGGG	0.00	0.00	0.00	0.68	0.00
GATCAACGTCTATGGCAGGA	0.00	0.00	0.00	0.00	0.00
GATCAACGTCTCACGCAAAC	0.00	0.00	0.00	0.00	0.00
GATCAACGTCTCCATGCGAC	0.00	0.00	0.00	0.00	0.00

GATCAACGTCTCCATGGAAA	0.00	0.00	0.00	0.00	0.00
GATCAACGTCTCCATGGAAC	0.23	0.65	0.00	0.00	0.00
GATCAACGTCTCCATGGACC	30.90	28.95	9.74	21.04	14.12
GATCAACGTCTCCATGGACN	0.23	0.00	0.00	0.00	0.00
GATCAACGTCTCCATGGACT	0.69	0.00	0.00	0.34	0.00
GATCAACGTCTCCATGGANN	0.23	0.00	0.00	0.00	0.00
GATCAACGTCTCCATGGNNN	0.23	0.00	0.00	0.00	0.00
GATCAACGTCTCCATTGACC	0.92	0.00	0.00	0.00	0.00
GATCAACGTCTCCCAGCTGC	0.00	0.33	0.00	0.34	0.34
GATCAACGTCTCCCATGTGC	0.00	0.00	0.00	0.68	1.38
GATCAACGTCTCCCGAGGGC	0.00	0.00	0.00	0.34	0.00
GATCAACGTCTCCGACGCCG	0.00	0.00	0.00	0.00	0.00
GATCAACGTCTCCGACTGAC	0.46	0.00	0.00	0.00	0.00
GATCAACGTCTCCGCCGGGA	0.00	0.00	0.00	0.00	0.34
GATCAACGTCTCCGTAGTGG	0.00	0.00	0.29	0.00	0.00
GATCAACGTCTCCGTGCCTC	0.23	0.00	0.00	0.00	0.00
GATCAACGTCTCCGTGTCTC	2.06	2.60	3.15	5.09	4.48
GATCAACGTCTCCGTTTCTC	0.00	0.00	0.29	0.00	0.00
GATCAACGTCTCCTTCTCGG	0.23	0.00	0.00	0.00	0.00
GATCAACGTCTCGAGGTCAA	0.00	0.00	0.00	0.00	0.00
GATCAACGTCTCGCGTCGAT	0.00	0.00	0.00	0.00	0.00
GATCAACGTCTCGCGTTTGA	0.00	0.00	0.00	0.00	0.00
GATCAACGTCTCGGACTTCG	0.46	0.33	0.29	0.34	0.00
GATCAACGTCTCGGCACTGA	0.00	0.00	0.00	0.00	0.00
GATCAACGTCTCGGCATTGA	0.00	0.65	0.00	0.00	0.00
GATCAACGTCTCGGGAGAGA	0.00	0.00	0.00	0.00	0.34
GATCAACGTCTCGTAAAAAA	0.23	0.00	0.00	0.00	0.00
GATCAACGTCTCGTGGCGCT	0.00	0.33	0.00	0.34	0.00
GATCAACGTCTCGTGTTAAA	0.00	0.00	0.00	0.68	0.00
GATCAACGTCTCGTGTTGAA	0.23	0.00	0.00	0.00	0.00
GATCAACGTCTCGTGTTGAC	1.60	1.30	1.43	2.38	2.07
GATCAACGTCTCGTGTTGAT	0.00	0.00	0.00	0.68	0.00
GATCAACGTCTCTGGTTTCA	0.00	0.33	0.00	0.00	0.00
GATCAACGTCTGACCTCCAT	0.00	0.33	0.00	0.00	0.00
GATCAACGTCTGACTTTCAC	0.00	0.33	0.00	0.00	0.00
GATCAACGTCTGAGAATTAT	0.00	0.33	0.00	0.00	0.00
GATCAACGTCTGCCTGCTTG	0.00	0.00	0.29	0.00	0.00

GATCAACGTCTGCCTGGTCG	0.00	0.00	0.00	0.00	0.00
GATCAACGTCTGCGAACAGC	0.00	0.00	0.00	0.00	0.00
GATCAACGTCTGCGAATTAN	0.00	0.00	0.00	0.00	0.00
GATCAACGTCTGCGAATTAT	14.88	18.22	37.80	18.33	31.00
GATCAACGTCTGCGAATTCT	0.00	0.00	0.29	0.00	0.00
GATCAACGTCTGCGACAGCT	0.00	0.00	0.00	0.00	0.34
GATCAACGTCTGCGACTTAC	0.00	0.00	0.00	0.00	0.00
GATCAACGTCTGCGCATTAT	0.00	0.00	0.29	0.00	0.00
GATCAACGTCTGCGCCGTGG	0.00	0.00	0.57	0.00	0.00
GATCAACGTCTGCTAATTAT	0.00	0.33	0.00	0.00	0.00
GATCAACGTCTGGATGGAGA	0.00	0.00	0.29	0.00	0.00
GATCAACGTCTGGCGCGTGG	0.00	0.00	0.00	0.00	0.34
GATCAACGTCTGGGCTGCTG	0.00	0.00	0.00	0.00	0.00
GATCAACGTCTGGGGCCGTG	0.00	0.00	0.29	0.00	0.00
GATCAACGTCTGTCCGCTGA	0.00	0.00	0.00	0.00	0.00
GATCAACGTCTGTGGACATG	0.00	0.00	0.29	0.00	0.00
GATCAACGTCTTATCTCCTA	0.00	0.00	0.00	0.00	1.03
GATCAACGTCTTCAACTTCG	0.00	0.00	0.00	0.00	0.00
GATCAACGTCTTCCAGGTGT	0.00	0.33	0.00	0.00	0.00
GATCAACGTCTTCCTGGTTG	0.00	0.33	0.00	0.00	0.00
GATCAACGTCTTCGTCTGCG	0.92	0.98	0.57	2.38	0.34
GATCAACGTCTTCTCTCTTG	0.00	0.00	0.00	0.00	0.00
GATCAACGTCTTCTTCCTGC	0.00	0.00	0.00	0.00	0.00
GATCAACGTCTTGAACATCA	0.00	0.00	0.29	0.00	0.00
GATCAACGTCTTGACCCGCA	0.23	0.00	0.29	0.00	0.00
GATCAACGTCTTGACCCTCA	0.00	0.00	0.00	0.34	0.00
GATCAACGTCTTGAGGAGAC	0.00	0.33	0.29	0.68	0.34
GATCAACGTCTTGAGGCAGA	0.00	0.00	0.00	0.00	0.00
GATCAACGTCTTGAGGGCAA	0.23	0.00	0.00	0.34	0.00
GATCAACGTCTTGAGGNCAA	0.00	0.00	0.00	0.00	0.34
GATCAACGTCTTGAGGTCAA	8.93	15.94	18.90	15.27	10.33
GATCAACGTCTTGAGGTCTT	0.00	0.33	0.00	0.34	0.00
GATCAACGTCTTGATGGGTC	0.00	0.00	0.00	1.02	0.34
GATCAACGTCTTGCTGGGCG	0.00	0.00	0.00	0.00	0.34
GATCAACGTCTTGGACAATG	0.00	0.33	0.00	0.00	0.00
GATCAACGTCTTGGTGAAGA	0.00	0.00	0.00	0.00	0.00
GATCAACGTCTTTCCGGATG	0.00	0.00	0.00	0.00	0.00

GATCAACGTCTTTGACGAGA	0.00	0.33	0.00	0.00	0.00
GATCAACGTCTTTTGAGAGA	0.00	0.00	0.00	0.00	0.34
GATCAACGTCTTTTGAGTGT	0.92	0.00	0.00	0.00	0.00
GATCAACGTGAAAAAAAAAA	1.14	0.65	0.00	1.02	0.34
GATCAACGTGAAACAGCTTA	0.00	0.33	0.00	0.00	0.00
GATCAACGTGAAACAGCTTC	0.00	0.33	0.00	0.00	0.00
GATCAACGTGAAACCGTTTC	0.69	2.60	0.86	2.72	0.00
GATCAACGTGAAACCGTTTN	0.23	0.00	0.00	0.00	0.00
GATCAACGTGAAAGAGTTCG	0.00	0.00	0.00	0.00	0.69
GATCAACGTGAACAAAAAAA	1.60	1.30	0.29	2.72	0.34
GATCAACGTGAACAAAAATG	2.29	0.98	0.00	0.00	0.69
GATCAACGTGAACAACCAGG	0.92	0.00	0.00	0.00	0.00
GATCAACGTGAACAAGTGCA	0.23	0.00	0.00	0.00	0.34
GATCAACGTGAACAAGTNNN	0.00	0.00	0.00	0.00	0.00
GATCAACGTGAACACAGTGG	0.00	0.00	0.00	0.00	0.34
GATCAACGTGAACATGTGCG	0.00	0.00	0.00	0.68	0.00
GATCAACGTGAACCACACGG	0.00	0.00	0.00	0.00	0.34
GATCAACGTGAACCCAGTGT	0.00	0.00	0.00	0.00	0.00
GATCAACGTGACAAACGGGA	0.46	0.00	0.00	1.02	0.00
GATCAACGTGACACACCTTC	0.00	0.00	0.00	0.00	0.34
GATCAACGTGACACAGATTA	0.00	0.33	0.00	0.00	0.00
GATCAACGTGACACAGATTC	0.00	0.00	0.00	0.00	0.00
GATCAACGTGACACAGCCTA	0.00	0.00	0.00	0.34	0.00
GATCAACGTGACACAGCCTC	0.00	0.33	0.00	0.34	0.00
GATCAACGTGACACAGCGTC	0.23	0.00	0.00	0.00	0.00
GATCAACGTGACACAGCTGA	0.00	0.00	0.00	0.00	0.00
GATCAACGTGACACAGCTNN	0.69	0.00	0.00	0.00	0.00
GATCAACGTGACACAGCTTA	0.00	0.00	0.29	0.00	0.34
GATCAACGTGACACAGCTTC	57.23	57.58	69.87	60.76	49.25
GATCAACGTGACACAGCTTT	0.00	0.00	0.00	1.02	0.00
GATCAACGTGACACAGGTGC	0.00	0.33	0.00	0.00	0.00
GATCAACGTGACACAGNNNN	0.46	0.00	0.00	0.00	0.00
GATCAACGTGACACAGNTNN	0.23	0.00	0.00	0.00	0.00
GATCAACGTGACACAGNTTC	0.00	0.00	0.00	0.34	0.00
GATCAACGTGACACAGTCGT	0.00	0.00	0.00	0.68	0.00
GATCAACGTGACACAGTTTC	0.00	0.33	0.00	0.00	0.00
GATCAACGTGACACATGTCC	0.46	0.33	0.00	0.00	1.03

GATCAACGTGACACCGCTGC	0.23	0.00	0.00	0.00	0.00
GATCAACGTGACACGGCTTC	0.00	0.33	0.00	0.00	0.00
GATCAACGTGACAGCGTCAC	0.00	0.00	0.00	0.00	0.00
GATCAACGTGACATAGCTTC	0.00	0.33	0.00	0.00	0.00
GATCAACGTGACATCAGGCT	0.00	0.00	0.00	0.00	0.34
GATCAACGTGACCACCGTAG	0.00	0.00	0.00	0.34	0.00
GATCAACGTGACCCAGCTTC	0.23	0.00	0.00	0.00	0.00
GATCAACGTGACCCCCTGGG	0.23	0.00	0.00	0.00	0.00
GATCAACGTGACCCTCCGGG	0.23	0.00	0.00	0.00	0.00
GATCAACGTGACCCTCGGGG	0.00	0.00	0.00	0.00	0.00
GATCAACGTGACCCTCTCGT	0.00	0.00	0.00	0.00	0.34
GATCAACGTGACCCTCTGGG	21.97	20.82	9.45	13.58	9.30
GATCAACGTGACCCTCTGGN	0.23	0.00	0.00	0.00	0.00
GATCAACGTGACCCTCTGTG	0.00	0.00	0.00	0.34	0.00
GATCAACGTGACCCTCTTGG	0.23	0.00	0.00	0.00	0.00
GATCAACGTGACCCTTCGTA	0.23	0.00	0.00	0.00	0.00
GATCAACGTGACCCTTTGGG	0.46	0.00	0.00	0.00	0.00
GATCAACGTGACCGAGAGGA	0.00	0.00	0.00	0.00	0.34
GATCAACGTGACCTGGGACT	0.00	0.00	0.29	0.00	0.00
GATCAACGTGACCTTCTCCA	0.00	0.00	0.00	0.68	0.00
GATCAACGTGACGCAGCTTC	0.00	0.00	0.29	0.00	0.00
GATCAACGTGACTCTCTGGG	0.00	0.00	0.29	0.00	0.00
GATCAACGTGAGCATCCCGG	0.00	0.00	0.00	0.00	0.00
GATCAACGTGAGCCAGGCGG	0.00	0.00	0.00	0.34	0.00
GATCAACGTGAGCCTGCGCT	0.00	0.00	0.29	0.00	0.00
GATCAACGTGAGCTTGTGCT	0.00	0.33	0.00	0.00	0.00
GATCAACGTGAGGTGGAACC	0.00	0.00	0.29	0.00	0.00
GATCAACGTGAGTGAGCAAG	0.00	0.00	0.00	0.34	0.00
GATCAACGTGAGTTCGATGA	0.00	0.00	0.00	0.00	0.34
GATCAACGTGAGTTGGCATC	0.00	0.00	0.29	0.00	0.00
GATCAACGTGATAACTGCAG	0.00	0.00	0.57	0.00	0.00
GATCAACGTGATACAGCTTC	0.23	0.00	0.00	0.00	0.00
GATCAACGTGATGCCGCGAA	0.00	0.00	0.00	0.00	0.00
GATCAACGTGATGCGGTGTT	0.00	0.00	0.29	0.00	0.00
GATCAACGTGATGGAGACGT	0.00	0.00	0.00	0.00	0.00
GATCAACGTGATGGAGCCGT	0.00	0.00	0.00	0.00	0.34
GATCAACGTGATGGTGGAGA	0.00	0.00	0.00	0.34	0.00

GATCAACGTGATGGTGGGGA	0.00	0.00	0.00	0.34	0.00
GATCAACGTGATTGGCTCCT	0.00	0.00	0.29	0.00	0.00
GATCAACGTGCAAAAAAAAA	0.00	0.00	0.00	0.34	0.00
GATCAACGTGCAACCACACT	0.00	0.00	0.00	0.00	0.34
GATCAACGTGCACATTGCAG	1.37	0.33	0.00	0.00	0.00
GATCAACGTGCACCCCAGCC	0.00	0.00	0.00	0.00	0.00
GATCAACGTGCACCTGGCCA	0.00	0.00	0.00	0.68	0.00
GATCAACGTGCACTCCCCTC	0.00	0.00	0.00	0.00	0.00
GATCAACGTGCACTCGACGA	0.00	0.33	0.00	0.00	0.00
GATCAACGTGCACTCTACAC	0.00	0.00	0.00	0.68	0.00
GATCAACGTGCACTGGAGTC	0.00	0.00	0.00	0.00	0.00
GATCAACGTGCACTTAAAGC	0.00	0.00	0.00	0.34	0.00
GATCAACGTGCACTTGCATG	0.23	0.00	0.00	0.00	0.00
GATCAACGTGCAGCACTACG	0.00	0.00	0.00	0.00	0.00
GATCAACGTGCAGCCCAGCC	0.00	0.00	0.00	0.00	0.34
GATCAACGTGCAGGACTTCA	0.00	0.00	0.00	0.00	0.00
GATCAACGTGCAGGTGGCGA	0.00	0.00	0.00	0.34	0.00
GATCAACGTGCATCCCCACT	0.00	0.00	0.00	0.00	0.69
GATCAACGTGCATCGTATGC	0.00	0.00	0.00	0.68	0.00
GATCAACGTGCATTGCCCCG	0.46	0.00	0.00	0.00	0.00
GATCAACGTGCATTTAAAGC	9.61	1.95	12.89	1.70	7.58
GATCAACGTGCATTTAACGC	0.00	0.00	0.00	0.34	0.00
GATCAACGTGCATTTAAGAT	0.46	0.33	0.00	0.00	0.00
GATCAACGTGCCAAACATGC	0.00	0.00	0.00	0.00	0.00
GATCAACGTGCCAACGCCAA	0.00	0.33	0.00	0.00	0.00
GATCAACGTGCCAAGGAATT	0.00	0.00	0.00	0.00	0.00
GATCAACGTGCCACAGCTTC	0.00	0.00	0.00	0.34	0.00
GATCAACGTGCCACATTTTC	0.00	0.33	0.00	0.00	0.00
GATCAACGTGCCCACCGTTG	0.00	0.33	0.00	0.00	0.00
GATCAACGTGCCCAGCGGGC	0.00	0.00	0.29	0.34	0.34
GATCAACGTGCCCAGCGGGG	0.00	0.00	0.00	0.00	0.00
GATCAACGTGCCCAGTCGTG	0.00	0.00	0.00	0.00	0.34
GATCAACGTGCCCCTCCAGG	0.00	0.00	0.00	0.00	0.00
GATCAACGTGCCCGAACTCG	0.23	0.00	0.00	0.00	0.00
GATCAACGTGCCCGCACTCG	0.00	0.33	0.57	0.00	0.00
GATCAACGTGCCCGCCATGA	0.00	0.00	0.00	0.00	0.00
GATCAACGTGCCCGCCCTCG	6.87	26.03	24.63	15.27	19.29

GATCAACGTGCCCGCCCTCN	0.23	0.00	0.00	0.00	0.00
GATCAACGTGCCCGCCCTCT	0.00	0.00	0.00	0.00	0.00
GATCAACGTGCCCTCGCACA	0.00	0.98	2.00	0.34	0.69
GATCAACGTGCCCTCGCACN	0.00	0.00	0.00	0.00	0.00
GATCAACGTGCCCTTCAAAA	0.00	0.00	0.29	0.00	0.00
GATCAACGTGCCGACACCCT	0.00	0.00	0.00	0.00	0.00
GATCAACGTGCCGCAGCGCG	0.00	0.33	0.00	0.00	0.00
GATCAACGTGCCGTGAGCCG	0.00	0.00	0.00	0.00	0.00
GATCAACGTGCCTTGCCTTC	0.00	0.00	0.00	0.00	0.00
GATCAACGTGCGAAAAAAAA	0.00	0.00	0.29	0.00	0.00
GATCAACGTGCGATGCAGCC	0.00	0.00	0.00	0.00	0.69
GATCAACGTGCGCGAAGTCA	0.00	0.00	0.00	0.34	0.00
GATCAACGTGCGCGAGAGTG	0.00	0.00	0.00	0.34	0.00
GATCAACGTGCGCGAGGCCC	0.46	0.00	0.00	0.00	0.00
GATCAACGTGCGCGTGTACG	0.00	0.33	0.00	0.00	0.00
GATCAACGTGCGCGTTTGGA	0.00	0.00	0.00	0.34	0.69
GATCAACGTGCGCTCAATAA	0.00	0.00	0.29	0.00	0.00
GATCAACGTGCGCTGCAAAA	0.00	0.33	0.00	0.00	0.00
GATCAACGTGCGCTTGGCAT	0.00	0.33	0.00	0.00	0.00
GATCAACGTGCGGAAGAAGG	0.00	0.33	0.00	0.34	0.00
GATCAACGTGCTCAGCGCGG	0.00	0.00	0.00	0.00	0.00
GATCAACGTGCTCAGCGGGG	0.46	0.33	0.00	2.38	0.34
GATCAACGTGCTCAGTGGGG	0.00	0.00	0.00	0.00	0.00
GATCAACGTGCTCTGCAGGG	0.00	0.00	0.29	0.00	0.00
GATCAACGTGCTGCGGAGCA	0.23	0.00	0.00	0.00	0.00
GATCAACGTGCTGCTGTGCG	0.00	0.00	0.00	0.00	0.34
GATCAACGTGCTGTCCTCGG	0.00	0.00	0.29	0.00	0.00
GATCAACGTGCTGTGGTGAT	0.46	0.33	0.57	1.02	0.34
GATCAACGTGCTTCGGACGG	0.23	0.33	0.86	0.34	0.34
GATCAACGTGCTTCTGTGCC	0.00	0.00	0.00	0.00	0.00
GATCAACGTGCTTGCCTGTT	0.00	0.00	0.00	0.00	0.34
GATCAACGTGCTTGTGCGCT	0.00	0.00	0.00	0.00	0.34
GATCAACGTGGAAAAAAAAA	0.00	0.00	0.00	0.00	0.00
GATCAACGTGGAAAACATGC	0.23	0.00	0.00	0.00	0.00
GATCAACGTGGAAATCGTAG	0.00	0.00	0.29	0.00	0.00
GATCAACGTGGAAGCATGAA	0.00	0.00	0.00	0.00	0.00
GATCAACGTGGAAGCATGTC	0.00	0.00	0.00	0.00	0.00

GATCAACGTGGAAGGCCACG	0.00	0.33	0.00	0.00	0.00
GATCAACGTGGAAGTGAGTA	0.00	0.00	0.29	0.00	0.00
GATCAACGTGGACAGCTCAG	0.00	0.33	0.00	0.00	0.00
GATCAACGTGGACCAGACTG	0.00	0.00	0.29	0.34	0.00
GATCAACGTGGACGGCGAGC	0.00	0.33	0.00	0.00	0.00
GATCAACGTGGACGGTTCTG	0.00	0.00	0.00	0.34	0.00
GATCAACGTGGACGTGCTCT	0.00	0.00	0.00	0.00	0.00
GATCAACGTGGAGACGGCAN	0.23	0.00	0.00	0.00	0.00
GATCAACGTGGAGCGCCAGC	0.23	0.00	0.00	0.34	0.00
GATCAACGTGGAGCGCCATG	0.00	0.00	0.00	0.00	0.00
GATCAACGTGGAGCGCCTCG	0.00	0.00	0.00	0.00	0.00
GATCAACGTGGAGGCCGGTC	0.23	0.00	0.00	0.00	0.00
GATCAACGTGGAGGCGAAGA	0.23	0.00	0.29	0.00	0.00
GATCAACGTGGATGAGACCA	0.00	0.00	0.29	0.00	0.00
GATCAACGTGGATGAGCCCA	0.00	0.00	0.29	0.00	0.00
GATCAACGTGGATGGAGGGG	0.00	0.00	0.00	0.34	0.00
GATCAACGTGGATGGATAAT	0.00	0.00	0.00	0.00	0.00
GATCAACGTGGATGGCCCAA	0.00	0.00	0.00	0.00	0.00
GATCAACGTGGATTGCTGGA	0.00	0.00	0.00	0.00	0.00
GATCAACGTGGCAAATCCAT	0.00	0.00	0.00	0.34	0.00
GATCAACGTGGCACGACTTT	0.23	0.00	0.00	0.00	0.00
GATCAACGTGGCACGCGGCC	0.00	0.00	0.00	0.34	0.34
GATCAACGTGGCAGAACAGG	0.00	0.65	0.00	0.00	0.00
GATCAACGTGGCATCGAGAT	0.00	0.00	0.00	0.00	0.00
GATCAACGTGGCCAGAGAAG	0.00	0.00	0.00	0.34	0.00
GATCAACGTGGCCATCGGTG	0.00	0.00	0.00	0.34	0.00
GATCAACGTGGCCCGCGTTG	0.00	0.33	0.00	0.00	0.00
GATCAACGTGGCCCTCTGGG	0.23	0.00	0.00	0.34	0.00
GATCAACGTGGCCCTTCGCA	0.00	0.00	0.00	0.34	0.00
GATCAACGTGGCCCTTCGCG	0.23	0.00	0.00	0.00	0.00
GATCAACGTGGCCGCGAGCG	0.00	0.00	0.00	0.34	0.00
GATCAACGTGGCCGGCAGAG	0.46	0.33	0.00	0.00	0.00
GATCAACGTGGCCGGGCATC	0.00	0.00	0.00	0.00	0.34
GATCAACGTGGCCTTCCAAA	0.00	0.00	0.00	0.34	0.00
GATCAACGTGGCCTTCCTCC	0.00	0.00	0.00	0.00	0.00
GATCAACGTGGCCTTCCTGG	0.00	0.00	0.00	0.00	1.03
GATCAACGTGGCCTTGTAGA	0.00	0.33	0.00	0.00	0.00

GATCAACGTGGCCTTGTTGG	0.00	0.33	0.29	0.34	0.00
GATCAACGTGGCGAAGGCGG	0.00	0.33	0.00	0.34	0.00
GATCAACGTGGCGAATTGAG	0.00	0.00	0.00	0.00	0.34
GATCAACGTGGCGCAAGAGA	0.23	0.00	0.29	1.02	0.00
GATCAACGTGGCGCAAGAGC	0.23	0.00	0.00	0.00	0.00
GATCAACGTGGCGGCCACGC	0.00	0.33	0.29	0.00	0.00
GATCAACGTGGCGGCCATGC	2.29	1.30	0.00	0.34	0.34
GATCAACGTGGCGGCCCTGC	0.23	0.00	0.00	0.00	0.00
GATCAACGTGGCGGCTATGC	0.23	0.00	0.00	0.00	0.00
GATCAACGTGGCGTCGAGAT	0.00	0.00	0.00	0.34	0.00
GATCAACGTGGCTCTTGCTC	0.00	0.00	0.00	0.00	0.00
GATCAACGTGGCTGCGGAGA	0.00	0.00	0.00	0.00	0.00
GATCAACGTGGGACCGCTGG	0.00	0.00	0.00	0.34	0.00
GATCAACGTGGGAGGCGTCG	0.00	0.33	0.00	0.00	0.00
GATCAACGTGGGCCTCCAGT	0.00	0.33	0.00	0.00	0.00
GATCAACGTGGGCGCTTACA	0.46	0.00	0.00	0.68	0.00
GATCAACGTGGGCGGCGCCA	0.00	0.00	0.00	0.00	0.34
GATCAACGTGGGCTGGAGGC	0.00	0.00	0.29	0.00	0.00
GATCAACGTGGGGAAAAAAA	0.00	0.00	0.29	0.00	0.00
GATCAACGTGGGGAAGCTCC	0.00	0.00	0.00	0.00	0.34
GATCAACGTGGGGAAGGAGG	0.23	0.00	0.00	0.00	0.00
GATCAACGTGGGGAAGGCGG	0.23	0.00	0.00	0.00	0.00
GATCAACGTGGGGACTCGCT	0.00	0.33	0.00	0.00	0.00
GATCAACGTGGGGAGGGGGG	0.23	0.00	0.00	0.00	0.00
GATCAACGTGGGGCAGGAGG	0.23	0.00	0.00	0.00	0.00
GATCAACGTGGGGCTTGGTG	0.00	0.00	0.00	0.00	0.00
GATCAACGTGGTCAAAAAAA	0.00	0.00	0.00	0.34	0.00
GATCAACGTGGTCACGCGCA	0.00	0.00	0.00	0.00	0.69
GATCAACGTGGTCAGAGTCC	0.00	0.00	0.00	0.34	0.00
GATCAACGTGGTCTAGATTG	0.23	0.00	0.00	0.00	0.00
GATCAACGTGGTCTGCAAAA	2.06	1.63	1.43	2.38	2.41
GATCAACGTGGTCTGCACAA	0.00	0.00	0.57	0.00	0.00
GATCAACGTGGTCTGCACCA	3.43	2.28	0.86	2.04	1.38
GATCAACGTGGTCTGCACCG	0.00	0.00	0.57	0.00	0.00
GATCAACGTGGTCTGCATCA	0.23	0.00	0.00	0.00	0.00
GATCAACGTGGTCTGCCAAA	0.23	0.00	0.00	0.00	0.00
GATCAACGTGGTCTGCCCCA	0.00	0.00	0.00	0.34	0.00

GATCAACGTGGTCTGCGAAA	0.23	0.00	0.00	0.00	0.00
GATCAACGTGGTCTGCGTGA	0.00	0.00	0.00	0.00	0.00
GATCAACGTGGTGAACACGT	0.00	0.33	0.57	0.68	0.00
GATCAACGTGGTGAAGGAGG	39.60	40.99	20.33	39.03	29.27
GATCAACGTGGTGAAGGANN	0.23	0.00	0.00	0.00	0.00
GATCAACGTGGTGAAGGATC	0.00	0.00	0.00	0.00	0.00
GATCAACGTGGTGAAGGATG	0.23	0.00	0.00	0.00	0.00
GATCAACGTGGTGAAGGCGG	0.92	0.33	0.29	0.34	0.34
GATCAACGTGGTGAAGGGGG	1.14	0.33	0.57	0.00	0.00
GATCAACGTGGTGAAGGTGC	0.00	0.00	0.00	0.00	0.00
GATCAACGTGGTGAAGNAGG	0.00	0.33	0.00	0.00	0.00
GATCAACGTGGTGAAGNANN	0.00	0.00	0.00	0.34	0.00
GATCAACGTGGTGAAGTAGG	0.00	0.00	0.00	0.34	0.00
GATCAACGTGGTGAGCCAGC	0.00	0.00	0.00	0.00	0.00
GATCAACGTGGTGAGCCTCG	0.00	0.00	0.29	0.00	0.00
GATCAACGTGGTGAGGGAGG	0.23	0.00	0.00	0.00	0.00
GATCAACGTGGTGCAAGTGC	0.00	0.00	0.00	0.34	0.00
GATCAACGTGGTGCAGAAGT	0.00	0.00	0.00	0.00	0.00
GATCAACGTGGTGCAGGTGG	0.23	0.00	0.00	0.00	0.00
GATCAACGTGGTGGAGGAGG	0.00	0.33	0.29	0.00	0.00
GATCAACGTGGTGGAGTTTG	0.00	0.00	0.00	0.00	0.00
GATCAACGTGGTGGTGGGCG	0.00	0.00	0.00	0.00	0.00
GATCAACGTGGTGGTGGGGG	0.00	0.00	0.00	0.00	0.34
GATCAACGTGGTGGTGGGNN	0.00	0.00	0.00	0.34	0.00
GATCAACGTGGTGGTGGGTG	1.60	1.63	0.86	1.36	1.72
GATCAACGTGGTGTGGCCAT	0.00	0.00	0.00	0.00	0.69
GATCAACGTGGTGTTCCCAG	0.23	0.33	0.00	0.34	0.00
GATCAACGTGGTGTTCCCCA	0.00	0.00	0.00	0.68	0.00
GATCAACGTGGTTACGAGCA	0.00	0.00	0.57	0.00	0.00
GATCAACGTGGTTGCATTCA	0.69	0.00	0.00	0.00	0.00
GATCAACGTGGTTGCTCGTA	0.23	0.00	0.00	0.00	0.00
GATCAACGTGGTTGTGTGAG	0.00	0.00	0.00	0.00	0.00
GATCAACGTGTAAAGCGTGA	0.00	0.00	0.00	0.00	0.00
GATCAACGTGTAAATTCACG	0.00	0.00	0.29	0.00	0.00
GATCAACGTGTAAGCGGCTG	0.00	0.00	0.29	0.00	0.00
GATCAACGTGTACAACTGCT	0.00	0.00	0.00	0.00	0.00
GATCAACGTGTACAGGAAGG	0.00	0.00	0.57	0.34	0.00

GATCAACGTGTACATCAGGC	0.00	0.00	0.29	0.00	0.00
GATCAACGTGTACTACAATG	0.00	0.33	0.00	0.34	0.00
GATCAACGTGTACTTCACAG	0.00	0.00	0.00	0.00	0.00
GATCAACGTGTACTTCGGAG	0.00	0.33	0.00	0.00	0.00
GATCAACGTGTAGGGCTCCT	0.00	0.00	0.57	0.00	0.00
GATCAACGTGTAGGTCAACC	0.00	0.00	0.29	0.00	0.00
GATCAACGTGTATACCGATG	0.92	1.30	0.00	0.68	0.34
GATCAACGTGTATACTCATG	0.00	0.00	0.00	0.00	0.34
GATCAACGTGTATTCCTCTG	0.00	0.00	0.57	0.00	0.00
GATCAACGTGTCAAACATCG	0.00	0.00	0.29	0.00	0.00
GATCAACGTGTCAACATTTT	0.00	0.00	0.29	0.00	0.34
GATCAACGTGTCCTGGGCTC	0.00	0.00	0.29	0.00	0.00
GATCAACGTGTCGCGAACCA	0.23	0.00	0.00	0.00	0.00
GATCAACGTGTCGCGACCCA	0.46	5.21	1.15	2.04	1.38
GATCAACGTGTCGCGACCCT	0.23	0.00	0.00	0.00	0.00
GATCAACGTGTCTCAGATAG	0.00	0.00	0.00	0.00	0.00
GATCAACGTGTCTCTGCCGG	0.00	0.33	0.00	0.00	0.00
GATCAACGTGTCTCTGCCTG	2.29	4.55	0.57	2.38	1.38
GATCAACGTGTCTGTCCGAG	0.00	0.00	0.00	0.34	0.00
GATCAACGTGTGACACGCAC	0.00	0.00	0.00	0.00	0.00
GATCAACGTGTGACCATGGC	0.00	0.00	0.00	0.34	0.00
GATCAACGTGTGAGATGTGG	0.23	0.00	0.00	0.00	0.00
GATCAACGTGTGAGATGTGT	0.46	0.33	0.00	0.00	0.34
GATCAACGTGTGATGGAGCG	0.23	0.00	0.00	0.00	0.00
GATCAACGTGTGCACCGTTT	0.00	0.00	0.29	0.00	0.00
GATCAACGTGTGCAGCAGAT	0.00	0.00	0.00	0.00	0.00
GATCAACGTGTGCAGTATGC	0.00	0.00	0.00	0.34	0.00
GATCAACGTGTGCCGTGTTA	0.00	0.00	0.29	0.00	0.00
GATCAACGTGTGCTGCATCA	0.00	0.00	0.00	0.00	0.00
GATCAACGTGTGGACAAAAC	0.00	0.00	0.00	0.00	0.00
GATCAACGTGTGTTCAGCGG	0.00	0.00	0.29	0.00	0.00
GATCAACGTGTTCCTCAGTA	0.00	0.65	0.00	0.00	0.00
GATCAACGTGTTCTCGGCGG	0.23	0.00	0.00	0.00	0.00
GATCAACGTGTTCTGCATCA	0.00	0.00	0.00	0.00	0.00
GATCAACGTGTTGAGGCTTC	0.00	0.33	0.29	0.00	0.00
GATCAACGTGTTGCAAACGA	0.00	0.00	0.29	0.00	0.00
GATCAACGTGTTGCAAGCGA	0.00	0.00	0.00	0.00	0.34

GATCAACGTGTTGCGCGCAA	0.00	0.00	0.29	0.00	0.00
GATCAACGTGTTGGCCTCAC	0.00	0.00	0.00	0.34	0.34
GATCAACGTGTTGGTAATAC	0.00	0.00	0.29	0.00	0.00
GATCAACGTGTTGGTTCGCC	0.00	0.00	0.00	0.00	0.00
GATCAACGTGTTGTGGTGAT	0.00	0.00	0.00	0.34	0.00
GATCAACGTGTTTCACTGTG	0.00	0.00	0.29	0.00	0.00
GATCAACGTGTTTGGGAACG	0.00	0.00	0.29	0.00	0.00
GATCAACGTTAAAAAAAAAA	0.00	0.00	0.00	2.72	0.00
GATCAACGTTAAACTCACGT	0.00	0.00	0.29	0.00	0.00
GATCAACGTTAAGCCGGAGT	1.14	0.00	0.57	0.68	0.69
GATCAACGTTAAGGCCTACA	0.46	0.33	0.00	0.34	0.69
GATCAACGTTACCCGGCGCT	0.00	0.00	0.00	0.00	0.34
GATCAACGTTAGGTTTGGAG	0.00	0.33	0.00	0.00	0.00
GATCAACGTTATGAGCAAGG	0.00	0.00	0.00	0.00	0.00
GATCAACGTTATGCGTCATG	0.00	0.00	0.00	0.00	0.00
GATCAACGTTCAAAAAAAAA	6.64	3.58	4.01	7.47	4.48
GATCAACGTTCAAAAAAAAC	0.00	0.00	0.29	0.00	0.00
GATCAACGTTCAAAAAAGAA	0.00	0.00	0.00	0.34	0.00
GATCAACGTTCAAAACCAAA	0.23	0.00	0.00	0.00	0.00
GATCAACGTTCAACGTTCAA	0.00	0.00	0.29	0.00	0.00
GATCAACGTTCAAGCGCGCG	0.00	0.00	0.00	0.00	0.00
GATCAACGTTCAAGGTGCTC	0.00	0.00	0.00	0.00	0.00
GATCAACGTTCACCAGATTG	0.00	0.00	0.00	0.00	0.00
GATCAACGTTCAGCCTCGGC	0.00	0.65	0.00	0.00	0.00
GATCAACGTTCAGGAACATG	0.00	0.33	0.00	0.00	0.00
GATCAACGTTCAGGCCGTAG	0.00	0.33	0.29	0.34	0.00
GATCAACGTTCAGGCCGTCG	0.23	1.63	0.00	1.02	0.00
GATCAACGTTCAGGCTCTCG	0.00	0.00	0.00	0.00	0.00
GATCAACGTTCAGTGGCATC	0.00	0.00	0.00	0.00	0.34
GATCAACGTTCAGTTCGAGC	0.00	0.00	0.00	0.00	0.00
GATCAACGTTCATCACTTCC	0.23	0.00	0.00	0.00	0.00
GATCAACGTTCATCTCTCGT	0.23	0.00	0.00	0.00	0.00
GATCAACGTTCATCTCTCTG	0.00	0.00	0.00	0.00	0.00
GATCAACGTTCATCTCTCTT	0.46	0.00	0.86	0.34	0.00
GATCAACGTTCCAAAAAAAA	5.49	2.60	3.72	3.73	8.61
GATCAACGTTCCAAAAAACA	0.23	0.00	0.00	0.00	0.00
GATCAACGTTCCAAAACAAA	0.69	0.33	0.29	0.34	0.00

GATCAACGTTCCAAAACAGC	0.00	0.00	0.00	0.00	0.00
GATCAACGTTCCAAAACAGT	0.23	0.00	0.57	0.00	0.00
GATCAACGTTCCAAAAGAAA	0.00	0.00	0.00	0.00	0.00
GATCAACGTTCCAAAGAAAA	0.00	0.00	0.00	0.34	0.00
GATCAACGTTCCAACAAAAA	0.23	0.00	0.00	0.00	0.00
GATCAACGTTCCATCCATCC	0.00	0.00	0.00	0.00	0.34
GATCAACGTTCCATCCCAGG	0.00	0.00	0.00	0.00	0.34
GATCAACGTTCCCATGGCCG	0.00	0.00	0.00	0.00	0.00
GATCAACGTTCCGTCCGTCC	0.92	0.00	2.58	3.73	0.34
GATCAACGTTCCTCACTAGC	0.00	0.00	0.29	0.00	0.00
GATCAACGTTCCTCTCCGTG	1.14	0.00	0.29	0.00	0.00
GATCAACGTTCCTCTCCGTT	0.46	0.00	0.00	0.00	0.00
GATCAACGTTCGACTGGTGG	0.23	0.00	0.00	0.00	0.00
GATCAACGTTCGCAGATGCA	0.00	0.00	0.00	0.00	0.00
GATCAACGTTCGCGAGGCTT	0.00	0.00	0.29	0.00	0.00
GATCAACGTTCGGAGGGAGG	0.23	5.86	2.58	9.50	6.20
GATCAACGTTCGGGCAGTGC	0.00	0.00	0.00	0.00	0.34
GATCAACGTTCGGTCCCGTG	0.00	0.00	0.00	0.00	0.00
GATCAACGTTCGGTTTGCCG	0.00	0.33	0.00	0.00	0.00
GATCAACGTTCGTATGCCGT	0.00	0.00	0.00	0.00	0.34
GATCAACGTTCGTGATAGTC	0.00	0.00	0.00	0.00	0.34
GATCAACGTTCTAAAAAAAA	0.00	0.00	0.00	0.00	0.00
GATCAACGTTCTATCGATGG	0.00	0.00	0.00	0.00	0.00
GATCAACGTTCTCCGTGCGG	0.00	0.00	0.29	0.00	0.00
GATCAACGTTCTCTTGGTCA	0.00	0.00	0.00	0.34	0.00
GATCAACGTTCTGAAAAAAA	0.69	0.33	0.00	0.00	0.69
GATCAACGTTCTGCAAAAAA	0.00	0.00	0.00	0.34	0.00
GATCAACGTTCTGCCCTGAA	0.00	0.00	0.00	0.00	0.00
GATCAACGTTCTGCGGACGG	0.00	0.00	0.00	0.00	0.00
GATCAACGTTCTGCGGGGAA	0.00	0.00	0.00	0.00	0.34
GATCAACGTTCTGCGTAAAA	0.00	0.00	0.00	0.00	0.00
GATCAACGTTCTGCGTGAAA	0.69	0.00	0.29	0.00	0.00
GATCAACGTTCTGCGTGAGA	0.00	0.00	0.00	0.00	0.00
GATCAACGTTCTGCGTGGAA	0.23	0.65	0.00	0.34	0.00
GATCAACGTTCTGCGTGGCT	0.00	0.00	0.00	0.00	0.00
GATCAACGTTCTGCGTGGGA	0.69	1.63	0.57	2.04	1.03
GATCAACGTTCTGCGTGGGC	5.04	6.18	4.01	5.43	2.41

GATCAACGTTCTGCGTGGTA	0.00	0.00	0.00	0.00	0.00
GATCAACGTTCTGCGTGGTC	0.00	0.00	0.00	0.68	0.00
GATCAACGTTCTTGTGCTGC	0.00	0.00	0.00	0.00	0.00
GATCAACGTTGAACGCCCCC	0.00	0.00	0.00	0.00	0.00
GATCAACGTTGAAGATCTGC	0.23	0.00	0.00	0.00	0.00
GATCAACGTTGAAGTGGACA	0.23	0.00	0.00	0.00	0.00
GATCAACGTTGAATAGAAAC	0.23	0.65	0.00	0.00	1.03
GATCAACGTTGACAGCTCCG	0.00	0.00	0.00	0.68	0.34
GATCAACGTTGACCTCGCCG	0.23	0.00	0.00	0.00	0.00
GATCAACGTTGAGGAAACGG	0.00	0.00	0.00	0.00	0.00
GATCAACGTTGAGGGATGAC	0.23	0.00	0.00	0.00	0.00
GATCAACGTTGAGGTATGAC	0.46	0.00	0.00	0.00	0.00
GATCAACGTTGATACGAACG	0.00	0.00	0.00	0.00	0.00
GATCAACGTTGATGAACGTC	0.92	0.33	1.43	0.68	0.69
GATCAACGTTGATGACTTGA	0.00	0.00	0.29	2.04	0.00
GATCAACGTTGATGACTTGT	0.00	0.00	0.00	0.00	0.00
GATCAACGTTGATGAGTGGT	0.00	0.65	0.00	0.00	0.00
GATCAACGTTGATGGACGTC	0.23	0.00	0.00	0.00	0.00
GATCAACGTTGATGGCCGTC	0.00	0.00	0.00	0.00	0.34
GATCAACGTTGATGGTCACG	0.92	0.00	0.00	0.00	0.00
GATCAACGTTGATTGGTTGT	0.00	0.00	0.29	0.00	0.00
GATCAACGTTGCAAAAAAAA	0.00	0.00	0.00	0.34	0.00
GATCAACGTTGCAAACAGGC	0.00	0.33	0.00	0.00	0.00
GATCAACGTTGCAAACAGTG	0.46	0.33	0.29	0.00	0.00
GATCAACGTTGCACGGCAGC	0.00	0.00	0.29	0.34	0.00
GATCAACGTTGCAGAGCGCG	0.23	0.00	0.00	0.00	0.00
GATCAACGTTGCAGAGTGAA	0.00	0.00	0.00	0.34	0.00
GATCAACGTTGCAGAGTGCA	5.95	5.86	6.30	4.41	4.13
GATCAACGTTGCCACCTGAA	0.00	0.00	0.00	0.34	0.00
GATCAACGTTGCCATGCAGC	0.00	0.00	0.00	0.00	0.00
GATCAACGTTGCCTGATGTG	0.00	0.00	0.00	0.00	0.34
GATCAACGTTGCGCGTGCCA	0.00	0.65	0.00	0.00	0.00
GATCAACGTTGCGCTGTCCG	0.00	0.00	0.00	0.00	0.00
GATCAACGTTGCGGCGGGGG	0.00	0.00	0.29	0.00	0.00
GATCAACGTTGCGTTGCACG	0.00	0.00	0.00	0.00	0.69
GATCAACGTTGCTGAAGACT	0.00	0.00	0.00	0.00	0.00
GATCAACGTTGCTGACAATA	0.00	0.00	0.29	0.00	0.00

GATCAACGTTGCTGCTTTGC	0.00	0.33	0.00	0.00	0.00
GATCAACGTTGCTGGAGCCG	0.00	0.33	0.00	0.00	0.00
GATCAACGTTGGAATCCAAG	0.00	0.00	0.00	0.00	0.00
GATCAACGTTGGACCTACAG	0.00	0.00	0.00	0.00	0.34
GATCAACGTTGGAGGAGGTA	0.00	0.00	0.00	0.00	0.69
GATCAACGTTGGCCTGATTC	0.00	0.00	0.29	0.00	0.00
GATCAACGTTGGCGAGCGAG	0.23	0.00	0.00	0.00	0.00
GATCAACGTTGGCGGCGAGG	0.00	0.00	0.00	0.00	0.00
GATCAACGTTGGCTGATTGC	0.23	0.00	0.57	0.34	0.00
GATCAACGTTGGGCAGGTTG	0.00	0.33	0.00	0.00	0.00
GATCAACGTTGGGCATGCAG	0.00	0.00	0.29	0.00	0.00
GATCAACGTTGGGCGTGGAA	0.00	0.00	0.00	0.00	0.00
GATCAACGTTGGGGAACTGC	0.00	0.00	0.00	0.00	0.34
GATCAACGTTGGTAGACTTT	0.00	0.00	0.00	0.00	0.00
GATCAACGTTGGTGCTCCGT	0.00	0.65	0.57	0.00	0.00
GATCAACGTTGGTGGCAGGC	0.00	0.00	0.00	0.00	0.00
GATCAACGTTGGTGGCCGGC	0.00	0.00	0.00	0.00	0.00
GATCAACGTTGTCTCGCAGG	0.23	0.33	0.29	2.38	1.03
GATCAACGTTGTCTTTGCGC	0.00	0.33	0.00	0.00	0.00
GATCAACGTTGTGAGCTGCA	0.00	0.00	0.29	0.00	0.00
GATCAACGTTGTGCACTGCC	0.23	0.00	0.00	0.00	0.00
GATCAACGTTGTGCTCTACA	0.00	0.00	0.00	0.00	0.00
GATCAACGTTGTGGCTTTGT	0.00	0.00	0.00	0.00	0.34
GATCAACGTTGTGTGGTTCC	0.00	0.00	0.00	0.00	0.00
GATCAACGTTGTTTTTTTCG	0.00	0.00	0.00	0.00	0.00
GATCAACGTTTAAAAAAAAA	0.46	0.00	0.86	0.34	0.00
GATCAACGTTTAGGACCAAT	0.00	0.00	0.00	0.00	0.00
GATCAACGTTTATCCTCTGC	0.00	0.00	0.00	0.00	0.34
GATCAACGTTTCAAAAAAAA	2.52	0.98	0.00	0.34	1.72
GATCAACGTTTCATCATTCC	0.00	0.00	0.00	0.00	0.00
GATCAACGTTTCCAAAAAAA	0.00	0.00	0.00	0.34	0.00
GATCAACGTTTCCACAGTTT	0.00	0.00	0.29	0.00	0.00
GATCAACGTTTCCGACAAGA	0.00	0.00	0.00	0.00	0.00
GATCAACGTTTCGGCAATGA	0.00	0.00	0.00	0.00	0.34
GATCAACGTTTCGTGTCCGG	0.00	0.00	0.00	0.00	0.34
GATCAACGTTTCTAACTGTG	0.00	0.00	0.29	0.00	0.00
GATCAACGTTTCTGTGTCCT	0.00	0.00	0.00	0.00	0.69

GATCAACGTTTGAACATGTG	0.00	0.33	0.00	0.00	0.00
GATCAACGTTTGAACCTATG	0.00	0.00	0.00	0.34	0.00
GATCAACGTTTGAACCTGTG	6.64	6.51	4.87	10.18	7.92
GATCAACGTTTGAGCCAGAC	0.23	0.00	0.00	0.00	0.00
GATCAACGTTTGAGCCTCAA	0.23	0.33	0.00	0.00	0.00
GATCAACGTTTGCGAATTAT	0.00	0.33	0.00	0.00	0.00
GATCAACGTTTGCGCTGTCC	0.92	0.00	0.00	0.00	0.69
GATCAACGTTTGGGTTCGGC	0.23	0.00	0.00	0.00	0.00
GATCAACGTTTTAAAAAAAA	0.00	0.00	0.00	0.00	0.00
GATCAACGTTTTCGACGGGG	0.00	0.00	0.00	0.68	0.34
GATCAACGTTTTGAGGTAAA	0.46	0.00	0.00	0.00	0.00
GATCAACGTTTTGAGGTCCA	0.23	0.00	0.86	0.00	0.00
GATCAACGTTTTGAGGTCCC	2.29	2.93	6.01	6.11	2.76
GATCAACGTTTTGAGGTCCT	0.00	0.00	0.29	0.00	0.00
GATCAACGTTTTGAGGTCNC	0.00	0.00	0.00	0.00	0.00
GATCAACGTTTTGAGGTCTC	0.00	0.00	0.00	0.00	0.34
GATCAACGTTTTGAGGTNNN	0.23	0.00	0.00	0.00	0.00
GATCAACGTTTTGCAGACTC	0.00	1.30	0.29	0.34	0.00
GATCAACGTTTTGCAGAGTA	0.23	0.00	0.00	0.00	0.00
GATCAACGTTTTGCGGTCCA	0.00	0.00	0.00	0.34	0.00
GATCAACGTTTTGGAATGGC	0.00	0.00	0.00	0.00	1.38
GATCAACGTTTTTTAAAGAC	0.00	0.00	0.00	0.00	0.00
GATCAACGTTTTTTTCCAGC	0.00	0.00	0.29	0.00	0.00
GATCAACTAAAAAAAAAAAA	3.89	1.95	0.57	1.70	0.00
GATCAACTAAAAAAAAAAAT	0.00	0.00	0.00	0.00	0.00
GATCAACTAAAAAGAATTAG	0.00	0.00	0.00	0.00	0.34
GATCAACTAAAATCAAGTTG	0.00	0.00	0.00	0.00	0.00
GATCAACTAAAATCCATAAG	0.23	0.00	0.00	0.00	0.00
GATCAACTAAACAGAAAAAA	0.23	0.00	0.00	0.00	0.00
GATCAACTAAACAGAATTGG	0.23	1.30	0.57	1.70	1.38
GATCAACTAAACAGAATTGT	0.00	0.00	0.00	0.00	0.34
GATCAACTAAACTGTTTTCT	0.23	0.00	0.00	0.00	0.00
GATCAACTAAATATAACTTT	0.00	0.00	0.29	0.00	0.00
GATCAACTAAATGACGGAGA	0.23	0.98	0.29	0.00	0.00
GATCAACTAAATTAGAATAT	0.00	0.00	0.00	0.00	0.34
GATCAACTAAATTGTGTGTG	0.00	0.00	0.00	0.00	0.00
GATCAACTAAATTTGCCAAT	0.00	0.00	0.57	0.00	0.00

GATCAACTAACAACAGGCAC	0.00	0.00	0.00	0.00	0.00
GATCAACTAACACGTGCGCC	0.00	0.00	0.29	0.00	0.00
GATCAACTAACAGGCGCGAG	0.00	0.33	0.00	0.00	0.00
GATCAACTAACAGTTAAATT	0.46	0.00	0.00	0.00	0.00
GATCAACTAACAGTTATATT	0.23	0.00	0.00	0.00	0.00
GATCAACTAACCATGTGTGG	0.00	0.00	0.29	0.00	0.00
GATCAACTAACCCACACATG	0.00	0.00	0.00	0.00	0.34
GATCAACTAACCGGCCTGCA	0.00	0.00	0.00	0.00	0.34
GATCAACTAACCGTGCTGGC	0.00	0.00	0.00	0.00	0.00
GATCAACTAACCTGCCCGTC	0.23	0.00	0.00	0.00	0.00
GATCAACTAACGAAAAAATT	0.00	0.00	0.00	0.34	0.00
GATCAACTAACGTACGGCCA	0.00	0.33	0.00	0.00	0.00
GATCAACTAACGTCCGGTCA	0.00	0.00	0.57	0.00	0.34
GATCAACTAACGTCCGGTTA	0.23	0.00	0.00	0.00	0.00
GATCAACTAACGTGGAATGC	0.23	0.00	0.00	0.00	0.00
GATCAACTAACGTGTTGCTG	0.00	0.00	0.29	0.00	0.00
GATCAACTAACTCATCGCTG	0.00	0.00	0.00	0.00	0.34
GATCAACTAAGAGGATATGT	1.60	0.33	0.57	2.38	1.72
GATCAACTAAGCAGCGCTTA	0.00	0.00	0.29	0.00	0.00
GATCAACTAAGGCACACGCG	0.00	0.00	0.29	0.00	0.00
GATCAACTAATACCTGCGGT	0.00	0.00	0.00	0.00	0.00
GATCAACTAATAGCTCTGCT	0.23	0.00	0.00	0.00	0.00
GATCAACTAATATAAGATGC	0.00	0.33	0.00	0.00	0.00
GATCAACTAATCGACCGGTC	0.00	0.00	0.29	0.00	0.00
GATCAACTAATCTCATGTTC	0.00	0.00	0.29	0.00	0.00
GATCAACTAATGAAAAAAAA	0.23	0.00	0.00	0.00	0.00
GATCAACTAATGACGCCAAA	0.23	0.00	0.00	0.00	0.00
GATCAACTAATGCAAATTGG	0.00	0.00	0.29	0.00	0.00
GATCAACTAATGGTCAAAGT	0.00	0.00	0.00	0.00	0.34
GATCAACTAATGTACAGATC	0.00	0.00	0.57	0.00	0.00
GATCAACTAATGTGCAATTC	0.00	0.00	0.29	0.00	0.00
GATCAACTAATTCTATTACA	0.00	0.00	0.29	0.00	0.00
GATCAACTAATTTTGCTGTG	0.00	0.00	0.00	0.00	0.00
GATCAACTACAAAAAAAAAA	0.00	0.33	0.00	0.00	0.00
GATCAACTACAACGCGAAGG	0.00	0.00	0.00	0.00	0.00
GATCAACTACAAGGGCAGCG	0.00	0.00	0.57	0.68	0.00
GATCAACTACAAGTATCCCA	0.00	0.00	0.29	0.00	0.00

GATCAACTACAAGTATCCGC	0.00	0.00	0.00	0.34	0.69
GATCAACTACAAGTGTCGTC	0.00	0.00	0.00	0.00	0.00
GATCAACTACAATCGAACCA	0.00	0.00	0.00	0.00	0.00
GATCAACTACACACGCAACA	0.00	0.00	0.00	0.00	0.00
GATCAACTACACACGGGTAA	0.00	0.00	0.00	0.00	0.34
GATCAACTACACAGGAGACT	0.00	0.33	0.00	0.00	0.34
GATCAACTACACATCACGAT	0.23	0.00	0.00	0.00	0.00
GATCAACTACACCGGCTACG	0.00	0.00	0.29	0.00	0.00
GATCAACTACACGAACTTCA	0.00	0.00	0.00	0.00	0.00
GATCAACTACAGAGGAAAAG	0.00	0.00	0.29	0.00	0.69
GATCAACTACAGCACTCCGT	0.23	0.65	0.00	0.34	0.00
GATCAACTACAGCAGGCACT	0.00	0.00	0.00	0.34	0.00
GATCAACTACAGCCTTGTTC	0.00	0.33	0.00	0.00	0.00
GATCAACTACAGCGATTGTG	0.00	0.00	0.00	0.34	0.00
GATCAACTACAGCGCCTCCA	0.00	0.33	0.00	0.00	0.00
GATCAACTACAGCTCTGGCG	0.00	0.00	0.00	0.34	0.00
GATCAACTACAGCTGCGAGG	0.46	0.33	0.29	0.00	0.69
GATCAACTACAGCTGCGCTC	0.00	0.00	0.00	0.00	0.00
GATCAACTACAGCTGCGTGG	0.00	0.00	0.57	0.00	0.00
GATCAACTACAGGCGGAGGG	0.00	0.00	0.00	0.34	0.00
GATCAACTACAGGCGGAGGT	0.00	0.00	0.00	1.02	0.00
GATCAACTACAGGCTGGAGT	0.00	0.00	0.00	0.00	0.00
GATCAACTACAGGTGTGTGA	0.00	0.00	0.00	0.00	0.00
GATCAACTACAGTCCGGTAA	0.00	0.33	0.00	0.00	0.00
GATCAACTACAGTCCGGTCA	0.00	0.00	0.29	0.00	0.00
GATCAACTACAGTGGAGACT	0.00	0.00	0.00	0.00	0.00
GATCAACTACATATGTGAGA	0.23	0.00	0.29	0.34	0.00
GATCAACTACATCACGCAGC	0.00	0.00	0.00	0.00	0.00
GATCAACTACATCATCTCGA	0.00	0.00	0.29	0.00	0.00
GATCAACTACATCTGACGGG	0.00	0.00	0.00	0.00	0.34
GATCAACTACATGGACCTCG	0.00	0.33	0.00	0.00	0.00
GATCAACTACATGGAGCGCG	0.00	0.00	0.29	0.00	0.34
GATCAACTACATGGAGCGGC	0.00	0.00	0.00	0.00	0.00
GATCAACTACATGGGCTTCA	0.23	0.00	1.15	0.34	0.34
GATCAACTACATTGACACGT	0.23	0.00	0.00	0.00	0.00
GATCAACTACCAAACACAAC	0.00	0.33	0.29	0.00	0.00
GATCAACTACCAAGGCCTCT	0.00	0.33	0.00	0.00	0.00

GATCAACTACCAAGTGCGCT	0.00	0.33	0.00	0.00	0.00
GATCAACTACCACACAACAC	0.00	0.00	0.00	0.00	0.34
GATCAACTACCACATGATGC	0.00	0.00	0.00	0.34	0.00
GATCAACTACCAGCAGGCAC	10.76	14.31	7.45	12.90	12.74
GATCAACTACCAGCAGGCCT	0.00	0.00	0.00	0.34	0.00
GATCAACTACCAGCAGGTAC	0.00	0.00	0.00	0.00	0.34
GATCAACTACCAGCAGGTCG	0.23	0.00	0.00	0.00	0.00
GATCAACTACCAGCAGTCAC	0.00	0.00	0.29	0.00	0.00
GATCAACTACCATAAGGCGC	0.00	0.00	0.00	0.34	0.00
GATCAACTACCCGGCCTGCA	0.00	0.00	0.00	0.00	0.34
GATCAACTACCCTACGAAAC	0.00	0.00	0.57	1.02	0.00
GATCAACTACCCTTTGACTT	0.00	0.00	0.29	0.00	0.00
GATCAACTACCGCATCGTCC	0.00	0.00	0.00	0.00	0.00
GATCAACTACCGCATCTCGA	0.00	0.00	0.00	0.34	0.00
GATCAACTACCGCGAGATTG	0.00	0.33	0.00	0.00	0.00
GATCAACTACCGCTCCGAGG	0.00	0.00	0.00	0.34	0.00
GATCAACTACCGCTCGGTAC	0.92	1.30	1.15	0.34	0.00
GATCAACTACCGGCCGGTCA	0.00	0.00	0.29	0.34	0.00
GATCAACTACCGGGAATTCT	0.00	0.33	0.00	0.00	0.00
GATCAACTACCGTAAGGTCC	0.00	0.00	0.29	0.00	0.00
GATCAACTACCGTACGGTCA	0.00	0.33	0.00	0.00	0.00
GATCAACTACCGTCCGACCA	0.00	0.00	0.00	0.00	0.00
GATCAACTACCGTCCGCACA	0.00	0.00	0.00	0.34	0.00
GATCAACTACCGTCCGGACA	0.00	0.33	0.29	0.34	0.00
GATCAACTACCGTCCGGGCA	0.00	0.33	0.00	0.00	0.00
GATCAACTACCGTCCGGTAA	0.00	0.00	0.29	0.00	0.00
GATCAACTACCGTCCGGTCA	0.69	1.63	2.86	0.68	1.72
GATCAACTACCGTCCGGTCC	0.00	0.98	0.00	0.34	0.00
GATCAACTACCGTCCTGCCA	0.00	0.00	0.00	0.00	0.34
GATCAACTACCGTCCTTTCC	0.00	0.33	0.00	0.00	0.00
GATCAACTACCGTCGGGTCA	0.00	0.00	0.29	0.00	0.00
GATCAACTACCGTCGTGGCA	0.00	0.00	0.00	0.00	0.00
GATCAACTACCGTCTGGACA	0.23	0.00	0.00	0.00	0.00
GATCAACTACCGTGCGCTCT	0.00	0.00	0.00	0.00	0.00
GATCAACTACCGTGCTGCGC	0.00	0.00	0.29	0.00	0.00
GATCAACTACCGTGTAGCGA	0.00	0.00	0.00	0.00	0.00
GATCAACTACCGTGTCGTGC	0.23	0.00	0.00	0.00	0.00

GATCAACTACCGTGTCTTGC	9.16	10.74	6.59	5.43	6.20
GATCAACTACCGTGTTGCCA	0.00	0.33	0.00	0.00	0.00
GATCAACTACCGTGTTGCTC	0.00	0.00	0.00	0.00	0.00
GATCAACTACCGTGTTGCTG	0.00	0.00	0.29	0.00	0.00
GATCAACTACCGTGTTGGAA	0.00	0.00	0.00	0.00	0.34
GATCAACTACCGTGTTGGCG	0.00	0.00	0.29	0.00	0.00
GATCAACTACCTCATCCCGA	0.00	0.00	0.00	0.34	0.00
GATCAACTACCTCATCTAGA	0.00	0.33	0.57	0.00	0.00
GATCAACTACCTCATCTCGA	30.90	25.05	14.89	27.15	17.56
GATCAACTACCTCATCTCGC	0.00	0.33	0.29	0.34	0.00
GATCAACTACCTCATCTCGG	0.00	0.00	0.00	0.00	0.34
GATCAACTACCTCATCTCGT	0.00	0.33	0.00	0.00	0.00
GATCAACTACCTCATCTCNA	0.00	0.00	0.00	0.34	0.00
GATCAACTACCTCATCTCNN	0.00	0.00	0.00	0.00	0.00
GATCAACTACCTCATCTTGA	0.00	0.33	0.00	0.34	0.00
GATCAACTACCTCTGCGGAG	0.00	0.00	0.00	0.00	0.00
GATCAACTACCTCTGGGGAG	0.00	0.33	0.00	0.00	0.00
GATCAACTACCTGCGCGAAG	0.00	0.33	0.00	0.00	0.00
GATCAACTACCTGGAGTTCC	0.23	0.33	0.29	0.00	0.00
GATCAACTACCTTCCGGGCA	0.00	0.00	0.00	0.34	0.00
GATCAACTACGAAAAAAGAG	0.23	0.00	0.00	0.00	0.00
GATCAACTACGACACTGGCG	0.23	0.00	0.00	0.00	0.00
GATCAACTACGACCTCCCTG	0.00	0.00	0.00	0.00	0.69
GATCAACTACGACCTGGACT	0.00	0.00	0.00	0.00	0.00
GATCAACTACGACCTTCCAA	0.00	0.00	0.00	0.00	0.00
GATCAACTACGACCTTCCAG	0.00	0.00	0.00	0.68	0.34
GATCAACTACGACCTTCCAT	0.00	0.00	0.29	0.68	1.03
GATCAACTACGACCTTTCAA	0.00	0.33	0.00	0.00	0.00
GATCAACTACGACGAGTCCA	0.00	0.33	0.00	0.00	0.00
GATCAACTACGACGAGTGCA	0.00	0.00	0.29	0.00	0.00
GATCAACTACGACGAGTGGC	0.00	0.00	0.00	0.00	0.00
GATCAACTACGACGAGTTCA	1.37	2.60	2.29	1.70	4.48
GATCAACTACGACGCGCGCG	0.00	0.33	0.00	0.00	0.00
GATCAACTACGACGCTCATC	0.00	0.33	0.00	0.00	0.00
GATCAACTACGACGGGCAAA	0.00	0.00	0.00	0.00	0.00
GATCAACTACGACGTGCCAC	0.00	0.00	0.00	0.00	0.00
GATCAACTACGACTTCTTCG	0.00	0.00	0.00	0.00	0.00

GATCAACTACGAGAAGGTCC	0.00	0.33	0.00	0.00	0.00
GATCAACTACGAGAAGGTCG	0.46	0.98	2.58	1.36	1.72
GATCAACTACGAGAAGGTNG	0.00	0.33	0.00	0.00	0.00
GATCAACTACGAGAATGTTA	0.00	0.00	0.00	0.00	0.34
GATCAACTACGAGACCACCA	0.00	0.65	0.29	0.68	1.03
GATCAACTACGAGCAGCTCC	0.46	0.00	0.00	0.00	0.00
GATCAACTACGAGCCGTTTG	0.00	0.33	0.00	0.00	0.00
GATCAACTACGAGGAGTACT	0.00	0.00	0.00	0.00	0.00
GATCAACTACGAGGGCCGAG	0.00	0.00	0.00	0.34	0.00
GATCAACTACGATGCTCCTG	0.00	0.00	0.00	0.00	0.34
GATCAACTACGCACGGATAA	0.00	0.00	0.00	0.00	0.34
GATCAACTACGCACGGATAT	0.00	0.00	0.00	0.00	0.34
GATCAACTACGCACGGCAAA	0.00	0.00	0.00	0.00	0.00
GATCAACTACGCACGGGCAA	0.00	0.33	0.29	0.34	0.00
GATCAACTACGCACGGGGAA	0.23	0.00	0.00	0.00	0.34
GATCAACTACGCACGGGGAC	0.00	0.00	0.00	0.00	0.00
GATCAACTACGCACGGGNNN	0.23	0.00	0.00	0.00	0.00
GATCAACTACGCACGGGTAA	8.93	34.81	38.09	29.53	48.56
GATCAACTACGCACGGGTAC	0.00	0.00	0.00	0.00	0.00
GATCAACTACGCACGGGTCA	0.23	0.00	0.29	0.00	0.00
GATCAACTACGCACGGGTGA	0.00	0.00	0.29	0.00	0.00
GATCAACTACGCACGGGTTA	0.23	0.00	0.29	0.00	0.00
GATCAACTACGCAGTCGTGG	2.06	0.65	2.00	1.02	1.38
GATCAACTACGCAGTGCAGG	0.00	0.33	0.29	0.00	0.00
GATCAACTACGCCAACGTCA	0.00	0.00	0.00	0.00	0.34
GATCAACTACGCCCTCACGA	0.00	0.00	0.00	0.00	0.34
GATCAACTACGCCCTCACGC	0.00	0.00	0.00	0.00	0.00
GATCAACTACGCCTTGTACC	0.00	0.00	0.00	0.00	0.00
GATCAACTACGCGGGCATAC	0.00	0.00	0.00	0.34	0.00
GATCAACTACGCGTGCCGAT	0.00	0.00	0.00	0.00	0.00
GATCAACTACGGACGGGTAG	0.00	0.33	0.00	0.00	0.00
GATCAACTACGGAGGGGACG	0.23	0.00	0.00	0.00	0.00
GATCAACTACGGCCAACCTC	0.92	0.00	0.00	2.04	0.34
GATCAACTACGGCCTGCGCG	0.00	0.00	0.00	0.00	0.00
GATCAACTACGGCTTCTATC	0.69	0.33	0.29	0.00	0.00
GATCAACTACGGCTTCTTTC	0.00	0.00	0.00	0.00	0.00
GATCAACTACGGGCCGTTCG	0.23	0.00	0.00	0.00	0.00

GATCAACTACGGGGGCAGGG	0.00	0.00	0.29	0.34	0.00
GATCAACTACGGGGGCGGCG	0.00	0.33	0.00	0.00	0.00
GATCAACTACGGTGGTACAC	0.00	0.00	0.29	0.00	0.00
GATCAACTACGTAGAGGTTG	0.46	0.33	0.00	0.00	0.34
GATCAACTACGTCATCTCGA	0.00	0.00	0.00	0.34	0.00
GATCAACTACGTCGGCCTGG	0.00	0.00	0.00	0.00	0.00
GATCAACTACTACCGGGAGT	0.00	0.00	0.00	0.00	0.00
GATCAACTACTACGAAGTTG	0.46	0.33	0.00	1.02	0.00
GATCAACTACTACGTCTTCA	0.23	0.00	0.00	0.00	0.00
GATCAACTACTATGAGGTCT	0.00	0.00	0.00	0.00	0.00
GATCAACTACTCACGGGTAA	0.00	0.00	0.00	0.00	0.00
GATCAACTACTCGCCGAAGG	0.00	0.00	0.29	0.00	0.00
GATCAACTACTCGTTCTACA	0.00	0.33	0.00	0.34	0.00
GATCAACTACTGACTGAAAA	0.00	0.33	0.00	0.00	0.00
GATCAACTACTGCAGCGGTC	0.46	0.00	0.00	0.00	0.00
GATCAACTACTGCCGCCGTA	0.23	0.00	0.00	0.00	0.00
GATCAACTACTGGAAGGTCA	0.00	0.33	0.00	0.00	0.00
GATCAACTACTGGAAGTTCA	0.00	0.00	0.00	0.00	0.00
GATCAACTACTGGAAGTTCC	0.00	0.00	0.29	0.68	0.00
GATCAACTACTGGAATTCAT	0.00	0.00	0.00	0.00	0.00
GATCAACTACTGTACGGTCT	0.00	0.00	0.00	0.00	0.34
GATCAACTACTTCACTGACA	0.23	0.00	0.00	0.00	0.00
GATCAACTACTTCACTGCCA	6.64	4.55	3.44	2.04	1.38
GATCAACTACTTCACTGCNN	0.23	0.00	0.00	0.00	0.00
GATCAACTACTTCACTGTCA	0.00	0.00	0.00	0.00	0.34
GATCAACTACTTCACTTCCA	0.00	1.30	0.00	0.34	0.00
GATCAACTACTTCATTTGGC	0.00	0.00	0.00	0.00	0.00
GATCAACTACTTCCTGTATT	0.92	1.63	0.00	0.68	0.00
GATCAACTACTTGCACTACC	0.00	0.00	0.29	0.00	0.00
GATCAACTACTTGCATGGGA	0.46	0.00	0.00	0.00	0.00
GATCAACTACTTGCGCACGC	0.00	0.00	0.00	0.00	0.00
GATCAACTACTTGCGCCACC	0.00	0.33	0.29	0.00	0.00
GATCAACTACTTGCGCNNNN	0.23	0.00	0.00	0.00	0.00
GATCAACTACTTGCGCTAAA	0.00	0.33	0.29	0.00	0.00
GATCAACTACTTGCGCTAAC	0.00	0.33	0.00	0.00	0.34
GATCAACTACTTGCGCTACA	0.00	0.00	0.00	0.00	0.69
GATCAACTACTTGCGCTACC	40.75	45.87	19.19	21.38	16.19

GATCAACTACTTGCGCTACN	0.46	0.00	0.00	0.00	0.00
GATCAACTACTTGCGCTCCC	0.23	0.33	0.00	0.00	0.00
GATCAACTACTTGCGCTGCC	0.23	0.00	0.00	0.00	0.00
GATCAACTACTTGCGCTNCC	0.00	0.33	0.00	0.00	0.00
GATCAACTACTTGCGGTTAA	0.00	0.00	0.00	0.00	0.00
GATCAACTACTTGCTCAAAA	0.00	0.00	0.00	0.34	0.00
GATCAACTACTTGTTTGCTA	0.00	0.00	0.00	0.00	0.34
GATCAACTACTTTGCCCGCG	0.00	0.65	0.00	0.00	0.00
GATCAACTAGAATGATGCGT	0.00	0.00	0.00	0.00	0.00
GATCAACTAGACAACATGAC	0.00	0.00	0.00	0.00	0.00
GATCAACTAGACCCTCGCAG	0.00	0.00	0.29	0.00	0.00
GATCAACTAGAGAAAAAAAA	0.00	0.00	0.00	0.34	0.00
GATCAACTAGAGAAGAAGAA	0.00	0.00	0.29	0.00	0.00
GATCAACTAGAGAAGTGCAT	0.00	0.00	0.00	0.68	0.00
GATCAACTAGATGAGCCGGT	0.00	0.00	0.29	0.00	0.00
GATCAACTAGATGCGCAGGT	0.00	0.00	0.00	0.00	0.34
GATCAACTAGCACATTGGGC	0.00	0.33	0.00	0.00	0.00
GATCAACTAGCAGTCTGCTG	0.00	0.00	0.00	0.00	0.34
GATCAACTAGCAGTGAGCCC	0.00	0.00	0.00	0.00	0.34
GATCAACTAGCATAATTCCA	0.00	0.00	0.00	0.00	0.34
GATCAACTAGCCACAGCATA	0.00	0.00	0.00	0.00	0.34
GATCAACTAGCCGCAACAAC	0.00	0.00	0.00	0.00	0.34
GATCAACTAGCGCACTCCAG	0.00	0.00	0.00	0.00	0.00
GATCAACTAGCGTCACTCAG	0.00	0.00	0.00	0.00	0.34
GATCAACTAGCGTCCGGGAA	0.00	0.00	0.29	0.00	0.00
GATCAACTAGCGTCGGGTCA	0.23	0.00	0.00	0.00	0.00
GATCAACTAGCGTGAATGCC	0.00	0.33	0.00	0.00	0.00
GATCAACTAGCTAGAGTATC	0.23	0.00	0.00	0.00	0.00
GATCAACTAGCTGCAGCATA	1.14	0.65	1.15	0.68	1.72
GATCAACTAGCTGCAGCNTC	0.00	0.00	0.29	0.00	0.00
GATCAACTAGGACTGCCATG	0.00	0.00	0.00	0.00	0.69
GATCAACTAGGATAGTGGCC	0.00	0.00	0.00	0.00	0.34
GATCAACTAGGCCAGAGAGG	0.00	0.00	0.00	0.00	0.00
GATCAACTAGGCGTGTACAT	0.00	0.00	0.00	0.00	0.34
GATCAACTAGGGCTGTGTTG	0.46	0.33	0.57	1.02	0.69
GATCAACTAGTAATGCTACA	0.00	0.00	0.29	0.00	0.00
GATCAACTAGTCAGGGCTCA	0.00	0.00	0.00	0.00	0.69

GATCAACTAGTCCTGTTGCT	0.00	0.00	0.00	0.00	0.34
GATCAACTAGTGGCAGAGGG	0.00	0.00	0.29	0.00	0.00
GATCAACTAGTGTAAACCAT	0.00	0.00	0.57	0.00	0.69
GATCAACTAGTGTGCATAAG	0.00	0.00	0.00	0.00	0.00
GATCAACTAGTTCAGGAATT	0.00	0.00	0.00	0.00	0.34
GATCAACTAGTTCAGGACTT	2.29	3.90	1.15	3.73	2.07
GATCAACTAGTTCAGGNNNN	0.23	0.00	0.00	0.00	0.00
GATCAACTAGTTTGCTGATT	0.00	0.00	0.00	0.34	0.00
GATCAACTATAAGCATGGTC	0.23	0.00	0.00	0.00	0.00
GATCAACTATACGAAATTTG	0.46	0.65	0.00	1.02	0.00
GATCAACTATAGAACAGCTT	0.00	0.00	0.00	0.00	0.00
GATCAACTATCAAACCTGCT	0.00	0.00	0.00	0.00	0.34
GATCAACTATCAACCGGAGA	0.00	0.00	0.29	0.00	0.00
GATCAACTATCAACGAGCAA	0.00	0.00	0.57	0.00	0.00
GATCAACTATCAGAGCGAAC	0.00	0.00	0.00	0.00	0.00
GATCAACTATCAGGCCGGCT	0.00	0.00	0.00	0.00	0.00
GATCAACTATCATGCTCCCA	0.00	0.00	0.00	0.68	0.34
GATCAACTATCCAGGGGCCC	0.00	0.00	0.00	0.00	0.34
GATCAACTATCCCTGTCATG	0.46	0.00	0.00	0.00	0.00
GATCAACTATCCTGACATCA	0.23	0.00	0.00	0.00	0.00
GATCAACTATCCTGGCTTCC	0.00	0.65	0.00	0.00	0.34
GATCAACTATCGAACAGCTT	0.00	0.33	0.00	0.00	0.00
GATCAACTATCGCCAAATGC	1.60	0.00	0.00	0.00	0.34
GATCAACTATCGCCACNNNN	0.23	0.00	0.00	0.00	0.00
GATCAACTATCGCGGCCGCC	0.00	0.00	0.00	0.00	0.00
GATCAACTATCGCGGTCTGC	0.69	0.00	0.00	0.00	0.00
GATCAACTATCGTATGCCGT	0.23	0.00	0.00	0.00	0.00
GATCAACTATCGTGCAGGTG	0.92	0.98	0.00	0.34	0.34
GATCAACTATCGTGCCGTCG	0.00	0.00	0.00	0.34	0.00
GATCAACTATCTCATCTCGA	0.23	0.00	0.00	0.00	0.69
GATCAACTATCTCGTTGACC	0.23	0.98	0.00	0.34	0.69
GATCAACTATCTGTTTCTTC	0.00	0.00	0.29	0.00	0.00
GATCAACTATCTTGTCGGTA	0.00	0.65	0.00	0.00	0.00
GATCAACTATCTTTCAGTTG	0.00	0.00	0.00	0.00	0.69
GATCAACTATGAAAGGTACA	0.00	0.00	0.00	0.34	0.69
GATCAACTATGACATGCCGC	0.00	0.33	0.00	0.34	2.07
GATCAACTATGACATGCCNN	0.23	0.00	0.00	0.00	0.00

GATCAACTATGACCGGTTCT	0.00	0.00	0.00	0.34	0.00
GATCAACTATGACCTTCCAG	0.00	0.00	0.00	0.68	0.00
GATCAACTATGACCTTCCAT	0.23	1.30	1.43	1.36	0.69
GATCAACTATGACCTTCCCT	0.00	0.00	0.00	0.00	0.00
GATCAACTATGAGAAGTGCC	0.00	0.33	0.00	0.00	0.00
GATCAACTATGAGCCTCGCT	0.00	0.00	0.00	0.34	0.00
GATCAACTATGATACATTTG	0.00	0.00	0.29	0.00	1.72
GATCAACTATGCAGTCATGG	0.00	0.00	0.00	0.00	0.00
GATCAACTATGCAGTCCTTG	0.00	0.00	0.00	0.00	0.00
GATCAACTATGCGGCGATGG	0.00	0.33	0.00	0.00	0.00
GATCAACTATGCTTTGTTCT	0.00	0.00	0.00	0.00	0.34
GATCAACTATGGACTTCGTA	0.00	0.00	0.00	0.68	0.00
GATCAACTATGGACTTCTTA	1.37	5.21	2.86	3.39	4.13
GATCAACTATGGACTTNTNN	0.23	0.00	0.00	0.00	0.00
GATCAACTATGGGACTGTGC	0.00	0.00	0.29	0.00	0.00
GATCAACTATGTCCCATTCG	0.00	0.00	0.00	0.34	0.00
GATCAACTATGTGACATCCA	0.00	0.00	0.00	0.00	0.00
GATCAACTATGTGACATCCT	0.00	0.00	0.00	0.00	0.00
GATCAACTATGTGTTTGCCT	0.00	0.33	0.00	0.00	0.00
GATCAACTATGTTCTGTTCT	0.00	0.00	0.29	0.00	1.72
GATCAACTATTAAAAAAAAA	0.00	0.00	0.00	0.34	0.00
GATCAACTATTGAACAAAAA	1.14	0.00	0.00	0.00	0.00
GATCAACTATTGAACAGATT	0.00	0.00	0.00	0.00	0.00
GATCAACTATTGAACAGCAA	0.23	0.00	0.00	0.00	0.00
GATCAACTATTGAACAGCAC	0.00	0.00	0.29	0.00	0.00
GATCAACTATTGAACAGCAT	0.00	0.00	0.29	0.00	0.34
GATCAACTATTGAACAGCCC	0.23	0.00	0.00	0.00	0.00
GATCAACTATTGAACAGCCT	0.69	0.33	0.29	0.00	0.34
GATCAACTATTGAACAGCGT	0.23	0.00	0.00	0.00	0.00
GATCAACTATTGAACAGCNN	0.46	0.00	0.00	0.00	0.00
GATCAACTATTGAACAGCTC	0.69	0.00	0.00	0.34	0.00
GATCAACTATTGAACAGCTG	0.00	0.33	0.29	0.00	0.00
GATCAACTATTGAACAGCTN	0.92	0.00	0.00	0.00	0.00
GATCAACTATTGAACAGCTT	128.19	44.57	32.93	56.01	52.69
GATCAACTATTGAACAGGTT	0.00	0.00	0.57	0.00	0.00
GATCAACTATTGAACAGTCG	0.00	0.00	0.00	0.00	0.00
GATCAACTATTGAACAGTTT	0.00	0.00	0.00	0.00	0.34

GATCAACTATTGAACANNNN	0.23	0.00	0.00	0.00	0.00
GATCAACTATTGAACATCTT	0.00	0.00	0.00	0.00	0.34
GATCAACTATTGAACCGCTT	0.23	0.00	0.00	0.00	0.00
GATCAACTATTGAACCNNNN	0.46	0.00	0.00	0.00	0.00
GATCAACTATTGAACGGCAA	0.00	0.00	0.00	0.00	0.69
GATCAACTATTGCACCGCTT	0.00	0.00	0.29	0.00	0.00
GATCAACTATTGCACCTCTT	0.00	0.00	0.00	0.00	0.34
GATCAACTATTGCCGCTGCG	0.00	0.00	0.00	0.00	0.00
GATCAACTATTGCCGCTTCA	0.00	0.00	0.00	0.00	0.00
GATCAACTATTGGTGCAACC	0.00	0.00	0.00	0.00	0.69
GATCAACTATTGTCCAGAGG	6.18	2.28	2.29	3.05	3.44
GATCAACTATTGTTCACCGC	0.00	0.00	0.86	0.00	0.00
GATCAACTATTTAACAGCTT	0.23	0.00	0.00	0.00	0.00
GATCAACTATTTCAAGGAGC	0.00	0.33	0.00	0.34	0.00
GATCAACTATTTGTTGTCGC	0.23	0.00	0.00	0.00	0.00
GATCAACTATTTTGACATAC	0.00	0.00	0.00	0.00	0.00
GATCAACTCAAAAAAAAAAA	19.69	4.55	5.15	8.15	4.82
GATCAACTCAAAAAAAAAAT	0.92	0.00	0.00	0.34	0.00
GATCAACTCAAAAAAAACAA	0.00	0.33	0.00	0.00	0.00
GATCAACTCAAAAAAAAGAA	0.00	0.00	0.00	0.00	0.00
GATCAACTCAAAAAAAGAAA	0.00	0.33	0.00	0.00	0.00
GATCAACTCAAAAAAGAAAA	0.69	0.00	0.00	0.00	0.00
GATCAACTCAAAAAGAGGTG	0.23	0.33	0.29	0.00	0.00
GATCAACTCAAAAAGCCAAA	0.00	0.33	0.00	0.00	0.69
GATCAACTCAAAAATAGAAA	0.00	0.00	0.00	0.00	0.00
GATCAACTCAAACAAAAAAA	0.00	0.00	0.00	0.00	0.00
GATCAACTCAAACAAACACA	0.00	0.00	0.29	0.00	0.69
GATCAACTCAAACCAAACCA	0.00	0.00	0.00	0.00	0.34
GATCAACTCAAAGAAGGAGA	0.00	0.00	0.00	0.00	0.00
GATCAACTCAAAGAAGGAGG	0.92	0.00	0.57	0.00	0.69
GATCAACTCAAAGAGATGTG	0.00	0.00	0.57	0.00	0.00
GATCAACTCAAAGCCACTGG	0.23	0.00	0.00	0.00	0.00
GATCAACTCAAAGCTGCCTT	0.00	0.00	0.29	0.00	0.00
GATCAACTCAAAGGGGTAAA	0.46	0.00	0.00	0.00	0.00
GATCAACTCAAAGGGGTAGA	0.92	0.33	0.00	1.36	0.00
GATCAACTCAAATTCTGAGG	0.46	0.00	0.00	0.34	0.00
GATCAACTCAACAACATGTT	0.00	0.00	0.00	0.00	0.69

GATCAACTCAACACAAAAAA	0.00	0.00	0.00	0.00	0.34
GATCAACTCAACACAACACA	0.00	0.00	0.00	0.00	0.34
GATCAACTCAACACTGGCGA	0.00	0.00	0.00	0.00	0.34
GATCAACTCAACCATCCGGC	1.37	0.65	0.29	0.00	0.34
GATCAACTCAACCCTCTTCG	0.00	0.00	0.00	0.34	0.00
GATCAACTCAACCCTCTTCT	0.46	0.00	0.00	0.34	0.34
GATCAACTCAACGACGCAGG	0.00	0.00	0.00	0.00	0.00
GATCAACTCAACGCAGCTCA	0.00	0.00	0.00	0.00	2.07
GATCAACTCAACGCAGNTCA	0.00	0.00	0.00	0.00	0.34
GATCAACTCAACGCAGTTCA	0.00	0.00	0.00	0.00	0.34
GATCAACTCAACGCGCATGA	0.69	0.00	0.00	0.00	0.69
GATCAACTCAACGGAGGTGG	0.00	0.00	0.00	0.00	0.34
GATCAACTCAACGTGCAAGT	0.00	0.33	0.00	0.00	0.00
GATCAACTCAACGTTGTCGC	0.00	0.00	0.57	0.68	0.00
GATCAACTCAACTACAACCC	0.00	0.00	0.29	0.00	0.00
GATCAACTCAACTCAACAAC	0.00	0.00	0.00	0.00	0.34
GATCAACTCAACTCAACTCA	0.00	0.00	0.00	0.00	0.34
GATCAACTCAACTCAACTCC	0.23	0.00	0.00	0.00	0.00
GATCAACTCAACTTCTGATT	0.00	0.00	0.00	0.68	0.00
GATCAACTCAAGAAGAAAGC	0.00	0.00	0.00	0.00	0.00
GATCAACTCAAGATAAATTG	0.00	0.00	0.29	0.00	0.00
GATCAACTCAAGCATGGTAC	2.06	1.30	0.00	0.34	1.03
GATCAACTCAAGCATGGTGC	0.00	0.00	0.00	0.00	0.00
GATCAACTCAAGGAAATGGC	0.00	0.33	0.00	0.00	0.00
GATCAACTCAAGGATGACAC	0.23	0.00	0.00	0.00	0.00
GATCAACTCAAGGATTGCCT	0.00	0.00	0.00	0.34	0.69
GATCAACTCAAGGCTGGTGG	0.00	0.00	0.00	1.02	0.00
GATCAACTCAAGTCAACACA	0.00	0.00	0.00	0.00	0.00
GATCAACTCAAGTGCAGGTG	0.00	0.00	0.00	0.00	0.34
GATCAACTCAAGTGCTGGTG	0.23	0.00	0.00	0.00	0.00
GATCAACTCAAGTGCTTGAT	0.23	0.98	0.00	0.00	0.00
GATCAACTCAAGTGGGCAGA	0.00	0.00	0.00	0.00	0.34
GATCAACTCAAGTTTGACAG	0.00	0.00	0.00	0.00	0.00
GATCAACTCAAGTTTGTCAA	0.00	0.00	0.57	0.00	0.00
GATCAACTCAAGTTTGTCAC	0.00	0.00	0.29	0.00	0.00
GATCAACTCAAGTTTGTCAG	1.60	0.98	2.00	1.36	3.10
GATCAACTCAAGTTTGTCGG	0.00	0.00	0.00	0.34	0.00

GATCAACTCAAGTTTGTCNN	0.23	0.00	0.00	0.00	0.00
GATCAACTCAATCACCTCGA	0.00	0.33	0.00	0.68	0.00
GATCAACTCAATCGCCTCGA	0.23	0.33	0.86	1.02	0.69
GATCAACTCAATGCAAGTAG	0.00	0.00	0.00	0.00	0.34
GATCAACTCAATGCCCACCG	0.00	0.00	0.00	0.00	0.34
GATCAACTCAATGTAATGGC	0.23	0.00	0.00	0.00	0.00
GATCAACTCAATGTCATGGC	10.30	5.21	0.57	4.75	3.10
GATCAACTCAATTGCTTGAT	0.00	0.00	0.00	0.34	0.00
GATCAACTCACAAAAAAAAA	0.00	0.00	0.00	0.68	0.00
GATCAACTCACAAAAACAAA	0.00	0.33	0.00	0.00	0.00
GATCAACTCACAACGCCGCC	0.00	0.00	0.00	0.00	0.00
GATCAACTCACAACGCCGGC	0.00	0.00	0.00	0.00	0.00
GATCAACTCACAACGCCGTC	0.00	0.00	0.00	0.00	0.00
GATCAACTCACAACGCCGTN	0.00	0.00	0.00	0.34	0.00
GATCAACTCACACATTGGCG	0.00	0.00	0.57	0.00	0.00
GATCAACTCACAGAAAGTTG	0.00	0.00	0.29	0.00	0.00
GATCAACTCACAGCCTGCTC	0.00	0.33	0.00	0.00	0.00
GATCAACTCACAGCGGCAGA	0.00	0.00	0.29	0.00	0.00
GATCAACTCACAGGCCGACT	0.00	0.33	0.00	0.00	0.34
GATCAACTCACAGTAGCAGA	0.00	0.00	0.29	0.00	0.00
GATCAACTCACAGTGACAGA	0.23	0.00	0.00	0.00	0.00
GATCAACTCACAGTGGCAGA	15.34	26.03	28.64	17.31	21.70
GATCAACTCACAGTGGCAGC	0.00	0.00	0.29	0.00	0.00
GATCAACTCACAGTGGCAGG	0.23	0.00	0.00	1.36	0.00
GATCAACTCACAGTGGCAGN	0.23	0.00	0.00	0.00	0.34
GATCAACTCACAGTGGCANA	0.00	0.00	0.00	0.00	0.00
GATCAACTCACAGTGGCATA	0.23	0.00	0.00	0.00	0.00
GATCAACTCACAGTGGCCGA	0.00	0.00	0.00	0.00	0.34
GATCAACTCACAGTGGCGGA	0.00	0.00	0.00	0.00	0.34
GATCAACTCACAGTGGCTCG	0.00	0.00	0.00	0.00	0.34
GATCAACTCACAGTGGNAAA	0.00	0.00	0.00	0.00	0.34
GATCAACTCACATCACAGCC	0.00	0.00	0.00	0.00	1.03
GATCAACTCACATCTGCTTT	1.14	0.00	0.00	0.34	0.00
GATCAACTCACATGGGGATT	0.00	0.00	0.00	0.68	0.69
GATCAACTCACATGTGTGGA	0.00	0.00	0.29	0.00	0.00
GATCAACTCACCAATAAACC	0.00	0.00	0.00	0.00	1.38
GATCAACTCACCGCATTCAC	0.00	0.00	0.00	0.00	0.00

GATCAACTCACCTGCAGATG	0.00	0.00	0.00	0.34	0.00
GATCAACTCACCTTGCAGCG	0.00	0.00	0.00	0.00	0.00
GATCAACTCACGAAGCCGTG	0.00	0.00	0.00	0.00	0.34
GATCAACTCACGCAGCCCAC	0.23	0.00	0.00	0.00	0.00
GATCAACTCACGCAGCTGTG	0.00	0.00	0.00	0.00	0.00
GATCAACTCACGCCCGTCGC	0.00	0.00	0.00	0.00	0.00
GATCAACTCACGCCCGTCGT	0.00	0.00	0.00	0.00	0.00
GATCAACTCACGCCTCAGTC	0.00	0.00	0.00	0.00	0.34
GATCAACTCACGGATGACAC	0.00	0.00	0.29	0.00	0.00
GATCAACTCACGGATGGCAC	0.23	0.00	0.00	0.00	0.00
GATCAACTCACGGGTGGTGT	0.23	0.00	0.00	0.00	0.00
GATCAACTCACGTGATGACC	0.92	1.63	0.29	2.04	0.34
GATCAACTCACTCCCTACCC	0.00	0.00	0.57	0.00	0.00
GATCAACTCACTCCTCGAGG	0.00	0.00	0.00	0.00	0.00
GATCAACTCACTCCTCGAGN	0.00	0.00	0.00	0.00	0.00
GATCAACTCACTCGTATGCC	0.00	0.00	0.29	0.00	0.00
GATCAACTCACTGACGCCTG	0.00	0.00	0.29	0.00	0.00
GATCAACTCACTGCTGTCGA	0.23	0.00	0.00	0.00	0.00
GATCAACTCACTGGAGGAGC	0.00	0.00	0.00	0.00	0.00
GATCAACTCACTGTGAGTGA	0.00	0.65	0.00	0.00	0.00
GATCAACTCACTGTTGGGGA	0.23	0.00	0.57	0.00	0.00
GATCAACTCACTGTTGGGTC	0.00	0.33	0.00	0.00	0.00
GATCAACTCACTGTTGGTGA	25.87	24.72	12.60	21.04	17.56
GATCAACTCACTGTTGGTGC	0.23	0.65	0.29	0.00	0.00
GATCAACTCACTGTTGGTGG	0.23	0.00	0.00	0.00	0.00
GATCAACTCACTGTTGGTGN	0.23	0.00	0.00	0.00	0.00
GATCAACTCACTGTTGGTNA	0.00	0.00	0.00	0.34	0.00
GATCAACTCACTGTTGGTNN	0.46	0.00	0.00	0.00	0.00
GATCAACTCACTGTTGGTTA	0.00	0.00	0.29	0.00	0.00
GATCAACTCACTTGTCCATT	0.00	0.00	0.00	0.00	0.00
GATCAACTCAGAAAAAAAAA	0.23	0.65	0.00	0.00	0.00
GATCAACTCAGACCACACCT	0.00	0.00	0.00	0.00	0.00
GATCAACTCAGACGCAAAAA	0.00	0.00	0.00	0.34	0.00
GATCAACTCAGACGCCAAAA	0.00	0.00	0.29	0.00	0.69
GATCAACTCAGACTCAGCGC	0.00	0.33	0.00	0.00	0.00
GATCAACTCAGACTCTGACT	0.00	0.00	0.00	0.00	1.03
GATCAACTCAGACTGGTCCA	1.14	0.00	0.00	0.34	0.00

GATCAACTCAGACTGGTCCG	0.00	0.33	0.29	0.00	0.00
GATCAACTCAGAGAGGCGCA	0.00	0.00	0.00	0.00	0.00
GATCAACTCAGAGCTCGGGC	6.64	13.34	6.87	16.63	10.33
GATCAACTCAGAGCTTGGGA	0.00	0.00	0.00	0.00	0.00
GATCAACTCAGAGCTTGGGC	0.00	0.00	0.00	0.00	0.00
GATCAACTCAGAGTCGTTGC	0.00	0.00	0.00	0.00	0.69
GATCAACTCAGATGAATGAT	0.00	0.00	0.29	0.00	0.34
GATCAACTCAGATGTGTGGA	0.23	0.00	0.00	0.00	0.34
GATCAACTCAGATGTGTGGG	0.23	0.00	0.00	0.00	0.00
GATCAACTCAGATGTGTGGT	0.23	0.00	0.00	0.00	0.00
GATCAACTCAGCAAGGCCGA	0.23	0.00	0.00	0.34	0.00
GATCAACTCAGCACACACAA	0.00	0.00	0.00	0.00	0.34
GATCAACTCAGCAGAGCTGA	0.00	0.00	0.00	0.00	0.34
GATCAACTCAGCAGCGCTGA	0.00	0.00	0.00	0.00	0.34
GATCAACTCAGCAGCTGCAC	0.00	0.00	0.00	0.34	0.00
GATCAACTCAGCAGGCTTGT	0.00	0.00	0.00	0.00	0.00
GATCAACTCAGCAGGGCTGA	0.00	0.00	0.29	0.00	0.34
GATCAACTCAGCAGTCAACA	13.73	8.46	7.73	6.45	3.10
GATCAACTCAGCAGTCAACC	0.23	0.00	0.00	0.00	0.00
GATCAACTCAGCAGTCCACA	0.23	0.00	0.29	0.00	0.00
GATCAACTCAGCAGTCCCCT	0.00	0.00	0.29	0.00	0.00
GATCAACTCAGCAGTCGATA	0.00	0.00	0.00	0.00	0.00
GATCAACTCAGCAGTGACCG	0.00	0.00	0.00	0.00	0.00
GATCAACTCAGCAGTGATGA	0.00	0.00	0.29	0.00	0.00
GATCAACTCAGCAGTGCCGA	0.00	0.00	0.00	0.34	0.00
GATCAACTCAGCAGTGCTGA	13.96	30.58	60.71	37.68	56.83
GATCAACTCAGCAGTGCTGC	0.00	0.33	0.57	0.00	0.00
GATCAACTCAGCAGTGCTGT	0.00	0.00	0.00	0.00	0.34
GATCAACTCAGCAGTGNTGA	0.00	0.00	0.00	0.00	1.03
GATCAACTCAGCAGTGTTGA	0.00	0.00	0.00	0.34	0.00
GATCAACTCAGCCAGACCAG	0.00	0.33	0.00	0.00	0.00
GATCAACTCAGCCGGCTCTG	0.00	0.00	0.00	0.00	0.34
GATCAACTCAGCCGTGCTGG	0.00	0.00	0.29	0.00	0.00
GATCAACTCAGCCTGGATGG	0.00	0.00	0.00	0.00	0.00
GATCAACTCAGCCTTCTCCA	0.23	0.00	0.00	0.00	0.00
GATCAACTCAGCGACGCACA	0.46	0.00	0.00	0.00	0.69
GATCAACTCAGCGACGCACG	0.00	0.00	0.29	0.00	0.00

GATCAACTCAGCGACGCGCA	0.23	0.00	0.00	0.00	0.00
GATCAACTCAGCGATCGGCC	0.00	0.00	0.00	0.34	0.34
GATCAACTCAGCGATGGTTG	0.00	0.00	0.00	0.34	0.00
GATCAACTCAGCGCAACGCA	0.00	0.00	0.29	0.00	0.00
GATCAACTCAGCGCAGCCGC	0.69	1.30	0.29	0.00	0.69
GATCAACTCAGCGCGCATGG	0.23	0.00	0.00	0.00	0.00
GATCAACTCAGCGCGGGGAT	0.00	0.33	0.00	0.00	0.00
GATCAACTCAGCGCGGGGCG	0.23	0.00	0.00	0.00	0.00
GATCAACTCAGCGCGGGGCN	0.23	0.00	0.00	0.00	0.00
GATCAACTCAGCGCGGGGCT	12.59	13.99	9.45	17.99	11.02
GATCAACTCAGCGCGGGGGT	0.23	0.00	0.00	0.00	0.00
GATCAACTCAGCGCGGGGNN	0.23	0.00	0.00	0.00	0.00
GATCAACTCAGCGCTCAGGG	0.23	0.00	0.00	0.00	0.00
GATCAACTCAGCGCTCATCG	0.00	0.00	0.00	0.34	0.00
GATCAACTCAGCGCTCATGG	16.71	10.09	7.73	17.31	7.58
GATCAACTCAGCGCTCATGN	0.23	0.00	0.00	0.00	0.00
GATCAACTCAGCGCTCGTGG	0.23	0.00	0.00	0.00	0.00
GATCAACTCAGCGGGCCCGC	0.00	0.00	0.29	0.00	0.00
GATCAACTCAGCTGCAGGCG	0.00	0.00	0.29	0.00	0.00
GATCAACTCAGCTTTTTCAA	0.00	0.00	0.00	0.00	0.00
GATCAACTCAGCTTTTTCTC	0.00	0.00	0.00	0.00	0.00
GATCAACTCAGGAGATGCAC	0.00	0.00	0.00	0.00	0.34
GATCAACTCAGGAGTAGGAA	0.00	0.65	0.00	0.00	0.00
GATCAACTCAGGCAGAATAA	0.00	0.00	0.00	0.00	0.00
GATCAACTCAGGCAGATTCA	0.00	0.00	0.00	0.00	0.34
GATCAACTCAGGCAGCCGGT	0.00	0.00	0.00	0.00	0.34
GATCAACTCAGGCAGCCTAA	5.49	7.81	2.58	5.77	2.07
GATCAACTCAGGCAGCCTAN	0.00	0.00	0.00	0.00	0.00
GATCAACTCAGGCAGCCTGC	0.23	0.33	0.00	1.02	0.00
GATCAACTCAGGCAGCGTCC	0.00	0.00	0.00	0.00	0.00
GATCAACTCAGGCAGCNTCC	0.00	0.00	0.00	0.00	0.34
GATCAACTCAGGCAGCTTCA	0.00	0.00	0.00	0.34	0.00
GATCAACTCAGGCAGCTTCC	43.49	31.56	27.49	34.96	24.11
GATCAACTCAGGCAGCTTCG	0.00	0.00	0.29	0.34	0.00
GATCAACTCAGGCAGCTTCN	0.46	0.00	0.00	0.00	0.00
GATCAACTCAGGCAGCTTNN	0.23	0.00	0.00	0.00	0.00
GATCAACTCAGGCAGTTTAG	0.00	0.00	0.00	0.00	0.00

GATCAACTCAGGCATCACGT	0.00	0.00	0.00	0.68	0.00
GATCAACTCAGGCATTGGAG	0.00	0.00	1.43	0.00	0.00
GATCAACTCAGGCATTGTTC	0.00	0.00	0.57	0.00	0.00
GATCAACTCAGGCCGTTTCC	0.23	0.00	0.00	0.00	0.00
GATCAACTCAGGCGGCCTGA	0.00	0.00	0.00	0.00	0.00
GATCAACTCAGGCGTGCGTG	0.00	0.00	0.00	0.34	0.00
GATCAACTCAGGGGAGGAGA	0.00	0.00	0.00	0.00	0.00
GATCAACTCAGGTGATTCAT	0.23	0.00	0.00	0.00	0.00
GATCAACTCAGGTTCTTCCC	0.00	0.33	0.00	0.00	0.00
GATCAACTCAGGTTGCGGCT	0.00	0.00	0.00	0.34	0.00
GATCAACTCAGTAGACACAC	0.46	0.33	0.00	0.00	0.34
GATCAACTCAGTCAATCTTC	0.92	0.65	0.86	0.68	0.34
GATCAACTCAGTCAATGTTC	0.00	0.00	0.00	0.34	0.00
GATCAACTCAGTCGGGATGA	0.00	0.33	0.00	0.00	0.00
GATCAACTCAGTGGCAAGGC	0.46	0.00	0.00	0.00	0.34
GATCAACTCATAACAAATCA	0.00	0.00	0.00	0.00	0.69
GATCAACTCATAACACACAC	0.00	0.00	0.29	0.00	0.00
GATCAACTCATAATTGAATT	0.00	0.00	0.00	0.00	0.00
GATCAACTCATACATCCAAG	0.46	0.33	0.00	0.34	0.00
GATCAACTCATACATCCACG	0.00	0.00	0.00	0.00	0.00
GATCAACTCATACCTGCGGT	0.23	0.00	0.00	0.00	0.00
GATCAACTCATAGAATGCAA	0.00	0.00	0.00	0.00	0.34
GATCAACTCATAGCAAAACA	0.00	0.00	0.00	0.00	0.34
GATCAACTCATAGTGGCAGA	0.00	0.00	0.00	0.00	0.34
GATCAACTCATATGGCAATA	0.00	0.00	0.00	0.00	0.69
GATCAACTCATCAAAATCAA	0.00	0.00	0.00	0.34	0.00
GATCAACTCATCACTTGACG	0.00	0.33	0.00	0.34	0.00
GATCAACTCATCACTTGTCC	0.00	0.00	0.00	0.34	0.00
GATCAACTCATCACTTGTCG	0.00	0.33	0.00	3.39	0.69
GATCAACTCATCCAAGTTTG	0.00	0.33	0.00	0.00	0.34
GATCAACTCATCGCGTGATA	0.00	0.00	0.00	0.00	0.00
GATCAACTCATCGGCACAGG	0.00	0.00	0.29	0.00	0.00
GATCAACTCATCGGGACATG	0.23	0.00	0.00	0.00	0.00
GATCAACTCATCGGGAGAGA	0.00	0.00	0.29	0.00	0.69
GATCAACTCATCGTCCTGCT	0.00	0.33	0.00	0.34	0.00
GATCAACTCATCGTCGACCA	0.00	0.33	0.00	0.00	0.00
GATCAACTCATCTCTTGGCC	0.00	0.00	0.00	0.00	0.00

GATCAACTCATGACGCCAAA	0.00	0.00	0.29	0.00	0.00
GATCAACTCATGCACACTTA	0.00	0.00	0.29	0.00	0.00
GATCAACTCATGCAGCTTCC	0.00	0.00	0.57	0.00	0.00
GATCAACTCATGCCAATAAT	0.00	0.00	0.00	0.00	1.03
GATCAACTCATGCTCAAGCC	0.00	0.00	0.29	0.00	0.00
GATCAACTCATGCTCCCACC	0.00	0.00	0.00	0.00	0.00
GATCAACTCATGGAACATGT	0.00	0.00	0.00	0.00	0.00
GATCAACTCATGTAATGAAT	0.23	0.00	0.00	0.00	0.00
GATCAACTCATGTGCAGTTC	0.00	0.00	0.00	0.00	0.34
GATCAACTCATGTGCCTGGC	0.69	2.60	3.44	1.70	3.44
GATCAACTCATGTTCAAATC	0.00	0.33	0.00	0.00	0.00
GATCAACTCATTCATGTTGC	0.00	0.00	0.29	0.00	0.00
GATCAACTCATTCCACAACC	0.23	0.00	0.00	1.36	0.00
GATCAACTCATTGCAGGGTG	0.00	0.00	0.29	0.00	0.00
GATCAACTCATTGGATGTTG	0.00	0.00	0.00	0.00	0.00
GATCAACTCATTGGATTTTG	0.00	0.00	0.29	0.00	0.00
GATCAACTCATTGGGCGAGA	0.00	0.00	0.29	0.00	0.00
GATCAACTCATTGTAGTCGT	0.00	0.33	0.00	0.00	0.00
GATCAACTCCAAAAAAAAAA	0.23	0.00	0.00	0.34	0.00
GATCAACTCCAAAAAAAGAA	0.00	0.00	0.00	0.00	0.00
GATCAACTCCAAAAAGAAAA	1.14	0.00	0.00	0.00	0.00
GATCAACTCCAAAAGATAAA	0.46	0.00	0.00	0.00	0.00
GATCAACTCCAAACTTCCCT	0.00	0.00	0.00	0.00	0.34
GATCAACTCCAAAGGTGAGC	5.95	1.30	1.43	1.36	0.00
GATCAACTCCAACAGGCCGT	0.00	0.00	0.29	0.00	0.00
GATCAACTCCAACATCGGCA	0.00	0.00	0.00	0.00	0.00
GATCAACTCCAACATGACGG	0.00	0.33	0.00	1.70	0.00
GATCAACTCCAACTACCGGG	0.92	0.33	0.00	0.34	0.69
GATCAACTCCAAGAGATTGA	0.00	0.00	0.29	0.00	0.00
GATCAACTCCAAGCTGTAGT	0.00	0.33	0.00	0.00	0.00
GATCAACTCCAAGGGGTCTC	0.00	0.00	0.00	0.00	0.34
GATCAACTCCAAGGTGGACT	0.00	0.00	0.57	0.00	0.00
GATCAACTCCAATGGTTCCG	0.00	0.00	0.00	0.00	0.34
GATCAACTCCACAAAGCCGG	0.00	0.00	0.00	0.00	0.34
GATCAACTCCACAACGTTGA	0.23	0.00	0.00	0.00	0.00
GATCAACTCCACACAACACA	0.23	0.00	0.00	0.00	0.00
GATCAACTCCACACGCCACC	0.00	0.00	0.00	0.00	0.34

GATCAACTCCACAGCCCCAA	0.00	0.00	0.29	0.00	0.00
GATCAACTCCACAGCCCCCA	0.00	0.33	0.00	0.00	0.00
GATCAACTCCACCAAGGCCA	0.00	0.00	0.00	0.00	0.00
GATCAACTCCACCCATGTGC	0.23	0.00	0.00	0.00	0.00
GATCAACTCCACCGCACAGA	0.00	0.00	0.00	0.68	0.00
GATCAACTCCACCGGACGAT	0.00	0.00	0.00	0.34	0.00
GATCAACTCCACGAACTGTG	0.00	0.00	0.00	0.00	0.00
GATCAACTCCACGCACATGG	0.00	0.00	0.00	0.00	0.00
GATCAACTCCACGCAGATGG	12.36	7.81	3.15	9.16	3.79
GATCAACTCCACGCAGATGN	0.23	0.00	0.00	0.00	0.00
GATCAACTCCACGCTCCTGA	0.00	0.65	0.00	0.00	0.00
GATCAACTCCACGCTCGTGA	0.00	0.00	0.00	0.00	0.69
GATCAACTCCACGCTGATGT	0.00	0.00	0.00	0.34	0.00
GATCAACTCCACGCTGCTCT	0.00	0.65	0.00	0.00	0.00
GATCAACTCCACGGCGGCTG	1.37	0.65	0.29	0.68	0.34
GATCAACTCCACGGGTGCGG	0.00	0.00	0.00	0.34	0.00
GATCAACTCCACGTGCCATT	0.00	0.00	0.29	0.00	0.00
GATCAACTCCACGTGGTTGC	0.00	0.00	0.57	0.00	0.00
GATCAACTCCACGTGGTTTG	0.23	1.30	1.72	0.34	0.34
GATCAACTCCACTGCGAGAG	0.00	0.00	0.00	0.00	0.00
GATCAACTCCAGATGTCCCT	0.00	0.33	0.00	0.34	0.00
GATCAACTCCAGCAAGCATG	0.00	0.98	0.86	0.00	0.34
GATCAACTCCAGCATCTGGA	0.00	0.33	0.00	0.00	0.00
GATCAACTCCAGCATGACCC	0.00	0.33	0.29	0.00	0.00
GATCAACTCCAGCCAACGGG	0.00	0.00	0.00	0.00	0.00
GATCAACTCCAGCCATGAGA	0.00	0.33	0.00	0.00	0.00
GATCAACTCCAGGATGCAAG	0.00	0.00	0.00	0.00	0.34
GATCAACTCCAGGATGCAGG	0.00	0.00	0.00	0.00	0.00
GATCAACTCCAGGCGCTTGG	0.00	0.00	0.29	0.34	0.00
GATCAACTCCAGGCGGAGAG	0.23	0.00	0.00	0.00	0.34
GATCAACTCCAGGCTGCAAC	0.00	0.33	0.00	0.00	0.00
GATCAACTCCAGGGCCTGAG	0.00	0.00	0.00	0.34	0.00
GATCAACTCCAGGGTGCAAG	0.00	0.00	0.00	0.00	0.00
GATCAACTCCAGGGTGCATG	0.00	0.33	0.00	0.00	0.34
GATCAACTCCAGGGTGTCGA	0.00	0.00	0.00	0.00	0.00
GATCAACTCCAGGGTGTCGG	0.00	0.00	0.00	0.00	0.00
GATCAACTCCAGGTGGGTAC	1.83	0.98	2.58	1.70	0.34

GATCAACTCCAGGTTCTGGC	0.00	0.33	0.00	0.00	0.00
GATCAACTCCAGTACCATGC	0.00	0.00	0.00	0.34	0.00
GATCAACTCCAGTCCACTCC	0.00	0.00	0.00	0.34	0.00
GATCAACTCCAGTCCGGTCC	0.23	0.00	0.00	0.00	0.00
GATCAACTCCAGTGCCAGGT	0.23	0.00	0.00	0.00	0.00
GATCAACTCCAGTGCCAGTT	0.23	0.00	0.00	0.00	0.00
GATCAACTCCAGTGGGACGA	0.00	0.00	0.29	0.00	0.00
GATCAACTCCAGTGGGACGC	0.00	0.33	1.15	0.00	0.00
GATCAACTCCAGTGGGCCAC	0.00	0.33	0.00	0.00	0.00
GATCAACTCCAGTGGGCCGC	7.10	10.09	8.88	5.43	13.09
GATCAACTCCAGTGGGTCGC	0.23	0.00	0.00	0.00	0.00
GATCAACTCCAGTGTCACTT	0.23	0.00	0.00	0.00	0.00
GATCAACTCCATATCCATTG	0.00	0.00	0.00	0.00	0.34
GATCAACTCCATCCAATCAA	0.00	0.00	0.00	0.00	0.00
GATCAACTCCATCCAATCGA	0.00	0.33	0.00	0.00	0.00
GATCAACTCCATCCAGTCGA	0.00	0.00	0.00	0.34	0.00
GATCAACTCCATCCCAAGCT	0.23	0.00	0.00	0.00	0.00
GATCAACTCCATCCCAAGTG	0.00	0.00	0.00	0.00	0.00
GATCAACTCCATCCGAGCTC	0.23	0.00	0.00	0.00	0.00
GATCAACTCCATCGAATTCC	0.00	0.00	0.29	0.00	0.00
GATCAACTCCATCTCCATCG	0.00	0.00	0.29	0.00	0.34
GATCAACTCCATCTCCTTCG	0.46	0.00	0.00	0.00	0.34
GATCAACTCCATCTGCATTT	0.00	0.00	0.00	0.00	0.34
GATCAACTCCATGAACACGC	0.00	0.00	0.29	0.00	0.00
GATCAACTCCATGAGGACGC	0.00	0.00	0.00	0.00	0.00
GATCAACTCCATGAGGGCGC	0.00	0.00	0.00	0.00	0.34
GATCAACTCCATGATAGATG	0.00	0.00	0.00	0.34	0.00
GATCAACTCCATGCAGTTCA	0.23	0.33	0.29	0.00	0.00
GATCAACTCCATGCATCAAA	1.14	1.63	0.57	2.04	2.41
GATCAACTCCATGGCCTCAT	0.69	0.00	0.00	0.34	0.00
GATCAACTCCATGGCGCTGC	0.00	0.33	0.00	0.00	0.00
GATCAACTCCATGGCTGGTC	0.00	0.00	0.00	0.00	0.00
GATCAACTCCATGGTGCTCG	0.00	0.00	0.29	0.00	0.00
GATCAACTCCATGTGCCAAT	0.00	0.33	0.00	0.00	0.00
GATCAACTCCATGTGCCATG	0.00	0.00	0.29	0.00	0.00
GATCAACTCCATGTGCCATN	0.23	0.00	0.00	0.34	0.00
GATCAACTCCATGTGCCATT	10.07	22.77	44.96	25.80	38.92

GATCAACTCCATGTGCCCTT	0.00	0.00	0.29	0.00	0.00
GATCAACTCCATGTGCGATT	0.00	0.00	0.29	0.00	0.00
GATCAACTCCATGTGCNATT	0.00	0.00	0.00	0.00	0.34
GATCAACTCCATGTTCCATT	0.00	0.00	0.00	0.00	0.34
GATCAACTCCATTGACCGCA	0.00	0.00	0.00	0.00	0.34
GATCAACTCCATTGGCAGCG	0.00	0.00	0.29	0.00	0.00
GATCAACTCCATTGGCGGAG	0.00	0.33	0.00	0.00	0.00
GATCAACTCCATTGGCGGCG	0.00	1.95	1.43	2.04	1.03
GATCAACTCCATTGGCGGGG	0.00	0.00	0.00	0.34	0.00
GATCAACTCCATTGTGCTCG	0.00	0.00	0.00	0.00	0.00
GATCAACTCCATTTGCCAAA	0.23	0.33	1.15	0.34	1.72
GATCAACTCCATTTGCCATG	0.00	0.65	0.00	0.00	0.00
GATCAACTCCATTTGCCATT	0.23	0.00	0.00	0.00	0.00
GATCAACTCCCAATCGAGAC	0.69	0.00	0.29	0.00	0.00
GATCAACTCCCAGAAAAAAA	0.00	0.33	0.00	0.00	0.00
GATCAACTCCCAGAAGTTTG	0.00	0.00	0.00	0.00	0.00
GATCAACTCCCAGAATTCGC	0.00	0.00	0.00	0.00	0.00
GATCAACTCCCAGATGACAC	0.00	0.00	0.29	0.00	0.00
GATCAACTCCCAGCGCCGGC	0.00	0.33	0.00	0.00	0.00
GATCAACTCCCAGGTCTATT	0.00	0.33	0.00	0.00	0.00
GATCAACTCCCAGGTGTGCA	0.00	0.00	0.29	0.00	0.00
GATCAACTCCCATAGCGCAT	0.00	0.00	0.00	0.00	0.34
GATCAACTCCCATGACTCGC	0.00	0.00	0.00	0.00	0.00
GATCAACTCCCCCAAGGACG	0.00	0.33	0.00	0.00	0.00
GATCAACTCCCCCAAGGCCC	0.23	0.00	0.00	0.00	0.00
GATCAACTCCCCCTGGTTTC	0.23	0.00	0.00	0.00	0.00
GATCAACTCCCGACAGCTTG	0.00	0.00	0.29	0.00	0.00
GATCAACTCCCGCAATTGGC	0.00	0.00	0.00	0.00	0.00
GATCAACTCCCGCACCTGGG	0.00	0.00	0.00	0.00	0.34
GATCAACTCCCGCATCGACC	0.00	0.00	0.29	0.00	0.00
GATCAACTCCCGCCGGCCGC	0.00	0.00	0.00	0.34	0.00
GATCAACTCCCGCGACCGCG	0.23	0.33	0.00	0.68	0.00
GATCAACTCCCGGATGAAAC	0.00	0.00	0.00	0.00	0.34
GATCAACTCCCGGATGACAC	59.29	40.99	35.51	39.03	51.32
GATCAACTCCCGGATGACAN	0.46	0.00	0.00	0.00	0.00
GATCAACTCCCGGATGACAT	0.00	0.00	0.29	0.00	0.00
GATCAACTCCCGGATGACCC	0.23	0.33	0.29	0.00	0.00

GATCAACTCCCGGATGACCN	0.46	0.00	0.00	0.00	0.00
GATCAACTCCCGGATGACGC	0.00	0.00	0.29	0.00	0.34
GATCAACTCCCGGATGACTC	0.00	0.33	0.00	0.00	0.00
GATCAACTCCCGGATGAGGC	0.23	0.00	0.00	0.00	0.00
GATCAACTCCCGGATGATAC	0.00	0.00	0.00	0.34	0.00
GATCAACTCCCGGATGCCAC	0.00	0.00	0.29	0.00	0.34
GATCAACTCCCGGATGGCAC	0.23	0.00	0.00	0.00	0.00
GATCAACTCCCGGATGNCAN	0.00	0.00	0.00	0.00	0.34
GATCAACTCCCGGATGNCNN	0.23	0.00	0.00	0.00	0.00
GATCAACTCCCGGATTACAC	0.00	0.00	0.00	0.34	0.00
GATCAACTCCCGGCTCGAGG	0.00	0.33	0.00	0.00	0.00
GATCAACTCCCGGCTGACAC	0.00	0.33	0.00	0.00	0.00
GATCAACTCCCGGGTGACAC	0.23	0.00	0.00	0.00	0.00
GATCAACTCCCGTACGGTAA	0.00	0.33	0.00	0.00	0.00
GATCAACTCCCGTCCCCCCA	0.00	0.00	0.29	0.00	0.00
GATCAACTCCCTAGGCGTCT	0.00	0.00	0.00	0.34	0.00
GATCAACTCCCTCGCAGCGA	0.00	0.65	0.00	0.00	0.00
GATCAACTCCCTCGGCGTCA	0.23	0.00	0.00	0.00	0.00
GATCAACTCCCTCTGGAAGC	0.00	0.00	0.00	0.00	0.34
GATCAACTCCCTCTGGTTCG	0.00	0.00	0.00	0.34	0.00
GATCAACTCCCTGAACTATG	0.00	0.33	0.00	0.00	0.00
GATCAACTCCCTGAAGAAGG	0.00	0.00	0.00	0.68	0.00
GATCAACTCCCTGCTCCTGC	0.23	0.00	0.00	0.34	0.34
GATCAACTCCCTGCTGACAC	0.00	0.00	0.00	0.00	0.34
GATCAACTCCCTGCTTTTGC	0.00	0.00	0.29	0.00	0.00
GATCAACTCCCTGGCGGAAG	0.23	0.00	0.00	0.00	0.00
GATCAACTCCCTGGTCTCCA	0.00	0.33	0.29	0.00	0.00
GATCAACTCCCTGTCGTTGC	0.23	0.00	0.00	0.00	0.00
GATCAACTCCCTGTGGCGTG	0.00	0.33	0.00	1.02	0.00
GATCAACTCCCTTAAGGCCA	0.00	0.00	0.00	0.34	0.34
GATCAACTCCGAACCTGATG	0.46	0.00	0.00	0.00	0.00
GATCAACTCCGAAGCGCCGG	0.00	0.33	0.00	0.00	0.00
GATCAACTCCGACGTGTCCA	0.00	0.33	0.00	0.00	0.00
GATCAACTCCGAGACTCTGT	0.00	0.33	0.00	0.00	0.00
GATCAACTCCGAGAGGCTCA	0.69	0.00	0.29	0.00	0.34
GATCAACTCCGAGAGGTGCC	0.00	0.00	0.00	0.00	0.00
GATCAACTCCGAGATGTTGT	0.00	0.33	0.00	0.00	0.00

GATCAACTCCGAGCGCTTCT	0.00	0.00	0.29	0.00	0.00
GATCAACTCCGAGCGGCGGG	0.23	0.00	0.00	0.00	0.00
GATCAACTCCGAGGCCCTGG	0.00	0.00	0.00	0.00	0.34
GATCAACTCCGAGGCGTCGG	0.00	0.00	0.00	0.00	0.00
GATCAACTCCGAGGGAGAGC	0.00	0.00	0.00	0.68	0.00
GATCAACTCCGAGTGGGACG	0.00	0.00	0.00	0.00	0.00
GATCAACTCCGCAAGGACCT	0.46	1.63	0.57	0.34	0.34
GATCAACTCCGCAGCGACGG	0.00	0.33	0.29	0.00	0.00
GATCAACTCCGCAGCGCACA	0.00	0.33	0.00	0.00	0.00
GATCAACTCCGCAGCGCAGG	0.00	0.00	0.00	0.00	0.00
GATCAACTCCGCAGCGCCAG	0.00	0.33	0.00	0.00	0.00
GATCAACTCCGCAGCGCCCA	0.23	0.00	0.00	0.00	0.00
GATCAACTCCGCAGCGCCGC	0.00	0.00	0.00	0.00	0.00
GATCAACTCCGCAGCGCCGG	16.25	30.26	14.89	18.67	16.53
GATCAACTCCGCAGCGCCGN	0.00	0.00	0.00	0.00	0.00
GATCAACTCCGCAGCGCCNN	0.00	0.00	0.00	0.00	0.00
GATCAACTCCGCAGCGCGGG	0.00	0.00	0.00	0.34	0.00
GATCAACTCCGCAGCGGCGG	0.00	0.00	0.00	0.00	0.00
GATCAACTCCGCAGGATTTC	0.00	0.00	0.57	0.00	0.00
GATCAACTCCGCAGTCAACC	0.00	0.00	0.29	0.00	0.00
GATCAACTCCGCCAAGGCCC	0.23	0.33	0.57	0.68	0.00
GATCAACTCCGCCACGGCCC	0.00	0.00	0.29	0.00	0.00
GATCAACTCCGCCAGCTCCG	0.00	0.33	0.00	0.00	0.00
GATCAACTCCGCCCGCGGGA	0.00	0.00	0.00	0.34	0.00
GATCAACTCCGCCCGCTCCG	0.00	0.00	0.00	0.00	0.00
GATCAACTCCGCCCGCTGGC	0.00	0.00	0.29	0.00	0.00
GATCAACTCCGCCGATTCAG	0.00	0.65	0.00	0.00	0.34
GATCAACTCCGCCGCGCCGC	0.00	0.00	0.00	0.00	0.00
GATCAACTCCGCCGTCTCGA	0.00	0.33	0.00	0.00	0.00
GATCAACTCCGCGACTGATG	0.00	0.00	0.00	0.00	0.34
GATCAACTCCGCGCAGTCTG	0.00	0.33	0.00	0.00	0.00
GATCAACTCCGCGCCTCGCA	2.06	0.65	0.86	3.73	2.07
GATCAACTCCGCGCCTCGCC	0.23	0.00	0.00	0.00	0.00
GATCAACTCCGCGCGGACCC	0.00	0.00	0.00	0.34	0.00
GATCAACTCCGCGCTCATGG	0.23	0.00	0.00	0.00	0.00
GATCAACTCCGCGCTGCTGG	0.23	0.00	0.00	0.00	0.00
GATCAACTCCGCTCACAGGC	0.00	0.00	0.00	0.00	0.00

GATCAACTCCGCTCAGATAA	0.00	0.00	0.00	0.00	0.34
GATCAACTCCGCTGTTGCAG	0.00	0.00	0.00	0.34	0.69
GATCAACTCCGGACGGAGCC	0.23	0.00	0.00	0.00	0.34
GATCAACTCCGGCAGCTTCC	0.23	0.00	0.00	0.00	0.00
GATCAACTCCGGCGTCAGCA	0.00	0.00	0.00	0.34	0.00
GATCAACTCCGGGACGCGGT	0.00	0.00	0.29	0.00	0.00
GATCAACTCCGGGCTCGCCG	0.00	0.00	0.00	0.00	0.34
GATCAACTCCGGGGCGCCAC	0.23	0.00	0.00	0.00	0.00
GATCAACTCCGGGGCTGACG	0.00	0.00	0.29	0.00	0.00
GATCAACTCCGGGGGGCCAC	0.00	0.00	0.00	0.34	0.00
GATCAACTCCGGGGGTACAC	0.00	0.00	0.00	0.00	0.00
GATCAACTCCGGGGGTACGC	0.00	0.00	0.29	0.00	0.00
GATCAACTCCGGTCCAGGCA	0.00	0.00	0.00	0.00	0.34
GATCAACTCCGGTCCAGGCC	3.66	3.90	6.01	3.39	4.13
GATCAACTCCGGTGCTACAC	0.00	0.00	0.00	0.00	0.34
GATCAACTCCGGTGGCACAC	0.00	0.00	0.00	0.00	0.00
GATCAACTCCGGTGGTAAAC	0.00	0.00	0.00	0.00	0.00
GATCAACTCCGGTGGTACAA	0.00	0.00	0.00	0.34	0.34
GATCAACTCCGGTGGTACAC	7.78	22.77	43.53	24.78	32.37
GATCAACTCCGGTGGTACAN	0.00	0.00	0.00	0.00	0.00
GATCAACTCCGGTGGTACAT	0.00	0.33	0.00	0.00	0.00
GATCAACTCCGGTGGTACCC	0.00	0.00	0.29	0.00	0.00
GATCAACTCCGGTGGTAGAC	0.00	0.00	0.00	0.00	0.00
GATCAACTCCGGTGGTATCC	0.00	0.00	0.00	0.34	0.00
GATCAACTCCGGTGGTATCG	0.23	0.00	0.00	0.00	0.00
GATCAACTCCGTACAGATAT	0.00	0.00	0.00	0.00	0.00
GATCAACTCCGTACCTTTGC	0.00	0.00	0.29	0.00	0.00
GATCAACTCCGTAGCCAGAA	0.46	0.00	0.00	0.34	0.00
GATCAACTCCGTAGCCAGAG	0.46	0.00	0.00	0.00	0.00
GATCAACTCCGTCCCTGCTG	0.00	0.00	0.00	0.34	0.00
GATCAACTCCGTCGCCATAA	0.23	0.00	0.00	0.00	0.00
GATCAACTCCGTCGCCTGAA	0.23	0.00	0.00	0.00	0.00
GATCAACTCCGTGCAAGTGC	0.00	0.00	0.00	0.00	0.00
GATCAACTCCGTGCATTTGC	0.00	0.00	0.00	0.34	0.00
GATCAACTCCGTGCCCTTGC	0.00	0.00	0.00	0.00	0.34
GATCAACTCCGTGCCGCACC	0.00	0.33	0.00	0.00	0.00
GATCAACTCCGTGCCTTTGC	9.61	17.89	9.16	15.27	9.99

GATCAACTCCGTGCCTTTGT	0.00	0.33	0.00	0.00	0.00
GATCAACTCCGTGCGGCAGA	0.00	0.00	0.00	0.00	0.00
GATCAACTCCGTGCGGCGGC	0.23	0.00	0.00	0.00	0.34
GATCAACTCCGTGGAGCCAC	0.00	0.00	0.00	0.00	0.00
GATCAACTCCGTGGCGCCAC	3.43	6.18	0.86	4.07	1.38
GATCAACTCCGTGGCGCCCC	2.75	1.63	0.57	1.02	1.03
GATCAACTCCGTGGCGCCCN	0.23	0.00	0.00	0.00	0.00
GATCAACTCCGTGGCTGGCC	0.00	0.00	0.00	0.00	0.00
GATCAACTCCGTGGTCAACC	0.00	0.00	0.00	0.00	0.34
GATCAACTCCGTGGTCAGCC	0.00	0.00	0.00	0.00	0.00
GATCAACTCCGTGGTCATGA	0.00	0.33	0.00	0.00	0.00
GATCAACTCCGTGTCTCTTA	0.00	0.00	0.00	0.34	0.00
GATCAACTCCGTGTGCCATT	0.00	0.00	0.00	0.34	0.00
GATCAACTCCTACATGCGAT	0.00	0.00	0.00	0.00	0.00
GATCAACTCCTACATGCGCN	0.00	0.00	0.00	0.00	0.00
GATCAACTCCTACATGCGCT	3.20	1.95	1.43	5.43	2.41
GATCAACTCCTACCGGACAG	0.00	0.00	0.00	0.68	0.00
GATCAACTCCTAGCCGAAGG	0.00	0.00	0.00	0.00	0.00
GATCAACTCCTAGTCGCAGG	0.23	0.00	0.00	0.00	0.00
GATCAACTCCTCAAAAAAAA	0.00	0.00	0.57	0.00	0.00
GATCAACTCCTCAAAAATCA	0.69	0.00	0.00	0.00	0.00
GATCAACTCCTCATCCCCGT	0.00	0.00	0.00	0.34	0.00
GATCAACTCCTCATGAATTG	0.00	0.00	0.00	0.00	0.00
GATCAACTCCTCCCCCGACC	0.23	0.00	0.00	0.00	0.69
GATCAACTCCTCCCTCGCTG	0.00	0.00	0.00	0.00	0.00
GATCAACTCCTCCGCCAAGG	0.00	0.33	0.00	0.00	0.00
GATCAACTCCTCGCCGAAGG	16.71	39.04	65.86	36.32	42.71
GATCAACTCCTCGCCGAAGN	0.00	0.00	0.00	0.00	0.00
GATCAACTCCTCGCCGAAGT	0.00	0.33	0.29	0.00	0.34
GATCAACTCCTCGCCGAANN	0.00	0.00	0.00	0.00	0.00
GATCAACTCCTCGCCGACGG	0.23	0.33	0.29	0.00	0.00
GATCAACTCCTCGCCGACTG	0.00	0.33	0.00	0.00	0.00
GATCAACTCCTCGCCGCAGG	0.00	0.98	0.57	0.34	0.34
GATCAACTCCTCGCCGGAGG	0.23	0.00	0.00	0.00	0.00
GATCAACTCCTCGCCGTAGG	0.00	0.00	0.00	0.00	0.00
GATCAACTCCTCGCTGAAGA	0.00	0.00	0.00	0.34	0.00
GATCAACTCCTCGCTGAAGG	0.00	0.00	0.00	0.00	0.34

GATCAACTCCTCGCTTGCGG	0.00	0.00	0.00	0.00	0.00
GATCAACTCCTCGGCTGGAC	0.00	0.00	0.00	0.34	0.00
GATCAACTCCTCGGGAGCGA	0.00	0.00	0.00	0.00	0.00
GATCAACTCCTCTTGGCCTC	0.00	0.33	0.00	0.00	0.00
GATCAACTCCTCTTGGTACA	0.00	0.65	0.00	0.34	0.00
GATCAACTCCTGACCAACTT	0.00	0.00	0.00	0.34	0.00
GATCAACTCCTGAGCCGAGA	0.23	0.00	0.00	0.00	0.00
GATCAACTCCTGAGGCCTCC	0.00	0.33	0.57	0.34	0.00
GATCAACTCCTGATACTGGG	0.00	0.00	0.29	0.00	0.00
GATCAACTCCTGCGCAAAAG	0.00	0.00	0.00	0.00	0.00
GATCAACTCCTGCGTCGTGA	0.00	0.33	0.00	0.00	0.00
GATCAACTCCTGCTCTGCAA	0.00	0.00	0.00	0.00	0.34
GATCAACTCCTGGAATGGAG	0.00	0.00	0.29	0.00	0.00
GATCAACTCCTGGCGACGCG	0.00	0.00	0.00	0.34	0.00
GATCAACTCCTGGCGCCAGC	0.00	0.00	0.29	0.00	0.69
GATCAACTCCTGGCTGGTGT	0.00	0.00	0.00	0.34	0.00
GATCAACTCCTTCACCGAAT	0.23	0.00	0.00	0.00	0.00
GATCAACTCCTTCCTGGCCA	0.00	0.00	0.00	0.68	0.00
GATCAACTCCTTCTACTTGC	0.00	0.00	0.00	0.00	1.03
GATCAACTCCTTCTGCATGG	0.00	0.00	0.00	0.00	0.00
GATCAACTCCTTCTTCTTCT	0.00	0.00	0.00	0.00	0.00
GATCAACTCCTTGAGTGCAG	0.00	0.33	0.29	0.00	0.00
GATCAACTCCTTGATGTAAT	0.00	0.00	0.00	0.00	0.00
GATCAACTCCTTGCACAGAG	0.23	0.33	0.00	0.00	0.00
GATCAACTCCTTGCCGCACC	0.23	0.00	0.00	0.34	0.00
GATCAACTCCTTGCTCCTTG	0.00	0.00	0.00	0.34	0.00
GATCAACTCCTTGGCGGCGG	0.23	0.00	0.00	0.00	0.00
GATCAACTCCTTTCCTCCAG	0.00	0.00	0.29	0.00	0.00
GATCAACTCCTTTGTTGCGC	0.00	0.00	0.57	0.00	0.00
GATCAACTCCTTTTTCGAGG	0.00	0.00	0.00	0.00	0.00
GATCAACTCCTTTTTCGGGG	0.00	0.33	0.00	0.00	0.00
GATCAACTCGAAAAAAAAAA	0.00	0.33	0.00	0.34	0.00
GATCAACTCGAAAAGCCATC	0.00	0.00	0.00	0.00	0.00
GATCAACTCGAAAGGGCCCA	0.00	0.00	0.00	0.00	0.34
GATCAACTCGAAATGTAAAA	0.00	0.00	0.00	0.00	0.00
GATCAACTCGAACATGCAAG	0.00	0.00	0.29	0.00	0.00
GATCAACTCGAACGCGCTTC	0.23	0.00	0.00	0.00	0.00

GATCAACTCGAAGGATGGAC	0.00	0.00	0.00	0.00	0.34
GATCAACTCGAAGGGTTTCT	0.00	0.00	0.00	0.34	0.00
GATCAACTCGAAGGTGAGCA	0.00	0.00	0.00	0.00	0.00
GATCAACTCGAAGGTGAGCC	0.23	0.00	0.00	0.34	0.00
GATCAACTCGAAGGTTTTTC	0.00	0.00	0.00	0.00	0.00
GATCAACTCGAATCCAGCGA	0.00	0.00	0.29	0.00	0.00
GATCAACTCGACCAGTTCCA	0.46	0.00	0.00	0.00	0.00
GATCAACTCGACCATCACGC	0.00	0.33	0.00	0.00	0.00
GATCAACTCGACCATCCCGA	0.00	0.33	0.00	0.00	0.00
GATCAACTCGACCATCCCGC	0.69	0.33	0.00	0.68	0.00
GATCAACTCGACCTCCTGCT	0.00	0.00	0.00	0.00	0.00
GATCAACTCGACGAAGCCTG	0.00	0.33	0.00	0.00	0.00
GATCAACTCGACGCGAAGGA	0.00	0.00	0.00	0.00	1.03
GATCAACTCGACGGATCGCA	0.00	0.33	0.00	0.00	0.00
GATCAACTCGACGGCATCCG	0.00	0.00	0.00	0.00	0.00
GATCAACTCGACTATGGGCA	0.00	0.00	0.00	0.68	0.00
GATCAACTCGACTGCGCTCC	0.00	0.00	0.00	0.34	0.00
GATCAACTCGACTGTTCATC	0.00	0.98	0.00	1.02	0.00
GATCAACTCGACTTGCATGG	0.00	0.00	0.00	0.00	0.34
GATCAACTCGACTTGCGTGG	8.01	7.81	2.58	7.47	6.89
GATCAACTCGAGAAGCTGAA	0.00	0.00	0.29	0.00	0.00
GATCAACTCGAGCAGACTGG	0.00	0.00	0.00	0.00	0.00
GATCAACTCGATCGAGTACC	0.00	0.00	0.00	0.00	0.00
GATCAACTCGATGACCGGCT	0.00	0.00	0.29	0.00	0.34
GATCAACTCGATGCAGTTCA	0.00	0.00	0.00	0.00	0.34
GATCAACTCGATTTTCAAAA	0.00	0.00	0.00	0.34	0.00
GATCAACTCGATTTTCAACA	0.00	0.00	0.29	0.00	0.00
GATCAACTCGCAAAACCAAG	0.92	0.33	0.00	0.68	0.00
GATCAACTCGCAAATGTGTA	0.00	0.00	0.00	0.00	0.34
GATCAACTCGCAACGTGAAC	0.00	0.00	0.29	0.00	0.00
GATCAACTCGCAAGCACCAA	0.00	0.33	0.00	0.00	0.00
GATCAACTCGCAAGGTCGAC	0.00	0.65	0.00	0.00	0.00
GATCAACTCGCACCAAGAAT	0.00	0.00	0.00	0.34	0.00
GATCAACTCGCACCAAGCAT	0.92	0.33	0.57	0.68	1.38
GATCAACTCGCACGTGCGCG	0.00	0.33	0.29	0.00	0.00
GATCAACTCGCACGTGCTCG	0.00	0.00	0.00	0.00	0.00
GATCAACTCGCACTTCAAGA	0.46	0.00	0.00	0.00	0.00

GATCAACTCGCAGCTCAAGG	0.00	0.00	0.00	0.34	0.00
GATCAACTCGCAGGCCGACT	0.00	0.00	0.00	0.00	0.34
GATCAACTCGCAGTATGCAA	0.00	0.33	0.00	0.00	0.00
GATCAACTCGCCAGATGCCG	0.00	0.00	0.00	0.00	0.00
GATCAACTCGCCGCACCACG	0.00	0.00	0.00	0.00	0.34
GATCAACTCGCCGCCGCAGA	0.23	0.65	0.57	0.34	1.03
GATCAACTCGCCGCGCGGCG	0.00	0.00	0.00	0.00	0.34
GATCAACTCGCCGGGCGACA	0.23	0.33	0.00	0.00	0.00
GATCAACTCGCCGTGGTTGG	0.00	0.00	0.00	0.34	0.00
GATCAACTCGCCTCGCCGGA	0.00	0.00	0.29	0.00	0.00
GATCAACTCGCCTGCGAACT	0.00	1.30	0.86	0.34	0.34
GATCAACTCGCCTGCGCACT	0.00	0.00	0.29	0.00	0.00
GATCAACTCGCCTGCGGACT	0.00	0.00	0.00	0.00	0.00
GATCAACTCGCCTGCTCAGC	0.00	0.00	0.00	0.34	0.00
GATCAACTCGCCTGTATCAC	0.00	0.00	0.00	0.00	0.34
GATCAACTCGCGAAGCTGGG	0.00	0.00	0.00	0.00	0.34
GATCAACTCGCGCAAAAAAA	0.00	0.65	0.86	0.34	0.00
GATCAACTCGCGCAGAAAAA	0.00	0.33	0.00	0.00	0.00
GATCAACTCGCGCAGCAAAA	1.14	0.65	1.43	2.72	2.07
GATCAACTCGCGCAGCAGAG	0.00	0.00	0.00	0.00	0.34
GATCAACTCGCGCAGCAGCG	0.00	0.00	0.00	0.00	0.00
GATCAACTCGCGCAGCCGCG	0.00	0.00	0.00	0.00	0.00
GATCAACTCGCGCAGCCGTG	0.46	0.33	0.00	0.34	0.00
GATCAACTCGCGCAGCGGGG	0.00	0.00	0.29	0.00	0.00
GATCAACTCGCGCAGCGGTG	0.23	0.00	0.00	0.00	0.00
GATCAACTCGCGCAGCNGTG	0.00	0.00	0.29	0.00	0.34
GATCAACTCGCGCAGCNNNN	0.46	0.00	0.00	0.00	0.00
GATCAACTCGCGCAGCTAAA	0.00	0.00	0.86	0.00	0.00
GATCAACTCGCGCAGCTATG	0.00	0.00	0.00	0.34	0.00
GATCAACTCGCGCAGCTCAA	0.00	0.00	0.57	0.00	0.00
GATCAACTCGCGCAGCTCCG	0.00	0.00	0.29	0.34	0.00
GATCAACTCGCGCAGCTGAA	0.00	0.00	0.29	0.68	0.69
GATCAACTCGCGCAGCTGAG	0.00	0.00	0.29	0.00	0.00
GATCAACTCGCGCAGCTGCG	0.46	0.33	0.29	0.00	0.00
GATCAACTCGCGCAGCTGCT	0.00	0.00	0.00	0.00	0.00
GATCAACTCGCGCAGCTGGG	0.46	0.33	0.00	0.00	0.34
GATCAACTCGCGCAGCTGNN	0.23	0.00	0.00	0.00	0.00

GATCAACTCGCGCAGCTGTC	0.00	0.00	0.29	0.00	0.00
GATCAACTCGCGCAGCTGTG	48.76	46.20	59.28	65.51	84.72
GATCAACTCGCGCAGCTGTN	0.00	0.00	0.00	0.00	0.00
GATCAACTCGCGCAGCTGTT	0.00	0.00	0.00	0.00	0.00
GATCAACTCGCGCAGCTNNN	0.23	0.00	0.00	0.00	0.34
GATCAACTCGCGCAGCTTCG	0.00	0.00	0.00	0.00	0.00
GATCAACTCGCGCAGCTTTG	0.23	0.00	0.00	0.00	0.34
GATCAACTCGCGCCATGCAA	0.46	0.98	0.57	0.68	0.00
GATCAACTCGCGCCCAAAAA	0.00	0.00	0.00	0.34	0.00
GATCAACTCGCGCCGCTGTG	0.00	0.00	0.00	0.00	0.00
GATCAACTCGCGCGACGGGT	0.00	0.00	0.29	0.34	0.00
GATCAACTCGCGCGGCTGCG	0.00	0.00	0.00	0.00	0.34
GATCAACTCGCGCGGCTGTG	0.00	0.00	0.00	0.00	0.34
GATCAACTCGCGCTACAGCT	0.00	0.00	0.00	0.00	0.00
GATCAACTCGCGCTACGGGA	0.00	0.00	0.00	0.00	0.00
GATCAACTCGCGCTATAGCT	0.00	0.65	0.00	0.00	0.00
GATCAACTCGCGGCACTTGC	0.69	0.00	0.00	0.68	0.00
GATCAACTCGCGGCCGCACC	0.23	0.00	0.00	0.00	0.00
GATCAACTCGCTCAAGCCGG	0.00	0.00	0.00	0.68	0.00
GATCAACTCGCTCATCAACA	0.23	0.65	0.00	0.34	0.00
GATCAACTCGCTCCTTCGCT	0.00	0.00	0.00	0.00	0.00
GATCAACTCGCTCGAGTAAC	0.00	0.00	0.29	0.00	0.00
GATCAACTCGCTCGAGTACC	4.81	2.60	3.72	1.70	4.13
GATCAACTCGCTCGCACTCG	0.00	0.00	0.00	0.00	0.00
GATCAACTCGCTCGCGCCAA	2.52	1.95	1.72	1.02	1.38
GATCAACTCGCTCGCGCCNN	0.46	0.00	0.00	0.00	0.00
GATCAACTCGCTCTATAACG	0.69	0.00	0.00	0.00	0.00
GATCAACTCGCTGAGGCCGT	0.00	0.00	0.00	0.00	0.00
GATCAACTCGCTGCAAACGG	0.00	1.30	0.86	0.68	1.72
GATCAACTCGCTGCATTCTG	0.00	0.00	0.00	0.00	0.00
GATCAACTCGCTGCCCCTGT	0.00	0.33	0.00	0.00	0.00
GATCAACTCGCTGCGACCTC	0.00	0.00	0.00	0.34	0.00
GATCAACTCGCTGGATGCAG	0.00	0.00	0.00	0.00	0.00
GATCAACTCGCTGGCGAGCT	0.00	0.98	0.00	0.00	0.00
GATCAACTCGCTGGTGACCA	0.00	0.65	0.29	0.00	0.00
GATCAACTCGCTGTGGATGA	0.00	0.00	0.00	0.00	0.34
GATCAACTCGCTTGTGCGGT	0.00	0.33	0.00	0.00	0.00

GATCAACTCGGAAAAAAAAA	0.00	0.00	0.00	0.34	0.00
GATCAACTCGGAAAAGTCAA	0.00	0.00	0.00	0.00	0.00
GATCAACTCGGACAGCTCAG	0.00	0.33	0.00	0.00	0.00
GATCAACTCGGACCAGCGAG	1.14	0.65	0.29	1.02	0.34
GATCAACTCGGACGACAAAG	0.00	0.33	0.00	0.00	0.00
GATCAACTCGGACGACAACT	0.00	0.00	0.00	0.34	0.00
GATCAACTCGGACGCAGCGG	0.00	0.00	0.00	0.00	0.00
GATCAACTCGGACGGCAGTG	0.23	0.00	0.00	0.00	0.00
GATCAACTCGGACTCCATGA	0.46	0.00	0.00	0.00	0.00
GATCAACTCGGACTCGGCAT	0.00	0.00	0.00	0.68	0.00
GATCAACTCGGACTCGGCGA	7.78	9.43	4.01	3.73	4.82
GATCAACTCGGACTCGGCGC	0.23	0.00	0.29	0.00	0.00
GATCAACTCGGACTGCGCCA	0.23	0.00	0.00	0.00	0.00
GATCAACTCGGAGGCTTCAG	0.00	0.00	0.29	0.00	0.00
GATCAACTCGGATACGTTCC	0.00	0.00	0.00	0.34	0.00
GATCAACTCGGATGGCATGC	0.23	0.00	0.00	0.00	0.00
GATCAACTCGGATGTTGCGG	0.00	0.33	0.00	0.00	0.00
GATCAACTCGGCAAAAAAAA	0.00	0.33	0.00	0.00	0.00
GATCAACTCGGCAAATGAAC	0.00	0.00	0.00	0.00	0.00
GATCAACTCGGCAACGCGAC	0.00	0.00	0.29	0.00	0.00
GATCAACTCGGCAAGGCGGC	0.00	0.00	0.29	0.00	0.00
GATCAACTCGGCAAGGGAGA	0.46	0.00	0.00	0.00	0.00
GATCAACTCGGCAAGGGGGA	0.23	0.00	0.00	0.00	0.00
GATCAACTCGGCAATCGTGC	0.00	0.00	0.00	0.00	0.00
GATCAACTCGGCAGCGCCGG	0.00	0.00	0.29	0.00	0.00
GATCAACTCGGCATCGGCTG	0.00	0.00	0.00	0.00	0.00
GATCAACTCGGCCAAGGCCG	0.00	0.33	0.00	0.00	0.00
GATCAACTCGGCCATCGCGG	0.00	0.00	0.00	0.00	0.00
GATCAACTCGGCCGCCGCGC	0.00	0.00	0.00	0.00	0.00
GATCAACTCGGCCGGCAGAG	0.00	0.65	0.00	0.34	0.00
GATCAACTCGGCCTGAAAAA	0.00	0.98	0.00	0.00	0.00
GATCAACTCGGCGAAAATCA	0.00	0.00	0.86	0.00	0.00
GATCAACTCGGCGAAACGGG	0.00	0.00	0.00	0.00	0.34
GATCAACTCGGCGAACGATT	0.00	0.00	0.00	0.00	0.00
GATCAACTCGGCGAAGCAGG	0.00	0.00	0.00	0.00	0.00
GATCAACTCGGCGACCCCGG	0.00	0.00	0.00	0.00	0.00
GATCAACTCGGCGACCGCAG	0.00	0.33	0.00	0.00	0.00

GATCAACTCGGCGAGTGGGC	0.00	0.00	0.00	0.00	0.00
GATCAACTCGGCGATAGGCC	0.00	0.00	0.00	0.00	0.34
GATCAACTCGGCGATATGCC	0.00	0.00	0.00	0.34	0.00
GATCAACTCGGCGATCAGCC	0.00	0.33	0.00	0.00	0.00
GATCAACTCGGCGATCGGCA	0.00	0.33	0.00	0.00	0.00
GATCAACTCGGCGATCGGCC	0.00	2.28	0.57	3.73	4.82
GATCAACTCGGCGATCTGCC	0.00	0.00	0.00	0.34	0.00
GATCAACTCGGCGATGGCTT	0.00	0.00	0.00	1.02	0.00
GATCAACTCGGCGGCCGAGG	0.46	0.65	0.57	2.04	0.00
GATCAACTCGGCGGCCGCGG	0.00	0.00	0.00	0.00	0.00
GATCAACTCGGCGGGCGACG	0.00	0.00	0.00	0.00	0.00
GATCAACTCGGCGTAGTTGA	0.00	0.00	0.00	0.00	0.00
GATCAACTCGGCGTCCTCTG	0.00	0.00	0.00	0.34	0.00
GATCAACTCGGCTAAGTTCC	0.00	0.00	0.00	0.00	0.34
GATCAACTCGGCTCTCGACG	0.00	0.00	0.00	0.34	0.00
GATCAACTCGGCTTTGACGG	0.00	0.00	0.00	0.34	0.00
GATCAACTCGGCTTTGTGCT	0.00	0.00	0.00	0.00	0.00
GATCAACTCGGGACGTTAAA	0.00	0.00	0.00	0.68	0.00
GATCAACTCGGGACGTTGTC	5.49	2.60	2.29	4.41	1.72
GATCAACTCGGGCAAGAGAG	0.46	0.00	0.00	0.00	0.00
GATCAACTCGGGCACGACGC	0.00	0.00	0.00	0.00	0.00
GATCAACTCGGGCACTGACC	0.00	0.00	0.29	0.00	0.00
GATCAACTCGGGCAGCTGTG	0.00	0.00	0.00	0.00	0.00
GATCAACTCGGGCATGGAGC	0.00	0.00	0.29	0.34	0.69
GATCAACTCGGGCATGGCGC	0.00	0.00	0.00	0.00	0.34
GATCAACTCGGGCCGTTGGC	0.23	0.00	0.00	0.00	0.00
GATCAACTCGGGCCGTTGTC	0.23	0.00	0.00	0.34	0.00
GATCAACTCGGGCGCGGTCG	0.00	0.00	0.00	0.00	0.00
GATCAACTCGGGCGGCAGCT	0.00	0.00	0.29	0.00	0.00
GATCAACTCGGGCTCCCTGC	0.00	0.00	0.29	0.00	0.00
GATCAACTCGGGCTGCAACG	0.00	0.33	0.00	0.00	0.00
GATCAACTCGGGCTGCACCG	3.89	5.86	2.29	5.09	4.48
GATCAACTCGGGCTGCACGG	0.00	0.00	0.00	0.34	0.00
GATCAACTCGGGCTGCAGCC	0.00	0.00	0.00	0.00	0.00
GATCAACTCGGGCTGCAGCT	0.00	0.00	0.00	0.34	0.00
GATCAACTCGGGCTTCTGGT	0.00	0.00	0.00	0.00	0.00
GATCAACTCGGGTAGATATT	0.00	0.00	0.29	0.00	0.00

GATCAACTCGGTCAGCTCCG	0.00	0.00	0.29	0.00	0.00
GATCAACTCGGTCGAGTTCC	0.23	0.00	0.00	0.00	0.00
GATCAACTCGGTGAACACAT	0.00	0.00	0.00	0.00	0.34
GATCAACTCGGTGGAGCTGT	0.00	0.00	0.00	0.00	0.34
GATCAACTCGGTGTCTGCTG	0.23	0.00	0.00	0.00	0.34
GATCAACTCGGTTCTGTTCT	0.00	0.00	0.00	0.00	0.69
GATCAACTCGGTTTCGTTGT	0.00	0.00	0.00	0.34	0.00
GATCAACTCGTACGCGTGGA	0.00	0.00	0.00	0.34	0.00
GATCAACTCGTAGAGGACGC	0.00	0.00	0.00	0.00	0.00
GATCAACTCGTATGAACTCT	0.23	0.00	0.00	0.00	0.00
GATCAACTCGTATGCGTCCT	0.00	0.33	0.00	0.00	0.00
GATCAACTCGTATGGCAACT	0.00	0.00	0.29	0.00	0.00
GATCAACTCGTATTTGAGAC	0.00	0.00	0.00	0.00	0.34
GATCAACTCGTCATGCCAGA	0.00	0.00	0.29	0.00	0.00
GATCAACTCGTCATTCTTCA	0.00	0.65	0.00	0.34	0.00
GATCAACTCGTCCAGACGAC	0.00	0.00	0.00	0.00	0.00
GATCAACTCGTCGCCAATGC	0.23	0.00	0.00	0.00	0.00
GATCAACTCGTCGCCAATGT	5.26	4.23	3.15	7.47	2.07
GATCAACTCGTCGTATGCCG	0.00	0.00	0.29	0.00	0.00
GATCAACTCGTCGTGCTTGC	0.00	0.00	0.00	0.00	0.00
GATCAACTCGTCTTCTGTCA	0.69	0.00	0.00	0.00	0.00
GATCAACTCGTGACACTTGC	0.00	0.00	0.00	0.00	0.00
GATCAACTCGTGCAAGCAGG	0.23	0.00	0.00	0.00	0.00
GATCAACTCGTGCCCGCACC	0.00	0.00	0.00	0.00	0.34
GATCAACTCGTGGCCGCACC	0.23	0.65	0.00	0.00	0.00
GATCAACTCGTGTTTCACTG	0.23	0.33	0.29	0.68	0.69
GATCAACTCGTTCATGCAGG	0.00	0.00	0.29	0.00	0.00
GATCAACTCGTTGAAGACGA	0.23	0.65	0.00	0.34	0.34
GATCAACTCGTTGAAGTCGA	1.60	2.28	0.86	1.36	0.00
GATCAACTCGTTGAATCGTA	0.00	0.00	0.00	0.68	0.00
GATCAACTCGTTGATGAGGA	0.00	0.65	0.00	0.00	0.00
GATCAACTCGTTGCAGTCGA	0.00	0.33	0.00	0.00	0.00
GATCAACTCGTTGGCGTACC	0.00	0.00	0.00	0.00	0.00
GATCAACTCGTTGTCTAGCA	0.00	0.00	0.00	0.00	0.69
GATCAACTCGTTTCTGGAAG	0.00	0.33	0.00	0.00	0.34
GATCAACTCTAAAAAAAAAA	0.00	0.33	0.00	0.00	0.00
GATCAACTCTAATTAATGTA	0.00	0.00	0.00	0.00	0.00

GATCAACTCTACCAGTGCGA	0.00	0.00	0.29	0.00	0.00
GATCAACTCTACGGAGGAGT	0.00	0.00	0.00	0.34	0.00
GATCAACTCTACGGAGGCGG	0.23	0.00	0.00	0.00	0.00
GATCAACTCTACGGAGGCGT	0.92	0.00	0.00	0.00	0.00
GATCAACTCTACGGAGGGGC	0.23	0.00	0.00	0.00	0.00
GATCAACTCTACGGAGGGGT	0.92	0.00	0.29	1.36	0.00
GATCAACTCTACGGAGGTGC	0.00	0.33	0.00	0.00	0.00
GATCAACTCTACGGAGGTGG	1.14	0.00	0.00	0.00	0.00
GATCAACTCTACGGAGGTGT	30.67	15.94	16.90	14.93	15.84
GATCAACTCTACGGAGNTGT	0.00	0.00	0.00	0.00	0.34
GATCAACTCTACGGCGGCGT	0.23	0.00	0.00	0.00	0.00
GATCAACTCTACGTGCTCGG	0.00	0.00	0.00	0.34	0.00
GATCAACTCTACTCGCTGGT	0.23	0.00	0.00	0.00	0.00
GATCAACTCTAGAAATTGGC	0.00	0.00	0.00	0.00	0.00
GATCAACTCTAGCACGTGTC	0.23	0.33	0.29	1.02	0.34
GATCAACTCTAGCAGGCTGC	0.23	0.00	0.00	0.00	0.00
GATCAACTCTAGCCTGGCGC	0.00	0.00	0.00	0.00	0.00
GATCAACTCTAGCGCCTCCC	0.23	0.33	0.00	0.34	0.00
GATCAACTCTAGCTTGNGTT	0.00	0.00	0.00	0.00	0.34
GATCAACTCTAGCTTGTGTT	0.00	0.00	0.00	0.00	0.00
GATCAACTCTATCTTTTTGA	0.92	0.00	0.00	0.00	0.00
GATCAACTCTATGCGTGGGT	0.23	0.00	0.00	0.00	0.00
GATCAACTCTATGGCCGTTC	0.00	0.00	0.29	0.00	0.00
GATCAACTCTATGGGTGTTC	0.00	0.00	0.00	0.00	0.34
GATCAACTCTCAAAAAAAAA	0.69	0.00	0.00	0.00	0.00
GATCAACTCTCAAACATGGC	0.00	0.33	0.00	0.00	0.00
GATCAACTCTCAACAGCCTG	0.00	0.00	0.29	0.00	0.00
GATCAACTCTCAAGCCGACT	0.00	0.00	0.00	0.00	0.00
GATCAACTCTCACAAAATGC	0.69	0.00	0.29	0.00	0.00
GATCAACTCTCACAATATCA	0.00	0.00	0.29	0.00	0.00
GATCAACTCTCACATGCGTG	0.00	0.00	0.00	0.00	0.34
GATCAACTCTCAGAATGTAC	1.60	1.30	0.86	2.04	1.03
GATCAACTCTCAGAGGTCTC	0.69	0.65	0.00	0.34	0.00
GATCAACTCTCAGCCCGACT	0.00	0.00	0.00	0.00	0.34
GATCAACTCTCAGCTGGCCA	0.00	0.33	0.00	0.00	0.00
GATCAACTCTCAGGAAAAAA	0.00	0.00	0.00	0.00	0.00
GATCAACTCTCAGGACGACT	0.00	0.00	0.29	0.00	0.34

GATCAACTCTCAGGAGTGAA	0.00	0.00	0.00	0.34	0.00
GATCAACTCTCAGGCAGAAA	0.23	0.00	0.00	0.00	0.00
GATCAACTCTCAGGCAGACT	0.00	0.33	0.00	0.34	0.34
GATCAACTCTCAGGCCAAAA	0.00	0.65	0.86	0.34	0.00
GATCAACTCTCAGGCCAACC	0.00	0.00	0.00	0.00	0.34
GATCAACTCTCAGGCCAACT	0.00	0.00	0.29	0.00	0.00
GATCAACTCTCAGGCCCACT	0.00	0.00	0.57	0.00	0.00
GATCAACTCTCAGGCCCGAA	0.00	0.00	0.29	0.00	0.00
GATCAACTCTCAGGCCGAAA	3.20	1.30	3.72	4.41	5.51
GATCAACTCTCAGGCCGAAT	0.00	0.00	0.00	0.00	0.69
GATCAACTCTCAGGCCGACA	0.00	0.33	0.00	0.00	0.00
GATCAACTCTCAGGCCGACC	0.00	0.33	0.00	0.34	0.00
GATCAACTCTCAGGCCGACG	0.23	0.00	0.57	0.00	0.69
GATCAACTCTCAGGCCGACN	0.00	0.00	0.00	0.00	0.00
GATCAACTCTCAGGCCGACT	62.72	87.19	98.22	105.22	110.21
GATCAACTCTCAGGCCGAGC	0.00	0.00	0.00	0.34	0.00
GATCAACTCTCAGGCCGAGG	0.00	0.33	0.00	0.00	0.00
GATCAACTCTCAGGCCGANN	0.23	0.00	0.00	0.00	0.00
GATCAACTCTCAGGCCGATT	0.00	0.00	0.00	0.00	0.34
GATCAACTCTCAGGCCGCAA	0.00	0.00	0.29	0.00	0.00
GATCAACTCTCAGGCCGCCG	0.00	0.00	0.00	0.00	0.00
GATCAACTCTCAGGCCGCCT	0.69	0.65	1.15	0.34	0.34
GATCAACTCTCAGGCCGGAA	0.00	0.33	0.00	0.00	0.00
GATCAACTCTCAGGCCGGCT	0.00	0.00	0.29	0.00	0.00
GATCAACTCTCAGGCCGTGA	0.00	0.00	0.00	0.34	0.00
GATCAACTCTCAGGCCNNNN	0.23	0.00	0.00	0.00	0.00
GATCAACTCTCAGGCCTACT	0.00	0.00	0.29	0.00	0.00
GATCAACTCTCAGGCCTCCG	0.00	0.00	0.00	0.00	0.00
GATCAACTCTCAGGCCTTCG	0.00	0.00	0.29	0.00	0.00
GATCAACTCTCAGGCGCTCT	0.00	0.00	0.29	0.00	0.00
GATCAACTCTCAGGCTGACT	0.00	0.33	0.00	0.34	0.00
GATCAACTCTCAGGCTGAGC	0.00	0.00	0.00	0.00	0.00
GATCAACTCTCAGGCTGTCT	0.00	0.00	0.29	0.00	0.00
GATCAACTCTCAGGCTGTTG	0.00	0.00	0.00	0.34	0.00
GATCAACTCTCAGGTAGTGA	0.00	0.00	0.00	0.00	0.00
GATCAACTCTCAGGTCAAAA	0.00	0.00	0.00	0.00	0.00
GATCAACTCTCAGGTCGACT	0.00	0.33	0.00	0.00	0.00

GATCAACTCTCATCTCTTGG	0.00	0.00	0.00	0.00	0.00
GATCAACTCTCATGAGCCGC	0.00	0.00	0.00	0.00	0.00
GATCAACTCTCATTGACTTG	0.00	0.00	0.29	0.00	0.00
GATCAACTCTCATTGTGAAA	0.46	0.00	0.00	0.00	0.69
GATCAACTCTCATTGTGAGC	0.00	0.00	0.00	0.00	0.00
GATCAACTCTCCATGGATGG	0.00	0.00	0.00	0.00	0.00
GATCAACTCTCCCGCTCTTC	0.23	0.00	0.00	0.00	0.00
GATCAACTCTCCCTTTGCAA	0.00	0.00	0.00	0.34	0.00
GATCAACTCTCCGCACCTTC	0.00	0.33	0.00	0.00	0.00
GATCAACTCTCCGCATTAGC	0.00	0.00	0.00	0.00	0.34
GATCAACTCTCCGCATTCGA	0.00	0.00	0.00	0.34	0.34
GATCAACTCTCCGCATTCGC	41.43	30.58	38.37	18.33	44.77
GATCAACTCTCCGCATTCGG	0.00	0.00	0.00	0.00	0.00
GATCAACTCTCCGCATTCGN	0.46	0.00	0.00	0.00	0.00
GATCAACTCTCCGCATTCNN	0.23	0.00	0.00	0.00	0.00
GATCAACTCTCCGCATTTGC	0.00	0.00	0.57	0.00	0.00
GATCAACTCTCCGCCTTCGC	0.00	0.00	0.00	0.00	0.34
GATCAACTCTCCGGCCGAAT	0.00	0.00	0.00	0.00	0.00
GATCAACTCTCCGGCCGACT	0.00	0.33	0.00	0.00	0.00
GATCAACTCTCCGGCCGCCT	0.23	0.00	0.00	0.00	0.00
GATCAACTCTCCGTGCATGT	0.00	0.00	0.00	0.00	0.00
GATCAACTCTCGATTGCGCT	0.00	0.33	0.00	0.00	0.00
GATCAACTCTCGCAGACTGC	0.00	0.00	0.00	0.00	0.00
GATCAACTCTCGCAGCTGTT	0.00	0.00	0.00	0.00	0.34
GATCAACTCTCGCAGGATGC	0.00	0.00	0.29	0.34	0.34
GATCAACTCTCGCAGGCGGC	0.00	0.00	0.00	0.00	0.00
GATCAACTCTCGCAGGCTGA	0.00	0.33	0.00	0.00	0.00
GATCAACTCTCGCAGGCTGC	28.16	19.84	19.19	31.23	22.39
GATCAACTCTCGCAGGCTGT	0.00	0.00	0.00	0.68	0.00
GATCAACTCTCGCATTCGCT	0.00	0.00	0.00	0.00	0.00
GATCAACTCTCGCGCATATC	0.23	0.00	0.00	0.00	0.00
GATCAACTCTCGGAATTGGA	0.23	0.00	0.00	0.00	0.00
GATCAACTCTCGGCCTGAAA	0.00	0.33	0.00	0.00	0.00
GATCAACTCTCGTATGCCGT	0.00	0.00	0.00	0.00	0.00
GATCAACTCTCGTGATGACC	0.00	0.00	0.00	0.00	0.00
GATCAACTCTCTCCCACGCT	0.23	0.00	0.00	0.00	0.00
GATCAACTCTCTGAAGGCGT	0.00	0.00	0.29	0.00	0.00

GATCAACTCTCTGAGCGAAG	0.23	0.33	0.00	0.34	0.00
GATCAACTCTCTGCGCAACA	0.00	0.00	0.00	0.00	0.34
GATCAACTCTCTGCGCCTTA	0.00	0.00	0.57	0.00	0.00
GATCAACTCTCTGCTGGTGC	0.46	0.00	0.29	0.00	0.00
GATCAACTCTCTGCTGGTNN	0.23	0.00	0.00	0.00	0.00
GATCAACTCTCTGGTTGACC	1.37	0.33	0.00	0.00	0.00
GATCAACTCTGAACAACTCT	0.00	0.00	0.00	0.00	0.34
GATCAACTCTGAACAGCTCT	0.00	0.00	0.00	0.00	0.34
GATCAACTCTGAAGAGGCCT	2.06	0.33	1.43	1.70	0.34
GATCAACTCTGAAGATGTGC	0.00	0.33	0.00	0.00	0.00
GATCAACTCTGAATTTGGCG	0.00	0.98	0.00	0.00	0.00
GATCAACTCTGAATTTGGTG	0.00	0.65	0.00	0.00	0.00
GATCAACTCTGACAAGAAGC	0.00	0.00	0.00	0.34	0.00
GATCAACTCTGACATGCTGG	0.69	0.33	2.86	1.02	0.00
GATCAACTCTGACCAACTCT	0.00	0.00	0.00	0.00	1.38
GATCAACTCTGAGACCGAGA	0.00	0.00	0.00	0.00	0.00
GATCAACTCTGAGCTCTTAA	0.23	0.00	0.00	0.00	0.00
GATCAACTCTGAGCTGAGGA	0.00	0.00	0.00	0.00	0.00
GATCAACTCTGAGGACCCAT	0.00	0.00	0.00	0.00	0.00
GATCAACTCTGAGGATAGTT	0.46	0.33	0.00	0.34	0.00
GATCAACTCTGAGGATTCAG	0.00	0.00	0.29	0.34	0.00
GATCAACTCTGAGGCTNCAG	0.00	0.00	0.00	0.00	0.34
GATCAACTCTGAGGCTTCAG	4.12	6.18	15.75	7.81	11.71
GATCAACTCTGAGGCTTCCG	0.00	0.00	0.29	0.00	0.69
GATCAACTCTGAGGTATGAC	0.23	0.33	0.00	0.00	0.00
GATCAACTCTGAGTATGTGC	0.00	0.00	0.00	0.00	0.00
GATCAACTCTGAGTCTCGGA	0.00	0.33	0.00	0.00	0.00
GATCAACTCTGATCAACTCT	0.00	0.00	0.00	0.00	0.34
GATCAACTCTGATTAACTCT	0.00	0.00	0.00	0.00	0.34
GATCAACTCTGATTAGCTCT	0.00	0.00	0.00	0.00	0.34
GATCAACTCTGCAATGGTGT	0.00	0.00	0.00	0.34	0.00
GATCAACTCTGCACAACAAC	0.00	0.00	0.00	0.00	0.34
GATCAACTCTGCCACATGCT	0.23	0.00	0.00	0.00	0.00
GATCAACTCTGCCATCGTAC	0.00	0.00	0.00	0.00	0.00
GATCAACTCTGCCATGGTGG	0.00	0.00	0.29	0.00	0.34
GATCAACTCTGCCCAAGGCC	0.00	0.00	0.29	0.00	0.00
GATCAACTCTGCCCACAAGA	0.00	0.00	0.00	0.00	0.00

GATCAACTCTGCCCGTTGAT	0.23	0.98	0.00	0.68	0.00
GATCAACTCTGCCGGGATAG	0.00	0.00	0.29	0.00	0.00
GATCAACTCTGCCTATCAAA	0.00	0.33	0.00	0.00	0.00
GATCAACTCTGCCTCGCGGC	0.00	0.00	0.00	0.68	0.00
GATCAACTCTGCGAAGCCTC	0.00	0.00	0.57	0.00	0.00
GATCAACTCTGCGAGCACGA	0.46	0.00	0.00	0.00	0.00
GATCAACTCTGCGGAGCTCG	0.00	0.00	0.00	0.00	0.00
GATCAACTCTGCGGCGCGGC	0.00	0.33	0.00	0.00	0.00
GATCAACTCTGCGTTGGGGC	0.00	0.00	0.00	0.00	0.69
GATCAACTCTGCTCTTGTCC	0.00	0.00	0.29	0.00	0.00
GATCAACTCTGCTGAGGTTG	0.00	0.33	0.00	0.34	0.34
GATCAACTCTGCTGGGATGC	0.00	0.00	0.00	0.00	0.69
GATCAACTCTGGAACCGCCC	0.23	0.00	0.00	0.00	0.00
GATCAACTCTGGAACCTCCC	1.37	0.33	0.29	0.00	1.03
GATCAACTCTGGAACCTCNN	0.23	0.00	0.00	0.00	0.00
GATCAACTCTGGAGGAGACG	0.00	0.00	0.00	0.00	0.00
GATCAACTCTGGCACGTCTG	0.00	0.00	0.00	0.34	0.00
GATCAACTCTGGCAGCTTCC	0.00	0.00	0.29	0.00	0.00
GATCAACTCTGGCCAACTCT	0.00	0.00	0.86	0.00	0.34
GATCAACTCTGGGAGCGGTC	0.00	0.00	0.00	0.00	0.00
GATCAACTCTGGTATGTTCT	0.00	0.00	0.00	0.00	0.69
GATCAACTCTGGTCAACTCG	0.00	0.00	0.00	0.00	0.69
GATCAACTCTGGTCAACTCT	0.00	0.00	0.00	0.00	2.41
GATCAACTCTGGTCACGTCG	0.00	0.33	0.00	0.00	0.00
GATCAACTCTGGTCTCTCAG	0.23	0.33	0.57	0.68	0.34
GATCAACTCTGGTCTGTTCT	0.00	0.00	0.00	0.00	0.34
GATCAACTCTGTACAACGCA	0.00	0.00	0.29	0.00	0.00
GATCAACTCTGTACGCGCAA	0.00	0.98	0.00	0.00	0.34
GATCAACTCTGTACTGTTCT	0.00	0.00	0.00	0.00	0.34
GATCAACTCTGTCACTCCGG	0.23	0.00	0.00	0.00	0.00
GATCAACTCTGTCCCAAGGC	0.00	0.00	0.00	0.34	0.00
GATCAACTCTGTCCTGGTCA	0.00	0.00	0.00	0.34	0.00
GATCAACTCTGTCCTGTTCT	0.00	0.00	0.00	0.00	0.69
GATCAACTCTGTCTCTGCCT	0.00	0.00	0.00	0.00	0.69
GATCAACTCTGTGACCTGCC	0.00	0.00	0.00	0.00	0.34
GATCAACTCTGTGATGTTCT	0.00	0.00	0.00	0.00	1.03
GATCAACTCTGTGCTGTTCT	0.00	0.00	0.00	0.00	0.34

GATCAACTCTGTGTTGTTCT	0.00	0.00	0.00	0.00	0.34
GATCAACTCTGTTATGTCCT	0.00	0.00	0.00	0.00	0.34
GATCAACTCTGTTATGTGAT	0.00	0.00	0.00	0.00	0.34
GATCAACTCTGTTATGTTAT	0.00	0.00	0.00	0.00	1.03
GATCAACTCTGTTATGTTCT	0.00	0.00	0.57	0.00	3.10
GATCAACTCTGTTATGTTGT	0.00	0.00	0.00	0.00	2.41
GATCAACTCTGTTCAACTCN	0.00	0.00	0.00	0.00	0.34
GATCAACTCTGTTCAACTCT	0.00	0.00	0.00	0.00	2.07
GATCAACTCTGTTCGGTTCT	0.00	0.00	0.00	0.00	0.34
GATCAACTCTGTTCGTTGAA	0.00	0.00	0.00	0.00	0.00
GATCAACTCTGTTCTGTCCT	0.00	0.00	0.57	0.00	1.72
GATCAACTCTGTTCTGTGCT	0.00	0.00	0.57	0.00	0.00
GATCAACTCTGTTCTGTTAT	0.00	0.00	0.57	0.00	3.10
GATCAACTCTGTTCTGTTCA	0.00	0.00	0.00	0.00	0.69
GATCAACTCTGTTCTGTTCG	0.00	0.00	0.00	0.00	1.38
GATCAACTCTGTTCTGTTCT	0.00	0.00	5.44	0.00	29.27
GATCAACTCTGTTCTTTTCT	0.00	0.00	0.00	0.00	0.34
GATCAACTCTGTTGGAGTCG	0.00	0.00	0.00	0.00	0.34
GATCAACTCTGTTTTGTTCT	0.00	0.00	0.00	0.00	1.38
GATCAACTCTTACGAGGAAG	0.00	0.00	0.29	0.00	0.00
GATCAACTCTTAGGCCGACT	0.00	0.33	0.00	0.00	0.00
GATCAACTCTTCAGCGTACA	0.46	0.33	0.29	0.00	0.34
GATCAACTCTTCAGCTTGAC	0.00	0.00	0.00	0.00	0.00
GATCAACTCTTCCAGCTGGT	0.00	0.33	0.29	0.00	0.00
GATCAACTCTTCCGTGCACA	0.00	0.00	0.00	0.00	0.00
GATCAACTCTTCGAAAGGGA	0.00	0.00	0.29	0.00	0.00
GATCAACTCTTCGCCGAAGG	0.00	0.00	0.00	0.00	0.00
GATCAACTCTTCGGCTGGTG	0.00	0.00	0.00	0.00	0.34
GATCAACTCTTCGTGGCCTT	0.00	0.00	0.00	0.00	0.00
GATCAACTCTTCTTTTTCTT	0.46	0.00	0.00	0.00	0.00
GATCAACTCTTGAACTCTAC	0.00	0.00	0.00	0.00	0.00
GATCAACTCTTGACATATGC	0.00	0.00	0.00	0.00	0.34
GATCAACTCTTGACCAGCTT	0.00	0.00	0.29	0.00	0.00
GATCAACTCTTGAGGCCACC	0.00	0.00	0.00	0.00	0.00
GATCAACTCTTGAGGCCTCC	0.00	0.00	0.00	0.00	0.00
GATCAACTCTTGCACATTAC	0.23	0.00	0.00	0.00	0.00
GATCAACTCTTGCAGCAAAA	0.00	0.00	0.57	0.00	0.00

GATCAACTCTTGCAGCAAAG	0.00	0.33	0.00	0.00	0.00
GATCAACTCTTGCAGGCTGC	0.00	0.00	0.00	0.00	0.34
GATCAACTCTTGGCGCGCGT	0.00	0.00	0.00	0.00	0.00
GATCAACTCTTGTCTGACCT	0.00	0.00	0.00	0.00	0.00
GATCAACTCTTGTGGCACCA	0.00	0.33	0.00	0.00	0.00
GATCAACTCTTGTTCCTGGC	0.00	0.33	0.00	0.34	0.00
GATCAACTCTTGTTGACAAC	0.00	0.00	0.00	0.00	0.00
GATCAACTCTTTACACGGCT	0.00	0.00	0.00	0.00	0.34
GATCAACTCTTTCGGCCTGA	0.00	0.00	0.29	0.00	0.00
GATCAACTCTTTCTTGGACA	0.23	0.00	0.00	0.68	0.00
GATCAACTCTTTGAACACGC	0.00	0.33	0.00	0.00	0.00
GATCAACTCTTTGAACACGT	0.00	0.33	0.00	0.00	0.00
GATCAACTCTTTTACAGTGC	0.00	0.00	0.00	0.00	0.00
GATCAACTGAAAAAAAAAAA	4.58	0.00	0.57	1.70	2.41
GATCAACTGAAAAAAAAAAT	0.23	0.00	0.00	0.00	0.00
GATCAACTGAAAAAAGAAAA	0.23	0.00	0.00	0.00	0.00
GATCAACTGAAAAGAAAAAA	0.00	0.00	0.00	0.00	0.34
GATCAACTGAAAAGCAAAAA	24.49	5.21	10.88	9.16	11.37
GATCAACTGAAAAGCAAAAN	0.46	0.00	0.00	0.00	0.00
GATCAACTGAAAAGCAAAAT	0.23	0.00	0.00	0.00	0.00
GATCAACTGAAAAGCAACAA	0.00	0.00	0.00	0.68	0.00
GATCAACTGAAAAGCAACGT	0.00	0.00	0.00	0.00	0.00
GATCAACTGAAAAGCAANNN	0.23	0.00	0.00	0.00	0.00
GATCAACTGAAAAGCACAAA	0.00	0.00	0.00	0.34	0.00
GATCAACTGAAAAGCTAAAA	0.00	0.00	0.00	0.00	0.00
GATCAACTGAAAAGTAAAAA	0.00	0.33	0.00	0.00	0.00
GATCAACTGAAACACTGCGG	0.23	0.00	0.00	0.00	0.00
GATCAACTGAAACCTTGAGT	0.00	0.00	0.00	0.00	0.00
GATCAACTGAAAGCACAGCG	0.00	0.00	0.00	0.34	0.00
GATCAACTGAAAGGCAAAAA	0.46	0.00	0.00	0.00	0.00
GATCAACTGAAAGTAAGGCA	0.00	0.00	0.29	0.00	0.00
GATCAACTGAAAGTCCGTTG	0.46	0.00	0.00	0.34	0.00
GATCAACTGAAATTCATGGA	0.46	0.00	0.00	0.00	0.00
GATCAACTGAACCAGACAAT	0.00	0.65	0.00	0.00	0.00
GATCAACTGAACCTCCGACT	0.00	0.00	0.00	0.00	0.00
GATCAACTGAAGACCACTCA	0.00	0.00	0.00	0.00	0.34
GATCAACTGAAGACCACTCG	0.00	0.00	0.29	0.00	0.00

GATCAACTGAAGACCACTTG	30.22	22.12	22.91	19.35	20.66
GATCAACTGAAGACCACTTT	0.46	0.00	0.00	0.34	0.00
GATCAACTGAAGACCATTAG	0.23	0.00	0.00	0.00	0.00
GATCAACTGAAGAGGCAAAG	0.00	0.00	0.00	0.00	0.00
GATCAACTGAAGAGGTTTGC	0.00	0.00	0.00	0.34	0.00
GATCAACTGAAGCACGCATT	0.69	0.00	0.00	0.00	0.00
GATCAACTGAAGCAGACACG	0.00	0.00	0.00	0.00	0.00
GATCAACTGAAGCCGCCTCC	0.00	0.00	0.00	0.00	0.00
GATCAACTGAAGCCGCCTTT	0.00	0.00	0.00	0.00	0.00
GATCAACTGAAGGCATGTCC	0.00	0.00	0.00	0.00	0.34
GATCAACTGAAGGCCACTTG	0.23	0.00	0.00	0.00	0.00
GATCAACTGAAGGGGATTGA	0.00	0.00	0.00	0.00	0.00
GATCAACTGAAGGGGTGCTT	0.00	0.00	0.86	0.00	0.34
GATCAACTGAAGTACCACTG	0.00	0.00	0.57	0.00	0.00
GATCAACTGAAGTTGTTGCT	0.92	0.00	1.15	0.00	0.69
GATCAACTGAATCTTCCTAT	0.00	0.00	0.00	0.68	0.00
GATCAACTGAATGTAGCCTG	4.81	3.58	1.43	4.07	2.07
GATCAACTGAATTCAAACCA	0.00	0.00	0.00	0.00	0.69
GATCAACTGAATTCCTACAG	0.00	0.00	0.29	0.00	0.00
GATCAACTGAATTCGTACAA	0.00	0.65	0.00	0.00	0.00
GATCAACTGAATTGTTGAAG	0.23	0.00	0.00	0.00	0.00
GATCAACTGACAAAGGATTA	0.23	0.00	0.00	0.00	0.00
GATCAACTGACAACCATGCG	0.00	0.00	0.00	0.00	0.34
GATCAACTGACAACTAAAAT	0.23	0.00	0.29	0.00	0.00
GATCAACTGACAAGGCCGCG	0.00	0.00	0.00	0.34	0.00
GATCAACTGACACGCCAACC	0.00	0.00	0.00	0.00	0.34
GATCAACTGACACTGGCATC	0.00	0.65	0.86	0.00	0.00
GATCAACTGACAGCCTTTCA	0.00	0.00	0.00	0.00	0.00
GATCAACTGACAGTCTGTGA	0.00	0.00	0.29	0.00	0.00
GATCAACTGACATGCCCTTG	0.00	0.00	0.00	0.00	0.00
GATCAACTGACATGCGAGAG	1.60	1.30	0.86	1.02	2.07
GATCAACTGACCACTGAATG	0.00	0.00	0.29	0.00	0.00
GATCAACTGACCAGCGCCCC	0.23	0.00	0.00	0.00	0.00
GATCAACTGACCTACGTTCG	0.46	1.30	0.00	0.00	0.34
GATCAACTGACGAAGAGGCG	0.00	0.00	0.29	0.00	0.00
GATCAACTGACGAATACAAG	0.00	0.00	0.29	0.00	0.00
GATCAACTGACGACCCCCTC	0.23	0.00	0.00	0.00	0.00

GATCAACTGACGACCTACTC	0.00	0.00	0.29	0.00	0.00
GATCAACTGACGACCTCCTC	0.23	0.33	0.00	0.68	0.00
GATCAACTGACGACCTCGTT	0.00	0.00	0.29	0.00	0.00
GATCAACTGACGCCATGGTA	0.23	0.00	0.00	0.00	0.00
GATCAACTGACGCGAAGCCT	0.00	0.65	0.00	0.00	0.34
GATCAACTGACGCGTTCCGG	0.23	0.00	0.00	0.00	0.00
GATCAACTGACGGCACATCT	0.23	0.00	0.00	0.00	0.00
GATCAACTGACGGTCAACAG	0.00	0.00	0.29	0.00	0.00
GATCAACTGACGTCAAGCAC	0.00	0.33	0.00	0.00	0.00
GATCAACTGACGTCAAGCAT	0.00	0.00	0.29	0.00	0.00
GATCAACTGACGTCGTAAGT	0.00	0.33	0.00	0.00	0.00
GATCAACTGACGTGCCCGGC	0.23	0.00	0.00	0.00	0.00
GATCAACTGACGTGCCCTTG	0.00	0.00	0.00	0.00	0.00
GATCAACTGACGTTGGGGTT	0.23	0.00	1.43	1.02	0.00
GATCAACTGACTACCGTTAT	0.00	0.00	0.00	0.00	0.34
GATCAACTGACTCCAAACTG	0.00	0.33	1.43	1.36	0.34
GATCAACTGACTCGTGCTAG	0.00	0.00	0.00	0.00	0.00
GATCAACTGACTTCGTCAGC	0.00	0.00	0.00	0.00	0.00
GATCAACTGACTTCTACAGA	0.00	0.00	0.00	0.00	0.00
GATCAACTGACTTCTCTTTG	0.00	0.00	0.00	0.34	0.00
GATCAACTGACTTCTGAAGT	0.69	0.00	0.00	0.00	0.00
GATCAACTGACTTCTGGAGT	0.23	0.00	0.00	0.00	0.00
GATCAACTGACTTTGCGCGG	0.00	0.00	0.00	0.00	0.00
GATCAACTGAGACAAAAGAA	0.23	0.00	0.00	0.00	0.00
GATCAACTGAGACACCGTCA	0.00	0.00	0.00	0.00	0.00
GATCAACTGAGACCCGATCT	0.23	0.00	0.00	0.00	0.00
GATCAACTGAGACCCGCTCG	0.00	0.00	0.00	0.34	0.00
GATCAACTGAGACCCGCTCN	0.00	0.00	0.00	0.00	0.00
GATCAACTGAGACCCGCTCT	18.08	22.12	10.31	17.31	16.88
GATCAACTGAGACCCGCTNN	0.23	0.00	0.00	0.00	0.00
GATCAACTGAGACCTCCTTG	0.00	0.00	0.00	0.00	0.00
GATCAACTGAGAGCTCATCG	0.00	0.00	0.00	0.00	0.00
GATCAACTGAGAGGAGAGCT	0.00	0.00	0.00	1.02	0.00
GATCAACTGAGAGGCTTGGG	0.00	0.00	0.00	0.00	0.00
GATCAACTGAGCAAAAAAAA	0.00	0.00	0.29	0.00	0.00
GATCAACTGAGCAAAAATCA	0.00	0.65	0.00	0.00	0.00
GATCAACTGAGCACGGNGTG	0.00	0.00	0.00	0.00	0.34

GATCAACTGAGCACGTCCCG	0.00	0.00	0.00	0.00	0.00
GATCAACTGAGCACGTCCTG	0.00	0.00	0.00	0.34	0.00
GATCAACTGAGCACGTGCTG	0.23	0.00	0.00	0.00	0.00
GATCAACTGAGCACGTTACG	0.00	0.00	0.00	0.00	0.00
GATCAACTGAGCAGAAAGTA	0.00	0.00	0.00	0.34	0.00
GATCAACTGAGCATAAGGCC	0.00	0.33	0.00	0.00	0.00
GATCAACTGAGCATGCTCCA	0.00	0.00	0.00	0.68	0.00
GATCAACTGAGCATGCTGCA	4.35	2.93	1.15	2.72	1.38
GATCAACTGAGCATGCTGCC	0.00	0.00	0.00	0.00	0.00
GATCAACTGAGCATTCCGGA	0.00	0.33	0.00	0.00	0.00
GATCAACTGAGCCACGCAGC	0.00	0.33	0.57	1.36	0.00
GATCAACTGAGCCTGTCCTC	0.00	0.00	0.29	0.00	0.00
GATCAACTGAGCCTGTGCTC	2.75	4.88	0.86	2.72	1.38
GATCAACTGAGCCTGTGCTG	0.00	0.00	0.00	0.00	0.00
GATCAACTGAGCGCGAGAGG	0.23	0.00	0.00	0.00	0.00
GATCAACTGAGCGCGANTCG	0.00	0.33	0.00	0.00	0.00
GATCAACTGAGCGGAGTCCT	0.00	0.00	0.00	0.00	0.00
GATCAACTGAGCGGAGTGCC	0.00	0.00	0.00	0.00	0.00
GATCAACTGAGCGTCCATCA	0.00	0.00	0.00	0.00	0.00
GATCAACTGAGCTCCCCCGC	0.23	0.00	0.00	0.00	0.00
GATCAACTGAGCTCCTCCGC	5.26	2.60	1.72	3.05	0.00
GATCAACTGAGCTGAATTCC	0.00	0.00	0.00	0.00	0.34
GATCAACTGAGCTGCAAGCC	0.00	0.33	0.00	0.00	0.34
GATCAACTGAGCTGCATCTG	0.00	0.00	0.29	0.00	0.00
GATCAACTGAGCTGCCTGAG	0.00	0.00	0.29	0.00	0.00
GATCAACTGAGCTGCTCGTG	0.00	0.00	0.00	0.00	0.00
GATCAACTGAGCTGTGATGC	0.00	0.00	0.00	0.00	0.34
GATCAACTGAGCTTGTGTTT	0.23	0.00	0.00	0.00	0.00
GATCAACTGAGGACGCCTCC	0.00	0.00	0.00	0.34	0.00
GATCAACTGAGGACGTTGTC	0.00	0.00	0.29	0.00	0.00
GATCAACTGAGGAGGAGGAA	0.00	0.00	0.00	0.00	0.69
GATCAACTGAGGAGGAGGAG	0.00	0.00	0.00	0.00	0.34
GATCAACTGAGGAGGCTCTG	0.00	0.00	0.00	0.00	0.00
GATCAACTGAGGCAAACGGC	0.00	0.00	0.00	0.00	0.00
GATCAACTGAGGCAACTTAA	0.00	0.65	0.29	0.00	0.69
GATCAACTGAGGCACGCGGC	0.00	0.00	0.00	0.00	0.00
GATCAACTGAGGCAGACTCA	0.00	0.00	0.00	0.34	0.00

GATCAACTGAGGCAGATGCA	0.00	0.00	0.00	0.00	0.34
GATCAACTGAGGCAGCAGGT	0.23	0.00	0.29	0.00	0.00
GATCAACTGAGGCAGCCTCC	0.00	0.00	0.29	0.00	0.34
GATCAACTGAGGCATCCTTG	0.00	0.00	0.00	0.00	0.00
GATCAACTGAGGCCAGCGGC	0.23	0.00	0.00	0.00	0.00
GATCAACTGAGGCCCCTCCT	0.00	0.00	0.00	0.00	0.00
GATCAACTGAGGCCGACTCC	0.00	0.00	0.00	0.00	0.34
GATCAACTGAGGCCGACTTC	0.00	0.00	0.29	0.00	0.00
GATCAACTGAGGCCGCACCT	0.00	0.00	0.00	0.34	0.00
GATCAACTGAGGCCGCCACC	0.00	0.33	0.00	0.00	0.00
GATCAACTGAGGCCGCCCCN	0.00	0.00	0.00	0.00	0.34
GATCAACTGAGGCCGCCTAC	0.00	0.00	0.00	0.00	0.34
GATCAACTGAGGCCGCCTCA	0.00	0.00	0.00	0.34	0.00
GATCAACTGAGGCCGCCTCC	27.70	28.30	41.81	32.92	29.62
GATCAACTGAGGCCGCCTCG	0.23	0.00	0.00	0.00	0.00
GATCAACTGAGGCCGCCTCT	0.00	0.00	0.00	0.34	0.00
GATCAACTGAGGCCGCCTNN	0.23	0.00	0.00	0.00	0.00
GATCAACTGAGGCCGTCTCC	0.23	0.00	0.29	0.00	0.00
GATCAACTGAGGCCTCCTGG	0.23	1.95	2.86	3.39	1.38
GATCAACTGAGGCCTCCTTG	0.00	0.00	0.00	0.00	0.00
GATCAACTGAGGCGCAATGA	0.00	0.00	0.29	0.00	0.00
GATCAACTGAGGCGCCTCGA	0.00	0.98	0.57	1.70	1.03
GATCAACTGAGGCGCGCGGC	0.00	0.00	0.29	0.00	0.00
GATCAACTGAGGGCTGCCAC	0.46	0.00	0.00	0.00	0.00
GATCAACTGAGGTCTTGCTG	0.00	0.00	0.00	0.00	0.34
GATCAACTGAGGTTCGCAGT	0.92	0.00	0.00	0.00	0.00
GATCAACTGAGTAAAGCCAC	0.23	0.00	0.00	0.00	0.00
GATCAACTGAGTATTGCTGC	0.00	0.00	0.00	0.34	0.00
GATCAACTGAGTCAAGCCCA	0.00	0.00	0.00	0.00	0.00
GATCAACTGAGTCCGCCTCC	0.00	0.00	0.29	0.00	0.00
GATCAACTGAGTGCAGCACA	0.23	0.00	0.00	0.00	0.00
GATCAACTGAGTGGCACAGC	0.46	0.98	0.00	0.00	0.00
GATCAACTGAGTTCAAAAAA	0.00	0.00	0.00	0.34	0.00
GATCAACTGATACTGGTCAA	0.23	0.00	0.00	0.00	0.00
GATCAACTGATACTGGTCCA	0.00	0.00	0.00	0.00	0.34
GATCAACTGATACTGGTCCG	0.00	0.00	0.00	0.34	0.34
GATCAACTGATACTTCCTCT	0.23	0.98	0.57	0.34	0.34

GATCAACTGATACTTGTCCA	0.23	0.00	0.00	0.00	0.00
GATCAACTGATATCGACAAG	0.46	0.00	0.00	0.00	0.00
GATCAACTGATATCGAGTGT	0.00	0.00	0.00	0.34	0.00
GATCAACTGATATGTTGGAT	0.00	0.00	0.29	0.00	0.00
GATCAACTGATGCAAATTGC	0.00	0.00	0.00	0.00	0.00
GATCAACTGATGCACCGGGT	0.00	0.00	0.00	0.00	0.00
GATCAACTGATGCACGCGTT	0.00	0.33	0.00	0.00	0.00
GATCAACTGATGCTCAACTG	0.00	0.00	0.00	0.00	1.38
GATCAACTGATGCTCAAGCC	0.00	0.00	0.00	0.00	0.00
GATCAACTGATGGACGTGCA	0.00	0.00	0.00	0.00	0.00
GATCAACTGATGGAGCAGGG	0.00	0.00	0.00	0.34	0.00
GATCAACTGATGGCTGTGTG	0.00	0.00	0.00	0.34	0.00
GATCAACTGATGGTTTGCCG	0.23	0.00	0.00	0.00	0.00
GATCAACTGATGTACCGGGG	0.23	0.00	0.29	0.00	0.00
GATCAACTGATGTACCGGGT	1.60	1.30	1.72	0.68	2.41
GATCAACTGATTAATGGGAA	0.00	0.00	0.00	1.02	0.00
GATCAACTGATTCAAAAGAG	0.00	0.33	0.00	0.00	0.00
GATCAACTGATTCCAAGAAA	0.00	0.00	0.00	0.34	0.00
GATCAACTGATTCTAAACCT	0.00	0.00	0.00	0.00	0.34
GATCAACTGATTGAAAAAAA	0.23	0.00	0.00	0.00	0.00
GATCAACTGATTGTTTGAAA	4.35	0.98	0.00	2.04	0.34
GATCAACTGATTGTTTGAAC	0.23	0.00	0.00	0.00	0.00
GATCAACTGATTTGCTAAGT	0.92	0.00	0.00	0.00	0.00
GATCAACTGATTTGTTAAGT	0.00	0.00	0.29	0.00	0.00
GATCAACTGATTTTGTTCCT	0.00	0.00	0.00	0.00	0.69
GATCAACTGCAAAAAAAAAA	0.00	0.33	0.29	0.34	0.34
GATCAACTGCAAAAGCGGTG	0.23	0.00	0.00	0.00	0.00
GATCAACTGCAAAAGCTGTG	0.92	0.00	0.29	0.34	0.34
GATCAACTGCAAAAGTGACA	0.00	0.00	0.29	0.00	0.00
GATCAACTGCAAAGCAGAAA	0.23	0.00	0.00	0.00	0.00
GATCAACTGCAACCTACGAC	0.00	0.00	0.00	0.00	0.34
GATCAACTGCAACGAAAGGC	0.00	0.00	0.29	0.00	0.00
GATCAACTGCAACGTGTGAG	0.23	0.00	0.00	0.00	0.00
GATCAACTGCAACTCGTCGC	0.23	0.00	0.00	0.00	0.00
GATCAACTGCAAGAAAAAAA	0.00	0.00	0.00	0.00	0.00
GATCAACTGCAAGCACAGCG	4.81	2.93	1.72	4.41	3.79
GATCAACTGCAAGCGACGAT	0.46	0.33	0.00	0.00	0.00

GATCAACTGCAAGGGAATGC	0.00	0.00	0.00	0.00	0.00
GATCAACTGCAAGGTTCCTC	0.00	0.00	0.00	1.02	0.00
GATCAACTGCAAGTCAAGTC	0.00	0.00	0.00	0.00	0.34
GATCAACTGCAAGTCTGGGC	0.00	0.00	0.00	0.34	0.00
GATCAACTGCAATAGGCCCG	0.00	0.00	0.00	0.00	0.00
GATCAACTGCAATCCCGAGA	0.00	0.00	0.00	0.34	0.00
GATCAACTGCAATCGCCATT	0.00	0.00	0.00	0.68	0.00
GATCAACTGCAATCGGCACG	0.23	0.00	0.00	0.00	0.00
GATCAACTGCAATCGGCAGC	0.46	0.00	0.00	0.00	0.00
GATCAACTGCAATCGGCCCG	0.46	0.65	0.00	0.68	0.00
GATCAACTGCAATGCGGCGG	0.00	0.00	0.00	0.00	0.00
GATCAACTGCACACGCCCAG	0.00	0.00	0.29	0.00	0.00
GATCAACTGCACACTGTAAA	0.23	0.00	0.00	0.00	0.00
GATCAACTGCACATCTGCTA	0.00	0.00	0.00	0.34	0.00
GATCAACTGCACCAAGCAGT	2.29	3.58	1.72	2.72	1.38
GATCAACTGCACCCGCGGGC	0.23	0.00	0.00	0.00	0.00
GATCAACTGCACCTCCGACT	0.00	0.33	0.00	0.00	0.34
GATCAACTGCACGGGCTTCC	0.23	1.30	0.29	0.34	0.34
GATCAACTGCACGGGGTTGT	0.00	0.00	0.00	0.34	0.00
GATCAACTGCACGTCGCCCG	0.00	0.00	0.00	0.00	0.00
GATCAACTGCACTGCCCTGC	0.46	0.33	1.15	0.00	0.00
GATCAACTGCACTGTATGGC	0.00	0.00	0.00	0.00	0.00
GATCAACTGCACTTAAGGAA	0.00	0.65	0.00	0.00	1.03
GATCAACTGCAGAACTGCAG	0.00	0.00	0.29	0.00	0.00
GATCAACTGCAGAAGTTCTC	0.00	0.00	0.29	0.00	0.00
GATCAACTGCAGACCTGCTC	0.00	0.00	0.00	0.68	0.00
GATCAACTGCAGCCCAGAGG	0.00	0.33	0.00	0.00	0.00
GATCAACTGCAGCCCTCTGG	0.23	0.00	0.00	0.00	0.34
GATCAACTGCAGCGAGGGAG	0.00	0.00	0.00	0.34	1.03
GATCAACTGCAGCGCATTCC	0.00	0.00	0.00	0.34	0.00
GATCAACTGCAGCTCACGAG	0.00	0.00	0.57	0.00	0.00
GATCAACTGCAGCTCATAGG	0.00	0.00	0.00	0.00	0.00
GATCAACTGCAGCTCATCGG	5.26	7.48	9.16	8.49	7.92
GATCAACTGCAGCTCATCNN	0.00	0.00	0.00	0.00	0.00
GATCAACTGCAGCTCCTGCC	0.00	0.00	0.00	0.00	0.34
GATCAACTGCAGCTCCTTCA	0.00	0.00	0.00	0.00	0.00
GATCAACTGCAGCTGCTTCA	0.00	0.00	0.29	0.00	0.34

GATCAACTGCAGGACAATGG	0.00	0.00	0.29	0.00	0.00
GATCAACTGCAGGACTGAGG	0.00	0.00	0.29	0.00	0.00
GATCAACTGCAGGACTGATG	0.46	0.00	0.57	0.68	0.34
GATCAACTGCAGGAGGGAGA	0.23	0.00	0.00	0.00	0.00
GATCAACTGCAGGAGGTCTT	0.00	0.00	0.29	0.00	0.00
GATCAACTGCAGGCACAGGA	0.00	0.00	0.00	0.34	0.00
GATCAACTGCAGGCGCCAGG	0.00	0.00	0.29	0.00	0.00
GATCAACTGCAGGCGTCCTG	0.00	0.00	0.00	0.34	0.00
GATCAACTGCAGGGAGGGAG	0.00	0.33	0.00	0.00	0.34
GATCAACTGCAGGGGTTTTT	0.00	0.00	0.29	0.00	0.00
GATCAACTGCAGTGGAAGAC	0.00	0.00	0.00	0.00	0.34
GATCAACTGCAGTGGCAATG	0.46	0.00	0.00	0.00	0.00
GATCAACTGCAGTGGCTGCA	0.23	0.00	0.00	0.00	0.00
GATCAACTGCAGTTGCCGCA	0.23	0.00	0.00	0.00	0.00
GATCAACTGCAGTTGCTGCA	13.96	18.54	4.01	14.26	5.51
GATCAACTGCAGTTGCTGCC	0.00	0.65	0.00	0.34	0.00
GATCAACTGCAGTTGCTGCN	0.23	0.00	0.00	0.00	0.00
GATCAACTGCAGTTGCTGGA	0.00	0.00	0.00	0.34	0.00
GATCAACTGCAGTTGCTGNN	0.23	0.00	0.00	0.00	0.00
GATCAACTGCATAATGAACC	0.00	0.00	0.00	0.00	0.00
GATCAACTGCATACAACTCG	0.00	0.00	0.00	0.00	0.69
GATCAACTGCATATGCTCCA	0.00	0.00	0.00	0.00	0.69
GATCAACTGCATCAGCGCGT	0.69	0.33	0.57	0.34	0.34
GATCAACTGCATCATCTAAC	0.00	0.00	0.00	0.00	0.34
GATCAACTGCATCATCTAAG	0.00	0.33	0.29	1.02	0.34
GATCAACTGCATCATGAACA	0.00	0.33	0.00	0.00	0.00
GATCAACTGCATCATGAACC	6.41	3.58	1.43	2.04	1.72
GATCAACTGCATCCAATCTC	0.00	0.00	0.00	0.00	0.00
GATCAACTGCATCCCCGTCT	0.00	0.00	0.00	0.34	0.00
GATCAACTGCATCTCATCGG	0.00	0.33	0.00	0.00	0.00
GATCAACTGCATGAACAGGA	0.00	0.00	0.00	0.00	0.00
GATCAACTGCATGCCCTTGG	0.00	0.00	0.00	0.00	0.00
GATCAACTGCATGCGGAACA	0.00	0.00	0.29	0.00	0.00
GATCAACTGCATGCGGACCA	0.00	0.00	0.29	0.00	0.00
GATCAACTGCATGTCCGCGC	0.00	0.00	0.00	0.34	0.00
GATCAACTGCATGTGGAAGA	0.00	0.33	0.29	3.05	2.07
GATCAACTGCATGTTTTTTA	0.00	0.00	0.00	0.34	0.00

GATCAACTGCATTCCCGTTG	0.00	0.00	0.00	1.36	0.00
GATCAACTGCATTGACCTCA	0.92	0.65	0.00	0.00	0.34
GATCAACTGCATTGACCTCT	0.00	0.33	0.00	0.00	0.00
GATCAACTGCATTGGCGCCG	0.00	0.00	0.29	0.00	0.00
GATCAACTGCATTGTAGAGT	0.23	0.33	0.00	0.00	0.00
GATCAACTGCATTTCTGTCA	0.00	0.33	0.00	0.00	0.00
GATCAACTGCATTTTTCGAG	0.00	0.00	0.00	0.68	0.00
GATCAACTGCCAATCAGGAG	0.00	0.00	0.29	0.00	0.00
GATCAACTGCCAATGCTCCC	0.00	0.00	0.00	0.34	0.00
GATCAACTGCCACATGTGGT	0.00	0.65	0.29	0.00	0.00
GATCAACTGCCACCGCATCA	0.00	0.00	0.86	0.00	0.00
GATCAACTGCCACCTGCAGG	0.00	0.00	0.00	0.00	0.34
GATCAACTGCCACGATGTGG	0.00	0.33	0.00	0.00	0.69
GATCAACTGCCAGAGGAAGC	0.00	0.65	0.00	0.00	0.00
GATCAACTGCCAGCAGTGTT	0.46	1.63	0.57	1.02	0.00
GATCAACTGCCAGCGAAGGA	0.00	0.00	0.00	0.00	0.00
GATCAACTGCCAGGCAATGA	0.00	0.00	0.00	1.36	0.00
GATCAACTGCCAGGTCCCCT	0.00	0.00	0.00	0.00	0.00
GATCAACTGCCAGTGCCAGA	0.00	0.00	0.00	0.00	0.34
GATCAACTGCCAGTGGCACG	0.23	0.00	0.00	0.00	0.00
GATCAACTGCCATCCTGACG	0.00	0.00	0.29	0.00	0.00
GATCAACTGCCATGCCCTTG	0.00	0.33	0.00	0.00	0.00
GATCAACTGCCCAAACAATC	0.00	0.00	0.29	0.00	0.00
GATCAACTGCCCAAGGAAAA	0.00	0.00	0.00	0.00	0.69
GATCAACTGCCCAAGGAAAT	0.00	0.33	0.00	0.00	0.00
GATCAACTGCCCAAGGAAGG	0.46	0.00	0.57	0.34	0.34
GATCAACTGCCCAAGTGGTG	0.00	0.00	0.00	0.34	0.00
GATCAACTGCCCACAGGAGG	0.23	0.33	0.00	0.00	0.00
GATCAACTGCCCAGATGGTT	0.00	0.00	0.00	0.34	0.00
GATCAACTGCCCATGCAGCA	0.00	0.00	0.00	0.00	0.34
GATCAACTGCCCATGGCCCG	0.46	0.00	0.00	0.00	0.00
GATCAACTGCCCCCCGCTTG	0.00	0.00	0.57	0.00	0.00
GATCAACTGCCCCGCTTCGC	0.00	0.00	0.00	0.34	0.00
GATCAACTGCCCCTTCAAGA	0.00	0.00	0.00	0.00	0.34
GATCAACTGCCCTCACCACT	0.23	0.33	0.29	0.34	0.34
GATCAACTGCCCTCGAGGAT	0.00	0.65	0.00	0.00	0.00
GATCAACTGCCCTCTGTCAG	0.46	0.00	0.00	1.36	0.69

GATCAACTGCCCTTTTCTCA	0.00	0.00	0.29	0.00	0.00
GATCAACTGCCGAGACATCT	0.00	0.00	0.00	0.00	0.00
GATCAACTGCCGAGACATTC	0.00	0.00	0.29	0.00	0.00
GATCAACTGCCGAGTCGACG	0.69	0.98	0.57	0.00	0.69
GATCAACTGCCGATGTCGCT	0.00	0.33	0.00	0.00	0.00
GATCAACTGCCGCAAACACC	0.00	0.00	0.29	0.00	0.00
GATCAACTGCCGCACCAGAA	0.00	0.00	0.00	0.00	0.34
GATCAACTGCCGCGGCTGCC	0.00	0.00	0.00	0.00	0.34
GATCAACTGCCGCGGGGCGA	0.23	0.00	0.00	0.00	0.00
GATCAACTGCCGCTGAGGAG	0.00	0.33	0.86	0.00	0.00
GATCAACTGCCGCTGGCAAG	0.00	0.00	0.00	0.00	0.00
GATCAACTGCCGGCCAGCCG	4.35	3.25	0.86	1.36	1.03
GATCAACTGCCGGTCGTCAG	0.00	0.00	0.00	0.00	0.00
GATCAACTGCCGGTGTGCGA	0.00	0.00	0.00	0.00	0.34
GATCAACTGCCGTCGGCGTG	0.00	0.00	0.29	0.00	0.00
GATCAACTGCCGTGAGGGCG	0.00	0.00	0.00	0.34	0.00
GATCAACTGCCGTGAGTGCG	0.23	0.00	0.00	0.00	0.00
GATCAACTGCCTACATTAGG	0.00	0.33	0.00	0.00	0.00
GATCAACTGCCTCAGCTTCC	0.00	0.00	0.00	0.00	0.00
GATCAACTGCCTCAGCTTCG	0.00	0.00	0.00	0.00	0.00
GATCAACTGCCTCCGCTGCC	0.00	0.33	0.00	0.00	0.00
GATCAACTGCCTCGATGGCC	0.00	0.00	0.00	0.00	0.00
GATCAACTGCCTCGCGCGAC	0.00	0.00	0.00	0.00	0.34
GATCAACTGCCTCTTTCAAG	0.00	0.00	0.00	0.00	0.00
GATCAACTGCCTGACGGCGC	0.00	0.33	0.00	0.00	0.00
GATCAACTGCCTGCTGCAAA	0.00	0.00	0.00	0.00	0.34
GATCAACTGCCTGCTGGGCA	0.00	0.00	0.00	0.34	0.00
GATCAACTGCCTGGACGGCT	0.00	0.00	0.00	0.34	0.00
GATCAACTGCCTGGTAATGG	0.00	0.00	0.29	0.00	0.00
GATCAACTGCCTGTGAGTGG	0.00	0.00	0.00	0.00	0.34
GATCAACTGCCTGTGTGCAA	0.00	0.00	0.29	0.00	0.00
GATCAACTGCCTTAACCATG	0.00	0.00	0.00	0.00	0.34
GATCAACTGCCTTGTCTTCT	0.00	0.00	0.00	0.68	0.00
GATCAACTGCCTTTGCCACT	0.00	0.00	0.00	0.00	0.00
GATCAACTGCGAAAAAAAAA	0.00	0.33	0.00	0.00	0.34
GATCAACTGCGAAACGCTCA	0.00	0.00	0.29	0.00	0.00
GATCAACTGCGAACTCATCG	0.00	0.00	0.00	0.00	0.00

GATCAACTGCGAACTGTATG	0.00	0.33	0.00	0.00	0.34
GATCAACTGCGACAAGGCCA	0.00	0.00	0.29	0.00	0.00
GATCAACTGCGACAGCGCCT	0.46	0.00	0.00	0.00	0.00
GATCAACTGCGACATCGTGN	0.23	0.00	0.00	0.00	0.00
GATCAACTGCGACATCGTGT	0.69	0.00	0.00	0.00	0.00
GATCAACTGCGACATGTATT	0.92	0.00	0.00	0.00	0.00
GATCAACTGCGACCGCGGGC	0.00	0.00	0.00	0.00	0.00
GATCAACTGCGACGAGTCGG	0.23	0.00	0.00	0.00	0.00
GATCAACTGCGACGGCTTCA	1.60	0.33	0.00	0.00	0.00
GATCAACTGCGACTCATGGG	0.00	0.00	0.00	0.00	0.00
GATCAACTGCGAGAAGAGAA	0.00	0.00	0.00	0.00	0.34
GATCAACTGCGAGATAATAG	0.00	0.00	0.00	0.34	0.00
GATCAACTGCGAGCCAATGC	0.23	0.00	0.00	0.00	0.34
GATCAACTGCGAGCCACTGC	0.00	0.33	0.00	0.00	0.00
GATCAACTGCGAGCTCAACG	0.23	0.00	0.00	0.34	0.00
GATCAACTGCGAGCTCATAG	0.00	0.00	0.00	0.34	0.00
GATCAACTGCGAGCTCATAT	0.00	0.00	0.00	0.34	0.00
GATCAACTGCGAGCTCATCG	50.82	43.27	20.33	42.09	28.24
GATCAACTGCGAGCTCATTG	0.00	0.00	0.00	0.34	0.00
GATCAACTGCGAGCTCCTCG	0.46	0.00	0.29	0.00	0.00
GATCAACTGCGAGCTCTTTG	0.23	0.00	0.00	0.00	0.00
GATCAACTGCGAGCTTCGTA	0.00	0.00	0.00	0.00	0.00
GATCAACTGCGAGGATTCCA	0.00	0.00	0.29	0.00	0.00
GATCAACTGCGAGGCGGTGT	0.00	0.00	0.29	0.00	0.00
GATCAACTGCGAGGGCAGCT	0.46	0.00	0.00	0.68	0.00
GATCAACTGCGAGGGTGAGC	0.00	0.00	0.57	0.00	0.00
GATCAACTGCGAGTGAGCTG	0.00	0.00	0.00	0.00	0.00
GATCAACTGCGCAAGTTCTT	0.46	0.65	0.29	0.68	1.38
GATCAACTGCGCACCTTTCC	0.23	0.00	0.00	0.00	0.00
GATCAACTGCGCACGCGGGC	0.00	0.00	0.57	0.00	0.34
GATCAACTGCGCACGCGGTC	0.00	0.00	0.29	0.00	0.00
GATCAACTGCGCACGGGTAA	0.00	0.00	0.29	0.00	0.00
GATCAACTGCGCAGAGTGCT	0.69	1.30	0.29	2.04	0.34
GATCAACTGCGCAGCTGGCC	0.00	0.00	0.00	0.34	0.00
GATCAACTGCGCCAGCGGAC	0.23	0.00	0.00	0.00	0.00
GATCAACTGCGCCAGCGGGC	0.00	0.00	0.00	0.00	0.34
GATCAACTGCGCCCAGCTCT	0.00	0.00	0.29	0.00	0.00

GATCAACTGCGCCCGCAAGC	0.00	0.00	0.00	0.34	0.00
GATCAACTGCGCCCGCAGGC	0.00	0.33	0.00	0.00	0.00
GATCAACTGCGCCCGCGGCT	0.00	0.00	0.00	0.00	0.00
GATCAACTGCGCCCGCGGGA	0.23	0.00	0.00	0.68	0.00
GATCAACTGCGCCCGCGGGC	35.71	28.63	36.65	26.48	19.98
GATCAACTGCGCCCGCGGGG	0.23	0.00	0.00	0.00	0.00
GATCAACTGCGCCCGCGGNN	0.23	0.00	0.00	0.00	0.00
GATCAACTGCGCCCGCNNNN	0.23	0.00	0.00	0.00	0.00
GATCAACTGCGCCCGCTGGC	0.00	0.00	0.00	0.00	0.00
GATCAACTGCGCCCGCTGTC	0.23	0.00	0.00	0.00	0.00
GATCAACTGCGCCCGGGGGC	0.23	0.00	0.00	0.00	0.00
GATCAACTGCGCCGTCACGC	0.00	0.00	0.00	0.34	0.00
GATCAACTGCGCCGTGACGT	0.92	0.00	0.29	0.34	0.00
GATCAACTGCGCCTCGTCCC	0.00	0.00	0.00	0.00	0.69
GATCAACTGCGCCTGCGGGC	0.00	0.33	0.00	0.00	0.00
GATCAACTGCGCCTTCAAAA	0.00	0.00	0.00	0.34	0.00
GATCAACTGCGCCTTCAAGA	0.92	0.33	0.29	0.68	0.00
GATCAACTGCGCCTTCAAGT	0.23	0.00	0.00	0.00	0.00
GATCAACTGCGCCTTTTCGT	0.00	0.00	0.00	0.00	0.34
GATCAACTGCGCGACAATAA	0.00	0.33	0.00	0.00	0.00
GATCAACTGCGCGCTCATCG	0.23	0.00	0.00	0.00	0.00
GATCAACTGCGCGGAAGAGT	0.00	0.00	0.29	0.00	0.00
GATCAACTGCGCGGTGACGC	0.00	0.00	0.00	0.68	0.69
GATCAACTGCGCTCACAGCA	0.23	0.00	0.00	0.00	0.00
GATCAACTGCGCTCACGGCA	0.23	0.00	0.00	0.00	0.00
GATCAACTGCGCTCGCGGGC	0.00	0.00	0.57	0.00	0.00
GATCAACTGCGCTCTGTTCG	0.00	0.00	0.00	1.02	0.00
GATCAACTGCGCTGCGCTTC	0.46	0.33	0.29	0.68	0.69
GATCAACTGCGCTGCGGTTC	0.00	0.00	0.29	0.00	0.00
GATCAACTGCGCTGCGTCGC	0.00	0.33	0.00	0.00	0.00
GATCAACTGCGCTTGTATCC	0.00	0.00	0.00	0.00	0.00
GATCAACTGCGGACAGAACC	0.23	0.00	0.00	0.00	0.00
GATCAACTGCGGAGTGGAAG	0.00	0.00	0.29	0.00	0.00
GATCAACTGCGGCACGTGGG	0.23	0.00	0.00	0.00	0.00
GATCAACTGCGGCAGGCTGG	0.23	0.33	0.00	0.00	0.00
GATCAACTGCGGCATATCAC	0.23	0.00	0.00	0.00	0.00
GATCAACTGCGGCATCAAGT	0.00	0.00	0.00	0.34	0.00

GATCAACTGCGGCATCACGT	0.00	0.00	0.00	0.00	0.34
GATCAACTGCGGCATCATTC	0.00	0.00	0.00	0.00	0.34
GATCAACTGCGGCCGCATCC	0.00	0.33	0.00	0.00	0.00
GATCAACTGCGGCCGCCTCC	0.00	0.00	0.00	0.00	0.34
GATCAACTGCGGCCGGCTGG	0.69	0.65	0.00	0.00	0.00
GATCAACTGCGGCGCGGCGC	0.00	0.00	0.00	0.00	0.00
GATCAACTGCGGCGGCTTCC	0.00	0.00	0.00	0.00	0.00
GATCAACTGCGGCGTTTTGT	0.00	0.00	0.00	0.34	0.00
GATCAACTGCGGCTCCGGTA	0.00	0.00	0.00	0.00	0.00
GATCAACTGCGGGATGAGTA	0.00	0.00	0.00	0.00	0.00
GATCAACTGCGGGCACACTC	0.46	0.65	0.57	1.02	0.00
GATCAACTGCGGGCTCATCG	0.00	0.00	0.00	0.00	0.00
GATCAACTGCGGGGAGGACG	0.00	0.33	0.00	0.00	0.00
GATCAACTGCGGGGCAGAGG	0.00	0.00	0.00	0.34	0.00
GATCAACTGCGGGGCGGGGG	0.00	0.00	0.29	0.00	0.00
GATCAACTGCGGGGCGTGGC	0.00	0.00	0.00	0.00	0.00
GATCAACTGCGGGTGTGTGT	0.00	0.00	0.29	0.00	0.00
GATCAACTGCGTAGACTTCG	0.00	0.00	0.00	0.00	0.00
GATCAACTGCGTAGATGCCT	0.23	0.00	0.00	0.00	0.69
GATCAACTGCGTCAGCTGTT	0.23	0.33	0.00	0.00	0.00
GATCAACTGCGTCGCGGTCA	0.00	0.00	0.29	0.00	0.00
GATCAACTGCGTGATGACCG	0.00	0.00	0.00	0.00	0.00
GATCAACTGCGTGCACACGC	0.00	0.00	0.86	0.00	0.00
GATCAACTGCGTGGCGCCAC	0.00	0.00	0.00	0.00	0.00
GATCAACTGCGTTTGCGCGG	0.23	0.33	0.29	0.00	0.00
GATCAACTGCTACATCGGAG	0.00	0.00	0.00	0.00	0.00
GATCAACTGCTACGCCGTCG	0.23	0.00	0.00	0.00	0.00
GATCAACTGCTACGCGCTGC	0.00	0.00	0.00	0.00	0.00
GATCAACTGCTATGGAACGT	0.23	0.33	0.00	0.00	0.00
GATCAACTGCTCAACTTCCG	0.23	0.00	0.00	0.00	0.00
GATCAACTGCTCACGAGCAA	0.00	0.65	0.29	0.00	0.00
GATCAACTGCTCAGAGGTTG	0.00	0.00	0.00	0.00	0.00
GATCAACTGCTCATAGAAAA	0.00	0.00	0.00	0.34	0.00
GATCAACTGCTCCAAAAAAA	0.00	0.00	0.00	0.00	0.00
GATCAACTGCTCCAGGTATG	0.23	0.00	0.29	0.00	0.00
GATCAACTGCTCCAGTCAAA	0.00	0.00	0.00	0.00	0.00
GATCAACTGCTCCAGTCGAA	0.00	0.00	0.00	0.34	0.00

GATCAACTGCTCCAGTCGCA	0.00	0.65	0.00	0.68	0.34
GATCAACTGCTCCAGTCGCG	0.69	0.65	0.00	0.68	0.34
GATCAACTGCTCCATCCCGG	0.00	0.00	0.00	0.34	0.00
GATCAACTGCTCCATCCCTG	0.69	0.33	0.86	0.68	0.00
GATCAACTGCTCCCTAATAT	0.00	0.00	0.29	0.00	0.00
GATCAACTGCTCCCTCATAT	0.00	0.00	0.29	0.00	0.00
GATCAACTGCTCCCTTCTAT	0.00	0.33	0.00	0.00	0.00
GATCAACTGCTCCGGCAGCA	0.00	0.00	0.00	0.00	0.34
GATCAACTGCTCCTTCCTTT	0.00	0.00	0.00	0.00	0.34
GATCAACTGCTCGCCGGATA	0.00	0.33	0.00	0.00	0.00
GATCAACTGCTCGCCTGAGC	6.41	2.60	3.44	4.07	4.82
GATCAACTGCTCGTCTTACC	0.00	0.00	0.00	0.00	0.00
GATCAACTGCTCTCATCACA	0.23	0.00	0.00	0.00	0.00
GATCAACTGCTCTCTGCACA	0.00	0.00	0.29	0.00	0.00
GATCAACTGCTCTGCAAACA	0.00	0.00	0.00	0.00	0.69
GATCAACTGCTCTGCGAATC	0.00	0.00	0.00	0.68	0.00
GATCAACTGCTCTTGGAGCG	0.00	0.00	0.00	0.00	0.00
GATCAACTGCTCTTGGAGCN	0.46	0.00	0.00	0.00	0.00
GATCAACTGCTCTTGGAGCT	16.48	1.30	0.86	1.02	2.07
GATCAACTGCTCTTGGNGCT	0.00	0.00	0.00	0.00	0.34
GATCAACTGCTCTTGGNNNN	0.23	0.00	0.00	0.00	0.00
GATCAACTGCTGCAATGGCT	0.00	0.00	0.29	0.00	0.00
GATCAACTGCTGCAGGGCGG	0.23	0.00	0.00	0.00	0.00
GATCAACTGCTGCCACTTGT	0.00	0.33	0.00	0.00	0.00
GATCAACTGCTGCTCGCCTC	0.00	0.00	0.00	0.00	0.34
GATCAACTGCTGCTGGAAGT	0.00	0.00	0.57	0.00	0.00
GATCAACTGCTGGAAACACG	0.00	0.00	0.00	0.00	0.00
GATCAACTGCTGGACAAGGA	0.00	0.00	0.00	0.00	0.00
GATCAACTGCTGGCCGCTTG	0.23	0.00	0.00	0.00	0.00
GATCAACTGCTGGTCTCACG	0.00	0.33	0.00	0.34	0.34
GATCAACTGCTGGTTTCAAT	0.00	0.33	0.00	0.00	0.00
GATCAACTGCTTAGCCTTGA	0.46	0.00	0.29	0.00	0.00
GATCAACTGCTTAGCTTTGG	0.00	0.00	0.00	0.00	0.00
GATCAACTGCTTCATCCCCT	0.00	0.00	0.00	0.00	1.03
GATCAACTGCTTCATGAAAA	0.00	0.00	0.00	0.00	0.34
GATCAACTGCTTCCAGAGCC	0.00	0.33	0.00	0.00	0.00
GATCAACTGCTTCCTAAAAA	0.00	0.00	0.00	0.00	0.00

GATCAACTGCTTCCTGAAAA	1.14	0.33	0.29	1.36	0.00
GATCAACTGCTTCGAACGGG	0.00	0.00	0.00	0.00	0.00
GATCAACTGCTTCGAAGCGG	0.00	0.00	0.29	0.00	0.00
GATCAACTGCTTCTGAGCTC	0.00	0.65	0.00	0.00	0.00
GATCAACTGCTTGAAAAAAA	0.46	0.00	0.00	0.00	0.00
GATCAACTGCTTGAGTACCA	0.00	0.33	0.00	0.00	0.00
GATCAACTGCTTGATGCTCA	0.00	0.00	0.00	0.00	0.34
GATCAACTGCTTGGAGCAAG	0.23	0.00	0.00	0.00	0.34
GATCAACTGCTTGTGAATCG	0.00	0.00	0.00	0.00	0.00
GATCAACTGCTTTTCTCAAC	0.46	0.33	0.00	0.00	0.34
GATCAACTGGAAAAAAAAAA	0.00	0.00	0.00	0.34	0.00
GATCAACTGGAAACATGTCG	0.69	0.33	0.57	0.00	0.00
GATCAACTGGAAAGCGTCAC	0.00	0.00	0.00	0.34	0.00
GATCAACTGGAACAAGCCTG	0.00	0.33	0.00	0.00	0.00
GATCAACTGGAACAATAAGG	0.00	0.00	0.00	0.34	0.00
GATCAACTGGAACAATGTAG	0.00	0.00	0.00	0.00	0.00
GATCAACTGGAACAATGTGC	0.00	0.00	0.00	0.34	0.00
GATCAACTGGAACAATGTGG	0.00	1.63	1.15	1.02	1.03
GATCAACTGGAACATACTTT	0.00	0.33	0.00	0.00	0.00
GATCAACTGGAACATATGTA	0.00	0.00	0.29	0.00	0.00
GATCAACTGGAACATATTNN	0.46	0.00	0.00	0.00	0.00
GATCAACTGGAACATATTTG	0.00	0.00	0.00	0.34	0.00
GATCAACTGGAACATATTTN	0.23	0.00	0.00	0.00	0.00
GATCAACTGGAACATATTTT	9.61	9.43	5.73	11.20	7.92
GATCAACTGGAACCATGTCG	0.00	0.00	0.29	0.00	0.00
GATCAACTGGAACGCCCTCG	0.00	0.00	0.29	0.00	0.00
GATCAACTGGAACTCCAAGA	0.00	0.00	0.29	0.00	0.00
GATCAACTGGAAGACAAGCT	1.14	0.00	0.57	0.34	0.00
GATCAACTGGAAGACAATTT	0.23	0.00	0.00	0.00	0.00
GATCAACTGGAAGCGAGAGA	0.00	0.00	0.00	0.00	0.34
GATCAACTGGAAGGACAAGA	0.00	0.33	0.00	0.00	0.00
GATCAACTGGAAGGACAAGC	8.93	10.41	2.29	5.09	2.76
GATCAACTGGAAGGACAANN	0.23	0.00	0.00	0.00	0.00
GATCAACTGGAAGGACGAGC	0.00	0.00	0.00	0.34	0.00
GATCAACTGGAAGGCACTGG	0.00	0.33	0.00	0.00	0.00
GATCAACTGGAAGGGTTTTG	0.00	0.00	0.00	0.00	0.00
GATCAACTGGAAGGGTTTTN	0.23	0.00	0.00	0.00	0.00

GATCAACTGGAAGGTGTTTG	0.00	0.00	0.00	0.34	0.00
GATCAACTGGAAGGTTTTTG	23.35	10.09	2.58	11.88	8.27
GATCAACTGGAAGGTTTTTN	0.23	0.00	0.00	0.00	0.00
GATCAACTGGAATATATTTT	0.00	0.00	0.00	0.00	0.00
GATCAACTGGAATGACCTTG	0.00	0.00	0.00	0.00	0.34
GATCAACTGGAATGCCAATG	0.00	0.00	0.00	0.34	0.00
GATCAACTGGAATGCCCCTG	0.00	0.00	0.00	0.00	0.00
GATCAACTGGAATGCCCTGG	0.00	0.00	0.00	0.00	0.00
GATCAACTGGAATGCCCTTG	0.00	0.00	0.00	0.00	0.00
GATCAACTGGAATGTCCGCC	0.00	0.00	0.00	0.00	0.00
GATCAACTGGAATTGATTCT	0.00	0.00	0.00	0.00	0.34
GATCAACTGGACAAAAATCT	0.00	0.00	0.00	0.34	0.00
GATCAACTGGACAACGCTGC	0.23	0.98	1.15	0.34	0.00
GATCAACTGGACACGTCGAA	0.23	0.00	0.00	0.00	0.00
GATCAACTGGACATGGCCAT	0.00	0.33	0.00	0.00	0.00
GATCAACTGGACCTATTCCA	0.00	0.00	0.29	0.00	0.00
GATCAACTGGACGAGTTCGT	0.00	0.00	0.00	0.00	0.34
GATCAACTGGACGGGGCGCT	0.23	0.00	0.00	0.00	0.00
GATCAACTGGACGTGTCATG	0.00	0.00	0.00	0.00	0.00
GATCAACTGGACTAAAAAAA	0.00	0.00	0.00	0.68	0.00
GATCAACTGGACTAATTGTA	0.00	0.00	0.00	0.00	0.00
GATCAACTGGACTATGAAGC	0.00	0.00	0.00	0.00	0.34
GATCAACTGGACTCATGACA	0.00	0.00	0.00	0.34	0.00
GATCAACTGGACTCCCGAGA	0.00	0.00	0.00	0.00	0.34
GATCAACTGGACTCCTCGTA	0.46	0.00	0.00	0.00	0.00
GATCAACTGGACTCCTNNNN	0.23	0.00	0.00	0.00	0.00
GATCAACTGGACTCCTTCTA	0.00	0.00	0.29	0.00	0.00
GATCAACTGGACTCCTTGGA	0.23	0.00	0.00	0.00	0.34
GATCAACTGGACTCCTTGNA	0.00	0.00	0.00	0.00	0.00
GATCAACTGGACTCCTTGNN	0.23	0.00	0.00	0.00	0.00
GATCAACTGGACTCCTTGTA	44.18	35.46	21.76	35.64	19.29
GATCAACTGGACTCCTTGTC	0.46	0.33	0.00	0.00	0.00
GATCAACTGGACTCCTTGTN	0.23	0.00	0.00	0.00	0.00
GATCAACTGGACTCCTTGTT	0.92	0.00	0.00	0.00	0.00
GATCAACTGGACTGTCAAAA	0.00	0.00	0.00	0.68	0.69
GATCAACTGGACTGTCACAA	0.00	0.33	0.00	0.00	0.00
GATCAACTGGAGAGCACTCG	0.92	1.95	1.43	1.36	0.34

GATCAACTGGAGATACGGCT	0.00	0.00	0.00	0.00	0.00
GATCAACTGGAGCAGGCCTG	0.00	0.00	0.00	0.00	0.00
GATCAACTGGAGCGGATTGA	0.00	0.00	0.00	0.34	0.00
GATCAACTGGAGCGTGAAAG	0.00	0.00	0.00	0.00	0.34
GATCAACTGGAGGCAGAAGC	0.00	0.33	0.00	0.00	0.00
GATCAACTGGAGGCAGATCC	0.00	0.00	0.29	0.00	0.00
GATCAACTGGAGGCAGATGA	0.23	0.00	0.00	0.00	0.00
GATCAACTGGAGGCAGATGC	47.38	36.76	27.49	31.23	19.63
GATCAACTGGAGGCAGATGG	0.00	0.00	0.29	0.00	0.00
GATCAACTGGAGGCAGATGN	0.23	0.00	0.00	0.00	0.00
GATCAACTGGAGGCAGATNC	0.00	0.00	0.00	0.34	0.00
GATCAACTGGAGGCAGATNN	0.46	0.00	0.00	0.00	0.00
GATCAACTGGAGGCAGATTC	0.00	0.00	0.29	0.00	0.34
GATCAACTGGAGGCAGCTNC	0.00	0.33	0.00	0.00	0.00
GATCAACTGGAGGCAGGTGC	0.92	0.00	0.00	0.00	0.00
GATCAACTGGAGGCAGNCNN	0.23	0.00	0.00	0.00	0.00
GATCAACTGGAGGCAGNNNN	0.23	0.00	0.00	0.00	0.00
GATCAACTGGAGGCCAAGGC	0.00	0.00	0.00	0.00	0.00
GATCAACTGGAGGCGCAACG	0.00	0.33	0.00	0.34	0.69
GATCAACTGGAGGCGCAGGA	0.00	0.00	0.00	0.00	0.00
GATCAACTGGAGGCGCCTTC	0.00	0.00	0.00	0.34	0.00
GATCAACTGGAGGGACATGT	0.00	0.00	0.00	0.00	0.34
GATCAACTGGAGGGACCTGN	0.00	0.00	0.00	0.00	0.00
GATCAACTGGAGGGACCTGT	5.49	11.71	4.87	7.81	8.61
GATCAACTGGAGGGATCTGT	0.00	0.00	0.29	0.00	0.00
GATCAACTGGAGGTCCGTAC	0.00	0.00	0.00	0.00	0.00
GATCAACTGGAGGTTTGAGA	0.00	0.00	0.00	0.34	0.00
GATCAACTGGAGTCAGCTCC	0.23	0.00	0.00	0.00	0.00
GATCAACTGGAGTCTGCGGG	0.00	0.00	0.00	0.00	0.00
GATCAACTGGAGTGCTGTCT	0.00	0.00	0.00	0.00	0.00
GATCAACTGGAGTTTTCCGA	0.00	0.33	0.00	0.00	0.00
GATCAACTGGATCTGCAAGC	0.00	0.00	0.00	0.00	0.00
GATCAACTGGATGATGTCCT	0.00	0.00	0.00	0.00	0.00
GATCAACTGGATGCAGACGT	0.00	0.00	0.00	0.34	0.00
GATCAACTGGATGCCAGCAG	0.00	0.00	0.00	0.00	0.00
GATCAACTGGATGCGCTGGC	0.00	0.00	0.00	0.00	0.34
GATCAACTGGATGCGTCACC	0.23	0.33	0.57	0.34	0.00

GATCAACTGGATGCGTCATC	0.23	0.00	0.00	0.00	0.00
GATCAACTGGATGCTTGGGG	0.00	0.00	0.00	0.00	0.34
GATCAACTGGATGGGGAATG	0.00	0.00	0.29	0.00	0.00
GATCAACTGGATGTCTGATG	0.00	0.00	0.00	0.00	0.00
GATCAACTGGATTAGAGCTC	0.00	0.00	0.00	0.00	0.00
GATCAACTGGATTTAAACAT	0.23	0.00	0.00	0.00	0.00
GATCAACTGGCAAAAAAAAA	0.00	0.00	0.00	0.00	0.34
GATCAACTGGCAACGCCATA	0.00	0.00	0.00	0.00	0.34
GATCAACTGGCAAGCCCTCG	0.00	0.00	0.00	0.00	0.34
GATCAACTGGCAAGCCCTTG	0.00	0.00	0.00	0.34	0.00
GATCAACTGGCAAGTTGACG	0.00	0.00	0.29	1.02	0.00
GATCAACTGGCACAGGGAGG	0.00	0.00	0.00	0.34	0.00
GATCAACTGGCACATTGCAA	0.00	0.00	0.29	0.00	0.00
GATCAACTGGCACGACCTCG	0.23	0.00	0.00	0.00	0.00
GATCAACTGGCACGCCATAC	0.00	0.00	0.00	0.00	0.34
GATCAACTGGCACGCCCCCG	0.23	0.33	0.00	0.00	0.34
GATCAACTGGCACGCCCGCG	0.23	0.65	0.00	0.00	0.34
GATCAACTGGCACGCCCNNN	0.00	0.00	0.00	0.00	0.00
GATCAACTGGCACGCCCTAG	0.00	0.00	0.00	0.34	0.00
GATCAACTGGCACGCCCTCG	38.68	55.31	25.77	46.84	33.06
GATCAACTGGCACGCCCTCN	0.23	0.00	0.00	0.00	0.00
GATCAACTGGCACGCCCTCT	0.23	0.00	0.00	0.34	0.00
GATCAACTGGCACGCCCTTG	0.00	0.00	0.00	0.34	0.00
GATCAACTGGCACGCGATTG	0.00	0.65	0.29	0.00	0.00
GATCAACTGGCACGCGCGTG	0.00	0.00	0.00	0.34	0.34
GATCAACTGGCACGCGCTAG	0.00	0.00	0.00	0.00	0.00
GATCAACTGGCACGCGCTTC	0.00	0.00	0.29	0.00	0.00
GATCAACTGGCACGCGCTTG	23.35	39.69	22.62	37.68	26.86
GATCAACTGGCACGCGCTTT	0.00	0.33	0.00	0.00	0.00
GATCAACTGGCACGCGGTTG	0.23	0.00	0.00	0.00	0.00
GATCAACTGGCACGCTTGCA	0.00	0.00	0.00	0.00	0.00
GATCAACTGGCACGGCCTCG	0.23	0.00	0.00	0.00	0.00
GATCAACTGGCACTACGCAG	0.00	0.33	0.29	0.00	0.00
GATCAACTGGCAGAACATGG	0.23	0.00	0.00	0.00	0.00
GATCAACTGGCAGAGAGCAG	0.00	0.00	0.29	0.00	0.00
GATCAACTGGCAGAGCGCCG	0.00	0.00	0.00	1.36	0.00
GATCAACTGGCAGAGCGTGG	0.00	0.33	0.00	0.00	0.00

GATCAACTGGCAGATTGGGC	0.00	0.00	0.29	0.00	0.00
GATCAACTGGCAGCGCAAGA	0.00	0.33	0.00	0.00	0.00
GATCAACTGGCAGCGCAGAA	0.00	0.00	0.00	0.00	0.00
GATCAACTGGCAGGAAAAAA	0.00	0.00	0.29	0.00	0.00
GATCAACTGGCAGGAGACAG	0.00	0.00	0.00	0.00	0.00
GATCAACTGGCAGGAGACCG	0.00	0.00	0.00	0.00	0.00
GATCAACTGGCAGGAGCAAC	1.14	0.00	0.29	0.00	0.00
GATCAACTGGCAGGCACTCG	0.00	0.00	0.00	0.00	0.00
GATCAACTGGCAGGTGGACG	0.00	0.65	0.29	0.00	0.00
GATCAACTGGCAGGTTTTTG	0.00	0.00	0.00	0.00	0.00
GATCAACTGGCATACCCTTG	0.23	0.00	0.29	0.00	0.00
GATCAACTGGCATACCGCTT	0.23	0.00	0.00	0.00	0.00
GATCAACTGGCATCACCATA	0.00	0.33	0.00	0.00	0.00
GATCAACTGGCATGAAGGCT	0.00	0.33	0.00	0.00	0.00
GATCAACTGGCATGACCTTG	0.00	0.00	0.29	0.34	0.34
GATCAACTGGCATGAGGCCC	0.23	0.00	0.00	0.00	0.00
GATCAACTGGCATGATTCAA	0.00	0.00	0.00	0.34	0.00
GATCAACTGGCATGCAATTG	0.00	0.00	0.00	0.34	0.00
GATCAACTGGCATGCACTGA	0.00	0.00	0.29	0.00	0.00
GATCAACTGGCATGCACTGG	0.00	0.00	0.00	0.00	0.34
GATCAACTGGCATGCACTTG	0.23	0.00	0.29	1.36	0.00
GATCAACTGGCATGCCACTG	0.00	0.00	0.00	0.34	0.00
GATCAACTGGCATGCCATCG	0.00	0.00	0.00	0.34	0.00
GATCAACTGGCATGCCATGG	0.23	0.00	0.00	0.34	0.00
GATCAACTGGCATGCCATTG	0.00	2.28	0.57	0.68	0.69
GATCAACTGGCATGCCCATG	0.00	0.00	0.00	0.00	0.34
GATCAACTGGCATGCCCCCC	0.00	0.00	0.57	0.00	0.00
GATCAACTGGCATGCCCCTG	0.23	0.33	0.29	0.68	0.69
GATCAACTGGCATGCCCGTG	0.69	0.33	0.00	0.34	0.00
GATCAACTGGCATGCCCNTG	0.00	0.33	0.00	0.00	0.00
GATCAACTGGCATGCCCTAG	0.00	0.00	0.00	0.68	0.00
GATCAACTGGCATGCCCTCG	0.00	0.33	0.00	0.34	0.00
GATCAACTGGCATGCCCTGG	2.98	15.94	11.74	14.93	8.95
GATCAACTGGCATGCCCTGN	0.00	0.00	0.00	0.00	0.00
GATCAACTGGCATGCCCTNG	0.00	0.00	0.00	0.34	0.00
GATCAACTGGCATGCCCTNN	0.46	0.00	0.00	0.00	0.00
GATCAACTGGCATGCCCTTA	0.00	0.00	0.29	0.00	0.00

GATCAACTGGCATGCCCTTC	0.69	0.33	0.29	0.00	0.00
GATCAACTGGCATGCCCTTG	268.05	369.90	170.38	391.36	160.15
GATCAACTGGCATGCCCTTN	1.37	0.00	0.00	0.00	0.00
GATCAACTGGCATGCCCTTT	1.60	1.95	0.29	0.68	0.34
GATCAACTGGCATGCCGTTG	0.23	0.00	0.00	0.00	0.00
GATCAACTGGCATGCCNNNN	0.23	0.00	0.00	0.00	0.00
GATCAACTGGCATGCCNTGG	0.00	0.00	0.00	0.00	0.00
GATCAACTGGCATGCCNTNN	0.46	0.00	0.00	0.00	0.00
GATCAACTGGCATGCCNTTG	0.00	0.00	0.00	0.00	0.69
GATCAACTGGCATGCCTTAC	0.00	0.00	0.00	0.00	0.00
GATCAACTGGCATGCCTTTG	0.00	0.33	0.00	0.00	0.34
GATCAACTGGCATGCGCTTG	0.23	0.00	0.00	0.00	0.00
GATCAACTGGCATGCTCGTA	0.46	0.00	0.00	0.00	0.00
GATCAACTGGCATGCTCTTG	0.46	0.00	0.29	0.00	0.00
GATCAACTGGCATGGCACCC	0.00	0.00	0.29	0.00	0.00
GATCAACTGGCATGGCTCCT	0.00	0.00	0.00	0.34	0.00
GATCAACTGGCATGTCCTTG	0.00	0.33	0.00	0.00	0.00
GATCAACTGGCATTCCCTTG	0.00	0.33	0.00	0.00	0.00
GATCAACTGGCATTGCTCGG	0.00	0.33	0.00	0.00	0.00
GATCAACTGGCCACGGCGCT	0.69	1.30	0.29	0.00	0.00
GATCAACTGGCCAGGCGCCC	0.00	0.33	0.00	0.00	0.00
GATCAACTGGCCATAGACAC	0.00	0.00	0.00	0.00	0.34
GATCAACTGGCCATGCAGCT	0.00	0.00	0.00	0.00	0.34
GATCAACTGGCCCCGGAAGG	0.00	0.00	0.00	0.34	0.00
GATCAACTGGCCCCGGACGG	0.23	0.98	0.86	1.70	0.34
GATCAACTGGCCCTATTTGT	0.00	0.33	0.00	0.00	0.00
GATCAACTGGCCGAACATCC	0.46	0.33	0.00	0.00	0.00
GATCAACTGGCCGCCCTCGG	0.46	0.00	0.00	0.00	0.00
GATCAACTGGCCGGCAGAGT	0.00	0.00	0.00	0.00	0.34
GATCAACTGGCCGGGCACCC	0.00	0.00	0.00	0.34	0.34
GATCAACTGGCCGGGCCCCC	0.00	0.00	0.00	0.00	0.34
GATCAACTGGCCGGGCGACA	0.00	0.33	0.00	0.00	0.00
GATCAACTGGCCGGGCGCAC	0.23	0.00	0.00	0.00	0.00
GATCAACTGGCCGGGCGCCA	0.23	0.33	0.00	0.00	0.34
GATCAACTGGCCGGGCGCCC	12.13	9.76	8.02	8.49	6.20
GATCAACTGGCCGGGCGCNC	0.00	0.33	0.00	0.00	0.00
GATCAACTGGCCGGGGGACC	0.00	0.00	0.00	0.00	0.00

GATCAACTGGCCTCCTTGTA	0.23	0.00	0.00	0.00	0.00
GATCAACTGGCCTGCCAGGC	0.00	0.00	0.00	0.00	0.00
GATCAACTGGCCTGCCCTTG	0.23	0.00	0.00	0.34	0.00
GATCAACTGGCCTGCCCTTT	0.00	0.00	0.29	0.00	0.00
GATCAACTGGCCTGTGCGCC	0.00	0.00	0.00	0.34	0.00
GATCAACTGGCGACACTCCA	0.23	0.00	0.00	0.00	0.00
GATCAACTGGCGAGTGGATA	0.00	0.00	0.29	0.00	0.00
GATCAACTGGCGCAAAGCTC	0.00	0.00	0.00	0.00	0.00
GATCAACTGGCGCCCTCTGC	0.00	0.00	0.00	0.34	0.00
GATCAACTGGCGCGAAGCCA	0.00	0.00	0.00	0.34	0.00
GATCAACTGGCGCGCACACG	0.00	0.00	0.00	0.00	0.00
GATCAACTGGCGCGCCATGG	0.00	0.00	0.00	0.00	0.00
GATCAACTGGCGCGCCCTGG	0.23	0.00	0.00	0.00	1.03
GATCAACTGGCGCTCCATGA	0.92	0.00	0.00	0.34	0.00
GATCAACTGGCGGCAGATGC	0.00	0.33	0.00	0.00	0.00
GATCAACTGGCGGCAGCTGC	0.00	0.00	0.00	0.00	0.00
GATCAACTGGCGGCGCAATG	0.00	0.00	0.00	0.68	0.00
GATCAACTGGCGGGAGTACG	0.00	0.00	0.00	0.34	0.00
GATCAACTGGCGTGCCCTTG	0.23	0.00	0.29	0.00	0.00
GATCAACTGGCGTGTGGATA	0.00	0.00	0.29	0.00	0.00
GATCAACTGGCGTTGTCTCC	1.83	0.33	0.00	0.00	1.03
GATCAACTGGCTCAGTCGAG	0.69	0.00	0.00	0.68	0.00
GATCAACTGGCTCCCCGGCC	0.00	0.00	0.00	0.00	0.00
GATCAACTGGCTCCGGAAGC	0.00	0.33	0.00	0.68	0.00
GATCAACTGGCTCTCATTCT	0.00	0.00	0.00	0.34	0.00
GATCAACTGGCTCTGTTTTG	0.00	0.00	0.00	0.00	0.34
GATCAACTGGCTCTTCCCTT	0.00	0.65	0.00	0.00	0.00
GATCAACTGGCTGCCCTTGG	0.46	0.00	0.00	0.00	0.00
GATCAACTGGCTGCGAGACT	0.00	0.00	0.00	0.00	0.00
GATCAACTGGCTGCGGAGAG	0.00	0.33	0.00	0.00	0.00
GATCAACTGGCTGCTCTCCC	0.00	0.00	0.00	0.00	0.00
GATCAACTGGCTGCTGCAAA	0.00	0.00	0.29	0.00	0.00
GATCAACTGGCTGCTGGAAA	0.23	0.00	0.00	0.00	0.00
GATCAACTGGCTGCTGGGAT	0.23	0.00	0.00	0.00	0.00
GATCAACTGGCTGCTGTTTG	0.00	0.00	0.29	0.00	0.00
GATCAACTGGCTGGACGTTC	0.00	0.00	0.00	0.00	0.00
GATCAACTGGCTGGAGAATG	0.23	0.00	0.00	0.34	0.00

GATCAACTGGCTGGAGAATT	0.00	0.33	0.00	0.00	0.00
GATCAACTGGCTGGCCGATG	0.00	0.33	0.00	0.00	0.00
GATCAACTGGCTGGCCTGGA	0.00	0.00	0.57	0.00	0.00
GATCAACTGGCTGGCGAAAC	0.00	0.00	0.00	0.34	0.00
GATCAACTGGCTGGCGAAAG	0.00	0.00	0.00	0.34	0.00
GATCAACTGGCTGGCGAAAT	0.00	0.00	0.00	0.00	0.00
GATCAACTGGCTGGCGAACT	0.00	0.00	0.29	0.00	0.00
GATCAACTGGCTGGCGAAGG	0.23	0.33	0.00	0.00	0.34
GATCAACTGGCTGGCGAATA	0.00	0.00	0.00	0.00	0.34
GATCAACTGGCTGGCGAATG	7.10	9.43	4.58	1.02	9.64
GATCAACTGGCTGGCGAATT	0.00	0.33	0.00	0.68	0.00
GATCAACTGGCTGGCGACTG	0.00	0.00	0.29	0.00	0.00
GATCAACTGGCTGGCGCAAA	0.00	0.00	0.00	0.00	0.34
GATCAACTGGCTGGCGCATG	0.00	0.98	0.00	0.00	0.00
GATCAACTGGCTGGCGCGTG	0.23	0.00	0.00	0.00	0.00
GATCAACTGGCTGGCGCTTG	0.00	0.00	0.29	0.00	0.00
GATCAACTGGCTGGCGGAGG	0.23	0.00	0.00	0.00	0.00
GATCAACTGGCTGGCGGATG	13.73	1.63	1.15	0.00	0.69
GATCAACTGGCTGGCGGGTG	0.23	0.00	0.00	0.00	0.00
GATCAACTGGCTGGCTCGTA	0.00	0.00	0.00	0.00	0.34
GATCAACTGGCTGGGCGCCC	0.00	0.00	0.00	0.34	0.00
GATCAACTGGCTGGGCTGCG	0.23	1.95	0.00	0.68	0.00
GATCAACTGGCTGGGCTGGG	0.23	0.00	0.00	0.00	0.00
GATCAACTGGCTGGGCTTCT	0.00	0.00	0.00	0.00	0.34
GATCAACTGGCTGGGGAAGA	0.23	0.00	0.00	0.00	0.00
GATCAACTGGCTGGGGAAGG	0.23	0.00	0.00	0.00	0.00
GATCAACTGGCTGGGGAATG	0.23	0.98	1.15	1.36	0.00
GATCAACTGGCTGGGGCATG	0.00	0.33	0.00	0.00	0.00
GATCAACTGGCTGGGGGANG	0.00	0.00	0.00	0.34	0.00
GATCAACTGGCTGGGGGATG	0.00	0.33	0.57	2.38	0.69
GATCAACTGGCTGGGGGCTG	0.00	0.33	0.00	0.00	0.00
GATCAACTGGCTGGTGGATG	0.00	0.00	0.00	0.00	0.34
GATCAACTGGCTTGATTCCG	0.00	0.33	0.00	0.00	0.00
GATCAACTGGCTTGCACCTG	0.00	0.00	0.29	0.00	0.00
GATCAACTGGCTTGCCCTTG	0.00	0.33	0.00	0.00	0.00
GATCAACTGGGAAGAATACA	0.00	0.00	0.00	0.34	0.00
GATCAACTGGGAAGCAGCGA	0.23	0.00	0.00	0.00	0.00

GATCAACTGGGAAGCAGCTA	11.67	9.76	10.02	8.15	7.58
GATCAACTGGGAAGCAGCTC	0.46	0.33	0.00	0.00	0.00
GATCAACTGGGAAGCAGCTN	0.23	0.00	0.00	0.00	0.00
GATCAACTGGGAAGCCGCTA	0.00	0.00	0.00	0.34	0.00
GATCAACTGGGAATATTGAA	0.00	0.00	0.00	0.00	0.34
GATCAACTGGGACACGGAAA	0.00	0.00	0.00	0.34	0.00
GATCAACTGGGACCACACTC	0.23	0.00	0.00	0.00	0.00
GATCAACTGGGACGACTGGA	0.00	0.00	0.00	0.00	0.34
GATCAACTGGGACGATTGGA	0.00	0.00	0.29	0.00	0.00
GATCAACTGGGACGTGGCCC	0.00	0.00	0.57	0.00	0.00
GATCAACTGGGACGTGGTCG	0.00	0.00	0.00	0.34	0.00
GATCAACTGGGACTTGCACA	0.00	0.00	0.00	0.00	0.00
GATCAACTGGGACTTTCGTA	0.00	0.33	0.00	0.00	0.00
GATCAACTGGGAGAGCTGCG	0.92	0.00	0.00	0.00	0.00
GATCAACTGGGAGGAGAGGC	0.00	0.00	0.00	0.00	0.00
GATCAACTGGGAGGAGTCTG	0.00	0.00	0.00	0.00	0.00
GATCAACTGGGATAACCGCG	0.00	0.00	0.29	0.00	0.00
GATCAACTGGGATGACTGGA	0.00	0.00	0.00	0.00	0.00
GATCAACTGGGATGACTGGG	0.00	0.33	0.00	0.00	0.00
GATCAACTGGGATGCTGCCC	0.92	0.00	0.00	0.34	0.00
GATCAACTGGGCAAGCGAGC	0.00	0.00	0.29	0.00	0.00
GATCAACTGGGCAAGTGTGA	0.00	0.00	0.00	0.34	0.00
GATCAACTGGGCACTCATGG	0.00	0.00	0.00	0.68	0.00
GATCAACTGGGCACTTATTT	0.00	0.00	0.00	0.34	0.00
GATCAACTGGGCAGCAGCGA	0.23	0.00	0.00	0.00	0.00
GATCAACTGGGCAGCTGGCC	0.00	0.33	0.00	0.00	0.00
GATCAACTGGGCAGGAACGA	0.00	0.33	0.00	0.00	0.00
GATCAACTGGGCAGGAACGC	5.04	4.55	3.44	5.09	2.76
GATCAACTGGGCAGGAAGGG	0.23	0.00	0.00	0.00	0.00
GATCAACTGGGCAGGAATGC	0.00	0.00	0.00	0.34	0.00
GATCAACTGGGCAGGACCNN	0.23	0.00	0.00	0.00	0.00
GATCAACTGGGCATCGGCAA	0.00	0.33	0.00	0.00	0.00
GATCAACTGGGCATCGGCTG	0.00	0.33	0.00	0.00	0.00
GATCAACTGGGCATTGGCCT	0.00	0.00	0.00	0.34	0.00
GATCAACTGGGCCATGCAGA	0.00	0.00	0.00	0.00	0.34
GATCAACTGGGCCCCGAGGC	0.00	0.00	0.29	0.00	0.00
GATCAACTGGGCCGCCGGCG	0.23	0.00	0.00	0.00	0.00

GATCAACTGGGCCGGAAGAG	0.00	0.00	0.00	0.00	0.00
GATCAACTGGGCCGGCTAAG	0.00	0.00	0.29	0.00	0.00
GATCAACTGGGCCGTGGGAT	0.00	0.33	0.00	0.00	0.00
GATCAACTGGGCCTCCCCAA	0.00	0.00	0.00	0.00	0.00
GATCAACTGGGCCTGTGCTC	0.00	0.00	0.00	0.00	0.34
GATCAACTGGGCGCTGCCAG	0.00	0.33	0.00	0.34	0.69
GATCAACTGGGCGGCAAGAG	0.00	0.00	0.00	0.34	0.00
GATCAACTGGGCGGGCGCAC	0.23	0.00	0.00	0.00	0.00
GATCAACTGGGCTCCTTGTA	0.23	0.00	0.00	0.00	0.00
GATCAACTGGGGAAGATAAG	0.00	0.00	0.29	0.00	0.00
GATCAACTGGGGAGCGGTCG	0.00	0.00	0.00	0.00	0.00
GATCAACTGGGGAGCGGTCT	1.60	4.88	3.15	3.39	1.03
GATCAACTGGGGCATAGGGA	0.00	0.33	0.00	0.00	0.00
GATCAACTGGGGCCTGGTCC	0.00	0.00	0.00	0.34	0.00
GATCAACTGGGGCCTGTCCT	1.37	0.33	0.57	3.05	1.72
GATCAACTGGGGCCTGTGGT	0.00	0.00	0.00	0.00	0.00
GATCAACTGGGGCTACTGGC	0.00	0.65	0.00	0.00	0.00
GATCAACTGGGGCTTTGTCT	0.23	0.00	0.00	0.00	0.00
GATCAACTGGGGGAATTTGT	0.00	0.33	0.00	0.68	0.00
GATCAACTGGGGGCGGAAGA	0.00	0.00	0.00	0.00	0.00
GATCAACTGGGGGTGCACGT	0.00	0.00	0.00	0.00	0.00
GATCAACTGGGGTCTTTTCT	0.00	0.33	0.29	0.00	0.00
GATCAACTGGGGTGCGGAGC	0.00	0.00	0.00	0.00	0.34
GATCAACTGGGGTGCGGTGG	0.23	0.00	0.00	0.00	0.00
GATCAACTGGGGTGGGGAGC	0.00	0.00	0.57	0.00	0.00
GATCAACTGGGGTTGAATTG	0.00	0.00	0.00	0.00	0.34
GATCAACTGGGTAAAGACAC	0.00	0.00	0.29	0.00	0.00
GATCAACTGGGTAAAGCAAC	0.00	0.00	0.29	0.00	0.00
GATCAACTGGGTAAAGCCAC	4.35	6.83	4.87	2.72	6.54
GATCAACTGGGTAAGGACGC	0.00	0.33	0.00	0.00	0.00
GATCAACTGGGTAATGCCAC	0.00	0.33	0.00	0.00	0.00
GATCAACTGGGTACTTATGT	0.23	0.00	0.00	0.00	0.00
GATCAACTGGGTAGACATAG	0.00	0.00	0.00	0.34	0.00
GATCAACTGGGTAGAGCCCC	0.00	0.00	0.29	0.00	0.00
GATCAACTGGGTAGCAAGAA	0.69	1.63	4.30	2.72	0.69
GATCAACTGGGTCAATGAGA	0.00	0.00	0.29	0.00	0.00
GATCAACTGGGTCACCCACA	1.37	0.33	0.00	0.68	0.34

GATCAACTGGGTCCCATTGC	0.00	0.00	0.00	0.00	0.00
GATCAACTGGGTCCGCCATG	0.23	0.00	0.00	0.00	0.00
GATCAACTGGGTCCGTCATG	0.00	0.00	0.57	0.34	0.69
GATCAACTGGGTCCTGTCCT	0.00	0.33	0.00	0.00	0.00
GATCAACTGGGTCGATTTCA	0.00	0.00	0.29	0.34	0.00
GATCAACTGGGTCTTGTGTA	0.00	0.00	0.00	0.34	0.00
GATCAACTGGGTCTTTCGTA	0.00	0.98	0.00	0.00	0.00
GATCAACTGGGTCTTTGTAG	0.23	0.00	0.00	0.34	0.00
GATCAACTGGGTCTTTNGTA	0.00	0.00	0.00	0.34	0.00
GATCAACTGGGTCTTTNNNN	0.23	0.00	0.00	0.00	0.00
GATCAACTGGGTCTTTTCTA	0.00	0.33	0.00	0.00	0.00
GATCAACTGGGTCTTTTGTA	20.14	17.89	16.32	22.74	17.56
GATCAACTGGGTCTTTTGTN	0.23	0.00	0.00	0.00	0.00
GATCAACTGGGTCTTTTGTT	0.00	0.00	0.00	0.00	0.34
GATCAACTGGGTGAAGCCCC	0.69	0.33	0.00	0.34	0.00
GATCAACTGGGTGAAGCCGC	0.00	0.33	0.29	1.02	1.03
GATCAACTGGGTGACCATGA	0.00	0.00	0.00	0.00	0.34
GATCAACTGGGTGCTTGAAA	0.00	0.00	0.29	0.00	0.00
GATCAACTGGGTGTGAGAGC	0.00	0.00	0.00	0.34	0.00
GATCAACTGGGTGTGAGCGC	2.29	1.30	0.00	2.04	0.34
GATCAACTGGGTGTGCGCAG	0.00	0.00	0.29	0.00	0.00
GATCAACTGGGTTGAGAGAA	0.00	0.00	0.00	0.00	0.34
GATCAACTGGGTTGCGGTGT	0.23	0.00	0.00	0.00	0.00
GATCAACTGGGTTTCCTGGC	0.23	0.00	0.00	0.00	0.00
GATCAACTGGTACCACAATC	0.00	0.00	0.00	0.34	0.00
GATCAACTGGTACCACACNC	0.00	0.00	0.00	0.34	0.00
GATCAACTGGTACCACACNN	0.00	0.00	0.00	0.00	0.00
GATCAACTGGTACCACACTA	0.00	0.00	0.00	0.00	0.34
GATCAACTGGTACCACACTC	11.22	9.76	1.43	8.15	4.13
GATCAACTGGTACCACACTN	0.23	0.00	0.00	0.00	0.00
GATCAACTGGTACCACCCTC	0.00	0.00	0.00	0.00	0.00
GATCAACTGGTACCATACTC	0.00	0.00	0.00	0.34	0.00
GATCAACTGGTACGCCCTCG	0.00	0.00	0.00	0.34	0.00
GATCAACTGGTACGCGCTTG	0.00	0.00	0.00	0.34	0.00
GATCAACTGGTACTATTACC	3.43	0.65	1.43	2.04	2.07
GATCAACTGGTAGAAGGCGT	0.23	0.00	0.00	0.34	0.34
GATCAACTGGTAGCAAGCAA	0.00	0.65	0.57	0.00	0.00

GATCAACTGGTAGGCGCCGG	0.46	0.00	0.00	0.00	0.00
GATCAACTGGTATACAAATA	0.00	0.00	0.00	0.00	0.69
GATCAACTGGTATGCCCTTG	0.00	0.00	0.00	0.34	0.00
GATCAACTGGTATGCCGGTC	0.23	1.30	2.00	1.70	1.38
GATCAACTGGTCAGACGGCG	0.00	0.00	0.00	0.00	0.34
GATCAACTGGTCAGGAAATC	0.00	0.33	0.00	0.00	0.00
GATCAACTGGTCCAGCAAAA	0.00	0.00	0.00	0.00	0.00
GATCAACTGGTCCAGCGGAA	0.00	0.00	0.00	0.34	0.00
GATCAACTGGTCCATACAGG	0.00	0.00	0.00	0.00	0.00
GATCAACTGGTCCGCCGCCG	0.00	0.00	0.00	0.00	0.34
GATCAACTGGTCCGCCGGAG	0.00	0.00	0.00	0.00	0.00
GATCAACTGGTCCGCCGGCC	0.00	0.00	0.00	0.00	0.34
GATCAACTGGTCCGCCGGCG	5.95	9.43	3.15	5.77	5.51
GATCAACTGGTCCGCCGGCT	0.00	0.00	0.00	0.00	0.00
GATCAACTGGTCCGGACAAC	0.46	0.00	0.00	0.00	0.00
GATCAACTGGTCCGGACACC	0.00	0.00	0.00	0.00	0.00
GATCAACTGGTCCGGACATC	9.16	7.16	2.00	7.81	3.10
GATCAACTGGTCCGGACGTC	0.00	0.33	0.00	0.00	0.00
GATCAACTGGTCCTGAGTGC	0.00	0.00	0.00	0.34	0.00
GATCAACTGGTCGCCCCAGG	0.00	0.33	0.00	0.00	0.00
GATCAACTGGTCGGGCTACG	0.00	0.65	0.29	0.00	1.03
GATCAACTGGTCGTACGAGC	0.00	0.00	0.00	0.00	0.00
GATCAACTGGTCGTCGCAGC	0.00	0.00	0.57	0.00	0.00
GATCAACTGGTCGTGGTGTC	0.00	0.00	0.00	0.00	0.34
GATCAACTGGTGAAATGTTG	0.00	0.33	2.86	0.00	1.72
GATCAACTGGTGAACACGAT	0.00	0.00	0.00	0.34	0.00
GATCAACTGGTGAAGCATCC	0.23	0.00	0.00	0.00	0.00
GATCAACTGGTGCATCATCG	0.92	0.65	0.00	2.04	1.03
GATCAACTGGTGCATCATTG	0.46	0.33	0.29	0.34	0.00
GATCAACTGGTGCCCTCCGG	0.00	0.00	0.00	0.34	0.00
GATCAACTGGTGCGAGCAGG	0.00	0.00	0.00	0.00	0.00
GATCAACTGGTGCTGTCCAG	0.00	0.00	0.00	0.00	0.00
GATCAACTGGTGGCATTTGC	0.00	0.00	0.29	0.00	0.00
GATCAACTGGTGGCGTTGGG	0.00	0.00	0.29	0.00	0.00
GATCAACTGGTGGGAGCTGA	0.00	0.00	0.00	0.00	0.00
GATCAACTGGTGGTCGCGCC	0.23	0.00	0.00	0.00	0.00
GATCAACTGGTGGTGGCGTG	0.00	0.00	0.00	0.00	0.00

GATCAACTGGTGTCGCACCC	0.00	0.33	0.00	0.00	0.00
GATCAACTGGTGTGAGGAGC	0.00	0.00	0.29	0.00	0.00
GATCAACTGGTGTGCGGAGC	3.66	8.13	11.45	7.47	6.54
GATCAACTGGTGTGCGGCGC	0.00	0.00	0.00	0.00	0.00
GATCAACTGGTGTGCGNAGC	0.00	0.00	0.00	0.00	0.69
GATCAACTGGTGTGCTGAGT	0.00	0.00	0.00	0.00	0.00
GATCAACTGGTTCCGCAGAC	0.00	0.00	0.00	0.00	0.00
GATCAACTGGTTCGAAGCGG	0.23	0.00	0.00	0.00	0.00
GATCAACTGGTTCTTTTATC	0.46	0.00	0.00	0.00	0.00
GATCAACTGGTTGAAAAAGG	0.00	0.00	0.00	0.00	0.69
GATCAACTGGTTGCGCCGGC	0.00	0.00	0.00	0.00	0.69
GATCAACTGGTTGCGCGATT	1.14	0.33	0.00	0.00	0.34
GATCAACTGGTTGCGTATGC	0.00	0.00	0.00	0.00	0.34
GATCAACTGGTTGGCGAATG	0.00	0.33	0.00	0.00	0.00
GATCAACTGGTTGGGCGGCA	0.00	0.00	0.29	0.00	0.00
GATCAACTGGTTGTACGAGC	0.00	0.00	0.00	0.00	0.00
GATCAACTGGTTGTACGCGC	0.00	0.00	0.00	0.00	0.00
GATCAACTGGTTTCCACCCA	0.00	0.00	0.29	0.00	0.00
GATCAACTGGTTTGACTTCA	0.00	0.00	0.29	0.00	0.00
GATCAACTGGTTTTGGGATG	0.00	0.00	0.00	0.00	0.00
GATCAACTGTAAGGTCAGCC	0.46	0.00	0.00	0.00	0.00
GATCAACTGTAATTCTATCT	0.23	0.00	0.00	0.00	0.00
GATCAACTGTACAGTCGGAG	0.00	0.00	0.57	0.00	0.00
GATCAACTGTACATCATGGA	0.23	0.33	0.86	0.00	0.69
GATCAACTGTACCAAGCATT	0.00	0.00	0.29	0.00	0.00
GATCAACTGTACCCGTGGGA	0.00	0.00	0.00	0.34	0.69
GATCAACTGTACCTTTGTGT	0.00	0.00	0.00	1.36	0.00
GATCAACTGTACCTTTGTNT	0.00	0.00	0.00	0.00	0.00
GATCAACTGTACTGTCACAC	0.00	0.00	0.00	0.00	0.34
GATCAACTGTACTTAACTAT	0.00	0.00	0.00	0.00	0.00
GATCAACTGTAGAGCTTTGC	0.69	0.00	0.57	0.34	0.00
GATCAACTGTAGCGCTGGAG	0.00	0.00	0.00	0.00	0.00
GATCAACTGTAGGGCGTGCG	0.00	0.00	0.00	0.00	0.00
GATCAACTGTATCTCGTCTA	0.00	0.00	0.29	0.00	0.00
GATCAACTGTATCTGTCACC	0.23	0.00	0.00	0.00	0.00
GATCAACTGTATGAGCCGCA	0.23	0.00	0.00	0.00	0.34
GATCAACTGTATGCGCGCAT	0.00	0.33	0.00	0.00	0.00

GATCAACTGTATGCTGCAAA	0.00	0.00	0.00	0.00	0.00
GATCAACTGTATGGCCTGGT	0.69	0.00	0.00	0.00	0.00
GATCAACTGTATGTGCCGCA	0.00	0.33	0.00	0.00	0.00
GATCAACTGTATGTGTCTTG	0.00	0.00	0.29	0.00	0.00
GATCAACTGTATTTAAACAT	2.75	0.65	0.00	0.34	1.72
GATCAACTGTATTTAAANNN	0.23	0.00	0.00	0.00	0.00
GATCAACTGTCAAGGCCGAG	0.00	0.33	0.00	0.00	0.00
GATCAACTGTCACCACGTGA	0.00	0.00	0.00	0.34	0.00
GATCAACTGTCACCAGTTGG	0.00	0.00	0.00	0.00	0.00
GATCAACTGTCACTGAGTCT	0.00	0.00	0.29	0.00	0.00
GATCAACTGTCATCATAGTG	0.23	0.33	0.00	0.00	0.00
GATCAACTGTCATCCTTGCC	0.00	0.00	0.29	0.00	0.00
GATCAACTGTCATTAAAAAA	0.00	0.00	0.00	0.00	0.34
GATCAACTGTCATTGCCATC	0.00	0.00	0.00	0.00	0.69
GATCAACTGTCCCGCCGTCG	0.00	0.00	0.00	0.00	0.34
GATCAACTGTCCGAGAGCCG	0.00	0.00	0.00	0.34	0.00
GATCAACTGTCCGCCTCAAC	0.00	0.00	0.00	0.00	0.34
GATCAACTGTCCGCTGCAAA	0.00	0.00	0.57	0.00	0.00
GATCAACTGTCCGCTGCGGT	0.00	0.33	0.00	0.00	0.00
GATCAACTGTCCGGCATGCA	0.00	0.00	0.00	0.00	0.00
GATCAACTGTCCGGGCACCC	0.00	0.33	0.00	0.00	0.00
GATCAACTGTCCGTGGAAGC	0.23	0.00	0.00	0.00	0.00
GATCAACTGTCCGTGTAAGA	0.00	0.00	0.00	0.34	0.00
GATCAACTGTCCGTGTAAGC	5.72	10.74	9.16	6.45	8.61
GATCAACTGTCCGTGTAAGG	0.00	0.00	0.00	0.34	0.00
GATCAACTGTCCGTGTAANN	0.23	0.00	0.00	0.00	0.00
GATCAACTGTCCGTGTACGC	0.23	0.00	0.00	0.00	0.00
GATCAACTGTCCGTGTCAGC	0.00	0.00	0.00	0.00	0.00
GATCAACTGTCCGTGTNNNN	0.23	0.00	0.00	0.00	0.00
GATCAACTGTCCGTGTTTGC	0.00	0.33	0.29	0.34	1.03
GATCAACTGTCGCGCTTCAG	0.00	0.00	0.00	0.00	0.34
GATCAACTGTCGGAGCTTCG	0.23	0.00	0.00	0.00	0.00
GATCAACTGTCGTATGAAAA	0.00	0.00	0.00	0.00	0.00
GATCAACTGTCGTGTAAGTA	0.00	0.00	0.86	0.00	0.00
GATCAACTGTCTAAGCTTCG	0.00	0.00	0.00	0.00	0.00
GATCAACTGTCTCGAAGCGG	0.00	0.00	0.00	0.34	0.00
GATCAACTGTCTCTTGCAAA	0.00	0.33	0.00	0.00	0.00

GATCAACTGTCTGAGCGGAT	0.23	0.33	0.00	0.00	0.00
GATCAACTGTCTGAGCGTAN	0.00	0.00	0.00	0.00	0.00
GATCAACTGTCTGAGCGTAT	15.57	13.99	5.15	11.88	6.20
GATCAACTGTCTGAGCGTCN	0.23	0.00	0.00	0.00	0.00
GATCAACTGTCTGAGCGTCT	0.23	0.00	0.00	0.00	0.00
GATCAACTGTCTGAGCTCCG	0.23	0.00	0.00	0.00	0.00
GATCAACTGTCTGAGCTGCG	0.23	0.00	0.00	0.00	0.00
GATCAACTGTCTGAGCTTCG	9.61	6.51	5.73	5.09	6.54
GATCAACTGTCTGATCGTAT	0.00	0.00	0.29	0.00	0.34
GATCAACTGTCTGATTGACC	0.00	0.00	0.29	0.00	0.00
GATCAACTGTCTGCAGGGCT	0.00	0.00	0.00	0.00	0.00
GATCAACTGTCTGCCGCAAA	0.00	0.00	0.00	0.00	0.00
GATCAACTGTCTGCCGCAAT	0.00	0.00	0.29	0.00	0.00
GATCAACTGTCTGCCGCAGA	0.00	0.00	0.29	0.00	0.00
GATCAACTGTCTGCGCGTAT	0.23	0.00	0.00	0.00	0.00
GATCAACTGTCTGCGGCAAT	0.00	0.33	0.00	0.00	0.00
GATCAACTGTCTGCGTGAAA	0.00	0.00	0.29	0.00	0.00
GATCAACTGTCTGCTACAAA	0.23	0.00	0.00	0.00	0.00
GATCAACTGTCTGCTGAAAA	0.00	0.00	0.00	0.00	0.00
GATCAACTGTCTGCTGAAAC	0.00	0.00	0.29	0.00	0.00
GATCAACTGTCTGCTGAAAG	0.00	0.00	0.00	0.00	0.34
GATCAACTGTCTGCTGAGAG	0.00	0.00	0.00	0.00	0.34
GATCAACTGTCTGCTGAGCT	0.00	0.00	0.29	0.00	0.00
GATCAACTGTCTGCTGATTA	0.00	0.00	0.00	0.00	0.00
GATCAACTGTCTGCTGCAAA	26.78	30.58	67.87	54.65	56.48
GATCAACTGTCTGCTGCAAC	0.00	0.00	0.29	0.00	0.00
GATCAACTGTCTGCTGCAAG	0.00	0.00	0.00	0.34	1.72
GATCAACTGTCTGCTGCAAN	0.00	0.00	0.00	0.00	0.00
GATCAACTGTCTGCTGCAAT	0.00	0.00	0.00	0.00	0.69
GATCAACTGTCTGCTGCACA	0.00	0.00	0.00	0.00	0.34
GATCAACTGTCTGCTGCACC	0.00	0.00	0.00	0.00	0.34
GATCAACTGTCTGCTGCAGA	0.00	0.00	0.29	0.34	0.00
GATCAACTGTCTGCTGCAGC	0.00	0.00	0.00	0.00	0.34
GATCAACTGTCTGCTGCANN	0.23	0.00	0.00	0.00	0.00
GATCAACTGTCTGCTGCATT	0.00	0.00	0.00	0.00	0.34
GATCAACTGTCTGCTGCCAA	0.46	0.33	0.86	0.00	0.34
GATCAACTGTCTGCTGCCAT	0.00	0.00	0.29	0.00	0.00

GATCAACTGTCTGCTGCCCG	0.23	0.00	0.00	0.00	0.00
GATCAACTGTCTGCTGCCTT	0.00	0.00	0.00	0.34	0.00
GATCAACTGTCTGCTGCGAA	0.00	0.33	0.29	0.00	0.69
GATCAACTGTCTGCTGCGAG	0.00	0.00	0.00	1.02	0.00
GATCAACTGTCTGCTGCGGA	0.00	0.00	0.29	0.00	0.00
GATCAACTGTCTGCTGCGGC	0.00	0.00	0.00	0.00	0.00
GATCAACTGTCTGCTGCNNN	0.23	0.00	0.00	0.00	0.00
GATCAACTGTCTGCTGCTCG	0.00	0.33	0.00	0.00	0.00
GATCAACTGTCTGCTGCTTC	0.00	0.33	0.29	0.00	0.00
GATCAACTGTCTGCTGGAAA	0.00	0.00	0.00	0.00	0.00
GATCAACTGTCTGCTGGGGA	0.00	0.00	0.00	0.34	0.00
GATCAACTGTCTGCTGNNNN	0.23	0.00	0.00	0.00	0.00
GATCAACTGTCTGCTGTAAA	0.00	0.00	0.57	0.68	0.00
GATCAACTGTCTGCTGTTAA	0.00	0.00	0.00	0.00	0.34
GATCAACTGTCTGCTTCATC	0.00	0.00	1.15	0.00	0.00
GATCAACTGTCTGCTTGTCG	0.00	0.33	0.00	0.00	0.00
GATCAACTGTCTGTCAACTC	0.92	0.00	0.00	0.00	0.00
GATCAACTGTCTGTCACCTC	0.23	0.00	0.00	0.00	0.00
GATCAACTGTCTTCTGCAAT	0.00	0.00	0.00	0.00	0.34
GATCAACTGTCTTTGGCGAT	0.00	0.00	0.00	0.00	0.34
GATCAACTGTCTTTGGCGGT	0.00	0.00	0.00	0.68	0.34
GATCAACTGTGAAAAAAAAA	0.00	0.00	0.00	0.00	0.00
GATCAACTGTGAATAAGTTG	0.00	0.00	0.29	0.00	0.00
GATCAACTGTGACAAATGGC	1.37	1.30	0.00	0.00	0.00
GATCAACTGTGACAGCTGCG	0.00	0.00	0.29	0.00	0.00
GATCAACTGTGACGCTGCGG	0.23	0.00	0.00	0.00	0.00
GATCAACTGTGAGAGCCCAT	0.00	0.00	0.00	0.34	0.00
GATCAACTGTGAGGGAGTCA	0.00	0.00	0.57	0.34	0.00
GATCAACTGTGAGGGGCAGA	0.23	0.00	0.00	0.00	0.00
GATCAACTGTGAGTCGTGCA	0.00	0.00	0.00	0.00	0.00
GATCAACTGTGAGTCTTCAT	0.00	0.00	0.00	0.34	0.00
GATCAACTGTGCAAAAAAAA	1.14	0.00	0.29	0.68	0.34
GATCAACTGTGCAACTGGCC	0.00	0.00	0.00	0.00	0.34
GATCAACTGTGCAGCAAGGC	0.00	0.00	0.00	0.00	1.38
GATCAACTGTGCAGCAGGCC	0.00	0.00	0.29	0.00	0.00
GATCAACTGTGCAGCGAGGT	0.00	0.00	0.00	0.00	0.34
GATCAACTGTGCAGCTGACC	0.00	0.00	0.00	0.00	0.34

GATCAACTGTGCAGCTGGAC	0.00	0.33	0.29	0.00	0.00
GATCAACTGTGCAGCTGGCA	0.00	0.00	0.00	0.00	0.34
GATCAACTGTGCAGCTGGCC	13.73	30.91	27.78	22.74	23.42
GATCAACTGTGCAGCTGGCT	0.00	0.98	1.15	0.00	0.34
GATCAACTGTGCAGCTGGGC	0.23	0.00	0.00	0.00	0.00
GATCAACTGTGCAGCTTGCC	0.00	0.00	0.00	0.34	0.00
GATCAACTGTGCATCAAAAA	0.92	0.00	0.00	0.00	0.34
GATCAACTGTGCATCATGCC	0.69	0.00	0.29	1.36	2.07
GATCAACTGTGCATGACGGT	0.00	0.00	0.00	0.00	0.34
GATCAACTGTGCCAAGCTCC	0.00	0.00	0.00	0.00	1.03
GATCAACTGTGCCAATATCG	0.00	1.63	0.29	1.36	0.00
GATCAACTGTGCCAATATNN	0.00	0.00	0.00	0.00	0.00
GATCAACTGTGCCATGCGTG	0.00	0.00	0.29	0.00	0.00
GATCAACTGTGCCGACGCTT	0.00	0.00	0.00	0.00	0.00
GATCAACTGTGCCGATATCG	0.23	0.00	0.00	0.00	0.00
GATCAACTGTGCCTGAGGAA	0.00	0.00	0.00	0.00	0.34
GATCAACTGTGCGGATTGCA	0.23	0.00	0.00	0.00	0.00
GATCAACTGTGCGGCCGTGC	0.00	0.00	0.29	0.00	0.00
GATCAACTGTGCGGTGACCC	0.46	0.00	0.00	0.00	0.00
GATCAACTGTGCGTTATGTC	0.00	0.00	0.00	0.00	0.00
GATCAACTGTGCTGGGGGCG	0.00	0.33	0.00	0.00	0.00
GATCAACTGTGCTGTGACCT	0.69	0.00	0.00	0.00	0.00
GATCAACTGTGCTGTTGACA	0.00	0.00	0.00	0.00	0.00
GATCAACTGTGGAAGAGCTG	0.46	0.00	0.00	0.00	0.00
GATCAACTGTGGACCTAGAG	0.23	0.00	0.00	0.00	0.00
GATCAACTGTGGAGAGAAAA	0.23	0.00	0.29	0.34	0.00
GATCAACTGTGGAGGAGTGT	0.00	0.00	0.00	0.34	0.00
GATCAACTGTGGATCGGTAA	0.23	0.00	0.00	0.00	0.00
GATCAACTGTGGATGCATGC	0.00	0.00	0.00	0.00	0.34
GATCAACTGTGGCATACACC	0.23	0.00	0.00	0.00	0.00
GATCAACTGTGGCATCAAGT	0.00	0.00	0.00	0.00	0.34
GATCAACTGTGGCCCCATAC	0.00	0.00	0.00	0.00	0.00
GATCAACTGTGGCCGGCCAC	0.00	0.00	0.29	0.00	0.00
GATCAACTGTGGCTGCTTGC	0.00	0.00	0.00	0.34	0.00
GATCAACTGTGGGCGATTTC	0.00	0.00	0.00	0.00	0.34
GATCAACTGTGGGTGACTTT	0.00	0.00	0.00	1.02	0.00
GATCAACTGTGGTCTAAAAA	0.00	0.00	0.00	0.00	0.00

GATCAACTGTGGTGGAAAGG	0.00	0.33	0.00	0.00	0.00
GATCAACTGTGGTTCACGCT	0.00	0.00	0.00	0.00	0.34
GATCAACTGTGTAATCTTTT	0.00	0.00	0.00	0.00	0.00
GATCAACTGTGTAGGCACAC	1.14	1.63	0.00	0.68	0.00
GATCAACTGTGTCGTTGTGG	0.23	0.00	0.00	0.00	0.00
GATCAACTGTGTCTACACCC	0.00	0.00	0.29	0.00	0.00
GATCAACTGTGTGCGCGTAC	0.00	0.00	0.00	0.00	0.00
GATCAACTGTGTGGCCCCTA	0.00	0.00	0.29	0.00	0.00
GATCAACTGTGTGTGCATGT	0.00	0.00	0.29	0.00	0.00
GATCAACTGTGTTGACTTTG	0.69	0.00	0.86	0.68	1.03
GATCAACTGTTAACAAGTGA	0.00	0.00	0.00	0.00	0.00
GATCAACTGTTAACTCTTGT	0.23	0.65	0.00	1.70	0.00
GATCAACTGTTCACGGATGC	2.06	0.00	0.00	0.68	0.00
GATCAACTGTTCACTGCTGG	0.00	0.00	0.29	0.00	0.00
GATCAACTGTTCCAGAGCGG	0.00	0.00	0.00	0.34	0.00
GATCAACTGTTCCGGAGAAA	0.00	0.00	0.00	0.34	0.00
GATCAACTGTTCCTTCCGCG	0.00	0.00	0.00	0.00	0.34
GATCAACTGTTCTCTTCGAG	0.00	0.00	0.29	1.02	0.00
GATCAACTGTTGAAAAAAAA	0.00	0.00	0.29	0.00	0.00
GATCAACTGTTGAAATGTTG	0.00	0.00	0.00	0.00	0.34
GATCAACTGTTGACCGTGCA	0.00	0.33	0.00	0.00	0.00
GATCAACTGTTGAGGGTCTA	0.00	0.00	0.00	0.00	0.00
GATCAACTGTTGCACTGGGC	0.23	0.00	0.00	0.00	0.69
GATCAACTGTTGCCAAAAAA	0.00	0.00	0.00	0.00	0.00
GATCAACTGTTGCCGCCATC	0.00	0.00	0.00	0.34	0.00
GATCAACTGTTGCCTGCAGA	0.46	0.00	0.00	0.00	0.69
GATCAACTGTTGCGACAGCA	0.00	0.00	0.00	0.00	0.00
GATCAACTGTTGTCAACTGT	0.69	0.00	0.00	0.00	0.00
GATCAACTGTTGTGCTTGCA	0.00	0.00	0.00	0.00	0.34
GATCAACTGTTTACTGATGT	0.00	0.00	0.00	0.00	0.00
GATCAACTGTTTAGAAACAG	0.00	0.00	0.29	0.00	0.00
GATCAACTGTTTAGACGCGG	0.00	0.00	0.29	0.00	0.00
GATCAACTGTTTATGCTGAA	0.00	0.00	0.00	0.00	0.00
GATCAACTGTTTATGCTGGA	0.46	0.00	0.00	0.00	0.00
GATCAACTGTTTCAAAAAAA	0.92	0.00	0.00	0.00	0.00
GATCAACTGTTTCGAAAAAA	0.00	0.33	0.00	0.00	0.34
GATCAACTGTTTCGAACTCG	0.00	0.00	0.00	0.00	0.00

GATCAACTGTTTCGAAGAAG	0.00	0.33	0.00	0.00	0.00
GATCAACTGTTTCGAAGAGA	0.00	0.00	0.57	0.00	0.34
GATCAACTGTTTCGAAGAGG	0.00	0.33	0.29	0.34	0.00
GATCAACTGTTTCGAAGCAA	0.46	0.33	0.29	1.02	0.00
GATCAACTGTTTCGAAGCAC	0.00	0.33	0.00	0.00	0.00
GATCAACTGTTTCGAAGCCA	0.00	0.33	0.00	0.00	0.00
GATCAACTGTTTCGAAGCCG	0.00	0.00	0.29	0.00	0.00
GATCAACTGTTTCGAAGCGA	4.58	3.25	6.59	5.77	4.48
GATCAACTGTTTCGAAGCGC	0.00	0.00	0.29	0.00	0.00
GATCAACTGTTTCGAAGCGG	41.89	29.28	44.67	53.63	43.74
GATCAACTGTTTCGAAGCGN	0.23	0.00	0.00	0.68	0.00
GATCAACTGTTTCGAAGCGT	0.00	0.00	0.00	0.34	0.00
GATCAACTGTTTCGAAGCNN	0.23	0.00	0.00	0.00	0.00
GATCAACTGTTTCGAAGGAA	0.00	0.00	0.00	0.00	0.00
GATCAACTGTTTCGAAGGGA	0.00	0.00	0.29	0.00	0.00
GATCAACTGTTTCGAAGGGG	0.00	0.00	0.00	0.00	0.00
GATCAACTGTTTCGAAGNGG	0.00	0.00	0.00	0.00	0.00
GATCAACTGTTTCGAAGTGC	0.00	0.00	0.00	0.00	0.00
GATCAACTGTTTCGAANCGG	0.00	0.00	0.00	0.00	0.34
GATCAACTGTTTCGACACGG	0.00	0.00	0.00	0.00	0.00
GATCAACTGTTTCGAGAAAA	0.00	0.00	0.29	0.00	0.00
GATCAACTGTTTCGAGGCAG	0.00	0.00	0.29	0.00	0.00
GATCAACTGTTTCGCANNNN	0.00	0.00	0.00	0.00	0.00
GATCAACTGTTTCGCTTCTG	0.00	0.00	0.00	0.34	0.00
GATCAACTGTTTCGGAGCGA	0.00	0.00	0.00	0.68	0.00
GATCAACTGTTTCGGTAGCC	0.00	0.00	0.29	0.00	0.00
GATCAACTGTTTCGTCCGGA	0.00	0.00	0.00	0.00	0.00
GATCAACTGTTTCTAAGCGG	0.23	0.00	0.00	0.00	0.00
GATCAACTGTTTCTACGTTA	0.00	0.00	0.00	0.00	0.34
GATCAACTGTTTCTCAACGG	0.00	0.00	0.29	0.00	0.00
GATCAACTGTTTGAGGAAAC	0.00	0.00	0.00	0.00	0.69
GATCAACTGTTTGAGGGACT	0.23	0.00	0.00	0.00	0.00
GATCAACTGTTTGAGGGTCT	5.49	8.13	2.00	3.39	2.76
GATCAACTGTTTGCAATGTT	0.69	0.98	0.00	2.38	1.03
GATCAACTGTTTGGGCGTCC	0.92	0.65	0.29	3.05	1.03
GATCAACTGTTTGGGCGTNN	0.23	0.00	0.00	0.00	0.00
GATCAACTGTTTTGTCGGGC	0.00	0.00	0.29	0.00	0.00

GATCAACTTAAAAAAAAAAA	2.52	1.95	2.29	0.00	1.72
GATCAACTTAAAAGGAAAAA	0.00	0.00	0.00	0.00	0.00
GATCAACTTAAAAGGGAAAA	0.00	1.30	0.29	1.36	0.34
GATCAACTTAAAAGGGAGAC	0.46	0.00	0.00	0.00	0.00
GATCAACTTAAATGTTAAAA	0.00	0.00	0.00	0.00	0.00
GATCAACTTAAGGACCGCCG	0.00	0.00	0.00	0.34	0.00
GATCAACTTAAGGTATCGGG	0.00	0.00	0.00	0.00	0.00
GATCAACTTAATCTCTTGAG	0.00	0.00	0.00	0.00	0.34
GATCAACTTAATGACATGTA	0.00	0.00	0.00	0.00	0.34
GATCAACTTAATGTACCCAT	0.00	0.00	0.00	0.00	0.34
GATCAACTTAATTTAGTTGT	0.23	0.00	0.00	0.00	0.00
GATCAACTTACACGAGACAC	0.00	0.00	0.00	0.00	0.34
GATCAACTTACAGCGGATGT	0.00	0.33	0.00	0.00	0.00
GATCAACTTACAGCGGCTGT	0.00	0.33	0.00	0.00	0.00
GATCAACTTACATTACACAG	0.00	0.00	0.29	0.00	0.00
GATCAACTTACCCGTGCAGA	0.00	0.00	0.29	0.00	0.00
GATCAACTTACGACATCACT	0.00	0.00	0.00	0.00	0.34
GATCAACTTACGGGCAAGTA	0.00	0.00	0.00	0.00	0.34
GATCAACTTACTCTGTGTGT	0.00	0.00	0.00	0.00	0.00
GATCAACTTACTCTTCCTGG	0.00	0.00	0.00	0.00	0.34
GATCAACTTACTGTCTTCGC	0.00	0.00	0.00	0.00	0.00
GATCAACTTACTTGTTCGTG	0.00	0.33	1.43	0.00	0.34
GATCAACTTAGACAGCAGCT	0.00	0.00	0.00	0.34	0.00
GATCAACTTAGAGGTACATG	0.00	0.00	0.00	0.00	0.34
GATCAACTTAGAGGTATGAC	0.00	0.00	0.00	0.34	0.00
GATCAACTTAGCACCGCTGC	0.00	0.00	0.00	0.00	0.34
GATCAACTTAGCAGGCAGAG	0.00	0.00	0.00	0.34	0.00
GATCAACTTAGCAGGTAAGG	0.00	0.00	0.00	0.34	0.00
GATCAACTTAGCCGAGCCAG	0.00	0.00	0.00	0.34	0.00
GATCAACTTAGCCGGCAGAG	0.00	0.00	0.00	0.34	0.00
GATCAACTTAGCCGTCGTAT	0.00	0.00	0.00	0.00	0.34
GATCAACTTAGCGCTGTTCC	0.00	0.00	0.00	0.00	0.00
GATCAACTTAGCGCTGTTCG	0.00	0.00	0.00	0.00	0.00
GATCAACTTAGCTGCAGTCC	0.00	0.00	0.00	0.68	0.00
GATCAACTTAGGGGATCTCA	0.00	0.00	0.00	0.00	0.00
GATCAACTTAGGGGTGAGGG	0.00	0.00	0.00	0.00	0.34
GATCAACTTAGTGCGGTGTG	0.00	0.00	0.00	0.00	0.34

GATCAACTTATAGGGCAACC	0.00	0.00	0.00	0.00	0.00
GATCAACTTATATTGGCATG	0.00	0.00	0.00	0.00	0.34
GATCAACTTATATTGGCTCA	0.00	0.00	0.00	0.00	0.00
GATCAACTTATCAGGCGGTC	0.00	0.33	0.00	0.00	0.00
GATCAACTTATCATTCCCCT	0.00	0.00	0.00	0.00	0.00
GATCAACTTATGTCCGCTGA	0.00	0.00	0.00	0.34	0.00
GATCAACTTATTATTTGTTT	4.12	1.63	2.58	3.05	1.72
GATCAACTTATTGTGTGCGC	0.00	0.00	0.00	0.00	0.00
GATCAACTTATTTAGCATGT	1.14	0.00	0.00	0.00	0.00
GATCAACTTATTTCAGATAT	0.00	0.00	0.00	0.00	0.69
GATCAACTTATTTCATATAT	0.00	0.00	0.00	0.00	0.34
GATCAACTTCAAAAAAAAAA	12.36	3.90	4.87	3.73	5.17
GATCAACTTCAAAAAAAAAN	0.00	0.00	0.00	0.00	0.00
GATCAACTTCAAAAAAAAAT	0.23	0.00	0.29	0.34	0.00
GATCAACTTCAAAAAACAAA	0.00	0.33	0.00	0.00	0.00
GATCAACTTCAAAAAACCNN	0.23	0.00	0.00	0.00	0.00
GATCAACTTCAAACAAAAAA	0.46	0.00	0.00	0.00	0.00
GATCAACTTCAAACATCCAA	0.00	0.65	0.29	0.00	0.00
GATCAACTTCAAACATGCAA	0.00	0.00	0.29	0.00	0.00
GATCAACTTCAAAGAGATGA	0.23	0.00	0.00	0.00	0.00
GATCAACTTCAAAGATGACT	0.00	0.00	0.00	0.68	0.00
GATCAACTTCAAATCCGGGG	0.00	0.00	0.29	0.00	0.00
GATCAACTTCAACAAGCACG	0.00	0.00	0.00	0.00	0.34
GATCAACTTCAACAAGCATG	0.00	0.00	0.00	0.34	0.00
GATCAACTTCAACAAGCCCG	0.00	0.00	0.00	0.00	0.34
GATCAACTTCAACAAGCCGG	0.23	0.00	0.00	0.34	0.00
GATCAACTTCAACAAGCCTG	0.00	0.00	0.57	0.00	0.34
GATCAACTTCAACCAGCTGC	0.00	0.33	0.29	0.00	0.34
GATCAACTTCAACTTTTTGA	0.00	0.00	0.00	0.34	0.00
GATCAACTTCAAGAAGGACA	0.00	0.33	0.00	0.00	0.00
GATCAACTTCAAGAAGGTCA	1.37	0.00	0.29	1.70	0.00
GATCAACTTCAAGAAGGTGC	0.00	0.00	0.00	0.34	0.00
GATCAACTTCAAGACGGTAC	2.06	1.63	1.15	0.34	0.00
GATCAACTTCAAGAGCAATA	0.00	0.00	0.00	0.00	0.00
GATCAACTTCAAGCAAAATA	0.00	0.00	0.00	0.00	0.34
GATCAACTTCAAGCCACCCA	0.92	0.33	0.00	0.68	0.00
GATCAACTTCAAGCCGCTCT	0.00	0.00	0.00	0.34	0.00

GATCAACTTCAAGCGCGGGG	0.46	0.00	0.00	0.00	0.00
GATCAACTTCAAGCGCGTGA	2.06	1.30	0.57	0.68	1.72
GATCAACTTCAAGCTTGGGG	0.00	0.00	0.00	0.00	0.00
GATCAACTTCAAGGAGAAGG	0.46	0.00	0.00	0.00	0.00
GATCAACTTCAAGGAGGAGT	0.00	0.00	0.29	0.00	0.00
GATCAACTTCAAGGAGGGAT	0.00	0.33	0.00	0.00	0.00
GATCAACTTCAAGGAGGTCC	0.00	0.00	0.00	0.00	0.00
GATCAACTTCAAGGGTAGCA	0.00	0.33	0.00	0.00	0.00
GATCAACTTCAAGTGTAGCA	2.52	2.60	0.29	0.34	0.00
GATCAACTTCAATAACATCA	0.00	0.00	0.00	0.00	0.34
GATCAACTTCAATCAAATCA	0.00	0.00	0.00	0.00	0.00
GATCAACTTCAATTCCAGCG	0.00	0.00	0.00	0.00	0.00
GATCAACTTCACAAAAAAAA	0.00	0.00	0.00	0.00	0.00
GATCAACTTCACAAAGATGA	0.00	0.00	0.00	0.34	0.00
GATCAACTTCACACTGGGCA	0.00	0.00	0.00	0.34	0.00
GATCAACTTCACAGGCCGGG	5.04	3.25	3.15	5.77	2.76
GATCAACTTCACATGGTGGG	0.00	0.65	0.00	0.34	0.69
GATCAACTTCACATGGTGNN	0.23	0.00	0.00	0.00	0.00
GATCAACTTCACATTCGCGG	0.00	0.33	0.00	0.00	0.00
GATCAACTTCACCATCATCA	0.00	0.00	0.57	0.00	0.00
GATCAACTTCACCCAGTGCA	0.00	0.00	0.00	0.00	0.00
GATCAACTTCACCCATGCCA	0.00	0.00	0.00	0.00	0.00
GATCAACTTCACCCCCCGCC	0.00	0.00	0.00	0.34	0.00
GATCAACTTCACCCTTATCT	0.00	0.33	0.29	0.34	0.00
GATCAACTTCACCCTTTGCT	0.00	0.00	0.00	0.00	0.00
GATCAACTTCACCGTGTCCT	0.46	0.00	0.00	0.00	0.00
GATCAACTTCACCTCCCCCC	1.14	2.28	1.43	1.70	0.34
GATCAACTTCACGAGGGTGG	0.00	0.00	0.57	0.00	0.00
GATCAACTTCACGCCTACAC	0.00	0.00	0.00	0.00	0.00
GATCAACTTCACGGGCGAGG	0.00	0.00	0.00	0.68	0.00
GATCAACTTCACGTCGTGAA	0.46	0.00	0.00	0.00	0.00
GATCAACTTCACGTCGTGCG	0.46	0.00	0.29	0.00	0.00
GATCAACTTCACTCGGGACC	8.70	11.71	19.76	9.84	7.58
GATCAACTTCACTCGGGCCC	0.00	0.00	0.00	0.00	0.34
GATCAACTTCACTCGGGCNN	0.23	0.00	0.00	0.00	0.00
GATCAACTTCACTCGGNACC	0.00	0.00	0.00	0.00	0.34
GATCAACTTCACTTTCGCGG	0.00	0.00	0.00	0.34	0.00

GATCAACTTCAGAATGTTCA	0.00	0.00	0.29	0.00	0.00
GATCAACTTCAGAGTGCTCT	0.00	0.00	0.00	0.00	0.00
GATCAACTTCAGAGTGCTGC	0.46	0.00	0.00	0.34	0.34
GATCAACTTCAGCAAGAAAC	0.00	0.33	0.00	0.00	0.00
GATCAACTTCAGCATTGAGG	0.69	0.33	0.00	0.00	0.00
GATCAACTTCAGCCCCTCTG	0.00	0.00	0.00	0.00	0.34
GATCAACTTCAGCCGAGTGG	0.00	0.00	0.00	0.34	0.00
GATCAACTTCAGCCTCCTCT	0.00	0.65	0.29	0.00	0.00
GATCAACTTCAGCGAGTGTC	0.00	0.33	0.00	0.00	0.00
GATCAACTTCAGCGCATGCC	0.00	0.00	0.00	0.68	0.00
GATCAACTTCAGCGGCCTAG	0.00	0.00	0.29	0.00	0.00
GATCAACTTCAGCGGCTATG	0.00	0.00	0.00	0.00	0.00
GATCAACTTCAGCGGCTTCA	0.00	0.00	0.00	0.00	0.00
GATCAACTTCAGCGTAAAAA	3.89	0.00	1.15	1.70	1.38
GATCAACTTCAGCGTAAAAG	1.14	0.33	0.57	1.02	0.69
GATCAACTTCAGCGTCACCC	0.23	0.00	0.00	0.00	0.00
GATCAACTTCAGCGTGAAAA	0.00	0.33	0.29	0.00	0.00
GATCAACTTCAGCGTGCTCC	0.00	0.00	0.00	0.00	0.00
GATCAACTTCAGCTCGTGGC	0.23	0.00	0.00	0.00	0.00
GATCAACTTCAGGAGCAAAA	0.00	0.00	0.00	0.00	0.00
GATCAACTTCAGGAGCTCAC	0.00	0.00	0.00	0.34	0.00
GATCAACTTCAGGCAGCGCG	0.23	0.00	0.00	0.00	0.00
GATCAACTTCAGGCAGCTCT	0.00	0.00	0.00	0.00	0.00
GATCAACTTCAGGCAGGTCT	0.00	0.00	0.29	0.00	0.00
GATCAACTTCAGGTGGTGCT	0.23	0.00	0.00	0.00	0.00
GATCAACTTCAGGTTCCCGG	0.00	0.00	0.00	0.00	0.00
GATCAACTTCAGTGAGCAGG	0.46	0.00	0.00	0.00	0.00
GATCAACTTCAGTGGAGTGG	0.00	0.00	0.00	0.34	0.00
GATCAACTTCATCAACTGTG	0.00	0.65	0.00	0.00	0.00
GATCAACTTCATCAGACGGG	0.23	0.00	0.00	0.00	0.00
GATCAACTTCATCAGCCAGT	0.00	0.00	0.00	0.34	0.00
GATCAACTTCATCCGCCACT	0.00	0.00	0.00	0.00	0.00
GATCAACTTCATCCGTGGCC	0.23	0.00	0.00	0.00	0.00
GATCAACTTCATCGCATTCA	0.00	0.00	0.29	0.00	0.00
GATCAACTTCATCGGACGGT	0.00	0.33	0.00	0.00	0.00
GATCAACTTCATCTAACGGG	0.00	0.00	0.00	0.00	0.69
GATCAACTTCATCTCAAGGA	0.00	0.00	0.57	0.00	0.00

GATCAACTTCATCTCACGGG	0.00	0.00	0.00	0.00	0.34
GATCAACTTCATCTCACTGG	0.23	0.00	0.00	0.00	0.00
GATCAACTTCATCTGAAGGC	0.00	0.00	0.00	0.00	0.00
GATCAACTTCATCTGAAGGG	0.00	0.00	0.00	0.34	0.00
GATCAACTTCATCTGACACG	0.00	0.00	0.00	0.00	0.69
GATCAACTTCATCTGACAGG	0.00	0.00	0.00	0.00	0.00
GATCAACTTCATCTGACGAG	0.00	0.33	0.00	0.00	1.72
GATCAACTTCATCTGACGAT	0.00	0.00	0.00	0.34	0.00
GATCAACTTCATCTGACGCG	0.23	0.33	0.00	0.00	0.00
GATCAACTTCATCTGACGGA	1.14	0.33	0.00	0.34	0.69
GATCAACTTCATCTGACGGG	182.67	93.37	76.17	79.43	90.92
GATCAACTTCATCTGACGGN	0.23	0.00	0.00	0.00	0.00
GATCAACTTCATCTGACGGT	0.00	0.65	0.29	0.34	0.34
GATCAACTTCATCTGACGNG	0.00	0.00	0.00	0.00	0.00
GATCAACTTCATCTGACGNN	0.46	0.00	0.00	0.00	0.00
GATCAACTTCATCTGACGTC	0.00	0.00	0.00	0.00	0.69
GATCAACTTCATCTGACGTG	0.00	0.00	0.29	0.34	1.03
GATCAACTTCATCTGACNGG	0.00	0.00	0.00	0.34	0.34
GATCAACTTCATCTGACNNN	0.23	0.00	0.00	0.00	0.00
GATCAACTTCATCTGACTCG	0.00	0.33	0.00	0.00	0.00
GATCAACTTCATCTGAGGGA	0.00	0.00	0.29	0.00	0.00
GATCAACTTCATCTGATAGA	0.00	0.00	0.00	0.00	0.00
GATCAACTTCATCTGATGGG	0.23	0.00	0.00	0.00	0.00
GATCAACTTCATCTGCCGGG	0.23	0.00	0.00	0.00	0.00
GATCAACTTCATCTTCTCAA	0.00	0.00	0.29	0.00	0.00
GATCAACTTCATCTTGGTCG	0.00	0.33	0.00	0.00	0.00
GATCAACTTCATGAACTGTG	0.46	0.00	0.00	0.00	0.34
GATCAACTTCATGAGCTTCA	0.00	0.65	0.00	0.00	0.34
GATCAACTTCATGCCCTTCA	0.00	0.00	0.29	0.00	0.00
GATCAACTTCATGCGCCCGG	0.00	0.00	0.29	0.00	0.00
GATCAACTTCATGCGGTGTG	0.00	0.00	0.00	0.68	0.34
GATCAACTTCATGGCCGTCT	0.00	0.65	0.57	0.00	0.00
GATCAACTTCATTCAGACTT	0.00	0.33	0.00	0.00	0.00
GATCAACTTCATTTCCTGCT	0.00	0.00	0.00	0.00	0.34
GATCAACTTCATTTGACGGG	0.46	0.00	0.00	0.00	0.00
GATCAACTTCCACAGCACTT	0.00	0.00	0.00	0.68	0.00
GATCAACTTCCACGGCCCCT	0.00	0.33	0.00	0.34	0.00

GATCAACTTCCACGGTCGGC	0.00	0.00	0.00	0.00	0.00
GATCAACTTCCAGATGCCAA	2.75	0.33	0.00	0.00	0.00
GATCAACTTCCAGCACCGAA	10.07	5.53	5.15	6.79	2.76
GATCAACTTCCAGCACCGAC	0.00	0.33	0.00	0.00	0.00
GATCAACTTCCAGCACCGAN	0.23	0.00	0.00	0.00	0.00
GATCAACTTCCAGCACCGCA	0.00	0.00	0.00	0.34	0.00
GATCAACTTCCAGCACCGNN	0.23	0.00	0.00	0.00	0.00
GATCAACTTCCAGCAGCTCG	0.46	0.65	0.86	0.34	0.69
GATCAACTTCCAGCCTGTAG	0.00	0.00	0.29	0.00	0.00
GATCAACTTCCAGCGGAGCG	0.00	0.00	0.00	0.00	0.00
GATCAACTTCCAGCGGATGA	0.00	0.00	0.29	0.68	0.00
GATCAACTTCCAGCGGATGT	0.00	0.00	0.00	0.34	0.00
GATCAACTTCCAGGAGGTGC	0.92	0.00	0.00	0.68	0.00
GATCAACTTCCAGGAGTGGG	0.00	0.65	0.00	0.00	0.00
GATCAACTTCCAGGATGCGG	0.00	0.00	0.00	0.00	0.69
GATCAACTTCCAGGTGGCGC	0.00	0.65	0.00	0.00	0.00
GATCAACTTCCAGTGAGCAG	0.00	0.00	0.00	0.00	0.00
GATCAACTTCCATCCGCACC	0.69	0.65	0.00	0.00	0.00
GATCAACTTCCATGAGAGGG	0.00	0.00	0.00	0.00	0.00
GATCAACTTCCCAAGAGCCG	0.00	0.00	0.00	0.00	0.00
GATCAACTTCCCAGCAGTCT	0.00	0.00	0.29	0.00	0.00
GATCAACTTCCCGCCCAAAC	0.00	0.00	0.29	0.00	0.00
GATCAACTTCCCGCGTGGCC	0.00	0.00	0.00	0.00	0.34
GATCAACTTCCCGGGAGATG	0.00	0.00	0.00	0.68	0.00
GATCAACTTCCCTGTCCTTC	0.00	0.00	0.00	0.00	0.34
GATCAACTTCCGAAGGACCC	0.00	0.00	0.00	0.00	0.00
GATCAACTTCCGATGGACCC	0.23	0.00	0.00	0.00	0.00
GATCAACTTCCGCTCGCCGG	0.00	0.00	0.00	0.00	0.00
GATCAACTTCCGGCAGCTCG	0.00	0.00	0.00	0.00	0.00
GATCAACTTCCGGGAGATTA	0.00	0.00	0.00	0.34	0.00
GATCAACTTCCGGGAGGGGA	0.00	0.33	0.00	0.00	0.00
GATCAACTTCCGTGTCCCCT	0.00	0.00	0.00	0.00	0.34
GATCAACTTCCTCGAGCGCT	0.00	0.00	0.57	0.00	0.34
GATCAACTTCCTCGAGCGGT	0.00	0.00	0.00	0.34	0.00
GATCAACTTCCTCTGACGGG	0.00	0.33	0.00	0.00	0.00
GATCAACTTCCTCTGACTGT	0.23	0.00	0.00	0.00	0.00
GATCAACTTCCTCTGGACGC	0.23	0.00	0.00	0.00	0.00

GATCAACTTCCTCTGTGTGC	0.00	0.00	0.00	0.34	0.00
GATCAACTTCCTGAACCCAT	0.00	0.00	0.00	0.00	0.00
GATCAACTTCCTGAAGTTCT	0.00	0.00	0.00	0.00	0.00
GATCAACTTCCTGACCTCCT	0.00	0.00	0.00	0.34	0.00
GATCAACTTCCTGAGCGAGC	0.00	0.33	0.00	0.00	0.00
GATCAACTTCCTGCACGAGC	0.00	0.00	0.00	0.34	0.00
GATCAACTTCCTGCTCGGCG	0.00	0.00	0.00	0.34	0.00
GATCAACTTCCTGGACACGA	0.00	0.65	0.00	0.34	0.00
GATCAACTTCCTGGACTTTG	0.00	0.00	0.00	0.00	0.00
GATCAACTTCCTGGCTCTCT	0.69	0.00	0.00	0.00	0.00
GATCAACTTCCTGGGCCCCG	0.46	0.00	0.00	0.00	0.00
GATCAACTTCCTGGGCGCGT	0.00	0.33	0.00	0.00	0.00
GATCAACTTCCTGTAGTCAT	1.37	1.95	0.00	1.02	1.72
GATCAACTTCCTGTAGTTAT	0.00	0.00	0.00	0.34	0.00
GATCAACTTCCTGTATTTAG	0.00	0.00	0.00	0.00	0.00
GATCAACTTCCTGTGGCCGA	0.00	0.00	0.00	0.00	0.34
GATCAACTTCCTTCAAAAGT	0.00	0.98	0.29	0.00	0.00
GATCAACTTCCTTGACCCAA	0.23	0.00	0.00	0.00	0.34
GATCAACTTCCTTGTGGAGG	0.69	0.00	0.00	0.00	0.00
GATCAACTTCGAAAAAAAAA	7.55	0.65	0.00	0.00	1.38
GATCAACTTCGAAAAAAAAC	0.23	0.00	0.00	0.00	0.00
GATCAACTTCGAAAAAAAAN	0.23	0.00	0.00	0.00	0.00
GATCAACTTCGAAAAAAGAG	13.51	6.51	3.15	4.75	7.23
GATCAACTTCGAAAAAAGAN	0.23	0.00	0.00	0.00	0.00
GATCAACTTCGAAGAAAGAG	0.00	0.33	0.00	0.00	0.00
GATCAACTTCGAAGAAAGCC	0.00	0.00	0.57	0.00	0.00
GATCAACTTCGAAGAAGAGG	0.00	0.33	0.57	0.00	0.00
GATCAACTTCGACAAAAAAA	0.23	0.00	0.00	0.00	0.00
GATCAACTTCGACAAAAGAG	0.00	0.00	0.00	0.00	0.34
GATCAACTTCGACAAGGACA	0.00	0.00	0.00	0.00	0.00
GATCAACTTCGACATCCAGT	0.00	0.33	0.00	0.00	0.00
GATCAACTTCGACCAGGTCA	0.00	0.00	0.00	0.34	0.00
GATCAACTTCGACCGGGCTG	0.00	0.00	0.00	0.68	0.00
GATCAACTTCGACCGGTAAA	0.23	0.00	0.00	0.00	0.00
GATCAACTTCGACCGGTCTG	0.00	0.00	0.00	0.00	0.34
GATCAACTTCGACCGGTCTT	3.20	0.98	0.29	2.38	0.69
GATCAACTTCGACCGGTTTG	0.00	0.00	0.00	0.00	0.00

GATCAACTTCGACCTGACCG	1.14	1.95	0.86	1.36	1.03
GATCAACTTCGACTGCCCCG	0.00	0.00	0.00	0.00	0.00
GATCAACTTCGAGACAAAAA	0.00	0.00	0.00	0.00	0.00
GATCAACTTCGAGACCATGG	0.00	0.00	0.00	0.00	0.69
GATCAACTTCGAGACGCACG	0.00	0.65	0.29	0.68	0.34
GATCAACTTCGAGCACTGTG	0.00	0.00	0.00	0.34	0.00
GATCAACTTCGAGCGCTCGA	0.00	0.00	0.00	0.00	0.00
GATCAACTTCGAGGAGGAGG	0.00	0.00	0.00	0.00	0.34
GATCAACTTCGAGGAGGTGG	0.00	0.00	0.00	0.00	0.00
GATCAACTTCGAGGCAGTGT	0.00	0.00	0.00	0.00	0.34
GATCAACTTCGAGGCATGTC	0.23	0.00	0.00	0.00	0.00
GATCAACTTCGAGGTATGAC	0.00	0.33	0.00	0.00	1.03
GATCAACTTCGCAAAAAAAA	0.00	0.33	0.29	0.00	0.00
GATCAACTTCGCAAACTTCA	0.00	0.00	0.57	0.00	0.00
GATCAACTTCGCAAAGCCCA	0.00	0.33	0.00	0.00	0.00
GATCAACTTCGCAACGCGCT	0.00	0.00	0.00	0.34	0.00
GATCAACTTCGCAGCGGAGG	0.00	0.00	0.00	0.34	0.00
GATCAACTTCGCAGTGAAGC	0.00	0.33	0.00	0.00	0.00
GATCAACTTCGCATTCAACA	0.00	0.00	0.29	0.34	0.00
GATCAACTTCGCCCGCGGGC	0.00	0.33	0.00	0.00	0.00
GATCAACTTCGCGAAGATGT	0.00	0.98	0.29	0.68	0.34
GATCAACTTCGCGCAAGCAG	0.00	0.33	0.00	0.00	0.00
GATCAACTTCGCGCCATGTG	2.06	0.00	0.57	0.68	0.00
GATCAACTTCGCGCCATGTT	0.00	0.33	0.00	0.00	0.00
GATCAACTTCGCGCGGGTGC	0.00	0.33	0.29	0.00	0.00
GATCAACTTCGCGGCTCTCA	0.00	0.00	0.00	0.34	0.00
GATCAACTTCGCGGCTCTCC	0.00	0.00	0.00	0.00	0.00
GATCAACTTCGCGTGACGCA	0.00	0.00	0.00	0.00	0.00
GATCAACTTCGCTAGTCTTG	0.00	0.00	0.00	0.34	0.00
GATCAACTTCGGAAAACTTT	0.00	0.00	0.29	0.00	0.00
GATCAACTTCGGAAATGGCC	0.00	0.00	0.00	0.00	0.69
GATCAACTTCGGAGGTGGAG	0.00	0.00	0.00	0.00	0.00
GATCAACTTCGGCAATCCTC	0.00	0.00	0.00	0.00	1.03
GATCAACTTCGGCATCATCT	0.00	0.00	0.00	0.00	0.00
GATCAACTTCGGCGCCCTCA	0.46	0.00	0.00	0.68	0.00
GATCAACTTCGGCGCGAACG	0.00	0.00	0.00	0.00	0.00
GATCAACTTCGGCGTGGCTT	0.00	0.00	0.00	0.00	0.00

GATCAACTTCGGGGAGCAGG	0.00	0.00	0.00	0.00	0.00
GATCAACTTCGGTTCCTGGC	0.92	0.00	0.29	0.34	0.00
GATCAACTTCGTACAGGCCT	0.00	0.00	0.00	0.34	0.00
GATCAACTTCGTCACAGAGG	0.00	0.00	0.00	0.00	0.00
GATCAACTTCGTCACCGAGG	16.25	6.83	2.58	8.83	2.76
GATCAACTTCGTCACGAACA	0.00	0.00	0.29	0.00	0.00
GATCAACTTCGTCACTGAGG	0.23	0.00	0.00	0.00	0.00
GATCAACTTCGTCAGTCCTC	0.00	0.00	0.29	0.00	0.00
GATCAACTTCGTCGCGGCTG	0.23	0.00	0.00	0.00	0.00
GATCAACTTCGTCGGCCTCA	0.00	0.33	0.00	0.00	0.00
GATCAACTTCGTCTCCTACA	0.23	0.33	1.15	0.00	1.03
GATCAACTTCGTGAAAAAAA	0.23	0.00	0.00	1.02	0.00
GATCAACTTCGTGACCTGCC	0.00	0.00	0.29	0.00	0.00
GATCAACTTCGTGACGAATA	0.00	0.00	0.00	0.00	0.00
GATCAACTTCGTGATCAAAG	0.00	0.00	0.00	0.00	0.00
GATCAACTTCGTGCAGGGGA	0.00	0.00	0.00	0.00	0.00
GATCAACTTCGTGCAGGGGC	0.00	0.33	0.00	0.00	0.00
GATCAACTTCGTGGAGGAGG	0.00	0.00	0.00	0.00	0.00
GATCAACTTCTAAAAAAAAA	0.69	0.00	0.00	0.00	0.00
GATCAACTTCTAAACAATAG	0.00	0.00	0.00	0.00	0.00
GATCAACTTCTACAGCCAGC	0.00	0.33	0.00	0.00	0.00
GATCAACTTCTACATTGAGC	0.00	0.00	0.00	0.00	0.00
GATCAACTTCTACCAAGGTC	1.14	0.65	0.00	0.00	0.69
GATCAACTTCTACCGACTCT	0.00	0.00	0.00	0.00	0.00
GATCAACTTCTACCGCTTTG	0.69	0.00	0.00	0.00	0.00
GATCAACTTCTACTTCAAGC	1.14	0.98	0.00	0.68	1.03
GATCAACTTCTCCATGTCGC	0.00	0.00	0.00	0.00	0.34
GATCAACTTCTCCATTGCTC	0.00	0.65	0.00	0.00	0.00
GATCAACTTCTCCATTGNNN	0.00	0.00	0.00	0.00	0.00
GATCAACTTCTCCCAGTTTT	0.00	0.00	0.00	0.34	0.00
GATCAACTTCTCCGTGAACT	0.00	0.00	0.29	0.00	0.00
GATCAACTTCTCCGTGGAGC	0.23	0.00	0.00	0.34	0.00
GATCAACTTCTCCGTGGGCG	0.46	0.00	0.00	0.00	0.00
GATCAACTTCTCCGTGTTGC	0.00	0.00	0.00	0.00	0.00
GATCAACTTCTCCTTGCTGC	0.00	0.00	0.29	0.00	0.00
GATCAACTTCTCGAAAAAAA	0.69	0.00	0.00	0.00	0.00
GATCAACTTCTCGAAACATA	0.00	0.00	0.00	0.00	0.00

GATCAACTTCTCGAAACATG	1.14	1.30	0.29	2.38	0.34
GATCAACTTCTCGACCTGCC	0.00	0.00	0.00	0.00	0.00
GATCAACTTCTCGAGCCACC	0.00	0.00	0.00	0.00	0.00
GATCAACTTCTCGAGCCGGA	0.00	0.00	0.00	0.00	0.00
GATCAACTTCTCGGAACATG	0.00	0.00	0.00	0.00	0.00
GATCAACTTCTCGTCGCAGG	0.00	0.00	0.00	0.00	0.34
GATCAACTTCTCTGAACAAG	0.23	0.00	0.00	0.00	0.00
GATCAACTTCTGAACCCAAG	0.00	0.00	0.00	0.34	0.00
GATCAACTTCTGAACCGAGA	0.00	0.00	0.29	0.00	0.00
GATCAACTTCTGAATCTCGT	0.23	0.00	0.00	0.00	0.00
GATCAACTTCTGAATGACTC	0.00	0.33	0.00	0.00	0.00
GATCAACTTCTGAATGCTGA	0.23	0.00	0.00	0.00	0.00
GATCAACTTCTGACACATGT	0.00	0.33	0.00	0.00	0.00
GATCAACTTCTGACGGGAAG	1.60	0.33	0.00	0.34	0.34
GATCAACTTCTGACTTGCGG	0.00	0.00	0.00	0.34	0.00
GATCAACTTCTGAGATGTGT	0.23	0.00	0.29	0.00	0.00
GATCAACTTCTGAGCAGAGA	0.00	0.00	0.29	0.00	0.34
GATCAACTTCTGAGCCGAGA	17.63	10.74	2.86	10.86	5.51
GATCAACTTCTGAGCCGAGT	0.23	0.00	0.00	0.00	0.00
GATCAACTTCTGAGCCGCGA	0.23	0.00	0.00	0.00	0.00
GATCAACTTCTGATACATCC	0.00	0.00	0.29	0.00	0.00
GATCAACTTCTGATGCAAGC	0.00	0.00	0.00	0.00	0.34
GATCAACTTCTGATGCATGC	0.00	0.33	0.57	0.00	0.00
GATCAACTTCTGCATCTCGT	0.23	0.00	0.00	0.00	0.00
GATCAACTTCTGCATCTTTT	0.00	0.00	0.29	0.00	0.00
GATCAACTTCTGCCGGAAGG	0.00	0.33	0.00	0.00	0.00
GATCAACTTCTGCGCCGAGA	0.46	0.00	0.00	0.00	0.00
GATCAACTTCTGGAAGTACC	0.23	0.00	0.00	0.00	0.00
GATCAACTTCTGGCAGTGCC	0.00	0.00	0.00	0.00	0.00
GATCAACTTCTGGCAGTGGG	0.00	0.00	0.00	0.34	0.00
GATCAACTTCTGGCATGACA	0.00	0.33	0.00	0.00	0.00
GATCAACTTCTGGCATGCCA	2.75	0.98	0.00	0.34	0.00
GATCAACTTCTGGCTCTCGC	0.00	0.00	0.00	0.00	0.00
GATCAACTTCTGGGAGATTA	0.00	0.00	0.00	0.00	0.00
GATCAACTTCTGGGATATCC	0.46	0.65	0.00	0.00	0.34
GATCAACTTCTGGGGCGCTC	0.00	0.00	0.00	0.00	0.00
GATCAACTTCTGTCCATCAG	0.00	0.00	0.00	0.00	0.34

GATCAACTTCTGTCCATCCG	0.23	0.33	0.00	0.00	0.34
GATCAACTTCTGTCGTATGC	0.00	0.00	0.00	0.00	0.00
GATCAACTTCTTCAACGGCC	0.00	0.00	0.00	0.00	0.00
GATCAACTTCTTCAACTGGG	0.23	0.00	0.00	0.00	0.00
GATCAACTTCTTCAAGTCAT	0.00	0.33	0.00	0.00	0.00
GATCAACTTCTTCACGTCCG	0.00	0.00	0.00	0.34	0.00
GATCAACTTCTTCATGTATG	0.00	0.00	0.29	0.00	0.00
GATCAACTTCTTCATTTGCA	0.69	0.00	0.00	0.00	0.00
GATCAACTTCTTCATTTGCT	2.75	0.65	0.00	1.70	0.00
GATCAACTTCTTCCAGTCGC	0.00	0.00	0.29	0.34	0.00
GATCAACTTCTTCGAGCTAC	0.23	0.00	0.00	0.00	0.00
GATCAACTTCTTCGAGGGCC	0.00	0.00	0.00	0.00	0.69
GATCAACTTCTTCGCAGAGT	0.00	0.98	0.00	0.34	0.00
GATCAACTTCTTCGGGCTGC	1.83	1.63	0.57	0.34	0.34
GATCAACTTCTTCGGTTGTC	0.00	0.00	0.00	0.00	0.34
GATCAACTTCTTGCAAAGCC	0.00	0.00	0.00	0.00	0.00
GATCAACTTCTTGCTGTTCG	0.00	0.00	0.29	0.00	0.34
GATCAACTTCTTTACATCAG	0.46	0.98	0.29	0.34	0.34
GATCAACTTCTTTACATCGC	0.00	0.00	0.29	0.00	0.00
GATCAACTTCTTTACATGAA	0.00	0.00	0.29	0.00	0.00
GATCAACTTCTTTACATTTG	0.00	0.00	0.57	0.00	0.00
GATCAACTTCTTTCACCATG	0.00	0.33	0.00	0.00	0.00
GATCAACTTGAAAAAAAAAA	4.81	1.30	1.43	2.72	1.72
GATCAACTTGAAAACAAAAA	0.00	0.00	0.29	0.00	0.00
GATCAACTTGAAACAAAAAA	0.00	0.00	0.29	0.00	0.00
GATCAACTTGAAACAACAAA	0.23	0.00	0.00	0.00	0.00
GATCAACTTGAAAGCTGGAA	0.00	0.33	0.00	0.00	0.00
GATCAACTTGAAATAAAAAA	2.06	0.33	0.57	0.34	1.03
GATCAACTTGAAATAGCAAA	0.00	0.33	0.86	0.00	0.00
GATCAACTTGAAATAGCTAA	0.92	0.33	0.29	0.00	0.00
GATCAACTTGAAATAGCTNN	0.23	0.00	0.00	0.00	0.00
GATCAACTTGAAATAGCTTA	0.00	0.00	0.29	0.34	0.00
GATCAACTTGAAATAGCTTG	5.95	1.30	1.15	2.04	2.07
GATCAACTTGAAATAGCTTN	0.23	0.00	0.00	0.00	0.00
GATCAACTTGAAATAGTAAA	0.00	0.00	0.29	0.00	0.00
GATCAACTTGAACATAAAAC	0.00	0.00	0.00	0.00	0.34
GATCAACTTGAACTCTGTAT	0.00	0.00	0.29	0.00	0.00

GATCAACTTGAAGACAATTC	0.00	0.00	0.00	0.34	0.00
GATCAACTTGAAGCACATGC	0.00	0.00	0.00	0.00	0.00
GATCAACTTGAAGGCCCCTT	0.00	0.00	0.00	0.00	0.34
GATCAACTTGAATGACACCG	0.00	0.00	0.00	0.00	0.00
GATCAACTTGAATGCGAAGA	0.00	0.00	0.00	0.00	0.00
GATCAACTTGAATGGCACCG	9.16	10.74	4.58	6.79	3.79
GATCAACTTGAATGGCACTG	0.00	0.33	0.00	0.00	0.00
GATCAACTTGAATTCACTTC	1.60	0.00	0.29	0.68	0.34
GATCAACTTGACACACCACA	0.00	0.00	0.00	0.00	0.69
GATCAACTTGACAGCGGCGT	0.00	0.00	0.00	0.00	0.34
GATCAACTTGACAGTGAACT	0.00	0.00	0.29	0.00	0.00
GATCAACTTGACATGCCTCC	0.00	0.00	0.29	0.00	0.00
GATCAACTTGACATGGGTGA	0.00	0.33	0.00	0.00	0.00
GATCAACTTGACCATCCCAG	0.00	0.33	0.00	0.00	0.00
GATCAACTTGACCGGCAGAG	0.00	0.00	0.00	0.00	0.34
GATCAACTTGACCGGGTGGC	0.00	0.00	0.00	0.00	0.00
GATCAACTTGACCTTTTGGA	0.00	0.00	0.00	0.34	0.00
GATCAACTTGACGAGGCTCA	0.23	0.65	0.00	0.00	0.00
GATCAACTTGACGATTGGCA	1.37	0.65	0.29	0.00	0.34
GATCAACTTGACGCGCAAGA	0.46	2.28	0.57	0.00	4.13
GATCAACTTGACGCTTGCGA	0.00	0.00	0.00	0.00	0.00
GATCAACTTGACGTGGCACT	0.00	0.65	0.00	0.00	0.00
GATCAACTTGACGTGGCGCT	7.10	5.53	2.29	8.83	3.44
GATCAACTTGACGTGGGGCT	0.23	0.00	0.00	0.00	0.00
GATCAACTTGACTGGCACCG	0.23	0.00	0.00	0.00	0.00
GATCAACTTGACTGGGAGGC	0.00	0.00	0.29	0.00	0.00
GATCAACTTGACTGGGGGGC	0.46	0.00	0.00	0.00	0.00
GATCAACTTGACTGGGTCGC	0.00	0.00	0.00	0.00	0.00
GATCAACTTGACTGGGTGGC	22.20	17.89	12.03	22.74	13.43
GATCAACTTGACTGGGTGGG	0.00	0.33	0.00	0.68	0.00
GATCAACTTGACTGGGTGGN	0.23	0.00	0.00	0.00	0.00
GATCAACTTGACTGGGTGTC	0.23	0.00	0.00	0.00	0.00
GATCAACTTGACTGGGTTCG	0.46	0.00	0.00	0.00	0.00
GATCAACTTGACTGGGTTGC	0.00	0.00	0.00	0.00	0.34
GATCAACTTGACTGTCATCA	2.75	2.28	0.57	1.02	1.03
GATCAACTTGACTGTCCNNN	0.23	0.00	0.00	0.00	0.00
GATCAACTTGACTGTCCTCC	0.23	0.00	0.00	0.00	0.00

GATCAACTTGACTTCGTATT	0.00	0.33	0.00	0.00	0.00
GATCAACTTGACTTGGTGGC	0.46	0.00	0.00	0.00	0.00
GATCAACTTGAGAAAAAAAA	0.69	0.00	0.00	0.00	0.34
GATCAACTTGAGACTCACGC	0.23	0.00	0.00	0.00	0.00
GATCAACTTGAGCAGATGGT	0.00	0.00	0.00	0.00	0.00
GATCAACTTGAGCGGGCGCG	0.00	0.00	0.00	0.00	0.34
GATCAACTTGAGCTGATTGC	0.23	0.00	0.00	0.34	0.00
GATCAACTTGAGGTATGACT	0.00	0.00	0.00	0.00	0.69
GATCAACTTGAGGTCCCGGA	0.46	1.30	0.57	0.34	0.00
GATCAACTTGAGTGTTCGTA	0.00	0.00	0.00	0.68	0.00
GATCAACTTGATAAGTTGGC	0.46	0.00	0.00	0.00	0.69
GATCAACTTGATAAGTTGGG	0.00	0.00	0.00	0.00	0.00
GATCAACTTGATGAGCATGT	0.46	0.00	0.29	0.00	0.00
GATCAACTTGATTCGTCAGG	0.00	0.00	0.00	0.00	0.00
GATCAACTTGCAAAAAAAAA	1.37	0.65	1.15	0.68	0.69
GATCAACTTGCAAAAAAAAT	0.23	0.00	0.00	0.00	0.34
GATCAACTTGCAAAAATCTA	0.00	0.00	0.29	0.00	0.00
GATCAACTTGCAAACGAATA	0.00	0.00	0.00	0.00	0.00
GATCAACTTGCAAAGCCTGC	0.00	0.00	0.00	0.00	0.34
GATCAACTTGCAAAGGCCTT	0.46	0.00	0.00	0.00	0.00
GATCAACTTGCAACCTTCCT	0.00	0.00	0.00	0.00	0.00
GATCAACTTGCAATCATGCA	0.00	0.00	0.29	0.34	0.00
GATCAACTTGCACCAACTTG	0.00	0.00	0.57	0.00	0.00
GATCAACTTGCACCACAGTA	0.00	0.00	0.29	0.00	0.00
GATCAACTTGCACCCCGGTG	0.00	0.00	0.86	0.68	0.34
GATCAACTTGCACGATAGGC	0.69	0.00	0.00	0.00	0.00
GATCAACTTGCACTTACATG	0.23	0.00	0.00	0.00	0.00
GATCAACTTGCACTTGAAGG	0.23	0.00	0.00	0.00	0.00
GATCAACTTGCACTTGCAGG	0.23	0.00	0.00	0.00	0.00
GATCAACTTGCACTTGCANN	0.23	0.00	0.00	0.00	0.00
GATCAACTTGCACTTGCATG	68.67	37.74	26.92	26.48	26.17
GATCAACTTGCACTTGCATN	0.46	0.00	0.00	0.00	0.00
GATCAACTTGCACTTGCATT	0.00	0.98	0.00	0.00	0.34
GATCAACTTGCACTTGCCAG	0.00	0.00	0.29	0.00	0.00
GATCAACTTGCACTTGCCTG	0.00	0.33	0.29	0.00	0.00
GATCAACTTGCACTTGCGTG	0.46	0.33	0.00	0.00	0.00
GATCAACTTGCACTTGNNNN	0.23	0.00	0.00	0.00	0.00

GATCAACTTGCAGAGGTGTG	5.72	6.51	2.86	7.13	4.82
GATCAACTTGCAGCAAGTGG	0.46	0.00	0.00	0.34	0.00
GATCAACTTGCAGCACCGAA	0.00	0.00	0.00	0.34	0.00
GATCAACTTGCAGCACCGCC	0.00	0.00	0.00	0.34	0.00
GATCAACTTGCAGCGTCCCC	0.00	0.33	0.00	0.00	0.00
GATCAACTTGCAGGATGAGG	0.23	0.00	0.00	0.00	0.00
GATCAACTTGCAGGCGGACG	0.00	0.33	0.00	0.00	0.00
GATCAACTTGCAGGTGGCGC	3.20	2.60	1.15	2.72	1.38
GATCAACTTGCAGGTGGCNC	0.00	0.00	0.00	0.00	0.00
GATCAACTTGCATGCCCTTG	0.00	0.00	0.00	0.00	0.34
GATCAACTTGCATGCGGTGC	0.23	0.00	0.00	0.00	0.00
GATCAACTTGCATGCTGCGG	0.00	0.00	0.29	0.00	0.00
GATCAACTTGCATGGCTTTG	0.00	0.00	0.00	0.34	0.00
GATCAACTTGCATTTCATGA	0.00	0.33	0.00	0.00	0.00
GATCAACTTGCCAAGCCGCG	0.00	0.00	0.29	0.00	0.00
GATCAACTTGCCACGTTCTT	0.00	0.00	0.00	0.00	0.69
GATCAACTTGCCACGTTGCG	0.00	0.00	0.00	0.00	0.00
GATCAACTTGCCAGATGCGC	0.00	0.00	0.00	0.00	0.34
GATCAACTTGCCAGTCAGCC	0.00	0.00	0.86	0.00	0.00
GATCAACTTGCCAGTGCAAG	0.00	0.00	0.00	0.00	0.00
GATCAACTTGCCATCATTCT	0.00	0.00	0.00	0.00	0.00
GATCAACTTGCCATGGGAAA	0.00	0.00	0.00	0.00	0.34
GATCAACTTGCCCAGCGGTT	0.00	0.00	0.00	0.00	0.34
GATCAACTTGCCCAGTTTAT	0.00	0.00	0.00	0.00	0.34
GATCAACTTGCCCGAGGCCG	0.00	0.00	0.00	0.34	0.00
GATCAACTTGCCCGCAGGCT	0.00	0.00	0.00	0.00	0.00
GATCAACTTGCCCGGCCTGA	0.00	0.00	0.00	0.00	0.00
GATCAACTTGCCCGGCCTGC	0.00	0.00	0.00	0.00	0.00
GATCAACTTGCCCTACCCAC	0.00	0.00	0.00	0.00	0.34
GATCAACTTGCCCTTCATAG	0.00	0.00	0.00	0.34	0.00
GATCAACTTGCCCTTCTTGC	0.00	0.33	0.29	0.00	0.34
GATCAACTTGCCGCATCCCA	0.23	0.65	0.57	0.00	1.38
GATCAACTTGCCGGAGGAGA	0.00	0.00	0.00	0.00	0.34
GATCAACTTGCCGTGCTCGG	0.46	1.30	0.00	0.34	0.34
GATCAACTTGCCGTTGTGGT	0.00	0.00	0.00	0.00	0.00
GATCAACTTGCCTGCTTGCA	0.00	0.00	0.00	0.00	0.00
GATCAACTTGCCTGTCGCGG	0.00	0.65	0.29	0.00	0.00

GATCAACTTGCCTGTTGCGG	0.00	0.00	0.00	0.34	0.00
GATCAACTTGCCTTCACAGT	0.00	0.00	0.00	0.00	0.00
GATCAACTTGCCTTCAGACC	0.92	0.65	0.00	1.02	1.38
GATCAACTTGCCTTCCTTCA	0.23	0.00	0.00	0.00	0.00
GATCAACTTGCCTTCTGCCG	0.00	0.00	0.29	0.00	0.00
GATCAACTTGCCTTCTTTGT	0.00	0.00	0.00	0.00	0.00
GATCAACTTGCCTTGATGGT	0.23	0.00	0.00	0.00	0.34
GATCAACTTGCCTTGCATGA	0.23	0.00	0.00	0.00	0.00
GATCAACTTGCCTTTCCATC	0.00	0.00	0.00	0.34	0.00
GATCAACTTGCGAGCAGCCC	0.00	0.00	0.00	0.34	0.00
GATCAACTTGCGCAAGGACT	0.23	0.00	0.29	0.34	0.69
GATCAACTTGCGCTGGTCAG	0.00	0.00	0.00	0.34	0.00
GATCAACTTGCGGCGAGTGC	0.00	0.00	0.00	0.00	0.00
GATCAACTTGCTATGGTGAA	0.23	0.00	0.00	0.00	0.00
GATCAACTTGCTCAAGTGGA	0.00	0.00	0.00	0.00	0.00
GATCAACTTGCTCATCGGGG	0.00	0.00	0.00	0.34	0.34
GATCAACTTGCTCATCGTGG	0.00	0.33	0.00	0.00	0.00
GATCAACTTGCTCGAGTACC	0.00	0.00	0.00	0.00	0.00
GATCAACTTGCTCGCATGAC	0.00	0.33	0.00	0.00	0.00
GATCAACTTGCTCTGCGACC	0.00	0.33	0.00	0.00	0.00
GATCAACTTGCTCTTCCTGG	0.69	0.33	0.00	0.68	0.69
GATCAACTTGCTCTTCGCGG	0.00	0.33	0.00	0.00	0.00
GATCAACTTGCTCTTCTTGG	0.00	0.33	0.00	0.34	0.00
GATCAACTTGCTGACCCCGC	0.00	0.33	0.00	0.00	0.00
GATCAACTTGCTGACCCGGG	0.00	0.00	0.00	0.34	0.00
GATCAACTTGCTGACCCTGC	3.89	4.55	4.30	6.11	6.54
GATCAACTTGCTGACCCTGG	0.00	0.00	0.00	0.00	0.00
GATCAACTTGCTGACCNNNN	0.23	0.00	0.00	0.00	0.00
GATCAACTTGCTGACCNTNN	0.23	0.00	0.00	0.00	0.00
GATCAACTTGCTGACCTCAA	0.00	0.33	0.00	0.00	0.00
GATCAACTTGCTGACCTCAC	0.69	0.65	0.57	0.00	0.00
GATCAACTTGCTGAGGCACT	0.00	0.00	0.00	1.02	0.00
GATCAACTTGCTGAGGTCCC	0.23	0.00	0.00	0.00	0.00
GATCAACTTGCTGCTGAGGC	0.00	0.00	0.29	0.00	0.00
GATCAACTTGCTGGTTGATG	0.00	0.33	0.00	0.00	0.00
GATCAACTTGCTGTGTCAGA	0.00	1.30	0.00	0.00	0.00
GATCAACTTGCTGTTCTTGG	0.23	0.00	1.15	0.68	0.34

GATCAACTTGCTGTTGCAGT	0.23	0.00	0.00	0.00	0.00
GATCAACTTGCTTCATGGCG	0.00	0.98	0.00	0.00	0.00
GATCAACTTGCTTCCGTGGC	0.23	0.00	0.00	0.00	0.00
GATCAACTTGCTTCTGGCGA	0.00	0.00	0.00	0.00	0.34
GATCAACTTGCTTGAAGCAT	0.00	0.00	0.00	0.00	0.00
GATCAACTTGCTTGCGAGCT	0.00	0.00	0.00	0.00	0.34
GATCAACTTGCTTGGATTGT	0.00	0.00	0.00	0.34	0.00
GATCAACTTGCTTGGCGTGT	0.00	0.33	0.29	0.00	0.00
GATCAACTTGCTTGTGGAGG	0.00	0.00	0.00	0.00	0.34
GATCAACTTGCTTTTGTGGG	0.00	0.33	0.00	0.00	0.00
GATCAACTTGCTTTTGTGGT	0.00	0.00	0.29	0.00	0.00
GATCAACTTGCTTTTGTTGT	0.00	0.00	0.00	0.00	0.34
GATCAACTTGCTTTTTTGGC	0.00	0.00	0.00	0.00	0.00
GATCAACTTGGAAAACAATC	0.23	0.00	0.29	0.68	0.34
GATCAACTTGGAACAGTGTT	0.00	0.00	0.00	0.34	0.00
GATCAACTTGGAAGCAGCTA	0.00	0.00	0.00	0.00	0.34
GATCAACTTGGAAGCCGGGA	0.00	0.65	0.29	0.00	0.00
GATCAACTTGGACAACAATC	0.00	0.33	0.00	0.00	0.00
GATCAACTTGGACAACATGC	0.00	0.00	0.29	0.68	0.00
GATCAACTTGGACACCATTC	0.00	0.65	0.57	0.68	0.34
GATCAACTTGGACAGATGCT	0.00	0.00	0.00	0.34	0.00
GATCAACTTGGACAGCAGCT	0.00	0.00	0.00	0.00	1.03
GATCAACTTGGACCGCATAG	0.00	0.00	0.00	0.34	0.00
GATCAACTTGGACGGAAGTC	0.00	0.00	0.29	0.00	0.00
GATCAACTTGGACGGCAGAG	0.00	0.65	0.00	0.34	0.69
GATCAACTTGGACGGCAGCG	0.00	0.00	0.00	0.00	0.34
GATCAACTTGGACGGCAGTG	0.00	0.33	0.00	0.00	0.00
GATCAACTTGGACTCCCTCA	0.23	0.00	0.00	0.00	0.00
GATCAACTTGGACTGCTAGT	0.00	0.33	0.00	0.00	0.00
GATCAACTTGGAGAGCCTCG	0.00	0.33	0.00	0.00	0.00
GATCAACTTGGAGCTGCATC	0.00	0.33	0.00	0.00	0.00
GATCAACTTGGAGGCTGCTG	0.23	0.00	0.00	0.00	0.00
GATCAACTTGGAGGGCAGAG	0.00	0.00	0.00	0.00	0.00
GATCAACTTGGAGGGCGGAG	6.87	3.90	2.29	1.70	5.51
GATCAACTTGGAGGGCGGGG	0.23	0.00	0.00	0.00	0.00
GATCAACTTGGAGGGCGGNN	0.23	0.00	0.00	0.00	0.00
GATCAACTTGGAGTCGTCTG	0.00	0.00	0.00	0.00	0.34

GATCAACTTGGAGTCTGATG	0.00	0.00	0.00	0.00	0.34
GATCAACTTGGAGTCTGCTG	10.07	19.52	17.75	28.85	26.17
GATCAACTTGGAGTCTGCTT	0.00	0.00	0.29	0.00	0.00
GATCAACTTGGAGTCTNNNN	0.23	0.00	0.00	0.00	0.00
GATCAACTTGGAGTGCAATG	0.00	0.00	0.00	0.00	0.00
GATCAACTTGGAGTGCAGAG	0.00	0.33	0.00	0.00	0.00
GATCAACTTGGAGTGGCCAC	0.00	0.00	0.00	0.34	0.00
GATCAACTTGGAGTTTGACA	0.69	0.00	0.00	0.68	0.00
GATCAACTTGGAGTTTGGGG	0.00	0.33	0.00	0.00	0.00
GATCAACTTGGATGAGGAGG	0.00	0.00	0.57	0.34	0.34
GATCAACTTGGCAAGGGGCG	0.00	0.00	0.00	0.00	0.34
GATCAACTTGGCACGCCCAA	0.00	0.00	0.29	0.00	0.00
GATCAACTTGGCACTTATGT	0.00	0.00	0.00	0.34	0.00
GATCAACTTGGCAGAGGAGT	0.00	0.00	0.00	0.00	0.34
GATCAACTTGGCAGGCACAG	0.00	0.00	0.00	0.00	0.34
GATCAACTTGGCAGGCAGAA	0.00	0.00	0.00	0.34	0.00
GATCAACTTGGCAGGCAGAG	0.00	0.98	0.00	0.00	0.69
GATCAACTTGGCAGGCAGGC	0.00	0.00	0.00	0.34	0.00
GATCAACTTGGCAGGTAGAG	0.00	0.00	0.00	0.00	0.34
GATCAACTTGGCATGGATGG	0.00	0.00	0.00	0.00	0.34
GATCAACTTGGCATTGTTGG	0.00	0.00	0.00	0.00	0.34
GATCAACTTGGCCACCAAAC	0.00	0.00	0.00	0.00	0.34
GATCAACTTGGCCAGCAGAG	0.00	0.00	0.00	0.34	0.34
GATCAACTTGGCCAGGGCTC	0.00	0.00	0.00	0.34	0.00
GATCAACTTGGCCCAGCCGC	0.00	0.00	0.29	0.00	0.00
GATCAACTTGGCCCAGTCAT	0.00	0.00	0.00	0.00	0.34
GATCAACTTGGCCCGCAGAG	0.23	0.00	0.00	0.00	0.00
GATCAACTTGGCCCTCCTCG	0.00	0.00	0.00	0.00	0.00
GATCAACTTGGCCCTGTACG	0.46	0.65	1.43	0.34	0.34
GATCAACTTGGCCCTTAGAA	0.00	0.00	0.00	0.00	0.34
GATCAACTTGGCCCTTCGCA	3.89	4.23	2.86	4.07	4.13
GATCAACTTGGCCCTTCGCT	0.00	0.00	0.00	0.00	0.00
GATCAACTTGGCCGACAGAG	0.00	0.00	0.00	0.00	0.34
GATCAACTTGGCCGACCAGA	0.00	0.00	0.00	0.00	0.00
GATCAACTTGGCCGGAAGAG	0.00	0.00	0.00	0.68	0.00
GATCAACTTGGCCGGAAGCG	0.00	0.00	0.00	0.34	0.00
GATCAACTTGGCCGGAAGTG	0.00	0.33	0.00	0.00	0.00

GATCAACTTGGCCGGCAAAG	0.00	0.65	0.00	0.00	0.00
GATCAACTTGGCCGGCACAG	0.00	0.00	0.00	0.00	0.00
GATCAACTTGGCCGGCAGAA	0.00	0.33	0.00	0.34	0.00
GATCAACTTGGCCGGCAGAC	0.00	0.00	0.29	0.00	0.00
GATCAACTTGGCCGGCAGAG	116.51	210.49	222.21	244.73	260.02
GATCAACTTGGCCGGCAGAT	0.00	0.00	0.29	0.34	0.00
GATCAACTTGGCCGGCAGCG	0.69	0.98	1.43	2.04	1.72
GATCAACTTGGCCGGCAGGG	1.14	0.33	0.57	0.68	0.34
GATCAACTTGGCCGGCAGNG	0.00	0.00	0.00	0.34	0.34
GATCAACTTGGCCGGCAGNN	0.23	0.00	0.00	0.00	0.34
GATCAACTTGGCCGGCAGTG	0.00	0.00	0.29	0.00	0.00
GATCAACTTGGCCGGCANNN	0.00	0.00	0.00	0.00	0.00
GATCAACTTGGCCGGCATAG	0.00	0.00	0.29	0.34	0.00
GATCAACTTGGCCGGCATAT	0.00	0.00	0.00	0.34	0.00
GATCAACTTGGCCGGCCGAG	0.46	1.63	1.15	1.36	0.34
GATCAACTTGGCCGGCCGCG	0.23	0.33	0.00	0.00	0.00
GATCAACTTGGCCGGCCNNN	0.46	0.00	0.00	0.00	0.00
GATCAACTTGGCCGGCGGAG	0.23	0.00	0.29	0.00	0.00
GATCAACTTGGCCGGCGGGG	0.23	0.00	0.00	0.00	0.00
GATCAACTTGGCCGGCNGNN	0.23	0.00	0.00	0.00	0.00
GATCAACTTGGCCGGCTCAC	0.00	0.00	0.00	0.00	0.34
GATCAACTTGGCCGGCTGAG	0.23	0.33	0.00	0.00	0.00
GATCAACTTGGCCGGGAGAG	0.23	0.00	0.00	0.00	0.00
GATCAACTTGGCCGGTAGAG	0.00	0.00	0.00	0.00	0.34
GATCAACTTGGCCGTCAGAG	0.00	0.00	0.00	0.00	0.00
GATCAACTTGGCCGTCTTCT	0.00	0.00	0.00	0.00	0.00
GATCAACTTGGCCTGCAGAG	0.23	0.00	0.00	0.00	0.00
GATCAACTTGGCCTGCTTTG	0.23	0.00	0.57	0.00	0.00
GATCAACTTGGCGATGGCGT	0.23	0.00	0.00	0.00	0.00
GATCAACTTGGCGATGTCGT	0.23	0.00	0.00	0.00	0.00
GATCAACTTGGCGCGCAATA	0.00	0.00	0.00	0.00	0.34
GATCAACTTGGCGCGGCTGC	0.00	0.00	0.29	0.00	0.00
GATCAACTTGGCGCTACGAT	0.00	0.00	0.00	0.00	0.00
GATCAACTTGGCGGCGTTAA	0.23	0.00	0.00	0.00	0.00
GATCAACTTGGCTAAAGCAC	0.23	0.00	0.00	0.00	0.00
GATCAACTTGGCTCAGCGTG	0.00	0.00	0.00	0.00	0.34
GATCAACTTGGCTGGCAGAG	0.00	0.00	0.00	0.34	0.00

GATCAACTTGGGAAAAAAAA	1.83	0.00	0.00	0.00	0.00
GATCAACTTGGGAAACAAAA	0.23	0.00	0.00	0.00	0.00
GATCAACTTGGGCAACGGAC	0.00	0.00	0.57	0.00	0.00
GATCAACTTGGGCAATGCAA	0.00	0.00	0.29	0.00	0.00
GATCAACTTGGGCATATTTC	0.00	0.00	0.29	0.00	0.00
GATCAACTTGGGCATGCACA	0.00	0.00	0.00	0.00	0.00
GATCAACTTGGGCCTGTGCG	0.23	0.00	0.00	0.00	0.00
GATCAACTTGGGCGGCAGAG	0.23	0.00	0.00	0.00	0.00
GATCAACTTGGGGAGCCTCG	0.23	0.00	0.00	0.00	0.00
GATCAACTTGGGGCCCGCAG	0.00	0.00	0.00	0.00	0.34
GATCAACTTGGGGCGCCTGC	0.00	0.33	0.00	0.00	0.00
GATCAACTTGGGGGAAGCCC	0.00	0.00	0.29	0.00	0.00
GATCAACTTGGGGTCTGCTG	0.23	0.00	0.00	0.00	0.00
GATCAACTTGGTAAAGCCAC	0.23	0.00	0.00	0.00	0.00
GATCAACTTGGTAGTCCCGT	0.23	0.33	0.00	0.68	0.69
GATCAACTTGGTCGCGTCAT	0.23	0.33	0.00	0.68	0.00
GATCAACTTGGTCGCGTGCG	0.00	0.00	0.29	0.00	0.00
GATCAACTTGGTCGGCAGAG	0.46	0.65	0.00	0.00	0.34
GATCAACTTGGTCGTGTTCT	0.00	0.00	0.00	0.00	0.00
GATCAACTTGGTCTCTGCTG	0.23	0.00	0.00	0.00	0.00
GATCAACTTGGTGACACTCG	0.00	0.00	0.00	0.00	0.00
GATCAACTTGGTGACCTCCC	0.00	0.00	0.00	0.00	0.00
GATCAACTTGGTGAGACTCG	0.00	0.00	0.29	0.00	0.00
GATCAACTTGGTGAGCATGT	0.00	0.00	0.00	0.00	0.00
GATCAACTTGGTGAGCCCCG	0.00	0.00	0.00	0.34	0.34
GATCAACTTGGTGAGCCTCG	18.77	21.15	16.32	22.06	16.19
GATCAACTTGGTGAGCCTCN	0.23	0.00	0.00	0.34	0.00
GATCAACTTGGTGAGTCTCG	0.00	0.00	0.00	0.00	0.00
GATCAACTTGGTGATCCTCG	0.00	0.00	0.29	0.00	0.00
GATCAACTTGGTGCCTGCTG	0.23	0.00	0.00	0.00	0.34
GATCAACTTGGTGGACAAGA	0.00	0.00	0.00	0.00	0.00
GATCAACTTGGTGGCTGCTG	0.00	0.00	0.00	0.34	0.00
GATCAACTTGGTGGGGCGTG	0.00	0.00	0.29	0.00	0.00
GATCAACTTGGTGTATGATG	0.00	0.00	0.00	0.00	0.00
GATCAACTTGGTGTCCGCTG	0.23	0.33	0.86	0.34	1.03
GATCAACTTGGTGTCGGCTG	0.23	0.00	0.00	0.00	0.00
GATCAACTTGGTGTCTGATG	0.00	0.00	0.29	0.00	0.34

GATCAACTTGGTGTCTGCAT	0.00	0.00	0.00	0.00	0.00
GATCAACTTGGTGTCTGCCG	0.00	0.00	0.00	0.34	0.00
GATCAACTTGGTGTCTGCTA	0.00	0.33	0.29	0.34	0.34
GATCAACTTGGTGTCTGCTG	63.86	119.72	74.45	99.79	78.87
GATCAACTTGGTGTCTGCTT	0.46	0.33	0.00	0.00	0.69
GATCAACTTGGTGTCTGNNN	0.23	0.00	0.00	0.00	0.00
GATCAACTTGGTGTCTGNTG	0.00	0.33	0.00	0.34	0.00
GATCAACTTGGTGTCTNNNN	0.92	0.00	0.00	0.00	0.00
GATCAACTTGGTGTGTGCTG	0.23	0.00	0.00	0.00	0.00
GATCAACTTGGTTGAGACAG	3.20	2.93	3.44	5.09	2.76
GATCAACTTGGTTGAGACCG	0.00	0.00	0.00	0.00	0.00
GATCAACTTGGTTGAGCAAG	0.00	0.00	0.00	0.00	0.34
GATCAACTTGGTTGGACAAG	0.92	0.00	0.00	0.00	0.00
GATCAACTTGGTTGGACACC	0.00	0.00	0.29	0.00	0.00
GATCAACTTGGTTTTCCCTT	0.00	0.00	0.00	0.00	0.00
GATCAACTTGTAACCTGTAA	0.00	0.00	0.29	0.00	0.00
GATCAACTTGTAATTGAGAC	0.23	0.00	0.00	0.00	0.00
GATCAACTTGTACAAAGTGG	0.00	0.33	0.00	0.00	0.00
GATCAACTTGTACCTGAAGA	0.00	0.00	0.00	0.00	0.00
GATCAACTTGTATTTGAGAA	0.00	0.00	0.00	0.00	0.00
GATCAACTTGTATTTGAGAC	12.36	28.95	30.07	24.44	29.27
GATCAACTTGTATTTGAGAN	0.00	0.00	0.00	0.00	0.00
GATCAACTTGTATTTGAGCC	0.00	0.00	0.29	0.00	0.00
GATCAACTTGTATTTGAGGC	0.46	0.00	0.00	0.00	0.34
GATCAACTTGTATTTGATAC	0.00	0.33	0.00	0.00	0.00
GATCAACTTGTATTTGCGAC	0.00	0.00	0.29	0.34	0.00
GATCAACTTGTATTTGCGNN	0.23	0.00	0.00	0.00	0.00
GATCAACTTGTATTTGGGAC	0.00	0.33	0.00	0.00	0.00
GATCAACTTGTATTTGTGAC	0.23	0.00	1.15	0.00	0.00
GATCAACTTGTCAACACCTC	0.00	0.33	0.00	0.00	0.00
GATCAACTTGTCAACGGTCC	0.69	0.65	0.00	0.68	0.69
GATCAACTTGTCACCCTTGT	0.00	0.65	0.00	0.00	0.00
GATCAACTTGTCAGGCGGTC	0.00	0.98	0.00	0.34	0.00
GATCAACTTGTCATCAAGCG	0.00	0.00	0.00	0.00	0.34
GATCAACTTGTCCACGGGCG	0.00	0.00	0.00	0.00	0.34
GATCAACTTGTCCGCACACG	0.00	0.00	0.00	0.00	0.00
GATCAACTTGTCCTTTTCCC	0.00	0.33	0.00	0.00	0.00

GATCAACTTGTCGCGGCTGC	0.92	0.00	0.00	0.34	0.34
GATCAACTTGTCGCGGCTGG	0.00	0.00	0.29	0.00	0.00
GATCAACTTGTCGTGTGAAA	0.23	0.00	0.00	0.00	0.00
GATCAACTTGTCGTGTGAAG	0.23	0.00	0.00	0.00	0.00
GATCAACTTGTCTTTGAGAC	0.23	0.00	0.00	0.00	0.00
GATCAACTTGTGAATGTTGA	0.00	0.00	0.00	0.34	0.00
GATCAACTTGTGACCAGCGG	0.00	0.00	0.00	0.00	0.34
GATCAACTTGTGATACGTCC	0.00	0.00	0.00	0.00	0.00
GATCAACTTGTGCAAAGGCA	0.00	0.00	0.00	0.00	0.00
GATCAACTTGTGCCTAGGGT	0.00	0.33	0.00	0.34	0.00
GATCAACTTGTGCGGTGGAA	0.00	0.33	0.00	0.00	0.00
GATCAACTTGTGCGTGCGAA	0.00	0.00	0.29	0.00	0.00
GATCAACTTGTGCTTCAGGA	0.00	0.00	0.00	0.00	0.00
GATCAACTTGTGGCTGGCCA	0.00	0.00	0.00	0.00	0.69
GATCAACTTGTGGGTAGTTC	0.00	0.00	0.00	0.00	0.34
GATCAACTTGTGTAGCTCGA	0.00	0.33	0.00	0.00	0.00
GATCAACTTGTGTGCCTCGT	0.23	0.00	0.00	0.00	0.00
GATCAACTTGTGTGTCCCAA	0.00	0.00	0.29	0.00	0.00
GATCAACTTGTGTTGTGTTT	0.00	0.00	0.29	0.00	0.00
GATCAACTTGTGTTTGAGAC	0.23	0.00	0.00	0.00	0.00
GATCAACTTGTTAATGCACC	0.00	0.00	0.00	0.00	0.00
GATCAACTTGTTCAAGTATT	0.00	0.00	0.00	0.00	0.00
GATCAACTTGTTCGTCCTCG	0.00	0.33	0.00	0.00	0.00
GATCAACTTGTTCGTCCTTG	0.00	0.00	0.00	0.00	1.03
GATCAACTTGTTGCAGTAGA	0.00	0.65	0.00	0.00	0.00
GATCAACTTGTTGGCTGCCC	0.00	0.65	0.00	0.00	0.34
GATCAACTTGTTGTCAACGC	0.00	0.33	0.00	0.00	0.00
GATCAACTTGTTGTTGGCCT	0.00	0.00	0.00	0.00	0.34
GATCAACTTGTTTATCTGGA	0.69	0.00	0.00	0.00	0.00
GATCAACTTGTTTGCATGCA	0.00	0.00	0.00	0.00	0.00
GATCAACTTGTTTGCGTCCA	0.00	0.00	0.00	0.00	0.00
GATCAACTTGTTTGTCCTGC	0.00	0.00	0.00	0.00	0.00
GATCAACTTGTTTTAATATT	0.00	0.00	0.29	0.00	0.00
GATCAACTTGTTTTTGAGAC	0.00	0.33	0.29	0.34	0.00
GATCAACTTTAAAAAAAAAA	0.00	0.00	0.00	0.00	0.00
GATCAACTTTACAAAGGATT	0.00	0.33	0.00	0.00	0.00
GATCAACTTTACAGATGCAC	0.00	0.00	0.00	0.00	0.34

GATCAACTTTACCATCACCA	0.00	0.00	0.00	0.00	0.69
GATCAACTTTACCTGCCACT	0.00	0.00	0.00	0.00	0.00
GATCAACTTTACCTGGCGCT	0.00	0.00	0.29	0.00	0.00
GATCAACTTTACTGAGTGCG	0.00	0.33	0.00	0.00	0.00
GATCAACTTTAGGCTTATGC	0.00	0.00	0.00	0.00	0.34
GATCAACTTTATCTCCCAAA	0.00	0.00	0.00	0.68	0.00
GATCAACTTTATCTCCCACA	8.70	8.78	11.45	5.77	5.85
GATCAACTTTATCTCCCANA	0.00	0.00	0.00	0.00	0.00
GATCAACTTTATCTCCCANN	0.00	0.00	0.00	0.00	0.00
GATCAACTTTATCTCCCATA	0.00	0.00	0.29	0.00	0.00
GATCAACTTTATCTGATGAG	0.00	0.00	0.00	0.00	0.34
GATCAACTTTATTGTGCCAG	1.60	5.21	1.15	4.75	4.13
GATCAACTTTATTGTGCCNN	0.00	0.00	0.00	0.00	0.00
GATCAACTTTCAAATGCGGA	0.23	0.00	0.00	0.00	0.00
GATCAACTTTCAAGACCAAG	0.00	0.33	0.00	0.34	0.00
GATCAACTTTCAGCGCGCTG	0.00	0.00	0.00	0.00	0.00
GATCAACTTTCAGGAGGCTC	0.00	0.00	0.00	1.36	0.00
GATCAACTTTCCACTCCAGA	0.00	0.00	0.00	0.68	0.00
GATCAACTTTCCACTCCAGC	0.46	1.30	0.57	0.00	2.07
GATCAACTTTCCCATCACCA	0.00	0.00	0.00	0.00	0.34
GATCAACTTTCCGCACAACC	0.46	0.33	0.29	1.36	1.03
GATCAACTTTCCGCCTGAGT	0.00	0.98	0.00	0.00	0.00
GATCAACTTTCCGGAGAAGC	1.83	3.25	2.00	1.36	1.72
GATCAACTTTCCGGAGCAGC	0.23	0.00	0.29	0.00	0.00
GATCAACTTTCGAGCTCGAC	0.00	0.00	0.00	0.00	0.00
GATCAACTTTCGAGCTCGCT	6.64	4.55	1.15	7.47	3.79
GATCAACTTTCGCAAGGCCC	0.00	0.00	0.00	0.00	0.00
GATCAACTTTCGCAGGGAAA	0.00	0.00	0.00	0.34	0.00
GATCAACTTTCGCAGGGCAA	0.00	0.00	0.00	0.34	0.00
GATCAACTTTCGCAGGGCAC	0.23	0.00	0.00	0.00	0.00
GATCAACTTTCGCAGGGCCA	0.46	0.00	0.00	0.00	0.00
GATCAACTTTCGCAGGGCCC	2.29	2.28	2.58	6.45	2.76
GATCAACTTTCGCAGGNCCC	0.00	0.00	0.00	0.00	0.34
GATCAACTTTCGCGAAAAAA	0.00	0.33	0.00	0.00	0.34
GATCAACTTTCGCGAAGTCT	0.00	0.00	0.00	0.00	0.00
GATCAACTTTCGCGTGAAAG	0.00	0.00	0.29	0.00	0.00
GATCAACTTTCGCTTCAAGG	0.00	0.00	0.00	0.00	0.00

GATCAACTTTCGGTGGGCTC	0.23	0.00	0.00	0.00	0.00
GATCAACTTTCGTCAATGCC	0.00	0.00	0.00	0.00	1.03
GATCAACTTTCGTTGTGCTT	0.00	0.00	0.00	0.00	0.34
GATCAACTTTCTCGGATGCA	0.46	0.00	0.00	0.00	0.00
GATCAACTTTCTGCGATTGG	0.23	0.00	0.00	0.00	0.00
GATCAACTTTCTGGCCCCGC	0.00	0.00	0.00	0.00	0.34
GATCAACTTTGAAAAAAAAA	0.00	0.00	0.00	0.34	0.00
GATCAACTTTGAAGAGGTGC	0.00	0.33	0.00	0.00	0.00
GATCAACTTTGAAGATATGC	0.00	0.00	0.00	0.00	0.00
GATCAACTTTGAAGATGAGC	0.00	0.33	0.29	0.00	0.34
GATCAACTTTGAAGATGGGC	0.00	0.00	0.29	0.00	0.00
GATCAACTTTGAAGATGTGA	0.00	0.33	0.57	0.00	0.00
GATCAACTTTGAAGATGTGC	13.05	66.04	62.71	93.34	73.01
GATCAACTTTGAAGATGTGN	0.23	0.00	0.00	0.00	0.00
GATCAACTTTGAAGATGTGT	0.00	0.00	0.29	0.00	0.00
GATCAACTTTGAAGATGTNN	0.23	0.00	0.00	0.00	0.00
GATCAACTTTGAAGATGTTC	0.00	0.33	0.00	0.00	0.00
GATCAACTTTGAAGATNTGC	0.00	0.00	0.00	0.34	0.69
GATCAACTTTGAAGATNTNC	0.00	0.00	0.29	0.00	0.00
GATCAACTTTGAAGCTGTGC	0.00	0.00	0.00	0.00	0.34
GATCAACTTTGAAGTTGTGC	0.00	0.00	0.00	0.00	0.69
GATCAACTTTGAATATGTGC	0.00	0.00	0.00	0.34	0.00
GATCAACTTTGACAACCTGG	0.00	0.00	0.00	1.70	0.00
GATCAACTTTGACAGGCAGA	0.00	0.00	0.00	0.34	0.00
GATCAACTTTGACATCATAG	0.00	0.00	0.00	0.00	0.34
GATCAACTTTGACATCATGA	0.00	0.33	0.00	0.00	0.34
GATCAACTTTGACATCCTAG	0.00	0.00	0.00	0.00	0.34
GATCAACTTTGACATGTGGA	0.46	0.00	0.00	0.00	0.00
GATCAACTTTGACATTGCAA	0.00	0.00	0.00	0.00	0.00
GATCAACTTTGACCTCATAG	8.01	8.78	9.45	8.15	7.23
GATCAACTTTGACCTCATAN	0.00	0.33	0.00	0.00	0.00
GATCAACTTTGACCTCATNN	0.23	0.00	0.00	0.00	0.00
GATCAACTTTGACGACCAGG	0.00	0.00	0.00	0.00	0.00
GATCAACTTTGACGTATGAC	0.00	0.00	0.00	0.34	0.00
GATCAACTTTGACTCAATGT	0.00	0.00	0.00	0.00	0.34
GATCAACTTTGACTGCCCAC	0.00	0.65	0.00	0.00	0.00
GATCAACTTTGACTGCCCTT	0.00	0.33	0.00	0.00	0.00

GATCAACTTTGAGCGATGCC	0.00	0.00	0.29	0.00	0.00
GATCAACTTTGAGCTCGGCT	0.00	0.33	0.00	0.00	0.34
GATCAACTTTGAGGAAACGG	0.00	0.65	0.00	0.00	0.00
GATCAACTTTGAGGAAAGAC	0.23	0.00	0.00	0.00	0.00
GATCAACTTTGAGGAGCGTG	0.00	0.00	0.00	0.00	0.00
GATCAACTTTGAGGCATGAC	0.00	0.00	0.00	0.34	0.00
GATCAACTTTGAGGGATGAC	0.92	0.00	0.00	0.00	0.00
GATCAACTTTGAGGGGTGAA	0.00	0.00	0.00	0.00	0.34
GATCAACTTTGAGGTACGAC	0.00	0.00	0.00	0.00	0.00
GATCAACTTTGAGGTAGGAC	0.46	0.00	0.00	0.00	0.00
GATCAACTTTGAGGTANGAC	0.00	0.00	0.00	0.00	0.34
GATCAACTTTGAGGTANNNN	0.46	0.00	0.00	0.00	0.00
GATCAACTTTGAGGTATAAC	0.46	0.00	0.00	0.00	0.00
GATCAACTTTGAGGTATGAA	0.23	0.33	0.29	1.02	0.00
GATCAACTTTGAGGTATGAC	310.17	185.44	60.42	180.58	95.05
GATCAACTTTGAGGTATGAN	1.14	0.00	0.00	0.00	0.00
GATCAACTTTGAGGTATGAT	0.46	0.00	0.00	0.68	0.00
GATCAACTTTGAGGTATGCC	0.23	0.00	0.00	0.00	0.00
GATCAACTTTGAGGTATGCN	0.69	0.00	0.00	0.00	0.00
GATCAACTTTGAGGTATGGC	1.37	0.33	0.00	0.00	0.00
GATCAACTTTGAGGTATGNC	0.00	0.00	0.00	0.34	0.00
GATCAACTTTGAGGTATGNN	1.60	0.00	0.00	0.00	0.00
GATCAACTTTGAGGTATGTC	0.23	0.00	0.00	0.00	0.00
GATCAACTTTGAGGTATTAC	0.00	0.33	0.00	0.00	0.00
GATCAACTTTGAGGTCTGAC	0.23	0.00	0.00	0.00	0.00
GATCAACTTTGAGGTGACTT	0.00	0.33	0.00	0.00	0.00
GATCAACTTTGAGTCATGTG	0.00	0.00	0.29	0.00	0.00
GATCAACTTTGAGTGCTTCC	0.00	0.98	0.00	0.34	0.34
GATCAACTTTGATAGACCGC	0.00	0.00	0.00	0.00	0.00
GATCAACTTTGATGATGTGC	0.00	0.00	0.29	0.00	0.00
GATCAACTTTGATGCACTCG	0.00	0.00	0.00	0.00	0.34
GATCAACTTTGATTCCTCGG	0.00	0.00	0.00	0.00	0.00
GATCAACTTTGATTGATGTT	0.00	0.00	0.00	0.34	0.00
GATCAACTTTGATTGGTTTG	0.00	0.00	0.29	0.68	0.00
GATCAACTTTGCAAAAAAAA	0.92	0.00	0.57	0.68	0.00
GATCAACTTTGCAAAGGTTC	0.00	0.00	0.29	0.00	0.00
GATCAACTTTGCAACGCTCA	0.00	0.00	0.00	0.00	0.00

GATCAACTTTGCAATGCCTT	0.00	0.00	0.00	0.34	0.00
GATCAACTTTGCACATGCGC	0.00	0.00	0.00	0.00	0.69
GATCAACTTTGCACATGCGG	0.00	0.00	0.00	0.00	0.34
GATCAACTTTGCACTGCACG	0.00	0.00	0.29	0.00	0.00
GATCAACTTTGCAGAGGAGG	0.00	0.65	0.29	1.02	0.00
GATCAACTTTGCAGATCAGG	0.00	0.00	0.00	0.00	0.00
GATCAACTTTGCAGATGTGC	0.00	0.33	0.00	0.00	0.00
GATCAACTTTGCAGGTCAAA	0.00	0.00	0.57	0.00	0.34
GATCAACTTTGCAGGTCTAA	0.23	0.00	0.00	0.00	0.00
GATCAACTTTGCAGGTCTAC	1.83	0.33	0.00	0.00	0.00
GATCAACTTTGCAGGTNNNN	0.23	0.00	0.00	0.00	0.00
GATCAACTTTGCAGTTTGTT	0.00	0.00	0.29	0.00	0.00
GATCAACTTTGCCAACCCCG	0.00	0.33	0.00	0.00	0.00
GATCAACTTTGCCAACGCGA	0.00	0.00	0.00	1.70	0.00
GATCAACTTTGCCAAGACTG	0.23	0.00	0.00	0.00	0.00
GATCAACTTTGCCACCCAAG	0.00	0.00	0.00	0.00	0.34
GATCAACTTTGCCCCTGCGC	0.00	0.00	0.29	0.00	0.00
GATCAACTTTGCCGCCGCTT	0.00	0.00	0.00	0.00	0.00
GATCAACTTTGCCGGGTGAG	2.29	0.98	0.86	1.02	0.34
GATCAACTTTGCCTCCCGAG	0.00	0.00	0.00	0.00	0.00
GATCAACTTTGCCTCCTCCC	1.14	0.98	0.57	0.68	0.34
GATCAACTTTGCGAAGATGT	0.00	0.33	0.29	0.00	0.00
GATCAACTTTGCGACCGATG	0.00	0.00	0.00	0.34	0.00
GATCAACTTTGCGAGGGAGT	0.23	0.00	0.00	0.00	0.00
GATCAACTTTGCGCAAGCAG	0.46	0.33	0.86	0.00	0.34
GATCAACTTTGCGCACGTGA	0.00	0.65	0.00	0.00	0.00
GATCAACTTTGCGCCGAAGG	0.00	0.00	0.00	0.00	0.00
GATCAACTTTGCGCGCAGGG	0.00	0.98	0.00	0.00	0.00
GATCAACTTTGCGCGCTTGC	0.00	0.00	0.29	0.00	0.00
GATCAACTTTGCGGAAATCT	0.23	0.00	0.00	0.00	0.00
GATCAACTTTGCGGACATCT	23.35	11.71	2.58	7.13	1.38
GATCAACTTTGCGGACATNN	0.23	0.00	0.00	0.00	0.00
GATCAACTTTGCGGACTCGT	0.23	0.00	0.00	0.00	0.00
GATCAACTTTGCGGCAAAGG	0.46	0.00	0.00	0.00	0.00
GATCAACTTTGCGGGCGCCT	0.00	0.98	0.00	0.00	0.00
GATCAACTTTGCGGTAACGG	0.00	0.00	0.00	0.68	0.00
GATCAACTTTGCGGTATGAC	0.23	0.00	0.00	0.00	0.00

GATCAACTTTGCGGTCAGCT	0.00	0.33	0.00	0.00	0.00
GATCAACTTTGCGGTCTGCC	0.23	0.00	0.00	0.00	0.00
GATCAACTTTGCGGTGTGCC	0.23	0.00	0.00	0.00	0.00
GATCAACTTTGCGTCCTACA	0.00	0.00	0.00	0.00	0.00
GATCAACTTTGCTTGCGAGC	2.98	4.23	2.86	4.07	1.38
GATCAACTTTGCTTGCGCGC	0.00	0.33	0.29	0.00	0.00
GATCAACTTTGCTTGCGGTC	0.00	0.33	0.00	0.00	0.00
GATCAACTTTGCTTGCNAGC	0.00	0.00	0.00	0.00	0.34
GATCAACTTTGCTTGCTGGC	0.00	0.33	0.00	0.00	0.00
GATCAACTTTGGACATAGAG	0.23	0.00	0.29	0.00	0.00
GATCAACTTTGGACCCAGAG	0.23	0.00	0.00	0.00	0.34
GATCAACTTTGGACCCNGNN	0.23	0.00	0.00	0.00	0.00
GATCAACTTTGGACCTAGAG	71.42	63.76	39.23	66.19	49.94
GATCAACTTTGGACCTAGAN	0.00	0.00	0.00	0.00	0.00
GATCAACTTTGGACCTAGAT	0.23	0.00	0.29	0.00	0.00
GATCAACTTTGGACCTAGCG	0.23	0.00	0.00	0.34	0.00
GATCAACTTTGGACCTAGNN	0.23	0.00	0.00	0.00	0.00
GATCAACTTTGGACCTANNN	0.00	0.00	0.00	0.00	0.00
GATCAACTTTGGACCTCGAG	0.23	0.33	0.00	0.00	0.34
GATCAACTTTGGACCTGGAG	0.23	0.00	0.00	0.00	0.34
GATCAACTTTGGACCTNNNN	0.23	0.00	0.00	0.00	0.00
GATCAACTTTGGACTGAGTC	0.00	0.33	0.00	0.00	0.00
GATCAACTTTGGACTTAGAG	0.00	0.00	0.00	0.34	0.00
GATCAACTTTGGACTTGTTC	0.00	0.00	0.00	0.34	0.00
GATCAACTTTGGAGAACTCT	0.00	0.00	0.00	0.34	0.00
GATCAACTTTGGAGAGGCCC	0.00	0.00	0.00	0.34	0.00
GATCAACTTTGGAGATGTGC	0.00	0.00	0.29	0.00	0.00
GATCAACTTTGGAGCAGCTG	0.00	0.00	0.00	0.00	0.00
GATCAACTTTGGCACGAAGC	0.00	0.00	0.00	0.00	0.00
GATCAACTTTGGCACTCCTG	0.23	0.00	0.00	0.00	0.00
GATCAACTTTGGCAGGCTCA	0.00	0.00	0.29	0.00	0.00
GATCAACTTTGGCATCAAGG	0.00	0.33	1.43	0.68	0.69
GATCAACTTTGGCCCATTTT	0.00	0.33	0.00	0.00	0.00
GATCAACTTTGGCCCTGCAG	0.00	0.00	0.00	0.00	0.00
GATCAACTTTGGCCTATTTT	0.00	0.00	0.57	0.00	0.00
GATCAACTTTGGCCTGTGCG	5.04	10.41	7.45	5.09	8.27
GATCAACTTTGGCTGATTGC	0.46	0.00	0.00	0.00	0.00

GATCAACTTTGGGACCTGCC	0.00	0.33	0.00	0.00	0.00
GATCAACTTTGGGCAGTCCC	0.00	0.65	0.29	0.00	0.00
GATCAACTTTGGGCCTAGAG	0.23	0.00	0.00	0.00	0.00
GATCAACTTTGGGGGCGTGC	0.00	0.00	0.57	0.00	0.00
GATCAACTTTGGGGTATGAC	0.46	0.00	0.00	0.00	0.00
GATCAACTTTGGGTATGTCA	0.00	0.00	0.00	0.34	0.00
GATCAACTTTGGTCGTATGC	0.00	0.00	0.00	0.00	0.34
GATCAACTTTGGTGAGGCCT	0.00	0.00	0.00	0.00	1.38
GATCAACTTTGGTGGACCAC	0.23	0.98	0.29	0.00	0.00
GATCAACTTTGTAACTAGAG	0.23	0.00	0.00	0.00	0.00
GATCAACTTTGTAGATGTGC	0.00	0.33	0.00	0.00	0.00
GATCAACTTTGTCATTGTCA	0.46	0.00	0.00	0.00	0.00
GATCAACTTTGTGAAGAACG	0.00	0.00	0.00	0.34	0.00
GATCAACTTTGTGACCAATA	0.00	0.33	0.00	0.00	1.03
GATCAACTTTGTGACCGGCC	0.00	0.33	0.00	0.00	0.00
GATCAACTTTGTGACCNGCC	0.00	0.00	0.00	0.00	0.34
GATCAACTTTGTGACCTACC	0.00	0.00	0.00	0.00	0.34
GATCAACTTTGTGACCTGAA	0.00	0.00	0.00	0.00	0.00
GATCAACTTTGTGACCTGCA	0.00	0.00	0.00	0.68	0.34
GATCAACTTTGTGACCTGCC	28.38	30.91	23.48	41.41	38.57
GATCAACTTTGTGACCTGCN	0.23	0.00	0.00	0.00	0.00
GATCAACTTTGTGACCTGCT	0.00	0.98	0.29	0.00	0.00
GATCAACTTTGTGACCTGNN	0.46	0.00	0.00	0.00	0.00
GATCAACTTTGTGACGAATA	0.00	0.00	0.00	0.34	0.00
GATCAACTTTGTGACTCGCG	0.00	0.00	0.00	0.68	0.00
GATCAACTTTGTGACTTGCC	0.00	0.00	0.29	0.00	0.00
GATCAACTTTGTGAGCTGCC	0.00	0.00	0.00	0.00	0.34
GATCAACTTTGTGCCCTGCC	0.23	0.00	0.00	0.00	0.00
GATCAACTTTGTGGAGTACC	0.23	0.00	0.29	0.00	0.34
GATCAACTTTGTGGCCTGCG	0.23	0.00	0.00	0.00	0.00
GATCAACTTTGTGGGATTGC	0.23	0.00	0.00	0.00	0.00
GATCAACTTTGTGGTATGAA	0.23	0.00	0.00	0.00	0.00
GATCAACTTTGTGGTATGAC	0.23	0.00	0.29	0.00	0.00
GATCAACTTTGTGTCTGCTG	0.00	0.00	0.00	0.00	0.00
GATCAACTTTGTTACCTGCC	0.00	0.33	0.00	0.00	0.00
GATCAACTTTGTTCTGTTCT	0.00	0.00	0.29	0.00	0.69
GATCAACTTTGTTGAGACAG	0.00	0.33	0.00	0.00	0.00

GATCAACTTTGTTGAGCCAG	0.00	0.00	0.29	0.00	0.00
GATCAACTTTGTTGGCTTAT	0.00	0.00	0.00	0.00	0.00
GATCAACTTTGTTGGCTTTA	0.00	0.00	0.29	0.00	0.00
GATCAACTTTTACCGGCGCT	0.23	0.00	0.00	0.00	0.00
GATCAACTTTTATTGGAGGA	0.00	0.00	0.00	0.00	0.34
GATCAACTTTTATTGGATGA	0.23	0.00	0.00	0.00	0.00
GATCAACTTTTATTTGAGTA	0.00	0.00	0.00	0.34	0.00
GATCAACTTTTCATGATTGC	0.00	0.00	0.00	0.34	0.00
GATCAACTTTTCCGCACCGC	0.00	0.00	0.00	0.00	0.00
GATCAACTTTTCCTCCTCCC	0.46	0.00	0.00	0.00	0.00
GATCAACTTTTCGCCGTCCG	0.00	0.00	0.00	0.00	0.00
GATCAACTTTTCGGCCATCT	0.00	0.00	0.00	0.00	0.00
GATCAACTTTTCTCTTCTTT	2.75	0.98	1.15	2.72	2.07
GATCAACTTTTCTTGGCGGA	0.00	0.00	0.00	0.00	0.00
GATCAACTTTTGAACTGCGG	0.00	0.65	0.00	0.00	0.00
GATCAACTTTTGAAGTTCTG	0.00	0.00	0.00	0.34	0.00
GATCAACTTTTGAATCCAGT	0.00	0.00	0.00	0.00	0.00
GATCAACTTTTGAATTCCCT	1.60	2.60	0.57	3.05	0.00
GATCAACTTTTGACCCCGGC	0.00	0.00	0.00	0.34	0.00
GATCAACTTTTGACCTAGAG	0.23	0.00	0.00	0.00	0.00
GATCAACTTTTGATGCATGG	1.37	2.28	1.15	0.00	2.41
GATCAACTTTTGATGCGGAG	0.00	0.00	0.29	0.00	0.00
GATCAACTTTTGCAACGTCG	0.00	0.00	0.00	0.00	0.00
GATCAACTTTTGGAACACGT	0.00	0.00	0.00	0.00	0.00
GATCAACTTTTGGCGAGGCG	0.23	0.00	0.00	0.00	0.00
GATCAACTTTTGGGGATGTT	0.00	0.00	0.00	0.00	0.00
GATCAACTTTTGGGTTCAAG	0.00	0.00	0.00	0.00	0.00
GATCAACTTTTGGGTTCCGC	1.83	2.28	2.00	1.70	0.00
GATCAACTTTTGGGTTNCGC	0.00	0.00	0.00	0.00	0.34
GATCAACTTTTGGTGGAGGG	0.00	0.00	0.00	0.00	0.00
GATCAACTTTTGTACCAGTT	0.92	0.33	0.00	0.68	0.34
GATCAACTTTTTAAACTGTT	0.00	0.00	0.00	0.00	1.03
GATCAACTTTTTCATGGACT	0.00	0.00	0.00	0.34	0.00
GATCAACTTTTTCCCCCTCC	0.00	0.00	0.29	0.00	0.00
GATCAACTTTTTCGAAGCGG	0.00	0.00	0.29	0.00	0.00
GATCAACTTTTTCGCACAGA	0.00	0.00	0.00	0.00	0.34
GATCAACTTTTTGAGACCAT	0.00	0.00	0.00	0.00	0.34

GATCAACTTTTTGCAAAGCC	0.00	0.33	0.00	0.00	0.00
GATCAACTTTTTGCAGCATG	0.00	0.33	0.29	0.00	0.00
GATCAACTTTTTGGAGGAAG	3.20	1.63	0.57	1.02	1.72
GATCAACTTTTTGGAGGAGC	0.00	0.00	0.00	0.34	0.00
GATCAACTTTTTGGGGGAAG	0.23	0.00	0.00	0.00	0.00
GATCAACTTTTTGTCTTGTT	0.00	0.00	0.00	0.00	0.34
GATCAACTTTTTTATAAAGG	0.00	0.00	0.00	0.00	0.00
GATCAACTTTTTTGAGAACC	0.00	0.00	0.00	0.00	0.00
GATCAACTTTTTTTCCATAG	0.00	0.00	0.00	0.00	0.00
GATCAAGAAAAAAAAAAAAA	96.83	25.70	36.65	49.56	56.83
GATCAAGAAAAAAAAAAAAC	0.23	0.33	0.00	0.00	0.00
GATCAAGAAAAAAAAAAAAG	0.46	0.00	0.00	0.00	0.00
GATCAAGAAAAAAAAAAAAN	0.46	0.00	0.00	0.00	0.00
GATCAAGAAAAAAAAAAAAT	8.93	2.60	5.15	6.11	6.54
GATCAAGAAAAAAAAAAACA	0.00	0.00	0.00	0.00	0.69
GATCAAGAAAAAAAAAAACN	0.23	0.00	0.00	0.00	0.00
GATCAAGAAAAAAAAAAAGA	0.23	0.00	0.29	0.00	0.00
GATCAAGAAAAAAAAAAAGT	0.23	0.00	0.00	0.00	0.00
GATCAAGAAAAAAAAAAANN	0.46	0.00	0.00	0.00	0.00
GATCAAGAAAAAAAAAAATC	0.46	0.65	0.29	0.00	0.00
GATCAAGAAAAAAAAAACAA	0.00	0.00	0.00	0.00	0.34
GATCAAGAAAAAAAAAAGAA	0.46	0.00	0.86	0.34	0.00
GATCAAGAAAAAAAAAAGAT	0.00	0.00	0.29	0.00	0.00
GATCAAGAAAAAAAAAATCG	0.00	0.00	0.00	0.00	0.34
GATCAAGAAAAAAAAACAAT	0.00	0.33	0.00	0.00	0.00
GATCAAGAAAAAAAAACACA	0.00	0.33	0.00	0.00	0.00
GATCAAGAAAAAAAAAGAAA	0.23	0.33	0.57	0.00	0.34
GATCAAGAAAAAAAAANNNN	0.23	0.00	0.00	0.00	0.00
GATCAAGAAAAAAAAATAAA	0.00	0.00	0.29	0.00	0.00
GATCAAGAAAAAAAACAAAA	0.00	0.00	0.00	0.00	0.34
GATCAAGAAAAAAAACATCC	0.23	0.00	0.00	0.00	0.00
GATCAAGAAAAAAAACNNNN	0.23	0.00	0.00	0.00	0.00
GATCAAGAAAAAAAAGAAAA	0.00	0.33	1.15	0.00	0.00
GATCAAGAAAAAAAGAAAAA	0.00	0.00	0.00	0.34	0.00
GATCAAGAAAAAACAAGAAT	0.23	0.00	0.00	0.00	0.00
GATCAAGAAAAAACAATGTC	0.00	0.00	0.00	0.00	0.00
GATCAAGAAAAAAGAAAAAA	0.00	0.00	0.00	0.00	0.00

GATCAAGAAAAAAGAAACAA	0.00	0.00	0.00	0.00	0.34
GATCAAGAAAAAAGGGCAGA	0.00	0.00	0.00	0.00	0.00
GATCAAGAAAAAATAAATGA	0.00	0.00	0.29	0.00	0.00
GATCAAGAAAAACAAAAAAA	1.37	0.00	0.00	0.00	0.34
GATCAAGAAAAACAAGGTGG	0.00	0.00	0.00	0.00	0.69
GATCAAGAAAAACATGTCTA	0.00	0.00	0.29	0.00	0.00
GATCAAGAAAAAGAAAAAAA	0.46	0.33	0.00	0.00	0.00
GATCAAGAAAAAGAAACAAG	0.00	0.00	0.00	0.00	0.34
GATCAAGAAAAAGAACAAGT	0.00	0.00	0.29	0.00	0.00
GATCAAGAAAAAGACTAAGA	0.00	0.00	0.00	0.00	0.00
GATCAAGAAAAAGAGAAGAG	0.00	0.00	0.57	0.00	0.34
GATCAAGAAAAAGATCGTAT	0.00	0.00	0.29	0.00	0.00
GATCAAGAAAAAGATGAAGG	0.00	0.00	0.00	0.34	0.00
GATCAAGAAAAAGATGAATG	0.00	0.00	0.00	0.00	0.00
GATCAAGAAAAAGATGTAGG	0.00	0.00	0.00	0.00	0.00
GATCAAGAAAAAGTGATTTT	0.00	0.00	0.29	0.00	0.00
GATCAAGAAAAATAAGGACA	0.46	0.00	0.00	0.00	0.00
GATCAAGAAAAATCAGCGCG	0.00	0.33	0.00	0.00	0.00
GATCAAGAAAACAAAAAAAA	8.47	2.28	3.15	5.09	3.44
GATCAAGAAAACAAAAAAAC	0.23	0.00	0.00	0.00	0.00
GATCAAGAAAACAAAAAACA	0.00	0.00	0.00	0.00	0.00
GATCAAGAAAACAAAAACAA	0.00	0.00	0.00	0.34	0.00
GATCAAGAAAACAAAANNNN	0.23	0.00	0.00	0.00	0.00
GATCAAGAAAACAAAATGAA	0.23	0.00	0.00	0.00	0.00
GATCAAGAAAACAAAATGGA	0.00	0.00	0.00	0.00	0.00
GATCAAGAAAACAAACAGCG	0.00	0.00	0.00	0.68	0.00
GATCAAGAAAACAAATTCGA	3.20	0.33	0.00	0.68	0.69
GATCAAGAAAACAAATTCGT	0.00	0.00	0.00	0.34	0.00
GATCAAGAAAACAAGAAAAA	1.83	0.33	0.00	0.34	1.38
GATCAAGAAAACAAGAAATA	0.00	0.00	0.00	0.00	0.34
GATCAAGAAAACAAGAATAA	0.00	0.00	0.29	0.00	0.00
GATCAAGAAAACAAGAATCC	0.00	0.65	0.00	0.00	1.03
GATCAAGAAAACAAGAATGA	0.00	0.00	0.29	0.00	0.00
GATCAAGAAAACAAGAATNN	0.23	0.00	0.00	0.00	0.00
GATCAAGAAAACAAGAATTA	54.02	12.04	22.62	19.35	59.58
GATCAAGAAAACAAGAATTC	0.46	0.00	0.29	0.34	0.34
GATCAAGAAAACAAGACTTA	0.00	0.00	0.00	0.00	0.34

GATCAAGAAAACAAGANTTA	0.00	0.00	0.00	0.00	1.03
GATCAAGAAAACAATAACTA	0.00	0.00	0.00	0.00	0.34
GATCAAGAAAACAATTGTCT	0.00	0.00	0.00	0.00	0.00
GATCAAGAAAACACAAACAA	0.23	0.00	0.00	0.00	0.00
GATCAAGAAAACACGCATCA	0.00	0.00	0.00	0.00	0.34
GATCAAGAAAACCCCATCTG	0.00	0.00	0.00	0.00	0.00
GATCAAGAAAACGCAACGAG	0.00	0.00	0.29	0.00	0.00
GATCAAGAAAAGAAAAAAAA	0.00	0.00	0.00	0.00	0.34
GATCAAGAAAAGAAAGAAAA	0.00	0.00	0.00	0.00	0.00
GATCAAGAAAAGAACAAAGT	1.83	0.00	0.00	0.00	0.00
GATCAAGAAAAGAAGAGAAA	0.00	0.33	0.00	0.00	0.00
GATCAAGAAAAGACGCAAAA	0.00	0.00	0.29	0.00	0.00
GATCAAGAAAAGACGCGATT	0.46	0.65	0.57	0.68	0.69
GATCAAGAAAAGACGCTCGT	0.23	0.00	0.00	0.00	0.00
GATCAAGAAAAGAGGCAAGG	0.00	0.00	0.00	0.00	0.69
GATCAAGAAAAGGAAAAAAA	0.00	0.00	0.00	0.34	1.03
GATCAAGAAAAGGAAGAGGA	0.00	0.00	0.29	0.00	2.76
GATCAAGAAAAGGAGAAGGA	0.00	0.00	0.29	0.00	2.07
GATCAAGAAAAGGAGCAGGC	0.00	0.00	0.00	0.00	0.34
GATCAAGAAAAGGCATGGAT	0.00	0.65	0.00	0.68	0.00
GATCAAGAAAAGGCGAAGGG	0.00	0.00	0.86	0.00	0.00
GATCAAGAAAAGGGGAGGAA	0.00	0.00	0.29	0.00	0.00
GATCAAGAAAAGGTGCCGTT	0.00	0.00	0.29	0.00	0.00
GATCAAGAAAAGGTGTCAGG	0.00	0.00	0.00	0.00	0.34
GATCAAGAAAAGTGGCGCGG	0.00	0.00	0.00	0.34	0.00
GATCAAGAAAAGTTCCGACC	2.75	2.60	0.00	1.36	0.00
GATCAAGAAAATAGGAAGAC	0.00	0.00	0.29	0.00	0.69
GATCAAGAAAATAGGAAGGC	0.00	0.00	0.29	0.00	0.00
GATCAAGAAAATCAAGAAAC	0.00	0.00	0.00	0.00	0.34
GATCAAGAAAATCAAGGGGG	0.00	0.00	0.00	0.00	0.34
GATCAAGAAAATCAAGGGNG	0.00	0.00	0.00	0.00	0.00
GATCAAGAAAATCAGCAAGT	0.00	0.00	0.00	0.34	0.00
GATCAAGAAAATCTGGCAAG	0.00	0.33	0.00	0.00	1.03
GATCAAGAAAATGAAGTCGA	0.00	0.00	0.00	0.00	0.34
GATCAAGAAAATGACTTTCG	0.00	0.00	0.00	0.00	0.00
GATCAAGAAAATGCACTTGG	0.00	0.00	0.00	0.00	0.00
GATCAAGAAAATGGAGGACC	0.00	0.33	0.00	0.00	0.00

GATCAAGAAAATGGTGGACG	0.00	0.00	0.29	0.00	0.00
GATCAAGAAACAAAAAAAAA	0.00	0.00	0.00	0.00	0.34
GATCAAGAAACAAAAAACAA	0.00	0.00	0.00	0.00	0.00
GATCAAGAAACAAAAACCAA	0.00	0.00	0.00	0.00	0.00
GATCAAGAAACAAACACAAA	0.00	0.00	0.00	0.00	0.00
GATCAAGAAACAAACTTGTT	0.00	0.00	0.00	0.00	0.34
GATCAAGAAACACAACACAA	0.00	0.00	0.00	0.00	0.34
GATCAAGAAACACAAGCCAA	0.00	0.00	0.00	0.00	0.34
GATCAAGAAACACTTTGACG	0.00	0.65	0.29	0.34	1.38
GATCAAGAAACAGAAGGACA	0.00	0.00	0.29	0.00	0.00
GATCAAGAAACAGCAGAGGG	0.00	0.00	0.29	0.00	0.00
GATCAAGAAACAGCTCAACC	0.00	0.00	0.00	0.00	0.34
GATCAAGAAACATGGCAGCG	0.00	0.00	0.00	0.34	0.00
GATCAAGAAACCAAGAAAAA	0.23	0.00	0.00	0.00	0.00
GATCAAGAAACCACCCGCGT	0.23	0.00	0.00	0.00	0.00
GATCAAGAAACCACGTGCAT	0.00	0.00	0.00	0.00	0.34
GATCAAGAAACCATCCAGAG	0.00	0.00	0.00	0.00	0.34
GATCAAGAAACCATTGCAAA	0.00	0.00	0.29	0.00	0.00
GATCAAGAAACCCCCACCCC	0.23	0.00	0.00	0.00	0.00
GATCAAGAAACCCGCTCGAG	0.23	0.00	0.00	0.00	0.00
GATCAAGAAACCGAGTGTTG	0.00	0.33	0.00	0.00	0.00
GATCAAGAAACCGGGCACTC	0.00	0.00	0.29	0.00	0.00
GATCAAGAAACCTCGACGAG	0.00	0.00	0.00	0.00	0.00
GATCAAGAAACCTGACAGCT	0.00	0.00	0.00	0.00	0.00
GATCAAGAAACCTGGTGCCA	0.00	0.00	0.29	0.00	0.00
GATCAAGAAACCTTGCCACA	0.00	0.33	0.00	0.00	0.00
GATCAAGAAACCTTGGTTTC	0.00	0.00	0.29	0.00	0.00
GATCAAGAAACGAATATCAT	0.23	0.00	0.00	0.00	0.00
GATCAAGAAACGCAGCAAAA	0.23	0.00	0.00	0.00	0.00
GATCAAGAAACGCAGCGCCG	0.00	0.00	0.00	0.00	0.00
GATCAAGAAACGCAGTGCCG	0.00	0.00	0.00	0.34	0.00
GATCAAGAAACGCCAACGCC	0.23	0.33	0.00	0.34	0.00
GATCAAGAAACGCCAACGGT	0.23	0.00	0.00	0.00	0.00
GATCAAGAAACGCCCTGGTA	0.00	0.00	0.29	0.00	0.00
GATCAAGAAACGGCGGGCCA	0.23	0.00	0.00	0.00	0.00
GATCAAGAAACGGGCAAGCT	0.69	0.33	0.29	0.68	0.34
GATCAAGAAACGGGCTGAGC	0.00	0.00	0.00	0.34	0.00

GATCAAGAAACGTAGGAAAA	0.00	0.00	0.00	0.34	0.00
GATCAAGAAACTACGAATAA	0.00	0.00	0.00	0.00	0.69
GATCAAGAAACTCATGTGAG	0.00	0.00	0.29	0.00	0.00
GATCAAGAAACTCTTGAAGA	0.69	0.00	0.29	0.00	0.00
GATCAAGAAACTGAAAGTGC	0.23	0.00	0.00	0.34	0.00
GATCAAGAAACTGAACGACC	0.00	0.00	0.00	0.00	0.00
GATCAAGAAACTGAACTTCC	0.00	0.00	0.00	0.34	0.00
GATCAAGAAACTGATGAAGA	0.00	0.33	0.00	0.00	0.00
GATCAAGAAACTGCAGGCTG	0.00	0.33	0.00	0.00	0.00
GATCAAGAAACTTAGGGCAG	0.00	0.65	0.00	0.00	0.00
GATCAAGAAAGAAAAAAAAA	0.00	0.00	0.00	0.34	0.00
GATCAAGAAAGAAAAGAAAA	0.00	0.00	0.00	0.00	0.00
GATCAAGAAAGAAAAGACAA	0.00	0.00	0.57	0.00	0.00
GATCAAGAAAGAACAGAAGG	0.69	0.00	0.57	0.00	0.00
GATCAAGAAAGAAGAAAAAA	0.00	0.00	0.29	0.00	0.00
GATCAAGAAAGAAGTGCGTG	0.00	0.33	0.00	0.00	0.00
GATCAAGAAAGACAAGGAGC	0.00	0.00	0.29	0.00	0.00
GATCAAGAAAGACAGGATGC	0.00	0.00	0.29	0.00	0.00
GATCAAGAAAGACCAAGAGG	0.00	0.00	0.29	0.00	0.00
GATCAAGAAAGACGAGTTGG	0.00	0.00	0.00	0.00	0.00
GATCAAGAAAGACGGGCAGC	0.00	0.00	0.00	0.00	0.00
GATCAAGAAAGACGTGCATG	0.00	0.65	0.00	0.00	0.00
GATCAAGAAAGACTAAGAGA	0.00	0.00	0.00	0.00	1.03
GATCAAGAAAGACTACTCGA	0.00	0.00	0.00	0.00	0.34
GATCAAGAAAGACTCCGCTC	0.00	0.00	0.00	0.34	0.00
GATCAAGAAAGACTTCGCAG	0.00	0.33	0.29	0.68	1.03
GATCAAGAAAGACTTCGCTG	0.00	0.00	0.00	0.34	0.00
GATCAAGAAAGACTTGCTTG	0.00	0.00	0.00	0.00	0.00
GATCAAGAAAGAGAAAGAGA	0.00	0.00	0.86	0.00	0.00
GATCAAGAAAGAGAAGAGAC	0.00	0.00	0.00	0.00	0.34
GATCAAGAAAGAGAGGCTGC	0.00	0.33	0.00	0.68	0.00
GATCAAGAAAGAGAGGCTGG	0.00	0.00	0.00	1.02	0.00
GATCAAGAAAGAGAGGCTTG	0.00	0.00	0.00	0.00	0.00
GATCAAGAAAGAGGAGTTGA	0.92	0.33	0.00	0.00	0.34
GATCAAGAAAGAGGGGATGG	0.00	0.00	0.00	0.00	1.03
GATCAAGAAAGATAAACAGG	0.00	0.00	0.00	0.00	0.00
GATCAAGAAAGATAAGAAGA	0.00	0.00	0.29	0.00	0.00

GATCAAGAAAGATAGAAAAA	0.00	0.00	0.29	0.00	0.00
GATCAAGAAAGATATCAAGA	0.00	0.00	0.29	0.00	0.00
GATCAAGAAAGATATTGACA	0.00	0.00	0.00	0.00	0.00
GATCAAGAAAGATATTGCCA	0.00	0.00	0.00	0.00	0.00
GATCAAGAAAGCAAGAATTA	0.23	0.00	0.00	0.00	0.00
GATCAAGAAAGCAGACTTGC	0.00	0.00	0.00	0.00	0.00
GATCAAGAAAGCAGCAGCAC	0.23	0.00	0.00	0.00	0.00
GATCAAGAAAGCAGCGGCAC	0.00	0.00	0.00	0.00	0.34
GATCAAGAAAGCAGCGGCCT	0.00	0.00	0.29	0.00	0.00
GATCAAGAAAGCAGCGGGCA	0.00	0.00	0.00	0.00	0.34
GATCAAGAAAGCAGCGGGCC	0.69	1.95	0.86	0.34	5.51
GATCAAGAAAGCAGCTGAGG	0.00	0.00	0.00	0.34	0.34
GATCAAGAAAGCAGGATAAG	0.00	0.00	0.00	0.00	0.00
GATCAAGAAAGCGACGTGTG	0.23	0.00	0.00	0.00	0.00
GATCAAGAAAGCGAGGAGGA	0.00	0.00	0.00	0.00	0.34
GATCAAGAAAGCGAGTGGCC	0.00	0.00	0.00	0.00	0.00
GATCAAGAAAGCGCGAGAGG	0.00	0.00	0.00	0.34	0.00
GATCAAGAAAGCGGAAGTGC	0.00	0.33	0.00	0.00	0.00
GATCAAGAAAGCTACGATGT	0.00	0.00	0.29	0.00	0.00
GATCAAGAAAGCTCATCCTC	0.00	0.65	0.00	0.68	0.69
GATCAAGAAAGCTCCCTCGG	0.00	0.00	0.00	0.00	0.69
GATCAAGAAAGCTGCCAGGC	0.00	0.00	0.00	0.34	0.00
GATCAAGAAAGCTGGCTGCG	0.00	0.00	0.00	0.34	0.00
GATCAAGAAAGCTTGTCTCT	0.23	0.00	0.00	0.00	0.00
GATCAAGAAAGGAAAAAAAA	0.00	0.00	1.72	0.00	0.00
GATCAAGAAAGGAACGCCAA	0.46	0.33	0.00	0.00	0.34
GATCAAGAAAGGACGCCTCG	0.00	0.00	0.00	0.00	0.34
GATCAAGAAAGGACGGGCGG	0.23	0.00	0.00	0.00	0.00
GATCAAGAAAGGAGGCCTCG	0.00	0.00	0.00	0.00	0.00
GATCAAGAAAGGATGAGGGA	0.00	0.00	0.29	0.00	0.00
GATCAAGAAAGGCAAAAAAA	0.46	0.00	0.00	1.36	0.69
GATCAAGAAAGGCAAGGGAA	0.92	0.98	0.57	1.36	1.72
GATCAAGAAAGGCAAGGGCA	0.23	0.00	0.00	0.00	0.00
GATCAAGAAAGGCAATCGTA	0.00	0.00	0.00	0.34	0.00
GATCAAGAAAGGCAGATTGG	0.00	0.00	0.29	0.00	0.00
GATCAAGAAAGGCAGGGAAC	0.00	0.33	0.00	0.00	0.00
GATCAAGAAAGGCATGCAAC	0.00	0.00	0.00	0.00	0.00

GATCAAGAAAGGCCAGGGAN	0.00	0.00	0.00	0.34	0.00
GATCAAGAAAGGCCAGTTGC	0.23	0.00	0.00	0.00	0.00
GATCAAGAAAGGCGAAAAAA	0.46	0.98	0.86	0.34	0.00
GATCAAGAAAGGCGAAAAAC	0.00	0.33	0.00	0.00	0.00
GATCAAGAAAGGCGAAAAAG	0.00	0.65	0.00	0.00	0.00
GATCAAGAAAGGCGAAGGGA	0.00	0.00	0.00	0.00	0.69
GATCAAGAAAGGCGAGTGGC	0.00	0.00	0.00	0.34	0.00
GATCAAGAAAGGCGAGTTGC	2.29	0.65	0.57	0.34	1.38
GATCAAGAAAGGCGATAAAA	0.00	0.00	0.00	0.00	0.00
GATCAAGAAAGGCGGAAAAA	0.00	0.00	0.00	0.34	0.00
GATCAAGAAAGGCGTGAAAA	0.00	0.65	0.00	0.00	0.00
GATCAAGAAAGGCGTGGTTG	0.00	0.00	0.29	0.00	0.00
GATCAAGAAAGGCTCAAATC	0.00	0.00	0.00	0.00	0.00
GATCAAGAAAGGCTTCAAAG	0.00	0.33	0.00	0.00	1.03
GATCAAGAAAGGGACGGATC	0.00	0.00	0.57	0.00	0.00
GATCAAGAAAGGGATAAAAA	0.00	0.00	0.00	0.34	0.00
GATCAAGAAAGGGGAGGACC	0.00	0.33	0.00	2.72	0.00
GATCAAGAAAGGGGAGGACT	0.00	0.00	0.00	0.00	0.00
GATCAAGAAAGGGGATTGCC	0.00	0.00	0.29	0.00	0.00
GATCAAGAAAGGGGTGGACG	0.00	0.00	0.00	0.00	0.00
GATCAAGAAAGGTGATTGGT	0.00	0.00	0.00	0.00	0.00
GATCAAGAAAGTCCTCATCA	0.00	0.00	0.00	0.00	0.00
GATCAAGAAAGTGATGCTTG	0.23	0.98	0.57	2.72	0.69
GATCAAGAAAGTGCGTGGCC	0.00	0.00	0.00	0.00	0.00
GATCAAGAAAGTGGCCGGAG	0.46	0.00	0.00	0.00	0.00
GATCAAGAAAGTGGTGGACG	3.43	3.58	3.15	4.07	2.07
GATCAAGAAAGTGGTGGAGG	0.23	0.00	0.00	0.00	0.00
GATCAAGAAAGTGGTGGGCG	0.23	0.00	0.00	0.00	0.00
GATCAAGAAAGTTGCACAAG	2.06	0.33	0.57	2.04	1.03
GATCAAGAAAGTTGGATGCG	0.00	0.00	0.29	0.00	0.00
GATCAAGAAATAAAAGAAGC	0.23	0.00	0.00	0.00	0.00
GATCAAGAAATAAGAAGAAA	0.00	0.00	0.00	0.00	2.07
GATCAAGAAATACACCACGG	0.00	0.00	0.00	0.00	0.34
GATCAAGAAATACCACTCCG	0.00	0.33	0.00	0.00	0.00
GATCAAGAAATACCCCACCC	0.46	0.00	0.00	0.00	0.00
GATCAAGAAATACCCCACCT	0.23	0.33	0.00	0.00	0.00
GATCAAGAAATACTGTGTTT	0.23	0.00	0.00	0.00	0.00

GATCAAGAAATACTTGCTTG	0.00	0.33	0.57	0.00	0.00
GATCAAGAAATAGAATCGGC	0.00	0.00	0.29	0.00	0.00
GATCAAGAAATATACATACT	0.23	0.00	0.00	0.00	0.00
GATCAAGAAATATCCATGTG	0.00	0.65	0.00	0.00	0.00
GATCAAGAAATATGGCGAGG	0.00	0.65	0.00	0.00	0.00
GATCAAGAAATCATGTGAGA	0.00	0.00	0.00	0.68	0.00
GATCAAGAAATCCACACCGC	0.00	0.00	0.29	0.00	0.00
GATCAAGAAATCCACGCCAT	0.23	0.00	0.57	0.34	1.03
GATCAAGAAATCCACGCCCC	0.00	0.33	0.00	0.00	0.00
GATCAAGAAATCCACGCCGC	0.00	0.33	0.00	0.00	0.00
GATCAAGAAATCGACGGTCA	0.00	0.00	0.00	0.00	0.00
GATCAAGAAATCGAGCTCAC	0.00	0.00	0.00	0.00	0.00
GATCAAGAAATCGAGGGGCG	0.00	0.00	0.00	0.00	0.00
GATCAAGAAATCGATCGAGC	0.00	0.33	0.00	0.00	0.00
GATCAAGAAATCGCAGATGC	0.00	0.00	0.00	0.00	0.00
GATCAAGAAATCGGGAGAGA	0.00	0.00	0.00	0.00	0.00
GATCAAGAAATGCAAGATTT	0.00	0.00	0.00	0.00	0.00
GATCAAGAAATGCTGCTGGT	0.23	0.00	0.00	0.00	0.00
GATCAAGAAATGGAAACTCA	0.23	0.00	0.00	0.00	0.34
GATCAAGAAATGGAAACTCG	0.00	0.00	0.00	0.00	0.34
GATCAAGAAATGGCCAAGCG	0.00	0.33	0.00	0.00	0.00
GATCAAGAAATGGGAAGATG	0.00	0.00	0.00	0.00	0.34
GATCAAGAAATGGTAGGGGC	0.00	0.33	0.00	0.00	0.00
GATCAAGAAATGTAGGAAAA	0.00	0.00	0.57	0.00	0.00
GATCAAGAAATGTGCGATAA	0.00	0.00	0.00	0.34	0.00
GATCAAGAAATTCTAAAAAA	0.46	0.00	0.00	0.00	0.00
GATCAAGAAATTTTAATTGG	0.00	0.00	0.00	0.00	0.00
GATCAAGAACAAAAAAAAAA	0.23	0.33	0.00	0.34	0.34
GATCAAGAACAAAAAAAACA	0.00	0.00	0.00	0.00	0.34
GATCAAGAACAAAAAACACT	0.00	0.00	0.00	0.00	0.00
GATCAAGAACAAAAAAGAGA	0.00	0.00	0.00	0.00	0.00
GATCAAGAACAAAAACAAAA	0.69	0.00	0.00	0.00	0.00
GATCAAGAACAAAAGCTGAG	0.00	0.33	0.00	0.00	0.00
GATCAAGAACAAACAAAAAA	0.00	0.33	0.00	0.00	0.00
GATCAAGAACAAACACATAA	0.00	0.00	0.29	0.00	0.00
GATCAAGAACAAACTCCATG	0.00	0.33	0.00	0.34	0.00
GATCAAGAACAAAGCACCCA	0.00	0.00	0.00	0.00	0.00

GATCAAGAACAACACCACGG	0.00	0.00	0.00	0.00	0.00
GATCAAGAACAACATCATCC	0.00	0.00	0.00	0.00	0.00
GATCAAGAACAACATCGAGG	0.00	0.00	0.00	0.00	0.34
GATCAAGAACAACCATTCAG	0.00	0.00	0.00	0.00	0.00
GATCAAGAACAACCTCAAGG	0.00	0.00	0.00	0.00	0.00
GATCAAGAACAACCTCGAGA	0.00	0.33	0.00	0.00	0.00
GATCAAGAACAACCTCGAGG	10.99	25.70	18.61	27.15	19.63
GATCAAGAACAACCTCGAGN	0.23	0.00	0.00	0.00	0.00
GATCAAGAACAACCTCGCGG	0.00	0.65	0.00	0.00	0.00
GATCAAGAACAACCTCGTGG	0.00	0.33	0.00	0.00	0.00
GATCAAGAACAACCTTGAGG	0.00	0.00	0.00	0.34	0.00
GATCAAGAACAACGCCAACG	0.00	0.00	0.29	0.00	0.00
GATCAAGAACAACGTGCTGA	0.00	0.00	0.57	0.34	0.00
GATCAAGAACAACTGTGAGG	0.00	0.00	0.29	0.00	0.00
GATCAAGAACAACTTCGCCG	0.46	0.00	0.00	0.00	0.34
GATCAAGAACAAGAACAAGA	0.00	0.00	0.00	0.00	0.34
GATCAAGAACAAGAACAATA	0.69	0.00	0.00	0.00	0.00
GATCAAGAACAAGAACAGAA	0.00	0.00	0.00	0.00	0.34
GATCAAGAACAAGAACAGAC	0.00	0.00	0.00	0.00	1.72
GATCAAGAACAAGAACATAA	0.00	0.00	0.00	0.00	0.34
GATCAAGAACAAGAACCGGC	0.00	0.00	0.00	0.00	0.34
GATCAAGAACAAGAAGATGA	0.23	0.33	0.00	0.00	0.00
GATCAAGAACAAGAAGCCAG	0.00	0.00	0.29	0.00	0.00
GATCAAGAACAAGAAGCTGA	30.67	30.26	24.91	17.31	22.04
GATCAAGAACAAGAAGCTGC	0.00	0.33	0.00	0.00	0.00
GATCAAGAACAAGAAGCTNN	0.23	0.00	0.00	0.00	0.00
GATCAAGAACAAGACCCGTG	0.00	0.33	0.00	0.00	0.00
GATCAAGAACAAGACGCTGA	0.23	0.00	0.00	0.00	0.00
GATCAAGAACAAGCAAGCAG	0.00	0.00	0.00	0.00	0.00
GATCAAGAACAAGCACACCT	0.23	0.00	0.00	0.00	0.00
GATCAAGAACAAGCCAGGCT	0.00	0.00	0.57	0.00	0.00
GATCAAGAACAAGGACACCA	0.00	0.00	0.00	0.34	0.00
GATCAAGAACAAGGACGAGA	0.00	0.00	0.29	0.00	0.69
GATCAAGAACAAGGCAAAAA	0.00	0.00	0.00	0.34	0.00
GATCAAGAACAAGGCCCCCA	0.00	0.33	0.00	0.00	0.00
GATCAAGAACAAGGGCAGGA	0.00	0.00	0.00	0.00	0.00
GATCAAGAACAAGGTGAATG	0.00	0.00	0.29	0.00	0.00

GATCAAGAACAAGTACGCAA	0.00	0.00	0.00	0.34	0.00
GATCAAGAACAAGTACGGAC	0.00	0.00	0.00	0.00	0.34
GATCAAGAACAAGTGGCGAT	0.00	0.00	0.00	0.00	0.34
GATCAAGAACAATACCAACA	0.00	0.00	0.00	0.00	0.69
GATCAAGAACAATAGCAAGA	0.00	0.00	0.00	0.00	0.34
GATCAAGAACAATGAGACCG	0.00	0.00	0.29	0.00	0.00
GATCAAGAACAATGCCTCCA	0.23	0.00	0.00	0.00	0.00
GATCAAGAACAATGGACGAC	0.00	0.00	0.00	0.00	0.34
GATCAAGAACAATTGCAGAG	0.00	0.33	0.00	0.00	0.00
GATCAAGAACAATTGGACGC	1.14	0.33	0.00	0.00	0.00
GATCAAGAACAATTTTGCCT	0.00	0.65	0.00	0.00	0.00
GATCAAGAACACAAAAAATC	0.00	0.00	0.29	0.00	0.00
GATCAAGAACACAACACGAC	0.00	0.00	0.00	0.00	0.34
GATCAAGAACACAACCGACA	0.00	0.00	0.00	0.34	0.00
GATCAAGAACACACCTCTGT	0.00	0.00	0.00	0.00	0.00
GATCAAGAACACACGAACCA	0.00	0.00	0.29	0.00	0.00
GATCAAGAACACACGCATGG	0.00	0.33	0.00	0.00	0.00
GATCAAGAACACAGCTCCTT	0.00	0.00	0.29	0.00	0.00
GATCAAGAACACATGCTGCG	0.00	0.00	0.00	0.00	0.00
GATCAAGAACACCAACAGCG	0.00	0.00	0.00	0.00	0.34
GATCAAGAACACCAAGCGCT	0.00	0.00	1.15	0.34	0.00
GATCAAGAACACCAGCAAGG	0.23	0.65	0.00	0.00	0.00
GATCAAGAACACCATTGGCC	0.00	0.00	0.00	0.00	0.00
GATCAAGAACACCCATGGCA	0.00	0.00	0.00	0.00	0.00
GATCAAGAACACCCTCGAGG	0.00	0.33	0.00	0.00	0.00
GATCAAGAACACCTGCGCAT	0.00	0.00	0.00	0.00	0.00
GATCAAGAACACCTGCGCCG	0.00	0.33	0.00	0.00	0.00
GATCAAGAACACCTTCATTG	0.00	0.00	0.00	0.34	0.00
GATCAAGAACACGACGCACA	0.00	0.00	0.29	0.00	0.00
GATCAAGAACACGAGCAAGA	0.00	0.00	0.00	0.00	1.03
GATCAAGAACACGAGGCACG	0.00	0.33	0.00	0.00	0.00
GATCAAGAACACGAGGCTGC	0.00	0.00	0.00	0.00	0.00
GATCAAGAACACGAGTCAGC	0.00	0.00	0.00	0.00	0.00
GATCAAGAACACGCGACAGC	0.00	0.00	0.00	0.00	0.00
GATCAAGAACACGCGATGCT	0.00	0.65	0.00	0.00	0.00
GATCAAGAACACGCGGCCGG	0.00	0.00	0.29	0.00	0.00
GATCAAGAACACGCGGTCGC	0.00	0.00	0.29	0.00	0.00

GATCAAGAACACGCGTCAGC	2.52	2.28	0.29	2.04	0.34
GATCAAGAACACGCGTCAGN	0.23	0.00	0.00	0.00	0.00
GATCAAGAACACGCTCCTGC	0.23	0.00	0.00	0.00	0.00
GATCAAGAACACGTGCGCCG	0.00	0.00	0.00	0.00	0.34
GATCAAGAACACTCCCTTCT	0.00	0.65	0.00	0.00	0.00
GATCAAGAACACTCGAGATG	0.00	0.00	0.00	0.34	0.00
GATCAAGAACACTTCTCAGG	0.00	0.00	0.29	0.00	0.00
GATCAAGAACACTTGGCTGG	0.00	0.00	0.29	0.00	0.00
GATCAAGAACACTTTTGCCA	0.00	0.00	0.29	0.34	0.00
GATCAAGAACAGAAAGTCAA	0.00	0.00	0.00	0.34	0.00
GATCAAGAACAGAACAGAAC	0.00	0.00	0.57	0.00	0.00
GATCAAGAACAGAACTCGAC	0.00	0.00	0.29	0.00	0.00
GATCAAGAACAGAGCAAAAA	0.00	0.00	0.00	0.34	0.00
GATCAAGAACAGAGCAAACA	0.00	0.00	0.00	0.34	0.00
GATCAAGAACAGAGCAATTG	0.00	0.00	0.00	0.34	0.00
GATCAAGAACAGCAGAACGG	0.00	0.00	0.29	0.00	0.00
GATCAAGAACAGCCACATAC	0.00	0.00	0.00	0.00	0.69
GATCAAGAACAGCCACGTCA	0.00	0.00	0.57	0.00	0.00
GATCAAGAACAGCCTGCTCG	0.00	1.63	1.43	0.68	1.03
GATCAAGAACAGCGCATCTG	0.23	0.00	0.00	0.00	0.00
GATCAAGAACAGGAACTTTG	0.00	0.00	0.00	0.00	0.34
GATCAAGAACAGGAAGAAGA	0.23	0.00	0.00	0.00	0.00
GATCAAGAACAGGAGCACAC	0.00	0.00	0.29	0.00	0.00
GATCAAGAACAGGAGCACCC	0.00	0.00	0.29	0.00	0.00
GATCAAGAACAGGATGACCA	0.00	0.00	0.00	0.00	0.00
GATCAAGAACAGGTACGGCC	0.23	0.00	0.00	0.34	0.00
GATCAAGAACAGGTTCCTAA	0.00	0.00	0.00	0.00	0.34
GATCAAGAACAGGTTCCTCA	0.00	0.33	0.00	0.00	1.03
GATCAAGAACAGTGCATGGT	0.00	0.00	0.00	0.00	0.00
GATCAAGAACAGTGTCGGGA	0.00	0.00	0.00	0.00	0.00
GATCAAGAACATAAAAAAAA	0.00	0.00	0.00	0.68	0.00
GATCAAGAACATAAAACACA	0.00	0.00	0.00	0.00	1.72
GATCAAGAACATAACATAAC	0.00	0.00	0.29	0.00	0.00
GATCAAGAACATCAAGAAGC	0.00	0.00	0.00	0.00	0.00
GATCAAGAACATCAAGAAGG	0.23	0.00	0.00	0.00	0.00
GATCAAGAACATCAAGGAGG	0.23	0.00	0.00	0.00	0.00
GATCAAGAACATCACTTGGC	0.00	0.33	0.00	0.00	0.00

GATCAAGAACATCAGGTACA	0.00	0.00	0.00	0.00	0.00
GATCAAGAACATCCCGCCGC	0.00	0.00	0.00	0.00	0.00
GATCAAGAACATCCGCTTCC	1.14	1.95	1.72	1.70	1.38
GATCAAGAACATCCGCTTCN	0.00	0.00	0.00	0.00	0.00
GATCAAGAACATCCTCATGG	0.00	0.33	0.00	0.00	0.00
GATCAAGAACATCGAGCTTG	0.00	0.00	0.57	0.34	0.34
GATCAAGAACATCGCACGCT	0.00	0.00	0.29	0.00	0.00
GATCAAGAACATCGTGAGCT	0.00	0.00	0.00	0.00	0.00
GATCAAGAACATCTTGGAGA	0.00	0.00	0.00	0.00	0.00
GATCAAGAACATGAAAAAAA	0.00	0.00	0.00	0.68	0.00
GATCAAGAACATGAACAGGA	0.00	0.00	0.00	0.00	0.69
GATCAAGAACATGAAGATGG	0.00	0.00	0.29	0.00	0.00
GATCAAGAACATGAAGTGAT	0.00	0.00	0.57	0.34	0.00
GATCAAGAACATGATGAAGC	0.00	0.33	0.00	0.00	0.00
GATCAAGAACATGATGGGCA	0.00	0.00	0.00	0.34	0.00
GATCAAGAACATGATGGGCG	0.00	0.00	0.00	0.00	0.34
GATCAAGAACATGATGTCCA	0.00	0.00	0.29	0.00	0.00
GATCAAGAACATGCACGGCT	0.00	0.33	0.00	0.00	0.00
GATCAAGAACATGCTCGCCT	0.00	0.00	0.00	0.00	0.00
GATCAAGAACATGCTCGGCT	0.46	1.63	0.29	2.38	0.00
GATCAAGAACATGCTCGGNN	0.00	0.00	0.00	0.00	0.00
GATCAAGAACATGGACAAGG	0.00	0.00	0.00	0.34	0.00
GATCAAGAACATGGCGAATG	0.00	0.00	0.00	0.34	0.00
GATCAAGAACATGGGTTCGA	0.00	0.00	0.00	0.34	0.00
GATCAAGAACATGGTGGACG	0.00	0.65	0.29	0.34	0.34
GATCAAGAACATGGTGGATG	0.00	0.00	1.43	0.00	0.34
GATCAAGAACATGTACTACT	0.00	0.33	0.00	0.00	0.00
GATCAAGAACATGTGGGCAA	0.23	0.00	0.00	0.00	0.00
GATCAAGAACATGTTTGCCG	0.00	0.00	0.00	0.00	0.34
GATCAAGAACATTGACGAGA	0.23	0.00	0.00	0.00	0.00
GATCAAGAACATTGAGCATG	0.92	2.60	2.86	6.11	5.51
GATCAAGAACATTGCCTCAA	1.60	1.30	0.00	0.68	0.34
GATCAAGAACATTGCGCATA	1.14	0.33	0.29	0.34	0.00
GATCAAGAACATTGCGCATG	0.00	0.00	0.29	0.00	0.00
GATCAAGAACATTGTTATGT	0.00	0.00	0.29	0.00	0.00
GATCAAGAACATTTACTCCC	0.00	0.33	0.00	0.00	0.00
GATCAAGAACCAACTGGCAG	0.00	0.00	0.00	0.00	0.69

GATCAAGAACCAAGAGGGAA	0.00	0.00	0.00	0.00	0.69
GATCAAGAACCAAGAGGGAG	0.00	0.00	0.00	0.00	0.00
GATCAAGAACCAAGCCATTA	0.00	0.98	0.00	0.00	0.00
GATCAAGAACCAAGCCTGTG	0.00	0.00	0.29	0.00	0.00
GATCAAGAACCAATTCAAGC	0.00	0.33	0.00	0.00	0.00
GATCAAGAACCACTGTGAGG	0.00	0.00	0.29	0.00	0.00
GATCAAGAACCAGAGGCGCA	0.00	0.33	0.00	0.00	0.00
GATCAAGAACCAGCAGTCAG	0.00	0.00	0.00	0.00	0.00
GATCAAGAACCAGCCGAAGG	0.00	0.00	0.00	0.00	0.00
GATCAAGAACCAGCCTGGTG	0.00	0.33	0.00	0.00	0.34
GATCAAGAACCAGCGGCGCA	0.00	0.33	0.00	0.00	0.00
GATCAAGAACCAGCGGCTCA	0.00	0.00	0.29	0.00	0.00
GATCAAGAACCAGCTCTGGT	0.23	0.00	0.00	0.00	0.00
GATCAAGAACCAGCTGAAGG	0.69	0.33	0.00	0.00	0.00
GATCAAGAACCAGGCCGCAA	0.00	0.00	0.00	0.00	0.00
GATCAAGAACCAGGCCTTCA	0.00	0.00	0.00	0.00	0.00
GATCAAGAACCAGGCGAAGC	0.00	0.33	0.00	0.00	0.00
GATCAAGAACCAGGGGCGCA	0.23	0.00	0.00	0.00	0.00
GATCAAGAACCAGGTGGCCG	0.00	0.00	0.00	0.34	0.00
GATCAAGAACCAGTACGTGG	0.00	0.00	0.00	0.68	0.00
GATCAAGAACCAGTCCGGTC	0.00	0.00	0.00	0.00	0.00
GATCAAGAACCAGTGACGCA	0.00	0.00	0.29	0.00	0.00
GATCAAGAACCAGTGGAGCA	0.00	0.00	0.29	0.34	0.00
GATCAAGAACCAGTGGCGAA	0.00	0.00	0.29	0.34	0.00
GATCAAGAACCAGTGGCGCA	35.71	40.34	39.23	57.02	36.16
GATCAAGAACCAGTGGCGCC	0.46	0.00	0.29	0.68	0.00
GATCAAGAACCAGTGGCGCG	0.23	0.00	0.29	0.00	0.34
GATCAAGAACCAGTGGCGGA	0.46	0.00	0.00	0.00	0.00
GATCAAGAACCAGTGGCGNA	0.00	0.00	0.00	0.00	0.00
GATCAAGAACCAGTGGCGNN	0.23	0.00	0.00	0.00	0.00
GATCAAGAACCAGTGGCTGC	0.00	0.00	0.00	0.34	0.00
GATCAAGAACCAGTGGGGCA	0.00	0.00	0.00	0.00	0.69
GATCAAGAACCAGTGGNGCA	0.00	0.00	0.00	0.00	0.34
GATCAAGAACCAGTGGTTGT	0.00	0.00	0.00	0.68	0.00
GATCAAGAACCCAAAGCGAA	0.00	0.00	0.00	0.00	0.34
GATCAAGAACCCAAAGCGAT	0.00	0.00	2.00	0.00	0.00
GATCAAGAACCCAAAGCGCG	0.00	0.00	0.00	0.00	0.00

GATCAAGAACCCAAAGCGCT	0.23	0.33	0.86	0.34	0.69
GATCAAGAACCCAAATTTTC	0.00	0.00	0.00	0.00	0.00
GATCAAGAACCCAGACAAGG	14.42	3.58	1.15	2.38	1.38
GATCAAGAACCCAGACGAGG	0.23	0.00	0.00	0.00	0.00
GATCAAGAACCCAGACNNNN	0.23	0.00	0.00	0.00	0.00
GATCAAGAACCCATTGGCTC	0.00	0.00	0.29	0.00	0.00
GATCAAGAACCCCAAGCGCT	0.00	1.30	0.29	0.00	0.00
GATCAAGAACCCCAAGTTCT	1.14	0.33	0.00	1.02	0.69
GATCAAGAACCCCAGAGTGA	0.00	0.33	0.00	0.00	0.00
GATCAAGAACCCCAGCGCCT	0.00	0.00	0.57	0.00	0.00
GATCAAGAACCCCAGCGGCC	0.00	0.00	0.00	0.00	0.00
GATCAAGAACCCCAGCGGCT	0.00	0.00	0.00	0.68	0.00
GATCAAGAACCCCCGCGTGG	0.00	0.65	0.29	0.68	0.00
GATCAAGAACCCCCTCGCGT	0.23	0.00	0.00	0.00	0.00
GATCAAGAACCCCGAGGTCT	0.00	0.33	0.00	0.00	0.00
GATCAAGAACCCCGTTCCGG	0.00	0.00	0.00	0.34	0.00
GATCAAGAACCCCTACTCCC	0.00	0.33	0.00	0.00	0.00
GATCAAGAACCCGAAGCATA	0.00	0.00	0.00	0.00	0.00
GATCAAGAACCCGAAGTTCG	0.00	0.65	0.00	0.00	0.00
GATCAAGAACCCGAGGGACT	0.00	0.65	0.00	0.00	0.00
GATCAAGAACCCGGACAAGG	0.00	0.00	0.00	0.00	0.34
GATCAAGAACCCGGACCCCG	0.00	0.00	0.00	0.00	0.00
GATCAAGAACCCGGGGGTGG	0.00	0.00	0.00	0.00	0.69
GATCAAGAACCCGGTACCTA	0.00	0.00	0.00	0.00	0.00
GATCAAGAACCCGTCCAAGT	0.00	0.00	0.00	0.00	0.34
GATCAAGAACCCTCAGAACA	0.00	0.00	0.00	0.00	0.00
GATCAAGAACCCTGGGTGGC	0.00	0.00	0.29	0.00	0.00
GATCAAGAACCGACGACTGG	0.00	0.00	0.00	0.00	0.00
GATCAAGAACCGACGCTACG	5.26	4.88	1.72	2.04	2.07
GATCAAGAACCGACGGCTGG	0.00	0.33	0.29	1.02	0.00
GATCAAGAACCGATATGGCA	0.00	0.33	0.00	0.00	0.00
GATCAAGAACCGCATCGCAT	0.23	0.33	0.00	0.00	0.00
GATCAAGAACCGCATGGAGT	0.92	0.00	0.00	0.00	0.00
GATCAAGAACCGCCGCCTCG	0.00	0.00	0.00	0.34	0.00
GATCAAGAACCGCGAGCTGG	0.92	0.65	0.57	0.34	0.00
GATCAAGAACCGCGCTCCTG	0.00	0.00	0.00	0.00	0.34
GATCAAGAACCGCGTCTTCG	0.00	0.00	0.00	0.68	0.00

GATCAAGAACCGGAAGCTCG	0.00	0.65	0.57	0.34	0.00
GATCAAGAACCGGAGAATGA	0.00	0.00	0.29	0.00	0.00
GATCAAGAACCGGCTATACA	0.00	0.00	0.29	0.00	0.00
GATCAAGAACCGGCTATACC	0.00	0.00	0.00	0.00	0.34
GATCAAGAACCGGGTTTGCC	0.00	0.00	0.00	0.34	0.00
GATCAAGAACCGGTGACCTC	0.00	0.00	0.00	0.00	0.34
GATCAAGAACCGGTGGCGCA	0.00	0.00	0.00	0.34	0.34
GATCAAGAACCGTGGCAGAC	0.00	0.00	0.00	0.34	0.00
GATCAAGAACCTAGAGGTCA	0.00	0.33	0.00	0.00	0.00
GATCAAGAACCTCATTCTGC	0.00	0.00	0.29	0.00	0.00
GATCAAGAACCTCGAGAAGG	0.00	0.00	0.00	0.00	0.00
GATCAAGAACCTCGGCAAGG	0.00	0.33	0.00	0.34	0.00
GATCAAGAACCTCTGACGAG	0.00	0.00	0.00	0.00	0.00
GATCAAGAACCTCTTGCCGA	0.00	0.00	0.00	0.00	0.69
GATCAAGAACCTGCCGCCGC	0.00	0.00	0.00	0.00	0.00
GATCAAGAACCTGCCGTTGA	0.00	0.00	0.00	0.34	0.00
GATCAAGAACCTGCGCTGCA	0.00	0.00	0.00	0.00	0.00
GATCAAGAACCTGGAGAAGG	0.23	0.00	0.00	0.00	0.00
GATCAAGAACCTGGCACAGG	0.46	0.65	0.00	0.00	0.00
GATCAAGAACCTGGCACCGA	0.00	0.00	0.00	0.68	0.00
GATCAAGAACCTGTTATCAC	0.00	0.00	0.29	0.00	0.00
GATCAAGAACCTGTTCCAGG	0.23	0.00	0.00	0.00	0.00
GATCAAGAACCTTGCCTCGT	0.00	0.00	0.00	0.34	0.00
GATCAAGAACGAAAAAAAAA	0.00	0.00	0.00	0.00	0.00
GATCAAGAACGAAAGAGCAA	0.00	0.00	0.00	0.00	0.00
GATCAAGAACGAAAGCAAGG	0.23	0.00	0.00	0.00	0.00
GATCAAGAACGAAAGCTTAG	0.00	0.00	0.00	0.00	0.00
GATCAAGAACGAAAGTCAGG	0.00	0.00	0.00	0.00	0.00
GATCAAGAACGAAAGTTAAA	0.00	0.00	0.00	0.00	0.00
GATCAAGAACGAAAGTTAAG	0.46	1.30	3.15	2.04	9.99
GATCAAGAACGAAAGTTAGG	0.00	0.00	0.00	0.00	0.34
GATCAAGAACGAACTGCGCG	0.00	0.00	0.00	0.00	0.00
GATCAAGAACGAAGACCTGT	0.00	0.00	0.00	0.00	0.00
GATCAAGAACGAAGGGTTTT	0.00	0.00	0.29	0.00	0.00
GATCAAGAACGACACCAAGG	0.00	0.00	0.29	0.00	0.00
GATCAAGAACGACACGAAAG	0.00	0.00	0.00	0.34	0.00
GATCAAGAACGACAGGCTCC	0.00	0.00	0.00	0.00	0.00

GATCAAGAACGACAGGCTGC	7.10	4.55	1.72	4.75	2.41
GATCAAGAACGACAGGCTGG	0.23	0.00	0.00	0.34	0.00
GATCAAGAACGACAGGTTGC	0.00	0.00	0.00	0.34	0.00
GATCAAGAACGACATCCCCG	0.00	0.00	0.00	0.00	0.00
GATCAAGAACGACCTCGAGG	0.00	0.00	0.00	0.00	0.00
GATCAAGAACGACCTCGCGG	0.00	0.00	0.00	0.00	0.00
GATCAAGAACGACCTTCCGC	0.00	0.00	0.00	0.00	0.00
GATCAAGAACGACGAGCAAC	0.00	0.00	0.00	0.34	0.00
GATCAAGAACGACGGGTCCC	0.00	0.33	0.00	0.00	0.00
GATCAAGAACGACGGGTTAG	0.23	0.33	0.00	0.00	0.00
GATCAAGAACGAGAACAACA	0.00	0.00	0.00	0.00	0.69
GATCAAGAACGAGAAGCTGA	0.23	0.00	0.00	0.00	0.00
GATCAAGAACGAGCATGTGC	0.00	0.00	0.00	0.00	0.00
GATCAAGAACGAGCGCTTCC	0.92	0.00	0.00	0.68	0.00
GATCAAGAACGAGGAGACTG	0.00	0.00	0.00	0.00	0.00
GATCAAGAACGATACTGACA	0.00	0.00	0.00	0.00	0.00
GATCAAGAACGATTTTTTCT	0.00	0.00	0.00	0.00	0.00
GATCAAGAACGCAAAAAAAA	0.00	0.00	0.29	0.00	0.00
GATCAAGAACGCAGAGGTCA	0.00	0.33	0.00	0.34	0.00
GATCAAGAACGCATTCCAAG	0.00	0.00	0.29	0.00	0.00
GATCAAGAACGCCAAAAAAA	0.00	0.00	0.29	0.00	0.00
GATCAAGAACGCCAACATGG	0.00	0.00	0.00	0.00	0.34
GATCAAGAACGCCAACGGCA	0.00	0.00	0.00	0.00	0.34
GATCAAGAACGCCAACTAAG	0.00	0.00	0.00	0.34	0.00
GATCAAGAACGCCAAGCCAA	0.00	0.00	0.00	0.34	0.00
GATCAAGAACGCCACCAAGG	0.23	0.00	0.29	0.00	0.00
GATCAAGAACGCCACCTCAA	0.00	0.33	0.00	0.00	0.00
GATCAAGAACGCCACGAAAA	0.00	0.00	0.29	0.00	0.00
GATCAAGAACGCCACGCAAA	8.24	16.59	19.19	17.99	14.81
GATCAAGAACGCCACGCAAC	0.00	0.00	0.00	0.00	0.00
GATCAAGAACGCCACGCAAG	0.23	0.00	0.00	0.00	0.00
GATCAAGAACGCCACGCAAT	0.00	0.00	0.29	0.00	0.00
GATCAAGAACGCCACGCCAC	0.00	0.00	0.00	0.00	0.00
GATCAAGAACGCCACGCTAA	0.00	0.00	0.57	0.00	0.00
GATCAAGAACGCCACGCTCG	0.23	0.33	0.00	0.00	0.00
GATCAAGAACGCCACGGAAA	0.00	0.00	0.00	0.00	0.00
GATCAAGAACGCCACGGACG	0.00	0.00	0.00	0.34	0.00

GATCAAGAACGCCACTTGGG	0.00	0.00	0.00	0.00	0.00
GATCAAGAACGCCATTTTGA	0.00	0.65	0.00	0.00	0.00
GATCAAGAACGCCCCGCCAC	0.00	0.00	0.29	0.00	0.00
GATCAAGAACGCCCTCGCGG	0.00	0.00	0.00	0.34	0.00
GATCAAGAACGCCGAAGGAG	0.00	0.00	0.00	0.00	0.34
GATCAAGAACGCCGACATGG	0.46	0.00	0.00	0.34	0.69
GATCAAGAACGCCGATACGG	0.00	0.00	0.00	0.00	0.00
GATCAAGAACGCCGATATGG	0.46	0.33	2.58	1.02	1.72
GATCAAGAACGCCGATATGN	0.00	0.00	0.00	0.00	0.00
GATCAAGAACGCCGATGGCA	0.23	0.00	0.00	0.00	0.00
GATCAAGAACGCCGGAGGCA	5.95	12.69	7.73	12.56	8.27
GATCAAGAACGCCGGGGGAA	0.00	0.00	0.00	0.00	0.00
GATCAAGAACGCCTTGCACA	0.00	0.00	0.00	0.00	0.00
GATCAAGAACGCCTTTGAGG	0.00	0.33	0.00	0.00	0.00
GATCAAGAACGCGAAAGTCA	0.00	0.00	0.00	0.34	0.00
GATCAAGAACGCGACGCATG	0.23	0.00	0.00	0.00	0.00
GATCAAGAACGCGGATGGCT	0.00	0.00	0.00	0.00	0.00
GATCAAGAACGCGGCTATTC	0.00	0.00	0.00	0.00	0.34
GATCAAGAACGCGGGCGAGG	0.92	0.33	0.29	0.00	0.69
GATCAAGAACGCGGTGGCCA	0.00	0.00	0.29	0.00	0.00
GATCAAGAACGCGTACAATG	8.47	4.23	1.72	3.39	0.69
GATCAAGAACGCGTACGATG	0.23	0.00	0.00	0.00	0.00
GATCAAGAACGCGTTCCAAG	0.00	0.00	0.00	0.00	0.00
GATCAAGAACGCTGACAACC	0.00	0.33	0.00	0.00	0.00
GATCAAGAACGCTGAGTCCG	0.00	0.00	0.00	0.00	0.69
GATCAAGAACGCTGCTCTGG	0.46	0.00	0.00	0.00	0.00
GATCAAGAACGCTGGATGGA	0.23	0.00	0.00	0.00	0.00
GATCAAGAACGGAAAAAAAA	0.00	0.00	0.00	0.00	0.00
GATCAAGAACGGAACAGAAC	0.00	0.00	0.29	0.00	0.00
GATCAAGAACGGAAGCCTCG	0.00	0.00	0.00	0.00	0.00
GATCAAGAACGGAAGTTCGA	0.00	0.00	0.00	0.00	0.00
GATCAAGAACGGACGACTAG	0.00	0.00	0.00	0.68	0.00
GATCAAGAACGGACGACTCG	0.23	0.65	0.00	0.34	0.00
GATCAAGAACGGACGCCACG	0.00	0.33	0.00	0.00	0.00
GATCAAGAACGGACGCCCCG	0.00	0.98	0.29	0.00	0.34
GATCAAGAACGGACGCCGCG	0.46	0.33	0.00	0.00	0.00
GATCAAGAACGGACGCCNNN	0.00	0.00	0.00	0.00	0.00

GATCAAGAACGGACGCCTAG	0.00	0.00	0.29	0.00	0.34
GATCAAGAACGGACGCCTCA	0.00	0.33	0.00	0.00	0.00
GATCAAGAACGGACGCCTCC	0.00	0.00	0.00	0.00	0.00
GATCAAGAACGGACGCCTCG	36.85	105.41	71.88	94.02	56.83
GATCAAGAACGGACGCCTCT	0.00	0.33	0.57	0.00	0.00
GATCAAGAACGGACGCCTNG	0.00	0.00	0.00	0.34	0.00
GATCAAGAACGGACGCCTTG	0.00	0.00	0.29	0.00	0.00
GATCAAGAACGGACGCGGGG	0.00	0.00	0.00	0.00	0.00
GATCAAGAACGGACGCGTAG	0.00	0.00	0.00	0.00	0.34
GATCAAGAACGGACGCTCGC	0.00	0.00	0.00	0.00	0.00
GATCAAGAACGGACGCTCGT	0.23	0.00	0.00	0.00	0.00
GATCAAGAACGGACGGAAAA	0.00	0.33	0.00	0.00	0.00
GATCAAGAACGGACGGCTCG	0.23	0.00	0.00	0.00	0.00
GATCAAGAACGGACGGCTTG	0.00	0.00	0.00	0.00	0.00
GATCAAGAACGGACGTCTCG	0.23	0.00	0.00	0.00	0.00
GATCAAGAACGGCAAAAAAA	0.00	0.00	0.00	0.34	0.00
GATCAAGAACGGCAAGAAGA	0.46	3.58	4.30	4.41	4.48
GATCAAGAACGGCAAGAAGC	0.00	0.00	0.00	0.00	0.00
GATCAAGAACGGCAAGAAGG	0.00	0.00	0.00	0.00	0.00
GATCAAGAACGGCAAGAATA	0.00	0.00	0.00	0.00	0.00
GATCAAGAACGGCAAGACGA	0.00	0.00	0.00	0.00	0.34
GATCAAGAACGGCAAGCTCA	0.00	0.00	0.00	0.00	0.34
GATCAAGAACGGCAGAGAGA	0.00	0.00	0.29	0.00	0.00
GATCAAGAACGGCAGGGTGG	0.00	0.00	0.00	0.34	0.00
GATCAAGAACGGCATCTTGA	0.00	0.33	0.00	0.00	0.00
GATCAAGAACGGCATGAAGG	0.00	0.00	0.00	0.00	0.00
GATCAAGAACGGCATTGCGG	0.00	0.00	0.86	0.34	0.00
GATCAAGAACGGCCGACTCG	0.23	0.00	0.00	0.00	0.00
GATCAAGAACGGCCGAGTGG	0.00	0.65	0.00	0.00	0.00
GATCAAGAACGGCCGCATGG	0.00	0.00	0.29	0.00	0.00
GATCAAGAACGGCCGTGGAG	0.00	0.00	0.00	0.34	0.00
GATCAAGAACGGCCGTGTAG	0.00	0.00	0.00	0.68	0.00
GATCAAGAACGGCCGTGTGG	0.23	1.30	0.29	0.00	0.34
GATCAAGAACGGCCGTGTTG	0.00	0.00	0.00	0.00	0.00
GATCAAGAACGGCCTCGCGG	0.23	0.00	0.00	0.00	0.00
GATCAAGAACGGCGCGGTCT	0.00	0.00	0.29	0.00	0.00
GATCAAGAACGGCTATATCT	0.00	0.00	0.29	0.00	0.00

GATCAAGAACGGCTTCCAGC	0.00	0.00	0.00	0.00	0.00
GATCAAGAACGGGCAGGGGA	0.00	0.00	0.29	0.00	0.00
GATCAAGAACGGGCGCCTCG	0.23	0.00	0.00	0.00	0.34
GATCAAGAACGGGCGCGCCC	0.00	0.00	0.57	0.00	0.00
GATCAAGAACGGGTTGAAAA	0.23	0.00	0.00	0.00	0.00
GATCAAGAACGGTGAAGCAC	0.00	0.00	0.00	0.00	1.03
GATCAAGAACGGTGACTTTT	0.00	0.00	0.00	0.34	0.00
GATCAAGAACGTACTCGCGG	0.00	0.33	0.00	0.00	0.00
GATCAAGAACGTCAAGGAGG	0.00	0.00	0.29	0.00	0.00
GATCAAGAACGTCACTCTGC	0.00	0.00	0.00	0.00	1.38
GATCAAGAACGTCCACGGGG	0.00	0.00	0.00	0.00	0.00
GATCAAGAACGTCCTCCCGG	0.00	0.00	0.00	0.00	0.34
GATCAAGAACGTCCTCGAGA	0.00	0.00	0.00	0.34	0.00
GATCAAGAACGTCCTCGAGG	0.00	0.00	0.29	0.00	0.69
GATCAAGAACGTCCTCGCGG	29.76	42.62	23.20	41.07	25.14
GATCAAGAACGTCCTCGCGN	0.00	0.00	0.00	0.34	0.00
GATCAAGAACGTCCTCGCGT	0.23	0.00	0.00	0.34	0.00
GATCAAGAACGTCCTCGCNN	0.23	0.00	0.00	0.00	0.00
GATCAAGAACGTCCTCGCTC	0.00	0.00	0.00	0.34	0.00
GATCAAGAACGTCCTCNCGG	0.00	0.00	0.00	0.00	0.69
GATCAAGAACGTCCTCNNNN	0.23	0.00	0.00	0.00	0.00
GATCAAGAACGTCCTGGCCA	0.00	0.00	0.00	0.34	0.00
GATCAAGAACGTCCTTGGGA	3.43	2.93	1.15	2.38	1.03
GATCAAGAACGTCCTTGGGG	0.23	0.00	0.00	0.00	0.00
GATCAAGAACGTCCTTGGGT	0.00	0.33	0.00	0.00	0.00
GATCAAGAACGTCCTTTAGT	0.00	0.00	0.29	0.00	0.00
GATCAAGAACGTCTTCTGCT	0.23	0.00	0.00	0.00	0.00
GATCAAGAACGTGACGTGTT	0.00	0.00	0.29	0.00	0.00
GATCAAGAACGTGAGTAAGC	0.00	0.00	0.00	0.34	0.00
GATCAAGAACGTGCCCTTCA	0.00	0.33	0.00	0.00	0.00
GATCAAGAACGTGCCGTGTT	0.23	0.00	0.29	0.00	0.00
GATCAAGAACGTGGACACCG	0.00	0.33	0.00	0.00	0.00
GATCAAGAACGTGGGTGCGC	0.00	0.00	0.00	0.00	0.34
GATCAAGAACGTGGGTGTGC	0.23	0.33	0.29	0.00	0.34
GATCAAGAACGTGGTCCGGG	0.00	0.00	0.29	0.00	0.34
GATCAAGAACGTGGTCGACG	0.00	0.00	0.29	0.00	0.00
GATCAAGAACGTGGTGAAGG	0.00	0.33	0.00	0.34	0.00

GATCAAGAACGTGGTGGACG	0.00	0.00	0.29	0.00	0.00
GATCAAGAACGTTCCATTAC	0.00	0.00	0.00	0.00	0.00
GATCAAGAACGTTCGGAAGG	0.00	0.33	0.00	0.00	0.00
GATCAAGAACGTTGATAAGG	0.00	0.00	0.00	0.00	0.00
GATCAAGAACGTTGGCAGCC	0.00	0.00	0.29	0.00	0.00
GATCAAGAACTAAAAAAAAA	0.00	0.00	0.29	0.00	0.00
GATCAAGAACTAAAAGACGC	0.00	0.00	0.00	0.00	0.00
GATCAAGAACTACAAGACGC	0.00	0.00	0.00	0.00	0.00
GATCAAGAACTACAAGACGG	0.00	0.65	0.00	0.00	0.00
GATCAAGAACTACCCTCACA	0.00	0.00	0.00	0.00	0.00
GATCAAGAACTACCTCAAGA	0.00	0.33	0.00	0.00	0.00
GATCAAGAACTACTCAGCGC	0.00	0.00	0.00	0.00	0.00
GATCAAGAACTACTCAGTGC	0.00	0.33	0.00	1.02	0.00
GATCAAGAACTAGTGGCGCA	0.23	0.00	0.00	0.00	0.00
GATCAAGAACTATCACTTCT	0.00	0.00	0.00	0.00	0.00
GATCAAGAACTATCCCCATA	0.00	0.00	0.00	0.00	0.00
GATCAAGAACTATTACAAGA	1.83	0.33	0.00	0.00	0.34
GATCAAGAACTATTACAAGG	0.00	0.00	0.29	0.00	0.00
GATCAAGAACTCATAGAGTG	0.00	0.00	0.00	0.00	0.00
GATCAAGAACTCATGGGGTG	0.00	0.00	0.00	0.34	0.00
GATCAAGAACTCGACGCCGC	0.00	0.00	0.00	0.00	0.00
GATCAAGAACTCGCGGAGAT	0.00	0.00	0.00	0.00	0.00
GATCAAGAACTCGCGGCTCA	0.00	0.00	0.00	0.00	0.69
GATCAAGAACTCTCGCCCCG	0.00	0.00	0.00	0.34	0.00
GATCAAGAACTCTGCGAAGG	0.23	0.00	0.00	0.00	0.00
GATCAAGAACTCTGTTAGTT	0.00	0.00	0.00	0.00	0.00
GATCAAGAACTGAACTGCAA	0.23	0.33	0.00	0.00	0.00
GATCAAGAACTGAAGCTGCT	0.23	0.00	0.00	0.00	0.00
GATCAAGAACTGCACCAAAG	0.00	0.00	0.00	0.34	0.00
GATCAAGAACTGCAGATATC	0.00	0.00	0.57	0.00	0.00
GATCAAGAACTGCAGGTTCG	0.00	0.00	0.00	0.00	0.00
GATCAAGAACTGCATCTTTG	0.00	0.00	0.57	0.00	0.00
GATCAAGAACTGCCTGAAGC	0.00	0.00	0.00	0.34	0.00
GATCAAGAACTGCGAGCTGG	0.00	0.33	0.00	0.00	0.00
GATCAAGAACTGCGAGGGTC	0.00	0.00	0.00	0.00	0.00
GATCAAGAACTGCGAGTCTC	0.00	0.33	0.29	0.00	0.00
GATCAAGAACTGCGATGATC	0.00	0.00	0.00	0.00	0.00

GATCAAGAACTGCGCCATTG	0.23	0.00	0.00	0.00	0.00
GATCAAGAACTGCGGGGAGT	0.00	0.33	0.00	0.00	0.00
GATCAAGAACTGCGTGGCCC	0.23	0.00	0.00	0.00	0.00
GATCAAGAACTGCTCAGTGA	0.00	0.00	0.00	0.34	0.00
GATCAAGAACTGCTCGCCGT	0.00	0.00	0.00	0.00	0.00
GATCAAGAACTGCTCGGACA	0.00	0.00	0.00	0.00	0.00
GATCAAGAACTGCTCGGACG	0.00	0.33	0.00	0.00	0.00
GATCAAGAACTGCTCGGACT	20.37	14.97	9.74	20.03	10.68
GATCAAGAACTGCTCGGCCT	0.23	0.00	0.00	0.00	0.00
GATCAAGAACTGCTCGGGCA	0.00	0.00	0.00	0.00	0.34
GATCAAGAACTGCTCGGGCT	0.23	0.00	0.00	0.00	0.00
GATCAAGAACTGCTCGGTCG	0.00	0.00	0.29	0.00	0.00
GATCAAGAACTGCTTGGTAC	0.00	0.00	0.00	0.34	0.00
GATCAAGAACTGCTTTCTGG	0.00	0.33	0.00	0.00	0.00
GATCAAGAACTGGAACAACG	0.00	0.00	0.00	0.00	0.00
GATCAAGAACTGGAACAGAA	0.00	0.00	0.00	0.34	0.00
GATCAAGAACTGGGGGCCTC	0.00	0.00	0.00	0.34	0.00
GATCAAGAACTGGTACGTGG	0.00	0.00	0.00	0.00	0.00
GATCAAGAACTTAAAGATTA	0.00	0.00	0.00	0.00	1.03
GATCAAGAACTTAGCCAACG	0.00	0.00	0.00	0.00	0.00
GATCAAGAACTTAGCCACTC	0.00	0.00	0.00	0.00	0.00
GATCAAGAACTTCACGGTCC	0.23	0.00	0.00	0.00	0.00
GATCAAGAACTTCCAAGCGA	0.00	0.00	0.00	0.34	0.00
GATCAAGAACTTCCTGCCTA	0.00	0.00	0.00	0.00	0.69
GATCAAGAACTTCGACGAGC	0.46	0.00	0.29	0.34	0.34
GATCAAGAACTTCGACGTGC	0.23	0.00	0.00	0.00	0.00
GATCAAGAACTTCGAGGCCT	0.00	0.00	0.00	0.00	0.00
GATCAAGAACTTCGGGGCGT	0.46	0.00	0.00	0.34	0.00
GATCAAGAACTTCGTGGCCC	5.95	0.33	1.43	1.36	0.69
GATCAAGAACTTCTCTGAGA	0.00	0.00	0.00	0.34	0.00
GATCAAGAACTTGAAGCGCG	0.00	0.33	0.00	0.00	0.00
GATCAAGAACTTGAATGAAT	0.00	0.33	0.00	0.00	0.00
GATCAAGAACTTGAGACACA	0.00	0.00	0.00	0.68	0.00
GATCAAGAACTTGAGGGGCT	0.00	0.33	0.00	0.00	0.00
GATCAAGAACTTGCAGACGG	0.00	0.00	0.00	0.00	0.00
GATCAAGAACTTGCCGCGGA	0.00	0.00	0.00	0.34	0.00
GATCAAGAACTTGCGCAGTT	0.00	0.33	0.00	0.34	0.00

GATCAAGAACTTGCGGCGAT	0.00	0.00	0.00	0.00	0.00
GATCAAGAACTTGCGTGCAC	0.00	0.65	0.29	1.02	0.69
GATCAAGAACTTGCGTGCCG	0.00	0.00	0.00	0.34	0.00
GATCAAGAACTTGGATAAAG	0.00	0.00	0.00	0.00	0.34
GATCAAGAACTTGTACAACA	0.00	0.00	0.29	1.02	0.34
GATCAAGAACTTGTTCTTCT	0.00	0.00	0.00	0.00	1.03
GATCAAGAACTTTGAGCATG	0.00	0.00	0.00	0.00	0.00
GATCAAGAACTTTGCCCTGG	0.46	0.00	0.00	0.34	0.00
GATCAAGAACTTTGCTTGCG	0.00	0.00	0.00	0.00	0.00
GATCAAGAACTTTTCTTGTT	0.00	0.00	0.00	0.00	0.00
GATCAAGAACTTTTTCAAAT	0.46	0.00	0.00	0.00	0.00
GATCAAGAAGAAAAAAAAAA	1.37	0.98	0.57	1.02	0.34
GATCAAGAAGAAAAAGAAAA	0.46	0.00	0.00	0.00	0.00
GATCAAGAAGAAAAAGAACA	0.00	0.00	0.29	0.00	0.00
GATCAAGAAGAAACAGACGC	0.00	0.00	1.15	0.00	0.00
GATCAAGAAGAAAGAAAAAA	0.00	0.00	0.00	0.00	0.00
GATCAAGAAGAAAGAAGAAG	0.00	0.00	0.00	0.00	0.34
GATCAAGAAGAAAGAATAAG	0.00	0.00	0.00	0.00	0.34
GATCAAGAAGAAAGAGTCCA	0.00	0.00	0.00	0.00	0.00
GATCAAGAAGAAAGGCCGAT	0.00	0.00	0.00	0.00	0.34
GATCAAGAAGAAAGTGGCAC	0.46	0.00	0.00	0.00	0.00
GATCAAGAAGAAAGTGGTCA	0.23	0.98	0.00	1.02	0.34
GATCAAGAAGAACAACATCC	0.00	0.00	0.00	0.00	1.03
GATCAAGAAGAACAAGATGG	0.00	0.00	0.29	0.00	0.00
GATCAAGAAGAACAAGCCTG	0.23	0.00	0.00	0.00	0.00
GATCAAGAAGAACAAGGCGA	0.00	0.00	0.00	0.34	0.00
GATCAAGAAGAACAAGGGCA	0.00	0.33	0.00	0.00	0.00
GATCAAGAAGAACACCCTAC	0.00	0.00	0.29	0.00	0.00
GATCAAGAAGAACAGCACGC	0.00	1.30	0.00	0.34	0.69
GATCAAGAAGAACAGCCTGC	0.00	0.00	0.86	0.00	0.00
GATCAAGAAGAACATCGCCG	0.00	0.98	0.00	1.02	0.69
GATCAAGAAGAACATCGCGC	0.00	0.00	0.00	0.34	0.00
GATCAAGAAGAACATTGAGC	0.46	0.00	0.86	0.34	1.38
GATCAAGAAGAACCTTGCTG	0.00	0.00	0.00	0.00	0.00
GATCAAGAAGAACGAGACCT	0.00	0.00	0.00	0.00	0.00
GATCAAGAAGAACGGAGCGC	0.00	0.00	0.29	0.00	0.00
GATCAAGAAGAACGGAGGCG	0.00	0.33	0.00	0.00	0.00

GATCAAGAAGAACGGCAAAA	0.00	0.00	0.00	0.00	0.00
GATCAAGAAGAACGGCACGC	0.23	0.98	1.43	1.02	1.38
GATCAAGAAGAACGGCACTG	0.00	0.00	0.00	0.00	0.00
GATCAAGAAGAACGGCTCAC	0.00	0.00	0.00	0.00	0.34
GATCAAGAAGAACGGCTCGC	0.00	0.33	0.29	0.00	1.38
GATCAAGAAGAACGGGACGC	0.00	1.63	1.72	0.68	2.76
GATCAAGAAGAACGGTCAAC	0.00	0.00	0.00	0.00	0.34
GATCAAGAAGAACTAGAAGA	0.00	0.00	2.58	0.00	0.34
GATCAAGAAGAACTCCGGCT	0.69	0.33	0.00	0.68	0.34
GATCAAGAAGAAGAAAAAAA	0.00	0.00	0.00	0.00	0.34
GATCAAGAAGAAGAAAAAGA	0.00	0.00	1.15	0.00	0.34
GATCAAGAAGAAGAAAACCA	0.00	0.00	0.00	0.00	1.03
GATCAAGAAGAAGAAAAGAC	0.00	0.00	0.29	0.00	0.69
GATCAAGAAGAAGAAAGACG	0.00	0.00	2.58	0.00	0.34
GATCAAGAAGAAGAAAGGCG	0.00	0.00	0.29	0.00	0.00
GATCAAGAAGAAGAACAAGA	0.00	0.00	0.00	0.00	0.69
GATCAAGAAGAAGAACGAGG	0.23	0.33	0.00	0.00	0.00
GATCAAGAAGAAGAAGAAAA	0.00	0.00	0.00	0.34	0.34
GATCAAGAAGAAGAAGAAGA	0.00	0.33	45.53	0.00	50.63
GATCAAGAAGAAGAAGAAGC	0.00	0.00	1.43	0.00	0.34
GATCAAGAAGAAGAAGAAGG	0.00	0.00	0.29	0.00	1.72
GATCAAGAAGAAGAAGAATA	0.00	0.00	0.00	0.00	1.03
GATCAAGAAGAAGAAGACAC	0.00	0.00	0.29	0.00	0.00
GATCAAGAAGAAGAAGACGA	0.00	0.00	0.86	0.00	0.34
GATCAAGAAGAAGAAGAGGA	0.00	0.00	2.58	0.00	0.69
GATCAAGAAGAAGAAGCAAC	0.00	0.00	0.00	0.00	0.69
GATCAAGAAGAAGAAGCAGA	0.00	0.00	0.00	0.00	0.69
GATCAAGAAGAAGAAGCAGC	0.00	0.00	0.29	0.00	0.69
GATCAAGAAGAAGAAGGAGG	0.23	0.00	1.72	0.00	2.07
GATCAAGAAGAAGAAGGAGT	0.23	0.00	0.00	0.00	0.00
GATCAAGAAGAAGAATAAGA	0.00	0.00	2.00	0.00	1.03
GATCAAGAAGAAGAATGAGA	0.00	0.00	0.00	0.00	1.03
GATCAAGAAGAAGAATGAGG	0.00	0.00	0.00	0.00	0.34
GATCAAGAAGAAGACACACC	0.00	0.00	0.29	0.00	0.00
GATCAAGAAGAAGACGAAGA	0.00	0.00	0.00	0.00	0.34
GATCAAGAAGAAGAGGAAGA	0.00	0.00	0.00	0.00	0.34
GATCAAGAAGAAGAGGAAGG	0.00	0.00	0.29	0.00	0.00

GATCAAGAAGAAGCACAAGG	0.00	0.00	0.00	0.00	0.00
GATCAAGAAGAAGCCCCTGG	0.00	0.00	0.00	0.34	0.00
GATCAAGAAGAAGCTCAAGA	0.00	0.00	0.29	0.00	0.00
GATCAAGAAGAAGCTGAATG	0.00	0.00	0.00	0.00	0.00
GATCAAGAAGAAGCTGGAGT	4.58	0.33	0.57	4.07	2.41
GATCAAGAAGAAGGAAGAAG	0.00	0.00	0.86	0.00	1.03
GATCAAGAAGAAGGAGAAGA	0.00	0.00	0.29	0.00	0.00
GATCAAGAAGAAGGAGAAGT	0.00	0.00	0.00	0.34	0.00
GATCAAGAAGAAGGAGAATA	0.00	0.00	0.29	0.00	1.38
GATCAAGAAGAAGGAGCACG	0.00	0.00	0.29	0.00	0.00
GATCAAGAAGAAGGAGGAGG	0.00	0.00	0.29	0.00	0.34
GATCAAGAAGAAGGCCAGCG	0.00	0.00	0.00	0.34	0.34
GATCAAGAAGAAGGCCGAGG	0.00	0.00	0.00	0.00	0.00
GATCAAGAAGAAGGCCGCGG	0.00	0.00	0.29	0.68	0.69
GATCAAGAAGAAGGCCGCTG	0.46	0.98	0.00	0.00	0.00
GATCAAGAAGAAGGCCTTCT	0.00	0.33	0.00	0.00	0.00
GATCAAGAAGAAGGCGAAGA	0.00	0.00	1.43	0.00	0.69
GATCAAGAAGAAGGGCACCA	0.00	0.00	0.00	0.00	0.34
GATCAAGAAGAAGGGCCTCT	0.46	2.28	1.15	1.70	2.07
GATCAAGAAGAAGGGTTGAG	0.00	0.33	0.57	0.00	0.00
GATCAAGAAGAAGGTCAAGG	0.00	1.30	0.29	0.00	0.34
GATCAAGAAGAAGTGATGAG	0.00	0.00	0.00	0.00	0.00
GATCAAGAAGAAGTGATGTG	0.00	0.00	0.00	0.34	0.00
GATCAAGAAGAATACAGACG	0.00	0.00	0.86	0.00	0.00
GATCAAGAAGAATGCAAACG	0.00	0.00	0.00	0.00	0.00
GATCAAGAAGAATGCGGCGT	0.23	0.00	0.57	0.00	0.34
GATCAAGAAGAATGCTGCGC	0.00	0.00	0.00	0.00	0.00
GATCAAGAAGAATGGCACGT	0.00	0.33	1.43	0.00	0.69
GATCAAGAAGAATGGCCCAA	0.00	0.00	0.29	0.00	0.00
GATCAAGAAGAATGGCCTCA	0.00	0.33	0.29	0.00	0.34
GATCAAGAAGACAAAAAAAA	0.00	0.00	0.00	0.34	0.00
GATCAAGAAGACAAAGGAGG	0.00	0.00	0.00	0.34	0.34
GATCAAGAAGACAAGAATTA	0.00	0.00	0.00	0.00	0.34
GATCAAGAAGACAAGTCATC	0.00	0.00	0.00	0.00	0.34
GATCAAGAAGACAATCGAGA	0.00	0.00	0.29	0.00	0.00
GATCAAGAAGACACAGGAGA	0.00	0.00	3.15	0.00	1.38
GATCAAGAAGACACAGTAAG	0.00	0.00	0.00	0.00	0.34

GATCAAGAAGACAGCACAGC	0.00	0.00	0.00	0.00	0.34
GATCAAGAAGACAGTAATCG	0.00	0.00	0.29	0.00	0.00
GATCAAGAAGACATGCCGGT	0.00	0.33	0.00	0.00	0.00
GATCAAGAAGACCAAGAAGG	0.00	0.00	0.00	0.00	0.00
GATCAAGAAGACCACACCGC	0.00	0.00	0.29	0.00	0.34
GATCAAGAAGACCACGCCGA	0.00	0.00	0.00	0.00	0.00
GATCAAGAAGACCACGCCGC	0.00	0.00	0.00	0.00	0.00
GATCAAGAAGACCAGCGACG	0.00	0.00	0.29	0.00	0.00
GATCAAGAAGACCCCGCCCC	0.00	0.00	0.00	0.00	0.00
GATCAAGAAGACCCGGAACC	0.92	0.00	0.00	0.68	0.00
GATCAAGAAGACCCGGAAGA	0.00	0.33	0.00	0.00	0.00
GATCAAGAAGACCGACATCG	0.00	0.00	0.00	0.00	0.00
GATCAAGAAGACCGGCATCG	0.23	0.00	0.00	0.00	0.00
GATCAAGAAGACCGTCACCG	0.00	0.00	0.00	0.00	0.69
GATCAAGAAGACCGTCANNN	0.00	0.00	0.00	0.00	0.00
GATCAAGAAGACCGTCATAG	0.23	0.00	0.57	0.00	0.00
GATCAAGAAGACCGTCATCG	8.93	21.47	23.48	14.93	15.50
GATCAAGAAGACCGTCATCT	0.00	0.00	0.57	0.00	0.00
GATCAAGAAGACCGTCATGG	0.00	0.33	0.00	0.00	0.00
GATCAAGAAGACCGTCATNN	0.23	0.00	0.00	0.00	0.00
GATCAAGAAGACCGTCCTCG	0.23	0.00	0.00	0.00	0.00
GATCAAGAAGACCTACACNG	0.00	0.00	0.00	0.00	0.00
GATCAAGAAGACCTACAGGA	0.00	0.00	0.00	0.00	0.00
GATCAAGAAGACGAAGAAGA	0.00	0.00	0.00	0.00	0.00
GATCAAGAAGACGAAGAAGG	0.00	0.33	0.00	0.00	0.00
GATCAAGAAGACGAAGACGA	0.00	0.00	0.29	0.00	0.00
GATCAAGAAGACGAAGGAAG	0.00	0.33	0.00	0.00	0.00
GATCAAGAAGACGAAGGACT	0.00	0.00	0.00	0.00	0.34
GATCAAGAAGACGAAGGAGA	0.23	0.00	0.29	0.00	0.00
GATCAAGAAGACGAAGGAGC	0.00	0.00	0.00	0.00	0.34
GATCAAGAAGACGAAGGAGG	160.46	140.87	92.49	61.10	59.58
GATCAAGAAGACGAAGGAGN	0.69	0.00	0.00	0.00	0.00
GATCAAGAAGACGAAGGAGT	0.46	0.00	0.00	0.68	0.00
GATCAAGAAGACGAAGGANG	0.00	0.00	0.29	0.00	0.00
GATCAAGAAGACGAAGGANN	0.23	0.00	0.00	0.00	0.00
GATCAAGAAGACGAAGGATG	0.46	0.00	0.00	0.00	0.00
GATCAAGAAGACGAAGGCGG	0.00	0.33	0.57	0.00	0.00

GATCAAGAAGACGAAGGCNN	0.23	0.00	0.00	0.00	0.00
GATCAAGAAGACGAAGGTGG	0.00	0.33	0.00	0.00	0.00
GATCAAGAAGACGAAGNAGG	0.00	0.00	0.00	0.00	0.00
GATCAAGAAGACGAAGNGNN	0.23	0.00	0.00	0.00	0.00
GATCAAGAAGACGAAGNNNN	0.23	0.00	0.00	0.00	0.00
GATCAAGAAGACGAATGAGG	0.00	0.00	0.00	0.00	0.34
GATCAAGAAGACGACGCCGC	0.00	0.00	0.00	0.00	0.34
GATCAAGAAGACGACGGAGG	0.46	0.00	0.29	0.00	0.00
GATCAAGAAGACGAGCCGGT	0.00	0.00	0.29	0.00	0.00
GATCAAGAAGACGCAGGAGG	0.23	0.00	0.00	0.00	0.00
GATCAAGAAGACGCCACTGA	0.23	0.00	0.00	0.34	0.69
GATCAAGAAGACGCCCAAGG	0.00	0.00	0.00	0.34	0.00
GATCAAGAAGACGCCGGCGG	0.23	0.00	0.00	0.00	0.00
GATCAAGAAGACGCGCCTGG	0.46	0.00	0.00	0.00	0.00
GATCAAGAAGACGCTTTGTT	0.00	0.33	0.00	0.00	0.00
GATCAAGAAGACGGAGAAGA	0.00	0.00	0.00	0.00	0.34
GATCAAGAAGACGGAGAAGG	0.00	0.00	0.00	0.34	0.00
GATCAAGAAGACGGAGGAGG	0.46	0.00	0.00	0.00	0.00
GATCAAGAAGACGTCGCCCG	0.00	0.00	0.00	0.00	0.34
GATCAAGAAGACGTCTCGCC	0.00	0.00	0.29	0.00	0.00
GATCAAGAAGACGTTCCAGC	0.00	0.00	0.00	0.00	0.34
GATCAAGAAGACTCCTAGCG	0.00	0.00	0.00	0.34	0.34
GATCAAGAAGACTGACTGAG	0.00	0.00	0.29	0.00	0.00
GATCAAGAAGACTGCTCCGC	0.00	0.00	0.00	0.00	0.69
GATCAAGAAGACTGCTGTCA	0.00	0.00	0.00	0.34	0.00
GATCAAGAAGAGAAAAAAAA	0.00	0.00	0.00	0.00	0.00
GATCAAGAAGAGAAAAAGCA	0.00	0.00	0.00	0.00	0.34
GATCAAGAAGAGAAAGAAGA	0.00	0.33	0.00	0.00	0.00
GATCAAGAAGAGAAGAAGAA	0.00	0.33	0.00	0.00	0.00
GATCAAGAAGAGAAGACGAA	0.00	0.00	0.57	0.00	0.00
GATCAAGAAGAGAAGGAATA	0.00	0.00	0.86	0.00	0.00
GATCAAGAAGAGAAGGAGAA	0.00	0.00	0.00	0.00	1.03
GATCAAGAAGAGAAGGGGGC	0.00	0.00	0.29	0.00	0.00
GATCAAGAAGAGAAGGGGTC	0.00	0.00	0.57	0.68	0.00
GATCAAGAAGAGAGGGGTTG	0.00	0.33	0.00	0.00	0.00
GATCAAGAAGAGCAAAAAAA	1.60	0.33	0.00	0.00	0.00
GATCAAGAAGAGCAAAAGCA	9.39	4.23	3.15	5.77	4.13

GATCAAGAAGAGCAAAAGTC	0.00	0.00	0.00	0.00	0.00
GATCAAGAAGAGCAAAGGCA	0.00	0.00	0.29	0.00	0.00
GATCAAGAAGAGCAAGAGGA	0.00	0.00	0.00	0.00	0.69
GATCAAGAAGAGCACGCCTC	0.00	0.33	0.00	0.68	0.34
GATCAAGAAGAGCATTCAGA	0.00	0.00	0.00	0.00	0.34
GATCAAGAAGAGCCACCAAA	0.00	0.00	0.00	0.68	0.00
GATCAAGAAGAGCCCCAATC	0.00	0.00	0.29	0.00	0.00
GATCAAGAAGAGCGAAAGCA	0.00	0.33	0.00	0.00	0.00
GATCAAGAAGAGCGGGATGA	0.00	0.00	0.29	0.00	0.00
GATCAAGAAGAGGAAAAGGA	0.00	0.00	0.57	0.00	0.00
GATCAAGAAGAGGAAGAAGA	0.00	0.00	0.86	0.00	0.34
GATCAAGAAGAGGAAGAAGC	0.00	0.00	0.29	0.00	0.00
GATCAAGAAGAGGAAGACGC	0.00	0.33	0.29	0.00	0.00
GATCAAGAAGAGGAAGAGAT	0.00	0.00	0.00	0.00	0.00
GATCAAGAAGAGGAAGAGGA	0.00	0.00	1.43	0.00	3.44
GATCAAGAAGAGGAAGCAGA	0.00	0.00	0.29	0.00	0.00
GATCAAGAAGAGGAAGCTTG	0.00	0.00	0.00	0.00	0.69
GATCAAGAAGAGGAAGGAGG	0.00	0.00	0.00	0.00	0.00
GATCAAGAAGAGGCAGTATG	0.00	0.33	0.00	0.00	0.00
GATCAAGAAGAGGCGCATGA	0.00	0.00	0.00	0.00	0.34
GATCAAGAAGAGGCGCGTGG	0.00	0.00	0.00	0.00	0.00
GATCAAGAAGAGGCGCTCAT	0.00	0.00	0.00	0.00	0.00
GATCAAGAAGAGGCGCTGGT	0.00	0.00	0.29	0.00	0.00
GATCAAGAAGAGGCTCAAGA	0.00	0.00	0.29	0.68	0.00
GATCAAGAAGAGGCTGGACG	0.00	0.33	0.00	0.00	0.00
GATCAAGAAGAGGGAGCAGC	0.69	0.33	0.00	0.34	0.00
GATCAAGAAGAGGGATGGGG	0.00	0.33	0.00	0.00	0.00
GATCAAGAAGAGGGCGATGA	0.00	0.33	0.00	0.00	0.00
GATCAAGAAGAGGGGGAACG	0.00	0.00	0.00	0.00	0.00
GATCAAGAAGAGGGGGTCCA	0.00	0.00	0.00	0.00	0.00
GATCAAGAAGAGGTTGCATC	0.00	0.33	0.57	0.00	0.00
GATCAAGAAGAGTCCCTGAG	0.00	0.00	0.00	0.00	0.00
GATCAAGAAGAGTGGGAGGA	0.00	0.00	0.00	1.70	0.00
GATCAAGAAGATAAGCACGA	0.00	0.00	0.00	0.00	0.00
GATCAAGAAGATAGCAATAA	0.00	0.00	0.00	0.00	0.34
GATCAAGAAGATAGCGAGGA	0.23	0.00	0.00	0.00	0.00
GATCAAGAAGATCAAGAAGA	0.00	0.00	1.15	0.00	0.69

GATCAAGAAGATCACGGACA	0.00	0.00	0.00	0.34	0.00
GATCAAGAAGATCATACCGA	0.00	0.00	0.29	0.00	0.00
GATCAAGAAGATCCAGTGAG	0.00	0.00	0.00	0.00	0.34
GATCAAGAAGATCTTGAAGA	0.00	0.00	0.57	0.00	0.00
GATCAAGAAGATGAAAAAAA	0.69	0.33	0.00	0.00	0.00
GATCAAGAAGATGAAACAGT	0.00	0.33	0.00	0.00	0.00
GATCAAGAAGATGAAGCCGA	0.23	0.00	0.00	0.00	0.00
GATCAAGAAGATGAAGGAGG	0.00	0.33	0.00	0.00	0.00
GATCAAGAAGATGAAGGCGG	0.00	0.00	0.00	0.00	0.00
GATCAAGAAGATGAAGTAGA	0.00	0.00	0.00	0.34	0.34
GATCAAGAAGATGAAGTCGA	12.36	15.94	12.31	8.15	8.61
GATCAAGAAGATGAAGTCGT	0.00	0.00	0.00	0.00	0.34
GATCAAGAAGATGAGCAGGA	0.00	0.00	0.00	0.34	0.00
GATCAAGAAGATGAGCAGGC	0.00	0.00	0.00	0.34	0.00
GATCAAGAAGATGAGCAGGT	0.23	0.33	0.57	0.00	0.00
GATCAAGAAGATGATGATGC	0.23	0.00	0.57	0.34	0.00
GATCAAGAAGATGATGCTGC	4.58	8.46	5.15	9.16	7.92
GATCAAGAAGATGATGCTTC	0.00	0.33	0.00	0.00	0.00
GATCAAGAAGATGCAAGGCT	0.00	0.00	0.00	0.00	0.69
GATCAAGAAGATGCCAATCC	0.00	0.00	0.29	0.00	0.00
GATCAAGAAGATGCCAGTGC	0.00	0.00	0.29	0.00	0.00
GATCAAGAAGATGCCTCCCT	0.00	0.00	0.00	0.00	0.34
GATCAAGAAGATGCCTCCGC	0.00	0.33	0.00	0.00	0.00
GATCAAGAAGATGCGCAGGA	0.00	0.00	0.00	0.00	0.34
GATCAAGAAGATGCGCAGGT	0.00	0.33	1.43	0.34	1.72
GATCAAGAAGATGCGCAGTA	0.00	0.00	0.00	0.00	0.34
GATCAAGAAGATGCGCCGGG	0.00	0.33	0.00	0.00	0.00
GATCAAGAAGATGCGCCGGT	0.00	0.00	0.86	0.00	0.00
GATCAAGAAGATGCGCGGGT	0.00	0.00	0.29	0.00	0.00
GATCAAGAAGATGCGTGCTG	0.00	0.33	0.29	1.36	1.72
GATCAAGAAGATGCTCGAGG	0.00	0.00	0.00	0.00	0.00
GATCAAGAAGATGCTGCTGC	0.23	0.00	0.00	0.00	0.00
GATCAAGAAGATGGACGAGA	0.00	0.00	0.00	0.00	0.34
GATCAAGAAGATGGCGCGCA	0.00	0.33	0.00	0.00	0.00
GATCAAGAAGATGGCTGAGT	0.00	0.00	0.29	0.00	0.00
GATCAAGAAGATGGGATGCA	0.00	0.00	0.00	0.34	0.00
GATCAAGAAGATGTACAAGG	0.00	0.00	1.15	1.36	0.00

GATCAAGAAGATGTGCAGGT	0.00	0.00	0.29	0.00	0.00
GATCAAGAAGATTACCAAGC	0.23	0.33	0.00	0.00	0.34
GATCAAGAAGATTACGGACA	0.00	0.00	0.29	0.00	0.00
GATCAAGAAGATTGCAAAGC	0.00	0.00	0.29	0.00	0.00
GATCAAGAAGATTGCCAAGA	0.23	0.00	0.29	0.00	0.00
GATCAAGAAGATTGCCAAGC	27.24	32.53	46.68	29.19	36.16
GATCAAGAAGATTGCCAAGG	0.46	0.00	0.00	0.00	0.00
GATCAAGAAGATTGCCAATC	0.00	0.00	0.57	0.00	0.00
GATCAAGAAGATTGCCACGC	0.00	0.00	0.00	0.00	0.69
GATCAAGAAGATTGCCAGGC	0.23	0.00	0.00	0.00	0.00
GATCAAGAAGATTGCCATGC	0.23	0.00	0.00	0.00	0.00
GATCAAGAAGATTGCCCAGC	0.00	0.00	0.57	0.34	0.34
GATCAAGAAGATTGCCCATG	0.00	0.00	0.00	0.00	0.00
GATCAAGAAGATTGCCTAGC	0.00	0.00	0.00	0.00	0.00
GATCAAGAAGATTGCCTCGT	0.00	0.33	0.00	0.00	0.00
GATCAAGAAGATTGCGGAGG	0.00	0.33	0.00	0.00	0.00
GATCAAGAAGATTGCGGCCG	0.00	0.33	0.29	0.34	0.00
GATCAAGAAGATTGCTAAGC	0.00	0.00	0.00	0.00	0.00
GATCAAGAAGATTGGCAAGC	0.00	0.00	0.00	0.00	0.00
GATCAAGAAGCAAAAAAAAA	0.92	0.98	0.00	0.68	0.34
GATCAAGAAGCAAACACGTC	0.00	0.00	0.00	0.34	0.00
GATCAAGAAGCAAACCTCCT	0.00	0.00	0.00	0.00	1.38
GATCAAGAAGCAAACTTCTT	0.00	0.00	0.00	0.00	0.00
GATCAAGAAGCAAATGATGC	0.00	0.00	0.00	0.00	0.34
GATCAAGAAGCAACAGATGC	0.00	0.00	0.00	0.00	0.00
GATCAAGAAGCAACCAAAAA	0.00	0.00	0.00	0.34	0.00
GATCAAGAAGCAACCTAACA	0.00	0.00	0.00	0.00	0.00
GATCAAGAAGCAACGTCGCT	0.00	0.00	0.00	0.00	0.34
GATCAAGAAGCAACGTGGGC	1.83	1.63	0.00	1.02	1.03
GATCAAGAAGCAAGAAGAAG	0.00	0.00	0.86	0.00	0.00
GATCAAGAAGCAAGAAGCAA	0.00	0.00	1.15	0.00	0.00
GATCAAGAAGCAAGCACCAT	0.00	0.00	0.00	0.00	0.00
GATCAAGAAGCAAGCTCAGC	0.00	0.00	0.00	0.68	0.00
GATCAAGAAGCAAGGGACAT	0.00	0.00	0.00	0.00	0.00
GATCAAGAAGCAATGAAGAA	0.00	0.00	0.00	0.00	0.00
GATCAAGAAGCAATTGTCGG	0.00	0.33	0.00	0.00	0.34
GATCAAGAAGCACAAAAAAA	0.00	0.00	0.00	0.00	0.34

GATCAAGAAGCACAAGAAGC	0.00	0.00	0.00	0.68	0.00
GATCAAGAAGCACAAGGAAA	0.00	0.00	0.00	0.00	0.00
GATCAAGAAGCACAAGGTTA	0.00	0.00	0.86	0.00	0.69
GATCAAGAAGCACCAGAGGG	0.00	0.00	0.29	0.00	0.00
GATCAAGAAGCACCTCAAGA	0.00	0.00	0.00	0.00	0.00
GATCAAGAAGCACCTGCAGC	0.00	0.98	0.00	0.34	0.00
GATCAAGAAGCACCTGCGAG	0.00	0.00	0.00	0.00	0.00
GATCAAGAAGCACCTGCGGG	0.00	0.00	0.00	0.00	0.00
GATCAAGAAGCACGCGCCAG	0.00	0.00	0.00	0.34	0.00
GATCAAGAAGCACGCGGATG	0.46	0.33	0.00	0.00	0.00
GATCAAGAAGCACTGCATTT	0.00	0.33	0.00	0.00	0.00
GATCAAGAAGCACTGGACAG	0.00	0.33	0.00	0.00	0.00
GATCAAGAAGCACTTGGGAG	0.00	0.00	0.29	0.00	0.00
GATCAAGAAGCAGAACCTCC	0.00	0.33	0.00	0.00	0.00
GATCAAGAAGCAGAAGAAGC	0.00	0.00	0.29	0.00	0.00
GATCAAGAAGCAGAAGATTA	0.00	0.00	0.00	0.00	0.34
GATCAAGAAGCAGAGAAACA	0.00	0.00	0.00	0.00	0.00
GATCAAGAAGCAGAGGACTT	0.00	0.00	0.00	0.00	0.00
GATCAAGAAGCAGAGGCAGG	0.00	0.00	0.00	0.34	0.00
GATCAAGAAGCAGAGGCTGG	0.00	0.00	0.57	0.00	0.00
GATCAAGAAGCAGCAAAAAA	0.00	0.00	0.57	0.00	0.00
GATCAAGAAGCAGCAAATTC	0.00	0.00	0.00	0.00	0.00
GATCAAGAAGCAGCAACACC	0.00	0.00	0.29	0.00	0.00
GATCAAGAAGCAGCAATTGG	0.00	0.00	0.00	0.34	0.00
GATCAAGAAGCAGCACACCC	0.00	0.00	0.00	0.00	0.00
GATCAAGAAGCAGCACGAGA	0.00	0.00	0.00	0.00	0.00
GATCAAGAAGCAGCAGAAAC	0.00	0.00	0.00	0.34	0.00
GATCAAGAAGCAGCAGAAAG	0.00	0.00	0.57	0.00	0.34
GATCAAGAAGCAGCAGAAGA	0.00	0.00	0.00	0.00	1.38
GATCAAGAAGCAGCAGAAGC	8.93	24.40	32.64	27.49	29.62
GATCAAGAAGCAGCAGAAGG	0.00	0.00	0.00	0.34	0.00
GATCAAGAAGCAGCAGAAGN	0.46	0.00	0.00	0.34	0.00
GATCAAGAAGCAGCAGAANC	0.00	0.00	0.00	0.00	0.00
GATCAAGAAGCAGCAGACGC	0.00	0.00	0.00	0.00	0.34
GATCAAGAAGCAGCAGAGAG	0.00	0.00	0.29	0.00	0.00
GATCAAGAAGCAGCAGAGGA	0.00	0.00	0.00	0.00	0.34
GATCAAGAAGCAGCAGAGGC	0.00	0.00	0.00	0.00	0.69

GATCAAGAAGCAGCAGAGGG	13.73	21.47	33.50	21.38	24.80
GATCAAGAAGCAGCAGAGTG	0.23	0.00	0.00	0.00	0.00
GATCAAGAAGCAGCAGATGC	0.00	0.00	0.00	0.00	0.00
GATCAAGAAGCAGCAGCACC	0.00	0.00	0.00	0.00	0.00
GATCAAGAAGCAGCAGCAGC	0.00	0.00	0.29	0.00	0.00
GATCAAGAAGCAGCAGCCGA	0.00	0.00	0.00	0.00	0.69
GATCAAGAAGCAGCAGCGGC	0.00	0.00	0.00	0.34	0.00
GATCAAGAAGCAGCAGCGGG	0.00	0.33	0.29	0.00	0.00
GATCAAGAAGCAGCAGGAGC	0.00	0.00	0.00	0.00	0.00
GATCAAGAAGCAGCAGGAGG	0.00	0.00	0.29	0.00	0.00
GATCAAGAAGCAGCAGGGAC	0.00	0.00	0.57	0.00	0.00
GATCAAGAAGCAGCAGGGTG	0.00	0.00	0.29	0.00	0.00
GATCAAGAAGCAGCAGGTGC	0.00	0.00	0.29	0.00	0.00
GATCAAGAAGCAGCAGNAGC	0.00	0.00	0.00	0.34	0.34
GATCAAGAAGCAGCAGTGCT	0.00	0.00	0.00	0.00	0.00
GATCAAGAAGCAGCAGTTGC	0.00	0.33	0.00	0.00	0.00
GATCAAGAAGCAGCAGTTGG	0.00	0.00	0.00	0.00	0.00
GATCAAGAAGCAGCATAAGC	0.00	0.00	0.29	0.00	0.00
GATCAAGAAGCAGCATCCTT	0.00	0.00	0.29	0.00	0.00
GATCAAGAAGCAGCATGAGA	0.00	0.00	0.00	0.68	0.34
GATCAAGAAGCAGCCATCCA	0.00	0.00	0.00	0.00	0.00
GATCAAGAAGCAGCCGAAAT	0.00	0.00	0.00	0.34	0.00
GATCAAGAAGCAGCCGAGGG	0.00	0.00	0.00	0.00	0.34
GATCAAGAAGCAGCCTCTGA	0.00	0.33	0.00	0.00	0.00
GATCAAGAAGCAGCGAAAAA	0.00	0.00	0.29	0.00	0.00
GATCAAGAAGCAGCGAAATC	0.00	0.65	0.00	0.00	0.00
GATCAAGAAGCAGCGAAGCA	0.00	0.33	0.00	0.00	0.00
GATCAAGAAGCAGCGCAAGA	0.00	0.00	0.00	0.00	0.00
GATCAAGAAGCAGCGCAGAA	0.00	0.00	0.00	0.34	0.00
GATCAAGAAGCAGCGCCAGG	0.00	0.00	0.00	0.68	0.00
GATCAAGAAGCAGCGGAAAA	0.00	0.00	0.00	0.34	0.00
GATCAAGAAGCAGCGGAAGA	0.00	0.00	0.00	0.00	0.00
GATCAAGAAGCAGCTCCCGC	0.00	0.00	0.00	0.00	0.34
GATCAAGAAGCAGCTCGAGG	0.69	0.65	0.00	0.34	0.34
GATCAAGAAGCAGCTCTACG	0.00	0.00	0.29	0.00	0.00
GATCAAGAAGCAGCTGAAGC	0.00	0.00	0.29	0.34	0.00
GATCAAGAAGCAGCTGAAGG	2.06	2.93	2.29	1.36	2.07

GATCAAGAAGCAGCTGAGAA	0.00	0.00	0.29	0.00	0.00
GATCAAGAAGCAGCTGAGGA	0.00	0.00	0.29	0.00	0.00
GATCAAGAAGCAGCTGCGGA	0.00	0.00	0.57	0.00	0.00
GATCAAGAAGCAGCTGCGTG	0.00	0.00	0.00	0.00	0.00
GATCAAGAAGCAGCTTCAGC	0.00	0.00	0.00	0.34	0.00
GATCAAGAAGCAGCTTTCGC	0.00	0.33	0.00	0.00	0.00
GATCAAGAAGCAGGAAGACC	0.00	0.00	0.00	0.00	0.00
GATCAAGAAGCAGGAGAGGA	0.00	0.00	0.00	0.34	0.00
GATCAAGAAGCAGGAGGACC	0.23	0.33	0.00	0.00	0.00
GATCAAGAAGCAGGCACGAC	0.00	0.00	0.29	0.00	0.00
GATCAAGAAGCAGGCAGAAA	0.00	0.00	0.00	0.00	0.34
GATCAAGAAGCAGGCATGAA	0.00	0.33	0.00	0.00	0.00
GATCAAGAAGCAGGCCAAGG	0.00	0.00	0.00	0.34	0.00
GATCAAGAAGCAGGCGAAAA	0.00	0.00	0.00	0.00	0.34
GATCAAGAAGCAGGCGAAAC	0.00	0.00	0.00	0.00	0.00
GATCAAGAAGCAGGCGAAGA	0.23	0.00	0.29	0.34	0.69
GATCAAGAAGCAGGCGAAGC	7.78	25.38	35.22	19.69	31.00
GATCAAGAAGCAGGCGAAGG	0.00	0.00	0.29	0.00	0.00
GATCAAGAAGCAGGCGAAGN	0.00	0.00	0.00	0.00	0.00
GATCAAGAAGCAGGCGACGC	0.23	0.00	0.00	0.00	0.00
GATCAAGAAGCAGGCGCCCA	0.00	0.00	0.00	0.68	0.34
GATCAAGAAGCAGGCGGAGC	0.00	0.00	0.00	0.00	0.34
GATCAAGAAGCAGGCGNCNN	0.23	0.00	0.00	0.00	0.00
GATCAAGAAGCAGGCTGCCC	0.00	0.00	0.29	0.00	0.00
GATCAAGAAGCAGGCTTAGG	0.00	0.00	0.00	0.00	0.00
GATCAAGAAGCAGGGCAGCG	0.00	0.00	0.00	0.34	0.00
GATCAAGAAGCAGGGCAGTG	0.23	0.00	0.00	0.00	0.00
GATCAAGAAGCAGGGCATCG	0.00	0.00	0.00	0.00	0.34
GATCAAGAAGCAGTACAACA	0.00	0.33	0.00	0.00	0.00
GATCAAGAAGCAGTACAAGA	0.00	0.00	0.00	0.00	0.34
GATCAAGAAGCAGTAGAGGG	0.00	0.33	0.00	0.00	0.00
GATCAAGAAGCAGTATATGC	0.00	0.00	0.00	0.00	0.00
GATCAAGAAGCAGTCCAAGG	0.23	6.83	5.73	4.07	5.17
GATCAAGAAGCAGTCCCAGG	0.00	0.00	0.29	0.00	0.00
GATCAAGAAGCAGTCGAAGC	0.00	0.00	0.00	0.00	0.00
GATCAAGAAGCAGTTCCCAA	0.46	0.33	0.57	0.00	0.00
GATCAAGAAGCATCAGAGCA	0.00	0.00	0.00	0.34	0.00

GATCAAGAAGCATCTGTGTG	0.00	0.33	0.00	0.00	0.00
GATCAAGAAGCCAACACTTC	0.23	0.00	0.00	0.00	0.69
GATCAAGAAGCCAACCGTCC	0.00	0.00	0.00	0.00	0.34
GATCAAGAAGCCAACTCGGA	0.00	0.00	0.00	0.00	0.00
GATCAAGAAGCCAAGTAGGA	0.00	0.00	0.00	0.00	0.00
GATCAAGAAGCCCAAGACCA	0.23	0.98	0.29	2.04	1.72
GATCAAGAAGCCCACGCACC	0.00	0.00	0.00	0.00	0.00
GATCAAGAAGCCCACGCCGC	0.00	0.00	0.29	0.00	0.34
GATCAAGAAGCCCACGCGCA	0.92	0.00	0.00	0.34	0.69
GATCAAGAAGCCCCTTTGAC	0.00	0.00	0.00	0.00	0.00
GATCAAGAAGCCCCTTTGCC	0.00	0.00	0.00	0.00	0.34
GATCAAGAAGCCGAAGGAGG	0.00	0.00	0.29	0.00	0.00
GATCAAGAAGCCGACGCCGC	0.00	0.00	0.00	0.00	0.34
GATCAAGAAGCCGCCAGTGA	0.00	0.00	0.29	0.00	0.00
GATCAAGAAGCCGGAATCAC	0.00	0.65	0.00	0.00	0.00
GATCAAGAAGCCGGAGTGTC	0.00	0.00	0.00	0.00	0.34
GATCAAGAAGCCGGCCTGAA	0.00	0.00	0.00	0.00	0.00
GATCAAGAAGCCGGCGGCGC	0.00	0.00	0.00	0.00	0.34
GATCAAGAAGCCTCCCTACT	0.00	0.00	0.00	0.00	0.00
GATCAAGAAGCCTGTGGAAG	0.00	0.33	0.00	0.00	0.00
GATCAAGAAGCCTTCGTTGC	0.00	0.00	0.29	0.00	0.00
GATCAAGAAGCGAAACCAAA	0.00	0.00	0.00	0.00	0.00
GATCAAGAAGCGAGAGCAGC	0.00	0.00	0.00	0.34	0.00
GATCAAGAAGCGAGGAGGCT	0.00	0.33	0.29	0.00	0.69
GATCAAGAAGCGATGGGTCA	0.00	0.33	0.00	0.00	0.00
GATCAAGAAGCGATTCGGCG	0.00	0.00	0.00	0.00	0.00
GATCAAGAAGCGCAATTAGT	0.00	0.00	0.00	0.00	0.00
GATCAAGAAGCGCACCGAGG	2.29	1.30	0.00	0.34	0.34
GATCAAGAAGCGCACCGTCT	0.00	0.65	0.00	0.00	0.00
GATCAAGAAGCGCAGCAAAA	0.00	0.00	0.00	0.00	0.34
GATCAAGAAGCGCAGCTTTT	0.46	0.33	0.29	0.68	0.00
GATCAAGAAGCGCCAGGACG	0.23	0.00	0.00	0.00	0.00
GATCAAGAAGCGCCCCCCCA	0.23	0.00	0.00	0.00	0.00
GATCAAGAAGCGCCTCCTCC	0.00	0.00	0.00	0.00	0.00
GATCAAGAAGCGCCTGCGCG	0.00	0.00	0.00	0.00	0.00
GATCAAGAAGCGCCTGGCGG	0.00	0.00	0.00	0.00	0.34
GATCAAGAAGCGCGATGTCG	0.00	0.33	0.00	0.00	0.00

GATCAAGAAGCGCGGGCTCG	0.46	0.65	0.57	0.34	1.38
GATCAAGAAGCGCGTGGAGG	0.46	0.65	0.00	1.36	0.34
GATCAAGAAGCGCTCAGAGG	0.46	0.33	1.72	2.72	1.72
GATCAAGAAGCGCTCTGATG	0.46	0.00	0.00	0.00	0.00
GATCAAGAAGCGCTGCTGCT	0.00	0.00	0.00	0.00	0.00
GATCAAGAAGCGCTGTCGGT	0.00	0.00	0.00	0.00	0.00
GATCAAGAAGCGGAAGAAGG	0.00	0.00	0.57	0.00	0.34
GATCAAGAAGCGGAAGGATG	0.00	0.00	0.00	0.00	0.00
GATCAAGAAGCGGAGCTCCA	0.00	0.00	0.29	0.00	0.00
GATCAAGAAGCGGCCATCGA	0.00	0.00	0.00	0.34	0.00
GATCAAGAAGCGGCGCGAGA	0.00	0.33	0.29	0.34	0.34
GATCAAGAAGCGGCTCCAGC	0.00	0.00	0.00	0.00	0.00
GATCAAGAAGCGGCTCCAGG	0.00	0.00	0.00	0.00	0.00
GATCAAGAAGCGGCTTCCGC	0.00	0.00	0.00	0.00	0.00
GATCAAGAAGCGGGAGAACG	0.00	0.65	0.00	0.00	0.00
GATCAAGAAGCGGGAGAATG	0.00	0.65	0.00	1.02	0.34
GATCAAGAAGCGGGATGCGA	0.00	0.00	0.00	0.00	0.00
GATCAAGAAGCGGGCACCGG	0.00	0.00	0.00	0.00	0.00
GATCAAGAAGCGGGCCTTCA	0.00	0.00	0.00	0.00	0.34
GATCAAGAAGCGGGGAAAAA	0.00	0.00	0.29	0.00	0.00
GATCAAGAAGCGGGGGAGCG	0.00	0.00	0.00	0.34	0.00
GATCAAGAAGCGGGGGGAGG	0.00	0.00	0.57	0.00	0.00
GATCAAGAAGCGGGTGGATT	0.00	0.00	0.00	0.00	0.00
GATCAAGAAGCGGTCCAAGG	0.00	0.00	0.00	0.00	0.00
GATCAAGAAGCGGTCTTCGG	0.00	0.00	0.00	0.00	0.34
GATCAAGAAGCGTAAACAAC	0.00	0.00	0.00	0.00	0.00
GATCAAGAAGCGTCAACGGC	0.92	0.33	0.29	0.00	0.00
GATCAAGAAGCGTGGCGTTG	0.00	0.00	0.00	0.00	0.00
GATCAAGAAGCGTTGACGGG	0.00	0.00	0.00	0.00	0.00
GATCAAGAAGCGTTGGGCCG	0.00	0.00	0.00	0.00	0.00
GATCAAGAAGCTAAAAAAAA	0.00	0.00	0.00	0.00	0.00
GATCAAGAAGCTAGAGAGGG	0.00	0.00	0.00	0.00	0.00
GATCAAGAAGCTAGGCGGGA	0.00	0.33	0.00	0.00	0.00
GATCAAGAAGCTATCTGAAG	0.00	0.33	0.00	0.00	0.00
GATCAAGAAGCTCAAGACCA	0.00	0.00	0.00	0.34	0.00
GATCAAGAAGCTCAAGCCGC	0.00	0.00	0.00	0.34	0.00
GATCAAGAAGCTCAAGGAGC	0.00	0.33	0.00	0.00	0.00

GATCAAGAAGCTCACAGGAC	0.00	0.00	0.29	0.00	0.00
GATCAAGAAGCTCACGGTCT	0.00	0.00	0.00	0.34	0.00
GATCAAGAAGCTCAGGCTAG	0.00	0.00	0.00	0.00	0.34
GATCAAGAAGCTCAGTTTGA	0.69	0.33	0.00	0.34	0.00
GATCAAGAAGCTCCCCAGGC	0.00	0.00	0.00	0.00	0.00
GATCAAGAAGCTCCTGAAGC	0.00	0.00	0.00	0.00	0.00
GATCAAGAAGCTCGAGAAGA	1.14	0.00	0.57	0.00	0.69
GATCAAGAAGCTCGAGAAGC	0.00	0.00	0.00	0.00	0.00
GATCAAGAAGCTCGAGCGCG	0.00	0.00	0.00	0.00	0.00
GATCAAGAAGCTCGATGAGA	0.00	0.33	0.00	0.68	0.34
GATCAAGAAGCTCGTCGACA	0.23	0.33	0.00	0.00	0.00
GATCAAGAAGCTCTACCAGA	0.00	0.00	0.00	0.68	0.00
GATCAAGAAGCTCTCCGAGC	0.00	0.33	0.00	0.00	0.00
GATCAAGAAGCTCTCGGAAG	0.00	0.00	0.00	0.00	0.34
GATCAAGAAGCTCTCGGAGC	0.00	0.00	0.00	0.00	0.00
GATCAAGAAGCTCTGCGAGG	0.23	0.00	0.00	0.00	0.00
GATCAAGAAGCTGAGCCCTC	0.00	0.00	0.29	0.68	0.00
GATCAAGAAGCTGAGTTTGA	0.46	0.00	0.00	0.00	0.00
GATCAAGAAGCTGCACCGGA	0.00	0.33	0.00	0.00	0.00
GATCAAGAAGCTGCACCGGC	0.00	0.00	0.57	0.00	0.00
GATCAAGAAGCTGCAGAACT	2.29	0.00	0.00	0.00	0.00
GATCAAGAAGCTGCAGAGGG	0.00	0.00	0.00	0.00	0.34
GATCAAGAAGCTGCAGATAT	0.00	0.00	0.29	0.00	0.00
GATCAAGAAGCTGCAGGCAA	0.00	0.33	0.00	0.00	0.00
GATCAAGAAGCTGCCAGGAC	0.00	0.00	0.00	0.00	0.00
GATCAAGAAGCTGCCGGGCT	0.46	1.30	1.43	0.00	2.41
GATCAAGAAGCTGCTGGACC	0.00	0.33	0.00	0.68	1.38
GATCAAGAAGCTGCTGGGCA	0.00	0.00	0.00	0.00	0.00
GATCAAGAAGCTGCTTGGCC	0.00	0.00	0.00	0.00	0.00
GATCAAGAAGCTGGACGAGA	0.69	0.98	1.72	1.70	1.72
GATCAAGAAGCTGGACGCGA	0.00	0.33	0.00	0.00	0.00
GATCAAGAAGCTGGACTGGA	0.00	0.00	0.00	0.00	0.00
GATCAAGAAGCTGGAGAACC	0.00	0.00	0.00	0.00	0.00
GATCAAGAAGCTGGAGAGGA	0.00	0.00	0.00	0.34	0.00
GATCAAGAAGCTGGAGCAGA	0.00	0.00	0.00	0.00	0.00
GATCAAGAAGCTGGAGGCAC	0.00	0.33	0.29	0.00	0.00
GATCAAGAAGCTGGATGCGC	0.00	0.00	0.00	0.34	0.00

GATCAAGAAGCTGGGCTTCA	0.00	0.00	0.00	0.68	0.00
GATCAAGAAGCTGGGTTCGG	0.00	0.00	0.00	0.00	0.00
GATCAAGAAGCTGGGTTTCA	0.00	0.98	0.29	1.02	0.00
GATCAAGAAGCTGGTCATCG	0.00	0.33	0.00	0.00	0.00
GATCAAGAAGCTGGTGAGGG	0.00	0.00	0.00	0.34	0.00
GATCAAGAAGCTGGTTAACT	0.00	0.65	0.00	0.34	0.00
GATCAAGAAGCTGTACAAGG	0.00	0.33	0.00	0.00	0.34
GATCAAGAAGCTGTATGTAA	0.00	0.00	0.00	0.00	0.00
GATCAAGAAGCTGTTGGACA	0.00	0.33	0.00	0.00	0.00
GATCAAGAAGCTGTTTGCGG	0.00	0.65	0.00	0.00	0.00
GATCAAGAAGCTTAGCACAG	0.00	0.00	0.00	0.34	0.00
GATCAAGAAGCTTCAAGACC	0.00	0.00	0.00	0.00	0.34
GATCAAGAAGCTTCTTAGGT	0.92	0.00	0.00	0.00	0.00
GATCAAGAAGCTTGACAAGA	0.00	0.33	0.00	0.00	0.00
GATCAAGAAGCTTGACCAGA	0.00	0.00	0.00	0.00	0.34
GATCAAGAAGCTTGACTTCT	0.00	0.00	0.00	0.00	0.00
GATCAAGAAGCTTGAGGACT	0.00	0.98	0.00	1.02	0.69
GATCAAGAAGCTTGCCAAGC	0.00	0.33	0.00	0.00	0.00
GATCAAGAAGCTTGCGGAAG	0.00	0.00	0.29	0.00	0.00
GATCAAGAAGCTTGGCTATC	0.00	0.00	0.00	0.00	0.00
GATCAAGAAGCTTGGCTTCA	0.69	1.95	1.43	1.70	1.72
GATCAAGAAGCTTGGGGAAG	0.00	0.00	0.00	0.00	0.00
GATCAAGAAGCTTGTGGAAG	13.73	16.92	11.74	10.86	8.95
GATCAAGAAGCTTGTGGAAN	0.00	0.00	0.00	0.00	0.00
GATCAAGAAGCTTGTGGACG	0.00	0.00	0.29	0.00	0.00
GATCAAGAAGCTTGTGGAGG	0.23	0.00	0.00	0.00	0.00
GATCAAGAAGCTTGTGGATT	0.00	0.00	0.00	0.00	0.00
GATCAAGAAGCTTGTGGCCG	0.23	0.00	0.00	0.00	0.00
GATCAAGAAGCTTGTGGTGA	0.00	0.00	0.00	0.00	0.34
GATCAAGAAGCTTTACCAGG	0.00	0.33	0.00	0.00	0.00
GATCAAGAAGCTTTCGCAGG	0.46	0.98	0.00	0.34	0.69
GATCAAGAAGGAAAAAAAAA	1.60	0.00	0.29	0.34	0.34
GATCAAGAAGGAAAGAAGGA	0.00	0.00	0.29	0.00	0.00
GATCAAGAAGGAAAGCCGGA	0.00	0.00	0.00	0.00	0.34
GATCAAGAAGGAACAGCCCG	0.00	0.33	0.00	0.00	0.00
GATCAAGAAGGAACCGTCCA	0.23	0.00	0.00	0.00	0.00
GATCAAGAAGGAACTCGAAG	0.23	2.28	0.29	0.34	0.34

GATCAAGAAGGAACTCGATT	0.00	0.00	0.00	0.00	0.00
GATCAAGAAGGAACTCGCAG	0.00	0.33	0.00	0.00	0.00
GATCAAGAAGGAACTCGGAG	0.23	0.00	0.00	0.00	0.00
GATCAAGAAGGAACTCTCGG	0.00	1.30	0.57	1.70	0.69
GATCAAGAAGGAAGAAGGAA	0.00	0.00	3.15	0.00	4.13
GATCAAGAAGGAAGAATATG	0.23	0.00	0.00	0.00	0.00
GATCAAGAAGGAATTTTTGT	0.00	0.00	0.29	0.00	0.69
GATCAAGAAGGACAAGAACG	0.00	0.33	0.00	0.00	0.00
GATCAAGAAGGACAAGCACG	0.23	0.00	0.00	0.00	0.34
GATCAAGAAGGACAAGCAGC	0.00	0.00	0.00	0.00	0.00
GATCAAGAAGGACAAGCATG	0.00	0.33	0.00	0.00	0.00
GATCAAGAAGGACAGCTGAG	0.00	0.00	0.00	0.34	0.00
GATCAAGAAGGACATCGAGG	8.24	3.58	3.72	5.43	6.89
GATCAAGAAGGACATCGAGT	0.00	0.00	0.00	0.00	0.00
GATCAAGAAGGACCCCGTCA	0.00	0.65	0.00	0.34	0.34
GATCAAGAAGGACCTGGAGA	0.00	0.00	0.00	0.00	0.00
GATCAAGAAGGACGAGTACG	0.00	0.00	0.00	0.00	0.00
GATCAAGAAGGACGCCCAAA	0.00	0.00	0.29	0.00	0.00
GATCAAGAAGGACGCGAAGA	0.00	0.98	0.29	0.68	1.03
GATCAAGAAGGACGGTGAGG	0.00	0.33	0.00	0.00	0.00
GATCAAGAAGGACTCCTCCG	0.00	0.00	0.00	0.34	0.00
GATCAAGAAGGACTGCAAGA	0.00	0.00	0.00	0.00	0.00
GATCAAGAAGGACTGGTGGG	0.00	0.33	0.29	0.00	0.00
GATCAAGAAGGAGAAAAAAA	0.00	0.00	0.00	0.00	0.34
GATCAAGAAGGAGAAGAAGA	0.00	0.00	0.86	0.00	2.41
GATCAAGAAGGAGAAGACGA	0.00	0.00	0.00	0.00	0.34
GATCAAGAAGGAGAAGCAGG	0.00	0.00	0.00	0.00	0.34
GATCAAGAAGGAGAAGGAGA	0.00	0.00	0.29	0.00	0.34
GATCAAGAAGGAGAAGGATG	0.00	0.00	0.29	0.00	0.00
GATCAAGAAGGAGACATGCG	0.23	0.00	0.00	0.00	0.00
GATCAAGAAGGAGAGCACTT	0.00	0.00	0.00	0.00	0.34
GATCAAGAAGGAGATGGAGG	0.00	0.98	0.00	0.68	0.00
GATCAAGAAGGAGATTCAAA	0.00	0.00	0.00	0.00	0.69
GATCAAGAAGGAGATTTTCA	0.00	0.00	0.00	0.00	0.00
GATCAAGAAGGAGCAGGCAG	0.23	0.00	0.00	0.00	0.00
GATCAAGAAGGAGCCCTCCG	0.23	0.00	0.00	0.00	0.00
GATCAAGAAGGAGCCTGCGG	0.00	0.00	0.00	0.00	0.69

GATCAAGAAGGAGCGCCAAG	0.23	0.00	0.00	0.68	0.34
GATCAAGAAGGAGCGCTTGC	3.66	3.25	3.44	3.39	3.10
GATCAAGAAGGAGCGGTGTG	0.00	0.00	0.00	0.00	0.00
GATCAAGAAGGAGCTGAAAA	0.46	0.98	0.57	0.00	2.41
GATCAAGAAGGAGCTGTTCG	0.00	0.00	0.00	0.00	0.00
GATCAAGAAGGAGGAAAAAA	0.00	0.00	0.00	0.34	0.00
GATCAAGAAGGAGGAGACAA	0.00	0.00	0.29	0.00	0.00
GATCAAGAAGGAGGAGCACC	0.00	0.33	0.29	0.34	0.00
GATCAAGAAGGAGGAGCCAA	3.66	1.95	1.72	2.72	3.10
GATCAAGAAGGAGGAGGAGG	0.00	0.00	0.29	0.00	0.00
GATCAAGAAGGAGGAGGGAG	0.00	0.00	0.29	0.00	0.34
GATCAAGAAGGAGGAGTACG	0.00	0.00	0.29	0.00	0.00
GATCAAGAAGGAGGATTCGT	0.00	0.00	0.00	0.00	0.34
GATCAAGAAGGAGGCAAAAA	0.46	0.00	0.00	0.34	0.00
GATCAAGAAGGAGGCCGAGA	0.00	0.00	0.00	0.34	0.00
GATCAAGAAGGAGGCCTCGG	0.00	0.00	0.00	0.00	0.00
GATCAAGAAGGAGGCGATGG	0.23	0.00	0.00	0.00	0.00
GATCAAGAAGGAGGCGGAAG	0.00	0.00	0.00	0.00	0.00
GATCAAGAAGGAGGCTGAGC	1.60	0.65	0.86	0.68	0.34
GATCAAGAAGGAGGGCGAGC	0.00	0.33	0.00	0.00	0.00
GATCAAGAAGGAGGTGATAA	0.23	0.00	0.00	0.00	0.00
GATCAAGAAGGAGGTGCCGC	0.23	0.65	0.00	1.02	0.00
GATCAAGAAGGAGGTGTGAG	1.37	0.00	0.00	0.00	0.00
GATCAAGAAGGAGGTTTTCA	0.23	0.00	0.00	0.00	0.00
GATCAAGAAGGAGTACGACG	0.00	0.00	0.00	0.00	0.00
GATCAAGAAGGAGTGCCAAG	0.00	0.00	0.00	0.00	0.34
GATCAAGAAGGAGTTCATGG	0.00	0.00	0.00	0.00	0.00
GATCAAGAAGGAGTTTATCA	0.00	0.33	0.00	0.00	0.00
GATCAAGAAGGAGTTTNNNN	0.23	0.00	0.00	0.00	0.00
GATCAAGAAGGAGTTTTCAA	0.23	0.00	0.00	0.00	0.00
GATCAAGAAGGAGTTTTCCA	0.00	0.00	0.00	0.00	0.00
GATCAAGAAGGAGTTTTTAA	0.00	0.00	0.00	0.34	0.00
GATCAAGAAGGAGTTTTTCA	32.28	49.12	35.51	41.75	37.88
GATCAAGAAGGAGTTTTTCC	0.46	0.00	0.29	0.00	0.00
GATCAAGAAGGAGTTTTTCG	0.23	0.00	0.00	0.00	0.00
GATCAAGAAGGAGTTTTTCN	0.00	0.00	0.00	0.00	0.00
GATCAAGAAGGAGTTTTTCT	1.37	0.00	0.00	0.00	0.00

GATCAAGAAGGAGTTTTTNN	0.00	0.00	0.00	0.34	0.00
GATCAAGAAGGAGTTTTTTA	0.23	0.00	0.00	0.00	0.00
GATCAAGAAGGAGTTTTTTC	0.00	0.00	0.00	0.34	0.00
GATCAAGAAGGATATCGAGG	0.00	0.33	0.00	0.00	0.00
GATCAAGAAGGATGAGGAAA	0.00	0.00	0.57	0.00	0.00
GATCAAGAAGGATGATGCCC	0.00	0.33	0.00	0.00	0.00
GATCAAGAAGGATGGAGAGG	0.00	0.00	0.00	0.00	0.69
GATCAAGAAGGATGGTTCTG	0.00	0.00	0.00	0.00	0.34
GATCAAGAAGGATGGTTGTG	0.92	0.33	0.00	0.68	1.03
GATCAAGAAGGATTCCAGCA	3.20	1.95	1.15	0.68	1.38
GATCAAGAAGGATTCCAGNN	0.00	0.00	0.00	0.34	0.00
GATCAAGAAGGATTGGACAA	0.00	0.33	0.00	0.00	0.00
GATCAAGAAGGCAAACGTAC	0.00	0.00	0.00	0.00	0.00
GATCAAGAAGGCAAACGTGA	0.00	0.00	0.29	0.00	0.00
GATCAAGAAGGCAAAGAAGA	0.00	0.00	0.00	0.00	0.00
GATCAAGAAGGCAACAGACC	0.00	0.00	0.29	0.00	0.00
GATCAAGAAGGCAACAGTCC	0.00	0.00	0.29	0.00	0.00
GATCAAGAAGGCAACCATCC	0.00	0.00	0.29	0.00	0.00
GATCAAGAAGGCAACCGACC	0.00	0.33	0.29	0.00	0.34
GATCAAGAAGGCAACCGCCC	0.23	0.00	0.00	0.00	0.34
GATCAAGAAGGCAACCGGCC	0.23	0.00	0.57	0.34	0.00
GATCAAGAAGGCAACCGTAC	0.00	0.33	0.00	0.68	0.00
GATCAAGAAGGCAACCGTCA	0.00	0.00	0.00	0.34	0.34
GATCAAGAAGGCAACCGTCC	54.25	29.60	24.63	44.47	23.42
GATCAAGAAGGCAACCGTCN	0.23	0.00	0.00	0.34	0.00
GATCAAGAAGGCAACCGTNN	0.46	0.00	0.00	0.00	0.00
GATCAAGAAGGCAACCGTTC	0.00	0.00	0.57	0.00	0.34
GATCAAGAAGGCAACCNNNN	0.23	0.00	0.00	0.00	0.00
GATCAAGAAGGCAACCNTCC	0.00	0.00	0.00	0.00	0.34
GATCAAGAAGGCAACCTGAC	0.00	0.00	0.00	0.00	0.00
GATCAAGAAGGCAACTCGTA	0.00	0.33	0.00	0.00	0.00
GATCAAGAAGGCAACTGTCC	0.23	0.00	0.00	0.00	0.00
GATCAAGAAGGCAATCGTCC	0.23	0.00	0.00	0.00	0.00
GATCAAGAAGGCAATGCAGA	0.00	0.33	0.00	0.00	0.00
GATCAAGAAGGCAATGCGGG	1.14	0.98	0.00	0.34	1.03
GATCAAGAAGGCAATTGCTG	0.00	0.00	0.29	0.00	0.00
GATCAAGAAGGCACAAGAGA	0.23	0.00	0.00	0.00	0.00

GATCAAGAAGGCACAGGAAG	0.00	0.00	0.00	0.34	0.00
GATCAAGAAGGCACCGTCCA	0.00	0.00	0.00	0.00	0.00
GATCAAGAAGGCACGCGGGA	0.00	0.33	0.00	0.00	0.00
GATCAAGAAGGCACGGGAAG	24.04	16.27	6.59	9.50	4.82
GATCAAGAAGGCACGGGAGG	0.23	0.00	0.00	0.00	0.00
GATCAAGAAGGCACGGGCAG	0.23	0.33	0.00	0.00	0.00
GATCAAGAAGGCACGGGGAG	0.23	0.00	0.00	0.00	0.00
GATCAAGAAGGCACGGGTAG	0.23	0.00	0.00	0.00	0.00
GATCAAGAAGGCACGTGAAG	0.46	0.00	0.00	0.00	0.00
GATCAAGAAGGCACTGAGGA	0.00	0.00	0.29	0.00	0.00
GATCAAGAAGGCAGAGATGG	0.00	0.00	0.29	0.00	0.00
GATCAAGAAGGCAGCAGAGG	0.92	0.00	0.00	0.00	0.00
GATCAAGAAGGCAGTGGCCA	0.00	0.33	0.29	0.00	0.34
GATCAAGAAGGCATCCCGCG	0.00	0.00	0.00	0.00	0.00
GATCAAGAAGGCATGGGAAG	0.00	0.00	0.00	0.00	0.00
GATCAAGAAGGCCAAGAAGA	0.46	0.00	0.00	0.00	0.00
GATCAAGAAGGCCACACCGC	0.00	0.00	0.29	0.00	0.00
GATCAAGAAGGCCACGCCCC	0.00	0.00	0.00	0.00	0.34
GATCAAGAAGGCCACGCCGC	0.23	0.00	1.15	0.68	0.34
GATCAAGAAGGCCACGCCGT	0.00	0.00	0.29	0.00	0.00
GATCAAGAAGGCCACGCCTC	0.00	0.00	0.00	0.00	0.34
GATCAAGAAGGCCATCAACC	0.00	0.00	0.29	0.00	0.00
GATCAAGAAGGCCATCCAGG	0.00	0.00	0.29	0.00	0.00
GATCAAGAAGGCCATGCGCC	0.00	0.00	0.00	0.34	0.00
GATCAAGAAGGCCATGTACA	0.00	0.33	0.00	0.00	0.00
GATCAAGAAGGCCCAGGCCG	0.00	0.00	0.29	0.00	0.00
GATCAAGAAGGCCCGAGCCG	0.23	0.00	0.00	0.00	0.00
GATCAAGAAGGCCGACCTCG	0.00	0.98	0.86	0.34	0.00
GATCAAGAAGGCCGAGGAGG	0.23	0.00	0.00	0.00	0.00
GATCAAGAAGGCCGAGGGGA	2.52	0.33	0.00	0.34	0.34
GATCAAGAAGGCCGAGGGGC	0.23	0.00	0.00	0.00	0.00
GATCAAGAAGGCCGCGGGGC	0.23	0.00	0.00	0.00	0.00
GATCAAGAAGGCCGCTCTCT	0.00	0.00	0.00	0.00	0.00
GATCAAGAAGGCCGGCGAGG	0.00	0.33	0.57	0.34	0.34
GATCAAGAAGGCCGGCGGCT	0.23	0.00	0.00	0.00	0.00
GATCAAGAAGGCCGTGAAGC	0.00	0.00	0.00	0.34	0.00
GATCAAGAAGGCCGTGGAGC	0.00	0.33	0.00	0.00	0.00

GATCAAGAAGGCCTACAGAG	0.23	0.00	0.00	0.00	0.00
GATCAAGAAGGCCTACAGCC	0.23	0.00	0.00	0.00	0.00
GATCAAGAAGGCCTACCGCC	5.72	4.55	1.72	3.73	3.10
GATCAAGAAGGCCTACCGCG	1.37	0.33	0.29	0.00	1.38
GATCAAGAAGGCCTACCGGA	0.00	0.00	0.00	0.34	0.00
GATCAAGAAGGCCTACCGTC	0.00	0.00	0.00	0.34	0.00
GATCAAGAAGGCCTACTACC	0.46	0.00	0.00	0.34	0.00
GATCAAGAAGGCCTATGAAG	0.00	0.00	0.00	0.00	0.00
GATCAAGAAGGCCTCTAGCC	0.00	0.33	0.00	0.00	0.00
GATCAAGAAGGCCTTCAAGA	1.14	1.30	0.57	1.02	0.00
GATCAAGAAGGCCTTCCGGA	0.69	0.33	0.00	0.68	0.34
GATCAAGAAGGCCTTGAACT	0.00	0.00	0.29	0.00	0.00
GATCAAGAAGGCCTTTGATG	0.00	0.00	0.00	0.00	0.34
GATCAAGAAGGCGAAGTAAG	0.23	0.00	0.00	0.00	0.00
GATCAAGAAGGCGACGCAGC	0.00	0.00	0.00	0.00	0.34
GATCAAGAAGGCGACGCCGC	0.46	0.33	0.00	0.00	0.00
GATCAAGAAGGCGAGCCAGG	0.00	0.00	0.00	0.34	0.00
GATCAAGAAGGCGCAACAGA	0.00	0.00	0.29	0.00	0.00
GATCAAGAAGGCGCACAAGG	0.00	0.00	0.00	0.00	0.00
GATCAAGAAGGCGCACCGGC	0.00	0.33	0.29	0.00	0.00
GATCAAGAAGGCGCGGGAAG	0.23	0.00	0.00	0.00	0.00
GATCAAGAAGGCGCTCCCTT	0.00	0.00	0.00	0.34	0.00
GATCAAGAAGGCGCTGCAGC	0.00	0.33	0.00	0.34	0.00
GATCAAGAAGGCGCTGCCGG	0.23	0.00	0.00	0.00	0.34
GATCAAGAAGGCGGACTTCG	0.00	0.00	0.00	0.00	0.00
GATCAAGAAGGCGGAGGCCA	0.00	0.00	0.00	0.00	0.34
GATCAAGAAGGCGGATATAT	0.00	0.00	0.57	0.00	0.00
GATCAAGAAGGCGGCGGACA	0.00	0.00	0.29	0.00	0.00
GATCAAGAAGGCGGCGGCCA	0.00	0.00	0.00	0.00	0.34
GATCAAGAAGGCGGCGGGCA	0.00	0.00	0.00	0.34	0.00
GATCAAGAAGGCGGGCAACA	0.00	0.00	0.00	0.34	0.00
GATCAAGAAGGCGGGGCCAT	0.00	0.00	0.00	0.00	0.00
GATCAAGAAGGCGGGGGCCA	0.46	0.33	0.29	0.34	0.00
GATCAAGAAGGCGGGGGCCC	0.00	0.00	0.00	0.34	0.00
GATCAAGAAGGCGGGGGGCA	0.00	0.00	0.00	0.00	0.34
GATCAAGAAGGCGGTAGCCA	0.00	0.00	0.00	0.00	0.00
GATCAAGAAGGCGGTGGAAA	0.00	0.00	0.00	0.00	0.00

GATCAAGAAGGCGGTGGACA	0.23	0.33	0.29	0.00	0.34
GATCAAGAAGGCGGTGGCAA	0.23	0.00	0.00	0.68	0.34
GATCAAGAAGGCGGTGGCCA	18.31	59.86	101.94	52.27	68.54
GATCAAGAAGGCGGTGGCCC	0.00	1.30	1.15	0.00	1.03
GATCAAGAAGGCGGTGGCCG	0.00	0.00	0.57	0.00	0.00
GATCAAGAAGGCGGTGGCCN	0.00	0.00	0.00	0.00	0.00
GATCAAGAAGGCGGTGGCCT	0.00	0.00	0.57	0.00	0.00
GATCAAGAAGGCGGTGGCGA	0.23	0.00	0.29	0.00	0.34
GATCAAGAAGGCGGTGGCTA	0.00	0.00	0.29	0.00	0.00
GATCAAGAAGGCGGTGGGCA	0.46	0.00	0.00	0.00	0.00
GATCAAGAAGGCGGTGGTCA	0.00	0.00	0.29	0.00	0.00
GATCAAGAAGGCGGTGNCCA	0.00	0.00	0.29	0.34	0.34
GATCAAGAAGGCGGTGTCCA	0.00	0.00	0.00	0.00	0.34
GATCAAGAAGGCGGTTGCCC	0.00	0.00	0.00	0.00	0.34
GATCAAGAAGGCGTACCGGC	0.00	0.00	0.00	0.68	0.00
GATCAAGAAGGCGTACTCCG	0.00	0.00	0.00	0.34	0.00
GATCAAGAAGGCGTACTTCG	8.70	14.97	16.32	9.16	10.68
GATCAAGAAGGCGTACTTNN	0.00	0.00	0.00	0.00	0.00
GATCAAGAAGGCGTCAACTG	0.00	0.00	0.00	0.34	0.00
GATCAAGAAGGCGTCTTCCG	0.00	0.00	0.00	0.68	0.00
GATCAAGAAGGCGTGCCTGG	0.69	0.33	0.29	0.68	0.69
GATCAAGAAGGCTAAGATGT	0.00	0.00	0.00	0.00	0.00
GATCAAGAAGGCTAGGCGCG	0.00	0.00	0.00	0.00	0.00
GATCAAGAAGGCTCGTATGC	0.00	0.00	0.00	0.00	0.34
GATCAAGAAGGCTGAGCCGC	0.00	0.00	0.29	0.00	0.00
GATCAAGAAGGCTGCCATGC	0.00	0.00	0.00	0.00	0.34
GATCAAGAAGGCTGCGGCCA	0.23	0.00	0.00	0.00	0.00
GATCAAGAAGGCTGCTCAGA	0.00	0.00	0.00	0.34	0.00
GATCAAGAAGGCTGGAACGG	0.00	0.00	0.00	0.00	0.00
GATCAAGAAGGCTGGCCACA	3.66	1.63	0.86	2.72	1.38
GATCAAGAAGGCTGGCCCCA	0.00	0.00	0.29	0.00	0.00
GATCAAGAAGGCTGGCGTGG	0.00	0.00	0.29	0.00	0.69
GATCAAGAAGGCTGTCAGCC	0.00	0.00	0.00	0.00	0.34
GATCAAGAAGGCTGTGAACA	0.00	0.00	0.00	0.00	0.00
GATCAAGAAGGCTGTGGACA	0.00	0.00	0.00	0.00	0.34
GATCAAGAAGGCTGTGGCCA	7.78	20.82	27.78	14.60	24.45
GATCAAGAAGGCTGTGGCCC	0.00	0.65	0.00	0.00	0.34

GATCAAGAAGGCTGTGGCCN	0.23	0.00	0.00	0.00	0.00
GATCAAGAAGGCTGTGGCTA	0.00	0.00	0.29	0.00	0.00
GATCAAGAAGGCTGTGGGCA	0.00	0.00	0.29	0.00	0.00
GATCAAGAAGGCTTACCTGG	0.46	0.00	0.00	0.34	0.00
GATCAAGAAGGCTTCCACGA	0.00	0.00	0.00	0.00	0.34
GATCAAGAAGGCTTTTAATA	0.00	0.00	0.00	0.34	0.00
GATCAAGAAGGGAAAAAAAA	0.00	0.00	0.00	0.00	0.34
GATCAAGAAGGGAAGCAGAT	0.00	0.00	0.29	0.68	0.00
GATCAAGAAGGGAAGCTGAC	0.00	0.00	0.00	0.00	0.34
GATCAAGAAGGGAAGCTGAT	14.19	21.47	14.60	17.31	8.95
GATCAAGAAGGGAAGCTGCT	0.00	0.33	0.00	0.00	0.00
GATCAAGAAGGGAAGGCATT	0.00	0.00	0.29	0.00	0.00
GATCAAGAAGGGAAGGGGCA	0.00	0.33	0.00	0.00	0.00
GATCAAGAAGGGAAGTTAAG	0.00	0.00	0.57	0.00	0.00
GATCAAGAAGGGAAGTTGAT	0.23	0.00	0.00	0.00	0.00
GATCAAGAAGGGACTGGCGA	0.23	0.00	0.00	0.00	0.00
GATCAAGAAGGGAGACAGAT	0.00	0.00	0.00	0.34	0.00
GATCAAGAAGGGAGACAGCT	2.98	5.86	9.74	10.18	9.99
GATCAAGAAGGGAGACAGTC	0.00	0.33	0.00	0.00	0.00
GATCAAGAAGGGAGAGGTTG	0.00	0.00	0.00	0.00	0.34
GATCAAGAAGGGAGATGCCT	0.00	0.00	0.00	0.34	0.00
GATCAAGAAGGGAGCACGTC	0.00	0.00	0.00	0.00	0.00
GATCAAGAAGGGAGCAGAGG	0.00	0.00	0.00	0.00	0.00
GATCAAGAAGGGAGCTTGTG	0.00	0.00	0.00	0.00	0.00
GATCAAGAAGGGAGGCTGAT	0.23	0.00	0.00	0.00	0.00
GATCAAGAAGGGATGGCCCA	0.00	0.00	0.00	0.00	0.00
GATCAAGAAGGGCAAAAGCA	0.00	0.00	0.00	0.00	0.00
GATCAAGAAGGGCAAACTCG	0.00	0.00	0.00	0.00	0.00
GATCAAGAAGGGCAAGGGCA	0.00	0.00	0.00	0.00	0.00
GATCAAGAAGGGCAAGGTCA	0.69	0.33	0.00	0.34	0.00
GATCAAGAAGGGCAAGTGAG	0.00	1.30	1.72	0.00	0.00
GATCAAGAAGGGCAGCGAGA	0.46	0.00	0.29	0.68	0.00
GATCAAGAAGGGCATCCAGG	0.00	0.00	0.00	0.00	0.34
GATCAAGAAGGGCATGATGT	0.00	0.00	0.00	0.34	0.00
GATCAAGAAGGGCATGTTCA	0.00	0.00	0.00	0.34	0.00
GATCAAGAAGGGCCAATGCA	0.00	0.00	0.29	0.34	0.00
GATCAAGAAGGGCCATGCCT	0.00	0.00	0.00	0.00	0.00

GATCAAGAAGGGCCCTCTTC	0.00	0.33	0.00	0.00	0.00
GATCAAGAAGGGCCTCATTG	0.00	0.00	0.29	0.00	0.69
GATCAAGAAGGGCCTCCGGA	0.00	0.00	0.00	0.34	0.00
GATCAAGAAGGGCCTCCTCT	0.00	0.00	0.29	0.00	0.00
GATCAAGAAGGGCCTGGTCG	0.00	0.33	0.00	0.00	0.00
GATCAAGAAGGGCCTTGCGT	0.00	0.00	0.00	0.00	0.00
GATCAAGAAGGGCGACTACT	0.46	0.00	0.00	0.34	0.00
GATCAAGAAGGGCGAGGCCT	0.00	0.00	0.29	0.00	0.34
GATCAAGAAGGGCGAGGCGT	0.00	0.00	0.00	0.34	0.00
GATCAAGAAGGGCGATACCT	0.00	0.00	0.00	0.00	0.34
GATCAAGAAGGGCGATGACT	0.46	0.65	0.00	0.00	0.34
GATCAAGAAGGGCGATGCAT	0.00	0.33	0.57	1.02	0.00
GATCAAGAAGGGCGATGCCC	0.00	0.00	0.00	0.00	0.00
GATCAAGAAGGGCGATGCCG	0.00	0.00	0.29	0.68	0.00
GATCAAGAAGGGCGATGCCN	0.00	0.00	0.00	0.00	0.34
GATCAAGAAGGGCGATGCCT	49.44	96.30	113.11	85.54	90.92
GATCAAGAAGGGCGATGCNN	0.23	0.00	0.00	0.00	0.00
GATCAAGAAGGGCGATGCNT	0.00	0.00	0.00	0.00	0.00
GATCAAGAAGGGCGATGCTC	0.00	0.00	0.86	0.00	0.00
GATCAAGAAGGGCGATGGCT	0.23	0.00	0.00	0.34	0.00
GATCAAGAAGGGCGATGTCT	0.00	0.00	0.00	0.00	0.34
GATCAAGAAGGGCGATNCCT	0.00	0.00	0.00	0.00	0.34
GATCAAGAAGGGCGATTCCT	0.00	0.00	0.29	0.00	0.00
GATCAAGAAGGGCGCAGCCC	0.00	0.00	0.00	0.00	0.00
GATCAAGAAGGGCGCAGCTG	0.00	0.33	0.57	1.36	1.72
GATCAAGAAGGGCGCCCCAT	0.00	0.00	0.00	0.00	0.34
GATCAAGAAGGGCGCCGCAT	0.00	0.00	0.00	0.00	0.00
GATCAAGAAGGGCGCGGCCT	0.23	0.00	0.00	0.00	0.00
GATCAAGAAGGGCGGCCTGA	0.00	0.33	0.00	0.00	0.00
GATCAAGAAGGGCGGCCTTG	0.00	0.00	0.00	0.00	0.00
GATCAAGAAGGGCGGCGCAC	0.00	0.00	0.00	0.00	0.34
GATCAAGAAGGGCGGTGCCT	0.23	0.00	0.00	0.00	0.00
GATCAAGAAGGGCGTCCAGC	1.14	1.95	1.15	1.36	0.00
GATCAAGAAGGGCGTGGAGG	0.00	0.00	0.00	0.34	0.00
GATCAAGAAGGGCTCCGAAG	0.23	0.00	0.29	1.02	0.69
GATCAAGAAGGGCTCCGCAG	0.00	0.33	0.00	0.00	0.00
GATCAAGAAGGGCTTCCTCA	0.00	0.00	0.00	0.00	0.00

GATCAAGAAGGGCTTTGCCG	0.69	0.65	0.86	0.68	0.69
GATCAAGAAGGGGAAGAAGA	0.00	0.00	0.29	0.00	0.00
GATCAAGAAGGGGAAGAAGG	0.00	0.00	0.29	0.00	0.00
GATCAAGAAGGGGAAGAGGA	0.00	0.00	0.86	0.00	1.03
GATCAAGAAGGGGAATAAGG	0.00	0.00	0.57	0.00	0.00
GATCAAGAAGGGGAGCGCCG	0.00	0.00	0.29	0.00	0.00
GATCAAGAAGGGGAGCTGCT	0.23	0.00	0.00	0.00	0.00
GATCAAGAAGGGGAGTGGGC	0.00	0.00	0.29	0.00	0.00
GATCAAGAAGGGGAGTGTGC	0.00	0.00	0.29	0.00	0.00
GATCAAGAAGGGGCATGCAT	0.00	0.00	0.00	0.34	0.00
GATCAAGAAGGGGCGCTTGC	0.00	0.00	0.29	0.00	0.00
GATCAAGAAGGGGGATGCCT	0.00	0.00	0.00	0.00	0.00
GATCAAGAAGGGGTCGCAAC	0.00	0.00	0.00	0.00	0.34
GATCAAGAAGGGGTGTGAGA	0.00	0.00	0.00	0.00	0.00
GATCAAGAAGGGGTTCCCGA	0.23	0.00	0.00	0.00	0.00
GATCAAGAAGGGTAAGAAGG	0.00	0.00	0.00	0.00	0.69
GATCAAGAAGGGTCATGAAA	0.00	0.00	0.00	0.34	0.00
GATCAAGAAGGGTCTCTTCC	0.00	0.00	0.00	0.00	0.00
GATCAAGAAGGGTGCAGGCC	0.00	0.00	0.00	0.00	0.00
GATCAAGAAGGGTGCGGCCT	0.00	0.00	0.00	0.34	0.00
GATCAAGAAGGGTGCTGGTG	0.23	0.65	0.00	0.00	0.00
GATCAAGAAGGGTGTGCAAG	0.00	0.00	0.00	0.00	0.34
GATCAAGAAGGGTTTGCCTC	0.00	0.00	0.29	0.00	0.00
GATCAAGAAGGTATACGAGA	0.00	0.00	0.57	0.00	0.00
GATCAAGAAGGTCAAGGCAG	0.00	0.33	0.00	1.02	0.00
GATCAAGAAGGTCAAGGCTG	0.00	0.33	0.00	0.00	0.00
GATCAAGAAGGTCCAAGAGT	0.00	0.00	0.00	0.00	0.00
GATCAAGAAGGTCCAAGCGT	0.46	0.65	0.00	0.00	0.00
GATCAAGAAGGTCCGAGCAC	0.00	0.00	0.57	0.00	0.34
GATCAAGAAGGTCCTCACCA	0.00	0.00	0.57	0.00	0.00
GATCAAGAAGGTCCTCGGCG	0.00	0.65	0.00	0.00	0.00
GATCAAGAAGGTCCTCTCAG	0.00	0.00	0.00	0.00	0.00
GATCAAGAAGGTCCTGGAGG	0.00	0.00	0.00	0.00	0.00
GATCAAGAAGGTCGAAGAAG	0.00	0.00	0.00	0.00	0.69
GATCAAGAAGGTCGATGCCT	0.00	0.00	0.00	0.00	0.00
GATCAAGAAGGTCTACAAGC	0.00	0.00	0.00	0.00	0.00
GATCAAGAAGGTCTGTGAGG	0.46	0.00	0.00	0.00	0.00

GATCAAGAAGGTCTTCAAGA	0.00	0.00	0.00	0.00	0.00
GATCAAGAAGGTCTTCGAGT	0.00	0.00	0.00	0.00	0.00
GATCAAGAAGGTGAACACGC	0.46	0.00	0.29	0.68	0.34
GATCAAGAAGGTGAAGCCGC	0.46	0.00	0.00	0.00	0.00
GATCAAGAAGGTGAAGCCNN	0.23	0.00	0.00	0.00	0.00
GATCAAGAAGGTGACTTGCC	0.00	0.98	0.00	0.00	0.00
GATCAAGAAGGTGAGGCCGT	0.00	0.33	0.00	0.00	0.00
GATCAAGAAGGTGAGGCTGG	0.00	0.00	0.00	0.34	0.00
GATCAAGAAGGTGAGGCTGT	3.66	5.21	1.15	2.38	1.38
GATCAAGAAGGTGCTCGAGG	0.00	0.00	0.00	0.00	0.34
GATCAAGAAGGTGCTTTTAA	0.00	0.00	0.00	0.00	0.34
GATCAAGAAGGTGGACACGG	0.69	0.00	0.00	0.00	0.00
GATCAAGAAGGTGGCCTCGA	0.23	0.00	0.00	0.00	0.00
GATCAAGAAGGTGGCGGCCA	0.00	0.00	0.00	0.00	0.34
GATCAAGAAGGTGGCTGCGG	0.00	0.00	0.00	0.00	0.00
GATCAAGAAGGTGTCGTGGG	0.00	0.33	0.00	0.00	0.00
GATCAAGAAGGTTATTCACG	0.00	0.00	0.00	0.00	0.00
GATCAAGAAGGTTGCCAAGC	0.00	0.00	0.29	0.00	0.00
GATCAAGAAGGTTTAGACGT	0.46	0.00	0.00	0.00	0.00
GATCAAGAAGGTTTGCCATA	0.69	0.65	1.43	3.73	1.03
GATCAAGAAGTACAAGCCGC	0.00	0.00	0.29	0.00	0.00
GATCAAGAAGTACACACCGC	0.00	0.00	0.00	0.00	0.34
GATCAAGAAGTACACGAAGA	0.00	0.00	0.29	0.00	0.34
GATCAAGAAGTACACGACCC	0.00	0.00	0.00	0.34	0.00
GATCAAGAAGTACACGCAGA	0.00	0.00	0.00	0.00	0.00
GATCAAGAAGTACACGCAGC	0.00	0.00	0.00	0.34	0.00
GATCAAGAAGTACACGCATC	0.00	0.00	0.29	0.00	0.00
GATCAAGAAGTACACGCCGC	0.00	0.00	0.00	0.00	0.00
GATCAAGAAGTACACGCCTC	0.00	0.00	0.00	0.00	0.00
GATCAAGAAGTACACGNATC	0.00	0.00	0.00	0.00	0.34
GATCAAGAAGTACATCGATG	0.00	0.00	0.00	0.00	0.00
GATCAAGAAGTACCCGTGAG	0.00	0.00	0.29	0.00	0.00
GATCAAGAAGTACCTCGAGG	0.00	0.00	0.00	0.34	0.00
GATCAAGAAGTACGAGAACT	0.00	0.00	0.00	0.34	0.00
GATCAAGAAGTACGAGACAT	0.00	0.00	0.00	0.00	0.34
GATCAAGAAGTACGAGACCT	14.65	17.89	15.46	17.65	16.19
GATCAAGAAGTACGAGCATG	0.00	0.33	0.00	0.34	0.00

GATCAAGAAGTACGAGGCCT	0.00	0.33	0.00	0.00	0.00
GATCAAGAAGTACGATAGGC	0.00	0.00	0.00	0.00	0.00
GATCAAGAAGTACGCGACCT	0.00	0.00	0.00	0.00	0.00
GATCAAGAAGTACGGGGACG	0.00	0.00	0.00	0.00	0.34
GATCAAGAAGTACGGTACTC	0.00	0.00	0.00	0.00	0.00
GATCAAGAAGTACGGTGACG	0.46	0.00	0.00	0.00	0.00
GATCAAGAAGTACGGTGACT	1.37	2.93	0.57	0.34	1.03
GATCAAGAAGTACGGTGGCT	0.00	0.33	0.29	0.00	0.00
GATCAAGAAGTACGGTGGTA	0.00	0.00	0.00	0.00	0.00
GATCAAGAAGTACGGTNACT	0.00	0.00	0.29	0.00	0.00
GATCAAGAAGTACTACCAGA	0.00	0.00	0.00	0.00	1.03
GATCAAGAAGTACTACCCCG	0.00	0.33	0.00	0.00	0.00
GATCAAGAAGTACTACCCGA	0.00	0.33	0.00	0.00	0.00
GATCAAGAAGTACTCGAAAG	0.00	0.00	0.00	0.00	0.00
GATCAAGAAGTACTCGACAG	0.00	0.00	0.00	0.00	0.00
GATCAAGAAGTACTTTCTGT	0.00	0.00	0.00	0.00	0.00
GATCAAGAAGTAGACGCCAN	0.00	0.00	0.29	0.00	0.00
GATCAAGAAGTAGACGCCGC	0.00	0.00	0.57	0.00	0.00
GATCAAGAAGTAGAGTGCTG	0.00	0.00	0.00	0.00	0.00
GATCAAGAAGTAGCAGTCAA	0.00	0.00	0.00	0.34	0.00
GATCAAGAAGTAGGCGCCGG	0.23	0.00	0.00	0.00	0.00
GATCAAGAAGTATGCTAACG	0.00	0.00	0.00	0.00	0.00
GATCAAGAAGTATGGCCTCC	0.00	0.00	0.00	0.00	0.00
GATCAAGAAGTATGGGCCCA	0.00	0.33	0.00	0.00	0.00
GATCAAGAAGTATGGGCCCG	0.23	0.00	0.00	0.00	0.00
GATCAAGAAGTATTTTGGCA	0.23	0.00	0.00	0.00	0.34
GATCAAGAAGTCAACACCCA	0.00	0.00	0.29	0.00	0.00
GATCAAGAAGTCAACACCGC	0.00	0.00	0.86	0.00	0.00
GATCAAGAAGTCAACCGTCC	0.00	0.00	0.00	0.34	0.00
GATCAAGAAGTCAACGCAGC	0.00	0.00	0.00	0.00	0.00
GATCAAGAAGTCAACGCCGC	0.00	0.00	0.29	0.00	0.00
GATCAAGAAGTCAACGCCNC	0.00	0.00	0.00	0.00	0.34
GATCAAGAAGTCAACGCCTC	0.00	0.00	0.29	1.02	0.00
GATCAAGAAGTCACAGCATC	0.00	1.30	1.72	1.70	2.07
GATCAAGAAGTCACGCCTCT	0.00	0.00	0.00	0.34	0.00
GATCAAGAAGTCAGACCAGG	0.00	0.00	0.00	0.00	0.00
GATCAAGAAGTCAGAGGACA	0.00	0.33	0.29	0.00	0.00

GATCAAGAAGTCAGCTGTGC	0.00	0.00	0.29	0.00	0.00
GATCAAGAAGTCAGCTTGTG	0.00	0.00	0.00	0.00	0.34
GATCAAGAAGTCATACCGGT	0.00	0.33	0.00	0.00	0.00
GATCAAGAAGTCATCGACGC	0.23	0.33	0.00	0.00	0.00
GATCAAGAAGTCATTTCGGT	0.00	0.00	0.00	0.00	0.00
GATCAAGAAGTCCAAACCGC	0.23	0.00	0.00	0.00	0.00
GATCAAGAAGTCCAAGCCCC	0.00	0.00	0.00	0.00	0.00
GATCAAGAAGTCCAAGCCGC	0.00	0.00	0.00	0.00	0.34
GATCAAGAAGTCCACAACGA	0.00	0.33	0.00	0.00	0.34
GATCAAGAAGTCCACAACGC	0.00	0.65	4.30	0.00	0.34
GATCAAGAAGTCCACACAAC	0.00	0.00	0.00	0.00	0.00
GATCAAGAAGTCCACACAGC	0.00	0.65	0.00	0.34	0.00
GATCAAGAAGTCCACACAGG	0.00	0.00	0.00	0.00	0.00
GATCAAGAAGTCCACACCAC	0.00	0.00	0.57	0.34	0.69
GATCAAGAAGTCCACACCGA	0.00	0.65	0.29	0.34	0.00
GATCAAGAAGTCCACACCGC	42.12	98.57	212.48	95.72	167.38
GATCAAGAAGTCCACACCGN	0.00	0.00	0.00	0.00	0.00
GATCAAGAAGTCCACACCNC	0.00	0.00	0.00	0.34	0.00
GATCAAGAAGTCCACACCNN	0.00	0.00	0.00	0.34	0.00
GATCAAGAAGTCCACACCTC	0.23	0.00	0.00	0.00	0.00
GATCAAGAAGTCCACACGGC	0.00	0.00	0.00	0.00	0.34
GATCAAGAAGTCCACACTCG	0.00	0.98	0.86	0.00	0.00
GATCAAGAAGTCCACAGCGC	0.00	0.33	0.00	0.00	0.00
GATCAAGAAGTCCACANCGC	0.00	0.33	0.00	0.00	0.34
GATCAAGAAGTCCACANNGC	0.00	0.00	0.00	0.00	0.34
GATCAAGAAGTCCACANNNN	0.23	0.00	0.00	0.00	0.00
GATCAAGAAGTCCACATCGC	0.00	0.33	0.29	0.00	0.00
GATCAAGAAGTCCACCGCTG	0.00	0.00	0.00	0.00	0.00
GATCAAGAAGTCCACCNNNN	0.23	0.00	0.00	0.00	0.00
GATCAAGAAGTCCACGACCC	0.00	0.00	0.29	0.00	0.00
GATCAAGAAGTCCACGACGC	0.23	0.33	1.43	0.34	0.69
GATCAAGAAGTCCACGACNC	0.00	0.00	0.00	0.00	0.34
GATCAAGAAGTCCACGACTC	0.00	0.00	0.00	0.00	0.34
GATCAAGAAGTCCACGCACC	0.00	0.00	0.29	0.00	0.00
GATCAAGAAGTCCACGCAGC	0.23	0.00	1.72	0.00	0.69
GATCAAGAAGTCCACGCAGG	0.00	0.00	0.00	0.00	0.00
GATCAAGAAGTCCACGCATC	0.00	0.00	0.29	0.00	0.00

GATCAAGAAGTCCACGCCAC	0.00	0.33	0.29	0.00	0.00
GATCAAGAAGTCCACGCCCA	0.00	0.00	0.00	0.00	0.69
GATCAAGAAGTCCACGCCCC	12.59	33.18	45.82	22.40	30.31
GATCAAGAAGTCCACGCCCN	0.00	0.00	0.00	0.00	0.34
GATCAAGAAGTCCACGCCCT	0.23	0.00	0.00	0.00	0.00
GATCAAGAAGTCCACGCCGA	0.00	0.65	1.15	0.34	0.34
GATCAAGAAGTCCACGCCGC	49.67	179.91	290.08	161.57	210.43
GATCAAGAAGTCCACGCCGG	0.23	0.00	0.00	0.00	0.69
GATCAAGAAGTCCACGCCGN	0.92	0.00	0.00	0.00	0.00
GATCAAGAAGTCCACGCCGT	0.23	0.00	0.00	0.00	0.34
GATCAAGAAGTCCACGCCNC	0.00	0.00	0.00	0.00	0.00
GATCAAGAAGTCCACGCCNN	0.46	0.00	0.00	0.00	0.00
GATCAAGAAGTCCACGCCTA	0.23	0.00	0.00	0.00	0.00
GATCAAGAAGTCCACGCCTC	18.31	47.50	62.14	37.00	45.46
GATCAAGAAGTCCACGCCTG	0.00	0.33	0.00	0.68	0.00
GATCAAGAAGTCCACGCGGC	0.23	0.00	0.00	0.00	0.00
GATCAAGAAGTCCACGCTCG	0.00	0.00	0.86	1.02	0.00
GATCAAGAAGTCCACGCTGC	0.00	0.00	0.00	0.00	0.34
GATCAAGAAGTCCACGNCGC	0.00	0.00	0.00	0.00	0.69
GATCAAGAAGTCCACGNCTC	0.00	0.00	0.00	0.00	0.34
GATCAAGAAGTCCACGTCGC	0.00	0.00	0.29	0.00	0.00
GATCAAGAAGTCCACGTTGA	0.00	0.00	0.00	0.00	0.00
GATCAAGAAGTCCACGTTGC	0.00	0.00	0.29	0.00	0.00
GATCAAGAAGTCCACTGCTG	0.00	0.00	0.00	0.00	0.00
GATCAAGAAGTCCAGGCGAC	0.00	0.00	0.00	0.00	0.00
GATCAAGAAGTCCAGGTCCC	0.00	0.98	0.00	0.00	0.00
GATCAAGAAGTCCATACCGC	0.00	0.33	0.00	0.00	0.34
GATCAAGAAGTCCATGCCCC	0.00	0.00	0.00	0.00	0.00
GATCAAGAAGTCCATGCCGC	0.00	0.00	0.00	0.00	0.00
GATCAAGAAGTCCATGTAAA	0.00	0.00	0.00	0.68	0.00
GATCAAGAAGTCCCAGTGAG	0.00	0.00	0.00	0.34	0.00
GATCAAGAAGTCCCCACCCC	0.00	0.00	0.00	0.00	0.00
GATCAAGAAGTCCCCACCGC	0.23	0.00	0.00	0.00	0.00
GATCAAGAAGTCCCCGCCCC	0.00	0.00	0.29	0.00	0.00
GATCAAGAAGTCCCCGCCGC	0.00	0.33	0.29	0.00	0.00
GATCAAGAAGTCCCCGCGCC	0.23	0.00	0.00	0.00	0.00
GATCAAGAAGTCCCCGCTGC	0.00	0.00	0.00	0.34	0.00

GATCAAGAAGTCCCGCGCTT	0.00	0.00	0.00	0.68	0.00
GATCAAGAAGTCCCGTGCAC	0.46	0.00	0.00	0.00	0.34
GATCAAGAAGTCCCTTCGCA	0.00	0.65	0.00	0.34	0.00
GATCAAGAAGTCCGCACCGC	0.23	0.00	0.00	0.00	0.00
GATCAAGAAGTCCGCGCCAC	0.00	0.00	0.00	0.00	0.00
GATCAAGAAGTCCGCGCCGC	0.23	0.00	0.00	0.00	0.34
GATCAAGAAGTCCGCGTGTT	0.00	0.00	0.29	0.00	0.00
GATCAAGAAGTCCGGCCACG	0.00	0.00	0.00	0.00	0.00
GATCAAGAAGTCCGGCCATG	0.00	0.00	0.00	0.34	0.69
GATCAAGAAGTCCTCAGGGC	0.00	0.33	0.00	0.00	0.00
GATCAAGAAGTCCTCATGAG	0.00	0.00	0.00	0.00	0.00
GATCAAGAAGTCCTTCAGGA	0.00	0.00	0.00	0.34	0.00
GATCAAGAAGTCCTTTGACA	0.00	0.33	0.57	0.00	0.00
GATCAAGAAGTCGAAGCCGC	0.00	0.00	0.00	0.68	0.00
GATCAAGAAGTCGACACCGC	0.00	0.00	0.00	0.34	0.34
GATCAAGAAGTCGACCACGC	0.00	0.00	0.29	0.00	0.00
GATCAAGAAGTCGACGAAGC	0.00	0.00	0.00	0.00	0.00
GATCAAGAAGTCGACGACGA	0.00	0.00	0.00	0.00	0.34
GATCAAGAAGTCGACGACGC	0.00	0.98	0.00	0.00	0.69
GATCAAGAAGTCGACGACGG	0.00	0.33	0.00	0.00	0.00
GATCAAGAAGTCGACGAGGA	0.00	0.00	0.29	0.00	0.00
GATCAAGAAGTCGACGCAGC	0.00	0.00	0.00	0.34	0.00
GATCAAGAAGTCGACGCCAC	0.00	0.00	0.00	0.34	0.00
GATCAAGAAGTCGACGCCGA	0.00	0.33	0.57	0.34	0.34
GATCAAGAAGTCGACGCCGC	20.14	98.57	117.98	103.53	86.79
GATCAAGAAGTCGACGCCGG	0.00	0.00	0.29	0.00	0.34
GATCAAGAAGTCGACGCCGN	0.00	0.00	0.29	0.00	0.00
GATCAAGAAGTCGACGCCGT	0.00	0.33	0.00	0.00	0.69
GATCAAGAAGTCGACGCCNC	0.00	0.00	0.00	0.34	0.00
GATCAAGAAGTCGACGCCNN	0.00	0.00	0.00	0.00	0.00
GATCAAGAAGTCGACGCCTC	0.00	0.00	0.00	0.00	0.34
GATCAAGAAGTCGACGCGGC	0.23	0.00	0.00	0.00	0.00
GATCAAGAAGTCGACGCTGC	0.23	0.00	0.00	0.00	0.00
GATCAAGAAGTCGACGNCGC	0.00	0.33	0.00	0.00	0.34
GATCAAGAAGTCGACGTCGC	0.00	0.00	0.00	0.00	0.34
GATCAAGAAGTCGCGGTTGA	0.00	0.00	0.29	0.00	0.00
GATCAAGAAGTCGGCCTTCT	0.23	0.00	0.00	0.00	0.00

GATCAAGAAGTCGGCGCCGC	0.00	0.00	0.29	0.00	0.00
GATCAAGAAGTCTACACCGC	0.00	0.00	0.00	0.00	0.34
GATCAAGAAGTCTACGCCCC	0.00	0.00	0.29	0.00	0.00
GATCAAGAAGTCTACGCCTC	0.00	0.33	0.00	0.00	0.34
GATCAAGAAGTCTATGCGCT	0.00	0.00	0.00	0.00	0.34
GATCAAGAAGTCTCATCGTG	0.00	0.00	0.00	0.00	0.00
GATCAAGAAGTCTGCAAAAA	0.23	0.00	0.00	0.00	0.00
GATCAAGAAGTCTGGCGACG	0.00	0.00	0.00	0.00	0.34
GATCAAGAAGTCTGTGGCCA	0.00	0.33	0.00	0.00	0.00
GATCAAGAAGTCTTCGAGGG	0.00	0.00	0.00	0.68	0.00
GATCAAGAAGTGAAAGCGCA	0.00	0.00	0.00	0.00	0.34
GATCAAGAAGTGAACAAAAA	0.00	0.00	0.00	0.68	0.00
GATCAAGAAGTGACCGCATC	0.00	0.00	0.29	0.00	0.00
GATCAAGAAGTGACTGGCAA	1.14	1.95	1.72	1.36	1.38
GATCAAGAAGTGAGAAGGCC	0.00	0.33	0.00	0.00	0.00
GATCAAGAAGTGAGATGGAT	0.00	0.33	0.00	0.00	0.00
GATCAAGAAGTGAGCAACAA	0.00	0.00	0.00	0.00	0.69
GATCAAGAAGTGAGCACGTC	3.20	3.25	0.57	1.70	2.76
GATCAAGAAGTGAGCAGGAC	0.00	0.00	0.00	0.00	0.00
GATCAAGAAGTGAGCAGGCC	1.14	3.58	0.29	3.73	0.34
GATCAAGAAGTGAGCAGGCT	0.00	0.00	0.00	0.34	0.00
GATCAAGAAGTGAGCAGGGA	0.46	0.00	0.00	0.00	0.00
GATCAAGAAGTGAGCCATCG	0.00	0.00	0.00	0.68	0.00
GATCAAGAAGTGAGCCCAGG	0.00	0.00	0.00	0.00	0.00
GATCAAGAAGTGAGCCTGTG	0.00	0.00	0.29	0.00	0.00
GATCAAGAAGTGAGCGACAA	0.69	0.00	0.57	0.68	0.00
GATCAAGAAGTGAGCTNGTG	0.00	0.00	0.00	0.00	0.34
GATCAAGAAGTGAGCTTGTG	1.60	4.55	6.87	3.73	7.58
GATCAAGAAGTGAGGCATGC	0.46	0.00	0.00	0.00	0.00
GATCAAGAAGTGAGGGCGGC	0.00	0.33	0.00	0.00	0.00
GATCAAGAAGTGATCCAAGT	0.00	0.33	0.00	0.00	0.00
GATCAAGAAGTGCAAGAGTG	0.23	0.00	0.00	0.00	0.00
GATCAAGAAGTGCAAGGCGA	0.00	0.00	0.00	0.34	0.00
GATCAAGAAGTGCACACCGC	0.00	0.00	0.57	0.00	0.00
GATCAAGAAGTGCACGCCGC	0.00	0.00	0.00	0.00	0.00
GATCAAGAAGTGCATGGAGG	0.00	0.33	0.00	0.00	0.00
GATCAAGAAGTGCCACTGGA	0.23	0.00	0.00	0.00	0.00

GATCAAGAAGTGCCATGGTG	0.00	0.00	0.00	0.00	0.00
GATCAAGAAGTGCCCAAGGT	0.00	0.00	0.00	0.00	0.69
GATCAAGAAGTGCCCCAAGT	0.00	0.00	0.00	0.00	0.00
GATCAAGAAGTGCCGCAGGC	0.00	0.00	0.00	0.00	0.00
GATCAAGAAGTGCCTCTTCA	0.00	0.00	0.00	0.00	0.00
GATCAAGAAGTGCCTCTTCG	0.00	0.00	0.00	0.34	0.34
GATCAAGAAGTGCGAATGGT	0.00	0.00	0.00	0.34	0.00
GATCAAGAAGTGCGAGACCT	0.00	0.00	0.00	0.00	0.00
GATCAAGAAGTGCGAGGCCA	0.00	0.00	0.00	0.00	0.00
GATCAAGAAGTGCGCGGATG	0.00	0.00	0.00	0.00	0.00
GATCAAGAAGTGCGTCGTGC	0.00	0.00	0.00	0.00	0.00
GATCAAGAAGTGCTCAAAGT	0.00	0.33	0.00	0.00	0.00
GATCAAGAAGTGCTCCAAGC	0.00	0.00	0.00	0.00	0.00
GATCAAGAAGTGCTCCAAGG	0.00	0.00	0.29	0.00	0.00
GATCAAGAAGTGCTCCAAGN	0.00	0.00	0.00	0.00	0.00
GATCAAGAAGTGCTCCAAGT	11.45	26.03	22.62	18.67	14.46
GATCAAGAAGTGCTCCAANT	0.00	0.00	0.29	0.00	0.00
GATCAAGAAGTGCTCCAATT	0.00	0.33	0.00	0.00	0.00
GATCAAGAAGTGCTCCACGT	0.00	0.00	0.00	0.00	0.00
GATCAAGAAGTGCTCCAGTC	0.00	0.00	0.00	0.00	0.00
GATCAAGAAGTGCTCCANGT	0.00	0.00	0.00	0.00	0.00
GATCAAGAAGTGCTCCGTGC	0.00	0.00	0.57	0.00	0.00
GATCAAGAAGTGCTGATGTA	0.00	0.00	0.29	0.00	0.00
GATCAAGAAGTGCTGGACAA	0.46	0.00	0.29	0.00	0.00
GATCAAGAAGTGCTTCAGGA	0.00	0.00	0.00	0.00	0.00
GATCAAGAAGTGGACGCCGA	0.00	0.00	0.00	0.00	0.00
GATCAAGAAGTGGACGCCGC	0.00	0.00	0.00	0.00	0.34
GATCAAGAAGTGGAGCGCCG	0.00	0.00	0.29	1.36	0.34
GATCAAGAAGTGGAGCGCGG	0.00	0.33	0.00	0.00	0.34
GATCAAGAAGTGGAGGGCTG	0.00	0.00	0.29	0.00	0.00
GATCAAGAAGTGGAGTACTG	0.00	0.00	0.00	0.00	0.00
GATCAAGAAGTGGAGTGATG	0.00	0.00	0.00	0.00	0.00
GATCAAGAAGTGGAGTGCAG	0.00	0.00	0.00	0.00	0.34
GATCAAGAAGTGGAGTGCGG	0.00	0.00	0.00	0.00	0.00
GATCAAGAAGTGGAGTGCTC	0.00	0.33	0.00	0.34	0.00
GATCAAGAAGTGGAGTGCTG	6.64	34.81	42.67	27.15	37.54
GATCAAGAAGTGGAGTGCTN	0.23	0.00	0.00	0.34	0.00

GATCAAGAAGTGGAGTGCTT	0.00	0.00	0.00	0.00	0.34
GATCAAGAAGTGGAGTGGTG	0.00	0.00	0.00	0.00	0.00
GATCAAGAAGTGGAGTGTCG	0.00	0.33	0.00	0.00	0.00
GATCAAGAAGTGGATCGTAT	0.00	0.00	0.00	0.00	0.34
GATCAAGAAGTGGCCAAAAA	0.00	0.33	0.00	0.00	0.00
GATCAAGAAGTGGCCAAATT	0.46	0.33	0.29	0.34	0.34
GATCAAGAAGTGGCGCGGTG	0.00	0.00	0.29	0.00	0.00
GATCAAGAAGTGGGAGGCGT	0.00	0.00	0.00	0.00	0.34
GATCAAGAAGTGGGCCATTG	0.00	0.00	0.29	0.00	0.00
GATCAAGAAGTGGGCGGCGG	0.00	0.00	0.29	0.00	0.00
GATCAAGAAGTGGTTCCTCC	0.00	0.33	0.00	0.68	0.00
GATCAAGAAGTGTCGCGACG	0.00	0.00	0.00	0.00	0.00
GATCAAGAAGTGTGCACGCA	0.00	0.00	0.00	0.00	0.34
GATCAAGAAGTGTGCCATAC	0.00	0.00	0.29	0.00	0.00
GATCAAGAAGTGTGGCTGAC	0.00	0.00	0.00	0.00	0.00
GATCAAGAAGTTAAACAGGC	0.00	0.00	0.00	0.00	0.34
GATCAAGAAGTTACAGTGAG	0.00	0.00	0.00	0.00	0.00
GATCAAGAAGTTCAAGAAAG	0.00	0.00	0.00	0.00	0.00
GATCAAGAAGTTCAAGAAGT	0.00	0.00	0.00	0.00	0.69
GATCAAGAAGTTCAAGGAAA	0.00	0.33	0.00	0.00	0.00
GATCAAGAAGTTCAAGGAAG	0.00	0.65	0.00	0.00	0.00
GATCAAGAAGTTCAAGGCCG	0.23	0.00	0.00	0.00	0.00
GATCAAGAAGTTCAAGGCNN	0.23	0.00	0.00	0.00	0.00
GATCAAGAAGTTCAAGGCTG	16.48	4.23	3.44	5.09	4.13
GATCAAGAAGTTCAAGGCTT	0.00	0.33	0.00	0.00	0.00
GATCAAGAAGTTCAAGGTTG	0.00	0.00	0.00	0.00	0.00
GATCAAGAAGTTCAAGNNNN	0.23	0.00	0.00	0.00	0.00
GATCAAGAAGTTCAAGTGAG	0.00	0.00	0.00	0.34	0.00
GATCAAGAAGTTCACACCGC	0.00	0.00	0.29	0.34	0.00
GATCAAGAAGTTCACCGCTG	0.23	0.00	0.00	0.00	0.00
GATCAAGAAGTTCACGCCGC	0.00	0.00	0.00	0.00	0.00
GATCAAGAAGTTCAGGAAGG	0.23	0.00	0.00	0.00	0.00
GATCAAGAAGTTCCAGAAAG	0.00	0.00	0.00	0.00	0.00
GATCAAGAAGTTCCAGAGAC	0.00	0.00	0.00	0.34	0.00
GATCAAGAAGTTCCAGAGAG	0.00	0.00	0.00	0.00	0.00
GATCAAGAAGTTCCAGCAAC	0.00	0.00	0.57	0.00	0.00
GATCAAGAAGTTCCAGCCGC	0.00	0.00	0.00	0.68	0.00

GATCAAGAAGTTCCAGCGAG	0.00	0.00	0.00	0.00	0.00
GATCAAGAAGTTCCAGNNNN	0.23	0.00	0.00	0.00	0.00
GATCAAGAAGTTCCAGTGAC	0.00	0.00	0.00	0.00	0.00
GATCAAGAAGTTCCAGTGAG	51.73	62.46	26.92	55.67	48.22
GATCAAGAAGTTCCAGTGAN	0.23	0.00	0.00	0.34	0.00
GATCAAGAAGTTCCAGTGCG	0.00	0.00	0.29	0.34	0.00
GATCAAGAAGTTCCAGTGGG	0.23	0.00	0.00	0.00	0.00
GATCAAGAAGTTCCAGTGNN	0.23	0.00	0.00	0.00	0.00
GATCAAGAAGTTCCAGTNNN	0.00	0.00	0.00	0.00	0.00
GATCAAGAAGTTCCAGTTCG	0.00	0.00	0.00	0.00	0.34
GATCAAGAAGTTCCATGCAC	0.00	0.00	0.29	0.00	0.00
GATCAAGAAGTTCCCGCCGC	0.00	0.00	0.00	0.00	0.00
GATCAAGAAGTTCCGTGCAC	0.00	0.00	0.00	0.00	1.03
GATCAAGAAGTTCCTGTGAG	0.00	0.00	0.00	0.34	0.00
GATCAAGAAGTTCGAGGAGC	0.23	0.00	0.00	0.00	0.00
GATCAAGAAGTTCGAGGAGT	0.23	0.00	0.00	0.00	0.00
GATCAAGAAGTTCGGACATG	0.00	0.00	0.29	0.00	0.00
GATCAAGAAGTTCGGGCCTG	0.46	0.00	0.00	0.00	0.00
GATCAAGAAGTTCTGGTTGT	0.00	0.00	0.00	0.00	0.00
GATCAAGAAGTTCTGGTTTA	0.00	0.00	0.00	0.34	0.00
GATCAAGAAGTTCTTGAACA	0.00	0.00	0.00	1.02	0.00
GATCAAGAAGTTGAAAACCT	0.00	0.00	0.00	0.00	0.34
GATCAAGAAGTTGACACAAG	0.00	0.00	0.00	0.00	0.34
GATCAAGAAGTTGACTATAA	1.37	0.33	0.00	0.68	0.00
GATCAAGAAGTTGACTATAT	1.14	0.00	0.00	0.00	0.00
GATCAAGAAGTTGAGCAAGG	0.00	0.00	0.29	0.00	0.00
GATCAAGAAGTTGAGCTAAA	0.00	0.00	0.00	0.00	0.00
GATCAAGAAGTTGAGTTTGA	0.23	0.00	0.00	0.00	0.00
GATCAAGAAGTTGCATGCAG	0.00	0.00	0.29	0.00	0.00
GATCAAGAAGTTGGACGCCT	0.00	0.00	0.00	0.34	0.00
GATCAAGAAGTTGGATCTCA	0.00	0.33	0.00	0.00	0.00
GATCAAGAAGTTGGGATTCA	2.98	6.83	1.15	3.39	1.72
GATCAAGAAGTTTACTACTC	0.69	0.00	0.00	0.68	0.00
GATCAAGAAGTTTATAGAAG	0.23	0.00	0.00	0.00	0.00
GATCAAGAAGTTTCGGTACA	0.00	0.00	0.00	0.00	0.00
GATCAAGAAGTTTCTTGTAT	0.00	0.00	0.29	0.00	0.00
GATCAAGAAGTTTCTTGTTA	8.93	8.13	6.59	8.15	13.43

GATCAAGAAGTTTCTTGTTN	0.23	0.00	0.00	0.00	0.00
GATCAAGAAGTTTCTTTGTG	0.00	0.00	0.00	0.00	0.00
GATCAAGAAGTTTGCTGAGG	0.00	0.00	0.00	0.00	0.00
GATCAAGAAGTTTGGAGAAC	0.00	0.00	0.86	0.00	0.00
GATCAAGAAGTTTGGAGATG	0.00	0.00	0.00	0.00	0.00
GATCAAGAAGTTTGTGGCAG	0.00	0.00	0.29	0.00	0.00
GATCAAGAAGTTTGTGGCCG	0.00	0.65	0.00	0.68	0.34
GATCAAGAAGTTTTCGGTAC	0.23	0.00	0.29	0.00	0.00
GATCAAGAAGTTTTCGNTNN	0.23	0.00	0.00	0.00	0.00
GATCAAGAAGTTTTTAGAAG	10.99	7.81	7.16	3.05	7.23
GATCAAGAAGTTTTTAGAAT	0.00	0.00	0.00	0.00	0.00
GATCAAGAAGTTTTTAGACN	0.23	0.00	0.00	0.00	0.00
GATCAAGAAGTTTTTAGCAG	0.00	0.00	0.00	0.00	0.00
GATCAAGAATAAAAAAAAAA	1.60	0.00	0.00	0.34	0.34
GATCAAGAATAAAAAAAAAT	0.23	0.00	0.00	0.00	0.00
GATCAAGAATAAAATGGCCT	0.00	0.00	0.00	0.00	0.34
GATCAAGAATAACTTTCTTT	0.46	0.00	0.00	0.00	0.00
GATCAAGAATAAGAAGAAGA	0.00	0.00	0.57	0.00	0.00
GATCAAGAATAAGAATAAGA	0.00	0.00	0.00	0.00	0.34
GATCAAGAATAAGAATAATA	0.00	0.00	1.43	0.00	0.00
GATCAAGAATAAGAATAGGA	0.00	0.00	4.01	0.00	2.07
GATCAAGAATAAGACTAGGA	0.00	0.00	0.86	0.00	2.76
GATCAAGAATAAGGAAGACG	0.00	0.00	0.29	0.00	0.00
GATCAAGAATAATAAAAAAA	0.23	0.00	0.00	0.00	0.00
GATCAAGAATACCAAGCGCT	0.00	0.00	0.29	1.02	0.00
GATCAAGAATACCTTCTTGG	0.46	0.33	0.00	0.00	0.00
GATCAAGAATACGTCGGAGC	0.00	0.00	0.00	0.00	0.34
GATCAAGAATACGTTCTTCC	0.00	0.00	0.00	0.00	0.00
GATCAAGAATACTGAAGGCA	0.00	0.00	0.00	0.34	0.00
GATCAAGAATAGACGGAATC	0.00	0.00	0.00	1.02	0.00
GATCAAGAATATCGACGGGA	0.23	0.00	0.00	0.00	0.00
GATCAAGAATATCGCAGAAG	0.00	0.00	0.00	0.00	0.69
GATCAAGAATATGAAGTGTT	0.00	0.00	0.00	0.00	0.34
GATCAAGAATATGAGTTGTG	0.00	0.00	0.00	0.00	1.72
GATCAAGAATATGAGTTGTT	0.00	0.00	0.00	0.00	0.34
GATCAAGAATATTCGGAAGG	0.00	0.00	0.00	0.00	0.00
GATCAAGAATCAAAAAAAAA	30.67	7.16	2.86	7.47	7.23

GATCAAGAATCAAAAAAAAN	0.23	0.00	0.00	0.00	0.00
GATCAAGAATCAAAAAAAAT	0.00	0.00	0.00	0.00	0.34
GATCAAGAATCAAAAAAANN	0.23	0.00	0.00	0.00	0.00
GATCAAGAATCAAAAAATAA	0.00	0.00	0.00	0.00	0.00
GATCAAGAATCAATGTGCGC	0.00	0.00	0.00	0.00	0.69
GATCAAGAATCACAAGAAGG	0.69	0.33	0.57	0.68	0.69
GATCAAGAATCACAAGCCGA	0.00	0.65	0.00	0.00	0.00
GATCAAGAATCACAAGGAAT	0.00	0.33	0.29	0.00	0.00
GATCAAGAATCAGAAAAAAA	0.23	0.00	0.00	0.00	0.00
GATCAAGAATCAGTGAAGTT	0.00	0.98	0.00	0.00	0.00
GATCAAGAATCAGTGCCGCA	0.23	0.00	0.00	0.00	0.00
GATCAAGAATCAGTGGTCGT	0.00	0.33	1.72	0.68	0.00
GATCAAGAATCATAAAAAAA	1.83	0.00	0.00	0.00	0.00
GATCAAGAATCATGAAAAAA	0.00	1.63	0.00	0.00	0.34
GATCAAGAATCATGACCAGG	1.14	1.63	0.86	1.36	0.69
GATCAAGAATCCAAAAAAAA	0.00	0.00	0.00	0.00	0.00
GATCAAGAATCCAAAGGGAG	0.00	0.00	0.00	0.34	0.00
GATCAAGAATCCAGAAACAG	0.69	0.00	0.00	0.00	0.00
GATCAAGAATCCAGATGTGA	0.69	0.65	0.57	0.34	0.00
GATCAAGAATCCCTGGCACC	0.00	0.00	0.00	0.00	0.00
GATCAAGAATCCGAAGCGAG	0.00	0.00	0.00	0.00	0.00
GATCAAGAATCCGAAGCGGT	0.69	0.00	0.29	0.00	0.00
GATCAAGAATCCGCTGACAG	0.00	0.00	0.00	0.00	0.69
GATCAAGAATCCTGCATGCG	0.00	0.00	0.00	0.00	0.00
GATCAAGAATCCTGGTGGCC	0.23	0.00	0.00	0.00	0.00
GATCAAGAATCGAAACACAG	0.00	0.00	0.00	0.00	0.00
GATCAAGAATCGCCGCGAGT	0.00	0.00	0.00	0.34	0.00
GATCAAGAATCGCTCAGAGG	0.46	1.95	2.29	0.00	0.00
GATCAAGAATCGGGAGTGGA	0.00	0.00	0.29	0.00	0.00
GATCAAGAATCGGTACAAAC	0.00	0.00	0.00	0.00	0.00
GATCAAGAATCGTATGCCGT	0.00	0.00	0.00	0.68	0.69
GATCAAGAATCGTTCGGCAC	0.00	0.00	0.29	0.00	0.00
GATCAAGAATCTAGTGGAAG	0.23	0.00	0.00	0.00	0.00
GATCAAGAATCTCAACGGCC	0.46	0.00	0.00	0.00	0.69
GATCAAGAATCTCAACGGGC	0.00	0.00	0.29	0.00	0.00
GATCAAGAATCTCAATAGAC	0.00	0.00	0.00	0.00	0.00
GATCAAGAATCTCAATGGCC	10.30	0.65	0.57	0.00	0.34

GATCAAGAATCTCATGGCAC	0.00	0.00	0.00	0.00	0.00
GATCAAGAATCTCGGCAAAG	0.00	0.33	0.29	0.34	0.34
GATCAAGAATCTCGGGCAAG	0.46	0.33	0.86	1.36	0.34
GATCAAGAATCTCGGGCGGA	0.00	0.00	0.00	0.68	0.00
GATCAAGAATCTGGCATGAG	0.00	0.00	0.00	0.00	0.00
GATCAAGAATCTGGCGTGGC	0.23	0.00	0.00	0.00	0.00
GATCAAGAATCTTAAGACTA	0.00	0.00	0.86	0.00	0.00
GATCAAGAATCTTGTGAAGC	13.28	5.86	4.30	3.05	2.07
GATCAAGAATCTTGTGCAGC	0.23	0.00	0.00	0.00	0.00
GATCAAGAATCTTTGAAGAG	0.00	0.00	0.00	0.34	0.00
GATCAAGAATGAAATGGCCT	0.00	0.00	0.00	0.00	0.34
GATCAAGAATGAATGTATAT	0.00	0.00	0.29	0.00	0.00
GATCAAGAATGAATTGAAAA	0.00	0.00	0.00	0.34	0.00
GATCAAGAATGACACAAGCA	0.00	0.00	0.00	0.00	0.00
GATCAAGAATGACCTTGTTC	0.00	0.00	0.00	0.34	0.00
GATCAAGAATGACGGGCAGA	0.00	0.00	0.00	0.34	0.00
GATCAAGAATGACGGGCAGC	2.06	4.88	1.72	2.72	0.34
GATCAAGAATGACGGGCANN	0.00	0.00	0.00	0.00	0.00
GATCAAGAATGACGGGCTGC	0.00	0.00	0.00	0.34	0.00
GATCAAGAATGACTTACCTG	0.00	0.00	0.29	0.00	0.00
GATCAAGAATGAGAAGGCTG	0.00	0.33	0.00	0.00	0.34
GATCAAGAATGAGACGCGGG	0.00	0.33	0.00	0.00	0.00
GATCAAGAATGAGCAGTTCT	0.00	0.33	0.00	0.00	0.00
GATCAAGAATGAGCAGTTGT	0.00	0.00	0.00	0.00	0.00
GATCAAGAATGAGCCGCCTC	0.23	0.00	0.00	0.00	0.00
GATCAAGAATGAGGCACAGC	0.00	0.00	0.29	0.00	0.00
GATCAAGAATGAGGCGAACA	0.00	0.00	0.00	0.00	0.00
GATCAAGAATGATACCTCCT	0.46	0.00	0.00	0.00	0.00
GATCAAGAATGATGATGAGT	0.23	0.00	0.00	0.00	0.00
GATCAAGAATGCACAACCAA	0.00	0.00	0.29	0.00	0.00
GATCAAGAATGCAGGCCTGC	0.23	0.33	0.00	1.02	0.00
GATCAAGAATGCCGACATGG	0.23	0.33	0.86	0.34	1.03
GATCAAGAATGCCTTCGGTA	0.00	0.00	0.00	1.36	0.00
GATCAAGAATGCCTTTGGCA	0.00	0.00	0.00	0.00	0.00
GATCAAGAATGCGAAAAAAA	0.00	0.00	0.00	0.34	0.00
GATCAAGAATGCGAAAGAAG	0.00	0.00	0.29	0.00	0.00
GATCAAGAATGCGCTCCAGC	0.00	0.65	0.29	1.36	0.34

GATCAAGAATGCGGGCCTTG	0.00	0.00	0.00	0.34	0.00
GATCAAGAATGCGTTCATTC	0.00	0.33	0.00	0.00	0.00
GATCAAGAATGCTAGCTCCG	0.00	0.00	0.00	0.00	0.34
GATCAAGAATGCTGACATGG	0.00	0.00	0.00	0.34	0.00
GATCAAGAATGCTGTTGGGA	0.00	0.00	0.00	0.00	0.00
GATCAAGAATGCTTGGGGGC	0.00	0.33	0.00	0.00	0.00
GATCAAGAATGGACAGTGAG	0.00	0.00	0.00	0.00	0.00
GATCAAGAATGGACCCAATA	0.00	0.00	0.00	0.00	1.03
GATCAAGAATGGACGCCTCG	0.00	0.00	0.29	0.00	0.00
GATCAAGAATGGCAAGAAGA	13.96	56.93	91.92	42.77	69.22
GATCAAGAATGGCAAGAAGG	0.00	0.00	0.29	0.00	0.00
GATCAAGAATGGCAAGAAGT	0.23	0.00	0.00	0.00	0.00
GATCAAGAATGGCAAGCAGA	0.23	0.00	0.00	0.34	0.00
GATCAAGAATGGCAAGGAGA	0.00	0.00	0.29	0.00	0.00
GATCAAGAATGGCAATCCCT	0.00	0.00	0.00	0.00	0.00
GATCAAGAATGGCACGACGA	0.00	0.00	0.00	0.00	0.34
GATCAAGAATGGCAGCAAGG	0.00	0.00	0.00	0.00	0.00
GATCAAGAATGGCAGCGTGA	0.23	0.00	0.00	0.00	0.00
GATCAAGAATGGCATGACAT	0.00	0.00	0.29	0.00	0.00
GATCAAGAATGGCCCCCGGC	0.23	0.00	0.00	0.00	0.00
GATCAAGAATGGCCGCAATG	0.00	0.00	0.29	0.00	0.00
GATCAAGAATGGCCGCACAG	0.00	0.00	0.00	0.00	0.34
GATCAAGAATGGCCGCATGG	11.90	27.33	16.90	18.67	16.88
GATCAAGAATGGCCGCATGT	0.00	0.00	0.00	0.00	0.69
GATCAAGAATGGCCGCCTGG	0.00	0.33	0.29	0.00	0.00
GATCAAGAATGGCCGCGTGT	0.00	0.00	0.00	0.00	0.34
GATCAAGAATGGCCTCAAGT	0.00	0.00	0.00	0.00	0.00
GATCAAGAATGGCCTGACGG	0.23	0.00	0.00	0.00	0.00
GATCAAGAATGGCGATGCCT	0.00	0.00	0.29	0.00	0.00
GATCAAGAATGGCGGGGGGG	0.00	0.00	0.29	0.00	0.00
GATCAAGAATGGCTAGAAGA	0.00	0.00	0.00	0.00	0.00
GATCAAGAATGGGCTCAATG	0.00	0.00	0.00	0.68	0.00
GATCAAGAATGGTACTATTA	0.00	0.00	0.00	0.00	0.00
GATCAAGAATGGTCGCCTTG	0.23	0.00	0.00	0.00	0.00
GATCAAGAATGTACTATTCC	0.00	0.00	0.00	0.00	0.34
GATCAAGAATGTCCGCAAAA	0.00	0.00	0.00	0.00	0.34
GATCAAGAATGTCGACATGG	0.46	3.58	5.15	3.05	3.10

GATCAAGAATGTCGACATGT	0.00	0.00	0.29	0.00	0.00
GATCAAGAATGTCGCCACGC	0.23	0.00	0.00	0.34	0.00
GATCAAGAATGTCGGGGGGC	0.23	0.00	0.00	0.00	0.00
GATCAAGAATGTCGGGGGTC	0.46	1.30	0.86	0.00	0.00
GATCAAGAATGTCGGGGTCT	0.00	0.33	0.00	0.00	0.00
GATCAAGAATGTCGTGCTGA	0.00	0.00	0.00	0.00	0.00
GATCAAGAATGTGCGAGGCC	0.00	0.00	0.00	0.00	0.00
GATCAAGAATGTGCGGAAGG	0.00	0.00	0.29	1.02	0.00
GATCAAGAATGTTCGAAAGG	0.00	0.00	0.00	0.00	0.00
GATCAAGAATGTTCGGAAGG	12.36	24.72	30.35	14.93	14.81
GATCAAGAATGTTCGGACGG	0.23	0.00	0.00	0.00	0.00
GATCAAGAATGTTCGGAGGG	0.23	0.00	0.00	0.00	0.00
GATCAAGAATGTTCGGCAGA	0.00	0.00	0.00	0.00	0.34
GATCAAGAATGTTCGGCAGG	0.00	0.00	0.00	0.00	0.34
GATCAAGAATGTTCGGGAGG	0.23	0.00	0.00	0.00	0.00
GATCAAGAATGTTCGGNAGG	0.00	0.00	0.00	0.00	0.34
GATCAAGAATGTTGAGTAAA	0.00	0.00	0.00	0.00	0.00
GATCAAGAATGTTGATTGTT	0.00	0.00	0.00	0.00	0.00
GATCAAGAATGTTTGGGTCA	0.00	0.00	0.29	0.00	0.00
GATCAAGAATTAGGGCCTTG	0.00	0.33	0.00	0.00	0.00
GATCAAGAATTCAAAATGGC	2.98	0.33	0.00	1.70	0.69
GATCAAGAATTCAAGATGCG	0.00	0.00	0.57	0.00	0.00
GATCAAGAATTCAGAATGGC	0.00	0.00	0.00	0.00	0.00
GATCAAGAATTCAGATGTCA	0.00	0.00	0.00	0.34	0.00
GATCAAGAATTCAGCATGGC	0.00	0.00	0.00	0.00	0.34
GATCAAGAATTCAGCCTGTG	0.00	0.33	0.29	0.00	0.69
GATCAAGAATTCAGCTCCAA	0.46	0.33	0.29	0.00	0.00
GATCAAGAATTCAGGCTCAA	0.23	0.98	0.86	1.02	0.00
GATCAAGAATTCAGGCTCTA	0.00	0.33	0.00	0.00	0.00
GATCAAGAATTCAGGCTCTG	0.00	0.00	1.72	1.02	0.34
GATCAAGAATTCCACACTGG	0.00	0.00	0.00	0.34	0.00
GATCAAGAATTCCACGCCGC	0.00	0.00	0.00	0.34	0.00
GATCAAGAATTCCTGGGGGA	13.96	3.90	2.58	4.75	2.41
GATCAAGAATTCCTGGGGGC	0.69	0.00	0.00	0.00	0.00
GATCAAGAATTCCTGGGGGG	1.37	0.00	0.00	0.00	0.00
GATCAAGAATTCCTGGGGGN	0.23	0.00	0.00	0.00	0.00
GATCAAGAATTCCTGGGGGT	0.46	0.00	0.00	0.00	0.00

GATCAAGAATTCCTGGTGGA	0.00	0.00	0.00	0.00	0.34
GATCAAGAATTCGAGCGCGG	0.00	0.00	0.00	0.00	0.00
GATCAAGAATTCGCCCGCAC	0.00	0.33	0.00	0.00	0.34
GATCAAGAATTCGGGCCTAG	0.00	0.00	0.00	0.00	0.00
GATCAAGAATTCGGGCCTCG	2.52	3.90	3.72	2.04	3.10
GATCAAGAATTCGGGCCTTG	1.14	2.28	5.15	1.70	3.44
GATCAAGAATTCGGTTGTAG	0.00	0.33	0.00	0.00	0.00
GATCAAGAATTCGTGCATTG	0.00	0.00	0.29	0.00	0.00
GATCAAGAATTCTGGCAAAA	0.00	0.00	0.57	0.00	0.00
GATCAAGAATTGAATTCAAG	0.00	0.00	0.00	0.00	0.34
GATCAAGAATTGAATTCATG	0.00	0.00	0.00	0.00	0.34
GATCAAGAATTGACTATCTA	0.00	0.00	0.00	0.00	0.00
GATCAAGAATTGCAAAAAAA	0.23	0.00	0.00	0.00	0.00
GATCAAGAATTGCAAGAAGA	0.00	0.00	0.29	0.00	0.00
GATCAAGAATTGCATTGGCC	0.00	0.00	0.00	0.68	0.00
GATCAAGAATTGCGAGGGCT	0.00	0.00	0.00	0.34	0.00
GATCAAGAATTGCGGTGGTG	0.00	0.00	0.00	0.00	0.00
GATCAAGAATTGCGTGGCCT	0.00	0.00	0.00	0.00	0.00
GATCAAGAATTGCTGGATGA	0.00	0.00	0.00	0.00	0.00
GATCAAGAATTGTCTTAAAA	0.23	0.00	0.00	0.00	0.00
GATCAAGAATTGTGAAGGTT	0.00	0.00	0.00	0.34	0.00
GATCAAGAATTGTGAGGGTC	0.00	0.00	0.00	0.00	0.00
GATCAAGAATTGTTGAAGAG	0.00	0.00	0.00	0.00	0.00
GATCAAGAATTTCACCATAC	0.23	0.00	0.00	0.00	0.00
GATCAAGAATTTCCAGCGAA	0.00	0.00	0.00	0.34	0.00
GATCAAGAATTTCCAGCGGA	0.00	0.00	0.00	0.00	0.00
GATCAAGAATTTCCATCAGA	0.00	0.00	0.00	0.00	0.00
GATCAAGAATTTCCTCTTCG	0.00	0.65	0.00	0.34	0.00
GATCAAGAATTTGAGGAGAT	0.00	0.00	0.00	0.00	0.00
GATCAAGAATTTGAGGGTTT	0.00	0.00	0.00	0.34	0.69
GATCAAGAATTTGGAGACAG	0.00	0.00	0.00	0.34	0.00
GATCAAGAATTTGGCAGACG	0.00	0.00	0.00	0.68	0.00
GATCAAGAATTTGGGTGGGA	0.23	0.00	0.00	0.00	0.00
GATCAAGAATTTGTGTTCGT	0.00	0.00	0.00	0.68	0.00
GATCAAGAATTTTGCCGCGC	0.00	0.00	0.00	0.34	0.00
GATCAAGACAAAAAAAAAAA	28.38	6.83	7.16	7.47	7.23
GATCAAGACAAAAAAAAAAC	0.23	0.00	0.57	0.00	0.00

GATCAAGACAAAAAAAAAAT	2.52	0.33	0.29	0.34	0.00
GATCAAGACAAAAAAAAACA	0.23	0.00	0.00	0.00	0.00
GATCAAGACAAAAAAAAAGA	0.00	0.00	0.00	0.34	0.00
GATCAAGACAAAAAAACAAT	0.23	0.00	0.00	0.00	0.00
GATCAAGACAAAAAAACNNN	0.23	0.00	0.00	0.00	0.00
GATCAAGACAAAAAAANAAA	0.00	0.00	0.00	0.00	0.34
GATCAAGACAAAAAACAAAA	0.23	0.00	0.00	0.00	0.00
GATCAAGACAAAAATCAAAA	0.00	0.00	0.00	0.34	0.00
GATCAAGACAAAATGTGAAA	0.23	0.00	0.00	0.34	0.34
GATCAAGACAAACAAAAAAA	0.00	0.00	0.00	0.00	0.00
GATCAAGACAAACAACGCAA	0.00	0.00	0.57	0.00	0.00
GATCAAGACAAACGGAGAAN	0.00	0.00	0.00	0.00	0.00
GATCAAGACAAACGTCCCTT	0.00	0.00	0.00	0.00	0.00
GATCAAGACAAACGTGTCAG	0.00	0.00	0.00	0.00	0.00
GATCAAGACAAAGAAAAAAA	0.00	0.00	0.00	0.00	0.34
GATCAAGACAAAGAATCCGC	0.00	0.00	0.57	0.00	0.69
GATCAAGACAAAGCAAAAAA	0.00	0.00	0.57	0.00	0.00
GATCAAGACAAAGCAAAAGG	0.00	0.65	0.00	0.34	0.34
GATCAAGACAAAGCAAAANG	0.00	0.00	0.00	0.00	0.00
GATCAAGACAAAGCGGGAAG	0.00	0.00	0.00	0.00	0.00
GATCAAGACAAAGCGGTAAG	0.00	0.00	0.00	0.00	0.00
GATCAAGACAAAGGCGTCGT	0.00	0.33	0.00	0.00	0.00
GATCAAGACAAAGTACGGAT	4.35	2.60	0.57	1.70	0.34
GATCAAGACAAAGTACGGGG	0.23	0.00	0.00	0.00	0.00
GATCAAGACAAAGTGACGGC	0.00	0.33	0.00	0.00	0.00
GATCAAGACAACACAAAACA	0.00	0.00	0.00	0.00	0.69
GATCAAGACAACACAACGTA	0.00	0.00	0.00	0.00	0.69
GATCAAGACAACACACAAAA	0.00	0.00	0.00	0.00	0.34
GATCAAGACAACACACACAC	0.00	0.00	0.00	0.00	0.69
GATCAAGACAACACCTCCCG	0.00	0.33	0.57	0.00	0.69
GATCAAGACAACAGGCACCC	0.00	0.00	0.00	0.68	0.00
GATCAAGACAACATCGTGGA	0.00	0.00	0.00	0.68	1.03
GATCAAGACAACATCTGACT	0.00	0.33	0.29	0.00	0.00
GATCAAGACAACATGATGGC	0.00	0.00	0.29	0.00	0.00
GATCAAGACAACCGAGCAAC	0.00	0.00	0.29	0.00	0.00
GATCAAGACAACCGGATTCG	0.00	0.00	0.00	0.00	0.00
GATCAAGACAACCTGCCACT	0.46	0.00	0.00	0.00	0.00

GATCAAGACAACGATGCTTC	0.46	0.00	0.00	0.00	0.00
GATCAAGACAACGCAAAAGA	0.00	0.00	0.29	0.00	0.00
GATCAAGACAACGCGGAGCC	0.00	0.00	0.00	0.00	0.69
GATCAAGACAACGCGGCGCC	0.00	0.00	0.00	0.00	0.34
GATCAAGACAACGGCGGGCT	0.23	0.00	0.00	0.00	0.00
GATCAAGACAACGGGATATA	0.00	0.00	0.00	0.00	0.00
GATCAAGACAACGTACACTT	0.00	0.00	0.00	0.00	0.34
GATCAAGACAACGTGATCGA	0.00	0.00	0.29	0.00	0.00
GATCAAGACAACGTGCAGGT	0.69	0.00	0.00	0.00	0.00
GATCAAGACAACTCGGCGCT	0.00	0.00	0.00	0.00	0.34
GATCAAGACAAGAAAGCCCA	0.00	0.00	0.29	0.00	0.00
GATCAAGACAAGAACAAGTG	0.00	0.00	0.29	0.00	0.00
GATCAAGACAAGAAGCAAAG	0.00	0.00	0.00	0.00	0.00
GATCAAGACAAGAATCGATG	0.00	0.00	0.00	0.00	0.34
GATCAAGACAAGAATGGATG	0.00	0.00	0.00	0.00	0.00
GATCAAGACAAGACAAGACA	0.00	0.00	0.29	0.00	0.69
GATCAAGACAAGACAAGACC	0.00	0.00	0.29	0.00	1.03
GATCAAGACAAGACAAGCCA	0.00	0.00	0.29	0.00	0.00
GATCAAGACAAGACAGAACA	0.00	0.00	0.00	0.00	0.34
GATCAAGACAAGACCGAGCA	0.00	0.00	0.00	0.00	0.69
GATCAAGACAAGAGACTCTG	0.00	0.65	0.00	0.00	0.00
GATCAAGACAAGAGTAAAAA	0.00	0.00	0.00	0.00	0.34
GATCAAGACAAGCAAGAGAA	0.00	0.00	0.00	0.34	0.00
GATCAAGACAAGCAAGTCTC	0.00	0.00	0.29	0.00	0.00
GATCAAGACAAGCAGACGCC	0.00	0.00	0.00	0.00	0.34
GATCAAGACAAGCAGCGCAC	0.00	0.00	0.00	0.00	0.00
GATCAAGACAAGCTACCGTA	0.00	0.00	0.00	0.00	0.00
GATCAAGACAAGCTCGTGCA	0.00	0.00	0.00	0.34	0.00
GATCAAGACAAGGAAAAAAA	0.46	0.00	0.00	0.00	0.00
GATCAAGACAAGGAAGAAAA	0.00	0.00	0.29	0.00	0.00
GATCAAGACAAGGAAGAAGC	0.00	0.33	0.57	0.00	0.00
GATCAAGACAAGGAAGTATG	0.00	0.33	0.00	0.00	0.00
GATCAAGACAAGGACAGAAA	0.00	0.00	0.00	0.00	0.34
GATCAAGACAAGGACCAGGT	0.00	0.00	0.00	0.00	0.00
GATCAAGACAAGGACCGGGA	0.23	0.00	0.00	1.36	0.00
GATCAAGACAAGGACCGGGC	0.00	0.00	0.00	0.34	0.00
GATCAAGACAAGGACTTCGA	0.00	0.00	0.00	0.00	0.00

GATCAAGACAAGGAGATGAG	0.00	0.00	0.00	0.00	0.00
GATCAAGACAAGGAGGAGTA	0.00	0.00	0.00	0.00	0.00
GATCAAGACAAGGAGTCGGA	0.00	0.33	0.00	0.00	0.00
GATCAAGACAAGGATGACCG	0.00	0.98	0.00	0.00	0.00
GATCAAGACAAGGATGCAAA	0.69	0.00	0.00	0.00	0.00
GATCAAGACAAGGATTGCCA	0.00	0.00	0.00	0.00	0.34
GATCAAGACAAGGCCAAGGC	0.00	0.00	0.29	0.00	0.00
GATCAAGACAAGGCGGTGGA	0.00	0.00	0.00	0.00	0.00
GATCAAGACAAGGCGGTTCA	0.00	0.00	0.00	0.00	0.00
GATCAAGACAAGGCTCTTCA	0.00	0.00	0.00	0.34	0.00
GATCAAGACAAGGCTTCTGC	0.00	0.00	0.00	0.00	0.00
GATCAAGACAAGGGAGAAAT	0.00	0.33	0.00	0.00	0.00
GATCAAGACAAGGTATTCTC	0.00	0.00	0.00	0.00	0.34
GATCAAGACAAGGTGCTGCC	0.00	0.00	0.00	0.68	0.00
GATCAAGACAAGTGAGAAGG	0.00	0.00	0.00	0.00	0.34
GATCAAGACAAGTGAGCGGA	0.00	0.33	0.00	0.00	0.00
GATCAAGACAAGTGAGCTCG	0.00	0.00	0.00	0.00	0.34
GATCAAGACAAGTGCCGTCG	0.00	0.33	0.00	0.00	0.00
GATCAAGACAATCAAGCTCC	0.00	0.33	0.00	0.68	1.03
GATCAAGACAATCACACACC	0.00	0.00	0.00	0.00	0.34
GATCAAGACAATCATGAAAG	0.00	0.00	0.29	0.00	0.00
GATCAAGACAATCATGAACG	0.00	0.00	0.29	0.00	0.00
GATCAAGACAATGACGCGCG	0.00	0.00	0.57	0.00	0.00
GATCAAGACAATGAGTCTCG	0.00	0.00	0.00	0.00	0.34
GATCAAGACAATGCCCCAAT	0.00	0.00	0.00	0.00	0.34
GATCAAGACAATGCGTTGTA	0.00	0.00	0.00	0.00	0.69
GATCAAGACAATGTGCCGTG	0.00	0.00	0.00	0.34	0.00
GATCAAGACAATTAATCTGG	0.00	0.00	0.57	0.00	0.00
GATCAAGACAATTGGAAGAC	0.00	0.00	0.00	0.00	0.34
GATCAAGACAATTGTTCTGT	0.00	0.00	0.00	0.34	0.00
GATCAAGACACAAAAAAAAA	0.00	0.00	0.00	0.34	0.69
GATCAAGACACAAAAANAAA	0.00	0.00	0.00	0.00	0.34
GATCAAGACACAAAGAAAAA	0.00	0.00	0.00	0.34	0.00
GATCAAGACACAACCAATGT	0.00	0.00	0.00	0.00	0.00
GATCAAGACACAACGACAAA	0.00	0.00	0.00	0.34	0.00
GATCAAGACACACAAAAAAA	0.00	0.00	0.29	0.00	0.00
GATCAAGACACACACGACTA	0.00	0.00	0.29	0.00	0.00

GATCAAGACACACCCGCTGT	0.00	0.00	0.00	0.00	0.00
GATCAAGACACACCTGGCCG	0.00	0.00	0.00	0.00	0.34
GATCAAGACACACGAACAAT	0.00	0.00	0.29	0.00	0.00
GATCAAGACACACGGCAGAA	0.00	0.00	0.00	0.00	0.69
GATCAAGACACAGAACAGAA	0.00	0.00	0.00	0.00	2.07
GATCAAGACACAGCCCTGGA	0.00	0.00	0.00	0.34	0.00
GATCAAGACACAGCTAATCG	0.00	0.33	0.29	0.34	0.00
GATCAAGACACAGGTGGAAC	0.23	0.00	0.00	0.00	0.00
GATCAAGACACAGTACGGAT	0.00	0.33	0.00	0.00	0.00
GATCAAGACACAGTTCCAGC	0.00	0.33	0.00	0.00	0.00
GATCAAGACACATCCATGAC	0.00	0.00	0.29	0.00	0.00
GATCAAGACACATGAGCAAA	0.00	0.00	0.00	0.00	0.00
GATCAAGACACATGCCCCGG	1.60	0.98	3.15	1.36	2.41
GATCAAGACACATGCCCGGG	0.00	0.00	0.00	0.00	0.00
GATCAAGACACATTGTTCGT	0.00	0.00	0.00	0.00	0.34
GATCAAGACACCACAACACA	0.00	0.00	0.29	0.00	0.00
GATCAAGACACCACCAAAGT	0.00	0.33	0.00	0.00	0.00
GATCAAGACACCACCAATGT	7.78	6.51	2.58	4.41	6.54
GATCAAGACACCACCAATNN	0.23	0.00	0.00	0.00	0.00
GATCAAGACACCACCANNNT	0.00	0.33	0.00	0.00	0.00
GATCAAGACACCACCANTGT	0.00	0.00	0.00	0.00	0.34
GATCAAGACACCATCTGAAT	0.00	0.00	0.00	0.34	0.00
GATCAAGACACCATGATGTC	0.00	0.00	0.00	0.34	0.00
GATCAAGACACCATGTGCTG	0.92	0.33	1.43	1.70	3.44
GATCAAGACACCGGAGCAAG	0.00	0.00	0.00	0.34	0.00
GATCAAGACACCGGTCCAAA	0.00	0.00	0.00	0.00	0.69
GATCAAGACACCGGTCCAGG	0.23	0.98	2.00	0.34	0.69
GATCAAGACACCGGTTCAGG	0.00	0.00	0.00	0.34	0.00
GATCAAGACACCTGACCTGG	0.00	0.00	0.00	0.00	0.34
GATCAAGACACCTGAGCCTG	2.52	1.30	0.57	0.68	0.69
GATCAAGACACCTGAGCGGT	0.23	0.00	0.00	0.00	0.00
GATCAAGACACCTGGCGAGT	0.00	0.00	0.57	0.00	0.00
GATCAAGACACGAAAAAAAA	6.41	1.95	3.15	6.79	8.61
GATCAAGACACGAAAAAAAG	4.58	0.33	0.86	1.36	1.38
GATCAAGACACGAAAAAGGC	0.00	0.00	0.00	0.34	0.00
GATCAAGACACGAGAAAAAA	0.00	0.00	0.29	0.00	0.00
GATCAAGACACGAGAAGCTC	0.23	0.00	0.00	0.00	0.34

GATCAAGACACGATAAAAAA	0.00	0.65	0.00	0.00	0.00
GATCAAGACACGATGGTTGC	0.00	0.00	0.00	0.34	0.00
GATCAAGACACGATTTTTGC	0.00	0.00	0.00	0.34	0.00
GATCAAGACACGCAAAAAAA	0.00	0.00	0.00	0.00	0.69
GATCAAGACACGCAGCCTTC	0.46	0.00	0.00	0.00	0.00
GATCAAGACACGCATGTGCA	0.00	0.00	0.00	0.00	0.00
GATCAAGACACGCCCAGAGA	0.00	0.33	0.00	0.00	0.00
GATCAAGACACGCCTGCCCG	0.00	0.00	0.00	0.00	0.34
GATCAAGACACGCCTGGACG	0.00	0.00	0.00	0.34	0.00
GATCAAGACACGCCTGGCAG	0.00	0.00	0.29	0.00	0.00
GATCAAGACACGCCTGGCCG	3.66	19.52	24.34	12.90	15.15
GATCAAGACACGCCTGGCNN	0.00	0.00	0.00	0.00	0.00
GATCAAGACACGCCTGGGCG	0.00	0.00	0.29	0.00	0.00
GATCAAGACACGCCTGGNNN	0.00	0.00	0.00	0.00	0.00
GATCAAGACACGCGGCGGCG	0.00	0.33	0.00	0.34	0.00
GATCAAGACACGCTTCAAAC	0.00	0.00	0.00	0.00	0.00
GATCAAGACACGCTTGCAGA	0.23	0.00	0.00	0.00	0.34
GATCAAGACACGGATAAGGT	0.00	0.00	0.00	0.00	0.34
GATCAAGACACGGCAGGAGC	0.00	0.00	0.57	0.00	0.00
GATCAAGACACGGGAAAAAA	0.00	0.65	0.00	0.00	0.00
GATCAAGACACGGGCTCCGC	0.00	0.00	0.29	0.00	0.00
GATCAAGACACGGGCTCCGG	0.00	0.00	0.29	0.00	0.69
GATCAAGACACGGTCAGGCA	0.23	0.33	0.00	0.00	0.00
GATCAAGACACGTGCCTGTG	0.00	0.00	0.29	0.00	0.00
GATCAAGACACGTTCCATCA	0.00	0.33	0.00	0.00	0.00
GATCAAGACACGTTCCCTCA	0.00	0.33	0.00	0.00	0.00
GATCAAGACACTCAAAAGGA	1.60	5.21	3.72	2.72	2.76
GATCAAGACACTCACAAGGA	0.23	0.00	0.00	0.00	0.00
GATCAAGACACTCCAAAGGA	0.00	0.33	0.00	0.00	0.00
GATCAAGACACTCCTCCTTG	0.00	0.00	0.00	0.00	0.34
GATCAAGACACTCGACTTTG	0.00	0.00	0.00	0.00	0.00
GATCAAGACACTCGAGCAGA	0.00	0.00	0.57	0.34	0.69
GATCAAGACACTCTACAGAG	0.00	0.00	0.00	0.00	0.34
GATCAAGACACTCTCGTGGA	0.00	0.00	0.29	0.00	0.00
GATCAAGACACTCTGGCNNN	0.00	0.00	0.00	0.00	0.00
GATCAAGACACTGAATGAAG	0.23	0.00	0.00	0.00	0.00
GATCAAGACACTGCAAGCCT	0.00	0.00	0.29	0.00	0.00

GATCAAGACACTGCGAGCCA	0.00	0.00	0.00	0.00	0.34
GATCAAGACACTGGCCGAAT	1.83	0.65	0.00	1.36	0.00
GATCAAGACACTGTGAATTG	0.00	0.00	0.00	0.00	0.34
GATCAAGACACTGTTCAAGC	0.46	0.00	0.00	0.00	0.00
GATCAAGACACTTGATGAGG	0.00	0.00	0.29	0.00	0.00
GATCAAGACAGAAAAAAAAA	0.00	0.33	0.00	0.68	0.00
GATCAAGACAGAACAGAACA	0.00	0.00	0.00	0.00	0.34
GATCAAGACAGAACCAAAAA	0.00	0.00	0.00	0.00	0.34
GATCAAGACAGAAGGGAACA	0.46	0.98	0.29	0.34	0.34
GATCAAGACAGAAGTGATGG	0.00	0.00	0.00	0.34	0.00
GATCAAGACAGAATTGCCGC	0.23	0.00	0.00	0.00	0.34
GATCAAGACAGACAGAAAGA	0.00	0.00	0.00	0.00	0.34
GATCAAGACAGACAGACAGC	0.00	0.00	0.57	0.00	0.00
GATCAAGACAGACATCGCCG	0.69	0.33	0.00	0.00	0.00
GATCAAGACAGACCAAGACA	0.00	0.00	0.29	0.00	0.00
GATCAAGACAGACCCAGTTC	0.00	0.00	0.00	0.00	0.00
GATCAAGACAGACTCTGCAA	0.69	0.33	0.00	0.00	0.00
GATCAAGACAGAGAAAAACN	0.23	0.00	0.00	0.00	0.00
GATCAAGACAGAGAACACAG	0.00	0.00	0.00	0.00	0.34
GATCAAGACAGAGAAGCAGA	0.00	0.00	0.57	0.00	0.00
GATCAAGACAGAGACAAAGA	0.00	0.00	0.29	0.00	0.00
GATCAAGACAGAGACACAGA	0.00	0.00	0.00	0.00	0.00
GATCAAGACAGAGACACGAT	0.00	0.00	0.00	0.00	0.00
GATCAAGACAGAGAGAGTTT	0.00	0.00	0.29	0.00	0.00
GATCAAGACAGAGAGGGAGA	0.00	0.00	0.00	0.34	0.00
GATCAAGACAGAGCAGAGAC	0.00	0.00	0.29	0.00	0.00
GATCAAGACAGAGCAGTGCC	0.23	0.00	0.00	0.00	0.00
GATCAAGACAGAGCTCTGGC	0.00	0.00	0.29	0.00	0.00
GATCAAGACAGAGCTTGGCA	0.00	0.00	0.57	0.00	0.00
GATCAAGACAGAGGAAGCTC	0.00	0.00	0.29	0.00	0.00
GATCAAGACAGAGGAATGCA	0.00	0.00	0.00	0.00	0.00
GATCAAGACAGAGGATGAAG	0.00	0.33	0.29	0.00	0.00
GATCAAGACAGAGGATGACG	9.39	20.17	7.16	11.88	12.74
GATCAAGACAGAGGATGACT	0.00	0.00	0.00	0.00	0.00
GATCAAGACAGAGGATGATN	0.00	0.00	0.00	0.00	0.00
GATCAAGACAGAGGCGCCCT	0.00	0.33	0.00	0.00	0.00
GATCAAGACAGAGGCGCTGC	0.00	0.65	0.00	0.00	0.00

GATCAAGACAGAGGGATTCT	0.92	0.00	0.00	0.00	0.00
GATCAAGACAGAGTGCCCCA	0.00	0.00	0.00	0.00	0.00
GATCAAGACAGAGTGCCCCC	0.00	0.00	0.00	0.34	0.00
GATCAAGACAGAGTGTACAA	0.00	0.00	0.29	0.00	0.00
GATCAAGACAGATATGAACA	0.23	0.00	0.00	0.00	0.00
GATCAAGACAGATATGCCCA	0.23	0.00	0.00	0.00	0.00
GATCAAGACAGATATGTCCA	2.98	2.60	0.00	2.38	0.69
GATCAAGACAGATATTGAGG	0.69	0.98	0.29	0.00	0.34
GATCAAGACAGATGTCAAGA	0.00	1.30	0.00	0.34	0.00
GATCAAGACAGATGTGTGCN	0.23	0.00	0.00	0.00	0.00
GATCAAGACAGCAAAAAAAA	0.00	0.00	0.29	0.00	0.00
GATCAAGACAGCAACAGCTG	0.00	0.00	0.00	0.68	0.34
GATCAAGACAGCACAGAACA	0.00	0.00	0.00	0.00	0.34
GATCAAGACAGCACAGAGCC	0.00	0.00	0.29	0.00	0.00
GATCAAGACAGCACCAACAC	0.00	0.00	0.00	0.00	0.34
GATCAAGACAGCAGATTTGC	0.00	0.33	0.00	0.00	0.00
GATCAAGACAGCAGGCACAA	0.00	0.33	0.57	0.68	0.34
GATCAAGACAGCATGGATGC	0.00	0.00	0.29	0.00	0.00
GATCAAGACAGCATTGACGG	0.00	0.00	0.29	0.00	0.00
GATCAAGACAGCATTGGCGA	0.00	0.00	0.29	0.00	0.00
GATCAAGACAGCCAACAAGG	0.00	0.33	0.00	0.00	0.00
GATCAAGACAGCCAAGAAGA	0.00	0.00	0.00	0.34	0.00
GATCAAGACAGCCACCAAGG	0.00	0.00	0.00	0.34	0.00
GATCAAGACAGCCACGTTGA	0.00	0.33	0.86	0.00	0.00
GATCAAGACAGCCGAAGCAA	0.00	0.00	0.29	0.00	0.00
GATCAAGACAGCCGCCAAGG	0.69	0.00	0.00	0.00	0.00
GATCAAGACAGCCTGCTCGC	0.00	0.00	0.29	0.00	0.00
GATCAAGACAGCGAAGAGCC	0.00	0.00	0.29	0.00	0.00
GATCAAGACAGCGACTAATG	0.00	0.00	0.00	0.00	0.00
GATCAAGACAGCGAGACACG	0.00	0.00	1.15	0.00	0.00
GATCAAGACAGCGCAAAAAA	0.00	0.00	0.29	0.00	1.03
GATCAAGACAGCGCAAAAGC	0.00	0.00	0.29	0.00	0.00
GATCAAGACAGCGCAAAGCC	0.23	0.00	0.00	0.00	0.00
GATCAAGACAGCGCAAGAGA	0.00	0.00	0.00	0.68	0.00
GATCAAGACAGCGCAGAAAA	0.00	0.00	0.00	0.34	0.00
GATCAAGACAGCGCAGAACT	0.00	0.00	0.00	0.00	0.00
GATCAAGACAGCGCAGAGAG	0.00	0.00	0.00	0.00	0.34

GATCAAGACAGCGCAGAGCA	1.60	0.65	2.29	2.38	1.38
GATCAAGACAGCGCAGAGCC	54.71	28.30	40.38	34.62	43.05
GATCAAGACAGCGCAGAGCG	0.46	0.00	0.00	0.00	0.34
GATCAAGACAGCGCAGAGCN	0.46	0.00	0.00	0.00	0.00
GATCAAGACAGCGCAGAGGC	0.23	0.00	0.00	0.00	0.00
GATCAAGACAGCGCAGAGNC	0.00	0.00	0.00	0.00	0.34
GATCAAGACAGCGCAGAGNN	0.46	0.00	0.00	0.00	0.00
GATCAAGACAGCGCAGAGTC	0.00	0.33	0.00	0.00	0.00
GATCAAGACAGCGCAGAGTG	0.00	0.00	0.00	0.00	0.34
GATCAAGACAGCGCAGCGCC	0.23	0.00	0.29	0.00	0.34
GATCAAGACAGCGCAGGAAA	0.00	0.33	0.00	0.00	0.00
GATCAAGACAGCGCAGGGCC	0.23	0.00	0.00	0.34	0.00
GATCAAGACAGCGCAGTATG	0.00	0.00	0.29	0.00	0.00
GATCAAGACAGCGCAGTGCC	0.23	0.00	0.00	0.00	0.00
GATCAAGACAGCGCATCTGA	0.00	0.00	0.00	0.34	0.00
GATCAAGACAGCGCCGCGCC	0.23	0.00	0.00	0.00	0.00
GATCAAGACAGCGCGAAAAG	0.00	0.00	0.00	0.34	0.00
GATCAAGACAGCGCGGAACC	0.00	0.00	0.00	0.00	0.34
GATCAAGACAGCGCGGAGAA	0.00	0.00	0.29	0.00	0.00
GATCAAGACAGCGCGGAGCC	0.23	0.00	0.00	0.00	0.00
GATCAAGACAGCGCGGAGTC	0.23	0.00	0.00	0.00	0.00
GATCAAGACAGCGCTCGAAA	0.00	0.00	0.00	0.34	0.00
GATCAAGACAGCGCTCGTAT	0.00	0.00	0.00	0.34	0.00
GATCAAGACAGCGGAGTGGA	0.00	0.00	0.29	0.00	0.00
GATCAAGACAGCGGCGAGAG	0.00	0.65	0.00	0.00	0.00
GATCAAGACAGCGGCTATTC	0.00	0.33	0.00	0.00	0.00
GATCAAGACAGCGGGAGAGA	0.00	0.00	0.00	0.34	0.00
GATCAAGACAGCGGTACTCG	0.23	0.00	0.00	0.00	0.00
GATCAAGACAGCGGTGGGTG	0.00	0.33	0.00	0.00	0.00
GATCAAGACAGCGGTTCCTT	0.69	0.00	0.00	0.00	0.00
GATCAAGACAGCGTACAAAT	0.00	0.00	0.00	0.00	0.34
GATCAAGACAGCTACCACTG	0.00	0.00	0.00	0.34	0.00
GATCAAGACAGCTCCCAAAA	0.00	0.00	0.29	0.00	0.00
GATCAAGACAGCTCCCACCG	0.00	0.00	0.29	0.00	0.00
GATCAAGACAGCTCCCACGG	0.00	0.00	0.00	0.00	0.00
GATCAAGACAGCTCCCACTG	2.75	1.30	0.57	1.02	0.69
GATCAAGACAGCTTCTGGCA	0.00	0.00	0.29	0.00	0.00

GATCAAGACAGCTTGACACA	0.00	0.00	0.00	0.00	0.00
GATCAAGACAGGAAAAAAAA	3.43	0.33	0.29	1.02	0.69
GATCAAGACAGGAAAAAGAA	0.23	0.00	0.00	0.00	0.00
GATCAAGACAGGAACAGCCT	0.00	0.33	0.00	0.00	0.00
GATCAAGACAGGAAGAAAAA	0.00	0.00	0.00	0.00	0.00
GATCAAGACAGGAAGAAGGA	0.00	0.33	0.00	0.68	0.00
GATCAAGACAGGAAGAGAGA	0.00	0.00	0.00	0.34	0.00
GATCAAGACAGGAAGAGAGC	0.00	0.00	0.00	0.00	0.00
GATCAAGACAGGAAGAGGAA	0.69	1.95	0.00	0.00	0.00
GATCAAGACAGGAAGAGGAG	0.00	0.00	0.00	0.00	0.00
GATCAAGACAGGAAGAGGCA	0.00	0.00	0.00	0.68	0.00
GATCAAGACAGGAAGAGGGA	20.60	19.84	12.31	30.55	16.19
GATCAAGACAGGAAGAGGGC	0.92	0.33	0.00	0.00	0.34
GATCAAGACAGGAAGAGGGG	2.06	0.00	0.29	0.00	0.00
GATCAAGACAGGAAGAGGGN	0.23	0.00	0.00	0.00	0.00
GATCAAGACAGGAAGAGGGT	0.00	0.00	0.29	0.00	0.00
GATCAAGACAGGAAGAGGNN	0.46	0.00	0.00	0.00	0.00
GATCAAGACAGGAAGAGNNN	0.46	0.00	0.00	0.00	0.00
GATCAAGACAGGAAGAGTAA	0.00	0.33	0.00	0.00	0.00
GATCAAGACAGGAAGGGGGA	0.00	0.00	0.29	0.00	0.00
GATCAAGACAGGAAGTGATG	0.00	0.00	0.00	0.00	0.34
GATCAAGACAGGAGCAGCAG	0.00	0.00	0.00	0.00	0.34
GATCAAGACAGGAGCCTCGC	0.00	0.00	0.00	0.34	0.00
GATCAAGACAGGAGGCGAAA	0.00	0.33	0.00	0.00	0.00
GATCAAGACAGGAGGCTCTT	0.23	0.00	0.00	0.00	0.00
GATCAAGACAGGCACCCCCG	0.23	0.00	0.00	0.00	0.00
GATCAAGACAGGCACGTCGC	0.00	0.00	0.00	0.00	0.00
GATCAAGACAGGCACGTTGC	2.98	1.30	0.57	0.34	0.34
GATCAAGACAGGCCAGTGAT	0.00	0.33	0.00	0.00	0.00
GATCAAGACAGGCGACCTGA	0.00	0.00	0.00	0.00	0.00
GATCAAGACAGGCGAGTCCG	0.00	0.00	0.29	0.00	0.00
GATCAAGACAGGCGCGCCAT	0.23	0.00	0.29	0.00	0.00
GATCAAGACAGGCGCGCCCT	0.23	0.00	0.00	0.00	0.00
GATCAAGACAGGCGGACACA	0.23	0.00	0.00	0.34	0.00
GATCAAGACAGGCGGATACA	0.23	0.00	0.00	0.00	0.00
GATCAAGACAGGCGGATGAC	0.00	0.65	0.00	0.34	0.00
GATCAAGACAGGCTCAGTTG	0.23	0.00	0.00	0.00	0.00

GATCAAGACAGGCTCTTTGG	0.00	0.00	0.00	0.34	0.00
GATCAAGACAGGCTGAGCCT	0.00	0.00	0.00	0.34	0.00
GATCAAGACAGGCTGCACCA	0.46	0.00	0.00	0.00	0.00
GATCAAGACAGGCTGGCAGA	0.00	0.00	0.00	0.00	0.00
GATCAAGACAGGGAAAAAAA	0.23	0.00	0.00	0.00	0.00
GATCAAGACAGGGAGAGGGA	0.23	0.00	0.00	0.00	0.00
GATCAAGACAGGGCCGTTCT	0.00	0.00	0.00	0.00	0.34
GATCAAGACAGGGGCTTGAC	0.00	0.33	0.00	0.68	0.00
GATCAAGACAGGGTTAGGGA	0.00	0.00	0.00	0.00	0.69
GATCAAGACAGGTAAAAAAA	0.00	0.00	0.00	0.34	0.00
GATCAAGACAGGTAAGTAGA	0.00	0.00	0.00	0.00	0.00
GATCAAGACAGGTCACGTCT	0.00	0.00	0.00	0.00	0.34
GATCAAGACAGGTCGGACTC	0.00	0.00	0.00	0.00	0.00
GATCAAGACAGGTGCCCCTT	0.00	0.00	0.00	0.34	0.00
GATCAAGACAGGTGGCTCTG	0.00	0.00	0.00	0.00	0.00
GATCAAGACAGGTGGCTCTT	2.29	0.98	0.00	1.70	0.69
GATCAAGACAGGTTCATTGG	0.23	0.00	0.00	0.00	0.00
GATCAAGACAGGTTCTGTAC	0.00	0.00	0.00	0.00	0.34
GATCAAGACAGGTTCTGTCC	2.75	9.11	3.44	2.04	2.41
GATCAAGACAGGTTCTTTGG	2.06	0.65	0.29	0.34	1.38
GATCAAGACAGTACCAACAC	0.00	0.33	0.00	0.00	0.00
GATCAAGACAGTCCGTGTTA	1.37	1.63	3.44	2.38	2.07
GATCAAGACAGTCCTGTGTC	0.00	0.33	0.00	0.68	0.00
GATCAAGACAGTCGAGTCCG	0.00	0.33	0.00	0.00	0.00
GATCAAGACAGTCGGAAAGA	0.00	0.00	0.29	0.00	0.00
GATCAAGACAGTCGGCACCG	0.00	0.00	0.00	0.34	0.00
GATCAAGACAGTCTAGTTGT	0.00	0.00	0.00	0.00	0.00
GATCAAGACAGTCTCAGGTG	0.23	0.00	0.00	0.00	0.00
GATCAAGACAGTCTCAGTTG	6.87	1.63	0.29	2.04	1.03
GATCAAGACAGTGAAGATGC	0.00	0.00	0.00	0.00	0.34
GATCAAGACAGTGAAGCTGC	0.00	0.00	0.00	0.00	0.00
GATCAAGACAGTGACTCGCA	0.00	0.00	0.29	0.00	0.00
GATCAAGACAGTGCCGTGTT	0.00	0.33	0.00	0.00	0.00
GATCAAGACAGTGCCTATTG	0.00	0.33	0.00	0.00	0.00
GATCAAGACAGTGCTGACCA	0.00	0.00	0.00	0.00	0.00
GATCAAGACAGTGCTGAGCA	0.46	0.00	0.00	0.00	0.00
GATCAAGACAGTGCTGAGCT	0.00	0.00	0.00	0.68	0.00

GATCAAGACAGTGCTGNGCT	0.00	0.00	0.00	0.34	0.00
GATCAAGACAGTGGAGTTGT	0.23	0.00	0.00	0.00	0.00
GATCAAGACAGTGGCGTTGT	0.00	0.00	0.00	0.00	0.00
GATCAAGACAGTGGCTTGGC	0.00	0.00	0.00	0.34	0.00
GATCAAGACAGTGGTTCATG	0.00	0.00	0.00	0.00	0.34
GATCAAGACAGTGTCTCGGA	0.00	0.00	0.29	0.00	0.00
GATCAAGACAGTGTCTTGAA	0.69	0.00	0.00	0.00	0.00
GATCAAGACAGTGTCTTGCA	0.00	0.00	0.00	0.00	0.34
GATCAAGACAGTGTCTTGGA	6.64	4.88	2.86	7.13	1.38
GATCAAGACAGTGTCTTGGC	0.46	0.33	0.00	0.00	0.00
GATCAAGACAGTGTCTTGGT	0.00	0.33	0.00	0.00	0.00
GATCAAGACAGTGTGCCTGG	0.00	0.00	0.00	0.00	0.00
GATCAAGACAGTTGGAATTG	0.00	0.00	0.00	0.68	0.00
GATCAAGACAGTTTTGGCCA	0.23	0.00	0.00	0.00	0.00
GATCAAGACATAAAAAAAAA	0.00	0.00	0.00	0.00	1.03
GATCAAGACATAAATGAAGA	0.23	0.00	0.00	0.00	0.00
GATCAAGACATAACAACACA	0.00	0.00	0.57	0.00	0.00
GATCAAGACATACAAGATGC	0.00	0.00	0.00	0.00	0.34
GATCAAGACATACATCGACT	0.00	0.33	0.00	0.00	0.00
GATCAAGACATACCAGCCGC	0.00	0.33	0.00	0.00	0.00
GATCAAGACATAGAAACATC	0.46	0.00	0.00	0.00	0.00
GATCAAGACATAGGAATGCT	0.00	0.00	0.29	0.00	0.00
GATCAAGACATAGGGAGAGC	0.00	0.00	0.00	0.34	0.00
GATCAAGACATAGGGAGNGA	0.00	0.00	0.00	0.00	0.00
GATCAAGACATATTGCAGAG	0.00	0.00	0.00	0.00	0.00
GATCAAGACATCAACGAAGA	0.00	0.00	0.00	0.00	0.34
GATCAAGACATCAATGAAGA	16.02	18.54	11.17	8.49	19.63
GATCAAGACATCAATGAAGC	0.00	0.00	0.00	0.34	0.00
GATCAAGACATCAATGAAGN	0.23	0.00	0.00	0.00	0.00
GATCAAGACATCAATGACGC	0.23	0.00	0.00	0.00	0.00
GATCAAGACATCAATGATCG	0.00	0.00	0.00	0.34	0.00
GATCAAGACATCAATGCAGA	0.00	0.00	0.00	0.00	0.34
GATCAAGACATCAATGGAGG	0.23	0.00	0.00	0.00	0.00
GATCAAGACATCACAACAGG	0.69	1.95	0.86	1.70	0.00
GATCAAGACATCCATCGGAT	5.04	8.78	5.44	12.56	8.61
GATCAAGACATCCATCGGCC	0.00	0.00	0.00	0.00	0.00
GATCAAGACATCCATCGGCT	0.00	0.00	0.00	0.34	0.00

GATCAAGACATCCATCGGNN	0.23	0.00	0.00	0.00	0.00
GATCAAGACATCCATCGGTT	0.00	0.33	0.00	0.00	0.00
GATCAAGACATCCATGCGCG	0.23	0.33	0.00	0.00	0.00
GATCAAGACATCCATGGAGG	0.00	0.00	0.00	0.34	0.00
GATCAAGACATCCCTGCAGC	0.00	0.00	0.00	0.00	0.34
GATCAAGACATCCCTGCCGC	0.23	0.00	0.00	0.00	0.00
GATCAAGACATCCGCAGCTC	0.00	0.00	0.29	0.00	0.00
GATCAAGACATCCTAGGATT	0.00	0.00	0.00	0.00	0.00
GATCAAGACATCCTTCGTGC	0.00	0.65	0.00	0.00	0.00
GATCAAGACATCGGGAAAAT	0.00	0.33	0.00	0.00	0.00
GATCAAGACATCGGGAGAAA	0.00	0.00	0.00	0.00	0.34
GATCAAGACATCGGGAGAGA	1.14	1.63	1.72	1.02	0.69
GATCAAGACATCGGGAGAGG	0.00	0.00	0.00	0.00	0.00
GATCAAGACATCGGGAGAGT	0.00	0.33	0.00	0.00	0.00
GATCAAGACATCGGGAGCGA	0.00	0.33	0.00	0.00	0.00
GATCAAGACATCGGGAGGGA	0.00	0.00	0.57	0.00	0.00
GATCAAGACATCGGGAGTGA	0.00	0.33	0.00	0.00	0.00
GATCAAGACATCGGGATATA	0.00	0.00	0.00	0.34	0.00
GATCAAGACATCGGGATTGA	0.00	0.00	0.29	0.00	0.00
GATCAAGACATCGGGCGAGA	0.00	0.33	0.00	0.00	0.00
GATCAAGACATCGGGCGAGT	0.00	0.00	0.29	0.00	0.00
GATCAAGACATCTCGCGGGA	0.23	0.00	0.00	0.00	0.00
GATCAAGACATCTCGCGTGA	0.23	1.30	0.86	0.68	0.69
GATCAAGACATCTGCTGATT	0.00	0.65	0.29	0.34	0.00
GATCAAGACATCTGCTTGTT	0.00	0.65	0.00	0.00	0.00
GATCAAGACATCTTCACATA	0.00	0.00	0.00	0.00	0.00
GATCAAGACATGACAGAACA	0.00	0.00	0.00	0.00	0.34
GATCAAGACATGACTTCGAA	0.00	0.00	0.29	0.00	0.00
GATCAAGACATGACTTCGCA	0.00	0.00	0.29	0.00	0.00
GATCAAGACATGATGGGTAT	0.23	0.00	0.00	0.34	0.00
GATCAAGACATGCAAGCGCA	0.00	0.00	0.00	0.00	0.34
GATCAAGACATGCACCAAGA	0.00	0.00	0.00	0.00	0.00
GATCAAGACATGCATGCGAT	0.00	0.00	0.00	0.00	0.34
GATCAAGACATGCATGCGCT	0.00	0.00	0.00	0.00	0.34
GATCAAGACATGCCGCCGAC	0.00	0.33	0.00	0.00	0.00
GATCAAGACATGCCGCCGTG	0.00	0.00	0.29	0.00	0.00
GATCAAGACATGCGCTTCAN	0.00	0.00	0.00	0.00	0.00

GATCAAGACATGCGCTTCAT	0.46	0.33	0.00	1.02	0.00
GATCAAGACATGCGCTTCGA	0.00	0.00	0.00	0.00	0.34
GATCAAGACATGCTGCAAAA	0.46	0.00	0.00	0.00	0.00
GATCAAGACATGCTGCAGAC	0.23	0.00	0.00	0.00	0.00
GATCAAGACATGCTGCCGAC	0.00	0.00	0.00	0.34	0.00
GATCAAGACATGCTGCCGAG	0.00	0.00	0.29	0.00	0.00
GATCAAGACATGCTTGAAAA	0.23	0.00	0.00	0.00	0.34
GATCAAGACATGGAACTCCA	0.00	0.00	0.00	0.00	0.00
GATCAAGACATGGAATTGGT	0.00	0.33	0.00	0.00	0.00
GATCAAGACATGGACGAAAC	0.00	0.00	0.29	0.00	0.00
GATCAAGACATGGCAATGCC	0.00	0.00	0.29	0.00	0.00
GATCAAGACATGGCCAACTC	0.00	0.00	0.00	0.00	0.34
GATCAAGACATGGCCAAGCG	8.01	6.83	4.01	8.15	6.89
GATCAAGACATGGCCAAGTG	0.00	0.00	0.00	0.00	0.34
GATCAAGACATGGCCGCCAA	0.00	0.00	0.00	0.00	0.00
GATCAAGACATGGCCTTGGG	0.00	0.00	0.29	0.00	0.00
GATCAAGACATGGTAGGCGC	0.00	0.00	0.00	0.00	0.69
GATCAAGACATGTGAGACCC	0.00	0.00	0.00	0.34	0.00
GATCAAGACATGTGAGACTA	0.00	0.00	0.00	0.34	0.00
GATCAAGACATGTGCGAGAA	0.23	0.00	0.00	0.00	0.00
GATCAAGACATGTGCGCCCA	0.00	0.98	0.00	0.00	0.00
GATCAAGACATGTGTCTTGT	0.00	0.00	0.00	0.34	0.00
GATCAAGACATTAAAAAAAA	0.00	0.00	0.00	0.34	0.00
GATCAAGACATTATACACTC	0.00	0.00	0.00	0.00	0.00
GATCAAGACATTCTTAGCTG	0.23	0.00	0.29	0.00	0.00
GATCAAGACATTGAAAAAAA	0.00	0.33	0.00	0.00	0.00
GATCAAGACATTGAAACCGG	0.00	0.00	0.57	0.00	0.00
GATCAAGACATTGAATGAAA	0.00	0.00	0.57	0.00	0.00
GATCAAGACATTGAATGAAG	22.43	21.80	24.91	22.40	27.55
GATCAAGACATTGAATGAAT	0.23	0.00	0.00	0.00	0.00
GATCAAGACATTGAATGAGA	0.00	0.00	0.00	0.00	0.00
GATCAAGACATTGAATGCAG	0.00	0.00	0.00	0.00	0.00
GATCAAGACATTGAATGNNN	0.23	0.00	0.00	0.00	0.00
GATCAAGACATTGAATGTGC	0.00	0.00	0.29	0.00	0.00
GATCAAGACATTGAATNAAG	0.00	0.00	0.00	0.00	0.34
GATCAAGACATTGAATNNNN	0.46	0.00	0.00	0.00	0.00
GATCAAGACATTGACTGACG	0.23	0.00	0.00	0.00	0.00

GATCAAGACATTGGATGAGT	0.00	0.00	0.00	0.00	0.00
GATCAAGACATTGGATGCCC	0.23	0.00	0.00	0.00	0.00
GATCAAGACATTGTAACTCC	0.69	0.98	0.86	1.36	2.07
GATCAAGACATTTATTGCCT	0.00	0.00	0.29	0.00	0.00
GATCAAGACCAAAAAAAAAA	2.98	0.33	0.29	1.70	0.69
GATCAAGACCAAAAAANAAA	0.00	0.00	0.00	0.00	0.00
GATCAAGACCAAAAGCGGCA	0.00	0.00	0.00	0.00	0.00
GATCAAGACCAACAACACAA	0.00	0.00	0.00	0.00	0.00
GATCAAGACCAACACCAAGG	0.00	0.00	0.29	0.00	0.00
GATCAAGACCAACACCTCCC	0.00	0.00	0.00	0.00	0.34
GATCAAGACCAACATTGACG	0.23	0.00	0.00	0.00	0.00
GATCAAGACCAACATTGACT	0.46	0.00	0.00	0.00	0.00
GATCAAGACCAACCTCCTGA	0.00	0.33	0.29	0.34	0.00
GATCAAGACCAACCTCTGGA	0.00	0.00	0.00	0.68	0.00
GATCAAGACCAACGTGCCCA	0.00	0.00	0.29	0.00	0.00
GATCAAGACCAACTCTGCAA	0.00	0.00	0.00	0.34	0.00
GATCAAGACCAACTCTGCGC	0.00	0.33	0.00	0.00	0.00
GATCAAGACCAACTCTGCGG	0.23	0.00	0.00	0.00	0.69
GATCAAGACCAACTCTGCTC	0.23	0.00	0.57	0.68	1.03
GATCAAGACCAACTGTGAGG	0.00	0.00	0.29	0.00	0.00
GATCAAGACCAACTGTNNGG	0.00	0.00	0.00	0.00	0.00
GATCAAGACCAACTTGTCGA	0.00	0.00	0.29	0.00	0.00
GATCAAGACCAAGAACAGAA	0.00	0.00	0.29	0.00	0.00
GATCAAGACCAAGAAGAACC	0.00	0.00	0.00	0.00	0.00
GATCAAGACCAAGAAGAACG	0.00	0.00	0.00	0.34	0.00
GATCAAGACCAAGAAGAACT	0.00	0.65	0.00	0.00	0.00
GATCAAGACCAAGAAGAATC	0.00	0.33	0.00	0.00	0.00
GATCAAGACCAAGAAGCTGA	0.00	0.33	0.00	0.00	0.00
GATCAAGACCAAGACCAAGA	0.00	0.33	0.29	0.34	0.34
GATCAAGACCAAGACCAAGC	0.00	0.00	0.86	0.00	0.34
GATCAAGACCAAGACCAAGG	0.00	0.00	0.00	0.00	0.69
GATCAAGACCAAGAGGAGAA	0.00	0.00	0.00	0.00	1.03
GATCAAGACCAAGATGGAGG	0.00	0.00	0.00	0.00	0.34
GATCAAGACCAAGCAAGTTG	0.00	0.33	0.00	0.00	0.00
GATCAAGACCAAGCGCGAAG	0.00	0.33	0.00	0.00	0.00
GATCAAGACCAAGCTCATCG	0.00	0.00	0.00	0.00	0.00
GATCAAGACCAAGCTGAGGG	0.00	0.00	0.00	0.00	0.00

GATCAAGACCAAGCTGGAAG	0.00	0.33	0.29	0.68	0.00
GATCAAGACCAAGGAGGAGG	0.00	0.00	0.29	0.00	0.00
GATCAAGACCAAGGCCAAGA	0.00	0.00	0.57	0.00	0.00
GATCAAGACCAAGTACTCGA	0.69	0.00	0.00	0.00	0.34
GATCAAGACCAAGTGGTTCT	0.00	0.33	0.00	0.34	0.00
GATCAAGACCAATACCAAGA	0.00	0.00	0.00	0.00	0.34
GATCAAGACCAATGATATGC	0.00	0.00	0.00	0.00	0.00
GATCAAGACCAATGATGATG	0.00	0.33	0.00	0.00	0.00
GATCAAGACCAATGCAAGTC	0.23	0.00	0.00	0.00	0.00
GATCAAGACCAATGCTGGCC	0.00	0.00	0.00	0.34	0.00
GATCAAGACCAATGGCCTCA	0.00	0.00	0.00	0.00	0.00
GATCAAGACCACAGGCTCTT	0.00	0.00	0.00	0.00	0.00
GATCAAGACCACCAAGGAGG	0.23	0.00	0.00	0.00	0.00
GATCAAGACCACCACCAAGG	0.46	1.30	0.29	1.36	0.00
GATCAAGACCACCACCATTC	0.00	0.65	0.00	0.34	0.00
GATCAAGACCACCAGTCCCT	0.00	0.00	0.00	0.34	0.00
GATCAAGACCACCGCATCTG	0.23	0.00	0.00	0.00	0.00
GATCAAGACCACCGCCAGGT	0.00	0.00	0.00	0.00	0.34
GATCAAGACCACCGCTCGTA	0.23	0.00	0.00	0.00	0.00
GATCAAGACCACCGTGTCAT	0.00	0.00	0.86	0.00	0.00
GATCAAGACCACCTTCGCAA	0.00	0.00	0.00	0.00	0.00
GATCAAGACCACCTTTGCCA	0.00	0.00	0.00	0.00	0.34
GATCAAGACCACGACCACGA	0.00	0.00	0.29	0.00	0.00
GATCAAGACCACGAGGAAGC	0.00	0.65	0.00	0.00	0.00
GATCAAGACCACGAGTGNGC	0.00	0.00	0.29	0.00	0.00
GATCAAGACCACGATGCACA	0.00	0.00	0.29	0.00	0.34
GATCAAGACCACGGCACAGG	0.00	0.33	0.00	0.00	0.00
GATCAAGACCACGGCTATTC	0.00	0.00	0.00	0.34	0.00
GATCAAGACCACGTCCATGC	0.46	0.00	0.00	0.00	0.00
GATCAAGACCACGTGCTCTT	0.00	0.33	0.00	0.00	0.34
GATCAAGACCACTCTTGCCA	0.00	0.00	0.00	0.34	0.00
GATCAAGACCACTGCTAGGT	0.00	0.00	0.00	1.02	0.00
GATCAAGACCACTGGTAATG	0.00	0.33	0.57	0.34	0.00
GATCAAGACCACTGGTACTG	0.00	0.33	0.57	0.00	0.00
GATCAAGACCACTGTTGCCA	0.00	0.00	0.00	0.00	0.00
GATCAAGACCACTGTTTCCA	0.00	0.00	0.29	0.00	0.00
GATCAAGACCACTTCACTCG	0.00	0.00	0.00	0.34	0.00

GATCAAGACCACTTCAGGCG	0.00	0.00	0.00	0.34	0.00
GATCAAGACCACTTCTGCCA	0.23	0.00	0.00	0.00	0.00
GATCAAGACCACTTGGGACC	0.00	0.00	0.00	0.00	0.00
GATCAAGACCACTTGTGCCA	0.00	0.33	0.00	0.00	0.00
GATCAAGACCACTTTTGCAA	0.00	0.00	0.00	0.34	0.00
GATCAAGACCACTTTTGCCA	32.50	18.54	23.48	22.74	19.98
GATCAAGACCACTTTTGCCC	0.00	0.33	0.00	0.00	0.00
GATCAAGACCACTTTTGCCN	0.46	0.00	0.00	0.00	0.00
GATCAAGACCACTTTTNCCA	0.00	0.00	0.29	0.00	0.00
GATCAAGACCACTTTTNNNN	0.23	0.00	0.00	0.00	0.00
GATCAAGACCAGAACAAGAC	0.00	0.00	0.00	0.00	0.69
GATCAAGACCAGAAGGCCCA	0.00	0.00	0.00	0.00	0.00
GATCAAGACCAGAATGCCCA	0.69	0.00	0.57	0.00	0.69
GATCAAGACCAGAATGTGCT	0.00	0.00	0.00	0.34	0.00
GATCAAGACCAGACAATGCA	0.23	0.33	0.00	0.00	0.00
GATCAAGACCAGACAGACCC	0.23	0.00	0.00	0.00	0.00
GATCAAGACCAGACGCCCAA	0.00	0.00	0.00	0.00	0.00
GATCAAGACCAGAGCTCCGG	0.00	0.00	0.00	0.00	0.00
[truncated: 490,149 more chars]
